# Locational memory of macrovessel vascular cells is transcriptionally imprinted

**DOI:** 10.1038/s41598-023-38880-6

**Published:** 2023-08-10

**Authors:** Talitha C. F. Spanjersberg, Loes A. Oosterhoff, Hedwig S. Kruitwagen, Noortje A. M. van den Dungen, Johannes C. M. Vernooij, Folkert W. Asselbergs, Michal Mokry, Bart Spee, Magdalena Harakalova, Frank G. van Steenbeek

**Affiliations:** 1 Department of Clinical Sciences, Faculty of Veterinary Medicine, Utrecht University, Yalelaan 104, Utrecht, The Netherlands; 2 Regenerative Medicine Centre Utrecht, University Medical Center Utrecht, Utrecht University, Uppsalalaan 8, Utrecht, The Netherlands; 3 Division Heart and Lungs, Department of Cardiology, Circulatory Health Research Center, University Medical Center Utrecht, Utrecht University, Heidelberglaan 100, Utrecht, The Netherlands; 4 Central Diagnostics Laboratory, University Medical Center Utrecht, Utrecht University, Utrecht, The Netherlands; 5 Department of Population Health Sciences, Faculty of Veterinary Medicine, Utrecht University, Yalelaan 7, Utrecht, The Netherlands; 6 Department of Cardiology, Amsterdam University Medical Centers, University of Amsterdam, Meibergdreef 9, Amsterdam, The Netherlands; 7 Health Data Research UK and Institute of Health Informatics, University College London, London, UK; 8 Laboratory of Experimental Cardiology, Department of Cardiology, University Medical Center Utrecht, University Utrecht, Utrecht, The Netherlands

**Keywords:** Gene ontology, Gene expression, Cell biology

## Abstract

Vascular pathologies show locational predisposition throughout the body; further insights into the transcriptomics basis of this vascular heterogeneity are needed. We analyzed transcriptomes from cultured endothelial cells and vascular smooth muscle cells from nine adult canine macrovessels: the aorta, coronary artery, vena cava, portal vein, femoral artery, femoral vein, saphenous vein, pulmonary vein, and pulmonary artery. We observed that organ-specific expression patterns persist in vitro, indicating that these genes are not regulated by blood flow or surrounding cell types but are likely fixed in the epigenetic memory. We further demonstrated the preserved location-specific expression of GATA4 protein in cultured cells and in the primary adult vessel. On a functional level, arterial and venous endothelial cells differed in vascular network morphology as the arterial networks maintained a higher complexity. Our findings prompt the rethinking of the extrapolation of results from single-origin endothelial cell systems.

## Introduction

Various types of vascular disorders with a spatial predisposition, such as atherosclerosis, deep vein thrombosis, and pulmonary artery embolism, are known to be influenced by vascular heterogeneity^1^. Nowadays, the emergence of large-scale transcriptome characterization technologies enables the identification of vascular specialization on a molecular level with increased specificity^2,3^. Although many studies focus on endothelial cells (ECs), other supporting cell types, such as vascular smooth muscle cells (VSMCs), also play a role in vascular pathologies^4^. To investigate how VSMCs compare to ECs in vascular heterogeneity, combining data from these cell types originating from various vessels is needed. Obtaining such data, however, is hampered by the limited variety of vascular cell types that can be isolated from humans due to legal and ethical constraints. Therefore, large animals mimicking human physiology pose an attractive alternative tissue source, particularly considering the increasing demand for suitable animal models for advancing vascular tissue engineering^5^.

In an extension of our previous study, we used our recently published isolation protocol to isolate and culture both ECs and VSMCs from nine different canine macrovessels^6^. Although endothelial heterogeneity has been reported to disappear after several passages in culture, accumulating evidence demonstrates that some organ-specific expression patterns persist in vitro^7–9^*.* In umbilical cord endothelial cells, the most widely used cell type for vascular research, it was discovered that more than half of the transcriptome remains unaltered following in vitro culture^10^. This epigenetic memory may be the foundation of vascular heterogeneity as they may explain the inadequate adaptations to hemodynamic forces in some vessels but not others. For instance, back in the 70s, a canine jugular vein segment was transplanted into the arterial circuit of the carotid artery. Instead of acquiring arterial properties, the tissue response of the venous grafts was reflected by intimal proliferation, a common consequence of vascular injury^11^. Nowadays, venous graft failure is still a frequent complication of bypass surgery, illustrating the need for a deeper insight into vascular heterogeneity^12^.

Studying the expression pattern of cultured cells from different anatomic locations may reveal an origin-dependent response to vascular injury. VSMCs are known to originate from various sources that may partially influence the disease susceptibility of vessels. For example, VSMCs in the aortic arch, embryonically derived from cardiac neural crest cells, are more prone to pathological calcification than VSMCs in the descending aorta originating from the mesoderm^13^. These differences may persist in vitro even though cultured VSMCs are known to switch mainly to a proliferative synthetic phenotype. This switch from the typical quiescent contractile phenotype to a proliferative synthetic phenotype, accompanied by increased migration and extracellular matrix synthesis, is also an eminent mechanism of the response to injury^14,15^.

This study aims to compare expression profiles of cultured ECs and VSMCs originating from various anatomical locations. A fundamental question relevant to vascular diseases and their complications is whether differential expression profiles in vitro can be linked to vascular functionality and disease susceptibility. Therefore, we generated a combined transcriptomic atlas for these nine vessel types and we assessed the strength of the association of co-expressed gene clusters to vessel origin and cell type. Our observations extend knowledge to the fragmentary characterization of vascular subtypes and, therefore, assert the significance of using relevant cell types in cardiovascular research.

## Results

### RNA sequencing identifies differentially expressed genes between vascular cells from different macrovessels

**Figure 1:**
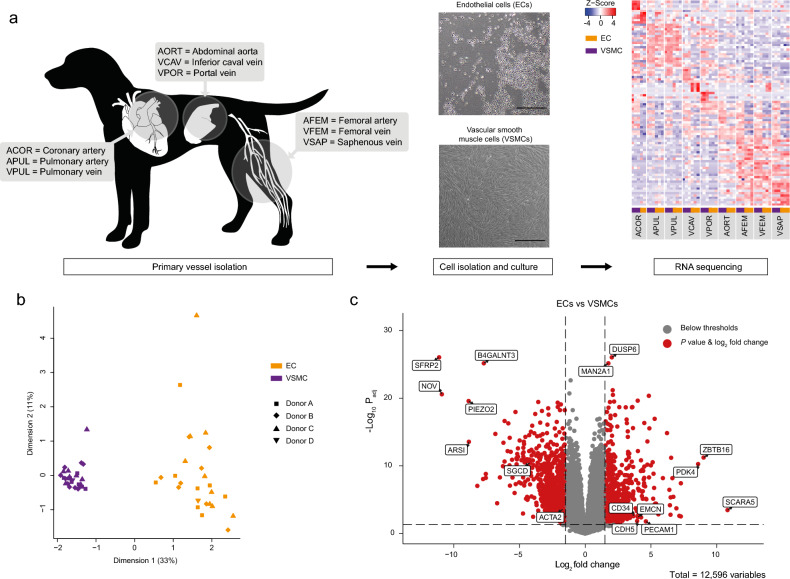
RNA sequencing identifies differentially expressed genes between vascular cells. (**a**) Vessel abbreviations and source location of the nine vessels used for endothelial and vascular smooth muscle cell isolation (left). After isolation of the vessels, cells were dissociated and cultured up to the third passage (middle). Scale bar: 500 μm. From these cells, RNA was extracted, and whole transcriptome sequencing was performed. The heatmap shows the top five differential expressed genes per cell type and vessel. (**b**) Multidimensional scaling plot of all samples. By default, 500 top genes were used to calculate the pairwise distances. Colors indicate the cell type and symbols represent the donor (Dog A, B, C, or D). (**c**) Vulcano plot of genes differentially expressed in ECs versus VSMCs (red dots: log_2_ fold change ≥ 1.5; *P*_adj_ ≤ 0.05). Labels were added to marker genes and top differentially expressed genes.

Recently, we have analyzed the transcriptomes of canine ECs and VSMCs originating from the aorta, vena cava, and portal vein obtained by a novel isolation technique^6,16^. To further test the hypothesis that transcription patterns of vascular cells are origin dependent, we performed RNA-sequencing of cultured ECs (*n* = 26) and VSMCs (*n* = 27) isolates originating from nine different macrovessels isolated from healthy dogs (*n* = 4, Fig. 1a). Three samples were used from each cell and vessel type, except for coronary artery ECs, since only two samples from this group had sufficient viable cells. A total of 18,374 genes were detected at least once, and after filtering, 12,596 robustly expressed genes were used in the analysis.

### ECs and VSMCs expression differences

Consistent with previous findings, principal component analysis, and donor profiles showed distinctive segregation of EC and VSMC cohorts (Fig. 1b) which was also confirmed by differential expression analysis as 3530 genes were found to be significantly upregulated (*P*_*adj*_ < 0.05) and 3350 genes downregulated (*P*_*adj*_ < 0.05) in ECs versus VSMCs with log_2_ fold changes (logFC) ranging from 10.83 to -11.12. The differential expressed genes contained classical markers, including *PECAM1, CD34, ACTA2*, and other genes associated with their phenotype, such as *EMCN* and *SGCD* (Fig. 1c). The expression of 5716 genes did not significantly differ between ECs and VSMCs.

### Arterial and venous endothelial cells show a functional difference

**Figure 2:**
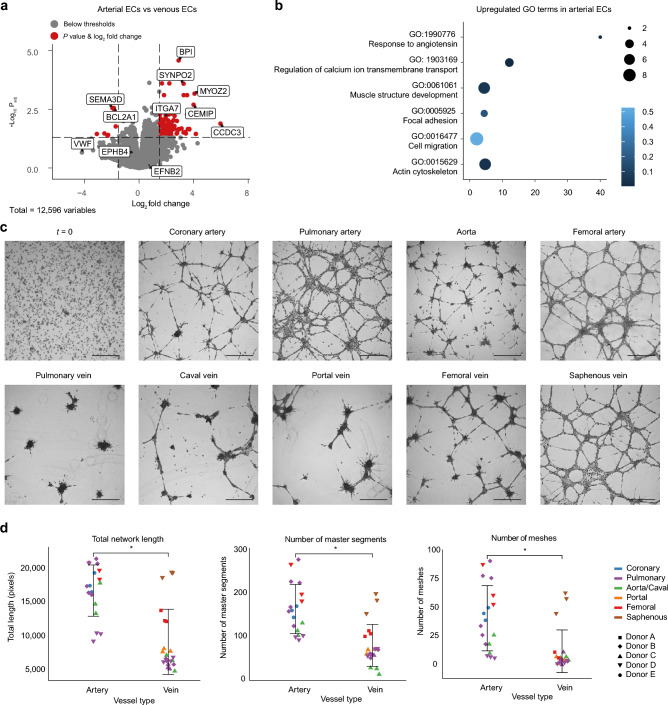
Arterial and venous endothelial cells show a functional difference. (**a**) Volcano plot illustrating genes differentially expressed in arterial versus venous ECs (red dots: log_2_ fold change ≥ 1.5; *p* ≤ 0.05). Labels were added to marker genes and top DEGs. (**b**) Upregulated gene ontology terms for significantly higher expressed genes in arterial ECs (*P*_adj_ < 0.05) compared to venous ECs. (**c**) Representative images from the tube formation assay of nine different EC types. Cells were seeded in a single-cell suspension (t = 0). After 24 h, cellular networks were present in all EC types. Scale bars: 500 μm. (**d**) Results from image analysis of the networks after 24 h. Icons colors by vessel location represent the parameter value per individual well (n = 3), grouped by arterial or venous origin. Poisson mixed-effects model (*p < 0.005).

The most widely used classification of ECs is based on arterial or venous origin. A differential expression analysis between arterial and venous ECs showed that 258 DEGs are upregulated in arterial ECs compared to venous ECs, and 78 DEGs are upregulated in venous ECs compared to arterial ECs (Supplemental Table 1). The venous marker *EPHB4* and arterial marker *EFNB2* did not belong to the group of genes with highest p-values; instead, other genes such as *CCDC3, BPI, SEMA3D*, and *BCL2A1* were among the top DEGs (Fig. 2a). Genes regulating the actin cytoskeleton and focal adhesion such as *ITGA7*, *MYOZ2* and *SYNPO2* were expressed at higher levels (logFC > 1.5) in arterial ECs (Fig. 2b). No DEGs were identified between arterial and venous VSMCs (Supplemental Table 2).

We performed a tube formation experiment to test for functional differences between EC types and analyzed the digital images using the ImageJ angiogenesis analyzer software^17^. After 6 h, we observed similar networks with only minor differences between the EC types. Differences in the networks became apparent after 24 h as most venous networks regressed. However, arterial networks were still visible (Fig. 2c). Arterial networks differed significantly from venous networks in nine out of 20 network parameters including the number of master segments (segments separated by two junctions connecting to more than one branch), the total network length and the number of meshes (*P* < 0.005) (Fig. 2d). The average number of master segments in venous networks was 0.50 (95% CI 0.30–0.81) times the number in arterial networks, the average total network length in pixels from venous ECs was 0.53 (95% CI 0.36–0.78) times the length of the total network from arterial ECs and the average number of meshes in venous networks was 0.27.

To test whether this difference would still be visible after several days, we made images of the tube formation assay from the pulmonary artery and vein from donor B and D after five days. Also, here, we observed higher network stability in the arterial ECs (Supplemental Fig. 1).

### Location-specific expression patterns

Comparing expression profiles based on vessel origin from the nine macrovessels in ECs and VSMCs revealed 3463 and 2247 DEGs (*P*_*adj*_ < 0.05), respectively. Each contrast consisted of samples from one vessel and cell type versus the average of the other vessels from the same cell type. The entire dataset with the DEGs for each contrast is provided in Supplemental Table 3, and the top 5 DEGs are provided in Supplemental Table 4. Groups with the highest number of location-specific genes are saphenous vein ECs, vena cava ECs, and coronary artery VSMCs with more than 1000 DEGs each. Less than 20 DEGs were detected in aortic VSMCs, pulmonary artery VSMCs, and femoral artery ECs.

Some overlap in ECs and VSMCs from the same vessel was observed as a total of 194 genes were DEGs in both cell types, of which 14 genes were DEGs in more than one vessel. Highly significant genes (*P*_*adj*_ < 5 × 10^–7^) expressed in both ECs and VSMCs were *CPQ* and *SH3TC2* for the coronary artery, *HOXC10* for the femoral vein, and *HOXA11*, *HOXA10*, *TBX15*, *LMX1B* for the saphenous vein with a logFC ranging from 2.25 to 6.14. Functional annotation showed that several up- or down-regulated DEGs were linked to Gene Ontology (GO) biological processes or KEGG pathways relevant to the specific location of the vessel (Supplemental Tables 5, 6). Genes involved in heart and coronary vasculature development, including *GATA4, SMAD6*, *BMP4,* and *NRP1,* were found in higher levels in coronary artery ECs, VSMCs, or both. In the upregulated DEGs of the femoral vein, femoral artery, and saphenous vein ECs and VSMCs, GO terms for biological processes linked to embryonic (limb/skeletal system) morphogenesis were identified. The annotated DEGs in these GO terms contained many homeobox genes, such as *SHOX2*, *HOXA9,* and *PITX1*.

### Vascular Hox expression in ECs and VSMCs depends on the position of the vessel on the anterior-posterior axis

**Figure 3:**
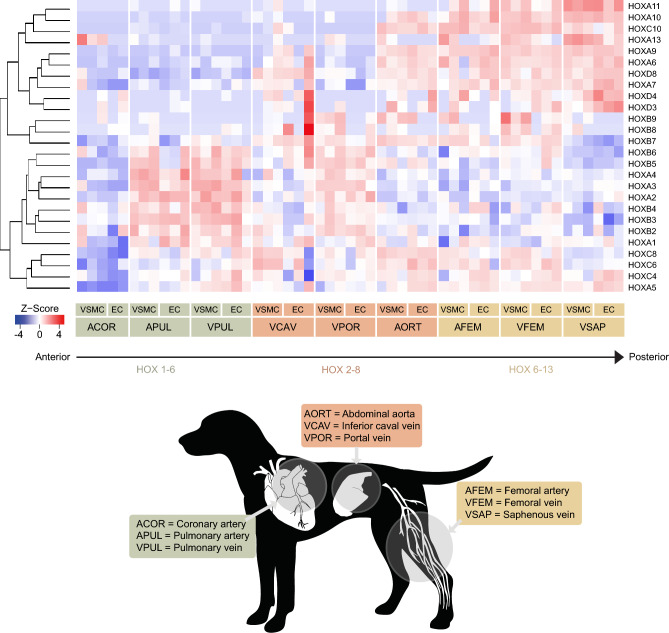
Heatmap of HOX gene expression in endothelial and vascular smooth muscle cells. Expression of HOX genes in ECs and VSMCs. Expression of Hox genes at the 3′ end (HOX1-6) can be found predominantly in the thoracic vessels, and the expression of Hox genes at the 5′ end (HOX6-13) more in the hindlimb vessels as indicated by the arrow.

Next, we hypothesized that homeobox genes are strongly linked to vascular heterogeneity as we observed collective differential expression in proximal vessels. To further investigate this tendency, the quantitative expression of genes containing a homeobox domain was displayed in a heatmap and sorted on sample location (Fig. 3, Supplemental Fig. 3). High fold changes for *HOXA1-4* and *HOXB2*-*6* could be observed for the portal vein, pulmonary artery, and pulmonary vein. HOX genes with high numbers, including *HOXA6*, *HOXA7*, *HOXD8*, HOXA9, *HOXA10*, *HOXC10*, *HOXA11,* and *HOXA13,* were higher expressed in both cell types for the saphenous vein, femoral artery, femoral vein, and variably enriched for the vena cava and aorta. *HOXC6*, *HOXC4*, and *HOXA5* were markedly down-regulated in the coronary artery compared to the other vessels. In other genes containing a homeobox domain, additional location-specific patterns were observed. In the femoral artery, femoral vein, and saphenous vein, *LMX1B*, *IRX2*, *IRX3*, *IRX5*, *ZFHX4*, *EMX2*, *SHOX2*, *TLX2*, and *PPRX1* were upregulated (*P*_*adj*_ < 0.05). Cell type patterns were observed for *PRRX2*, *PBX1*, *CUX2*, *PITX2*, *NKX2-5*, *TSHZ3*, *LHX9*, and *POU6F1,* as these genes were enriched in most VSMCs.

### Co-expression network analysis identifies several modules related to vascular cell types

The EC and VSMC types datasets were used to find groups of co-expressed genes that could be related to the different vessels. This analysis aimed to investigate common control mechanisms or activity in related biological processes by studying the correlation between differentially expressed genes with other genes that show a similar pattern across all samples. The weighted co-expression network analysis (WGCNA) is a well-known method that requires a topological overlap matrix to reveal trends of co-expression in the transcriptome. Groups of co-expressed genes are organized in modules and summarized by eigengenes, the typical expression pattern of genes within the module (Supplemental Tables 7, 8). The MDS plot (Fig. 1b) showed that cell type is a significant source of variation between the expression sets; therefore, WGCNA analysis was performed separately for ECs and VSMCs to identify vessel origin as the primary driver of heterogeneity. A total of 10 modules for ECs and VSMCs were classified based on hierarchical clustering. Genes not linked to co-expression modules were assigned to M10 (grey module). Subsequently, to ease the comparison of ECs and VSMCs, module names of co-expressed clusters in VSMCs were matched to the most congruent module in ECs (*P* < 0.05).

### Module construction of co-expressed genes linked to EC subtypes confirms location-specific imprinting

**Figure 4:**
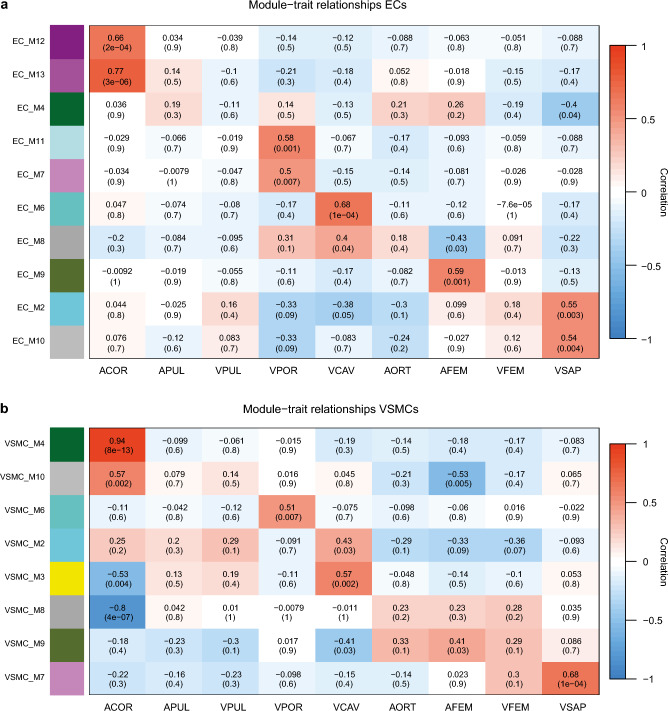
Module-trait relationships of the co-expression analysis in the EC (**a**) and VSMC (**b**) datasets. Rows correspond to the modules; columns correspond to the traits (vessel sources). Each cell of the matrix contains the Pearson correlation coefficient (top) and the associated *P* value (bottom). *ACOR* coronary artery, *APUL* pulmonary artery, *VPUL* pulmonary vein, *VPOR* portal vein, *VCAV* caval vein, *AORT* aorta, *AFEM* femoral artery, *VFEM* femoral vein, *VSAP* saphenous vein.

In the EC dataset, all ten identified modules were significantly correlated to at least one vessel (Fig. 4a, Supplemental Fig. 4d). For a selection of modules, the genes with the highest connectivity degree were used to construct hub gene networks (Supplemental Fig. 5).

**Figure 5:**
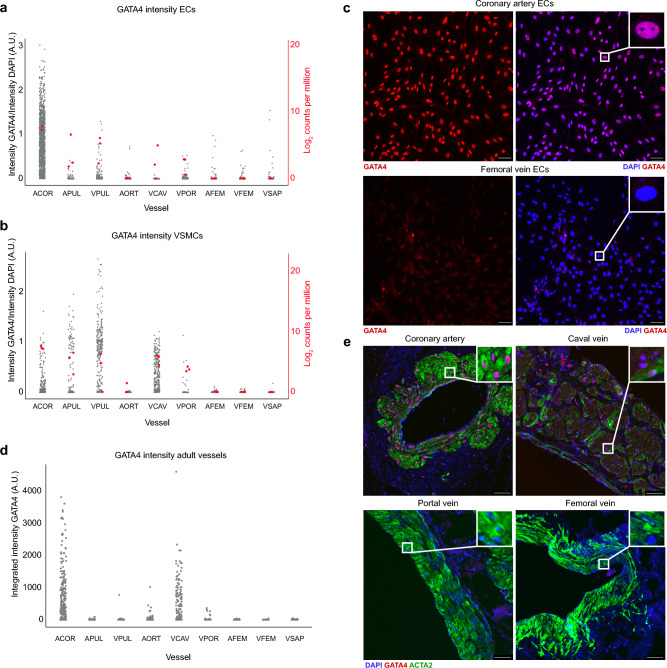
GATA4 expression validated on protein level. (**a**), (**b**), Expression values for GATA4 RNA and protein in cultured ECs and VSMCs. Red dots represent RNA sequencing GATA4 expression values. Grey dots represent the GATA4 immunocytochemistry protein signal corrected for DAPI intensity measured per nucleus. (**c**) Representative images of cells stained for GATA4 by immunocytochemistry. Scale bars: 50 μm. (**d**) GATA4 protein intensity in adult vessels originating from two additional donors (merged data). Each dot represents a nucleus. (**e**) Representative pictures of GATA4 immunofluorescent staining of paraffin-embedded adult canine vessels. VSMCs are stained with ACTA2. Scale bars: 50 μm.

Module M13 had a high positive correlation to the coronary artery ECs (correlation = 0.77, *P* = 3 × 10^–6^). In the rest of the samples, this module was upregulated for arterial ECs. This module includes the hub gene *GNAO1* and other genes, such as *TBX18*, *NOS1AP, ATP1B1, SLC4A3, ATP1B2*, and *GATA4*, which were annotated in GO terms such as GO: 1903522 Regulation of blood circulation and GO: 0060047 Heart contraction.

Another module with higher expression in arterial ECs was M4. This module was also higher in portal vein ECs and significantly downregulated in the saphenous vein ECs (correlation = − 0.4, *P* = 4 × 10^–2^). Gene ontology terms such as GO: 0007155 Cell adhesion and GO: 0030036 Actin cytoskeleton organization were highly significant and contained many hub genes like *PDLIM5, ITGB1, MYH9 and ACTN1*.

Module M7 and M11 were significantly upregulated in portal vein ECs, although their expression was particularly high in two different samples. Both modules contained genes linked to intestinal function and development, including the hub genes *BARX1* in M11 and *IL18RAP*, *ABCG5* and *ABCG8* in module M7.

Module M8 was upregulated in the ECs from the trunk vessels (aorta correlation = 0.18, *P* = 4 × 10^–1^, caval vein correlation = 0.4, *P* = 4 × 10^–2^, portal vein correlation = 0.31, *P* = 1 × 10^–1^) and close to zero or down in the other vessels. Significant GO terms linked to this module were GO: 1901989 Positive regulation of cell cycle process and related GO terms like GO: 0051276 chromosome organization and GO: 0009059 macromolecule biosynthetic process. Many hub genes were annotated to these GO terms, including *CENPE*, *MCM3*, *MCM4* and *BUB1*.

The opposite expression pattern of module M8 was found in module M2, as there was an upregulation in the hindlimbs and downregulation in the aorta, caval vein, and portal vein. Genes in M2 were significantly correlated to GO: 0006629 Lipid metabolic process with hub genes *MPST* and *ACAA1* and GO: 0009058 Biosynthetic process with hub genes *TSHZ1*, *KLF9*, *MED25* and *ZHX3*.

Module M12, M6, and M9 were all highly expressed in just one sample. Module M6 contained *PECAM1*, *CD34*, *VWF*, and many other genes linked to GO: 0001944 vasculature development.

### Module construction of co-expressed genes linked to VSMCs subtypes confirms location-specific imprinting

In the VSMC dataset, eight modules were significantly correlated to at least one vessel (Fig. 4b). A very high positive correlation was found between module M4 and the coronary artery VSMCs (correlation = 0.94, *P* = 8 × 10^–13^). Many significantly upregulated GO terms such as GO: 0030335 Positive regulation of cell migration, GO: 0006936 Muscle contraction, GO: 0097746 Blood vessel diameter maintenance, and GO: 0009725 Response to hormone were linked to this module. In addition, several genes specifically regulating smooth muscle contraction, such as *EDN1*, *EDN2*, *RGS2*, and genes involved in cardiac development, including *CRIM1*, *SORBS2*, *TBX20*, *TBX18*, and *GATA4*, were identified in M4.

Module M8 showed the opposite pattern for the coronary artery VSMCs as it was significantly down in these cells (correlation = − 0.8, *P* = 4 × 10^–7^). Nearly all hub genes were involved in the GO: 0007049 Cell cycle.

An upper/front body expression pattern can be observed in module M2 as it is upregulated in the coronary artery (correlation = 0.25, *P* = 2 × 10^–1^), pulmonary artery (correlation = 0.2, *P* = 3 × 10^–2^), pulmonary vein (correlation = 0.29, *P* = 1 × 10^–1^), and caval vein (correlation = 0.43, *P* = 3 × 10^–2^) VSMCs. One-third of the genes in this module (315/1060) was linked to GO: 0048731 System development represented by the hub genes *RHBDD3*, *GRN*, *PSAP*, *GPRC5B*, and *PTPRS* and many embryonal transcription factors including *FOXC2*, *LRP4*, *HOXB1*, *HOXB2*, and *HOXB4*. Also, GO: 0006952 Defense response was significantly associated with module M2.

Module M3 had a moderate positive correlation to caval vein VSMCs (correlation = 0.57, *P* = 2 × 10^–3^) and a moderate negative correlation to coronary artery VSMCs (correlation = − 0.53, *P* = 4 × 10^–3^). GO terms GO: 0006695 Cholesterol biosynthetic process, and GO: 1902652 Secondary alcohol metabolic process was significantly associated with the genes in module M3. In line with this, the hub genes *PHB2*, *ILVBL*, and *AKT1* were linked to GO: 0006629 Lipid metabolic process.

Module M7 was significantly upregulated in the saphenous vein VSMCs (correlation = 0.68, *P* = 1 × 10^–4^) and had a decreasing expression towards the abdominal and thoracal VSMCs. Significant associated GO terms are GO: 0030198 extracellular matrix organization including the hub genes *VIT*, *COMP* and *EGFLAM* and GO: 0007275 multicellular organism development including most of the hub genes and additional developmental genes such as *HOXD11*, *LMX1A*, *EMX2*, *LMX1B*.

Module M9 follows the opposite pattern in comparison with module M2. Module M9 is upregulated in the femoral artery (correlation = 0.41, *P* = 3 × 10^–2^), femoral vein (correlation = 0.29, *P* = 1 × 10^–1^), and saphenous vein (correlation = 0.086, *P* = 7 × 10^–1^) VSMCs and downregulated in the caval vein VMSCs (correlation = -0.41, *P* = 3 × 10^–2^). Only GO: 0002062 Chondrocyte differentiation containing *HOXA11* and *SHOX2* was significantly associated. Several hub genes, *IPO7*, *MAN1A2*, *EPRS*, *FMN2*, *MSN*, and *PTPN12*, were linked to GO: 0019538 Protein metabolic process. Also, this module contained 13 genes linked to GO: 0048706 Embryonic skeletal system development, such as *HOXA9*, *RDH10*, *IRX5*, *TBX15*, and *PRRX1*.

### Confirmation of locational memory of GATA4

To confirm in vitro locational memory, we mimicked the vessel-specific microenvironment by co-culturing location- and donor-specific ECs and VSMCs and measured *GATA4* mRNA expression by qPCR. Vessels with high (pulmonary artery and vena cava) and low (femoral artery and saphenous vein) expression of *GATA4* were selected based on the whole transcriptome data (Fig. 5a, b). Cells from the same donor were co-cultured in fibrin gel for 48 h before harvesting and mRNA quantification. In concordance with the *GATA4* expression differences detected in the transcriptome data (Fig. 5a, b, Supplemental Fig. 2a) we detected no *GATA4* mRNA expression for both the femoral artery and saphenous vein, whereas the pulmonary artery and vena cava co-cultures did reveal *GATA4* mRNA expression (Supplemental Fig. 2).

GATA4 was also strongly linked to the coronary artery cells in the differential expression and co-expression analyses. To confirm a differential expression on protein level, we performed immunocytochemistry on all EC and VSMC types for GATA4. The nuclear GATA4 protein level concurs with the RNA expression values (Fig. 5a–c). GATA4 protein was present at high levels in the coronary artery cells and almost absent in cells from the hindlimb vessels (Fig. 5c). As a complementary approach, we also stained GATA4 in adult canine vessels to see whether this gene has a persistent or reactivated expression in cultured cells in comparison to vascular cells in situ. The caval vein and coronary artery VSMCs showed a positive protein signal, whereas the protein signal in the other vessels was minimal or absent (Fig. 5d+e).

## Discussion

Our prior work documented the presence of diversity in EC and VSMC subtypes^6,16^. In this study, we adopted a simple and rapid method for isolating and characterizing ECs and VSMCs from the same vessel to identify the persistent vessel and cell type-specific expression patterns that define the basis of vascular heterogeneity in cultured canine ECs and VSMCs upon removal from the natural environment. In this present study, we, for the first time, present a comprehensive characterization of the transcriptomic landscape of donor- and vessel-specific, both primary endothelial cells and smooth muscle cells originating from nine major vessels (the aorta, coronary artery, vena cava, portal vein, femoral artery, femoral vein, saphenous vein, pulmonary vein, and pulmonary artery). As a proof of concept, we use the example of GATA4 to manifest the locational memory throughout the longitudinal body axis, which has never been shown before. We confirm the conservation of this locational memory in adult vasculature ex vivo and cultured cells in vitro.

Among the differentially expressed genes, several genes with a role in embryonic development were identified. These genes may be the basis of positional memory because once cell fate is determined, the expression of several of these genes was shown to be maintained in adult tissues to regulate regeneration in response to injury^18^. Key players in the positional memory of many cell types, including ECs and VSMCs, are the Hox genes, a subset of the homeobox domain genes^19,20^. Hox genes, the most well-characterized group of homeobox genes, are known for their collinearity (correspondence between the sequence of Hox genes along the chromosome and their expression along the anterior–posterior axis) during embryonic development in all bilateral animals^21^. In line with this, our analyses suggested that vascular Hox expression in ECs and VSMCs depends on the position of the vessel on the anterior–posterior axis, as the expression of Hox genes at the 3' end (*HOX1-6*) was found predominantly in the thoracic vessels and the expression of Hox genes at the 5′ end (*HOX6-13*) more in the hindlimb vessels^21^. Vascular spatial collinearity has been previously described in several endothelial and supporting vascular cell types in different species^19,20,22,23^. Our results thus lend further credence to the earlier findings that one may expect clustering of Hox genes analogous to the vessel′s position on the body axis. The function of Hox genes in the adult vasculature has not been entirely elucidated, although some studies suggest a role for Hox in angiogenesis and vascular remodeling^24–26^.

We also identified the expression of other embryonal transcription factors and homeobox domain genes that do not belong to the subgroup of Hox genes. For example, our data showed that *LMX1B* (associated with hindlimb VSMCs) was co-expressed with *EMX2*, a known target of *LMX1B* during limb development^27^. *TBX15,* another gene differentially enriched for all limb samples, regulates the formation of the skeleton elements of the limb, vertebral column, and head. However, their functional impact on vascular homeostasis is yet to be discovered. Still, as several genes in the co-expression module were associated with extracellular matrix formation in VSMCs, these genes may also play a role in these processes.

GATA4 mRNA and protein were expressed at various levels in cultured cells from the coronary artery, pulmonary artery, caval vein, and portal vein. At the same time, expression in the aorta and hindlimb vessels was almost absent. In adult vessels, high protein expression was detected only in the coronary artery and caval vein. Our results are consistent with what is reported in the literature, as GATA4 is known to be expressed in the developing heart, gut, lung, and gonads^28^. In adult tissues, multiple functions of GATA4 are described. For example, an increase in capillary density after GATA4 overexpression was reported, and a GATA4-dependent increase in VSMC proliferation after blood vessel injury was observed^29,30^.

Coronary artery VSMCs displayed a highly divergent expression pattern compared to the other VSMC types. The proepicardial origin of these VSMCs may, at least partially, account for this difference. In line with this, the co-expressed genes *CRIM1* and *TBX18*, known to be highly expressed in the proepicardial organ, were found to have sustained transcription in coronary artery VSMCs in our study^31–33^. We also found a higher expression of genes regulating vasotone and a strong down-regulation of co-expressed genes involved in cell cycle regulation suggesting a lower proliferation rate in these samples^4,34,35^.

Several highly differentially expressed genes were identified for the pulmonary artery and vein. For example, *TBX5,* a gene crucial in cardiac morphogenesis and lung development, was enriched for the pulmonary vessels and the coronary artery^36,37^. *TCF21*, enriched for the portal vein, pulmonary artery, and pulmonary vein in ECs and the coronary artery in VSMCs, has also been found to be expressed in tissue contributing to the developing lung, heart, and gut^38^. In the positional memory of portal vein samples, we identified several genes involved in gastrointestinal development.

Vena cava VSMCs share a similar co-expression pattern with pulmonary VSMCs, while vena cava ECs do not share this pattern. The mesothelial origin of both vena cava and pulmonary VSMCs may explain the comparable co-expression patterns of these VSMCs. Lineage tracing of *WT1* (the number one DEG in our vena cava VSMCs) in mesothelial cells showed that most of the VSMCs in the gut and approximately one-third of the VSMCs in the lung vasculature had a mesothelial origin^39,40^.

Among the vena cava EC samples, we detected one outlier that expressed several typical EC markers, such as *VWF* and *CD34,* at very high levels. Since the other two vena cava samples expressed these genes at levels comparable to the entire set of ECs, these results are probably not representative of the positional memory of vena cava ECs but rather a result of fewer divisions due to a higher yield after isolation. In line with observations by others, these endothelial markers most likely faded out in the other EC samples during culture^7^.

On a functional level, in the tube formation assay, we observed more stable and extensive networks in arterial ECs, although saphenous vein ECs also formed many cellular connections. We speculate that blood pressure in the vessel of origin could influence endothelial behavior in the tube formation assay.

To conclude, this study shows that adult vascular cells in culture express a remarkably high amount of transcription factors crucial to organ development in the embryo. The persistent expression of these genes in culture indicates that these genes are not regulated by flow or surrounding cell types but are rather fixed in the molecular memory. We show for the first time that GATA4 expression confirms the locational memory over the longitudinal body axis in 2D and 3D cell cultures and in primary adult vasculature. Identifying genes that are part of the positional memory of vascular cell types provides us with better tools for tissue transplantation, genetic (re)programming, and regeneration. Matching this basic expression profile comprising Hox genes and other embryonic transcription factors to the site of transplanted tissue, for example, may increase the success rate of tissue implementation. This study shows that we are still at the beginning of understanding angiodiversity. Expanding the data collection may reveal additional genes that are important to the key position of the cardiovascular system in homeostasis and disease.

## Methods

### Ethics statement

Blood vessels from healthy male dogs (n = 4; age 12–14 months, with an average of 26 kg) were collected for this study. The vessels were obtained as surplus material from fresh canine cadavers used in unrelated research on pacemakers which was performed according to the Dutch Experiments on Animals Act and conformed to the EU standards (European Directive 2010/63/EU)^41^. The study was approved by the Central Authority for Scientific Procedures on Animals (CCD) under license AVD #115,002,016,531. The animals were killed by removal of the heart under general anesthesia. Acquiring this tissue did not influence the method and moment of euthanasia.

### Whole transcriptome sequencing of vascular cells

ECs and VSMCs were isolated from the aorta, coronary artery, caudal vena cava, vena porta, femoral artery, femoral vein, saphenous vein, pulmonary vein, and pulmonary artery (Fig. 1a) according to a previously published protocol^6^. The isolation of these two different cell types was confirmed on gene and protein expression and morphological and functional levels^16^. In short, a vessel of approximately 5 cm in length was inverted inside out to bring the endothelial cell layer to the outside of the vessel. Both ends of the vessel were closed by placing purse string sutures to prevent exposure of the non-endothelial vascular tissue to the digestion medium consisting of collagenase II (0.15 U/mL, Life Technologies) and dispase (0.15 U/mL, Life Technologies) in Dulbecco’s Modified Eagle's Medium (DMEM) GlutaMAX (Life Technologies). After each vessel was digested for one hour, the vessel was removed, and the cell suspension was centrifuged at 1500 rpm for 5 min. The cell pellet consisting of ECs was suspended in Canine Endothelial Growth Medium (CECGM, Cell Applications, San Diego, CA, United States) and added to gelatin (Sigma–Aldrich, Saint Louis, MO, United States) coated 6-wells plates. To isolate the VSMCs, the remaining tissue was chopped finely and digested with collagenase type II (0.09 U/mL) in DMEM GlutaMAX. After 4 h, a single cell suspension was obtained by filtration through a 70 μM strainer. The cell suspension was centrifuged at 1500 rpm for 5 min and resolved in a culture medium consisting of DMEM GlutaMAX, 10% Fetal Calf Serum (FCS, Life Technologies), and 100 μg/mL Primocin (Invivogen, Toulouse, France) before adding the cells to the culture plates. All cells were maintained in a humidified incubator with 5% CO^2^ at 37 °C and passaged when 70–80% confluence was reached.

Three samples were selected per vessel and cell type. RNA was extracted from a cell pellet with approximately one million ECs or VSMCs at the third passage using an RNeasy Mini Kit (Qiagen, Venlo, Netherlands). The remaining genomic DNA traces were removed with an on-column DNase digestion (Qiagen).

Poly(A) Beads (NEXTflex) were used to isolate polyadenylated mRNA, from which sequencing libraries were made using the Rapid Directional RNA-seq kit (NEXTflex). Libraries were sequenced using the Nextseq500 platform (Illumina), producing single-end reads of 75 bp. Reads were aligned to the canine reference genome CanFam3.1 using STAR version 2.4.2a (https://github.com/UMCUGenetics/RNASeq). The raw and analyzed files were uploaded to Gene Expression Omnibus under accession number GSE171437.

### Differential expression analysis

The RNA-sequencing data was merged with the publically available dataset GSE118029^16^ and analyzed by the workflow outlined by Law et al.^42^ R version 4.1.2 and R Studio version 2022.02.0 were used^43^. Briefly, the dataset, consisting of 56 samples, was imported, organized, filtered, and normalized for composition bias using trimmed mean of M-values (TMM) in the edgeR package^44^. Data quality was assessed by including three technical replicates, which were excluded from further analysis after testing for reproducibility (Spearman R^2^ > 0.9). In our analyses, counts per million (CPM) and log_2_-counts per million (log-CPM) transformations were applied to account for library size differences. Genes were filtered on worthwhile counts using the *filterByExpr* function that filters genes based on the median library size and the number of samples per group. The limma package was used to apply the voom method, which estimates the mean–variance relationship of log counts, generates a precision weight for each observation, and enters these into the limma empirical Bayes moderation pipeline to assess differential gene expression^45,46^. Differential expression was calculated for contrasts across all sample groups within the same cell type. The model matrix was processed by *contrast.fit* before being passed to *eBayes* in the limma package, which resulted in an adjusted *P* value and a logarithm-transformed fold change (logFC) for each contrast. A multidimensional scaling plot was generated from the top 500 most variable genes by using the *plotMDS* function^45^. Volcano plots were made using the R package EnhancedVulcano^47^.

### Functional enrichment

Gene annotations were obtained from the Ensembl BioMart database^48–50^. Gene set enrichment analysis (GSEA) was performed using the GOseq Bioconductor package and the *enrichKEGG* function of the clusterProfiler package^51–53^.

### Co-expression network construction

Preprocessing of the datasets was performed by the variance stabilizing transformation as implemented by the package DESeq2^54^. Co-expression was calculated using the Weighted Gene Co-expression Analysis (WGCNA) R package^55,56^. Network construction with the WGCNA algorithm entails the calculation of an adjacency matrix by raising the connectivity between genes to a soft thresholding power. The optimal soft thresholding power was chosen using the scale-free topology criterion (Supplemental Fig. 4). Subsequently, a signed network resulting in the separation of inversely correlated genes to different modules was constructed. By using a topological overlap matrix, the network interconnectedness required for hierarchical clustering was calculated. For the detection of modules in the cluster tree using the *cutreeHybrid* function, the minimum module size was set at 27^57^. Modules with similar eigengenes were merged using *mergeCloseModules*, as measured by a maximum dissimilarity of 0.7 for ECs and 0.4 for VSMCs^55^.

### Functional annotation

The correlations of the module eigengenes with vessel origin were calculated and displayed in a module-trait heatmap. Modules without significant correlation with any vessel were excluded from this plot. Functional annotation was performed by utilizing the GOseq function in the Bioconductor package and the enrichKEGG function of the clusterProfiler package on genes assigned to each module^51–53^. For all functional terms with more than three genes per term, the adjusted *P* value was calculated using the Benjamini and Hochberg false discovery rate^58^. Besides significance, relevant gene ontology terms and KEGG pathways were selected based on the number of annotated genes and their role in vascular functioning.

### Hub gene selection and visualization

Hub genes are the most influential genes within a module and, therefore, possibly exert strong biological importance. In this study, intramodular hub genes are defined as genes with a high correlation to the module connections with other genes in the module. To define the hub genes, 20 genes with the highest intramodular connectivity were selected for each module using the softConnectivity function^55^. Secondly, the number and weight of edges between these genes were extracted from the topological overlap matrix and exported to Cytoscape (Version 3.9.1)^59,60^. Exporting the edges to Cytoscape requires an adjacency threshold in the *exportNetworkToCytoscape* function^55^. To obtain consistent results from all modules, the average adjacency value of the hub genes was used as a cut-off threshold for the edges. Node size and edge transparency were scaled to intramodular connectivity and edge weight, respectively.

### mRNA expression in co-culture

ECs and VSMCs originating from four vessels (pulmonary artery, caval vein, femoral artery, and saphenous vein) and two different donors were selected for co-cultures. 500,000 donor- and vessel-specific ECs and VSMCs were collected and mixed 1:1. After spinning down the cells and removing the supernatant, cells were resuspended in 82.5 ul Endothelial Cell Growth Medium-2 (EGM-2; Lonza, Geleen, the Netherlands) and 3.3 ul thrombin (100 U/mL). 85.8 ul of fibrinogen (5 mg/mL) was added and plated in triplicate in a 96-well plate. After 10 min of polymerization, EGM-2 medium was added to the co-cultures and replaced after 24 h. After 48 h, medium was removed, the cell pellet was washed with HBSS (Gibco) and the pellet was resuspended in RLT-buffer (Qiagen). Total RNA was isolated using a RNeasy Micro Kit (Qiagen) and on-column DNase digestion. Perlprimer v1.1.21 was used for primer design on Ensembl annotated transcripts and the amplicon was tested for secondary structures using MFold^61^. Gradient PCRs were performed to determine the optimum temperature for obtaining 95–105% PCR efficiency. Primer specificity was validated in silico (BLAST specificity analysis) and empirically (DNA sequencing, gel electrophoresis, and melting profiles). qPCR reactions were performed in 10-µl duplicates containing 0.5 × SYBR Green-Supermix (BioRad, Veenendaal, the Netherlands), 0.4 µM primer, and 1 µl cDNA. Four reference genes were used for normalization, namely, tyrosine 3-monooxygenase/tryptophan 5-monooxygenase activation protein zeta (*YWHAZ*), glyceraldehyde-3-phosphate dehydrogenase (*GAPDH*), succinate dehydrogenase Complex flavoprotein subunit A (*SDHA*), and ribosomal protein L13 (*RPL13*). Primers for reference genes and *GATA4*, including their optimum temperature, are listed in Supplemental Table 13. Cycling conditions were a 3-min Taq polymerase activation step at 95 °C, followed by 45 cycles of 10 s at 95 °C for denaturation, and 30 s at Tm for annealing and elongation. All experiments were conducted with a CFX384 Touch Real-Time PCR Detection System (BioRad). A fourfold standard dilution series of a pool containing all samples was used to determine relative expression. Data analysis was performed with IQ5 Real-Time PCR detection system software (BioRad). Expression levels were normalized by using the average relative amount of the reference genes.

### Tube formation assay

The tube formation assay was performed using the µ-Slide Angiogenesis (Ibidi, Munich, Germany) according to the manufacturer's protocol. In short, 10 µl Matrigel (Corning, New York, USA) was applied to each inner well. Per EC type, a suspension containing 2 × 10^5^ cells/ml of passage 2–4 in EGM-2 (Lonza, Basel, Switzerland) was added to triplicates. Microscopy images were made after 6 and 24 h. Images were analyzed using the Angiogenesis Analyzer software for ImageJ. For the images after 6 h, the same settings could be applied to all EC types, but the settings had to be optimized per EC type in the images after 24 h because of the high variability in networks.

The network parameters were analyzed separately for each parameter with a linear mixed effects Poisson regression with log link^62^. The type of vessel was the explanatory variable (Artery as reference), and batch, donor and vessel within donor were taken as random effects to account for the correlation between repeated measurements. The results (exponentiated model estimates) were presented as ratios with 95% confidence intervals.

### GATA4 staining

Paraffin-embedded vessels from two additional donors were used for immunofluorescence. All vessels were used for both donors except for one femoral vein where paraffin-embedding failed. After antigen retrieval using a sodium citrate buffer containing 0.5% Tween 20 (Sigma–Aldrich, Saint Louis, USA), non-specific binding was reduced by using 10% normal goat serum as a blocking agent. The mounted whole-vessel slices of 4 µm were stained with rabbit anti-GATA-4 (D3A3M, Cell Signal, Danvers, USA) at a dilution of 1:150 and Alexa Fluor 488 mouse anti-alpha smooth muscle actin antibody (ab184675, Abcam, Cambridge, UK) at a dilution of 1:200. A nuclear counterstain was performed using 1:1000 DAPI fluorescent dye (Thermo Scientific, Waltham, USA). Negative controls lacking the primary antibody were made for each sample.

For the immunocytochemistry, ECs and VSMCs from different donors were seeded at a concentration of 5 × 10^4^ cells/ml in Nunc Lab-Tek II glass bottom chamber slides (Thermo Scientific, Waltham, USA). When 70–80% confluency was reached, the cells were fixated with 4% PFA and permeabilized with 0.5% Triton X-100 (Sigma–Aldrich, Saint Louis, USA). Non-specific binding was blocked using 1% bovine serum albumin (Sigma–Aldrich, Saint Louis, USA). The same concentration of anti-GATA-4 antibody and DAPI fluorescent dye were used for the immunocytochemistry.

Quantification of GATA4 nuclear signal was performed using Cell Profiler. The integrated intensity of GATA4 protein signal inside the nuclei was used for the graphs. The GATA4 signal in the immunocytochemistry was corrected by dividing GATA4 integrated intensity by DAPI integrated intensity inside the nuclei. Because of the higher background signal of the immunofluorescence compared to the immunocytochemistry, a different correction method was applied: the illumination was corrected using the *Background illumination* function with block size 60, and subsequently, a global threshold using the method *Robust background* was applied. Nuclei were identified as objects and filtered on their proximity to alpha smooth muscle actin protein signal to select only the nuclei inside VSMCs.

### Supplementary Information

Supplementary Figure 1.

Supplementary Figure 2.

Supplementary Figure 3.

Supplementary Figure 4.

Supplementary Figure 5.

Supplementary Table 1.

Supplementary Table 10.

Supplementary Table 11.

Supplementary Table 12.

Supplementary Table 13.

Supplementary Table 2.

Supplementary Table 3.

Supplementary Table 4.

Supplementary Table 5.

Supplementary Table 6.

Supplementary Table 7.

Supplementary Table 8.

Supplementary Table 9.

## Data Availability

The RNA sequencing datasets are publicly available in the Gene Expression Omnibus under accession GSE171437. All other datasets used or analyzed during the current study are available from the corresponding author upon reasonable request.
