# Supplementary material for: Locational memory of macrovessel vascular cells is transcriptionally imprinted
Source: Sci Rep. 2023 Aug 10;13:13028. doi: 10.1038/s41598-023-38880-6 (PMC10415317; doi:10.1038/s41598-023-38880-6)
Supplement: Supplementary file 2 — Supplementary Figure 2. [file 41598_2023_38880_MOESM2_ESM.pdf]

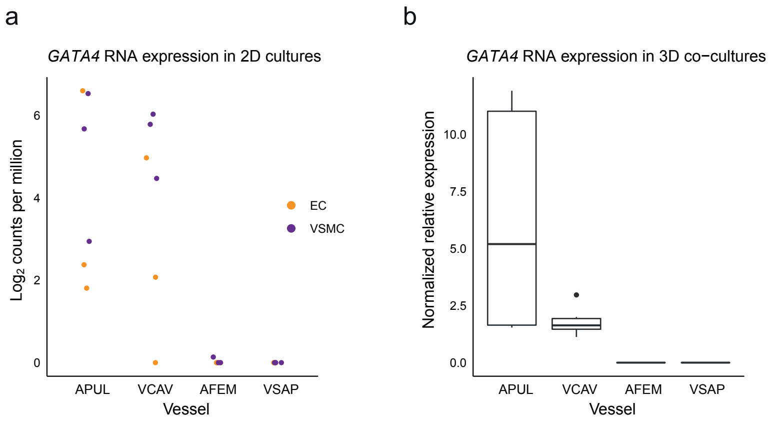

Supplemental Figure 2. *GATA4* RNA expression in co-cultured vascular cells
a, Expression values in $\log_2$ counts per million for *GATA4* RNA in ECs and VSMCs cultured separately.
b, Normalized relative expression of *GATA4* RNA in co-cultures with ECs and VSMCs originating from the same vessel after 48 hours.
APUL, pulmonary artery; VCAV, caval vein; AFEM, femoral artery; VSAP, saphenous vein.
