# Supplementary material for: Locational memory of macrovessel vascular cells is transcriptionally imprinted
Source: Sci Rep. 2023 Aug 10;13:13028. doi: 10.1038/s41598-023-38880-6 (PMC10415317; doi:10.1038/s41598-023-38880-6)
Supplement: Supplementary file 3 — Supplementary Figure 3. [file 41598_2023_38880_MOESM3_ESM.pdf]

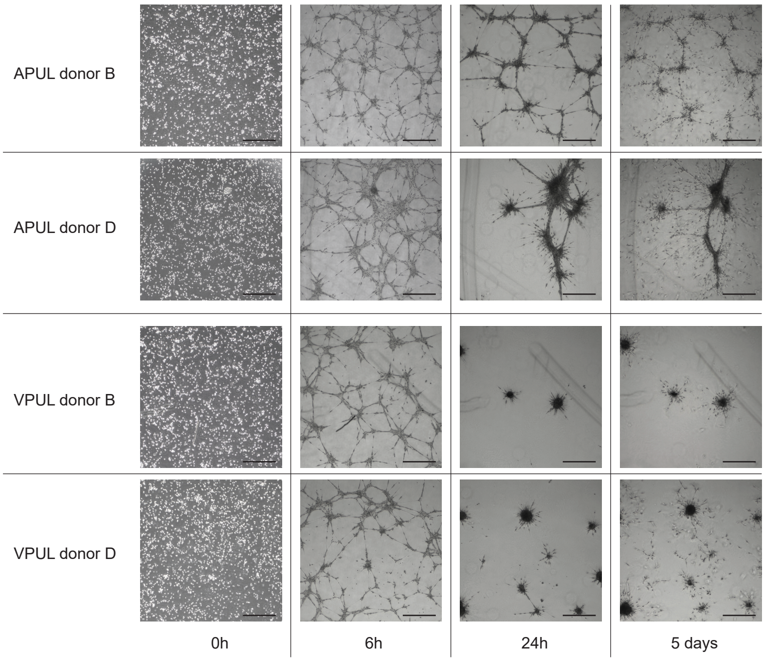

Supplemental Figure 3. Networks in the tube formation assay persist for several days
Representative images from the tube formation assay for pulmonary artery and vein ECs from donors B and D (n = 3). Images were taken directly upon seeding, after 6 hours, 24 hours, and 5 days. Scale bars, 500 µm. APUL, pulmonary artery; VPUL, pulmonary vein.
