# Supplementary material for: Locational memory of macrovessel vascular cells is transcriptionally imprinted
Source: Sci Rep. 2023 Aug 10;13:13028. doi: 10.1038/s41598-023-38880-6 (PMC10415317; doi:10.1038/s41598-023-38880-6)
Supplement: Supplementary file 4 — Supplementary Figure 4. [file 41598_2023_38880_MOESM4_ESM.pdf]

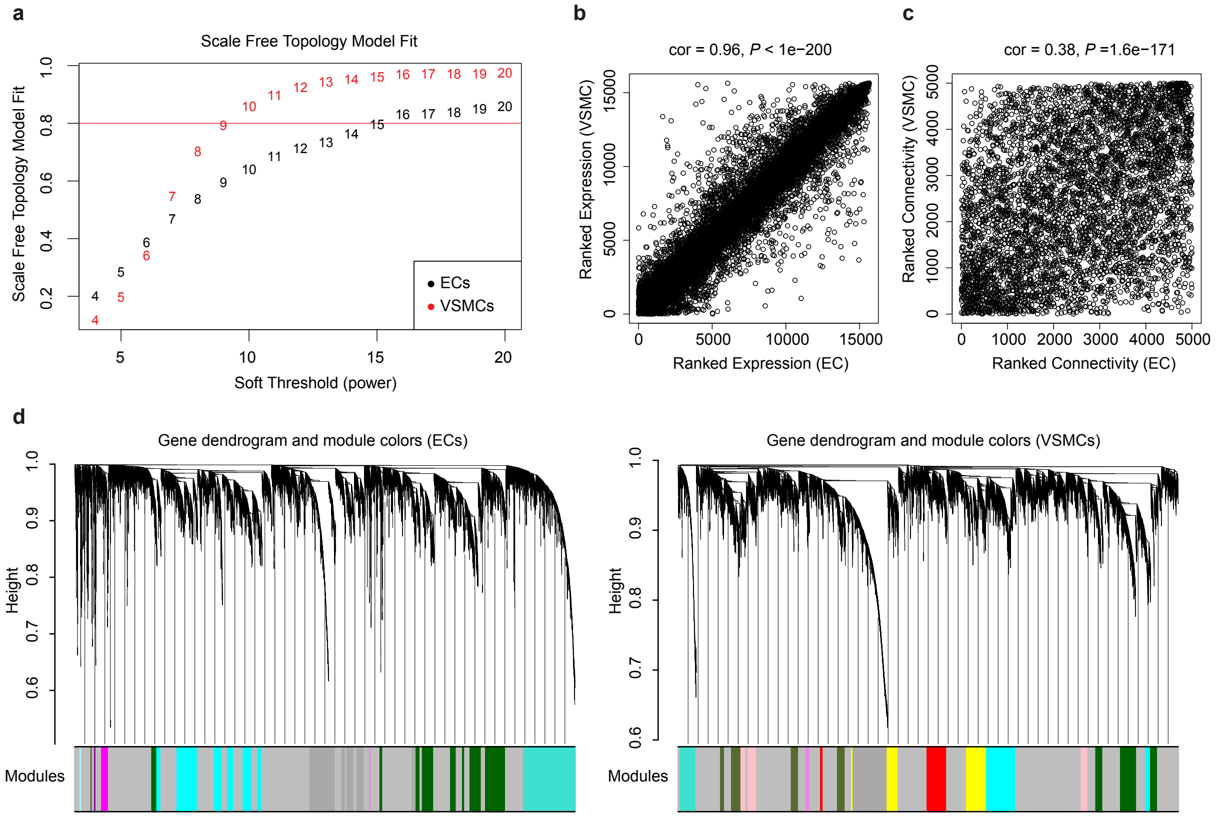

Supplemental Figure 4. Statistics used for the weighted gene co-expression analysis.
a, A scale-free topology fit index was made to determine the optimal value for the soft threshold for the EC and VSMC datasets. Numbers in the plots illustrate the soft thresholding powers. The approximate scale-free topology can be achieved for ECs at the soft-thresholding power of 16 and for VSMCs of 10. **b**,**c**, The correlations between the average gene expression and overall connectivity between the EC and VSMC datasets were used to assess the comparability. **d**, Gene dendrograms based on the topological overlap dissimilarity index showing the final modules identified by WGCNA in the EC and VSMC datasets.
