# Supplementary material for: Locational memory of macrovessel vascular cells is transcriptionally imprinted
Source: Sci Rep. 2023 Aug 10;13:13028. doi: 10.1038/s41598-023-38880-6 (PMC10415317; doi:10.1038/s41598-023-38880-6)
Supplement: Supplementary file 5 — Supplementary Figure 5. [file 41598_2023_38880_MOESM5_ESM.pdf]

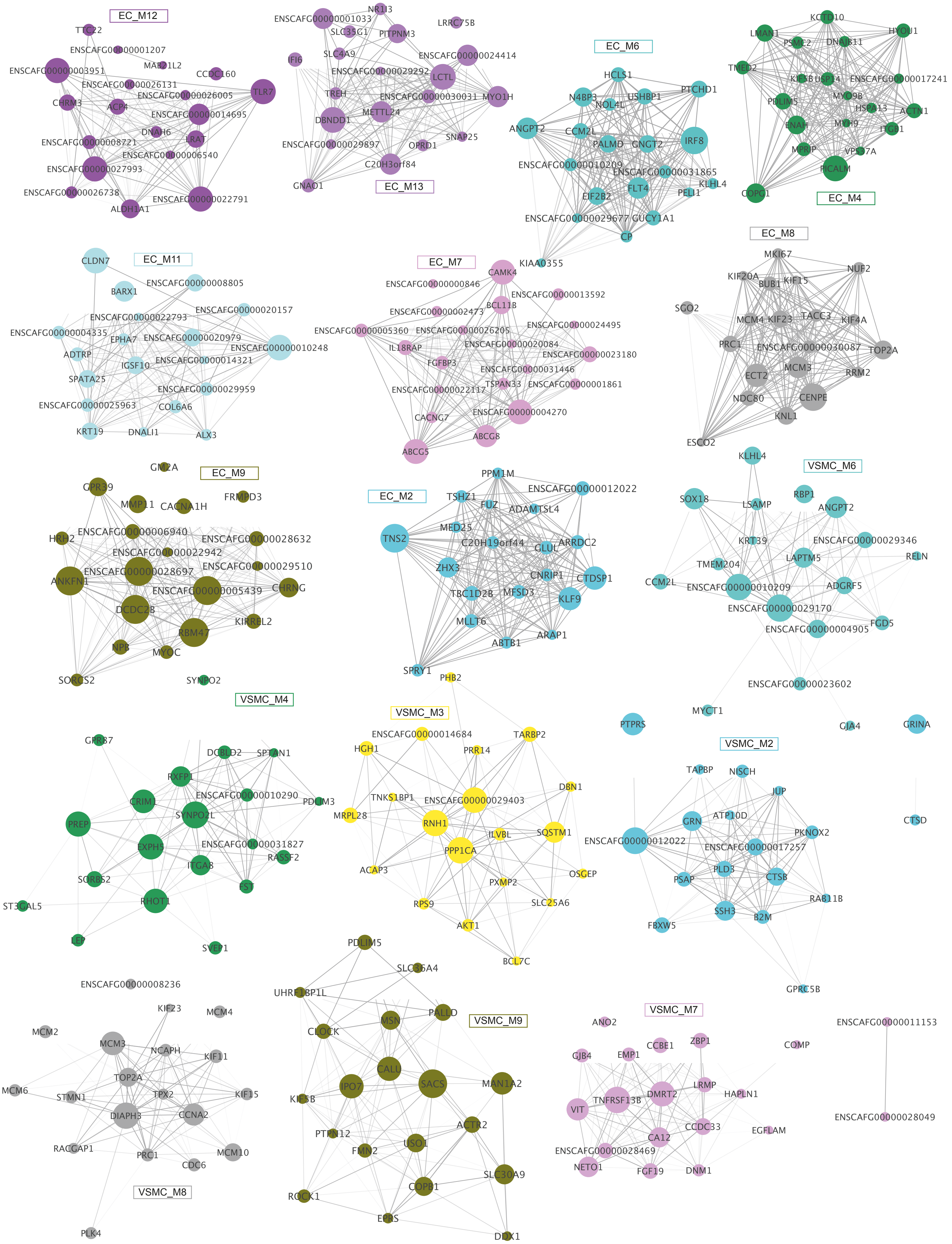

Supplemental Figure 5. Hub gene networks generated by Cytoscape.
Hub gene networks for a selection of modules significantly correlated to one or more vessel types in ECs or VSMCs. Each network consists of 20 genes with the highest intramodular connectivity in the module. Node size was scaled to intramodular connectivity and line transparency to edge weight.
