# Supplementary material for: Locational memory of macrovessel vascular cells is transcriptionally imprinted
Source: Sci Rep. 2023 Aug 10;13:13028. doi: 10.1038/s41598-023-38880-6 (PMC10415317; doi:10.1038/s41598-023-38880-6)
Supplement: Supplementary file 6 — Supplementary Table 1. [file 41598_2023_38880_MOESM6_ESM.pdf]

Supplemental Table 1. Differential expression analysis of arterial versus venous endothelial cells (ECs). Positive log2 fold changes (logFC) indicate higher expression in arterial ECs, while the negative values represent a higher expression in venous ECs. AveExpr, average log2-expression; t, moderated t-statistic; p, raw P value; adj.P.Val, adjusted P value; B, log-odds that the gene is differentially expressed

Gene	logFC	AveExpr	t	P.Value	adj.P.Val	B
BPI	2.89978976	-1.5353168	7.22330147	2.06932E-09	2.6065E-05	9.78076596
SLC39A14	0.75095302	6.24932834	6.43923858	3.72687E-08	0.00023472	8.43220989
CRYAB	2.22063654	6.93762489	6.24814803	7.52025E-08	0.00024778	7.76551057
KIAA0895	1.67446095	3.81353058	6.23581016	7.86843E-08	0.00024778	7.85315367
SYNPO2	3.26361525	3.78256141	6.16760113	1.01036E-07	0.00025453	7.60485508
BRSK1	1.47808021	1.49853431	5.90335137	2.653E-07	0.00055695	6.64487428
MYOZ2	4.06798271	-0.4882076	5.80732164	3.76212E-07	0.00067697	5.70036473
MYBL1	1.82757825	4.79635515	5.72281723	5.11182E-07	0.00078676	5.95470851
BICD1	3.5192425	1.1217649	5.68993671	5.75822E-07	0.00078676	5.68874583
HTR4	3.27118711	-2.537413	5.66745895	6.24608E-07	0.00078676	4.69794497
CEMIP	3.99641102	7.02484538	5.3793547	1.76108E-06	0.0020166	4.89154443
MARK1	1.25565505	4.28255676	5.2984918	2.35064E-06	0.00246738	4.53946082
CD59	1.25692044	5.37213331	5.23980933	2.8967E-06	0.00266393	4.20337801
ARHGEF17	0.98827013	6.55386119	5.2153241	3.15995E-06	0.00266393	4.09646018
PCSK1N	2.63583599	1.01462649	5.21306791	3.18536E-06	0.00266393	4.38202975
C23H3orf58	0.94441254	4.55255713	5.1802009	3.57923E-06	0.00266393	4.03955658
FAIM2	2.36323605	0.62168627	5.17893465	3.59533E-06	0.00266393	3.95455536
ANKH	1.45706083	3.85378106	5.14775507	4.01504E-06	0.00269146	3.98023492
MAPT	2.31856863	1.25336477	5.13779863	4.15897E-06	0.00269146	4.1501391
CHSY3	2.88445944	0.30142533	5.11627681	4.48768E-06	0.00269146	3.53728924
SGSM1	1.91432096	0.15800155	5.11460287	4.51429E-06	0.00269146	4.03633766
DMPK	1.71028538	4.32014598	5.09248305	4.88092E-06	0.00269146	3.72861403
RFTN1	1.61251426	5.84524204	5.0905365	4.91455E-06	0.00269146	3.67407132
ATP6V1A	0.39672181	5.81309011	5.05830428	5.5058E-06	0.0027705	3.55501595
ABHD1	1.41647146	2.04533147	5.05025866	5.66398E-06	0.0027705	3.85478738
CALY	2.22110539	-1.6317736	5.04248607	5.82104E-06	0.0027705	3.2958151
BCL2A1	-1.8520779	1.93752457	-5.0334714	6.00857E-06	0.0027705	3.74511613
SEMA3D	-1.9576337	6.21127443	-5.0264556	6.15862E-06	0.0027705	3.54625656
PPME1	0.53384218	4.49265775	4.96107974	7.74636E-06	0.00336459	3.31216812
SFMBT2	1.57469762	2.57480551	4.93242839	8.56314E-06	0.00353765	3.48927657
GALNT2	0.52002439	7.82860762	4.9276781	8.7065E-06	0.00353765	3.13498649
ENSCAFG00000008063	-1.802435	0.13883419	-4.9172011	9.03108E-06	0.00355486	2.81252512
UNC45B	2.23417777	1.41458604	4.87956914	1.0298E-05	0.00385945	3.27145589
AK7	2.4223869	-1.8804281	4.87625179	1.04177E-05	0.00385945	2.77391778
SRPK3	1.48857236	3.58770203	4.86787372	1.07261E-05	0.00386018	3.24995966
MAP7D2	2.15902659	-1.3438447	4.85345372	1.12782E-05	0.0039461	2.76723143
HSPA2	1.79038708	3.16340862	4.83456339	1.20437E-05	0.00410005	3.07408275
OPRD1	3.5755614	-2.1475516	4.82317332	1.25296E-05	0.00415324	2.54127939
RASSF3	0.96051826	6.53636059	4.78158482	1.44729E-05	0.00467439	2.61810937
XKRX	2.31872873	-1.3280533	4.76042795	1.5572E-05	0.00490363	2.75724732
ANKRD44	2.18381175	0.42043247	4.72614876	1.75288E-05	0.00525912	2.6152044
VWA1	1.27123305	1.62638892	4.72082297	1.78537E-05	0.00525912	2.78163975
ABCC4	-0.6440047	5.5200015	-4.7134889	1.83107E-05	0.00525912	2.45397344
PLCL1	1.36325451	4.94639192	4.70447465	1.88882E-05	0.00525912	2.36669881

ACOT7	1.53504086	3.94508046	4.70200107	1.90498E-05	0.00525912	2.57757464
PKIB	3.52043781	-0.6971211	4.69962832	1.92061E-05	0.00525912	2.69365922
ENSCAFG00000004676	1.21322641	2.9781164	4.68019721	2.05338E-05	0.00550305	2.57765452
ZNF853	1.96068268	2.1479973	4.67033185	2.12419E-05	0.00557422	2.52981844
SH3GL2	2.60344977	-2.0771849	4.63930145	2.36284E-05	0.00603086	1.88676089
EIF3E	-0.4275398	7.81829473	-4.6354835	2.39396E-05	0.00603086	2.17080495
ITGA7	1.9488488	4.53744476	4.59265895	2.77167E-05	0.00684548	2.00731133
ENSCAFG00000029756	1.58264789	2.18637631	4.5429432	3.28355E-05	0.00795377	2.23696931
ATP1B1	1.62379391	4.14989916	4.52125358	3.5348E-05	0.00840081	2.04541672
UBASH3B	1.57826642	1.61689016	4.50232754	3.76929E-05	0.00872912	2.06078945
RPS12	-0.5396545	8.19672879	-4.4990405	3.81154E-05	0.00872912	1.73347674
CLIC4	0.71669296	8.35604301	4.48564171	3.98858E-05	0.00897145	1.71150351
ESM1	1.85846702	4.50133142	4.47599057	4.12104E-05	0.00910679	1.62522393
ENSCAFG00000008917	2.53843923	-2.0383221	4.46921639	4.21658E-05	0.00915724	1.39889058
PDE1A	2.12368017	6.22474319	4.45713567	4.39234E-05	0.00937726	1.69773543
CAP2	2.77549238	4.13583661	4.43503325	4.73257E-05	0.00993525	1.86347967
UHMK1	0.45304387	5.24092201	4.42610237	4.87722E-05	0.01001913	1.47823414
ARL4A	-1.1477431	3.13455525	-4.4228109	4.93162E-05	0.01001913	1.6399737
SERPINI1	2.42986412	0.33359256	4.39615657	5.3944E-05	0.01062549	1.80268456
ZFYVE28	1.35316532	2.45630215	4.39591438	5.39879E-05	0.01062549	1.78258904
NACC2	0.8168604	5.49235507	4.36267984	6.03587E-05	0.0115772	1.25458407
ATRIP	-0.4534941	3.7140232	-4.3611854	6.06617E-05	0.0115772	1.49218737
PCSK7	0.50951924	6.08046077	4.35403463	6.21324E-05	0.01168089	1.21156653
FAM178B	2.23865577	-1.4799205	4.33364769	6.65184E-05	0.01227137	1.23967192
SPINK2	1.76212973	0.55652791	4.33050075	6.72217E-05	0.01227137	1.5395999
SNX25	0.52238117	4.52204067	4.32015071	6.95863E-05	0.01252156	1.20813741
ENSCAFG00000018078	0.57152263	4.68135047	4.30940959	7.21258E-05	0.01279573	1.14528293
CCDC3	5.96931814	2.33575779	4.2946113	7.57726E-05	0.01289302	1.46717572
NXPE3	0.40089019	5.46361856	4.29276922	7.62389E-05	0.01289302	1.02598149
FRY	1.17615633	4.29632613	4.28923938	7.71403E-05	0.01289302	1.22100469
CPEB4	0.77655784	4.03959486	4.28836056	7.73663E-05	0.01289302	1.13086124
ANKRD34A	1.50901373	0.10918618	4.28671184	7.77921E-05	0.01289302	1.35110611
TBX2	1.74685844	2.544722	4.28125448	7.92177E-05	0.01295878	1.42451584
AHCYL2	0.86329321	3.8211383	4.26947535	8.23819E-05	0.01327559	1.16978661
SIL1	0.50589601	5.41518963	4.26627601	8.32623E-05	0.01327559	0.95289572
APLP1	1.16460863	4.98633349	4.2588643	8.53371E-05	0.01343633	0.95782462
LZTS1	2.34642307	-1.4409582	4.25013303	8.78459E-05	0.01366058	1.30892137
RCSD1	2.03432464	-1.6367331	4.24178793	9.03106E-05	0.01387259	1.1997758
TNR	2.59175352	-1.2666492	4.18791086	0.000107918	0.01637749	0.97713667
FAM20B	0.41200876	6.99323561	4.18212015	0.000109997	0.01649438	0.66557191
ACTB	0.58152945	11.3109618	4.17531853	0.00011249	0.01666969	0.87725943
TSPAN3	0.38820728	5.98201885	4.16975674	0.000114569	0.01677829	0.62016161
DBNDD2	0.98251897	4.19025224	4.16628065	0.000115887	0.01677829	0.85253864
LIPG	-1.7107825	3.94878975	-4.1576457	0.000119225	0.0169763	0.76127499
MRVI1	1.75443467	4.66900763	4.15580159	0.00011995	0.0169763	0.76514941
ENSCAFG00000003845	2.07983316	-2.1353286	4.14530822	0.000124157	0.01726266	0.63082536
LPP	0.82723653	5.2871209	4.14090923	0.000125963	0.01726266	0.5633613
PLEKHA5	-0.5595969	4.82621606	-4.1379862	0.000127177	0.01726266	0.60155952
SUMF1	0.45466674	6.2767229	4.13313516	0.000129217	0.01726266	0.50564518
HIPK2	0.6974545	5.56501128	4.13154421	0.000129893	0.01726266	0.51782938

CDO1	2.36103943	4.71328893	4.13083193	0.000130196	0.01726266	0.55381584
TEX2	0.46584284	5.27924283	4.1214168	0.000134276	0.01761812	0.49646183
FBXO27	1.5657087	3.43837163	4.1157683	0.000136783	0.01771973	0.91742853
SMCHD1	-0.6040801	5.03027608	-4.1102357	0.000139282	0.01771973	0.50343126
GDF11	0.5911762	4.93128843	4.10786714	0.000140365	0.01771973	0.45824553
ACTN4	0.60828959	9.72455889	4.10492867	0.000141721	0.01771973	0.56518067
ENSCAFG00000008335	2.44269745	-0.9369048	4.10161349	0.000143265	0.01771973	0.69139646
CAMK2G	0.37345317	5.76154624	4.10113119	0.000143491	0.01771973	0.41422176
ARMCX1	-0.5939169	5.07905601	-4.0946382	0.000146568	0.01792396	0.44980146
DNASE2	0.73065583	6.0250438	4.08795643	0.000149801	0.01806058	0.36533364
PANX2	0.79347394	3.33132735	4.08642262	0.000150553	0.01806058	0.65039515
TMTC2	1.26032768	4.30323364	4.06988096	0.000158896	0.01888168	0.47743764
EIF3M	-0.3930831	6.87968562	-4.0626114	0.000162703	0.01915335	0.28778383
NEDD4	-0.5384896	6.63006997	-4.0550481	0.000166757	0.01944885	0.26154034
EMC1	0.48904522	7.52871134	4.04880826	0.000170176	0.01966543	0.2605112
CTNNAL1	2.17921597	3.89894198	4.04589762	0.000171793	0.01967188	0.56549427
PRDM16	3.11615128	1.12332262	4.02634598	0.000183051	0.02077212	0.50966788
NSD1	-0.5516222	5.47260594	-4.0129084	0.000191198	0.02150292	0.20903866
FST	1.70072018	5.55497904	4.00227484	0.000197892	0.0218487	0.15034954
ENSCAFG00000017326	2.27259047	7.85306207	4.00031834	0.000199148	0.0218487	0.11956413
KIAA0319L	0.46538975	5.95657343	3.99264032	0.000204153	0.0218487	0.07160526
ART3	1.39977128	1.78028244	3.98913956	0.000206475	0.0218487	0.46602564
CYP19A1	2.57195313	-2.3081872	3.98776324	0.000207394	0.0218487	0.22244251
RELL1	-0.7219548	4.729813	-3.9871285	0.00020782	0.0218487	0.23976319
GJA5	3.58047565	-2.5328712	3.98611085	0.000208504	0.0218487	0.28231317
TPM4	0.76100888	9.50547894	3.98511336	0.000209177	0.0218487	0.16880442
SAMM50	-0.4815719	6.35115118	-3.9819656	0.000211313	0.0218487	0.03495172
ARMCX2	0.97286296	3.37476914	3.98151814	0.000211618	0.0218487	0.42991863
CFI	0.84484036	4.63267111	3.9746448	0.000216363	0.02185011	0.08364519
PDIA4	0.41370615	8.67251706	3.97212396	0.000218128	0.02185011	0.10675752
SDC3	1.18385939	5.96265916	3.97209986	0.000218145	0.02185011	0.02042888
DDR1	1.27733954	3.49815992	3.97149573	0.000218571	0.02185011	0.40921203
DTNA	2.94420612	1.45070899	3.9667383	0.000221947	0.02195333	0.53205828
GLDN	3.68759959	-0.8643975	3.96422713	0.00022375	0.02195333	0.35963859
ENSCAFG00000031808	-0.9412319	4.44028941	-3.9627299	0.000224832	0.02195333	0.14107793
ENSCAFG00000029877	0.80286007	3.13389753	3.9544151	0.00023093	0.02221923	0.3324415
ENSCAFG00000010570	1.5332492	-0.8869214	3.95342342	0.000231668	0.02221923	0.35302254
PPP1R26	1.2045463	3.62395567	3.95184596	0.000232847	0.02221923	0.34047103
JPH2	1.91806489	-1.8348631	3.94863418	0.000235264	0.02228112	0.13331481
EIF3D	-0.3260731	8.13144617	-3.9455635	0.000237598	0.02230106	-0.0224626
GALNT16	1.1867546	5.49571427	3.94371258	0.000239016	0.02230106	-0.0564839
NPTX2	2.86468197	0.87437517	3.93409019	0.000246518	0.02265957	0.39209751
RPL4	-0.320926	8.93719962	-3.9337311	0.000246802	0.02265957	-0.0166083
ARG1	1.77277853	0.26208258	3.93190239	0.000248255	0.02265957	0.28847711
ENSCAFG00000031952	-0.3769717	7.29282348	-3.9269127	0.000252261	0.0226772	-0.1207348
BTD	0.48266693	6.06402216	3.92650908	0.000252588	0.0226772	-0.1347274
IFI6	2.02531751	-1.5872643	3.92164089	0.000256562	0.0226772	0.22859578
VAT1L	3.6826208	-2.3912192	3.91993335	0.00025797	0.0226772	0.13980845
TYRO3	0.41321113	4.4111249	3.91903373	0.000258714	0.0226772	-0.0361835
SLC24A3	2.92495142	1.74916414	3.91838783	0.00025925	0.0226772	0.37429153

HAGH	0.50620246	4.8660844	3.91602245	0.000261222	0.02269213	-0.1095326
NMNAT2	1.42493145	1.27397396	3.9095728	0.000266674	0.02300699	0.07540248
SIAE	1.1887467	4.83739232	3.90237604	0.000272886	0.02328092	-0.0894801
BAZ1B	-0.3439944	6.09290799	-3.9013059	0.000273821	0.02328092	-0.2120618
RGMA	1.45566891	2.49150395	3.89951527	0.000275394	0.02328092	0.11851292
MCTS1	-0.4138011	3.83452527	-3.8937181	0.000280544	0.02355821	-0.0472618
OBSL1	0.81820619	7.28425361	3.88823883	0.000285497	0.02375374	-0.2462929
ANGPT4	2.21403956	2.05296357	3.88698315	0.000286644	0.02375374	0.02893371
EPOR	2.21662959	-1.5548312	3.87972818	0.000293358	0.02415123	0.05248534
SORBS1	2.02808174	2.86986641	3.87717407	0.000295758	0.02419066	0.01065416
TUBB4A	1.17060679	1.77094214	3.86742563	0.000305091	0.0247326	0.16618905
DYNLRB1	0.51512775	6.02151855	3.86617332	0.00030631	0.0247326	-0.3149479
FHL1	1.12131087	7.73702252	3.84896512	0.000323548	0.02595604	-0.356889
PGPEP1	0.744505	3.01080173	3.84699178	0.000325584	0.02595604	-0.1169123
SDF4	0.38615904	7.06110348	3.84060128	0.00033226	0.02632171	-0.3808625
EPS8L2	2.33872947	-0.4819632	3.83521636	0.000337988	0.02646746	-0.0389014
GGT7	1.41728139	5.04829167	3.83492342	0.000338303	0.02646746	-0.3441849
CHPF2	0.43955326	6.45767886	3.83046758	0.000343119	0.02660096	-0.4279151
ENSCAFG00000009876	-0.3334461	6.88852653	-3.8278518	0.000345976	0.02660096	-0.433289
CRELD2	0.62191202	4.66996916	3.82640094	0.000347571	0.02660096	-0.3502454
PPP3CC	0.43001177	4.58927574	3.82460039	0.00034956	0.02660096	-0.3330848
EPN3	1.950241	-1.6650239	3.82304187	0.000351291	0.02660096	-0.0366996
CTSZ	0.83910252	5.35154113	3.82179606	0.00035268	0.02660096	-0.4011095
HNRNPF	-0.3927452	7.52104398	-3.8153779	0.000359922	0.02698557	-0.4467515
C1QTNF1	1.55802145	2.77507676	3.81299683	0.000362645	0.02702882	-0.0140713
RPS6	-0.3816583	8.04865449	-3.8084825	0.000367861	0.02718278	-0.4401596
XK	2.35599805	-1.0791777	3.80590884	0.000370867	0.02718278	-0.0679451
ADGRL1	0.76804647	4.63156735	3.80563861	0.000371184	0.02718278	-0.4151956
MCMBP	-0.444459	3.64251007	-3.7833245	0.00039828	0.02899848	-0.2939247
SYNC	1.23981498	4.51034285	3.77713897	0.000406121	0.02931035	-0.3780537
KCTD20	-0.495724	3.6121414	-3.7762396	0.000407274	0.02931035	-0.2557429
ENSCAFG00000032016	1.24756233	2.48254449	3.77395595	0.000410214	0.02931035	-0.2671903
KIAA1217	1.02427608	5.11812891	3.77267562	0.000411871	0.02931035	-0.4890341
NDRG2	1.79944152	4.57030117	3.76735342	0.00041883	0.0295534	-0.450574
MMS19	-0.5020666	6.17430928	-3.7653674	0.000421456	0.0295534	-0.6186023
SPEG	3.39217511	2.34725162	3.76471202	0.000422325	0.0295534	-0.0357412
KLF3	-0.6207871	6.00996465	-3.7573473	0.00043222	0.02971268	-0.6407983
PIPOX	2.2346096	-0.3320083	3.75715223	0.000432485	0.02971268	-0.0533144
SIGIRR	-0.9386285	2.62384739	-3.7562382	0.000433729	0.02971268	-0.244476
TTC34	1.12282771	1.64244527	3.75513346	0.000435237	0.02971268	-0.1448814
GEM	1.1674866	5.30895619	3.75428705	0.000436396	0.02971268	-0.3703022
KCNMB1	0.91014695	5.46575854	3.75065672	0.000441401	0.02989186	-0.6618572
CHAD	2.13691945	-0.9956066	3.74090061	0.000455125	0.03065645	-0.1863285
DOCK8	3.26253049	1.86111978	3.73755535	0.000459925	0.03078642	-0.1261283
CD68	1.89800371	0.64055387	3.7351978	0.000463336	0.03078642	-0.1135269
ATP6AP1	0.56183853	6.68383138	3.7344748	0.000464387	0.03078642	-0.7133727
B3GAT3	0.49302007	5.38449404	3.73083731	0.00046971	0.03079909	-0.6839365
SLC10A7	-0.4686475	3.69060803	-3.7302151	0.000470626	0.03079909	-0.4675988
KCTD10	0.64573417	8.40784715	3.72934301	0.000471914	0.03079909	-0.6689304
FAM83H	1.16856892	3.55358702	3.7227456	0.000481762	0.03112182	-0.4330052

REM1	1.33057899	5.15250792	3.72272015	0.0004818	0.03112182	-0.6917715
ENSCAFG00000002517	-1.2128306	0.26076318	-3.7151922	0.000493279	0.03153149	-0.2655557
FXR2	0.37394061	5.22293304	3.71444513	0.000494432	0.03153149	-0.7378442
P2RX4	0.49565106	4.80848013	3.7099932	0.000501359	0.03153149	-0.7006021
ENSCAFG00000019979	0.31571477	5.40766442	3.70952947	0.000502086	0.03153149	-0.7624479
SRC	0.54155613	6.22477326	3.70903404	0.000502864	0.03153149	-0.7898909
RAB6B	1.13841851	2.85765237	3.70884416	0.000503162	0.03153149	-0.5060529
PPM1A	-0.3189172	5.09230581	-3.7052137	0.000508899	0.03173314	-0.7302536
ENSCAFG00000029395	0.29567095	6.50646409	3.69782873	0.000520764	0.03185127	-0.8193153
PLEKHH1	0.82583312	2.45698643	3.69692285	0.000522237	0.03185127	-0.3154712
ENSCAFG00000007840	-1.0210101	1.5367743	-3.6959707	0.00052379	0.03185127	-0.2219014
MYH14	2.13302066	-1.2735824	3.69576378	0.000524128	0.03185127	-0.3273935
NTN1	1.8573772	2.15813434	3.69534214	0.000524818	0.03185127	-0.304572
NFASC	2.78574227	-0.414796	3.69393135	0.000527132	0.03185127	-0.2936247
EIF2S3	-0.3176079	7.30224912	-3.6924843	0.000529515	0.03185127	-0.822632
UBE4B	-0.4278123	6.36299781	-3.6915718	0.000531023	0.03185127	-0.8415279
RASL11A	1.59871678	5.26947373	3.68393367	0.00054381	0.03246364	-0.6952191
ENSCAFG00000028877	-0.5550389	3.10597231	-3.6820468	0.000547014	0.03250087	-0.503067
ENAH	0.66582945	6.69404439	3.6751149	0.00055894	0.03305355	-0.8892877
PABPC1	-0.3037384	10.3672601	-3.6695606	0.000568675	0.03347213	-0.7040418
KCNK5	-2.5386131	-0.7554383	-3.6654636	0.00057596	0.03374324	-0.4518279
COL11A1	3.43047242	9.076536	3.66102492	0.000583954	0.03392842	-0.8678146
LRPAP1	0.59388024	7.21746283	3.66071903	0.000584508	0.03392842	-0.9064583
FZD5	1.5958812	0.20525826	3.65871368	0.000588158	0.03398365	-0.3416252
EAF1	0.3970566	5.21309808	3.65415805	0.000596529	0.03420023	-0.9054486
DYNC1I1	2.75963381	1.21481335	3.65372204	0.000597337	0.03420023	-0.3351952
NTRK2	2.32016743	-1.48999	3.65034035	0.000603633	0.03426883	-0.4632457
LACTB	-0.574772	4.59737661	-3.650157	0.000603976	0.03426883	-0.8783574
ENSCAFG00000030873	0.50377639	6.96889741	3.64345467	0.00061665	0.03468257	-0.9757719
IRF1	-0.6610794	5.05051633	-3.6433891	0.000616775	0.03468257	-0.9471515
CCNYL1	0.74925038	4.98500287	3.63976522	0.000623734	0.03491039	-0.9507666
ENSCAFG00000017241	0.89940524	5.42754772	3.63840318	0.000626369	0.03491039	-0.9247265
ENSCAFG00000002230	1.550887	1.24871086	3.63161226	0.000639667	0.03512101	-0.5079198
ENSCAFG00000015116	0.72449692	7.3229379	3.62845138	0.000645949	0.03512101	-1.0121532
POLR1E	-0.3934701	5.30770855	-3.6283212	0.000646209	0.03512101	-0.9922245
CLU	1.32773179	8.27240443	3.6282497	0.000646351	0.03512101	-0.9026748
CYLD	0.37617617	6.07272223	3.62809447	0.000646662	0.03512101	-1.0274652
ASB1	0.60796216	2.74433474	3.62798629	0.000646878	0.03512101	-0.5880899
CCND1	0.9249967	7.71075404	3.62394053	0.000655015	0.0354053	-0.9651134
GP2	4.16222147	0.83692146	3.62259841	0.000657736	0.0354053	-0.4591263
CCDC171	-1.2246632	0.4855636	-3.6190856	0.000664909	0.03541123	-0.4330009
FKBP1B	1.25991169	1.57970419	3.61773102	0.000667695	0.03541123	-0.4850967
SBSPON	1.89215302	0.04639123	3.61553368	0.000672237	0.03541123	-0.4811827
GM2A	2.33635177	1.11267245	3.61500947	0.000673326	0.03541123	-0.4581823
PNCK	2.09135774	2.31061802	3.61485043	0.000673656	0.03541123	-0.559914
HSPB2	1.01955233	3.22070017	3.61434178	0.000674714	0.03541123	-0.5807495
FBXL5	0.36424066	5.54071318	3.61020401	0.000683379	0.03554645	-1.0669791
RPL23A	-0.3650311	6.00345343	-3.610104	0.00068359	0.03554645	-1.0787925
EPHB1	-3.095688	-1.3389676	-3.6090776	0.000685756	0.03554645	-0.729813
PLCB4	2.33909126	-0.5418063	3.60737079	0.000689374	0.03558751	-0.4638858

HAPLN3	0.97252546	6.42118692	3.60373645	0.000697137	0.03584138	-1.0947502
SGCA	1.25381641	4.72819184	3.59747641	0.000710705	0.03639042	-1.0457835
PLOD1	0.53685348	8.39772801	3.59143462	0.00072404	0.03687643	-1.0296114
ENSCAFG00000006102	-0.3786978	7.68409347	-3.5905319	0.000726052	0.03687643	-1.1036701
B4GALT7	0.4449039	5.14850711	3.58770981	0.000732379	0.03697442	-1.0809006
PNMA1	0.64283559	2.93667572	3.58705588	0.000733852	0.03697442	-0.7638256
RPS4X	-0.3633293	9.47981217	-3.5853069	0.000737807	0.03702558	-1.0182349
NRIP1	-0.5805533	3.97491422	-3.5798047	0.000750383	0.03749003	-0.9806087
KCTD18	-0.4723248	3.32966185	-3.578664	0.000753015	0.03749003	-0.827651
RNF150	1.82804693	0.6368432	3.57559354	0.000760145	0.03763678	-0.5473546
MRTFA	0.76484371	7.50859739	3.57382615	0.000764278	0.03763678	-1.1586795
NFKB1	-0.3475577	5.65335798	-3.5735498	0.000764927	0.03763678	-1.172914
ETAA1	-0.4414421	4.34928273	-3.5710098	0.000770909	0.03778355	-1.0238562
FAM129A	2.07596075	4.46518808	3.5675012	0.000779247	0.03799326	-0.6971432
RPS8	-0.3365186	9.08164692	-3.5666758	0.000781221	0.03799326	-1.0962796
NIT1	0.45863364	4.54562949	3.56532062	0.000784472	0.03800464	-1.061976
ENSCAFG00000031588	0.40095203	6.80344217	3.55892916	0.00079998	0.0385223	-1.2208183
NCDN	0.45007141	5.14953242	3.55840144	0.000801274	0.0385223	-1.1805612
MYL9	1.55785353	9.65936881	3.55485005	0.00081003	0.03879521	-1.1028833
FLVCR1	-0.5609088	4.0680137	-3.553426	0.000813567	0.03881702	-1.1258972
SPINT1	2.030532	0.01065618	3.55079772	0.000820134	0.03896038	-0.6434127
B3GNT7	1.9558413	-0.775346	3.54887415	0.000824972	0.03896038	-0.7753803
CUL1	-0.2290712	6.92470497	-3.5485256	0.000825851	0.03896038	-1.2570205
SNTA1	0.9605879	6.47187664	3.54709488	0.000829471	0.03896418	-1.2529067
PPFIA4	0.87434946	4.34898576	3.54548999	0.000833549	0.03896418	-1.1532501
CCDC117	-0.453895	4.20155764	-3.5429819	0.000839961	0.03896418	-1.0794002
SULT4A1	1.07474158	1.191117	3.54128031	0.000844338	0.03896418	-0.6935037
SPRED3	0.93559679	1.64990895	3.54066786	0.000845918	0.03896418	-0.6840709
ENSCAFG00000018181	0.29343401	8.73277832	3.54005846	0.000847494	0.03896418	-1.1931168
ADAP1	0.92768855	2.90836418	3.54002314	0.000847585	0.03896418	-0.7945928
APC2	1.17252116	1.23198628	3.53530965	0.000859868	0.03907903	-0.6582835
TSHZ3	0.80114837	3.88072249	3.53327193	0.000865231	0.03907903	-0.9881214
ENSCAFG00000031564	0.59278556	6.72467317	3.53254721	0.000867145	0.03907903	-1.2705668
KRT39	-2.2676209	-2.5091528	-3.5305763	0.000872373	0.03907903	-0.9461738
HIP1	0.74208748	7.37547318	3.53022172	0.000873317	0.03907903	-1.2904524
MRAS	0.7412424	3.89400372	3.52936753	0.000875594	0.03907903	-1.0531091
SLC16A2	2.12545459	-0.8244123	3.528295	0.000878462	0.03907903	-0.7337908
TRMT10B	-0.3578849	4.00062353	-3.5274854	0.000880633	0.03907903	-1.1075661
PTCD3	-0.4428756	5.31014662	-3.5274136	0.000880825	0.03907903	-1.2816872
ATAD2B	-0.6190208	3.47463606	-3.5273081	0.000881109	0.03907903	-0.9717669
OSBPL2	0.31508899	4.69562113	3.51784672	0.00090687	0.04008047	-1.2520518
ENSCAFG00000005922	1.39639789	0.25190484	3.51568664	0.000912851	0.04020376	-0.7166088
SLC25A4	0.4445544	6.23759016	3.51429519	0.000916724	0.04023365	-1.3576618
NEFH	1.73925169	-1.4460486	3.51286959	0.000920708	0.0402682	-0.7591481
STN1	1.30696188	1.02874024	3.51129947	0.000925115	0.04032095	-0.7296176
ELF2	-0.3951614	5.39342723	-3.5002239	0.000956779	0.0415572	-1.3683916
ACTG1	0.53949946	12.2254327	3.49838111	0.000962146	0.04159583	-1.0912992
ENSCAFG00000018360	1.70384861	0.29495725	3.49765373	0.000964273	0.04159583	-0.7517066
ENSCAFG00000000562	1.35707579	3.82699993	3.49164407	0.000982014	0.04217466	-1.2060484
SDC1	1.19421198	5.55387874	3.48861339	0.000991079	0.04217466	-1.3073622

PLXNB3	0.88806014	3.89559633	3.48852291	0.000991351	0.04217466	-1.0346087
SKIV2L	0.30023238	5.45072499	3.48814929	0.000992474	0.04217466	-1.4099653
TBX18	1.85920981	5.32044357	3.48749869	0.000994433	0.04217466	-1.3160776
TOX	-2.3051969	2.00502152	-3.483531	0.001006459	0.04247387	-0.7996938
PHIP	-0.485512	5.01130959	-3.4829499	0.001008232	0.04247387	-1.3882929
ERP44	0.27258127	6.54884439	3.47799788	0.001023461	0.04297172	-1.4617587
CHPT1	1.75927487	1.75753825	3.47600635	0.001029647	0.04308784	-0.8095875
KLF7	-0.572674	3.38191198	-3.4734061	0.001037778	0.04328429	-1.1523089
NAP1L1	-0.5285455	7.56507618	-3.4707056	0.001046287	0.04349515	-1.4462311
ENSCAFG00000014255	-1.4584941	1.08211717	-3.4664985	0.001059675	0.04390681	-0.8361884
IFIH1	-0.579258	4.26400575	-3.4645043	0.001066078	0.04402729	-1.3309742
PLPP1	0.74453987	6.18647913	3.46021116	0.001079988	0.04435471	-1.5105764
PRKCD	0.46710219	5.32900975	3.45988575	0.001081049	0.04435471	-1.452553
TUBA8	0.65728668	2.36787815	3.45503336	0.001096993	0.04486276	-1.0079312
KCNK1	0.92435119	1.8268027	3.45335385	0.001102564	0.04494464	-0.8713152
MAN2A2	0.85751751	7.18103692	3.44645946	0.001125714	0.0454379	-1.5487246
NPAS1	1.32873468	1.97486368	3.44252344	0.001139137	0.0454379	-0.932302
MYL6B	0.87096056	2.7170479	3.44247123	0.001139316	0.0454379	-1.0770221
PCBP4	0.47488016	5.45335818	3.44174321	0.001141816	0.0454379	-1.5389341
WFDC1	1.57941101	4.40282081	3.44153701	0.001142525	0.0454379	-1.5369033
SLC25A15	-0.5671527	3.38877518	-3.4407764	0.001145144	0.0454379	-1.1074452
PARP6	-0.4602556	3.93831179	-3.4404863	0.001146144	0.0454379	-1.3512133
LDOC1	0.71588384	3.57093315	3.44044303	0.001146293	0.0454379	-1.296205
POLR1D	-0.4332505	3.96809113	-3.4397175	0.001148799	0.0454379	-1.3574741
C4H1orf198	0.6122593	8.34576045	3.43915713	0.001150738	0.0454379	-1.5236963
SLC45A4	0.49190172	3.22464812	3.43485154	0.001165739	0.04588639	-1.2402372
PPP4R3B	-0.263881	6.19524458	-3.4311157	0.001178905	0.04626009	-1.5935895
XRCC4	0.55167692	2.12925569	3.42891457	0.001186729	0.04642248	-1.0932449
PTH1R	1.67638406	-0.069387	3.42731	0.001192463	0.04650238	-0.993721
FOXL1	1.98718584	0.04699082	3.42043484	0.001217333	0.04732569	-0.9480922
DCAF6	-0.3597187	5.29066229	-3.4158916	0.001234037	0.04782746	-1.6120772
CLSTN1	0.48596857	7.53699375	3.40975612	0.00125694	0.04840878	-1.6150315
RPL18	-0.3454473	8.23272883	-3.4093515	0.001258465	0.04840878	-1.5888528
ANKRD13A	0.45200041	6.00931158	3.40796826	0.00126369	0.04840878	-1.6543423
ADCY4	-1.0719167	3.60501672	-3.4076322	0.001264963	0.04840878	-1.3344037
CTCF	-0.3710944	5.33796559	-3.4067651	0.001268252	0.04840878	-1.6034309
DNM2	0.32335782	6.78072633	3.40571953	0.001272228	0.04841386	-1.6618027
ENSCAFG00000023746	-0.5300649	3.01167154	-3.4037072	0.001279916	0.0485597	-1.2672115
DNAL1	0.75430858	1.78355314	3.40160317	0.001288	0.04871968	-1.119876
ZPLD1	1.65561834	1.05017625	3.39495071	0.001313882	0.04954988	-1.0599654
LARP1	-0.1962017	7.08549689	-3.3927847	0.001322415	0.04972282	-1.6948826
PEX2	-0.4097487	4.28112093	-3.3916149	0.001327045	0.04974841	-1.5263996
INHBB	2.53054407	1.41216517	3.38597824	0.001349573	0.05033318	-1.0435261
ARHGEF4	1.84637531	-1.8696454	3.38571442	0.001350636	0.05033318	-1.2402416
ENSCAFG00000012963	3.04106208	7.29068058	3.38138651	0.001368192	0.05077473	-1.6015757
CTIF	0.77970628	6.0348902	3.38081006	0.001370547	0.05077473	-1.7304964
TIA1	-0.3835755	4.59583257	-3.3791984	0.00137715	0.05080027	-1.5899693
NXPH3	1.11890644	4.24909183	3.3786747	0.001379302	0.05080027	-1.577516
TBX20	0.59009052	5.52965799	3.37680315	0.00138702	0.05081513	-1.7214117
NAALADL2	-1.377171	3.09525716	-3.3766208	0.001387774	0.05081513	-1.1043471

SENP1	-0.4473216	3.96224506	-3.3755663	0.001392143	0.05082734	-1.5201422
ANKFY1	-0.1926189	6.35355237	-3.3735102	0.001400698	0.0509919	-1.7573551
ATP6V0C	0.56912586	7.80672612	3.37087525	0.001411736	0.0512456	-1.7139785
SMPD1	0.47810219	6.184272	3.36913361	0.001419076	0.05127884	-1.7690158
OTUD4	-0.328325	6.52752062	-3.368106	0.001423425	0.05127884	-1.7684062
DISC1	1.71076043	2.58256753	3.3677663	0.001424865	0.05127884	-1.160284
GTDC1	0.49045186	2.83230626	3.36488582	0.001437132	0.0514757	-1.36103
XYLT2	0.41113734	4.10606609	3.36366496	0.001442361	0.0514757	-1.5965802
ENSCAFG00000017848	-1.124335	1.12418875	-3.3635855	0.001442703	0.0514757	-1.0957588
GINS3	-0.4226716	3.57876426	-3.3617339	0.00145067	0.0514757	-1.5348324
C1QL1	2.04844561	0.3669359	3.36140457	0.001452091	0.0514757	-1.1305658
POLR2K	0.84267662	1.10023804	3.36076521	0.001454855	0.0514757	-1.1199419
ENSCAFG00000016997	1.99219578	-0.6181019	3.35385612	0.001485037	0.05239642	-1.155588
BGN	1.00063353	11.9070578	3.34895098	0.001506823	0.05280663	-1.519453
OLFM1	2.29936475	-0.1840374	3.34855088	0.001508614	0.05280663	-1.1597544
NPTN	0.59390003	8.3338408	3.34816428	0.001510346	0.05280663	-1.7627721
AP3S1	0.35351986	5.01749722	3.34747633	0.001513432	0.05280663	-1.7823363
PEA15	0.56767924	5.86165597	3.34638861	0.001518325	0.05283099	-1.8113119
CDH6	1.85815762	5.01190264	3.34389699	0.001529588	0.0530613	-1.6668877
TMED1	0.5834134	3.52157818	3.34301036	0.001533615	0.0530613	-1.5154942
PRSS35	1.70641652	0.78791706	3.34213928	0.001537581	0.0530613	-1.1479206
DIXDC1	0.41108351	5.21684937	3.33999018	0.001547408	0.0531245	-1.8202177
TBC1D1	1.68059998	5.19490913	3.33898003	0.001552048	0.0531245	-1.7036902
PRELID2	-0.3239804	5.03865067	-3.3388436	0.001552675	0.0531245	-1.7744484
ENSCAFG00000003668	-1.3642523	0.40747232	-3.3380604	0.001556283	0.0531245	-1.1844497
ITFG1	0.37272459	7.81757452	3.33281976	0.001580628	0.05371341	-1.8276989
CAMK2A	2.03568578	3.07311306	3.33251301	0.001582064	0.05371341	-1.1709308
ENSCAFG00000028613	-0.3549847	5.08716099	-3.3313084	0.001587715	0.05376038	-1.8116971
FAM71F2	1.53891774	-0.5604458	3.32712389	0.001607496	0.05428423	-1.1849839
ACTN1	0.85203951	10.3015653	3.32560235	0.001614746	0.05438326	-1.7126155
SH3BP5L	0.48391988	5.34949848	3.32388551	0.001622964	0.05451428	-1.8627169
ANKRD1	2.37498684	4.68031013	3.320902	0.001637339	0.0547621	-1.7381665
FNDC4	1.26810335	3.03332316	3.32055125	0.001639037	0.0547621	-1.310736
MAP1B	0.97333304	9.89006704	3.31930973	0.00164506	0.05477331	-1.7527121
ELF4	-0.5785527	5.72711381	-3.3180661	0.001651115	0.05477331	-1.8708533
GALNT5	2.42545009	0.1978423	3.31779902	0.001652418	0.05477331	-1.2156221
ZNF521	-0.908555	5.3170181	-3.3121766	0.001680075	0.05554389	-1.8734374
COL11A2	1.08380733	0.51304682	3.30534247	0.001714282	0.05652644	-1.2453491
ANO2	-3.4940431	-0.578664	-3.3043828	0.001719139	0.05653856	-1.2925674
PDRG1	0.59958955	2.6588765	3.29964733	0.001743291	0.05718357	-1.4952987
SLC6A8	0.76819145	5.38182032	3.2966107	0.001758947	0.05754727	-1.9061639
IL11RA	0.87533031	3.02099594	3.29552414	0.001764582	0.05758205	-1.6164901
PCYOX1	0.31102941	7.3391228	3.29428053	0.001771051	0.05764383	-1.959867
TMEM64	1.18122516	3.34533934	3.29204897	0.001782717	0.05787398	-1.4708901
ENSCAFG00000015433	-0.4103235	4.27134046	-3.2900829	0.001793055	0.05805995	-1.8425162
ENSCAFG00000032311	-1.955539	-0.3235318	-3.2877886	0.00180519	0.05817319	-1.3261991
RPL15	-0.3494864	8.04493916	-3.2875721	0.00180634	0.05817319	-1.9451412
ENSCAFG00000010333	1.68407607	-0.2019079	3.28680674	0.001810407	0.05817319	-1.3412895
HOPX	1.7422287	-2.3288397	3.28453071	0.001822556	0.05835768	-1.4545731
SMG9	-0.4708546	5.13007602	-3.2839972	0.001825415	0.05835768	-1.9351219

DOK4	0.35481903	5.03563745	3.28181745	0.001837139	0.0585838	-1.9400564
GNPDA1	-0.6635127	4.53548929	-3.2808462	0.001842386	0.05860276	-1.8404074
PHF11	-0.412943	3.26271535	-3.2783681	0.001855838	0.05888194	-1.637134
LUM	1.13801452	11.4346401	3.27722562	0.00186207	0.05893125	-1.7229974
CLPTM1L	0.39113708	7.43183804	3.27298043	0.001885404	0.05952016	-2.0147931
EPHB6	1.82001369	-0.149539	3.26745104	0.00191621	0.06034144	-1.3421318
DSTYK	0.360401	3.55359423	3.26313524	0.001940584	0.06095661	-1.7930966
LMOD1	1.08417966	5.72562325	3.26057468	0.001955184	0.0611944	-2.0533106
DNAJA4	1.37169631	2.46951998	3.26010532	0.001957871	0.0611944	-1.4256707
CDC42EP5	-0.9712951	3.0778215	-3.2562779	0.001979916	0.06159373	-1.5669313
MMP15	0.89565932	2.08183415	3.25618958	0.001980427	0.06159373	-1.5075965
SLC27A3	-0.7751266	3.1030235	-3.253615	0.001995391	0.06190628	-1.6770305
PCK2	-0.5217325	7.35566834	-3.2509228	0.002011153	0.06224197	-2.0898853
ADGRE5	0.65232467	5.90531662	3.24780625	0.002029546	0.06255695	-2.1012914
ATP10A	0.8779053	4.43163393	3.2475166	0.002031263	0.06255695	-1.807533
SESN3	1.13730505	1.72240541	3.2435407	0.002054977	0.06313291	-1.5651701
POLR3C	-0.3151755	4.38621575	-3.2394856	0.002079433	0.06372879	-1.9706586
RAC2	0.80052504	0.93922274	3.23769507	0.002090318	0.06373759	-1.4206956
TMED10	0.25567716	7.03397084	3.23729021	0.002092787	0.06373759	-2.1180463
TAGLN	1.26713734	10.6537944	3.23694395	0.0020949	0.06373759	-1.9007077
SRF	0.32593896	6.04872766	3.23552986	0.002103553	0.06384663	-2.1355756
FBXW12	2.00701593	-0.847417	3.23222173	0.002123927	0.06431005	-1.4184833
TANGO2	0.40435023	4.58362844	3.22992824	0.002138161	0.06458579	-2.0454431
RPL3	-0.331085	9.33848412	-3.2284314	0.002147499	0.06471268	-2.0184185
FMR1	-0.3646471	5.47538574	-3.2274887	0.0021534	0.06473562	-2.1060375
TPM2	1.53428258	9.5512953	3.22417243	0.00217428	0.0652077	-2.0025695
CLEC16A	0.38811377	5.26633479	3.21987727	0.002201608	0.06587044	-2.148773
ENSCAFG00000016333	0.83717256	7.7411453	3.21878392	0.002208616	0.06592352	-2.1604158
RGS2	1.30187441	3.89391885	3.21560389	0.002229118	0.06637818	-2.0611104
BTG1	-0.6534162	4.2023154	-3.2139601	0.002239786	0.06653853	-2.0819991
ENSCAFG00000013974	-1.1204807	0.83522094	-3.2094791	0.002269112	0.06717189	-1.4805029
ROBO3	1.27858905	4.07300966	3.2090755	0.002271771	0.06717189	-1.9348959
CSRP1	0.78527131	9.87740483	3.20299214	0.002312207	0.0681059	-2.0833837
EEPD1	1.31128894	1.14638788	3.20269896	0.002314173	0.0681059	-1.5581036
RHBDL3	-1.5015569	-1.7665823	-3.1979791	0.002346038	0.06888272	-1.5436956
CSF3	-1.8187608	-2.1604358	-3.1968937	0.002353424	0.06891576	-1.582878
EPB41L1	0.82953076	6.14986635	3.19552184	0.00236279	0.06891576	-2.2426818
LRRC4	1.22618031	1.283148	3.19540693	0.002363576	0.06891576	-1.6052914
MFSD6	1.85112463	-0.092146	3.19102205	0.00239376	0.06963466	-1.5218148
COL18A1	-1.4109531	4.74625314	-3.1875684	0.002417789	0.07012479	-2.2475033
EEF1A1	-0.4057622	11.0729466	-3.1870031	0.002421744	0.07012479	-2.0161599
MLEC	0.42156354	4.75688898	3.18600625	0.002428732	0.07016585	-2.1657362
SP3	-0.3556162	5.90855374	-3.1838017	0.002444255	0.07031512	-2.2663283
ANXA3	0.85128011	3.78274847	3.18368716	0.002445064	0.07031512	-1.9812955
SMAGP	-0.7070325	2.2133011	-3.1815044	0.00246053	0.07054884	-1.6432628
ELMO2	0.32509378	5.3746793	3.18096123	0.002464393	0.07054884	-2.2618852
CNOT8	-0.3769645	4.05516437	-3.1784743	0.002482152	0.07089611	-2.0768495
PRKACB	0.34938878	6.58239071	3.17617417	0.002498685	0.07120686	-2.298556
ENSCAFG00000006580	-0.5302795	7.34804727	-3.1749963	0.002507191	0.07125612	-2.2836108
MARCKSL1	1.72421801	3.17939428	3.1741816	0.00251309	0.07125612	-1.8762785

IGHMBP2	0.36366802	5.10221442	3.17317094	0.002520427	0.07125612	-2.2467647
BCAP31	0.36309546	6.45682306	3.17281147	0.002523041	0.07125612	-2.3074628
TUBA4A	1.63394307	5.08781599	3.17072068	0.002538298	0.07152662	-1.9765892
PRR5	1.15201903	1.29637247	3.16636959	0.002570326	0.07226706	-1.6986347
RP2	-0.4017335	2.75714948	-3.1654333	0.002577267	0.07226706	-1.8055621
JAKMIP1	1.47072689	-1.4825824	3.16416545	0.002586695	0.07226706	-1.5825928
SMARCD3	0.56181947	5.09861364	3.1640199	0.00258778	0.07226706	-2.2082494
NSD3	-0.2815567	5.78418854	-3.1632851	0.002593261	0.07226706	-2.3236348
RHEB	0.34088236	6.68809248	3.1624205	0.002599724	0.07228725	-2.3330805
EML2	0.62718858	5.89027447	3.16093508	0.002610863	0.07230934	-2.3232555
ENSCAFG00000016639	-0.3867465	3.67986687	-3.1607839	0.002612	0.07230934	-2.0365456
TNFSF9	1.47954486	0.37276461	3.15861674	0.002628339	0.07255597	-1.6507755
ENSCAFG00000006545	-0.3008013	7.42930009	-3.1580761	0.002632429	0.07255597	-2.3269057
IPO5	-0.3143163	7.91441548	-3.1541273	0.002662492	0.07314361	-2.3154996
CAPRIN2	0.40665973	4.5771004	3.15327209	0.002669046	0.07314361	-2.2473831
PCYOX1L	0.55961509	3.85263579	3.15246515	0.002675243	0.07314361	-2.1209888
NYNRIN	-1.2138171	4.19896985	-3.1508604	0.002687608	0.07314361	-1.8499854
TSPYL2	0.47576216	4.80183417	3.1499751	0.002694452	0.07314361	-2.3393388
FAM167B	-1.8857634	-2.7353953	-3.1496663	0.002696843	0.07314361	-1.7915632
RBM6	-0.2703436	4.94295736	-3.1493941	0.002698953	0.07314361	-2.3011488
ENSCAFG00000013802	-1.0902311	0.77224456	-3.1492326	0.002700205	0.07314361	-1.6223006
PPFIBP2	2.10468409	0.20900941	3.14743606	0.002714172	0.07336419	-1.6361667
SNRPD1	-0.424144	4.2935639	-3.1453595	0.002730401	0.07352629	-2.18934
INTS10	-0.3095888	5.53862595	-3.1451754	0.002731844	0.07352629	-2.3613899
DAPP1	2.53257966	-0.9878935	3.14274388	0.002750973	0.07375831	-1.6667176
ATP6AP2	0.38527044	7.61299001	3.14259144	0.002752176	0.07375831	-2.3481219
TMOD1	1.61276441	1.54885855	3.14108478	0.002764097	0.07392051	-1.6915138
ADIPOR1	0.3843069	7.4197627	3.13909195	0.002779938	0.07418666	-2.3716642
ADRA2A	1.44954819	0.26681124	3.13585656	0.002805839	0.07471956	-1.6733185
KCNG1	2.15941372	-0.5006424	3.13346741	0.002825111	0.07507405	-1.7013095
PREP	0.59806475	7.00914069	3.13129611	0.002842733	0.07526939	-2.4081329
ADGRB1	2.10401341	-1.0886018	3.13011171	0.002852389	0.07526939	-1.7488739
ATG4A	0.45741398	3.28358898	3.12993433	0.002853838	0.07526939	-2.0816486
ERLIN1	-0.3919848	4.12591634	-3.1293275	0.0028588	0.07526939	-2.2495065
PIBF1	-0.3892094	3.86887635	-3.1288951	0.00286234	0.07526939	-2.1912484
CA11	0.9327007	1.42293215	3.1212417	0.002925689	0.07669079	-1.9525677
WNT10B	0.31673731	5.81470082	3.1208973	0.00292857	0.07669079	-2.4414834
PTPN5	1.72681277	-1.8184413	3.11910705	0.002943591	0.07692421	-1.7941403
DUSP26	2.18063542	0.16953366	3.11829331	0.002950442	0.07694363	-1.7105955
SMC6	-0.5460026	6.30195703	-3.1142136	0.002985019	0.0776845	-2.4549899
GTF3C5	-0.3100107	4.94390118	-3.1130569	0.002994891	0.07774827	-2.4037866
TNFRSF12A	0.77994868	5.04364561	3.11248136	0.002999814	0.07774827	-2.3601955
EHMT1	-0.2315101	5.95335443	-3.1103471	0.003018138	0.07790011	-2.4657091
SAP30	-0.446821	3.16378786	-3.1099514	0.003021547	0.07790011	-2.0582375
MYO5B	1.63985478	-0.6452891	3.10898296	0.003029905	0.07790011	-1.7929695
DNAJC7	-0.3130565	4.98027909	-3.1089245	0.003030411	0.07790011	-2.4141229
ENSCAFG00000029013	0.52660255	4.2130548	3.1073053	0.003044436	0.07810125	-2.255973
ENSCAFG00000005160	-0.5390986	4.67104121	-3.1014238	0.003095894	0.07908738	-2.3701909
FCHSD2	0.57543093	3.85601618	3.10123694	0.003097543	0.07908738	-2.2645751
CHST2	1.71543762	3.46497103	3.10076466	0.003101712	0.07908738	-2.0731647

DACT3	1.02312204	1.32010062	3.0982561	0.003123946	0.07930611	-1.7606004
USP4	-0.2385079	6.25052996	-3.0982088	0.003124367	0.07930611	-2.5053599
TMEM63B	0.38277701	6.25886525	3.0965284	0.003139346	0.07930611	-2.5104243
NEXN	1.26728968	6.24874004	3.09578912	0.003145958	0.07930611	-2.4945413
DPH5	-0.4063363	4.32524472	-3.0955946	0.003147699	0.07930611	-2.3353806
CSPG5	0.96326594	1.49327719	3.09555353	0.003148067	0.07930611	-1.9048646
CRY2	0.6467812	4.26545749	3.09292568	0.003171689	0.07954916	-2.2909531
ENSCAFG00000025328	-0.5930145	2.79438682	-3.09241	0.003176344	0.07954916	-1.9959432
FAM174A	0.69397823	4.83695119	3.09237482	0.003176661	0.07954916	-2.4194173
TMEM59L	0.99661115	4.78158905	3.09149415	0.003184626	0.07959039	-2.4611423
ENSCAFG00000018296	-0.5442716	2.34372372	-3.0903262	0.003195219	0.07966887	-1.9503554
SMAD1	-0.6345196	6.09105977	-3.0895112	0.00320263	0.07966887	-2.5211146
METAP1	-0.3225818	5.26240467	-3.0884326	0.003212462	0.07966887	-2.4829715
MAGEL2	1.71761715	0.23433872	3.08816042	0.003214948	0.07966887	-1.7961101
BMP7	1.63965725	-2.0828003	3.08767431	0.003219391	0.07966887	-1.8192993
RPL5	-0.3043091	9.2704842	-3.0846291	0.003247361	0.07996441	-2.4110297
NCAPH2	-0.3816405	5.77320293	-3.0837793	0.003255207	0.07996441	-2.5232631
HYOU1	0.58936663	8.85885314	3.08334812	0.003259194	0.07996441	-2.4475068
FAM222B	0.41121931	4.59727585	3.08285598	0.003263751	0.07996441	-2.4299076
ERLEC1	0.37278492	6.60051741	3.08233594	0.003268573	0.07996441	-2.544619
TSEN54	-0.5291751	2.38347951	-3.0822442	0.003269425	0.07996441	-1.9506151
ENSCAFG00000024183	-0.385173	5.07165647	-3.0801936	0.003288507	0.0801072	-2.4917447
LARGE1	0.59004421	6.22081528	3.07959157	0.00329413	0.0801072	-2.5515236
ZCCHC8	-0.4480569	4.18111519	-3.0785338	0.00330403	0.0801072	-2.3582904
STK39	0.40477057	5.50029482	3.07821239	0.003307044	0.0801072	-2.5374409
WDR1	0.36511126	9.29480325	3.07821052	0.003307061	0.0801072	-2.43708
ARHGDIB	1.75065435	2.50667046	3.07308292	0.003355491	0.08106445	-2.2298459
ENSCAFG00000018094	-0.2771877	8.77849895	-3.0722811	0.003363124	0.08106445	-2.4735942
CDK5RAP3	-0.3268172	5.06248003	-3.0719913	0.003365886	0.08106445	-2.5011506
MAP2	2.17918476	1.98112396	3.06943766	0.003390323	0.08149716	-1.8251699
NFATC4	-0.5701622	5.16498227	-3.0685181	0.003399163	0.08155402	-2.5186954
FAM19A4	1.42538779	-0.936743	3.0657824	0.003425591	0.081925	-1.8145988
FHL2	0.62747459	7.07217315	3.06557178	0.003427634	0.081925	-2.5802582
PPP1R15B	-0.2903763	6.30257087	-3.0643339	0.003439661	0.08202224	-2.5905441
ST3GAL6	-1.0381892	3.41539742	-3.0635811	0.003446995	0.08202224	-2.118403
IL3RA	-2.6249641	-2.4954707	-3.0627756	0.003454859	0.08202224	-1.9335901
REEP2	1.47001837	-0.2197468	3.06247999	0.003457749	0.08202224	-1.82227
KCTD1	0.49259164	4.214122	3.06093115	0.003472929	0.08222746	-2.447251
DIS3L	-0.2182107	6.50572729	-3.056681	0.003514904	0.08290881	-2.6154275
ENSCAFG00000019807	2.67010863	3.4180528	3.05592104	0.003522459	0.08290881	-2.0503441
RPL19	-0.3306965	8.4519715	-3.0555543	0.00352611	0.08290881	-2.5387153
DYNC1LI2	0.34813626	6.03052329	3.05510143	0.003530624	0.08290881	-2.618153
ACVR1B	0.46936812	3.73552969	3.05424462	0.003539179	0.08290881	-2.4743203
ENSCAFG00000007307	-2.8738415	1.48190794	-3.0535708	0.003545921	0.08290881	-2.1119425
PLEKHG5	1.21331938	4.36788347	3.05338507	0.003547781	0.08290881	-2.4142201
TRIM46	1.2116593	-0.5239129	3.05219702	0.003559702	0.08303335	-1.8492263
EDEM2	0.41472313	5.83862672	3.05019057	0.003579921	0.08335062	-2.6321108
USP1	-0.6291929	5.57433051	-3.0483926	0.003598129	0.08356767	-2.5683665
TRIM21	-0.3931734	3.43439149	-3.0476398	0.00360578	0.08356767	-2.2919953
SLC22A17	0.78545833	4.59428035	3.04730891	0.003609147	0.08356767	-2.5191613

LZTR1	0.2922868	4.74579885	3.04584385	0.003624091	0.08375972	-2.5466585
TCOF1	-0.4190305	4.57416295	-3.0446964	0.003635836	0.08387726	-2.4904255
ICE1	-0.47119	5.60303359	-3.0429504	0.003653776	0.08413703	-2.6422986
STBD1	0.61393613	4.22481289	3.04096285	0.003674298	0.08430743	-2.4592652
ZNF740	-0.2592561	4.75753177	-3.0409374	0.003674561	0.08430743	-2.5682398
PLS3	0.5378696	10.1457644	3.03736469	0.003711724	0.08477075	-2.4884563
FAM184B	1.87672542	-1.1754082	3.0371812	0.003713642	0.08477075	-1.8865112
NUDT2	-0.5630889	2.46694574	-3.0368971	0.003716614	0.08477075	-2.1235061
RPS18	-0.3021078	8.73611766	-3.0364137	0.003721675	0.08477075	-2.563902
MYO1C	0.47567848	9.5672026	3.03564541	0.003729733	0.08479045	-2.5407897
DDOST	0.30935752	8.74308776	3.03460216	0.003740701	0.08479045	-2.5759363
RPSA	-0.3292246	8.39286945	-3.034409	0.003742735	0.08479045	-2.6037456
SCAMP5	0.82522676	2.8236233	3.03285513	0.003759135	0.08500908	-2.2497413
SLC33A1	0.26361324	5.83447679	3.03119065	0.003776776	0.08511708	-2.6760138
PALLD	1.13452782	8.6711231	3.03030342	0.003786212	0.08511708	-2.5781056
OGT	-0.4196573	7.70454251	-3.0284911	0.003805553	0.08511708	-2.6476084
SLC9A7	1.87526705	-1.8070383	3.0282582	0.003808046	0.08511708	-1.9559374
SFXN2	-0.5606987	4.08879525	-3.0275354	0.00381579	0.08511708	-2.5030146
ABCB9	0.44481016	3.80364342	3.02731531	0.003818151	0.08511708	-2.5011172
ENSCAFG00000029004	-0.470797	4.902639	-3.0271633	0.003819783	0.08511708	-2.5963921
PTPN14	0.77737064	4.192138	3.02678845	0.003823809	0.08511708	-2.4123234
LRIG2	-0.4281582	3.96373437	-3.026703	0.003824727	0.08511708	-2.4853522
ANKRD37	0.68149569	2.6537341	3.0252866	0.003839979	0.08530578	-2.3577177
ENSCAFG00000004233	-0.5471906	3.34373552	-3.0245713	0.003847703	0.08532688	-2.3258191
PPP1R14A	1.40457764	-1.1816139	3.023055	0.003864125	0.08554045	-1.9311864
TXNL4B	-0.3432789	3.03453739	-3.0184324	0.003914592	0.08650562	-2.31956
CHST10	0.49759311	6.45700405	3.01766139	0.00392307	0.08654114	-2.7160622
ENSCAFG00000031320	0.37956923	5.74285369	3.01579442	0.003943668	0.08662529	-2.6802886
KLF4	-0.9996307	6.09052916	-3.0156587	0.00394517	0.08662529	-2.7133825
DAAM2	1.83776703	2.22962609	3.01544666	0.003947517	0.08662529	-1.9334544
PTPMT1	-0.441457	3.09656296	-3.0143493	0.003959682	0.08674113	-2.2423132
CCAR1	-0.3130291	6.40653835	-3.0117093	0.003989092	0.08718471	-2.7317424
SNRK	-0.8202509	3.61595864	-3.0112908	0.003993774	0.08718471	-2.5570179
RASAL2	0.43595599	6.47401809	3.00958082	0.004012954	0.08745184	-2.7376338
ABCA3	1.58676225	0.85011868	3.0089106	0.004020495	0.08746485	-1.9645456
TMCO3	0.3114155	5.26654002	3.00771782	0.004033948	0.08757687	-2.7185816
MIB2	-0.5798643	3.52059578	-3.0066944	0.004045524	0.08757687	-2.3707763
ADAMTS8	1.86095332	-1.940853	3.0066081	0.004046502	0.08757687	-1.9897981
KANK3	-2.7171698	-1.1962799	-3.0057263	0.004056503	0.08764273	-1.9535456
FAM193A	-0.257641	4.65278265	-3.0049928	0.00406484	0.08767248	-2.6544426
PCBP2	-0.2333276	7.61593757	-3.0041222	0.004074757	0.08773614	-2.7219526
PLEKHA2	0.77665847	3.11548315	3.00250763	0.004093207	0.08798299	-2.4399082
SETD3	0.22511416	5.66199415	2.99875977	0.004136335	0.08860727	-2.7543283
MB21D2	-0.5453679	3.67941728	-2.9986159	0.004137999	0.08860727	-2.4764603
DSTN	0.51891396	9.46959331	2.9969665	0.00415712	0.08860727	-2.6413407
ACTA2	1.53461064	11.2105169	2.99649447	0.004162608	0.08860727	-2.5401914
DPY19L3	0.51973501	6.72818639	2.99627085	0.00416521	0.08860727	-2.7562611
CCKBR	2.41080217	-0.837193	2.99471748	0.004183326	0.08860727	-1.9782269
ATP11A	-0.356	6.73362186	-2.9939823	0.004191926	0.08860727	-2.7759444
FMOD	2.43328272	1.00265118	2.9936265	0.004196094	0.08860727	-1.9942004

PLXDC2	1.24788063	3.65234689	2.99298084	0.004203667	0.08860727	-2.370722
PAK3	0.51334971	4.80584453	2.99195466	0.00421573	0.08860727	-2.7031334
SEMA4F	0.43473694	4.82317349	2.99180826	0.004217454	0.08860727	-2.7392922
OSTM1	0.38289078	5.25664012	2.99168493	0.004218906	0.08860727	-2.7090854
EEF1B2	-0.3719606	8.02690746	-2.9914202	0.004222026	0.08860727	-2.713632
CLK2	-0.2944252	4.57439115	-2.9913891	0.004222392	0.08860727	-2.6645821
CS	-0.2391696	7.46317593	-2.9909333	0.004227768	0.08860727	-2.7654423
ENSCAFG00000000341	-0.4427369	3.30071323	-2.9889752	0.004250937	0.08861012	-2.4600622
PARD6B	1.19330068	-0.2529845	2.98850939	0.004256466	0.08861012	-1.9920424
DAPK3	0.53826513	7.50119337	2.98793784	0.00426326	0.08861012	-2.7693655
TNS3	4.28952094	2.24970269	2.98765942	0.004266572	0.08861012	-2.0059716
VXN	1.74103596	-1.8157534	2.98755297	0.00426784	0.08861012	-2.0634034
PQBP1	-0.4057997	5.35157982	-2.9873621	0.004270113	0.08861012	-2.7651114
WDR5	-0.3096605	6.05080329	-2.9855289	0.004292003	0.08885609	-2.7930556
IMPDH1	0.30950313	6.63088849	2.98518886	0.004296075	0.08885609	-2.8008248
RPL13A	-0.3119515	9.63046497	-2.9838298	0.004312384	0.08900457	-2.6512695
BCL2L2	0.44354834	2.7537509	2.98312579	0.004320856	0.08900457	-2.399719
DNHD1	1.32548971	-0.7256076	2.98282738	0.004324452	0.08900457	-2.0049025
ENSCAFG00000014378	-0.8588161	1.75988087	-2.9820539	0.004333784	0.08905114	-2.0497341
TMED8	0.49464326	2.27768212	2.9799192	0.004359639	0.0894365	-2.2366333
VCL	0.62388247	9.17594654	2.97717324	0.004393108	0.08983059	-2.6977638
CPAMD8	2.16982188	1.16126414	2.97717291	0.004393112	0.08983059	-2.0996433
ACVRL1	-0.9101411	6.05783564	-2.9765109	0.004401217	0.08985046	-2.816789
INA	1.55105121	1.61985633	2.97481851	0.004422	0.09006561	-2.3167239
ENSCAFG00000010867	-0.204096	5.58822349	-2.974489	0.004426057	0.09006561	-2.8106006
RPL14	-0.3335126	6.91246967	-2.9703717	0.004477043	0.09063277	-2.8294315
KTI12	-0.4252001	3.01723477	-2.9695172	0.004487693	0.09063277	-2.3595341
ITGB1BP2	1.13468308	1.16876353	2.96924989	0.00449103	0.09063277	-2.0951642
NEMF	-0.287036	5.42089933	-2.9690528	0.004493492	0.09063277	-2.8056042
CRIP2	-0.5093821	5.50058606	-2.9686346	0.004498718	0.09063277	-2.8243885
P4HTM	0.54006925	4.34639145	2.96859234	0.004499247	0.09063277	-2.7315513
ATOX1	0.42027602	5.14815168	2.9676949	0.004510485	0.09063277	-2.8115089
PPP4R3A	-0.3351181	5.93463922	-2.9676146	0.004511491	0.09063277	-2.8362627
TMEM150B	1.14344389	-0.4509708	2.96667531	0.004523283	0.09072496	-2.0413622
PRPF38A	-0.3944931	3.70779806	-2.9648	0.004546912	0.09095097	-2.5578155
RACK1	-0.3165036	9.95820427	-2.9646353	0.004548993	0.09095097	-2.6742389
RELT	1.41875247	0.63867708	2.96367254	0.004561174	0.09101044	-2.0529736
COL13A1	1.95462348	-0.7909121	2.96325881	0.004566418	0.09101044	-2.0591903
ABHD12	0.40101196	4.72384176	2.96074597	0.004598387	0.09150282	-2.7175754
WIPI1	0.2723488	6.45353238	2.95962969	0.004612656	0.09164197	-2.8666961
FAM19A5	1.42536976	-0.2877282	2.9588888	0.004622149	0.09168596	-2.059259
AP1B1	0.40640771	6.62498683	2.95740124	0.004641264	0.09192037	-2.8681341
SEMA3A	-1.8728901	-0.8700701	-2.954389	0.004680197	0.09249215	-2.1114049
IGFBP4	1.01568599	5.52095386	2.9535219	0.00469146	0.09249215	-2.7857814
BNIP3	0.80240963	6.4203651	2.95336345	0.004693521	0.09249215	-2.8763168
CALD1	0.76944213	10.8256872	2.95263942	0.004702949	0.09249215	-2.6805996
SAMD9L	-0.5572634	7.0005207	-2.9523403	0.004706849	0.09249215	-2.8502328
ENSCAFG00000007079	-1.1090705	0.51524526	-2.9492541	0.004747266	0.09314107	-2.0805265
APOL5	-0.921712	4.66342223	-2.9485209	0.004756916	0.09318524	-2.839163
HIRA	-0.28066	5.36495289	-2.9474769	0.004770687	0.09320296	-2.8533754

SMCO2	2.05233839	-2.3419129	2.94733061	0.004772619	0.09320296	-2.1081662
MTPAP	-0.3129703	4.66408461	-2.9457261	0.004793864	0.09347293	-2.7995125
EFHD1	1.58476786	5.3332405	2.94336632	0.00482527	0.09393988	-2.8301967
HSPB7	1.86304649	2.18329688	2.94232462	0.004839195	0.09406558	-2.2455307
ENSCAFG00000013587	-0.2883491	4.83504646	-2.9385984	0.00488931	0.09489329	-2.7925921
LCMT1	-0.2903544	4.63170551	-2.9353005	0.004934067	0.09544186	-2.7902652
AVPR1A	-2.6661637	0.66512271	-2.9349816	0.004938416	0.09544186	-2.6079856
SHANK1	1.27356072	1.54953789	2.93474457	0.00494165	0.09544186	-2.2118179
IFT80	-0.3415495	5.19707564	-2.9336524	0.004956577	0.09544186	-2.8792072
EIF3L	-0.2820709	5.64497204	-2.9334471	0.004959388	0.09544186	-2.9170024
ENSCAFG00000002897	-0.4101536	4.55307191	-2.9328782	0.004967183	0.09544186	-2.7941499
ZCCHC3	-0.9856775	0.76258438	-2.9322792	0.004975405	0.09544186	-2.1561
STARD9	-0.4664366	5.23557682	-2.9320764	0.004978192	0.09544186	-2.9135806
TMEM225B	-0.655487	3.50074914	-2.9299872	0.005006979	0.09584788	-2.5083804
SLC29A1	-0.3052116	9.60385031	-2.9284001	0.005028952	0.09602277	-2.7823758
CLIC3	1.48692353	1.20117447	2.92735361	0.005043489	0.09602277	-2.3184717
ZC3HAV1L	-0.5994035	1.45237718	-2.9261062	0.005060868	0.09602277	-2.22214
ENSCAFG00000002945	-0.6943713	2.57832851	-2.9256391	0.005067389	0.09602277	-2.3920494
EXOSC5	-0.4377491	2.64933704	-2.9252889	0.005072285	0.09602277	-2.4868311
ARL6IP6	-0.3842606	4.11698489	-2.9250879	0.005075096	0.09602277	-2.720761
PLXNB1	0.81429631	3.32886797	2.92465021	0.005081223	0.09602277	-2.4591873
ADCY5	1.59371289	-0.1139211	2.92462737	0.005081543	0.09602277	-2.1358608
KIF3B	0.29015399	5.1275757	2.92440032	0.005084725	0.09602277	-2.9218766
ANKRD6	2.19880613	-0.4706389	2.92023271	0.005143449	0.09698636	-2.1803509
DNAJC1	0.43435927	4.52529035	2.9196536	0.005151659	0.09699596	-2.8342581
B3GNT4	0.94276506	0.41109718	2.91909231	0.005159628	0.097001	-2.2012881
TMEM106C	0.33209006	4.73194371	2.91795212	0.005175851	0.09712682	-2.8646735
KERA	1.47822995	1.40106153	2.91753889	0.005181742	0.09712682	-2.2013747
STEAP2	-0.9395359	4.49254694	-2.9167761	0.005192633	0.09718633	-2.9517743
GABPA	-0.2824758	6.12628481	-2.915903	0.005205125	0.09723543	-2.9747767
NDST1	-0.3887213	6.00836967	-2.9155143	0.005210695	0.09723543	-2.9643779
MAPRE3	0.46421541	4.23879473	2.91475241	0.00522163	0.09729535	-2.8612767
RPS27A	-0.3424514	7.80292358	-2.9128917	0.005248426	0.09765018	-2.9476892
ENSCAFG00000026287	-1.115765	0.10750605	-2.9117647	0.005264717	0.09767274	-2.1685272
CTTNBP2NL	-0.4036445	5.00190032	-2.9116692	0.005266099	0.09767274	-2.9354754
LRIF1	-0.3104195	5.12442656	-2.9111997	0.005272901	0.09767274	-2.9501875
DARS	-0.2333036	6.41131655	-2.9102187	0.005287141	0.09776221	-2.991207
EIF4B	-0.3477296	8.85412773	-2.9097083	0.005294563	0.09776221	-2.8789765
ENSCAFG00000003714	-0.6673893	1.87089063	-2.9092652	0.005301016	0.09776221	-2.274825
CPQ	1.82766034	2.92366911	2.90779257	0.005322511	0.09801512	-2.3007552
SMTN	1.05689142	5.05943788	2.90552222	0.005355808	0.09845005	-2.958156
TTLL7	0.42184432	4.36787864	2.90471458	0.0053677	0.09845005	-2.8048481
EIF3H	-0.2251154	7.15294699	-2.9045873	0.005369576	0.09845005	-2.9924364
NAPA	0.24565815	5.31182146	2.90284413	0.005395334	0.09875406	-2.9724979
ENSCAFG00000024806	-0.6754473	1.99920563	-2.9022957	0.005403462	0.09875406	-2.2860869
ENSCAFG00000009523	-0.3485158	7.79567428	-2.9014975	0.005415312	0.09875406	-2.9732904
PPARGC1A	2.07568235	0.06882813	2.90134904	0.005417518	0.09875406	-2.1870831
MAML3	1.13444403	-1.3549592	2.90036321	0.005432192	0.09878878	-2.1893288
CDH18	1.75775702	-0.178652	2.90016757	0.005435108	0.09878878	-2.1953507
ITGA2B	1.18312838	-0.8548918	2.89892912	0.005453604	0.09891502	-2.1934226

ENSCAFG00000012395	-0.291382	7.65355057	-2.8976677	0.005472502	0.09891502	-2.986489
SEC11C	0.4712511	5.18393794	2.897413	0.005476326	0.09891502	-2.9645538
ENSCAFG00000028871	2.31746272	-1.0674847	2.89731435	0.005477807	0.09891502	-2.2064744
ENSCAFG00000028782	0.33902777	4.98820417	2.89708065	0.005481318	0.09891502	-2.9623824
SAT1	0.8949642	4.6254412	2.89567997	0.005502406	0.09898273	-2.9944242
NACA	-0.3749092	7.56950308	-2.8948895	0.00551434	0.09898273	-2.9917437
PDZD8	-0.2861399	5.83563173	-2.8946676	0.005517694	0.09898273	-3.0244519
ITGB7	1.18497701	0.05408684	2.89420033	0.005524764	0.09898273	-2.2360009
WDR41	-0.3906399	3.33600076	-2.8938851	0.005529538	0.09898273	-2.6379086
SDF2L1	0.55386733	3.68595781	2.89370815	0.00553222	0.09898273	-2.8323879
TMC6	1.17126986	0.19819133	2.89234871	0.005552862	0.09909159	-2.2640754
PIN1	0.34406394	4.0680618	2.89227142	0.005554038	0.09909159	-2.8352238
ENSCAFG00000020219	-0.5056083	2.79162829	-2.8908598	0.005575553	0.09933474	-2.5452601
C2CD5	-0.3937475	5.14310509	-2.8903176	0.005583836	0.09934181	-2.9933831
PRR13	0.51461089	4.63853875	2.8892558	0.005600092	0.09938189	-2.9182022
EVI5L	0.43447686	4.21840138	2.88913995	0.005601869	0.09938189	-2.8460425
TNFAIP3	-1.1514449	2.44900369	-2.8884636	0.005612249	0.099426	-2.6022173
EFCAB7	-0.6415043	2.01678798	-2.8872083	0.005631564	0.09958616	-2.4533158
PACSIN1	0.52893264	4.58070358	2.88684909	0.005637102	0.09958616	-2.9397751
GALK2	-0.3341551	5.13069772	-2.8855197	0.005657642	0.09980904	-3.0055156
PTCH2	1.15540567	2.37526824	2.88323432	0.005693115	0.10021385	-2.3309162
PQLC3	0.59142252	4.89224306	2.88273189	0.005700941	0.10021385	-3.0148418
NFXL1	-0.3228028	3.96530151	-2.8825064	0.005704456	0.10021385	-2.8547332
LMO4	0.60598636	7.65512699	2.87954582	0.005750802	0.10088029	-3.028957
BTF3	-0.3403507	6.34432307	-2.8783474	0.005769661	0.10088029	-3.0716945
BCAR3	0.82409646	6.25096017	2.87825413	0.005771131	0.10088029	-3.0514786
TUSC3	0.45352572	6.86153188	2.87777182	0.005778739	0.10088029	-3.0700472
PGAM1	0.57262979	8.63671466	2.87753767	0.005782436	0.10088029	-2.9730301
MMP1	1.64386193	-1.7197248	2.87539602	0.005816352	0.10116549	-2.244293
ENSCAFG00000019044	-0.4002765	8.63999953	-2.8736714	0.005843797	0.10116549	-2.9910494
ARAP2	2.22644253	1.53399744	2.87302828	0.005854062	0.10116549	-2.2716336
TRPC1	0.57277822	2.48418957	2.87296946	0.005855002	0.10116549	-2.5196736
C8H14orf93	-0.761615	1.95192934	-2.8726995	0.005859317	0.10116549	-2.4298618
GCFC2	-0.3254669	3.42813327	-2.8725353	0.005861941	0.10116549	-2.7495671
TMEM245	0.29711345	4.45384938	2.87243962	0.005863472	0.10116549	-2.9747393
OAS2	-0.390628	6.04142535	-2.8723479	0.00586494	0.10116549	-3.0781452
NBL1	0.68071487	3.90298396	2.8717572	0.005874399	0.10116549	-2.9996616
ICAM5	1.60127219	1.15539573	2.871464	0.0058791	0.10116549	-2.3738851
SIAH1	-0.3467763	3.4244671	-2.8709096	0.005887997	0.10118037	-2.7417092
TGFB3	1.23260414	5.26630412	2.86858467	0.005925446	0.10164424	-3.0125295
SYVN1	0.3409406	7.39297745	2.86814693	0.005932522	0.10164424	-3.068386
PRKD3	-0.2343464	6.37039489	-2.8677342	0.0059392	0.10164424	-3.0979998
RTN4	0.4561522	7.93649256	2.86590034	0.005968959	0.10201494	-3.0679338
RPL10L	-1.1317225	-0.2253949	-2.8635407	0.006007453	0.10236729	-2.2808276
TET2	-0.4181071	4.92455052	-2.8633097	0.006011233	0.10236729	-3.0219769
RAB2B	0.31901911	4.15569087	2.86314343	0.006013956	0.10236729	-2.9106443
PHC2	0.29815842	7.61632825	2.86110305	0.006047461	0.1026951	-3.0836649
DAG1	0.31658106	8.46101805	2.86097795	0.006049521	0.1026951	-3.0259452
CABIN1	0.31365027	5.81731464	2.8592584	0.0060779	0.10303799	-3.0984451
CX3CL1	1.0528371	4.57752648	2.85765993	0.006104392	0.10334801	-3.1172875

PRRT4	1.04612477	1.32501503	2.85715676	0.006112753	0.10335065	-2.324
EVI2B	1.35637948	1.47933415	2.85574175	0.006136323	0.10361009	-2.4456883
NPM3	-0.4665949	5.39806351	-2.8545586	0.006156097	0.10380481	-3.0792663
FAM110D	-1.8594324	-2.4868743	-2.8537872	0.006169019	0.10388364	-2.3652905
VOPP1	0.61811272	2.53034542	2.85227132	0.006194488	0.10417326	-2.6045393
ICE2	-0.4005046	4.55255433	-2.8517399	0.00620344	0.1041847	-2.9903954
ILK	0.38212268	7.96480151	2.85059585	0.006222752	0.10436988	-3.0952278
MYH11	3.20830381	0.78545231	2.84864936	0.006255737	0.10478359	-2.3043641
IDH2	0.68828466	6.51197163	2.84780551	0.006270087	0.10486801	-3.1461949
HCN2	1.01057785	2.4655468	2.84737453	0.006277428	0.10486801	-2.7862335
TOM1L2	0.2853784	6.35272574	2.84581228	0.006304104	0.10517417	-3.152791
PLXNA3	0.31805666	7.36180442	2.84517095	0.006315086	0.10519853	-3.1337521
RPL35A	-0.3073775	7.63492533	-2.844752	0.006322268	0.10519853	-3.1214301
ZBTB47	0.55967583	6.00487903	2.84401218	0.006334972	0.10527085	-3.1566741
PHLDB2	1.06804715	6.90396256	2.8424324	0.006362177	0.10558364	-3.1559632
PFKFB3	0.68412993	6.69196008	2.84148029	0.006378625	0.10571732	-3.151632
LMCD1	1.40900134	5.14904036	2.84046319	0.00639624	0.10586995	-3.048579
SLC41A1	0.4976433	5.09700217	2.83929347	0.006416553	0.1060668	-3.1029822
HSPB1	1.07019049	8.59048529	2.83799543	0.006439164	0.106203	-3.0374159
CNOT2	-0.2064788	6.07817896	-2.8377786	0.006442949	0.106203	-3.1696272
ZBTB7B	0.3685307	4.73803927	2.83622401	0.006470141	0.106203	-3.0730082
MEA1	0.35929117	3.77698088	2.83595126	0.006474923	0.106203	-2.9355897
YWHAH	0.65231329	7.04220005	2.83547862	0.006483217	0.106203	-3.1775392
DPYSL3	0.75311496	11.4773152	2.8350279	0.006491135	0.106203	-2.9104325
FXN	-0.5667057	2.82761449	-2.8349953	0.006491709	0.106203	-2.7107367
ENSCAFG00000029170	-2.1766946	-2.3456163	-2.8345745	0.00649911	0.106203	-2.4073843
GLRX5	-0.5992076	2.49181616	-2.8337328	0.006513938	0.106203	-2.7027053
CHID1	0.42152518	5.82443657	2.83357003	0.006516809	0.106203	-3.1804226
CDR2L	0.41925664	6.98076363	2.83332962	0.006521052	0.106203	-3.1722431
BDNF	2.55834652	0.58452761	2.83305112	0.00652597	0.106203	-2.336368
TRIM13	-0.4131376	3.29819912	-2.8317258	0.006549421	0.10631761	-2.7903023
EMILIN1	0.77028972	7.41248396	2.83169915	0.006549894	0.10631761	-3.1285136
EPHA3	1.87138075	5.57146989	2.82992953	0.006581331	0.1063303	-3.0062049
STX1B	1.31279717	-1.027338	2.82989108	0.006582016	0.1063303	-2.3658858
WFS1	0.53359789	6.46216441	2.82934068	0.006591823	0.1063303	-3.1934002
MYH9	0.54916103	11.7432146	2.82932482	0.006592105	0.1063303	-2.9048548
ENSCAFG00000000528	-0.2974969	3.51094118	-2.8292812	0.006592884	0.1063303	-2.8882959
ENSCAFG00000031488	-0.9343507	0.52765725	-2.8287032	0.006603198	0.10636047	-2.3449806
ENSCAFG00000013899	2.54270034	-0.6122888	2.82789204	0.006617699	0.10645791	-2.3507462
PAN2	-0.434547	3.47544466	-2.8265174	0.006642341	0.10671802	-2.8994161
PGD	-0.3538545	8.63174073	-2.825433	0.00666184	0.10689495	-3.0985081
SYT11	0.52836629	4.68366948	2.8246647	0.006675687	0.10698085	-3.090882
DUS4L	-0.410485	3.9973268	-2.8212144	0.006738201	0.10771602	-2.9895101
FAM177A1	0.46875642	3.16887811	2.82118885	0.006738665	0.10771602	-2.7838295
NTHL1	-0.6654171	1.52386688	-2.820284	0.00675515	0.10784267	-2.4812061
ENSCAFG00000024864	1.12390744	2.80564648	2.81912866	0.006776252	0.10804261	-2.7390342
SWAP70	-0.2529814	6.46249574	-2.8183105	0.006791232	0.10814457	-3.2214279
LY6E	2.10479355	-1.097823	2.81656046	0.006823378	0.10825335	-2.4143004
PARP2	-0.7328969	3.52144334	-2.8144423	0.006862473	0.10825335	-2.8277894
PFKM	0.29517583	5.22226063	2.81434489	0.006864277	0.10825335	-3.2058008

GYG1	0.53991494	5.1017747	2.8143234	0.006864675	0.10825335	-3.1388587
TMEFF2	-0.9213366	4.23503089	-2.8138445	0.006873545	0.10825335	-3.2185706
TBC1D16	1.88997927	-0.8597779	2.81383918	0.006873644	0.10825335	-2.3902421
DUSP14	0.55088738	4.2194654	2.8136928	0.006876357	0.10825335	-3.0568377
SRP9	0.40164265	3.90443609	2.81327157	0.006884171	0.10825335	-2.963892
PRKAG2	0.56154225	4.68933722	2.81306984	0.006887916	0.10825335	-3.07709
SLC2A4	1.54594112	-0.8127179	2.81293783	0.006890368	0.10825335	-2.3819092
LDB3	1.39563666	-2.672518	2.81281771	0.0068926	0.10825335	-2.441495
LMO7	0.83201856	9.15741534	2.81115418	0.006923575	0.10849311	-3.1477731
ENSCAFG00000023724	0.79939976	2.46541364	2.81107286	0.006925092	0.10849311	-2.6800189
TCIM	1.59802194	0.52346857	2.81056852	0.00693451	0.1085057	-2.4098647
TENT2	-0.2535456	5.55375141	-2.8083235	0.006976577	0.10885743	-3.2272172
FAM120A	-0.2716262	8.1961598	-2.8081493	0.006979851	0.10885743	-3.1920391
RTN3	0.23635667	7.89436482	2.80756845	0.006990778	0.10885743	-3.1914005
TAF4	-0.3507063	3.75535066	-2.8075271	0.006991557	0.10885743	-2.9866286
PTPRU	-1.4054812	3.50213017	-2.8067526	0.007006153	0.10895	-3.0883798
OLFML2B	0.94331925	6.00078489	2.80449352	0.007048888	0.1094794	-3.2436407
ABTB1	-0.7699789	3.15133945	-2.8022647	0.007091287	0.10983427	-2.8747028
CD151	0.33980328	8.6677613	2.80203662	0.007095639	0.10983427	-3.1674364
PCMT1	0.2614242	5.75391868	2.80152542	0.007105402	0.10983427	-3.251887
HAPLN4	1.08436261	-0.1164107	2.80146189	0.007106616	0.10983427	-2.4661804
LATS1	-0.2768694	5.69603322	-2.8000381	0.007133877	0.10989279	-3.2515646
CEP170B	0.34934832	5.30594047	2.79997994	0.007134994	0.10989279	-3.1945306
SERTAD4	-1.6006118	0.98920955	-2.799466	0.007144859	0.10989279	-2.4186603
SYCP2	0.99738713	0.89338566	2.79944306	0.0071453	0.10989279	-2.4515115
JPH1	1.88454856	0.28187977	2.79790111	0.007174977	0.11015951	-2.4264926
TSG101	0.25935768	5.97415286	2.79763382	0.007180133	0.11015951	-3.2670138
PIGO	-0.3076488	5.82884089	-2.797154	0.007189398	0.11016747	-3.2640429
SP110	-0.3273062	4.57940951	-2.7957418	0.007216728	0.1104519	-3.1781677
RPL31	-0.2985311	5.40636334	-2.795273	0.007225822	0.11045687	-3.242107
NPL	-1.3876218	0.76148621	-2.7938945	0.007252623	0.11066241	-2.4495045
PLA2G15	0.33653214	4.18466756	2.79367807	0.007256839	0.11066241	-3.1385682
NDUFA4L2	1.24014482	3.90545523	2.79239893	0.007281805	0.11083924	-3.2249332
ANKHD1	-0.2971399	7.02528847	-2.7919427	0.007290728	0.11083924	-3.273948
PHTF2	0.60140361	5.51923917	2.79173296	0.007294834	0.11083924	-3.2285367
ARHGAP22	-0.6526998	2.62253166	-2.7894652	0.007339362	0.11138146	-2.9043198
AGPAT3	1.56838764	0.37697054	2.78887512	0.007350991	0.11142368	-2.4647822
RHBDL2	1.05959781	1.1481726	2.78658905	0.007396199	0.11197418	-2.5281246
FILIP1	2.21284599	2.33543965	2.78243228	0.007479059	0.11309271	-2.7475633
ZNF32	-0.3833268	2.8687613	-2.7800136	0.007527666	0.11369122	-2.8086396
C3	2.13941264	1.67458846	2.77912207	0.007545655	0.1137393	-3.1411915
SLC35B2	0.35600283	4.63970103	2.77896104	0.007548909	0.1137393	-3.1874844
TREX2	1.05043729	1.92726163	2.77810345	0.007566258	0.1138645	-2.566354
USP20	0.45591045	4.18639495	2.7768797	0.007591078	0.1141017	-3.1014771
PIGS	0.28187012	6.57856712	2.77536763	0.00762185	0.11416448	-3.3260802
ATIC	-0.2330558	6.55975936	-2.7750753	0.007627813	0.11416448	-3.3278151
AKIRIN1	0.51430536	3.54112866	2.77446062	0.007640364	0.11416448	-3.0371651
SLC2A8	0.3811791	4.26228337	2.77342228	0.007661609	0.11416448	-3.1681928
ENSCAFG00000002166	-0.6010161	2.63320015	-2.7730808	0.007668609	0.11416448	-2.7138485
MPHOSPH10	-0.3696723	4.79479008	-2.7723938	0.007682706	0.11416448	-3.2323839

EVA1A	1.66274496	1.99728069	2.77223839	0.007685899	0.11416448	-2.6107934
ARMH4	0.71241585	5.86549765	2.77221808	0.007686316	0.11416448	-3.3081103
PON2	0.54578997	5.88396739	2.77218365	0.007687023	0.11416448	-3.3195936
SCUBE3	1.7256932	-1.9168458	2.77030241	0.007725772	0.11416448	-2.5183508
MAP3K4	-0.3376931	3.76508546	-2.7699245	0.007733576	0.11416448	-3.0652013
CSRNP2	0.49832598	2.72200965	2.76910615	0.007750505	0.11416448	-2.8324391
ITGA2	1.06878922	6.70264087	2.76909944	0.007750644	0.11416448	-3.3190024
PIGK	0.36191652	6.88246894	2.76848088	0.007763462	0.11416448	-3.3420028
SART3	-0.2542906	5.09856603	-2.7684296	0.007764524	0.11416448	-3.2812275
PCBD1	1.02218055	2.95405492	2.76788934	0.007775738	0.11416448	-2.9133446
GLRB	0.72082898	6.49072648	2.76761994	0.007781335	0.11416448	-3.3342509
IKBIP	0.35963476	6.11704476	2.76749903	0.007783848	0.11416448	-3.3452881
FLNC	0.67164521	9.75173228	2.76744557	0.007784959	0.11416448	-3.1953813
SLC8A1	1.72467847	2.36164934	2.76723346	0.00778937	0.11416448	-2.7280892
DEPDC7	1.20014681	2.83882838	2.76625323	0.007809785	0.11416448	-2.7139515
PPP1R3D	0.67913061	1.94203388	2.76617643	0.007811387	0.11416448	-2.6332953
PLCG1	-0.351405	6.30405126	-2.7660809	0.007813379	0.11416448	-3.3489002
TSEN34	-0.4616533	3.91558165	-2.7649753	0.007836474	0.11416448	-3.072685
MAP7D1	0.36519888	8.04092227	2.76490616	0.00783792	0.11416448	-3.2938883
SRRD	0.39617938	2.60698866	2.76484883	0.00783912	0.11416448	-2.8635094
RPS15A	-0.3153546	8.30121294	-2.7648082	0.007839971	0.11416448	-3.2836012
GABARAPL2	0.42759565	5.81947241	2.76403858	0.007856091	0.11418322	-3.3493641
FRMD6	-0.4053424	5.87527874	-2.7638813	0.007859388	0.11418322	-3.3460677
SMC3	-0.3892869	6.30974763	-2.7627174	0.007883835	0.11440644	-3.3538427
TRANK1	-0.6540877	3.80793647	-2.7616309	0.007906718	0.11448532	-3.1640268
PLOD3	0.35135684	8.36964954	2.76159626	0.007907449	0.11448532	-3.2843022
RTL5	0.53543026	6.24679871	2.76070537	0.007926261	0.11462592	-3.3607785
RAB3A	0.7628797	0.75486531	2.75970479	0.007947437	0.11480036	-2.5703177
CNTNAP4	1.79992713	-0.278338	2.75814103	0.007980637	0.11504946	-2.563735
ZNF622	-0.2591695	4.36799099	-2.7580324	0.007982949	0.11504946	-3.1995604
ENSCAFG00000024681	-0.6306155	3.23896974	-2.7564125	0.008017484	0.11526928	-2.8211347
TRIM36	0.88671777	2.55640085	2.75623097	0.008021363	0.11526928	-2.8203503
DUSP22	1.19954963	1.89637211	2.75572329	0.008032219	0.11526928	-2.683972
MSL2	-0.3113937	4.01150102	-2.7555458	0.008036018	0.11526928	-3.1781146
ADAMTS15	2.25411645	-0.9308036	2.75517507	0.008043958	0.11526928	-2.5021834
SPART	-0.2186995	5.56981651	-2.7545169	0.008058073	0.11531773	-3.3577349
DEFB124	1.50063251	-1.4326222	2.754164	0.00806565	0.11531773	-2.5275415
TINAGL1	1.97525013	3.21685361	2.753313	0.008083948	0.11535254	-2.8563352
AKAP12	-0.7031714	8.2607104	-2.7531991	0.0080864	0.11535254	-3.3270698
RPL12	-0.2983684	9.07001245	-2.752211	0.008107701	0.1153579	-3.2656786
RPL24	-0.2555048	8.13857574	-2.7518428	0.008115651	0.1153579	-3.320888
MPI	0.27951744	4.22201141	2.7515059	0.008122933	0.1153579	-3.2234621
NCSTN	0.38535153	7.55957506	2.75127032	0.008128028	0.1153579	-3.3534011
LRFN3	0.83587939	1.06128112	2.75097048	0.008134517	0.1153579	-2.6435504
ERN1	0.53971037	5.76174014	2.75039959	0.008146885	0.1153579	-3.3837118
INTS7	-0.4528207	4.56783417	-2.7500008	0.008155535	0.1153579	-3.2488929
ENSCAFG00000003386	0.24835037	5.7731446	2.74935859	0.008169482	0.1153579	-3.378116
PPP6C	0.2901228	3.36489015	2.74922626	0.008172358	0.1153579	-3.0359631
FN1	0.95569171	14.6851343	2.74825132	0.008193581	0.1153579	-2.8871959
FAM111A	-0.310979	5.88102094	-2.748101	0.008196858	0.1153579	-3.3805149

ENSCAFG00000003477	0.33797834	8.97318099	2.74788953	0.008201469	0.1153579	-3.268898
CCDC93	-0.3113045	5.52726015	-2.7474184	0.008211752	0.1153579	-3.362468
ENSCAFG00000006533	0.96183767	0.89235657	2.74727004	0.008214992	0.1153579	-2.580933
EFL1	-0.2448195	5.14432543	-2.7468079	0.008225093	0.11537113	-3.3404312
KCNN4	-0.3719998	4.53507336	-2.7457034	0.008249284	0.11544962	-3.3493723
COMTD1	-0.7737592	2.0554519	-2.7456946	0.008249476	0.11544962	-2.7827019
SH2D3C	-2.5653563	-0.8468748	-2.7450489	0.008263647	0.11544962	-2.5336624
ABTB2	1.09459586	1.21927963	2.7448803	0.008267352	0.11544962	-2.7712757
ARPC5	0.29029218	5.90196424	2.74377903	0.008291584	0.11553733	-3.4000239
ENSCAFG00000007151	-0.2580702	5.0636132	-2.7434411	0.008299032	0.11553733	-3.3432304
CXHXorf36	-2.6511469	-1.2146473	-2.7433451	0.00830115	0.11553733	-2.5345203
NDUFB3	0.23551369	4.69817304	2.74121345	0.008348291	0.1160652	-3.3015897
CA13	1.05306929	0.60208131	2.73980926	0.008379478	0.11637035	-2.6560895
OVGP1	-1.0982873	0.05494712	-2.7383846	0.00841123	0.11668266	-2.5527255
KAT14	-0.215519	5.73815943	-2.737527	0.008430396	0.11681987	-3.398848
ENSCAFG00000018235	1.34735176	2.25109543	2.73672262	0.008448411	0.11694086	-2.7182914
ENSCAFG00000001210	-0.8210628	1.96337168	-2.7359581	0.008465565	0.11698162	-2.7257065
ENSCAFG00000030886	0.81926894	5.57093225	2.7356692	0.008472055	0.11698162	-3.3687305
DUSP2	1.07436306	-0.9687508	2.73479439	0.008491737	0.11698162	-2.566025
RNF139	-0.2850557	5.34802282	-2.7344897	0.008498602	0.11698162	-3.3871817
SETDB1	-0.2742731	5.18630785	-2.7343846	0.008500971	0.11698162	-3.3635433
MTHFD1L	-0.456422	7.06860025	-2.7339853	0.008509977	0.11698162	-3.4220416
MOSPD3	0.46206579	3.23871497	2.73370232	0.008516366	0.11698162	-3.1035062
EHBP1	-0.5426453	6.04479139	-2.7317621	0.008560285	0.11745681	-3.4302902
C2CD2	0.40325039	4.26676628	2.72961222	0.008609193	0.11796793	-3.2895285
HACD2	0.54545534	5.48039218	2.72901288	0.008622873	0.11796793	-3.3781063
ENSCAFG00000014799	-2.0427795	0.98324054	-2.7288921	0.008625632	0.11796793	-2.6770549
ZFYVE9	0.23768151	6.84306659	2.72699512	0.008669076	0.11813157	-3.4238399
RNF145	0.40907184	6.1705898	2.72686108	0.008672153	0.11813157	-3.4345061
P3H4	0.3390711	6.4196449	2.72682767	0.008672921	0.11813157	-3.4426534
ANGPTL6	0.64233972	1.57220679	2.7265065	0.008680299	0.11813157	-2.7291019
SPIN1	-0.2152994	6.48234647	-2.7259403	0.00869332	0.11813157	-3.4454335
P4HB	0.36216629	10.7456073	2.72591647	0.008693869	0.11813157	-3.2073363
STAG2	-0.3412638	6.5456617	-2.7243949	0.008728952	0.11841814	-3.4493612
NCK1	-0.3642958	4.798509	-2.7238548	0.008741438	0.11841814	-3.3845907
ENSCAFG00000029897	2.03455617	-2.3062978	2.72302257	0.008760707	0.11841814	-2.5978248
ACTA1	1.84878012	3.68923503	2.72291621	0.008763172	0.11841814	-2.8443826
ZNF639	-0.2758281	4.12465672	-2.722545	0.008771782	0.11841814	-3.2447183
EIF4G3	0.2610069	7.95969969	2.72226748	0.008778224	0.11841814	-3.3869089
PRAP1	1.80640604	-1.2040318	2.72213339	0.008781338	0.11841814	-2.5740706
SPAG9	-0.303161	6.82195226	-2.7217534	0.008790168	0.11841814	-3.4529078
PITRM1	0.75051618	6.37831386	2.71914544	0.008850994	0.11911017	-3.4487892
C12H6orf203	-0.341274	3.88859527	-2.7181511	0.008874285	0.11924967	-3.2582266
EXOSC10	-0.2146593	6.39769859	-2.717895	0.008880295	0.11924967	-3.4658673
CTDSPL2	-0.3371321	5.37436092	-2.7168762	0.008904235	0.11944381	-3.4258258
EMC7	0.37944174	5.59781924	2.71545815	0.008937652	0.11976454	-3.4496753
ANXA2	0.36636853	11.1557781	2.71373592	0.008978395	0.12014991	-3.2054542
DLA-DMB	1.27919234	0.37976087	2.71343678	0.008985489	0.12014991	-2.6244728
NRCAM	1.40591637	0.61208347	2.71303361	0.008995058	0.12015032	-2.7073593
OSMR	0.63372018	8.59913557	2.71261585	0.009004983	0.12015548	-3.3712763

FBXO9	0.22703005	5.34695714	2.71141254	0.009033628	0.12041014	-3.4418102
ENSCAFG00000032355	-1.4167256	1.53755049	-2.7104197	0.009057326	0.12051531	-2.7356103
ZBTB2	-0.3343721	5.15959894	-2.7102805	0.009060654	0.12051531	-3.4196411
RPL11	-0.2887663	8.02448102	-2.7077823	0.009120557	0.12113032	-3.4320306
PLIN2	0.33077634	5.89618654	2.70755079	0.009126125	0.12113032	-3.4907337
TRIM59	-0.4687145	3.54244611	-2.7071128	0.00913667	0.12114263	-3.1192296
CHMP4B	0.23906206	7.43563729	2.70572398	0.009170183	0.12130348	-3.4729157
FAM189B	0.39059475	7.41573691	2.70529286	0.009180609	0.12130348	-3.4758827
DCTN3	0.24721034	5.40221001	2.70526115	0.009181376	0.12130348	-3.4657703
GALK1	-0.5034145	3.98977356	-2.7045726	0.009198053	0.12130348	-3.1827606
SLC26A7	1.97530495	-1.9653927	2.70425437	0.009205768	0.12130348	-2.6159294
TLR4	-2.157749	-1.3982327	-2.7042207	0.009206584	0.12130348	-2.6065534
OSGIN1	0.68969544	3.22477252	2.70345683	0.009225132	0.12142086	-3.1732082
CYP39A1	-0.4268531	4.0486787	-2.7021475	0.009257004	0.12171317	-3.2456978
DNAH5	1.05207299	1.97461399	2.70126085	0.009278645	0.1218705	-2.8226645
WARS2	-0.5252493	3.4963183	-2.7007946	0.009290043	0.12187421	-3.1841657
SCAF8	-0.2013433	6.02371687	-2.700458	0.009298279	0.12187421	-3.5027576
TMPO	-0.8071656	4.63408801	-2.6999552	0.009310596	0.1219088	-3.2780088
HSPB6	0.84057193	6.00751686	2.69876962	0.009339697	0.12216285	-3.5054034
FAM78B	-1.274274	-0.9136366	-2.697712	0.009365727	0.12216823	-2.6199155
RBM12	-0.2768839	5.7012993	-2.6976289	0.009367775	0.12216823	-3.4936398
PHF3	-0.2819356	6.59652075	-2.6975709	0.009369205	0.12216823	-3.5133899
SOX9	1.29066416	2.67022967	2.69532313	0.009424767	0.1224956	-3.2333515
PTPRN	1.14637003	4.85120613	2.69494905	0.009434043	0.1224956	-3.4246596
PTGES2	-0.4811225	2.47042785	-2.6946045	0.009442594	0.1224956	-3.0013385
QSER1	-0.3122555	6.57499638	-2.6943505	0.009448903	0.1224956	-3.5224195
MAPKAPK2	0.27126762	6.75672096	2.69420819	0.009452439	0.1224956	-3.5218342
BBS9	-0.3625167	3.82396481	-2.6936991	0.009465098	0.1224956	-3.2888278
ACE	-2.9194204	-0.1074593	-2.6936013	0.009467532	0.1224956	-2.6948784
ASCC1	-0.4484089	2.63883356	-2.6930539	0.009481167	0.1224956	-2.9813533
IDS	0.27886793	8.68823421	2.69266111	0.00949096	0.1224956	-3.4200801
ALMS1	-0.3927876	4.85969076	-2.6925686	0.009493267	0.1224956	-3.406213
BICDL1	1.24703905	-1.6639379	2.69224736	0.009501286	0.1224956	-2.6317235
CEP295	-0.3794368	4.98259482	-2.6912757	0.009525578	0.12257073	-3.4427859
ENSCAFG00000006540	0.74536475	5.50964111	2.69031416	0.009549672	0.12257073	-3.517467
HP1BP3	-0.1974996	6.04711654	-2.6898323	0.009561767	0.12257073	-3.525922
ERRFI1	0.76950246	5.82247194	2.68948262	0.009570554	0.12257073	-3.4805072
NELFA	-0.2632962	4.57892599	-2.6892314	0.009576871	0.12257073	-3.4006028
FLNA	0.50714547	12.6995302	2.68899024	0.009582938	0.12257073	-3.1839747
CIAO2A	-0.2969583	4.1497717	-2.6886631	0.009591174	0.12257073	-3.3441342
MORC3	-0.2720687	4.80848546	-2.688002	0.00960784	0.12257073	-3.4531204
ENSCAFG00000012367	-0.4149808	3.74197089	-2.6878664	0.00961126	0.12257073	-3.249521
ZNF146	-0.3567407	4.18652626	-2.6877977	0.009612995	0.12257073	-3.3390143
TVP23B	0.36029328	3.23453659	2.68775178	0.009614154	0.12257073	-3.154349
ZNF236	-0.3538735	3.4485869	-2.6863649	0.009649219	0.12289339	-3.2527618
ENSCAFG00000021568	-1.1360678	-1.0921401	-2.6858185	0.009663067	0.12294545	-2.6445311
GDPD5	-0.74089	3.08412649	-2.6851292	0.009680563	0.12304376	-3.0800231
CASP6	-0.2917427	4.04661041	-2.6834532	0.009723223	0.12346141	-3.3112732
ENSCAFG00000008503	-0.3098415	3.15287012	-2.6827502	0.009741169	0.12346417	-3.1117822
TCFL5	0.39184133	4.30296309	2.68267678	0.009743044	0.12346417	-3.3778131

PEG3	0.82962716	5.25848552	2.6803543	0.009802556	0.12409346	-3.4691353
ENSCAFG00000003564	-0.3979897	4.85222134	-2.6787402	0.00984411	0.12449439	-3.4668725
ENSCAFG00000014970	-0.8240983	0.7802103	-2.6768605	0.00989271	0.12498352	-2.6709598
ERCC6L2	-0.2832485	6.31280954	-2.6746607	0.009949862	0.12557962	-3.5694433
KHSRP	-0.22282	6.27489711	-2.6709797	0.010046173	0.12666827	-3.5773584
PDE4DIP	0.4626372	6.31690537	2.67032695	0.01006334	0.12675276	-3.5784188
GNAQ	0.35525969	5.48678734	2.6699601	0.010073	0.12675276	-3.5482956
MATR3	-0.2394216	8.1874894	-2.66909	0.010095947	0.12682242	-3.5219053
ADAMTSL2	1.56057879	-2.3316856	2.66869798	0.010106301	0.12682242	-2.7082046
NECTIN1	0.62261005	4.64674901	2.66860561	0.010108742	0.12682242	-3.4331862
NRAS	-0.3656464	4.73641804	-2.6663097	0.010169588	0.12733241	-3.4794707
AQR	-0.1923413	6.87745318	-2.6663089	0.01016961	0.12733241	-3.5827368
RPS9	-0.3239835	8.36228488	-2.6655643	0.010189418	0.12737671	-3.5081318
TP53BP2	0.24572271	5.70644475	2.66512652	0.010201078	0.12737671	-3.5804755
NF1	0.34701923	6.79563029	2.66503619	0.010203486	0.12737671	-3.5854618
RNF115	-0.2128418	4.8965406	-2.6628284	0.010262494	0.12798651	-3.4994878
ENSCAFG00000007112	-0.2403009	6.99132502	-2.6621115	0.010281721	0.12809946	-3.5933337
ENSCAFG00000004331	0.80750384	2.18518626	2.6606602	0.010320748	0.12826075	-3.0420621
ITGA3	0.97110501	5.90946896	2.66013451	0.010334917	0.12826075	-3.5816332
RPS20	-0.3724124	8.73526655	-2.659997	0.010338628	0.12826075	-3.4930147
FAM49A	-2.287099	1.09243382	-2.6599416	0.010340121	0.12826075	-2.7513309
STARD13	0.57590072	6.41001635	2.65945447	0.010353274	0.12826075	-3.5935067
SMARCAD1	-0.3986472	4.47740032	-2.6592331	0.010359256	0.12826075	-3.479532
ENSCAFG00000008873	-0.3645237	8.23833657	-2.6588285	0.010370197	0.12826075	-3.5386243
WDCP	0.38538649	3.37813997	2.65847481	0.01037977	0.12826075	-3.2417103
TMEM100	1.01248716	2.15816097	2.65823333	0.010386311	0.12826075	-2.987502
ZNF317	-0.3399061	3.55243776	-2.6567879	0.010425539	0.12861909	-3.2737977
LARS	-0.2722098	6.9370501	-2.6563014	0.010438774	0.12865635	-3.6103766
PDLIM5	0.73892904	8.99965582	2.65533634	0.010465073	0.12885441	-3.5226284
C16H8orf48	0.54339698	1.56077974	2.65260885	0.010539726	0.12964686	-2.9026566
CRELD1	0.49755354	4.17874607	2.65166755	0.010565603	0.12983837	-3.4778605
MKNK2	-0.3950803	5.53869072	-2.6512055	0.010578325	0.12986802	-3.5738375
INTS14	-0.3253092	4.14465447	-2.6495409	0.01062428	0.13030519	-3.3914363
SLC22A23	-1.2092787	0.34651742	-2.6488472	0.010643485	0.13036882	-2.931367
FAM189A1	1.17812883	3.69354073	2.64853397	0.010652166	0.13036882	-2.9921993
ENSCAFG00000007877	-0.7184809	1.13860358	-2.6480574	0.010665387	0.13036882	-2.777545
NDUFB4	0.31607326	4.61554657	2.64786001	0.010670868	0.13036882	-3.4861588
PARP8	0.53884966	4.73370257	2.64730235	0.010686366	0.13043165	-3.5838551
GABPB2	-0.3775279	2.97623484	-2.6467878	0.010700684	0.13047998	-3.1880222
ENSCAFG00000017697	-0.7515134	2.77348314	-2.6425959	0.010817983	0.13162504	-3.2008715
AQP1	2.80805939	2.97135612	2.64168139	0.010843729	0.13162504	-3.213062
CREB3	0.3437604	6.7907859	2.64165958	0.010844343	0.13162504	-3.6449374
VWA5B2	0.85008213	1.24097358	2.64161109	0.01084571	0.13162504	-3.0267574
DPY19L1	-0.3678498	5.31600054	-2.641571	0.010846839	0.13162504	-3.6005161
FAM206A	-0.3585437	4.06018401	-2.6407621	0.010869665	0.13177507	-3.4110057
FGFRL1	1.09156085	1.93550797	2.63957579	0.01090322	0.13205477	-2.9392056
WDR70	-0.2177225	5.89506438	-2.6383394	0.01093829	0.13235226	-3.6433702
RGS3	0.59571968	5.32851123	2.63636166	0.010994607	0.13287929	-3.5287589
CEP95	-0.3184012	4.98593996	-2.6360119	0.011004595	0.13287929	-3.5841377
RREB1	-0.3291468	4.92415779	-2.6357004	0.011013495	0.13287929	-3.575392

WSCD1	-2.4633976	-2.0059804	-2.6345615	0.011046096	0.1331451	-2.7554721
EXD1	0.73516947	1.57782434	2.63279561	0.011096819	0.13358441	-3.0802072
A2M	1.16733313	7.4625274	2.63232422	0.011110395	0.13358441	-3.6255462
EGR3	1.11078023	0.8842015	2.63218671	0.011114358	0.13358441	-3.0939303
ISLR2	1.37132935	-2.7601904	2.63097934	0.011149211	0.13387556	-2.789342
ENSCAFG00000018792	-0.5610445	2.15112866	-2.6297838	0.01118382	0.13404666	-2.9983223
RPLP0	-0.2604398	8.8112781	-2.6296497	0.011187709	0.13404666	-3.5702244
PEX19	0.26369813	4.09620781	2.62938504	0.011195386	0.13404666	-3.4619851
IRS2	0.85910623	5.73999291	2.62850358	0.011220991	0.13410565	-3.6536499
VPS26C	0.26488823	4.2911058	2.62848239	0.011221607	0.13410565	-3.5202292
SNX1	0.22093101	5.69214396	2.62793719	0.011237471	0.13411822	-3.6743925
LGI4	0.82628994	3.78110534	2.6277146	0.011243954	0.13411822	-3.3579958
DDX41	0.26821217	5.55106452	2.62585717	0.011298184	0.13463758	-3.6718241
PPP2R5A	-0.4544599	5.27152049	-2.6246204	0.011334425	0.13482215	-3.6400594
EHBP1L1	0.48296811	7.86209658	2.62449032	0.011338244	0.13482215	-3.6298193
DOC2B	1.63941575	2.48505508	2.62373098	0.011360555	0.13482215	-3.105394
SYNGR2	0.51788586	4.1664899	2.6232274	0.011375373	0.13482215	-3.5222379
CDKN1A	0.69537144	8.35061174	2.62317807	0.011376825	0.13482215	-3.6293961
ZFYVE27	0.33952871	4.33457928	2.62314181	0.011377893	0.13482215	-3.5199568
NCS1	0.60544426	2.07456817	2.62182505	0.011416731	0.13515521	-2.9777274
ENSCAFG00000028808	-0.5719512	2.38143253	-2.6212672	0.011433222	0.13522334	-2.9706424
RPL7A	-0.2958137	9.65991161	-2.619772	0.011477528	0.13550528	-3.5278208
ACIN1	-0.1941544	6.95831685	-2.6194989	0.011485637	0.13550528	-3.6908406
EGLN3	0.60597428	3.27780823	2.6193652	0.011489608	0.13550528	-3.6883374
WWC3	0.40557174	6.17551886	2.61890122	0.011503402	0.13550528	-3.6994611
SLC38A10	0.30049529	7.53583937	2.61865094	0.011510849	0.13550528	-3.6630179
PPARG	-2.0058208	-2.2013405	-2.6180754	0.011527991	0.13558037	-2.7974244
SIN3B	0.45116834	7.15651028	2.61750849	0.011544898	0.13565255	-3.6798606
PKD1	0.3747546	8.5779997	2.61624092	0.011582783	0.13597086	-3.6132945
ADCY7	1.41894975	4.91858224	2.61548066	0.01160556	0.13611139	-3.4585168
AP3M2	0.78772161	1.2069978	2.61509815	0.011617036	0.13611924	-2.9558695
PDLIM2	0.95382213	4.88853129	2.61397659	0.011650742	0.13617859	-3.5813203
DOCK11	-0.8135624	4.53612826	-2.613938	0.011651903	0.13617859	-3.575472
CEP70	-0.5013239	3.26724671	-2.6138506	0.011654535	0.13617859	-3.27479
ENSCAFG00000032146	0.35439796	4.62632201	2.61293725	0.011682057	0.13637367	-3.599947
SLC10A4	1.38354622	-2.8922576	2.61202993	0.011709458	0.1364707	-2.8198756
ENSCAFG00000013221	1.67957746	-1.3678109	2.61194459	0.011712038	0.1364707	-2.8081672
SMAD5	-0.3138045	5.17175277	-2.610103	0.011767844	0.13699302	-3.6660401
ATG16L1	0.24251911	5.04430252	2.60972381	0.011779365	0.13699302	-3.6493225
UBE2L6	-0.427327	3.47541863	-2.6093908	0.011789492	0.13699302	-3.3982746
ENSCAFG00000029134	0.3431007	7.2642678	2.6068725	0.011866325	0.13757398	-3.7067391
TFAP4	-0.8113194	1.8003859	-2.6067652	0.011869607	0.13757398	-3.0487036
PUS1	-0.2608202	4.16265203	-2.6066788	0.011872255	0.13757398	-3.5472043
ABI3	-1.5748646	-2.5224818	-2.6049624	0.011924907	0.13803752	-2.8385719
TOP2B	-0.3019887	6.96378321	-2.6045345	0.011938065	0.13803752	-3.7245787
PSME2	-0.2965329	4.72809883	-2.6043049	0.011945133	0.13803752	-3.6222825
NOTCH3	1.29679879	6.29304284	2.60364216	0.011965552	0.13810606	-3.6991867
OXNAD1	0.39333482	2.14447188	2.60305686	0.011983611	0.13810606	-3.1275228
CASP8	-0.6768842	2.43150553	-2.6026454	0.01199632	0.13810606	-3.2333247
ACKR1	-2.1657878	-2.285047	-2.6024594	0.01200207	0.13810606	-2.8238905

PLS1	1.42035839	-0.0726909	2.60233606	0.012005886	0.13810606	-2.9853025
RGL1	-0.4587059	5.82876282	-2.6004395	0.012064675	0.13865569	-3.7426399
COMMD1	-0.212987	6.09773649	-2.5996519	0.012089167	0.13874529	-3.7402292
TBCB	0.30922359	5.67032931	2.59895661	0.012110824	0.13874529	-3.7308646
EVI5	-0.3632428	5.45356776	-2.598779	0.012116361	0.13874529	-3.7096321
UBE2D1	-0.2903768	4.41003785	-2.5987736	0.01211653	0.13874529	-3.5631264
ENSCAFG00000013905	-0.4345496	3.84739188	-2.5973188	0.012161981	0.13909591	-3.4430705
ZNF277	-0.2664143	4.76151173	-2.597087	0.012169236	0.13909591	-3.6204224
AKR1B1	0.45692774	5.42334756	2.59561743	0.012215332	0.13931998	-3.7219876
ENSCAFG00000030587	1.24183511	-0.0896092	2.5955915	0.012216146	0.13931998	-2.934295
FBLN5	0.77554476	6.89881509	2.59540459	0.012222021	0.13931998	-3.7504416
FARP1	0.68441335	6.60315428	2.59496742	0.01223577	0.13933498	-3.7373458
SDC4	0.44374069	8.18313949	2.59465959	0.012245461	0.13933498	-3.6714744
ENSCAFG00000006177	0.29236736	3.99059066	2.59418891	0.012260291	0.13937782	-3.5489593
NUDT16	0.70604507	3.39077317	2.59351073	0.012281688	0.13943518	-3.2823622
GFOD1	0.77972418	2.80602276	2.593175	0.012292293	0.13943518	-3.2306879
RGS1	1.674597	-0.194429	2.59269571	0.012307448	0.13943518	-2.9040766
ZMYM1	-0.3265281	4.27566929	-2.5926272	0.012309616	0.13943518	-3.591577
NEDD8	0.37545733	4.12727919	2.59198353	0.012329997	0.13954056	-3.5762899
TRMO	-0.5149827	2.92637721	-2.5910306	0.012360228	0.13975712	-3.2890659
ENSCAFG00000030323	-0.4750818	3.11813847	-2.5893056	0.012415125	0.14014187	-3.3596199
BHLHE41	0.61212175	3.6732107	2.58926224	0.012416507	0.14014187	-3.5022921
CRNKL1	-0.2555209	5.24773366	-2.587492	0.01247308	0.14065436	-3.735481
ADPGK	0.25484509	6.34630115	2.5867188	0.012497863	0.14080776	-3.7761211
AMOT	-2.5855151	0.25041863	-2.5857423	0.012529225	0.14084274	-2.8479007
RPL9	-0.3598317	8.12969482	-2.585655	0.012532033	0.14084274	-3.7231318
ACTG2	2.41196194	8.67138471	2.58530733	0.01254322	0.14084274	-3.6524765
MRPL37	-0.3922259	5.34416643	-2.5851867	0.012547103	0.14084274	-3.7102686
MRPS30	-0.3262188	4.20360806	-2.5848833	0.012556875	0.14084274	-3.5866247
HPSE	-1.5685863	-1.1658849	-2.5845243	0.012568448	0.14084712	-2.8526448
PARP14	-0.8893794	3.00550425	-2.5832425	0.012609843	0.14104432	-3.3434988
NR2C2	-0.3241018	4.06275308	-2.5823838	0.012637644	0.14104432	-3.5362051
BEGAIN	1.18119029	-0.9935217	2.5819226	0.012652598	0.14104432	-2.8672883
C27H12orf10	-0.3204928	5.34451275	-2.5814578	0.012667687	0.14104432	-3.7247895
OASL2	-0.3115919	3.7554549	-2.5813492	0.012671215	0.14104432	-3.4964618
ANGEL2	-0.2745345	4.25775607	-2.5813111	0.012672451	0.14104432	-3.569962
IFI44L	-1.2104103	0.16575205	-2.580888	0.012686206	0.14104432	-2.9157599
SCP2	0.26960895	6.95241178	2.58055575	0.012697014	0.14104432	-3.7754301
DOK7	1.46591914	-0.9174661	2.58049218	0.012699083	0.14104432	-2.8539942
ODC1	-0.5358879	6.55919895	-2.5803836	0.012702618	0.14104432	-3.7886076
RCAN1	0.65260772	6.70589978	2.58018095	0.012709218	0.14104432	-3.750469
SGMS2	0.4174526	3.39507201	2.57959792	0.012728223	0.1411309	-3.4562686
SLC35G1	0.72058285	3.24036688	2.57888391	0.012751533	0.14120302	-3.4112453
AOC2	0.96261197	-0.4896079	2.57845451	0.012765571	0.14120302	-2.882317
MSRB3	0.43305251	8.73038959	2.5783693	0.012768358	0.14120302	-3.6872567
ASPHD1	1.40720262	-0.368191	2.57764135	0.012792192	0.1413425	-2.89299
EXOSC7	-0.4515743	3.66903722	-2.5766723	0.012823983	0.14154886	-3.5486893
SLC38A2	-0.3368425	8.91012585	-2.5763874	0.012833343	0.14154886	-3.6896987
SULT1A1	-0.5872209	4.17525247	-2.5751441	0.012874263	0.14178221	-3.7078622
RWDD3	-0.5392177	2.22131611	-2.5750607	0.012877012	0.14178221	-3.1253795

ZNF462	-0.300745	5.86087395	-2.5736971	0.012922038	0.14211414	-3.8015028
TRMT5	-0.2678045	4.02133156	-2.5734647	0.012929724	0.14211414	-3.5852023
LSM14A	-0.1779066	6.13892198	-2.573066	0.012942925	0.1421352	-3.804219
CORO6	0.60372169	3.23746544	2.57253058	0.012960667	0.14218503	-3.4236154
ERO1B	0.38845434	4.08875402	2.57224809	0.012970038	0.14218503	-3.6841401
PRADC1	0.49939434	4.4567243	2.57176693	0.012986014	0.14220937	-3.5849533
SERINC3	0.3230797	7.22858487	2.57134284	0.013000109	0.14220937	-3.7757944
CCNY	-0.182238	6.79602614	-2.5711619	0.013006129	0.14220937	-3.8067092
HELB	-0.3798874	2.69475158	-2.5701868	0.013038602	0.14244079	-3.3248322
EPHX1	0.68497405	5.14796628	2.56963088	0.013057151	0.14251982	-3.771991
MAPK8IP2	0.78663234	1.91794745	2.56917986	0.013072216	0.14256073	-3.1072303
TRAF3IP2	0.46670844	4.21684881	2.566373	0.013166331	0.14337025	-3.6345754
FOLR2	0.44328043	6.56388277	2.56628741	0.013169211	0.14337025	-3.8223846
RPN2	0.25234183	9.00487687	2.56517725	0.013206612	0.14365327	-3.7124676
RNASE4	1.39851259	1.71465918	2.56467859	0.013223443	0.14367003	-3.0770815
THAP12	-0.3046646	5.1173698	-2.5644559	0.013230965	0.14367003	-3.7690371
PLEKHG2	0.55244021	4.66851678	2.5640832	0.013243565	0.14368298	-3.7127428
ARHGAP1	0.41995033	7.19979158	2.56316935	0.013274503	0.14388654	-3.8205277
ENSCAFG00000015438	-0.8340339	1.00736504	-2.5627148	0.013289916	0.14388654	-2.9297509
ENSCAFG00000012071	0.20746981	6.40117075	2.56251794	0.013296597	0.14388654	-3.8321157
ZNF609	-0.3112675	4.9720912	-2.5621436	0.013309309	0.14390048	-3.7792301
HIC1	-0.6191937	4.85285793	-2.5612682	0.013339079	0.1439898	-3.6725926
PDLIM3	3.32471449	1.37599791	2.56122837	0.013340434	0.1439898	-2.9348752
RPS5	-0.3061616	8.71325754	-2.5601972	0.01337558	0.14424555	-3.7382463
FARS2	-0.364692	4.31145902	-2.5596362	0.013394737	0.14425718	-3.6696802
PLEC	-0.4690132	10.5069828	-2.5591457	0.013411509	0.14425718	-3.647139
MNDA	-0.3682075	6.3334411	-2.5588709	0.01342091	0.14425718	-3.8378566
CD3EAP	-0.4553562	3.38013535	-2.5585495	0.013431916	0.14425718	-3.462846
ENSCAFG00000029710	-0.8567446	0.06093066	-2.558491	0.013433921	0.14425718	-2.9308068
LPCAT2	0.79213599	5.26187185	2.55723181	0.013477125	0.14459785	-3.7340958
CPNE4	1.54794277	-1.560803	2.55623001	0.013511588	0.144787	-2.9004791
SLC2A1	0.68275164	6.38348089	2.55572219	0.013529088	0.144787	-3.8452779
PLXNA2	1.11113656	6.07491359	2.55571781	0.013529239	0.144787	-3.8400353
ENSCAFG00000031899	-0.9370885	0.78165445	-2.5548022	0.013560845	0.14494568	-2.9630201
ADRM1	0.23225222	6.44512919	2.55462176	0.013567081	0.14494568	-3.8501674
EEF2	-0.2809298	11.5757767	-2.5535148	0.013605397	0.14523185	-3.5401686
CCL22	1.54080359	-1.8927155	2.55171531	0.013667894	0.14577544	-2.9110364
MALL	-2.1856567	-2.0247891	-2.5504537	0.013711864	0.14592381	-2.913849
LNX2	-0.427235	4.25869073	-2.5504459	0.013712137	0.14592381	-3.6186706
ESD	0.28194831	5.43172434	2.55031922	0.01371656	0.14592381	-3.8317305
NR1H4	1.82424743	0.67823224	2.54955239	0.013743358	0.14608552	-3.2493945
NPFFR2	1.43600585	-2.3616668	2.54894068	0.013764769	0.14618974	-2.9144538
PLTP	1.14660492	0.54696458	2.54797113	0.013798767	0.14642736	-2.9464451
GPR82	1.60987333	-2.0015875	2.54753112	0.013814222	0.14646796	-2.9275284
DOP1A	-0.281618	4.78752449	-2.5464667	0.013851673	0.14674153	-3.7551132
CRKL	-0.1822278	6.55283195	-2.5456823	0.01387933	0.14687237	-3.8700317
SLC45A3	0.88666447	3.76774704	2.54545527	0.013887344	0.14687237	-3.6018698
ARV1	0.52562689	3.69382754	2.54503748	0.013902104	0.14690512	-3.5997908
LSM1	0.33125415	2.96590773	2.54413229	0.01393413	0.14700151	-3.3805168
HNRNPU	-0.2439572	8.19341685	-2.5438409	0.013944455	0.14700151	-3.8201708

DR1	-0.2709754	3.60551063	-2.5437906	0.013946237	0.14700151	-3.5372443
TMED3	0.31549041	6.64688369	2.54315513	0.013968778	0.147116	-3.8750676
HNRNPR	-0.3353116	6.78419236	-2.5428157	0.013980832	0.14711993	-3.8751225
SMARCA1	1.54703297	2.213966	2.5423162	0.013998587	0.14714988	-2.9965329
AP1G2	-0.3993471	4.72129191	-2.5420785	0.014007042	0.14714988	-3.7513338
PSD2	1.04005177	-0.2177061	2.54021217	0.014073602	0.147668	-2.9611658
VKORC1	0.42764658	5.00340689	2.54003857	0.014079808	0.147668	-3.8257122
LRRC15	1.64713943	1.69533988	2.53902218	0.014116191	0.14792641	-3.1828563
ENSCAFG00000009998	-0.7215092	0.82034431	-2.5354801	0.014243651	0.14913801	-2.9847839
RALGPS2	1.57273692	1.87796718	2.53419573	0.014290125	0.14940675	-3.0017752
ENSCAFG00000023151	0.90647327	3.8238338	2.53411531	0.01429304	0.14940675	-3.5444168
KIAA1324L	1.47379274	-1.2099941	2.53353792	0.014313981	0.14950158	-2.9452107
TCF7L2	-0.5368139	5.06138942	-2.5329157	0.014336582	0.14951828	-3.8361725
LRRC46	0.54486554	2.09669157	2.53284029	0.014339321	0.14951828	-3.3462272
ACAN	1.33522812	-2.2068472	2.53229148	0.014359283	0.14960259	-2.9684269
ZNF398	0.43365834	3.43833319	2.5315264	0.014387155	0.14976909	-3.5899372
ENSCAFG00000032036	-0.2265354	4.89612464	-2.5311099	0.014402347	0.1497842	-3.8250918
MVB12A	-0.3934257	4.39052647	-2.5305777	0.014421785	0.1497842	-3.7390212
ENSCAFG00000003012	0.32917796	8.37438485	2.53050937	0.014424281	0.1497842	-3.8252154
CCDC136	1.36441331	1.17186459	2.52996006	0.014444369	0.14982109	-3.066445
ATP6V0E1	0.29420349	4.93978425	2.5297619	0.014451622	0.14982109	-3.8420853
PANK3	-0.3455151	4.1303055	-2.5283466	0.014503518	0.15016652	-3.7178591
HMGB2	-0.7592699	4.7107839	-2.5281884	0.014509333	0.15016652	-3.7586401
ANTXR1	1.15339605	7.29617263	2.52787891	0.014520707	0.15016652	-3.9023136
TFCP2	-0.2308505	5.22340943	-2.5275083	0.014534339	0.15018419	-3.8725548
MAFB	1.55567863	-0.6736826	2.52647196	0.014572522	0.15024552	-2.957626
COQ3	-0.4079044	3.52557813	-2.5263929	0.01457544	0.15024552	-3.5707586
KIAA0930	0.47590754	4.93982049	2.52637611	0.014576058	0.15024552	-3.8453146
ZNF135	-0.2840082	4.47468043	-2.5250633	0.014624566	0.15044808	-3.765879
ENSCAFG00000031673	-0.5161914	3.42718836	-2.5249807	0.014627623	0.15044808	-3.5574887
HTR2B	1.70345881	0.10815564	2.52487488	0.014631542	0.15044808	-3.1876979
MPRIP	0.50503725	9.50431679	2.52440386	0.01464899	0.15050463	-3.7686968
PIR	-0.6623858	8.15576323	-2.5234622	0.014683929	0.15065062	-3.8594795
RTKN	0.91256533	0.61379184	2.52337629	0.01468712	0.15065062	-3.1377332
MED17	-0.2144059	5.25176333	-2.5226045	0.014715816	0.15082215	-3.8497917
ITPR2	-0.3140736	6.41115483	-2.5219948	0.014738524	0.15093207	-3.9229627
RPS11	-0.2724477	9.42483406	-2.5211701	0.014769288	0.15100823	-3.7697788
CALM1	0.36771932	9.06689049	2.52115269	0.014769938	0.15100823	-3.7939813
FAM214B	0.34914301	5.77116191	2.51914125	0.014845219	0.15165481	-3.9217247
GNA12	0.34380394	5.8994755	2.51812011	0.014883569	0.15166328	-3.9262861
POMGNT1	0.21832507	5.44359599	2.51766728	0.014900605	0.15166328	-3.8975219
JOSD1	-0.3247816	4.40619507	-2.5176614	0.014900827	0.15166328	-3.7633856
AIG1	0.39950814	4.35315515	2.51757393	0.014904119	0.15166328	-3.843788
UBE3B	0.19018112	7.00728723	2.51731729	0.014913784	0.15166328	-3.9293782
TRIM33	-0.2573715	5.06566217	-2.5171976	0.014918292	0.15166328	-3.8706035
RPS24	-0.264452	7.58329245	-2.5162673	0.014953383	0.15184596	-3.9001036
MRS2	0.30591566	3.48982416	2.51593472	0.014965947	0.15184596	-3.6155556
PCDH7	1.13455811	6.51589269	2.51516254	0.014995153	0.15184596	-3.9218581
PRAF2	0.29817318	6.42100948	2.5151438	0.014995862	0.15184596	-3.9394863
KAT6A	-0.2176798	6.00878225	-2.5149229	0.015004225	0.15184596	-3.9369318

TMEM173	-1.5750402	-2.3739168	-2.5143542	0.015025781	0.15184596	-2.988164
PSIP1	-0.3874136	6.03380028	-2.5143426	0.015026222	0.15184596	-3.9389523
VHL	-0.4028504	4.36103283	-2.5140805	0.015036167	0.15184596	-3.8106356
LYZ	-2.2281507	-2.0455588	-2.513777	0.015047687	0.15184596	-2.9815388
ENSCAFG00000008776	-0.6810138	0.43900478	-2.5135368	0.015056812	0.15184596	-3.0241335
MAP3K7	0.33444853	6.19697715	2.51280687	0.015084569	0.15200419	-3.9421147
SLC25A6	-0.3350219	9.15525183	-2.5119307	0.015117949	0.15221877	-3.8212588
CILP2	1.00524778	2.55725369	2.5115049	0.015134196	0.15226065	-3.4279706
SNRPN	0.30224733	6.46143377	2.51013839	0.015186443	0.15266435	-3.9513218
ZNF407	-0.2289818	4.36952713	-2.5096705	0.015204368	0.15271869	-3.7859312
STX6	0.23678412	3.61559943	2.50936468	0.015216097	0.15271869	-3.6942348
HACE1	-0.1754351	6.09132052	-2.5087228	0.015240739	0.15284423	-3.9501087
RCAN2	1.51734955	2.00597723	2.50807828	0.015265517	0.15297092	-3.0705959
FAM160A2	-0.2336131	3.99634077	-2.507551	0.015285815	0.15305256	-3.7403552
LRCH4	0.29902488	4.53483448	2.50595877	0.015347259	0.15339844	-3.8313766
METAP1D	-0.5766669	2.61865163	-2.5059552	0.015347397	0.15339844	-3.3314579
VPS36	-0.2884607	6.3687331	-2.5057096	0.015356893	0.15339844	-3.9602863
SRSF6	-0.2167271	6.71837264	-2.5032915	0.015450691	0.15421308	-3.9648244
LYSMD4	-0.2740028	4.54252149	-2.5024021	0.015485319	0.15443633	-3.8277621
MAPK8IP1	0.60427254	3.01826664	2.50166757	0.015513972	0.15459967	-3.4979783
SLC2A10	0.32937609	5.10755742	2.50134044	0.015526748	0.15460467	-3.9268733
GAMT	0.58791528	4.00128827	2.50033804	0.015565955	0.15487265	-3.7370132
ACTR3B	0.31954464	3.14132603	2.49947174	0.015599912	0.15503425	-3.577675
ITPRID2	0.31596948	6.92993153	2.49929585	0.015606814	0.15503425	-3.9505799
RHBDF2	0.53796167	3.42101488	2.49837993	0.015642803	0.15526931	-3.6533275
MTREX	-0.2687586	6.09369992	-2.4978204	0.015664826	0.15536547	-3.9754811
JMJD1C	-0.2763867	5.88001838	-2.4967293	0.015707852	0.15556789	-3.9803894
BCL6B	-2.3578311	-1.4139101	-2.4966765	0.015709937	0.15556789	-3.0249695
IPO9	-0.1512866	6.28961112	-2.49567	0.015749723	0.15565017	-3.9824837
BTBD11	1.72306951	0.65401889	2.49550287	0.015756339	0.15565017	-3.0157559
LYAR	-0.5137319	3.52572536	-2.495213	0.01576782	0.15565017	-3.6587199
AFAP1L2	1.2709984	2.82977506	2.49494464	0.015778454	0.15565017	-3.371273
GNPTG	0.29551581	5.59114518	2.4946089	0.015791769	0.15565017	-3.9735885
ICAM1	0.72049713	8.29426381	2.49414318	0.015810255	0.15565017	-3.9191023
HSCB	-0.3585104	3.21014552	-2.4935283	0.01583469	0.15565017	-3.553461
TEAD1	0.44048617	6.13931412	2.49348017	0.015836605	0.15565017	-3.9850811
DOCK9	-0.3963165	6.18850057	-2.4931331	0.015850417	0.15565017	-3.9732133
ANLN	1.01763796	6.43922139	2.4931115	0.015851275	0.15565017	-3.9597837
KIF5C	0.59099961	5.38857135	2.49303869	0.015854174	0.15565017	-3.9841212
ENSCAFG00000011780	-0.3435225	3.07196184	-2.4916928	0.015907842	0.15605543	-3.5851735
ZNF365	0.45141133	5.41289133	2.49033643	0.015962092	0.15638875	-3.9214385
ENSCAFG00000017688	-0.3739649	7.13365004	-2.4901374	0.015970069	0.15638875	-3.9778118
FAU	-0.2737148	7.74924294	-2.4896782	0.015988481	0.15638875	-3.9541789
ENSCAFG00000009806	1.82644927	-1.2390727	2.48960338	0.015991482	0.15638875	-3.1095751
ENSCAFG00000023230	1.27433527	-2.4433913	2.48806356	0.016053376	0.15675031	-3.0298121
PTGER4	1.48307901	-0.7973964	2.48764413	0.016070273	0.15675031	-3.0566872
FOXC2	1.11669775	2.74633688	2.4875128	0.016075567	0.15675031	-3.3739652
GSTZ1	-0.4937142	2.30265056	-2.4874467	0.016078231	0.15675031	-3.3200059
ATXN7L1	-0.555388	1.73035068	-2.4863656	0.016121878	0.15692859	-3.342391
C7H18orf32	0.32227857	3.89935182	2.4862865	0.016125076	0.15692859	-3.7496837

KIAA1191	0.25725584	7.37495269	2.48606845	0.016133894	0.15692859	-3.9866681
ROGDI	0.68602062	2.02869481	2.48522238	0.016168149	0.15714044	-3.386531
ATP6V0B	0.31366589	5.07622186	2.4846247	0.016192387	0.15725467	-3.9431644
RPL8	-0.2831967	9.48076795	-2.4829295	0.016261314	0.15780006	-3.8652964
TMEM248	0.25163482	6.76452472	2.48262796	0.016273601	0.15780006	-4.0089676
PRELID3B	0.33428215	4.45425702	2.48102675	0.016338993	0.15831228	-3.8708578
ELL3	-1.1336613	-0.1556579	-2.4799903	0.016381449	0.15848252	-3.056553
PKN1	-0.2934779	6.5798841	-2.4797728	0.01639037	0.15848252	-4.0190113
HIPK3	0.40839575	7.15355254	2.47967678	0.016394309	0.15848252	-3.9950977
YBX3	-0.3675689	7.07778623	-2.4784631	0.016444183	0.15872115	-3.9971644
CD40LG	1.07388847	0.3653973	2.47846282	0.016444197	0.15872115	-3.100478
TUBB2A	0.74864082	5.09528515	2.47737421	0.01648905	0.15903221	-3.9975382
MTMR9	-0.2727719	5.05895667	-2.476343	0.01653164	0.15925907	-3.9765522
HECW2	-0.5702069	4.51349731	-2.4761926	0.016537858	0.15925907	-3.8723793
RABEPK	-0.310287	3.98098573	-2.4751753	0.016579988	0.1595428	-3.7765936
DHX16	-0.1976894	5.58565227	-2.4746172	0.016603138	0.1596436	-4.0000946
ADGRB2	1.26190908	2.21923231	2.4739239	0.01663194	0.15968406	-3.1792959
DDX10	-0.2580888	4.71961301	-2.4739056	0.0166327	0.15968406	-3.9377791
BCL6	0.46229776	5.66752219	2.47334651	0.016655961	0.15975324	-4.0242341
JKAMP	0.24761363	5.743128	2.47312291	0.016665272	0.15975324	-4.0151947
SLC6A3	1.69128356	-1.6975551	2.47123968	0.016743876	0.16038469	-3.0857376
RNF219	-0.3440246	4.70263011	-2.4706847	0.016767105	0.16048515	-3.8997846
HIST1H2AC	0.8294013	0.30377041	2.46992318	0.016799025	0.16055518	-3.0860035
NAGLU	0.54910969	6.3349734	2.46990198	0.016799914	0.16055518	-4.0382706
SNCG	-1.715854	-2.4693619	-2.4686017	0.016854545	0.16088849	-3.0752195
CTNNBL1	0.25274816	5.05960054	2.46846412	0.016860337	0.16088849	-4.0005787
SLC7A1	-0.3685374	5.72226509	-2.4672431	0.016911799	0.1612574	-4.0133249
ODF2	-0.2640873	5.41558612	-2.466435	0.016945937	0.16134123	-4.0041867
CREBBP	-0.2931563	6.0895171	-2.466005	0.016964131	0.16134123	-4.048187
NFE2L1	0.373833	8.34182664	2.46586715	0.016969966	0.16134123	-3.9605535
TRIM8	0.34313598	6.01410648	2.4658232	0.016971826	0.16134123	-4.0500132
AGPAT1	0.30826562	4.33204939	2.46548122	0.016986312	0.16135715	-3.9049544
CDC37	0.27463172	7.41196524	2.46360075	0.017066165	0.16199353	-4.0252583
APLNR	-1.145139	-2.9893611	-2.4629982	0.017091821	0.1621149	-3.1018963
ITGB1	0.5219117	11.5186835	2.46206385	0.017131679	0.16234984	-3.7549186
SERINC2	1.3027714	0.48663273	2.46181357	0.01714237	0.16234984	-3.1367566
COL4A3BP	-0.2821307	6.07411217	-2.460888	0.017181955	0.16260248	-4.0534505
PTGS1	-1.8694579	-1.4919182	-2.4595261	0.017240354	0.16303265	-3.0898254
SNX10	0.47354289	6.2944721	2.45826886	0.017294425	0.1630599	-4.0655695
ALDOC	0.59467711	5.09262422	2.45731157	0.017335698	0.1630599	-4.0664664
TMEM39B	-0.3996609	2.50908148	-2.4570548	0.017346781	0.1630599	-3.5555261
SELENOW	0.36867691	6.47184517	2.45693492	0.017351961	0.1630599	-4.0655335
SNX18	0.40854931	5.80440416	2.45684971	0.017355642	0.1630599	-4.0605848
SUSD2	1.82471185	2.47036203	2.45684498	0.017355846	0.1630599	-3.5007823
RPL32	-0.2342595	8.09656292	-2.4567269	0.01736095	0.1630599	-4.0133124
SAMD4A	0.57408092	5.25461772	2.45661187	0.01736592	0.1630599	-4.0059848
GRIK4	1.38211074	-1.2586893	2.45648464	0.017371421	0.1630599	-3.0893864
SLC2A6	0.62406773	2.17677359	2.45645532	0.017372689	0.1630599	-3.5089879
TDRP	1.17956943	0.76735002	2.4560686	0.017389419	0.1630954	-3.1359909
RNF114	0.34387823	4.88400876	2.45573502	0.017403862	0.16310941	-3.9738411

PDZRN3	0.79701853	4.48578814	2.45483473	0.017442896	0.16335369	-3.7043307
CCND3	0.50082795	4.68547384	2.45409574	0.017474996	0.16344595	-4.0067379
PBXIP1	0.30970657	6.23602671	2.45371932	0.017491367	0.16344595	-4.0777485
CCND2	0.89665374	6.49480035	2.45371222	0.017491676	0.16344595	-4.0027005
GLB1	0.26274144	7.41341868	2.45267387	0.017536907	0.16374713	-4.0539749
DPM1	0.21563034	4.83232005	2.45130886	0.017596528	0.16418213	-4.0097083
ENSCAFG00000009631	-0.5024747	2.73035623	-2.4497606	0.017664376	0.16469318	-3.6280904
HOXA5	-1.3895283	2.08668437	-2.448329	0.017727319	0.16515777	-3.4366684
IGFBP3	-1.6926307	-2.4323249	-2.4477971	0.017750757	0.16516974	-3.1087681
USP34	-0.2318688	6.71997946	-2.4476737	0.017756199	0.16516974	-4.08685
SYTL1	1.02576513	1.92344458	2.44740754	0.017767941	0.16516974	-3.4273985
ETNK2	1.19766752	0.69632676	2.4470948	0.017781748	0.16517617	-3.156635
ILKAP	-0.2080662	4.59390002	-2.4464736	0.017809197	0.16530925	-3.9898308
UBE2A	0.46065569	4.28028001	2.44577661	0.017840045	0.16534978	-3.8582697
ITGB8	1.87945395	0.50962338	2.44573679	0.017841809	0.16534978	-3.1277044
FAM189A2	1.68388674	-0.0680034	2.44548543	0.017852946	0.16534978	-3.1639679
VSIR	-0.8824251	3.69288468	-2.4451221	0.017869053	0.16537736	-3.8276511
CCDC18	-0.5414946	3.47986932	-2.4437283	0.017930974	0.16575896	-3.8221132
TRPV3	-1.2413911	-1.5856284	-2.4436018	0.017936604	0.16575896	-3.1262517
BTF3L4	-0.2861261	3.62099018	-2.4425996	0.01798126	0.16604982	-3.805122
CARM1	-0.2744305	5.55461529	-2.4417923	0.0180173	0.16626074	-4.0798076
MIER1	-0.3507931	4.83129003	-2.4404091	0.01807921	0.16670991	-4.0182303
YEATS4	-0.3058729	3.36497535	-2.4362596	0.018266075	0.16818203	-3.7363628
SEC22B	0.27634344	5.2396711	2.43601375	0.018277202	0.16818203	-4.0766858
BOC	0.75030016	3.78354094	2.43597592	0.018278914	0.16818203	-3.8827579
LPIN3	-0.4043788	4.39663435	-2.4351883	0.018314604	0.16830523	-4.0242083
IL27RA	0.40117567	5.0323357	2.43502189	0.01832215	0.16830523	-4.0755136
TMEM30A	0.27169049	7.43827285	2.43467028	0.018338108	0.16830523	-4.0993417
PTPRM	-0.3522519	7.89950764	-2.434502	0.018345751	0.16830523	-4.0572586
LGALS3BP	0.67030319	7.68162607	2.43416452	0.018361084	0.16831004	-4.0612872
MAGI2	0.79497038	2.80977842	2.43390245	0.018373	0.16831004	-3.5645758
MAML1	-0.2646817	5.72918095	-2.4328588	0.018420523	0.16850924	-4.1056245
DMD	1.15104547	4.4715981	2.43283732	0.0184215	0.16850924	-3.9561021
KIF21B	-1.4441857	0.28507584	-2.4321151	0.018454453	0.16859905	-3.194023
PLAC1	1.23634863	-0.8418322	2.43203545	0.01845809	0.16859905	-3.1383023
DNAJB11	0.37887152	6.47353308	2.43097646	0.018506511	0.16889417	-4.1272253
TRAPPC3L	1.07643197	0.79668524	2.43074269	0.018517216	0.16889417	-3.2351355
SYNRG	-0.2271691	5.0191533	-2.4304485	0.018530693	0.16889479	-4.0476225
MTSS1L	0.58338888	3.82661793	2.42851388	0.018619554	0.16956176	-3.6785077
B9D1	-0.4597006	3.52922539	-2.4282698	0.018630794	0.16956176	-3.7971001
FAM192A	-0.2120531	4.21785878	-2.4268419	0.018696656	0.16978323	-3.9444283
NUP153	-0.2519498	6.13829777	-2.426827	0.018697345	0.16978323	-4.1350369
ZYX	0.42455479	9.091933	2.42679917	0.018698633	0.16978323	-4.0143016
KXD1	0.44558638	4.88899627	2.42642902	0.018715743	0.16978323	-4.0725744
GPC1	0.46865971	6.78070459	2.4262824	0.018722524	0.16978323	-4.1366525
TTI1	0.20494774	3.86710618	2.42489683	0.018786718	0.17015057	-3.9200016
DERA	-0.2163393	4.5798217	-2.4248251	0.018790048	0.17015057	-3.9872284
KPNA1	0.26146166	5.73471877	2.42378511	0.018838367	0.17042123	-4.1293813
POMGNT2	0.43825339	3.87580658	2.42359962	0.018846997	0.17042123	-3.9096727
ATP7B	0.36751158	3.32386763	2.4223627	0.018904637	0.170788	-3.7214558

MED15	0.29350968	6.17344501	2.42214761	0.018914677	0.170788	-4.1463321
P3H1	0.22979447	6.86336484	2.42179774	0.018931017	0.1708131	-4.1451621
C18H11orf24	0.43688206	6.6516499	2.41994463	0.019017776	0.17147309	-4.151126
ENSCAFG00000020576	0.92106112	-0.3265351	2.41923691	0.019051005	0.17164983	-3.1866234
XXYLT1	0.39704927	4.3762192	2.41737942	0.019138466	0.17231459	-3.9649877
TRA2A	-0.2980135	4.24939902	-2.4168239	0.019164693	0.17234089	-3.9703088
PKNOX2	1.8890359	2.09540491	2.41673801	0.019168751	0.17234089	-3.3329062
KDM6A	-0.2583913	4.57936133	-2.4157463	0.019215659	0.17249718	-4.0653591
TMEM127	0.40788894	4.63802511	2.41574499	0.019215722	0.17249718	-4.051073
TGFB2	1.05069925	6.70713497	2.41527381	0.019238046	0.17249718	-4.14737
LMOD2	0.98143708	0.07734732	2.41521332	0.019240913	0.17249718	-3.2640568
THUMPD2	-0.6395431	1.797351	-2.4144962	0.019274939	0.1725532	-3.4179349
TBC1D15	-0.2920351	5.07461808	-2.4142518	0.019286545	0.1725532	-4.1033807
SERINC1	0.2962451	7.71738439	2.41415809	0.019290999	0.1725532	-4.1201757
RTN1	1.47546124	-0.0989145	2.41392754	0.019301958	0.1725532	-3.1796508
RAB31	0.51929092	6.20076242	2.4130349	0.01934444	0.17281033	-4.1615125
NACC1	0.31213267	5.08859976	2.41175429	0.019405533	0.17292795	-4.097158
ENSCAFG00000015834	-0.2440775	7.16759609	-2.4115379	0.019415873	0.17292795	-4.1568635
LSG1	0.26221435	5.34031363	2.41133267	0.019425685	0.17292795	-4.1355934
PPP2R3A	-0.4581392	5.02621633	-2.4112487	0.019429701	0.17292795	-4.0615656
PCDH12	-2.2093973	-2.0374583	-2.4110361	0.019439873	0.17292795	-3.170814
ENSCAFG00000032678	0.79202402	-0.3900857	2.41075953	0.019453109	0.17292795	-3.1826035
PRKAA2	1.31611264	-1.4526008	2.41057393	0.019461997	0.17292795	-3.1717417
TARBP1	-0.3779946	4.44778479	-2.4104252	0.019469122	0.17292795	-4.0133357
ZNF169	-0.227276	5.08510957	-2.4101739	0.019481166	0.17292795	-4.1059416
GBGT1	0.25621478	5.8314676	2.40934967	0.019520717	0.17308028	-4.1704555
AGRN	0.60149306	4.51715093	2.40924369	0.019525808	0.17308028	-3.975315
HSPA13	0.35915649	6.802485	2.40871974	0.019550993	0.17318165	-4.1730799
FILIP1L	0.85826182	6.12924165	2.40788867	0.019591	0.17341408	-4.1681393
PIP4K2C	0.28568136	6.04387466	2.40691421	0.019638004	0.17370808	-4.180892
ENSCAFG00000008221	0.27427996	9.87055995	2.40515595	0.019723072	0.17433811	-3.9720017
LHX6	-2.0106952	-1.3083615	-2.4046383	0.019748179	0.17443763	-3.1873783
RMI1	-0.3180761	3.63392015	-2.4043008	0.019764564	0.17444022	-3.8733672
C1H19orf12	0.49608414	2.12837685	2.40406192	0.019776169	0.17444022	-3.5416059
ADCY6	0.38267418	6.45533442	2.40361124	0.01979808	0.17451128	-4.186616
KLC2	0.29023373	4.14770392	2.40320547	0.019817826	0.17454063	-3.9107873
SRSF11	-0.2290603	6.50147372	-2.4028605	0.019834626	0.17454063	-4.1894691
ENSCAFG00000032154	1.37141987	2.36214174	2.40268908	0.019842981	0.17454063	-3.5225366
INPP4A	0.26470104	5.38502649	2.401999	0.019876641	0.1747147	-4.1668824
MYO6	0.27451125	6.27374014	2.400255	0.019961938	0.1753421	-4.1955389
ENSCAFG00000030283	-1.0151664	-0.664724	-2.3995989	0.01999411	0.1755023	-3.2262769
CREB3L2	0.29654448	5.31727927	2.39897016	0.020024987	0.17553745	-4.1552436
ENSCAFG00000000747	-0.7332776	0.15447047	-2.3986964	0.020038443	0.17553745	-3.2364397
CHD3	-0.2501233	7.7644632	-2.3985913	0.020043612	0.17553745	-4.1700589
CAPZB	0.2459514	6.97259683	2.39838306	0.020053857	0.17553745	-4.1848222
INTU	-0.7072062	1.2989404	-2.398077	0.020068923	0.17554733	-3.3644828
BCOR	-0.3088702	5.20508477	-2.3964296	0.020150186	0.17613584	-4.0801194
ANKZF1	-0.2683519	4.40543876	-2.395667	0.020187901	0.17634313	-4.0683957
RPS7	-0.2419011	9.00269464	-2.3952625	0.020207935	0.1763958	-4.0832209
DOCK5	1.43800412	1.72850864	2.39449972	0.020245756	0.17640322	-3.284105

CDC42EP4	-0.5329597	3.66432697	-2.3944716	0.020247152	0.17640322	-3.9730032
UTP3	-0.2304303	5.25376463	-2.3940721	0.020266987	0.17640322	-4.1632139
ZNF496	-0.2127855	5.37109412	-2.3939733	0.020271898	0.17640322	-4.1585186
LMAN2	0.213277	7.14960831	2.39383422	0.020278807	0.17640322	-4.1960931
MCC	-0.5369834	4.62101885	-2.3933698	0.020301899	0.17643253	-3.9521804
FOSL2	0.4427848	6.10631506	2.3932032	0.020310192	0.17643253	-4.1990755
CCDC22	-0.3179008	3.61800354	-2.3925955	0.020340456	0.17657366	-3.8665471
NGEF	0.9862812	3.89880296	2.39166992	0.02038663	0.17669728	-3.7673353
DACT1	1.08465703	4.42606406	2.3914238	0.020398924	0.17669728	-3.8994253
ACSS3	1.06577552	3.75204865	2.39128099	0.020406061	0.17669728	-3.773631
ABHD2	0.64112686	6.86320625	2.39104544	0.020417837	0.17669728	-4.213627
ATG13	0.2444557	5.84222653	2.39063043	0.020438599	0.17669728	-4.2116436
MT-CO1	-0.3605158	13.6886202	-2.3901849	0.020460911	0.17669728	-3.759212
UBA7	-0.6298349	4.10198301	-2.3899781	0.020471273	0.17669728	-4.0471366
ALCAM	0.5010325	9.35560602	2.38988762	0.020475808	0.17669728	-4.0866237
RPS3A	-0.288407	8.45073055	-2.3893812	0.020501209	0.17669728	-4.1352121
SYDE2	1.00194436	1.9413267	2.38865355	0.02053776	0.17669728	-3.4149113
PLVAP	-3.3702437	-0.9724757	-2.3885723	0.020541844	0.17669728	-3.2345534
GALT	0.37348287	4.16620792	2.38843542	0.020548727	0.17669728	-4.0542682
TAB1	-0.2468284	4.72712077	-2.3883784	0.020551595	0.17669728	-4.1179607
TRPV2	0.61246372	6.37634098	2.38826371	0.020557365	0.17669728	-4.219447
IPO7	-0.2354114	8.2004584	-2.3881097	0.020565117	0.17669728	-4.1608542
PRKAG3	1.45560284	-0.9904854	2.38762764	0.02058939	0.17677067	-3.219142
TNFRSF25	1.29745117	-0.3027327	2.3872127	0.020610305	0.17677067	-3.2370615
TP53	-0.3009712	6.45994328	-2.386869	0.020627646	0.17677067	-4.2240195
PRPF39	-0.3409141	4.55130714	-2.3868264	0.020629794	0.17677067	-4.0901369
ENSCAFG00000008119	0.4477705	3.2948708	2.3862973	0.020656513	0.17678522	-3.8842833
DOCK7	0.32538595	7.5253655	2.38592066	0.020675553	0.17678522	-4.1855975
TOLLIP	0.2756987	6.73075868	2.38569889	0.020686771	0.17678522	-4.223207
P2RY1	1.32765122	-2.0123882	2.38568188	0.020687632	0.17678522	-3.2166302
ACYP2	0.80613636	0.70210143	2.38475429	0.020734614	0.17706658	-3.3789563
EPHA4	0.93748063	1.74139116	2.38427115	0.020759123	0.17715577	-3.5744159
STOML1	0.49289558	3.44473457	2.38321906	0.020812583	0.17746379	-3.8637325
NPHP3	-0.2269818	4.15173141	-2.3830066	0.020823395	0.17746379	-4.0228756
PPP1R10	0.19459026	6.04085208	2.38135975	0.020907363	0.17797883	-4.2361691
PAICS	-0.238184	5.86242254	-2.3812672	0.020912089	0.17797883	-4.2113459
IMPDH2	-0.4248052	7.4668371	-2.3794997	0.021002566	0.17859521	-4.2096142
ENSCAFG00000007135	-0.3257215	3.79474914	-2.3792989	0.02101287	0.17859521	-3.9717942
RAD52	-0.4893552	2.37751295	-2.3786659	0.021045372	0.17875085	-3.6186723
ECE1	0.55357061	9.39737994	2.37767804	0.021096184	0.17906168	-4.0385252
BACE1	0.39854872	5.76462899	2.37639796	0.021162192	0.17932399	-4.2321296
TNRC6A	-0.2618055	6.76005084	-2.3762815	0.021168204	0.17932399	-4.2418924
ENSCAFG00000024456	-0.6624741	0.65442082	-2.3760729	0.021178981	0.17932399	-3.3251763
AOC3	1.21837365	-1.8113024	2.37597515	0.021184035	0.17932399	-3.2394846
ARHGAP35	-0.1783819	7.08940049	-2.3745786	0.021256328	0.17971387	-4.2388199
HACD1	0.87187726	2.76152348	2.37446627	0.02126215	0.17971387	-3.590638
RPL21	-0.2328615	8.35388568	-2.373968	0.021288	0.17971387	-4.1797334
PIDD1	-0.4079005	4.29876738	-2.3736936	0.021302253	0.17971387	-4.0868186
SSR2	0.24767813	8.12881136	2.37361541	0.021306314	0.17971387	-4.1892637
TCEA1	-0.2425483	6.22992952	-2.3734348	0.021315698	0.17971387	-4.2494103

SHISA2	1.56565805	-1.3991736	2.3712032	0.021431964	0.18057326	-3.2420188
FKBP8	0.29606083	7.88478726	2.37033426	0.021477389	0.18083502	-4.2034586
CCNL1	-0.3833048	5.06713212	-2.3699696	0.021496477	0.18087483	-4.2136653
THADA	-0.2426868	4.48730866	-2.3693017	0.021531479	0.18104841	-4.1073831
ENSCAFG00000031478	0.31075725	5.15532994	2.36901291	0.02154663	0.18105494	-4.1709673
DDA1	0.30746502	3.83806595	2.36840723	0.021578435	0.18120131	-4.0294514
NUP37	-0.4031319	3.16047385	-2.3677769	0.021611578	0.18124306	-3.8368743
QSOX2	-0.2828937	5.10489371	-2.3677654	0.021612184	0.18124306	-4.2054317
SLC22A13	1.23329258	-0.6866664	2.36744561	0.021629018	0.1812451	-3.2484309
YDJC	1.01992836	-1.2880352	2.36721421	0.021641205	0.1812451	-3.274444
SDC2	0.58305236	7.35925269	2.36661511	0.021672789	0.18131179	-4.2474031
LIF	1.13880828	4.31019104	2.36628705	0.021690101	0.18131179	-3.9134889
CEP63	-0.2128137	4.67514155	-2.3662444	0.021692352	0.18131179	-4.1500968
B3GALT1	1.2065383	1.50958169	2.36589591	0.021710758	0.18134529	-3.5367728
LIMCH1	-1.489339	4.83940477	-2.3648831	0.021764326	0.18167227	-3.9296481
CEP85	-0.7245371	2.95281991	-2.3642677	0.021796935	0.18182397	-3.8855114
LARS2	-0.2992013	3.78347005	-2.3634862	0.021838407	0.18204935	-3.967922
GTF3C4	-0.2580363	5.55460229	-2.3625386	0.021888788	0.18234866	-4.2476872
KRCC1	-0.2263157	4.40370599	-2.3616175	0.021937863	0.1826367	-4.093132
TFAP2B	1.13243941	-0.9788351	2.36108694	0.021966173	0.1827516	-3.2773835
ZC3HAV1	-0.5425195	3.71620878	-2.3599496	0.022026974	0.18313648	-4.0945968
SORT1	0.43959656	6.36927488	2.35912683	0.022071048	0.18335607	-4.273975
C9orf3	0.39441292	4.79340651	2.35891332	0.022082499	0.18335607	-4.2211894
BLM	-0.9977049	2.77052977	-2.3575695	0.022154694	0.18383434	-3.6335066
CD109	-0.397122	7.44392816	-2.3563806	0.02221874	0.1842444	-4.2538279
PCDH19	-1.1291826	-0.7758202	-2.3550632	0.0222899	0.18459632	-3.3376981
PTGDS	1.42781336	1.05816204	2.35505231	0.022290489	0.18459632	-3.564954
GUF1	-0.381784	2.80798012	-2.353852	0.022355503	0.18487976	-3.7425186
ENSCAFG00000007984	-0.7234225	0.95572641	-2.3538458	0.022355839	0.18487976	-3.3537974
SEPT10	-0.2079177	6.68509	-2.3535669	0.022370972	0.18487976	-4.2950226
SLC35F6	0.24640958	5.77719463	2.35333749	0.022383426	0.18487976	-4.287516
FAM20C	0.43100916	7.81801694	2.35272724	0.022416583	0.18503229	-4.2499502
HSDL2	0.27848723	4.33557596	2.35238967	0.022434943	0.18506257	-4.1840348
KIAA2026	-0.2505324	4.31578679	-2.3512163	0.022498868	0.18546842	-4.1753589
MID2	-0.4442383	4.64839114	-2.350709	0.022526553	0.18557263	-4.263723
AGO4	-0.4471751	4.30882395	-2.350445	0.022540975	0.18557263	-4.1740195
C18H7orf57	-0.4844159	4.06609155	-2.3499352	0.022568846	0.18568072	-4.0778991
TCEAL1	-0.2837233	3.6316036	-2.3495119	0.022592008	0.18574996	-3.9564595
CBARP	0.87505745	1.61655371	2.34876262	0.022633064	0.18596613	-3.5154605
KIAA1549	0.51037801	2.57052292	2.34823328	0.022662109	0.18603998	-3.7738826
CANT1	0.2765746	4.46269318	2.34806059	0.022671592	0.18603998	-4.1822451
IFT20	0.30693813	3.75515168	2.34738942	0.02270848	0.18622137	-4.0167421
RAB5B	0.22113315	4.69166224	2.34694643	0.022732858	0.18625935	-4.2184191
DEK	-0.4404621	3.80652642	-2.3465468	0.022754867	0.18625935	-4.0278187
ENSCAFG00000031145	1.24166746	0.43676123	2.34649953	0.022757473	0.18625935	-3.3704668
ITPRIPL1	0.70403044	1.67055158	2.34575728	0.02279841	0.18647323	-3.6091702
PSME1	-0.54324	4.74987768	-2.3445876	0.022863053	0.18676058	-4.2019127
RBM4	-0.1904057	4.83537965	-2.344585	0.022863196	0.18676058	-4.2064332
IPO4	-0.2649114	6.16756516	-2.3436508	0.022914947	0.18692312	-4.3117634
VASH2	1.0811997	5.98955165	2.34352523	0.022921908	0.18692312	-4.2858113

PSMD2	0.22571122	8.45579007	2.34342237	0.022927614	0.18692312	-4.233799
HAUS7	0.83004547	2.2996031	2.34315184	0.022942626	0.18692453	-3.5948659
ZNF182	0.45343987	2.40231184	2.34265209	0.022970381	0.18701243	-3.6940714
PTPN11	0.2900453	7.07599805	2.34242309	0.022983109	0.18701243	-4.3151792
SOS2	-0.2914934	4.28799462	-2.3418863	0.02301297	0.18705512	-4.1749706
U2AF1	-0.2756985	5.43555222	-2.3417455	0.023020809	0.18705512	-4.2649978
EMP2	-0.5735936	3.39608123	-2.3415155	0.023033616	0.18705512	-4.0388212
PRDX2	0.30663345	6.48199128	2.34126173	0.023047757	0.18705512	-4.3229491
LSM7	-0.5280216	2.24985985	-2.3406249	0.023083273	0.18722274	-3.6250483
DLAT	-0.2711153	5.2911637	-2.3401112	0.02311196	0.18724964	-4.2741051
LOXL1	0.5370627	8.18591154	2.34003317	0.023116322	0.18724964	-4.2888902
SURF2	-0.2925572	3.33541054	-2.3396575	0.023137322	0.1872993	-3.987637
HNRNPL	-0.1996518	7.88525077	-2.3393789	0.023152911	0.18730512	-4.2798292
TPM1	0.64350708	9.42993132	2.339052	0.023171212	0.18733285	-4.1910036
RPL26	-0.4796714	2.11907789	-2.3385402	0.023199891	0.18744441	-3.6728057
TRAPPC3	0.20429811	5.18522267	2.33815771	0.023221342	0.18749745	-4.2688788
PORCN	0.52601632	2.69966581	2.33760856	0.023252173	0.18750609	-3.8867719
GATAD1	-0.3857218	4.21493284	-2.3376084	0.023252185	0.18750609	-4.1506435
GPR176	1.10687827	1.01353175	2.33668138	0.023304311	0.18780621	-3.4009646
PLSCR4	-0.6811062	2.96270194	-2.3363767	0.023321468	0.1878243	-3.884699
EDNRB	-2.1256861	-1.2536943	-2.3357428	0.023357199	0.18799187	-3.3218718
CENPS	0.24127125	4.91048204	2.33508413	0.023394374	0.18817084	-4.2517178
CHMP7	0.22351658	4.01653801	2.33381121	0.023466371	0.18862949	-4.119418
RALGPS1	0.50347845	2.02011896	2.33350977	0.02348345	0.18864639	-3.5941882
EFNA1	-0.5365536	2.61344189	-2.3301389	0.02367519	0.19006545	-3.8381392
TMEM63A	0.3105972	5.76960425	2.32966241	0.023702406	0.19016274	-4.3283871
GGACT	-0.6603707	0.95949267	-2.3290387	0.02373807	0.19030091	-3.4559676
OTUD5	0.23388674	6.75412061	2.32883306	0.023749843	0.19030091	-4.3451097
N4BP2	-0.3919839	4.04340337	-2.3282066	0.023785733	0.19045412	-4.1390359
IFT122	-0.2505998	4.74081199	-2.3279716	0.023799205	0.19045412	-4.2497198
CMAS	0.30660124	4.81510665	2.32752821	0.023824649	0.19053668	-4.2592606
PTGS2	1.11279237	5.03281494	2.3269998	0.023855002	0.19065838	-4.3447288
CEP192	-0.2408972	5.55459168	-2.3267094	0.023871696	0.19067082	-4.3318952
MFAP4	1.60496586	4.05942136	2.32552275	0.02394003	0.19085836	-3.8744787
TRMT1	-0.2740449	3.71992266	-2.3254815	0.023942411	0.19085836	-4.0563623
VCAM1	2.06450485	3.23809529	2.32536197	0.023949302	0.19085836	-3.8277512
TTC33	-0.4330206	2.4386195	-2.3252496	0.023955785	0.19085836	-3.7818262
RBM28	0.33796566	4.52202874	2.3228451	0.024094862	0.19184506	-4.2056387
ENSCAFG00000005298	-0.642191	1.77249039	-2.3219757	0.024145324	0.1921254	-3.5047869
TMEM164	-0.4443554	4.44394377	-2.3211626	0.024192607	0.19229636	-4.2557024
ENSCAFG00000017892	-0.4497172	6.51728383	-2.3210812	0.024197342	0.19229636	-4.3644062
FPGT	-0.3709661	3.42217671	-2.3206437	0.024222823	0.19233064	-3.9856563
CD81	0.4990895	8.03363885	2.32048289	0.024232195	0.19233064	-4.2861689
PPP6R2	-0.2043846	5.77452353	-2.3192096	0.024306512	0.1926192	-4.3543532
USP25	-0.2738408	5.02664111	-2.319209	0.024306544	0.1926192	-4.2988313
IL33	1.12560395	2.14652763	2.31907414	0.024314428	0.1926192	-4.2750082
SF3B1	-0.2600514	8.26387065	-2.3185213	0.024346767	0.19275416	-4.3038787
RGS12	0.28676962	4.46942902	2.31815787	0.024368046	0.19280145	-4.1817973
CMTM4	0.64073631	1.77598766	2.31757702	0.024402091	0.19289669	-3.5721744
BAHCC1	-0.459116	4.17551752	-2.3174301	0.024410712	0.19289669	-4.1262056

NOV	2.78172883	4.40002868	2.3165197	0.024464172	0.19319794	-3.3522609
FLT1	1.89705979	4.35648471	2.31617173	0.024484635	0.19323838	-4.032963
TGM2	2.12725375	5.97927445	2.31570846	0.0245119	0.1932796	-4.3397284
DUS2	-0.2351238	3.76723952	-2.3153198	0.024534796	0.1932796	-4.0811707
GMFB	0.38100107	6.58178169	2.31530121	0.024535891	0.1932796	-4.3775092
SLC30A3	1.44298443	-0.9838493	2.31312723	0.024664315	0.19416982	-3.3485499
ENSCAFG00000014227	-1.1329006	0.10690533	-2.3116879	0.02474967	0.19472008	-3.3619424
NAALAD2	-1.6428812	-0.226347	-2.3112335	0.024776672	0.19481083	-3.3698649
ENSCAFG00000016847	-0.4536254	2.78541808	-2.3104551	0.024822988	0.19505325	-3.8047991
TSPAN17	0.26046226	6.23207489	2.30899403	0.024910128	0.19561594	-4.3917313
MEX3C	-0.1315758	6.10186447	-2.3083654	0.024947707	0.19568835	-4.3913939
DIS3L2	-0.2371876	3.52937098	-2.30832	0.02495042	0.19568835	-4.0671558
TMEM62	-0.3577621	2.69646236	-2.307975	0.024971065	0.1957284	-3.8565274
RSU1	0.25120506	7.82625616	2.30728371	0.025012482	0.19592667	-4.350666
SIRT1	-0.3208109	4.08643618	-2.3070338	0.02502747	0.19592667	-4.154959
ST6GALNAC4	0.37906205	6.27802572	2.30649739	0.025059665	0.19605686	-4.3855625
KHNYN	-0.2435517	4.63936463	-2.3060804	0.025084718	0.19613105	-4.2789456
NONO	-0.2098518	6.15635451	-2.3052753	0.025133152	0.19631458	-4.3949563
ENPP5	1.10244615	-0.7060167	2.30517218	0.025139363	0.19631458	-3.4314289
NIPA1	0.55853318	2.55365491	2.30475217	0.02516467	0.19639045	-3.8421028
SIGMAR1	0.46213572	6.96838558	2.30401414	0.025209193	0.1966161	-4.3909028
THOC2	-0.260182	5.95947549	-2.3034924	0.025240708	0.19674007	-4.3953556
VEZF1	-0.3626738	5.34534698	-2.3021432	0.025322373	0.19725456	-4.3406251
TEX9	0.67585962	1.18475683	2.30157116	0.02535707	0.19740276	-3.550091
NAXD	0.33989056	4.10317078	2.30109125	0.025386211	0.19747884	-4.2288965
TYW3	-0.4165378	1.99516156	-2.300894	0.025398199	0.19747884	-3.6843108
EXOSC2	-0.2936682	3.87504616	-2.2997196	0.02546966	0.19782254	-4.1518827
LRP5	0.43152551	6.29288949	2.29965147	0.025473813	0.19782254	-4.4106539
GSTT4	1.00921032	-1.5357785	2.29922576	0.025499767	0.19790207	-3.380768
CXXC5	0.36294054	5.36532302	2.29732908	0.025615686	0.19858129	-4.3815964
ZNF792	-0.7377154	1.71342681	-2.2970919	0.025630216	0.19858129	-3.6756046
TP53INP2	0.68510962	1.47113349	2.29676178	0.025650448	0.19858129	-3.6532734
L3MBTL3	-0.4262733	3.87052133	-2.2966672	0.025656247	0.19858129	-4.0990149
PPP1R3C	0.77519498	2.04397474	2.29650638	0.025666112	0.19858129	-3.9058979
PRUNE2	1.01259207	7.13296126	2.29561462	0.025720871	0.19879481	-4.4045083
CACNA1H	1.3526512	-2.1279442	2.29554299	0.025725274	0.19879481	-3.3725146
BCHE	1.14742976	-1.043487	2.29494159	0.025762267	0.19895863	-3.4130246
CCSER2	0.29929366	7.12439722	2.29454196	0.025786876	0.19902665	-4.4093925
ENSCAFG00000032728	-0.3279702	5.99870463	-2.2935053	0.025850806	0.19927838	-4.4098673
EDA	0.85841743	1.96079129	2.2928231	0.025892957	0.19927838	-3.551945
TMEM209	-0.3058955	4.83639619	-2.2922471	0.025928593	0.19927838	-4.3129405
ENSCAFG00000028262	-1.1114348	-0.4371639	-2.2922443	0.025928767	0.19927838	-3.4114402
THSD4	-1.6447126	-1.3361152	-2.2921953	0.025931799	0.19927838	-3.3809335
ENSCAFG00000020852	-1.0507166	-0.891458	-2.2920863	0.025938547	0.19927838	-3.3869846
DOK1	0.40138754	5.65991394	2.29150524	0.025974554	0.19927838	-4.4203535
TMTC4	-0.4330801	3.61827154	-2.2909648	0.026008082	0.19927838	-4.0027182
CLTCL1	0.76657113	2.51859605	2.29088443	0.026013071	0.19927838	-3.7157624
NEU1	0.25428317	6.52109517	2.29084339	0.026015619	0.19927838	-4.4287044
C9H17orf75	-0.3372596	3.13337875	-2.2907251	0.026022964	0.19927838	-3.9672302
NOB1	-0.3306753	4.86860926	-2.2905614	0.026033136	0.19927838	-4.3206426

THOC1	-0.3515678	3.45382931	-2.2905404	0.026034439	0.19927838	-4.0457358
TMEM41A	0.72988572	1.63680395	2.29043507	0.026040982	0.19927838	-3.6120417
ATP2C1	0.30355305	6.38697805	2.29017213	0.026057327	0.19928239	-4.4245424
ENSCAFG00000000081	0.97880779	-0.6936317	2.28926393	0.026113852	0.19950549	-3.4060583
ITPR1	0.31707421	5.71548753	2.2891828	0.026118906	0.19950549	-4.4240244
ACSL3	-0.3545739	6.83385787	-2.288602	0.026155119	0.19950549	-4.4257365
GEMIN6	-0.2695673	4.22801981	-2.288308	0.026173464	0.19950549	-4.2869639
CFAP46	1.25750345	-0.9568705	2.28830569	0.026173609	0.19950549	-3.3995864
ENSCAFG00000029967	-0.9238092	0.16582015	-2.2881788	0.026181532	0.19950549	-3.4352469
EBAG9	-0.3139721	3.19241259	-2.2875511	0.026220749	0.19953335	-4.0556572
FAM126A	0.37525588	6.96943306	2.28736319	0.026232504	0.19953335	-4.4321682
RBM20	1.63368749	-0.0248797	2.28735987	0.026232711	0.19953335	-3.4409401
FBL	-0.4152733	6.33485077	-2.2866164	0.026279252	0.19971593	-4.4355104
ENSCAFG00000001866	-1.036503	3.63300784	-2.28647	0.026288426	0.19971593	-4.4147229
PTGFR	-1.1186942	1.11022633	-2.2861402	0.026309099	0.19975251	-3.4578269
DCAF17	-0.4458771	2.02528062	-2.2852205	0.026366832	0.20001615	-3.818536
ENSCAFG00000023188	-0.9847072	1.26090393	-2.2850813	0.026375582	0.20001615	-3.4686558
IL4R	-0.4256638	4.85229738	-2.2846221	0.026404454	0.20011462	-4.3567302
PDE5A	1.61792251	1.69704763	2.28429375	0.026425122	0.20013927	-3.4921537
RPS3	-0.2355548	8.62876126	-2.284	0.026443623	0.20013927	-4.3441343
VMP1	0.36917959	6.61205023	2.28381349	0.026455373	0.20013927	-4.4421692
MPP4	1.25993618	0.23161064	2.28289544	0.026513287	0.200457	-3.435511
EPC2	-0.2532055	5.38713246	-2.2811345	0.026624685	0.20117848	-4.4142653
LIMD1	-0.4567257	5.06317433	-2.2801719	0.026685758	0.20151907	-4.3565981
ZNF879	-0.2956009	3.16848951	-2.2793454	0.026738293	0.20179481	-4.0508943
MBTPS1	0.16370034	8.24937645	2.27866626	0.026781534	0.20181928	-4.3709591
PCLO	0.91797057	0.42674306	2.27845999	0.026794678	0.20181928	-3.572347
ABCC5	0.45333635	5.67413048	2.27843027	0.026796573	0.20181928	-4.4492906
CAMSAP1	0.17989593	6.19091308	2.27809814	0.026817752	0.20181928	-4.4542843
PDP1	-0.1947909	6.94542299	-2.2778401	0.026834215	0.20181928	-4.455335
ATG4D	0.37937892	3.58304602	2.27778597	0.02683767	0.20181928	-4.0596631
RICTOR	-0.3115786	5.04984597	-2.2757462	0.02696815	0.20267949	-4.3988631
NOL8	-0.2351888	5.70749829	-2.2752923	0.026997262	0.20273278	-4.441538
RFX2	-0.4586666	2.99407379	-2.2750025	0.027015865	0.20273278	-4.0551958
SCRN1	0.62871104	5.96827891	2.27484898	0.027025722	0.20273278	-4.4333609
ANKRD11	-0.2180979	7.61971844	-2.2743914	0.027055127	0.20273278	-4.4378756
CHST7	-1.4492934	-1.5762672	-2.2743822	0.027055717	0.20273278	-3.4477818
SFR1	-0.4634007	2.44738327	-2.2740464	0.027077311	0.20277396	-3.9344941
RMDN3	0.26530491	4.45929348	2.27177331	0.027223906	0.20375064	-4.3238221
SCAF11	-0.2610552	6.73920228	-2.2713306	0.027252542	0.20384383	-4.4678421
VAV3	-0.797593	2.71178861	-2.271055	0.02727038	0.20385621	-4.2308168
FAAP100	-0.3867051	3.8469488	-2.2700305	0.027336779	0.20423136	-4.1965346
ACBD6	-0.2666219	4.55290184	-2.2692058	0.027390331	0.20451014	-4.32641
TEAD3	0.76639601	5.65314987	2.2684315	0.027440701	0.20476485	-4.445414
ZNF618	-0.4183242	2.78855624	-2.2679187	0.027474102	0.20486636	-3.9141077
POLG2	-0.4067704	1.95213162	-2.2676913	0.027488924	0.20486636	-3.7419326
QARS	-0.2215099	6.74176749	-2.267474	0.027503097	0.20486636	-4.4725649
PREB	0.28700659	5.17960578	2.26602842	0.027597543	0.20544838	-4.4268789
ENSCAFG00000000626	-0.7627382	1.44776662	-2.2636567	0.027753118	0.20638079	-3.7045843
BTAF1	-0.2654809	5.29767166	-2.2636195	0.027755562	0.20638079	-4.4463822

IL1R2	1.06241683	-1.0473804	2.2630997	0.027789766	0.20651321	-3.5143093
CCL16	1.25439142	-1.9795404	2.26229179	0.027843002	0.20656718	-3.4654989
NPNT	2.1419124	3.33009893	2.26219885	0.027849131	0.20656718	-3.7071821
SAP30BP	0.20711269	4.7521626	2.26206803	0.027857761	0.20656718	-4.3918164
CCDC97	-0.2584542	4.78272161	-2.2619943	0.027862626	0.20656718	-4.3875782
NEDD1	-0.2515413	5.64027739	-2.2611845	0.027916109	0.20684195	-4.4629208
GPR146	-1.1004233	-0.7935952	-2.2599165	0.028000039	0.20734185	-3.5103183
CCM2L	-1.6940341	-2.2582964	-2.2589332	0.028065274	0.20760656	-3.4336525
SYNGR1	1.16561897	0.87866775	2.2588809	0.02806875	0.20760656	-3.5840683
P2RX2	0.9531776	-1.3051823	2.25859185	0.028087955	0.20762669	-3.4781652
UBE2Z	0.18520585	5.59254205	2.258316	0.028106294	0.2076404	-4.4659758
YPEL2	0.68305464	1.9691284	2.25700744	0.028193434	0.20816207	-4.0501149
ARHGEF15	-2.1995177	-1.2707203	-2.256692	0.028214476	0.2081954	-3.4561492
APOD	-1.928994	-1.5945548	-2.2557106	0.02828003	0.20855601	-3.4709379
ENSCAFG00000019200	-1.5139403	-2.4907749	-2.2554649	0.028296461	0.20855601	-3.4393038
ENSCAFG00000005772	0.51325599	4.60063619	2.25509438	0.028321259	0.20861672	-4.3485372
PHLDA1	0.68120669	3.01445128	2.25448009	0.028362414	0.20879776	-4.1905297
UIMC1	-0.3202929	3.16035108	-2.253158	0.028451164	0.20932878	-4.0775033
ACOT8	0.27589164	3.35485073	2.25194633	0.028532722	0.20980629	-4.1609485
EMC4	0.31801385	4.67722731	2.25137849	0.028571014	0.2098873	-4.3790647
NWD1	1.08312782	-0.7219904	2.25126831	0.028578449	0.2098873	-3.5084542
EDEM1	0.28476774	7.10539853	2.25099171	0.028597122	0.2098873	-4.5008911
RNF11	0.23021364	4.57080511	2.25077632	0.02861167	0.2098873	-4.3977595
ATP6V1B2	0.20586213	7.09176034	2.25042516	0.028635404	0.2098873	-4.5023463
PIAS3	0.26516996	5.20139936	2.25027059	0.028645855	0.2098873	-4.477463
UBN1	-0.2670147	5.0974746	-2.2497552	0.02868073	0.2098873	-4.4441367
MAP2K3	0.20731304	6.43463629	2.24965374	0.028687601	0.2098873	-4.5143268
ENSCAFG00000018879	0.46671752	5.71099138	2.24956358	0.028693706	0.2098873	-4.5034231
RAD23A	0.19196266	6.59744195	2.24853343	0.028763555	0.2102005	-4.5183828
TEX10	-0.2626961	4.623447	-2.24844	0.0287699	0.2102005	-4.3725351
MEIOB	-0.6629394	1.37416871	-2.2480482	0.028796505	0.21022951	-3.6958328
SETD2	-0.1989055	6.36041439	-2.2478901	0.028807252	0.21022951	-4.5193298
GNGT2	-1.6383813	-2.2544939	-2.247086	0.028861947	0.21043189	-3.4530386
CNTFR	1.14361342	-0.8427204	2.24699136	0.028868395	0.21043189	-3.4687717
UBE2Q2	0.27655922	4.64587028	2.24617953	0.028923724	0.21069633	-4.4434908
PCYT1A	0.20797365	5.22985475	2.24596841	0.028938128	0.21069633	-4.4866972
USP16	-0.217173	5.55059718	-2.2447701	0.029020004	0.21108817	-4.494379
ENSCAFG00000004924	0.40308178	2.00600443	2.24459166	0.029032215	0.21108817	-3.7371618
OLFML2A	-1.3772863	4.32933087	-2.2440386	0.029070089	0.21108817	-4.4153969
RAB7A	0.17006084	7.66070726	2.24400992	0.029072052	0.21108817	-4.4884905
SPOPL	0.29998978	5.34259477	2.24395615	0.029075737	0.21108817	-4.4998123
SPCS2	-0.1605977	6.04716909	-2.243352	0.029117161	0.21114881	-4.5221276
USP14	0.37481951	6.6702279	2.24334544	0.029117615	0.21114881	-4.5249651
CLYBL	-0.4406151	2.6596525	-2.242148	0.029199884	0.21162355	-3.9947057
NR1D1	0.33885846	5.75433307	2.24168552	0.029231709	0.21173238	-4.508448
P2RX1	1.23681954	-2.214232	2.24065503	0.02930274	0.21212489	-3.4657712
TNC	2.09949307	7.25738736	2.24018067	0.029335487	0.21223595	-4.4121155
CHEK1	-0.4038066	3.5902025	-2.2399448	0.02935178	0.21223595	-4.1243665
ENSCAFG00000000340	-0.8419594	0.02691359	-2.2393085	0.029395785	0.21243219	-3.5375411
SLC25A28	-0.323366	2.75601859	-2.2382217	0.029471068	0.21285411	-4.0612974

CDC26	-0.3494011	2.56177606	-2.2377276	0.029505358	0.21297965	-3.9664396
PRXL2A	-0.4715698	3.6270672	-2.237267	0.029537349	0.2130271	-4.2289456
DCTD	-0.2415015	4.21997312	-2.2367946	0.029570189	0.2130271	-4.3234638
CACNB2	1.37598281	-0.9166937	2.23672059	0.02957534	0.2130271	-3.4708324
TRIM28	-0.2098739	7.40767371	-2.2365107	0.029589949	0.2130271	-4.5296592
MGST3	-0.4126886	3.95432601	-2.2354508	0.029663794	0.2130271	-4.3556013
ENSCAFG00000025953	-1.0583525	-1.1678195	-2.2354346	0.029664925	0.2130271	-3.5070926
CAMTA2	0.22160928	5.81968324	2.23542482	0.029665608	0.2130271	-4.5384311
LRPPRC	-0.2620151	7.25749583	-2.2354179	0.029666089	0.2130271	-4.5250074
ENSCAFG00000011367	-0.3576137	4.12875217	-2.2353758	0.029669027	0.2130271	-4.3098693
SH3BGRL	0.37861299	4.07078832	2.23520345	0.029681054	0.2130271	-4.3304975
ENSCAFG00000031848	-0.9102747	2.52012118	-2.234316	0.029743043	0.21335044	-4.0857532
ORMDL1	0.28027962	2.92356019	2.23332804	0.029812195	0.21365265	-4.0476296
ENSCAFG00000029405	0.88240003	3.61405053	2.23307079	0.029830223	0.21365265	-4.1064194
ENSCAFG00000000600	-0.3141777	3.05378964	-2.2328869	0.029843118	0.21365265	-4.1350637
NFATC2IP	-0.2077985	4.97603993	-2.2326205	0.029861801	0.21365265	-4.4447927
PLIN3	0.24963774	4.42646043	2.23250392	0.029869984	0.21365265	-4.4259576
PHLPP1	-0.2949188	4.82520679	-2.231516	0.029939389	0.21388675	-4.4618576
CACNB1	0.52353167	1.01962349	2.23141071	0.029946796	0.21388675	-3.6302722
ENSCAFG00000004800	-0.5439516	1.1914738	-2.2310898	0.029969379	0.21388675	-3.6552856
MDFIC	0.46060796	5.64525091	2.23084254	0.029986786	0.21388675	-4.5472192
HNRNPK	-0.1913546	8.85464211	-2.2308308	0.029987616	0.21388675	-4.4480811
ZNF200	-0.2892021	2.7660875	-2.2300384	0.030043464	0.21416382	-4.0917104
KLHDC3	-0.1894996	5.08327937	-2.2279642	0.030190101	0.21492198	-4.4817761
SLC25A46	0.21461548	6.53378176	2.22774075	0.030205934	0.21492198	-4.5602688
ZNF699	-0.4995271	1.86866728	-2.2275343	0.030220571	0.21492198	-3.9098296
ENSCAFG00000031930	-0.3320867	3.4210413	-2.2271376	0.030248714	0.21492198	-4.14609
ENSCAFG00000019914	-0.4494859	1.95718413	-2.2271137	0.030250411	0.21492198	-3.8235941
TC2N	-1.7354155	-2.0404074	-2.2267477	0.030276391	0.21492198	-3.4862457
AGPAT2	0.46011732	4.18821912	2.22651847	0.03029268	0.21492198	-4.1371553
FNDC3B	0.35328344	7.77152016	2.22641606	0.030299958	0.21492198	-4.5295481
PNO1	-0.2640793	4.97816647	-2.2263679	0.030303384	0.21492198	-4.4741276
SLC1A5	-0.2955983	7.29937115	-2.2237031	0.030493328	0.21614742	-4.5649931
MT-ND4	-0.379395	12.4706059	-2.223207	0.030528803	0.21627717	-4.1890355
SORBS2	0.63650214	4.44185373	2.22137561	0.030660086	0.21673027	-4.5688373
SSRP1	-0.2792961	7.02968645	-2.2213144	0.030664482	0.21673027	-4.5682779
SMAP2	0.19475519	5.02040021	2.22127806	0.030667093	0.21673027	-4.5122231
GYS1	0.34237472	5.70729838	2.22119036	0.030673393	0.21673027	-4.5732656
UST	0.36802105	6.89015025	2.2210686	0.030682142	0.21673027	-4.5563225
DDB1	0.10952174	8.17302128	2.22079583	0.03070175	0.21673027	-4.5072123
PEMT	0.41018837	3.28896822	2.22063656	0.030713205	0.21673027	-4.0990373
ASXL1	-0.2006652	6.24040501	-2.2192632	0.030812133	0.21723636	-4.578002
ZNF217	-0.386846	4.34547695	-2.2188782	0.030839914	0.21723636	-4.4244485
PKN2	-0.2260087	5.6010184	-2.2187271	0.030850823	0.21723636	-4.5512796
CGGBP1	-0.2232518	4.61214894	-2.2186844	0.03085391	0.21723636	-4.4479625
CDYL	-0.350909	5.28900241	-2.2180624	0.030898861	0.21739334	-4.5384612
DYNLL1	0.3329106	5.02428257	2.21773407	0.030922611	0.21739334	-4.5247396
AGK	-0.2144133	4.72541263	-2.2176599	0.030927982	0.21739334	-4.4502891
PRICKLE2	0.77706757	4.22521049	2.21686009	0.030985913	0.21767906	-4.4252942
UXS1	0.26771014	5.59494529	2.21529741	0.031099382	0.21821896	-4.5613172

PPP1R21	-0.2511409	5.59509291	-2.215173	0.031108435	0.21821896	-4.5484189
CLCC1	0.52934994	6.14008268	2.21508631	0.031114738	0.21821896	-4.5820285
VWF	-4.1874631	0.51666074	-2.2147134	0.031141882	0.21828778	-3.6634911
LEP	2.07785215	0.81288688	2.21413245	0.03118421	0.21846291	-3.7824284
MFAP3L	1.18333676	-1.4049874	2.21348175	0.031231681	0.21867385	-3.5501446
GPRASP2	0.40753846	3.26235319	2.21179649	0.031354922	0.21941478	-4.1485608
CHP1	0.23189736	5.23916683	2.21151381	0.031375637	0.21943782	-4.5634378
DGKG	0.9516483	0.05748936	2.21100734	0.03141278	0.21957568	-3.5791533
NOP53	-0.3683453	6.90806113	-2.2102078	0.031471491	0.21986406	-4.5883935
ABCG4	0.78775523	0.2264876	2.20990068	0.031494074	0.2198962	-3.626017
TRIP10	-0.3833556	5.22688799	-2.2096469	0.031512744	0.2198962	-4.5744294
PGK1	0.40070302	8.02389169	2.20943329	0.031528464	0.2198962	-4.5158905
HTRA2	-0.5438135	2.9040839	-2.2083475	0.031608485	0.2203323	-4.0050483
POLR1C	-0.288871	4.18164295	-2.2078334	0.031646434	0.22047483	-4.3656411
BARD1	-0.8782663	2.99029557	-2.2072589	0.031688886	0.22060169	-3.9416551
SF3A1	-0.1562402	7.32229415	-2.2071131	0.031699671	0.22060169	-4.5809213
EIF1B	-0.2858101	3.16950216	-2.2063022	0.031759702	0.22079463	-4.1650635
STRN3	0.2973156	5.74041908	2.20626504	0.031762453	0.22079463	-4.5873045
PTP4A3	0.99586397	-0.514082	2.20525668	0.031837246	0.22117073	-3.5882491
SPCS1	0.23152431	5.45072008	2.20506237	0.031851676	0.22117073	-4.5677209
GON7	0.32108041	3.50967747	2.20468303	0.031879864	0.2212445	-4.308417
HEBP2	-0.610009	2.74883865	-2.2037567	0.031948788	0.22147683	-3.8234683
ENSCAFG00000025105	0.37572784	3.82060265	2.20362439	0.031958646	0.22147683	-4.442743
HERC6	-0.4956288	3.15119223	-2.2035245	0.03196609	0.22147683	-4.189072
SUN2	0.19519061	7.08001777	2.20286402	0.032015337	0.22169609	-4.6053593
MGA	-0.2667357	4.82652279	-2.202209	0.032064242	0.22191274	-4.5242318
HSD17B6	0.57548224	1.5953512	2.20152664	0.032115264	0.22203776	-3.841518
NCR3LG1	0.34481327	6.67203163	2.20136339	0.03212748	0.22203776	-4.6021067
LHFPL1	-1.2910271	-2.8103333	-2.2010815	0.032148585	0.22203776	-3.5278753
RPL7	-0.2530648	8.68817584	-2.201025	0.032152816	0.22203776	-4.5141445
SMARCE1	-0.3158412	4.35459653	-2.2000044	0.032229334	0.22240611	-4.4376196
RUNX1	0.89763584	3.86579593	2.19984277	0.03224147	0.22240611	-4.1653042
EHHADH	-0.341345	4.19670756	-2.1988077	0.032319266	0.22282073	-4.4614422
ENSCAFG00000031852	0.3030455	5.68005395	2.19805248	0.032376132	0.22309068	-4.6118239
MTMR4	-0.2821725	5.8077587	-2.197333	0.032430389	0.22334236	-4.6134342
GPR107	0.23694862	5.75450371	2.19629745	0.032508623	0.22351428	-4.6098926
GAPDH	0.26964454	10.6911976	2.19599534	0.032531478	0.22351428	-4.3639579
RBMX	-0.3512592	4.91019417	-2.1955339	0.032566411	0.22351428	-4.5026509
R3HDM1	-0.2088849	5.77936303	-2.1947285	0.032627473	0.22351428	-4.6101023
SUPT20H	-0.1799464	5.48394237	-2.1942777	0.032661687	0.22351428	-4.5890112
DNASE1L1	0.29901031	5.32758487	2.19409378	0.032675659	0.22351428	-4.5987742
YIPF1	0.2011746	4.4133345	2.19404623	0.032679272	0.22351428	-4.4907891
BAHD1	0.334967	4.13203296	2.19402218	0.032681099	0.22351428	-4.4818011
ERF	0.35500052	5.26572899	2.19399281	0.032683331	0.22351428	-4.5870315
PRSS23	0.51760294	8.33735277	2.19396786	0.032685227	0.22351428	-4.537293
CDKN2A	0.64503196	5.54109181	2.19362874	0.032711005	0.22351428	-4.6274529
PNP	-0.5165804	7.7815213	-2.1933834	0.032729669	0.22351428	-4.5467339
RITA1	0.26847141	4.70694518	2.19328186	0.032737392	0.22351428	-4.5152507
ENSCAFG00000008484	0.41273089	4.48238286	2.19318648	0.032744651	0.22351428	-4.5271871
TIMM22	0.27335713	4.95073824	2.19317725	0.032745354	0.22351428	-4.5606385

TCTN2	0.3201221	5.25364795	2.19275433	0.032777557	0.22351428	-4.5995409
CD63	0.22338663	9.44983837	2.19256924	0.032791659	0.22351428	-4.4622697
CES2	0.87257374	0.3924751	2.19231581	0.032810978	0.22351428	-3.8041545
ENSCAFG00000007260	0.2218061	4.62025123	2.19222674	0.03281777	0.22351428	-4.5230517
SLC26A2	-0.4474975	2.52083306	-2.1921118	0.032826535	0.22351428	-4.0295043
MAP1A	0.72640515	8.26467407	2.19207689	0.0328292	0.22351428	-4.5337603
NDUFAF6	0.26440907	3.63669508	2.19180765	0.032849744	0.22351428	-4.3122983
LDLR	0.31091838	8.88852787	2.19162767	0.032863484	0.22351428	-4.515238
MYO9A	-0.2631292	5.36482671	-2.1906261	0.03294004	0.22377506	-4.5994925
PCSK5	0.76187554	4.93155421	2.19026654	0.032967557	0.22377506	-4.5992649
AKAP2	0.38783556	8.43564498	2.19024152	0.032969473	0.22377506	-4.5243156
MAPK10	1.29068594	-1.2028343	2.19019691	0.032972889	0.22377506	-3.663632
CYFIP2	0.78746493	3.52184346	2.18961698	0.033017326	0.22395597	-4.2145741
ENSCAFG00000013060	0.38166241	4.76307204	2.18916267	0.033052173	0.22406109	-4.5671379
RBM38	0.45270741	2.85485275	2.18883135	0.033077608	0.22406109	-4.2308406
PTGIS	0.40430193	10.4861094	2.18871962	0.033086189	0.22406109	-4.3918271
KAT5	-0.2102993	4.88290664	-2.1883481	0.033114738	0.22413393	-4.5582383
TDRKH	-0.3732371	3.33086605	-2.1877836	0.033158154	0.22424702	-4.2560267
MINDY3	0.33875661	4.69185078	2.18743828	0.033184738	0.22424702	-4.5259826
ATP6V1F	0.32932693	5.31029956	2.18733515	0.033192681	0.22424702	-4.5925036
SAV1	-0.4453093	4.92643201	-2.1872056	0.033202659	0.22424702	-4.5885513
TCP11	-0.702811	0.66529014	-2.1866965	0.033241907	0.22439178	-3.6625394
TTC28	-0.2414184	5.26469914	-2.1859728	0.033297764	0.22464844	-4.589238
ENSCAFG00000015122	-0.2490913	8.38429279	-2.1855389	0.033331296	0.22475428	-4.5649911
TSPAN15	-1.4148647	-2.482014	-2.1851988	0.0333576	0.2248113	-3.5543421
UROD	0.25526394	5.78312304	2.18433641	0.033424376	0.22514088	-4.6411632
FAM89B	0.56599167	4.51626218	2.18405214	0.033446414	0.22516891	-4.5760033
CYTH1	-0.2868551	4.94571455	-2.1837012	0.033473635	0.22523179	-4.6039843
EMB	1.30771799	-2.0526989	2.18320197	0.033512397	0.22537221	-3.5694359
KNOP1	-0.3101364	3.3554475	-2.1823547	0.033578269	0.2256947	-4.2463904
RGS16	-1.3806507	-2.3622269	-2.1817772	0.033623239	0.22587643	-3.5583879
ZBTB14	-0.2970563	3.39346943	-2.1813134	0.033659389	0.22599875	-4.2962692
PHACTR2	-0.5311768	4.69403208	-2.1800986	0.033754229	0.2265148	-4.5280486
CDC37L1	0.26632487	4.29094296	2.17692672	0.034002991	0.22797625	-4.5267178
PSMB4	0.14471379	6.49665446	2.17686045	0.034008206	0.22797625	-4.6648268
EHD4	-0.4462412	8.50884408	-2.1765692	0.034031133	0.22800859	-4.5628724
MT-ND3	-0.4124623	8.73123184	-2.1760999	0.034068102	0.22801936	-4.563084
SP1	-0.2143309	6.80312632	-2.1758316	0.034089251	0.22801936	-4.660891
NTNG2	0.98814192	1.83895764	2.17577486	0.034093726	0.22801936	-3.898057
PACSIN3	0.499193	3.73647082	2.17563002	0.03410515	0.22801936	-4.3275803
PDZRN4	1.5874133	-1.0770693	2.17498906	0.034155747	0.22823649	-3.5869173
UBE2H	0.30977616	4.74756948	2.17457424	0.034188528	0.22833441	-4.6005671
CPSF6	-0.2115562	5.38794178	-2.1739466	0.034238183	0.22854486	-4.6231303
EPHB4	-0.4384457	5.29651418	-2.1735434	0.034270111	0.22863682	-4.6231455
ENSCAFG00000000225	1.09436853	-0.6247329	2.17276633	0.034331719	0.22874207	-3.6026372
SF3B3	-0.1873769	7.23131152	-2.1726912	0.034337683	0.22874207	-4.6618116
ENSCAFG00000006238	-0.3321256	3.42508828	-2.1726574	0.034340366	0.22874207	-4.3120547
ENSCAFG00000017563	0.58298837	2.82399602	2.1721195	0.034383078	0.22884857	-4.108935
ENSCAFG00000015899	-0.9500146	0.71034182	-2.1719985	0.034392692	0.22884857	-3.6359799
ERGIC2	0.18863332	4.97010483	2.17148029	0.034433897	0.22900178	-4.5931429

SIPA1L1	0.38162887	7.90634617	2.17088485	0.034481297	0.229196	-4.6124309
ENSCAFG00000005538	0.60716772	3.44548275	2.17062266	0.034502187	0.22921389	-4.3079786
ENSCAFG00000016072	1.09880411	4.13832829	2.17038722	0.034520954	0.22921768	-4.2747154
PPA2	-0.291182	3.37787364	-2.1700811	0.034545368	0.2292254	-4.3086052
PCF11	-0.2745178	4.48383202	-2.169912	0.034558862	0.2292254	-4.5854336
MSMP	-1.6013363	-2.4494956	-2.1696884	0.034576712	0.2292254	-3.5774285
DSG2	0.55690002	4.54405359	2.16940131	0.034599642	0.22925675	-4.4293803
TMEM156	-1.3931249	-0.9392814	-2.1683986	0.034679829	0.22966726	-3.6283762
TAP1	-0.2996097	4.84550871	-2.1676329	0.034741178	0.22995264	-4.5565977
CSDC2	0.94136436	0.26196036	2.16726913	0.034770355	0.23002489	-3.7180616
TNK2	0.48636279	4.6082484	2.16701604	0.034790668	0.23003845	-4.5922028
DCLRE1A	-0.2169028	4.82441085	-2.1649407	0.034957628	0.23082956	-4.576816
STARD3	0.19912194	5.33602135	2.16487953	0.034962557	0.23082956	-4.6421947
TINF2	-0.3118051	4.44705372	-2.1648456	0.034965291	0.23082956	-4.4969936
SUCLG2	-0.1931494	6.82676077	-2.1644491	0.034997275	0.23085628	-4.6858137
PTK2B	0.37438825	4.43495429	2.16434111	0.035005993	0.23085628	-4.678036
MX2	-1.4858413	-0.9177766	-2.1634223	0.035080223	0.23119986	-3.5924258
GNG10	0.42540113	4.30821263	2.16324206	0.035094802	0.23119986	-4.5317109
CTSA	0.33688921	7.36463722	2.16236932	0.035165465	0.23154428	-4.6664582
ENDOD1	0.29810636	5.10290072	2.16175409	0.035215354	0.23175162	-4.6228823
SLC4A2	0.25499538	7.24025457	2.16142446	0.035242109	0.23180658	-4.6828196
PEX5	0.18939822	5.42682674	2.16109403	0.035268947	0.23186203	-4.6589945
ZCCHC9	0.49507777	1.01848694	2.16065571	0.035304575	0.23195788	-3.8220164
ENSCAFG00000011119	0.88423695	-0.3686976	2.16046167	0.035320357	0.23195788	-3.6458699
SLC44A2	0.3805109	8.20395886	2.15943954	0.035403595	0.23232425	-4.6217177
CCDC138	0.40418295	1.50962547	2.15932379	0.035413032	0.23232425	-3.9411995
IMP4	-0.3033461	5.42616318	-2.1588449	0.035452099	0.23245947	-4.6502213
KREMEN1	0.47059073	3.5893987	2.15814897	0.03550894	0.23271103	-4.4023136
ENSCAFG00000031402	0.29059852	5.61704641	2.15602418	0.035682979	0.23368826	-4.676001
COL8A2	1.04582248	1.2560848	2.15579678	0.035701649	0.23368826	-3.7655027
USHBP1	-1.3587837	-2.5091245	-2.1555662	0.035720586	0.23368826	-3.601812
LHX9	1.59345101	-0.1351529	2.15503026	0.035764645	0.23368826	-3.6254966
MAZ	-0.3663097	4.65251962	-2.1550008	0.035767066	0.23368826	-4.5029655
CYP1B1	0.77344192	8.40251439	2.15497284	0.035769369	0.23368826	-4.6124103
SFXN4	-0.5170565	2.37843753	-2.1546459	0.035796268	0.23373647	-4.0573863
FGFR1	0.20445736	7.46807002	2.15443227	0.03581386	0.23373647	-4.672233
NT5C	-0.3849004	4.12400491	-2.1539804	0.03585109	0.23377746	-4.4304094
NPPB	1.91817835	-1.2375324	2.15381616	0.035864627	0.23377746	-3.6070655
METTL18	-0.3431393	2.59059149	-2.1536804	0.035875819	0.23377746	-4.1574134
ENSCAFG00000018611	0.24606754	6.02724995	2.15334156	0.035903777	0.23383866	-4.7115509
ZBTB38	-0.2925489	6.22868304	-2.1530008	0.035931906	0.23390092	-4.7061938
RIOK3	-0.2605044	5.4680917	-2.1523065	0.035989286	0.23415344	-4.6729789
IGSF9	-0.7272789	2.48179915	-2.1519794	0.036016348	0.23420853	-3.8923212
CFL2	0.39950471	8.05506371	2.15078112	0.036115636	0.23454516	-4.6525684
CDC42EP3	0.46705365	7.11017506	2.15065335	0.036126237	0.23454516	-4.7023508
ARL6	0.37770561	4.39921325	2.15065248	0.036126308	0.23454516	-4.5269401
VPS13C	-0.2582165	6.57022125	-2.1503245	0.036153532	0.23454516	-4.7167396
NRF1	-0.2210143	3.72702309	-2.150232	0.036161217	0.23454516	-4.4189324
CETN3	-0.3167212	3.9082097	-2.1498888	0.036189726	0.23460926	-4.5556459
CYB5R1	0.31668529	4.94484122	2.14914855	0.036251291	0.23488748	-4.6826813

FAM162A	0.34259421	5.0299856	2.14808277	0.036340091	0.23534079	-4.6362587
TMEM200A	1.19870887	1.98290607	2.14772024	0.036370341	0.23534079	-3.7791469
MYLK	1.31350874	7.11314112	2.14759934	0.036380433	0.23534079	-4.6840333
PDGFA	0.77826523	5.43523877	2.14741307	0.036395988	0.23534079	-4.6068446
MFSD11	-0.2256534	5.92514803	-2.1462862	0.036490209	0.23582897	-4.7140378
ENSCAFG00000002290	0.95676295	-0.3290737	2.14497227	0.036600347	0.23641947	-3.672371
SYPL2	0.92353956	-0.802916	2.14418643	0.036666356	0.23672446	-3.6625402
LDB2	-0.6329969	5.36109251	-2.1436951	0.036707678	0.23686983	-4.6897759
SRRM3	1.69858277	-1.0323056	2.14297076	0.036768676	0.2371234	-3.782089
AGFG1	0.13925833	6.79830327	2.14278155	0.036784625	0.2371234	-4.7280807
POMT2	0.19310039	5.09809976	2.14233341	0.036822421	0.23723761	-4.6592394
NIPBL	-0.2264327	6.58791588	-2.142125	0.03684001	0.23723761	-4.7327446
EBF3	-1.6025715	1.57656284	-2.1411596	0.036921584	0.23764021	-3.9761553
AFAP1	0.36884367	6.94097067	2.14093879	0.036940261	0.23764021	-4.7327662
GLYR1	-0.1563979	6.06039931	-2.1404249	0.036983764	0.23779862	-4.7347091
KIT	-2.2561237	-1.4325999	-2.1399834	0.037021174	0.23791771	-3.6704782
HSPBP1	0.23184819	4.97596671	2.13957459	0.03705585	0.23801912	-4.6580562
ENSCAFG00000031003	0.34640464	6.57300091	2.13835931	0.037159088	0.23856059	-4.7384544
DNAJB4	0.36376482	6.58221462	2.13787476	0.037200321	0.23870134	-4.7388991
PCSK4	0.65826101	-0.3545024	2.13765641	0.037218914	0.23870134	-3.8082193
TXLNA	-0.1281933	6.88926033	-2.1359936	0.037360774	0.23942542	-4.7411887
MFAP3	-0.259877	5.18696724	-2.1358877	0.037369829	0.23942542	-4.6902239
ILF2	-0.2740548	6.24938437	-2.1355954	0.037394819	0.23943827	-4.7428961
TRIM68	-0.3866982	2.62631707	-2.1351186	0.037435622	0.23943827	-4.1890428
SERPINE1	1.31736414	10.7482684	2.1350541	0.037441141	0.23943827	-4.5336196
ZNF22	-0.4571046	2.69427461	-2.134959	0.037449283	0.23943827	-4.2529832
TBC1D8	-0.7879685	3.60165328	-2.1346503	0.037475729	0.23943827	-4.5697144
CEP57	-0.233405	4.86495849	-2.1344011	0.037497084	0.23943827	-4.6367782
SEPT5	0.55638462	6.00021542	2.13430998	0.037504898	0.23943827	-4.737917
KLHL21	0.31964789	4.85088164	2.13345935	0.037577899	0.23978278	-4.7059818
XPO1	-0.2602094	6.69266382	-2.1321585	0.037689775	0.24037489	-4.7520557
NME7	-0.3822863	2.31510035	-2.1313687	0.037757843	0.24068714	-4.1151463
MRPS9	-0.1628666	5.30565183	-2.1309531	0.037793709	0.24079391	-4.7112468
ENSCAFG00000007340	-0.1900807	5.26485585	-2.1299941	0.037876572	0.24110839	-4.7167159
CD34	-2.4110371	1.01024607	-2.1299388	0.037881351	0.24110839	-4.0775706
CTRL	-0.8215509	-0.2868209	-2.1294596	0.037922821	0.24125043	-3.6999542
PLEKHB2	0.19995778	7.43778777	2.1290454	0.037958701	0.24125556	-4.7268592
USP31	-0.3482213	4.1089729	-2.1287553	0.037983842	0.24125556	-4.55316
SPATA7	-0.2277036	4.65145827	-2.1285877	0.037998382	0.24125556	-4.6377392
APBB3	-0.9305113	1.67809756	-2.1285663	0.03800024	0.24125556	-4.1704673
IL12A	-0.7357273	1.1271824	-2.1269714	0.038138789	0.24201319	-3.9648395
ENSCAFG00000031645	0.89848206	0.51568937	2.12614444	0.038210808	0.24207577	-3.841848
TAX1BP3	0.2061371	6.17467949	2.1261241	0.038212581	0.24207577	-4.7659054
LIN7B	0.72766352	0.79795794	2.12589162	0.038232848	0.24207577	-3.8662556
C18H11orf49	0.37432754	3.69239039	2.12586357	0.038235294	0.24207577	-4.4434435
CDKAL1	-0.2413159	3.62206211	-2.1255141	0.03826578	0.24207577	-4.4077701
ZNF451	-0.2420251	4.70271137	-2.1252967	0.038284754	0.24207577	-4.656212
GPS1	0.23284933	6.46373168	2.12528242	0.038286004	0.24207577	-4.7675766
YTHDC2	-0.211674	5.2529279	-2.1250947	0.038302399	0.24207577	-4.6974874
TGFBR1	0.30937699	7.60939922	2.12448777	0.038355436	0.24228746	-4.7492131

RNASEL	-0.3729921	3.60465068	-2.1242714	0.038374363	0.24228746	-4.598116
NAP1L3	1.05098427	-0.7475436	2.12390832	0.038406136	0.24236658	-3.6750597
MYOM1	1.29225212	0.8905568	2.12114437	0.038648776	0.24370713	-3.783757
ENSCAFG00000024406	-0.4731105	2.45528032	-2.121048	0.038657259	0.24370713	-4.1229987
EVPL	0.4677533	1.62697804	2.12001797	0.038748044	0.24414799	-3.9928663
AGPAT5	-0.1786878	5.91006272	-2.119815	0.038765955	0.24414799	-4.768863
KIF1B	0.22977225	6.73212913	2.11800736	0.038925789	0.24493143	-4.7748003
PSMD8	0.20490235	7.10928678	2.1179684	0.038929241	0.24493143	-4.7726776
GRK7	1.15808751	-0.9490629	2.11721417	0.038996106	0.24522963	-3.6902204
ENSCAFG00000005575	0.80855327	5.83084676	2.11650618	0.039058964	0.24550235	-4.7804534
ENSCAFG00000000215	-0.1987964	7.02890483	-2.1158679	0.039115709	0.2457364	-4.7752993
ITGB5	0.53716306	8.41864705	2.11526169	0.039169668	0.24595271	-4.7015901
NQO1	-0.4333584	8.45451983	-2.1147455	0.039215663	0.24601795	-4.6797433
SLC25A26	-0.38523	2.3106038	-2.1147068	0.03921912	0.24601795	-4.166549
RBM12B	-0.2661908	3.29558221	-2.1141327	0.03927033	0.24615565	-4.3917983
IQCK	-0.7562683	0.87987494	-2.1140227	0.039280157	0.24615565	-3.8396132
APMAP	0.195641	4.98172911	2.11328445	0.039346118	0.2464464	-4.7138566
LZIC	-0.2108362	5.31614624	-2.1127995	0.039389502	0.24659551	-4.7512651
UBE2V1	0.20692282	5.88260353	2.11222575	0.039440881	0.24669429	-4.7870825
TTL	-0.2306566	5.41216601	-2.1121442	0.039448189	0.24669429	-4.7372168
TENT4A	-0.1721159	5.84659044	-2.1118325	0.039476135	0.24669429	-4.782472
VTA1	-0.1955348	5.03417455	-2.1113275	0.039521441	0.24669429	-4.7232117
WSB2	0.28248054	6.46563784	2.1109229	0.039557773	0.24669429	-4.7922956
RCN3	0.52306185	6.60524034	2.11091337	0.039558629	0.24669429	-4.7885796
ATF1	-0.1962994	4.28319902	-2.1109029	0.039559574	0.24669429	-4.6098049
PSMB10	-0.363634	3.57105999	-2.1107965	0.039569126	0.24669429	-4.4809764
SYNPO	0.57996768	7.54785587	2.11037052	0.039607423	0.24669429	-4.7935012
METTL3	-0.2200862	4.73252098	-2.1102782	0.039615731	0.24669429	-4.6616906
TAB3	0.28466189	4.69039439	2.11017359	0.039625138	0.24669429	-4.6709366
ARHGAP12	-0.2871294	4.95988558	-2.1100016	0.039640616	0.24669429	-4.7436133
CDKN1B	-0.490114	4.51926819	-2.1097875	0.039659887	0.24669429	-4.617916
SYNE3	-0.8085033	4.57829124	-2.1093116	0.039702758	0.24683906	-4.7390705
KLHL23	0.28867084	3.39106285	2.10896855	0.039733683	0.24689843	-4.4577053
MAP4K4	-0.3013446	8.47828571	-2.1087709	0.039751509	0.24689843	-4.6873912
TUBGCP3	-0.162879	5.40023538	-2.1080361	0.039817853	0.24708977	-4.74344
KDM2B	-0.3157759	3.97715464	-2.1078659	0.039833234	0.24708977	-4.5257422
CADM4	0.86552938	1.89295003	2.10748161	0.039867979	0.24708977	-3.9479239
DEPTOR	-1.1139807	1.61451455	-2.1074545	0.039870428	0.24708977	-3.8620592
STT3B	0.29188309	8.80494503	2.10734432	0.039880399	0.24708977	-4.7245421
AMFR	0.13085142	7.41007196	2.10585123	0.040015686	0.24780609	-4.7870912
MTMR6	0.2074454	5.9282548	2.10439411	0.040148099	0.24850391	-4.7973861
ENSCAFG00000032160	0.80056736	1.78372913	2.10335828	0.040242462	0.24862769	-3.9507228
BGLAP	0.99375962	0.83859313	2.10324377	0.040252905	0.24862769	-4.0697718
STAG1	-0.259932	5.58030262	-2.1031917	0.040257656	0.24862769	-4.7667072
RPS19	-0.2500866	9.11983009	-2.1030173	0.040273565	0.24862769	-4.678162
LGALSL	1.32954002	-0.3973356	2.10298841	0.040276203	0.24862769	-3.7094854
SYAP1	0.30962758	5.56655823	2.10287527	0.040286529	0.24862769	-4.7754697
FZD9	1.17680371	-0.3078046	2.10211863	0.040355645	0.2488824	-3.7184263
ZFP64	-0.2319712	4.74293888	-2.101991	0.040367318	0.2488824	-4.6896478
ENSCAFG00000009915	-0.6703873	0.12473576	-2.1016076	0.040402387	0.24897674	-3.7835586

BAZ2B	-0.3231055	3.72963061	-2.1009892	0.040459006	0.24914415	-4.5076821
DHX30	0.17150051	6.37603325	2.10087892	0.040469112	0.24914415	-4.8140824
ENSCAFG00000029292	1.17705365	1.54039865	2.09934378	0.040610004	0.2498894	-4.3156009
CSTF3	-0.2739385	4.35207574	-2.0987996	0.040660053	0.2500173	-4.679511
EXOC6B	0.49123521	6.09902279	2.09868619	0.040670487	0.2500173	-4.81634
NCOA2	-0.3000438	5.14836463	-2.0980404	0.040729963	0.25026079	-4.7502642
GDF6	1.23011366	4.13756539	2.0973969	0.0407893	0.25050318	-4.5863228
TSEN2	-0.3606342	3.45097427	-2.0971011	0.0408166	0.25053897	-4.461885
ENSCAFG00000031879	1.02403091	-1.009688	2.09690285	0.040834908	0.25053897	-3.7094367
STK32B	-1.1103639	-0.5236849	-2.096461	0.040875735	0.25066736	-3.7637663
ZNF582	-0.2973376	3.12339841	-2.0960928	0.040909789	0.25075411	-4.371306
C20H19orf44	-0.5876968	4.44422765	-2.095107	0.041001065	0.25119135	-4.7624987
MYCBPAP	0.75522585	0.65953867	2.09427809	0.041077961	0.25141034	-3.9453603
PFAS	-0.3033571	5.7787013	-2.0941089	0.041093672	0.25141034	-4.8025773
MRPS35	-0.2734177	4.31575666	-2.093815	0.041120978	0.25141034	-4.6349132
DHRS9	0.83599197	2.89643423	2.093739	0.041128038	0.25141034	-4.6132413
HDHD2	0.20858972	4.57217204	2.0936468	0.041136607	0.25141034	-4.6792268
CTTN	0.18743765	8.47982679	2.09321704	0.041176572	0.25153254	-4.7411245
RIF1	-0.2513333	6.0617686	-2.0927911	0.041216213	0.25165265	-4.8254106
GRIK5	1.40886707	1.92791777	2.0921532	0.041275649	0.25181985	-3.8700428
FBXO31	0.23025229	4.02644754	2.09206812	0.041283582	0.25181985	-4.592398
ENSCAFG00000005629	-0.3894513	2.54596453	-2.0917692	0.04131146	0.25183472	-4.2184469
SSNA1	-0.3484966	2.46417963	-2.0915583	0.041331144	0.25183472	-4.2207222
ZNF70	-0.2695945	3.90204742	-2.0911927	0.041365279	0.25183472	-4.5677523
PHTF1	-0.2212517	5.16469866	-2.0911851	0.041365992	0.25183472	-4.7754847
MRPS6	0.52253698	4.43632595	2.09010057	0.041467401	0.25213876	-4.5551971
IGDCC4	1.11086936	4.92270757	2.09009267	0.041468141	0.25213876	-4.4824965
DTD2	-0.4585845	3.32194654	-2.0900089	0.041475985	0.25213876	-4.4377683
ENSCAFG00000013214	0.23236846	9.80771008	2.0895871	0.04151549	0.25225717	-4.6506639
GABRA3	-1.5144969	-1.0644946	-2.0891348	0.041557886	0.25233025	-3.7070984
ING5	-0.4904156	1.16051023	-2.0890315	0.041567582	0.25233025	-4.0068646
WIPI2	0.1679336	5.94506918	2.08872272	0.041596552	0.25238447	-4.8364804
DFFB	-0.3950004	2.20855043	-2.0881084	0.041654242	0.25261282	-4.2297301
ENSCAFG00000006946	0.18128322	6.04763338	2.08723577	0.04173632	0.25298878	-4.839651
SEPT2	-0.1141031	7.95028673	-2.0858615	0.041865859	0.2534611	-4.7898881
ENSCAFG00000031225	1.07191753	-0.2525833	2.08581367	0.041870376	0.2534611	-3.7788277
SH3KBP1	0.71402128	6.53585106	2.08576886	0.041874607	0.2534611	-4.8315902
DGAT2	0.70997158	0.50952168	2.08503014	0.041944397	0.25376159	-4.0773522
TRPS1	0.57674645	3.5111013	2.08436675	0.042007157	0.25396409	-4.5890312
SPTAN1	0.27076399	9.80947261	2.08416234	0.042026511	0.25396409	-4.6490154
PHACTR1	0.88519864	2.82280078	2.08403728	0.042038356	0.25396409	-4.4210428
TARBP2	-0.2904445	4.8521096	-2.0836102	0.042078825	0.25408025	-4.7189172
ZNF34	-0.3179638	2.96341332	-2.0833416	0.042104297	0.25408025	-4.3762597
KHDRBS1	-0.2745161	5.6736118	-2.0828376	0.042152132	0.25408025	-4.8244459
SOCS5	0.33405851	6.58672035	2.08279523	0.042156156	0.25408025	-4.8467001
TUFM	-0.259412	6.1474339	-2.0827712	0.042158441	0.25408025	-4.8480841
HCLS1	-1.7088174	-1.7005796	-2.0824745	0.042186625	0.25412852	-3.7542908
SP4	-0.3718936	3.50077697	-2.0822189	0.042210915	0.25415329	-4.5382264
SLC9A5	0.35042141	3.008812	2.0817587	0.042254684	0.25420812	-4.4952517
PCBP1	-0.1854312	6.51081298	-2.0816988	0.042260385	0.25420812	-4.8526102

SWT1	0.40379352	3.09730485	2.08029218	0.042394426	0.25477413	-4.4284928
CFL1	0.25927323	7.6585593	2.08028686	0.042394934	0.25477413	-4.8182069
FMO4	0.33699914	3.27267203	2.07965339	0.042455421	0.25493278	-4.518574
UBE3D	-0.3466771	3.15280917	-2.0794638	0.042473542	0.25493278	-4.4004194
SLC39A3	0.28985957	3.36902276	2.07890165	0.042527299	0.25493278	-4.418915
PAXX	0.37794228	4.77350966	2.07881536	0.042535556	0.25493278	-4.7694054
TTYH2	0.67923178	4.19851406	2.07879658	0.042537353	0.25493278	-4.5903137
HSPA12B	-1.2820851	-2.3268282	-2.078668	0.042549666	0.25493278	-3.7204701
IL1RAP	-0.583881	2.88685407	-2.0785286	0.042563008	0.25493278	-4.2654648
C10H22orf23	-0.8079673	-0.6652833	-2.0781801	0.042596386	0.25501144	-3.8007475
SYNE2	-1.0634942	3.43358945	-2.0777126	0.042641204	0.25509159	-4.3557315
FNBP1	0.4157592	6.73208914	2.07754374	0.042657404	0.25509159	-4.8598387
G6PC3	0.25096511	4.78179424	2.07690254	0.042718959	0.25509159	-4.7605538
CDH3	0.90826503	-1.4501793	2.07676371	0.042732298	0.25509159	-3.8003137
C14H1orf35	0.27835733	3.18613762	2.07668869	0.042739506	0.25509159	-4.4029255
SZRD1	0.2406311	3.81229298	2.076524	0.042755336	0.25509159	-4.5582219
SLC10A6	-0.8715054	0.55953506	-2.0763891	0.042768306	0.25509159	-3.9504732
ENSCAFG00000003645	-0.7004045	0.41349227	-2.0763422	0.042772816	0.25509159	-3.8159528
C7H1orf21	0.39269241	4.52737004	2.07614234	0.042792039	0.25509159	-4.5913824
SPNS3	-1.3366568	-0.2886098	-2.0756362	0.042840751	0.25526116	-3.7541701
SLC20A2	0.37964815	3.9133812	2.075045	0.042897721	0.25547976	-4.6727827
ENTPD4	0.26115798	4.92858729	2.07334536	0.043061855	0.25602434	-4.7930346
RALB	-0.2393214	5.63223486	-2.0731818	0.043077681	0.25602434	-4.8523594
PARVA	0.21765513	6.82703924	2.0731748	0.043078356	0.25602434	-4.8565297
ENSCAFG00000002523	-0.8471637	0.94924161	-2.0731296	0.043082727	0.25602434	-3.8383686
HM13	0.26857634	7.52996544	2.07303281	0.043092097	0.25602434	-4.8346074
FSTL3	0.6637184	6.19069632	2.07283634	0.043111117	0.25602434	-4.856219
ENSCAFG00000032585	-0.3700658	2.31673364	-2.0724276	0.043150714	0.25613873	-4.2359999
PARP16	-0.498691	2.1152211	-2.0713234	0.04325783	0.25665362	-4.2648663
CPLX2	-1.4777076	0.26205595	-2.0706509	0.043323177	0.25692031	-3.7671732
ARID5B	0.31188821	6.3374242	2.0696145	0.043424061	0.2573974	-4.8746542
CDC25B	-1.3334034	4.92963499	-2.0691849	0.043465935	0.25752442	-4.7487926
INHBA	1.26496974	7.27957303	2.06616833	0.043760978	0.25915058	-4.8728792
EPB41L5	0.58964857	1.53206546	2.0658271	0.043794461	0.25922699	-4.0574531
EDC4	-0.1693449	6.25393693	-2.0655269	0.043823936	0.25927961	-4.8817552
NBAS	-0.2703826	6.53943235	-2.0646207	0.043913021	0.2596847	-4.8852615
ABR	0.22323186	7.68223355	2.06422163	0.043952299	0.25979501	-4.8329461
FASTKD5	-0.3073039	3.38622944	-2.0638198	0.043991879	0.25982469	-4.5226435
RUBCNL	1.37874751	-1.5556299	2.06355734	0.044017753	0.25982469	-3.7415698
PAIP2	-0.2225773	5.58395237	-2.0634945	0.044023948	0.25982469	-4.8635221
TRIM63	0.94104213	1.74534154	2.06333345	0.044039832	0.25982469	-4.1500357
ENSCAFG00000008815	0.26822391	3.33344245	2.0628236	0.044090146	0.25996802	-4.5549907
ENSCAFG00000023251	1.26475303	-1.4904763	2.06266909	0.044105404	0.25996802	-3.755542
AKAP11	0.17582694	6.29715838	2.0621764	0.044154088	0.26013325	-4.890618
ZNF366	-0.6872407	2.51422823	-2.0615884	0.044212249	0.26035413	-4.5586514
ESYT3	1.00133406	-1.3175732	2.06132733	0.044238095	0.2603846	-3.8163206
CSGALNACT1	-0.5982382	6.79909972	-2.0611114	0.044259483	0.26038881	-4.8831072
KCNJ2	-1.3863985	-1.2443892	-2.0608954	0.044280881	0.26039308	-3.7694743
MDGA1	1.01788563	2.50688425	2.06001803	0.04436791	0.26066231	-4.456744
POMT1	0.22588884	5.02958669	2.05993109	0.044376541	0.26066231	-4.7929359

SMAD3	-0.4196155	5.55659356	-2.0598082	0.044388747	0.26066231	-4.8547582
YAP1	0.19488218	7.46939136	2.05802415	0.044566235	0.26152369	-4.8716588
RHOF	0.4399754	1.57760039	2.05791656	0.044576958	0.26152369	-4.1327039
EEA1	-0.2687245	7.82751107	-2.0567393	0.044694446	0.26209089	-4.8518424
TACO1	0.25829027	2.62865685	2.05593643	0.044774718	0.26243943	-4.3463738
ZNF655	0.28309153	5.02129234	2.05531144	0.044837294	0.26268398	-4.8205159
SMG8	-0.2503743	4.14475585	-2.054546	0.044914041	0.26301128	-4.6900819
DKKL1	0.99760411	-0.927874	2.05342967	0.045026164	0.26354534	-3.8309743
PEX11G	0.7315273	0.71323495	2.05286416	0.045083058	0.26365143	-3.9479592
BSG	0.36483613	8.91767941	2.05283342	0.045086153	0.26365143	-4.764917
SEPHS1	-0.2134653	6.43563289	-2.0525485	0.045114841	0.26369677	-4.9075004
SLC39A9	0.17237437	6.05426037	2.05227039	0.045142862	0.26373817	-4.9095498
MRPL38	-0.2189693	4.28972992	-2.051137	0.045257211	0.26428365	-4.7093797
USP28	-0.2715621	4.42426144	-2.0508948	0.04528168	0.264304	-4.7752687
CALHM5	0.58044803	4.9613307	2.04987007	0.045385322	0.26478625	-4.7612925
NUP98	-0.1603977	6.79225905	-2.0495795	0.045414755	0.2648353	-4.9080478
HIST2H2AC	-0.8454849	0.27900842	-2.0492407	0.045449081	0.26491283	-3.8611221
TTC17	0.18122571	6.2270643	2.04874252	0.045499607	0.26494232	-4.9163109
HECTD4	0.29344012	5.86813068	2.0487182	0.045502075	0.26494232	-4.9110731
SLC25A39	-0.2932263	6.34961064	-2.0485687	0.045517242	0.26494232	-4.9156085
ITGA1	0.56351892	8.07629681	2.04806388	0.045568512	0.26511824	-4.8608306
CELSR3	0.59481318	1.30358935	2.04740796	0.045635196	0.26533683	-4.2708202
UBP1	-0.1754285	6.69260251	-2.0472352	0.045652778	0.26533683	-4.9166027
UBIAD1	0.34402997	3.88151408	2.04707303	0.04566928	0.26533683	-4.6705265
UQCR10	0.22093209	4.8675899	2.0462859	0.045749466	0.26568017	-4.8213394
USP38	-0.2235778	4.62848266	-2.0458219	0.045796797	0.26571008	-4.7861777
OVOL1	1.35902	-2.0371874	2.04582176	0.045796807	0.26571008	-3.783733
IFT88	-0.4110752	3.54531349	-2.0450781	0.045872748	0.26577503	-4.4923522
COX18	0.29067878	4.22944235	2.04499621	0.045881115	0.26577503	-4.6792443
RAD21	-0.2932964	7.30420961	-2.0448106	0.045900087	0.26577503	-4.8994599
NPM1	-0.2535946	8.12078766	-2.0447335	0.045907973	0.26577503	-4.8612268
NMUR2	1.20812543	0.98303505	2.04467942	0.045913502	0.26577503	-4.161221
TNXB	-1.2380689	3.58182137	-2.0440294	0.04598002	0.26603782	-4.6299764
XPO5	-0.1638425	5.81150252	-2.0429605	0.046089581	0.26654929	-4.91873
EFTUD2	-0.224843	6.40740427	-2.0420701	0.046181025	0.26695557	-4.9278856
ZRSR2	0.32563364	4.15310534	2.04134067	0.046256047	0.26704775	-4.736696
CXHXorf57	-1.3701694	-1.5967137	-2.0412299	0.046267454	0.26704775	-3.785437
RAB12	-0.1834421	4.51983092	-2.0410737	0.04628353	0.26704775	-4.7776351
FADS3	0.2819664	7.94336659	2.04094367	0.046296925	0.26704775	-4.880555
MYD88	-0.2012515	5.03302517	-2.0408849	0.046302976	0.26704775	-4.8526888
ENSCAFG00000007622	-0.3842202	2.34946298	-2.0405784	0.046334567	0.26705526	-4.2604026
FAM198B	0.42454208	7.89310281	2.04043604	0.04634924	0.26705526	-4.8820253
RNF144B	1.49388565	1.17017349	2.04008142	0.046385815	0.26705526	-3.9712948
GNMT	-0.9946129	-1.1828202	-2.0400497	0.046389085	0.26705526	-3.7941855
ATF6	0.24613163	6.34293171	2.03952719	0.04644303	0.26723407	-4.9326061
ITPRIP	0.52325785	5.22097744	2.03933798	0.046462577	0.26723407	-4.8753112
CDYL2	0.86542863	0.99708649	2.03862875	0.046535908	0.26731586	-3.9405578
TMEM206	0.47818843	2.53940598	2.03854181	0.046544905	0.26731586	-4.3600944
TIAM1	-1.3430357	1.78488371	-2.0384165	0.046557873	0.26731586	-4.4300213
ENSCAFG00000030508	0.5483636	4.80530911	2.03837968	0.046561685	0.26731586	-4.7943598

GAB1	-0.3538553	5.89837716	-2.0377867	0.046623107	0.26745056	-4.9162336
ND4L	-0.3520725	7.95121468	-2.0377432	0.046627615	0.26745056	-4.8877199
TTC23	-0.2706504	3.40519161	-2.0374548	0.04665751	0.26750023	-4.5473937
ENSCAFG00000000523	-0.8585891	-0.2311015	-2.0371905	0.046684928	0.26753565	-3.8385118
RPGR	0.6403868	7.21298543	2.03620207	0.046787576	0.26767752	-4.9244917
BABAM2	0.23001384	5.49842788	2.03591535	0.046817388	0.26767752	-4.913006
SARAF	0.18988224	7.80985097	2.03591171	0.046817768	0.26767752	-4.8879424
NOLC1	-0.280494	6.00189351	-2.0359038	0.046818592	0.26767752	-4.9278223
KITLG	-0.4671432	7.74868341	-2.0358919	0.046819828	0.26767752	-4.9056576
ENSCAFG00000030822	0.85272278	1.71623186	2.03539461	0.046871577	0.26767752	-4.0068826
ZSCAN18	0.56296266	4.33661218	2.03523936	0.046887743	0.26767752	-4.6548754
ENSCAFG00000006478	-0.3929316	6.12531961	-2.0351879	0.046893105	0.26767752	-4.9403708
METTL15	-0.3432398	3.72837152	-2.0351126	0.046900944	0.26767752	-4.5841822
VWCE	-0.8799356	2.06747789	-2.0344548	0.046969509	0.26794743	-4.3592012
AMZ2	0.20043093	6.42317996	2.03422063	0.04699394	0.26796545	-4.9439479
PLOD2	0.78791098	9.54284326	2.03191356	0.047235212	0.26921933	-4.8298664
MED6	-0.2384043	3.93273112	-2.0309755	0.047333615	0.26945536	-4.7306742
ENSCAFG00000014958	-0.8340101	-0.5108803	-2.0308179	0.04735017	0.26945536	-3.865758
ZKSCAN2	-0.2356682	4.61052842	-2.0306285	0.047370065	0.26945536	-4.8239143
ENSCAFG00000005456	-0.7293587	0.69916566	-2.0303295	0.047401493	0.26945536	-3.9007965
ADNP2	-0.2139773	5.03836244	-2.0303051	0.047404055	0.26945536	-4.8739955
BRWD1	-0.3458529	3.95745302	-2.0301274	0.047422744	0.26945536	-4.7138368
RAB5C	0.18351474	7.06034311	2.03009292	0.047426368	0.26945536	-4.9392123
C1GALT1C1	0.29028446	3.36523203	2.02957512	0.047480856	0.26964331	-4.559452
STX4	-0.2244259	5.04430556	-2.0291285	0.047527901	0.26977197	-4.8763595
MRPL3	-0.192035	5.08184977	-2.0289535	0.047546345	0.26977197	-4.884944
ARHGAP45	1.03381922	-0.2662474	2.02687968	0.047765373	0.27089268	-3.857159
OXSR1	-0.16866	6.73778408	-2.0246049	0.048006636	0.27183162	-4.960994
KCNQ5	1.78011288	1.21350709	2.02452933	0.048014665	0.27183162	-4.0994822
IBTK	-0.2566418	6.68432745	-2.0242727	0.048041957	0.27183162	-4.9587267
MYO1E	0.41899146	8.1737437	2.02410951	0.048059312	0.27183162	-4.9135129
DBP	0.68998106	2.64340758	2.02396856	0.04807431	0.27183162	-4.2733728
FGF1	1.13084842	-0.4031366	2.02383001	0.048089057	0.27183162	-3.920809
APAF1	-0.2391416	4.17155197	-2.0236901	0.048103952	0.27183162	-4.7778721
SLC1A1	1.71895625	1.30443762	2.02362916	0.04811044	0.27183162	-3.9502795
FAM193B	-0.3136753	4.02610014	-2.0234909	0.04812516	0.27183162	-4.8016304
ELK3	-0.3875528	7.12782594	-2.0230195	0.048175402	0.27189455	-4.9507007
KBTBD3	-0.2845801	3.23561015	-2.0229813	0.048179473	0.27189455	-4.5892263
EIF2AK2	-0.3614271	3.15769721	-2.0223377	0.04824813	0.27216008	-4.5413748
DENND5B	-0.5273528	4.08929491	-2.0216302	0.048323718	0.27241005	-4.6715511
SH2B1	-0.3037707	4.38481936	-2.0211585	0.048374156	0.27241005	-4.8052392
C24H20orf194	0.32781527	3.3476329	2.02107188	0.048383427	0.27241005	-4.6840723
IFT57	0.29295517	3.93399101	2.02087266	0.048404749	0.27241005	-4.742063
FBXL7	-0.3532584	5.80165032	-2.0207903	0.048413565	0.27241005	-4.9410489
SNX31	1.57996154	-0.5411017	2.02070962	0.048422206	0.27241005	-3.8968239
C4H5orf51	-0.2244848	6.7787239	-2.0203726	0.048458302	0.27249141	-4.968951
ARHGAP32	-0.4110104	3.77339615	-2.019378	0.048564978	0.27283141	-4.5668159
CHRDL1	0.61498787	9.31791062	2.01935761	0.048567172	0.27283141	-4.7935859
PCNX4	-0.3725317	3.85352152	-2.0192033	0.048583745	0.27283141	-4.7166124
ATRAID	0.2937996	4.806848	2.018842	0.048622556	0.27284412	-4.8764541

FUT8	0.25868294	5.81332503	2.01860703	0.048647813	0.27284412	-4.9497835
ACSL1	-0.2134442	6.15351725	-2.0184361	0.048666196	0.27284412	-4.9626459
CD38	1.09249218	-1.8527898	2.01837603	0.048672654	0.27284412	-3.8474679
LCAT	0.52295663	5.41057397	2.01710277	0.048809776	0.27349107	-4.9463156
NFKBIZ	-0.4808648	4.74946591	-2.016402	0.04888539	0.27375711	-4.9348244
TMEM119	0.96266478	4.65021539	2.0162097	0.048906151	0.27375711	-4.5668446
SEMA6B	-1.7027769	-1.4049434	-2.0159954	0.048929308	0.27375711	-3.8736147
SLC44A1	-0.5072586	5.74263896	-2.0156921	0.04896208	0.27375711	-4.9606663
ENSCAFG00000025142	-0.6244596	1.06684395	-2.0156566	0.048965924	0.27375711	-3.9984465
ASTE1	-0.5103269	1.78015652	-2.0149008	0.049047703	0.27402323	-4.1866201
GRK3	0.68286672	3.02103603	2.01481461	0.049057033	0.27402323	-4.4976589
ENSCAFG00000029851	-0.3236755	2.99235866	-2.0140701	0.049137714	0.27435224	-4.5060893
PABPC5	-0.3975398	1.79730092	-2.0134826	0.049201469	0.27458649	-4.2126813
ENSCAFG00000010854	-0.6895804	0.09227171	-2.0131175	0.049241121	0.27468607	-3.9589667
FMN1	1.26124476	0.45938254	2.01183756	0.04938035	0.27534081	-3.9047932
ACADM	-0.3102963	6.31976144	-2.0113284	0.049435827	0.27552676	-4.9861287
ENSCAFG00000012895	-0.2108759	4.32829713	-2.0109568	0.049476358	0.27552676	-4.8563702
DERL2	0.22520233	4.66899888	2.01079256	0.049494276	0.27552676	-4.8698551
PLEKHH2	0.55229414	3.30649613	2.01066929	0.04950773	0.27552676	-4.655774
ATAD3A	-0.2895205	5.28586916	-2.0104287	0.049534	0.27552676	-4.9389805
TMEM9B	0.28025669	4.98971706	2.01032847	0.049544944	0.27552676	-4.9201816
S100A5	-1.12184	-0.8476991	-2.0099149	0.049590136	0.27565638	-3.9233816
LMNA	0.29485564	9.85301017	2.00962854	0.049621444	0.27570874	-4.8052115
DQX1	0.90337921	-0.7140945	2.00900711	0.04968945	0.27594794	-3.9088776
SOD1	0.19254098	5.7874321	2.00880514	0.049711569	0.27594794	-4.9909361
GPBP1	-0.2025484	5.25818686	-2.0086349	0.049730218	0.27594794	-4.9439818
ENSCAFG00000004091	-1.0269119	-0.6243392	-2.0083169	0.049765076	0.27601977	-3.8429463
SRPRA	0.1767897	8.08038666	2.00767073	0.049835969	0.27628866	-4.9404306
PDS5A	-0.1700488	7.02429882	-2.0074753	0.049857425	0.27628866	-4.9901593
SEC61B	0.2851211	5.82460262	2.00690624	0.049919954	0.27651352	-4.9595858
CRYZ	-0.6041259	1.84812086	-2.0066809	0.049944733	0.27652917	-4.2564352
HECA	-0.240945	3.72501266	-2.0060496	0.050014202	0.27679213	-4.7438594
ENSCAFG00000014648	-0.1983274	5.05452607	-2.0054431	0.050081036	0.27704029	-4.9286107
TMEM161A	0.30088124	4.91453005	2.0050995	0.050118923	0.27712816	-4.9341794
RPL28	-0.2848723	8.56224119	-2.0047785	0.050154342	0.27720232	-4.9088651
FOXJ3	-0.1651747	5.81192626	-2.0045054	0.050184501	0.27724736	-4.9902496
NBR1	0.19781161	7.40376074	2.00406347	0.050233325	0.27739542	-4.9643197
ADGRF5	-2.3672265	-0.9591881	-2.0033665	0.050310412	0.27769936	-3.9427953
ENSCAFG00000001129	-0.9226078	0.14347745	-2.0031371	0.050335805	0.27771783	-3.8820037
HSD17B10	0.19242614	5.27743891	2.0022965	0.05042896	0.27810997	-4.9563859
PBRM1	-0.2094596	5.17135221	-2.0014424	0.050523758	0.27851083	-4.9487321
PRICKLE3	-0.2256309	4.76277595	-2.0009867	0.050574396	0.27860697	-4.9165947
P3H2	-1.2247854	-0.2430172	-2.0008875	0.050585434	0.27860697	-3.9354994
TM4SF18	-2.4562147	-1.0827401	-2.0004222	0.050637201	0.27877018	-3.9270269
AKAP8	-0.2065332	4.59781006	-1.9996038	0.050728354	0.27909273	-4.8763259
ECD	-0.1347596	5.4281947	-1.9994984	0.050740105	0.27909273	-4.9699999
CHAMP1	-0.1881611	5.16247313	-1.9984259	0.0508598	0.27956409	-4.9610343
PDE2A	-2.4092289	-0.5519324	-1.9983329	0.050870188	0.27956409	-3.9802602
GNB1	0.12897097	8.14382824	1.99813134	0.050892717	0.27956592	-4.9537822
KATNB1	-0.2275953	4.24775832	-1.9974075	0.050973682	0.27988862	-4.8052091

MCPH1	-0.207284	4.43258248	-1.9971176	0.051006145	0.27994484	-4.8814028
METTL4	-0.3831891	3.36677298	-1.9966664	0.051056695	0.28008468	-4.5614671
RBPMS	0.35995275	5.91391169	1.99649336	0.051076097	0.28008468	-4.9965122
CALU	0.41935161	10.4810156	1.99530308	0.051209712	0.28069518	-4.7863913
ENSCAFG00000001846	0.18794032	5.38859633	1.9948518	0.051260448	0.28078096	-4.9971202
MMP24	1.0183467	-2.5942154	1.9945701	0.051292142	0.28078096	-3.8563434
DNER	1.25160381	-0.0758267	1.99456927	0.051292236	0.28078096	-3.9601345
ELMO1	-0.5466785	5.88398721	-1.9936044	0.051400917	0.28121667	-4.9711461
EFHD2	-0.3559874	3.94220624	-1.99332	0.051432986	0.28121667	-4.6804319
PTMA	-0.366893	8.30298408	-1.993129	0.051454543	0.28121667	-4.9526781
ENSCAFG00000003595	-0.2692952	6.40657931	-1.9930706	0.051461133	0.28121667	-5.0203681
ZFYVE21	-0.27356	4.3693875	-1.9923771	0.05153945	0.28139253	-4.8686878
E2F5	0.22918728	4.94282071	1.99236859	0.051540408	0.28139253	-4.9846403
SSH2	-0.4523778	4.04494643	-1.9921923	0.051560334	0.28139253	-4.9431072
FKBP2	0.30264462	4.43246389	1.99172726	0.051612927	0.28155757	-4.8939889
DUSP5	0.52254295	5.06458088	1.99130717	0.051660475	0.28169495	-5.0228017
RIN3	-0.3866743	4.91806305	-1.9910853	0.051685602	0.28171001	-4.9117013
PXK	-0.2910773	5.41278337	-1.9905122	0.05175056	0.28194206	-4.9940336
SPATA2	0.25433913	3.00307349	1.98876627	0.051948878	0.28258899	-4.6064465
LEPROTL1	0.35275747	3.74227148	1.98862214	0.051965279	0.28258899	-4.7441263
ENSCAFG00000002977	0.39858631	3.70056032	1.98853415	0.051975293	0.28258899	-4.63933
NCAPD2	-0.44172	5.69309494	-1.9885167	0.051977275	0.28258899	-4.981435
ZYG11B	-0.1899193	5.07579066	-1.9880939	0.052025429	0.28258899	-4.9671981
FBXO6	-0.3202512	4.65745343	-1.9880728	0.052027835	0.28258899	-4.8822964
LAP3	0.22966311	7.0811973	1.98793934	0.052043036	0.28258899	-5.0160072
MFSD4B	0.54274991	0.55684749	1.98788892	0.052048782	0.28258899	-4.1041986
CP	-1.9743501	-1.0602812	-1.9872468	0.052122011	0.28286465	-3.9320274
HNRNPLL	-0.2011932	4.93618516	-1.9867143	0.052182799	0.28307258	-4.9125756
PARP3	-0.2579765	6.09373736	-1.9860937	0.052253723	0.2833353	-5.0202709
MYL12B	0.23227626	6.7115563	1.98561009	0.052309045	0.28351322	-5.0354155
GAD2	0.95178303	-0.160548	1.98513153	0.052363843	0.28360226	-3.9212778
GABARAP	0.19934054	7.01880589	1.9850734	0.052370503	0.28360226	-5.0278541
CALML6	-1.5266235	-1.5972411	-1.9847252	0.052410409	0.2836964	-3.8655104
CTNS	-0.6574961	5.67997369	-1.9845245	0.052433426	0.28369907	-5.033639
CASC4	0.21141974	5.95439105	1.98346127	0.052555486	0.28421119	-5.0386015
GPATCH4	-0.266847	5.85892296	-1.9833071	0.052573204	0.28421119	-5.022443
IRX5	1.27994423	0.72761158	1.98290823	0.052619072	0.28427731	-4.1364491
ENSCAFG00000030770	-0.3985828	5.83860081	-1.9828083	0.052630572	0.28427731	-5.0359761
PTPRK	1.00529903	5.46232461	1.98197918	0.052726042	0.28467091	-4.8994052
EYA2	0.8988814	4.47849485	1.98109132	0.052828443	0.28498556	-5.0163823
NFKBIA	-0.5502301	5.57350655	-1.9810815	0.052829572	0.28498556	-5.0361395
SHBG	0.7913477	0.55827846	1.98042436	0.052905479	0.28509919	-4.0859098
ZNF597	-0.3197642	2.18202072	-1.9803097	0.052918732	0.28509919	-4.4518121
EPM2A	0.43689913	1.55999469	1.98003467	0.052950534	0.28509919	-4.1924533
ANAPC5	-0.1988343	4.45389138	-1.9800141	0.052952915	0.28509919	-4.8884712
CABYR	-0.4587207	3.09914938	-1.9799199	0.052963805	0.28509919	-4.456701
PAQR3	0.36080386	2.08538765	1.97946924	0.053015967	0.28525806	-4.3595577
NUPR1	-0.4393443	6.01802043	-1.979009	0.053069281	0.285423	-5.0433196
ZNF384	-0.2058532	5.65130122	-1.978681	0.053107301	0.28550557	-5.0266309
ANKRD35	0.60773318	-0.1913761	1.9782519	0.053157076	0.28565125	-4.0537312

DPH1	-0.2374908	4.37803603	-1.9774622	0.053248789	0.28602206	-4.86935
TSPAN5	-0.4496566	5.7323286	-1.9767602	0.053330422	0.2863121	-5.0495519
IRAK4	-0.2407365	5.38587843	-1.9764605	0.053365319	0.2863121	-5.0247939
ENSCAFG00000002399	-0.4826527	2.60884616	-1.9762455	0.053390356	0.2863121	-4.6107972
PSMB8	-0.9751724	0.09930866	-1.9762167	0.053393706	0.2863121	-3.9947234
GTF3C6	-0.3878161	2.53054717	-1.9751659	0.053516233	0.28684701	-4.3993983
EDIL3	0.7241036	7.58666188	1.97495845	0.053540456	0.28685478	-4.9742788
TXNDC5	0.28236749	8.59076148	1.97460597	0.053581628	0.28688644	-4.9440835
PITPNC1	0.52630368	0.79080805	1.97451792	0.053591917	0.28688644	-4.1250215
ZNF354A	-0.2451218	4.59654448	-1.9739136	0.053662575	0.28706921	-4.9439888
BMI1	0.28297354	4.34975203	1.97376876	0.053679527	0.28706921	-4.8553332
CAVIN3	0.37293148	6.05958869	1.97364142	0.053694431	0.28706921	-5.0522114
WRNIP1	-0.1563268	5.18810282	-1.971681	0.053924335	0.28817604	-5.0231988
RPL10A	-0.285161	7.70047874	-1.9711917	0.05398184	0.28836101	-5.024463
ENSCAFG00000016929	-0.1544783	5.92225057	-1.9709113	0.054014824	0.28841489	-5.0572465
ENSCAFG00000011612	0.22979016	5.85020765	1.97007908	0.054112815	0.28860976	-5.0552919
PASK	-0.4186293	2.27971751	-1.9699879	0.054123556	0.28860976	-4.3583599
POGZ	-0.2406504	4.79202032	-1.9698106	0.054144464	0.28860976	-4.9623029
MS4A13	-1.1748219	-0.6086769	-1.9696317	0.054165558	0.28860976	-3.9361634
ENSCAFG00000016356	0.64526835	2.23565891	1.96962891	0.054165885	0.28860976	-4.312327
NDUFAF7	0.29214913	3.19934003	1.96925316	0.054210215	0.28872384	-4.6525353
COMMD2	-0.2277185	4.2430031	-1.9684017	0.054310779	0.28913718	-4.833606
SRSF5	-0.2047624	6.32209934	-1.9680955	0.054346987	0.28916215	-5.0677406
URB1	-0.2200089	4.2456038	-1.9678864	0.054371721	0.28916215	-4.9225633
ABCD4	-0.3429361	3.29469198	-1.9677798	0.054384339	0.28916215	-4.67268
SCARF2	0.62092515	4.86342087	1.9671934	0.054453776	0.28927182	-4.9075163
ENSCAFG00000023004	-1.2701287	2.01226251	-1.9671605	0.054457672	0.28927182	-4.2454833
ACOX3	0.22550386	5.31321351	1.9670239	0.054473861	0.28927182	-5.0233354
FGFR2	1.82015979	2.30673931	1.96681769	0.054498303	0.28927966	-4.0533376
MEIS3	0.33188992	4.53570132	1.96575216	0.054624757	0.28982874	-4.9757888
SH3RF2	1.5339321	-0.5314314	1.96510452	0.054701739	0.2899622	-3.9273607
ENSCAFG00000025410	-1.4039613	-1.7122828	-1.9650594	0.0547071	0.2899622	-3.9013577
RPL37	-0.4523934	2.22482638	-1.9648589	0.05473096	0.2899622	-4.5531658
ATXN2L	-0.1195898	7.31093369	-1.9647662	0.05474199	0.2899622	-5.0565526
WDR33	-0.1611299	5.5218645	-1.96404	0.054828482	0.29015592	-5.0568792
ZNF711	-0.4804504	2.23334877	-1.9636417	0.054875963	0.29015592	-4.4239154
LTBP1	0.51750384	9.87197653	1.96333516	0.05491253	0.29015592	-4.8468516
MYO16	1.33342462	2.43832718	1.96324537	0.054923246	0.29015592	-4.3780294
C16H4orf47	-0.8901334	-1.3443578	-1.9631358	0.054936321	0.29015592	-3.9293899
TMEM40	1.16125376	0.06857003	1.96294802	0.054958745	0.29015592	-3.9542086
MANBAL	0.31741327	3.19424653	1.96291366	0.054962848	0.29015592	-4.6043284
ZFP62	-0.2625672	3.07877102	-1.9627643	0.054980692	0.29015592	-4.6531826
ENSCAFG00000009692	0.26086187	4.92332482	1.9627208	0.054985884	0.29015592	-5.0284782
MCU	-0.2250759	4.37395633	-1.9624803	0.05501462	0.29018599	-4.8930453
RHBDD3	0.35633892	4.61895801	1.96181795	0.055093836	0.29041861	-4.9000296
YIPF2	0.34039119	4.6568433	1.96172606	0.055104833	0.29041861	-4.9323304
ENSCAFG00000001149	0.31761978	4.72873799	1.96128454	0.055157699	0.29054997	-4.9720267
PYGB	0.22258367	7.73307933	1.9611327	0.055175891	0.29054997	-5.0360368
INSR	-0.3906009	3.85954007	-1.9603134	0.055274131	0.29081907	-4.9519293
IFI35	-0.7761685	1.00233649	-1.9603009	0.055275633	0.29081907	-4.2245841

PKHD1L1	-1.6398899	-2.0161209	-1.9601291	0.055296259	0.29081907	-3.894202
BFAR	-0.131696	6.13742677	-1.9597675	0.055339678	0.29092596	-5.0813955
RIPK1	-0.1249779	6.12150343	-1.9590578	0.055424989	0.29116299	-5.0826146
RASGRP3	-2.0263609	-0.5413941	-1.9588488	0.055450139	0.29116299	-4.0235974
SLC43A1	-0.9654885	2.08104913	-1.9586939	0.055468774	0.29116299	-4.3382591
MANF	0.34598078	5.38990367	1.9585243	0.055489193	0.29116299	-5.0463995
OBSCN	0.97706545	-0.9729788	1.95828814	0.055517634	0.29116299	-3.925288
MCFD2	0.32197894	7.80978374	1.95818995	0.055529462	0.29116299	-5.0079447
FAM81A	-1.075135	2.9539037	-1.9580479	0.055546576	0.29116299	-4.3773168
CASQ1	1.29657856	0.94943296	1.95756239	0.055605115	0.2913486	-4.0417629
TPI1	0.30053475	8.86800956	1.95734251	0.055631643	0.29136639	-4.9562661
PRKAA1	0.25737178	5.54579889	1.95595477	0.055799316	0.29210605	-5.0697931
SUFU	0.25017863	3.34505466	1.95579003	0.055819249	0.29210605	-4.747216
ENSCAFG00000015903	-0.4567941	1.72038497	-1.9550682	0.055906658	0.29244197	-4.2038504
PRKDC	-0.2139437	5.74875533	-1.9540639	0.056028478	0.29295754	-5.0719782
FAM3C	-0.255508	6.19583025	-1.9534972	0.056097315	0.29307821	-5.0919536
ENSCAFG00000009766	0.65603268	0.34370467	1.95349081	0.056098092	0.29307821	-4.0911546
MFSD1	-0.2062109	5.04980863	-1.9531165	0.056143599	0.29307996	-5.0122111
PHB2	-0.2638775	6.23060654	-1.9529246	0.056166944	0.29307996	-5.093308
ARHGAP24	1.09091943	2.07704339	1.95291402	0.056168229	0.29307996	-4.4599626
M6PR	0.18296207	6.51994088	1.95218061	0.056257521	0.29342432	-5.0970173
ZNF143	-0.2093754	3.2588663	-1.9516466	0.056322615	0.2936359	-4.7219966
HIF1AN	0.2491386	5.06507884	1.95146543	0.05634471	0.2936359	-5.0008015
DLX1	0.56677475	1.42730275	1.95111849	0.056387048	0.29373501	-4.2464518
CNTNAP1	1.22377971	1.09838637	1.9507887	0.056427319	0.29382328	-4.177818
SNX6	-0.1965151	4.85582804	-1.9503783	0.056477471	0.29383203	-5.0276537
RABGGTB	-0.2528899	4.51680807	-1.9502649	0.056491324	0.29383203	-4.9396696
AMACR	0.35515769	4.96458326	1.95020231	0.056498983	0.29383203	-5.013128
ARFGEF3	0.96184631	2.92013657	1.94977289	0.056551513	0.29389645	-4.3722219
CXCR4	-2.4307725	-0.6287164	-1.9496188	0.056570377	0.29389645	-4.0818647
ABCA7	0.94304659	0.40041002	1.94952899	0.056581367	0.29389645	-4.069454
TMEM63C	1.0936216	-0.9664915	1.94871422	0.056681196	0.29419122	-3.9566831
COMMD10	0.22146913	4.11589692	1.94868461	0.056684827	0.29419122	-4.8666036
QSOX1	0.49561836	9.61782416	1.94812273	0.056753763	0.29442767	-4.9341172
MED19	-0.211981	3.75101853	-1.9476283	0.056814476	0.29455555	-4.7988591
DOCK3	1.07367795	2.53722327	1.94742568	0.056839381	0.29455555	-4.3948318
ENSCAFG00000012703	0.53267033	1.12600768	1.94735095	0.056848567	0.29455555	-4.2570542
CACNA2D1	-0.2230172	5.7098598	-1.9471203	0.056876926	0.29458132	-5.0867446
GLIPR1	0.3091388	5.23172614	1.94680586	0.056915606	0.29466049	-5.0085832
HTRA3	-0.9356464	2.89396758	-1.9461063	0.057001743	0.29491857	-4.556138
PPP2R2D	-0.1887857	4.56911906	-1.9460208	0.057012284	0.29491857	-4.9627994
ZNF280D	-0.3103161	4.16279664	-1.9450176	0.05713602	0.29543732	-4.9147795
EVL	0.54862879	2.44781687	1.94462085	0.057185017	0.29556934	-4.5197822
GREB1	2.06475463	-0.7346542	1.94412196	0.057246683	0.2957667	-3.9428908
FNTA	-0.1906857	6.17387632	-1.943537	0.057319065	0.29590961	-5.1093874
COTL1	0.58499967	6.03462324	1.94351868	0.057321328	0.29590961	-5.1118191
CEP135	-0.3930796	3.86063782	-1.9432531	0.057354212	0.29595807	-4.8313218
SH3TC2	1.68195242	-1.0906466	1.94291356	0.057396285	0.29605389	-4.0092915
MAMLD1	0.90709772	2.09430988	1.94221055	0.057483472	0.29625116	-4.3959701
MME	-1.8762759	2.97990586	-1.9418456	0.057528781	0.29625116	-4.1798823

VLDLR	0.90273549	4.84478496	1.94169021	0.057548077	0.29625116	-4.8953098
RYR1	0.87829783	-1.3780214	1.94140954	0.057582951	0.29625116	-3.9828682
APP	0.31868198	10.6442741	1.94130026	0.057596534	0.29625116	-4.8740834
SLC25A11	0.2004935	5.46160626	1.94116412	0.05761346	0.29625116	-5.0654195
SNX5	-0.1693354	6.30499105	-1.9411226	0.057618626	0.29625116	-5.1173508
TOMM70	-0.1959631	5.67005693	-1.940891	0.057647424	0.29625116	-5.1067495
C27H12orf4	-0.2044522	4.62726124	-1.9407824	0.057660944	0.29625116	-4.9571171
LRRC75B	1.22370312	-1.3669564	1.94063376	0.057679437	0.29625116	-3.9870021
USP3	-0.1710054	6.30521831	-1.9403807	0.057710944	0.29625116	-5.1189429
KAT7	-0.2574084	3.48047533	-1.9403339	0.057716763	0.29625116	-4.7460656
PCDHGB1	0.58641261	0.89079879	1.93945613	0.057826166	0.29637689	-4.243024
SH2D4A	-0.6787933	2.98402202	-1.9389853	0.057884922	0.29637689	-4.6972772
DOCK1	-0.1684233	7.49048944	-1.9389656	0.057887382	0.29637689	-5.08124
RBBP6	-0.2007728	6.35063991	-1.9389261	0.057892309	0.29637689	-5.1214793
L3MBTL4	1.02904579	-0.2610959	1.93892315	0.057892679	0.29637689	-3.9896238
NFE2L2	-0.1678114	7.85416789	-1.9387913	0.057909144	0.29637689	-5.0712925
ZNF791	0.35685909	1.93942939	1.93870012	0.057920532	0.29637689	-4.4618951
SCRIB	-0.2103808	6.57003458	-1.9385847	0.057934948	0.29637689	-5.1207846
RECQL5	-0.2686477	3.25134292	-1.93844	0.057953024	0.29637689	-4.7551096
RBM44	-1.2060845	-0.8968907	-1.9377159	0.058043573	0.29648869	-3.9446116
RADIL	-0.702675	2.67757457	-1.9377074	0.058044645	0.29648869	-4.5102683
GPCPD1	-0.2864037	5.37322149	-1.9377005	0.058045499	0.29648869	-5.0970142
IFT74	-0.2593259	4.69302152	-1.9372482	0.05810213	0.29655597	-5.0090673
PNISR	-0.2712733	5.63623078	-1.9370789	0.058123332	0.29655597	-5.1148082
PSMC2	0.26501408	6.61823489	1.93703127	0.058129302	0.29655597	-5.1248402
RPS6KL1	-1.0152217	-2.2329549	-1.9368292	0.058154624	0.29656504	-3.9442839
CCNI	-0.2044836	7.39990692	-1.9366057	0.058182638	0.29658782	-5.1070405
SLC11A2	-0.2369868	3.78673059	-1.9358095	0.058282547	0.29697692	-4.8822872
KCTD15	0.32987471	4.65848396	1.93555268	0.058314809	0.29702116	-4.9221116
GK5	-0.249154	4.43217383	-1.9343094	0.05847118	0.29769725	-4.9435953
IKZF5	-0.1820628	4.4751391	-1.9333828	0.058587966	0.29817132	-4.9591837
AKT1S1	0.21860925	6.05880537	1.93300339	0.058635837	0.29829443	-5.1297324
CNOT10	-0.2001003	5.87346147	-1.9323583	0.05871731	0.29858831	-5.107378
THRAP3	-0.1690988	7.84511564	-1.9319799	0.058765149	0.29871098	-5.0884634
ABCD1	0.2784983	3.96358385	1.93158275	0.05881539	0.29884577	-4.8553952
SYTL4	0.23084799	5.0990443	1.93135051	0.058844787	0.29887457	-5.0704026
TOB1	0.24958917	6.25884439	1.92933088	0.059100964	0.29995345	-5.1349653
RNF169	-0.2515721	4.03178562	-1.9293004	0.059104832	0.29995345	-4.8665493
FAM131B	0.4675412	3.18995596	1.92900448	0.059142454	0.3000235	-4.6862685
NEK7	0.39762912	6.03216079	1.92809384	0.059258343	0.30047838	-5.135891
GPATCH2L	0.22936882	3.70238127	1.92792515	0.059279832	0.30047838	-4.868323
ZNF613	-0.6010589	-0.0762538	-1.9273816	0.059349119	0.30056869	-4.1075929
ENSCAFG00000004540	-0.2575327	2.71637017	-1.9271148	0.059383159	0.30056869	-4.5859634
SEMA4C	0.33823233	3.7998275	1.92704394	0.059392194	0.30056869	-5.0011551
CD200	1.42942576	0.23689277	1.92703686	0.059393098	0.30056869	-4.1170972
ELOVL4	0.80542779	5.00900873	1.92677642	0.059426342	0.30060496	-4.8504223
AMPH	1.59535125	-1.320902	1.92660684	0.059447997	0.30060496	-3.9680609
TSSC4	0.23519205	3.98242255	1.92619652	0.05950042	0.30074932	-4.8409988
TTC19	0.28565509	3.4381701	1.9257291	0.059560188	0.30093066	-4.7699302
ZNF837	0.46308038	1.22647517	1.92546644	0.059593795	0.30097973	-4.2681514

POLQ	1.19429492	2.77436304	1.92507171	0.059644332	0.30111423	-4.5586566
PALMD	-1.3627209	-2.5305246	-1.9244653	0.059722043	0.30138576	-3.9503801
C7	1.45061306	-2.2244404	1.92325369	0.059877564	0.30198929	-3.9628289
ARL6IP5	0.27072084	6.66143262	1.92316013	0.059889587	0.30198929	-5.1503473
CDON	-1.5853313	4.8674569	-1.9229623	0.059915021	0.30199664	-4.6099013
ENSCAFG00000018018	0.29439748	6.40688186	1.92257169	0.059965256	0.30212895	-5.1500287
ACTR1A	0.162125	7.76182632	1.92201078	0.060037461	0.3023718	-5.1079341
PICALM	0.24859368	8.82253975	1.92159739	0.060090725	0.30251909	-5.0412846
PDE8A	-0.1835618	6.33132054	-1.921119	0.060152411	0.30270866	-5.1502247
SPRTN	-0.2707128	2.86304453	-1.9208417	0.060188196	0.30276778	-4.6397561
PRG4	1.2941801	0.96690121	1.92056393	0.060224055	0.30281777	-5.0004316
ACTR10	0.16044926	6.56327053	1.92039236	0.060246215	0.30281777	-5.1553555
UTP25	-0.157414	5.3461077	-1.9201037	0.060283514	0.30288438	-5.1185598
CCDC9B	0.3606074	5.19844418	1.91976151	0.060327753	0.30292647	-5.120058
TMBIM1	0.30602805	7.54428538	1.91966689	0.060339991	0.30292647	-5.1184258
SIN3A	-0.1702131	5.82421207	-1.9186053	0.060477434	0.30349552	-5.1517665
DGCR8	-0.205712	4.60771611	-1.9182555	0.060522781	0.30360213	-5.0223922
DNAJB9	0.29868552	6.14941302	1.91775982	0.060587098	0.30380378	-5.1601342
CCDC120	-0.294658	3.35440968	-1.9173664	0.060638186	0.30393896	-4.7381637
ZNF350	-0.3654232	2.42955975	-1.916906	0.060698008	0.30411778	-4.5738733
FICD	0.35609319	3.84612225	1.91623891	0.060784785	0.30443147	-4.8964619
ZNF35	-0.3478939	1.66866288	-1.9158085	0.060840832	0.30459107	-4.3808747
PTBP1	-0.2321036	8.10506742	-1.9154878	0.060882612	0.30467914	-5.1168754
INPP5D	-1.6530759	-1.582793	-1.9151963	0.060920628	0.3047483	-4.0146247
ENSCAFG00000030307	-1.5651592	3.27769642	-1.9148461	0.06096631	0.30485575	-4.2095114
OSTF1	-0.2781951	5.22579754	-1.9143658	0.06102901	0.30497129	-5.1381791
ENSCAFG00000022718	-0.9271794	-1.3140875	-1.9141455	0.061057791	0.30497129	-4.038756
PDP2	-0.2827727	4.4004688	-1.9138547	0.061095796	0.30497129	-4.9902813
TRPV1	-0.6965219	0.97673394	-1.9137752	0.06110618	0.30497129	-4.592324
PRKAR2B	0.68827692	3.67621397	1.91374239	0.061110475	0.30497129	-4.8578223
COIL	-0.2088894	3.95545698	-1.91344	0.061150023	0.3050478	-4.931687
ZC3H4	-0.1701206	5.06435651	-1.9130812	0.061196975	0.30516116	-5.0904795
COX3	-0.2757484	12.6092666	-1.9128664	0.061225105	0.30518062	-4.7642172
ST5	0.48013907	6.2584621	1.91238816	0.061287766	0.30519634	-5.1686179
UBXN2A	-0.218794	7.11193598	-1.9123162	0.061297194	0.30519634	-5.1416943
ZNF449	-0.4862096	2.76052703	-1.9120323	0.061334427	0.30519634	-4.658765
FAT1	0.35867014	8.73661498	1.9117786	0.061367711	0.30519634	-5.0937533
SLC25A24	-0.179434	6.32490752	-1.9117498	0.06137149	0.30519634	-5.1706183
PPIL4	-0.2086681	5.58252916	-1.9117334	0.061373636	0.30519634	-5.1557297
PPP1R12A	0.25452235	7.34845562	1.91153023	0.06140031	0.30520849	-5.1587881
TRAPPC6B	0.27218356	2.64254972	1.91110859	0.061455685	0.30536324	-4.6610866
MARCH9	0.37271454	3.44456335	1.91079238	0.061497243	0.30544924	-4.7258566
HGFAC	0.73796204	0.00381717	1.91037054	0.061552718	0.30553595	-4.0805871
PDK4	0.83076128	3.11916294	1.91029077	0.061563213	0.30553595	-5.1355341
ZBTB44	-0.2582872	4.43962125	-1.9099719	0.061605177	0.30559325	-5.0327977
KARS	-0.1921541	9.03557909	-1.9098344	0.061623281	0.30559325	-5.0281985
SPSB1	0.41343653	5.31974778	1.90938224	0.061682856	0.3057683	-5.0960648
ENSCAFG00000023039	-0.5787382	1.64011629	-1.9081344	0.0618475	0.30646385	-4.2028847
WIPF2	0.18121661	3.80024026	1.9072178	0.061968686	0.3069436	-4.9317216
DCTN1	0.13552087	7.94628318	1.90615216	0.062109824	0.30752176	-5.1258842

SIPA1L3	-0.2907899	6.70917796	-1.9058293	0.062152643	0.30761285	-5.1520529
FAM184A	1.04265274	-0.3389503	1.90540091	0.062209488	0.30777326	-4.042559
KANK1	0.4932458	7.39776852	1.90412627	0.062378899	0.30838928	-5.1500994
SPRYD7	0.2731913	3.52822895	1.90409567	0.06238297	0.30838928	-4.8539311
MOCOS	-0.4534637	1.96580652	-1.9037062	0.062434816	0.3085245	-4.5674952
ZNF569	-0.3448303	2.10377559	-1.9028007	0.0625555	0.30889353	-4.4565667
SERPINE3	1.10045902	-1.4126346	1.90277785	0.062558542	0.30889353	-3.9847744
EFNB1	-0.3102987	3.9957071	-1.9025876	0.062583923	0.30889776	-4.9869412
ANKRD52	0.25893918	5.57910667	1.9020553	0.062654985	0.30909039	-5.1348685
TCF25	-0.1377634	6.8358207	-1.9017717	0.062692867	0.30909039	-5.185027
CCDC107	0.36244766	2.44809915	1.90165534	0.062708424	0.30909039	-4.5951941
PPP1R3F	0.68711164	0.55738223	1.90156047	0.062721105	0.30909039	-4.1762081
LAPTM5	-2.6833293	-0.6170092	-1.8998548	0.062949471	0.30998644	-4.1220854
ENTPD1	-1.6871434	-1.6707684	-1.899719	0.062967693	0.30998644	-4.0497158
SLC4A4	-0.7192812	1.62371928	-1.8994907	0.062998318	0.30998644	-4.2913876
AGTPBP1	-0.2020203	4.10681222	-1.8994679	0.063001372	0.30998644	-4.9956597
PARN	-0.1766119	5.61844323	-1.8990347	0.063059527	0.31001379	-5.1774792
PSEN2	0.48343642	2.07083477	1.89885434	0.063083757	0.31001379	-4.458896
BMPR1B	1.44644463	0.9255068	1.89859116	0.063119121	0.31001379	-4.0309653
TUT7	-0.1739158	5.23934294	-1.8985142	0.063129472	0.31001379	-5.1599157
TFEB	0.27483543	3.74245024	1.89824311	0.063165915	0.31001379	-4.9487027
EXT1	0.21348365	6.84768183	1.89806886	0.063189354	0.31001379	-5.1873514
PSEN1	-0.1887314	5.34717706	-1.8978825	0.063214425	0.31001379	-5.1677495
SARDH	0.74219681	2.44653295	1.89768133	0.063241507	0.31001379	-4.5733294
RBKS	0.35664686	3.48916736	1.89744029	0.063273964	0.31001379	-4.8320544
A4GALT	0.74368029	2.33759476	1.89736698	0.063283838	0.31001379	-4.7737494
MT2A	0.79131904	4.20496631	1.89735455	0.063285513	0.31001379	-5.1920988
ZNF516	0.27942916	3.81245348	1.89723011	0.063302276	0.31001379	-4.8341915
GGA2	-0.2622636	4.25110894	-1.8966186	0.063384711	0.31009202	-5.0007228
PCNT	-0.1430016	6.33412402	-1.8965215	0.063397811	0.31009202	-5.1982032
ENSCAFG00000025074	-0.3655085	2.30436944	-1.8963998	0.063414238	0.31009202	-4.5628484
IER3	0.45102452	5.32785481	1.89638134	0.063416724	0.31009202	-5.1692282
LAMC2	-0.6401417	2.76545382	-1.8959674	0.063472601	0.31013482	-5.0380802
TRAPPC8	-0.1549495	5.87390424	-1.8959517	0.06347472	0.31013482	-5.1921847
ENSCAFG00000028650	-0.2236875	2.87343295	-1.8950874	0.063591536	0.31058511	-4.6770993
ZDHHC6	-0.1543024	6.03578804	-1.8940529	0.063731592	0.31106128	-5.1965097
RPRD2	-0.1908347	4.97777483	-1.8940025	0.063738421	0.31106128	-5.1466589
SPRY2	-0.4509674	3.84603218	-1.8932387	0.063842011	0.31137692	-5.1068546
ZNF518B	-0.1984574	4.96081829	-1.8931611	0.063852538	0.31137692	-5.1046922
EIF3F	-0.1846365	7.14568725	-1.8923012	0.063969338	0.31155129	-5.1917395
CEP162	-0.2888764	2.68097297	-1.8921892	0.063984575	0.31155129	-4.6808858
PARP4	-0.2027838	6.18401731	-1.8920438	0.064004343	0.31155129	-5.1999584
FNBP4	-0.2035544	5.00343248	-1.8920403	0.064004817	0.31155129	-5.1536864
ZZZ3	-0.1790768	6.29510223	-1.8918195	0.064034855	0.31155129	-5.204409
HSPA12A	0.91734857	1.01786688	1.89180592	0.064036702	0.31155129	-4.3021585
ENSCAFG00000019944	0.57353373	0.39807836	1.89139043	0.064093257	0.31170605	-4.1226746
ENSCAFG00000030871	-0.8063551	-0.1098915	-1.8911038	0.064132294	0.31177552	-4.1104245
SFXN3	-0.252829	5.16160013	-1.890823	0.064170557	0.31184118	-5.1209352
FAF1	-0.1421419	5.63228652	-1.8903945	0.064228989	0.31189813	-5.1839442
GYPC	-1.6039672	-0.1576448	-1.8902317	0.0642512	0.31189813	-4.1492512

CCL7	0.7531523	0.85058472	1.89019241	0.064256561	0.31189813	-4.738436
ZNF592	-0.1825485	5.48986191	-1.8898763	0.064299715	0.31198737	-5.1741327
ZNF358	-0.3102385	5.53191319	-1.8894373	0.064359678	0.31215807	-5.1624506
SLC4A3	0.66234994	1.76095567	1.88901003	0.06441808	0.31232107	-4.3489859
RCL1	-0.4499163	5.23068372	-1.8887035	0.064460005	0.31237961	-5.1789884
NAA10	0.24300563	6.40644914	1.88839723	0.064501927	0.31237961	-5.2122179
ALKBH4	0.21940242	3.59979599	1.88825166	0.064521858	0.31237961	-4.9043596
GJB5	-0.8923656	2.89866811	-1.8881969	0.064529354	0.31237961	-4.6876817
TSR2	0.32297299	2.71997782	1.88696519	0.064698228	0.31307679	-4.65007
ADGRL4	-2.5682795	-0.7017578	-1.8865459	0.064755805	0.31323507	-4.1457227
TMEM65	0.27096065	4.14982687	1.8855625	0.064890998	0.31376853	-4.9717577
ENSCAFG00000031995	0.27605413	4.13351276	1.88512279	0.064951527	0.31387135	-4.9941232
TGFB1I1	0.26270386	7.77011881	1.88492155	0.064979245	0.31387135	-5.1929882
ENSCAFG00000023994	1.09519349	-0.6147012	1.88481267	0.064994245	0.31387135	-4.0717748
TNFAIP8L1	-0.9980006	-0.8995916	-1.8846843	0.065011937	0.31387135	-4.0881895
ENSCAFG00000009211	0.35410212	9.60017388	1.88432747	0.065061129	0.3139885	-5.0342868
MPHOSPH9	-0.180351	5.57201924	-1.8841031	0.065092074	0.31401753	-5.1999889
FGF18	-1.0254032	-1.0959812	-1.8829739	0.065248019	0.31455921	-4.0570649
TNKS2	-0.1871557	6.38256645	-1.8828514	0.065264962	0.31455921	-5.2227145
GSKIP	0.28475369	2.88521511	1.88274789	0.065279277	0.31455921	-4.7160456
ZIC2	-0.9530949	-1.415745	-1.8823844	0.065329568	0.31468116	-4.0780305
HDAC2	-0.1740071	6.63460311	-1.8818706	0.065400702	0.31484473	-5.2240016
ENSCAFG00000025209	-0.5583208	0.52129526	-1.881656	0.065430438	0.31484473	-4.2720438
CHCHD4	0.40489888	1.60155164	1.8815977	0.065438512	0.31484473	-4.4106918
APBB2	-0.2909146	6.06168602	-1.8809942	0.0655222	0.31512701	-5.2247431
FBXO42	0.16801179	5.16870086	1.88061265	0.065575149	0.31526129	-5.178218
SPATA1	0.28382005	3.25387294	1.88016989	0.065636642	0.31535906	-4.8309273
SEC24D	0.2699632	8.23769515	1.88010571	0.06564556	0.31535906	-5.1657264
JAK2	-0.2094998	5.8144424	-1.8798541	0.06568053	0.31540677	-5.225789
KDM3B	-0.1687713	6.32579072	-1.8794331	0.065739082	0.31556763	-5.2287576
TMUB1	0.23332275	3.87886115	1.87889261	0.065814314	0.31580842	-4.9490576
ENSCAFG00000017072	0.34996188	2.86192946	1.87859231	0.065856146	0.31588881	-4.7819989
ENSCAFG00000028851	0.51870118	0.85196326	1.87815521	0.065917073	0.31598016	-4.3502372
MLLT11	0.96261598	2.00360011	1.87809577	0.065925363	0.31598016	-4.5391904
ABHD16A	-0.2090404	5.41426704	-1.8775768	0.065997771	0.3162069	-5.2094406
DOCK6	-0.3479432	6.22158705	-1.8772274	0.066046555	0.3163203	-5.2318264
S100A11	0.24897626	8.32528707	1.87686929	0.066096596	0.31643965	-5.1445704
ENSCAFG00000024474	-0.5264148	1.04478844	-1.8762737	0.066179883	0.31661706	-4.1947171
GIGYF2	-0.1191519	6.55357681	-1.8759474	0.06622556	0.31661706	-5.2335608
PODN	1.25264591	6.98686117	1.87580396	0.066245636	0.31661706	-5.2165859
NAMPT	0.25748878	6.44273113	1.87557317	0.066277962	0.31661706	-5.231703
CEP85L	-0.491873	1.48352751	-1.8755297	0.066284049	0.31661706	-4.4214562
GPR161	0.52154692	1.82463585	1.87549105	0.066289468	0.31661706	-4.3733622
USP53	0.50491921	5.31980884	1.87534734	0.066309606	0.31661706	-5.2329675
CLN8	0.32171772	3.63914288	1.87516168	0.06633563	0.3166213	-4.9144627
FOXN2	-0.2729287	4.85312851	-1.8738955	0.06651334	0.31724893	-5.1345259
GTF2IRD1	0.43213402	2.91187499	1.87366484	0.066545763	0.31724893	-4.608226
ATP6V0A2	0.15728704	5.32206157	1.87337959	0.066585871	0.31724893	-5.1972039
CIAO1	0.18632054	4.01154146	1.87316038	0.066616707	0.31724893	-5.0325258
ZMYND8	0.20907305	6.10731426	1.87315591	0.066617336	0.31724893	-5.2399551

PTCHD1	-1.6916302	-1.7336028	-1.8731121	0.066623496	0.31724893	-4.0193914
CFAP45	0.8660469	0.79232939	1.87297047	0.066643432	0.31724893	-4.2596601
FAM208A	-0.154317	6.03593307	-1.8726063	0.066694698	0.31734266	-5.2356653
TMEM109	0.23011203	7.34839002	1.8724728	0.066713509	0.31734266	-5.2181516
ENSCAFG00000017103	-0.2042594	6.11179498	-1.8715418	0.066844767	0.31784699	-5.2423751
SIPA1	-0.3458734	4.4931708	-1.869997	0.067063054	0.31862764	-5.136987
EPC1	-0.2018773	4.58076488	-1.8696831	0.067107479	0.31862764	-5.1279738
ARHGAP17	-0.1908292	5.70064012	-1.8696667	0.0671098	0.31862764	-5.2296742
TAF5L	0.18058319	4.43535179	1.86935799	0.067153521	0.31862764	-5.1008274
KIAA0232	0.23431961	4.61513162	1.86916436	0.067180956	0.31862764	-5.0925143
CRAMP1	-0.2304376	4.76753854	-1.8688882	0.067220107	0.31862764	-5.1653379
COLEC12	1.51701756	0.39624789	1.8688565	0.067224595	0.31862764	-4.5760422
COG3	-0.2022868	4.58660197	-1.8687772	0.067235836	0.31862764	-5.1280831
NAP1L5	0.68366491	-0.2602987	1.86874603	0.067240259	0.31862764	-4.1446205
CTSD	0.40799452	8.49077369	1.86844432	0.067283058	0.31862764	-5.1498848
PAPPA	0.83411348	6.79784846	1.86841515	0.067287196	0.31862764	-5.2297767
ZNF276	-0.2700448	3.35929048	-1.8681107	0.067330412	0.31871247	-4.8803087
THNSL2	0.63763184	1.50198249	1.86729346	0.06744652	0.31914214	-4.3621377
ZNF385A	-0.693861	4.81395569	-1.8666274	0.067541275	0.31947048	-5.1802828
ETV6	0.33174057	3.91852734	1.86619649	0.067602643	0.31953898	-4.9793552
MMP28	0.86230556	2.45354688	1.86616947	0.067606492	0.31953898	-4.8295904
LMAN2L	0.49339212	3.86911802	1.86521546	0.067742521	0.31987067	-4.8404102
HDAC10	-0.358799	4.16302583	-1.865129	0.067754856	0.31987067	-5.0255865
PAK1	0.63935552	4.48367227	1.86498528	0.067775377	0.31987067	-5.0161226
PNKP	-0.2410195	4.03101161	-1.864771	0.06780597	0.31987067	-5.0271295
MATN4	1.19075767	-0.7837623	1.86472809	0.067812104	0.31987067	-4.0989537
HTR7	0.87808648	1.94753896	1.86460955	0.067829038	0.31987067	-4.7162846
GADD45G	-0.5233502	2.61374288	-1.8641674	0.067892226	0.319949	-4.5879726
PDXK	0.3266449	6.53424349	1.86413789	0.067896449	0.319949	-5.2477099
NUDT13	-0.4631577	1.72777917	-1.8636604	0.067964745	0.32015106	-4.4879963
ENSCAFG00000009311	0.41664965	1.71776931	1.86311144	0.068043349	0.3204015	-4.4785187
PPM1K	-0.4994383	2.70535461	-1.8628912	0.06807491	0.320415	-4.6971028
PLEKHA4	0.46998703	5.29610619	1.8627364	0.06809709	0.320415	-5.1963965
ARID2	-0.2362944	5.37532259	-1.8622198	0.068171174	0.32064381	-5.2038768
CRIM1	0.40601355	9.51422093	1.86183411	0.06822653	0.32078439	-5.1030807
MED23	-0.1615939	5.32699543	-1.8613259	0.068299527	0.32085067	-5.20347
WDR47	0.30545551	4.8648505	1.86121677	0.068315211	0.32085067	-5.1878351
STXBP2	0.21982447	5.43222541	1.86093181	0.068356178	0.32085067	-5.2254724
SERPINB8	-1.3902307	1.65203138	-1.8608581	0.068366778	0.32085067	-4.1322029
TNIK	0.6947172	5.58238035	1.8604979	0.0684186	0.32085067	-5.2077965
GALNT6	-0.8992743	2.52314551	-1.8603056	0.068446277	0.32085067	-4.921122
ENSCAFG00000023653	-0.7415266	-0.1133055	-1.8602572	0.068453252	0.32085067	-4.1592201
ATP23	-0.3668066	2.4115919	-1.8602101	0.068460025	0.32085067	-4.6497269
RPS27	-0.2445462	7.52853784	-1.8601417	0.06846988	0.32085067	-5.2335508
DLA88	0.60563137	4.34101722	1.85967911	0.06853652	0.32094865	-5.1079591
ANXA7	-0.1410118	6.95755856	-1.8596428	0.06854175	0.32094865	-5.2558069
DCLK2	0.5136979	3.74806013	1.85930455	0.06859052	0.32105767	-4.7494221
CUX2	1.19834645	1.7172648	1.85895228	0.068641341	0.32113161	-4.2752707
CHKB	-0.3022307	3.33068491	-1.8586877	0.068679527	0.32113161	-4.8801101
CCDC17	-0.6302035	1.50232273	-1.8584422	0.068714979	0.32113161	-4.3389129

CNPY3	0.18803157	4.29186501	1.8582755	0.068739065	0.32113161	-5.1027443
BAZ1A	-0.2729628	5.97192744	-1.8582742	0.068739254	0.32113161	-5.2535621
LTO1	0.28982735	3.19504305	1.85813557	0.068759285	0.32113161	-4.8129521
ATP6V0D2	1.22245406	-2.3270099	1.85767141	0.068826393	0.32118991	-4.0525621
RBM5	-0.1759635	5.94863865	-1.8575428	0.068845003	0.32118991	-5.2649341
RNF144A	-0.4746225	3.20360759	-1.8574939	0.068852069	0.32118991	-4.9002087
MRI1	-0.3897341	3.56064632	-1.8572968	0.068880601	0.32118991	-4.9310045
TXN2	0.18675957	5.11092382	1.85700029	0.068923521	0.32118991	-5.2080443
ENSCAFG00000002101	-0.4716728	2.19828467	-1.8567997	0.068952576	0.32118991	-4.6583576
ENSCAFG00000013406	1.46468438	0.44541746	1.85666689	0.068971815	0.32118991	-4.1911981
MPPE1	-0.3880548	2.68775224	-1.8566092	0.06898018	0.32118991	-4.7508658
SURF4	0.18865734	6.82977582	1.85646369	0.069001263	0.32118991	-5.2663186
CTSC	-1.297179	2.50380619	-1.855815	0.069095348	0.32138854	-4.5591425
PALD1	-2.1592704	0.3196714	-1.8557232	0.069108669	0.32138854	-4.3261629
TMEM94	-0.2084702	4.68803574	-1.8554425	0.069149412	0.32138854	-5.1219669
FBXO32	0.62112409	3.74243664	1.85519312	0.069185639	0.32138854	-4.9500863
ENSCAFG00000001540	-0.308811	3.86866872	-1.8549636	0.069218982	0.32138854	-5.0385101
ENSCAFG00000018632	-0.33005	2.87211317	-1.8549129	0.069226353	0.32138854	-4.7566552
PPIC	-0.2364787	5.32010943	-1.8548692	0.069232716	0.32138854	-5.2108218
F5	1.48695824	-0.5053961	1.85476365	0.069248054	0.32138854	-4.0686357
EPN2	-0.3479062	4.7143847	-1.8543373	0.069310068	0.32155787	-5.2265272
HCFC1	-0.1655208	6.81474872	-1.8536783	0.069405996	0.32188436	-5.2737556
COA5	0.28389724	2.57725531	1.85261563	0.069560943	0.32248422	-4.7052606
PSMG2	-0.1992887	4.34948296	-1.8509318	0.069807054	0.3233289	-5.1079886
EIF5A2	0.2367554	5.08092895	1.85074639	0.069834194	0.3233289	-5.1970512
ENSCAFG00000011218	0.5975396	1.23622267	1.85071827	0.069838312	0.3233289	-4.3285824
PKD1L1	1.17284367	-1.4272776	1.85066702	0.069845818	0.3233289	-4.0556655
MARCH6	0.18233348	6.41475695	1.85032231	0.069896314	0.32344378	-5.2798762
MFSD14A	-0.1869681	4.9517706	-1.8500752	0.069932532	0.32349253	-5.2126421
SRD5A3	-0.1787945	4.76221033	-1.8493792	0.070034633	0.32374764	-5.1735415
CDK14	0.31020698	7.14360571	1.84934884	0.070039085	0.32374764	-5.2491294
PCGF5	0.42564546	4.81012176	1.8490902	0.070077059	0.32375925	-5.19744
ACKR4	-1.1664634	4.30587052	-1.8489816	0.070093004	0.32375925	-5.1890751
MUC1	1.04543846	0.13740967	1.84814987	0.070215267	0.3241153	-4.1224477
GIPC2	0.77768533	0.01250285	1.84804897	0.07023011	0.3241153	-4.3348327
CWF19L2	-0.2383055	3.89711612	-1.8479323	0.070247282	0.3241153	-5.0684605
CHKA	-0.2879911	3.06376001	-1.8457741	0.070565477	0.3254642	-4.8545291
CA9	1.38221532	1.15183191	1.84550902	0.070604643	0.32552565	-4.3848721
ENSCAFG00000029671	-0.3192728	2.16109095	-1.8438237	0.070854087	0.3265562	-4.6063229
MBD4	-0.2512369	3.30845658	-1.8425929	0.071036729	0.32727821	-4.8873902
DCAF5	0.1455914	5.6826092	1.8422842	0.071082592	0.32736977	-5.2818238
NHP2	0.26307501	4.61136828	1.84205658	0.071116429	0.3274059	-5.1390529
IRAK1BP1	-0.4595439	1.135397	-1.8411328	0.071253903	0.32791895	-4.4270063
SSR1	0.29683385	7.2649159	1.84058337	0.071335768	0.32800528	-5.2783923
TP53RK	0.24192516	3.43736709	1.84052573	0.071344361	0.32800528	-4.9054583
MYOCD	0.67704331	5.72089662	1.84047471	0.071351968	0.32800528	-5.2936388
CPM	0.93672348	-1.83967	1.84030804	0.071376823	0.32800528	-4.1138633
MMS22L	-0.4217147	3.54450897	-1.8397406	0.071461493	0.3282746	-4.8306489
WASF1	0.58865212	1.26684319	1.8388447	0.071595362	0.32876465	-4.4776417
MSL1	-0.181294	5.15614193	-1.8386775	0.071620372	0.32876465	-5.2557196

DDX23	-0.1801616	5.87764325	-1.838483	0.071649458	0.32877835	-5.2837882
HOXB5	-1.4198697	0.1205839	-1.8380983	0.071707054	0.32882391	-4.2090228
TBC1D31	-0.3345305	2.71991289	-1.8379467	0.071729744	0.32882391	-4.7149119
ATN1	0.22812203	7.79247422	1.83786793	0.071741549	0.32882391	-5.2618792
GPR153	0.25327968	3.95916961	1.83755166	0.071788936	0.32882391	-4.9982062
ASAH1	0.43553565	7.35676958	1.83754514	0.071789914	0.32882391	-5.2762244
TRMT6	0.22585599	4.72730352	1.83692099	0.071883511	0.32911897	-5.1793001
HSPA4	0.25568428	8.2717527	1.8367672	0.071906589	0.32911897	-5.2484544
CNOT1	-0.1359558	7.91032303	-1.8361121	0.072004961	0.32937539	-5.2542787
PCDHB2	0.67659866	2.01253273	1.83604591	0.072014911	0.32937539	-4.5670338
ENSCAFG00000032283	-0.5266305	1.15878882	-1.8355333	0.072091976	0.32942313	-4.4616262
SLTM	-0.2154969	5.89040648	-1.8355067	0.072095973	0.32942313	-5.2961641
TM7SF3	0.1625803	5.68188731	1.83545464	0.072103809	0.32942313	-5.2914055
SOCS2	0.71443903	2.37326511	1.83515433	0.072148997	0.32949067	-4.4934288
SLC25A23	0.4322074	3.755967	1.83500876	0.072170909	0.32949067	-5.0828834
AIDA	0.1335773	6.51192108	1.83463673	0.072226934	0.32962698	-5.3072845
GP1BB	0.74755379	1.31427558	1.83413996	0.072301804	0.32984916	-4.4044458
MINPP1	0.15278014	6.41012108	1.83386591	0.072343135	0.32991822	-5.3081418
ENSCAFG00000013855	0.34547251	3.51627339	1.83361289	0.07238131	0.32997285	-5.0398906
LRRC8A	0.23744004	6.86392086	1.83300415	0.072473229	0.33021398	-5.3016322
RASIP1	-1.4141498	1.20333546	-1.8329154	0.072486634	0.33021398	-4.4818914
DENND6B	0.22032705	4.70907788	1.83226333	0.072585223	0.33047017	-5.2391217
SLAMF9	-1.1424125	-0.4937764	-1.8320766	0.072613473	0.33047017	-4.214836
DDX51	-0.2068531	4.37987026	-1.832023	0.072621581	0.33047017	-5.1424558
SRBD1	-0.2264446	4.29324093	-1.8312368	0.072740656	0.33079271	-5.0979883
XPOT	-0.1870209	8.34740268	-1.8312082	0.072744984	0.33079271	-5.2581858
TERF2IP	0.18960885	6.46647661	1.83020795	0.072896712	0.33102179	-5.3132879
DERL1	0.21736661	6.74638919	1.83018305	0.072900493	0.33102179	-5.3138482
BST1	0.59485073	4.46921286	1.83009947	0.072913182	0.33102179	-5.2338621
VCAN	0.63932041	11.2860939	1.83002881	0.072923914	0.33102179	-5.0614218
IL15RA	0.46788825	3.0605132	1.82989386	0.072944411	0.33102179	-4.9026229
UFC1	-0.170009	5.19108114	-1.8298371	0.072953039	0.33102179	-5.2655716
NUAK1	0.80539711	7.3002731	1.82948683	0.073006262	0.33105971	-5.3015524
NUP205	-0.217292	6.43530858	-1.8294362	0.073013964	0.33105971	-5.314439
LRTOMT	-0.4509786	1.68342952	-1.8291691	0.073054576	0.33112466	-4.5003194
ENSCAFG00000006899	-0.7090275	0.67660895	-1.8288612	0.073101423	0.33121782	-4.2262135
TFPI	-0.5534002	6.67418327	-1.8283041	0.073186243	0.33148289	-5.2357674
PLCXD2	0.94986316	-0.7278054	1.82806033	0.07322338	0.33149276	-4.1441817
BBS7	-0.2454509	3.7215546	-1.8277746	0.073266938	0.33149276	-5.0473514
MEI1	0.84768567	0.75925777	1.82777172	0.073267373	0.33149276	-4.2634247
LRRC75A	-0.7901585	2.8986669	-1.8274757	0.073312512	0.33153705	-4.7669758
CDKL4	1.02412609	-1.1072158	1.8273624	0.073329806	0.33153705	-4.2163475
ENSCAFG00000019644	0.29589274	7.64993428	1.82691654	0.073397861	0.33172568	-5.2927477
SPECC1L	0.19489358	6.4398234	1.82639397	0.073477694	0.331884	-5.3214761
CCDC125	-0.2656267	3.6804955	-1.8263423	0.07348559	0.331884	-5.0259599
ENSCAFG00000029649	-0.4420496	1.4420256	-1.8258115	0.073566757	0.33189228	-4.4700132
ENSCAFG00000030428	-0.3519082	2.19114369	-1.8257392	0.073577825	0.33189228	-4.5833227
AP3B2	0.86936899	-1.6858867	1.82572226	0.073580417	0.33189228	-4.1914672
ALDH1A3	-2.0846549	1.32837458	-1.8255681	0.073604017	0.33189228	-4.317027
TIMM8A	-0.4207024	2.18313255	-1.8254691	0.073619168	0.33189228	-4.6162667

KIAA2013	0.21146369	6.50557838	1.82524434	0.073653579	0.33192862	-5.3236536
B4GALT4	0.32947112	5.30616605	1.82425894	0.073804618	0.33246495	-5.2914887
ENSCAFG00000031105	-0.8997304	-0.1944861	-1.8241236	0.073825379	0.33246495	-4.1851552
MMRN1	-2.2153492	-0.9569766	-1.8226783	0.074047435	0.33319236	-4.192313
HNRNPH1	-0.2865114	7.58431323	-1.8225887	0.074061228	0.33319236	-5.3050947
COL21A1	1.14533466	-0.0196484	1.82255598	0.074066261	0.33319236	-4.3934934
WDR45	0.25365064	4.63310018	1.8217596	0.074188884	0.33358833	-5.2396606
ENSCAFG00000005099	-0.3698141	3.49115537	-1.8214806	0.074231876	0.33358833	-4.9813227
GUCY1B1	1.43388603	0.5145515	1.82146861	0.074233731	0.33358833	-4.3747945
TADA2A	-0.204465	4.97766711	-1.8209994	0.074306101	0.33379445	-5.2436432
MAX	0.17358601	5.13019468	1.82050737	0.07438204	0.33401646	-5.2699949
ZDHHC21	0.39425669	1.63862275	1.82019452	0.074430363	0.33411434	-4.5478687
ABCA2	0.27020574	5.38144236	1.81907342	0.074603747	0.33477335	-5.3167453
ZBTB48	0.27101004	3.11695313	1.81861225	0.074675167	0.33488442	-4.9440963
RHBDL1	0.52748679	2.22791441	1.81857027	0.074681671	0.33488442	-4.6451312
TTLL5	-0.2011628	4.49099867	-1.8182331	0.074733934	0.33496998	-5.1770795
EPB41L3	1.10990408	3.64463161	1.81810405	0.074753938	0.33496998	-4.5634411
THAP11	-0.3026016	3.38426449	-1.8177148	0.074814311	0.33512129	-4.9049019
ZNF518A	-0.227025	4.03863424	-1.8173611	0.074869215	0.335248	-5.1090248
DEF8	0.19027241	4.92837549	1.81696085	0.074931388	0.33531118	-5.2454138
CCNG2	-0.3405399	4.70148413	-1.8165354	0.074997518	0.33531118	-5.2676827
KIF18A	-0.484109	3.26292163	-1.816485	0.075005358	0.33531118	-4.8279648
NKRF	0.20564705	4.46432508	1.8164844	0.075005448	0.33531118	-5.1967924
CSMD2	1.00271823	1.73883934	1.81631935	0.075031118	0.33531118	-4.681889
RILPL1	0.13581496	6.01157839	1.81624267	0.075043046	0.33531118	-5.3361941
L1CAM	2.30883276	3.10991188	1.81594505	0.07508936	0.33532524	-4.5615745
ESYT2	0.18689523	8.22076284	1.81588031	0.075099437	0.33532524	-5.2750206
MEAF6	-0.1875903	3.60114492	-1.8154811	0.075161604	0.3354839	-5.0229367
MYDGF	0.24315183	5.24161506	1.81511353	0.075218881	0.33562062	-5.2864369
GPD2	0.22904936	5.77582524	1.81458714	0.075300971	0.33566302	-5.3267686
COA6	0.42388348	1.16314336	1.81449403	0.0753155	0.33566302	-4.444247
CTGF	0.78962334	10.2739849	1.81445743	0.07532121	0.33566302	-5.1278985
FXYD1	0.54481524	5.32437898	1.81436923	0.075334976	0.33566302	-5.2616786
GDF15	1.11177001	-1.9539139	1.81403411	0.075387297	0.33576251	-4.1391949
NOD2	0.69499412	0.41216427	1.8138848	0.075410617	0.33576251	-4.4213221
ENSCAFG00000031661	-0.5469448	0.95957923	-1.8134172	0.075483687	0.33582516	-4.448469
ENSCAFG00000010377	0.13501516	8.04038324	1.8133113	0.075500249	0.33582516	-5.2783269
RAB32	-0.3164677	5.34508787	-1.8131271	0.075529064	0.33582516	-5.2639812
IRF3	-0.3402705	6.0756129	-1.8130243	0.075545138	0.33582516	-5.3438743
FAIM	0.27147805	3.05155098	1.81294213	0.075557994	0.33582516	-4.8813398
NAB1	0.22303986	4.20438426	1.81130597	0.075814364	0.33684576	-5.0703988
TMOD3	-0.2432802	6.43781242	-1.8109269	0.075873869	0.33699128	-5.3416612
PLCB1	0.34763248	6.24096531	1.81035524	0.07596367	0.3372712	-5.3493465
PDK3	0.31520232	4.74671352	1.81001143	0.076017722	0.33739226	-5.2127292
CDK10	-0.2458241	3.29392553	-1.8096973	0.076067143	0.33749269	-4.9680836
CBLB	0.44434388	6.72806398	1.80919654	0.076145967	0.33766372	-5.3478633
TMA7	0.35172242	3.03457636	1.80911185	0.076159306	0.33766372	-4.9173218
TMEM88	0.63680204	2.32206913	1.80876416	0.076214089	0.33778771	-4.8256818
MOV10	-0.400443	4.69072411	-1.8079128	0.076348361	0.33820751	-5.1685987
USP42	-0.2045955	4.13555075	-1.8078232	0.076362508	0.33820751	-5.1505749

DHRS7	0.32326882	6.17191937	1.80760073	0.076397638	0.33824416	-5.3541955
PNMA8A	0.62969465	0.53958342	1.80736972	0.076434129	0.33828682	-4.3810437
PEAR1	0.95514587	4.33036622	1.80698081	0.076495592	0.33843993	-5.1009154
GNAZ	0.66305547	1.81513835	1.80648087	0.076574665	0.33857664	-4.637662
ENSCAFG00000032314	0.88907055	-1.0262096	1.80639354	0.076588484	0.33857664	-4.2182318
AKAP1	0.15651599	5.90089821	1.80617148	0.076623633	0.33857664	-5.3531687
FBXO48	-0.8792712	0.49905563	-1.8057913	0.076683839	0.33857664	-4.3352454
TWF2	-0.2376605	4.19496994	-1.8056023	0.076713788	0.33857664	-5.1820381
SMCR8	-0.2144902	3.81925917	-1.8055799	0.076717335	0.33857664	-5.0967094
UNC13B	0.29874093	6.48300021	1.80527134	0.076766251	0.33857664	-5.3542638
SPINT2	0.33148468	3.61943203	1.80523042	0.076772741	0.33857664	-4.9495771
MVD	-0.2831976	6.36873157	-1.8051472	0.076785941	0.33857664	-5.3572252
NBEAL2	-0.6518291	3.16651316	-1.8050213	0.076805905	0.33857664	-4.9736503
NUP133	-0.1568979	5.1711746	-1.8048476	0.076833459	0.33857664	-5.3146429
MOCS3	0.29785152	1.77695041	1.80474942	0.076849048	0.33857664	-4.6037168
WDR19	-0.2772467	4.63203735	-1.8043436	0.076913474	0.338742	-5.2628898
ENSCAFG00000014143	0.33421648	2.59807485	1.80417071	0.076940939	0.33874452	-4.7122499
TIE1	-2.141431	-0.6941665	-1.8039403	0.076977554	0.33878731	-4.3241003
FAM180B	-1.2637442	-1.6502539	-1.8037404	0.077009326	0.33880876	-4.1613826
PHC1	-0.3359478	3.83954244	-1.803217	0.07709257	0.33902164	-5.0180304
ITGAV	0.33047328	10.7568944	1.80309783	0.077111543	0.33902164	-5.1580321
ENSCAFG00000010687	-0.2861921	4.88353896	-1.8020981	0.077270799	0.33960327	-5.2371313
POC1B	0.23254982	3.12956739	1.80145955	0.077372665	0.33986003	-4.9910826
MGAT5	0.47441447	5.42264704	1.80139366	0.077383182	0.33986003	-5.3642353
MTA1	-0.1579458	5.76255916	-1.8008363	0.077472197	0.34013238	-5.3532189
PRDX3	0.15654788	4.87370676	1.80062754	0.07750556	0.34016029	-5.2852796
NPR1	-1.426187	-0.2306568	-1.8004466	0.077534485	0.34016871	-4.3008831
ENSCAFG00000013310	-0.6569546	-0.007241	-1.7999573	0.077612753	0.34029681	-4.2410935
UBXN6	-0.2144739	5.87592819	-1.7997044	0.077653242	0.34029681	-5.3633619
MXRA8	0.67610411	6.96465466	1.79964722	0.07766239	0.34029681	-5.3632286
CPE	0.49876293	6.98858253	1.79958879	0.077671747	0.34029681	-5.3508166
FUT11	0.32795122	3.69309464	1.79879083	0.077799613	0.3407385	-5.0723724
POLR2C	0.15254918	5.1655239	1.79840376	0.0778617	0.34089189	-5.3026531
PLCD1	-0.242721	7.32062318	-1.7973211	0.07803558	0.34145191	-5.3451906
PLEKHA3	0.20271214	5.53446537	1.79726983	0.078043827	0.34145191	-5.3430717
PYCR1	0.302409	6.3802591	1.79690527	0.078102456	0.34158482	-5.3717886
TAF1A	-0.3057019	2.6755924	-1.7966049	0.07815079	0.34158482	-4.9420698
C20H19orf66	-0.2548439	3.49459051	-1.7965753	0.078155561	0.34158482	-5.0478276
KLHL15	-0.2906258	2.90653985	-1.7958863	0.078266522	0.34195113	-4.9348336
LNPEP	0.33445033	3.71130552	1.79502923	0.078404751	0.34243629	-5.0683272
NUP54	-0.1619321	4.97517204	-1.7945596	0.078480576	0.34264864	-5.2989747
HNRNPM	-0.2363757	7.25719877	-1.7943677	0.078511588	0.34266527	-5.365357
POT1	-0.1572133	5.15495777	-1.7938869	0.078589296	0.34266739	-5.3311131
ANP32B	-0.2708117	6.37006518	-1.7937743	0.078607509	0.34266739	-5.3770823
RAB9A	-0.1678596	5.21634415	-1.7934002	0.078668031	0.34266739	-5.324576
LOXL3	0.61827168	7.40096144	1.79325724	0.07869118	0.34266739	-5.3151388
RPS23	-0.2342502	7.95870082	-1.7931871	0.078702539	0.34266739	-5.3344677
CHD1	-0.2709493	5.57709704	-1.7930359	0.078727026	0.34266739	-5.3460711
ENSCAFG00000016252	-1.0746849	-1.2909956	-1.7929946	0.078733702	0.34266739	-4.1441507
AFTPH	-0.2080129	4.73738163	-1.7929449	0.07874176	0.34266739	-5.2795537

TRPM7	-0.1853251	6.45073537	-1.7928513	0.078756914	0.34266739	-5.3795252
ATG2B	-0.1561516	5.78419035	-1.7926678	0.078786648	0.34267839	-5.3733064
OAS1	-0.2905016	3.75831191	-1.7924286	0.07882543	0.34269117	-5.0061363
CEP78	-0.3838349	3.0570213	-1.792314	0.078843998	0.34269117	-4.8393307
NIN	-0.3284827	5.2018592	-1.7914253	0.078988213	0.34319956	-5.3431502
WDR37	0.23141079	5.52516	1.79111035	0.07903938	0.34330346	-5.3734727
ENSCAFG00000012839	-0.4210205	1.37966799	-1.7899525	0.079227707	0.34374914	-4.3865702
SLC16A5	0.32128372	4.46050781	1.78994906	0.079228264	0.34374914	-5.1924239
ENSCAFG00000030825	-1.0224716	-0.9131369	-1.7899103	0.079234573	0.34374914	-4.1747467
SH3BGRL3	-0.5632611	3.98614635	-1.7898085	0.079251152	0.34374914	-5.2807948
FITM2	0.25044164	3.02913318	1.78887389	0.079403478	0.34429129	-4.9849931
ENSCAFG00000024792	-1.2022032	-2.0008497	-1.7884418	0.07947399	0.34447104	-4.1631108
RUSC2	0.3861378	7.18040941	1.78828472	0.079499628	0.34447104	-5.3462483
NGF	-0.7757004	1.49089959	-1.7879948	0.079546976	0.34448365	-4.3846136
NIPAL2	0.93142859	3.41552677	1.78778875	0.079580644	0.34448365	-4.6671233
HYAL1	0.37591974	4.28593703	1.78773582	0.079589294	0.34448365	-5.3007522
CAMK2D	0.36449165	7.53052725	1.78759731	0.079611934	0.34448365	-5.3402823
TANC2	-0.5403075	4.95236702	-1.7873263	0.079656253	0.34455706	-5.2192988
TMX4	0.33625024	3.96013577	1.78699774	0.079709998	0.34467117	-5.1436636
RASSF7	0.81231648	0.22549307	1.78663544	0.079769303	0.34480925	-4.3331459
ME3	0.41446423	6.46393101	1.78620345	0.079840065	0.34499673	-5.3888099
ACOX1	-0.1465878	6.55982379	-1.786034	0.079867828	0.34499834	-5.3905386
FSTL1	0.28249015	11.9700783	1.78545458	0.079962855	0.34524099	-5.0241874
ENSCAFG00000018225	0.53478862	0.97211725	1.78499539	0.080038226	0.34524099	-4.384425
MIER2	0.23898818	4.45089555	1.78499131	0.080038896	0.34524099	-5.2540356
SNX9	-0.2273485	6.19232886	-1.7849842	0.080040071	0.34524099	-5.3919832
HEPH	-1.3865384	-2.3338748	-1.7847398	0.080080201	0.34524099	-4.164967
MMD	0.5034035	4.14193653	1.78468958	0.080088455	0.34524099	-5.2141115
AFAP1L1	-1.6757325	-1.7989179	-1.7842161	0.080166273	0.34535894	-4.197129
GEMIN4	-0.2362988	4.61034519	-1.7841895	0.080170653	0.34535894	-5.2673122
WDR35	-0.2396218	4.87671767	-1.7836996	0.080251243	0.345374	-5.2905147
IGSF8	0.37454444	5.18659918	1.78369779	0.080251534	0.345374	-5.3390635
BLZF1	0.2354981	4.72941152	1.78355712	0.080274686	0.345374	-5.2839302
TMCO1	0.20687953	5.4022478	1.78342664	0.080296167	0.345374	-5.3512854
RAD50	-0.2022535	6.78572461	-1.7833351	0.080311245	0.345374	-5.3935674
ENSCAFG00000000641	-0.7313005	0.44369287	-1.7830846	0.080352505	0.3454335	-4.4095157
NCOA4	-0.1441395	7.2465669	-1.7825525	0.080440197	0.34559249	-5.3761903
FOLH1	-0.7659012	4.09061513	-1.7825273	0.080444361	0.34559249	-5.0945496
RPL35	-0.1654732	8.14298519	-1.7822255	0.080494131	0.3456884	-5.3291345
ENSCAFG00000016098	0.94421661	0.25550451	1.78183997	0.080557761	0.34584375	-4.3185149
STK38L	0.5022454	6.50343051	1.78131681	0.080644169	0.3459803	-5.398131
RCC2	-0.1753703	5.92140073	-1.7812971	0.080647426	0.3459803	-5.3848178
ENSCAFG00000011635	0.33330366	2.52632903	1.78114859	0.080671971	0.3459803	-4.8364246
TCP11L2	-0.3472626	4.459431	-1.7804872	0.080781354	0.34603316	-5.3197522
DNAJC13	-0.1483793	7.0250994	-1.7804249	0.080791661	0.34603316	-5.3972044
FKBP11	0.34580452	5.18656769	1.78032114	0.080808837	0.34603316	-5.3565972
FXR1	-0.1522446	6.94078107	-1.7803051	0.080811497	0.34603316	-5.3941699
MED24	-0.1545877	5.61249285	-1.7800905	0.080847012	0.34603316	-5.3813854
PSMA5	-0.2130304	5.19528259	-1.7800778	0.080849126	0.34603316	-5.3488271
ENSCAFG00000002440	0.63314914	0.6701969	1.77905343	0.0810189	0.34660119	-4.3451363

CHD9	-0.2046226	5.65796079	-1.7788185	0.081057875	0.34660119	-5.3962788
MSH6	-0.2135279	5.29991648	-1.7787792	0.081064395	0.34660119	-5.3328416
VPS26B	0.15388694	5.974709	1.77815705	0.081167707	0.34692516	-5.3991108
CTU2	-0.230248	3.59226081	-1.7778787	0.081213971	0.34700515	-5.0508215
OOEP	-1.2794981	-1.1399215	-1.7776856	0.081246059	0.34702454	-4.1593522
ANKRD45	0.73049981	-0.5159195	1.77750354	0.081276341	0.3470362	-4.3957971
XPNPEP2	-1.1516797	-0.3689654	-1.7770242	0.081356105	0.34725906	-4.3774075
BMF	-0.7701811	2.62863826	-1.7767988	0.081393633	0.34726531	-4.703852
RNF38	-0.248052	5.80583165	-1.7766842	0.081412706	0.34726531	-5.4042611
TMEM184B	0.23771444	7.24263814	1.77628642	0.081478987	0.34743037	-5.3857764
USP7	-0.1294609	6.89996651	-1.7757758	0.081564132	0.34767574	-5.4010713
ENSCAFG00000032448	-0.1699775	5.41045581	-1.7755575	0.081600551	0.34771331	-5.3658439
GNG5	0.53805823	0.78594359	1.77506414	0.081682916	0.34794657	-4.4240703
RBM26	-0.1930127	5.12187952	-1.774801	0.081726879	0.34795998	-5.3433064
ELL2	0.39123951	4.77474292	1.77462813	0.081755763	0.34795998	-5.170117
CEP68	-0.3529378	3.09242194	-1.7745493	0.081768938	0.34795998	-5.0103444
ZBTB10	-0.2551394	4.23855517	-1.7740476	0.081852839	0.34819938	-5.1758661
ZNF507	-0.2122946	4.93129385	-1.7738238	0.081890286	0.34824107	-5.347171
TNRC6B	-0.1636345	5.69188262	-1.7730917	0.082012897	0.34862312	-5.3969436
SLC43A3	-0.5894627	4.95069009	-1.7724079	0.08212756	0.34862312	-5.2968825
COX7A1	0.36902701	2.39647811	1.77239371	0.08212994	0.34862312	-4.8501403
RHOB	0.44702924	5.88194845	1.77233618	0.082139594	0.34862312	-5.3965456
KLHDC9	0.81018061	-0.7516143	1.77230299	0.082145163	0.34862312	-4.2657229
SLC35G2	0.54832123	1.36870671	1.77229686	0.082146191	0.34862312	-4.5917269
THOC6	-0.3079362	4.24897285	-1.7718776	0.082216574	0.3488043	-5.1227421
ITM2B	0.29460981	9.00851858	1.77157417	0.082267538	0.348903	-5.2565539
ZNF777	-0.155632	4.74565447	-1.7713781	0.08230049	0.34892527	-5.3093224
SPDL1	-0.4800149	4.39171476	-1.7711404	0.082340442	0.34897719	-5.1902812
ANO4	0.88872313	1.54893905	1.77096831	0.082369384	0.34898243	-4.6117245
MTSS1	-0.8599261	-0.0689433	-1.7703516	0.082473166	0.34916059	-4.5531394
PIP4K2B	-0.1351812	6.63270142	-1.7702389	0.082492143	0.34916059	-5.4156822
LRRC28	-0.2198484	5.08400403	-1.7702243	0.082494596	0.34916059	-5.3463132
SRRT	-0.1718648	6.6099321	-1.7699634	0.082538545	0.3491895	-5.4180949
MKI67	-1.6719354	5.08001107	-1.7697423	0.082575794	0.3491895	-4.9696991
MTMR14	0.25588466	5.06354391	1.76969013	0.082584592	0.3491895	-5.3560481
PARK7	0.19011824	6.07684846	1.76923903	0.082660656	0.34939383	-5.4187302
PRPF38B	-0.1926575	5.3651576	-1.7686511	0.082759882	0.3496959	-5.3815949
NFATC2	-1.3026563	-0.889985	-1.7683629	0.082808549	0.3497842	-4.2190201
ZDHHC17	-0.1870858	4.37704523	-1.7680662	0.082858695	0.34984389	-5.2654354
RFNG	0.26321052	5.78695329	1.7679506	0.08287823	0.34984389	-5.4046692
ENSCAFG00000031561	0.55812704	2.50160956	1.76766982	0.082925708	0.34992704	-4.7198469
OSGIN2	1.01834231	0.89686687	1.76729597	0.082988958	0.35007027	-4.4688364
SLC31A2	0.68476366	0.29692928	1.76711568	0.083019476	0.35007027	-4.3656267
TMEM45A	0.60131031	2.16087331	1.76681465	0.083070451	0.35007027	-4.7922195
HECTD3	-0.1722364	5.58984213	-1.7668125	0.083070819	0.35007027	-5.3820664
EPB41L4B	-0.8824844	0.33760605	-1.7665887	0.083108738	0.35011293	-4.4323292
ELF1	-0.2098732	5.9373042	-1.7662763	0.083161672	0.35020654	-5.4170967
SCN4B	0.87962848	2.65257022	1.76612953	0.083186564	0.35020654	-4.8916612
CD70	0.85560991	-0.7705579	1.76575347	0.083250356	0.35035799	-4.3766484
PATL1	0.43659206	7.1070075	1.76535795	0.083317493	0.35052343	-5.4009091

MMP11	0.47390051	2.64416596	1.7649835	0.083381095	0.35058592	-4.8990559
AKTIP	0.20873982	4.34391248	1.76494278	0.083388013	0.35058592	-5.2652639
FNDC11	0.86170303	-0.7081366	1.76392419	0.083561242	0.351197	-4.2736983
GTPBP4	-0.2249518	6.32153224	-1.7634248	0.083646273	0.35137892	-5.4288975
SLC35B1	0.19811216	6.07266157	1.7633424	0.083660319	0.35137892	-5.4258012
ENSCAFG00000025170	-0.5238282	1.75275857	-1.7624361	0.08381486	0.35191066	-4.5083732
ARMCX3	0.20580104	5.95475211	1.76210455	0.083871447	0.3520309	-5.4243427
HACD4	-0.6730634	1.90800438	-1.7618864	0.083908699	0.35206184	-4.8665878
TGS1	-0.2022204	4.62805621	-1.7616368	0.083951336	0.35206184	-5.336199
SF1	-0.1205737	7.40278439	-1.7615705	0.083962667	0.35206184	-5.4115577
ADAM11	0.42295683	1.86477115	1.76132878	0.08400399	0.35211789	-4.559524
EPB41	-0.2937366	4.53959018	-1.7610214	0.08405656	0.35222103	-5.3470335
NCOA5	-0.1563274	4.62064674	-1.7602388	0.084190508	0.35264829	-5.2845588
ENSCAFG00000011993	-0.19796	5.14980297	-1.7599834	0.084234269	0.35264829	-5.3738699
CCL17	0.97386841	-0.9513943	1.75993526	0.084242513	0.35264829	-4.3068174
ERGIC3	0.2138648	6.65637296	1.75893836	0.084413495	0.35324664	-5.431356
SEMA6D	0.99987228	2.17509082	1.758257	0.084530522	0.35343414	-4.9319785
FBXL2	0.42802357	2.16545225	1.75816503	0.08454633	0.35343414	-4.679437
TPC3	0.93556509	5.37529098	1.75813032	0.084552296	0.35343414	-5.415131
PTPN22	1.25477181	0.82344559	1.75796697	0.084580379	0.35343414	-4.3295563
ENSCAFG00000017708	0.21759894	6.51112625	1.75786102	0.084598597	0.35343414	-5.4346455
RBM33	-0.1420446	4.72428717	-1.7569135	0.084761681	0.35399806	-5.3391996
ND5	-0.3267375	11.5468331	-1.75669	0.084800183	0.35404147	-5.119537
ECI2	0.18122755	4.89484958	1.7563321	0.084861867	0.35406495	-5.3911776
DLA-64	0.6531343	5.02548334	1.75627665	0.084871428	0.35406495	-5.3744353
SYNE1	-0.3369023	7.16689863	-1.7561682	0.084890136	0.35406495	-5.4144511
ZNF593	0.26914914	3.71709659	1.75585612	0.084943966	0.35417219	-5.0910433
ENSCAFG00000028464	0.40875095	1.31988583	1.75555355	0.084996191	0.35427267	-4.6574829
TMEM204	-1.0915471	-2.274816	-1.7542353	0.085224025	0.35484059	-4.2036967
ST6GALNAC2	1.02682211	-1.6169704	1.75415429	0.085238049	0.35484059	-4.2799529
MRE11	-0.2426031	5.11800105	-1.7541471	0.085239289	0.35484059	-5.3764952
PPFIBP1	0.29735338	8.57149592	1.75411339	0.085245128	0.35484059	-5.2822772
CDH2	0.58402713	8.7018933	1.7537979	0.085299741	0.35495062	-5.38409
ENSCAFG00000006456	-0.1734033	5.8989118	-1.7535835	0.085336871	0.35498785	-5.4373084
CLSTN3	1.03223305	-0.5273745	1.75324468	0.08539558	0.35511145	-4.2297864
DAD1	0.31782106	4.6905932	1.75308669	0.085422966	0.35511145	-5.3192576
PODXL2	0.4834858	0.35374641	1.75282877	0.08546769	0.35518015	-4.4955717
ENSCAFG00000010539	0.47753524	2.95771728	1.75235718	0.085549514	0.35538464	-4.8532213
CIZ1	-0.2304449	4.84097434	-1.7520464	0.085603467	0.35538464	-5.3554597
PRR12	-0.2379794	5.05116211	-1.7518304	0.085640991	0.35538464	-5.382395
DBR1	-0.2636027	3.73929815	-1.7517061	0.085662592	0.35538464	-5.152633
SDF2	0.17834026	5.29419997	1.75162888	0.085676009	0.35538464	-5.407679
ZFP2	-0.3708536	2.02045723	-1.7514981	0.085698742	0.35538464	-4.7780917
RAPGEF3	-1.0079825	0.82558482	-1.751408	0.085714395	0.35538464	-4.8179449
SLC25A17	0.19379894	4.26480812	1.75117254	0.085755346	0.35541574	-5.2704619
EFEMP2	0.79992458	6.17139586	1.75100155	0.085785091	0.35541574	-5.4345008
NUP43	-0.2499628	4.50740229	-1.7508782	0.085806546	0.35541574	-5.2590555
SH3BP5	-0.4028212	5.55220422	-1.7502655	0.085913216	0.35565181	-5.4289085
TENM4	0.84980754	3.59842784	1.75022654	0.085920012	0.35565181	-5.4322617
POFUT2	0.23564203	4.90330288	1.74996133	0.085966222	0.35565849	-5.3742395

TPR	-0.1448488	7.9694418	-1.7498932	0.085978096	0.35565849	-5.3999188
TBC1D12	-0.2057952	3.1983202	-1.7496609	0.086018597	0.35567407	-5.0324812
RNFT1	-0.2833152	4.11818627	-1.7495477	0.086038336	0.35567407	-5.1865676
SLC41A3	-0.2292455	4.88903312	-1.7493039	0.086080862	0.35570362	-5.3757357
RAB34	0.55477843	6.24042566	1.74895925	0.086141012	0.35570362	-5.4521191
PPP3CB	-0.179595	5.6052164	-1.7488129	0.086166563	0.35570362	-5.4168357
TAMM41	-0.2922367	3.2187606	-1.7483664	0.086244557	0.35570362	-5.0489463
ENSCAFG00000007078	-0.7521673	-0.8260503	-1.7482926	0.086257465	0.35570362	-4.271716
BIRC2	-0.1064335	6.11407265	-1.7481963	0.086274288	0.35570362	-5.4530621
DOK5	1.14886792	-0.9416729	1.74788196	0.08632925	0.35570362	-4.2673165
DLA-79	1.26528129	-0.0965412	1.74780186	0.08634326	0.35570362	-4.4616991
IZUMO1	-0.6137055	0.11959792	-1.7477414	0.086353839	0.35570362	-4.3936235
LHPP	0.46667776	1.85514443	1.7477214	0.086357335	0.35570362	-4.7541578
AKAP5	0.72452847	1.29279039	1.7475936	0.086379695	0.35570362	-4.6624335
PATZ1	-0.3278354	3.10639629	-1.7475632	0.086385021	0.35570362	-4.9217579
SYNJ1	-0.1397307	5.73464225	-1.7474056	0.086412596	0.35570362	-5.4378938
LAMC1	-0.3457088	10.771959	-1.7469604	0.086490556	0.35590821	-5.1324934
ENSCAFG00000030031	0.98409254	-0.4712561	1.74641236	0.086586588	0.35610539	-4.459964
SGCD	1.54874709	2.08860167	1.74636431	0.086595014	0.35610539	-4.3857803
STOM	0.47997459	6.91649125	1.74590287	0.086675954	0.35622946	-5.450008
TIAM2	0.34301215	4.65729264	1.7456159	0.086726322	0.35622946	-5.2414735
SUPT6H	0.15270297	7.38296137	1.74543656	0.086757812	0.35622946	-5.4405717
ENSCAFG00000031458	0.2025859	6.4763955	1.7453748	0.086768657	0.35622946	-5.4595742
ENSCAFG00000015023	-0.5014555	0.64599836	-1.7451163	0.08681406	0.35622946	-4.4065488
ENSCAFG00000022282	-0.7522185	-0.4130013	-1.7450298	0.086829274	0.35622946	-4.3407171
EFCAB1	0.58369196	0.57227358	1.7448285	0.086864649	0.35622946	-4.5009188
KDM4A	-0.1071346	7.25275251	-1.7446861	0.086889685	0.35622946	-5.446646
L3HYPDH	-0.3595302	3.12557439	-1.7445883	0.086906885	0.35622946	-5.0588813
RBP4	0.83648753	5.04063148	1.74446643	0.086928317	0.35622946	-5.1128666
CDK2AP2	0.24173794	3.44044772	1.74429049	0.086959269	0.35622946	-5.1018327
TBC1D2B	-0.5919781	4.10772919	-1.7441613	0.086982008	0.35622946	-5.3315952
WWTR1	0.22732162	6.64313428	1.74385832	0.087035338	0.35622946	-5.4538531
HNRNPD	-0.2770514	6.89728592	-1.7436249	0.087076454	0.35622946	-5.4611126
TMEM183A	-0.1499592	5.82705261	-1.7433915	0.08711756	0.35622946	-5.4519249
ZNF773	-0.3067622	2.20790202	-1.7433751	0.087120449	0.35622946	-4.7696142
ENSCAFG00000017632	0.66987129	1.83564511	1.74332247	0.087129731	0.35622946	-4.6596601
FKBP15	-0.1607989	5.82005386	-1.7432969	0.087134246	0.35622946	-5.4550019
ZDHHC15	-1.0391794	0.58170341	-1.7428784	0.087208026	0.35627833	-4.3122891
MIOS	-0.1781196	4.92661122	-1.7428423	0.087214383	0.35627833	-5.3772392
DYRK3	-0.3279206	2.07787107	-1.7427478	0.087231055	0.35627833	-4.7912696
FLT3	0.77527933	-0.6959773	1.74255396	0.087265259	0.3563025	-4.2989402
LRFN1	0.57219074	1.49451329	1.74230007	0.087310071	0.35636995	-4.5201683
ZMYM4	-0.1631063	5.5988633	-1.7415474	0.087443024	0.35654169	-5.4427315
PAMR1	0.9041944	3.66191292	1.74127832	0.087490606	0.35654169	-5.4471472
CISH	-0.3406575	4.64124122	-1.7412637	0.087493197	0.35654169	-5.3379262
BLMH	-0.1444314	5.29510628	-1.7410161	0.087536981	0.35654169	-5.4049656
FBXW2	0.17587126	5.54681163	1.74096807	0.087545487	0.35654169	-5.4519379
EEF1D	-0.2514966	8.12532472	-1.7409432	0.087549894	0.35654169	-5.3924395
EIF4A3	-0.1686705	6.3694956	-1.7409409	0.087550291	0.35654169	-5.4666619
LONRF2	0.76768797	-0.1005748	1.74060504	0.087609739	0.35666848	-4.3606655

ENSCAFG00000016132	-0.1810682	6.39988308	-1.7402902	0.0876655	0.35678017	-5.4657099
DCTN4	-0.1180949	7.02993794	-1.7399218	0.08773077	0.35681559	-5.4584336
ANAPC2	-0.255116	5.04005647	-1.7399213	0.087730857	0.35681559	-5.3992577
SMC1A	-0.3111851	5.84408411	-1.7393254	0.087836541	0.35713011	-5.435358
CD86	-1.4402921	-2.0598218	-1.7382504	0.088027441	0.35779079	-4.2465713
USP2	0.73284289	0.94220372	1.73750963	0.088159184	0.35814751	-4.4671711
DYNLT3	0.19119386	5.98497752	1.73740726	0.088177403	0.35814751	-5.4694611
SYNPO2L	1.84748504	0.53060693	1.73696616	0.088255945	0.35814751	-4.3138734
COX11	-0.3472438	1.84815412	-1.7369232	0.088263602	0.35814751	-4.6834243
LNPK	0.29267664	4.70341632	1.73687474	0.08827223	0.35814751	-5.3071635
WHAMM	-0.3564566	2.29996754	-1.7367985	0.088285807	0.35814751	-4.8529153
PREPL	0.25594121	4.76276397	1.73657712	0.088325265	0.35819222	-5.3785692
PCDHB4	0.46478365	1.17742634	1.73615058	0.08840132	0.35834007	-4.5664808
UHRF1BP1L	0.22995504	6.68900202	1.73586385	0.088452477	0.35834007	-5.4743073
ZNF362	-0.2556757	4.79747607	-1.7357912	0.088465448	0.35834007	-5.3359013
PDLIM4	0.48163274	5.29347597	1.73573474	0.088475519	0.35834007	-5.4711631
CHCHD10	0.63025652	3.10037194	1.73529714	0.088553656	0.35847032	-4.9959719
TTC13	0.18557216	4.5857346	1.73510501	0.088587981	0.35847032	-5.3221867
FBXO4	-0.3461917	2.80932037	-1.7349417	0.088617172	0.35847032	-4.9484197
B4GALT1	0.22671249	7.95785389	1.73491738	0.088621513	0.35847032	-5.4105236
TNPO3	-0.1318715	6.83250484	-1.7342013	0.088749583	0.35858187	-5.4768663
CUX1	0.2667445	5.3363276	1.73394128	0.088796119	0.35858187	-5.4288905
TMEM260	0.23307928	5.04780914	1.73378248	0.088824554	0.35858187	-5.43356
ZNF862	-0.2206289	3.99413742	-1.7337301	0.08883394	0.35858187	-5.2440904
SELENOS	0.16456346	5.05253869	1.73354638	0.088866842	0.35858187	-5.4020347
ARL1	0.17972526	4.91537365	1.73344017	0.08888587	0.35858187	-5.3945922
HTATSF1	0.15308394	5.95734353	1.73338439	0.088895866	0.35858187	-5.4720256
MVK	-0.3324159	4.48886867	-1.7333412	0.088903604	0.35858187	-5.3312533
ACKR3	-1.7533548	1.83695606	-1.7333317	0.088905301	0.35858187	-4.974223
OTUD3	0.31996233	4.03978476	1.73309162	0.088948343	0.35864063	-5.2718304
MT-ATP8	-0.4567766	5.02814057	-1.7327333	0.089012596	0.35878485	-5.3860768
ENSCAFG00000018628	-0.5274152	1.5741441	-1.7320993	0.089126389	0.35904309	-4.5406906
HDAC3	0.13569979	5.5477257	1.73205878	0.089133673	0.35904309	-5.4573744
YRDC	0.20559192	4.48783679	1.73166774	0.089203923	0.35914399	-5.3330562
NME6	0.23942763	2.91846737	1.73160195	0.089215747	0.35914399	-5.0070142
BRD9	-0.1760884	5.48698772	-1.7310654	0.089312223	0.3594175	-5.4523577
AHRR	-0.6535016	2.4993674	-1.7307945	0.089360968	0.3594988	-4.7273323
TAOK3	-0.155984	5.51127944	-1.7304344	0.089425793	0.3595612	-5.4582132
TMEM259	0.21806172	6.93665427	1.73039123	0.08943357	0.3595612	-5.4768658
GAS2L3	-0.9918036	1.28590047	-1.7301929	0.08946929	0.35959004	-4.4394183
DUSP16	0.44720374	3.25493573	1.72965184	0.089566812	0.35986717	-4.9250838
MT-ATP6	-0.3061954	12.7068994	-1.729271	0.089635512	0.36002835	-5.0585694
EHD3	0.50398337	2.30714182	1.72901885	0.089681011	0.36009627	-4.9290204
LMBR1L	-0.2232735	3.79259561	-1.7282997	0.089810902	0.36046464	-5.2533216
NT5C3B	0.11766857	5.68021219	1.72819409	0.089829987	0.36046464	-5.4605574
RMND5A	-0.297008	4.75487107	-1.7277343	0.089913135	0.36068339	-5.4134094
DISP3	0.59788934	2.12154898	1.72750061	0.089955409	0.36073809	-4.7822494
OGN	-1.062736	6.69475934	-1.7272323	0.090003974	0.36078195	-5.4500525
RPS16	-0.2098824	8.22136812	-1.7271237	0.090023632	0.36078195	-5.4149406
POLR1A	-0.1851246	5.61253594	-1.7268099	0.090080465	0.36087798	-5.4555664

DLL4	-1.5269595	-1.7701092	-1.7266751	0.090104894	0.36087798	-4.3301831
ANKRD27	-0.1600331	5.0209543	-1.7264321	0.090148944	0.36093964	-5.4305121
ENSCAFG00000011673	-0.5152699	0.2671794	-1.7259697	0.090232809	0.36105566	-4.3775001
LTA4H	-0.1375066	6.46518822	-1.7258093	0.090261905	0.36105566	-5.4921186
SMNDC1	-0.2164485	3.77275983	-1.7257982	0.090263915	0.36105566	-5.1920169
PRDM10	-0.2493889	3.10595332	-1.7254184	0.090332858	0.36106452	-5.0074389
RGS22	-0.5025883	1.93259572	-1.7253259	0.090349663	0.36106452	-4.6562278
SERPING1	1.39851589	4.1251347	1.72531235	0.090352126	0.36106452	-5.2678644
NMNAT1	-0.2120054	3.25687968	-1.7250543	0.090398999	0.36109274	-5.1107919
ACER3	0.31887737	2.45586273	1.7249579	0.090416522	0.36109274	-4.9843992
FAM149B1	-0.2779006	3.11818306	-1.7241914	0.090555907	0.36149718	-4.9507086
RPL13	-0.2296802	8.27836316	-1.7238733	0.090613802	0.36149718	-5.4205926
AKNA	-0.3301668	2.99505333	-1.7237127	0.090643051	0.36149718	-4.953486
ABCA8	-0.5400819	7.07689942	-1.723521	0.090677955	0.36149718	-5.4761684
SERHL2	0.41822825	1.06569577	1.72349134	0.090683366	0.36149718	-4.5951319
RPS17	-0.1890797	8.05787056	-1.723455	0.090689989	0.36149718	-5.4307482
ORAI1	-0.3360835	3.09905564	-1.7231826	0.090739617	0.36158058	-5.1480802
TMEM92	0.92489027	-1.0318032	1.72224268	0.090911076	0.36214925	-4.2957776
PPP2R1A	0.15513727	7.75515503	1.72204416	0.090947323	0.3621791	-5.4593554
VPS39	0.1357274	6.75228543	1.72186144	0.090980695	0.36219748	-5.4947022
PIP4P1	-0.2647866	3.94113827	-1.7213236	0.091078982	0.36230915	-5.2854728
RHOD	0.39940372	2.71147918	1.72131225	0.091081062	0.36230915	-4.9473477
PPA1	-0.214429	6.21134257	-1.7211949	0.091102511	0.36230915	-5.4989375
LAMA4	-0.3330566	10.0593272	-1.7210785	0.091123801	0.36230915	-5.2804823
AGO3	0.22803687	4.90761458	1.72072455	0.091188566	0.36245225	-5.4057344
GPSM1	0.20505081	5.12709881	1.72032014	0.091262604	0.3625699	-5.4475784
TPCN2	-0.3979445	4.23069407	-1.7202484	0.091275735	0.3625699	-5.3186283
FBXO10	-0.6019249	2.01715603	-1.7197825	0.091361116	0.36279465	-4.8868123
ENSCAFG00000024014	-0.4319515	1.42212999	-1.719358	0.091438943	0.36293288	-4.5869164
ADA	0.7704135	6.81510298	1.71903931	0.09149742	0.36293288	-5.4995745
RSL1D1	-0.2575252	5.64788876	-1.7188072	0.091540022	0.36293288	-5.4837319
ABCB7	-0.1919569	5.07232776	-1.7187679	0.09154724	0.36293288	-5.4352339
PODXL	-2.3915113	1.1863166	-1.7187378	0.091552763	0.36293288	-4.4731679
PMS2	0.16167901	3.91730116	1.71865046	0.091568808	0.36293288	-5.2779909
IGFBP7	0.56885992	9.452131	1.71787486	0.091711334	0.36332574	-5.3182398
ARAP1	-0.2944746	5.90368755	-1.7177972	0.091725615	0.36332574	-5.5033988
NYX	0.94466161	-1.7372675	1.71725149	0.091826019	0.3636091	-4.2977054
MOB1A	-0.1513035	5.20674025	-1.7166016	0.09194571	0.36387312	-5.4445917
SLC39A8	-0.9224436	0.09820864	-1.7165526	0.091954747	0.36387312	-4.3539295
ZNF215	-0.2364028	2.71273966	-1.716419	0.09197936	0.36387312	-5.0342389
CAAP1	-0.2126844	3.46818602	-1.7159993	0.092056742	0.36398656	-5.0937221
FAM131C	-0.773064	0.36134282	-1.7159501	0.09206583	0.36398656	-4.509527
ZMAT3	0.323719	4.10497276	1.7150947	0.092223732	0.36449643	-5.3669865
MMP2	0.73838403	10.3238785	1.71418845	0.092391268	0.36493841	-5.2993827
ARL5A	-0.2015191	3.734779	-1.7141764	0.092393505	0.36493841	-5.2367251
PPP1R12B	0.61409306	2.94209506	1.71371305	0.092479255	0.3651626	-4.9262983
C30H15orf41	-0.1877299	4.24035512	-1.7134051	0.092536294	0.36520821	-5.3057323
SERBP1	-0.1570876	7.76941589	-1.7130909	0.092594498	0.36520821	-5.4713247
ENSCAFG00000017874	0.53830179	1.44690261	1.71298608	0.092613936	0.36520821	-4.7999147
ZNF76	-0.2406541	4.05733037	-1.712749	0.092657896	0.36520821	-5.2583001

MED13	-0.1713208	7.08553034	-1.7127189	0.092663467	0.36520821	-5.5017579
ZBTB46	0.86479683	-1.7733035	1.71271192	0.09266477	0.36520821	-4.3246183
OLFML1	1.76225068	0.48674733	1.71208441	0.092781208	0.36538993	-4.2685968
FRA10AC1	-0.241378	3.93433337	-1.7120592	0.092785885	0.36538993	-5.2563878
ENSCAFG00000011856	0.38651018	1.76642923	1.71199449	0.092797903	0.36538993	-4.7475701
FBXW4	-0.2977665	2.91211742	-1.7113006	0.09292682	0.36551323	-5.0457834
FASTKD3	-0.1838699	3.79578806	-1.7110185	0.092979279	0.36551323	-5.2558861
ARRDC3	-0.4041333	6.2911321	-1.7109453	0.092992894	0.36551323	-5.5052182
CMIP	-0.3422026	4.65413617	-1.7108806	0.093004925	0.36551323	-5.4402485
NSDHL	-0.34887	5.02241458	-1.7108004	0.093019843	0.36551323	-5.4861907
ZMYND11	-0.1131892	6.44094053	-1.7107477	0.093029647	0.36551323	-5.5169369
SNRNP48	0.30342766	4.46149134	1.71049075	0.093077464	0.36551323	-5.3850747
FDPS	-0.3395504	6.92196888	-1.7103763	0.093098777	0.36551323	-5.499727
TOPORS	-0.1831961	5.23853586	-1.7101107	0.093148229	0.36551323	-5.4775216
INSIG2	-0.300729	3.92086362	-1.7100976	0.093150662	0.36551323	-5.3031708
CPZ	0.83555831	-0.7117012	1.71003469	0.093162387	0.36551323	-4.2713908
KLF12	-0.3406624	3.46718681	-1.7099539	0.093177436	0.36551323	-5.1732181
MT-CYB	-0.304498	10.2952687	-1.7096005	0.093243294	0.3656577	-5.2853655
SH3BP4	-0.2610716	5.30967679	-1.7088378	0.09338557	0.36610166	-5.366302
STK33	-0.33105	2.31350186	-1.7086274	0.093424847	0.36614168	-4.7896475
ENSCAFG00000010224	-0.1718088	6.10247741	-1.7083459	0.093477411	0.36623374	-5.5182023
FBP2	1.09449862	-2.1260203	1.70786503	0.093567278	0.36637004	-4.2362424
RBM23	-0.2295508	3.83924894	-1.7078485	0.093570374	0.36637004	-5.209165
TSPO	0.32121168	5.70871741	1.70660646	0.093802811	0.36674795	-5.4840595
SLC27A1	0.32242725	5.22527666	1.70647734	0.093827003	0.36674795	-5.5182821
MEOX2	-1.4459087	1.08787729	-1.7061742	0.093883818	0.36674795	-4.8227167
TNPO2	0.16780964	6.06770438	1.70616769	0.093885038	0.36674795	-5.5211542
TRAPPC9	0.15315898	5.13904346	1.70609755	0.093898188	0.36674795	-5.4534547
TESK1	0.2021165	5.05232456	1.70607074	0.093903215	0.36674795	-5.444582
PAFAH1B3	-0.3524938	4.97435435	-1.7060474	0.093907596	0.36674795	-5.4341844
DLX4	0.96676898	-1.4644946	1.70584033	0.093946426	0.36674795	-4.2723544
ENOX2	-0.3586347	2.23909208	-1.7057003	0.093972697	0.36674795	-4.8793489
STC2	1.16186892	5.61055012	1.70569895	0.093972948	0.36674795	-5.4257341
LIG3	-0.1491334	5.3160103	-1.7055641	0.093998246	0.36674795	-5.4595131
PPWD1	-0.1792924	4.63395668	-1.7051837	0.094069668	0.36674795	-5.4103398
RAB20	0.48143548	1.83939033	1.70516626	0.094072935	0.36674795	-5.0000726
GMPS	-0.1450476	6.57345693	-1.7051578	0.094074518	0.36674795	-5.5246252
HAUS6	-0.2501932	4.39704899	-1.7048204	0.094137891	0.36677579	-5.3292814
PRUNE1	0.17556401	4.87272429	1.70480977	0.094139897	0.36677579	-5.4366752
MLYCD	0.16807776	4.21000901	1.70461533	0.094176436	0.3668047	-5.3508037
UNG	-0.4221081	2.13670798	-1.7042482	0.094245453	0.36696004	-4.7613275
HMGN3	-0.2876962	3.19262465	-1.7039177	0.094307637	0.36708869	-5.0822426
HSPA9	-0.1804346	8.06120897	-1.7033286	0.094418528	0.36740679	-5.4836021
CNNM4	0.54349392	4.58229475	1.70314201	0.094453686	0.36743009	-5.2688391
XRN1	-0.1954805	5.38289182	-1.7025262	0.094569772	0.36751442	-5.4901233
FMO5	-0.4451612	2.56488215	-1.702525	0.094570003	0.36751442	-5.0540962
ENSCAFG00000008984	-1.2089743	-0.3297401	-1.7019189	0.094684369	0.36751442	-4.3623069
PSMB9	-0.6171526	1.38203173	-1.7019053	0.09468694	0.36751442	-4.6231243
NOL9	-0.2103154	4.79462913	-1.7018767	0.094692327	0.36751442	-5.4214424
ENSCAFG00000016285	-1.1189262	1.45416881	-1.7017567	0.09471499	0.36751442	-4.5438833

FLCN	-0.329838	5.16484571	-1.7017041	0.094724922	0.36751442	-5.4329867
ZNF570	-0.5238872	1.08061928	-1.7016462	0.094735861	0.36751442	-4.5195822
CFAP69	-0.8670883	0.21864858	-1.7016351	0.094737959	0.36751442	-4.8738264
EDEM3	0.22459121	6.90745985	1.70125458	0.094809847	0.36768006	-5.5281309
ARHGDIG	0.63257489	1.73185518	1.70085724	0.094884966	0.36779146	-4.6686569
ENSCAFG00000004905	-2.1584573	-0.441071	-1.7007938	0.09489697	0.36779146	-4.4705188
MTHFR	-0.3575849	3.37944356	-1.7004935	0.094953786	0.36789846	-5.1932554
UBE2S	0.43965381	5.03546516	1.69964056	0.095115293	0.36840933	-5.3812931
TRRAP	-0.17449	6.35395015	-1.6994884	0.095144138	0.36840933	-5.5351379
SESTD1	-0.2406259	5.7696479	-1.699149	0.095208478	0.36841117	-5.523336
TMEM108	1.26428912	1.41817969	1.6990796	0.095221641	0.36841117	-4.4482907
PAK1IP1	-0.2050615	4.25858653	-1.6990231	0.095232357	0.36841117	-5.3342254
ENSCAFG00000006487	-0.7081485	-0.8643481	-1.6988306	0.095268881	0.36843931	-4.3203959
TRIB2	0.47889434	4.96691331	1.69854935	0.095322258	0.36846629	-5.5329963
DEXI	0.42797682	2.36419335	1.69843506	0.095343956	0.36846629	-4.9847931
NHS	0.63531967	4.41131855	1.69833152	0.095363617	0.36846629	-5.3635677
ENSCAFG00000003087	0.81065263	-0.5103055	1.69813396	0.09540114	0.36849824	-4.3416645
RALBP1	-0.1363468	5.86062721	-1.6970891	0.095599796	0.36915237	-5.5327282
ENSCAFG00000023540	-0.6664917	1.17327061	-1.6965386	0.095704593	0.36944378	-4.4730403
PCTP	0.33640848	1.82624233	1.6960364	0.09580029	0.36967602	-4.7339881
FNDC5	1.0531453	-1.5617878	1.6959149	0.095823453	0.36967602	-4.2703325
ZNF638	-0.1970925	5.70608058	-1.6956571	0.095872605	0.3697524	-5.5301153
ENSCAFG00000023313	-0.9576253	-0.4440103	-1.695304	0.095939975	0.36989897	-4.4074426
CKAP4	0.35251414	8.53356749	1.6947034	0.096054663	0.37006351	-5.4628862
FAM3A	0.21354365	4.9247517	1.69464465	0.096065888	0.37006351	-5.4352692
GNB1L	0.21109093	4.54345294	1.69461899	0.09607079	0.37006351	-5.3782378
RARRES3	-0.4174725	3.39137788	-1.6943452	0.096123107	0.37015184	-5.251142
ENSCAFG00000014246	0.22538058	3.75485901	1.69296885	0.096386496	0.37105266	-5.2600297
PRPSAP1	-0.2407029	5.41448295	-1.6923107	0.096512645	0.37139508	-5.5057107
REPS1	-0.1586854	4.61919233	-1.6921972	0.096534416	0.37139508	-5.4384448
ENSCAFG00000013770	-1.3175045	0.10392772	-1.6917338	0.096623345	0.37162371	-4.4949401
CSDE1	0.11369402	9.08890049	1.69153843	0.096660854	0.37165449	-5.4153357
DNPH1	-0.5151867	2.02931578	-1.6913224	0.096702353	0.37170059	-4.7097431
NAA35	-0.1607202	4.56332164	-1.6908234	0.096798249	0.37195569	-5.3987527
SCN9A	-0.3883801	5.71617412	-1.6905937	0.096842411	0.3720119	-5.5187778
NTMT1	0.23356697	3.21528728	1.69017285	0.096923378	0.37220941	-5.1140196
CNPY4	0.23043991	3.73881769	1.68989624	0.096976626	0.37229088	-5.2953021
ADAM19	0.36418124	8.86103879	1.68975561	0.097003705	0.37229088	-5.4346127
NNAT	0.86410831	-1.525471	1.68914708	0.097120959	0.37260312	-4.348556
CREB5	-0.8398509	0.65608476	-1.6890264	0.097144223	0.37260312	-4.349391
ENSCAFG00000028034	-0.4908869	1.30886287	-1.6888481	0.097178605	0.37260679	-4.6498077
TPRG1L	0.13994817	4.90628234	1.6887147	0.097204343	0.37260679	-5.4850118
ANXA9	0.64541742	1.42854183	1.68838914	0.097267165	0.37273417	-4.6390434
NEK6	-0.2363768	6.71600534	-1.6875815	0.097423152	0.37321838	-5.5477094
RPS26	-0.2347735	7.35932271	-1.6868147	0.097571445	0.37367283	-5.5361468
ENSCAFG00000012879	-0.3557547	3.69457629	-1.6863457	0.097662251	0.37370098	-5.2097067
FKBP10	0.25551531	9.43020414	1.68628131	0.097674715	0.37370098	-5.3940911
CST6	1.09026022	-1.4152273	1.68612745	0.097704519	0.37370098	-4.3715159
APPL1	-0.1914835	5.53275762	-1.68608	0.097713705	0.37370098	-5.524563
DES	1.33631661	1.50782457	1.685998	0.097729599	0.37370098	-4.5610594

GAS2L1	0.21743767	6.00828702	1.68576218	0.097775304	0.37370098	-5.5542515
EFCAB11	-0.5224819	0.46527204	-1.6857007	0.097787226	0.37370098	-4.4811223
ARHGEF10L	0.63701346	3.47792709	1.68555155	0.097816142	0.37370098	-5.0505582
FAAH	-0.434605	2.54718111	-1.6850686	0.097909833	0.37392892	-4.7334849
SYNE4	-0.5716333	0.40874615	-1.6849102	0.097940567	0.37392892	-4.5367704
NPEPL1	-0.1786145	4.92648769	-1.6847851	0.097964864	0.37392892	-5.4530288
PECAM1	-2.4695048	1.09794492	-1.6842948	0.098060092	0.37406791	-4.9511473
ENSCAFG00000000492	1.50958421	2.16646166	1.68429181	0.098060672	0.37406791	-4.8277991
C19H4orf33	-0.2240262	3.95503232	-1.6838342	0.09814963	0.37428832	-5.2993323
ENSCAFG00000024028	-0.4103525	2.2814571	-1.6836889	0.098177883	0.37428832	-4.9884189
POLR3D	0.25482451	5.07113418	1.6832177	0.098269567	0.3745245	-5.5068083
VTI1B	-0.5022063	0.47158995	-1.6830306	0.098305994	0.37455	-4.4487223
TNS1	0.29941397	9.21503788	1.68276641	0.098357443	0.37463271	-5.4016107
RGS17	-0.8954129	0.29636278	-1.6825015	0.098409049	0.37469993	-4.5697386
ENSCAFG00000018717	-0.1918931	5.62216824	-1.6823705	0.098434588	0.37469993	-5.5340394
ENSCAFG00000010021	0.57241171	0.51786913	1.68180552	0.098544767	0.3749988	-4.5297362
SREBF1	-0.2649373	6.73460128	-1.6816627	0.098572644	0.3749988	-5.5634342
KIAA0319	0.50870135	0.72121525	1.68121549	0.098659937	0.37512842	-4.6765277
ENSCAFG00000022714	-0.4752417	1.37868889	-1.6811559	0.098671567	0.37512842	-4.85138
HDAC11	0.90288856	-1.1154196	1.68080726	0.098739684	0.37512842	-4.3787208
VANGL1	-0.2224685	3.82295599	-1.6807289	0.098754994	0.37512842	-5.2636263
MFSD2A	0.67109094	3.57612774	1.68068194	0.098764177	0.37512842	-5.1614094
LSM4	-0.3182973	2.23724706	-1.6805733	0.098785405	0.37512842	-4.8704133
PHF23	0.1505957	5.28718404	1.6799546	0.098906424	0.37545406	-5.5252661
KLC4	0.19628897	4.40886912	1.67983019	0.098930773	0.37545406	-5.4380129
HMGXB3	0.12988553	6.03442227	1.67863701	0.099164537	0.37603734	-5.5640279
FAM168B	0.23858674	4.87736627	1.67863637	0.099164662	0.37603734	-5.4625028
UTP20	-0.2131529	5.85260295	-1.6785886	0.099174028	0.37603734	-5.5506702
CDH5	-2.711121	0.5517619	-1.6782104	0.09924822	0.37606594	-4.7409345
ZNF23	-0.2253255	3.62838081	-1.677762	0.099336244	0.37606594	-5.2156898
RNF103	0.13876807	6.2778336	1.67771904	0.099344691	0.37606594	-5.5706984
INPP5F	0.25539835	5.17647598	1.67763957	0.099360299	0.37606594	-5.5344379
GRHPR	-0.2078634	3.65692973	-1.6773498	0.099417232	0.37606594	-5.2150203
PTPRA	0.17065609	6.41414695	1.6771998	0.099446712	0.37606594	-5.5667289
RBM19	-0.1618779	5.91630457	-1.6771557	0.099455377	0.37606594	-5.556473
ZNF333	-0.3370521	3.42751409	-1.6771143	0.099463519	0.37606594	-5.2364985
CMTR2	-0.1781901	4.26825486	-1.676885	0.099508602	0.37606594	-5.3677544
GRIA3	-1.6131932	1.93233345	-1.6767491	0.099535337	0.37606594	-4.6291887
SOBP	0.39276331	3.84192441	1.67670221	0.09954456	0.37606594	-5.1805645
PI3	0.91309002	-2.3655657	1.67644602	0.099594968	0.37606594	-4.3428422
GCH1	-0.5170128	1.55941953	-1.6764346	0.09959722	0.37606594	-4.8401005
BLOC1S3	-0.7825565	-0.1772615	-1.6764227	0.099599553	0.37606594	-4.4090743
TP53INP1	-0.4358552	1.45983865	-1.6761201	0.099659133	0.37617814	-4.900961
ENSCAFG00000009769	-0.414971	1.2639053	-1.6751442	0.099851444	0.37679113	-4.5815272
ENSCAFG00000011075	-0.1895348	5.19831784	-1.6748519	0.099909112	0.37682783	-5.5117941
NOP58	-0.2770328	5.78750861	-1.6747916	0.099921004	0.37682783	-5.5556709
PJA1	-0.2668824	4.01987102	-1.6746044	0.099957956	0.37684972	-5.3119011
IDH1	-0.315053	4.96179142	-1.6744591	0.099986644	0.37684972	-5.5245217
TRAF5	0.2528642	3.40350863	1.67427922	0.100022155	0.37686954	-5.2017253
CPSF4	-0.2028055	3.45946345	-1.6741294	0.100051742	0.37686954	-5.2057777

ATG5	-0.1129316	5.6594345	-1.6738883	0.100099376	0.37693625	-5.5584824
KIF5A	-0.3328817	3.31950803	-1.6734094	0.100194073	0.37718008	-5.0837191
SORBS3	0.5063351	6.86379427	1.67318367	0.100238715	0.37723539	-5.5749115
ARRDC5	0.56084497	0.75120408	1.67282872	0.100308961	0.377387	-4.5339743
ENSCAFG00000028864	-0.3629523	1.85866532	-1.6721229	0.10044877	0.37780015	-4.7676912
NOTCH1	-0.5952519	4.59637677	-1.6716225	0.100547977	0.37797221	-5.4778309
GOPC	-0.1632971	4.32041142	-1.6715702	0.100558347	0.37797221	-5.4126349
ENSCAFG00000010893	-0.1970358	4.97386471	-1.6714382	0.100584539	0.37797221	-5.5163069
NGRN	0.1656047	4.39829522	1.67086855	0.100697616	0.37828428	-5.4365817
KSR1	-0.4835412	1.9730676	-1.6706921	0.100732657	0.37830308	-4.889782
NTAN1	0.13646602	5.16718645	1.67030565	0.100809454	0.37847865	-5.5132277
EXOC1	0.23284222	5.70502305	1.66949151	0.100971387	0.37865618	-5.5649211
SLCO3A1	0.60566254	4.37574886	1.6694258	0.100984467	0.37865618	-5.2921432
BLCAP	0.30446823	4.06063356	1.66937663	0.100994254	0.37865618	-5.3898371
DDX24	0.12200365	7.05720789	1.66932581	0.101004372	0.37865618	-5.5726656
VEGFA	0.52957034	7.91686559	1.66924298	0.101020863	0.37865618	-5.5485365
SP2	-0.2255473	4.35540375	-1.6691614	0.101037109	0.37865618	-5.3944328
SYN1	0.59709283	0.10322106	1.66889484	0.101090201	0.37874247	-4.6766768
DCTN5	0.19722385	3.38718641	1.66841037	0.101186757	0.37899149	-5.2021728
ARSD	0.48755527	2.96422761	1.66719775	0.101428768	0.37978501	-5.0659142
ZNF536	1.05213655	-1.2552833	1.66678194	0.101511863	0.37988901	-4.2926309
ASCC2	0.16279687	6.56470389	1.66675692	0.101516863	0.37988901	-5.5882693
TACC1	0.17850017	7.67380347	1.66632311	0.101603619	0.38001205	-5.5687475
AHI1	0.27570764	4.64499526	1.66629081	0.101610081	0.38001205	-5.4695568
HPS1	-0.1991573	4.93286614	-1.6661033	0.101647595	0.38003951	-5.4890209
WNK1	0.18232343	7.65549728	1.66586342	0.101695616	0.38010623	-5.5511114
PRDM6	1.61069986	-1.0343872	1.66514254	0.101840021	0.38053305	-4.2982426
GNA14	0.97530289	-1.5099714	1.66486837	0.101894986	0.38062552	-4.3340509
USP5	0.16916221	6.72307845	1.66384896	0.10209957	0.38127666	-5.5910696
EZH2	-0.3906844	3.75326582	-1.6635122	0.102167221	0.38141622	-5.1781639
SMIM7	0.25840976	2.88367576	1.66304808	0.102260531	0.38165145	-5.0654733
SPARC	0.28556685	11.6865039	1.66280178	0.102310075	0.38170265	-5.2581928
B4GALT5	0.2897547	4.97178781	1.66260061	0.102350555	0.38170265	-5.5209437
SLC25A37	0.26988149	5.41901168	1.66252805	0.102365159	0.38170265	-5.556358
LGR6	1.28001448	-0.9989534	1.66234477	0.102402055	0.38172722	-4.333142
CITED2	0.33257118	5.10041772	1.6618585	0.102499998	0.38187465	-5.5666458
HOGA1	0.68557019	-0.2383467	1.6618474	0.102502237	0.38187465	-4.5306476
ENSCAFG00000014766	0.77209151	-0.8586733	1.66154075	0.102564042	0.38199192	-4.4612533
PIGB	0.23961089	3.52830697	1.66085414	0.102702539	0.38211402	-5.2347034
ENSCAFG00000012627	0.36360187	0.84284102	1.6607254	0.102728524	0.38211402	-4.6682574
AHNAK	-0.2983603	10.7390471	-1.6606579	0.102742148	0.38211402	-5.3134309
BATF2	-0.6258393	1.02105903	-1.6606284	0.102748107	0.38211402	-4.7498258
ENSCAFG00000010850	-0.2698426	3.07076919	-1.6603685	0.102800593	0.38211402	-5.0516554
XDH	-0.7965541	2.62962393	-1.6601833	0.102838003	0.38211402	-5.5903263
ALG2	-0.2345762	3.95686517	-1.6601768	0.102839316	0.38211402	-5.3206608
KCTD21	0.66594335	-0.2636377	1.66006305	0.102862301	0.38211402	-4.5268483
MICAL2	0.33515701	7.98961399	1.66002569	0.10286985	0.38211402	-5.5703194
UBAC1	0.1614753	6.49043086	1.65930472	0.103015636	0.38254273	-5.599817
GPR34	1.2891507	1.36953868	1.6588005	0.103117695	0.38280887	-4.7640523
GALNS	0.26679335	3.93653663	1.6580578	0.103268175	0.3831812	-5.3588448

ENSCAFG00000029378	0.31249956	2.10039522	1.65800523	0.103278833	0.3831812	-4.8194108
ALKBH3	-0.1299082	4.18833814	-1.657755	0.103329576	0.38325658	-5.3704111
RNF152	-0.7529577	-0.2496215	-1.6568607	0.10351109	0.38375282	-4.4451159
ENSCAFG00000017861	0.16064636	6.05771898	1.6567957	0.103524301	0.38375282	-5.5997716
HMGB1	-0.2742444	7.43143909	-1.6553834	0.103811525	0.38470432	-5.5936021
ABHD17A	0.18132949	5.78704456	1.65515856	0.103857312	0.38476079	-5.5975177
NHLRC2	0.20139882	5.13650904	1.65499043	0.103891562	0.38476193	-5.540652
TMEM185A	0.17658625	4.21269632	1.65485717	0.103918712	0.38476193	-5.4365101
CTDSP1	-0.2926632	5.73537308	-1.6539631	0.104101033	0.38522913	-5.5950664
CCDC57	-0.4558475	1.16961645	-1.6539385	0.104106063	0.38522913	-4.711447
PTHLH	0.79465364	4.61515878	1.65376985	0.104140476	0.3852433	-5.5507042
GAS6	0.76812644	5.32839974	1.653447	0.104206396	0.38537398	-5.5383029
LRP12	0.4038233	5.16119675	1.65303043	0.104291502	0.38546158	-5.5537783
ENSCAFG00000003353	-0.3518034	1.11478812	-1.6530216	0.104293298	0.38546158	-4.664525
POSTN	1.53824548	3.94390444	1.65288177	0.10432189	0.38546158	-5.5278334
BCLAF3	-0.4384409	2.57531511	-1.6525845	0.104382673	0.38557307	-4.9001204
WDPCP	-0.6435232	0.42928544	-1.6517111	0.104561413	0.38612007	-4.4971386
DNAJB12	0.14315653	5.01135423	1.65146785	0.104611251	0.38619089	-5.5480344
NOL11	-0.1705855	4.95756575	-1.6512926	0.104647157	0.38621025	-5.5118935
ENSCAFG00000011121	-0.202835	4.10181088	-1.6507511	0.10475818	0.38646251	-5.3973703
CYR61	0.29227793	9.60012266	1.6506435	0.10478026	0.38646251	-5.4408934
BNC2	-0.183849	5.98857227	-1.6505105	0.104807552	0.38646251	-5.6032474
KIZ	-0.1571194	5.42458408	-1.650062	0.104899604	0.38668873	-5.5749308
HCK	0.88684936	-2.4934683	1.64940549	0.105034494	0.38704485	-4.3112173
SINHCAF	-0.5857744	0.2928493	-1.6491703	0.105082859	0.38704485	-4.5651147
PCYT1B	0.50699139	0.3179664	1.64914335	0.105088391	0.38704485	-4.7389423
ISM1	0.9261776	3.18128609	1.64889334	0.105139816	0.38712105	-5.3846298
VPS35L	-0.1583925	5.53710994	-1.6486678	0.105186233	0.38717141	-5.5819292
LRRK1	-0.2379423	5.27756796	-1.6485281	0.105214969	0.38717141	-5.5593704
PLK2	-0.8417448	3.78230173	-1.6482373	0.105274858	0.38727865	-5.4013621
LRRC1	0.38957871	2.35622541	1.64771549	0.105382362	0.38756094	-4.9362755
ZNF624	-0.3658433	1.36141325	-1.6470038	0.105529142	0.38798747	-4.7353067
KRT10	-0.2073679	3.3093816	-1.6465713	0.105618421	0.3882024	-5.1589208
PLPP3	-0.7197842	6.10851328	-1.6459202	0.105752939	0.38858344	-5.5954363
PDE3A	0.48262191	4.79122342	1.6454965	0.105840544	0.38861307	-5.5592801
MTUS2	1.13222593	1.34522117	1.64545486	0.105849158	0.38861307	-4.5138782
PTEN	-0.1349836	6.5832787	-1.6451666	0.105908791	0.38861307	-5.6218222
ENSCAFG00000032762	-0.2417068	3.4693299	-1.6451556	0.105911071	0.38861307	-5.3507514
SLC25A36	-0.2205492	6.46959731	-1.6451354	0.105915264	0.38861307	-5.6194411
NDN	0.29982119	4.95597374	1.64469686	0.106006053	0.38882937	-5.5522091
ENSCAFG00000014021	-0.2404885	3.07190572	-1.6445525	0.106035955	0.38882937	-5.2098414
ZNF614	-0.2801467	2.84349305	-1.6442075	0.106107453	0.38897686	-5.1352098
RHBDD2	0.22792619	5.12653122	1.6440604	0.106137938	0.38897686	-5.5854264
DTNBP1	-0.238383	4.31226877	-1.6436678	0.106219361	0.38916203	-5.423311
LRRC8C	-0.3165631	6.81520396	-1.6426973	0.106420841	0.38973648	-5.6234303
ENSCAFG00000011174	0.16570868	3.54911964	1.64261458	0.106438034	0.38973648	-5.2774413
PDGFD	-0.954142	4.13214087	-1.6422537	0.106513038	0.38983896	-5.5785813
STAC2	0.87845975	1.27909241	1.64205061	0.106555279	0.38983896	-4.5407596
DARS2	-0.1995151	3.72585329	-1.6419112	0.106584286	0.38983896	-5.3415255
CDR2	0.2434413	4.57843666	1.64188456	0.106589821	0.38983896	-5.4919627

EXOC3L2	-1.049077	-2.6020725	-1.6415246	0.106664733	0.3899997	-4.3221375
WRAP73	-0.2264344	3.13563426	-1.6413214	0.106707023	0.39004111	-5.1830223
GNA13	-0.1363573	4.61222363	-1.6409175	0.106791168	0.39023544	-5.4997467
DDX50	-0.1584333	5.9582824	-1.6405189	0.106874243	0.39042574	-5.6231032
RSPH6A	0.56100796	0.86096635	1.63889308	0.107213655	0.3915521	-4.5981766
GRPEL1	0.16739507	4.2902695	1.63826282	0.107345466	0.39191985	-5.4684896
ENSCAFG00000029032	-0.9909176	-1.4858243	-1.6379723	0.107406271	0.39198721	-4.3383893
ENSCAFG00000015396	0.33603696	1.88020259	1.63787637	0.107426354	0.39198721	-4.797164
ITGA5	0.30777406	11.6721025	1.6377287	0.107457276	0.39198721	-5.2843464
GIT1	-0.1905899	4.91416704	-1.6371267	0.107583396	0.3922369	-5.5574345
REXO2	0.22958783	5.66017811	1.63710477	0.107588003	0.3922369	-5.6209356
CARD6	-0.2632816	6.17719871	-1.6369182	0.107627128	0.392266	-5.6335924
SCAF4	-0.1450046	5.27277017	-1.6364131	0.107733082	0.39248122	-5.5887801
CIAPIN1	0.15277444	5.51202173	1.63624594	0.107768161	0.39248122	-5.605712
MAGED1	0.24027763	8.44168904	1.63612967	0.107792569	0.39248122	-5.5439662
ENSCAFG00000028659	-0.1885295	5.11244753	-1.6358598	0.107849247	0.39248122	-5.5404487
LCN2	-0.9643693	0.54460346	-1.6357731	0.107867445	0.39248122	-4.876004
DNAJC10	0.11238398	8.71223774	1.63554043	0.10791634	0.39248122	-5.5218906
ANXA11	0.21425661	7.76381979	1.63547762	0.107929541	0.39248122	-5.5781021
PFN1	0.30360791	8.01919316	1.6354495	0.107935451	0.39248122	-5.5938873
NLN	-0.2225905	3.27475692	-1.6347353	0.10808564	0.39291392	-5.2136786
VEZT	0.22595544	4.84477223	1.63450061	0.108135037	0.39296428	-5.5484925
GAA	0.31166314	6.76713438	1.63433124	0.108170693	0.39296428	-5.6342473
FKTN	-0.2337479	2.80881654	-1.6342249	0.108193086	0.39296428	-5.1199879
AKT2	0.22205321	5.28615221	1.63317414	0.108414547	0.39357238	-5.6062062
ENSCAFG00000017001	-0.4589829	1.60851416	-1.6329953	0.108452267	0.39357238	-4.7949422
DDIT4	-0.4479764	6.83067607	-1.6329859	0.108454251	0.39357238	-5.6265865
RBMX2	-0.19656	4.29768477	-1.6323258	0.108593623	0.39395669	-5.4708625
STRADB	-0.1979437	3.75875382	-1.6321881	0.108622704	0.39395669	-5.3220489
MCM4	-0.4993586	5.99669172	-1.6319633	0.108670214	0.39401555	-5.5872335
FGD5	-1.6364489	-0.8208096	-1.6313111	0.108808105	0.39440198	-4.5822195
WFIKKN2	1.21661563	2.79794143	1.63062089	0.108954216	0.39456967	-4.8837486
APLP2	0.25587588	9.42552846	1.63026926	0.109028708	0.39456967	-5.4403231
ZBED6	-0.2097162	4.59668376	-1.6301443	0.109055186	0.39456967	-5.5279133
DVL2	-0.23142	4.63838118	-1.630117	0.109060978	0.39456967	-5.4994399
STAT3	0.19334883	7.71698995	1.63000818	0.109084046	0.39456967	-5.5993438
KCNC4	1.00181628	0.12803595	1.62986308	0.10911481	0.39456967	-4.6031706
LEPROT	0.23703057	4.44102197	1.629859	0.109115675	0.39456967	-5.446413
EMC10	0.28253292	4.25122171	1.62976751	0.109135077	0.39456967	-5.4412464
TAF4B	-0.2881477	2.90756418	-1.6296355	0.10916308	0.39456967	-5.2751618
SLMAP	0.31417059	7.03805579	1.62952405	0.109186719	0.39456967	-5.6383615
F3	0.90758763	7.55545442	1.62945812	0.109200708	0.39456967	-5.6376792
HNRNPDL	-0.2652171	6.66690147	-1.6293188	0.109230266	0.39456967	-5.6466906
ALDH16A1	-0.2864763	4.39194838	-1.62894	0.10931068	0.39474694	-5.4316196
CCDC146	-0.5758253	1.08724082	-1.6285724	0.109388767	0.39491571	-4.7214546
MIS18BP1	-0.5275682	3.50378794	-1.6279578	0.109519424	0.39527412	-5.1795771
GTF3C2	-0.1911173	5.85159707	-1.6277547	0.109562627	0.39528092	-5.6338163
VPS26A	-0.1388389	6.26622465	-1.6276539	0.109584071	0.39528092	-5.648966
LMAN1	0.24338538	7.33214291	1.62748452	0.109620124	0.39529776	-5.6336409
TNIP1	-0.2438609	6.38133538	-1.6261551	0.109903388	0.3962058	-5.6515494

C11H5orf15	0.16110188	6.58202444	1.62569476	0.110001605	0.39644641	-5.6506314
SUN1	0.21074153	6.91218641	1.62520625	0.110105916	0.39670884	-5.6429592
ENSCAFG00000030608	0.88926536	-2.250137	1.6247837	0.110196207	0.39692062	-4.374683
DALRD3	-0.2920774	3.42523108	-1.6231874	0.110537858	0.3980076	-5.3143007
PSMD6	0.14761711	6.3041801	1.62307856	0.110561177	0.3980076	-5.6562182
CAPN2	-0.1504065	9.1736468	-1.6228305	0.110614357	0.39808527	-5.5309713
RCN2	0.20662342	5.79807012	1.62263379	0.110656538	0.39812332	-5.6510508
PFDN5	-0.1279517	5.34480423	-1.6220412	0.110783686	0.39846696	-5.6205263
PKN3	-0.5293143	4.00451283	-1.6218038	0.110834669	0.39853654	-5.4210035
LEF1	1.02025624	-0.9388119	1.62108789	0.110988505	0.3989758	-4.530478
USP54	0.59535066	3.95093797	1.62021645	0.111175994	0.39929558	-5.3909586
ENSCAFG00000004229	-0.5106862	1.06332555	-1.6201726	0.111185428	0.39929558	-4.615106
POU6F1	-0.2743542	4.04189979	-1.6199451	0.111234421	0.39929558	-5.3326268
RBP1	-0.9820207	-2.3496512	-1.619899	0.111244361	0.39929558	-4.3670322
OSGEP	-0.2692631	4.59742565	-1.6198113	0.111263247	0.39929558	-5.5138922
CCAR2	-0.1164675	6.18090987	-1.6197908	0.111267663	0.39929558	-5.6585748
SEPSECS	-0.194885	4.19808444	-1.6194649	0.111337899	0.39940137	-5.4493673
TCF20	-0.1261459	5.80644603	-1.6192609	0.111381879	0.39940137	-5.6550448
FBXO5	-0.7498053	2.31375465	-1.619198	0.111395447	0.39940137	-4.8077904
UFL1	-0.1650034	5.94492338	-1.6190657	0.111423977	0.39940137	-5.65699
HOXD3	-0.9612789	-1.9828867	-1.6188297	0.111474877	0.39945072	-4.3790135
GZF1	-0.1947249	4.23680407	-1.6187079	0.111501169	0.39945072	-5.4379853
ZNF503	0.57339075	4.15239872	1.61832472	0.111583883	0.39963338	-5.4693113
ENSCAFG00000003181	0.1991581	4.28639363	1.6181565	0.111620213	0.39964986	-5.5172883
IMMT	-0.1300808	6.68226978	-1.6177952	0.111698283	0.39981574	-5.6616516
ENSCAFG00000019863	-0.5692219	0.43074072	-1.6174715	0.111768251	0.39995253	-4.4897789
RASL12	0.92187106	-0.322066	1.61713846	0.111840279	0.40009661	-4.5391373
NCLN	0.23486446	6.79772482	1.61694725	0.111881653	0.40013098	-5.665514
CORO1C	0.17029679	7.75498899	1.61679313	0.11191501	0.40013666	-5.630141
VSIG10	-0.2319154	5.33715598	-1.6162768	0.112026811	0.40033153	-5.5486702
ENSCAFG00000010097	0.65318172	-0.3065238	1.61599439	0.112088011	0.40033153	-4.4576832
RPS6KA2	-0.42849	6.90589128	-1.6159657	0.112094227	0.40033153	-5.6576979
ACAA2	0.20734929	6.12400418	1.61592532	0.112102981	0.40033153	-5.6680102
CEMIP2	0.44939355	5.51719909	1.61567032	0.112158262	0.40033153	-5.6459515
TBX1	-1.2921843	-2.082806	-1.6156614	0.112160206	0.40033153	-4.3573439
LONRF1	-0.2487568	3.55841893	-1.6153952	0.112217926	0.40042408	-5.2652159
CARS2	0.20912112	6.01349816	1.61523384	0.112252941	0.40043135	-5.658913
CD47	0.27334038	5.20542087	1.61509282	0.112283544	0.40043135	-5.6223893
SMIM20	-0.181967	5.29635732	-1.6148115	0.112344615	0.40053574	-5.6375077
ACSL5	-0.4715732	5.04620938	-1.6142599	0.112464443	0.40070408	-5.660554
PLPP2	0.60868264	4.40054242	1.61423769	0.112469262	0.40070408	-5.3899987
ERCC4	0.12793282	5.42631907	1.61415485	0.112487268	0.40070408	-5.6383605
ENSCAFG00000005550	-0.1442671	5.42208027	-1.6139898	0.112523152	0.40071858	-5.6333729
TRUB1	-0.1690563	3.8889087	-1.6130581	0.112725847	0.40132696	-5.4316263
DOK2	0.68389426	0.57730374	1.61257417	0.112831262	0.40158875	-4.6766278
DHX40	-0.1596014	5.7535741	-1.6123593	0.112878092	0.40164193	-5.6663861
PLEKHA7	1.06855733	-1.4651832	1.61188129	0.112982317	0.4018645	-4.4781716
SLC30A6	0.20526361	4.04082219	1.61177983	0.11300445	0.4018645	-5.4537312
EIF2A	-0.1594459	6.42077587	-1.6115038	0.113064677	0.40190484	-5.6748617
RRP12	0.18371351	6.10754727	1.61120559	0.113129785	0.40190484	-5.6705291

HSPBAP1	-0.2422369	3.94424152	-1.6111009	0.113152655	0.40190484	-5.4484367
ARL16	0.28391557	2.49611215	1.61101551	0.113171299	0.40190484	-5.1456031
ZNF263	-0.2106996	2.8099026	-1.610997	0.113175331	0.40190484	-5.1808355
JAK1	-0.153061	8.25388562	-1.6108371	0.113210268	0.4019156	-5.5914135
ERO1A	0.52743486	8.67675141	1.61010338	0.113370672	0.40237165	-5.5628485
SLC3A2	0.21667707	6.31776527	1.60957093	0.113487188	0.40267172	-5.6762192
PRKG1	0.5526451	6.06119108	1.60887	0.113640719	0.40306661	-5.6576213
RNF13	0.18547895	6.08744744	1.60877072	0.11366248	0.40306661	-5.6710244
HGS	0.13948646	6.23055825	1.60852891	0.113715493	0.40314111	-5.6785154
F8	-1.0767422	-2.1553321	-1.6077846	0.113878792	0.40360643	-4.370462
NMD3	-0.172855	5.5063088	-1.6072266	0.114001355	0.40380575	-5.6317787
ENSCAFG00000031469	0.35095167	2.83807459	1.60707259	0.114035193	0.40380575	-5.4427651
PLXND1	-0.6294882	6.42182977	-1.6069884	0.1140537	0.40380575	-5.6777434
IDO2	-0.7278564	-0.4102762	-1.6069449	0.114063263	0.40380575	-4.5320505
MYO9B	0.21711952	8.20458465	1.60666662	0.114124443	0.40383483	-5.640658
ENSCAFG00000029427	-0.3660187	2.53859419	-1.6066159	0.114135598	0.40383483	-5.0683996
ATP8B1	0.33742943	7.48391897	1.60602864	0.114264815	0.40407864	-5.658917
ABI1	-0.1087717	6.18890116	-1.6060111	0.114268667	0.40407864	-5.6816247
ENSCAFG00000016314	0.72139251	1.0476502	1.60570083	0.114336995	0.40414743	-4.69542
ING3	-0.2674723	2.60974272	-1.6056017	0.114358827	0.40414743	-5.1681356
ENSCAFG00000023602	-1.988944	-0.7163565	-1.6054713	0.114387562	0.40414743	-4.5692236
NUP160	-0.2310596	4.61462713	-1.6053401	0.114416462	0.40414743	-5.5382978
ENSCAFG00000003862	1.02110355	-0.9565075	1.60519006	0.114449536	0.40415093	-4.4598407
CDK17	-0.2689856	3.01370102	-1.604614	0.114576579	0.40440195	-5.2634986
HMGN2	-0.3765777	4.9759265	-1.6045765	0.114584832	0.40440195	-5.5507029
ENKD1	-0.2493065	2.40293519	-1.6037437	0.114768716	0.40493747	-5.1168705
RAB7B	0.57988718	2.49169676	1.60349465	0.114823736	0.40501814	-5.1387175
TMED7	0.16021091	4.72849389	1.60315871	0.114897999	0.40516663	-5.5725773
DSC2	-1.1966123	-2.2411999	-1.6026235	0.115016405	0.40535742	-4.3796653
ENSCAFG00000008683	0.59020716	5.08135436	1.6025072	0.115042135	0.40535742	-5.6232221
ENSCAFG00000013496	0.41705351	3.04780591	1.60247778	0.115048648	0.40535742	-5.230747
RARRES2	0.59114153	1.80844145	1.60222399	0.115104837	0.40544198	-5.5140839
NFATC3	-0.1983696	4.89668229	-1.6019947	0.115155622	0.40546701	-5.600299
PRELP	0.87064977	6.89938074	1.60190126	0.115176322	0.40546701	-5.682377
PIM1	-0.5194572	4.07124167	-1.6016247	0.115237617	0.40551259	-5.0880017
ENSCAFG00000011368	0.66670887	3.20542114	1.60140973	0.115285265	0.40551259	-5.1745388
RAMAC	0.15983998	3.73493401	1.60139564	0.115288388	0.40551259	-5.4329743
PNPLA7	-0.250389	5.52129544	-1.6012619	0.115318045	0.40551259	-5.6513557
CTC1	-0.2288651	4.7259389	-1.6009753	0.115381631	0.40562295	-5.5558945
QKI	-0.176227	6.28250396	-1.6003595	0.115518319	0.40594758	-5.6886099
CNBP	-0.1347145	7.6188248	-1.6002689	0.115538431	0.40594758	-5.6582584
GLO1	0.30903693	7.1340115	1.59997136	0.115604541	0.40606659	-5.667717
DNAAF5	0.14929552	5.1181979	1.59957736	0.115692124	0.40626094	-5.6364867
ENSCAFG00000028993	-1.2831951	1.93063661	-1.599338	0.115745347	0.40627922	-4.75947
HSP90B1	0.29271412	10.3161924	1.59913081	0.115791451	0.40627922	-5.4664231
TIMELESS	-0.4047137	3.49432417	-1.5990671	0.115805629	0.40627922	-5.2001468
NDUFS2	-0.1788461	6.48854166	-1.598974	0.115826349	0.40627922	-5.6938107
ARVCF	0.40184926	3.63661667	1.59821873	0.115994543	0.4067054	-5.2488122
ACHE	-0.9160687	1.04913081	-1.5980607	0.116029756	0.4067054	-4.574062
ZNF446	-0.2680983	2.15406201	-1.5977917	0.116089725	0.4067054	-5.0074489

CEP170	-0.1799094	7.25522474	-1.5977459	0.11609995	0.4067054	-5.6783319
FGD1	-0.1784195	4.36055667	-1.5975929	0.11613407	0.4067054	-5.5102907
ATP5IF1	0.17260717	5.27677287	1.5975592	0.116141579	0.4067054	-5.6485342
PRCP	-0.3112532	4.76956828	-1.5973075	0.116197731	0.40678894	-5.5829442
CCDC84	-0.3361521	1.52154833	-1.5959755	0.116495261	0.40761347	-4.8608315
ENSCAFG00000015445	-0.5529333	2.6676055	-1.5957648	0.116542376	0.40761347	-4.9303384
ENSCAFG00000017228	-0.2145488	4.09050241	-1.5956284	0.116572893	0.40761347	-5.4838231
SCAMP4	-0.1597678	6.52778896	-1.5954869	0.116604569	0.40761347	-5.6993996
COPZ1	-0.2039768	3.81206205	-1.5954842	0.11660516	0.40761347	-5.3914581
SUZ12	-0.1949658	5.29337632	-1.5953848	0.116627416	0.40761347	-5.6442622
ADRA1B	1.33783947	2.31619888	1.59522415	0.116663369	0.40762602	-4.7414121
HNRNPA0	-0.2119403	5.06927099	-1.594955	0.116723641	0.40772351	-5.6278255
MLX	0.14135321	4.53336028	1.59473589	0.116772714	0.40778184	-5.5506476
RAB30	-0.4497534	2.40918583	-1.5944435	0.116838228	0.40789754	-5.0673688
DRC1	0.83946951	-1.0064635	1.59393323	0.116952649	0.40812081	-4.4021359
PAFAH2	-0.2120819	3.9577275	-1.5938693	0.116966984	0.40812081	-5.4935384
ENSCAFG00000017257	-0.3332131	6.99106191	-1.5933164	0.117091084	0.40840982	-5.6831051
ZNF250	-0.2862209	3.22985942	-1.5932114	0.117114661	0.40840982	-5.3040577
KLHL2	-0.2239874	5.47584432	-1.5929425	0.117175056	0.40850734	-5.6475538
TFPI2	1.912483	4.87605859	1.59260026	0.117251959	0.40866233	-4.927691
EDARADD	-0.7460102	-0.2281261	-1.5920634	0.117372685	0.40894522	-4.4688949
UBR5	-0.1413422	6.97174858	-1.591607	0.117475384	0.40894522	-5.6945056
TMEM241	-0.2780952	2.13295781	-1.5913889	0.117524507	0.40894522	-4.9647427
PIKFYVE	-0.1620738	4.86161622	-1.5913863	0.117525075	0.40894522	-5.6281837
CST3	0.49153834	7.58308324	1.59114351	0.117579772	0.40894522	-5.6536073
RPS6KB2	0.20060964	4.6153225	1.59108461	0.117593042	0.40894522	-5.589686
VPS18	0.18357052	5.54155989	1.59083404	0.117649506	0.40894522	-5.6816886
ENSCAFG00000018000	-0.1829007	4.16515703	-1.5907686	0.117664263	0.40894522	-5.5046428
MRPL44	-0.179719	3.90624404	-1.5907312	0.117672685	0.40894522	-5.40952
SNX3	0.1526987	5.42186772	1.59040873	0.117745399	0.40894522	-5.6870108
TEX264	0.24463092	4.80424506	1.59036885	0.117754395	0.40894522	-5.6038937
CLIC1	-0.2107442	7.10338485	-1.5903027	0.117769318	0.40894522	-5.7005511
OAS3	-0.3490921	3.07672524	-1.5901119	0.117812369	0.40894522	-5.0936388
REEP5	0.19545385	5.36005845	1.59010482	0.117813959	0.40894522	-5.6657828
JAG2	0.55715989	1.85035907	1.58975191	0.117893615	0.40894522	-5.0605213
ENSCAFG00000028578	0.41792398	0.84518105	1.58970482	0.117904247	0.40894522	-4.7169184
ZC3H3	0.1826281	4.32260341	1.58954846	0.117939554	0.40894522	-5.5636758
TOGARAM1	-0.158703	5.55870218	-1.5895041	0.117949576	0.40894522	-5.6882855
C20H3orf14	0.61438009	0.04479291	1.58937187	0.11797944	0.40894522	-4.5838231
TRUB2	-0.2409262	3.79231853	-1.5893201	0.117991133	0.40894522	-5.3821165
NOP56	-0.2094542	6.72578782	-1.5891927	0.118019925	0.40894522	-5.7056274
PCCB	-0.232415	4.74964975	-1.5890712	0.118047381	0.40894522	-5.6110469
FAM83D	-0.565868	3.21287378	-1.5889098	0.118083859	0.40895911	-5.1552092
ENSCAFG00000007436	-0.2349351	2.55421427	-1.5876104	0.118377908	0.4098274	-5.0601752
HSPA5	0.27956323	10.0359357	1.58751442	0.118399644	0.4098274	-5.515248
JCAD	0.45411295	7.0872516	1.58725679	0.118458023	0.40991683	-5.7110607
PRMT3	-0.1592964	6.86002904	-1.5865709	0.118613556	0.41025835	-5.7035074
ENSCAFG00000030979	-0.3247161	3.68106466	-1.5865343	0.118621857	0.41025835	-5.351267
SRA1	-0.2418586	4.89748985	-1.5862266	0.11869168	0.41028954	-5.6368522
CLPTM1	0.18468402	7.31217919	1.58620752	0.118696021	0.41028954	-5.7015076

SETD1B	-0.1766243	4.63753753	-1.5855772	0.118839175	0.41058299	-5.5873479
YIPF3	0.14157848	6.65080453	1.58530125	0.118901895	0.41058299	-5.7124544
SMAD4	-0.1666362	5.38051781	-1.5852333	0.118917338	0.41058299	-5.6836658
SMPDL3A	1.33143999	-1.4209703	1.58519481	0.118926095	0.41058299	-4.6291439
RASSF4	0.62252581	2.95696596	1.58511651	0.118943897	0.41058299	-5.0764034
HVCN1	0.37993045	4.04804992	1.58492479	0.1189875	0.41062097	-5.5361878
COMMD3	-0.1377124	4.53823048	-1.584617	0.119057531	0.4107139	-5.5538891
OS9	0.16570014	7.37550648	1.58451982	0.119079642	0.4107139	-5.6944828
ADGRA3	0.2505007	5.6794305	1.58426058	0.119138657	0.4107475	-5.661802
P4HA2	0.33531193	8.31277586	1.58407049	0.119181943	0.4107475	-5.6284637
ZNF12	-0.2155338	3.92412301	-1.5840474	0.119187212	0.4107475	-5.4807268
IMPACT	-0.1763279	5.57835765	-1.5834443	0.119324628	0.41107843	-5.6785304
GLA	0.27029626	2.62503678	1.58330069	0.119357374	0.41107843	-5.1638278
TRAPPC13	0.15637641	4.69572828	1.58319646	0.119381145	0.41107843	-5.6181929
TSPAN11	0.73567836	1.75257784	1.58295007	0.119437349	0.41115956	-4.7807854
MBNL1	0.28394172	9.19968948	1.58250243	0.119539513	0.41139883	-5.5580369
LCOR	-0.4004176	1.49895316	-1.5818285	0.11969346	0.41168055	-4.8540644
ZNF367	-0.4435416	2.23656997	-1.5818057	0.119698674	0.41168055	-5.0464524
WISP1	1.43654116	4.63252454	1.58157731	0.119750876	0.41168055	-5.2992262
JCHAIN	0.23439237	5.27039681	1.58157192	0.119752108	0.41168055	-5.6811424
RAB28	-0.3997028	1.08252205	-1.5811267	0.11985393	0.41191817	-4.714541
TJP1	-0.1976456	8.2262962	-1.580678	0.11995664	0.41215871	-5.6749279
ST7L	-0.2294932	3.23368895	-1.5805162	0.119993671	0.41217351	-5.2975118
MAFG	0.33050001	1.65605907	1.58026938	0.120050211	0.41225531	-4.8849832
MAP4	0.12010708	9.07177076	1.57987489	0.120140613	0.41231309	-5.5851306
ORC4	-0.1352032	5.28959993	-1.5796921	0.120182513	0.41231309	-5.6746439
TTLL12	-0.2713826	6.35841965	-1.579653	0.120191483	0.41231309	-5.7231162
PFDN6	-0.2152662	3.31362776	-1.5796247	0.120197972	0.41231309	-5.3168906
VWA8	-0.1419287	5.42253798	-1.5791309	0.120311262	0.41233513	-5.670092
NUDT15	-0.2542398	2.56442176	-1.5790816	0.120322575	0.41233513	-5.0977696
PTGIR	0.50479044	3.19959584	1.57904103	0.120331883	0.41233513	-5.2829663
ACAP1	-0.33942	2.49281919	-1.579026	0.12033534	0.41233513	-5.254658
UNC119B	0.12599055	4.30626206	1.57877697	0.120392504	0.41238689	-5.580546
GYG2	0.98412838	-0.5038054	1.57861337	0.120430075	0.41238689	-4.5250184
UBXN1	-0.1621004	5.29606862	-1.5782999	0.120502094	0.41238689	-5.688858
FGF22	0.78727137	-1.4364846	1.57822003	0.120520444	0.41238689	-4.5004092
ENSCAFG00000032015	0.13127654	5.35682137	1.57794846	0.120582868	0.41238689	-5.6940513
GORASP2	0.12064047	7.25576254	1.5779356	0.120585824	0.41238689	-5.7094705
TOP2A	-1.2271618	5.05662049	-1.577868	0.120601371	0.41238689	-5.3865826
ZBED9	-0.1460201	5.23138747	-1.5778202	0.12061236	0.41238689	-5.6758051
CCDC113	0.40159228	4.60976602	1.57740031	0.120708947	0.41260513	-5.5844974
ZNF395	-0.2795478	5.68054842	-1.5771777	0.120760185	0.41266828	-5.7269429
CMTM6	0.24942336	2.58241434	1.57681714	0.120843197	0.41283995	-5.1899355
USP12	0.26246366	7.27683508	1.57658647	0.120896333	0.41290949	-5.7231765
ASPSCR1	0.21038129	6.21386618	1.57601469	0.121028124	0.41302093	-5.7286189
TRIM14	-0.6851783	1.63027179	-1.5760041	0.121030562	0.41302093	-4.844772
KCNU1	1.04443879	-0.9977661	1.57585196	0.121065653	0.41302093	-4.5508404
PRAM1	0.45049474	1.61058288	1.5756802	0.121105275	0.41302093	-4.8697376
YTHDC1	-0.1831191	5.44947024	-1.5756352	0.121115663	0.41302093	-5.7016099
ENSCAFG00000016263	0.39937864	5.98971706	1.57552632	0.121140781	0.41302093	-5.7268611

ETFDH	0.12355962	6.46199855	1.57544959	0.12115849	0.41302093	-5.729664
FAT2	1.07224522	-1.4050915	1.57504225	0.121252531	0.41322968	-4.4368797
ACTR2	0.21515945	7.6939128	1.57487583	0.121290967	0.41324039	-5.6957577
DKK2	1.33659806	0.08728014	1.5746236	0.121349244	0.41324039	-4.9043289
ZFP1	-0.207629	2.4766289	-1.5746026	0.121354096	0.41324039	-5.1348582
PXMP2	-0.4500419	1.15178028	-1.5736249	0.121580207	0.41389846	-4.7039825
GHITM	0.14248327	6.61686263	1.57305842	0.12171137	0.41418567	-5.7326382
BVES	1.54403221	3.03862171	1.57297653	0.121730339	0.41418567	-4.7572109
OSBPL10	0.50547969	3.98130098	1.57266277	0.121803047	0.41432114	-5.2874746
GRSF1	-0.1389337	5.9626099	-1.572128	0.121927049	0.41454137	-5.7241163
ARL4D	0.4598664	2.43365085	1.57197616	0.121962274	0.41454137	-5.2657695
CSGALNACT2	0.27794771	6.02352177	1.57195785	0.121966522	0.41454137	-5.7258116
BACE2	-0.736932	4.06418202	-1.571798	0.122003625	0.41455561	-5.5361732
ENSCAFG00000004198	-0.560343	0.0379329	-1.5709913	0.122190958	0.41508018	-4.5712005
MRM3	-0.1665923	3.63807582	-1.5708178	0.122231289	0.41510523	-5.4378233
CYB5R4	-0.1679289	3.7552852	-1.5705226	0.122299905	0.41519802	-5.427482
SLC45A1	0.37854799	3.39786548	1.57041672	0.122324537	0.41519802	-5.5369564
SLC9A9	0.32474575	4.25074613	1.57001771	0.122417365	0.41540117	-5.6464814
NUP93	-0.1940691	5.29558751	-1.5695236	0.122532389	0.4154767	-5.7024062
FUT4	0.43670435	1.73132965	1.56947455	0.122543824	0.4154767	-4.8173798
IFT43	0.29546954	3.59254099	1.56917692	0.122613162	0.4154767	-5.3709048
SKP2	-0.4297896	3.37923238	-1.5691455	0.122620481	0.4154767	-5.3783578
PFKL	0.27175332	6.25764844	1.56901013	0.122652032	0.4154767	-5.7399336
ENSCAFG00000008661	0.38539848	6.75758863	1.56890951	0.122675485	0.4154767	-5.7396968
FAM107B	0.41278882	2.66807502	1.56877583	0.122706652	0.4154767	-5.1187506
GAS8	-0.2346693	2.77798814	-1.5683077	0.122815841	0.4154767	-5.2570313
KIAA1958	0.70975715	-0.6189739	1.56829206	0.12281949	0.4154767	-4.5446277
ZNF641	0.62178367	0.29572382	1.56828502	0.122821133	0.4154767	-4.6502982
QDPR	0.20955368	5.10510222	1.56819735	0.122841592	0.4154767	-5.6911514
ATG12	0.21073303	3.84843321	1.56812553	0.122858354	0.4154767	-5.5058698
ANAPC15	0.25392112	3.76839051	1.56790984	0.122908704	0.4154767	-5.4279582
SAMD11	1.5877279	3.23328579	1.56782411	0.122928719	0.4154767	-4.8249913
ARHGEF3	-0.8705474	1.7553276	-1.5677998	0.122934396	0.4154767	-4.8893437
UGGT2	0.21308494	6.90486891	1.56682652	0.123161835	0.41613371	-5.7306979
DDX11	-0.2736667	3.95363771	-1.5666448	0.123204329	0.41614264	-5.4934997
DNAJC3	0.15275808	8.07535159	1.56653275	0.123230552	0.41614264	-5.6755726
PARD3	-0.1895909	6.89704344	-1.5661248	0.12332603	0.41629119	-5.7314168
KDELC1	0.30589174	5.68651439	1.56606237	0.123340642	0.41629119	-5.7269669
HDAC9	0.87975336	3.59036673	1.5644019	0.123729902	0.41749313	-4.978306
GAP43	-0.7815037	4.30948182	-1.5640685	0.123808176	0.4175115	-5.5376315
DNMT3A	-0.3137354	2.64252318	-1.5639722	0.123830799	0.4175115	-5.1145788
CSTF1	0.16417766	4.41362304	1.56395521	0.123834786	0.4175115	-5.5776849
NFYC	-0.1513074	4.18233193	-1.5636252	0.123912317	0.41763445	-5.5361076
ATF7IP	-0.1666262	5.79565316	-1.5634582	0.123951577	0.41763445	-5.738446
ENSCAFG00000006873	0.63692767	2.20539378	1.56337673	0.123970721	0.41763445	-4.810515
SPAST	-0.1681094	3.78229985	-1.5630793	0.124040659	0.41775833	-5.4655472
SAFB2	-0.151959	4.90124386	-1.5622689	0.124231387	0.41824253	-5.6698793
CDH13	0.48443008	9.04652322	1.56218629	0.124250837	0.41824253	-5.6042936
GNPAT	-0.1171287	5.45943211	-1.5616717	0.124372075	0.4185097	-5.7162076
TMED2	0.17629669	8.40802113	1.56156743	0.124396658	0.4185097	-5.6681832

ENSCAFG00000010507	-0.6807265	-0.5734331	-1.5612858	0.124463058	0.41857135	-4.518817
FGL2	0.74543526	6.55268757	1.56119481	0.124484523	0.41857135	-5.6903939
FBXO17	0.77724988	0.7888266	1.561067	0.124514674	0.41857135	-4.5693022
PTGES3	-0.1650025	7.32945763	-1.5601502	0.124731117	0.41918707	-5.7355817
CC2D1B	0.15943825	5.91965872	1.55990089	0.124790023	0.41924691	-5.7505189
RTCB	0.12769158	5.77989321	1.55979314	0.12481549	0.41924691	-5.7325489
IQCJ-SCHIP1	0.43272284	5.07121244	1.55958755	0.124864096	0.41929836	-5.6391577
LRBA	-0.2227684	4.68370862	-1.5593085	0.124930085	0.41940814	-5.6354655
UHRF2	-0.213307	4.24869982	-1.558713	0.125071021	0.41968159	-5.6305169
FAM122B	0.26105232	3.4899919	1.55859506	0.125098946	0.41968159	-5.3122314
INPP1	0.17262245	5.00992151	1.55854208	0.125111492	0.41968159	-5.6948018
QPCT	0.56076364	4.83390647	1.55814813	0.125204816	0.41988282	-5.5638439
DMAC1	-0.1687891	4.26379266	-1.5579211	0.125258629	0.41995147	-5.5424046
HDDC3	-0.2154736	3.79932604	-1.5577368	0.125302312	0.41998614	-5.459025
CRADD	-0.4334227	2.08295576	-1.557544	0.125348043	0.42002765	-5.1360233
UNK	-0.2128339	4.48593504	-1.5572897	0.125408373	0.42006414	-5.587015
MAU2	-0.1835173	4.90935165	-1.557217	0.12542563	0.42006414	-5.6957789
CYB561D2	-0.2682576	2.32227915	-1.5570517	0.125464861	0.42007655	-4.9653723
MDM4	-0.2468308	3.92109204	-1.5569203	0.125496035	0.42007655	-5.5011665
LPL	-0.8740074	-2.1729438	-1.5563608	0.125628939	0.4204097	-4.4408229
SLC39A13	0.2885107	4.80830273	1.55582861	0.125755434	0.42072123	-5.6613303
PLEKHF1	-0.5426423	2.50211671	-1.555647	0.125798635	0.42075401	-5.0404736
ST6GALNAC5	0.96969815	0.82256194	1.55534227	0.125871128	0.42088472	-4.6222211
TRIM25	-0.260929	6.14548369	-1.554959	0.125962372	0.42107803	-5.7579382
BANK1	-0.3107533	4.05684151	-1.5546286	0.126041068	0.42122932	-5.6012659
PRPF40B	-0.2070357	3.47208833	-1.5544121	0.126092643	0.4212899	-5.3553174
ISCU	0.18713443	5.1653951	1.55405412	0.126177977	0.42146322	-5.6964693
NOP2	-0.2397531	5.2773313	-1.5539125	0.12621176	0.4214643	-5.6890144
ADAM10	-0.1598856	6.0124434	-1.5536734	0.126268774	0.42152328	-5.7586853
NDRG3	0.1908886	4.37735367	1.55355785	0.126296352	0.42152328	-5.6162637
FAS	0.24822492	5.50749246	1.55314093	0.12639587	0.42166499	-5.7110914
RAPGEF5	0.9325787	1.72716142	1.55309949	0.126405764	0.42166499	-4.9578485
PHF8	-0.1500019	4.06889848	-1.5525856	0.126528528	0.42196276	-5.5581983
TACC2	-0.227716	6.37765432	-1.5522071	0.126618987	0.42209329	-5.7646217
SLC16A3	0.4986086	4.78719449	1.55214148	0.12663469	0.42209329	-5.7235338
NCOR1	-0.1173892	7.31357413	-1.5517676	0.126724116	0.42227962	-5.7440685
NINJ2	0.44721076	1.44367312	1.55155894	0.126774056	0.42233431	-4.8283927
HELZ2	-0.3762429	3.81092917	-1.5511644	0.126868513	0.42253723	-5.5160929
COPS2	-0.1396347	6.84593944	-1.5504576	0.12703787	0.42290681	-5.7567058
DVL1	0.16706759	5.17216115	1.55042107	0.127046633	0.42290681	-5.6997259
ENSCAFG00000030354	-0.3849361	1.86376996	-1.5499056	0.12717026	0.4232065	-4.9492394
OAF	-0.4423855	6.60125865	-1.5495728	0.127250128	0.423349	-5.7374084
SALL2	0.24016584	3.6685482	1.54944717	0.127280302	0.423349	-5.4656781
DNPEP	-0.2496114	4.18797389	-1.549059	0.127373532	0.42336355	-5.5807229
ARHGAP21	-0.1988282	6.9430013	-1.5490337	0.127379617	0.42336355	-5.7602129
ARID5A	0.41813736	2.93524012	1.54900915	0.127385508	0.42336355	-5.1669874
PGRMC1	0.14579421	6.69203784	1.54865926	0.127469598	0.42343596	-5.7633783
PBX1	-0.5816303	5.08202869	-1.5486387	0.127474529	0.42343596	-5.5097838
ENSCAFG00000006911	0.33697362	1.56289679	1.54841171	0.127529118	0.42349005	-4.8917214
TGFBRAP1	-0.1792246	4.61277332	-1.5481925	0.127581855	0.42349005	-5.6406179

ZBTB12	-0.3294687	2.32749709	-1.5481516	0.127591674	0.42349005	-5.0632888
BICD2	0.12158807	5.49753561	1.54761921	0.127719816	0.42380369	-5.7500826
MLLT10	-0.2039172	4.67839064	-1.5474046	0.127771507	0.42386355	-5.6360223
NOL7	-0.2162698	4.25022403	-1.5470343	0.127860705	0.42404777	-5.5416429
ILF3	-0.1547676	7.29681159	-1.5465873	0.127968471	0.42409907	-5.7608273
ATP6V1H	0.12651662	6.47164071	1.54644757	0.128002162	0.42409907	-5.7732779
PLD3	0.30999427	6.5366681	1.5464121	0.128010717	0.42409907	-5.756425
EFCAB6	-0.8634099	-0.4510961	-1.5464116	0.128010849	0.42409907	-4.5965222
BCAT1	-0.2801859	6.03790505	-1.5458718	0.128141099	0.42441896	-5.7726373
UBTF	-0.1476629	6.22917893	-1.545704	0.128181609	0.42444152	-5.7744004
ZNF629	0.25125396	3.26044183	1.54529784	0.128279715	0.42462993	-5.3761296
DHX9	-0.1715687	6.93879956	-1.545062	0.128336707	0.42462993	-5.7715587
ZNF425	0.2462915	3.56950519	1.54504985	0.128339644	0.42462993	-5.4480871
PTPRE	0.65307419	1.5735228	1.54479818	0.128400484	0.42469645	-5.2325165
ELOC	0.28106921	4.28538672	1.54468778	0.128427181	0.42469645	-5.5631585
ENSCAFG00000014154	-0.880135	0.47915416	-1.5444016	0.128496404	0.4247681	-5.0587545
SLC35E1	0.20334647	4.56806748	1.5443194	0.128516294	0.4247681	-5.6425615
LTBP3	0.36692579	7.53074674	1.54410037	0.128569303	0.42483183	-5.7131886
MLXIP	-0.1616364	5.67692433	-1.5435194	0.128709986	0.42508541	-5.7743991
HBEGF	0.73310794	1.80092799	1.54332728	0.128756538	0.42508541	-5.0466875
MINK1	0.17881792	6.18584376	1.5431895	0.12878993	0.42508541	-5.7787775
TRIOBP	-0.1947298	6.37358525	-1.543165	0.128795881	0.42508541	-5.77638
SNX8	-0.1792689	5.84675219	-1.543087	0.128814785	0.42508541	-5.750683
STAP1	-0.5454329	1.07954143	-1.5429271	0.128853556	0.42510199	-4.8295247
ASH2L	-0.189289	4.07953141	-1.5427771	0.128889924	0.42511063	-5.5203445
ENSCAFG00000010433	0.11306989	6.4444272	1.54253058	0.128949721	0.42519651	-5.7800673
SLC36A1	0.22485313	4.43190573	1.54205796	0.12906443	0.42546338	-5.5673961
INTS12	-0.2812067	3.44956965	-1.5415848	0.129179362	0.42565116	-5.4557241
REST	0.20370183	4.54239604	1.54154518	0.129188979	0.42565116	-5.6508706
ENSCAFG00000016281	0.41539466	1.12549714	1.54129639	0.129249443	0.42573901	-4.8094971
LURAP1	0.41530031	1.66194673	1.54071247	0.129391441	0.42607496	-5.0817084
IL4I1	-0.6751223	-0.8471784	-1.5405988	0.129419086	0.42607496	-4.5849019
PITPNM1	0.51915295	5.1604212	1.54002511	0.129558753	0.42642332	-5.714266
HSD17B8	-0.2678272	3.92260731	-1.5394353	0.129702461	0.42663747	-5.5158307
PPIB	0.27236158	9.09239507	1.53942866	0.129704077	0.42663747	-5.6270401
CBLC	0.52591583	3.03361988	1.53934107	0.12972543	0.42663747	-5.156231
ENSCAFG00000023499	-0.3383635	2.29856747	-1.5390252	0.129802458	0.42664732	-5.1421717
ABHD17B	-0.310244	3.40426852	-1.5389162	0.129829032	0.42664732	-5.4192571
PDE4B	-0.7581647	3.01280613	-1.5389121	0.12983004	0.42664732	-5.6185851
MTA2	-0.1407309	6.60097312	-1.5387505	0.12986947	0.42666558	-5.7854475
PRPH	1.15243252	0.61840752	1.53841794	0.129950634	0.42682091	-4.7574557
KIFC3	0.23178257	5.11856065	1.53825924	0.129989382	0.42683688	-5.6737622
DLG4	0.19099022	3.55334089	1.53810132	0.130027948	0.42685005	-5.5364019
ENSCAFG00000010412	-1.2050934	1.46810782	-1.5378041	0.130100563	0.42685005	-4.6889966
DNAH10	0.76354575	-0.3739359	1.53775592	0.130112332	0.42685005	-4.6408407
FBXO45	0.43072759	0.96717141	1.537528	0.130168039	0.42685005	-4.8468479
CDC14B	-0.3594569	2.8931499	-1.5374595	0.130184788	0.42685005	-5.3253168
NLGN2	0.27878701	2.85040171	1.53741067	0.130196722	0.42685005	-5.3989995
TIMP4	-0.693241	-1.8056745	-1.5371567	0.130258831	0.42694255	-4.5614212
FBXL3	-0.1560783	5.31238983	-1.5368348	0.130337591	0.42708474	-5.7476279

SSH3	0.23630619	6.10267505	1.53658238	0.130399362	0.42708474	-5.7879562
HMGCL	-0.2188452	4.42905008	-1.5364397	0.130434298	0.42708474	-5.6176456
MYRF	1.69285818	3.55266253	1.53639439	0.130445388	0.42708474	-5.122399
LIPT1	-0.2496264	3.1243151	-1.5362868	0.130471744	0.42708474	-5.3631562
EPHA2	0.31751328	6.94462431	1.53600777	0.130540087	0.42719744	-5.78197
EZH1	-0.3445893	3.26732304	-1.5351024	0.130762064	0.42781272	-5.4628325
VEGFC	-0.6458169	5.01346403	-1.5349203	0.130806751	0.42784779	-5.6430581
ENSCAFG00000010899	0.20457199	5.03512273	1.53462159	0.130880068	0.42797646	-5.712566
P2RY2	0.74962566	-1.927151	1.53427142	0.130966065	0.42814652	-4.5096961
PRMT5	-0.170994	5.71375479	-1.5336415	0.131120877	0.42843328	-5.7679139
TMEM132A	0.25542319	6.14659436	1.53363771	0.131121807	0.42843328	-5.7916489
INTS2	-0.1475739	4.7564203	-1.5334721	0.131162533	0.4284552	-5.6912685
JMJD7	-0.3645992	2.34554482	-1.5330247	0.131272608	0.4287036	-5.1085051
ENSCAFG00000026614	0.49486925	0.65771663	1.53281026	0.131325387	0.42876479	-4.7857294
CTR9	-0.1218807	6.13370038	-1.5320508	0.131512474	0.42926435	-5.7944029
CELSR1	-1.1583901	-0.7619613	-1.53178	0.131579209	0.42937091	-4.5735382
TMEM37	-0.8441575	-2.9803844	-1.5315893	0.131626237	0.42941313	-4.4396542
IGF2BP1	-1.0634114	0.18218211	-1.530864	0.131805228	0.42988572	-4.5495348
ELAC1	-0.1958542	3.07284822	-1.5305522	0.131882223	0.43002549	-5.3287717
JDP2	-0.2476481	3.57380733	-1.5304093	0.131917528	0.43002929	-5.3821588
BTG3	0.26601989	4.34114789	1.52912371	0.13223547	0.4309542	-5.5784075
USE1	-0.3689108	2.80194771	-1.5280922	0.132491011	0.43160757	-5.3533252
RAB38	-0.9098903	-1.1311008	-1.5280379	0.132504483	0.43160757	-4.5619012
ANKRD17	-0.1204082	7.43536983	-1.5278138	0.132560039	0.4316769	-5.768516
AK5	1.06688893	5.17965379	1.52651938	0.132881411	0.43258872	-5.362485
ACAD9	0.1450685	5.94554897	1.52640946	0.13290873	0.43258872	-5.8007719
SCARA5	1.43178768	2.00249223	1.52609563	0.132986749	0.43273084	-5.7551209
LPGAT1	-0.2678588	7.53947679	-1.5257678	0.133068302	0.43273774	-5.7609874
YEATS2	-0.1773224	7.30154433	-1.5257542	0.133071673	0.43273774	-5.7575975
BDP1	-0.2346014	5.16612512	-1.5256608	0.133094902	0.43273774	-5.7588267
CCPG1	0.24576516	6.39866249	1.52532064	0.133179573	0.43273774	-5.7996164
OCIAD2	0.58037229	4.61771842	1.52526508	0.133193405	0.43273774	-5.6727567
FAM171A2	0.38848249	2.43209178	1.52524827	0.133197592	0.43273774	-5.1187403
PRDM4	-0.1329294	5.18507783	-1.5250028	0.13325872	0.43273774	-5.7432392
PTPRB	-1.0123305	4.98751877	-1.5248844	0.133288215	0.43273774	-5.5127728
NOX4	-0.436693	4.11649058	-1.5246997	0.133334222	0.43273774	-5.5553328
PRKAR2A	-0.1531097	5.92951608	-1.5245802	0.133364016	0.43273774	-5.8005146
RAB4B	0.19725897	4.24786049	1.5241714	0.133465923	0.43273774	-5.6394301
WDR54	0.25450195	2.5821635	1.52408268	0.133488051	0.43273774	-5.2990421
LY96	0.23057775	3.30091229	1.52401054	0.133506044	0.43273774	-5.4653589
UCP1	0.79951038	-1.5752366	1.52376607	0.133567035	0.43273774	-4.5247184
RNASEH1	0.17869938	4.0667487	1.52358584	0.133612012	0.43273774	-5.5550641
LDAH	0.17057881	5.32782922	1.52346949	0.133641056	0.43273774	-5.7794224
CDC5L	-0.1277768	6.44615637	-1.5233232	0.133677571	0.43273774	-5.8082431
PLPP7	0.46964857	1.06625127	1.52328217	0.133687825	0.43273774	-4.9204955
SHROOM3	0.88448838	-0.1227345	1.52324383	0.133697399	0.43273774	-4.6321244
ECI1	0.31283141	3.39112823	1.5231965	0.133709219	0.43273774	-5.4335941
ENSCAFG00000022711	-0.4955838	13.4529699	-1.5231921	0.133710327	0.43273774	-5.3128973
RDX	0.12657368	7.50490588	1.52264179	0.133847809	0.43307141	-5.782679
SLC36A4	0.23006013	6.2550681	1.52214106	0.13397301	0.43336518	-5.8096353

RTL6	0.20167845	3.65175178	1.52143386	0.134149998	0.4336657	-5.4754055
ZNF134	-0.192915	3.53341969	-1.521041	0.134248402	0.4336657	-5.5030753
MAP2K7	0.1575519	5.00790756	1.52097756	0.13426429	0.4336657	-5.7717353
IQGAP2	-0.9428766	-0.3742561	-1.5209749	0.134264955	0.4336657	-4.5681579
P4HA3	-0.4341616	5.60830146	-1.5209668	0.134266976	0.4336657	-5.7516767
RPUSD4	-0.18934	3.48324294	-1.5209449	0.134272485	0.4336657	-5.4567599
ATG2A	-0.2431366	5.28474682	-1.5206825	0.134338238	0.43376684	-5.7948138
MBTD1	-0.2735965	2.58143124	-1.5204895	0.134386625	0.43381187	-5.2877578
TMEM38A	0.5822383	1.62101914	1.52007363	0.134490936	0.43394366	-4.927334
ZBTB11	-0.1733238	4.54896798	-1.5198492	0.134547253	0.43394366	-5.6969237
TBL1XR1	-0.2135003	3.93037342	-1.5197983	0.134560022	0.43394366	-5.5748001
SAP18	0.16989483	3.76278525	1.51968232	0.134589144	0.43394366	-5.5044789
CAPG	0.33640392	9.34559939	1.51958091	0.134614605	0.43394366	-5.6773731
FAM91A1	0.17995477	4.9999408	1.51950304	0.134634156	0.43394366	-5.7349473
SOX5	0.47614927	0.88351956	1.51925916	0.134695411	0.43399495	-5.2245129
STX10	-0.2333645	3.57011815	-1.5191653	0.13471898	0.43399495	-5.5071068
CACNA1A	-0.6242873	2.01340326	-1.5183829	0.134915674	0.43451747	-5.1449929
RBM17	-0.1430772	5.29010223	-1.5179024	0.135036578	0.43479569	-5.7650321
CCDC134	0.25597811	3.11329642	1.5173185	0.135183612	0.43515788	-5.3410378
TNMD	0.68368951	-2.9499742	1.51681797	0.135309757	0.43545266	-4.451602
GLI3	-0.535969	5.11045926	-1.5160639	0.135499981	0.43592317	-5.7778643
ARHGEF25	0.51382959	5.17722002	1.51596405	0.135525178	0.43592317	-5.7604042
BUB1	-1.4905912	2.87869941	-1.5156084	0.135614983	0.43610067	-4.8854123
SLC25A44	0.18123892	4.09208424	1.51541013	0.135665063	0.43615037	-5.5740501
UBXN4	0.11248313	7.10654441	1.5145811	0.135874638	0.43666816	-5.8052318
SLC23A2	0.19405375	4.81990279	1.51418127	0.135975807	0.43666816	-5.7726633
ENSCAFG00000029346	-1.7828352	-0.1565775	-1.5141554	0.135982352	0.43666816	-4.7855921
SCUBE2	0.82053095	2.76706016	1.5140075	0.136019794	0.43666816	-5.253202
TAX1BP1	0.22161721	7.50752749	1.51394197	0.136036384	0.43666816	-5.8003274
CCL5	0.6542709	0.4533868	1.51383564	0.136063308	0.43666816	-5.0147111
DUSP11	-0.1458339	5.07977542	-1.5136661	0.136106246	0.43666816	-5.7497426
PERP	-0.8857138	-0.2091265	-1.5134459	0.136162031	0.43666816	-4.5658787
TBC1D9B	0.10577863	7.12196611	1.51340925	0.136171315	0.43666816	-5.8064274
ACAD11	0.12381192	5.95391285	1.51340341	0.136172795	0.43666816	-5.811665
FBLIM1	0.40019637	6.24991976	1.51291895	0.136295597	0.4369256	-5.8229381
PPP1CA	-0.1482757	7.08712697	-1.5123963	0.136428173	0.4369256	-5.8140799
ENSCAFG00000030486	-1.5334085	2.07220023	-1.5123847	0.136431115	0.4369256	-4.7778394
ADAMTS9	-1.1896509	-1.6179798	-1.5123562	0.13643836	0.4369256	-4.6538859
OPTC	0.85926358	-0.8244774	1.51233173	0.136444565	0.4369256	-4.5692024
FRZB	1.4004756	-0.4107256	1.51226617	0.136461203	0.4369256	-4.5051279
IFI30	0.32806367	5.78263628	1.51208284	0.136507744	0.43694747	-5.8194542
HIF1A	0.25173203	8.48693154	1.51194858	0.136541834	0.43694747	-5.7566927
GNG11	0.83687256	3.55030932	1.51170158	0.136604567	0.43694747	-5.2498229
NUP35	-0.2232105	4.4536451	-1.5116928	0.136606792	0.43694747	-5.635962
UNC5A	0.339583	2.36465863	1.51144422	0.136669956	0.43703853	-5.3671181
KCTD3	-0.1389463	7.16165306	-1.510884	0.136812373	0.43727751	-5.8200573
ADCY1	-0.6460361	-0.1189364	-1.5108772	0.13681412	0.43727751	-4.6820068
ARF6	-0.210547	5.41003468	-1.5106351	0.136875707	0.43728771	-5.8033152
FNIP1	-0.1812728	4.94459979	-1.510494	0.136911594	0.43728771	-5.7431056
MMRN2	-1.8496194	-0.3544173	-1.5104552	0.136921462	0.43728771	-4.8463599

NUDC	0.17662207	6.22647039	1.50979004	0.137090838	0.43771767	-5.8274096
HJURP	-1.5984496	1.77239066	-1.509531	0.137156832	0.43779481	-4.704449
ENSCAFG00000012303	0.16258776	7.17775564	1.50942243	0.137184511	0.43779481	-5.8117237
ABCB10	-0.1976112	5.15048044	-1.5092221	0.137235579	0.43784685	-5.782913
DNAJC6	0.81272111	-0.6117048	1.50905765	0.137277511	0.43786972	-4.621743
C30H15orf39	-0.3789142	3.10220431	-1.5084329	0.137436918	0.43826719	-5.3877477
ATF6B	-0.1431423	5.91396339	-1.5079152	0.137569102	0.43845614	-5.8227017
TBC1D30	-0.7906827	-1.3274785	-1.5078457	0.137586869	0.43845614	-4.6200135
MTMR3	-0.1377693	5.30362805	-1.5077919	0.137600597	0.43845614	-5.7732253
CD82	0.85419222	3.68240665	1.50747093	0.137682633	0.43859399	-5.1728157
NBEA	0.28495893	4.12654322	1.50728131	0.13773111	0.43859399	-5.5523538
GPATCH8	0.14761939	6.9037468	1.50721401	0.137748319	0.43859399	-5.822439
GTF2A1	-0.1799375	5.2145985	-1.5069314	0.137820599	0.43861413	-5.7816938
SOAT2	-0.6122648	0.64634703	-1.5068647	0.13783767	0.43861413	-4.7954485
CNPPD1	-0.2274095	4.99200946	-1.5066762	0.137885909	0.43861413	-5.7493824
PPP1CC	-0.1525544	6.45709987	-1.5065755	0.137911682	0.43861413	-5.8315523
CDKN2AIP	-0.1934604	3.88624051	-1.5064677	0.137939267	0.43861413	-5.5864806
SMC5	-0.1731091	5.93449436	-1.5062147	0.13800405	0.43861413	-5.8276041
SKP1	0.1063589	6.46488332	1.50612826	0.138026185	0.43861413	-5.8319034
ENSCAFG00000008941	0.79139338	1.65466795	1.50610079	0.138033219	0.43861413	-4.9686041
DPH3	0.47539036	0.65557306	1.50507626	0.138295834	0.43919617	-4.7972084
SBF2	-0.17086	6.80773464	-1.5049981	0.138315877	0.43919617	-5.823135
ALPL	0.70420663	-0.5895369	1.5049782	0.13832099	0.43919617	-4.7561013
ENSCAFG00000007230	0.76868056	0.86010977	1.50478771	0.138369869	0.43924064	-4.7515088
GTF2A1L	0.34627729	4.32005099	1.50449848	0.138444109	0.43936558	-5.6282555
CLDN6	0.71559173	0.07832792	1.50402182	0.13856653	0.43964333	-4.7667653
ENSCAFG00000003207	-0.4691784	0.8464081	-1.5038658	0.138606613	0.43965976	-4.7743873
IQCG	-0.4283976	0.81382929	-1.5034828	0.138705065	0.43982524	-4.8616102
ENSCAFG00000031112	0.58603075	-0.3885909	1.50339122	0.138728619	0.43982524	-4.6470348
TOP1	-0.1684838	6.11614447	-1.5030603	0.138813748	0.43993077	-5.8368087
CABLES2	0.16784222	4.6323195	1.50294991	0.13884214	0.43993077	-5.69144
PIGL	0.26701854	2.19784373	1.50285454	0.138866684	0.43993077	-5.1388297
B3GNT5	-1.0788902	-0.5577032	-1.5023906	0.138986129	0.44019846	-4.5288469
ZNF829	-0.3209939	2.59851339	-1.5020581	0.139071764	0.44035896	-5.218946
ASPM	-0.9171629	4.36273533	-1.5019166	0.139108241	0.44036376	-5.2669189
LCP1	1.14896843	3.16692169	1.50170686	0.139162297	0.44042419	-4.94888
MFGE8	0.26648156	9.33390146	1.50119672	0.139293857	0.44072982	-5.6728781
CSF1	0.49856935	7.20115758	1.50077661	0.139402274	0.44096209	-5.7654062
ATP2A2	0.17830685	7.92687424	1.50047501	0.139480147	0.44109765	-5.7914763
TRIM45	-0.4529803	2.12375555	-1.499992	0.139604938	0.44138148	-5.0615797
ENSCAFG00000000683	-0.5409672	0.60932836	-1.4996504	0.139693241	0.44154308	-4.7284964
NPAS2	-0.8036885	1.06212872	-1.4995231	0.139726161	0.44154308	-4.9212294
COL12A1	0.78508007	10.7680335	1.49928077	0.139788841	0.44163036	-5.5985273
ENSCAFG00000017317	-0.5170882	3.35468339	-1.4990041	0.139860444	0.44174578	-5.1731392
ENSCAFG00000000072	-0.1761863	5.80487646	-1.4983386	0.140032755	0.44212768	-5.8401116
CCNE2	-0.9143465	0.2678163	-1.4982023	0.140068065	0.44212768	-4.6603072
U2SURP	-0.178151	6.16307823	-1.4981306	0.140086661	0.44212768	-5.8426887
MSN	0.25104372	9.75651315	1.49772574	0.140191605	0.44233188	-5.6576816
G6PD	-0.2308851	7.04589372	-1.4974906	0.140252574	0.44233188	-5.8372507
LAMA3	0.59355165	6.15636237	1.4974747	0.140256712	0.44233188	-5.8433431

ARHGEF10	-0.1784528	5.96519942	-1.4970295	0.140372238	0.44258541	-5.8424711
ENSCAFG00000031092	-0.2077622	3.4456549	-1.4968515	0.140418436	0.44262028	-5.4518511
NUP214	-0.1554755	5.73317719	-1.4964513	0.140522381	0.44283711	-5.8280185
ZMYM2	-0.187181	5.4439436	-1.4960115	0.140636666	0.44308056	-5.8046433
ISOC2	0.21569966	4.05397688	1.49583255	0.140683193	0.44308056	-5.6680772
CRLF3	0.1780438	5.24295437	1.4956562	0.140729053	0.44308056	-5.8098411
DCTN6	0.16161221	5.25734368	1.49538947	0.140798439	0.44308056	-5.7958635
CSPP1	0.19034822	4.24120868	1.49534313	0.140810497	0.44308056	-5.6687026
NRXN2	0.8881938	-1.4583275	1.49534237	0.140810694	0.44308056	-4.5342057
ENSCAFG00000010098	0.23524346	3.58917426	1.49507262	0.1408809	0.44319076	-5.487838
PDLIM1	0.28454564	7.00579487	1.49471993	0.140972731	0.44336892	-5.8438623
ENSCAFG00000030449	0.34828457	1.19021085	1.49437973	0.141061357	0.44348768	-4.8743415
CCM2	0.14882679	5.30216003	1.49429319	0.141083908	0.44348768	-5.8104676
HS6ST1	0.31017108	2.52071623	1.49414313	0.14112302	0.44348768	-5.2424184
NETO2	-0.658894	1.30168859	-1.4940345	0.141151328	0.44348768	-4.763683
TAF9	-0.1617081	4.36518232	-1.4935534	0.141276801	0.44377122	-5.6477245
WDR59	-0.1322666	4.25718223	-1.4928403	0.141462954	0.44424517	-5.6924114
TBC1D13	0.1321605	3.77897957	1.49230284	0.141603361	0.44441811	-5.5873757
YARS	-0.1966939	6.60574575	-1.4922748	0.1416107	0.44441811	-5.853347
THSD1	-0.4539487	3.98727323	-1.4922244	0.141623873	0.44441811	-5.4897814
SURF1	0.19833224	3.85217455	1.49179426	0.141736335	0.44466024	-5.6029114
EIF4A1	-0.1743728	8.51564981	-1.4916338	0.141778321	0.44468121	-5.7592148
ZNF785	-0.2691541	2.06546549	-1.4913371	0.141855955	0.44481394	-5.0872875
ZNF786	-0.214734	3.14499747	-1.4909297	0.141962607	0.44502522	-5.3975207
TRIM47	-0.7288478	3.55974823	-1.4908099	0.141993993	0.44502522	-5.5453071
ENSCAFG00000008814	-0.3222844	1.35600365	-1.4904396	0.142091018	0.44510242	-4.8351457
SLC2A9	-0.998017	0.29703675	-1.4903481	0.142114984	0.44510242	-4.820269
CDH23	0.80819022	-2.1360186	1.49031129	0.142124637	0.44510242	-4.6126242
DCXR	0.32849985	3.54314233	1.4901652	0.142162936	0.44511169	-5.5740969
AGTR1	-1.6481605	-0.7211992	-1.4898381	0.142248702	0.44520153	-4.5069742
ZNF382	-0.3120329	3.27685383	-1.4897623	0.142268601	0.44520153	-5.391778
ZCCHC7	-0.1942165	3.71249439	-1.4896515	0.142297661	0.44520153	-5.5777635
HDAC6	-0.2092633	4.97074231	-1.4894876	0.142340666	0.44522548	-5.7875452
BHLHB9	-0.1863912	3.93065495	-1.489214	0.142412484	0.4452377	-5.6157512
RTN2	0.27174966	3.24123007	1.4890954	0.142443635	0.4452377	-5.5439903
VPS37A	0.20953213	5.75029164	1.48906882	0.142450614	0.4452377	-5.833956
PEX11B	0.26970341	2.28887242	1.48891051	0.14249219	0.44525716	-5.2222169
MRPL15	-0.2149668	4.07916943	-1.4887022	0.142546906	0.44531766	-5.6157793
BCL7A	0.35933494	1.84938551	1.4881802	0.142684116	0.44551783	-5.052071
BOD1L1	-0.157596	5.95428669	-1.4880098	0.142728924	0.44551783	-5.8572727
PTPRD	2.04111087	2.41713513	1.48800113	0.142731208	0.44551783	-4.7424646
TOMM40L	0.17582309	3.40960449	1.48792034	0.142752458	0.44551783	-5.4986982
ENSCAFG00000013391	-0.2577197	2.44862942	-1.4875029	0.142862299	0.44575019	-5.2689798
FASTKD2	-0.1962519	4.04172717	-1.4872819	0.142920462	0.44582123	-5.6018685
MAP1LC3A	0.23055244	4.98276683	1.48656493	0.143109334	0.44629987	-5.7833872
ATG4C	-0.1662936	4.11083333	-1.4863173	0.143174605	0.4463929	-5.6638934
BAG1	0.19940598	6.7127323	1.48543537	0.143407282	0.44681935	-5.8590259
ENSCAFG00000000640	-0.4999348	1.34482532	-1.4852136	0.143465831	0.44681935	-5.0131673
RNPEP	-0.1681989	6.22548804	-1.485182	0.143474184	0.44681935	-5.8626053
SUSD6	0.20377233	6.37698888	1.4850836	0.143500169	0.44681935	-5.8600796

PFDN2	0.18915297	4.02284718	1.48494079	0.143537895	0.44681935	-5.5920332
NAA16	-0.1940919	3.82407732	-1.4849379	0.143538663	0.44681935	-5.6314336
RBM22	-0.1403629	4.7843584	-1.4847479	0.143588846	0.44681935	-5.7493701
CFAP70	-0.4365437	1.77316199	-1.4846839	0.143605768	0.44681935	-4.9932892
ENSCAFG00000031865	-1.1529167	-0.1052852	-1.4845335	0.143645525	0.44681935	-4.8715967
ENSCAFG00000004444	-0.2899231	2.50762125	-1.4844556	0.143666115	0.44681935	-5.288555
THBS1	0.59341849	12.6771152	1.48348101	0.143923948	0.44751075	-5.4375785
ERCC2	0.17638003	3.78904481	1.48284215	0.14409316	0.44792632	-5.5662245
KNL1	-1.6316272	3.85372243	-1.482374	0.144217254	0.44820146	-5.1042543
ENSCAFG00000031218	0.2165215	5.17711324	1.48123075	0.144520656	0.44893574	-5.8360059
BEND6	-0.534034	3.52078309	-1.4812151	0.144524805	0.44893574	-5.2812948
CEP41	0.30709498	4.00225532	1.4804269	0.144734289	0.44938764	-5.5875024
FAM208B	-0.1550944	6.60799072	-1.4803993	0.144741636	0.44938764	-5.8701158
ADGRA2	-0.3949613	6.28867137	-1.4798538	0.144886747	0.44972732	-5.8695945
BICRAL	-0.2289184	4.64213756	-1.4789201	0.14513542	0.45038821	-5.7659995
MICU1	-0.1207357	5.54412292	-1.4785662	0.145229737	0.45047335	-5.8426057
AHCY	0.28917015	5.59560309	1.47854881	0.14523438	0.45047335	-5.8536492
EIF3G	-0.1712529	6.4443459	-1.4780649	0.145363453	0.45076269	-5.8737289
TTF2	-0.1284109	6.42755011	-1.4774226	0.145534907	0.45118329	-5.8742433
ANAPC10	0.36009268	1.77029602	1.47671253	0.145724656	0.45166038	-5.0240374
AUP1	0.1533776	5.79488466	1.47627242	0.145842355	0.45188683	-5.8534401
CLIP1	-0.1507672	7.26922504	-1.4761711	0.145869472	0.45188683	-5.8668574
RERE	0.19417171	6.24827734	1.47580077	0.145968573	0.45199092	-5.8750672
ENSCAFG00000019232	0.08556277	6.03512606	1.47577737	0.145974837	0.45199092	-5.8739866
TUBGCP5	-0.1484175	4.83454146	-1.4754378	0.146065754	0.45207441	-5.7855035
RNF14	0.15003531	5.10742287	1.4753825	0.146080578	0.45207441	-5.7977429
MAPKBP1	0.17954919	5.05518034	1.47526244	0.146112741	0.45207441	-5.8542169
BMPR1A	0.62592443	4.90490334	1.47511915	0.146151132	0.45207441	-5.7853792
SUSD1	0.72446415	0.85894769	1.47496322	0.146192922	0.45207441	-5.071431
MMAA	-0.2528171	2.18077311	-1.4747858	0.146240469	0.45207441	-5.1923472
FTSJ3	-0.2234701	5.09564065	-1.4746987	0.146263825	0.45207441	-5.7909141
ITPR3	-0.3805926	6.49561707	-1.4745959	0.146291391	0.45207441	-5.8487676
NFYB	-0.2083634	3.79430403	-1.4744713	0.146324814	0.45207441	-5.5779707
GRASP	-0.4192672	2.41715802	-1.4739076	0.146476081	0.45232094	-5.2099457
RRH	0.61299515	-0.6623784	1.47376317	0.146514842	0.45232094	-4.6891093
FGFR1OP2	-0.2043426	3.639917	-1.4736154	0.146554526	0.45232094	-5.5647613
ORMDL3	0.22411889	5.36737109	1.47357738	0.146564732	0.45232094	-5.8454405
REV1	-0.1632594	4.563903	-1.4734446	0.146600385	0.45232094	-5.732103
MYO19	-0.2099676	4.22627267	-1.4732882	0.146642405	0.45232094	-5.5564596
BID	-0.1511227	5.49609435	-1.4732377	0.146655981	0.45232094	-5.8327613
ILVBL	-0.1862844	5.31331043	-1.4729498	0.146733343	0.45244876	-5.8467244
SP7	0.79202848	0.14166847	1.47265804	0.146811794	0.45246591	-4.7275991
HOXB8	-0.951016	-2.0823089	-1.4723009	0.146907872	0.45246591	-4.5282084
VAMP7	0.16629785	6.35241486	1.47229553	0.146909304	0.45246591	-5.8825107
TPD52L2	0.13175217	5.32933363	1.4722826	0.146912784	0.45246591	-5.8396424
ENSCAFG00000014627	-0.6126585	-0.65965	-1.4722613	0.146918511	0.45246591	-4.5781476
SLC39A10	0.16633685	5.87263565	1.47135441	0.147162692	0.45278822	-5.8821871
TFPT	0.18522924	2.61384984	1.4711409	0.147220226	0.45278822	-5.2859818
COL28A1	1.14744279	-0.7289434	1.47088748	0.147288534	0.45278822	-5.1450528
CCNB2	-1.6655733	2.08877982	-1.4708747	0.147291971	0.45278822	-4.7731958

BCLAF1	-0.1624096	7.14421343	-1.4708587	0.147296285	0.45278822	-5.8743997
NPAT	-0.2338721	4.10052393	-1.470842	0.147300791	0.45278822	-5.6702855
DENND4B	-0.2470655	5.10936863	-1.4707992	0.147312339	0.45278822	-5.8223047
ENSCAFG00000029779	-0.7423925	-0.2847138	-1.4705302	0.147384877	0.45278822	-4.7279625
TRIM9	-0.7850622	-1.0409808	-1.4705291	0.147385167	0.45278822	-4.7280763
SCCPDH	0.14547085	5.10921207	1.47010266	0.147500246	0.45278822	-5.8121579
PRMT9	-0.1709259	3.81547626	-1.4698062	0.147580277	0.45278822	-5.6345536
SRP14	0.18560692	4.96381397	1.46977069	0.147589867	0.45278822	-5.797783
EXOSC1	-0.1865421	3.00226287	-1.4697176	0.1476042	0.45278822	-5.3458657
GSDMD	-0.2265445	4.75010134	-1.4696235	0.147629623	0.45278822	-5.7955914
TBCA	0.16393453	5.05892196	1.46958905	0.147638924	0.45278822	-5.8108461
GPATCH11	0.19236778	3.27656947	1.46948617	0.147666715	0.45278822	-5.5341007
COL8A1	1.19953588	6.85462986	1.46946767	0.147671713	0.45278822	-5.851464
ACSL6	1.27143054	0.7756333	1.46943028	0.147681815	0.45278822	-4.6771487
ENSCAFG00000018285	-0.137882	7.0681854	-1.469272	0.147724573	0.45278822	-5.8776906
DAGLA	0.29925235	2.94491278	1.46918039	0.147749339	0.45278822	-5.3488578
CA5B	-0.3979067	4.32092865	-1.4690741	0.147778055	0.45278822	-5.7066019
ASXL2	-0.1566683	4.51376211	-1.4685574	0.147917789	0.45310615	-5.7402073
STC1	-1.7647325	0.32623926	-1.4681528	0.148027258	0.45323259	-5.0012822
TBC1D9	0.62044854	4.55491746	1.46807528	0.14804825	0.45323259	-5.4524171
SRRM2	-0.1242473	9.29334299	-1.468006	0.148067013	0.45323259	-5.7320717
NCAPG2	-0.4240026	4.65959816	-1.4677496	0.148136423	0.45333488	-5.6575562
ENSCAFG00000009957	-0.5993397	0.76722704	-1.4672976	0.148258892	0.45338456	-4.72538
ENSCAFG00000016140	-0.1680311	5.13030719	-1.4672217	0.14827944	0.45338456	-5.8174028
KIF11	-1.4305961	3.96598798	-1.4671877	0.148288666	0.45338456	-5.2240459
TLN1	0.12359054	10.153491	1.46706196	0.148322751	0.45338456	-5.683892
DPP9	0.1755803	5.79447664	1.46702554	0.148332626	0.45338456	-5.8765971
DOK6	1.55857677	0.81217194	1.46670427	0.148419742	0.45354077	-4.6659648
TCF4	-0.265822	5.97604042	-1.4663394	0.14851874	0.45373322	-5.8795966
PALB2	-0.2644104	3.31488552	-1.466076	0.14859022	0.45384152	-5.492867
PSMB2	0.16880987	6.47181593	1.46529491	0.148802389	0.45437937	-5.892071
DIABLO	0.18581585	4.89916151	1.46507879	0.148861135	0.45444858	-5.789371
EIF4EBP1	0.21365542	4.67559152	1.46455251	0.149004265	0.45477532	-5.7649071
RIPK2	0.2082193	4.52165898	1.46406142	0.149137924	0.45479651	-5.7407496
AGBL5	0.20505333	3.59017661	1.46400329	0.149153749	0.45479651	-5.5409602
ZNF623	-0.2351808	2.44094343	-1.463935	0.149172356	0.45479651	-5.2722259
PLCE1	0.86126601	2.5050189	1.46392506	0.149175053	0.45479651	-5.0119135
TEF	0.23749212	3.23457532	1.46376473	0.149218716	0.45479651	-5.4393889
PYGM	0.5943746	0.10652529	1.4637067	0.149234524	0.45479651	-4.8114383
ENSCAFG00000013990	-0.5845211	-0.646353	-1.4635987	0.149263956	0.45479651	-4.6207103
FAM111B	-0.1758261	6.02873852	-1.463212	0.149369335	0.45500753	-5.8919349
COBL	1.2997425	0.44935707	1.46251498	0.149559424	0.45547643	-4.5947994
STK17A	-0.2366007	5.57651081	-1.4621008	0.149672484	0.45564707	-5.8816378
GLRX2	0.23188403	3.4805532	1.46204465	0.149687803	0.45564707	-5.5394696
EIF2B5	-0.1217517	5.71006925	-1.461654	0.149794496	0.45586167	-5.8791013
CDIPT	0.14738389	6.38342091	1.46137122	0.149871771	0.45598667	-5.8974665
SH3PXD2A	0.26226742	6.11909422	1.46108932	0.149948833	0.45611096	-5.8968758
PM20D2	0.35289799	3.91465556	1.46092747	0.14999309	0.45613543	-5.5383961
MARCH2	0.27001539	3.82393907	1.46074022	0.150044307	0.45618105	-5.6241726
GPR137	0.23095571	4.22730793	1.46034085	0.150153589	0.45640314	-5.6827837

STAT5A	-0.1769371	4.4667562	-1.460135	0.150209948	0.45646336	-5.7584318
MLH1	-0.1314004	4.76381834	-1.4598788	0.150280095	0.45646336	-5.7835088
TCEANC2	-0.2800611	3.47829737	-1.4597679	0.150310475	0.45646336	-5.551476
RPRD1A	0.19469239	3.57909658	1.45973914	0.150318356	0.45646336	-5.6242318
HECTD2	0.27721248	5.37918611	1.45917073	0.150474135	0.45678083	-5.8681303
PPRC1	-0.1515312	5.95416433	-1.4590931	0.150495432	0.45678083	-5.8840255
PROCA1	-0.4508863	1.40728418	-1.4587168	0.150598633	0.45691268	-5.0319758
TTC39A	0.21375689	4.51995105	1.45867019	0.150611421	0.45691268	-5.7408526
XPR1	-0.1845867	4.14764597	-1.4578404	0.150839224	0.45749359	-5.7172147
CLASRP	0.1739264	3.60362259	1.45752891	0.150924807	0.45753345	-5.5948779
MTERF1	-0.2273616	2.88806021	-1.4575164	0.15092825	0.45753345	-5.4777572
PCDH9	0.92449805	0.1342254	1.45739599	0.150961339	0.45753345	-4.6319691
ADAMTS7	-1.1903312	2.98035825	-1.4572133	0.151011568	0.45757559	-5.2558269
CHSY1	0.27415938	6.4653296	1.45607685	0.15132427	0.4583185	-5.9053524
ZNF532	0.22072117	5.70244743	1.45605778	0.151329522	0.4583185	-5.9046334
DHRS4	-0.2172806	5.27844814	-1.4556713	0.151435989	0.4585307	-5.8584596
ENSCAFG00000018552	-0.5645665	-0.3584212	-1.4554364	0.151500709	0.45860519	-4.6547646
DLX3	0.84490217	-1.5065369	1.45531783	0.151533406	0.45860519	-4.7190345
CLIP3	0.19794729	5.79738972	1.45419475	0.151843269	0.45933389	-5.9041462
RNF2	0.25783605	3.12451894	1.45418081	0.15184712	0.45933389	-5.3694481
SNX19	0.13508459	6.53715602	1.4540038	0.151896004	0.45937144	-5.9085083
ZSCAN16	-0.2154372	3.29307085	-1.4532641	0.152100421	0.45987924	-5.5161399
KCNK6	-0.3041916	3.53766494	-1.4529752	0.152180307	0.46001036	-5.6156198
VIRMA	-0.1098136	7.34081591	-1.4524748	0.152318775	0.46031845	-5.8839784
PSRC1	-0.4353223	0.98912484	-1.4519144	0.15247397	0.46067693	-4.922663
ZNF205	-0.1911053	3.76931422	-1.4515185	0.152583655	0.46089777	-5.6309968
LRRC24	0.60114044	0.16107176	1.45100872	0.152725015	0.46121417	-4.8544677
RECK	0.444761	5.87151268	1.45007656	0.152983748	0.46188478	-5.9130487
NPRL3	-0.2518669	3.96252966	-1.4498087	0.15305816	0.4619987	-5.6931872
AJUBA	-0.135827	4.96980683	-1.4495884	0.153119388	0.46203163	-5.7570085
MAFF	0.31361808	5.63190088	1.44950547	0.153142431	0.46203163	-5.9106543
RFXANK	-0.2450052	2.44662802	-1.4492366	0.153217175	0.4621092	-5.261393
MPND	-0.2663675	2.85507897	-1.4491491	0.153241514	0.4621092	-5.3186648
DBI	-0.1901868	5.1145561	-1.4489029	0.153309989	0.46211818	-5.8407924
OGFRL1	-0.1876336	4.01559428	-1.4488746	0.153317869	0.46211818	-5.7184753
OPTN	0.17224276	6.4185252	1.44830558	0.153476254	0.4624849	-5.9163479
ENSCAFG00000030409	-0.1995967	3.25476767	-1.4479994	0.153561521	0.46263117	-5.518996
SHE	-1.5326072	-0.8213408	-1.4476583	0.153656568	0.46280682	-4.8053408
BCAP29	-0.2527113	3.16620687	-1.4474752	0.1537076	0.46284985	-5.4253453
SNRNP70	-0.1411589	6.38574508	-1.4470252	0.153833087	0.46311701	-5.917774
TDP1	-0.2745403	2.29091591	-1.4461023	0.15409071	0.46378174	-5.2337377
PPP2R1B	-0.1763554	5.88046316	-1.4455852	0.154235189	0.46402897	-5.9093214
GALM	0.29393269	4.65602289	1.44554467	0.15424653	0.46402897	-5.7298351
MACO1	0.1781913	5.49875211	1.44528001	0.154320528	0.46414073	-5.9005818
TUT1	-0.1595685	4.87921205	-1.4449925	0.154400954	0.46427176	-5.8411182
ENSCAFG00000019051	0.41175491	1.43314755	1.44477088	0.154462956	0.46434735	-5.0721558
ARID4B	-0.1883683	5.30130956	-1.4444415	0.154555146	0.46451363	-5.8938318
SELENOT	0.10607229	7.22331197	1.44383445	0.154725185	0.46477364	-5.9062923
ADH5	0.37276274	2.052532	1.44382136	0.154728854	0.46477364	-5.213482
SLC35A5	0.22465915	3.54339117	1.44373751	0.154752352	0.46477364	-5.6269062

CLIP4	0.83277464	-1.9543732	1.44358859	0.15479409	0.46478817	-4.6075454
IGBP1	-0.1106118	5.63891902	-1.4428364	0.155005059	0.4652497	-5.9016873
DCP1B	-0.1827302	4.26396942	-1.4427772	0.155021674	0.4652497	-5.6997322
SRPK1	-0.2393964	5.30913559	-1.442569	0.155080118	0.46531424	-5.8753299
GAS1	0.69636532	2.01075041	1.44215498	0.155196363	0.4654437	-5.3499891
ATXN7L2	-0.2711763	2.45933614	-1.442046	0.155226983	0.4654437	-5.388967
CNP	0.13887376	6.01262652	1.44194543	0.15525523	0.4654437	-5.9200698
SMIM8	-0.3520293	1.60037085	-1.4418891	0.155271071	0.4654437	-5.0757331
RBM15B	-0.1582145	4.82083403	-1.4414659	0.155390019	0.46559702	-5.8115008
PTK2	-0.1893857	6.8038169	-1.4414353	0.155398609	0.46559702	-5.9256017
ENSCAFG00000018277	0.55858875	7.06522178	1.44131261	0.155433111	0.46559702	-5.9135005
H2AFY	-0.1087384	6.09826133	-1.4409383	0.155538411	0.46562928	-5.9206596
SWSAP1	0.29821055	1.68960247	1.44082613	0.155569965	0.46562928	-5.2155661
USP33	-0.1758123	5.85677395	-1.4407971	0.155578133	0.46562928	-5.9009107
PRPF4B	-0.1554569	5.90380935	-1.4407487	0.155591746	0.46562928	-5.9174712
RGS4	0.84360237	-1.5947788	1.4402787	0.155724079	0.46591461	-4.669553
CD99	0.2029457	9.89654693	1.43986673	0.155840134	0.46615111	-5.7356939
SLC25A34	0.60140872	-0.1644866	1.43949876	0.155943853	0.46635061	-4.7088109
GALNT10	0.31835498	4.13006669	1.43910815	0.156054012	0.4664642	-5.6889852
EEF1AKMT1	-0.184128	2.81966758	-1.439042	0.156072663	0.4664642	-5.4021501
ENSCAFG00000019472	-0.2096214	9.5032056	-1.4389702	0.156092933	0.4664642	-5.7696296
TCEANC	-0.4461438	1.02966074	-1.4381399	0.15632734	0.46695751	-4.9613114
TIGD5	0.31373398	3.09768196	1.4381053	0.156337112	0.46695751	-5.4311494
CLDN15	0.46989924	0.39140635	1.43799162	0.156369228	0.46695751	-5.1006311
XRCC5	-0.1454572	5.19955919	-1.4376164	0.156475262	0.46707362	-5.8656838
CEND1	0.26280441	3.41446957	1.43759165	0.156482269	0.46707362	-5.5296546
RASSF8	0.26109418	5.46206727	1.43714412	0.156608824	0.46721184	-5.9082583
CLOCK	0.29668129	5.16321657	1.43699838	0.156650055	0.46721184	-5.8083753
ENSCAFG00000018651	0.23699911	2.52612471	1.43694484	0.156665204	0.46721184	-5.4150536
SUPT4H1	0.17495066	3.43526818	1.43680487	0.156704814	0.46721184	-5.576061
GATAD2B	-0.1404645	4.50657105	-1.4367723	0.15671404	0.46721184	-5.8233621
NID1	-0.5070018	9.38526021	-1.436104	0.15690327	0.46765774	-5.8131813
RIPOR1	-0.1027243	7.26897633	-1.4359819	0.156937858	0.46765774	-5.9147386
MRPL14	0.1448501	4.58864724	1.43583852	0.156978489	0.46766818	-5.8000505
OGG1	-0.359895	1.23238783	-1.4356909	0.15702033	0.46768221	-4.9784126
SYNJ2BP	0.13773378	4.32238374	1.43539527	0.157104144	0.46782123	-5.7942914
ENSCAFG00000001000	-0.2943187	1.60043764	-1.4351353	0.157177877	0.46793017	-5.1039236
SMYD2	0.17330043	5.20589975	1.43482295	0.157266506	0.46808339	-5.8941295
MUTYH	-0.2027813	4.61364217	-1.4334747	0.157649499	0.46900517	-5.7645236
POLR3K	0.23903538	3.1037191	1.43343262	0.157661471	0.46900517	-5.4527191
KIAA0040	0.35499968	1.80996794	1.43318527	0.157731821	0.46900517	-5.131258
OGA	-0.112993	6.19508856	-1.4328807	0.157818475	0.46900517	-5.9378861
PXYLP1	0.30950123	3.42330662	1.43281085	0.157838356	0.46900517	-5.5056333
KIAA1109	-0.1999151	4.92125844	-1.4328051	0.157839995	0.46900517	-5.8495282
TMEM147	0.21023129	5.62939773	1.43275193	0.157855126	0.46900517	-5.9179938
TADA3	0.12708604	5.78478901	1.43268533	0.157874082	0.46900517	-5.9261796
SSX2IP	0.47175954	3.46115248	1.43245643	0.157939253	0.46908815	-5.5258864
SHQ1	0.2121911	5.69008373	1.43225833	0.15799567	0.46914509	-5.8981446
PUS7	-0.2805301	4.8350015	-1.432077	0.158047316	0.46918784	-5.8275587
HIST1H1C	0.48430718	1.28620681	1.43163586	0.158173045	0.46945028	-4.917902

DYNC2H1	-0.2089179	5.21127494	-1.4314067	0.15823839	0.46945028	-5.8969527
MPP3	0.76006035	-1.8092285	1.43137462	0.15824753	0.46945028	-4.6334825
CSNK1A1	-0.1052479	8.40447213	-1.4310238	0.158347609	0.46955191	-5.8554926
STAT6	-0.1487154	6.94326067	-1.4309932	0.158356343	0.46955191	-5.9278395
RBM10	-0.1301437	6.17966168	-1.4306027	0.1584678	0.46977181	-5.9386536
LRIG1	-0.507916	5.59631253	-1.4303314	0.158545269	0.46987524	-5.9170959
TASP1	-0.252005	2.16826928	-1.4302192	0.158577297	0.46987524	-5.2772313
SMARCA5	-0.1833991	7.23445739	-1.4297534	0.1587104	0.47005733	-5.9259694
HTRA1	0.36879145	9.81276809	1.42974295	0.158713387	0.47005733	-5.7665351
ENSCAFG00000032470	0.16586204	5.15758783	1.42939275	0.158813513	0.4702433	-5.8917885
NXF1	-0.0935609	6.14107541	-1.4289782	0.158932092	0.47048381	-5.9401255
ENSCAFG00000017655	0.18759541	9.95159209	1.42860269	0.159039575	0.47063182	-5.7424764
TRDMT1	-0.2798438	2.93376803	-1.4284827	0.159073922	0.47063182	-5.3720827
ARSA	0.32580648	7.51676168	1.42835286	0.159111112	0.47063182	-5.9074999
RPS10	-0.1821917	6.19521322	-1.4282815	0.159131545	0.47063182	-5.9437841
S1PR1	-0.8837201	3.61059256	-1.4278415	0.159257607	0.47074021	-5.7222447
TIMM21	-0.1854797	3.57136593	-1.4277449	0.159285307	0.47074021	-5.602551
PRKCI	0.26491443	7.1743322	1.42762127	0.159320744	0.47074021	-5.9380888
ENSCAFG00000010704	-0.2358107	3.93845468	-1.4275827	0.159331806	0.47074021	-5.74935
ETS2	0.36305507	5.09738154	1.42750161	0.159355053	0.47074021	-5.8237848
MAP3K6	-0.2820603	3.29964783	-1.4270464	0.159485618	0.47096838	-5.4372241
ADAM32	0.65869215	-1.1579637	1.42697162	0.159507073	0.47096838	-4.737277
WDR44	0.20762024	3.88642077	1.42656264	0.159624465	0.47118022	-5.7169636
MAPRE2	-0.2314514	5.8554769	-1.4264611	0.159653634	0.47118022	-5.9352045
UCK1	-0.216787	3.09257889	-1.4260901	0.159760188	0.47137516	-5.4495106
PSMC4	0.11585153	6.00016282	1.42584085	0.159831804	0.47137516	-5.9396619
GNB4	0.18299725	6.42273221	1.4256974	0.159873035	0.47137516	-5.9468319
NFKBIL1	-0.2714624	3.44990975	-1.4254861	0.159933772	0.47137516	-5.5713864
KLHL34	-0.513723	0.88923509	-1.4254671	0.159939262	0.47137516	-5.0408834
PDPK1	-0.1401214	4.27010485	-1.4254498	0.159944224	0.47137516	-5.762534
BRD2	0.09228885	8.25110303	1.42501315	0.160069825	0.47163497	-5.8766137
ATP6V0D1	0.13945363	7.07990584	1.42463358	0.16017907	0.47169886	-5.9378295
JMJD6	0.21990948	4.10514886	1.42455927	0.160200463	0.47169886	-5.7224114
ENSCAFG00000000491	-0.5997798	0.16884211	-1.4245475	0.160203853	0.47169886	-4.7218806
GLRX3	-0.0968178	6.80947385	-1.4240759	0.160339693	0.47183878	-5.946802
ZNF436	-0.1652916	5.26174822	-1.4238448	0.160406277	0.47183878	-5.9086455
KATNA1	-0.1707125	4.04778353	-1.4236512	0.160462087	0.47183878	-5.6986627
DMC1	0.83086815	-0.9553972	1.42347017	0.160514275	0.47183878	-4.5797549
SSBP3	0.16303911	5.37668689	1.42321504	0.160587854	0.47183878	-5.9090295
ZFP36L1	-0.2886194	6.55676878	-1.42319	0.16059508	0.47183878	-5.9506539
KIAA1841	0.32707024	2.40191045	1.42303617	0.160639459	0.47183878	-5.245642
ENSCAFG00000015284	-0.1194338	4.86763884	-1.4230075	0.160647719	0.47183878	-5.8512015
CLCA2	-0.8052377	-1.5005782	-1.4229368	0.160668128	0.47183878	-4.7073231
COPA	0.13092504	9.19028961	1.42292368	0.160671919	0.47183878	-5.8263757
ENSCAFG00000024531	0.18329593	5.78061941	1.4228865	0.160682648	0.47183878	-5.9345885
C4orf48	0.26402649	3.67352748	1.42277447	0.16071498	0.47183878	-5.6908644
GRB2	0.11055059	5.94663904	1.42269352	0.160738348	0.47183878	-5.9493683
NCKAP5	-0.6235522	1.7065621	-1.4218671	0.160977054	0.47233835	-5.5458183
DNAJC4	-0.293468	4.89883142	-1.4216997	0.161025434	0.47233835	-5.8646395
ENSCAFG00000023674	-0.1145647	5.32517992	-1.4215229	0.161076558	0.47233835	-5.9108083

ZNF646	-0.1859429	4.14085224	-1.4214943	0.161084818	0.47233835	-5.7875647
FAM50A	-0.1384822	5.04449506	-1.4214555	0.161096025	0.47233835	-5.8728088
CALR3	-0.5769227	-0.0189588	-1.4210717	0.161207058	0.47244889	-4.9999983
FBXL6	-0.1907191	3.74625921	-1.4210658	0.161208743	0.47244889	-5.661182
RRM1	-0.2958641	6.20348913	-1.4208656	0.161266678	0.47245504	-5.939454
LIN54	-0.2634017	3.72297326	-1.4207994	0.161285858	0.47245504	-5.5485392
ENSCAFG00000032235	-0.7670668	-0.9155718	-1.4205165	0.16136775	0.47255616	-4.6031487
PUM1	-0.1030326	6.77143878	-1.4204209	0.161395412	0.47255616	-5.9504056
ENSCAFG00000001588	0.20319849	5.35788908	1.42027935	0.161436419	0.47256638	-5.9016189
TDP2	0.202678	3.09802285	1.41958712	0.161637015	0.47302716	-5.4873572
PAN3	-0.1466426	4.94026104	-1.419477	0.161668938	0.47302716	-5.9043117
ISCA2	0.29239601	1.83721814	1.41922637	0.161741632	0.47312996	-5.1912586
ENSCAFG00000018512	-0.5420908	0.23120401	-1.4185403	0.161940735	0.47355109	-4.7815097
ENSCAFG00000017940	-0.8061393	-2.3330853	-1.4183715	0.161989752	0.47355109	-4.6042677
WDHD1	-0.3654969	3.96898951	-1.4183418	0.161998384	0.47355109	-5.5878877
SLC17A9	0.32756844	3.78110255	1.41730257	0.162300414	0.4743239	-5.7340341
ENSCAFG00000005454	-0.4178765	1.44890297	-1.4169403	0.162405818	0.47452185	-5.0831862
ZSWIM4	0.33369445	4.77554241	1.41668621	0.162479755	0.47462778	-5.8810229
SOX18	-1.2561867	-1.6460676	-1.4161778	0.162627815	0.47488816	-4.7207827
MAGIX	0.26344621	3.59205788	1.4161212	0.162644292	0.47488816	-5.7069137
SH3GLB2	0.24757847	4.37492713	1.4155687	0.162805311	0.47524813	-5.8454301
HHIPL1	0.36234921	5.32079304	1.41538592	0.162858605	0.47527348	-5.8699888
NRBF2	-0.1744921	4.35624505	-1.4152801	0.162889459	0.47527348	-5.8148776
TMEM222	0.17401005	4.06525876	1.41470033	0.163058637	0.47565692	-5.7369754
LRRC73	0.491637	0.4460068	1.41407548	0.163241109	0.47590063	-4.8749171
ENSCAFG00000013986	-0.3221945	1.96719908	-1.4140685	0.163243136	0.47590063	-5.3659008
NDOR1	-0.1839957	4.51781417	-1.4140261	0.163255526	0.47590063	-5.7897474
ENSCAFG00000031958	-0.2111432	4.67358672	-1.41349	0.163412231	0.47624722	-5.8508416
CDK20	-0.2735065	3.46365313	-1.412921	0.163578658	0.47662197	-5.5718636
POLI	-0.2105296	2.98393988	-1.4125819	0.163677929	0.47680093	-5.4925696
MTR	-0.1953145	4.12768787	-1.4121291	0.163810515	0.47707682	-5.7527989
LRP1B	0.68191465	-1.8349279	1.41159452	0.163967177	0.47742269	-4.6687032
MTMR1	-0.1624816	5.15121659	-1.4112582	0.164065809	0.47759947	-5.9018391
ANO5	-1.1554796	2.11352784	-1.4110471	0.164127713	0.47762472	-4.8094457
MDFI	-0.9612766	-1.6437441	-1.4108549	0.164184125	0.47762472	-4.6168824
NAV1	-0.1941238	7.57201324	-1.4108409	0.164188237	0.47762472	-5.945662
AASDHPPT	0.11382622	5.65369777	1.41052304	0.164281527	0.47778576	-5.9562309
STAB1	-1.5653286	0.33084218	-1.4102609	0.164358505	0.47789929	-5.0263846
ENSCAFG00000010209	-1.2151774	-2.0638862	-1.4092227	0.164663653	0.47862405	-4.6767134
ENSCAFG00000008934	-0.6147592	-0.8784087	-1.4091543	0.164683758	0.47862405	-4.6548048
SATB2	0.17408114	4.49993072	1.40890407	0.164757384	0.47872757	-5.8670334
OTUD7B	0.13202201	4.51664466	1.40850176	0.164875797	0.47882898	-5.8530812
KRIT1	-0.1573821	4.57226156	-1.4084783	0.164882698	0.47882898	-5.8813988
ENSCAFG00000013452	-0.8247313	-1.9572234	-1.4083981	0.16490633	0.47882898	-4.6295558
TMEM59	0.16619473	7.59045043	1.40816638	0.164974563	0.47891671	-5.9223142
DHX32	-0.106406	5.14558069	-1.4074741	0.165178568	0.47939844	-5.9197162
DIDO1	-0.0871785	5.22306521	-1.4069184	0.165342469	0.47976359	-5.9171315
PRKRA	0.1999288	3.30982954	1.40675228	0.1653915	0.4797855	-5.4952287
MAP3K20	0.26597104	4.6670574	1.40663471	0.1654262	0.4797855	-5.8834527
TMEM150A	0.22985121	5.01307808	1.4064589	0.165478103	0.47982555	-5.9168115

NOSIP	-0.1223154	4.6510994	-1.4062216	0.165548164	0.47991822	-5.8612194
COMT	0.19718342	6.18279947	1.40577442	0.165680292	0.48009408	-5.9739366
NDNF	0.93114792	-2.5511	1.40549715	0.165762248	0.48009408	-4.605619
ARPC2	0.16402859	7.44431992	1.40547372	0.165769177	0.48009408	-5.9497296
HMG20A	-0.1283082	5.11212093	-1.4053545	0.165804424	0.48009408	-5.9262452
TMEM80	-0.5367422	0.38561138	-1.4052749	0.165827952	0.48009408	-4.8847491
CDK4	-0.1960297	5.91930233	-1.4052426	0.165837515	0.48009408	-5.9529787
MRM1	0.33694476	1.14859754	1.40509007	0.165882635	0.48011435	-5.0320244
PRKAG1	-0.1287585	5.33507122	-1.4046889	0.166001334	0.48034753	-5.9362299
SMARCC2	-0.1310864	6.70734193	-1.4042863	0.166120525	0.48054504	-5.9768159
UQCRC2	-0.1129469	5.80306629	-1.4042007	0.166145891	0.48054504	-5.9693519
LZTS3	0.40817442	2.47434489	1.40382775	0.166256366	0.48075417	-5.2830404
ENSCAFG00000004573	0.34009098	1.68637132	1.40360754	0.16632163	0.48080247	-5.2366131
NLGN3	0.54879609	0.54178632	1.40351382	0.166349411	0.48080247	-4.984952
GSTCD	-0.3527883	3.12291119	-1.4027538	0.166574842	0.48109716	-5.6339229
ENSCAFG00000032642	0.16154068	4.81827932	1.40272402	0.166583676	0.48109716	-5.8939928
NTN4	-0.4178644	6.77785762	-1.4027109	0.166587564	0.48109716	-5.9771643
RELCH	0.12913328	5.22966817	1.40265506	0.166604144	0.48109716	-5.9148327
POLR1B	-0.1811229	5.82702655	-1.4023347	0.166699258	0.48126148	-5.9652856
HOXA7	-0.9307986	0.84443617	-1.4020157	0.166793989	0.48142463	-4.9276326
WEE1	0.23963382	3.29092861	1.40184723	0.166844043	0.48145166	-5.599086
PRDX1	-0.1780542	7.44209525	-1.4015293	0.166938519	0.48145166	-5.9522475
KIAA1671	-0.3759903	3.50362049	-1.4014793	0.166953404	0.48145166	-5.5463108
BRD4	-0.0937412	6.37254859	-1.4014697	0.166956244	0.48145166	-5.9805768
PPIE	-0.1364344	4.44067685	-1.4012816	0.167012173	0.48147243	-5.8378866
COPE	0.13745042	6.53666122	1.40118839	0.167039896	0.48147243	-5.981761
GPAT3	-0.28328	4.15873801	-1.4010162	0.167091111	0.48150987	-5.9045488
NSMCE4A	-0.231172	3.7189942	-1.4007896	0.167158534	0.48153651	-5.6718013
IRX3	0.67404685	2.74080169	1.40070362	0.167184118	0.48153651	-5.4694916
PPM1B	-0.1308973	4.76686522	-1.4005834	0.167219904	0.48153651	-5.8760285
RRM2	-1.4618995	3.84028169	-1.4004713	0.167253272	0.48153651	-5.2245531
PTPDC1	0.44078182	0.96290027	1.40002101	0.167387359	0.48176302	-4.9021214
RGL2	-0.2544672	5.34512743	-1.3999502	0.167408444	0.48176302	-5.9445889
DST	0.20554806	8.66783897	1.39931673	0.167597253	0.48204865	-5.9085132
ATL1	0.43761034	0.86145682	1.39927674	0.167609179	0.48204865	-5.0070928
GBA	0.19903481	5.90626846	1.39923205	0.167622505	0.48204865	-5.9775854
ATP2B1	-0.2529903	6.70950643	-1.3985561	0.167824175	0.48212214	-5.9801493
KMT2E	-0.2233481	7.0753808	-1.3983828	0.167875893	0.48212214	-5.9533639
MYBPC2	0.85707779	-0.3541523	1.39832127	0.167894275	0.48212214	-4.9025589
HHAT	-0.8230664	-0.7251539	-1.3980518	0.167974736	0.48212214	-4.6813564
TTBK1	0.90242138	-1.3103493	1.39787579	0.168027326	0.48212214	-4.727949
ENSCAFG00000017325	-0.2370938	5.56010432	-1.3978213	0.168043615	0.48212214	-5.9679804
MINDY4	0.41724535	5.07696638	1.3977086	0.168077283	0.48212214	-5.9446241
SPATA24	-0.4617223	0.98958996	-1.3976812	0.168085456	0.48212214	-4.9753748
CLK1	-0.2641302	4.27564617	-1.3976127	0.168105943	0.48212214	-5.8447045
PDK2	0.31830468	5.2937964	1.39755078	0.16812445	0.48212214	-5.9535068
ENSCAFG00000018430	-0.1477906	3.69557805	-1.3975219	0.168133074	0.48212214	-5.7006705
CAPNS1	0.12701416	9.29118737	1.39748561	0.16814393	0.48212214	-5.8405445
F2R	-0.2503444	6.29930399	-1.3972206	0.168223149	0.48212214	-5.9858152
PAXIP1	-0.1568673	4.2345907	-1.3972199	0.168223365	0.48212214	-5.8056569

CDS1	0.88275324	-0.1225358	1.39715174	0.168243751	0.48212214	-4.8499615
ZNF7	-0.1370886	5.59521603	-1.397012	0.168285545	0.48212214	-5.9737039
COPG1	0.14808434	8.36235615	1.39695344	0.168303064	0.48212214	-5.9129899
SLCO1A2	1.49977765	-0.2382512	1.39679255	0.168351198	0.48212214	-4.7172507
CNOT11	-0.1053406	5.01449339	-1.3966951	0.168380357	0.48212214	-5.9104966
KDM4B	0.12244058	5.87098329	1.3965841	0.168413576	0.48212214	-5.9849712
GEMIN8	-0.1930838	3.03033918	-1.3964434	0.168455697	0.48213314	-5.475141
CCDC158	0.31311592	1.74690757	1.3962105	0.168525421	0.48218939	-5.2122543
ENO3	0.25960255	2.8615036	1.39612204	0.168551911	0.48218939	-5.4171872
SDHC	0.13812304	5.32484887	1.39598808	0.168592034	0.48219466	-5.9507032
MAGEE2	0.5787106	-0.2581473	1.39532492	0.168790765	0.48265346	-4.7798267
TRMT13	-0.2587906	1.73623005	-1.3949057	0.168916478	0.48268499	-5.1967522
ENSCAFG00000000288	-0.4561221	1.18621971	-1.3947608	0.168959955	0.48268499	-4.9055942
RET	-0.6923922	0.73535954	-1.3947599	0.168960215	0.48268499	-4.9515613
GTPBP1	-0.1482225	4.51347371	-1.3946946	0.168979826	0.48268499	-5.8309985
VGLL2	-0.8956453	3.12657868	-1.3946494	0.168993396	0.48268499	-5.2266669
IPO13	0.13543357	6.06806469	1.39387844	0.169224863	0.4830615	-5.985181
PLEKHN1	-0.5245202	-0.0045588	-1.3938293	0.169239641	0.4830615	-4.810489
ARMC2	0.47892316	-0.3918831	1.39368011	0.16928445	0.4830615	-4.9057207
MANEA	-0.2148984	4.85129999	-1.3936211	0.169302186	0.4830615	-5.8643511
CAMK1D	0.37005196	3.44300891	1.3935719	0.169316969	0.4830615	-5.5037189
MCUB	-0.2007216	5.08681022	-1.3932974	0.169399483	0.48311689	-5.8883207
WIPF1	0.20946609	5.83996814	1.39315424	0.169442523	0.48311689	-5.9785679
VPS29	0.14452822	4.64942825	1.39302412	0.169481655	0.48311689	-5.8728744
RPL27A	-0.2077989	6.99430652	-1.392997	0.1694898	0.48311689	-5.9835488
CCDC80	0.45079323	9.55834484	1.39284947	0.169534187	0.48312042	-5.872871
ENSCAFG00000022709	-0.3659011	13.5942207	-1.3927379	0.16956775	0.48312042	-5.491911
GLG1	0.21066276	9.67864367	1.39245228	0.169653708	0.48325602	-5.8459345
ENSCAFG00000024501	-0.1841624	3.94118052	-1.3919766	0.169796916	0.4834605	-5.7554175
RYR3	1.09971266	0.07645692	1.3919589	0.169802259	0.4834605	-4.6601692
GKAP1	-0.2233625	3.03317521	-1.3917277	0.16987192	0.48354954	-5.5725588
ZC4H2	-0.3524149	2.83626382	-1.3902981	0.170303053	0.48457334	-5.3406743
TBL2	-0.1448064	3.97031387	-1.3901765	0.170339757	0.48457334	-5.7547711
EFNA4	0.43469126	0.65899822	1.39010702	0.170360742	0.48457334	-4.9962887
HERPUD2	-0.1406354	6.19514705	-1.3900252	0.170385466	0.48457334	-5.9966215
ST7	0.18307348	5.08939484	1.38960082	0.170513648	0.48482842	-5.9258268
MGST1	0.73955308	0.3798117	1.38913448	0.170654605	0.48503766	-5.6755621
DDX21	-0.224127	5.4579242	-1.3889917	0.17069778	0.48503766	-5.9629125
ENSCAFG00000014234	-0.5573515	0.0077134	-1.3889752	0.170702757	0.48503766	-4.7606892
TIMP1	0.50645021	9.85668164	1.388682	0.170791459	0.48518025	-5.6537446
ENSCAFG00000012787	1.38725337	1.69637363	1.38828639	0.170911183	0.48533901	-4.753485
PRELID1	-0.1556981	6.68232502	-1.3882427	0.170924409	0.48533901	-5.9981069
GMPR	0.49141504	1.06609272	1.38807263	0.170975897	0.4853758	-4.9340432
SCOC	0.30808922	2.04115342	1.38778371	0.1710634	0.48551478	-5.2063976
ANKRD13D	0.17880172	3.9210572	1.38749134	0.171151983	0.48558734	-5.7430019
PIK3R1	-0.3300321	6.33339976	-1.3874449	0.171166068	0.48558734	-5.9886313
COQ10B	0.2006051	4.25903813	1.38720229	0.171239593	0.48561112	-5.7728687
GINS4	-0.4852788	3.2500334	-1.3869521	0.171315466	0.48561112	-5.4427633
ZNF697	0.29518827	3.19528694	1.38694991	0.171316117	0.48561112	-5.5559986
NUP62	-0.1720168	5.25700562	-1.3869085	0.171328663	0.48561112	-5.9318624

ENO1	0.19628736	9.71462445	1.3866208	0.171415945	0.48574921	-5.8173843
ZNF598	-0.1579943	5.16141021	-1.3858763	0.171641934	0.48628021	-5.9494149
NDUFS3	-0.1522436	5.3930254	-1.3853883	0.171790213	0.48659085	-5.9574386
PGF	0.86508005	2.49456993	1.38436147	0.172102478	0.48736574	-5.8692661
ZCRB1	-0.1449045	5.21675964	-1.3838592	0.172255379	0.4876347	-5.9505228
BTBD7	-0.1686239	4.39197648	-1.3837952	0.172274881	0.4876347	-5.8287524
KIAA1147	-0.9655806	-1.5593615	-1.3833573	0.172408303	0.48790271	-4.7159902
CAMSAP2	-0.1647407	6.46129781	-1.3829035	0.172546623	0.48807981	-6.0060806
PMEPA1	0.74904275	6.00531841	1.38284807	0.172563531	0.48807981	-5.9559113
MARCH7	-0.1642394	5.95983958	-1.3827707	0.17258713	0.48807981	-6.0007139
FAM210B	0.26096213	6.96216325	1.38254338	0.172656469	0.4881663	-5.9719219
SHOX2	-0.7112906	4.93774611	-1.3823365	0.172719604	0.48823522	-5.7842025
LETM2	-0.4462927	-0.5552253	-1.3822008	0.172761026	0.48824274	-4.8117893
ENSCAFG00000009222	0.20066616	4.25410614	1.38190963	0.172849903	0.48827827	-5.844027
MSTO1	0.19483027	4.34607113	1.38189914	0.172853105	0.48827827	-5.7748414
APH1B	-0.2911384	2.67378178	-1.3817787	0.172889891	0.48827827	-5.5080371
GTF3C3	-0.1681441	4.29819504	-1.3812893	0.173039388	0.48852737	-5.8383227
GNL1	-0.1298878	5.15842454	-1.3812361	0.173055662	0.48852737	-5.9426198
DEPP1	-0.9012495	-1.4569014	-1.3806269	0.173241924	0.48879623	-4.8470737
MPST	-0.2329991	4.72635398	-1.3804597	0.173293067	0.48879623	-5.8781708
TCTA	0.28654273	2.76427071	1.38045148	0.17329559	0.48879623	-5.4873857
RABGAP1	-0.1424537	6.21782085	-1.380417	0.173306126	0.48879623	-6.0084743
SMG5	0.13482335	6.00100808	1.38024372	0.173359165	0.48883636	-5.9993203
GPD1L	0.27326074	3.61365663	1.37952183	0.1735802	0.48924292	-5.8041383
IDUA	0.23776464	4.88421542	1.37951914	0.173581025	0.48924292	-5.9244615
APPBP2	-0.1225565	6.29207696	-1.379009	0.173737365	0.48957402	-6.0113897
FAM216A	0.14423078	4.23476619	1.37851836	0.173887814	0.48988837	-5.8307421
TEP1	-0.2133322	5.7308841	-1.3781674	0.173995499	0.49008213	-5.9802167
OCRL	0.14216518	5.86774174	1.37795379	0.174061068	0.49012066	-6.0061839
PIGT	0.19900675	7.45080468	1.37786932	0.174086998	0.49012066	-5.9900498
TM2D1	0.17247022	3.447889	1.37702645	0.174345933	0.49073997	-5.6525034
LMBRD2	0.18327013	4.82457861	1.37648139	0.174513534	0.49107371	-5.911516
NCK2	0.19961097	5.77489302	1.37638732	0.174542473	0.49107371	-5.9771268
SELE	-1.3384137	-1.4001075	-1.376176	0.174607498	0.49114695	-4.8255794
C12H6orf136	0.16289702	5.15684161	1.37591073	0.174689143	0.4912669	-5.9496478
ENSCAFG00000017503	-0.2088538	6.75006869	-1.3754781	0.174822365	0.49132594	-6.0154249
PKDCC	0.51434635	4.20445007	1.37542489	0.174838753	0.49132594	-5.6011697
RPN1	0.15013128	8.68740992	1.37539539	0.174847841	0.49132594	-5.9104885
NSMAF	-0.1239975	5.36802865	-1.3751489	0.17492378	0.49132594	-5.9752283
ENSCAFG00000030659	-0.1513082	3.80484719	-1.3751097	0.174935865	0.49132594	-5.7238141
PUSL1	0.48275908	0.8472337	1.37487199	0.175009135	0.49132594	-5.0072827
ENSCAFG00000025115	-0.6145725	2.35604266	-1.3748366	0.175020057	0.49132594	-5.601991
AGTRAP	0.31603487	2.6194262	1.37477754	0.175038255	0.49132594	-5.5012956
ENSCAFG00000014670	0.463102	7.68886115	1.3745867	0.1750971	0.49132594	-5.864332
CHM	-0.1294739	4.94043255	-1.3743799	0.17516088	0.49132594	-5.9536631
CTTNBP2	-0.2645815	4.15702054	-1.3743556	0.175168384	0.49132594	-5.819912
GCLM	-0.2551392	3.96708453	-1.3743237	0.175178213	0.49132594	-5.6944062
PIMREG	-1.3533437	1.58845818	-1.3736201	0.175395382	0.49172025	-4.8265985
NEDD4L	-0.2478962	6.4614002	-1.3736153	0.17539688	0.49172025	-6.0183474
SMN	-0.1332186	5.1569911	-1.3726414	0.175697814	0.49245431	-5.954063

GLT8D1	-0.1696074	5.01211153	-1.3723025	0.175802638	0.49260399	-5.9369041
SLC37A1	0.75552032	-1.7755175	1.37209796	0.175865911	0.49260399	-4.6950883
GNPDA2	-0.1150927	4.75928258	-1.3720895	0.175868541	0.49260399	-5.9226814
LMNB2	-0.2476694	4.07867914	-1.3718545	0.175941272	0.49263622	-5.755175
PTDSS1	0.12167965	6.05537148	1.37179954	0.17595827	0.49263622	-6.0180504
PRKACA	0.13209349	6.17233363	1.37144922	0.176066743	0.49282933	-6.0219598
CNRIP1	-0.5611137	3.46186628	-1.3712206	0.176137546	0.49282933	-5.7744674
TTYH3	-0.2777123	4.17010335	-1.3710799	0.176181148	0.49282933	-5.864937
EWSR1	-0.1747621	5.77376167	-1.3709632	0.176217307	0.49282933	-5.9936639
SF3B6	-0.1227788	4.83302741	-1.3708449	0.176253989	0.49282933	-5.9083774
MKLN1	0.15918753	5.55758425	1.37081902	0.176262	0.49282933	-5.9999453
SNF8	-0.11727	4.16867905	-1.3706028	0.176329026	0.49290733	-5.7758117
PFKP	0.18256059	8.49862413	1.37031116	0.176419477	0.49305075	-5.9339317
RBM41	-0.2392473	1.95803895	-1.3700437	0.176502455	0.49313409	-5.241051
ABCA4	0.78206497	-1.4428712	1.36989288	0.176549255	0.49313409	-4.7247299
SLC38A1	-0.2502435	6.32412222	-1.3698365	0.176566747	0.49313409	-6.0128665
PDGFC	0.35486268	4.5107187	1.36941661	0.176697117	0.49337453	-5.841108
ARHGAP6	0.6740668	2.22379388	1.36930694	0.176731175	0.49337453	-5.5490152
COQ2	0.11721355	5.2374873	1.36908261	0.176800864	0.49345971	-5.9890319
OFD1	-0.1772049	4.96566204	-1.3688971	0.176858499	0.49351122	-5.9600083
MAP7D3	-0.1437145	6.55742657	-1.3686386	0.176938857	0.4936261	-6.0253075
GJB3	-0.6221338	4.14150571	-1.3682564	0.177057699	0.49375953	-5.6506469
PTPN3	0.22762375	3.46464396	1.36823269	0.177065083	0.49375953	-5.7296857
B3GALT6	0.36876611	1.63177968	1.36806407	0.177117536	0.49376324	-5.179757
FASTKD1	-0.2229652	3.50368305	-1.3678125	0.177195816	0.49376324	-5.6863642
ENSCAFG00000031486	-0.2332581	4.63959544	-1.3677572	0.177213028	0.49376324	-5.930888
CLCN6	-0.1932678	5.49387051	-1.3676349	0.177251089	0.49376324	-5.9768783
TOP3A	-0.1578598	4.76182781	-1.3675986	0.177262416	0.49376324	-5.9005423
DYRK1A	-0.1055413	5.72320963	-1.3674517	0.177308148	0.49378144	-6.012248
PRKCH	-1.2805384	-0.274035	-1.3671558	0.177400298	0.4938857	-4.8213278
JMY	-0.1922216	4.82779481	-1.3670213	0.177442197	0.4938857	-5.9581336
SLC30A9	0.17936213	7.12631917	1.366701	0.177542008	0.4938857	-6.0180977
ACP6	-0.2483988	2.57223597	-1.3666661	0.177552892	0.4938857	-5.4898469
EDF1	0.11906513	5.98988768	1.36663364	0.177563003	0.4938857	-6.0211758
DLA-DMA	0.49950071	0.99374401	1.36655306	0.177588123	0.4938857	-5.07118
SERPINB5	0.48237231	1.19225041	1.36637061	0.177645009	0.4938857	-5.0900112
MTHFD2L	0.33101474	1.33694125	1.36620654	0.177696178	0.4938857	-5.0742543
C6H7orf26	-0.1481545	4.59303222	-1.3661992	0.177698474	0.4938857	-5.8686701
HSD17B7	-0.2283752	3.50363801	-1.3657162	0.177849169	0.49392019	-5.7696775
RXFP1	1.49974978	-0.513752	1.36562386	0.177877986	0.49392019	-4.7984261
ENSCAFG00000009085	0.49050742	-0.3584398	1.36560917	0.177882572	0.49392019	-4.8321841
ZNF180	-0.343519	1.1114463	-1.3654595	0.177929312	0.49392019	-5.071002
LIAS	-0.1757487	2.71282771	-1.3654101	0.177944715	0.49392019	-5.4858078
GMNN	-0.4164156	2.96312441	-1.3653869	0.177951955	0.49392019	-5.3604613
PVR	0.29307819	4.87234995	1.36522072	0.178003858	0.49392019	-5.8810855
ZSCAN21	-0.1368083	3.53940054	-1.3650959	0.178042846	0.49392019	-5.6913171
PANX1	0.2690755	5.04088292	1.36502883	0.178063796	0.49392019	-5.9393173
PEX11A	-0.1791213	2.98602921	-1.3647106	0.17816323	0.4940872	-5.6236935
STAT1	-0.1105043	7.092521	-1.3645579	0.17821095	0.49411075	-6.0197785
FBXO33	0.17783806	3.62916586	1.36437429	0.178268363	0.49414316	-5.7741395

PRR5L	-0.8986429	-1.5849708	-1.3642696	0.1783011	0.49414316	-4.8054457
PPP1R35	-0.3127691	2.74036666	-1.3641435	0.178340534	0.49414372	-5.4064771
CD3G	1.07529506	-0.9491827	1.36396664	0.178395856	0.4941883	-4.8327687
TM9SF2	0.12148512	7.95064951	1.36379635	0.178449136	0.4942272	-5.9784248
ENSCAFG00000013064	0.4383217	3.01088346	1.36363358	0.178500073	0.4942596	-5.4708084
ENSCAFG00000000399	0.24521803	3.36474782	1.36332871	0.178595511	0.49435541	-5.7920773
PPIL2	-0.1056099	4.72656416	-1.3632708	0.178613656	0.49435541	-5.923666
SCN8A	0.79036737	1.99452549	1.363147	0.178652414	0.49435541	-4.986573
ENSCAFG00000029256	-0.4585804	1.90712784	-1.3628973	0.178730615	0.49446317	-5.2602095
TMEM39A	-0.1476508	4.88483518	-1.3625649	0.178834781	0.49462364	-5.9595154
NCAM2	-0.8073679	-1.3502151	-1.3624616	0.178867156	0.49462364	-4.8887956
MGRN1	0.15170954	6.75696414	1.36204545	0.178997655	0.49483937	-6.0249314
ENSCAFG00000031693	0.34105712	1.7049538	1.3619623	0.179023739	0.49483937	-5.2870181
VPS53	0.12374572	4.68815025	1.36179322	0.179076781	0.49487739	-5.912326
GPAA1	0.17894264	6.45518073	1.36161481	0.179132764	0.49492351	-6.0333734
ARHGAP39	0.23488378	3.24921098	1.36141042	0.179196917	0.49499219	-5.6869706
HAUS3	-0.225491	4.5602354	-1.3610923	0.179296785	0.49515946	-5.9014707
WRN	-0.1859222	4.82212729	-1.3608256	0.179380572	0.49516184	-5.9231494
ENSCAFG00000031423	0.38382048	1.75412242	1.36077797	0.179395535	0.49516184	-5.1842945
EDN1	1.18438743	3.66055366	1.36070363	0.179418894	0.49516184	-5.4312253
MFHAS1	-0.1761807	6.67909324	-1.3604313	0.179504492	0.49516184	-6.0357601
PRRX2	0.4126152	4.00626613	1.360418	0.179508659	0.49516184	-5.5048284
ENSCAFG00000005880	-0.2527084	2.24587706	-1.3603389	0.179533512	0.49516184	-5.2957036
ENSCAFG00000012234	-0.3240327	1.06674685	-1.3602003	0.179577088	0.4951736	-5.07045
NOD1	0.29281093	4.81940775	1.35985861	0.179684558	0.4953615	-5.8369035
SGO1	-1.0719175	1.79582218	-1.3593522	0.179843915	0.49569233	-4.9664741
PABPN1	-0.0837268	6.34223829	-1.3590934	0.179925386	0.49580839	-6.0381986
NMT1	0.10781436	5.880383	1.35896086	0.179967132	0.49581496	-6.0283409
NKTR	-0.1896005	5.35974623	-1.3583987	0.180144245	0.49619438	-6.0093484
TRIB1	-0.3994265	4.722287	-1.3581166	0.180233184	0.49633027	-5.9340158
DHRS7B	0.21018337	3.4504197	1.35789012	0.180304599	0.49633027	-5.643922
UBR1	-0.1591182	6.18298589	-1.3578673	0.180311792	0.49633027	-6.0389803
MT-CO2	-0.2035407	12.3025927	-1.3576803	0.180370796	0.49638421	-5.6543493
ATG16L2	0.286065	2.60385186	1.3570729	0.180562488	0.49671508	-5.4023923
ALG6	-0.1686358	4.12899872	-1.3570495	0.180569892	0.49671508	-5.8569816
CROCC	-0.3174201	2.23475877	-1.3567017	0.180679712	0.49679481	-5.3117735
ENSCAFG00000013408	-0.1388603	5.51304552	-1.3565864	0.180716154	0.49679481	-6.0143171
COMMD8	-0.1952667	4.19623257	-1.3565831	0.180717197	0.49679481	-5.8280116
COG6	0.14776538	5.67121194	1.35629499	0.180808235	0.49693662	-5.9960559
ASPHD2	0.67351219	-0.5465675	1.35612722	0.180861266	0.49697393	-4.8497784
C1H18orf54	-0.2288367	3.42584846	-1.3559042	0.18093178	0.49705926	-5.704405
EBF4	0.87506035	1.31631961	1.35541683	0.181085958	0.49725045	-4.8958152
UACA	0.42912234	7.72625129	1.35533317	0.181112431	0.49725045	-5.9993846
NR2F6	0.36615537	2.51314402	1.35518939	0.181157938	0.49725045	-5.5243491
POP5	-0.2108957	2.92702153	-1.3551851	0.181159283	0.49725045	-5.5042304
GOLGA7	-0.0961441	5.97606342	-1.354631	0.181334756	0.49754853	-6.038824
C1RL	0.75793363	3.00978031	1.35437781	0.181414974	0.49754853	-5.45428
CDH24	-0.486493	1.35784928	-1.354323	0.181432353	0.49754853	-4.9880931
ZBTB24	-0.1146571	5.35892951	-1.3539652	0.181545756	0.49754853	-6.0037268
PHETA2	-0.3467715	3.16505193	-1.3538113	0.181594567	0.49754853	-5.5162349

GARS	-0.1832229	8.81519476	-1.3536267	0.181653108	0.49754853	-5.9546439
ENSCAFG00000011131	0.21692433	3.17525547	1.35356218	0.181673571	0.49754853	-5.6037598
INPP5E	-0.2748511	3.30271725	-1.353417	0.181719643	0.49754853	-5.6778162
ZNF550	0.40784944	0.40760288	1.35339355	0.18172707	0.49754853	-4.9918127
ENSCAFG00000013321	-0.3218468	1.91628789	-1.3533752	0.181732897	0.49754853	-5.3508235
TIMM9	-0.2059124	3.27514069	-1.3533585	0.181738189	0.49754853	-5.6388729
TSHZ2	-0.259891	5.0680453	-1.353256	0.181770707	0.49754853	-6.0179135
LRRC8B	0.19637631	3.45424261	1.35315555	0.181802599	0.49754853	-5.6637729
ENSCAFG00000012222	-0.408549	0.81829617	-1.352965	0.181863082	0.49754853	-4.9446878
ENSCAFG00000020060	-0.495555	2.44855589	-1.3529543	0.181866494	0.49754853	-5.2708776
AFDN	-0.5654104	4.75884506	-1.3528491	0.181899886	0.49754853	-5.8529604
PTGER2	0.57663127	0.38549787	1.35260616	0.181977035	0.49765148	-5.2251604
PM20D1	0.52362534	0.73208619	1.35241846	0.182036664	0.49770649	-4.9924867
DSCAML1	-1.0426338	-0.8507673	-1.3520905	0.182140871	0.49780361	-5.1104436
GHDC	-0.2052894	3.96978575	-1.3517782	0.182240159	0.49780361	-5.859398
ITPRIPL2	0.25619854	3.48281695	1.35171285	0.182260947	0.49780361	-5.7796924
H3F3B	-0.2027175	6.83722073	-1.3514522	0.18234385	0.49780361	-6.0446676
GPX7	-0.5446647	2.71166915	-1.3513718	0.182369443	0.49780361	-5.17193
RBM39	-0.1595438	6.81669319	-1.3512711	0.182401479	0.49780361	-6.0430129
EREG	0.85925875	5.25229497	1.35111179	0.182452162	0.49780361	-6.0036293
RNF181	0.17420859	5.24282397	1.35105531	0.18247014	0.49780361	-6.0013973
PAPLN	-0.8859415	-0.5342001	-1.3508427	0.182537816	0.49780361	-4.896383
MANEAL	0.48624941	1.93128968	1.35072834	0.182574232	0.49780361	-5.2769428
MITD1	-0.5071447	0.10709404	-1.3506854	0.182587914	0.49780361	-4.9547117
ENSCAFG00000019620	-0.2499573	5.5273945	-1.3506289	0.182605902	0.49780361	-6.0037273
ENSCAFG00000018724	-1.5734713	2.72844051	-1.3505865	0.182619409	0.49780361	-5.0440772
ENSCAFG00000016181	0.19917688	3.85066849	1.35056743	0.182625475	0.49780361	-5.7727418
ENSCAFG00000003424	-0.5837794	0.55339742	-1.3504351	0.182667617	0.49781075	-4.869616
FAM171B	0.32893311	5.4837647	1.35000109	0.182805917	0.49807989	-6.0223467
SLC35A2	0.24209148	4.31819882	1.349328	0.18302055	0.49827875	-5.8419227
ENSCAFG00000011389	-0.176592	6.96162839	-1.3492612	0.183041851	0.49827875	-6.0407388
KAT8	-0.1753439	4.09366248	-1.3492473	0.183046285	0.49827875	-5.8303617
ANKRA2	0.15559226	5.02961886	1.3491106	0.183089912	0.49827875	-5.9750089
SUPT5H	0.10816481	7.23054457	1.34902845	0.183116131	0.49827875	-6.0340265
ATP8A1	-0.7965674	-0.85103	-1.3490281	0.183116255	0.49827875	-4.7660694
LPIN1	-0.2843354	3.23148603	-1.3486721	0.183229874	0.49844323	-5.8330433
SPECC1	0.30431518	7.34745596	1.3485908	0.183255845	0.49844323	-6.0404418
MRPL39	-0.1364191	5.01749524	-1.3483537	0.18333156	0.49854152	-5.9762585
KMT2A	-0.1736578	5.42555799	-1.3481822	0.183386371	0.49858293	-6.0263919
ENSCAFG00000030021	-0.8269182	-1.4372332	-1.3476745	0.183548617	0.4987598	-4.7954252
ENSCAFG00000014034	0.86382261	0.03666879	1.34764864	0.183556891	0.4987598	-4.714643
CYP2U1	0.20523946	2.89728834	1.34760697	0.183570215	0.4987598	-5.6454229
CAMK1	0.35531556	3.37891874	1.34747456	0.183612557	0.49876726	-5.7275005
DHX37	-0.1381239	5.92694481	-1.3465372	0.183912519	0.49909992	-6.0456316
FUS	-0.2369642	7.16934013	-1.3464366	0.183944724	0.49909992	-6.0468921
ENSCAFG00000032288	-0.4454697	0.94467652	-1.3462855	0.18399313	0.49909992	-5.0480068
COL1A2	0.42548546	13.6859788	1.34595495	0.184099023	0.49909992	-5.6055677
ENSCAFG00000027581	-0.6204488	-0.8788866	-1.3459528	0.184099726	0.49909992	-4.8371909
CCDC61	-0.3608598	2.29834612	-1.3459079	0.184114092	0.49909992	-5.4113676
ENSCAFG00000028596	0.74872889	-1.1470597	1.34580214	0.184147995	0.49909992	-4.7266182

CD2AP	-0.1774358	5.12076671	-1.3456316	0.18420265	0.49909992	-5.9762056
FAM117A	-0.2666351	2.58730862	-1.3456173	0.184207247	0.49909992	-5.5501642
CDC42	0.14025643	4.74762473	1.34556921	0.184222663	0.49909992	-5.9282653
ENSCAFG00000002395	-0.1322849	4.4615093	-1.3455156	0.184239852	0.49909992	-5.8921874
ENSCAFG00000032618	0.31951535	2.90490901	1.34545856	0.184258143	0.49909992	-5.6568394
IRF2BP2	-0.2062336	5.66272314	-1.3453601	0.184289706	0.49909992	-6.0368297
ENSCAFG00000004189	-0.5261465	-0.1660509	-1.34536	0.184289754	0.49909992	-4.8355539
IPP	-0.2255816	3.54157308	-1.3448369	0.184457562	0.499447	-5.7226076
LHFPL4	0.88908159	0.95886411	1.34396901	0.184736252	0.50008754	-4.9307734
ENSCAFG00000010918	-0.1065809	6.18354067	-1.3437384	0.18481037	0.50008754	-6.0568153
ENSCAFG00000031559	0.32014743	0.56390203	1.34361715	0.184849329	0.50008754	-4.9950014
TM6SF1	0.77273073	-1.3951329	1.34339692	0.184920132	0.50008754	-4.7588777
GRM8	0.73679305	-2.2385978	1.34335817	0.184932593	0.50008754	-4.7282156
SGTA	0.15273776	6.33633422	1.34332348	0.184943747	0.50008754	-6.0590554
TTC21B	-0.2379893	4.64750311	-1.3432355	0.184972043	0.50008754	-5.9746856
PPARGC1B	-0.4375204	2.20688743	-1.3430049	0.185046212	0.5001807	-5.483793
NUDT8	-0.2884498	2.73861966	-1.3428265	0.185103601	0.50022848	-5.4821312
FAM168A	0.16225656	3.93706572	1.34241752	0.185235247	0.50047687	-5.8024968
SUCLA2	0.12936747	5.7380089	1.3422394	0.185292601	0.50052447	-6.0518435
UBAP1	0.12849828	4.55714172	1.3418991	0.185402213	0.50071318	-5.9193523
MAP2K6	-0.9678991	-0.9931269	-1.3417059	0.185464462	0.50077393	-4.8420906
ACADS	-0.270835	3.73367319	-1.341398	0.185563704	0.50093451	-5.7825378
CHIC1	-0.3388972	1.21516608	-1.3411478	0.185644375	0.50100168	-5.1200295
HMCES	-0.1706735	3.7739357	-1.3410394	0.185679358	0.50100168	-5.7632242
NDUFA10	-0.1231581	6.06515104	-1.3409508	0.185707911	0.50100168	-6.0568106
DEPDC1B	-1.1001334	1.35768853	-1.3407454	0.185774179	0.50105719	-4.8988713
AP1S3	-1.2236088	0.02512366	-1.3405817	0.185827012	0.50105719	-4.6633236
PRMT6	0.40651005	1.68524718	1.34051722	0.185847824	0.50105719	-5.1049101
ACAT2	-0.285947	6.06883676	-1.339855	0.186061656	0.50146384	-6.0628393
COG7	-0.1139566	5.69533973	-1.3398036	0.186078278	0.50146384	-6.0456604
ENSCAFG00000015889	-0.3480443	1.58898209	-1.3395187	0.186170312	0.50154225	-5.1050206
DUSP10	0.66236682	-1.1729363	1.3394657	0.186187454	0.50154225	-4.8821096
TSKU	0.31766237	3.32649745	1.339163	0.186285308	0.50154225	-5.6608038
HDGFL3	-0.1277553	5.77305201	-1.3390675	0.186316183	0.50154225	-6.0415025
TRIR	-0.1334998	5.75780818	-1.3389856	0.186342675	0.50154225	-6.0430149
HIVEP1	-0.3400351	3.9756984	-1.3389745	0.186346278	0.50154225	-5.7520518
LIX1L	-0.1298404	4.68501368	-1.3387606	0.186415458	0.50161954	-5.9542183
NKAPD1	-0.1294905	4.63848084	-1.3386395	0.186454644	0.50161954	-5.9359127
GATB	-0.2376134	2.20116697	-1.3380654	0.186640468	0.50201224	-5.3161701
PTGES	-0.8583314	1.48248599	-1.3377978	0.186727117	0.50208107	-5.1722317
ENSCAFG00000009800	-0.7243589	-1.1385418	-1.3377402	0.186745779	0.50208107	-4.7681518
RHOQ	0.30742489	6.62406567	1.33730542	0.186886675	0.50235266	-6.0658513
TMEM50A	0.15910154	5.60925703	1.33711006	0.186950008	0.50240716	-6.0590871
FAM129B	0.20778876	8.34580213	1.33699321	0.186987894	0.50240716	-5.9438192
ENSCAFG00000029571	0.5460891	-0.565874	1.33687382	0.18702661	0.50240716	-4.8166206
RBM4B	-0.2104679	2.96822514	-1.3365891	0.187118981	0.50254812	-5.5692994
SECISBP2	-0.1088015	4.94117641	-1.3358952	0.187344187	0.5030457	-5.98598
LRRC8D	-0.5037071	2.41706017	-1.3354861	0.1874771	0.50311247	-5.4833877
DCTN2	-0.090809	6.44210799	-1.3354491	0.187489122	0.50311247	-6.0693251
MAPK9	0.12436702	6.44002413	1.33537745	0.18751239	0.50311247	-6.0695423

ACTL6A	-0.1232041	4.55322055	-1.3353269	0.187528821	0.50311247	-5.9219287
EBF1	-0.5087602	4.75337333	-1.3347761	0.187707889	0.50348564	-6.0415871
RNF138	-0.3424765	2.05402275	-1.3338922	0.187995492	0.50414972	-5.3538282
ERC1	0.15686499	5.60728836	1.33365534	0.188072636	0.50424924	-6.0488633
ZNF502	-0.2145063	2.2870573	-1.3334841	0.188128414	0.50429145	-5.4141309
KLHL18	0.19340425	3.08095042	1.33321553	0.18821592	0.50431534	-5.6218349
LDHD	-0.4817322	1.85208125	-1.333211	0.188217402	0.50431534	-5.394639
ENSCAFG00000030939	0.54633348	0.86644707	1.33271691	0.188378467	0.50445076	-5.3681717
HOXA3	-0.9696122	1.08788004	-1.3326737	0.188392569	0.50445076	-5.0679556
COMP	0.77637603	0.650462	1.33258192	0.18842249	0.50445076	-5.1855488
FEM1B	-0.1167191	5.2686194	-1.3323844	0.18848692	0.50445076	-6.0194571
ENSCAFG00000019909	0.16372183	4.74446182	1.33210821	0.188577041	0.50445076	-5.9960729
C35H6orf62	-0.1064451	5.66892762	-1.3320993	0.188579959	0.50445076	-6.0474142
NCAPG	-1.3264193	3.94267794	-1.3320949	0.188581381	0.50445076	-5.3616744
ENSCAFG00000029731	-0.68771	0.06838708	-1.3320736	0.188588331	0.50445076	-4.7822422
PLPPR5	0.96915813	1.31189924	1.3317407	0.188697009	0.50463429	-4.9309807
MIA3	-0.101194	7.54277217	-1.3309456	0.188956757	0.50511251	-6.0483255
OSTC	-0.4144483	2.00753709	-1.3309003	0.188971567	0.50511251	-5.2413585
ENSCAFG00000030758	0.62641017	-0.9027192	1.33082514	0.188996132	0.50511251	-4.8295668
SBNO2	0.1818488	5.72774215	1.33056684	0.189080588	0.50511251	-6.0535124
PREX1	0.5154064	4.95838255	1.33034784	0.189152214	0.50511251	-6.0743048
UFSP2	-0.1325395	4.19969773	-1.3301327	0.189222614	0.50511251	-5.8809189
TMEM208	0.15436855	3.36519752	1.33011829	0.189227313	0.50511251	-5.6812836
ENSCAFG00000031581	0.40055336	3.67632797	1.33006446	0.189244927	0.50511251	-5.7794284
RASGRP1	0.7420171	-0.990017	1.33002743	0.189257048	0.50511251	-4.8367778
PRIM2	-0.255033	3.61706384	-1.329967	0.189276838	0.50511251	-5.6735189
ENSCAFG00000006913	0.18586238	5.87016944	1.32955184	0.189412732	0.50522758	-6.0707312
ZNF514	-0.2353575	3.11682593	-1.3294313	0.189452202	0.50522758	-5.6501513
MLH3	-0.2140795	4.46759947	-1.3291812	0.189534128	0.50522758	-5.9606031
ZNF664	-0.1386683	4.62105971	-1.3290929	0.189563073	0.50522758	-5.9589843
WWOX	0.26620259	2.79017295	1.32908708	0.189564965	0.50522758	-5.5221595
BRINP2	-0.9139375	-1.25678	-1.3290456	0.189578565	0.50522758	-4.7498233
COQ5	-0.1743664	2.87094669	-1.3289779	0.189600728	0.50522758	-5.5746172
VPS45	0.11724965	5.06607004	1.32876195	0.189671516	0.50529552	-5.9940476
KIAA0753	-0.1362133	4.68796061	-1.3285895	0.189728048	0.50529552	-5.9653812
C7H1orf53	-0.5094726	-0.080018	-1.3284999	0.189757431	0.50529552	-4.8907453
SCARB2	0.20233039	7.41347424	1.32840512	0.189788511	0.50529552	-6.042492
POLG	0.12526551	5.90910631	1.32819064	0.18985886	0.50529552	-6.0733071
SLC26A6	-0.1979385	4.50851899	-1.3281661	0.189866916	0.50529552	-5.9797282
RHOBTB3	0.2641068	6.21408383	1.32776114	0.189999792	0.50554233	-6.0457065
PRTG	0.63414605	-0.2819632	1.32754418	0.190071012	0.50562502	-4.8824391
RASSF2	-0.630377	1.02311616	-1.3269675	0.190260422	0.50598807	-5.5853725
ADGRG6	-0.6504773	6.47187663	-1.3268841	0.190287828	0.50598807	-6.0683971
ENSCAFG00000002087	-0.5040723	-0.5707678	-1.3265338	0.190402944	0.50618731	-4.7708449
COL6A3	0.98244174	8.31029764	1.32624113	0.190499188	0.5063019	-5.8556817
ATMIN	-0.1106232	6.17788239	-1.3261583	0.190526437	0.5063019	-6.0805417
SHROOM1	0.29717575	3.1614383	1.32578985	0.190647648	0.50632548	-5.5136973
CEACAM20	0.86772002	-1.0735287	1.32559903	0.190710449	0.50632548	-4.7954923
ZSCAN25	-0.2665232	2.43939824	-1.3255784	0.190717233	0.50632548	-5.4603653
BTBD8	-0.3734937	1.11971012	-1.3254158	0.190770777	0.50632548	-5.1284023

PRRX1	0.4767273	8.57263949	1.32541433	0.190771251	0.50632548	-6.0119751
ACAD8	0.1023551	4.67232645	1.32537051	0.190785678	0.50632548	-5.9655286
PPP3CA	-0.193489	6.47575787	-1.3252763	0.190816694	0.50632548	-6.0765533
ZRANB3	-0.1795474	3.58707243	-1.3250312	0.190897417	0.50638602	-5.756643
SSR3	0.19162532	5.07958901	1.32494949	0.190924338	0.50638602	-6.0128822
ADAMTS10	-0.25135	4.52230952	-1.3248409	0.190960113	0.50638602	-5.8682976
RSRC1	-0.1707159	4.64562636	-1.3242939	0.191140406	0.50666257	-5.9213316
ABCF1	-0.0988037	6.54968866	-1.3242782	0.191145566	0.50666257	-6.0838035
ASPA	-0.6336088	5.16885631	-1.3241584	0.191185076	0.50666257	-6.0739949
ENSCAFG00000019368	0.2093332	2.793517	1.32369706	0.191337266	0.5069543	-5.6292387
RASA1	-0.2000845	7.45825288	-1.3233654	0.191446717	0.5069543	-6.0629227
SPATA6	-0.3051848	2.92644589	-1.3232754	0.191476431	0.5069543	-5.5403272
PROSER2	-1.0374286	-0.692787	-1.3232636	0.191480327	0.5069543	-4.7141182
CIAO3	-0.1597028	3.26170428	-1.323215	0.191496391	0.5069543	-5.6726602
ZNF821	0.29544739	2.21708278	1.32279735	0.191634317	0.50721283	-5.3741339
CACNB4	0.7329047	-1.1040764	1.32229916	0.191798952	0.50750339	-4.8483977
ENSCAFG00000012371	-0.1939657	3.21429687	-1.3222043	0.191830303	0.50750339	-5.6207809
PIGH	-0.243286	2.65367347	-1.3220323	0.19188717	0.50750339	-5.4677735
SAR1B	0.1285976	7.01468778	1.32197765	0.191905258	0.50750339	-6.0762775
FRRS1	0.75566983	-1.2200915	1.32185314	0.191946438	0.50750574	-4.8138977
ENSCAFG00000017763	-0.2381909	3.65938683	-1.3214777	0.192070648	0.50768893	-5.7459428
INIP	0.22203253	2.23149487	1.32140009	0.192096337	0.50768893	-5.4527915
PRICKLE1	0.46092627	5.48559743	1.3210435	0.19221438	0.50785196	-6.0102086
PRDM2	-0.1123096	5.67085932	-1.3209702	0.192238658	0.50785196	-6.074385
RUSC1	0.22216722	4.82134633	1.3206923	0.192330696	0.50798856	-5.9897696
RSBN1L	-0.1868076	3.97197061	-1.3203559	0.19244217	0.50803421	-5.8259764
GARNL3	0.35291446	3.10990116	1.3203048	0.192459093	0.50803421	-5.6367691
TLR2	-0.8644277	0.22594976	-1.3201056	0.192525136	0.50803421	-4.8293455
UBR2	-0.1208425	6.30967962	-1.3199904	0.192563322	0.50803421	-6.0891205
ZFAND1	-0.1747643	3.30590043	-1.3199258	0.192584726	0.50803421	-5.6781342
ADAM33	-0.4807543	5.36852499	-1.3197984	0.192626996	0.50803421	-6.0128644
ZFP37	-0.5789699	0.96168748	-1.3197884	0.19263031	0.50803421	-4.9995007
TXNDC16	-0.2700843	3.45156728	-1.3194391	0.192746174	0.50817804	-5.8920678
FSD1L	-0.3169131	2.19957869	-1.3193808	0.192765534	0.50817804	-5.3910795
IQGAP3	-1.5277194	2.54814344	-1.3190898	0.192862102	0.50821657	-5.0364553
CEP250	-0.2485519	3.39118133	-1.3190831	0.192864313	0.50821657	-5.6931998
UTRN	-0.1954879	8.46023842	-1.3188415	0.192944542	0.50821657	-5.9790674
TRAK2	0.1728078	5.13331561	1.31876	0.192971606	0.50821657	-6.0236973
GLIS1	0.6440167	-0.9470765	1.31867004	0.193001482	0.50821657	-4.802608
TEX14	0.42420388	0.44938086	1.31848639	0.193062488	0.50821657	-5.0968764
KIF16B	0.15266021	6.28533673	1.31841854	0.19308503	0.50821657	-6.0915433
TMEM189	0.20585144	6.70861106	1.31836467	0.193102929	0.50821657	-6.0812648
IQGAP1	0.14539926	9.6036462	1.31819644	0.193158833	0.5082575	-5.9134513
ENSCAFG00000019461	-0.1885842	2.85387065	-1.318007	0.193221804	0.50831701	-5.5387206
KIAA1549L	0.95012163	-0.632862	1.3175689	0.193367478	0.5085155	-4.7747486
KIF18B	-1.3375793	0.99062424	-1.3174216	0.193416471	0.5085155	-4.8821091
BAD	0.23533535	2.48700801	1.31741592	0.193418366	0.5085155	-5.4505453
SIX5	-0.4314493	1.82152985	-1.3172889	0.193460618	0.50852044	-5.2723937
CRABP2	0.83779061	3.5940927	1.31706116	0.193536415	0.50861354	-5.8488409
PCNA	-0.2751935	5.09915568	-1.3168728	0.193599104	0.50867216	-5.9908229

TENT5A	-0.2784036	5.88720957	-1.3166012	0.193689564	0.5088037	-6.0754754
MARC1	-0.8203475	-0.0300083	-1.316077	0.193864184	0.50909883	-4.8408504
ENSCAFG00000025678	0.52250463	-0.0671574	1.31599902	0.193890176	0.50909883	-4.9527262
PCOLCE2	0.5881802	3.69848304	1.31590005	0.193923165	0.50909883	-5.9431893
PLAC8B	1.07542003	-0.8974607	1.31567408	0.193998501	0.50917617	-4.7159917
GTPBP10	-0.321451	2.57223881	-1.3155692	0.194033473	0.50917617	-5.5587415
ENSCAFG00000018228	-0.3623242	1.63751088	-1.315353	0.194105575	0.50925928	-5.0739292
DDRGK1	0.12451947	5.21439971	1.31477958	0.194296932	0.50965517	-6.0421865
BAG4	0.14907319	3.79348349	1.31397774	0.194564741	0.5102514	-5.898896
ENSCAFG00000005837	-0.2074087	2.34724975	-1.3134526	0.194740292	0.51048726	-5.4076897
OTUD1	-0.3168633	2.6706301	-1.3134448	0.194742882	0.51048726	-5.4063713
ENSCAFG00000017118	-0.2767134	2.50466483	-1.313345	0.19477626	0.51048726	-5.4824865
RFWD3	-0.1469044	4.64835602	-1.312985	0.194896713	0.51069669	-5.9507856
USP10	-0.0989134	6.71420112	-1.3125761	0.195033537	0.51094892	-6.0972071
RTF1	-0.0870266	5.91371721	-1.3123783	0.19509977	0.51097578	-6.096918
NINL	-0.2141805	3.34408815	-1.3120789	0.195200065	0.51097578	-5.7150876
SCN1B	0.33988289	2.83947512	1.31200101	0.195226151	0.51097578	-5.6665368
HNRNPUL1	-0.113808	8.2363349	-1.3118515	0.195276245	0.51097578	-6.0302329
PPEF1	-0.6994229	-1.2161665	-1.3118415	0.195279605	0.51097578	-4.7805085
MEST	0.63808444	2.52589579	1.31170073	0.195326773	0.51097578	-5.2661342
ETV4	-0.3238806	4.22302006	-1.3116978	0.195327754	0.51097578	-6.0448314
BPNT1	0.23005501	4.59991123	1.31135759	0.195441802	0.51114339	-6.0120775
ENSCAFG00000018385	0.22335441	3.63417599	1.31126461	0.195472983	0.51114339	-5.7626166
KLF5	0.3108402	4.25173949	1.31105416	0.195543564	0.51114464	-5.8430549
HMCN1	0.71346356	1.39934834	1.31094338	0.195580729	0.51114464	-5.3171208
FAXC	0.28096432	2.12685327	1.31090024	0.195595202	0.51114464	-5.4677113
SYTL2	1.17021198	1.99144669	1.30968648	0.196002741	0.51207895	-4.9116116
VAMP8	0.23411299	4.27921326	1.30959336	0.196034036	0.51207895	-5.9098429
OGFOD2	-0.21398	2.88816985	-1.3090493	0.196216949	0.51241202	-5.6332006
KMT2C	-0.194139	5.80606967	-1.3089189	0.196260803	0.51241202	-6.095269
TAB2	0.14537399	6.82728458	1.30885118	0.19628358	0.51241202	-6.0950775
ZNF581	0.44943405	0.30337018	1.30806964	0.196546618	0.5128342	-4.9968534
FAM53C	0.13181057	5.21680916	1.30803948	0.196556774	0.5128342	-6.0623081
AGPAT4	0.1641283	4.27645835	1.30791147	0.196599883	0.5128342	-5.9835762
PIP5KL1	0.60047199	-0.7004553	1.30788691	0.196608156	0.5128342	-4.7945895
GRIPAP1	-0.1122927	5.04867297	-1.3074632	0.196750893	0.51301598	-6.0422006
ABHD6	-0.3709991	1.72695873	-1.3074383	0.196759306	0.51301598	-5.2662432
ENSCAFG00000017901	-0.3829881	0.78121686	-1.3069691	0.196917482	0.51332214	-5.0625793
EPS15L1	-0.2797799	6.12194498	-1.3067536	0.196990145	0.51340531	-6.1039809
ZNF667	-0.272104	2.33731862	-1.3064192	0.197102969	0.51359309	-5.6086025
RAB33B	-0.1657816	2.89718294	-1.3062131	0.197172519	0.51366806	-5.6189393
RNF213	-0.5159495	3.4394205	-1.3059306	0.197267909	0.5138103	-5.8115761
MTOR	-0.1267339	7.16484121	-1.30524	0.197501192	0.51402208	-6.0977643
STMN1	-1.161668	3.70774434	-1.3052249	0.197506302	0.51402208	-5.4244003
DRAM1	-0.5160319	2.59219408	-1.3052111	0.197510973	0.51402208	-5.4345059
N4BP2L1	-0.6735855	0.11912805	-1.3051849	0.197519831	0.51402208	-5.0765195
ZNF782	-0.2599028	2.34520496	-1.3050859	0.197553264	0.51402208	-5.474599
IRF8	-0.8443888	-2.5416965	-1.3049196	0.19760951	0.51406225	-4.7320202
ZMYM6	-0.1309466	5.3247538	-1.3047593	0.197663701	0.51409704	-6.0754624
TAF7	-0.1485267	5.25442867	-1.304557	0.197732128	0.51416885	-6.0659045

TNKS1BP1	-0.1359801	7.7644093	-1.3044207	0.197778227	0.51418257	-6.060322
ATXN7L3	-0.1283168	4.04688271	-1.3039602	0.19793409	0.51428733	-5.8795708
C28H10orf90	0.57206286	3.16323875	1.30386421	0.197966573	0.51428733	-5.722319
TWSG1	0.18507279	5.03523934	1.30383363	0.197976928	0.51428733	-6.0829368
ENSCAFG00000017221	-0.3058542	1.22557689	-1.3038126	0.197984057	0.51428733	-5.1127883
PPM1H	0.53674001	3.79816821	1.30369854	0.19802267	0.51428733	-5.6816445
FAAP24	0.14197034	3.76283717	1.30346044	0.198103309	0.51432334	-5.8007377
INTS1	0.16036425	6.43262224	1.30341648	0.198118201	0.51432334	-6.1103637
ENSCAFG00000004260	-0.1765945	5.03238781	-1.3029322	0.198282309	0.51464331	-6.0552723
UQCC3	-0.4247296	1.04185201	-1.3026741	0.19836981	0.51476434	-5.0748692
FOXP4	0.230342	6.56097278	1.30238029	0.198469455	0.51491684	-6.1060175
HDAC5	0.18347191	6.30994541	1.30191748	0.198626492	0.51521814	-6.1123651
CPA6	-0.8616146	-1.9971713	-1.3015796	0.198741192	0.51540952	-4.8538925
ROBO1	0.43709396	7.66896332	1.30136229	0.198814997	0.51541202	-6.0940933
TRIM65	-0.4121158	2.27666882	-1.3012972	0.198837113	0.51541202	-5.6201516
NAT9	0.17945538	2.98099701	1.30095555	0.198953183	0.51541202	-5.6327234
PLSCR3	0.1729199	4.50683844	1.30089962	0.198972193	0.51541202	-5.9885647
C8orf33	-0.2844615	1.95032875	-1.3008679	0.198982955	0.51541202	-5.3148025
MERTK	1.0605944	2.55778374	1.30085409	0.198987665	0.51541202	-5.1880757
GOSR1	-0.1511566	3.69190181	-1.3003917	0.199144883	0.51571319	-5.8280629
CENPP	-0.3692199	3.0393783	-1.2998999	0.199312181	0.51604033	-5.6352889
NUMA1	-0.1115132	8.19390911	-1.2996977	0.199381	0.51611243	-6.0283342
SCNM1	0.18141021	3.16169065	1.29950701	0.1994459	0.51612905	-5.6135881
CCDC65	-0.4379437	-0.5177533	-1.2994381	0.199469374	0.51612905	-4.947357
FOPNL	0.13632634	4.57324403	1.29898672	0.199623102	0.51642074	-5.9813042
ENSCAFG00000002833	-0.2308287	2.66436747	-1.2986264	0.199745874	0.51655683	-5.5060464
MLST8	-0.255621	2.85509461	-1.298521	0.199781812	0.51655683	-5.5441792
ARFGAP2	-0.0977127	5.13110491	-1.2984714	0.199798737	0.51655683	-6.0519369
SSBP1	0.21227343	2.45324363	1.29810176	0.199924772	0.51677661	-5.4529031
BLVRA	-0.1464808	5.41564232	-1.2979717	0.199969136	0.51678524	-6.0669444
AZIN1	0.25506703	6.6846078	1.29756126	0.200109188	0.51704109	-6.1154309
CSNK1D	-0.0974997	6.29647409	-1.2970341	0.200289164	0.51739998	-6.118267
SAFB	-0.1402221	5.39427099	-1.2965485	0.200455091	0.51750729	-6.079979
RRAGD	0.53555972	3.16824735	1.29653545	0.200459543	0.51750729	-5.4331036
OXA1L	-0.1050999	5.81205464	-1.2965344	0.200459901	0.51750729	-6.1094215
LBR	-0.24409	5.33677576	-1.2959258	0.200667972	0.51750729	-6.040495
N4BP3	-0.55621	1.71093928	-1.2957763	0.200719116	0.51750729	-5.4745397
THAP1	-0.1684822	2.67774537	-1.2957187	0.200738824	0.51750729	-5.5284453
NSUN6	0.15086582	3.81630476	1.29570115	0.200744829	0.51750729	-5.8225837
NHLRC3	0.26868036	2.9407063	1.29558176	0.200785676	0.51750729	-5.5843537
EIF4A2	0.14768575	7.49104076	1.29554394	0.200798621	0.51750729	-6.0772497
RNF185	-0.1818244	2.72766387	-1.2954743	0.200822456	0.51750729	-5.5277305
TENT5C	0.55487812	0.72040528	1.29532307	0.20087421	0.51750729	-5.3878056
MBIP	0.16699684	4.19720496	1.29525746	0.200896668	0.51750729	-5.9126392
ZKSCAN5	0.27810108	2.09803335	1.29525568	0.200897279	0.51750729	-5.388143
TRPC4AP	0.08760895	7.23657443	1.29523051	0.200905895	0.51750729	-6.1020996
TNFRSF13C	0.39660012	0.03279303	1.29477104	0.201063236	0.51780669	-4.9687026
ENSCAFG00000009910	0.19687413	7.08545099	1.29363822	0.201451548	0.51864244	-6.110554
SCLY	-0.1644804	4.35298424	-1.2935841	0.201470105	0.51864244	-5.9278927
TLR3	-0.3675106	3.44498405	-1.2934609	0.201512368	0.51864524	-5.7358279

PATJ	0.56457711	-0.5985813	1.29326708	0.201578891	0.51871046	-4.9330121
RHOU	0.7001341	0.66196481	1.292938	0.201691854	0.51889514	-5.1054758
USF1	-0.1093986	4.65557075	-1.2927868	0.20174376	0.51892269	-5.9989988
TUBB4B	0.22083675	7.86506786	1.29222064	0.201938262	0.51918234	-6.0866543
KLHDC8B	0.49292559	1.83677859	1.29217103	0.201955314	0.51918234	-5.4549389
TRPM3	1.13923931	-0.6813145	1.29199398	0.202016167	0.51918234	-4.8160071
GNAS	0.09273058	9.40431221	1.29189216	0.20205117	0.51918234	-5.9789829
MDC1	-0.0897509	6.89505483	-1.2917947	0.202084667	0.51918234	-6.1208119
TRIM41	0.163615	2.83189383	1.29174063	0.202103272	0.51918234	-5.6858818
ZC2HC1A	0.24302843	4.28640844	1.2916535	0.202133233	0.51918234	-5.9695419
NPLOC4	0.11554487	6.60298784	1.29143361	0.202208866	0.51922718	-6.125535
ENSCAFG00000029259	-0.365357	-0.1084564	-1.2913631	0.202233132	0.51922718	-4.9555217
FES	-0.1905033	4.85393202	-1.2908172	0.202421008	0.51946702	-5.970806
ZBTB4	0.21290217	6.83284219	1.29081044	0.20242332	0.51946702	-6.1125406
ZNF189	-0.1987625	3.13650232	-1.2907322	0.202450269	0.51946702	-5.7400787
ENSCAFG00000007115	0.62330941	-0.6631117	1.29024908	0.20261665	0.51978805	-4.8438421
WDFY3	-0.1573319	6.89537902	-1.2898794	0.202744053	0.51987635	-6.1212156
CSTB	0.26295252	7.03718961	1.28969666	0.202807035	0.51987635	-6.1178145
CLEC11A	0.64033444	3.23185799	1.28958048	0.202847094	0.51987635	-5.7197253
KLF9	-0.5000709	2.11429284	-1.2895329	0.2028635	0.51987635	-5.7709492
PACRGL	-0.1994934	2.87178134	-1.2894699	0.202885228	0.51987635	-5.6096214
HEBP1	0.19165846	5.39627397	1.28926953	0.202954335	0.51987635	-6.0886528
HBS1L	0.10991294	6.27812426	1.28917136	0.202988198	0.51987635	-6.1283673
NFIA	-0.2225893	4.33073645	-1.2891578	0.202992886	0.51987635	-5.9396207
ENSCAFG00000002706	0.4642216	1.20012678	1.28899458	0.203049193	0.51987635	-5.1775524
CYYR1	-1.007148	-2.1717505	-1.2888684	0.203092722	0.51987635	-4.7957222
RNASEK	0.18422561	6.33145746	1.28883266	0.203105074	0.51987635	-6.1286154
ZNF75D	-0.2857156	1.60574277	-1.2886009	0.203185065	0.51994539	-5.2475035
ARHGAP29	-0.2138082	7.19187942	-1.2884945	0.203221796	0.51994539	-6.0922038
BBS10	-0.130042	3.57218311	-1.2883897	0.203258012	0.51994539	-5.8125112
B4GALT2	0.17890239	6.02079807	1.28822672	0.203314289	0.51994539	-6.1182024
CASP8AP2	-0.1798052	3.99086259	-1.2881332	0.203346585	0.51994539	-5.936944
ENSCAFG00000014771	-0.2001959	2.83678608	-1.2880373	0.203379719	0.51994539	-5.5896754
CCDC191	0.24485551	3.05448247	1.2876188	0.20352435	0.52020956	-5.7330388
NEGR1	0.63744035	3.61451285	1.28729301	0.203636992	0.52039187	-5.5668846
OPHN1	-0.2435147	2.52692602	-1.2871211	0.203696463	0.52043826	-5.513717
MCL1	-0.1487524	7.72291207	-1.2869429	0.203758078	0.52047429	-6.0740859
PTP4A2	-0.1504467	7.30985654	-1.2866972	0.203843095	0.52047429	-6.1181632
MRRF	-0.1715716	4.56069209	-1.2866114	0.203872819	0.52047429	-5.9808634
ENSCAFG00000009857	0.14834496	4.97540268	1.28657613	0.203885016	0.52047429	-6.0743885
CCDC141	1.03760754	-0.5671774	1.28645181	0.203928049	0.52047429	-4.8609381
GPD1	0.5342377	-1.3520313	1.28636389	0.203958488	0.52047429	-4.872048
NQO2	-0.1993436	3.43946395	-1.2855681	0.204234137	0.52107215	-5.8083601
SSSCA1	0.18886805	3.45563634	1.2850154	0.204425772	0.52145545	-5.7717693
ENSCAFG00000009894	-0.6204214	1.03312166	-1.2841745	0.204717573	0.52202532	-5.0247339
TBRG1	-0.0875921	5.40308171	-1.2841327	0.204732066	0.52202532	-6.095105
FEZ1	0.79686174	-1.7575913	1.28334247	0.205006592	0.52247393	-4.7271851
COL3A1	0.36762657	13.9057832	1.28330678	0.205018996	0.52247393	-5.707733
VMA21	0.17464259	2.59967428	1.2832681	0.205032443	0.52247393	-5.5046131
NSFL1C	0.10698557	5.13060299	1.28269752	0.205230841	0.52262402	-6.0564185

ATRN	0.10855474	6.29628981	1.28261214	0.205260541	0.52262402	-6.1361837
ENSCAFG00000030734	0.64654318	0.12931019	1.28258543	0.205269834	0.52262402	-4.9231527
RALY	-0.1009944	6.47440167	-1.2825741	0.205273765	0.52262402	-6.1367776
NOTCH2	0.32406851	9.48758046	1.28250218	0.205298796	0.52262402	-5.9709627
RAD17	-0.1144964	5.15893752	-1.2822227	0.205396054	0.52266074	-6.0774946
SLC11A1	0.46356007	1.04819114	1.28222224	0.205396212	0.52266074	-5.183431
NDUFV2	-0.0837351	6.05972062	-1.2813239	0.205709063	0.52319112	-6.1365258
CTH	-0.403981	3.35477546	-1.2811787	0.205759652	0.52319112	-5.6791446
WDR18	-0.2202017	2.96496226	-1.2811033	0.205785939	0.52319112	-5.6002182
MINDY1	-0.2534922	4.6287717	-1.2810614	0.205800538	0.52319112	-6.0310716
KRT23	-0.787779	-2.7918558	-1.2810276	0.205812321	0.52319112	-4.7127338
RILPL2	-0.2575917	3.35154793	-1.2802328	0.206089526	0.52370143	-5.8517412
ENSCAFG00000010596	-0.3693297	3.27882817	-1.2800422	0.206156026	0.52370143	-5.7023825
FUZ	-0.3524438	2.2399964	-1.279994	0.206172861	0.52370143	-5.4104335
DPP4	-0.3192696	8.43340978	-1.2799753	0.206179374	0.52370143	-6.0497355
ENSCAFG00000025524	-0.3129167	6.18427384	-1.2795646	0.206322776	0.52396002	-6.1336701
ENSCAFG00000025787	-0.5900466	-0.1105506	-1.2792989	0.206415584	0.52409004	-4.9992183
ZBTB41	-0.1785061	4.00101728	-1.2788599	0.206568965	0.52437378	-5.8709712
AP1S2	-0.2334477	7.66221825	-1.2786576	0.206639701	0.52444765	-6.132864
GLUL	-0.4294103	4.10773052	-1.277947	0.206888232	0.52497264	-6.1004668
SAMD4B	0.16970027	6.13377476	1.27744804	0.207062909	0.52531005	-6.1411515
NEK2	-1.2682077	1.72857142	-1.2772761	0.207123121	0.52535699	-4.9978503
COL4A1	0.33011072	11.1930822	1.27706849	0.207195842	0.52536332	-5.8008065
KIF21A	0.66943933	0.57563459	1.27703084	0.207209034	0.52536332	-5.0095346
NXPH4	0.59406834	0.81580901	1.27684429	0.207274397	0.52542329	-5.1938683
ADAMTS12	-0.3804161	4.62111284	-1.2765612	0.207373625	0.52555743	-6.1181941
RIC8B	-0.1915264	2.65022493	-1.2764552	0.207410763	0.52555743	-5.5540582
DPF1	0.51804323	0.42344352	1.27617262	0.207509867	0.52561498	-5.2146145
SOWAHC	0.30942127	2.04742444	1.27591105	0.207601621	0.52561498	-5.4608777
TUBGCP6	-0.1288852	4.5454671	-1.2758611	0.207619131	0.52561498	-5.9798264
USB1	0.13630622	4.22693428	1.27584307	0.20762547	0.52561498	-5.9497666
ABCB4	0.7382594	1.64702361	1.27579562	0.207642121	0.52561498	-5.0949172
METAP2	-0.1209697	6.3642232	-1.2756052	0.20770895	0.52567851	-6.1456561
AMOTL2	-0.2686856	8.24591041	-1.2752887	0.207820045	0.52585402	-6.0678753
MCAM	0.8658977	3.05517692	1.27485235	0.207973301	0.52613611	-5.5262528
ENSCAFG00000002646	0.2337412	7.2254461	1.27471351	0.208022082	0.52615291	-6.1190845
LIFR	-0.3629228	4.07103139	-1.2745957	0.208063485	0.52615291	-5.9019035
TP53I3	-0.2989473	1.37126911	-1.2744012	0.208131854	0.52622016	-5.2767113
PSMB5	0.13057591	6.18110702	1.274118	0.208231402	0.52636619	-6.1386758
BDH2	-0.3220526	2.35479677	-1.2739162	0.208302374	0.52643995	-5.5009759
PRPF31	-0.098954	5.45882076	-1.2736069	0.208411169	0.52652333	-6.1056831
SERPINE2	-0.4671394	8.85253751	-1.2735848	0.208418967	0.52652333	-6.0065901
SELENOO	0.1743302	4.17238291	1.27331429	0.208514155	0.52653672	-5.9188407
KIFAP3	0.18646288	6.48720103	1.27294252	0.208645045	0.52653672	-6.1477389
WDR12	-0.126106	4.21261187	-1.2728718	0.208669945	0.52653672	-5.9486812
SRSF2	-0.2320893	4.84627563	-1.2728122	0.208690938	0.52653672	-6.00333
PRR14L	-0.1445953	5.89495152	-1.2727972	0.208696237	0.52653672	-6.1433725
HNRNPA3	-0.1749334	6.34580802	-1.2727034	0.208729283	0.52653672	-6.1478084
TPPP	0.69003139	-1.2745072	1.27266689	0.208742126	0.52653672	-4.8284128
ICAM2	-1.0342727	-0.0065665	-1.272538	0.208787519	0.52653672	-5.0221205

PRR11	-1.0042113	1.77020153	-1.2723323	0.208860019	0.52653672	-5.0917085
PYROXD2	0.35462402	3.33571957	1.27228976	0.208875012	0.52653672	-5.7235199
KCNE3	-0.8143809	-0.1242871	-1.2721974	0.208907558	0.52653672	-5.1268906
RCOR1	0.17003436	3.0096432	1.27214541	0.208925892	0.52653672	-5.6273404
CSNK1G2	0.11397577	5.85737127	1.27200263	0.208976226	0.52655822	-6.1448092
SLC25A21	0.37217167	0.76476598	1.27147804	0.209161241	0.526919	-5.1253438
TIMM44	-0.1523655	4.16265287	-1.271271	0.209234281	0.5269217	-5.9230994
WTAP	-0.1199629	4.83203231	-1.2712379	0.209245977	0.5269217	-6.0627979
RIBC1	0.38725446	0.38037949	1.2704764	0.209514844	0.5274933	-5.070412
TTPAL	0.15035158	5.2521393	1.27014608	0.209631553	0.52768166	-6.1198784
MALT1	0.23667384	4.86165164	1.26952383	0.209851539	0.52812987	-6.0617614
ENSCAFG00000007675	-0.1140863	6.05116025	-1.2691346	0.209989231	0.52837083	-6.1483426
DCLK1	0.31926423	5.41378668	1.26894232	0.210057278	0.52843648	-6.0186885
ELOA	0.12248042	5.49040402	1.2682549	0.210300679	0.52894316	-6.1280506
BAG2	0.29372897	4.28807026	1.26813386	0.210343559	0.52894539	-5.9562296
ENSCAFG00000002619	0.30883687	1.02860724	1.26796486	0.210403437	0.52899036	-5.201651
BAP1	0.10290317	6.06346994	1.26741182	0.210599484	0.52928157	-6.1496758
ZNF287	-0.193715	2.50956121	-1.267401	0.210603306	0.52928157	-5.5984904
AUTS2	0.28050434	5.43011041	1.26693717	0.210767847	0.52958943	-6.1112197
DHCR7	-0.2527192	6.28825722	-1.2666517	0.210869148	0.52973829	-6.1557958
ENSCAFG00000005722	-0.3538618	1.82813059	-1.2663826	0.210964674	0.52987259	-5.4319112
CXCL16	0.65185392	3.52159222	1.26596293	0.211113736	0.53002724	-5.5056164
ENSCAFG00000002015	-0.1984797	7.38246155	-1.2657926	0.211174245	0.53002724	-6.1469467
FTH1	0.14925922	8.07143931	1.26578435	0.211177184	0.53002724	-6.0887192
MLLT6	-0.2532282	4.65573042	-1.2657354	0.211194564	0.53002724	-6.0594024
ATP6V1D	0.13115162	5.8532988	1.26551103	0.21127432	0.53003543	-6.1492883
CCDC149	-0.8104799	-0.1865643	-1.2653868	0.21131849	0.53003543	-4.845878
CRY1	-0.1206571	4.15741717	-1.2653711	0.211324067	0.53003543	-5.9681245
C5H1orf123	-0.1871483	3.56857811	-1.2652124	0.211380472	0.53007136	-5.8211079
MORC2	0.11201771	5.8816631	1.26498675	0.211460738	0.53016709	-6.1525804
MCRIP1	0.23786795	3.45744512	1.26472781	0.211552854	0.53029249	-5.7899169
TMCO4	0.41038891	0.93600803	1.26428147	0.211711708	0.53053258	-5.2878368
HIC2	-0.2121207	2.0950312	-1.2641578	0.211755742	0.53053258	-5.6059291
UBQLN4	0.16614741	4.03152541	1.26406962	0.211787136	0.53053258	-5.8890342
CUL4A	-0.1058009	7.12529801	-1.2639854	0.211817114	0.53053258	-6.1449753
ZC3H7A	-0.1266493	6.41121068	-1.2631923	0.212099728	0.53113483	-6.1606912
ZFAT	0.15703724	3.20973232	1.26293174	0.212192617	0.53121455	-5.7496781
RTTN	-0.2952557	3.32014306	-1.2628664	0.212215909	0.53121455	-5.6767057
NBN	-0.1345271	6.29754901	-1.2625087	0.212343525	0.53134464	-6.1616715
HNRNPA2B1	-0.1844078	8.37843248	-1.2623785	0.212389981	0.53134464	-6.0934913
PTPN21	0.16421368	7.78492993	1.26230286	0.212416963	0.53134464	-6.1208741
ROCK2	0.17135816	7.13338715	1.26222225	0.212445732	0.53134464	-6.1481515
AKT3	0.1866474	5.22238749	1.26212961	0.212478798	0.53134464	-6.1003171
LMNB1	-0.4567482	5.31693115	-1.2619486	0.212543421	0.53135624	-6.0320386
PLEKHO1	0.31329588	4.59562019	1.26176324	0.212609609	0.53135624	-5.987519
BMP2K	-0.1571917	6.11919296	-1.2617622	0.21260999	0.53135624	-6.1542644
CRTC2	-0.174954	4.55174521	-1.261572	0.212677926	0.53136904	-6.0429327
KIAA1551	-0.2836639	4.94643156	-1.2615116	0.212699484	0.53136904	-6.1282631
CTDNEP1	-0.0887525	5.72982597	-1.2611728	0.212820554	0.53153531	-6.1512129
DLGAP4	0.10734312	6.73065004	1.26108917	0.212850435	0.53153531	-6.1618898

SSR4	0.18464418	7.07821212	1.26088416	0.21292372	0.53158403	-6.1502565
ANGPT1	0.56318425	5.47350132	1.26079847	0.212954353	0.53158403	-6.1555529
ACP5	0.79797876	0.26352158	1.25994292	0.213260422	0.53224258	-5.0326162
ABHD12B	-0.8134623	0.30402178	-1.259725	0.213338416	0.53227831	-5.052152
C10H12orf66	-0.1488077	3.82454039	-1.2596111	0.213379215	0.53227831	-5.9154576
DDHD2	-0.1146382	5.22366071	-1.2595488	0.213401515	0.53227831	-6.1365536
MIEN1	0.17599026	3.15554001	1.25926164	0.213504378	0.53242945	-5.6483698
APEX1	-0.1409896	7.0286347	-1.2590058	0.213596045	0.53255261	-6.1622943
NDC80	-0.7060339	2.93254923	-1.2588637	0.213646975	0.53257417	-5.3736446
ADAP2	0.83297358	0.64791705	1.25858478	0.213746957	0.53267023	-5.1422847
IL6ST	-0.1737841	8.20554034	-1.2585203	0.213770086	0.53267023	-6.0807984
CDH17	0.79882552	0.24966536	1.25751239	0.214131706	0.53336342	-4.8346573
KLHL4	-0.9234618	-2.4574168	-1.2573139	0.21420299	0.53336342	-4.7682704
DRAM2	-0.1794438	3.32879449	-1.2571057	0.214277752	0.53336342	-5.7850093
EFR3B	0.37549328	1.89140291	1.25706653	0.214291823	0.53336342	-5.2252587
ABRACL	0.30214443	5.3255541	1.25697238	0.214325645	0.53336342	-6.1443404
BBS12	-0.2177589	2.94186474	-1.2569253	0.214342552	0.53336342	-5.6485209
ZNF41	-0.2438535	1.81158601	-1.2569194	0.214344683	0.53336342	-5.3743674
DENND4A	-0.1634352	5.57213221	-1.2567768	0.214395913	0.53338553	-6.1499639
LRRC6	0.63188345	0.00913578	1.25636011	0.214545692	0.53359284	-4.8898334
ADH4	-0.5866223	2.2343662	-1.2563093	0.214563967	0.53359284	-5.6743302
VPS33B	-0.1338108	4.50570082	-1.2561312	0.214627989	0.53364669	-6.0406707
KANSL1	-0.116437	6.12751302	-1.2559394	0.214697001	0.53371293	-6.1688736
IKBKE	-0.1612368	5.75451119	-1.2556683	0.214794521	0.5337747	-6.1605029
SRP54	0.1341895	6.74609817	1.25563474	0.214806603	0.5337747	-6.1669568
RAD51	-1.1934736	2.08094161	-1.2552003	0.21496297	0.53397677	-5.1102151
OGFOD1	0.155151	4.35279397	1.25511563	0.214993468	0.53397677	-6.0116843
PDE1B	0.75029864	-0.796105	1.25505557	0.215015097	0.53397677	-4.7901886
PSMD4	-0.0856589	5.87506791	-1.2547742	0.215116427	0.53403195	-6.1586856
DDB2	-0.4916662	2.44130916	-1.2545991	0.215179526	0.53403195	-5.2965083
TNFRSF1A	-0.1600435	6.12463935	-1.2543265	0.215277752	0.53403195	-6.1707911
PQLC2	-0.2727382	2.1500264	-1.2542542	0.215303825	0.53403195	-5.4382077
IRAK3	-0.3653027	2.43540964	-1.2541984	0.215323952	0.53403195	-5.6269644
ARHGEF28	-0.8375937	1.69703046	-1.2541837	0.215329257	0.53403195	-5.2123331
PRTFDC1	-0.536178	2.51014357	-1.2541702	0.215334095	0.53403195	-5.5319171
LMBR1	-0.1201283	3.20021018	-1.2540218	0.215387604	0.5340595	-5.7194463
SLC46A3	0.47849433	3.70830219	1.25378636	0.21547252	0.5341649	-5.8370615
ENSCAFG00000019687	0.23614881	6.28715992	1.25322144	0.215676347	0.53456499	-6.1734275
COP1	-0.1161409	5.24169235	-1.2530058	0.215754188	0.5346103	-6.1223985
APOPT1	0.24404449	5.78940911	1.25293566	0.215779513	0.5346103	-6.1593069
NOVA1	-0.2029493	2.86735394	-1.2527777	0.215836541	0.5346138	-5.5886475
MINDY2	-0.2522305	2.77168284	-1.2526711	0.215875057	0.5346138	-5.777882
PIF1	0.52204368	-0.2912591	1.25257917	0.215908257	0.5346138	-4.9479425
NRG1	1.00842643	1.03217007	1.25229048	0.216012557	0.53476694	-4.8474482
PCDH1	-1.1560055	0.5198464	-1.2521245	0.216072537	0.53481031	-4.9833766
DGKA	0.11011025	6.78416254	1.25178567	0.21619503	0.53499819	-6.1723377
SLC37A2	0.93818501	3.337454	1.2516103	0.216258447	0.53499819	-5.3498747
NLRX1	-0.1466269	4.7211342	-1.2515621	0.216275866	0.53499819	-6.0458642
ZSWIM6	0.21703727	4.90977409	1.25089131	0.216518592	0.53549346	-6.0874084
ENSCAFG00000010233	-0.1448162	4.3309968	-1.2507676	0.21656338	0.53549908	-6.0004361

TMEM237	-0.1436601	4.70300501	-1.2505405	0.216645589	0.53559722	-6.0337759
ZNF770	-0.160181	4.76159159	-1.2504034	0.216695243	0.53561485	-6.06345
LSM8	-0.2336057	3.13826125	-1.2499428	0.216862118	0.53590113	-5.7153168
TSGA10	-0.6767865	-0.6956902	-1.249846	0.216897225	0.53590113	-4.9079581
ENSCAFG00000032682	0.21861525	2.54115565	1.24973156	0.216938698	0.53590113	-5.6103379
ENSCAFG00000005635	-0.6094416	-0.9155572	-1.2495888	0.21699046	0.53591356	-4.9208896
ENSCAFG00000024219	-0.1447744	4.99218211	-1.2492998	0.217095268	0.53591356	-6.1137159
ENSCAFG00000012637	0.16496581	3.38223249	1.24896109	0.217218125	0.53591356	-5.8330074
CLPX	-0.1566534	5.6120701	-1.2488411	0.217261658	0.53591356	-6.1621394
DPEP2	0.52106939	-0.5640641	1.24863697	0.217335757	0.53591356	-5.0026165
TMEM54	-0.589568	-0.7746729	-1.2485511	0.217366917	0.53591356	-4.9050803
LRP6	-0.1371549	4.96081991	-1.2483644	0.2174347	0.53591356	-6.1361002
ENSCAFG00000007359	-0.1089738	5.28233623	-1.2483618	0.217435656	0.53591356	-6.1307567
IVD	-0.2276902	5.68961028	-1.2481928	0.217497017	0.53591356	-6.1683443
EPB41L2	-0.2024286	6.87387807	-1.2481753	0.21750338	0.53591356	-6.1786715
CLPB	0.12671251	4.64783257	1.24805165	0.217548297	0.53591356	-6.0666217
LIN52	-0.2355636	2.37321157	-1.2480064	0.217564747	0.53591356	-5.4839909
CDA	-0.5664382	-2.9072364	-1.2480049	0.217565268	0.53591356	-4.7394894
GAR1	-0.1889766	3.65564796	-1.2479206	0.217595922	0.53591356	-5.8354614
SUOX	0.22128266	4.47188966	1.24785763	0.217618786	0.53591356	-6.0796573
MDGA2	1.16473079	0.35526247	1.24782681	0.217629983	0.53591356	-4.7796631
ENSCAFG00000015798	0.19510077	7.28229881	1.24772489	0.217667018	0.53591356	-6.1566699
RAB3GAP1	-0.1474375	6.25649279	-1.2474362	0.217771929	0.53606707	-6.1777495
SMC4	-0.3196512	6.16743097	-1.2472178	0.217851335	0.53611311	-6.1642356
RAVER2	-0.4081587	2.01466901	-1.2471507	0.217875757	0.53611311	-5.5855838
SDK2	-0.9612194	-0.0785517	-1.2470238	0.217921896	0.53612192	-4.9004286
PLK4	-1.2330886	2.17253853	-1.2467588	0.218018307	0.53616748	-5.132657
ZNF346	-0.1437034	3.15862276	-1.2467281	0.218029484	0.53616748	-5.7641964
EXOSC8	-0.2925016	3.1997727	-1.2466219	0.218068119	0.53616748	-5.5461132
ADNP	-0.1542499	6.14299402	-1.2464359	0.218135796	0.53622647	-6.1807271
TPCN1	0.22473545	4.19781482	1.24622137	0.218213903	0.53622647	-6.0225127
ENSCAFG00000031317	-0.2575221	1.10438934	-1.2462051	0.218219822	0.53622647	-5.1885763
MRPL36	0.24279546	3.19527176	1.24591662	0.218324878	0.53636306	-5.7431
TNFRSF1B	-0.7527924	-0.246225	-1.2458186	0.218360572	0.53636306	-4.9850082
HABP4	0.20818363	3.18596411	1.24550132	0.218476173	0.53654238	-5.7779836
ZCCHC14	0.20696817	4.4799203	1.24517659	0.218594525	0.53672839	-6.0432627
CA4	0.76375793	-0.9359724	1.24489885	0.218695793	0.53687238	-5.1416317
EP300	-0.1043453	7.26510935	-1.2443689	0.218889106	0.5371635	-6.1645745
SLC50A1	-0.3200833	2.5862627	-1.2442377	0.21893701	0.5371635	-5.5066146
ATL2	-0.151309	4.31984914	-1.2442231	0.218942317	0.5371635	-5.9960102
RNF126	0.15095956	5.13066388	1.24392274	0.219051965	0.53728807	-6.0993854
TYK2	-0.1442987	6.02403465	-1.2436789	0.219140987	0.53728807	-6.177979
TAL1	-0.7858199	-0.7777109	-1.2436776	0.219141472	0.53728807	-4.8798968
FUNDC2	0.12444351	5.13967041	1.24354309	0.219190607	0.53728807	-6.1370901
TMTC1	0.48973523	5.11744681	1.24349995	0.219206368	0.53728807	-6.1060553
PPARD	0.19931015	6.55585612	1.24330016	0.21927936	0.53736242	-6.1825722
ACKR2	-0.8066289	-0.7423362	-1.2430881	0.219356853	0.53737197	-4.974443
MTRF1	-0.2322062	2.34107513	-1.2429614	0.219403153	0.53737197	-5.5319357
ENSCAFG00000032401	-0.1926504	2.36174024	-1.2428736	0.219435277	0.53737197	-5.4584102
ENSCAFG00000015964	-0.1608662	4.44808871	-1.2428226	0.219453907	0.53737197	-5.9747393

ENSCAFG00000028721	0.55522143	-0.4418746	1.24259351	0.219537673	0.5374726	-4.9840036
IQCD	0.74408859	-0.4875113	1.24235478	0.219624989	0.53758188	-4.8834535
CD74	0.81821003	-1.5569588	1.24216028	0.219696149	0.53765158	-4.9535276
LHFPL6	0.24982133	6.17956079	1.24165106	0.219882532	0.53800318	-6.1811302
DHRS2	0.72397978	1.30716119	1.24126208	0.22002498	0.53824711	-5.4049558
DYNLL2	0.17219273	4.69982391	1.2411455	0.220067688	0.53824711	-6.0760507
TRIM7	-0.2987002	3.12768123	-1.2407124	0.220226391	0.5385307	-5.7949566
ARHGAP5	-0.1331693	6.01024313	-1.2400922	0.220453809	0.53898218	-6.1887289
TST	0.45669773	0.45522117	1.23958638	0.22063943	0.53912769	-5.1289006
ZNF432	-0.3856065	2.05863324	-1.2394785	0.220679019	0.53912769	-5.4844164
PHF14	-0.1747779	4.67393159	-1.2394781	0.220679184	0.53912769	-6.0667093
MIPOL1	-0.190965	4.79098773	-1.2394304	0.220696693	0.53912769	-6.0877172
PADI2	0.81284819	-0.5401628	1.23934694	0.220727332	0.53912769	-5.0091243
STARD7	-0.119882	4.84478065	-1.2391385	0.220803863	0.53921006	-6.1129434
RB1CC1	-0.1424606	7.27442254	-1.2387451	0.220948393	0.53945841	-6.1593075
ENSCAFG00000000733	0.55845874	0.14663008	1.23796949	0.221233511	0.53991844	-4.95021
ENSCAFG00000023760	-0.3194666	3.64064604	-1.2379021	0.221258317	0.53991844	-5.8347985
DDX17	-0.1189115	8.51542397	-1.2378828	0.2212654	0.53991844	-6.1021841
SCN2B	0.66083399	2.35071337	1.23740121	0.221442593	0.54024615	-5.758225
MTBP	-0.1904748	2.96692246	-1.2370713	0.22156404	0.54042996	-5.6938912
APOL6	-0.2127125	3.31543066	-1.2369635	0.221603741	0.54042996	-5.8897805
ENSCAFG00000004177	-0.1628967	2.90223577	-1.235894	0.221997831	0.54119212	-5.7036673
CLTB	0.14693092	6.12278094	1.23578973	0.22203627	0.54119212	-6.1911973
PNPO	-0.2562627	3.41181666	-1.2356292	0.22209546	0.54119212	-5.9171778
EDRF1	-0.1728853	4.21054798	-1.2355125	0.222138515	0.54119212	-6.01772
KMT5B	-0.1267676	4.1365892	-1.2354987	0.222143615	0.54119212	-5.9863791
PTPN4	-0.2684548	2.5161631	-1.2354162	0.222174058	0.54119212	-5.4865334
CDC42EP2	-0.4622524	2.45475495	-1.2352376	0.222239936	0.54124792	-5.6422513
AHCYL1	0.17801316	7.52179565	1.23501995	0.222320279	0.54129242	-6.1366173
NLK	0.23804573	3.08361792	1.23495526	0.222344156	0.54129242	-5.8470666
C1H19orf54	-0.2493139	1.57395636	-1.2347662	0.222413972	0.54129919	-5.3067577
ARNTL	0.18924974	4.09704188	1.23471493	0.222432886	0.54129919	-6.1278627
WNT2	-0.6913826	2.93770673	-1.2341285	0.222649521	0.541683	-5.7339457
SENP6	-0.1153237	6.25140674	-1.2340552	0.222676611	0.541683	-6.1960072
HINT2	-0.206192	2.56579915	-1.2337441	0.2227916	0.54185438	-5.600141
SYT7	-0.621838	0.22014342	-1.2336318	0.222833095	0.54185438	-5.1650181
SLC25A32	-0.1469197	4.28298863	-1.2334661	0.222894383	0.54189879	-6.0229993
CYB5R3	0.20369282	8.85223663	1.23298947	0.223070694	0.54222278	-6.0579761
COL27A1	0.45186598	3.92957974	1.2324117	0.223284566	0.54236787	-5.8223644
PODNL1	0.44397214	1.08791534	1.23233559	0.223312752	0.54236787	-5.1743736
ENSCAFG00000018258	-0.5662577	0.78962785	-1.2322963	0.223327296	0.54236787	-4.9790101
XKR8	-0.230211	2.62545643	-1.2322431	0.223347014	0.54236787	-5.6921088
STON2	-0.8629766	3.9316105	-1.2321054	0.223397991	0.54236787	-5.8480079
AACS	-0.214626	5.60679649	-1.2320812	0.223406989	0.54236787	-6.1783273
ELMO3	-0.1556691	4.54376332	-1.2319935	0.22343947	0.54236787	-6.0760682
CASD1	-0.220066	2.97236527	-1.2316158	0.223579423	0.54236787	-5.6317746
SF3B2	-0.1030796	7.47905754	-1.2315585	0.223600678	0.54236787	-6.1754983
GAB2	0.23496571	6.24409799	1.23130445	0.223694854	0.54236787	-6.1957932
GEN1	-0.5052292	2.88736082	-1.2312927	0.223699225	0.54236787	-5.5340755
THUMPD3	0.15507832	3.7836224	1.23123394	0.223721001	0.54236787	-5.8824237

COMMD7	0.08091138	5.36666761	1.23121246	0.223728966	0.54236787	-6.1743867
ENSCAFG00000018374	0.579295	-0.7133856	1.23120103	0.223733204	0.54236787	-4.9963286
SGK3	-0.163471	4.71503566	-1.2309905	0.22381129	0.54245276	-6.0824875
POMK	-0.1187685	5.26121769	-1.230773	0.223891961	0.54254389	-6.1525274
CDC45	-1.2141597	1.36577256	-1.2305383	0.223979087	0.54265062	-5.0165589
KIF1C	0.13780681	7.18332402	1.22994181	0.224200535	0.54295506	-6.1821416
MDN1	-0.2061726	5.82082755	-1.2298582	0.224231577	0.54295506	-6.1925057
CCDC71	-0.1365316	3.68076584	-1.2298516	0.224234061	0.54295506	-5.9085739
COMMD4	-0.1429855	4.40055782	-1.2295334	0.224352245	0.54313682	-5.9835238
CNOT9	-0.1503806	5.35215376	-1.2290548	0.224530174	0.54336425	-6.1361894
TLN2	0.44683201	7.20698043	1.22897072	0.224561424	0.54336425	-6.1511179
MT-ND6	0.16392187	5.89530935	1.22893259	0.224575603	0.54336425	-6.1985446
CTSB	0.20424989	9.44573498	1.22831168	0.224806591	0.54377089	-6.0260193
SSPN	0.28185095	3.75108704	1.22824875	0.224830011	0.54377089	-5.8401796
ENSCAFG00000016776	0.19709042	2.5562234	1.22806605	0.224898012	0.54383094	-5.5456606
MPIG6B	0.53051376	-1.2641385	1.22790053	0.224959638	0.54384681	-4.9724653
BCAR1	0.22081551	7.27655091	1.22770736	0.22503157	0.54384681	-6.1875418
IL7R	-1.0989222	-1.6317383	-1.2277006	0.225034104	0.54384681	-4.9062503
TTK	-1.0983117	1.89257687	-1.2272806	0.225190561	0.54409787	-5.099299
EGLN1	0.15069808	6.23070851	1.22718981	0.22522438	0.54409787	-6.2048374
RFTN2	0.45493577	2.05447439	1.22678816	0.225374094	0.54429866	-5.5095252
RFX3	-0.2896881	1.70650162	-1.226735	0.225393921	0.54429866	-5.3348995
CUEDC2	-0.1074249	5.03769273	-1.2265577	0.225460019	0.54435392	-6.1391149
C1S	0.86422624	5.06811884	1.22584311	0.225726649	0.54486374	-6.1050726
ENSCAFG00000007550	-0.5857978	-0.7308348	-1.225717	0.225773709	0.54486374	-4.9822113
INTS3	-0.1097283	6.14907871	-1.2255325	0.225842597	0.54486374	-6.2062787
ALDH5A1	-0.4477945	2.25908882	-1.2255282	0.225844203	0.54486374	-5.5111246
MON1B	-0.2176895	3.61840419	-1.2253222	0.225921161	0.54492446	-5.8683958
ENSCAFG00000028773	0.39934848	0.46657533	1.22522918	0.225955897	0.54492446	-5.0247616
NUPL2	-0.1613669	3.98087615	-1.2244844	0.226234227	0.54537719	-5.9326238
PMPCA	-0.1543342	5.80366233	-1.224319	0.226296092	0.54537719	-6.1929229
HMGXB4	-0.1034036	4.60466775	-1.2243065	0.226300758	0.54537719	-6.0712201
ACO1	-0.1531402	6.16471722	-1.2242636	0.226316813	0.54537719	-6.2038346
ZHX3	-0.2769044	4.53392391	-1.2239226	0.226444337	0.54558012	-6.1535639
FAM204A	0.16447959	5.35420057	1.22366078	0.226542325	0.54571182	-6.1584518
SCPEP1	0.24032484	7.56774127	1.22352605	0.226592755	0.54572894	-6.1707545
TRNT1	0.17885358	4.03997675	1.22336238	0.226654028	0.54577215	-5.9761299
ENSCAFG00000030360	-0.1778944	3.69446317	-1.2226752	0.226911424	0.54628752	-5.9095214
PHKG1	0.475529	1.78712928	1.22250979	0.226973397	0.5463323	-5.4816206
TRMT12	0.22914155	2.16053642	1.22217296	0.22709966	0.54637826	-5.4814015
ENSCAFG00000020954	-0.511263	0.87843127	-1.2219953	0.227166286	0.54637826	-5.1142731
GCAT	-0.4280792	2.84292069	-1.2218454	0.227222489	0.54637826	-5.7346888
SNX7	-0.1539968	4.85308354	-1.2218175	0.227232956	0.54637826	-6.0737272
FCGRT	-0.3804883	3.9861336	-1.2217885	0.227243856	0.54637826	-6.0246307
RWDD1	-0.1015516	6.03515006	-1.2217647	0.227252754	0.54637826	-6.2059614
NAIF1	0.41652802	0.6645194	1.22147443	0.227361673	0.54653581	-5.1557115
NFE2L3	-0.6637268	-0.1178528	-1.2210906	0.227505758	0.54675005	-5.0412812
AK1	0.19484666	4.52227704	1.22098749	0.227544451	0.54675005	-6.0942672
FGF2	0.25954589	2.32105953	1.22081861	0.227607869	0.54675005	-5.6477612
MTG2	0.18565699	3.09086948	1.22077452	0.227624426	0.54675005	-5.7421285

HOXB6	-0.4121111	1.52336972	-1.2203391	0.227787983	0.54690381	-5.3280651
AP3M1	-0.1290356	4.91133825	-1.2203172	0.22779623	0.54690381	-6.1397303
ENSCAFG00000030144	-0.2482913	2.80795962	-1.2202307	0.227828737	0.54690381	-5.8269206
ENSCAFG00000014844	0.6621392	-0.6529729	1.22014189	0.227862116	0.54690381	-4.9311415
UBE2G2	0.10197553	5.92182277	1.21947757	0.228111904	0.54739904	-6.2119347
PYGO2	0.15837965	3.70925133	1.21922513	0.228206875	0.54742073	-5.9431442
SPOP	0.13783397	5.97422167	1.21922251	0.228207863	0.54742073	-6.2120365
TMEM132B	-0.8032755	0.44643222	-1.2187629	0.228380854	0.54773139	-5.1088314
IPO11	-0.1254561	5.47139698	-1.2181739	0.228602674	0.54815901	-6.185295
XRN2	-0.1039902	6.59930854	-1.2177886	0.22874788	0.5484028	-6.216346
MYC	-0.2005101	6.81914157	-1.2175209	0.228848796	0.54842887	-6.2022843
DENND3	-0.2720747	4.53713584	-1.217191	0.228973218	0.54842887	-6.0375713
C1H9orf85	-0.3848931	0.70147551	-1.2171703	0.228981006	0.54842887	-5.1613768
NCOA6	-0.1085397	6.22586384	-1.2171588	0.228985365	0.54842887	-6.216393
FBXL19	-0.1648438	3.38655536	-1.2171296	0.228996371	0.54842887	-5.8190553
BRMS1L	-0.1690942	3.62057658	-1.2168921	0.229085986	0.54842887	-5.8676129
MAP2K2	-0.1112871	6.488166	-1.2168077	0.229117836	0.54842887	-6.2174108
TMEM178A	0.79644841	-1.5603024	1.21664526	0.229179127	0.54842887	-4.7995061
AMIGO1	-0.4024443	1.74493041	-1.2166164	0.229190025	0.54842887	-5.4536751
TBK1	-0.1250588	5.59653759	-1.2166054	0.229194155	0.54842887	-6.1895847
EHD2	-0.1295982	8.47995369	-1.2163654	0.229284773	0.54844243	-6.1382147
MBOAT2	0.58328928	0.38748535	1.21635977	0.229286904	0.54844243	-5.1758707
IL17RB	-0.8914308	-0.684391	-1.2154373	0.229635408	0.54893565	-4.8195192
ENSCAFG00000022498	-0.4890226	-0.17491	-1.2154342	0.229636574	0.54893565	-5.0600316
C10H12orf45	0.18164614	3.44934914	1.21538106	0.229656659	0.54893565	-5.8701842
BMS1	-0.139566	6.42194637	-1.2153526	0.229667423	0.54893565	-6.219166
ACTN2	1.14569967	0.61094167	1.21486694	0.22985107	0.54918722	-4.9165925
PPP2R3C	-0.2129319	3.6066315	-1.2147652	0.229889541	0.54918722	-5.8824116
FTO	0.09696192	4.47358832	1.2147284	0.229903478	0.54918722	-6.0653888
DCUN1D4	0.12951701	5.61185574	1.21432141	0.230057489	0.54933544	-6.1985414
SLC35B3	-0.1180107	4.6178721	-1.2142601	0.230080681	0.54933544	-6.0824647
E2F7	-0.5539141	3.51484851	-1.2142187	0.230096363	0.54933544	-5.5798753
ENSCAFG00000025748	-0.6607048	-0.6460874	-1.2137008	0.230292479	0.54969946	-4.9618825
DACH1	-0.9319166	-1.7107025	-1.213348	0.230426138	0.54986525	-4.9220905
ENSCAFG00000000142	-0.4129921	1.81774148	-1.2131263	0.230510172	0.54986525	-5.2541278
GRK6	0.15822401	4.88124271	1.21300032	0.230557927	0.54986525	-6.1256417
L2HGDH	-0.2207593	2.14098542	-1.212993	0.230560686	0.54986525	-5.5164999
CHIC2	-0.1257911	3.66358282	-1.2129416	0.230580202	0.54986525	-5.9435794
NINJ1	-0.2238134	3.77430216	-1.2125624	0.230724005	0.54995275	-5.915443
GABBR2	-0.7787915	-0.6231428	-1.2124152	0.230779817	0.54995275	-4.9312606
MSH2	-0.2778036	4.10503201	-1.2123033	0.230822295	0.54995275	-5.9471905
FXYD5	0.17904704	7.0031447	1.21229149	0.230826763	0.54995275	-6.2150808
AMER1	-0.266245	2.2404141	-1.2122693	0.230835201	0.54995275	-5.6366646
HEATR6	-0.1887123	4.37181103	-1.2119375	0.230961101	0.55012114	-6.0622012
TMEM219	0.17251438	3.80176679	1.21184221	0.23099728	0.55012114	-5.93282
ENSCAFG00000003936	-0.086805	6.52086445	-1.2116521	0.231069465	0.55012114	-6.223444
ENSCAFG00000012233	0.20495106	5.69545731	1.21162282	0.231080578	0.55012114	-6.2076364
ENSCAFG00000009113	0.93677241	1.92251091	1.21135057	0.231183981	0.55023952	-5.9665131
ANXA5	-0.155277	8.83048103	-1.2112619	0.231217669	0.55023952	-6.0768751
RUFY1	-0.1934775	4.25975656	-1.2111164	0.231272939	0.55026708	-6.0159639

UBLCP1	0.11729911	4.95168999	1.2107855	0.231398698	0.55046232	-6.1297397
CMBL	0.43867352	2.21154534	1.21058215	0.231476007	0.55046586	-5.3396797
ENSCAFG00000030808	-0.3112163	1.13549065	-1.2105517	0.23148759	0.55046586	-5.3657894
MDH1	0.09082614	6.31608215	1.21036976	0.23155677	0.55052644	-6.2254493
ZFP3	-0.2837093	1.43956906	-1.2101457	0.231642005	0.55062515	-5.4378109
GNPNAT1	-0.2182255	4.71707419	-1.2098653	0.231748663	0.5507013	-6.1600847
SMIM3	-0.2797457	2.24516761	-1.2098317	0.231761477	0.5507013	-5.6752576
ABCG1	-1.0436429	-0.5976442	-1.2091947	0.232003972	0.55116738	-4.8395654
LAS1L	-0.1068362	4.90320178	-1.2090866	0.232045144	0.55116738	-6.1364224
ENSCAFG00000007270	-0.2150865	3.96429904	-1.208868	0.232128415	0.55126122	-5.9948975
DHODH	-0.2564431	2.994319	-1.2078173	0.232528965	0.55210836	-5.729997
SMAD2	-0.1293886	4.87375682	-1.2075377	0.232635641	0.55225755	-6.1350964
ENSCAFG00000000580	0.15297864	6.0964964	1.20726026	0.232741541	0.55240483	-6.2242782
KIF14	-1.3339983	1.92821224	-1.2071423	0.232786575	0.55240763	-5.094461
PDE7A	0.42896968	0.29011626	1.20699337	0.232843439	0.55243849	-5.172589
RPGRIP1L	-0.233008	2.71146958	-1.2063627	0.233084341	0.55273916	-5.6737353
ENSCAFG00000030445	-0.2857572	2.25471604	-1.2060905	0.233188377	0.55273916	-5.5673597
MARVELD2	0.61062493	-1.0539339	1.20599756	0.233223912	0.55273916	-4.9050067
URI1	-0.1341714	5.83092759	-1.2059521	0.23324129	0.55273916	-6.2196759
TRAPPC6A	-0.5531034	0.05916388	-1.2059474	0.233243085	0.55273916	-5.045024
ENSCAFG00000017475	0.26925149	1.0644551	1.20587745	0.233269836	0.55273916	-5.3083673
TBC1D24	0.20316583	3.22553569	1.20567489	0.233347296	0.55273916	-5.8410809
C6H7orf50	0.21315746	2.11212389	1.20559108	0.23337935	0.55273916	-5.4928007
NAT10	-0.1026314	5.30327607	-1.205549	0.233395449	0.55273916	-6.1811649
ENSCAFG00000010717	-0.1653629	4.40880203	-1.2054715	0.233425106	0.55273916	-6.1366065
WDR83OS	0.1712807	4.96607525	1.20539889	0.233452867	0.55273916	-6.1461593
ENSCAFG00000031887	0.19835468	2.09298533	1.20473729	0.233706083	0.5532347	-5.4836923
OXR1	-0.1857204	5.05688452	-1.2042584	0.233889478	0.5535648	-6.1275
ENSCAFG00000012894	0.10890895	5.86907075	1.20394309	0.234010311	0.55374674	-6.2222019
CH25H	-0.5554245	2.95126965	-1.2036992	0.234103813	0.55386394	-5.8280973
ENSCAFG00000028124	-0.4214703	0.18839337	-1.2032616	0.234271607	0.55407791	-5.0887274
HOMER3	-0.3065478	4.36738407	-1.2032339	0.23428223	0.55407791	-6.0098575
MROH8	-0.4407187	0.22270899	-1.2030962	0.234335037	0.55409877	-5.0670766
CRYM	-0.7001528	-2.1141719	-1.2028216	0.234440415	0.55419001	-4.7901206
MAN1A2	0.16927154	6.15012365	1.20276631	0.234461621	0.55419001	-6.2337683
PREX2	-0.7988585	4.40379786	-1.2024108	0.234598081	0.55434908	-5.9223826
TYSND1	0.07297394	5.98980878	1.20236172	0.234616936	0.55434908	-6.2255079
DNAH7	0.90230582	-0.8578284	1.20199222	0.234758843	0.55450045	-4.7892025
SNAP91	-0.8982218	0.99164164	-1.2019657	0.234769045	0.55450045	-5.0496527
MORF4L1	-0.0806619	7.81188667	-1.2009966	0.235141516	0.55518208	-6.1977943
KDM6B	0.21435583	4.30741445	1.2009855	0.235145794	0.55518208	-6.035528
SEMA6C	0.35654636	2.62331698	1.20054375	0.235315734	0.5554792	-5.6713147
WDR43	-0.150498	5.87861537	-1.2003108	0.235405391	0.55557359	-6.2281099
NLRC5	-0.902007	1.1482854	-1.2002106	0.235443936	0.55557359	-5.0730005
NAV3	0.154917	6.84602616	1.19998977	0.235528974	0.55565927	-6.2336139
TMEM126A	-0.1698115	3.26778053	-1.1998268	0.235591744	0.55565927	-5.7053952
APOLD1	0.56935205	-1.1742918	1.19977265	0.23561259	0.55565927	-5.0012538
RNF135	-0.2177671	2.66178216	-1.1992107	0.2358291	0.55595915	-5.6502993
CHCHD1	0.19248945	3.00875719	1.19911743	0.235865047	0.55595915	-5.6789091
CCP110	-0.1668696	4.15612081	-1.199064	0.235885637	0.55595915	-6.0140796

ARMT1	0.18295742	5.22514225	1.19898449	0.235916294	0.55595915	-6.1926949
PCGF2	-0.1525111	4.93334159	-1.1983999	0.236141733	0.55638632	-6.1566844
ENSCAFG00000014497	-0.3004868	0.83392779	-1.1981358	0.236243641	0.55647262	-5.0935225
SSU72	0.10851014	4.84985979	1.19796845	0.236308228	0.55647262	-6.1594639
ATF4	-0.0906679	8.70471116	-1.1978073	0.236370436	0.55647262	-6.1243606
CKAP5	-0.112092	6.91783263	-1.1978006	0.236373032	0.55647262	-6.2369625
RARRES1	0.27956925	5.51892155	1.19766667	0.236424731	0.55647262	-6.2279515
DDX59	-0.1831633	3.72677144	-1.1976182	0.236443431	0.55647262	-5.9527542
CREB3L1	-0.2872734	7.54885651	-1.1974198	0.236520079	0.55654642	-6.237617
ZDHHC16	0.12859687	4.20203886	1.19721095	0.236600744	0.55654642	-6.0082327
KCNH3	-0.4942223	0.56140193	-1.1971939	0.236607343	0.55654642	-5.220474
SLC25A20	0.1880242	3.81867892	1.19693067	0.236709041	0.55660287	-5.9911389
PIP5K1C	-0.1728826	4.14151847	-1.1968948	0.236722911	0.55660287	-6.0968847
ENSCAFG00000006639	-0.1676794	5.61298189	-1.1967887	0.236763909	0.55660287	-6.2118516
LAMTOR2	0.17634594	3.33997506	1.19625161	0.236971573	0.55698711	-5.8332432
STRIP2	-0.7518715	-1.1111366	-1.1960576	0.237046618	0.55705955	-4.8613521
CYP51A1	-0.1939587	6.3890704	-1.1958915	0.237110897	0.55710667	-6.2360076
PARP15	-0.4518143	3.64601509	-1.1957414	0.237168986	0.55713923	-5.7457392
CFAP97	-0.129238	4.3256917	-1.1946789	0.237580405	0.55800164	-6.0638085
RRP15	-0.1630251	3.76463181	-1.1941093	0.237801207	0.55841611	-5.9572247
DDX55	-0.1344475	4.18146245	-1.193858	0.237898679	0.55854087	-6.0463709
ADD2	-0.4125005	4.20498401	-1.1936596	0.237975635	0.55861742	-5.789031
ZNF606	-0.2337587	2.77985179	-1.193047	0.238213382	0.55874035	-5.713709
LRRC45	-0.1864034	4.32144673	-1.1929742	0.23824164	0.55874035	-6.0817185
ENSCAFG00000023425	0.4683792	0.9244874	1.19294373	0.23825347	0.55874035	-5.1805559
ENSCAFG00000032037	-0.365402	1.52820531	-1.1929258	0.238260419	0.55874035	-5.3580363
FAM151B	-0.1202528	4.21916188	-1.1928878	0.238275177	0.55874035	-6.0665888
NFX1	-0.127541	5.1247351	-1.1927527	0.238327645	0.55874035	-6.145741
MCM7	-0.2802818	5.86995376	-1.1927247	0.238338511	0.55874035	-6.2043322
ZNF84	-0.2263819	3.2039445	-1.1921968	0.238543624	0.55905758	-5.8627934
ENSCAFG00000018271	-0.5803244	0.79322048	-1.1921335	0.238568207	0.55905758	-5.1384433
THYN1	0.14855472	4.53374736	1.19203379	0.238606981	0.55905758	-6.0781615
RAB27A	-0.4396795	2.3235636	-1.191836	0.238683883	0.55913375	-5.4936534
KCTD5	0.21365015	2.66705964	1.19124704	0.238912935	0.55938629	-5.7538059
DAGLB	-0.0937096	5.63008104	-1.1911854	0.238936903	0.55938629	-6.2345975
THBD	-0.3125944	4.7067387	-1.1911827	0.238937961	0.55938629	-6.0875668
TSPAN7	0.5485109	1.19491108	1.19110212	0.238969325	0.55938629	-5.3245258
ENSCAFG00000003457	0.84989663	-0.520032	1.19095962	0.239024783	0.55939953	-4.8971006
EMX2	-1.0863204	0.61535807	-1.1908594	0.239063802	0.55939953	-5.082728
SLC45A2	0.68149894	-0.2302449	1.19055746	0.239181339	0.55955594	-5.0569636
ZNF580	-0.2672149	2.933656	-1.1903367	0.239267305	0.55955594	-5.5687349
GPSM2	-0.2063382	5.34830432	-1.1903112	0.239277261	0.55955594	-6.1363109
KCNJ4	0.74802636	-1.7714102	1.19023137	0.23930834	0.55955594	-4.8091974
ITM2C	0.21307456	7.10112541	1.18985948	0.239453236	0.55977241	-6.2211108
RAP2B	-0.2767494	3.01084872	-1.1897657	0.239489799	0.55977241	-5.7245439
BLVRB	-0.2064485	5.05722693	-1.1896455	0.23953662	0.55977797	-6.1739327
UBOX5	0.12548136	3.96147679	1.18893385	0.239814156	0.56018772	-5.969449
GIPC3	0.65391846	-0.8297826	1.18889019	0.23983119	0.56018772	-4.968649
ERCC6	-0.1496736	4.09839327	-1.1888538	0.239845379	0.56018772	-6.0808997
DCPS	-0.1695282	4.23402031	-1.188376	0.240031844	0.5603872	-5.9967267

ZNF445	-0.2021351	4.31935693	-1.1883229	0.24005261	0.5603872	-6.0744412
USP47	-0.1242486	7.18249552	-1.1881287	0.240128432	0.5603872	-6.2313071
LAPTM4A	0.11596989	7.92302392	1.18810931	0.240135994	0.5603872	-6.1907713
CENPI	-0.5847675	1.1635249	-1.1880652	0.24015323	0.5603872	-5.1526932
EED	0.13673169	4.2575289	1.18769401	0.240298212	0.56062165	-6.0575005
ZCCHC10	-0.2036659	2.17117537	-1.1874123	0.240408288	0.56077459	-5.4545816
RFC2	-0.2393882	3.78584673	-1.1872531	0.24047052	0.5608159	-5.8894038
PLAGL1	-0.1059655	5.00154584	-1.1867771	0.24065665	0.56114609	-6.1998402
PRKD2	-0.1870838	4.49160849	-1.1863794	0.24081224	0.5613068	-6.108598
HEXIM1	-0.1720988	5.27299097	-1.18624	0.240866796	0.5613068	-6.1938951
ZNF140	-0.3245078	1.58702861	-1.1862228	0.240873531	0.5613068	-5.4057713
TFAP2C	0.4405322	0.39830847	1.18608584	0.240927126	0.5613068	-5.6457207
WDR76	-0.4027246	3.41672795	-1.1860315	0.240948385	0.5613068	-5.7802367
GTF2H1	0.11411431	5.56622048	1.18586857	0.241012186	0.56132607	-6.2342165
COL5A1	0.42592071	11.5584985	1.18566618	0.241091438	0.56132607	-6.0051918
GCDH	-0.2320454	2.90574607	-1.1856377	0.2411026	0.56132607	-5.7607396
NOS3	-1.1542022	-1.5065541	-1.1855552	0.241134915	0.56132607	-5.0330709
RAP2C	-0.1113728	5.74858343	-1.1852955	0.241236636	0.5614591	-6.2244223
FER	-0.1548265	4.64507734	-1.184829	0.241419488	0.56169012	-6.1377525
KIF1A	0.65371081	4.11264317	1.18481469	0.241425081	0.56169012	-5.8501833
CXXC1	-0.1222181	5.16709846	-1.1841515	0.241685166	0.56215278	-6.1811725
SLC49A3	-0.6740179	-2.6403215	-1.1840801	0.241713198	0.56215278	-4.816285
GAREM2	0.43638398	0.42924924	1.18366802	0.241874914	0.56238631	-5.1272784
RSBN1	-0.1936771	2.23279705	-1.1835967	0.241902908	0.56238631	-5.651911
LRRTM2	0.4165291	2.26471713	1.18308375	0.242104353	0.56275077	-6.0200519
TNRC18	-0.1071527	6.82802781	-1.1828302	0.24220397	0.56287845	-6.2527128
CDK5RAP1	-0.1253044	4.21353155	-1.1823473	0.242393793	0.5631684	-6.0681608
TBCE	-0.1276985	5.90085237	-1.1821689	0.242463941	0.5631684	-6.2449806
SAAL1	-0.1522336	5.22711184	-1.1820719	0.242502078	0.5631684	-6.1909845
PRNP	0.24577228	6.62068056	1.18205791	0.242507574	0.5631684	-6.2486236
MCCC1	-0.1594898	4.76616308	-1.181843	0.242592116	0.56326088	-6.157137
RDH10	0.3603773	6.39390103	1.18170121	0.242647894	0.56328656	-6.2562423
SEMA7A	0.68438837	4.34511041	1.18126151	0.242820946	0.56358442	-6.1642727
TUBG1	0.13966807	4.94431123	1.18110576	0.242882263	0.5636189	-6.1604671
HYAL2	-0.3350678	4.55375508	-1.1809965	0.242925295	0.5636189	-6.1216613
ACTC1	0.72159303	-1.5843184	1.18071578	0.243035852	0.56377156	-4.8729651
CHD6	0.15754849	5.11554056	1.18017285	0.24324979	0.56398026	-6.2246953
UPRT	-0.119766	3.5491364	-1.180098	0.243279311	0.56398026	-5.8931536
ACVR1	0.15831903	6.50978619	1.18009533	0.243280349	0.56398026	-6.2609673
AGBL2	-0.3016369	1.97949711	-1.180033	0.243304917	0.56398026	-5.5421919
ZNF827	0.14074062	4.75725648	1.17953179	0.243502573	0.56433457	-6.1176346
RPP38	-0.1461499	3.72695575	-1.1791647	0.243647427	0.56456641	-5.9476832
RPF1	-0.1173345	4.90553204	-1.1788746	0.243761906	0.56472778	-6.1633598
TBC1D10A	-0.1692935	2.91959389	-1.1785309	0.243897623	0.5649159	-5.7766328
SPTLC3	0.77693993	-0.3519401	1.17844182	0.243932803	0.5649159	-5.0792385
C21H11orf54	0.12567688	4.26728235	1.17826443	0.244002876	0.56493077	-6.1430193
CBWD2	-0.1114522	4.76593601	-1.178113	0.244062695	0.56493077	-6.1505414
TRIM3	-0.1248078	4.57372313	-1.178085	0.244073775	0.56493077	-6.1275262
TRA2B	-0.14744	5.28917455	-1.1778163	0.244179964	0.56497793	-6.1839517
CPLANE2	0.30458872	1.40329988	1.17780644	0.244183855	0.56497793	-5.3841307

ENSCAFG00000012316	0.34556605	0.51279424	1.17715155	0.244442811	0.56544632	-5.1644694
CLEC14A	-0.9688279	-0.3045347	-1.1770544	0.244481239	0.56544632	-5.0971154
INTS11	0.1308397	5.18965207	1.17690449	0.244540551	0.56544632	-6.1975874
MTCH1	0.10514386	7.4617253	1.17684053	0.24456586	0.56544632	-6.232801
IKZF4	0.25336688	3.21141928	1.176482	0.244707767	0.56567059	-5.7946947
ENC1	-0.2565644	2.27457373	-1.1760398	0.244882863	0.56586691	-5.6330276
IFNLR1	-0.7402094	-1.2739873	-1.1760093	0.24489494	0.56586691	-4.8185754
ZDHHC13	-0.1193098	4.04687085	-1.1758973	0.244939307	0.56586691	-6.0137212
SLC16A13	0.39964135	2.45098261	1.1758138	0.244972394	0.56586691	-5.6383173
KCNAB2	0.36656055	1.01173612	1.17546749	0.245109625	0.56608009	-5.2991586
TRAF7	-0.1617079	5.92596894	-1.1751731	0.245226324	0.56624579	-6.2610153
DNAJB1	-0.1186316	5.70191629	-1.1748477	0.245355358	0.5664399	-6.2588843
ABHD3	0.20329531	3.13198239	1.17463072	0.245441439	0.56653479	-5.9332647
ENSCAFG00000001208	-0.2547312	2.6582642	-1.1741839	0.245618745	0.56661975	-5.6939737
GOT1	-0.15355	5.1674917	-1.1741665	0.245625677	0.56661975	-6.1978732
RNF41	0.14121659	4.61341346	1.17409089	0.245655673	0.56661975	-6.1115166
IFT46	0.09526294	5.80739589	1.17408457	0.245658182	0.56661975	-6.2575769
SPR	0.14401927	5.14235092	1.17388924	0.245735734	0.56669486	-6.1857101
ENSCAFG00000000701	0.22256694	5.38471752	1.17351324	0.24588507	0.56693545	-6.2147261
NOX5	0.71723172	-1.467007	1.17336937	0.245942227	0.56696345	-5.0313151
CERK	-0.2107034	7.28511296	-1.1731279	0.246038165	0.56708083	-6.248603
SOCS6	0.12850026	4.00283079	1.17232171	0.246358733	0.56771581	-6.0232875
LRP10	0.17460866	7.65984723	1.17213936	0.24643128	0.56777911	-6.229593
DPM2	0.22721151	2.42997187	1.17189033	0.24653038	0.56790356	-5.6039495
ENSCAFG00000010290	0.980663	1.4037325	1.17131887	0.246757896	0.56832373	-5.7002157
ZW10	-0.153026	5.56834276	-1.1710207	0.246876656	0.5684933	-6.2366696
RIDA	0.16833943	3.59937637	1.17088742	0.246929775	0.56851169	-5.9757653
PPP2R5E	0.13648882	5.01691744	1.17029678	0.247165203	0.56894973	-6.2019966
PDPN	-0.8032937	7.63650921	-1.1700333	0.247270287	0.56900124	-6.1838056
RCOR3	-0.1392143	4.66666614	-1.1700141	0.247277928	0.56900124	-6.1731545
VARS	-0.1376665	7.43137476	-1.169509	0.24747948	0.56936101	-6.2583851
TMSB10	0.23623851	6.55423435	1.16907018	0.247654649	0.56965996	-6.2721766
TUBE1	-0.1627508	3.32833981	-1.1687314	0.247789948	0.56986711	-5.9568957
SNX4	0.12539374	5.8779043	1.16837582	0.247932026	0.56999395	-6.2564149
PXDNL	1.4862954	0.40708791	1.16836687	0.247935603	0.56999395	-5.0030805
AMMECR1L	-0.0830655	6.16018927	-1.1672866	0.24836757	0.57088283	-6.2751917
NDUFV1	-0.1315848	6.01374764	-1.1667771	0.248571528	0.57100607	-6.2705902
SETX	-0.1125928	5.95201212	-1.1666784	0.248611042	0.57100607	-6.2756426
TBC1D8B	-0.2426361	2.9475168	-1.1664928	0.248685349	0.57100607	-5.6892241
CEP290	-0.2543468	2.84041969	-1.1664055	0.248720309	0.57100607	-5.7496961
PUM2	-0.083845	6.91407175	-1.1662976	0.248763563	0.57100607	-6.2690695
ENSCAFG00000009307	0.34181736	2.16034545	1.16625366	0.248781151	0.57100607	-5.4934694
CAMTA1	0.69548892	1.35527619	1.16616584	0.248816333	0.57100607	-5.3544573
TIMM17A	-0.130493	5.03079847	-1.1661621	0.248817814	0.57100607	-6.1961818
ENSCAFG00000031671	-0.2708118	1.8409401	-1.1661338	0.248829176	0.57100607	-5.4903125
EMD	0.12817702	5.9037388	1.16596252	0.248897807	0.57105952	-6.2683045
COL4A5	-1.2670107	-0.4143502	-1.1656867	0.24900837	0.57120915	-4.8701285
HOXA2	-0.7985589	-0.7891397	-1.1653657	0.249137049	0.57130134	-5.0187691
GCHFR	0.34325536	1.34889335	1.16518478	0.249209624	0.57130134	-5.329257
LBH	0.60643729	3.77232213	1.16514843	0.249224207	0.57130134	-5.9784922

RHPN2	0.49137012	1.1058866	1.16513403	0.249229981	0.57130134	-5.2727406
PUDP	-0.1380984	4.08099912	-1.164998	0.249284543	0.57132244	-6.0419051
ENSCAFG00000011327	-0.6822675	-0.9080865	-1.164408	0.249521348	0.57149344	-4.90732
AKT1	0.11090608	8.10198925	1.16423333	0.249591473	0.57149344	-6.2251572
TSEN15	-0.1488716	4.60902492	-1.1641353	0.249630829	0.57149344	-6.0733271
SNPH	0.41253864	0.90487444	1.16401754	0.249678137	0.57149344	-5.2741463
WDFY1	-0.1252643	4.38946446	-1.164004	0.249683558	0.57149344	-6.0701404
GCN1	-0.1172782	7.14726194	-1.1639972	0.249686298	0.57149344	-6.2710778
VAPB	0.11855664	6.02200034	1.16397872	0.249693728	0.57149344	-6.271148
NISCH	0.17708532	6.93294535	1.16388046	0.249733197	0.57149344	-6.2560345
NEIL2	-0.2738732	2.78439198	-1.1637951	0.249767496	0.57149344	-5.9148723
SLC17A5	0.24494114	6.03917395	1.16366382	0.24982024	0.57151031	-6.2797311
ADCY9	0.10511979	5.69805256	1.16338733	0.249931355	0.57162153	-6.2613689
ENSCAFG00000000033	-0.2894474	1.1691067	-1.1632227	0.249997539	0.57162153	-5.2281241
F2RL1	-0.7663446	1.04894685	-1.1631182	0.250039533	0.57162153	-5.2596848
STX8	0.10452601	4.39941807	1.16309124	0.250050385	0.57162153	-6.1308891
MAGI1	0.32065908	3.61813834	1.16231185	0.25036391	0.57223441	-5.8710087
GORAB	0.18696819	4.60596181	1.1621532	0.250427762	0.57227559	-6.1475051
ZNF391	-0.2947437	1.82426147	-1.1620413	0.250472795	0.57227559	-5.473944
ENSCAFG00000028814	0.88755262	3.21884269	1.16172287	0.250601021	0.57242422	-5.8659777
ASB15	-0.8585724	-0.1909579	-1.161654	0.250628739	0.57242422	-5.0360533
CASP3	0.15213815	5.38726152	1.16149097	0.250694425	0.57247045	-6.2472747
ENSCAFG00000013760	-0.1620124	2.74030411	-1.1612472	0.250792647	0.57259093	-5.7631011
EMP3	-0.2454719	6.90693016	-1.1607074	0.2510102	0.57297265	-6.2709455
SYMPK	-0.0953645	5.89879488	-1.1606067	0.251050816	0.57297265	-6.2747715
AK2	-0.1899525	2.99433755	-1.1600806	0.251263018	0.57328724	-5.7867675
CELF1	0.15413655	5.32027175	1.15988983	0.251340007	0.57328724	-6.2326431
LRRC14	0.17066461	3.48655543	1.1598693	0.251348292	0.57328724	-5.9393673
NOP14	-0.1347798	6.13058916	-1.1597227	0.25140747	0.57328724	-6.2779695
C10H2orf42	-0.1012981	5.44422378	-1.159701	0.251416221	0.57328724	-6.2459928
ENSCAFG00000023283	-0.1284688	4.86655658	-1.1590872	0.251664071	0.57365614	-6.187311
CUL7	-0.1844698	6.52739065	-1.1588992	0.251740008	0.57365614	-6.2799721
RASL11B	0.62092026	4.0405893	1.15889575	0.251741416	0.57365614	-5.6514542
REX1BD	-0.2296156	3.74310787	-1.1587155	0.251814251	0.57365614	-5.9316802
YME1L1	-0.0806185	7.29919809	-1.1586739	0.251831052	0.57365614	-6.2683965
CKAP2	-0.271613	4.62321665	-1.1586239	0.251851259	0.57365614	-6.0321965
CALR	0.16828998	9.53753792	1.15845015	0.251921502	0.57369169	-6.1174722
ENSCAFG00000029457	-0.2177963	3.40914135	-1.15836	0.251957958	0.57369169	-5.8689228
EML5	0.4073127	-0.4811452	1.15815368	0.252041367	0.57377789	-5.0822766
ENSCAFG00000019976	-0.3322971	1.07473965	-1.1579672	0.252116795	0.57384589	-5.1846544
RDH13	-0.2837085	2.36860389	-1.1576312	0.252252726	0.57402063	-5.6015928
TCF12	-0.1942742	6.70043183	-1.1575424	0.252288635	0.57402063	-6.2803581
LINS1	0.13301677	4.3401398	1.15743951	0.252330281	0.57402063	-6.1206803
ZNF423	0.44822407	3.0811471	1.15724025	0.252410934	0.57410042	-5.8126619
SSBP4	0.17063971	4.88668871	1.157092	0.252470952	0.57412811	-6.2346221
MAP4K5	-0.1249174	5.99241486	-1.1569017	0.252548016	0.57412811	-6.2731265
CD83	-0.7625249	-0.3026577	-1.1568725	0.252559851	0.57412811	-4.9913277
TMEM47	-0.1936989	7.66007517	-1.1560529	0.252891888	0.57477918	-6.2511327
CHORDC1	-0.1600912	5.66883703	-1.1553096	0.253193326	0.57536048	-6.2430979
ENSCAFG00000030404	-0.3170653	1.62702423	-1.1547276	0.253429527	0.57579335	-5.4361439

PDGFB	-1.1469727	-0.0825475	-1.1543601	0.253578756	0.5760285	-5.1556803
GLCCI1	0.36112461	3.04586163	1.15405593	0.253702326	0.57614731	-5.6467843
ENSCAFG00000002078	-0.8377971	1.27751136	-1.1540062	0.253722541	0.57614731	-5.4166575
ZNF3	-0.1678555	3.35818397	-1.1535935	0.253890244	0.57637025	-5.9307367
PGM1	-0.1209644	6.68238521	-1.1535394	0.253912237	0.57637025	-6.2868443
KIAA0556	0.11741046	4.58516136	1.15320965	0.254046327	0.5764566	-6.1713729
TENM2	1.04879668	1.03188769	1.15309597	0.254092559	0.5764566	-5.0847646
TMEM67	-0.3294508	3.48268166	-1.1529954	0.254133459	0.5764566	-6.0145188
AIPL1	-0.9642288	-0.4834242	-1.1528938	0.254174808	0.5764566	-4.8583569
AKAP7	-0.2071113	3.18438809	-1.1528832	0.2541791	0.5764566	-5.8762226
PAXBP1	-0.1490567	4.24614575	-1.152502	0.254334246	0.57670462	-6.1050181
ENSCAFG00000032625	-0.6973283	4.16682466	-1.152056	0.254515799	0.57690566	-5.9721985
LRP11	0.11953521	4.82502154	1.1519703	0.254550707	0.57690566	-6.2404478
STAM2	0.10442294	7.13887769	1.15194672	0.254560308	0.57690566	-6.2786953
ENSCAFG00000013864	-0.1861706	3.39640841	-1.1516345	0.254687508	0.57693338	-5.8980859
GCC2	-0.1379278	6.55700239	-1.1516068	0.254698792	0.57693338	-6.293112
TTC26	-0.2057606	3.177673	-1.1515794	0.254709948	0.57693338	-5.9519164
ANKRD13B	-0.2815201	2.85331612	-1.1513876	0.25478808	0.57700659	-5.7766297
FAR1	0.14631695	6.99319197	1.15126786	0.254836897	0.5770134	-6.2912026
NOS2	-0.6157429	-1.5621625	-1.1508652	0.25500104	0.57707153	-4.9057878
BEND7	-0.2792359	3.02924587	-1.1508346	0.255013531	0.57707153	-5.6980122
COPS7B	-0.1617856	3.20273251	-1.1507151	0.25506228	0.57707153	-5.8208795
PDE3B	0.52502843	2.86626419	1.15063945	0.25509312	0.57707153	-5.7330058
PNPLA6	0.12575688	5.54883801	1.15056694	0.255122697	0.57707153	-6.2859913
SPIN4	-0.3746264	0.95847247	-1.1505308	0.255137452	0.57707153	-5.3305803
PPP1R14B	-0.1756534	5.23200115	-1.1502668	0.255245165	0.57716316	-6.1929552
KDR	-0.6285413	3.3146084	-1.1502008	0.255272094	0.57716316	-5.9293954
GNL3L	0.07966233	6.63664902	1.15009459	0.255315429	0.57716316	-6.2918269
YIPF4	0.11071925	4.74828577	1.14994388	0.255376943	0.57719863	-6.2106001
N6AMT1	-0.2902395	1.13875603	-1.1497229	0.255467162	0.57729895	-5.3419207
ENSCAFG00000013365	-0.1228526	2.96252888	-1.1494847	0.255564419	0.57741514	-5.8065313
GAK	0.08961306	6.47576178	1.14896224	0.255777882	0.57779379	-6.296541
SGCE	0.19450088	4.88866712	1.14874826	0.255865336	0.57788771	-6.2320569
DHRS11	0.46618323	1.58651615	1.14847887	0.255975471	0.57789926	-5.2291636
ENSCAFG00000007285	-0.5028727	0.05669933	-1.14837	0.25601998	0.57789926	-5.0113735
ALDH6A1	-0.1698137	4.88134988	-1.1482603	0.256064869	0.57789926	-6.2333763
ENSCAFG00000012900	-0.1582759	3.10380054	-1.1482066	0.256086829	0.57789926	-5.8505057
CEP83	-0.1911782	4.12152935	-1.1480655	0.256144549	0.57789926	-6.0415452
PAQR4	0.31226271	2.3752637	1.14806258	0.256145727	0.57789926	-5.688472
STX2	0.11703864	4.86478575	1.14781889	0.256245429	0.57791354	-6.2030567
ENSCAFG00000016482	-0.8713636	0.88552958	-1.147704	0.25629243	0.57791354	-5.076804
ABT1	0.13218266	5.2781699	1.14760274	0.256333889	0.57791354	-6.2481201
RFC1	-0.1093378	5.55278218	-1.1474733	0.256386877	0.57791354	-6.2648284
PDCD2	-0.094729	6.17654671	-1.1474061	0.256414382	0.57791354	-6.2897724
SCLT1	-0.1869634	4.13869917	-1.1473744	0.256427342	0.57791354	-6.0840131
ENSCAFG00000020713	-0.5417505	-1.3069798	-1.1470015	0.256580063	0.57815429	-4.9539065
TUBA1B	0.33450202	9.80865135	1.14665131	0.256723519	0.57827891	-6.1063921
ENOX1	-0.7230742	1.20582211	-1.1466424	0.25672719	0.57827891	-5.3502418
GTF2E2	-0.0897154	5.54979947	-1.1463844	0.256832914	0.5783311	-6.2726624
ZRANB2	-0.1132423	4.68635561	-1.1463251	0.256857187	0.5783311	-6.1803619

NAB2	0.21366486	5.04006786	1.14624973	0.2568881	0.5783311	-6.2464907
ZNF608	-0.2339656	4.40196999	-1.1458483	0.257052681	0.57841659	-6.2775892
IQSEC2	0.25712019	2.32045174	1.14569942	0.257113761	0.57841659	-5.6533015
POU2F1	-0.2372276	1.73477117	-1.1456834	0.25712032	0.57841659	-5.5036662
GNAO1	0.60004129	-2.8944377	1.14562162	0.257145677	0.57841659	-4.8201327
GPT	-0.2362724	1.5385227	-1.1455972	0.257155677	0.57841659	-5.4470577
KIF20B	-0.2898362	4.75641835	-1.1452563	0.25729557	0.57845685	-6.1261326
LSP1	-0.7299902	7.26409735	-1.1451539	0.257337591	0.57845685	-6.1980388
SULF1	0.51043111	8.78043527	1.14512541	0.257349288	0.57845685	-6.245907
HK1	0.10261995	7.92640323	1.14510596	0.257357272	0.57845685	-6.2377211
IGLON5	0.73549631	-2.0988856	1.14456118	0.257580957	0.57885633	-4.8220648
ZNF185	-0.7580478	2.0054001	-1.1442417	0.25771219	0.5789916	-5.2587232
MPC2	-0.1405671	3.56630718	-1.1441909	0.257733083	0.5789916	-5.9585519
SLC19A1	0.21371848	4.97116282	1.14393479	0.257838318	0.57912472	-6.1967016
SPAG7	-0.1176589	4.57773813	-1.143603	0.257974724	0.57928502	-6.183113
STARD6	-0.2913125	2.17698391	-1.1435375	0.258001662	0.57928502	-5.630864
USP15	-0.1144883	5.24621339	-1.1433509	0.258078378	0.57935399	-6.2569347
TARSL2	0.38754708	1.87885352	1.14278585	0.258310855	0.57960567	-5.5280713
EIF3K	-0.0863061	7.36943202	-1.1426868	0.258351602	0.57960567	-6.2807741
BANP	-0.334731	2.36000263	-1.1426392	0.258371194	0.57960567	-5.6188345
KLHL24	-0.2738615	4.62875717	-1.1425454	0.258409809	0.57960567	-6.2529884
CRAT	-0.1664043	6.0438159	-1.1423853	0.258475754	0.57960567	-6.3025329
FMNL2	-0.1788004	5.77392353	-1.1422228	0.258542654	0.57960567	-6.2919221
SLC35D2	-0.1878314	2.70816565	-1.1421141	0.258587437	0.57960567	-5.8141135
SLC22A4	0.46145424	-0.6680487	1.14207395	0.258603955	0.57960567	-5.0988376
SPG21	-0.2052445	5.05873716	-1.1420723	0.258604627	0.57960567	-6.2160784
EIF3A	-0.1103226	9.08256673	-1.141556	0.258817361	0.57992419	-6.1781817
POLD1	-0.2663177	3.6013827	-1.141331	0.258910094	0.57992419	-5.8611329
CENPJ	-0.3206642	3.59562333	-1.1412832	0.258929787	0.57992419	-5.8842522
ENSCAFG00000031198	-0.3353835	0.83989264	-1.1412805	0.258930902	0.57992419	-5.3200683
ENSCAFG00000015742	-0.5621329	-0.8851693	-1.141055	0.259023884	0.58002931	-5.0261725
KANK4	0.95254977	4.06878424	1.14078687	0.25913447	0.58007509	-5.8043904
ERBB2	0.1312022	5.96768952	1.1407821	0.259136436	0.58007509	-6.2839855
COL7A1	-0.442301	3.00165812	-1.1401798	0.259384947	0.58052821	-5.9278809
EXO1	-0.8460606	2.1216952	-1.1394873	0.259670915	0.58090487	-5.266039
GBP1	0.63587601	-1.4157779	1.13947369	0.259676537	0.58090487	-5.1555012
H6PD	0.30325017	6.7377853	1.13943724	0.259691596	0.58090487	-6.28508
TGFB1	0.17027379	5.80692887	1.13827222	0.260173202	0.58177899	-6.3079055
ENSCAFG00000012668	0.47175984	4.01324816	1.1382685	0.260174741	0.58177899	-6.0000736
POLR2F	0.16821864	4.03505938	1.13782583	0.260357902	0.58208522	-6.0589241
PFN2	0.17209509	7.2994598	1.13762055	0.260442871	0.5821008	-6.2927638
EFCAB8	0.44877581	-1.1096184	1.1375857	0.260457299	0.5821008	-5.0268279
DCAF10	-0.110576	4.34401683	-1.1371976	0.260617982	0.58223745	-6.119103
CIT	-0.9060444	3.27813893	-1.1369447	0.260722744	0.58223745	-5.5174367
MYEF2	0.25624538	3.0768774	1.13688399	0.260747911	0.58223745	-5.8460369
ENSCAFG00000000842	0.17459869	6.0079363	1.13687945	0.260749794	0.58223745	-6.3094268
SERF2	-0.3834259	0.68209013	-1.1368561	0.260759457	0.58223745	-5.1885185
TUB	0.71879575	-0.1611449	1.13671714	0.260817044	0.58223745	-5.0609207
HK2	0.28849411	4.63956098	1.13664603	0.260846514	0.58223745	-6.2573918
ARHGDIA	0.11291537	7.75096648	1.13654537	0.260888233	0.58223745	-6.2747258

HRH1	0.88999454	-1.0816163	1.1362548	0.261008686	0.58224733	-4.8960439
ENSCAFG00000008336	-0.1437079	6.08776331	-1.1362478	0.261011581	0.58224733	-6.3071596
TEPSIN	0.15675501	2.82277938	1.13609091	0.26107664	0.58224733	-5.786547
ZFP30	-0.3294952	1.9605296	-1.1360887	0.261077557	0.58224733	-5.4766333
SPATA5L1	-0.1786031	3.04046907	-1.1357638	0.261212326	0.58244476	-5.840655
GIGYF1	-0.1692044	4.30301906	-1.1355701	0.261292655	0.58252076	-6.1797305
FSD1	0.39447217	1.26575182	1.13525826	0.26142209	0.58268989	-5.3942427
PCOLCE	0.46494631	8.34794461	1.13512263	0.261478391	0.58268989	-6.2241893
RASSF5	0.47656811	1.21257281	1.135053	0.261507299	0.58268989	-5.2293311
ENSCAFG00000024665	-0.4094896	-0.0526219	-1.1347692	0.261625141	0.58284936	-5.0655745
PSMB6	0.12618889	6.00473737	1.13460548	0.26169314	0.58289775	-6.2984145
HAPLN1	1.05472244	-0.0968556	1.13408369	0.261909946	0.58327752	-5.1091744
ENSCAFG00000025712	-0.5565367	-1.7808939	-1.1338544	0.262005248	0.58335285	-4.945378
ZFC3H1	-0.1446121	5.69311741	-1.1336763	0.262079299	0.58335285	-6.2988831
AMIGO3	0.41196873	0.66940191	1.13355738	0.262128759	0.58335285	-5.2289574
ENSCAFG00000016467	-0.6417796	2.56776953	-1.1335435	0.262134526	0.58335285	-5.3973634
PAFAH1B2	0.09441732	5.77806081	1.13344539	0.262175332	0.58335285	-6.3070045
AHCTF1	-0.1292682	7.19981803	-1.1331241	0.26230899	0.58353184	-6.2844649
KIF26A	-0.6588131	-2.2972604	-1.1330211	0.262351848	0.58353184	-4.9411717
PCGF1	-0.2273591	3.43622999	-1.132918	0.262394758	0.58353184	-5.9559752
PARPBP	-0.69567	1.92461551	-1.1326806	0.262493551	0.5836485	-5.2838232
RNF32	-0.4000579	0.46004271	-1.1325666	0.262541012	0.583651	-5.2103327
ANK1	-0.7217642	0.37929854	-1.1319314	0.262805591	0.58394538	-5.0939797
ENSCAFG00000032349	-0.332858	1.62284368	-1.131916	0.262811978	0.58394538	-5.3376536
CCHCR1	-0.1685313	4.40775378	-1.1319148	0.262812507	0.58394538	-6.1412995
XAB2	-0.1161754	5.10933581	-1.1317093	0.262898108	0.58403255	-6.2452442
COBLL1	-0.2816303	6.83886529	-1.1313834	0.26303398	0.58417001	-6.3136751
RUFY2	-0.1205626	4.5706709	-1.1313384	0.263052739	0.58417001	-6.1867593
THG1L	-0.2034713	2.26238732	-1.1311427	0.263134327	0.58424819	-5.6307987
ENSCAFG00000000415	-0.117648	7.49582964	-1.1309514	0.263214082	0.58432227	-6.2804003
RGS19	-0.1705571	3.58650233	-1.1308076	0.263274063	0.58435244	-5.9362973
MAGED2	0.11751753	6.93069097	1.13020574	0.263525241	0.5848069	-6.3115487
TANGO6	-0.1127368	4.01849243	-1.1300473	0.263591369	0.5848506	-6.0584877
CCDC180	0.9328913	-0.3601743	1.12973505	0.263721778	0.58491035	-4.9988359
MAP2K1	0.12566547	5.2894945	1.12970627	0.2637338	0.58491035	-6.2742004
PDF	0.21216971	2.66487912	1.12964928	0.263757605	0.58491035	-5.6718168
PLPPR2	0.65676477	-1.225495	1.1295346	0.263805509	0.58491361	-4.9943292
SCFD1	0.14805688	5.95919723	1.12935567	0.263880265	0.58496932	-6.3060356
TOMM7	0.13837108	3.83423223	1.12925216	0.26392352	0.58496932	-6.0588728
OLFML3	-0.5235614	5.38709119	-1.1291029	0.263985893	0.58500463	-6.1967115
STK24	-0.1444356	6.04393098	-1.1286792	0.26416304	0.58529422	-6.3133095
CHD8	-0.0947508	6.61257189	-1.1285174	0.264230689	0.58534115	-6.3181534
CATSPER2	-0.4103779	-0.4282717	-1.1283228	0.264312076	0.58541848	-5.0741069
PDE4A	0.1624894	4.63118579	1.12813103	0.264392324	0.58544298	-6.18952
ENSCAFG00000013477	0.09174327	6.64268784	1.12807423	0.264416094	0.58544298	-6.3174901
SPESP1	0.20729971	2.60394607	1.12742465	0.264688003	0.58587301	-5.8149658
RPS15	-0.1587618	8.00385491	-1.1273878	0.264703427	0.58587301	-6.2656304
ZBTB25	-0.2596836	2.23080364	-1.1272393	0.264765646	0.58587301	-5.606586
AAGAB	0.13441789	4.42386374	1.12716591	0.264796369	0.58587301	-6.1693632
PIK3CA	0.17199171	5.50347974	1.12693312	0.26489389	0.58598585	-6.3184611

PKNOX1	0.14725555	3.12645794	1.12663465	0.265018963	0.58615959	-5.9210555
TLDC1	0.24052018	2.32321393	1.12645817	0.265092938	0.58618458	-5.6734291
ENSCAFG00000021068	-0.4842741	-1.3274356	-1.1263091	0.265155422	0.58618458	-4.959078
ANKS4B	-0.5363109	-0.7998727	-1.126116	0.265236387	0.58618458	-5.0796398
PTPRJ	0.34152734	5.17633814	1.12605687	0.265261201	0.58618458	-6.2761947
ZBTB18	0.21691442	3.22665524	1.1260527	0.265262949	0.58618458	-5.8831416
CCDC47	-0.0977549	6.14550896	-1.1254376	0.265520994	0.58652902	-6.3210476
HYLS1	-0.1704127	2.65658766	-1.1253717	0.265548652	0.58652902	-5.6689999
ENSCAFG00000004735	0.12091671	4.55710352	1.12534825	0.265558509	0.58652902	-6.2069474
PPIL1	-0.1281227	4.95163739	-1.1249263	0.265735662	0.58661895	-6.2239749
USP19	-0.1407023	6.30361175	-1.1249062	0.265744077	0.58661895	-6.3231976
SAP130	-0.0919318	5.28652939	-1.1248217	0.265779584	0.58661895	-6.2633106
ZPR1	0.16258951	5.71591216	1.12480757	0.265785515	0.58661895	-6.3095096
RNFT2	0.30822691	1.49756351	1.12435561	0.265975375	0.58693515	-5.4466938
FAM32A	0.23579275	1.4492399	1.12404487	0.266105967	0.58712047	-5.4201481
TFAM	-0.211037	2.40967983	-1.1234782	0.266344237	0.58749944	-5.6232082
KCTD12	0.6880091	0.13628565	1.12336162	0.266393274	0.58749944	-5.2991213
TNFSF15	0.75475792	2.3815456	1.12330366	0.266417656	0.58749944	-5.4836982
ZNF322	0.1463641	3.47536184	1.1231452	0.266484321	0.58754359	-5.9716663
IL17RA	0.09674324	7.97744461	1.12294165	0.266569977	0.58762958	-6.2702823
ASNSD1	-0.0985407	6.21501784	-1.1227986	0.26663017	0.58765943	-6.3251309
BUB1B	-1.1101055	3.27301826	-1.1224095	0.26679401	0.58791766	-5.4733724
NKX2-5	0.75596444	-1.0358053	1.12207502	0.266934881	0.5881252	-4.8747128
MACROD1	-0.277071	2.00891196	-1.1216661	0.267107179	0.58830883	-5.5789334
UAP1	-0.2035368	5.86223038	-1.1216555	0.267111639	0.58830883	-6.3250978
ENSCAFG00000019817	-0.1021583	5.82694266	-1.121524	0.267167075	0.58832806	-6.315887
PABPC4	-0.1317984	7.14345513	-1.1206502	0.267535599	0.58895486	-6.3107575
TSPYL5	-0.4274676	0.60564438	-1.1206273	0.26754523	0.58895486	-5.1131453
DIAPH1	-0.0985136	7.0102465	-1.1202852	0.267689607	0.58916972	-6.3146243
OSBPL7	-0.1739461	4.0625247	-1.1200628	0.267783502	0.58923677	-6.1577362
POLH	0.21216003	4.0426142	1.11991077	0.267847706	0.58923677	-6.064927
UBTD1	0.25187895	3.95813535	1.11988068	0.26786041	0.58923677	-6.0510812
DDAH1	-0.2844604	4.99237217	-1.1194759	0.268031388	0.58929962	-6.2204448
EIF5A	0.16640533	7.46219816	1.11936382	0.268078741	0.58929962	-6.3051056
MTO1	0.11581897	4.73802436	1.11929127	0.268109398	0.58929962	-6.1919042
DCN	0.42821374	11.1452653	1.11920934	0.268144021	0.58929962	-6.0316193
ZNF687	-0.1597253	4.44376359	-1.1191603	0.268164728	0.58929962	-6.1511877
TSC22D3	-0.1722672	6.01667717	-1.1190587	0.268207708	0.58929962	-6.3187803
TLNRD1	-0.2637826	2.93965212	-1.1189353	0.268259835	0.58929962	-5.6849185
ZNF599	-0.1928527	2.32377947	-1.1186434	0.26838328	0.58929962	-5.7113971
SCD5	0.55171322	0.27585708	1.11863663	0.268386134	0.58929962	-5.1771024
RABGEF1	0.10163834	4.9904061	1.11850123	0.268443395	0.58929962	-6.247891
PRPF8	-0.090819	8.69543702	-1.118264	0.268543747	0.58929962	-6.2317176
BLOC1S6	-0.2168313	1.9747654	-1.1181709	0.26858314	0.58929962	-5.5635296
C26H12orf65	0.26143188	0.80032598	1.118136	0.268597898	0.58929962	-5.3578126
RAB11FIP5	-0.1286719	6.77570599	-1.1181265	0.268601925	0.58929962	-6.3274338
TMEM134	0.20909006	3.78517426	1.11812449	0.268602769	0.58929962	-6.0666947
PRKCA	0.21432071	4.69103067	1.11804052	0.268638302	0.58929962	-6.2498047
SASH1	0.17102271	7.98891365	1.11793178	0.26868432	0.58929962	-6.2627706
ZNF688	-0.2440949	1.76917077	-1.1177268	0.268771071	0.58938726	-5.5316149

ENSCAFG00000019285	0.19237684	1.95136898	1.11725422	0.268971181	0.58972341	-5.4955073
TXNRD1	0.18770896	8.9102684	1.11695931	0.269096103	0.5897475	-6.2154677
BACH1	0.14371403	7.09312247	1.11692913	0.26910889	0.5897475	-6.3075061
PRMT2	0.13539065	5.11698625	1.11689671	0.269122628	0.5897475	-6.2598788
TNPO1	-0.1063385	6.98918684	-1.1164514	0.269311338	0.59000765	-6.3266179
ENSCAFG00000029785	-0.2128331	2.04612171	-1.1163905	0.269337189	0.59000765	-5.5957715
RPF2	-0.1203608	5.19469634	-1.1162851	0.269381868	0.59000765	-6.2893224
ENSCAFG00000008590	-0.3165863	1.40674324	-1.1160569	0.269478628	0.59011697	-5.4068209
DCUN1D2	0.13386084	3.76247647	1.11592652	0.269533918	0.59013545	-6.0408805
NFIL3	-0.2165566	4.46863541	-1.1156885	0.269634874	0.59025389	-6.1862753
TBCEL	-0.1428059	4.39178681	-1.1155764	0.269682433	0.59025542	-6.1606713
HMOX1	-0.3413112	6.35625347	-1.1151722	0.269854023	0.59043799	-6.3288677
GTF2I	-0.1196329	7.02544771	-1.115159	0.269859597	0.59043799	-6.3177135
SMYD4	0.156098	3.85988178	1.11472714	0.270042984	0.59073662	-6.037913
NOTCH4	-0.939148	-1.8277868	-1.1138289	0.270424677	0.59146887	-5.0339478
ENSCAFG00000004186	-0.1357137	5.05779216	-1.1136786	0.270488585	0.59150594	-6.2477475
STK11	-0.1278377	5.78537416	-1.1133804	0.270615413	0.5916192	-6.3237778
MAT2B	0.11924108	4.62845406	1.11333594	0.270634315	0.5916192	-6.2191815
SRSF12	0.27738969	1.07898402	1.11312302	0.270724901	0.59171453	-5.3596732
ZDHHC20	-0.1106319	5.63365717	-1.1129616	0.270793574	0.59174855	-6.3052612
ATG4B	0.09531948	5.30545182	1.11284472	0.270843328	0.59174855	-6.2985472
BRF2	0.17845093	2.53190682	1.11275527	0.270881402	0.59174855	-5.6714684
P2RX7	-0.4685237	0.50690595	-1.1123709	0.271045035	0.59197216	-5.5012686
NADSYN1	-0.1426212	3.99788713	-1.1121998	0.271117908	0.59197216	-6.1187569
PHLDB3	0.22339675	2.10614686	1.11218374	0.271124751	0.59197216	-5.6146853
CINP	-0.1528477	3.45544875	-1.1119804	0.271211381	0.59197734	-5.9971225
ENTPD6	0.15149899	4.29402075	1.11195751	0.27122112	0.59197734	-6.137444
ENSCAFG00000025112	-0.1333309	3.90139446	-1.1115638	0.271388907	0.59224093	-6.0625947
FZD6	0.76081602	4.24666217	1.11142402	0.27144847	0.59226831	-5.9344486
CBR4	-0.3195282	1.34099887	-1.111005	0.271627152	0.59239107	-5.4172396
PLIN4	0.33801359	1.1002831	1.1110025	0.271628198	0.59239107	-5.3507785
SYDE1	0.17478113	5.06026752	1.11096117	0.271645825	0.59239107	-6.2381837
ENSCAFG00000031537	-0.3794574	2.89665255	-1.1107384	0.271740838	0.59249569	-5.7412121
CERS4	-0.2729407	4.7229704	-1.1106152	0.271793409	0.59250237	-6.2970342
GOLPH3L	-0.1157299	5.13353254	-1.1104212	0.271876187	0.59250237	-6.2797602
KIF7	-0.1951554	3.85782984	-1.1103162	0.271920992	0.59250237	-6.0568995
KPNA3	0.10222493	6.02553265	1.11029029	0.27193206	0.59250237	-6.3373968
CYGB	0.73162694	0.11676569	1.10994707	0.27207858	0.59257751	-5.7976215
HSD11B1L	0.24192705	2.45762522	1.10994421	0.272079801	0.59257751	-5.6595213
GJC1	-0.4278631	1.9274373	-1.1098698	0.272111596	0.59257751	-5.3946812
XRCC3	0.42882342	0.03627554	1.10976868	0.272154757	0.59257751	-5.2006889
HADHA	0.07889826	7.23603978	1.10965861	0.272201766	0.59257751	-6.3092388
AP1AR	0.19999825	2.86480177	1.1088216	0.272559429	0.5932536	-5.8020825
GPC4	0.58703535	4.23678492	1.10835638	0.272758369	0.59337928	-5.7850278
PCBD2	0.1894859	2.44362246	1.1082848	0.272788987	0.59337928	-5.6111955
IFT172	-0.1783718	4.67108569	-1.1081049	0.272865932	0.59337928	-6.2664906
VTI1A	-0.1210308	3.86530164	-1.1080732	0.272879506	0.59337928	-6.0356305
SH3GL1	-0.1546931	6.39517208	-1.1080181	0.272903083	0.59337928	-6.3406426
ACOT13	-0.1496059	4.46172014	-1.1079516	0.272931531	0.59337928	-6.1831257
CNOT6L	-0.1585016	4.63023329	-1.1078511	0.27297454	0.59337928	-6.2581319

PDCD7	-0.1185232	3.02143231	-1.1078056	0.272994039	0.59337928	-5.9105253
ENSCAFG00000016914	0.34148045	1.71651159	1.1076027	0.273080871	0.59338626	-5.4801275
ADD3	-0.3634168	6.16854452	-1.1075779	0.27309147	0.59338626	-6.3266977
NDFIP1	0.13351713	6.71987025	1.10712954	0.273283478	0.59358901	-6.3415422
CNKSR1	0.24939657	2.20833762	1.10710362	0.273294582	0.59358901	-5.6882158
TRPV4	-0.1506993	4.50188671	-1.1069317	0.273368242	0.59358901	-6.1248288
SEC23B	0.18699233	6.81746926	1.106802	0.273423789	0.59358901	-6.3393592
ANKIB1	-0.1004071	6.94273143	-1.1067579	0.273442668	0.59358901	-6.3346886
CWC25	-0.133172	3.29762605	-1.1066999	0.273467531	0.59358901	-5.9800429
EMG1	-0.1942721	3.89005504	-1.106407	0.273593062	0.59372832	-6.0369627
ENSCAFG00000004288	-0.2802888	1.19069528	-1.1063302	0.273625986	0.59372832	-5.4934115
CNTRL	-0.1160999	4.81780879	-1.1060246	0.273757012	0.59383311	-6.2425203
PEX7	-0.2146054	3.81795466	-1.1059103	0.273806052	0.59383311	-6.0757635
PNRC1	0.20440965	5.38199022	1.10588775	0.273815712	0.59383311	-6.3444153
ENSCAFG00000019111	-0.1327654	4.11525802	-1.1055837	0.273946141	0.5940137	-6.1116809
C30H15orf62	0.48946834	-0.1173912	1.10525652	0.274086534	0.59421583	-5.095821
SEMA3C	0.39514585	9.6000928	1.10474568	0.274305848	0.59458896	-6.1880556
SEC24C	0.08416849	6.9140429	1.10411174	0.274578176	0.59496009	-6.3368466
C31H21orf91	-0.1994418	2.18774827	-1.1040987	0.274583785	0.59496009	-5.6362187
SH3D19	-0.2166512	5.77774365	-1.1039992	0.274626536	0.59496009	-6.3277789
BRAT1	-0.1412217	4.71749603	-1.1036995	0.274755383	0.59496009	-6.2516263
FMNL1	-0.8454381	1.18193586	-1.1036158	0.274791377	0.59496009	-5.32918
ZSWIM7	-0.4883323	0.20689944	-1.1034653	0.27485606	0.59496009	-5.099596
CDK1	-0.560075	3.6134057	-1.1033685	0.274897694	0.59496009	-5.7929217
TCHP	-0.188999	3.89102145	-1.1033637	0.274899747	0.59496009	-6.0604173
ALS2	-0.1298839	4.74211079	-1.1033119	0.274922055	0.59496009	-6.1989003
CRTAC1	0.90740964	-0.4720131	1.10317257	0.274981976	0.59496009	-4.9267207
ENSCAFG00000025131	0.76822367	1.92110699	1.10313849	0.274996639	0.59496009	-5.372175
AADAT	-0.9070128	0.04163067	-1.102878	0.275108701	0.595073	-5.0145359
TTC39B	0.31108163	2.882168	1.10269069	0.275189325	0.595073	-5.7724996
FAM149A	-0.7393691	-0.0179641	-1.1026411	0.275210692	0.595073	-5.0391823
SCN3A	-1.2918801	0.71326076	-1.1025159	0.275264583	0.595073	-5.0863992
DNAAF3	0.33393186	1.42319735	1.10246834	0.27528504	0.595073	-5.5973295
EYA3	-0.1049709	3.96597538	-1.102238	0.275384231	0.59510691	-6.1046187
ZNF214	-0.3932654	0.41533458	-1.1022124	0.275395221	0.59510691	-5.2939802
RNF40	-0.0977946	5.65414696	-1.1016087	0.275655303	0.59556676	-6.3335583
SKI	-0.1136994	6.86253975	-1.100768	0.276017733	0.59624753	-6.3358661
PCIF1	-0.1327495	4.71665793	-1.1002771	0.276229532	0.59645836	-6.2054171
PDGFRB	0.45570491	8.54252208	1.10014099	0.276288266	0.59645836	-6.2355457
ENSCAFG00000007173	-0.4123342	-0.9313717	-1.100038	0.276332714	0.59645836	-4.9797858
VAMP5	-0.3198297	1.72094188	-1.1000075	0.276345908	0.59645836	-5.6221117
MYRFL	1.0259332	0.20525505	1.09999312	0.276352096	0.59645836	-5.0105534
CDK13	-0.0872706	5.97950191	-1.0998212	0.276426334	0.59651638	-6.3470735
MIS18A	-0.6764791	0.05472122	-1.0996727	0.276490445	0.59655252	-5.0691154
ENSCAFG00000010830	0.48122059	-0.2615955	1.09945983	0.276582372	0.59664867	-5.176175
ENPP2	-0.8277136	-2.2937701	-1.0992281	0.276682464	0.59676238	-4.9272222
ENSCAFG00000031903	-0.123059	3.58363564	-1.0987251	0.276899849	0.597129	-6.0311865
CPT2	0.19778719	5.97281119	1.09846418	0.277012663	0.59727003	-6.3482288
UBQLN1	0.10111216	7.10221729	1.09830312	0.277082312	0.59728349	-6.3438507
CYB5A	0.25405736	6.9092199	1.09813543	0.277154838	0.59728349	-6.3525068

CDC25C	-0.5075475	0.32981913	-1.0980935	0.277172976	0.59728349	-5.1110644
TATDN3	-0.1656971	3.30472945	-1.0979371	0.27724065	0.59728349	-5.958666
BRIP1	-0.4490272	1.77014548	-1.0979016	0.277255997	0.59728349	-5.4078529
ENSCAFG00000000102	-0.1411076	7.13707975	-1.0975268	0.277418176	0.59745081	-6.3454026
RHOBTB2	-0.168628	2.92716313	-1.0974241	0.277462638	0.59745081	-5.8893303
ENSCAFG00000031282	0.36520492	-0.0562472	1.09739335	0.277475962	0.59745081	-5.2023486
KRI1	-0.189878	3.95096217	-1.0971418	0.277584865	0.59758314	-6.1062616
CPT1B	-0.2533341	2.4644837	-1.0968367	0.277717023	0.59776549	-5.7519511
EML1	0.21578454	6.37063865	1.09659621	0.277821205	0.59788756	-6.3543853
PTPN1	0.13811385	4.29488781	1.09645445	0.27788263	0.5979176	-6.2076027
PMM1	-0.1688242	3.74096603	-1.0957889	0.278171166	0.59833562	-6.0520453
TRIM37	-0.1387239	5.37351576	-1.0957872	0.278171909	0.59833562	-6.3104246
FAM186A	0.66008812	-1.4273452	1.09559715	0.278254321	0.59834386	-4.9782341
MYO18B	1.17237003	-0.1431355	1.09544993	0.278318183	0.59834386	-4.9141049
MEF2D	0.14438869	5.28868149	1.09540953	0.278335713	0.59834386	-6.321558
ZNF467	0.45198104	-1.6924263	1.09534029	0.278365751	0.59834386	-5.0809821
RNF217	0.21064009	2.46176122	1.09522435	0.27841606	0.59834989	-5.7177585
ADGRE2	0.41786763	0.96878126	1.0950267	0.278501838	0.59843213	-5.3718929
HSF4	0.27335613	2.57163726	1.09476484	0.27861551	0.59857427	-5.7921788
ENSCAFG00000025882	-0.5629121	-1.1800188	-1.0942036	0.278859255	0.59899577	-5.0343267
ENSCAFG00000029155	0.10376344	5.56642483	1.09406278	0.27892043	0.59902502	-6.3325691
CHRAC1	0.13044248	2.84451355	1.0936681	0.27909195	0.59928475	-5.9029379
MSRA	0.22057547	2.46982831	1.09340189	0.27920768	0.59928475	-5.7402578
WBP2	0.13206924	6.17281216	1.09336393	0.279224187	0.59928475	-6.356643
MAGT1	0.11261681	4.31862598	1.09331503	0.279245447	0.59928475	-6.2147336
AP3D1	-0.0616441	7.60843712	-1.0932373	0.279279253	0.59928475	-6.3265346
TMEM177	-0.2125611	1.87758042	-1.0929672	0.279396743	0.59943474	-5.5293771
PCDH18	0.3220936	6.20310367	1.09278913	0.279474199	0.59949881	-6.352484
GTF2H4	-0.1333329	5.07813011	-1.0926157	0.279549649	0.59955855	-6.3011508
VASP	0.19896986	6.77633829	1.09240371	0.279641927	0.59965436	-6.3555153
FAM76B	-0.2700931	1.82603738	-1.0921821	0.279738405	0.59975914	-5.5770589
ENSCAFG00000011263	-0.5098236	-0.3176185	-1.0919957	0.279819569	0.59983106	-5.0490927
RIMBP2	0.51813031	-1.6592253	1.09145923	0.280053245	0.60022982	-5.1150803
PDE9A	0.39171383	0.93771644	1.0910667	0.280224314	0.60049429	-5.5309487
NOXA1	0.33605938	1.49739028	1.09050113	0.280470925	0.60092053	-5.4275481
SHF	0.49224248	1.52911749	1.09023019	0.280589118	0.60099448	-5.2026803
SCAP	0.13323446	6.09421157	1.09017453	0.280613404	0.60099448	-6.3578564
UBE2D2	-0.098946	6.18096034	-1.0900939	0.28064858	0.60099448	-6.3556999
MRGPRF	0.50577043	4.76354217	1.08985271	0.280753852	0.60109673	-6.0763209
TNFSF13B	-0.3970126	1.33551063	-1.0897658	0.280791771	0.60109673	-5.6557108
ENSCAFG00000014370	0.31454413	2.82611662	1.08963351	0.280849538	0.60111823	-5.8522545
ENSCAFG00000016628	0.08118274	5.35064516	1.08891924	0.281161501	0.6016837	-6.3110747
PKIA	0.56275499	1.65784595	1.08864947	0.281279388	0.60183373	-5.1854992
NR6A1	-0.3810149	0.24370878	-1.0884196	0.281379875	0.60191782	-5.2526575
CNNM3	-0.1985435	3.55976037	-1.0882549	0.28145185	0.60191782	-6.0229175
DOCK10	0.30984379	6.12969012	1.08823163	0.281462047	0.60191782	-6.3631078
ENSCAFG00000017792	0.28585552	1.81570513	1.08781062	0.281646175	0.6021201	-5.5158111
GPNMB	0.5237483	7.90586867	1.08779676	0.28165224	0.6021201	-6.3343272
TMEM200B	-0.4512216	1.28918133	-1.0873832	0.281833186	0.60240469	-5.3919261
CPLANE1	-0.1147843	6.11172621	-1.0871269	0.281945381	0.60243997	-6.3634667

FHOD1	-0.1933132	5.18013351	-1.0871214	0.281947793	0.60243997	-6.3325588
ACTL7A	-0.3480717	0.74862725	-1.0870177	0.281993175	0.60243997	-5.3549398
ITGB3BP	-0.3741136	0.62661735	-1.0868771	0.282054745	0.60246932	-5.2506028
KAT6B	-0.1236124	5.11425401	-1.0862639	0.282323365	0.60281463	-6.3259047
ENSCAFG00000016475	0.38824734	2.33621149	1.08611266	0.282389634	0.60281463	-5.8668334
ARL2BP	0.10082408	5.81919835	1.08607286	0.282407076	0.60281463	-6.3525751
STK17B	-0.2159782	4.88821312	-1.0859731	0.282450799	0.60281463	-6.2730972
EXOSC4	0.14312047	3.84268623	1.08581046	0.282522094	0.60281463	-6.0921902
CLP1	0.12929855	3.39610533	1.08577177	0.282539058	0.60281463	-5.9809606
ARHGEF5	-0.5147251	1.20386934	-1.0857436	0.282551411	0.60281463	-5.346678
NCL	-0.1707473	8.98919283	-1.0854794	0.282667279	0.60293999	-6.2397659
ANXA4	-0.11573	6.2528194	-1.0853913	0.282705905	0.60293999	-6.3664803
MARC2	-0.1865873	3.04021068	-1.0848893	0.282926114	0.60324181	-5.8639673
ANKRD28	-0.2246511	6.50320564	-1.0848504	0.282943207	0.60324181	-6.3562912
ENSCAFG00000030087	-1.2645804	2.24417987	-1.0846209	0.283043919	0.60335441	-5.3308528
MAPK1	0.08250539	8.1153694	1.08405977	0.283290324	0.60377748	-6.3208307
RMDN1	0.10703444	4.09373683	1.0837847	0.283411159	0.60383369	-6.1630889
KIF27	-0.3140223	1.93344844	-1.0837173	0.283440753	0.60383369	-5.6393361
ZRANB1	0.16593269	4.97726806	1.08348879	0.283541192	0.60383369	-6.2809831
EPAS1	0.2043307	7.56597169	1.08344732	0.28355942	0.60383369	-6.3305579
RHOJ	-0.5534825	3.42966005	-1.0833683	0.283594155	0.60383369	-5.7764867
MTMR10	-0.1971863	4.95033379	-1.0833451	0.283604329	0.60383369	-6.3013481
GABBR1	0.22968661	4.31999545	1.08312234	0.283702279	0.60391236	-6.1781404
CLN3	-0.1552756	4.20936965	-1.0829378	0.283783445	0.60391236	-6.1098257
TMEM246	0.49675316	0.47337196	1.08293397	0.28378511	0.60391236	-5.2569131
FLT4	-0.6944406	2.64417272	-1.0825616	0.283948911	0.60409469	-5.6905942
EFNA5	0.78403899	-1.0502482	1.08252112	0.283966708	0.60409469	-5.019789
OXCT1	-0.4119743	4.10668755	-1.0822114	0.284103013	0.6042826	-6.1756699
SLC38A6	-0.2771352	2.83186175	-1.0815033	0.284414747	0.60475878	-5.7871502
GSTP1	0.22937731	4.93801963	1.08148477	0.284422914	0.60475878	-6.3145445
MPP7	0.54759088	2.6885263	1.08120115	0.284547853	0.604849	-5.5536581
PSD	0.28080386	1.46394274	1.08117045	0.284561381	0.604849	-5.622308
DCAF1	-0.0893277	5.29132694	-1.0809571	0.284655384	0.60493397	-6.3298594
ETFBKMT	-0.477319	0.21089308	-1.0808618	0.284697411	0.60493397	-5.2316347
PKD1L3	-0.3638892	0.91622821	-1.0806522	0.284789766	0.60499087	-5.3736399
ENSCAFG00000028589	-0.7922785	1.35051719	-1.0805831	0.28482025	0.60499087	-5.6422282
GPR162	0.52879035	-0.3462464	1.08027916	0.284954274	0.6051735	-5.1357321
SPSB2	-0.3724405	1.07106025	-1.0796602	0.285227348	0.60565133	-5.3496977
CDKL5	-0.2377977	1.48062851	-1.0793251	0.285375232	0.60586321	-5.4779859
PRKAB2	0.1297292	5.52072768	1.07917654	0.285440847	0.60589195	-6.3542224
KANSL1L	0.20590532	3.90374789	1.07907661	0.285484971	0.60589195	-6.1568125
API5	-0.0821768	6.09709399	-1.0789222	0.285553145	0.60593454	-6.3684913
C32H4orf36	-0.4969542	-0.6169121	-1.0787855	0.285613552	0.60596064	-5.1190279
FAM217B	0.42336508	-0.3135057	1.07849232	0.285743073	0.60613334	-5.1465145
SDR42E1	0.2152614	3.20148249	1.07804852	0.285939222	0.6063972	-5.9548938
ZDHHC24	-0.4359662	0.53142778	-1.077993	0.285963748	0.6063972	-5.270265
THY1	0.49295587	7.56655599	1.07768333	0.286100701	0.60648298	-6.3465913
ENSCAFG00000029520	-0.3913337	0.71433465	-1.0774071	0.286222863	0.60648298	-5.3058447
ENSCAFG00000011954	-0.0899229	6.72957592	-1.0773716	0.2862386	0.60648298	-6.373049
SCO1	-0.0920147	5.16662245	-1.0773674	0.286240461	0.60648298	-6.3265994

STAC	0.48712048	4.27412901	1.07735723	0.286244945	0.60648298	-6.272306
PPCDC	-0.1678805	2.61384016	-1.0770963	0.286360388	0.60662554	-5.8085611
TCIRG1	0.13568706	6.13771455	1.0764543	0.286644607	0.60712552	-6.371285
ENSCAFG00000029334	0.37318328	1.04042412	1.07624592	0.286736896	0.60720013	-5.2835081
CHRM2	0.86496006	0.96490504	1.07615709	0.286776243	0.60720013	-5.3366304
PNMA2	0.53925375	-0.7936355	1.07602894	0.286833016	0.60720125	-5.1161532
TMEM175	0.13932989	4.72900559	1.07588243	0.286897933	0.60720125	-6.2535207
NPC1	0.14738903	6.67509863	1.07572249	0.286968809	0.60720125	-6.3766676
SNAPC5	-0.4863981	0.21643192	-1.0755541	0.287043455	0.60720125	-5.1659115
PTCH1	-0.1796916	4.4013757	-1.0755178	0.287059541	0.60720125	-6.2166513
ATP5F1A	-0.0948324	8.57694944	-1.0754525	0.287088477	0.60720125	-6.2754831
CPNE8	-0.5073195	3.24751514	-1.0753945	0.287114215	0.60720125	-5.4548505
ELL	0.1287143	4.53846433	1.07495193	0.287310455	0.60742257	-6.2901135
ECHS1	-0.1152936	5.89377045	-1.0748773	0.28734357	0.60742257	-6.3752891
LRRC59	0.24065816	6.41094659	1.07456592	0.287481708	0.60742257	-6.3695024
KIAA0100	-0.0696554	5.64259061	-1.0745186	0.287502694	0.60742257	-6.3520426
GNB5	0.2277467	3.94060919	1.07448209	0.287518908	0.60742257	-6.187916
TPST2	0.21477051	4.56995217	1.07448076	0.287519498	0.60742257	-6.2395693
TRMT2A	-0.131328	5.44106798	-1.0743976	0.287556428	0.60742257	-6.3493444
ARL4C	0.47142922	4.75913527	1.07405967	0.287706413	0.60763749	-6.2216965
XIAP	-0.1405101	5.31127984	-1.0739324	0.287762921	0.60765495	-6.3175002
NES	0.37474466	7.89437553	1.07350373	0.287953315	0.60795507	-6.2945978
ATXN2	0.12100461	6.23684424	1.07326548	0.288059166	0.60797009	-6.3788626
ZNF45	-0.1956819	2.29725006	-1.0730976	0.28813377	0.60797009	-5.7462701
KIF20A	-1.1755848	3.64193971	-1.0730836	0.288139994	0.60797009	-5.6111448
DTX3L	-0.3297546	3.23234668	-1.0730532	0.288153498	0.60797009	-6.0223437
COL15A1	0.58266959	9.11514194	1.07251906	0.288390977	0.60828246	-6.2683354
ENSCAFG00000010494	-0.3474024	1.3255624	-1.0724532	0.288420288	0.60828246	-5.3681524
THAP5	0.24057014	3.87277534	1.07230425	0.288486517	0.60828246	-6.098837
ENSCAFG00000008935	-0.2376447	2.50150107	-1.0722858	0.288494712	0.60828246	-5.8456561
TPGS2	0.09254458	4.50106741	1.07156154	0.28881702	0.60886011	-6.2476865
ENSCAFG00000031575	-0.6035646	-1.4549271	-1.071327	0.288921423	0.60891037	-4.9753899
AFG1L	-0.2616073	1.25160006	-1.0712908	0.288937543	0.60891037	-5.445663
ENSCAFG00000023943	-0.4055997	-0.1381762	-1.0711487	0.289000832	0.60894187	-5.2605675
ENSCAFG00000025983	-0.294231	5.20012778	-1.0708661	0.289126743	0.60910528	-6.2254314
UCHL1	0.29813105	6.28767717	1.07059297	0.289248421	0.60925972	-6.3821565
CENPN	-0.6416571	2.07443513	-1.0701958	0.289425433	0.60953064	-5.3773565
CEP104	0.13578423	4.28805995	1.07006968	0.289481677	0.60954717	-6.2264759
ADGRG1	-1.0722169	1.33603691	-1.0697943	0.289604481	0.60963048	-5.2776449
PIH1D1	-0.1638422	3.91487019	-1.0697639	0.289618038	0.60963048	-6.1514894
BCCIP	-0.1296234	4.06088483	-1.0693593	0.289798528	0.6098995	-6.1545909
BRK1	0.1036664	4.91028472	1.06926038	0.289842682	0.6098995	-6.2960071
CHRNE	0.27376685	2.53860263	1.06904771	0.289937602	0.60999733	-5.7271923
ATP11C	-0.1436337	5.84969776	-1.0685369	0.290165663	0.6103752	-6.3794087
GEMIN5	-0.1269807	6.15789887	-1.0681393	0.290343301	0.6106384	-6.3791266
ZFAND4	-0.2214486	4.55322846	-1.06785	0.290472571	0.6106384	-6.1716213
HSPB8	0.23144215	6.36305177	1.06781432	0.290488504	0.6106384	-6.3848053
BMPER	0.70594541	-1.1978604	1.06761847	0.290576049	0.6106384	-4.9657234
NCALD	0.29188505	5.74314161	1.06757695	0.29059461	0.6106384	-6.3832481
RBCK1	-0.1281314	6.24449991	-1.0675251	0.290617791	0.6106384	-6.3848043

ENSCAFG00000004857	-0.4844698	-0.3676906	-1.0674975	0.290630138	0.6106384	-5.0598007
ENSCAFG00000029831	-0.1696861	2.36862701	-1.0672777	0.290728401	0.61074299	-5.7837248
YAF2	0.13009832	3.14367569	1.0670876	0.290813437	0.61081975	-5.9495466
ZNF621	-0.1791908	2.89990709	-1.0669	0.290897357	0.61089415	-5.8978082
ABCA9	-0.6040103	4.60210957	-1.0664397	0.291103327	0.61122479	-6.3774224
SLC41A2	0.15300452	4.79582849	1.06604868	0.291278401	0.61149046	-6.3115323
MRC1	-0.4275654	-0.4994329	-1.0652398	0.29164078	0.61207387	-5.2570716
HCFC1R1	-0.2482792	4.16438113	-1.0652114	0.29165349	0.61207387	-6.0557379
ENSCAFG00000028692	0.24043503	2.32863028	1.06491394	0.291786838	0.61219056	-5.7276907
COL5A2	0.24532789	13.3695441	1.06480384	0.291836201	0.61219056	-5.9322287
MYO1D	0.23808156	8.45969652	1.06467022	0.291896121	0.61219056	-6.3245874
NUDCD1	-0.1539168	4.48497214	-1.0646538	0.2919035	0.61219056	-6.2345691
PAPOLG	-0.155812	3.84021026	-1.0644318	0.292003066	0.61229742	-6.1342512
ENSCAFG00000029677	-0.7803359	-1.832291	-1.064166	0.292122276	0.61244544	-5.0049682
NDUFAF5	0.14459015	3.35028226	1.06390329	0.292240188	0.61253939	-6.0010548
TANK	0.17535627	4.68855076	1.06380673	0.29228353	0.61253939	-6.2776381
ENSCAFG00000004935	0.6165379	-1.2367366	1.06374112	0.292312979	0.61253939	-5.3549367
AEN	-0.1209452	6.16656266	-1.0636199	0.292367399	0.61254922	-6.3863964
ENSCAFG00000014885	-0.3877833	-0.5178467	-1.063514	0.292414931	0.61254922	-5.1440883
CD99L2	0.19876917	1.68518234	1.06333968	0.292493219	0.61261134	-5.6898103
SLIT2	-0.6769665	5.52233809	-1.0629432	0.292671321	0.61288245	-6.3825038
DCAF4	-0.1676449	3.20349947	-1.0623443	0.292940453	0.61334407	-5.9139446
SLC7A2	0.53282529	2.33800787	1.06205906	0.293068717	0.61340754	-5.7120476
ENSCAFG00000008376	0.18668158	2.63221329	1.06203641	0.293078901	0.61340754	-5.7735019
PPP1R12C	0.1148344	5.56970692	1.061952	0.293116862	0.61340754	-6.3624108
IQCB1	-0.2115449	2.72432168	-1.0614116	0.293359986	0.61381435	-5.7319657
TMEM141	-0.3495643	0.91929501	-1.061202	0.2934543	0.61382371	-5.4138431
HAS1	-0.6381735	0.93055932	-1.0611851	0.293461923	0.61382371	-5.6037436
NDUFAF1	-0.1141149	3.88785711	-1.0609439	0.293570481	0.61387837	-6.1094583
TXNL4A	0.12560168	3.85896869	1.0609105	0.293585527	0.61387837	-6.115532
PPIP5K2	-0.1289715	6.6122358	-1.06076	0.293653276	0.61391812	-6.3925838
ENSA	0.11092151	5.08854999	1.06064313	0.293705925	0.61392629	-6.3375307
SOCS3	-0.4228464	2.54128476	-1.0597946	0.294088269	0.6146235	-5.6341029
CUL4B	-0.1057766	6.21790066	-1.0594255	0.294254661	0.61464344	-6.3938453
PSD4	-0.3742817	4.0933111	-1.0593511	0.294288234	0.61464344	-5.9327387
MCM2	-0.4795673	5.00333041	-1.0592856	0.294317761	0.61464344	-6.185744
SLC25A13	0.19864254	3.90963024	1.05915012	0.294378858	0.61464344	-6.0543795
PPP2R2B	0.51303922	-1.1224114	1.05908419	0.2944086	0.61464344	-5.1972243
ENSCAFG00000014560	0.21028836	2.36756135	1.05902628	0.294434726	0.61464344	-5.6743171
ZFYVE1	-0.1090764	5.45768519	-1.0590159	0.294439387	0.61464344	-6.3667027
THEM4	-0.1866764	4.77720302	-1.0581159	0.294845625	0.61535355	-6.2777439
TCF19	-0.3936722	2.12130167	-1.0580458	0.294877266	0.61535355	-5.5189372
ZXDC	-0.0995438	4.70632244	-1.0574948	0.2951262	0.61539949	-6.2692844
ZNF704	0.30930413	2.14853987	1.05748099	0.295132436	0.61539949	-5.4488362
CERS2	0.07399052	6.55354504	1.05747928	0.295133206	0.61539949	-6.3958921
RBMS3	-0.2147106	3.39098962	-1.0573612	0.295186582	0.61539949	-6.1383624
EIF2B2	-0.2664373	4.43392788	-1.0573076	0.295210772	0.61539949	-6.2711114
CNTROB	0.14957334	3.25595704	1.05721241	0.295253817	0.61539949	-5.98062
TBX15	0.6717447	3.31365775	1.05717461	0.295270903	0.61539949	-5.9817801
MYO5A	0.16601283	6.85418267	1.05713206	0.295290134	0.61539949	-6.3903188

GSTM3	-0.2123032	4.20264799	-1.0568911	0.295399086	0.61541019	-6.2626583
RCAN3	0.16180415	3.25496843	1.05680761	0.295436824	0.61541019	-5.929822
ENSCAFG00000029945	-0.2102961	2.27885463	-1.0565972	0.295531961	0.61541019	-5.6634214
SLITRK5	0.888188	-1.0369321	1.05652971	0.295562509	0.61541019	-5.0397869
CTHRC1	-1.023891	1.97363601	-1.0564489	0.295599045	0.61541019	-5.0981566
STXBP3	-0.1048024	5.12010927	-1.0562749	0.295677783	0.61541019	-6.3322656
GAN	0.21123344	3.16233945	1.05622183	0.295701795	0.61541019	-5.8616309
ENSCAFG00000015189	-0.1335061	4.75270152	-1.0561945	0.295714164	0.61541019	-6.2940976
CAVIN1	-0.1123726	10.0113288	-1.0561244	0.295745898	0.61541019	-6.1675237
DNAJC2	-0.1330832	5.29723979	-1.0560405	0.295783844	0.61541019	-6.3493877
JRKL	0.12261595	3.66857381	1.05580593	0.295890018	0.61548355	-6.1355963
ENSCAFG00000000172	-0.131014	3.57079613	-1.055639	0.295965574	0.61548355	-6.0861493
MAP3K11	-0.3157606	2.90045672	-1.0556388	0.295965692	0.61548355	-5.8968488
TMCO6	-0.1699025	3.33026603	-1.0554748	0.296039933	0.61553631	-6.0157917
ATP5S	-0.178549	2.06798431	-1.0551116	0.296204428	0.61568951	-5.5845186
CRYBA4	0.65775606	-0.5448826	1.0550963	0.29621137	0.61568951	-5.0441522
WLS	0.20222882	8.0994217	1.05484651	0.296324544	0.61582312	-6.3446203
UBE2M	0.11035708	5.45032744	1.0545161	0.296474291	0.61596391	-6.3542308
BRI3	-0.2122792	3.15115814	-1.0544179	0.296518826	0.61596391	-5.8972384
CDCA2	-0.7419428	2.27881887	-1.0543734	0.296538994	0.61596391	-5.4551504
MXD1	0.22076238	1.85797959	1.05423438	0.296602007	0.61599322	-5.6564057
MFAP2	0.58884165	3.17876196	1.05398642	0.296714456	0.61612517	-6.0673511
HMGA1	-0.4166702	7.73834716	-1.0535873	0.296895531	0.61639956	-6.3035845
ENSCAFG00000008682	0.28919796	1.75970756	1.0533005	0.29702566	0.61640239	-5.5580163
ENSCAFG00000031706	0.80343599	1.57543318	1.05329743	0.297027051	0.61640239	-5.2825034
EXT2	0.10134494	7.9606589	1.05326074	0.297043704	0.61640239	-6.3437345
CAMKK2	-0.1624057	4.5973404	-1.0530839	0.297123968	0.61646739	-6.3016473
ENSCAFG00000032749	-0.481407	-1.0600395	-1.0525499	0.29736648	0.61686894	-5.0123004
IMPA2	-0.3012226	1.24690804	-1.0518129	0.297701366	0.61746195	-5.4586124
MED28	-0.0937436	5.78445595	-1.0516471	0.297776747	0.61751661	-6.38267
RARG	-0.3340306	4.13471623	-1.0511874	0.29798581	0.61776139	-6.0705705
ARL5B	-0.234682	2.04574887	-1.0511718	0.29799287	0.61776139	-5.6660838
AKIP1	0.1918013	4.71621905	1.05095327	0.298092307	0.61786584	-6.3686067
GCA	-0.396722	0.29789263	-1.0507495	0.298185046	0.61795637	-5.2810687
MED30	-0.1135787	3.62628558	-1.0504849	0.298305446	0.61810419	-6.0884713
HOXA11	-1.0739386	-1.3653077	-1.0502796	0.298398944	0.61819623	-4.9969947
KIF15	-0.9021685	2.66993304	-1.0498415	0.298598429	0.61850778	-5.5127526
LLPH	-0.1308152	4.16179789	-1.0490819	0.298944609	0.61875313	-6.201192
RANBP9	0.10729115	6.18644748	1.04900503	0.29897964	0.61875313	-6.4035619
NUFIP1	0.1626672	3.72244504	1.0489377	0.299010339	0.61875313	-6.0805698
RPL36AL	-0.09985	6.04181798	-1.0488885	0.299032759	0.61875313	-6.4018029
HMMR	-0.3772635	3.07851368	-1.0487765	0.299083834	0.61875313	-5.754529
BCAS4	0.55198969	2.85991351	1.04875997	0.299091384	0.61875313	-5.8068388
DUSP12	0.10799661	4.95222186	1.04875692	0.299092772	0.61875313	-6.3317515
ENSCAFG00000003715	-0.6935093	-2.3241724	-1.0487195	0.29910986	0.61875313	-4.9668052
GPRC5B	-0.3170689	4.22066034	-1.0483561	0.299275598	0.61899432	-6.2803615
FIGN	-0.5255117	-0.2843656	-1.0478615	0.299501287	0.61935942	-5.1553813
KHDRBS3	0.29918942	5.32054561	1.04737946	0.299721399	0.61971286	-6.2551021
RNF130	0.13303369	5.67525135	1.04725506	0.299778215	0.6197286	-6.4049983
ESAM	-0.9029579	-0.3414123	-1.0470413	0.299875874	0.61978899	-5.4416018

RPH3AL	0.62448033	-0.8483418	1.0469757	0.299905833	0.61978899	-5.2287721
CDC14A	-0.2162754	3.23368171	-1.0467123	0.300026216	0.61993606	-6.0838499
PROSER1	-0.1149686	6.10252654	-1.0461381	0.3002887	0.62037666	-6.4005417
DHX29	-0.1119868	5.8660975	-1.0459845	0.300358956	0.62042004	-6.3973705
CXADR	0.49308323	1.7274725	1.04485776	0.300874564	0.62136791	-5.6311879
TSPAN31	0.09095518	4.18126762	1.04466962	0.300960724	0.62136791	-6.2231005
IER5	-0.2938242	1.61917127	-1.0445958	0.300994519	0.62136791	-5.5547797
CHST11	0.48191939	4.5217534	1.04444162	0.301065157	0.62136791	-6.2404655
SEZ6L	0.86069499	-1.6984945	1.04441809	0.301075933	0.62136791	-5.2239821
PLEK	-0.5316985	-1.0092032	-1.0443354	0.301113824	0.62136791	-5.1728874
MSH3	-0.1393086	5.14342405	-1.0439455	0.301292507	0.62163479	-6.3244948
CHD2	-0.1114988	6.03091372	-1.043507	0.301493505	0.62194762	-6.4098282
PPP1R15A	-0.2119965	5.97397542	-1.0431733	0.301646548	0.62210399	-6.4099923
LRRC47	-0.0860109	5.84407812	-1.0431264	0.301668081	0.62210399	-6.3963271
CDH10	0.76145642	-1.3334104	1.04282237	0.301807555	0.62228973	-4.9668035
CPT1A	0.14009134	7.36442965	1.0425489	0.301933063	0.62240955	-6.3897262
ENSCAFG00000008456	-0.1248831	5.11124588	-1.0424804	0.301964493	0.62240955	-6.3482119
ETFRF1	0.22188228	1.63074331	1.041838	0.302259487	0.62291566	-5.6185048
TMEM242	-0.1869097	2.94224814	-1.0416115	0.302363549	0.62302818	-5.958126
ENSCAFG00000003530	-0.2643523	2.9471984	-1.0407773	0.302746977	0.62371621	-5.9581436
RPS6KA4	-0.1295164	5.05835719	-1.0404726	0.302887122	0.62390289	-6.3107901
GRK5	-0.1734672	5.57965498	-1.0401056	0.303055985	0.62414866	-6.410564
SPA17	0.32423994	0.666195	1.03983499	0.303180511	0.62424097	-5.4105579
SUMF2	0.09492026	5.16620339	1.03979282	0.303199924	0.62424097	-6.3575138
SYNGAP1	0.17923455	2.42375611	1.03965281	0.303264379	0.62427163	-5.8255371
ERCC6L	-0.9593735	0.91111004	-1.039359	0.30339967	0.62436471	-5.1474366
GIPC1	0.12569583	5.25452954	1.03933931	0.30340873	0.62436471	-6.373924
MRTFB	-0.1751177	4.72225271	-1.0389517	0.303587269	0.62463006	-6.3392163
BRMS1	-0.1385403	3.32926691	-1.0387198	0.303694121	0.62474786	-5.9724614
ENSCAFG00000005717	0.86611724	0.82795679	1.03849592	0.303797312	0.62485809	-5.4320202
CNTNAP5	0.63458164	-1.1482531	1.0375186	0.304248023	0.62560897	-4.9805109
SLC35B4	0.11638583	4.10365402	1.03748894	0.304261712	0.62560897	-6.1835725
GADD45A	0.20208927	5.76250786	1.03735958	0.304321403	0.62561734	-6.3911221
SLC25A14	0.26117358	2.41410269	1.03726486	0.304365119	0.62561734	-5.7613752
RHOBTB1	-0.1862696	3.58762614	-1.0364361	0.304747784	0.62630169	-5.9157279
SRGN	0.49810094	1.57428248	1.03620959	0.304852421	0.62641453	-5.6358865
ATAD5	-0.485902	2.70932514	-1.0355013	0.305179772	0.62695026	-5.6402171
ENSCAFG00000026308	-0.3963033	0.76155217	-1.0354302	0.305212684	0.62695026	-5.2665841
LMF1	-0.1823605	3.61145642	-1.0352937	0.305275787	0.62697763	-6.0685768
SH3BGRL2	0.61355604	-1.3153317	1.03466116	0.305568418	0.62744053	-5.0685039
KAT2A	-0.1200965	4.81763988	-1.0345912	0.305600797	0.62744053	-6.3232442
TENT4B	-0.1272893	4.84648421	-1.0343804	0.305698348	0.62753853	-6.3066514
ASB8	-0.1137628	3.48773553	-1.0341405	0.305809432	0.62759464	-6.0528985
REL	-0.2129001	2.70020701	-1.0341061	0.305825334	0.62759464	-5.7287265
BAZ2A	-0.116211	6.42802852	-1.0338421	0.305947595	0.62774327	-6.4200715
ITGA10	0.33921751	3.50549859	1.03372422	0.306002217	0.62775308	-6.4067177
SRP19	-0.2540504	1.19199019	-1.0335446	0.30608541	0.62778035	-5.4363393
ENSCAFG00000008308	0.08451684	5.07321455	1.03342134	0.306142539	0.62778035	-6.3588145
METTL26	0.17081269	4.18604104	1.03327336	0.306211112	0.62778035	-6.2620475
CRK	0.08813496	4.88769663	1.03325855	0.306217973	0.62778035	-6.3347532

COA7	-0.1776875	2.97722032	-1.0331577	0.306264708	0.62778035	-5.9001935
TPK1	0.15539397	4.25229933	1.03298816	0.306343305	0.62783929	-6.2133824
SERPINB6	-0.1590775	7.03981694	-1.0327885	0.306435853	0.62792679	-6.4186656
ENSCAFG00000030168	0.11104694	4.70502461	1.0326808	0.30648581	0.62792701	-6.3076822
CEP55	-1.0149717	1.88216827	-1.03255	0.306546457	0.62794913	-5.3171229
CDK8	0.20965157	5.02146807	1.03197602	0.306812747	0.62839242	-6.245526
FAM167A	-0.5720077	-1.7383792	-1.0315768	0.306998036	0.62866969	-5.0535641
SNX33	-0.2223696	6.53793795	-1.0311405	0.307200631	0.62888906	-6.4193596
CYP27C1	0.29519499	4.43888859	1.03105378	0.307240922	0.62888906	-6.1353258
POR	-0.1033795	5.39499835	-1.030829	0.307345352	0.62888906	-6.3973973
PTOV1	0.15282966	4.68508866	1.03077424	0.307370785	0.62888906	-6.2938715
DOT1L	-0.1524575	4.70060373	-1.030599	0.307452232	0.62888906	-6.2541368
TBCC	0.16036889	5.1762559	1.03058905	0.307456837	0.62888906	-6.364494
PDHA1	-0.0995814	7.57271292	-1.0305321	0.307483286	0.62888906	-6.3877356
DECR1	0.27800343	3.63776684	1.03048632	0.307504584	0.62888906	-6.0959015
ENSCAFG00000000713	0.10456239	3.86513503	1.0298702	0.307791026	0.62932078	-6.1835286
COA1	0.15011616	4.38734178	1.02981735	0.307815604	0.62932078	-6.2457569
SCYL3	0.13181944	4.00631732	1.02940383	0.308007971	0.62948837	-6.2172253
AGL	0.19625404	6.29665902	1.02935174	0.308032206	0.62948837	-6.4199103
FDFT1	-0.2033693	6.33989262	-1.0293189	0.3080475	0.62948837	-6.4175136
IK	-0.0479492	6.90210456	-1.0290174	0.308187805	0.62967293	-6.4186242
ENSCAFG00000024530	-0.200607	1.88294126	-1.0287779	0.308299297	0.62976394	-5.6693389
FERMT1	-0.7219119	-1.5671616	-1.0287069	0.308332344	0.62976394	-4.9518386
BEND3	0.17448341	3.20151303	1.02816992	0.308582456	0.63007708	-5.92952
GCNT7	0.69793586	0.3277087	1.02816295	0.308585703	0.63007708	-5.3820427
CBY1	0.21200294	2.66292295	1.02785708	0.308728226	0.63026592	-5.7798969
ENSCAFG00000029800	-0.4907048	0.07075847	-1.0277359	0.30878469	0.63027904	-5.2813195
ENSCAFG00000018434	-0.3224081	0.08185418	-1.02726	0.309006577	0.63048104	-5.2123323
ENSCAFG00000012593	-1.1447192	2.8561408	-1.0271931	0.309037761	0.63048104	-5.47449
STOX1	0.93588592	-1.0908918	1.02718227	0.309042815	0.63048104	-4.9399082
TLE1	-0.2406898	5.96561085	-1.027083	0.309089106	0.63048104	-6.402043
KIDINS220	0.11421026	7.96278709	1.02698692	0.309133923	0.63048104	-6.37098
SLC2A12	0.35534632	1.81297361	1.02630051	0.309454207	0.63098957	-5.5535013
NFAT5	-0.1726095	4.84414819	-1.0262379	0.309483451	0.63098957	-6.3630433
NDST3	0.73571721	-0.6920199	1.026053	0.309569754	0.63106338	-5.0227097
NMT2	-0.1171821	4.98830429	-1.0257366	0.3097175	0.63126149	-6.3993669
CNOT7	-0.1048081	5.80663038	-1.0256303	0.309767169	0.63126149	-6.4135215
ISLR	0.51061955	2.10660697	1.02548329	0.309835822	0.63126748	-5.873334
PRRT3	0.51817299	-0.0248299	1.0254094	0.309870343	0.63126748	-5.3187334
TMEM136	0.51795292	-1.3201682	1.02509273	0.310018316	0.6314668	-5.0020727
FABP3	-0.5494891	6.52977842	-1.0248644	0.310125045	0.63158206	-6.4049112
SQLE	-0.2937836	7.17496977	-1.0246988	0.310202446	0.63163757	-6.3821643
DCP2	-0.2826834	1.31176844	-1.0245538	0.310270236	0.63167349	-5.5376252
NCOA7	-0.2087905	4.22365725	-1.0243795	0.310351769	0.63173738	-6.2742176
PES1	-0.1008313	5.61192903	-1.0241491	0.310459538	0.63183877	-6.4143161
GSTO1	0.11568215	5.39642436	1.02405853	0.310501904	0.63183877	-6.3956143
ACSF2	0.22750115	2.40917381	1.02379637	0.310624569	0.63198628	-6.0210306
CNEP1R1	-0.1431325	2.66974265	-1.0236661	0.310685552	0.63200827	-5.8366912
DNAJC19	-0.1227081	4.58438657	-1.0234333	0.310794505	0.63212782	-6.2754748
MELK	-0.5517096	3.060461	-1.0230319	0.310982445	0.63240796	-5.6803481

CECR2	-0.2433994	1.85993973	-1.0227851	0.31109806	0.63254095	-5.7019158
ENSCAFG00000030184	-0.2255325	2.94909404	-1.022574	0.311196977	0.63257986	-5.9769523
CCDC160	0.9843209	-1.0627929	1.02247066	0.311245381	0.63257986	-4.9552287
DTX4	0.42655705	0.98594626	1.0224227	0.311267858	0.63257986	-5.325272
TSPAN6	0.21628298	4.83742799	1.02152991	0.311686436	0.63332833	-6.3341005
CASC1	-0.2273154	1.30129988	-1.0213276	0.311781328	0.63341897	-5.4575446
NUP58	-0.1158469	5.62903494	-1.0209531	0.311957066	0.63367379	-6.3851605
GOLM1	0.26817977	6.25731834	1.02073751	0.312058263	0.63377715	-6.4098733
FGF10	0.94195987	-0.5108844	1.02052147	0.31215969	0.63387227	-5.0537146
RASGEF1B	-0.539162	1.28053752	-1.0204234	0.312205747	0.63387227	-5.7337957
FBXW7	0.14538572	3.6125328	1.02016891	0.312325262	0.63401273	-6.1195956
ENSCAFG00000010493	0.10660963	6.71690484	1.01996428	0.312421388	0.63410567	-6.4342706
MIB1	-0.1129349	5.69642579	-1.0198446	0.312477596	0.63411758	-6.4126157
RAB11FIP4	0.53628966	-1.0306189	1.01902975	0.312860646	0.63461253	-5.1154914
CEP164	0.10173782	5.65834274	1.01901849	0.312865937	0.63461253	-6.4159132
RAB11FIP1	0.27352398	6.89460102	1.01877351	0.312981157	0.63461253	-6.4319516
COPB2	0.09611653	8.23668695	1.0187671	0.312984172	0.63461253	-6.3623803
TOR1A	0.13580015	4.00540975	1.01862821	0.313049509	0.63461253	-6.147186
CCDC32	-0.1764842	1.96487669	-1.0185024	0.313108682	0.63461253	-5.7315587
FANCA	-0.5395635	1.67519394	-1.0184404	0.313137887	0.63461253	-5.380048
ERG	0.49475467	4.79146538	1.01837198	0.313170066	0.63461253	-6.1570872
STK10	-0.1702478	4.79264554	-1.0182738	0.31321629	0.63461253	-6.3221872
TSC2	0.10675092	6.44352892	1.01811764	0.313289765	0.63461253	-6.4366595
SOX13	0.64838318	1.37717036	1.01804245	0.313325157	0.63461253	-5.4841376
STARD5	-0.2537441	1.42972869	-1.0180405	0.313326081	0.63461253	-5.5148502
ARFGEF1	-0.1137308	5.85718997	-1.0177102	0.313481577	0.63482539	-6.4325997
IL2RG	-0.5659798	-2.3344466	-1.0174917	0.313584485	0.63483928	-5.0055266
PROS1	0.39604823	6.27795821	1.01736711	0.313643163	0.63483928	-6.3917185
ENSCAFG00000013781	0.83916279	0.30418088	1.01719928	0.313722227	0.63483928	-5.106178
BRPF1	-0.1046766	4.45532744	-1.0170318	0.313801161	0.63483928	-6.2756989
SGPL1	0.15051132	6.0470342	1.01698729	0.313822112	0.63483928	-6.4186824
ETS1	-0.2296101	5.95281965	-1.0169525	0.313838507	0.63483928	-6.4272133
ZNF283	-0.2390774	1.36713309	-1.0169467	0.313841234	0.63483928	-5.5331738
ENSCAFG00000032123	0.17200746	3.5167827	1.01649795	0.314052764	0.63516516	-6.0448948
FBLN1	0.62910302	4.25980957	1.01591017	0.314329964	0.635401	-6.4199487
DLD	-0.0921458	6.33118543	-1.0158519	0.314357435	0.635401	-6.4386974
CCNL2	-0.0947609	4.52648872	-1.0156252	0.314464414	0.635401	-6.3015587
FBF1	-0.2115255	2.76174151	-1.0154349	0.314554248	0.635401	-5.9232209
ASTN1	-1.0527867	0.15382447	-1.0153874	0.314576653	0.635401	-5.5258025
PSMB1	0.09696899	6.57716184	1.01534013	0.314598955	0.635401	-6.4391147
ANP32A	-0.1558661	5.3825945	-1.0153108	0.314612806	0.635401	-6.4091587
TRAK1	0.23229794	6.77048796	1.01525204	0.314640539	0.635401	-6.4335635
PNN	-0.1203441	5.66520832	-1.0151434	0.314691836	0.635401	-6.4154556
C1R	0.7417214	4.94705875	1.0149452	0.314785408	0.635401	-6.2912078
CYP2J2	0.97761674	0.3510108	1.0149234	0.314795703	0.635401	-5.0039327
KLHL28	-0.1490781	4.3907547	-1.0148807	0.314815848	0.635401	-6.3024632
MAN1C1	0.41478031	3.08858153	1.01478593	0.314860624	0.635401	-6.1378847
WWC2	0.12080964	7.03334786	1.01471592	0.314893689	0.635401	-6.4352522
TMEM131L	-0.1298941	5.23125617	-1.0146474	0.314926045	0.635401	-6.4080747
ESYT1	0.15145154	8.51296574	1.01446291	0.315013206	0.63547507	-6.3609373

UBE2V2	0.15729886	5.22334815	1.01389525	0.315281466	0.63584283	-6.3653
MFSD12	0.18467051	4.77957413	1.01377819	0.315336807	0.63584283	-6.3665839
RABL3	0.14000787	2.42585986	1.01375673	0.31534695	0.63584283	-5.8299209
STAU1	0.06562474	7.02681184	1.01360513	0.31541863	0.63588557	-6.4314382
XRCC1	-0.124307	4.7184082	-1.0134278	0.315502472	0.63595281	-6.3370514
ENSCAFG00000031973	-0.2555157	3.88438731	-1.0127396	0.315828069	0.63650726	-6.2012614
SMUG1	-0.1094584	4.7079469	-1.0124734	0.315954064	0.63663039	-6.3177494
AURKB	-0.7464316	2.36099139	-1.012397	0.315990249	0.63663039	-5.5101114
FBXO11	-0.100463	6.37563671	-1.0120966	0.316132504	0.63681513	-6.4418586
KMT2B	-0.0863994	6.29536562	-1.0114256	0.31645036	0.63721059	-6.4429662
KLHL26	0.20270058	2.96091659	1.01136296	0.316480043	0.63721059	-6.1056791
TTC7B	0.111669	5.24079255	1.01126057	0.316528573	0.63721059	-6.3770472
RFX7	-0.1277329	5.14488692	-1.0112551	0.316531175	0.63721059	-6.4046067
TCEA2	0.26764748	2.54525449	1.01094729	0.316677084	0.63740245	-5.9976341
MEI4	0.4694137	-0.731203	1.01067281	0.316807239	0.63752262	-5.1338744
FAM13A	0.1284285	3.79652075	1.01051968	0.316879868	0.63752262	-6.3344337
AP4E1	-0.1020674	5.54728759	-1.0104469	0.316914399	0.63752262	-6.4238498
RING1	-0.1029966	5.53780792	-1.0103945	0.31693924	0.63752262	-6.4042033
ASF1A	-0.1994933	2.65511186	-1.0101869	0.317037723	0.63760128	-5.843759
TKFC	-0.1193896	5.00564286	-1.0100869	0.31708521	0.63760128	-6.3851486
HOXD8	-0.7591159	1.36661568	-1.0099921	0.317130202	0.63760128	-5.5736832
KIF5B	0.18989967	8.5576518	1.0097617	0.317239533	0.6377193	-6.3671663
UXT	-0.1364817	3.20073907	-1.0096188	0.317307397	0.63775394	-5.9606879
PELI2	0.57060601	0.56511266	1.00948531	0.317370754	0.63777952	-5.2597283
GCNT2	0.80270253	-1.5469191	1.00923904	0.317487705	0.63791277	-5.0468724
RASA2	-0.1771937	3.3849058	-1.0086185	0.317782503	0.63830397	-6.1673635
ALPK3	0.73195798	-0.360464	1.00861589	0.317783756	0.63830397	-5.0276436
ENSCAFG00000028494	-0.1207361	3.35789891	-1.0082272	0.317968525	0.63850572	-6.063114
ENSCAFG00000010650	0.12380875	4.05915347	1.0081913	0.31798558	0.63850572	-6.1772268
ENSCAFG00000016848	-0.2259341	2.51102615	-1.0079565	0.318097252	0.63862815	-5.852327
TIMP3	0.41956478	9.77982555	1.00727485	0.318421505	0.63917726	-6.1657825
HOMEZ	0.18357819	2.8952021	1.00602452	0.319016888	0.64027035	-5.9520859
AFF2	0.52167184	-1.0199488	1.00562143	0.319208995	0.64055385	-5.1613226
ENSCAFG00000002074	-0.2401266	1.63032561	-1.0055035	0.319265198	0.64056458	-5.5759037
RBM48	-0.1314156	3.06444413	-1.004883	0.319561098	0.64094198	-6.0123676
ANKS1B	0.3347296	1.5528949	1.00488061	0.319562255	0.64094198	-5.7438242
ARMC1	-0.0859867	4.82362896	-1.004789	0.319605953	0.64094198	-6.3482239
ZFAND2B	-0.1295502	4.12797281	-1.0045396	0.319724964	0.64107858	-6.2053601
CARMIL1	-0.3099678	4.71707572	-1.0042428	0.31986662	0.64126054	-6.3636867
SLC25A19	0.20698419	3.49959702	1.00355906	0.320193094	0.64181289	-6.0774348
ACAP2	-0.093807	6.04083014	-1.0034308	0.320254362	0.64183356	-6.4472338
ZDHHC22	-0.5802552	-1.6714846	-1.0031641	0.320381774	0.64193277	-5.0237229
POLA1	-0.2254778	4.61957797	-1.002917	0.320499882	0.64193277	-6.254689
SPHK1	-0.3296823	4.83652505	-1.0029121	0.320502226	0.64193277	-6.3847107
TJP3	-0.5341483	-2.1658848	-1.0029006	0.320507718	0.64193277	-5.0383714
ENSCAFG00000005953	-0.3488872	0.92108945	-1.0025548	0.320673042	0.64207608	-5.3261052
E2F4	-0.1261475	4.29938514	-1.0024425	0.320726711	0.64207608	-6.2860713
EGR2	0.33210675	4.36018588	1.00230866	0.320790739	0.64207608	-6.426144
CAVIN4	-0.6232236	-0.3856723	-1.0022899	0.320799726	0.64207608	-5.0320073
PXMP4	0.19310081	2.98066796	1.00221792	0.320834141	0.64207608	-5.9819624

UQCRB	0.09829426	5.4589748	1.00131366	0.321266848	0.64283991	-6.4137562
SCAMP2	0.13882105	6.31676867	1.00050053	0.321656286	0.64341713	-6.4543034
IMMP1L	0.10735629	3.19489386	1.00049483	0.32165902	0.64341713	-5.9752673
SNX17	0.0851539	6.60980592	1.00039143	0.321708563	0.64341713	-6.4544186
TOB2	-0.1806584	4.17310086	-0.9995807	0.322097206	0.64409214	-6.321318
GNB3	-0.4367675	1.76277897	-0.9993455	0.322210021	0.64419699	-5.4337571
LRRC9	-0.3738081	0.71594171	-0.9990248	0.322363893	0.64419699	-5.3785686
AP2A2	0.09028795	7.85311332	0.99899189	0.322379666	0.64419699	-6.397898
SEPT11	-0.1565642	5.08517736	-0.9989704	0.322389964	0.64419699	-6.4078897
SPTBN1	0.14345803	9.71595261	0.99893837	0.322405351	0.64419699	-6.2388715
JAZF1	0.372533	1.66778256	0.99824154	0.322739862	0.64453308	-5.4556933
CYB5D1	0.29972378	0.01799932	0.99818356	0.322767703	0.64453308	-5.3233271
ZNF227	-0.2537552	2.34667254	-0.9981412	0.322788044	0.64453308	-5.7666199
ENSCAFG00000031288	-0.2104222	5.51633425	-0.9980377	0.322837755	0.64453308	-6.4195001
ENSCAFG00000024573	-0.4661401	0.01990416	-0.9980172	0.32284762	0.64453308	-5.2000364
NDUFA13	0.1157083	4.79355268	0.9978996	0.322904093	0.64453308	-6.3627075
CHL1	-0.9110057	0.37227892	-0.9978363	0.322934489	0.64453308	-5.129304
CCT4	-0.0915642	7.5472218	-0.9977355	0.322982914	0.64453308	-6.4305251
MATN3	0.59037772	0.26883642	0.99744142	0.323124242	0.64471297	-5.1601553
BUB3	-0.1033492	6.29193176	-0.9972262	0.323227699	0.64481586	-6.4552475
ABL2	0.13470106	4.23949364	0.99712113	0.323278197	0.64481586	-6.24284
SLC46A1	0.1513146	4.04912178	0.99675663	0.323453462	0.64497947	-6.1994509
SLC5A6	0.18891199	5.55885027	0.99660614	0.323525844	0.64497947	-6.4308167
DEAF1	-0.1716254	3.74729563	-0.9964592	0.323596522	0.64497947	-6.1948473
PRKAB1	-0.0802492	5.49616918	-0.9963722	0.323638394	0.64497947	-6.4346436
POGK	0.0829483	6.29462641	0.99629297	0.323676501	0.64497947	-6.4567398
XPC	-0.1219337	4.51372865	-0.996218	0.32371255	0.64497947	-6.3415529
TLK1	-0.0936438	5.5440572	-0.9961647	0.323738233	0.64497947	-6.4334467
ESCO2	-1.0889842	2.72533096	-0.9960989	0.323769862	0.64497947	-5.4694814
NUBPL	-0.207321	2.2396784	-0.9959613	0.323836085	0.64500938	-5.7540226
ACACB	0.51423861	0.12233191	0.99579319	0.323917029	0.6450686	-5.607251
RBM11	0.63384529	1.8521771	0.99558505	0.324017232	0.64516615	-5.4953895
ENSCAFG00000004548	0.21852385	1.19823327	0.99531954	0.324145092	0.64531873	-5.5346714
MAPK8	-0.0980627	6.07725722	-0.9949527	0.324321779	0.64556845	-6.4543463
ASAP1	-0.1503446	7.09242465	-0.9946639	0.324460972	0.64574347	-6.4518524
CEBPZ	-0.1234723	5.9878847	-0.9945481	0.324516758	0.64575246	-6.4554719
ENSCAFG00000004543	0.43317287	-0.0654039	0.99410776	0.32472905	0.64607283	-5.2320951
APBB1IP	0.36289762	6.15214018	0.99353525	0.325005191	0.64633497	-6.4238882
MSX2	0.40436933	1.4575407	0.99348139	0.32503118	0.64633497	-5.4516656
BICRA	-0.1872034	3.99297077	-0.9934779	0.325032847	0.64633497	-6.2643255
TSHZ1	-0.2113392	5.17338793	-0.9934091	0.325066057	0.64633497	-6.3922193
CRACR2B	-0.5654671	-0.5427277	-0.9931452	0.325193433	0.64648619	-5.2769473
RNF170	-0.1844561	2.83389846	-0.9929078	0.325308032	0.64656982	-5.8979256
SMARCAL1	0.08769873	4.85239588	0.99284535	0.325338162	0.64656982	-6.3688245
SLC35A1	-0.1600145	3.78850425	-0.9922117	0.325644165	0.64698116	-6.0355851
MBNL2	0.13344667	7.64751337	0.99220407	0.32564787	0.64698116	-6.4386577
IL6	-0.9002421	-0.9117922	-0.9906044	0.326421303	0.64838806	-5.152852
MPDZ	0.12193535	7.34358355	0.99052657	0.326458959	0.64838806	-6.4511749
ABI2	0.10166617	4.87837441	0.99015714	0.32663776	0.64851069	-6.3667113
ENSCAFG00000029903	-0.4901162	-0.6455266	-0.9901282	0.326651759	0.64851069	-5.1339817

ENSCAFG00000030381	-0.5955797	-1.7484528	-0.9900799	0.326675162	0.64851069	-5.05815
DNMT3B	-0.4556444	-0.3599158	-0.989825	0.326798588	0.64852786	-5.2000683
WNT5B	0.38264203	3.48875626	0.98955969	0.326927066	0.64852786	-6.1414553
RMC1	0.13196372	4.26164886	0.98946232	0.326974234	0.64852786	-6.2331925
ENSCAFG00000006508	-0.1345355	3.9477721	-0.9894436	0.326983319	0.64852786	-6.2272665
OVCA2	-0.1416206	3.42083712	-0.9894302	0.32698978	0.64852786	-6.0724422
HSBP1	0.11782174	4.42665521	0.98942414	0.32699273	0.64852786	-6.3238401
DDX5	-0.0845188	9.10195021	-0.9888326	0.327279376	0.64890604	-6.3222083
ELK4	-0.0971784	4.18903325	-0.988818	0.327286443	0.64890604	-6.3221038
DNAJC27	-0.4170374	-0.2525896	-0.9884343	0.327472485	0.64917271	-5.2308428
CSKMT	-0.2317104	2.68744415	-0.987981	0.327692349	0.64950635	-5.8242954
F11R	-0.9862095	0.9126235	-0.9876267	0.32786428	0.64974488	-5.4663375
LRSAM1	0.10783313	4.48189233	0.98736838	0.327989638	0.64989106	-6.2853545
SH2B3	-0.1584038	6.54982222	-0.9871938	0.328074404	0.64995678	-6.4578051
PPP2R5D	-0.0794453	5.94083233	-0.9865718	0.328376475	0.65042473	-6.4569147
ENSCAFG00000008655	-0.4321035	0.0082954	-0.9863932	0.328463244	0.65042473	-5.183134
UFD1	0.09621464	5.11789707	0.9863885	0.328465524	0.65042473	-6.4054426
COPS4	-0.094083	5.78763353	-0.9861813	0.328566218	0.65049196	-6.4529275
AARS2	0.14767425	3.0209945	0.9861061	0.328602761	0.65049196	-5.9571718
TSPAN4	0.15836044	5.17208451	0.98561734	0.32884037	0.65086004	-6.417514
ENSCAFG00000001728	-0.106088	4.31726216	-0.9853183	0.328985815	0.65090313	-6.2838322
HERC3	0.0929443	5.93031061	0.98513719	0.32907391	0.65090313	-6.4655266
LAMB1	-0.162563	9.29820033	-0.9849655	0.329157453	0.65090313	-6.2588415
TMEM140	-0.4122381	0.02941825	-0.9849287	0.329175368	0.65090313	-5.3594756
ALG12	0.17607137	5.4700451	0.98491694	0.329181069	0.65090313	-6.4382815
SPRYD3	-0.1430068	5.47801777	-0.9849011	0.329188756	0.65090313	-6.4454042
LDLRAP1	0.15006865	4.22269306	0.98482899	0.329223867	0.65090313	-6.315312
SLC35A3	0.21877144	3.63165639	0.98461258	0.329329193	0.65095228	-6.1247551
TEX15	0.39402525	0.01087401	0.98456555	0.329352087	0.65095228	-5.2819316
OCLN	-0.6239714	-2.5492486	-0.9841955	0.329532263	0.65120621	-4.9919452
ENSCAFG00000010117	-0.2372459	1.64187154	-0.9838233	0.329713547	0.65146225	-5.6277077
DCAF11	-0.1109633	4.65467928	-0.9836093	0.329817795	0.65156602	-6.3457196
PARD6G	-0.5772565	-0.7047458	-0.9831882	0.330022989	0.65171558	-5.1508351
TNFAIP2	-0.3734367	3.46094956	-0.9831683	0.330032671	0.65171558	-6.2249031
LOX	0.33748729	10.7642102	0.98313543	0.330048719	0.65171558	-6.2434855
CXCL12	0.41315313	4.27505792	0.98294476	0.330141669	0.65177783	-6.468669
CAV1	-0.2347212	6.86145557	-0.9827198	0.330251356	0.65177783	-6.4465404
CREB1	-0.181774	2.83698745	-0.9826361	0.330292201	0.65177783	-5.9088498
SIRT2	-0.1349076	4.98945169	-0.9824957	0.330360647	0.65177783	-6.3650268
STAU2	0.08306145	5.46432727	0.98247779	0.330369391	0.65177783	-6.4311245
INO80	-0.0825995	5.3001308	-0.9823511	0.330431194	0.65177783	-6.4292807
CCNK	-0.0802229	4.39915163	-0.982328	0.330442457	0.65177783	-6.2853445
GLMN	-0.1334555	4.39180325	-0.982069	0.330568824	0.65192499	-6.2718205
PUS7L	-0.2220857	3.08923622	-0.9817871	0.330706413	0.65209424	-6.0484556
HEATR5A	-0.108897	6.67954847	-0.9816563	0.330770234	0.65211799	-6.4725655
RUVBL2	-0.1210051	5.25972829	-0.9812876	0.330950256	0.65227022	-6.4176702
TSPYL4	0.16187044	2.91574569	0.98128609	0.330951012	0.65227022	-5.9676093
ENSCAFG00000006763	-0.6817662	-2.3306515	-0.9810803	0.33105152	0.65236623	-5.0758068
ENSCAFG00000004386	0.10922262	5.42548784	0.98097101	0.331104902	0.65236936	-6.4242768
L3MBTL2	0.11131282	5.17481073	0.98052321	0.331323696	0.65269835	-6.4000539

SGSM2	0.10362624	5.46686239	0.98031627	0.331424843	0.65279552	-6.4443551
FAT4	0.20300692	7.00532165	0.97987525	0.33164046	0.65311472	-6.4612568
ZNF529	-0.2078096	2.48731781	-0.9797727	0.331690605	0.65311472	-5.8269341
ROBO4	-0.7531916	2.94725396	-0.9793975	0.331874159	0.65337401	-6.1119506
PPCS	-0.1035264	3.859732	-0.9792679	0.33193754	0.65339666	-6.2010038
RAB10	0.05853271	6.88484838	0.97913733	0.332001448	0.65342035	-6.4700043
ATP6AP1L	0.79740233	-0.7183946	0.9788009	0.332166117	0.65359797	-5.0416269
COG1	-0.0868084	6.35913511	-0.9787409	0.332195473	0.65359797	-6.4754693
LATS2	-0.1206489	6.97056367	-0.9785223	0.332302501	0.65365873	-6.4621187
ENSCAFG00000004388	0.19537399	2.64706561	0.97846589	0.332330143	0.65365873	-5.900912
ENSCAFG00000029415	-0.6736629	-0.4298036	-0.9779181	0.332598443	0.65399947	-5.1091647
YWHAG	0.10762685	6.34499482	0.97790022	0.332607225	0.65399947	-6.4762543
ARHGAP20	-0.449468	3.70684271	-0.9774794	0.332813439	0.65406494	-6.2723335
HS1BP3	-0.146759	5.81040316	-0.9774386	0.332833457	0.65406494	-6.4598703
CCDC86	-0.1490596	3.80215725	-0.9773429	0.332880352	0.65406494	-6.144239
BMP4	0.46425514	4.19880514	0.97733563	0.332883931	0.65406494	-6.3645887
SDHB	0.10469525	4.35068869	0.97730254	0.332900152	0.65406494	-6.2836333
MTA3	0.14565468	3.65520741	0.97717895	0.332960749	0.65408197	-6.0976263
SLC39A6	0.12342596	6.24105441	0.97690243	0.33309635	0.65414096	-6.4759021
PELP1	0.12082881	5.56461709	0.97674603	0.333173061	0.65414096	-6.4455109
SNX29	-0.1465901	4.57081467	-0.9767357	0.333178128	0.65414096	-6.278998
ENSCAFG00000013076	0.11888414	4.11111875	0.97657993	0.333254541	0.65414096	-6.2324695
PCNP	-0.0926599	6.06642367	-0.9765754	0.333256742	0.65414096	-6.4730596
MIEF2	0.1967919	3.10430144	0.97641527	0.33333533	0.65414096	-5.9219417
SLC40A1	0.58419781	1.23617283	0.9763766	0.333354304	0.65414096	-5.8612869
ENSCAFG00000019210	-0.2045355	3.7685408	-0.9761312	0.333474728	0.65427534	-6.0368751
HSPA8	0.14099362	9.57172727	0.97558221	0.333744263	0.65435016	-6.3217026
ENSCAFG00000030568	-0.2016938	2.04697671	-0.9755464	0.333761848	0.65435016	-5.6958926
LIPT2	-0.1915932	3.4538448	-0.9754998	0.333784714	0.65435016	-6.0181467
TP53BP1	0.0959867	5.93107651	0.97541399	0.33382688	0.65435016	-6.4696426
GRAMD1C	0.40131422	-0.1419079	0.97525263	0.333906137	0.65435016	-5.2572706
ST8SIA5	0.66092493	-1.2527317	0.97518614	0.333938799	0.65435016	-5.0710506
GBP6	-0.5132906	0.45181596	-0.9750622	0.333999692	0.65435016	-5.5637849
TMEM205	-0.1954068	3.11532619	-0.9749337	0.334062832	0.65435016	-6.0121542
TMEM243	0.1561818	2.10085109	0.97492084	0.334069149	0.65435016	-5.6846198
ENSCAFG00000028760	0.14647024	4.15723492	0.97481383	0.334121737	0.65435016	-6.2460936
ENSCAFG00000006187	0.12604079	5.58787777	0.97478703	0.334134911	0.65435016	-6.4472958
RASD2	-0.7991808	0.89714886	-0.9747843	0.334136253	0.65435016	-6.1445596
MGLL	-0.6583862	-1.7462477	-0.9746649	0.334194912	0.6543633	-5.0700357
RFC4	-0.2567969	3.99638211	-0.9743606	0.334344531	0.65440429	-6.1229426
RANBP3L	0.11651352	3.35468943	0.97430799	0.334370393	0.65440429	-6.0559011
TUT4	-0.1109605	4.78040631	-0.9741308	0.334457527	0.65440429	-6.3826419
HAUS1	-0.2349666	2.28338053	-0.9741287	0.334458531	0.65440429	-5.7114052
SGMS1	0.11297392	6.34752563	0.97401881	0.334512601	0.65440429	-6.4800948
PPP1R13L	0.24949874	4.73315886	0.97398838	0.334527566	0.65440429	-6.3100698
MRPL58	-0.1478012	3.49226938	-0.9737093	0.334664871	0.65451573	-6.0506427
MAP2K5	-0.1117906	4.54735643	-0.9736613	0.334688459	0.65451573	-6.3602404
FLII	0.06205822	7.74178831	0.97347203	0.334781594	0.65451806	-6.444654
TCF7L1	0.14455279	6.32416456	0.97344768	0.334793574	0.65451806	-6.480729
ENSCAFG00000023820	0.27252439	1.36250777	0.97328746	0.334872426	0.65453632	-5.7423971

C3AR1	-0.7043189	-2.1272067	-0.9731779	0.334926357	0.65453632	-4.9874281
PPP5C	-0.0936293	5.16226654	-0.973112	0.334958808	0.65453632	-6.4289557
ZNF674	-0.2322975	1.29179212	-0.9729892	0.335019231	0.65455285	-5.6036715
MSC	-0.5130116	4.90473729	-0.9728377	0.335093833	0.65459707	-6.2487714
LIN7A	0.81710407	-0.5712892	0.97271358	0.335154959	0.65461496	-5.0108135
LYST	-0.1549111	4.53698265	-0.9717618	0.335623882	0.65541595	-6.3549102
PHOSPHO2	-0.1491115	2.98439495	-0.9716569	0.335675582	0.65541595	-5.9858601
RAPGEF1	-0.1652989	5.93860852	-0.9715645	0.335721161	0.65541595	-6.4693236
SWI5	-0.1168199	3.59086345	-0.9713103	0.33584649	0.65545358	-6.1875443
MRGBP	0.21588754	1.31989611	0.97127755	0.335862627	0.65545358	-5.5018113
QPCTL	0.13322211	3.34924491	0.97120879	0.335896541	0.65545358	-6.1042673
SH3BP1	0.29104818	4.54593116	0.97107119	0.335964407	0.65547418	-6.4225288
FLI1	-0.8896124	2.08282782	-0.9709422	0.336028012	0.65547418	-5.4697879
ATG10	-0.3404934	0.4066417	-0.9708709	0.336063213	0.65547418	-5.3277141
CD44	0.17149912	8.95755514	0.9705876	0.336202986	0.65564527	-6.3622673
WDR66	0.3009932	1.80487437	0.97010204	0.336442655	0.65594205	-6.0901211
ENSCAFG00000001367	0.20074151	4.68309642	0.97006829	0.336459318	0.65594205	-6.4159049
KYAT1	0.20435039	2.76103187	0.96972206	0.336630289	0.6561738	-6.0892851
GNE	0.11535358	5.45139751	0.96951937	0.336730405	0.6562239	-6.4406365
IRF2	-0.1408167	4.71478302	-0.9694591	0.336760185	0.6562239	-6.3953959
RBPJ	-0.1252263	4.98744254	-0.9685859	0.337191743	0.65696322	-6.4619798
ARL9	0.52129094	1.52514243	0.96845893	0.337254517	0.6569839	-5.3671967
PPP1R18	-0.0901185	6.82356872	-0.9682004	0.337382387	0.65713137	-6.4811287
DAZAP1	-0.142466	5.48528904	-0.967714	0.337623017	0.65742483	-6.4284425
PIAS2	-0.124636	4.62921868	-0.9676848	0.337637441	0.65742483	-6.3524457
PLA2G4B	-0.3380984	0.64942373	-0.9675764	0.337691132	0.65742774	-5.368914
CEP76	-0.1690227	3.24101638	-0.9672335	0.337860862	0.65765653	-5.9933207
EFR3A	-0.0779574	6.20070185	-0.9669609	0.337995801	0.65781754	-6.4867426
LYN	-0.1628019	6.49023511	-0.9657916	0.338575207	0.6588434	-6.4777218
BRD7	0.12632858	6.31774415	0.96551192	0.338713878	0.65893552	-6.487935
LGALS1	0.19729031	8.62081572	0.96544515	0.33874699	0.65893552	-6.392779
ENSCAFG00000032192	-0.2486046	4.58445275	-0.9653796	0.338779487	0.65893552	-6.3407778
CSNK1G3	-0.0912275	6.09513229	-0.9651024	0.338917	0.65895222	-6.4864663
ZEB2	0.16773864	6.93087025	0.96509975	0.338918314	0.65895222	-6.4776217
ELP5	-0.1217767	4.02586968	-0.9650459	0.338945019	0.65895222	-6.260289
ENOSF1	0.25178572	0.91234719	0.96490044	0.339017203	0.65899085	-5.5973585
CENPU	-0.8084312	0.4220799	-0.9646913	0.339121009	0.65909092	-5.1576564
ENSCAFG00000030398	0.50082284	-0.4578446	0.96453657	0.339197786	0.65909791	-5.1770224
ENSCAFG00000009702	0.14000297	3.94183251	0.96440114	0.339265015	0.65909791	-6.265418
VDAC3	-0.1230326	5.12729318	-0.96431	0.339310268	0.65909791	-6.4192337
CNOT3	-0.0946279	5.08685115	-0.9641754	0.339377077	0.65909791	-6.4191049
CUL5	-0.1130331	6.08414929	-0.964157	0.339386235	0.65909791	-6.4855369
CRYBG1	-0.3378889	5.88627852	-0.9638992	0.339514259	0.6592155	-6.4872134
MRPS23	-0.137313	3.25073614	-0.9638237	0.339551746	0.6592155	-6.0552272
ENSCAFG00000003222	-0.1058089	4.66987362	-0.9637189	0.339603793	0.6592155	-6.3500647
RANGRF	-0.2323998	1.61888673	-0.9635838	0.339670909	0.65924419	-5.5433367
HELQ	0.10193376	4.25992791	0.96303848	0.339941939	0.65946135	-6.320467
RIN2	-0.205038	5.31203648	-0.9630315	0.339945396	0.65946135	-6.4881468
TMEM251	-0.1825938	2.02343213	-0.9629625	0.339979687	0.65946135	-5.6656569
ARHGAP25	-0.5849548	-2.2240503	-0.9629373	0.33999222	0.65946135	-5.0593852

CNOT6	-0.1257899	4.68291358	-0.9625715	0.340174132	0.65968458	-6.3509809
ENSCAFG00000018740	-0.3198892	1.59556987	-0.9624952	0.340212055	0.65968458	-5.4522141
COPS6	-0.0802445	5.49200929	-0.9619847	0.340466006	0.66007539	-6.4574591
FAM102A	0.25503521	3.56187113	0.9618579	0.340529131	0.66009617	-6.1708841
NEO1	0.37291908	5.35349387	0.96159366	0.340660648	0.66021797	-6.329668
NDUFA12	-0.0916589	4.32727723	-0.9615211	0.340696791	0.66021797	-6.3118599
KLHL5	-0.1684321	6.51513014	-0.9608681	0.341021961	0.66065154	-6.4849575
EIF4E	0.14528845	5.29421434	0.9608611	0.341025431	0.66065154	-6.4491451
TMEM42	0.36186852	0.11846557	0.96042466	0.341242885	0.66082694	-5.3539359
ZDHHC4	0.10745697	3.95239122	0.9603893	0.341260506	0.66082694	-6.2161449
ATP2A1	0.29058719	0.90411635	0.96027661	0.341316669	0.66082694	-5.526425
MRPS21	0.10606918	4.02810215	0.96021383	0.341347962	0.66082694	-6.2627727
BSDC1	0.09152222	6.07720656	0.96015132	0.341379121	0.66082694	-6.4933755
ARL10	0.23292345	1.53203891	0.96004775	0.34143075	0.66082694	-5.5209321
ENSCAFG00000026225	0.29640405	-0.1989667	0.95954817	0.341679865	0.66120749	-5.2564697
ENSCAFG00000031019	-0.1203428	4.53765282	-0.9592717	0.341817773	0.66137276	-6.3231116
ENSCAFG00000019758	-0.1611888	3.01216071	-0.9588902	0.34200815	0.66163948	-5.9910591
TIMM17B	0.11928764	3.7479193	0.95847874	0.342213534	0.66189793	-6.1473145
CCDC137	0.16553775	3.06569953	0.95841203	0.342246843	0.66189793	-6.0394153
S100P	0.40697903	-1.195817	0.95793939	0.342482887	0.66225275	-5.329469
UQCRFS1	-0.1088766	5.46164077	-0.957498	0.342703443	0.6625096	-6.4651042
ENSCAFG00000016002	-0.2616155	1.972373	-0.957463	0.342720906	0.6625096	-5.5630332
CALHM2	-0.2139831	3.52570427	-0.9571483	0.342878225	0.662712	-6.2616638
TBL3	0.08560501	5.65364242	0.9570421	0.34293131	0.66271291	-6.4599125
DEPDC1	-0.9604063	1.74539171	-0.9568191	0.34304283	0.66282674	-5.3212502
PCCA	-0.1359049	3.85840974	-0.9563544	0.343275255	0.66294748	-6.2410602
GPRIN3	0.74066437	-2.0335444	0.95630903	0.343297949	0.66294748	-5.0069079
BASP1	-0.5598734	3.06471381	-0.9562805	0.343312233	0.66294748	-5.5911124
SCAF1	0.08265887	6.37954269	0.95627326	0.343315845	0.66294748	-6.4967897
PI4KA	0.09628162	7.23209493	0.955654	0.343625775	0.66344425	-6.4731465
NDRG4	0.15271088	5.96510362	0.95529087	0.343807601	0.66365743	-6.4951312
ENSCAFG00000024878	-0.3677893	-0.6505059	-0.9552231	0.343841566	0.66365743	-5.2857605
ZNFX1	0.08273794	7.67022489	0.95503765	0.343934431	0.66373496	-6.4560141
ENSCAFG00000020257	-0.2332723	1.29342049	-0.9549242	0.34399126	0.66374294	-5.5500051
FAM220A	-0.1336925	3.94700571	-0.9542452	0.344331562	0.66423292	-6.3144299
ARAP3	-0.2394475	5.38190667	-0.9541233	0.344392662	0.66423292	-6.4573675
ENSCAFG00000000564	-0.2655911	1.43130544	-0.9540058	0.34445156	0.66423292	-5.6203471
RAB18	-0.0667149	6.41067126	-0.9539967	0.344456134	0.66423292	-6.4992534
CAPN1	0.11824285	7.30455274	0.9537354	0.344587172	0.6643839	-6.4714523
ZNF354C	0.48708746	-1.0399656	0.95352016	0.344695134	0.66449034	-5.0548725
EMSY	-0.1165063	4.60589105	-0.9531068	0.344902517	0.66472389	-6.3845455
TMEM14A	0.23876842	1.55684516	0.95306837	0.344921828	0.66472389	-5.4967575
NSMCE1	-0.101622	4.32096729	-0.9525893	0.345162337	0.66506765	-6.3280723
MROH1	-0.1678703	4.73574964	-0.9524069	0.3452539	0.66506765	-6.3862348
ENSCAFG00000029798	-0.2349815	4.44381308	-0.9523975	0.345258601	0.66506765	-6.3098847
PTDSS2	-0.1227828	4.91061791	-0.952152	0.345381941	0.66520351	-6.3813593
HTT	0.11542687	6.34382658	0.95203232	0.345442044	0.66521755	-6.5003865
ENSCAFG00000030495	-0.1380933	3.62246759	-0.9518184	0.345549542	0.66532284	-6.1029073
PTX3	0.35936585	8.73552927	0.95141904	0.345750229	0.6656075	-6.3480428
ITPK1	0.10026107	4.58218485	0.95127835	0.345820954	0.66564192	-6.3772366

TCAP	0.37740724	0.08738283	0.95084029	0.346041226	0.66587068	-5.3201033
S100A2	-0.2634668	-0.0537232	-0.9508317	0.346045527	0.66587068	-5.3565335
RNF125	0.47602841	0.66246088	0.95052068	0.346201991	0.66602168	-5.3566049
COX8A	0.16117395	5.03835696	0.9504655	0.346229754	0.66602168	-6.4248338
SPHK2	0.15110082	4.19748494	0.9501731	0.346376883	0.66609473	-6.2296978
POLD3	-0.2167591	2.66447904	-0.9501496	0.346388706	0.66609473	-5.8259975
SPRED2	0.17615252	4.98356029	0.95007477	0.346426373	0.66609473	-6.4845278
RAI1	0.13107768	5.29270093	0.94910585	0.34691426	0.66689764	-6.4514194
HCN3	-0.3573726	-0.1529598	-0.9489333	0.34700117	0.66689764	-5.2470659
UMPS	-0.0928182	5.29362568	-0.9489301	0.347002787	0.66689764	-6.4576545
RAC1	-0.0892468	6.8345529	-0.9484366	0.347251519	0.6672001	-6.4983491
N4BP1	-0.0923612	5.70337341	-0.9482852	0.347327826	0.6672001	-6.4805738
CMTM3	0.44476356	0.08084319	0.94823419	0.347353556	0.6672001	-5.2978983
CDK19	0.08577846	6.10398725	0.94809729	0.347422584	0.6672001	-6.5012256
RPUSD1	0.15675412	4.12486845	0.94809248	0.347425012	0.6672001	-6.3077422
IFNGR1	-0.1520372	5.65213029	-0.947902	0.347521086	0.66728287	-6.4965345
GRAMD2B	-0.1666896	5.08865301	-0.9477898	0.347577655	0.66728976	-6.4561948
LLGL1	-0.1154232	5.2676831	-0.9475554	0.3476959	0.66741505	-6.4799202
PPP1R11	0.12486351	4.53822061	0.94728118	0.347834271	0.66743814	-6.3497406
ALOXE3	0.58933044	-0.4832732	0.94721242	0.347868969	0.66743814	-5.121894
ZFP28	-0.1513616	3.43279006	-0.947211	0.3478697	0.66743814	-6.1680667
PBX2	-0.1407835	3.77778813	-0.9470803	0.34793564	0.66743814	-6.2513606
VPS54	0.11633903	5.09997334	0.94700656	0.347972871	0.66743814	-6.4185173
UTP15	-0.1171219	4.33097734	-0.9463362	0.348311371	0.6678768	-6.3012631
ENSCAFG00000032075	-0.1471873	2.87580857	-0.9462945	0.348332444	0.6678768	-5.9405632
NR2C2AP	0.17929367	1.55982909	0.94623865	0.348360634	0.6678768	-5.5905215
SLC10A3	0.13116822	5.47661251	0.94611198	0.348424628	0.66789783	-6.4766423
SPATA6L	0.25879749	1.21536238	0.94583443	0.348564866	0.66806498	-5.6731135
MOB2	0.12511776	4.79783419	0.94531414	0.348827852	0.66846731	-6.4392558
ZNF571	0.50656551	-0.7675341	0.94520864	0.348881192	0.66846783	-5.1667385
RC3H1	-0.1566773	4.2533027	-0.9451001	0.348936058	0.66847127	-6.3445742
TRAF3IP1	-0.1644741	2.34177813	-0.9448361	0.349069604	0.66859349	-5.9768157
STIM1	0.2157689	4.7352015	0.94467126	0.349152979	0.66859349	-6.4937488
ARHGEF37	-0.6763501	1.81848531	-0.9445883	0.349194974	0.66859349	-5.3156403
PIP4P2	-0.3383088	1.00760958	-0.9445543	0.349212176	0.66859349	-5.5233161
TCF7	-0.6260025	-2.1715833	-0.9440719	0.349456254	0.66895911	-5.0078506
ANO3	-0.5475923	-2.8476093	-0.9439524	0.349516776	0.6689733	-4.9934864
EIF4H	-0.0556563	8.42238007	-0.9437419	0.349623331	0.66907558	-6.4259494
UBL3	-0.111228	6.66444386	-0.9436227	0.349683686	0.66908943	-6.5075878
MED12	-0.0759722	6.12680535	-0.9431593	0.349918442	0.66939757	-6.5052699
TMUB2	0.10909753	3.90991936	0.94286237	0.35006887	0.66939757	-6.2714018
SYS1	0.1984223	3.06375184	0.94277666	0.350112305	0.66939757	-6.0459528
BLNK	0.9190231	-0.6839966	0.9427736	0.350113855	0.66939757	-5.048853
TMEM230	0.12530494	4.84171176	0.94276127	0.350120102	0.66939757	-6.4032339
PIGW	-0.2446537	1.4631683	-0.942657	0.350172958	0.66939757	-5.540916
ESPL1	-0.7765379	3.09332204	-0.9424547	0.350275477	0.66939757	-5.7001632
ENSCAFG00000009225	-0.2504637	2.02838789	-0.9424159	0.350295145	0.66939757	-5.5926783
APOE	0.63719317	7.0498555	0.94226903	0.350369631	0.66939757	-6.4625253
GPR155	0.12143058	4.75567718	0.94216112	0.350424348	0.66939757	-6.4263211
CHMP2B	-0.1271262	3.98627689	-0.9421513	0.350429308	0.66939757	-6.2846603

ZNF789	-0.3518542	0.36279594	-0.941849	0.350582644	0.66958893	-5.3148814
FBLN7	0.9555657	1.54813876	0.94149349	0.350763008	0.66983185	-5.1399426
ID3	0.3356705	5.28451769	0.94110601	0.350959659	0.67010578	-6.4250489
TBC1D14	-0.1281663	4.20186309	-0.9409611	0.351033238	0.67014469	-6.346174
ENSCAFG00000016809	-0.1558352	3.84733671	-0.9407376	0.351146682	0.67025968	-6.2682505
C5AR2	0.49210336	-1.6458423	0.94059073	0.351221278	0.67030049	-5.2077028
ENSCAFG00000003676	-0.2020282	2.92305277	-0.9401845	0.351427638	0.67047415	-5.919159
NOS1AP	0.55011678	-1.3894006	0.93996456	0.351539371	0.67047415	-5.0924524
EIF2B1	0.12123478	4.98046668	0.93974332	0.351651806	0.67047415	-6.4267214
VRK1	-0.2164449	3.16773849	-0.9396281	0.35171037	0.67047415	-6.0523742
LRRK2	0.1313826	7.17560262	0.93962235	0.351713289	0.67047415	-6.5006327
LAMTOR4	0.09977892	4.49759311	0.93950368	0.351773614	0.67047415	-6.3173761
ENSCAFG00000006000	0.09322861	6.48250307	0.93949074	0.351780193	0.67047415	-6.5096243
SLC8B1	0.25162099	4.75470181	0.93935006	0.351851715	0.67047415	-6.4182076
EFNB2	0.67934271	2.14178716	0.93934952	0.351851991	0.67047415	-5.6152718
CSF1R	-0.5722045	-0.8433924	-0.9393169	0.351868587	0.67047415	-5.256608
UBL5	0.12861512	5.62891475	0.93925462	0.351900245	0.67047415	-6.4997133
GCKR	0.51971862	1.58591201	0.93913077	0.351963223	0.67047415	-5.3582422
NAGPA	0.1340093	5.39759444	0.9389852	0.352037257	0.67047415	-6.4704514
RRAS	0.10148035	5.37173459	0.93894544	0.352057479	0.67047415	-6.4736066
ANGPTL2	0.26645724	6.41508378	0.93877733	0.352142993	0.67051213	-6.5056226
PLAGL2	0.11453825	4.38222654	0.93869694	0.352183889	0.67051213	-6.3927369
ENSCAFG00000029455	0.36778869	2.21753115	0.93849959	0.352284302	0.67060194	-5.6225272
MAP3K21	-0.2255362	2.17385093	-0.9379258	0.352576357	0.67085967	-5.8772823
RYBP	0.12034138	3.07941527	0.93782019	0.352630124	0.67085967	-6.051974
ENSCAFG00000031711	-0.1293539	4.96628153	-0.9377452	0.352668313	0.67085967	-6.4368143
DET1	-0.1269507	4.14450989	-0.9374533	0.352816974	0.67085967	-6.2682084
ENSCAFG00000029083	0.60050356	0.35945969	0.93744703	0.352820159	0.67085967	-5.2533757
ZMAT5	0.15003074	1.87751319	0.93740141	0.352843397	0.67085967	-5.7099869
NCKAP1	-0.0724289	8.86058113	-0.9373088	0.352890581	0.67085967	-6.4015238
ZC3H13	-0.0761661	5.8741557	-0.9372965	0.352896842	0.67085967	-6.5097735
CIB1	0.07836641	5.43350031	0.93721617	0.352937763	0.67085967	-6.4629088
BIN3	-0.1849312	2.36251218	-0.9371877	0.352952288	0.67085967	-5.8695053
HSP90AA1	0.07972297	8.83434082	0.93704519	0.353024875	0.6708964	-6.3987637
NLE1	-0.1358201	3.30288266	-0.9368135	0.353142951	0.67099528	-6.0739448
COLGALT2	0.69902062	-2.335406	0.93673403	0.35318345	0.67099528	-4.9910632
ACTR1B	0.09990287	6.08626439	0.93659265	0.353255514	0.67103098	-6.4965944
PSMF1	0.09754652	5.64936992	0.93631727	0.353395913	0.67119646	-6.4945712
ACTR5	-0.1660823	2.39374421	-0.9359312	0.353592825	0.67137991	-5.9200341
ENSCAFG00000000121	0.06623185	6.12315631	0.93591884	0.353599106	0.67137991	-6.5114928
LAMB2	-0.2537322	8.63740052	-0.9345556	0.354294903	0.67259964	-6.4035708
CLK4	-0.1434688	3.14006837	-0.9341665	0.354493662	0.67287555	-6.1537167
NIPSNAP3B	-0.1445744	3.34296201	-0.9339498	0.354604385	0.67287854	-6.1353098
MND1	-0.7327511	-0.2296693	-0.9338254	0.354667971	0.67287854	-5.1486837
ENSCAFG00000029067	0.21368982	3.26295164	0.93377208	0.354695231	0.67287854	-6.0831921
ZNF275	0.17018877	2.05083982	0.93365243	0.354756392	0.67287854	-5.7771251
RSRC2	-0.0958659	5.44427993	-0.9336408	0.354762335	0.67287854	-6.490033
AZIN2	0.3078415	1.13332538	0.93315221	0.355012148	0.67306765	-5.5896049
SARS	-0.1129626	7.37338095	-0.9331254	0.355025839	0.67306765	-6.5078345
DHCR24	-0.2750593	5.45802816	-0.9330506	0.355064091	0.67306765	-6.516779

ENSCAFG00000018753	0.13157808	4.94894779	0.93302779	0.355075779	0.67306765	-6.4230366
ITPKC	-0.1475293	2.39413889	-0.9326328	0.355277836	0.67334933	-5.9438287
TMEM128	-0.0832512	4.32930927	-0.9317206	0.355744755	0.67399606	-6.3557083
ENSCAFG00000032488	0.32764434	2.87662961	0.93166314	0.355774198	0.67399606	-5.9038365
ENSCAFG00000016350	0.27071541	0.5617538	0.93165259	0.355779599	0.67399606	-5.401155
WNK4	0.07016522	5.44211431	0.93144263	0.355887135	0.674051	-6.4796946
DYNC1LI1	0.10783397	4.81176378	0.93138702	0.355915623	0.674051	-6.4265792
CUEDC1	-0.1414599	5.36059098	-0.9308024	0.356215155	0.67451685	-6.4615961
KANK2	0.25015306	7.26408744	0.93041331	0.356414633	0.67473053	-6.4857307
HPDL	0.28926936	1.51461938	0.93031699	0.356464018	0.67473053	-5.6226076
ENSCAFG00000011110	0.08544325	5.08381294	0.93026885	0.356488704	0.67473053	-6.4269319
PRAG1	-0.5996416	1.12555844	-0.9300588	0.356596402	0.67482096	-5.5965089
ENSCAFG00000028991	0.18864638	1.93448642	0.92996677	0.356643627	0.67482096	-5.7071615
TMEM218	0.19355211	3.84669712	0.92940945	0.356929568	0.67526056	-6.2164463
PRKAR1A	0.0743225	7.39008282	0.92843906	0.357427793	0.67610159	-6.4952147
JAG1	0.37019414	4.67424778	0.92817364	0.357564146	0.67625796	-6.358505
RPTOR	-0.08063	5.74833815	-0.9276707	0.357822601	0.67656362	-6.5047367
ENSCAFG00000023940	-0.2322666	1.14452372	-0.9276501	0.357833189	0.67656362	-5.5813164
NUB1	0.12702251	6.62963173	0.92719758	0.358065861	0.67659321	-6.5232519
ASB7	-0.0924808	3.62183095	-0.9271833	0.358073227	0.67659321	-6.2332591
ENSCAFG00000010825	-0.4313817	0.48755319	-0.9270844	0.358124046	0.67659321	-5.278387
MRPS2	-0.1023779	4.87274145	-0.9270719	0.358130502	0.67659321	-6.438181
ZNF174	0.09412106	4.09085688	0.92701198	0.358161316	0.67659321	-6.2951768
OTULIN	-0.0995637	3.86564053	-0.9268911	0.358223495	0.67659321	-6.2308685
MAMDC2	-0.789315	-0.8987645	-0.9268885	0.35822484	0.67659321	-5.0813875
GGTA1P	0.09239644	8.62067965	0.9267263	0.358308275	0.67664933	-6.4148277
ENSCAFG00000023300	-0.395941	-0.3579022	-0.9264402	0.35845548	0.67682585	-5.2388331
HIVEP3	0.31858247	3.22593766	0.92602684	0.358668249	0.67697892	-6.0694527
VAMP3	-0.0716932	6.6814752	-0.925987	0.358688733	0.67697892	-6.5230011
R3HDM4	-0.0973448	3.04509395	-0.9259175	0.358724563	0.67697892	-6.1143888
MBTPS2	-0.1018527	4.71539171	-0.9257908	0.358789771	0.67697892	-6.3798199
FIBIN	-0.4934766	3.84015194	-0.9257607	0.358805278	0.67697892	-6.2240405
MAPK1IP1L	0.08854943	6.93412907	0.92536744	0.359007819	0.67710279	-6.5160698
MZF1	-0.1429076	3.03803499	-0.9253233	0.359030575	0.67710279	-6.0758309
KIF4A	-0.7291122	2.85983707	-0.9253201	0.359032194	0.67710279	-5.6485699
WDR75	-0.1243201	5.87567602	-0.9249484	0.359223719	0.67736257	-6.5188162
CLSPN	-0.8090546	2.20153996	-0.9247741	0.359313552	0.67743055	-5.4780351
TRABD2B	0.97697946	1.62105529	0.92440813	0.359502208	0.6776848	-5.4788833
PGLS	0.14472784	5.50461066	0.92409543	0.359663455	0.67773894	-6.4992018
ALG5	0.13782741	5.60353884	0.92403465	0.359694802	0.67773894	-6.4804362
TMEM176B	1.01408932	0.91831587	0.92402623	0.359699145	0.67773894	-5.3639007
RSC1A1	-0.1533573	3.88636221	-0.9239351	0.359746154	0.67773894	-6.2351958
HYDIN	0.32959936	-0.0968813	0.92373781	0.359847922	0.67782928	-5.3650547
TMEM254	0.15479061	3.04397197	0.92344951	0.359996679	0.6780081	-6.0626758
PRDX4	0.11296037	6.67516658	0.92333311	0.36005675	0.67801986	-6.5279192
MT-ND1	-0.1251061	11.3833239	-0.9232013	0.360124788	0.67804661	-6.2019907
AP2A1	0.13864971	7.64657397	0.92242105	0.360527664	0.6787037	-6.4727066
SMOX	0.14195852	3.11546181	0.92229315	0.360593735	0.67872664	-6.3026915
DNAH8	0.56399024	-2.5811498	0.92215861	0.36066324	0.67875604	-5.0813209
SFRP5	0.55614961	-2.0883914	0.92198868	0.360751043	0.67881986	-5.0416059

ZBED2	0.71122339	0.79096934	0.92183177	0.360832132	0.67887103	-5.320491
SUPT16H	-0.0759964	6.60759211	-0.9217154	0.360892285	0.6788828	-6.5281542
SLC6A9	-0.1865576	5.72383691	-0.9213749	0.361068294	0.67903894	-6.4652942
ARMC6	0.17115523	4.72677252	0.92134627	0.361083106	0.67903894	-6.3551664
HDGF	-0.1155387	7.57444346	-0.921183	0.361167517	0.67909629	-6.5024904
ULK3	-0.1312135	3.99819241	-0.9210345	0.361244338	0.67913936	-6.3423457
AKR1A1	0.08615592	4.9349499	0.92068801	0.361423562	0.67922123	-6.4368496
PRR16	0.61157143	0.92222824	0.92050594	0.361517768	0.67922123	-5.8257786
ENSCAFG00000011225	-0.431404	-0.3042085	-0.9205044	0.361518555	0.67922123	-5.1561962
ZNF318	-0.1004989	5.48850876	-0.9204804	0.361530957	0.67922123	-6.496843
CDNF	0.30949036	0.11771208	0.92042914	0.361557508	0.67922123	-5.4950462
FAM110B	0.45247855	2.13733983	0.92018223	0.361685297	0.67927337	-5.6273435
SEPT6	0.22264351	7.30007186	0.92016712	0.361693115	0.67927337	-6.5112003
SEPT7	0.09444395	8.33948045	0.91912679	0.362231864	0.67989822	-6.4512014
LIMA1	-0.1389734	7.37349867	-0.919103	0.362244168	0.67989822	-6.5039133
NDUFS7	-0.1505105	4.4649986	-0.9190964	0.3622476	0.67989822	-6.3913444
CALM3	0.11554425	6.87940086	0.91906239	0.362265233	0.67989822	-6.5315141
C11H9orf72	-0.121316	4.10760153	-0.9190036	0.362295718	0.67989822	-6.3093797
ARHGEF2	-0.1385034	8.13644697	-0.9187996	0.362401385	0.67999521	-6.4873753
SLC30A5	-0.0784117	6.47730806	-0.9185404	0.362535775	0.68014606	-6.5321127
RBPMS2	-0.3432593	1.12648883	-0.9182177	0.362703054	0.68035855	-5.3608335
PEX10	0.26585877	0.93438006	0.91804906	0.362790523	0.6804213	-5.4865974
GLIS2	-0.2112297	4.83042127	-0.9176923	0.362975564	0.68054843	-6.3663498
TWISTNB	-0.1538672	3.93932725	-0.9176231	0.363011471	0.68054843	-6.2518446
KLKB1	-0.606037	-2.0396507	-0.9176059	0.363020397	0.68054843	-5.092448
PLXNA1	0.11015238	6.99414898	0.91704167	0.363313225	0.68081305	-6.5244999
AIFM2	-0.1410664	5.09920153	-0.9169876	0.363341308	0.68081305	-6.4517809
ZNF558	-0.1427603	3.47255095	-0.9169254	0.36337357	0.68081305	-6.1907728
DDX42	-0.0787853	5.98211451	-0.9169174	0.363377752	0.68081305	-6.5276625
MCCC2	0.09025523	6.21562116	0.91677234	0.363453055	0.68085287	-6.5324319
UCHL3	0.11990159	5.03438477	0.91622144	0.36373917	0.68128752	-6.4312192
TEFM	-0.1481874	2.6786225	-0.915314	0.364210804	0.68206894	-5.8967146
UNC45A	0.06454971	6.7229391	0.91514843	0.364296871	0.68206894	-6.5333486
SNRPA1	0.1803143	4.21169148	0.91499874	0.364374721	0.68206894	-6.31947
KIAA1614	0.18375546	2.63060429	0.91473453	0.364512145	0.68206894	-5.9881715
PRC1	-0.6418495	4.31818703	-0.9146956	0.364532408	0.68206894	-6.0144946
HBP1	-0.1060956	5.44635238	-0.9146544	0.364553815	0.68206894	-6.5232412
PMPCB	-0.0772012	5.33675468	-0.9146315	0.364565757	0.68206894	-6.4882314
ENSCAFG00000013668	0.28995045	5.63848091	0.91458571	0.364589567	0.68206894	-6.5110936
TRMT10A	-0.1164445	4.23380294	-0.9139243	0.364933781	0.68261151	-6.3493284
SACM1L	-0.072829	4.96067292	-0.9136588	0.365072016	0.68276869	-6.4705826
GANC	-0.1499829	3.18588345	-0.9134774	0.365166484	0.68284398	-6.1065193
CFAP299	-0.6755879	1.05453903	-0.9132721	0.365273434	0.68294258	-5.2626237
TTI2	0.11792441	3.72802168	0.91313495	0.365344871	0.68297477	-6.253
ACSL4	-0.1369314	7.19389533	-0.9127343	0.365553633	0.68319261	-6.517337
FAM237B	-0.3616898	-1.3168054	-0.9125281	0.36566111	0.68319261	-5.3804644
GMPPA	-0.0929754	5.63996526	-0.9124337	0.365710309	0.68319261	-6.5200962
PRRC2C	-0.0858316	8.31316721	-0.9123941	0.365730981	0.68319261	-6.4592689
ENSCAFG00000012763	0.12930024	6.07722068	0.9121213	0.365873201	0.68319261	-6.5340966
MFAP1	-0.0628776	5.35329017	-0.9120135	0.365929413	0.68319261	-6.4869051

COL16A1	0.2366088	8.13382579	0.91200589	0.365933384	0.68319261	-6.5265428
ABHD4	-0.1216685	5.36185612	-0.9119747	0.365949652	0.68319261	-6.4663893
TMEM68	-0.1236645	4.15923756	-0.9119508	0.365962107	0.68319261	-6.3459226
TPBG	0.27215484	4.7705371	0.91187089	0.366003789	0.68319261	-6.4025927
SLC39A7	0.10335476	7.43627907	0.91148359	0.366205831	0.68346846	-6.5222977
SMARCB1	-0.0819173	5.58373276	-0.9110443	0.366435065	0.68374865	-6.5089267
SIK3	-0.0835362	5.93265934	-0.9109468	0.366485964	0.68374865	-6.5235606
SULT1C4	-0.1416589	7.02324463	-0.9108839	0.366518808	0.68374865	-6.532662
POLR3E	-0.0906565	4.49279694	-0.9105372	0.366699812	0.68377692	-6.3884902
LPCAT4	0.14717369	4.80926384	0.91052049	0.366708562	0.68377692	-6.4465308
FLVCR2	0.46007408	-1.4584546	0.91033031	0.366807882	0.68377692	-5.2295737
RALGAPB	0.09375419	7.15036285	0.91020033	0.366875777	0.68377692	-6.5251619
SHC4	0.23570915	1.42863083	0.91015556	0.366899162	0.68377692	-5.8065238
RTN4RL1	0.53075119	-2.1085276	0.90996997	0.366996126	0.68377692	-5.1599858
EMC8	-0.1076378	3.77724706	-0.9099542	0.367004356	0.68377692	-6.226761
IST1	-0.0476728	6.66793081	-0.9099361	0.367013824	0.68377692	-6.5375467
UGP2	-0.1625366	6.85705833	-0.9099194	0.367022528	0.68377692	-6.5320481
SNRPB2	-0.119838	4.46373844	-0.909739	0.367116812	0.68385143	-6.3722552
SGO2	-0.2633713	4.14636091	-0.909511	0.367235967	0.68397224	-6.1635909
PIGG	0.10515851	5.25942036	0.90897905	0.367514096	0.6843374	-6.4895872
REPIN1	0.12497623	4.41083121	0.9089282	0.367540686	0.6843374	-6.3666495
ARNT2	0.1706399	4.41983449	0.90867128	0.367675065	0.68448642	-6.1757933
DNM1	-0.8097694	1.43972328	-0.9083329	0.367852081	0.68452367	-6.1855612
HOXA6	-0.6353292	-0.7051624	-0.9083261	0.367855676	0.68452367	-5.2504417
SZT2	-0.0921242	5.20383365	-0.9083214	0.367858109	0.68452367	-6.493016
TIMMDC1	0.11301658	4.05197874	0.90746243	0.368307765	0.68507026	-6.2771787
ZNF148	0.08655968	5.11861372	0.90745096	0.368313769	0.68507026	-6.4739709
EGR1	0.34615125	8.71392041	0.90744705	0.368315821	0.68507026	-6.4025444
SETMAR	0.26354276	1.52937572	0.90734475	0.368369392	0.68507026	-5.7479317
UBA6	0.15429595	5.98500058	0.90687833	0.368613724	0.68517535	-6.5249318
ADGRG2	-0.485992	2.97843728	-0.9068668	0.368619794	0.68517535	-6.1416065
CANX	0.0840373	9.53345858	0.90668835	0.368713276	0.68517535	-6.3718218
TRIP11	-0.104796	6.46310225	-0.9066295	0.368744094	0.68517535	-6.5429651
TBC1D2	-0.1820831	3.63448248	-0.9066217	0.368748214	0.68517535	-6.1940365
RSPO3	0.63149922	-1.6413206	0.90660064	0.368759244	0.68517535	-5.0252092
AGFG2	0.15745683	4.89588601	0.90640964	0.368859351	0.68517535	-6.5004566
PYURF	-0.0983484	5.43161217	-0.9064016	0.36886359	0.68517535	-6.500626
CDKN2AIPNL	-0.2921758	0.58851627	-0.9063026	0.368915466	0.68517535	-5.4312286
RPS19BP1	0.09251245	4.98873195	0.90583928	0.369158399	0.68552546	-6.4473041
ANKLE1	-0.4751558	1.50716755	-0.9053385	0.369421088	0.685693	-5.493239
HLX	-0.1999122	1.98180113	-0.905275	0.369454433	0.685693	-5.9199092
PFKFB2	0.12809562	2.73297198	0.90525352	0.369465684	0.685693	-6.0373536
HAND2	-1.0571604	-0.2698417	-0.9052522	0.369466369	0.685693	-5.1051704
UBTD2	-0.1174833	4.61660942	-0.9049366	0.369632013	0.6858057	-6.4083452
TFDP1	0.07086303	5.78605069	0.90492901	0.369635986	0.6858057	-6.5234697
ENSCAFG00000012184	-0.2473902	1.76549518	-0.9045497	0.369835111	0.68607409	-5.7390398
PHF12	0.09593498	4.52330933	0.90432575	0.369952717	0.68616592	-6.4385766
NKIRAS1	0.25402994	0.44156427	0.90424797	0.369993565	0.68616592	-5.4253493
ZWILCH	0.22678451	3.50035111	0.90353022	0.37037066	0.68676415	-6.1196583
BARX1	-0.6012165	-2.4314814	-0.9031626	0.370563915	0.68702135	-5.0451432

IGFBP2	0.61450087	8.17801094	0.90286071	0.370722625	0.68705907	-6.5367666
KIFC2	0.13611679	4.26423307	0.90283744	0.370734865	0.68705907	-6.4424445
FANCL	-0.1310387	3.43741073	-0.9028127	0.370747894	0.68705907	-6.080419
ENSCAFG00000013154	0.09973769	3.8494681	0.9026014	0.370859006	0.68716388	-6.2328797
UBE2E1	-0.0896171	6.00961051	-0.9023028	0.371016103	0.68735385	-6.5378578
CRIPT	0.13826642	2.67596727	0.90200897	0.371170704	0.68744581	-5.9880557
MICU3	0.20611871	2.62920242	0.90200101	0.371174893	0.68744581	-5.8584181
SNRNP25	0.10264216	3.72382755	0.90158349	0.371394668	0.68775173	-6.2250849
B4GALT6	-0.78097	-0.6834206	-0.9011855	0.371604216	0.68793829	-5.0909139
ZNF239	-0.2299404	1.80390629	-0.9011847	0.371604648	0.68793829	-5.774853
C1H9orf64	-0.0661409	5.54924407	-0.9007706	0.37182277	0.68824094	-6.526423
ENSCAFG00000002561	-0.0878075	4.22465332	-0.9005252	0.371952089	0.6882999	-6.3156654
USP37	-0.1589685	3.05516037	-0.9004318	0.372001333	0.6882999	-6.0866065
RNF4	0.06073335	6.09520174	0.90039911	0.372018553	0.6882999	-6.5451552
CPLX1	-0.2390303	1.17597479	-0.9000418	0.372206909	0.68854725	-5.5282876
NASP	-0.1401485	4.94845198	-0.8998327	0.372317147	0.68856198	-6.4547126
CLCN3	0.09522989	5.92331722	0.89981933	0.372324201	0.68856198	-6.5302089
ENSCAFG00000018497	0.16398174	4.26427716	0.89944324	0.372522553	0.68882767	-6.3416559
PLA2G4A	0.24883088	4.65848033	0.89922481	0.372637787	0.68889943	-6.5024268
PARG	-0.0737952	4.86417724	-0.8991623	0.372670744	0.68889943	-6.4581975
DZANK1	0.20705873	1.63840336	0.89885521	0.37283282	0.68903274	-5.7211176
CERCAM	0.26895565	7.13398859	0.89881836	0.37285227	0.68903274	-6.5158911
SMS	-0.1419102	5.70345871	-0.8985476	0.372995192	0.68919575	-6.5345863
KCNMA1	-0.6770993	-0.8445589	-0.8984165	0.373064426	0.68922257	-5.056473
MOK	-0.2020966	1.70957435	-0.8981089	0.373226843	0.68937468	-5.791409
MAPK7	0.13755856	3.87483575	0.89805328	0.373256217	0.68937468	-6.2634632
MASP1	-0.6617656	-2.3637125	-0.8977278	0.373428123	0.68959106	-5.0171591
EIF4G1	0.08243585	9.33560172	0.89732338	0.373641844	0.68964854	-6.3989786
CASP14	0.48671556	1.99309286	0.8973037	0.373652246	0.68964854	-5.9410465
H2AFZ	-0.1856549	5.47021635	-0.8972137	0.373699833	0.68964854	-6.4871134
DYNC1I2	0.06376073	7.64336502	0.89714328	0.373737035	0.68964854	-6.5191472
RRM2B	0.13255483	4.65840173	0.89692641	0.373851683	0.68964854	-6.4024121
RECQL	0.18862167	4.27366875	0.8968748	0.373878969	0.68964854	-6.3947525
DSEL	0.18981898	4.53111672	0.89680122	0.373917877	0.68964854	-6.2304853
TRAM1L1	-0.1982466	2.57869805	-0.8967709	0.373933914	0.68964854	-5.8930214
IRGQ	0.11430048	5.70869794	0.8966084	0.374019838	0.68964854	-6.5297991
RHBG	0.25501716	1.07519731	0.89660315	0.374022613	0.68964854	-5.6129325
EPS8	0.20143628	8.30256924	0.8965296	0.374061511	0.68964854	-6.4597479
ENSCAFG00000029673	-0.1170176	4.3780523	-0.8961304	0.374272669	0.68988808	-6.3880507
CCDC130	-0.1143099	3.02354505	-0.8960769	0.374300981	0.68988808	-6.076389
MGAT2	0.08374827	6.00186207	0.89546165	0.374626609	0.69038724	-6.5437845
ENSCAFG00000017667	0.11824036	4.13855844	0.89531121	0.374706257	0.690433	-6.3641726
VRK3	-0.181392	3.78007053	-0.8950828	0.374827209	0.69051405	-6.285027
CCNF	-0.7942316	2.2004665	-0.8950211	0.374859883	0.69051405	-5.4940237
BBS4	-0.1427122	3.38291121	-0.8946614	0.375050431	0.69064885	-6.195623
MYH7B	0.16083425	2.90018459	0.89464568	0.375058736	0.69064885	-6.1477321
ZFP14	-0.2213506	2.44111205	-0.8944363	0.375169676	0.69064885	-5.8329975
NIPAL1	0.47202022	0.8070948	0.89432742	0.375227366	0.69064885	-5.4119382
ENSCAFG00000031828	0.61580972	-1.4978827	0.89414996	0.375321417	0.69064885	-5.0385987
SFSWAP	-0.0801995	4.81500482	-0.894098	0.375348935	0.69064885	-6.4322187

ENSCAFG00000009315	-0.284836	1.0121828	-0.8940673	0.375365239	0.69064885	-5.4832787
RIOK1	0.09113898	4.58465125	0.89403729	0.375381137	0.69064885	-6.4563434
SERPINB1	0.10725164	5.87781927	0.89389577	0.375456156	0.69064885	-6.5396044
ENSCAFG00000007808	0.08913084	3.98272938	0.8936463	0.375588424	0.69064885	-6.3299879
ARNTL2	0.15689704	5.87554305	0.89360088	0.375612511	0.69064885	-6.5484223
MELTF	0.41230214	-0.1935801	0.89351901	0.375655924	0.69064885	-5.3362703
HUWE1	-0.0990986	7.91663448	-0.8934985	0.375666804	0.69064885	-6.5054846
SLC43A2	0.32355739	0.8479031	0.8934346	0.375700692	0.69064885	-5.7153023
AURKAIP1	-0.1225324	4.52579057	-0.8928376	0.376017377	0.69113014	-6.368878
TRERF1	-0.2648214	2.91635054	-0.8924751	0.376209814	0.69138296	-6.002706
CPTP	0.15206801	4.34361098	0.89236919	0.376266007	0.69138536	-6.423204
MRPS18A	0.11528832	3.50010517	0.89211869	0.376399006	0.69152886	-6.186657
ENSCAFG00000028473	0.23108539	3.2534964	0.89182606	0.376554405	0.69171347	-6.0856402
PDSS2	-0.1047761	2.57155707	-0.8917015	0.376620568	0.69173413	-5.9933916
CYBA	0.18617542	4.93283975	0.89136604	0.376798786	0.69196056	-6.494389
MSANTD2	-0.1247843	3.72117766	-0.8911775	0.376898965	0.69204364	-6.214613
IDO1	0.63287003	2.55785399	0.89038184	0.377321962	0.69271935	-6.0095041
ENSCAFG00000030459	-0.1194373	4.51388362	-0.890015	0.377517081	0.69288427	-6.4460648
LSAMP	-0.4137989	-3.0389348	-0.8900061	0.377521813	0.69288427	-5.0106876
ADARB1	0.20307903	4.85370066	0.88981219	0.377624984	0.69291308	-6.4326763
RAB3B	0.37625922	0.04389905	0.88967795	0.377696412	0.69291308	-5.3966722
AKIRIN2	-0.0882755	4.01308655	-0.8896664	0.377702542	0.69291308	-6.3400605
ENSCAFG00000032152	-0.1541995	5.64112667	-0.8893463	0.377872907	0.69298331	-6.5405187
SUGP2	0.12982226	4.42113119	0.88932605	0.377883702	0.69298331	-6.3825102
BCDIN3D	0.12429776	2.70670559	0.88928441	0.377905871	0.69298331	-5.9839812
SASS6	-0.7094714	1.94085396	-0.8890457	0.378032964	0.69309495	-5.5105562
GPER1	0.61624822	0.62614261	0.88886364	0.378129901	0.69309495	-5.4567369
ENSCAFG00000032009	0.0876327	4.93837612	0.88881447	0.378156087	0.69309495	-6.4533215
SORCS1	-1.243739	1.69735071	-0.8887219	0.378205385	0.69309495	-5.3228029
ZFP69B	-0.3066628	0.46051078	-0.8886115	0.378264203	0.69309495	-5.3931942
GMEB1	-0.0823311	4.09026589	-0.8885501	0.378296904	0.69309495	-6.3762074
GRAMD4	-0.1522642	4.01828102	-0.8881607	0.378504377	0.69337422	-6.3629999
ENSCAFG00000002412	-0.2527362	1.03019135	-0.8880035	0.378588139	0.69342681	-5.5782015
RPL23	-0.1196401	7.71134339	-0.887468	0.378873589	0.69376353	-6.5119763
NT5C3A	-0.1230211	2.89740667	-0.887315	0.378955182	0.69376353	-6.0876607
AKAP9	-0.1166039	5.29165223	-0.8871204	0.379058961	0.69376353	-6.5247138
JPT2	0.1100473	4.25604582	0.88699862	0.379123906	0.69376353	-6.3382805
CCDC90B	-0.0872645	5.49960556	-0.8869657	0.37914149	0.69376353	-6.5354502
PPIL6	-0.156728	3.17422099	-0.8869497	0.379150016	0.69376353	-6.1421331
YLPM1	-0.0627378	6.1038175	-0.8869356	0.379157519	0.69376353	-6.5584821
UHRF1	-0.7170089	3.76952247	-0.8863277	0.379481915	0.69425624	-5.905761
EIF4E2	0.18574542	1.61637193	0.88600592	0.379653662	0.69446958	-5.6962112
UBE3A	-0.0962872	5.71223597	-0.8855874	0.379877124	0.69468412	-6.5456918
CCDC186	-0.1027379	4.56655731	-0.8855797	0.379881249	0.69468412	-6.4517566
CCL2	0.29920889	6.38299607	0.88545408	0.379948356	0.69470598	-6.54826
WDR62	-0.4273838	2.73324683	-0.8853459	0.380006126	0.69471076	-5.7598627
TBC1D25	0.10687492	4.29724018	0.88516682	0.380101817	0.69478486	-6.3785055
PRPF19	-0.0710515	6.87727457	-0.8848786	0.380255828	0.69496553	-6.5571083
PAX8	-0.4237152	0.02939457	-0.8846465	0.380379888	0.6950017	-5.2204955
PHKA2	-0.2266893	3.66594016	-0.8846351	0.380385972	0.6950017	-6.2453391

ENOPH1	0.12692384	5.13243688	0.88446859	0.380474985	0.69506351	-6.4787569
COX16	0.26192165	0.63221471	0.88427739	0.380577212	0.69508934	-5.5330235
ADAMTS1	0.31043348	5.63363149	0.88423573	0.38059949	0.69508934	-6.5616197
BAX	-0.1316273	3.26760798	-0.8840258	0.380711777	0.69515318	-6.1996906
ENSCAFG00000032099	-0.443817	-0.2449551	-0.883964	0.380744823	0.69515318	-5.3647065
KNSTRN	-0.597238	0.59597314	-0.883833	0.380814896	0.69518035	-5.3078695
DNLZ	-0.2432135	1.26161827	-0.8835886	0.380945613	0.69531821	-5.6014782
KMT2D	-0.128507	5.41372365	-0.8832754	0.381113226	0.69550211	-6.5421905
ENSCAFG00000029422	0.17713334	6.22769314	0.88315732	0.381176425	0.69550211	-6.5635014
LURAP1L	-0.5450548	1.3231444	-0.8830908	0.381212016	0.69550211	-5.3897892
SDR16C5	-0.2833235	1.85268669	-0.882852	0.381339853	0.69562747	-5.9048572
METTL7A	-0.467204	3.41928974	-0.8827562	0.381391182	0.69562747	-6.3424046
STK32C	0.40852443	-0.3141814	0.88252851	0.381513083	0.6956335	-5.3099334
DAZAP2	-0.0959682	7.47642367	-0.8824703	0.381544244	0.6956335	-6.5306264
ENSCAFG00000002748	-0.3657812	-0.3162375	-0.8823876	0.381588535	0.6956335	-5.2414176
KLHL3	0.19860112	4.39437422	0.88233748	0.381615393	0.6956335	-6.4533472
ENSCAFG00000028127	-0.2398441	3.08465746	-0.8818706	0.381865528	0.69592736	-6.0318197
CTPS2	-0.2533122	3.86189954	-0.8818303	0.381887101	0.69592736	-6.1684743
AMN1	0.12270909	2.76157914	0.88167336	0.381971223	0.69597997	-6.0699077
GEMIN7	-0.2104576	1.85863347	-0.8814712	0.382079568	0.6960272	-5.7097769
IDE	-0.0779378	6.08021322	-0.8813881	0.382124138	0.6960272	-6.5493966
IRX2	0.60125891	-0.4466286	0.88131574	0.382162918	0.6960272	-5.2235703
ZNF396	-0.2484623	0.84394647	-0.8810083	0.382327772	0.69622678	-5.4312435
ZNF510	-0.1513705	2.50211581	-0.8808713	0.382401243	0.69625991	-5.9893661
SNX15	-0.0950508	3.91336168	-0.8806452	0.382522533	0.69638009	-6.3020091
ENSCAFG00000023527	0.11358574	3.84495827	0.88039065	0.382659074	0.69639893	-6.2725184
TTC5	0.11025843	3.45160779	0.88032796	0.382692709	0.69639893	-6.1880472
PPM1J	-0.3060177	-0.5956036	-0.8803167	0.382698746	0.69639893	-5.2932677
TENT5B	-0.2377163	3.62354915	-0.8800041	0.382866539	0.69648182	-6.3166706
CCNT2	-0.1119909	4.24710503	-0.8799292	0.382906702	0.69648182	-6.3842713
NHEJ1	0.13944576	3.02815187	0.87984688	0.382950905	0.69648182	-6.0899285
SMAD7	-0.2060321	3.08920343	-0.8797425	0.383006951	0.69648182	-6.0058704
ZNF454	-0.2372437	1.56922485	-0.8797167	0.383020766	0.69648182	-5.6963966
GCNT4	0.4790063	-0.0084322	0.87938438	0.38319923	0.69661191	-5.3285606
TXLNG	-0.1196297	2.73730676	-0.8792799	0.383255363	0.69661191	-6.0886721
ENSCAFG00000010861	-0.2092699	2.20200227	-0.8791812	0.38330834	0.69661191	-5.7593758
C30H15orf61	0.35533545	0.24452413	0.87912509	0.383338496	0.69661191	-5.4148332
RBAK	-0.1013555	4.47241091	-0.8790675	0.383369424	0.69661191	-6.4189708
TMEM120A	-0.092693	4.7319856	-0.8789393	0.383438282	0.69661191	-6.4678391
BDKRB2	-0.5813137	4.26340923	-0.8788627	0.383479436	0.69661191	-6.232965
ABCE1	-0.1129827	5.8895221	-0.8782899	0.383787269	0.69698747	-6.5537961
CBFB	-0.1383102	3.35197441	-0.8782721	0.383796848	0.69698747	-6.1361742
TLK2	-0.0794038	4.92729715	-0.8777886	0.384056803	0.69735901	-6.4962627
GPRASP1	-0.2330056	1.53197588	-0.8770112	0.384475034	0.69797295	-5.7755504
CBX8	0.24284713	1.96879726	0.87686356	0.384554494	0.69797295	-5.8437716
CDCA7L	-0.1681591	5.06504798	-0.8765941	0.384699525	0.69797295	-6.469826
MRNIP	0.19770689	0.63322046	0.8765873	0.384703207	0.69797295	-5.4656399
ENSCAFG00000029384	0.51124743	-1.5419801	0.87653118	0.384733417	0.69797295	-5.1488585
CUL3	-0.0835442	6.4886146	-0.8765266	0.384735869	0.69797295	-6.5695274
TMX1	-0.0949299	4.97767101	-0.8764395	0.384782802	0.69797295	-6.484151

NPR3	1.53977181	1.24811849	0.87626157	0.384878589	0.69804618	-5.075301
ARHGAP23	0.17502779	5.18708469	0.87612501	0.38495213	0.69807904	-6.4907012
ENSCAFG00000029162	-0.1066939	4.58292694	-0.87586	0.385094899	0.69817563	-6.4668821
NEURL3	-0.482184	-2.4106318	-0.8758089	0.385122389	0.69817563	-5.0934935
ARSB	0.14294138	5.38968611	0.87569071	0.385186078	0.69817563	-6.514681
FHDC1	-0.538824	0.06721632	-0.8756146	0.385227106	0.69817563	-5.3848655
SLC25A35	-0.2020264	1.67067338	-0.8753941	0.385345934	0.69828961	-5.7629053
CEP152	-0.2486152	3.01141926	-0.8752906	0.385401677	0.69828961	-6.0151121
USP49	-0.2416742	0.55149756	-0.8751893	0.38545631	0.69828961	-5.5406725
JADE3	-0.355051	1.51586693	-0.8749608	0.385579483	0.69832291	-5.4683214
ENSCAFG00000030302	0.32505155	0.44468448	0.87494948	0.385585572	0.69832291	-5.5348751
TMEM234	0.15890298	2.14556127	0.87480933	0.385661135	0.69835935	-5.8454024
PGS1	0.09278989	4.93756711	0.87432856	0.38592042	0.69862924	-6.4451844
STARD10	0.25206206	1.91371154	0.87424733	0.38596424	0.69862924	-5.7721775
ENSCAFG00000001985	0.30366516	0.84478127	0.87422447	0.385976572	0.69862924	-5.5389444
SKA2	0.1405824	4.9209116	0.87328573	0.38648321	0.69935664	-6.4392332
SELL	-0.3982913	-2.9833131	-0.8732575	0.386498452	0.69935664	-5.0399214
COPS7A	0.10203764	6.15215906	0.87311784	0.386573865	0.69935664	-6.5688713
FAM131A	0.17877586	1.7067872	0.87302039	0.386626492	0.69935664	-5.6904361
EXOC2	0.09863411	5.88208319	0.87295052	0.386664225	0.69935664	-6.5691073
FOSB	-0.4844646	4.77456875	-0.8728628	0.386711576	0.69935664	-6.521247
ITPA	0.10402467	4.23800889	0.87264935	0.386826903	0.699384	-6.3421039
ATR	-0.1233021	5.1984676	-0.87247	0.386923797	0.699384	-6.5187316
CHN1	-0.1488684	4.61380102	-0.8724127	0.38695477	0.699384	-6.3154052
RAB3IP	0.20088767	4.08984349	0.87237354	0.386975916	0.699384	-6.2175555
CPSF7	-0.07439	5.94367008	-0.872321	0.387004325	0.699384	-6.5624508
ACADVL	0.07361719	6.58251586	0.87205825	0.387146307	0.69954022	-6.5719061
ENSCAFG00000007663	-0.19243	1.39421453	-0.87177	0.387302119	0.69954354	-5.635259
GTSE1	-0.8680833	2.41712049	-0.8716719	0.387355167	0.69954354	-5.4864798
MRPL28	-0.1321911	4.69856666	-0.8716546	0.387364495	0.69954354	-6.459821
ERBIN	-0.0902596	6.5081016	-0.8716439	0.387370292	0.69954354	-6.5737526
ADORA2B	0.24679082	4.98330774	0.87145586	0.387471986	0.69962688	-6.5403472
ENSCAFG00000019891	-0.3797298	0.38041332	-0.8710264	0.387704273	0.69994597	-5.4695125
TNFAIP1	0.11349776	5.89708615	0.87082247	0.387814612	0.70004483	-6.5584851
ZNF574	0.1134938	4.29271664	0.8706212	0.387923525	0.7000631	-6.4010614
PROM1	-0.8995786	-1.0387402	-0.8705897	0.38794058	0.7000631	-5.1370412
PEF1	-0.0664034	5.33090576	-0.8704957	0.387991465	0.7000631	-6.5171156
ENSCAFG00000024271	0.13009745	2.78375689	0.87009857	0.388206428	0.70024298	-6.0616702
DHX35	-0.1918306	2.22118217	-0.8698351	0.388349094	0.70024298	-5.8297999
CBX1	-0.1059716	3.69328215	-0.8697495	0.388395447	0.70024298	-6.2453551
ENSCAFG00000030329	-0.7420703	1.39079646	-0.8697094	0.388417182	0.70024298	-5.315275
ENSCAFG00000011251	0.73634828	-1.5893632	0.8696258	0.388462456	0.70024298	-5.2021723
AP1M1	0.08856035	5.81392722	0.86962286	0.388464048	0.70024298	-6.5649775
LONRF3	0.14104129	2.49241081	0.86959285	0.388480304	0.70024298	-6.1239218
VAC14	-0.081827	5.70465993	-0.8692806	0.388649437	0.7004476	-6.550283
RNF19B	-0.1334988	4.33046541	-0.8691167	0.388738288	0.70050751	-6.3756626
CPNE1	-0.0860847	7.01283699	-0.8689819	0.388811339	0.70053892	-6.56761
DDX28	-0.1264942	3.31571162	-0.8686272	0.38900361	0.70067534	-6.1570769
ATRNL1	0.77453751	3.62426113	0.86859203	0.389022653	0.70067534	-5.8644967
FCHO2	-0.1236524	5.60170215	-0.8685343	0.389053933	0.70067534	-6.5606513

HPS6	0.13773727	2.94034141	0.86812054	0.389278319	0.70097923	-6.1033242
FAM98C	0.11167966	4.30832429	0.86771375	0.389498987	0.7011925	-6.3931571
MTPN	0.09289619	9.05409033	0.86769697	0.38950809	0.7011925	-6.4223905
RSF1	-0.0951728	5.2138576	-0.8673925	0.389673286	0.70127226	-6.5417034
PPP1R8	0.07383302	5.59117984	0.86723489	0.389758852	0.70127226	-6.5474186
PLA2G16	0.36126358	2.22498822	0.86722252	0.389765569	0.70127226	-6.0543682
IARS	-0.0780587	8.07480799	-0.8671495	0.389805181	0.70127226	-6.529371
PRSS12	0.53328899	0.50212146	0.86696817	0.389903641	0.70127226	-5.3059094
HARS	0.09183315	5.7748322	0.8668654	0.389959436	0.70127226	-6.5597529
SMIM4	0.19335459	2.3915021	0.86679908	0.389995447	0.70127226	-5.9425966
RNF167	0.10268409	4.69995328	0.86679477	0.389997791	0.70127226	-6.4555349
C18H11orf74	-0.1299026	2.70498069	-0.8665912	0.39010833	0.7013709	-6.0490195
PDCD1LG2	-0.4188065	-2.8637328	-0.8663746	0.390225968	0.70148227	-5.0647668
PGAP3	0.17348219	2.09953659	0.86586171	0.390504637	0.70188305	-5.7865628
STX1A	0.2290298	1.00470069	0.86560305	0.390645218	0.70200897	-5.5404711
HNF4A	-0.5423279	-1.7563022	-0.8654513	0.390727733	0.70200897	-5.3238251
SERP2	0.2907747	0.49961334	0.86533533	0.390790756	0.70200897	-5.423775
PLAU	-0.2374261	5.38987808	-0.8652366	0.390844459	0.70200897	-6.5304345
PARP9	-0.3047785	2.38074567	-0.8651743	0.390878324	0.70200897	-5.8810338
NCOA1	-0.1065196	4.86659759	-0.8651177	0.390909092	0.70200897	-6.4516501
UBE2I	-0.0876768	4.73382025	-0.864665	0.391155326	0.70235103	-6.4595984
INPP5A	0.0699779	6.04254702	0.86441369	0.391292041	0.70249637	-6.5483569
FGD6	0.26373574	3.79469083	0.86426157	0.391374815	0.70254484	-6.3047444
ENSCAFG00000030295	0.26221718	0.38040256	0.86363692	0.391714835	0.70305501	-5.4340431
CALCRL	0.60505565	0.02542572	0.86351354	0.391782018	0.70307541	-5.4702329
FAP	-0.5266897	6.3392795	-0.8633072	0.391894394	0.70317689	-6.5304363
CCDC6	-0.101109	5.24726934	-0.8629993	0.392062112	0.70332535	-6.516012
MOXD1	0.63544189	4.82919122	0.8629503	0.392088804	0.70332535	-6.3943438
ZNF517	-0.0640408	5.09763368	-0.8624392	0.392367342	0.70359158	-6.5088749
FRMD4A	0.17959676	6.09281248	0.86240377	0.392386625	0.70359158	-6.5769089
RPL22L1	-0.1698555	4.46264683	-0.8623477	0.392417214	0.70359158	-6.4143664
ZSCAN22	-0.0950488	3.75939283	-0.862154	0.392522794	0.70359158	-6.2755281
POP7	-0.1843722	2.14294413	-0.8620775	0.392564499	0.70359158	-5.8180416
RAB8A	-0.0748249	5.10788732	-0.8619825	0.392616265	0.70359158	-6.51493
LPAR6	-0.4155994	-0.9731942	-0.8619385	0.392640266	0.70359158	-5.321358
NFIB	-0.2258504	5.40291236	-0.8618582	0.392684093	0.70359158	-6.5512907
TTC37	-0.0837865	6.95100439	-0.8616851	0.392778492	0.70363817	-6.574393
SLC20A1	0.24315085	8.74847637	0.86160421	0.392822593	0.70363817	-6.4377283
CD2BP2	-0.0790581	5.92976766	-0.8615032	0.392877682	0.70363817	-6.5736796
ENSCAFG00000024449	0.324768	-0.5182005	0.86120479	0.39304049	0.703684	-5.3463441
ZNF470	-0.3867302	0.16017798	-0.8611173	0.393088233	0.703684	-5.3445132
ENTR1	0.10081619	4.06115078	0.86102619	0.393137952	0.703684	-6.3432426
NUDT3	-0.0764131	4.75475092	-0.8610231	0.393139657	0.703684	-6.4911678
SH3GL3	0.47399846	-0.5208578	0.86079134	0.393266128	0.703684	-5.1872102
CTNNB1	0.15318846	9.18397703	0.86077922	0.393272742	0.703684	-6.4322983
ARPC4	0.0847048	5.9366223	0.86073967	0.393294327	0.703684	-6.5711214
TMEM19	0.07711711	4.66446693	0.86042026	0.393468702	0.703896	-6.4740152
ENSCAFG00000001530	-0.3355322	1.23574091	-0.8598163	0.393798573	0.70426315	-5.4276663
PLA2G7	0.29516859	7.42563784	0.85980572	0.393804336	0.70426315	-6.5812889
ENSCAFG00000026443	-0.3505208	-0.3493823	-0.8596678	0.393879673	0.70426315	-5.3829642

ZNF79	-0.1430224	2.09969039	-0.859635	0.393897579	0.70426315	-5.923141
OGDHL	0.13559619	3.6811384	0.85923193	0.394117869	0.70455701	-6.3069759
MEGF8	0.0911326	5.83773306	0.8586918	0.394413157	0.70489206	-6.5725917
GLCE	-0.1193344	4.48337008	-0.8586844	0.394417216	0.70489206	-6.3112583
UBE2J1	-0.0918221	7.38161765	-0.8585594	0.394485569	0.7049142	-6.5666453
SOX4	0.32223406	1.83309293	0.85792307	0.394833651	0.70543612	-5.6984279
HECTD1	-0.0823373	7.49907262	-0.857379	0.395131429	0.70584226	-6.5612247
MED4	-0.0942488	2.93734041	-0.857303	0.395173042	0.70584226	-6.0873241
ALDH18A1	0.12844852	7.38735185	0.85668803	0.395509805	0.70634361	-6.5706964
SMIM30	0.15257926	3.45620147	0.85638361	0.395676579	0.70654128	-6.2008609
ANO1	-0.5801674	-2.1123457	-0.8558972	0.395943133	0.70688926	-5.073591
BMX	-0.5497477	-0.7237673	-0.8558232	0.395983696	0.70688926	-5.340518
TRIM26	-0.1727385	3.45839191	-0.8556688	0.396068349	0.70694019	-6.2099979
JMJD8	-0.1148432	4.94950451	-0.8552239	0.396312285	0.70719139	-6.4669348
PLBD1	-0.5293018	-1.8710217	-0.8550811	0.396390619	0.70719139	-5.2145649
PPP2CA	-0.0859757	6.19846539	-0.8549778	0.396447291	0.70719139	-6.5835455
TRMT44	-0.1013238	3.16765413	-0.8549473	0.396464059	0.70719139	-6.1304846
GPR157	0.30935716	1.36185443	0.85474654	0.396574179	0.70719139	-5.6263019
PDCD6	0.06338035	5.7361961	0.85473905	0.396578286	0.70719139	-6.576573
IL1R1	-0.2374853	5.30297048	-0.8545969	0.396656277	0.70719139	-6.5811461
ATM	-0.0898079	5.17294555	-0.8545392	0.396687971	0.70719139	-6.5325491
TEX30	-0.1558146	2.88781932	-0.854491	0.39671438	0.70719139	-6.0427661
BOLA1	0.13845112	2.80505755	0.8543784	0.396776198	0.7072015	-6.0435396
ENO2	0.26093087	5.22257966	0.85413619	0.396909154	0.70727382	-6.5386114
INO80D	-0.1390912	2.52545386	-0.8540289	0.396968073	0.70727382	-6.0033836
TMEM231	0.14207744	3.70017704	0.85394793	0.397012509	0.70727382	-6.3080015
PGM2L1	-0.2287234	1.41657242	-0.8536922	0.397152922	0.70727382	-5.6974655
GAS7	-0.5990589	1.44053771	-0.8536071	0.397199683	0.70727382	-6.2254194
SORCS2	-0.6862724	-1.3961597	-0.8535527	0.397229572	0.70727382	-5.0827736
SHMT2	0.14412771	8.41496285	0.85350147	0.39725769	0.70727382	-6.5409832
C15H1orf216	0.44504929	-0.0849684	0.85340507	0.397310637	0.70727382	-5.4039881
PDS5B	-0.0842125	5.37375382	-0.8533842	0.39732213	0.70727382	-6.5662882
CLEC3B	0.85552307	0.98037806	0.85307188	0.397493691	0.70747923	-5.8454633
PPP1R3E	-0.3676991	-0.3198771	-0.8520143	0.398075058	0.70841388	-5.2890926
MBOAT1	0.4053416	1.69923953	0.85152292	0.398345365	0.70879131	-5.7963558
FPGS	0.10350221	4.55192947	0.85142419	0.39839969	0.70879131	-6.4189618
SMTNL2	-0.456193	-0.7003132	-0.8511175	0.398568456	0.70893781	-5.3222615
MTRF1L	-0.1884187	2.09149144	-0.85107	0.398594597	0.70893781	-5.7824016
TIMM10	0.11322425	2.99390567	0.85075704	0.398766892	0.70914412	-6.0616849
PINK1	0.10327629	3.94766504	0.8506119	0.398846804	0.7091861	-6.3828553
ZGPAT	0.10376035	4.74194756	0.85047029	0.398924784	0.70922464	-6.4575943
ENSCAFG00000010945	-0.2515924	1.08039383	-0.8503032	0.399016778	0.70928808	-5.5805115
PITPNA	0.07694581	5.60272965	0.85009758	0.399130065	0.70930468	-6.5736235
CAND1	-0.0806357	7.71060796	-0.8500668	0.399147009	0.70930468	-6.5617304
TRIO	-0.0706868	6.97161919	-0.8499796	0.399195055	0.70930468	-6.5855322
NRDC	-0.0676031	6.86799462	-0.8496378	0.399383366	0.70953919	-6.5832435
CEACAM1	0.2674872	3.46614839	0.84933255	0.399551636	0.70962025	-6.1634748
SNX2	-0.082513	6.28150856	-0.8492451	0.399599832	0.70962025	-6.5902366
DLC1	-0.1090519	7.25143772	-0.8491669	0.399642935	0.70962025	-6.5614784
ARIH1	-0.0988018	4.94111581	-0.8491462	0.399654339	0.70962025	-6.4970148

GOLGA5	-0.0811007	5.92039237	-0.8489491	0.399763018	0.70962919	-6.5885147
TIMP2	0.32178273	7.93174678	0.84890243	0.399788772	0.70962919	-6.5070312
RNF7	-0.099405	4.2574542	-0.8488306	0.399828388	0.70962919	-6.3908687
KCND1	0.30081643	0.52767203	0.84832776	0.400105745	0.7098955	-6.0595929
SLC25A42	0.21157882	1.44986483	0.84825457	0.400146125	0.7098955	-5.735437
UBL4A	0.14151047	2.66428026	0.848161	0.40019775	0.7098955	-5.9654564
SLC48A1	0.26194106	2.5013419	0.84808825	0.400237894	0.7098955	-5.8650121
ENSCAFG00000009016	-0.1712021	2.64526613	-0.8479131	0.400334537	0.7098955	-5.893274
CENPV	-0.1516271	3.5203652	-0.84789	0.400347295	0.7098955	-6.2545839
CARD10	-0.2848481	3.59451803	-0.8478435	0.400372945	0.7098955	-6.3686526
PPIG	-0.1001694	6.07113266	-0.8471803	0.400739116	0.71044474	-6.5934512
ENSCAFG00000004690	-0.2575874	3.67329115	-0.8467913	0.400953977	0.71069048	-6.0957685
SYT16	0.51963454	1.27537759	0.84671404	0.400996655	0.71069048	-5.3366964
MYNN	-0.1194816	3.76238124	-0.8466229	0.401046995	0.71069048	-6.25815
HOXA13	-0.7823871	-1.4962939	-0.8462236	0.401267668	0.71087665	-5.0714679
SLCO1C1	0.43534696	-1.0648391	0.84620128	0.401279997	0.71087665	-5.2938844
GPR63	0.53029562	-1.6520123	0.84589775	0.401447777	0.71087665	-5.2147091
CCL27	-0.263329	-0.4599433	-0.8458204	0.401490521	0.71087665	-5.368067
INF2	-0.1353213	6.58757229	-0.8457915	0.401506494	0.71087665	-6.5943088
TOX4	0.0749578	6.13747514	0.84573388	0.401538382	0.71087665	-6.5931156
ENSCAFG00000017339	-0.50819	-0.986903	-0.8455355	0.401648069	0.71087665	-5.1710279
CRTAP	0.07457977	6.74849963	0.84552992	0.401651163	0.71087665	-6.5936309
RCN1	-0.0973308	8.30365614	-0.845514	0.40165998	0.71087665	-6.4999186
F2RL2	-0.5448092	1.19259364	-0.8453131	0.401771065	0.71097335	-6.2981948
TXNRD3	-0.1663274	4.3116265	-0.8451488	0.401861974	0.71103433	-6.4941987
CCDC159	-0.4354852	-1.3992458	-0.8449762	0.40195747	0.71110341	-5.2703627
ENSCAFG00000000069	0.18406554	2.11942306	0.84471466	0.402102177	0.71125952	-5.9147729
EPHB2	1.13570949	1.43622388	0.84402025	0.402486575	0.71175171	-5.1850643
LCMT2	-0.1251291	2.71572758	-0.8440078	0.402493444	0.71175171	-6.0066023
FBRS	0.09690883	5.1349323	0.84370061	0.402663598	0.71195265	-6.5509599
ENSCAFG00000001499	-0.1724756	2.10571335	-0.8433234	0.402872537	0.7122221	-5.7496507
DLA-12	0.21747203	5.46586552	0.84310467	0.402993757	0.71223732	-6.5811526
GGPS1	-0.1285434	2.73290564	-0.8431038	0.402994237	0.71223732	-6.0371439
GSTA4	-0.0934652	4.8147088	-0.8428763	0.403120324	0.71236021	-6.5085953
ADAT2	0.19506113	1.56232184	0.84256729	0.403291618	0.71240221	-5.7323782
ENSCAFG00000017868	-0.1984681	1.70154647	-0.8424972	0.403330493	0.71240221	-5.6375615
CC2D2A	-0.149081	4.73801867	-0.8424612	0.403350448	0.71240221	-6.4842839
ENSCAFG00000029900	-0.2525414	0.92961476	-0.8424253	0.403370321	0.71240221	-5.4904795
TTC21A	-0.2147054	2.76572972	-0.8423232	0.403426954	0.71240234	-6.0424028
SLC6A4	-0.6992581	-2.5916995	-0.842168	0.403513051	0.7124545	-5.1149182
ECHDC1	-0.1061244	4.72841184	-0.8420094	0.403600994	0.71250487	-6.5103517
E2F8	-0.8859341	1.58622104	-0.8417984	0.403718023	0.71250487	-5.3676011
SPRY1	-0.3434328	3.27606936	-0.841679	0.403784302	0.71250487	-6.590168
ZHX2	-0.2076637	2.54639999	-0.8415764	0.403841213	0.71250487	-5.942369
ARHGEF40	0.10973814	7.7021462	0.84149515	0.403886299	0.71250487	-6.5663905
MCM3AP	-0.0509393	6.73766037	-0.841437	0.403918552	0.71250487	-6.5990646
CCDC82	-0.1263047	2.81891882	-0.841377	0.403951838	0.71250487	-6.1980894
YWHAB	0.07178235	7.10696532	0.84130088	0.403994106	0.71250487	-6.5897153
BANF1	0.11632523	4.5162012	0.84105431	0.404130973	0.71256371	-6.4171606
SLC9A6	0.07376662	5.44359673	0.84103694	0.404140614	0.71256371	-6.5777908

ENSCAFG00000017264	-0.5729303	2.46290096	-0.8408893	0.404222559	0.71260845	-5.632046
GNG12	0.09892359	3.65098644	0.84054876	0.404411673	0.71284207	-6.2170777
UCP3	-0.0982893	3.99303719	-0.8402599	0.404572117	0.71284476	-6.3116463
CDKL2	0.1346602	5.96760278	0.84022004	0.404594258	0.71284476	-6.6008708
NOL4L	-0.2545081	3.2839833	-0.8402194	0.404594636	0.71284476	-6.3433684
ENSCAFG00000014978	0.15524858	5.98965281	0.84012615	0.404646417	0.71284476	-6.5999594
ZNF37A	-0.1486112	2.581601	-0.8400366	0.404696165	0.71284476	-5.9935606
CCDC50	-0.1188709	4.80528633	-0.8396688	0.404900567	0.71306197	-6.4846657
RAP1GDS1	-0.0831756	5.18213818	-0.8395704	0.404955244	0.71306197	-6.5467051
GPR156	-0.4377103	-0.3326749	-0.8395091	0.404989309	0.71306197	-5.2868706
CDK16	-0.0773885	6.4694118	-0.8391811	0.40517163	0.71328328	-6.6014824
RPL27	-0.0769122	7.02008315	-0.8389633	0.405292744	0.71339679	-6.5880647
CERS5	0.07499831	5.69349725	0.83867708	0.405451951	0.71351444	-6.5812284
ARL14EP	0.09956217	4.16917743	0.83863946	0.405472875	0.71351444	-6.4720485
GGN	0.20128782	2.66194882	0.83849966	0.405550645	0.7135516	-5.8702681
ENSCAFG00000013158	-0.5363088	-0.1303482	-0.8380258	0.405814293	0.71391576	-5.2734975
ENSCAFG00000014657	-0.3437298	0.01107664	-0.8378978	0.405885567	0.71394143	-5.2482755
METTL14	-0.092121	3.85135125	-0.8376614	0.406017167	0.7140732	-6.3235266
PCED1A	0.17845895	4.33394389	0.83750814	0.406102478	0.71412353	-6.4516613
RAPGEF4	-0.3383137	3.24152714	-0.8370694	0.40634679	0.71427206	-5.9647134
PAPOLA	-0.0529231	7.19814406	-0.837034	0.406366529	0.71427206	-6.5860168
CCNA2	-1.0482259	2.95979731	-0.8370271	0.406370345	0.71427206	-5.6300765
LRRCC1	-0.136276	4.6501013	-0.8369492	0.406413771	0.71427206	-6.4526542
KCP	0.40115765	-0.5182972	0.83576371	0.407074465	0.71523815	-5.3276232
LBX2	-0.3176569	0.38543423	-0.8357344	0.407090796	0.71523815	-5.4453442
ZNF234	-0.107856	3.59603031	-0.8355833	0.407175058	0.71523815	-6.3263844
AFG3L2	-0.0890847	5.68972715	-0.8355314	0.407204024	0.71523815	-6.5960158
MAP3K3	-0.1071241	4.64737043	-0.8354359	0.407257255	0.71523815	-6.5264205
ENSCAFG00000011362	-0.0625117	7.11580171	-0.8353518	0.407304162	0.71523815	-6.5982383
AMDHD1	-0.326047	1.13367293	-0.8350232	0.407487498	0.71546035	-5.7068132
CHRD	-0.5566629	1.53283547	-0.8341387	0.407981178	0.71622731	-5.4574598
SUGP1	0.07853103	4.90363277	0.83399312	0.40806249	0.71627022	-6.5163589
DTL	-0.4030107	3.0417621	-0.8333742	0.408408221	0.7166228	-5.8718011
CD164	0.08944803	9.05782229	0.83329721	0.408451224	0.7166228	-6.4730834
PLGRKT	-0.1549963	2.41804893	-0.8329989	0.4086179	0.7166228	-5.9298181
DLGAP5	-0.7901167	3.98761	-0.8329973	0.408618823	0.7166228	-5.9795849
TMEM161B	-0.1616342	2.38559227	-0.832996	0.408619567	0.7166228	-5.9303019
FGFR3	0.31266545	1.4249508	0.83297472	0.408631441	0.7166228	-5.7271298
TRPT1	0.3303982	1.03215508	0.8328939	0.408676613	0.7166228	-5.6003237
SPTLC1	-0.0792587	5.91913582	-0.832819	0.408718497	0.7166228	-6.6010849
ENSCAFG00000004882	0.09892329	5.57002483	0.83243323	0.408934153	0.71673403	-6.5817397
WDR89	-0.1880101	1.45855929	-0.8324047	0.408950085	0.71673403	-5.728144
PSMA6	-0.0967978	4.65945495	-0.8324002	0.408952644	0.71673403	-6.4924589
RALGAPA2	-0.211034	3.87698932	-0.832159	0.409087493	0.71686429	-6.3274668
ENSCAFG00000018518	0.34512339	0.25117409	0.83197666	0.409189502	0.71686429	-5.3900337
UBA2	-0.0993244	6.30568855	-0.8318227	0.409275615	0.71686429	-6.6061614
NUCB2	0.11147928	6.90079789	0.83178913	0.409294407	0.71686429	-6.6030342
SCD	-0.2022561	9.55027775	-0.8317585	0.409311524	0.71686429	-6.4099651
ENSCAFG00000002360	-0.1365423	2.58706407	-0.8314812	0.409466697	0.71703636	-6.0463536
NDUFB6	0.14692507	3.78794842	0.83100615	0.4097326	0.71740226	-6.2917244

LIPC	-0.577561	0.27595524	-0.8308027	0.409846496	0.71750194	-5.3163915
CDKN2B	0.31768019	2.46307325	0.83065129	0.409931294	0.71751181	-6.201866
SPACA6	0.19058028	1.91135259	0.8305892	0.409966063	0.71751181	-5.9189877
STRN	-0.0953824	5.0176039	-0.8304132	0.410064624	0.7175422	-6.5152017
GPC3	-0.5470404	-0.7738471	-0.8303548	0.410097356	0.7175422	-5.317222
TM7SF2	-0.2357618	3.89378528	-0.8302214	0.410172092	0.71757329	-6.4893511
WDR34	-0.2752778	3.20719055	-0.8295241	0.410562804	0.71815707	-6.0542932
PGAM5	-0.1023183	4.6619143	-0.829254	0.410714237	0.71832221	-6.4664845
TRMT11	0.10711887	3.2150666	0.82882873	0.410952698	0.71863948	-6.2530166
MED10	0.13869558	2.50251126	0.82861126	0.411074681	0.71875301	-6.0206965
SHPRH	-0.0941802	4.72830045	-0.8284003	0.411193024	0.71886014	-6.5006143
SOS1	-0.0810501	5.54669435	-0.8278031	0.411528191	0.71927453	-6.5842669
CYP4F8	0.36137776	2.0086532	0.82777444	0.411544261	0.71927453	-5.6872856
CDC16	0.08476038	5.14943453	0.82762713	0.411626953	0.71931924	-6.5335591
CXCL8	0.54255986	0.69913413	0.82724839	0.411839619	0.71959104	-5.9018191
NDUFA2	0.140883	2.68781442	0.82709646	0.411924946	0.71964031	-6.0230093
LUC7L2	-0.0645997	5.84755127	-0.8269135	0.412027725	0.71969379	-6.6023876
ENSCAFG00000032230	0.26073416	0.56700199	0.82683853	0.412069832	0.71969379	-5.5270532
NR1D2	-0.1014328	5.89023337	-0.8266768	0.412160691	0.71975268	-6.575546
FRS3	-0.1629683	1.78522056	-0.8260445	0.41251607	0.7202493	-5.8000273
ENSCAFG00000028156	-0.359996	-0.0811985	-0.8258872	0.412604485	0.7202493	-5.3741216
ZNF335	-0.0734047	5.08310786	-0.8258656	0.412616618	0.7202493	-6.5403474
ARMC5	0.11207198	3.52600222	0.82560209	0.412764797	0.72037117	-6.2676021
ENSCAFG00000029095	0.28569257	0.08938255	0.82553804	0.412800818	0.72037117	-5.4488142
ZNF484	0.23998578	1.26686016	0.82538922	0.412884516	0.72038555	-5.6922752
ADIPOR2	-0.0726306	6.3243382	-0.8252187	0.412980444	0.72038555	-6.6123669
DNMBP	-0.0997063	5.26884808	-0.8252183	0.412980631	0.72038555	-6.5766551
ZNHIT1	-0.1133201	3.43693575	-0.8251094	0.413041909	0.72039267	-6.272362
CCDC173	-0.3576842	-0.1587695	-0.8248477	0.413189193	0.72040199	-5.4269395
SYCE1L	-0.2601029	0.10689339	-0.8248344	0.413196647	0.72040199	-5.4300515
ENPP1	0.21296046	5.79410957	0.82465803	0.413295906	0.72040199	-6.6118324
RHOT1	0.12996426	6.26032954	0.82463244	0.413310304	0.72040199	-6.6099516
FAM228B	-0.2553451	-0.3198945	-0.8245104	0.413378969	0.72040199	-5.4668267
KLHL41	-0.1761298	2.11211608	-0.8244105	0.413435215	0.72040199	-5.8203723
IZUMO4	0.28324742	0.16510433	0.82438852	0.4134476	0.72040199	-5.4848533
SLC25A33	0.14962957	3.32256943	0.82403864	0.413644588	0.72064554	-6.2114904
IER2	-0.2402715	5.43331018	-0.823056	0.414198131	0.72144415	-6.5898572
USP35	0.15767409	2.18265548	0.82302155	0.414217539	0.72144415	-5.9014112
KDELC2	-0.2275732	5.86487839	-0.8223985	0.414568729	0.72194638	-6.608698
WASHC5	-0.0661242	7.50291645	-0.8223067	0.414620521	0.72194638	-6.5946147
ENSCAFG00000001830	-0.4245292	-1.2045764	-0.8220227	0.414780675	0.72212542	-5.1255016
FUK	-0.1457099	3.52257234	-0.8218015	0.414905443	0.72217239	-6.226641
KCNJ8	-0.4861255	-1.9241512	-0.8217149	0.41495432	0.72217239	-5.070394
C20H19orf38	0.33963104	-1.7348549	0.82166999	0.414979656	0.72217239	-5.3014069
HOXA1	-0.3514466	1.12250579	-0.8205954	0.415586211	0.72303659	-5.6633964
THBS3	0.20566927	7.4731549	0.82058681	0.415591052	0.72303659	-6.5935623
MPPED2	0.53283255	-1.6177844	0.82043871	0.415674685	0.7230598	-5.1068635
HGH1	-0.1428349	3.10581297	-0.8203599	0.4157192	0.7230598	-6.1218306
KCNIP2	0.30211805	-0.2582298	0.82024902	0.415781828	0.72306888	-5.4615677
CCS	-0.2018865	3.37139028	-0.8198417	0.416011951	0.72331336	-6.3271259

CCDC15	0.17917731	4.61647996	0.81966659	0.416110893	0.72331336	-6.5537657
EIF4G2	0.10618366	10.8280125	0.81966121	0.416113937	0.72331336	-6.349565
LOXL2	0.32586038	9.36606187	0.81955916	0.416171608	0.72331336	-6.5314446
TMEM104	0.10380019	4.19087932	0.81949207	0.41620953	0.72331336	-6.3691738
PTPN13	-0.2319696	6.7373035	-0.8193176	0.416308144	0.72338493	-6.6151964
DUS3L	-0.1457963	3.52671526	-0.8192021	0.416373453	0.72339862	-6.2008624
ENSCAFG00000031933	-0.2377795	3.1470828	-0.8190099	0.416482094	0.72343518	-6.1498797
LRRC41	0.08243388	6.55641811	0.8189617	0.416509362	0.72343518	-6.6176754
TMEM187	0.24840852	0.87510893	0.81871534	0.416648681	0.72357739	-5.6084997
ENSCAFG00000030172	-0.1717383	2.3539926	-0.8184189	0.416816373	0.72360882	-5.9858973
PIEZO2	-0.8781926	4.01823677	-0.8183506	0.416854979	0.72360882	-5.3097403
OAZ2	0.08820545	4.34549107	0.81828338	0.416893031	0.72360882	-6.4864738
METTL23	-0.2095921	1.74709074	-0.8182771	0.41689657	0.72360882	-5.7235373
HRCT1	-0.2979181	1.21866316	-0.8179823	0.417063374	0.72378701	-6.0485412
SLF1	0.21317853	2.74443965	0.81781186	0.417159856	0.72378701	-5.9714548
RNF157	-0.19214	5.54250915	-0.8176749	0.417237353	0.72378701	-6.5912537
ATOH8	0.49468755	-0.766381	0.81762801	0.41726392	0.72378701	-5.2860058
WBP1L	0.14193487	4.22743002	0.81758805	0.417286542	0.72378701	-6.4290665
USP22	0.0625967	6.27472405	0.81744515	0.417367443	0.72382766	-6.6170322
ENSCAFG00000014120	-0.114946	4.3244863	-0.8171549	0.417531774	0.72399086	-6.3877968
ASIC3	-0.2747206	0.19008905	-0.8170759	0.417576502	0.72399086	-5.4584323
PIGX	0.11711886	3.72445702	0.81690431	0.417673714	0.72405974	-6.3282757
ENSCAFG00000016695	-0.4810605	2.63124694	-0.8164475	0.417932527	0.72435055	-5.929087
NELFE	-0.1019666	3.21802671	-0.8164052	0.417956476	0.72435055	-6.1302343
CUTA	0.10504595	4.55763609	0.81629954	0.418016349	0.72435465	-6.4612123
ENSCAFG00000005848	-0.2174788	1.11106235	-0.8160385	0.418164273	0.72449387	-5.6917135
MLLT1	0.0853991	6.37274875	0.81588508	0.41825126	0.72449387	-6.6208536
PRRG2	-0.3403924	-1.0223714	-0.8158533	0.418269249	0.72449387	-5.3146107
ERAL1	-0.0597438	5.40749191	-0.8155457	0.418443658	0.72469632	-6.5725277
SEC14L1	0.14426142	6.2730738	0.81535202	0.418553515	0.7247439	-6.615123
PKD2	0.11502074	5.55370176	0.81523092	0.418622199	0.7247439	-6.5778825
TRAFD1	-0.1068474	6.39098998	-0.8151929	0.418643748	0.7247439	-6.6205861
SMURF1	0.14322603	5.11996928	0.81498641	0.418760892	0.72484708	-6.5529072
MMP23	0.3632078	4.43915386	0.81443089	0.419076119	0.72521222	-6.5386638
ENSCAFG00000013366	-0.1121564	5.31537517	-0.8143251	0.419136189	0.72521222	-6.5741687
IL16	-0.4932429	2.69554059	-0.8143103	0.419144568	0.72521222	-5.9536652
TIAL1	-0.0898932	5.46988032	-0.8141823	0.419217228	0.72523832	-6.5855528
STAMBPL1	-0.0920108	4.32484457	-0.8139123	0.419370519	0.72540388	-6.4009762
CASKIN2	-0.1555202	4.09499792	-0.8135336	0.419585589	0.72567624	-6.3789298
ENSCAFG00000019141	-0.2811606	0.37441653	-0.8131892	0.41978124	0.72574531	-5.5415525
SEC31B	-0.2961852	0.71788501	-0.8131659	0.41979444	0.72574531	-5.5151399
ENSCAFG00000001098	-0.29853	0.15654136	-0.8129599	0.419911513	0.72574531	-5.4578064
SMIM10L1	0.20135923	2.57328027	0.81295706	0.419913114	0.72574531	-6.0536326
ACBD5	-0.0899316	5.25612021	-0.8129562	0.41991361	0.72574531	-6.5789139
MSANTD4	-0.0977807	3.16767101	-0.8126872	0.420066499	0.72590995	-6.2109853
CXHXorf38	-0.1933826	2.51752301	-0.8118601	0.420536757	0.72662291	-6.1295481
NFIX	0.22126823	7.68961347	0.81138434	0.420807393	0.72664564	-6.5914783
KBTBD12	0.25530099	1.88701851	0.81129438	0.420858579	0.72664564	-5.9083488
TICRR	-0.2965837	2.77150277	-0.8111929	0.420916307	0.72664564	-5.9297096
EPB41L4A	0.54437038	-1.0701385	0.81116818	0.420930391	0.72664564	-5.1922105

SPINK5	-0.8272395	-1.0728539	-0.811132	0.420951004	0.72664564	-5.1005952
ZNF691	-0.1718593	1.5740074	-0.8109806	0.421037165	0.72664564	-5.752872
ERCC3	-0.0614986	5.63824298	-0.810971	0.421042613	0.72664564	-6.5970952
AVPI1	-0.3250606	1.52954691	-0.8109272	0.421067546	0.72664564	-5.6155184
POLRMT	0.10487086	4.91603986	0.8108115	0.421133403	0.72664564	-6.5474439
NCOA3	0.10819474	5.31171978	0.8107303	0.421179626	0.72664564	-6.5578207
RAB5A	-0.0795995	5.25868892	-0.8106972	0.421198496	0.72664564	-6.5857589
IQCC	-0.2473981	1.71314229	-0.8105725	0.421269462	0.72664564	-5.7551085
KRTCAP2	0.10752361	5.68520001	0.81051911	0.421299865	0.72664564	-6.6134013
PAG1	-0.5050783	-1.0117599	-0.8103545	0.421393579	0.72665669	-5.1828491
FAR2	-0.2549846	4.18706364	-0.8103052	0.421421651	0.72665669	-6.3657866
TMEM223	-0.1993034	2.81283075	-0.8098039	0.421707202	0.72700029	-6.035891
PNKD	0.20948336	2.23751611	0.80967736	0.421779311	0.72700029	-5.857486
CLASP1	0.06618061	5.98748589	0.80965145	0.42179407	0.72700029	-6.6221978
ENTPD7	-0.1687263	3.59721451	-0.8094081	0.421932757	0.72713983	-6.2312422
CEP350	-0.1110695	6.17270506	-0.8092875	0.422001462	0.72715874	-6.6250065
NGLY1	-0.0916263	4.00787613	-0.809057	0.422132866	0.72728568	-6.4167683
ENSCAFG00000005665	-0.1036278	3.70563625	-0.8084305	0.422490093	0.72772423	-6.3316894
PIGC	0.07754633	4.21373094	0.80840795	0.422502962	0.72772423	-6.4138019
C15H1orf50	-0.1226962	3.31578867	-0.8082944	0.422567754	0.72773632	-6.2105728
FLYWCH2	0.13250573	2.75526834	0.80678917	0.423426864	0.72903253	-6.0753599
ENSCAFG00000007705	-0.9920693	2.22689234	-0.8067729	0.423436172	0.72903253	-5.4833671
ELMSAN1	-0.0895095	5.0188536	-0.8065237	0.423578465	0.72913103	-6.5715583
BYSL	-0.112468	4.59824046	-0.80647	0.423609152	0.72913103	-6.4482629
HEG1	-0.1668952	7.02427798	-0.8062341	0.423743962	0.72926342	-6.6132656
GPI	0.08769771	8.31565414	0.80603276	0.423858989	0.72929859	-6.5271128
FLRT2	0.2242074	6.09326429	0.80581444	0.423983763	0.72929859	-6.6282891
ENSCAFG00000006376	-0.0792077	5.18615938	-0.8056057	0.4241031	0.72929859	-6.5862098
SETD1A	-0.0903767	4.73581267	-0.8055891	0.424112592	0.72929859	-6.528104
TKT	-0.1191136	8.25737675	-0.8055735	0.424121501	0.72929859	-6.5327645
PTPRR	-0.5156751	0.10055388	-0.8055287	0.424147101	0.72929859	-5.3456841
MBD5	0.08010674	4.67963942	0.80544585	0.42419447	0.72929859	-6.5548374
PIGU	0.11177511	4.72521436	0.80538792	0.424227594	0.72929859	-6.4920391
SPOCK1	0.47170415	-1.661037	0.80507927	0.42440409	0.72950245	-5.3671968
ENSCAFG00000006373	-0.1825372	3.99139384	-0.8049452	0.424480791	0.72953473	-6.1164814
ZFR	0.07391137	7.3208064	0.80450441	0.424732932	0.72971673	-6.6157681
ENSCAFG00000032575	-0.2334297	0.74480523	-0.8044904	0.424740972	0.72971673	-5.4931389
ENSCAFG00000026010	-0.2813488	1.43315976	-0.8044563	0.424760482	0.72971673	-5.6532783
HSD11B2	0.44961096	-0.9739792	0.80424551	0.424881081	0.72982437	-5.5255679
LRRFIP2	0.09749251	6.05142994	0.80410806	0.42495975	0.72985997	-6.6167301
TEAD4	-0.144196	4.28238889	-0.803729	0.425176729	0.73001625	-6.4398348
TSPAN9	-0.1620913	5.49823104	-0.8036148	0.425242102	0.73001625	-6.6211278
GHR	-0.1825452	5.15575636	-0.8035834	0.425260126	0.73001625	-6.5776088
ADAM9	0.10970403	8.7380388	0.80352024	0.425296272	0.73001625	-6.5102598
ALDH3A2	-0.1152484	4.67637236	-0.803443	0.425340527	0.73001625	-6.4982299
ENSCAFG00000019479	-0.122099	2.76239379	-0.8029521	0.425621702	0.73039931	-6.0719543
RPP30	-0.089127	4.74808695	-0.8026257	0.425808678	0.73056136	-6.5153114
ZNF252	-0.1499673	3.45619007	-0.8025738	0.42583842	0.73056136	-6.2981836
E2F6	-0.1154831	3.80623	-0.8024836	0.42589013	0.73056136	-6.3368293
BPTF	-0.0799852	6.36649157	-0.801895	0.426227523	0.73096318	-6.6316678

HPS5	0.07584488	4.91760708	0.80187252	0.426240436	0.73096318	-6.5366359
ENSCAFG00000031526	0.13841718	3.70575076	0.80163149	0.426378654	0.73107604	-6.3193946
POC1A	-0.2907576	2.79407029	-0.8015553	0.426422331	0.73107604	-5.9467332
NDUFB11	0.0973067	4.41772913	0.80130175	0.426567786	0.73113387	-6.4777722
PDHX	0.09025542	4.06448298	0.8012028	0.426624551	0.73113387	-6.4167065
AFF1	-0.099196	5.59723084	-0.8011414	0.426659788	0.73113387	-6.628359
TRIL	-0.4981336	0.83766574	-0.8010591	0.426706981	0.73113387	-5.4964317
ARID1B	-0.073153	6.24690209	-0.8009906	0.426746284	0.73113387	-6.6327843
TOR1B	0.08468058	4.57243156	0.80076938	0.426873247	0.73116575	-6.4817792
ZNF385D	0.63805019	1.55874568	0.80075589	0.42688099	0.73116575	-5.5562921
POLE	-0.4057707	4.39838281	-0.8003842	0.427094317	0.73143168	-6.2689567
MECOM	0.41238169	2.91550661	0.80024034	0.427176931	0.73147371	-6.2746687
FAM83G	0.5218529	-0.4715043	0.80000518	0.42731196	0.73160547	-5.2283212
ENSCAFG00000029340	-0.1234131	2.98736985	-0.7998394	0.42740717	0.73166903	-6.1078376
ACOT9	-0.0589811	6.37068549	-0.7990556	0.427857472	0.73234036	-6.6315119
ZFP36L2	-0.2308102	6.61114318	-0.7987145	0.428053508	0.73257593	-6.6035192
FAM118B	0.13516591	3.26529172	0.79851518	0.428168116	0.73257593	-6.2151914
GLI1	-0.3820712	1.78248665	-0.7985126	0.428169575	0.73257593	-5.7572367
STIL	-0.3726706	2.23134393	-0.7981998	0.428349478	0.73275838	-5.7435376
GTF2A2	-0.1179393	2.99607687	-0.7980062	0.428460824	0.73275838	-6.0720103
FAM43A	-0.3834404	0.51969341	-0.7980006	0.428464059	0.73275838	-5.774467
ENSCAFG00000001604	-0.2204255	0.56530101	-0.7977154	0.428628106	0.73275838	-5.6696449
TBC1D32	-0.1265453	3.0645833	-0.7976336	0.428675162	0.73275838	-6.137424
HIRIP3	-0.1202505	4.07486725	-0.7975927	0.428698707	0.73275838	-6.4133945
NSUN4	-0.1204835	3.1272454	-0.7975758	0.428708415	0.73275838	-6.1937913
EIF4EBP2	0.10398442	4.170772	0.79743828	0.428787548	0.73275838	-6.432849
AMMECR1	-0.1114064	3.14174272	-0.7973881	0.428816408	0.73275838	-6.1980891
PLA2R1	-0.1673083	6.7183216	-0.7971874	0.428931927	0.73275838	-6.6311433
ENSCAFG00000015774	-0.3810292	-0.8866356	-0.7971734	0.42893999	0.73275838	-5.1713044
ENSCAFG00000029003	0.39558231	-0.88705	0.79711378	0.428974301	0.73275838	-5.2170918
RNPS1	-0.0852828	6.07710643	-0.7968151	0.429146221	0.73295265	-6.6283522
BCS1L	0.0999588	3.62984929	0.79664116	0.429246392	0.73302434	-6.2416491
NELFCD	0.04999606	5.65134969	0.79617796	0.429513156	0.73330341	-6.6162011
SCEL	0.38565465	2.35937756	0.79615524	0.429526242	0.73330341	-5.9964063
IFIT2	0.4433304	-2.3913403	0.79567435	0.429803305	0.73367698	-5.1498402
SENP5	0.10272262	4.91752945	0.79499975	0.430192158	0.73424125	-6.5703048
ZSWIM3	-0.1451716	1.99672001	-0.7948054	0.43030421	0.7342573	-5.9271691
ENSCAFG00000007479	0.0584261	6.2021243	0.79478025	0.430318725	0.7342573	-6.6374675
CDH11	0.23636676	8.63574738	0.79468017	0.430376439	0.7342573	-6.5738482
ENSCAFG00000030556	0.16877288	1.64546874	0.79448726	0.4304877	0.73434765	-5.8140142
SEC23IP	0.05881972	6.66804903	0.79430643	0.430592014	0.73442613	-6.6344944
ARNT	-0.0839994	5.86434894	-0.7939688	0.430786822	0.73450584	-6.6196226
AXL	0.28950348	8.43908994	0.79392164	0.430814032	0.73450584	-6.5776006
ENSCAFG00000004431	-0.308367	-0.2522023	-0.7937244	0.430927831	0.73450584	-5.333738
HSPA1L	0.3038012	1.03166094	0.79354469	0.431031586	0.73450584	-5.5930107
NIFK	-0.095272	5.93406727	-0.7934579	0.43108167	0.73450584	-6.6283629
ZZEF1	-0.0626715	6.01263136	-0.7934182	0.431104604	0.73450584	-6.6329493
UBE2Q1	0.05877983	6.83931954	0.79336713	0.431134089	0.73450584	-6.6320286
ENSCAFG00000029777	-0.0795705	4.78749028	-0.7931467	0.431261347	0.73450584	-6.5425949
PLCXD1	0.27818139	0.63057387	0.79308249	0.43129844	0.73450584	-5.4963067

SH3TC1	-0.5033225	2.80270151	-0.7930354	0.431325603	0.73450584	-5.9733836
SIGLEC1	0.09357311	5.34084357	0.79300918	0.431340771	0.73450584	-6.6044629
ORC1	-0.6049831	1.92365252	-0.7929873	0.43135341	0.73450584	-5.5346752
TNFAIP8	-0.4390469	0.5325183	-0.7929121	0.431396808	0.73450584	-5.4548004
ENSCAFG00000013913	0.11483923	4.01768165	0.79267474	0.431533931	0.73464	-6.4036229
ANK3	-0.4785023	2.81723627	-0.7920283	0.431907426	0.73517648	-5.8616769
TXNDC11	0.08101219	6.22305363	0.79159261	0.432159285	0.73550579	-6.6397973
CD9	-0.2010038	7.3309108	-0.7911411	0.432420358	0.73578921	-6.5955443
NFKB2	-0.0806662	5.47156298	-0.7910282	0.432485644	0.73578921	-6.6043163
ENSCAFG00000014790	0.1765339	2.22899243	0.79095792	0.432526323	0.73578921	-5.863157
GDA	-0.4093443	-2.8467324	-0.7906588	0.432699377	0.73578921	-5.0992921
UBAC2	-0.086724	5.08868524	-0.7906552	0.432701416	0.73578921	-6.6065696
NUF2	-0.8314554	2.145945	-0.7906016	0.432732479	0.73578921	-5.5111528
NUP50	-0.0785832	5.27931383	-0.7905977	0.432734718	0.73578921	-6.5853888
KIFC1	-0.468399	3.95171078	-0.790236	0.432944018	0.73604573	-6.1182659
ENSCAFG00000012747	0.15880007	2.99967914	0.79012079	0.433010708	0.73605977	-6.1372441
PLBD2	0.1746913	7.07615299	0.78993783	0.433116614	0.73607211	-6.626495
KLHL20	-0.0918444	5.22345576	-0.7895891	0.433318504	0.73607211	-6.5967001
SRSF3	-0.1052445	5.82501238	-0.7895696	0.433329798	0.73607211	-6.6170699
HTR1D	0.65797119	1.69755989	0.78929105	0.433491136	0.73607211	-5.3384021
STMN3	-0.3908981	-1.3478152	-0.7892633	0.433507218	0.73607211	-5.339054
TAL2	0.3091855	0.08347129	0.78925215	0.433513667	0.73607211	-5.3996382
PRPS2	-0.1507812	3.38850686	-0.789209	0.433538653	0.73607211	-6.190672
PARP12	-0.0928309	4.69748225	-0.7891643	0.433564571	0.73607211	-6.545182
DLG5	-0.0688052	6.23220138	-0.7891576	0.433568403	0.73607211	-6.637679
WNK2	0.50131844	0.65309868	0.78909906	0.43360234	0.73607211	-5.4795546
USP45	-0.2658714	1.8227534	-0.7885724	0.433907495	0.73649088	-5.7493602
IVNS1ABP	-0.1091684	6.2794001	-0.7884483	0.43397944	0.73651375	-6.6397247
ZBTB40	-0.1110654	4.61624652	-0.7882652	0.434085549	0.73659458	-6.5188426
CYHR1	0.0891839	4.41856613	0.78806349	0.434202485	0.73669376	-6.4667043
TAF5	-0.1769302	1.66829808	-0.7878639	0.434318221	0.73679088	-5.8114064
ENSCAFG00000002828	0.08058946	5.2495218	0.78770397	0.43441095	0.73679854	-6.5876516
CHAF1A	-0.3091036	3.7155544	-0.7875801	0.43448278	0.73679854	-6.1606125
EGFL7	-0.5600092	1.05429051	-0.7874676	0.434548056	0.73679854	-5.8143031
DESI2	-0.0647127	6.24351207	-0.7874527	0.434556714	0.73679854	-6.64319
SLC1A4	0.13796247	6.4191015	0.78720375	0.434701104	0.73694416	-6.6406166
CEP120	-0.0914121	5.53960467	-0.7870784	0.434773837	0.73696827	-6.6240219
ENSCAFG00000013858	-0.3503173	-0.517585	-0.7867655	0.434955383	0.7371768	-5.3097265
CDC42BPB	-0.0605185	7.41078008	-0.7866002	0.435051339	0.73720108	-6.6123165
CCDC69	-0.4257473	-2.4326739	-0.7865392	0.435086758	0.73720108	-5.0965903
ALDH7A1	-0.1305395	5.01558423	-0.7864105	0.435161478	0.73722851	-6.57369
COG5	-0.0686591	4.82021804	-0.7856275	0.435616145	0.73779077	-6.5169152
HMGCLL1	-0.4488391	-2.4231643	-0.7856055	0.435628961	0.73779077	-5.1976671
ENSCAFG00000017087	-0.1592888	1.20159489	-0.7855364	0.435669083	0.73779077	-5.6481507
ENSCAFG00000009117	-0.1711301	5.80082879	-0.7851463	0.43589574	0.73799845	-6.6413753
GGNBP2	0.06063109	5.88845072	0.78512364	0.435908897	0.73799845	-6.6394125
ATAT1	-0.1590461	2.38854076	-0.7849159	0.436029626	0.73810364	-5.939721
CCDC43	0.11066176	3.08638554	0.7845167	0.436261689	0.73839724	-6.2085291
ENSCAFG00000002783	-0.1947047	0.98125648	-0.7841236	0.43649028	0.7384629	-5.570044
ESF1	-0.0930878	4.77411902	-0.7840561	0.436529539	0.7384629	-6.5604777

DNAAF4	0.22524119	2.04223163	0.7839863	0.436570124	0.7384629	-5.854282
ENSCAFG00000028770	-0.0675183	4.89226004	-0.7838201	0.43666681	0.7384629	-6.5569308
CCDC148	0.22474783	0.91833624	0.78381112	0.436672022	0.7384629	-5.5795025
MYO18A	-0.1278826	6.22697217	-0.7836452	0.436768571	0.7384629	-6.6419447
CYB5RL	0.10512576	2.89959705	0.78361562	0.436785758	0.7384629	-6.1475105
ENSCAFG00000018136	-0.1664306	4.01563315	-0.7835827	0.436804922	0.7384629	-6.3721317
ASPN	-0.4573389	8.21081441	-0.7835428	0.436828126	0.7384629	-6.6444048
TUSC2	0.19115877	1.159453	0.78282621	0.437245188	0.73906876	-5.6518585
ELMOD3	-0.1368174	4.51372077	-0.7825012	0.437434449	0.73928946	-6.4584981
GPBP1L1	-0.0713716	5.53516762	-0.7823174	0.437541465	0.73932286	-6.6277131
MIF4GD	-0.1565213	4.05600774	-0.7822164	0.437600295	0.73932286	-6.3982132
ATE1	-0.0472903	6.43562126	-0.7821649	0.437630298	0.73932286	-6.6474154
SNAPC1	0.09506788	3.96562202	0.7818953	0.437787342	0.73942049	-6.4025778
LRIG3	-0.5621108	1.70668332	-0.7818641	0.437805496	0.73942049	-5.5255446
ENSCAFG00000031375	0.48300531	-1.5882772	0.78167415	0.437916192	0.73947719	-5.1869393
RHNO1	0.17370696	2.65381946	0.78160501	0.437956483	0.73947719	-5.9470112
ENSCAFG00000003316	-0.3334646	0.68646894	-0.7814186	0.438065135	0.73956151	-5.3821104
WDR27	-0.3511613	0.66151682	-0.7809694	0.438326936	0.73990433	-5.4715848
POLR3B	-0.1054252	5.04527939	-0.7805583	0.438566664	0.74003818	-6.5455117
RGS11	0.15299571	3.53125123	0.78055588	0.4385681	0.74003818	-6.3992457
RCOR2	-0.2846821	1.03906829	-0.7805312	0.438582485	0.74003818	-5.7066217
LRRN2	0.54551602	-0.367953	0.78029273	0.438721589	0.74013946	-5.3059288
PPP1R3B	0.15436843	3.452893	0.78019132	0.438780743	0.74013946	-6.3854695
CCNH	-0.08087	4.38275375	-0.7801261	0.438818789	0.74013946	-6.4314167
PSD3	-0.1227072	3.70815739	-0.7798897	0.438956698	0.7401925	-6.3108883
ENSCAFG00000031827	0.18730002	5.85279012	0.77980815	0.439004316	0.7401925	-6.622696
CCDC8	0.27758147	2.84332142	0.77977008	0.439026529	0.7401925	-6.0107585
FBXO34	-0.0915398	5.2744092	-0.7794843	0.439193315	0.74031107	-6.5919328
DNAJB2	0.08726683	5.10540615	0.77944819	0.439214401	0.74031107	-6.5617806
CNNM2	0.34872156	3.91009208	0.77905572	0.439443532	0.74040048	-6.0669988
SELENBP1	-0.3505659	3.80655688	-0.778996	0.439478409	0.74040048	-6.3010379
ENSCAFG00000031840	-0.3287243	-0.0546887	-0.7787256	0.439636333	0.74040048	-5.4348637
MET	0.19046684	6.78301503	0.7786256	0.439694721	0.74040048	-6.6499849
TERF2	-0.068911	4.20955205	-0.7784001	0.439826467	0.74040048	-6.4504471
USF2	0.08587423	6.23175991	0.77839532	0.43982924	0.74040048	-6.650185
CENPM	-0.5281941	1.0826575	-0.7781451	0.439975422	0.74040048	-5.4186824
SLC38A9	-0.1265242	3.17547837	-0.7779398	0.440095424	0.74040048	-6.2107866
C6H7orf43	0.12257537	3.79433243	0.77791938	0.440107334	0.74040048	-6.2904063
EIF1AX	-0.0785014	6.63879282	-0.7778255	0.440162188	0.74040048	-6.6497881
ENSCAFG00000012643	0.35417578	3.49357345	0.77768977	0.440241537	0.74040048	-6.2096802
COL6A2	0.27447924	9.9184793	0.77768665	0.440243364	0.74040048	-6.4293736
AJM1	0.3139623	-0.968432	0.77752889	0.440335585	0.74040048	-5.4284338
ENSCAFG00000022727	-0.3199528	-1.4238345	-0.7775283	0.440335931	0.74040048	-5.3281465
FEN1	0.22535759	3.3508986	0.7774927	0.440356739	0.74040048	-6.1070106
TCERG1	-0.1106068	5.72752434	-0.7773437	0.440443845	0.74040048	-6.6307787
ICK	-0.1051469	6.01503388	-0.7772948	0.440472452	0.74040048	-6.6431168
LMBRD1	0.11712287	5.77274755	0.77727758	0.440482512	0.74040048	-6.6229183
LRRC58	0.10309518	5.45311753	0.777257	0.44049455	0.74040048	-6.6220249
DDX1	-0.0749199	7.29522762	-0.7772345	0.440507694	0.74040048	-6.6325707
HIBCH	-0.1184619	4.74531593	-0.7772152	0.440518988	0.74040048	-6.5760408

SCARA3	0.27596293	7.70125713	0.77714401	0.44056062	0.74040048	-6.5895406
ALPK1	-0.1808076	3.08651155	-0.7769488	0.4406748	0.74040524	-6.1842023
EXOSC9	-0.1147611	4.43684321	-0.7767691	0.44077988	0.74040524	-6.4718854
ENSCAFG00000030310	0.25748127	2.97057534	0.77673123	0.440802041	0.74040524	-6.1186916
KIAA0408	0.65920384	-1.4407712	0.77662957	0.440861516	0.74040524	-5.2110952
ECH1	0.09205676	4.84765969	0.77656009	0.44090216	0.74040524	-6.5775627
TMEM35A	0.27398651	-0.6168435	0.77653619	0.440916141	0.74040524	-5.4131392
TMEM115	0.08665026	4.6894153	0.77639068	0.441001279	0.7404495	-6.544236
LYSMD2	0.4098482	-1.4167079	0.77613089	0.441153306	0.74060603	-5.2599575
IL6R	-0.4235683	-0.7122283	-0.7758547	0.441314962	0.74068613	-5.4412708
ASMTL	-0.1510828	3.75001549	-0.7758484	0.441318624	0.74068613	-6.3510467
OARD1	-0.1076334	2.80137612	-0.7752343	0.441678186	0.74119084	-6.1192257
ENSCAFG00000012422	0.38967063	-0.5924056	0.77512501	0.441742222	0.74119955	-5.2771751
ZFAND3	0.09690071	5.79833481	0.77488497	0.441882822	0.74126189	-6.6451174
MARK3	-0.0732727	5.78988392	-0.7748606	0.441897074	0.74126189	-6.6412581
CSK	0.06510643	5.46248635	0.77445997	0.442131835	0.74155694	-6.6256818
LGR4	-0.2054955	4.57077728	-0.7739368	0.442438506	0.74180691	-6.6389668
CACNA1C	0.23587812	4.60184084	0.77380281	0.442517037	0.74180691	-6.293149
C5H16orf70	0.06121579	4.05249721	0.77376058	0.442541794	0.74180691	-6.4367283
THOC7	-0.0739545	5.27085301	-0.7737289	0.442560368	0.74180691	-6.6134571
KCND3	0.41724602	-1.6482198	0.77370337	0.442575337	0.74180691	-5.2987303
LRP1	0.27304602	11.3261019	0.7734851	0.442703333	0.74192272	-6.337776
ROM1	0.20849533	1.04459776	0.77330907	0.442806572	0.74199702	-5.7315639
TRNAU1AP	0.08653071	3.8371425	0.77282504	0.443090527	0.74237407	-6.392598
ACACA	-0.1074043	6.8027735	-0.7722934	0.443402542	0.74273609	-6.6522048
C1QTNF6	-0.1944739	5.34535882	-0.7722115	0.443450623	0.74273609	-6.5026904
MFNG	-0.4385383	-2.2991419	-0.7721555	0.443483495	0.74273609	-5.2397351
RELB	-0.1312382	3.9421783	-0.7720487	0.443546209	0.74274236	-6.3717859
RRP1	-0.0872177	4.94327025	-0.7714906	0.443873923	0.74319233	-6.5601163
RNASEH2B	-0.1170444	4.94594885	-0.7713255	0.443970924	0.74325595	-6.5983954
ENSCAFG00000028982	-0.3855931	2.74731918	-0.7707271	0.444322507	0.74358843	-5.8318473
ENSCAFG00000029416	0.30079109	0.58381252	0.7706984	0.444339373	0.74358843	-5.4627869
RMND5B	-0.0764746	5.81098563	-0.7706861	0.444346628	0.74358843	-6.6406359
BTBD10	0.0754474	4.85854632	0.77020641	0.444628586	0.74396143	-6.5394338
SETD6	-0.106643	4.33059572	-0.7697521	0.444895767	0.74430961	-6.4967284
NDE1	-0.1664908	2.79091867	-0.7695246	0.445029556	0.74443457	-6.1419341
ENSCAFG00000022269	-0.2400059	1.03777044	-0.769241	0.445196384	0.74461475	-5.5758081
ZCCHC24	0.15815873	6.26860903	0.76912435	0.445265048	0.74463071	-6.6577391
MCAT	-0.1274371	4.70992116	-0.7689048	0.445394275	0.74473254	-6.5311425
C5H1orf174	-0.0827435	3.54433317	-0.7687175	0.445504507	0.74473254	-6.3287869
ENSCAFG00000000112	0.21625917	1.24786803	0.7686426	0.445548583	0.74473254	-5.653406
FURIN	0.08223718	6.5069857	0.76847308	0.445648381	0.74473254	-6.6571861
SELP	-0.5919467	-2.0639863	-0.7684196	0.445679865	0.74473254	-5.2422283
CIAO2B	0.09190593	4.18156588	0.76841055	0.445685197	0.74473254	-6.4266329
PLEKHM3	0.09294049	4.3710216	0.76831779	0.44573981	0.74473254	-6.4846866
NNT	0.07355461	7.64444366	0.76806569	0.445888262	0.74474279	-6.6376771
SIRT3	-0.0834953	3.84184501	-0.7680642	0.445889126	0.74474279	-6.3673825
ENSCAFG00000005319	-0.5745743	0.22724766	-0.7679609	0.445950002	0.74474279	-5.2084125
SESN1	-0.2532314	3.71747274	-0.7679058	0.445982445	0.74474279	-6.5290381
ENSCAFG00000011904	-0.3705632	-0.6648977	-0.7677062	0.446099981	0.7447687	-5.282406

ATP5PB	0.06553586	6.87372166	0.76767869	0.446116215	0.7447687	-6.6537829
ENSCAFG00000003077	0.16071467	2.80155542	0.76732018	0.446327442	0.74502259	-6.0019771
ZNF385B	1.08370303	-0.1537146	0.76661314	0.446744188	0.74561942	-5.1466349
SHISAL1	0.51182471	-1.8598827	0.76613672	0.447025132	0.74588087	-5.1797051
GMFG	-0.2700778	0.59593208	-0.7661051	0.447043759	0.74588087	-5.6072719
ENSCAFG00000029055	0.14746467	2.79278233	0.76604012	0.447082107	0.74588087	-6.0407412
EML4	-0.13956	5.54023332	-0.7659459	0.447137701	0.74588087	-6.6139609
ENSCAFG00000002350	-0.2766398	0.40197703	-0.7657931	0.447227816	0.74590085	-5.5818899
ENSCAFG00000016246	-0.0871664	4.37562391	-0.7657129	0.447275166	0.74590085	-6.5049955
ENSCAFG00000016728	0.08860324	5.60094612	0.76559282	0.447345993	0.74590085	-6.632444
SLC7A5	0.23891787	7.66991262	0.7655241	0.447386542	0.74590085	-6.6482603
DTX2	-0.1184084	4.69470417	-0.7650403	0.447672082	0.74627813	-6.5681409
UBR4	0.09712412	7.69880943	0.7648215	0.447801246	0.74639467	-6.6223542
ENSCAFG00000013638	0.175438	1.79220285	0.76448868	0.447997773	0.74662344	-5.8284885
TSR3	-0.081186	4.89296954	-0.7643649	0.448070875	0.74664648	-6.5508017
XPA	-0.1382127	3.13979999	-0.7635068	0.44857783	0.74728674	-6.2544943
DHDH	-0.4128684	-0.5144266	-0.7634261	0.448625567	0.74728674	-5.5580378
TMEM43	0.10100652	7.16114335	0.76330575	0.448696673	0.74728674	-6.6465991
ENSCAFG00000015459	-0.0700889	4.57030842	-0.763253	0.448727884	0.74728674	-6.4951338
SEC16A	-0.0606029	7.38438859	-0.7632126	0.448751736	0.74728674	-6.6455736
TMED9	0.07318373	6.51390885	0.76301553	0.448868239	0.74737106	-6.6622536
ATP8B2	0.08975075	8.03310584	0.7628994	0.448936906	0.74737106	-6.5975939
RORA	0.20865363	3.38227871	0.76271432	0.449046343	0.74737106	-6.3064449
ENSCAFG00000006021	-0.0868283	4.68247439	-0.762637	0.449092059	0.74737106	-6.5712099
ABHD10	-0.1594706	3.14590787	-0.7625481	0.449144673	0.74737106	-6.1871231
ADAM12	0.19465889	8.74796798	0.76245375	0.44920045	0.74737106	-6.5782939
LRRN3	-0.4785482	-1.9485995	-0.7623583	0.449256911	0.74737106	-5.2383151
MYO10	0.18928599	8.7029785	0.76232426	0.449277045	0.74737106	-6.6013071
AVL9	-0.0892771	4.36342629	-0.7622072	0.449346299	0.74738756	-6.4968972
WASF2	0.09130396	7.397331	0.76208732	0.449417218	0.74740682	-6.6236949
DDX6	-0.0774818	5.12534002	-0.7616053	0.449702468	0.74766496	-6.5919272
SH3GLB1	-0.0621105	6.25800768	-0.7613444	0.449856899	0.74766496	-6.6609306
EVA1C	-0.1561836	1.94510241	-0.7612336	0.449922509	0.74766496	-6.5313309
C15H4orf46	-0.2538017	1.05477083	-0.7610681	0.450020489	0.74766496	-5.6234662
CASP2	-0.1103204	5.20226375	-0.7609997	0.450060947	0.74766496	-6.5766086
KIAA0586	-0.0866939	5.35436446	-0.760984	0.450070256	0.74766496	-6.6215596
IGDCC3	0.30961584	0.66929941	0.76095999	0.450084488	0.74766496	-5.5397087
DBT	-0.0988117	3.83511873	-0.7609171	0.45010989	0.74766496	-6.3842568
ENSCAFG00000020536	-0.3691823	0.406951	-0.7608411	0.450154917	0.74766496	-5.3958549
PTER	-0.2151279	1.66041977	-0.7608223	0.450166011	0.74766496	-5.9065421
DHX33	-0.0778755	5.53410893	-0.7605954	0.45030041	0.74773744	-6.6202773
ENSCAFG00000005024	-0.0599001	5.81855906	-0.7605482	0.450328373	0.74773744	-6.654033
JADE2	-0.2483685	4.8216867	-0.7598243	0.450757274	0.74824826	-6.6501053
PTPA	0.06375221	5.86728242	0.75953365	0.450929567	0.74824826	-6.6552554
PHF6	-0.0789959	5.21621691	-0.7594646	0.450970486	0.74824826	-6.6141846
ISG15	-0.4296191	-1.744622	-0.759419	0.450997539	0.74824826	-5.1933633
ENSCAFG00000006734	0.09274866	3.03183393	0.75920408	0.451124968	0.74824826	-6.2124434
PLD2	-0.1227199	5.06139331	-0.7591699	0.451145217	0.74824826	-6.562621
SLC25A40	-0.1127867	2.8625034	-0.759133	0.451167142	0.74824826	-6.1793208
COQ6	-0.0859001	4.25701281	-0.7590874	0.451194131	0.74824826	-6.4198864

POP1	-0.1521786	3.92462477	-0.759077	0.451200321	0.74824826	-6.3263765
ZNF48	-0.093997	4.11249961	-0.7589825	0.451256369	0.74824826	-6.4394636
NRP2	-0.2899988	8.66052319	-0.7588858	0.451313689	0.74824826	-6.6095805
DUOX1	0.57703696	-1.9408292	0.75881745	0.451354257	0.74824826	-5.0957748
MGAT4B	0.12630838	6.88492718	0.75870206	0.451422699	0.74824826	-6.6449777
SNRNP40	-0.1018828	4.63506824	-0.7584823	0.451553096	0.74824826	-6.5236287
COX17	0.13875508	2.47135827	0.75843452	0.451581416	0.74824826	-5.96504
CSNK2A2	0.0576291	5.22501754	0.75842599	0.451586477	0.74824826	-6.6133798
FCHO1	0.41639453	-1.396738	0.75822076	0.451708253	0.74835159	-5.231979
AGAP1	-0.1055279	4.25025002	-0.757902	0.451897447	0.74839765	-6.4711945
TBC1D22B	-0.119317	2.9422708	-0.7575287	0.452119001	0.74839765	-6.1198185
ZWINT	-0.3809124	2.3638377	-0.7574901	0.452141934	0.74839765	-5.7755865
RALA	0.10255569	5.67713765	0.75746032	0.452159626	0.74839765	-6.6502362
CPSF2	-0.0689798	6.14293041	-0.7573581	0.45222035	0.74839765	-6.6634953
IFI44	0.34727488	2.67638463	0.75731339	0.452246873	0.74839765	-6.2741664
GNS	0.07577007	6.77180426	0.75723508	0.452293375	0.74839765	-6.6653334
ISG20L2	0.08005535	4.9238536	0.75716721	0.452333679	0.74839765	-6.5459789
MAP6D1	0.27204529	0.43570948	0.75715342	0.45234187	0.74839765	-5.5833281
C7H1orf112	0.1937444	3.13561879	0.75706041	0.45239711	0.74839765	-6.1770865
TTC31	-0.0865727	4.25324342	-0.7570152	0.452423977	0.74839765	-6.4521588
PHYHD1	-0.1262075	1.98485788	-0.7569171	0.452482235	0.74839765	-6.0587321
ERI3	0.07325924	4.645544	0.75687295	0.452508458	0.74839765	-6.564266
ENSCAFG00000025332	0.19177338	4.22929781	0.75673606	0.452589775	0.74843387	-6.5566072
UQCRQ	0.11063452	4.42901702	0.75639836	0.45279042	0.7484667	-6.4759287
ARFGAP3	-0.0692999	7.02088209	-0.7563541	0.452816729	0.7484667	-6.6607979
INTS8	-0.0726017	4.80633825	-0.7563303	0.452830869	0.7484667	-6.5920885
SETD5	-0.0753788	7.08278657	-0.7563026	0.452847308	0.7484667	-6.6524221
LENG1	-0.1351056	2.36994291	-0.7560587	0.452992306	0.74860812	-6.0681567
PPM1G	-0.0834944	6.25186631	-0.7557664	0.453166028	0.74879697	-6.6660225
ZNF16	-0.1400495	2.60480579	-0.7556023	0.453263616	0.74880176	-6.0203748
AKAP13	0.06473675	8.59196963	0.7555193	0.453312957	0.74880176	-6.552397
NPEPPS	0.0908614	7.42894372	0.75538413	0.453393336	0.74880176	-6.6455747
C18H11orf68	-0.1011826	5.32132364	-0.7553616	0.453406718	0.74880176	-6.6027625
YIPF5	0.07477139	5.67809961	0.7548313	0.453722165	0.74922449	-6.6412017
SPPL3	-0.1077911	4.04872018	-0.7546789	0.453812841	0.74926217	-6.4239699
PHKA1	-0.0811291	4.70499434	-0.7543458	0.454011084	0.74926217	-6.5448679
ZNF554	-0.2505739	-0.2634637	-0.7543166	0.454028419	0.74926217	-5.4661075
EYA4	1.05445306	1.99135264	0.75425336	0.45406608	0.74926217	-5.4328736
PPM1D	-0.133189	3.36111421	-0.7542318	0.45407894	0.74926217	-6.3303596
PLRG1	-0.056148	5.35089985	-0.7541061	0.454153737	0.74926217	-6.6277654
CDK5RAP2	-0.0743029	5.65903876	-0.7540748	0.45417235	0.74926217	-6.6418517
ABAT	-0.2088688	2.39213997	-0.7539933	0.45422086	0.74926217	-6.1088825
TSC22D4	-0.1134969	5.27924975	-0.7535833	0.454464974	0.74938914	-6.6382017
POLR3H	0.10198925	3.66275756	0.75356695	0.454474738	0.74938914	-6.3501571
ENSCAFG00000003315	0.11354998	4.74020508	0.75353289	0.454495021	0.74938914	-6.5498219
TMEM126B	-0.0915216	3.86018754	-0.7534223	0.454560893	0.74938914	-6.314161
STPG2	-0.3501157	-0.6247397	-0.7532945	0.454636998	0.74938914	-5.37872
CWC22	-0.082999	5.04790679	-0.7532646	0.454654799	0.74938914	-6.6054403
COQ8B	-0.1022586	3.90136945	-0.7530947	0.454756043	0.74945795	-6.368217
NUDT6	-0.2216458	1.43787126	-0.7526221	0.455037595	0.74982386	-5.8327474

DMAP1	-0.0560581	5.81239221	-0.7521502	0.455318887	0.75016046	-6.6427958
ENSCAFG00000031969	0.3893959	-2.4163279	0.75199724	0.455410073	0.75016046	-5.2300802
RIOX1	-0.1042343	3.5566636	-0.7519253	0.455452943	0.75016046	-6.3351042
ADAMTS6	0.26055296	4.75646485	0.75187982	0.455480088	0.75016046	-6.5984965
SLC4A7	0.16866886	5.56667474	0.75170353	0.455585212	0.7502355	-6.664126
KLHL36	-0.1039585	4.56596485	-0.7515296	0.455688931	0.75030821	-6.5431224
PPP4R2	-0.0845825	5.84124188	-0.751321	0.455813375	0.75031974	-6.6613432
ENSCAFG00000015648	-0.1176323	2.69177993	-0.7513181	0.455815075	0.75031974	-6.1884324
ENSCAFG00000013203	-0.0793636	3.80714726	-0.7511438	0.455919097	0.75038995	-6.3993516
MTFMT	0.13279528	2.80009463	0.75096477	0.456025895	0.75038995	-6.1859384
PMAIP1	-0.4366269	-1.876937	-0.7509471	0.456036446	0.75038995	-5.1905321
ENSCAFG00000017554	0.44689926	-1.5227278	0.75079106	0.45612955	0.75044512	-5.1831685
GABRE	0.55022254	-0.972141	0.75054378	0.456277136	0.75049186	-5.3171156
MLNR	-0.2550178	0.16329771	-0.7504514	0.456332256	0.75049186	-5.6217389
EXOC4	0.07639195	6.41386311	0.75044397	0.456336709	0.75049186	-6.6721519
DENND1B	0.17125905	2.22485025	0.7499438	0.456635331	0.75079871	-5.8616835
ENSCAFG00000025482	0.30638817	-0.810576	0.74993179	0.456642498	0.75079871	-5.3650788
JUP	0.20406373	7.20016204	0.74954318	0.456874593	0.75108227	-6.6384015
GFPT1	0.08841103	6.61732255	0.7489788	0.457211789	0.75153852	-6.6694379
AGO1	0.07398224	4.99411184	0.74863569	0.457416851	0.75177749	-6.6011977
ELP2	-0.0643117	5.66532938	-0.7485135	0.457489919	0.75179948	-6.6446933
ABCF3	-0.0782316	5.01112141	-0.7483707	0.45757528	0.75184167	-6.5962518
SOX12	-0.1557821	2.15742621	-0.7482281	0.457660529	0.75188366	-5.8675729
HYAL3	0.14193286	3.1238131	0.74799112	0.45780223	0.75201837	-6.2542695
KLK4	0.3664948	-0.6484782	0.74776425	0.457937917	0.75214317	-5.3956631
SELENOI	0.17446905	4.42009833	0.7472008	0.458275004	0.75248699	-6.5205732
ADPRM	0.15058439	2.52312893	0.74693434	0.458434465	0.75248699	-6.1108827
MAP3K5	-0.2218116	3.49505676	-0.7466769	0.458588531	0.75248699	-6.5992178
TBC1D10C	-0.3917973	-1.4674525	-0.7465238	0.458680237	0.75248699	-5.326995
CYCS	0.12468132	6.07640143	0.74652339	0.458680459	0.75248699	-6.6664929
DLL1	-0.39144	0.81898117	-0.7464433	0.458728411	0.75248699	-6.0321898
PHF5A	-0.0695439	5.44373828	-0.7464085	0.458749267	0.75248699	-6.626472
RFLNB	-0.3174863	1.11709849	-0.7464002	0.458754216	0.75248699	-5.5941187
LEMD2	-0.089863	4.35434104	-0.7462258	0.458858665	0.75248699	-6.5003061
UCHL5	0.09134434	6.31881473	0.74616036	0.458897831	0.75248699	-6.6751841
LAPTM4B	-0.6890864	-1.3643943	-0.7461133	0.458926037	0.75248699	-5.1615698
MYOF	-0.0917616	9.1556083	-0.746005	0.458990848	0.75248699	-6.5435746
CDKN2C	-0.3541545	2.91043433	-0.7459181	0.459042952	0.75248699	-5.9186867
SOCS4	0.10769308	3.46525764	0.7458608	0.459077242	0.75248699	-6.3582011
DNTTIP2	-0.0984841	6.24170958	-0.7457664	0.459133793	0.75248699	-6.6708592
TAS1R2	0.08549092	6.55900265	0.74565225	0.459202172	0.75248699	-6.6747746
GALNT17	0.62122133	1.434169	0.74563203	0.459214288	0.75248699	-5.6025742
COQ8A	-0.1722329	3.24556251	-0.7456182	0.459222573	0.75248699	-6.3901592
THRA	-0.1240413	3.64739781	-0.7454551	0.459320266	0.75254918	-6.3908257
HECW1	0.46422832	0.03110437	0.74528633	0.459421418	0.75261701	-5.728563
GREM2	0.29147774	3.70099984	0.74513954	0.459509387	0.75266323	-6.4108883
KPTN	0.17600162	1.58824389	0.74501548	0.459583745	0.75268715	-5.8143908
SRSF4	-0.0753697	4.78889874	-0.74475	0.459742855	0.75283817	-6.5681248
DKK1	0.52839716	6.24010502	0.74466224	0.459795492	0.75283817	-6.6478179
FAM135A	-0.1188904	5.15027763	-0.7442785	0.460025589	0.75311702	-6.6120565

DCUN1D5	-0.1245689	3.92947414	-0.7441758	0.460087181	0.75311996	-6.4046465
TCTEX1D4	0.30561808	0.76281855	0.74404556	0.460165298	0.75314996	-5.5716806
ENSCAFG00000015454	-0.0731194	5.75506354	-0.7437557	0.46033916	0.75333663	-6.660794
AMT	-0.1011431	4.56121723	-0.7432236	0.46065847	0.75357984	-6.5603672
PARL	0.0794857	5.65424739	0.74315152	0.460701727	0.75357984	-6.6587408
HMBOX1	-0.1392623	2.26731364	-0.7431223	0.460719289	0.75357984	-5.9753967
NDUFS8	0.1556996	5.35656304	0.74310928	0.460727083	0.75357984	-6.6417231
DIAPH2	0.10639235	6.0438913	0.74280603	0.460909122	0.75359175	-6.6778427
MEPCE	-0.0753765	4.76212111	-0.742645	0.4610058	0.75359175	-6.5767518
ERAP1	-0.139059	5.4623419	-0.7423577	0.461178302	0.75359175	-6.6671485
LRP8	-0.1524543	4.97876045	-0.7423502	0.461182837	0.75359175	-6.6218266
TEX28	0.52308358	-0.6917002	0.74232947	0.461195281	0.75359175	-5.3406916
ENSCAFG00000005387	-0.2175633	0.00640434	-0.742234	0.461252609	0.75359175	-5.4445544
NDUFV3	0.05398007	5.71207773	0.74210944	0.461327433	0.75359175	-6.6591816
GDI2	-0.0473144	7.73809642	-0.7420724	0.461349659	0.75359175	-6.6413931
VASN	0.36302874	6.27938838	0.74204305	0.461367317	0.75359175	-6.6729777
ACO2	0.06968765	7.56598839	0.7419346	0.461432468	0.75359175	-6.6395977
PKP2	0.58055463	4.20664144	0.74190191	0.461452103	0.75359175	-6.167119
COL1A1	0.2561962	13.7530172	0.74183558	0.461491957	0.75359175	-6.2531572
INKA1	-0.1893019	1.70559254	-0.741802	0.461512128	0.75359175	-5.9713059
KIAA1328	-0.0917243	3.36496647	-0.741282	0.461824599	0.75393101	-6.3300591
KLHL22	0.08150734	4.17560532	0.74125706	0.461839608	0.75393101	-6.4941471
ZNHIT6	0.09609684	4.03196181	0.74114732	0.461905567	0.75394098	-6.4699967
GMEB2	0.09557885	3.65400746	0.74098191	0.462005004	0.75400558	-6.3593257
ENSCAFG00000009798	0.11745169	3.09188314	0.74083914	0.46209084	0.75404796	-6.3250193
GLT8D2	0.26678559	4.70541704	0.74043479	0.46233399	0.75434701	-6.6260872
NCAPH	-0.5970559	3.05574958	-0.7402168	0.462465092	0.75439444	-5.8501628
KDM5A	-0.0789875	5.08953565	-0.740145	0.462508305	0.75439444	-6.6411672
TMEM165	0.07574268	5.83364475	0.73998415	0.462605066	0.75439444	-6.6643004
ABCA1	-0.4279196	5.00358648	-0.7399791	0.462608089	0.75439444	-6.5184043
ADSS	-0.0930366	6.47923639	-0.7398781	0.462668844	0.75439444	-6.6787164
DCHS1	-0.2467143	5.2954497	-0.7397134	0.462767979	0.75439444	-6.5480373
WDR74	0.08424616	3.68359343	0.73949791	0.462897654	0.75439444	-6.3456852
CDC42EP1	0.18291885	5.14345844	0.73927074	0.46303439	0.75439444	-6.5370191
TET1	0.34229125	0.83787146	0.73924559	0.463049529	0.75439444	-5.5038498
NPDC1	-0.3738703	1.41999711	-0.7392252	0.463061815	0.75439444	-5.9165667
MCF2L	0.28680601	0.39862411	0.73915911	0.463101587	0.75439444	-5.6578783
HIST2H2BF	0.19729604	1.15426969	0.73901589	0.463187812	0.75439444	-5.5875432
HID1	0.36676077	-0.1018076	0.73900032	0.463197188	0.75439444	-5.5629658
NR3C1	0.09195454	6.95410648	0.73899308	0.463201542	0.75439444	-6.6742847
SRXN1	0.17296088	2.66390456	0.7388407	0.463293292	0.75441654	-6.0977487
AEBP2	-0.0896275	5.84079392	-0.7387716	0.463334896	0.75441654	-6.6612376
DMTN	0.37247149	-0.5168721	0.73861898	0.463426815	0.75446868	-5.4062981
ZNF410	-0.09536	4.12858846	-0.7384381	0.463535742	0.7545476	-6.4462812
CCDC170	0.45029578	-0.6194424	0.73833598	0.463597268	0.7545476	-5.5503694
SNRNP27	0.08780532	4.05850928	0.73819257	0.463683655	0.7545476	-6.43747
RELN	-0.8519857	-0.0345753	-0.7381407	0.463714909	0.7545476	-5.6152238
NRL	0.31206411	-0.420481	0.73786301	0.463882221	0.75472235	-5.5154123
DHX58	-0.3011992	2.06072844	-0.7375934	0.464044694	0.75484593	-5.9948906
TES	0.18622716	5.89975111	0.73741944	0.464149551	0.75484593	-6.6792477

FAM20A	0.32773749	5.77692047	0.73738115	0.464172636	0.75484593	-6.6788119
MAP3K2	0.09296956	4.36758656	0.73733925	0.464197889	0.75484593	-6.4841305
MYPOP	0.24413177	0.97193296	0.73715706	0.464307724	0.75491089	-5.5997891
SLC14A1	-1.0140203	1.93313843	-0.7370031	0.464400527	0.75491089	-5.5077997
ADORA2A	0.34939522	0.32939106	0.73695254	0.464431043	0.75491089	-5.4249033
RAD51AP1	-0.4756393	1.23678465	-0.7368754	0.464477565	0.75491089	-5.4930568
VGLL3	0.32833515	6.39995691	0.73663563	0.464622155	0.75504847	-6.6818475
ENSCAFG00000003956	-0.3291237	-0.0378381	-0.7360139	0.464997236	0.75548294	-5.2863901
SEC61A1	0.09645386	10.099166	0.73598891	0.465012313	0.75548294	-6.4877239
DDX46	-0.1077212	5.79023993	-0.735804	0.465123917	0.75548294	-6.6653289
NCKIPSD	-0.1032567	3.44669536	-0.7357948	0.465129423	0.75548294	-6.3763004
ENSCAFG00000008026	0.62885737	2.29386102	0.73512647	0.465532892	0.75604078	-5.7125859
DIRC2	0.09743983	4.5115051	0.73497016	0.465627279	0.75609658	-6.5951619
RAB11FIP2	-0.1247136	5.60681757	-0.7347318	0.465771214	0.75623282	-6.6597305
RNF8	0.08353083	4.03659093	0.73458556	0.465859564	0.75627878	-6.4455791
SPICE1	-0.0983905	4.33712744	-0.73409	0.466158966	0.75657787	-6.4920948
CNST	-0.1705201	2.55947806	-0.7340818	0.466163928	0.75657787	-5.9898968
KLHL42	0.07932627	4.71737915	0.73375798	0.466359621	0.75679796	-6.5697773
ENSCAFG00000015713	-0.2947843	-0.6401719	-0.7335785	0.466468125	0.75687653	-5.4157182
TTC38	-0.1637581	3.39310162	-0.7332671	0.466656355	0.75703941	-6.2697916
TMEM267	0.1451499	2.90762925	0.73321362	0.46668871	0.75703941	-6.1525631
DSC3	-0.3943519	-1.0925912	-0.7325796	0.467072145	0.75745744	-5.3280184
KIF23	-0.6745478	3.88064763	-0.7324922	0.467125023	0.75745744	-5.9768877
PHYKPL	-0.1132185	3.57785323	-0.7324893	0.467126819	0.75745744	-6.345164
PLEK2	0.34384308	0.63209732	0.73216098	0.467325457	0.757682	-5.5585251
TBX5	0.69765613	-0.8897474	0.73187391	0.467499192	0.75786613	-5.3331334
UNC79	0.33500873	-1.8506891	0.73175686	0.467570037	0.75788344	-5.345218
ENSCAFG00000015055	0.07727582	4.06786138	0.73154077	0.467700854	0.75799794	-6.4765175
LSM6	-0.0935513	3.98412408	-0.7312835	0.467856598	0.7580831	-6.436101
BHLHE40	0.18507314	8.36933735	0.73117212	0.46792407	0.7580831	-6.6289632
ST6GAL1	-0.6352896	0.44953505	-0.7310653	0.467988754	0.7580831	-5.7622984
C18H11orf95	-0.2193844	1.48220189	-0.7310564	0.467994139	0.7580831	-5.8565569
WBP1	0.12443619	4.80681355	0.7307842	0.468159021	0.75821981	-6.585709
GPN3	0.06981528	5.00809788	0.73067038	0.46822797	0.75821981	-6.5993245
CSRP2	0.21509227	3.3057319	0.73056149	0.468293937	0.75821981	-6.3216294
PEX26	-0.1091634	3.45672439	-0.7305196	0.468319316	0.75821981	-6.3331275
NIF3L1	-0.1357814	3.3068121	-0.7300141	0.468625637	0.75849959	-6.2688029
CYB561	0.10162806	5.2001592	0.72991224	0.46868738	0.75849959	-6.6254984
BCL2L13	-0.0749273	5.04522854	-0.7297829	0.46876581	0.75849959	-6.6093085
ERG28	-0.139813	3.89073991	-0.7297703	0.468773419	0.75849959	-6.4894084
CLBA1	-0.1993408	1.04247176	-0.7295755	0.468891536	0.75849959	-5.7083227
CACNB3	0.15920279	6.35122	0.72956967	0.468895049	0.75849959	-6.68435
ADAMTS20	0.47813717	-2.2417769	0.729539	0.468913648	0.75849959	-5.1596421
THAP2	-0.1784545	1.12950803	-0.7293804	0.469009841	0.75855777	-5.7556931
SIPA1L2	-0.6885071	-0.62438	-0.7291271	0.469163413	0.75870874	-5.2939347
POLB	0.09302405	3.61742111	0.7288772	0.469314999	0.75885645	-6.3322609
C17H2orf68	0.08959207	6.39721965	0.72865622	0.469449057	0.75897578	-6.687693
TLR6	-0.3957304	-2.6694739	-0.7285182	0.469532811	0.75901377	-5.1414181
ABHD13	0.07879016	3.76216253	0.72813117	0.469767669	0.75912449	-6.4134796
EMC6	0.13356314	3.79617732	0.72803794	0.469824252	0.75912449	-6.4085831

ADAR	-0.0871596	6.70774219	-0.7280351	0.469825984	0.75912449	-6.6842093
DCUN1D3	0.11092383	3.03730649	0.7278162	0.469958859	0.75912449	-6.3314118
MRPL21	-0.0980432	5.4780679	-0.727766	0.469989339	0.75912449	-6.6500751
ACPP	0.36279005	2.98092096	0.72764445	0.470063129	0.75912449	-6.0144246
NDC1	-0.1283002	5.44486356	-0.7276193	0.470078369	0.75912449	-6.6303771
ARSI	0.98412351	1.72018033	0.72761099	0.470083442	0.75912449	-5.1463689
CNIH4	0.14043552	2.68639374	0.7274965	0.47015296	0.75913943	-6.0647241
DHX15	-0.0804322	7.00006559	-0.7271334	0.470373471	0.75939813	-6.6821388
CDC42BPA	0.11864994	8.13182192	0.72674767	0.470607792	0.75953643	-6.6356858
GLS2	0.33681052	-0.7293362	0.72657998	0.470709675	0.75953643	-5.3789793
ZNF184	-0.1066683	3.63401626	-0.7265334	0.470737996	0.75953643	-6.3861648
SLC26A11	0.18959306	3.60181909	0.72650574	0.470754789	0.75953643	-6.3234886
HGSNAT	0.0736129	6.10419458	0.72649612	0.470760631	0.75953643	-6.6802526
SV2C	0.53663915	-0.1672486	0.72625553	0.470906844	0.75967503	-5.5064272
TYW5	0.14445002	3.10885566	0.72605027	0.471031603	0.75968757	-6.1985496
INSYN1	0.33338072	1.75600761	0.72589776	0.471124313	0.75968757	-5.5968365
CPD	-0.0710666	8.59044776	-0.7258566	0.471149344	0.75968757	-6.5742922
SRSF1	-0.0795523	5.44132339	-0.7258362	0.471161719	0.75968757	-6.6382306
ENSCAFG00000023721	0.07113181	6.62694341	0.72569126	0.471249859	0.75968757	-6.6904156
BRIX1	-0.0949318	4.38611931	-0.7256475	0.471276488	0.75968757	-6.5237517
NOP9	-0.0697213	4.87843088	-0.725335	0.47146653	0.75989299	-6.5981758
CSTF2T	-0.1219029	3.59200113	-0.725199	0.471549233	0.75989299	-6.3114353
FGFR1OP	-0.1516497	2.98112523	-0.7250095	0.471664468	0.75989299	-6.2118432
APH1A	-0.0805236	5.23556939	-0.7249609	0.471694026	0.75989299	-6.6467974
ZBTB49	-0.1115252	2.54841366	-0.7249347	0.471710006	0.75989299	-6.1021283
PAPPA2	0.62372333	-1.4847225	0.72471248	0.471845198	0.75989299	-5.1456245
ENSCAFG00000021670	0.3793759	-0.7138106	0.7247078	0.471848046	0.75989299	-5.3044523
ANGEL1	-0.1346921	2.20178658	-0.7246445	0.471886546	0.75989299	-6.0889258
RDH5	0.14945283	2.02118535	0.7240074	0.47227432	0.76042021	-5.9753026
MICAL1	-0.0964725	6.3943791	-0.7235279	0.472566266	0.76070025	-6.6915515
SDHAF1	0.16182227	1.81879451	0.72351399	0.472574746	0.76070025	-5.8726377
ENSCAFG00000011354	0.11936188	4.16512699	0.72342422	0.472629417	0.76070025	-6.4527836
RLF	-0.086446	5.25007994	-0.7230961	0.472829267	0.76085379	-6.6584299
CSNK2B	-0.0768458	6.14612207	-0.723045	0.47286043	0.76085379	-6.6883222
ENSCAFG00000018189	-0.2120047	1.49724717	-0.722909	0.472943239	0.76085379	-5.7419594
ENSCAFG00000016971	-0.1046094	3.13053658	-0.7226666	0.473090942	0.76085379	-6.1812814
PPID	-0.0997888	5.57389175	-0.7226467	0.473103095	0.76085379	-6.6659158
JUN	0.17955254	5.69964719	0.72261876	0.473120113	0.76085379	-6.6729207
EIF2AK1	-0.0767513	4.83954537	-0.7225736	0.473147643	0.76085379	-6.5974841
SOX6	0.56547163	-0.7868932	0.72206746	0.473456133	0.76125267	-5.1905196
TMEM236	-0.352904	-0.4864429	-0.721877	0.473572247	0.76134219	-5.5239908
BNIP2	-0.0854392	5.04952016	-0.7216934	0.473684207	0.761425	-6.6280474
STAC3	-0.1722558	1.50696686	-0.7214291	0.473845393	0.76157187	-5.7542748
SEMA3F	0.27604141	4.48443897	0.72134531	0.473896498	0.76157187	-6.5818349
TICAM1	0.11056631	4.14505833	0.7207946	0.474232477	0.7619339	-6.4885071
TSPAN18	-0.2963766	4.0840178	-0.7207031	0.474288313	0.7619339	-6.248068
AGA	-0.1135431	3.84496529	-0.7206419	0.474325673	0.7619339	-6.4126397
MTX1	0.09380977	4.70554833	0.72051665	0.474402095	0.7619339	-6.5498403
RAB39B	-0.170647	1.37488715	-0.7203908	0.474478884	0.7619339	-5.7780744
TMCC1	0.09189551	4.96724402	0.72038128	0.474484718	0.7619339	-6.6303936

TIMM50	-0.0772137	5.25863653	-0.7201682	0.474614776	0.76198866	-6.6446956
ZNF800	-0.0879397	4.0499849	-0.7201272	0.474639806	0.76198866	-6.4955943
ENSCAFG00000030475	-0.1127497	3.74628665	-0.7198979	0.474779796	0.76210316	-6.426007
SMARCA4	0.08262474	7.11001732	0.71974849	0.474871054	0.76210316	-6.6879794
TMEM38B	0.1154512	2.96017101	0.71967572	0.474915489	0.76210316	-6.1430003
MON2	-0.0630568	6.65422818	-0.7194589	0.475047924	0.76210316	-6.6925702
GNG7	0.26345194	-0.2089582	0.71920526	0.475202847	0.76210316	-5.4455769
TIFA	-0.2220238	2.00685155	-0.7192049	0.475203039	0.76210316	-5.9217364
TBC1D5	-0.1036089	6.86941182	-0.719201	0.47520545	0.76210316	-6.6919038
ELMOD2	-0.120533	3.6540694	-0.7191842	0.475215703	0.76210316	-6.3786947
BMP1	0.134296	7.66059912	0.71908118	0.475278655	0.76210316	-6.6532264
RIC8A	-0.0748344	4.51199086	-0.7190198	0.475316166	0.76210316	-6.5296205
NCEH1	0.13582122	4.52986731	0.71890918	0.475383745	0.7621145	-6.547971
ARL6IP4	0.07513956	4.55917395	0.71874125	0.475486364	0.76218201	-6.5522019
DPP8	-0.0621759	6.4846446	-0.718465	0.475655172	0.76235558	-6.6952083
ZDHHC3	-0.1006313	5.87327177	-0.7182909	0.475761632	0.7624292	-6.6889884
CHMP1A	0.07429162	4.1394466	0.71797469	0.475954945	0.76264196	-6.4796933
MRPS27	0.0562978	4.49191678	0.7175799	0.476196371	0.76269321	-6.5327425
ENSCAFG00000015206	-0.5863598	2.83375759	-0.7175688	0.476203132	0.76269321	-6.4250249
HERC2	-0.0808716	6.96693567	-0.7175461	0.476217026	0.76269321	-6.6832883
ZNF235	-0.141382	2.11633998	-0.7175263	0.476229129	0.76269321	-5.9899298
HSPA14	-0.0861531	4.78013071	-0.7172024	0.476427273	0.76282361	-6.6098769
TAPBP	0.11619987	7.74501532	0.71719524	0.476431673	0.76282361	-6.6542789
EBPL	0.12074791	3.62411194	0.71631994	0.476967346	0.76358423	-6.376545
SDE2	0.07066212	5.05046814	0.71621962	0.477028763	0.7635855	-6.6163198
RGS9	0.4615157	-0.3765027	0.71590041	0.477224215	0.76370767	-5.3481499
CENPE	-0.3309438	4.56911856	-0.7158969	0.477226347	0.76370767	-6.4162938
SPTBN4	-0.393224	-2.195512	-0.7156757	0.477361807	0.7638274	-5.2359089
ERMARD	-0.1195287	3.27644348	-0.7154342	0.477509744	0.76390454	-6.255269
RNF151	-0.2778933	0.03139209	-0.7152626	0.477614849	0.76390454	-5.4636157
CAPN7	-0.0859655	4.95819217	-0.7152161	0.477643357	0.76390454	-6.6244613
NUCB1	0.10452628	8.44892997	0.71520103	0.477652599	0.76390454	-6.5922582
ATAD1	0.07495143	5.98056513	0.71485416	0.477865144	0.76414744	-6.6930208
FANCG	-0.1010765	3.60554586	-0.7143745	0.478159155	0.76443277	-6.3579763
TRIM39	0.08600691	2.47494085	0.71436502	0.478164957	0.76443277	-6.0866647
C4H5orf22	-0.113583	4.81959929	-0.7141339	0.478306689	0.76456232	-6.5660332
REEP3	0.09776849	7.91379761	0.7137883	0.478518589	0.76467516	-6.6400473
FOXF1	-0.3686638	4.39287181	-0.7137592	0.478536413	0.76467516	-6.5393664
TNFRSF19	0.43140592	1.35597795	0.71372175	0.478559405	0.76467516	-5.6623064
CPEB3	-0.3247209	-0.646982	-0.7134614	0.478719121	0.76483334	-5.411406
B3GNT2	-0.095079	5.26350117	-0.7131471	0.478911931	0.76492866	-6.6568684
ENSCAFG00000022725	-0.2174551	-0.9310643	-0.7131145	0.478931942	0.76492866	-5.4868371
INPPL1	0.05334474	7.74665229	0.71305842	0.478966343	0.76492866	-6.6670222
ABCB11	-0.4078977	-0.8514175	-0.7128914	0.479068813	0.76492866	-5.5150912
ENSCAFG00000028483	0.17791629	3.29614335	0.71286926	0.479082424	0.76492866	-6.2985885
KPNA6	-0.0736131	5.01930993	-0.7124409	0.479345384	0.76501297	-6.6193679
TRAPPC2	0.09944034	2.93249743	0.71241115	0.47936362	0.76501297	-6.1626253
ENSCAFG00000028550	-0.1667499	2.05312982	-0.7123341	0.479410929	0.76501297	-5.9349885
ZNF219	-0.2260475	2.43516175	-0.7123007	0.479431433	0.76501297	-6.1386669
ARPC1B	0.12595656	7.91554864	0.71223056	0.479474487	0.76501297	-6.6111845

MX1	-0.5020312	0.37770304	-0.7121338	0.479533922	0.76501297	-5.5024238
VIT	-0.6850289	1.58375932	-0.711944	0.479650469	0.76501297	-6.0596834
CPSF1	-0.0763898	6.58253149	-0.7118163	0.479728887	0.76501297	-6.6986598
CSRNP1	0.14584321	4.69872805	0.71181085	0.479732225	0.76501297	-6.4942993
C20H3orf67	-0.0840883	4.69905276	-0.711794	0.479742572	0.76501297	-6.6153274
NT5DC3	0.08542494	2.77862039	0.71156369	0.479884039	0.7650692	-6.1295878
TBXA2R	0.34146804	1.91775174	0.71153883	0.479899311	0.7650692	-6.2007001
CBL	-0.0846142	4.18810041	-0.7113542	0.480012742	0.76507503	-6.5313312
ENSCAFG00000013514	-0.0965284	3.23647809	-0.7113351	0.480024446	0.76507503	-6.2568797
GOLT1B	0.12977286	5.62955703	0.71090942	0.480286039	0.76533171	-6.6848661
KPNA4	0.11710934	6.27820046	0.7108753	0.480307014	0.76533171	-6.7004673
PLEKHA8	-0.1064355	4.02247834	-0.710588	0.480483572	0.76536477	-6.4438716
CCDC28A	-0.1415374	3.360279	-0.7105832	0.480486524	0.76536477	-6.379733
NDUFAF8	0.29812219	-0.0267353	0.71054498	0.480510048	0.76536477	-5.5070852
SHCBP1	-0.8648487	2.77692621	-0.7102882	0.480667888	0.76551937	-5.6678684
ZNF292	-0.1061233	4.73512762	-0.7098127	0.48096032	0.76588827	-6.614724
ENSCAFG00000003792	-0.4369959	-0.4210249	-0.7095337	0.481131932	0.76590612	-5.3006791
TSPOAP1	0.39354771	2.68275072	0.70945831	0.481178317	0.76590612	-6.0484583
GDAP2	0.0791391	4.49454046	0.70942773	0.481197131	0.76590612	-6.552597
PUS10	-0.1278876	2.94624191	-0.7093991	0.481214755	0.76590612	-6.1989303
MEF2A	0.077697	5.35656534	0.70915576	0.481364469	0.76604761	-6.6522617
PARD3B	-0.1536478	7.65482009	-0.709001	0.481459685	0.76610235	-6.6726016
ENSCAFG00000019170	-0.0678298	5.01042382	-0.7088556	0.481549209	0.76614802	-6.6621645
SBNO1	-0.0741836	5.38630966	-0.7086977	0.481646391	0.76620585	-6.6627291
TSNAX-DISC1	-0.0862556	4.8774134	-0.7084402	0.481804895	0.76636121	-6.6060091
NSD2	-0.1473747	6.02834709	-0.7083347	0.481869829	0.76636772	-6.6826339
NMRK1	0.1983933	1.4415723	0.70814599	0.481986054	0.76645579	-5.8505795
BROX	-0.0657177	5.90345593	-0.7079034	0.482135423	0.76659654	-6.6957552
ALYREF	-0.1137802	4.99102436	-0.707732	0.482241032	0.76666768	-6.6082613
RPRD1B	0.0983102	3.67984916	0.70736541	0.482466857	0.76668896	-6.4510802
PRR14	-0.0842794	4.78926676	-0.7073373	0.482484171	0.76668896	-6.5807334
ZNF485	0.15614858	1.84173763	0.70732882	0.482489402	0.76668896	-5.854765
EHMT2	0.09577403	6.00903886	0.70722132	0.482555641	0.76668896	-6.6960569
ANXA1	-0.0965992	10.0836114	-0.7072091	0.482563178	0.76668896	-6.4642978
FRYL	0.09614167	6.58172728	0.70710917	0.48262475	0.76668896	-6.699592
MAFK	0.1180062	2.60546168	0.70701873	0.482680489	0.76668896	-6.1440163
TMEM199	-0.1072115	3.0102308	-0.7068177	0.48280437	0.76672347	-6.2143841
UQCC1	-0.0993646	3.01767841	-0.7067859	0.482823958	0.76672347	-6.2583429
SPNS1	0.08921925	5.53629228	0.70668326	0.482887259	0.76672733	-6.64965
LRCH2	-0.351661	2.56848246	-0.7063801	0.483074143	0.76681048	-5.8101751
ATXN7	0.0822505	3.86230961	0.70634796	0.483093973	0.76681048	-6.4864329
NOG	0.32635434	1.26480178	0.70630209	0.483122258	0.76681048	-5.4611218
NAE1	0.08373645	5.06934657	0.70597132	0.483326234	0.7669363	-6.6300389
GBF1	0.05738826	6.83674654	0.70593451	0.483348934	0.7669363	-6.6983014
TECPR2	0.06363277	4.71182758	0.70578545	0.483440875	0.7669363	-6.5883949
PUS3	-0.0794242	3.39533153	-0.7057786	0.483445078	0.7669363	-6.3262839
NEK10	0.1435886	2.25840699	0.70551042	0.483610538	0.76710217	-6.0861906
HEXB	0.11429583	7.47180555	0.70515674	0.483828767	0.76735169	-6.6633191
ADAL	-0.1149451	2.70574259	-0.7048552	0.484014848	0.76754476	-6.1596724
GPX1	0.12574295	7.9554538	0.70462485	0.484157059	0.76754476	-6.6462741

BCAT2	0.10425576	5.0965718	0.70461622	0.484162391	0.76754476	-6.66559
FAM57A	0.09397485	4.53640757	0.70456462	0.484194243	0.76754476	-6.5400307
TSTD1	0.46143346	-0.3981761	0.70421924	0.484407493	0.76778618	-5.3359704
ZFPL1	-0.0724618	5.59706778	-0.7040777	0.484494909	0.76782812	-6.6707346
RNF220	-0.0836508	5.04061623	-0.7038431	0.484639817	0.76790406	-6.6158945
CASP12	-0.3690609	-0.8963088	-0.7038027	0.48466476	0.76790406	-5.5107653
HOMER1	0.13878745	3.21275953	0.70299469	0.485164004	0.76827826	-6.2851282
UVRAG	-0.0758173	4.69000336	-0.702851	0.485252829	0.76827826	-6.6064147
CCDC184	0.14885976	1.43432007	0.70284986	0.485253519	0.76827826	-5.7653014
ENSCAFG00000014028	-0.4418749	-1.6932979	-0.7028396	0.485259884	0.76827826	-5.1672636
DIS3	-0.1064028	4.76539229	-0.7028384	0.485260629	0.76827826	-6.5951265
CCDC127	0.06667767	3.33940532	0.70282822	0.4852669	0.76827826	-6.3464063
ARHGAP10	0.10310921	6.87592142	0.70253563	0.485447776	0.76841872	-6.6952143
ESRP1	-0.5744542	-0.8761479	-0.7021707	0.485673395	0.76841872	-5.1691661
MRPL19	0.08687088	3.69818056	0.70214573	0.485688864	0.76841872	-6.3830292
RHEBL1	-0.1953201	0.05174752	-0.7021196	0.485705001	0.76841872	-5.5796248
DDX27	-0.080251	5.78607079	-0.7021049	0.485714122	0.76841872	-6.6934277
PTPRG	0.17773135	7.12250536	0.70209272	0.485721646	0.76841872	-6.6796918
ENSCAFG00000028761	-0.3175568	0.2754707	-0.7018814	0.485852377	0.76852048	-5.5093425
NXPH2	0.71124393	-0.6277903	0.70176038	0.485927209	0.76852048	-5.373902
KIAA0355	-0.1234038	5.00141063	-0.7016928	0.485969009	0.76852048	-6.6492348
ZNF706	0.0945426	4.10189101	0.7009114	0.48645254	0.76918858	-6.459097
ENSCAFG00000032208	-0.0723799	5.06544947	-0.7006171	0.486634723	0.76938006	-6.6295427
ENSCAFG00000010466	-0.1224422	2.27659761	-0.7004956	0.486709968	0.76940245	-5.9389794
C20H3orf62	0.1333126	1.09616706	0.70034784	0.486801437	0.76945048	-5.7868897
ARFRP1	0.08519645	5.74644385	0.69987502	0.487094258	0.76981672	-6.6949286
TONSL	-0.3491591	2.25002464	-0.6997342	0.487181487	0.76985799	-5.8704875
MTERF3	-0.0719127	4.41148077	-0.6993313	0.487431113	0.77015583	-6.5538793
ENSCAFG00000013536	-0.1690853	1.62832342	-0.699095	0.48757753	0.77026919	-5.8759896
MORN4	0.13130162	2.34319723	0.6990134	0.48762813	0.77026919	-6.0572146
ANKRD9	0.29900812	1.42609208	0.69891952	0.487686314	0.77026919	-5.79874
COL4A2	0.15740879	10.767491	0.69854077	0.487921109	0.77038342	-6.3981647
ATP8B4	-0.519592	1.30708631	-0.6984414	0.487982701	0.77038342	-5.7496906
CWF19L1	-0.1179127	3.11533184	-0.6984156	0.487998731	0.77038342	-6.2179867
ZDHHC1	0.13992256	4.25882294	0.69840824	0.488003282	0.77038342	-6.5644798
STAM	-0.0672933	5.59066038	-0.6980803	0.488206621	0.77051927	-6.6822312
ENSCAFG00000016569	0.37581862	-0.0431045	0.69799309	0.488260738	0.77051927	-5.4356658
FAM160A1	-0.4411275	0.21672461	-0.6979736	0.488272847	0.77051927	-5.4574366
GNG4	-0.3550309	0.84632029	-0.6977453	0.488414418	0.77064612	-5.5612193
RAB11B	0.09732288	6.44372071	0.69725824	0.488716643	0.7709216	-6.7093412
CDCA3	-0.6270146	2.94442187	-0.6972007	0.488752326	0.7709216	-5.7828327
SS18L2	-0.1444937	2.00888119	-0.697168	0.48877262	0.7709216	-5.8614162
SPPL2B	0.0722215	4.32123964	0.69676265	0.489024244	0.77120176	-6.5308219
ENSCAFG00000028771	0.25644282	0.17480797	0.69665151	0.489093243	0.77120176	-5.5452309
DNAJC5	0.07218223	5.37319942	0.69657606	0.489140082	0.77120176	-6.6567431
C3H5orf30	0.1344968	2.57796853	0.69648737	0.489195147	0.77120176	-6.0446214
GPR180	0.11388062	3.591617	0.6962614	0.489335468	0.77132644	-6.3068534
PROKR2	0.57291928	-1.2406325	0.69596629	0.489518751	0.77137583	-5.2339708
ENSCAFG00000026794	-0.3995827	2.8437105	-0.6959438	0.489532702	0.77137583	-5.9728857
MED11	-0.1681823	1.95589432	-0.6959151	0.489550522	0.77137583	-5.9150024

PURA	-0.0844548	3.09709056	-0.6957399	0.489659391	0.77142433	-6.2604152
FAM185A	-0.1645191	2.05578372	-0.6956684	0.489703792	0.77142433	-5.9663662
FAM76A	-0.0841444	3.57667168	-0.695193	0.489999219	0.77179319	-6.3539101
PAQR5	-0.3861685	-2.4098398	-0.6950436	0.490092041	0.77180579	-5.2360457
TMEM167B	-0.1079213	2.3119498	-0.6949829	0.490129763	0.77180579	-6.0190352
LAMP2	0.09582832	7.71433875	0.69468175	0.490316972	0.77200407	-6.6700704
TM2D2	0.10051276	3.68999564	0.69446667	0.490450694	0.7721181	-6.3853522
SDHA	-0.0705893	6.97922945	-0.6940444	0.490713303	0.77243499	-6.6959809
TMEM87B	0.10365038	4.74471874	0.69355379	0.491018492	0.77281881	-6.561024
PYROXD1	0.08242126	4.99077243	0.69299013	0.491369262	0.77327427	-6.6211607
KLHL11	-0.0718118	4.88457873	-0.692756	0.491514992	0.77335222	-6.6241812
ABHD8	0.1037503	3.40851418	0.6926432	0.491585222	0.77335222	-6.3308068
DNAJB6	0.09372698	4.52038239	0.69248164	0.491685811	0.77335222	-6.5739835
EGFLAM	-0.520494	1.8312268	-0.6924612	0.491698538	0.77335222	-5.9169639
ENSCAFG00000019344	-0.1796309	1.87605508	-0.6920753	0.491938847	0.77335222	-6.0604714
CPSF3	-0.0562225	5.12179865	-0.692048	0.491955867	0.77335222	-6.6550347
HOXB7	-0.5334014	1.30859471	-0.6919112	0.492041081	0.77335222	-5.6518924
CHRNB2	-0.230469	-0.03963	-0.6918808	0.492059995	0.77335222	-5.5447424
UHRF1BP1	0.07567597	4.90509622	0.69173423	0.492151305	0.77335222	-6.6157146
JAM3	-0.1245151	5.37420111	-0.6917294	0.492154289	0.77335222	-6.6887346
ENSCAFG00000002743	0.3093097	0.03079799	0.69170964	0.492166627	0.77335222	-5.4602843
PDCD10	0.08540949	4.75137675	0.69170866	0.49216724	0.77335222	-6.6104022
SKAP2	0.09003745	5.82826563	0.69162885	0.492216956	0.77335222	-6.7103365
PIK3C2B	0.20092505	4.36047564	0.69141565	0.492349797	0.77346446	-6.4260494
ENSCAFG00000017707	0.23269104	-0.3620258	0.69131098	0.49241502	0.77347046	-5.3757156
CCDC58	-0.0966141	3.4118191	-0.6911294	0.492528173	0.77355173	-6.3179034
ENSCAFG00000031934	-0.2084368	1.29146586	-0.6907729	0.492750418	0.7738043	-5.7297571
SKA1	0.06551291	4.88893442	0.69037621	0.492997721	0.77393187	-6.6150807
USP30	0.05484699	5.03605913	0.69036208	0.493006528	0.77393187	-6.6448954
CDC34	0.06945062	5.67133829	0.69034692	0.493015985	0.77393187	-6.6949783
RPS6KC1	0.06463374	6.19189504	0.69020107	0.493106937	0.77397819	-6.7137881
UBN2	-0.1092638	2.76713601	-0.6899672	0.493252827	0.77411072	-6.20023
PUM3	-0.0874332	5.85910185	-0.6896282	0.493464248	0.77434604	-6.7038576
XYLT1	0.3098426	2.39285299	0.68924047	0.493706207	0.77454083	-5.8192908
TFB2M	-0.0728662	5.38961522	-0.6891891	0.493738277	0.77454083	-6.670267
ELK1	0.10003739	4.08062778	0.68913368	0.493772853	0.77454083	-6.4776689
METTL1	0.19286214	2.70601222	0.6889499	0.493887555	0.77462429	-5.9660114
FBXO46	0.10898473	3.12799932	0.68882978	0.493962535	0.77463782	-6.192402
USP24	0.07152016	7.07272271	0.68873904	0.494019182	0.77463782	-6.6969475
PURG	0.21055139	0.76305078	0.6886151	0.494096561	0.77466272	-5.6527099
FAM198A	0.45134039	0.17007251	0.68838102	0.494242712	0.77479542	-5.6499247
WDR36	-0.0799916	6.30086448	-0.6882638	0.494315889	0.77481371	-6.7160318
NOL10	-0.0822327	6.0415373	-0.6880235	0.494465969	0.77495251	-6.7123031
CABCOCO1	0.25024875	0.54727147	0.68791803	0.494531863	0.77495936	-5.5835469
ATP9B	-0.0590259	5.01434784	-0.6878185	0.494594021	0.77496035	-6.6364293
PINX1	-0.1496088	2.80628116	-0.6871707	0.494998822	0.77515653	-6.1768455
MTMR2	-0.1026297	5.38280707	-0.6871434	0.495015836	0.77515653	-6.667305
ZFYVE16	-0.0774858	5.5691497	-0.6869958	0.495108101	0.77515653	-6.6802315
ENSCAFG00000031454	-0.2219225	1.77145541	-0.6869095	0.49516202	0.77515653	-5.8102209
B4GALT3	0.09933398	4.31974515	0.6868901	0.495174171	0.77515653	-6.4894103

ARF4	0.06902205	8.63133998	0.68688173	0.495179401	0.77515653	-6.6288881
ALKBH5	0.06534214	4.3225602	0.6868747	0.495183799	0.77515653	-6.5617767
ADK	0.11947267	4.92500666	0.68683032	0.495211541	0.77515653	-6.6320967
C1QBP	0.08696549	5.60776592	0.68671853	0.49528142	0.77516958	-6.6967179
INTS6	-0.0694082	5.7475977	-0.6864752	0.495433541	0.77531133	-6.7008271
FAM227B	0.26435099	0.1414312	0.68619645	0.495607858	0.77532869	-5.4678899
NAT14	0.20915195	1.4874961	0.68617111	0.495623705	0.77532869	-5.8377835
THSD7A	0.40064146	-2.3835555	0.6861141	0.495659361	0.77532869	-5.2575521
ABI3BP	0.65805403	2.47393901	0.68606375	0.49569085	0.77532869	-6.2597633
XRRA1	-0.2037686	0.1513173	-0.6859064	0.495789276	0.77538636	-5.5520009
DIMT1	-0.0831064	4.61996711	-0.6856732	0.495935139	0.77551819	-6.6103794
MARK2	0.0521759	5.30759217	0.68502676	0.496339655	0.77605441	-6.6698996
RNF123	-0.0787367	4.89019521	-0.6849071	0.496414528	0.77607514	-6.6377802
ENSCAFG00000025619	-0.3142813	0.23598284	-0.6846479	0.496576808	0.7762325	-5.440099
USP48	-0.0739026	6.19743999	-0.684535	0.496647471	0.77624662	-6.7177558
RIT1	-0.0986439	3.56839861	-0.6843703	0.496750612	0.7763115	-6.3805152
DDIAS	0.39872523	0.47004729	0.68395914	0.49700811	0.77653264	-5.4752514
ENSCAFG00000010185	-0.1580335	1.75548056	-0.6838855	0.497054263	0.77653264	-5.8353529
MFAP5	0.40046759	7.54046896	0.68370735	0.497165831	0.77653264	-6.7184735
KCNB1	0.26862858	-0.0513736	0.6836611	0.497194803	0.77653264	-5.6577245
ADAMTSL5	0.17141092	4.24265334	0.68365223	0.49720036	0.77653264	-6.5609635
ST8SIA6	0.42301221	-1.8726575	0.68349116	0.497301276	0.77659396	-5.3183715
RTKN2	0.28847368	1.67126127	0.68338023	0.497370782	0.77660622	-5.5971572
NHSL1	0.27050089	6.40118963	0.68324215	0.497457309	0.77664505	-6.7199608
PRDM5	0.07911445	3.63417491	0.68274546	0.497768613	0.77698547	-6.3806368
IL34	0.20376999	4.80372675	0.68264726	0.497830174	0.77698547	-6.6188872
ASB13	-0.149694	1.89630155	-0.682599	0.497860413	0.77698547	-6.0291155
GDPGP1	-0.1135441	2.0764049	-0.682174	0.498126902	0.77724018	-6.0169416
PRMT1	0.07798919	6.9440835	0.68214193	0.498147028	0.77724018	-6.7160351
CENPH	-0.3420956	2.7969691	-0.6818682	0.498318707	0.77741175	-5.8977656
SREK1	-0.0865615	5.54894551	-0.6817633	0.498384528	0.77741814	-6.7054073
SLC30A1	0.12454226	6.65113742	0.6815839	0.498497056	0.77749739	-6.7174031
KBTBD8	-0.2506392	0.95471031	-0.6813411	0.498649383	0.77761272	-5.6210142
DGUOK	-0.1133505	2.65149314	-0.6811845	0.498747691	0.77761272	-6.0863021
MXI1	0.14273227	5.748848	0.6811028	0.498798933	0.77761272	-6.7199044
NFU1	-0.0640409	4.63159093	-0.6810725	0.498817938	0.77761272	-6.5645893
EID1	0.075441	6.07067155	0.68087591	0.498941341	0.77765102	-6.719556
CORO2A	0.15591639	2.4669925	0.68083665	0.498965984	0.77765102	-6.2263
EME1	-0.2106198	1.52252345	-0.6805413	0.499151416	0.77783	-5.8291303
TMX3	-0.0688522	5.56971547	-0.6804568	0.499204451	0.77783	-6.6919499
MTF2	-0.1389011	2.13976879	-0.6802575	0.499329563	0.77783	-6.0089567
FXYD6	0.57350093	-0.3943601	0.68024689	0.499336245	0.77783	-5.7274918
FAM104A	-0.1023843	3.07378157	-0.6801609	0.499390218	0.77783	-6.2537739
CCNC	-0.0914484	4.37320579	-0.6800636	0.499451336	0.77783	-6.5452299
PECR	0.15795532	1.79689795	0.67970136	0.499678875	0.77798821	-5.9721131
AIFM1	-0.0641144	5.05785592	-0.6796509	0.499710577	0.77798821	-6.6510907
INPP5B	-0.0622143	5.8029148	-0.6796069	0.499738221	0.77798821	-6.7106398
ZNF319	-0.1810613	2.26061003	-0.6793846	0.499877864	0.77810944	-6.0215587
TRAPPC1	0.08383413	4.12611498	0.67902954	0.500101005	0.77831806	-6.4950649
RPS21	-0.1051141	7.41555487	-0.6789747	0.500135473	0.77831806	-6.6977188

RNF25	0.08144022	4.48900816	0.67826788	0.500579814	0.77891332	-6.5486215
ZNF331	-0.118942	3.63064037	-0.6780046	0.500745388	0.77899637	-6.4232935
ENSCAFG00000019273	0.08500103	4.28983083	0.67798632	0.500756877	0.77899637	-6.5522945
KBTBD6	-0.1113768	2.40138375	-0.6778059	0.500870336	0.77900282	-6.1058339
ZNF316	0.08453036	4.1093227	0.67768357	0.500947303	0.77900282	-6.4776331
ENSCAFG00000008369	-0.7706023	1.26067942	-0.677657	0.500963989	0.77900282	-5.4743844
MECR	-0.0799745	4.23801339	-0.6775864	0.501008402	0.77900282	-6.4912489
ANKS3	-0.0768286	4.03896026	-0.6774219	0.501111919	0.77902805	-6.4387528
ZNF710	0.09409572	2.6417538	0.67736405	0.501148322	0.77902805	-6.1350414
ZNF304	-0.1316172	1.79884781	-0.677145	0.501286136	0.77914612	-5.916675
SMARCD2	-0.1265185	5.83424612	-0.676811	0.501496332	0.77937666	-6.7201203
ITGB1BP1	-0.0968532	3.84563411	-0.6765372	0.501668735	0.77946293	-6.3956182
CCDC59	-0.0841336	3.27863499	-0.6765263	0.50167561	0.77946293	-6.3022214
DNAJC15	0.12082442	3.24348102	0.6760469	0.501977437	0.77976612	-6.2953981
ZNF248	-0.1028814	3.93205621	-0.6760197	0.50199456	0.77976612	-6.503122
GALNT3	0.4886189	-2.1762742	0.67582762	0.50211554	0.77977974	-5.1615277
SIMC1	-0.0893366	3.29577913	-0.6756962	0.50219831	0.77977974	-6.2686941
CIRBP	-0.1037245	3.93004102	-0.6756268	0.502242016	0.77977974	-6.4094383
ENSCAFG00000019431	0.16042991	2.12074194	0.67543228	0.502364586	0.77977974	-5.9719388
ADCK2	-0.1384294	3.18986568	-0.6754219	0.502371133	0.77977974	-6.3843463
JAGN1	-0.0702698	4.54647893	-0.6754161	0.502374767	0.77977974	-6.5828898
OLA1	-0.0736534	6.42298029	-0.6751779	0.502524856	0.77982393	-6.7247329
ENSCAFG00000032173	0.09011511	8.18018609	0.67515647	0.502538366	0.77982393	-6.6627329
ASIC2	-0.5879559	2.89512667	-0.6750762	0.502588971	0.77982393	-5.7667186
NEDD9	0.59951175	3.6259599	0.67482164	0.502749379	0.77990156	-6.0453891
ENSCAFG00000008732	0.09414612	3.20200452	0.6748003	0.502762835	0.77990156	-6.2705284
CCDC126	-0.1884311	2.60053583	-0.6745141	0.502943263	0.7800041	-6.0163393
SAT2	0.1458084	2.90749522	0.67449896	0.502952784	0.7800041	-6.2636852
FREM2	0.32261633	-0.2640065	0.67389757	0.503331992	0.78049609	-5.5091203
ENSCAFG00000011151	0.08507103	4.86551059	0.67356055	0.503544574	0.78072962	-6.6493413
GRB10	0.14190777	4.90120438	0.67334881	0.503678151	0.78075743	-6.5537194
PSME4	-0.0789499	6.9875604	-0.6733356	0.503686478	0.78075743	-6.7186643
CYSTM1	0.14509378	4.29978838	0.6729606	0.503923116	0.78096119	-6.4560155
CLCN7	0.07515938	5.79002737	0.67293079	0.503941929	0.78096119	-6.716218
HGF	-0.2592202	3.70924098	-0.6723977	0.50427842	0.78131055	-6.6964748
CD274	-0.2878604	2.39051539	-0.6723771	0.504291427	0.78131055	-6.2506453
PROX1	-0.435265	-1.1931803	-0.6721676	0.504423727	0.78136148	-5.3442723
ENSCAFG00000013769	0.13736822	6.03782824	0.67204951	0.504498268	0.78136148	-6.7195391
GAREM1	0.27437271	1.40394802	0.67203032	0.504510391	0.78136148	-6.0192345
MRPL10	-0.0779544	4.32181228	-0.67188	0.504605286	0.78137809	-6.5210378
TSC22D1	-0.1267358	7.12326368	-0.6718169	0.504645187	0.78137809	-6.7090968
METTL9	-0.0434443	6.6141206	-0.6717114	0.504711789	0.78138516	-6.7259082
ETFA	-0.0610023	5.81329937	-0.6715111	0.504838297	0.78148497	-6.7194366
TEK	-0.5343755	2.45620555	-0.6712294	0.505016291	0.78161671	-6.0161751
XRCC2	0.24996119	0.06649105	0.67118	0.505047507	0.78161671	-5.5163441
MMAB	-0.1107074	2.2300642	-0.6709504	0.505192561	0.78174515	-6.0101417
KLF15	-0.2706747	1.47998159	-0.6706349	0.505391984	0.78188338	-5.906288
RAD51B	0.25168361	0.33388985	0.67061267	0.505406041	0.78188338	-5.5663949
PISD	0.1024578	4.17649499	0.67022415	0.505651656	0.78216729	-6.4999374
RIT2	0.49934896	-1.421733	0.66998792	0.505801027	0.78230228	-5.304155

ENSCAFG00000013683	0.06127341	4.83567054	0.66969798	0.505984392	0.7824898	-6.6172883
ENSCAFG00000018211	-0.0865495	4.12290616	-0.6693514	0.50620361	0.78266371	-6.5165938
SPC24	-0.3630618	2.34282854	-0.6692058	0.506295728	0.78266371	-5.7433265
MAML2	-0.1441236	5.65801788	-0.6692016	0.506298408	0.78266371	-6.7221155
ENSCAFG00000028699	-0.3845736	-1.2208166	-0.6688902	0.506495467	0.78266371	-5.3360559
FAM173B	-0.1036376	2.72539232	-0.6688819	0.506500691	0.78266371	-6.1522521
GXYLT1	0.12378873	3.1520813	0.6688517	0.506519803	0.78266371	-6.3025449
CCL24	0.65096504	-1.6674753	0.66883275	0.5065318	0.78266371	-5.5266928
USP39	-0.0609306	4.95850478	-0.6686619	0.506639896	0.78267519	-6.6154171
GCSH	0.11989595	3.00811415	0.66851903	0.506730356	0.78267519	-6.2536545
INCA1	0.26766127	-1.0295694	0.6684756	0.50675785	0.78267519	-5.444437
DUT	-0.0994077	4.60647352	-0.668386	0.506814565	0.78267519	-6.5876802
NXT2	-0.0923885	3.0076535	-0.6683302	0.506849918	0.78267519	-6.1765099
MAST2	0.06597895	6.06887784	0.66821616	0.506922089	0.78269069	-6.7174118
IRF5	0.18981458	1.14201043	0.6680069	0.507054587	0.78279931	-5.851391
ENSCAFG00000030875	-0.2411899	4.02331336	-0.6667965	0.507821317	0.78385654	-6.3248
LRRC36	0.20217104	0.29821156	0.66672939	0.507863865	0.78385654	-5.5617106
HDAC1	0.06716682	4.59702119	0.66651445	0.508000093	0.78397074	-6.6263322
CBX5	0.10931244	4.79094085	0.66604499	0.508297706	0.78416081	-6.5883877
LRCH3	0.05773692	5.20631008	0.66603958	0.508301133	0.78416081	-6.684426
ENSCAFG00000001231	-0.1518828	1.90486072	-0.6660256	0.508310023	0.78416081	-5.8426927
TUBGCP4	0.07082443	4.28545895	0.66583213	0.508432678	0.78418415	-6.4938724
CORO1A	-0.2689013	0.39280816	-0.6658053	0.508449664	0.78418415	-5.5532029
ENSCAFG00000029061	0.14626063	2.08150407	0.66559703	0.508581778	0.78429188	-5.9310968
CDC42BPG	-0.2382494	1.50401749	-0.6654633	0.508666612	0.78432668	-5.8320347
SFRP1	0.47986994	-2.4520505	0.66496912	0.508980106	0.78464263	-5.2022175
TRMT61A	0.11794046	4.37296273	0.66494391	0.508996102	0.78464263	-6.5049599
AASS	-0.3731405	-2.3218381	-0.6647889	0.509094447	0.7846982	-5.2341854
FRAS1	0.38452813	3.64898311	0.66461356	0.509205735	0.7847737	-6.6763683
KLHL13	0.36008186	-1.6280735	0.66433933	0.509379791	0.78494591	-5.1570707
ENSCAFG00000029110	-0.3769728	-1.0000007	-0.6634715	0.509930831	0.7855076	-5.3453569
SPTY2D1	0.08132491	4.00478763	0.66346579	0.509934453	0.7855076	-6.5512245
PDZD11	-0.0649986	4.54432115	-0.6634376	0.509952356	0.7855076	-6.5627959
NAA25	-0.0980746	4.24742645	-0.6632909	0.510045562	0.7855076	-6.5335479
AUH	-0.0706	3.94759274	-0.6632743	0.510056104	0.7855076	-6.501856
ALG3	-0.0811796	4.69903758	-0.6630385	0.510205909	0.78564225	-6.5850275
UBE2R2	0.05308091	5.43269139	0.6629138	0.510285113	0.78566817	-6.7020395
RAB11FIP3	-0.0759985	5.41403281	-0.6625537	0.510513943	0.7858381	-6.7098145
ZDHHC23	0.35756863	-1.6808491	0.66246145	0.510572571	0.7858381	-5.2981179
NRDE2	0.06315806	3.99164975	0.66230931	0.510669271	0.7858381	-6.5179166
UBA5	0.09727278	5.4443536	0.66219972	0.510738935	0.7858381	-6.6808961
ENSCAFG00000032664	-0.2483052	-0.3171812	-0.6620783	0.510816125	0.7858381	-5.4157845
UTP18	-0.0701444	4.6874609	-0.6620544	0.510831346	0.7858381	-6.5971592
CARS	-0.0895845	6.95764824	-0.6620461	0.510836621	0.7858381	-6.7313747
HOOK3	-0.1032556	6.1946631	-0.6619414	0.51090315	0.7858381	-6.7307972
ENSCAFG00000030859	0.26601515	-0.0785697	0.66168858	0.511063913	0.7858381	-5.4741006
RWDD2B	-0.1385688	2.49217268	-0.6614807	0.51119612	0.7858381	-6.113384
HSD17B12	0.08709114	6.71847887	0.66128449	0.51132091	0.7858381	-6.7256681
ST8SIA4	0.40913413	1.86717131	0.66105612	0.511466179	0.7858381	-5.6128928
ENSCAFG00000001017	-0.0889907	3.28338083	-0.6610068	0.511497535	0.7858381	-6.3108859

CDK7	0.07690948	3.49151626	0.66098021	0.511514474	0.7858381	-6.4038429
APLF	0.06894021	5.53110323	0.66093936	0.511540464	0.7858381	-6.712539
COL6A1	0.25557298	11.1176766	0.66077112	0.511647506	0.7858381	-6.4317992
P4HA1	0.11502936	8.48760528	0.66075231	0.511659478	0.7858381	-6.6331665
S100A6	-0.1412639	7.90896856	-0.6606931	0.511697173	0.7858381	-6.6534869
PLEKHH3	-0.1898405	2.5590272	-0.6606923	0.511697679	0.7858381	-6.2374173
ARAF	0.06631649	6.55496531	0.66066936	0.511712261	0.7858381	-6.7344563
PLXNB2	0.07924179	8.41677536	0.66056051	0.511781526	0.7858381	-6.6576843
MYO3A	0.51792323	1.08677342	0.66051009	0.511813613	0.7858381	-5.6738765
RANBP10	-0.0860969	4.4403989	-0.6604837	0.511830403	0.7858381	-6.5524437
NIT2	0.09179245	4.05306627	0.66023136	0.511991011	0.78593153	-6.5658822
URM1	-0.0981691	2.7435847	-0.6601247	0.512058923	0.78593153	-6.1043506
RFX1	-0.1525083	2.61903277	-0.660094	0.51207844	0.78593153	-6.1520377
LARP4	0.07028104	5.10586895	0.6599561	0.512166239	0.78597051	-6.6683438
ARL15	-0.1501759	1.52804811	-0.6593643	0.512543083	0.78632509	-5.8476716
LEO1	-0.1006956	4.39299471	-0.6593475	0.512553777	0.78632509	-6.5549738
HLA-DRB1	0.41767693	-1.7038769	0.65926464	0.51260655	0.78632509	-5.3979345
SLC25A43	-0.1359386	3.20374486	-0.6591679	0.512668155	0.78632509	-6.4059668
SLC35C2	0.09504101	4.80496307	0.65910313	0.51270943	0.78632509	-6.6202451
DPF2	-0.0479229	6.03720206	-0.6588335	0.512881192	0.78647818	-6.7305302
GSE1	0.08828709	4.54151861	0.65875043	0.512934123	0.78647818	-6.6298468
FZR1	0.07096648	4.80078869	0.65859279	0.513034566	0.78653644	-6.6268521
FKRP	0.16013419	2.28611489	0.65823341	0.513263598	0.78679181	-6.0369388
SLC1A3	0.64581185	0.68154012	0.65785023	0.513507858	0.78698219	-5.4552569
BCORL1	-0.0850752	4.05314821	-0.6578426	0.513512749	0.78698219	-6.5353025
UBE2G1	0.0588272	5.4638095	0.65755771	0.513694363	0.78706865	-6.7033749
STS	0.21701014	1.56429635	0.65752973	0.513712207	0.78706865	-5.81537
DPYSL2	-0.0791946	8.81771631	-0.6574389	0.5137701	0.78706865	-6.6019326
TSC22D2	-0.0980934	4.91023911	-0.6573621	0.513819109	0.78706865	-6.5609068
ZNF133	0.08733265	3.60691133	0.65686856	0.514133904	0.78737414	-6.4277653
FANCB	0.24990845	1.01577972	0.65685342	0.514143562	0.78737414	-5.6453265
CAB39	0.05420809	6.60224784	0.65650703	0.514364572	0.78761684	-6.7301418
CCT5	-0.0880357	8.02909054	-0.6563196	0.51448417	0.78770422	-6.6826075
TMEM25	0.17011593	2.49136945	0.65617442	0.51457683	0.78772391	-6.1436046
CD93	-0.7271071	1.2748697	-0.6561035	0.514622108	0.78772391	-5.8952903
RNF5	0.09051571	3.67495683	0.65578133	0.514827752	0.78784839	-6.4083266
ENSCAFG00000020291	-0.090555	5.06694594	-0.6557801	0.514828522	0.78784839	-6.6630043
DIO2	0.42228637	-1.9886462	0.65545611	0.515035396	0.78796327	-5.1515992
CCT6A	0.07706485	6.61641782	0.65538916	0.515078146	0.78796327	-6.7360455
FZD8	-0.4462483	-0.6556337	-0.6553686	0.515091267	0.78796327	-5.3883264
CLDN12	0.08368882	3.85216191	0.65501212	0.515318949	0.78813486	-6.5559707
TAGLN3	0.44303594	2.21234489	0.65485057	0.515422138	0.78813486	-5.7330627
WDR4	0.09966015	3.64883225	0.65480935	0.515448472	0.78813486	-6.3564159
SGCB	0.06948345	6.67642128	0.65480114	0.515453717	0.78813486	-6.7376632
LRCH1	0.10554347	4.24842796	0.65460765	0.515577331	0.78821548	-6.61269
SGK1	0.14779969	5.45863321	0.65452271	0.515631597	0.78821548	-6.7360328
MUM1	-0.0863851	4.05495852	-0.6543984	0.515711007	0.78824121	-6.5018841
DDIT3	0.13372095	4.3224317	0.65415359	0.515867474	0.78833043	-6.4983652
RIIAD1	0.17312895	0.33793943	0.6540246	0.515949912	0.78833043	-5.642263
RCE1	-0.1305041	2.79057873	-0.6540133	0.515957133	0.78833043	-6.1383742

SMIM19	-0.0806461	5.6175396	-0.6537093	0.51615146	0.78853169	-6.7147223
MAPK6	-0.0994039	6.6752826	-0.6532763	0.516428309	0.78866526	-6.7394525
ART4	0.45106832	-1.4311944	0.6532727	0.516430613	0.78866526	-5.4672676
WNK3	0.06840138	4.78125389	0.65318859	0.516484401	0.78866526	-6.6615208
SERPINB9	-0.1562801	4.9684067	-0.6531809	0.51648934	0.78866526	-6.6507757
MEN1	-0.0823328	3.78376931	-0.6529475	0.516638604	0.78876808	-6.4988032
RABAC1	0.08072651	7.07590219	0.65287976	0.516681918	0.78876808	-6.7283488
ENSCAFG00000032012	0.12133302	3.79706863	0.65262546	0.516844585	0.78892079	-6.4728612
SGSM3	-0.0833686	4.72028804	-0.6524809	0.516937062	0.78895849	-6.6327301
SPPL2A	-0.0670258	7.02212048	-0.6523911	0.516994554	0.78895849	-6.7294416
TMEM132E	0.8951681	1.29220235	0.65204928	0.517213263	0.78904475	-5.4712996
GLE1	-0.0899831	4.44375067	-0.6520128	0.51723658	0.78904475	-6.5844581
IL1RL1	0.44217462	4.22272228	0.65200906	0.517239007	0.78904475	-6.7401673
NUCKS1	-0.0728291	6.31102739	-0.6517791	0.517386186	0.7891737	-6.7406533
ENSCAFG00000016065	-0.4783122	3.55493247	-0.6515304	0.517545404	0.78932097	-6.3589893
ENSCAFG00000004917	-0.2109189	0.97303295	-0.6513589	0.517655182	0.78939282	-5.5536072
FZD10	-0.3625237	-1.7155915	-0.651097	0.517822931	0.78951449	-5.3043643
ENSCAFG00000020261	-0.0871346	3.55907237	-0.6509579	0.517911996	0.78951449	-6.4043517
SPATA2L	0.1461689	1.97337438	0.65094068	0.517923008	0.78951449	-5.9912844
STYX	-0.1014867	2.88893385	-0.6508313	0.517993047	0.7895257	-6.2673667
OTUB2	0.23215985	-0.7083066	0.65065232	0.518107708	0.78960492	-5.4861107
ENSCAFG00000010422	-0.4373281	-1.7810131	-0.6498675	0.518610561	0.79019106	-5.2550582
EXO5	0.08771877	4.71237583	0.64977794	0.518667975	0.79019106	-6.6504835
CBX7	0.15241882	3.50274451	0.64975839	0.518680506	0.79019106	-6.4971627
TMEM176A	0.6400032	0.63121571	0.64950056	0.518845776	0.79029922	-5.5160387
ENSCAFG00000016321	-0.2405825	0.57266505	-0.6493732	0.51892744	0.79029922	-5.460292
ENSCAFG00000002332	0.36487932	-1.6233724	0.6492957	0.518977108	0.79029922	-5.2279018
SYT9	0.45490098	-1.5751443	0.64923395	0.519016704	0.79029922	-5.2138944
ETNK1	-0.0991579	3.4131951	-0.6489965	0.519168981	0.79029922	-6.3601624
SMOC1	-0.616225	3.12559624	-0.6489472	0.519200578	0.79029922	-6.0136392
ITGA9	-0.2592906	3.89871581	-0.6487229	0.519344454	0.79029922	-6.4314324
C26H22orf39	0.18847278	0.00730174	0.64869589	0.519361752	0.79029922	-5.4552901
SEMA5A	-0.5023485	1.59614366	-0.6486871	0.51936739	0.79029922	-5.5524904
RHOT2	-0.0790012	4.73890568	-0.6486691	0.519378925	0.79029922	-6.6397199
MST1	0.29494247	-0.0861125	0.64820559	0.51967628	0.79065617	-5.5385628
ARHGEF6	-0.1879892	4.59295802	-0.6476501	0.520032716	0.79110291	-6.496431
OSBP	-0.0500434	5.80330008	-0.6471913	0.520327249	0.79130627	-6.7332445
MANBA	-0.0880951	6.44678438	-0.6470882	0.520393484	0.79130627	-6.7432825
SMAD9	0.16530896	5.98570241	0.64705435	0.520415197	0.79130627	-6.699557
ENSCAFG00000031764	0.15501705	1.80723514	0.64699164	0.52045546	0.79130627	-5.9200711
NAA20	-0.0608027	5.2435515	-0.6469224	0.520499906	0.79130627	-6.6862689
DNAJC30	-0.1868739	1.56536441	-0.6467833	0.52058924	0.79130627	-5.9303165
PITX1	0.51216084	-1.145302	0.64666704	0.520663913	0.79130627	-5.3656358
FASTK	-0.0681075	5.38041058	-0.6465425	0.520743909	0.79130627	-6.6932763
RABGGTA	-0.0905958	4.28120618	-0.6464851	0.520780782	0.79130627	-6.5448928
AAMDC	-0.1647162	1.60008337	-0.6464098	0.520829119	0.79130627	-5.8519744
ZFPM2	-0.1901339	5.56432564	-0.6461479	0.520997397	0.79130627	-6.7161289
MIGA2	-0.1067493	3.48443993	-0.6461306	0.52100852	0.79130627	-6.4545642
AKR1E2	-0.1665029	1.95880029	-0.6458446	0.521192262	0.79130627	-6.0292588
CFAP298	-0.0747757	5.22708695	-0.6458276	0.521203209	0.79130627	-6.6966568

MED9	0.22547419	1.38782128	0.64575978	0.521246775	0.79130627	-5.7768463
ENSCAFG00000009480	0.24702651	0.43603011	0.64564864	0.521318196	0.79130627	-5.6110831
TAF15	-0.0584236	6.42519299	-0.6453941	0.521481831	0.79130627	-6.7426842
TXNL1	-0.064661	6.10275378	-0.6452066	0.521602344	0.79130627	-6.7384865
RNASEH2C	0.15315493	2.18163965	0.64517273	0.521624106	0.79130627	-6.0423087
EXPH5	0.72460425	-0.9480312	0.64513504	0.521648335	0.79130627	-5.2507292
ENSCAFG00000008104	-0.4469237	-2.0219504	-0.6450675	0.521691735	0.79130627	-5.3376388
UBA1	-0.0488595	8.55009821	-0.645036	0.52171202	0.79130627	-6.6606276
SLC9B1	-0.2529206	1.42563308	-0.644823	0.521848982	0.79130627	-5.8248425
TAF13	-0.142779	1.13128806	-0.6447754	0.521879545	0.79130627	-5.7498628
SYPL1	-0.0603307	6.25922782	-0.6447628	0.521887694	0.79130627	-6.7433924
TRMT9B	-0.4675824	0.50427503	-0.6446882	0.521935628	0.79130627	-5.4219686
PLCL2	0.20035782	3.11326138	0.64457728	0.522006979	0.79130627	-6.3084642
IER5L	0.14690562	4.2922249	0.64455882	0.522018849	0.79130627	-6.5378249
PTS	0.10187689	2.95520285	0.64452502	0.522040588	0.79130627	-6.2185582
ENSCAFG00000005006	-0.0786159	5.26950008	-0.6445087	0.522051054	0.79130627	-6.7168554
TAF10	0.10193081	4.87878396	0.64436125	0.522145925	0.79135484	-6.6284386
ADAMTSL1	0.21845124	6.44255466	0.64424328	0.522221805	0.79137462	-6.7310686
ENSCAFG00000006206	0.46043863	-1.0615163	0.64383416	0.522485015	0.79167824	-5.2788251
TNFSF10	-0.1559134	5.54340094	-0.6437076	0.522566441	0.79170639	-6.693158
SVEP1	0.39139994	8.23665495	0.64360431	0.522632921	0.79171188	-6.6832986
PER3	-0.1156366	4.29997473	-0.6435043	0.522697267	0.79171414	-6.533541
NUSAP1	-0.2453727	2.97437187	-0.6432825	0.52284002	0.79183514	-6.1281529
STEAP3	0.30963662	0.92289249	0.64300592	0.523018076	0.79186427	-5.9700358
SMC2	-0.2881825	4.16502832	-0.6429754	0.523037707	0.79186427	-6.3698124
ATL3	-0.0462207	8.49712571	-0.6429012	0.523085525	0.79186427	-6.655045
DMAC2	-0.0849246	3.55786631	-0.642862	0.523110717	0.79186427	-6.3299822
CHST14	-0.1452589	3.23969233	-0.6424523	0.523374575	0.79199691	-6.2450955
OSR2	-0.2659129	2.52982543	-0.6424456	0.523378874	0.79199691	-6.352493
SH3RF1	-0.0967621	4.51913764	-0.642433	0.523386968	0.79199691	-6.5810677
ZMYND19	0.10317182	2.98997842	0.64230479	0.523469558	0.79202673	-6.2204696
PLPP6	0.144888	2.6214589	0.6416834	0.52386986	0.7925372	-6.1346924
RARA	-0.0934773	5.43466913	-0.6415276	0.523970269	0.79259391	-6.727032
CCDC36	0.17256592	1.47497798	0.64117468	0.524197702	0.79284273	-5.9367638
FZD7	0.23922336	3.73686149	0.64096752	0.524331236	0.79289524	-6.3289907
IBA57	-0.2140901	1.32737505	-0.6409255	0.524358315	0.79289524	-5.8418933
ENSCAFG00000017211	-0.0573219	5.54311769	-0.6405771	0.524582977	0.79313974	-6.7026784
PRDM1	-0.3502509	-2.3633501	-0.6398169	0.525073274	0.7937439	-5.313393
SGTB	-0.0762036	3.8351818	-0.6397124	0.525140693	0.7937439	-6.4744365
IGF2BP3	0.44145677	-0.2882953	0.6395171	0.52526667	0.7937439	-5.4910024
ENSCAFG00000019360	0.09410414	4.7510099	0.6393346	0.525384437	0.7937439	-6.6265926
NUDT22	0.14330117	2.4216581	0.63926407	0.525429951	0.7937439	-6.0513481
ZSCAN2	-0.1762537	1.83586262	-0.6392409	0.525444919	0.7937439	-5.870587
ZNF689	-0.1463018	1.84359435	-0.639191	0.525477077	0.7937439	-5.9479043
G3BP1	-0.0578612	7.31493389	-0.6391293	0.525516927	0.7937439	-6.7334422
ENSCAFG00000030662	-0.2416942	5.64453218	-0.6388732	0.525682222	0.7937439	-6.7116145
SMIM14	0.09591287	4.87272203	0.6388442	0.525700946	0.7937439	-6.6850053
PTGFRN	-0.2153016	6.28706513	-0.6385334	0.525901617	0.7937439	-6.7493312
UTP6	0.09773356	5.33379106	0.63850735	0.525918415	0.7937439	-6.6994896
ENSCAFG00000016452	-0.0553149	7.00067829	-0.6384988	0.525923944	0.7937439	-6.739375

TRAF3	0.09430388	3.70375938	0.63849472	0.525926572	0.7937439	-6.4074942
R3HCC1L	0.11713538	2.67970642	0.63841257	0.525979614	0.7937439	-6.2339207
ENSCAFG00000032541	-0.340894	-2.5665156	-0.6383138	0.526043386	0.7937439	-5.2114431
PSTPIP1	0.33569812	-1.0953914	0.63829763	0.526053834	0.7937439	-5.3818013
ZDHHC7	-0.0655305	5.54559212	-0.6381726	0.526134595	0.79377067	-6.7213696
BAIAP2	0.11357047	4.70224385	0.63802525	0.526229737	0.79381913	-6.6851781
PMS1	-0.0604347	5.56865443	-0.6375324	0.526548093	0.79414321	-6.7137323
AGAP2	0.28657706	-0.2863162	0.63749748	0.526570663	0.79414321	-5.5291421
HEATR1	-0.0567145	6.17370345	-0.6372611	0.526723373	0.79426851	-6.7487615
SMARCA2	0.09915838	7.39980186	0.63697669	0.526907196	0.79426851	-6.7305545
ITGA11	-0.5205096	5.0079142	-0.6369477	0.526925917	0.79426851	-6.6205109
CBR3	-0.2271627	1.93825823	-0.6368498	0.526989196	0.79426851	-6.090291
BCL2L12	0.10274651	3.44344048	0.63676443	0.527044389	0.79426851	-6.3398051
ATF2	0.07131629	5.34967964	0.6367638	0.527044798	0.79426851	-6.7163719
LYRM1	-0.0918403	4.31987086	-0.6366651	0.527108583	0.79426851	-6.5173421
PHF19	0.19111535	4.45594713	0.63658836	0.527158206	0.79426851	-6.4265563
ADAM17	0.06809541	6.41123577	0.63629548	0.527347559	0.79431002	-6.7510538
ATRX	-0.0749704	6.92662791	-0.6360948	0.527477353	0.79431002	-6.7462523
SMG1	-0.090139	6.10093623	-0.6360409	0.527512201	0.79431002	-6.7489415
FZD3	-0.3024511	0.69591938	-0.6359511	0.527570285	0.79431002	-5.5348607
INTS4	0.07412721	4.75843911	0.63585773	0.527630639	0.79431002	-6.623145
EXOC8	0.08364623	3.4896498	0.63580652	0.527663763	0.79431002	-6.4154119
UTP23	-0.120574	2.44894355	-0.6357634	0.527691677	0.79431002	-6.1445306
DPM3	0.17749534	1.68419638	0.63572409	0.527717078	0.79431002	-5.8748093
GINS2	-0.2452829	0.80882401	-0.6356681	0.527753297	0.79431002	-5.5548566
USPL1	-0.0966118	3.492265	-0.6352835	0.528002071	0.79453217	-6.3997426
ENSCAFG00000014207	-0.0779463	6.89738354	-0.6352449	0.528027058	0.79453217	-6.7469971
HSPA4L	0.09290176	5.56947053	0.63476878	0.528335182	0.79490085	-6.7295028
CBFA2T3	-0.3772075	-1.2927255	-0.6345288	0.528490547	0.79503964	-5.3814706
ZNF619	-0.175155	2.05947604	-0.6340583	0.528795119	0.79540283	-5.9202731
PAX1	-0.5953123	-0.2208942	-0.6337319	0.529006503	0.79555675	-5.3380737
CUL9	-0.0882305	5.91276861	-0.6337052	0.529023765	0.79555675	-6.7437726
ENSCAFG00000026245	-0.2568178	0.03560899	-0.6333438	0.529257912	0.79557965	-5.453853
NATD1	0.21495042	0.61314119	0.63327974	0.529299381	0.79557965	-5.6442585
CCNJ	-0.1358691	4.51688626	-0.633259	0.529312785	0.79557965	-6.5995996
LRP4	0.22178211	3.60103632	0.63307813	0.529429999	0.79557965	-6.4643165
UBE2E3	0.07044359	4.29440145	0.6330605	0.529441423	0.79557965	-6.5695716
CDS2	-0.0905048	4.53953362	-0.6330492	0.529448771	0.79557965	-6.6354139
TNIP2	0.09384741	2.48626962	0.63299923	0.529481119	0.79557965	-6.1119389
UBL7	0.06832139	5.39836345	0.63268215	0.529686596	0.79579346	-6.7217498
ENSCAFG00000003455	0.26711317	-0.4211466	0.63219094	0.530004992	0.79597477	-5.3540681
MCM3	-0.31062	4.91320548	-0.6321892	0.530006105	0.79597477	-6.5010398
CENPA	-0.4901363	-0.1372319	-0.6321824	0.530010502	0.79597477	-5.3295421
ENSCAFG00000020526	0.17226993	0.45977337	0.63208317	0.530074863	0.79597477	-5.6054959
NYAP1	0.11962564	3.52009596	0.63200855	0.530123242	0.79597477	-6.4282735
CYB561A3	0.10960354	3.19849166	0.63119956	0.530647897	0.79666757	-6.2475689
ZFHX4	-0.3763574	4.41599922	-0.6310793	0.530725894	0.79668971	-6.4460275
MFSD9	-0.1455795	1.80458186	-0.6308376	0.530882749	0.79683021	-5.834706
PPHLN1	-0.0693319	4.35528655	-0.6306027	0.531035161	0.796964	-6.5776104
TIGD6	-0.1041019	1.91905028	-0.6303135	0.531222831	0.79715068	-6.0329181

DENND4C	-0.0664225	6.28484081	-0.6301017	0.531360329	0.79726203	-6.7541275
ARHGEF11	-0.0576994	6.24074166	-0.6299323	0.531470306	0.79733206	-6.7542321
CHST3	0.14631579	3.70170876	0.62955261	0.531716836	0.79758288	-6.3391832
MED8	0.06412558	3.50100413	0.62947978	0.531764131	0.79758288	-6.4155097
TGFBR2	-0.2075589	9.40746645	-0.6292144	0.531936492	0.7977464	-6.5358229
LUC7L3	-0.0713261	5.08677062	-0.6289555	0.532104665	0.79788197	-6.6792988
ZHX1	0.07183355	4.67312967	0.62866302	0.532294678	0.79788197	-6.6473326
NPR2	0.08013485	5.4332869	0.62865095	0.532302521	0.79788197	-6.7298163
TCN2	0.13431719	5.08609663	0.62854423	0.532371867	0.79788197	-6.6884248
AP5S1	0.12699191	2.14051981	0.62853976	0.532374771	0.79788197	-5.9686475
ZNF584	-0.1557992	1.28094336	-0.6284415	0.532438609	0.79788197	-5.8599562
ENSCAFG00000019501	0.16853828	2.07533267	0.62832255	0.532515926	0.79788197	-6.0471477
SNX12	0.0672954	3.61799317	0.62826502	0.532553316	0.79788197	-6.4462368
MAATS1	0.24794616	-0.5607924	0.62819783	0.532596984	0.79788197	-5.4591038
ENSCAFG00000023152	-0.3270283	-0.6366087	-0.6278946	0.532794068	0.79804864	-5.4469042
IFFO1	-0.0987443	3.4094141	-0.6278317	0.532834953	0.79804864	-6.3679916
ENSCAFG00000002541	-0.06245	6.55113914	-0.6277192	0.532908082	0.79806327	-6.7538601
ENSCAFG00000023585	0.58973804	-2.1665897	0.62761822	0.532973761	0.79806675	-5.2190751
FNIP2	-0.1065148	6.09647605	-0.6274848	0.533060486	0.79810173	-6.7566588
CARHSP1	-0.186624	1.77419223	-0.6271905	0.533251909	0.79823417	-6.0404388
SAE1	-0.0749766	6.35181528	-0.6271539	0.533275689	0.79823417	-6.7546946
RENBP	-0.2762843	3.57292258	-0.6270017	0.533374688	0.7982875	-6.4268368
CDC123	-0.0528509	5.69385661	-0.626668	0.533591773	0.79845827	-6.7449533
FAM98B	-0.0749331	4.03521252	-0.6265416	0.533674017	0.79845827	-6.5353557
LSS	-0.1576325	6.98265932	-0.6264814	0.533713157	0.79845827	-6.7402869
DDX39B	-0.0560512	6.65474438	-0.6264366	0.533742347	0.79845827	-6.7567917
CD248	0.31266626	7.72235696	0.62623644	0.533872565	0.79852073	-6.6391795
PEBP4	0.3074165	-2.7119005	0.62617754	0.533910893	0.79852073	-5.2432871
ZBED4	-0.0735869	4.82490886	-0.6259659	0.534048631	0.79863191	-6.6398926
LRRC27	0.19649164	0.51058875	0.6257742	0.53417341	0.79872368	-5.7221896
ACCS	0.09374189	4.4633628	0.6256245	0.53427086	0.79877457	-6.5767576
ENSCAFG00000032623	-0.1328021	2.14065415	-0.6254098	0.534410621	0.79881068	-5.9934002
ENSCAFG00000010311	0.10324483	2.44606754	0.62538584	0.534426242	0.79881068	-6.1433091
ELP4	0.05834232	4.18840702	0.62529519	0.534485267	0.79881068	-6.52041
DCBLD1	0.19669473	1.82568889	0.6248345	0.534785279	0.7989885	-5.9602216
USP36	-0.0561271	5.20365604	-0.6246191	0.534925609	0.7989885	-6.693854
ENSCAFG00000029336	-0.1954361	1.89926387	-0.6245261	0.534986178	0.7989885	-5.944093
PSKH1	0.08744259	3.76033548	0.62451397	0.53499407	0.7989885	-6.4637741
SVBP	0.09140262	2.55531377	0.62445111	0.535035022	0.7989885	-6.1500954
GPR65	-0.4157855	-0.2289498	-0.6244112	0.535061057	0.7989885	-5.422477
CSNK1E	-0.0558984	5.28650795	-0.6243328	0.535112077	0.7989885	-6.7249914
ENSCAFG00000008773	0.12254632	2.73650794	0.624156	0.535227301	0.7989885	-6.182496
ZNF280B	0.13371469	2.8884958	0.6239697	0.535348702	0.7989885	-6.2590977
MYCL	0.26073543	-0.5251184	0.62378049	0.535472016	0.7989885	-5.4891738
TEN1	-0.1140658	2.66491904	-0.6237221	0.535510052	0.7989885	-6.0813334
TADA2B	0.09686098	2.38079917	0.62343797	0.535695281	0.7989885	-6.0724677
POU3F3	-0.3412059	-2.9081363	-0.6233737	0.535737168	0.7989885	-5.186346
NUP88	-0.0552726	5.34478672	-0.6233042	0.535782475	0.7989885	-6.7097902
WDR6	-0.0683947	5.97883667	-0.6232218	0.535836189	0.7989885	-6.7481669
ZNF865	-0.1016493	3.32141622	-0.623178	0.5358648	0.7989885	-6.339546

MIPEP	-0.0686831	5.07998619	-0.6230819	0.535927452	0.7989885	-6.698632
BORCS5	0.06207178	4.78918482	0.62286809	0.536066855	0.7989885	-6.641183
CLCN5	0.12113403	2.03347069	0.62284441	0.536082296	0.7989885	-5.9251882
ANKRD46	0.08765676	5.5014016	0.62281268	0.536102992	0.7989885	-6.7491066
ZNRF3	0.16540855	2.02047371	0.62273266	0.536155175	0.7989885	-5.9778075
CDIP1	0.09732046	2.74144249	0.6226994	0.536176867	0.7989885	-6.1528512
NFKBID	-0.2189506	-0.4865799	-0.6226933	0.536180871	0.7989885	-5.4915429
CHAF1B	-0.2373855	2.593173	-0.6226864	0.53618535	0.7989885	-5.9557357
ANAPC13	0.22238945	0.22145581	0.6226792	0.536190044	0.7989885	-5.5900102
CYBRD1	0.2362763	7.95094686	0.62255378	0.536271847	0.79901587	-6.6235677
ENSCAFG00000029063	-0.110612	2.28554565	-0.6223589	0.536398957	0.79903086	-6.0774211
CASZ1	-0.3327852	-0.5980385	-0.6223439	0.536408779	0.79903086	-5.3577633
LONP2	-0.0753772	6.06377082	-0.6220615	0.536592972	0.79912015	-6.7530656
DCLRE1B	0.14646346	3.9454339	0.6220575	0.536595602	0.79912015	-6.3847819
COG4	-0.0455094	5.2594216	-0.6219421	0.536670927	0.79913784	-6.7014362
COLEC11	0.55755541	-0.8019878	0.62180487	0.536760453	0.79917667	-5.3863304
TP53I13	0.10784758	3.82931036	0.62146878	0.536979797	0.79940876	-6.4457662
PER1	-0.1220594	5.40506273	-0.6212783	0.537104162	0.79942946	-6.7605504
MMP19	0.14730117	4.8685653	0.62125302	0.537120633	0.79942946	-6.7481108
KCTD2	0.11165212	2.63443822	0.62111948	0.537207817	0.79946475	-6.1454661
GPR173	0.24406598	0.55128257	0.62092125	0.537337241	0.79956289	-5.6606938
WIF1	-0.5909461	0.17701333	-0.6206961	0.537484273	0.79960538	-6.430608
XPO6	-0.051705	7.27664409	-0.6206831	0.537492753	0.79960538	-6.7432238
HOXB2	-0.2399112	1.25571454	-0.620577	0.537562021	0.79961398	-5.7762496
GRIP1	0.48104331	0.05773429	0.62032394	0.537727314	0.79971912	-5.3388427
SLC9A1	-0.0802605	4.9019169	-0.6202744	0.537759683	0.79971912	-6.6861852
HRH2	0.48052501	0.48478851	0.62009991	0.537873658	0.79977775	-5.2203889
ACBD3	-0.0545108	6.46996112	-0.6199642	0.537962345	0.79977775	-6.761442
SLC22A5	0.18065341	0.50736775	0.61988094	0.538016713	0.79977775	-5.6689056
ENSCAFG00000005488	0.25983674	-0.4075383	0.61982528	0.538053081	0.79977775	-5.4563776
ZNF329	0.12601334	2.05129215	0.61961587	0.538189915	0.79988675	-6.0087783
MANSC1	-0.0762034	6.36697646	-0.6193016	0.538395293	0.79996419	-6.7617107
TUBB6	0.11326197	7.95998083	0.61921785	0.538450046	0.79996419	-6.7284378
PEX13	0.06036121	4.09977013	0.61912067	0.538513566	0.79996419	-6.5052226
CUTC	0.12496276	1.61957512	0.6190941	0.538530933	0.79996419	-5.8836839
TAF11	0.08200968	4.0099911	0.61894987	0.538625218	0.79996419	-6.486422
LANCL2	0.06769551	4.62815419	0.61887516	0.538674064	0.79996419	-6.640742
HOOK2	0.14565895	1.78725122	0.618856	0.53868659	0.79996419	-5.9492618
ANKRD12	-0.083343	6.61714165	-0.6187252	0.538772106	0.79997692	-6.7540294
SFXN1	-0.0737923	6.15618729	-0.6186486	0.538822178	0.79997692	-6.7576437
ATP13A3	0.10281246	6.24440837	0.61839971	0.538984947	0.80012427	-6.7618727
RBM34	0.07255239	4.25307034	0.61807951	0.539194369	0.80034083	-6.5897925
MYL4	0.31747296	-1.4800108	0.61758691	0.539516627	0.8005771	-5.2620201
ENSCAFG00000017231	-0.2000575	3.21055377	-0.6175631	0.539532179	0.8005771	-6.2148961
FAM53A	0.20541867	0.28923961	0.61749029	0.539579847	0.8005771	-5.6363174
MRPS28	0.10999583	3.31112342	0.61739039	0.539645216	0.8005771	-6.2843451
ENSCAFG00000026374	-0.2573433	-1.2045321	-0.6173505	0.539671338	0.8005771	-5.4142254
ENSCAFG00000018563	-0.0771721	2.51740419	-0.6170811	0.539847612	0.80061456	-6.1257338
CLTA	0.04641085	6.34733564	0.61703562	0.539877398	0.80061456	-6.7622238
ENSCAFG00000014991	-0.1060177	2.59101256	-0.6170205	0.539887272	0.80061456	-6.213612

SECISBP2L	-0.0799683	5.13475219	-0.6165688	0.540183019	0.80095884	-6.716535
UBXN11	0.23178119	0.07878835	0.61636361	0.540317335	0.8010637	-5.481531
MED22	-0.1213262	3.18359825	-0.6161927	0.540429271	0.80109871	-6.2827287
NEPRO	-0.0602751	3.87854085	-0.6161244	0.540473955	0.80109871	-6.5143213
SLC16A12	-0.6607141	-1.396313	-0.6159882	0.540563214	0.80109871	-5.2064482
CNTLN	-0.0890536	4.59344941	-0.615926	0.540603937	0.80109871	-6.6283821
ENSCAFG00000000293	0.05677512	5.28513909	0.61573037	0.540732066	0.80109871	-6.6916755
ZNF81	0.16719684	1.66590208	0.6156442	0.540788511	0.80109871	-5.9299117
CLCF1	0.14027092	3.08873904	0.61559746	0.540819131	0.80109871	-6.3022962
MRPL2	-0.0735806	4.16561702	-0.6154723	0.540901152	0.80109871	-6.5636151
MARS2	0.11237409	1.46439472	0.61535966	0.54097493	0.80109871	-5.9035285
PSMD14	0.07034807	5.70474063	0.61535658	0.54097695	0.80109871	-6.7402408
ANKRD54	-0.0756653	4.31333623	-0.6152222	0.541065	0.80113492	-6.5654368
GDF9	-0.2883574	-0.4497231	-0.615084	0.541155553	0.80115011	-5.4670548
RAB23	0.10596875	3.99534081	0.61475961	0.541368163	0.80115011	-6.467833
ALKBH1	-0.0807324	3.34453167	-0.6147421	0.541379667	0.80115011	-6.388764
VPS52	-0.0513616	5.26451417	-0.614706	0.541403317	0.80115011	-6.7233312
NFATC1	-0.1453773	5.85835536	-0.6145897	0.541479533	0.80115011	-6.7378341
CA12	0.57561262	0.46009075	0.6145348	0.541515525	0.80115011	-5.7200278
HPCAL1	-0.2103999	5.69268715	-0.6145272	0.541520485	0.80115011	-6.7340967
FAM161A	-0.0950931	2.7814026	-0.6142001	0.541734985	0.80137333	-6.1457522
REXO5	-0.2146301	3.46974603	-0.6139958	0.541868914	0.80147732	-6.271338
MFSD4A	0.45387537	-1.4696641	0.61372413	0.542047091	0.80159799	-5.4720991
SEC22C	-0.1134934	1.86643105	-0.6136773	0.542077777	0.80159799	-5.9534397
MICALL1	0.10385811	5.29434757	0.61347091	0.542213188	0.80170411	-6.6590981
MGMT	0.25142535	-0.0711427	0.61319493	0.542394238	0.80187768	-5.6306917
ZC3H14	-0.0562241	6.77331373	-0.612882	0.542599576	0.80192721	-6.7641686
DCAF15	0.07695957	4.02188809	0.61287532	0.542603956	0.80192721	-6.4854904
IKZF2	-0.2513748	0.92646313	-0.6125965	0.542786911	0.80192721	-5.8946243
RNF122	0.31715532	-1.2701202	0.61258591	0.542793892	0.80192721	-5.2400434
KIAA0513	-0.1431135	2.2796016	-0.6125791	0.542798343	0.80192721	-5.9648376
S100A16	-0.1504759	5.70695536	-0.6124745	0.542867034	0.80192721	-6.7609948
RPP40	0.14438725	2.76976982	0.61246477	0.542873399	0.80192721	-6.1660046
ZBTB33	-0.0641969	4.43568536	-0.6121941	0.543051063	0.80196967	-6.6120329
ARID3A	-0.1004485	2.55894495	-0.6120952	0.543115999	0.80196967	-6.2425629
NUS1	-0.0595593	6.05795505	-0.6119526	0.543209663	0.80196967	-6.7618239
PMVK	0.10299509	2.28787704	0.61188101	0.543256643	0.80196967	-6.157176
VAMP4	0.08861534	3.88377797	0.61184404	0.543280918	0.80196967	-6.4778183
MRPL34	0.10940931	2.1769909	0.61183912	0.543284153	0.80196967	-6.0374312
IGSF11	0.41892121	-1.425066	0.61139848	0.543573535	0.80227732	-5.2534574
FAM160B2	0.08868224	4.90267507	0.61132781	0.543619951	0.80227732	-6.6806668
UVSSA	-0.0932125	4.87009461	-0.6111343	0.543747063	0.8023709	-6.6994381
SIX2	-0.4145204	-0.3102929	-0.6109452	0.543871304	0.80246023	-5.4397246
ENSCAFG00000032711	-0.0722051	6.76067718	-0.6104953	0.544166898	0.80270683	-6.7640256
ENSCAFG00000006633	0.10982171	3.94808137	0.61043643	0.544205623	0.80270683	-6.4554265
SAA1	-0.4878611	2.15373441	-0.6103999	0.544229624	0.80270683	-6.2567996
GIT2	0.06140871	6.42372071	0.61011698	0.544415589	0.8028871	-6.7673761
TNS2	-0.1426309	7.37713145	-0.6098366	0.544599898	0.80306489	-6.7322627
AP4B1	-0.0610586	4.22757706	-0.6093034	0.544950499	0.80348782	-6.5412279
ABCA12	-0.236869	0.86292964	-0.6090989	0.545085003	0.80359208	-5.736442

SIDT2	0.10465804	8.28067667	0.60894929	0.545183437	0.80361222	-6.6736159
GORASP1	0.07765802	5.29172956	0.60880473	0.545278539	0.80361222	-6.681339
SH3RF3	0.13047847	4.60906705	0.60878721	0.545290062	0.80361222	-6.6686899
CCT8	0.06613949	7.66535548	0.60859088	0.545419232	0.80364074	-6.7328491
PSMB3	0.06183196	5.63300559	0.60856385	0.545437016	0.80364074	-6.7372933
ATP2B4	0.09856898	7.23306208	0.60829318	0.545615129	0.80380914	-6.7451284
MFSD13A	0.23305863	0.52241059	0.60813287	0.54572063	0.80382605	-5.6915516
G2E3	-0.1068641	4.61190761	-0.6080066	0.545803738	0.80382605	-6.6074579
ENSCAFG00000022743	0.2980596	2.53920327	0.60798485	0.545818051	0.80382605	-6.112099
ENSCAFG00000002306	-0.0685624	4.80140252	-0.6075067	0.54613281	0.80419557	-6.6587804
DYNC2LI1	-0.1024444	3.71985296	-0.6072133	0.546326032	0.80433516	-6.4601225
HDGFL2	-0.0587282	5.1720761	-0.6071688	0.546355322	0.80433516	-6.720005
TRIM35	0.12180737	2.72520993	0.60679875	0.546599037	0.80459992	-6.2012419
TSPAN12	0.11600207	5.21336175	0.60651688	0.546784718	0.80477919	-6.7332081
SLC12A6	-0.0810771	5.08016818	-0.6063281	0.546909097	0.80480504	-6.7244174
ENSCAFG00000016095	-0.0678592	7.81679451	-0.6062963	0.546930067	0.80480504	-6.7209843
STIM2	-0.0999017	5.63676673	-0.6060514	0.54709142	0.80489371	-6.7513313
RGP1	0.05820232	4.05339221	0.60601088	0.547118126	0.80489371	-6.5085365
MRPL1	0.08502265	3.66064546	0.60568689	0.547331656	0.80493584	-6.4637586
EZR	0.11018869	8.40784569	0.60550222	0.547453388	0.80493584	-6.6776778
MPZL1	0.07212029	6.9573145	0.60541881	0.547508371	0.80493584	-6.7651334
EGFL8	-0.2404741	-0.3349693	-0.6053706	0.547540138	0.80493584	-5.527121
ENSCAFG00000029124	-0.2167145	0.16078262	-0.6052431	0.547624208	0.80493584	-5.5108955
ZC3H15	0.05838584	6.48089195	0.60521487	0.547642827	0.80493584	-6.7696448
SAMD1	-0.1035229	3.7915128	-0.605207	0.547647998	0.80493584	-6.5052405
ZNF10	-0.1848169	0.78271366	-0.6051919	0.547658001	0.80493584	-5.6787799
ENSCAFG00000028442	-0.4482868	0.19390985	-0.604946	0.547820105	0.80505306	-5.452588
HELLS	-0.3907025	2.56580799	-0.6048771	0.547865581	0.80505306	-5.8833175
NKX2-3	-0.399721	-2.8575074	-0.6047132	0.547973635	0.80511792	-5.1744315
ZNF804B	-0.5451715	-0.1984831	-0.6044243	0.548164182	0.80530395	-5.3500514
ENSCAFG00000004650	-0.2278741	0.74125295	-0.6041958	0.54831491	0.80543144	-5.5219042
RNASEH2A	0.09465658	3.34998755	0.60366361	0.548666102	0.80585334	-6.3476772
VDAC1	-0.0591335	7.18898233	-0.6033802	0.548853127	0.8059713	-6.753607
C2CD2L	0.08270099	4.33648374	0.60334803	0.548874392	0.8059713	-6.618136
ENSCAFG00000032647	-0.1808617	0.24291198	-0.6031685	0.548992914	0.80605138	-5.7159956
DBF4	-0.1581972	3.55613337	-0.603004	0.549101483	0.80611682	-6.3169511
DCUN1D1	0.06207595	3.9320501	0.60269441	0.54930592	0.80632297	-6.5075893
ENSCAFG00000005852	0.2953914	0.07909552	0.6024035	0.549498035	0.80651098	-5.8949594
NRBP1	0.04515839	6.61743932	0.60212785	0.54968011	0.80668422	-6.7700986
NPTX1	-0.42533	5.30481201	-0.6019252	0.54981395	0.80669337	-6.762945
ENSCAFG00000003574	0.0827181	4.62570989	0.60186959	0.549850722	0.80669337	-6.6151008
NF2	0.05042447	7.08040868	0.60182758	0.549878479	0.80669337	-6.7695464
PLCD3	-0.1036399	4.81784769	-0.6013255	0.550210274	0.80708613	-6.6775783
C15H12orf73	-0.1462802	1.5897415	-0.6011129	0.550350736	0.80717457	-5.8562741
ZNF555	0.13963646	1.32512497	0.60104034	0.550398728	0.80717457	-5.706051
TATDN1	0.08698194	4.08218681	0.6008683	0.55051245	0.80724736	-6.5524462
MADD	0.16053084	4.13651152	0.60061471	0.550680109	0.80739921	-6.4969086
RAB3IL1	-0.1983055	6.57747389	-0.6001339	0.550998073	0.80777138	-6.7685045
CCDC91	-0.0722675	5.62491108	-0.599875	0.551169328	0.80792841	-6.7482274
ENSCAFG00000032347	0.05151859	5.94023771	0.59968951	0.551292006	0.80801421	-6.7691228

TMEM70	0.08208049	4.74512806	0.59949962	0.551417632	0.8081043	-6.6886587
ENSCAFG00000023441	-0.0962866	7.19527865	-0.5992679	0.551570958	0.80822769	-6.7565489
KCNF1	-0.3782933	0.82684841	-0.599175	0.551632412	0.80822769	-5.7176135
NAXE	-0.0857606	4.36242615	-0.599036	0.551724417	0.80822769	-6.5887561
SHC1	0.08360322	8.24841364	0.59898453	0.551758485	0.80822769	-6.6671277
SLIRP	0.07921944	3.65917094	0.59881812	0.551868632	0.8082762	-6.4635995
CPOX	-0.0806842	4.50645994	-0.5981907	0.552284016	0.8082762	-6.6655896
ADM	-0.1775995	6.56382928	-0.5981091	0.552338014	0.8082762	-6.7569065
CPNE2	-0.139328	2.99530825	-0.598088	0.552352008	0.8082762	-6.3580589
MYCBP	0.21707665	0.17948228	0.59805185	0.552375945	0.8082762	-5.6363437
RBBP5	-0.0499393	4.74321049	-0.5980376	0.552385393	0.8082762	-6.6972685
ENSCAFG00000007435	0.32827192	2.7250538	0.59792069	0.552462799	0.8082762	-6.0648793
ENSCAFG00000018671	0.12024824	3.09699808	0.59786336	0.552500771	0.8082762	-6.2837465
LENG8	-0.0859695	5.49558384	-0.5978241	0.552526744	0.8082762	-6.7460305
CADM3	-0.5263358	-0.4243994	-0.5977638	0.552566709	0.8082762	-5.4855993
MCM5	-0.5437205	3.84631382	-0.5977298	0.552589261	0.8082762	-6.0283846
ANP32E	-0.0834968	5.79286147	-0.5976497	0.552642304	0.8082762	-6.7596024
SIK2	-0.0750206	4.25131	-0.5976476	0.552643683	0.8082762	-6.6019629
COQ7	-0.1458353	1.85197601	-0.5975395	0.552715297	0.8082762	-5.855312
ENSCAFG00000027802	-0.1758759	2.61556075	-0.5974808	0.552754146	0.8082762	-6.1898083
ARSK	-0.1185046	2.95196491	-0.5973618	0.552833015	0.8082977	-6.1628331
CHAC2	-0.1222643	0.86045239	-0.5971778	0.552954916	0.80838209	-5.7323817
ZNF229	0.13432924	2.31938543	0.59688416	0.553149471	0.80857267	-6.058258
SCAMP1	-0.0754727	5.41711443	-0.5967484	0.553239456	0.80861037	-6.7354308
MRPS15	-0.0563782	5.18233157	-0.5961562	0.553631959	0.80894137	-6.7120561
IGF1	-0.3577251	-2.2762051	-0.5961242	0.553653198	0.80894137	-5.2715607
FAM210A	0.07987111	3.31835285	0.59611608	0.553658584	0.80894137	-6.3653397
ANAPC16	-0.0852259	3.07287567	-0.5950458	0.554368422	0.80952777	-6.2301559
ENSCAFG00000008345	0.30117602	-1.0829584	0.59495849	0.554426317	0.80952777	-5.4354034
SHKBP1	0.07360652	5.01264013	0.59486671	0.554487206	0.80952777	-6.6608773
ENSCAFG00000024323	-0.2393615	-0.4453897	-0.5948105	0.554524506	0.80952777	-5.3943706
ENSCAFG00000031583	-0.0618439	4.86894552	-0.5947928	0.554536258	0.80952777	-6.6921121
KIF13A	-0.0672623	6.21427516	-0.5947466	0.554566933	0.80952777	-6.7757623
TTC4	-0.0464265	5.33365966	-0.5946708	0.554617218	0.80952777	-6.7312255
MMP27	-0.3072528	-3.0291078	-0.5945671	0.554686015	0.80952777	-5.1931265
RIPOR3	-0.5095897	-0.2372848	-0.5945491	0.554697972	0.80952777	-5.649796
TOMM34	-0.0885169	3.47101126	-0.5945421	0.554702616	0.80952777	-6.3916682
IKBKB	-0.0567422	4.90817617	-0.5940879	0.555004067	0.80982575	-6.6954095
EPG5	-0.063534	4.77686976	-0.5940407	0.555035383	0.80982575	-6.6837791
SBF1	0.05337009	6.23811072	0.59376854	0.555216067	0.80995232	-6.7770873
ENSCAFG00000016577	-0.1937895	3.02977079	-0.5936153	0.555317778	0.80995232	-6.0209441
FAM92A	-0.0603918	4.11147117	-0.5935785	0.555342268	0.80995232	-6.5474288
TANC1	-0.1315794	4.31421463	-0.5935226	0.555379339	0.80995232	-6.6225969
PRRC1	0.07289905	6.56985951	0.59323538	0.555570101	0.81006812	-6.7773485
FKBP3	0.06273672	4.93583195	0.59320938	0.555587371	0.81006812	-6.6776394
PCGF6	-0.1013511	2.58818138	-0.5925725	0.556010416	0.81058909	-6.1046904
HOXC4	-0.260564	2.00132154	-0.5924733	0.556076368	0.81058909	-6.0267757
C7H1orf74	-0.0872231	2.66978673	-0.5923809	0.556137739	0.81058909	-6.1815681
ENSCAFG00000005816	-0.1200584	3.14354301	-0.5921293	0.556304933	0.81073897	-6.2527958
LEMD3	-0.1069776	3.60202763	-0.5919679	0.556412215	0.81080151	-6.5285292

SCRN3	0.06619975	4.38733466	0.59174008	0.556563631	0.81081804	-6.6272043
HOXC6	-0.3133022	1.73759728	-0.5916674	0.556611926	0.81081804	-5.9263415
ENSCAFG00000016640	-0.0492047	7.0712297	-0.5916603	0.556616668	0.81081804	-6.7657296
CSAD	0.22412371	0.3465426	0.59154186	0.5566954	0.81083895	-5.6706731
ALKBH2	0.11419994	1.1087255	0.59121917	0.556909951	0.81097512	-5.8494923
DDX47	0.06416628	4.71962247	0.59111753	0.556977535	0.81097512	-6.6564168
GRPEL2	-0.0917132	3.73952692	-0.5911108	0.55698204	0.81097512	-6.4861207
OSBPL3	0.14372156	2.34663038	0.59084193	0.557160821	0.81112165	-6.7534602
TMEM192	-0.057523	4.65008606	-0.5907658	0.557211466	0.81112165	-6.6569746
PLCB3	0.04792441	7.02007897	0.59036133	0.557480507	0.81129194	-6.7654943
CBX2	0.15184363	1.37371141	0.59034397	0.557492054	0.81129194	-5.8222066
RASGRP2	-0.225302	2.38187692	-0.5902994	0.557521678	0.81129194	-6.3076758
DXO	-0.0652456	4.10855667	-0.5900881	0.557662292	0.81140282	-6.5574618
THOC5	0.04003816	6.56509756	0.58951298	0.55804504	0.81184783	-6.7794544
ITGBL1	0.36445334	5.26818108	0.58935844	0.55814791	0.81184783	-6.7700898
EIF3I	0.05015842	7.04673268	0.58927548	0.558203136	0.81184783	-6.7700801
DGKH	0.17398734	2.29975293	0.58924122	0.558225946	0.81184783	-6.0388437
MBD6	-0.0956837	4.95840866	-0.588655	0.558616249	0.81232167	-6.6658615
ATP6V0A1	0.11994903	6.47323546	0.58848412	0.558730077	0.8123934	-6.76562
KRAS	-0.1003203	3.39999112	-0.5882884	0.558860446	0.81248917	-6.3187967
BCL2L1	0.07287624	4.94934218	0.58806978	0.559006075	0.8126071	-6.7470435
CCDC56	-0.0623528	4.34556511	-0.5877309	0.559231868	0.81276958	-6.5802245
MYORG	0.12791566	2.39208981	0.58770832	0.559246899	0.81276958	-6.2572892
SNAPC3	-0.0778262	3.63195346	-0.5875195	0.559372731	0.81285867	-6.3954029
ATG14	-0.1161383	2.5050139	-0.5872858	0.559528454	0.81299116	-6.1758937
DNAJC9	-0.1858582	1.3524649	-0.5868307	0.55983182	0.81333072	-5.7001307
DNAJC25	0.0886676	4.83811047	0.5866591	0.559946247	0.81333072	-6.6913635
DDX54	0.05994809	6.46146471	0.58664469	0.559955859	0.81333072	-6.781776
MARK4	0.07228578	4.42503657	0.58650937	0.560046084	0.81336743	-6.6097912
GPKOW	0.06441365	4.20541668	0.58634894	0.560153066	0.81336743	-6.5505562
ASRGL1	0.0795933	4.78878429	0.58620666	0.560247957	0.81336743	-6.6530924
SLC31A1	-0.0519151	5.82829952	-0.5861675	0.560274045	0.81336743	-6.7746956
CCNB3	-0.478046	0.59648511	-0.5860377	0.560360662	0.81336743	-5.4962966
NEU3	0.17442744	0.68900582	0.58567418	0.560603137	0.81336743	-5.7678768
TROAP	-0.5913167	1.76990755	-0.5854124	0.560777801	0.81336743	-5.6141643
ENSCAFG00000011833	-0.0613488	5.31495896	-0.5854085	0.560780426	0.81336743	-6.7200822
HEATR3	-0.0747521	5.42103241	-0.5853787	0.560800272	0.81336743	-6.7282512
ENSCAFG00000031011	-0.3244093	-1.1706022	-0.5853102	0.560845959	0.81336743	-5.3581023
RXRB	-0.075979	4.99439267	-0.58523	0.560899525	0.81336743	-6.6995667
ABHD18	0.13141328	3.65655562	0.58520605	0.56091549	0.81336743	-6.4526244
RPAP1	-0.0521803	5.28487556	-0.5848851	0.561129715	0.81336743	-6.7293645
ENSCAFG00000015864	0.17988229	1.4129465	0.58485677	0.561148597	0.81336743	-5.8281158
RRN3	0.05166686	5.42820868	0.58469766	0.561254799	0.81336743	-6.7635625
ENSCAFG00000010782	-0.1354267	1.83060036	-0.584632	0.561298641	0.81336743	-6.0278158
AAK1	0.06036342	5.26724528	0.58458205	0.561331978	0.81336743	-6.7327883
DELE1	-0.065016	5.5544983	-0.5844697	0.561406999	0.81336743	-6.7614725
NUDT16L1	-0.2403611	0.99358845	-0.5844284	0.56143455	0.81336743	-5.6720249
HELZ	-0.0685215	5.26743583	-0.5843636	0.561477819	0.81336743	-6.7364941
DAPK1	0.12904142	5.08406055	0.58435111	0.561486157	0.81336743	-6.6917783
ENSCAFG00000001397	-0.3348185	0.90886716	-0.5843334	0.561497968	0.81336743	-5.6816003

C9H17orf53	-0.2622395	1.05696346	-0.5841563	0.561616238	0.81336743	-5.6698415
HDHD5	-0.0680231	5.52272048	-0.5841449	0.561623817	0.81336743	-6.7427796
DUSP3	0.07507652	5.21244107	0.58407509	0.561670456	0.81336743	-6.7062776
CTDP1	-0.0532528	4.6892026	-0.5840312	0.56169979	0.81336743	-6.6554398
ENSCAFG00000023007	0.11138672	2.29949642	0.58399399	0.561724617	0.81336743	-6.1470616
SHARPIN	0.07493806	5.43847539	0.58374794	0.561888939	0.81351185	-6.7291694
WRAP53	-0.087892	3.87846704	-0.5836361	0.561963627	0.81352647	-6.4943918
ENSCAFG00000031908	-0.0981082	2.40878155	-0.5833756	0.562137654	0.81360917	-6.055037
ZFP90	-0.0862428	4.99958387	-0.5833572	0.562149934	0.81360917	-6.7585116
NR1H2	0.07387294	5.50998924	0.58301859	0.562376176	0.8138431	-6.7664078
ATP6V1C1	0.06966051	5.42250727	0.58282403	0.562506188	0.81393773	-6.7346066
PIK3IP1	0.21555292	1.89879931	0.58267162	0.562608044	0.81399161	-6.2705497
ZNF768	-0.0649818	4.97689033	-0.5825186	0.562710294	0.81399326	-6.7024703
COASY	0.0474355	4.78460116	0.58240631	0.56278537	0.81399326	-6.6856995
ZNF772	-0.1442001	1.7339585	-0.5823433	0.562827489	0.81399326	-5.9517737
FAM129C	0.28617028	-2.1445261	0.58213961	0.562963654	0.81399326	-5.3576491
ENSCAFG00000022770	-0.1839091	0.01926487	-0.5820223	0.563042097	0.81399326	-5.6506696
HMG20B	-0.0724853	4.64440084	-0.5818361	0.563166557	0.81399326	-6.6742986
ALG13	-0.1059274	3.09129316	-0.5817693	0.563211253	0.81399326	-6.2723105
AMBRA1	-0.0704471	4.75927674	-0.5815878	0.563332633	0.81399326	-6.7005949
ENSCAFG00000031249	-0.2479592	2.84802215	-0.581552	0.563356588	0.81399326	-6.269208
DPP3	0.05417459	6.05617164	0.58153194	0.563369972	0.81399326	-6.7813098
SATB1	0.2416363	3.42619816	0.58150008	0.563391282	0.81399326	-5.9922094
DRG1	-0.0486612	5.05017165	-0.581425	0.563441491	0.81399326	-6.7118857
IQSEC1	0.08366373	4.89267905	0.58139596	0.563460917	0.81399326	-6.6889984
PRPF4	0.07982028	3.53454838	0.58131673	0.563513909	0.81399326	-6.4052868
TAF3	0.05858481	4.26458754	0.58104604	0.563694979	0.81410482	-6.5939901
FLT3LG	0.1668154	2.76028738	0.58100803	0.563720406	0.81410482	-6.2360629
TMEM11	0.06029216	4.69316527	0.58061373	0.563984217	0.81435548	-6.6446292
RPS6KB1	0.05105584	4.96318185	0.58055536	0.564023277	0.81435548	-6.7083344
CDK12	0.05814613	5.63023766	0.58032506	0.564177395	0.81448464	-6.7693967
UBQLN2	-0.0599677	5.609029	-0.5800987	0.564328926	0.81461003	-6.7598383
HYI	-0.1800603	2.00972401	-0.5798852	0.56447183	0.81472295	-6.0144507
PRRG1	-0.0926503	4.19201388	-0.5797434	0.564566745	0.81476658	-6.5600754
TMEM74	0.31957372	0.26288949	0.57936238	0.564821845	0.81501614	-5.4093338
RAB8B	-0.1039646	4.9448576	-0.5792105	0.564923578	0.81501614	-6.7517685
TMEM143	-0.082503	2.95690361	-0.5791287	0.564978318	0.81501614	-6.3714691
GOLGA3	-0.0466557	5.90116915	-0.5790986	0.564998486	0.81501614	-6.7811188
ZNF524	-0.1787804	1.88341803	-0.578946	0.565100717	0.81505404	-5.9648536
ENSCAFG00000029533	-0.2969632	-1.6073569	-0.5788643	0.565155451	0.81505404	-5.2509068
IKBKG	0.04863815	5.2874458	0.57876177	0.565224101	0.81505404	-6.7311443
APBA3	0.10904585	2.94539894	0.57859079	0.565338643	0.81505404	-6.3175241
GTPBP8	0.09649399	2.39012665	0.57857638	0.565348299	0.81505404	-6.1201649
PRKRIP1	-0.0648298	3.03462094	-0.5784434	0.565437386	0.81508919	-6.2942087
MAPRE1	0.1641997	1.54475929	0.57816433	0.565624376	0.81526544	-5.8727158
ADGRV1	0.26801669	-0.4099286	0.57779497	0.565871912	0.81536435	-5.4904411
LAMA2	-0.3412494	9.11359841	-0.5777471	0.565904012	0.81536435	-6.610736
MEX3B	0.14026926	2.50863593	0.57769555	0.565938548	0.81536435	-6.0464867
DENND2A	0.14100498	5.54221575	0.57767559	0.565951927	0.81536435	-6.7870096
TELO2	-0.0756149	3.95394023	-0.5773743	0.566153864	0.81546887	-6.5638801

SPON1	-0.8779113	-0.6861866	-0.577192	0.566276144	0.81546887	-5.2505055
WDFY4	0.22143679	1.05492284	0.57714685	0.566306391	0.81546887	-6.1145818
ENSCAFG00000019739	-0.250446	-0.3908717	-0.5770974	0.566339516	0.81546887	-5.5111653
RAD51C	-0.0898932	2.61753714	-0.5770845	0.566348179	0.81546887	-6.1445834
PHLDB1	0.07349765	8.06624691	0.57685731	0.566500545	0.81552954	-6.7216289
DNAJC18	0.06829254	4.78374559	0.57681723	0.566527423	0.81552954	-6.7050468
SUDS3	-0.0434264	5.76490859	-0.576732	0.566584549	0.81552954	-6.7764264
MEOX1	0.22963088	3.31426039	0.57646269	0.566765206	0.81563137	-6.6055585
SPOUT1	0.08067529	3.5176118	0.57643348	0.566784802	0.81563137	-6.442134
ENSCAFG00000030696	-0.3354275	0.39996861	-0.5757532	0.567241213	0.81619492	-5.5463746
SMYD5	-0.0838472	4.33464859	-0.5755099	0.567404472	0.81633658	-6.4969868
C11H9orf43	-0.1941753	0.09419882	-0.5753756	0.567494593	0.81634578	-5.6093514
GJA1	0.10356349	7.32405851	0.5750046	0.567743662	0.81634578	-6.7573517
NEMP1	-0.076931	5.33692198	-0.5747534	0.567912312	0.81634578	-6.7634375
PCM1	-0.0559273	7.08719066	-0.5746276	0.567996809	0.81634578	-6.7743734
EVC	0.16880029	6.1477358	0.57451233	0.568074188	0.81634578	-6.7739891
SETBP1	-0.2911382	1.89086488	-0.5744697	0.568102793	0.81634578	-5.8294372
ABCG2	-0.1192203	2.96249223	-0.574341	0.568189266	0.81634578	-6.1948136
ENSCAFG00000009128	0.07595513	4.9714147	0.57422558	0.568266762	0.81634578	-6.7214276
TRIM4	-0.0474969	6.00639643	-0.5741254	0.568334079	0.81634578	-6.7834007
LHFPL2	-0.1069359	3.40849533	-0.5740133	0.568409343	0.81634578	-6.3928243
ABRAXAS2	0.0629867	4.96489277	0.57398116	0.568430935	0.81634578	-6.7162631
AP1S1	0.05323306	4.62789444	0.57390664	0.568480994	0.81634578	-6.6651774
CYB561D1	-0.1991871	0.87924733	-0.573884	0.568496192	0.81634578	-5.7063275
RTN4IP1	-0.0692388	3.45866393	-0.5737892	0.568559883	0.81634578	-6.3916562
LYVE1	0.35667323	3.31228287	0.57377034	0.568572559	0.81634578	-6.3065391
POLR2D	0.08924855	3.17894951	0.57372194	0.568605079	0.81634578	-6.3333047
PEX16	0.07916502	3.23010235	0.57369932	0.568620273	0.81634578	-6.3503839
TRADD	-0.0612637	4.53751703	-0.5734587	0.568781957	0.81634578	-6.6179263
THAP3	-0.1107197	1.4715559	-0.5734048	0.568818161	0.81634578	-5.8448704
PAK4	0.05939825	4.48957193	0.57340412	0.568818618	0.81634578	-6.6295751
SPCS3	0.11059269	4.61971362	0.57335983	0.568848384	0.81634578	-6.6310789
FBH1	0.05833018	5.49512445	0.57329074	0.568894812	0.81634578	-6.7514482
UBB	0.06374169	4.70422472	0.57321962	0.568942602	0.81634578	-6.6710987
ITGA6	0.25959173	5.38042839	0.57316677	0.568978118	0.81634578	-6.7756159
PXDC1	0.19251226	4.37360463	0.57298952	0.569097246	0.81634578	-6.6015596
ZNF696	-0.1364195	1.29192518	-0.5729847	0.569100507	0.81634578	-5.8219503
COL5A3	-0.300754	4.37166208	-0.5727959	0.569227391	0.81634578	-6.7870408
SMAD6	0.27871439	3.02412822	0.57277012	0.569244722	0.81634578	-6.0917945
MORF4L2	-0.0797976	6.70354759	-0.5727022	0.569290353	0.81634578	-6.7888688
ACSM3	-0.4347966	-0.9383832	-0.5722712	0.569580181	0.81666841	-5.8224984
ACTR3	0.07445328	8.43196169	0.57198935	0.569769682	0.81680724	-6.7206661
RAB13	-0.0696433	5.43081667	-0.5719343	0.5698067	0.81680724	-6.7799698
HPS4	-0.0541882	4.83874219	-0.5718106	0.569889921	0.81681168	-6.6925653
MAB21L1	-0.4918469	-1.6584775	-0.5717369	0.569939492	0.81681168	-5.3276689
POFUT1	0.05979735	6.67061545	0.5711507	0.570333818	0.81697822	-6.783112
LTBP4	-0.2802897	5.5577178	-0.5708552	0.570532661	0.81697822	-6.7834084
IDH3G	-0.055431	6.33950122	-0.5708463	0.570538663	0.81697822	-6.790823
TMEM8A	0.0864821	5.39093732	0.57072915	0.570617486	0.81697822	-6.7491971
MRPL4	-0.081693	4.51988342	-0.5706893	0.570644313	0.81697822	-6.6207015

SH3BP2	-0.0975909	5.12437883	-0.570656	0.570666687	0.81697822	-6.752229
EPDR1	0.5389518	0.83792267	0.57064442	0.570674513	0.81697822	-5.5143458
DDX49	-0.0747684	5.01900103	-0.5703748	0.57085598	0.81697822	-6.6930407
MED31	-0.0906055	2.41205817	-0.5702819	0.570918524	0.81697822	-6.116729
SPATS2L	0.10564785	6.74814854	0.5702575	0.570934953	0.81697822	-6.7898016
MARCH5	0.05297989	5.04240316	0.57021926	0.570960697	0.81697822	-6.7255382
SNX27	0.07222077	5.7012665	0.57013104	0.571020087	0.81697822	-6.7660949
NR3C2	0.18266687	1.66208777	0.56995321	0.571139817	0.81697822	-5.8504468
ANGPT2	-0.5391021	-1.002193	-0.5699381	0.571149993	0.81697822	-5.5179879
CCDC114	-0.2213964	0.10887197	-0.5699132	0.571166767	0.81697822	-5.6456766
REPS2	0.36264245	2.94484063	0.56982398	0.571226829	0.81697822	-6.1430427
PI16	0.44449444	-0.520284	0.5698035	0.571240622	0.81697822	-5.5610743
RFXAP	-0.1192722	3.80834488	-0.5697669	0.571265273	0.81697822	-6.3586024
WNT9A	0.20572216	3.76908096	0.56973308	0.571288042	0.81697822	-6.3624764
CPNE7	0.24926384	0.25534016	0.56948619	0.571454304	0.81699743	-6.1585576
ARMCX5	-0.1208106	1.12112233	-0.5694319	0.571490837	0.81699743	-5.8625261
GPR4	-0.471524	0.62621392	-0.5694242	0.57149606	0.81699743	-5.5708421
COL4A6	-0.3983969	-2.5797783	-0.5690362	0.571757379	0.81699765	-5.1898942
ST8SIA1	-0.3429185	-1.5436863	-0.5690016	0.571780725	0.81699765	-5.3888762
ST3GAL1	0.10287419	5.233813	0.56872521	0.571966925	0.81699765	-6.7693331
ADGRA1	-0.3077164	-2.3550836	-0.5687233	0.571968235	0.81699765	-5.2604121
TTC1	0.05648865	4.79535056	0.56866084	0.5720103	0.81699765	-6.696599
HSD17B1	0.18233246	0.35239858	0.56859004	0.572058005	0.81699765	-5.7604194
ZNRD1	-0.0998428	2.53457927	-0.5685842	0.572061909	0.81699765	-6.1514791
LZTS2	0.07681119	5.54307158	0.5685111	0.572111199	0.81699765	-6.7541032
ENTPD5	-0.1388444	2.83012686	-0.5684437	0.572156593	0.81699765	-6.2025137
CCT2	-0.0750025	6.95243426	-0.5684188	0.572173416	0.81699765	-6.7883138
SGF29	-0.1004641	2.84788137	-0.5683649	0.572209694	0.81699765	-6.2878743
SCAPER	0.06933674	4.22945509	0.56799279	0.572460521	0.81717322	-6.5751577
ENSCAFG00000029695	0.11127512	2.19269293	0.56798999	0.572462405	0.81717322	-6.0188901
HSD11B1	-0.4328333	-0.0919274	-0.5676901	0.572664577	0.81736918	-6.3060159
MFSD5	0.07069529	5.07084705	0.56754593	0.572761771	0.81741528	-6.7079375
BLOC1S4	0.13515134	2.63172754	0.56729748	0.572929297	0.81749298	-6.1619599
CIC	0.09076371	6.86086681	0.56727269	0.572946018	0.81749298	-6.7905222
GNAI2	0.0717327	8.79651691	0.56713895	0.573036205	0.81752906	-6.6728444
STX5	0.04430749	4.87663895	0.56685115	0.573230313	0.81755948	-6.698131
ENSCAFG00000012125	-0.0561641	6.20179992	-0.5667735	0.573282688	0.81755948	-6.7930938
CELF2	-0.4766727	3.05578419	-0.5667638	0.573289207	0.81755948	-5.9806691
BBX	0.07486959	6.21312983	0.56667694	0.573347827	0.81755948	-6.7905903
GIN1	0.07289095	3.68159548	0.56662619	0.573382062	0.81755948	-6.5100156
UPF3A	0.08321751	2.49164589	0.5665172	0.573455585	0.81756764	-6.2162548
ENSCAFG00000030118	-0.1176452	2.52777006	-0.566237	0.573644611	0.81756764	-6.1192337
UMAD1	-0.2136093	0.28515492	-0.5662357	0.573645536	0.81756764	-5.5653582
PRMT7	0.06152232	5.02350876	0.56623288	0.573647413	0.81756764	-6.7100887
COX15	0.06887186	3.78723844	0.56577538	0.57395615	0.81780669	-6.4696812
GLI4	-0.1516133	2.04365185	-0.5656953	0.574010168	0.81780669	-6.0653084
EIF2D	-0.0485224	5.03999329	-0.5656038	0.574071975	0.81780669	-6.693028
JOSD2	0.08908236	4.49495832	0.56543777	0.574184035	0.81780669	-6.6567055
PPP2R5B	-0.0905871	3.51256525	-0.5654305	0.574188954	0.81780669	-6.4870076
CREB3L4	-0.2534985	0.35306927	-0.5653299	0.574256853	0.81780669	-5.5859589

MST1R	-0.3725001	-0.0249648	-0.565311	0.574269624	0.81780669	-5.61054
ENSCAFG00000019510	-0.2017399	4.07963673	-0.5650373	0.574454413	0.81797184	-6.5135605
OSR1	-0.2388176	4.82457627	-0.5648945	0.574550819	0.81797184	-6.5531465
AAR2	-0.0532693	4.60614345	-0.564752	0.574647043	0.81797184	-6.6497278
ENSCAFG00000008385	-0.0935666	2.67662873	-0.5646711	0.574701673	0.81797184	-6.2489696
HAUS2	-0.0651891	4.57828785	-0.5646584	0.574710286	0.81797184	-6.6666191
PSENEN	0.08476452	5.00794651	0.56426121	0.574978545	0.81819444	-6.6986751
SLC30A7	-0.0734549	4.61452963	-0.5642047	0.575016723	0.81819444	-6.6414988
CHST12	0.08782893	3.95442275	0.56409568	0.575090367	0.81819444	-6.5968007
MAN2A1	-0.0671146	7.94630121	-0.5639587	0.575182919	0.81819444	-6.6787627
RABIF	-0.0771388	3.21341729	-0.563946	0.575191471	0.81819444	-6.3338741
DZIP3	-0.0633385	5.0850497	-0.5638372	0.575265026	0.81820667	-6.7351367
RAD9A	-0.0747642	4.63201467	-0.5636539	0.575388828	0.81829035	-6.6187351
CDK2	-0.187854	2.3208061	-0.5631196	0.575749987	0.81863222	-5.9438398
ARGLU1	-0.0577959	5.48945517	-0.5631059	0.575759195	0.81863222	-6.7602668
SLC29A3	0.10019393	2.64319507	0.562712	0.576025514	0.81891567	-6.2000048
POLR3G	0.10320186	3.28244376	0.56261873	0.576088577	0.81891567	-6.4470943
ENSCAFG00000000177	-0.1519015	1.47401477	-0.5620212	0.576492676	0.81921894	-5.8445165
ENSCAFG00000003149	-0.1339747	1.02826963	-0.5617281	0.576690933	0.81921894	-5.7852252
LIN9	-0.146222	2.89810133	-0.5616818	0.576722262	0.81921894	-6.2251921
TTC9	0.30802799	-0.4134229	0.56168134	0.576722556	0.81921894	-5.4874434
TM9SF3	0.04356559	8.33941342	0.56160909	0.576771433	0.81921894	-6.7201401
ENSCAFG00000006856	0.10025757	2.24054076	0.56131637	0.576969486	0.81921894	-6.0559655
ITPKB	-0.1875901	3.42511365	-0.5613129	0.576971842	0.81921894	-6.3022717
ABHD14A	0.09050024	3.73415757	0.56125596	0.57701036	0.81921894	-6.5257298
EXOSC3	-0.104534	2.02765653	-0.5611833	0.57705953	0.81921894	-6.0312662
TMEM60	-0.1248633	2.19188112	-0.5611457	0.577084952	0.81921894	-6.0774451
MBP	0.33770958	-1.4086174	0.56114238	0.57708722	0.81921894	-5.2956454
CFAP20	-0.0646292	3.89700364	-0.5609413	0.577223295	0.81921894	-6.5668108
AGBL3	0.26910181	-1.6125247	0.56092879	0.577231763	0.81921894	-5.391839
DHPS	0.06744663	3.94181087	0.56074277	0.577357672	0.81921894	-6.5002331
TROVE2	-0.0733913	5.39011922	-0.5606264	0.577436431	0.81921894	-6.7484834
NAPG	0.0687195	4.26625092	0.56054159	0.577493849	0.81921894	-6.5960619
BCL2	0.21102241	3.25155641	0.56050633	0.57751772	0.81921894	-6.2086621
FOXP2	-0.5133966	1.10276346	-0.5604706	0.577541884	0.81921894	-5.5153472
PPP1R13B	-0.1031242	6.12939663	-0.5603588	0.577617598	0.81921894	-6.7869771
CASP9	0.0592461	4.87398707	0.56031822	0.577645066	0.81921894	-6.6643026
RHOC	0.05605211	6.32145762	0.56020677	0.577720524	0.81921894	-6.7970068
RDH14	-0.0648	5.02448581	-0.5601656	0.577748404	0.81921894	-6.7173507
TMEM117	-0.1304146	3.67350418	-0.5600485	0.577827672	0.81921894	-6.6652968
FAM49B	0.07983928	4.88543153	0.5599131	0.577919376	0.81921894	-6.6844105
IFT27	0.12276575	1.78164167	0.55988932	0.57793548	0.81921894	-6.0070567
CCDC112	-0.0754812	3.82970664	-0.5598045	0.577992909	0.81921894	-6.5573873
CBLN3	0.27238631	0.1088545	0.55960012	0.578131339	0.81928135	-5.5577926
GALNT18	-0.364239	-2.0490124	-0.5595474	0.578167031	0.81928135	-5.3118818
PPP1R16B	0.2535057	2.28464075	0.55914997	0.578436267	0.81957067	-6.1990884
ANKRD2	0.29460298	0.46077058	0.55904087	0.578510183	0.81957734	-5.6770602
SNAPIN	0.13160326	2.34225431	0.5588305	0.578652717	0.81957734	-6.0895671
CCDC34	-0.0594538	5.00316657	-0.5588154	0.578662933	0.81957734	-6.7473027
PARP1	0.0573023	6.55896636	0.55871421	0.578731524	0.81957734	-6.7948145

ORC6	-0.2404269	1.71838711	-0.5585825	0.578820755	0.81957734	-5.8005221
FOS	-0.2767915	6.70273312	-0.5584916	0.578882348	0.81957734	-6.7929793
TRAPPC11	0.03812164	7.06621524	0.55842444	0.578927899	0.81957734	-6.7879175
KDELR2	-0.0565655	8.21216667	-0.5583207	0.578998178	0.81957734	-6.7346957
SLC35F2	0.23069759	3.39974139	0.55827885	0.579026577	0.81957734	-6.3032853
MAD2L1BP	-0.1532085	2.8384634	-0.5580801	0.579161279	0.8196759	-6.279005
WDR5B	-0.0911343	3.45158198	-0.5576627	0.579444263	0.81998426	-6.3921774
ENY2	-0.092169	2.06389129	-0.5574027	0.579620617	0.820092	-6.0276049
MTX2	0.06329525	5.34755495	0.55730493	0.57968689	0.820092	-6.7503374
GTF2H5	0.08270171	2.98240255	0.55726243	0.579715715	0.820092	-6.256445
MBLAC2	-0.0819231	3.59204973	-0.5571391	0.579799376	0.82011824	-6.4452579
ENSCAFG00000003095	0.12806524	1.75584658	0.55677306	0.580047652	0.8203393	-5.9041008
REC8	-0.190346	2.27702477	-0.5567167	0.580085912	0.8203393	-6.1521004
WNT5A	0.35979851	2.8090384	0.55646951	0.580253595	0.82043713	-6.3647671
ANGPTL4	0.24372444	3.25198697	0.55642269	0.580285363	0.82043713	-6.6129725
MICU2	-0.0489923	5.54145817	-0.5560619	0.580530208	0.82069119	-6.7805351
RPP21	0.06995631	3.64687152	0.55592195	0.580625168	0.82073103	-6.4403947
NDST2	-0.0998349	4.73049659	-0.5558283	0.580688704	0.82073103	-6.6932957
DNAJB5	0.21027989	0.1485985	0.55566313	0.580800846	0.82077759	-5.5397191
NVL	-0.0610491	4.90872747	-0.5555878	0.580851969	0.82077759	-6.6923633
ENSCAFG00000030159	-0.1213694	1.77093386	-0.5546095	0.581516282	0.82162413	-5.9833321
GLI2	0.18787442	4.48332999	0.5545051	0.581587186	0.82163214	-6.6959657
TMEM132C	0.59432228	0.11037489	0.55416752	0.581816511	0.8217878	-5.2497551
L3MBTL1	-0.1004828	2.78642812	-0.5541508	0.581827847	0.8217878	-6.2108169
GRN	0.10678001	8.18432835	0.55390305	0.581996203	0.82193342	-6.7199145
DHX57	-0.0619032	4.24726347	-0.5537514	0.582099276	0.82198683	-6.6302353
PDCD11	0.06631584	6.4460496	0.55351454	0.58226022	0.82212193	-6.8007525
GATA2	-0.3902438	1.22700674	-0.5530755	0.58255866	0.82245112	-5.7260861
DHRS13	0.08342827	6.60914804	0.55296238	0.58263554	0.82246747	-6.7948491
ARRB1	-0.3137644	0.506759	-0.5528662	0.582700921	0.82246759	-6.0383101
CIPC	0.06843752	3.10540361	0.55269433	0.582817785	0.82254037	-6.4159839
TMEM253	-0.1994576	0.21883729	-0.5524791	0.582964138	0.82260671	-5.7288123
ZC3H8	0.15402748	1.28583762	0.55243313	0.582995404	0.82260671	-5.8612997
CHFR	0.05899484	5.5505291	0.55189761	0.583359631	0.82295536	-6.7463969
RUNDC1	0.06339606	4.32653579	0.55187772	0.583373165	0.82295536	-6.636669
MEMO1	-0.0413004	5.81203944	-0.5513004	0.583765973	0.82330602	-6.7865437
ENSCAFG00000001369	-0.1400119	2.54724227	-0.5512088	0.583828258	0.82330602	-6.1746561
CCDC88C	0.28606222	-0.4563733	0.55119254	0.583839346	0.82330602	-5.6306449
SLC8A3	0.52087077	1.76230508	0.55090895	0.584032354	0.82330602	-5.7588559
TRIM66	-0.2500761	-0.0067919	-0.5508904	0.584044947	0.82330602	-5.5761396
CMTM7	0.11720008	2.5144098	0.55088657	0.584047586	0.82330602	-6.1910866
AMPD2	0.06110844	6.73053669	0.55084	0.584079282	0.82330602	-6.7943706
KDELR3	-0.0685919	6.11217722	-0.5506576	0.58420342	0.82338886	-6.7841013
PDE10A	0.30285105	2.4720711	0.55045918	0.584338514	0.82341913	-6.1195803
CHERP	-0.0500248	5.57107129	-0.550434	0.584355638	0.82341913	-6.7736965
ENSCAFG00000007577	-0.26963	0.01105121	-0.5502679	0.584468751	0.8234864	-5.5055796
ENSCAFG00000015869	-0.2187831	-0.2629923	-0.54986	0.584746523	0.82378562	-5.5081884
CHD7	-0.2287957	2.69187724	-0.5491843	0.585206722	0.82428015	-6.2407911
ZNF202	-0.0617948	3.63056359	-0.5491525	0.585228435	0.82428015	-6.4881195
TIPRL	-0.0598625	4.94236483	-0.5489197	0.585387033	0.82441134	-6.7097349

FUCA1	0.08349583	6.54132379	0.54878463	0.585479068	0.82444878	-6.7931208
NAPB	-0.1080118	2.63834848	-0.548459	0.585700982	0.82466908	-6.2490028
STK3	-0.0747158	4.81752266	-0.5482512	0.585842619	0.82477631	-6.7284191
BTBD1	-0.0561266	5.52293114	-0.54811	0.585938869	0.8247897	-6.7854386
UTP11	0.0597909	4.90797897	0.54804513	0.585983091	0.8247897	-6.7256151
SIRT4	-0.127132	1.14982152	-0.5478042	0.586147314	0.82482788	-5.7986536
ENSCAFG00000032043	0.10478857	3.85434322	0.54760093	0.586285946	0.82482788	-6.4846026
TCAF1	-0.3097729	3.76247415	-0.5474956	0.58635775	0.82482788	-6.4404496
DCAF13	-0.0461002	4.56294198	-0.5474936	0.586359148	0.82482788	-6.6425387
MITF	0.10991858	5.41198247	0.54747874	0.586369265	0.82482788	-6.74848
SF3B4	-0.0617239	5.45350889	-0.5474255	0.586405576	0.82482788	-6.74922
ENSCAFG00000013888	-0.085945	4.92103528	-0.5472594	0.58651885	0.82482788	-6.7254683
CASK	-0.0877431	6.09296243	-0.5472371	0.586534082	0.82482788	-6.7712521
NKIRAS2	0.06795034	5.46223194	0.54661233	0.586960234	0.82533502	-6.7522085
RPUSD2	-0.1087239	2.46794745	-0.5460935	0.587314278	0.82574067	-6.1457743
THAP7	0.09255214	2.91908509	0.54592189	0.587431378	0.82577151	-6.211813
GALNT1	0.17790746	9.01895152	0.5457954	0.587517708	0.82577151	-6.7236126
EIF2B4	-0.0556906	5.28628821	-0.5457481	0.587550002	0.82577151	-6.728271
BMP6	0.3319551	3.37665763	0.54557948	0.587665098	0.82577151	-6.5752637
ZNF106	0.05929632	7.41686317	0.54556796	0.587672959	0.82577151	-6.7813892
RIMKLB	0.30502649	-1.4184316	0.54529107	0.587861995	0.82577151	-5.2256369
ENSCAFG00000032613	-0.1391641	1.68088823	-0.5452054	0.587920471	0.82577151	-5.8603784
PGP	0.09079401	3.57412234	0.54517024	0.587944493	0.82577151	-6.4743193
CLDN1	-0.4988109	3.52451949	-0.5450229	0.588045128	0.82577151	-5.9128112
MTRR	-0.0505704	5.14798683	-0.5449934	0.588065237	0.82577151	-6.7491139
RAI14	-0.1323797	7.73395509	-0.5448944	0.588132818	0.82577151	-6.7553465
PHKB	-0.0671203	5.44453416	-0.5447648	0.588221371	0.82577151	-6.7673046
IGFBP5	-1.4536895	1.19583724	-0.5446542	0.588296923	0.82577151	-5.2589253
CDCA8	0.3432571	3.49285204	0.54460944	0.588327463	0.82577151	-6.2626807
CFAP53	0.13811743	0.9122199	0.54458882	0.588341548	0.82577151	-5.7995498
LFNG	0.34993619	0.516915	0.54452499	0.588385147	0.82577151	-5.8007247
BMP2	0.30746466	3.78590303	0.54438815	0.588478614	0.82581068	-6.3021012
BCL3	-0.1722488	2.57064395	-0.5441866	0.588616298	0.82590959	-6.0233032
TMEM26	-0.7112109	0.12074761	-0.544093	0.588680243	0.82590959	-5.3150736
CCDC85B	0.13333141	2.61248266	0.54333825	0.589195996	0.82641964	-6.2681937
IDH3A	-0.0591044	5.83510541	-0.5433053	0.589218532	0.82641964	-6.7963562
CSTF2	0.07214618	3.63823871	0.54327298	0.589240613	0.82641964	-6.458836
CCDC77	-0.150285	1.45797895	-0.5431367	0.589333731	0.82645578	-5.8487225
C37H2orf69	-0.0955292	2.30732222	-0.5429861	0.589436724	0.82645578	-6.1143919
SERINC5	-0.1111347	3.10932616	-0.5429473	0.589463218	0.82645578	-6.4809775
TRAPPC12	-0.05229	4.70786504	-0.5427377	0.589606528	0.82655757	-6.7052121
BRAP	0.04332545	5.34051302	0.54264917	0.589667062	0.82655757	-6.7717113
ABCD3	0.05244798	5.54789784	0.54248233	0.589781144	0.82662549	-6.7837576
RNF34	0.08758812	2.49125068	0.54229904	0.589906489	0.82670918	-6.1775038
ATF5	-0.181193	5.29103268	-0.5421537	0.590005857	0.82675646	-6.6762772
HSDL1	0.07965339	4.1114545	0.54188686	0.590188398	0.82692025	-6.5496633
UBE2B	0.05000143	4.15014553	0.54174527	0.590285252	0.82696397	-6.6306136
KATNBL1	0.06658355	3.54605421	0.54156141	0.590411035	0.82697742	-6.4899456
MTIF2	-0.0713024	4.01858298	-0.5414746	0.590470397	0.82697742	-6.5420654
KIAA1211	-0.3172039	-1.4279154	-0.5413489	0.590556411	0.82697742	-5.3278975

QRICH1	-0.0335379	6.15937861	-0.5410817	0.590739281	0.82697742	-6.8062369
ZNF74	-0.1139176	2.85840207	-0.5410044	0.590792194	0.82697742	-6.3109896
ZNHIT3	-0.0916834	2.41934006	-0.5408903	0.590870252	0.82697742	-6.1235483
TECPR1	-0.0529853	4.62099683	-0.5405054	0.591133691	0.82697742	-6.6624026
NCAM1	-0.3536932	4.58226935	-0.5405029	0.591135425	0.82697742	-6.3358161
AXIN1	-0.0472397	5.23419334	-0.5404069	0.591201142	0.82697742	-6.7561872
ACOT11	-0.353958	2.14407091	-0.5403682	0.591227652	0.82697742	-5.9795197
PDE4D	-0.2865252	0.42954233	-0.5403528	0.591238174	0.82697742	-5.529943
FGF5	-0.5175434	-1.0781096	-0.5403395	0.591247325	0.82697742	-5.2249946
KIF2C	0.23449824	2.6161793	0.54030197	0.591272986	0.82697742	-5.9741128
ENSCAFG00000015277	-0.1012384	2.33056636	-0.5402583	0.591302906	0.82697742	-6.1326933
VDAC2	0.06578506	7.15208265	0.54003184	0.591457936	0.82697742	-6.7973047
IL17RC	-0.1043153	4.69760074	-0.5400031	0.591477629	0.82697742	-6.7108101
MAP3K1	-0.1186849	3.80503201	-0.5399694	0.591500662	0.82697742	-6.5786938
SLC2A3	0.192367	3.81296539	0.53996535	0.591503468	0.82697742	-6.7260476
MGAT4A	0.34096104	0.44566447	0.53968494	0.591695491	0.82697742	-5.6058961
RHBDD1	-0.0667496	4.65560978	-0.5396782	0.591700133	0.82697742	-6.6586116
ENDOG	0.11762693	1.11372064	0.5396228	0.59173805	0.82697742	-5.9575193
PSTPIP2	-0.0975711	3.33715321	-0.5395939	0.591757876	0.82697742	-6.4137771
SEL1L	0.06233398	7.30520127	0.5395252	0.591804897	0.82697742	-6.7993395
PTRH1	0.10761871	2.49588595	0.53940284	0.591888707	0.82700279	-6.1520907
AIP	-0.0577785	4.6360244	-0.539014	0.592155049	0.82724695	-6.6955167
TBC1D4	-0.1138296	6.03995074	-0.538956	0.592194802	0.82724695	-6.7781532
ARMC9	-0.1022787	3.53836929	-0.5388208	0.592287456	0.82728463	-6.5164746
GUCY1A1	-0.3830169	-2.2865639	-0.538664	0.592394882	0.82734293	-5.2860341
TWNK	-0.1039382	1.74102922	-0.5385122	0.592498913	0.82739649	-6.0668252
VPS13B	-0.0500024	6.46088064	-0.5381328	0.592758917	0.82755965	-6.8085757
MAPK8IP3	0.05687557	5.54081903	0.53802819	0.592830641	0.82755965	-6.7909923
HEXIM2	-0.1869028	-0.1871007	-0.537992	0.592855483	0.82755965	-5.6655043
ROR2	0.37430407	4.3605026	0.53795829	0.592878557	0.82755965	-6.5489209
WDR91	-0.0912905	4.26723946	-0.5377754	0.593003966	0.82764299	-6.6760517
ROCK1	0.07594788	6.75153544	0.53761926	0.593110985	0.82770064	-6.8032263
SLC9A8	-0.0966804	2.39486389	-0.5370095	0.593529149	0.8280594	-6.1196305
ANAPC1	-0.0687611	5.74950509	-0.536969	0.593556873	0.8280594	-6.7893101
TDRD7	-0.2310053	2.82115454	-0.5369568	0.593565283	0.8280594	-6.1404032
GPR108	-0.068843	6.09689314	-0.5364825	0.593890616	0.82842151	-6.8070258
KDM4C	-0.0699782	4.66614825	-0.5359393	0.594263338	0.82863411	-6.7052795
COX10	-0.0592264	4.67538781	-0.5359231	0.594274457	0.82863411	-6.6738921
TXNDC15	0.04727848	6.00081953	0.53591926	0.594277098	0.82863411	-6.8043588
SART1	-0.055573	5.48634371	-0.5358769	0.594306173	0.82863411	-6.7696249
MAPKAPK3	0.06167982	5.59601386	0.53564455	0.594465636	0.82874143	-6.7908536
ZNF527	0.10561603	1.99061911	0.53557302	0.594514731	0.82874143	-6.0697099
ENSCAFG00000014066	-0.0939771	3.54498444	-0.5352207	0.594756599	0.82898685	-6.4310463
NRBP2	0.07834177	5.12728215	0.53491041	0.594969627	0.82911145	-6.72368
LRP2	0.31893404	-2.6689362	0.53480441	0.595042409	0.82911145	-5.2021939
CDC27	-0.0503454	6.85874906	-0.5347669	0.595068171	0.82911145	-6.8061337
H2AFY2	-0.3153164	1.23744875	-0.534707	0.595109292	0.82911145	-5.4636252
CD276	0.08305746	6.75933071	0.53458538	0.595192822	0.82913612	-6.8096133
AZI2	-0.0497513	4.55831024	-0.5343637	0.595345042	0.82924623	-6.6845845
EPHB3	0.23760405	4.87674106	0.53427859	0.595403532	0.82924623	-6.3031041

SUGCT	0.15598661	3.68316824	0.53417385	0.595475476	0.82925474	-6.6305032
FH	-0.0531292	6.30299098	-0.5338496	0.595698207	0.829287	-6.8112802
SALL1	0.37135476	-1.3452963	0.53381962	0.595718829	0.829287	-5.3324402
AKAP3	0.2357155	-0.7157493	0.5336222	0.59585447	0.829287	-5.5403225
ENSCAFG00000018511	0.18326594	0.88746265	0.53360254	0.595867982	0.829287	-5.7320347
ACSS2	0.07391697	6.85737746	0.53356337	0.595894892	0.829287	-6.7892954
PQLC1	0.07311676	4.21186425	0.53352636	0.595920322	0.829287	-6.5927436
ENSCAFG00000013651	-0.3986367	2.18260929	-0.5334693	0.595959503	0.829287	-6.1380962
ENSCAFG00000016594	-0.1760008	2.68337566	-0.5331736	0.596162739	0.82947817	-6.202031
IL13RA1	-0.1156751	8.33850915	-0.5327166	0.596476907	0.82982363	-6.7331352
ENSCAFG00000013278	-0.2362658	1.84057376	-0.532521	0.596611322	0.82987851	-5.7803095
CDC6	-0.3025287	4.02300777	-0.5324675	0.596648124	0.82987851	-6.3637383
COCH	0.41955802	-0.1191814	0.53219386	0.596836289	0.83002575	-5.5974846
SPRY4	-0.1469729	3.32131534	-0.5321219	0.596885774	0.83002575	-6.7233134
VPS51	-0.0798911	5.17228583	-0.5319094	0.597031912	0.83013732	-6.7585074
OSBPL11	-0.0553701	6.02544851	-0.53179	0.597114018	0.83015984	-6.7977129
SETD9	0.09646938	1.47266617	0.53151458	0.597303481	0.83020166	-5.902517
COQ4	0.04644754	4.81054347	0.5314358	0.597357675	0.83020166	-6.6864657
HAGHL	-0.1014633	2.67456549	-0.5313769	0.597398213	0.83020166	-6.2245575
MED7	0.06180773	3.24229871	0.53134391	0.597420893	0.83020166	-6.3694103
ENSCAFG00000018939	-0.0539876	5.13735846	-0.5311598	0.59754758	0.83020166	-6.7418839
ABHD11	0.12223599	2.08807581	0.53112236	0.597573319	0.83020166	-5.922861
HSF2	0.07597837	3.02983175	0.53107564	0.597605466	0.83020166	-6.3108258
MSL3	0.04949128	5.33170041	0.53096203	0.597683639	0.83021869	-6.7637836
ENSCAFG00000024514	-0.1742448	1.88256093	-0.5308279	0.597775915	0.83025531	-6.104972
SHB	-0.1526547	2.83093698	-0.5305015	0.598000588	0.83047579	-6.2642988
KNTC1	-0.3859382	3.41163548	-0.5302315	0.598186442	0.83050971	-6.0923958
NOC2L	0.05527959	5.61496038	0.53015867	0.598236557	0.83050971	-6.7788425
PCDH17	0.22726815	5.99581683	0.5301099	0.598270129	0.83050971	-6.7957618
ENSCAFG00000030647	0.05968937	3.73462307	0.53005466	0.598308155	0.83050971	-6.5467098
FRMD4B	0.24081577	2.80072591	0.52993144	0.598392989	0.83050971	-6.2769812
ENSCAFG00000014869	0.11255805	1.82720227	0.52975129	0.598517026	0.83050971	-5.8323639
DNAJC17	-0.1086268	1.94325935	-0.5296033	0.598618906	0.83050971	-5.9764325
NECTIN3	-0.1295999	5.09894505	-0.5295823	0.598633359	0.83050971	-6.6871741
ENSCAFG00000003468	0.05311118	4.68266534	0.52953029	0.598669208	0.83050971	-6.6714316
STARD4	0.05971866	3.22321042	0.52928196	0.598840224	0.83050971	-6.4462649
TRIP12	0.04120247	8.29152199	0.52921146	0.598888782	0.83050971	-6.7359455
RNPEPL1	0.06323393	6.18062128	0.52919571	0.598899628	0.83050971	-6.8065307
ENSCAFG00000031543	-0.3193534	-1.1616737	-0.5291809	0.598909819	0.83050971	-5.4908741
HDAC7	-0.0785514	6.98314533	-0.5290913	0.598971554	0.83050971	-6.7962217
PACS2	0.04681239	6.67089497	0.5289454	0.599072046	0.83050971	-6.8135733
ZNF408	0.07815063	2.77464694	0.52893391	0.599079964	0.83050971	-6.3039039
ENSCAFG00000020278	0.05373587	4.98404086	0.527924	0.599775829	0.83128978	-6.734105
DUSP19	0.15538646	2.7626894	0.52780796	0.599855815	0.83128978	-6.3172306
CNIH1	-0.0545376	6.02551017	-0.5276807	0.599943542	0.83128978	-6.8052205
FOXJ2	-0.0561386	4.75061112	-0.5276118	0.599990996	0.83128978	-6.6777512
ACLY	-0.1065537	8.7160456	-0.5275967	0.600001437	0.83128978	-6.676735
ENSCAFG00000029341	-0.0697701	4.36708826	-0.5275136	0.600058696	0.83128978	-6.6781732
RNF146	0.03928729	5.22386947	0.527447	0.600104637	0.83128978	-6.7604987
TAOK1	-0.0608932	5.12153167	-0.5271027	0.600342013	0.83152716	-6.7638686

DPCD	0.12426059	3.26370333	0.52700483	0.600409505	0.8315292	-6.4048889
EMP1	-0.0955138	10.3635437	-0.5268687	0.600503414	0.83156783	-6.5543115
CENPC	-0.052064	6.22992865	-0.5265448	0.600726735	0.83178564	-6.8142442
ENSCAFG00000011003	-0.080077	2.86401559	-0.5261992	0.600965135	0.8319527	-6.271308
ENSCAFG00000032163	0.29441686	0.16558932	0.52606884	0.601055096	0.8319527	-5.4632333
XPNPEP3	0.04701465	4.72822327	0.52601404	0.601092903	0.8319527	-6.7305202
GGA1	0.04932278	4.27230841	0.52598696	0.601111587	0.8319527	-6.632698
B4GAT1	0.08273356	3.95279417	0.52578833	0.601248637	0.83196209	-6.6155138
DCAKD	-0.0921226	4.00763085	-0.5257276	0.601290573	0.83196209	-6.5039284
CDKN3	-0.4192214	-0.3713124	-0.52569	0.601316518	0.83196209	-5.3406244
SAYSD1	0.12897069	1.6396012	0.52542323	0.601500587	0.83206365	-5.9526819
RBM14	-0.0753821	5.22307944	-0.5253922	0.601522036	0.83206365	-6.7251173
GALNT15	-0.4381914	1.80698786	-0.5252356	0.601630086	0.83212173	-6.7445905
NR1H3	0.15722995	2.22359256	0.52505299	0.601756136	0.83220469	-6.2164146
PFKFB4	0.08529626	2.08876544	0.52480485	0.601927436	0.83232209	-6.360898
ENSCAFG00000000118	0.05562482	5.41558504	0.52469397	0.602003982	0.83232209	-6.7753328
CASC3	-0.0311682	6.53034869	-0.5246424	0.60203957	0.83232209	-6.8161615
ENSCAFG00000032034	-0.1748522	-0.2537344	-0.5245472	0.602105345	0.83232209	-5.617302
SPATA17	-0.1769728	1.22274586	-0.5243179	0.60226366	0.83244958	-5.7903521
MED1	-0.0365731	6.85119082	-0.5242101	0.602338088	0.83246111	-6.8131319
EIPR1	0.05633739	5.28664488	0.52399266	0.60248827	0.83257732	-6.7699523
THAP8	-0.1430324	0.77811549	-0.5235514	0.602793085	0.83290716	-5.669329
MED25	-0.0770799	5.28133111	-0.5233938	0.602901941	0.8329662	-6.7750117
NAF1	-0.0561739	4.87028972	-0.52296	0.603201714	0.83318591	-6.7226048
PI15	0.33243846	2.47770477	0.52290106	0.60324243	0.83318591	-5.9839848
MARCH8	-0.0629485	4.07531729	-0.5228419	0.603283301	0.83318591	-6.5621558
SLC2A13	-0.1261481	2.71124949	-0.5227808	0.603325558	0.83318591	-6.1568503
THAP4	-0.0536538	4.29919039	-0.5226585	0.603410058	0.83321126	-6.6201289
RNF214	-0.0349418	4.57704532	-0.5224629	0.603545291	0.83322137	-6.6838743
UBE2C	-0.4751163	3.06218269	-0.5223471	0.603625285	0.83322137	-6.0106435
GPATCH3	-0.0941953	2.63752111	-0.5222727	0.603676757	0.83322137	-6.2334316
THBS2	0.27052918	11.3278722	0.52226513	0.603681978	0.83322137	-6.6492137
AKAP8L	-0.0472352	4.79628163	-0.5219842	0.603876192	0.83339811	-6.737206
FBXO21	0.03786368	6.76748626	0.52180423	0.604000643	0.83347854	-6.8149134
LYSMD3	-0.0664993	2.88252704	-0.5212462	0.604386552	0.83389867	-6.3165086
MKKS	0.05853204	5.9907705	0.52117255	0.604437507	0.83389867	-6.8100586
ZSCAN23	-0.1676458	-0.045203	-0.5208315	0.604673425	0.83413278	-5.5817425
ENSCAFG00000004830	-0.0432997	5.1103192	-0.5202921	0.605046637	0.83447356	-6.732716
HLCS	-0.070667	2.99079218	-0.5201911	0.605116593	0.83447356	-6.3786339
GPAM	0.1004261	6.57932408	0.52012204	0.605164355	0.83447356	-6.816319
CNTF	-0.2157635	-0.3575102	-0.5199315	0.605296218	0.83447356	-5.5614805
CPPED1	0.07713566	5.28968072	0.51971279	0.605447627	0.83447356	-6.7648717
NOA1	-0.0691715	3.24635884	-0.5197035	0.605454044	0.83447356	-6.3696865
C27H12orf57	0.07238061	4.82828565	0.51966677	0.60547948	0.83447356	-6.71743
RETREG1	0.08072318	5.31230157	0.51959634	0.60552824	0.83447356	-6.7357258
STARD8	0.18511911	1.47158562	0.51951752	0.605582805	0.83447356	-5.8342843
CSNK1G1	-0.0563951	3.46264406	-0.5195173	0.60558295	0.83447356	-6.4975921
ENSCAFG00000029381	0.07475459	4.5446769	0.51937817	0.605679283	0.83450168	-6.6926343
TNNC1	0.17253324	0.45634581	0.51929646	0.60573586	0.83450168	-5.8194895
ARG2	-0.2386306	2.57272484	-0.518927	0.605991673	0.83476281	-6.1074835

FUCA2	-0.085449	5.35138297	-0.518617	0.606206394	0.83481829	-6.7673784
TOX2	-0.3751252	-1.3008599	-0.5185023	0.606285876	0.83481829	-5.5422103
JARID2	0.08990966	3.32148006	0.51846768	0.606309821	0.83481829	-6.4243839
ENSCAFG00000028627	0.10777089	2.55177132	0.51843712	0.606330993	0.83481829	-6.2712112
ENSCAFG00000007610	0.09328949	2.00537825	0.51837965	0.606370805	0.83481829	-6.0385574
SEC61G	-0.075881	4.33580621	-0.5182948	0.606429611	0.83481829	-6.6210824
PRKX	-0.1612331	5.32101181	-0.5179719	0.606653301	0.83502586	-6.7784345
WISP2	0.34448837	5.51667292	0.51779698	0.606774495	0.83502586	-6.7863007
DYNC1H1	0.05924123	9.69402527	0.51777304	0.606791088	0.83502586	-6.6337256
GPAT4	-0.0434757	6.76449319	-0.5176944	0.606845562	0.83502586	-6.8178528
EXTL2	-0.0685625	5.4317242	-0.5172891	0.607126465	0.83532113	-6.8092818
HOXB3	0.21716545	2.638084	0.51692251	0.607380588	0.83545396	-6.1942841
MAN1A1	0.15575971	6.75534846	0.51686562	0.607420031	0.83545396	-6.7934877
FBXL14	-0.0685141	3.3815457	-0.5167619	0.607491962	0.83545396	-6.4689945
FRMD8	0.08205106	5.12442612	0.51650916	0.607667181	0.83545396	-6.8036489
SOD2	0.10917512	5.43630734	0.51648739	0.607682271	0.83545396	-6.8186274
CYP26B1	-0.3960807	2.13742116	-0.5164451	0.607711577	0.83545396	-5.9254955
ZNF668	0.08560349	2.77043901	0.51644292	0.607713108	0.83545396	-6.2935595
MTM1	0.07897168	3.65440953	0.51621168	0.607873472	0.83545396	-6.5063832
HPGD	0.3340871	3.93965391	0.51582474	0.608141845	0.83545396	-6.604123
ODR4	-0.0483363	5.01189874	-0.5157334	0.608205235	0.83545396	-6.7543849
PDIK1L	0.08900689	2.20735317	0.51569975	0.608228549	0.83545396	-6.1090626
CBFA2T2	0.06224657	3.7729849	0.51564889	0.60826383	0.83545396	-6.4854772
ASNS	-0.122802	7.94207394	-0.5156275	0.608278665	0.83545396	-6.8028144
RASSF1	0.11055213	2.74342989	0.51553174	0.608345102	0.83545396	-6.1747939
CDK6	0.08553683	4.54469891	0.51552371	0.608350675	0.83545396	-6.7281616
KIF22	-0.3540338	2.93932997	-0.5154874	0.608375849	0.83545396	-6.0587405
C1QTNF7	0.22689091	1.55334221	0.51544709	0.608403826	0.83545396	-5.9816354
EDNRA	0.31131961	2.63387083	0.51540442	0.608433433	0.83545396	-6.6118792
CASP7	0.1159959	4.13937493	0.51503112	0.608692454	0.83545396	-6.5416059
EFEMP1	0.37224576	7.07339179	0.51492003	0.608769547	0.83545396	-6.818153
MRPL54	0.06204514	2.88539188	0.51482874	0.608832908	0.83545396	-6.2407238
LDLRAD3	0.12622006	3.02716943	0.51479855	0.60885386	0.83545396	-6.39062
MCEE	-0.0672495	3.43382147	-0.514642	0.608962487	0.83545396	-6.4471554
YOD1	0.14510883	1.13102188	0.51456317	0.609017227	0.83545396	-5.875727
RNF166	0.06168779	4.35113733	0.51454862	0.609027325	0.83545396	-6.63262
RBMS2	-0.0600038	4.75326017	-0.5145332	0.609037999	0.83545396	-6.7117477
ANKMY2	0.05126172	5.75731669	0.51448224	0.609073405	0.83545396	-6.8037866
PIAS4	-0.0525308	4.21691917	-0.5143924	0.609135755	0.83545396	-6.6091923
RBSN	0.05145073	6.28006397	0.51437194	0.609149968	0.83545396	-6.8212422
HOOK1	0.11065466	2.46021771	0.51415377	0.609301428	0.83545396	-6.1760037
PSMC1	0.05023538	6.07192982	0.5141125	0.609330082	0.83545396	-6.8153642
RBM8A	0.05554939	4.43922537	0.51409034	0.609345467	0.83545396	-6.6513127
ENSCAFG00000012363	0.05800177	3.87837238	0.51392869	0.609457698	0.83551689	-6.5710481
KIF3C	0.07747746	4.59187532	0.51376859	0.609568872	0.83557836	-6.7099623
TUBB	0.09889972	8.76245243	0.51349651	0.609757815	0.8357464	-6.7362046
C15orf48	0.32068318	-2.7535672	0.51337653	0.609841144	0.83576967	-5.2138102
AP5Z1	-0.0812382	4.76676387	-0.5132637	0.609919486	0.8357861	-6.7116143
SRP68	0.04436133	6.57422407	0.51302132	0.610087873	0.83592591	-6.8221399
ENSCAFG00000009353	0.24259358	-0.7382751	0.51265788	0.610340367	0.83612596	-5.3925438

RNF19A	0.0890369	6.97991733	0.5125929	0.610385515	0.83612596	-6.8085762
TFRC	0.10226519	7.12801709	0.51252453	0.610433023	0.83612596	-6.8009115
MRPL55	0.07750078	3.95671943	0.51211329	0.610718806	0.83634682	-6.5339922
TSNAXIP1	-0.1915504	-0.0645357	-0.5121014	0.610727057	0.83634682	-5.6182917
IWS1	0.05284569	6.21603352	0.51194843	0.610833389	0.83636484	-6.822854
DIP2C	0.05340425	5.68123882	0.51183377	0.610913089	0.83636484	-6.7977462
ENSCAFG00000010373	-0.1912539	0.79381706	-0.5117959	0.61093942	0.83636484	-5.6149685
KLHL12	0.05722023	3.99198357	0.51154491	0.61111389	0.83651277	-6.5513204
ATXN1	0.0709995	4.73053815	0.51140256	0.611212855	0.83655733	-6.7302468
ANAPC11	0.06821334	4.32284893	0.51103631	0.611467513	0.83681495	-6.6665379
GPRIN1	-0.1345544	1.15757503	-0.5107069	0.611696601	0.83701283	-6.0278589
HADH	0.08504139	6.47591554	0.5106373	0.611745006	0.83701283	-6.819218
ENSCAFG00000013515	0.07357126	5.52837117	0.51031682	0.611967929	0.83706558	-6.801238
C6H1orf52	0.10100053	2.85358851	0.51029653	0.611982043	0.83706558	-6.2382998
ERCC1	0.06524288	3.40613523	0.51029527	0.611982924	0.83706558	-6.4305656
VPS72	-0.0547911	4.8898291	-0.5101815	0.612062054	0.83708291	-6.7112671
NSUN3	-0.0829536	1.99431398	-0.5099297	0.612237263	0.83723163	-6.0643524
ENSCAFG00000028936	-0.160275	1.56339457	-0.5094998	0.612536395	0.83723501	-6.6157635
NR4A2	0.2184702	3.26756673	0.50947557	0.612553267	0.83723501	-6.5862261
TRIM6	0.27629142	0.54495017	0.50926791	0.612697801	0.83723501	-5.7194114
SLC25A29	-0.1542149	2.54630215	-0.509251	0.612709538	0.83723501	-6.2849598
ADGRL3	0.55532074	2.74296482	0.50923634	0.612719774	0.83723501	-6.1316594
PGAP1	-0.1135229	2.3373749	-0.5091285	0.612794826	0.83723501	-5.9606914
NEURL2	-0.1743494	0.02754444	-0.5090771	0.612830607	0.83723501	-5.581813
ENSCAFG00000022137	-0.358195	0.49858729	-0.5089737	0.612902568	0.83723501	-5.4979778
DNAJC8	0.03583629	5.12038061	0.50896635	0.612907705	0.83723501	-6.7456448
ENSCAFG00000021718	-0.222093	0.67462483	-0.5088887	0.612961743	0.83723501	-5.6997301
EMC9	0.14698152	2.01082709	0.50878178	0.613036198	0.83723501	-6.0871466
ENSCAFG00000017975	0.09453775	2.55428334	0.50878012	0.613037352	0.83723501	-6.1748253
STIP1	0.05767142	6.29914522	0.50859149	0.613168685	0.83727086	-6.8243635
CTSF	0.0896333	5.75088735	0.50855148	0.613196542	0.83727086	-6.8246323
FBLN2	0.19074382	10.1503532	0.50817928	0.613455721	0.83753395	-6.6146688
CAPN5	-0.1421839	3.1587222	-0.5080751	0.613528275	0.83754223	-6.4033338
TBC1D23	0.04845619	6.12258523	0.50788937	0.613657635	0.83762804	-6.8217498
ATP7A	-0.0535692	5.56374161	-0.5071496	0.614173019	0.83824069	-6.8080025
SHROOM2	0.14873779	6.42450542	0.50699294	0.614282161	0.83829882	-6.8248748
PSMB7	-0.0417138	5.967879	-0.5066814	0.614499272	0.83842352	-6.8139425
SENP7	-0.0836093	3.83850458	-0.5065428	0.614595889	0.83842352	-6.5599232
GPR137B	0.10060693	3.13186341	0.50648156	0.614638554	0.83842352	-6.4728329
SGSH	0.16582246	5.26703999	0.50641963	0.614681725	0.83842352	-6.7903742
PIANP	-0.3148229	-1.0336175	-0.5063245	0.614748027	0.83842352	-5.250442
ENSCAFG00000010086	-0.0667937	3.59727544	-0.506115	0.614894057	0.83842352	-6.4983658
SBDS	0.04039167	6.1409165	0.50608681	0.614913731	0.83842352	-6.8257259
TBX3	-0.27052	2.56654654	-0.5060242	0.614957369	0.83842352	-6.2108894
ANKRD10	0.0535623	5.27628884	0.50578533	0.615123929	0.83842352	-6.7582805
EIF3B	-0.0546664	7.84451107	-0.5057602	0.615141452	0.83842352	-6.7756223
EPHX2	0.24991667	-0.3764546	0.505737	0.615157629	0.83842352	-5.5430871
KHK	-0.1765183	0.46789199	-0.5055583	0.615282236	0.83842352	-5.8046184
ENSCAFG00000016151	0.04810469	5.79942312	0.50534525	0.615430818	0.83842352	-6.8186055
TCF15	-0.2755924	-0.9391241	-0.5052839	0.615473592	0.83842352	-5.4503346

WDR25	0.04587007	4.29647743	0.50509433	0.615605829	0.83842352	-6.6406366
SYBU	-0.2035622	0.57076436	-0.5050489	0.615637503	0.83842352	-6.0744605
TULP4	0.07705133	4.82750398	0.5050292	0.615651261	0.83842352	-6.7599777
ARMC7	0.11394771	1.28782199	0.5049236	0.615724923	0.83842352	-5.9017427
SPSB4	0.36348858	-0.1978061	0.50490323	0.615739132	0.83842352	-5.3902735
HSD17B14	-0.1781425	2.51436314	-0.5048319	0.615788895	0.83842352	-6.2684909
TNFRSF11B	0.32197827	-1.9137826	0.50481851	0.615798236	0.83842352	-5.2464018
ZNF444	-0.099878	2.38372465	-0.5046136	0.615941183	0.83842352	-6.1292222
EXOC3L1	0.39272596	-0.9607347	0.50459565	0.615953711	0.83842352	-5.6956256
MRPL18	0.05914801	5.18960124	0.50457081	0.615971046	0.83842352	-6.756099
ENSCAFG00000019223	0.09761298	2.47891972	0.50442234	0.616074637	0.83847392	-6.1884615
JADE1	0.12080191	7.10851512	0.5040304	0.61634814	0.8386788	-6.8267453
DSE	-0.1337066	6.29338474	-0.5039337	0.616415651	0.8386788	-6.8232942
ENSCAFG00000013253	0.05497811	4.28171512	0.5039002	0.616439015	0.8386788	-6.6386801
TOMM20	-0.046494	5.01511152	-0.503718	0.616566187	0.8386788	-6.740912
HCN4	0.72060734	-0.8245458	0.50361241	0.616639889	0.8386788	-5.226897
MXD4	-0.090577	3.97271394	-0.5035608	0.616675938	0.8386788	-6.689415
C7H1orf43	-0.0528715	4.49411769	-0.50352	0.616704363	0.8386788	-6.6581573
IMPA1	0.05877775	5.77806072	0.50344345	0.616757837	0.8386788	-6.8129115
BRCA2	-0.1344713	3.95892861	-0.5032862	0.616867611	0.83868937	-6.4846954
WBP11	-0.0484609	4.97208615	-0.5032416	0.616898776	0.83868937	-6.7495054
GOLPH3	-0.043101	4.98713743	-0.5029244	0.617120268	0.83889995	-6.7281529
PRPS1	-0.0791787	4.45703499	-0.5026636	0.617302339	0.83903743	-6.6648513
ETFB	0.05723162	4.76510777	0.50258877	0.617354625	0.83903743	-6.7502519
DCLRE1C	-0.0710352	2.97529896	-0.5024878	0.61742516	0.83904276	-6.3759491
ATF3	0.18629767	4.34452016	0.50233444	0.61753226	0.83909777	-6.5820701
DDIT4L	-0.260463	-1.6258408	-0.5021132	0.617686834	0.83921728	-5.4057606
ENSCAFG00000007873	-0.364574	-2.2240514	-0.5019569	0.617796016	0.83927509	-5.221755
PSMG1	-0.0688361	5.55100359	-0.5018182	0.617892908	0.83931619	-6.7849728
SLC22A15	0.25893633	-0.5006164	0.50161577	0.618034351	0.8394178	-5.5240955
LSM10	-0.0866014	3.68002524	-0.5013519	0.618218715	0.83956217	-6.4995552
ACADSB	0.05076362	4.99816052	0.50127291	0.618273948	0.83956217	-6.7378206
CCDC62	-0.0974191	2.40907176	-0.5011708	0.6183453	0.83956855	-6.1397234
DUSP6	0.06235025	6.64721868	0.50081276	0.618595571	0.83980656	-6.7892112
FANCM	-0.350578	1.70260788	-0.5007293	0.61865394	0.83980656	-5.7319198
B4GALNT3	0.31668032	1.26309035	0.5004805	0.618827854	0.83995212	-5.2555366
SF3B5	-0.0811168	3.64871753	-0.4998545	0.619265624	0.84045575	-6.465585
TMEM167A	0.07310344	3.5274749	0.49888429	0.61994432	0.84121068	-6.4873121
NELFB	-0.0376499	5.68491185	-0.4987757	0.620020337	0.84121068	-6.8136466
CNNM1	0.27755164	-0.4639612	0.49868568	0.620083295	0.84121068	-5.5323706
FBXW9	-0.103049	2.039829	-0.4986775	0.620089006	0.84121068	-5.9983974
BICC1	0.10466994	7.93010877	0.4984012	0.620282387	0.8413824	-6.7540644
ANO8	0.07392487	3.9380031	0.49829357	0.620357719	0.84139397	-6.6370599
KCTD19	0.18412075	-0.6156903	0.49766506	0.620797709	0.84190008	-5.6219107
TLX2	0.15741763	3.77803496	0.49730315	0.621051126	0.84215308	-6.4020877
EGLN2	-0.0545463	4.87396746	-0.4966688	0.621495437	0.84266486	-6.7149554
ENSCAFG00000012020	-0.0368041	5.85666021	-0.4963739	0.621702028	0.84275695	-6.8157001
ZNF397	-0.0859139	2.29820661	-0.4963166	0.621742152	0.84275695	-6.2055657
DPY19L4	0.06544146	6.41330624	0.49604858	0.621929966	0.84275695	-6.8310505
RPAIN	0.11289384	2.1999291	0.49603536	0.621939224	0.84275695	-6.0794007

RABL6	0.03687559	6.59186917	0.49603523	0.621939317	0.84275695	-6.8309607
CPXM1	0.74998189	1.7390715	0.49599887	0.621964799	0.84275695	-5.5454818
DSCC1	-0.1545233	1.74838873	-0.4954206	0.62237007	0.84317573	-5.8839637
ASB3	-0.0419314	5.22122265	-0.4953669	0.62240774	0.84317573	-6.7795039
QTRT1	-0.086936	3.306272	-0.4950509	0.622629258	0.84320167	-6.4068354
PLEKHG4	0.18343421	2.95872513	0.49486901	0.62275677	0.84320167	-6.3462623
ELOVL6	-0.1354501	3.07797467	-0.4948485	0.622771146	0.84320167	-6.3872959
TBP	-0.0518377	4.07298253	-0.4947236	0.622858729	0.84320167	-6.5895726
PLK3	0.10081374	4.77913558	0.49471139	0.622867286	0.84320167	-6.6285726
PHAX	-0.0747305	3.29340783	-0.4947019	0.622873931	0.84320167	-6.4073185
ENSCAFG00000023832	0.35107906	-0.5628535	0.4946619	0.622901989	0.84320167	-5.4095324
JMJD4	-0.0669276	3.82599854	-0.4945636	0.622970924	0.84320167	-6.5153933
FBXO7	0.04238831	5.79576619	0.49448025	0.623029368	0.84320167	-6.8220218
GRID2	-0.375753	-0.7281409	-0.494021	0.623351464	0.84354371	-5.3960968
ENSCAFG00000030366	0.07142557	4.0586319	0.49388207	0.623448924	0.84354371	-6.6080767
PPM1L	-0.1901921	0.58717707	-0.4937849	0.623517117	0.84354371	-5.6846792
CCDC181	-0.0979956	2.23472318	-0.493738	0.623549976	0.84354371	-6.2036702
TRAP1	0.05358757	6.2240982	0.4935627	0.623672977	0.8436195	-6.8321574
SLC2A11	0.15763693	0.94788379	0.49345013	0.623751961	0.84363575	-5.8929827
EIF2B3	0.05525335	4.86664771	0.49329345	0.6238619	0.84369385	-6.7185764
CENPL	0.15977734	3.14311832	0.49286889	0.624159844	0.84393885	-6.2752311
RIMS2	-0.281529	-1.9911337	-0.4928444	0.624177063	0.84393885	-5.3250852
SEMA4B	-0.0928233	3.01954512	-0.4925894	0.62435602	0.84397712	-6.2863612
CCNQ	0.11612446	1.86611944	0.49252122	0.624403875	0.84397712	-6.029287
MAP3K12	0.06607813	4.56235642	0.49249027	0.624425604	0.84397712	-6.7293866
TMEM120B	0.07144905	2.77318876	0.4924222	0.624473384	0.84397712	-6.2897819
MRM2	-0.0667817	2.4419378	-0.4920874	0.624708451	0.84419712	-6.2120673
SEPT4	0.22713342	1.17156772	0.4918574	0.624869932	0.84419712	-6.1397657
MRC2	-0.103884	8.26843982	-0.4918521	0.624873639	0.84419712	-6.7896083
UNC119	0.13594726	1.86323369	0.49180853	0.624904252	0.84419712	-6.2050447
ENSCAFG00000003519	-0.0698211	3.29360458	-0.4914376	0.625164758	0.84444204	-6.3743649
ASAP2	0.17465294	4.00408641	0.49135947	0.625219628	0.84444204	-6.5607493
GET4	0.04708916	4.53516388	0.49112953	0.625381139	0.84456962	-6.6667214
FOCAD	0.06178072	6.55627267	0.49085509	0.625573933	0.84470346	-6.833601
CENPQ	-0.184318	2.42184379	-0.4907259	0.625664728	0.84470346	-6.0047484
ACD	-0.0601839	3.62120232	-0.4907021	0.625681429	0.84470346	-6.4748519
CPXM2	0.42668004	1.43544655	0.49051745	0.625811168	0.84472132	-5.8647182
SCAI	-0.1324236	1.14632621	-0.4904924	0.625828783	0.84472132	-5.8646144
MMP14	0.10301384	9.57996585	0.49037559	0.625910847	0.84474157	-6.6893949
SLC25A16	0.07322069	5.28668356	0.48979381	0.626319735	0.84491551	-6.7642327
GNAI1	0.25012359	3.46869472	0.48961801	0.626443314	0.84491551	-6.5429291
MED16	0.07637185	5.66752824	0.4896016	0.62645485	0.84491551	-6.7716727
MKS1	-0.1046179	1.96927553	-0.4895717	0.626475838	0.84491551	-6.0733357
SARNP	-0.0746435	4.88791968	-0.4895584	0.626485247	0.84491551	-6.731318
ULK4	-0.0891245	3.79704349	-0.4895378	0.626499735	0.84491551	-6.5969496
TMLHE	0.07253917	3.66411363	0.48952419	0.626509275	0.84491551	-6.4747865
ZBTB7A	0.08016538	4.31571669	0.48924948	0.626702411	0.84508549	-6.6662184
ENSCAFG00000018825	0.29916909	7.77089186	0.48915098	0.626771669	0.84508841	-6.8229986
MATK	-0.2487653	-2.4078318	-0.4888768	0.626964448	0.84517647	-5.3228303
IDNK	-0.1520399	2.42373995	-0.4888491	0.62698395	0.84517647	-6.1279336

SPATS2	0.0726469	6.11811357	0.48877186	0.627038271	0.84517647	-6.8344002
CCDC51	0.07670355	2.43391825	0.48863638	0.627133558	0.84521446	-6.2208836
ENSCAFG00000032500	-0.11362	1.14005412	-0.4882575	0.62740006	0.84541401	-5.818068
COPG2	-0.0414071	4.59861522	-0.488235	0.627415861	0.84541401	-6.7132742
TPT1	-0.0594994	11.1915105	-0.4880154	0.627570399	0.84550465	-6.533641
WSB1	0.10762237	7.6257817	0.48791235	0.627642881	0.84550465	-6.8117237
PTAR1	-0.0918059	3.14484738	-0.4878525	0.627685002	0.84550465	-6.4702357
PLK1	-0.3575792	3.96393411	-0.4877578	0.627751625	0.84550465	-6.236607
TMEM106A	0.22879453	0.25434509	0.48753694	0.627907033	0.8455422	-5.6501726
PCDHGA1	0.18785665	1.10511604	0.48744187	0.627973935	0.8455422	-5.7857299
PIP5K1A	0.03548701	6.92322414	0.48743199	0.627980888	0.8455422	-6.8206071
GPR1	-0.3898003	-1.6763171	-0.4871784	0.628159395	0.84569215	-5.587681
MAMSTR	0.19659187	-0.6195647	0.48690218	0.628353791	0.84580901	-5.5723157
PJA2	-0.0465826	7.30495981	-0.4868642	0.628380493	0.84580901	-6.8130126
AHSA1	0.04562082	6.11907701	0.4866863	0.628505764	0.84588723	-6.8324508
TRIM32	-0.0638542	3.23787203	-0.4865128	0.628627946	0.84596128	-6.4267572
PC	0.11135859	4.95503679	0.48628548	0.628787976	0.84601239	-6.7420688
RAP1A	-0.0559587	5.43173473	-0.486215	0.628837628	0.84601239	-6.8033998
TRMU	-0.0903351	2.43939055	-0.4861727	0.628867421	0.84601239	-6.1966984
TMEM41B	-0.1172422	4.61077178	-0.4860029	0.628986943	0.84608282	-6.6717259
ENSCAFG00000013709	0.07591831	4.95586442	0.48565689	0.629230662	0.84632028	-6.7088191
EFCAB5	-0.1313899	0.81848923	-0.4854548	0.629372992	0.84642133	-5.7809855
JAKMIP2	-0.3317964	0.4644903	-0.4853546	0.629443611	0.84642593	-5.4666968
STK40	0.04652027	5.13356358	0.48518083	0.629566023	0.84650017	-6.7919846
CLASP2	-0.0469001	5.33059702	-0.4844215	0.630101124	0.84706183	-6.8032899
TAF6	0.03573113	5.47427736	0.48435397	0.630148686	0.84706183	-6.7973807
TSN	-0.0522646	4.46990218	-0.4843018	0.630185487	0.84706183	-6.674743
MVP	-0.0559202	7.43052326	-0.4840345	0.630373856	0.84722461	-6.8028616
ENSCAFG00000019511	0.069873	3.09205691	0.4838753	0.630486102	0.84728507	-6.4158062
C5H16orf74	0.13927614	0.8030538	0.48313444	0.631008489	0.84789662	-5.7277034
ST6GAL2	-0.5225435	0.8340419	-0.4827865	0.631253861	0.84804799	-5.2806382
LSM14B	-0.0465961	4.81217489	-0.4827838	0.631255794	0.84804799	-6.7345384
IP6K2	-0.0520109	4.18696798	-0.4826193	0.631371861	0.84807248	-6.7024818
ZNF652	-0.0752729	2.91702612	-0.4825671	0.631408679	0.84807248	-6.3200597
LCLAT1	0.0746938	5.43161674	0.48238496	0.631537144	0.84815459	-6.8148194
LYRM7	-0.2175565	-0.2116795	-0.4818569	0.631909746	0.84856452	-5.5405411
SMARCC1	-0.0491245	7.45643471	-0.4817452	0.631988584	0.84857992	-6.8088245
HSF1	-0.0520359	5.44352986	-0.48151	0.632154554	0.8487123	-6.7996782
ENSCAFG00000014968	0.05502136	6.6784405	0.48141321	0.632222878	0.84871356	-6.8355584
ZBED8	-0.0908023	2.40034593	-0.481196	0.632376232	0.84882897	-6.1579628
MED27	0.05260891	3.95022274	0.48093476	0.632560623	0.848986	-6.5765002
MIER3	-0.0700559	3.34533705	-0.4807271	0.63270726	0.84909233	-6.4378736
PLLP	-0.2853356	-2.4774415	-0.480525	0.632849915	0.8491933	-5.2602974
FUT10	-0.126315	2.28947046	-0.4801809	0.633092963	0.8493377	-6.0242145
PLPP4	0.30184147	-1.3373715	0.48013899	0.63312254	0.8493377	-5.2394955
ACER2	0.08533639	4.97649405	0.48008622	0.633159812	0.8493377	-6.6883453
MTAP	-0.0460881	6.25152232	-0.4799041	0.63328846	0.84941981	-6.8387707
ENSCAFG00000029103	-0.0999598	2.45702677	-0.4796399	0.633475111	0.84945013	-6.1579309
NCKAP5L	-0.0558709	5.88637395	-0.4794845	0.633584881	0.84945013	-6.8332275
ENSCAFG00000017277	0.06780708	3.99244556	0.47942604	0.633626162	0.84945013	-6.7765658

SCG5	-0.2724005	-0.1594498	-0.4793772	0.633660672	0.84945013	-5.4749113
FAM78A	0.25409845	-0.0303127	0.47932335	0.63369872	0.84945013	-5.6413497
PRCC	-0.0452181	4.75003453	-0.4792466	0.633752926	0.84945013	-6.7272495
ENSCAFG00000029365	-0.1179929	0.92563836	-0.4791909	0.633792283	0.84945013	-5.7778958
PMM2	-0.0516806	4.42048258	-0.4791084	0.63385057	0.84945013	-6.6470723
NFIC	0.04617986	7.31357386	0.47898707	0.633936336	0.84947469	-6.8289164
PGRMC2	0.08525804	7.71585505	0.47839729	0.634353174	0.84994283	-6.8013493
MPDU1	-0.0568552	5.29517919	-0.4781689	0.634514618	0.84999326	-6.7799093
NOP10	0.09930602	4.14127739	0.47811703	0.634551297	0.84999326	-6.5186348
NUDCD2	-0.0816465	2.81969468	-0.4780577	0.634593251	0.84999326	-6.3335864
MPV17	0.12331642	2.05254257	0.47755324	0.63494993	0.85025912	-6.0617786
FMNL3	0.11427789	6.67188475	0.47749398	0.634991836	0.85025912	-6.8336561
ELOVL5	-0.0456678	6.3108249	-0.4774367	0.635032371	0.85025912	-6.8397231
OSER1	-0.0497862	4.73829853	-0.4773951	0.635061747	0.85025912	-6.7359187
IL20RB	-0.228237	0.11548293	-0.4765223	0.635679121	0.85091767	-5.4751731
RNF20	0.03348939	6.04684339	0.4764876	0.635703701	0.85091767	-6.8395774
C6H16orf72	0.05440852	4.87950942	0.47641327	0.635756291	0.85091767	-6.7630854
ZC3H6	-0.0752014	3.86707313	-0.4762896	0.635843817	0.8509444	-6.6307641
MOCS2	0.06105043	4.24276339	0.47609407	0.635982158	0.85100996	-6.6628442
GMPPB	-0.0708079	4.73637727	-0.4759858	0.636058771	0.85100996	-6.6949936
C34H3orf70	0.12636158	1.34934116	0.47593393	0.636095489	0.85100996	-5.7839643
RNF149	0.06990721	3.1443309	0.47581271	0.636181279	0.85103434	-6.5151716
ZNF260	0.11894037	2.06056428	0.47509577	0.636688784	0.8516228	-6.0670425
KIAA0895L	0.08662183	1.98474634	0.47487588	0.636844474	0.8517406	-6.0594963
ENSCAFG00000017208	0.0520524	5.46338257	0.47442733	0.637162116	0.85202912	-6.8044672
RPS6KA5	0.13313006	2.22558818	0.47438022	0.637195483	0.85202912	-6.1066502
EGFR	-0.1336578	4.50264146	-0.474048	0.637430813	0.85217548	-6.5886727
ENSCAFG00000001352	0.14051193	0.75768761	0.47403465	0.637440246	0.85217548	-5.7739999
ENSCAFG00000030498	-0.2071827	-0.7040241	-0.4738027	0.637604585	0.85230472	-5.4285705
NOC4L	0.08796746	4.97301739	0.47364484	0.637716404	0.8523626	-6.7092833
TJAP1	0.04785722	4.41457141	0.47355052	0.637783228	0.8523626	-6.6733817
SYNJ2	0.10143426	4.09263887	0.47328918	0.637968403	0.85250308	-6.5000061
FKBP7	0.06795954	5.41089223	0.47321115	0.638023698	0.85250308	-6.8249146
ENSCAFG00000019261	-0.1640357	0.66773832	-0.4730148	0.638162838	0.85259855	-5.6469742
METTL8	-0.0759759	2.73943431	-0.4726684	0.638408396	0.85278192	-6.3175608
ENSCAFG00000029179	0.0489937	6.29563015	0.47261729	0.638444592	0.85278192	-6.8421203
ZUP1	-0.0537308	3.97955127	-0.4725346	0.638503202	0.85278192	-6.6154935
TRABD	0.05262497	5.24390527	0.47210297	0.638809212	0.85290517	-6.7960722
TRAPPC2L	0.06303281	3.05935961	0.47200916	0.638875723	0.85290517	-6.349123
APOOL	0.05225131	2.79868854	0.4719934	0.638886901	0.85290517	-6.2992735
ENSCAFG00000003159	0.1604717	1.10699496	0.4719739	0.638900727	0.85290517	-5.7260506
TPX2	-0.2542515	4.52303022	-0.4719269	0.638934045	0.85290517	-6.5131464
DHX38	-0.0367317	5.12390791	-0.4716511	0.639129662	0.85302828	-6.7708469
MRPL35	-0.0610497	3.38250472	-0.4716059	0.639161708	0.85302828	-6.3903149
FBXW5	-0.0648599	5.47850533	-0.4715093	0.639230173	0.85302927	-6.7938614
LPIN2	0.0494713	5.55251613	0.4713914	0.639313816	0.85305051	-6.7960333
ENSCAFG00000020100	-0.2429585	-0.2491652	-0.4711581	0.639479292	0.85314263	-5.4744722
ADAM15	-0.1089745	6.5693862	-0.4711031	0.639518314	0.85314263	-6.8316806
COPZ2	0.05540213	5.84530229	0.47094671	0.639629245	0.85314437	-6.8255814
NOVA2	-0.2611542	-2.0632027	-0.4708969	0.639664567	0.85314437	-5.4091697

ENSCAFG00000029663	-0.1022761	1.25273632	-0.4708148	0.639722818	0.85314437	-5.8555848
RAB3D	-0.1995489	1.37968031	-0.4700852	0.640240551	0.85374444	-5.9565698
KCNIP1	-0.3168765	0.7562579	-0.4698654	0.640396552	0.85386207	-5.6446194
SDHAF3	-0.1087998	1.73727875	-0.4695091	0.640649429	0.85410883	-5.9626807
TIGD7	-0.0651946	4.52898556	-0.4693341	0.640773682	0.85411832	-6.6780107
RIMKLA	0.2150622	0.29572882	0.46929904	0.64079858	0.85411832	-5.7337026
ZEB1	-0.0802723	6.30631163	-0.4692126	0.640859976	0.85411832	-6.8438061
LPCAT3	-0.0670087	5.17943705	-0.4688145	0.641142655	0.85440466	-6.7737464
MCOLN1	-0.0736145	4.89399176	-0.4687045	0.641220715	0.85441094	-6.7496107
ALDH9A1	-0.0939663	6.72288805	-0.4686168	0.641283026	0.85441094	-6.8402214
HMGCR	-0.0968241	6.97980541	-0.4676523	0.641968189	0.85497142	-6.8215419
ABCA5	-0.1001748	4.48732205	-0.4675472	0.642042882	0.85497142	-6.7562959
CLCN4	0.05623981	4.7129585	0.46753177	0.642053817	0.85497142	-6.6912388
ENSCAFG00000032330	0.10506968	2.00210015	0.4675255	0.642058273	0.85497142	-6.0609913
TMEM101	0.07813356	3.14013735	0.46740982	0.642140472	0.85497142	-6.4145318
APEX2	-0.0781934	3.03643828	-0.4673795	0.642162025	0.85497142	-6.3440224
MRAP2	0.2493633	-1.5098514	0.46733292	0.642195117	0.85497142	-5.4136411
B2M	0.11608179	8.01668887	0.46717076	0.642310355	0.85497142	-6.7674533
LRRC32	-0.3443704	3.19170531	-0.4671648	0.642314588	0.85497142	-6.1489314
CHCHD3	-0.0550415	5.6200915	-0.4670278	0.642411997	0.85501073	-6.8236182
MEX3D	0.08564011	3.6603844	0.46691352	0.642493189	0.85502844	-6.509504
SLC12A7	0.40216207	1.32904756	0.46666624	0.64266896	0.85516962	-5.68368
SRGAP1	0.0590816	4.46575174	0.46651808	0.642774282	0.85516962	-6.6958368
ENSCAFG00000012457	0.11507547	2.18607357	0.46647775	0.642802952	0.85516962	-6.0757885
BZW2	0.05225511	7.07499423	0.46637117	0.642878725	0.85518011	-6.8373378
SH3PXD2B	-0.074235	6.40176296	-0.4662634	0.642955312	0.85519167	-6.8451191
CDT1	-0.3111718	2.52163037	-0.4659938	0.64314705	0.85535638	-5.9757239
SC5D	-0.0659469	5.75793416	-0.4658177	0.643272242	0.85543256	-6.8442837
TNN	0.24778258	-2.4204493	0.4655643	0.643452484	0.85556595	-5.2967205
KEAP1	-0.0491123	5.56837736	-0.4654154	0.643558395	0.85556595	-6.8107916
FAM161B	0.10530426	1.79063061	0.46537515	0.643587014	0.85556595	-5.895887
KCTD17	-0.0709987	3.10074502	-0.4652947	0.643644247	0.85556595	-6.3820703
S100A4	-0.16767	7.50032601	-0.4651144	0.643772465	0.85559339	-6.7608029
CLPP	0.0532791	4.15621449	0.46490604	0.643920727	0.85559339	-6.5989408
CLIC5	0.32121834	-0.607578	0.46483506	0.64397123	0.85559339	-5.6114706
PPIH	-0.0741305	3.54863036	-0.4647923	0.644001667	0.85559339	-6.4837912
TM9SF4	0.03375439	6.79935342	0.46478827	0.644004517	0.85559339	-6.8436473
SLC35F5	0.04872526	6.24160838	0.4644516	0.644244077	0.85582139	-6.8447475
PELI3	0.12172491	2.02587672	0.46394115	0.644607353	0.85621367	-6.0786622
OTUD6B	-0.0554003	5.75946512	-0.4636577	0.644809125	0.85631839	-6.8384256
TACC3	-0.3529905	3.43882406	-0.4636394	0.644822156	0.85631839	-6.1713276
ENSCAFG00000030682	-0.2039201	3.99664844	-0.463406	0.644988281	0.85641761	-6.5143175
MNS1	-0.088044	2.64455599	-0.4632553	0.645095575	0.85641761	-6.2226592
PEX1	0.05334741	4.98684134	0.46324793	0.645100848	0.85641761	-6.7706597
R3HCC1	-0.0398318	4.76732745	-0.4630686	0.645228515	0.85643517	-6.7209068
RACGAP1	-0.2111243	5.38590242	-0.4630384	0.645250064	0.85643517	-6.701245
GTPBP3	0.07075974	2.8014865	0.46273162	0.645468506	0.85663484	-6.3231234
ZFYVE26	-0.0454777	5.07835112	-0.462438	0.645677606	0.85682207	-6.7709353
MFSD10	0.07671089	4.71494551	0.46229568	0.645779003	0.85683609	-6.7545969
MYBL2	-0.4643852	4.34721564	-0.4622322	0.645824213	0.85683609	-6.2486014

RINT1	0.04833834	6.40858406	0.46196035	0.646017889	0.85700277	-6.8474083
BLOC1S2	0.06491798	3.99867892	0.46164889	0.6462398	0.85720688	-6.5680319
CHAC1	0.18164187	4.82859664	0.46122682	0.646540572	0.85751554	-6.7216321
ENSCAFG00000015452	0.12552736	1.06005924	0.46077288	0.646864119	0.85785433	-5.7746987
BTBD19	0.0965495	5.25806251	0.46063412	0.646963035	0.85788194	-6.8031271
ZBTB17	-0.0749877	3.23597878	-0.4605091	0.64705213	0.85788194	-6.380691
ENSCAFG00000005785	-0.157562	0.65212639	-0.4604571	0.647089263	0.85788194	-5.8396799
ENSCAFG00000022575	-0.3057281	-0.45051	-0.4603023	0.647199576	0.85793789	-5.4282327
FBXO8	-0.0561964	3.44224405	-0.4600741	0.64736229	0.85799407	-6.5136584
NRIP3	0.23727479	-0.7553783	0.46005183	0.647378189	0.85799407	-5.467207
ZNF326	0.04525379	5.68094714	0.45965538	0.647660918	0.85827848	-6.8300679
FLNB	-0.1044268	9.71457116	-0.4594668	0.6477954	0.85836639	-6.6395244
SLC23A3	0.13955033	0.15281197	0.45909308	0.648062004	0.85862273	-5.7672947
CEP128	0.15078272	2.60882473	0.45900451	0.648125189	0.85862273	-6.1638104
RIOK2	-0.0600185	5.5925566	-0.4587229	0.648326123	0.85874802	-6.8145627
FCMR	-0.1559603	-0.1713453	-0.4586808	0.648356119	0.85874802	-5.6294605
WDR11	-0.0381573	5.99914544	-0.4584676	0.648508264	0.85881231	-6.8422683
FZD1	-0.2936204	2.72469032	-0.4584083	0.648550579	0.85881231	-5.8375352
ADAMTSL3	0.29583257	3.91200973	0.45832618	0.648609199	0.85881231	-6.2774201
PPM1M	-0.1138516	4.00793104	-0.4581027	0.648768718	0.85893323	-6.6633928
PPP2R5C	-0.0325552	6.87090708	-0.4576252	0.649109505	0.8592941	-6.8362497
ENSCAFG00000016517	-0.0709127	3.25148813	-0.4571954	0.649416389	0.8594615	-6.4374854
COL14A1	-0.3507155	3.45767758	-0.4571782	0.6494287	0.8594615	-6.3660229
STK38	-0.0647684	5.33273892	-0.4570997	0.649484735	0.8594615	-6.8170984
GREM1	0.25702092	7.33920337	0.45696867	0.649578285	0.8594615	-6.8467511
GLMP	0.07454399	6.39131603	0.45669232	0.649775641	0.8594615	-6.849808
CHCHD2	0.04410873	6.36486916	0.45666439	0.649795583	0.8594615	-6.8497588
ENSCAFG00000028833	-0.1161793	3.25503124	-0.4566184	0.649828426	0.8594615	-6.4062697
ZDHHC8	-0.0719088	5.37201892	-0.4565102	0.649905691	0.8594615	-6.821593
SNX21	0.07934396	4.16668755	0.45644483	0.649952404	0.8594615	-6.5769391
GTF2B	0.03661245	4.46225429	0.4563666	0.650008282	0.8594615	-6.7021629
KLHDC10	0.0659667	4.14129298	0.45632453	0.650038334	0.8594615	-6.5955576
ENSCAFG00000022716	0.21430986	-0.4779462	0.45623834	0.650099897	0.8594615	-5.6062377
SNX16	-0.0599984	3.93998468	-0.456206	0.65012299	0.8594615	-6.6275852
SIRT7	-0.057923	3.99821996	-0.4558487	0.650378283	0.85970877	-6.6182011
TMEM181	0.05255445	6.11872605	0.45544794	0.650664615	0.85991475	-6.8496302
ENSCAFG00000002008	0.16473963	5.94030562	0.4553358	0.65074475	0.85991475	-6.849123
BTBD3	0.05625647	5.14319696	0.45532668	0.650751271	0.85991475	-6.7944705
ENSCAFG00000015098	0.04849383	4.27765093	0.45521085	0.650834047	0.85991475	-6.6388379
ENSCAFG00000006785	-0.0989095	2.26448087	-0.4551099	0.650906195	0.85991475	-6.1819744
ZNF131	0.0519489	3.82587132	0.45505739	0.650943724	0.85991475	-6.588234
ZC3H10	-0.0849356	1.67576051	-0.4548269	0.651108507	0.85992671	-6.050206
ANPEP	0.24925339	7.57278366	0.45473775	0.651172196	0.85992671	-6.8256503
MGAT1	-0.0622464	5.7034636	-0.4547096	0.651192291	0.85992671	-6.8281392
PRDX6	-0.0503302	7.08028672	-0.45454	0.651313561	0.85992671	-6.8364539
ST3GAL5	0.16667631	3.93105332	0.4544721	0.651362104	0.85992671	-6.3769195
MAP3K8	0.13052282	1.79014717	0.45447169	0.651362397	0.85992671	-5.9776755
ENSCAFG00000005299	-0.2844168	-0.1286251	-0.4541682	0.651579396	0.86012304	-5.4812483
MCOLN3	0.25614345	-1.3054759	0.45398819	0.651708101	0.86013009	-5.2664079
MAD2L2	0.07969833	3.52546679	0.45391089	0.65176338	0.86013009	-6.4742444

CAMLG	-0.0509438	3.76098083	-0.4538742	0.651789593	0.86013009	-6.5848844
OXLD1	0.08903813	2.27498646	0.45328772	0.652209072	0.86059349	-6.1547643
ENSCAFG00000028519	0.12992955	0.56901414	0.45302598	0.65239631	0.86075038	-5.7434077
SETDB2	-0.070194	4.35245085	-0.4529288	0.652465857	0.86075198	-6.6602167
SREBF2	-0.081694	7.74476374	-0.4527646	0.65258332	0.86081679	-6.794124
TRIM24	-0.0571876	5.17220753	-0.4523255	0.652897484	0.86100365	-6.8100257
ZNF311	0.04996302	3.39794992	0.45229035	0.652922667	0.86100365	-6.494463
BTK	0.20416762	2.06227302	0.45225142	0.652950524	0.86100365	-5.787265
H2AFJ	-0.1042257	2.58079361	-0.4521845	0.652998405	0.86100365	-6.388557
RER1	0.0569987	3.79921306	0.45166745	0.653368498	0.86140147	-6.6063909
VPS41	0.03824983	6.16712984	0.4514579	0.653518514	0.86144228	-6.8498425
CFAP206	-0.1900566	-0.2915539	-0.4513836	0.653571701	0.86144228	-5.5617238
KDM3A	-0.0532124	5.82632901	-0.4512791	0.653646522	0.86144228	-6.8495662
ABHD5	0.06168173	5.86764582	0.4512421	0.653673015	0.86144228	-6.8389845
TOP3B	-0.0364607	6.71308333	-0.4510536	0.653807963	0.86152998	-6.8520002
CLUAP1	-0.0801277	2.33281967	-0.4508729	0.653937377	0.86154706	-6.2210863
ABL1	0.05657809	7.69835129	0.45084449	0.653957721	0.86154706	-6.7906544
ATP5F1D	-0.0561266	6.1528558	-0.4501359	0.654465259	0.86209441	-6.8510889
DPAGT1	0.04323388	4.75590067	0.4500214	0.654547257	0.86209441	-6.702587
CHEK2	-0.0748523	3.20353203	-0.4499778	0.654578509	0.86209441	-6.4643726
CYTH3	-0.0694256	6.0745033	-0.4498121	0.654697224	0.86216061	-6.8528763
ENSCAFG00000030829	-0.0791528	3.16686095	-0.4496013	0.654848216	0.8622693	-6.4463169
RFX5	-0.0706929	3.23379465	-0.4493603	0.655020901	0.86240653	-6.509003
TMEM98	0.07905047	4.59167402	0.44910766	0.655201983	0.86255478	-6.6522272
RETSAT	0.08195806	4.4478135	0.4488083	0.655416544	0.86274708	-6.6997228
KDM8	0.13826273	0.14508997	0.44857728	0.655582144	0.86283826	-5.6366353
SEPT8	0.04928779	6.54168827	0.44852054	0.655622817	0.86283826	-6.8533185
RBMS1	-0.0463688	8.1864348	-0.4482461	0.655819597	0.86291583	-6.7785637
HDHD3	-0.1555886	-0.0918569	-0.4481498	0.655888598	0.86291583	-5.6032878
TMEM145	-0.1604847	-1.0297323	-0.4480689	0.65594665	0.86291583	-5.5578062
RRP9	-0.0603845	4.34918685	-0.447981	0.656009623	0.86291583	-6.6470972
VARS2	0.04087701	6.39545936	0.44796058	0.656024289	0.86291583	-6.8528581
SLCO2B1	-0.2628932	-2.8782709	-0.4477475	0.65617711	0.86293389	-5.2525734
SMDT1	0.06544207	3.56797566	0.44773541	0.656185756	0.86293389	-6.4388054
RB1	-0.0525793	5.61072012	-0.4476548	0.656243547	0.86293389	-6.8277175
WDR86	0.51146033	0.19255002	0.44750446	0.656351382	0.8629856	-5.6019715
ENSCAFG00000031217	-0.1127037	2.34123216	-0.4471175	0.656628936	0.8631444	-6.1033927
BDKRB1	0.27139145	-0.4471751	0.4470577	0.656671835	0.8631444	-5.5282527
XPNPEP1	-0.0260699	6.49737255	-0.4470495	0.65667774	0.8631444	-6.8540491
IFRD1	-0.100545	5.68255943	-0.446424	0.657126516	0.86359561	-6.8193367
CLIP2	-0.0718175	7.52901455	-0.4463799	0.657158142	0.86359561	-6.8116264
LNX1	-0.2846793	-0.6960893	-0.44617	0.657308788	0.86362498	-5.4353533
BAK1	0.05304949	4.39033863	0.44615765	0.657317615	0.86362498	-6.6769119
CHRM1	0.23152951	-1.3950655	0.44603103	0.65740849	0.86365429	-5.6394344
ALAS1	0.08491359	5.70495251	0.44592381	0.657485441	0.86366531	-6.8462268
PGR	-0.3301577	3.76092405	-0.4457341	0.65762163	0.8637501	-6.5522989
FKBP4	-0.0572107	5.85803371	-0.4456428	0.657687137	0.8637501	-6.8309005
PDAP1	0.06044439	5.29803498	0.44475246	0.658326361	0.86449642	-6.7921522
CYP1A1	-0.4098658	0.29385167	-0.4445474	0.658473602	0.86449642	-5.4053296
GPC2	0.26242459	-1.106233	0.4445257	0.658489209	0.86449642	-5.3117882

TMEM151B	-0.208084	-1.1822027	-0.444469	0.658529945	0.86449642	-5.5105324
GSAP	0.08674903	4.07203625	0.4442542	0.658684203	0.86460882	-6.6768677
TPMT	0.12116326	3.70158539	0.44406963	0.658816782	0.86469274	-6.6599374
RHBDF1	-0.0495896	6.42016933	-0.4436584	0.659112188	0.86488728	-6.8556934
RIN1	0.0762174	4.03641926	0.44356508	0.659179248	0.86488728	-6.6966826
DHX36	0.05346693	5.28743289	0.44351909	0.659212291	0.86488728	-6.7818338
ACVR2A	-0.1001821	3.82248491	-0.443481	0.659239661	0.86488728	-6.6001351
PAK2	0.03302294	6.60344872	0.44338188	0.659310882	0.86489063	-6.8533212
C1QTNF5	0.16107519	2.64161305	0.44303069	0.659563246	0.86513159	-5.9484888
SLC1A7	0.33895161	0.46807388	0.44264561	0.659840012	0.8654045	-5.5895325
ASB9	-0.2683464	-1.3434072	-0.442465	0.659969824	0.86548463	-5.3735996
FANCC	-0.1149158	1.82341391	-0.4422698	0.66011019	0.86556575	-5.9297312
ENSCAFG00000030681	-0.2065089	-0.7426994	-0.4421878	0.660169118	0.86556575	-5.3705372
SOCS1	-0.2486105	-2.0868792	-0.4419727	0.660323729	0.86567836	-5.3646994
NHSL2	0.11664613	3.51867834	0.44147024	0.660685066	0.86606193	-6.4511978
ADPRH	-0.0554222	2.95468619	-0.4413195	0.66079351	0.86607096	-6.3395741
NDUFS6	0.06895654	4.06142996	0.44126945	0.660829472	0.86607096	-6.5886885
ZFAND5	0.08710386	5.70316613	0.44082711	0.661147645	0.86630382	-6.8540054
MRPL49	0.05811868	3.90817004	0.44066344	0.661265387	0.86630382	-6.5687253
KCTD16	0.19122722	1.88900067	0.44065497	0.66127148	0.86630382	-5.9098682
TIRAP	0.10362686	1.74424389	0.44064	0.661282249	0.86630382	-5.9587418
SHOC2	-0.0412537	6.2938323	-0.4403293	0.661505757	0.8664331	-6.8567979
CD55	0.35654553	5.62117431	0.44031163	0.661518506	0.8664331	-6.7584494
FAM172A	0.03999464	5.51220807	0.44008231	0.66168352	0.86655912	-6.8367598
UNKL	-0.1036317	1.91583955	-0.4396624	0.661985719	0.86686476	-6.1770538
ADCK5	-0.0603318	3.70231089	-0.4393349	0.662221435	0.86694925	-6.527968
MIIP	-0.0939368	2.60695332	-0.4392935	0.66225125	0.86694925	-6.171967
ENSCAFG00000031956	-0.1074739	2.91437589	-0.4392859	0.662256727	0.86694925	-6.3084359
PKP4	-0.0681324	6.26400692	-0.439015	0.662451779	0.86711448	-6.8490245
MTDH	-0.0395176	7.75157199	-0.4384033	0.662892197	0.86754305	-6.8191903
KIAA1143	0.06482715	2.51036572	0.43836473	0.662919976	0.86754305	-6.23591
HS3ST3B1	0.19976246	3.20587196	0.43820236	0.663036912	0.86754305	-6.8571542
GABPB1	0.06798908	3.87595524	0.43801125	0.663174555	0.86754305	-6.5712751
SEMA4D	-0.2375899	-1.6498309	-0.4379876	0.663191565	0.86754305	-5.3029153
TIPIN	0.06337565	4.220759	0.43798642	0.663192442	0.86754305	-6.6428803
UEVLD	-0.0951555	3.0317321	-0.4377627	0.663353618	0.86766378	-6.3484325
GLRX	-0.0887399	3.48972467	-0.4365303	0.664241589	0.86854447	-6.5012227
ENSCAFG00000018377	-0.1698533	-0.1224603	-0.4364117	0.664327094	0.86854447	-5.5260044
ENSCAFG00000028601	-0.2078502	-0.6589229	-0.4364026	0.664333603	0.86854447	-5.4955455
PDPR	-0.0546711	5.97653514	-0.4362856	0.664417977	0.86854447	-6.8570549
DCBLD2	0.08827026	6.10778965	0.43627473	0.664425802	0.86854447	-6.8399323
LUC7L	-0.0694158	4.33634339	-0.4361317	0.664528898	0.86854447	-6.6862757
VEGFD	-0.713501	-0.3850808	-0.4359255	0.664677527	0.86854447	-5.2995922
EN1	-0.2564938	1.57758585	-0.4358794	0.664710802	0.86854447	-5.9796535
LIMK2	0.05755013	4.4752527	0.43564724	0.66487818	0.86854447	-6.6973665
HMGN4	-0.0606039	2.97146802	-0.4356336	0.664887996	0.86854447	-6.3947912
PHF20	0.05529121	5.75167473	0.43562731	0.664892552	0.86854447	-6.8497783
ENSCAFG00000030005	-0.0572288	6.33494527	-0.435626	0.664893521	0.86854447	-6.8549827
CDC42SE2	0.11250974	1.69959525	0.43558461	0.664923336	0.86854447	-5.9904854
NFYA	-0.0669294	3.14941377	-0.4352061	0.665196319	0.86881095	-6.3956466

PTPRF	0.07299991	5.79043013	0.43505601	0.665304525	0.86881958	-6.8497371
MSMO1	0.13385191	2.01070052	0.43500562	0.665340872	0.86881958	-6.3604167
ESRRA	-0.0699473	3.77095873	-0.4348505	0.665452746	0.86887559	-6.5903828
MKNK1	0.05415906	4.30573659	0.43457574	0.665650941	0.86898854	-6.6664512
PSTK	0.05211894	2.97594564	0.43453929	0.665677236	0.86898854	-6.3657198
WWP1	-0.0542134	6.22991437	-0.4344263	0.665758771	0.86900492	-6.8597543
PAQR8	0.16306034	1.09858696	0.43393876	0.666110503	0.86923491	-5.7174297
KLHL17	-0.0687188	3.48218269	-0.4337641	0.666236574	0.86923491	-6.4968252
BBS2	-0.0858106	5.08455027	-0.4336719	0.666303053	0.86923491	-6.8452811
RUVBL1	0.06007987	5.9629281	0.43365618	0.66631442	0.86923491	-6.8476252
RANBP3	-0.0305097	6.04616647	-0.4335962	0.666357717	0.86923491	-6.8523324
KBTBD2	0.0542176	4.75116134	0.43351898	0.666413437	0.86923491	-6.7576467
SPATA5	0.0656888	3.97595415	0.43351261	0.666418029	0.86923491	-6.6206318
ADAMTS13	0.19623818	-2.0178834	0.43340488	0.666495781	0.86924631	-5.4246098
DAAM1	0.09822455	5.40201975	0.43317803	0.666659515	0.86936984	-6.7857673
IFT22	0.09126925	2.47328816	0.43278183	0.666945527	0.86965278	-6.1798409
TGFBI	1.0223116	1.75145622	0.43239855	0.66722225	0.86985619	-5.3150783
TFB1M	0.11297477	1.80584297	0.43228225	0.667306229	0.86985619	-6.0227134
SOAT1	0.06886954	5.34412873	0.43220656	0.667360884	0.86985619	-6.8487985
SLC35A4	-0.0649304	4.69126936	-0.432162	0.667393099	0.86985619	-6.7198521
ENSCAFG00000032253	0.20593861	-0.8415835	0.43208757	0.667446814	0.86985619	-5.4768576
MRPL52	-0.0685781	3.45668521	-0.4317446	0.667694479	0.86994016	-6.4458703
RAD23B	0.03657511	7.48345627	0.43171868	0.667713231	0.86994016	-6.8432442
ZBTB6	0.05157691	4.31673184	0.43161858	0.667785534	0.86994016	-6.7015927
PUF60	-0.0320747	6.67158339	-0.4316159	0.667787501	0.86994016	-6.8597048
GPATCH2	-0.0566481	3.81046352	-0.4314173	0.667930925	0.86995784	-6.6268826
IGSF3	-0.2457328	3.26842036	-0.4314058	0.667939209	0.86995784	-6.22751
FUBP1	-0.0413504	6.46578176	-0.4309922	0.668238045	0.87024474	-6.8605614
ZNF394	-0.0457536	3.56121625	-0.4309071	0.668299506	0.87024474	-6.5639035
FAM133B	-0.1009916	2.08713725	-0.4308141	0.668366754	0.87024474	-6.1624727
TGFBR3	0.26154206	7.19898778	0.43050651	0.668588985	0.87041717	-6.7440237
ATP9A	0.24715179	3.77210304	0.43037322	0.668685314	0.87041717	-6.2358968
XPO7	0.02923774	6.39127876	0.43034392	0.668706488	0.87041717	-6.8616353
EIF4E3	0.13002889	0.55196142	0.43020621	0.668806018	0.87045677	-5.7568014
ENSCAFG00000012507	-0.1381402	0.97452028	-0.430081	0.668896522	0.87048461	-5.691746
ERCC8	0.07587663	2.86951484	0.42964384	0.669212521	0.87066022	-6.2709726
GNL2	-0.0502774	5.61093886	-0.4296231	0.669227533	0.87066022	-6.8389619
FERMT2	-0.0567524	8.80969864	-0.4296075	0.669238824	0.87066022	-6.7428662
YY1	-0.0295693	6.48590426	-0.4295039	0.669313665	0.87066766	-6.8608432
HR	0.15558816	5.86634712	0.42933111	0.669438616	0.87074027	-6.8512528
P3H3	-0.1716829	5.43206357	-0.4290635	0.669632145	0.87081437	-6.6987615
ENSCAFG00000031829	0.03504255	4.80831918	0.4290611	0.669633853	0.87081437	-6.7491202
ERI2	-0.0616365	3.32902321	-0.4287781	0.669838481	0.87091554	-6.4440227
ENSCAFG00000030164	0.08959093	4.9464136	0.42876231	0.669849929	0.87091554	-6.7454645
BRD8	-0.0440184	5.20393148	-0.4284368	0.67008535	0.87106042	-6.8086434
CHD4	0.03754272	8.0447834	0.42835599	0.670143809	0.87106042	-6.7859175
MRPL27	-0.0739838	3.60669007	-0.4283214	0.670168829	0.87106042	-6.5049256
RABEP2	-0.0525754	4.53473115	-0.4281663	0.670281029	0.87111637	-6.6846941
CAPZA2	-0.0462119	7.11693727	-0.4279291	0.670452606	0.8711779	-6.8504269
BORA	-0.0678937	3.26494777	-0.4279096	0.670466703	0.8711779	-6.4065307

PBX3	-0.0782402	6.24277569	-0.4277984	0.670547194	0.87119262	-6.8612426
ENSCAFG00000028441	-0.199745	0.00527248	-0.427441	0.670805765	0.87143868	-5.5624352
ENSCAFG00000028801	-0.291559	-1.0292495	-0.4270568	0.671083819	0.87170999	-5.4438388
LRRC42	-0.0582772	3.69337691	-0.4268547	0.671230112	0.87174842	-6.5943095
HAS2	-0.378214	1.76904726	-0.4267462	0.671308615	0.87174842	-5.9679954
GNAI3	0.05721698	6.57448065	0.42670836	0.671336026	0.87174842	-6.8631945
ENSCAFG00000000548	-0.0904822	2.05588411	-0.4266335	0.671390234	0.87174842	-6.1363075
CCT3	-0.046528	7.89874674	-0.4264485	0.671524106	0.87183237	-6.8140086
GSS	-0.0448042	4.12423609	-0.4262912	0.67163799	0.87189036	-6.6617071
ESR1	0.36459718	-0.5103058	0.42615455	0.67173695	0.8719158	-5.3204611
DLG1	-0.0480648	7.02216363	-0.426018	0.671835816	0.8719158	-6.8578288
ENSCAFG00000032226	0.03459512	6.6470894	0.42597735	0.671865252	0.8719158	-6.8617845
ZNF330	0.04340427	4.74631426	0.42581698	0.671981383	0.87192682	-6.7414697
CREG1	-0.0954136	7.03483437	-0.4257744	0.672012189	0.87192682	-6.8486693
GRAMD1B	0.20001103	-1.7534737	0.42565118	0.672101445	0.87193512	-5.52242
TAF12	-0.1104165	1.3722429	-0.4255744	0.672157032	0.87193512	-5.9377138
PTP4A1	-0.0568865	4.8402035	-0.4252118	0.67241966	0.87213845	-6.7552965
BTG2	0.13317062	3.29584726	0.42516681	0.672452259	0.87213845	-6.4048729
DDAH2	-0.0812919	3.9700773	-0.4246905	0.672797319	0.87232519	-6.5794815
MAPK14	0.04438869	4.85347331	0.42466971	0.672812361	0.87232519	-6.7982763
FOXRED1	0.04999266	5.09532846	0.42461524	0.672851826	0.87232519	-6.7742104
ENSCAFG00000006937	-0.1482017	0.50504479	-0.4243832	0.673019923	0.87232519	-5.6128776
DCX	-0.2787367	-1.2197225	-0.4243662	0.673032295	0.87232519	-5.8094398
ARL13B	-0.0595246	5.18667461	-0.4242679	0.673103508	0.87232519	-6.800787
SEL1L3	0.28863242	0.72357999	0.42423915	0.673124331	0.87232519	-5.5523753
RNH1	0.05731819	7.4367759	0.42411162	0.673216744	0.87232519	-6.8423368
ENSCAFG00000006342	0.19394003	-0.5608665	0.42409153	0.673231305	0.87232519	-5.4229138
XRCC6	0.0300818	5.43665623	0.42401222	0.673288781	0.87232519	-6.8359545
RUFY3	-0.0524902	5.8988275	-0.4235533	0.673621353	0.87265524	-6.8490934
C8H14orf28	-0.10191	1.47925026	-0.4234696	0.673682085	0.87265524	-6.0283664
SMIM26	0.10284387	3.17612074	0.4233295	0.673783618	0.87269701	-6.4153088
UBE2T	0.15723801	2.26249694	0.42319863	0.673878489	0.87273015	-5.9869175
IGF2R	0.05497362	7.60879364	0.42304003	0.673993469	0.87274355	-6.8406827
GRHL1	-0.1202978	0.43955718	-0.4229914	0.67402876	0.87274355	-5.8684189
TAF1C	-0.0511744	4.72792206	-0.4228104	0.674159957	0.87274355	-6.7572488
STRN4	0.03499148	5.9189278	0.42274475	0.674207558	0.87274355	-6.8515342
GATA3	-0.2880686	0.78112251	-0.4227065	0.67423527	0.87274355	-5.6033241
CDCA4	-0.1084901	2.40060869	-0.4225228	0.674368526	0.87282634	-6.0898044
GFM2	-0.0552003	4.22056372	-0.4224239	0.67444022	0.87282945	-6.6741784
DMXL2	0.05247281	4.71931529	0.42210361	0.674672511	0.87304037	-6.7695866
SEC31A	-0.0334823	8.61841438	-0.4216776	0.674981513	0.8733505	-6.7683176
ENSCAFG00000004887	0.0759623	3.37497534	0.42134573	0.675222288	0.8735723	-6.4698817
SIKE1	-0.0468368	4.10989967	-0.4208434	0.675586789	0.87395411	-6.6485812
NDFIP2	0.04431372	5.67794404	0.4207118	0.67568228	0.87398788	-6.852324
DPEP1	0.28423172	-1.9351777	0.42032292	0.675964523	0.87420788	-5.5513281
TMEM185B	-0.0572326	4.25751482	-0.4199906	0.676205756	0.87420788	-6.6607673
INPP4B	0.36835002	1.57731853	0.4199257	0.676252873	0.87420788	-5.7184077
MED13L	-0.071383	7.35316064	-0.4196418	0.676458968	0.87420788	-6.8216458
ISL1	0.27793949	-2.0328845	0.41962917	0.676468154	0.87420788	-5.3644855
JPT1	0.06204051	5.1652444	0.41960847	0.676483182	0.87420788	-6.7677203

THRB	0.15241162	3.13971542	0.4195512	0.676524764	0.87420788	-6.4204988
AMD1	0.05865368	4.98451483	0.41954734	0.67652757	0.87420788	-6.7668098
ENSCAFG00000007159	0.16179302	-0.4633292	0.41947037	0.676583458	0.87420788	-5.6101175
SFRP4	0.36015173	-0.7771983	0.41945946	0.676591378	0.87420788	-5.3874142
BACH2	-0.2283372	-1.1003665	-0.4194258	0.676615802	0.87420788	-5.3428066
ENSCAFG00000018099	-0.2374794	-1.9888008	-0.4192043	0.676776644	0.87432601	-5.2950711
MID1	0.1752968	3.47780228	0.41892232	0.676981445	0.87445228	-6.4267999
ALAD	-0.1152972	1.98764204	-0.4188786	0.677013233	0.87445228	-6.0013357
HINT3	0.13522977	1.14259404	0.41874393	0.677111014	0.87448891	-5.925239
ENSCAFG00000022470	-0.1496488	1.00454361	-0.4183477	0.677398818	0.87477092	-5.7489012
GPALPP1	-0.0433453	4.8816181	-0.4181786	0.677521675	0.87478422	-6.7821348
SLC16A14	0.19014636	-2.9896149	0.4180727	0.677598619	0.87478422	-5.2450723
ENSCAFG00000031337	0.16701897	2.33304866	0.41798301	0.677663786	0.87478422	-6.1457778
ENSCAFG00000002106	0.16701197	0.14039352	0.41787584	0.677741651	0.87478422	-5.5896363
NAPRT	-0.129759	2.22797694	-0.4177814	0.677810291	0.87478422	-6.2895119
FAHD2A	-0.0677603	2.86687076	-0.41776	0.67782582	0.87478422	-6.4146626
NUDT19	-0.0530651	3.24892633	-0.4169277	0.678430689	0.87540011	-6.4914062
LIPE	-0.0824201	2.52230312	-0.4169121	0.678442034	0.87540011	-6.3781919
EBF2	-0.2714015	0.99802253	-0.4167491	0.67856056	0.87546336	-6.3658427
BOLA3	-0.0649037	2.96230379	-0.4165489	0.678706071	0.87556142	-6.3934376
ZBTB8A	-0.0792804	4.04620055	-0.4163145	0.678876471	0.87558261	-6.5709894
AP5B1	-0.0789324	4.05549442	-0.4161797	0.678974532	0.87558261	-6.6266145
ENSCAFG00000015053	-0.0714298	2.45560405	-0.4160283	0.679084594	0.87558261	-6.2127501
ENSCAFG00000001038	-0.0605077	4.46438625	-0.4159446	0.679145504	0.87558261	-6.6607883
LRRN4CL	-0.206925	4.57835753	-0.4159147	0.679167237	0.87558261	-6.8432622
YTHDF2	-0.0224163	6.19238817	-0.4158914	0.679184161	0.87558261	-6.8668505
FRMPD1	0.31628922	-1.4759254	0.41585712	0.679209089	0.87558261	-5.3852357
C4H5orf34	0.27857834	-1.0330073	0.41564753	0.679361509	0.87563757	-5.3704397
SNAP23	0.05506745	3.49543635	0.41560732	0.679390754	0.87563757	-6.5695361
UCP2	-0.1887958	5.63954679	-0.4151596	0.679716389	0.87591228	-6.8442433
NANS	0.05532663	5.23882942	0.41512309	0.679742974	0.87591228	-6.8117579
FZD4	-0.0968467	4.22588535	-0.4144326	0.680245353	0.87645099	-6.6437577
ASB6	-0.0534252	5.03290324	-0.41432	0.680327282	0.87645099	-6.7914733
ALDH3B1	0.2754653	-0.031419	0.41420115	0.680413767	0.87645099	-5.7628635
ENSCAFG00000011124	0.19438341	-0.5009396	0.41416598	0.680439362	0.87645099	-5.5481702
WARS	-0.0465125	8.51227679	-0.4139187	0.680619341	0.87659317	-6.7808186
HLTF	0.04762235	7.23617018	0.41375644	0.680737425	0.87665562	-6.8589607
PYGO1	0.06556932	3.5823973	0.4135648	0.68087692	0.876692	-6.4613779
RAB43	-0.0732351	2.63554534	-0.4135264	0.680904877	0.876692	-6.2514235
PXDN	-0.0941381	7.5787346	-0.4132489	0.681106913	0.8768625	-6.8640443
CASTOR1	-0.082453	3.15299783	-0.413114	0.681205068	0.87689924	-6.5082517
SLF2	-0.0600179	3.31467468	-0.4128098	0.681426566	0.87709066	-6.5740503
COQ10A	0.05825451	3.89786245	0.41260197	0.681577933	0.87709066	-6.5734575
HINFP	-0.0373225	5.32507622	-0.4123749	0.681743299	0.87709066	-6.8126626
BIRC3	-0.1043064	2.93767072	-0.4123521	0.681759868	0.87709066	-6.4409915
ST3GAL2	0.04501274	4.55134817	0.41233972	0.681768915	0.87709066	-6.7260968
SULF2	-0.3075084	5.42183034	-0.4123361	0.681771565	0.87709066	-6.7859404
RETREG2	0.04248681	4.87366071	0.41213776	0.681916008	0.87709307	-6.7795531
ETHE1	-0.0925148	5.03987361	-0.4120894	0.681951202	0.87709307	-6.783608
ANAPC4	-0.0284233	6.69544152	-0.411831	0.682139473	0.87709307	-6.8692841

GTPBP6	0.06700893	2.28131562	0.41175171	0.682197214	0.87709307	-6.1647056
MUC20	0.17075383	-0.5536925	0.41169103	0.682241421	0.87709307	-5.6560085
NUBP1	0.03555612	4.08898353	0.41168969	0.682242396	0.87709307	-6.6103894
ARF5	-0.0660949	3.77117248	-0.4115446	0.682348098	0.87709307	-6.566269
DDX39A	-0.0889968	4.66539723	-0.4114644	0.68240652	0.87709307	-6.7136673
ENSCAFG00000030782	-0.0455941	3.56910522	-0.4113979	0.682454994	0.87709307	-6.4495123
FAM98A	0.04284509	6.35667152	0.4113776	0.682469766	0.87709307	-6.8696487
OIP5	-0.2990198	-0.9170596	-0.411143	0.682640676	0.87713879	-5.3362368
ARHGEF19	-0.1483052	1.3026585	-0.4111376	0.682644618	0.87713879	-5.923798
NOL3	0.12017312	2.34496116	0.4109385	0.682789725	0.87716307	-6.1537402
ENSCAFG00000016940	0.0280649	4.77229166	0.41092057	0.68280279	0.87716307	-6.7697305
TMEM121	0.13340611	1.95793715	0.41070137	0.682962534	0.87719067	-6.0739197
PMP22	-0.0684822	8.58852657	-0.4106238	0.68301905	0.87719067	-6.7475256
LPCAT1	0.05208676	4.9952826	0.41060443	0.68303319	0.87719067	-6.7810388
CHMP6	0.04393838	3.89381574	0.41010101	0.683400135	0.87751284	-6.5790034
ESCO1	-0.0550345	4.04244532	-0.4100691	0.683423384	0.87751284	-6.6517115
METTL25	-0.0971766	1.67945455	-0.4099463	0.683512887	0.87753831	-6.0254833
ENSCAFG00000010865	-0.0600898	7.69833011	-0.409646	0.683731852	0.87769348	-6.8419996
ECE2	0.08852448	2.51285392	0.40952461	0.683820369	0.87769348	-6.230534
TCP1	0.04960332	7.61734658	0.40949385	0.683842793	0.87769348	-6.8421426
ENSCAFG00000031146	0.04064622	6.13527612	0.40933443	0.683959047	0.87775325	-6.8675987
DCK	-0.2076485	3.13742056	-0.409104	0.684127059	0.87782106	-6.2421837
KLHL8	-0.0989245	2.28193271	-0.4089576	0.684233877	0.87782106	-6.1070439
PNPLA2	0.1086908	6.78427371	0.40874003	0.684392543	0.87782106	-6.8425534
THOP1	0.05035108	5.72690142	0.40867724	0.684438341	0.87782106	-6.8352746
BORCS8	0.07695576	2.98793712	0.40851969	0.684553262	0.87782106	-6.2694922
ERAP2	-0.2636539	-0.7630399	-0.4084717	0.684588289	0.87782106	-5.5032925
RFC3	-0.174805	2.66177747	-0.4084281	0.684620101	0.87782106	-6.0800155
SLC46A2	0.19126931	-0.7198	0.40840553	0.684636541	0.87782106	-5.4705763
RANBP1	-0.07137	5.95441716	-0.4080778	0.684875663	0.87782106	-6.8547559
KCNH1	0.19949128	-0.9397845	0.40806702	0.684883503	0.87782106	-5.5586927
CFDP1	0.04001387	5.88925492	0.408039	0.684903941	0.87782106	-6.8606923
EPS15	0.04162471	6.92870079	0.40801964	0.684918067	0.87782106	-6.8550498
ENSCAFG00000027901	-0.1614817	-0.0607055	-0.4079413	0.684975192	0.87782106	-5.5830322
LPAR1	0.25034309	3.87695405	0.40789478	0.685009166	0.87782106	-6.5705995
ENSCAFG00000014689	-0.2084812	0.07431293	-0.4077906	0.68508515	0.87782106	-5.5556863
ENSCAFG00000030944	0.1147381	0.49009225	0.40744014	0.685340929	0.87782106	-5.712228
TNNI1	-0.2252822	-0.9330709	-0.4073176	0.685430354	0.87782106	-5.4080035
MEF2B	0.16077342	-0.4209331	0.4071139	0.685579031	0.87782106	-5.6671341
NREP	-0.2919317	1.342136	-0.4070993	0.685589684	0.87782106	-5.5810707
GPANK1	0.05934068	2.64764115	0.40704864	0.68562666	0.87782106	-6.3204695
ENSCAFG00000030810	-0.083992	6.03494983	-0.4068823	0.685748096	0.87782106	-6.8711101
CLCN2	-0.1278624	1.86130229	-0.4068261	0.685789122	0.87782106	-6.1135158
AHR	-0.1210489	5.1065552	-0.4066994	0.685881613	0.87782106	-6.8106774
CRLS1	-0.0633744	3.96566609	-0.4066898	0.685888603	0.87782106	-6.5972817
KIN	-0.0499158	3.48991148	-0.4066833	0.685893356	0.87782106	-6.516695
SERTAD1	-0.0764302	4.01550364	-0.4066731	0.685900769	0.87782106	-6.6353997
EML6	-0.2186614	-1.5553114	-0.4066162	0.685942321	0.87782106	-5.4213954
ENSCAFG00000028533	0.20122548	-0.6414239	0.4064939	0.686031617	0.87782106	-5.4966192
PSMD11	0.04424475	5.73903171	0.40639808	0.686101573	0.87782106	-6.8516261

FSCN1	0.08889517	8.00763656	0.40638712	0.686109577	0.87782106	-6.8057365
WWP2	-0.0427496	5.32361613	-0.4063012	0.686172289	0.87782106	-6.7858996
KDM7A	-0.057871	3.69420687	-0.4060416	0.686361895	0.87789195	-6.7515657
SAMHD1	0.10386002	2.76521889	0.40603443	0.686367095	0.87789195	-6.4774128
ENSCAFG00000020072	-0.0320713	6.53313311	-0.4058237	0.686520969	0.87799961	-6.8707013
SEC23A	-0.0592186	7.08236801	-0.4057116	0.68660282	0.87801514	-6.8659034
THAP9	-0.1016858	1.40043699	-0.4055312	0.686734575	0.87809448	-6.0640394
CAMKK1	0.07061988	4.28881074	0.40527759	0.686919832	0.87824221	-6.6346722
ENSCAFG00000016148	-0.03913	4.26728082	-0.405026	0.687103626	0.87838803	-6.6696326
POLR2M	-0.0362012	5.42020081	-0.4048206	0.687253687	0.87849071	-6.838214
GATA4	0.81797282	1.25685148	0.40456205	0.687442568	0.87857669	-5.7343122
SUV39H1	-0.1416404	2.69182648	-0.4045376	0.687460449	0.87857669	-6.163747
ZBTB22	-0.0630314	3.15924233	-0.4042318	0.687683858	0.87877304	-6.4331111
ARL8B	0.04555864	4.11347832	0.40407976	0.687794991	0.8788259	-6.6158307
FECH	0.04198645	4.49011479	0.40385736	0.687957532	0.87884362	-6.7438766
CMPK1	0.04894036	5.58153888	0.40384218	0.687968623	0.87884362	-6.8486025
UBXN7	-0.0424884	4.04125953	-0.4037283	0.688051881	0.87884362	-6.6615783
FZD2	0.14334116	4.90298227	0.40367893	0.688087945	0.87884362	-6.7181833
APOC1	0.19968845	-1.5976179	0.40278014	0.688745006	0.87959364	-5.5090058
TP53I11	0.25276809	-1.0290795	0.40258602	0.688886947	0.87968572	-5.472666
BST2	-0.274534	0.48735289	-0.4021861	0.689179439	0.87997002	-5.6703292
NECAP1	0.06001483	3.08551449	0.40202806	0.689295	0.88002836	-6.4265452
RECQL4	-0.2506737	1.09553026	-0.4015923	0.689613778	0.88034612	-5.7033837
CRTC3	0.04474417	5.47220995	0.40139898	0.689755181	0.88035169	-6.8464936
ENSCAFG00000026367	-0.1787756	-0.7053524	-0.4013952	0.689757924	0.88035169	-5.5339074
GCC1	0.04069371	3.7354749	0.40001925	0.690764872	0.88154755	-6.606116
ENSCAFG00000031286	-0.0615204	5.0567364	-0.3998232	0.690908403	0.88156406	-6.7746139
LIMS2	0.10719829	5.37902378	0.39981036	0.690917787	0.88156406	-6.796689
MRPS10	-0.0732542	3.2414652	-0.3994259	0.691199258	0.88167862	-6.4561223
ANKRD50	-0.0937582	5.32428868	-0.3993619	0.691246091	0.88167862	-6.8685201
MRPL46	-0.0527176	3.387607	-0.3993295	0.691269884	0.88167862	-6.4380981
DEGS1	0.03703636	7.263136	0.39930532	0.691287553	0.88167862	-6.8561218
LRRN4	0.84456205	-0.8924889	0.3991834	0.691376832	0.88170321	-5.2697934
MAOA	0.16694794	7.58332005	0.39903097	0.691488449	0.88173713	-6.7107853
ENSCAFG00000009420	0.05681965	3.3081265	0.39895589	0.691543433	0.88173713	-6.523494
MT-ND2	-0.0672437	11.2107945	-0.3987725	0.691677738	0.88181911	-6.5457202
MNAT1	-0.0643021	4.05168506	-0.3984074	0.691945126	0.88207072	-6.6310018
ENSCAFG00000024413	0.09066916	3.2770188	0.39829369	0.692028444	0.88208766	-6.4516651
NDUFB8	-0.035649	5.45339884	-0.3977517	0.692425538	0.88244067	-6.8297381
PKMYT1	-0.4187805	1.46784496	-0.3977244	0.692445504	0.88244067	-5.5913805
ENSCAFG00000003909	0.07142132	2.81752233	0.39748441	0.692621363	0.88257549	-6.3163804
GRWD1	-0.0812007	2.81753422	-0.3972501	0.692793043	0.88270495	-6.2214955
PPT2	0.0856721	2.68837625	0.39710502	0.692899383	0.88275115	-6.374274
TOPAZ1	-0.2662072	-0.3055034	-0.3968194	0.69310875	0.88292858	-5.5255612
ENSCAFG00000019869	-0.092795	4.06738722	-0.396383	0.693428619	0.88320629	-6.7608817
RNF168	-0.0339067	5.55460078	-0.3963307	0.693466988	0.88320629	-6.8544954
NAA60	0.04415818	4.50159984	0.39617827	0.693578703	0.88325926	-6.7221889
PPP6R1	-0.0312289	6.25698387	-0.3958627	0.69381008	0.88340899	-6.8739498
MOSPD2	-0.0757782	5.08149252	-0.3958266	0.69383655	0.88340899	-6.8202534
UCK2	-0.0531762	6.1595985	-0.3952916	0.694228907	0.88373924	-6.8745448

DEPDC5	-0.0508756	3.68396426	-0.3952816	0.694236245	0.88373924	-6.558804
ATP1B3	-0.0485672	6.0221998	-0.3950775	0.694385912	0.88374574	-6.8757604
CUL2	0.04010671	5.47588684	0.39498801	0.694451549	0.88374574	-6.8495884
DKK3	0.1656232	6.02533587	0.39487653	0.694533322	0.88374574	-6.8561393
B3GLCT	0.05623378	4.36987379	0.39485405	0.694549814	0.88374574	-6.6472424
BET1L	0.04717532	3.65996882	0.39470376	0.694660065	0.88374574	-6.5178939
PLAUR	0.19307084	6.8222721	0.39470069	0.694662316	0.88374574	-6.8442013
ETV5	0.0529061	5.43502306	0.39445318	0.694843891	0.88388746	-6.8645708
RAB21	0.04620516	4.78853603	0.3941056	0.695098911	0.88412258	-6.7745451
MCRS1	-0.0363007	5.33042482	-0.393866	0.695274742	0.88425693	-6.8303051
CELSR2	0.11958003	1.40786486	0.39356739	0.695493874	0.88441127	-6.1343917
ZNF420	-0.0823692	2.23335123	-0.3934306	0.695594294	0.88441127	-6.1435531
MAP2K4	-0.0539784	3.66482919	-0.3934136	0.695606734	0.88441127	-6.5784771
TNFSF18	0.23430217	-0.6892093	0.39301538	0.695899048	0.88469362	-5.8574231
ATP13A1	0.03786948	6.3124991	0.39289878	0.695984646	0.88471315	-6.8750526
ERMP1	0.04252811	5.49482531	0.39200071	0.69664405	0.88540468	-6.8552273
H2AFX	-0.158555	1.93201588	-0.3918082	0.696785417	0.88540468	-5.9170717
C12H6orf89	0.04839361	5.00074236	0.39174899	0.696828915	0.88540468	-6.7769988
SEMA3B	0.20594336	2.99006182	0.39156723	0.696962415	0.88540468	-6.2799194
ETV3	0.03548672	5.14587858	0.3915242	0.696994019	0.88540468	-6.837957
NOP16	-0.0472057	3.91829019	-0.3915239	0.696994227	0.88540468	-6.6073967
TNFSF13	0.10556083	3.0329984	0.39137006	0.697107241	0.88540468	-6.4722925
MSANTD3-TMEFF1	-0.0864054	3.45520391	-0.391163	0.697259348	0.88540468	-6.2150812
ENSCAFG00000013185	0.08976335	3.24480633	0.39100189	0.697377706	0.88540468	-6.4298699
MICALL2	0.08166599	4.38406553	0.39096569	0.697404303	0.88540468	-6.6272341
HCFC2	0.03537946	4.7551692	0.39090829	0.69744647	0.88540468	-6.7686994
ITGA8	0.36207391	3.20679604	0.39089308	0.697457649	0.88540468	-6.3472746
NAP1L4	-0.0320899	6.89538458	-0.3907357	0.69757329	0.88540468	-6.8687015
RUNX1T1	-0.114984	5.3013215	-0.3907048	0.697595989	0.88540468	-6.8097
FEM1C	-0.068444	4.07290305	-0.3906822	0.69761259	0.88540468	-6.713561
APTX	0.05610771	2.81022125	0.39049466	0.69775039	0.88540468	-6.3144705
ENSCAFG00000002703	0.07591401	3.81150755	0.39047962	0.69776144	0.88540468	-6.6088917
ZNF566	0.10295149	1.38679718	0.39025483	0.697926628	0.88540468	-5.9177386
MLF2	0.03224195	6.92951165	0.3902194	0.697952666	0.88540468	-6.8753733
ENSCAFG00000030356	-0.1533876	0.54292895	-0.3901704	0.697988672	0.88540468	-5.7635769
SHLD2	0.08564295	2.59102247	0.39005406	0.698074178	0.88540468	-6.3217229
TPGS1	0.08647727	3.28984323	0.39002323	0.698096836	0.88540468	-6.4558545
TMEM179B	0.06308948	4.21806859	0.38995717	0.698145384	0.88540468	-6.6824737
ENSCAFG00000014614	-0.0427898	4.68564393	-0.3894565	0.698513413	0.88564121	-6.7110136
DENND5A	-0.0430609	7.05234119	-0.3894395	0.698525919	0.88564121	-6.8690691
PSMD13	-0.0333802	6.61060357	-0.3894165	0.698542828	0.88564121	-6.8766894
PRKCZ	0.17869349	0.00653376	0.38913564	0.69874929	0.88569369	-5.6856145
ZMIZ1	0.05730733	6.25374722	0.38912345	0.698758253	0.88569369	-6.8786629
ENSCAFG00000017108	-0.0676294	4.73393044	-0.3890732	0.698795163	0.88569369	-6.7990995
WBP4	-0.0267529	5.57965181	-0.3889473	0.698887755	0.88572192	-6.8546789
OTUB1	0.05251532	3.15067353	0.38863652	0.699116284	0.88592241	-6.38122
POLR2E	-0.0468498	5.07575481	-0.3884187	0.699276473	0.88602411	-6.7653897
TNFAIP6	0.27649238	3.15515391	0.38833609	0.699337223	0.88602411	-6.5923045
NSL1	0.0703697	3.53319379	0.38808409	0.699522561	0.88616979	-6.4438527
GALC	-0.0502055	3.10238605	-0.3875028	0.699950162	0.88659416	-6.4936106

SSC5D	0.16193066	5.66639084	0.38736567	0.700051049	0.88659416	-6.8375652
COX5B	-0.0386198	5.55981593	-0.3873417	0.700068716	0.88659416	-6.8510319
HUS1	-0.0528764	5.708614	-0.3872071	0.700167732	0.88663042	-6.8582725
ENSCAFG00000012867	0.03079031	5.53912794	0.38690544	0.700389678	0.88682231	-6.8527703
AP2S1	0.03890983	6.1452274	0.38670343	0.700538339	0.88692139	-6.8783084
MAK16	-0.0469862	6.20338804	-0.3865743	0.700633389	0.88695258	-6.8752817
ENSCAFG00000022715	-0.1831928	-0.9656539	-0.3863547	0.700794994	0.88705828	-5.4959312
ZNF839	-0.0916518	1.66315614	-0.3862695	0.700857729	0.88705828	-6.100801
TMEM250	-0.129325	0.73670186	-0.3859025	0.701127818	0.8871911	-5.79915
DZIP1	0.06379549	7.83882059	0.38588127	0.701143474	0.8871911	-6.8350258
RPA1	-0.047278	6.47489143	-0.3858398	0.701173975	0.8871911	-6.8789553
SLC4A10	-0.411403	0.20621082	-0.3854937	0.701428835	0.88741458	-5.4059923
BAG3	0.05568674	5.64149107	0.38540855	0.701491507	0.88741458	-6.8730718
GLIPR2	0.08691458	6.86134228	0.38512764	0.701698346	0.88758509	-6.8557357
PAM16	-0.049463	3.73326685	-0.3850341	0.70176722	0.88758509	-6.5394005
HASPIN	-0.2424118	1.76436867	-0.384681	0.702027254	0.88780195	-5.8241714
SOGA1	0.04038724	5.28072767	0.38460989	0.702079644	0.88780195	-6.8156126
CDADC1	0.04029595	3.74376722	0.38443461	0.702208748	0.88787607	-6.6196624
ADAMTSL4	-0.1345987	5.10392515	-0.3840624	0.702482932	0.8881336	-6.874733
FKBP5	0.11545394	5.69999514	0.38377292	0.702696193	0.88830077	-6.8062292
ECT2	-0.2233724	4.32306595	-0.3833662	0.702995861	0.88830077	-6.3933667
DCP1A	-0.0498035	3.52831647	-0.3833294	0.703023011	0.88830077	-6.5566439
CHDH	-0.2243776	1.22267281	-0.3832826	0.703057494	0.88830077	-5.6157709
MZT1	0.08814435	5.2603807	0.38326948	0.703067142	0.88830077	-6.8187875
CEP72	-0.2457967	0.21347802	-0.3832691	0.703067434	0.88830077	-5.6065419
MDM1	-0.0784966	2.12930601	-0.3832129	0.703108816	0.88830077	-6.148414
PITX2	0.23714999	1.19728678	0.383101	0.703191303	0.88831588	-5.6675905
NIPAL3	0.06294289	3.61698099	0.38281394	0.703402865	0.88849403	-6.5874745
LYRM2	0.13292581	1.00390381	0.38256838	0.703583856	0.88863354	-5.7843284
TTLL1	-0.0598286	3.72889184	-0.3823881	0.703716726	0.88870944	-6.5354982
LGALS3	0.13346848	9.40339579	0.38229543	0.703785065	0.88870944	-6.7190257
ANKRD66	0.26095782	-1.0990603	0.38188317	0.704089003	0.88900412	-5.4724407
HSPB11	0.0828504	1.05890446	0.3815946	0.704301773	0.88918364	-5.9259301
VDR	0.12961528	3.98226463	0.38131255	0.70450977	0.88935709	-6.6824246
HYKK	0.16067933	1.17784774	0.38101422	0.704729787	0.88954569	-5.9964107
GFM1	0.0415379	6.75120888	0.38083259	0.704863754	0.88962564	-6.8791927
PNPLA4	-0.1399403	1.5827077	-0.3806113	0.705027015	0.88974254	-6.0490152
TNKS	-0.0394478	4.86462998	-0.3803223	0.705240152	0.88992236	-6.7893866
BCAS3	0.04892286	5.03736274	0.38002242	0.705461433	0.89005249	-6.8344311
SLC38A7	-0.0491177	5.14826032	-0.379991	0.705484601	0.89005249	-6.8013155
CDC20	-0.2836249	4.1247562	-0.379762	0.705653568	0.8901765	-6.4163835
CARNMT1	0.04261659	3.90303196	0.3794663	0.705871801	0.8902928	-6.6475134
EXOG	-0.046066	3.34271567	-0.3794189	0.705906762	0.8902928	-6.4684503
ABLIM1	0.11202313	6.61169312	0.37934978	0.7059578	0.8902928	-6.8612907
WDR24	0.04817813	4.22703357	0.37925321	0.706029067	0.89029354	-6.6828325
METTL17	0.05439586	3.59659921	0.37913212	0.706118445	0.89030255	-6.5696532
MYSM1	-0.0611591	3.78732497	-0.379052	0.706177577	0.89030255	-6.6222725
NAA15	-0.0450922	6.41753636	-0.378659	0.70646768	0.89049016	-6.8825861
KLHL31	0.22672369	0.11922372	0.37853971	0.706555742	0.89049016	-5.5394529
DLX2	0.14342461	1.8623413	0.37830665	0.706727808	0.89049016	-6.1022996

YPEL3	0.09348563	4.08031301	0.37830624	0.706728115	0.89049016	-6.7991084
VPS11	-0.0358929	5.75468851	-0.3782457	0.70677282	0.89049016	-6.868515
TRIP13	-0.1226705	4.26660096	-0.3782007	0.706806058	0.89049016	-6.5867351
OPN3	-0.1184301	2.67002514	-0.3781801	0.706821263	0.89049016	-6.331744
FOXK1	-0.05069	5.53390757	-0.3780722	0.706900918	0.89050145	-6.8307555
RAB22A	0.0390657	4.17852758	0.37791702	0.707015504	0.89055673	-6.6733911
ENSCAFG00000018380	0.07705832	1.59687593	0.37781883	0.70708801	0.890559	-5.9577724
ENSCAFG00000006726	-0.0482128	5.0392092	-0.3768354	0.707814375	0.89138471	-6.8283913
RYK	-0.0496532	7.46653782	-0.376455	0.708095411	0.89164949	-6.8429975
ENSCAFG00000016371	-0.1405252	0.27071028	-0.3761077	0.708352004	0.89175519	-5.5736123
WDR61	0.03036944	4.98800107	0.37610385	0.708354877	0.89175519	-6.7947962
LRFN4	0.11988079	3.62595923	0.37597869	0.708447363	0.89175519	-6.5074885
NABP1	0.05434622	3.70900577	0.37595815	0.708462545	0.89175519	-6.6529076
GTF2E1	0.04917672	3.35268778	0.37572555	0.708634442	0.89188244	-6.4790983
TEDC1	0.05881788	3.17959911	0.37553954	0.708771917	0.89196634	-6.4293079
RRAGC	-0.0411912	6.78313409	-0.3752155	0.709011413	0.89217604	-6.8828775
SCMH1	-0.0440774	5.0086037	-0.3750925	0.709102334	0.89217604	-6.7945649
RTF2	0.03248266	5.33575722	0.37496882	0.709193783	0.89217604	-6.838036
PLXNA4	-0.2090633	1.56029544	-0.3749308	0.709221872	0.89217604	-5.9297523
CCDC30	0.08760371	2.011257	0.37466752	0.709416542	0.89233181	-6.0318601
TNNT2	0.26635597	-2.3898169	0.3745288	0.709519107	0.89237171	-5.3017084
ENSCAFG00000005812	-0.0428649	4.9378976	-0.3742744	0.709707208	0.89251917	-6.8107662
APEH	-0.0373638	5.55134985	-0.3739417	0.709953236	0.89273944	-6.8657721
UBXN8	-0.0342842	4.20450563	-0.3738357	0.71003165	0.89274892	-6.7128645
COPRS	-0.070937	4.5493309	-0.3734247	0.710335662	0.89295666	-6.6139863
CCDC142	-0.0915602	1.24937118	-0.3734206	0.710338658	0.89295666	-5.9669831
RRP1B	0.03564688	5.82425623	0.37330945	0.710420889	0.89297091	-6.8760508
CFAP36	0.06344129	4.77836117	0.37289216	0.710729599	0.89311923	-6.761327
OSCP1	-0.0902452	2.65348967	-0.3728773	0.710740566	0.89311923	-6.3329122
RAB29	-0.1160087	2.97307665	-0.3727931	0.710802902	0.89311923	-6.3920277
ARPP19	-0.041083	4.07173095	-0.3726076	0.710940164	0.89311923	-6.6142508
MYBBP1A	-0.0545857	7.41515193	-0.3725344	0.710994318	0.89311923	-6.8639505
GID8	-0.0414959	3.92723483	-0.3725006	0.711019283	0.89311923	-6.6328006
SLC16A1	-0.0573523	5.39829836	-0.3724791	0.711035221	0.89311923	-6.852463
ENSCAFG00000010718	0.11581363	1.78314784	0.37215967	0.711271605	0.89332707	-6.0943716
KLHL29	0.10663341	2.58577682	0.37121378	0.711971743	0.89411726	-6.2375045
DIAPH3	-0.217292	4.93910765	-0.3710904	0.712063055	0.89414278	-6.584967
SIRT5	-0.0890286	5.2918444	-0.370817	0.712265498	0.89429754	-6.8255366
ENSCAFG00000012004	0.06255939	3.76054486	0.3707322	0.712328299	0.89429754	-6.534165
MYRIP	0.17480062	-1.4377541	0.37062852	0.712405074	0.89430479	-5.6026393
GPN2	-0.0635746	3.05398829	-0.3704639	0.712526992	0.8943129	-6.3903459
BCKDK	0.02993864	6.47731504	0.37027626	0.712665936	0.8943129	-6.8858283
SERPINH1	0.05207423	9.93055425	0.37021585	0.71271067	0.8943129	-6.7185842
ENSCAFG00000002916	0.0689131	5.44751168	0.37015455	0.712756073	0.8943129	-6.8411478
PEAK1	0.04230318	6.92044158	0.37014043	0.712766531	0.8943129	-6.8693139
CEP57L1	-0.0674319	2.78514206	-0.3699982	0.712871895	0.89435601	-6.3318262
MORN1	0.13881974	-0.2304967	0.36981065	0.713010788	0.89444118	-5.6087148
GOLGA1	-0.0332868	4.46298163	-0.3696566	0.713124909	0.89448024	-6.6940942
CLNS1A	-0.0643492	2.73645959	-0.3695769	0.713183948	0.89448024	-6.3247007
SLC25A30	0.06681008	3.59836693	0.36944393	0.713282438	0.89448283	-6.5199489

YJU2	-0.0605259	2.90370802	-0.3693482	0.713353385	0.89448283	-6.4142075
MTHFD1	0.04044195	7.19809497	0.36902205	0.713594996	0.89448283	-6.8642913
TIGAR	-0.0730942	6.39099582	-0.3690159	0.713599555	0.89448283	-6.8856863
ENSCAFG00000008060	0.14778488	-0.2504367	0.36889828	0.713686707	0.89448283	-5.5081833
CHST9	-0.2358507	-1.7713271	-0.368802	0.713758077	0.89448283	-5.2942349
ENSCAFG00000017795	0.07943275	2.35668622	0.36879101	0.713766189	0.89448283	-6.342561
MALSU1	0.04319635	3.83045821	0.36868327	0.713846027	0.89448283	-6.5796159
RILP	-0.1480737	0.54575663	-0.3684307	0.714033161	0.89448283	-5.9586761
ERH	0.03405892	5.6873431	0.36827171	0.714151026	0.89448283	-6.8659187
GSN	0.20628763	10.8260509	0.36825987	0.7141598	0.89448283	-6.4703736
GUK1	0.04010919	4.81818222	0.36821816	0.714190714	0.89448283	-6.7612883
ENSCAFG00000003577	0.09920855	0.58600471	0.36812562	0.714259301	0.89448283	-5.8219662
ENSCAFG00000031010	0.02437627	6.9456786	0.36806103	0.714307179	0.89448283	-6.8825003
IL18R1	0.30503343	-0.5361677	0.36802206	0.714336065	0.89448283	-5.4550478
GNG2	-0.2006018	3.75337496	-0.3679803	0.714366982	0.89448283	-6.7734384
OSBPL6	0.07592918	5.44650146	0.36794492	0.714393238	0.89448283	-6.7926668
GALNT11	0.03397949	4.94754323	0.36774038	0.714544855	0.89458374	-6.8146236
NDUFB10	-0.037095	4.55302024	-0.3672161	0.714933558	0.89498143	-6.7493631
CCDC102A	-0.0462921	5.30413819	-0.3669466	0.71513336	0.89507855	-6.8363002
RBBP8	0.06533962	6.25560584	0.36691976	0.715153265	0.89507855	-6.8700602
VSTM4	-0.0747002	3.23946697	-0.3666405	0.715360367	0.89524731	-6.3421246
ENSCAFG00000004842	0.05694258	4.47616806	0.36652514	0.715445906	0.89524731	-6.7283422
TNRC6C	-0.0351963	4.9262002	-0.3664452	0.71550522	0.89524731	-6.8029363
ADAMTS5	0.23108702	6.31505443	0.36635458	0.715572398	0.89524731	-6.8788293
C6H16orf91	-0.116587	1.0259863	-0.3662477	0.715651638	0.89525753	-5.7858755
TERF1	-0.0796653	3.30861837	-0.3661104	0.715753528	0.89529607	-6.4085372
BCL9L	0.06223871	6.66548341	0.36590471	0.715906083	0.89539798	-6.8866797
ENSCAFG00000031244	0.06676025	2.88008364	0.36551547	0.716194841	0.8956702	-6.3384874
LAMTOR3	0.03120927	5.16596977	0.36529306	0.71635985	0.89578762	-6.8280503
HAUS5	-0.076921	3.01574352	-0.3650224	0.716560661	0.89588385	-6.3288443
FTH1_version2	0.1550116	6.44077213	0.36499764	0.716579055	0.89588385	-6.8871398
TGIF2-RAB5IF	0.03225364	4.98138189	0.36478755	0.716734955	0.89598983	-6.8093958
FGF13	0.53862496	0.86517161	0.3646104	0.716866425	0.89599682	-5.3961898
DCAF12	0.03106024	5.42992281	0.36458831	0.716882819	0.89599682	-6.8495491
ENSCAFG00000005585	-0.0656682	2.6637389	-0.364242	0.717139879	0.89622918	-6.197358
ENSCAFG00000018572	0.0907812	6.50556048	0.36346388	0.717717495	0.89678097	-6.8695781
SRSF7	-0.0585902	4.72471607	-0.3634554	0.717723798	0.89678097	-6.7302232
ENSCAFG00000031664	-0.2475251	-1.1486464	-0.36335	0.717802031	0.89678976	-5.3125456
ENSCAFG00000030331	-0.1617149	-0.4930304	-0.3630675	0.718011849	0.89690641	-5.5761266
FEZ2	-0.0481598	5.07322639	-0.3630325	0.718037809	0.89690641	-6.8040868
C1D	-0.0532923	4.45401487	-0.362699	0.718285466	0.89699945	-6.7284538
ENSCAFG00000016514	-0.056699	2.21706862	-0.3624109	0.718499485	0.89699945	-6.1579335
NEBL	0.22497196	-2.1560643	0.36223528	0.7186299	0.89699945	-5.2536555
GOLGB1	0.038136	7.85803136	0.36223526	0.718629912	0.89699945	-6.8262476
ENSCAFG00000000331	-0.111301	0.1656913	-0.3622221	0.718639653	0.89699945	-5.655608
TRAPPC5	0.05184634	3.55801831	0.36197937	0.718819999	0.89699945	-6.4867045
TP73	0.2066115	0.2963893	0.36186387	0.718905799	0.89699945	-5.5148735
STXBP4	-0.0609988	3.46716852	-0.3617332	0.719002889	0.89699945	-6.5233901
CBLL1	0.03307237	4.82500937	0.36165718	0.719059355	0.89699945	-6.8019465
SPP2	-0.2098707	1.84458871	-0.3615311	0.71915302	0.89699945	-5.893697

CAV2	-0.0697304	7.28283064	-0.3614078	0.719244642	0.89699945	-6.8492536
CYP4V2	-0.2204376	2.65856728	-0.3613761	0.719268178	0.89699945	-6.4184103
RAP2A	0.03909842	6.70700127	0.36130615	0.719320178	0.89699945	-6.8872885
CCDC14	0.04477861	5.10777067	0.36121459	0.719388215	0.89699945	-6.817232
RAPH1	-0.0750606	4.94782326	-0.3612126	0.719389687	0.89699945	-6.82061
AR	0.22793536	-1.8925291	0.36111616	0.719461352	0.89699945	-5.3219047
REXO4	0.03831366	5.75921675	0.36084597	0.719662143	0.89699945	-6.8671316
ENSCAFG00000030466	0.06576689	6.79407045	0.36080985	0.719688989	0.89699945	-6.8775889
GPR39	0.23241939	-2.60841	0.36074828	0.719734745	0.89699945	-5.2586723
IRAK1	0.04991227	6.33368023	0.36066498	0.719796657	0.89699945	-6.8701398
TOMM6	-0.0649122	2.56733788	-0.3606186	0.719831126	0.89699945	-6.2487216
LIN37	-0.0538637	2.13718215	-0.3605706	0.719866773	0.89699945	-6.1748613
NEK11	-0.1457587	0.30233712	-0.3605369	0.719891846	0.89699945	-5.7752083
PDXDC1	0.02662766	6.17094078	0.36052353	0.719901789	0.89699945	-6.888385
ULK2	-0.04077	6.98576383	-0.3603676	0.720017703	0.89699945	-6.8723764
DNTTIP1	-0.0314959	4.36165152	-0.3602643	0.720094458	0.89699945	-6.6961494
UGDH	0.12294062	8.06225367	0.36026256	0.720095771	0.89699945	-6.8505406
DDX25	0.18814801	-1.2477927	0.36017124	0.720163652	0.89699945	-5.4965141
MYH10	0.20134585	6.92170295	0.36015265	0.72017747	0.89699945	-6.8233513
ZFAND2A	0.04764631	4.28452722	0.35990941	0.720358301	0.89713183	-6.6879003
PEPD	0.05149691	5.06182212	0.35981807	0.720426204	0.89713183	-6.7894665
TIGD4	-0.0947406	0.75250704	-0.3595452	0.720629119	0.89713353	-5.9121578
RNF215	-0.0639338	2.72362009	-0.3594381	0.720708749	0.89713353	-6.3545591
ACAA1	-0.045052	5.155433	-0.359426	0.720717688	0.89713353	-6.84324
IMPG2	0.13020577	0.212098	0.35933731	0.720783663	0.89713353	-5.7569127
PRKCSH	0.03963897	7.67886256	0.35933728	0.720783689	0.89713353	-6.848959
GMPR2	0.0346074	4.35995011	0.35912253	0.720943381	0.89719677	-6.720941
PTPN2	0.04978333	4.56221659	0.35907738	0.720976954	0.89719677	-6.7483904
STXBP6	0.21909296	6.35119308	0.35888766	0.721118048	0.89721438	-6.8844404
NFRKB	-0.033569	4.38853647	-0.3588668	0.721133563	0.89721438	-6.7256886
ITSN2	0.04536319	6.1045797	0.35867669	0.721274952	0.89730166	-6.8874116
SRM	0.08316909	6.01292147	0.35814005	0.721674129	0.89761997	-6.8719933
DISP1	0.05061242	5.30253876	0.35812617	0.72168446	0.89761997	-6.83625
NIPA2	-0.0360291	6.94737219	-0.3579272	0.721832453	0.89761997	-6.8837387
PSMD1	0.03765478	7.06635514	0.35786019	0.721882332	0.89761997	-6.8812272
FBXO22	0.03024111	5.57290031	0.35785374	0.721887132	0.89761997	-6.8542562
ORC2	-0.0372335	5.299339	-0.3575913	0.722082433	0.89777419	-6.8373467
CDC42SE1	-0.0567628	3.95140749	-0.3572523	0.722334676	0.89791725	-6.6410051
ENSCAFG00000005346	0.03321499	3.8441156	0.35724503	0.722340064	0.89791725	-6.6105927
PEX12	-0.0395176	3.79573369	-0.3567769	0.722688445	0.89811302	-6.5694332
ENSCAFG00000008074	-0.1704867	-0.712953	-0.3566204	0.722804954	0.89811302	-5.501721
PICK1	0.06127637	2.94839109	0.35658903	0.72282829	0.89811302	-6.4002052
TSC1	0.03668232	4.99554828	0.35657766	0.722836751	0.89811302	-6.8118922
AIF1L	0.2094844	-0.4667838	0.3565544	0.722854064	0.89811302	-5.5171338
PRKCE	-0.0617643	4.16777639	-0.3562165	0.723105603	0.89826973	-6.7335541
RND1	0.14309071	0.04799534	0.35617482	0.723136622	0.89826973	-5.686336
ENSCAFG00000029553	-0.2766703	2.79873173	-0.3559546	0.723300571	0.89826973	-6.5522675
GPM6A	-0.191548	-2.7239132	-0.3559259	0.723321913	0.89826973	-5.2848715
FOXRED2	-0.1732514	0.70501313	-0.3558243	0.723397563	0.89826973	-5.8875783
ENSCAFG00000007461	0.1259496	0.61967385	0.35581021	0.723408074	0.89826973	-5.7099526

TPP2	-0.0305332	6.24983743	-0.3555294	0.723617186	0.89837702	-6.8888012
YBEY	0.04529978	3.10984951	0.35537937	0.723728878	0.89837702	-6.4335396
CAMKMT	-0.0828903	1.86797384	-0.3552912	0.723794507	0.89837702	-6.0121204
ELP6	0.07056077	1.91826078	0.35521882	0.723848441	0.89837702	-6.0612799
STAT5B	0.03251325	5.27435923	0.35517443	0.723881498	0.89837702	-6.8568708
AP3S2	-0.0307206	5.93344435	-0.3551195	0.723922413	0.89837702	-6.8737814
IRF9	0.06368693	3.44866411	0.35472361	0.724217254	0.89861553	-6.5482361
ENSCAFG00000001921	0.11581485	0.188193	0.35445406	0.724418036	0.89861553	-5.7059206
STUB1	0.03010973	5.59937513	0.35435041	0.724495253	0.89861553	-6.8782369
WDR60	-0.0428001	3.33171127	-0.3543356	0.724506261	0.89861553	-6.5154228
TMED5	0.03032912	5.27870521	0.35425544	0.724566	0.89861553	-6.8541763
PACS1	-0.0296437	6.01335748	-0.3541552	0.724640681	0.89861553	-6.8868233
DBN1	-0.0418585	7.36409768	-0.3540659	0.724707239	0.89861553	-6.8758881
ENSCAFG00000030202	0.06149921	2.51874404	0.3539477	0.724795267	0.89861553	-6.2722821
TM2D3	-0.0651247	3.1339937	-0.3539341	0.724805369	0.89861553	-6.4240608
SRSF9	-0.0272724	5.38515	-0.3538486	0.724869125	0.89861553	-6.8420573
PSMA7	-0.0333316	6.40763379	-0.3537157	0.724968121	0.89861553	-6.8915945
VCP	0.03090437	8.44660682	0.35371223	0.724970708	0.89861553	-6.8061092
HPF1	-0.0582847	3.26021801	-0.3535614	0.725083069	0.89866637	-6.425594
DEDD2	0.05733689	3.36340251	0.35325261	0.725313207	0.89873576	-6.5740186
ENSCAFG00000030097	-0.0741193	1.8504125	-0.3531806	0.725366893	0.89873576	-6.0593629
ENSCAFG00000012089	-0.0378503	4.57253555	-0.3530757	0.72544507	0.89873576	-6.7556595
JUND	0.08658662	5.48230974	0.35287866	0.725591907	0.89873576	-6.8610072
ENSCAFG00000029709	-0.043427	2.96727921	-0.3526758	0.725743116	0.89873576	-6.325869
SNAI2	0.10615284	6.39763582	0.35262338	0.72578218	0.89873576	-6.8867864
TCTN1	0.05044413	4.7976388	0.35260656	0.725794718	0.89873576	-6.7295594
ZNF280C	0.04420746	4.36428716	0.35260197	0.725798138	0.89873576	-6.7301567
CYP20A1	-0.0351132	5.72484093	-0.3525578	0.725831036	0.89873576	-6.8728608
SLC12A2	-0.0539715	5.24914372	-0.3524342	0.725923179	0.89873576	-6.8145931
ENSCAFG00000018403	0.02573886	6.84532412	0.35235364	0.725983257	0.89873576	-6.889477
ENSCAFG00000006577	-0.0767528	4.26404855	-0.3520843	0.726184087	0.89873576	-6.7324294
ENSCAFG00000030154	0.15523958	-0.9060881	0.35208281	0.726185159	0.89873576	-5.4257124
WDR26	0.02835868	7.12003916	0.35202858	0.726225592	0.89873576	-6.8716191
KLHDC2	-0.0280144	5.45656866	-0.3520072	0.7262415	0.89873576	-6.8561286
SLC25A38	0.05727639	4.11515577	0.35195471	0.72628067	0.89873576	-6.6153847
MRPL33	0.03008965	4.63071902	0.35172631	0.726450963	0.89885819	-6.7541282
DNAJA3	0.02248817	5.35910885	0.35154717	0.726584544	0.89893516	-6.8546765
UBXN2B	0.03491197	6.64398807	0.35126563	0.726794495	0.89901456	-6.8921098
NOC3L	0.04402742	4.57388692	0.35121411	0.726832919	0.89901456	-6.7699477
ENSCAFG00000030603	-0.0546989	2.75974742	-0.351174	0.726862834	0.89901456	-6.2773918
CHCHD5	-0.0677301	2.05835297	-0.3509434	0.727034815	0.89913898	-6.1264301
ALG11	0.04736241	3.32254534	0.35071985	0.727201558	0.89917114	-6.5132971
SHROOM4	-0.0844833	4.47375052	-0.3505383	0.727337018	0.89917114	-6.7511001
SLC6A17	0.0471394	5.1451757	0.35048132	0.727379487	0.89917114	-6.8829532
TPRA1	-0.0449014	4.06208468	-0.3504028	0.727438093	0.89917114	-6.6835508
EXD2	-0.0743523	2.17234215	-0.3503483	0.72747872	0.89917114	-6.1279709
RFK	0.07167972	4.94891945	0.35033434	0.727489132	0.89917114	-6.7907586
C1GALT1	-0.0577501	4.73818067	-0.3500445	0.72770537	0.89935016	-6.8072829
ENSCAFG00000020503	-0.1636432	-0.3978757	-0.3495071	0.728106321	0.8997574	-5.5523411
TXNDC12	-0.0339053	6.42547302	-0.3492508	0.72829765	0.89989591	-6.8919877

FGGY	-0.0697702	2.6912644	-0.3491655	0.728361288	0.89989591	-6.2647225
ENSCAFG00000018353	-0.0412365	3.98346503	-0.3490549	0.72844382	0.89990961	-6.5987151
SLCO4A1	-0.2321797	0.38994596	-0.3488459	0.728599824	0.90001406	-5.7656565
SMPD4	-0.029678	5.86723948	-0.3480356	0.729204736	0.90060373	-6.8740694
TMEM159	-0.0929429	2.22433116	-0.3480149	0.729220185	0.90060373	-6.1717832
DLEC1	-0.0816844	1.65941047	-0.3476583	0.729486472	0.90077533	-6.1839926
SPAG4	0.0866055	1.0088875	0.34763726	0.729502151	0.90077533	-5.9106562
ENSCAFG00000002800	-0.0963714	1.91184413	-0.3473062	0.729749372	0.90099226	-6.1737296
BZW1	-0.0513938	6.79570921	-0.3470814	0.729917287	0.90099357	-6.8869763
ENSCAFG00000029412	-0.1263533	0.44948707	-0.3470653	0.729929258	0.90099357	-5.7379004
EIF5	-0.0441248	7.54377087	-0.3470175	0.729965016	0.90099357	-6.8632937
SLC25A25	0.04865587	4.35714498	0.34687701	0.730069925	0.90103476	-6.6867153
ELOF1	0.12089049	1.38269808	0.34676702	0.730152082	0.90103576	-5.877784
VCPKMT	0.09740179	0.30663006	0.34635093	0.730462904	0.90103576	-5.7680437
RNF26	0.05072603	4.05451454	0.34633744	0.730472986	0.90103576	-6.6555022
TSPAN8	0.20749944	-2.1995426	0.34631424	0.730490312	0.90103576	-5.3523059
ENSCAFG00000011681	-0.161026	0.27743801	-0.3463119	0.730492075	0.90103576	-5.6619915
BOP1	0.04624134	5.89856558	0.34622494	0.730557028	0.90103576	-6.8836787
MCM6	-0.0721817	6.72201187	-0.3462056	0.730571466	0.90103576	-6.8922077
OAZ1	-0.0240081	7.67965446	-0.3458745	0.730818893	0.90125267	-6.8435682
STK4	-0.0322922	5.34118993	-0.3456904	0.730956454	0.90133407	-6.8623594
LRP3	0.24299951	-0.1340837	0.34549416	0.731103065	0.90142661	-5.44999
ZSCAN12	0.04794291	3.01466975	0.34535508	0.731207	0.90146651	-6.3700822
GMIP	-0.0428351	4.48106656	-0.345133	0.731372986	0.90158291	-6.7277091
LIMK1	0.0640252	2.66645716	0.34491897	0.73153294	0.90161614	-6.3428894
SYNCRIP	-0.0449663	7.29374605	-0.3448377	0.731593682	0.90161614	-6.879658
G3BP2	0.0304638	5.97819734	0.34478415	0.73163371	0.90161614	-6.8925465
ITIH5	0.24748436	4.16411073	0.34465411	0.731730919	0.90161614	-6.1150252
MFSD14B	0.02730335	5.31395084	0.34461809	0.731757846	0.90161614	-6.8614051
RBFOX2	0.02811837	6.11499171	0.34440168	0.731919617	0.90163771	-6.8911297
ENSCAFG00000015941	0.14934283	-0.1909041	0.34436477	0.731947214	0.90163771	-5.5757176
ID4	0.19604418	-2.1498309	0.34430741	0.731990095	0.90163771	-5.3042823
TCEA3	-0.1282961	3.69188207	-0.3439554	0.732253292	0.90183461	-6.3443225
BIN1	-0.0636618	5.70252407	-0.3438306	0.732346574	0.90183461	-6.8748978
PITPNM2	-0.0698641	4.29198284	-0.3438061	0.732364896	0.90183461	-6.7041338
CEP89	-0.0647775	4.37747412	-0.3436935	0.732449069	0.90183461	-6.7419635
ENSCAFG00000013713	-0.0948619	3.19897417	-0.3436148	0.732507935	0.90183461	-6.2374545
DGAT1	0.05674808	3.3634403	0.34334757	0.732707794	0.90199251	-6.490263
EMC3	0.02793515	5.32284493	0.34279573	0.733120524	0.9024124	-6.8513891
ENSCAFG00000023364	-0.1902108	-1.3523497	-0.342593	0.733272201	0.90249587	-5.4792199
AXIN2	-0.1374337	1.29670051	-0.3423758	0.733434662	0.90249587	-6.0616562
TLE4	0.0478798	5.39782978	0.34234146	0.733460345	0.90249587	-6.8477823
CREM	-0.060093	2.8101157	-0.342322	0.733474932	0.90249587	-6.351168
IQUB	0.08468412	2.7516066	0.34170298	0.733938057	0.90297751	-6.3226444
AGGF1	-0.0345195	4.52797706	-0.3414015	0.73416363	0.90309975	-6.7603333
HAVCR1	0.09891019	0.911516	0.34137565	0.734183001	0.90309975	-5.8499226
ZNF787	0.05759527	3.12553585	0.34128278	0.734252504	0.90309975	-6.3645733
ENSCAFG00000031436	0.08361951	0.96593086	0.34092109	0.734523204	0.90315848	-5.9257906
FGF7	0.22441974	2.38912617	0.34086549	0.734564821	0.90315848	-6.8007694
ARHGEF12	-0.0323807	8.10175567	-0.3407954	0.734617297	0.90315848	-6.8380069

ENSCAFG00000009779	-0.1437422	-0.8546883	-0.3407554	0.734647243	0.90315848	-5.5001023
ALG8	-0.0476237	4.32362189	-0.3406064	0.734758763	0.90315848	-6.663581
PYM1	-0.0453793	3.37508157	-0.3405062	0.734833786	0.90315848	-6.4517778
IP6K1	0.03653046	4.88900852	0.34045834	0.734869594	0.90315848	-6.8243546
SLC35E3	-0.058187	2.89637761	-0.3404526	0.734873871	0.90315848	-6.387318
NSG1	0.04968922	4.27759833	0.34032935	0.734966163	0.90316979	-6.6908745
MPLKIP	0.11156733	4.15290838	0.34024877	0.735026482	0.90316979	-6.745348
GTF2F1	0.0312772	5.76709256	0.33979164	0.735368745	0.90341396	-6.8800656
TLE2	0.16342265	0.99925067	0.33977923	0.73537804	0.90341396	-6.4580317
RSRP1	0.10615311	2.19123228	0.33969601	0.735440354	0.90341396	-6.0475956
LGALS9	-0.222969	0.06511081	-0.3395539	0.735546755	0.90345655	-5.6758763
MYO1B	0.05242236	7.94462072	0.33936416	0.735688858	0.90354299	-6.8477559
ZNRF1	0.10655371	1.6826319	0.33924457	0.735778418	0.90356488	-5.9905066
PLA2G4E	-0.2096369	-1.9393248	-0.339057	0.7359189	0.90364929	-5.4139514
PRIMPOL	-0.0510203	3.97543061	-0.3388225	0.736094512	0.90373961	-6.6682911
DIP2B	-0.0493985	6.56260925	-0.338747	0.736151064	0.90373961	-6.8965713
TMEM240	-0.1192231	0.04782601	-0.3385489	0.736299515	0.90373961	-5.7050784
IPO8	-0.029646	5.3662422	-0.3384389	0.736381856	0.90373961	-6.8617051
MCM9	-0.0361499	3.92998775	-0.3383855	0.736421852	0.90373961	-6.5700846
ARL3	0.04726713	4.33118174	0.3383841	0.736422937	0.90373961	-6.7617188
ENSCAFG00000015686	-0.0244289	7.63235479	-0.3381608	0.736590187	0.90385163	-6.8579934
ZC3H11A	-0.0237552	7.1399049	-0.3380707	0.736657734	0.90385163	-6.8804649
KIF1BP	0.03361829	5.47043462	0.3378755	0.736803983	0.90393349	-6.8652532
TMEM160	0.06807317	3.21855085	0.33772863	0.736914028	0.90393349	-6.4043514
ARHGAP26	-0.1230339	0.46059635	-0.3376544	0.736969663	0.90393349	-5.840154
ZNF473	-0.0523659	3.2280258	-0.3375985	0.737011507	0.90393349	-6.5012031
TAGLN2	0.05396644	9.41789977	0.33732601	0.737215732	0.90403684	-6.7597913
BCL7B	0.03306256	4.5430574	0.33729454	0.737239318	0.90403684	-6.736511
ASPH	0.03723331	7.45232182	0.33700751	0.737454435	0.90413377	-6.8630163
GRIN2C	0.2144782	-1.9504483	0.33681229	0.737600759	0.90413377	-5.3065225
CABLES1	-0.1268617	2.40087119	-0.3367187	0.737670925	0.90413377	-5.9952438
HNRNPH3	-0.0445772	5.89449177	-0.3366696	0.737707685	0.90413377	-6.882691
KDM1A	0.03065366	6.36892274	0.33666271	0.737712879	0.90413377	-6.8970249
ECHDC3	-0.0456259	4.12230682	-0.3365541	0.737794281	0.90413377	-6.6892487
THOC3	0.04901517	4.20448008	0.33651871	0.737820821	0.90413377	-6.7083699
THTPA	0.04503039	3.41963051	0.33639135	0.737916296	0.90416281	-6.4476586
C19H2orf76	-0.0957776	1.84611287	-0.3362501	0.738022168	0.90420457	-6.0616829
RRP7	0.06010006	3.08968734	0.33577493	0.738378443	0.90455309	-6.4076374
ACADL	-0.0688659	3.07347066	-0.3354992	0.738585191	0.9047001	-6.3256846
COMMD9	-0.0353462	4.62655932	-0.3353401	0.738704492	0.9047001	-6.7377949
NUP85	0.04044962	4.92672669	0.33526178	0.738763243	0.9047001	-6.7878606
GBE1	-0.0434492	6.21165719	-0.3352318	0.738785747	0.9047001	-6.8971367
HSPH1	-0.0622423	6.3858396	-0.3350457	0.738925261	0.90478299	-6.8969922
TBX4	-0.2013013	-2.7479095	-0.3346922	0.739190451	0.90498011	-5.2757109
ZNF414	0.06901362	2.97801103	0.33463953	0.739229939	0.90498011	-6.3588698
ANKRD29	-0.0672252	5.11849735	-0.3344844	0.739346337	0.90503125	-6.8220204
CLIC6	-0.3771896	0.33171477	-0.3343923	0.739415421	0.90503125	-5.8013179
FNDC3A	0.05379972	7.26092989	0.33429647	0.739487286	0.90503127	-6.8475337
CRISPLD2	0.21683469	4.38373398	0.33405627	0.739667484	0.90516386	-6.885511
PRPF6	-0.0295705	6.95304542	-0.3336645	0.739961413	0.90535636	-6.8877308

DUS1L	0.04266394	5.31502963	0.33365503	0.739968541	0.90535636	-6.8470706
GPS2	-0.0446657	4.4387714	-0.3334967	0.740087371	0.9054138	-6.7095714
PIH1D2	-0.109689	-0.5361791	-0.3332633	0.740262505	0.90554011	-5.6388865
ZNF644	0.04093521	4.92693693	0.33235618	0.740943342	0.90620236	-6.810963
ENSCAFG00000031030	0.07628396	1.93506987	0.33224462	0.741027093	0.90620236	-6.2253901
ATXN7L3B	-0.1437977	-0.5901109	-0.332244	0.741027535	0.90620236	-5.6060265
CTSO	0.07319435	2.42373495	0.33211391	0.741125223	0.90620236	-6.1688978
YAE1	0.02937557	4.59699275	0.33201101	0.74120247	0.90620236	-6.7426737
TOPBP1	-0.044342	6.23043684	-0.3319504	0.741248001	0.90620236	-6.891787
SGPP1	0.05584535	5.15581897	0.33172371	0.741418183	0.90620236	-6.8239661
CEBPB	0.10557553	3.12674277	0.33167344	0.741455926	0.90620236	-6.3738559
KLHL25	0.08618767	1.61186179	0.33126914	0.741759514	0.90620236	-6.2413296
MUS81	-0.0291146	4.84640011	-0.3311983	0.741812743	0.90620236	-6.7867563
ENSCAFG00000009583	-0.0482802	4.8894343	-0.3311096	0.741879295	0.90620236	-6.786797
NDUFA9	0.0405553	5.50720153	0.33106192	0.741915136	0.90620236	-6.8670573
ELOVL7	0.14777989	3.94205736	0.33099067	0.741968642	0.90620236	-6.4650321
ZFP36	-0.1043585	6.6222716	-0.3309469	0.742001513	0.90620236	-6.8844352
ZMAT2	0.05554212	3.55081975	0.33081666	0.742099336	0.90620236	-6.561628
TMOD4	-0.2003645	-1.8608049	-0.3307883	0.742120646	0.90620236	-5.4112056
MXD3	-0.1569108	-0.8864779	-0.3307859	0.74212243	0.90620236	-5.4689442
EHD1	-0.0446891	6.59689377	-0.3307504	0.74214911	0.90620236	-6.8995622
HDLBP	-0.0262381	9.89877006	-0.3306698	0.742209658	0.90620236	-6.6954817
CFD	-0.1979045	5.03729679	-0.3304041	0.742409242	0.90620236	-6.8241459
PTRH2	-0.048007	3.40228286	-0.3303738	0.742431995	0.90620236	-6.5168656
ENSCAFG00000030317	0.11417186	0.48330478	0.33026965	0.742510212	0.90620236	-5.8469425
STXBP1	-0.0392732	5.66755462	-0.3302158	0.742550636	0.90620236	-6.8990066
ENSCAFG00000004823	0.04824557	3.46310188	0.33017693	0.74257987	0.90620236	-6.4565966
RASSF9	-0.1134318	2.35721933	-0.3301468	0.742602477	0.90620236	-6.1282182
NDRG1	0.05919894	8.45819078	0.32998451	0.742724424	0.90621658	-6.7683102
SSH1	-0.0462299	4.56619593	-0.3299398	0.74275802	0.90621658	-6.7871073
POLA2	-0.1466768	4.0099608	-0.3298346	0.742837084	0.90622527	-6.4537606
CENPW	-0.1589497	0.84673751	-0.3295247	0.74306987	0.90633516	-5.6422647
MRPS5	0.0310145	5.13957465	0.32940413	0.743160492	0.90633516	-6.8298288
GMDS	-0.068975	3.32854343	-0.3292592	0.743269431	0.90633516	-6.4762771
SNN	-0.1201462	-0.5317783	-0.3291991	0.743314541	0.90633516	-5.6602479
ZNF461	0.12358274	0.48062117	0.32919347	0.743318798	0.90633516	-5.6860765
PCYT2	-0.0465349	4.68615595	-0.3289674	0.743488672	0.90633516	-6.8021263
CDC7	-0.0654884	4.23964319	-0.3289325	0.743514894	0.90633516	-6.6457575
BLOC1S5	0.03717274	5.69127815	0.32890119	0.743538451	0.90633516	-6.8888667
ENSCAFG00000004704	0.07027285	1.90739496	0.32885289	0.743574751	0.90633516	-6.1601664
PHF20L1	-0.0359648	5.88319553	-0.3285331	0.743815114	0.90645721	-6.8794226
MRPS7	-0.0400707	4.54471598	-0.3285186	0.743826041	0.90645721	-6.7129757
CTSS	-0.1562553	1.58731832	-0.3284324	0.743890772	0.90645721	-6.1351143
CEPT1	-0.0239478	4.25608961	-0.3282327	0.744040946	0.9065525	-6.7234158
ENSCAFG00000024295	-0.1769577	-0.5777194	-0.3280034	0.74421328	0.9066356	-5.5285661
ZBTB3	0.10821468	0.52779702	0.32795044	0.744253104	0.9066356	-5.7979753
FUOM	-0.0813097	1.77640796	-0.3278062	0.744361535	0.90664895	-6.1382342
C6	0.79859941	0.52368939	0.32774438	0.744408024	0.90664895	-5.4018912
LRWD1	0.03945542	4.32041622	0.32729396	0.744746695	0.90680876	-6.7041542
ENSCAFG00000011014	-0.0364318	4.7756367	-0.3271464	0.744857666	0.90680876	-6.7774172

DICER1	-0.0422321	6.32248044	-0.3270177	0.74495442	0.90680876	-6.900952
PDE6D	0.0293333	4.3298799	0.32685765	0.745074807	0.90680876	-6.7075251
ENSCAFG00000030667	-0.0954498	0.82178004	-0.3267042	0.745190194	0.90680876	-5.8815691
GRAMD1A	-0.0623625	5.53968542	-0.3265876	0.745277906	0.90680876	-6.8680305
PPP4R1	-0.0353848	5.92825322	-0.3262951	0.745497951	0.90680876	-6.8840274
NICN1	-0.0452671	4.03481088	-0.3261907	0.745576449	0.90680876	-6.6897878
GPR160	-0.1261574	2.71742588	-0.3260665	0.745669881	0.90680876	-6.0361401
HIPK1	0.03863984	6.25339557	0.3260461	0.745685228	0.90680876	-6.8970832
NEK1	-0.0337919	5.27050218	-0.3260295	0.74569775	0.90680876	-6.8455909
ENSCAFG00000023394	0.0437686	3.20097375	0.32592461	0.745776618	0.90680876	-6.4634627
PDGFRA	-0.1143762	8.47254146	-0.3259051	0.745791282	0.90680876	-6.8109111
SRPX2	-0.1243431	6.584893	-0.325773	0.745890664	0.90680876	-6.9008231
TAF6L	0.03865848	4.16318546	0.32554337	0.746063446	0.90680876	-6.6688757
ENSCAFG00000030348	-0.0527506	1.65060729	-0.325494	0.746100585	0.90680876	-6.0380238
GTF3C1	-0.034551	5.9782754	-0.3254539	0.746130787	0.90680876	-6.8991594
PCDHGC5	0.04193446	5.28048252	0.32541121	0.746162884	0.90680876	-6.8570296
RAD18	-0.0961558	2.05614048	-0.325405	0.746167518	0.90680876	-6.0379033
C33H3orf38	0.02954957	4.42053407	0.32527444	0.746265795	0.90680876	-6.7420116
ENSCAFG00000008862	-0.2343375	-2.1077991	-0.3252583	0.746277946	0.90680876	-5.3469243
DOLPP1	0.05081773	3.56872565	0.32523828	0.746293001	0.90680876	-6.5218507
C15H1orf122	-0.1226231	1.03434309	-0.3251429	0.746364778	0.90680876	-5.8530164
RAB24	0.03701925	4.16695546	0.32512392	0.746379061	0.90680876	-6.7125851
GBA2	0.04366938	4.61235138	0.32510872	0.746390497	0.90680876	-6.7561901
TAF2	-0.0278069	6.22368008	-0.3250814	0.746411018	0.90680876	-6.8980031
LRR1	-0.0966402	1.79812432	-0.3247659	0.746648452	0.90700973	-5.974717
APRT	-0.0603782	3.8357184	-0.3246611	0.746727382	0.90701814	-6.6464175
FBXO38	-0.0293182	5.03991391	-0.3243356	0.746972328	0.90722818	-6.8387469
ADCK1	-0.0766488	1.6152187	-0.3241708	0.747096426	0.90729142	-5.9227605
ENSCAFG00000006193	-0.0374402	4.70550839	-0.3239666	0.747250087	0.90739054	-6.781874
ZNF703	0.0545619	5.15950596	0.32373594	0.747423751	0.90751393	-6.8390305
ZDHHC9	0.0459883	5.64204009	0.32329398	0.747756501	0.90783045	-6.8882835
KTN1	0.04766579	8.23082012	0.32267805	0.748220314	0.90828352	-6.842983
MED20	0.03918519	2.55333937	0.32260689	0.748273907	0.90828352	-6.3294987
RBBP7	0.04044806	7.33344287	0.32217055	0.748602542	0.90830222	-6.8916513
ZNF483	0.12486846	-0.3419989	0.3220502	0.748693196	0.90830222	-5.6036345
MXRA5	-0.6165027	2.82567345	-0.3220126	0.7487215	0.90830222	-5.4870869
MAOB	0.3471906	-1.9853191	0.32199724	0.74873309	0.90830222	-5.2876738
SEPT9	0.03576502	8.10153288	0.32196822	0.748754953	0.90830222	-6.8530195
FAM114A1	-0.0413723	7.05240889	-0.3217351	0.748930571	0.90830222	-6.8856243
FANCI	-0.1550969	3.43696579	-0.3216474	0.748996642	0.90830222	-6.3118297
ENSCAFG00000023462	-0.119372	0.77807568	-0.3214136	0.749172768	0.90830222	-5.7068572
CACUL1	0.03734496	3.56886824	0.32140708	0.749177689	0.90830222	-6.5701848
ANKS6	0.04121096	3.60938574	0.3210892	0.749417193	0.90830222	-6.4425042
SLC35C1	0.04228873	4.85148293	0.32108171	0.749422842	0.90830222	-6.7852062
XPO4	-0.0399083	4.31720728	-0.3209072	0.749554304	0.90830222	-6.7288924
BDH1	-0.1444092	1.62351902	-0.3209049	0.749556055	0.90830222	-5.9835363
ERI1	0.06148524	2.84275454	0.32076317	0.749662872	0.90830222	-6.3364719
EYA1	-0.2976622	-1.7940801	-0.3207239	0.749692444	0.90830222	-5.3563763
C4H1orf131	-0.033514	3.91976181	-0.3206457	0.749751363	0.90830222	-6.645973
GABARAPL1	0.06030713	4.38956991	0.32054438	0.749827752	0.90830222	-6.7759675

ENSCAFG00000029845	-0.046844	2.8547518	-0.3204673	0.749885831	0.90830222	-6.3673654
ENSCAFG00000007154	-0.3173168	4.41665869	-0.3204498	0.749899041	0.90830222	-6.3103434
MAP3K10	0.06685387	2.57923995	0.32044568	0.749902138	0.90830222	-6.2401866
SEC16B	0.12835569	0.98127865	0.32044124	0.749905481	0.90830222	-5.6748627
SLC6A6	0.11470467	7.02594217	0.32041692	0.749923813	0.90830222	-6.8412654
TESK2	-0.0795118	0.54106429	-0.3203291	0.749989975	0.90830222	-5.909082
FOXO1	-0.0821762	4.11082739	-0.3201885	0.750095968	0.90830222	-6.7734657
RAD51D	-0.0587416	2.61633142	-0.3201871	0.750097039	0.90830222	-6.2678262
ENSCAFG00000014043	0.22356104	-0.5886494	0.31988913	0.750321619	0.90830222	-5.3876416
ENSCAFG00000017933	0.06798758	2.44042383	0.31986803	0.750337528	0.90830222	-6.2523436
INO80E	0.04171789	3.83747973	0.3197286	0.750442633	0.90830222	-6.5495209
ZMYM3	-0.0326531	4.7637456	-0.3196476	0.750503711	0.90830222	-6.7813495
RCHY1	-0.0348868	3.63102505	-0.3196369	0.750511748	0.90830222	-6.5789859
USP46	-0.0731733	2.05466355	-0.3195905	0.750546767	0.90830222	-5.9802939
EBNA1BP2	-0.0612862	5.72308947	-0.319524	0.750596839	0.90830222	-6.8840472
SNX30	0.04926443	3.65393624	0.31933499	0.750739364	0.90838742	-6.7047547
CBX4	0.04118413	4.3806969	0.3190714	0.750938102	0.90847572	-6.7353823
ANOS1	0.44565509	0.6971804	0.31894371	0.751034379	0.90847572	-5.3843927
EPRS	0.04638319	8.95991842	0.3189225	0.751050372	0.90847572	-6.7824363
PDE7B	-0.2697642	-0.0105961	-0.3187952	0.751146369	0.90847572	-5.5994997
FLRT3	-0.1058888	3.75815184	-0.3187599	0.751172965	0.90847572	-6.5623093
RP9	0.06179474	1.45919803	0.31855091	0.751330581	0.90849399	-5.9968866
MTFP1	-0.1312329	-0.2088735	-0.3184015	0.751443257	0.90849399	-5.6644602
MN1	-0.2180049	1.30881457	-0.31839	0.751451899	0.90849399	-5.6398809
SEC11A	-0.0255779	6.1101687	-0.3183573	0.751476573	0.90849399	-6.8986775
SS18L1	0.03850007	3.49739822	0.31825309	0.751555183	0.90850183	-6.5635655
AGMO	0.17823795	-0.2839229	0.3180362	0.751718767	0.90861238	-5.9043551
INCENP	-0.0627909	5.4923989	-0.3178423	0.751865009	0.90866949	-6.823959
PCNX3	0.02927673	6.40210247	0.31778227	0.751910295	0.90866949	-6.9039013
ENSCAFG00000000687	-0.1110537	-0.5051318	-0.3176448	0.752013979	0.9087076	-5.7414574
ENSCAFG00000004703	0.03345717	4.39063062	0.31744029	0.752168269	0.90879574	-6.7471745
HEATR5B	-0.0332623	5.5509012	-0.3172115	0.752340884	0.90879574	-6.8741026
KALRN	0.16153125	1.207602	0.31717539	0.752368111	0.90879574	-5.7840933
CSPG4	0.13632095	6.50680467	0.31716559	0.752375512	0.90879574	-6.8978887
DHRS1	-0.0442438	3.81572402	-0.3165031	0.752875389	0.90927665	-6.6336539
RAB3GAP2	0.02686557	6.30012652	0.31644659	0.752918027	0.90927665	-6.9030835
ZNF341	-0.0576239	1.98800224	-0.3163051	0.753024821	0.90931844	-6.1705551
ACTR6	0.04334798	3.88107949	0.31611863	0.753165526	0.90940117	-6.6501736
IRF2BP1	-0.0470506	3.50166936	-0.3158984	0.753331737	0.90947124	-6.512963
LMF2	-0.0472979	5.8149493	-0.315827	0.753385672	0.90947124	-6.8792115
ADAMTS2	0.13764039	6.80620218	0.31575475	0.753440169	0.90947124	-6.9010246
HSF2BP	-0.034082	3.1742414	-0.3155318	0.753608472	0.90958723	-6.4648153
ENSCAFG00000010669	-0.0295225	5.63043376	-0.3154186	0.753693875	0.90960315	-6.8766711
STK11IP	-0.0459942	3.22661217	-0.3151324	0.753909996	0.90977681	-6.5130738
RAB11A	0.02792927	6.12035027	0.31449922	0.754388031	0.9100484	-6.9046333
RHPN1	-0.0618023	2.81697249	-0.3144673	0.75441216	0.9100484	-6.3956246
ENSCAFG00000030187	-0.0538661	4.2400731	-0.3144567	0.754420164	0.9100484	-6.6964521
ALDH1L2	-0.054092	7.96374324	-0.3144515	0.754424054	0.9100484	-6.8581923
PGAP2	-0.0573994	2.69314362	-0.3141615	0.754643076	0.91022543	-6.369013
SEMA3G	-0.1599664	0.33017853	-0.3137828	0.754929056	0.91040566	-6.5468635

ENSCAFG00000025725	0.16621059	-1.4668539	0.31377223	0.754937052	0.91040566	-5.4531225
CLINT1	0.03079981	7.07298578	0.31360191	0.755065692	0.91047362	-6.8953889
AFF3	0.31960356	0.83354572	0.31270305	0.755744715	0.91115795	-5.5285466
XKR5	-0.2618427	-1.4552389	-0.3126591	0.755777891	0.91115795	-5.2726775
KRBA2	-0.0667973	2.24262788	-0.3124186	0.755959622	0.91128983	-6.1386776
MGARP	0.04361789	4.17317611	0.31219028	0.756132159	0.91141059	-6.7479362
ZNF175	-0.0504269	3.17547053	-0.3118748	0.756370586	0.91157417	-6.4967559
ECM2	0.12812308	5.85412254	0.31167464	0.756521841	0.91157417	-6.9006229
NETO1	-0.2632418	-2.0465051	-0.3115413	0.756622632	0.91157417	-5.3296971
ENSCAFG00000031628	-0.0450187	3.45324597	-0.3114392	0.756699786	0.91157417	-6.503235
EEF2K	-0.0817492	6.83063567	-0.3113926	0.756735037	0.91157417	-6.8844653
STK25	-0.0277862	5.96891316	-0.3112846	0.75681661	0.91157417	-6.8910411
ENSCAFG00000016006	0.02897373	5.43645498	0.31126439	0.756831923	0.91157417	-6.8592357
CEP97	0.0536176	3.13536594	0.31120104	0.756879806	0.91157417	-6.467774
TAPT1	0.03833824	4.9763893	0.31114893	0.756919198	0.91157417	-6.8136114
COX19	-0.0842253	0.84900105	-0.3109802	0.757046714	0.91164057	-5.8315598
ENSCAFG00000029288	0.05942702	2.51647193	0.31072721	0.757237999	0.91178375	-6.198175
ADAMDEC1	0.17545439	-2.6534402	0.31056283	0.757362277	0.91184623	-5.3874111
PIK3CD	0.10923641	2.1679155	0.31045976	0.757440206	0.91185289	-6.1805845
ANKRD33B	-0.1095867	1.44864302	-0.3102579	0.75759286	0.91189532	-5.9016986
RTCA	0.03052261	5.20924628	0.31019366	0.757641402	0.91189532	-6.8524291
SFI1	0.03091576	3.44568379	0.3101259	0.757692637	0.91189532	-6.5756796
TMEM184C	0.02983387	5.46036902	0.30985537	0.757897213	0.9120348	-6.8760944
TNFRSF14	-0.089731	2.02291801	-0.309722	0.757998077	0.9120348	-6.2165602
TAF8	0.05395869	2.76370335	0.30966522	0.758041014	0.9120348	-6.4618854
FAM13C	0.0542483	4.10129814	0.30945402	0.758200736	0.9120348	-6.6429505
SLC25A1	-0.0268948	5.85777614	-0.3092144	0.75838197	0.9120348	-6.8935023
C28H10orf71	0.18547311	-1.9679268	0.30909818	0.758469876	0.9120348	-5.2708114
AP4M1	0.04387997	3.57925974	0.30908979	0.75847623	0.9120348	-6.61682
FAM69A	-0.2202741	1.30150033	-0.3090572	0.758500846	0.9120348	-5.6491585
ENSCAFG00000022040	0.12635124	-0.9405767	0.30905047	0.758505966	0.9120348	-5.594576
ENSCAFG00000024887	-0.140039	-0.0787079	-0.308957	0.758576699	0.9120348	-5.6358237
PARP11	0.04986886	2.37949052	0.30885292	0.758655403	0.9120348	-6.2383941
TMBIM6	-0.0251346	8.38650984	-0.3087399	0.758740906	0.9120348	-6.8244754
LUZP1	0.05303616	6.41562501	0.30872811	0.758749815	0.9120348	-6.9066453
ENSCAFG00000001383	0.04928508	3.56562094	0.30855001	0.758884551	0.91210971	-6.5225511
ARRDC2	-0.0868862	3.44328973	-0.3084074	0.75899242	0.91215233	-6.8424869
BAG5	-0.0470836	6.22601386	-0.3082297	0.759126854	0.91220454	-6.9069621
CDCA5	-0.2495783	1.1686823	-0.3080407	0.759269889	0.91220454	-5.6855316
WDTC1	0.03143858	6.38651833	0.3079	0.759376349	0.91220454	-6.9063423
MAD1L1	-0.0405935	3.91974685	-0.3078311	0.759428488	0.91220454	-6.6278964
MVB12B	-0.0428706	4.23961796	-0.3077889	0.759460455	0.91220454	-6.6447564
ENSCAFG00000031862	0.04728957	2.4447902	0.30777572	0.759470391	0.91220454	-6.3000924
RGS7	-0.0928037	4.21567471	-0.3075456	0.759644515	0.91231206	-6.761153
EIF2S1	0.03705993	6.42080377	0.30746601	0.759704765	0.91231206	-6.9072954
THAP6	-0.0432205	3.39795431	-0.3073229	0.759813101	0.91234997	-6.5431063
ENGASE	-0.106732	0.82393245	-0.3071651	0.75993249	0.91234997	-5.8408807
OAT	-0.0406531	6.09762402	-0.3071028	0.759979652	0.91234997	-6.9072848
ALOX15B	0.10453138	4.0368592	0.30704149	0.760026058	0.91234997	-6.8039976
COPS3	0.03513572	5.53782701	0.30660736	0.760354667	0.91260516	-6.8706908

RETREG3	0.03805887	5.07287677	0.30656921	0.760383548	0.91260516	-6.861518
WDR90	-0.0389661	5.78363491	-0.3063465	0.760552141	0.9126346	-6.9037414
LACTB2	0.0496147	6.00948341	0.30624933	0.760625704	0.9126346	-6.9059452
RANBP17	-0.0366591	3.1817709	-0.3061424	0.76070663	0.9126346	-6.4412452
SNRPG	0.16415412	-1.2439366	0.30603452	0.760788337	0.9126346	-5.461093
COL9A2	0.15386757	1.71075269	0.3059695	0.760837567	0.9126346	-5.9372021
PTPRQ	0.21533484	-1.9368187	0.30581853	0.760951877	0.9126346	-5.3148188
RMND1	0.02638456	3.90818832	0.30578835	0.760974725	0.9126346	-6.6444985
LGMN	0.05415133	6.8359929	0.30577121	0.760987708	0.9126346	-6.9059029
COX5A	-0.1262975	0.80379051	-0.3053528	0.761304559	0.91292767	-5.8076915
ETF1	0.03600704	5.04115605	0.30525172	0.761381084	0.91293252	-6.8316715
VWA5A	-0.2088605	0.02830419	-0.304957	0.761604263	0.912934	-5.8209236
TTC8	0.05619194	3.38491117	0.30487296	0.761667938	0.912934	-6.5105103
LARP7	0.0300831	6.04576594	0.30473035	0.761775956	0.912934	-6.9041045
ZDHHC12	0.05886504	4.28392854	0.30467253	0.761819753	0.912934	-6.7154837
NIPSNAP1	-0.0540293	3.00124855	-0.3046392	0.761845029	0.912934	-6.4019318
PHB	-0.0333941	5.17743729	-0.3046161	0.761862518	0.912934	-6.8407823
PRRC2B	-0.0371937	6.86792729	-0.3044893	0.761958576	0.912934	-6.9016336
VPS4B	0.0225419	5.38015715	0.30444511	0.761992011	0.912934	-6.8670921
ENSCAFG00000014647	-0.0425945	3.5289512	-0.3043066	0.762096901	0.912934	-6.6436579
ZNF620	-0.0342408	3.55409315	-0.3042541	0.762136715	0.912934	-6.6308558
BMT2	-0.0391199	4.05007817	-0.3041635	0.762205316	0.912934	-6.7228654
ALOX15	0.16600071	0.96896776	0.30410184	0.762252055	0.912934	-6.2698653
PSMD3	-0.0283889	6.88402213	-0.3038142	0.762469987	0.91310819	-6.9051674
NECTIN2	0.05913942	4.96818153	0.30366794	0.76258079	0.91315407	-6.8195134
ATP5MC1	0.04820723	4.97847755	0.30334732	0.762823726	0.91331315	-6.8040746
ENSCAFG00000031354	-0.1015087	-0.3464266	-0.3033012	0.762858654	0.91331315	-5.6171448
ANKRD13C	-0.0307181	7.218334	-0.3031669	0.762960436	0.9133176	-6.8999099
ARHGAP18	-0.0827659	3.05541667	-0.3030779	0.763027908	0.9133176	-6.41276
ARRDC1	0.05485542	4.04490099	0.30300927	0.763079899	0.9133176	-6.697384
ENSCAFG00000032479	-0.1443776	-0.8728396	-0.3027587	0.7632698	0.91340284	-5.5346702
FOXF2	-0.1991982	2.05839304	-0.3026854	0.763325318	0.91340284	-6.668332
FAN1	-0.023323	5.24104348	-0.302466	0.763491641	0.91340284	-6.8562062
ENSCAFG00000000686	0.09671367	5.65542926	0.30240557	0.763537448	0.91340284	-6.8408699
SLC7A7	-0.1501308	-0.0719874	-0.3023923	0.763547478	0.91340284	-5.6481465
PAM	0.06350321	7.53365053	0.3023362	0.763590024	0.91340284	-6.8284508
ENG	-0.0792996	6.51497974	-0.3022456	0.763658724	0.91340284	-6.908588
ENSCAFG00000019542	0.04474213	3.60576003	0.30206572	0.763795057	0.91347916	-6.622247
PTPN9	-0.0223999	7.33595109	-0.3016132	0.764138125	0.91372057	-6.8782449
RAE1	-0.0229906	5.63712708	-0.3016081	0.764141989	0.91372057	-6.8869533
RWDD4	0.04833675	2.73308238	0.3012809	0.764390068	0.91381463	-6.3336767
ZBTB8OS	0.04130754	2.67183204	0.3011881	0.764460427	0.91381463	-6.3311014
C10H12orf56	-0.1249885	-0.3473251	-0.3010539	0.764562196	0.91381463	-5.634918
ARHGEF26	-0.1992753	1.66583259	-0.3010095	0.764595882	0.91381463	-5.7493326
ENSCAFG00000012274	-0.0390886	3.36072564	-0.3010017	0.764601768	0.91381463	-6.5633303
AP1G1	0.02394392	6.75015238	0.30081493	0.764743408	0.91381463	-6.9053095
SLC27A4	-0.0348481	4.85639595	-0.3007096	0.764823283	0.91381463	-6.7747608
MPV17L2	0.06114253	2.41031705	0.3003046	0.765130439	0.91381463	-6.1779792
ACAT1	0.03187308	5.92276185	0.30026891	0.765157513	0.91381463	-6.9065771
TGDS	-0.0404685	3.99355265	-0.3000671	0.765310547	0.91381463	-6.6827497

LETM1	-0.0317457	6.25913505	-0.2999964	0.765364237	0.91381463	-6.9075925
TOR4A	0.0710046	1.59198143	0.29995957	0.765392148	0.91381463	-5.9925672
STK16	-0.0326382	4.77961447	-0.2998484	0.765476513	0.91381463	-6.8049072
GALNT12	-0.1155185	2.3549773	-0.2997845	0.765524954	0.91381463	-6.1795939
ARHGEF7	-0.0341304	5.95528716	-0.2997016	0.765587823	0.91381463	-6.9063519
PYGL	0.05516857	4.70748599	0.29966556	0.765615179	0.91381463	-6.8685
SMG7	0.03197507	6.71148865	0.29964133	0.765633558	0.91381463	-6.8991774
FHOD3	0.33512405	1.61845531	0.29961677	0.765652189	0.91381463	-5.586055
ENSCAFG00000011658	-0.1617836	-0.4401498	-0.2995534	0.765700266	0.91381463	-5.452798
TTC39C	-0.0703325	4.38537179	-0.2995155	0.765729011	0.91381463	-6.7488101
ZNF512B	0.03996956	4.56519956	0.29938785	0.765825858	0.91381463	-6.7451687
AP5M1	-0.0370496	4.49197771	-0.2992433	0.765935538	0.91381463	-6.7414706
ZNF684	-0.0833725	1.48049562	-0.2992366	0.765940625	0.91381463	-6.025123
NUMBL	0.0505484	5.45026884	0.29920867	0.765961804	0.91381463	-6.8791092
LTBR	-0.0263798	7.30759943	-0.2990572	0.766076699	0.91386515	-6.8938599
TRMT1L	0.04856152	4.58563204	0.29873801	0.766318925	0.91405013	-6.7972666
GFRA2	0.31849037	-0.0332757	0.29861875	0.766409422	0.91405013	-5.2890897
CLDN34	-0.1284707	-0.1857844	-0.2984952	0.7665032	0.91405013	-5.7250882
ROR1	0.12634532	4.8883898	0.29844404	0.766542002	0.91405013	-6.7709652
CA2	-0.2822612	-1.0671979	-0.2983747	0.766594597	0.91405013	-5.4608053
SUMO2	0.0220502	5.64767193	0.29793602	0.766927569	0.91430183	-6.8935555
ENSCAFG00000000370	0.03043001	5.67402038	0.29790532	0.766950867	0.91430183	-6.8852196
MEF2C	-0.2713335	2.51541845	-0.2977397	0.767076613	0.91434409	-5.9066516
ENSCAFG00000031202	-0.0515341	2.57659629	-0.2975533	0.767218081	0.91434409	-6.3592333
CDC23	0.04546947	4.23038872	0.29750589	0.767254065	0.91434409	-6.681546
CISD2	0.05512191	2.84003715	0.29747609	0.76727668	0.91434409	-6.3115303
CACNA2D2	-0.1012325	4.41896862	-0.2971643	0.767513362	0.91447848	-6.80365
ZADH2	-0.0415686	4.15503075	-0.2971218	0.76754564	0.91447848	-6.7187769
GCLC	0.03282132	4.97520873	0.29704066	0.76760725	0.91447848	-6.8053808
CSE1L	0.04131826	6.72060215	0.29634913	0.768132319	0.91501747	-6.9093794
ENSCAFG00000030257	0.0503236	2.18764113	0.29611346	0.768311292	0.91514412	-6.1579614
PSMD9	0.02813392	5.76351011	0.29587049	0.768495814	0.91527735	-6.8960233
UBA3	0.02559915	6.33305881	0.2956599	0.768655756	0.91538129	-6.9102126
COG2	0.02060643	5.77838703	0.29552342	0.768759424	0.9154182	-6.8969176
PALM3	0.15715935	2.3939057	0.29531463	0.768918016	0.9155205	-6.1865396
TRAF1	-0.1541331	0.8646167	-0.2951884	0.769013878	0.91554809	-5.7788878
MSX1	0.09476346	3.98124877	0.29492046	0.769217448	0.91563319	-6.7243958
HEY1	-0.2081246	1.96485765	-0.294903	0.769230739	0.91563319	-5.8803174
DGKQ	-0.0573022	2.90786256	-0.2946519	0.769421499	0.91577371	-6.4181583
CFAP300	-0.0694265	1.62752346	-0.2945049	0.769533142	0.91582005	-6.0083386
LDHB	-0.0400949	4.41217866	-0.2941828	0.769777908	0.91600394	-6.7670551
ENSCAFG00000019677	-0.0483065	4.21681825	-0.294028	0.769895527	0.91600394	-6.7526022
CCSAP	0.06991124	2.55095519	0.29395552	0.769950615	0.91600394	-6.1808966
TOR2A	0.03761106	3.26884113	0.29391878	0.76997854	0.91600394	-6.44879
ENSCAFG00000026605	0.0926601	0.77218528	0.29368874	0.770153359	0.91601268	-5.7763347
FAM107A	-0.1699427	-1.2756894	-0.2936197	0.770205861	0.91601268	-5.5936518
KCNJ12	0.15835861	-1.6970313	0.29355344	0.770256184	0.91601268	-5.4195254
HDAC4	-0.0415676	3.54412088	-0.2934245	0.770354214	0.91601268	-6.5765492
IMPAD1	0.04691018	5.11792179	0.293384	0.770384962	0.91601268	-6.861007
YTHDF1	-0.0267016	5.96030071	-0.293335	0.77042222	0.91601268	-6.905501

PIK3C3	-0.0249003	5.45330928	-0.2930063	0.770672035	0.91622321	-6.8929503
ELOB	0.02799591	5.12373666	0.29276516	0.770855359	0.91635467	-6.8438354
ELOVL1	-0.0360825	5.25754825	-0.2926494	0.770943378	0.91637282	-6.8651342
C9H9orf16	0.07954698	4.41649888	0.29191365	0.771502746	0.91687209	-6.7946136
FAM171A1	0.05417808	7.60824504	0.29189648	0.771515803	0.91687209	-6.8468928
USP27X	-0.0816173	0.70103479	-0.2917827	0.771602301	0.91687209	-5.8265788
DPP7	-0.0598006	6.05777395	-0.291714	0.771654578	0.91687209	-6.9116595
TRAM2	-0.0354782	6.88369888	-0.2914905	0.771824554	0.91697282	-6.9114633
CAPRIN1	0.02021235	8.29140451	0.29133756	0.771940833	0.91697282	-6.8401074
PSAT1	-0.0930686	6.82075534	-0.2913153	0.771957746	0.91697282	-6.9101101
ACP2	-0.029898	6.16159986	-0.2910659	0.772147411	0.91701633	-6.9063945
STRAP	0.03071377	7.21180916	0.2910016	0.772196345	0.91701633	-6.895882
PLEKHM2	0.04865208	7.47244001	0.29094682	0.772238012	0.91701633	-6.9048034
GANAB	-0.019351	8.08822681	-0.2908843	0.772285589	0.91701633	-6.8691668
CARD9	-0.1371141	-0.0555542	-0.2907095	0.772418505	0.91708771	-5.7175094
TENM3	0.45528904	0.84911758	0.29052053	0.772562266	0.91711998	-5.3395086
SDHAF2	-0.0243713	5.17782384	-0.2904824	0.772591311	0.91711998	-6.8473829
PTK7	0.0956993	6.75670385	0.29026087	0.772759799	0.91719358	-6.9124143
FJX1	0.15244973	1.20991045	0.29020942	0.77279894	0.91719358	-6.5329752
ZNF71	0.08770524	1.20907216	0.28997398	0.77297806	0.91722174	-5.9535035
PTPN12	-0.0531799	7.00602949	-0.2899631	0.772986314	0.91722174	-6.8904977
ENSCAFG00000020027	0.25694008	0.61711444	0.28988293	0.773047333	0.91722174	-5.41692
IGSF10	-0.1573108	-2.1986623	-0.2897954	0.773113941	0.91722174	-5.2751972
MTURN	-0.1314805	-0.2879536	-0.2896404	0.773231896	0.91727528	-5.6352481
DNAJC16	-0.0363253	3.48297927	-0.2892852	0.773502137	0.91750946	-6.5465961
PGM3	0.03963495	6.28613109	0.28902184	0.773702572	0.91766079	-6.9103911
HARS2	-0.0380449	3.33665781	-0.2886625	0.773976023	0.91789856	-6.5574842
TTC14	0.04552344	6.3700382	0.28856694	0.774048786	0.91789856	-6.9113053
DAP3	0.02520284	5.55064071	0.28825907	0.774283123	0.91795037	-6.8672118
PHF21A	-0.036082	5.01442244	-0.2881518	0.774364752	0.91795037	-6.8578656
NT5C2	0.03512987	5.49018311	0.28808271	0.774417378	0.91795037	-6.8726928
PIEZO1	-0.0456057	7.68794863	-0.28806	0.774434652	0.91795037	-6.8760532
SPRYD4	0.05881592	1.79804933	0.28800727	0.774474805	0.91795037	-6.1193718
ENSCAFG00000029818	-0.1345749	-0.9504914	-0.2879351	0.774529732	0.91795037	-5.5646301
SLC25A12	-0.0379724	5.62931401	-0.2877578	0.774664748	0.91802401	-6.888362
ZSCAN29	0.04197872	3.51213691	0.2872402	0.775058812	0.91819623	-6.5821065
THNSL1	-0.0482229	3.67289184	-0.2872368	0.775061415	0.91819623	-6.5729541
DAXX	0.0384018	4.58116178	0.2871339	0.775139749	0.91819623	-6.7504685
EVC2	0.07479587	6.37490032	0.2871243	0.775147059	0.91819623	-6.9090174
ARMH3	0.03503457	3.653918	0.2870865	0.775175843	0.91819623	-6.5399528
NPHP4	0.04839957	4.36199028	0.28699246	0.775247455	0.91819623	-6.7617349
ENSCAFG00000023983	-0.0946272	0.51043744	-0.2868126	0.775384426	0.91827212	-5.7785875
PHPT1	0.03866359	4.46666747	0.28653579	0.775595221	0.91843541	-6.7719867
SLC9A3R1	-0.0866049	2.57988681	-0.2863331	0.775749581	0.91852676	-6.2947936
GATAD2A	-0.0243332	6.97641534	-0.286243	0.775818212	0.91852676	-6.9059173
ENSCAFG00000014256	-0.2490778	-1.3975863	-0.2860419	0.775971355	0.91856447	-5.4808519
FIBP	0.03492099	5.87048119	0.2860097	0.775995912	0.91856447	-6.898323
SYT4	-0.3410789	0.87557215	-0.2857577	0.776187834	0.91864649	-5.6129943
PDE1C	0.18250012	-1.8861705	0.28572725	0.776211067	0.91864649	-5.4259252
ZSWIM8	-0.0230433	7.23803533	-0.2854717	0.776405749	0.91874846	-6.8989176

ELP3	-0.0203312	5.273587	-0.2852202	0.776597349	0.91874846	-6.8692429
ZBTB5	0.05176385	2.53326055	0.28520499	0.77660894	0.91874846	-6.2821734
TMEM86A	0.11276098	2.42113507	0.28514147	0.776657329	0.91874846	-6.021203
TULP3	-0.0314963	5.94244158	-0.2850933	0.776694002	0.91874846	-6.9111303
ENSCAFG00000030425	-0.1051735	1.82952446	-0.2850397	0.776734867	0.91874846	-5.8801956
STX11	-0.1194587	1.99438312	-0.2848719	0.776862699	0.91875205	-6.0962288
IL11	-0.129523	0.01190317	-0.2848443	0.776883782	0.91875205	-5.7111197
HMGB3	-0.098336	3.53902155	-0.2844356	0.7771952	0.91889806	-6.4404208
ENSCAFG00000025389	0.02708376	4.12804209	0.28436216	0.777251155	0.91889806	-6.683176
LYPD6	-0.2013312	-1.8905332	-0.2843362	0.77727097	0.91889806	-5.4040202
ENSCAFG00000001339	0.11329039	-0.9378118	0.28420614	0.777370051	0.91889806	-5.6185319
FOXO4	-0.0500821	3.04777079	-0.2841781	0.777391444	0.91889806	-6.529118
ENSCAFG00000016149	0.19933913	5.6951819	0.28410786	0.777444949	0.91889806	-6.7382618
HOXC10	0.35001966	-0.0424242	0.2837071	0.777750399	0.91917283	-5.6358044
DNM3	-0.1516027	-0.0851515	-0.283481	0.77792276	0.91928369	-5.7965632
ROMO1	0.10811831	-0.3700941	0.28339255	0.777990162	0.91928369	-5.542027
ENSCAFG00000025811	0.12632659	3.34248249	0.28305374	0.778248447	0.91932333	-6.3355297
ATF7IP2	0.18564249	-2.1109788	0.28302665	0.778269095	0.91932333	-5.4812035
TMEM220	-0.0900794	1.16575271	-0.2828911	0.778372447	0.91932333	-5.9092128
REV3L	-0.0475723	7.18644672	-0.2827311	0.778494443	0.91932333	-6.8955854
SLC7A11	-0.0861393	3.81055033	-0.28267	0.778541043	0.91932333	-6.5347133
SESN2	0.061123	5.92767666	0.2826344	0.778568147	0.91932333	-6.8940425
HS3ST2	0.25739379	-0.8438936	0.28259302	0.778599701	0.91932333	-5.3671485
SNAP29	-0.0170894	6.77093051	-0.2825827	0.778607598	0.91932333	-6.9099713
ENSCAFG00000014638	0.01695851	6.9636302	0.28233642	0.778795351	0.91933276	-6.9066571
MAPKAP1	0.02307322	5.64288741	0.28226207	0.778852044	0.91933276	-6.8963771
NKAP	0.02984489	3.65783231	0.28225522	0.77885727	0.91933276	-6.6267371
PGGHG	-0.0471877	4.20865598	-0.2821893	0.778907529	0.91933276	-6.7701323
HDX	-0.0991148	0.00856289	-0.2820335	0.779026336	0.91933497	-5.6592627
ALG14	-0.0921493	1.53280483	-0.2819111	0.779119716	0.91933497	-5.9341029
RBL1	-0.0492861	4.06811837	-0.2818324	0.779179672	0.91933497	-6.6454667
CDPF1	-0.0864988	0.62508113	-0.281804	0.779201342	0.91933497	-5.8720927
NUP155	-0.0446392	5.40088765	-0.2813694	0.779532828	0.91963993	-6.8547829
PTN	-0.378956	0.78759304	-0.2810034	0.779811995	0.91988311	-5.4648653
AURKA	0.12671648	2.59319552	0.28088547	0.779901954	0.91990308	-6.1952971
SLC19A2	0.07594932	3.67272177	0.28066165	0.780072696	0.92001832	-6.6616541
MAT2A	-0.033884	6.00724019	-0.2803771	0.780289764	0.92010758	-6.9102407
FANCD2	-0.1443089	3.67492864	-0.2801402	0.780470557	0.92010758	-6.3089121
ARRDC4	0.04781529	2.71036158	0.28012136	0.780484898	0.92010758	-6.6077869
C12H6orf120	0.05892814	4.07134663	0.27997566	0.780596073	0.92010758	-6.6814219
KRBA1	-0.0502587	3.05201597	-0.2799484	0.780616848	0.92010758	-6.4592638
ENSCAFG00000005637	0.02729446	6.80411634	0.27992492	0.780634783	0.92010758	-6.9106435
VIM	0.03488816	13.2209984	0.27989225	0.780659712	0.92010758	-6.4607644
TET3	-0.0533281	3.53232645	-0.2797394	0.780776311	0.92015891	-6.655456
TSPAN13	0.16425631	-1.2679293	0.27947761	0.780976122	0.92017652	-5.6722794
ENSCAFG00000009973	0.0445251	2.00663751	0.2794646	0.780986053	0.92017652	-6.196592
ENSCAFG00000012755	-0.0305648	3.4556906	-0.2794327	0.781010417	0.92017652	-6.5825651
SV2A	-0.0740434	2.00522708	-0.2792472	0.781151939	0.92025718	-6.1120787
PHEX	0.19038057	0.88767219	0.27912679	0.781243861	0.9202794	-5.7697425
IREB2	-0.024081	5.92769881	-0.2790235	0.781322721	0.92028624	-6.8997473

MFN2	-0.025113	6.34427025	-0.278538	0.781693298	0.92063663	-6.9157638
ENSCAFG00000015771	0.10069089	-0.045495	0.27831831	0.781860974	0.9206637	-5.8623729
B3GALNT1	-0.0543308	3.65908047	-0.2783164	0.781862461	0.9206637	-6.5979912
ENSCAFG00000030502	-0.030907	5.14744638	-0.2780419	0.782071965	0.92067633	-6.8687663
SLC35E4	0.05932192	2.7616628	0.27797705	0.782121495	0.92067633	-6.3934282
CCNE1	-0.0674414	2.29305757	-0.2779341	0.782154302	0.92067633	-6.0883164
DSP	0.17132418	-1.835979	0.27791934	0.782165559	0.92067633	-5.4215139
USF3	-0.0294498	4.99764106	-0.2777015	0.782331909	0.92078609	-6.8516681
PPT1	-0.0203776	6.4387687	-0.2775858	0.782420218	0.92080399	-6.9159705
ENSCAFG00000032308	0.03843199	4.14190413	0.27722396	0.782696512	0.92104309	-6.6823729
ENSCAFG00000000314	-0.0402248	5.26530387	-0.2768387	0.782990705	0.92125173	-6.8718077
ABCC10	-0.0368279	4.8426603	-0.2767926	0.783025924	0.92125173	-6.7404236
ENSCAFG00000012933	0.07271944	2.03702332	0.27654754	0.783213094	0.92125173	-6.135196
TOR1AIP1	0.01978254	6.67228944	0.27644816	0.783288995	0.92125173	-6.91498
EPHX3	-0.0498295	2.65766489	-0.2764172	0.783312624	0.92125173	-6.3685628
MYCT1	0.22601803	-0.2444229	0.27634572	0.783367237	0.92125173	-5.9970626
TLCD1	0.07592664	1.73629031	0.27632144	0.783385783	0.92125173	-6.0966049
ADAT1	-0.0483766	3.14470249	-0.2756607	0.783890544	0.92162683	-6.4102904
PPP2CB	-0.0242699	6.81210117	-0.2755744	0.783956473	0.92162683	-6.9158111
ADD1	-0.0208528	7.7233389	-0.2754636	0.78404106	0.92162683	-6.8753236
VPS13A	-0.0280054	7.18060586	-0.2753727	0.784110558	0.92162683	-6.9031259
GRINA	-0.0462021	6.44132044	-0.2753169	0.784153166	0.92162683	-6.9166045
MAGEH1	-0.0336728	4.41738094	-0.2752441	0.784208816	0.92162683	-6.7837157
ZFP82	0.05336153	2.73307856	0.27523347	0.784216925	0.92162683	-6.3495567
MAEA	-0.0208505	5.50216011	-0.2749251	0.784452538	0.92180028	-6.898161
TSPAN14	-0.0931614	3.44272764	-0.274751	0.784585575	0.92180028	-6.5147426
AKAP10	-0.0263528	4.08376638	-0.274704	0.784621464	0.92180028	-6.7094616
RNLS	0.05778708	3.8451035	0.27465722	0.784657243	0.92180028	-6.591343
TLL1	-0.234553	-0.7324166	-0.2744635	0.784805261	0.92186004	-5.5698957
NR4A1	0.12171468	5.23508488	0.27439912	0.784854479	0.92186004	-6.822425
AQP3	0.1943072	-1.4111568	0.27425117	0.784967549	0.92186409	-5.3134114
SLC18B1	0.07695407	1.77469533	0.27409915	0.785083738	0.92186409	-6.0865578
PLPP5	0.04133182	3.02399152	0.27407167	0.785104742	0.92186409	-6.4144018
HSD17B4	-0.0177211	6.15555406	-0.2740116	0.785150676	0.92186409	-6.914415
CALCOCO1	0.0568206	5.52112632	0.27387067	0.785258367	0.92189374	-6.9123523
MACF1	0.03896172	8.01885401	0.27378701	0.785322312	0.92189374	-6.8420723
PDCL	-0.0259281	4.56179695	-0.2734272	0.785597357	0.92204591	-6.7918109
WDR46	0.04152533	4.61819641	0.27342591	0.785598339	0.92204591	-6.7695338
SEC22A	-0.0446399	3.22393687	-0.2732972	0.785696722	0.92207546	-6.5003455
ANK2	-0.0740713	6.11746334	-0.2729951	0.785927655	0.92221828	-6.9031495
TK2	-0.0656426	2.33191891	-0.2729465	0.785964851	0.92221828	-6.2904384
KMT5A	0.02152762	5.31720958	0.27278685	0.7860869	0.92224148	-6.8710847
LTBP2	0.11907933	10.4774857	0.2727291	0.786131053	0.92224148	-6.6452708
TDG	-0.0399012	4.58984972	-0.2722894	0.786467286	0.92255	-6.7783659
GSK3B	0.02780871	6.24720169	0.27218326	0.786548424	0.92255927	-6.91686
STAMBP	0.03341928	3.07973046	0.27197685	0.786706273	0.92265849	-6.4405794
UBAP2	0.02378269	6.76966034	0.27161965	0.786979457	0.92281255	-6.9153119
NCBP3	-0.0330599	4.50408146	-0.2716135	0.786984156	0.92281255	-6.7672892
BPGM	-0.0505344	2.23041367	-0.2713768	0.787165232	0.92289092	-6.1163033
ENTPD3	0.10659219	6.27078348	0.27128541	0.787235098	0.92289092	-6.8853527

NUFIP2	-0.0250543	5.74398804	-0.2712387	0.787270792	0.92289092	-6.9081875
CD40	0.13535627	1.52445777	0.27065338	0.787718577	0.92332991	-5.8833147
BORCS6	0.11497582	0.30768162	0.27053856	0.787806419	0.92334695	-5.7419349
FHIT	0.11308541	-0.6272819	0.27038607	0.787923076	0.92339775	-5.636269
STARD3NL	0.03005309	4.74307587	0.27017908	0.788081449	0.92345807	-6.8155738
ENSCAFG00000032088	0.10591706	2.32908797	0.27010133	0.788140935	0.92345807	-6.2269096
USP6NL	0.04881559	5.24259633	0.26997059	0.788240973	0.92345807	-6.8060098
ENSCAFG00000021239	-0.1146329	1.19526637	-0.2699355	0.788267795	0.92345807	-5.9356281
CIART	0.07604446	2.13589389	0.26959137	0.788531158	0.92368069	-6.1585195
TMEM51	-0.1554787	0.36111115	-0.2689469	0.789024388	0.92412313	-5.5691364
MAGI3	-0.0481851	3.48990802	-0.2689058	0.7890558	0.92412313	-6.5849799
ENSCAFG00000019517	0.03291545	5.33681243	0.26878388	0.789149151	0.92412313	-6.8763515
ENSCAFG00000000451	-0.3027436	-0.3934548	-0.2687144	0.789202331	0.92412313	-5.3104375
ENSCAFG00000025660	0.10375139	0.13265438	0.26853546	0.789339303	0.92419761	-5.7113793
DENND1A	-0.0313466	4.61841964	-0.2682739	0.789539561	0.92430377	-6.7856414
PCDHB15	0.12521453	0.56509441	0.26813454	0.789646212	0.92430377	-5.7952137
SNRPA	-0.0496272	3.8298988	-0.2681044	0.789669263	0.92430377	-6.5450235
SERAC1	-0.0471716	3.48907961	-0.2680022	0.789747515	0.92430377	-6.5459869
PEG10	-0.11838	3.84245025	-0.2679078	0.789819775	0.92430377	-6.6386241
SLC5A2	0.15944586	-1.542234	0.26784189	0.789870258	0.92430377	-5.4759647
EIF2AK4	-0.0221497	5.77476782	-0.2677025	0.789976977	0.92434278	-6.9128952
SLC27A6	-0.1597618	-2.7255714	-0.2668956	0.790594872	0.9248568	-5.3517629
NR2C1	0.03615748	4.25205204	0.26688696	0.790601462	0.9248568	-6.7058735
THUMPD1	-0.0357528	3.45233578	-0.2668411	0.790636556	0.9248568	-6.6036051
SLC4A11	0.15141862	0.25435433	0.26655398	0.790856473	0.92490755	-5.743517
KIF24	-0.1252997	1.90713511	-0.2665067	0.790892697	0.92490755	-5.9547218
ENSCAFG00000018194	0.07119575	4.28756302	0.26649685	0.790900228	0.92490755	-6.6389708
TM4SF1	0.09865985	6.95599589	0.26631081	0.791042715	0.92498831	-6.8863361
UBE2N	0.11348077	-1.0445684	0.26592251	0.791340144	0.9252312	-5.5644646
CAT	0.05270639	5.14299573	0.26584784	0.791397342	0.9252312	-6.8838598
GPR68	-0.1993753	0.32467405	-0.265645	0.791552748	0.92528547	-5.7266367
PPP4R4	-0.0638235	1.29705435	-0.2655955	0.791590685	0.92528547	-5.9599467
ZNF583	-0.0538962	1.50132265	-0.2654702	0.791686636	0.92531176	-6.0135355
SLBP	0.046082	3.42741686	0.26527743	0.791834327	0.92537738	-6.5339992
ENSCAFG00000007015	0.08568337	0.43413495	0.26512033	0.791954685	0.92537738	-5.8041758
SETD7	-0.0216715	5.89748233	-0.2651093	0.791963176	0.92537738	-6.9072101
POLR2G	0.04688767	3.3040622	0.26494535	0.792088758	0.92543827	-6.484027
ENSCAFG00000008287	-0.0932388	0.28386217	-0.2643787	0.792522983	0.92583588	-5.6173297
SNAI1	-0.0718569	3.63105143	-0.2643094	0.792576081	0.92583588	-6.672254
TCF21	0.30736337	-0.1695043	0.26421305	0.792649913	0.92583627	-5.5590726
CAST	-0.0237697	8.29155335	-0.263855	0.792924295	0.92600193	-6.841972
CCDC92	0.03749628	3.41993477	0.26378487	0.792978075	0.92600193	-6.5377011
DUSP8	0.08712072	2.31561001	0.26365696	0.793076111	0.92600193	-6.0711722
PCGF3	-0.0301042	4.54363328	-0.2636443	0.793085803	0.92600193	-6.7642733
BIRC5	-0.1411317	3.11767438	-0.2634361	0.793245412	0.92610244	-6.1929532
ARSE	0.14671482	1.27706586	0.26310264	0.793501018	0.926315	-6.0354852
PDCD5	-0.0288255	4.49807356	-0.2624736	0.793983311	0.92679212	-6.7574093
ENSCAFG00000028220	0.10822604	2.61126173	0.26214145	0.794237954	0.92697638	-6.0778635
CPNE3	-0.0301653	6.69281676	-0.2620757	0.794288353	0.92697638	-6.9165046
POGLUT1	-0.0206895	6.13727504	-0.2617607	0.794529897	0.92709843	-6.918637

RPA3	0.05955725	1.14910398	0.26167738	0.794593823	0.92709843	-5.8708492
AGAP3	0.03716515	4.56169113	0.26147066	0.794752351	0.92709843	-6.8366581
BAALC	0.19390878	-0.2674506	0.26137214	0.794827914	0.92709843	-5.3975261
CPEB2	-0.0469035	5.10033398	-0.2611421	0.795004324	0.92709843	-6.8566663
CCDC28B	-0.072557	1.94186538	-0.2610256	0.795093725	0.92709843	-6.0618326
FYTTD1	0.05293167	1.84469691	0.26097293	0.7951341	0.92709843	-6.0009577
ERICH5	0.11743009	-0.0997491	0.26094498	0.795155544	0.92709843	-5.6359585
TIMM29	0.05698282	1.55057002	0.26088018	0.795205243	0.92709843	-6.0927851
ATG9A	-0.0289161	5.85524114	-0.2607368	0.795315207	0.92709843	-6.9091381
IL18BP	0.08197907	2.9701468	0.26066764	0.795368275	0.92709843	-6.6898594
PDIA5	-0.0450753	5.25449298	-0.2606652	0.795370133	0.92709843	-6.8975385
D2HGDH	0.05668972	4.03227668	0.2605525	0.795456605	0.92709843	-6.6620073
GLOD4	0.02425009	4.76057275	0.26051431	0.795485895	0.92709843	-6.8172654
WDR83	0.03304331	2.78767187	0.26049987	0.795496974	0.92709843	-6.3371896
TMCC3	0.16117894	0.44868959	0.26032007	0.795634909	0.92711113	-5.6738628
CYTH2	-0.035705	4.45657453	-0.2602938	0.795655074	0.92711113	-6.7489715
SFRP2	0.21617333	7.08059388	0.25997606	0.79589883	0.9271901	-5.9412933
ENSCAFG00000031494	0.17935657	0.02596232	0.25989025	0.795964666	0.9271901	-5.6347574
RNPC3	0.03563006	4.36075578	0.25988676	0.795967349	0.9271901	-6.7744014
MRPL20	-0.0336383	4.72347502	-0.2596437	0.796153856	0.9271901	-6.778265
ABCC9	-0.3300191	0.4365155	-0.2596182	0.796173431	0.9271901	-5.3346428
CNDP2	0.02483255	6.16297859	0.25953488	0.796237338	0.9271901	-6.9178494
FBXO3	-0.0257357	4.23469532	-0.2595339	0.796238114	0.9271901	-6.7047173
ENSCAFG00000029403	0.02231904	6.57649021	0.25909748	0.796572982	0.92745022	-6.9205904
DUSP7	0.04098473	5.44298866	0.25905086	0.796608759	0.92745022	-6.8860727
ZNF18	0.02555756	4.71619278	0.25890976	0.796717045	0.92749056	-6.8020001
TRIM23	-0.0356783	3.72704743	-0.25872	0.796862693	0.92757439	-6.6443579
ZCCHC4	-0.0343364	3.08818341	-0.2584757	0.797050208	0.92761294	-6.4295997
NAPEPLD	0.06715504	2.26400164	0.25840992	0.797100674	0.92761294	-6.3952818
ENSCAFG00000005131	0.02529723	5.0653963	0.25838898	0.797116742	0.92761294	-6.8436618
ENSCAFG00000013811	-0.0322699	3.38219942	-0.2582432	0.797228671	0.92765749	-6.5213568
DNAL4	-0.0672727	1.29291832	-0.2580297	0.797392491	0.92776241	-6.0283547
ARFGEF2	-0.0228346	6.01187395	-0.2579084	0.797485601	0.92778504	-6.9190552
LRGUK	0.06845006	0.96638115	0.25769903	0.797646367	0.92788637	-5.9325291
RABEP1	0.0267399	5.51758815	0.25751933	0.797784321	0.92796115	-6.9009204
PPP1R9B	0.03417255	8.08723843	0.25724425	0.797995521	0.92812111	-6.8541579
DHRSX	0.03321737	3.89811475	0.25689754	0.798261729	0.92822868	-6.6126162
RIMS1	-0.1023442	0.97000767	-0.2568611	0.798289678	0.92822868	-5.7288827
FYN	-0.0450583	7.69169102	-0.2568359	0.798309091	0.92822868	-6.9067985
TRPM4	-0.0507338	3.20372798	-0.2564691	0.798590745	0.92841847	-6.4961199
YPEL5	0.03373278	6.1513452	0.25634251	0.798687941	0.92841847	-6.9197279
AMOTL1	-0.0310755	5.17935543	-0.2562676	0.798745481	0.92841847	-6.8828228
MPP6	0.09488651	3.17532067	0.25618026	0.798812545	0.92841847	-6.4571404
ZSCAN30	0.0679935	1.24760478	0.25600337	0.7989484	0.92841847	-5.9681321
BBOF1	0.07712405	1.09070584	0.25596565	0.798977368	0.92841847	-5.9208427
UGGT1	-0.0267014	7.32131984	-0.2559515	0.798988264	0.92841847	-6.9000672
WAPL	-0.0245096	6.71846725	-0.255765	0.799131488	0.92849924	-6.917832
DHTKD1	-0.1017594	1.89953257	-0.2555962	0.79926113	0.92856421	-5.9242539
FAM200A	0.05389687	1.94709148	0.25516654	0.799591182	0.92884244	-6.0693531
SQSTM1	-0.0397247	7.32504682	-0.2550924	0.799648099	0.92884244	-6.8964273

FADS1	0.06151693	7.71206618	0.25466003	0.799980303	0.92899276	-6.8698004
PLB1	0.23507585	-0.846441	0.25465209	0.799986411	0.92899276	-5.381302
MMP16	0.12553177	-1.5975644	0.25458367	0.800038977	0.92899276	-5.2997899
RNF141	0.04266421	3.51053616	0.25454001	0.800072522	0.92899276	-6.6599457
YPEL4	0.10576763	-0.3113235	0.25399633	0.800490269	0.92933688	-5.8050029
TUBGCP2	-0.0190531	7.0653204	-0.2539623	0.800516443	0.92933688	-6.9110975
ADHFE1	-0.0651873	2.23196735	-0.253602	0.800793303	0.92957189	-6.3061762
MTMR12	0.04663356	2.22491509	0.25343477	0.800921821	0.92957189	-6.2759365
ENSCAFG00000018701	-0.1217977	0.16079616	-0.2534101	0.800940749	0.92957189	-5.6130666
ZDHHC14	0.14179888	-1.7078186	0.25331473	0.80101408	0.92957189	-5.4647807
RNF24	-0.068843	1.827471	-0.2529919	0.801262217	0.92972281	-6.2885005
ICA1L	-0.0480249	2.59514195	-0.2529535	0.801291749	0.92972281	-6.2032576
DESI1	0.0463214	3.98125196	0.25270617	0.801481839	0.92985772	-6.6281403
FOXN3	-0.1013559	-0.3931148	-0.2525272	0.8016194	0.92993166	-5.6671183
RPP14	-0.0351663	2.372206	-0.252419	0.801702567	0.92993466	-6.2206376
GGCX	0.02550275	6.24645092	0.25223439	0.801844511	0.92993466	-6.9217787
SNRPD2	-0.0283551	5.75584229	-0.2520976	0.801949702	0.92993466	-6.9105232
RBBP9	0.04173967	1.99411103	0.25198006	0.802040039	0.92993466	-6.1900403
TBC1D7	-0.0276213	4.64335564	-0.2519658	0.802050988	0.92993466	-6.7831854
STX18	-0.0341223	4.14383231	-0.2518173	0.802165186	0.92993466	-6.7209826
ZNF142	0.0193468	5.41179433	0.25173704	0.802226892	0.92993466	-6.8630212
MTIF3	-0.0267353	4.42009355	-0.2516974	0.802257347	0.92993466	-6.7529229
ENSCAFG00000004640	-0.1032938	-0.307969	-0.2516478	0.802295519	0.92993466	-5.4926257
NUDT9	-0.0230586	5.72744987	-0.2515636	0.802360265	0.92993466	-6.902241
NOL6	-0.040656	4.79063095	-0.2514208	0.802470032	0.92997631	-6.8008708
BSCL2	0.02199545	6.00006247	0.25096432	0.802821077	0.93025938	-6.9101407
RXRA	-0.0430478	5.99203506	-0.2509111	0.802861995	0.93025938	-6.9222047
ENSCAFG00000032629	0.0934283	0.69035882	0.25060582	0.803096789	0.93044584	-5.8099549
ABRAXAS1	-0.0351121	4.01497584	-0.2502477	0.803372249	0.93055317	-6.6294562
TMEM201	0.04511723	2.78644408	0.25017034	0.803431732	0.93055317	-6.3740909
DNA2	-0.1647172	1.51190913	-0.2500935	0.803490842	0.93055317	-5.7906543
RNGTT	0.02299649	4.98851961	0.25008774	0.803495267	0.93055317	-6.841088
ZC2HC1C	-0.111378	0.30241024	-0.2500051	0.803558815	0.93055317	-5.7650987
NAGA	0.02692127	6.14001092	0.24986712	0.803664976	0.93059055	-6.9201907
TRIB3	-0.0463299	5.55981559	-0.2496094	0.803863209	0.93073453	-6.807822
MRPL51	0.05522397	4.73324255	0.24949294	0.803952822	0.93075273	-6.8051558
PACSIN2	0.03120786	5.85656787	0.24888789	0.804418338	0.93115252	-6.9159488
STRBP	0.04308103	3.36724124	0.24878328	0.804498827	0.93115252	-6.4744828
ENSCAFG00000008311	-0.0388846	3.81592425	-0.2487559	0.804519916	0.93115252	-6.602392
CORO2B	-0.1500409	4.52965415	-0.2485261	0.804696709	0.93116487	-6.7812776
CACTIN	0.02933219	4.28968898	0.24852307	0.804699059	0.93116487	-6.7405321
CPED1	0.14369496	2.03083168	0.2484538	0.804752365	0.93116487	-6.5974691
ENSCAFG00000002179	-0.0285548	4.57275424	-0.2481601	0.804978405	0.93134086	-6.7544736
ENSCAFG00000005758	0.09855334	0.51594399	0.24806297	0.805053131	0.93134112	-5.6766793
SIX4	-0.0969255	3.82710122	-0.2479676	0.805126501	0.93134112	-6.6203829
VEGFB	0.0498878	4.99204313	0.24773168	0.805308101	0.93146564	-6.7904396
ENPP4	-0.05886	3.06905706	-0.2475754	0.805428361	0.9315192	-6.519439
ATP5PF	0.02646482	4.61669784	0.24741707	0.805550254	0.93157464	-6.7809834
CNOT4	0.02686766	4.57570486	0.24729022	0.805647892	0.93159965	-6.8029272
ORC5	0.03359723	4.14438542	0.24710596	0.80578973	0.93159965	-6.7395268

CMSS1	0.05677892	3.05903806	0.24710073	0.805793758	0.93159965	-6.4181244
ERP29	-0.0305417	5.31232757	-0.246851	0.80598598	0.93166846	-6.882078
ENSCAFG00000025044	0.02527446	6.07026941	0.24681764	0.806011684	0.93166846	-6.9169633
SEMA4G	0.03480934	3.2393052	0.24651049	0.806248147	0.93166846	-6.4675707
NGDN	-0.0345206	4.10202114	-0.2464493	0.806295222	0.93166846	-6.6967331
TNNI3	-0.098051	-0.7627335	-0.2464267	0.806312628	0.93166846	-5.7036006
FNDC1	0.24573616	4.30871776	0.24642496	0.806313997	0.93166846	-6.03323
ENSCAFG00000030169	0.12138123	-0.5792466	0.24635088	0.806371034	0.93166846	-5.6499566
BCL7C	-0.044148	3.87711044	-0.2460185	0.806626931	0.93178768	-6.5917866
TSTD2	-0.0311355	3.95957409	-0.2460027	0.806639129	0.93178768	-6.6294455
SOX17	0.14331111	-1.6499029	0.24592864	0.806696149	0.93178768	-5.6584337
TSPYL1	-0.0184503	4.04806072	-0.2458202	0.806779624	0.93179866	-6.6715957
ORC3	-0.0262693	4.76861833	-0.2456988	0.80687314	0.93182122	-6.8249022
ENSCAFG00000029633	0.02560268	6.95702073	0.2455715	0.806971161	0.93184899	-6.9082808
ENSCAFG00000022297	-0.0578811	6.0897769	-0.2454119	0.807094065	0.93190282	-6.9230066
MBD1	0.02365729	4.43916022	0.24531882	0.80716575	0.93190282	-6.7858451
KCTD6	-0.0845554	0.71586757	-0.2448114	0.807556584	0.9322686	-5.8646748
TBC1D17	0.03598068	5.61735257	0.24468439	0.807654384	0.93229606	-6.9007033
SLC39A1	-0.0195054	6.53597266	-0.2444155	0.80786149	0.93238568	-6.9247877
C12H6orf132	0.10834195	-1.0973021	0.2443914	0.807880065	0.93238568	-5.3514867
NTRK3	-0.1616092	-1.7530522	-0.2441596	0.808058646	0.93250634	-5.3611308
SLC2A4RG	0.04795852	3.78179995	0.24400329	0.808179044	0.93255984	-6.6365758
ENSCAFG00000026748	0.09670852	-0.5165165	0.24305865	0.808906863	0.93331417	-5.6753602
ELAC2	0.04202659	4.74167209	0.2428632	0.809057472	0.93340245	-6.8501313
RIC1	0.03761954	7.55049681	0.24274251	0.809150475	0.93342425	-6.8927018
DYNLT1	0.03344702	4.42146886	0.24250096	0.809336626	0.93355349	-6.6982862
NT5E	-0.1648475	-0.2708938	-0.2423042	0.8094883	0.9336063	-5.7194425
SLC2A5	0.16321226	-0.2443436	0.24224921	0.809530649	0.9336063	-5.6958319
PSMC5	-0.0197111	5.82718071	-0.2420136	0.809712219	0.93373021	-6.90792
AKAP6	-0.1439982	-1.0559573	-0.2418232	0.80985903	0.93381402	-5.6006813
C10H2orf49	0.02618071	4.13129855	0.24155239	0.81006774	0.93388342	-6.7287761
ENSCAFG00000031771	-0.0402185	3.28064046	-0.2415126	0.810098378	0.93388342	-6.4665498
FAM53B	-0.0405258	6.36738259	-0.2414565	0.810141645	0.93388342	-6.925356
PHRF1	0.02265421	6.03124955	0.24121814	0.810325406	0.93393921	-6.9218857
ZBTB45	0.08074579	1.36696438	0.24114514	0.810381688	0.93393921	-5.9428243
ENSCAFG00000006771	0.05171689	1.00120724	0.2411052	0.81041248	0.93393921	-5.9479916
SDR39U1	0.03871123	3.31352423	0.24044587	0.810920821	0.93414554	-6.4217262
CPT1C	-0.0570705	3.80474827	-0.2404049	0.810952375	0.93414554	-6.7951261
ATG7	0.0299029	4.46338849	0.24034327	0.810999926	0.93414554	-6.7993757
ADGRG4	-0.123481	-2.6493299	-0.2402402	0.811079383	0.93414554	-5.388005
FAM110A	-0.0688534	1.17376923	-0.2402308	0.811086653	0.93414554	-5.9958561
MTF1	-0.0318038	3.54139603	-0.2401674	0.811135574	0.93414554	-6.6497659
KANSL2	-0.0196379	4.91914912	-0.2400819	0.811201468	0.93414554	-6.8399258
SFMBT1	-0.055847	2.75510127	-0.2400812	0.811201996	0.93414554	-6.4163184
POLR3F	0.03781675	3.35545354	0.24000733	0.811258974	0.93414554	-6.425501
ABLIM3	0.17362813	3.02423546	0.23906116	0.81198869	0.93490032	-6.4211791
POC5	0.02829778	3.99276044	0.23870998	0.812259576	0.93508201	-6.6393117
SENP8	-0.0516841	1.63744905	-0.2386641	0.812294966	0.93508201	-6.0300249
PELI1	-0.0505123	3.49146854	-0.2383582	0.812530912	0.93526815	-6.623848
PPIP5K1	0.03053285	4.17418893	0.23801177	0.812798208	0.93544063	-6.7229967

DNAAF2	0.03391113	4.49268074	0.23797148	0.812829287	0.93544063	-6.7295167
STOX2	-0.1709413	0.62130388	-0.2377872	0.812971485	0.93547204	-5.5802228
CHURC1	-0.0417511	2.7339153	-0.2375804	0.813131035	0.93547204	-6.2870478
TMEM131	-0.0239444	7.23204359	-0.2375388	0.813163099	0.93547204	-6.9186716
NABP2	0.0242054	6.8390907	0.23722005	0.813409085	0.93547204	-6.9244843
ENSCAFG00000004578	0.07187486	1.88826711	0.23710183	0.813500314	0.93547204	-5.9489494
C11H5orf24	0.0210997	4.57302807	0.23706251	0.813530659	0.93547204	-6.7991024
INPP5K	0.03082688	4.92755781	0.23701314	0.813568754	0.93547204	-6.8475386
SCFD2	-0.0331951	3.40943321	-0.2369589	0.813610587	0.93547204	-6.5821306
RRAGB	0.09799306	-0.2716661	0.2369456	0.813620873	0.93547204	-5.6371563
DDX19A	0.01701246	5.82106317	0.23692353	0.813637909	0.93547204	-6.9135369
ENSCAFG00000023355	-0.1258265	-1.0128961	-0.2368149	0.813721764	0.93547204	-5.5152127
ENSCAFG00000032029	0.07082642	2.62495842	0.23677672	0.813751206	0.93547204	-6.3392203
ENSCAFG00000002874	-0.099904	-0.6485109	-0.2366849	0.813822053	0.93547204	-5.5992344
TCP11L1	0.04249686	4.15193878	0.23593122	0.814403786	0.93605531	-6.6205349
VWA7	-0.0975073	-0.1290595	-0.2354224	0.8147966	0.93616864	-5.697937
TMEM102	-0.0487691	2.03994121	-0.2353652	0.814840745	0.93616864	-6.1078031
ENSCAFG00000002155	0.11196887	-2.8484493	0.23518136	0.814982653	0.93616864	-5.3151512
LRRC7	0.29954371	-0.8968235	0.23514112	0.815013722	0.93616864	-5.284758
TCTN3	-0.0232804	3.35951319	-0.2350708	0.815067982	0.93616864	-6.5427879
MTFR2	-0.0618094	3.52720247	-0.2349383	0.81517032	0.93616864	-6.3918092
CLN5	0.02042413	6.13151727	0.23486743	0.81522503	0.93616864	-6.9227115
TMA16	-0.0290441	3.65344734	-0.234859	0.815231501	0.93616864	-6.634026
RPS6KA1	-0.0761783	1.65073347	-0.2348534	0.815235866	0.93616864	-5.9756454
NAV2	-0.1064257	4.24946627	-0.2348408	0.815245621	0.93616864	-6.5548551
WNT2B	0.13482036	-1.9075177	0.2346061	0.815426809	0.93629135	-5.3493524
CCDC12	0.04152304	2.22884565	0.23450978	0.815501179	0.93629139	-6.2317729
TRIM62	-0.0405757	2.76943847	-0.2340504	0.815855879	0.93657468	-6.41811
MOGS	0.03233112	4.04462435	0.23399768	0.815896631	0.93657468	-6.661923
ACSS1	-0.0469499	5.74014382	-0.2335075	0.816275232	0.93661554	-6.8960897
FAM214A	-0.0439959	5.25776627	-0.2333755	0.816377133	0.93661554	-6.8825463
SPIDR	-0.0329056	3.65448489	-0.2333236	0.81641722	0.93661554	-6.6213413
IL17RD	-0.0891873	1.87636001	-0.2332772	0.81645306	0.93661554	-6.3558167
FAM180A	0.16134891	2.53721955	0.23325237	0.816472256	0.93661554	-6.5986195
LRRC8E	0.05384494	2.5374616	0.23320129	0.816511709	0.93661554	-6.1775779
ENSCAFG00000029869	0.06002151	1.19686579	0.23307086	0.816612454	0.93661554	-5.930738
ENSCAFG00000025091	-0.0774487	0.60455619	-0.2330651	0.816616903	0.93661554	-5.8207634
ENSCAFG00000031912	-0.1212577	-1.8670267	-0.2330188	0.816652692	0.93661554	-5.557932
MRPL50	-0.0403358	3.54987999	-0.2329888	0.816675811	0.93661554	-6.5432485
POLR2B	0.02122827	6.56910268	0.23282201	0.816804681	0.93667806	-6.9269045
PDCL3	-0.028578	5.09857934	-0.2325498	0.817014982	0.93683393	-6.8300708
DNAJB14	-0.0402643	3.12751949	-0.2324164	0.817118007	0.93686678	-6.4312867
MOB3B	-0.1458452	0.04586718	-0.231825	0.817574946	0.93711021	-5.688432
FIP1L1	-0.0172254	6.29487238	-0.2318105	0.817586152	0.93711021	-6.9275702
ZNF354B	0.02913389	3.67018231	0.23178539	0.817605538	0.93711021	-6.6160016
ENSCAFG00000004551	0.03164439	2.66611333	0.23175644	0.817627911	0.93711021	-6.3242836
SIX1	-0.0980938	4.02359944	-0.2316127	0.817738995	0.93715225	-6.7357293
CMC1	-0.0424602	2.04193749	-0.231412	0.81789409	0.93717159	-6.139173
TTLL11	0.04631325	2.15920407	0.23139825	0.817904678	0.93717159	-6.2023083
TPP1	-0.0489796	5.95927012	-0.2306767	0.818462297	0.93772522	-6.9227865

ANGPTL1	0.25177407	1.19236063	0.23043684	0.818647681	0.93781727	-5.3683061
PLAA	-0.0237881	5.86871232	-0.2303801	0.818691545	0.93781727	-6.9153984
ENSCAFG00000028476	0.02502433	5.82537392	0.22998081	0.819000171	0.93808549	-6.9186781
SEC24B	-0.0180073	6.30387468	-0.2297722	0.819161418	0.9381227	-6.9279393
FDXR	-0.0492372	1.67198772	-0.229726	0.819197151	0.9381227	-6.0662511
FBXO28	-0.0174949	5.54920975	-0.229643	0.819261293	0.9381227	-6.9117968
BRPF3	0.03657886	3.73434189	0.2295534	0.819330569	0.9381227	-6.6413627
MLLT3	-0.0503978	3.35865038	-0.2289722	0.819779872	0.93855183	-6.5918732
ENSCAFG00000002455	-0.0924359	-0.074666	-0.2288382	0.819883542	0.93858521	-5.5738431
C5H11orf87	-0.1810492	-0.3309535	-0.228733	0.81996488	0.93859302	-5.312024
SNUPN	0.03774113	4.22857968	0.22851402	0.820134176	0.93870151	-6.7734642
ALKAL1	-0.1199709	-2.9395916	-0.2281849	0.820388716	0.93890753	-5.2862235
SKIL	-0.0539303	5.61092074	-0.2280867	0.820464636	0.93890911	-6.8797332
CLIC2	0.04883167	2.58424165	0.22796322	0.820560117	0.93893307	-6.6137974
PPAT	-0.0357178	5.18484113	-0.2276595	0.820795033	0.93895843	-6.8652608
MDM2	0.02715511	6.74383846	0.22749703	0.820920665	0.93895843	-6.9238177
NCBP2	0.02002327	4.98812237	0.22749361	0.82092331	0.93895843	-6.8466155
MPHOSPH6	0.04002566	3.24738554	0.22729754	0.821074962	0.93895843	-6.443522
ENSCAFG00000000153	-0.0591967	1.56090604	-0.2270775	0.821245134	0.93895843	-6.0701491
ENSCAFG00000015065	-0.0250864	5.36738407	-0.2270711	0.82125015	0.93895843	-6.8590437
RSL24D1	-0.0221815	4.29749185	-0.2270309	0.821281235	0.93895843	-6.7620734
GLB1L	-0.0484058	4.67394407	-0.2268656	0.821409091	0.93895843	-6.8180023
ISCA1	0.02816781	3.46306314	0.22679377	0.821464641	0.93895843	-6.594182
PPOX	-0.036687	4.0901926	-0.2267149	0.821525659	0.93895843	-6.6834887
DDI2	-0.0516641	2.38801295	-0.2266015	0.821613354	0.93895843	-6.2280613
PCBP3	-0.0710368	3.59288259	-0.2265414	0.82165985	0.93895843	-6.580494
ST8SIA2	0.1976536	-0.4129297	0.22653368	0.821665845	0.93895843	-5.4580642
UPF3B	-0.0412718	3.40549805	-0.2264372	0.821740469	0.93895843	-6.5179642
ATP10D	-0.0598106	6.39205223	-0.2261767	0.821942012	0.93895843	-6.9249568
OPLAH	-0.0506881	4.71935589	-0.2260753	0.822020463	0.93895843	-6.8311575
ENSCAFG00000026428	0.07662194	-0.0840418	0.22599838	0.822079983	0.93895843	-5.7139857
PRPSAP2	-0.0485366	2.54216104	-0.2259897	0.822086665	0.93895843	-6.3606001
CFAP410	0.05908246	2.82541018	0.22573313	0.822285213	0.93895843	-6.4560834
ENSCAFG00000029966	-0.1576847	0.22984614	-0.2257193	0.822295884	0.93895843	-5.5410692
TK1	-0.1251437	4.22638765	-0.2256406	0.822356811	0.93895843	-6.5262475
OSBPL8	0.02188799	6.29017741	0.22561065	0.822379985	0.93895843	-6.9279429
SLC7A6OS	-0.0407915	2.54888704	-0.2255086	0.822458943	0.93895843	-6.293019
GFPT2	0.08929418	6.99008966	0.22546856	0.822489934	0.93895843	-6.9154739
DHDDS	0.02613781	5.46719981	0.22543206	0.822518173	0.93895843	-6.8850137
TAOK2	-0.0252788	6.04919673	-0.2254291	0.822520426	0.93895843	-6.9264781
NAA50	-0.0290022	4.42693512	-0.2252921	0.822626457	0.93899437	-6.7453913
OTULINL	-0.0921643	1.4107021	-0.2250693	0.8227989	0.93903933	-6.0217093
CADPS2	0.16644643	2.67677759	0.22504197	0.822820043	0.93903933	-6.3790288
DTX3	0.03478838	6.22697999	0.22495222	0.822889501	0.93903933	-6.9285892
ANKRD16	-0.0412432	3.98349808	-0.2246198	0.82314673	0.93924778	-6.6226413
PGM2	-0.0261531	6.46099859	-0.2243372	0.823365507	0.93926615	-6.9279822
HACL1	0.04634932	3.85236461	0.22431728	0.823380906	0.93926615	-6.7278504
MON1A	0.02822363	3.84826701	0.22423466	0.82344486	0.93926615	-6.6599974
TMEM69	-0.0269299	3.90811005	-0.2242137	0.823461105	0.93926615	-6.6594116
OMD	0.13603124	4.80077218	0.22382526	0.823761756	0.93946042	-6.9295528

GCNT1	0.14034182	-0.4508658	0.22380093	0.82378059	0.93946042	-5.4040358
KPNA5	0.03244101	3.27761048	0.22369085	0.823865801	0.93947254	-6.5028479
CYP2S1	-0.1897662	-0.8588856	-0.223322	0.824151334	0.93968909	-5.4425795
GRK2	-0.0217084	5.26973909	-0.2232401	0.824214716	0.93968909	-6.8908307
CTDSP2	-0.0359997	5.64411161	-0.2231073	0.824317572	0.93968909	-6.9130452
CEP126	0.0864761	0.18688115	0.22306009	0.824354116	0.93968909	-5.8470309
EXOC7	-0.0162968	6.31579559	-0.2225521	0.824747438	0.93999971	-6.9294703
SRP72	-0.023653	8.26777434	-0.2223694	0.824888886	0.93999971	-6.8612866
RCC1L	-0.0274505	3.76274754	-0.2223384	0.824912952	0.93999971	-6.5857372
MOSPD1	-0.0337382	2.94037144	-0.2221588	0.82505202	0.93999971	-6.3939247
SACS	0.05271577	7.02731052	0.22215629	0.825053937	0.93999971	-6.9300995
MAST3	0.02752742	3.88180308	0.2221299	0.825074372	0.93999971	-6.7793277
HMCN2	0.08504516	-1.2241643	0.22167254	0.825428566	0.94031819	-5.6338547
SEZ6	0.09848533	-0.5743356	0.22153699	0.825533545	0.94035273	-5.7267445
ASCC3	0.02387362	6.62345216	0.22113472	0.825845118	0.94056662	-6.9297286
PPP2R2A	0.02681442	6.49704808	0.22110175	0.825870656	0.94056662	-6.9291891
TIGD2	0.04389257	2.33028428	0.22080584	0.826099863	0.94072989	-6.2476551
IGF1R	0.03358393	6.95291617	0.22072383	0.826163391	0.94072989	-6.8986942
IRS1	0.08361115	3.40958874	0.2202999	0.8264918	0.94081701	-6.5042804
IGFALS	-0.153765	-1.0833036	-0.2202222	0.826552028	0.94081701	-5.7080056
ARFIP2	0.02353529	4.45913925	0.22018251	0.826582741	0.94081701	-6.7836222
TBC1D22A	-0.0228812	5.42890865	-0.2201415	0.826614536	0.94081701	-6.8937464
WASF3	-0.0843759	4.58485416	-0.2198058	0.826874572	0.94081701	-6.7439607
ZC3H12C	-0.04058	2.6146805	-0.219725	0.826937186	0.94081701	-6.4544135
RAB35	-0.0180117	6.42887071	-0.2196486	0.826996366	0.94081701	-6.9305874
ENSCAFG00000000061	-0.0300031	3.502437	-0.2194548	0.827146588	0.94081701	-6.5920282
SLC16A7	0.15083653	-1.3801964	0.21941716	0.827175735	0.94081701	-5.5817379
ANO6	0.01966419	8.37409462	0.21930947	0.827259176	0.94081701	-6.8573025
ZSWIM1	-0.0408633	2.41803559	-0.2192865	0.827277011	0.94081701	-6.2250866
DGCR2	0.03516445	4.73108839	0.21923536	0.827316607	0.94081701	-6.8125581
POLDIP3	-0.0240315	5.25367951	-0.2192303	0.82732054	0.94081701	-6.8706744
YIF1A	-0.0224041	5.10025115	-0.2191421	0.827388901	0.94081701	-6.8604682
SMOC2	-0.2267329	2.59855917	-0.2191332	0.827395779	0.94081701	-5.787124
ZNF771	-0.0514127	2.02279361	-0.2186757	0.827750337	0.94081701	-6.1296605
STX17	0.04536341	2.28101094	0.21859763	0.827810812	0.94081701	-6.2245512
CLUH	-0.0278652	6.31141211	-0.2185728	0.827830031	0.94081701	-6.93083
TAF9B	-0.0809473	2.97810428	-0.2184602	0.827917334	0.94081701	-6.2469505
SLC30A4	-0.0266796	3.55043509	-0.2182987	0.828042474	0.94081701	-6.5653086
ENSCAFG00000007343	-0.0256622	7.09575884	-0.2182926	0.82804719	0.94081701	-6.9178919
NDUFB9	0.02652786	4.8274291	0.21813875	0.828166461	0.94081701	-6.8344268
FBN1	0.05617517	10.8263102	0.21812238	0.82817915	0.94081701	-6.663859
NEK9	0.02138653	6.14014177	0.21804966	0.828235518	0.94081701	-6.9264647
ENSCAFG00000028797	0.08714864	-1.906398	0.21804776	0.828236989	0.94081701	-5.5342925
CDK5	0.02709274	3.52413884	0.21804715	0.828237459	0.94081701	-6.6017067
NUDT5	-0.0181604	4.64287702	-0.2180225	0.828256573	0.94081701	-6.8302982
ENSCAFG00000008236	-0.1086762	4.28066974	-0.2175136	0.828651062	0.94118023	-6.5576249
ZNF692	0.03381958	3.29279427	0.21734305	0.82878325	0.94118285	-6.5611549
ENPEP	-0.2208837	1.93058688	-0.2172927	0.828822283	0.94118285	-5.5640037
NDEL1	0.02224257	4.92732293	0.21722144	0.828877525	0.94118285	-6.8531638
NET1	-0.0632953	3.06291912	-0.2167411	0.829249938	0.94152084	-6.4993441

ADCY3	-0.128294	2.84175145	-0.2163768	0.829532375	0.94158774	-6.0185769
METTL27	-0.0842595	0.75296813	-0.2163336	0.829565865	0.94158774	-5.6925756
CTBP1	-0.021927	5.96520311	-0.2162761	0.829610491	0.94158774	-6.9200725
SLCO2A1	0.16016818	-0.4258053	0.21614531	0.829711886	0.94158774	-5.706276
VPS9D1	0.037982	3.0812108	0.21596212	0.829853937	0.94158774	-6.5003075
CYP7B1	0.08260967	3.49168013	0.21595465	0.82985973	0.94158774	-6.4157052
IGSF9B	0.07044251	1.12439566	0.21589429	0.829906541	0.94158774	-6.233652
CAND2	-0.1285764	0.13199035	-0.2156012	0.830133794	0.94158774	-5.496393
VRK2	-0.034612	3.5007723	-0.2155931	0.830140082	0.94158774	-6.5084035
LEPR	-0.1159332	4.1865463	-0.2154802	0.830227679	0.94158774	-6.344091
GAL3ST4	0.08790516	1.88153842	0.21548004	0.83022779	0.94158774	-5.7272831
ENSCAFG00000023972	0.10047586	1.23213753	0.21546982	0.830235713	0.94158774	-5.8240203
MAVS	0.02903187	5.35475761	0.21541188	0.830280649	0.94158774	-6.884618
ENSCAFG00000029758	-0.0380911	3.40010543	-0.2152557	0.830401772	0.94164032	-6.4708882
MTFR1L	-0.0212399	4.60504791	-0.2150688	0.83054676	0.94171177	-6.7982467
PITHD1	-0.02221	4.29338398	-0.2149817	0.830614302	0.94171177	-6.769316
RFFL	-0.0367041	2.83821586	-0.2147733	0.830775922	0.94181023	-6.4207377
COLGALT1	-0.0228221	7.73431493	-0.2143802	0.83108084	0.94207112	-6.9062408
SF3A2	0.02123715	6.05563246	0.21403378	0.831349597	0.94222921	-6.922804
NOTUM	0.14014938	-1.7115986	0.21400758	0.831369918	0.94222921	-5.4126348
MRPL47	0.02740408	3.44799851	0.21351097	0.831755207	0.94239456	-6.5642323
BEX4	-0.0423494	2.08549309	-0.2134888	0.831772383	0.94239456	-6.2391353
DPY30	-0.0259685	3.35816507	-0.2133299	0.831895697	0.94239456	-6.5253988
ENSCAFG00000017143	0.0218105	9.17185137	0.21322705	0.831975499	0.94239456	-6.8170512
ARFGAP1	0.01901976	5.53286528	0.21320631	0.831991592	0.94239456	-6.9029596
MFSD3	-0.0599247	3.39315642	-0.213059	0.832105868	0.94239456	-6.5549466
RASA3	-0.0302635	6.24185218	-0.2130543	0.832109572	0.94239456	-6.9318006
SNAPC4	-0.0235424	4.64221783	-0.2127989	0.832307701	0.94239456	-6.8174655
TAPBPL	-0.0584195	2.42126308	-0.212763	0.832335544	0.94239456	-6.2798839
ING2	0.03244279	2.69993007	0.21274053	0.832353014	0.94239456	-6.4001975
HKR1	0.11227482	-1.2269175	0.21260123	0.832461118	0.94239456	-5.4835131
PAPSS2	0.06931061	6.15172573	0.21257086	0.832484688	0.94239456	-6.8999034
TIMM10B	-0.0582537	1.98779244	-0.212556	0.832496235	0.94239456	-6.091145
ENSCAFG00000001608	-0.0871745	-0.6732515	-0.2124434	0.832583583	0.94239456	-5.5048874
UBE2J2	-0.0167893	4.98875993	-0.2123732	0.832638063	0.94239456	-6.8550019
ZNF572	-0.0732678	0.48619098	-0.212025	0.83290835	0.94259486	-5.7820249
ENSCAFG00000002139	0.06297655	3.46764366	0.2117534	0.83311911	0.94259486	-6.3097218
MED29	0.0257763	4.05955978	0.21167421	0.833180577	0.94259486	-6.6806193
NCKAP1L	0.12779293	-1.633106	0.21159692	0.833240566	0.94259486	-5.4088777
METTL5	-0.0231398	4.51987266	-0.2115898	0.833246104	0.94259486	-6.7620904
TTC9C	0.0294135	3.0606914	0.21156669	0.833264037	0.94259486	-6.426312
MTMR11	0.05130925	3.77060708	0.2111561	0.833582746	0.94270817	-6.7444466
FAH	-0.0847981	2.38817825	-0.2111392	0.833595832	0.94270817	-6.2856267
ENSCAFG00000028195	-0.060833	2.43394856	-0.2111121	0.833616908	0.94270817	-6.3190013
TAF1B	0.02593651	3.63243128	0.21105199	0.833663568	0.94270817	-6.6054227
ENSCAFG00000022570	0.15143613	-2.483821	0.21093196	0.833756743	0.9427289	-5.3261782
CTNNA1	-0.0182315	8.57012563	-0.2105337	0.834065943	0.94274927	-6.819725
HIST1H2BB	-0.1441856	-0.8726181	-0.2105139	0.834081285	0.94274927	-5.4112897
ENSCAFG00000031732	-0.0619857	1.11196679	-0.210341	0.834215571	0.94274927	-5.9419302
ENSCAFG00000023074	-0.1691983	1.24757997	-0.2102599	0.834278484	0.94274927	-5.8633931

EIF1	0.02013802	8.59115861	0.2102392	0.834294572	0.94274927	-6.8350384
BMPR2	-0.0447637	6.39055312	-0.210122	0.834385606	0.94274927	-6.9319787
SPIRE1	0.03319836	3.53363079	0.21010877	0.834395844	0.94274927	-6.643459
HERC1	-0.0236776	5.8859268	-0.209884	0.834570332	0.94274927	-6.9248901
CLN6	0.03501648	4.25788358	0.20977858	0.834652223	0.94274927	-6.6799745
OSBPL9	-0.0295154	6.52975143	-0.2097196	0.834698042	0.94274927	-6.9326418
MASTL	0.08904295	2.53255405	0.20969102	0.834720218	0.94274927	-6.1854579
CLEC2L	-0.1291578	-1.1130781	-0.2096617	0.83474296	0.94274927	-5.5865117
CHCHD7	-0.0312247	2.40810545	-0.2096029	0.834788646	0.94274927	-6.4722959
PSME3	-0.0227555	5.26023821	-0.2095592	0.834822589	0.94274927	-6.8685876
SCYL1	0.02132114	5.53144253	0.20930624	0.835019018	0.94288656	-6.9068588
ATXN1L	-0.0271165	4.07122324	-0.2088835	0.835347323	0.94312078	-6.6445027
FAM199X	0.04711312	1.85832009	0.20884631	0.835376199	0.94312078	-6.1803943
TTF1	0.02511867	3.97199105	0.20871295	0.83547978	0.94315319	-6.7290491
SCG3	-0.0629745	1.75401889	-0.2084118	0.835713708	0.94316963	-6.2288198
LMX1B	-0.2081317	-0.9875692	-0.2083914	0.835729552	0.94316963	-5.4982109
ZDHHC5	-0.0173841	8.02891041	-0.2082427	0.835845003	0.94316963	-6.8666093
PDCD2L	-0.0220101	3.87722264	-0.2080848	0.835967652	0.94316963	-6.6194891
KANSL3	-0.0222301	7.11238176	-0.2080753	0.835975032	0.94316963	-6.9264077
UBE2K	-0.0246111	5.05898209	-0.2079151	0.836099514	0.94316963	-6.8359675
CLDND1	0.01991046	5.43349473	0.20778238	0.836202597	0.94316963	-6.9057301
FGF23	0.10447121	-3.0296274	0.20774915	0.836228412	0.94316963	-5.3062951
ENSCAFG00000008627	-0.0617738	2.34784355	-0.2076402	0.836313073	0.94316963	-6.3397103
BCL2L11	0.04519517	4.47985218	0.20754327	0.836388347	0.94316963	-6.742485
GOT2	-0.0268676	5.74512784	-0.2075343	0.836395292	0.94316963	-6.9217805
ENSCAFG00000032636	0.03596536	2.04502158	0.20747925	0.83643808	0.94316963	-6.1884825
ENSCAFG00000003969	0.03243382	2.79978301	0.20744105	0.836467759	0.94316963	-6.3993391
NIP7	0.02700111	3.18022327	0.20710754	0.836726869	0.94326526	-6.4858407
FAM117B	-0.0499861	4.50612753	-0.2070116	0.836801388	0.94326526	-6.7956278
WASL	0.02030075	5.93044368	0.2069599	0.836841576	0.94326526	-6.9294164
VAPA	0.02324164	6.6120745	0.20693548	0.836860546	0.94326526	-6.9331277
TKTL1	0.27622394	3.29494786	0.20684995	0.836927003	0.94326526	-6.2714128
ENSCAFG00000003752	0.18547311	-0.2958707	0.20648735	0.837208742	0.94349837	-5.2912526
SUPV3L1	-0.0203062	3.65040632	-0.2057864	0.837753478	0.94397413	-6.63222
LIPA	-0.0311429	5.59136837	-0.2057512	0.83778079	0.94397413	-6.9257809
SNED1	0.0923399	2.58473137	0.20532272	0.838113813	0.94415633	-6.491313
ENSCAFG00000012527	0.09830519	-0.453359	0.20530561	0.838127116	0.94415633	-5.5031244
HAUS8	-0.0607444	2.52165623	-0.2052538	0.83816736	0.94415633	-6.0966894
LPXN	-0.0444886	3.24207443	-0.2048836	0.838455161	0.94436493	-6.431739
HOXB9	-0.133921	-2.0162674	-0.2048227	0.838502488	0.94436493	-5.3310116
ENSCAFG00000011431	-0.0964088	0.06002137	-0.2046916	0.838604338	0.94439519	-5.5707117
S100PBP	0.03222133	3.33392045	0.20420185	0.838985091	0.94473951	-6.4803479
DECR2	-0.037716	2.12885758	-0.2038444	0.839263004	0.94496798	-6.233469
SLIT3	-0.0830578	7.69396959	-0.203505	0.839526841	0.94510182	-6.8507325
ABHD14B	0.04235354	3.0993317	0.20349848	0.839531938	0.94510182	-6.4850541
ENSCAFG00000014151	0.03723892	3.04187981	0.20338048	0.83962368	0.94512063	-6.4069862
DGKD	0.02997752	4.55454942	0.20303589	0.839891627	0.94527117	-6.7714588
MOB3C	-0.0618405	1.12171902	-0.2029486	0.839959502	0.94527117	-5.9114349
ENSCAFG00000030140	0.04419708	11.414126	0.20291897	0.839982547	0.94527117	-6.5937774
LSM12	0.01951208	4.50465048	0.20281194	0.840065773	0.94528037	-6.7828914

VGLL4	0.03231168	5.45065426	0.20251293	0.840298297	0.94540914	-6.8409185
ENSCAFG00000000823	-0.0175624	5.4751747	-0.2024717	0.840330324	0.94540914	-6.8938078
LTN1	0.02244384	5.51381985	0.20212631	0.840598975	0.94562121	-6.9062682
EI24	-0.0188533	5.94227236	-0.2018457	0.840817197	0.94562121	-6.9250743
DOP1B	-0.1035979	3.9804485	-0.2018455	0.840817401	0.94562121	-6.5270611
ACAP3	0.02463845	4.30639452	0.20183491	0.840825616	0.94562121	-6.7467978
WWC1	-0.1480915	-1.2863048	-0.2017468	0.840894188	0.94562121	-5.4649823
SRFBP1	0.02678546	4.03552016	0.20157374	0.841028762	0.94568812	-6.6762996
DPH6	-0.0371436	3.54159799	-0.2014707	0.841108939	0.94569385	-6.5719376
ATAD2	-0.0553696	5.9572499	-0.2013285	0.841219488	0.94573373	-6.8851847
RFC5	-0.0477009	4.04430785	-0.2011348	0.841370167	0.94581871	-6.5990946
MAP4K2	0.04705161	2.84536346	0.20090028	0.841552637	0.94587975	-6.4589869
SPTLC2	-0.0287265	5.55159904	-0.2007925	0.841636509	0.94587975	-6.8960915
ERLIN2	-0.014963	5.14052884	-0.2007686	0.841655073	0.94587975	-6.8718987
DNM1L	0.02017134	6.40474446	0.20058892	0.841794856	0.94587975	-6.9344913
MTUS1	-0.0668483	4.31055078	-0.2005824	0.841799937	0.94587975	-6.88946
CDC25A	-0.0527502	2.7397374	-0.2003806	0.841956971	0.94597181	-6.3379804
PANK2	0.02771028	2.99451202	0.20023617	0.842069302	0.94601364	-6.4522827
ENSCAFG00000005305	0.03313886	2.36951979	0.19980633	0.842403753	0.94630044	-6.2908989
LGALS8	-0.0186936	7.34895586	-0.199715	0.842474847	0.94630044	-6.9076278
PRDM11	0.05731212	1.38116032	0.19959981	0.842564455	0.94631259	-6.0531461
RAB1B	0.01124011	6.29491383	0.19950798	0.842635913	0.94631259	-6.9344122
MEIS2	0.0375746	5.66410992	0.19926925	0.842821681	0.94634286	-6.9129013
SLU7	-0.0154863	6.36378248	-0.1992405	0.842844029	0.94634286	-6.9339205
ZFHX2	0.03848856	2.78546325	0.19912143	0.842936719	0.94634286	-6.5563692
KCMF1	-0.0197085	5.5064017	-0.1989029	0.843106756	0.94634286	-6.8958584
TOM1	-0.0261087	5.79765243	-0.198854	0.843144822	0.94634286	-6.9130809
GADD45B	-0.0614624	5.27683536	-0.1987669	0.843212596	0.94634286	-6.8226521
ENSCAFG00000030774	0.0625973	0.28895006	0.19871049	0.843256537	0.94634286	-5.7128716
HSD3B7	0.02857555	5.87750427	0.19865724	0.843297979	0.94634286	-6.9204799
IFT81	0.03670699	3.38073875	0.19860447	0.843339047	0.94634286	-6.586345
HFE	0.04214234	1.74076442	0.19831941	0.843560915	0.94639064	-6.129511
ERGIC1	-0.0310811	7.05811808	-0.1981734	0.843674569	0.94639064	-6.916742
TNFRSF11A	-0.1067401	-1.5832242	-0.198157	0.843687321	0.94639064	-5.4751762
CCDC66	0.02751169	4.25949752	0.19809115	0.843738588	0.94639064	-6.756499
NDUFB2	-0.0294017	3.71566272	-0.1980671	0.843757295	0.94639064	-6.5671788
SLC35D1	0.02017038	5.80221098	0.19774915	0.844004803	0.94651723	-6.9262705
NMI	0.02049286	6.05422586	0.19772906	0.844020442	0.94651723	-6.932109
CWC15	-0.022742	4.19170798	-0.1972656	0.844381198	0.94657174	-6.7119046
AGPS	-0.0215704	4.70095525	-0.1972247	0.84441308	0.94657174	-6.8194913
MORC4	-0.0249145	5.30207899	-0.1971943	0.844436765	0.94657174	-6.8936319
RRNAD1	-0.0183807	4.79496152	-0.1970353	0.844560549	0.94657174	-6.8120753
SSB	0.02753583	6.47900285	0.19678139	0.844758211	0.94657174	-6.935352
CEP44	-0.0317904	2.82118434	-0.1967688	0.844767992	0.94657174	-6.3682113
RGS10	-0.0894735	0.33766123	-0.1967	0.844821549	0.94657174	-5.7820622
ENSCAFG00000019000	-0.0373833	2.05538216	-0.1966306	0.844875612	0.94657174	-6.2020337
ENSCAFG00000013404	0.0332206	4.38239494	0.19661951	0.844884255	0.94657174	-6.7561227
MAP3K14	-0.0606081	2.61733822	-0.1965741	0.844919575	0.94657174	-6.388603
SLK	-0.0283494	6.1173225	-0.1965495	0.844938757	0.94657174	-6.9353821
LAMP1	0.01925053	8.57487189	0.19650831	0.84497084	0.94657174	-6.8317189

KIF9	0.05125791	0.78648597	0.19635378	0.845091161	0.94662234	-5.8928712
HMGN1	-0.0295857	4.94007954	-0.1959245	0.845425438	0.94683601	-6.8036276
HSPG2	-0.0306547	11.6873701	-0.1959158	0.845432246	0.94683601	-6.5832201
C12H6orf141	-0.081458	-0.2213676	-0.1950566	0.846101356	0.94750113	-5.6603455
FHL3	-0.0381849	6.54081902	-0.1946431	0.846423423	0.94765963	-6.934634
PAFAH1B1	0.01818299	6.73418302	0.19461139	0.846448115	0.94765963	-6.9344443
TLE3	-0.0423235	4.61637108	-0.1945851	0.846468598	0.94765963	-6.85482
LMTK2	0.02239465	5.22134725	0.19397109	0.846946892	0.94811083	-6.8701017
MCM8	0.03085125	3.01826467	0.19368683	0.847168344	0.94827446	-6.4682309
RDH11	0.02179665	4.24991048	0.1934934	0.84731904	0.94835886	-6.7717231
ATP1A1	0.02014958	9.1838159	0.19291994	0.847765844	0.94870451	-6.7919255
RHOG	0.02959486	4.55532806	0.19290371	0.847778494	0.94870451	-6.8326465
LINGO2	0.13075847	-2.1276468	0.19273321	0.847911349	0.94876889	-5.3003335
POP4	-0.0291244	4.67297714	-0.1923242	0.848230053	0.94898301	-6.784657
ENSCAFG00000029150	0.07248677	-0.5869895	0.19229426	0.848253394	0.94898301	-5.6067964
ENSCAFG00000020381	-0.0495942	1.5165412	-0.1920956	0.848408202	0.94907191	-5.9450157
SSBP2	-0.0505892	6.02558881	-0.1917066	0.848711393	0.94924963	-6.9363056
NT5DC1	-0.0171437	5.73523915	-0.1916984	0.848717797	0.94924963	-6.9198344
COPB1	0.01968998	7.95792301	0.19126504	0.849055528	0.94953355	-6.8822873
UNC93B1	0.04081503	4.01368002	0.19117923	0.849122409	0.94953355	-6.6988233
ENSCAFG00000028725	-0.0368116	2.03670276	-0.1909012	0.849339119	0.94962231	-6.2107162
LCA5	-0.0358778	3.38675144	-0.1908141	0.849407011	0.94962231	-6.5833371
MOCS1	0.0463625	2.73251574	0.19078724	0.849427954	0.94962231	-6.2701395
SYNM	0.07968798	1.61053057	0.18993209	0.850094606	0.95007708	-6.117449
FAM120B	-0.016977	5.36597299	-0.1898954	0.850123246	0.95007708	-6.9126629
SLC15A4	0.02222562	5.95352924	0.18987778	0.850136953	0.95007708	-6.9275026
EDC3	0.02543185	4.11255386	0.18986525	0.850146724	0.95007708	-6.6803737
RARS2	-0.0248313	3.84222248	-0.1897012	0.850274598	0.95007708	-6.6379987
FUNDC1	-0.0354724	2.55167032	-0.1895992	0.850354147	0.95007708	-6.3892948
KDSR	0.02447077	5.47299382	0.1894438	0.850475319	0.95007708	-6.9049767
CCNG1	0.0325211	7.88207533	0.18929981	0.850587586	0.95007708	-6.8962351
EIF3J	0.02376256	4.98640837	0.18922564	0.850645425	0.95007708	-6.8410582
STXBP5	-0.0269227	5.8511128	-0.1891689	0.85068969	0.95007708	-6.9158944
SERTAD3	-0.0295934	3.20022196	-0.1891173	0.850729895	0.95007708	-6.5015788
ARID4A	-0.0231655	4.18383282	-0.1891045	0.850739866	0.95007708	-6.7633912
TTC32	0.0358783	1.95217484	0.1888821	0.850913303	0.95011462	-6.1845354
EFCAB2	0.07169417	-0.006052	0.18886794	0.850924348	0.95011462	-5.6219542
ENSCAFG00000030092	-0.1048771	-1.4610266	-0.1882024	0.851443341	0.95060985	-5.4376828
TFDP2	0.02417235	3.07424772	0.18774169	0.851802688	0.95092676	-6.6503584
TOR1AIP2	-0.018411	6.26853304	-0.1872062	0.852220338	0.95123792	-6.937264
RCBTB2	0.03207671	4.38827202	0.18719072	0.852232448	0.95123792	-6.7244862
ENSCAFG00000020283	-0.0125667	5.42946155	-0.1867508	0.852575655	0.95134602	-6.9044283
ENSCAFG00000032089	0.06762217	3.27584505	0.18673482	0.852588087	0.95134602	-6.4320205
SERPINF1	-0.074717	9.40887986	-0.1865873	0.852703152	0.95134602	-6.7489162
TXNDC9	0.02180892	4.47274557	0.18651765	0.852757513	0.95134602	-6.7749422
SAP30L	-0.0252125	3.05672502	-0.186497	0.852773633	0.95134602	-6.4526669
THBS4	0.11772863	-2.7767064	0.18648566	0.852782464	0.95134602	-5.3257109
ZNF296	-0.0574575	1.67353703	-0.1862111	0.852996644	0.95150068	-6.0994479
ZSWIM9	-0.0582327	0.25690412	-0.185864	0.853267503	0.95167388	-5.8188694
AVIL	0.10036332	-0.2384352	0.18581846	0.853303021	0.95167388	-5.6379746

ZNF2	-0.0494929	1.66687712	-0.1856678	0.853420586	0.95172073	-6.1410942
DNAJC11	0.02186495	4.95196988	0.18542462	0.853610325	0.95184806	-6.8468169
UNC5B	0.07459408	4.61944362	0.18484304	0.85406416	0.95201859	-6.8217709
HEMK1	-0.0261523	2.61001782	-0.184787	0.854107902	0.95201859	-6.2980597
CRIP1	0.05360126	6.37450707	0.18469806	0.854177304	0.95201859	-6.933477
ZNF438	-0.0405856	1.92903683	-0.184684	0.854188272	0.95201859	-6.0819486
NSRP1	-0.0227383	4.75919003	-0.1846677	0.854200992	0.95201859	-6.8603223
TTC7A	0.03468327	7.42416354	0.18458979	0.854261801	0.95201859	-6.8851659
PI4KB	-0.0153257	5.39883763	-0.1845507	0.854292326	0.95201859	-6.903297
RNF44	0.03122167	3.50758603	0.18383025	0.854854611	0.95226927	-6.6931054
ELP1	0.01739967	5.74094693	0.18366587	0.854982921	0.95226927	-6.9174979
TLR1	0.1143058	-2.5774995	0.18355202	0.85507179	0.95226927	-5.3252967
NDUFA8	0.01864136	5.83768974	0.18340031	0.855190212	0.95226927	-6.9264475
LRRC71	-0.046881	0.85008832	-0.1833101	0.85526062	0.95226927	-5.8568221
ENSCAFG00000032358	-0.1433345	-2.1518295	-0.1832308	0.855322509	0.95226927	-5.325627
GBP5	0.08839938	1.09374249	0.18320621	0.85534173	0.95226927	-6.1038589
PBLD	-0.0629161	0.01511537	-0.1831212	0.855408123	0.95226927	-5.7540378
MYLIP	-0.0677068	2.51372229	-0.1829432	0.855547035	0.95226927	-6.1236134
SENP2	-0.0145794	5.13683746	-0.1828816	0.85559512	0.95226927	-6.8843343
ALX1	-0.1141972	-2.3297662	-0.1828687	0.855605224	0.95226927	-5.3408766
ENSCAFG00000013213	-0.0803122	-0.2113297	-0.1828041	0.855655669	0.95226927	-5.5741078
KDM1B	-0.0430018	6.02199676	-0.182782	0.855672915	0.95226927	-6.9380626
ENSCAFG00000016171	0.03397675	2.28410284	0.18273999	0.855705694	0.95226927	-6.3171953
SNX13	0.01692196	5.20234508	0.18265539	0.855771741	0.95226927	-6.8794976
CCDC157	0.03925979	2.06625233	0.18261056	0.855806743	0.95226927	-6.2260893
GLT1D1	0.0645285	4.0236172	0.18254532	0.855857676	0.95226927	-6.4796298
MPP5	0.02899725	3.36690027	0.18251918	0.855878086	0.95226927	-6.5848864
BCR	-0.0356487	6.18552652	-0.1824119	0.855961848	0.95227835	-6.9374796
AATF	-0.0234216	6.33146581	-0.1822011	0.856126415	0.95237731	-6.9368905
SKA3	-0.0746986	2.52196811	-0.181908	0.85635525	0.95254775	-6.1595659
ABHD15	0.04551345	2.23639479	0.18165308	0.856554334	0.95268507	-6.2339118
LMO2	0.09318471	2.07954193	0.1812909	0.856837152	0.952831	-6.6034965
RARS	-0.0204778	6.17520374	-0.181111	0.856977659	0.952831	-6.9355938
MT3	0.16173063	-1.1394101	0.18109213	0.856992378	0.952831	-5.9323146
ZNF654	0.02198274	4.17527818	0.18099525	0.85706804	0.952831	-6.7664154
SNX14	-0.0144614	6.90943738	-0.1809808	0.857079333	0.952831	-6.9241548
CRYL1	-0.0407205	4.01815836	-0.1809039	0.857139411	0.952831	-6.6985343
MED18	-0.0250772	2.76712876	-0.1804284	0.857510778	0.95306177	-6.3867986
LRRC40	-0.0173093	4.85169528	-0.1803713	0.857555324	0.95306177	-6.8310701
PIK3R4	-0.0158061	5.57174738	-0.1802789	0.857627527	0.95306177	-6.9165123
ENSCAFG00000030359	0.04456493	4.97175089	0.18025054	0.85764966	0.95306177	-6.8562558
TMEM71	-0.0803836	0.89358349	-0.1801475	0.857730163	0.95306714	-5.6901147
CYC1	-0.0236813	5.94543938	-0.1799954	0.857848987	0.9530695	-6.9257548
DSN1	-0.0950716	2.89534636	-0.1798849	0.857935279	0.9530695	-6.1807053
ENSCAFG00000006797	-0.0491445	1.23348267	-0.1798239	0.857982902	0.9530695	-5.8892897
TRMT10C	0.02448413	3.36314214	0.17974247	0.858046517	0.9530695	-6.5719757
LRRC74A	0.09799955	-1.0385427	0.1795037	0.858233034	0.9530695	-5.6387433
SUMO1	-0.0165995	4.03500832	-0.1793716	0.858336233	0.9530695	-6.7044746
CLTC	0.01892745	9.3362959	0.17933783	0.858362607	0.9530695	-6.7955441
ADORA1	0.08664064	-1.9026081	0.17933746	0.858362892	0.9530695	-5.2897212

P2RY11	-0.0217196	4.69370618	-0.1792074	0.858464535	0.9530695	-6.8066607
POPDC3	0.14187832	-0.786792	0.17917612	0.858488933	0.9530695	-5.4101109
ALKBH7	-0.0355719	2.62380888	-0.1784526	0.859054162	0.95349002	-6.2183703
ZCCHC2	-0.0202349	4.55762814	-0.178417	0.859082008	0.95349002	-6.8033218
PPP1R16A	0.02275383	3.15762349	0.17837391	0.859115677	0.95349002	-6.4903445
MIEF1	0.01249148	6.01094593	0.17830374	0.859170508	0.95349002	-6.934457
PRR7	-0.0886866	-0.1135051	-0.1782008	0.859250919	0.95349525	-5.604102
SNX24	-0.0692702	0.53026077	-0.1779979	0.859409504	0.95353869	-5.7306891
ZNF653	-0.0432009	1.49002482	-0.1778898	0.859493983	0.95353869	-6.0301751
UPF1	-0.0142994	5.71834357	-0.1778601	0.859517167	0.95353869	-6.9266685
HIST1H1A	-0.1133637	-1.6158942	-0.1774165	0.859863834	0.95383926	-5.3420901
ANKRD42	-0.027307	3.81156902	-0.1772958	0.859958112	0.95385276	-6.6635741
LXN	0.04457651	4.95569594	0.17720708	0.860027455	0.95385276	-6.8365057
ENSCAFG00000013797	0.03188867	4.00390909	0.17674321	0.860389985	0.95417083	-6.6948418
VWDE	-0.0928243	0.55991528	-0.1766106	0.860493636	0.95420176	-5.8527617
ENSCAFG00000032467	-0.0519722	2.00617597	-0.1761867	0.860824982	0.95448516	-6.1757897
BRWD3	-0.0225449	3.98169419	-0.1759376	0.861019628	0.95460627	-6.6779982
PIGM	0.03643009	3.0264859	0.17585302	0.861085773	0.95460627	-6.445906
GOLGA4	-0.0176545	6.65949615	-0.1755839	0.861296168	0.95465066	-6.9318487
ME1	-0.0239711	6.99999857	-0.1755181	0.861347567	0.95465066	-6.9302055
PRDM15	-0.047018	1.83525427	-0.1754714	0.861384115	0.95465066	-6.1696678
STRADA	0.02215777	4.00563952	0.17539508	0.86144375	0.95465066	-6.7016587
ENSCAFG00000001272	0.08664064	-3.0621571	0.17531703	0.86150477	0.95465066	-5.2902706
ALPK2	0.11692275	3.54207641	0.17497244	0.861774166	0.95486518	-6.2724976
LYSMD1	0.03924857	1.30767116	0.17467975	0.862002995	0.95487709	-5.936197
SDK1	-0.0996968	1.11329973	-0.1744359	0.86219363	0.95487709	-5.5776465
PNPT1	-0.0206027	4.8731292	-0.1743427	0.862266519	0.95487709	-6.8416641
CUBN	0.05373385	4.03999697	0.17423735	0.862348902	0.95487709	-6.7881596
PTBP2	0.03030775	3.52384851	0.17422962	0.862354944	0.95487709	-6.4545089
YTHDF3	0.01368237	6.44591829	0.17398475	0.862546413	0.95487709	-6.9390312
TMEM255A	0.16864647	1.97550148	0.17395066	0.862573072	0.95487709	-5.6440939
CEP19	-0.0522523	1.63698817	-0.1739093	0.862605414	0.95487709	-6.1135607
FGD4	-0.0689886	0.57045451	-0.1738167	0.862677803	0.95487709	-5.9872799
JUNB	-0.0499867	6.92248632	-0.1738079	0.862684728	0.95487709	-6.9242071
ZNF471	-0.0640731	0.77629063	-0.1737525	0.862728022	0.95487709	-5.8793304
URB2	-0.0151915	6.0559803	-0.1737191	0.862754156	0.95487709	-6.9345114
EXD3	0.09266636	0.28644135	0.17369829	0.862770419	0.95487709	-5.7508424
CREBL2	0.02676692	2.78251818	0.17323283	0.863134417	0.95515049	-6.464726
RAB6A	-0.0209793	7.57365194	-0.1731067	0.863233029	0.95515049	-6.9135
DGKE	0.04562079	1.04431124	0.17309152	0.86324493	0.95515049	-5.9925576
TIPARP	-0.0273111	5.23812031	-0.1727712	0.863495459	0.95534377	-6.88446
RAN	-0.0189383	7.63851419	-0.1724063	0.863780826	0.95546495	-6.9121981
ANAPC7	-0.0169045	4.72689974	-0.1722932	0.863869283	0.95546495	-6.7951937
ENSCAFG00000006614	-0.0648115	-0.0688836	-0.1721235	0.864002069	0.95546495	-5.6057619
PWP1	0.02357039	4.99634727	0.17212178	0.864003398	0.95546495	-6.851087
FAXDC2	-0.0482011	4.76736332	-0.1720326	0.864073126	0.95546495	-6.8470892
BIRC6	-0.0231742	7.01229471	-0.1718108	0.864246622	0.95546495	-6.9285602
ENSCAFG00000024834	-0.1040793	-1.1526063	-0.1718081	0.864248799	0.95546495	-5.5366165
DUSP1	0.03271757	8.23874217	0.17138857	0.864576952	0.95546495	-6.8525657
SPTBN5	0.10868347	-1.0393876	0.17136455	0.864595744	0.95546495	-5.4823423

BTBD9	-0.0260073	3.3309551	-0.1713102	0.864638299	0.95546495	-6.5184651
CERS6	0.02555111	4.42857803	0.17129249	0.864652114	0.95546495	-6.7505778
MTCP1	-0.0810362	-1.3483427	-0.1712793	0.864662458	0.95546495	-5.4680643
IARS2	0.01469456	7.08816356	0.17126538	0.864673324	0.95546495	-6.9248246
CC2D1A	-0.0119908	5.83183511	-0.1712509	0.864684688	0.95546495	-6.923201
ZNF713	0.0295217	4.65445483	0.17117657	0.864742805	0.95546495	-6.7732008
MSRB2	0.03101308	2.27096647	0.17099232	0.864886948	0.95546854	-6.2899745
FUBP3	-0.0139065	5.1838026	-0.1709242	0.864940224	0.95546854	-6.8794928
ENSCAFG00000030220	0.01185438	5.27758465	0.17088154	0.864973619	0.95546854	-6.8860153
PHF10	0.01847875	4.54802965	0.17044066	0.865318566	0.95576575	-6.7972315
DDX18	0.01984617	5.72283075	0.17030419	0.865425342	0.95579988	-6.9222555
WDR20	-0.0204057	3.36518144	-0.1700163	0.865650626	0.95596487	-6.5795783
BCKDHB	-0.028431	3.75156889	-0.1697675	0.865845262	0.95609099	-6.6152863
FTL	0.03492611	8.42419227	0.16967633	0.86591664	0.95609099	-6.8263183
ANKRD39	-0.0263091	3.25338967	-0.1694922	0.866060766	0.95609331	-6.5165593
C20H19orf53	0.01699319	4.35201094	0.16939282	0.866138503	0.95609331	-6.7135554
RHOA	0.01336914	8.39268503	0.16935274	0.866169866	0.95609331	-6.8647897
NUDT14	-0.0463097	1.80817802	-0.1692857	0.866222358	0.95609331	-6.0964152
EIF5B	0.02008692	7.54514758	0.16854229	0.866804151	0.95665163	-6.9046492
GNB2	0.01594514	7.26353518	0.16830308	0.866991385	0.95672514	-6.9275639
ENSCAFG00000009917	-0.0217425	4.04687434	-0.1682631	0.867022659	0.95672514	-6.6687472
MAP4K3	-0.0253876	5.51938576	-0.1679884	0.8672377	0.9568786	-6.9134101
NDUFB7	0.0244146	3.89276306	0.16765239	0.867500716	0.95703755	-6.6389503
UCKL1	0.02661494	4.38258798	0.16761023	0.867533723	0.95703755	-6.8322208
ENSCAFG00000011616	0.05220582	1.11316494	0.16729986	0.867776687	0.95722175	-5.9591341
NSUN2	0.01909579	6.25396568	0.16706414	0.867961232	0.95728949	-6.9397819
VPS28	-0.0195455	5.90808987	-0.1670273	0.867990093	0.95728949	-6.9306004
SDAD1	-0.0163714	5.16548467	-0.1668116	0.868158918	0.95736094	-6.8832826
ENSCAFG00000030080	-0.0503328	5.43666115	-0.1667504	0.868206896	0.95736094	-6.9128743
NSUN5	-0.0208906	3.76350133	-0.1663999	0.868481312	0.95757971	-6.60624
MRPS31	-0.0176169	6.07177832	-0.1656575	0.869062574	0.95813673	-6.9298005
ENSCAFG00000028878	0.10050701	2.64944009	0.16532124	0.869325918	0.95815828	-6.4036762
XIRP1	0.12761126	-0.9267881	0.16521484	0.869409242	0.95815828	-5.3565621
ADAM22	0.10071061	-1.3795888	0.16511027	0.869491137	0.95815828	-5.4458414
SNRPB	-0.0196988	5.53376062	-0.1650075	0.869571625	0.95815828	-6.9091304
ATP13A2	0.02768662	4.78468509	0.16499003	0.869585304	0.95815828	-6.819375
SHLD1	-0.0464637	1.15215572	-0.1649693	0.869601539	0.95815828	-5.9178542
DFFA	-0.0208229	3.51919729	-0.164707	0.86980698	0.95815828	-6.5947791
KIF13B	0.03035767	4.83188652	0.16465296	0.869849294	0.95815828	-6.864356
HCCS	-0.0159382	3.65649288	-0.1646053	0.869886587	0.95815828	-6.6105172
ZKSCAN1	-0.0188647	4.29617587	-0.1645657	0.869917638	0.95815828	-6.7897362
LOXL4	0.16255926	3.08826733	0.16456412	0.869918873	0.95815828	-5.7826351
NECAP2	-0.0160203	5.06745199	-0.1642268	0.870183058	0.9582572	-6.8779818
FBXW8	-0.0161702	5.79279445	-0.1641914	0.870210775	0.9582572	-6.9206892
RPUSD3	-0.0241403	3.41721889	-0.1641127	0.870272433	0.9582572	-6.5753529
IMP3	-0.032787	3.16863287	-0.1640285	0.8703384	0.9582572	-6.4162274
METTL16	0.02035938	4.54577227	0.16396382	0.870389058	0.9582572	-6.8050411
CISD3	0.02447948	2.94498163	0.16371176	0.870586504	0.95839081	-6.2743258
ADAMTS14	-0.1184712	2.93861095	-0.163495	0.870756282	0.95849394	-5.9348397
FDX1	-0.0240155	3.31398366	-0.1632639	0.870937335	0.95860946	-6.5308368

TMEM168	-0.0159767	4.83038185	-0.1630235	0.871125672	0.95865069	-6.8468757
ENSCAFG00000012283	-0.0153338	5.79487962	-0.1629107	0.871214003	0.95865069	-6.9217353
PRELID3A	-0.0457514	0.79704572	-0.1629077	0.871216421	0.95865069	-5.9134486
ASH1L	-0.0135022	7.19093169	-0.1628275	0.87127922	0.95865069	-6.9246986
REXO1	-0.0154132	4.94148435	-0.1624848	0.871547729	0.95879378	-6.8643206
ENSCAFG00000030953	-0.1396405	-1.7493859	-0.1622032	0.871768344	0.95879378	-5.4741509
C23H3orf33	0.02155895	3.70951097	0.16217751	0.871788474	0.95879378	-6.6826894
PPP1R2	-0.0233985	5.94597073	-0.1620994	0.871849667	0.95879378	-6.9350669
SLC25A5	-0.0217097	6.79937018	-0.1620954	0.871852841	0.95879378	-6.9370648
ZNF790	0.04435229	1.21008116	0.16203647	0.871898986	0.95879378	-5.9772361
QRSL1	-0.0197264	4.04480685	-0.1619814	0.871942101	0.95879378	-6.675825
ZNF19	-0.0618699	-0.4143186	-0.1616534	0.872199131	0.9589791	-5.614783
LIG1	-0.0264794	5.16860233	-0.161572	0.872262908	0.9589791	-6.8691123
ARHGAP28	0.17457832	1.69419337	0.16099258	0.872716994	0.95932649	-5.7741123
MARF1	-0.0164806	5.89309648	-0.1609744	0.8727312	0.95932649	-6.9298277
NUP107	0.02344939	4.56747098	0.16071454	0.87293489	0.95940327	-6.808175
PRSS53	0.13805494	0.35785794	0.16066932	0.872970327	0.95940327	-5.6090633
NUDT1	-0.0515633	1.72406525	-0.1605028	0.873100868	0.95940327	-6.0449267
ENSCAFG00000030446	0.03981743	1.19166894	0.16049656	0.873105725	0.95940327	-5.8728373
ARHGAP31	0.03114388	7.80515126	0.16027131	0.873282266	0.95951356	-6.9122332
WASHC1	0.02195452	4.85558292	0.16012201	0.873399288	0.95955843	-6.8396012
SMPD2	-0.0208402	4.57405964	-0.1597572	0.873685208	0.95970925	-6.7901343
EIF6	0.02082015	5.1862491	0.15975246	0.873688949	0.95970925	-6.879286
PSAP	0.02704178	9.75349904	0.15955703	0.873842134	0.95979382	-6.7394895
GALNT14	0.08694441	-2.6463336	0.15931306	0.874033375	0.95980631	-5.3687626
PRKD1	0.0635989	2.92344083	0.15926444	0.874071489	0.95980631	-6.1902501
ENSCAFG00000032445	0.14714309	1.47559336	0.15920107	0.874121165	0.95980631	-5.5748686
DEF6	-0.0343956	3.70920014	-0.1590011	0.874277926	0.95980631	-6.7006474
PML	-0.0297749	5.39424613	-0.158909	0.874350166	0.95980631	-6.906364
ISPD	0.01957429	4.10314021	0.15885652	0.874391272	0.95980631	-6.7107902
B3GALT4	0.03721769	2.36009019	0.15884331	0.874401625	0.95980631	-6.2456791
EIF1AD	0.01555798	5.15072981	0.15876489	0.874463105	0.95980631	-6.8788432
ENSCAFG00000029477	0.01779073	3.65452368	0.15853902	0.874640186	0.95991703	-6.5745785
NADK	-0.0182059	6.35001585	-0.1583226	0.874809886	0.95993364	-6.9421455
RAB2A	0.01276034	7.58497657	0.15828779	0.874837147	0.95993364	-6.9088712
NDUFS4	0.01781861	3.90408047	0.1582281	0.874883951	0.95993364	-6.6513952
BEND2	0.08664064	-2.7069303	0.1579947	0.875066945	0.96004026	-5.2924956
CFLAR	-0.021799	4.59533865	-0.157823	0.875201547	0.96004026	-6.8527256
EFS	0.0874922	4.46684408	0.157709	0.875290953	0.96004026	-6.8013053
DOHH	0.02863256	3.28789209	0.15747302	0.875475993	0.96004026	-6.4379156
TMF1	-0.0192455	5.712589	-0.1574654	0.875481948	0.96004026	-6.9286527
GINM1	0.01752984	5.77169409	0.15742368	0.875514679	0.96004026	-6.9325721
EML3	-0.0207571	5.28753292	-0.1572863	0.875622375	0.96004026	-6.9207113
KMT5C	0.04971958	0.65653275	0.15719077	0.875697318	0.96004026	-5.8769371
FANCE	-0.019571	3.99293062	-0.1570726	0.875789978	0.96004026	-6.7430793
ISYNA1	0.03515112	3.96806836	0.15703794	0.875817158	0.96004026	-6.8107793
INTS5	0.01549633	5.01295375	0.15703494	0.875819515	0.96004026	-6.8670528
ULK1	0.02313653	5.91845035	0.15643866	0.876287126	0.96046925	-6.9402512
METTL21A	-0.0499715	1.009242	-0.156313	0.876385702	0.96049372	-5.946267
TMEM97	-0.0374088	3.71999808	-0.1560048	0.876627436	0.96052514	-6.7545741

ITCH	-0.0144247	5.84947144	-0.1559496	0.876670709	0.96052514	-6.9328834
HOXA4	0.08189648	-0.691941	0.15579877	0.876788996	0.96052514	-5.5729563
UBR3	0.01391971	6.26512019	0.15579165	0.876794578	0.96052514	-6.9427122
SLC37A4	0.02459899	3.02169506	0.15574289	0.876832818	0.96052514	-6.4397175
CAPN15	0.02384174	5.02328771	0.15569305	0.876871909	0.96052514	-6.881438
CDK15	0.10812179	-1.4424823	0.15544659	0.877065229	0.96057934	-5.3703506
ENSCAFG00000000496	0.02215777	3.03319345	0.15543552	0.877073913	0.96057934	-6.4356563
TEAD2	-0.0199148	4.81948001	-0.1552438	0.877224316	0.96066054	-6.8359815
ENSCAFG00000015313	-0.0620009	0.41193495	-0.1546309	0.877705088	0.96099186	-5.9474034
RBM18	0.0239515	5.37634714	0.15445813	0.877840614	0.96099186	-6.8882706
POLK	-0.0227167	5.13474772	-0.1544181	0.877872045	0.96099186	-6.8741579
MLF1	-0.0295387	4.76329523	-0.1542745	0.877984663	0.96099186	-6.8555198
ACTR8	0.01609621	5.15560784	0.15426025	0.877995855	0.96099186	-6.8541124
WNT7A	-0.1120536	-1.8780992	-0.1542277	0.878021402	0.96099186	-5.3390799
ENSCAFG00000010051	0.02672309	2.72088929	0.15402059	0.878183882	0.96099186	-6.3649448
ANKRD49	0.03203996	2.73306347	0.15397026	0.878223368	0.96099186	-6.345523
C33H3orf52	0.09388362	-1.6075834	0.15384232	0.878323747	0.96099186	-5.3746572
OSBPL5	-0.0247824	5.89449559	-0.1538075	0.878351036	0.96099186	-6.9417325
RWDD2A	-0.0201748	2.19475044	-0.153554	0.878549948	0.96099186	-6.2721837
OSBPL1A	0.02301374	5.11135886	0.15322774	0.878805958	0.96099186	-6.8617198
LRRC20	-0.0458347	1.98290448	-0.1532005	0.878827352	0.96099186	-6.2138719
MTX3	0.03919797	0.80079858	0.15310876	0.878899318	0.96099186	-5.9142991
PPM1F	0.04841468	3.89124871	0.15304634	0.878948292	0.96099186	-6.7171634
SMG6	0.02964963	5.82351151	0.15289688	0.879065575	0.96099186	-6.931449
NIPSNAP2	0.01382404	6.1234482	0.15273369	0.879193629	0.96099186	-6.939632
ABCB6	0.02102763	5.89119551	0.15270928	0.879212786	0.96099186	-6.9357564
CACHD1	-0.0434966	2.11033734	-0.1526468	0.879261826	0.96099186	-6.2303795
CCDC189	-0.0471944	0.98525603	-0.1526099	0.879290767	0.96099186	-5.8909614
MED21	-0.0180238	3.82490378	-0.1525845	0.879310663	0.96099186	-6.597301
MBD3	-0.0132486	6.1283969	-0.1525682	0.879323488	0.96099186	-6.9348646
ADAMTS17	0.13703173	2.5771749	0.15251646	0.879364092	0.96099186	-6.0147478
UFM1	0.02721316	2.63751277	0.15236368	0.879483991	0.96099186	-6.3444042
AMPD3	0.0472379	4.64411408	0.15212051	0.879674825	0.96099186	-6.9269873
CTSH	0.04650408	5.86467226	0.15201662	0.879756354	0.96099186	-6.9420894
CLK3	0.01443011	4.96656033	0.1520166	0.879756375	0.96099186	-6.8720334
TMEM214	-0.0169057	7.67718783	-0.1516983	0.880006188	0.96099186	-6.905637
CGRRF1	0.01665481	3.66897143	0.15166253	0.880034254	0.96099186	-6.654602
ZSCAN31	-0.0192041	3.61505954	-0.151392	0.880246595	0.96099186	-6.6347198
BTBD6	0.03843648	4.6068445	0.15136058	0.880271242	0.96099186	-6.830138
PIP5K1B	0.11533174	-1.1767443	0.15126389	0.880347136	0.96099186	-5.4863947
WT1	-0.1138664	-1.5915956	-0.1510845	0.880487973	0.96099186	-5.3308016
ENSCAFG00000031971	-0.0501253	-0.1255029	-0.1510814	0.880490364	0.96099186	-5.751168
TPM3	-0.0251984	6.61982414	-0.1510808	0.880490839	0.96099186	-6.9392911
STAP2	-0.0636028	-0.5157274	-0.1510622	0.880505434	0.96099186	-5.6659038
TMEM268	0.0248092	3.95271305	0.15102907	0.880531442	0.96099186	-6.628999
ENSCAFG00000020269	-0.0532565	0.07607593	-0.1509896	0.880562436	0.96099186	-5.8097057
EMCN	-0.128648	1.34591164	-0.1509612	0.880584685	0.96099186	-6.5558186
FKBP9	-0.0157161	7.29742582	-0.1509073	0.88062703	0.96099186	-6.920625
U2AF2	0.01381992	6.69666348	0.15081847	0.880696751	0.96099186	-6.9424027
CHMP2A	-0.0115349	5.88127078	-0.1507401	0.880758289	0.96099186	-6.9361049

BRF1	0.01676249	4.1668225	0.15053631	0.88091824	0.96099186	-6.7370585
KAZN	-0.0423946	1.74071269	-0.150289	0.881112346	0.96099186	-5.9979387
TTBK2	0.02079029	3.52914104	0.15028894	0.881112419	0.96099186	-6.5803332
TMOD2	0.08761849	-1.252721	0.15027969	0.881119685	0.96099186	-5.5558528
ENSCAFG00000019635	-0.039698	0.91471223	-0.1502769	0.881121872	0.96099186	-5.9085484
ENDOV	0.02533547	2.02829237	0.15012277	0.881242872	0.96099186	-6.1496335
SRPK2	-0.0156068	6.14097667	-0.1500884	0.881269867	0.96099186	-6.9392385
ZNF511	0.01831136	3.24477588	0.14999709	0.881341534	0.96099186	-6.4716867
ZNF383	-0.0367616	2.03924893	-0.1496597	0.881606384	0.96104857	-6.1930286
SOD3	0.11508672	-0.3353125	0.14963141	0.881628614	0.96104857	-5.5655477
ZBED5	-0.0156913	5.04360895	-0.1495653	0.881680488	0.96104857	-6.8906626
UBE2O	-0.0125533	5.72660461	-0.1495421	0.881698739	0.96104857	-6.9374676
DDR2	0.02136381	8.07546149	0.14923187	0.881942298	0.96121552	-6.8692683
PCNX1	-0.0142999	6.07813139	-0.1491526	0.882004527	0.96121552	-6.9418213
CHUK	0.01698139	3.96630504	0.14892341	0.882184495	0.96132848	-6.7054209
ENSCAFG00000031334	-0.0662082	-1.0329811	-0.1485746	0.882458397	0.96154377	-5.7255368
CAPS	0.10263807	0.78142679	0.14837152	0.882617842	0.96158047	-5.6134259
HOMER2	-0.101735	-0.1846912	-0.1483372	0.882644756	0.96158047	-5.428255
BFSP1	-0.0340104	3.05466557	-0.1482336	0.882726141	0.96158596	-6.4211096
AP3B1	0.01225125	7.45008885	0.14801517	0.882897669	0.96168964	-6.9214068
MRPS12	0.02231772	3.08767041	0.14779225	0.883072724	0.96179715	-6.4247298
LETMD1	-0.0138041	4.28542856	-0.147116	0.883603798	0.96209743	-6.7615145
USP8	-0.0121458	6.49926021	-0.1470643	0.883644431	0.96209743	-6.9435478
PLD1	-0.0462464	7.10603949	-0.1470635	0.88364503	0.96209743	-6.9249295
FAM8A1	-0.022784	6.04141976	-0.1467733	0.883872941	0.96209743	-6.9438594
SUSD5	0.10224918	-0.0834731	0.14673837	0.88390041	0.96209743	-5.3692515
PALM	-0.0766988	2.11801266	-0.146715	0.883918768	0.96209743	-6.428258
TMEM8B	0.02883551	1.94848635	0.14669427	0.883935046	0.96209743	-6.1610012
TMC7	0.05654356	1.72387577	0.14663765	0.883979521	0.96209743	-6.1174302
WDR7	0.01097119	5.61770307	0.14653344	0.884061372	0.96209743	-6.917275
ENSCAFG00000014664	-0.0548084	0.70456689	-0.1464687	0.884112238	0.96209743	-5.6633057
KCTD13	-0.0269833	2.28030878	-0.1458251	0.884617802	0.962549	-6.2187124
HOXA9	0.15858587	0.13193816	0.14574583	0.88468004	0.962549	-5.7467799
UBALD1	0.03037009	2.82092553	0.14562719	0.884773238	0.96256726	-6.5302618
HDDC2	0.01407438	4.11873249	0.14552183	0.884856005	0.96257416	-6.7486332
CKS2	-0.042604	2.99395449	-0.1452206	0.88509269	0.96273565	-6.3803635
ADGRF3	-0.0584599	-0.1773496	-0.1451383	0.885157313	0.96273565	-5.7229981
PDCD4	0.02438109	5.18501676	0.14499308	0.885271399	0.9627766	-6.9321873
ENSCAFG00000028941	0.02115167	3.27452297	0.14442775	0.885715562	0.96310271	-6.5035186
ENSCAFG00000019300	0.07212554	-1.4531836	0.14441678	0.885724179	0.96310271	-5.7107446
PCMTD2	-0.0226407	4.57060559	-0.1439759	0.886070594	0.96325834	-6.8488437
AP2B1	-0.0099876	7.45803	-0.1439342	0.886103325	0.96325834	-6.909977
ENSCAFG00000023888	0.04527881	0.35379144	0.14388389	0.886142886	0.96325834	-5.8384864
C15H12orf29	0.01423555	4.20368437	0.14384531	0.886173202	0.96325834	-6.7642276
SPG7	-0.0155994	4.95789472	-0.1434791	0.886460956	0.96348798	-6.8783579
SETD4	-0.0267445	2.3770594	-0.1433626	0.886552487	0.96350178	-6.2556569
RMDN2	-0.0196458	3.54492427	-0.1431812	0.886695078	0.96350178	-6.5734301
TCAIM	-0.0240199	3.35824974	-0.1431138	0.886748028	0.96350178	-6.5271005
FNBP1L	0.07459461	3.48978317	0.1430736	0.886779621	0.96350178	-6.2707832
RAD54L	-0.0961672	1.77278521	-0.1426953	0.88707695	0.9636113	-5.8874477

UQCRC1	0.01482202	6.49956292	0.14266021	0.887104495	0.9636113	-6.9445455
ZNHIT2	-0.0303173	2.74143881	-0.1426149	0.887140113	0.9636113	-6.2771329
ENSCAFG00000025140	-0.0170542	5.22026099	-0.1425144	0.887219087	0.9636113	-6.8676164
USP11	0.01425237	6.43052481	0.14245863	0.887262926	0.9636113	-6.9447407
MECP2	0.01276527	4.46129052	0.14217621	0.887484891	0.96371026	-6.7694629
FAM69C	0.08664064	-2.0451935	0.14214799	0.88750707	0.96371026	-5.2943287
ADGRL2	0.07265455	3.78227622	0.14204344	0.88758924	0.96371607	-6.5582271
POLR2I	0.01862867	3.43429407	0.1419465	0.887665436	0.96371607	-6.5319712
ENSCAFG00000032172	-0.0683364	-0.7320529	-0.1418033	0.887777969	0.96375518	-5.5032891
CADM1	0.04133215	4.8276592	0.14154004	0.887984915	0.96376853	-6.8548862
PLEKHG3	0.04279141	4.09005854	0.14150859	0.888009637	0.96376853	-6.6199147
DEDD	-0.0119915	4.55045504	-0.1413866	0.888105552	0.96376853	-6.796963
ENSCAFG00000032688	0.03018681	1.46564125	0.14114751	0.888293469	0.96376853	-6.0775987
ENSCAFG00000031148	0.0346261	1.96130306	0.14111359	0.888320129	0.96376853	-6.1489668
ZNF830	-0.0214748	2.70010104	-0.1409648	0.888437104	0.96376853	-6.4257022
ENSCAFG00000026256	-0.046225	-0.2393795	-0.1409419	0.888455101	0.96376853	-5.7225615
ENSCAFG00000025886	0.03606699	1.30814676	0.14087944	0.888504196	0.96376853	-5.9223006
ENSCAFG00000008439	0.04679125	0.2751852	0.14080872	0.888559791	0.96376853	-5.7606348
DMRT2	-0.1659422	-0.8655674	-0.1406504	0.888684214	0.96376853	-5.4379048
SLC38A5	-0.0212073	8.02855104	-0.1405717	0.888746097	0.96376853	-6.9188082
TPRKB	0.02592756	2.7952626	0.14056744	0.888749467	0.96376853	-6.3493142
RSPH3	-0.0336406	1.36837042	-0.1404363	0.888852597	0.96376853	-6.0017299
GGA3	-0.0162493	4.93008384	-0.140425	0.888861464	0.96376853	-6.8662866
METTL22	0.01890538	4.12746849	0.14031815	0.888945444	0.96377662	-6.6903127
MBNL3	0.10674119	-2.2395174	0.13979942	0.889353275	0.96406382	-5.3065474
OASL	-0.0726459	0.21181098	-0.1397865	0.889363415	0.96406382	-5.7968783
ENSCAFG00000013249	0.08097405	0.52073242	0.13967348	0.88945229	0.96407342	-5.9547582
ARIH2	0.01349262	5.84434916	0.13958056	0.889525345	0.96407342	-6.9300825
TMEM107	0.04155871	1.11691837	0.13927991	0.889761741	0.96421047	-5.9898506
MARCH3	-0.0251015	4.91740933	-0.139225	0.889804896	0.96421047	-6.8719289
C1QTNF2	0.03900811	3.25477773	0.13896338	0.890010627	0.96435044	-6.4566255
PYCR3	0.02105662	3.30162686	0.13860066	0.890295849	0.96457651	-6.5733484
TRIM52	-0.0512144	-0.5323061	-0.1380745	0.890709658	0.96494185	-5.5975724
AAMP	-0.0139776	5.49009833	-0.1377053	0.891000007	0.96503634	-6.9230416
ENSCAFG00000004589	-0.0245211	5.85542512	-0.1376734	0.891025076	0.96503634	-6.9395242
DRG2	0.01012394	5.46906608	0.13757193	0.891104867	0.96503634	-6.9136997
DAB2IP	0.01920479	5.45581387	0.1375224	0.891143816	0.96503634	-6.9099966
GDNF	0.06335778	5.40804963	0.13747645	0.891179957	0.96503634	-6.8386733
ENSCAFG00000002216	-0.0176853	4.12647439	-0.1373009	0.891318023	0.96510288	-6.7322344
ENSCAFG00000005494	-0.0514579	1.4741341	-0.1370596	0.891507798	0.96510482	-5.8531051
MRPS18B	-0.014015	5.08744205	-0.1370394	0.89152374	0.96510482	-6.8635988
CLDN4	0.08664064	-2.2018049	0.13696595	0.891581473	0.96510482	-5.2948862
CARF	0.03098682	1.30383206	0.13690897	0.891626293	0.96510482	-5.9355321
RAF1	0.00918204	6.5565502	0.13676364	0.891740605	0.96512431	-6.9455288
RPA2	-0.0331646	3.09895619	-0.1366422	0.891836151	0.96512431	-6.3617776
NME1	0.01480219	7.35580287	0.13653939	0.891916988	0.96512431	-6.9213382
ENSCAFG00000013972	-0.0151957	4.89883594	-0.1364452	0.891991073	0.96512431	-6.8825494
TAZ	-0.0195545	4.91680383	-0.136399	0.892027406	0.96512431	-6.8226075
ARCN1	0.01294663	8.62235995	0.13621186	0.892174633	0.96518447	-6.8449909
FCHSD1	0.01650676	3.60581898	0.13603908	0.892310542	0.96518447	-6.6426512

EOGT	0.01434244	6.29754413	0.13591842	0.892405464	0.96518447	-6.9455552
ENSCAFG00000018144	0.04664026	0.75309314	0.13587898	0.892436486	0.96518447	-5.7428851
CCDC106	0.03079792	2.63227891	0.13584128	0.892466143	0.96518447	-6.3640793
VPS16	0.01476096	5.22075948	0.13533787	0.892862175	0.96531384	-6.8980111
ZBTB20	0.03025705	2.68185624	0.13525287	0.892929046	0.96531384	-6.5231566
FLAD1	0.0175121	5.20611688	0.13524642	0.892934117	0.96531384	-6.9096995
ZBTB1	-0.0110029	5.60323562	-0.1352042	0.892967328	0.96531384	-6.939083
DPYD	0.04905697	7.07920796	0.13520215	0.89296895	0.96531384	-6.8913418
VBP1	0.0128313	3.72902103	0.1348633	0.89323554	0.96534777	-6.6426491
TMEM263	-0.0202754	4.3829487	-0.1348368	0.893256358	0.96534777	-6.7651683
ENSCAFG00000013883	-0.0121315	4.96131001	-0.1348011	0.893284468	0.96534777	-6.8751471
TSTD3	-0.1008433	-0.8608628	-0.1346488	0.893404335	0.96534777	-5.5372765
SNCAIP	-0.0786213	1.58818427	-0.1345196	0.893505931	0.96534777	-5.6220453
MTERF2	-0.0261115	2.14395525	-0.1344534	0.893558085	0.96534777	-6.2289517
TPPP3	-0.0413174	1.601602	-0.1343879	0.893609586	0.96534777	-6.308185
IRF2BPL	-0.0242078	6.16361924	-0.1343769	0.893618215	0.96534777	-6.9426129
TRAF6	-0.0160917	5.95215707	-0.1342239	0.893738592	0.96534777	-6.9400773
PLEKHO2	0.01610181	6.58751577	0.13410858	0.893829365	0.96534777	-6.9389007
SEC24A	-0.0151022	6.25174086	-0.1340908	0.893843368	0.96534777	-6.9444709
RRS1	0.02421884	3.04392437	0.13388984	0.894001478	0.96543575	-6.4444537
GGH	-0.0164623	5.86750691	-0.1336981	0.894152387	0.96551594	-6.9429484
LONP1	0.01267758	6.36832786	0.13338328	0.894400095	0.96553017	-6.9450267
TRIP6	0.01406598	5.00696966	0.133228	0.89452229	0.96553017	-6.8407387
NAA30	0.0243125	2.54755886	0.13315265	0.894581586	0.96553017	-6.2750899
OSGEPL1	-0.0222358	3.85681662	-0.1331144	0.894611725	0.96553017	-6.7456349
C6H16orf58	-0.0111437	5.04350347	-0.1331042	0.894619679	0.96553017	-6.8449748
PLSCR1	0.07523696	-0.9717516	0.13305046	0.89466201	0.96553017	-5.5315662
MIGA1	-0.0171433	4.88487279	-0.1329567	0.89473583	0.96553017	-6.8431108
SCYL2	-0.0139523	6.38777963	-0.1329021	0.894778797	0.96553017	-6.945049
ORAI2	-0.0290533	3.21417971	-0.1325808	0.895031613	0.96565852	-6.4981679
PER2	0.04532297	4.12441198	0.13255609	0.895051069	0.96565852	-6.753731
RBM45	0.01239481	4.69771888	0.13235367	0.895210381	0.96566702	-6.8163481
ADGRD1	0.16807005	2.08274581	0.13235127	0.89521227	0.96566702	-5.8311039
CCDC88A	-0.0244041	7.74907579	-0.1321765	0.895349822	0.96573269	-6.9229344
ENSCAFG00000018971	-0.0388077	0.65536179	-0.1317827	0.895659744	0.9658128	-5.7802081
ME2	0.01329158	6.26060027	0.13134958	0.896000699	0.9658128	-6.9460401
RAB36	-0.0481623	-0.2155975	-0.1313076	0.89603372	0.9658128	-5.6524307
CTSK	0.04065511	9.00194754	0.13127205	0.896061727	0.9658128	-6.8266115
ENSCAFG00000008716	0.03044228	5.53598873	0.13122193	0.896101174	0.9658128	-6.8669033
TMEM203	0.03295331	2.09822256	0.13115458	0.896154188	0.9658128	-6.1908873
LAYN	0.04110282	2.40919253	0.13115108	0.896156947	0.9658128	-6.1582054
ACBD4	0.0289996	2.65175038	0.13099441	0.896280276	0.9658128	-6.3903377
AGO2	-0.0174715	4.6243163	-0.130977	0.896294004	0.9658128	-6.8108417
PPP1R37	-0.0136156	5.25800053	-0.1308396	0.896402146	0.9658128	-6.8970236
MRPL17	-0.0126496	4.88224106	-0.1307528	0.896470491	0.9658128	-6.8776802
FIG4	0.01237974	4.88222198	0.13066901	0.896536428	0.9658128	-6.8384977
PRPF40A	0.01389656	7.17156002	0.13065726	0.89654568	0.9658128	-6.9323113
LAMTOR1	0.02174347	4.65193479	0.13062873	0.896568139	0.9658128	-6.7740675
SMU1	-0.0086745	6.58724625	-0.130571	0.896613599	0.9658128	-6.9460634
ORAI3	0.01856345	5.92387106	0.13052358	0.896650915	0.9658128	-6.9401511

BRD1	-0.0105883	4.81387652	-0.130334	0.896800183	0.96589099	-6.8477964
BCAS2	-0.0143657	4.20535888	-0.1296878	0.897308935	0.9663563	-6.7443997
TMEM154	-0.0209634	3.41029868	-0.1292832	0.897627441	0.96657317	-6.5605899
OMA1	-0.0212308	3.48565113	-0.1291517	0.897731014	0.96657317	-6.6167263
ENSCAFG00000029129	-0.0466718	4.81507429	-0.1291396	0.897740519	0.96657317	-6.8996888
WDR53	0.0210361	2.32830315	0.12879375	0.898012853	0.96667126	-6.2862867
ENSCAFG00000022659	-0.0609037	-1.0249716	-0.1287361	0.898058258	0.96667126	-5.5849144
CEBPG	0.02122239	3.70191907	0.12864993	0.898126099	0.96667126	-6.5309774
ENSCAFG00000016869	0.02241267	2.696443	0.12860802	0.898159097	0.96667126	-6.4396051
DMTF1	-0.0150356	4.42001137	-0.1285275	0.898222492	0.96667126	-6.8026426
NDUFA6	0.01730206	3.85510627	0.12838313	0.898336186	0.96667126	-6.6491928
RANBP2	0.01358797	7.55422442	0.12834168	0.898368827	0.96667126	-6.9173499
ASL	-0.0183444	4.09338672	-0.1280735	0.898579975	0.9668054	-6.7378952
MEIS1	0.04744264	3.60241432	0.12798843	0.898647004	0.9668054	-6.7358928
CYP2C18	-0.0668749	0.8145104	-0.1276622	0.898903934	0.9668817	-5.6999151
DRC3	0.04647171	-0.0272647	0.12751878	0.899016859	0.9668817	-5.7374705
FBXL18	-0.009517	6.23032599	-0.1274766	0.899050044	0.9668817	-6.9464626
HHEX	-0.0676843	0.1751361	-0.1274125	0.899100592	0.9668817	-5.948883
C20H19orf70	-0.023614	3.35552047	-0.127411	0.899101725	0.9668817	-6.599778
CD320	-0.0270743	2.01397736	-0.1273025	0.899187177	0.96689104	-6.2133422
ENSCAFG00000003032	-0.0549431	-0.6989911	-0.1271391	0.899315851	0.96691505	-5.4717592
IPPK	-0.0162162	3.75401814	-0.1270792	0.899363031	0.96691505	-6.6384434
ZNF672	0.04533764	0.4515771	0.12636464	0.899925866	0.96743759	-5.7376527
PHF1	-0.0210156	4.17713669	-0.1254818	0.90062125	0.96810251	-6.73613
FYCO1	-0.0227708	6.25079509	-0.1253246	0.900745136	0.9681444	-6.9456709
GSDME	0.06022577	-0.1276943	0.12523725	0.900813936	0.9681444	-5.7539288
NDUFAF3	0.01373961	3.83043318	0.12486416	0.901107853	0.96837766	-6.6620979
BNC1	0.24501383	-1.3280382	0.12462317	0.901297711	0.96843874	-5.2982489
RIOX2	-0.0158927	4.51026522	-0.1245669	0.901342023	0.96843874	-6.7519275
COQ9	-0.0115221	4.88869284	-0.1244797	0.90141071	0.96843874	-6.8630888
TRIM11	-0.0182981	3.31730323	-0.1244017	0.901472232	0.96843874	-6.5334124
PTPN23	-0.0101489	6.51592003	-0.12428	0.901568083	0.96844801	-6.9470507
ENSCAFG00000024837	0.0502172	0.15900886	0.12419552	0.901634633	0.96844801	-5.6644967
NCOR2	-0.0142915	7.68635223	-0.1238727	0.90188902	0.96863865	-6.9082589
DAB2	0.02519649	9.81534215	0.12348514	0.902194357	0.96888397	-6.8395953
PLEKHA1	-0.0153824	4.40470704	-0.1233624	0.902291097	0.96890526	-6.8321328
KHDC4	-0.0172396	3.67975148	-0.1230835	0.902510834	0.96903484	-6.6044596
STT3A	-0.0143842	8.35599512	-0.1229352	0.902627722	0.96903484	-6.8831629
NAA40	0.01607113	2.8546713	0.12290086	0.902654757	0.96903484	-6.3913057
MUM1L1	0.02717136	2.6775386	0.1228187	0.902719501	0.96903484	-6.3314438
BCAM	0.03989052	4.19263728	0.12271036	0.902804876	0.9690439	-6.8469035
SCAND1	0.01867703	4.41241583	0.12259804	0.902893386	0.96905304	-6.7511997
INSIG1	-0.053909	7.89348401	-0.1225043	0.902967258	0.96905304	-6.8209508
ZNF24	-0.014135	3.96038125	-0.1222491	0.903168333	0.9691388	-6.6975954
PANK4	-0.0144739	3.65124194	-0.1221031	0.903283423	0.9691388	-6.6475747
ENSCAFG00000005269	0.0443457	-0.4681269	0.12205166	0.903323971	0.9691388	-5.6458959
MIDN	0.0242874	5.02878319	0.12198982	0.903372703	0.9691388	-6.8317888
COX14	0.01708484	2.98643213	0.12191475	0.903431864	0.9691388	-6.4417901
ANKLE2	-0.0086923	6.14115216	-0.1216038	0.903676954	0.96931916	-6.9442833
BUD13	0.01523959	4.23927694	0.12134549	0.903880519	0.96941904	-6.7649926

RCC1	0.02526425	4.12948656	0.12121999	0.903979435	0.96941904	-6.6870492
ARMCX6	0.01902299	2.69254428	0.12117308	0.904016406	0.96941904	-6.3066135
XG	0.02057992	7.7204179	0.12109504	0.904077916	0.96941904	-6.9068827
DYRK1B	0.01917061	4.39097802	0.12083919	0.904279579	0.96946475	-6.8106854
CCDC89	-0.0438839	0.03560701	-0.1207757	0.904329622	0.96946475	-5.6839125
BAIAP2L1	-0.0954464	-1.461397	-0.120748	0.904351445	0.96946475	-5.4240494
DYRK2	-0.020186	5.60630172	-0.1206221	0.904450685	0.96948862	-6.9024384
EXOC5	-0.0151513	5.72388323	-0.1204068	0.904620364	0.96950813	-6.9330787
SNTB2	-0.0134075	4.35740046	-0.1204037	0.904622825	0.96950813	-6.8111394
TSTA3	-0.0112044	5.23454543	-0.120105	0.904858306	0.96960845	-6.8920827
GNL3	-0.0153336	5.66530894	-0.1200897	0.904870382	0.96960845	-6.9328415
MCM10	0.06882763	3.64727217	0.11998142	0.904955715	0.9696174	-6.3706743
MAP1S	-0.014627	5.95314669	-0.1196554	0.905212706	0.96970327	-6.9336323
FOXO3	0.02680331	5.68366279	0.11962997	0.905232767	0.96970327	-6.9477676
DDX3X	0.00857632	8.23551731	0.11958679	0.905266807	0.96970327	-6.8754662
TARS	-0.0158712	6.75108336	-0.1193267	0.90547183	0.96977327	-6.947174
PCMTD1	0.014668	6.18721604	0.11930194	0.90549137	0.96977327	-6.9401812
ST13	0.00933673	6.29810524	0.11921092	0.905563127	0.96977327	-6.9476046
CRLF1	0.06777263	5.06065829	0.11899943	0.905729855	0.96986936	-6.9124892
CHST1	0.05234075	2.79943828	0.11862532	0.906024805	0.97003726	-6.4735541
C7H18orf21	0.0146587	3.54786511	0.11856952	0.906068805	0.97003726	-6.5365573
BNIP1	-0.0202165	2.75207498	-0.1185075	0.906117685	0.97003726	-6.3658449
ENSCAFG00000008243	0.02017965	2.80890498	0.11835817	0.906235441	0.97004075	-6.4471709
SLC7A6	0.02159096	3.83137304	0.11821251	0.906350284	0.97004075	-6.6211961
IAH1	0.02798043	3.34480863	0.1181868	0.906370554	0.97004075	-6.5767084
SLC12A9	-0.0154546	4.41492316	-0.1181127	0.906428991	0.97004075	-6.7530138
ADCYAP1R1	0.10619444	-1.0324017	0.11780376	0.906672578	0.970219	-5.9908339
EPN1	0.01228452	6.64859795	0.11743123	0.906966327	0.97045089	-6.9475335
CAD	-0.0137902	7.47998122	-0.1170357	0.90727819	0.9706333	-6.9264501
TMTC3	-0.0144223	5.58334656	-0.1169379	0.907355371	0.9706333	-6.9106188
TADA1	-0.0217694	2.85923235	-0.1169219	0.90736798	0.9706333	-6.4643858
LIMD2	-0.0344553	1.62936073	-0.1166388	0.907591243	0.97069503	-6.0314673
PRPF3	0.01164508	5.28665532	0.11658543	0.907633305	0.97069503	-6.9026258
ENSCAFG00000032522	-0.0213534	3.18944076	-0.1164811	0.907715543	0.97069503	-6.4699209
NXN	0.01959991	5.82800989	0.11635017	0.907818831	0.97069503	-6.913708
DENND6A	-0.0166588	3.71617369	-0.1162491	0.907898553	0.97069503	-6.6340892
OCEL1	0.01827351	2.33286988	0.11615553	0.90797234	0.97069503	-6.3217124
CDK9	-0.0112259	5.66443378	-0.1160852	0.908027802	0.97069503	-6.9351703
UROS	0.01578872	4.65832553	0.11587793	0.908191271	0.97069503	-6.8381537
COPS5	0.01108956	5.19999432	0.11568683	0.90834199	0.97069503	-6.8876716
AHDC1	-0.0255034	5.35727926	-0.1156409	0.908378203	0.97069503	-6.9191576
MRPS11	0.01361585	3.90642238	0.11556941	0.908434599	0.97069503	-6.6441032
PLEKHG1	0.06901868	1.98880093	0.11556523	0.908437896	0.97069503	-5.9773226
STX3	-0.0470875	1.11816982	-0.1154199	0.908552542	0.97069503	-5.8569922
COX7A2L	-0.0102952	5.89317674	-0.115397	0.908570587	0.97069503	-6.9383459
SEC61A2	0.0136177	3.03471987	0.11535418	0.908604357	0.97069503	-6.4360467
MOB1B	-0.0337103	1.65217092	-0.1151439	0.908770229	0.97069503	-6.0897134
GPHN	-0.0137456	4.2472161	-0.1151305	0.908780807	0.97069503	-6.6854079
KYAT3	0.01756553	2.76270334	0.11508986	0.908812838	0.97069503	-6.4735411
NRG2	0.07325779	-1.1359316	0.11472562	0.909100136	0.97089668	-5.5719121

CREG2	0.06697615	-1.9201147	0.11456963	0.909223186	0.97089668	-5.6043378
PEX6	0.01301496	4.43176222	0.11444438	0.909321978	0.97089668	-6.8115881
ARHGAP44	-0.019323	3.16231962	-0.1143922	0.909363106	0.97089668	-6.4989415
SUCLG1	-0.010129	6.07756004	-0.1143585	0.90938975	0.97089668	-6.9463611
ARID1A	-0.0099875	6.20450214	-0.1142402	0.909483046	0.97089668	-6.9478532
TFIP11	0.00911269	5.6031647	0.11416649	0.909541188	0.97089668	-6.9209221
PEX3	0.01347141	4.06986256	0.11389424	0.909755957	0.97098016	-6.6976617
CDV3	-0.0148416	7.95537987	-0.1138719	0.909773563	0.97098016	-6.9049215
ENSCAFG00000002083	0.02351196	2.68146438	0.11364903	0.909949394	0.97103219	-6.4459487
ADPRHL2	-0.0200245	3.68608333	-0.1136147	0.909976502	0.97103219	-6.639227
TTLL4	0.02380402	4.01482153	0.11341102	0.910137156	0.97112136	-6.7207162
PKP3	-0.0716702	-1.1761199	-0.1132833	0.910237895	0.97114658	-5.5342155
CYFIP1	0.00709461	7.86969925	0.1131618	0.910333773	0.97116661	-6.903122
RBM3	0.01892086	6.27975772	0.11280811	0.910612815	0.97126595	-6.9422422
CAPN10	0.01582519	4.06361687	0.11266089	0.910728968	0.97126595	-6.6698394
CXHXorf56	-0.0250042	1.73801027	-0.1126604	0.910729317	0.97126595	-6.1095301
IFIT3	0.07484126	0.28629187	0.11265284	0.910735322	0.97126595	-5.6932954
SRI	-0.0143705	5.15948386	-0.1123364	0.910985025	0.9714486	-6.8959251
MEDAG	0.04734439	7.57469783	0.11210623	0.911166601	0.9714486	-6.9090973
FARP2	-0.0143827	3.87353387	-0.1120333	0.911224134	0.9714486	-6.7203431
PHACTR4	0.00961615	6.20445761	0.11200857	0.911243652	0.9714486	-6.9486502
TBC1D19	-0.0119535	4.39940864	-0.111947	0.911292209	0.9714486	-6.8047441
PTGDR	0.03597904	3.69936316	0.1118424	0.911374773	0.9714544	-6.8580583
ENSCAFG00000031206	0.04183008	-1.2319603	0.11173859	0.911456681	0.9714595	-5.6322899
ARID3B	-0.0291484	1.3232676	-0.111635	0.911538387	0.97146438	-6.0851717
SPSB3	-0.0162855	3.18292412	-0.1115265	0.911624035	0.97147346	-6.5957226
HNRNPAB	-0.0146001	5.96193744	-0.1113709	0.911746829	0.97152213	-6.936487
WDR3	0.01381519	6.44575613	0.111261	0.911833539	0.97153233	-6.9480895
PGGT1B	0.02218265	2.65076316	0.1111411	0.911928154	0.97155096	-6.2858666
ENSCAFG00000001388	0.03351469	0.73270055	0.11085024	0.912157678	0.97156293	-5.8338297
CREBZF	-0.0223441	2.64375403	-0.1108349	0.912169752	0.97156293	-6.2902554
UBE4A	0.00904098	7.08073843	0.11083363	0.912170783	0.97156293	-6.9313514
HEPHL1	-0.0772198	-2.1137427	-0.1105762	0.912373955	0.97157671	-5.3066279
ENSCAFG00000032225	0.0397847	-0.1106271	0.11049717	0.912436303	0.97157671	-5.7200428
YARS2	0.01263205	2.85445873	0.11046828	0.912459107	0.97157671	-6.3708646
SCRN2	-0.032147	1.65904306	-0.1104263	0.912492258	0.97157671	-6.0117736
ENSCAFG00000029044	0.0146946	3.87604029	0.10997835	0.912845756	0.97187094	-6.6517258
RANGAP1	-0.0230026	4.42110247	-0.1096853	0.913077051	0.97203504	-6.7178936
NENF	-0.0148897	4.64466395	-0.1095733	0.913165415	0.97204695	-6.8612837
MED14	-0.0165195	5.8104352	-0.1094651	0.913250801	0.97205569	-6.9368608
ENSCAFG00000030958	0.13253811	4.95382318	0.10911309	0.913528663	0.97219763	-6.7084706
ENSCAFG00000011703	-0.0288913	2.39360666	-0.1091006	0.913538514	0.97219763	-6.2433763
RAB14	-0.0070964	7.15577326	-0.108666	0.913881585	0.97248057	-6.9337095
SLAIN1	-0.0442779	-0.0936654	-0.1084553	0.914047887	0.97255795	-6.011132
GADD45GIP1	-0.0219871	3.29934773	-0.1083782	0.914108732	0.97255795	-6.4168273
TGIF2	-0.0191347	2.34813109	-0.1079384	0.914455883	0.97284513	-6.2897232
TRAF4	0.01990258	3.55619736	0.1076871	0.91465428	0.97297401	-6.6323526
TFE3	-0.0132725	6.50459651	-0.1075328	0.914776078	0.97302141	-6.9489532
ATXN10	0.01030952	6.19803847	0.1069631	0.915225842	0.97340439	-6.9466381
MRPL11	-0.0117526	4.41962878	-0.106881	0.915290696	0.97340439	-6.775387

ENSCAFG00000002956	0.0422995	1.29028409	0.1064841	0.915604011	0.97363658	-6.0169306
ZSCAN20	-0.0220102	2.13381512	-0.1064086	0.915663613	0.97363658	-6.311638
RBBP4	-0.0078079	5.77943405	-0.1062288	0.915805618	0.97366178	-6.9308727
ADAMTS4	0.04116866	2.62042956	0.10618278	0.915841917	0.97366178	-6.365585
C29H8orf37	0.02308764	1.71861554	0.10605908	0.915939585	0.97368343	-6.1811252
NRP1	0.02505347	7.22398334	0.1057161	0.916210389	0.97384116	-6.9173092
PIGQ	0.01564941	5.35130825	0.10538882	0.916468809	0.97384116	-6.8759899
HDAC8	-0.0161943	2.57539767	-0.1053321	0.916513579	0.97384116	-6.2989625
GPX8	-0.0160663	5.89287907	-0.1053045	0.916535428	0.97384116	-6.9284821
NUDT18	0.01597922	2.7018124	0.1052764	0.916557579	0.97384116	-6.3365697
FADD	-0.0175131	2.88502023	-0.1052729	0.916560365	0.97384116	-6.4351013
OLR1	0.10309926	-0.218807	0.10518576	0.916629154	0.97384116	-5.4988561
GEMIN2	-0.0150523	2.91476332	-0.1049595	0.916807815	0.9739392	-6.4066017
NECAB3	0.02987668	3.71759278	0.10487305	0.916876077	0.9739392	-6.7416387
TBC1D20	-0.0096923	5.89746223	-0.1047196	0.916997277	0.97398581	-6.9421907
ZKSCAN8	-0.0139089	3.43131758	-0.1043683	0.917274704	0.97416002	-6.6006977
ENSCAFG00000024756	-0.0137773	4.62091552	-0.104316	0.917315975	0.97416002	-6.8079851
PCSK6	0.05518545	6.01140731	0.10373251	0.917776773	0.97444544	-6.9468239
TCF3	0.01238469	4.89238672	0.10360684	0.917876023	0.97444544	-6.837567
ENSCAFG00000015024	0.04802268	-0.2689951	0.10351493	0.917948606	0.97444544	-5.614538
UBA52	0.0167712	7.92626146	0.10346976	0.917984284	0.97444544	-6.8885443
ENSCAFG00000031506	0.01632147	4.05889283	0.1034142	0.918028166	0.97444544	-6.7413287
USP32	0.00940781	5.4654046	0.10338662	0.918049942	0.97444544	-6.9334503
CALCOCO2	0.01029209	7.24107527	0.10328998	0.918126269	0.97444544	-6.9243966
FAM126B	0.01506255	3.05533071	0.10281738	0.918499533	0.97475947	-6.4487826
ZNF526	0.0169162	2.72855016	0.10227762	0.918925866	0.97503334	-6.4303364
ANKS1A	0.01314336	4.4538365	0.10218604	0.9189982	0.97503334	-6.8245924
TMEM129	0.01502808	3.66127736	0.10213384	0.919039436	0.97503334	-6.6037606
RAB15	0.04779874	-0.7223462	0.10188036	0.919239655	0.97503334	-5.5685199
REEP4	0.02430614	3.16752338	0.10187134	0.91924678	0.97503334	-6.4239554
ENSCAFG00000006595	-0.0715761	-1.8850211	-0.1016882	0.919391455	0.97503334	-5.3261229
DNAJC21	-0.0108244	3.87582774	-0.1016285	0.919438631	0.97503334	-6.666279
SUPT3H	0.0141435	3.67529227	0.10156946	0.919485244	0.97503334	-6.6227107
SCNN1A	0.01761393	2.98132528	0.10152402	0.91952114	0.97503334	-6.5057853
TFG	-0.0068566	7.3473992	-0.1013601	0.919650633	0.97503334	-6.9281601
RRP8	0.0225403	3.36237376	0.10125675	0.919732268	0.97503334	-6.5139707
COMMD6	0.01722321	1.70029861	0.10118812	0.919786486	0.97503334	-6.0847843
ZNF575	-0.030223	1.62154159	-0.1011302	0.91983221	0.97503334	-6.1188813
HIVEP2	0.01355306	5.65743125	0.10111871	0.919841313	0.97503334	-6.9428614
HNRNPH2	-0.0075719	5.85188557	-0.1008601	0.920045643	0.97514719	-6.9409769
EPOP	-0.036055	0.34611096	-0.1007868	0.920103551	0.97514719	-5.8203654
ENSCAFG00000030548	-0.0350238	2.23479781	-0.1006115	0.920241982	0.97521185	-6.0623054
TGM1	0.0311705	0.1342025	0.10034187	0.92045502	0.97532646	-5.7858465
FGL1	-0.0881789	-1.6448821	-0.1000969	0.920648563	0.97532646	-5.7116912
UBE3C	-0.0064917	5.94940266	-0.1000593	0.920678278	0.97532646	-6.9459863
RARB	-0.0673694	-0.6693702	-0.1000114	0.920716127	0.97532646	-5.5905559
ALKBH6	0.01277722	3.54335709	0.09998458	0.920737293	0.97532646	-6.6329869
TNFRSF6B	0.03276987	1.53363529	0.09944852	0.921160824	0.97569305	-6.1118535
ENSCAFG00000011656	0.04198537	5.40231159	0.09934927	0.921239244	0.97569407	-6.9406863
TMEM150C	-0.0518816	-0.2243974	-0.0991613	0.921387731	0.9757652	-5.8095475

EPM2AIP1	-0.0133032	3.42789402	-0.0989962	0.92151819	0.9757652	-6.616526
HARBI1	-0.0262102	1.48888272	-0.0988175	0.921659433	0.9757652	-6.1003071
APPL2	-0.0157413	5.26196638	-0.0987866	0.92168385	0.9757652	-6.846317
CCDC150	-0.0607377	1.79541714	-0.0987741	0.921693743	0.9757652	-5.9269014
LASP1	0.00753087	8.64793036	0.09858954	0.921839536	0.97583753	-6.840152
CCDC115	0.01150324	4.60439053	0.0982842	0.922080814	0.97595074	-6.8167295
MRPL22	-0.0098817	3.95985743	-0.0982581	0.922101445	0.97595074	-6.6747651
ENSCAFG00000008380	0.0380611	0.48750394	0.09802633	0.922284587	0.97600559	-5.7637897
ENSCAFG00000023591	0.03392559	4.07495289	0.09787243	0.922406198	0.97600559	-6.8753367
TTC3	-0.0100245	8.25323996	-0.0978501	0.922423857	0.97600559	-6.8736381
RBL2	-0.0136917	5.80247213	-0.0978003	0.922463207	0.97600559	-6.9469031
DTWD1	-0.014886	4.90932722	-0.0975661	0.922648276	0.97611941	-6.889645
HERC4	0.0089641	5.59017479	0.09713916	0.922985675	0.97632073	-6.9272817
NDUFAF2	-0.0248795	2.05703449	-0.0970532	0.92305364	0.97632073	-6.0040723
FAM160B1	-0.0075749	5.06895258	-0.0970311	0.923071102	0.97632073	-6.8635449
ENSCAFG00000024769	0.05971839	-2.3961346	0.09692756	0.923152899	0.97632527	-5.3311864
KIAA0825	-0.0193524	2.37641646	-0.0964485	0.923531505	0.97664368	-6.3160017
FARSA	0.01338657	4.94810072	0.09625403	0.923685214	0.97666828	-6.8496862
ENSCAFG00000025063	-0.0173651	6.82236224	-0.0960635	0.923835834	0.97666828	-6.9386075
TRIM44	0.0141152	2.91334372	0.09592648	0.923944094	0.97666828	-6.3071656
SEC13	0.01060603	6.4016812	0.09585291	0.924002243	0.97666828	-6.9501104
MDH1B	0.03883127	0.29545006	0.09584517	0.92400836	0.97666828	-5.8071612
GNPTAB	-0.0102415	6.97494899	-0.0958304	0.924019997	0.97666828	-6.9454675
VPS35	0.00674493	7.61364096	0.09530835	0.924432666	0.97695314	-6.9146367
FANK1	-0.0561148	0.86889436	-0.0952333	0.924491995	0.97695314	-5.9364864
ZER1	-0.0123634	5.2237418	-0.0951951	0.92452218	0.97695314	-6.9020836
SAC3D1	0.02079035	2.12162158	0.095027	0.924655063	0.97701159	-6.2042004
MKRN2	-0.0109135	3.93913632	-0.0946569	0.92494765	0.97706801	-6.7166976
ENSCAFG00000032717	-0.0365856	1.95659089	-0.0946381	0.924962486	0.97706801	-6.1350984
NSF	-0.0084744	6.37407241	-0.094483	0.925085082	0.97706801	-6.9504184
POLE3	0.01010372	5.50040218	0.09429286	0.925235383	0.97706801	-6.9143716
EIF2AK3	0.01056564	5.4367957	0.09422599	0.925288241	0.97706801	-6.9314994
ENSCAFG00000031129	-0.0129522	4.74082916	-0.0939785	0.925483863	0.97706801	-6.8395699
FOXM1	0.04959317	3.00310077	0.09397698	0.925485091	0.97706801	-6.1267907
ALKBH8	-0.0150955	2.48795865	-0.0939762	0.925485685	0.97706801	-6.32803
FAM155B	-0.0461233	1.45320341	-0.0939294	0.925522708	0.97706801	-5.7860404
ZBTB43	0.0198213	1.70959261	0.09368628	0.925714899	0.97706801	-6.1408092
ENSCAFG00000004113	-0.0519102	0.0849655	-0.0936514	0.925742481	0.97706801	-5.6635805
KLF10	-0.0139674	6.78637283	-0.0933699	0.925965047	0.97706801	-6.9417099
ATG101	0.01015781	5.347599	0.09314094	0.926146035	0.97706801	-6.881713
MICAL3	0.01778815	4.33161063	0.09310875	0.926171482	0.97706801	-6.7748675
WNT11	-0.0558621	-2.0321218	-0.0930695	0.926202497	0.97706801	-5.5463885
ENSCAFG00000016965	-0.0395749	1.59214989	-0.0929259	0.926316033	0.97706801	-6.1049372
SMYD3	-0.0138064	2.87423254	-0.0928874	0.926346512	0.97706801	-6.4123842
IL22RA1	-0.0596892	-2.0218124	-0.0928248	0.926395971	0.97706801	-5.3015352
AUNIP	-0.0630483	-0.9242294	-0.092503	0.926650379	0.97706801	-5.4792731
HMOX2	0.00868302	5.26040071	0.09241914	0.926716701	0.97706801	-6.8889755
PROCR	0.02359056	3.19028218	0.09215346	0.926926765	0.97706801	-6.8611965
PHLPP2	0.01098202	4.17238113	0.09212498	0.926949283	0.97706801	-6.8075261
HMBS	0.00990775	4.15882315	0.09210006	0.926968982	0.97706801	-6.7061048

MARS	-0.0092428	6.1264233	-0.09205	0.927008533	0.97706801	-6.941446
ZBTB34	0.01750564	1.93353474	0.09202626	0.927027337	0.97706801	-6.2042859
HTATIP2	-0.012344	3.84036823	-0.0918971	0.927129443	0.97706801	-6.7181967
ARFIP1	0.01003574	5.29954878	0.09165854	0.92731809	0.97706801	-6.9189206
FAM71E1	-0.0335836	-0.8141914	-0.0916527	0.927322683	0.97706801	-5.6654433
PSMC3IP	-0.0495113	-0.7197002	-0.0914816	0.927458	0.97706801	-5.4963829
ZNF226	0.01727406	2.08965808	0.09145118	0.927482054	0.97706801	-6.2304737
STK36	0.01061314	4.51063402	0.09144162	0.927489614	0.97706801	-6.813357
CKAP2L	0.06388981	3.12352555	0.09141431	0.927511207	0.97706801	-6.1489867
DNAH11	0.0318479	0.00731149	0.09131447	0.927590147	0.97706801	-5.8408481
AAAS	-0.0121482	5.10608982	-0.0912821	0.927615778	0.97706801	-6.8568125
RAPGEF2	-0.0108431	5.78927372	-0.0912251	0.927660812	0.97706801	-6.942644
FOXP1	0.01204197	6.03534854	0.09097649	0.927857409	0.97706801	-6.9395843
SLX4IP	-0.0240445	0.52326814	-0.0909302	0.927893986	0.97706801	-5.8536028
BTBD2	0.01354326	5.79318927	0.09088611	0.927928873	0.97706801	-6.9399677
LRRC17	-0.0936917	0.85013018	-0.0908738	0.927938645	0.97706801	-5.6054979
CCDC78	0.02155161	1.810528	0.09084133	0.927964284	0.97706801	-6.2676761
IL17RE	-0.0348676	0.08236917	-0.090751	0.928035736	0.97706801	-5.7121429
PIGF	0.01565674	2.3073507	0.09067733	0.928093968	0.97706801	-6.2275725
TBPL1	0.03115958	-0.198727	0.09056057	0.928186303	0.97706801	-5.6780852
KDM5B	0.01196188	6.33159788	0.09053649	0.928205342	0.97706801	-6.9506527
PAIP2B	0.02147109	1.25119514	0.09052447	0.928214852	0.97706801	-6.0666902
FBXW11	-0.0080303	6.41341909	-0.0904463	0.928276661	0.97706801	-6.950817
ENSCAFG00000030304	0.02017459	0.97160532	0.09013503	0.92852282	0.97720258	-5.98834
HPS3	-0.0155712	4.09963559	-0.0900203	0.928613529	0.97720258	-6.7059756
TMX2	-0.0111615	4.61764532	-0.0899903	0.928637254	0.97720258	-6.8116311
SNTB1	-0.0588648	-2.1383923	-0.0897125	0.92885697	0.97732039	-5.3335213
ENSCAFG00000020250	0.01290532	4.14759999	0.08956255	0.92897556	0.97732039	-6.7052793
PDSS1	-0.0219868	3.51312249	-0.0895544	0.928981984	0.97732039	-6.4643804
RFX8	0.04731645	-1.5988673	0.08942374	0.929085335	0.97734749	-5.5956749
LYPLA1	0.01091242	3.93411456	0.08925043	0.929222403	0.97741005	-6.6549204
ENSCAFG00000017791	0.01818191	1.56969725	0.08908708	0.929351592	0.97746432	-6.1354087
ZFYVE19	0.01524404	2.50114479	0.08895345	0.929457282	0.97746877	-6.2636577
OXSM	-0.0118797	3.40140872	-0.0887528	0.929615957	0.97746877	-6.5897455
ENSCAFG00000015158	0.02691324	0.7533447	0.08869488	0.929661783	0.97746877	-5.8861198
ENSCAFG00000032245	-0.0145223	2.6524941	-0.0886893	0.929666229	0.97746877	-6.3282191
KBTBD7	0.02434261	0.87115383	0.0885412	0.929783333	0.9775103	-5.9355782
ENSCAFG00000020075	0.03731325	1.78681217	0.08830732	0.92996832	0.97757872	-5.9875205
ANXA6	0.00922914	8.94190331	0.08819611	0.930056284	0.97757872	-6.8201199
KIF2A	-0.0100645	6.43358776	-0.0881646	0.930081244	0.97757872	-6.9509278
SFT2D1	0.00796925	3.97856058	0.08793641	0.930261693	0.97765694	-6.7292594
FMO3	-0.0498603	1.08588132	-0.087857	0.930324499	0.97765694	-6.0786503
ENSCAFG00000031490	-0.0197285	14.3903636	-0.0877761	0.930388514	0.97765694	-6.3882873
ENSCAFG00000019017	-0.0220875	0.82187126	-0.087579	0.930544389	0.97773917	-5.8138722
GLS	-0.0122716	6.889966	-0.0870505	0.930962485	0.97791017	-6.9508337
ARHGAP42	0.00909806	4.9793194	0.08704521	0.930966643	0.97791017	-6.7737763
ZNF605	0.02036389	2.22448259	0.0870013	0.931001373	0.97791017	-6.263315
ARPC5L	-0.0123244	4.38561025	-0.0868918	0.931087994	0.97791017	-6.7781765
ARSJ	0.02162724	6.28109641	0.08679888	0.931161494	0.97791017	-6.9507306
RAD54B	-0.0371018	0.7577782	-0.0867564	0.931195073	0.97791017	-5.7583719

ZNF428	0.01041788	3.45316721	0.08660212	0.931317148	0.97791017	-6.5051856
CCBE1	-0.0655859	-2.0993781	-0.0864637	0.931426685	0.97791017	-5.4187541
ITGAE	-0.0224477	1.27424196	-0.0863201	0.931540281	0.97791017	-6.0157013
POLL	-0.0109164	3.33765181	-0.0862091	0.931628072	0.97791017	-6.5986705
CTBP2	0.00964501	5.15002544	0.08620209	0.9316336	0.97791017	-6.9159898
SDCCAG8	0.01382717	3.31866193	0.08617929	0.931651643	0.97791017	-6.5562461
LAMC3	0.06732024	-1.1794361	0.08609741	0.931716412	0.97791017	-5.4861683
PIK3CB	-0.0121641	4.28310713	-0.0856281	0.932087716	0.97802774	-6.726558
CEP131	-0.0118023	5.14617252	-0.0855383	0.932158778	0.97802774	-6.9013976
NEIL3	-0.0574017	-1.3035454	-0.0852477	0.932388666	0.97802774	-5.3629395
KDM2A	0.00641541	6.66833201	0.08513862	0.932474949	0.97802774	-6.9467735
FBXL15	-0.0249324	1.35219597	-0.0850744	0.932525734	0.97802774	-5.9702364
ARPIN	0.00865781	4.58255326	0.08496262	0.932614194	0.97802774	-6.8049959
CHST15	-0.0240385	5.6557594	-0.0849438	0.932629091	0.97802774	-6.9095532
ST3GAL4	-0.0140779	5.21973979	-0.0849159	0.932651182	0.97802774	-6.8581241
IQCE	0.0085939	4.19914692	0.08488207	0.932677924	0.97802774	-6.7526643
ANKRD40	0.00915919	4.13499426	0.08477353	0.932763802	0.97802774	-6.6882785
LDB1	-0.0072674	5.43741865	-0.0847227	0.932804024	0.97802774	-6.9309352
KIRREL1	-0.0140704	6.9949629	-0.0846938	0.932826843	0.97802774	-6.9513079
ENSCAFG00000023205	0.01850401	3.14103484	0.08461542	0.932888895	0.97802774	-6.449207
PPFIA1	-0.0060321	5.8210271	-0.084507	0.932974676	0.97802774	-6.9381538
ENSCAFG00000011417	-0.0872989	2.37548666	-0.0844038	0.933056362	0.97802774	-6.1460287
VAT1	-0.0161006	6.65317273	-0.0843856	0.933070762	0.97802774	-6.9500791
WDR48	0.01030976	5.02418416	0.08426369	0.933167188	0.97804742	-6.8933559
ENSCAFG00000014399	-0.0096672	4.18248802	-0.0839874	0.933385831	0.97819518	-6.7241088
PCID2	-0.0070992	5.33432554	-0.08376	0.933565764	0.97830236	-6.9224681
SVIL	-0.0343591	4.59260243	-0.0834736	0.93379235	0.9784584	-6.8432152
DDHD1	0.01587349	4.00999807	0.08310255	0.934085963	0.97862463	-6.7595002
FAM13B	0.01274633	4.10556653	0.0830415	0.934134268	0.97862463	-6.7031893
AASDH	-0.0083548	4.30356054	-0.0829786	0.934184066	0.97862463	-6.7614322
ENSCAFG00000014684	0.01514936	5.73566695	0.08241259	0.934631946	0.97893598	-6.9364657
DMXL1	-0.0092934	5.56183788	-0.0824066	0.934636718	0.97893598	-6.9263152
RBM15	-0.0091308	3.1541643	-0.0820212	0.934941647	0.97917394	-6.5326575
PPL	0.04805688	7.1138253	0.08179893	0.935117578	0.97918656	-6.78028
ARRB2	-0.0122994	2.83594653	-0.0817233	0.935177419	0.97918656	-6.5125705
ARHGEF39	-0.04179	2.95600224	-0.0817113	0.935186906	0.97918656	-6.1995556
ENSCAFG00000016649	0.03836081	-0.327509	0.08153321	0.935327873	0.97925275	-5.5560334
SND1	0.00831227	8.48999145	0.0812862	0.935523362	0.97937602	-6.8596283
ENSCAFG00000011598	-0.018952	5.13427795	-0.0811146	0.935659133	0.97938188	-6.8640187
TUBD1	0.01991166	1.59337451	0.08101907	0.935734778	0.97938188	-6.0614594
APIP	0.00858084	4.59659843	0.0809844	0.935762216	0.97938188	-6.8291728
CCDC174	0.0085769	3.58470175	0.0806501	0.936026802	0.97951866	-6.6532159
TARS2	0.00901131	4.71041666	0.08062277	0.936048433	0.97951866	-6.8063888
ODF2L	0.02592443	1.33904291	0.08049115	0.936152609	0.97954629	-5.9226211
CENPK	0.04256412	1.07875038	0.08033687	0.936274716	0.97959268	-5.8081806
FIGNL1	-0.0171517	2.45716355	-0.0801443	0.936427149	0.97967079	-6.1636239
PPP4C	-0.0083314	5.77931843	-0.0799513	0.936579919	0.97972707	-6.9323478
CTPS1	0.01523454	5.31266088	0.07981317	0.936689224	0.97972707	-6.8909244
KAZALD1	0.04399358	2.26901159	0.07978152	0.93671428	0.97972707	-6.5849449
DUSP18	0.02080464	2.88091881	0.07954868	0.936898576	0.97983846	-6.4427585

ENSCAFG00000030272	-0.0395562	-0.7358798	-0.0792661	0.937122238	0.97986211	-5.4166105
TWF1	0.01170823	7.04383398	0.07924999	0.937135004	0.97986211	-6.9442326
KCNE4	-0.070707	1.69862666	-0.0791953	0.937178307	0.97986211	-5.7255501
PDGFRL	0.03919647	2.93352008	0.07912701	0.937232353	0.97986211	-6.5393639
TMEM33	0.00954234	4.97116528	0.07895512	0.937368418	0.97989852	-6.8378812
SLC37A3	-0.0118876	4.96508498	-0.0787954	0.937494879	0.97989852	-6.8357387
SNRPF	-0.0135452	4.09972969	-0.0787687	0.937515976	0.97989852	-6.7072956
MTHFSD	0.00783029	3.71627107	0.07863358	0.937622944	0.97989852	-6.6516827
NHLRC1	0.0298031	0.37140767	0.07841245	0.937797995	0.97989852	-5.7641306
ACOT6	-0.0383273	-0.9733958	-0.0783653	0.937835353	0.97989852	-5.6035489
VPS8	0.00605388	6.24396825	0.07833488	0.937859397	0.97989852	-6.9504233
ENSCAFG00000006506	0.01906923	1.52141166	0.07829681	0.937889532	0.97989852	-6.0426569
TRMT112	0.00810762	5.31928987	0.07791981	0.938187977	0.98005629	-6.9054381
TNFAIP8L3	0.0760874	0.26342232	0.07789183	0.938210127	0.98005629	-5.6129853
DHX8	-0.0052356	5.85062852	-0.0778112	0.938273955	0.98005629	-6.9403364
GALE	0.01950309	3.9802772	0.07769051	0.938369504	0.98007482	-6.636129
NKAIN1	-0.016811	4.39941029	-0.0775719	0.938463421	0.98009164	-6.696106
PID1	-0.0453293	5.05598479	-0.0772601	0.938710282	0.98026817	-6.9255439
RNF6	0.01055217	6.24228909	0.07706595	0.938863951	0.98034737	-6.9477918
ST6GALNAC6	-0.0114234	5.67437584	-0.0764015	0.939390023	0.98064525	-6.9387459
ENSCAFG00000012580	0.01520579	2.37790763	0.07633236	0.939444753	0.98064525	-6.2843916
POLR2A	0.00646502	7.33078357	0.07619704	0.939551886	0.98064525	-6.9312643
ENSCAFG00000032459	0.0741718	1.73491513	0.07605147	0.939667143	0.98064525	-5.9858658
ENSCAFG00000002375	-0.0357342	-0.5133666	-0.0760337	0.939681242	0.98064525	-5.563241
ENSCAFG00000000071	0.00971866	3.55728603	0.07601755	0.939693998	0.98064525	-6.6073426
ALS2CL	0.0583117	0.74513082	0.0760173	0.939694199	0.98064525	-5.6954878
ENSCAFG00000014496	-0.0109853	3.27995492	-0.0755005	0.940103391	0.980991	-6.5307552
NUP188	-0.0083498	6.5796527	-0.0751269	0.94039921	0.98110195	-6.9520436
GID4	0.00812099	4.58554693	0.07511825	0.940406054	0.98110195	-6.8645547
FAM114A2	0.01022844	6.06701985	0.0750711	0.940443388	0.98110195	-6.9478485
ZNF596	-0.0250637	1.05608644	-0.0749153	0.940566791	0.98112133	-5.9148213
ENSCAFG00000004560	-0.0139981	2.17898779	-0.074776	0.940677036	0.98112133	-6.1417771
CENPO	-0.0283219	1.64749823	-0.0747525	0.940695638	0.98112133	-5.9110391
ANTXR2	-0.0082103	6.43620265	-0.0745498	0.940856137	0.98120748	-6.9480271
NPHP1	0.01380912	2.47660231	0.07439139	0.940981615	0.98125709	-6.3712897
CREBRF	-0.0181594	3.25697378	-0.0741674	0.941159011	0.98136084	-6.7003832
KCNQ2	0.03588848	-0.8031661	0.07382109	0.941433219	0.9815655	-5.7710001
TPST1	0.0185574	4.86293562	0.073601	0.941607505	0.98166596	-6.9256148
YIF1B	-0.0160115	5.59016538	-0.0733499	0.941806323	0.98179198	-6.9360591
DENR	0.01035143	5.71051171	0.07294318	0.94212845	0.9820465	-6.9216493
PAPSS1	-0.008378	6.73062488	-0.0725382	0.942449172	0.98227934	-6.9493293
ENSCAFG00000007165	0.01927387	1.50226389	0.07228372	0.942650723	0.98227934	-6.056188
NT5DC2	0.017727	6.15164898	0.07222015	0.942701068	0.98227934	-6.9518942
LMLN	-0.0090621	5.19761026	-0.0721795	0.942733239	0.98227934	-6.8902849
ENSCAFG00000006847	0.00689675	6.71347678	0.0721688	0.94274174	0.98227934	-6.9497174
FASN	0.01222816	9.04505082	0.07183762	0.943004035	0.98240916	-6.8130282
CYB5D2	0.01389113	2.50944375	0.07176753	0.943059547	0.98240916	-6.3396945
TRAM1	0.00969852	7.96842935	0.07162856	0.943169614	0.98240916	-6.9020073
ENSCAFG00000017016	-0.0607821	0.09363467	-0.0716176	0.943178312	0.98240916	-5.5625225
C5H1orf159	-0.024183	1.10641664	-0.0714741	0.943291913	0.98244625	-5.890287

RCBTB1	0.01119313	3.59243054	0.07126742	0.94345565	0.98249736	-6.676601
ENSCAFG00000000554	-0.0090759	3.69863072	-0.0712152	0.943496993	0.98249736	-6.6673016
PSPH	-0.0104152	5.86639798	-0.0710541	0.943624615	0.98250555	-6.9317293
RAB1A	-0.0047912	7.03144563	-0.0710083	0.943660855	0.98250555	-6.9414749
SLC9A3R2	0.01431191	4.72609612	0.07053323	0.944037172	0.98274378	-6.8891443
EFHC1	-0.0196541	1.44274061	-0.0705224	0.944045712	0.98274378	-6.1181195
ZC3H12A	0.01697441	3.29089907	0.07020989	0.944293284	0.98292027	-6.5444164
SNIP1	-0.0095684	2.80439664	-0.0699867	0.944470104	0.98299169	-6.3814104
SLAIN2	0.00557137	5.86745904	0.06992623	0.944517974	0.98299169	-6.9385176
ENSCAFG00000019388	-0.012035	2.72490972	-0.0698111	0.944609189	0.9830054	-6.4231825
ENSCAFG00000028505	0.00826628	3.51541122	0.06958697	0.944786718	0.98310892	-6.5652349
SMARCD1	-0.0122402	4.3423337	-0.0694257	0.944914448	0.98313882	-6.7696883
PTBP3	-0.0086614	5.18355118	-0.0693239	0.944995126	0.98313882	-6.919261
SMIM15	0.00777878	4.02184438	0.06925511	0.945049602	0.98313882	-6.686038
TMCC2	-0.0117196	3.38016036	-0.0691518	0.945131454	0.98314277	-6.5139821
GPRC5A	0.02868028	4.83102176	0.06904388	0.945216923	0.98315048	-6.595681
TMEM53	-0.0152074	2.14291266	-0.0688166	0.945396935	0.98318424	-6.228302
TWIST2	-0.0311196	2.03349663	-0.0688059	0.945405482	0.98318424	-6.1402721
ENSCAFG00000032731	-0.0358276	-2.6547685	-0.0685353	0.945619824	0.98332596	-5.4056795
SFT2D2	-0.0097245	2.71728955	-0.0683098	0.945798451	0.98343052	-6.3873186
CDC73	0.0079023	4.91541656	0.06818863	0.945894448	0.98344915	-6.8587437
PSMD7	-0.0071737	6.36513386	-0.068006	0.946039156	0.98349098	-6.9525417
CASP10	0.02902083	1.99364889	0.06794074	0.946090836	0.98349098	-6.0710857
NPC2	0.01567588	6.6891091	0.0678181	0.946187997	0.98351081	-6.9423594
GTF3A	0.00856746	6.56309543	0.0676972	0.946283779	0.98352868	-6.9523724
IFT52	0.00917607	4.15109769	0.0674459	0.946482865	0.98352868	-6.7449321
ZNF282	-0.007058	4.23283402	-0.0673727	0.946540824	0.98352868	-6.7711806
CROT	-0.0092215	5.21865746	-0.0672615	0.946628924	0.98352868	-6.9173211
DENND2C	0.0125027	4.56542282	0.06711746	0.946743079	0.98352868	-6.7648
MOSMO	-0.0106417	2.00994653	-0.067092	0.946763267	0.98352868	-6.2023597
ENSCAFG00000018187	0.03147701	3.23084371	0.06708313	0.946770277	0.98352868	-6.410597
ARMC8	-0.0093202	3.40168119	-0.0669952	0.946839913	0.98352868	-6.5881394
CARD19	0.01552552	3.08677829	0.06690938	0.946907934	0.98352868	-6.4145269
PSMD12	0.00823639	5.85953657	0.06672481	0.947054167	0.98356936	-6.9406114
CMTR1	0.00430549	5.52121365	0.06659815	0.947154524	0.98356936	-6.9208841
EEFSEC	-0.0068677	3.62118438	-0.0665643	0.947181357	0.98356936	-6.5029545
AFF4	-0.0077869	6.9490843	-0.066419	0.947296502	0.98360784	-6.9484158
SPG11	-0.0060392	5.53494314	-0.0662683	0.9474159	0.98363598	-6.9257653
EIF4ENIF1	0.00573565	5.37679797	0.06609792	0.947550863	0.98363598	-6.9176716
DCAF7	0.00498787	4.88207405	0.06608908	0.947557869	0.98363598	-6.859591
USO1	0.0070698	7.2898393	0.06596137	0.947659059	0.98365995	-6.935555
NEK4	-0.0068266	4.19606657	-0.0655949	0.947949438	0.98388028	-6.7720253
GSPT1	-0.0087556	6.91698997	-0.0653573	0.948137677	0.98399458	-6.9471841
CCL14	-0.0351744	1.91605552	-0.0650857	0.948352893	0.98405815	-6.9082717
TRIT1	-0.0089559	3.13476726	-0.0650828	0.948355182	0.98405815	-6.5339533
SPRED1	-0.0074828	4.82796836	-0.0647644	0.948607495	0.98411095	-6.9101157
SHMT1	-0.0316488	1.39539234	-0.0647366	0.948629562	0.98411095	-5.9846674
PLA2G3	0.02547124	2.91327538	0.06472281	0.948640451	0.98411095	-6.6244044
TXNRD2	0.00857453	3.12490098	0.06461046	0.948729482	0.98412226	-6.5006616
PRX	-0.0163655	1.62518902	-0.0643261	0.94895482	0.98427494	-6.0938702

BRAF	0.00704445	4.05948248	0.06410454	0.949130379	0.98430378	-6.7079323
DVL3	0.0073211	7.41794646	0.06404676	0.949176173	0.98430378	-6.9260139
PHKG2	-0.0090537	3.74934212	-0.0639126	0.9492825	0.98430378	-6.6293447
SNX11	0.00742026	3.2355517	0.06386266	0.949322062	0.98430378	-6.4800314
FBXL12	-0.0069811	4.67984671	-0.0637315	0.949425966	0.98430378	-6.7820075
MID1IP1	-0.0117573	3.4746205	-0.0636927	0.949456746	0.98430378	-6.4902684
ENSCAFG00000000136	-0.0054501	9.0687853	-0.0635074	0.949603617	0.98430378	-6.8160518
RELA	0.00708826	6.41762738	0.06350212	0.949607774	0.98430378	-6.9529452
PDXP	-0.0163603	1.17331256	-0.0630689	0.949951049	0.98435189	-6.0090638
FAM45A	0.00604299	5.21370386	0.06285245	0.950122623	0.98435189	-6.8819414
LIG4	0.01022189	4.14659788	0.0628442	0.950129163	0.98435189	-6.7417068
ENSCAFG00000004247	0.00547712	5.40977274	0.06283415	0.950137123	0.98435189	-6.9061089
PLA2G6	0.01306743	2.76567454	0.06280077	0.950163581	0.98435189	-6.4646362
PDE12	-0.0065314	5.44302688	-0.0627499	0.95020389	0.98435189	-6.9154797
PIAS1	0.00728475	5.29850286	0.06266053	0.950274717	0.98435189	-6.9111242
TIMM8B	0.01484462	2.43226304	0.06265466	0.95027937	0.98435189	-6.2320865
PGM5	0.05525081	2.52017323	0.06249054	0.950409438	0.98440566	-5.7353724
SLC4A8	-0.0101695	2.73231764	-0.0623904	0.950488791	0.98440691	-6.2742049
ENSCAFG00000013419	0.00707051	3.95515905	0.06196872	0.950822995	0.98446168	-6.6759966
LCORL	-0.0104134	2.58963473	-0.06187	0.950901266	0.98446168	-6.3674458
PFDN1	0.01085154	1.92211552	0.06185668	0.950911793	0.98446168	-6.0884414
KLF11	-0.0158666	3.34272284	-0.0618121	0.950947101	0.98446168	-6.606652
RERG	-0.0678548	1.19095148	-0.061582	0.951129456	0.98446168	-5.7016642
CTNNBIP1	-0.022334	-0.2563232	-0.0614534	0.951231381	0.98446168	-5.668958
NARS	-0.0071966	7.73831257	-0.0614406	0.951241591	0.98446168	-6.924323
INTS13	-0.0102165	5.00273481	-0.0613778	0.951291349	0.98446168	-6.9052877
FAM221A	0.01961721	1.04579181	0.06132695	0.951331639	0.98446168	-5.8201602
CHN2	-0.0537142	-1.2612531	-0.0612941	0.951357642	0.98446168	-5.4622383
AP2M1	-0.0047626	7.93633018	-0.0611433	0.951477174	0.98446168	-6.9041097
CYP27A1	0.03692777	3.067818	0.06114032	0.951479556	0.98446168	-6.7474816
ABCG8	-0.0326421	-2.8555467	-0.0609485	0.95163162	0.98448524	-5.3090082
TRIAP1	0.02128909	0.26063186	0.06085677	0.951704297	0.98448524	-5.8250696
ENSCAFG00000013040	-0.0094347	4.24007992	-0.0608158	0.951736807	0.98448524	-6.7757873
INO80C	-0.0092759	3.47483481	-0.060553	0.951945048	0.98450568	-6.5008822
ZMPSTE24	0.00667521	6.38143352	0.06051398	0.95197599	0.98450568	-6.9519056
RAB4A	0.00566042	3.62244643	0.06049499	0.951991046	0.98450568	-6.6431535
CTXN1	-0.0342001	0.71276778	-0.0602134	0.952214239	0.98452218	-5.889151
PXN	-0.0098986	8.15478685	-0.0602119	0.952215445	0.98452218	-6.8777808
LANCL1	0.00549351	7.82528809	0.06014272	0.952270263	0.98452218	-6.895745
PPP1CB	-0.0048881	6.29172353	-0.0599535	0.952420255	0.98452218	-6.9495357
PNPLA8	-0.0054362	6.01292693	-0.0599269	0.952441355	0.98452218	-6.9526979
ENSCAFG00000001559	-0.0383376	-1.1634435	-0.0598832	0.952475969	0.98452218	-5.3214851
ENSCAFG00000004696	-0.0094181	2.51982163	-0.0597241	0.952602042	0.9845717	-6.4097548
TRAF2	0.01005921	3.17027831	0.0586668	0.953440183	0.98532141	-6.5098425
MUT	-0.0044262	5.37580576	-0.0585184	0.953557795	0.98532141	-6.9233684
PPP1R9A	-0.0364458	-1.3959218	-0.058513	0.953562084	0.98532141	-5.4981437
TJP2	0.0136738	5.80628996	0.05810738	0.95388364	0.98557283	-6.9430457
ABLIM2	-0.0438653	-0.0955441	-0.0578037	0.954124366	0.98567699	-5.9023647
ZFPM1	0.01617873	3.58621781	0.05761021	0.954277765	0.98567699	-6.585909
PAQR6	0.02692033	1.21924244	0.05758974	0.954293994	0.98567699	-6.0824999

HPRT1	-0.0068247	5.71916114	-0.0575854	0.95429747	0.98567699	-6.9392668
ENSCAFG00000012022	-0.029238	6.86797916	-0.0574433	0.954410066	0.9856845	-6.9051915
ENSCAFG00000023349	0.02480237	2.17599004	0.05731023	0.954515578	0.9856845	-6.2786698
MRPS25	-0.0073292	4.27721685	-0.0571964	0.954605808	0.9856845	-6.7528759
MRPL41	-0.0132495	2.81678068	-0.0571414	0.954649411	0.9856845	-6.3175662
GATM	-0.0668419	-0.6922685	-0.0570279	0.954739442	0.9856845	-5.3171791
BAG6	-0.0045894	7.76126174	-0.0569839	0.954774262	0.9856845	-6.9156311
CRCP	-0.0067106	3.27366088	-0.0566322	0.955053137	0.9858916	-6.5631545
VEPH1	-0.0257077	1.06533883	-0.0564454	0.955201191	0.9858954	-6.299205
LRRC49	-0.0124078	4.75765299	-0.0563821	0.955251394	0.9858954	-6.8538257
CCNT1	0.0060097	5.1781121	0.0562584	0.95534946	0.9858954	-6.9221698
CDCP1	-0.0227913	6.37226587	-0.0561815	0.955410409	0.9858954	-6.9505404
ESS2	0.00664093	3.79394311	0.0561339	0.95544817	0.9858954	-6.7019954
RPS6KA3	-0.0108901	5.8615507	-0.0560152	0.955542282	0.98591175	-6.9254258
DNAAF1	-0.0287323	-0.8046446	-0.0558011	0.955712013	0.9860061	-5.4337939
RLIM	0.00549974	4.82697084	0.05546165	0.955981157	0.98606113	-6.8467813
AARS	-0.0073066	8.02468129	-0.0553359	0.956080854	0.98606113	-6.9177106
VAV2	-0.0083401	5.76339546	-0.0553068	0.95610391	0.98606113	-6.9197888
SDHAF4	-0.0077272	2.96442781	-0.0552834	0.956122467	0.98606113	-6.3714474
DYSF	-0.0408467	4.28409856	-0.0552402	0.956156763	0.98606113	-6.5256102
NOCT	0.00996781	2.84029896	0.0548657	0.956453662	0.98616691	-6.3962445
DLG2	0.03331887	-1.0372691	0.0547928	0.956511464	0.98616691	-5.6989127
ANO10	0.00605949	6.09790807	0.05460086	0.956663652	0.98616691	-6.9484092
FAF2	-0.0043634	5.05148796	-0.0545927	0.956670158	0.98616691	-6.8836457
B3GNT9	-0.0169041	3.47959665	-0.0545328	0.956717622	0.98616691	-6.5080428
BTRC	0.00733787	4.75823859	0.05447151	0.956766213	0.98616691	-6.8686257
ENSCAFG00000007869	-0.0226165	0.47957227	-0.0543098	0.956894455	0.98616691	-5.6792264
CHTF18	0.02219282	2.62367941	0.05430654	0.956897011	0.98616691	-6.2441359
EARS2	0.00829461	2.69612434	0.0542221	0.95696397	0.98616691	-6.4057221
TPD52L1	-0.0367914	0.27292045	-0.0538531	0.957256566	0.98626669	-5.7578077
PSMD10	0.00544519	4.53345211	0.05378277	0.95731232	0.98626669	-6.7962508
MAGEF1	0.01423804	2.40073783	0.05373099	0.95735338	0.98626669	-6.1501436
DYNLRB2	0.01940436	0.77832115	0.05369336	0.957383219	0.98626669	-5.8587629
ENSCAFG00000019779	-0.0166083	0.91177441	-0.0534909	0.957543716	0.98626669	-5.80701
CYP2R1	0.01988884	2.73428925	0.05344507	0.957580093	0.98626669	-6.229318
ENSCAFG00000002336	-0.0157086	1.8808439	-0.0533243	0.957675818	0.98626669	-6.0199606
LYPLA2	-0.006466	6.09526038	-0.05331	0.95768719	0.98626669	-6.9501351
TRPC4	0.07453178	0.86298581	0.05315272	0.957811912	0.98627542	-5.3796305
MBOAT7	-0.0078568	5.09940607	-0.0531018	0.957852275	0.98627542	-6.8533678
GATA5	-0.0868209	1.17663761	-0.0526316	0.958225156	0.98657872	-5.4459863
PHYHIPL	0.03260293	1.18183442	0.05219602	0.958570548	0.9867047	-6.5121555
HERPUD1	0.00587985	6.92578749	0.05211492	0.958634862	0.9867047	-6.9394899
ENSCAFG00000003544	0.02393239	-0.6123392	0.05208985	0.958654744	0.9867047	-5.4733396
FAM122A	0.01034934	2.28751604	0.05208215	0.958660849	0.9867047	-6.2383687
COMMD5	-0.0107933	3.17168086	-0.0518965	0.958808099	0.98671344	-6.4276576
SMO	-0.0166164	4.17503812	-0.0518309	0.958860065	0.98671344	-6.5724216
PIK3C2A	-0.0079354	6.87043233	-0.0516824	0.958977856	0.98671344	-6.9483974
PSMA3	-0.0040137	6.30102594	-0.0515658	0.959070323	0.98671344	-6.9531718
GAPVD1	0.0049216	6.01745724	0.05145487	0.959158289	0.98671344	-6.9493066
PDCD6IP	0.00430584	7.93193219	0.05124835	0.959322063	0.98671344	-6.9028079

FBRSL1	0.00780754	4.01208634	0.05122263	0.959342459	0.98671344	-6.6956314
NCAPD3	-0.0116095	4.61922918	-0.0511781	0.959377752	0.98671344	-6.7681122
NUAK2	0.02300083	1.79413174	0.05113088	0.959415226	0.98671344	-6.0968841
TOR3A	0.0062633	5.10395782	0.05093329	0.959571919	0.98671344	-6.9048676
VPS37C	-0.0067525	3.07096756	-0.0508043	0.959674238	0.98671344	-6.4696847
ECPAS	0.00324944	7.73535593	0.05079508	0.959681529	0.98671344	-6.9094374
DIO3	-0.0297226	0.57439323	-0.0507873	0.959687706	0.98671344	-6.2258064
SS18	0.00407077	6.29808112	0.0503751	0.960014598	0.98691935	-6.9534321
RAD1	-0.0076688	2.82471852	-0.0503372	0.960044679	0.98691935	-6.3044594
FAM186B	-0.0219617	-0.428509	-0.0500481	0.960273952	0.98707448	-5.6433537
GULP1	0.01402304	5.65850816	0.04987683	0.960409768	0.98713353	-6.8507026
ENSCAFG00000015181	-0.0078071	4.61491903	-0.0491523	0.960984359	0.98746707	-6.8043387
ABCC1	0.01261659	4.65313114	0.04914669	0.960988844	0.98746707	-6.849038
C9H17orf80	-0.0071342	2.73417779	-0.0490903	0.961033604	0.98746707	-6.3819814
TBKBP1	-0.0103686	2.93084718	-0.0490723	0.961047853	0.98746707	-6.4829285
HOXB4	-0.0167476	1.32979065	-0.0486678	0.961368699	0.98771015	-5.9779004
C15H16orf87	-0.0142562	0.37187867	-0.0485315	0.961476774	0.98771015	-5.7900335
KLHL7	0.00579694	4.73678942	0.04834866	0.961621788	0.98771015	-6.851419
ENSCAFG00000017239	-0.0169998	0.26189758	-0.0482959	0.961663672	0.98771015	-5.7360526
WDYHV1	-0.007034	2.24769898	-0.0482797	0.961676512	0.98771015	-6.1720115
ENSCAFG00000030437	0.01997472	-0.3372032	0.04796286	0.96192779	0.98786083	-5.6641247
BRCA1	0.01914053	3.55443054	0.04789694	0.96198007	0.98786083	-6.4621485
CALML4	0.02381726	-0.6755144	0.04752051	0.962278648	0.98798254	-5.6304151
ARPC1A	0.00348951	7.55172313	0.04743306	0.962348009	0.98798254	-6.9160981
MTCL1	0.00885955	5.6371378	0.04743185	0.962348975	0.98798254	-6.9139394
DPH2	0.00860033	2.61364681	0.04719146	0.962539648	0.98798254	-6.3880262
FBXL20	-0.0065064	4.29874379	-0.0471852	0.962544587	0.98798254	-6.7507272
ENSCAFG00000031877	0.00672609	2.42884684	0.0471542	0.962569203	0.98798254	-6.292444
LLGL2	0.02501134	0.14564568	0.04692092	0.962754238	0.98799508	-5.6886911
CAPN3	-0.0202784	-0.2450089	-0.0468677	0.962796423	0.98799508	-5.7156197
ETV1	-0.0073256	5.26085838	-0.04667	0.962953261	0.98799508	-6.940241
RUBCN	0.0071014	3.73345606	0.04651344	0.963077454	0.98799508	-6.616026
PLAG1	0.00771032	1.46269253	0.04629429	0.963251291	0.98799508	-6.4545738
ENSCAFG00000019220	0.01099932	3.76017281	0.04606419	0.963433813	0.98799508	-6.6189616
H3F3A	0.00439704	4.83167447	0.04591016	0.963555995	0.98799508	-6.858442
PAAF1	-0.0106937	2.0818657	-0.045896	0.963567208	0.98799508	-6.2376376
WDR13	0.00426428	5.05500982	0.04584303	0.96360925	0.98799508	-6.8679224
ENSCAFG00000007919	-0.0042612	4.42943612	-0.0458027	0.963641259	0.98799508	-6.7716178
LARP1B	0.00577296	4.51476974	0.04574008	0.963690914	0.98799508	-6.8181929
ABCB8	0.00638791	4.75817506	0.04556512	0.963829702	0.98799508	-6.8417465
C15H1orf109	0.0050025	4.39468016	0.04553067	0.963857029	0.98799508	-6.7483716
ZNF268	-0.0074861	2.68652008	-0.0455167	0.963868116	0.98799508	-6.4282973
DDX58	0.01345921	3.6144921	0.04542401	0.963941634	0.98799508	-6.6000528
ENSCAFG00000006088	-0.0054511	3.62240282	-0.0452038	0.96411629	0.98799508	-6.6445764
NPAS3	-0.0288644	-1.4048391	-0.0450572	0.964232588	0.98799508	-5.3090435
STK35	-0.0038015	5.03799952	-0.0450473	0.964240468	0.98799508	-6.9031699
KATNAL1	-0.0127431	2.39224359	-0.0450364	0.964249109	0.98799508	-6.2458153
ENSCAFG00000007327	0.01104232	1.60610146	0.04490795	0.964351016	0.98799508	-6.0199808
SMIM12	0.00803937	2.80077484	0.04490236	0.964355449	0.98799508	-6.3628807
CHTOP	0.00383841	5.23936865	0.04487475	0.964377348	0.98799508	-6.8985749

NUDT12	-0.0061274	4.83638639	-0.0448645	0.964385478	0.98799508	-6.8584403
CACYBP	-0.0045122	6.40543053	-0.044637	0.964565956	0.98809487	-6.9538904
USP21	0.00757925	3.22606155	0.04449036	0.964682283	0.98809487	-6.5323463
BCL9	-0.0051659	4.95685149	-0.0444451	0.964718218	0.98809487	-6.8689689
ENSCAFG00000004223	0.02719919	0.56610942	0.04424785	0.964874662	0.98814744	-5.6193582
GJA4	0.02897427	-2.6067085	0.04418258	0.964926442	0.98814744	-5.3366608
ENSCAFG00000030024	0.01928784	0.64687911	0.04376541	0.965257389	0.98834302	-5.8714488
ENSCAFG00000007199	-0.0478421	-1.0762296	-0.043744	0.965274364	0.98834302	-5.3976867
MFN1	-0.0037778	5.52631192	-0.0436291	0.965365527	0.98835603	-6.9262359
POLR3A	-0.0046916	5.46002065	-0.0432772	0.965644662	0.98845321	-6.9184497
YES1	0.00941173	5.45316222	0.04325884	0.965659269	0.98845321	-6.936345
B3GALNT2	0.00614441	4.14371976	0.04308118	0.96580021	0.98845321	-6.7476307
GSTM4	0.01248154	6.0151218	0.04303605	0.965836018	0.98845321	-6.9509401
PGBD1	-0.012578	0.99209484	-0.0430149	0.965852822	0.98845321	-5.8794374
SNAPC2	0.00586323	3.80553927	0.04269992	0.966102685	0.9886286	-6.6463504
ENSCAFG00000024216	0.02597623	-2.0606058	0.04224685	0.966462134	0.98886822	-5.3382536
MUL1	0.00319036	5.20695648	0.04220686	0.966493864	0.98886822	-6.8894861
MRPL13	0.00470043	3.93955024	0.04204676	0.966620881	0.98891785	-6.6748129
FRS2	-0.0094052	4.24540204	-0.0415653	0.967002833	0.98922827	-6.7664947
OTUD7A	0.02034077	-1.4418127	0.04139535	0.967137705	0.98924841	-5.6541394
NPRL2	-0.0038529	4.65763773	-0.0413426	0.967179594	0.98924841	-6.8449984
BAMBI	-0.0387786	0.23022716	-0.0409986	0.967452477	0.98940705	-5.5901069
SOCS7	0.01049532	1.43622963	0.04086674	0.967557108	0.98940705	-6.032548
ENSCAFG00000030940	0.0186231	2.19570597	0.04085007	0.967570341	0.98940705	-6.1771333
INTS9	0.0053949	4.32180335	0.0407021	0.967687737	0.98944677	-6.7777423
FBP1	-0.0331024	-1.2220774	-0.0404667	0.96787451	0.98954069	-5.4799424
ECSIT	0.00626553	3.65593083	0.04038831	0.967936711	0.98954069	-6.5764445
SLC25A27	0.00862278	1.44815299	0.03976386	0.968432177	0.98982138	-6.14401
MPP1	-0.0058755	4.91408053	-0.0396543	0.968519076	0.98982138	-6.8621917
KIF3A	0.0054944	4.05450496	0.03964683	0.968525034	0.98982138	-6.7306584
ENSCAFG00000008186	0.00529581	3.97069304	0.03964611	0.968525608	0.98982138	-6.6827742
POLDIP2	-0.0043079	5.55307723	-0.0393257	0.968779851	0.99000089	-6.9198114
ACSF3	0.00606987	2.98958263	0.03908527	0.968970619	0.99009346	-6.5185034
WDSUB1	-0.0048524	4.23836705	-0.0389354	0.969089552	0.99009346	-6.7242792
PIWIL4	0.01523646	0.9314054	0.03882074	0.969180514	0.99009346	-5.6951513
ZNF300	0.00663696	2.95975854	0.03881527	0.969184853	0.99009346	-6.4575267
ATPAF2	-0.0051602	3.5308841	-0.0384628	0.969464565	0.99018453	-6.5923686
TMEM17	-0.0062915	2.78747873	-0.0382495	0.969633805	0.99018453	-6.478903
UGCG	-0.0061781	6.70345468	-0.0381729	0.96969455	0.99018453	-6.9445584
MRPS34	0.01264921	2.8720261	0.038088	0.969761945	0.99018453	-6.3807191
MATN2	-0.0331623	2.83305801	-0.0380816	0.969767028	0.99018453	-5.6659443
KLHDC1	0.00875293	1.66447511	0.03802179	0.969814481	0.99018453	-6.1614439
ENSCAFG00000005504	0.00290194	6.18451062	0.03800944	0.969824278	0.99018453	-6.9532677
LYPLAL1	-0.0073852	2.59633991	-0.0377797	0.970006578	0.99029039	-6.3775101
OCIAD1	-0.0030793	7.00819649	-0.037513	0.970218233	0.9904262	-6.9472843
PIGV	0.00591414	2.93610351	0.03735249	0.970345589	0.9904451	-6.4944757
APBB1	-0.0047834	5.89996025	-0.0372267	0.97044537	0.9904451	-6.9499253
ENSCAFG00000030711	-0.0116572	0.29026772	-0.0370585	0.970578894	0.9904451	-5.7938469
TRAPPC4	0.00484222	3.22482734	0.03699618	0.970628333	0.9904451	-6.4906889
ZDHHC18	-0.0098379	1.84746502	-0.036898	0.970706252	0.9904451	-6.2335567

MAST4	-0.0085875	5.36138935	-0.0367318	0.970838141	0.9904451	-6.9226929
GOLIM4	-0.0053994	5.95599248	-0.0367236	0.970844653	0.9904451	-6.951549
FAM234B	0.0071756	2.691551	0.03669692	0.970865803	0.9904451	-6.5287633
BPHL	0.01317374	2.50299168	0.03597406	0.971439437	0.99095004	-6.4243777
ENSCAFG00000003953	-0.0041225	4.2485713	-0.0357376	0.971627081	0.9909621	-6.8132516
TRIP4	-0.0033386	4.58557152	-0.0356933	0.971662267	0.9909621	-6.8120157
MEGF6	-0.0305904	3.38518092	-0.0356618	0.971687274	0.9909621	-5.6830385
RAD54L2	-0.0035476	4.69053383	-0.0352226	0.972035779	0.99120582	-6.8505054
ENSCAFG00000008701	-0.0035251	5.58802384	-0.0351623	0.972083636	0.99120582	-6.921385
PLEKHA6	-0.0111348	5.59607415	-0.0349128	0.972281651	0.99128223	-6.9440264
ENSCAFG00000018145	-0.0096933	2.48296972	-0.0348695	0.97231597	0.99128223	-6.2802961
FOSL1	-0.0090737	4.94975264	-0.0345704	0.972553412	0.99144406	-6.8236572
RRBP1	0.00469662	9.36355574	0.03438369	0.972701553	0.99151483	-6.8029906
GUSB	0.00571293	6.78398731	0.03423103	0.972822702	0.99155808	-6.924149
WDR81	-0.0048093	5.169403	-0.0339849	0.973018053	0.9916549	-6.8834965
FADS2	0.00783704	8.64331684	0.03391294	0.973075146	0.9916549	-6.8292213
ENSCAFG00000020158	0.01199351	0.19233806	0.03278852	0.973967539	0.99241669	-5.7502044
DPH7	0.00440568	4.25157403	0.03273023	0.974013802	0.99241669	-6.7944665
PSMG3	0.00784716	1.42123356	0.03267325	0.974059028	0.99241669	-5.9520849
CCNDBP1	0.00497402	4.31842284	0.03247917	0.974213057	0.99249334	-6.8431799
PRXL2C	-0.0042843	3.85382531	-0.0323786	0.974292845	0.99249435	-6.6687207
GTPBP2	-0.0050958	4.92527285	-0.0319576	0.974627012	0.99266379	-6.825408
FRMD3	0.02266059	1.2521285	0.03193168	0.974647591	0.99266379	-5.8681216
GFOD2	-0.005582	3.18519106	-0.0318099	0.97474427	0.99266379	-6.6100451
NDUFAF4	0.0047061	4.71859151	0.03171997	0.974815623	0.99266379	-6.8337897
ENSCAFG00000011847	-0.0132739	1.10823595	-0.0316726	0.974853212	0.99266379	-5.8899411
NR5A2	0.0167623	-2.5764496	0.03126528	0.975176506	0.99291272	-5.4120619
MYCBP2	0.0064731	6.97087706	0.03113124	0.975282896	0.99294078	-6.9500108
YIPF6	0.00413933	4.36299346	0.03099032	0.975394739	0.99297439	-6.7075575
VPS13D	0.00513942	6.70882058	0.03078542	0.97555737	0.99305969	-6.9506671
SCARB1	-0.0060447	6.46553959	-0.030659	0.975657718	0.9930603	-6.9456655
UBR7	-0.0026346	5.67373362	-0.030586	0.975715645	0.9930603	-6.934104
ENSCAFG00000030732	-0.0043904	2.79885525	-0.0301069	0.976095902	0.99311622	-6.4222069
ENSCAFG00000031737	0.0197462	-2.0849742	0.03008264	0.976115182	0.99311622	-5.3285577
CMKLR1	0.0209635	1.04951238	0.03007334	0.976122565	0.99311622	-6.1521594
SGIP1	0.01754222	-2.1324984	0.02995293	0.976218139	0.99311622	-5.3861896
MORN2	-0.0073099	2.25848359	-0.0299504	0.976220112	0.99311622	-6.2198161
NUDT7	-0.0048618	2.89778409	-0.0299208	0.976243656	0.99311622	-6.4581526
ITGA4	0.03324869	-1.307274	0.02951622	0.976564771	0.99331348	-5.3209363
IGF2BP2	-0.0086102	6.91759107	-0.0292652	0.976764039	0.99331348	-6.9391965
TXNIP	-0.0140438	5.73675626	-0.0290431	0.976940332	0.99331348	-6.9099745
PIGN	0.00313732	4.46085572	0.02901975	0.976958842	0.99331348	-6.8031382
HOXA10	0.02789097	-0.8876733	0.02893129	0.977029058	0.99331348	-5.5635921
MFSD8	-0.0046775	1.86131728	-0.0289158	0.977041375	0.99331348	-6.1170566
SRD5A1	-0.0062427	2.25837396	-0.0286937	0.977217651	0.99331348	-6.1978314
KLHDC4	0.00247813	4.96978343	0.02850246	0.977369453	0.99331348	-6.8682159
WASHC4	-0.0030977	5.55753391	-0.0284397	0.977419236	0.99331348	-6.9332751
LHX8	-0.0206567	-2.5231878	-0.0283968	0.977453345	0.99331348	-5.35671
PRRC2A	0.00185361	8.62735788	0.02816273	0.977639115	0.99331348	-6.8574251
DYM	0.00330314	7.62507249	0.02812929	0.977665664	0.99331348	-6.926173

ENSCAFG00000029335	-0.0151975	-0.9878321	-0.0280865	0.977699605	0.99331348	-5.4656366
MAN2C1	-0.0027108	5.45362219	-0.027994	0.977773016	0.99331348	-6.9112993
CDC40	-0.0034054	4.50263015	-0.0279937	0.977773256	0.99331348	-6.819135
CGNL1	-0.0208542	6.75262227	-0.027846	0.977890511	0.99331348	-6.8254448
MAPKAPK5	0.00239407	4.3018883	0.02784436	0.977891834	0.99331348	-6.7886886
EXTL3	-0.0051136	5.18057942	-0.0277445	0.977971074	0.99331348	-6.9418201
C1H19orf47	-0.0028559	3.2635935	-0.0277181	0.97799209	0.99331348	-6.5431547
FAM102B	-0.0060298	4.42169315	-0.0276477	0.978047917	0.99331348	-6.7904528
ATP11B	-0.0021536	7.32132143	-0.0275902	0.978093605	0.99331348	-6.9336097
ISY1	-0.002703	5.3667869	-0.0271803	0.978418973	0.99343324	-6.9094079
ENSCAFG00000032656	-0.0064795	2.05324432	-0.0271361	0.978454079	0.99343324	-6.3319797
STX7	-0.0028177	6.17019712	-0.0270908	0.978489977	0.99343324	-6.9496132
C12H6orf106	0.00274657	6.53512292	0.02704418	0.978527006	0.99343324	-6.9544319
HIKESHI	-0.0023931	4.6392849	-0.026596	0.978882774	0.99363594	-6.8006897
PLET1	-0.0139678	-3.0496324	-0.0265939	0.978884438	0.99363594	-5.3034496
KAT2B	0.00664637	6.49424993	0.02646344	0.978988003	0.99366099	-6.9524481
UBAP2L	-0.0020896	7.87407351	-0.0262119	0.979187696	0.99371727	-6.9055023
DHRS3	-0.0167032	2.40472693	-0.0261948	0.979201234	0.99371727	-6.2690302
EP400	0.00279377	6.15843388	0.02600703	0.979350307	0.99374152	-6.9530011
ICA1	-0.0202754	0.40552169	-0.0259262	0.979414462	0.99374152	-5.5884609
SMIM1	-0.0132114	0.99268712	-0.0258589	0.979467893	0.99374152	-5.7843281
KCTD9	0.00357462	5.4743906	0.02571892	0.979579015	0.99374152	-6.9314045
DHX34	-0.0034612	4.06896085	-0.0254588	0.979785493	0.99374152	-6.7471914
ERICH6	0.00572044	2.99944087	0.02531845	0.979896924	0.99374152	-6.4904467
BCL10	-0.0037354	4.42792021	-0.0252525	0.979949257	0.99374152	-6.8084849
HIP1R	0.00378031	4.24197893	0.0252509	0.979950545	0.99374152	-6.7225146
MRPS26	-0.004547	2.59555469	-0.0249338	0.980202294	0.99374152	-6.3212335
C24H20orf96	-0.0115226	-0.5582792	-0.0248974	0.980231196	0.99374152	-5.6369369
ATPAF1	-0.0039208	3.39552805	-0.0248826	0.980242911	0.99374152	-6.6003039
STRIP1	0.00219963	5.25645989	0.02485678	0.980263413	0.99374152	-6.8750118
MPG	-0.004292	3.48010674	-0.0247984	0.980309739	0.99374152	-6.5770645
KDM5C	-0.0014002	6.74384385	-0.0247734	0.980329635	0.99374152	-6.9527329
ENSCAFG00000014568	0.00546935	1.30395308	0.0243877	0.980635798	0.99388279	-5.9499211
VPS50	-0.0027439	4.26413483	-0.0243409	0.980672916	0.99388279	-6.7409025
TMEM135	-0.0039232	2.968362	-0.0242996	0.980705715	0.99388279	-6.4275168
KCNJ3	0.01958843	-1.5758898	0.02414403	0.980829233	0.993928	-5.4258708
SPC25	-0.0159374	1.72872669	-0.0240417	0.980910479	0.99393037	-5.8985456
OCA2	0.01396311	0.34380605	0.02350461	0.981336847	0.99416862	-5.6121677
ZNF513	-0.0035163	3.30523761	-0.0234295	0.981396495	0.99416862	-6.5295636
ENSCAFG00000007865	0.00690758	1.49069795	0.02336822	0.981445125	0.99416862	-5.9741942
S100A13	0.00374005	2.76442657	0.02314334	0.981623647	0.99416862	-6.3304516
TARDBP	-0.0019928	4.83382894	-0.0231411	0.981625441	0.99416862	-6.8323116
SLC12A4	0.00186335	7.31982728	0.02304413	0.981702411	0.99416862	-6.9390266
NBEAL1	0.00326245	5.18397524	0.02295515	0.981773054	0.99416862	-6.9058474
EPYC	-0.0191657	0.68785787	-0.0229	0.981816835	0.99416862	-5.7991967
MRPL43	-0.0036226	3.09956643	-0.0228507	0.981855957	0.99416862	-6.4878995
ENSCAFG00000000042	0.0065512	0.75410544	0.02260394	0.982051872	0.99421573	-5.8752929
PAGR1	-0.0044112	0.99948917	-0.0225933	0.98206035	0.99421573	-5.9114149
FAM118A	-0.0039621	2.25087546	-0.0222719	0.982315491	0.99428953	-6.2872202
C9H17orf58	0.00919725	0.17248399	0.02215931	0.982404862	0.99428953	-5.7641443

TBCCD1	0.00261908	3.96578603	0.02203502	0.982503536	0.99428953	-6.7195406
CWC27	-0.0021716	4.64931967	-0.0219736	0.982552276	0.99428953	-6.8471719
ILDR2	-0.01679	-0.0961148	-0.0219693	0.982555695	0.99428953	-5.8005481
ENSCAFG00000014182	-0.0074945	0.6921173	-0.0217525	0.982727855	0.99428953	-5.9289803
MNT	0.00322352	5.1794015	0.02172411	0.982750366	0.99428953	-6.9000659
UQCC2	0.00286645	3.6454523	0.02158262	0.982862695	0.99428953	-6.6156973
ENSCAFG00000015518	-0.013181	-2.5983569	-0.0215726	0.982870663	0.99428953	-5.3035429
VASH1	-0.0054533	3.26528029	-0.0215071	0.982922614	0.99428953	-6.4663164
MRPL32	-0.0023909	3.71129151	-0.0210695	0.983270101	0.99456117	-6.5970141
GPATCH1	-0.0041376	2.48122469	-0.0208128	0.98347386	0.99468739	-6.2904918
NUDT17	-0.0074563	0.07387697	-0.0206547	0.983599408	0.9946894	-5.7518882
DPT	0.01739098	2.39260891	0.02059841	0.983644072	0.9946894	-6.3334443
NCBP1	-0.0017795	6.413071	-0.0205119	0.983712757	0.9946894	-6.9546305
ENSCAFG00000000576	-0.0055563	1.57615102	-0.020131	0.984015141	0.99491529	-6.0617204
NR2F2	0.00675612	5.29586586	0.01984497	0.984242252	0.99499135	-6.8928226
TOE1	0.00260121	3.82005717	0.01983729	0.984248355	0.99499135	-6.6276172
QTRT2	0.0033445	3.40673562	0.01955515	0.984472351	0.99509545	-6.5727277
ENSCAFG00000031302	0.00914191	2.50928174	0.01950858	0.984509325	0.99509545	-6.550237
SNRNP35	-0.003261	3.29216157	-0.0193174	0.984661129	0.99514584	-6.5069193
WDFY2	-0.003232	4.6791598	-0.0192468	0.984717192	0.99514584	-6.847094
HNMT	-0.0031361	4.15769743	-0.0189184	0.984977885	0.99532944	-6.8110574
UPF2	0.00210512	5.02773471	0.01864457	0.985195309	0.99546929	-6.9040113
CDH1	-0.0097178	-2.2014076	-0.0183401	0.985437052	0.99551478	-5.4478882
ELN	-0.0114758	6.99509032	-0.0183103	0.985460664	0.99551478	-6.8562819
CNN3	-0.0021853	7.43544024	-0.0182892	0.985477433	0.99551478	-6.9158101
ENSCAFG00000030887	-0.0035591	2.48963021	-0.0181509	0.985587294	0.99554591	-6.2912055
ENSCAFG00000032375	-0.0047905	1.32545283	-0.0179441	0.985751432	0.99558121	-5.9994238
TEC	-0.0114621	-0.0691801	-0.0179077	0.985780316	0.99558121	-5.5012166
XYLB	0.00446959	1.95047434	0.01767423	0.985965721	0.99559046	-6.1092872
DGKZ	-0.0021403	6.23339404	-0.0176344	0.985997348	0.99559046	-6.9508581
FAM207A	0.00355784	2.32029373	0.01759756	0.986026593	0.99559046	-6.2334966
ITFG2	0.00248399	3.31861409	0.01737735	0.986201435	0.99568718	-6.4625139
PTGR1	0.00341231	8.54628055	0.01716797	0.986367679	0.99573566	-6.819218
RSPRY1	0.00138214	4.85960471	0.01711774	0.986407555	0.99573566	-6.8589127
WDR92	0.00175409	5.58209339	0.01700492	0.986497133	0.99574629	-6.9319377
ENSCAFG00000032635	-0.0019035	5.39901274	-0.0167868	0.986670288	0.99584126	-6.8959079
NUBP2	-0.0018896	4.4088075	-0.0158685	0.987399425	0.99649733	-6.7910825
RRAS2	-0.0018376	5.08903379	-0.0153936	0.987776491	0.99667257	-6.8708383
FAM219A	0.00291624	1.86701972	0.01533583	0.987822377	0.99667257	-6.1662474
ENSCAFG00000002042	-0.013181	-0.9415512	-0.0151971	0.987932511	0.99667257	-5.3036334
VIPAS39	-0.0017019	3.52736456	-0.0151158	0.98799708	0.99667257	-6.5870151
ASS1	-0.006798	0.59859318	-0.0150508	0.988048677	0.99667257	-5.9535842
COX4I1	0.00157248	7.17526921	0.01495696	0.988123194	0.99667257	-6.9411594
DDX20	-0.0015833	5.44793732	-0.0149429	0.988134385	0.99667257	-6.9202333
FLOT1	0.00202641	6.36566987	0.01482687	0.988226489	0.99667257	-6.9548073
RNF216	0.00139756	5.51936402	0.01472124	0.98831036	0.99667257	-6.9349859
SORD	-0.0050833	2.24808665	-0.0146386	0.98837597	0.99667257	-6.1248449
ZC3H18	0.00119245	5.84133518	0.01447119	0.9885089	0.99667257	-6.9427032
DUSP4	-0.003845	4.73022148	-0.0144314	0.988540504	0.99667257	-6.896095
GLIS3	0.00259594	4.50350752	0.01434846	0.988606351	0.99667257	-6.794718

AREL1	-0.0018376	4.49954972	-0.0140673	0.988829576	0.99667257	-6.809216
PLCG2	0.00984502	0.50450186	0.01405954	0.988835758	0.99667257	-5.6580412
RND3	-0.0049774	6.47319782	-0.0139905	0.988890549	0.99667257	-6.9547318
ENSCAFG00000018652	0.00292942	4.38339701	0.0139557	0.988918211	0.99667257	-6.7322518
NEIL1	-0.0059804	0.46304159	-0.0136194	0.989185206	0.99680877	-5.9081852
SLX4	0.00155597	4.36465289	0.01358617	0.989211621	0.99680877	-6.7465853
SMAP1	-0.0010416	4.90707876	-0.0134364	0.989330579	0.99681564	-6.8585338
TRAIP	0.00385447	2.62379817	0.01335035	0.989398866	0.99681564	-6.2302296
ENSCAFG00000008879	-0.0015628	6.4244517	-0.0132786	0.989455854	0.99681564	-6.9548708
USP13	0.0019254	3.58884103	0.01235369	0.990190242	0.99747571	-6.5879976
KPNB1	-0.0015101	8.33635183	-0.0120076	0.990465089	0.99767279	-6.8837877
ITGB3	-0.0039607	7.33057829	-0.0118602	0.990582067	0.99768385	-6.9310271
ZFHX3	0.00214217	5.94136963	0.01179422	0.990634485	0.99768385	-6.9541715
PHC3	-0.0011827	4.98459426	-0.0115841	0.990801303	0.99770599	-6.861878
TBCK	-0.001236	4.51019722	-0.011567	0.99081488	0.99770599	-6.8121037
UBFD1	-0.0011091	4.2665422	-0.0112426	0.991072527	0.99788566	-6.762334
GXYLT2	0.00372136	5.52180345	0.0109982	0.99126656	0.99800125	-6.7176128
PHF2	0.00095461	5.45215834	0.01081939	0.991408543	0.99803108	-6.9195603
RNF111	-0.0012632	5.47311593	-0.0107613	0.991454662	0.99803108	-6.9202916
IGFLR1	0.00353503	0.03759584	0.01063907	0.991551723	0.99804903	-5.7691282
ENSCAFG00000015635	0.0011456	5.05929506	0.01035764	0.991775192	0.99811862	-6.871772
NFKBIB	-0.0013851	4.29658287	-0.0102429	0.991866323	0.99811862	-6.766352
ENSCAFG00000000132	0.00451649	-1.1547631	0.01014968	0.991940325	0.99811862	-5.5088992
FREM1	0.00812914	1.85327127	0.01004414	0.992024133	0.99811862	-6.0598893
EFCAB14	-0.0012028	6.4590906	-0.0100314	0.992034276	0.99811862	-6.9509449
MOB3A	-0.0020757	3.84966453	-0.0099532	0.992096306	0.99811862	-6.6557574
ENSCAFG00000019092	0.00126648	4.46127131	0.00965189	0.992335597	0.99815253	-6.7898393
TECR	-0.0013864	6.22405753	-0.0095799	0.992392724	0.99815253	-6.9547264
SIRT6	0.00160224	2.84351026	0.00955981	0.992408714	0.99815253	-6.4376717
ENSCAFG00000029058	0.00174518	2.71586994	0.00949021	0.992463983	0.99815253	-6.4581613
VCPIP1	0.00097155	5.49829384	0.00931984	0.992599264	0.99815253	-6.9300037
TYMS	-0.0026794	3.96056554	-0.009312	0.992605473	0.99815253	-6.5635129
ENSCAFG00000007045	0.0050315	4.22264213	0.00916031	0.992725947	0.99815832	-6.7191064
WDR78	-0.0014369	2.88229262	-0.008991	0.992860419	0.99815832	-6.4597715
PITPNB	0.0009361	6.63671636	0.0089054	0.992928355	0.99815832	-6.9529571
DLST	-0.0008913	7.18003096	-0.0088739	0.992953383	0.99815832	-6.9352602
ENSCAFG00000004287	-0.0045219	-1.6828654	-0.0087084	0.99308478	0.99815832	-5.3259919
FTSJ1	0.00098401	4.7128379	0.00845084	0.99328931	0.99815832	-6.812027
FMN2	0.00352749	5.94584789	0.00838518	0.993341443	0.99815832	-6.9540879
NDUFS1	0.00074337	6.76238494	0.00820283	0.993486247	0.99815832	-6.9474205
EXOC6	-0.0026893	2.14297267	-0.0081379	0.993537795	0.99815832	-6.2341931
ISOC1	-0.0017497	4.63016225	-0.0081233	0.993549431	0.99815832	-6.8188345
EID2	0.0017892	1.52721668	0.00804287	0.993613262	0.99815832	-6.0719068
ZNF662	-0.0029483	1.26864043	-0.0080304	0.993623191	0.99815832	-5.9607504
WDR73	0.00094873	4.27580039	0.00800742	0.99364141	0.99815832	-6.7514813
SUCO	-0.0010821	5.14838696	-0.0078791	0.993743311	0.99818108	-6.8835959
NEMP2	-0.0014656	2.48759024	-0.0076439	0.99393004	0.99822886	-6.2948601
LARP6	-0.0024675	6.80541717	-0.0076196	0.993949381	0.99822886	-6.95397
ENSCAFG00000016094	-0.0008626	6.41442078	-0.0073659	0.994150863	0.99830771	-6.9536173
ABCB1	-0.0062342	0.94649627	-0.0073109	0.994194509	0.99830771	-6.1300899

HAT1	0.00109197	4.85393586	0.00718956	0.994290855	0.99830771	-6.8335082
RPAP3	0.00059839	5.92550573	0.00707968	0.994378103	0.99830771	-6.9488742
ENSCAFG00000004412	-0.0020746	0.74633993	-0.006934	0.994493818	0.99830771	-5.8563307
FBXO25	-0.000994	3.62629812	-0.0069219	0.994503427	0.99830771	-6.7005493
APBA1	-0.0031833	0.0298347	-0.0066118	0.994749664	0.99834402	-5.7907928
MAPK13	0.00345437	0.96106511	0.00660566	0.994754517	0.99834402	-5.859763
HACD3	-0.000984	3.99476857	-0.0065769	0.994777377	0.99834402	-6.6823046
FBXL4	-0.0008321	4.62030142	-0.0062988	0.994998184	0.99844745	-6.8083681
PPP6R3	0.00063237	6.43561179	0.00601457	0.995223885	0.99844745	-6.9547594
RNF121	-0.0007042	3.90164998	-0.0059171	0.995301275	0.99844745	-6.6459781
SREK1IP1	-0.0008119	2.66235847	-0.0058752	0.995334578	0.99844745	-6.4817003
SH3D21	-0.0013676	3.98663472	-0.0057828	0.995407921	0.99844745	-6.7465422
FBXO30	0.00071169	3.92353329	0.00574872	0.99543499	0.99844745	-6.6570202
ENSCAFG00000031306	-0.0024273	3.28283731	-0.0056639	0.995502321	0.99844745	-6.7511215
ENSCAFG00000015719	-0.001053	3.53178544	-0.0056485	0.995514567	0.99844745	-6.575419
PTCD2	-0.0005082	3.89257601	-0.0050214	0.996012581	0.99878155	-6.6209946
PPTC7	-0.0006668	5.35169195	-0.0050021	0.996027834	0.99878155	-6.9025372
ENSCAFG00000004602	0.00058398	4.09671923	0.00492944	0.996085571	0.99878155	-6.683921
SPARCL1	0.00209542	2.97887633	0.00477438	0.996208704	0.99882551	-6.7153818
FRMD5	-0.0029099	-2.0599334	-0.0045438	0.996391804	0.99889429	-5.3249127
C7H18orf25	-0.0005601	5.52281738	-0.0044883	0.996435908	0.99889429	-6.92227
PTRHD1	-0.001246	1.6765098	-0.0042891	0.996594045	0.9988984	-6.1044054
CHRNB1	0.00116881	2.31274962	0.00428335	0.996598622	0.9988984	-6.2888432
HRAS	0.00060307	3.56359545	0.004031	0.996799009	0.99901976	-6.6237682
EVA1B	0.00103081	3.21045129	0.00375529	0.997017947	0.99912584	-6.5246764
GPR19	-0.0017805	-0.483806	-0.0036979	0.997063494	0.99912584	-5.6118595
CIP2A	0.00109057	3.92750156	0.00347101	0.997243695	0.99918579	-6.6016891
CAB39L	-0.0006782	3.33363448	-0.0033137	0.997368614	0.99918579	-6.5434093
KBTBD4	0.00038073	3.05297583	0.00291185	0.99768772	0.99918579	-6.5026129
EIF2S2	-0.0003584	7.01033358	-0.0029095	0.997689567	0.99918579	-6.9503307
DNAJA1	-0.0004152	4.60780179	-0.002909	0.997689983	0.99918579	-6.8592348
MRPL24	-0.0004996	3.91475979	-0.0028444	0.997741293	0.99918579	-6.6611228
TOMM40	0.00045261	4.9622326	0.00273259	0.997830068	0.99918579	-6.8499865
WDR82	-0.0002327	5.28401442	-0.0026624	0.997885837	0.99918579	-6.9277926
NUMB	0.0002593	5.97618575	0.00245993	0.998046583	0.99918579	-6.952791
INVS	-0.0001908	6.06355629	-0.0023678	0.99811973	0.99918579	-6.9500409
TNK1	0.00146669	-0.6240887	0.00229361	0.998178653	0.99918579	-5.6443549
ENSCAFG00000029636	0.00071459	0.53893998	0.00222303	0.9982347	0.99918579	-5.8513681
FBN2	-0.0043116	1.29245355	-0.0021616	0.9982835	0.99918579	-5.3477703
SAMD8	0.0004002	4.22398035	0.00212708	0.998310898	0.99918579	-6.7786837
ENSCAFG00000031730	0.00041178	2.58880692	0.00187491	0.998511146	0.99918579	-6.3101952
KLF6	0.00028394	6.70204524	0.00177353	0.998591649	0.99918579	-6.9465301
FIS1	0.0001867	5.8055469	0.00168678	0.998660534	0.99918579	-6.9497512
ZBTB16	-0.0005226	1.13167081	-0.0016032	0.998726881	0.99918579	-6.9367234
E2F3	-0.0001539	4.55285769	-0.0015906	0.998736945	0.99918579	-6.8301867
IGFBP6	0.00082514	6.97734273	0.0015854	0.998741039	0.99918579	-6.8954913
CHD1L	-0.0002123	4.76184559	-0.0015248	0.998789159	0.99918579	-6.830963
PTPRS	-0.0001634	6.83704815	-0.000926	0.99926468	0.99958211	-6.9460687
KIF26B	-0.0003119	2.41047575	-0.0007836	0.999377719	0.9996158	-6.3487169
VSIG10L	0.00015295	1.19873241	0.00057409	0.999544119	0.99970285	-6.0812822

INTS6L	-4.947E-05	4.48094715	-0.0002325	0.999815359	0.99989474	-6.6520867
DOCK4	-2.764E-05	4.07109657	-6.838E-05	0.999945702	0.9999457	-6.6044252
