# Supplementary material for: Locational memory of macrovessel vascular cells is transcriptionally imprinted
Source: Sci Rep. 2023 Aug 10;13:13028. doi: 10.1038/s41598-023-38880-6 (PMC10415317; doi:10.1038/s41598-023-38880-6)
Supplement: Supplementary file 7 — Supplementary Table 10. [file 41598_2023_38880_MOESM7_ESM.pdf]

Supplemental Table 10. Module hub genes defined by intramodular connectivity.

Intramodular connectivity	name	module	module_num
111.6	LRP12	grey	EC_M10
99.2	DERL1	grey	EC_M10
100.2	MROH1	grey	EC_M10
106.1	CEMIP2	grey	EC_M10
112.7	CKAP4	grey	EC_M10
100.6	P4HB	grey	EC_M10
120.7	SSR1	grey	EC_M10
101.4	PNPLA2	grey	EC_M10
117.8	MANF	grey	EC_M10
103.7	UNC93B1	grey	EC_M10
107.6	NUDC	grey	EC_M10
130.1	SEC24D	grey	EC_M10
103.7	VPS11	grey	EC_M10
109.2	ZNF687	grey	EC_M10
98.2	UCKL1	grey	EC_M10
103.0	AFAP1	grey	EC_M10
101.9	LRRC59	grey	EC_M10
106.8	YPEL3	grey	EC_M10
105.7	ENSCAFG00000018825	grey	EC_M10
105.9	SLC7A5	grey	EC_M10
211.6	N4BP3	turquoise	EC_M6
214.5	FLT4	turquoise	EC_M6
199.3	PELI1	turquoise	EC_M6
207.1	CCM2L	turquoise	EC_M6
202.8	NOL4L	turquoise	EC_M6
198.0	KIAA0355	turquoise	EC_M6
201.7	CP	turquoise	EC_M6
206.0	GUCY1A1	turquoise	EC_M6
214.8	ANGPT2	turquoise	EC_M6
203.3	ENSCAFG00000010209	turquoise	EC_M6
205.6	HCLS1	turquoise	EC_M6
208.3	PTCHD1	turquoise	EC_M6
207.5	USHBP1	turquoise	EC_M6
209.3	GNGT2	turquoise	EC_M6
209.5	EIF2B2	turquoise	EC_M6
200.6	KLHL4	turquoise	EC_M6
222.7	IRF8	turquoise	EC_M6
209.3	PALMD	turquoise	EC_M6
198.6	ENSCAFG00000029677	turquoise	EC_M6
203.4	ENSCAFG00000031865	turquoise	EC_M6

135.1	TSHZ1	cyan	EC_M2
137.4	MFSD3	cyan	EC_M2
153.6	KLF9	cyan	EC_M2
133.0	MED25	cyan	EC_M2
129.9	FUZ	cyan	EC_M2
135.1	SPRY1	cyan	EC_M2
134.4	ABTB1	cyan	EC_M2
131.0	ARAP1	cyan	EC_M2
163.5	TNS2	cyan	EC_M2
148.6	ZHX3	cyan	EC_M2
133.1	PPM1M	cyan	EC_M2
138.3	ENSCAFG00000012022	cyan	EC_M2
131.6	ADAMTSL4	cyan	EC_M2
137.0	GLUL	cyan	EC_M2
139.3	TBC1D2B	cyan	EC_M2
154.3	CTDSP1	cyan	EC_M2
137.8	ARRDC2	cyan	EC_M2
135.2	C20H19orf44	cyan	EC_M2
137.7	MLLT6	cyan	EC_M2
135.2	CNRIP1	cyan	EC_M2
160.6	LMAN1	darkgreen	EC_M4
149.4	MYH9	darkgreen	EC_M4
155.9	ITGB1	darkgreen	EC_M4
150.6	KIF5B	darkgreen	EC_M4
154.3	PSMC2	darkgreen	EC_M4
163.7	COPG1	darkgreen	EC_M4
171.5	PICALM	darkgreen	EC_M4
150.0	VPS37A	darkgreen	EC_M4
156.1	TMED2	darkgreen	EC_M4
159.7	PDLIM5	darkgreen	EC_M4
156.3	KCTD10	darkgreen	EC_M4
156.8	HYOU1	darkgreen	EC_M4
152.9	DNAJB11	darkgreen	EC_M4
150.3	MYO9B	darkgreen	EC_M4
162.5	ENAH	darkgreen	EC_M4
157.7	ACTN1	darkgreen	EC_M4
151.4	ENSCAFG00000017241	darkgreen	EC_M4
156.0	USP14	darkgreen	EC_M4
156.3	MPRIP	darkgreen	EC_M4
151.9	HSPA13	darkgreen	EC_M4
145.8	KIF20A	darkgrey	EC_M8
158.8	MCM3	darkgrey	EC_M8
147.2	RRM2	darkgrey	EC_M8

149.7	MCM4	darkgrey	EC_M8
150.6	BUB1	darkgrey	EC_M8
144.7	ESCO2	darkgrey	EC_M8
154.4	KNL1	darkgrey	EC_M8
165.0	CENPE	darkgrey	EC_M8
149.5	SGO2	darkgrey	EC_M8
152.8	PRC1	darkgrey	EC_M8
147.0	MKI67	darkgrey	EC_M8
148.4	NUF2	darkgrey	EC_M8
144.8	KIF15	darkgrey	EC_M8
148.7	TACC3	darkgrey	EC_M8
160.6	ECT2	darkgrey	EC_M8
155.5	TOP2A	darkgrey	EC_M8
147.2	KIF4A	darkgrey	EC_M8
153.5	KIF23	darkgrey	EC_M8
151.7	NDC80	darkgrey	EC_M8
148.9	ENSCAFG00000030087	darkgrey	EC_M8
2.9	ENSCAFG00000001207	darkmagenta	EC_M12
6.4	ALDH1A1	darkmagenta	EC_M12
5.5	ACP4	darkmagenta	EC_M12
8.5	ENSCAFG00000003951	darkmagenta	EC_M12
3.0	ENSCAFG00000006540	darkmagenta	EC_M12
4.1	DNAH6	darkmagenta	EC_M12
2.9	MAB21L2	darkmagenta	EC_M12
4.8	ENSCAFG00000008721	darkmagenta	EC_M12
5.5	CHRM3	darkmagenta	EC_M12
8.5	TLR7	darkmagenta	EC_M12
7.2	ENSCAFG00000014695	darkmagenta	EC_M12
4.1	TTC22	darkmagenta	EC_M12
8.5	ENSCAFG00000022791	darkmagenta	EC_M12
4.5	CCDC160	darkmagenta	EC_M12
3.6	ENSCAFG00000026005	darkmagenta	EC_M12
3.6	ENSCAFG00000026131	darkmagenta	EC_M12
4.1	ENSCAFG00000026738	darkmagenta	EC_M12
8.5	ENSCAFG00000027993	darkmagenta	EC_M12
5.9	LRAT	darkmagenta	EC_M12
6.3	GPR39	darkolivegreen	EC_M9
10.4	ENSCAFG00000005439	darkolivegreen	EC_M9
4.9	KIRREL2	darkolivegreen	EC_M9
4.9	ENSCAFG00000006940	darkolivegreen	EC_M9
6.1	MMP11	darkolivegreen	EC_M9
4.0	SORCS2	darkolivegreen	EC_M9
4.3	MYOC	darkolivegreen	EC_M9

10.4	RBM47	darkolivegreen	EC_M9
4.3	HRH2	darkolivegreen	EC_M9
10.4	ANKFN1	darkolivegreen	EC_M9
2.8	GM2A	darkolivegreen	EC_M9
3.8	FRMPD3	darkolivegreen	EC_M9
5.2	CACNA1H	darkolivegreen	EC_M9
2.7	ENSCAFG00000022942	darkolivegreen	EC_M9
10.4	DCDC2B	darkolivegreen	EC_M9
6.7	CHRNG	darkolivegreen	EC_M9
4.9	ENSCAFG00000028632	darkolivegreen	EC_M9
10.4	ENSCAFG00000028697	darkolivegreen	EC_M9
5.0	NPB	darkolivegreen	EC_M9
2.8	ENSCAFG00000029510	darkolivegreen	EC_M9
23.6	ENSCAFG00000001033	magenta	EC_M13
23.8	METTL24	magenta	EC_M13
17.8	SNAP25	magenta	EC_M13
19.0	SLC4A9	magenta	EC_M13
19.1	SLC35G1	magenta	EC_M13
19.8	GNAO1	magenta	EC_M13
23.8	C20H3orf84	magenta	EC_M13
18.7	OPRD1	magenta	EC_M13
20.0	IFI6	magenta	EC_M13
19.0	TREH	magenta	EC_M13
19.0	NR1I3	magenta	EC_M13
18.9	LRRC75B	magenta	EC_M13
22.7	PITPNM3	magenta	EC_M13
26.2	LCTL	magenta	EC_M13
26.2	DBNDD1	magenta	EC_M13
23.8	MYO1H	magenta	EC_M13
23.8	ENSCAFG00000024414	magenta	EC_M13
16.9	ENSCAFG00000029292	magenta	EC_M13
17.7	ENSCAFG00000029897	magenta	EC_M13
19.8	ENSCAFG00000030031	magenta	EC_M13
3.5	ENSCAFG00000000846	violet	EC_M7
4.6	TSPAN33	violet	EC_M7
4.4	ENSCAFG00000001861	violet	EC_M7
5.9	IL18RAP	violet	EC_M7
4.2	ENSCAFG00000002473	violet	EC_M7
9.9	ABCG5	violet	EC_M7
8.9	ABCG8	violet	EC_M7
9.9	ENSCAFG00000004270	violet	EC_M7
5.3	ENSCAFG00000005360	violet	EC_M7
5.3	FGFBP3	violet	EC_M7

9.9	CAMK4	violet	EC_M7
4.6	ENSCAFG00000013592	violet	EC_M7
7.2	BCL11B	violet	EC_M7
3.4	ENSCAFG00000020084	violet	EC_M7
3.3	ENSCAFG00000022117	violet	EC_M7
6.3	ENSCAFG00000023180	violet	EC_M7
3.1	ENSCAFG00000024495	violet	EC_M7
3.6	ENSCAFG00000026205	violet	EC_M7
4.4	CACNG7	violet	EC_M7
3.6	ENSCAFG00000031446	violet	EC_M7
7.3	BARX1	paleturquoise	EC_M11
4.1	DNALI1	paleturquoise	EC_M11
4.8	EPHA7	paleturquoise	EC_M11
5.1	ENSCAFG00000004335	paleturquoise	EC_M11
5.1	COL6A6	paleturquoise	EC_M11
6.4	IGSF10	paleturquoise	EC_M11
6.3	ENSCAFG00000008805	paleturquoise	EC_M11
6.3	SPATA25	paleturquoise	EC_M11
5.3	ADTRP	paleturquoise	EC_M11
8.6	ENSCAFG00000010248	paleturquoise	EC_M11
3.7	ENSCAFG00000014321	paleturquoise	EC_M11
8.6	CLDN7	paleturquoise	EC_M11
4.6	ALX3	paleturquoise	EC_M11
5.1	ENSCAFG00000020157	paleturquoise	EC_M11
6.4	ENSCAFG00000020979	paleturquoise	EC_M11
3.7	ENSCAFG00000022793	paleturquoise	EC_M11
6.2	KRT19	paleturquoise	EC_M11
5.1	ENSCAFG00000025963	paleturquoise	EC_M11
5.1	ENSCAFG00000029959	paleturquoise	EC_M11
53.9	SQSTM1	yellow	VSMC_M3
46.7	HGH1	yellow	VSMC_M3
43.4	RPS9	yellow	VSMC_M3
40.6	PXMP2	yellow	VSMC_M3
47.3	TARBP2	yellow	VSMC_M3
40.4	TNKS1BP1	yellow	VSMC_M3
40.2	SLC25A6	yellow	VSMC_M3
59.8	PPP1CA	yellow	VSMC_M3
41.9	PHB2	yellow	VSMC_M3
46.1	ENSCAFG00000014684	yellow	VSMC_M3
44.6	ILVBL	yellow	VSMC_M3
41.9	DBN1	yellow	VSMC_M3
41.5	PRR14	yellow	VSMC_M3
40.6	BCL7C	yellow	VSMC_M3

44.4	AKT1	yellow	VSMC_M3
40.7	ACAP3	yellow	VSMC_M3
47.7	MRPL28	yellow	VSMC_M3
59.5	RNH1	yellow	VSMC_M3
58.7	ENSCAFG00000029403	yellow	VSMC_M3
41.7	OSGEP	yellow	VSMC_M3
56.6	KDSR	grey	VSMC_M10
57.2	TDRD7	grey	VSMC_M10
55.1	NR1H2	grey	VSMC_M10
57.1	LHFPL6	grey	VSMC_M10
56.9	ULK1	grey	VSMC_M10
50.9	HPS5	grey	VSMC_M10
57.3	USP19	grey	VSMC_M10
52.4	PARP9	grey	VSMC_M10
54.7	PARP14	grey	VSMC_M10
50.6	ADAMTSL4	grey	VSMC_M10
52.9	TMEM25	grey	VSMC_M10
64.2	PNPLA6	grey	VSMC_M10
62.5	DAB1	grey	VSMC_M10
50.5	MGRN1	grey	VSMC_M10
61.5	ENSCAFG00000019687	grey	VSMC_M10
61.0	BSG	grey	VSMC_M10
53.6	CELSR2	grey	VSMC_M10
62.5	ENSCAFG00000020220	grey	VSMC_M10
62.5	ENSCAFG00000020512	grey	VSMC_M10
50.9	RAB6B	grey	VSMC_M10
64.8	MYCT1	turquoise	VSMC_M6
68.0	ADGRF5	turquoise	VSMC_M6
63.8	GJA4	turquoise	VSMC_M6
66.4	FGD5	turquoise	VSMC_M6
68.5	ENSCAFG00000004905	turquoise	VSMC_M6
64.5	PDE2A	turquoise	VSMC_M6
66.9	CCM2L	turquoise	VSMC_M6
70.5	ANGPT2	turquoise	VSMC_M6
73.1	ENSCAFG00000010209	turquoise	VSMC_M6
65.0	LSAMP	turquoise	VSMC_M6
69.5	LAPTM5	turquoise	VSMC_M6
68.3	KLHL4	turquoise	VSMC_M6
65.3	TMEM204	turquoise	VSMC_M6
63.2	KRT39	turquoise	VSMC_M6
65.7	ENSCAFG00000023602	turquoise	VSMC_M6
63.4	RELN	turquoise	VSMC_M6
73.6	ENSCAFG00000029170	turquoise	VSMC_M6

70.2	SOX18	turquoise	VSMC_M6
67.4	ENSCAFG00000029346	turquoise	VSMC_M6
68.5	RBP1	turquoise	VSMC_M6
245.3	MCM3	darkgrey	VSMC_M8
233.6	PLK4	darkgrey	VSMC_M8
234.6	MCM2	darkgrey	VSMC_M8
243.8	CCNA2	darkgrey	VSMC_M8
239.7	MCM10	darkgrey	VSMC_M8
248.1	DIAPH3	darkgrey	VSMC_M8
233.9	MCM6	darkgrey	VSMC_M8
234.3	NCAPH	darkgrey	VSMC_M8
234.2	MCM4	darkgrey	VSMC_M8
232.8	TPX2	darkgrey	VSMC_M8
235.2	KIF11	darkgrey	VSMC_M8
232.3	ENSCAFG00000008236	darkgrey	VSMC_M8
232.5	RACGAP1	darkgrey	VSMC_M8
236.3	MYBL2	darkgrey	VSMC_M8
234.7	PRC1	darkgrey	VSMC_M8
234.1	KIF15	darkgrey	VSMC_M8
240.6	TOP2A	darkgrey	VSMC_M8
234.8	CDC6	darkgrey	VSMC_M8
230.3	KIF23	darkgrey	VSMC_M8
234.3	STMN1	darkgrey	VSMC_M8
68.3	TAPBP	cyan	VSMC_M2
79.8	GRINA	cyan	VSMC_M2
67.7	ATP10D	cyan	VSMC_M2
75.7	PLD3	cyan	VSMC_M2
78.3	CTSB	cyan	VSMC_M2
72.2	NISCH	cyan	VSMC_M2
69.4	CTSD	cyan	VSMC_M2
72.6	PKNOX2	cyan	VSMC_M2
80.4	SSH3	cyan	VSMC_M2
88.9	ENSCAFG00000012022	cyan	VSMC_M2
71.8	RHBDD3	cyan	VSMC_M2
72.9	B2M	cyan	VSMC_M2
80.4	GRN	cyan	VSMC_M2
74.0	PSAP	cyan	VSMC_M2
67.8	JUP	cyan	VSMC_M2
76.8	ENSCAFG00000017257	cyan	VSMC_M2
66.7	GPRC5B	cyan	VSMC_M2
83.7	PTPRS	cyan	VSMC_M2
75.1	FBXW5	cyan	VSMC_M2
67.2	RAB11B	cyan	VSMC_M2

99.5	LEP	darkgreen	VSMC_M4
100.5	SVEP1	darkgreen	VSMC_M4
116.1	PREP	darkgreen	VSMC_M4
109.7	ITGA8	darkgreen	VSMC_M4
114.2	CRIM1	darkgreen	VSMC_M4
102.0	RASSF2	darkgreen	VSMC_M4
104.2	SORBS2	darkgreen	VSMC_M4
98.1	ST3GAL5	darkgreen	VSMC_M4
98.7	GPR87	darkgreen	VSMC_M4
109.9	RXFP1	darkgreen	VSMC_M4
101.0	DCBLD2	darkgreen	VSMC_M4
100.3	ENSCAFG00000010290	darkgreen	VSMC_M4
96.9	SYNPO2	darkgreen	VSMC_M4
116.6	EXPH5	darkgreen	VSMC_M4
118.5	SYNPO2L	darkgreen	VSMC_M4
101.5	FST	darkgreen	VSMC_M4
115.2	RHOT1	darkgreen	VSMC_M4
95.1	SPTAN1	darkgreen	VSMC_M4
94.8	PDLIM3	darkgreen	VSMC_M4
97.2	ENSCAFG00000031827	darkgreen	VSMC_M4
12.2	NETO1	violet	VSMC_M7
10.6	CCBE1	violet	VSMC_M7
13.7	DMRT2	violet	VSMC_M7
10.0	GJB4	violet	VSMC_M7
12.7	VIT	violet	VSMC_M7
10.6	FGF19	violet	VSMC_M7
8.6	ENSCAFG00000011153	violet	VSMC_M7
10.5	LRMP	violet	VSMC_M7
8.4	COMP	violet	VSMC_M7
9.0	ANO2	violet	VSMC_M7
12.1	CA12	violet	VSMC_M7
11.0	CCDC33	violet	VSMC_M7
14.1	TNFRSF13B	violet	VSMC_M7
8.3	EGFLAM	violet	VSMC_M7
9.5	DNM1	violet	VSMC_M7
10.5	ZBP1	violet	VSMC_M7
8.6	ENSCAFG00000028049	violet	VSMC_M7
10.6	ENSCAFG00000028469	violet	VSMC_M7
8.7	HAPLN1	violet	VSMC_M7
10.0	EMP1	violet	VSMC_M7
56.7	CALU	darkolivegreen	VSMC_M9
54.1	SLC30A9	darkolivegreen	VSMC_M9
51.1	CLOCK	darkolivegreen	VSMC_M9

51.9	ACTR2	darkolivegreen	VSMC_M9
47.5	DDX1	darkolivegreen	VSMC_M9
47.9	KIF5B	darkolivegreen	VSMC_M9
48.1	SLC36A4	darkolivegreen	VSMC_M9
48.4	UHRF1BP1L	darkolivegreen	VSMC_M9
59.4	SACS	darkolivegreen	VSMC_M9
56.7	IPO7	darkolivegreen	VSMC_M9
52.1	PALLD	darkolivegreen	VSMC_M9
53.5	COPB1	darkolivegreen	VSMC_M9
53.2	USO1	darkolivegreen	VSMC_M9
55.6	MAN1A2	darkolivegreen	VSMC_M9
51.1	PDLIM5	darkolivegreen	VSMC_M9
48.2	EPRS	darkolivegreen	VSMC_M9
50.5	FMN2	darkolivegreen	VSMC_M9
53.5	MSN	darkolivegreen	VSMC_M9
50.0	ROCK1	darkolivegreen	VSMC_M9
47.8	PTPN12	darkolivegreen	VSMC_M9
35.4	TAB2	pink	VSMC_M5
31.1	SERINC1	pink	VSMC_M5
28.5	WASL	pink	VSMC_M5
28.4	HSPA4L	pink	VSMC_M5
34.6	TMTC3	pink	VSMC_M5
33.4	TRIP12	pink	VSMC_M5
29.6	SHOC2	pink	VSMC_M5
32.4	UBR1	pink	VSMC_M5
27.8	SCFD1	pink	VSMC_M5
28.8	ARHGAP5	pink	VSMC_M5
27.9	COPS2	pink	VSMC_M5
31.7	USP8	pink	VSMC_M5
30.2	ASH1L	pink	VSMC_M5
31.4	WDR78	pink	VSMC_M5
28.7	CPD	pink	VSMC_M5
27.9	SULT1C4	pink	VSMC_M5
31.6	ENSCAFG00000025044	pink	VSMC_M5
33.0	NCR3LG1	pink	VSMC_M5
28.0	BMT2	pink	VSMC_M5
29.6	ENSCAFG00000032635	pink	VSMC_M5
27.4	CSNK2B	red	VSMC_M1
20.9	RPL10A	red	VSMC_M1
25.6	RPS5	red	VSMC_M1
18.1	ALDH1B1	red	VSMC_M1
21.5	RPL28	red	VSMC_M1
24.4	EEF1A1	red	VSMC_M1

18.6	UTP11	red	VSMC_M1
20.3	SNRPD2	red	VSMC_M1
20.3	RPS3	red	VSMC_M1
19.8	RPL7	red	VSMC_M1
19.3	NPM1	red	VSMC_M1
18.8	WDR5	red	VSMC_M1
25.9	COX4I1	red	VSMC_M1
21.2	RPL5	red	VSMC_M1
20.0	ENSCAFG00000022470	red	VSMC_M1
18.7	ENSCAFG00000023283	red	VSMC_M1
20.0	ENSCAFG00000023724	red	VSMC_M1
18.9	ENSCAFG00000028808	red	VSMC_M1
22.5	ENSCAFG00000031952	red	VSMC_M1
23.8	RPS26	red	VSMC_M1
