# Supplementary material for: Locational memory of macrovessel vascular cells is transcriptionally imprinted
Source: Sci Rep. 2023 Aug 10;13:13028. doi: 10.1038/s41598-023-38880-6 (PMC10415317; doi:10.1038/s41598-023-38880-6)
Supplement: Supplementary file 10 — Supplementary Table 13. [file 41598_2023_38880_MOESM10_ESM.pdf]

Supplemental Table 13. Primers for reference genes and GATA4.

Gene	Orientation	Primer sequence	Product size	Melting Temperature (Tm)
GATA4	fwd	ACGGAAGCCCAAGAACCT	104	60
	rev	GCCACATTGCTGGAGTTG		
RPL13	fwd	GCCGGAAGGTTGTAGTCGT	87	61
	rev	GGAGGAAGGCCAGGTAATTC		
SDHA	fwd	GCCTTGGATCTCTTGATGGA	92	61
	rev	TTCTTGGCTCTTATGCGATG		
YWHAZ	fwd	CGAAGTTGCTGCTGGTGA	94	58
	rev	TTGCATTTCCTTTTTGCTGA		
GAPDH	fwd	TGTCCCCACCCCCAATGTATC	100	58
	rev	CTCCGATGCCTGCTTCACTACCTT		
