# Supplementary material for: Locational memory of macrovessel vascular cells is transcriptionally imprinted
Source: Sci Rep. 2023 Aug 10;13:13028. doi: 10.1038/s41598-023-38880-6 (PMC10415317; doi:10.1038/s41598-023-38880-6)
Supplement: Supplementary file 11 — Supplementary Table 2. [file 41598_2023_38880_MOESM11_ESM.pdf]

Supplemental Table 2. Differential expression analysis of arterial versus venous vascular smooth muscle cells (VSMCs). Positive log fold changes (logFC) indicate higher expression in arterial VSMCs, while the negative values represent a higher expression in venous VSMCs. AveExpr, average log2-expression; t, moderated t-statistic; p, raw P value; adj.P.Val, adjusted P value; B, log-odds that the gene is differentially expressed

Gene	logFC	AveExpr	t	P.Value	adj.P.Val	B
SORBS2	1.91538539	4.44185373	3.30717445	0.00170505	0.99968241	-3.9260897
ENSCAFG00000010290	1.88130973	1.4037325	1.22596939	0.22567952	0.99978775	-4.5797719
WDR86	1.84518411	0.19255002	1.94423925	0.05723218	0.99978775	-4.4968078
RXFP1	1.73920859	-0.513752	1.79840024	0.07786227	0.99978775	-4.5354947
DIO2	1.65840364	-1.9886462	3.65641561	0.00059237	0.99968241	-4.3729774
GP2	1.65658091	0.83692146	2.43390804	0.01837275	0.99978775	-4.2878872
NDUFA4L2	1.6045604	3.90545523	2.15416299	0.03583604	0.99978775	-4.2702927
TCF21	1.56546361	-0.1695043	1.76478564	0.08341472	0.99978775	-4.5240833
PIP5K1B	1.51955664	-1.1767443	2.26141445	0.02790091	0.99978775	-4.5115798
WWC1	1.50581499	-1.2863048	2.30550476	0.02511934	0.99978775	-4.5120395
SLC1A1	1.4769823	1.30443762	3.19971233	0.00233429	0.99968241	-3.8869238
COLGALT2	1.47219665	-2.335406	2.41469269	0.01926561	0.99978775	-4.5192106
ENSCAFG00000029553	1.45686011	2.79873173	1.21796126	0.2286828	0.99978775	-4.5666547
ITGA8	1.43437741	3.20679604	1.86666319	0.06753618	0.99978775	-4.2866788
VCAM1	1.43172197	3.23809529	2.15512593	0.03575678	0.99978775	-4.1084508
BMP6	1.42308319	3.37665763	1.66996827	0.10087653	0.99978775	-4.4329742
HTR2B	1.41698755	0.10815564	1.14679676	0.25666393	0.99978775	-4.5895126
RGS1	1.40033551	-0.194429	1.64616455	0.10570243	0.99978775	-4.5592138
TNNT2	1.39164213	-2.3898169	2.16218712	0.03518023	0.99978775	-4.5437009
FRMPD1	1.38685931	-1.4759254	2.06944769	0.04344032	0.99978775	-4.5333147
EPB41L4A	1.36874219	-1.0701385	2.61438821	0.01163836	0.99978775	-4.459187
GATA4	1.34486065	1.25685148	1.00522384	0.31939856	0.99978775	-4.6001261
FILIP1	1.34423185	2.33543965	1.63242509	0.10857264	0.99978775	-4.4727281
EPYC	1.32944315	0.68785787	1.81696679	0.07493047	0.99978775	-4.5027338
SLC26A7	1.32447908	-1.9653927	1.67996003	0.09890536	0.99978775	-4.5693136
GLIS1	1.31879779	-0.9470765	3.01582562	0.00394332	0.99978775	-4.4293147
MFSD6	1.28720541	-0.092146	2.41836429	0.01909205	0.99978775	-4.4558118
SLC14A1	1.259614	1.93313843	2.15874008	0.03546066	0.99978775	-4.1591993
ENSCAFG00000018099	1.25114163	-1.9888008	2.65097257	0.01058475	0.99978775	-4.4980264
AGBL3	1.24656978	-1.6125247	2.40304474	0.01982565	0.99978775	-4.5256086
EXPH5	1.22896403	-0.9480312	1.66588196	0.1016919	0.99978775	-4.5407451
HCN4	1.22889814	-0.8245458	1.93630519	0.05822034	0.99978775	-4.4591073
PRDM6	1.22144298	-1.0343872	2.14412183	0.03667179	0.99978775	-4.4735026
KERA	1.20939082	1.40106153	3.18434242	0.00244044	0.99968241	-4.0523286
CD200	1.20925293	0.23689277	1.73480333	0.0886419	0.99978775	-4.5291605
MAOB	1.20172914	-1.9853191	1.52400005	0.13350866	0.99978775	-4.568408
ENSCAFG00000001272	1.19429376	-3.0621571	2.67865475	0.00984632	0.99978775	-4.5225301
F11R	1.19191889	0.9126235	1.11103931	0.2716125	0.99978775	-4.5878782
NPR3	1.18873067	1.24811849	3.37622708	0.0013894	0.99968241	-2.8405891
C15orf48	1.1663216	-2.7535672	2.11130545	0.03952342	0.99978775	-4.5493646
SEMA6D	1.15813026	2.17509082	1.4386111	0.15619428	0.99978775	-4.5375625
EDN1	1.15593452	3.66055366	2.27407927	0.0270752	0.99978775	-3.8807854
GCNT7	1.13908707	0.3277087	1.44790514	0.15358779	0.99978775	-4.5644425
SGIP1	1.12536647	-2.1324984	2.0938391	0.04111874	0.99978775	-4.5454018

TLR1	1.11768405	-2.5774995	1.97862163	0.05311418	0.99978775	-4.5559111
DNAJC6	1.11644901	-0.6117048	2.25295348	0.02846492	0.99978775	-4.4981663
ADGRB1	1.11356112	-1.0886018	2.16302881	0.03511206	0.99978775	-4.5033919
PDLIM3	1.11241567	1.37599791	1.65439047	0.10401385	0.99978775	-4.4402424
ZPLD1	1.10210409	1.05017625	1.87663224	0.06612974	0.99978775	-4.502378
ENSCAFG00000007154	1.10010388	4.41665869	2.04164978	0.04622424	0.99978775	-3.9980235
SYNPO2L	1.07042018	0.53060693	1.85326001	0.06946695	0.99978775	-4.4422084
SLCO1A2	1.06762263	-0.2382512	1.47235694	0.14689278	0.99978775	-4.5506739
MYH11	1.06687843	0.78545231	2.09752583	0.0407774	0.99978775	-4.3148016
CRYM	1.06640909	-2.1141719	2.14118102	0.03691977	0.99978775	-4.539188
PLET1	1.04647209	-3.0496324	2.19190324	0.03284245	0.99978775	-4.5491201
LHX9	1.03421761	-0.1351529	2.25832879	0.02810544	0.99978775	-4.4215821
RASL11B	1.02647561	4.0405893	3.66316088	0.00058009	0.99968241	-2.3901821
ZNF483	1.02148252	-0.3419989	2.79263551	0.00727718	0.99978775	-4.4316403
PRPH	1.01898154	0.61840752	1.43688304	0.15668269	0.99978775	-4.5553105
DSP	1.00821592	-1.835979	1.66106589	0.10265981	0.99978775	-4.5679858
ALPK3	1.00259104	-0.360464	2.52844861	0.01449977	0.99978775	-4.3616162
NPPB	0.99898628	-1.2375324	1.50414616	0.13853459	0.99978775	-4.5634779
SORCS1	0.99385631	1.69735071	1.81542981	0.07516959	0.99978775	-4.3268741
CLDN4	0.99336442	-2.2018049	2.19696263	0.03245835	0.99978775	-4.5219295
TNFRSF11B	0.99250616	-1.9137826	2.03661422	0.04674475	0.99978775	-4.5352094
NPNT	0.98839813	3.33009893	2.48780353	0.01606385	0.99978775	-3.6616123
LMCD1	0.98393417	5.14904036	2.23962126	0.02937415	0.99978775	-3.8395388
EYA4	0.97211309	1.99135264	2.1328286	0.03763211	0.99978775	-4.055663
ANKRD1	0.97068697	4.68031013	1.51572325	0.13558598	0.99978775	-4.3967638
XDH	0.96599458	2.62962393	0.49378797	0.62351493	0.99978775	-4.612605
GALNT3	0.96239501	-2.1762742	1.64079457	0.10681678	0.99978775	-4.5662497
MARVELD2	0.95864412	-1.0539339	2.1327364	0.03764004	0.99978775	-4.5195147
CEMIP	0.95024591	7.02484538	1.58947738	0.11795561	0.99978775	-4.3717465
MUC20	0.94880801	-0.5536925	1.88499494	0.06496914	0.99978775	-4.546551
ENSCAFG00000023251	0.93643204	-1.4904763	1.77216488	0.08216834	0.99978775	-4.5528301
NDRG2	0.92857614	4.57030117	2.17930055	0.03381667	0.99978775	-3.9456942
MYO18B	0.92124519	-0.1431355	2.13207089	0.03769732	0.99978775	-4.3570033
TMEM176B	0.92110683	0.91831587	1.24737605	0.21779381	0.99978775	-4.5651714
MYOM1	0.91784617	0.8905568	2.17528636	0.03413227	0.99978775	-4.388218
ENSCAFG00000028814	0.91334252	3.21884269	1.21349232	0.23037146	0.99978775	-4.5553542
CD55	0.90221742	5.62117431	1.24782776	0.21762964	0.99978775	-4.5451107
CCDC180	0.89860089	-0.3601743	1.51459334	0.13587154	0.99978775	-4.5510452
SUSD1	0.89696137	0.85894769	1.26170373	0.21263086	0.99978775	-4.5781572
BMP4	0.89668488	4.19880514	1.58170087	0.11972263	0.99978775	-4.401644
TMEM176A	0.89607943	0.63121571	1.29014813	0.20265143	0.99978775	-4.5637428
ADA	0.89412192	6.81510298	1.98235817	0.05268238	0.99978775	-4.0537919
ENSCAFG00000031706	0.89139782	1.57543318	2.26500767	0.0276644	0.99978775	-4.2061967
CD93	0.89098528	1.2748697	0.62571	0.5342152	0.99978775	-4.6202298
GPR39	0.88633364	-2.60841	1.63887047	0.10721838	0.99978775	-4.5702354
ENSCAFG00000032314	0.8836285	-1.0262096	1.64776523	0.10537211	0.99978775	-4.5636327
ENSCAFG00000032016	0.88353884	2.48254449	1.75713611	0.08472334	0.99978775	-4.4713058
ABCB11	0.87468332	-0.8514175	1.1018725	0.27554164	0.99978775	-4.5919782
COCH	0.8733739	-0.1191814	1.08342801	0.28356791	0.99978775	-4.5911627
CPNE7	0.87201156	0.25534016	0.78483408	0.43607719	0.99978775	-4.6016082

FRZB	0.87022883	-0.4107256	1.83143025	0.07271134	0.99978775	-4.4823157
GPR65	0.86941596	-0.2289498	1.61942221	0.1113471	0.99978775	-4.5453384
EFNB2	0.86706589	2.14178716	1.80659871	0.07655602	0.99978775	-4.3768788
CHPT1	0.86657673	1.75753825	2.79550944	0.00722123	0.99978775	-4.0086731
STC2	0.86593184	5.61055012	1.42946764	0.1587921	0.99978775	-4.4391503
LRP2	0.86551598	-2.6689362	1.68188649	0.09852897	0.99978775	-4.5693176
PIPOX	0.86295274	-0.3320083	0.94150091	0.35075925	0.99978775	-4.5975714
TESK2	0.86217589	0.54106429	2.70489936	0.00919013	0.99978775	-4.4331183
ENSCAFG00000025131	0.86066365	1.92110699	2.01612258	0.04891556	0.99978775	-4.307412
GCNT2	0.85337647	-1.5469191	1.15245803	0.25435213	0.99978775	-4.589398
ENSCAFG00000029384	0.85322571	-1.5419801	1.57410124	0.12147	0.99978775	-4.5686446
EFHD1	0.85061723	5.3332405	1.70636832	0.09384743	0.99978775	-4.2596829
GRIK4	0.8456468	-1.2586893	1.56081697	0.12457367	0.99978775	-4.567538
MYOZ2	0.84239973	-0.4882076	1.1596318	0.25144415	0.99978775	-4.5874386
DNAH7	0.8384621	-0.8578284	2.20547424	0.0318211	0.99978775	-4.4358012
TENM2	0.83765075	1.03188769	1.80293188	0.07713796	0.99978775	-4.4172979
CCDC141	0.83469569	-0.5671774	1.22849014	0.22474018	0.99978775	-4.5816605
GPR63	0.83325311	-1.6520123	1.22955834	0.22434299	0.99978775	-4.5874442
AGMO	0.83024984	-0.2839229	0.88441841	0.38050181	0.99978775	-4.5991661
ACTC1	0.82464927	-1.5843184	1.52770292	0.13258755	0.99978775	-4.5702572
MFAP4	0.81324305	4.05942136	1.9269939	0.05939858	0.99978775	-4.1130817
PLAC8B	0.81189993	-0.8974607	1.58611501	0.11871702	0.99978775	-4.5454579
BNIP3	0.80805568	6.4203651	3.03249866	0.00376291	0.99978775	-3.0369941
SLC37A1	0.80511342	-1.7755175	1.46194555	0.14971486	0.99978775	-4.577256
EPOR	0.80119535	-1.5548312	1.34387056	0.18476789	0.99978775	-4.5818986
CAMTA1	0.79773239	1.35527619	1.4138797	0.16329831	0.99978775	-4.5449771
PCSK1N	0.79484157	1.01462649	1.17852981	0.24389805	0.99978775	-4.5829615
CPNE4	0.79424744	-1.560803	1.24631136	0.21818114	0.99978775	-4.586249
B3GNT7	0.79272474	-0.775346	1.90369697	0.06243604	0.99978775	-4.5212605
ENSCAFG00000015206	0.79238729	2.83375759	0.54697535	0.58671259	0.99978775	-4.6440141
ENSCAFG00000014256	0.79206178	-1.3975863	0.90406673	0.37008876	0.99978775	-4.5987173
PLXNA2	0.79013079	6.07491359	1.83114741	0.0727542	0.99978775	-4.1662405
ERICH5	0.78559869	-0.0997491	1.94853574	0.05670308	0.99978775	-4.5136279
GYG2	0.78507065	-0.5038054	1.45402932	0.15188896	0.99978775	-4.5651181
NXPH2	0.78386784	-0.6277903	0.8596566	0.3938858	0.99978775	-4.6026594
DOCK8	0.77675629	1.86111978	1.07259193	0.28835857	0.99978775	-4.590287
SAA1	0.76882353	2.15373441	0.62181665	0.53675276	0.99978775	-4.6300932
CA9	0.76814749	1.15183191	1.09242221	0.27963387	0.99978775	-4.5881664
FST	0.76504855	5.55497904	1.92717368	0.05937564	0.99978775	-4.0909275
ENSCAFG00000032459	0.76425434	1.73491513	1.01945195	0.31266214	0.99978775	-4.5988138
ZNF385B	0.75655013	-0.1537146	1.38981569	0.17044873	0.99978775	-4.5312409
EPHA3	0.75581007	5.57146989	1.42220198	0.16088029	0.99978775	-4.4437935
BLNK	0.75562698	-0.6839966	1.35955471	0.17978018	0.99978775	-4.5628322
MARCKSL1	0.75558885	3.17939428	1.61411518	0.11249589	0.99978775	-4.4048349
ENSCAFG00000007230	0.75382152	0.86010977	2.01645467	0.0488797	0.99978775	-4.4366123
CD68	0.7470812	0.64055387	1.34443428	0.18458681	0.99978775	-4.5681649
AQP3	0.74679086	-1.4111568	1.66250665	0.10236947	0.99978775	-4.5470407
FEZ1	0.74621873	-1.7575913	1.5276922	0.13259021	0.99978775	-4.568577
SLC16A3	0.74618351	4.78719449	2.04475413	0.04590586	0.99978775	-4.095976
ADAMTS20	0.74203885	-2.2417769	1.24588031	0.2183381	0.99978775	-4.5869297

GRIP1	0.73519279	0.05773429	1.77562319	0.08158959	0.99978775	-4.4817234
LYPD6	0.73423773	-1.8905332	1.06111718	0.29349248	0.99978775	-4.5934029
PNCK	0.72916055	2.31061802	1.266468	0.21093437	0.99978775	-4.5535473
CHRM1	0.72891953	-1.3950655	0.91874681	0.36242877	0.99978775	-4.5972844
IFI44	0.72611976	2.67638463	1.09829055	0.27708775	0.99978775	-4.5861558
SERPINE3	0.72610663	-1.4126346	1.66934263	0.10100102	0.99978775	-4.5537849
ENSCAFG00000002042	0.72248851	-0.9415512	1.92022411	0.06026795	0.99978775	-4.4636771
RIMKLB	0.72158628	-1.4184316	2.02970966	0.04746669	0.99978775	-4.5118829
CRYBA4	0.71720694	-0.5448826	1.58323338	0.11937272	0.99978775	-4.5477954
ACHE	0.71659178	1.04913081	2.10790147	0.03983002	0.99978775	-4.3720619
SH3TC2	0.7161404	-1.0906466	0.74125041	0.4618436	0.99978775	-4.6038611
SPINK2	0.71500812	0.55652791	1.88046315	0.06559591	0.99978775	-4.5008303
MMP1	0.71440701	-1.7197248	0.92182432	0.36083598	0.99978775	-4.5972417
BMP7	0.7072388	-2.0828003	1.16174828	0.25059079	0.99978775	-4.59066
FBLN7	0.70297664	1.54813876	2.71083926	0.00904731	0.99978775	-3.5851417
IGFBP2	0.700469	8.17801094	0.89948616	0.37249991	0.99978775	-4.6771265
IDO1	0.69868624	2.55785399	0.89340076	0.37571864	0.99978775	-4.6212568
INHBA	0.69550024	7.27957303	1.07900773	0.28551539	0.99978775	-4.6208586
ESYT3	0.69063397	-1.3175732	1.17401328	0.24568649	0.99978775	-4.5894375
SCG5	0.68806528	-0.1594498	1.63078205	0.10892009	0.99978775	-4.5382101
SERHL2	0.68664201	1.06569577	2.71027545	0.00906077	0.99978775	-4.3502088
ABCA1	0.68654424	5.00358648	1.37043361	0.1763815	0.99978775	-4.475711
ACACB	0.68566724	0.12233191	0.73128761	0.46785413	0.99978775	-4.6044601
NEBL	0.6845144	-2.1560643	1.54700642	0.12786743	0.99978775	-4.5672725
C33H3orf52	0.6809438	-1.6075834	1.44073996	0.15559422	0.99978775	-4.5727941
ENSCAFG00000031899	0.67721443	0.78165445	2.11456106	0.03923211	0.99978775	-4.4567244
LEP	0.67289619	0.81288688	0.56137442	0.57693021	0.99978775	-4.619513
UNC79	0.66619586	-1.8506891	1.13063568	0.26334581	0.99978775	-4.5917576
COL8A1	0.66600124	6.85462986	0.8018557	0.42625008	0.99978775	-4.7299967
WNT7A	0.66488684	-1.8780992	1.19406369	0.23781889	0.99978775	-4.5870026
AFAP1L2	0.66386818	2.82977506	1.84402433	0.07082435	0.99978775	-4.3029961
ICA1	0.663768	0.40552169	1.45487644	0.15165513	0.99978775	-4.5356177
TNS3	0.66255768	2.24970269	1.22433607	0.2262897	0.99978775	-4.5492115
GNA14	0.66176375	-1.5099714	1.25881737	0.21366358	0.99978775	-4.5841785
LBH	0.66019547	3.77232213	1.33154119	0.18876216	0.99978775	-4.506569
CABCOCO1	0.65918579	0.54727147	1.9468894	0.05690533	0.99978775	-4.4950705
SULT4A1	0.6567792	1.191117	1.64566955	0.10580475	0.99978775	-4.5331495
THBS1	0.65638518	12.6771152	1.72240193	0.09088201	0.99978775	-4.3682892
FOXP2	0.65551939	1.10276346	1.4374114	0.15653323	0.99978775	-4.5132661
ARL9	0.65448537	1.52514243	2.23706303	0.02955153	0.99978775	-4.2423793
ENSCAFG00000023591	0.65293116	4.07495289	1.31134483	0.19544608	0.99978775	-4.5260793
TRPM3	0.65235851	-0.6813145	0.95574968	0.34357788	0.99978775	-4.5986207
CHST2	0.64835869	3.46497103	1.53648892	0.13042224	0.99978775	-4.4123244
ANGPT1	0.64558811	5.47350132	1.38006263	0.17341459	0.99978775	-4.471527
MDGA2	0.64531168	0.35526247	2.18329475	0.03350519	0.99978775	-4.2169063
ST8SIA6	0.64530299	-1.8726575	0.90765885	0.36820491	0.99978775	-4.5977655
HMCN2	0.64076736	-1.2241643	1.29899474	0.19962037	0.99978775	-4.5850523
MAOA	0.63989654	7.58332005	1.80812425	0.076315	0.99978775	-4.1826448
TNR	0.63971746	-1.2666492	0.86095396	0.39317737	0.99978775	-4.5996697
TBX18	0.6397081	5.32044357	1.38767885	0.17109517	0.99978775	-4.4648651

FOXL1	0.63713849	0.04699082	0.97514019	0.33396137	0.99978775	-4.5972609
SEMA5A	0.63632963	1.59614366	1.73682756	0.08828064	0.99978775	-4.3948222
LONRF2	0.63400264	-0.1005748	1.55111555	0.12688021	0.99978775	-4.5542982
COL21A1	0.63149233	-0.0196484	0.70934479	0.48124816	0.99978775	-4.6065479
FNBP1	0.63123208	6.73208914	3.18522382	0.00243423	0.99968241	-2.8767523
CCL5	0.63009324	0.4533868	0.86685318	0.38996607	0.99978775	-4.6013996
DKK2	0.62977652	0.08728014	0.41184825	0.68212689	0.99978775	-4.6115517
ENSCAFG00000005852	0.62899188	0.07909552	0.69937508	0.48740399	0.99978775	-4.604884
ENSCAFG00000003577	0.62727134	0.58600471	2.1205737	0.03869904	0.99978775	-4.4866103
KCNE3	0.62540102	-0.1242871	0.68660587	0.49535186	0.99978775	-4.606194
ENSCAFG00000003862	0.62483757	-0.9565075	1.07705466	0.28637883	0.99978775	-4.5919994
ENSCAFG00000028801	0.62465332	-1.0292495	1.01306591	0.31567367	0.99978775	-4.5950543
PRKAA2	0.62293716	-1.4526008	1.29958845	0.19941818	0.99978775	-4.5816133
ENSCAFG00000031469	0.62180422	2.83807459	1.58536902	0.11888649	0.99978775	-4.5050039
PALD1	0.62133432	0.3196714	0.39251324	0.6962677	0.99978775	-4.6151635
ENTPD1	0.62113536	-1.6707684	0.55480998	0.58138011	0.99978775	-4.6043229
ARHGAP28	0.62099934	1.69419337	1.1701969	0.24720503	0.99978775	-4.5690295
ESR1	0.61912584	-0.5103058	1.4381614	0.15632126	0.99978775	-4.5459239
CNNM2	0.61773998	3.91009208	2.38951651	0.02049442	0.99978775	-3.7437883
HRH2	0.61761088	0.48478851	2.9437157	0.00482061	0.99978775	-3.7128225
PCGF5	0.61702833	4.81012176	2.65687504	0.01042317	0.99978775	-3.5606447
MPP3	0.6157509	-1.8092285	1.12259859	0.26671438	0.99978775	-4.5913323
ENSCAFG00000014958	0.61517843	-0.5108803	1.54948682	0.12727078	0.99978775	-4.5626504
CSPG5	0.61505359	1.49327719	1.53582003	0.13058609	0.99978775	-4.5376394
CDKN2B	0.61503123	2.46307325	1.03289937	0.30638447	0.99978775	-4.5952137
ENSCAFG00000031828	0.61429401	-1.4978827	1.32953493	0.18941827	0.99978775	-4.5758647
MECOM	0.61421731	2.91550661	0.89037743	0.3773243	0.99978775	-4.621837
DMPK	0.61408401	4.32014598	1.44499929	0.15439904	0.99978775	-4.4658914
CALHM5	0.6136818	4.9613307	2.5076679	0.01528131	0.99978775	-3.6038189
TNNI3	0.61366621	-0.7627335	1.17769199	0.2442291	0.99978775	-4.5887949
ANKRD37	0.61353314	2.6537341	1.86690799	0.06750135	0.99978775	-4.4301149
C5AR2	0.61088889	-1.6458423	0.92480967	0.35929522	0.99978775	-4.5973016
EPDR1	0.61030619	0.83792267	1.17536012	0.24515218	0.99978775	-4.5733224
RAB3B	0.60807189	0.04389905	1.39129183	0.17000327	0.99978775	-4.5695838
HTR7	0.60795817	1.94753896	0.86719733	0.38977924	0.99978775	-4.6101705
TPC3	0.60694511	5.37529098	1.11491746	0.26996216	0.99978775	-4.6065439
CD59	0.60654456	5.37213331	2.55322982	0.01361528	0.99978775	-3.5769667
SERPINE1	0.60468339	10.7482684	0.90180692	0.37127705	0.99978775	-4.6506015
ENOSF1	0.60454192	0.91234719	1.79481436	0.07843944	0.99978775	-4.5245232
SERPING1	0.60428884	4.1251347	0.78119179	0.43819732	0.99978775	-4.7043349
KCNC4	0.6041626	0.12803595	0.90533285	0.36942406	0.99978775	-4.6011966
SYPL2	0.60414365	-0.802916	1.46776785	0.14813149	0.99978775	-4.5694499
ESAM	0.60203661	-0.3414123	0.39237425	0.69636975	0.99978775	-4.6085979
PDGFB	0.60149499	-0.0825475	0.52863762	0.59928408	0.99978775	-4.6130619
TBX1	0.60020536	-2.082806	0.81701275	0.4176123	0.99978775	-4.6005229
DMC1	0.59710607	-0.9553972	1.7626703	0.0837749	0.99978775	-4.5220475
DMTN	0.59571238	-0.5168721	1.18095324	0.24294232	0.99978775	-4.5862399
TBX5	0.59355874	-0.8897474	0.66143889	0.51122271	0.99978775	-4.6085315
DAPP1	0.59345061	-0.9878935	0.77217111	0.44347432	0.99978775	-4.6046498
PKNOX2	0.59204345	2.09540491	1.33482967	0.18769046	0.99978775	-4.5200781

MYRF	0.59187802	3.55266253	1.00447454	0.319756	0.99978775	-4.6484271
BTBD11	0.59090714	0.65401889	2.2710271	0.02727218	0.99978775	-4.2068198
DLG2	0.58741943	-1.0372691	0.72592771	0.47110611	0.99978775	-4.6028454
ENSCAFG00000030939	0.58545413	0.86644707	0.80164619	0.42637022	0.99978775	-4.6052952
ENSCAFG00000028578	0.58453993	0.84518105	2.33462789	0.02342016	0.99978775	-4.4220508
TBX2	0.58168224	2.544722	1.76850048	0.08278531	0.99978775	-4.3839971
CRYAB	0.58104785	6.93762489	1.70871082	0.09340927	0.99978775	-4.2541217
MYH14	0.57959328	-1.2735824	0.87834522	0.38375755	0.99978775	-4.5993237
GLRB	0.5792621	6.49072648	2.26047508	0.02796304	0.99978775	-3.8117265
SYNPO	0.57871534	7.54785587	1.97085773	0.05402113	0.99978775	-4.0927797
ENTPD3	0.57853683	6.27078348	1.53171797	0.13159451	0.99978775	-4.3747953
MOB3C	0.57708793	1.12171902	1.98569576	0.05229924	0.99978775	-4.4654203
CUX2	0.57609745	1.7172648	1.71954964	0.0914038	0.99978775	-4.4000857
ENSCAFG00000003087	0.57408228	-0.5103055	1.50154649	0.13920364	0.99978775	-4.5588758
ST3GAL5	0.57326945	3.93105332	2.29800168	0.02557452	0.99978775	-3.8600724
IGFBP7	0.57300166	9.452131	1.86137571	0.06829237	0.99978775	-4.1933151
TMEM178A	0.57267981	-1.5603024	1.1505544	0.25512781	0.99978775	-4.5883529
LRRN4	0.57149431	-0.8924889	0.56604225	0.57377605	0.99978775	-4.6300229
CCDC136	0.56984384	1.17186459	1.24691493	0.21796151	0.99978775	-4.5677896
SPINT1	0.56973915	0.01065618	1.14197114	0.2586463	0.99978775	-4.5860783
MEIS1	0.56965231	3.60241432	1.19198904	0.23862438	0.99978775	-4.5626967
CASQ1	0.56924856	0.94943296	1.42109041	0.16120163	0.99978775	-4.5300195
APBB1IP	0.56807501	6.15214018	1.63321138	0.10840669	0.99978775	-4.3058501
ENSCAFG00000023940	0.5667172	1.14452372	2.10822551	0.03980074	0.99978775	-4.4586392
ENSCAFG00000024014	0.5661818	1.42212999	2.58915233	0.01242001	0.99978775	-4.2980365
TBC1D16	0.56460557	-0.8597779	1.03628098	0.30481944	0.99978775	-4.5938987
ENSCAFG00000030958	0.56347096	4.95382318	0.59458228	0.55467594	0.99978775	-4.8107859
FRRS1	0.56340955	-1.2200915	0.95172475	0.34559658	0.99978775	-4.597247
EXOC3L1	0.56168124	-0.9607347	0.44990351	0.65463172	0.99978775	-4.6065776
SESN1	0.5614207	3.71747274	1.13878412	0.25996151	0.99978775	-4.5771517
F2RL1	0.56127859	1.04894685	0.91649816	0.36359543	0.99978775	-4.6046817
ATOH8	0.56062274	-0.766381	0.99869892	0.32252027	0.99978775	-4.5957815
TNFRSF25	0.55898828	-0.3027327	1.18438433	0.24159384	0.99978775	-4.5839698
TBX20	0.55884133	5.52965799	3.2689401	0.00190787	0.99968241	-2.8185794
IGF2BP3	0.55841721	-0.2882953	0.82672014	0.41213635	0.99978775	-4.604373
C16H8orf48	0.55798981	1.56077974	2.64756979	0.01067893	0.99978775	-4.2972855
TSTD1	0.55721638	-0.3981761	1.12883137	0.26409941	0.99978775	-4.5872041
ALDOC	0.55699614	5.09262422	1.88510279	0.06495428	0.99978775	-4.2131338
TCAF1	0.55628332	3.76247415	1.12045917	0.2676162	0.99978775	-4.5885206
ENSCAFG00000030886	0.55596018	5.57093225	1.99128353	0.05166315	0.99978775	-4.0362647
CTGF	0.5555979	10.2739849	1.27428704	0.20817197	0.99978775	-4.5288278
ENSCAFG00000031664	0.55337744	-1.1486464	1.29929562	0.19951788	0.99978775	-4.5750605
PCDHB4	0.55228803	1.17742634	2.13011992	0.03786569	0.99978775	-4.4359553
CLDN34	0.55044949	-0.1857844	1.10066022	0.27606422	0.99978775	-4.590767
ENSCAFG00000006771	0.54784581	1.00120724	2.31970198	0.02427775	0.99978775	-4.4356712
VXN	0.54557358	-1.8157534	0.99733164	0.323177	0.99978775	-4.5955226
ZNF454	0.54359156	1.56922485	1.98761279	0.05208026	0.99978775	-4.4444781
MYO3A	0.54347369	1.08677342	0.81530867	0.4185781	0.99978775	-4.615827
PLCL1	0.54074993	4.94639192	1.47425885	0.14638181	0.99978775	-4.4435168
ENSCAFG00000013781	0.54030444	0.30418088	1.22447883	0.22623631	0.99978775	-4.5686562

CFAP300	0.53926362	1.62752346	2.42773377	0.01865549	0.99978775	-4.3262352
ANTXR1	0.53834116	7.29617263	1.13908123	0.2598387	0.99978775	-4.5955353
FAM69A	0.53818858	1.30150033	1.57489884	0.12128565	0.99978775	-4.4645252
JPH1	0.53791139	0.28187977	0.87612387	0.38495275	0.99978775	-4.6049651
ENSCAFG00000018144	0.53748873	0.75309314	2.0681702	0.04356499	0.99978775	-4.4420186
SLC8A1	0.53709525	2.36164934	1.00927568	0.3174703	0.99978775	-4.6042199
ANGPTL4	0.53695952	3.25198697	0.74504281	0.45956736	0.99978775	-4.6400788
GAD2	0.53289665	-0.160548	1.53241595	0.13142249	0.99978775	-4.5466986
PROKR2	0.53265284	-1.2406325	0.84029802	0.40455094	0.99978775	-4.6030199
SLC24A3	0.5322981	1.74916414	1.48389441	0.14381454	0.99978775	-4.4701676
HOPX	0.53187936	-2.3288397	0.97711061	0.33299426	0.99978775	-4.5959388
MYBL1	0.53007855	4.79635515	1.76608017	0.08319494	0.99978775	-4.238508
MOXD1	0.53006605	4.82919122	0.85100148	0.39863232	0.99978775	-4.7204444
CPAMD8	0.52991868	1.16126414	0.6934375	0.49109085	0.99978775	-4.6214603
COL13A1	0.52916404	-0.7909121	0.56293385	0.57587553	0.99978775	-4.6068233
ENSCAFG00000008935	0.52778274	2.50150107	1.9927436	0.05149805	0.99978775	-4.3851535
CRIM1	0.52583503	9.51422093	2.428003	0.01864308	0.99978775	-3.8121077
SINHCAF	0.52533771	0.2928493	1.4230699	0.16062973	0.99978775	-4.5655648
RFTN2	0.52501209	2.05447439	1.31904125	0.19287822	0.99978775	-4.5506968
RCAN2	0.52497786	2.00597723	1.98665679	0.05218936	0.99978775	-4.2213051
PDE10A	0.52305624	2.4720711	1.0605628	0.2937421	0.99978775	-4.5940393
DES	0.52247417	1.50782457	1.06084696	0.29361414	0.99978775	-4.5929018
C10H12orf56	0.52104837	-0.3473251	1.24168821	0.21986893	0.99978775	-4.5822824
C12H6orf141	0.51979164	-0.2213676	1.28084543	0.20587583	0.99978775	-4.5786653
CYBRD1	0.51973349	7.95094686	1.5920232	0.11738173	0.99978775	-4.3338343
FZD8	0.51945334	-0.6556337	0.83847647	0.40556355	0.99978775	-4.6031413
TUBA4A	0.51779711	5.08781599	1.51600065	0.13551594	0.99978775	-4.3840591
KCNE4	0.51725176	1.69862666	1.37209218	0.1758677	0.99978775	-4.5042247
MXD1	0.51703409	1.85797959	2.25367773	0.02841625	0.99978775	-4.3686688
DDIT4L	0.51694061	-1.6258408	0.92541422	0.35898372	0.99978775	-4.5976085
PPP4R4	0.51622237	1.29705435	2.15047056	0.03614141	0.99978775	-4.4306957
CD40	0.51299983	1.52445777	1.37414878	0.17523219	0.99978775	-4.5386388
JPH2	0.51045616	-1.8348631	1.05875771	0.2945559	0.99978775	-4.5934296
FREM2	0.50940943	-0.2640065	1.00164187	0.32110975	0.99978775	-4.5956912
ALOXE3	0.50916207	-0.4832732	1.1951641	0.23739246	0.99978775	-4.5813001
EYA2	0.50829946	4.47849485	0.81176016	0.42059359	0.99978775	-4.6675954
ENSCAFG00000025091	0.50746953	0.60455619	1.50006583	0.13958585	0.99978775	-4.5531526
C16H4orf47	0.50684564	-1.3443578	1.10948768	0.27227478	0.99978775	-4.5913854
DNAJB4	0.50565442	6.58221462	3.03537434	0.00373258	0.99978775	-3.0254566
C20H3orf62	0.50536117	1.09616706	2.30566921	0.02510945	0.99978775	-4.4368363
CCDC3	0.5050411	2.33575779	0.28633044	0.77575162	0.99978775	-4.6450018
NRL	0.50462669	-0.420481	0.96153086	0.34069191	0.99978775	-4.5971176
STT3B	0.50436081	8.80494503	3.47484764	0.00103326	0.99968241	-2.8569074
PRKCH	0.50414139	-0.274035	0.56574124	0.5739792	0.99978775	-4.6138155
FOXO1	0.50358588	4.11082739	1.62196967	0.11079905	0.99978775	-4.3903122
SLC7A2	0.50242418	2.33800787	1.05493698	0.29628355	0.99978775	-4.5940446
MAP7D2	0.50210058	-1.3438447	0.96202792	0.34044453	0.99978775	-4.5966087
PFKFB3	0.50176815	6.69196008	2.15086512	0.03610867	0.99978775	-3.897763
SEMA3C	0.49993734	9.6000928	1.37978597	0.1734993	0.99978775	-4.4790731
IMPG2	0.49898453	0.212098	1.23896219	0.22086863	0.99978775	-4.58097

ADRA1B	0.49870048	2.31619888	1.29287174	0.2017146	0.99978775	-4.523927
KCNJ3	0.49844124	-1.5758898	0.71467683	0.47797383	0.99978775	-4.6051821
INPP4B	0.49744542	1.57731853	1.06295666	0.29266526	0.99978775	-4.5939611
IGDCC4	0.49741127	4.92270757	1.39420234	0.16912758	0.99978775	-4.4606316
LGR6	0.49693253	-0.9989534	0.88122438	0.3822119	0.99978775	-4.6023246
PDE1A	0.49625049	6.22474319	1.22582069	0.22573502	0.99978775	-4.5533944
ENSCAFG00000020503	0.4952893	-0.3978757	1.19624379	0.2369746	0.99978775	-4.5840102
GRAMD1B	0.4949067	-1.7534737	0.79464628	0.43039598	0.99978775	-4.6000008
ENSCAFG00000030310	0.49460452	2.97057534	1.64167638	0.10663314	0.99978775	-4.414112
PM20D1	0.49422154	0.73208619	1.40387783	0.16624153	0.99978775	-4.5567528
ANO5	0.49401499	2.11352784	1.81470347	0.07528282	0.99978775	-4.2593441
ABCG1	0.4921428	-0.5976442	0.8942226	0.37528292	0.99978775	-4.603542
CSF1	0.48933843	7.20115758	1.61838849	0.11157011	0.99978775	-4.3151833
ENSCAFG00000008917	0.48912223	-2.0383221	0.85932344	0.39406786	0.99978775	-4.5994353
GCNT4	0.48689362	-0.0084322	1.01862001	0.31305337	0.99978775	-4.595032
VEGFD	0.48591113	-0.3850808	0.63868286	0.5258051	0.99978775	-4.6311565
DOK5	0.48570816	-0.9416729	0.80252929	0.42586395	0.99978775	-4.6038736
ENSCAFG00000030953	0.48526606	-1.7493859	0.53889874	0.59223404	0.99978775	-4.6064401
CNTNAP5	0.48495526	-1.1482531	1.11004113	0.27203842	0.99978775	-4.5895913
ABCC9	0.48468696	0.4365155	1.39940135	0.16757203	0.99978775	-4.4943784
PDLIM2	0.4843824	4.88853129	1.477499	0.14551451	0.99978775	-4.4138251
HAS1	0.48390171	0.93055932	0.50602665	0.61495567	0.99978775	-4.6170106
GJA1	0.48320816	7.32405851	2.78129963	0.00750178	0.99978775	-3.3121225
CDH6	0.48245892	5.01190264	1.0583269	0.29475036	0.99978775	-4.6389126
SVEP1	0.48153583	8.23665495	0.78948022	0.43338158	0.99978775	-4.7305418
ITGA1	0.48049689	8.07629681	1.7590948	0.08438664	0.99978775	-4.2359575
FBLIM1	0.48028944	6.24991976	1.84051691	0.07134568	0.99978775	-4.1540915
SNAP91	0.47807062	0.99164164	1.06425859	0.29208075	0.99978775	-4.5912884
ENSCAFG00000029800	0.47701148	0.07075847	0.94062789	0.35120241	0.99978775	-4.5990972
LRRC24	0.47700777	0.16107176	1.04046894	0.3028888	0.99978775	-4.5936346
ENSCAFG00000016350	0.47641341	0.5617538	1.77584976	0.08155179	0.99978775	-4.51655
EBF2	0.47602345	0.99802253	0.29259537	0.77098443	0.99978775	-4.6157181
ASAH1	0.47587987	7.35676958	2.05753887	0.04461462	0.99978775	-3.9892741
RIMKLA	0.47531378	0.29572882	0.93392601	0.35461656	0.99978775	-4.5998214
ENSCAFG00000018235	0.47506629	2.25109543	1.3730829	0.17556134	0.99978775	-4.5151348
HSD11B2	0.4748689	-0.9739792	0.5192956	0.60573646	0.99978775	-4.6054664
TMEM64	0.47407267	3.34533934	2.10676399	0.03993293	0.99978775	-4.0636243
IL18R1	0.47325257	-0.5361677	0.78078572	0.43843406	0.99978775	-4.6090121
ENSCAFG00000012963	0.47309738	7.29068058	0.62190216	0.53669697	0.99978775	-4.806045
PKP2	0.47075633	4.20664144	0.99801136	0.32285041	0.99978775	-4.6631133
IL6	0.47045108	-0.9117922	0.50642405	0.61467864	0.99978775	-4.6105402
PERP	0.47026693	-0.2091265	1.16259339	0.25025062	0.99978775	-4.583574
NCEH1	0.46998557	4.52986731	2.54692154	0.01383566	0.99978775	-3.6856961
DACT1	0.46994452	4.42606406	1.41504023	0.16295944	0.99978775	-4.4505418
ENSCAFG00000030608	0.46941058	-2.250137	0.80642018	0.42363763	0.99978775	-4.6000213
UGCG	0.4689838	6.70345468	2.97714939	0.0043934	0.99978775	-3.0880856
CADM4	0.46877021	1.89295003	1.69023774	0.09691089	0.99978775	-4.4367545
FLI1	0.46813334	2.08282782	0.90735418	0.36836446	0.99978775	-4.6297764
MAN2A2	0.46757118	7.18103692	1.81382162	0.07542049	0.99978775	-4.1933803
CPE	0.46718574	6.98858253	1.73975262	0.08776076	0.99978775	-4.2293941

FBXO27	0.46486966	3.43837163	2.23191689	0.02991121	0.99978775	-3.9270238
LPCAT2	0.46486247	5.26187185	1.69885904	0.09526348	0.99978775	-4.2612531
DISP3	0.46476973	2.12154898	1.41083658	0.16418949	0.99978775	-4.5260474
SLC2A5	0.46451132	-0.2443436	0.64284914	0.52311901	0.99978775	-4.6105123
GCNT1	0.46398479	-0.4508658	1.34607529	0.18406046	0.99978775	-4.5611886
DHRS2	0.46252358	1.30716119	0.60753073	0.546117	0.99978775	-4.6224942
LRRN3	0.46142884	-1.9485995	0.66438531	0.50935061	0.99978775	-4.6030624
C1RL	0.46128149	3.00978031	1.07413145	0.28767455	0.99978775	-4.5979795
PCDHGB1	0.46101936	0.89079879	1.34392474	0.18475048	0.99978775	-4.5669798
ENSCAFG00000029058	0.46094434	2.71586994	2.16778192	0.03472923	0.99978775	-4.2977373
ABCG8	0.46066919	-2.8555467	0.94513107	0.34892042	0.99978775	-4.5967099
CH25H	0.45977895	2.95126965	0.8110924	0.42097352	0.99978775	-4.6363946
LIMCH1	0.45968649	4.83940477	1.05820646	0.29480474	0.99978775	-4.6386875
BCL2	0.45879783	3.25155641	1.70036612	0.09497788	0.99978775	-4.338617
SPRY4	0.45855283	3.32131534	0.78669193	0.4349981	0.99978775	-4.6242575
ICAM5	0.45852011	1.15539573	0.71828287	0.47576653	0.99978775	-4.6177775
CDKN2A	0.45810901	5.54109181	1.46007151	0.15022733	0.99978775	-4.4274125
SRSF12	0.45799153	1.07898402	1.86719871	0.06745999	0.99978775	-4.4890454
TMEM86A	0.45613132	2.42113507	1.88453378	0.06503268	0.99978775	-4.3077909
CPQ	0.45585735	2.92366911	1.5017137	0.13916053	0.99978775	-4.4191434
TMOD1	0.45512098	1.54885855	1.06290162	0.29268999	0.99978775	-4.5914786
CCND1	0.45501894	7.71075404	1.95698585	0.05567469	0.99978775	-4.0668135
STOX1	0.45359398	-1.0908918	0.87979114	0.38298083	0.99978775	-4.6048061
PXDNL	0.4533697	0.40708791	0.62222048	0.53648927	0.99978775	-4.6366261
ADGRA1	0.45283799	-2.3550836	0.83551141	0.40721517	0.99978775	-4.5993691
PATJ	0.45279217	-0.5985813	0.95427801	0.34431509	0.99978775	-4.5975712
CD274	0.45237501	2.39051539	0.72522208	0.47153518	0.99978775	-4.62846
GDF6	0.45235876	4.13756539	0.84004951	0.404689	0.99978775	-4.691927
ABLIM1	0.45181609	6.61169312	1.5431173	0.12880743	0.99978775	-4.3700906
TPRKB	0.45117294	2.7952626	2.70917132	0.0090872	0.99978775	-3.9723128
TRIM52	0.45113299	-0.5323061	1.31346136	0.19473736	0.99978775	-4.5775159
GALNT1	0.45111225	9.01895152	1.27774117	0.20696029	0.99978775	-4.5260532
NEK7	0.44806181	6.03216079	2.21532565	0.03109733	0.99978775	-3.8437982
PGAM1	0.44680908	8.63671466	2.32293243	0.0240898	0.99978775	-3.8242291
PLEKHG5	0.44630936	4.36788347	1.28822326	0.20331549	0.99978775	-4.5206449
ATRNL1	0.44622811	3.62426113	0.98450686	0.32938066	0.99978775	-4.6607772
F3	0.44567208	7.55545442	0.76117586	0.44995667	0.99978775	-4.7415451
CHN2	0.44524508	-1.2612531	0.59830907	0.55220562	0.99978775	-4.6098424
ACTA1	0.44512218	3.68923503	1.29954152	0.19943415	0.99978775	-4.5150325
PDE5A	0.44509484	1.69704763	1.37114597	0.17616068	0.99978775	-4.507133
PDLIM5	0.44499094	8.99965582	1.52972231	0.13208735	0.99978775	-4.3991188
TMEM200A	0.44482721	1.98290607	1.74997554	0.08596374	0.99978775	-4.3471128
ENSCAFG00000030331	0.44438952	-0.4930304	1.01637714	0.31410972	0.99978775	-4.5949406
PIEZO2	0.44426329	4.01823677	1.61148958	0.11306779	0.99978775	-4.3348223
L1CAM	0.44389804	3.10991188	0.71742703	0.47628988	0.99978775	-4.7320329
CHST15	0.44272079	5.6557594	1.64498917	0.10594553	0.99978775	-4.2982862
SH3TC1	0.44262222	2.80270151	0.8741702	0.38600585	0.99978775	-4.6403414
SPATA6L	0.44251598	1.21536238	1.27355617	0.20842903	0.99978775	-4.572722
LRRC15	0.44112227	1.69533988	0.66034794	0.51191681	0.99978775	-4.6332165
RERG	0.44106767	1.19095148	0.76067674	0.45025224	0.99978775	-4.640223

SCN8A	0.4398328	1.99452549	1.64999338	0.1049137	0.99978775	-4.3924034
VWA7	0.4387325	-0.1290595	1.01632798	0.31413291	0.99978775	-4.5949841
JAKMIP2	0.43858232	0.4644903	1.18187242	0.24258054	0.99978775	-4.574722
ZNF662	0.4380605	1.26864043	1.26345464	0.21200621	0.99978775	-4.5678904
ECM2	0.43791467	5.85412254	1.06984127	0.28958353	0.99978775	-4.6338105
CD86	0.43780458	-2.0598218	0.45213007	0.65303737	0.99978775	-4.6052963
PCBD1	0.43729063	2.95405492	1.18718702	0.24049635	0.99978775	-4.5645397
ENSCAFG00000018611	0.4358855	6.02724995	3.78521402	0.00039591	0.99968241	-2.2346142
ENO2	0.43509135	5.22257966	1.44031645	0.15571345	0.99978775	-4.4362495
MAMLD1	0.43502289	2.09430988	1.03498771	0.30541732	0.99978775	-4.5968967
SLC45A2	0.43482208	-0.2302449	0.77206622	0.4435359	0.99978775	-4.6069851
BDNF	0.43373304	0.58452761	0.90190684	0.37122446	0.99978775	-4.6138916
ENSCAFG00000031764	0.43359917	1.80723514	1.84465639	0.07073075	0.99978775	-4.4510469
WDR66	0.43357535	1.80487437	0.63745702	0.5265968	0.99978775	-4.6166471
C30H15orf62	0.43262377	-0.1173912	1.17321626	0.24600307	0.99978775	-4.583909
BCL7A	0.43240762	1.84938551	1.96297563	0.05495545	0.99978775	-4.4091534
ENSCAFG00000029966	0.43225286	0.22984614	1.04325738	0.30160799	0.99978775	-4.5935254
CYP19A1	0.43104219	-2.3081872	0.60412669	0.54836053	0.99978775	-4.6037061
ENSCAFG00000009211	0.43081039	9.60017388	2.42327186	0.01886226	0.99978775	-3.7992022
TRIM63	0.43048843	1.74534154	0.92160006	0.36095189	0.99978775	-4.6085718
ENSCAFG00000028589	0.43025624	1.35051719	0.38517823	0.7016611	0.99978775	-4.6258871
STX1B	0.42952872	-1.027338	1.14939938	0.25559928	0.99978775	-4.5876415
CDH17	0.42932959	0.24966536	1.45847786	0.1506642	0.99978775	-4.5221009
ACSM3	0.42881944	-0.9383832	0.27164854	0.78695736	0.99978775	-4.6064612
ARHGAP18	0.42861874	3.05541667	1.6446749	0.1060106	0.99978775	-4.4143664
KANK1	0.42858371	7.39776852	1.71666084	0.09193479	0.99978775	-4.249715
ENSCAFG00000018360	0.42807485	0.29495725	0.70736819	0.48246515	0.99978775	-4.6097684
MFAP5	0.42781829	7.54046896	0.66516251	0.5088574	0.99978775	-4.7595425
INPP5D	0.42758233	-1.582793	0.42018136	0.67606728	0.99978775	-4.6069162
MAGEE2	0.42725296	-0.2581473	1.18585837	0.24101618	0.99978775	-4.5838793
CCDC126	0.42724225	2.60053583	1.89609743	0.06345504	0.99978775	-4.3379009
SYNPO2	0.42556366	3.78256141	0.72981647	0.46874543	0.99978775	-4.6797565
SLITRK5	0.42538228	-1.0369321	0.58218823	0.56293115	0.99978775	-4.6113361
NINJ2	0.42493846	1.44367312	1.80148513	0.07736858	0.99978775	-4.4618475
AP1S3	0.42438459	0.02512366	1.2562075	0.21460057	0.99978775	-4.5565648
TNFSF10	0.42302968	5.54340094	1.84918235	0.07006353	0.99978775	-4.1511555
GFRA2	0.42302056	-0.0332757	1.35752388	0.18042014	0.99978775	-4.5198124
NXPH4	0.42297153	0.81580901	0.7974051	0.42880664	0.99978775	-4.6096501
ZNF467	0.42242573	-1.6924263	0.78080226	0.43842442	0.99978775	-4.6004209
ZNF653	0.42234798	1.49002482	1.73371951	0.08883583	0.99978775	-4.4941996
VAV3	0.42184272	2.71178861	0.59718308	0.55295141	0.99978775	-4.6316688
GATA2	0.42184196	1.22700674	0.74372943	0.46035493	0.99978775	-4.6243578
CASP10	0.42161988	1.99364889	1.22010298	0.22787674	0.99978775	-4.5630549
ANAPC13	0.42108888	0.22145581	1.183007	0.2421345	0.99978775	-4.5837141
FAM214A	0.42097548	5.25776627	2.23750964	0.02952049	0.99978775	-3.8668906
FZD6	0.4207432	4.24666217	0.89420553	0.37529197	0.99978775	-4.6999309
NMUR2	0.42053077	0.98303505	0.50914547	0.61278302	0.99978775	-4.6210679
PODN	0.41748747	6.98686117	0.64188985	0.52373684	0.99978775	-4.7981758
CNTNAP1	0.41735035	1.09838637	0.74789692	0.45785856	0.99978775	-4.620887
MEOX2	0.41714552	1.08787729	0.28791612	0.7745442	0.99978775	-4.6215808

CCDC138	0.41689485	1.50962547	2.03559304	0.04685092	0.99978775	-4.4459703
CXCL16	0.41527271	3.52159222	1.43040396	0.15852453	0.99978775	-4.4482126
LAMC3	0.41506614	-1.1794361	0.60631386	0.54691848	0.99978775	-4.6096334
ENSCAFG00000013060	0.41409261	4.76307204	2.26090437	0.02793463	0.99978775	-3.9121722
NAP1L5	0.41393454	-0.2602987	1.23063424	0.22394347	0.99978775	-4.5813397
ENSCAFG00000005298	0.4137385	1.77249039	2.16671507	0.03481484	0.99978775	-4.3147796
ADAMTS8	0.41178413	-1.940853	0.58962545	0.55797018	0.99978775	-4.6045914
ENSCAFG00000023820	0.41099737	1.36250777	1.03441832	0.30568081	0.99978775	-4.594019
TDRP	0.40980128	0.76735002	1.24218512	0.21968706	0.99978775	-4.5679166
THBS4	0.40846621	-2.7767064	0.66878577	0.50656153	0.99978775	-4.6023185
RFK	0.40815629	4.94891945	2.09336533	0.04116278	0.99978775	-3.9915769
DPEP2	0.40765141	-0.5640641	0.86380934	0.39162096	0.99978775	-4.6010333
ENSCAFG00000020075	0.4073524	1.78681217	1.27343866	0.20847038	0.99978775	-4.5535175
ZNF280B	0.40573404	2.8884958	1.85144598	0.06973182	0.99978775	-4.3607261
CDK6	0.40437557	4.54469891	2.22577547	0.03034552	0.99978775	-3.9818518
ITGAV	0.40311971	10.7568944	1.99999197	0.0506851	0.99978775	-4.1984586
ETS2	0.40284479	5.09738154	1.77408381	0.08184678	0.99978775	-4.2122883
ANXA3	0.40265915	3.78274847	1.70554781	0.09400131	0.99978775	-4.3221876
DISC1	0.402437	2.58256753	1.28456054	0.20458357	0.99978775	-4.5307369
TGFBR1	0.40186993	7.60939922	2.72294083	0.0087626	0.99978775	-3.4213003
FGFRL1	0.40116654	1.93550797	1.14370412	0.25793314	0.99978775	-4.5780081
PITX2	0.40035244	1.19728678	1.14573842	0.25709776	0.99978775	-4.5773917
RAD51B	0.40004763	0.33388985	1.10952856	0.27225732	0.99978775	-4.5885343
NIPAL1	0.39964542	0.8070948	0.98025745	0.33145359	0.99978775	-4.599989
FERMT1	0.39786107	-1.5671616	0.75071694	0.45617378	0.99978775	-4.6053198
ENSCAFG00000000733	0.39778877	0.14663008	1.23371986	0.22280055	0.99978775	-4.5750238
SHANK1	0.3970704	1.54953789	1.0185712	0.31307633	0.99978775	-4.5971617
FBXO33	0.39692284	3.62916586	2.71713236	0.00889821	0.99978775	-3.8419111
B3GNT5	0.3968897	-0.5577032	0.85514457	0.39635582	0.99978775	-4.6059403
P4HA1	0.39670041	8.48760528	2.37982918	0.02098568	0.99978775	-3.7608281
ALS2CL	0.39623895	0.74513082	0.77646017	0.44096062	0.99978775	-4.6215029
CASTOR1	0.39584377	3.15299783	1.66769139	0.10133019	0.99978775	-4.4263588
FAM162A	0.39528762	5.0299856	2.59925824	0.01210142	0.99978775	-3.5488903
F8	0.39490726	-2.1553321	0.61651879	0.54021573	0.99978775	-4.6044388
ENSCAFG00000018701	0.39390277	0.16079616	1.0686793	0.29010208	0.99978775	-4.5912117
LEPR	0.39321226	4.1865463	1.3185219	0.19305069	0.99978775	-4.5050806
PHTF2	0.39206153	5.51923917	1.95822273	0.05552551	0.99978775	-4.0635536
PLS3	0.39166276	10.1457644	2.15589726	0.0356934	0.99978775	-4.0573203
CASZ1	0.39121637	-0.5980385	0.93161002	0.3558014	0.99978775	-4.599869
OTULINL	0.39111176	1.4107021	0.91187304	0.36600267	0.99978775	-4.6062386
KIT	0.39074862	-1.4325999	0.28939444	0.77341902	0.99978775	-4.6076528
HRCT1	0.39039486	1.21866316	0.51696194	0.60735325	0.99978775	-4.6156683
ENSCAFG00000008934	0.38992896	-0.8784087	1.15005942	0.25532978	0.99978775	-4.5873542
PPP1R12B	0.3892547	2.94209506	1.38261163	0.17263565	0.99978775	-4.4979053
KIF21A	0.3870827	0.57563459	0.92248033	0.36049704	0.99978775	-4.6040094
LMO4	0.38670358	7.65512699	1.88889311	0.06443407	0.99978775	-4.1292706
TRMT9B	0.38666298	0.50427503	0.89178836	0.37657443	0.99978775	-4.6104421
LIN7A	0.38653214	-0.5712892	0.90344006	0.37041804	0.99978775	-4.6064808
RECK	0.38602577	5.87151268	1.23301298	0.223062	0.99978775	-4.5508797
TINAGL1	0.38556362	3.21685361	0.75702604	0.45241752	0.99978775	-4.6934392

FZD4	0.38480479	4.22588535	1.74787311	0.0863308	0.99978775	-4.2810063
AKIP1	0.38478648	4.71621905	1.75189976	0.08562894	0.99978775	-4.2961871
ENSCAFG00000029150	0.38404268	-0.5869895	1.03775	0.30414127	0.99978775	-4.5938888
TPI1	0.38310806	8.86800956	2.63918051	0.01091442	0.99978775	-3.558109
REEP2	0.38296294	-0.2197468	0.7868415	0.4349113	0.99978775	-4.6062373
RNF24	0.38171717	1.827471	0.97949832	0.33182482	0.99978775	-4.5994936
WISP2	0.38153693	5.51667292	0.58889655	0.55845542	0.99978775	-4.8078721
DSTN	0.38145579	9.46959331	2.14550008	0.03655607	0.99978775	-4.0278967
DPP4	0.38127355	8.43340978	1.58255472	0.11952758	0.99978775	-4.3527426
ENSCAFG00000031375	0.38111585	-1.5882772	0.70033565	0.48680898	0.99978775	-4.6054124
WFIKKN2	0.38107649	2.79794143	0.87317081	0.38654526	0.99978775	-4.6608853
ZDHHC22	0.38096075	-1.6714846	0.71324139	0.47885408	0.99978775	-4.6041023
KCP	0.38062311	-0.5182972	0.81042914	0.4213511	0.99978775	-4.6042616
FBXW12	0.3805407	-0.847417	0.42414651	0.67319146	0.99978775	-4.6087099
ACSL6	0.38023159	0.7756333	0.90142981	0.37147558	0.99978775	-4.6183285
SPEG	0.38000778	2.34725162	0.79750134	0.42875126	0.99978775	-4.6686561
PROS1	0.37960646	6.27795821	1.02313075	0.31093617	0.99978775	-4.65567
SEMA7A	0.37907415	4.34511041	0.5611169	0.57710446	0.99978775	-4.740183
SERINC1	0.37849142	7.71738439	3.18153432	0.00246032	0.99968241	-2.9286111
GPR176	0.37813517	1.01353175	1.11323231	0.2706784	0.99978775	-4.5847358
ENSCAFG00000014970	0.3780765	0.7802103	1.91019112	0.06157633	0.99978775	-4.4514953
ENSCAFG00000031971	0.37766849	-0.1255029	1.01033268	0.31696857	0.99978775	-4.5952458
ERO1A	0.37735094	8.67675141	1.20140413	0.23498483	0.99978775	-4.5634221
MANEA	0.37615575	4.85129999	2.61520885	0.01161371	0.99978775	-3.5554618
CHAD	0.37573451	-0.9956066	0.58091751	0.56378096	0.99978775	-4.6080721
HMGCLL1	0.37568965	-2.4231643	0.56949902	0.57144566	0.99978775	-4.6033385
MAGI2	0.37567539	2.80977842	1.38531458	0.1718126	0.99978775	-4.5034283
SIAE	0.3753261	4.83739232	1.36194228	0.17903002	0.99978775	-4.4812135
CCL2	0.37531998	6.38299607	1.12468803	0.26583572	0.99978775	-4.6075191
P4HTM	0.37525968	4.34639145	1.89012408	0.06426589	0.99978775	-4.2140587
ZHX1	0.37485236	4.67312967	3.24231225	0.00206236	0.99968241	-3.0630927
SERINC2	0.37452142	0.48663273	0.83315906	0.40852842	0.99978775	-4.6097855
ADAMTS5	0.37423255	6.31505443	0.60793521	0.54585073	0.99978775	-4.8110646
LRRC8C	0.37422317	6.81520396	1.94008084	0.05774829	0.99978775	-4.0839595
PCDHB2	0.37409759	2.01253273	1.09564895	0.27823185	0.99978775	-4.5864925
SMARCD3	0.37393804	5.09861364	2.40691359	0.01963803	0.99978775	-3.6867293
RRAGB	0.37388894	-0.2716661	0.94719375	0.34787839	0.99978775	-4.5987146
ENSCAFG00000029877	0.37200815	3.13389753	2.0985921	0.04067915	0.99978775	-4.1856721
FAM71F2	0.37171453	-0.5604458	0.6303186	0.53121954	0.99978775	-4.6074212
ROBO3	0.3715309	4.07300966	1.12071804	0.26750696	0.99978775	-4.5951923
TAGLN3	0.37152216	2.21234489	1.0544599	0.29649977	0.99978775	-4.6016257
CFI	0.37036966	4.63267111	1.6302819	0.10902603	0.99978775	-4.3450307
LUM	0.37023694	11.4346401	1.13204829	0.26275688	0.99978775	-4.5820289
ITFG1	0.36989649	7.81757452	3.37871824	0.00137912	0.99968241	-2.7377602
SAMD11	0.36974889	3.23328579	0.97283246	0.33509642	0.99978775	-4.6704823
NACC2	0.36857734	5.49235507	1.97610099	0.05340719	0.99978775	-4.0642288
ENSCAFG00000030822	0.36824271	1.71623186	1.49511701	0.14086934	0.99978775	-4.4876777
MITF	0.36811671	5.41198247	1.9259339	0.05953399	0.99978775	-4.0963804
PRKG1	0.36782375	6.06119108	1.11492786	0.26995775	0.99978775	-4.6149672
TLR3	0.36780546	3.44498405	1.34184438	0.18541984	0.99978775	-4.5103914

PPTC7	0.36780105	5.35169195	2.85511617	0.00614677	0.99978775	-3.2697949
RNASE4	0.36760681	1.71465918	0.77420982	0.44227844	0.99978775	-4.6288603
DNER	0.3668037	-0.0758267	0.64083769	0.52441493	0.99978775	-4.6147827
TMEM167B	0.36599432	2.3119498	2.40867767	0.01955302	0.99978775	-4.238816
PLXNB3	0.36591344	3.89559633	2.15827254	0.03549884	0.99978775	-3.9648801
HIPK2	0.36575551	5.56501128	2.19189281	0.03284324	0.99978775	-3.8821905
MMP2	0.36548613	10.3238785	0.83579012	0.40705974	0.99978775	-4.6799833
SKP2	0.36396604	3.37923238	1.27537167	0.20779092	0.99978775	-4.5363588
CDC42EP3	0.36378359	7.11017506	1.71161511	0.09286837	0.99978775	-4.2518573
PLCB4	0.36361479	-0.5418063	0.40670133	0.68588018	0.99978775	-4.6111777
EPHB3	0.36348373	4.87674106	1.39430305	0.16909735	0.99978775	-4.4606104
RGS22	0.3634448	1.93259572	1.53835836	0.12996518	0.99978775	-4.4976164
MED4	0.36332601	2.93734041	3.29605582	0.00176182	0.99968241	-3.6839832
PLCXD1	0.36327706	0.63057387	1.19575043	0.23716547	0.99978775	-4.5790427
SH3KBP1	0.36301638	6.53585106	1.07596045	0.28686336	0.99978775	-4.6311646
ENSCAFG00000006614	0.36294873	-0.0688836	1.20645975	0.23304726	0.99978775	-4.580732
TGM2	0.36291357	5.97927445	0.41740828	0.67808142	0.99978775	-4.8551907
KCNJ4	0.36274773	-1.7714102	0.75195302	0.45543644	0.99978775	-4.6048559
ARHGEF26	0.36253594	1.66583259	1.06705222	0.29082926	0.99978775	-4.5936795
CCDC36	0.36227003	1.47497798	1.11802081	0.26864664	0.99978775	-4.5859802
SLC20A2	0.3621373	3.9133812	1.78805462	0.07953721	0.99978775	-4.3072583
CMTM4	0.36200442	1.77598766	1.64726701	0.10547483	0.99978775	-4.4738954
LTBP1	0.36137087	9.87197653	1.52045017	0.13439649	0.99978775	-4.4032677
RBMS3	0.36126148	3.39098962	1.36520842	0.1780077	0.99978775	-4.5177069
STK38L	0.36115517	6.50343051	1.30328218	0.1981637	0.99978775	-4.514213
SMARCA1	0.36109969	2.213966	1.51408757	0.13599952	0.99978775	-4.4231836
CCDC50	0.36039115	4.80528633	2.64280747	0.01081203	0.99978775	-3.5531204
MAP1B	0.36017739	9.89006704	1.18964239	0.23953785	0.99978775	-4.5632468
LRP5	0.35993511	6.29288949	1.95338565	0.05611087	0.99978775	-4.063369
GNB4	0.35981755	6.42273221	2.85487032	0.00615088	0.99978775	-3.2185334
ENSCAFG00000025678	0.35929114	-0.0671574	0.89134617	0.37680934	0.99978775	-4.6017264
ENSCAFG00000018225	0.35884807	0.97211725	1.51701469	0.13526017	0.99978775	-4.5301212
ENSCAFG00000031827	0.35855971	5.85279012	1.55773931	0.12530172	0.99978775	-4.3564482
DUSP26	0.3582702	0.16953366	0.78894073	0.43369407	0.99978775	-4.6161994
PCDH17	0.35813106	5.99581683	0.78276468	0.43728101	0.99978775	-4.7286522
NMNAT2	0.35803432	1.27397396	0.32493571	0.74652069	0.99978775	-4.6147757
HSPB1	0.3577357	8.59048529	1.05399633	0.29670996	0.99978775	-4.6342146
RAC2	0.357053	0.93922274	1.50958335	0.1371435	0.99978775	-4.5417014
ENSCAFG00000014670	0.35654239	7.68886115	1.25828833	0.21385327	0.99978775	-4.539453
DTX4	0.35628352	0.98594626	1.12697236	0.26487745	0.99978775	-4.5835333
NID1	0.35625162	9.38526021	0.96683979	0.3380558	0.99978775	-4.6504301
PITPNC1	0.35609696	0.79080805	1.31521726	0.19415086	0.99978775	-4.5685793
RAB29	0.35600113	2.97307665	1.14211039	0.25858894	0.99978775	-4.5762439
FBLN5	0.354941	6.89881509	1.20100898	0.23513676	0.99978775	-4.5695301
ZNF654	0.35337724	4.17527818	2.73332545	0.00852488	0.99978775	-3.6661417
SUGCT	0.35293659	3.68316824	0.91767838	0.3629828	0.99978775	-4.6312297
MN1	0.35223699	1.30881457	1.0918064	0.279902	0.99978775	-4.5877289
CTNNAL1	0.35221509	3.89894198	0.89198425	0.37647039	0.99978775	-4.6815287
FAM198B	0.35204867	7.89310281	1.72452557	0.09049512	0.99978775	-4.253923
FAM53A	0.35202487	0.28923961	0.99389753	0.32483043	0.99978775	-4.5964738

EBF1	0.35187347	4.75337333	0.73891381	0.46324928	0.99978775	-4.7021638
F2R	0.35159978	6.29930399	1.98679917	0.0521731	0.99978775	-4.0393312
PNMA2	0.35136193	-0.7936355	0.66991158	0.5058493	0.99978775	-4.6073208
CFD	0.35125498	5.03729679	0.28485504	0.77687557	0.99978775	-4.6642948
DOC2B	0.35044935	2.48505508	0.62145893	0.53698622	0.99978775	-4.667116
PTPRD	0.35014882	2.41713513	0.83797117	0.40584472	0.99978775	-4.7102123
ENSCAFG00000010718	0.34993359	1.78314784	1.04015786	0.30303192	0.99978775	-4.5941122
CHST3	0.34950352	3.70170876	1.86557375	0.06769141	0.99978775	-4.2271398
PRKCA	0.34926454	4.69103067	1.71405068	0.09241676	0.99978775	-4.2935804
FSD1	0.34905616	1.26575182	0.98772789	0.32781515	0.99978775	-4.5988543
ENSCAFG00000007865	0.34878204	1.49069795	1.36566967	0.17786369	0.99978775	-4.5470921
RHOT1	0.3486406	6.26032954	2.26124155	0.02791234	0.99978775	-3.7992437
ENSCAFG00000000132	0.3479063	-1.1547631	0.8539818	0.39699391	0.99978775	-4.6013043
TMEM59L	0.34763498	4.78158905	1.01550832	0.31451957	0.99978775	-4.6378227
SFRP2	0.34763059	7.08059388	0.76683494	0.44661343	0.99978775	-4.6695954
ENSCAFG00000005575	0.34748115	5.83084676	0.90133184	0.37152717	0.99978775	-4.7036841
KCNB1	0.34723863	-0.0513736	0.64919208	0.51904355	0.99978775	-4.6083535
ENSCAFG00000018385	0.34713799	3.63417599	2.15979787	0.03537439	0.99978775	-4.0983812
GXYLT2	0.34612818	5.52180345	1.31650355	0.19372208	0.99978775	-4.5065519
TAB3	0.34595296	4.69039439	2.67819141	0.00985828	0.99978775	-3.5329675
WASF3	0.34579418	4.58485416	0.99999771	0.32189726	0.99978775	-4.6507201
AK5	0.34523296	5.17965379	0.78863512	0.43387115	0.99978775	-4.7503107
PTX3	0.34511737	8.73552927	1.01714623	0.31374722	0.99978775	-4.6493463
ENSCAFG00000032160	0.34465468	1.78372913	1.28765056	0.20351337	0.99978775	-4.5474082
PSTPIP2	0.34384892	3.33715321	1.89791069	0.06321064	0.99978775	-4.292504
ANO4	0.34372491	1.54893905	0.67070053	0.50535051	0.99978775	-4.6295406
CYP4V2	0.34280664	2.65856728	0.44138374	0.66074727	0.99978775	-4.6659756
PLEKHA2	0.34276422	3.11548315	1.1131469	0.27071474	0.99978775	-4.5831931
ELL2	0.34260154	4.77474292	1.97994148	0.05296131	0.99978775	-4.0558357
HACD4	0.34258437	1.90800438	0.59186225	0.55648242	0.99978775	-4.6275507
EPM2A	0.34257293	1.55999469	1.79847325	0.07785055	0.99978775	-4.4617193
CGNL1	0.34241416	6.75262227	0.47713232	0.63524762	0.99978775	-4.8051152
MYOCD	0.34226827	5.72089662	0.90942567	0.36728058	0.99978775	-4.697465
PTGIS	0.34223662	10.4861094	1.95198948	0.05628081	0.99978775	-4.1782978
AGPAT3	0.34198276	0.37697054	0.59879804	0.55188192	0.99978775	-4.6183041
RTKN2	0.34156552	1.67126127	1.53882562	0.12985114	0.99978775	-4.4677169
TATDN1	0.34118913	4.08218681	2.36497168	0.02175964	0.99978775	-3.9140484
ENSCAFG00000010333	0.34098077	-0.2019079	0.95070048	0.34611154	0.99978775	-4.600536
RMDN2	0.3408583	3.54492427	2.62569859	0.01130283	0.99978775	-3.835545
OMD	0.34013603	4.80077218	0.38155391	0.70433178	0.99978775	-4.7328748
CALD1	0.34013545	10.8256872	1.20173222	0.23485873	0.99978775	-4.5577529
ENSCAFG00000007199	0.34013054	-1.0762296	0.45838368	0.64856816	0.99978775	-4.6190536
ADGRE2	0.3401186	0.96878126	0.87031193	0.38809092	0.99978775	-4.6071714
RGS4	0.33977985	-1.5947788	0.50971343	0.61238774	0.99978775	-4.6067519
ARMC2	0.3388257	-0.3918831	0.76860964	0.44556798	0.99978775	-4.6038793
ENSCAFG00000032488	0.33874547	2.87662961	1.19257692	0.23839593	0.99978775	-4.5617899
MX1	0.33813572	0.37770304	0.54102762	0.59077629	0.99978775	-4.6225651
ENSCAFG00000023151	0.33726629	3.8238338	1.17129824	0.24676612	0.99978775	-4.5718879
ZKSCAN5	0.33692026	2.09803335	1.62364992	0.11043877	0.99978775	-4.4816241
MAP4K3	0.33674643	5.51938576	2.25279841	0.02847535	0.99978775	-3.8334484

ENSCAFG00000000562	0.33670352	3.82699993	0.84089811	0.40421769	0.99978775	-4.6701781
DCBLD2	0.33635118	6.10778965	1.72560615	0.09029878	0.99978775	-4.2383906
LCAT	0.33629326	5.41057397	1.30630133	0.19714275	0.99978775	-4.5116486
SLC35G2	0.33621562	1.36870671	0.99983611	0.32197474	0.99978775	-4.5976329
SAT1	0.33596517	4.6254412	0.87705713	0.38445032	0.99978775	-4.6658051
LRRC75B	0.33588532	-1.3669564	0.48575702	0.62916013	0.99978775	-4.6085473
SLC2A1	0.33542411	6.38348089	1.28186919	0.20551912	0.99978775	-4.5264472
NPAS1	0.33539692	1.97486368	1.16914379	0.24762526	0.99978775	-4.5715862
CTIF	0.33511273	6.0348902	1.48519645	0.14347037	0.99978775	-4.404065
KIAA0040	0.33507041	1.80996794	1.42807735	0.15919003	0.99978775	-4.5320498
HIPK3	0.33495349	7.15355254	2.11305891	0.03936629	0.99978775	-3.9339745
AIG1	0.33485775	4.35315515	1.81363271	0.07545001	0.99978775	-4.2728299
MAGI1	0.33420342	3.61813834	1.45892459	0.15054163	0.99978775	-4.448767
GABBR1	0.33394996	4.31999545	1.64242505	0.10647742	0.99978775	-4.3380102
TNFRSF19	0.33390677	1.35597795	0.70331883	0.48496369	0.99978775	-4.631723
ENSCAFG00000032629	0.33380808	0.69035882	0.93145992	0.35587828	0.99978775	-4.601896
ZNF688	0.33312797	1.76917077	1.46480062	0.14893678	0.99978775	-4.5326799
DLX1	0.33298547	1.42730275	1.23102648	0.22379794	0.99978775	-4.5697195
AR	0.33294851	-1.8925291	0.63593083	0.52758337	0.99978775	-4.6066219
GATA5	0.33236103	1.17663761	0.79917496	0.42778888	0.99978775	-4.6920295
TLR6	0.33235086	-2.6694739	0.62517614	0.53456278	0.99978775	-4.6030963
TM4SF1	0.3319856	6.95599589	0.87016057	0.38817286	0.99978775	-4.7041084
C4H1orf198	0.33191756	8.34576045	1.79875217	0.07780581	0.99978775	-4.2229235
ENSCAFG00000012787	0.33118728	1.69637363	1.17673141	0.24460904	0.99978775	-4.5695916
HSF4	0.33067388	2.57163726	1.25451246	0.21521074	0.99978775	-4.5535604
MPP7	0.33054479	2.6885263	1.11367127	0.2704917	0.99978775	-4.5870334
UACA	0.33025884	7.72625129	1.06757857	0.29059388	0.99978775	-4.631619
ENSCAFG00000002101	0.32983839	2.19828467	1.11747498	0.26887769	0.99978775	-4.5835909
MT-ND3	0.32962062	8.73123184	1.78687373	0.07973029	0.99978775	-4.2272008
TRPV1	0.32947812	0.97673394	0.42280112	0.67416669	0.99978775	-4.6150861
HYDIN	0.32904226	-0.0968813	0.8459393	0.40142481	0.99978775	-4.6031721
GPC4	0.32888118	4.23678492	1.10430198	0.27449643	0.99978775	-4.6172632
ENSCAFG00000015518	0.32858527	-2.5983569	0.65342257	0.51633478	0.99978775	-4.6045021
ENSCAFG00000030873	0.32850422	6.96889741	2.42495416	0.01878406	0.99978775	-3.6554114
ZNF786	0.32848039	3.14499747	2.37716851	0.02112244	0.99978775	-4.0834362
NOS3	0.32827445	-1.5065541	0.23707609	0.81352018	0.99978775	-4.6072088
FOXC2	0.32805991	2.74633688	1.02210388	0.31141729	0.99978775	-4.6103819
RGS2	0.32763318	3.89391885	0.63605309	0.5275043	0.99978775	-4.696191
SLC6A3	0.32755427	-1.6975551	0.33013082	0.74261451	0.99978775	-4.6064485
PRRX1	0.32752983	8.57263949	0.88061954	0.38253628	0.99978775	-4.6910373
EXOC6	0.32723839	2.14297267	0.96089621	0.34100795	0.99978775	-4.6066027
REEP3	0.32710069	7.91379761	2.46851214	0.01685832	0.99978775	-3.6505971
KCNMB1	0.32667529	5.46575854	1.25308715	0.21572482	0.99978775	-4.5382562
WDR78	0.32636517	2.88229262	1.94612445	0.05699951	0.99978775	-4.3327112
PGPEP1	0.32633808	3.01080173	1.22963275	0.22431534	0.99978775	-4.5590896
PAPPA2	0.32616352	-1.4847225	0.56572141	0.57399258	0.99978775	-4.6140669
GRIN2C	0.32598249	-1.9504483	0.6464973	0.52077294	0.99978775	-4.6067625
RAPH1	0.32598244	4.94782326	1.53655376	0.13040637	0.99978775	-4.3860067
GAMT	0.32515171	4.00128827	1.45059876	0.15283876	0.99978775	-4.4490166
RPAIN	0.32499462	2.1999291	1.55213391	0.1266365	0.99978775	-4.4893273

TMEM9B	0.32498272	4.98971706	2.33972861	0.02313335	0.99978775	-3.8025182
TIGD6	0.32474543	1.91905028	1.71028421	0.09311592	0.99978775	-4.4888968
ENSCAFG00000031673	0.32456524	3.42718836	1.61882976	0.11147487	0.99978775	-4.4060554
STARD10	0.32392589	1.91371154	1.16766183	0.24821748	0.99978775	-4.5755412
ZDHHC15	0.32381617	0.58170341	0.89218344	0.37636462	0.99978775	-4.6102749
ENSCAFG00000009973	0.32346323	2.00663751	1.82546904	0.07361917	0.99978775	-4.4571296
ENSCAFG00000029709	0.32323808	2.96727921	3.00651202	0.00404759	0.99978775	-3.7441471
LMO7	0.323134	9.15741534	1.01788761	0.31339805	0.99978775	-4.6317154
HACD2	0.32248337	5.48039218	1.73800326	0.08807137	0.99978775	-4.2325334
PDE9A	0.32244118	0.93771644	0.63071928	0.5309595	0.99978775	-4.6153343
SYDE2	0.32203502	1.9413267	1.12082248	0.2674629	0.99978775	-4.5814131
ARHGDIG	0.32202147	1.73185518	1.06586743	0.29135957	0.99978775	-4.5912557
TMEM151B	0.32165096	-1.1822027	0.60600756	0.54712031	0.99978775	-4.6063163
ENSCAFG00000019261	0.32147135	0.66773832	1.16189729	0.25053079	0.99978775	-4.5815134
ENSCAFG00000008119	0.32141717	3.2948708	1.61670994	0.11193302	0.99978775	-4.4190413
DYNLRB1	0.32139338	6.02151855	2.46222179	0.01712494	0.99978775	-3.6164671
ENSCAFG00000032154	0.32123073	2.36214174	0.69664796	0.48909544	0.99978775	-4.6595833
PLSCR1	0.32121088	-0.9717516	0.61571547	0.54074183	0.99978775	-4.6095754
SLC1A7	0.32078774	0.46807388	0.58399439	0.56172435	0.99978775	-4.6294831
PGK1	0.320763	8.02389169	1.89432473	0.06369476	0.99978775	-4.1260234
ATP1B1	0.32028688	4.14989916	1.27259909	0.20876601	0.99978775	-4.5287658
MAPT	0.32025748	1.25336477	0.54116789	0.5906803	0.99978775	-4.6248041
MYBPC2	0.32005339	-0.3541523	0.41181462	0.68215139	0.99978775	-4.6131149
IFI6	0.31997443	-1.5872643	0.5107014	0.61170042	0.99978775	-4.6062566
BST1	0.31985536	4.46921286	0.86246545	0.39235301	0.99978775	-4.6757829
GYG1	0.31925318	5.1017747	1.80332612	0.07707521	0.99978775	-4.1944285
FGF1	0.31849026	-0.4031366	0.52816783	0.59960779	0.99978775	-4.612968
ZNF365	0.31846918	5.41289133	1.91480693	0.06097142	0.99978775	-4.0993918
ENSCAFG00000013406	0.31843846	0.44541746	0.5375925	0.59312933	0.99978775	-4.6306319
GLDN	0.31832805	-0.8643975	0.33997223	0.73523353	0.99978775	-4.6152488
ENSCAFG00000001846	0.31813408	5.38859633	3.30774442	0.00170219	0.99968241	-2.8446101
VCAN	0.31754363	11.2860939	0.83959625	0.40494087	0.99978775	-4.6616037
JADE1	0.31719739	7.10851512	1.28428454	0.20467937	0.99978775	-4.5234511
GTF2A1L	0.31659963	4.32005099	1.48906588	0.14245139	0.99978775	-4.4195967
TMEM59	0.31589559	7.59045043	2.81300285	0.00688916	0.99978775	-3.2882313
C15H16orf87	0.31527421	0.37187867	1.07726065	0.28628768	0.99978775	-4.5910027
ENSCAFG00000013769	0.31525248	6.03782824	1.57962212	0.12019857	0.99978775	-4.3416963
ANKRD34A	0.31509899	0.10918618	0.95511038	0.343898	0.99978775	-4.5991502
C5H16orf74	0.31506728	0.8030538	1.21280925	0.23063037	0.99978775	-4.5768561
ENSCAFG00000016065	0.3141561	3.55493247	0.45714176	0.64945469	0.99978775	-4.7383827
PDSS1	0.31412375	3.51312249	1.64442252	0.10606288	0.99978775	-4.3598165
ABCA5	0.31367092	4.48732205	1.28462479	0.20456128	0.99978775	-4.5242906
USP6NL	0.31320562	5.24259633	1.93694825	0.0581397	0.99978775	-4.085285
ENSCAFG00000008683	0.31299364	5.08135436	0.86491102	0.39102149	0.99978775	-4.7087481
RASSF2	0.31284249	1.02311616	0.26272177	0.793793	0.99978775	-4.6156876
PRDX4	0.31266459	6.67516658	2.58899796	0.01242494	0.99978775	-3.4943708
RFTN1	0.31247565	5.84524204	0.99618957	0.32372625	0.99978775	-4.6655432
MYO16	0.31246444	2.43832718	0.59447981	0.55474394	0.99978775	-4.6796759
RHOD	0.31224324	2.71147918	1.40051508	0.16724023	0.99978775	-4.5101458
OSMR	0.31205676	8.59913557	1.40620913	0.16555186	0.99978775	-4.4576782

TCP11L2	0.31184836	4.459431	1.35504037	0.18120512	0.99978775	-4.4960281
NKIRAS1	0.31173598	0.44156427	1.12994839	0.26363268	0.99978775	-4.5868724
ZNF711	0.3116297	2.23334877	1.23461135	0.22247114	0.99978775	-4.56283
FAM227B	0.3112811	0.1414312	0.95903325	0.34193675	0.99978775	-4.5993538
GLG1	0.3112315	9.67864367	1.97680678	0.05332501	0.99978775	-4.1514778
ENSCAFG00000005758	0.31115474	0.51594399	1.02942662	0.30799737	0.99978775	-4.5946456
TRPS1	0.31086364	3.5111013	0.96953536	0.3367225	0.99978775	-4.622031
GSDME	0.31067196	-0.1276943	0.57522422	0.56759623	0.99978775	-4.6124972
ID3	0.31037615	5.28451769	0.9457774	0.34859368	0.99978775	-4.6878389
GRIK5	0.3103615	1.92791777	1.02465901	0.31022106	0.99978775	-4.6110456
CACNB3	0.31033712	6.35122	1.45204137	0.15243879	0.99978775	-4.424815
KANK3	0.31021225	-1.1962799	0.24141657	0.81017244	0.99978775	-4.607626
ENSCAFG00000017795	0.31004721	2.35668622	1.12268623	0.26667749	0.99978775	-4.5826709
SLC25A29	0.30975034	2.54630215	0.87748826	0.38421836	0.99978775	-4.6210815
ZNF853	0.30847373	2.1479973	1.84228837	0.07108197	0.99978775	-4.2612003
ENSCAFG00000016285	0.3080952	1.45416881	0.64253099	0.52332388	0.99978775	-4.6397868
ACTA2	0.30806531	11.2105169	0.54857775	0.58562005	0.99978775	-4.7176552
CHRDL1	0.30756629	9.31791062	1.09810691	0.27716718	0.99978775	-4.6075622
CAMK2D	0.30748173	7.53052725	1.5883936	0.1182006	0.99978775	-4.3381746
ARAP2	0.30691624	1.53399744	0.73686792	0.46448207	0.99978775	-4.6535849
ETFRF1	0.30672109	1.63074331	1.28347286	0.20496128	0.99978775	-4.5644753
ENSCAFG00000029346	0.30641204	-0.1565775	0.18725406	0.85218304	0.99978775	-4.6141754
ENSCAFG00000019807	0.30639184	3.4180528	0.61031774	0.54428363	0.99978775	-4.756037
ZC3H12C	0.30629268	2.6146805	1.29905159	0.199601	0.99978775	-4.5494138
SDR42E1	0.30603812	3.20148249	1.53178457	0.13157809	0.99978775	-4.4507105
APLP1	0.3052154	4.98633349	1.1091895	0.27240218	0.99978775	-4.6059364
HADH	0.30497834	6.47591554	1.86320605	0.0680298	0.99978775	-4.1365576
KLHL25	0.30486012	1.61186179	0.77526873	0.44165805	0.99978775	-4.6134279
FAM186B	0.30472891	-0.428509	0.71306731	0.47896089	0.99978775	-4.608317
MFSD13A	0.30451355	0.52241059	0.73587597	0.46508046	0.99978775	-4.6115041
DKK1	0.3038895	6.24010502	0.44591706	0.65749029	0.99978775	-4.8482462
BMPR1B	0.30381756	0.9255068	1.18402468	0.24173493	0.99978775	-4.5643512
PLCG2	0.30362338	0.50450186	0.63935109	0.52537379	0.99978775	-4.6283091
ADGRL1	0.30238137	4.63156735	1.45508788	0.15159681	0.99978775	-4.437328
RHEB	0.30231776	6.68809248	2.86075725	0.00605316	0.99978775	-3.2128261
ENSCAFG00000031879	0.30214043	-1.009688	0.76489562	0.44775749	0.99978775	-4.6064049
MOCOS	0.30211183	1.96580652	0.98374008	0.32975407	0.99978775	-4.6001092
KCNH1	0.30204736	-0.9397845	0.54986157	0.58474543	0.99978775	-4.6087176
SUSD5	0.30197995	-0.0834731	1.12887466	0.26408131	0.99978775	-4.580854
SATB1	0.30174937	3.42619816	1.508355	0.1374568	0.99978775	-4.4017184
RUSC2	0.30165168	7.18040941	1.48317089	0.14400607	0.99978775	-4.4050982
LETMD1	0.30148009	4.28542856	3.22010127	0.00220017	0.99968241	-3.1937529
CYR61	0.30145528	9.60012266	1.74940146	0.08606384	0.99978775	-4.2751493
SDHB	0.30089056	4.35068869	2.96231853	0.00457836	0.99978775	-3.3557669
PPIL6	0.30038829	3.17422099	1.64313055	0.10633086	0.99978775	-4.4163548
ITGBL1	0.30003766	5.26818108	0.44305264	0.65954747	0.99978775	-4.8068559
ENSCAFG00000016263	0.29905452	5.98971706	1.19405127	0.23782371	0.99978775	-4.573501
TMX4	0.29904622	3.96013577	1.63100017	0.10887391	0.99978775	-4.3640982
PREP	0.29895222	7.00914069	1.59716384	0.11622979	0.99978775	-4.331073
MYO10	0.29893097	8.7029785	1.12201987	0.26695811	0.99978775	-4.5949485

ENSCAFG00000008345	0.29867403	-1.0829584	0.55164957	0.58352838	0.99978775	-4.6087001
ENSCAFG00000014227	0.29860877	0.10690533	0.88793208	0.37862619	0.99978775	-4.6057859
ANGPTL6	0.29834347	1.57220679	1.26557509	0.21125155	0.99978775	-4.5646573
MBNL2	0.29805143	7.64751337	2.19906097	0.03230022	0.99978775	-3.8905485
C1QTNF7	0.2979302	1.55334221	0.61357353	0.54214587	0.99978775	-4.6302809
CLIC3	0.29759688	1.20117447	0.40134064	0.68979786	0.99978775	-4.6263345
PON2	0.29750322	5.88396739	1.55805087	0.12522786	0.99978775	-4.3565102
PRRT3	0.29732933	-0.0248299	0.5046559	0.61591168	0.99978775	-4.6146885
EFNA1	0.29691874	2.61344189	1.15517758	0.25324691	0.99978775	-4.5753013
CCNT2	0.29678451	4.24710503	2.30170735	0.02534881	0.99978775	-3.9400058
PLXDC2	0.29672978	3.65234689	0.96351908	0.33970309	0.99978775	-4.6498141
TPM2	0.2963915	9.5512953	0.64492626	0.52178257	0.99978775	-4.7476526
DOK6	0.29576452	0.81217194	0.62189213	0.53670351	0.99978775	-4.6612004
GDF11	0.29572812	4.93128843	1.88510551	0.06495391	0.99978775	-4.1796123
FXYD1	0.29540531	5.32437898	1.07013164	0.28945405	0.99978775	-4.6337206
MST1R	0.29514223	-0.0249648	0.40794525	0.68497234	0.99978775	-4.6174457
TMEM38A	0.29509272	1.62101914	0.90467374	0.36976999	0.99978775	-4.6123792
PPARGC1A	0.29496245	0.06882813	0.49663889	0.62151638	0.99978775	-4.6242436
KCTD1	0.29488657	4.214122	1.7805303	0.08077422	0.99978775	-4.2751721
ZNF596	0.29393536	1.05608644	0.91223143	0.36581578	0.99978775	-4.6049464
HEBP1	0.29378107	5.39627397	2.01930635	0.04857268	0.99978775	-4.0292281
NEDD9	0.29355256	3.6259599	0.61467459	0.54142389	0.99978775	-4.7773018
CYGB	0.2934533	0.11676569	0.14805802	0.88286402	0.99978775	-4.6089281
PEX7	0.2932665	3.81795466	1.48387691	0.14381917	0.99978775	-4.4458233
LIMS2	0.29318827	5.37902378	1.18313061	0.24208595	0.99978775	-4.5783875
TSPYL5	0.2930956	0.60564438	1.12831086	0.26431709	0.99978775	-4.5839759
DLL1	0.29243328	0.81898117	0.26075608	0.79530043	0.99978775	-4.6162946
ENSCAFG00000002977	0.29232811	3.70056032	1.80749122	0.07641494	0.99978775	-4.2595201
LRCH1	0.29209951	4.24842796	1.59140797	0.11752021	0.99978775	-4.3897329
IQCJ-SCHIP1	0.29207687	5.07121244	1.16447175	0.24949575	0.99978775	-4.5859482
IFNGR1	0.29204601	5.65213029	1.78983121	0.07924745	0.99978775	-4.2054908
ENSCAFG00000012234	0.2917178	1.06674685	1.22699644	0.22529645	0.99978775	-4.5753352
GNG5	0.29152334	0.78594359	1.02875397	0.30831044	0.99978775	-4.5945794
MAMDC2	0.29123252	-0.8987645	0.48194415	0.63184817	0.99978775	-4.6183565
BCHE	0.29092032	-1.043487	0.50041952	0.61887049	0.99978775	-4.6090237
EML6	0.29088029	-1.5553114	0.53764268	0.59309493	0.99978775	-4.6075509
PDIK1L	0.29059705	2.20735317	1.68525725	0.09787322	0.99978775	-4.4644369
WLS	0.29054442	8.0994217	1.5172603	0.13519828	0.99978775	-4.3920754
ARL14EP	0.28942552	4.16917743	2.08505192	0.04194234	0.99978775	-4.139574
MYL9	0.28929113	9.65936881	0.63663999	0.52712483	0.99978775	-4.7379857
FAXDC2	0.28912562	4.76736332	1.01191776	0.31621719	0.99978775	-4.6415987
CTSS	0.28870826	1.58731832	0.47553195	0.63638	0.99978775	-4.6331481
DAB2	0.28865747	9.81534215	1.21814616	0.22861313	0.99978775	-4.5516037
FRMD5	0.28864088	-2.0599334	0.59343073	0.55544037	0.99978775	-4.6082391
DUSP10	0.28849851	-1.1729363	0.44480701	0.65828719	0.99978775	-4.6077934
STN1	0.28839244	1.02874024	0.71324424	0.47885233	0.99978775	-4.6175918
ENSCAFG00000023039	0.28835922	1.64011629	1.5698184	0.12246376	0.99978775	-4.4778918
SIPA1L1	0.28829925	7.90634617	1.71858491	0.09158085	0.99978775	-4.254407
DHRS7	0.28828867	6.17191937	1.6267696	0.10977238	0.99978775	-4.3107751
ENSCAFG00000010494	0.28789609	1.3255624	1.07684986	0.28646948	0.99978775	-4.5895954

ADGRL3	0.2876199	2.74296482	0.34456178	0.73179994	0.99978775	-4.7225095
TP53RK	0.28686393	3.43736709	2.38560774	0.02069138	0.99978775	-3.991396
MTMR10	0.2867853	4.95033379	1.53682119	0.13034091	0.99978775	-4.3864834
SCN2B	0.28658277	2.35071337	0.34676527	0.73015339	0.99978775	-4.6512606
SORT1	0.28652413	6.36927488	1.55913668	0.12497074	0.99978775	-4.357448
ENSCAFG00000022498	0.28649777	-0.17491	0.6628194	0.51034509	0.99978775	-4.6098374
GEM	0.28613513	5.30895619	1.32647461	0.19042242	0.99978775	-4.5007625
ELOVL7	0.28609776	3.94205736	0.88955933	0.37775954	0.99978775	-4.6864089
TRABD2B	0.2858893	1.62105529	0.43656645	0.66421554	0.99978775	-4.6786109
FOXO3	0.28567634	5.68366279	1.19738271	0.23653439	0.99978775	-4.5662057
SMAD6	0.28565321	3.02412822	0.89924091	0.37262929	0.99978775	-4.6542092
LIPT2	0.28537044	3.4538448	1.74746857	0.08640157	0.99978775	-4.3216361
FGFR2	0.28534259	2.30673931	0.90960121	0.36718883	0.99978775	-4.6722853
SLC41A1	0.28519131	5.09700217	1.67831379	0.09922794	0.99978775	-4.2876466
ZSCAN23	0.28504183	-0.045203	0.90932715	0.36733209	0.99978775	-4.6009812
CRELD1	0.28500831	4.17874607	1.40150641	0.16694534	0.99978775	-4.4750178
SLC22A23	0.28499513	0.34651742	0.32781881	0.74435207	0.99978775	-4.6124568
HRH1	0.28468629	-1.0816163	0.52228658	0.60366715	0.99978775	-4.6172503
FMOD	0.28465562	1.00265118	0.91998339	0.36178823	0.99978775	-4.6287118
RASSF3	0.28455219	6.53636059	1.45364355	0.15199553	0.99978775	-4.4237564
FAM83H	0.28452825	3.55358702	1.01766961	0.31350069	0.99978775	-4.6201674
MAT2B	0.28430553	4.62845406	2.64049005	0.01087735	0.99978775	-3.6068505
LEPROT	0.28429644	4.44102197	2.16785469	0.0347234	0.99978775	-3.9670425
TARSL2	0.2835534	1.87885352	0.8293955	0.4106349	0.99978775	-4.6204105
IDO2	0.28321734	-0.4102762	0.60707211	0.54641899	0.99978775	-4.6107219
DYNLT3	0.28319918	5.98497752	2.60596496	0.01189413	0.99978775	-3.4825503
PLA2G16	0.28282365	2.22498822	0.48348998	0.63075777	0.99978775	-4.6453436
DMD	0.28253705	4.4715981	0.64053491	0.52461015	0.99978775	-4.7610867
UBLCP1	0.28195181	4.95168999	3.02598226	0.00383248	0.99978775	-3.160613
ENSCAFG00000016569	0.28180755	-0.0431045	0.61704413	0.53987183	0.99978775	-4.6171919
ENSCAFG00000008063	0.28160053	0.13883419	1.17187878	0.24653498	0.99978775	-4.5812037
ENSCAFG00000012233	0.2805555	5.69545731	1.6816504	0.09857503	0.99978775	-4.2752675
CAPRIN2	0.28043369	4.5771004	2.17067202	0.03449825	0.99978775	-3.9813531
UBE2V2	0.28015343	5.22334815	1.91050068	0.0615356	0.99978775	-4.1144009
C1R	0.27941139	4.94705875	0.43559153	0.66491835	0.99978775	-4.8373854
THAP5	0.279392	3.87277534	1.30148454	0.19877348	0.99978775	-4.5181775
C30H15orf61	0.27919996	0.24452413	0.70133017	0.48619338	0.99978775	-4.6126847
CCNT1	0.27919585	5.1781121	2.4906542	0.01594937	0.99978775	-3.6845166
RIMS2	0.27876943	-1.9911337	0.49447604	0.62303232	0.99978775	-4.6068071
ADAP2	0.27848302	0.64791705	0.40905672	0.68416156	0.99978775	-4.6277128
C23H3orf58	0.27842545	4.55255713	1.42443879	0.16023515	0.99978775	-4.4568199
NEXN	0.27832846	6.24874004	0.70573844	0.48346987	0.99978775	-4.7821383
PKIA	0.27773585	1.65784595	1.29926247	0.19952917	0.99978775	-4.5277413
ENSCAFG00000006540	0.27746495	5.50964111	0.98761912	0.32786794	0.99978775	-4.6651086
ENO1	0.27737479	9.71462445	2.0190931	0.04859558	0.99978775	-4.1069436
TMEM173	0.27715963	-2.3739168	0.39702222	0.69296007	0.99978775	-4.6054903
INKA1	0.27676557	1.70559254	0.81919865	0.41637539	0.99978775	-4.6130926
LAPTM4A	0.27655634	7.92302392	2.94044651	0.00486439	0.99978775	-3.193147
GULP1	0.27649221	5.65850816	1.11145041	0.27143722	0.99978775	-4.6167501
ENSCAFG00000003032	0.27606301	-0.6989911	0.88258448	0.38148311	0.99978775	-4.6029205

SDC2	0.27596742	7.35925269	1.13674891	0.26080388	0.99978775	-4.6007816
CHRM2	0.27579478	0.96490504	0.38931614	0.69861659	0.99978775	-4.639779
ATP8B1	0.2755084	7.48391897	1.32626056	0.1904928	0.99978775	-4.500688
SCARB2	0.27549878	7.41347424	1.88233304	0.06533667	0.99978775	-4.1258557
TSPAN12	0.27537002	5.21336175	1.40631378	0.16552095	0.99978775	-4.4572082
THRB	0.27489089	3.13971542	0.76167468	0.4496614	0.99978775	-4.6643666
ZNF425	0.2745668	3.56950519	1.74183029	0.08739304	0.99978775	-4.3381135
PLOD2	0.27434266	9.54284326	0.65236266	0.51701273	0.99978775	-4.7327474
FNDC3B	0.27398417	7.77152016	1.72996872	0.08950969	0.99978775	-4.2503495
ENSCAFG00000004917	0.27379987	0.97303295	1.24718895	0.21786184	0.99978775	-4.5657845
CSRP1	0.27378955	9.87740483	1.05248752	0.2973948	0.99978775	-4.614012
RNF38	0.27365327	5.80583165	1.92920385	0.05911711	0.99978775	-4.0994003
ENSCAFG00000032401	0.27341793	2.36174024	2.05723226	0.04464521	0.99978775	-4.3195438
LOX	0.27265768	10.7642102	0.74914536	0.45711226	0.99978775	-4.6920372
PDGFC	0.27264393	4.5107187	1.15360414	0.25388593	0.99978775	-4.5841291
HIF1A	0.27249435	8.48693154	1.60170479	0.11521986	0.99978775	-4.3477752
ENSCAFG00000029155	0.27249409	5.56642483	2.91579761	0.00520664	0.99978775	-3.1959623
TERF2IP	0.27237674	6.46647661	2.68115164	0.00978209	0.99978775	-3.3974475
BMPR1A	0.27216042	4.90490334	0.66141257	0.51123944	0.99978775	-4.7693733
NDUFA2	0.2721123	2.68781442	1.66765833	0.10133679	0.99978775	-4.4320873
C9orf3	0.27196433	4.79340651	1.53245054	0.13141397	0.99978775	-4.3946394
ASPHD1	0.27194926	-0.368191	0.43596559	0.66464866	0.99978775	-4.6144614
MEOX1	0.27166266	3.31426039	0.4202036	0.67605114	0.99978775	-4.6746781
RPH3AL	0.27153092	-0.8483418	0.34765225	0.72949096	0.99978775	-4.6105836
ITM2C	0.27146142	7.10112541	1.58553506	0.11884875	0.99978775	-4.3376357
SUMF1	0.27138565	6.2767229	2.51957717	0.01482887	0.99978775	-3.5564377
ENSCAFG00000018628	0.27131658	1.5741441	1.19429621	0.23772874	0.99978775	-4.5706663
COL11A1	0.27118025	9.076536	0.2253458	0.82258492	0.99978775	-4.7613136
ENSCAFG00000008221	0.27109039	9.87055995	2.5830529	0.01261598	0.99978775	-3.6798388
HACD1	0.27090079	2.76152348	1.10074154	0.27602915	0.99978775	-4.589657
ENSCAFG00000020526	0.27069821	0.45977337	1.04707923	0.29985853	0.99978775	-4.5930427
CMC1	0.27043082	2.04193749	1.54955311	0.12725487	0.99978775	-4.5006875
SPAG4	0.2701945	1.0088875	0.99719699	0.32324173	0.99978775	-4.5970635
ENSCAFG00000032123	0.27016541	3.5167827	1.7683968	0.08280283	0.99978775	-4.31549
HOXB3	0.26988495	2.638084	0.69343495	0.49109243	0.99978775	-4.6601287
DIO3	0.26978186	0.57439323	0.22703017	0.82128177	0.99978775	-4.6154667
LDB3	0.26966763	-2.672518	0.51773485	0.60681755	0.99978775	-4.6044426
FRY	0.26964645	4.29632613	1.20318881	0.23429951	0.99978775	-4.5618968
EVL	0.26957208	2.44781687	0.95296996	0.34497122	0.99978775	-4.6121416
PSMG1	0.26956997	5.55100359	2.05193552	0.04517662	0.99978775	-3.9922282
HIP1	0.26932912	7.37547318	1.30562685	0.19737048	0.99978775	-4.5122995
RCAN1	0.26923683	6.70589978	1.06871457	0.29008633	0.99978775	-4.628406
ENSCAFG00000014255	0.26900578	1.08211717	0.62612612	0.53394436	0.99978775	-4.6222075
LIF	0.26892774	4.31019104	0.79397876	0.43078107	0.99978775	-4.7363322
ZDHHC21	0.26883398	1.63862275	1.20361495	0.2341361	0.99978775	-4.5733568
SYNGR2	0.26882553	4.1664899	1.31959443	0.19269464	0.99978775	-4.5091584
C1GALT1	0.26882141	4.73818067	1.55862367	0.12509217	0.99978775	-4.3805726
PPM1H	0.26873576	3.79816821	0.87606348	0.38498527	0.99978775	-4.6834958
PBLD	0.26841437	0.01511537	0.71642211	0.4769048	0.99978775	-4.6089233
SRPK3	0.26793471	3.58770203	1.38352028	0.17235863	0.99978775	-4.4745457

ADCY6	0.26790468	6.45533442	1.71366051	0.09248898	0.99978775	-4.2475697
ZC2HC1A	0.26786115	4.28640844	1.36739703	0.17732516	0.99978775	-4.4868773
ZRANB1	0.26775255	4.97726806	1.80365926	0.07702223	0.99978775	-4.2072352
ANKRD49	0.2677524	2.73306347	1.37680212	0.1744149	0.99978775	-4.5155441
ADCY5	0.26732913	-0.1139211	0.52652098	0.60074319	0.99978775	-4.618254
STARD13	0.26717951	6.41001635	1.2572629	0.21422129	0.99978775	-4.5394605
DDA1	0.26700745	3.83806595	1.98479049	0.05240293	0.99978775	-4.1971219
ITGA6	0.26697863	5.38042839	0.56288341	0.57590963	0.99978775	-4.7961186
TTC1	0.26684171	4.79535056	2.67684845	0.00989302	0.99978775	-3.5459579
SPACA6	0.26631697	1.91135259	0.95809925	0.34240304	0.99978775	-4.6033439
PPP1R3D	0.26622433	1.94203388	1.35068187	0.18258903	0.99978775	-4.5358335
B3GLCT	0.2654947	4.36987379	2.1001987	0.04053149	0.99978775	-4.0223727
HSPA13	0.26526243	6.802485	1.81198022	0.07570863	0.99978775	-4.175398
BRMS1L	0.2650381	3.62057658	2.01812322	0.04869985	0.99978775	-4.1863028
MXI1	0.26495652	5.748848	1.172741	0.24619198	0.99978775	-4.5769137
SYN1	0.26474012	0.10322106	0.48854681	0.62719656	0.99978775	-4.6121759
ZFP82	0.26463584	2.73307856	1.37731065	0.17425859	0.99978775	-4.5182458
ZNF445	0.26449507	4.31935693	1.55718383	0.12543349	0.99978775	-4.3906813
ENSCAFG00000003012	0.2644848	8.37438485	2.08370166	0.04207016	0.99978775	-4.0032115
TMX2	0.26422521	4.61764532	2.19204363	0.03283174	0.99978775	-3.9535947
GPM6A	0.26417472	-2.7239132	0.51609291	0.60795584	0.99978775	-4.6048593
PDGFRA	0.2639768	8.47254146	0.77182634	0.44367675	0.99978775	-4.7366
ENSCAFG00000017326	0.26386583	7.85306207	0.42651879	0.67147325	0.99978775	-4.8029114
NECAP1	0.2636543	3.08551449	1.7136295	0.09249473	0.99978775	-4.3959811
TMEM47	0.26357149	7.66007517	1.60903679	0.11360417	0.99978775	-4.3275599
ENSCAFG00000000842	0.26356222	6.0079363	1.72334945	0.09070922	0.99978775	-4.2441026
CD47	0.26341775	5.20542087	1.56165478	0.12437607	0.99978775	-4.3634979
POLQ	0.26339729	2.77436304	0.50871149	0.61308513	0.99978775	-4.7011997
SPRYD7	0.26332162	3.52822895	1.85348418	0.06943428	0.99978775	-4.2885002
BCAR3	0.26322087	6.25096017	0.95414199	0.34438329	0.99978775	-4.6883536
EXTL3	0.26314834	5.18057942	1.27938995	0.20638376	0.99978775	-4.5249769
INTS9	0.26309949	4.32180335	1.99049695	0.05175229	0.99978775	-4.1340069
DNM3	0.26304365	-0.0851515	0.38034104	0.70522636	0.99978775	-4.6147407
ARHGEF3	0.26286746	1.7553276	0.52099819	0.60455812	0.99978775	-4.6467893
STX11	0.26276995	1.99438312	0.680327	0.49928595	0.99978775	-4.6411695
SFT2D2	0.26276226	2.71728955	1.8389937	0.07157308	0.99978775	-4.3802651
SORBS3	0.26264121	6.86379427	0.87667792	0.38465442	0.99978775	-4.7184326
ENSCAFG00000000370	0.26256102	5.67402038	2.63926554	0.01091201	0.99978775	-3.4584865
ENSCAFG00000005772	0.26253275	4.60063619	1.24879309	0.21727909	0.99978775	-4.5401324
TIMP1	0.26243437	9.85668164	0.9887299	0.32732916	0.99978775	-4.6627456
ENSCAFG00000028441	0.26240123	0.00527248	0.67633153	0.50179821	0.99978775	-4.6150209
CSTF2	0.26232173	3.63823871	2.09291098	0.04120505	0.99978775	-4.1390804
CPEB4	0.26231404	4.03959486	1.29210916	0.20197658	0.99978775	-4.5237303
GALM	0.26137697	4.65602289	1.48001929	0.14484271	0.99978775	-4.4145042
ENSCAFG00000025170	0.26129225	1.75275857	1.24040503	0.22033909	0.99978775	-4.5595386
ITGA3	0.26109037	5.90946896	0.7450287	0.45957582	0.99978775	-4.7689836
ENSCAFG00000022570	0.260992	-2.483821	0.39982159	0.69090957	0.99978775	-4.6075968
ENSCAFG00000026605	0.26086787	0.77218528	0.93769864	0.35269202	0.99978775	-4.6024239
ENSCAFG00000019909	0.26079267	4.74446182	2.01278303	0.04927747	0.99978775	-4.0960717
MERTK	0.26076009	2.55778374	0.66944774	0.50614268	0.99978775	-4.7161547

SAMD8	0.26050624	4.22398035	1.33618852	0.18724897	0.99978775	-4.5017513
ITGB1BP2	0.26031986	1.16876353	0.7213081	0.4739192	0.99978775	-4.6220455
KIF1A	0.26029324	4.11264317	0.66781288	0.50717745	0.99978775	-4.7656909
EFEMP1	0.26014045	7.07339179	0.34760419	0.72952685	0.99978775	-4.8459686
SAR1B	0.25983851	7.01468778	2.72879137	0.00862793	0.99978775	-3.3576424
FHIT	0.25969789	-0.6272819	0.56285969	0.57592567	0.99978775	-4.6101116
ENSCAFG00000030466	0.2595675	6.79407045	1.46755021	0.14819044	0.99978775	-4.4149413
ARMH4	0.25954602	5.86549765	1.05784047	0.29497003	0.99978775	-4.6423901
HHEX	0.25911917	0.1751361	0.32899916	0.74346482	0.99978775	-4.6153985
EIF4E2	0.25887025	1.61637193	1.25023607	0.21675587	0.99978775	-4.566449
ASIC2	0.25873997	2.89512667	0.67637108	0.5017733	0.99978775	-4.7346268
SULT1C4	0.25852491	7.02324463	1.68469066	0.0979832	0.99978775	-4.2707934
SLC8B1	0.25822709	4.75470181	0.95312549	0.34489316	0.99978775	-4.6647536
KCTD10	0.25817887	8.40784715	1.47531303	0.14609919	0.99978775	-4.420401
NMRK1	0.25813169	1.4415723	0.81798737	0.41706053	0.99978775	-4.6134572
TMTC1	0.25810258	5.11744681	0.68804184	0.49445453	0.99978775	-4.7718191
SMNDC1	0.25779287	3.77275983	2.13104676	0.03778562	0.99978775	-4.1073949
UNC45B	0.25770995	1.41458604	0.65308468	0.51655085	0.99978775	-4.6348183
NAPRT	0.25754634	2.22797694	0.6371123	0.52681955	0.99978775	-4.6368453
LIPE	0.25749906	2.52230312	0.99027837	0.32657908	0.99978775	-4.6022882
SFR1	0.25735804	2.44738327	1.0866901	0.28213665	0.99978775	-4.5879612
HOGA1	0.25722536	-0.2383467	0.5346713	0.59513382	0.99978775	-4.6125538
GGTA1P	0.2571925	8.62067965	2.69301763	0.00948207	0.99978775	-3.4929936
CDPF1	0.2569217	0.62508113	0.73083468	0.46812844	0.99978775	-4.6114199
SLC44A2	0.25676464	8.20395886	1.51480973	0.13581682	0.99978775	-4.3921288
ZNF772	0.2567075	1.7339585	1.00819666	0.31798303	0.99978775	-4.597956
ENSCAFG00000031581	0.25670524	3.67632797	0.8486492	0.39992843	0.99978775	-4.6645141
TNS1	0.25613504	9.21503788	1.53244157	0.13141618	0.99978775	-4.3908593
HSPA4L	0.25570436	5.56947053	1.76534714	0.08331933	0.99978775	-4.218712
ENSCAFG00000008984	0.25562007	-0.3297401	0.40019277	0.69063786	0.99978775	-4.6187761
PDE1B	0.2551826	-0.796105	0.66314894	0.51013572	0.99978775	-4.6157254
GADD45G	0.25511516	2.61374288	1.12484194	0.26577108	0.99978775	-4.5804981
ABHD18	0.25506832	3.65655562	1.17448078	0.24550093	0.99978775	-4.5673076
CIRBP	0.25492845	3.93004102	1.86757215	0.06740691	0.99978775	-4.2239443
ENSCAFG00000031543	0.25490878	-1.1616737	0.35880392	0.72118033	0.99978775	-4.6099581
DHX58	0.2547363	2.06072844	0.53684565	0.59364151	0.99978775	-4.6438432
SERPINI1	0.25467468	0.33359256	0.29303241	0.7706522	0.99978775	-4.6170686
C7H18orf32	0.25451164	3.89935182	2.04319232	0.0460658	0.99978775	-4.1361716
ENSCAFG00000007343	0.25448424	7.09575884	2.21146989	0.03137886	0.99978775	-3.8509364
ITGB1	0.25434355	11.5186835	1.21647776	0.22924236	0.99978775	-4.5526579
TTBK2	0.2541912	3.52914104	1.9012604	0.06276123	0.99978775	-4.2614347
F5	0.25383754	-0.5053961	0.53385398	0.59569522	0.99978775	-4.6273596
SRP19	0.25371585	1.19199019	1.0893637	0.28096735	0.99978775	-4.5886569
RCAN3	0.25352492	3.25496843	1.86612903	0.06761225	0.99978775	-4.290601
CLK1	0.253511	4.27564617	1.2221492	0.22710857	0.99978775	-4.550038
FAM122A	0.25347261	2.28751604	1.32944549	0.18944756	0.99978775	-4.5403934
NOCT	0.25347141	2.84029896	1.46020041	0.15019203	0.99978775	-4.488774
AHCYL2	0.25346034	3.8211383	1.33928137	0.18624704	0.99978775	-4.5021073
LMF1	0.25339862	3.61145642	1.51429124	0.13594798	0.99978775	-4.4351278
VAT1L	0.25338887	-2.3912192	0.21587807	0.82991912	0.99978775	-4.6066545

TRIM39	0.25337962	2.47494085	2.02403208	0.04806755	0.99978775	-4.3521796
SPINT2	0.25330246	3.61943203	1.69156783	0.09665521	0.99978775	-4.3322353
BRK1	0.2532693	4.91028472	2.64958553	0.01062305	0.99978775	-3.5403104
NPTN	0.2532293	8.3338408	1.43317387	0.15773506	0.99978775	-4.4429105
BIRC6	0.25321728	7.01229471	1.92168727	0.06007914	0.99978775	-4.0912196
B3GALT1	0.25300654	1.50958169	0.45084684	0.65395604	0.99978775	-4.6394935
GABRA3	0.25284018	-1.0644946	0.47458947	0.63704729	0.99978775	-4.6161205
BEND2	0.25276508	-2.7069303	0.54754236	0.58632588	0.99978775	-4.6060067
FKBP7	0.25267523	5.41089223	1.70381236	0.09432746	0.99978775	-4.272405
CDH1	0.25264348	-2.2014076	0.43785709	0.6632856	0.99978775	-4.6060005
GAPDH	0.25242664	10.6911976	2.16075418	0.03529657	0.99978775	-4.0582012
CD38	0.25240115	-1.8527898	0.40275643	0.68876234	0.99978775	-4.607142
ENSCAFG00000031249	0.25212984	2.84802215	0.57304301	0.5690613	0.99978775	-4.6768004
ENSCAFG00000013310	0.25210945	-0.007241	0.82800004	0.41141763	0.99978775	-4.6068763
BBS4	0.25184382	3.38291121	1.50150022	0.13921558	0.99978775	-4.4588761
RARRES1	0.25181966	5.51892155	1.05554634	0.29600754	0.99978775	-4.6351219
ENSCAFG00000020027	0.25177445	0.61711444	0.85631458	0.3957144	0.99978775	-4.6406472
INA	0.25165015	1.61985633	0.29357569	0.77023928	0.99978775	-4.6329206
RFX8	0.25152685	-1.5988673	0.37175715	0.71156952	0.99978775	-4.6071575
QSOX1	0.25138638	9.61782416	1.0104842	0.3168967	0.99978775	-4.6360711
NCR3LG1	0.25135167	6.67203163	1.65568273	0.1037506	0.99978775	-4.2887552
IMPAD1	0.25134333	5.11792179	1.54834591	0.12754494	0.99978775	-4.375102
PRSS23	0.25130012	8.33735277	1.12036258	0.26765696	0.99978775	-4.6039288
ENSCAFG00000024756	0.25115628	4.62091552	1.96678079	0.05450268	0.99978775	-4.1156591
CFLAR	0.25061541	4.59533865	1.66596978	0.10167432	0.99978775	-4.3305155
ENSCAFG00000032731	0.2505958	-2.6547685	0.43833611	0.66294059	0.99978775	-4.6048892
HNF4A	0.2505958	-1.7563022	0.27209704	0.78661436	0.99978775	-4.606321
FAM221A	0.25045685	1.04579181	1.00667777	0.31870573	0.99978775	-4.5977205
NUAK1	0.25040559	7.3002731	0.56998668	0.57111728	0.99978775	-4.8104374
TMED8	0.25010261	2.27768212	1.55510616	0.12592733	0.99978775	-4.487915
ENSCAFG00000006873	0.24991415	2.20539378	1.16715786	0.2484191	0.99978775	-4.5691736
SCUBE3	0.24971176	-1.9168458	0.46005892	0.64737314	0.99978775	-4.6099645
BCAS3	0.24955127	5.03736274	1.85932603	0.06858742	0.99978775	-4.1810422
NAB1	0.24945021	4.20438426	2.41051773	0.01946469	0.99978775	-3.7895235
CREBL2	0.24878863	2.78251818	1.39057471	0.17021956	0.99978775	-4.5210461
ARHGEF17	0.24878333	6.55386119	1.33675188	0.18706616	0.99978775	-4.4948201
SPRED3	0.24868267	1.64990895	0.93871799	0.35217318	0.99978775	-4.6060758
ZNF239	0.24868197	1.80390629	0.88707345	0.37908399	0.99978775	-4.6110449
TTC19	0.24852358	3.4381701	1.78155042	0.08060558	0.99978775	-4.3222764
SLC35F5	0.24845724	6.24160838	2.41378318	0.01930882	0.99978775	-3.6587538
FRS3	0.24845453	1.78522056	1.23031696	0.22406123	0.99978775	-4.5683271
CCDC146	0.2484393	1.08724082	0.67919966	0.4999941	0.99978775	-4.6200554
SGTB	0.24834879	3.8351818	2.08317435	0.04212017	0.99978775	-4.1356057
SMIM14	0.24786874	4.87272203	1.57498071	0.12126674	0.99978775	-4.3676934
TENT5C	0.24784098	0.72040528	0.32086224	0.74958821	0.99978775	-4.6178484
SDC3	0.2477042	5.96265916	0.86205984	0.39257412	0.99978775	-4.7263986
ENSCAFG00000007285	0.24757165	0.05669933	0.80504176	0.42442554	0.99978775	-4.611306
VGLL2	0.24754266	3.12657868	0.70267265	0.48536307	0.99978775	-4.7186229
ASB13	0.24750373	1.89630155	0.91168169	0.36610248	0.99978775	-4.6077019
HFE	0.24712021	1.74076442	1.07412005	0.2876796	0.99978775	-4.5899636

ATP6V0D2	0.24672012	-2.3270099	0.36160188	0.71910045	0.99978775	-4.607152
CFL2	0.24668214	8.05506371	1.36857544	0.17695849	0.99978775	-4.4772801
RPL26	0.24646604	2.11907789	1.16347449	0.24989632	0.99978775	-4.5760764
BNC2	0.24636446	5.98857227	2.26730451	0.02751416	0.99978775	-3.7972597
SOS2	0.24595822	4.28799462	1.85612258	0.06905072	0.99978775	-4.2373542
ERLEC1	0.24571668	6.60051741	2.07858309	0.04255779	0.99978775	-3.9590063
TRIM6	0.24564119	0.54495017	0.44259472	0.65987659	0.99978775	-4.6247077
TPK1	0.24563713	4.25229933	1.71462386	0.09231074	0.99978775	-4.2993161
PTPN21	0.24543476	7.78492993	1.91513411	0.06092873	0.99978775	-4.1139467
DIAPH2	0.24542589	6.0438913	1.7119727	0.09280195	0.99978775	-4.2533854
ERO1B	0.24504722	4.08875402	1.35999031	0.17964313	0.99978775	-4.4991163
VPS13A	0.24447099	7.18060586	2.44433562	0.01790397	0.99978775	-3.6438037
ENSCAFG00000031282	0.24444686	-0.0562472	0.69612792	0.48941836	0.99978775	-4.6099637
EPB41L5	0.24421078	1.53206546	0.93022953	0.35650887	0.99978775	-4.6072395
TNFSF15	0.2439928	2.3815456	0.56183862	0.57661616	0.99978775	-4.6958157
ADAMTS9	0.24394319	-1.6179798	0.21338928	0.83184962	0.99978775	-4.6068712
ENSCAFG00000001210	0.24370094	1.96337168	0.88638087	0.37945351	0.99978775	-4.6158639
CTNNBIP1	0.24348846	-0.2563232	0.69011947	0.49315783	0.99978775	-4.6100954
CTXN1	0.24342286	0.71276778	0.40326299	0.68839198	0.99978775	-4.6269145
TMEM150A	0.24318742	5.01307808	1.45912075	0.15048784	0.99978775	-4.4290535
ENSCAFG00000002087	0.24317908	-0.5707678	0.84880725	0.39984126	0.99978775	-4.6046425
VGLL3	0.2431316	6.39995691	0.55580864	0.58070208	0.99978775	-4.825785
CNKSR1	0.24310282	2.20833762	0.99986483	0.32196097	0.99978775	-4.601181
ENSCAFG00000032470	0.24282664	5.15758783	2.09057694	0.04142283	0.99978775	-3.9927906
PPME1	0.24270924	4.49265775	2.19173479	0.03285531	0.99978775	-3.9851629
FNDC4	0.24263478	3.03332316	1.14526884	0.25729042	0.99978775	-4.5805991
FAM133B	0.24237318	2.08713725	0.92868388	0.35730205	0.99978775	-4.6089796
LAPTM4B	0.24225757	-1.3643943	0.35779649	0.72192973	0.99978775	-4.6170699
GFOD2	0.24222658	3.18519106	1.14350471	0.25801513	0.99978775	-4.5760376
MID1	0.24200242	3.47780228	0.67821484	0.50061317	0.99978775	-4.7084494
PLPP1	0.24186275	6.18647913	1.13879724	0.25995608	0.99978775	-4.60191
ZBTB6	0.24182496	4.31673184	1.9613209	0.05515334	0.99978775	-4.1614653
PRKAG3	0.24180169	-0.9904854	0.66992616	0.50584008	0.99978775	-4.6165728
KSR1	0.24173474	1.9730676	0.74064849	0.46220547	0.99978775	-4.6266741
CDC7	0.24170077	4.23964319	1.36639074	0.17763873	0.99978775	-4.4832063
CPLANE2	0.24169866	1.40329988	0.90306546	0.37061497	0.99978775	-4.6072666
PDK3	0.24161924	4.74671352	1.49694345	0.14039457	0.99978775	-4.4060192
NEGR1	0.24123909	3.61451285	0.76984236	0.44484266	0.99978775	-4.7201863
SPARCL1	0.24122379	2.97887633	0.31770507	0.75196853	0.99978775	-4.6639403
BMX	0.24091649	-0.7237673	0.3246022	0.74677168	0.99978775	-4.6129178
ENSCAFG00000010782	0.24061652	1.83060036	0.9101634	0.36689507	0.99978775	-4.6083333
ENSCAFG00000020219	0.24044779	2.79162829	1.42526255	0.15999808	0.99978775	-4.5032407
KAT2B	0.2404009	6.49424993	0.97634564	0.33336949	0.99978775	-4.6796618
HK2	0.2398591	4.63956098	0.80926054	0.42201684	0.99978775	-4.6924629
SLC18B1	0.23983225	1.77469533	0.84483495	0.40203561	0.99978775	-4.6168947
WFS1	0.23965116	6.46216441	1.29666153	0.20041646	0.99978775	-4.5180786
SMIM26	0.23948705	3.17612074	1.01493766	0.31478897	0.99978775	-4.6104599
GPHN	0.23947834	4.2472161	2.29806081	0.02557091	0.99978775	-3.8867384
PRRG1	0.23939495	4.19201388	1.56933415	0.12257653	0.99978775	-4.3847469
RSPH3	0.23934296	1.36837042	0.99913767	0.32230972	0.99978775	-4.5980068

DCLK2	0.23916412	3.74806013	1.35134794	0.18237702	0.99978775	-4.489222
ATP13A3	0.23901194	6.24440837	1.45217864	0.15240077	0.99978775	-4.4252993
IL13RA1	0.23894192	8.33850915	1.12401526	0.26611841	0.99978775	-4.6010875
COL4A2	0.23862059	10.767491	1.18001054	0.24331377	0.99978775	-4.5671324
EREG	0.23860301	5.25229497	0.37820459	0.70680316	0.99978775	-4.8409405
ENSCAFG00000025105	0.23815037	3.82060265	1.13501906	0.26152139	0.99978775	-4.5789
DSG2	0.23810732	4.54405359	1.16242595	0.25031799	0.99978775	-4.5846246
GNE	0.23802348	5.45139751	2.07162246	0.04322879	0.99978775	-3.9814948
DCLRE1C	0.23798256	2.97529896	1.54501625	0.12834776	0.99978775	-4.4668435
EBF4	0.23795202	1.31631961	0.75137094	0.45578358	0.99978775	-4.6500427
ALCAM	0.23794674	9.35560602	1.12028781	0.26768852	0.99978775	-4.5939706
PGRMC1	0.23781225	6.69203784	2.59334978	0.01228677	0.99978775	-3.4837826
PCYOX1	0.23773249	7.3391228	2.55435982	0.01357614	0.99978775	-3.5487972
NTRK2	0.23772197	-1.48999	0.32560833	0.74601457	0.99978775	-4.609759
ACPP	0.23759928	2.98092096	0.68838689	0.49423905	0.99978775	-4.6984516
ENSCAFG00000025328	0.23737176	2.79438682	1.43378163	0.15756225	0.99978775	-4.4943886
HSPB7	0.23728018	2.18329688	0.48763702	0.62783661	0.99978775	-4.6809612
DDR1	0.23718647	3.49815992	1.07182425	0.28870009	0.99978775	-4.6101207
MT-ATP6	0.23703785	12.7068994	1.43432355	0.15740829	0.99978775	-4.4788031
CDK8	0.23696724	5.02146807	1.39344269	0.1693558	0.99978775	-4.4617427
PIGK	0.23680279	6.88246894	1.82923783	0.07304412	0.99978775	-4.1647686
ZFYVE28	0.23680036	2.45630215	0.93396593	0.35459616	0.99978775	-4.6211765
COL4A1	0.23660462	11.1930822	1.02258732	0.31119072	0.99978775	-4.6221194
AP3B2	0.23658274	-1.6858867	0.37064541	0.71239257	0.99978775	-4.6066423
ENSCAFG00000001369	0.23618295	2.54724227	0.948341	0.34729971	0.99978775	-4.6142343
PHKG1	0.23612237	1.78712928	0.5245697	0.60208979	0.99978775	-4.6395273
NAV3	0.23600768	6.84602616	1.85962483	0.06854434	0.99978775	-4.1392946
ENSCAFG00000005538	0.23579428	3.44548275	0.90474844	0.36973077	0.99978775	-4.6476457
HEG1	0.23578536	7.02427798	1.17362774	0.24583959	0.99978775	-4.5847256
JKAMP	0.23565034	5.743128	2.41775339	0.01912083	0.99978775	-3.6659436
IL17RD	0.23545614	1.87636001	0.3910309	0.69735639	0.99978775	-4.635915
UQCR10	0.23533269	4.8675899	2.25758764	0.02815477	0.99978775	-3.8709993
NEO1	0.2351734	5.35349387	0.72825365	0.46969333	0.99978775	-4.7749877
ENSCAFG00000031354	0.23516778	-0.3464266	0.71556813	0.47742772	0.99978775	-4.6084246
OOEP	0.23477244	-1.1399215	0.4434513	0.659261	0.99978775	-4.6163788
BIRC3	0.23427869	2.93767072	0.78590945	0.43545239	0.99978775	-4.6425349
CERS2	0.23427694	6.55354504	3.41077077	0.00125313	0.99968241	-2.6033271
PALLD	0.2341567	8.6711231	0.64817086	0.51969856	0.99978775	-4.7675603
QPCT	0.23375013	4.83390647	0.77844869	0.43979806	0.99978775	-4.7471568
FAM172A	0.23374379	5.51220807	2.56182266	0.01332022	0.99978775	-3.5625065
MOCS2	0.23354655	4.24276339	1.78363434	0.08026198	0.99978775	-4.271665
ANO1	0.23352347	-2.1123457	0.39206742	0.69659506	0.99978775	-4.6092785
CCND3	0.23339198	4.68547384	1.05746824	0.29513819	0.99978775	-4.6188735
ATP6AP2	0.23310059	7.61299001	1.97184608	0.05390494	0.99978775	-4.0613781
ZNF621	0.2327208	2.89990709	1.3270445	0.19023512	0.99978775	-4.5299077
TPST2	0.23257186	4.56995217	1.18810266	0.24013859	0.99978775	-4.5671925
ZNF226	0.23255729	2.08965808	1.15364462	0.25386948	0.99978775	-4.5778808
EGLN1	0.23252476	6.23070851	1.92512247	0.05963783	0.99978775	-4.0865385
METTL8	0.23251191	2.73943431	1.34668937	0.18386379	0.99978775	-4.5297306
ENSCAFG00000032635	0.23229563	5.39901274	2.15638011	0.03565377	0.99978775	-3.9099068

ZNF292	0.23226054	4.73512762	1.48575942	0.14332176	0.99978775	-4.4208873
PGRMC2	0.23211962	7.71585505	1.32332907	0.19145872	0.99978775	-4.502293
LPL	0.2319318	-2.1729438	0.41557868	0.67941158	0.99978775	-4.6070874
ENSCAFG00000018377	0.23162717	-0.1224603	0.73673061	0.46456487	0.99978775	-4.6116802
ENSCAFG00000016321	0.23138181	0.57266505	0.96941944	0.33677977	0.99978775	-4.6016854
C15H1orf50	0.23137974	3.31578867	1.57701428	0.1207978	0.99978775	-4.4254188
PTPRK	0.23112504	5.46232461	0.53981886	0.59160378	0.99978775	-4.8298845
MED10	0.23110013	2.50251126	1.29753699	0.20011747	0.99978775	-4.5459012
PRKAG2	0.23107695	4.68933722	1.29885647	0.19966748	0.99978775	-4.5153158
ENSCAFG00000006937	0.23094204	0.50504479	0.87856163	0.38364123	0.99978775	-4.6077274
EFEMP2	0.23091259	6.17139586	0.52340868	0.60289167	0.99978775	-4.833764
SNX24	0.23078032	0.53026077	0.70587021	0.48338859	0.99978775	-4.6177742
CXHXorf36	0.2307693	-1.2146473	0.23122972	0.81803491	0.99978775	-4.6115546
MBNL1	0.23072237	9.19968948	1.37339586	0.17546465	0.99978775	-4.4774788
LXN	0.23046259	4.95569594	0.96290283	0.34000937	0.99978775	-4.6708287
SGCA	0.23041587	4.72819184	0.62906	0.53203677	0.99978775	-4.7572702
MTERF1	0.23031347	2.88806021	1.2425432	0.21955607	0.99978775	-4.5551557
C24H20orf194	0.23022897	3.3476329	1.18518891	0.24127841	0.99978775	-4.5648663
ANKRD29	0.23021367	5.11849735	1.18626202	0.24085817	0.99978775	-4.5730844
CALM1	0.23005917	9.06689049	1.63711967	0.10758488	0.99978775	-4.3295743
CLEC14A	0.22992321	-0.3045347	0.24632623	0.80639001	0.99978775	-4.6168565
ENSCAFG00000030304	0.22991504	0.97160532	0.88466479	0.38037011	0.99978775	-4.6047066
TMEM181	0.22983412	6.11872605	2.01586488	0.04894341	0.99978775	-4.0154564
TSPAN3	0.22971932	5.98201885	2.48009976	0.01637696	0.99978775	-3.6098433
CECR2	0.22969126	1.85993973	0.82906444	0.41082051	0.99978775	-4.6161354
STRN3	0.22927163	5.74041908	1.74110085	0.087522	0.99978775	-4.2310558
PSRC1	0.22921247	0.98912484	0.79318581	0.43123878	0.99978775	-4.6133166
MED30	0.22911894	3.62628558	2.09549998	0.04096466	0.99978775	-4.1605871
SPA17	0.22900412	0.666195	0.65033484	0.5183111	0.99978775	-4.6159604
TEAD1	0.22874148	6.13931412	1.32271179	0.19166258	0.99978775	-4.503062
MBTPS2	0.22873293	4.71539171	2.22597507	0.03033132	0.99978775	-3.9041759
TPM1	0.22864175	9.42993132	0.81764662	0.41725339	0.99978775	-4.6993708
HSPBAP1	0.22845799	3.94424152	1.45714175	0.15103123	0.99978775	-4.4551701
CCSER2	0.22835075	7.12439722	1.78427303	0.08015691	0.99978775	-4.1990864
BCDIN3D	0.22832787	2.70670559	1.67859229	0.09917331	0.99978775	-4.4290996
PPA1	0.22822136	6.21134257	1.84392682	0.0708388	0.99978775	-4.1542193
SEPT7	0.22816445	8.33948045	2.27340578	0.02711856	0.99978775	-3.8487875
ENSCAFG00000030172	0.22803632	2.3539926	1.00652813	0.31877699	0.99978775	-4.6008514
PRR13	0.22801945	4.63853875	1.33728535	0.18689318	0.99978775	-4.495928
ENSCAFG00000006487	0.22767469	-0.8643481	0.62155344	0.53692454	0.99978775	-4.6102712
GALNT10	0.22748557	4.13006669	1.10670711	0.27346445	0.99978775	-4.5977621
ZNF516	0.22739276	3.81245348	1.87919372	0.06577239	0.99978775	-4.2110725
PDE4D	0.22738504	0.42954233	0.59281	0.55585265	0.99978775	-4.6271939
ENSCAFG00000016997	0.22711773	-0.6181019	0.40169535	0.68953837	0.99978775	-4.617002
ENSCAFG00000015903	0.22694407	1.72038497	1.29741195	0.20016015	0.99978775	-4.5504368
PRKCI	0.22669977	7.1743322	1.22342179	0.22663179	0.99978775	-4.5569014
ICAM2	0.22656178	-0.0065665	0.24986025	0.80367026	0.99978775	-4.6192438
PPP1R2	0.22651578	5.94597073	1.59287652	0.11718988	0.99978775	-4.3339486
LY96	0.2265079	3.30091229	1.35673651	0.18066872	0.99978775	-4.5132576
YAE1	0.22648147	4.59699275	2.70747877	0.00912786	0.99978775	-3.5216363

MDFIC	0.22605764	5.64525091	1.07889623	0.28556464	0.99978775	-4.6263118
MYO1D	0.22591024	8.45969652	0.98273188	0.33024547	0.99978775	-4.65608
ENSCAFG00000028771	0.22586312	0.17480797	0.60384842	0.54854414	0.99978775	-4.6155441
NBEAL1	0.22584965	5.18397524	1.58171188	0.11972011	0.99978775	-4.3524509
LRIF1	0.22582635	5.12442656	2.03566276	0.04684367	0.99978775	-4.0522538
RPP14	0.22579834	2.372206	1.73315888	0.08893628	0.99978775	-4.4335897
CUX1	0.22578342	5.3363276	1.51598208	0.13552063	0.99978775	-4.3882449
DYNC1I2	0.22565055	7.64336502	3.21537186	0.00223062	0.99968241	-2.8982614
GPR146	0.22553888	-0.7935952	0.34443454	0.73189506	0.99978775	-4.6097822
GXYLT1	0.22512875	3.1520813	1.18775812	0.24027317	0.99978775	-4.5637463
ATP5PF	0.22508986	4.61669784	2.18210234	0.03359791	0.99978775	-3.9584416
YIPF5	0.22499157	5.67809961	2.34465453	0.02285935	0.99978775	-3.7352425
USP8	0.22496573	6.49926021	2.77626664	0.00760354	0.99978775	-3.2982889
FBXL20	0.2245775	4.29874379	1.70441361	0.09421436	0.99978775	-4.3039669
PCDH1	0.22449526	0.5198464	0.32134731	0.74922272	0.99978775	-4.6368374
KIAA1671	0.22443618	3.50362049	0.98753667	0.32790795	0.99978775	-4.6288787
EIF5A2	0.22442671	5.08092895	1.84087823	0.07129182	0.99978775	-4.1733435
ADAM9	0.22441288	8.7380388	1.72280338	0.09080877	0.99978775	-4.2674089
UBE2E1	0.22428034	6.00961051	2.31140895	0.02476624	0.99978775	-3.75693
ARF4	0.22414192	8.63133998	2.22876058	0.03013373	0.99978775	-3.9098744
TTC34	0.22412938	1.64244527	0.56020339	0.57772281	0.99978775	-4.6299027
LCOR	0.22410701	1.49895316	0.89860252	0.3729662	0.99978775	-4.6086178
RANBP2	0.22410028	7.55422442	2.14936632	0.03623317	0.99978775	-3.9198559
NRG1	0.22389958	1.03217007	0.89692836	0.37385065	0.99978775	-4.6458575
PICALM	0.22373927	8.82253975	1.7716381	0.0822568	0.99978775	-4.2412087
MYO5B	0.22339199	-0.6452891	0.63894548	0.52563557	0.99978775	-4.6175788
ENSCAFG00000032358	0.22329704	-2.1518295	0.34802689	0.72921122	0.99978775	-4.6109143
RSBN1L	0.22329047	3.97197061	1.64471632	0.10600202	0.99978775	-4.3564387
NCALD	0.2232217	5.74314161	0.75652512	0.4527151	0.99978775	-4.7379342
CCDC159	0.22306564	-1.3992458	0.36733048	0.71484873	0.99978775	-4.608746
PLEKHA3	0.22289661	5.53446537	2.01864045	0.04864422	0.99978775	-4.0242278
NHSL2	0.22274486	3.51867834	0.9439812	0.34950219	0.99978775	-4.6405302
RNF34	0.22270536	2.49125068	1.38043073	0.17330194	0.99978775	-4.524925
WBP1	0.22266588	4.80681355	1.32432459	0.19113028	0.99978775	-4.5023757
TRNAU1AP	0.22260388	3.8371425	1.95579914	0.05581815	0.99978775	-4.209723
MOSMO	0.22243342	2.00994653	1.33668683	0.18708726	0.99978775	-4.5483752
GP1BB	0.22225123	1.31427558	0.61330494	0.54232207	0.99978775	-4.6337955
C1H9orf85	0.22221607	0.70147551	0.71640743	0.47691379	0.99978775	-4.6151918
ENSCAFG00000010117	0.22219745	1.64187154	0.89265558	0.37611399	0.99978775	-4.6098917
ANKRD16	0.22202504	3.98349808	1.3661821	0.1777038	0.99978775	-4.486551
PKP4	0.22198118	6.26400692	1.46952744	0.14765557	0.99978775	-4.4136922
MALT1	0.22196977	4.86165164	1.20523245	0.23351655	0.99978775	-4.5613191
ADAMTS13	0.22175838	-2.0178834	0.41871723	0.67713041	0.99978775	-4.6061513
CCDC148	0.22144946	0.91833624	0.84994682	0.39921312	0.99978775	-4.6100316
EML4	0.22115249	5.54023332	1.27170964	0.20907955	0.99978775	-4.5312807
OLFML2B	0.2210551	6.00078489	0.67639269	0.5017597	0.99978775	-4.7907717
E2F5	0.22097231	4.94282071	1.76110018	0.08404308	0.99978775	-4.2593784
TM7SF3	0.22077113	5.68188731	2.51772621	0.01489839	0.99978775	-3.5834429
CAP2	0.22075452	4.13583661	0.64029565	0.52476444	0.99978775	-4.7904781
TAX1BP1	0.22073345	7.50752749	1.51409545	0.13599753	0.99978775	-4.38929

A2M	0.22043983	7.4625274	0.52024512	0.60507918	0.99978775	-4.8265959
TRAF3	0.22012373	3.70375938	1.6082364	0.11377965	0.99978775	-4.3849287
MZT1	0.22004876	5.2603807	0.9929053	0.32530922	0.99978775	-4.6638097
TUSC2	0.220034	1.159453	0.93994021	0.35155174	0.99978775	-4.6031744
NDUFS4	0.21995078	3.90408047	2.07890461	0.04252702	0.99978775	-4.1097993
GTF2H5	0.21974705	2.98240255	1.64121282	0.10672965	0.99978775	-4.4140659
OXLD1	0.21955284	2.27498646	1.12731195	0.2647352	0.99978775	-4.5808289
ZEB2	0.21950964	6.93087025	1.2964271	0.20049657	0.99978775	-4.5180834
NCAM1	0.21949104	4.58226935	0.54783671	0.58612518	0.99978775	-4.8244423
SEPT5	0.21944396	6.00021542	0.86637428	0.39022615	0.99978775	-4.7248793
JUP	0.2194169	7.20016204	0.84666838	0.40102188	0.99978775	-4.7319982
ENSCAFG00000019944	0.21926197	0.39807836	0.97660372	0.33324287	0.99978775	-4.5994169
CEMIP2	0.21915758	5.51719909	0.79249388	0.43163842	0.99978775	-4.7427812
ENSCAFG00000012527	0.21913638	-0.453359	0.62853299	0.53237917	0.99978775	-4.616702
MT-ATP8	0.21896532	5.02814057	0.86982893	0.38835244	0.99978775	-4.7064995
FSTL1	0.21858698	11.9700783	1.4584596	0.15066921	0.99978775	-4.4627363
CRLF3	0.21844629	5.24295437	1.81918318	0.07458676	0.99978775	-4.1928789
ENSCAFG00000010098	0.21817633	3.58917426	1.50880227	0.13734265	0.99978775	-4.4348523
CYP2U1	0.21814381	2.89728834	1.18745386	0.24039205	0.99978775	-4.5672105
PLXNA3	0.21799413	7.36180442	1.98937111	0.0518801	0.99978775	-4.044913
SIPA1L2	0.21792084	-0.62438	0.28899965	0.77371946	0.99978775	-4.6220708
RPL21	0.21790643	8.35388568	2.26038499	0.027969	0.99978775	-3.8613965
BABAM2	0.21789893	5.49842788	1.95673878	0.05570454	0.99978775	-4.0761482
SERINC3	0.21788012	7.22858487	1.82889727	0.07309593	0.99978775	-4.1623675
N4BP2L1	0.21783324	0.11912805	0.32834129	0.74395928	0.99978775	-4.6170859
ENSCAFG00000019017	0.21780977	0.82187126	0.99756328	0.32306568	0.99978775	-4.5975379
WDSUB1	0.21768937	4.23836705	1.88308384	0.06523283	0.99978775	-4.1941496
DCTN5	0.21762883	3.38718641	1.91160548	0.06139043	0.99978775	-4.270533
SDHAF3	0.21759896	1.73727875	1.01310721	0.31565413	0.99978775	-4.5980591
RHOBTB3	0.21741271	6.21408383	1.14465938	0.25754062	0.99978775	-4.5992316
ITGAE	0.21739222	1.27424196	0.78263292	0.43735772	0.99978775	-4.6151914
AHCYL1	0.21724379	7.52179565	1.61601801	0.11208289	0.99978775	-4.3180575
ENSCAFG00000016072	0.21655763	4.13832829	0.62632255	0.53381653	0.99978775	-4.7812804
NPAS2	0.21632762	1.06212872	0.36254917	0.71839675	0.99978775	-4.6306112
FAM131B	0.21631752	3.18995596	0.9902208	0.32660695	0.99978775	-4.6197437
PSMD12	0.21587927	5.85953657	1.78333413	0.0803114	0.99978775	-4.199105
PIK3IP1	0.21578907	1.89879931	0.33936256	0.73569005	0.99978775	-4.63528
ENSCAFG00000028692	0.21565326	2.32863028	0.95312557	0.34489312	0.99978775	-4.6104001
DAG1	0.21544006	8.46101805	2.02345449	0.04812904	0.99978775	-4.0487525
EFCAB5	0.21539637	0.81848923	0.78160615	0.43795581	0.99978775	-4.6119455
ENSCAFG00000025044	0.21503634	6.07026941	2.14941045	0.0362295	0.99978775	-3.8997652
PUSL1	0.21463247	0.8472337	0.63263949	0.52971425	0.99978775	-4.6222999
ENSCAFG00000000713	0.21454538	3.86513503	2.02051932	0.04844259	0.99978775	-4.1778835
RET	0.21452995	0.73535954	0.44094301	0.66106427	0.99978775	-4.6275412
FBXL5	0.21424774	5.54071318	2.11271543	0.03939703	0.99978775	-3.955621
ENSCAFG00000017231	0.21421724	3.21055377	0.82223364	0.41466172	0.99978775	-4.666399
TMEM30A	0.21404481	7.43827285	1.93735348	0.05808894	0.99978775	-4.0899548
IDH2	0.21359341	6.51197163	0.90045615	0.37198849	0.99978775	-4.7111369
ENSCAFG00000016649	0.21350014	-0.327509	0.59316926	0.55561401	0.99978775	-4.617951
MAP1LC3A	0.21330106	4.98276683	1.3992452	0.16761858	0.99978775	-4.4613576

ENSCAFG00000004602	0.21319776	4.09671923	1.98709404	0.05213944	0.99978775	-4.1360395
MT-CO2	0.21308995	12.3025927	1.46241147	0.14958767	0.99978775	-4.4665385
POLR2K	0.21305262	1.10023804	0.8503058	0.39901537	0.99978775	-4.6101541
NSUN4	0.21271176	3.1272454	1.38533743	0.17180566	0.99978775	-4.5055078
SLC25A17	0.21264598	4.26480812	1.93230933	0.0587235	0.99978775	-4.1746152
ENSCAFG00000009480	0.21241495	0.43603011	0.5594431	0.57823769	0.99978775	-4.6200594
BCAM	0.21239785	4.19263728	0.52007416	0.6051975	0.99978775	-4.7278645
MBP	0.21194872	-1.4086174	0.43776757	0.66335008	0.99978775	-4.6138268
SYT11	0.2119074	4.68366948	1.16845395	0.2479008	0.99978775	-4.5775045
ENSCAFG00000022770	0.21189697	0.01926487	0.56553101	0.57412109	0.99978775	-4.6126633
IQCD	0.2118437	-0.4875113	0.4638821	0.64464938	0.99978775	-4.621588
GABARAPL2	0.21184344	5.81947241	1.37494306	0.17498723	0.99978775	-4.4726376
SLC25A23	0.21164899	3.755967	0.80551878	0.42415277	0.99978775	-4.6698909
GOLGA1	0.21162871	4.46298163	2.54377543	0.01394677	0.99978775	-3.6766461
JAZF1	0.21157419	1.66778256	0.82773792	0.41156476	0.99978775	-4.6292668
LMBRD1	0.21119197	5.77274755	1.46017065	0.15020018	0.99978775	-4.4201104
CLOCK	0.21117451	5.16321657	1.15845961	0.25191768	0.99978775	-4.5905338
SRGAP1	0.21107784	4.46575174	1.66974766	0.10092042	0.99978775	-4.3212211
LNPK	0.21079128	4.70341632	1.40320446	0.16644114	0.99978775	-4.4588818
ENSCAFG00000009631	0.21057181	2.73035623	0.88533547	0.38001172	0.99978775	-4.6223687
NOX5	0.21041928	-1.467007	0.26101142	0.79510458	0.99978775	-4.6084483
UXS1	0.21035931	5.59494529	1.78012155	0.08084188	0.99978775	-4.2053605
ENSCAFG00000032075	0.21019688	2.87580857	1.48412689	0.14375304	0.99978775	-4.4768072
ABHD12	0.21014906	4.72384176	1.71971917	0.09137272	0.99978775	-4.2649685
CALR3	0.21011204	-0.0189588	0.33378842	0.73986845	0.99978775	-4.6129859
CTSC	0.20991478	2.50380619	0.35518334	0.72387486	0.99978775	-4.6873899
KANSL1L	0.20987835	3.90374789	1.01975692	0.31251882	0.99978775	-4.6182349
MAPK8IP1	0.20980809	3.01826664	0.96403337	0.33944762	0.99978775	-4.6229088
C37H2orf69	0.20961671	2.30732222	1.20420141	0.23391133	0.99978775	-4.56642
CCPG1	0.20949379	6.39866249	1.3216848	0.19200212	0.99978775	-4.5035381
GOSR1	0.20931146	3.69190181	1.73436664	0.08872	0.99978775	-4.3422049
RPL10L	0.20915566	-0.2253949	0.71876744	0.47547036	0.99978775	-4.6131288
FUT8	0.20914761	5.81332503	1.69595012	0.09581674	0.99978775	-4.2611545
INTS6L	0.20911344	4.48094715	1.27658739	0.20736444	0.99978775	-4.5271597
PIGC	0.20878088	4.21373094	2.26343179	0.02776791	0.99978775	-3.950315
HEXB	0.20871753	7.47180555	1.3470954	0.18373384	0.99978775	-4.4888173
UNKL	0.20848458	1.91583955	0.70055724	0.48667178	0.99978775	-4.6263257
CPXM1	0.20843971	1.7390715	0.41110787	0.6826663	0.99978775	-4.7599535
ENSCAFG00000032283	0.20831338	1.15878882	0.62075419	0.53744632	0.99978775	-4.6212145
ZMAT5	0.20825569	1.87751319	1.33154274	0.18876165	0.99978775	-4.5495015
PELI3	0.20817147	2.02587672	0.80369698	0.42519507	0.99978775	-4.6262157
SHISAL1	0.20807978	-1.8598827	0.33963214	0.73548818	0.99978775	-4.6107232
CHPF2	0.20796121	6.45767886	1.84811381	0.07022057	0.99978775	-4.1460334
ENSCAFG00000031092	0.2078037	3.4456549	1.60442746	0.11461773	0.99978775	-4.4035093
ENSCAFG00000032375	0.2078033	1.32545283	0.77980773	0.43900456	0.99978775	-4.6174202
SCEL	0.2077718	2.35937756	0.41762244	0.67792578	0.99978775	-4.6722752
OSBPL1A	0.20772083	5.11135886	1.44431529	0.15459049	0.99978775	-4.4337987
CSRNP2	0.20771934	2.72200965	1.22691949	0.22532513	0.99978775	-4.5554688
DHRS7B	0.20761601	3.4504197	1.46039152	0.15013972	0.99978775	-4.4606445
CMTM7	0.20759446	2.5144098	0.95679573	0.3430545	0.99978775	-4.6117225

HEATR5A	0.20740894	6.67954847	1.89624271	0.06343543	0.99978775	-4.1103743
ATG13	0.20736055	5.84222653	2.03569244	0.04684058	0.99978775	-4.0061885
ARMT1	0.20733721	5.22514225	1.36208568	0.17898504	0.99978775	-4.4811058
ARHGAP20	0.20722614	3.70684271	0.35677549	0.72268951	0.99978775	-4.7214565
SMAD9	0.20720842	5.98570241	0.86396459	0.39153645	0.99978775	-4.726227
ICAM1	0.20714747	8.29426381	0.72126441	0.47394585	0.99978775	-4.7515314
HMCES	0.20707562	3.7739357	1.67244618	0.10038471	0.99978775	-4.3563657
GLIS3	0.20693407	4.50350752	1.18091092	0.24295899	0.99978775	-4.5699304
GLCCI1	0.206885	3.04586163	0.95003845	0.34644465	0.99978775	-4.6375231
HCN2	0.20684094	2.4655468	0.37256913	0.71096861	0.99978775	-4.6526712
KPNA5	0.2068329	3.27761048	1.47001577	0.1475237	0.99978775	-4.4681657
DKKL1	0.20676141	-0.927874	0.39111674	0.69729333	0.99978775	-4.6123669
MIEN1	0.20675002	3.15554001	1.76237333	0.08382557	0.99978775	-4.3394481
MBD5	0.20671649	4.67963942	1.90543337	0.06220518	0.99978775	-4.1823059
DCAF10	0.2065068	4.34401683	2.20998979	0.03148752	0.99978775	-3.9718827
ENSCAFG00000025063	0.20625926	6.82236224	1.17583159	0.24496535	0.99978775	-4.5840196
EMSY	0.20624898	4.60589105	1.64877188	0.10516481	0.99978775	-4.3311797
CST3	0.20605413	7.58308324	0.70429007	0.48436376	0.99978775	-4.7770474
KIAA0895	0.20591441	3.81353058	0.80183321	0.42626298	0.99978775	-4.6848907
RBM11	0.20572543	1.8521771	0.47863846	0.63418271	0.99978775	-4.6760041
MT-ND2	0.20564547	11.2107945	1.31009519	0.19586544	0.99978775	-4.5154754
LAP3	0.20562521	7.0811973	1.82810843	0.07321605	0.99978775	-4.16436
PRDM11	0.20547418	1.38116032	0.63374086	0.5290007	0.99978775	-4.6250265
MYO6	0.20546246	6.27374014	1.82689767	0.07340074	0.99978775	-4.1631081
MAP3K11	0.20542312	2.90045672	0.66361687	0.5098385	0.99978775	-4.6658427
ENSCAFG00000010051	0.20539061	2.72088929	1.20887116	0.23212721	0.99978775	-4.5609703
RBBP9	0.20524086	1.99411103	1.13480882	0.26160869	0.99978775	-4.5813473
NUDT17	0.2052047	0.07387697	0.55218744	0.58316249	0.99978775	-4.616108
SPRY1	0.20518529	3.27606936	0.16551788	0.86917194	0.99978775	-4.6347524
FCGRT	0.20501618	3.9861336	0.58028375	0.56420504	0.99978775	-4.720579
PSD	0.20491318	1.46394274	0.59499944	0.55439915	0.99978775	-4.6247889
SASH1	0.20489231	7.98891365	1.39935739	0.16758513	0.99978775	-4.4592363
ADAM11	0.20479388	1.86477115	1.16556876	0.24905564	0.99978775	-4.573088
ENSCAFG00000028550	0.20451904	2.05312982	0.88376362	0.380852	0.99978775	-4.616325
TIMP2	0.20437222	7.93174678	0.58398766	0.56172884	0.99978775	-4.8089243
DCTN6	0.20431144	5.25734368	1.9356866	0.05829799	0.99978775	-4.1002187
CYYR1	0.20427009	-2.1717505	0.21944772	0.82715205	0.99978775	-4.6069103
ZBTB25	0.20416106	2.23080364	0.91741787	0.36311797	0.99978775	-4.6147825
ENSCAFG00000030859	0.20415327	-0.0785697	0.56194969	0.57654103	0.99978775	-4.6174217
TTC13	0.20396437	4.5857346	2.02844299	0.04760018	0.99978775	-4.0668608
KDELR3	0.20385521	6.11217722	1.69815446	0.09539725	0.99978775	-4.2581871
CD99	0.20375109	9.89654693	1.47436902	0.14635225	0.99978775	-4.433241
ZNF148	0.20371239	5.11861372	2.18526053	0.03335282	0.99978775	-3.9124053
PYROXD2	0.20356666	3.33571957	0.79921551	0.42776558	0.99978775	-4.6702081
RAB28	0.20356127	1.08252205	0.9527888	0.34506215	0.99978775	-4.602646
PPP1R3B	0.20342174	3.452893	0.83919613	0.4051633	0.99978775	-4.6463216
CD151	0.20338698	8.6677613	1.70875337	0.09340133	0.99978775	-4.2793915
NDRG4	0.20329213	5.96510362	1.28250303	0.2052985	0.99978775	-4.5255625
MAN1A2	0.203147	6.15012365	1.46358402	0.14926794	0.99978775	-4.417854
LRRC4	0.20308649	1.283148	0.42916184	0.66956101	0.99978775	-4.6315015

ADCY3	0.20304656	2.84175145	0.70294083	0.4851973	0.99978775	-4.7180156
HTRA1	0.20277766	9.81276809	0.78354124	0.43682904	0.99978775	-4.7045556
DNASE2	0.20271094	6.0250438	1.14682136	0.25665385	0.99978775	-4.5975859
ZSCAN16	0.20262763	3.29307085	1.34818084	0.18338679	0.99978775	-4.5131823
ENSCAFG00000032717	0.20261961	1.95659089	0.55399786	0.58193178	0.99978775	-4.650441
CTSZ	0.20258623	5.35154113	0.97410636	0.33446954	0.99978775	-4.6750431
ENSCAFG00000005387	0.20257345	0.00640434	0.74211423	0.46132456	0.99978775	-4.6099003
CYFIP2	0.20254602	3.52184346	0.70148466	0.48609778	0.99978775	-4.7126888
MST1	0.20249156	-0.0861125	0.43184958	0.66761869	0.99978775	-4.6183758
MSANTD2	0.20229527	3.72117766	1.56728955	0.12305359	0.99978775	-4.4035492
AFF4	0.20220608	6.9490843	1.74658243	0.08655677	0.99978775	-4.2258663
RGS11	0.20218993	3.53125123	0.84967699	0.3993618	0.99978775	-4.6467715
ENSCAFG00000000112	0.20206448	1.24786803	0.80378578	0.42514423	0.99978775	-4.617189
SUSD6	0.20203209	6.37698888	1.50387756	0.1386036	0.99978775	-4.3917115
LAMP2	0.20200708	7.71433875	1.50046379	0.13948304	0.99978775	-4.3978101
GPRIN3	0.20183492	-2.0335444	0.30847051	0.75894469	0.99978775	-4.6117189
TRAPPC2L	0.20179355	3.05935961	1.59021245	0.11778967	0.99978775	-4.4331323
CDH13	0.20172514	9.04652322	0.68732178	0.49490438	0.99978775	-4.7530742
EXT1	0.20171952	6.84768183	1.83944546	0.07150557	0.99978775	-4.1535333
XKRX	0.20167249	-1.3280533	0.25569067	0.79918857	0.99978775	-4.6072849
ATP2A1	0.20157564	0.90411635	0.59450105	0.55472984	0.99978775	-4.6210564
ENSCAFG00000001231	0.20148537	1.90486072	1.06885047	0.29002565	0.99978775	-4.5908963
PLXNB1	0.20139683	3.32886797	1.0339999	0.30587453	0.99978775	-4.6191943
SIAH1	0.20133525	3.4244671	1.70720987	0.09368983	0.99978775	-4.3673782
PREX1	0.20089389	4.95838255	0.38981614	0.69824904	0.99978775	-4.7568019
RNF145	0.20087172	6.1705898	1.38013519	0.17339238	0.99978775	-4.4691112
SLC25A27	0.20086613	1.44815299	0.74282591	0.46089719	0.99978775	-4.6169929
ND5	0.2005666	11.5468331	1.11121383	0.27153808	0.99978775	-4.5884859
BAALC	0.20017056	-0.2674506	0.53442595	0.59530232	0.99978775	-4.6363491
TMEM205	0.20002166	3.11532619	1.02599276	0.30959788	0.99978775	-4.6063433
AGA	0.1999706	3.84496529	1.2890222	0.20303966	0.99978775	-4.5243265
PTPN9	0.19962781	7.33595109	2.78984978	0.00733179	0.99978775	-3.3012148
LCMT2	0.19959425	2.71572758	1.42001271	0.16151366	0.99978775	-4.5050929
ENSCAFG00000019976	0.19959378	1.07473965	0.94737245	0.34778821	0.99978775	-4.6048462
MAP2	0.19948363	1.98112396	0.58536979	0.56080622	0.99978775	-4.7035146
LRGUK	0.19944449	0.96638115	0.68386574	0.49706661	0.99978775	-4.6177023
FCHSD2	0.19942503	3.85601618	1.07840324	0.28578244	0.99978775	-4.6017963
CYP2C18	0.19931774	0.8145104	0.57363268	0.56866505	0.99978775	-4.6385142
DUSP16	0.19905339	3.25493573	1.07517057	0.28721349	0.99978775	-4.6026442
HSDL2	0.19901955	4.33557596	1.51977591	0.13456565	0.99978775	-4.417769
ENSCAFG00000009702	0.19901561	3.94183251	1.31052794	0.19572014	0.99978775	-4.5159246
ITM2B	0.19895579	9.00851858	1.31005634	0.19587849	0.99978775	-4.5095209
MANSC1	0.1988126	6.36697646	1.64360471	0.10623245	0.99978775	-4.2973872
BCL6	0.19860587	5.66752219	1.05917072	0.29436957	0.99978775	-4.636686
PDE4DIP	0.19833134	6.31690537	1.16889676	0.2477239	0.99978775	-4.5876651
ZNF438	0.1982757	1.92903683	1.02397725	0.31053993	0.99978775	-4.597759
OVOL1	0.19819383	-2.0371874	0.28400503	0.77752332	0.99978775	-4.6088007
ENSCAFG00000029405	0.19818939	3.61405053	0.67534222	0.50242132	0.99978775	-4.7314169
ARHGAP45	0.1981327	-0.2662474	0.49881366	0.61999374	0.99978775	-4.6221002
FUT11	0.19810093	3.69309464	1.10137302	0.27575687	0.99978775	-4.5920972

ENSCAFG00000029129	0.19808546	4.81507429	0.4869496	0.62832041	0.99978775	-4.7778569
NR2F2	0.19801512	5.29586586	0.60610446	0.54705646	0.99978775	-4.7985339
KRAS	0.19792166	3.39999112	1.35509563	0.18118762	0.99978775	-4.5013142
PQLC3	0.19790026	4.89224306	0.8991052	0.3727009	0.99978775	-4.6815013
C6H1orf52	0.19787082	2.85358851	1.12198228	0.26697395	0.99978775	-4.5814069
YARS2	0.19768936	2.85445873	1.90165442	0.06270855	0.99978775	-4.3267517
IL6ST	0.19762155	8.20554034	1.51269919	0.13635133	0.99978775	-4.3920082
IFT57	0.1975936	3.93399101	1.33645397	0.18716281	0.99978775	-4.504619
SOD2	0.19756298	5.43630734	0.85082833	0.39872764	0.99978775	-4.7033207
TRAF6	0.19749565	5.95215707	1.66996282	0.10087762	0.99978775	-4.2808967
BGN	0.19740325	11.9070578	0.66468336	0.50916144	0.99978775	-4.6968044
ATP9B	0.19736917	5.01434784	2.34766859	0.02269313	0.99978775	-3.7890249
PROX1	0.1973317	-1.1931803	0.30102694	0.76458264	0.99978775	-4.6126329
DEGS1	0.19731746	7.263136	2.17034367	0.03452443	0.99978775	-3.8915071
CD109	0.19728313	7.44392816	1.22407087	0.22638888	0.99978775	-4.5573845
AFF1	0.19691811	5.59723084	1.52364088	0.13359828	0.99978775	-4.3872369
MTPN	0.19675402	9.05409033	1.97143403	0.05395335	0.99978775	-4.1014309
ENSCAFG00000031995	0.19667497	4.13351276	1.41533775	0.16287266	0.99978775	-4.4629378
MARCH2	0.19657782	3.82393907	1.08120447	0.28454639	0.99978775	-4.6005481
ENSCAFG00000021670	0.1963208	-0.7138106	0.45810892	0.64876425	0.99978775	-4.6177526
CHSY3	0.19628149	0.30142533	0.53980142	0.59161572	0.99978775	-4.6349355
TMEM106C	0.19621186	4.73194371	1.7741314	0.08183882	0.99978775	-4.2387613
ELMOD3	0.19616451	4.51372077	1.22505517	0.2260209	0.99978775	-4.5509717
ACVR1B	0.19615161	3.73552969	0.96212678	0.34039534	0.99978775	-4.6219413
SLC30A7	0.1959251	4.61452963	1.58551412	0.11885351	0.99978775	-4.3596522
BORCS5	0.19554273	4.78918482	2.0418656	0.04620204	0.99978775	-4.0459309
SULF1	0.19523196	8.78043527	0.39630435	0.69348627	0.99978775	-4.7956839
LMBRD2	0.19514561	4.82457861	1.50363937	0.13866482	0.99978775	-4.4038257
VPS53	0.19484311	4.68815025	2.19597037	0.03253337	0.99978775	-3.9435502
REC8	0.19473532	2.27702477	0.51581041	0.60815179	0.99978775	-4.6553232
ADAP1	0.19440153	2.90836418	0.96231135	0.34030351	0.99978775	-4.6266914
TOB1	0.19436621	6.25884439	1.53783571	0.13009283	0.99978775	-4.3692313
ENSCAFG00000024323	0.19427948	-0.4453897	0.6146472	0.54144185	0.99978775	-4.6154033
ZNF329	0.19404199	2.05129215	0.95522215	0.34384202	0.99978775	-4.6071117
LDHB	0.19395499	4.41217866	1.37913615	0.17369838	0.99978775	-4.4796004
FAM210B	0.19373903	6.96216325	1.08374621	0.28342807	0.99978775	-4.6302909
ZNF45	0.19328094	2.29725006	0.96088701	0.34101253	0.99978775	-4.6068534
B4GALT6	0.19328015	-0.6834206	0.35215331	0.7261326	0.99978775	-4.6272164
CARD6	0.19314398	6.17719871	1.22004535	0.2278984	0.99978775	-4.5602085
BTD	0.19309086	6.06402216	1.55829277	0.12517055	0.99978775	-4.3599999
MEF2C	0.193087	2.51541845	0.44813914	0.65589625	0.99978775	-4.7491329
SMOC2	0.19303851	2.59855917	0.53668229	0.59375357	0.99978775	-4.7791279
PAK3	0.19266122	4.80584453	1.11281249	0.27085705	0.99978775	-4.6019712
KLHDC2	0.19265432	5.45656866	2.46865566	0.01685228	0.99978775	-3.6409878
FBXO28	0.19241021	5.54920975	2.50301257	0.01546154	0.99978775	-3.6180722
CFAP206	0.19232177	-0.2915539	0.47912821	0.6338366	0.99978775	-4.6167975
TMEM185A	0.19232073	4.21269632	1.73916182	0.08786556	0.99978775	-4.3009582
SHB	0.19224749	2.83093698	0.68200502	0.49823289	0.99978775	-4.6649201
PRADC1	0.19215395	4.4567243	1.17863829	0.24385522	0.99978775	-4.5744503
WDR47	0.19212411	4.8648505	1.14618806	0.25691338	0.99978775	-4.5873867

ENSCAFG00000028782	0.19212335	4.98820417	1.63690707	0.10762945	0.99978775	-4.3217414
PLPP6	0.19211776	2.6214589	0.89403733	0.37538112	0.99978775	-4.626432
ENSCAFG00000017791	0.19194215	1.56969725	0.80683367	0.42340145	0.99978775	-4.6149466
INPP5K	0.19190626	4.92755781	1.47219268	0.14693698	0.99978775	-4.4219785
FYN	0.19190298	7.69169102	1.05824935	0.29478537	0.99978775	-4.6319033
APLP2	0.19186426	9.42552846	1.37901793	0.17373462	0.99978775	-4.4740644
GCLC	0.19183066	4.97520873	1.83935965	0.07151839	0.99978775	-4.1783406
ZBED8	0.19172427	2.40034593	1.05792767	0.29493064	0.99978775	-4.5935003
MRVI1	0.19156976	4.66900763	0.55723161	0.57973662	0.99978775	-4.8059273
STOM	0.19153426	6.91649125	0.71228481	0.47944118	0.99978775	-4.7787286
GGPS1	0.19151288	2.73290564	1.25901094	0.2135942	0.99978775	-4.549163
MPLKIP	0.1914603	4.15290838	0.51966445	0.60548109	0.99978775	-4.7427623
PRKX	0.19126229	5.32101181	0.61711793	0.53982352	0.99978775	-4.7901628
B3GALNT2	0.19106719	4.14371976	1.33075526	0.18901898	0.99978775	-4.5042315
ITIH5	0.19106302	4.16411073	0.60153303	0.5500731	0.99978775	-4.813
SCAMP1	0.19091591	5.41711443	1.54067709	0.12940005	0.99978775	-4.3727547
IKBKB	0.19091043	4.90817617	1.99114939	0.05167834	0.99978775	-4.0867133
ENSCAFG00000028864	0.19067707	1.85866532	0.96450937	0.33921129	0.99978775	-4.6051115
ENSCAFG00000001129	0.19058024	0.14347745	0.64986467	0.51861238	0.99978775	-4.6240669
PARD6B	0.19054961	-0.2529845	0.47560707	0.63632683	0.99978775	-4.6167572
VEZT	0.19043774	4.84477223	1.38813853	0.17095595	0.99978775	-4.4687861
SARAF	0.19041308	7.80985097	2.11974236	0.03877237	0.99978775	-3.9471353
SPTBN5	0.18977418	-1.0393876	0.35305064	0.72546373	0.99978775	-4.6166952
FLT3	0.18974247	-0.6959773	0.45364604	0.65195278	0.99978775	-4.6152276
MOB2	0.18968985	4.79783419	1.34824238	0.18336713	0.99978775	-4.491706
GNG12	0.18965895	3.65098644	1.84801558	0.07023502	0.99978775	-4.2564036
PSMB9	0.18960298	1.38203173	0.56320297	0.57569361	0.99978775	-4.6347525
ACTR3B	0.18959145	3.14132603	1.46933842	0.14770663	0.99978775	-4.4759766
ATXN7L1	0.18943389	1.73035068	0.69579417	0.48962566	0.99978775	-4.6237886
ACKR4	0.18941044	4.30587052	0.2319282	0.8174952	0.99978775	-4.7526202
TUBA8	0.18924717	2.36787815	1.0262365	0.30948409	0.99978775	-4.5992282
RNF2	0.18911983	3.12451894	1.3161154	0.19385139	0.99978775	-4.5189311
GPATCH2L	0.1890742	3.70238127	1.54213411	0.12904594	0.99978775	-4.4251946
SLC37A2	0.18905058	3.337454	0.56349042	0.57549933	0.99978775	-4.7910096
FBXL2	0.18898176	2.16545225	0.94574172	0.34861172	0.99978775	-4.6139091
PPIP5K1	0.18895624	4.17418893	1.47637962	0.14581368	0.99978775	-4.4355867
ENSCAFG00000026256	0.1889143	-0.2393795	0.52006369	0.60520474	0.99978775	-4.6133395
MSN	0.18889285	9.75651315	1.1648184	0.24935662	0.99978775	-4.5751814
EXOSC9	0.18881796	4.43684321	1.34143188	0.18555279	0.99978775	-4.4952533
ZDHHC24	0.18881504	0.53142778	0.47908656	0.63386603	0.99978775	-4.6239458
TRIM41	0.18879086	2.83189383	1.26602456	0.21109184	0.99978775	-4.5499364
NT5C3B	0.18870998	5.68021219	2.86703365	0.00595055	0.99978775	-3.2233588
TMF1	0.18870717	5.712589	1.55629773	0.12564392	0.99978775	-4.360553
DPYSL3	0.18869712	11.4773152	0.68160006	0.49848692	0.99978775	-4.6962458
JAK2	0.18860511	5.8144424	1.66519164	0.10183018	0.99978775	-4.2905425
ZNF275	0.18858726	2.05083982	1.02701119	0.30912261	0.99978775	-4.5968892
UBA5	0.18851488	5.4443536	1.34845529	0.18329912	0.99978775	-4.4880679
RBM48	0.18847998	3.06444413	1.36189334	0.17904537	0.99978775	-4.5165264
ENSCAFG00000030097	0.18835546	1.8504125	0.92711788	0.35810685	0.99978775	-4.6091843
ENSCAFG00000030548	0.18829416	2.23479781	0.77760978	0.44028829	0.99978775	-4.649221

EIF4E3	0.1882689	0.55196142	0.60571586	0.54731256	0.99978775	-4.6185229
ENSCAFG00000013899	0.1882542	-0.6122888	0.18506046	0.85389449	0.99978775	-4.6166347
MAP3K7	0.18816152	6.19697715	1.44478712	0.15445841	0.99978775	-4.4293472
ENSCAFG00000002523	0.1880903	0.94924161	0.7190167	0.47531805	0.99978775	-4.6296962
PHLPP2	0.18798779	4.17238113	1.38526706	0.17182705	0.99978775	-4.4853357
DCBLD1	0.18795984	1.82568889	0.57322005	0.56894231	0.99978775	-4.6407124
ETNK2	0.18774344	0.69632676	0.52588551	0.60118159	0.99978775	-4.636405
DPH3	0.18771937	0.65557306	0.62782964	0.53283631	0.99978775	-4.62078
GADD45B	0.18761137	5.27683536	0.68066516	0.49907363	0.99978775	-4.7849679
FGFR1	0.18759905	7.46807002	2.05867535	0.04450138	0.99978775	-3.9858892
WNK1	0.18756435	7.65549728	1.75592115	0.08493275	0.99978775	-4.2264029
OSTC	0.18754812	2.00753709	0.72044304	0.47444702	0.99978775	-4.6405497
DNAJB9	0.18741428	6.14941302	1.21644604	0.22925433	0.99978775	-4.561864
ORMDL1	0.18732095	2.92356019	1.62669249	0.10978881	0.99978775	-4.4239675
ENSCAFG00000012363	0.18711103	3.87837238	1.61659276	0.11195839	0.99978775	-4.3826689
CALU	0.18705762	10.4810156	0.90030887	0.37206612	0.99978775	-4.6619444
ACYP2	0.18703331	0.70210143	0.48892462	0.62693084	0.99978775	-4.6229808
DNAJB6	0.18696409	4.52038239	1.38421931	0.17214575	0.99978775	-4.4743488
ENSCAFG00000030354	0.18660925	1.86376996	0.82624214	0.41240495	0.99978775	-4.6222601
DLA-64	0.18649603	5.02548334	0.50552776	0.61530354	0.99978775	-4.8083266
TLR2	0.18644524	0.22594976	0.43831962	0.66295246	0.99978775	-4.6357009
FAM45A	0.18626037	5.21370386	2.02368055	0.04810497	0.99978775	-4.0304455
RAB33B	0.1861119	2.89718294	1.41309562	0.16352757	0.99978775	-4.5059475
PTP4A1	0.18600467	4.8402035	1.43346596	0.15765199	0.99978775	-4.4434675
SEC22B	0.18586071	5.2396711	1.63565021	0.10789327	0.99978775	-4.3162525
PPP1R12A	0.1857104	7.34845562	1.3944681	0.1690478	0.99978775	-4.4615347
ENSCAFG00000000071	0.18569124	3.55728603	1.46597718	0.14861706	0.99978775	-4.4602545
SHROOM2	0.18554788	6.42450542	0.64427851	0.52219914	0.99978775	-4.8014181
SGCE	0.18541557	4.88866712	1.05286486	0.29722343	0.99978775	-4.6263588
WDR83	0.18517794	2.78767187	1.56870108	0.12272408	0.99978775	-4.4549539
ALG5	0.18512161	5.60353884	1.31199381	0.19522856	0.99978775	-4.5090369
DENND1B	0.18511296	2.22485025	1.01913137	0.31281286	0.99978775	-4.6017239
PTPN11	0.18502295	7.07599805	1.50288522	0.13885879	0.99978775	-4.394201
PCMT1	0.18493208	5.75391868	2.0063017	0.04998645	0.99978775	-4.0294646
DCAF5	0.18488809	5.6826092	2.34990632	0.02257042	0.99978775	-3.7414829
ALPK1	0.18471503	3.08651155	0.8020745	0.42612463	0.99978775	-4.6539209
ENSCAFG00000001608	0.18464015	-0.6732515	0.55722014	0.57974439	0.99978775	-4.6152315
ENSCAFG00000005305	0.18461084	2.36951979	1.06044263	0.29379623	0.99978775	-4.5924983
UNC119	0.18457532	1.86323369	0.46859744	0.64129678	0.99978775	-4.635831
TLK1	0.18440158	5.5440572	1.98009234	0.05294387	0.99978775	-4.058477
ZC3H6	0.18435471	3.86707313	1.03317027	0.30625889	0.99978775	-4.6113349
ATP6AP1	0.18418586	6.68383138	1.24812908	0.21752017	0.99978775	-4.5451136
FAAH	0.1841421	2.54718111	1.2029405	0.23439478	0.99978775	-4.5581083
KIAA1191	0.18411949	7.37495269	1.80206918	0.07727541	0.99978775	-4.1903717
ESYT2	0.18411849	8.22076284	1.81016252	0.07599396	0.99978775	-4.2019649
IRF2	0.18405963	4.71478302	1.20861513	0.23222477	0.99978775	-4.5573335
ENSCAFG00000030887	0.18381368	2.48963021	0.99190573	0.32579202	0.99978775	-4.6065471
TAPT1	0.18378577	4.9763893	1.54549919	0.12823107	0.99978775	-4.3755042
UBE2A	0.18369918	4.28028001	1.12507591	0.26567283	0.99978775	-4.5951249
MTUS1	0.18355263	4.31055078	0.39028	0.69790813	0.99978775	-4.7303093

TM9SF2	0.18341064	7.95064951	2.10294657	0.04028002	0.99978775	-3.9708014
ENAH	0.18337344	6.69404439	1.02122326	0.31183029	0.99978775	-4.6573407
ENSCAFG00000031337	0.18324487	2.33304866	0.50208018	0.61770988	0.99978775	-4.6709999
CXHXorf56	0.18323583	1.73801027	0.80619954	0.42376369	0.99978775	-4.6197853
TTC7B	0.18323307	5.24079255	1.73217944	0.089112	0.99978775	-4.2459477
CDC27	0.18317872	6.85874906	1.98249256	0.05266691	0.99978775	-4.0413879
ATXN2	0.18312941	6.23684424	1.65336551	0.10422304	0.99978775	-4.2904961
CXADR	0.18308163	1.7274725	0.35250715	0.72586882	0.99978775	-4.6485627
TXNDC9	0.18304254	4.47274557	1.60862813	0.11369374	0.99978775	-4.3534042
PPM1D	0.18294706	3.36111421	0.93199577	0.35560388	0.99978775	-4.6286366
ENSCAFG00000015445	0.18291462	2.6676055	0.80458885	0.42468462	0.99978775	-4.6603087
FN1	0.18290011	14.6851343	0.58799056	0.55905885	0.99978775	-4.6874287
GNG10	0.18250829	4.30821263	0.92043807	0.36155289	0.99978775	-4.6642494
PRTFDC1	0.18250212	2.51014357	0.43922687	0.66229922	0.99978775	-4.6769395
MAPRE3	0.18232781	4.23879473	1.01815816	0.31327069	0.99978775	-4.6230384
ZNF350	0.18231287	2.42955975	0.89431146	0.37523583	0.99978775	-4.6183998
FTH1_version2	0.18231214	6.44077213	0.43741593	0.66360341	0.99978775	-4.8526841
PTGIR	0.18229458	3.19959584	0.61086905	0.54392133	0.99978775	-4.6990559
HPSE	0.18228313	-1.1658849	0.34868657	0.72871875	0.99978775	-4.6145362
CYTH3	0.18223854	6.0745033	1.17931172	0.2435894	0.99978775	-4.5801934
RCHY1	0.18208309	3.63102505	1.67333726	0.10020833	0.99978775	-4.3681151
KIAA0319L	0.18199169	5.95657343	1.58745987	0.118412	0.99978775	-4.3373147
DPH6	0.18196811	3.54159799	1.02057776	0.31213326	0.99978775	-4.6151041
ENSCAFG00000032500	0.181835	1.14005412	0.81748944	0.41734237	0.99978775	-4.6132257
C1GALT1C1	0.18179233	3.36523203	1.35718221	0.18052798	0.99978775	-4.5041708
ACTN4	0.18167015	9.72455889	1.24575908	0.21838226	0.99978775	-4.5403465
NFRKB	0.18165811	4.38853647	1.94265969	0.05742776	0.99978775	-4.1600391
EMC7	0.18157941	5.59781924	1.32499486	0.19090939	0.99978775	-4.5014546
NAPEPLD	0.1815576	2.26400164	0.4977287	0.62075315	0.99978775	-4.6448613
UHRF1BP1L	0.18152464	6.68900202	1.39452805	0.1690298	0.99978775	-4.460463
ROR1	0.18133627	4.8883898	0.47325754	0.63799083	0.99978775	-4.8226231
SLC9A6	0.18120997	5.44359673	2.04301871	0.04608361	0.99978775	-4.0184823
AFDN	0.18114507	4.75884506	0.50192358	0.61781928	0.99978775	-4.8122382
ENSCAFG00000028761	0.18113793	0.2754707	0.43392365	0.66612141	0.99978775	-4.6241492
EXT2	0.18102066	7.9606589	1.93014904	0.05899707	0.99978775	-4.104842
PIAS1	0.18085797	5.29850286	1.56151054	0.12441007	0.99978775	-4.362397
IFT74	0.18073956	4.69302152	1.35835764	0.18015719	0.99978775	-4.4859236
MSRB3	0.18071787	8.73038959	1.11422813	0.27025499	0.99978775	-4.6029693
BTF3L4	0.18062244	3.62099018	1.51293554	0.13629139	0.99978775	-4.4420532
TSPAN13	0.18055356	-1.2679293	0.21218921	0.83278087	0.99978775	-4.6084416
ENSCAFG00000012879	0.1804501	3.69457629	0.9343361	0.35440703	0.99978775	-4.6459431
DLA88	0.18041473	4.34101722	0.54946681	0.5850143	0.99978775	-4.7640212
MED13L	0.18039413	7.35316064	1.12354692	0.26631533	0.99978775	-4.6099612
ZDHHC1	0.18023763	4.25882294	0.82979026	0.41041364	0.99978775	-4.6831828
RGS7	0.18014024	4.21567471	0.53799751	0.59285168	0.99978775	-4.7442238
PHF21A	0.17999724	5.01442244	1.39119025	0.1700339	0.99978775	-4.4672629
ENSCAFG00000028797	0.17996678	-1.906398	0.35742749	0.72220429	0.99978775	-4.6064535
ENSCAFG00000018078	0.17995391	4.68135047	1.33101577	0.18893382	0.99978775	-4.4998732
RHOBTB2	0.17982984	2.92716313	1.07846671	0.28575439	0.99978775	-4.5907567
CHRNE	0.17971835	2.53860263	0.78144042	0.4380524	0.99978775	-4.6454762

CHMP2B	0.17969359	3.98627689	1.28918022	0.20298514	0.99978775	-4.5239405
PXDC1	0.17969155	4.37360463	0.56101737	0.57717181	0.99978775	-4.7702137
BTBD1	0.1795917	5.52293114	1.73687486	0.08827221	0.99978775	-4.2436579
ARV1	0.17956622	3.69382754	0.86184783	0.39268973	0.99978775	-4.6616119
IGFALS	0.17941589	-1.0833036	0.1783485	0.85913554	0.99978775	-4.6094476
PRKD1	0.1793785	2.92344083	0.70763527	0.4823006	0.99978775	-4.6969993
POLR2B	0.17933009	6.56910268	2.00469623	0.05016343	0.99978775	-4.0208813
HELB	0.17928117	2.69475158	1.12523134	0.26560758	0.99978775	-4.5806399
TMEM100	0.17925219	2.15816097	0.47921948	0.63377211	0.99978775	-4.6627837
TNFAIP8L1	0.17909662	-0.8995916	0.31727719	0.75229131	0.99978775	-4.6129723
ENSCAFG00000031559	0.17889039	0.56390203	0.77415704	0.44230937	0.99978775	-4.6114074
SPATA24	0.17881881	0.98958996	0.5842558	0.56154979	0.99978775	-4.6270877
ENSCAFG00000032311	0.17871351	-0.3235318	0.33863817	0.73623261	0.99978775	-4.6197332
RWDD2A	0.1786799	2.19475044	1.22826771	0.22482295	0.99978775	-4.5653415
SLC35E1	0.17865094	4.56806748	1.37771899	0.17413316	0.99978775	-4.4765714
RFLNB	0.17861556	1.11709849	0.51012236	0.61210321	0.99978775	-4.6379079
FAT1	0.17856182	8.73661498	0.91676356	0.36345761	0.99978775	-4.6759733
ENSCAFG00000003845	0.17850322	-2.1353286	0.32251835	0.74834059	0.99978775	-4.6076167
SH3GLB2	0.17845768	4.37492713	0.92874902	0.3572686	0.99978775	-4.6561475
FICD	0.17842525	3.84612225	0.98249961	0.33035875	0.99978775	-4.6334741
PLOD1	0.17816409	8.39772801	1.30579046	0.19731522	0.99978775	-4.5118272
ENSCAFG00000004412	0.17804927	0.74633993	0.61113252	0.54374824	0.99978775	-4.6216506
ENSCAFG00000016002	0.17786413	1.972373	0.92857444	0.35735826	0.99978775	-4.6173354
ELOVL4	0.17785199	5.00900873	0.59147999	0.55673653	0.99978775	-4.8161801
GINM1	0.17783396	5.77169409	1.606652	0.11412766	0.99978775	-4.3268924
TEX9	0.17773459	1.18475683	0.65629875	0.51449748	0.99978775	-4.627233
TLDC1	0.17766322	2.32321393	0.80796436	0.42275601	0.99978775	-4.630407
LYST	0.17762154	4.53698265	1.08846657	0.28135933	0.99978775	-4.6068271
ATRX	0.17754403	6.92662791	1.53103283	0.13176355	0.99978775	-4.3746911
TOMM20	0.17743655	5.01511152	1.98310101	0.0525969	0.99978775	-4.0766306
APP	0.17742998	10.6442741	1.09241888	0.27963532	0.99978775	-4.5980677
OPN3	0.17741453	2.67002514	0.53490881	0.59497072	0.99978775	-4.6719708
STXBP5	0.17741052	5.8511128	1.28902891	0.20303735	0.99978775	-4.5221767
PRDM16	0.17734507	1.12332262	0.60814979	0.54570949	0.99978775	-4.6896535
ADGRD1	0.17730712	2.08274581	0.31043512	0.75745883	0.99978775	-4.7467305
NT5C2	0.17730531	5.49018311	1.50179773	0.13913887	0.99978775	-4.3958157
HMCN1	0.17705565	1.39934834	0.26179811	0.79450124	0.99978775	-4.6384684
ENSCAFG00000003316	0.17701745	0.68646894	0.66878587	0.50656147	0.99978775	-4.6319634
ENSCAFG00000023721	0.17696371	6.62694341	1.83233822	0.0725739	0.99978775	-4.1595412
NXPH3	0.17679051	4.24909183	0.54152725	0.59043441	0.99978775	-4.7639235
ENSCAFG00000002440	0.17655701	0.6701969	0.63440064	0.52857348	0.99978775	-4.6262925
DICER1	0.176498	6.32248044	1.38922691	0.17062666	0.99978775	-4.4636678
PARL	0.17645565	5.65424739	1.67106274	0.10065906	0.99978775	-4.2834668
NCSTN	0.17634543	7.55957506	1.28971453	0.20280088	0.99978775	-4.520999
TACC1	0.17626278	7.67380347	1.6160563	0.11207459	0.99978775	-4.3276386
SPATA6	0.17616393	2.92644589	0.83652811	0.40664837	0.99978775	-4.6459909
TAB2	0.17610846	6.82728458	1.62759509	0.10959659	0.99978775	-4.3084823
TCP11L1	0.17605738	4.15193878	1.17274784	0.24618926	0.99978775	-4.5736029
CREM	0.17580452	2.8101157	1.00414375	0.31991389	0.99978775	-4.6068259
MYNN	0.17575208	3.76238124	1.36189563	0.17904465	0.99978775	-4.4935283

NBR1	0.17572893	7.40376074	1.8605472	0.0684115	0.99978775	-4.1415971
SACS	0.17572513	7.02731052	0.72964174	0.46885136	0.99978775	-4.7645521
SOBP	0.17557995	3.84192441	0.9666705	0.33813965	0.99978775	-4.6516518
CCNK	0.17556259	4.39915163	2.26184979	0.02787216	0.99978775	-3.9220258
DCP1A	0.17550972	3.52831647	1.28694275	0.20375814	0.99978775	-4.5302752
ENSCAFG00000004887	0.17540682	3.37497534	0.99485975	0.32436658	0.99978775	-4.6188725
ENSCAFG00000030446	0.17538844	1.19166894	0.84334353	0.4028614	0.99978775	-4.6142006
AGO4	0.17531196	4.30882395	0.82998638	0.41030375	0.99978775	-4.6804413
CEACAM1	0.17520188	3.46614839	0.64091466	0.52436531	0.99978775	-4.7136978
NUBPL	0.17519025	2.2396784	0.85276835	0.39766049	0.99978775	-4.6235031
DLC1	0.17515214	7.25143772	1.42613198	0.15974815	0.99978775	-4.4412824
TBC1D10A	0.1750579	2.91959389	1.17145006	0.24670565	0.99978775	-4.5694019
HTR1D	0.17497198	1.69755989	0.68310396	0.49754391	0.99978775	-4.7205715
CAMK1D	0.17488902	3.44300891	0.85486246	0.39651058	0.99978775	-4.6723609
DUSP12	0.17485631	4.95222186	1.68992913	0.09697029	0.99978775	-4.2898917
RBPMS	0.17485356	5.91391169	1.00513744	0.31943976	0.99978775	-4.6665181
MFGE8	0.17470301	9.33390146	1.04278453	0.30182492	0.99978775	-4.6290698
RBM41	0.17460703	1.95803895	1.04879418	0.29907578	0.99978775	-4.5936114
PLGRKT	0.17460076	2.41804893	0.99046053	0.32649092	0.99978775	-4.6058731
UST	0.17457962	6.89015025	1.0944812	0.27873867	0.99978775	-4.6250862
VAMP7	0.17451677	6.35241486	1.57246244	0.12184949	0.99978775	-4.3462006
FANCL	0.17450944	3.43741073	1.4489051	0.15330939	0.99978775	-4.4605665
NFU1	0.17444147	4.63159093	1.9790496	0.05306457	0.99978775	-4.0996656
GNB1	0.17439437	8.14382824	2.72749151	0.00865769	0.99978775	-3.4396432
SLC9A7	0.17433585	-1.8070383	0.27268717	0.78616311	0.99978775	-4.6101951
ACTN1	0.17419761	10.3015653	0.65166382	0.51745998	0.99978775	-4.7227536
CCNYL1	0.17407976	4.98500287	0.81047963	0.42132234	0.99978775	-4.7169392
HSPB2	0.17385391	3.22070017	0.93891907	0.35207089	0.99978775	-4.6499595
LGMN	0.17379067	6.8359929	0.9940261	0.32476843	0.99978775	-4.6704857
TVP23B	0.17351692	3.23453659	1.32450909	0.19106946	0.99978775	-4.5199822
RIOK2	0.17340055	5.5925566	1.36994581	0.17653283	0.99978775	-4.4755503
EOGT	0.17337301	6.29754413	1.67142676	0.10058681	0.99978775	-4.2776904
SLC36A4	0.17328858	6.2550681	1.16726765	0.24837517	0.99978775	-4.588359
CTSB	0.17322709	9.44573498	1.1043277	0.27448538	0.99978775	-4.603087
MTA3	0.17322349	3.65520741	1.32941293	0.18945822	0.99978775	-4.5071466
ANKRD12	0.1730684	6.61714165	1.31844945	0.19307476	0.99978775	-4.5055395
VPS29	0.17306838	4.64942825	1.68167142	0.09857093	0.99978775	-4.304862
ATP6V1F	0.17304034	5.31029956	1.18704947	0.24055013	0.99978775	-4.5743792
GLRX2	0.17301481	3.4805532	1.11707357	0.2690477	0.99978775	-4.5852077
UBR7	0.17281124	5.67373362	2.04154412	0.04623511	0.99978775	-4.0022555
CACYBP	0.1727227	6.40543053	1.74098378	0.08754271	0.99978775	-4.2269196
SSR1	0.17267627	7.2649159	1.09329069	0.27925604	0.99978775	-4.6230235
DHRS9	0.17266029	2.89643423	0.23713238	0.81347674	0.99978775	-4.661861
OCIAD1	0.17242089	7.00819649	2.13247388	0.03766263	0.99978775	-3.9195097
ATP11B	0.1722792	7.32132143	2.25299893	0.02846186	0.99978775	-3.8209567
ENSCAFG00000000341	0.17221066	3.30071323	1.07414261	0.28766959	0.99978775	-4.5939907
SH3GL2	0.17211339	-2.0771849	0.28492108	0.77682525	0.99978775	-4.608553
SEC24C	0.17210155	6.9140429	2.31438405	0.02459	0.99978775	-3.7533626
FRMD4B	0.17207387	2.80072591	0.37578025	0.70859401	0.99978775	-4.696882
NOXA1	0.17189754	1.49739028	0.63163383	0.53036623	0.99978775	-4.6360115

SPTAN1	0.17182922	9.80947261	1.40024105	0.16732182	0.99978775	-4.4675217
CD81	0.17178859	8.03363885	0.85304192	0.39751016	0.99978775	-4.7203157
PLIN2	0.1717725	5.89618654	1.39471721	0.16897304	0.99978775	-4.4612102
TM2D2	0.17164541	3.68999564	1.23741398	0.22143789	0.99978775	-4.5446713
TSG101	0.17149703	5.97415286	1.88376326	0.06513898	0.99978775	-4.1211688
EPAS1	0.17145932	7.56597169	0.93788909	0.35259504	0.99978775	-4.6904012
ENSCAFG00000029756	0.17144471	2.18637631	0.87623852	0.384891	0.99978775	-4.6431763
CLCN3	0.17138009	5.92331722	1.67687999	0.09950959	0.99978775	-4.2741698
ENSCAFG00000013802	0.17131665	0.77224456	0.75206763	0.45536811	0.99978775	-4.6230745
MARCH6	0.17125421	6.41475695	1.77016091	0.08250527	0.99978775	-4.2055819
MARCH5	0.17121113	5.04240316	1.8413024	0.07122864	0.99978775	-4.1843897
CACNA1C	0.17119117	4.60184084	0.80973454	0.42174673	0.99978775	-4.7408506
C7H1orf74	0.17116747	2.66978673	1.19917058	0.23584456	0.99978775	-4.5636583
SKIL	0.17102679	5.61092074	0.76595378	0.44713304	0.99978775	-4.7593487
ENSCAFG00000020250	0.1709117	4.14759999	1.2730501	0.20860716	0.99978775	-4.5283602
FGD6	0.17086237	3.79469083	0.55556653	0.58086642	0.99978775	-4.7316864
FAM131A	0.17079744	1.7067872	0.91738769	0.36313363	0.99978775	-4.6101759
FAM126A	0.17076697	6.96943306	1.05357413	0.29690149	0.99978775	-4.6426416
MT-CO1	0.17074052	13.6886202	1.1644767	0.24949376	0.99978775	-4.5703609
RMC1	0.17070339	4.26164886	1.40573774	0.16569113	0.99978775	-4.4638042
ZFYVE27	0.17060668	4.33457928	1.36064816	0.17943632	0.99978775	-4.487004
RNF19A	0.17059023	6.97991733	1.01106338	0.31662205	0.99978775	-4.6636548
ENSCAFG00000024769	0.17042	-2.3961346	0.31692459	0.75255734	0.99978775	-4.6092776
MPP5	0.17036126	3.36690027	0.99589894	0.32386612	0.99978775	-4.6149809
STXBP2	0.17034524	5.43222541	1.47262596	0.14682042	0.99978775	-4.414852
STARD8	0.17027207	1.47158562	0.56394206	0.57519415	0.99978775	-4.6421554
ENSCAFG00000017239	0.17011128	0.26189758	0.52219092	0.60373328	0.99978775	-4.6212722
CCDC88C	0.17005683	-0.4563733	0.25678924	0.79834488	0.99978775	-4.6142784
MYEF2	0.17002288	3.0768774	0.77108905	0.44410982	0.99978775	-4.6610207
B3GNT4	0.16999171	0.41109718	0.4228226	0.67415112	0.99978775	-4.6194492
RNF149	0.16994212	3.1443309	0.89903151	0.37273978	0.99978775	-4.625298
YDJC	0.16990322	-1.2880352	0.32237763	0.74844657	0.99978775	-4.6096996
ISG15	0.16987684	-1.744622	0.32314378	0.7478696	0.99978775	-4.6109155
UBE2G2	0.16961164	5.92182277	2.0337899	0.0470389	0.99978775	-4.0068289
ANO6	0.16931465	8.37409462	1.89697372	0.06333683	0.99978775	-4.1460388
SLC41A2	0.16914647	4.79582849	1.12834948	0.26430094	0.99978775	-4.5932961
TM2D1	0.16910298	3.447889	1.36749526	0.17729457	0.99978775	-4.5008812
ENSCAFG00000028699	0.16906588	-1.2208166	0.2963159	0.76815756	0.99978775	-4.6129328
LPIN2	0.16877069	5.55251613	1.69102348	0.09675978	0.99978775	-4.2672848
KLHDC8B	0.16876675	1.83677859	0.35002297	0.72772143	0.99978775	-4.6457674
FGL2	0.16871537	6.55268757	0.36182576	0.71893411	0.99978775	-4.8429254
HERPUD2	0.16870197	6.19514705	1.6820716	0.09849287	0.99978775	-4.2722051
SLC4A8	0.16858571	2.73231764	1.29463108	0.20111118	0.99978775	-4.5332152
UBIAD1	0.16851399	3.88151408	1.00816998	0.31799572	0.99978775	-4.6253034
ENSCAFG00000029395	0.16845269	6.50646409	2.15442041	0.03581484	0.99978775	-3.895475
HSPB11	0.16830518	1.05890446	0.68974359	0.49339228	0.99978775	-4.6178342
GPBP1	0.16827893	5.25818686	1.68573572	0.09778043	0.99978775	-4.2822215
FAM107A	0.16826949	-1.2756894	0.23166446	0.81769898	0.99978775	-4.6098975
IRS2	0.1682574	5.73999291	0.53374395	0.59577082	0.99978775	-4.8276603
LTN1	0.16823053	5.51381985	1.53366941	0.13111401	0.99978775	-4.3766023

CGRRF1	0.16809486	3.66897143	1.4570066	0.1510684	0.99978775	-4.4641016
C11H5orf15	0.16803893	6.58202444	1.73216066	0.08911538	0.99978775	-4.2334064
ATAD1	0.16798337	5.98056513	1.62612358	0.10991011	0.99978775	-4.3110069
MRPS31	0.16798107	6.07177832	1.62596804	0.10994329	0.99978775	-4.3095472
ASAP1	0.1679241	7.09242465	1.12519934	0.26562101	0.99978775	-4.6080274
GCA	0.16781341	0.29789263	0.43046	0.6686226	0.99978775	-4.621321
CSGALNACT2	0.16774106	6.02352177	0.97430823	0.33437027	0.99978775	-4.6802635
IFT43	0.16772277	3.59254099	0.99075187	0.32634994	0.99978775	-4.628692
STOML1	0.16772002	3.44473457	0.87503405	0.38553998	0.99978775	-4.6552568
PIGG	0.16771741	5.25942036	1.47577804	0.14597466	0.99978775	-4.4146704
MPP4	0.16766445	0.23161064	0.42166217	0.67499271	0.99978775	-4.6340065
ELF1	0.16763292	5.9373042	1.43282209	0.15783516	0.99978775	-4.4374364
ENSCAFG00000031526	0.16752743	3.70575076	0.99707432	0.3233007	0.99978775	-4.6256396
DHX36	0.16745447	5.28743289	1.47600659	0.14591348	0.99978775	-4.4124912
SLC25A36	0.16726042	6.46959731	1.27210147	0.20894138	0.99978775	-4.5318438
ABHD1	0.16720798	2.04533147	0.4395572	0.66206144	0.99978775	-4.644469
SPOPL	0.16717749	5.34259477	1.23594728	0.22197818	0.99978775	-4.5482844
ENSCAFG00000013213	0.16709963	-0.2113297	0.48120775	0.6323679	0.99978775	-4.622141
ATP7A	0.16705052	5.56374161	1.57349292	0.12161075	0.99978775	-4.3520929
MED6	0.16687775	3.93273112	1.3439638	0.18473793	0.99978775	-4.5035634
SLC2A6	0.16674589	2.17677359	0.55757122	0.57950631	0.99978775	-4.6472481
ENSCAFG00000010850	0.16670809	3.07076919	1.24012555	0.22044159	0.99978775	-4.5464238
ENSCAFG00000002874	0.16667403	-0.6485109	0.39074216	0.69756853	0.99978775	-4.61503
HOXC10	0.1663986	-0.0424242	0.15390872	0.87827165	0.99978775	-4.6287392
TPT1	0.16633133	11.1915105	1.39128118	0.17000648	0.99978775	-4.4840249
ENSCAFG00000030425	0.16632957	1.82952446	0.71123644	0.48008509	0.99978775	-4.6519187
WDR53	0.16619719	2.32830315	0.97068075	0.33615702	0.99978775	-4.6066132
DHRSX	0.1661278	3.89811475	1.41135157	0.16403841	0.99978775	-4.4683438
ENSCAFG00000014207	0.16612536	6.89738354	1.37762353	0.17416248	0.99978775	-4.4707355
MICU3	0.16612528	2.62920242	0.90083482	0.37178896	0.99978775	-4.631303
ENSCAFG00000020158	0.16609536	0.19233806	0.46328459	0.64507475	0.99978775	-4.621061
HAVCR1	0.16600229	0.911516	0.57097419	0.57045259	0.99978775	-4.6248416
RFX3	0.16598456	1.70650162	0.76607489	0.4470616	0.99978775	-4.625937
MRPL13	0.16585777	3.93955024	1.56139502	0.12443731	0.99978775	-4.3987044
GCKR	0.16560028	1.58591201	0.62165282	0.53685968	0.99978775	-4.6778534
TRAPPC2	0.16553383	2.93249743	1.27143558	0.20917622	0.99978775	-4.5408035
RNF146	0.16552027	5.22386947	2.25710899	0.02818666	0.99978775	-3.8474143
TOB2	0.16543152	4.17310086	0.78364777	0.43676705	0.99978775	-4.6838103
DOK4	0.16518413	5.03563745	1.57532807	0.12118654	0.99978775	-4.3558566
EAF1	0.16518203	5.21309808	1.5488064	0.12743423	0.99978775	-4.3701472
KATNBL1	0.16509599	3.54605421	1.28046933	0.20600699	0.99978775	-4.5320912
MMP11	0.16500909	2.64416596	0.58660451	0.55998265	0.99978775	-4.6673061
MED14	0.16500906	5.8104352	1.10977174	0.27215345	0.99978775	-4.6153462
RBM3	0.16473495	6.27975772	1.00680083	0.31864714	0.99978775	-4.6662823
SNTB2	0.16470503	4.35740046	1.38076624	0.17319931	0.99978775	-4.4812076
AKTIP	0.16458871	4.34391248	1.38914795	0.17065053	0.99978775	-4.4743207
PEMT	0.16455154	3.28896822	1.10898953	0.27248764	0.99978775	-4.5893214
ENSCAFG00000000523	0.16453109	-0.2311015	0.4843633	0.63014211	0.99978775	-4.6205674
TEC	0.16442458	-0.0691801	0.44058786	0.66131977	0.99978775	-4.6369116
VPS4B	0.16438374	5.38015715	2.25865657	0.02808365	0.99978775	-3.8343702

CHSY1	0.164358	6.4653296	0.88937192	0.37785929	0.99978775	-4.7163567
UCP1	0.16435652	-1.5752366	0.322592	0.74828512	0.99978775	-4.611543
CPNE3	0.1643441	6.69281676	1.45960128	0.15035613	0.99978775	-4.4199946
USP14	0.16413434	6.6702279	1.00476501	0.31961741	0.99978775	-4.6671406
ENSCAFG00000018181	0.16383522	8.73277832	2.00695019	0.04991512	0.99978775	-4.0777272
ENSCAFG00000006946	0.16376302	6.04763338	1.91188687	0.0613535	0.99978775	-4.099238
FMNL2	0.16376301	5.77392353	1.05673708	0.29546872	0.99978775	-4.6396649
ENSCAFG00000007359	0.16373584	5.28233623	1.90254675	0.06258937	0.99978775	-4.1270479
CYB5R1	0.16364956	4.94484122	1.01738473	0.31363487	0.99978775	-4.6375314
APC2	0.16330327	1.23198628	0.53654568	0.59384728	0.99978775	-4.6362234
NDUFB3	0.16323029	4.69817304	1.92939689	0.05909258	0.99978775	-4.1390627
ENSCAFG00000031217	0.1630523	2.34123216	0.7468815	0.45846609	0.99978775	-4.6455808
GPR137B	0.16301983	3.13186341	0.67389044	0.50333649	0.99978775	-4.6639761
THAP9	0.16290853	1.40043699	0.50341717	0.61677618	0.99978775	-4.6280204
KIDINS220	0.16274948	7.96278709	1.5004841	0.1394778	0.99978775	-4.3994811
PEX3	0.1627362	4.06986256	1.44852678	0.15341467	0.99978775	-4.4489209
SMDT1	0.16272936	3.56797566	1.29435168	0.20120692	0.99978775	-4.5222363
PRDX3	0.16270337	4.87370676	1.86232154	0.06815658	0.99978775	-4.1789493
COX3	0.16247699	12.6092666	1.17783138	0.244174	0.99978775	-4.5659061
CCNI	0.16247219	7.39990692	1.55831308	0.12516573	0.99978775	-4.3594581
SH3GLB1	0.16227238	6.25800768	2.03210857	0.04721478	0.99978775	-3.998127
SPTLC1	0.16205988	5.91913582	1.72096301	0.09114493	0.99978775	-4.2454828
FBXO8	0.16196122	3.44224405	1.23960823	0.22063141	0.99978775	-4.5470472
VLDLR	0.16182501	4.84478496	0.43733427	0.66366224	0.99978775	-4.8430436
ENSCAFG00000032749	0.16181615	-1.0600395	0.4273789	0.67085072	0.99978775	-4.615208
BRD7	0.16176157	6.31774415	1.25975303	0.2133284	0.99978775	-4.5388427
AMOTL2	0.1616957	8.24591041	0.7960498	0.42958698	0.99978775	-4.7344705
GLA	0.16169304	2.62503678	0.92545084	0.35896486	0.99978775	-4.6187546
CBX7	0.16167157	3.50274451	0.55096212	0.58399617	0.99978775	-4.6936486
CDADC1	0.16166387	3.74376722	1.43885878	0.15612437	0.99978775	-4.4702415
SEC61A2	0.16159457	3.03471987	1.46516676	0.14883722	0.99978775	-4.4772632
FKBP1B	0.16154787	1.57970419	0.41550636	0.67946418	0.99978775	-4.6421112
PDCD1LG2	0.16154081	-2.8637328	0.32196863	0.74875464	0.99978775	-4.6058551
GORASP2	0.16152202	7.25576254	2.15272075	0.03595504	0.99978775	-3.9069119
UGP2	0.16151022	6.85705833	0.92688797	0.35822511	0.99978775	-4.7005519
SDHC	0.1614802	5.32484887	1.63928621	0.1071315	0.99978775	-4.3115642
MIEF2	0.16146452	3.10430144	0.98632689	0.32849546	0.99978775	-4.6221929
TUSC3	0.16143486	6.86153188	1.04048928	0.30287945	0.99978775	-4.6494849
ASPH	0.1613863	7.45232182	1.51284151	0.13631524	0.99978775	-4.3878191
GYS1	0.16122873	5.70729838	1.00984279	0.31720104	0.99978775	-4.6546368
DNAJC4	0.16114135	4.89883142	0.78505708	0.43594758	0.99978775	-4.7271292
RAB1A	0.16109743	7.03144563	2.4399553	0.01809956	0.99978775	-3.6405175
UBE2J1	0.16095322	7.38161765	1.52245475	0.13389456	0.99978775	-4.3825898
IKBIP	0.160945	6.11704476	1.25729865	0.21420845	0.99978775	-4.539906
ENSCAFG00000005922	0.16080823	0.25190484	0.45612107	0.65018367	0.99978775	-4.6256529
KLHL7	0.16078037	4.73678942	1.32774628	0.19000467	0.99978775	-4.5012309
ENSCAFG00000028473	0.16072586	3.2534964	0.70054235	0.486681	0.99978775	-4.6894957
NFIA	0.16064898	4.33073645	0.95837133	0.34226716	0.99978775	-4.6536319
TOLLIP	0.16062341	6.73075868	1.4205355	0.16136224	0.99978775	-4.4444912
KIF5C	0.16056331	5.38857135	0.62557364	0.53430397	0.99978775	-4.7725382

OAZ2	0.16053122	4.34549107	1.37824076	0.17397299	0.99978775	-4.4828228
TRPC4	0.16043004	0.86298581	0.48331953	0.63087796	0.99978775	-4.7551925
ENSCAFG00000030944	0.16042004	0.49009225	0.60773751	0.54598087	0.99978775	-4.6200115
MT-ND4	0.16039371	12.4706059	0.97072431	0.33613553	0.99978775	-4.6249266
ENSCAFG00000004735	0.16039227	4.55710352	1.44449116	0.15454125	0.99978775	-4.4448991
CNIH4	0.16032815	2.68639374	0.92590216	0.35873244	0.99978775	-4.6237123
RNF144B	0.16023086	1.17017349	0.30363548	0.76260539	0.99978775	-4.6594145
PTPRN	0.16012762	4.85120613	0.38920319	0.69869963	0.99978775	-4.8261691
TAGLN	0.16012257	10.6537944	0.41741312	0.6780779	0.99978775	-4.7635263
FAM114A1	0.16007254	7.05240889	1.28425847	0.20468842	0.99978775	-4.5249253
MSTO1	0.15998293	4.34607113	1.29115459	0.20230486	0.99978775	-4.5192534
C12H6orf89	0.15993209	5.00074236	1.36879377	0.17689062	0.99978775	-4.4774218
NFATC2	0.15978404	-0.889985	0.24385471	0.80829351	0.99978775	-4.6171912
GOLGA7	0.15968092	5.97606342	2.28181946	0.02658131	0.99978775	-3.7881835
ALKBH1	0.15957109	3.34453167	1.17142758	0.24671461	0.99978775	-4.5679659
ENSCAFG00000031478	0.15947936	5.15532994	1.31942515	0.19275081	0.99978775	-4.5044884
DDX3X	0.15920108	8.23551731	2.26706557	0.02752975	0.99978775	-3.8488701
WDR6	0.15916504	5.97883667	1.4877707	0.14279182	0.99978775	-4.402399
PRELP	0.15909679	6.89938074	0.29703404	0.76761227	0.99978775	-4.8714731
PCMTD2	0.1589895	4.57060559	0.93302875	0.35507529	0.99978775	-4.6604435
TMEM248	0.15898696	6.76452472	1.60276275	0.11498558	0.99978775	-4.3257345
AMPH	0.15874443	-1.320902	0.22602723	0.82205766	0.99978775	-4.6169431
PGAP3	0.15869123	2.09953659	0.89618097	0.37424593	0.99978775	-4.6185432
ENSCAFG00000017475	0.15867914	1.0644551	0.63947079	0.52529655	0.99978775	-4.6210333
ENSCAFG00000001921	0.15857457	0.188193	0.48057419	0.63281521	0.99978775	-4.6197772
TMEM33	0.15853703	4.97116528	1.40889953	0.16475872	0.99978775	-4.4546864
FBXO21	0.15851442	6.76748626	2.2254402	0.03036939	0.99978775	-3.8331744
ENSCAFG00000007869	0.15851215	0.47957227	0.51697096	0.607347	0.99978775	-4.6314552
CSPP1	0.15843922	4.24120868	1.23656656	0.22174994	0.99978775	-4.5442263
ENSCAFG00000005099	0.15838023	3.49115537	0.77747948	0.44036447	0.99978775	-4.6717748
ENSCAFG00000003159	0.15812173	1.10699496	0.60767239	0.54602373	0.99978775	-4.6355052
ENSCAFG00000030603	0.15808554	2.75974742	1.13513153	0.2614747	0.99978775	-4.5779964
TMEM87B	0.15808046	4.74471874	1.15582042	0.25298616	0.99978775	-4.5856938
SEC14L1	0.15804951	6.2730738	0.9156779	0.36402161	0.99978775	-4.7056413
TEX264	0.15802752	4.80424506	1.05163091	0.2977841	0.99978775	-4.6296506
PTPRG	0.15798601	7.12250536	0.6515962	0.51750327	0.99978775	-4.7983344
ENSCAFG00000014066	0.15795534	3.54498444	0.96440512	0.33926304	0.99978775	-4.6328191
MRTFA	0.15789848	7.50859739	0.75286219	0.45489454	0.99978775	-4.7574605
BBX	0.15782527	6.21312983	1.19736274	0.23654211	0.99978775	-4.5709771
DHRS11	0.15779831	1.58651615	0.66439025	0.50934747	0.99978775	-4.6570184
KLHL28	0.15772076	4.3907547	1.01591952	0.31432555	0.99978775	-4.6295337
ENSCAFG00000029067	0.15766874	3.26295164	0.72678732	0.4705837	0.99978775	-4.6789781
TMEM70	0.15756374	4.74512806	1.10515221	0.27413131	0.99978775	-4.6025052
ZMYM2	0.15753068	5.4439436	1.29807113	0.19993522	0.99978775	-4.5163381
B4GALT3	0.15751838	4.31974515	1.20727861	0.23273454	0.99978775	-4.5582427
ENSCAFG00000029412	0.15744744	0.44948707	0.44361302	0.6591448	0.99978775	-4.6245072
NTN4	0.15743808	6.77785762	0.53244489	0.59666368	0.99978775	-4.8265338
MXRA8	0.1574201	6.96465466	0.41073646	0.68293696	0.99978775	-4.8405104
SRRM3	0.15727879	-1.0323056	0.1202736	0.9047254	0.99978775	-4.6088981
ZNF821	0.1572463	2.21708278	0.74255792	0.46105809	0.99978775	-4.6391409

GOT2	0.1572243	5.74512784	1.22203187	0.22715256	0.99978775	-4.5576881
ANKRD9	0.157087	1.42609208	0.36340641	0.71776017	0.99978775	-4.6436725
DEPP1	0.15695745	-1.4569014	0.16085373	0.87282581	0.99978775	-4.6074763
GMFG	0.15694732	0.59593208	0.40169357	0.68953967	0.99978775	-4.6239193
FIG4	0.15691101	4.88222198	1.72530737	0.09035303	0.99978775	-4.2634727
ZNF619	0.15690415	2.05947604	0.66323232	0.51008276	0.99978775	-4.6478344
FUNDC2	0.15680369	5.13967041	1.54751675	0.12774449	0.99978775	-4.3749212
MYO1B	0.15675799	7.94462072	1.0269535	0.30914951	0.99978775	-4.647135
SLCO3A1	0.15670666	4.37574886	0.55109962	0.58390259	0.99978775	-4.7981243
H6PD	0.15670026	6.7377853	0.61220289	0.54304531	0.99978775	-4.8100072
ENSCAFG00000007159	0.15667745	-0.4633292	0.37265817	0.71090272	0.99978775	-4.6152982
TMEM127	0.15667469	4.63802511	0.92727671	0.35802517	0.99978775	-4.672056
SEMA3G	0.15664044	0.33017853	0.0804508	0.93618454	0.99978775	-4.6080887
UBXN2B	0.15661596	6.64398807	1.60355999	0.1148093	0.99978775	-4.3251499
KRBA2	0.15653271	2.24262788	0.82590641	0.41259368	0.99978775	-4.6310394
LCLAT1	0.156516	5.43161674	0.99835216	0.32268674	0.99978775	-4.6597049
TSPYL2	0.15639812	4.80183417	0.88516831	0.38010102	0.99978775	-4.6736568
TMEM132E	0.15637524	1.29220235	0.26569991	0.79151066	0.99978775	-4.7146246
FAM177A1	0.15622155	3.16887811	1.052339	0.29746227	0.99978775	-4.6027604
LIPA	0.15617238	5.59136837	1.00277085	0.32056974	0.99978775	-4.6576882
SUMO2	0.15609218	5.64767193	2.122697	0.03851231	0.99978775	-3.9396066
TRAPPC3	0.15603888	5.18522267	1.83831246	0.07167499	0.99978775	-4.1743066
MARK1	0.15603464	4.28255676	0.74022107	0.46246254	0.99978775	-4.7303525
ZNF785	0.15589945	2.06546549	0.94781752	0.34756368	0.99978775	-4.6095209
INPP5F	0.15588897	5.17647598	0.9940479	0.32475792	0.99978775	-4.6558997
UBE2V1	0.15574843	5.88260353	1.60712886	0.11402283	0.99978775	-4.3250471
SNX10	0.15564771	6.2944721	0.82534574	0.41290897	0.99978775	-4.7412223
SYAP1	0.15559218	5.56655823	1.0983919	0.27704391	0.99978775	-4.6205243
ANKRD28	0.15554161	6.50320564	0.76695439	0.44654302	0.99978775	-4.7595619
PLAA	0.15550863	5.86871232	1.53833079	0.12997191	0.99978775	-4.3702566
ENSCAFG00000017975	0.15539625	2.55428334	0.90264977	0.37083356	0.99978775	-4.6242463
ENSCAFG00000032642	0.15535172	4.81827932	1.33058861	0.18907347	0.99978775	-4.4994767
FAM160A1	0.15532063	0.21672461	0.29488889	0.76924143	0.99978775	-4.630113
SNAPC1	0.15529876	3.96562202	1.28309736	0.20509179	0.99978775	-4.5257345
TPRG1L	0.15529162	4.90628234	1.82466925	0.0737417	0.99978775	-4.2073798
ABCA9	0.15524559	4.60210957	0.17781377	0.85955336	0.99978775	-4.7335761
UBR1	0.15517899	6.18298589	1.34356506	0.18486607	0.99978775	-4.4908782
SPTBN1	0.1551352	9.71595261	1.18505705	0.24133008	0.99978775	-4.5681324
CBLB	0.1550436	6.72806398	0.64448248	0.52206795	0.99978775	-4.8008451
FZD7	0.15474131	3.73686149	0.52440071	0.60220648	0.99978775	-4.7628631
CLIC2	0.15462742	2.58424165	0.39640085	0.69341553	0.99978775	-4.6459743
CD164	0.15451554	9.05782229	1.47963679	0.14494451	0.99978775	-4.4209903
PRPF4	0.15439674	3.53454838	1.20548189	0.23342112	0.99978775	-4.5564644
MAML2	0.15436656	5.65801788	0.7031507	0.48506759	0.99978775	-4.7690931
C1S	0.15431996	5.06811884	0.23636452	0.81406934	0.99978775	-4.8642694
KDM7A	0.15419082	3.69420687	0.69674257	0.48903671	0.99978775	-4.6599649
RIBC1	0.15417033	0.38037949	0.49659504	0.6215471	0.99978775	-4.6212604
FAM184A	0.15412486	-0.3389503	0.36590827	0.71590344	0.99978775	-4.6256516
ENSCAFG00000024878	0.15411245	-0.6505059	0.33471023	0.73917691	0.99978775	-4.6126799
BACH2	0.15405354	-1.1003665	0.39635192	0.6934514	0.99978775	-4.6191498

UBE2D2	0.1540356	6.18096034	1.73851465	0.08798048	0.99978775	-4.2289015
NXPE3	0.15400891	5.46361856	1.62984594	0.10911845	0.99978775	-4.3171381
TCF4	0.1539681	5.97604042	0.87007254	0.38822052	0.99978775	-4.7224267
PRKAA1	0.15394118	5.54579889	1.1763872	0.2447453	0.99978775	-4.5802181
ENSCAFG00000019170	0.15391392	5.01042382	1.49581703	0.14068723	0.99978775	-4.4127137
COX18	0.15380438	4.22944235	1.22934943	0.22442063	0.99978775	-4.5481757
MIER3	0.15374144	3.34533705	1.06370144	0.29233079	0.99978775	-4.5990144
MMD	0.15369769	4.14193653	0.52464017	0.60204113	0.99978775	-4.7534469
TRAF3IP2	0.15359992	4.21684881	0.86612377	0.39036224	0.99978775	-4.6811775
ENSCAFG00000024834	0.15347147	-1.1526063	0.25095754	0.80282629	0.99978775	-4.61372
PIGT	0.15336167	7.45080468	1.0759082	0.28688651	0.99978775	-4.6294855
SGMS2	0.15335502	3.39507201	0.93619427	0.35345863	0.99978775	-4.6334252
MICAL2	0.15334598	7.98961399	0.72952695	0.46892095	0.99978775	-4.7484885
FAR1	0.15330442	6.99319197	1.21233973	0.23080846	0.99978775	-4.5633603
CSNK1G3	0.153253	6.09513229	1.6445292	0.10604078	0.99978775	-4.2977844
IFNLR1	0.15316153	-1.2739873	0.3669267	0.71514812	0.99978775	-4.6201794
PPP1R26	0.15315738	3.62395567	0.74227204	0.46122977	0.99978775	-4.7230667
CCDC32	0.15315177	1.96487669	0.76501735	0.44768563	0.99978775	-4.623896
ENSCAFG00000006913	0.15313046	5.87016944	1.10567338	0.27390766	0.99978775	-4.6173446
PSMC2	0.15308639	6.61823489	1.13844384	0.26010222	0.99978775	-4.6030194
ANXA1	0.15298371	10.0836114	1.21233034	0.23081202	0.99978775	-4.5549614
SLC45A3	0.15295074	3.76774704	0.4476406	0.65625374	0.99978775	-4.7518718
SERPINF1	0.15288486	9.40887986	0.41448524	0.68020704	0.99978775	-4.8091513
ZNF791	0.1528711	1.93942939	0.71850939	0.47562807	0.99978775	-4.6283949
NDFIP2	0.15285382	5.67794404	1.45589005	0.15137572	0.99978775	-4.4245101
ENSCAFG00000003645	0.15281163	0.41349227	0.59061945	0.55730879	0.99978775	-4.6247445
ENSCAFG00000022137	0.15279646	0.49858729	0.35694198	0.72256559	0.99978775	-4.6484299
MMP23	0.15275679	4.43915386	0.29340472	0.77036922	0.99978775	-4.7814967
CRAMP1	0.15261292	4.76753854	1.18456211	0.24152412	0.99978775	-4.5683064
VPS8	0.15254721	6.24396825	2.01181408	0.04938291	0.99978775	-4.0154234
FUCA2	0.15243357	5.35138297	0.95500006	0.34395326	0.99978775	-4.6805671
RAP2A	0.1524075	6.70700127	1.43454341	0.15734586	0.99978775	-4.4358382
RETREG3	0.15234611	5.07287677	1.18175117	0.24262824	0.99978775	-4.5722213
TGFBR2	0.15229663	9.40746645	0.53368525	0.59581115	0.99978775	-4.7966127
GPATCH8	0.1521783	6.9037468	1.59376513	0.11699036	0.99978775	-4.3320998
VWA5B2	0.15209697	1.24097358	0.30066926	0.76485388	0.99978775	-4.6274876
ENSCAFG00000023394	0.15208289	3.20097375	1.14800661	0.25616862	0.99978775	-4.5747079
ACAD11	0.15208056	5.95391285	1.90691117	0.06200927	0.99978775	-4.102049
CCNG2	0.15204589	4.70148413	0.73559247	0.46525157	0.99978775	-4.7204785
POP7	0.1520297	2.14294413	0.76186429	0.44954919	0.99978775	-4.634804
DERL2	0.15199585	4.66899888	1.38219271	0.17276348	0.99978775	-4.473169
ENSCAFG00000003956	0.15199549	-0.0378381	0.5552069	0.58111058	0.99978775	-4.6292524
GORAB	0.15197345	4.60596181	0.96610909	0.33841782	0.99978775	-4.658772
RNF6	0.15169693	6.24228909	1.13345299	0.26217217	0.99978775	-4.6057934
C10H12orf45	0.15161404	3.44934914	0.99704902	0.32331286	0.99978775	-4.6184033
RNF217	0.15146507	2.46176122	0.81247302	0.42018823	0.99978775	-4.6354421
SLC26A2	0.15145557	2.52083306	0.78205478	0.43769443	0.99978775	-4.6407043
ADAM22	0.15140805	-1.3795888	0.28443044	0.77719912	0.99978775	-4.6146822
MAFF	0.15123331	5.63190088	0.67671554	0.50155645	0.99978775	-4.7739252
ENSCAFG00000011225	0.1506526	-0.3042085	0.46679545	0.64257711	0.99978775	-4.6255712

POLK	0.15063866	5.13474772	1.04886389	0.29904399	0.99978775	-4.6364074
LRRC8E	0.15057686	2.5374616	0.84357601	0.40273261	0.99978775	-4.6416034
PODXL2	0.15053833	0.35374641	0.44013456	0.66164592	0.99978775	-4.6183782
MPZL1	0.15036485	6.9573145	1.28086933	0.2058675	0.99978775	-4.5266266
ZNF248	0.15036287	3.93205621	0.9235151	0.35996283	0.99978775	-4.6470387
PUS7L	0.15036065	3.08923622	0.61499923	0.54121112	0.99978775	-4.6781871
UBE2G1	0.15032972	5.4638095	1.70972934	0.09321928	0.99978775	-4.2594469
TMED10	0.1503005	7.03397084	1.9638815	0.05484737	0.99978775	-4.0564861
CCDC107	0.15029278	2.44809915	0.78018786	0.43878276	0.99978775	-4.6379387
ENSCAFG00000018740	0.1501775	1.59556987	0.66360422	0.50984654	0.99978775	-4.6461984
ZNF629	0.15013856	3.26044183	0.92794236	0.35768299	0.99978775	-4.6328095
NCK1	0.15008183	4.798509	1.04959783	0.29870946	0.99978775	-4.6233824
VPS26A	0.15006205	6.26622465	1.79183143	0.07892229	0.99978775	-4.1894829
MRPS21	0.1500363	4.02810215	1.36236083	0.17889876	0.99978775	-4.4915445
ZNF471	0.15003359	0.77629063	0.3904175	0.69780709	0.99978775	-4.6287273
MGAT5	0.15002804	5.42264704	0.50245079	0.617451	0.99978775	-4.7872108
DNAJB5	0.14992559	0.1485985	0.46983999	0.64041457	0.99978775	-4.6246128
WDR1	0.14977179	9.29480325	1.2503729	0.21670631	0.99978775	-4.5384161
CREB3	0.14966551	6.7907859	1.17046497	0.24709815	0.99978775	-4.586339
WDFY3	0.14963877	6.89537902	1.25307322	0.21572985	0.99978775	-4.5422934
PTPN1	0.14962073	4.29488781	1.11580896	0.26958379	0.99978775	-4.5917129
TM9SF3	0.1495603	8.33941342	1.95689185	0.05568605	0.99978775	-4.0991243
SLC17A5	0.14954281	6.03917395	0.70838941	0.48183617	0.99978775	-4.7749263
OCRL	0.14952362	5.86774174	1.46455705	0.14900303	0.99978775	-4.4180383
RUNDC1	0.14950984	4.32653579	1.27861174	0.20665573	0.99978775	-4.5260218
RPS6KA5	0.1494232	2.22558818	0.56162228	0.57676251	0.99978775	-4.658639
UTP11	0.14942063	4.90797897	1.35652626	0.18073515	0.99978775	-4.485652
BRF2	0.14934733	2.53190682	1.05602394	0.29579135	0.99978775	-4.5954232
ENSCAFG00000007877	0.14931831	1.13860358	0.6995077	0.48732182	0.99978775	-4.6267999
SHOC2	0.14930306	6.2938323	1.62243242	0.11069974	0.99978775	-4.3120116
PITPNB	0.14929421	6.63671636	1.44933691	0.15318929	0.99978775	-4.4264706
EPS15	0.14929405	6.92870079	1.5134641	0.13615742	0.99978775	-4.3854069
TMEM41A	0.14915335	1.63680395	0.56744111	0.57283245	0.99978775	-4.648204
TLE4	0.14910463	5.39782978	1.10205379	0.27546355	0.99978775	-4.6165571
UGGT2	0.14903765	6.90486891	1.12938161	0.26386943	0.99978775	-4.6075949
TNFRSF1B	0.14882894	-0.246225	0.24584293	0.80676215	0.99978775	-4.6201241
LYSMD1	0.14875348	1.30767116	0.72487869	0.47174407	0.99978775	-4.6236007
PIGL	0.14864878	2.19784373	0.86928945	0.38864467	0.99978775	-4.6218286
ADAMTS15	0.14861719	-0.9308036	0.16114455	0.8725979	0.99978775	-4.6145378
HECTD4	0.14856782	5.86813068	1.04502254	0.30079912	0.99978775	-4.645762
CPNE8	0.14849739	3.24751514	0.77769415	0.44023898	0.99978775	-4.7324157
ZDHHC20	0.14845697	5.63365717	1.54326797	0.12877091	0.99978775	-4.3680845
ENSCAFG00000006342	0.14845063	-0.5608665	0.45228893	0.65292368	0.99978775	-4.6225894
LANCL1	0.14838705	7.82528809	1.69326611	0.09632956	0.99978775	-4.2706706
SLC30A6	0.14838311	4.04082219	1.16617738	0.24881171	0.99978775	-4.5718905
AQP1	0.1483443	2.97135612	0.12609653	0.90013705	0.99978775	-4.7020282
STX7	0.14832335	6.17019712	1.45892804	0.15054069	0.99978775	-4.4204506
TMCC1	0.14809567	4.96724402	1.13383479	0.26201341	0.99978775	-4.5937159
ZNF461	0.1480906	0.48062117	0.47062347	0.63985858	0.99978775	-4.6288724
POLB	0.14804692	3.61742111	1.23876578	0.22094079	0.99978775	-4.5442919

MBTPS1	0.14803386	8.24937645	2.1675719	0.03474607	0.99978775	-3.9219688
LMAN2L	0.14800004	3.86911802	0.76300959	0.44887176	0.99978775	-4.7224752
RABGGTB	0.14779091	4.51680807	1.19043728	0.23922814	0.99978775	-4.5658733
CDH2	0.14774292	8.7018933	0.41331054	0.68106201	0.99978775	-4.8001431
FAM129A	0.1475634	4.46518808	0.51262698	0.61036184	0.99978775	-4.8365251
FAM171A2	0.14754663	2.43209178	0.67516873	0.50253064	0.99978775	-4.6603413
KRIT1	0.14752445	4.57226156	1.20387464	0.23403655	0.99978775	-4.5580508
G3BP2	0.14751155	5.97819734	1.68128124	0.0986471	0.99978775	-4.2737103
GNAI3	0.14750256	6.57448065	1.11905949	0.26820735	0.99978775	-4.6128059
URI1	0.14727555	5.83092759	1.34179757	0.18543493	0.99978775	-4.4918834
ENSCAFG00000032711	0.14718353	6.76067718	1.26945186	0.20987699	0.99978775	-4.5333401
WHAMM	0.1471067	2.29996754	0.66084949	0.51159764	0.99978775	-4.6434764
GTF2A2	0.14700129	2.99607687	1.15517143	0.2532494	0.99978775	-4.5726804
PCDH7	0.14698306	6.51589269	0.33335986	0.74019003	0.99978775	-4.8647668
TNKS2	0.1469656	6.38256645	1.50597302	0.13806595	0.99978775	-4.3902348
GTDC1	0.14693893	2.83230626	0.97802239	0.33254737	0.99978775	-4.6122051
ZBTB14	0.14675764	3.39346943	1.06750939	0.29062482	0.99978775	-4.5979632
ATP2B1	0.14672795	6.70950643	0.83067682	0.409917	0.99978775	-4.7391663
DACT3	0.14667592	1.32010062	0.5933156	0.55551683	0.99978775	-4.6434102
SLC31A2	0.14658022	0.29692928	0.40670208	0.68587964	0.99978775	-4.6259084
SLC25A28	0.14651651	2.75601859	0.92540389	0.35898904	0.99978775	-4.6183611
FRMD4A	0.14640601	6.09281248	0.7180883	0.47588548	0.99978775	-4.7779144
DSEL	0.14628458	4.53111672	0.9358421	0.35363825	0.99978775	-4.6873291
USP46	0.14624217	2.05466355	0.89900245	0.37275511	0.99978775	-4.623567
GOLGA5	0.14608728	5.92039237	1.54171196	0.12914846	0.99978775	-4.3687579
CMIP	0.14607609	4.65413617	0.65822451	0.51326927	0.99978775	-4.737138
MED21	0.14600119	3.82490378	1.40714606	0.16527531	0.99978775	-4.4704389
ENSCAFG00000006376	0.14590749	5.18615938	1.45629125	0.15126524	0.99978775	-4.428984
ENSCAFG00000008311	0.14584877	3.81592425	1.00940912	0.31740693	0.99978775	-4.6262102
CHRNB1	0.14568678	2.31274962	0.51142616	0.61119644	0.99978775	-4.6604211
ENSCAFG00000010899	0.14568234	5.03512273	1.12556388	0.26546802	0.99978775	-4.6010441
BNIP1	0.14564902	2.75207498	0.89252254	0.37618461	0.99978775	-4.6290504
ABHD4	0.14559752	5.36185612	1.16631177	0.24875787	0.99978775	-4.5862382
PYGL	0.14549679	4.70748599	0.66475743	0.50911443	0.99978775	-4.727943
TBC1D14	0.14533525	4.20186309	0.99604805	0.32379436	0.99978775	-4.6315629
RMND1	0.14531028	3.90818832	1.69893079	0.09524987	0.99978775	-4.3361949
SH3PXD2A	0.14531013	6.11909422	0.82185245	0.41487672	0.99978775	-4.7410563
CCDC15	0.14515867	4.61647996	0.57862181	0.56531786	0.99978775	-4.7495276
SPSB1	0.1451209	5.31974778	0.72645281	0.47078695	0.99978775	-4.7692841
FHL1	0.14511051	7.73702252	0.47356741	0.63777126	0.99978775	-4.8123004
RDH10	0.14506513	6.39390103	0.4854816	0.62935413	0.99978775	-4.8422544
ENSCAFG00000032678	0.14505621	-0.3900857	0.53514575	0.59480804	0.99978775	-4.6181188
PSMC1	0.14504631	6.07192982	1.51780137	0.13506201	0.99978775	-4.3827741
WDR37	0.14496382	5.52516	1.08782305	0.28164074	0.99978775	-4.6197347
ZFYVE9	0.14491403	6.84306659	1.7330438	0.08895692	0.99978775	-4.2328113
DUS4L	0.14490482	3.9973268	0.99607346	0.32378213	0.99978775	-4.6300371
TXNDC5	0.14484046	8.59076148	1.07253933	0.28838196	0.99978775	-4.6238712
TMEM255A	0.14475974	1.97550148	0.48163224	0.63206829	0.99978775	-4.7727949
PLEKHB2	0.14469388	7.43778777	1.59654756	0.11636741	0.99978775	-4.3328639
NOV	0.14464122	4.40002868	0.44171144	0.66051161	0.99978775	-4.7870002

SLC25A44	0.14457419	4.09208424	1.29508999	0.200954	0.99978775	-4.5187813
MAP2K4	0.14450803	3.66482919	1.01780285	0.31343796	0.99978775	-4.6157007
FAM214B	0.14449313	5.77116191	1.05122191	0.2979701	0.99978775	-4.6423053
IGBP1	0.14448848	5.63891902	1.91692218	0.06069591	0.99978775	-4.1029913
VEGFC	0.14446955	5.01346403	0.38843402	0.6992652	0.99978775	-4.8424061
DLX4	0.14440164	-1.4644946	0.29825865	0.7666827	0.99978775	-4.6148411
RAPGEF4	0.14438858	3.24152714	0.53533617	0.59467732	0.99978775	-4.7439013
PIP5K1A	0.1443571	6.92322414	2.04732062	0.04564408	0.99978775	-3.9860791
ATF6	0.14426649	6.34293171	1.21995291	0.22793315	0.99978775	-4.5606548
SFT2D1	0.14420085	3.97856058	1.5226241	0.13385223	0.99978775	-4.424179
ST6GALNAC5	0.14418236	0.82256194	0.38385114	0.70263856	0.99978775	-4.657816
ATP6V0A2	0.14413774	5.32206157	1.74230107	0.08730989	0.99978775	-4.2403787
KLHDC10	0.1440674	4.14129298	1.08812527	0.28150856	0.99978775	-4.6054504
BTG3	0.1439816	4.34114789	0.92323815	0.36010576	0.99978775	-4.6732673
ENSCAFG00000009222	0.14391073	4.25410614	0.95421967	0.34434434	0.99978775	-4.6490265
PIK3C2A	0.14387402	6.87043233	0.9550763	0.34391507	0.99978775	-4.688027
PNRC1	0.14382124	5.38199022	0.67416505	0.50316331	0.99978775	-4.7437236
SORBS1	0.14378646	2.86986641	0.19867352	0.8432853	0.99978775	-4.6811297
RTCA	0.1437253	5.20924628	1.4771004	0.14562099	0.99978775	-4.4148082
ENSCAFG00000008776	0.14366811	0.43900478	0.59900509	0.55174488	0.99978775	-4.6205198
MINDY3	0.14364395	4.69185078	0.95161749	0.34565048	0.99978775	-4.666868
PLPP2	0.14360183	4.40054242	0.4802133	0.63307006	0.99978775	-4.8135719
ARL1	0.14338394	4.91537365	1.40196701	0.16680846	0.99978775	-4.4605522
COPS2	0.14338289	6.84593944	1.64070362	0.10683574	0.99978775	-4.2991328
FAM160B1	0.14335721	5.06895258	1.91826255	0.06052187	0.99978775	-4.1190067
PJA2	0.14331759	7.30495981	1.53625359	0.13047987	0.99978775	-4.3723927
MRPS23	0.14325445	3.25073614	1.03986627	0.30316611	0.99978775	-4.6047857
IDS	0.14318581	8.68823421	1.44211929	0.15520639	0.99978775	-4.4382283
ENSCAFG00000029365	0.14315192	0.92563836	0.60384449	0.54854674	0.99978775	-4.6243872
ENSCAFG00000029945	0.1430548	2.27885463	0.79465285	0.43039219	0.99978775	-4.6352558
CASD1	0.14295344	2.97236527	0.93512547	0.35400394	0.99978775	-4.6291154
SUN1	0.14276908	6.91218641	1.13115021	0.26313119	0.99978775	-4.6065619
ASH1L	0.14275171	7.19093169	1.76137347	0.08399635	0.99978775	-4.2157615
GOLT1B	0.14269968	5.62955703	0.78486046	0.43606185	0.99978775	-4.7469543
ACTR10	0.14269685	6.56327053	1.73963192	0.08778216	0.99978775	-4.2280929
SLC41A3	0.14266269	4.88903312	1.06946418	0.28975175	0.99978775	-4.6203831
UBXN4	0.14263136	7.10654441	1.97287533	0.05378417	0.99978775	-4.0509785
DIRC2	0.14259762	4.5115051	0.95695929	0.34297271	0.99978775	-4.6481775
TMEM259	0.14257107	6.93665427	1.15510182	0.25327765	0.99978775	-4.5940537
USP39	0.14256046	4.95850478	1.67673453	0.0995382	0.99978775	-4.289788
ENSCAFG00000011110	0.14252952	5.08381294	1.65272113	0.10435473	0.99978775	-4.3023464
CCDC160	0.14246879	-1.0627929	0.24854495	0.80468222	0.99978775	-4.6291513
FEZ2	0.14237128	5.07322639	1.11616657	0.26943212	0.99978775	-4.6060981
MOB3B	0.14235816	0.04586718	0.23619177	0.81420267	0.99978775	-4.6250392
PSKH1	0.14235471	3.76033548	1.02656067	0.30933279	0.99978775	-4.6167337
EFCAB14	0.14226588	6.4590906	1.20937503	0.2319353	0.99978775	-4.5660221
CDH11	0.14224558	8.63574738	0.45331128	0.65219222	0.99978775	-4.7990029
DUSP11	0.14219272	5.07977542	1.50696366	0.13781235	0.99978775	-4.3982889
BAHD1	0.14217669	4.13203296	0.83489337	0.40755995	0.99978775	-4.6750767
BDH2	0.14216036	2.35479677	0.54732112	0.58647675	0.99978775	-4.6590985

PAIP2	0.14213907	5.58395237	1.33566192	0.18741997	0.99978775	-4.4953962
ENSCAFG00000012004	0.14205224	3.76054486	0.94656006	0.3481983	0.99978775	-4.6470962
STAM2	0.14189315	7.13887769	1.59980142	0.11564231	0.99978775	-4.3295022
ENSCAFG00000018187	0.14187168	3.23084371	0.3765769	0.70800535	0.99978775	-4.7464149
DPP8	0.1417083	6.4846446	1.669007	0.10106786	0.99978775	-4.279038
ARPP19	0.14169923	4.07173095	1.40414235	0.16616316	0.99978775	-4.4684081
UEVLD	0.14156225	3.0317321	0.69052567	0.49290452	0.99978775	-4.674916
AFAP1	0.14146837	6.94097067	0.82963319	0.41050167	0.99978775	-4.7361394
RSBN1	0.1414644	2.23279705	0.71697097	0.47656889	0.99978775	-4.6323884
SPATA2	0.14126415	3.00307349	1.0346673	0.30556557	0.99978775	-4.6014378
KIF1B	0.14120977	6.73212913	1.33605882	0.18729107	0.99978775	-4.4953094
GPI	0.14116924	8.31565414	1.38825903	0.17091947	0.99978775	-4.466064
PTCH1	0.14112093	4.4013757	0.8419803	0.40361714	0.99978775	-4.691149
ZNF16	0.14111609	2.60480579	0.83885926	0.40535062	0.99978775	-4.6367439
MTMR6	0.14103503	5.9282548	1.46639022	0.14850494	0.99978775	-4.4161238
SIMC1	0.14092788	3.29577913	1.17697877	0.24451116	0.99978775	-4.566122
IMPA1	0.14092366	5.77806072	1.22777892	0.22500492	0.99978775	-4.5552074
PLAG1	0.14089169	1.46269253	0.39421461	0.69501893	0.99978775	-4.6209437
EML2	0.14083804	5.89027447	0.73340316	0.46657411	0.99978775	-4.772306
CDC40	0.14078562	4.50263015	1.13701175	0.26069498	0.99978775	-4.5871437
B2M	0.14075942	8.01668887	0.59945597	0.55144652	0.99978775	-4.8000948
SLC35A2	0.14073665	4.31819882	0.84673125	0.40098714	0.99978775	-4.6952844
LATS2	0.14059893	6.97056367	1.17468887	0.24541837	0.99978775	-4.5843399
PPP2R5A	0.1405093	5.27152049	0.81740179	0.41739199	0.99978775	-4.7269719
RAB5C	0.1404884	7.06034311	1.59159805	0.11747741	0.99978775	-4.3344613
ENSCAFG00000030696	0.14034693	0.39996861	0.30136603	0.76432552	0.99978775	-4.6345729
RMDN1	0.14024433	4.09373683	1.3904272	0.17026408	0.99978775	-4.4790332
PPP6R3	0.1401855	6.43561179	1.3586205	0.18007435	0.99978775	-4.4819938
CPEB2	0.14011207	5.10033398	0.78549241	0.43569463	0.99978775	-4.734322
SPPL2A	0.14004273	7.02212048	1.39551083	0.16873503	0.99978775	-4.4599931
KMT2A	0.14004195	5.42555799	1.07784931	0.2860273	0.99978775	-4.6249442
ENSCAFG00000031887	0.14002077	2.09298533	0.89461033	0.37507746	0.99978775	-4.6168249
CSAD	0.13975543	0.3465426	0.3362399	0.73802983	0.99978775	-4.6231298
MARC1	0.13970598	-0.0300083	0.29873157	0.76632381	0.99978775	-4.6303549
DDAH1	0.13969727	4.99237217	0.58592051	0.56043881	0.99978775	-4.7946773
RAP2B	0.13968345	3.01084872	0.65412776	0.51588398	0.99978775	-4.680872
C18H11orf24	0.13966875	6.6516499	0.78739002	0.43459305	0.99978775	-4.7542821
SIRT5	0.13962906	5.2918444	0.60311019	0.5490314	0.99978775	-4.7982822
ENSCAFG00000011151	0.13955462	4.86551059	1.08067328	0.28478049	0.99978775	-4.6154374
MTFR1L	0.13955106	4.60504791	1.43008727	0.15861499	0.99978775	-4.4493372
COL11A2	0.13952955	0.51304682	0.38713725	0.70021911	0.99978775	-4.6245569
UCHL5	0.13952655	6.31881473	1.16050223	0.25109295	0.99978775	-4.5919258
ENSCAFG00000000580	0.13949505	6.0964964	1.12398424	0.26613145	0.99978775	-4.610174
CAPN7	0.13948017	4.95819217	1.15080619	0.25502512	0.99978775	-4.5865551
ENSCAFG00000011218	0.13940639	1.23622267	0.5349297	0.59495638	0.99978775	-4.641305
PIK3C3	0.13937286	5.45330928	1.60548338	0.1143849	0.99978775	-4.3347133
MYL12B	0.13934411	6.7115563	1.20709039	0.23280639	0.99978775	-4.5670382
FAT4	0.13919712	7.00532165	0.68997058	0.49325069	0.99978775	-4.7859322
ZNF770	0.1390721	4.76159159	1.10699509	0.27334107	0.99978775	-4.6046383
CHCHD5	0.13905223	2.05835297	0.72871935	0.46941076	0.99978775	-4.6346442

YEATS2	0.13901628	7.30154433	1.27690936	0.2072516	0.99978775	-4.5291668
EIF4E	0.13889592	5.29421434	0.9271108	0.35811049	0.99978775	-4.6887689
NDUFB10	0.13880582	4.55302024	1.37874885	0.17381712	0.99978775	-4.4767988
HARS	0.13869336	5.7748322	1.34201074	0.18536625	0.99978775	-4.4917639
SYNC	0.13868296	4.51034285	0.51629098	0.60781847	0.99978775	-4.8080756
ZC3HAV1L	0.13856143	1.45237718	0.69537005	0.48988917	0.99978775	-4.6255159
MAP3K2	0.13853534	4.36758656	1.16927022	0.24757478	0.99978775	-4.5745172
WDR82	0.13847469	5.28401442	1.53133514	0.13168894	0.99978775	-4.3846496
KCMF1	0.13840648	5.5064017	1.4414336	0.15539909	0.99978775	-4.4331609
PEG3	0.13836421	5.25848552	0.48968461	0.6263965	0.99978775	-4.8329529
MPIG6B	0.13819736	-1.2641385	0.258146	0.79730325	0.99978775	-4.6101256
ENSCAFG00000025142	0.13807936	1.06684395	0.57683738	0.56651391	0.99978775	-4.6352674
APOOL	0.13795599	2.79868854	1.24687806	0.21797492	0.99978775	-4.5511057
KPNA1	0.1379423	5.73471877	1.29992436	0.19930385	0.99978775	-4.5156772
ENSCAFG00000031320	0.13789098	5.74285369	1.16640303	0.24872132	0.99978775	-4.5886732
ENSCAFG00000019200	0.13788007	-2.4907749	0.192062	0.8484344	0.99978775	-4.6070092
SAP30L	0.13784303	3.05672502	1.04363906	0.30143296	0.99978775	-4.6013196
TTLL11	0.13743618	2.15920407	0.68297413	0.49762528	0.99978775	-4.6417407
RAB3A	0.13741266	0.75486531	0.45385799	0.65180121	0.99978775	-4.6255531
GNB5	0.13740814	3.94060919	0.5771164	0.56632681	0.99978775	-4.722781
PRICKLE2	0.13737202	4.22521049	0.37563675	0.70870007	0.99978775	-4.7808942
VPS52	0.13735087	5.26451417	1.63564002	0.10789541	0.99978775	-4.3166944
HPRT1	0.13728192	5.71916114	1.16905084	0.24766237	0.99978775	-4.5850433
GLIPR1	0.1372678	5.23172614	0.95459834	0.34415454	0.99978775	-4.6841677
TBC1D12	0.13723744	3.1983202	1.17038855	0.24712861	0.99978775	-4.5684132
DNAJC19	0.13719456	4.58438657	1.20136955	0.23499812	0.99978775	-4.5616992
S100PBP	0.13718757	3.33392045	0.9700602	0.33646331	0.99978775	-4.6279406
PLA2G6	0.13713298	2.76567454	0.57865837	0.56529337	0.99978775	-4.6664146
ENSCAFG00000015713	0.13706399	-0.6401719	0.31430813	0.75453233	0.99978775	-4.6148699
ENSCAFG00000018552	0.13692747	-0.3584212	0.45061774	0.65412011	0.99978775	-4.6215078
LYPLA2	0.13685148	6.09526038	1.14808599	0.25613615	0.99978775	-4.5977969
HADHA	0.13685041	7.23603978	2.01147361	0.04942	0.99978775	-4.0185082
TXNRD1	0.13680726	8.9102684	0.82732812	0.41179485	0.99978775	-4.7093993
ENSCAFG00000025112	0.1367263	3.90139446	1.17611332	0.24485375	0.99978775	-4.5675454
BCAP29	0.13670064	3.16620687	0.87825567	0.38380569	0.99978775	-4.6456782
ATP6V1A	0.13662652	5.81309011	1.75249724	0.08552521	0.99978775	-4.2244885
ATP1A1	0.13661987	9.1838159	1.34631575	0.18398343	0.99978775	-4.4920605
BAZ2B	0.13655467	3.72963061	0.91962439	0.36197411	0.99978775	-4.647879
CEP120	0.13649296	5.53960467	1.17108272	0.24685196	0.99978775	-4.582168
MFSD2A	0.13648125	3.57612774	0.40762884	0.68520322	0.99978775	-4.7646447
ENSCAFG00000013972	0.1364265	4.89883594	1.16711808	0.24843502	0.99978775	-4.5770627
ENSCAFG00000017708	0.13642222	6.51112625	1.12623273	0.26518745	0.99978775	-4.6092084
AMIGO3	0.13622006	0.66940191	0.4069073	0.68572983	0.99978775	-4.6310649
SPECC1	0.13621874	7.34745596	0.60153448	0.55007215	0.99978775	-4.8016987
TMEM165	0.13611569	5.83364475	1.36247175	0.17886399	0.99978775	-4.4797893
SCAND1	0.13587315	4.41241583	0.95728262	0.34281107	0.99978775	-4.659935
TMEM208	0.1358352	3.36519752	1.214556	0.22996871	0.99978775	-4.5541356
ATRN	0.1358134	6.29628981	1.63593262	0.10783395	0.99978775	-4.302435
NFAT5	0.13580647	4.84414819	0.75892779	0.45128881	0.99978775	-4.7244707
HIPK1	0.13563193	6.25339557	1.17110344	0.24684371	0.99978775	-4.5865315

GGA3	0.1354624	4.93008384	1.16542532	0.24911316	0.99978775	-4.5797855
TSTD3	0.13532568	-0.8608628	0.19945464	0.84267742	0.99978775	-4.6184487
RTN4	0.13522469	7.93649256	0.839037	0.40525178	0.99978775	-4.7176291
IGDCC3	0.13519229	0.66929941	0.36850222	0.71398019	0.99978775	-4.6328785
SSR4	0.13518884	7.07821212	0.94562507	0.34867067	0.99978775	-4.6911379
CALCOCO2	0.13518407	7.24107527	1.4037324	0.16628462	0.99978775	-4.455131
GSK3B	0.1351115	6.24720169	1.34595662	0.18409849	0.99978775	-4.4894868
MBNL3	0.13509773	-2.2395174	0.22743626	0.82096766	0.99978775	-4.6128653
TMEM243	0.13498408	2.10085109	0.96606207	0.33844113	0.99978775	-4.6082216
SMAD1	0.13495564	6.09105977	0.67172978	0.50470019	0.99978775	-4.7913752
CBARP	0.13492345	1.61655371	0.40715414	0.68554966	0.99978775	-4.6542891
COL4A5	0.13472968	-0.4143502	0.21380194	0.83152946	0.99978775	-4.6363755
ARL13B	0.1347173	5.18667461	0.98315563	0.33003887	0.99978775	-4.6651279
ENSCAFG00000030508	0.13455621	4.80530911	0.5525216	0.58293524	0.99978775	-4.8018692
VAMP4	0.13439671	3.88377797	0.9781382	0.33249064	0.99978775	-4.6369648
TBC1D23	0.13439638	6.12258523	1.43520782	0.15715731	0.99978775	-4.4354924
CABLES2	0.134371	4.6323195	1.27354882	0.20843162	0.99978775	-4.5278873
DEPDC7	0.1343655	2.83882838	0.48998379	0.6261862	0.99978775	-4.7353918
NUAK2	0.13407602	1.79413174	0.31195392	0.75631078	0.99978775	-4.6590114
ND4L	0.13399583	7.95121468	0.79096857	0.43252016	0.99978775	-4.7391908
ERG	0.13398933	4.79146538	0.36722634	0.71492595	0.99978775	-4.8581303
TMEM65	0.13394628	4.14982687	1.02849259	0.30843215	0.99978775	-4.6289206
NGF	0.13388233	1.49089959	0.48190615	0.63187498	0.99978775	-4.6628256
UBE2Q2	0.13387862	4.64587028	1.00916071	0.3175249	0.99978775	-4.6363695
SRC	0.13383298	6.22477326	0.92811835	0.35759256	0.99978775	-4.6985621
ZC2HC1C	0.13378304	0.30241024	0.28965211	0.77322296	0.99978775	-4.6247955
SLC39A6	0.13372497	6.24105441	1.07907441	0.28548594	0.99978775	-4.6324682
TXNDC11	0.13364946	6.22305363	1.32806265	0.18990085	0.99978775	-4.4999454
ABCC5	0.13351246	5.67413048	0.59621484	0.55359311	0.99978775	-4.7676771
ERCC6L2	0.13347886	6.31280954	1.28112021	0.20578004	0.99978775	-4.526756
SLC2A8	0.13343013	4.26228337	0.96519642	0.33887036	0.99978775	-4.6481378
ENSCAFG00000015899	0.13342746	0.71034182	0.49563242	0.62222161	0.99978775	-4.6439071
CEND1	0.13330734	3.41446957	0.79142498	0.4322562	0.99978775	-4.674305
PTGER4	0.13328803	-0.7973964	0.19209324	0.84841005	0.99978775	-4.6144947
PDCL	0.13322277	4.56179695	1.37836353	0.17393531	0.99978775	-4.4776786
CDKL2	0.13307398	5.96760278	0.82224783	0.41465372	0.99978775	-4.7344975
FAM76A	0.13300957	3.57667168	1.14946549	0.25557228	0.99978775	-4.5755264
FAM171B	0.13296972	5.4837647	0.55182556	0.58340864	0.99978775	-4.8135818
WDR44	0.13287761	3.88642077	0.86638624	0.39021966	0.99978775	-4.6630503
COP1	0.13273911	5.24169235	1.44806744	0.15354258	0.99978775	-4.4318304
ARMC7	0.13267897	1.28782199	0.55078111	0.58411937	0.99978775	-4.6299534
RPS11	0.13262748	9.42483406	1.28118443	0.20575766	0.99978775	-4.5241415
ZMAT3	0.13252476	4.10497276	0.60917217	0.54503683	0.99978775	-4.7215482
SEPT2	0.13250187	7.95028673	2.49315497	0.01584954	0.99978775	-3.6306527
FCHO2	0.13234229	5.60170215	0.92634728	0.35850331	0.99978775	-4.6924246
DTWD1	0.13230206	4.90932722	0.82523925	0.41296887	0.99978775	-4.7084517
NIT2	0.13229534	4.05306627	0.84531039	0.40177258	0.99978775	-4.668545
BBS12	0.13216851	2.94186474	0.80301526	0.4255855	0.99978775	-4.6510442
ENSCAFG00000009876	0.13216466	6.88852653	1.54075639	0.12938076	0.99978775	-4.3682448
PLA2R1	0.13213591	6.7183216	0.64436737	0.52214198	0.99978775	-4.8015231

FUCA1	0.13204764	6.54132379	0.88895344	0.37808208	0.99978775	-4.7153036
ENSCAFG00000008814	0.13200541	1.35600365	0.84839513	0.40006858	0.99978775	-4.6189964
TMEM63B	0.13198351	6.25886525	1.08353044	0.28352289	0.99978775	-4.6296982
ITCH	0.13195954	5.84947144	1.44615452	0.15407613	0.99978775	-4.4294433
TTC33	0.13194756	2.4386195	0.67710937	0.50130858	0.99978775	-4.6472403
ENSCAFG00000024216	0.13194607	-2.0606058	0.26987543	0.78831379	0.99978775	-4.6128312
ENSCAFG00000029633	0.13189661	6.95702073	1.30794041	0.19659014	0.99978775	-4.5115788
GSAP	0.13181495	4.07203625	0.61489534	0.5412792	0.99978775	-4.7243453
DHX9	0.13176572	6.93879956	1.204627	0.23374831	0.99978775	-4.5681083
DHX40	0.13171104	5.7535741	1.32387044	0.19128006	0.99978775	-4.5020359
SHQ1	0.13165039	5.69008373	0.93791291	0.35258292	0.99978775	-4.6948707
INTS6	0.13158779	5.7475977	1.32582113	0.19063736	0.99978775	-4.5010468
PSME4	0.13150356	6.9875604	1.14372946	0.25792272	0.99978775	-4.5996518
BLOC1S5	0.13149994	5.69127815	1.16364142	0.24982924	0.99978775	-4.5870827
SAMD4A	0.13149052	5.25461772	0.59288321	0.55580402	0.99978775	-4.8030983
KANSL2	0.13140722	4.91914912	1.61828997	0.11159139	0.99978775	-4.3348946
TMEM128	0.13138356	4.32930927	1.44108771	0.15549637	0.99978775	-4.4507882
OVGP1	0.13126783	0.05494712	0.31090915	0.75710046	0.99978775	-4.6203974
TEK	0.13123699	2.45620555	0.1818845	0.85637363	0.99978775	-4.6931688
ARSD	0.13121268	2.96422761	0.49933386	0.61962978	0.99978775	-4.7049326
ITSN2	0.13118417	6.1045797	1.0545696	0.29645004	0.99978775	-4.6434518
TOM1L2	0.13115564	6.35272574	1.32970933	0.18936116	0.99978775	-4.498973
GIGYF2	0.13113166	6.55357681	2.10747576	0.03986851	0.99978775	-3.9342084
PIK3CB	0.13112139	4.28310713	0.99903724	0.32235791	0.99978775	-4.6415893
ENSCAFG00000029422	0.13102118	6.22769314	0.66432412	0.50938945	0.99978775	-4.7948925
SCFD1	0.1310126	5.95919723	1.02719644	0.3090362	0.99978775	-4.6562699
ZNF582	0.13097649	3.12339841	0.96920804	0.33688422	0.99978775	-4.6205201
DAPK3	0.13094489	7.50119337	0.73568341	0.46519668	0.99978775	-4.762399
SNRPN	0.13094445	6.46143377	1.10835054	0.27276087	0.99978775	-4.618329
FAM92A	0.13084872	4.11147117	1.32151884	0.19205704	0.99978775	-4.5078389
DGUOK	0.13081198	2.65149314	0.86925662	0.38866246	0.99978775	-4.6329702
HMBOX1	0.13068157	2.26731364	0.70358796	0.48479741	0.99978775	-4.6423927
OTUD6B	0.13061241	5.75946512	1.09215823	0.27974879	0.99978775	-4.6221039
ABCD3	0.13059861	5.54789784	1.36015374	0.17959174	0.99978775	-4.481502
PRRC2C	0.13046909	8.31316721	1.42311004	0.16061815	0.99978775	-4.447284
NUDT5	0.13046034	4.64287702	1.49904198	0.13985063	0.99978775	-4.4155787
GMFB	0.13034844	6.58178169	0.80704453	0.42328103	0.99978775	-4.7476684
SLC46A2	0.1302095	-0.7198	0.32677017	0.74514059	0.99978775	-4.6195288
ARHGEF25	0.13012239	5.17722002	0.39238135	0.69636454	0.99978775	-4.8383907
WBP2	0.12985273	6.17281216	1.09363763	0.27910519	0.99978775	-4.6250884
MARCH9	0.12980046	3.44456335	0.82015117	0.4158371	0.99978775	-4.6782335
ENSCAFG00000023230	0.12979271	-2.4433913	0.20740288	0.83649741	0.99978775	-4.6065849
RPP21	0.12978156	3.64687152	1.11950339	0.26801977	0.99978775	-4.5867859
IFIH1	0.12965653	4.26400575	0.77381921	0.44250742	0.99978775	-4.7038275
MATN2	0.12961081	2.83305801	0.5736063	0.56868277	0.99978775	-4.8155355
ZSWIM6	0.1295462	4.90977409	0.75996272	0.45067525	0.99978775	-4.7382027
ATL3	0.12945653	8.49712571	1.84879443	0.07012051	0.99978775	-4.1789823
FAM174A	0.1294073	4.83695119	0.60484976	0.54788358	0.99978775	-4.7836527
LSM1	0.12920802	2.96590773	1.11667724	0.26921563	0.99978775	-4.5832369
MYO1C	0.12919392	9.5672026	0.79407637	0.43072474	0.99978775	-4.7020549

TGIF2	0.12918705	2.34813109	0.70090809	0.48645459	0.99978775	-4.643118
VCPIP1	0.12910851	5.49829384	1.24167785	0.21987272	0.99978775	-4.5462969
UBE2R2	0.12910515	5.43269139	1.62494308	0.11016214	0.99978775	-4.3188226
DBNDD2	0.12901591	4.19025224	0.67713567	0.50129203	0.99978775	-4.7537997
ZNF518B	0.12887078	4.96081829	1.28825377	0.20330495	0.99978775	-4.5208506
PDE4A	0.12885484	4.63118579	0.91446386	0.36465297	0.99978775	-4.6780939
GNAI1	0.12883662	3.46869472	0.21845945	0.8279179	0.99978775	-4.7322737
ACVR2A	0.12880435	3.82248491	0.54303784	0.58940134	0.99978775	-4.7293117
ZSCAN20	0.12877686	2.13381512	0.50982849	0.61230768	0.99978775	-4.6464897
NETO2	0.12857406	1.30168859	0.47673239	0.63553052	0.99978775	-4.6608914
PIGN	0.12850613	4.46085572	1.1893083	0.2396681	0.99978775	-4.565251
ADAM32	0.1284243	-1.1579637	0.2370665	0.81352758	0.99978775	-4.6115848
ENSCAFG00000013496	0.12831122	3.04780591	0.50225094	0.61759059	0.99978775	-4.7012459
DLX2	0.12825563	1.8623413	0.32154614	0.74907292	0.99978775	-4.6563825
DIP2B	0.12824515	6.56260925	0.89368342	0.37556874	0.99978775	-4.7138912
CPT1A	0.12822812	7.36442965	0.97228697	0.33536508	0.99978775	-4.6769127
PDE1C	0.12814599	-1.8861705	0.19482698	0.84628019	0.99978775	-4.6100091
ENSCAFG00000003668	0.12814515	0.40747232	0.43040808	0.66866012	0.99978775	-4.6327148
GAB2	0.12809576	6.24409799	0.68713474	0.49502126	0.99978775	-4.7887105
DCTN4	0.12808522	7.02993794	1.92948175	0.05908179	0.99978775	-4.0852906
RAF1	0.12806635	6.5565502	1.94041437	0.05770675	0.99978775	-4.0738424
ARG1	0.12805571	0.26208258	0.42917013	0.66955502	0.99978775	-4.6383587
RPP30	0.12804106	4.74808695	1.17698737	0.24450776	0.99978775	-4.5735363
FBXO3	0.12800303	4.23469532	1.35194907	0.18218584	0.99978775	-4.4921491
ENSCAFG00000029288	0.12784609	2.51647193	0.78566298	0.43559555	0.99978775	-4.6463274
ZNF280C	0.12780279	4.36428716	1.00925269	0.31748122	0.99978775	-4.6344905
CCNG1	0.12775306	7.88207533	0.74548212	0.4593041	0.99978775	-4.7524796
ENSCAFG00000030202	0.12773809	2.51874404	0.72625585	0.47090665	0.99978775	-4.6465448
CDK14	0.12771423	7.14360571	0.80120682	0.42662225	0.99978775	-4.7494517
TMTC4	0.12757136	3.61827154	0.85315956	0.39744551	0.99978775	-4.6753034
GPNMB	0.1275467	7.90586867	0.25720011	0.7980294	0.99978775	-4.8489001
PSMD3	0.12753744	6.88402213	1.38436338	0.1721019	0.99978775	-4.4667314
DNMT3B	0.12744543	-0.3599158	0.29503959	0.76912695	0.99978775	-4.6203385
ENSCAFG00000029695	0.12727272	2.19269293	0.73170606	0.46760079	0.99978775	-4.6426103
EXOC1	0.12724037	5.70502305	0.93130338	0.35595847	0.99978775	-4.695089
TTC8	0.12712357	3.38491117	0.71339728	0.47875844	0.99978775	-4.6855961
CACNB4	0.12699784	-1.1040764	0.20578045	0.83775807	0.99978775	-4.6130193
EXD1	0.12699756	1.57782434	0.30896578	0.75857003	0.99978775	-4.6351019
TMEM106A	0.12697983	0.25434509	0.27843245	0.78177384	0.99978775	-4.6265597
RPS6KA3	0.12696621	5.8615507	0.68220183	0.49810947	0.99978775	-4.7891348
MYCBP	0.12690965	0.17948228	0.32012479	0.75014399	0.99978775	-4.6218321
COA1	0.12685957	4.38734178	0.90429976	0.36996637	0.99978775	-4.6754812
ARPC5	0.1268326	5.90196424	1.20849052	0.23227226	0.99978775	-4.5654719
S100P	0.12660357	-1.195817	0.19628529	0.84514449	0.99978775	-4.6084143
UHMK1	0.12653128	5.24092201	1.2482389	0.21748029	0.99978775	-4.5421002
HSD17B10	0.12650685	5.27743891	1.34146189	0.18554311	0.99978775	-4.4922748
SSBP2	0.1263675	6.02558881	0.47515499	0.63664686	0.99978775	-4.8346427
DENND5A	0.12632335	7.05234119	1.16298606	0.25009268	0.99978775	-4.5895472
HERC1	0.12622965	5.8859268	1.13297461	0.2623712	0.99978775	-4.6042053
CDNF	0.1261988	0.11771208	0.28275685	0.77847479	0.99978775	-4.6177406

ZBTB4	0.12615982	6.83284219	0.79064841	0.43270537	0.99978775	-4.7538842
MGAT2	0.12601592	6.00186207	1.38021864	0.17336684	0.99978775	-4.4691369
ENSCAFG00000029134	0.12581418	7.2642678	0.98440347	0.32943099	0.99978775	-4.6734278
TRAK2	0.12575302	5.13331561	0.9847727	0.32925126	0.99978775	-4.6639322
NOP10	0.12575132	4.14127739	0.74360328	0.46043062	0.99978775	-4.7308931
PPP2R5E	0.12574703	5.01691744	1.08341464	0.28357378	0.99978775	-4.6182992
FBXL3	0.12572818	5.31238983	1.24195497	0.21977128	0.99978775	-4.5453256
WASL	0.1256749	5.93044368	1.29022401	0.20262529	0.99978775	-4.5211743
ENSCAFG00000030940	0.12557546	2.19570597	0.30733958	0.75980045	0.99978775	-4.6794508
IRGQ	0.12553667	5.70869794	1.01039659	0.31693826	0.99978775	-4.6616612
GFM1	0.125531	6.75120888	1.17256241	0.246263	0.99978775	-4.5854719
MAPRE2	0.12551953	5.8554769	0.78636512	0.43518781	0.99978775	-4.7511997
ARRDC5	0.12548086	0.75120408	0.44997189	0.65458272	0.99978775	-4.6348211
MRPL35	0.12545864	3.38250472	1.10380257	0.27471106	0.99978775	-4.5900109
NR3C1	0.1253155	6.95410648	1.02435933	0.3103612	0.99978775	-4.6568069
FLNA	0.12515979	12.6995302	0.62961739	0.53167476	0.99978775	-4.686678
PHF20	0.12515492	5.75167473	0.98905669	0.32717077	0.99978775	-4.6691431
GHITM	0.12513381	6.61686263	1.40920382	0.1646692	0.99978775	-4.4514282
NCKAP1	0.12513186	8.86058113	1.65012122	0.10488745	0.99978775	-4.319309
RNF141	0.12507824	3.51053616	0.61947741	0.5382804	0.99978775	-4.6877612
CEBPZ	0.1250563	5.9878847	1.02013603	0.31234071	0.99978775	-4.6582159
ZBED6	0.12501768	4.59668376	0.95159716	0.3456607	0.99978775	-4.6593377
ITPRIPL2	0.12483913	3.48281695	0.55778432	0.57936182	0.99978775	-4.6981548
DDIT4	0.12480454	6.83067607	0.47088066	0.6396761	0.99978775	-4.8459592
TMTC3	0.12479159	5.58334656	1.05051032	0.29829389	0.99978775	-4.642912
ENSCAFG00000032448	0.1247413	5.41045581	1.33720981	0.18691767	0.99978775	-4.4945448
PRKAB1	0.12468772	5.49616918	1.54581913	0.12815381	0.99978775	-4.3702677
TWSG1	0.12467609	5.03523934	0.79697956	0.42905156	0.99978775	-4.7139682
FOLR2	0.12462555	6.56388277	0.73530052	0.46542781	0.99978775	-4.7727804
DGCR8	0.12459167	4.60771611	1.19041474	0.23923692	0.99978775	-4.5662163
WDR26	0.12456277	7.12003916	1.59932876	0.11574741	0.99978775	-4.3287865
ETV3	0.12455861	5.14587858	1.32710686	0.19021463	0.99978775	-4.5006552
MCFD2	0.12439418	7.80978374	0.82124582	0.41521901	0.99978775	-4.7373811
ENSCAFG00000009998	0.1243608	0.82034431	0.56957698	0.57139316	0.99978775	-4.6315838
RHBDL1	0.12432336	2.22791441	0.4640904	0.64450113	0.99978775	-4.6700265
LRCH2	0.12418823	2.56848246	0.43745696	0.66357384	0.99978775	-4.7302875
SCNM1	0.1240981	3.16169065	1.02974812	0.3078478	0.99978775	-4.6095635
SIN3B	0.12408959	7.15651028	0.74855467	0.45746528	0.99978775	-4.7665763
SPCS1	0.12402511	5.45072008	1.21870058	0.22840431	0.99978775	-4.5590222
ENSCAFG00000022709	0.12399715	13.5942207	0.50666148	0.61451315	0.99978775	-4.7028582
FAF2	0.12395836	5.05148796	1.56324301	0.12400216	0.99978775	-4.3654228
WWP1	0.12395664	6.22991437	1.00675973	0.3186667	0.99978775	-4.6653004
ITGA9	0.12388394	3.89871581	0.33818681	0.73657073	0.99978775	-4.7796212
HSPA4	0.12384774	8.2717527	0.87579606	0.38512932	0.99978775	-4.6995376
USP20	0.12378764	4.18639495	0.82653651	0.41223952	0.99978775	-4.6982761
PQLC2	0.12364848	2.1500264	0.59047084	0.55740765	0.99978775	-4.651656
ATE1	0.12364415	6.43562126	2.08333872	0.04210457	0.99978775	-3.9551443
SRD5A1	0.12361307	2.25837396	0.63444936	0.52854194	0.99978775	-4.6556834
TRAPPC3L	0.12351426	0.79668524	0.31386219	0.7548691	0.99978775	-4.6383829
NAMPT	0.12336055	6.44273113	0.91609278	0.36380602	0.99978775	-4.7045933

BLOC1S2	0.12334258	3.99867892	0.94886395	0.34703614	0.99978775	-4.651045
POLR2D	0.12328453	3.17894951	0.82442995	0.41342428	0.99978775	-4.6547321
SEC61G	0.12326322	4.33580621	0.88309601	0.38120924	0.99978775	-4.6808601
AUH	0.12321221	3.94759274	1.11288958	0.27082423	0.99978775	-4.589415
ATP6V0E1	0.12312069	4.93978425	1.0414582	0.30243398	0.99978775	-4.6335876
MRAS	0.12310998	3.89400372	0.65388895	0.51603662	0.99978775	-4.7323766
CAPZA2	0.12309599	7.11693727	1.16204463	0.25047147	0.99978775	-4.5898707
ACTB	0.12309157	11.3109618	0.88760516	0.37880046	0.99978775	-4.6549756
EMC1	0.12304106	7.52871134	1.0222502	0.3113487	0.99978775	-4.6520248
ZNF398	0.12303783	3.43833319	0.68686716	0.49518851	0.99978775	-4.6870185
IQCB1	0.12285537	2.72432168	0.75147959	0.45571877	0.99978775	-4.6604979
SRP72	0.12284566	8.26777434	1.16706825	0.24845497	0.99978775	-4.5809982
ZNF19	0.12278943	-0.4143186	0.3425542	0.7333012	0.99978775	-4.6193009
THSD1	0.12268384	3.98727323	0.50447676	0.61603667	0.99978775	-4.7774039
CCNY	0.12264482	6.79602614	1.77116171	0.08233686	0.99978775	-4.2049702
TMEM154	0.1226186	3.41029868	0.76914165	0.44525487	0.99978775	-4.6734831
PSENEN	0.12260384	5.00794651	0.85725612	0.3951987	0.99978775	-4.7121112
TMEM50A	0.12258389	5.60925703	1.00880572	0.31769355	0.99978775	-4.6563662
NECTIN2	0.12255382	4.96818153	0.64588548	0.521166	0.99978775	-4.774064
ENSCAFG00000032009	0.12255333	4.93837612	1.3099645	0.19590934	0.99978775	-4.5094903
CRKL	0.12254412	6.55283195	1.74561694	0.08672614	0.99978775	-4.2235083
ZNF773	0.12253698	2.20790202	0.71658781	0.47680338	0.99978775	-4.6398323
ERGIC3	0.12252973	6.65637296	1.03321715	0.30623716	0.99978775	-4.6542791
ARHGAP29	0.12249434	7.19187942	0.78036593	0.43867889	0.99978775	-4.7571203
FHL2	0.12244456	7.07217315	0.61023004	0.54434127	0.99978775	-4.8070039
NT5C3A	0.12225242	2.89740667	0.80936165	0.42195921	0.99978775	-4.6415563
CHD2	0.12197767	6.03091372	1.12423997	0.26602397	0.99978775	-4.6058637
SLC25A24	0.12190878	6.32490752	1.32308845	0.19153817	0.99978775	-4.5028601
SETD3	0.12190447	5.66199415	1.62696075	0.10973165	0.99978775	-4.3147359
DNAJC3	0.12187815	8.07535159	1.2957062	0.2007431	0.99978775	-4.5172423
CTSF	0.12180014	5.75088735	0.66180438	0.51099028	0.99978775	-4.776104
FRYL	0.12174875	6.58172728	0.91592471	0.36389334	0.99978775	-4.7055541
ABI2	0.12165669	4.87837441	1.21455615	0.22996865	0.99978775	-4.5572176
TMX3	0.12164568	5.56971547	1.23003589	0.22416559	0.99978775	-4.5533028
ROBO4	0.12164423	2.94725396	0.11230744	0.91100784	0.99978775	-4.6814924
PTBP3	0.12160667	5.18355118	0.93250712	0.35534215	0.99978775	-4.6782723
UBE2Z	0.12158371	5.59254205	1.5337281	0.13109958	0.99978775	-4.3743869
DAP3	0.12157127	5.55064071	1.4585485	0.15064481	0.99978775	-4.4219245
PIAS3	0.12154384	5.20139936	1.01149739	0.31641634	0.99978775	-4.650139
CSDE1	0.12153311	9.08890049	1.85951513	0.06856016	0.99978775	-4.1894901
PRNP	0.12153298	6.62068056	0.60088709	0.55050003	0.99978775	-4.8135397
MCTS1	0.12152908	3.83452527	1.02034066	0.3122446	0.99978775	-4.614253
RASL11A	0.12147248	5.26947373	0.34907141	0.72843151	0.99978775	-4.8683114
CYLD	0.12145547	6.07272223	1.18963247	0.23954171	0.99978775	-4.5762218
ZDHHC23	0.12144618	-1.6808491	0.22161229	0.82547523	0.99978775	-4.6110987
STAT5B	0.12140413	5.27435923	1.30349085	0.19809301	0.99978775	-4.5129196
WAPL	0.12138267	6.71846725	1.29501246	0.20098055	0.99978775	-4.5190009
HDGFL3	0.12135764	5.77305201	1.31257058	0.1950354	0.99978775	-4.5087017
RAB2B	0.12132011	4.15569087	1.12668476	0.26499796	0.99978775	-4.5890292
ZNF362	0.12107945	4.79747607	0.89004619	0.37750049	0.99978775	-4.6960287

JADE3	0.1209803	1.51586693	0.48771088	0.62778464	0.99978775	-4.6676434
CTSK	0.12093677	9.00194754	0.39318839	0.69577205	0.99978775	-4.8106006
SEMA6B	0.12092665	-1.4049434	0.11610075	0.90801554	0.99978775	-4.609358
GSKIP	0.12089285	2.88521511	0.83615363	0.40685708	0.99978775	-4.6437314
TEAD2	0.12088731	4.81948001	0.96299557	0.33996327	0.99978775	-4.6648553
PRDM2	0.12088345	5.67085932	1.42400491	0.16036014	0.99978775	-4.4439766
ENSCAFG00000013419	0.12079895	3.95515905	1.1169267	0.26910992	0.99978775	-4.5904866
TMEM240	0.12069692	0.04782601	0.32360401	0.74752307	0.99978775	-4.6208615
KCTD18	0.12067555	3.32966185	0.93625164	0.35342938	0.99978775	-4.631631
PDZRN3	0.12047996	4.48578814	0.55630577	0.5803647	0.99978775	-4.816634
GLCE	0.12046486	4.48337008	1.07984763	0.28514463	0.99978775	-4.6207684
NSFL1C	0.12045334	5.13060299	1.51592727	0.13553446	0.99978775	-4.3901619
NCK2	0.12040566	5.77489302	0.87692476	0.38452156	0.99978775	-4.720539
ADIPOR1	0.12039247	7.4197627	1.00889448	0.31765138	0.99978775	-4.6609973
ENSCAFG00000031583	0.12029823	4.86894552	1.14699718	0.25658183	0.99978775	-4.5873049
PIP5KL1	0.12027869	-0.7004553	0.33581894	0.73834544	0.99978775	-4.6219922
ENSCAFG00000030568	0.12012396	2.04697671	0.61961544	0.53819019	0.99978775	-4.6473419
TSPAN11	0.12007347	1.75257784	0.38486012	0.70189535	0.99978775	-4.6808566
L3MBTL1	0.11989002	2.78642812	0.71514176	0.47768891	0.99978775	-4.6619556
OSTF1	0.11983199	5.22579754	0.78406102	0.43652666	0.99978775	-4.728951
IL6R	0.11982006	-0.7122283	0.17371883	0.86275436	0.99978775	-4.6130382
CPD	0.11977323	8.59044776	1.29987023	0.19932227	0.99978775	-4.5147994
TRPV2	0.11974081	6.37634098	0.47699213	0.63534678	0.99978775	-4.8446131
JCHAIN	0.11971034	5.27039681	0.80720444	0.42318973	0.99978775	-4.7311498
ENSCAFG00000011856	0.11962568	1.76642923	0.51621763	0.60786934	0.99978775	-4.6442259
PNPLA7	0.11961912	5.52129544	0.7905849	0.43274212	0.99978775	-4.7461623
ENSCAFG00000019051	0.1196152	1.43314755	0.39734031	0.69272695	0.99978775	-4.6407683
ENSCAFG00000031402	0.11960901	5.61704641	0.91809235	0.36276807	0.99978775	-4.7006566
STX8	0.11954139	4.39941807	1.30603075	0.19723409	0.99978775	-4.513394
TRMT11	0.11951454	3.2150666	0.80470452	0.42461844	0.99978775	-4.6492901
RMND5A	0.11948206	4.75487107	0.65055843	0.51816785	0.99978775	-4.7491966
STRIP1	0.11934022	5.25645989	1.43801702	0.15636205	0.99978775	-4.4358018
PPIL1	0.11932676	4.95163739	1.09176276	0.27992101	0.99978775	-4.6154107
TCTN1	0.1192792	4.7976388	0.93630383	0.35340277	0.99978775	-4.682358
DDI2	0.11918154	2.38801295	0.5602107	0.57771786	0.99978775	-4.6656075
ENSCAFG00000000415	0.11912177	7.49582964	1.1876754	0.24030548	0.99978775	-4.575952
NBL1	0.11911581	3.90298396	0.3462956	0.73050424	0.99978775	-4.7161125
ST6GALNAC4	0.11906412	6.27802572	0.74575443	0.45914096	0.99978775	-4.7689355
ATXN7	0.11904185	3.86230961	0.92156793	0.36096851	0.99978775	-4.6437256
ATN1	0.1190376	7.79247422	0.97221436	0.33540086	0.99978775	-4.6719446
ARL6	0.11902533	4.39921325	0.73555735	0.46527277	0.99978775	-4.7330611
C1D	0.11894137	4.45401487	0.82007325	0.41588112	0.99978775	-4.7016088
ENSCAFG00000011431	0.11893354	0.06002137	0.35805619	0.72173651	0.99978775	-4.6332403
EHD4	0.11891459	8.50884408	0.61445562	0.54156743	0.99978775	-4.7864144
ENSCAFG00000004388	0.11890216	2.64706561	0.59417005	0.55494953	0.99978775	-4.668636
RLF	0.11880946	5.25007994	0.96765608	0.33765167	0.99978775	-4.6676152
ENSCAFG00000018228	0.11878631	1.63751088	0.66865787	0.50664248	0.99978775	-4.6499532
NDUFB2	0.11874107	3.71566272	0.90557559	0.36929671	0.99978775	-4.6581555
SELENOS	0.11855774	5.05253869	1.28707406	0.20371272	0.99978775	-4.5215679
BMPR2	0.11850038	6.39055312	0.56717475	0.57301206	0.99978775	-4.8230585

RAB2A	0.11849587	7.58497657	1.50132793	0.13926001	0.99978775	-4.3965831
CLPTM1	0.11849148	7.31217919	1.02125187	0.31181687	0.99978775	-4.6544947
ENSCAFG00000018353	0.11841342	3.98346503	1.10385235	0.27468966	0.99978775	-4.5969111
YAP1	0.11826745	7.46939136	1.27506332	0.2078992	0.99978775	-4.5291441
MARCH7	0.11822814	5.95983958	1.00865921	0.31776317	0.99978775	-4.6632124
RICTOR	0.1181801	5.04984597	0.85678249	0.39545807	0.99978775	-4.7061417
RAB31	0.11808897	6.20076242	0.56287073	0.5759182	0.99978775	-4.8241444
ENSCAFG00000012895	0.11800978	4.32829713	1.0333129	0.30619279	0.99978775	-4.6204393
SYTL4	0.11791575	5.0990443	1.00460476	0.31969386	0.99978775	-4.6544804
REV3L	0.11789273	7.18644672	0.7200768	0.47467059	0.99978775	-4.774748
ENSCAFG00000022711	0.11789209	13.4529699	0.39622308	0.69354585	0.99978775	-4.7197206
ERAP1	0.1178565	5.4623419	0.60068131	0.55063607	0.99978775	-4.7878481
SLC49A3	0.11783394	-2.6403215	0.20194976	0.84073629	0.99978775	-4.607136
STAC	0.11769717	4.27412901	0.21999463	0.8267283	0.99978775	-4.777253
PEAR1	0.11767002	4.33036622	0.26467955	0.79229243	0.99978775	-4.8343143
PI3	0.1176294	-2.3655657	0.17384492	0.86265576	0.99978775	-4.6067391
CSF3	0.1176294	-2.1604358	0.16864758	0.86672175	0.99978775	-4.6067605
AGTRAP	0.11751259	2.6194262	0.45440382	0.65141092	0.99978775	-4.6735255
ENSCAFG00000030080	0.11750529	5.43666115	0.38861861	0.69912945	0.99978775	-4.8425168
CLINT1	0.11737968	7.07298578	1.21606027	0.22940001	0.99978775	-4.5618075
SLC43A1	0.11734868	2.08104913	0.25998753	0.79589003	0.99978775	-4.6718011
TICAM1	0.1173088	4.14505833	0.76192003	0.4495162	0.99978775	-4.7045827
PAQR3	0.11728347	2.08538765	0.64692012	0.52050139	0.99978775	-4.6441242
NIPSNAP2	0.11728158	6.1234482	1.3207509	0.19231128	0.99978775	-4.5041707
ZNF696	0.11720031	1.29192518	0.50475925	0.61583957	0.99978775	-4.6353697
VIM	0.11710015	13.2209984	0.93892972	0.35206548	0.99978775	-4.6262067
N4BP1	0.11709705	5.70337341	1.23500749	0.22232488	0.99978775	-4.5513299
DAZAP2	0.11700495	7.47642367	1.11225395	0.27109485	0.99978775	-4.6135872
ENSCAFG00000015053	0.11699586	2.45560405	0.69371619	0.49091746	0.99978775	-4.6499315
XIAP	0.11679502	5.31127984	0.93063211	0.35630246	0.99978775	-4.690443
SLC25A46	0.11677702	6.53378176	1.23644098	0.22179621	0.99978775	-4.5517112
KLHL3	0.11671322	4.39437422	0.46665287	0.64267846	0.99978775	-4.7653642
SMURF1	0.11664413	5.11996928	0.67979676	0.49961895	0.99978775	-4.7701554
GNPAT	0.11649121	5.45943211	1.58070325	0.11995085	0.99978775	-4.3467486
TMEM241	0.11637431	2.13295781	0.72087433	0.47418382	0.99978775	-4.6397022
YIPF4	0.11628753	4.74828577	1.16129478	0.25077346	0.99978775	-4.5787207
TBRG1	0.11628173	5.40308171	1.73580738	0.08846255	0.99978775	-4.242866
HSD11B1L	0.11625647	2.45762522	0.59964695	0.55132016	0.99978775	-4.668359
SLC25A35	0.11625015	1.67067338	0.45757983	0.64914192	0.99978775	-4.6412637
NPAT	0.11622953	4.10052393	0.72703427	0.47043368	0.99978775	-4.710204
RPS6KB1	0.11607138	4.96318185	1.32263063	0.1916894	0.99978775	-4.503003
ENSCAFG00000002008	0.11605213	5.94030562	0.32060753	0.74978016	0.99978775	-4.8645926
OMA1	0.11603953	3.48565113	0.66495553	0.50898873	0.99978775	-4.6904494
CASC4	0.11602477	5.95439105	1.08934716	0.28097457	0.99978775	-4.6247019
NSG1	0.11597908	4.27759833	0.82930618	0.41068497	0.99978775	-4.6954621
ZNF268	0.11597723	2.68652008	0.63404103	0.52880631	0.99978775	-4.6583621
RMI1	0.11596032	3.63392015	0.89312252	0.37586623	0.99978775	-4.6513311
PGM3	0.11593747	6.28613109	0.85334625	0.39734295	0.99978775	-4.727429
NAA10	0.11587371	6.40644914	0.91806889	0.36278024	0.99978775	-4.7046335
ARPC2	0.11586549	7.44431992	1.01308435	0.31566495	0.99978775	-4.6586389

VPS37A	0.11583702	5.75029164	0.85167074	0.39826404	0.99978775	-4.7285609
PFKL	0.11582431	6.25764844	0.67806347	0.50070836	0.99978775	-4.7897514
DCAF13	0.11575589	4.56294198	1.44760897	0.15367032	0.99978775	-4.4389055
LEPROTL1	0.115687	3.74227148	0.66331361	0.51003112	0.99978775	-4.7116049
ENSCAFG00000000701	0.11566857	5.38471752	0.63717512	0.52677896	0.99978775	-4.7934518
MCUB	0.11559642	5.08681022	0.86694015	0.38991886	0.99978775	-4.7129524
PIGS	0.11559466	6.57856712	1.16158858	0.25065511	0.99978775	-4.5914427
RTN3	0.11556504	7.89436482	1.43018136	0.15858811	0.99978775	-4.4408677
ENSCAFG00000031458	0.11549756	6.4763955	1.01434464	0.31506908	0.99978775	-4.6629338
ITPR1	0.11544325	5.71548753	0.83468826	0.40767442	0.99978775	-4.7302461
CTSO	0.11537692	2.42373495	0.60973891	0.54466414	0.99978775	-4.6670865
KDM6A	0.1153768	4.57936133	1.00616051	0.3189521	0.99978775	-4.6357025
SETD4	0.11535628	2.3770594	0.64096251	0.52433446	0.99978775	-4.6544652
PMEPA1	0.11522562	6.00531841	0.22779429	0.82069076	0.99978775	-4.8839414
LARP4	0.11517676	5.10586895	1.10051726	0.2761259	0.99978775	-4.6126554
EPC1	0.11513692	4.58076488	1.042001	0.30218462	0.99978775	-4.6255706
RAP1GDS1	0.11510407	5.18213818	1.16825595	0.24797993	0.99978775	-4.5811787
ENC1	0.11510006	2.27457373	0.46991583	0.64036074	0.99978775	-4.6568874
SBSPON	0.1150693	0.04639123	0.11305302	0.9104196	0.99978775	-4.6131298
ENSCAFG00000001017	0.11498504	3.28338083	0.87814666	0.38386429	0.99978775	-4.645368
FASTKD5	0.11478429	3.38622944	0.76115493	0.44996906	0.99978775	-4.6703704
ANKMY2	0.11471998	5.75731669	1.17651481	0.24469478	0.99978775	-4.5821144
UBR4	0.1147175	7.69880943	0.92159077	0.3609567	0.99978775	-4.6944048
ENSCAFG00000004560	0.1146933	2.17898779	0.72752316	0.47013678	0.99978775	-4.6450823
ENSCAFG00000012668	0.11453332	4.01324816	0.32189723	0.74880843	0.99978775	-4.8026715
MEIS2	0.11447793	5.66410992	0.619025	0.5385761	0.99978775	-4.8014214
PYM1	0.11445252	3.37508157	0.96076027	0.34107566	0.99978775	-4.6315379
ENSCAFG00000015771	0.11440272	-0.045495	0.22182378	0.82531144	0.99978775	-4.6157885
ENSCAFG00000030587	0.1144	-0.0896092	0.16094728	0.87275249	0.99978775	-4.615744
TPM4	0.11428184	9.50547894	0.58847459	0.55873642	0.99978775	-4.7565529
NAIF1	0.11428069	0.6645194	0.34344158	0.73263748	0.99978775	-4.6307734
DDR2	0.11426932	8.07546149	0.83525718	0.40735697	0.99978775	-4.7245392
FRS2	0.11416756	4.24540204	0.50164339	0.61801505	0.99978775	-4.7662632
BLZF1	0.11412506	4.72941152	0.88118937	0.38223067	0.99978775	-4.6925972
FBXO11	0.11409794	6.37563671	1.17225821	0.24638399	0.99978775	-4.5859374
CHD7	0.11396901	2.69187724	0.26024687	0.79569107	0.99978775	-4.6917754
RLIM	0.11395543	4.82697084	1.17724173	0.24440714	0.99978775	-4.5743596
PNPLA4	0.11391049	1.5827077	0.27784644	0.78222122	0.99978775	-4.6459197
PARK7	0.11384216	6.07684846	1.07175181	0.28873233	0.99978775	-4.634741
TRIM23	0.11383304	3.72704743	0.78673658	0.43497219	0.99978775	-4.6769101
SNX27	0.11377979	5.7012665	0.92525994	0.3590632	0.99978775	-4.6983034
ARSB	0.11359104	5.38968611	0.7293063	0.46905474	0.99978775	-4.7661465
ENSCAFG00000029061	0.11356605	2.08150407	0.58085338	0.56382387	0.99978775	-4.6555967
NFE2L1	0.11351514	8.34182664	0.78731189	0.43463837	0.99978775	-4.7365216
DOK2	0.11346955	0.57730374	0.25456037	0.80005688	0.99978775	-4.6294371
SELENOW	0.11346667	6.47184517	0.77242631	0.44332452	0.99978775	-4.7594266
RMDN3	0.11342827	4.45929348	0.98301769	0.33010612	0.99978775	-4.6479802
PSMD8	0.1134242	7.10928678	1.18850525	0.23998141	0.99978775	-4.5759472
ENSCAFG00000012839	0.11342247	1.37966799	0.70541651	0.48366848	0.99978775	-4.6366335
MED7	0.11338201	3.24229871	1.0234628	0.3107807	0.99978775	-4.6097137

TMED7	0.11337528	4.72849389	1.16124873	0.25079202	0.99978775	-4.580753
RIC1	0.11329861	7.55049681	0.74786464	0.45787787	0.99978775	-4.7600482
VPS13B	0.11314519	6.46088064	1.24099073	0.2201244	0.99978775	-4.5491968
ACOT7	0.11311417	3.94508046	0.39791296	0.69230736	0.99978775	-4.7854836
GPR180	0.11284651	3.591617	0.79935512	0.42768536	0.99978775	-4.6845812
QKI	0.1128255	6.28250396	1.04808908	0.29939744	0.99978775	-4.6473399
ENSCAFG00000031561	0.11278349	2.50160956	0.46307839	0.64522157	0.99978775	-4.6975012
SERPINB5	0.11278055	1.19225041	0.32793868	0.74426194	0.99978775	-4.6415642
ZNF234	0.11272668	3.59603031	0.78212002	0.43765643	0.99978775	-4.6677436
DUSP3	0.11263356	5.21244107	0.92510008	0.35914556	0.99978775	-4.6920613
NLK	0.11263079	3.08361792	0.47087491	0.63968018	0.99978775	-4.6848506
ENSCAFG00000014766	0.11260168	-0.8586733	0.2062607	0.83738487	0.99978775	-4.6135796
DPM2	0.11252079	2.42997187	0.62456059	0.5349637	0.99978775	-4.6613988
PARG	0.11239761	4.86417724	1.37575156	0.17473815	0.99978775	-4.4755138
EPHX1	0.11234339	5.14796628	0.41591441	0.67916743	0.99978775	-4.8262023
ACTG1	0.11229907	12.2254327	0.72236657	0.47327381	0.99978775	-4.6806047
NXT2	0.11229366	3.0076535	0.92955974	0.35685245	0.99978775	-4.6308261
STC1	0.11221499	0.32623926	0.05602266	0.95553636	0.99978775	-4.6154031
OPTN	0.11212619	6.4185252	0.96053968	0.34118557	0.99978775	-4.6867131
GCH1	0.11212313	1.55941953	0.28173293	0.77925556	0.99978775	-4.6379272
RBM4B	0.11208397	2.96822514	0.72676047	0.47060001	0.99978775	-4.663255
CORO6	0.1120748	3.23746544	0.48819249	0.62744579	0.99978775	-4.7143196
CRYBG1	0.11204182	5.88627852	0.31619096	0.75311094	0.99978775	-4.860369
ENSCAFG00000032043	0.11203699	3.85434322	0.6456621	0.52130955	0.99978775	-4.7292554
HOMER1	0.11203417	3.21275953	0.56381075	0.57528287	0.99978775	-4.6986391
TMED2	0.1120288	8.40802113	1.01495713	0.31477977	0.99978775	-4.647958
RB1	0.11192807	5.61072012	0.97273413	0.33514483	0.99978775	-4.6762605
RAB5A	0.11192773	5.25868892	1.12690837	0.26490426	0.99978775	-4.6005236
RALGAPB	0.11189293	7.15036285	1.10982635	0.27213013	0.99978775	-4.615846
LAMB2	0.11183536	8.63740052	0.43439387	0.66578215	0.99978775	-4.8219575
ENSCAFG00000015798	0.11182542	7.28229881	0.73575636	0.46515265	0.99978775	-4.7683912
SSH3	0.1117928	6.10267505	0.73575718	0.46515216	0.99978775	-4.7705445
FBXO46	0.11175943	3.12799932	0.82113177	0.41528338	0.99978775	-4.6605636
AGRN	0.11174718	4.51715093	0.51566375	0.60825352	0.99978775	-4.8014125
ENSCAFG00000031840	0.11174287	-0.0546887	0.26653706	0.79086943	0.99978775	-4.6223295
HECTD1	0.11169086	7.49907262	1.17951718	0.24350834	0.99978775	-4.5791096
ZFYVE21	0.11165709	4.3693875	0.79555895	0.42986981	0.99978775	-4.701217
POGK	0.11164082	6.29462641	1.36849306	0.17698411	0.99978775	-4.4761035
COBL	0.11162969	0.44935707	0.32592238	0.7457783	0.99978775	-4.6899089
USP16	0.11153648	5.55059718	1.18080795	0.24299955	0.99978775	-4.5786151
SH3RF2	0.11152517	-0.5314314	0.19740304	0.84427424	0.99978775	-4.628874
SMG7	0.11151988	6.71148865	1.07598154	0.28685402	0.99978775	-4.6340813
ENSCAFG00000002399	0.11144062	2.60884616	0.33927794	0.73575343	0.99978775	-4.6651052
SPRTN	0.11139215	2.86304453	0.81019877	0.42148229	0.99978775	-4.6452627
EFCAB8	0.11138227	-1.1096184	0.26301267	0.79356999	0.99978775	-4.6130022
ENSCAFG00000023540	0.11137159	1.17327061	0.44247775	0.65996067	0.99978775	-4.6561769
ENSCAFG00000028494	0.11136888	3.35789891	0.91628155	0.36370794	0.99978775	-4.6361067
ZBTB48	0.11136687	3.11695313	0.68743676	0.49483253	0.99978775	-4.6696377
HDDC2	0.11128342	4.11873249	1.10560688	0.27393619	0.99978775	-4.5939711
GPR4	0.11126464	0.62621392	0.17502961	0.86172947	0.99978775	-4.6426811

YPEL4	0.11105727	-0.3113235	0.19238198	0.84818504	0.99978775	-4.6144231
PYGO1	0.11104745	3.5823973	0.80938535	0.42194571	0.99978775	-4.681354
NFASC	0.11097567	-0.414796	0.15159463	0.88008754	0.99978775	-4.6219706
FBXO25	0.11095298	3.62629812	0.66907547	0.50637821	0.99978775	-4.6884011
UBE3A	0.11084317	5.71223597	1.03187966	0.30685746	0.99978775	-4.6506843
GOLGB1	0.110812	7.85803136	1.10373561	0.27473985	0.99978775	-4.6157172
DPY19L1	0.11080971	5.31600054	0.81023299	0.4214628	0.99978775	-4.7323125
PTPN12	0.110635	7.00602949	0.62774988	0.53288816	0.99978775	-4.806098
ILK	0.11063093	7.96480151	0.83341087	0.40838772	0.99978775	-4.7221808
PLA2G4A	0.11056262	4.65848033	0.3381575	0.7365927	0.99978775	-4.7843984
PTOV1	0.11049351	4.68508866	0.77249692	0.44328307	0.99978775	-4.7283214
AGO3	0.11049128	4.90761458	0.8609559	0.39317631	0.99978775	-4.7064101
OTUD7A	0.1104573	-1.4418127	0.16402529	0.87034091	0.99978775	-4.6085688
MDH1	0.11044462	6.31608215	1.49686139	0.14041587	0.99978775	-4.3962549
NR3C2	0.11038318	1.66208777	0.41009657	0.68340337	0.99978775	-4.6578657
ENSCAFG00000025140	0.11025238	5.22026099	0.97277195	0.33512621	0.99978775	-4.6727022
PIK3R4	0.11017639	5.57174738	1.26564106	0.21122811	0.99978775	-4.5339475
CHD9	0.11013792	5.65796079	0.93744283	0.3528223	0.99978775	-4.6860165
ANKH	0.1100631	3.85378106	0.31635096	0.75299019	0.99978775	-4.7394007
PTPRE	0.1100162	1.5735228	0.14426892	0.88584035	0.99978775	-4.6316576
ASRGL1	0.11000005	4.78878429	0.85800775	0.39478732	0.99978775	-4.7059544
PACS1	0.10992245	6.01335748	1.33404734	0.187945	0.99978775	-4.4964075
ENSCAFG00000008376	0.10983856	2.63221329	0.67747966	0.50107558	0.99978775	-4.6627113
CRLS1	0.10979879	3.96566609	0.74222902	0.46125561	0.99978775	-4.7067641
ENSCAFG00000006734	0.10976616	3.03183393	0.86025819	0.3935572	0.99978775	-4.638642
DDX6	0.10969255	5.12534002	1.10760676	0.27307914	0.99978775	-4.6100528
MCEE	0.10961777	3.43382147	0.8428604	0.40312914	0.99978775	-4.6570034
NRP1	0.10957485	7.22398334	0.48396609	0.63042209	0.99978775	-4.8409693
PPP4R2	0.10953797	5.84124188	0.98550844	0.32889333	0.99978775	-4.6725109
PPAT	0.10952707	5.18484113	0.71462579	0.47800511	0.99978775	-4.7614098
PRKACB	0.10945087	6.58239071	1.01064955	0.31681827	0.99978775	-4.6638831
ENSCAFG00000025811	0.10938282	3.34248249	0.34750453	0.72960127	0.99978775	-4.7753854
ENSCAFG00000009692	0.10935122	4.92332482	0.77252599	0.44326601	0.99978775	-4.723746
PHF6	0.10928965	5.21621691	1.05610716	0.29575368	0.99978775	-4.6330412
BCS1L	0.10925446	3.62984929	1.0057019	0.31917064	0.99978775	-4.6261281
PRUNE2	0.10921408	7.13296126	0.25153848	0.80237956	0.99978775	-4.8739508
COPS7A	0.10918961	6.15215906	0.95418464	0.3443619	0.99978775	-4.6891347
CAB39L	0.10913412	3.33363448	0.54457205	0.588353	0.99978775	-4.7094616
ENSCAFG00000023313	0.10912775	-0.4440103	0.18406164	0.85467401	0.99978775	-4.6179045
P4HA2	0.10905979	8.31277586	0.5387033	0.59236796	0.99978775	-4.8060929
SHROOM4	0.10887425	4.47375052	0.44333413	0.65934519	0.99978775	-4.7863796
NUCB1	0.10882371	8.44892997	0.78893326	0.43369839	0.99978775	-4.735362
TUBA1B	0.10881252	9.80865135	0.38436701	0.70225854	0.99978775	-4.7936024
PRKAB2	0.1087809	5.52072768	0.90010133	0.37217551	0.99978775	-4.7016145
AKAP9	0.10877664	5.29165223	0.81684905	0.41770501	0.99978775	-4.7259572
ZNF570	0.10873713	1.08061928	0.44052998	0.66136141	0.99978775	-4.6417231
CELSR3	0.1087122	1.30358935	0.23545094	0.81477454	0.99978775	-4.6291486
ENSCAFG00000029710	0.10865548	0.06093066	0.31522673	0.75383875	0.99978775	-4.6211685
SEC24B	0.10864977	6.30387468	1.41174865	0.163922	0.99978775	-4.4498833
URM1	0.10864911	2.7435847	0.84173303	0.40375431	0.99978775	-4.642218

PSD2	0.1086352	-0.2177061	0.25755443	0.79775738	0.99978775	-4.6207998
RNF139	0.10853614	5.34802282	1.05053701	0.29828174	0.99978775	-4.6373841
DERA	0.10850338	4.5798217	1.28891309	0.20307732	0.99978775	-4.5203296
YIPF6	0.10844531	4.36299346	0.93790354	0.35258769	0.99978775	-4.6707955
BTF3	0.10838718	6.34432307	0.93382351	0.35466895	0.99978775	-4.6978165
ZNF655	0.1082671	5.02129234	0.81671797	0.41777927	0.99978775	-4.7259463
PTPRB	0.10826701	4.98751877	0.22349329	0.82401874	0.99978775	-4.8812581
IMPACT	0.10825842	5.57835765	1.01265107	0.31586997	0.99978775	-4.6600043
EEF1A1	0.10814765	11.0729466	0.8659996	0.39042971	0.99978775	-4.6656107
RPL5	0.10807129	9.2704842	1.12106989	0.26735854	0.99978775	-4.5960659
LAMA3	0.10804074	6.15636237	0.27754875	0.78244851	0.99978775	-4.8776136
ENSCAFG00000023602	0.10803794	-0.7163565	0.06572283	0.94784806	0.99978775	-4.6117197
ANAPC10	0.10793184	1.77029602	0.50936014	0.61263361	0.99978775	-4.6531704
KAT6A	0.10784254	6.00878225	1.26020569	0.21316638	0.99978775	-4.5379673
SPATA7	0.10783951	4.65145827	1.0211786	0.31185125	0.99978775	-4.6373595
SF3B5	0.10783893	3.64871753	0.724158	0.47218264	0.99978775	-4.6992798
IL17RA	0.10765296	7.97744461	1.27680184	0.20728927	0.99978775	-4.527305
PNPLA2	0.1075837	6.78427371	0.42253328	0.6743609	0.99978775	-4.8543229
KXD1	0.10747256	4.88899627	0.56925639	0.57160908	0.99978775	-4.782249
ARFGEF3	0.10742916	2.92013657	0.38211294	0.70391959	0.99978775	-4.7673761
ENSCAFG00000012022	0.10742314	6.86797916	0.22343381	0.82406479	0.99978775	-4.8809619
CCP110	0.10742073	4.15612081	0.79965683	0.42751203	0.99978775	-4.6978612
FOXP4	0.10737305	6.56097278	0.6151197	0.54113217	0.99978775	-4.8058915
LAMTOR3	0.10721689	5.16596977	1.27171218	0.20907865	0.99978775	-4.5296573
ENSCAFG00000032682	0.10721559	2.54115565	0.57325853	0.56891645	0.99978775	-4.6625033
C12H6orf132	0.10712195	-1.0973021	0.38367312	0.70276972	0.99978775	-4.6233123
SUOX	0.10710074	4.47188966	0.54852955	0.5856529	0.99978775	-4.7564267
ENSCAFG00000019635	0.10707391	0.91471223	0.39112403	0.69728797	0.99978775	-4.6309378
SIL1	0.1070658	5.41518963	0.91335423	0.36523064	0.99978775	-4.6969448
STX1A	0.10705793	1.00470069	0.43599856	0.66462489	0.99978775	-4.6351408
DALRD3	0.10704683	3.42523108	0.56626826	0.57362355	0.99978775	-4.703674
ZNF174	0.1069995	4.09085688	1.07927798	0.28539605	0.99978775	-4.6054085
TM9SF4	0.10699883	6.79935342	1.49780903	0.14017001	0.99978775	-4.3959409
MYO1E	0.10688886	8.1737437	0.50817698	0.61345732	0.99978775	-4.805527
ACSS3	0.10681164	3.75204865	0.34004545	0.73517871	0.99978775	-4.8085126
TBC1D13	0.10660759	3.77897957	1.17428699	0.24557783	0.99978775	-4.5673793
ENSCAFG00000012283	0.10659147	5.79487962	1.16426571	0.24957847	0.99978775	-4.5888258
ENSCAFG00000031306	0.10650447	3.28283731	0.15761747	0.87536273	0.99978775	-4.6893193
TMED3	0.10649766	6.64688369	0.87477325	0.38568059	0.99978775	-4.72183
ENSCAFG00000031206	0.10646134	-1.2319603	0.22699916	0.82130576	0.99978775	-4.610178
DCUN1D4	0.1064519	5.61185574	1.01304127	0.31568533	0.99978775	-4.6584577
FILIP1L	0.10641118	6.12924165	0.30768909	0.75953595	0.99978775	-4.8750479
HDX	0.10637445	0.00856289	0.3227855	0.74813939	0.99978775	-4.6236224
RPS27	0.106266	7.52853784	0.82706776	0.41194106	0.99978775	-4.7331968
STAT3	0.10622524	7.71698995	0.92713451	0.3580983	0.99978775	-4.6933994
STRADB	0.10622307	3.75875382	0.93797063	0.35255353	0.99978775	-4.6460148
CLYBL	0.10620457	2.6596525	0.52772157	0.59991536	0.99978775	-4.6722594
CCDC30	0.10610665	2.011257	0.53862271	0.59242318	0.99978775	-4.6602158
CYBA	0.10603808	4.93283975	0.49014859	0.62607038	0.99978775	-4.798885
DGKH	0.10603777	2.29975293	0.40340622	0.68828728	0.99978775	-4.6793582

BZW2	0.10600437	7.07499423	0.95851206	0.3421969	0.99978775	-4.6846137
LRCH3	0.10600092	5.20631008	1.22007146	0.22788859	0.99978775	-4.555819
RPF2	0.10594319	5.19469634	0.96729304	0.33783137	0.99978775	-4.6678605
SYNRG	0.10588151	5.0191533	1.16581338	0.24895758	0.99978775	-4.5815722
CDKN1A	0.10587951	8.35061174	0.39383216	0.69529957	0.99978775	-4.8222153
ENSCAFG00000011121	0.10577454	4.10181088	0.8629892	0.39206761	0.99978775	-4.6746336
PMM1	0.10568524	3.74096603	0.69665409	0.48909164	0.99978775	-4.702763
AKT3	0.10564893	5.22238749	0.73848594	0.46350694	0.99978775	-4.7569699
EFR3A	0.10561915	6.20070185	1.32712201	0.19020966	0.99978775	-4.5004403
RHBDL2	0.10559843	1.1481726	0.28157739	0.77937418	0.99978775	-4.6417935
ZBED5	0.10559712	5.04360895	0.97586325	0.33360627	0.99978775	-4.6601981
ENOX2	0.10546742	2.23909208	0.46659002	0.64272314	0.99978775	-4.6579114
KIAA1551	0.10546432	4.94643156	0.41756682	0.6779662	0.99978775	-4.7938108
RWDD4	0.10537898	2.73308238	0.67069808	0.50535206	0.99978775	-4.6631733
SH3RF1	0.10526244	4.51913764	0.73169897	0.46760508	0.99978775	-4.7336926
ACOT9	0.10522949	6.37068549	1.4563208	0.1512571	0.99978775	-4.4220636
GCC2	0.10516716	6.55700239	0.89494258	0.37490147	0.99978775	-4.7141185
MSL2	0.105108	4.01150102	0.88059952	0.38254701	0.99978775	-4.66168
SRFBP1	0.10506026	4.03552016	0.83154333	0.40943194	0.99978775	-4.6859057
ENSCAFG00000008503	0.10498527	3.15287012	0.98335861	0.32993994	0.99978775	-4.6186672
CHRD	0.10492088	1.53283547	0.29032238	0.77271301	0.99978775	-4.6927105
ENSCAFG00000028773	0.10474804	0.46657533	0.42580907	0.67198711	0.99978775	-4.6347805
HIVEP3	0.10472211	3.22593766	0.32909262	0.74339458	0.99978775	-4.7361136
TIGAR	0.10468922	6.39099582	0.53777494	0.59300425	0.99978775	-4.8297295
OTUD5	0.10467759	6.75412061	1.06564359	0.29145984	0.99978775	-4.6387911
SEMA4G	0.10466122	3.2393052	0.79298045	0.43135736	0.99978775	-4.6651965
ARL15	0.10464355	1.52804811	0.44819512	0.65585612	0.99978775	-4.6409474
ZMYM4	0.10458688	5.5988633	1.13175951	0.2628772	0.99978775	-4.6028497
EPB41L1	0.10437657	6.14986635	0.40942264	0.68389472	0.99978775	-4.856157
SEC61B	0.10435821	5.82460262	0.77629053	0.44105988	0.99978775	-4.7585507
SMAD7	0.10432737	3.08920343	0.54276432	0.58958834	0.99978775	-4.7130133
STAC3	0.10432388	1.50696686	0.47375752	0.63763657	0.99978775	-4.6439943
RPS6KB2	0.10423444	4.6153225	0.82112674	0.41528622	0.99978775	-4.7058351
ENSCAFG00000029967	0.10418028	0.16582015	0.30441866	0.76201205	0.99978775	-4.6281864
CLDN1	0.10414617	3.52451949	0.27740495	0.7825583	0.99978775	-4.8580577
ENTPD7	0.10414427	3.59721451	0.56434936	0.574919	0.99978775	-4.7318828
ACAA2	0.10411209	6.12400418	0.82004495	0.4158971	0.99978775	-4.7408178
NFKBIB	0.1040808	4.29658287	0.7814035	0.43807391	0.99978775	-4.7072424
FXR1	0.10407175	6.94078107	1.2418619	0.21980535	0.99978775	-4.5483372
TRIM62	0.10407139	2.76943847	0.54630667	0.58716879	0.99978775	-4.6718108
NR4A2	0.10403671	3.26756673	0.16700722	0.86800579	0.99978775	-4.6973293
JMY	0.10402669	4.82779481	0.69866866	0.48784182	0.99978775	-4.7412141
NPEPPS	0.10400755	7.42894372	0.87759802	0.38415932	0.99978775	-4.7142491
ANKRD13A	0.10397516	6.00931158	0.80171609	0.42633014	0.99978775	-4.7486702
ACADS	0.10396358	3.73367319	0.49432251	0.62314	0.99978775	-4.731704
ERCC3	0.10391895	5.63824298	1.40746307	0.16518183	0.99978775	-4.453307
WSB1	0.10390536	7.6257817	0.46864432	0.64126348	0.99978775	-4.8280973
GNS	0.10389758	6.77180426	1.05468799	0.29639638	0.99978775	-4.6434089
NHLRC2	0.10380727	5.13650904	0.87532071	0.38538546	0.99978775	-4.7067401
C4H5orf22	0.10379255	4.81959929	0.69834719	0.48804114	0.99978775	-4.7585288

CNOT2	0.10377678	6.07817896	1.45206515	0.1524322	0.99978775	-4.4251113
KDM3A	0.10376217	5.82632901	0.87065011	0.38790788	0.99978775	-4.7156285
HIP1R	0.10374497	4.24197893	0.74863876	0.45741502	0.99978775	-4.7204513
ARL6IP5	0.10371658	6.66143262	0.74759652	0.45803824	0.99978775	-4.7675656
CAPRIN1	0.10367273	8.29140451	1.51493922	0.13578408	0.99978775	-4.3941291
UBA6	0.10365801	5.98500058	0.63139056	0.530524	0.99978775	-4.804999
RBMS1	0.10354316	8.1864348	1.03579094	0.30504589	0.99978775	-4.6426535
GTPBP10	0.10352864	2.57223881	0.3679751	0.71437087	0.99978775	-4.6732997
FAM189B	0.10348009	7.41573691	0.7262951	0.4708828	0.99978775	-4.7671529
THSD7A	0.10344228	-2.3835555	0.15561657	0.8769319	0.99978775	-4.6073097
SPART	0.10341742	5.56981651	1.30765126	0.19668754	0.99978775	-4.5109929
SMIM1	0.10334078	0.99268712	0.27337575	0.78563668	0.99978775	-4.6512512
PLEKHA1	0.10321653	4.40470704	0.74980684	0.45671712	0.99978775	-4.7071628
ARHGEF28	0.10319847	1.69703046	0.20243494	0.84035895	0.99978775	-4.671587
MALSU1	0.10314951	3.83045821	0.93544758	0.35383954	0.99978775	-4.6490485
GBA	0.10301587	5.90626846	0.73410077	0.46615246	0.99978775	-4.7696707
EIF3L	0.10294294	5.64497204	1.07531191	0.28715081	0.99978775	-4.6292707
NUP54	0.10265967	4.97517204	1.14620729	0.2569055	0.99978775	-4.589423
PIP4P2	0.10263832	1.00760958	0.27259695	0.7862321	0.99978775	-4.6350501
DYNC1H1	0.10261041	9.69402527	0.9277495	0.35778211	0.99978775	-4.6657892
CNEP1R1	0.10259214	2.66974265	0.76205932	0.44943378	0.99978775	-4.6483286
AGO2	0.10255221	4.6243163	0.78526081	0.43582919	0.99978775	-4.7197812
FAM149A	0.10254437	-0.0179641	0.2182357	0.82809132	0.99978775	-4.6347123
ENSCAFG00000017848	0.10252852	1.12418875	0.46399841	0.64456659	0.99978775	-4.6504911
UBA3	0.10249636	6.33305881	1.20675127	0.23293589	0.99978775	-4.5677331
ENSCAFG00000015116	0.10248183	7.3229379	0.51830185	0.6064247	0.99978775	-4.8245111
CDC42	0.10229701	4.74762473	1.0319759	0.3068128	0.99978775	-4.6382013
LRBA	0.10226284	4.68370862	0.72178667	0.47362733	0.99978775	-4.7384096
CLK2	0.10220856	4.57439115	1.02890857	0.30823847	0.99978775	-4.6309741
GALT	0.10219907	4.16620792	0.62381869	0.53544712	0.99978775	-4.7339305
VAMP3	0.10215059	6.6814752	1.3466891	0.18386388	0.99978775	-4.4890589
ATG12	0.10212544	3.84843321	0.72016087	0.47461926	0.99978775	-4.6970935
ENSCAFG00000028505	0.10205312	3.51541122	0.92194055	0.36077591	0.99978775	-4.6440038
ELP4	0.10197569	4.18840702	1.17665229	0.24464036	0.99978775	-4.5699776
HMGXB3	0.10196944	6.03442227	1.34263251	0.18516604	0.99978775	-4.4914232
SETD5	0.10191339	7.08278657	1.0506527	0.29822908	0.99978775	-4.6448939
USP3	0.10182599	6.30521831	1.17333365	0.24595642	0.99978775	-4.5849639
PSAP	0.10179396	9.75349904	0.63514905	0.5280891	0.99978775	-4.7518062
ENSCAFG00000026374	0.10175845	-1.2045321	0.22002182	0.82670723	0.99978775	-4.6119758
ARHGAP5	0.10174569	6.01024313	0.94809106	0.34742573	0.99978775	-4.6878573
ISM1	0.10169756	3.18128609	0.11943468	0.90538672	0.99978775	-4.6907848
DYNC1LI2	0.10166022	6.03052329	0.90150221	0.37143746	0.99978775	-4.7089618
FUOM	0.1015199	1.77640796	0.35141523	0.72668293	0.99978775	-4.6464403
TMEM251	0.10149056	2.02343213	0.61756224	0.53953276	0.99978775	-4.650948
SCP2	0.10148521	6.95241178	1.00383219	0.32006265	0.99978775	-4.6672307
VPS45	0.10141776	5.06607004	1.19908672	0.23587688	0.99978775	-4.5666365
WIPI2	0.10140317	5.94506918	1.26948377	0.20986571	0.99978775	-4.5326812
SLC35A3	0.10138811	3.63165639	0.48329249	0.63089703	0.99978775	-4.7415232
KDELC1	0.10134442	5.68651439	0.52735077	0.60017098	0.99978775	-4.8251334
ENSCAFG00000032585	0.10129365	2.31673364	0.56241044	0.57622942	0.99978775	-4.6567471

MED13	0.10125669	7.08553034	1.03497479	0.3054233	0.99978775	-4.6517764
ENSCAFG00000017688	0.10123783	7.13365004	0.69664632	0.48909646	0.99978775	-4.7836923
DERL1	0.10121806	6.74638919	0.86662176	0.39009174	0.99978775	-4.724405
INPP5A	0.10119802	6.04254702	1.31536781	0.19410064	0.99978775	-4.5073436
CNBP	0.10108805	7.6188248	1.22782551	0.22498757	0.99978775	-4.5541015
MLH1	0.1009249	4.76381834	1.15333109	0.25399694	0.99978775	-4.5846457
RDH11	0.10092053	4.24991048	0.85071636	0.39878929	0.99978775	-4.6791331
PGM2	0.10090811	6.46099859	0.88224234	0.38166635	0.99978775	-4.7189546
MOSPD2	0.10090703	5.08149252	0.51875686	0.60610952	0.99978775	-4.8019826
ENSCAFG00000012755	0.10081814	3.4556906	0.85922012	0.39412432	0.99978775	-4.6493601
SBDS	0.10072575	6.1409165	1.27555395	0.20772693	0.99978775	-4.5296439
TXNL4B	0.10066174	3.03453739	0.833976	0.40807205	0.99978775	-4.6419103
ZNF420	0.1006053	2.23335123	0.50351389	0.61670867	0.99978775	-4.6632714
CACUL1	0.10055115	3.56886824	0.8589591	0.39426701	0.99978775	-4.6573278
RASSF9	0.1005248	2.35721933	0.35368772	0.72498897	0.99978775	-4.690397
KDM5B	0.1005232	6.33159788	0.77302793	0.44297149	0.99978775	-4.7592827
PIK3CA	0.10048588	5.50347974	0.61479189	0.54134701	0.99978775	-4.7797775
COX14	0.10045324	2.98643213	0.7381843	0.46368864	0.99978775	-4.6634937
RBAK	0.10042953	4.47241091	0.86871088	0.38895822	0.99978775	-4.6854104
ENSCAFG00000013477	0.10034709	6.64268784	1.26017415	0.21317767	0.99978775	-4.5386164
RAB7A	0.1003432	7.66070726	1.36093024	0.1793477	0.99978775	-4.4810157
PCNP	0.10027845	6.06642367	1.0760532	0.28682227	0.99978775	-4.633178
OXR1	0.10026891	5.05688452	0.69864063	0.4878592	0.99978775	-4.7677146
SEC31A	0.10011658	8.61841438	1.28500692	0.20442871	0.99978775	-4.5223084
ENSCAFG00000030734	0.10009226	0.12931019	0.25557822	0.79927494	0.99978775	-4.6335324
NRBP2	0.10005514	5.12728215	0.72341493	0.47263507	0.99978775	-4.7616123
FBXO31	0.10002633	4.02644754	0.9506713	0.34612622	0.99978775	-4.6495462
POLR3D	0.10001711	5.07113418	0.65301828	0.51659332	0.99978775	-4.7706403
VAPB	0.10000488	6.02200034	1.00530525	0.31935973	0.99978775	-4.6662805
ARNT	0.09999298	5.86434894	0.97476232	0.33414705	0.99978775	-4.6790756
STBD1	0.09999129	4.22481289	0.52712173	0.6003289	0.99978775	-4.7716685
GNPTG	0.099947	5.59114518	0.83804098	0.40580587	0.99978775	-4.7258745
ENSCAFG00000013064	0.09993201	3.01088346	0.37953812	0.7058188	0.99978775	-4.731337
FMN2	0.09988958	5.94584789	0.23084967	0.81832861	0.99978775	-4.8642472
SNX2	0.09984471	6.28150856	1.05020783	0.29843161	0.99978775	-4.6463399
NUMB	0.09983689	5.97618575	0.95229579	0.3453097	0.99978775	-4.6868676
DBT	0.0997891	3.83511873	0.77584123	0.44132285	0.99978775	-4.6886236
ZBTB47	0.09974845	6.00487903	0.50871595	0.61308203	0.99978775	-4.8308381
VPS33B	0.09974364	4.50570082	0.90883877	0.36758746	0.99978775	-4.6708086
TRAF5	0.09974122	3.40350863	0.67923671	0.49997081	0.99978775	-4.6927448
FUZ	0.099741	2.2399964	0.39191926	0.69670386	0.99978775	-4.6730553
ENSCAFG00000029869	0.09965251	1.19686579	0.39665444	0.69322963	0.99978775	-4.6383687
ENSCAFG00000016094	0.09953606	6.41442078	0.86678756	0.3900017	0.99978775	-4.725255
ZNF507	0.09953424	4.93129385	0.80331632	0.42541305	0.99978775	-4.7173373
NHLRC3	0.09952725	2.9407063	0.52804572	0.59969194	0.99978775	-4.6981152
CYB561D2	0.09946489	2.32227915	0.78332572	0.43695444	0.99978775	-4.6473478
SYCE1L	0.09941681	0.10689339	0.31526744	0.75380802	0.99978775	-4.6229493
BAG3	0.0993341	5.64149107	0.67297666	0.50391298	0.99978775	-4.7776152
RPL32	0.09922469	8.09656292	1.06230947	0.29295612	0.99978775	-4.6310618
TMCC2	0.09922035	3.38016036	0.6482231	0.51966504	0.99978775	-4.7031006

ZNF346	0.09921766	3.15862276	0.82412582	0.4135955	0.99978775	-4.6487775
PHKG2	0.09910403	3.74934212	0.73836383	0.46358049	0.99978775	-4.6979635
DRC1	0.09901066	-1.0064635	0.27813994	0.78199714	0.99978775	-4.6245302
FOXP1	0.09899734	6.03534854	0.76902658	0.44532258	0.99978775	-4.7609217
PSMB2	0.09895475	6.47181593	0.87469746	0.38572146	0.99978775	-4.7220089
PRDM5	0.09893557	3.63417491	0.88994676	0.37755339	0.99978775	-4.6547356
DAB2IP	0.09873552	5.45581387	0.71960401	0.47495928	0.99978775	-4.7667144
AVIL	0.09872625	-0.2384352	0.1968871	0.84467591	0.99978775	-4.623536
IQGAP1	0.09865218	9.6036462	0.92845714	0.35741851	0.99978775	-4.6669748
ATP6V0A1	0.0986057	6.47323546	0.49317892	0.62394227	0.99978775	-4.8353098
SLC31A1	0.09856061	5.82829952	1.11833187	0.26851503	0.99978775	-4.6104825
KIFAP3	0.09845794	6.48720103	0.68564796	0.49595094	0.99978775	-4.7890575
TMEM222	0.09841745	4.06525876	0.8133654	0.41968112	0.99978775	-4.6896861
RPP38	0.09834116	3.72695575	0.82027681	0.41576613	0.99978775	-4.6749733
ZFAND3	0.09833678	5.79833481	0.79104792	0.43247426	0.99978775	-4.7475968
METAP1	0.09830255	5.26240467	0.95557474	0.34366546	0.99978775	-4.6764972
RHOQ	0.09815879	6.62406567	0.43499963	0.66534519	0.99978775	-4.8528544
TNK2	0.09812987	4.6082484	0.41363688	0.68082445	0.99978775	-4.7940303
TMCO1	0.09812949	5.4022478	0.8729967	0.38663929	0.99978775	-4.7143369
CLN3	0.09810845	4.20936965	0.77104638	0.44413488	0.99978775	-4.7166315
ENSCAFG00000032762	0.09806625	3.4693299	0.55411547	0.58185187	0.99978775	-4.6937374
KCTD13	0.09801603	2.28030878	0.56084426	0.57728898	0.99978775	-4.6607681
SEC24A	0.09791628	6.25174086	0.88616442	0.37956905	0.99978775	-4.7174431
ENSCAFG00000032089	0.09786873	3.27584505	0.32396732	0.74724957	0.99978775	-4.7516537
MINPP1	0.09786815	6.41012108	1.19606878	0.23704229	0.99978775	-4.5732421
TADA1	0.09779612	2.85923235	0.47913465	0.63383205	0.99978775	-4.6829894
TANC1	0.09773718	4.31421463	0.41936437	0.67666043	0.99978775	-4.7760621
RERE	0.09773102	6.24827734	0.74810837	0.45773211	0.99978775	-4.7644225
ABCC4	0.09763007	5.5200015	0.78851037	0.43394344	0.99978775	-4.7517236
ADAM15	0.09760905	6.5693862	0.43266813	0.66702761	0.99978775	-4.8517427
TLK2	0.09754709	4.92729715	1.05808797	0.29485824	0.99978775	-4.625861
BACH1	0.09746074	7.09312247	0.78919594	0.43354622	0.99978775	-4.7535386
NKAP	0.09743845	3.65783231	0.87142156	0.38749054	0.99978775	-4.6538882
RNPC3	0.09742357	4.36075578	0.67838816	0.50050419	0.99978775	-4.7296315
TNFAIP3	0.09730473	2.44900369	0.17144059	0.86453626	0.99978775	-4.6583349
TOPORS	0.09710751	5.23853586	0.89462554	0.3750694	0.99978775	-4.6967989
SLC35A5	0.09707252	3.54339117	0.57949551	0.56473271	0.99978775	-4.7072113
KANSL3	0.09700893	7.11238176	0.9148074	0.36447424	0.99978775	-4.701756
MTMR12	0.09700771	2.22491509	0.46221659	0.64583535	0.99978775	-4.6558189
TNPO2	0.09697233	6.06770438	1.00279057	0.32056032	0.99978775	-4.6670719
GRB10	0.09696288	4.90120438	0.5361606	0.59411148	0.99978775	-4.815406
FBXO32	0.09687975	3.74243664	0.31701204	0.75249135	0.99978775	-4.7758878
LAMTOR4	0.0967292	4.49759311	1.00634305	0.31886514	0.99978775	-4.645769
LAMP1	0.0967285	8.57487189	1.02508229	0.31002319	0.99978775	-4.6431771
SLC2A10	0.09667417	5.10755742	0.71826513	0.47577737	0.99978775	-4.7518962
ISCU	0.09660692	5.1653951	0.82752801	0.41168261	0.99978775	-4.7252742
CNOT6L	0.09659975	4.63023329	0.61721278	0.53976145	0.99978775	-4.7485951
SGCD	0.096596	2.08860167	0.31438479	0.75447444	0.99978775	-4.7903098
ENSCAFG00000010650	0.09655486	4.05915347	0.85941493	0.39401786	0.99978775	-4.6821134
BCL7B	0.09655464	4.5430574	1.03221246	0.30670304	0.99978775	-4.6332698

AP2B1	0.09642093	7.45803	1.4389332	0.15610337	0.99978775	-4.4341327
EXOC4	0.09639171	6.41386311	0.96465957	0.33913674	0.99978775	-4.6849676
FKBP3	0.09636845	4.93583195	0.94902125	0.34695688	0.99978775	-4.6749536
RNF123	0.09633298	4.89019521	0.82910817	0.410796	0.99978775	-4.7111899
PNMA1	0.09631315	2.93667572	0.51733389	0.60709543	0.99978775	-4.6887135
PAPOLG	0.09629787	3.84021026	0.63235292	0.52989999	0.99978775	-4.7149671
CNIH1	0.09612299	6.02551017	0.95327209	0.3448196	0.99978775	-4.689075
UBASH3B	0.09609494	1.61689016	0.15323916	0.87879699	0.99978775	-4.6322815
RNF13	0.09600456	6.08744744	0.85483344	0.3965265	0.99978775	-4.7296981
WDFY1	0.095904	4.38946446	0.97376662	0.33463665	0.99978775	-4.6541002
NUCB2	0.09582372	6.90079789	0.72703614	0.47043255	0.99978775	-4.7736534
SRD5A3	0.09580879	4.76221033	1.0037811	0.32008705	0.99978775	-4.6466243
HCFC2	0.09580549	4.7551692	1.06965658	0.28966591	0.99978775	-4.6201334
TMEM136	0.09570788	-1.3201682	0.23836514	0.81252559	0.99978775	-4.6178166
MXD4	0.0956507	3.97271394	0.4169676	0.67840171	0.99978775	-4.7290881
ARSK	0.09564614	2.95196491	0.59703132	0.55305196	0.99978775	-4.6980909
ARFGEF1	0.09564455	5.85718997	0.85516946	0.39634217	0.99978775	-4.7235267
FAM216A	0.09553547	4.23476619	0.90386313	0.37019572	0.99978775	-4.6665346
CBLL1	0.09550257	4.82500937	1.02820075	0.30856809	0.99978775	-4.6365762
DDB1	0.09548885	8.17302128	1.99061914	0.05173843	0.99978775	-4.0650202
NAPA	0.09548483	5.31182146	1.13888386	0.25992028	0.99978775	-4.5968748
ENSCAFG00000019461	0.0952448	2.85387065	0.71168347	0.47981047	0.99978775	-4.6640361
SLC8A3	0.09523619	1.76230508	0.16142545	0.87237777	0.99978775	-4.6974498
LSM14A	0.09512764	6.13892198	1.40285959	0.16654345	0.99978775	-4.4554135
CLN8	0.09511643	3.63914288	0.57774494	0.56590544	0.99978775	-4.7250856
NDUFA6	0.09511631	3.85510627	0.74297967	0.46080488	0.99978775	-4.7016572
SPPL3	0.09508678	4.04872018	0.68533161	0.49614887	0.99978775	-4.7217965
GLMP	0.09490913	6.39131603	0.59204315	0.55636219	0.99978775	-4.8161891
CHAC2	0.09489021	0.86045239	0.45013289	0.65446739	0.99978775	-4.627995
HP1BP3	0.09487946	6.04711654	1.3231496	0.19151798	0.99978775	-4.5027712
GSS	0.09475259	4.12423609	0.87771563	0.38409606	0.99978775	-4.6690671
MAPK7	0.0947152	3.87483575	0.64975931	0.51867992	0.99978775	-4.7242124
MEGF8	0.09469007	5.83773306	0.90948372	0.36725024	0.99978775	-4.7052504
SERTAD4	0.09466985	0.98920955	0.23824058	0.81262168	0.99978775	-4.6531585
SLC25A11	0.09462154	5.46160626	0.96590165	0.33852064	0.99978775	-4.6799811
SLC12A7	0.09456816	1.32904756	0.18259275	0.85582065	0.99978775	-4.6812641
MED23	0.09455565	5.32699543	1.13324108	0.26226032	0.99978775	-4.6010003
PARN	0.09452007	5.61844323	1.01684628	0.31388857	0.99978775	-4.6551146
GPR162	0.09440993	-0.3462464	0.20148753	0.84109581	0.99978775	-4.6217649
MORN4	0.0944029	2.34319723	0.48530805	0.62947639	0.99978775	-4.66459
RINT1	0.09438434	6.40858406	0.91909832	0.36224662	0.99978775	-4.7043109
RHBDD2	0.09429804	5.12653122	0.65575251	0.51484615	0.99978775	-4.7674414
AGL	0.09428808	6.29665902	0.50647159	0.6146455	0.99978775	-4.8378315
UIMC1	0.09427747	3.16035108	0.68334354	0.49739377	0.99978775	-4.6787801
SDF4	0.09420201	7.06110348	0.96924567	0.33686562	0.99978775	-4.6818948
COMMD2	0.09412268	4.2430031	0.89480903	0.3749722	0.99978775	-4.6771369
DNAJC10	0.09405038	8.71223774	1.4348834	0.15724935	0.99978775	-4.4421365
VPS13D	0.09398467	6.70882058	0.57543272	0.56745628	0.99978775	-4.8206349
NDRG1	0.09395071	8.45819078	0.58124913	0.56355913	0.99978775	-4.8023308
WWC2	0.09392074	7.03334786	0.795404	0.42995912	0.99978775	-4.7477492

GAA	0.09391174	6.76713438	0.50366975	0.61659986	0.99978775	-4.8379233
MRAP2	0.09390326	-1.5098514	0.1728482	0.86343523	0.99978775	-4.6122668
EIF2S1	0.09386143	6.42080377	0.79331967	0.43116149	0.99978775	-4.752946
MSANTD3-TMEFF1	0.0938523	3.45520391	0.71546971	0.47748801	0.99978775	-4.7297459
SALL2	0.09383003	3.6685482	0.60887674	0.54523116	0.99978775	-4.7169923
PLCL2	0.09379928	3.11326138	0.29138451	0.77190513	0.99978775	-4.7180378
COG5	0.09371528	4.82021804	1.14050115	0.25925234	0.99978775	-4.5925195
AKAP11	0.09369787	6.29715838	1.11591097	0.26954051	0.99978775	-4.6140881
RAMAC	0.09361848	3.73493401	0.88160381	0.3820085	0.99978775	-4.6534689
YWHAG	0.0936074	6.34499482	0.86476061	0.3911033	0.99978775	-4.7255595
TAX1BP3	0.09359854	6.17467949	0.9769747	0.33306091	0.99978775	-4.6781428
ENSCAFG00000018403	0.09357114	6.84532412	1.30072367	0.199032	0.99978775	-4.5155664
TBC1D1	0.09343957	5.19490913	0.22290492	0.82447426	0.99978775	-4.8816137
RBL2	0.0933563	5.80247213	0.659256	0.51261206	0.99978775	-4.7860197
TOR1B	0.09331394	4.57243156	0.91766897	0.36298769	0.99978775	-4.6763684
SLC35B2	0.0932052	4.63970103	0.76298121	0.44888853	0.99978775	-4.7311738
ABI1	0.09320303	6.18890116	1.40149959	0.16694737	0.99978775	-4.4562204
RDX	0.09317925	7.50490588	1.1395474	0.25964609	0.99978775	-4.5990361
ARID5A	0.09317023	2.93524012	0.44421629	0.65871143	0.99978775	-4.7241685
CLPTM1L	0.09315231	7.43183804	0.79046306	0.43281262	0.99978775	-4.7456278
ENSCAFG00000014627	0.09314619	-0.65965	0.31538529	0.75371905	0.99978775	-4.6246058
CHD6	0.09314171	5.11554056	0.66432035	0.50939184	0.99978775	-4.7624072
ME3	0.09305388	6.46393101	0.40938478	0.68392233	0.99978775	-4.8580959
ADAM17	0.09302347	6.41123577	0.88591444	0.37970251	0.99978775	-4.7178197
CHMP4B	0.09299425	7.43563729	1.07063134	0.28923132	0.99978775	-4.6323435
ZNF513	0.09293734	3.30523761	0.64259482	0.52328278	0.99978775	-4.6943028
CYP20A1	0.09289126	5.72484093	0.95201045	0.34545303	0.99978775	-4.6864759
ENSCAFG00000029055	0.09285603	2.79278233	0.56598419	0.57381523	0.99978775	-4.6908901
ENSCAFG00000002139	0.092847	3.46764366	0.49516322	0.6225505	0.99978775	-4.7740779
ARL8B	0.0928446	4.11347832	0.89251297	0.37618968	0.99978775	-4.6731264
LYSMD3	0.09284416	2.88252704	0.69709744	0.48881644	0.99978775	-4.6608056
RPS15	0.09278426	8.00385491	0.67229902	0.50434072	0.99978775	-4.7753301
SUPT4H1	0.09273259	3.43526818	0.75847317	0.45155849	0.99978775	-4.6754989
ZFAND1	0.09270861	3.30590043	0.71640746	0.47691377	0.99978775	-4.6791219
UBR3	0.09264903	6.26512019	1.05299145	0.29716595	0.99978775	-4.6444516
RPS5	0.09250129	8.71325754	0.79420042	0.43065317	0.99978775	-4.7254752
ADGRE5	0.09248679	5.90531662	0.461641	0.64624542	0.99978775	-4.8398222
HERC3	0.09247226	5.93031061	0.98745497	0.3279476	0.99978775	-4.6718601
OSBPL8	0.09241844	6.29017741	0.9716109	0.33569827	0.99978775	-4.6819001
ENSCAFG00000014869	0.09236142	1.82720227	0.62161123	0.53688682	0.99978775	-4.6573629
ALG3	0.09231737	4.69903758	0.80632703	0.42369084	0.99978775	-4.7208195
GDPGP1	0.09230497	2.0764049	0.502978	0.61708281	0.99978775	-4.6496289
AZIN1	0.09222837	6.6846078	0.47187879	0.63896817	0.99978775	-4.8395022
RPS3A	0.09216536	8.45073055	0.78612698	0.43532607	0.99978775	-4.7332034
PIGX	0.09212802	3.72445702	0.6346752	0.52839575	0.99978775	-4.7124604
ERMP1	0.09211266	5.49482531	0.84570175	0.40155615	0.99978775	-4.7216328
AP1B1	0.09210218	6.62498683	0.68638119	0.49549234	0.99978775	-4.788958
USO1	0.09209984	7.2898393	0.87326815	0.3864927	0.99978775	-4.7176899
NSMCE1	0.09209526	4.32096729	0.85764245	0.39498722	0.99978775	-4.6834456
GALNT11	0.09195816	4.94754323	0.98246137	0.3303774	0.99978775	-4.6573917

HELZ	0.09194588	5.26743583	0.78859403	0.43389496	0.99978775	-4.7376041
CLN5	0.09190171	6.13151727	1.07914817	0.28545337	0.99978775	-4.6322151
BMT2	0.09183338	4.05007817	0.65112938	0.51780216	0.99978775	-4.7158256
SLC37A3	0.09175611	4.96508498	0.65363827	0.51619687	0.99978775	-4.7777842
MAPRE1	0.09172234	1.54475929	0.33824673	0.73652585	0.99978775	-4.6502838
MFSD9	0.09166135	1.80458186	0.48423934	0.63022948	0.99978775	-4.6582978
NAALADL2	0.09153322	3.09525716	0.47825947	0.6344506	0.99978775	-4.7747969
HGSNAT	0.09147471	6.10419458	0.92809664	0.35760371	0.99978775	-4.7003877
IARS2	0.09145134	7.08816356	1.09456713	0.27870136	0.99978775	-4.6239407
GSTZ1	0.09144551	2.30265056	0.55089375	0.5840427	0.99978775	-4.6687802
ENSCAFG00000009117	0.09138226	5.80082879	0.41470761	0.68004524	0.99978775	-4.8437802
ENSCAFG00000005837	0.09136287	2.34724975	0.61544035	0.54092206	0.99978775	-4.6571436
LHX6	0.09119306	-1.3083615	0.09653386	0.92346405	0.99978775	-4.6107759
SMIM19	0.09115721	5.6175396	0.75336111	0.45459733	0.99978775	-4.7593755
CYB5D1	0.09102659	0.01799932	0.27431178	0.78492123	0.99978775	-4.6207147
FMN1	0.09096445	0.45938254	0.23049596	0.81860199	0.99978775	-4.6518325
ATOX1	0.09087939	5.14815168	0.62009426	0.53787735	0.99978775	-4.7780566
PCMTD1	0.09086684	6.18721604	0.75877415	0.45137994	0.99978775	-4.7651245
ISCA1	0.09083741	3.46306314	0.6878554	0.49457099	0.99978775	-4.6851284
TBCA	0.09082498	5.05892196	0.83570784	0.40710562	0.99978775	-4.7188341
MACF1	0.0908038	8.01885401	0.67277257	0.50404179	0.99978775	-4.7794235
MTCP1	0.0907252	-1.3483427	0.20884692	0.83537573	0.99978775	-4.6145868
PER2	0.09071753	4.12441198	0.25493599	0.79976829	0.99978775	-4.7866958
RANBP9	0.09068038	6.18644748	0.90244824	0.37093957	0.99978775	-4.7105064
UBE2B	0.09066207	4.15014553	0.92025693	0.36164663	0.99978775	-4.6544726
BRCA2	0.09065586	3.95892861	0.40289806	0.68865878	0.99978775	-4.788137
STRN4	0.09062553	5.9189278	1.12350038	0.26633491	0.99978775	-4.6099298
RNF126	0.09052936	5.13066388	0.79001898	0.43306964	0.99978775	-4.7401431
LYPLAL1	0.09050756	2.59633991	0.43889554	0.66253775	0.99978775	-4.6775838
ARVCF	0.09040919	3.63661667	0.4721616	0.63876764	0.99978775	-4.7702137
MLH3	0.09039614	4.46759947	0.52188544	0.60394449	0.99978775	-4.7632613
KCND3	0.09021195	-1.6482198	0.14120661	0.88824701	0.99978775	-4.6095894
ZYG11B	0.09020006	5.07579066	0.94678053	0.34808698	0.99978775	-4.6751322
NAF1	0.09016839	4.87028972	0.84838938	0.40007175	0.99978775	-4.7065588
ADCY9	0.09016611	5.69805256	1.01622256	0.31418262	0.99978775	-4.6582936
TWF1	0.09011023	7.04383398	0.61876797	0.53874414	0.99978775	-4.8048239
MEF2A	0.08998264	5.35656534	0.84694635	0.40086832	0.99978775	-4.7228376
TOX4	0.08993075	6.13747514	1.03420813	0.30577811	0.99978775	-4.6532904
GRAMD1A	0.08988367	5.53968542	0.4822935	0.63160167	0.99978775	-4.8326323
VCL	0.08987186	9.17594654	0.43229036	0.66730038	0.99978775	-4.7986618
HDAC1	0.08979899	4.59702119	0.85014129	0.39910599	0.99978775	-4.691342
VGLL4	0.08979763	5.45065426	0.62495988	0.53470362	0.99978775	-4.8043825
MAGT1	0.08978977	4.31862598	0.8190056	0.41648454	0.99978775	-4.6897835
GABPB1	0.08974498	3.87595524	0.60256194	0.5493934	0.99978775	-4.7326108
DYRK1A	0.08966678	5.72320963	1.17462268	0.24544463	0.99978775	-4.5822751
OSR1	0.08966097	4.82457627	0.26746901	0.79015576	0.99978775	-4.8669848
ZNF571	0.08965207	-0.7675341	0.18999981	0.85004181	0.99978775	-4.6202093
ENSCAFG00000029673	0.08964909	4.3780523	0.68297531	0.49762454	0.99978775	-4.7338854
ENSCAFG00000015024	0.08960808	-0.2689951	0.22184918	0.82529177	0.99978775	-4.6246415
USP54	0.08946609	3.95093797	0.26124675	0.79492408	0.99978775	-4.7931818

SLC39A9	0.08938369	6.05426037	1.06877029	0.29006145	0.99978775	-4.6351366
MFN1	0.08931633	5.52631192	1.04449937	0.3010387	0.99978775	-4.6430904
ERC1	0.08929548	5.60728836	0.77161733	0.44379949	0.99978775	-4.7529373
OSTM1	0.08927592	5.25664012	0.75014702	0.45651399	0.99978775	-4.7590111
STX6	0.08923534	3.61559943	0.86631142	0.3902603	0.99978775	-4.6520863
ENSCAFG00000001388	0.08922269	0.73270055	0.31078713	0.7571927	0.99978775	-4.6333324
VTA1	0.08918534	5.03417455	0.97250199	0.33525917	0.99978775	-4.6647408
AOC3	0.0891158	-1.8113024	0.1464317	0.88414129	0.99978775	-4.6090222
RAB10	0.08905228	6.88484838	1.51756873	0.13512059	0.99978775	-4.3832145
MARC2	0.08901854	3.04021068	0.5503176	0.5844349	0.99978775	-4.6962621
DDX24	0.08899808	7.05720789	1.24425124	0.21893205	0.99978775	-4.5468267
SMYD4	0.08899727	3.85988178	0.67924555	0.49996525	0.99978775	-4.7189623
EIF2AK1	0.08896182	4.83954537	0.84042665	0.40447949	0.99978775	-4.7074416
HNRNPH2	0.08894015	5.85188557	1.19854553	0.23608557	0.99978775	-4.5706347
PIP4K2C	0.08893051	6.04387466	0.75135301	0.45579427	0.99978775	-4.7626734
DSTYK	0.08892566	3.55359423	0.76559443	0.44734504	0.99978775	-4.6754873
NOL4L	0.08892093	3.2839833	0.20809104	0.83596283	0.99978775	-4.6973252
CERS5	0.08882794	5.69349725	1.01458867	0.31495379	0.99978775	-4.6592072
COA5	0.08872891	2.57725531	0.58383183	0.56183291	0.99978775	-4.6679011
ENSCAFG00000006373	0.088698	3.99139384	0.61747985	0.53958668	0.99978775	-4.7794578
DPY19L4	0.08868635	6.41330624	0.68486021	0.4964439	0.99978775	-4.7893856
ENSCAFG00000007327	0.08868031	1.60610146	0.39846481	0.6919031	0.99978775	-4.6518924
RPL27	0.08862863	7.02008315	0.99412624	0.32472014	0.99978775	-4.6710297
TMED5	0.08860731	5.27870521	1.02484617	0.31013356	0.99978775	-4.6463982
PDGFRL	0.08853717	2.93352008	0.14885643	0.88223708	0.99978775	-4.7004607
ENSCAFG00000031202	0.08853602	2.57659629	0.4521441	0.65302733	0.99978775	-4.6701487
MAP6D1	0.08850609	0.43570948	0.22681469	0.82144846	0.99978775	-4.6267303
CXHXorf38	0.08849822	2.51752301	0.28509647	0.77669161	0.99978775	-4.6677529
PHF12	0.08848177	4.52330933	0.77938939	0.43924872	0.99978775	-4.7076135
CCDC189	0.08847697	0.98525603	0.29523838	0.76897593	0.99978775	-4.6373947
SEC22A	0.08842642	3.22393687	0.53051794	0.59798926	0.99978775	-4.7019582
MICU2	0.08836601	5.54145817	0.99713724	0.32327045	0.99978775	-4.6621929
CSNK1A1	0.08835917	8.40447213	1.23688862	0.22163131	0.99978775	-4.5470088
SNX25	0.0882737	4.52204067	0.73188036	0.46749529	0.99978775	-4.7301637
NIT1	0.08822054	4.54562949	0.74225557	0.46123966	0.99978775	-4.7372725
JAM3	0.08819462	5.37420111	0.47954349	0.63354319	0.99978775	-4.818918
PGLS	0.08819247	5.50461066	0.56999781	0.57110978	0.99978775	-4.80957
ENSCAFG00000018879	0.08814152	5.71099138	0.42859447	0.66997131	0.99978775	-4.8452338
POGLUT1	0.08800599	6.13727504	1.13089486	0.26323768	0.99978775	-4.6064398
COPG1	0.08799013	8.36235615	0.83886624	0.40534674	0.99978775	-4.714259
DDX1	0.08794118	7.29522762	0.9307976	0.35621763	0.99978775	-4.695235
ENSCAFG00000028770	0.08786987	4.89226004	1.02713947	0.30906277	0.99978775	-4.6397955
EIF4G3	0.08780646	7.95969969	0.96963278	0.33667438	0.99978775	-4.6750017
ENSCAFG00000015459	0.08772065	4.57030842	1.01939984	0.31268664	0.99978775	-4.6398536
FAM20B	0.08767773	6.99323561	0.9070509	0.36852331	0.99978775	-4.7069966
IL17RB	0.08767411	-0.684391	0.19128854	0.84903721	0.99978775	-4.6307604
UBE2H	0.0876738	4.74756948	0.5750955	0.56768264	0.99978775	-4.7678955
ENSCAFG00000003149	0.08755264	1.02826963	0.36046749	0.71994344	0.99978775	-4.6343595
ANKRA2	0.08754601	5.02961886	0.77509985	0.44175695	0.99978775	-4.7376418
SEMA6C	0.08751837	2.62331698	0.28972417	0.77316813	0.99978775	-4.6933387

EVI2B	0.08738638	1.47933415	0.16428427	0.87013806	0.99978775	-4.646858
SP7	0.08738145	0.14166847	0.19245252	0.84813006	0.99978775	-4.6318699
TTC39A	0.08734306	4.51995105	0.62659504	0.53363923	0.99978775	-4.7631148
VDAC2	0.0872279	7.15208265	0.72578273	0.47119425	0.99978775	-4.7711497
RNF103	0.08718921	6.2778336	1.06989538	0.2895594	0.99978775	-4.6363782
GLMN	0.08716547	4.39180325	0.68801175	0.49447332	0.99978775	-4.7424384
ARHGAP23	0.08712777	5.18708469	0.4610535	0.6466641	0.99978775	-4.8309254
E2F3	0.08712082	4.55285769	0.87945331	0.38316221	0.99978775	-4.682829
IGHMBP2	0.08700244	5.10221442	0.77292399	0.44303247	0.99978775	-4.7405901
ENSCAFG00000019687	0.08689251	6.28715992	0.46845345	0.64139905	0.99978775	-4.8452219
ATP6V1B2	0.08685304	7.09176034	0.9693986	0.33679007	0.99978775	-4.6806014
RABEP1	0.08684644	5.51758815	0.83269048	0.40879032	0.99978775	-4.7266499
LMTK2	0.08677198	5.22134725	0.77862148	0.43969712	0.99978775	-4.7437693
LRRC28	0.08674187	5.08400403	0.71206967	0.47957328	0.99978775	-4.7577852
NMNAT1	0.08673926	3.25687968	0.67143504	0.50488637	0.99978775	-4.6790456
SLC23A2	0.08665835	4.81990279	0.61123233	0.54368267	0.99978775	-4.7565906
LMAN1	0.0866417	7.33214291	0.58548354	0.56073033	0.99978775	-4.8090169
FBXW11	0.08658005	6.41341909	0.99359153	0.32497804	0.99978775	-4.6722654
BCAS2	0.08644739	4.20535888	0.78571212	0.435567	0.99978775	-4.7013503
PKDCC	0.08631875	4.20445007	0.33937875	0.73567793	0.99978775	-4.8433347
RCBTB2	0.08631134	4.38827202	0.55264482	0.58285145	0.99978775	-4.7782875
ENSCAFG00000031112	0.08618869	-0.3885909	0.24059715	0.81080417	0.99978775	-4.6220597
RNF121	0.08618446	3.90164998	0.79373583	0.43092126	0.99978775	-4.6944282
HYAL1	0.08617095	4.28593703	0.33530265	0.73873259	0.99978775	-4.7615532
ENSCAFG00000003315	0.08608354	4.74020508	0.58816976	0.55893947	0.99978775	-4.7795981
CDKAL1	0.08594095	3.62206211	0.83699854	0.40638628	0.99978775	-4.6711868
DPYD	0.08590308	7.07920796	0.25442467	0.80016114	0.99978775	-4.881331
FAAP24	0.08589853	3.76283717	0.83075414	0.4098737	0.99978775	-4.6759092
SYTL2	0.08578762	1.99144669	0.30736185	0.75978359	0.99978775	-4.7996928
JMJD1C	0.08578065	5.88001838	0.76210595	0.44940619	0.99978775	-4.752942
SLC39A7	0.08577087	7.43627907	0.75661846	0.45265964	0.99978775	-4.7552882
HBS1L	0.08571923	6.27812426	1.02379413	0.31062562	0.99978775	-4.6584059
ANKRD17	0.08547936	7.43536983	1.12507051	0.2656751	0.99978775	-4.6077996
PFDN5	0.08543951	5.34480423	1.08764166	0.28172009	0.99978775	-4.6205266
DENND4C	0.08543506	6.28484081	0.82609265	0.41248898	0.99978775	-4.7407494
EXOG	0.08515982	3.34271567	0.72649198	0.47076315	0.99978775	-4.6803767
ARID4B	0.08515905	5.30130956	0.63283869	0.52958515	0.99978775	-4.77864
ENSCAFG00000014978	0.0850957	5.98965281	0.46175907	0.64616129	0.99978775	-4.8411864
MRPL21	0.08497406	5.4780679	0.64734067	0.52023137	0.99978775	-4.7898795
TIAM2	0.08483834	4.65729264	0.52112949	0.6044673	0.99978775	-4.812587
RHOJ	0.08482982	3.42966005	0.24538674	0.80711344	0.99978775	-4.7905325
ADORA2B	0.08478711	4.98330774	0.27291175	0.78599141	0.99978775	-4.8216109
ENSCAFG00000000225	0.08476174	-0.6247329	0.20099697	0.84147741	0.99978775	-4.6230462
TGFB1I1	0.08458652	7.77011881	0.59809679	0.55234618	0.99978775	-4.7935475
PDCD6IP	0.08452136	7.93193219	1.02386176	0.31059397	0.99978775	-4.6491454
TANGO2	0.08451731	4.58362844	0.66744831	0.50740836	0.99978775	-4.7479745
TIPARP	0.08444168	5.23812031	0.54381656	0.58886912	0.99978775	-4.8082311
ATAT1	0.08442719	2.38854076	0.45735163	0.64930484	0.99978775	-4.6766491
ZMYND11	0.08442069	6.44094053	1.30042622	0.19913313	0.99978775	-4.5159413
SSX2IP	0.08438856	3.46115248	0.29155431	0.771776	0.99978775	-4.7629943

HSPG2	0.084363	11.6873701	0.56107163	0.5771351	0.99978775	-4.7241355
EIF3E	0.08430135	7.81829473	0.95573653	0.34358446	0.99978775	-4.6819363
SLC25A34	0.08425294	-0.1644866	0.2552723	0.79950994	0.99978775	-4.6290332
MEMO1	0.08423681	5.81203944	1.14843347	0.25599403	0.99978775	-4.5966035
DENND2A	0.08420843	5.54221575	0.3107378	0.75722999	0.99978775	-4.8255319
TSPAN6	0.08419453	4.83742799	0.40451041	0.6874803	0.99978775	-4.8194729
ANKIB1	0.08415604	6.94273143	0.94934787	0.34679235	0.99978775	-4.6905264
SLC4A2	0.08407184	7.24025457	0.72046255	0.47443511	0.99978775	-4.771197
SEC24D	0.08406837	8.23769515	0.58728804	0.55952698	0.99978775	-4.7896909
LPXN	0.08403638	3.24207443	0.4485999	0.65556593	0.99978775	-4.7300772
HMGCL	0.08397382	4.42905008	0.60850157	0.545478	0.99978775	-4.7588011
WWP2	0.08397045	5.32361613	0.86665388	0.3900743	0.99978775	-4.71943
SCNN1A	0.08391361	2.98132528	0.43752126	0.66352752	0.99978775	-4.6924125
CORO2B	0.0836855	4.52965415	0.13930967	0.88973834	0.99978775	-4.8278165
SCYL2	0.08357314	6.38777963	0.81194597	0.4204879	0.99978775	-4.7462099
ANGPTL1	0.08357138	1.19236063	0.35811564	0.72169229	0.99978775	-4.8097704
ME2	0.08352588	6.26060027	0.8391938	0.4051646	0.99978775	-4.7353626
INO80D	0.08348274	2.52545386	0.47584423	0.63615897	0.99978775	-4.6690492
GTF3A	0.08342495	6.56309543	0.67154979	0.50481388	0.99978775	-4.7933781
ENSCAFG00000006911	0.08330553	1.56289679	0.42471041	0.67278287	0.99978775	-4.6502988
YIPF1	0.08325248	4.4133345	0.88934953	0.37787121	0.99978775	-4.6760446
KIFC3	0.08320465	5.11856065	0.61113522	0.54374646	0.99978775	-4.7992818
QDPR	0.08319415	5.10510222	0.61348353	0.54220491	0.99978775	-4.7815899
ENSCAFG00000007112	0.08317705	6.99132502	0.93743689	0.35282533	0.99978775	-4.6946243
SPCS2	0.08312824	6.04716909	1.18628645	0.24084861	0.99978775	-4.5781104
NAXD	0.08307506	4.10317078	0.53647967	0.59389257	0.99978775	-4.747546
ENSCAFG00000018652	0.08296223	4.38339701	0.44029329	0.6615317	0.99978775	-4.8017924
RNF135	0.0829398	2.66178216	0.4664685	0.64280953	0.99978775	-4.6825507
MBLAC2	0.08293173	3.59204973	0.58768813	0.55926035	0.99978775	-4.7193153
KIF5B	0.08288271	8.5576518	0.43545815	0.66501452	0.99978775	-4.811554
ENSCAFG00000003574	0.08281655	4.62570989	0.64317382	0.52290999	0.99978775	-4.7654146
BCAP31	0.08276462	6.45682306	0.73745954	0.46412538	0.99978775	-4.77242
PFN2	0.08274441	7.2994598	0.55438493	0.58166882	0.99978775	-4.8180717
FAM200A	0.08253912	1.94709148	0.44256668	0.65989674	0.99978775	-4.662011
C6H7orf43	0.0825283	3.79433243	0.60994259	0.54453023	0.99978775	-4.7395554
ADAMTS1	0.08249914	5.63363149	0.22335513	0.8241257	0.99978775	-4.8554308
SLC30A9	0.0824094	7.12631917	0.63662035	0.52713752	0.99978775	-4.7985802
EVI5L	0.08240386	4.21840138	0.58100816	0.56372032	0.99978775	-4.7596205
SEC23A	0.08240274	7.08236801	0.56934586	0.57154882	0.99978775	-4.8164534
ABHD17B	0.08238692	3.40426852	0.40052145	0.6903973	0.99978775	-4.7260468
CRY2	0.0823257	4.26545749	0.45518047	0.65085576	0.99978775	-4.7991973
TNFRSF11A	0.0822567	-1.5832242	0.14979045	0.88150375	0.99978775	-4.611762
CUL4A	0.08224312	7.12529801	1.00772644	0.31820665	0.99978775	-4.6641222
NME6	0.08221286	2.91846737	0.58517408	0.56093682	0.99978775	-4.6807754
APOD	0.08212705	-1.5945548	0.07165171	0.94315127	0.99978775	-4.6078333
ENSCAFG00000015284	0.08205784	4.86763884	1.00132834	0.32125982	0.99978775	-4.6509092
HMG20A	0.08205598	5.11212093	0.87984365	0.38295264	0.99978775	-4.6984091
ENSCAFG00000011904	0.08192835	-0.6648977	0.20354126	0.83949867	0.99978775	-4.6218801
ST7L	0.0819098	3.23368895	0.57692367	0.56645604	0.99978775	-4.6989425
ACBD5	0.08187024	5.25612021	0.73923952	0.46305319	0.99978775	-4.7522333

ENSCAFG00000031003	0.08182485	6.57300091	0.51649691	0.60767567	0.99978775	-4.8356376
VPS41	0.08181752	6.16712984	0.98387375	0.32968895	0.99978775	-4.6760583
LRP6	0.08180311	4.96081991	0.68677242	0.49524774	0.99978775	-4.7453382
ST3GAL1	0.0817996	5.233813	0.42696983	0.67114677	0.99978775	-4.8169385
ZNF605	0.08179068	2.22448259	0.33223143	0.74103699	0.99978775	-4.6683479
MLF1	0.08175021	4.76329523	0.41280071	0.68143321	0.99978775	-4.8047114
TCN2	0.08174026	5.08609663	0.38766768	0.69982886	0.99978775	-4.8329323
SPRED1	0.08168713	4.82796836	0.62361851	0.5355776	0.99978775	-4.7491202
RSU1	0.08160435	7.82625616	0.76513085	0.44761863	0.99978775	-4.7493985
DNAJB11	0.08158683	6.47353308	0.53405897	0.59555439	0.99978775	-4.8313938
NDN	0.08156874	4.95597374	0.44529041	0.65794011	0.99978775	-4.8143574
ENSCAFG00000016356	0.08153018	2.23565891	0.32245761	0.74838633	0.99978775	-4.6947564
PRDX2	0.08151722	6.48199128	0.6341754	0.5287193	0.99978775	-4.8045341
ENSCAFG00000004924	0.08147904	2.00600443	0.60455124	0.54808046	0.99978775	-4.6613584
ENSCAFG00000020852	0.08139868	-0.891458	0.19704469	0.84455322	0.99978775	-4.6175401
SAP30BP	0.08135842	4.7521626	0.88972105	0.37767348	0.99978775	-4.6883543
SDHAF4	0.08127266	2.96442781	0.68177608	0.4983765	0.99978775	-4.6808449
NADK	0.0811589	6.35001585	0.71762974	0.47616589	0.99978775	-4.7782295
C7H18orf25	0.08111904	5.52281738	0.66417428	0.50948457	0.99978775	-4.7856591
CAAP1	0.08097354	3.46818602	0.74052228	0.46228138	0.99978775	-4.6898094
KIF13B	0.08096997	4.83188652	0.42354467	0.67362764	0.99978775	-4.8057565
SMIM15	0.08090037	4.02184438	0.76441244	0.4480428	0.99978775	-4.7044377
FLRT2	0.08078433	6.09326429	0.28938977	0.77342257	0.99978775	-4.8679319
CNPPD1	0.0807798	4.99200946	0.54525161	0.58788894	0.99978775	-4.7976982
RPN2	0.08077612	9.00487687	0.8284743	0.41115151	0.99978775	-4.7065961
GTF3C3	0.0807315	4.29819504	0.65036106	0.5182943	0.99978775	-4.7359694
TFRC	0.0806823	7.12801709	0.41862045	0.6772007	0.99978775	-4.854154
MAPK1IP1L	0.08062649	6.93412907	0.86383017	0.39160962	0.99978775	-4.7254813
ERI3	0.0805946	4.645544	0.80417902	0.42491914	0.99978775	-4.708545
CDR2	0.08056117	4.57843666	0.55451152	0.58158283	0.99978775	-4.7784074
SPTY2D1	0.08050744	4.00478763	0.58550417	0.56071656	0.99978775	-4.72385
WDR83OS	0.08049911	4.96607525	0.57951163	0.56472191	0.99978775	-4.7907429
MRTFB	0.0804932	4.72225271	0.4419864	0.66031391	0.99978775	-4.7888909
ENSCAFG00000015438	0.08044962	1.00736504	0.35015221	0.72762501	0.99978775	-4.6502094
BAG1	0.08044282	6.7127323	0.61313878	0.54243108	0.99978775	-4.8104273
AP1G1	0.0804316	6.75015238	1.03222179	0.30669871	0.99978775	-4.6544547
USP33	0.08034065	5.85677395	0.68584412	0.49582823	0.99978775	-4.787507
WDCP	0.08033631	3.37813997	0.58482615	0.56116903	0.99978775	-4.7109862
ZNF391	0.08033421	1.82426147	0.31621133	0.75309556	0.99978775	-4.6560827
VPS54	0.08031236	5.09997334	0.68988395	0.49330473	0.99978775	-4.7705023
HIKESHI	0.08025988	4.6392849	0.94699636	0.34797802	0.99978775	-4.669223
SUCO	0.0801779	5.14838696	0.60220426	0.54962963	0.99978775	-4.7928992
HNRNPLL	0.08014705	4.93618516	0.85737551	0.39513334	0.99978775	-4.7121892
ESD	0.08011234	5.43172434	0.72902308	0.46922652	0.99978775	-4.7617882
ENSCAFG00000016281	0.08005594	1.12549714	0.33235212	0.74094639	0.99978775	-4.6436007
STOX2	0.08002349	0.62130388	0.19979151	0.84241528	0.99978775	-4.6629165
RABGEF1	0.07995062	4.9904061	0.8999662	0.37224676	0.99978775	-4.6933701
SMYD2	0.07994915	5.20589975	0.65344715	0.51631906	0.99978775	-4.774646
UTP6	0.07992436	5.33379106	0.53683464	0.59364906	0.99978775	-4.8151181
CAB39	0.07982701	6.60224784	0.99171074	0.32588626	0.99978775	-4.6728103

EBPL	0.07978967	3.62411194	0.47290085	0.63824361	0.99978775	-4.73526
MED27	0.07977677	3.95022274	0.7473812	0.45816706	0.99978775	-4.7023237
PMS1	0.07975745	5.56865443	0.8707947	0.38782964	0.99978775	-4.7182223
ENSCAFG00000003936	0.07970199	6.52086445	1.13410708	0.26190023	0.99978775	-4.6054895
ALDH18A1	0.0796999	7.38735185	0.53401238	0.5955864	0.99978775	-4.8199489
PQLC1	0.07966827	4.21186425	0.60882029	0.5452683	0.99978775	-4.7504674
FURIN	0.07963006	6.5069857	0.7590254	0.45123092	0.99978775	-4.765017
SDK2	0.07961211	-0.0785517	0.14074231	0.888612	0.99978775	-4.6329004
GSTA4	0.07959488	4.8147088	0.70729679	0.48250914	0.99978775	-4.7449163
C3AR1	0.07955902	-2.1272067	0.12396743	0.90181435	0.99978775	-4.6111964
ZNF451	0.07951718	4.70271137	0.69935822	0.48741443	0.99978775	-4.7443471
TMEM39A	0.07951642	4.88483518	0.7136694	0.47859152	0.99978775	-4.7437067
NAA25	0.07948621	4.24742645	0.54663445	0.58694514	0.99978775	-4.7603859
TMBIM6	0.07944584	8.38650984	0.99759634	0.32304979	0.99978775	-4.6555034
WWC3	0.07937566	6.17551886	0.52221666	0.60371549	0.99978775	-4.8333637
BACE1	0.07934481	5.76462899	0.48303089	0.63108152	0.99978775	-4.8376246
ATG10	0.07926616	0.4066417	0.24560632	0.80694435	0.99978775	-4.6305063
PYGB	0.07919652	7.73307933	0.71932669	0.47512867	0.99978775	-4.7671628
FBXO9	0.0791808	5.34695714	0.9634665	0.33972921	0.99978775	-4.6761641
FNTA	0.07910398	6.17387632	0.82352516	0.41393378	0.99978775	-4.7413827
ENSCAFG00000014399	0.07900886	4.18248802	0.72003696	0.47469491	0.99978775	-4.7222775
SGSM2	0.07898598	5.46686239	0.75420296	0.45409608	0.99978775	-4.7547555
UFSP2	0.07897412	4.19969773	0.78916229	0.43356571	0.99978775	-4.6986946
TSN	0.07892721	4.46990218	0.7485994	0.45743854	0.99978775	-4.7244461
ATP10A	0.07891985	4.43163393	0.40165513	0.68956779	0.99978775	-4.8396679
ZNF569	0.0788672	2.10377559	0.4661288	0.64305105	0.99978775	-4.6611395
SAP130	0.07880704	5.28652939	1.00015325	0.32182271	0.99978775	-4.6608409
GBE1	0.07880088	6.21165719	0.61120007	0.54370386	0.99978775	-4.8059925
TCIRG1	0.07878987	6.13771455	0.6391573	0.52549886	0.99978775	-4.8026878
ATP2B4	0.07873352	7.23306208	0.50164161	0.6180163	0.99978775	-4.8349227
PSMD14	0.07866596	5.70474063	0.7077183	0.48224946	0.99978775	-4.7772619
CMTM3	0.07861071	0.08084319	0.18422284	0.85454819	0.99978775	-4.6281678
RTL5	0.07849908	6.24679871	0.40959605	0.68376828	0.99978775	-4.8543713
SLC30A5	0.07848837	6.47730806	0.93707854	0.35300788	0.99978775	-4.6968038
XXYLT1	0.07848236	4.3762192	0.52020839	0.60510459	0.99978775	-4.7844874
ACAD8	0.07841454	4.67232645	1.02246385	0.31124858	0.99978775	-4.6374811
ENSCAFG00000028533	0.07840652	-0.6414239	0.1789495	0.85866597	0.99978775	-4.6210875
ENSCAFG00000008655	0.07836887	0.0082954	0.23115572	0.8180921	0.99978775	-4.63122
ADAMTSL3	0.07832601	3.91200973	0.20588319	0.83767823	0.99978775	-4.8559666
BTBD10	0.07831331	4.85854632	0.84441181	0.4022698	0.99978775	-4.7126507
ABCB7	0.07826961	5.07232776	0.70783511	0.48217751	0.99978775	-4.757347
ASAP2	0.07823757	4.00408641	0.23564877	0.81462182	0.99978775	-4.796783
RC3H1	0.07813253	4.2533027	0.44621964	0.65727313	0.99978775	-4.7678767
GAS6	0.0781136	5.32839974	0.17985968	0.85795496	0.99978775	-4.8784615
ENSCAFG00000023832	0.07807892	-0.5628535	0.14625338	0.88428135	0.99978775	-4.6269032
ENSCAFG00000031302	0.07803219	2.50928174	0.10699184	0.91520315	0.99978775	-4.6621397
IFT46	0.07801922	5.80739589	0.97291911	0.33505375	0.99978775	-4.6776632
KDM2A	0.07795549	6.66833201	1.0589622	0.29446364	0.99978775	-4.6422296
SMIM7	0.07795269	2.88367576	0.53924134	0.59199933	0.99978775	-4.6919651
UXT	0.07792301	3.20073907	0.64206874	0.5236216	0.99978775	-4.6947888

PDPK1	0.07787767	4.27010485	0.79535405	0.4299879	0.99978775	-4.7005935
LRIG2	0.07784956	3.96373437	0.52601967	0.60108902	0.99978775	-4.7386992
DDOST	0.07784736	8.74308776	0.77773038	0.4402178	0.99978775	-4.7283227
PURA	0.07781974	3.09709056	0.62932406	0.53186526	0.99978775	-4.681608
IZUMO4	0.07773451	0.16510433	0.20785764	0.83614413	0.99978775	-4.6235181
SMIM30	0.07759929	3.45620147	0.4571286	0.64946409	0.99978775	-4.7326392
CIAO1	0.07758045	4.01154146	0.7514474	0.45573797	0.99978775	-4.6983343
ENSCAFG00000014143	0.0775802	2.59807485	0.50465978	0.61590897	0.99978775	-4.6910179
FKTN	0.07754568	2.80881654	0.53674923	0.59370765	0.99978775	-4.6802822
TROVE2	0.0774512	5.39011922	0.60975958	0.54465055	0.99978775	-4.7984686
TMEM184B	0.0774435	7.24263814	0.59587095	0.55382111	0.99978775	-4.8108434
EWSR1	0.07743965	5.77376167	0.63226517	0.52995687	0.99978775	-4.8022408
ULK2	0.07741328	6.98576383	0.70784875	0.48216911	0.99978775	-4.7814383
FLRT3	0.07731938	3.75815184	0.25447173	0.80012498	0.99978775	-4.7796354
TTC28	0.07730665	5.26469914	0.71997938	0.47473007	0.99978775	-4.7622901
LSM7	0.07722535	2.24985985	0.39810355	0.69216773	0.99978775	-4.6770254
ATP11C	0.07722505	5.84969776	0.57454103	0.56805491	0.99978775	-4.8123849
RFX7	0.07720203	5.14488692	0.58715099	0.55961833	0.99978775	-4.7839294
MAP1A	0.07717375	8.26467407	0.25058736	0.80311099	0.99978775	-4.8604382
CDK13	0.07716849	5.97950191	0.9827963	0.33021406	0.99978775	-4.6744787
TMEM26	0.07715461	0.12074761	0.13408665	0.89384662	0.99978775	-4.6692622
RHNO1	0.07713766	2.65381946	0.44482655	0.65827316	0.99978775	-4.7057335
CWC25	0.07713518	3.29762605	0.58860786	0.55864767	0.99978775	-4.6908993
KARS	0.07710429	9.03557909	0.82132655	0.41517345	0.99978775	-4.7157284
DAAM2	0.07708144	2.22962609	0.37408525	0.70984709	0.99978775	-4.7960769
TPGS2	0.07699337	4.50106741	0.87652157	0.38473859	0.99978775	-4.6829783
PEAK1	0.07692664	6.92044158	0.696845	0.48897313	0.99978775	-4.7853895
SGCB	0.0768965	6.67642128	0.73861726	0.46342785	0.99978775	-4.7715793
CUL5	0.07680196	6.08414929	0.66678211	0.50783045	0.99978775	-4.793184
TKT	0.0767978	8.25737675	0.55545703	0.58094076	0.99978775	-4.8079782
PARP8	0.07670438	4.73370257	0.3357503	0.73839691	0.99978775	-4.7990949
EMC4	0.07667543	4.67722731	0.57228393	0.56957159	0.99978775	-4.7843146
ORC2	0.07666178	5.299339	0.75461688	0.45384975	0.99978775	-4.752274
COL3A1	0.07647398	13.9057832	0.23862285	0.81232679	0.99978775	-4.7053738
PBRM1	0.07644959	5.17135221	0.73777141	0.46393742	0.99978775	-4.7514851
MT-ND1	0.07636464	11.3833239	0.58943576	0.55809644	0.99978775	-4.7257474
FLNC	0.07622692	9.75173228	0.30584043	0.76093529	0.99978775	-4.7949549
DUSP14	0.07610344	4.2194654	0.39198905	0.69665261	0.99978775	-4.7861533
PIGM	0.0760749	3.0264859	0.37590345	0.70850296	0.99978775	-4.7121075
ACKR1	0.07597363	-2.285047	0.08060627	0.93606149	0.99978775	-4.6072326
GNPTAB	0.07591785	6.97494899	0.71956225	0.47498479	0.99978775	-4.7748663
USP22	0.07591014	6.27472405	1.01270532	0.3158443	0.99978775	-4.6636607
DYNC1I1	0.07580437	1.21481335	0.13130034	0.89603946	0.99978775	-4.6623843
MARF1	0.0757077	5.89309648	0.75557451	0.45328013	0.99978775	-4.7633687
PTPRM	0.0756989	7.89950764	0.55237779	0.58303304	0.99978775	-4.814928
GNL3L	0.07559186	6.63664902	1.11605619	0.26947892	0.99978775	-4.6145292
ENSCAFG00000019979	0.07557317	5.40766442	0.89280234	0.37603611	0.99978775	-4.7039521
EXOC6B	0.07552219	6.09902279	0.32037587	0.74995475	0.99978775	-4.8608842
EIF3H	0.07548842	7.15294699	0.99778609	0.32295862	0.99978775	-4.6682906
B4GALT5	0.07537697	4.97178781	0.43468317	0.66557345	0.99978775	-4.8187428

ENSCAFG00000018094	0.07524123	8.77849895	0.85794713	0.39482049	0.99978775	-4.7033415
DEDD	0.07522005	4.55045504	0.9067579	0.36867683	0.99978775	-4.6778453
RPL24	0.07520499	8.13857574	0.83493578	0.40753629	0.99978775	-4.722189
MPPE1	0.07512225	2.68775224	0.33128128	0.7417504	0.99978775	-4.6851195
MED18	0.07511332	2.76712876	0.53458897	0.59519035	0.99978775	-4.6790345
RND3	0.07505757	6.47319782	0.21500329	0.83059755	0.99978775	-4.8863414
PIP5K1C	0.0749769	4.14151847	0.44806967	0.65594607	0.99978775	-4.7469813
PHLDB3	0.07495968	2.10614686	0.37151486	0.71174886	0.99978775	-4.6648801
EPM2AIP1	0.07489916	3.42789402	0.51207212	0.61074742	0.99978775	-4.708578
TM4SF18	0.07477705	-1.0827401	0.0463958	0.96317077	0.99978775	-4.609704
PMPCB	0.074767	5.33675468	0.90456081	0.36982928	0.99978775	-4.6992726
ENSCAFG00000015834	0.07470271	7.16759609	0.75458932	0.45386614	0.99978775	-4.7629521
KREMEN1	0.07469391	3.5893987	0.33771159	0.7369268	0.99978775	-4.7490067
ZNF641	0.07466662	0.29572382	0.20406742	0.8390896	0.99978775	-4.6305731
CHMP1A	0.07465405	4.1394466	0.73193809	0.46746035	0.99978775	-4.7138357
PHB	0.07464557	5.17743729	0.70099121	0.48640314	0.99978775	-4.7660606
ROCK2	0.07462985	7.13338715	0.56181246	0.57663385	0.99978775	-4.8201043
DQX1	0.07462848	-0.7140945	0.16045338	0.87313957	0.99978775	-4.6174474
NUDT2	0.07462072	2.46694574	0.40188851	0.68939707	0.99978775	-4.6755881
CHCHD7	0.07457964	2.40810545	0.33486316	0.7390622	0.99978775	-4.6537112
NPC1	0.0745344	6.67509863	0.55246606	0.58297301	0.99978775	-4.8254557
TMEM245	0.07443384	4.45384938	0.681958	0.49826239	0.99978775	-4.7327425
ZNF444	0.0742636	2.38372465	0.39887829	0.69160026	0.99978775	-4.6785281
ANK2	0.07423747	6.11746334	0.28279088	0.77844884	0.99978775	-4.8785136
ENSCAFG00000002783	0.07423485	0.98125648	0.33839234	0.73641676	0.99978775	-4.6397064
TMEM161B	0.07400966	2.38559227	0.39170486	0.69686133	0.99978775	-4.676166
ETV6	0.07397948	3.91852734	0.44268948	0.65980848	0.99978775	-4.7666691
CUEDC2	0.07389838	5.03769273	0.84181214	0.40371042	0.99978775	-4.7121314
SLC37A4	0.07388742	3.02169506	0.48875517	0.62705001	0.99978775	-4.7025715
OCEL1	0.07378932	2.33286988	0.42065648	0.67572243	0.99978775	-4.6639099
VPS36	0.07372096	6.3687331	0.65332246	0.51639879	0.99978775	-4.7989734
LRRC40	0.07371976	4.85169528	0.79269903	0.4315199	0.99978775	-4.726933
SLC35F6	0.07370718	5.77719463	0.71038137	0.48061063	0.99978775	-4.7748368
DDX42	0.07368746	5.98211451	0.87135073	0.38752885	0.99978775	-4.7212271
ENSCAFG00000011174	0.07365824	3.54911964	0.76202314	0.44945519	0.99978775	-4.6832279
ENSCAFG00000030187	0.07363555	4.2400731	0.44353031	0.65920423	0.99978775	-4.780802
SPTLC2	0.07357591	5.55159904	0.53282927	0.59639943	0.99978775	-4.8233422
KMT2C	0.07354249	5.80606967	0.49764575	0.62081123	0.99978775	-4.83085
RAB18	0.07347134	6.41067126	1.07049364	0.28929268	0.99978775	-4.6367251
RCN2	0.07344286	5.79807012	0.57838176	0.56547869	0.99978775	-4.8117414
ARHGAP12	0.0734393	4.95988558	0.51073587	0.61167645	0.99978775	-4.7902688
UBB	0.07339698	4.70422472	0.66954424	0.50608164	0.99978775	-4.7553046
ETNK1	0.07335496	3.4131951	0.48409177	0.6303335	0.99978775	-4.7209399
ENSCAFG00000001098	0.07334429	0.15654136	0.19461014	0.84644909	0.99978775	-4.6246252
NDUFA8	0.07333367	5.83768974	0.73331554	0.46662709	0.99978775	-4.7693362
CNN3	0.07327824	7.43544024	0.64103254	0.52428933	0.99978775	-4.7978744
ENSCAFG00000015964	0.07323537	4.44808871	0.63114448	0.53068363	0.99978775	-4.7643638
STK39	0.07317126	5.50029482	0.55877631	0.57868944	0.99978775	-4.8109973
ATXN1	0.07315245	4.73053815	0.5112493	0.61131941	0.99978775	-4.7862617
FADS3	0.0731168	7.94336659	0.53888484	0.59224357	0.99978775	-4.8103088

ORMDL3	0.07309568	5.36737109	0.48348432	0.63076176	0.99978775	-4.824413
ZNF280D	0.07307318	4.16279664	0.45085831	0.65394783	0.99978775	-4.7666995
P2RX4	0.07306504	4.80848013	0.55654862	0.58019992	0.99978775	-4.7890364
COG7	0.07304633	5.69533973	0.87109567	0.3876668	0.99978775	-4.7177275
NOTCH2	0.07304251	9.48758046	0.29758725	0.7671923	0.99978775	-4.813521
ZBTB43	0.07304193	1.70959261	0.31325962	0.75532424	0.99978775	-4.6486211
ERN1	0.07300765	5.76174014	0.36851793	0.71396855	0.99978775	-4.851951
ATP10D	0.07298653	6.39205223	0.28021023	0.78041709	0.99978775	-4.8754989
TCF25	0.0729462	6.8358207	1.02664998	0.30929111	0.99978775	-4.656576
ELOA	0.07292725	5.49040402	0.76092419	0.45010568	0.99978775	-4.7528255
ZNF800	0.07292036	4.0499849	0.5593792	0.57828098	0.99978775	-4.7364022
SRP68	0.07289388	6.57422407	0.85858932	0.3944692	0.99978775	-4.7284029
SKP1	0.07288524	6.46488332	1.05231999	0.29747091	0.99978775	-4.6451442
MEN1	0.07284939	3.78376931	0.52494771	0.60182881	0.99978775	-4.7243376
PNPLA8	0.07283017	6.01292693	0.80313433	0.42551729	0.99978775	-4.7440212
ENSCAFG00000022727	0.07280349	-1.4238345	0.14528259	0.88504395	0.99978775	-4.6099518
PTPRA	0.07277122	6.41414695	0.7275352	0.47012947	0.99978775	-4.7735974
KIAA0319	0.0726927	0.72121525	0.18857065	0.85115618	0.99978775	-4.6267642
CCNC	0.07267827	4.37320579	0.55092405	0.58402208	0.99978775	-4.767011
DSE	0.07266055	6.29338474	0.280106	0.78049662	0.99978775	-4.8789142
ALDH7A1	0.07263262	5.01558423	0.43693221	0.66395194	0.99978775	-4.8177471
ETF1	0.07262047	5.04115605	0.62695274	0.53340654	0.99978775	-4.7806426
BAP1	0.07254646	6.06346994	0.91344208	0.36518489	0.99978775	-4.7058542
ENSCAFG00000031930	0.07251343	3.4210413	0.55103542	0.58394627	0.99978775	-4.7227002
TCAIM	0.07246345	3.35824974	0.45442327	0.65139702	0.99978775	-4.7270282
MTRF1	0.0724429	2.34107513	0.3786649	0.70646332	0.99978775	-4.6714245
ALG11	0.0723439	3.32254534	0.51226127	0.61061597	0.99978775	-4.7070078
SESTD1	0.07233612	5.7696479	0.51560183	0.60829648	0.99978775	-4.8272502
BSG	0.07225849	8.91767941	0.43872488	0.66266063	0.99978775	-4.8164712
DDRGK1	0.07221673	5.21439971	0.77389323	0.44246402	0.99978775	-4.7427036
B3GALT4	0.07220753	2.36009019	0.32388613	0.74731068	0.99978775	-4.6822504
CAMK1	0.0721801	3.37891874	0.24795968	0.80513262	0.99978775	-4.7310281
PHF10	0.0721402	4.54802965	0.67358347	0.50353011	0.99978775	-4.7468654
DDX41	0.07210872	5.55106452	0.69744064	0.48860346	0.99978775	-4.7709055
C32H4orf36	0.07191016	-0.6169121	0.15135904	0.88027245	0.99978775	-4.6174957
ARCN1	0.0718677	8.62235995	0.77526002	0.44166315	0.99978775	-4.7326336
PRPF40A	0.07185249	7.17156002	0.68849439	0.49417192	0.99978775	-4.7836672
DPM3	0.0718435	1.68419638	0.27084234	0.78757402	0.99978775	-4.6576148
UFL1	0.07178316	5.94492338	0.71366348	0.47859515	0.99978775	-4.77627
ELMO2	0.07178206	5.3746793	0.69388258	0.49081394	0.99978775	-4.7681053
HSDL1	0.07170843	4.1114545	0.52913622	0.59894061	0.99978775	-4.7656689
ATP2C1	0.07164029	6.38697805	0.54884075	0.58544083	0.99978775	-4.8236572
GUSB	0.0716396	6.78398731	0.4485916	0.65557187	0.99978775	-4.8485782
MAP4K5	0.07154541	5.99241486	0.68320828	0.49747853	0.99978775	-4.7889457
PPP1R16A	0.07149585	3.15762349	0.56391363	0.57521336	0.99978775	-4.6963293
LSG1	0.07149086	5.34031363	0.66135378	0.51127684	0.99978775	-4.7796613
GPR155	0.07144524	4.75567718	0.5333908	0.59601348	0.99978775	-4.7817952
NGLY1	0.07144385	4.00787613	0.59538412	0.55414397	0.99978775	-4.72876
FAM110B	0.07139026	2.13733983	0.21702756	0.82902783	0.99978775	-4.7086051
REPIN1	0.07135148	4.41083121	0.53675466	0.59370392	0.99978775	-4.7749405

CFAP97	0.07133191	4.3256917	0.66646789	0.50802961	0.99978775	-4.7375629
SRF	0.07127035	6.04872766	0.71843345	0.47567449	0.99978775	-4.7765149
GTF2H1	0.07125958	5.56622048	0.74318656	0.4606807	0.99978775	-4.7595552
ENSCAFG00000029110	0.07117126	-1.0000007	0.13412175	0.893819	0.99978775	-4.6170504
RYR3	0.07116047	0.07645692	0.20050572	0.84185959	0.99978775	-4.6674134
CLSTN1	0.07115723	7.53699375	0.52273407	0.60335784	0.99978775	-4.826613
ATG2A	0.07114532	5.28474682	0.41511742	0.6797471	0.99978775	-4.8164316
DCTN3	0.07111246	5.40221001	0.78437802	0.43634232	0.99978775	-4.743215
RBMS2	0.07110084	4.75326017	0.61445587	0.54156727	0.99978775	-4.7699686
SEMA4F	0.0710405	4.82317349	0.44232435	0.66007094	0.99978775	-4.7905604
ALKBH6	0.07102222	3.54335709	0.52512225	0.60170832	0.99978775	-4.7162439
CAND1	0.07099746	7.71060796	0.75134335	0.45580003	0.99978775	-4.7537916
CBFA2T3	0.07096721	-1.2927255	0.11036475	0.91254081	0.99978775	-4.6124938
ACTR2	0.07091356	7.6939128	0.52666864	0.60064135	0.99978775	-4.8178594
PEX5	0.07088887	5.42682674	0.82700956	0.41197375	0.99978775	-4.7307371
HACE1	0.07082073	6.09132052	1.03241525	0.30660897	0.99978775	-4.6537959
BMP2K	0.07081895	6.11919296	0.58376031	0.56188068	0.99978775	-4.81825
REST	0.0707526	4.54239604	0.53365301	0.5958333	0.99978775	-4.7772312
BOC	0.07075023	3.78354094	0.21735523	0.82877381	0.99978775	-4.7664169
IMPDH1	0.07058723	6.63088849	0.69239576	0.49173929	0.99978775	-4.7862008
ERP44	0.07056748	6.54884439	0.91903609	0.36227886	0.99978775	-4.7043514
RB1CC1	0.07049509	7.27442254	0.64086706	0.524396	0.99978775	-4.8002774
CCDC14	0.07045127	5.10777067	0.58292366	0.56243961	0.99978775	-4.7956503
SNRNP27	0.07043961	4.05850928	0.61408126	0.54181288	0.99978775	-4.7397847
TMEM204	0.07039525	-2.274816	0.10186224	0.91925397	0.99978775	-4.6076634
PUM1	0.07037534	6.77143878	0.99235381	0.32557554	0.99978775	-4.6725602
CFL1	0.07032484	7.6585593	0.57739299	0.56614137	0.99978775	-4.8076536
AMZ2	0.0702611	6.42317996	0.72660183	0.4706964	0.99978775	-4.7759045
ATG16L1	0.07023853	5.04430252	0.77655049	0.44090778	0.99978775	-4.7385565
ENSCAFG00000009769	0.07023206	1.2639053	0.36500986	0.71656998	0.99978775	-4.6514806
UBTD2	0.07022568	4.61660942	0.5520108	0.58328264	0.99978775	-4.7796362
FERMT2	0.07017585	8.80969864	0.55062971	0.58422242	0.99978775	-4.7908495
MSL3	0.07011658	5.33170041	0.77094592	0.44419391	0.99978775	-4.7479989
MED9	0.07010927	1.38782128	0.21926197	0.82729599	0.99978775	-4.6520434
OSGEPL1	0.07009192	3.85681662	0.35982533	0.72042081	0.99978775	-4.7417925
CYB5R3	0.0699997	8.85223663	0.45544715	0.65066518	0.99978775	-4.8148774
ANKRD13C	0.06999037	7.218334	0.69327078	0.49119459	0.99978775	-4.7789747
PDP1	0.06984549	6.94542299	0.82300429	0.41422726	0.99978775	-4.7383737
DNAJC18	0.06981176	4.78374559	0.56843404	0.57216313	0.99978775	-4.7755745
ENSCAFG00000016517	0.06979576	3.25148813	0.44169547	0.6605231	0.99978775	-4.7139735
ALAS1	0.06977972	5.70495251	0.36419382	0.71717561	0.99978775	-4.8526239
SAP18	0.06977779	3.76278525	0.65963244	0.51237232	0.99978775	-4.7167929
GLB1	0.06975404	7.41341868	0.66766281	0.50727249	0.99978775	-4.7873397
GPALPP1	0.06967872	4.8816181	0.66797966	0.50707183	0.99978775	-4.7596837
PPP2R1A	0.06967139	7.75515503	0.78583052	0.43549823	0.99978775	-4.743126
TPP2	0.06962619	6.24983743	0.82767227	0.41160162	0.99978775	-4.7402335
UBXN6	0.06960901	5.87592819	0.58471121	0.56124575	0.99978775	-4.8101846
ABR	0.0696085	7.68223355	0.67800063	0.50074787	0.99978775	-4.7837431
ENSCAFG00000014497	0.06957155	0.83392779	0.38295684	0.70329754	0.99978775	-4.644516
SLC27A6	0.06955317	-2.7255714	0.11037898	0.91252958	0.99978775	-4.6071719

PITPNM2	0.06947422	4.29198284	0.34794053	0.72927571	0.99978775	-4.795812
PRELID2	0.06945749	5.03865067	0.74177719	0.46152704	0.99978775	-4.7488259
CDC123	0.06945073	5.69385661	0.82462981	0.41331179	0.99978775	-4.7332425
CDYL2	0.06944001	0.99708649	0.23219595	0.81728833	0.99978775	-4.6576761
ZNF555	0.06943491	1.32512497	0.39276795	0.69608069	0.99978775	-4.6540815
CIB1	0.06943151	5.43350031	0.86915513	0.38871745	0.99978775	-4.7176912
KPNA6	0.06935744	5.01930993	0.68471234	0.49653647	0.99978775	-4.763934
MORC4	0.06930373	5.30207899	0.55017307	0.58453331	0.99978775	-4.8063828
AMACR	0.06930028	4.96458326	0.39393198	0.69522631	0.99978775	-4.8308832
INIP	0.06923935	2.23149487	0.3709715	0.71215112	0.99978775	-4.6634013
BLCAP	0.06919771	4.06063356	0.36188704	0.71888859	0.99978775	-4.7708678
BBS2	0.06914755	5.08455027	0.30489644	0.76165016	0.99978775	-4.8093734
ENSCAFG00000008308	0.06912208	5.07321455	0.84494775	0.40197319	0.99978775	-4.7127115
HAUS2	0.06910482	4.57828785	0.59254808	0.55602665	0.99978775	-4.7643276
SEC11C	0.06901557	5.18393794	0.43837531	0.66291236	0.99978775	-4.8321133
ZNF252	0.06899392	3.45619007	0.34277085	0.73313914	0.99978775	-4.7290597
HEATR6	0.06890715	4.37181103	0.43619247	0.6644851	0.99978775	-4.7819115
KDM4C	0.06889814	4.66614825	0.51275847	0.61027048	0.99978775	-4.7825436
PSMA6	0.06888593	4.65945495	0.5865722	0.56000419	0.99978775	-4.7695098
MINDY1	0.0688541	4.6287717	0.33501521	0.73894816	0.99978775	-4.8066508
ENSCAFG00000007165	0.06880156	1.50226389	0.25061425	0.80309031	0.99978775	-4.6482726
VKORC1	0.06857884	5.00340689	0.40108733	0.6899832	0.99978775	-4.8222964
GPD2	0.06849452	5.77582524	0.55442852	0.58163921	0.99978775	-4.8211765
TRIP12	0.06842754	8.29152199	0.90181628	0.37127212	0.99978775	-4.6945938
ECI2	0.06838004	4.89484958	0.61612919	0.54047085	0.99978775	-4.7630557
RRM2B	0.06832866	4.65840173	0.49224069	0.62460082	0.99978775	-4.8015258
CSNK1E	0.06830769	5.28650795	0.74903509	0.45717815	0.99978775	-4.7473441
ARRDC2	0.06827619	3.44328973	0.11261237	0.91076725	0.99978775	-4.6647307
MARK2	0.06824532	5.30759217	0.91454962	0.36460835	0.99978775	-4.6949742
RPS15A	0.06821227	8.30121294	0.61161398	0.543432	0.99978775	-4.7862627
COPB2	0.06815661	8.23668695	0.74059904	0.46223521	0.99978775	-4.7506713
RRN3	0.06810922	5.42820868	0.75257613	0.45506501	0.99978775	-4.7488228
CNOT8	0.0680738	4.05516437	0.58317642	0.56227072	0.99978775	-4.7409393
SHROOM3	0.06803085	-0.1227345	0.13683582	0.89168383	0.99978775	-4.6283775
CCDC106	0.06802412	2.63227891	0.29236472	0.77115979	0.99978775	-4.6913056
ENDOV	0.06798557	2.02829237	0.42353134	0.67363731	0.99978775	-4.6617869
ENSCAFG00000002350	0.06798299	0.40197703	0.16568268	0.86904288	0.99978775	-4.6251265
TRPC4AP	0.06797252	7.23657443	1.02946105	0.30798135	0.99978775	-4.6533825
PSEN1	0.06797116	5.34717706	0.67048945	0.50548394	0.99978775	-4.7718737
NRP2	0.06795625	8.66052319	0.16598198	0.86880851	0.99978775	-4.8311552
FLOT1	0.06790418	6.36566987	0.50559244	0.61525843	0.99978775	-4.8376644
UBXN2A	0.06786779	7.11193598	0.62182288	0.5367487	0.99978775	-4.8076789
POLR3K	0.06781911	3.1037191	0.44351106	0.65921806	0.99978775	-4.7172007
CLP1	0.06779526	3.39610533	0.58176118	0.56321668	0.99978775	-4.7080793
BICC1	0.06779516	7.93010877	0.34398813	0.7322288	0.99978775	-4.8564553
MVB12B	0.06776991	4.23961796	0.55409938	0.58186281	0.99978775	-4.7738738
KDELR2	0.06771699	8.21216667	0.67381485	0.50338417	0.99978775	-4.7687788
TNIP1	0.06768832	6.38133538	0.46020547	0.64726864	0.99978775	-4.8481256
PSMD6	0.06768237	6.3041801	0.75891535	0.45129619	0.99978775	-4.7650442
TRUB1	0.06765606	3.8889087	0.62070162	0.53748065	0.99978775	-4.7193933

DEFB124	0.06765367	-1.4326222	0.07502239	0.94048196	0.99978775	-4.6074744
ESCO1	0.0676512	4.04244532	0.48998724	0.62618377	0.99978775	-4.7529025
ACOX3	0.06761696	5.31321351	0.60317508	0.54898856	0.99978775	-4.7974153
GALNS	0.06760121	3.93653663	0.42154984	0.67507421	0.99978775	-4.7628395
TRAM1	0.06746407	7.96842935	0.5044294	0.61606971	0.99978775	-4.8169479
ENSCAFG00000016848	0.06745931	2.51102615	0.28402255	0.77750997	0.99978775	-4.6823018
PRPF39	0.06732186	4.55130714	0.47256036	0.63848495	0.99978775	-4.7877523
AGFG1	0.06731709	6.79830327	1.05839892	0.29471784	0.99978775	-4.6420817
SUMF2	0.0672899	5.16620339	0.74466019	0.45979672	0.99978775	-4.7500253
ENSCAFG00000017503	0.06728069	6.75006869	0.44985174	0.65466881	0.99978775	-4.8484893
CDA	0.06719396	-2.9072364	0.14621352	0.88431266	0.99978775	-4.6069105
LZTS1	0.06719396	-1.4409582	0.06871859	0.94547461	0.99978775	-4.6071125
IRF8	0.06719396	-2.5416965	0.09334687	0.92598323	0.99978775	-4.6070641
INTS11	0.06716278	5.18965207	0.62569112	0.53422749	0.99978775	-4.7891847
KLHL18	0.06715832	3.08095042	0.46220932	0.64584053	0.99978775	-4.7048055
CSRP2	0.06705084	3.3057319	0.21144619	0.83335757	0.99978775	-4.730827
FBXW2	0.06701722	5.54681163	0.6568363	0.51415449	0.99978775	-4.7830585
MFAP3L	0.06700456	-1.4049874	0.10211114	0.91905737	0.99978775	-4.6105702
ENSCAFG00000013683	0.06700316	4.83567054	0.7609847	0.45006986	0.99978775	-4.7374158
ZSCAN18	0.06699289	4.33661218	0.30610129	0.76073779	0.99978775	-4.8381211
CIAPIN1	0.06698125	5.51202173	0.73165685	0.46763058	0.99978775	-4.7646639
ARHGEF11	0.06689381	6.24074166	0.7435252	0.46047747	0.99978775	-4.7697479
ENSCAFG00000001038	0.0668719	4.46438625	0.50969206	0.61240261	0.99978775	-4.7923899
COMMD6	0.06684192	1.70029861	0.3960663	0.6936608	0.99978775	-4.6500571
GOLIM4	0.06682538	5.95599248	0.4568782	0.64964289	0.99978775	-4.842473
FAM89B	0.06679084	4.51626218	0.22491094	0.82292145	0.99978775	-4.7955007
ENSCAFG00000004830	0.06677459	5.1103192	0.84301959	0.40304091	0.99978775	-4.7198005
IP6K2	0.06662752	4.18696798	0.5340615	0.59555266	0.99978775	-4.7368733
ATRAID	0.06661	4.806848	0.46363441	0.6448257	0.99978775	-4.8071776
SDF2L1	0.06657859	3.68595781	0.29300894	0.77067004	0.99978775	-4.7375203
SOS1	0.06655855	5.54669435	0.68861068	0.49409932	0.99978775	-4.7774145
KLHL26	0.06654561	2.96091659	0.24176627	0.80990288	0.99978775	-4.6858889
ACBD3	0.06653358	6.46996112	0.77099247	0.44416656	0.99978775	-4.7608546
HIF1AN	0.06653185	5.06507884	0.56006102	0.57781921	0.99978775	-4.8068169
PAPPA	0.06652438	6.79784846	0.15486843	0.87751874	0.99978775	-4.8914699
TMEM119	0.06652427	4.65021539	0.21999527	0.82672781	0.99978775	-4.8833238
ACTR3	0.06639928	8.43196169	0.50473103	0.61585926	0.99978775	-4.8019024
TSPAN17	0.06633467	6.23207489	0.59645922	0.55343111	0.99978775	-4.8135437
FAM3C	0.06633124	6.19583025	0.51734621	0.60708689	0.99978775	-4.8345391
ATL2	0.06631802	4.31984914	0.55887012	0.57862588	0.99978775	-4.76288
ZBTB18	0.06629268	3.22665524	0.3602189	0.72012823	0.99978775	-4.7289963
VOPP1	0.06627585	2.53034542	0.31270512	0.75574315	0.99978775	-4.6906721
ANTXR2	0.06626908	6.43620265	0.61254403	0.54282137	0.99978775	-4.8089971
ZC3H14	0.06625703	6.77331373	0.73411194	0.46614571	0.99978775	-4.7721649
ENSCAFG00000002155	0.06620974	-2.8484493	0.14196887	0.88764785	0.99978775	-4.6074662
ATP9A	0.06618854	3.77210304	0.20349301	0.83953619	0.99978775	-4.849766
PRPS2	0.06617322	3.38850686	0.39483495	0.69456382	0.99978775	-4.7443065
RPL4	0.06616522	8.93719962	0.8234594	0.41397083	0.99978775	-4.7107966
BUD13	0.06611257	4.23927694	0.51672147	0.60751997	0.99978775	-4.7613246
MED1	0.06607892	6.85119082	0.96293788	0.33999195	0.99978775	-4.6844973

OSBPL9	0.06598846	6.52975143	0.47768784	0.63485475	0.99978775	-4.8443755
FBXO22	0.0659725	5.57290031	0.80797414	0.42275043	0.99978775	-4.7417258
ENSCAFG00000025712	0.06590553	-1.7808939	0.12564111	0.90049579	0.99978775	-4.6100203
DARS2	0.06588325	3.72585329	0.53203717	0.59694404	0.99978775	-4.7286001
NFKB2	0.06585349	5.47156298	0.65937717	0.51253488	0.99978775	-4.7854737
PRKAG1	0.06582883	5.33507122	0.72323143	0.47274684	0.99978775	-4.7605267
MDM2	0.06581456	6.74383846	0.56420657	0.57501546	0.99978775	-4.8235214
IVNS1ABP	0.06579493	6.2794001	0.4857019	0.62919896	0.99978775	-4.8426662
CTC1	0.06578714	4.7259389	0.48455643	0.63000599	0.99978775	-4.8031799
YIPF2	0.06578709	4.6568433	0.4071832	0.68552845	0.99978775	-4.8189233
UQCRB	0.06578038	5.4589748	0.68886399	0.49394118	0.99978775	-4.7778477
ZFAND2A	0.06576364	4.28452722	0.51602545	0.60800263	0.99978775	-4.7722937
AP3S1	0.06570324	5.01749722	0.60394922	0.54847763	0.99978775	-4.7784254
EPS15L1	0.06566073	6.12194498	0.30397378	0.76234907	0.99978775	-4.8629272
KIZ	0.06556622	5.42458408	0.70301784	0.4851497	0.99978775	-4.7711051
KLF12	0.06547659	3.46718681	0.3210712	0.74943075	0.99978775	-4.7376945
OS9	0.06547464	7.37550648	0.63815183	0.52614799	0.99978775	-4.7956666
MITD1	0.06545407	0.10709404	0.15859916	0.87459304	0.99978775	-4.6225693
KDM4B	0.06545266	5.87098329	0.7455203	0.45928123	0.99978775	-4.7624981
ENSCAFG00000000118	0.06544542	5.41558504	0.63132184	0.53056858	0.99978775	-4.7924817
DCUN1D2	0.06541766	3.76247647	0.55197156	0.58330933	0.99978775	-4.7324421
GM2A	0.06539284	1.11267245	0.08899427	0.92942499	0.99978775	-4.6387451
TAF7	0.06539024	5.25442867	0.57159879	0.57003237	0.99978775	-4.7977455
ICK	0.06537901	6.01503388	0.4941769	0.62324212	0.99978775	-4.8387908
ARPC5L	0.0652676	4.38561025	0.46646592	0.64281136	0.99978775	-4.7828232
AKIRIN1	0.06523984	3.54112866	0.34182703	0.73384523	0.99978775	-4.7431719
NUFIP2	0.0652396	5.74398804	0.70702602	0.482676	0.99978775	-4.7735802
RNF8	0.06522305	4.03659093	0.5829011	0.56245468	0.99978775	-4.7424098
YWHAH	0.06514308	7.04220005	0.27803497	0.78207728	0.99978775	-4.8624142
ENSCAFG00000030360	0.06511764	3.69446317	0.4421788	0.66017558	0.99978775	-4.7406615
EXO5	0.06501548	4.71237583	0.46371944	0.64476517	0.99978775	-4.7929559
RNF20	0.06492625	6.04684339	0.93030876	0.35646824	0.99978775	-4.6966726
NTAN1	0.06488491	5.16718645	0.82390707	0.41371868	0.99978775	-4.7271655
CAMSAP2	0.06485497	6.46129781	0.55492519	0.58130188	0.99978775	-4.8261544
TTC17	0.06481541	6.2270643	0.7443253	0.45999752	0.99978775	-4.7690479
SRI	0.0647827	5.15948386	0.5046657	0.61590484	0.99978775	-4.8104229
UBR5	0.06476635	6.97174858	0.74893426	0.45723841	0.99978775	-4.7672828
ENSCAFG00000003544	0.06476332	-0.6123392	0.2036018	0.8394516	0.99978775	-4.6284761
CPPED1	0.06474662	5.28968072	0.44803546	0.65597059	0.99978775	-4.8324693
SOCS4	0.06469747	3.46525764	0.41131059	0.68251859	0.99978775	-4.7235927
LRP12	0.06467396	5.16119675	0.26811355	0.78966228	0.99978775	-4.8533657
ATP6V1D	0.06465781	5.8532988	0.63250237	0.52980312	0.99978775	-4.8001157
SRPX2	0.06465474	6.584893	0.17213055	0.86399654	0.99978775	-4.8886779
TXNL1	0.06463938	6.10275378	0.66056895	0.51177615	0.99978775	-4.7961587
KDELC2	0.06460329	5.86487839	0.23468213	0.8153681	0.99978775	-4.8753532
PFDN1	0.06450576	1.92211552	0.41902476	0.67690705	0.99978775	-4.6629308
ECE1	0.06449355	9.39737994	0.3179184	0.75180761	0.99978775	-4.8317903
CEP162	0.06433969	2.68097297	0.39781761	0.69237722	0.99978775	-4.6829843
HLTF	0.0642911	7.23617018	0.560933	0.57722892	0.99978775	-4.8157039
ANXA2	0.06428411	11.1557781	0.48295174	0.63113734	0.99978775	-4.7437397

PATZ1	0.06426416	3.10639629	0.4103865	0.68319203	0.99978775	-4.727592
FAS	0.06418973	5.50749246	0.42553086	0.67218859	0.99978775	-4.8488846
ENSCAFG00000018136	0.06415774	4.01563315	0.32504653	0.74643729	0.99978775	-4.7885708
ZSCAN31	0.06414668	3.61505954	0.48657136	0.62858669	0.99978775	-4.7277151
ARID5B	0.06408941	6.3374242	0.4341602	0.66595073	0.99978775	-4.8536064
FAM118A	0.06404649	2.25087546	0.33325853	0.74026607	0.99978775	-4.6675364
CAMLG	0.06401541	3.76098083	0.5397349	0.59166128	0.99978775	-4.725812
COMMD7	0.0640107	5.36666761	0.9572011	0.34285182	0.99978775	-4.6750094
MAPK8IP3	0.06389534	5.54081903	0.60080075	0.55055711	0.99978775	-4.7988655
BAZ1A	0.06389033	5.97192744	0.44677162	0.65687707	0.99978775	-4.8488105
TRAF2	0.06383654	3.17027831	0.37214154	0.71128503	0.99978775	-4.718366
TPST1	0.06383288	4.86293562	0.21479288	0.83076075	0.99978775	-4.8042443
TRANK1	0.06381563	3.80793647	0.22458745	0.8231718	0.99978775	-4.7415402
PLD3	0.06380488	6.5366681	0.32663626	0.74524131	0.99978775	-4.8676052
DYNLL2	0.06367445	4.69982391	0.46152727	0.64632646	0.99978775	-4.8005657
ABCA4	0.0635378	-1.4428712	0.1112242	0.91186258	0.99978775	-4.6133961
ENSCAFG00000008336	0.06352815	6.08776331	0.51081138	0.61162393	0.99978775	-4.8347212
ERH	0.06352322	5.6873431	0.7002331	0.48687248	0.99978775	-4.7779589
APBB2	0.06352145	6.06168602	0.41303895	0.68125974	0.99978775	-4.8516578
LRCH4	0.06351408	4.53483448	0.53562936	0.59447606	0.99978775	-4.7782877
ADPGK	0.06349883	6.34630115	0.65583978	0.51479044	0.99978775	-4.7977109
DEPDC5	0.06348387	3.68396426	0.49943088	0.61956191	0.99978775	-4.7358684
MATN3	0.06336232	0.26883642	0.16992393	0.86572289	0.99978775	-4.6481415
MORN2	0.06332291	2.25848359	0.27556494	0.78396367	0.99978775	-4.6802222
SUPV3L1	0.06322683	3.65040632	0.61850933	0.53891326	0.99978775	-4.7090503
BNIP2	0.06313707	5.04952016	0.53122074	0.59750563	0.99978775	-4.8000134
ENSCAFG00000010717	0.06309219	4.40880203	0.39543982	0.69412018	0.99978775	-4.7672299
ENSCAFG00000029416	0.06307984	0.58381252	0.20482591	0.83849997	0.99978775	-4.6411448
VCP	0.06306698	8.44660682	0.73641361	0.46475608	0.99978775	-4.7473338
DNAJA3	0.06304072	5.35910885	0.99194975	0.32577075	0.99978775	-4.6630846
PUS3	0.06301218	3.39533153	0.55667008	0.58011751	0.99978775	-4.7085794
TCTEX1D4	0.06298028	0.76281855	0.16929055	0.86621853	0.99978775	-4.6389729
TMEM45A	0.06294939	2.16087331	0.16690702	0.86808424	0.99978775	-4.6695698
ZFPM1	0.06293736	3.58621781	0.23859959	0.81234472	0.99978775	-4.765183
TIMM9	0.06287281	3.27514069	0.41335726	0.681028	0.99978775	-4.7201579
NR2C1	0.06282077	4.25205204	0.48598637	0.62899861	0.99978775	-4.7770534
BATF2	0.06279333	1.02105903	0.12993626	0.89711329	0.99978775	-4.6304379
ST13	0.06272438	6.29810524	0.81502236	0.4187405	0.99978775	-4.7446758
ARMCX3	0.06262159	5.95475211	0.54647914	0.58705111	0.99978775	-4.8254433
PDK2	0.06262072	5.2937964	0.2717973	0.78684359	0.99978775	-4.8529747
ENSCAFG00000002748	0.06250151	-0.3162375	0.18433575	0.85446006	0.99978775	-4.6261139
NDUFS6	0.06249282	4.06142996	0.43389647	0.66614102	0.99978775	-4.7790641
ZC3H11A	0.06245032	7.1399049	0.91264472	0.36560033	0.99978775	-4.7047299
ZNF200	0.06239891	2.7660875	0.4253517	0.67231834	0.99978775	-4.6793967
AEBP2	0.06231006	5.84079392	0.52935491	0.59878999	0.99978775	-4.8293623
MORF4L1	0.06226176	7.81188667	0.93569127	0.3537152	0.99978775	-4.6864268
RIN3	0.06225229	4.91806305	0.33961772	0.73549898	0.99978775	-4.8376662
SNX14	0.06220815	6.90943738	0.80391865	0.42506817	0.99978775	-4.7488445
ARID2	0.06218486	5.37532259	0.51125044	0.61131862	0.99978775	-4.8237083
PTPMT1	0.06204128	3.09656296	0.51252953	0.61042955	0.99978775	-4.7151355

ANKRD40	0.06203287	4.13499426	0.63221659	0.52998836	0.99978775	-4.7468169
HSF2	0.06195361	3.02983175	0.45390083	0.65177057	0.99978775	-4.7069512
CREB3L2	0.06190538	5.31727927	0.50988909	0.61226552	0.99978775	-4.8194667
SURF4	0.06187748	6.82977582	0.61946553	0.53828817	0.99978775	-4.8070374
XPNPEP1	0.06183916	6.49737255	1.08078664	0.28473052	0.99978775	-4.6318112
SF3B1	0.06182938	8.26387065	0.5619497	0.57654102	0.99978775	-4.7987879
RPS16	0.06177965	8.22136812	0.52690725	0.6004768	0.99978775	-4.8105452
PISD	0.06172732	4.17649499	0.42574193	0.67203573	0.99978775	-4.7835572
NIPBL	0.06172584	6.58791588	0.59599992	0.55373559	0.99978775	-4.8153954
GPATCH3	0.06171745	2.63752111	0.3356862	0.73844498	0.99978775	-4.6889372
TBC1D4	0.06163954	6.03995074	0.30671609	0.76027236	0.99978775	-4.8753155
RNF44	0.06163217	3.50758603	0.2877942	0.77463701	0.99978775	-4.7187221
ZMPSTE24	0.06162296	6.38143352	0.56990406	0.57117291	0.99978775	-4.8223429
ENSCAFG00000006000	0.06160396	6.48250307	0.63338072	0.52923396	0.99978775	-4.8041532
AUP1	0.06159451	5.79488466	0.61401564	0.54185592	0.99978775	-4.8076333
ACVR1	0.06158719	6.50978619	0.46745936	0.64210527	0.99978775	-4.8464445
MTREX	0.06153965	6.09369992	0.58155197	0.56335658	0.99978775	-4.8169589
YTHDF3	0.0615358	6.44591829	0.7973169	0.4288574	0.99978775	-4.7513613
KMT5C	0.06153455	0.65653275	0.17901499	0.85861481	0.99978775	-4.6300324
ENSCAFG00000028941	0.06152047	3.27452297	0.44144092	0.66070615	0.99978775	-4.7227642
ZNF276	0.06151706	3.35929048	0.41619121	0.67896615	0.99978775	-4.7217534
ZPR1	0.06149137	5.71591216	0.42996808	0.66897814	0.99978775	-4.8453612
ENSCAFG00000003792	0.06148363	-0.4210249	0.13811545	0.89067741	0.99978775	-4.6296275
CLASP1	0.0614648	5.98748589	0.76088711	0.45012765	0.99978775	-4.7611251
CPLANE1	0.06146246	6.11172621	0.58913107	0.55829928	0.99978775	-4.8142804
ECH1	0.06143805	4.84765969	0.50097452	0.6184825	0.99978775	-4.7930408
PEX1	0.06140685	4.98684134	0.53665027	0.59377553	0.99978775	-4.7986184
PRMT7	0.06140246	5.02350876	0.5815874	0.56333289	0.99978775	-4.7931243
RPL18	0.06137885	8.23272883	0.62767385	0.5329376	0.99978775	-4.7856193
CWC15	0.06135712	4.19170798	0.55587803	0.58065498	0.99978775	-4.7597358
DPM1	0.06134181	4.83232005	0.67763731	0.50097641	0.99978775	-4.7525932
ENSCAFG00000005456	0.06131148	0.69916566	0.24676499	0.80605222	0.99978775	-4.6478295
NF1	0.06128443	6.79563029	0.48288098	0.63118725	0.99978775	-4.8428213
GLO1	0.06127774	7.1340115	0.33017597	0.74258059	0.99978775	-4.8697282
MIEF1	0.0612595	6.01094593	0.88741209	0.3789034	0.99978775	-4.7150577
ENSCAFG00000030024	0.0612494	0.64687911	0.13161032	0.89579546	0.99978775	-4.631324
NFIB	0.0612459	5.40291236	0.23281433	0.81681061	0.99978775	-4.8613181
TRIM33	0.06119905	5.06566217	0.60062897	0.55067068	0.99978775	-4.7849174
ST7	0.06116715	5.08939484	0.47608319	0.63598986	0.99978775	-4.8192075
ENSCAFG00000016371	0.06108267	0.27071028	0.22505119	0.82281291	0.99978775	-4.6385614
SCCPDH	0.06106872	5.10921207	0.63741574	0.52662348	0.99978775	-4.7827768
PNO1	0.06105962	4.97816647	0.53127949	0.59746521	0.99978775	-4.8020668
GOLPH3L	0.06102129	5.13353254	0.5884898	0.55872629	0.99978775	-4.7913734
COPB1	0.06098211	7.95792301	0.60503919	0.54775867	0.99978775	-4.7946579
RPL15	0.06092286	8.04493916	0.58412942	0.56163418	0.99978775	-4.7983112
ENSCAFG00000018651	0.06090759	2.52612471	0.31044944	0.757448	0.99978775	-4.6743002
PEA15	0.06089364	5.86165597	0.37387856	0.70999994	0.99978775	-4.8634176
RYR1	0.06087864	-1.3780214	0.12318968	0.90242717	0.99978775	-4.6122857
RPL7	0.06086381	8.68817584	0.54600126	0.5873772	0.99978775	-4.7943681
ACP2	0.06083551	6.16159986	0.6064905	0.5468021	0.99978775	-4.8121931

ZNF839	0.06079582	1.66315614	0.21393446	0.83142665	0.99978775	-4.6461385
DNTTIP2	0.06077161	6.24170958	0.47110224	0.63951892	0.99978775	-4.84586
WDR75	0.06075797	5.87567602	0.456486	0.649923	0.99978775	-4.842382
NDUFB8	0.06073437	5.45339884	0.7017	0.48596456	0.99978775	-4.7744882
SMIM3	0.06069181	2.24516761	0.19522223	0.84597234	0.99978775	-4.6582851
ENSCAFG00000002306	0.0606856	4.80140252	0.55030737	0.58444187	0.99978775	-4.7895333
ENSCAFG00000002897	0.06067144	4.55307191	0.44411624	0.65878329	0.99978775	-4.7956313
DDX19A	0.06065433	5.82106317	0.85990653	0.39374927	0.99978775	-4.7243303
SOWAHC	0.06063035	2.04742444	0.22587692	0.82217396	0.99978775	-4.6632033
ENSCAFG00000022716	0.06049398	-0.4779462	0.11511642	0.90879189	0.99978775	-4.6176046
HIVEP2	0.06047333	5.65743125	0.44251535	0.65993364	0.99978775	-4.834156
ENSCAFG00000031588	0.06036471	6.80344217	0.55202974	0.58326976	0.99978775	-4.8267303
ZNF395	0.06034174	5.68054842	0.31891945	0.75105267	0.99978775	-4.8389229
EFCAB11	0.06031919	0.46527204	0.21972617	0.8269363	0.99978775	-4.6328922
UCHL1	0.06029428	6.28767717	0.22023296	0.82654366	0.99978775	-4.8849041
SLCO2A1	0.06027928	-0.4258053	0.06952269	0.94483763	0.99978775	-4.6171618
YLPM1	0.06024955	6.1038175	0.8642983	0.39135483	0.99978775	-4.724671
FAU	0.06024004	7.74924294	0.56528892	0.57428452	0.99978775	-4.8110318
ZNF623	0.06022918	2.44094343	0.36791346	0.71441656	0.99978775	-4.6767848
ARMC1	0.06020645	4.82362896	0.71254883	0.4792791	0.99978775	-4.7475862
DCXR	0.06018947	3.54314233	0.24671414	0.80609136	0.99978775	-4.7413237
SLC35G1	0.0600527	3.24036688	0.21803119	0.82824984	0.99978775	-4.7382021
USP24	0.06003739	7.07272271	0.59825053	0.55224438	0.99978775	-4.8131765
MAPKBP1	0.05995251	5.05518034	0.44493405	0.65819597	0.99978775	-4.7978172
DMXL1	0.05994311	5.56183788	0.53805402	0.59281294	0.99978775	-4.8184963
DNAJC15	0.059905	3.24348102	0.34415661	0.73210283	0.99978775	-4.7295389
RNLS	0.05987875	3.8451035	0.31511521	0.75392294	0.99978775	-4.782848
PTPRJ	0.05975581	5.17633814	0.19551157	0.845747	0.99978775	-4.856861
NIPSNAP3B	0.05970083	3.34296201	0.37626308	0.70823722	0.99978775	-4.7253437
JAGN1	0.05969045	4.54647893	0.57934023	0.56483668	0.99978775	-4.7686111
VEGFA	0.05965447	7.91686559	0.18506846	0.85388824	0.99978775	-4.8584698
RTF1	0.05962541	5.91371721	0.89757516	0.37350879	0.99978775	-4.7074685
RPS10	0.059504	6.19521322	0.47256357	0.63848268	0.99978775	-4.8430152
MTPAP	0.05949688	4.66408461	0.54803917	0.58598715	0.99978775	-4.7751799
LAS1L	0.05938943	4.90320178	0.68012457	0.49941306	0.99978775	-4.7596054
ZNF566	0.05934597	1.38679718	0.23525387	0.81492668	0.99978775	-4.6488312
USP32	0.05933626	5.4654046	0.63506002	0.52814671	0.99978775	-4.7836763
BRPF3	0.05918288	3.73434189	0.3619878	0.71881374	0.99978775	-4.7520426
PPP1R10	0.05915882	6.04085208	0.72861087	0.46947657	0.99978775	-4.7711824
THOC7	0.05913494	5.27085301	0.61525248	0.54104516	0.99978775	-4.7875066
SKA1	0.05911432	4.88893442	0.64150159	0.52398702	0.99978775	-4.7725775
EIF3I	0.05911032	7.04673268	0.70813949	0.48199005	0.99978775	-4.7789635
DUSP6	0.05906887	6.64721868	0.49119602	0.62533443	0.99978775	-4.8322167
RNGTT	0.05903298	4.98851961	0.65340636	0.51634514	0.99978775	-4.7715169
DDX51	0.0590037	4.37987026	0.53003973	0.59831843	0.99978775	-4.7701885
TBCK	0.05898315	4.51019722	0.55148472	0.58364053	0.99978775	-4.7715881
ENSCAFG00000012422	0.05895776	-0.5924056	0.14695399	0.88373106	0.99978775	-4.6248366
GABPA	0.05889482	6.12628481	0.61861768	0.53884241	0.99978775	-4.8072627
PCGF3	0.0588784	4.54363328	0.5329241	0.59633424	0.99978775	-4.7818452
SNX11	0.05885976	3.2355517	0.55609277	0.58050924	0.99978775	-4.7097318

CCDC88A	0.05885557	7.74907579	0.31292782	0.7555749	0.99978775	-4.8493098
SS18	0.05884717	6.29808112	0.74123649	0.46185197	0.99978775	-4.7707236
EGFR	0.05878908	4.50264146	0.25183232	0.80215363	0.99978775	-4.846298
ENSCAFG00000014689	0.05876343	0.07431293	0.14658123	0.88402384	0.99978775	-4.6331586
ENSCAFG00000009307	0.05867655	2.16034545	0.23642194	0.81402502	0.99978775	-4.6866874
ENSCAFG00000016695	0.05827923	2.63124694	0.11849641	0.90612645	0.99978775	-4.7148349
PPP3CC	0.05813159	4.58927574	0.52486464	0.60188615	0.99978775	-4.7848691
FAM8A1	0.05810861	6.04141976	0.37478764	0.70932773	0.99978775	-4.8572785
ENSCAFG00000002083	0.05800041	2.68146438	0.24065728	0.81075781	0.99978775	-4.6860852
SOD1	0.05799366	5.7874321	0.59251288	0.55605004	0.99978775	-4.8016987
PICK1	0.05794616	2.94839109	0.32888998	0.74354688	0.99978775	-4.7064737
ERBIN	0.05793364	6.5081016	0.57028669	0.5709153	0.99978775	-4.8222742
HUWE1	0.05789612	7.91663448	0.53170031	0.59717572	0.99978775	-4.8132605
GSPT1	0.05787498	6.91698997	0.43984182	0.66185659	0.99978775	-4.8498372
KIF1C	0.05781446	7.18332402	0.53104026	0.59762981	0.99978775	-4.8282314
TJAP1	0.05779978	4.41457141	0.58309781	0.56232325	0.99978775	-4.7629845
RFWD3	0.05769804	4.64835602	0.54333054	0.58920127	0.99978775	-4.7874885
RAD23B	0.05760003	7.48345627	0.679221	0.49998069	0.99978775	-4.7787928
TMEM234	0.05758544	2.14556127	0.31998172	0.75025183	0.99978775	-4.6700858
C10H12orf66	0.05756753	3.82454039	0.46172387	0.64618637	0.99978775	-4.7407656
ZNF710	0.05756028	2.6417538	0.41656585	0.67869376	0.99978775	-4.685942
BRSK1	0.05754377	1.49853431	0.21912367	0.82740315	0.99978775	-4.6497086
C7H18orf21	0.0575385	3.54786511	0.52958053	0.59863461	0.99978775	-4.7364332
TRAFD1	0.05750929	6.39098998	0.44632284	0.65719907	0.99978775	-4.8500744
ENSCAFG00000005816	0.05745692	3.14354301	0.32883924	0.74358501	0.99978775	-4.7367181
ZFHX3	0.05742729	5.94136963	0.30724562	0.75987156	0.99978775	-4.8551187
ENSCAFG00000014648	0.0573795	5.05452607	0.58586927	0.56047299	0.99978775	-4.7893911
PACRGL	0.05728932	2.87178134	0.37235614	0.71112622	0.99978775	-4.7007456
OARD1	0.05724248	2.80137612	0.40488789	0.68720451	0.99978775	-4.6925578
RIMS1	0.05721264	0.97000767	0.20421191	0.83897727	0.99978775	-4.65572
CTSA	0.05719514	7.36463722	0.3782351	0.70678064	0.99978775	-4.8566403
PHLDB2	0.05715298	6.90396256	0.15189508	0.87985174	0.99978775	-4.8796427
ENSCAFG00000012627	0.05711899	0.84284102	0.2350852	0.81505689	0.99978775	-4.6317996
RUFY3	0.05710042	5.8988275	0.47356014	0.63777641	0.99978775	-4.8426366
KATNB1	0.05709701	4.24775832	0.52562139	0.60136384	0.99978775	-4.7683807
ADAT2	0.05706744	1.56232184	0.24053287	0.81085374	0.99978775	-4.6505611
ADGRL2	0.05703818	3.78227622	0.13399499	0.89391874	0.99978775	-4.8034357
PCM1	0.05703324	7.08719066	0.60107767	0.55037405	0.99978775	-4.8111608
USP53	0.05691826	5.31980884	0.18742809	0.85204729	0.99978775	-4.8304187
SGMS1	0.05685943	6.34752563	0.49938032	0.61959727	0.99978775	-4.8394812
ENSCAFG00000029427	0.05685331	2.53859419	0.25604137	0.79891921	0.99978775	-4.690754
PHC2	0.05684122	7.61632825	0.55601708	0.58056061	0.99978775	-4.813092
LAMC1	0.05682341	10.771959	0.33853622	0.73630898	0.99978775	-4.7963402
PRCC	0.05671771	4.75003453	0.60851315	0.54547038	0.99978775	-4.7721147
EDEM3	0.05670708	6.90745985	0.43620076	0.66447913	0.99978775	-4.8499341
DDX18	0.05668891	5.72283075	0.49523961	0.62249695	0.99978775	-4.8331713
PPP3CA	0.05665159	6.47575787	0.39561738	0.69398997	0.99978775	-4.8585993
JMJD7	0.05664696	2.34554482	0.25354579	0.8008365	0.99978775	-4.6840201
ENSCAFG00000019511	0.05663085	3.09205691	0.36151154	0.71916756	0.99978775	-4.7067759
KIRREL1	0.05661084	6.9949629	0.33784144	0.7368295	0.99978775	-4.859245

ZNF473	0.05659886	3.2280258	0.34264767	0.73323128	0.99978775	-4.7176903
ENSCAFG00000030404	0.05658755	1.62702423	0.20412373	0.83904582	0.99978775	-4.6529463
ACTR8	0.05657355	5.15560784	0.57736197	0.56616216	0.99978775	-4.8040373
RRAS	0.05644207	5.37173459	0.52967174	0.5985718	0.99978775	-4.8156679
XRN1	0.0563625	5.38289182	0.49787492	0.62065078	0.99978775	-4.8222785
CRTAP	0.05634447	6.74849963	0.65112699	0.5178037	0.99978775	-4.7988502
THAP6	0.05629535	3.39795431	0.38717961	0.70018794	0.99978775	-4.7276306
SUPT3H	0.05629483	3.67529227	0.4152026	0.67968514	0.99978775	-4.748906
LTO1	0.05627494	3.19504305	0.39609377	0.69364065	0.99978775	-4.7289949
ENSCAFG00000012071	0.05626218	6.40117075	0.70793946	0.48211324	0.99978775	-4.7820014
ENSCAFG00000018258	0.05625958	0.78962785	0.20931401	0.83501298	0.99978775	-4.6626605
ELAC1	0.05616996	3.07284822	0.45057696	0.65414932	0.99978775	-4.7067633
PUM3	0.05609244	5.85910185	0.45055626	0.65416414	0.99978775	-4.8451704
FARS2	0.05607976	4.31145902	0.39223488	0.69647209	0.99978775	-4.7860718
ENSCAFG00000029831	0.0560771	2.36862701	0.31285287	0.75563153	0.99978775	-4.6701705
PITHD1	0.05606093	4.29338398	0.52116319	0.60444399	0.99978775	-4.760128
SIKE1	0.05603912	4.10989967	0.50228085	0.6175697	0.99978775	-4.7583973
SLC39A14	0.05587474	6.24932834	0.48619485	0.62885179	0.99978775	-4.8391893
SLC6A8	0.05585194	5.38182032	0.25793959	0.79746169	0.99978775	-4.8734057
MRPL17	0.05583132	4.88224106	0.55405984	0.58188967	0.99978775	-4.7818834
CHMP2A	0.05581996	5.88127078	0.73745447	0.46412844	0.99978775	-4.7676065
SLK	0.05575806	6.1173225	0.38905366	0.69880956	0.99978775	-4.8569906
TFB1M	0.05571071	1.80584297	0.21932	0.82725102	0.99978775	-4.6621278
RAB8A	0.05570453	5.10788732	0.65150712	0.5175603	0.99978775	-4.7757693
SMC5	0.0556497	5.93449436	0.48946351	0.62655193	0.99978775	-4.8363611
CCDC186	0.05562357	4.56655731	0.45544153	0.6506692	0.99978775	-4.7839826
DCAF7	0.05558622	4.88207405	0.74506359	0.45955491	0.99978775	-4.7403814
SUPT6H	0.05558165	7.38296137	0.64332767	0.52281096	0.99978775	-4.7929889
ENSCAFG00000022470	0.05557516	1.00454361	0.18943467	0.85048244	0.99978775	-4.6479527
GIN1	0.05556409	3.68159548	0.40985653	0.68357837	0.99978775	-4.740291
VDAC3	0.05549611	5.12729318	0.4453946	0.65786531	0.99978775	-4.8254929
ENSCAFG00000030323	0.05546421	3.11813847	0.29421367	0.76975445	0.99978775	-4.7160294
EPHX3	0.05545053	2.65766489	0.28400549	0.77752297	0.99978775	-4.6883508
UBE4A	0.05543862	7.08073843	0.70067234	0.48660053	0.99978775	-4.7825211
AK7	0.05542493	-1.8804281	0.08668468	0.93125184	0.99978775	-4.6082119
TRAM2	0.05540769	6.88369888	0.45295424	0.65244763	0.99978775	-4.8401037
STYX	0.05535829	2.88893385	0.32385462	0.7473344	0.99978775	-4.6967325
HMOX1	0.05531611	6.35625347	0.18514324	0.85382989	0.99978775	-4.888971
ENSCAFG00000032036	0.05522023	4.89612464	0.61165325	0.54340621	0.99978775	-4.7735991
EVI5	0.05521469	5.45356776	0.40376785	0.68802295	0.99978775	-4.8438349
UBAC2	0.05519968	5.08868524	0.47035557	0.64004867	0.99978775	-4.8012955
FAM220A	0.05517127	3.94700571	0.34113114	0.73436599	0.99978775	-4.7500029
RAPGEF2	0.05513823	5.78927372	0.46518648	0.64372122	0.99978775	-4.8376922
AKAP2	0.05512937	8.43564498	0.33769727	0.73693753	0.99978775	-4.8468994
ELOVL5	0.05508133	6.3108249	0.58639721	0.56012088	0.99978775	-4.8176229
ZFP62	0.05502431	3.07877102	0.39235248	0.69638573	0.99978775	-4.705239
MRPS18B	0.05502042	5.08744205	0.55861837	0.57879647	0.99978775	-4.8023139
IGF2BP2	0.05499608	6.91759107	0.1932514	0.84750759	0.99978775	-4.8879592
ME1	0.05496237	6.99999857	0.41084247	0.6828597	0.99978775	-4.8550973
ACADVL	0.05496109	6.58251586	0.66465211	0.50918127	0.99978775	-4.7956611

ENSCAFG00000012125	0.05493952	6.20179992	0.56144661	0.57688136	0.99978775	-4.8221883
XRCC6	0.05490794	5.43665623	0.77363491	0.44261548	0.99978775	-4.746015
HACL1	0.05488081	3.85236461	0.22914515	0.81964619	0.99978775	-4.7540427
ODR4	0.05472231	5.01189874	0.57802984	0.5657145	0.99978775	-4.7868704
ENSCAFG00000001000	0.05459902	1.60043764	0.25619827	0.79879871	0.99978775	-4.6494016
CALCOCO1	0.05457755	5.52112632	0.24869676	0.80456541	0.99978775	-4.8493973
HDAC2	0.05457688	6.63460311	0.60124863	0.55026106	0.99978775	-4.8135329
MORF4L2	0.0545713	6.70354759	0.39832825	0.69200313	0.99978775	-4.8592621
TP53BP1	0.05453349	5.93107651	0.5651997	0.57434476	0.99978775	-4.8204492
ENSCAFG00000013760	0.05452488	2.74030411	0.36227053	0.71860371	0.99978775	-4.6872739
TCP1	0.05448136	7.61734658	0.45316807	0.65229466	0.99978775	-4.8341181
KDM3B	0.05444153	6.32579072	0.61703166	0.53987999	0.99978775	-4.8089956
MPC2	0.05442032	3.56630718	0.44974371	0.6547462	0.99978775	-4.7356496
ATXN1L	0.05441471	4.07122324	0.47158762	0.63917465	0.99978775	-4.7778774
DARS	0.05437684	6.41131655	0.69225053	0.49182973	0.99978775	-4.7871093
ATP8B2	0.05436622	8.03310584	0.47781149	0.63476732	0.99978775	-4.8247209
COMT	0.05434171	6.18279947	0.39437842	0.69489874	0.99978775	-4.860103
FAM186A	0.05431951	-1.4273452	0.09782383	0.92244461	0.99978775	-4.6150453
ENSCAFG00000013713	0.0542873	3.19897417	0.29637787	0.7681105	0.99978775	-4.7744607
COX15	0.05427916	3.78723844	0.48120427	0.63237036	0.99978775	-4.7533004
SLC35B4	0.0541108	4.10365402	0.50075282	0.61863747	0.99978775	-4.7646149
LIN54	0.05400504	3.72297326	0.35840273	0.72147873	0.99978775	-4.7813424
SLC35D2	0.05400016	2.70816565	0.28200126	0.77905093	0.99978775	-4.6846064
ZBTB41	0.05395756	4.00101728	0.41183768	0.68213459	0.99978775	-4.7744209
INO80	0.05393905	5.3001308	0.64431678	0.52217453	0.99978775	-4.7822635
UBQLN1	0.05391538	7.10221729	0.59350354	0.55539202	0.99978775	-4.8108245
PRUNE1	0.05391328	4.87272429	0.52960932	0.59861478	0.99978775	-4.7962726
POMK	0.05389696	5.26121769	0.56284405	0.57593623	0.99978775	-4.8024024
TENT2	0.05383794	5.55375141	0.59499051	0.55440507	0.99978775	-4.8006398
ENSCAFG00000011014	0.05379433	4.7756367	0.50293863	0.6171103	0.99978775	-4.8010703
C5H11orf87	0.05378857	-0.3309535	0.1806405	0.85734509	0.99978775	-4.6716723
ENSCAFG00000030871	0.0537836	-0.1098915	0.13712525	0.89145618	0.99978775	-4.6247872
ZBTB11	0.05376773	4.54896798	0.45239809	0.65284556	0.99978775	-4.7849544
COQ7	0.05368677	1.85197601	0.27435487	0.7848883	0.99978775	-4.6737786
C2CD2L	0.05367902	4.33648374	0.37647201	0.70808285	0.99978775	-4.7858241
SETD7	0.05366498	5.89748233	0.67121731	0.50502393	0.99978775	-4.7906067
GRK3	0.05358398	3.02103603	0.17172755	0.86431177	0.99978775	-4.7338596
ZNF106	0.05354054	7.41686317	0.50159412	0.61804948	0.99978775	-4.8296391
MED29	0.05351066	4.05955978	0.46143815	0.64638996	0.99978775	-4.769989
HDAC9	0.05346375	3.59036673	0.2112597	0.83350232	0.99978775	-4.8637209
SPAG7	0.05346267	4.57773813	0.50624467	0.61480368	0.99978775	-4.7795671
RASA3	0.05343996	6.24185218	0.3803039	0.70525376	0.99978775	-4.860267
SCFD2	0.0533231	3.40943321	0.354319	0.72451865	0.99978775	-4.726637
C18H11orf95	0.05332022	1.48220189	0.15059055	0.88087566	0.99978775	-4.6428743
DGKA	0.05323908	6.78416254	0.6164536	0.54025841	0.99978775	-4.8084769
ZKSCAN1	0.05319475	4.29617587	0.43576005	0.66479684	0.99978775	-4.7709809
COPS3	0.05319213	5.53782701	0.47904083	0.63389835	0.99978775	-4.834657
HSPA14	0.05318087	4.78013071	0.42811175	0.67032049	0.99978775	-4.8024005
HTRA2	0.05318069	2.9040839	0.27451796	0.78476366	0.99978775	-4.7313664
IKZF5	0.05314506	4.4751391	0.59559517	0.55400399	0.99978775	-4.7669712

RPAP3	0.05309243	5.92550573	0.63562044	0.52778413	0.99978775	-4.7997001
CD320	0.05300989	2.01397736	0.22812893	0.82043197	0.99978775	-4.6613642
EEA1	0.0530028	7.82751107	0.41918994	0.67678709	0.99978775	-4.8413635
P3H4	0.05299791	6.4196449	0.43480715	0.66548402	0.99978775	-4.8527228
TMCO6	0.0529008	3.33026603	0.31919251	0.75084679	0.99978775	-4.7292242
ENSCAFG00000008484	0.05286217	4.48238286	0.26171169	0.79456751	0.99978775	-4.8034142
ENSCAFG00000019044	0.05283871	8.63999953	0.39061666	0.69766074	0.99978775	-4.8255247
RAB11A	0.05275775	6.12035027	0.60013912	0.55099461	0.99978775	-4.8110001
CHEK1	0.05275609	3.5902025	0.35314342	0.72539458	0.99978775	-4.7680626
CHD8	0.05267339	6.61257189	0.64000806	0.52494993	0.99978775	-4.8028785
SCAF8	0.05265145	6.02371687	0.71897546	0.47534324	0.99978775	-4.7761833
CD82	0.05262177	3.68240665	0.17606173	0.86092263	0.99978775	-4.8548688
ERCC1	0.05256456	3.40613523	0.42478341	0.67272999	0.99978775	-4.7318378
EMC10	0.05253063	4.25122171	0.31800226	0.75174436	0.99978775	-4.8028315
GLT8D1	0.05251393	5.01211153	0.43581697	0.66475581	0.99978775	-4.8224819
ENSCAFG00000029477	0.05250445	3.65452368	0.51410995	0.60933185	0.99978775	-4.7413709
PHACTR4	0.05247025	6.20445761	0.61801298	0.53923788	0.99978775	-4.8065856
ENSCAFG00000031628	0.05243035	3.45324597	0.38725266	0.7001342	0.99978775	-4.7421186
EIF2A	0.05237524	6.42077587	0.53936391	0.59191537	0.99978775	-4.8299468
SLMAP	0.05228884	7.03805579	0.27565815	0.78389247	0.99978775	-4.8744725
RASAL2	0.05225988	6.47401809	0.36791733	0.71441369	0.99978775	-4.8657629
ITGA2	0.05225926	6.70264087	0.14141113	0.88808624	0.99978775	-4.8917574
MTERF3	0.0521901	4.41148077	0.50208475	0.61770668	0.99978775	-4.7742046
ATXN7L3	0.05214558	4.04688271	0.53207501	0.59691802	0.99978775	-4.7500592
PHF5A	0.05213661	5.44373828	0.58178031	0.56320388	0.99978775	-4.8086697
NCBP2	0.05210324	4.98812237	0.60189982	0.54983075	0.99978775	-4.7846349
GLRX3	0.05209031	6.80947385	0.78073731	0.43846229	0.99978775	-4.7564144
NPAS3	0.05205262	-1.4048391	0.14281218	0.88698507	0.99978775	-4.628497
ERGIC2	0.05197322	4.97010483	0.61262079	0.542771	0.99978775	-4.7828013
SMIM10L1	0.05196649	2.57328027	0.1966649	0.84484892	0.99978775	-4.6899187
GTF2B	0.05194572	4.46225429	0.64394256	0.52241527	0.99978775	-4.7480173
SELENBP1	0.05190935	3.80655688	0.13048755	0.89667928	0.99978775	-4.7951791
NEDD8	0.05187301	4.12727919	0.35342746	0.72518291	0.99978775	-4.7814247
CD2AP	0.05183858	5.12076671	0.40926777	0.68400765	0.99978775	-4.8343494
NKTR	0.05179552	5.35974623	0.3658262	0.71596432	0.99978775	-4.8403775
BBS10	0.0517496	3.57218311	0.49459797	0.62294682	0.99978775	-4.7235628
ARHGAP31	0.05171726	7.80515126	0.26321297	0.79341644	0.99978775	-4.8557996
CEP63	0.05168163	4.67514155	0.58238564	0.56279919	0.99978775	-4.7741017
SYS1	0.0515639	3.06375184	0.24643175	0.80630877	0.99978775	-4.7231675
APPBP2	0.05154695	6.29207696	0.59001033	0.55771404	0.99978775	-4.816259
RBSN	0.05153895	6.28006397	0.52458763	0.60207741	0.99978775	-4.8332185
DOCK3	0.05153764	2.53722327	0.13060903	0.89658364	0.99978775	-4.7316192
NEURL3	0.05153675	-2.4106318	0.08906682	0.92936762	0.99978775	-4.6079946
TCTN2	0.05152917	5.25364795	0.34664868	0.73024048	0.99978775	-4.8401677
FAM57A	0.05149601	4.53640757	0.40764892	0.68518857	0.99978775	-4.8084069
ZUP1	0.05149378	3.97955127	0.43644361	0.66430408	0.99978775	-4.7562342
SMU1	0.05147389	6.58724625	0.78907779	0.43361466	0.99978775	-4.7542782
DCN	0.05144837	11.1452653	0.13775602	0.89096008	0.99978775	-4.7791537
SNX4	0.05135579	5.8779043	0.49381895	0.6234932	0.99978775	-4.8386337
BOLA3	0.0513396	2.96230379	0.31237274	0.75599429	0.99978775	-4.7053112

ZNF133	0.05127808	3.60691133	0.37251894	0.71100575	0.99978775	-4.7419338
SLC25A16	0.05122978	5.28668356	0.36157159	0.71912295	0.99978775	-4.8521195
ZNHIT3	0.05116744	2.41934006	0.31743673	0.75217095	0.99978775	-4.6842037
NOL3	0.05114864	2.34496116	0.19012392	0.84994506	0.99978775	-4.6903141
CPT2	0.05108609	5.97281119	0.28735507	0.77497134	0.99978775	-4.8734444
KLHL8	0.05108096	2.28193271	0.2416156	0.81001902	0.99978775	-4.6885444
ATP5PB	0.0510766	6.87372166	0.6098879	0.54456618	0.99978775	-4.8098051
FBXL4	0.05104778	4.62030142	0.40274083	0.68877375	0.99978775	-4.8116646
SRPRA	0.05102783	8.08038666	0.5844796	0.56140037	0.99978775	-4.7949592
FAM149B1	0.05098803	3.11818306	0.39135531	0.69711807	0.99978775	-4.734955
WDR46	0.05098226	4.61819641	0.35046174	0.72739409	0.99978775	-4.8196404
ATXN10	0.05098172	6.19803847	0.53950553	0.59181837	0.99978775	-4.8294757
ENSCAFG00000007151	0.05096322	5.0636132	0.54081048	0.59092489	0.99978775	-4.7981164
SMPD1	0.05089791	6.184272	0.36443512	0.71699651	0.99978775	-4.8645854
HSCB	0.05087882	3.21014552	0.38364497	0.70279046	0.99978775	-4.7268261
ZNF526	0.05078203	2.72855016	0.27909262	0.78126994	0.99978775	-4.6910702
COX8A	0.0507817	5.03835696	0.3067845	0.76022057	0.99978775	-4.844871
COX4I1	0.05078131	7.17526921	0.49188771	0.62484865	0.99978775	-4.8357325
FAM160A2	0.05076189	3.99634077	0.53211403	0.59689119	0.99978775	-4.742972
DNM2	0.05073145	6.78072633	0.54734068	0.58646341	0.99978775	-4.8275627
NAA50	0.05072434	4.42693512	0.41400464	0.68055678	0.99978775	-4.7995586
CPSF2	0.050722	6.14293041	0.56767999	0.57267139	0.99978775	-4.8218022
NT5DC3	0.05064902	2.77862039	0.44322085	0.65942659	0.99978775	-4.6944481
ROCK1	0.05064535	6.75153544	0.36736922	0.71482001	0.99978775	-4.8656429
C12H6orf136	0.05063959	5.15684161	0.43993964	0.66178619	0.99978775	-4.8294773
ENSCAFG00000027802	0.05063595	2.61556075	0.16773615	0.86743515	0.99978775	-4.6955315
ALS2	0.05060575	4.74211079	0.46485684	0.64395573	0.99978775	-4.8124193
GIT2	0.05055143	6.42372071	0.51160077	0.61107505	0.99978775	-4.8365586
CASP9	0.05050441	4.87398707	0.51898949	0.60594842	0.99978775	-4.809181
SATB2	0.05045326	4.49993072	0.377713	0.70716616	0.99978775	-4.7888424
FAM126B	0.05037921	3.05533071	0.36205899	0.71876085	0.99978775	-4.7177633
FOSL2	0.05033306	6.10631506	0.28076298	0.77999539	0.99978775	-4.8787747
FAM20A	0.05029047	5.77692047	0.111513	0.91163469	0.99978775	-4.8780842
TMTC2	0.05028261	4.30323364	0.17437424	0.86224187	0.99978775	-4.8244471
TAF2	0.05025903	6.22368008	0.60061347	0.55068093	0.99978775	-4.8139602
ABRAXAS1	0.05017679	4.01497584	0.39806223	0.692198	0.99978775	-4.7843347
GLYR1	0.05014187	6.06039931	0.69535838	0.48989642	0.99978775	-4.783256
EMC3	0.05013664	5.32284493	0.62310472	0.53591255	0.99978775	-4.7903855
QARS	0.0501319	6.74176749	0.52642752	0.60080766	0.99978775	-4.8332002
SYVN1	0.05002836	7.39297745	0.43565036	0.66487593	0.99978775	-4.8464313
GNAQ	0.05002524	5.48678734	0.38535582	0.70153033	0.99978775	-4.8505692
UQCRC2	0.04999307	5.80306629	0.62399182	0.53533429	0.99978775	-4.7994255
NCOA4	0.04998746	7.2465669	0.63648121	0.52722747	0.99978775	-4.7999059
RAPGEF3	0.04996399	0.82558482	0.04077986	0.96762605	0.99978775	-4.6170132
KIAA0355	0.04996364	5.00141063	0.27472956	0.78460196	0.99978775	-4.8342369
ZNF304	0.04992531	1.79884781	0.24547766	0.80704343	0.99978775	-4.6557155
TUBG1	0.0498893	4.94431123	0.44069624	0.66124179	0.99978775	-4.8226117
NUDCD1	0.04988855	4.48497214	0.34911154	0.72840155	0.99978775	-4.8057715
STK24	0.0498699	6.04393098	0.39723655	0.69280299	0.99978775	-4.858235
PPIL4	0.04986743	5.58252916	0.45268919	0.65263727	0.99978775	-4.832518

HEATR5B	0.04983975	5.5509012	0.48493122	0.62974189	0.99978775	-4.8316129
TBXA2R	0.04980518	1.91775174	0.05970581	0.95261658	0.99978775	-4.6403235
PSMG2	0.04976054	4.34948296	0.46565922	0.64338498	0.99978775	-4.7794212
METTL5	0.04972156	4.51987266	0.48028162	0.63302181	0.99978775	-4.7945199
SLC2A4RG	0.04966354	3.78179995	0.25306488	0.80120611	0.99978775	-4.7696533
RIOK1	0.04964488	4.58465125	0.45535991	0.65072752	0.99978775	-4.7829015
ZMYM3	0.04955827	4.7637456	0.50241422	0.61747654	0.99978775	-4.7998773
CBWD2	0.04951317	4.76593601	0.53285064	0.59638474	0.99978775	-4.7904876
ZZZ3	0.04949719	6.29510223	0.53421683	0.59544596	0.99978775	-4.8313046
SECISBP2L	0.04949112	5.13475219	0.37283195	0.71077415	0.99978775	-4.8293835
LGALS8	0.04948577	7.34895586	0.54518823	0.58793221	0.99978775	-4.822855
MAP2K1	0.04948547	5.2894945	0.44864608	0.65553283	0.99978775	-4.8291226
ZNF620	0.04944742	3.55409315	0.38357023	0.70284553	0.99978775	-4.7247447
REEP5	0.04943098	5.36005845	0.41005842	0.68343118	0.99978775	-4.8409872
MYL6B	0.04941223	2.7170479	0.2194084	0.82718252	0.99978775	-4.7155695
SLC23A3	0.04937937	0.15281197	0.12894417	0.89789441	0.99978775	-4.6205028
PANX1	0.04935112	5.04088292	0.26358676	0.79312992	0.99978775	-4.855264
LRRC41	0.04934895	6.55641811	0.49992383	0.61921712	0.99978775	-4.8394802
EIF2S2	0.04932993	7.01033358	0.40449821	0.68748922	0.99978775	-4.8538206
BDP1	0.04924468	5.16612512	0.31406677	0.7547146	0.99978775	-4.8392105
UFC1	0.04924361	5.19108114	0.52956286	0.59864678	0.99978775	-4.8059966
HK1	0.0492308	7.92640323	0.57336885	0.56884232	0.99978775	-4.8067636
ENSCAFG00000029378	0.04922717	2.10039522	0.31727116	0.75229586	0.99978775	-4.6824412
HBP1	0.04920498	5.44635238	0.40599751	0.68639406	0.99978775	-4.829339
RAB6B	0.04917393	2.85765237	0.11130295	0.91180044	0.99978775	-4.6810587
PEX12	0.04917061	3.79573369	0.47561156	0.63632365	0.99978775	-4.7531985
ZNF558	0.04913687	3.47255095	0.3028412	0.76320727	0.99978775	-4.7379123
PRDX6	0.04911049	7.08028672	0.45509289	0.65091835	0.99978775	-4.8462996
ACADM	0.04909128	6.31976144	0.32441056	0.74691592	0.99978775	-4.8722918
CDC42SE2	0.0490701	1.69959525	0.19527672	0.84592991	0.99978775	-4.6586504
MTCH1	0.04904941	7.4617253	0.56587452	0.57388925	0.99978775	-4.8156291
TNPO1	0.04899187	6.98918684	0.52258503	0.60346085	0.99978775	-4.8306032
ENSCAFG00000032347	0.04898618	5.94023771	0.57622986	0.5669214	0.99978775	-4.8158604
ARID4A	0.04898287	4.18383282	0.37609601	0.70836067	0.99978775	-4.7730755
CCDC17	0.04890383	1.50232273	0.18380604	0.85487351	0.99978775	-4.6640709
POC5	0.04889531	3.99276044	0.45071491	0.65405052	0.99978775	-4.7729123
SDF2	0.04885334	5.29419997	0.48297534	0.63112069	0.99978775	-4.8220659
ENSCAFG00000019758	0.04884131	3.01216071	0.30307367	0.7630311	0.99978775	-4.7182225
NDUFV2	0.04880652	6.05972062	0.75540515	0.45338084	0.99978775	-4.7633633
KDSR	0.04876439	5.47299382	0.3824322	0.70368424	0.99978775	-4.8478268
KANK2	0.04874844	7.26408744	0.19040525	0.84972573	0.99978775	-4.8859112
PTGES3	0.04874461	7.32945763	0.46858226	0.64130756	0.99978775	-4.8382377
CS	0.04869913	7.46317593	0.61772547	0.53942597	0.99978775	-4.799588
KPNB1	0.04865194	8.33635183	0.3892626	0.69865595	0.99978775	-4.8287962
MRPL50	0.04865064	3.54987999	0.30361152	0.76262354	0.99978775	-4.7589975
ENSCAFG00000013203	0.04860283	3.80714726	0.45133496	0.65360653	0.99978775	-4.7462318
PSMD2	0.04859961	8.45579007	0.51704147	0.60729813	0.99978775	-4.8044219
CLCN2	0.04850346	1.86130229	0.13782915	0.89090257	0.99978775	-4.6571211
SLC35D1	0.04840854	5.80221098	0.47779912	0.63477607	0.99978775	-4.8362256
ARFIP1	0.04836724	5.29954878	0.4331289	0.66669498	0.99978775	-4.8263489

ENSCAFG00000004823	0.04835152	3.46310188	0.38459079	0.70209371	0.99978775	-4.7538542
LHPP	0.0483179	1.85514443	0.16055667	0.87305862	0.99978775	-4.6572244
ENSCAFG00000010893	0.04831412	4.97386471	0.39969053	0.69100552	0.99978775	-4.8174057
ENSCAFG00000003181	0.04828828	4.28639363	0.36981192	0.71300985	0.99978775	-4.7812635
ZNF396	0.04828157	0.84394647	0.21235853	0.83264946	0.99978775	-4.6445669
TMEM160	0.04823267	3.21855085	0.27241879	0.78636832	0.99978775	-4.7455821
TMEM41B	0.04821774	4.61077178	0.21395283	0.8314124	0.99978775	-4.838054
ENSCAFG00000004233	0.04819606	3.34373552	0.27567792	0.78387736	0.99978775	-4.7376136
ZBTB1	0.04818393	5.60323562	0.57397011	0.56843836	0.99978775	-4.8011031
NOD1	0.0481126	4.81940775	0.26882387	0.78911854	0.99978775	-4.8629704
PCBP2	0.04804119	7.61593757	0.63107598	0.53072807	0.99978775	-4.794337
PSD3	0.04802485	3.70815739	0.32510559	0.74639285	0.99978775	-4.765355
PPP1R3E	0.04798533	-0.3198771	0.12723757	0.89923833	0.99978775	-4.6246581
MAPK9	0.04797792	6.44002413	0.52490229	0.60186016	0.99978775	-4.8336151
RP2	0.04795375	2.75714948	0.43266276	0.66703149	0.99978775	-4.6991526
SF3B6	0.04789155	4.83302741	0.55647265	0.58025146	0.99978775	-4.7917416
GALNT2	0.04777506	7.82860762	0.45342535	0.65211063	0.99978775	-4.8275688
MTSS1L	0.04777255	3.82661793	0.29657065	0.76796411	0.99978775	-4.825343
ST6GALNAC6	0.04774569	5.67437584	0.31965328	0.75049941	0.99978775	-4.8603651
CDK12	0.04767124	5.63023766	0.47695655	0.63537195	0.99978775	-4.8320121
ENSCAFG00000019914	0.04766664	1.95718413	0.25290722	0.8013273	0.99978775	-4.6669709
SPATS2L	0.04757654	6.74814854	0.25758824	0.79773142	0.99978775	-4.8742619
ITGA5	0.04755394	11.6721025	0.26842791	0.78942163	0.99978775	-4.7631344
DHPS	0.04754781	3.94181087	0.43134957	0.66797986	0.99978775	-4.7722641
DYNC2H1	0.04753721	5.21127494	0.32088476	0.74957125	0.99978775	-4.841358
STRADA	0.0475187	4.00563952	0.37599585	0.70843469	0.99978775	-4.7718055
PRPF31	0.04747837	5.45882076	0.63015648	0.53132476	0.99978775	-4.7948403
WDR45	0.04746739	4.63310018	0.32200924	0.74872405	0.99978775	-4.8072475
UBL3	0.04746588	6.66444386	0.41053056	0.68308703	0.99978775	-4.8577539
USP13	0.04738605	3.58884103	0.32319715	0.74782941	0.99978775	-4.7576393
TMEM220	0.04736638	1.16575271	0.15259498	0.87930248	0.99978775	-4.6440997
PDXDC1	0.04726813	6.17094078	0.64987901	0.5186032	0.99978775	-4.7985273
RAB11B	0.04723612	6.44372071	0.3448219	0.7316055	0.99978775	-4.869045
RAB12	0.04718962	4.51983092	0.54131345	0.59058069	0.99978775	-4.7777402
SELENOT	0.04712635	7.22331197	0.65459583	0.51558488	0.99978775	-4.7933674
UBA52	0.04700041	7.92626146	0.30116023	0.76448156	0.99978775	-4.8582366
SLC48A1	0.04693257	2.5013419	0.18993077	0.85009564	0.99978775	-4.7126721
CEP97	0.04685435	3.13536594	0.26576156	0.79146343	0.99978775	-4.7226905
DPYSL2	0.0468536	8.81771631	0.41634249	0.67885615	0.99978775	-4.8227414
ZDHHC17	0.04683648	4.37704523	0.43610785	0.6645461	0.99978775	-4.7824111
NTN1	0.04680131	2.15813434	0.09567847	0.92414012	0.99978775	-4.6807732
WDR13	0.04676207	5.05500982	0.52264073	0.60342235	0.99978775	-4.8095838
ENSCAFG00000024681	0.04663742	3.23896974	0.29657115	0.76796373	0.99978775	-4.7690231
SAMD9L	0.04662808	7.0005207	0.26145363	0.79476541	0.99978775	-4.8808
ENSCAFG00000002646	0.04660653	7.2254461	0.26438618	0.79251724	0.99978775	-4.8774321
PAK4	0.04660017	4.48957193	0.46258883	0.6455702	0.99978775	-4.7921847
POLR3G	0.04654075	3.28244376	0.22404141	0.82359444	0.99978775	-4.7227498
AMFR	0.04651921	7.41007196	0.75601607	0.45301762	0.99978775	-4.7573772
COL12A1	0.04651204	10.7680335	0.08696826	0.93102751	0.99978775	-4.7830633
MRPS5	0.04643123	5.13957465	0.50715877	0.61416661	0.99978775	-4.8146979

ENSCAFG00000024806	0.04641366	1.99920563	0.26744659	0.79017292	0.99978775	-4.6840789
ENSCAFG00000004857	0.04637952	-0.3676906	0.13194656	0.8955308	0.99978775	-4.6277092
BRPF1	0.04632841	4.45532744	0.4586239	0.64839675	0.99978775	-4.7887512
ENDOD1	0.04629713	5.10290072	0.34729294	0.72975928	0.99978775	-4.8441606
RRP15	0.04629226	3.76463181	0.33624429	0.73802654	0.99978775	-4.7580371
DKK3	0.04626439	6.02533587	0.11468281	0.90913391	0.99978775	-4.893857
CA5B	0.04624896	4.32092865	0.17163422	0.86438478	0.99978775	-4.8105333
GINS2	0.04622547	0.80882401	0.16386732	0.87046465	0.99978775	-4.6498959
SLC25A40	0.04613054	2.8625034	0.2865269	0.77560199	0.99978775	-4.6987359
RAB1B	0.04605268	6.29491383	0.83242936	0.40893632	0.99978775	-4.7383029
LGALS3BP	0.04604412	7.68162607	0.17774006	0.85961095	0.99978775	-4.8800199
SMARCE1	0.04603914	4.35459653	0.32997157	0.74273414	0.99978775	-4.8020513
CAV2	0.04596724	7.28283064	0.25163735	0.80230354	0.99978775	-4.8805032
RAB13	0.04582318	5.43081667	0.35598055	0.72328125	0.99978775	-4.8338209
PDCD4	0.04572604	5.18501676	0.24281029	0.80909825	0.99978775	-4.8251935
MMRN1	0.04571101	-0.9569766	0.03163696	0.97488151	0.99978775	-4.6117825
CETN3	0.04566417	3.9082097	0.25184635	0.80214285	0.99978775	-4.7446494
FKRP	0.04564629	2.28611489	0.19249136	0.8480998	0.99978775	-4.6827073
RPL14	0.04564297	6.91246967	0.41793675	0.67769739	0.99978775	-4.8559002
PLOD3	0.04563793	8.36964954	0.36664642	0.71535596	0.99978775	-4.8336664
EZH1	0.04558094	3.26732304	0.16928887	0.86621985	0.99978775	-4.7151504
ENSCAFG00000020278	0.04556492	4.98404086	0.45385975	0.65179995	0.99978775	-4.8166568
PID1	0.04553555	5.05598479	0.07026756	0.94424761	0.99978775	-4.8355324
PRKCD	0.04549482	5.32900975	0.35888092	0.72112306	0.99978775	-4.8561736
RSPRY1	0.04548354	4.85960471	0.56941892	0.57149961	0.99978775	-4.7861024
ENSCAFG00000012371	0.04548043	3.21429687	0.33671711	0.7376721	0.99978775	-4.7323906
ENSCAFG00000006847	0.0454674	6.71347678	0.48539256	0.62941686	0.99978775	-4.8422291
GOLGA3	0.04546292	5.90116915	0.56782675	0.57257245	0.99978775	-4.816409
C6H7orf50	0.04540654	2.11212389	0.26647368	0.79091797	0.99978775	-4.6728545
ZNHIT6	0.04540191	4.03196181	0.33455406	0.73929405	0.99978775	-4.7715283
TRIM36	0.0453373	2.55640085	0.15229525	0.87953769	0.99978775	-4.7046819
SPIRE1	0.04533515	3.53363079	0.25478518	0.79988415	0.99978775	-4.7367716
ZNF529	0.04526409	2.48731781	0.22053277	0.8263114	0.99978775	-4.6909901
RORA	0.04514179	3.38227871	0.1570612	0.87579892	0.99978775	-4.7416807
OGA	0.04512057	6.19508856	0.57873032	0.56524517	0.99978775	-4.8172417
ENSCAFG00000032288	0.04504844	0.94467652	0.14129903	0.88817436	0.99978775	-4.6394415
PDS5A	0.04503397	7.02429882	0.5370282	0.5935163	0.99978775	-4.825534
ELOC	0.04502659	4.28538672	0.26701498	0.79050342	0.99978775	-4.8153629
ENSCAFG00000001383	0.0449811	3.56562094	0.30739793	0.75975629	0.99978775	-4.7614639
KIAA2013	0.04496318	6.50557838	0.39573612	0.6939029	0.99978775	-4.8609448
TCIM	0.04495777	0.52346857	0.08607095	0.93173735	0.99978775	-4.636231
ENSCAFG00000012637	0.04488662	3.38223249	0.31932451	0.75074727	0.99978775	-4.7299141
ENSCAFG00000028650	0.04488243	2.87343295	0.39988526	0.69086296	0.99978775	-4.7022013
ZNF580	0.04487634	2.933656	0.28600637	0.77599845	0.99978775	-4.748767
CDK7	0.0448549	3.49151626	0.3663794	0.71555399	0.99978775	-4.7331609
FUT10	0.04481223	2.28947046	0.21664806	0.82932206	0.99978775	-4.6987069
CEP350	0.04471599	6.17270506	0.33080995	0.74210438	0.99978775	-4.8697089
ARIH2	0.04470517	5.84434916	0.47337152	0.63791006	0.99978775	-4.8412223
DCAF17	0.04470429	2.02528062	0.19247481	0.8481127	0.99978775	-4.6568741
LRFN1	0.04467672	1.49451329	0.18280585	0.85565428	0.99978775	-4.6703836

FEM1B	0.04464973	5.2686194	0.52058098	0.60484677	0.99978775	-4.8149036
RAPGEF5	0.04457373	1.72716142	0.07647521	0.93933165	0.99978775	-4.6631702
WDTC1	0.04453958	6.38651833	0.44484473	0.6582601	0.99978775	-4.851453
SLC35B1	0.04452359	6.07266157	0.40371855	0.68805898	0.99978775	-4.8576099
RTF2	0.04451743	5.33575722	0.52340196	0.60289632	0.99978775	-4.8164768
XRCC3	0.04451107	0.03627554	0.10910449	0.91353545	0.99978775	-4.6238078
FGF10	0.04450805	-0.5108844	0.07100475	0.94366369	0.99978775	-4.632425
LONRF3	0.04448851	2.49241081	0.19308015	0.84764101	0.99978775	-4.6652347
KLHL11	0.04448199	4.88457873	0.43081155	0.66836856	0.99978775	-4.813981
PYROXD1	0.04439245	4.99077243	0.38782416	0.69971376	0.99978775	-4.8328495
TAOK2	0.04433251	6.04919673	0.4005277	0.69039272	0.99978775	-4.8565089
GPR108	0.04431486	6.09689314	0.350902	0.72706569	0.99978775	-4.8661703
ENSCAFG00000031711	0.04431293	4.96628153	0.32133319	0.74923336	0.99978775	-4.8337595
LONP2	0.04425327	6.06377082	0.37354212	0.71024878	0.99978775	-4.863383
TBK1	0.04419629	5.59653759	0.43957552	0.66204825	0.99978775	-4.8422782
ENSCAFG00000025332	0.04405603	4.22929781	0.14550519	0.88486908	0.99978775	-4.7764062
ANO10	0.04400779	6.09790807	0.40481761	0.68725585	0.99978775	-4.8579267
IDH3A	0.04384048	5.83510541	0.40782683	0.68505875	0.99978775	-4.851791
MTF2	0.04379131	2.13976879	0.2052028	0.83820703	0.99978775	-4.6697338
CLU	0.04377768	8.27240443	0.13730153	0.89131754	0.99978775	-4.879506
AREL1	0.04371831	4.49954972	0.33495988	0.73898966	0.99978775	-4.807516
DEF8	0.04367367	4.92837549	0.43079223	0.66838252	0.99978775	-4.8219283
SH3D19	0.04355364	5.77774365	0.22703341	0.82127926	0.99978775	-4.878446
ASPN	0.04353442	8.21081441	0.06247879	0.95041875	0.99978775	-4.8159855
ENSCAFG00000004431	0.04352663	-0.2522023	0.13035107	0.89678672	0.99978775	-4.6258115
ARHGEF12	0.04351887	8.10175567	0.46368228	0.64479162	0.99978775	-4.8224077
SUCLA2	0.04343547	5.7380089	0.45057565	0.65415025	0.99978775	-4.8392181
CUL1	0.0434337	6.92470497	0.68328596	0.49742985	0.99978775	-4.7872741
RPF1	0.04342877	4.90553204	0.44883356	0.65539844	0.99978775	-4.8156364
TAF13	0.04333248	1.13128806	0.19567299	0.8456213	0.99978775	-4.6410204
MKLN1	0.04332378	5.55758425	0.37644297	0.7081043	0.99978775	-4.8507414
XKR8	0.04326741	2.62545643	0.19517235	0.84601119	0.99978775	-4.681945
CARS2	0.04325229	6.01349816	0.34319004	0.73282561	0.99978775	-4.8686879
DDX47	0.04324282	4.71962247	0.40913321	0.68410578	0.99978775	-4.8138165
NOTCH3	0.04318555	6.29304284	0.09104853	0.92780044	0.99978775	-4.890224
ENSCAFG00000011003	0.04318481	2.86401559	0.2940489	0.76987965	0.99978775	-4.7097037
AP3S2	0.04312714	5.93344435	0.51506082	0.60867184	0.99978775	-4.834467
TIA1	0.04312519	4.59583257	0.40822724	0.68476661	0.99978775	-4.8118237
MAP3K8	0.04310695	1.79014717	0.16428834	0.87013487	0.99978775	-4.6666851
ZNF777	0.04309577	4.74565447	0.49221696	0.62461747	0.99978775	-4.7952592
DNAAF5	0.04308643	5.1181979	0.46265864	0.64552048	0.99978775	-4.8188588
PITPNM1	0.04304478	5.1604212	0.13268397	0.89495043	0.99978775	-4.8701509
FAM222B	0.04303565	4.59727585	0.32586409	0.74582215	0.99978775	-4.8174459
ENSCAFG00000032226	0.04302366	6.6470894	0.54036328	0.59123101	0.99978775	-4.8296033
QPCTL	0.04293164	3.34924491	0.30723263	0.75988139	0.99978775	-4.7339418
TTC32	0.04293147	1.95217484	0.20952494	0.83484918	0.99978775	-4.6609962
TEAD3	0.04292568	5.65314987	0.13210464	0.89540638	0.99978775	-4.8870139
NUDT12	0.04290762	4.83638639	0.31532987	0.75376089	0.99978775	-4.8293371
LRRC20	0.04289879	1.98290448	0.12812896	0.89853634	0.99978775	-4.661239
CAST	0.04284216	8.29155335	0.48812389	0.62749405	0.99978775	-4.8153227

IL11RA	0.04284078	3.02099594	0.1456792	0.88473239	0.99978775	-4.7146017
RPGR	0.04279895	7.21298543	0.1382419	0.89057798	0.99978775	-4.8837861
LINS1	0.04267349	4.3401398	0.36954252	0.71320941	0.99978775	-4.7931846
THTPA	0.04261392	3.41963051	0.36803303	0.71432793	0.99978775	-4.7524078
FAM206A	0.04255199	4.06018401	0.33010347	0.74263505	0.99978775	-4.7862188
CFAP70	0.04252236	1.77316199	0.16814762	0.86711306	0.99978775	-4.6679733
KRT10	0.04248574	3.3093816	0.38671796	0.70052765	0.99978775	-4.7399889
RNF167	0.04248059	4.69995328	0.36668998	0.71532365	0.99978775	-4.818654
SH3BP5L	0.042455	5.34949848	0.29368774	0.77015412	0.99978775	-4.8564211
ZNF189	0.0422804	3.13650232	0.24291436	0.80901805	0.99978775	-4.7119996
CLCC1	0.04219886	6.14008268	0.18066053	0.85732945	0.99978775	-4.8888374
ENSCAFG00000001149	0.04210868	4.72873799	0.26371824	0.79302914	0.99978775	-4.832494
KBTBD2	0.04208275	4.75116134	0.33471392	0.73917414	0.99978775	-4.8207785
ATM	0.04207533	5.17294555	0.40273983	0.68877448	0.99978775	-4.831958
AMDHD1	0.04202882	1.13367293	0.08972961	0.92884343	0.99978775	-4.6355082
FNDC3A	0.04198115	7.26092989	0.27962522	0.78086348	0.99978775	-4.87858
ENSCAFG00000011612	0.04183821	5.85020765	0.36402754	0.71729904	0.99978775	-4.8606905
C6H16orf58	0.04182337	5.04350347	0.53178389	0.59711823	0.99978775	-4.8104979
ENSCAFG00000015023	0.0418172	0.64599836	0.17055184	0.86523157	0.99978775	-4.6382312
FBXO34	0.04179305	5.2744092	0.36627898	0.71562847	0.99978775	-4.8461758
ZNF354B	0.04176371	3.67018231	0.33028767	0.74249667	0.99978775	-4.7538378
MLYCD	0.04172959	4.21000901	0.41120193	0.68259777	0.99978775	-4.7755374
B4GALT4	0.04172698	5.30616605	0.22977582	0.81915863	0.99978775	-4.8592228
SKI	0.04172502	6.86253975	0.41754716	0.67798049	0.99978775	-4.85676
ACTR1A	0.04170207	7.76182632	0.50804924	0.61354629	0.99978775	-4.8230664
PDCL3	0.04168266	5.09857934	0.36265157	0.7183207	0.99978775	-4.8460968
YME1L1	0.0416733	7.29919809	0.6089058	0.54521205	0.99978775	-4.8047872
ENSCAFG00000031019	0.04163992	4.53765282	0.35290399	0.72557303	0.99978775	-4.8167068
VTI1A	0.04162293	3.86530164	0.41082666	0.68287122	0.99978775	-4.7674985
NINJ1	0.04159149	3.77430216	0.23351971	0.81626577	0.99978775	-4.7738797
KIF9	0.04158334	0.78648597	0.14773594	0.88311694	0.99978775	-4.632653
KRTCAP2	0.04155437	5.68520001	0.31336293	0.7552462	0.99978775	-4.861538
ZNF34	0.04151192	2.96341332	0.27102422	0.78743489	0.99978775	-4.7112121
DMTF1	0.04150701	4.42001137	0.34407054	0.73216719	0.99978775	-4.7962226
TRAPPC11	0.04144245	7.06621524	0.61826682	0.53907186	0.99978775	-4.8054416
ENSCAFG00000014034	0.04143368	0.03666879	0.13783064	0.8909014	0.99978775	-4.6645807
UROD	0.04142297	5.78312304	0.35659593	0.72282315	0.99978775	-4.8586272
MYRIP	0.04137402	-1.4377541	0.06284687	0.95012705	0.99978775	-4.6086798
TRPM7	0.04135858	6.45073537	0.40789697	0.68500757	0.99978775	-4.8586963
TIPRL	0.0413378	4.94236483	0.38906703	0.69879973	0.99978775	-4.827683
CNOT1	0.04131257	7.91032303	0.57230344	0.56955847	0.99978775	-4.8049343
VPS28	0.0413082	5.90808987	0.35978444	0.72045121	0.99978775	-4.8629397
TMCC3	0.04130153	0.44868959	0.0845511	0.93293979	0.99978775	-4.640013
MYCBP2	0.04127743	6.97087706	0.2009687	0.84149941	0.99978775	-4.8828406
PSMD9	0.04126359	5.76351011	0.44079548	0.6611704	0.99978775	-4.8450488
PLCXD2	0.0412552	-0.7278054	0.09531982	0.9244236	0.99978775	-4.6229873
DNM1L	0.04124821	6.40474446	0.41804017	0.67762226	0.99978775	-4.856767
GCSH	0.04120507	3.00811415	0.22675225	0.82149676	0.99978775	-4.7178777
ULK1	0.04119028	5.91845035	0.2785164	0.78170976	0.99978775	-4.870059
NIP7	0.04111555	3.18022327	0.32036422	0.74996353	0.99978775	-4.7254882

SLC7A7	0.04110351	-0.0719874	0.08660901	0.93131169	0.99978775	-4.625115
MRPS2	0.04101266	4.87274145	0.36934564	0.71335526	0.99978775	-4.8216059
PHKA1	0.04098287	4.70499434	0.38993393	0.69816247	0.99978775	-4.8148987
EVPL	0.04097992	1.62697804	0.18931087	0.85057897	0.99978775	-4.6563976
FAIM2	0.04094559	0.62168627	0.11866453	0.9059939	0.99978775	-4.6475278
CNTFR	0.04093774	-0.8427204	0.0932958	0.9260236	0.99978775	-4.6211016
KDM5A	0.04090471	5.08953565	0.36234599	0.71854767	0.99978775	-4.821928
POMGNT1	0.04087426	5.44359599	0.48330108	0.63089097	0.99978775	-4.8302883
ENSCAFG00000031877	0.04085548	2.42884684	0.29007749	0.77289931	0.99978775	-4.6840531
ANAPC15	0.04083599	3.76839051	0.27054928	0.78779821	0.99978775	-4.777991
ST5	0.04077505	6.2584621	0.16553387	0.86915941	0.99978775	-4.8900949
DIABLO	0.04076439	4.89916151	0.33533075	0.73871152	0.99978775	-4.8374228
ARSA	0.04074464	7.51676168	0.18470494	0.85417194	0.99978775	-4.8789126
COPS5	0.04073927	5.19999432	0.43389492	0.66614214	0.99978775	-4.8304829
SYT16	0.04072264	1.27537759	0.15401317	0.8781897	0.99978775	-4.7175026
CDK19	0.0407214	6.10398725	0.45861311	0.64840445	0.99978775	-4.8470783
KCTD19	0.04067084	-0.6156903	0.08747761	0.9306246	0.99978775	-4.6145373
CIAO2B	0.04058713	4.18156588	0.36051459	0.71990843	0.99978775	-4.7943217
EPG5	0.04057788	4.77686976	0.37255611	0.71097825	0.99978775	-4.8140558
CALM3	0.04046831	6.87940086	0.3232021	0.74782568	0.99978775	-4.8665491
PPP1R8	0.04046344	5.59117984	0.48832267	0.62735421	0.99978775	-4.8333386
RNF169	0.04045163	4.03178562	0.33982063	0.73534704	0.99978775	-4.7895043
CAMK2G	0.04044094	5.76154624	0.45031216	0.65433898	0.99978775	-4.8430571
DNAJC11	0.04042693	4.95196988	0.35139133	0.72670076	0.99978775	-4.8340904
MACO1	0.04030561	5.49875211	0.32485352	0.74658254	0.99978775	-4.8536185
TMEM192	0.04027568	4.65008606	0.41463891	0.68009522	0.99978775	-4.804565
ENSCAFG00000030437	0.0402541	-0.3372032	0.09754476	0.92266514	0.99978775	-4.6215812
PIGH	0.04025154	2.65367347	0.23928715	0.81181438	0.99978775	-4.7045286
TMEM126A	0.04012603	3.26778053	0.35874768	0.72122215	0.99978775	-4.7516804
GAPVD1	0.04009717	6.01745724	0.42543463	0.67225828	0.99978775	-4.8519124
ACVRL1	0.04007612	6.05783564	0.12901534	0.89783837	0.99978775	-4.8746578
ENSCAFG00000030808	0.04001411	1.13549065	0.12385526	0.90190272	0.99978775	-4.6335115
RAB6A	0.04001324	7.57365194	0.33302712	0.74043974	0.99978775	-4.8561485
THAP8	0.04000959	0.77811549	0.17302927	0.86329361	0.99978775	-4.6417054
POLR2M	0.03995271	5.42020081	0.45044025	0.65424723	0.99978775	-4.8326472
CDK17	0.03994136	3.01370102	0.21238301	0.83263046	0.99978775	-4.7060973
ENSCAFG00000019501	0.03991253	2.07533267	0.13767105	0.89102691	0.99978775	-4.667542
NUDT7	0.03988527	2.89778409	0.23448415	0.81552097	0.99978775	-4.7071228
CAPZB	0.03984535	6.97259683	0.40115385	0.68993453	0.99978775	-4.8587357
NFE2L2	0.03983666	7.85416789	0.47519981	0.63661512	0.99978775	-4.8295036
TGIF2-RAB5IF	0.03983264	4.98138189	0.45413241	0.65160497	0.99978775	-4.8153191
PAFAH1B2	0.03982095	5.77806081	0.47798046	0.63464785	0.99978775	-4.8342455
HPS6	0.03972133	2.94034141	0.23770321	0.81303627	0.99978775	-4.7092645
FAM43A	0.03965792	0.51969341	0.05004245	0.96027842	0.99978775	-4.6189151
TMCO3	0.03965354	5.26654002	0.36966081	0.71312178	0.99978775	-4.833011
PEBP4	0.0396383	-2.7119005	0.07412598	0.94119178	0.99978775	-4.6071031
C4orf48	0.03960096	3.67352748	0.19600022	0.84536647	0.99978775	-4.7548963
TMEM230	0.03951712	4.84171176	0.30699726	0.76005954	0.99978775	-4.8364802
DNAJC13	0.03950525	7.0250994	0.47765188	0.63488018	0.99978775	-4.8385318
KIF1BP	0.03947755	5.47043462	0.40205023	0.68927879	0.99978775	-4.8444424

PPP1R13L	0.03945839	4.73315886	0.17342175	0.86298667	0.99978775	-4.8584231
SSR2	0.03941785	8.12881136	0.38738345	0.70003797	0.99978775	-4.83744
ARNTL	0.03937885	4.09704188	0.17828346	0.85918635	0.99978775	-4.73525
PPP2R3C	0.03936195	3.6066315	0.22860278	0.82006554	0.99978775	-4.7598069
OSBPL11	0.03931819	6.02544851	0.38996692	0.69813822	0.99978775	-4.8612962
CYB5A	0.03930178	6.9092199	0.16994232	0.8657085	0.99978775	-4.8820193
MTHFD2L	0.03927693	1.33694125	0.18398372	0.85473482	0.99978775	-4.6535556
MRPS6	0.03920661	4.43632595	0.20109562	0.84140067	0.99978775	-4.8572831
APMAP	0.03920038	4.98172911	0.43015178	0.66884536	0.99978775	-4.8216091
EIF2S3	0.03917579	7.30224912	0.46631678	0.64291739	0.99978775	-4.840586
RPUSD2	0.03914672	2.46794745	0.20242086	0.8403699	0.99978775	-4.6904732
ETAA1	0.0390964	4.34928273	0.32914612	0.74335438	0.99978775	-4.8025028
PSMD11	0.03906112	5.73903171	0.36732543	0.71485248	0.99978775	-4.8594551
RBBP8	0.03894916	6.25560584	0.22619187	0.82193028	0.99978775	-4.8841557
ETFA	0.03890017	5.81329937	0.43104705	0.66819841	0.99978775	-4.8457506
AKAP5	0.03888914	1.29279039	0.07691831	0.93898084	0.99978775	-4.639226
PUM2	0.03888746	6.91407175	0.55330385	0.58240342	0.99978775	-4.8250648
TNFRSF12A	0.03888258	5.04364561	0.17149964	0.86449006	0.99978775	-4.8724752
ENSCAFG00000017108	0.03888062	4.73393044	0.20963765	0.83476166	0.99978775	-4.8208592
AMN1	0.03887233	2.76157914	0.25051992	0.80316285	0.99978775	-4.693403
ENSCAFG00000004223	0.03886397	0.56610942	0.10595008	0.91602564	0.99978775	-4.6584471
SMS	0.03883011	5.70345871	0.24807062	0.80504724	0.99978775	-4.8716352
ASCC3	0.03880856	6.62345216	0.36605751	0.71579274	0.99978775	-4.8656272
MTDH	0.03880495	7.75157199	0.43717761	0.66377511	0.99978775	-4.8361329
TECR	0.03877509	6.22405753	0.27191125	0.78675644	0.99978775	-4.8781023
HHIPL1	0.0387603	5.32079304	0.16675512	0.86820316	0.99978775	-4.8829218
RELB	0.03874157	3.9421783	0.24139012	0.81019283	0.99978775	-4.7886188
RAB11FIP1	0.03871519	6.89460102	0.1463117	0.88423554	0.99978775	-4.8883371
ENSCAFG00000000548	0.03871428	2.05588411	0.17006771	0.86561038	0.99978775	-4.6657349
SLC16A1	0.03868542	5.39829836	0.25133332	0.80253732	0.99978775	-4.8606422
MRPL1	0.03865541	3.66064546	0.27116748	0.7873253	0.99978775	-4.757693
BPGM	0.03863412	2.23041367	0.24800629	0.80509675	0.99978775	-4.6892293
GPN3	0.03847811	5.00809788	0.41614157	0.67900224	0.99978775	-4.8278665
KTN1	0.03842991	8.23082012	0.25928689	0.79642763	0.99978775	-4.8472838
ENSCAFG00000013214	0.03838467	9.80771008	0.35693986	0.72256717	0.99978775	-4.7982312
AZI2	0.03837409	4.55831024	0.40440691	0.68755593	0.99978775	-4.7974844
SLCO4A1	0.03833086	0.38994596	0.05355137	0.9574958	0.99978775	-4.6270255
NR1H3	0.03828463	2.22359256	0.10306977	0.9183002	0.99978775	-4.6652481
ITGB8	0.03826776	0.50962338	0.08098067	0.93576517	0.99978775	-4.6576501
RCOR1	0.03823564	3.0096432	0.31685376	0.75261079	0.99978775	-4.7246396
SOAT1	0.03822735	5.34412873	0.22434877	0.82335653	0.99978775	-4.8445853
ENSCAFG00000017001	0.03822127	1.60851416	0.13986807	0.8892993	0.99978775	-4.655389
LRRC8B	0.03822061	3.45424261	0.27817821	0.78196792	0.99978775	-4.7528007
NRDE2	0.03814476	3.99164975	0.38048627	0.70511922	0.99978775	-4.7627693
SNX6	0.03813836	4.85582804	0.36455591	0.71690687	0.99978775	-4.8146947
SIDT2	0.03812579	8.28067667	0.23527998	0.81490652	0.99978775	-4.8604113
DPY19L3	0.03810435	6.72818639	0.2278122	0.82067691	0.99978775	-4.8847271
LTBR	0.03809472	7.30759943	0.43732565	0.66366845	0.99978775	-4.8440312
PSMC5	0.03801862	5.82718071	0.47906281	0.63388282	0.99978775	-4.8400654
SFRP4	0.0380004	-0.7771983	0.06313745	0.94989676	0.99978775	-4.6278883

MARCH8	0.03795664	4.07531729	0.33420782	0.73955379	0.99978775	-4.7894522
MAST4	0.03791145	5.36138935	0.160771	0.87289064	0.99978775	-4.8654275
ITGB5	0.03788731	8.41864705	0.15318188	0.87884194	0.99978775	-4.8560937
RNF11	0.03787497	4.57080511	0.36405186	0.71728099	0.99978775	-4.8056122
ENSCAFG00000025787	0.03786124	-0.1105506	0.07574661	0.93990852	0.99978775	-4.6211272
SMAP2	0.0378589	5.02040021	0.42928445	0.66947235	0.99978775	-4.8196415
MAPK8	0.03773506	6.07725722	0.3905487	0.69771068	0.99978775	-4.860033
PGAP2	0.03768612	2.69314362	0.19010324	0.84996118	0.99978775	-4.6942709
TSPYL4	0.03767974	2.91574569	0.23011747	0.81889453	0.99978775	-4.7147301
CEPT1	0.03766618	4.25608961	0.50413938	0.61627209	0.99978775	-4.7635982
NISCH	0.03766598	6.93294535	0.25841888	0.79709379	0.99978775	-4.8816067
ENOX1	0.03766076	1.20582211	0.05882969	0.95331105	0.99978775	-4.643449
GNAO1	0.03765036	-2.8944377	0.07545707	0.94013778	0.99978775	-4.6079955
ART3	0.0375403	1.78028244	0.06378558	0.94938314	0.99978775	-4.6385436
ENSCAFG00000023499	0.03750748	2.29856747	0.16157325	0.87226195	0.99978775	-4.6764133
MPRIP	0.03749675	9.50431679	0.18904389	0.85078714	0.99978775	-4.8189674
ENSCAFG00000001728	0.03745933	4.31726216	0.35335647	0.72523581	0.99978775	-4.7958112
AHCTF1	0.03745383	7.19981803	0.34343842	0.73263985	0.99978775	-4.8679733
TBC1D15	0.03743122	5.07461808	0.30835429	0.75903262	0.99978775	-4.8390193
ANKRD10	0.03743022	5.27628884	0.37101209	0.71212107	0.99978775	-4.8493585
ZHX2	0.03740462	2.54639999	0.16506512	0.8695265	0.99978775	-4.701352
ZNF703	0.03733101	5.15950596	0.22564284	0.82235507	0.99978775	-4.8580883
MIER2	0.0373016	4.45089555	0.27471107	0.78461609	0.99978775	-4.8097702
JPT2	0.03727886	4.25604582	0.3224567	0.74838702	0.99978775	-4.8056174
ENSCAFG00000008456	0.03723697	5.11124588	0.31295093	0.75555744	0.99978775	-4.8428994
GRPEL1	0.03723589	4.2902695	0.35620463	0.72311443	0.99978775	-4.7887214
FOXK1	0.03721781	5.53390757	0.29327225	0.77046991	0.99978775	-4.8701115
POLG2	0.03721711	1.95213162	0.22232289	0.82492493	0.99978775	-4.6678666
LCA5	0.03716263	3.38675144	0.18495775	0.85397464	0.99978775	-4.7376409
TMEM168	0.03712532	4.83038185	0.37890545	0.70628575	0.99978775	-4.8194393
TMEM237	0.03706327	4.70300501	0.34200239	0.73371402	0.99978775	-4.8272844
PDCD7	0.03705014	3.02143231	0.31532105	0.75376755	0.99978775	-4.7043216
RCN1	0.03698712	8.30365614	0.34172619	0.73392068	0.99978775	-4.8473839
HOOK3	0.03696038	6.1946631	0.24206132	0.80967546	0.99978775	-4.8829269
TFB2M	0.03689152	5.38961522	0.35806526	0.72172977	0.99978775	-4.8512057
NAP1L4	0.03689146	6.89538458	0.46077713	0.64686109	0.99978775	-4.8470478
MED28	0.03681725	5.78445595	0.42389452	0.67337408	0.99978775	-4.8504164
LDB1	0.03681112	5.43741865	0.42038122	0.67592221	0.99978775	-4.8326532
ENSCAFG00000004551	0.03675472	2.66611333	0.27957191	0.78090416	0.99978775	-4.6994245
AKR1A1	0.03675137	4.9349499	0.40409296	0.68778535	0.99978775	-4.8259118
KIAA1217	0.03668031	5.11812891	0.15363179	0.87848893	0.99978775	-4.8806833
ENSCAFG00000010493	0.03668021	6.71690484	0.35614093	0.72316186	0.99978775	-4.8658465
ZC3H13	0.03664422	5.8741557	0.45261366	0.65269131	0.99978775	-4.8417374
MAP2K5	0.03655775	4.54735643	0.30697857	0.76007368	0.99978775	-4.8064215
UBALD1	0.03655576	2.82092553	0.13608868	0.89227152	0.99978775	-4.6878931
TNFAIP1	0.03652607	5.89708615	0.28865635	0.77398073	0.99978775	-4.8752957
TDP2	0.03650808	3.09802285	0.26934779	0.78871756	0.99978775	-4.7290745
TOP3B	0.03645401	6.71308333	0.45770759	0.64905071	0.99978775	-4.8470263
NRBF2	0.03645082	4.35624505	0.28225327	0.77885876	0.99978775	-4.7957404
RPS27A	0.03641693	7.80292358	0.31498956	0.7540178	0.99978775	-4.8557615

PRPS1	0.0364052	4.45703499	0.2352556	0.81492534	0.99978775	-4.8179729
TTC3	0.03637278	8.25323996	0.36520883	0.71642235	0.99978775	-4.8389427
RNF181	0.03632456	5.24282397	0.28386824	0.77762758	0.99978775	-4.8533036
PHACTR2	0.03632096	4.69403208	0.15281362	0.87913091	0.99978775	-4.8366555
IPO7	0.03631361	8.2004584	0.3737203	0.71011699	0.99978775	-4.8367451
ENSCAFG00000009128	0.03628471	4.9714147	0.26999495	0.78822233	0.99978775	-4.8379553
ENSCAFG00000014790	0.0362822	2.22899243	0.19099931	0.84926265	0.99978775	-4.6911007
CUL2	0.03627124	5.47588684	0.3581874	0.72163891	0.99978775	-4.8497651
PLBD2	0.0361661	7.07615299	0.1679666	0.86725476	0.99978775	-4.8871492
EME1	0.03610221	1.52252345	0.11461744	0.90918547	0.99978775	-4.6513677
ADAMTSL4	0.03602964	5.10392515	0.08561387	0.93209895	0.99978775	-4.8120304
NEMP2	0.03602896	2.48759024	0.1960439	0.84533246	0.99978775	-4.6935438
ENSCAFG00000030782	0.03593825	3.56910522	0.37856562	0.70653662	0.99978775	-4.762354
CCDC184	0.03592532	1.43432007	0.17967286	0.85810089	0.99978775	-4.6526115
SNX30	0.03583165	3.65393624	0.1790829	0.85856176	0.99978775	-4.7290254
STX17	0.03578252	2.28101094	0.17544723	0.86140299	0.99978775	-4.6817978
SLC5A2	0.03577096	-1.542234	0.05812523	0.95386949	0.99978775	-4.6122099
UBXN11	0.03577092	0.07878835	0.11703091	0.907282	0.99978775	-4.6326175
AASDH	0.03576488	4.30356054	0.36212058	0.7187151	0.99978775	-4.7950047
ENSCAFG00000030164	0.03561463	4.9464136	0.18295202	0.85554016	0.99978775	-4.8609142
NSUN3	0.03560187	1.99431398	0.21066175	0.83396652	0.99978775	-4.6642799
LTBP3	0.0355937	7.53074674	0.16188937	0.87201425	0.99978775	-4.8874942
DIP2C	0.0355678	5.68123882	0.35030388	0.72751186	0.99978775	-4.8615779
CCNDBP1	0.03553117	4.31842284	0.20085789	0.84158561	0.99978775	-4.7838142
EIF4G2	0.03553022	10.8280125	0.27853638	0.78169451	0.99978775	-4.7771602
MTMR2	0.03549544	5.38280707	0.24546986	0.80704943	0.99978775	-4.8670529
NECAP2	0.035488	5.06745199	0.36264724	0.71832392	0.99978775	-4.832386
KLHL21	0.03544065	4.85088164	0.21449618	0.83099089	0.99978775	-4.820236
TPM3	0.03543717	6.61982414	0.21739666	0.82874169	0.99978775	-4.8860849
FOXN2	0.03543023	4.85312851	0.24963229	0.80384562	0.99978775	-4.8404001
GABARAPL1	0.03540569	4.38956991	0.17417339	0.86239891	0.99978775	-4.8020317
PSTK	0.03539746	2.97594564	0.29485363	0.76926822	0.99978775	-4.7129764
AASDHPPT	0.03536904	5.65369777	0.43775857	0.66335656	0.99978775	-4.8397304
NCBP3	0.03536668	4.50408146	0.29337899	0.77038877	0.99978775	-4.8138276
PIGW	0.03534362	1.4631683	0.15692698	0.87590418	0.99978775	-4.65773
ING3	0.03533891	2.60974272	0.18422066	0.8545499	0.99978775	-4.6839478
TMEM242	0.03532635	2.94224814	0.17828048	0.85918868	0.99978775	-4.7059091
CMTR2	0.0352928	4.26825486	0.34586481	0.7308261	0.99978775	-4.7971338
SLC33A1	0.03527584	5.83447679	0.40834301	0.68468215	0.99978775	-4.8508075
SPAST	0.03522552	3.78229985	0.3280452	0.74418187	0.99978775	-4.7616751
SEMA4C	0.0352233	3.7998275	0.14893874	0.88217245	0.99978775	-4.7333915
CERS4	0.03517253	4.7229704	0.11988156	0.90503443	0.99978775	-4.7999537
IL2RG	0.03514599	-2.3344466	0.05796264	0.95399838	0.99978775	-4.6078937
ZC3H15	0.03513279	6.48089195	0.37132471	0.71188962	0.99978775	-4.8650798
VMA21	0.03508763	2.59967428	0.28413745	0.7774224	0.99978775	-4.7010982
TMEM107	0.03507026	1.11691837	0.10606066	0.91593833	0.99978775	-4.6382498
CDS2	0.03505635	4.53953362	0.23447245	0.81553001	0.99978775	-4.8119405
NPLOC4	0.03503653	6.60298784	0.39878517	0.69166846	0.99978775	-4.8599351
ZNF503	0.03503515	4.15239872	0.09839072	0.92199665	0.99978775	-4.8048283
IDE	0.03502293	6.08021322	0.41015853	0.6833582	0.99978775	-4.8581432

TMEM108	0.03501335	1.41817969	0.09855356	0.92186797	0.99978775	-4.7144692
VPS39	0.03497508	6.75228543	0.45340552	0.65212481	0.99978775	-4.8490753
STAP1	0.03496821	1.07954143	0.09847068	0.92193346	0.99978775	-4.6405845
EIF2D	0.0349529	5.03999329	0.43335459	0.66653208	0.99978775	-4.8305307
PODNL1	0.03493057	1.08791534	0.11288129	0.91055508	0.99978775	-4.6497191
MINDY4	0.03491424	5.07696638	0.11226966	0.91103765	0.99978775	-4.8504387
NKAPD1	0.03488319	4.63848084	0.36592474	0.71589122	0.99978775	-4.8130601
WASF2	0.03482474	7.397331	0.30495028	0.76160938	0.99978775	-4.8708508
ARHGEF7	0.03482018	5.95528716	0.30775808	0.75948374	0.99978775	-4.8685335
HOXB4	0.03478797	1.32979065	0.10480998	0.91692588	0.99978775	-4.6490834
TUBB4A	0.03476662	1.77094214	0.08532532	0.93232724	0.99978775	-4.6472411
ZNF41	0.0347341	1.81158601	0.17458946	0.86207359	0.99978775	-4.6588776
ENSCAFG00000007270	0.03468254	3.96429904	0.18911915	0.85072846	0.99978775	-4.7805904
INSYN1	0.03458011	1.75600761	0.13891538	0.89004837	0.99978775	-4.7131236
RAB3GAP2	0.03456716	6.30012652	0.41535052	0.67957754	0.99978775	-4.8571688
MAP3K10	0.03456053	2.57923995	0.18540006	0.85362949	0.99978775	-4.7064007
ENSCAFG00000030409	0.03448436	3.25476767	0.24288903	0.80903757	0.99978775	-4.7294196
ENSCAFG00000003207	0.03438103	0.8464081	0.13118058	0.89613373	0.99978775	-4.6432897
PBXIP1	0.03436741	6.23602671	0.27616907	0.78350217	0.99978775	-4.8772647
PABPC5	0.0343282	1.79730092	0.17691225	0.86025787	0.99978775	-4.6602726
CHM	0.03421732	4.94043255	0.35105204	0.72695379	0.99978775	-4.8217674
YIPF3	0.03420691	6.65080453	0.39145559	0.69704441	0.99978775	-4.8616098
AGO1	0.03419772	4.99411184	0.34617627	0.73059339	0.99978775	-4.832597
CMAS	0.03413808	4.81510665	0.26168685	0.79458656	0.99978775	-4.8366874
SAV1	0.03411976	4.92643201	0.15654994	0.87619986	0.99978775	-4.8314706
ENSCAFG00000031146	0.03403613	6.13527612	0.34910262	0.72840821	0.99978775	-4.8674876
ENSCAFG00000004703	0.03398555	4.39063062	0.3179591	0.75177691	0.99978775	-4.8005313
ENSCAFG00000002015	0.03398282	7.38246155	0.21569478	0.83006125	0.99978775	-4.8711468
RPL35A	0.03391169	7.63492533	0.32269388	0.74820839	0.99978775	-4.8605652
ENSCAFG00000006545	0.03388771	7.42930009	0.36186881	0.71890213	0.99978775	-4.8565516
CLASRP	0.03383968	3.60362259	0.27725807	0.78267047	0.99978775	-4.7526856
ENSCAFG00000019092	0.03380631	4.46127131	0.26442897	0.79248444	0.99978775	-4.8175119
CNNM3	0.03379821	3.55976037	0.18607884	0.85309986	0.99978775	-4.7581692
ARL2BP	0.03377804	5.81919835	0.37082687	0.7122582	0.99978775	-4.8597251
SKIV2L	0.03374171	5.45072499	0.3929356	0.69595761	0.99978775	-4.8436266
THAP12	0.0337299	5.1173698	0.28400544	0.77752301	0.99978775	-4.8446215
EFHC1	0.03371038	1.44274061	0.10024709	0.9205299	0.99978775	-4.6420181
TCEA3	0.03369735	3.69188207	0.13830714	0.89052667	0.99978775	-4.8292801
HSD17B6	0.03364871	1.5953512	0.12812603	0.89853864	0.99978775	-4.6555096
SGPP1	0.03363321	5.15581897	0.20810121	0.83595493	0.99978775	-4.8637249
USP19	0.03361241	6.30361175	0.27268176	0.78616725	0.99978775	-4.8778662
HOXD3	0.03361034	-1.9828867	0.05554635	0.955914	0.99978775	-4.6098967
CLDND1	0.03357663	5.43349473	0.34917173	0.72835663	0.99978775	-4.8483873
CEP95	0.03356495	4.98593996	0.28046527	0.78022251	0.99978775	-4.8405087
LRRCC1	0.0335635	4.6501013	0.21811618	0.82818396	0.99978775	-4.8381485
CYTH1	0.03352067	4.94571455	0.23588533	0.8144392	0.99978775	-4.8248056
THUMPD3	0.03350798	3.7836224	0.28664655	0.77551087	0.99978775	-4.7777955
NOX4	0.0334958	4.11649058	0.12475207	0.90119615	0.99978775	-4.8078074
CUL3	0.03334425	6.4886146	0.35658574	0.72283074	0.99978775	-4.8677053
REV1	0.03329378	4.563903	0.30974822	0.75797824	0.99978775	-4.8187142

TMEM184C	0.03325877	5.46036902	0.34820571	0.72907772	0.99978775	-4.8519341
MICALL1	0.03325155	5.29434757	0.2191198	0.82740615	0.99978775	-4.8783476
NKRF	0.03324332	4.46432508	0.29306396	0.77062822	0.99978775	-4.8107622
CDH3	0.03320792	-1.4501793	0.05700143	0.95476039	0.99978775	-4.6093327
RHOF	0.03316977	1.57760039	0.14488867	0.88535342	0.99978775	-4.6512489
AGPAT1	0.03316789	4.33204939	0.25793079	0.79746845	0.99978775	-4.8020625
PLEKHG2	0.03315677	4.66851678	0.15711011	0.87576057	0.99978775	-4.8397594
GGNBP2	0.03314649	5.88845072	0.4331174	0.66670329	0.99978775	-4.8473988
EDARADD	0.03313168	-0.2281261	0.09974623	0.92092561	0.99978775	-4.6327686
MRPL58	0.03310138	3.49226938	0.25141326	0.80247585	0.99978775	-4.7670535
NDUFAF5	0.03306241	3.35028226	0.24458034	0.80773453	0.99978775	-4.7417065
OSBPL6	0.03287788	5.44650146	0.17703768	0.86015984	0.99978775	-4.8860379
TIMM17B	0.03286786	3.7479193	0.29506112	0.76911059	0.99978775	-4.77943
IGFBP4	0.03283172	5.52095386	0.10810798	0.91432204	0.99978775	-4.8935122
PAPOLA	0.03282841	7.19814406	0.53202299	0.59695379	0.99978775	-4.8272903
ERCC4	0.03277058	5.42631907	0.41764664	0.6779082	0.99978775	-4.8398185
USP11	0.03268395	6.43052481	0.33286637	0.74056039	0.99978775	-4.8714508
IK	0.03262327	6.90210456	0.71538298	0.47754113	0.99978775	-4.7782987
ADAM19	0.03256734	8.86103879	0.15504815	0.87737776	0.99978775	-4.8434803
CNOT11	0.03255869	5.01449339	0.43765939	0.66342801	0.99978775	-4.8203104
CYHR1	0.03252551	4.41856613	0.29854039	0.76646889	0.99978775	-4.8135578
TXLNG	0.03251405	2.73730676	0.20164136	0.84097616	0.99978775	-4.6878479
CCDC66	0.03244486	4.25949752	0.22919137	0.81961046	0.99978775	-4.8002114
TGFB1	0.03237072	5.80692887	0.21151715	0.83330249	0.99978775	-4.8683897
METTL9	0.03236	6.6141206	0.51094595	0.61153035	0.99978775	-4.8369447
SOX17	0.03234926	-1.6499029	0.03678233	0.97079803	0.99978775	-4.6073445
ADAMTSL5	0.0323451	4.24265334	0.12159041	0.90368748	0.99978775	-4.7997745
VWCE	0.03233229	2.06747789	0.0554597	0.9559827	0.99978775	-4.6521648
UTP23	0.032298	2.44894355	0.15651753	0.87622528	0.99978775	-4.6818416
BMP2	0.03228066	3.78590303	0.08418515	0.93322934	0.99978775	-4.8348743
MTF1	0.03217427	3.54139603	0.21011478	0.83439118	0.99978775	-4.7362414
LDLRAP1	0.0321444	4.22269306	0.19513782	0.84603809	0.99978775	-4.7908955
RBM15	0.03212877	3.1541643	0.27196942	0.78671196	0.99978775	-4.7187451
ATP2A2	0.03204905	7.92687424	0.27471102	0.78461613	0.99978775	-4.8575051
OGN	0.03204358	6.69475934	0.05355094	0.95749615	0.99978775	-4.8819153
ZSCAN21	0.03200746	3.53940054	0.3183364	0.75149235	0.99978775	-4.7460603
NUS1	0.03199058	6.05795505	0.33453788	0.73930619	0.99978775	-4.8686973
TPGS1	0.03196898	3.28984323	0.14879954	0.88228175	0.99978775	-4.7459929
FAM184B	0.03195839	-1.1754082	0.04379905	0.9652307	0.99978775	-4.6126632
FAM98C	0.03194684	4.30832429	0.25227269	0.80181506	0.99978775	-4.8078004
ANKRD52	0.0318025	5.57910667	0.24948672	0.80395761	0.99978775	-4.8785623
WDR61	0.03170926	4.98800107	0.4044463	0.68752714	0.99978775	-4.8282305
NUDT9	0.03167518	5.72744987	0.35363375	0.72502919	0.99978775	-4.8612081
TRIT1	0.03160448	3.13476726	0.2138155	0.83151893	0.99978775	-4.7193994
ZNF593	0.03159863	3.71709659	0.23405035	0.81585596	0.99978775	-4.7852249
FNBP1L	0.03157827	3.48978317	0.10483717	0.91690442	0.99978775	-4.8296732
ARHGAP42	0.03156046	4.9793194	0.35705775	0.72247943	0.99978775	-4.8550253
TUBGCP4	0.03143029	4.28545895	0.32877708	0.74363172	0.99978775	-4.8121263
MPDZ	0.03142437	7.34358355	0.25571809	0.79916751	0.99978775	-4.8689492
KIF13A	0.03130888	6.21427516	0.28161751	0.77934358	0.99978775	-4.8774758

C5H16orf70	0.03128998	4.05249721	0.38809498	0.69951455	0.99978775	-4.7704041
ZNF134	0.03125109	3.53341969	0.22949431	0.81937625	0.99978775	-4.7430493
BCL2L11	0.03120264	4.47985218	0.15528776	0.87718981	0.99978775	-4.8369504
TTPAL	0.03118384	5.2521393	0.25702678	0.79816249	0.99978775	-4.8495927
TWISTNB	0.03104049	3.93932725	0.19514193	0.84603488	0.99978775	-4.7910888
MRPS12	0.03103902	3.08767041	0.22811194	0.82044511	0.99978775	-4.7355551
CANX	0.03102775	9.53345858	0.34553252	0.73107439	0.99978775	-4.8073126
TMEM38B	0.03102136	2.96017101	0.21703868	0.82901921	0.99978775	-4.7297252
CALY	0.0309941	-1.6317736	0.05508715	0.95627808	0.99978775	-4.6092849
CDK5	0.03096654	3.52413884	0.23921325	0.81187138	0.99978775	-4.7472419
NDFIP1	0.03094713	6.71987025	0.26117091	0.79498225	0.99978775	-4.8800347
GFPT1	0.03093991	6.61732255	0.26810316	0.78967023	0.99978775	-4.8804327
ENSCAFG00000015433	0.03091516	4.27134046	0.23406994	0.81584082	0.99978775	-4.791349
DNAJB2	0.03084464	5.10540615	0.2909788	0.77221369	0.99978775	-4.8549989
ENSCAFG00000032575	0.0308278	0.74480523	0.12417646	0.90164966	0.99978775	-4.6409066
COG6	0.03082117	5.67121194	0.30020773	0.76520391	0.99978775	-4.873358
UNC13B	0.03080842	6.48300021	0.18951826	0.85041726	0.99978775	-4.8862346
CITED2	0.03080387	5.10041772	0.14359029	0.88637359	0.99978775	-4.8424782
RMND5B	0.03079343	5.81098563	0.31714842	0.75238847	0.99978775	-4.8681413
CMBL	0.03076241	2.21154534	0.13191751	0.89555367	0.99978775	-4.7208824
TNFSF13	0.03075561	3.0329984	0.09848297	0.92192375	0.99978775	-4.7101614
PTHLH	0.03073567	4.61515878	0.05520115	0.95618769	0.99978775	-4.8062376
ZNF622	0.03070995	4.36799099	0.33508436	0.7388963	0.99978775	-4.8018047
OVCA2	0.0307075	3.42083712	0.22298438	0.82441274	0.99978775	-4.7508424
AJM1	0.03066034	-0.968432	0.05799469	0.95397297	0.99978775	-4.6118207
RHBDD1	0.03064345	4.65560978	0.26211027	0.79426186	0.99978775	-4.8345551
C14H1orf35	0.03063837	3.18613762	0.25566645	0.79920718	0.99978775	-4.7425927
DUSP7	0.0306366	5.44298866	0.19651344	0.84496684	0.99978775	-4.8709857
CLTC	0.03063549	9.3362959	0.2915022	0.77181562	0.99978775	-4.8147528
RNASEH1	0.03062521	4.0667487	0.28006604	0.78052711	0.99978775	-4.7977729
N4BP3	0.03061155	1.71093928	0.04801578	0.96188581	0.99978775	-4.6394363
PDZD8	0.03058567	5.83563173	0.31048915	0.75741799	0.99978775	-4.8648554
OBSL1	0.03052386	7.28425361	0.14289895	0.88691688	0.99978775	-4.8736582
GNAI2	0.03048748	8.79651691	0.25066592	0.80305056	0.99978775	-4.8406455
ZSCAN2	0.03043508	1.83586262	0.12451608	0.90138208	0.99978775	-4.6701301
MAGED2	0.03038117	6.93069097	0.29753486	0.76723207	0.99978775	-4.8736162
TAF1B	0.03036545	3.63243128	0.24912215	0.80423809	0.99978775	-4.7605346
PARP4	0.0303653	6.18401731	0.2905641	0.77252913	0.99978775	-4.8772901
ENSCAFG00000006088	0.03034291	3.62240282	0.24295654	0.80898554	0.99978775	-4.754043
NUB1	0.03033166	6.62963173	0.22566396	0.82233873	0.99978775	-4.8848713
ETFB	0.0302895	4.76510777	0.25165925	0.8022867	0.99978775	-4.8213162
ARHGAP1	0.03022796	7.19979158	0.18579793	0.85331904	0.99978775	-4.8790667
PPP6C	0.03019169	3.36489015	0.29170373	0.77166237	0.99978775	-4.7412438
DNAJC5	0.03007037	5.37319942	0.30212308	0.76375157	0.99978775	-4.8617892
C6H7orf26	0.03006494	4.59303222	0.29447104	0.76955889	0.99978775	-4.8273275
FMR1	0.03004405	5.47538574	0.28159933	0.77935745	0.99978775	-4.8690649
ENSCAFG00000022743	0.03003985	2.53920327	0.06655514	0.9471886	0.99978775	-4.7041112
ENSCAFG00000006506	0.02985909	1.52141166	0.12252845	0.90294823	0.99978775	-4.6524493
FAM3A	0.02983792	4.9247517	0.25108183	0.80273071	0.99978775	-4.8519855
ANKLE2	0.02982469	6.14115216	0.42523098	0.67240578	0.99978775	-4.8541187

NAGLU	0.02980201	6.3349734	0.13627439	0.89212544	0.99978775	-4.8898798
WDR12	0.02979645	4.21261187	0.30334674	0.76282417	0.99978775	-4.793789
IRAK4	0.02975139	5.38587843	0.24094404	0.81053672	0.99978775	-4.8574195
CLN6	0.02971833	4.25788358	0.20104311	0.84144152	0.99978775	-4.8255334
TGS1	0.02969053	4.62805621	0.24368224	0.80842638	0.99978775	-4.8125697
RXRB	0.02966288	4.99439267	0.23268317	0.81691193	0.99978775	-4.8490545
PTPRF	0.02958033	5.79043013	0.17769823	0.85964364	0.99978775	-4.8805886
PCYOX1L	0.02955228	3.85263579	0.1693037	0.86620825	0.99978775	-4.7843269
PCDHGC5	0.02954689	5.28048252	0.23055305	0.81855786	0.99978775	-4.8599597
NRBP1	0.0295023	6.61743932	0.40180715	0.68945659	0.99978775	-4.8598059
ENSCAFG00000031145	0.02949356	0.43676123	0.06146505	0.95122218	0.99978775	-4.635149
ECPAS	0.02948493	7.73535593	0.47360832	0.63774228	0.99978775	-4.8313187
THEM4	0.02945308	4.77720302	0.17193451	0.86414988	0.99978775	-4.8442054
STK4	0.02937336	5.34118993	0.31117111	0.75690243	0.99978775	-4.8490668
ENSCAFG00000011362	0.02933323	7.11580171	0.39503242	0.69441898	0.99978775	-4.8537535
METTL16	0.02931139	4.54577227	0.2354115	0.81480498	0.99978775	-4.820386
POFUT2	0.02928729	4.90330288	0.2163435	0.82955821	0.99978775	-4.8423872
ANKRD46	0.02927355	5.5014016	0.20081219	0.84162116	0.99978775	-4.8600412
OSER1	0.02925459	4.73829853	0.27814968	0.7819897	0.99978775	-4.8258529
THBS2	0.02923801	11.3278722	0.04315852	0.96573885	0.99978775	-4.7381516
RBM18	0.02914434	5.37634714	0.19562412	0.84565936	0.99978775	-4.8734138
TES	0.02911107	5.89975111	0.1152383	0.90869576	0.99978775	-4.8843135
MCU	0.02909989	4.37395633	0.26557993	0.79160257	0.99978775	-4.8139529
ARMC8	0.02906261	3.40168119	0.20187946	0.84079097	0.99978775	-4.7419997
RXRA	0.02906077	5.99203506	0.16948312	0.86606784	0.99978775	-4.8819071
SLU7	0.02899668	6.36378248	0.37899821	0.70621728	0.99978775	-4.8622431
TPR	0.02899284	7.9694418	0.35773754	0.72197359	0.99978775	-4.8461643
WDR36	0.02898871	6.30086448	0.25413973	0.80038008	0.99978775	-4.8817296
TRIO	0.02897533	6.97161919	0.35489259	0.7240914	0.99978775	-4.8647925
CDC42BPA	0.02895487	8.13182192	0.17707263	0.86013253	0.99978775	-4.8577267
THUMPD1	0.02891064	3.45233578	0.19349209	0.84732006	0.99978775	-4.7361701
MYCL	0.02887175	-0.5251184	0.0657137	0.9478553	0.99978775	-4.6187304
TCERG1	0.028848	5.72752434	0.20731966	0.83656207	0.99978775	-4.8793914
SSH2	0.02879571	4.04494643	0.08684211	0.9311273	0.99978775	-4.7315683
GAK	0.02875681	6.47576178	0.37574256	0.70862187	0.99978775	-4.8644822
RAE1	0.0285635	5.63712708	0.38062198	0.70501911	0.99978775	-4.8531627
XPR1	0.02851353	4.14764597	0.21488847	0.83068661	0.99978775	-4.7883318
TEX30	0.02850197	2.88781932	0.16443357	0.87002112	0.99978775	-4.7182865
ZNF449	0.0284619	2.76052703	0.10600907	0.91597907	0.99978775	-4.7002264
METTL14	0.02844347	3.85135125	0.26235114	0.79407717	0.99978775	-4.7747084
SRP54	0.02840315	6.74609817	0.27136631	0.78717322	0.99978775	-4.8792774
CCT8	0.02830223	7.66535548	0.26510405	0.79196716	0.99978775	-4.8649418
C20H19orf53	0.02829688	4.35201094	0.31319988	0.75536937	0.99978775	-4.8177996
ENSCAFG00000017339	0.02828873	-0.986903	0.05869876	0.95341485	0.99978775	-4.6208797
MTX2	0.02825196	5.34755495	0.25516967	0.79958877	0.99978775	-4.8640025
ENSCAFG00000032479	0.02824403	-0.8728396	0.06046792	0.9520125	0.99978775	-4.6173519
ZNF214	0.0282111	0.41533458	0.06937727	0.94495283	0.99978775	-4.6256992
PARP6	0.02820177	3.93831179	0.20686046	0.83691883	0.99978775	-4.7774016
TMEM126B	0.02820041	3.86018754	0.27287064	0.78602284	0.99978775	-4.7957384
ZNF287	0.02819185	2.50956121	0.16271797	0.87136503	0.99978775	-4.681433

SERAC1	0.02812959	3.48907961	0.16388236	0.87045287	0.99978775	-4.7573928
SYPL1	0.02807878	6.25922782	0.30640199	0.76051013	0.99978775	-4.8751667
CCNJ	0.02805184	4.51688626	0.1331348	0.89459563	0.99978775	-4.8286231
COG1	0.02804494	6.35913511	0.32200615	0.74872638	0.99978775	-4.8727835
LATS1	0.02804383	5.69603322	0.28596973	0.77602636	0.99978775	-4.8663827
MRPS27	0.0280328	4.49191678	0.36998676	0.71288034	0.99978775	-4.8080229
UBFD1	0.02798473	4.2665422	0.28673199	0.7754458	0.99978775	-4.8002128
ATP5IF1	0.02796416	5.27677287	0.2630216	0.79356314	0.99978775	-4.8588071
CCM2	0.02790572	5.30216003	0.28142596	0.77948968	0.99978775	-4.8552126
KLHL2	0.0278978	5.47584432	0.20956107	0.83482113	0.99978775	-4.8771226
ENSCAFG00000000177	0.02787816	1.47401477	0.11129583	0.91180605	0.99978775	-4.6551577
RPS18	0.02783734	8.73611766	0.29181825	0.77157529	0.99978775	-4.8390575
TOR1AIP1	0.02783584	6.67228944	0.39638307	0.69342856	0.99978775	-4.8602369
ITPRID2	0.02783491	6.92993153	0.23057934	0.81853754	0.99978775	-4.8847564
ABHD17A	0.02782755	5.78704456	0.25590085	0.79902714	0.99978775	-4.8728808
KCNK1	0.02779849	1.8268027	0.16624462	0.86860286	0.99978775	-4.7022281
TKTL1	0.0277715	3.29494786	0.03301142	0.97379064	0.99978775	-4.804769
PIK3R1	0.02776562	6.33339976	0.11701405	0.9072953	0.99978775	-4.8812586
TUBGCP5	0.02769241	4.83454146	0.27443714	0.78482543	0.99978775	-4.8319902
ZNF646	0.02768672	4.14085224	0.19250503	0.84808915	0.99978775	-4.7811718
EIF2B3	0.02764703	4.86664771	0.25885945	0.79675566	0.99978775	-4.8456642
ZDHHC4	0.02753639	3.95239122	0.26141778	0.79479291	0.99978775	-4.7896924
ENSCAFG00000017257	0.02752553	6.99106191	0.13682956	0.89168875	0.99978775	-4.892324
TNRC6C	0.02750384	4.9262002	0.28781363	0.77462222	0.99978775	-4.8375036
SPRYD4	0.02747609	1.79804933	0.12489212	0.90108583	0.99978775	-4.657628
DLG1	0.02737632	7.02216363	0.24557293	0.80697006	0.99978775	-4.877657
ATP6V1H	0.02735516	6.47164071	0.34090653	0.7345341	0.99978775	-4.870042
DOCK1	0.02734882	7.49048944	0.32984787	0.74282708	0.99978775	-4.8656642
RARS	0.02726652	6.17520374	0.24586841	0.80674252	0.99978775	-4.8820769
HOMEZ	0.02726496	2.8952021	0.14609442	0.88440621	0.99978775	-4.7137943
ANKRD44	0.02716884	0.42043247	0.07045596	0.94409838	0.99978775	-4.6387598
MRPS7	0.02715647	4.54471598	0.24388823	0.80826768	0.99978775	-4.8351691
ZNF713	0.02714667	4.65445483	0.17173946	0.86430246	0.99978775	-4.847896
YY1	0.02714192	6.48590426	0.40200406	0.68931256	0.99978775	-4.8595966
USP7	0.02713183	6.89996651	0.38104354	0.70470817	0.99978775	-4.8621395
NRIP3	0.02711391	-0.7553783	0.0584768	0.95359079	0.99978775	-4.6204924
ARL5A	0.02705045	3.734779	0.22154384	0.82552824	0.99978775	-4.7615814
HNRNPK	0.0268975	8.85464211	0.31919066	0.75084818	0.99978775	-4.8276513
TRIM68	0.02688207	2.62631707	0.1483541	0.88263152	0.99978775	-4.6970236
MYLK	0.02678505	7.11314112	0.04193568	0.96670901	0.99978775	-4.8522808
ATP6V0D1	0.02677063	7.07990584	0.27917862	0.7812043	0.99978775	-4.874622
PHC3	0.02672108	4.98459426	0.27143021	0.78712435	0.99978775	-4.8482682
DR1	0.02671778	3.60551063	0.26809777	0.78967436	0.99978775	-4.7632552
ITPRIPL1	0.02671288	1.67055158	0.07969847	0.93678001	0.99978775	-4.6530374
C11H5orf24	0.02665552	4.57302807	0.29587155	0.76849501	0.99978775	-4.8140773
PDCD11	0.0265995	6.4460496	0.22622113	0.82190764	0.99978775	-4.8852135
ZCCHC4	0.02656888	3.08818341	0.21042309	0.8341518	0.99978775	-4.7304052
CDC42BPG	0.02653879	1.50401749	0.0729493	0.94212361	0.99978775	-4.65118
CTNNB1	0.02643984	9.18397703	0.15522922	0.87723573	0.99978775	-4.8378784
CYP27C1	0.02643294	4.43888859	0.11723736	0.9071192	0.99978775	-4.8612694

ALG8	0.02642588	4.32362189	0.2099556	0.83451477	0.99978775	-4.8261766
ENSCAFG00000013797	0.02631667	4.00390909	0.14719805	0.88353938	0.99978775	-4.7928245
ENSCAFG00000004260	0.02628246	5.03238781	0.18812053	0.85150722	0.99978775	-4.8442151
TSTA3	0.0262315	5.23454543	0.28612279	0.77590978	0.99978775	-4.8540554
ZNF317	0.02619373	3.55243776	0.21319086	0.83200358	0.99978775	-4.7585707
ANKFY1	0.02618667	6.35355237	0.46751425	0.64206627	0.99978775	-4.8465164
CLIC4	0.02616266	8.35604301	0.17198517	0.86411025	0.99978775	-4.8605359
AP2S1	0.02616072	6.1452274	0.26385974	0.79292069	0.99978775	-4.8786473
NAE1	0.02612769	5.06934657	0.22526613	0.82264657	0.99978775	-4.8548856
LYRM1	0.02608625	4.31987086	0.20102993	0.84145178	0.99978775	-4.8265243
GNA12	0.02603768	5.8994755	0.19351897	0.84729912	0.99978775	-4.8830966
STAU1	0.02603589	7.02681184	0.41035128	0.6832177	0.99978775	-4.8547394
PTH1R	0.02581898	-0.069387	0.07308925	0.94201277	0.99978775	-4.6367636
TIMM22	0.02576215	4.95073824	0.20593175	0.83764049	0.99978775	-4.8462399
MICAL3	0.02574637	4.33161063	0.13485594	0.89324133	0.99978775	-4.8142855
SEMA3F	0.02573798	4.48443897	0.06266787	0.9502689	0.99978775	-4.8143502
STT3A	0.0256641	8.35599512	0.21711887	0.82895704	0.99978775	-4.8469025
GRPEL2	0.02559189	3.73952692	0.16275678	0.87133462	0.99978775	-4.7693743
ENSCAFG00000015396	0.02556896	1.88020259	0.15106867	0.88050036	0.99978775	-4.6785418
HMOX2	0.02552787	5.26040071	0.28103339	0.77978912	0.99978775	-4.8588364
ELF2	0.02550098	5.39342723	0.22540688	0.82253766	0.99978775	-4.8611094
NR1H4	0.02549092	0.67823224	0.01768625	0.98595618	0.99978775	-4.6180116
DNAJC1	0.02548862	4.52529035	0.17510111	0.86167357	0.99978775	-4.8299336
DDX17	0.02548004	8.51542397	0.27161603	0.78698223	0.99978775	-4.8438197
CACNA2D1	0.02547309	5.7098598	0.22669729	0.82153928	0.99978775	-4.8760873
IDH3G	0.0254644	6.33950122	0.26693145	0.79056739	0.99978775	-4.8800713
BFAR	0.02541356	6.13742677	0.38435625	0.70226647	0.99978775	-4.8608762
PANK4	0.02538952	3.65124194	0.20546722	0.83800151	0.99978775	-4.7573295
ZNF182	0.02538583	2.40231184	0.14495378	0.88530227	0.99978775	-4.6976425
ZNF512B	0.02533572	4.56519956	0.20105811	0.84142986	0.99978775	-4.8351495
MPV17L2	0.02525412	2.41031705	0.143297	0.88660406	0.99978775	-4.7001387
RUBCN	0.02524357	3.73345606	0.17807463	0.85934952	0.99978775	-4.7816327
BTBD9	0.02523926	3.3309551	0.17326465	0.86310953	0.99978775	-4.7478828
ATG5	0.02518304	5.6594345	0.3771025	0.70761707	0.99978775	-4.8529094
CCT3	0.02517341	7.89874674	0.23524604	0.81493271	0.99978775	-4.8632176
RNF41	0.02515353	4.61341346	0.22291537	0.82446617	0.99978775	-4.8379446
ENSCAFG00000009857	0.0251293	4.97540268	0.2107585	0.8338914	0.99978775	-4.8405004
OXA1L	0.02511599	5.81205464	0.31274282	0.75571467	0.99978775	-4.866068
EGLN2	0.02510668	4.87396746	0.24046042	0.8109096	0.99978775	-4.8479739
ATRIP	0.02509851	3.7140232	0.25798794	0.79742458	0.99978775	-4.7699057
TMSB10	0.02507239	6.55423435	0.12594681	0.90025498	0.99978775	-4.8909411
PRICKLE3	0.02504239	4.76277595	0.21605741	0.82978005	0.99978775	-4.8286374
PSMD1	0.02502281	7.06635514	0.24175473	0.80991178	0.99978775	-4.8784843
ATP6V0B	0.02501721	5.07622186	0.2016336	0.8409822	0.99978775	-4.8568886
ENSCAFG00000018285	0.02501291	7.0681854	0.27117575	0.78731898	0.99978775	-4.875314
NDEL1	0.02499391	4.92732293	0.24215743	0.80960139	0.99978775	-4.8399305
PARP11	0.024908	2.37949052	0.15438216	0.87790022	0.99978775	-4.6859801
MAT2A	0.02488008	6.00724019	0.20906196	0.83520872	0.99978775	-4.8831749
UBL5	0.02486255	5.62891475	0.18093562	0.85711461	0.99978775	-4.8737558
HDAC3	0.02485385	5.5477257	0.32101582	0.74947249	0.99978775	-4.8595879

PRPF8	0.02480564	8.69543702	0.31030713	0.75755561	0.99978775	-4.8326449
EIF2B5	0.02478707	5.71006925	0.30251333	0.76345577	0.99978775	-4.8667947
ENSCAFG00000017874	0.02478654	1.44690261	0.05787045	0.95407147	0.99978775	-4.6384068
ENSCAFG00000008661	0.0247406	6.75758863	0.10173064	0.91935793	0.99978775	-4.8903343
ENSCAFG00000020283	0.02469368	5.42946155	0.36960736	0.71316138	0.99978775	-4.8472254
GPATCH1	0.02466949	2.48122469	0.13022446	0.89688639	0.99978775	-4.6958469
CDC16	0.02464672	5.14943453	0.25165611	0.80228911	0.99978775	-4.8598471
PQBP1	0.02461897	5.35157982	0.17840166	0.85909399	0.99978775	-4.8603522
SLIRP	0.02460426	3.65917094	0.18428298	0.85450126	0.99978775	-4.764122
PLA2G15	0.02455694	4.18466756	0.18679581	0.85254051	0.99978775	-4.7879016
ENSCAFG00000018792	0.02454197	2.15112866	0.12812979	0.89853569	0.99978775	-4.6815711
RETREG2	0.02450388	4.87366071	0.23845761	0.81245425	0.99978775	-4.8399503
ZNFX1	0.02447129	7.67022489	0.29117664	0.77206322	0.99978775	-4.8640745
SFXN1	0.02445577	6.15618729	0.20955782	0.83482365	0.99978775	-4.8861021
TMEM11	0.02430535	4.69316527	0.24669811	0.8061037	0.99978775	-4.8382033
ATG16L2	0.02426353	2.60385186	0.12993454	0.89711465	0.99978775	-4.7105248
ABCB10	0.02425462	5.15048044	0.18121289	0.85689807	0.99978775	-4.851955
R3HDM4	0.02423391	3.04509395	0.20283566	0.84004732	0.99978775	-4.7085683
SMIM20	0.02421223	5.29635732	0.2104424	0.83413681	0.99978775	-4.8556999
CCT2	0.02421012	6.95243426	0.18579184	0.85332379	0.99978775	-4.8844871
LMBR1	0.02420225	3.20021018	0.26634424	0.79101711	0.99978775	-4.7343815
ENSCAFG00000020576	0.02410441	-0.3265351	0.06522757	0.94824049	0.99978775	-4.622856
VPS13C	0.02406119	6.57022125	0.20443776	0.8388017	0.99978775	-4.8873538
PIGQ	0.02405259	5.35130825	0.17326577	0.86310865	0.99978775	-4.8788732
CELF1	0.02401249	5.32027175	0.18595757	0.85319448	0.99978775	-4.8704395
MPV17	0.02400297	2.05254257	0.09706508	0.92304422	0.99978775	-4.6754448
TAF10	0.02388491	4.87878396	0.16019793	0.87333978	0.99978775	-4.857332
ENSCAFG00000004540	0.02387735	2.71637017	0.18497115	0.85396418	0.99978775	-4.7048907
TRADD	0.02386363	4.53751703	0.23751099	0.81318459	0.99978775	-4.8305112
BRAF	0.02383596	4.05948248	0.22428242	0.82340789	0.99978775	-4.7957274
ZFYVE26	0.02378342	5.07835112	0.24790931	0.80517139	0.99978775	-4.8529174
MRPL10	0.02377022	4.32181228	0.2173454	0.82878143	0.99978775	-4.8180776
SYCP2	0.02374505	0.89338566	0.07623656	0.9395206	0.99978775	-4.6455161
RAC1	0.02372033	6.8345529	0.25789618	0.79749502	0.99978775	-4.8806508
PTCH2	0.0236286	2.37526824	0.10514067	0.91666476	0.99978775	-4.7511875
FGF22	0.02356375	-1.4364846	0.04287719	0.96596204	0.99978775	-4.6120148
MARS2	0.02355545	1.46439472	0.11591619	0.9081611	0.99978775	-4.6460114
JAK1	0.02353468	8.25388562	0.26028438	0.79566229	0.99978775	-4.8572678
FTSJ1	0.02348662	4.7128379	0.21419171	0.83122708	0.99978775	-4.8434766
UBE3B	0.0234621	7.00728723	0.31480954	0.75415372	0.99978775	-4.869215
CHD3	0.02345288	7.7644632	0.22466984	0.82310804	0.99978775	-4.8634692
BAG4	0.02343939	3.79348349	0.17362419	0.86282837	0.99978775	-4.7500164
ABHD2	0.0233919	6.86320625	0.08716393	0.93087273	0.99978775	-4.8854364
ATP6V1C1	0.02337968	5.42250727	0.20325993	0.83971742	0.99978775	-4.8742291
CCL16	0.02328255	-1.9795404	0.02973004	0.97639506	0.99978775	-4.6071591
GRM8	0.02323818	-2.2385978	0.03688568	0.97071601	0.99978775	-4.6080097
CASC1	0.02315539	1.30129988	0.11291393	0.91052933	0.99978775	-4.65033
LRP10	0.02313946	7.65984723	0.15970787	0.8737239	0.99978775	-4.8768561
SLC40A1	0.02313523	1.23617283	0.02070625	0.98355846	0.99978775	-4.6260324
DENR	0.02313252	5.71051171	0.16935675	0.86616673	0.99978775	-4.8853001

SRPK2	0.02312032	6.14097667	0.22704266	0.82127211	0.99978775	-4.8841193
CSE1L	0.02311736	6.72060215	0.16863912	0.86672836	0.99978775	-4.8890544
ENSCAFG00000003455	0.02296171	-0.4211466	0.07474581	0.94070097	0.99978775	-4.6304991
ENSCAFG00000004247	0.02295185	5.40977274	0.27168365	0.7869305	0.99978775	-4.8651681
IZUMO1	0.02291763	0.11959792	0.06286595	0.95011192	0.99978775	-4.6245768
AMOT	0.02291456	0.25041863	0.04336923	0.96557169	0.99978775	-4.6537189
SYNGAP1	0.02288527	2.42375611	0.11969266	0.90518335	0.99978775	-4.6814279
ECHDC3	0.02288363	4.12230682	0.16629895	0.86856032	0.99978775	-4.7957667
RNF25	0.02280563	4.48900816	0.2010406	0.84144347	0.99978775	-4.8305061
CENPS	0.02279152	4.91048204	0.22402949	0.82360367	0.99978775	-4.8462639
TMEM60	0.02277453	2.19188112	0.10185079	0.91926301	0.99978775	-4.6772951
CDV3	0.02272458	7.95537987	0.17441444	0.86221043	0.99978775	-4.8631654
TMEM94	0.0227245	4.68803574	0.21711939	0.82895664	0.99978775	-4.8425509
VEZF1	0.02269965	5.34534698	0.15215432	0.87964829	0.99978775	-4.876596
ENG	0.02266364	6.51497974	0.08804625	0.93017482	0.99978775	-4.8952314
RAD1	0.02264384	2.82471852	0.18388172	0.85481444	0.99978775	-4.7318094
LRP11	0.0224685	4.82502154	0.19718922	0.8444407	0.99978775	-4.820992
KLHL23	0.02245733	3.39106285	0.15901419	0.87426766	0.99978775	-4.7450531
ZBTB7A	0.02242911	4.31571669	0.13460952	0.89343521	0.99978775	-4.8107018
MEIOB	0.02236802	1.37416871	0.07531541	0.94024994	0.99978775	-4.6472144
VHL	0.02234346	4.36103283	0.12910049	0.89777132	0.99978775	-4.7996981
CCT4	0.02228739	7.5472218	0.24597793	0.80665819	0.99978775	-4.8688647
CDO1	0.02220397	4.71328893	0.03251338	0.97418591	0.99978775	-4.7931215
SCO1	0.02220367	5.16662245	0.25690988	0.79825225	0.99978775	-4.8482907
TPRA1	0.02208745	4.06208468	0.16613737	0.86868684	0.99978775	-4.7883431
LENG8	0.02207569	5.49558384	0.15491364	0.87748328	0.99978775	-4.8743455
INVS	0.02204264	6.06355629	0.27867338	0.78158993	0.99978775	-4.8768942
ATG4D	0.02200381	3.58304602	0.15954315	0.87385302	0.99978775	-4.7891702
BCL9	0.02198279	4.95685149	0.19179922	0.84863919	0.99978775	-4.8503049
ENSCAFG00000028124	0.02194936	0.18839337	0.06366393	0.94947954	0.99978775	-4.6270016
KCTD9	0.02194317	5.4743906	0.15690847	0.87591869	0.99978775	-4.8702747
UCK2	0.02194156	6.1595985	0.16572847	0.86900703	0.99978775	-4.8886773
KIAA0232	0.02189298	4.61513162	0.18718336	0.85223819	0.99978775	-4.8422013
ABCA3	0.02185606	0.85011868	0.04766202	0.9621664	0.99978775	-4.6451939
APAF1	0.02183684	4.17155197	0.17930063	0.85839167	0.99978775	-4.7945025
TSPAN31	0.02174174	4.18126762	0.24291027	0.8090212	0.99978775	-4.7928861
IFT88	0.02172385	3.54531349	0.13186908	0.89559179	0.99978775	-4.7840585
CASP2	0.02171594	5.20226375	0.16022184	0.87332104	0.99978775	-4.8734304
H3F3A	0.02168915	4.83167447	0.22685143	0.82142004	0.99978775	-4.8383214
STK17A	0.02164644	5.57651081	0.13212404	0.89539111	0.99978775	-4.8724321
RILPL1	0.02156803	6.01157839	0.29239969	0.7711332	0.99978775	-4.8734644
DPH1	0.02156652	4.37803603	0.18657018	0.85271653	0.99978775	-4.8195272
WDR92	0.02153098	5.58209339	0.21077694	0.83387709	0.99978775	-4.8728136
GPD1	0.02151705	-1.3520313	0.04479641	0.9644395	0.99978775	-4.6116204
SLC22A17	0.02148199	4.59428035	0.08493531	0.9326358	0.99978775	-4.8386839
LSAMP	0.02145367	-3.0389348	0.04777397	0.96207761	0.99978775	-4.60737
ARHGEF4	0.02144654	-1.8696454	0.04443725	0.96472442	0.99978775	-4.6138448
USP12	0.02140496	7.27683508	0.12702062	0.8994092	0.99978775	-4.877088
ZNF511	0.02136109	3.24477588	0.19238855	0.84817992	0.99978775	-4.7474997
ZNF706	0.02131746	4.10189101	0.16822585	0.86705183	0.99978775	-4.8075712

ZFAND4	0.02129259	4.55322846	0.11786149	0.90662706	0.99978775	-4.8513512
RSL24D1	0.02121956	4.29749185	0.21055598	0.83404863	0.99978775	-4.8016706
GPS1	0.02121952	6.46373168	0.1973432	0.84432082	0.99978775	-4.8879511
TGFB3	0.02115812	5.26630412	0.05368239	0.95739191	0.99978775	-4.8848678
SLC25A4	0.02113193	6.23759016	0.17038676	0.86536074	0.99978775	-4.8898261
RBFOX2	0.02112096	6.11499171	0.26379562	0.79296983	0.99978775	-4.8795188
DNMBP	0.02111589	5.26884808	0.17188068	0.86419199	0.99978775	-4.8590937
ENSCAFG00000028721	0.02106961	-0.4418746	0.04614174	0.9633723	0.99978775	-4.6203461
LARGE1	0.02088936	6.22081528	0.11158476	0.91157807	0.99978775	-4.8939892
PHEX	0.02086186	0.88767219	0.03747595	0.97024761	0.99978775	-4.6475416
ADNP2	0.0208584	5.03836244	0.20205279	0.84065616	0.99978775	-4.8537612
CTHRC1	0.02081943	1.97363601	0.07886157	0.93744247	0.99978775	-4.8289602
ENSCAFG00000029103	0.02077406	2.45702677	0.10691008	0.9152677	0.99978775	-4.6965764
KLHL29	0.02077401	2.58577682	0.0790636	0.93728255	0.99978775	-4.707657
METAP1D	0.0205668	2.61865163	0.11081902	0.91218231	0.99978775	-4.7177337
MRPL19	0.02053726	3.69818056	0.17271491	0.86353947	0.99978775	-4.7749855
RASGRP1	0.02050174	-0.990017	0.03459904	0.97253065	0.99978775	-4.6154244
FAM32A	0.02047928	1.4492399	0.1006747	0.92019207	0.99978775	-4.6530625
TTLL7	0.02043528	4.36787864	0.15727376	0.87563224	0.99978775	-4.836047
MAP4K4	0.02038202	8.47828571	0.15461875	0.87771462	0.99978775	-4.8665991
G3BP1	0.02035623	7.31493389	0.22764678	0.82080485	0.99978775	-4.8751899
ARPC1A	0.02031199	7.55172313	0.2853509	0.77649777	0.99978775	-4.8680206
PRKACA	0.02030202	6.17233363	0.21271359	0.83237392	0.99978775	-4.8823334
ENSCAFG00000018018	0.02026258	6.40688186	0.13477455	0.89330537	0.99978775	-4.8918153
BIRC2	0.02021713	6.11407265	0.33702134	0.73744407	0.99978775	-4.8681624
ENSCAFG00000016728	0.02019021	5.60094612	0.17869927	0.85886147	0.99978775	-4.8793715
OAZ1	0.02012431	7.67965446	0.30338518	0.76279504	0.99978775	-4.8651882
PHF20L1	0.02012053	5.88319553	0.19035523	0.84976473	0.99978775	-4.8864169
DIS3L	0.02009073	6.50572729	0.28687597	0.77533616	0.99978775	-4.8779622
RPS6KC1	0.02000707	6.19189504	0.21755239	0.82862097	0.99978775	-4.885027
NUMBL	0.01996595	5.45026884	0.11901478	0.90571776	0.99978775	-4.8749593
PSMB4	0.0199348	6.49665446	0.3054645	0.76121995	0.99978775	-4.8753734
ENSCAFG00000005637	0.01992811	6.80411634	0.20873725	0.8354609	0.99978775	-4.8858494
MYSM1	0.0199094	3.78732497	0.11634562	0.90782242	0.99978775	-4.7680056
GSTM3	0.0198871	4.20264799	0.08571825	0.93201637	0.99978775	-4.7814281
MBIP	0.0198813	4.19720496	0.15869463	0.87451819	0.99978775	-4.8097559
NEK6	0.01980935	6.71600534	0.14517617	0.88512756	0.99978775	-4.8921631
ENSCAFG00000017655	0.01975759	9.95159209	0.15511271	0.87732712	0.99978775	-4.8127855
NAA20	0.01973303	5.2435515	0.21500646	0.83059509	0.99978775	-4.8632136
ENSCAFG00000018211	0.01973068	4.12290616	0.15155342	0.88011989	0.99978775	-4.7974611
ENSCAFG00000014378	0.01970715	1.75988087	0.10069607	0.92017519	0.99978775	-4.6859545
GPR82	0.01967904	-2.0015875	0.03219709	0.97443694	0.99978775	-4.6114145
COMMD3	0.01967665	4.53823048	0.2381838	0.81266549	0.99978775	-4.8282942
TBC1D31	0.01965201	2.71991289	0.1182683	0.9063063	0.99978775	-4.7123474
SETD6	0.01964211	4.33059572	0.13745938	0.89119338	0.99978775	-4.8080386
SOCS7	0.01959716	1.43622963	0.07477054	0.94068138	0.99978775	-4.6495004
AP1G2	0.01955056	4.72129191	0.12949788	0.89745844	0.99978775	-4.8437908
ENSCAFG00000005131	0.01953211	5.0653963	0.20463768	0.83864629	0.99978775	-4.8568296
ARID3A	0.01942744	2.55894495	0.09816018	0.92217881	0.99978775	-4.680517
DYRK2	0.01941919	5.60630172	0.12214577	0.9032498	0.99978775	-4.8877148

NFKBIA	0.01939046	5.57350655	0.0663008	0.94739011	0.99978775	-4.861836
B4GALT2	0.01938928	6.02079807	0.14368763	0.88629711	0.99978775	-4.891279
FAM167B	0.01938902	-2.7353953	0.03095455	0.97542313	0.99978775	-4.6071582
RUNX1	0.01932491	3.86579593	0.07018574	0.94431242	0.99978775	-4.8442137
TRAPPC13	0.01930301	4.69572828	0.19171995	0.84870097	0.99978775	-4.8303308
MRPL54	0.01929843	2.88539188	0.18105524	0.85702119	0.99978775	-4.7267402
MAPKAP1	0.0192974	5.64288741	0.23817477	0.81267245	0.99978775	-4.8716198
DHDDS	0.01927027	5.46719981	0.17210298	0.8640181	0.99978775	-4.8776139
FIS1	0.01924229	5.8055469	0.17314546	0.86320274	0.99978775	-4.877771
NCOR2	0.0192279	7.68635223	0.17043334	0.86532429	0.99978775	-4.8746936
NFATC1	0.01920776	5.85835536	0.08467054	0.93284528	0.99978775	-4.8933843
NPTX2	0.01916281	0.87437517	0.0385081	0.96942859	0.99978775	-4.6610281
TMEM69	0.01908955	3.90811005	0.16191733	0.87199234	0.99978775	-4.7866356
DMAC2	0.01902345	3.55786631	0.16635829	0.86851386	0.99978775	-4.7782055
RIOK3	0.01896726	5.4680917	0.16124688	0.8725177	0.99978775	-4.8762803
MORN1	0.01894892	-0.2304967	0.05227838	0.95850524	0.99978775	-4.6234496
USP42	0.01885731	4.13555075	0.16509613	0.86950222	0.99978775	-4.7968037
ADAL	0.01880619	2.70574259	0.10949515	0.91322711	0.99978775	-4.6995835
SUGP1	0.01880491	4.90363277	0.2022268	0.84052082	0.99978775	-4.8467285
SNX19	0.01880359	6.53715602	0.20598512	0.83759902	0.99978775	-4.8866934
POLR3C	0.01879103	4.38621575	0.19569565	0.84560365	0.99978775	-4.8143869
PSMC4	0.01876728	6.00016282	0.23614006	0.81424258	0.99978775	-4.8818969
REL	0.01865174	2.70020701	0.11555107	0.90844906	0.99978775	-4.7274399
UHRF1BP1	0.01854124	4.90509622	0.17465684	0.86202091	0.99978775	-4.852302
ENPP2	0.01853202	-2.2937701	0.02322614	0.98155791	0.99978775	-4.6083017
GMPR	0.01849274	1.06609272	0.06985514	0.94457429	0.99978775	-4.6575828
GTF2H4	0.01846374	5.07813011	0.14926052	0.88191981	0.99978775	-4.8530088
SYNCRIP	0.01843549	7.29374605	0.14316242	0.88670983	0.99978775	-4.8826611
DCTN2	0.01842154	6.44210799	0.27607289	0.78357564	0.99978775	-4.8794099
RPL35	0.01840916	8.14298519	0.20496249	0.83839381	0.99978775	-4.8627102
SUFU	0.01840654	3.34505466	0.13550115	0.89273372	0.99978775	-4.7389878
ENSCAFG00000000683	0.01837951	0.60932836	0.06312767	0.94990451	0.99978775	-4.6409419
NKX2-5	0.01837282	-1.0358053	0.04507524	0.96421831	0.99978775	-4.6305315
INTS14	0.01832109	4.14465447	0.1620759	0.87186809	0.99978775	-4.8116015
OBSCN	0.018215	-0.9729788	0.04629347	0.96325194	0.99978775	-4.6224915
CEP41	0.01813649	4.00225532	0.09715347	0.92297436	0.99978775	-4.8114812
DRG1	0.01808701	5.05017165	0.21868454	0.82774345	0.99978775	-4.8519177
ENSCAFG00000001530	0.01808348	1.23574091	0.07450791	0.94088934	0.99978775	-4.6750739
JCAD	0.01807447	7.0872516	0.06230322	0.95055789	0.99978775	-4.8809155
PPP2CB	0.01793144	6.81210117	0.20595982	0.83761868	0.99978775	-4.8836449
RBM5	0.01790861	5.94863865	0.1895596	0.85038503	0.99978775	-4.880287
SIRT4	0.01782908	1.14982152	0.07896118	0.93736362	0.99978775	-4.6444548
ENSCAFG00000030381	0.01779387	-1.7484528	0.02905156	0.9769336	0.99978775	-4.6111407
ENSCAFG00000002336	0.01777915	1.8808439	0.07785867	0.93823638	0.99978775	-4.6837944
PAFAH1B1	0.01773191	6.73418302	0.19300556	0.84769913	0.99978775	-4.8869234
ENSCAFG00000015277	0.01771735	2.33056636	0.09318886	0.92610814	0.99978775	-4.6831579
FAM234B	0.0176712	2.691551	0.06644938	0.94727239	0.99978775	-4.678413
NFYB	0.01765653	3.79430403	0.12978176	0.89723493	0.99978775	-4.7816027
WARS	0.01763723	8.51227679	0.15954043	0.87385515	0.99978775	-4.852411
JOSD1	0.01762403	4.40619507	0.14120551	0.88824788	0.99978775	-4.8225849

RASA2	0.01758703	3.3849058	0.07961564	0.93684558	0.99978775	-4.7198451
WWTR1	0.01752219	6.64313428	0.1381266	0.89066864	0.99978775	-4.8923584
ATMIN	0.01744785	6.17788239	0.21219391	0.83277722	0.99978775	-4.8842257
ENSCAFG00000007984	0.01740177	0.95572641	0.07601095	0.93969923	0.99978775	-4.6526228
ENSCAFG00000000153	0.01732675	1.56090604	0.06134862	0.95131446	0.99978775	-4.6501621
GNPNAT1	0.01731055	4.71707419	0.08610439	0.9317109	0.99978775	-4.8165988
BVES	0.01730531	3.03862171	0.05033389	0.96004728	0.99978775	-4.868902
SLAIN2	0.0172492	5.86745904	0.2215485	0.82552463	0.99978775	-4.8813815
KLHL36	0.01722452	4.56596485	0.12279981	0.90273438	0.99978775	-4.8267704
PTS	0.01713324	2.95520285	0.11527902	0.90866363	0.99978775	-4.7273694
TRIP4	0.01708724	4.58557152	0.18682946	0.85251426	0.99978775	-4.8314678
PSMD4	0.01705764	5.87506791	0.2552868	0.7994988	0.99978775	-4.8773526
ERI1	0.01702273	2.84275454	0.09470893	0.92490648	0.99978775	-4.7199897
PTCD3	0.01700551	5.31014662	0.13570977	0.8925696	0.99978775	-4.8655701
FGD5	0.01697566	-0.8208096	0.01100164	0.99126382	0.99978775	-4.6095593
AP2M1	0.01696421	7.93633018	0.2208248	0.82608517	0.99978775	-4.8625049
NELFE	0.01695428	3.21802671	0.15324346	0.87879362	0.99978775	-4.7500226
FECH	0.01693098	4.49011479	0.15722154	0.87567319	0.99978775	-4.8171323
IST1	0.01692176	6.66793081	0.32982089	0.74284735	0.99978775	-4.8717217
DAGLA	0.01690328	2.94491278	0.0930705	0.92620172	0.99978775	-4.7339002
KBTBD12	0.01688927	1.88701851	0.04620214	0.96332439	0.99978775	-4.6580474
GNAS	0.01688671	9.40431221	0.23634626	0.81408343	0.99978775	-4.8183286
ENSCAFG00000028601	0.01688438	-0.6589229	0.0384249	0.96949461	0.99978775	-4.6203969
EDA	0.01686623	1.96079129	0.08027201	0.93632606	0.99978775	-4.7240166
LRPAP1	0.01684353	7.21746283	0.10841678	0.91407828	0.99978775	-4.890855
BTBD7	0.01677087	4.39197648	0.14133903	0.88814292	0.99978775	-4.8215838
PPP2R3A	0.01675056	5.02621633	0.09518357	0.9245313	0.99978775	-4.8685421
ENSCAFG00000017901	0.01671124	0.78121686	0.05935329	0.95289601	0.99978775	-4.6369645
UCHL3	0.01666216	5.03438477	0.1362114	0.89217499	0.99978775	-4.8681923
SNX12	0.01664538	3.61799317	0.15075552	0.88074617	0.99978775	-4.7599503
ZNF644	0.016633	4.92693693	0.13691357	0.89162267	0.99978775	-4.8525753
ENSCAFG00000003714	0.01660659	1.87089063	0.0880371	0.93018205	0.99978775	-4.675802
ENSCAFG00000032173	0.01656567	8.18018609	0.12548123	0.90062174	0.99978775	-4.8624064
RBBP7	0.01655501	7.33344287	0.1316425	0.89577013	0.99978775	-4.8791127
ACD	0.01651643	3.62120232	0.14328581	0.88661286	0.99978775	-4.7728319
UBA2	0.01650978	6.30568855	0.14109481	0.88833489	0.99978775	-4.8923012
ZFC3H1	0.01650959	5.69311741	0.13020134	0.8969046	0.99978775	-4.8806829
GORASP1	0.01634434	5.29172956	0.13902959	0.88995857	0.99978775	-4.8812907
CORO1C	0.01625061	7.75498899	0.15600728	0.87662545	0.99978775	-4.8714132
BEGAIN	0.01621357	-0.9935217	0.0284659	0.97739847	0.99978775	-4.6127441
ELMOD2	0.01617974	3.6540694	0.09596631	0.92391261	0.99978775	-4.7669472
GEMIN2	0.016152	2.91476332	0.11871634	0.90595305	0.99978775	-4.7224467
TKFC	0.0160847	5.00564286	0.1322524	0.89529009	0.99978775	-4.8473202
C8H14orf28	0.01603626	1.47925026	0.05669004	0.95500726	0.99978775	-4.6442223
TRAPPC1	0.01602235	4.12611498	0.13392521	0.89397365	0.99978775	-4.8061142
MOSPD1	0.01600694	2.94037144	0.11140394	0.91172075	0.99978775	-4.7247369
ENSCAFG00000011703	0.0159989	2.39360666	0.06510529	0.94833738	0.99978775	-4.6940449
ENSCAFG00000001397	0.01599127	0.90886716	0.03151249	0.9749803	0.99978775	-4.6433783
SF3A2	0.01594006	6.05563246	0.16476955	0.86975798	0.99978775	-4.8895959
RENBP	0.01589025	3.57292258	0.03524198	0.97202041	0.99978775	-4.7593106

ENSCAFG00000011954	0.01588748	6.72957592	0.19398705	0.84693445	0.99978775	-4.8873915
PRKD3	0.01587011	6.37039489	0.19820057	0.84365342	0.99978775	-4.8879305
RASSF5	0.01581129	1.21257281	0.05389426	0.95722392	0.99978775	-4.667512
FBXO7	0.01578132	5.79576619	0.18558465	0.85348545	0.99978775	-4.8800062
NPHP3	0.01576038	4.15173141	0.16931661	0.86619814	0.99978775	-4.8027806
REX1BD	0.01572783	3.74310787	0.08859444	0.92974122	0.99978775	-4.7896265
RWDD1	0.01568846	6.03515006	0.19224695	0.84829026	0.99978775	-4.8856617
IDUA	0.01565543	4.88421542	0.09133698	0.92757235	0.99978775	-4.8514444
NAGPA	0.01558235	5.39759444	0.11190349	0.91132657	0.99978775	-4.8773925
LRRC9	0.01557552	0.71594171	0.04291712	0.96593037	0.99978775	-4.6356023
EXOSC10	0.01556612	6.39769859	0.20106371	0.8414255	0.99978775	-4.8876865
ENSCAFG00000007663	0.01556025	1.39421453	0.07255895	0.94243275	0.99978775	-4.6505371
CDH24	0.01555149	1.35784928	0.06274073	0.95021116	0.99978775	-4.6700448
TASP1	0.0155421	2.16826928	0.08055131	0.93610499	0.99978775	-4.6698305
OPRD1	0.0154294	-2.1475516	0.01650084	0.98689736	0.99978775	-4.6076769
SHMT2	0.01536565	8.41496285	0.08632934	0.93153294	0.99978775	-4.8443122
ENSCAFG00000000215	0.01525042	7.02890483	0.16620627	0.86863289	0.99978775	-4.8876838
ENSCAFG00000031671	0.0152334	1.8409401	0.06349571	0.94961285	0.99978775	-4.6612928
RAB15	0.01519627	-0.7223462	0.03512899	0.97211008	0.99978775	-4.6201029
ASXL2	0.01518204	4.51376211	0.14397764	0.88606923	0.99978775	-4.8268346
ENTPD4	0.01507531	4.92858729	0.11966111	0.90520822	0.99978775	-4.8514772
SMTN	0.01507102	5.05943788	0.04020333	0.96808348	0.99978775	-4.8538877
ZDHHC12	0.0150507	4.28392854	0.07953362	0.9369105	0.99978775	-4.8174419
SP3	0.01504852	5.90855374	0.13766679	0.89103026	0.99978775	-4.8878198
ENSCAFG00000016639	0.01502463	3.67986687	0.12967367	0.89732003	0.99978775	-4.7743083
PAXX	0.0150162	4.77350966	0.08151852	0.9353395	0.99978775	-4.8424438
GTF3C1	0.01499928	5.9782754	0.14206422	0.88757291	0.99978775	-4.8853827
B4GALT1	0.01498097	7.95785389	0.12017638	0.90480203	0.99978775	-4.8755949
ARHGAP44	0.01486732	3.16231962	0.0887979	0.92958031	0.99978775	-4.7353749
FANCC	0.01483324	1.82341391	0.07063706	0.94395493	0.99978775	-4.6776779
ACAA1	0.01475647	5.155433	0.11551031	0.90848121	0.99978775	-4.857522
RNF115	0.01470436	4.8965406	0.19047201	0.84967369	0.99978775	-4.8500472
SMPD2	0.01462173	4.57405964	0.11622455	0.9079179	0.99978775	-4.8371871
PFKP	0.01460521	8.49862413	0.11208926	0.91117998	0.99978775	-4.85612
FNIP2	0.01459865	6.09647605	0.08594415	0.93183766	0.99978775	-4.8865188
ENSCAFG00000006206	0.01459002	-1.0615163	0.02666555	0.97882757	0.99978775	-4.6222138
TRAF4	0.01458519	3.55619736	0.07460528	0.94081224	0.99978775	-4.7538195
ZNF81	0.01457573	1.66590208	0.0511926	0.95936627	0.99978775	-4.6558778
FAM53C	0.01452208	5.21680916	0.14276301	0.88702371	0.99978775	-4.8613778
SEMA4B	0.01443708	3.01954512	0.08619924	0.93163586	0.99978775	-4.7372795
PPCS	0.01434885	3.859732	0.13684548	0.89167623	0.99978775	-4.7823437
LRRC1	0.01434536	2.35622541	0.0674773	0.94645799	0.99978775	-4.6966439
TOMM7	0.01434286	3.83423223	0.11448174	0.90929251	0.99978775	-4.7780444
MFSD4B	0.01428857	0.55684749	0.04613422	0.96337826	0.99978775	-4.6288129
MRS2	0.01422022	3.48982416	0.11653188	0.90767553	0.99978775	-4.7571927
SNRPD2	0.01421253	5.75584229	0.12747228	0.89905348	0.99978775	-4.8832766
TMEM63A	0.01418079	5.76960425	0.10943104	0.91327771	0.99978775	-4.8892192
LARP7	0.01417616	6.04576594	0.14590156	0.88455771	0.99978775	-4.889007
ZNF2	0.01408773	1.66687712	0.04528582	0.96405126	0.99978775	-4.6493754
TMEM223	0.01408509	2.81283075	0.06294465	0.95004955	0.99978775	-4.7207126

EIF3M	0.01398514	6.87968562	0.14749211	0.88330843	0.99978775	-4.8900004
PIGB	0.01394213	3.52830697	0.10298941	0.91836366	0.99978775	-4.7696393
PMPCA	0.01387386	5.80366233	0.11227361	0.91103453	0.99978775	-4.8873888
ENSCAFG00000007840	0.01377564	1.5367743	0.05966167	0.95265156	0.99978775	-4.6608701
MOSPD3	0.01373515	3.23871497	0.072605	0.94239628	0.99978775	-4.7281733
SNTA1	0.01362412	6.47187664	0.05151866	0.9591077	0.99978775	-4.8923454
TEFM	0.01361239	2.6786225	0.09508268	0.92461105	0.99978775	-4.7145655
STAU2	0.01356837	5.46432727	0.16536242	0.86929367	0.99978775	-4.8773079
MTERF2	0.01353928	2.14395525	0.06668359	0.94708683	0.99978775	-4.6728916
BRIX1	0.01348013	4.38611931	0.10308649	0.91828699	0.99978775	-4.8185396
TOR1AIP2	0.01341042	6.26853304	0.13834353	0.89049805	0.99978775	-4.890649
ENSCAFG00000013905	0.01337997	3.84739188	0.08870525	0.92965359	0.99978775	-4.7948326
CCDC92	0.0133702	3.41993477	0.09521329	0.92450781	0.99978775	-4.75373
HGS	0.01336929	6.23055825	0.15708224	0.87578242	0.99978775	-4.8906632
EED	0.01332486	4.2575289	0.11816069	0.90639114	0.99978775	-4.8142442
ISLR2	0.01331632	-2.7601904	0.02448253	0.98056051	0.99978775	-4.6076304
NELFA	0.01330805	4.57892599	0.13851629	0.8903622	0.99978775	-4.8317403
ENSCAFG00000017241	0.01330725	5.42754772	0.05924147	0.95298464	0.99978775	-4.8915781
HECTD2	0.01320249	5.37918611	0.06973987	0.9446656	0.99978775	-4.8742006
TRIM8	0.0131978	6.01410648	0.0952883	0.92444852	0.99978775	-4.888075
ORAI1	0.01318332	3.09905564	0.05405095	0.95709968	0.99978775	-4.7042499
STARD7	0.01316671	4.84478065	0.13171546	0.8957127	0.99978775	-4.8382734
ZNF282	0.01311728	4.23283402	0.1219336	0.90341701	0.99978775	-4.803384
ATF7IP	0.01307758	5.79565316	0.12358737	0.9021138	0.99978775	-4.8836151
ENSCAFG00000019891	0.01301385	0.38041332	0.02782513	0.97790709	0.99978775	-4.6270875
PRDM10	0.01301181	3.10595332	0.09768749	0.92255234	0.99978775	-4.7379711
PCGF6	0.01299364	2.58818138	0.08676456	0.93118865	0.99978775	-4.710413
DLA-12	0.01298463	5.46586552	0.04931524	0.96085516	0.99978775	-4.8714926
SNX5	0.01286798	6.30499105	0.15014445	0.88122585	0.99978775	-4.8910382
ENSCAFG00000019517	0.01281205	5.33681243	0.10573903	0.91619228	0.99978775	-4.8722087
SOCS5	0.01274628	6.58672035	0.08132947	0.93548912	0.99978775	-4.895447
TMEM147	0.01273456	5.62939773	0.08797153	0.93023392	0.99978775	-4.8836117
FITM2	0.01268407	3.02913318	0.08110331	0.93566811	0.99978775	-4.7148655
USP15	0.01268329	5.24621339	0.12664516	0.89970492	0.99978775	-4.8647336
MAP4	0.01267011	9.07177076	0.17378113	0.86270564	0.99978775	-4.839744
APOPT1	0.01258688	5.78940911	0.06589685	0.94771018	0.99978775	-4.8896227
ENSCAFG00000030647	0.01255056	3.73462307	0.1044692	0.91719499	0.99978775	-4.7646375
AIP	0.01249182	4.6360244	0.11400428	0.90966915	0.99978775	-4.8299869
MCOLN1	0.01236999	4.89399176	0.07997888	0.93655807	0.99978775	-4.8531384
RAB14	0.01236851	7.15577326	0.19376029	0.84711111	0.99978775	-4.8833039
ENSCAFG00000032522	0.01236689	3.18944076	0.07285694	0.94219675	0.99978775	-4.7454486
TTF2	0.01235971	6.42755011	0.14502383	0.88524724	0.99978775	-4.8920962
PI4KB	0.01229597	5.39883763	0.14881535	0.88226934	0.99978775	-4.870746
SPATS2	0.0122873	6.11811357	0.08339598	0.93385377	0.99978775	-4.8908085
USP37	0.01223775	3.05516037	0.0674901	0.94644785	0.99978775	-4.7230161
TSEN54	0.01223648	2.38347951	0.08120476	0.93558782	0.99978775	-4.6962404
CDIP1	0.0121731	2.74144249	0.08579991	0.93195177	0.99978775	-4.7174109
PIR	0.01215748	8.15576323	0.04750017	0.96229478	0.99978775	-4.870121
BSDC1	0.01214878	6.07720656	0.12806825	0.89858414	0.99978775	-4.8869455
MTAP	0.01212022	6.25152232	0.12813965	0.89852792	0.99978775	-4.8915531

ENSCAFG00000018971	0.0121023	0.65536179	0.04658841	0.96301799	0.99978775	-4.6386456
DBR1	0.01209756	3.73929815	0.08102395	0.93573092	0.99978775	-4.7743753
PGAP1	0.01203403	2.3373749	0.0786791	0.93758691	0.99978775	-4.7200795
RPLP0	0.01202225	8.8112781	0.12485371	0.90111608	0.99978775	-4.8485183
CNNM4	0.01189964	4.58229475	0.04736731	0.96240016	0.99978775	-4.8732128
DNAJC2	0.01187204	5.29723979	0.09550625	0.92427625	0.99978775	-4.8710893
ENSCAFG00000029179	0.0118588	6.29563015	0.11646075	0.90773163	0.99978775	-4.8933462
DDX5	0.01182289	9.10195021	0.14463117	0.88555573	0.99978775	-4.8416476
ST6GAL2	0.01177427	0.8340419	0.04571901	0.96370763	0.99978775	-4.7893845
RAP2C	0.01173464	5.74858343	0.13006624	0.89701096	0.99978775	-4.8889352
CLCN7	0.01169017	5.79002737	0.1057932	0.91614951	0.99978775	-4.8858314
DCAF12	0.011648	5.42992281	0.13932506	0.88972624	0.99978775	-4.8755336
TECPR2	0.01164648	4.71182758	0.13071207	0.89650253	0.99978775	-4.840782
FTO	0.01164564	4.47358832	0.14839711	0.88259774	0.99978775	-4.8262556
OLA1	0.01164464	6.42298029	0.1086808	0.91386988	0.99978775	-4.8934448
COMMD8	0.01164138	4.19623257	0.08312381	0.93406914	0.99978775	-4.810557
ENSCAFG00000007260	0.01162501	4.62025123	0.11322696	0.91028236	0.99978775	-4.8316571
TIGD7	0.01161667	4.52898556	0.08774721	0.93041135	0.99978775	-4.8372919
ENSCAFG00000031129	0.01160717	4.74082916	0.08471412	0.93281081	0.99978775	-4.8427551
ANKS1A	0.01160006	4.4538365	0.08487753	0.93268151	0.99978775	-4.8127013
RNF216	0.01156107	5.51936402	0.12096929	0.90417703	0.99978775	-4.8736864
SHBG	0.01153484	0.55827846	0.02806487	0.97771679	0.99978775	-4.6322068
SSB	0.01152677	6.47900285	0.08394445	0.93341979	0.99978775	-4.895397
BZW1	0.01150114	6.79570921	0.07965263	0.9368163	0.99978775	-4.8951421
TAPBP	0.0114857	7.74501532	0.07245603	0.94251426	0.99978775	-4.878219
PTCD2	0.01146836	3.89257601	0.12929717	0.89761646	0.99978775	-4.804333
NXN	0.01144709	5.82800989	0.07147626	0.94329024	0.99978775	-4.8944873
GSTP1	0.01138897	4.93801963	0.05121414	0.9593492	0.99978775	-4.8441903
REPS2	0.01137528	2.94484063	0.02410549	0.98085983	0.99978775	-4.7576487
MGAT4A	0.01136147	0.44566447	0.02104732	0.98328768	0.99978775	-4.6368548
ZNF260	0.01135063	2.06056428	0.04704036	0.9626595	0.99978775	-4.6756825
RAB27A	0.0113468	2.3235636	0.03578056	0.971593	0.99978775	-4.6946017
OXNAD1	0.01128171	2.14447188	0.07110936	0.94358084	0.99978775	-4.6738462
IPO5	0.01128014	7.91441548	0.11490522	0.90895847	0.99978775	-4.8715192
AKAP1	0.01127217	5.90089821	0.13075504	0.8964687	0.99978775	-4.8851629
GAL3ST4	0.01122458	1.88153842	0.0677822	0.94621644	0.99978775	-4.7606597
TIE1	0.01118276	-0.6941665	0.00621894	0.9950616	0.99978775	-4.6099894
SRP14	0.01116101	4.96381397	0.09093755	0.9278882	0.99978775	-4.8599856
ASB7	0.01103473	3.62183095	0.10309445	0.9182807	0.99978775	-4.7554423
TUT4	0.01101898	4.78040631	0.09586497	0.92399272	0.99978775	-4.8414924
NUP153	0.0109117	6.13829777	0.10673654	0.9154047	0.99978775	-4.8916663
TRPC1	0.01090954	2.48418957	0.05977132	0.95256465	0.99978775	-4.7029908
TAF8	0.01088411	2.76370335	0.04997863	0.96032903	0.99978775	-4.6889995
PRG4	0.01078749	0.96690121	0.00358035	0.99715687	0.99978775	-4.6093285
ZRANB2	0.01077515	4.68635561	0.10987772	0.91292518	0.99978775	-4.8384498
ENSCAFG00000016514	0.01075308	2.21706862	0.07055531	0.94401968	0.99978775	-4.6810617
ZNF330	0.01070802	4.74631426	0.10839216	0.91409771	0.99978775	-4.8473216
MYH9	0.01066005	11.7432146	0.05304251	0.9578993	0.99978775	-4.7582987
JARID2	0.01064928	3.32148006	0.06076203	0.95177938	0.99978775	-4.7445895
SPATA5	0.01062596	3.97595415	0.06952126	0.94483876	0.99978775	-4.791275

AKAP13	0.01061652	8.59196963	0.13154957	0.89584328	0.99978775	-4.8600568
HSD17B12	0.01059991	6.71847887	0.08279308	0.93433085	0.99978775	-4.8954149
B3GNT2	0.01059329	5.26350117	0.07909018	0.93726151	0.99978775	-4.8666097
ELP3	0.0105373	5.273587	0.14823418	0.88272569	0.99978775	-4.8654823
PLA2G4B	0.01051018	0.64942373	0.03289736	0.97388116	0.99978775	-4.6367159
ATIC	0.01047096	6.55975936	0.12706882	0.89937123	0.99978775	-4.8930626
ANAPC4	0.01045892	6.69544152	0.15373973	0.87840423	0.99978775	-4.8895214
WDR27	0.01041677	0.66151682	0.02880879	0.9771263	0.99978775	-4.6430675
SPG7	0.01041387	4.95789472	0.09330721	0.92601458	0.99978775	-4.8476195
CHST10	0.01040844	6.45700405	0.06436925	0.94892062	0.99978775	-4.8956625
ENSCAFG00000030168	0.01036528	4.70502461	0.09714072	0.92298444	0.99978775	-4.8412581
RAP1A	0.01027712	5.43173473	0.08983753	0.92875809	0.99978775	-4.875099
DHX32	0.01025204	5.14558069	0.13478794	0.89329483	0.99978775	-4.8585498
SGK3	0.0102383	4.71503566	0.07815356	0.93800293	0.99978775	-4.8423925
C27H12orf4	0.01022487	4.62726124	0.10480078	0.91693315	0.99978775	-4.8470236
LPAR1	0.0102101	3.87695405	0.01798305	0.98572053	0.99978775	-4.8001668
SLC25A25	0.01014166	4.35714498	0.0776678	0.93838748	0.99978775	-4.8305816
ENSCAFG00000018753	0.01009943	4.94894779	0.07426502	0.94108168	0.99978775	-4.8610714
ENSCAFG00000002106	0.0100967	0.14039352	0.03053612	0.97575525	0.99978775	-4.6332179
INSIG2	0.00999967	3.92086362	0.05222828	0.95854497	0.99978775	-4.7733674
TAOK1	0.0099516	5.12153167	0.08467542	0.93284143	0.99978775	-4.8576323
ENSCAFG00000031852	0.00994517	5.68005395	0.07191502	0.94294273	0.99978775	-4.8813885
USP5	0.00985826	6.72307845	0.09871455	0.92174076	0.99978775	-4.8935037
NRDC	0.00982463	6.86799462	0.12686618	0.89953084	0.99978775	-4.8926027
GPAA1	0.00980949	6.45518073	0.07605165	0.939667	0.99978775	-4.8948713
IPPK	0.00980764	3.75401814	0.07873494	0.93754271	0.99978775	-4.7794779
UAP1	0.00970237	5.86223038	0.05274788	0.95813294	0.99978775	-4.8810525
TSPAN4	0.00969729	5.17208451	0.06034907	0.9521067	0.99978775	-4.8651837
CDC37L1	0.00967311	4.29094296	0.07359771	0.94161011	0.99978775	-4.8021768
ZNF517	0.00965085	5.09763368	0.13300377	0.89469875	0.99978775	-4.8626792
PRR14L	0.00959835	5.89495152	0.08507741	0.93252337	0.99978775	-4.8872994
TSEN15	0.00959813	4.60902492	0.08652968	0.93137445	0.99978775	-4.8574249
PTPN3	0.00955343	3.46464396	0.05089082	0.9596056	0.99978775	-4.74078
MRPL47	0.00936135	3.44799851	0.07282757	0.94222001	0.99978775	-4.7545699
ENSCAFG00000029851	0.00929369	2.99235866	0.05855548	0.95352842	0.99978775	-4.7224848
FXR2	0.00928161	5.22293304	0.091737	0.92725605	0.99978775	-4.8656075
C20H19orf70	0.00923863	3.35552047	0.04549422	0.96388594	0.99978775	-4.7371546
PDZD11	0.00921005	4.54432115	0.10002223	0.92070755	0.99978775	-4.8401434
RYK	0.00920652	7.46653782	0.07302846	0.94206092	0.99978775	-4.889272
FUBP3	0.00917614	5.1838026	0.11469647	0.90912313	0.99978775	-4.8664843
LBR	0.00916451	5.33677576	0.05237753	0.95842662	0.99978775	-4.8843297
SUGP2	0.00914626	4.42113119	0.06511745	0.94832775	0.99978775	-4.8300234
LCMT1	0.00912465	4.63170551	0.09677461	0.92327378	0.99978775	-4.842221
RGL1	0.00911846	5.82876282	0.0497117	0.96054073	0.99978775	-4.8701762
SERTAD3	0.00911688	3.20022196	0.05822835	0.95378774	0.99978775	-4.7371268
PIAS2	0.0090949	4.62921868	0.07193238	0.94292898	0.99978775	-4.8385499
AGPAT2	0.00903028	4.18821912	0.06702881	0.94681331	0.99978775	-4.8703779
PYGO2	0.00894035	3.70925133	0.06519567	0.94826577	0.99978775	-4.7660219
RPS4X	0.00893888	9.47981217	0.08961387	0.92893497	0.99978775	-4.8282117
COPS4	0.0088897	5.78763353	0.09490956	0.92474789	0.99978775	-4.8877128

BIN3	0.00888196	2.36251218	0.04286556	0.96597128	0.99978775	-4.6819681
MROH1	0.00876952	4.73574964	0.05025433	0.96011037	0.99978775	-4.8434325
AQR	0.00871825	6.87745318	0.1237856	0.90195761	0.99978775	-4.8922554
FH	0.00870742	6.30299098	0.08900222	0.92941871	0.99978775	-4.8943493
XRN2	0.00865774	6.59930854	0.10316707	0.91822334	0.99978775	-4.8938376
TPMT	0.00865088	3.70158539	0.02501745	0.98013587	0.99978775	-4.7392841
SMO	0.00857657	4.17503812	0.03625002	0.97122044	0.99978775	-4.8533301
ENSCAFG00000008186	0.00853185	3.97069304	0.06699691	0.94683859	0.99978775	-4.7994764
ENSCAFG00000030359	0.00852595	4.97175089	0.03496217	0.97224246	0.99978775	-4.8587052
TSHZ3	0.00845023	3.88072249	0.04659521	0.9630126	0.99978775	-4.8221194
RASA1	0.00844191	7.45825288	0.05660068	0.9550781	0.99978775	-4.8838094
UBQLN2	0.00832888	5.609029	0.0821055	0.93487497	0.99978775	-4.8838991
ENSCAFG00000013408	0.00831934	5.51304552	0.08195026	0.93499782	0.99978775	-4.878386
GBF1	0.00823762	6.83674654	0.1036287	0.91785876	0.99978775	-4.8933371
NDUFB7	0.0082114	3.89276306	0.06080602	0.95174452	0.99978775	-4.7984969
CHID1	0.00820434	5.82443657	0.05498603	0.95635825	0.99978775	-4.8845998
PRKCE	0.00819751	4.16777639	0.04246003	0.966293	0.99978775	-4.7868837
NSRP1	0.0081633	4.75919003	0.06236945	0.95050541	0.99978775	-4.8307244
MEX3C	0.00816102	6.10186447	0.14502161	0.88524898	0.99978775	-4.8886167
ASNS	0.00814687	7.94207394	0.03235709	0.97430995	0.99978775	-4.8568338
DBP	0.00813945	2.64340758	0.0336876	0.97325399	0.99978775	-4.7410229
INPPL1	0.00812967	7.74665229	0.10892277	0.91367889	0.99978775	-4.8726552
CUTA	0.00811369	4.55763609	0.06623635	0.94744118	0.99978775	-4.8406826
COG3	0.00807432	4.58660197	0.07310402	0.94200108	0.99978775	-4.8279682
TTI2	0.00802021	3.72802168	0.06043534	0.95203832	0.99978775	-4.770873
HERC4	0.00801709	5.59017479	0.08784827	0.93033141	0.99978775	-4.8815656
ZNF12	0.00801177	3.92412301	0.05686983	0.95486472	0.99978775	-4.7820068
SNRK	0.0079904	3.61595864	0.02081442	0.98347258	0.99978775	-4.7145702
UNC45A	0.00796351	6.7229391	0.11502828	0.9088614	0.99978775	-4.8929559
SLC12A4	0.00789272	7.31982728	0.09866193	0.92178234	0.99978775	-4.8841433
TENM4	0.00788901	3.59842784	0.00676543	0.99462764	0.99978775	-4.6575095
POLR2G	0.00786157	3.3040622	0.04684281	0.96281619	0.99978775	-4.7509806
PRXL2C	0.00784635	3.85382531	0.06087971	0.95168611	0.99978775	-4.7878129
P2RX7	0.00783258	0.50690595	0.01091058	0.99133613	0.99978775	-4.6180736
IKBKG	0.00778278	5.2874458	0.09519192	0.9245247	0.99978775	-4.8741817
NEDD4L	0.00772004	6.4614002	0.04361824	0.96537414	0.99978775	-4.8965634
CNOT4	0.00767589	4.57570486	0.06908557	0.9451839	0.99978775	-4.8284677
XAB2	0.0076601	5.10933581	0.0761707	0.93957275	0.99978775	-4.8650035
LZTS2	0.00764255	5.54307158	0.0585581	0.95352635	0.99978775	-4.8860356
ENSCAFG00000016333	0.00756398	7.7411453	0.02844076	0.97741842	0.99978775	-4.8673602
FBXO38	0.00752255	5.03991391	0.08226877	0.93474576	0.99978775	-4.8551385
ZNF135	0.00749205	4.47468043	0.06772053	0.94626529	0.99978775	-4.8272217
ZCCHC24	0.00749144	6.26860903	0.03704823	0.97058703	0.99978775	-4.8957133
BYSL	0.00748725	4.59824046	0.05893563	0.95322708	0.99978775	-4.8497739
RBM12	0.00743709	5.7012993	0.07406327	0.94124144	0.99978775	-4.8865606
WDYHV1	0.00741116	2.24769898	0.05904642	0.95313926	0.99978775	-4.6932434
VASP	0.00738713	6.77633829	0.04136063	0.96716525	0.99978775	-4.8953095
BRWD1	0.007386	3.95745302	0.04274431	0.96606747	0.99978775	-4.786644
NMT1	0.00738536	5.880383	0.09476922	0.92485882	0.99978775	-4.8898359
MORC3	0.00736203	4.80848546	0.07134635	0.94339313	0.99978775	-4.8408384

NCOA3	0.00734164	5.31171978	0.05804565	0.95393258	0.99978775	-4.8813491
IFI30	0.00732408	5.78263628	0.03363766	0.97329362	0.99978775	-4.8842218
USP34	0.00731399	6.71997946	0.07903392	0.93730604	0.99978775	-4.8953976
SLX4IP	0.00730077	0.52326814	0.02548409	0.97976543	0.99978775	-4.6290744
LIMK2	0.00723648	4.4752527	0.0562085	0.95538903	0.99978775	-4.8312605
PTDSS1	0.00722488	6.05537148	0.08274066	0.93437233	0.99978775	-4.8924657
TXNDC15	0.00712222	6.00081953	0.08215222	0.93483799	0.99978775	-4.8921267
HSP90B1	0.00704892	10.3161924	0.03954467	0.9686061	0.99978775	-4.8053549
FAM98A	0.00699185	6.35667152	0.06836372	0.94575574	0.99978775	-4.8956601
IKZF4	0.00698835	3.21141928	0.03607256	0.97136128	0.99978775	-4.7530231
ENSCAFG00000029013	0.00695667	4.2130548	0.04630667	0.96324147	0.99978775	-4.8313626
CFAP36	0.00689965	4.77836117	0.04237817	0.96635795	0.99978775	-4.8538746
GNL2	0.00688865	5.61093886	0.05964659	0.95266352	0.99978775	-4.8833732
IARS	0.00688305	8.07480799	0.07624601	0.93951311	0.99978775	-4.8648177
ENSCAFG00000018939	0.00685765	5.13735846	0.06930732	0.94500824	0.99978775	-4.8680705
MAPK6	0.0068362	6.6752826	0.04542453	0.96394122	0.99978775	-4.8926307
KLHL24	0.00680634	4.62875717	0.02354626	0.98130378	0.99978775	-4.7960055
N4BP2	0.00678898	4.04340337	0.03927125	0.96882305	0.99978775	-4.7906463
PPP2CA	0.00677515	6.19846539	0.06894381	0.9452962	0.99978775	-4.8955999
NLE1	0.00675018	3.30288266	0.0499723	0.96033405	0.99978775	-4.7525822
PRAF2	0.00673497	6.42100948	0.05789868	0.95404908	0.99978775	-4.8959198
SCAMP2	0.00669913	6.31676867	0.04907995	0.96104178	0.99978775	-4.8953897
ZNF664	0.0066982	4.62105971	0.06333583	0.94973955	0.99978775	-4.8320396
AMPD2	0.00667414	6.73053669	0.06178977	0.95096482	0.99978775	-4.8961346
AP5Z1	0.00666485	4.76676387	0.04264571	0.96614569	0.99978775	-4.8465065
TTC21A	0.00665525	2.76572972	0.02635278	0.97907585	0.99978775	-4.7100632
TRIM32	0.0066144	3.23787203	0.04892035	0.96116836	0.99978775	-4.7364202
NDUFAF7	0.0065569	3.19934003	0.04495228	0.96431585	0.99978775	-4.7409563
FAM104A	0.00653812	3.07378157	0.0434953	0.96547168	0.99978775	-4.7288317
EDNRB	0.00648129	-1.2536943	0.00585932	0.99534717	0.99978775	-4.610123
TSPAN8	0.00642833	-2.1995426	0.01093221	0.99131896	0.99978775	-4.6098671
PRTG	0.006409	-0.2819632	0.01410853	0.98879686	0.99978775	-4.6236571
ENSCAFG00000024474	0.00640257	1.04478844	0.03351607	0.97339012	0.99978775	-4.6615399
UBE2L6	0.00636302	3.47541863	0.03770766	0.97006375	0.99978775	-4.7502141
PRAM1	0.00634997	1.61058288	0.02426686	0.98073172	0.99978775	-4.6623999
HDAC4	0.00633043	3.54412088	0.04365092	0.96534822	0.99978775	-4.7581479
NIFK	0.00627186	5.93406727	0.05334195	0.95766186	0.99978775	-4.8925063
TP53BP2	0.0062455	5.70644475	0.06822079	0.94586897	0.99978775	-4.8847668
KIAA0556	0.00623155	4.58516136	0.06023683	0.95219567	0.99978775	-4.830751
RNF166	0.00622128	4.35113733	0.05382441	0.9572793	0.99978775	-4.8250391
UROS	0.00617198	4.65832553	0.04442854	0.96473132	0.99978775	-4.8344032
ATP1B3	0.00610058	6.0221998	0.04964989	0.96058975	0.99978775	-4.8880155
HSD3B7	0.00604985	5.87750427	0.04312319	0.96576689	0.99978775	-4.8927575
NAPG	0.00604111	4.26625092	0.05069374	0.9597619	0.99978775	-4.8177322
ENSCAFG00000008026	0.00597385	2.29386102	0.01302482	0.98965735	0.99978775	-4.7506762
ENSCAFG00000012316	0.00588534	0.51279424	0.02113421	0.9832187	0.99978775	-4.6338077
ZNF277	0.00582793	4.76151173	0.06054207	0.95195373	0.99978775	-4.8539103
NPL	0.0057591	0.76148621	0.01249044	0.99008166	0.99978775	-4.6363967
ABRACL	0.00575168	5.3255541	0.02325868	0.98153209	0.99978775	-4.8653745
SLC25A12	0.00573472	5.62931401	0.04432289	0.96481514	0.99978775	-4.8857678

ENSCAFG00000007919	0.00569826	4.42943612	0.06415976	0.94908662	0.99978775	-4.8312182
SLC35A1	0.00567472	3.78850425	0.04557528	0.96382164	0.99978775	-4.8159265
STXBP1	0.00566912	5.66755462	0.04523647	0.9640904	0.99978775	-4.865186
ZNF536	0.00566793	-1.2552833	0.0136569	0.98915546	0.99978775	-4.6256055
WSB2	0.00563115	6.46563784	0.04287	0.96596775	0.99978775	-4.8947477
TCEAL1	0.0056096	3.6316036	0.05080043	0.95967728	0.99978775	-4.7785556
DUS2	0.00552173	3.76723952	0.05668684	0.95500979	0.99978775	-4.7820346
TAS1R2	0.00551921	6.55900265	0.04909566	0.96102932	0.99978775	-4.8964445
ENSCAFG00000019677	0.00551779	4.21681825	0.03096862	0.97541197	0.99978775	-4.7951551
ARID1B	0.00550551	6.24690209	0.06100235	0.95158891	0.99978775	-4.8929557
SEC23IP	0.00546757	6.66804903	0.07551287	0.9400936	0.99978775	-4.8956287
ENSCAFG00000012395	0.00545952	7.65355057	0.0560241	0.95553522	0.99978775	-4.8834196
ENSCAFG00000013587	0.00544364	4.83504646	0.06006392	0.95233272	0.99978775	-4.8608842
NHP2	0.00542931	4.61136828	0.04079096	0.96761724	0.99978775	-4.8490134
FMO4	0.00542245	3.27267203	0.03021775	0.97600795	0.99978775	-4.7317503
DHRS1	0.00540084	3.81572402	0.03790926	0.96990378	0.99978775	-4.778635
ENSCAFG00000006856	0.00539408	2.24054076	0.03255672	0.97415151	0.99978775	-4.6872463
MICAL1	0.00535879	6.3943791	0.04096491	0.96747922	0.99978775	-4.8965911
MIGA1	0.00535286	4.88487279	0.04277001	0.96604708	0.99978775	-4.8566179
MGARP	0.00528567	4.17317611	0.03427316	0.97278927	0.99978775	-4.7896444
ENSCAFG00000029162	0.00519204	4.58292694	0.04006619	0.96819229	0.99978775	-4.8208359
OSBPL2	0.0051352	4.69562113	0.0573312	0.95449896	0.99978775	-4.8411331
ENSCAFG00000013913	0.00510411	4.01768165	0.03532354	0.97195568	0.99978775	-4.7963888
TXN2	0.0050932	5.11092382	0.05111786	0.95942554	0.99978775	-4.8646969
DDX58	0.00508957	3.6144921	0.01797998	0.98572296	0.99978775	-4.77289
TBCE	0.00506388	5.90085237	0.04801583	0.96188577	0.99978775	-4.8925609
TLN1	0.00496047	10.153491	0.05938867	0.95286797	0.99978775	-4.8070809
RPS26	0.0049433	7.35932271	0.03617381	0.97128093	0.99978775	-4.8872732
DPF2	0.00492709	6.03720206	0.06906065	0.94520364	0.99978775	-4.8933613
MIB1	0.00489916	5.69642579	0.04521991	0.96410354	0.99978775	-4.8877442
TMUB2	0.00486866	3.90991936	0.04054108	0.9678155	0.99978775	-4.7824501
FXYD5	0.00486352	7.0031447	0.03352065	0.97338649	0.99978775	-4.8925982
EIF3J	0.00485238	4.98640837	0.04028755	0.96801666	0.99978775	-4.8645352
GPBP1L1	0.00474751	5.53516762	0.05177721	0.95890266	0.99978775	-4.8769095
SBNO1	0.00472351	5.38630966	0.04572849	0.96370011	0.99978775	-4.8762731
RACK1	0.00470359	9.95820427	0.0450499	0.96423841	0.99978775	-4.8163395
APIP	0.00464479	4.59659843	0.043201	0.96570515	0.99978775	-4.8314679
BRAP	0.00452682	5.34051302	0.05649279	0.95516364	0.99978775	-4.8709425
ILKAP	0.00451466	4.59390002	0.05065763	0.95979054	0.99978775	-4.823658
OTUD4	0.004482	6.52752062	0.04698779	0.9627012	0.99978775	-4.8953846
PDCD10	0.00447666	4.75137675	0.03619695	0.97126256	0.99978775	-4.8435324
CCDC127	0.00441707	3.33940532	0.0434129	0.96553704	0.99978775	-4.7385482
EHD1	0.00434636	6.59689377	0.03274707	0.97400044	0.99978775	-4.8962381
RUBCNL	0.00429488	-1.5556299	0.00882833	0.99298956	0.99978775	-4.620279
DRAM1	0.00428873	2.59219408	0.01269611	0.98991835	0.99978775	-4.7121252
PKD2	0.00421742	5.55370176	0.03127797	0.97516643	0.99978775	-4.8888268
ABHD3	0.00418714	3.13198239	0.01929037	0.98468257	0.99978775	-4.7085903
C17H2orf68	0.00404962	6.39721965	0.03352202	0.9733854	0.99978775	-4.8959991
CAT	0.00404928	5.14299573	0.01947839	0.9845333	0.99978775	-4.8532595
NAA60	0.00401661	4.50159984	0.03668781	0.97087304	0.99978775	-4.8318503

HNMT	0.00398517	4.15769743	0.02092532	0.98338454	0.99978775	-4.7816722
TEF	0.00398033	3.23457532	0.02704228	0.97852852	0.99978775	-4.753623
DSC2	0.00392015	-2.2411999	0.00523799	0.99584056	0.99978775	-4.6090063
ABHD13	0.00389866	3.76216253	0.0348955	0.97229537	0.99978775	-4.7729448
EIF4ENIF1	0.00389356	5.37679797	0.04498656	0.96428865	0.99978775	-4.873842
C18H11orf68	0.00387441	5.32132364	0.03039349	0.97586845	0.99978775	-4.8808694
EIF2AK3	0.00382093	5.4367957	0.03324199	0.97360764	0.99978775	-4.8693201
FAN1	0.0037692	5.24104348	0.04950245	0.96070668	0.99978775	-4.8704724
PEX11B	0.00361289	2.28887242	0.01900757	0.9849071	0.99978775	-4.6808952
VIPAS39	0.00352355	3.52736456	0.03241398	0.9742648	0.99978775	-4.7654989
ASCC2	0.00342468	6.56470389	0.03567021	0.97168057	0.99978775	-4.8959929
CRIP1	0.00342423	6.37450707	0.01196467	0.99049914	0.99978775	-4.8928494
TMEM209	0.00342263	4.83639619	0.02678967	0.97872904	0.99978775	-4.8551073
ZNF574	0.0033818	4.29271664	0.02562473	0.97965379	0.99978775	-4.8130079
CLEC16A	0.00329685	5.26633479	0.02699122	0.97856905	0.99978775	-4.867088
FBXW7	0.00323225	3.6125328	0.02229783	0.98229489	0.99978775	-4.7648101
USP25	0.00322838	5.02664111	0.0274892	0.97817375	0.99978775	-4.8582405
ARHGAP10	0.00322428	6.87592142	0.02262405	0.98203591	0.99978775	-4.8963332
ENSCAFG00000000102	0.00317141	7.13707975	0.02491959	0.98021355	0.99978775	-4.8892451
PSMB3	0.00316626	5.63300559	0.03219821	0.97443605	0.99978775	-4.888778
PCIF1	0.00309153	4.71665793	0.0273345	0.97829655	0.99978775	-4.8528632
BSCL2	0.00307115	6.00006247	0.03607503	0.97135932	0.99978775	-4.8954108
ABCB6	0.00304865	5.89119551	0.02240307	0.98221134	0.99978775	-4.8903838
MBD4	0.00299806	3.30845658	0.02260981	0.98204721	0.99978775	-4.7468215
KATNA1	0.00297664	4.04778353	0.02602257	0.97933797	0.99978775	-4.8043781
SEPT10	0.00295844	6.68509	0.03415055	0.97288657	0.99978775	-4.8961999
FKBP9	0.00295829	7.29742582	0.02917904	0.97683241	0.99978775	-4.8900825
ENSCAFG00000004444	0.00294523	2.50762125	0.01388256	0.98897628	0.99978775	-4.6861231
BICD2	0.00286145	5.49753561	0.03635187	0.97113962	0.99978775	-4.8773297
NEU1	0.00284625	6.52109517	0.02616779	0.97922269	0.99978775	-4.8968347
NR1H2	0.00269245	5.50998924	0.02096295	0.98335466	0.99978775	-4.8748555
ENSCAFG00000006456	0.00264989	5.8989118	0.02719212	0.97840957	0.99978775	-4.8907728
SLC36A1	0.00256306	4.43190573	0.01978976	0.98428608	0.99978775	-4.8452616
SMG8	0.00255618	4.14475585	0.02138503	0.98301957	0.99978775	-4.8060574
TLL1	0.00255254	-0.7324166	0.00296722	0.99764375	0.99978775	-4.6176773
UNC5B	0.00254419	4.61944362	0.00618437	0.99508905	0.99978775	-4.8325556
SCN9A	0.00244955	5.71617412	0.01106237	0.99121561	0.99978775	-4.8905542
EML1	0.0023811	6.37063865	0.01233162	0.99020777	0.99978775	-4.8969259
ENY2	0.0023704	2.06389129	0.01470675	0.98832187	0.99978775	-4.6744534
ANXA4	0.00230031	6.2528194	0.02185035	0.98265015	0.99978775	-4.8940712
ENSCAFG00000000293	0.00228316	5.28513909	0.02622884	0.97917423	0.99978775	-4.8814267
POFUT1	0.00218841	6.67061545	0.02146059	0.98295957	0.99978775	-4.8968327
HMGN3	0.0021826	3.19262465	0.01347478	0.98930007	0.99978775	-4.7401794
ENSCAFG00000019863	0.00215041	0.43074072	0.00933187	0.99258972	0.99978775	-4.6487358
NUP98	0.00213752	6.79225905	0.0280122	0.97775861	0.99978775	-4.896479
PCSK7	0.00200378	6.08046077	0.01751663	0.98609085	0.99978775	-4.8957098
ST8SIA4	0.00199847	1.86717131	0.00674665	0.99464255	0.99978775	-4.7371649
CHN1	0.0019857	4.61380102	0.01451411	0.98847482	0.99978775	-4.8701168
MATR3	0.00195864	8.1874894	0.02224306	0.98233838	0.99978775	-4.8676328
PDCD6	0.00193105	5.7361961	0.02621781	0.97918299	0.99978775	-4.8860008

ZBTB17	0.00188333	3.23597878	0.01253404	0.99004704	0.99978775	-4.7495691
COPZ2	0.00188133	5.84530229	0.01644568	0.98694116	0.99978775	-4.8930498
TRIM3	0.00187633	4.57372313	0.01780997	0.98585794	0.99978775	-4.8335487
ENSCAFG00000031423	0.00186857	1.75412242	0.00740623	0.9941188	0.99978775	-4.6691513
NOL9	0.00185238	4.79462913	0.01532475	0.98783117	0.99978775	-4.8495441
DUS3L	0.00181991	3.52671526	0.01164167	0.99075561	0.99978775	-4.7774931
TAF5L	0.00169549	4.43535179	0.01747171	0.98612651	0.99978775	-4.8244461
CD44	0.00167374	8.95755514	0.00965018	0.99233696	0.99978775	-4.8446123
COG2	0.0016664	5.77838703	0.0242662	0.98073225	0.99978775	-4.8889652
PCNX1	0.0016351	6.07813139	0.01726604	0.98628981	0.99978775	-4.8931546
MLLT10	0.00162514	4.67839064	0.01283458	0.9898084	0.99978775	-4.8458752
CAPN1	0.00158189	7.30455274	0.01321905	0.98950312	0.99978775	-4.8915497
SGTA	0.00154764	6.33633422	0.01386249	0.98899222	0.99978775	-4.8966844
TAP1	0.00153809	4.84550871	0.01191053	0.99054213	0.99978775	-4.8600386
SLC35E3	0.0015194	2.89637761	0.00873673	0.99306229	0.99978775	-4.7160255
PSMF1	0.00149973	5.64936992	0.01464167	0.98837354	0.99978775	-4.8864194
C10H2orf49	0.00149429	4.13129855	0.01331697	0.98942537	0.99978775	-4.7979292
ENSCAFG00000015452	0.00145862	1.06005924	0.00612676	0.9951348	0.99978775	-4.6482369
APLF	0.00141567	5.53110323	0.01361299	0.98919033	0.99978775	-4.8797406
LUZP1	0.00140562	6.41562501	0.00833951	0.99337771	0.99978775	-4.8970052
THNSL2	0.00136732	1.50198249	0.00478025	0.99620404	0.99978775	-4.6647524
AHCY	0.00133037	5.59560309	0.00686231	0.99455072	0.99978775	-4.8833891
HCLS1	0.00117683	-1.7005796	0.00113824	0.99909613	0.99978775	-4.6081465
HTR4	0.00105136	-2.537413	0.00148327	0.99882214	0.99978775	-4.6070067
ADAMDEC1	0.00105136	-2.6534402	0.00164582	0.99869306	0.99978775	-4.6070067
IL3RA	0.00105136	-2.4954707	0.00110915	0.99911923	0.99978775	-4.6070068
COL28A1	0.00105136	-0.7289434	0.00051705	0.99958941	0.99978775	-4.6070068
DCX	0.00105136	-1.2197225	0.00083049	0.99934051	0.99978775	-4.6070068
GJA5	0.00105136	-2.5328712	0.00094547	0.99924921	0.99978775	-4.6070068
ADCYAP1R1	0.00105136	-1.0324017	0.00054171	0.99956983	0.99978775	-4.6070068
WIF1	0.00105136	0.17701333	0.00026835	0.9997869	0.99983746	-4.6070068
APLNR	0.00105136	-2.9893611	0.00222508	0.99823308	0.99978775	-4.6070067
SLC10A4	0.00105136	-2.8922576	0.00182853	0.99854797	0.99978775	-4.6070067
CPA6	0.00105136	-1.9971713	0.00116424	0.99907548	0.99978775	-4.6070068
CCL24	0.00105136	-1.6674753	0.00067097	0.99946719	0.99978775	-4.6070068
MT3	0.00105136	-1.1394101	0.00057651	0.9995422	0.99978775	-4.6070068
LHFPL1	0.00105136	-2.8103333	0.00170806	0.99864364	0.99978775	-4.6070067
SCARA5	0.00105136	2.00249223	0.00020468	0.99983746	0.99983746	-4.6070068
NPFFR2	0.00105136	-2.3616668	0.0014638	0.9988376	0.99978775	-4.6070067
TMEM37	0.00105136	-2.9803844	0.00187864	0.99850818	0.99978775	-4.6070067
PHKB	0.0009599	5.44453416	0.00794793	0.99368865	0.99978775	-4.8803472
ARHGEF10L	0.00095625	3.47792709	0.00351115	0.99721182	0.99978775	-4.8049663
MAPK1	0.00093998	8.1153694	0.01220328	0.99030967	0.99978775	-4.8626822
AFG1L	0.00092484	1.25160006	0.00368539	0.99707345	0.99978775	-4.6443686
GPR107	0.00090071	5.75450371	0.00850838	0.99324361	0.99978775	-4.8896636
DNAJC8	0.00089199	5.12038061	0.01317718	0.98953637	0.99978775	-4.8709953
DAGLB	0.00077048	5.63008104	0.00973306	0.99227115	0.99978775	-4.8797965
ORC4	0.00075729	5.28959993	0.0089889	0.99286205	0.99978775	-4.8731759
MARK4	0.00056183	4.42503657	0.00469453	0.99627211	0.99978775	-4.8292685
ENSCAFG00000017563	0.00052502	2.82399602	0.00231985	0.99815782	0.99978775	-4.7336925

LCP1	0.00051186	3.16692169	0.00165223	0.99868797	0.99978775	-4.8580946
WRAP73	0.00050558	3.13563426	0.00370464	0.99705817	0.99978775	-4.7331439
ARHGAP21	0.00048286	6.9430013	0.00386008	0.99693473	0.99978775	-4.8954284
EYA3	0.00045052	3.96597538	0.00470603	0.99626298	0.99978775	-4.7907891
EML5	0.00043901	-0.4811452	0.00117024	0.99907072	0.99978775	-4.6189763
EPHB4	0.00035915	5.29651418	0.00179604	0.99857378	0.99978775	-4.8702092
NDUFS3	0.00034524	5.3930254	0.00322425	0.99743964	0.99978775	-4.8793231
BAZ2A	0.00033144	6.42802852	0.00300312	0.99761524	0.99978775	-4.8965218
PSMB7	0.00032738	5.967879	0.00407811	0.9967616	0.99978775	-4.8945655
ENSCAFG00000031010	0.00031152	6.9456786	0.00476599	0.99621537	0.99978775	-4.892576
WIPF2	0.00027628	3.80024026	0.00277783	0.99779414	0.99978775	-4.7744699
ENSCAFG00000010433	0.00026488	6.4444272	0.00368192	0.99707621	0.99978775	-4.8969792
CDC73	0.00026203	4.91541656	0.00230886	0.99816655	0.99978775	-4.8571169
NR2F6	0.00025334	2.51314402	0.00078462	0.99937694	0.99978775	-4.681428
UBE2O	0.00024306	5.72660461	0.00286081	0.99772825	0.99978775	-4.8805429
UPRT	0.00024039	3.5491364	0.00250112	0.99801387	0.99978775	-4.7691853
UGGT1	0.00019025	7.32131984	0.00186655	0.99851778	0.99978775	-4.8891191
PAXIP1	0.00012001	4.2345907	0.00104638	0.99916907	0.99978775	-4.8063924
AP4E1	0.00010576	5.54728759	0.00104497	0.99917019	0.99978775	-4.8785686
CASP3	7.9216E-05	5.38726152	0.00060918	0.99951625	0.99978775	-4.8760664
ARAF	5.1648E-05	6.55496531	0.00052484	0.99958323	0.99978775	-4.897021
KCTD3	4.2765E-05	7.16165306	0.0004672	0.999629	0.99978775	-4.8875758
BRD8	-0.0001244	5.20393148	-0.0012191	0.99903192	0.99978775	-4.868076
GTF2I	-0.0001292	7.02544771	-0.0012437	0.99901239	0.99978775	-4.895511
DENND6A	-0.0001318	3.71617369	-0.000937	0.99925592	0.99978775	-4.7770708
AMMECR1L	-0.0001769	6.16018927	-0.002515	0.99800288	0.99978775	-4.8936388
PPP1R13B	-0.0001849	6.12939663	-0.0010332	0.99917951	0.99978775	-4.8967211
FTH1	-0.0001999	8.07143931	-0.0017606	0.99860193	0.99978775	-4.8741027
STXBP3	-0.0002085	5.12010927	-0.0021276	0.99831048	0.99978775	-4.8651927
WDR33	-0.0002318	5.5218645	-0.0027974	0.99777857	0.99978775	-4.8755767
ZNF142	-0.0002585	5.41179433	-0.0035494	0.99718145	0.99978775	-4.8855359
SNIP1	-0.0002899	2.80439664	-0.0022248	0.99823333	0.99978775	-4.7166391
APOC1	-0.0003539	-1.5976179	-0.0005781	0.99954097	0.99978775	-4.6094472
ZNF219	-0.0004681	2.43516175	-0.0012351	0.99901921	0.99978775	-4.6765968
LMOD1	-0.000507	5.72562325	-0.0015477	0.998771	0.99978775	-4.886732
MLNR	-0.0005244	0.16329771	-0.0011709	0.99907019	0.99978775	-4.6197454
CAMTA2	-0.0005638	5.81968324	-0.0057201	0.99545771	0.99978775	-4.8878332
SERPINB1	-0.0005686	5.87781927	-0.0048642	0.99613738	0.99978775	-4.8934573
MAGED1	-0.0005781	8.44168904	-0.0040878	0.99675389	0.99978775	-4.8633805
VPS35	-0.0005916	7.61364096	-0.0085576	0.99320451	0.99978775	-4.8830103
CD3EAP	-0.0006334	3.38013535	-0.003681	0.99707695	0.99978775	-4.7512155
AGGF1	-0.0007107	4.52797706	-0.0069516	0.99447983	0.99978775	-4.8280121
TMEM132A	-0.0007165	6.14659436	-0.0043728	0.99652762	0.99978775	-4.8951323
CTDSP2	-0.0007218	5.64411161	-0.004494	0.99643134	0.99978775	-4.8831375
ENSCAFG00000013536	-0.0007241	1.62832342	-0.0027392	0.99782482	0.99978775	-4.6512942
NSL1	-0.0007263	3.53319379	-0.0047446	0.99623237	0.99978775	-4.7851584
FUK	-0.0007346	3.52257234	-0.0044736	0.99644751	0.99978775	-4.7702546
MAX	-0.000791	5.13019468	-0.0084344	0.99330238	0.99978775	-4.8675479
LSM12	-0.0008212	4.50465048	-0.0086487	0.99313223	0.99978775	-4.8312489
COASY	-0.000829	4.78460116	-0.0101398	0.99194816	0.99978775	-4.8451791

KIAA0586	-0.0008898	5.35436446	-0.0079139	0.99371565	0.99978775	-4.8754442
C34H3orf70	-0.0008986	1.34934116	-0.0043718	0.99652842	0.99978775	-4.6640994
PNPLA6	-0.0009036	5.54883801	-0.0080093	0.99363993	0.99978775	-4.8708844
HNRNPDL	-0.0009239	6.66690147	-0.0057705	0.99541771	0.99978775	-4.8956387
TESK1	-0.0009273	5.05232456	-0.0081009	0.99356719	0.99978775	-4.8673847
SLC25A20	-0.0009983	3.81867892	-0.0060522	0.99519402	0.99978775	-4.7749114
ENSCAFG00000024530	-0.0010385	1.88294126	-0.0048827	0.99612271	0.99978775	-4.6597052
DHX8	-0.0010622	5.85062852	-0.0160558	0.98725072	0.99978775	-4.890848
FOPNL	-0.0010783	4.57324403	-0.0103894	0.99174997	0.99978775	-4.8357847
SUPT5H	-0.0011153	7.23054457	-0.0142203	0.98870811	0.99978775	-4.8902799
SH3BGRL	-0.0011298	4.07078832	-0.0067536	0.99463701	0.99978775	-4.803004
RPUSD4	-0.0011375	3.48324294	-0.0091561	0.99272932	0.99978775	-4.756642
LARP1B	-0.0011388	4.51476974	-0.0088894	0.99294104	0.99978775	-4.8267052
ENSCAFG00000008590	-0.0011551	1.40674324	-0.00422	0.99664894	0.99978775	-4.6514604
MPI	-0.0011905	4.22201141	-0.0113574	0.99098134	0.99978775	-4.8059213
SOX4	-0.0012085	1.83309293	-0.0038299	0.99695874	0.99978775	-4.6765202
RGS3	-0.0012308	5.32851123	-0.0063784	0.99493499	0.99978775	-4.8948915
TOR3A	-0.0013022	5.10395782	-0.0103184	0.99180637	0.99978775	-4.856556
RPL13A	-0.0013193	9.63046497	-0.0128188	0.98982094	0.99978775	-4.8251538
C12H6orf106	-0.0014467	6.53512292	-0.0145146	0.98847443	0.99978775	-4.8968748
LRFN3	-0.0014581	1.06128112	-0.0040856	0.99675567	0.99978775	-4.6363587
VPS11	-0.0014856	5.75468851	-0.0158583	0.98740753	0.99978775	-4.887819
NMI	-0.0014977	6.05422586	-0.0146583	0.98836035	0.99978775	-4.8935014
AAR2	-0.0015075	4.60614345	-0.0164985	0.98689926	0.99978775	-4.8407581
NFKBIZ	-0.0015136	4.74946591	-0.0053535	0.99574886	0.99978775	-4.8040631
PTEN	-0.0015835	6.5832787	-0.0196738	0.98437817	0.99978775	-4.8967747
DEDD2	-0.0016301	3.36340251	-0.0086746	0.99311162	0.99978775	-4.7310878
WDR11	-0.0016377	5.99914544	-0.0200417	0.98408606	0.99978775	-4.8936465
ACADSB	-0.0016432	4.99816052	-0.0168116	0.98665066	0.99978775	-4.8643445
ENSCAFG00000020072	-0.0016682	6.53313311	-0.0215349	0.98290054	0.99978775	-4.8968787
AGPAT4	-0.0017262	4.27645835	-0.0120778	0.99040934	0.99978775	-4.7909481
CARMIL1	-0.0017551	4.71707572	-0.0053572	0.99574588	0.99978775	-4.828125
BTBD3	-0.0017901	5.14319696	-0.0144936	0.98849111	0.99978775	-4.8643172
INTS8	-0.0018085	4.80633825	-0.0179325	0.98576067	0.99978775	-4.8364148
ZBED9	-0.0018323	5.23138747	-0.0199505	0.98415848	0.99978775	-4.8689094
CABLES1	-0.0018364	2.40087119	-0.0079336	0.99370001	0.99978775	-4.7377668
EIF3K	-0.0018375	7.36943202	-0.0248776	0.98024692	0.99978775	-4.8879422
ADGRA3	-0.0018606	5.6794305	-0.0126521	0.98995333	0.99978775	-4.8953872
ENSCAFG00000031908	-0.0018926	2.40878155	-0.0129731	0.98969845	0.99978775	-4.7015346
ZDHHC7	-0.0018948	5.54559212	-0.0187488	0.98511258	0.99978775	-4.8826923
USB1	-0.001896	4.22693428	-0.0179856	0.98571849	0.99978775	-4.813515
PIK3C2B	-0.0019293	4.36047564	-0.0082281	0.99346621	0.99978775	-4.8549205
GRB2	-0.0019775	5.94663904	-0.0255909	0.97968065	0.99978775	-4.8896031
MON2	-0.0020003	6.65422818	-0.0233	0.98149932	0.99978775	-4.8967026
AKAP10	-0.0020112	4.08376638	-0.0202832	0.98389437	0.99978775	-4.7948224
RANBP10	-0.0020322	4.4403989	-0.0164337	0.98695068	0.99978775	-4.8336676
ENSCAFG00000008815	-0.0020418	3.33344245	-0.0144745	0.98850629	0.99978775	-4.737983
ARHGAP35	-0.0021808	7.08940049	-0.0297392	0.97638781	0.99978775	-4.8930878
ZFP36L1	-0.002198	6.55676878	-0.0110478	0.99122721	0.99978775	-4.8967281
TXLNA	-0.0022084	6.88926033	-0.0375204	0.97021237	0.99978775	-4.8944638

ENSCAFG00000000641	-0.002209	0.44369287	-0.0047618	0.99621871	0.99978775	-4.625973
RPN1	-0.0022092	8.68740992	-0.020851	0.9834435	0.99978775	-4.8547381
MADD	-0.0022148	4.13651152	-0.0093933	0.99254097	0.99978775	-4.8253318
EPHB6	-0.0022328	-0.149539	-0.0043037	0.99658245	0.99978775	-4.6262763
PPP1CC	-0.0022529	6.45709987	-0.0226723	0.98199758	0.99978775	-4.8962107
TIMM17A	-0.0022559	5.03079847	-0.0206159	0.98363019	0.99978775	-4.8625669
POLR2C	-0.0023329	5.1655239	-0.0284301	0.97742687	0.99978775	-4.872094
AAK1	-0.0023667	5.26724528	-0.0232558	0.9815344	0.99978775	-4.8726951
ENSCAFG00000028659	-0.0024523	5.11244753	-0.0228023	0.9818944	0.99978775	-4.8748266
AMBRA1	-0.0024807	4.75927674	-0.0195675	0.98446254	0.99978775	-4.8346181
NDUFA10	-0.0025283	6.06515104	-0.0280605	0.97772027	0.99978775	-4.8944672
CYSTM1	-0.0025498	4.29978838	-0.0140228	0.98886494	0.99978775	-4.844367
LDAH	-0.0025562	5.32782922	-0.0224436	0.98217913	0.99978775	-4.8682104
LPGAT1	-0.0026325	7.53947679	-0.0157315	0.98750822	0.99978775	-4.8896774
MTO1	-0.0026548	4.73802436	-0.0272985	0.97832511	0.99978775	-4.85482
KMT5B	-0.0027061	4.1365892	-0.0262392	0.97916603	0.99978775	-4.8024449
ANXA6	-0.0027247	8.94190331	-0.027086	0.97849382	0.99978775	-4.8493427
SUCLG2	-0.0027429	6.82676077	-0.031336	0.97512038	0.99978775	-4.8951405
C7H1orf21	-0.002758	4.52737004	-0.0187507	0.98511106	0.99978775	-4.8712878
KPNA3	-0.0027829	6.02553265	-0.0305446	0.97574848	0.99978775	-4.8919956
RPS3	-0.0028006	8.62876126	-0.028192	0.97761586	0.99978775	-4.8587116
LZTR1	-0.0028383	4.74579885	-0.0297816	0.97635409	0.99978775	-4.845715
DAD1	-0.0028431	4.6905932	-0.0163025	0.98705483	0.99978775	-4.8486964
FUT4	-0.002885	1.73132965	-0.0142033	0.98872158	0.99978775	-4.6838683
LDB2	-0.0029335	5.36109251	-0.0099393	0.99210738	0.99978775	-4.8698261
CWC22	-0.0029741	5.04790679	-0.026812	0.97871128	0.99978775	-4.8576568
ACAD9	-0.0029867	5.94554897	-0.0316424	0.97487719	0.99978775	-4.8899421
LIMD2	-0.0030082	1.62936073	-0.0109241	0.99132539	0.99978775	-4.6605685
BRD2	-0.0030955	8.25110303	-0.048891	0.96119167	0.99978775	-4.865668
DPY30	-0.0031289	3.35816507	-0.0263136	0.97910693	0.99978775	-4.751218
GAN	-0.0031385	3.16233945	-0.0191737	0.98477522	0.99978775	-4.7607396
MORC2	-0.0031489	5.8816631	-0.0359029	0.97149588	0.99978775	-4.8896063
HMGXB4	-0.0031688	4.60466775	-0.0382508	0.96963274	0.99978775	-4.8378297
LACTB2	-0.0031838	6.00948341	-0.0197842	0.98429048	0.99978775	-4.8907122
PNISR	-0.003219	5.63623078	-0.0225972	0.98205726	0.99978775	-4.8761939
ENSCAFG00000010377	-0.0032778	8.04038324	-0.0457522	0.96368131	0.99978775	-4.8746471
ASPSCR1	-0.0033251	6.21386618	-0.0253537	0.97986898	0.99978775	-4.8958566
USP36	-0.0033459	5.20365604	-0.0383039	0.96959059	0.99978775	-4.8720098
ANKS6	-0.0034371	3.60938574	-0.0341443	0.97289157	0.99978775	-4.8006626
TYRO3	-0.0034381	4.4111249	-0.0320085	0.97458663	0.99978775	-4.8207253
XPO6	-0.0035444	7.27664409	-0.0433837	0.96556021	0.99978775	-4.8887372
ARFGAP1	-0.0035875	5.53286528	-0.0408436	0.9675755	0.99978775	-4.8821254
TMEM40	-0.0036534	0.06857003	-0.0095226	0.99243826	0.99978775	-4.6443204
PFN1	-0.0036791	8.01919316	-0.0196708	0.98438052	0.99978775	-4.8656489
PSMD10	-0.0037017	4.53345211	-0.0378058	0.96998588	0.99978775	-4.8364348
TRIM24	-0.0037078	5.17220753	-0.0285555	0.97732731	0.99978775	-4.858972
ENSCAFG00000031952	-0.0037255	7.29282348	-0.0395384	0.96861104	0.99978775	-4.8885859
MMP24	-0.0037392	-2.5942154	-0.0064727	0.99486007	0.99978775	-4.6072216
KRT39	-0.0037392	-2.5091528	-0.0053948	0.99571601	0.99978775	-4.6072218
LARP1	-0.0037428	7.08549689	-0.0659244	0.94768837	0.99978775	-4.8911492

NT5DC1	-0.0037987	5.73523915	-0.043144	0.96575041	0.99978775	-4.8876611
STAT6	-0.0038203	6.94326067	-0.0378766	0.96992968	0.99978775	-4.8957192
NPM1	-0.003833	8.12078766	-0.0317272	0.97480987	0.99978775	-4.870978
ABRAXAS2	-0.0038451	4.96489277	-0.0347709	0.97239427	0.99978775	-4.8537855
ENSCAFG00000023674	-0.0038479	5.32517992	-0.0481968	0.96174224	0.99978775	-4.8728944
ENSCAFG00000016452	-0.0038949	7.00067829	-0.0460198	0.96346903	0.99978775	-4.8937654
ASB6	-0.0039482	5.03290324	-0.0311069	0.97530218	0.99978775	-4.8617941
CHURC1	-0.0039646	2.7339153	-0.0260985	0.9792777	0.99978775	-4.7224038
EIPR1	-0.0039927	5.28664488	-0.0375435	0.97019401	0.99978775	-4.8724553
JPT1	-0.0040771	5.1652444	-0.0298762	0.97627902	0.99978775	-4.8798256
MTMR14	-0.0041511	5.06354391	-0.0288077	0.97712717	0.99978775	-4.8616785
PELI1	-0.004165	3.49146854	-0.01749	0.98611202	0.99978775	-4.7423828
CD40LG	-0.0042164	0.3653973	-0.0108304	0.99139976	0.99978775	-4.6342516
ATP13A2	-0.0042974	4.78468509	-0.026671	0.97882325	0.99978775	-4.8534811
VPS26B	-0.0043321	5.974709	-0.0508716	0.95962085	0.99978775	-4.8924897
PAK2	-0.0043391	6.60344872	-0.0595187	0.95276491	0.99978775	-4.8962045
RPL27A	-0.0043585	6.99430652	-0.0298697	0.97628417	0.99978775	-4.893944
RPL31	-0.0043977	5.40636334	-0.041658	0.96692934	0.99978775	-4.8757062
ATP6V0C	-0.0043988	7.80672612	-0.0271106	0.97847425	0.99978775	-4.8802504
MAPKAPK2	-0.0044066	6.75672096	-0.0444878	0.96468433	0.99978775	-4.8945502
GSTCD	-0.0044235	3.12291119	-0.0140869	0.98881402	0.99978775	-4.7064683
RARS2	-0.0044403	3.84222248	-0.0354997	0.97181592	0.99978775	-4.7900514
RNF4	-0.0044494	6.09520174	-0.0671512	0.94671631	0.99978775	-4.8939021
ZNF131	-0.004467	3.82587132	-0.0378858	0.96992238	0.99978775	-4.7770686
STX5	-0.0044702	4.87663895	-0.0580346	0.95394136	0.99978775	-4.8532712
ENSCAFG00000018671	-0.0044887	3.09699808	-0.0237956	0.98110583	0.99978775	-4.7386836
FZD2	-0.0045333	4.90298227	-0.0144786	0.98850305	0.99978775	-4.8751431
SELENOI	-0.0045722	4.42009833	-0.0195035	0.98451334	0.99978775	-4.8235164
CCDC174	-0.0045954	3.58470175	-0.0400142	0.96823351	0.99978775	-4.7538071
RGL2	-0.0046581	5.34512743	-0.0256479	0.97963539	0.99978775	-4.8714381
TFIP11	-0.0046606	5.6031647	-0.0596714	0.95264388	0.99978775	-4.8851339
INPP4A	-0.0047327	5.38502649	-0.0424287	0.96631785	0.99978775	-4.8712917
NEK9	-0.0047576	6.14014177	-0.0495453	0.96067271	0.99978775	-4.895474
DOCK7	-0.0048381	7.5253655	-0.0370624	0.97057577	0.99978775	-4.8880811
ALKBH5	-0.0048977	4.3225602	-0.0494684	0.96073371	0.99978775	-4.8095739
UBAP2	-0.0049338	6.76966034	-0.0573656	0.95447167	0.99978775	-4.8947422
ZCCHC10	-0.0049558	2.17117537	-0.0346837	0.97246348	0.99978775	-4.6914356
SBF2	-0.0049734	6.80773464	-0.0452061	0.96411449	0.99978775	-4.8965488
ZRSR2	-0.0050067	4.15310534	-0.0312601	0.97518061	0.99978775	-4.8058863
CEP295	-0.0050547	4.98259482	-0.0369362	0.97067593	0.99978775	-4.8601015
ELP6	-0.0050926	1.91826078	-0.0274009	0.97824386	0.99978775	-4.6718127
CBLN3	-0.0051133	0.1088545	-0.011677	0.99072757	0.99978775	-4.6298416
ENSCAFG00000016151	-0.0051164	5.79942312	-0.053919	0.95720432	0.99978775	-4.8858776
ENSCAFG00000014885	-0.0051768	-0.5178467	-0.0138659	0.98898948	0.99978775	-4.619004
CLK3	-0.0051873	4.96656033	-0.0540127	0.95712998	0.99978775	-4.8525702
PSMD7	-0.005201	6.36513386	-0.0502248	0.96013379	0.99978775	-4.8962521
HPS5	-0.0052304	4.91760708	-0.0568077	0.954914	0.99978775	-4.857929
GTF3C6	-0.0052376	2.53054717	-0.0308483	0.9755075	0.99978775	-4.7065841
ADSS	-0.0052795	6.47923639	-0.0428055	0.96601891	0.99978775	-4.8962672
CTPS2	-0.0053261	3.86189954	-0.0233647	0.98144791	0.99978775	-4.8180726

CRAT	-0.0053382	6.0438159	-0.036871	0.97072769	0.99978775	-4.8902849
ENSCAFG00000019223	-0.0053653	2.47891972	-0.0279399	0.97781601	0.99978775	-4.6942962
MTHFSD	-0.0053771	3.71627107	-0.0534948	0.95754067	0.99978775	-4.7723761
NOA1	-0.0053848	3.24635884	-0.0425797	0.96619804	0.99978775	-4.7464484
GLOD4	-0.0054342	4.76057275	-0.0582206	0.95379386	0.99978775	-4.8430947
SCPEP1	-0.0054377	7.56774127	-0.0286178	0.9772779	0.99978775	-4.8854508
ALKBH7	-0.0054606	2.62380888	-0.0347558	0.9724062	0.99978775	-4.7242689
SDHA	-0.0054688	6.97922945	-0.0556315	0.95584645	0.99978775	-4.8954713
TOR1A	-0.0055012	4.00540975	-0.0458314	0.96361849	0.99978775	-4.8121183
EIF2B1	-0.0055261	4.98046668	-0.0439064	0.96514558	0.99978775	-4.8612066
TYSND1	-0.005527	5.98980878	-0.0932066	0.92609409	0.99978775	-4.8926953
CERS6	-0.0055773	4.42857803	-0.0397879	0.9684131	0.99978775	-4.8348509
RPL19	-0.0056133	8.4519715	-0.0533379	0.95766509	0.99978775	-4.8617334
MFSD10	-0.0056177	4.71494551	-0.0326244	0.97409777	0.99978775	-4.8344757
RAD9A	-0.005668	4.63201467	-0.047044	0.96265659	0.99978775	-4.8531019
RNFT2	-0.0056804	1.49756351	-0.0207412	0.9835307	0.99978775	-4.6537117
ACOX1	-0.0057225	6.55982379	-0.0711231	0.94356999	0.99978775	-4.8958512
IER5L	-0.00582	4.2922249	-0.0270038	0.97855907	0.99978775	-4.8252519
LPP	-0.0059908	5.2871209	-0.0301385	0.97607083	0.99978775	-4.8719288
FNDC5	-0.0060096	-1.5617878	-0.0118963	0.99055345	0.99978775	-4.6170773
LASP1	-0.006127	8.64793036	-0.0834925	0.9337774	0.99978775	-4.8568758
SMAD4	-0.0061919	5.38051781	-0.0586228	0.95347509	0.99978775	-4.8713792
TSKU	-0.0062159	3.32649745	-0.027158	0.97843668	0.99978775	-4.752475
TNC	-0.006238	7.25738736	-0.0065136	0.99482763	0.99978775	-4.8262223
ENSCAFG00000024413	-0.0062527	3.2770188	-0.0281041	0.97768564	0.99978775	-4.7463639
SLC9A8	-0.0063013	2.39486389	-0.0366869	0.97087378	0.99978775	-4.6915443
HMBS	-0.0063581	4.15882315	-0.0633993	0.94968927	0.99978775	-4.816761
C26H12orf65	-0.0064132	0.80032598	-0.0240404	0.98091147	0.99978775	-4.6319607
HSPA5	-0.0064815	10.0359357	-0.0371218	0.97052864	0.99978775	-4.8104905
TCF7L1	-0.0065064	6.32416456	-0.0445419	0.96464143	0.99978775	-4.895488
CENPC	-0.0065112	6.22992865	-0.0670074	0.9468303	0.99978775	-4.8948647
CCT6A	-0.0065387	6.61641782	-0.0568298	0.95489642	0.99978775	-4.8962754
RPL9	-0.0066376	8.12969482	-0.0483669	0.96160732	0.99978775	-4.868095
CSNK2B	-0.0066904	6.14612207	-0.0642285	0.94903216	0.99978775	-4.8948384
ECD	-0.006761	5.4281947	-0.1025438	0.91871566	0.99978775	-4.8775309
RELCH	-0.0067925	5.22966817	-0.0766467	0.9391959	0.99978775	-4.8747475
WDR70	-0.0069149	5.89506438	-0.0856742	0.93205125	0.99978775	-4.8907142
ENSCAFG00000008335	-0.0069199	-0.9369048	-0.0117944	0.99063431	0.99978775	-4.617655
SACM1L	-0.0069836	4.96067292	-0.085453	0.93222625	0.99978775	-4.8482053
ERLIN2	-0.007028	5.14052884	-0.0954481	0.9243222	0.99978775	-4.8645366
CRELD2	-0.0070765	4.66996916	-0.0428901	0.96595182	0.99978775	-4.8374228
DYSF	-0.0070847	4.28409856	-0.0141862	0.98873521	0.99978775	-4.8705423
PSME3	-0.0071256	5.26023821	-0.0681316	0.94593966	0.99978775	-4.8756267
WIPI1	-0.0071507	6.45353238	-0.0792088	0.93716762	0.99978775	-4.8955594
SLC35B3	-0.0071734	4.6178721	-0.075617	0.94001118	0.99978775	-4.8385682
CKAP4	-0.0072212	8.53356749	-0.0346548	0.97248641	0.99978775	-4.8522495
ENSCAFG00000025660	-0.0072343	0.13265438	-0.0188242	0.98505272	0.99978775	-4.6266258
MRPL22	-0.0072409	3.95985743	-0.0755929	0.94003026	0.99978775	-4.7982374
MEIS3	-0.0073708	4.53570132	-0.0404634	0.9678771	0.99978775	-4.8170108
MIDN	-0.0074579	5.02878319	-0.0407307	0.96766505	0.99978775	-4.8737869

EXOC2	-0.0074634	5.88208319	-0.0661696	0.94749404	0.99978775	-4.886648
ZMYND8	-0.0074646	6.10731426	-0.0668866	0.94692597	0.99978775	-4.8874581
LYZ	-0.0075136	-2.0455588	-0.0068819	0.99453516	0.99978775	-4.6072006
TK2	-0.0075373	2.33191891	-0.0278706	0.97787103	0.99978775	-4.6760526
RGS19	-0.0075531	3.58650233	-0.0549362	0.95639774	0.99978775	-4.7762742
ZC4H2	-0.0075675	2.83626382	-0.0388014	0.96919586	0.99978775	-4.7398304
ENSCAFG00000020536	-0.007581	0.406951	-0.0216889	0.98277835	0.99978775	-4.6432901
INTU	-0.0075925	1.2989404	-0.0269962	0.97856509	0.99978775	-4.6477463
PLAGL1	-0.0076277	5.00154584	-0.0817244	0.93517658	0.99978775	-4.8457189
SEC13	-0.0076294	6.4016812	-0.0702745	0.94424214	0.99978775	-4.8958731
SEC23B	-0.0076304	6.81746926	-0.0460391	0.96345373	0.99978775	-4.8947807
LRP1	-0.0076511	11.3261019	-0.0223241	0.98227402	0.99978775	-4.7786646
APPL1	-0.0076998	5.53275762	-0.0692741	0.94503452	0.99978775	-4.8820675
DOCK5	-0.0077573	1.72850864	-0.0275025	0.97816316	0.99978775	-4.7350984
TAF6L	-0.007811	4.16318546	-0.0691423	0.945139	0.99978775	-4.8136312
AIDA	-0.0078273	6.51192108	-0.1096235	0.91312581	0.99978775	-4.8942377
GK5	-0.0078414	4.43217383	-0.0649025	0.94849808	0.99978775	-4.833023
SIGMAR1	-0.0078724	6.96838558	-0.0402518	0.96804505	0.99978775	-4.8940324
ZNF22	-0.0078858	2.69427461	-0.0328373	0.97392885	0.99978775	-4.6919237
FIP1L1	-0.0079763	6.29487238	-0.1092336	0.91343353	0.99978775	-4.8935468
EIF4H	-0.0080188	8.42238007	-0.1388303	0.89011525	0.99978775	-4.8572031
LANCL2	-0.008021	4.62815419	-0.0730061	0.94207864	0.99978775	-4.8348461
PPIG	-0.008022	6.07113266	-0.0685018	0.94564634	0.99978775	-4.8910994
CDC37	-0.0080327	7.41196524	-0.0745477	0.94085787	0.99978775	-4.8877513
ENSCAFG00000018434	-0.0080811	0.08185418	-0.0287773	0.97715131	0.99978775	-4.6290196
UBAP2L	-0.0081132	7.87407351	-0.1040025	0.91756355	0.99978775	-4.8740101
YIF1A	-0.0081563	5.10025115	-0.0811978	0.93559332	0.99978775	-4.8641942
SCOC	-0.0081797	2.04115342	-0.042358	0.96637396	0.99978775	-4.6830378
ENSCAFG00000017317	-0.008203	3.35468339	-0.0402699	0.96803067	0.99978775	-4.812827
SCMH1	-0.0082039	5.0086037	-0.0722292	0.94269391	0.99978775	-4.8632004
EIF1AD	-0.0082546	5.15072981	-0.0856055	0.93210557	0.99978775	-4.8663154
NFX1	-0.0083144	5.1247351	-0.0838949	0.93345902	0.99978775	-4.8752569
CLCN4	-0.0083539	4.7129585	-0.0751973	0.94034349	0.99978775	-4.8550842
ENSCAFG00000001367	-0.0083606	4.68309642	-0.0364757	0.97104132	0.99978775	-4.8192861
SLC45A1	-0.0083654	3.39786548	-0.0239198	0.98100728	0.99978775	-4.7064656
TMEM225B	-0.0083682	3.50074914	-0.0474793	0.96231135	0.99978775	-4.785571
FKBP10	-0.0084142	9.43020414	-0.0575888	0.95429478	0.99978775	-4.8330743
SMG1	-0.0084257	6.10093623	-0.0603401	0.95211382	0.99978775	-4.8932239
SRRM2	-0.0084919	9.29334299	-0.1051251	0.91667703	0.99978775	-4.8384501
CREBBP	-0.0085088	6.0895171	-0.072413	0.9425483	0.99978775	-4.8913598
ZDHHC5	-0.008531	8.02891041	-0.1060381	0.91595614	0.99978775	-4.8729477
H2AFJ	-0.0085406	2.58079361	-0.0274496	0.97820522	0.99978775	-4.6738139
FASTKD3	-0.008564	3.79578806	-0.0765566	0.9392672	0.99978775	-4.7717822
PPIC	-0.0085805	5.32010943	-0.0702515	0.94426034	0.99978775	-4.8780008
BCLAF1	-0.008582	7.14421343	-0.0788518	0.93745021	0.99978775	-4.8889255
WNK4	-0.0085974	5.44211431	-0.1171408	0.90719537	0.99978775	-4.8787318
FAM204A	-0.0086232	5.35420057	-0.0663081	0.94738432	0.99978775	-4.8791545
H2AFY	-0.0086361	6.09826133	-0.1169943	0.90731089	0.99978775	-4.8924674
SFXN2	-0.0086505	4.08879525	-0.0445279	0.96465254	0.99978775	-4.7887388
PDE12	-0.0086696	5.44302688	-0.0848329	0.93271684	0.99978775	-4.8784183

MED19	-0.0087121	3.75101853	-0.0835131	0.93376112	0.99978775	-4.7804834
ENSCAFG00000032618	-0.0087268	2.90490901	-0.0299231	0.97624183	0.99978775	-4.6987534
PCSK6	-0.0089225	6.01140731	-0.0169431	0.98654621	0.99978775	-4.8918367
ARMCX2	-0.0089811	3.37476914	-0.0492424	0.96091295	0.99978775	-4.7915091
TUT7	-0.0090018	5.23934294	-0.0956297	0.92417863	0.99978775	-4.8592633
MGAT4B	-0.0090466	6.88492718	-0.0565587	0.95511139	0.99978775	-4.8962798
GPRASP2	-0.0090828	3.26235319	-0.0564713	0.9551807	0.99978775	-4.7608762
ACER3	-0.009151	2.45586273	-0.0400497	0.96820542	0.99978775	-4.6760875
HS6ST1	-0.0091957	2.52071623	-0.0451968	0.96412186	0.99978775	-4.6977102
NUBP1	-0.0092189	4.08898353	-0.1147399	0.9090889	0.99978775	-4.8104328
TMED1	-0.0093004	3.52157818	-0.0579017	0.95404666	0.99978775	-4.7735709
ENSCAFG00000006102	-0.0093018	7.68409347	-0.0907044	0.92807256	0.99978775	-4.8810154
SPARC	-0.0093039	11.6865039	-0.0557887	0.95572184	0.99978775	-4.7690785
AMIGO1	-0.0093269	1.74493041	-0.0245086	0.9805398	0.99978775	-4.6517075
COQ3	-0.0093764	3.52557813	-0.0598885	0.95247177	0.99978775	-4.7613388
RAB35	-0.0094009	6.42887071	-0.1168005	0.90746373	0.99978775	-4.8937909
FKBP8	-0.0094098	7.88478726	-0.0783744	0.93782815	0.99978775	-4.8777313
SP4	-0.0094176	3.50077697	-0.0493489	0.96082845	0.99978775	-4.7473224
CEP85	-0.0094279	2.95281991	-0.0249888	0.98015863	0.99978775	-4.6960546
KDM2B	-0.009446	3.97715464	-0.0681499	0.94592515	0.99978775	-4.8024111
STEAP3	-0.0094581	0.92289249	-0.0121204	0.99037552	0.99978775	-4.6248184
FAM98B	-0.0094937	4.03521252	-0.0774092	0.93859224	0.99978775	-4.7913307
PIP4P1	-0.0095588	3.94113827	-0.0575866	0.95429647	0.99978775	-4.7761694
NOSIP	-0.0095701	4.6510994	-0.108549	0.9139739	0.99978775	-4.832436
RHOT2	-0.0096467	4.73890568	-0.0783779	0.93782532	0.99978775	-4.8392975
NAA35	-0.0096526	4.56332164	-0.1052103	0.91660978	0.99978775	-4.835976
WDR25	-0.0097453	4.29647743	-0.1085014	0.91401149	0.99978775	-4.8153278
MYO9A	-0.0097471	5.36482671	-0.0814741	0.93537463	0.99978775	-4.8716356
CHD1	-0.0097748	5.57709704	-0.0665172	0.94721863	0.99978775	-4.8844811
DNAAF1	-0.0098721	-0.8046446	-0.0285531	0.97732924	0.99978775	-4.6287158
SENP7	-0.0098893	3.83850458	-0.058605	0.95348918	0.99978775	-4.7787675
CDC23	-0.0099069	4.23038872	-0.0693109	0.94500541	0.99978775	-4.8211776
FPGS	-0.0099177	4.55192947	-0.0877251	0.93042883	0.99978775	-4.8438542
BPTF	-0.0099383	6.36649157	-0.1012828	0.9197117	0.99978775	-4.8933041
ENSCAFG00000032613	-0.0100457	1.68088823	-0.0470885	0.96262127	0.99978775	-4.6697362
ZEB1	-0.0100705	6.30631163	-0.0597448	0.95258571	0.99978775	-4.8943071
RHOA	-0.010088	8.39268503	-0.1288217	0.89799084	0.99978775	-4.8558946
BUB3	-0.0101699	6.29193176	-0.1001997	0.9205673	0.99978775	-4.8945874
ENSCAFG00000013811	-0.0102119	3.38219942	-0.0839262	0.93343426	0.99978775	-4.7529936
GDA	-0.0102232	-2.8467324	-0.0196852	0.9843691	0.99978775	-4.6073871
RANBP3L	-0.0102692	3.35468943	-0.0924586	0.92668552	0.99978775	-4.7574654
POLR1C	-0.0102956	4.18164295	-0.0851872	0.93243652	0.99978775	-4.8172054
HTT	-0.0103366	6.34382658	-0.0869412	0.9310489	0.99978775	-4.895248
COMMD10	-0.0103501	4.11589692	-0.0972903	0.9228662	0.99978775	-4.8128634
TBC1D22A	-0.0104308	5.42890865	-0.1018341	0.91927618	0.99978775	-4.876437
CHST12	-0.0105155	3.95442275	-0.0607071	0.95182296	0.99978775	-4.7739784
DST	-0.0105176	8.66783897	-0.0694781	0.94487298	0.99978775	-4.8424678
POLR3A	-0.0106108	5.46002065	-0.0995884	0.92105034	0.99978775	-4.878223
NAPB	-0.0106355	2.63834848	-0.0497537	0.96050738	0.99978775	-4.6935908
ZDHHC16	-0.0106905	4.20203886	-0.1071561	0.91507349	0.99978775	-4.819614

ADCY7	-0.0107059	4.91858224	-0.0262639	0.97914643	0.99978775	-4.8924001
SHLD2	-0.0107113	2.59102247	-0.0443456	0.96479711	0.99978775	-4.6911502
BASP1	-0.0107863	3.06471381	-0.0410299	0.96742762	0.99978775	-4.8261212
COL4A3BP	-0.0108037	6.07411217	-0.0965929	0.92341742	0.99978775	-4.8933434
PPT1	-0.0108535	6.4387687	-0.1506333	0.88084213	0.99978775	-4.8916707
COPA	-0.0108832	9.19028961	-0.1177698	0.90669932	0.99978775	-4.8308333
NYAP1	-0.01089	3.52009596	-0.0551317	0.95624278	0.99978775	-4.7549082
VBP1	-0.0109046	3.72902103	-0.1148678	0.90898803	0.99978775	-4.7733412
NCOA1	-0.0109157	4.86659759	-0.095172	0.92454046	0.99978775	-4.8611472
GAS2L1	-0.0109229	6.00828702	-0.08579	0.93195962	0.99978775	-4.8910839
PPP2R2A	-0.0109793	6.49704808	-0.0923241	0.92679186	0.99978775	-4.8949137
RAB3GAP1	-0.0109836	6.25649279	-0.0949209	0.92473893	0.99978775	-4.8946644
NEMF	-0.0110135	5.42089933	-0.1157942	0.90825729	0.99978775	-4.874792
TUBB	-0.0110784	8.76245243	-0.0561058	0.95547043	0.99978775	-4.8413368
ITFG2	-0.0111025	3.31861409	-0.0919913	0.92705499	0.99978775	-4.7664098
MFN2	-0.0111942	6.34427025	-0.1263861	0.89990894	0.99978775	-4.8928502
TRMT6	-0.0113037	4.72730352	-0.0958805	0.92398048	0.99978775	-4.8493232
CASP8AP2	-0.01142	3.99086259	-0.0737261	0.94150846	0.99978775	-4.7750356
GABARAP	-0.0114495	7.01880589	-0.1162175	0.90792343	0.99978775	-4.8898299
WBP4	-0.0114728	5.57965181	-0.1687069	0.86667529	0.99978775	-4.876568
OGFOD2	-0.0114866	2.88816985	-0.0642635	0.94900442	0.99978775	-4.7064367
PSMA3	-0.011648	6.30102594	-0.1523883	0.87946468	0.99978775	-4.8911713
NFYA	-0.0116483	3.14941377	-0.0777987	0.93828389	0.99978775	-4.736071
NSF	-0.0116636	6.37407241	-0.1324576	0.89512856	0.99978775	-4.8927809
UCK1	-0.0117061	3.09257889	-0.0842661	0.93316529	0.99978775	-4.7390695
TARS	-0.011804	6.75108336	-0.0899866	0.92864018	0.99978775	-4.8926562
C26H22orf39	-0.0118117	0.00730174	-0.0493646	0.96081601	0.99978775	-4.6314046
SNX17	-0.0119094	6.60980592	-0.1422092	0.88745893	0.99978775	-4.8912359
OXSM	-0.0119316	3.40140872	-0.0857769	0.93196996	0.99978775	-4.7462328
UNC119B	-0.0119604	4.30626206	-0.1409628	0.8884387	0.99978775	-4.8019178
USP49	-0.0119899	0.55149756	-0.0370383	0.97059492	0.99978775	-4.6269236
TMEM104	-0.0120447	4.19087932	-0.1045899	0.91709965	0.99978775	-4.8222503
YAF2	-0.0120716	3.14367569	-0.0981453	0.92219057	0.99978775	-4.7322402
YES1	-0.0120734	5.45316222	-0.054088	0.95707027	0.99978775	-4.8691187
ZNF37A	-0.012097	2.581601	-0.0685022	0.94564603	0.99978775	-4.697781
EBAG9	-0.0121447	3.19241259	-0.0825719	0.93450584	0.99978775	-4.7261247
RBMX2	-0.0121549	4.29768477	-0.099224	0.9213382	0.99978775	-4.8090574
SYNJ2BP	-0.0122066	4.32238374	-0.1189875	0.90573924	0.99978775	-4.8029176
PRPF19	-0.0123014	6.87727457	-0.1562592	0.87642783	0.99978775	-4.8893627
IQSEC2	-0.0123299	2.32045174	-0.0527445	0.95813562	0.99978775	-4.6820077
MRPL14	-0.0124669	4.58864724	-0.1254672	0.90063282	0.99978775	-4.8345339
PKN2	-0.0124674	5.6010184	-0.1252476	0.90080578	0.99978775	-4.8819323
PRKCSH	-0.0125059	7.67886256	-0.1164332	0.90775333	0.99978775	-4.8791199
TRNT1	-0.0125197	4.03997675	-0.0869579	0.9310357	0.99978775	-4.7994822
FBXL14	-0.0125243	3.3815457	-0.0879194	0.93027515	0.99978775	-4.7398857
IGF1R	-0.0125726	6.95291617	-0.0869091	0.93107427	0.99978775	-4.8951346
MTM1	-0.0126406	3.65440953	-0.0821948	0.93480428	0.99978775	-4.7684239
FAM111B	-0.0126517	6.02873852	-0.1065218	0.91557421	0.99978775	-4.8895774
MET	-0.0126833	6.78301503	-0.0524333	0.95838238	0.99978775	-4.8926849
EIF1	-0.0127161	8.59115861	-0.1359836	0.8923542	0.99978775	-4.8529912

UBE2E3	-0.0127501	4.29440145	-0.1153498	0.90860778	0.99978775	-4.8141187
RNF111	-0.012751	5.47311593	-0.1105595	0.91238709	0.99978775	-4.8782518
PDRG1	-0.0127559	2.6588765	-0.0693866	0.94494543	0.99978775	-4.7033356
LSM8	-0.0128056	3.13826125	-0.0703896	0.94415092	0.99978775	-4.7341648
DLD	-0.0128078	6.33118543	-0.143449	0.88648462	0.99978775	-4.8908191
MGA	-0.0128228	4.82652279	-0.1047724	0.91695557	0.99978775	-4.8424223
SENP6	-0.0128727	6.25140674	-0.1401746	0.88905833	0.99978775	-4.8914389
SETDB1	-0.0128971	5.18630785	-0.1321794	0.89534755	0.99978775	-4.8667222
HAGH	-0.0129028	4.8660844	-0.0963353	0.92362094	0.99978775	-4.8423036
PBX3	-0.0129714	6.24277569	-0.0722531	0.942675	0.99978775	-4.8950361
ENSCAFG00000016776	-0.012982	2.5562234	-0.0914325	0.92749679	0.99978775	-4.7081815
MYL4	-0.0130217	-1.4800108	-0.0310221	0.97536949	0.99978775	-4.6171641
EMD	-0.0130227	5.9037388	-0.1205115	0.90453784	0.99978775	-4.8888377
TJP1	-0.0130332	8.2262962	-0.101925	0.91920441	0.99978775	-4.8558459
KHDRBS3	-0.0130793	5.32054561	-0.054261	0.95693309	0.99978775	-4.8945067
UNC93B1	-0.013085	4.01368002	-0.0613449	0.95131741	0.99978775	-4.7953531
AP2A2	-0.0130892	7.85311332	-0.1508598	0.88066434	0.99978775	-4.8755528
TOE1	-0.0131142	3.82005717	-0.1092299	0.91343646	0.99978775	-4.7928313
MYOF	-0.0131411	9.1556083	-0.1081698	0.91427326	0.99978775	-4.8359258
PKD1	-0.0131592	8.5779997	-0.0946351	0.92496486	0.99978775	-4.8556911
DDX49	-0.0131664	5.01900103	-0.1056748	0.91624299	0.99978775	-4.8651757
QRICH1	-0.0131865	6.15937861	-0.2159679	0.82984947	0.99978775	-4.8840199
ZFR	-0.0132075	7.3208064	-0.144863	0.8853736	0.99978775	-4.8808024
SCARF2	-0.0132132	4.86342087	-0.0483052	0.96165625	0.99978775	-4.8757198
TBC1D8	-0.0132286	3.60165328	-0.024849	0.9802696	0.99978775	-4.7114003
NEK1	-0.0132535	5.27050218	-0.130967	0.89630183	0.99978775	-4.8707097
MEST	-0.0133035	2.52589579	-0.0455635	0.96383096	0.99978775	-4.7529497
PPP1R21	-0.0134024	5.59509291	-0.1231003	0.90249758	0.99978775	-4.8849871
PDCD5	-0.0134048	4.49807356	-0.1260395	0.90018198	0.99978775	-4.8309452
TMEM134	-0.0134947	3.78517426	-0.0697761	0.94463686	0.99978775	-4.7744576
CADPS2	-0.0135153	2.67677759	-0.0170445	0.9864657	0.99978775	-4.6972491
PLEKHA6	-0.0135549	5.59607415	-0.0416416	0.9669423	0.99978775	-4.8747468
SSU72	-0.0136069	4.84985979	-0.1471572	0.88357149	0.99978775	-4.8410372
GRK6	-0.0136639	4.88124271	-0.1070954	0.91512136	0.99978775	-4.8535087
SLC9A1	-0.0137131	4.9019169	-0.103608	0.91787514	0.99978775	-4.8449254
NRF1	-0.0137624	3.72702309	-0.1369999	0.8915548	0.99978775	-4.7742129
ENSCAFG00000008060	-0.0137788	-0.2504367	-0.0452975	0.96404199	0.99978775	-4.6310559
ZNF782	-0.0137889	2.34520496	-0.0648166	0.94856617	0.99978775	-4.6793234
ENSCAFG00000020381	-0.0138352	1.5165412	-0.0638175	0.94935786	0.99978775	-4.6633494
UBE2K	-0.0138622	5.05898209	-0.1241048	0.90170612	0.99978775	-4.8678245
ENSCAFG00000014182	-0.0139116	0.6921173	-0.0346807	0.97246587	0.99978775	-4.6294202
CLDN12	-0.0139229	3.85216191	-0.0902692	0.92841671	0.99978775	-4.7541643
TXNIP	-0.0139288	5.73675626	-0.022667	0.9820018	0.99978775	-4.7814694
ZNF10	-0.0139683	0.78271366	-0.0477304	0.96211218	0.99978775	-4.6373522
TIPIN	-0.0139936	4.220759	-0.100578	0.92026845	0.99978775	-4.8150087
ENSCAFG00000029334	-0.0140077	1.04042412	-0.0528043	0.95808818	0.99978775	-4.6555359
ENSCAFG00000031934	-0.0140091	1.29146586	-0.0492729	0.96088875	0.99978775	-4.6493839
ENSCAFG00000028936	-0.0140399	1.56339457	-0.0129582	0.98971027	0.99978775	-4.616694
PAN3	-0.0140788	4.94026104	-0.1277394	0.89884311	0.99978775	-4.8361394
FAM168B	-0.0140821	4.87736627	-0.1036871	0.91781267	0.99978775	-4.858301

CCDC47	-0.0141074	6.14550896	-0.1649898	0.86958547	0.99978775	-4.8884695
WASHC5	-0.014135	7.50291645	-0.1767793	0.8603618	0.99978775	-4.8747114
AJUBA	-0.014178	4.96980683	-0.1742193	0.86236305	0.99978775	-4.8722053
ZFAND2B	-0.0142223	4.12797281	-0.1170771	0.90724558	0.99978775	-4.8101726
YWHAB	-0.0142241	7.10696532	-0.1694104	0.86612473	0.99978775	-4.8845827
ENSCAFG00000006899	-0.0142374	0.67660895	-0.0524246	0.95838931	0.99978775	-4.6495891
WNT9A	-0.0143242	3.76908096	-0.0513011	0.95928023	0.99978775	-4.8159785
ENSCAFG00000030502	-0.0143635	5.14744638	-0.126699	0.8996625	0.99978775	-4.8564418
UBAP1	-0.0144548	4.55714172	-0.1539502	0.87823912	0.99978775	-4.8317998
RPS24	-0.01446	7.58329245	-0.1426195	0.88713649	0.99978775	-4.8820053
LRRC14	-0.0144777	3.48655543	-0.0971098	0.92300889	0.99978775	-4.7555018
BICD1	-0.0145418	1.1217649	-0.0309604	0.97541852	0.99978775	-4.66063
YTHDF2	-0.0145766	6.19238817	-0.2746752	0.78464351	0.99978775	-4.8778006
JMJD6	-0.0146272	4.10514886	-0.097911	0.92237569	0.99978775	-4.8068548
DDX46	-0.0146416	5.79023993	-0.1022534	0.918945	0.99978775	-4.8881083
ZNF3	-0.0146856	3.35818397	-0.0946393	0.92496152	0.99978775	-4.7379008
TRMT12	-0.0147096	2.16053642	-0.0823155	0.93470882	0.99978775	-4.6809172
COPS7B	-0.0147898	3.20273251	-0.1149211	0.90894592	0.99978775	-4.74597
C11H9orf72	-0.0147903	4.10760153	-0.1130206	0.91044516	0.99978775	-4.8008539
CAPNS1	-0.0148049	9.29118737	-0.1666367	0.86829586	0.99978775	-4.8302741
TTBK1	-0.0148418	-1.3103493	-0.021445	0.98297195	0.99978775	-4.6132629
MMS19	-0.0149234	6.17430928	-0.1144282	0.90933476	0.99978775	-4.8925656
BHLHB9	-0.0149756	3.93065495	-0.1170136	0.90729564	0.99978775	-4.7826161
LHFPL6	-0.0149772	6.17956079	-0.076351	0.93942995	0.99978775	-4.8955758
ENSCAFG00000000528	-0.0150155	3.51094118	-0.1374688	0.89118601	0.99978775	-4.7497898
MUT	-0.0150621	5.37580576	-0.1972409	0.84440044	0.99978775	-4.8629397
FES	-0.015069	4.85393202	-0.1145148	0.90926644	0.99978775	-4.8659677
ENSCAFG00000008879	-0.0150793	6.4244517	-0.1305504	0.89662978	0.99978775	-4.8930568
BMI1	-0.0151051	4.34975203	-0.1142523	0.9094735	0.99978775	-4.8312435
ZCCHC2	-0.0151131	4.55762814	-0.133108	0.89461673	0.99978775	-4.8285
ENSCAFG00000002395	-0.0151137	4.4615093	-0.1577342	0.87527122	0.99978775	-4.8251664
ZNF175	-0.0151183	3.17547053	-0.0874205	0.93066974	0.99978775	-4.726961
EIF4A2	-0.0151865	7.49104076	-0.1398244	0.88933366	0.99978775	-4.8858072
ZZEF1	-0.0152044	6.01263136	-0.1958915	0.84545111	0.99978775	-4.8848978
KEAP1	-0.0154167	5.56837736	-0.1506326	0.88084268	0.99978775	-4.8813375
SLC16A13	-0.0155241	2.45098261	-0.0457718	0.96366578	0.99978775	-4.6926735
HECW2	-0.0155433	4.51349731	-0.0691121	0.94516285	0.99978775	-4.8284992
ZNF184	-0.015548	3.63401626	-0.1018021	0.91930151	0.99978775	-4.7609233
EPN3	-0.0156044	-1.6650239	-0.0253202	0.97989557	0.99978775	-4.6098117
FBXL18	-0.0156092	6.23032599	-0.2122896	0.83270295	0.99978775	-4.8849156
PRRX2	-0.0156473	4.00626613	-0.0860401	0.93176172	0.99978775	-4.8670357
ARIH1	-0.0156802	4.94111581	-0.1390207	0.88996556	0.99978775	-4.855936
TEX2	-0.0157126	5.27924283	-0.1375207	0.89114519	0.99978775	-4.86434
SIRT2	-0.0159262	4.98945169	-0.1232481	0.90238114	0.99978775	-4.8644367
RGS12	-0.0159306	4.46942902	-0.1426395	0.88712081	0.99978775	-4.8413956
TIMM10	-0.0159401	2.99390567	-0.1309301	0.89633086	0.99978775	-4.7320311
BID	-0.0159418	5.49609435	-0.1625509	0.87149593	0.99978775	-4.880431
P3H3	-0.0159699	5.43206357	-0.0479536	0.9619351	0.99978775	-4.8961701
IQUB	-0.0160047	2.7516066	-0.066361	0.94734245	0.99978775	-4.7110072
TRIR	-0.0160234	5.75780818	-0.1651064	0.86949415	0.99978775	-4.8844221

ADAM10	-0.0160277	6.0124434	-0.1579253	0.87512132	0.99978775	-4.8869018
TMEM135	-0.0160414	2.968362	-0.1045871	0.91710186	0.99978775	-4.7261887
FKBP2	-0.0161137	4.43246389	-0.1021577	0.91902057	0.99978775	-4.8164606
PLXNB2	-0.0161338	8.41677536	-0.1357167	0.89256415	0.99978775	-4.854912
ENSCAFG00000002455	-0.0162638	-0.074666	-0.0525002	0.95832934	0.99978775	-4.6327432
NR1D2	-0.0162763	5.89023337	-0.1401419	0.88908399	0.99978775	-4.8920278
ENSCAFG00000031956	-0.0162959	2.91437589	-0.0711414	0.94355546	0.99978775	-4.7245973
COQ10A	-0.0163198	3.89786245	-0.1235404	0.90215085	0.99978775	-4.795462
FAM171A1	-0.0163458	7.60824504	-0.0947703	0.924858	0.99978775	-4.8899254
RAD51D	-0.0163489	2.61633142	-0.0952246	0.9244989	0.99978775	-4.7054914
MTMR1	-0.0163489	5.15121659	-0.1448665	0.88537085	0.99978775	-4.8632919
MED15	-0.0163489	6.17344501	-0.1372637	0.89134726	0.99978775	-4.8912494
GMPPA	-0.0163521	5.63996526	-0.1613737	0.87241831	0.99978775	-4.8772676
UBAC1	-0.0163642	6.49043086	-0.171405	0.86456407	0.99978775	-4.8902322
LRRC36	-0.0163848	0.29821156	-0.0558961	0.95563667	0.99978775	-4.6298596
NIPA2	-0.0164098	6.94737219	-0.1660923	0.86872211	0.99978775	-4.8876198
MFSD5	-0.01643	5.07084705	-0.1374219	0.89122283	0.99978775	-4.8650582
RPL23A	-0.0164842	6.00345343	-0.1642602	0.87015694	0.99978775	-4.8839398
ZDHHC6	-0.0165185	6.03578804	-0.2067768	0.83698383	0.99978775	-4.8844654
ACO1	-0.016564	6.16471722	-0.1353782	0.89283044	0.99978775	-4.8919946
SLC10A3	-0.0165664	5.47661251	-0.120848	0.90427261	0.99978775	-4.8768314
SLC12A9	-0.0165815	4.41492316	-0.1352618	0.89292204	0.99978775	-4.8307265
HDAC5	-0.0166098	6.30994541	-0.1200394	0.90491	0.99978775	-4.8933203
CLTB	-0.0166299	6.12278094	-0.1426293	0.88712876	0.99978775	-4.8909714
MAPKAPK3	-0.016667	5.59601386	-0.1455166	0.88486011	0.99978775	-4.8774831
CAMSAP1	-0.0166794	6.19091308	-0.2155074	0.83020657	0.99978775	-4.8856519
NOP16	-0.0167012	3.91829019	-0.1413711	0.88811769	0.99978775	-4.7880982
EPHB2	-0.016724	1.43622388	-0.050497	0.95991794	0.99978775	-4.8244964
ENSCAFG00000012867	-0.0167708	5.53912794	-0.2132689	0.83194305	0.99978775	-4.8718448
SLC38A5	-0.016822	8.02855104	-0.1062725	0.9157711	0.99978775	-4.8545344
VPS18	-0.0168834	5.54155989	-0.1480902	0.88283876	0.99978775	-4.8774544
CCDC134	-0.0169231	3.11329642	-0.1079393	0.91445519	0.99978775	-4.7395888
ANP32E	-0.0169363	5.79286147	-0.123529	0.90215978	0.99978775	-4.8864646
ABCE1	-0.0169922	5.8895221	-0.1353782	0.89283044	0.99978775	-4.8887904
SLC43A3	-0.0169947	4.95069009	-0.0545219	0.95672628	0.99978775	-4.8629788
RCOR3	-0.017046	4.66666614	-0.1374602	0.89119273	0.99978775	-4.8263929
ENSCAFG00000004650	-0.0170748	0.74125295	-0.0686712	0.9455122	0.99978775	-4.6559722
LPCAT4	-0.0171561	4.80926384	-0.105609	0.91629499	0.99978775	-4.8444657
ADNP	-0.0171955	6.14299402	-0.1406951	0.88864915	0.99978775	-4.8890837
ENSCAFG00000004573	-0.0171976	1.68637132	-0.0602449	0.95218927	0.99978775	-4.650697
TMEM109	-0.0172367	7.34839002	-0.1436517	0.88632533	0.99978775	-4.8836993
CASC3	-0.0172378	6.53034869	-0.2956632	0.76865328	0.99978775	-4.8767789
IPO11	-0.017359	5.47139698	-0.1699602	0.86569449	0.99978775	-4.8720458
SDHAF2	-0.0173756	5.17782384	-0.2120854	0.8328614	0.99978775	-4.8607171
ENSCAFG00000013855	-0.0173836	3.51627339	-0.0805004	0.93614529	0.99978775	-4.7420516
ARMH3	-0.0174227	3.653918	-0.1583042	0.87482427	0.99978775	-4.7803656
ENSCAFG00000005953	-0.017454	0.92108945	-0.0646556	0.9486937	0.99978775	-4.650366
ZNF70	-0.0174635	3.90204742	-0.1393829	0.88968079	0.99978775	-4.7872026
ZFYVE16	-0.0174779	5.5691497	-0.1604598	0.87313456	0.99978775	-4.8813992
DTNBP1	-0.0174796	4.31226877	-0.1263989	0.89989892	0.99978775	-4.819813

CTSH	-0.0175688	5.86467226	-0.0547217	0.95656784	0.99978775	-4.8665577
B9D1	-0.0175915	3.52922539	-0.0936797	0.92572012	0.99978775	-4.7572197
CNDP2	-0.0176733	6.16297859	-0.1883651	0.8513165	0.99978775	-4.8877819
UBE2M	-0.0176818	5.45032744	-0.1750711	0.86169706	0.99978775	-4.8771093
DCUN1D1	-0.017715	3.9320501	-0.1756641	0.86123344	0.99978775	-4.7881658
NUP35	-0.0178061	4.4536451	-0.1294619	0.89748678	0.99978775	-4.8333438
NRAS	-0.0178082	4.73641804	-0.1312281	0.89609632	0.99978775	-4.8392886
USP4	-0.0178199	6.25052996	-0.2356079	0.81465333	0.99978775	-4.8830463
ESF1	-0.017828	4.77411902	-0.1447002	0.88550147	0.99978775	-4.83308
TUBGCP6	-0.0178526	4.5454671	-0.186717	0.85260201	0.99978775	-4.8336752
SPIN4	-0.0178643	0.95847247	-0.0497772	0.96048879	0.99978775	-4.635033
PM20D2	-0.0179202	3.91465556	-0.0917974	0.92720831	0.99978775	-4.8211254
ARMC6	-0.0180201	4.72677252	-0.1095845	0.91315657	0.99978775	-4.8618693
TTC21B	-0.0180352	4.64750311	-0.093544	0.92582735	0.99978775	-4.8179708
CINP	-0.0180372	3.45544875	-0.1243079	0.9015461	0.99978775	-4.7450321
CYFIP1	-0.0180702	7.86969925	-0.2925156	0.77104505	0.99978775	-4.8563768
ZNF414	-0.0180767	2.97801103	-0.0958778	0.92398255	0.99978775	-4.7321864
ZNF384	-0.0180865	5.65130122	-0.1766685	0.86044834	0.99978775	-4.8784568
MEPCE	-0.0181112	4.76212111	-0.1771174	0.8600975	0.99978775	-4.8363919
NACC1	-0.0181139	5.08859976	-0.1448314	0.88539842	0.99978775	-4.8648043
LGI4	-0.0181553	3.78110534	-0.0664236	0.94729278	0.99978775	-4.8018389
PRRC1	-0.0181739	6.56985951	-0.1501935	0.88118732	0.99978775	-4.8900767
TRMT5	-0.0181969	4.02133156	-0.174143	0.86242271	0.99978775	-4.7884103
SLC39A10	-0.0182087	5.87263565	-0.1598372	0.87362249	0.99978775	-4.8787147
ENSCAFG00000012089	-0.018295	4.57253555	-0.1714611	0.86452019	0.99978775	-4.8280387
VIRMA	-0.0183104	7.34081591	-0.2500633	0.80351404	0.99978775	-4.8764038
CEP78	-0.0183283	3.0570213	-0.1012014	0.91977601	0.99978775	-4.7431591
MAP3K3	-0.0183495	4.64737043	-0.1295434	0.89742257	0.99978775	-4.8137105
ENSCAFG00000010669	-0.0183609	5.63043376	-0.2012658	0.84126831	0.99978775	-4.8782425
SCLT1	-0.0183833	4.13869917	-0.113216	0.91029101	0.99978775	-4.8014986
ZNF217	-0.0183936	4.34547695	-0.1021309	0.91904177	0.99978775	-4.808335
ZNF667	-0.0184143	2.33731862	-0.0620393	0.95076707	0.99978775	-4.6599096
PTBP2	-0.0184242	3.52384851	-0.1358861	0.8924309	0.99978775	-4.7915879
RPS7	-0.0184827	9.00269464	-0.1893425	0.85055433	0.99978775	-4.8399455
SLC38A9	-0.0185466	3.17547837	-0.1139223	0.90973381	0.99978775	-4.7336456
SNX1	-0.0186485	5.69214396	-0.218671	0.82775392	0.99978775	-4.8688887
ENSCAFG00000000121	-0.0186927	6.12315631	-0.2698387	0.78834193	0.99978775	-4.8791882
ARHGDIA	-0.0187176	7.75096648	-0.1901576	0.8499188	0.99978775	-4.8686739
HELQ	-0.0187571	4.25992791	-0.1717628	0.86428419	0.99978775	-4.8024852
C23H3orf33	-0.0187773	3.70951097	-0.1282012	0.89847943	0.99978775	-4.7571947
SHKBP1	-0.0188469	5.01264013	-0.1655479	0.8691484	0.99978775	-4.8676877
PRKRA	-0.0188625	3.30982954	-0.1579449	0.87510597	0.99978775	-4.7648003
GNPDA2	-0.0188634	4.75928258	-0.2221089	0.82509063	0.99978775	-4.8315005
ACO2	-0.0188669	7.56598839	-0.2077909	0.83619596	0.99978775	-4.8764348
ERAL1	-0.0189003	5.40749191	-0.2668333	0.79064255	0.99978775	-4.865819
ENSCAFG00000032664	-0.018908	-0.3171812	-0.0576007	0.95428529	0.99978775	-4.6250241
RAPGEF1	-0.0189175	5.93860852	-0.1140396	0.90964132	0.99978775	-4.8908885
PDE6D	-0.0189725	4.3298799	-0.2193604	0.82721968	0.99978775	-4.8151393
MATN4	-0.0189735	-0.7837623	-0.0334153	0.97347012	0.99978775	-4.6209677
GPATCH11	-0.0189781	3.27656947	-0.1312981	0.89604121	0.99978775	-4.729525

TTC23	-0.0189833	3.40519161	-0.1509408	0.88060077	0.99978775	-4.7543236
TOPAZ1	-0.0189863	-0.3055034	-0.0335649	0.97335133	0.99978775	-4.6263459
TBKBP1	-0.019052	2.93084718	-0.082923	0.93422807	0.99978775	-4.7098092
PRDM4	-0.0190908	5.18507783	-0.2237746	0.82380094	0.99978775	-4.8590112
NARS	-0.0191807	7.73831257	-0.1630382	0.87111414	0.99978775	-4.8682509
PCF11	-0.0192208	4.48383202	-0.1345624	0.89347227	0.99978775	-4.7993264
SNTB1	-0.0193376	-2.1383923	-0.0361575	0.97129389	0.99978775	-4.6131862
TRIM13	-0.0194534	3.29819912	-0.1416667	0.88788536	0.99978775	-4.7461431
GRK2	-0.0194672	5.26973909	-0.198082	0.84374572	0.99978775	-4.8586363
FAF1	-0.0195099	5.63228652	-0.2651762	0.79191189	0.99978775	-4.8705322
DMAP1	-0.0195218	5.81239221	-0.2729001	0.78600035	0.99978775	-4.8771489
ENSCAFG00000005717	-0.0195237	0.82795679	-0.0213333	0.98306063	0.99978775	-4.6343519
TSPAN18	-0.0195317	4.0840178	-0.0689291	0.94530787	0.99978775	-4.8522495
SUPT20H	-0.0195416	5.48394237	-0.2458512	0.80675574	0.99978775	-4.8699457
DOP1A	-0.0195504	4.78752449	-0.1828029	0.85565655	0.99978775	-4.8438797
HSP90AA1	-0.0195755	8.83434082	-0.236623	0.81386985	0.99978775	-4.838923
TENT4B	-0.0196185	4.84648421	-0.1659487	0.86883459	0.99978775	-4.8506331
POLR1B	-0.0196589	5.82702655	-0.1552882	0.87718947	0.99978775	-4.8851949
RARA	-0.0196593	5.43466913	-0.1320396	0.89545756	0.99978775	-4.8662871
PTPN2	-0.0196624	4.56221659	-0.143712	0.88627796	0.99978775	-4.8309926
DTX3	-0.0197158	6.22697999	-0.1296851	0.89731102	0.99978775	-4.8919893
WBP1L	-0.0197349	4.22743002	-0.1133768	0.91016417	0.99978775	-4.8083225
ENSCAFG00000016628	-0.0197562	5.35064516	-0.2738107	0.78530417	0.99978775	-4.8631571
UBXN7	-0.0197824	4.04125953	-0.1799003	0.85792325	0.99978775	-4.7847129
SUCLG1	-0.0198148	6.07756004	-0.2265252	0.82167243	0.99978775	-4.8815291
RNF214	-0.0198223	4.57704532	-0.296653	0.76790158	0.99978775	-4.816316
SLC15A4	-0.0198528	5.95352924	-0.1731122	0.86322877	0.99978775	-4.8866889
CCDC56	-0.0198562	4.34556511	-0.1973713	0.84429898	0.99978775	-4.8198033
ALDH6A1	-0.0198723	4.88134988	-0.1271647	0.89929572	0.99978775	-4.8353911
PWP1	-0.019878	4.99634727	-0.1496835	0.8815877	0.99978775	-4.8584582
HYAL3	-0.0198829	3.1238131	-0.0987605	0.92170444	0.99978775	-4.7254249
MAK16	-0.01989	6.20338804	-0.1673471	0.86773967	0.99978775	-4.8902022
EPRS	-0.0199213	8.95991842	-0.1394168	0.88965411	0.99978775	-4.840808
ITPR2	-0.0199408	6.41115483	-0.1634804	0.87076776	0.99978775	-4.8908458
SLF1	-0.0199524	2.74443965	-0.0925785	0.9265907	0.99978775	-4.7278643
MYH7B	-0.019964	2.90018459	-0.0900553	0.92858588	0.99978775	-4.6971535
ZNF436	-0.0200708	5.26174822	-0.1717022	0.86433162	0.99978775	-4.8606487
SLC38A10	-0.0201106	7.53583937	-0.1823707	0.85599398	0.99978775	-4.8799983
CD276	-0.020155	6.75933071	-0.130058	0.89701742	0.99978775	-4.8852531
COQ5	-0.0201892	2.87094669	-0.1495543	0.88168919	0.99978775	-4.7096331
ENSCAFG00000007436	-0.020195	2.55421427	-0.1536661	0.87846199	0.99978775	-4.7043644
ZNF614	-0.0202375	2.84349305	-0.1113883	0.91173306	0.99978775	-4.7049643
SYNJ1	-0.0202747	5.73464225	-0.2585052	0.79702752	0.99978775	-4.8734596
RUFY2	-0.0202921	4.5706709	-0.1886546	0.85109072	0.99978775	-4.8236127
PROSER1	-0.0203231	6.10252654	-0.1894571	0.85046497	0.99978775	-4.8877382
MED8	-0.0203613	3.50100413	-0.1943247	0.84667144	0.99978775	-4.7500078
ECHS1	-0.0204056	5.89377045	-0.1894065	0.8505044	0.99978775	-4.8774099
PITRM1	-0.0204227	6.37831386	-0.0752471	0.94030405	0.99978775	-4.8874851
ZNF32	-0.0204311	2.8687613	-0.1628464	0.87126443	0.99978775	-4.7205087
ENSCAFG00000018194	-0.0204765	4.28756302	-0.0916699	0.9273091	0.99978775	-4.8430581

RNASEK	-0.0204889	6.33145746	-0.1461296	0.88437862	0.99978775	-4.8920076
COG4	-0.0205	5.2594216	-0.2880955	0.77440762	0.99978775	-4.856638
COLGALT1	-0.0205123	7.73431493	-0.1906034	0.8495713	0.99978775	-4.8647751
BAG5	-0.0205396	6.22601386	-0.1360094	0.89233391	0.99978775	-4.8894309
CHIC2	-0.0206005	3.66358282	-0.1843092	0.85448081	0.99978775	-4.7538469
PGR	-0.0206251	3.76092405	-0.0281515	0.97764805	0.99978775	-4.7778824
PDS5B	-0.0206752	5.37375382	-0.2038886	0.83922858	0.99978775	-4.8576835
COX17	-0.0206879	2.47135827	-0.1295172	0.89744321	0.99978775	-4.703574
RANBP17	-0.0207177	3.1817709	-0.1818151	0.85642779	0.99978775	-4.7377759
LRRC6	-0.0208021	0.00913578	-0.062038	0.95076808	0.99978775	-4.6411643
BRD1	-0.0208301	4.81387652	-0.2568637	0.79828769	0.99978775	-4.8341973
GMEB1	-0.0208422	4.09026589	-0.2072622	0.83660673	0.99978775	-4.7796631
PDPR	-0.0209589	5.97653514	-0.1678318	0.86736029	0.99978775	-4.8828505
MBTD1	-0.0209632	2.58143124	-0.100635	0.92022347	0.99978775	-4.6839591
KANSL1	-0.020989	6.12751302	-0.229285	0.81953806	0.99978775	-4.8815866
SNRNP35	-0.021003	3.29216157	-0.1331127	0.89461299	0.99978775	-4.7494865
AVL9	-0.0210664	4.36342629	-0.1785383	0.85898722	0.99978775	-4.8119388
CCDC6	-0.0210893	5.24726934	-0.1868156	0.85252506	0.99978775	-4.8680706
CCNH	-0.0211337	4.38275375	-0.2248174	0.82299386	0.99978775	-4.8273298
YPEL5	-0.0212471	6.1513452	-0.1642344	0.87017713	0.99978775	-4.8890701
RBM23	-0.021252	3.83924894	-0.1722438	0.86390791	0.99978775	-4.7897895
CLTA	-0.0212616	6.34733564	-0.2883753	0.77419468	0.99978775	-4.8778258
ENSCAFG00000008701	-0.0212848	5.58802384	-0.2187013	0.82773045	0.99978775	-4.8762195
SOX5	-0.0213062	0.88351956	-0.0312474	0.97519071	0.99978775	-4.6186309
AKIRIN2	-0.0213568	4.01308655	-0.2066902	0.83705111	0.99978775	-4.7813353
CPSF4	-0.0214004	3.45946345	-0.1811047	0.85698259	0.99978775	-4.7530187
ZNF704	-0.0214051	2.14853987	-0.1230229	0.90255856	0.99978775	-4.7250972
STK35	-0.0214233	5.03799952	-0.2440397	0.80815103	0.99978775	-4.8382099
SC5D	-0.0214304	5.75793416	-0.1466693	0.88395469	0.99978775	-4.8695461
ENSCAFG00000004113	-0.0214501	0.0849655	-0.045338	0.9640099	0.99978775	-4.6309107
BTBD6	-0.0215042	4.6068445	-0.082207	0.93479462	0.99978775	-4.8283761
RAB22A	-0.0215987	4.17852758	-0.2114931	0.83332118	0.99978775	-4.8014806
KIF3C	-0.0216125	4.59187532	-0.1381808	0.89062603	0.99978775	-4.8238556
TMBIM1	-0.0216142	7.54428538	-0.1405089	0.88879547	0.99978775	-4.8820808
CCDC43	-0.0216187	3.08638554	-0.1459008	0.88455832	0.99978775	-4.7220819
ENSCAFG00000023355	-0.0216331	-1.0128961	-0.0431441	0.9657503	0.99978775	-4.617213
WDR74	-0.0216681	3.68359343	-0.1999783	0.84226995	0.99978775	-4.7732442
ACOT8	-0.0216995	3.35485073	-0.1723337	0.86383767	0.99978775	-4.742184
ENSCAFG00000014657	-0.0217017	0.01107664	-0.0803573	0.93625857	0.99978775	-4.6404476
SPATA1	-0.0217139	3.25387294	-0.1442158	0.88588209	0.99978775	-4.7401756
METAP2	-0.0217366	6.3642232	-0.2332776	0.81645275	0.99978775	-4.8838383
RDH14	-0.0217859	5.02448581	-0.1917669	0.8486644	0.99978775	-4.8540264
U2SURP	-0.0217904	6.16307823	-0.1864914	0.85277798	0.99978775	-4.8872291
GOLGA4	-0.0218809	6.65949615	-0.2233688	0.82411513	0.99978775	-4.8853562
ELOB	-0.0219015	5.12373666	-0.2334423	0.81632553	0.99978775	-4.8555559
ZBTB24	-0.0219111	5.35892951	-0.2617608	0.79452986	0.99978775	-4.8601186
FANCG	-0.0219993	3.60554586	-0.1585575	0.87462571	0.99978775	-4.7647867
PIANP	-0.0221038	-1.0336175	-0.061168	0.95145761	0.99978775	-4.6313253
WDR60	-0.0221476	3.33171127	-0.1747564	0.86194306	0.99978775	-4.7367378
ZNF485	-0.0221532	1.84173763	-0.1083021	0.91416882	0.99978775	-4.668483

MGAT1	-0.022171	5.7034636	-0.1657632	0.86897985	0.99978775	-4.8825226
NDUFB9	-0.0222384	4.8274291	-0.1833208	0.85525229	0.99978775	-4.841794
U2AF1	-0.0222622	5.43555222	-0.199417	0.84270672	0.99978775	-4.876203
SOCS6	-0.022288	4.00283079	-0.2084923	0.83565118	0.99978775	-4.7922301
BNC1	-0.0222954	-1.3280382	-0.0211277	0.98322387	0.99978775	-4.6326821
SEL1L	-0.0222989	7.30520127	-0.1923113	0.84824008	0.99978775	-4.8747199
S100A11	-0.0223823	8.32528707	-0.1768998	0.86026757	0.99978775	-4.861957
ENSCAFG00000017792	-0.0224333	1.81570513	-0.093878	0.92556336	0.99978775	-4.6691511
RPL12	-0.0224804	9.07001245	-0.2113585	0.83342562	0.99978775	-4.833553
LARP6	-0.0224857	6.80541717	-0.0703045	0.94421835	0.99978775	-4.8925754
PREPL	-0.0225224	4.76276397	-0.151866	0.87987458	0.99978775	-4.8395638
AXIN1	-0.0225356	5.23419334	-0.2600964	0.79580647	0.99978775	-4.8551929
AP1S2	-0.0225563	7.66221825	-0.1183501	0.90624181	0.99978775	-4.8630177
ENSCAFG00000010097	-0.0226691	-0.3065238	-0.0730903	0.94201191	0.99978775	-4.6301052
TNRC6A	-0.0226833	6.76005084	-0.2110281	0.8336821	0.99978775	-4.8865071
XG	-0.0226857	7.7204179	-0.1364795	0.89196408	0.99978775	-4.8761651
FDX1	-0.0227019	3.31398366	-0.1553699	0.87712535	0.99978775	-4.742616
UPF2	-0.0227057	5.02773471	-0.1928563	0.84781547	0.99978775	-4.8418179
ENSCAFG00000030556	-0.0227133	1.64546874	-0.0992159	0.92134465	0.99978775	-4.6530149
CCDC82	-0.0227243	2.81891882	-0.1142309	0.90949036	0.99978775	-4.6850305
MINK1	-0.0227506	6.18584376	-0.1990552	0.84298823	0.99978775	-4.8854955
ZBTB8A	-0.0227881	4.04620055	-0.135597	0.89265828	0.99978775	-4.8146216
ENSCAFG00000032088	-0.0228188	2.32908797	-0.0599212	0.95244584	0.99978775	-4.6886029
MFAP1	-0.0228468	5.35329017	-0.3414862	0.73410026	0.99978775	-4.8531546
NAA16	-0.0228687	3.82407732	-0.1596558	0.87376474	0.99978775	-4.7630911
PRDX1	-0.0228739	7.44209525	-0.1852894	0.85371586	0.99978775	-4.8803056
UQCRQ	-0.0228794	4.42901702	-0.1674607	0.86765077	0.99978775	-4.8307233
BTBD19	-0.0229406	5.25806251	-0.1096678	0.91309085	0.99978775	-4.8671815
EDEM1	-0.0230603	7.10539853	-0.1860332	0.8531355	0.99978775	-4.8839048
ENSCAFG00000012507	-0.0230653	0.97452028	-0.0973829	0.92279307	0.99978775	-4.6544603
RALB	-0.0230698	5.63223486	-0.2001666	0.84212343	0.99978775	-4.8724528
CNOT6	-0.0230741	4.68291358	-0.1844712	0.85435438	0.99978775	-4.8400628
UBR2	-0.0230746	6.30967962	-0.2557274	0.79916037	0.99978775	-4.8798764
PTPA	-0.02308	5.86728242	-0.2793318	0.78108743	0.99978775	-4.8733357
NOB1	-0.0230834	4.86860926	-0.1667955	0.86817159	0.99978775	-4.8507728
MRPS9	-0.0231245	5.30565183	-0.3059998	0.76081463	0.99978775	-4.8524369
WDR5B	-0.0231653	3.45158198	-0.1519268	0.87982685	0.99978775	-4.7603428
XK	-0.0231691	-1.0791777	-0.0361805	0.9712756	0.99978775	-4.6155893
KLHDC1	-0.023257	1.66447511	-0.086529	0.93137501	0.99978775	-4.6495011
NDRG3	-0.0232609	4.37735367	-0.1840976	0.85464592	0.99978775	-4.8100951
SMAP1	-0.0233001	4.90707876	-0.3076203	0.75958804	0.99978775	-4.8374104
HDHD2	-0.0233442	4.57217204	-0.246547	0.80622004	0.99978775	-4.8311028
ENSCAFG00000002833	-0.0233793	2.66436747	-0.1409607	0.88844031	0.99978775	-4.7062899
FBXL12	-0.0234947	4.67984671	-0.2368182	0.8137192	0.99978775	-4.8455483
MSL1	-0.0235005	5.15614193	-0.2329862	0.81667783	0.99978775	-4.8474293
GSTM4	-0.0235326	6.0151218	-0.0788006	0.93749077	0.99978775	-4.873118
MPDU1	-0.0235374	5.29517919	-0.2050535	0.83832308	0.99978775	-4.8682592
GPSM1	-0.0236645	5.12709881	-0.1979547	0.84384477	0.99978775	-4.8550001
DHODH	-0.0236717	2.994319	-0.1139634	0.90970142	0.99978775	-4.7232189
TMA7	-0.023725	3.03457636	-0.1176858	0.90676559	0.99978775	-4.7222773

ENSCAFG00000019141	-0.0237417	0.37441653	-0.0605477	0.95194926	0.99978775	-4.625552
CREB5	-0.0237879	0.65608476	-0.0994398	0.92116768	0.99978775	-4.6766212
ABT1	-0.023825	5.2781699	-0.2105482	0.83405466	0.99978775	-4.8643576
MYO5A	-0.0238484	6.85418267	-0.1552002	0.87725846	0.99978775	-4.8899118
RBM44	-0.0238675	-0.8968907	-0.0516741	0.95898446	0.99978775	-4.6234286
ZNF202	-0.023893	3.63056359	-0.2079592	0.83606521	0.99978775	-4.7583462
FAP	-0.0239282	6.3392795	-0.0414687	0.96707952	0.99978775	-4.8899887
GDAP2	-0.0239827	4.49454046	-0.2168263	0.82918389	0.99978775	-4.8209891
CEP70	-0.0239834	3.26724671	-0.1405774	0.8887416	0.99978775	-4.7508548
MKRN2	-0.0240291	3.93913632	-0.1999689	0.8422773	0.99978775	-4.7772829
SIX5	-0.0240659	1.82152985	-0.0761514	0.93958803	0.99978775	-4.6639049
THAP1	-0.0240781	2.67774537	-0.1934139	0.847381	0.99978775	-4.7034574
SEC61A1	-0.0240797	10.099166	-0.1816118	0.85658657	0.99978775	-4.8001743
POLG	-0.0241589	5.90910631	-0.2586866	0.79688831	0.99978775	-4.8752119
SLC2A12	-0.02416	1.81297361	-0.0814681	0.93537943	0.99978775	-4.6740251
FSTL3	-0.0241719	6.19069632	-0.0780267	0.93810339	0.99978775	-4.89541
ZNF592	-0.0241887	5.48986191	-0.2562928	0.79872613	0.99978775	-4.8674317
AGBL2	-0.0243029	1.97949711	-0.0862742	0.93157652	0.99978775	-4.6623427
RAB21	-0.0243396	4.78853603	-0.2082727	0.83582173	0.99978775	-4.8378762
SULT1A1	-0.0243462	4.17525247	-0.081582	0.93528929	0.99978775	-4.7549749
CASK	-0.0243518	6.09296243	-0.1598567	0.87360724	0.99978775	-4.8907103
FKBP15	-0.0243532	5.82005386	-0.2654207	0.79172453	0.99978775	-4.8711824
ITPA	-0.0243584	4.23800889	-0.2212211	0.8257782	0.99978775	-4.8158888
GANAB	-0.0243648	8.08822681	-0.3599403	0.7203353	0.99978775	-4.8351641
VAPA	-0.0243867	6.6120745	-0.2209821	0.82596333	0.99978775	-4.8851135
ZNF229	-0.0244079	2.31938543	-0.1165862	0.9076327	0.99978775	-4.689678
TRABD	-0.0244477	5.24390527	-0.219681	0.82697128	0.99978775	-4.8585388
MRPL55	-0.024463	3.95671943	-0.1747839	0.86192161	0.99978775	-4.7988868
COMMD1	-0.0245597	6.09773649	-0.3064075	0.76050593	0.99978775	-4.8738352
TMEM199	-0.0246001	3.0102308	-0.1633831	0.87084393	0.99978775	-4.7222377
ALKBH8	-0.0246737	2.48795865	-0.1483703	0.88261879	0.99978775	-4.6883632
ARNT2	-0.0247092	4.41983449	-0.1853834	0.85364247	0.99978775	-4.8672521
MCMBP	-0.0247607	3.64251007	-0.2134246	0.83182225	0.99978775	-4.7606719
SCYL1	-0.0248012	5.53144253	-0.2456649	0.80689921	0.99978775	-4.8676145
CHTOP	-0.0248471	5.23936865	-0.2956473	0.76866533	0.99978775	-4.8530349
DHX33	-0.0248654	5.53410893	-0.2532384	0.80107277	0.99978775	-4.8728262
EDRF1	-0.0248704	4.21054798	-0.1703065	0.8654235	0.99978775	-4.7958482
MRPL36	-0.0248778	3.19527176	-0.1321215	0.89539312	0.99978775	-4.7404239
TBC1D17	-0.0249418	5.61735257	-0.172531	0.86368334	0.99978775	-4.8787086
NONO	-0.0249678	6.15635451	-0.280255	0.78038296	0.99978775	-4.8778861
ENSCAFG00000017228	-0.02505	4.09050241	-0.1848034	0.85409507	0.99978775	-4.7923972
TRIM21	-0.025121	3.43439149	-0.2003071	0.84201412	0.99978775	-4.7492209
ATG4B	-0.0251622	5.30545182	-0.2931004	0.77060049	0.99978775	-4.8520864
ACOT13	-0.0251628	4.46172014	-0.1890823	0.85075717	0.99978775	-4.8212656
PPP3CB	-0.0252886	5.6052164	-0.2560692	0.79889781	0.99978775	-4.8738584
HDAC7	-0.0252912	6.98314533	-0.176447	0.86062147	0.99978775	-4.8891138
SAMD4B	-0.025294	6.13377476	-0.1937522	0.8471174	0.99978775	-4.886653
HCFC1R1	-0.0252997	4.16438113	-0.1362824	0.89211912	0.99978775	-4.8374105
EPC2	-0.0253502	5.38713246	-0.229731	0.81919325	0.99978775	-4.8628701
CCNL2	-0.0253552	4.52648872	-0.2695563	0.78855796	0.99978775	-4.8141879

FAM111A	-0.0253746	5.88102094	-0.2291441	0.81964701	0.99978775	-4.879737
ZNF652	-0.0253799	2.91702612	-0.1635821	0.87068805	0.99978775	-4.7158934
EP400	-0.0254237	6.15843388	-0.2405121	0.81086978	0.99978775	-4.8817797
ZNF354A	-0.0254547	4.59654448	-0.1986897	0.8432727	0.99978775	-4.8197357
ZNF575	-0.0254759	1.62154159	-0.076921	0.93897871	0.99978775	-4.6508386
AKT1S1	-0.0255316	6.05880537	-0.2292264	0.81958336	0.99978775	-4.8819376
MMP28	-0.0255499	2.45354688	-0.037484	0.97024121	0.99978775	-4.6642462
KLHL17	-0.0255807	3.48218269	-0.1619752	0.87194697	0.99978775	-4.7539257
NUP58	-0.0256423	5.62903494	-0.239433	0.81170193	0.99978775	-4.8791713
LCORL	-0.0256526	2.58963473	-0.1456331	0.8847686	0.99978775	-4.6931811
MFNG	-0.02566	-2.2991419	-0.0372677	0.97041284	0.99978775	-4.6071843
CFAP298	-0.0256779	5.22708695	-0.2218594	0.82528382	0.99978775	-4.856976
EP300	-0.0257326	7.26510935	-0.31458	0.75432701	0.99978775	-4.8681079
ERCC2	-0.0257397	3.78904481	-0.2271266	0.82120717	0.99978775	-4.7792702
RIOX2	-0.0257966	4.51026522	-0.2221731	0.82504092	0.99978775	-4.835493
FAHD2A	-0.0258667	2.86687076	-0.1366672	0.89181648	0.99978775	-4.69829
SPCS3	-0.0258927	4.61971362	-0.143095	0.8867628	0.99978775	-4.84378
ENSCAFG00000002703	-0.0259041	3.81150755	-0.1302501	0.8968662	0.99978775	-4.7755042
LRRFIP2	-0.0259225	6.05142994	-0.2206961	0.82618486	0.99978775	-4.8853265
ENSCAFG00000010466	-0.0260658	2.27659761	-0.1731961	0.86316312	0.99978775	-4.6916837
AKR1B1	-0.0260834	5.42334756	-0.1505735	0.88088905	0.99978775	-4.8746421
TRAPPC12	-0.0261467	4.70786504	-0.2647392	0.79224671	0.99978775	-4.8222939
HEATR1	-0.0261751	6.17370345	-0.2992249	0.76594946	0.99978775	-4.8748583
AKAP7	-0.0262678	3.18438809	-0.1397008	0.88943082	0.99978775	-4.7271675
ASB8	-0.0262708	3.48773553	-0.2434808	0.80858157	0.99978775	-4.7512389
SGK1	-0.0263481	5.45863321	-0.1077563	0.91459965	0.99978775	-4.8509962
AP3B1	-0.0263823	7.45008885	-0.3229645	0.74800457	0.99978775	-4.8610607
TRERF1	-0.0264	2.91635054	-0.0966435	0.92337737	0.99978775	-4.7257114
PI16	-0.0264119	-0.520284	-0.0296126	0.97648825	0.99978775	-4.617307
NPR2	-0.0264175	5.4332869	-0.2061	0.83750974	0.99978775	-4.8652818
HSPA8	-0.0264605	9.57172727	-0.1825552	0.85585	0.99978775	-4.8163924
HYOU1	-0.0266128	8.85885314	-0.1393539	0.88970358	0.99978775	-4.839187
FOXJ3	-0.0266351	5.81192626	-0.3261873	0.74557899	0.99978775	-4.8638764
ARFRP1	-0.0267131	5.74644385	-0.2220496	0.82513652	0.99978775	-4.8764793
VMP1	-0.0267185	6.61205023	-0.1688799	0.86653993	0.99978775	-4.8903404
ENSCAFG00000004690	-0.0267286	3.67329115	-0.1222127	0.90319703	0.99978775	-4.8126508
ETFDH	-0.0267614	6.46199855	-0.3477999	0.7293807	0.99978775	-4.8688641
TMEM101	-0.0267803	3.14013735	-0.1544049	0.87788238	0.99978775	-4.7268594
TAF1C	-0.0268098	4.72792206	-0.2202638	0.82651974	0.99978775	-4.8313589
ENSCAFG00000030169	-0.0268761	-0.5792466	-0.0492552	0.96090278	0.99978775	-4.6171531
RPL10A	-0.0268844	7.70047874	-0.1903682	0.84975463	0.99978775	-4.8735261
UBA1	-0.0268984	8.55009821	-0.3573585	0.72225562	0.99978775	-4.8283629
NGDN	-0.0269151	4.10202114	-0.1946312	0.8464327	0.99978775	-4.7968472
ENSCAFG00000016640	-0.0269193	7.0712297	-0.3316065	0.74150618	0.99978775	-4.8683377
SNRNP48	-0.0269289	4.46149134	-0.1480838	0.88284378	0.99978775	-4.8186269
ENSCAFG00000012900	-0.0269464	3.10380054	-0.1911721	0.84912801	0.99978775	-4.7230193
ENSCAFG00000003530	-0.0269493	2.9471984	-0.0960705	0.92383024	0.99978775	-4.7080468
ARID1A	-0.0269587	6.20450214	-0.3129773	0.75553753	0.99978775	-4.8725177
KIF3A	-0.0270672	4.05450496	-0.1929221	0.84776415	0.99978775	-4.7901297
C9H17orf75	-0.02713	3.13337875	-0.20042	0.84192628	0.99978775	-4.7358365

RAD23A	-0.027168	6.59744195	-0.3234641	0.74762839	0.99978775	-4.8718161
RFXANK	-0.0271772	2.44662802	-0.1688759	0.86654304	0.99978775	-4.691749
ADRM1	-0.0272245	6.44512919	-0.3051826	0.76143343	0.99978775	-4.8755144
HPGD	-0.0272907	3.93965391	-0.0394448	0.96868535	0.99978775	-4.7808952
CCDC80	-0.0273287	9.55834484	-0.0780797	0.9380614	0.99978775	-4.8075944
YTHDF1	-0.0273801	5.96030071	-0.3050297	0.76154925	0.99978775	-4.8709016
ZCCHC14	-0.027391	4.4799203	-0.1634832	0.87076558	0.99978775	-4.8216846
ACTR1B	-0.0274046	6.08626439	-0.2669237	0.79057333	0.99978775	-4.8805708
MAPK14	-0.0274598	4.85347331	-0.2484042	0.80479055	0.99978775	-4.8257736
CTNNA1	-0.0274796	8.57012563	-0.3358983	0.73828595	0.99978775	-4.8401284
ENSCAFG00000000823	-0.0275479	5.4751747	-0.3267069	0.7451882	0.99978775	-4.8592765
LAMTOR2	-0.0275676	3.33997506	-0.1956011	0.84567731	0.99978775	-4.7489255
TRMU	-0.0276043	2.43939055	-0.1446577	0.8855349	0.99978775	-4.6864343
KDM1A	-0.0276474	6.36892274	-0.3086045	0.75884334	0.99978775	-4.8736094
PARP12	-0.0276662	4.69748225	-0.2266683	0.82156171	0.99978775	-4.8229427
PLEKHH1	-0.0276837	2.45698643	-0.1412425	0.88821878	0.99978775	-4.7038377
PRMT1	-0.0277543	6.9440835	-0.2464637	0.80628416	0.99978775	-4.8792077
PPP1R11	-0.0278106	4.53822061	-0.2190584	0.82745375	0.99978775	-4.8288078
PARD3B	-0.0278666	7.65482009	-0.1297272	0.8972779	0.99978775	-4.875506
BCL2L12	-0.0278942	3.44344048	-0.1883113	0.85135846	0.99978775	-4.761498
ATP5F1A	-0.0279073	8.57694944	-0.3286824	0.74370291	0.99978775	-4.8376385
SENP2	-0.0279101	5.13683746	-0.347409	0.72967261	0.99978775	-4.8366193
TERF2	-0.0279598	4.20955205	-0.3164881	0.75288667	0.99978775	-4.791098
TMEM129	-0.0279604	3.66127736	-0.2005801	0.84180172	0.99978775	-4.7716344
CTU2	-0.0280236	3.59226081	-0.2291054	0.81967691	0.99978775	-4.7634106
VWA8	-0.028116	5.42253798	-0.3278804	0.74430577	0.99978775	-4.8602616
FGF13	-0.0281505	0.86517161	-0.0657412	0.9478335	0.99978775	-4.7586913
EIF1AX	-0.0281827	6.63879282	-0.2847731	0.77693798	0.99978775	-4.8780339
NKIRAS2	-0.028201	5.46223194	-0.2380613	0.81275998	0.99978775	-4.8737405
RETREG1	-0.0282205	5.31230157	-0.1964268	0.84503428	0.99978775	-4.8772298
MAEA	-0.0282883	5.50216011	-0.3678534	0.7144611	0.99978775	-4.8448767
ZNF316	-0.0283331	4.1093227	-0.2407411	0.81069316	0.99978775	-4.8016834
VASH2	-0.0283742	5.98955165	-0.0648812	0.94851491	0.99978775	-4.8960396
PABPC1	-0.0283921	10.3672601	-0.3572727	0.72231948	0.99978775	-4.7854222
FER	-0.0284207	4.64507734	-0.2153397	0.83033662	0.99978775	-4.8257737
TRMT10C	-0.0284397	3.36314214	-0.1984707	0.84344313	0.99978775	-4.737475
CELF2	-0.0284558	3.05578419	-0.063385	0.94970055	0.99978775	-4.8053405
APBB1	-0.0284599	5.89996025	-0.2224055	0.82486094	0.99978775	-4.8772994
PPM1K	-0.0284698	2.70535461	-0.1080306	0.91438309	0.99978775	-4.7034658
TGFBRAP1	-0.0285236	4.61277332	-0.2486041	0.80463671	0.99978775	-4.824163
TSNAX-DISC1	-0.0285316	4.8774134	-0.2377303	0.81301537	0.99978775	-4.8415011
NFIC	-0.0286714	7.31357386	-0.2971632	0.76751422	0.99978775	-4.8638958
MARS	-0.0286954	6.1264233	-0.2937137	0.7701344	0.99978775	-4.8768018
PRPF4B	-0.0286981	5.90380935	-0.2703623	0.78794129	0.99978775	-4.874826
ENSCAFG00000010086	-0.0287103	3.59727544	-0.2146341	0.83088392	0.99978775	-4.7564583
CLPB	-0.0287577	4.64783257	-0.2804265	0.78025211	0.99978775	-4.8210738
MVP	-0.0287638	7.43052326	-0.2584071	0.7971028	0.99978775	-4.8746672
RIPK1	-0.0288193	6.12150343	-0.4587942	0.64827525	0.99978775	-4.8462108
ENSCAFG00000032349	-0.0288399	1.62284368	-0.1284426	0.89828938	0.99978775	-4.6717764
ENSCAFG00000006633	-0.0288651	3.94808137	-0.1805968	0.85737925	0.99978775	-4.8039621

PEX13	-0.028894	4.09977013	-0.3195675	0.75056404	0.99978775	-4.7960287
ENSCAFG00000004186	-0.0289221	5.05779216	-0.247595	0.8054133	0.99978775	-4.854753
UTP25	-0.0289723	5.3461077	-0.3540406	0.72472607	0.99978775	-4.84513
PRKDC	-0.0290212	5.74875533	-0.2722901	0.78646671	0.99978775	-4.87336
ICA1L	-0.0290445	2.59514195	-0.1909572	0.84929544	0.99978775	-4.7167611
ANAPC5	-0.0290647	4.45389138	-0.2939937	0.76992157	0.99978775	-4.8108172
CLK4	-0.0290733	3.14006837	-0.1584542	0.87470668	0.99978775	-4.7100003
HINFP	-0.0291073	5.32507622	-0.332865	0.74056145	0.99978775	-4.8542051
RABIF	-0.0292816	3.21341729	-0.2257796	0.82224928	0.99978775	-4.7373336
SAAL1	-0.0292978	5.22711184	-0.2361539	0.81423191	0.99978775	-4.862507
CSNK2A2	-0.0293041	5.22501754	-0.3898807	0.6982016	0.99978775	-4.8374236
EIF2B4	-0.0293104	5.28628821	-0.3057437	0.7610085	0.99978775	-4.8611192
SSBP3	-0.0293265	5.37668689	-0.2614742	0.79474965	0.99978775	-4.8636288
ZBTB33	-0.0293406	4.43568536	-0.2780324	0.78207922	0.99978775	-4.808309
TRIM4	-0.0293628	6.00639643	-0.3607647	0.71972253	0.99978775	-4.8635898
P4HB	-0.0294335	10.7456073	-0.224352	0.823354	0.99978775	-4.7828976
PRPF38A	-0.0294853	3.70779806	-0.2292451	0.81956894	0.99978775	-4.7674408
RUSC1	-0.0295061	4.82134633	-0.1774449	0.8598416	0.99978775	-4.8441863
MRPL18	-0.029509	5.18960124	-0.2614092	0.79479949	0.99978775	-4.8590172
C6H16orf72	-0.0295324	4.87950942	-0.2532758	0.80104397	0.99978775	-4.8340237
CCDC86	-0.0295435	3.80215725	-0.2126036	0.83245924	0.99978775	-4.7856529
PSMA5	-0.0295529	5.19528259	-0.2473154	0.80562852	0.99978775	-4.8525176
ENSCAFG00000013709	-0.0296196	4.95586442	-0.2073628	0.83652856	0.99978775	-4.8624508
PHF3	-0.0296561	6.59652075	-0.2898416	0.77307876	0.99978775	-4.8776362
EXTL2	-0.029666	5.4317242	-0.2119788	0.83294416	0.99978775	-4.8511733
BOD1L1	-0.0296784	5.95428669	-0.2808685	0.77991487	0.99978775	-4.8705516
OSBP	-0.02968	5.80330008	-0.3879391	0.69962918	0.99978775	-4.854384
NUP50	-0.029693	5.27931383	-0.3063595	0.76054233	0.99978775	-4.8543911
FAM168A	-0.0296967	3.93706572	-0.2517556	0.80221265	0.99978775	-4.7844797
PNN	-0.0297917	5.66520832	-0.2568066	0.79833156	0.99978775	-4.8723994
DCP1B	-0.029831	4.26396942	-0.2537076	0.80071215	0.99978775	-4.8123122
CEP170	-0.0298444	7.25522474	-0.2711802	0.78731555	0.99978775	-4.8737278
FZD3	-0.0298564	0.69591938	-0.0851441	0.93247064	0.99978775	-4.6477086
BCL10	-0.0299105	4.42792021	-0.1970242	0.84456914	0.99978775	-4.8116738
PSPH	-0.0299366	5.86639798	-0.211194	0.83355334	0.99978775	-4.8840117
PREB	-0.0299736	5.17960578	-0.2402217	0.8110937	0.99978775	-4.8572511
FGFR1OP2	-0.0299934	3.639917	-0.2148679	0.83070257	0.99978775	-4.7594024
SLC50A1	-0.0300238	2.5862627	-0.1375737	0.89110347	0.99978775	-4.711175
ARSJ	-0.0300683	6.28109641	-0.1221668	0.90323326	0.99978775	-4.8904581
SNF8	-0.0300802	4.16867905	-0.3820461	0.70396886	0.99978775	-4.7923546
TADA3	-0.0301025	5.78478901	-0.3438602	0.73232445	0.99978775	-4.8621506
TIMMDC1	-0.0301587	4.05197874	-0.2607817	0.79528078	0.99978775	-4.798406
CALCRL	-0.0301778	0.02542572	-0.0365473	0.97098453	0.99978775	-4.6214247
STIM2	-0.0303219	5.63676673	-0.1852246	0.85376644	0.99978775	-4.8755863
DLST	-0.0303309	7.18003096	-0.3109265	0.75708732	0.99978775	-4.8706734
PLCE1	-0.0303935	2.5050189	-0.0975624	0.92265124	0.99978775	-4.767719
EFNA4	-0.0304143	0.65899822	-0.0902679	0.92841774	0.99978775	-4.6315308
VPS50	-0.030419	4.26413483	-0.2835517	0.77786885	0.99978775	-4.8064623
NDUFA12	-0.0304309	4.32727723	-0.3217897	0.74888944	0.99978775	-4.7989565
NCOA2	-0.0304629	5.14836463	-0.2193673	0.82721434	0.99978775	-4.8585811

TTF1	-0.0305197	3.97199105	-0.2321048	0.81735875	0.99978775	-4.7699812
COQ9	-0.0305523	4.88869284	-0.3279756	0.7442342	0.99978775	-4.8282473
ENSCAFG00000016971	-0.0305558	3.13053658	-0.2365948	0.81389161	0.99978775	-4.7384604
SMAD2	-0.0305636	4.87375682	-0.2878362	0.774605	0.99978775	-4.8349117
OSBPL7	-0.0305777	4.0625247	-0.1741766	0.86239637	0.99978775	-4.7726885
MIER1	-0.0305965	4.83129003	-0.210728	0.83391507	0.99978775	-4.8354002
ETV5	-0.0306374	5.43502306	-0.2179473	0.82831486	0.99978775	-4.853201
MT-CYB	-0.0306399	10.2952687	-0.18119	0.85691595	0.99978775	-4.80609
ENSCAFG00000029340	-0.0307016	2.98736985	-0.211676	0.83317917	0.99978775	-4.7235467
LZTS3	-0.0307085	2.47434489	-0.1253845	0.90069794	0.99978775	-4.7076941
GPSM2	-0.0307325	5.34830432	-0.2001907	0.8421047	0.99978775	-4.8821086
KMT2E	-0.0307552	7.0753808	-0.202776	0.8400937	0.99978775	-4.887424
RAD50	-0.0307681	6.78572461	-0.2761954	0.78348206	0.99978775	-4.8778687
EXOSC4	-0.0308302	3.84268623	-0.2359136	0.81441742	0.99978775	-4.7762928
XPNPEP3	-0.0308432	4.72822327	-0.3279599	0.74424596	0.99978775	-4.8116453
PLCB1	-0.03085	6.24096531	-0.163337	0.87088007	0.99978775	-4.8895564
NEK4	-0.0308651	4.19606657	-0.2842737	0.77731853	0.99978775	-4.7869766
EIF4A1	-0.0309287	8.51564981	-0.2744002	0.78485364	0.99978775	-4.8460632
RBM34	-0.0309505	4.25307034	-0.2551119	0.79963315	0.99978775	-4.7950728
ARL16	-0.0309609	2.49611215	-0.1504998	0.88094686	0.99978775	-4.6801905
MARCH3	-0.0309843	4.91740933	-0.1679199	0.86729129	0.99978775	-4.842074
TAF9	-0.0309857	4.36518232	-0.3073117	0.75982153	0.99978775	-4.8128833
ACTR6	-0.031056	3.88107949	-0.2210097	0.82594195	0.99978775	-4.7745671
RIC8B	-0.0310684	2.65022493	-0.210396	0.83417283	0.99978775	-4.6979183
DYNC2LI1	-0.0311043	3.71985296	-0.1859902	0.85316903	0.99978775	-4.7697163
TDG	-0.031122	4.58984972	-0.2156168	0.83012173	0.99978775	-4.8275967
OGT	-0.0311432	7.70454251	-0.2329639	0.81669509	0.99978775	-4.8718899
PPP2R2D	-0.0311948	4.56911906	-0.3311881	0.74182036	0.99978775	-4.8157108
KLF10	-0.0312305	6.78637283	-0.2145726	0.83093161	0.99978775	-4.8862486
ENSCAFG00000015454	-0.0312333	5.75506354	-0.3229849	0.74798925	0.99978775	-4.8650087
PTP4A2	-0.0312809	7.30985654	-0.2699319	0.78827058	0.99978775	-4.8699168
GPR161	-0.031304	1.82463585	-0.1497266	0.88155389	0.99978775	-4.6837964
RBKS	-0.0314022	3.48916736	-0.1774485	0.85983877	0.99978775	-4.7635237
IL18BP	-0.0314932	2.9701468	-0.0563854	0.95524875	0.99978775	-4.6685264
CTDSPL2	-0.031541	5.37436092	-0.2590608	0.79660114	0.99978775	-4.8615311
EXOSC8	-0.0316898	3.1997727	-0.1995989	0.84256513	0.99978775	-4.7777494
VPS16	-0.0317004	5.22075948	-0.2901671	0.77283116	0.99978775	-4.8490996
CNOT9	-0.0317434	5.35215376	-0.2743234	0.78491237	0.99978775	-4.8662269
SDC4	-0.0317694	8.18313949	-0.1978901	0.84389512	0.99978775	-4.8667337
VAMP8	-0.0317873	4.27921326	-0.1828663	0.85560706	0.99978775	-4.8136171
KIF3B	-0.0318466	5.1275757	-0.3076728	0.75954824	0.99978775	-4.834894
ATF2	-0.0319439	5.34967964	-0.2845713	0.77709175	0.99978775	-4.854802
VRK2	-0.0319458	3.5007723	-0.2257296	0.82228796	0.99978775	-4.7679452
ANKZF1	-0.0319826	4.40543876	-0.273336	0.78566709	0.99978775	-4.7990684
MFAP3	-0.031991	5.18696724	-0.2651315	0.79194614	0.99978775	-4.8514679
RAB7B	-0.0320789	2.49169676	-0.0792144	0.93716316	0.99978775	-4.6849599
MYD88	-0.0321624	5.03302517	-0.3333231	0.74021761	0.99978775	-4.8388787
SUDS3	-0.032174	5.76490859	-0.4300364	0.66892877	0.99978775	-4.8449473
NUP214	-0.0322786	5.73317719	-0.3171992	0.75235012	0.99978775	-4.8660195
DUSP19	-0.0323136	2.7626894	-0.0981187	0.92221157	0.99978775	-4.6982285

SMARCC2	-0.0323423	6.70734193	-0.3520833	0.72618481	0.99978775	-4.8669752
GAREM2	-0.0323707	0.42924924	-0.0969581	0.92312875	0.99978775	-4.634056
PACS2	-0.0324483	6.67089497	-0.3727922	0.71080352	0.99978775	-4.8638803
DOCK4	-0.0325537	4.07109657	-0.1005363	0.92030139	0.99978775	-4.831814
RPL8	-0.0326815	9.48076795	-0.2909242	0.77225521	0.99978775	-4.813403
TSR3	-0.0326865	4.89296954	-0.3220588	0.74868674	0.99978775	-4.8382108
NUDT16	-0.0326877	3.39077317	-0.1617421	0.87212963	0.99978775	-4.788897
PJA1	-0.0326921	4.01987102	-0.2179616	0.82830373	0.99978775	-4.7957901
SLC29A3	-0.0327209	2.64319507	-0.1859356	0.8532116	0.99978775	-4.699902
PLIN3	-0.0327468	4.42646043	-0.2790046	0.7813371	0.99978775	-4.8015987
TBC1D25	-0.0327539	4.29724018	-0.2739483	0.785199	0.99978775	-4.8036866
MED24	-0.0327595	5.61249285	-0.380004	0.70547502	0.99978775	-4.8504284
IL1RAP	-0.0327838	2.88685407	-0.1411248	0.88831134	0.99978775	-4.7290627
SLC46A1	-0.0328242	4.04912178	-0.2306081	0.8185153	0.99978775	-4.7999072
NDUFS1	-0.0328243	6.76238494	-0.3717669	0.71156229	0.99978775	-4.8650277
ZNF169	-0.0328243	5.08510957	-0.3527439	0.72569232	0.99978775	-4.8367151
ANAPC16	-0.0328614	3.07287567	-0.2667731	0.79068864	0.99978775	-4.7368193
CCDC51	-0.0328782	2.43391825	-0.1940081	0.84691809	0.99978775	-4.681189
RBBP6	-0.0328991	6.35063991	-0.3231013	0.74790162	0.99978775	-4.871869
CSNK1D	-0.032931	6.29647409	-0.4460191	0.65741703	0.99978775	-4.850617
SNAPC3	-0.0329467	3.63195346	-0.2792513	0.78114885	0.99978775	-4.7714049
SNAP29	-0.0330008	6.77093051	-0.5578564	0.57931293	0.99978775	-4.8249167
VDAC1	-0.0330108	7.18898233	-0.3458066	0.73086959	0.99978775	-4.8648738
EIF3F	-0.0330617	7.14568725	-0.3469505	0.730015	0.99978775	-4.8649407
RBM19	-0.0330832	5.91630457	-0.3523023	0.72602153	0.99978775	-4.8654556
ENSCAFG00000004386	-0.0330925	5.42548784	-0.3090557	0.75850201	0.99978775	-4.8623984
SNRPB2	-0.0330984	4.46373844	-0.2587566	0.79683462	0.99978775	-4.8176506
HLX	-0.0331079	1.98180113	-0.1101517	0.91270894	0.99978775	-4.650464
ZNF18	-0.033109	4.71619278	-0.3405643	0.73479027	0.99978775	-4.8214201
MSRA	-0.0331111	2.46982831	-0.1615047	0.87231564	0.99978775	-4.6895784
FKBP11	-0.0331706	5.18656769	-0.1692673	0.8662367	0.99978775	-4.8586779
SMYD5	-0.0332022	4.33464859	-0.2798664	0.78067943	0.99978775	-4.8344342
KBTBD6	-0.03322	2.40138375	-0.185453	0.85358821	0.99978775	-4.6788265
METTL26	-0.0332201	4.18604104	-0.1845544	0.85428938	0.99978775	-4.7875937
SCYL3	-0.033238	4.00631732	-0.245075	0.8073535	0.99978775	-4.7763458
TOGARAM1	-0.0332383	5.55870218	-0.3330249	0.74044138	0.99978775	-4.8548374
FBLN2	-0.0332479	10.1503532	-0.0893129	0.92917296	0.99978775	-4.8063343
PIEZO1	-0.0332872	7.68794863	-0.2142695	0.8311667	0.99978775	-4.8702939
EPB41L2	-0.0333067	6.87387807	-0.2071425	0.83669972	0.99978775	-4.882129
IPO8	-0.0333079	5.3662422	-0.3805551	0.70506848	0.99978775	-4.8419136
SNX13	-0.0334232	5.20234508	-0.3668499	0.71520508	0.99978775	-4.8412106
FAM208A	-0.0334248	6.03593307	-0.4128309	0.68141124	0.99978775	-4.8548907
ENSCAFG00000012303	-0.0334557	7.17775564	-0.3183842	0.75145629	0.99978775	-4.8686488
FNDC11	-0.0334855	-0.7081366	-0.0705215	0.94404645	0.99978775	-4.619193
R3HCC1	-0.0334995	4.76732745	-0.4061146	0.68630854	0.99978775	-4.8186342
ABHD5	-0.0335618	5.86764582	-0.2510097	0.80278617	0.99978775	-4.8780377
NR1D1	-0.0335619	5.75433307	-0.2298424	0.81910717	0.99978775	-4.8805643
ENSCAFG00000014614	-0.0335959	4.68564393	-0.3359806	0.73822421	0.99978775	-4.8331502
SPATA17	-0.0336556	1.22274586	-0.1109186	0.91210375	0.99978775	-4.6497679
PTPN14	-0.0337117	4.192138	-0.1634459	0.87079476	0.99978775	-4.8399833

STARD3NL	-0.0337372	4.74307587	-0.3003233	0.76511624	0.99978775	-4.8234895
TTL	-0.0337552	5.41216601	-0.3243998	0.74692402	0.99978775	-4.8601923
SS18L1	-0.0337659	3.49739822	-0.2697479	0.78841138	0.99978775	-4.7438774
PEX6	-0.03378	4.43176222	-0.2847959	0.77692061	0.99978775	-4.8016586
CUL4B	-0.033801	6.21790066	-0.3432098	0.73281085	0.99978775	-4.86755
MAGEH1	-0.0338048	4.41738094	-0.2607271	0.79532265	0.99978775	-4.7998727
MED12	-0.0338385	6.12680535	-0.4285298	0.67001806	0.99978775	-4.8533952
DLG4	-0.0338695	3.55334089	-0.228445	0.82018752	0.99978775	-4.7320607
KCTD20	-0.0340529	3.6121414	-0.3013886	0.76430842	0.99978775	-4.7693084
TBP	-0.0340961	4.07298253	-0.3358718	0.73830585	0.99978775	-4.7850016
TTC31	-0.034118	4.25324342	-0.3126714	0.7557686	0.99978775	-4.8014468
SFI1	-0.034179	3.44568379	-0.3163007	0.75302811	0.99978775	-4.7311007
DNAJB12	-0.0341792	5.01135423	-0.390835	0.69750033	0.99978775	-4.8247543
CLTCL1	-0.0342255	2.51859605	-0.1498208	0.88147994	0.99978775	-4.7333988
ENSCAFG00000015122	-0.0342692	8.38429279	-0.3107219	0.75724199	0.99978775	-4.844668
LIG4	-0.0343066	4.14659788	-0.2109668	0.83372969	0.99978775	-4.7970608
DCUN1D5	-0.0343764	3.92947414	-0.2107715	0.83388132	0.99978775	-4.7848025
NAGA	-0.0343863	6.14001092	-0.3252624	0.74627489	0.99978775	-4.8712881
ENSCAFG00000024531	-0.0344034	5.78061941	-0.2734112	0.7856096	0.99978775	-4.8740846
FLII	-0.034434	7.74178831	-0.5461116	0.58730188	0.99978775	-4.8120661
PPFIA1	-0.0344355	5.8210271	-0.4908898	0.62554953	0.99978775	-4.8358306
TSR2	-0.0344915	2.71997782	-0.2167485	0.82924421	0.99978775	-4.7096992
MFSD14A	-0.0346087	4.9517706	-0.3346824	0.73919778	0.99978775	-4.826186
HAPLN3	-0.034645	6.42118692	-0.1316421	0.89577044	0.99978775	-4.8926231
ENSCAFG00000025389	-0.0346804	4.12804209	-0.3767478	0.70787912	0.99978775	-4.7843745
CARNMT1	-0.0347712	3.90303196	-0.2931565	0.77055784	0.99978775	-4.7656388
IFT20	-0.0347959	3.75515168	-0.2789636	0.78136841	0.99978775	-4.772918
TBC1D20	-0.0348068	5.89746223	-0.3804141	0.70517247	0.99978775	-4.8576304
SLC39A3	-0.0348138	3.36902276	-0.2930453	0.7706424	0.99978775	-4.7586673
ENSCAFG00000023188	-0.0348788	1.26090393	-0.1390068	0.88997648	0.99978775	-4.6785647
ZNF608	-0.034893	4.40196999	-0.1130933	0.91038785	0.99978775	-4.7375553
ZNF382	-0.0349192	3.27685383	-0.1867046	0.85261169	0.99978775	-4.7499626
PHPT1	-0.0349406	4.46666747	-0.2539533	0.80052334	0.99978775	-4.8108039
CCDC8	-0.0349695	2.84332142	-0.1236299	0.9020803	0.99978775	-4.7391125
PI4KA	-0.0350013	7.23209493	-0.3595375	0.72063481	0.99978775	-4.8632225
DIXDC1	-0.0350049	5.21684937	-0.2769013	0.78294288	0.99978775	-4.8458557
CARF	-0.035058	1.30383206	-0.1715275	0.86446828	0.99978775	-4.6510933
DYNLT1	-0.0350712	4.42146886	-0.287975	0.77449938	0.99978775	-4.8279718
CSTB	-0.0350826	7.03718961	-0.175549	0.86132346	0.99978775	-4.8857447
ENSCAFG00000023724	-0.0350906	2.46541364	-0.1293909	0.89754264	0.99978775	-4.6969619
GGT7	-0.0351295	5.04829167	-0.0979374	0.92235489	0.99978775	-4.8632008
FAM13A	-0.035215	3.79652075	-0.1838958	0.85480342	0.99978775	-4.7151785
PEX19	-0.0352213	4.09620781	-0.3622992	0.71858241	0.99978775	-4.7844988
CD63	-0.035228	9.44983837	-0.366421	0.7155231	0.99978775	-4.8110061
DDX59	-0.0352879	3.72677144	-0.2249048	0.82292616	0.99978775	-4.7625833
MANBAL	-0.0354021	3.19424653	-0.251544	0.80237531	0.99978775	-4.7468158
MVB12A	-0.0354983	4.39052647	-0.2315469	0.81778985	0.99978775	-4.8113883
ZDHHC9	-0.035549	5.64204009	-0.2493978	0.804026	0.99978775	-4.8676818
GDI2	-0.0356734	7.73809642	-0.5672534	0.57295904	0.99978775	-4.8076548
PRMT6	-0.0357544	1.68524718	-0.1636537	0.870632	0.99978775	-4.6805228

SKA2	-0.0357725	4.9209116	-0.244343	0.80791735	0.99978775	-4.8579342
CCDC71	-0.035773	3.68076584	-0.3114878	0.75666303	0.99978775	-4.7516265
AP5B1	-0.0358287	4.05549442	-0.192771	0.84788192	0.99978775	-4.7947976
GRN	-0.0358861	8.18432835	-0.1946005	0.84645663	0.99978775	-4.8641892
SEMA3B	-0.0359052	2.99006182	-0.0847239	0.93280307	0.99978775	-4.7490494
PPP1R14B	-0.035932	5.23200115	-0.2586039	0.79695182	0.99978775	-4.8691369
PNP	-0.0359986	7.7815213	-0.1688683	0.86654903	0.99978775	-4.8864711
EIF5B	-0.0360472	7.54514758	-0.3118213	0.756411	0.99978775	-4.8641814
NTNG2	-0.0361087	1.83895764	-0.0894498	0.92906476	0.99978775	-4.6730417
GTF2A1	-0.0362307	5.2145985	-0.3040843	0.76226532	0.99978775	-4.8471444
RBMX	-0.0362816	4.91019417	-0.2450164	0.8073987	0.99978775	-4.8521921
ZNF326	-0.036338	5.68094714	-0.3741513	0.70979826	0.99978775	-4.8549375
USF2	-0.0363723	6.23175991	-0.3352569	0.73876689	0.99978775	-4.8699792
RHBDD3	-0.0364106	4.61895801	-0.2255603	0.82241893	0.99978775	-4.8473088
TFG	-0.0364598	7.3473992	-0.550279	0.58446117	0.99978775	-4.8199996
GCFC2	-0.0364894	3.42813327	-0.3200972	0.75016475	0.99978775	-4.7366553
C7H1orf43	-0.0365187	4.49411769	-0.3628071	0.71820518	0.99978775	-4.8098038
NELFCD	-0.0365528	5.65134969	-0.5906778	0.55727	0.99978775	-4.8078395
CLDN15	-0.0365767	0.39140635	-0.0665557	0.94718816	0.99978775	-4.6179648
BARX1	-0.0366268	-2.4314814	-0.0561894	0.95540413	0.99978775	-4.6087655
IFRD1	-0.0366429	5.68255943	-0.1696386	0.86594616	0.99978775	-4.8847999
BTBD2	-0.0366501	5.79318927	-0.2482974	0.80487268	0.99978775	-4.8740385
RBBP4	-0.0366794	5.77943405	-0.5107729	0.61165071	0.99978775	-4.8315761
EXOC7	-0.0367249	6.31579559	-0.5085851	0.61317314	0.99978775	-4.8354524
RNF130	-0.0367266	5.67525135	-0.2778909	0.78218724	0.99978775	-4.8539269
ENSCAFG00000024271	-0.036764	2.78375689	-0.231153	0.81809424	0.99978775	-4.6994509
ENSCAFG00000009523	-0.0367792	7.79567428	-0.3138548	0.75487472	0.99978775	-4.8577533
KIAA0895L	-0.0367952	1.98474634	-0.204062	0.83909382	0.99978775	-4.668031
RPSA	-0.0368147	8.39286945	-0.3442629	0.73202337	0.99978775	-4.8364729
ZFHX2	-0.0369711	2.78546325	-0.1343277	0.89365691	0.99978775	-4.6776159
PRKAR2A	-0.0370126	5.92951608	-0.372461	0.7110486	0.99978775	-4.8589892
DCTD	-0.037053	4.21997312	-0.364446	0.71698846	0.99978775	-4.7939256
SIN3A	-0.0370943	5.82421207	-0.4188982	0.676999	0.99978775	-4.8467541
PITPNA	-0.0371301	5.60272965	-0.4121903	0.68187777	0.99978775	-4.8445133
MAP1S	-0.0371566	5.95314669	-0.3126405	0.755792	0.99978775	-4.8723972
ZSWIM8	-0.0371589	7.23803533	-0.4679563	0.64175219	0.99978775	-4.8397269
ABCG2	-0.0371726	2.96249223	-0.2131222	0.83205685	0.99978775	-4.7338535
KLHDC4	-0.0371846	4.96978343	-0.4356805	0.66485419	0.99978775	-4.819683
TCEANC2	-0.0372226	3.47829737	-0.190797	0.84942034	0.99978775	-4.7485931
NTMT1	-0.0372376	3.21528728	-0.2837744	0.77769907	0.99978775	-4.7349084
ELP5	-0.0372448	4.02586968	-0.2907478	0.77238937	0.99978775	-4.778948
ZNF335	-0.0372545	5.08310786	-0.4265816	0.67142781	0.99978775	-4.825965
KIAA0100	-0.0372747	5.64259061	-0.5887741	0.55853695	0.99978775	-4.8094554
TOM1	-0.0372957	5.79765243	-0.2929515	0.77071367	0.99978775	-4.8728877
AIFM1	-0.0373255	5.05785592	-0.3996948	0.69100242	0.99978775	-4.828212
CAMKK2	-0.0374062	4.5973404	-0.2264112	0.8217606	0.99978775	-4.8106045
ENSCAFG00000019000	-0.037422	2.05538216	-0.1849307	0.85399573	0.99978775	-4.6658801
RBM28	-0.037435	4.52202874	-0.2715245	0.78705224	0.99978775	-4.826371
SNX16	-0.0374661	3.93998468	-0.2664983	0.79089914	0.99978775	-4.7680585
PFKM	-0.0375088	5.22226063	-0.3424036	0.73341387	0.99978775	-4.8334636

PCSK5	-0.0375261	4.93155421	-0.0984447	0.92195399	0.99978775	-4.8322754
SRPK1	-0.0375385	5.30913559	-0.229827	0.81911909	0.99978775	-4.8619465
ENSCAFG00000017221	-0.0375386	1.22557689	-0.184171	0.85458868	0.99978775	-4.6500816
ENSCAFG00000018632	-0.0375546	2.87211317	-0.2149028	0.83067546	0.99978775	-4.7105103
CYP39A1	-0.0375621	4.0486787	-0.2594481	0.79630391	0.99978775	-4.7972975
TNNC1	-0.0375753	0.45634581	-0.0863095	0.93154867	0.99978775	-4.6232694
ZNF331	-0.0375787	3.63064037	-0.2036576	0.83940822	0.99978775	-4.7543125
TP53INP2	-0.0376236	1.47113349	-0.1180633	0.90646796	0.99978775	-4.6489628
TRIP11	-0.037653	6.46310225	-0.3319771	0.74122794	0.99978775	-4.8715948
CCT5	-0.0376546	8.02909054	-0.2856631	0.77625994	0.99978775	-4.8539861
ZFP28	-0.0377167	3.43279006	-0.2186211	0.82779259	0.99978775	-4.7362962
RASSF8	-0.0377424	5.46206727	-0.2069855	0.83682166	0.99978775	-4.866853
C7H1orf112	-0.0377754	3.13561879	-0.1614801	0.87233492	0.99978775	-4.7417571
ENSCAFG00000028993	-0.0378038	1.93063661	-0.0681976	0.94588732	0.99978775	-4.6894482
IVD	-0.0379235	5.68961028	-0.2067377	0.83701422	0.99978775	-4.8715114
CCL22	-0.0379338	-1.8927155	-0.0486088	0.96141544	0.99978775	-4.6080896
SSPN	-0.038012	3.75108704	-0.1926136	0.84800452	0.99978775	-4.7943379
ALKBH4	-0.0380598	3.59979599	-0.323682	0.74746437	0.99978775	-4.7496478
SLC35A4	-0.0381014	4.69126936	-0.2662446	0.79109342	0.99978775	-4.8346353
PEX11A	-0.03811	2.98602921	-0.2473085	0.80563382	0.99978775	-4.6996939
SS18L2	-0.038139	2.00888119	-0.2166606	0.82931233	0.99978775	-4.6783961
UBE2I	-0.0381509	4.73382025	-0.3844481	0.70219882	0.99978775	-4.8165353
SCAMP5	-0.0381781	2.8236233	-0.1318705	0.89559069	0.99978775	-4.7070747
CDIPT	-0.0381956	6.38342091	-0.3861346	0.70095703	0.99978775	-4.8626395
BOLA1	-0.0381958	2.80505755	-0.2394784	0.81166686	0.99978775	-4.7077745
MED25	-0.0382193	5.28133111	-0.2588698	0.79674769	0.99978775	-4.8547167
FAM13B	-0.0382773	4.10556653	-0.2635127	0.79318665	0.99978775	-4.7984476
ENSCAFG00000001352	-0.038304	0.75768761	-0.1266206	0.89972423	0.99978775	-4.6344145
CHAMP1	-0.0383164	5.16247313	-0.4036315	0.68812263	0.99978775	-4.8279122
GTF3C4	-0.038325	5.55460229	-0.3576373	0.72204818	0.99978775	-4.8544663
HNRNPD	-0.0383561	6.89728592	-0.2437002	0.80841255	0.99978775	-4.8784618
SRSF2	-0.0383656	4.84627563	-0.2314334	0.8178775	0.99978775	-4.8535969
ENSCAFG00000003077	-0.0383766	2.80155542	-0.2312407	0.81802645	0.99978775	-4.7302667
ATAD5	-0.0384354	2.70932514	-0.1236498	0.90206458	0.99978775	-4.7452859
LSM6	-0.0384877	3.98412408	-0.3039498	0.76236726	0.99978775	-4.7789384
DOLPP1	-0.0384943	3.56872565	-0.2663121	0.79104172	0.99978775	-4.7636303
ENSCAFG00000008732	-0.0387103	3.20200452	-0.2913811	0.77190774	0.99978775	-4.7329872
SDAD1	-0.0387702	5.16548467	-0.3976649	0.6924891	0.99978775	-4.8326889
IER3	-0.0387862	5.32785481	-0.1609385	0.87275937	0.99978775	-4.8638464
COX7A2L	-0.0388056	5.89317674	-0.4423608	0.66004471	0.99978775	-4.8471743
KYAT3	-0.0388208	2.76270334	-0.2151133	0.83051219	0.99978775	-4.6894251
GLUL	-0.0388235	4.10773052	-0.0703493	0.94418286	0.99978775	-4.7146093
HPF1	-0.0388588	3.26021801	-0.2577676	0.79759374	0.99978775	-4.7433393
PER3	-0.038865	4.29997473	-0.2296889	0.81922585	0.99978775	-4.8151904
LRP8	-0.0389412	4.97876045	-0.1812473	0.85687122	0.99978775	-4.8391308
ORC5	-0.038959	4.14438542	-0.2709737	0.78747355	0.99978775	-4.7826935
JOSD2	-0.0389599	4.49495832	-0.2432726	0.80874199	0.99978775	-4.8142767
USP10	-0.0390158	6.71420112	-0.5275329	0.60004539	0.99978775	-4.8322638
ENSCAFG00000016914	-0.0390228	1.71651159	-0.1375446	0.89112636	0.99978775	-4.6645811
SLC7A1	-0.0391173	5.72226509	-0.273817	0.78529938	0.99978775	-4.874833

PGF	-0.0391201	2.49456993	-0.0225353	0.98210635	0.99978775	-4.6327806
NUP85	-0.0391417	4.92672669	-0.3424779	0.73335824	0.99978775	-4.8390713
ENSCAFG00000003468	-0.0391665	4.68266534	-0.4111628	0.68262628	0.99978775	-4.8152843
SUZ12	-0.0391785	5.29337632	-0.3295121	0.74307936	0.99978775	-4.8516357
GMPS	-0.0391838	6.57345693	-0.4701665	0.64018287	0.99978775	-4.8461207
PDIA4	-0.0392091	8.67251706	-0.3996872	0.69100795	0.99978775	-4.8271012
PHF23	-0.0392266	5.28718404	-0.4392121	0.66230983	0.99978775	-4.8297531
FBXO17	-0.0392878	0.7888266	-0.1523927	0.87946118	0.99978775	-4.6775347
GNB1L	-0.0392962	4.54345294	-0.3366092	0.73775296	0.99978775	-4.8211337
ENSCAFG00000000600	-0.0393286	3.05378964	-0.259439	0.7963109	0.99978775	-4.7098915
HIBCH	-0.0393772	4.74531593	-0.2405692	0.81082576	0.99978775	-4.8171802
CCDC58	-0.0394107	3.4118191	-0.2914448	0.7718593	0.99978775	-4.7440859
PRRC2B	-0.0394419	6.86792729	-0.3301168	0.74262504	0.99978775	-4.8704896
CEP19	-0.0394621	1.63698817	-0.1182039	0.90635707	0.99978775	-4.6506129
FBXW8	-0.0394963	5.79279445	-0.4125714	0.68160022	0.99978775	-4.8531911
ENSCAFG00000005024	-0.0395001	5.81855906	-0.5069353	0.6143223	0.99978775	-4.8307183
ENSCAFG00000023007	-0.0395295	2.29949642	-0.1856582	0.85342803	0.99978775	-4.6726827
FLYWCH2	-0.0396352	2.75526834	-0.2423451	0.80945676	0.99978775	-4.7038841
PHLPP1	-0.0397122	4.82520679	-0.2987203	0.76633237	0.99978775	-4.8274437
ENSCAFG00000012894	-0.0397288	5.86907075	-0.4470784	0.65665698	0.99978775	-4.846173
ZNF428	-0.0398051	3.45316721	-0.3830909	0.70319873	0.99978775	-4.752433
SNX18	-0.0398537	5.80440416	-0.2428566	0.80906256	0.99978775	-4.8761006
CLIP4	-0.0399027	-1.9543732	-0.0662202	0.947454	0.99978775	-4.6101689
CCDC120	-0.0399081	3.35440968	-0.2837227	0.77773851	0.99978775	-4.7464717
RPL13	-0.039923	8.27836316	-0.3084198	0.75898305	0.99978775	-4.8470422
PAICS	-0.0399541	5.86242254	-0.4162284	0.67893911	0.99978775	-4.8550305
NASP	-0.0399568	4.94845198	-0.2633992	0.79327371	0.99978775	-4.8442913
CCAR2	-0.0399834	6.18090987	-0.5677883	0.57259837	0.99978775	-4.8222201
FCHSD1	-0.0400255	3.60581898	-0.3114651	0.75668022	0.99978775	-4.7443846
TRAPPC9	-0.040058	5.13904346	-0.4615953	0.64627797	0.99978775	-4.8252246
CWF19L2	-0.0400598	3.89711612	-0.2836921	0.77776182	0.99978775	-4.7591088
POU2F1	-0.0401753	1.73477117	-0.1791071	0.85854289	0.99978775	-4.6529018
SLC25A26	-0.040201	2.3106038	-0.2110112	0.83369526	0.99978775	-4.6752947
CEP57	-0.0402902	4.86495849	-0.3864867	0.70069783	0.99978775	-4.826929
RPS17	-0.0402982	8.05787056	-0.3811759	0.70461055	0.99978775	-4.8433518
XPO5	-0.0403641	5.81150252	-0.5060978	0.61490605	0.99978775	-4.8294288
GKAP1	-0.0403788	3.03317521	-0.2263955	0.82177277	0.99978775	-4.7082882
MUC1	-0.0403809	0.13740967	-0.1197847	0.90511082	0.99978775	-4.6498828
ENSCAFG00000015635	-0.040385	5.05929506	-0.3773874	0.70740666	0.99978775	-4.8362689
ARL3	-0.0404041	4.33118174	-0.2660296	0.79125815	0.99978775	-4.7901034
STARD9	-0.0404381	5.23557682	-0.2374427	0.81323729	0.99978775	-4.837514
MEF2D	-0.0404827	5.28868149	-0.3028627	0.76319101	0.99978775	-4.8478071
PAXBP1	-0.040602	4.24614575	-0.3109678	0.75705614	0.99978775	-4.7916546
ENSCAFG00000031317	-0.0406221	1.10438934	-0.2084262	0.83570251	0.99978775	-4.6426409
IPO9	-0.040637	6.28961112	-0.6839527	0.49701217	0.99978775	-4.7895023
TAOK3	-0.0406493	5.51127944	-0.4535947	0.65198953	0.99978775	-4.8337748
CRCP	-0.0406681	3.27366088	-0.3256251	0.74600197	0.99978775	-4.7228873
ENSCAFG00000008873	-0.040694	8.23833657	-0.3056245	0.76109881	0.99978775	-4.8487552
ENSCAFG00000005504	-0.0407159	6.18451062	-0.5415701	0.59040506	0.99978775	-4.8279168
HERC2	-0.0407337	6.96693567	-0.3720084	0.71138355	0.99978775	-4.8638498

KCNK6	-0.0407514	3.53766494	-0.1745491	0.86210516	0.99978775	-4.7404779
FBXO42	-0.0407708	5.16870086	-0.4541876	0.65156548	0.99978775	-4.8209097
ZNF597	-0.0408707	2.18202072	-0.2089549	0.83529188	0.99978775	-4.6611497
ITPRIP	-0.0408773	5.22097744	-0.1643033	0.87012319	0.99978775	-4.868969
ATP13A1	-0.0409101	6.3124991	-0.4333831	0.66651151	0.99978775	-4.8537381
CCNE1	-0.0410069	2.29305757	-0.2205604	0.82629003	0.99978775	-4.7014043
CNOT7	-0.0410079	5.80663038	-0.4093179	0.68397113	0.99978775	-4.8523722
AHSA1	-0.0410128	6.11907701	-0.4454398	0.65783283	0.99978775	-4.8496836
ABCF1	-0.0410179	6.54968866	-0.5599185	0.5779157	0.99978775	-4.8246931
CPSF7	-0.0410571	5.94367008	-0.4923529	0.62452205	0.99978775	-4.8382597
RAB3D	-0.0410817	1.37968031	-0.0865484	0.93135968	0.99978775	-4.6435444
EIF5	-0.0412212	7.54377087	-0.3312299	0.74178895	0.99978775	-4.8597719
WBP11	-0.0412349	4.97208615	-0.4290354	0.6696524	0.99978775	-4.8178858
MAST2	-0.0412356	6.06887784	-0.4308275	0.66835705	0.99978775	-4.8539116
MSANTD4	-0.0412389	3.16767101	-0.3220156	0.74871929	0.99978775	-4.7151325
SLC30A4	-0.0412647	3.55043509	-0.3500278	0.72771781	0.99978775	-4.7484261
NDUFAF6	-0.0412896	3.63669508	-0.3589202	0.72109384	0.99978775	-4.7566283
RSF1	-0.0412948	5.2138576	-0.3645481	0.71691262	0.99978775	-4.831579
DNAJA1	-0.0413403	4.60780179	-0.2700904	0.78814932	0.99978775	-4.8078884
CHMP6	-0.0413532	3.89381574	-0.4078234	0.68506122	0.99978775	-4.7678398
ARFGAP3	-0.0413688	7.02088209	-0.4584135	0.64854685	0.99978775	-4.8444283
VASH1	-0.0414015	3.26528029	-0.1862657	0.85295408	0.99978775	-4.7531913
ANKRD50	-0.0414067	5.32428868	-0.158347	0.8747907	0.99978775	-4.8362242
CNPY3	-0.0414101	4.29186501	-0.4004946	0.69041695	0.99978775	-4.7830182
ADH4	-0.0414177	2.2343662	-0.0572105	0.95459467	0.99978775	-4.6508597
STRBP	-0.0414276	3.36724124	-0.2681125	0.78966306	0.99978775	-4.7538022
SZRD1	-0.0414336	3.81229298	-0.3889159	0.69891085	0.99978775	-4.7696817
DUSP5	-0.0414518	5.06458088	-0.1249352	0.90105187	0.99978775	-4.7941944
RARRES3	-0.0415063	3.39137788	-0.1387605	0.89017012	0.99978775	-4.7213707
MTIF3	-0.0415758	4.42009355	-0.3990878	0.69144684	0.99978775	-4.796511
ZRANB3	-0.0415875	3.58707243	-0.3030718	0.76303248	0.99978775	-4.748624
ENSCAFG00000005488	-0.0416193	-0.4075383	-0.1072738	0.91498056	0.99978775	-4.6228081
ENSCAFG00000015648	-0.0416466	2.69177993	-0.2192865	0.82727694	0.99978775	-4.6828984
ZNF79	-0.0417509	2.09969039	-0.212061	0.83288039	0.99978775	-4.6600421
DELE1	-0.0417931	5.5544983	-0.3762469	0.70824917	0.99978775	-4.8484075
ADM	-0.0418264	6.56382928	-0.1443819	0.88575156	0.99978775	-4.8867064
ZBTB7B	-0.0418548	4.73803927	-0.3281525	0.74410122	0.99978775	-4.8261212
NUP43	-0.0418786	4.50740229	-0.3221041	0.74865258	0.99978775	-4.8231203
SLC16A5	-0.0418932	4.46050781	-0.2564651	0.79859377	0.99978775	-4.8305335
ENSCAFG00000013391	-0.0420307	2.44862942	-0.2225104	0.82477977	0.99978775	-4.6787212
NPRL2	-0.0420398	4.65763773	-0.4403977	0.66145655	0.99978775	-4.7960403
RTCB	-0.0420722	5.77989321	-0.5299129	0.59840575	0.99978775	-4.8284222
ENSCAFG00000015719	-0.0420747	3.53178544	-0.2392909	0.81181151	0.99978775	-4.760425
HVCN1	-0.0420762	4.04804992	-0.1610469	0.87267445	0.99978775	-4.7809704
DHX29	-0.042112	5.8660975	-0.3996096	0.69106477	0.99978775	-4.8544825
STK3	-0.042162	4.81752266	-0.2950602	0.7691113	0.99978775	-4.8204476
MID1IP1	-0.042191	3.4746205	-0.2757516	0.78382109	0.99978775	-4.7703211
ZDHHC13	-0.0424242	4.04687085	-0.4365671	0.66421507	0.99978775	-4.7708311
NUDT22	-0.0424406	2.4216581	-0.2080878	0.83596535	0.99978775	-4.6943098
CBL	-0.042503	4.18810041	-0.3333696	0.7401827	0.99978775	-4.7767257

ALDH9A1	-0.0425509	6.72288805	-0.2169692	0.8290731	0.99978775	-4.8858359
CREG2	-0.0425612	-1.9201147	-0.0523093	0.95848075	0.99978775	-4.6072045
GTF2E1	-0.0425681	3.35268778	-0.3323832	0.74092307	0.99978775	-4.7361652
MKNK1	-0.0425743	4.30573659	-0.3508623	0.72709529	0.99978775	-4.7977164
ENSCAFG00000000288	-0.0425871	1.18621971	-0.1910801	0.84919969	0.99978775	-4.6636516
LRRC58	-0.0427196	5.45311753	-0.3238434	0.74734283	0.99978775	-4.8547774
MAN2A1	-0.042733	7.94630121	-0.4077429	0.68512	0.99978775	-4.854889
TRAK1	-0.0427353	6.77048796	-0.1912394	0.84907554	0.99978775	-4.8880124
ENSCAFG00000000314	-0.0427624	5.26530387	-0.2941579	0.76979683	0.99978775	-4.8503573
NMD3	-0.0427706	5.5063088	-0.4175407	0.67798517	0.99978775	-4.8480025
CBFA2T2	-0.0427886	3.7729849	-0.3871219	0.70023041	0.99978775	-4.7668814
USP38	-0.0428342	4.62848266	-0.4027269	0.68878395	0.99978775	-4.8082103
API5	-0.0428889	6.09709399	-0.5745446	0.56805255	0.99978775	-4.8197101
ZKSCAN8	-0.0429002	3.43131758	-0.3056341	0.76109152	0.99978775	-4.7345757
ZFP3	-0.0429212	1.43956906	-0.1443961	0.88574046	0.99978775	-4.6389909
SEC22C	-0.0429439	1.86643105	-0.2307317	0.8184198	0.99978775	-4.66042
PARP9	-0.0429623	2.38074567	-0.1286714	0.89810922	0.99978775	-4.6885926
GPATCH2	-0.0429867	3.81046352	-0.2948932	0.76923819	0.99978775	-4.7516915
TENT5A	-0.0430321	5.88720957	-0.2096092	0.83478377	0.99978775	-4.8833062
PLAC1	-0.0431003	-0.8418322	-0.0928443	0.92638059	0.99978775	-4.619671
DUT	-0.0431486	4.60647352	-0.2976526	0.76714272	0.99978775	-4.8217309
VPS72	-0.0431844	4.8898291	-0.4216236	0.67502067	0.99978775	-4.8229326
DGCR2	-0.0432046	4.73108839	-0.2743157	0.78491825	0.99978775	-4.8310501
RHOG	-0.0432094	4.55532806	-0.2627787	0.79374935	0.99978775	-4.8060242
TTC37	-0.0432916	6.95100439	-0.45507	0.65093474	0.99978775	-4.8472057
NCS1	-0.0433	2.07456817	-0.2356401	0.81462852	0.99978775	-4.6881636
APTX	-0.0433215	2.81022125	-0.3126019	0.75582111	0.99978775	-4.7047869
ENSCAFG00000010373	-0.0434041	0.79381706	-0.159675	0.8737497	0.99978775	-4.6499526
RIPK2	-0.0434047	4.52165898	-0.3154513	0.75366925	0.99978775	-4.8172373
B3GNT9	-0.0434083	3.47959665	-0.1647821	0.86974816	0.99978775	-4.7758495
PGM2L1	-0.0434407	1.41657242	-0.1525861	0.87930947	0.99978775	-4.6458817
STK38	-0.0434741	5.33273892	-0.3029176	0.76314939	0.99978775	-4.849119
RBM4	-0.0435174	4.83537965	-0.5559426	0.58061113	0.99978775	-4.790961
PDF	-0.043555	2.66487912	-0.2714837	0.78708346	0.99978775	-4.7116778
HEBP2	-0.0435637	2.74883865	-0.2920076	0.77143131	0.99978775	-4.7629605
EEF2	-0.0436052	11.5757767	-0.4126589	0.68153647	0.99978775	-4.7488916
NAA15	-0.0436242	6.41753636	-0.3732724	0.71044828	0.99978775	-4.8648627
EFNB1	-0.0436558	3.9957071	-0.2464751	0.80627541	0.99978775	-4.7690717
PRMT2	-0.0437397	5.11698625	-0.3716876	0.71162102	0.99978775	-4.8396493
MEA1	-0.0437434	3.77698088	-0.3341178	0.73962132	0.99978775	-4.75809
TIMM50	-0.0437453	5.25863653	-0.4116677	0.6822584	0.99978775	-4.8341167
GRSF1	-0.0437783	5.9626099	-0.5075469	0.61389616	0.99978775	-4.8352751
BAX	-0.0438086	3.26760798	-0.2634528	0.79323256	0.99978775	-4.7196112
POLR1E	-0.0438333	5.30770855	-0.402245	0.68913632	0.99978775	-4.8334181
NDUFAF2	-0.0439487	2.05703449	-0.2504709	0.80320054	0.99978775	-4.6979214
UQCRFS1	-0.0439819	5.46164077	-0.3894073	0.69854956	0.99978775	-4.8444643
TSC22D4	-0.0439875	5.27924975	-0.283984	0.77753934	0.99978775	-4.8457922
FAM210A	-0.0440281	3.31835285	-0.3345764	0.73927732	0.99978775	-4.7332199
MLF2	-0.0440633	6.92951165	-0.5390802	0.59210972	0.99978775	-4.8260964
ENSCAFG00000000331	-0.0440716	0.1656913	-0.1550778	0.87735452	0.99978775	-4.6283851

FBXW5	-0.0441077	5.47850533	-0.3348185	0.73909568	0.99978775	-4.8606174
NR2C2	-0.0441188	4.06275308	-0.3777701	0.70712396	0.99978775	-4.7830892
PPFIBP1	-0.0441887	8.57149592	-0.3039289	0.76238305	0.99978775	-4.8606303
CDK9	-0.0442179	5.66443378	-0.4563677	0.65000749	0.99978775	-4.8357797
PANK3	-0.0442527	4.1303055	-0.3145912	0.7543186	0.99978775	-4.779639
ZC3H7A	-0.0442979	6.41121068	-0.4497889	0.6547138	0.99978775	-4.8498082
ASNSD1	-0.0444351	6.21501784	-0.5142114	0.60926143	0.99978775	-4.8345966
PSMA7	-0.0444524	6.40763379	-0.4807886	0.6326638	0.99978775	-4.8437962
FBXL7	-0.0444959	5.80165032	-0.2650549	0.79200485	0.99978775	-4.8771511
PMP22	-0.0444995	8.58852657	-0.287199	0.77509017	0.99978775	-4.8492741
PRELID3B	-0.044603	4.45425702	-0.335067	0.73890931	0.99978775	-4.8075688
CRIP2	-0.0446051	5.50058606	-0.2546953	0.79995321	0.99978775	-4.8564086
MRPS11	-0.0446593	3.90642238	-0.4113987	0.68245443	0.99978775	-4.7715441
CHST14	-0.0446698	3.23969233	-0.229024	0.81973986	0.99978775	-4.7502805
ENSCAFG00000005665	-0.0447486	3.70563625	-0.340409	0.73490652	0.99978775	-4.7515658
NMT2	-0.0447641	4.98830429	-0.3489252	0.72854063	0.99978775	-4.8057729
TPP1	-0.0447663	5.95927012	-0.213332	0.83189406	0.99978775	-4.8810802
ENSCAFG00000006177	-0.0448008	3.99059066	-0.3883678	0.69931389	0.99978775	-4.7665006
SNAPC4	-0.0448012	4.64221783	-0.3976121	0.69252779	0.99978775	-4.8029453
PORCN	-0.044823	2.69966581	-0.1679444	0.8672721	0.99978775	-4.6887588
LYN	-0.0448502	6.49023511	-0.2712854	0.7872351	0.99978775	-4.8757394
CA13	-0.0448888	0.60208131	-0.0859782	0.9318107	0.99978775	-4.6245881
ENSCAFG00000030770	-0.0449075	5.83860081	-0.2212967	0.82571967	0.99978775	-4.8711879
ENSCAFG00000003477	-0.044937	8.97318099	-0.3810578	0.70469763	0.99978775	-4.8189911
TOR4A	-0.0449373	1.59198143	-0.2009907	0.8414823	0.99978775	-4.6562497
NDUFA9	-0.0450715	5.50720153	-0.3749926	0.70917621	0.99978775	-4.8511928
NYNRIN	-0.0451026	4.19896985	-0.2186573	0.82776457	0.99978775	-4.870335
CDKN1B	-0.0451865	4.51926819	-0.2090048	0.83525308	0.99978775	-4.8303038
ENSCAFG00000018277	-0.0451896	7.06522178	-0.1190611	0.90568124	0.99978775	-4.8887733
NOC3L	-0.0452612	4.57388692	-0.3534207	0.72518793	0.99978775	-4.8053678
STAG2	-0.0453233	6.5456617	-0.3694283	0.71329405	0.99978775	-4.8655129
NCOA6	-0.0453456	6.22586384	-0.5168177	0.60745322	0.99978775	-4.8342207
C18H11orf74	-0.0453652	2.70498069	-0.2777781	0.78227339	0.99978775	-4.6898762
LIX1L	-0.045409	4.68501368	-0.4632601	0.64509217	0.99978775	-4.7951979
CC2D1A	-0.0454247	5.83183511	-0.6652413	0.50880743	0.99978775	-4.7918029
ADAM12	-0.0455226	8.74796798	-0.1738443	0.86265622	0.99978775	-4.8361563
SENP5	-0.0455838	4.91752945	-0.3418352	0.73383915	0.99978775	-4.8231308
C35H6orf62	-0.0456212	5.66892762	-0.5863215	0.56017136	0.99978775	-4.8110377
COQ10B	-0.0456298	4.25903813	-0.3444096	0.73191369	0.99978775	-4.8056621
ZNF263	-0.0456468	2.8099026	-0.3251887	0.74633035	0.99978775	-4.6932602
CEP104	-0.0456584	4.28805995	-0.3429409	0.73301193	0.99978775	-4.7859855
ENSCAFG00000000492	-0.0456773	2.16646166	-0.0577977	0.95412914	0.99978775	-4.6893745
RPS20	-0.0456932	8.73526655	-0.3440944	0.73214937	0.99978775	-4.8343865
COQ2	-0.0456939	5.2374873	-0.5226842	0.6033923	0.99978775	-4.8060768
EDC3	-0.0457081	4.11255386	-0.3676513	0.71461088	0.99978775	-4.790315
NUP62	-0.0457474	5.25700562	-0.385963	0.70108333	0.99978775	-4.8445807
ELP2	-0.0457709	5.66532938	-0.5491139	0.58525475	0.99978775	-4.8210597
FOCAD	-0.045783	6.55627267	-0.370282	0.71266167	0.99978775	-4.865007
ZFP90	-0.0457951	4.99958387	-0.2723714	0.78640456	0.99978775	-4.8119743
POLDIP3	-0.0458308	5.25367951	-0.4302684	0.66876107	0.99978775	-4.8343721

UBE2Q1	-0.0458442	6.83931954	-0.6336072	0.52908729	0.99978775	-4.8040714
AHI1	-0.0459057	4.64499526	-0.2793547	0.7810699	0.99978775	-4.824478
MAP7D1	-0.0459125	8.04092227	-0.3572915	0.72230549	0.99978775	-4.8447723
XRRA1	-0.0459393	0.1513173	-0.1476927	0.88315087	0.99978775	-4.6244716
UBTF	-0.0459407	6.22917893	-0.4886892	0.6270964	0.99978775	-4.8406041
TSEN34	-0.0459539	3.91558165	-0.2971369	0.76753415	0.99978775	-4.7818016
RANBP3	-0.0460045	6.04616647	-0.6688288	0.50653429	0.99978775	-4.7930144
FUS	-0.0460821	7.16934013	-0.2639414	0.7928581	0.99978775	-4.8726685
SEC11A	-0.0461392	6.1101687	-0.5863804	0.56013211	0.99978775	-4.8168572
GDF9	-0.046188	-0.4497231	-0.1005897	0.92025922	0.99978775	-4.6204243
RBM45	-0.0462442	4.69771888	-0.5091142	0.61280479	0.99978775	-4.7944369
RRP12	-0.0462487	6.10754727	-0.4144801	0.6802108	0.99978775	-4.8564133
ENSCAFG00000020291	-0.0462606	5.06694594	-0.3415617	0.73404375	0.99978775	-4.8392438
CLPX	-0.0462885	5.6120701	-0.3683466	0.7140955	0.99978775	-4.8502385
PARP1	-0.0462895	6.55896636	-0.4611246	0.64661343	0.99978775	-4.8479177
GET4	-0.0463122	4.53516388	-0.5093045	0.61267233	0.99978775	-4.7893462
SF3B3	-0.0463323	7.23131152	-0.5432768	0.58923799	0.99978775	-4.8218015
SCAF4	-0.0463353	5.27277017	-0.5272539	0.6002378	0.99978775	-4.8110101
RAB5B	-0.046409	4.69166224	-0.4771349	0.63524578	0.99978775	-4.7906575
MYH10	-0.0464364	6.92170295	-0.0820884	0.93488847	0.99978775	-4.8537685
LPCAT3	-0.0464679	5.17943705	-0.3361276	0.73811402	0.99978775	-4.8476468
CDS1	-0.0465309	-0.1225358	-0.0720244	0.94285613	0.99978775	-4.6234567
DCUN1D3	-0.0465403	3.03730649	-0.2386855	0.81227847	0.99978775	-4.6952206
ENSCAFG00000031732	-0.0465688	1.11196679	-0.1519553	0.87980445	0.99978775	-4.6398373
C15H12orf29	-0.0465923	4.20368437	-0.45243	0.65282271	0.99978775	-4.7666608
ELL	-0.0466501	4.53846433	-0.3485915	0.72878974	0.99978775	-4.7876486
ZBTB5	-0.0466724	2.53326055	-0.2620658	0.79429598	0.99978775	-4.6915567
DDX21	-0.0468502	5.4579242	-0.2943753	0.76963162	0.99978775	-4.8595753
NFIX	-0.0468669	7.68961347	-0.1734885	0.86293445	0.99978775	-4.8713607
SPP2	-0.04688	1.84458871	-0.1162483	0.90789915	0.99978775	-4.689819
GNB2	-0.0469677	7.26353518	-0.500858	0.61856394	0.99978775	-4.8309174
SH2B1	-0.0470324	4.38481936	-0.3130972	0.75544697	0.99978775	-4.8011228
EDF1	-0.0470327	5.98988768	-0.5504227	0.58436337	0.99978775	-4.8248221
ENSCAFG00000017208	-0.047093	5.46338257	-0.4384797	0.66283715	0.99978775	-4.8388783
ENSCAFG00000030428	-0.0471538	2.19114369	-0.27952	0.78094374	0.99978775	-4.6808306
MAPKAPK5	-0.0471661	4.3018883	-0.5309708	0.5976776	0.99978775	-4.7595246
ENSCAFG00000029259	-0.0471774	-0.1084564	-0.1671694	0.86787881	0.99978775	-4.62272
AFF3	-0.0472177	0.83354572	-0.1063581	0.91570352	0.99978775	-4.6976218
TOMM40	-0.0472345	4.9622326	-0.3000205	0.76534595	0.99978775	-4.8456997
SPECC1L	-0.0472471	6.4398234	-0.451215	0.65369239	0.99978775	-4.8498392
IFT80	-0.0472692	5.19707564	-0.409583	0.68377776	0.99978775	-4.8309571
WNT10B	-0.0473155	5.81470082	-0.466223	0.64298405	0.99978775	-4.8373552
SRSF5	-0.047361	6.32209934	-0.4620886	0.6459265	0.99978775	-4.8460779
GFM2	-0.0474033	4.22056372	-0.3585422	0.72137498	0.99978775	-4.7850807
MAGIX	-0.04742	3.59205788	-0.2211378	0.82584277	0.99978775	-4.7403678
NOL11	-0.0474606	4.95756575	-0.4802393	0.6330517	0.99978775	-4.8140784
EIF4EBP2	-0.0475515	4.170772	-0.3645578	0.71690548	0.99978775	-4.7836936
DNTTIP1	-0.0475556	4.36165152	-0.5683097	0.5722469	0.99978775	-4.7660211
TOP1	-0.0476163	6.11614447	-0.4295078	0.6693109	0.99978775	-4.8508148
FAM49B	-0.0476724	4.88543153	-0.3513987	0.72669529	0.99978775	-4.8351037

ENSCAFG00000018430	-0.0476737	3.69557805	-0.4320199	0.6674957	0.99978775	-4.7378244
REM1	-0.04768	5.15250792	-0.1367639	0.89174042	0.99978775	-4.8653087
DUSP8	-0.0477125	2.31561001	-0.1996509	0.84252468	0.99978775	-4.7093859
NUDT15	-0.0477164	2.56442176	-0.3163985	0.75295429	0.99978775	-4.6920808
DVL3	-0.0477325	7.41794646	-0.4280813	0.67034252	0.99978775	-4.8463369
SNAPC2	-0.0477975	3.80553927	-0.3638665	0.71741859	0.99978775	-4.7655762
POP4	-0.0478049	4.67297714	-0.3360626	0.73816274	0.99978775	-4.827357
STARD3	-0.0478238	5.33602135	-0.533036	0.5962573	0.99978775	-4.8155997
PPP2R5D	-0.0478749	5.94083233	-0.6079275	0.54585577	0.99978775	-4.8093358
DZIP3	-0.0478955	5.0850497	-0.4229575	0.67405332	0.99978775	-4.8219916
MBD1	-0.04793	4.43916022	-0.4794442	0.63361335	0.99978775	-4.7759043
ADCK5	-0.0479525	3.70231089	-0.3634779	0.7177071	0.99978775	-4.7578321
SLC25A30	-0.04798	3.59836693	-0.283748	0.77771923	0.99978775	-4.7630197
NIN	-0.0480208	5.2018592	-0.2537449	0.80068347	0.99978775	-4.8443184
EIF3D	-0.0480357	8.13144617	-0.5972004	0.55293993	0.99978775	-4.7945551
PEF1	-0.0480406	5.33090576	-0.6542525	0.51580429	0.99978775	-4.7854605
ZNF410	-0.0480712	4.12858846	-0.3860402	0.70102647	0.99978775	-4.7822328
GANC	-0.0481041	3.18588345	-0.2886192	0.77400904	0.99978775	-4.7237079
HKR1	-0.0481128	-1.2269175	-0.0981612	0.92217804	0.99978775	-4.6159191
DIAPH1	-0.0481149	7.0102465	-0.5633532	0.57559205	0.99978775	-4.822567
RFC1	-0.0481358	5.55278218	-0.5183215	0.60641111	0.99978775	-4.824851
RAB4B	-0.0481489	4.24786049	-0.3596437	0.72055582	0.99978775	-4.7840188
LACTB	-0.0483227	4.59737661	-0.2816767	0.77929848	0.99978775	-4.7996725
MRPL33	-0.0483403	4.63071902	-0.5807759	0.56387573	0.99978775	-4.7751211
ENSCAFG00000005006	-0.0483499	5.26950008	-0.3824778	0.70365066	0.99978775	-4.8300087
EIF6	-0.0484842	5.1862491	-0.380091	0.70541082	0.99978775	-4.8392996
NBAS	-0.0485001	6.53943235	-0.3774488	0.70736131	0.99978775	-4.8639201
PAPSS2	-0.048591	6.15172573	-0.1564551	0.87627425	0.99978775	-4.890157
RBPMS2	-0.0486	1.12648883	-0.2085344	0.83561846	0.99978775	-4.6685247
SMCR8	-0.0488438	3.81925917	-0.3998911	0.6908587	0.99978775	-4.7511736
XPO7	-0.0488652	6.39127876	-0.7323904	0.46718666	0.99978775	-4.7739019
GPRC5A	-0.0489107	4.83102176	-0.171157	0.86475808	0.99978775	-4.887433
PPP4R1	-0.0489251	5.92825322	-0.4655969	0.64342926	0.99978775	-4.845248
HPS4	-0.0490107	4.83874219	-0.5206021	0.60483215	0.99978775	-4.7947539
URB1	-0.0490364	4.2456038	-0.4002444	0.69060009	0.99978775	-4.767305
TCTN3	-0.0490697	3.35951319	-0.4865063	0.62863252	0.99978775	-4.7149462
SUPT16H	-0.0491236	6.60759211	-0.6077025	0.54600393	0.99978775	-4.8120852
MLEC	-0.0491266	4.75688898	-0.3837317	0.70272658	0.99978775	-4.8215717
MTIF2	-0.0491584	4.01858298	-0.393321	0.69567474	0.99978775	-4.7767402
MTOR	-0.0491977	7.16484121	-0.5138061	0.60954283	0.99978775	-4.8302348
PDCD2	-0.0492156	6.17654671	-0.612662	0.54274394	0.99978775	-4.8107145
CCDC90B	-0.0492192	5.49960556	-0.5006175	0.6187321	0.99978775	-4.8227458
TBC1D9B	-0.0492486	7.12196611	-0.7243258	0.47208053	0.99978775	-4.7740989
CDC42BPB	-0.0492616	7.41078008	-0.6633958	0.50997889	0.99978775	-4.7906551
PDXK	-0.0493123	6.53424349	-0.2882389	0.77429847	0.99978775	-4.8762179
DHX30	-0.0493589	6.37603325	-0.6169947	0.5399042	0.99978775	-4.8095023
GTF3C2	-0.0494931	5.85159707	-0.4319161	0.66757066	0.99978775	-4.8495556
PPM1B	-0.0495273	4.76686522	-0.532859	0.59637901	0.99978775	-4.7882843
ENSCAFG00000016246	-0.0495613	4.37562391	-0.4238078	0.67343694	0.99978775	-4.7822662
ANKS4B	-0.0496123	-0.7998727	-0.0935673	0.92580899	0.99978775	-4.6149701

SMYD3	-0.0496873	2.87423254	-0.3415658	0.73404071	0.99978775	-4.7052562
CD99L2	-0.0496877	1.68518234	-0.1997392	0.84245601	0.99978775	-4.6428682
TUBGCP3	-0.049773	5.40023538	-0.6777911	0.50087967	0.99978775	-4.7819254
PTRH2	-0.0498314	3.40228286	-0.3422849	0.73350267	0.99978775	-4.7349324
ANXA7	-0.0498855	6.95755856	-0.6727135	0.50407907	0.99978775	-4.7914179
ENSCAFG00000031488	-0.0498866	0.52765725	-0.2256302	0.82236483	0.99978775	-4.6461021
SEPSECS	-0.049899	4.19808444	-0.4271596	0.67100943	0.99978775	-4.779335
MAGEL2	-0.0499404	0.23433872	-0.0681902	0.94589322	0.99978775	-4.6215141
ILF2	-0.0499442	6.24938437	-0.3981852	0.69210794	0.99978775	-4.8602425
NFYC	-0.050003	4.18233193	-0.528605	0.59930654	0.99978775	-4.7605529
BLVRA	-0.0500241	5.41564232	-0.4600573	0.64737431	0.99978775	-4.8350911
CASP7	-0.0501277	4.13937493	-0.2531752	0.80112135	0.99978775	-4.8134817
TANGO6	-0.0501331	4.01849243	-0.5255336	0.60142439	0.99978775	-4.7545015
SLC1A3	-0.0501412	0.68154012	-0.0860244	0.93177417	0.99978775	-4.663221
ENSCAFG00000018511	-0.0501453	0.88746265	-0.156188	0.87648372	0.99978775	-4.6398933
LSM4	-0.0501463	2.23724706	-0.2736985	0.78538995	0.99978775	-4.6754844
ENSCAFG00000002916	-0.0501799	5.44751168	-0.2785461	0.7816871	0.99978775	-4.8658684
TMEM115	-0.0502339	4.6894153	-0.4445371	0.65848101	0.99978775	-4.7993287
COPZ1	-0.0502399	3.81206205	-0.4182181	0.67749298	0.99978775	-4.760577
ECHDC1	-0.0502509	4.72841184	-0.3809111	0.70480582	0.99978775	-4.8047543
PCDHB15	-0.0502752	0.56509441	-0.1075796	0.91473912	0.99978775	-4.6319729
GNMT	-0.050324	-1.1828202	-0.1200415	0.90490834	0.99978775	-4.6171416
CPSF3	-0.0503614	5.12179865	-0.6197319	0.53811408	0.99978775	-4.7820716
SESN3	-0.0503794	1.72240541	-0.108141	0.91429594	0.99978775	-4.6465146
NSUN2	-0.0504719	6.25396568	-0.449765	0.65473093	0.99978775	-4.8498349
SPRYD3	-0.0504982	5.47801777	-0.3455754	0.73104238	0.99978775	-4.8489993
SMARCA2	-0.0505442	7.39980186	-0.3289606	0.74349379	0.99978775	-4.8610308
UBOX5	-0.0505492	3.96147679	-0.515782	0.6081715	0.99978775	-4.7579719
PUDP	-0.0506153	4.08099912	-0.4383314	0.66294398	0.99978775	-4.7703635
ECI1	-0.050684	3.39112823	-0.2511247	0.80269774	0.99978775	-4.7462608
C1H18orf54	-0.0506962	3.42584846	-0.2803119	0.78033949	0.99978775	-4.7329032
TMX1	-0.050698	4.97767101	-0.4770061	0.6353369	0.99978775	-4.8119572
ZNF74	-0.0507193	2.85840207	-0.2247779	0.82302444	0.99978775	-4.7024786
GMDS	-0.0507472	3.32854343	-0.2527497	0.80144837	0.99978775	-4.7427994
UTRN	-0.0507762	8.46023842	-0.3692178	0.71344998	0.99978775	-4.8417475
BCKDK	-0.0507791	6.47731504	-0.6397932	0.52508855	0.99978775	-4.8029683
C9H9orf16	-0.0507985	4.41649888	-0.1705906	0.86520128	0.99978775	-4.8017864
ASCC1	-0.0508088	2.63883356	-0.3168867	0.7525859	0.99978775	-4.6927627
ADHFE1	-0.0508582	2.23196735	-0.1635664	0.87070038	0.99978775	-4.6654971
PDE2A	-0.0508668	-0.5519324	-0.0314034	0.9750669	0.99978775	-4.6121991
ENSCAFG00000031958	-0.0508886	4.67358672	-0.3374663	0.73711061	0.99978775	-4.8139441
USP31	-0.0509103	4.1089729	-0.3064791	0.76045175	0.99978775	-4.7810527
YPEL3	-0.0509195	4.08031301	-0.1477066	0.88313999	0.99978775	-4.7409796
PPIP5K2	-0.0509444	6.6122358	-0.4259036	0.67191862	0.99978775	-4.8541375
ENSCAFG00000031737	-0.0509622	-2.0849742	-0.0994346	0.92117184	0.99978775	-4.6142252
TMEM219	-0.0509838	3.80176679	-0.3688458	0.71372561	0.99978775	-4.7640672
TRMT2A	-0.0509967	5.44106798	-0.4166026	0.67866704	0.99978775	-4.8372001
RAB11FIP2	-0.0510164	5.60681757	-0.3048184	0.76170926	0.99978775	-4.8632908
CAPN2	-0.0510187	9.1736468	-0.5536305	0.58218138	0.99978775	-4.7770449
ALG12	-0.0510723	5.4700451	-0.2893126	0.77348133	0.99978775	-4.8615955

PRMT3	-0.0510869	6.86002904	-0.5233658	0.60292131	0.99978775	-4.8336075
ADAMTS4	-0.0511064	2.62042956	-0.1271883	0.89927713	0.99978775	-4.6957501
USP47	-0.0511182	7.18249552	-0.504214	0.61622001	0.99978775	-4.8352126
RREB1	-0.0511746	4.92415779	-0.4146081	0.68011764	0.99978775	-4.8183431
CFAP53	-0.0512251	0.9122199	-0.1872053	0.85222109	0.99978775	-4.6343909
USP48	-0.0512277	6.19743999	-0.4827842	0.63125551	0.99978775	-4.8421271
EIF3G	-0.0512654	6.4443459	-0.4508681	0.65394078	0.99978775	-4.8499867
ENSCAFG00000023527	-0.0512705	3.84495827	-0.415869	0.67920043	0.99978775	-4.7624835
ABHD8	-0.0512906	3.40851418	-0.3468211	0.73011165	0.99978775	-4.7376306
TAF3	-0.0513492	4.26458754	-0.5130855	0.61004332	0.99978775	-4.7668348
ENSCAFG00000014496	-0.0514389	3.27995492	-0.3579799	0.72179328	0.99978775	-4.7277005
CEP89	-0.0515219	4.37747412	-0.2648925	0.79212928	0.99978775	-4.8014656
ST3GAL4	-0.0515306	5.21973979	-0.3362971	0.73798694	0.99978775	-4.8567169
ZYX	-0.0515611	9.091933	-0.2987307	0.76632449	0.99978775	-4.8215948
ENSCAFG00000019273	-0.0515826	4.28983083	-0.4025431	0.68891836	0.99978775	-4.7819543
COL15A1	-0.0516031	9.11514194	-0.0922495	0.92685083	0.99978775	-4.8281563
REXO1	-0.0516238	4.94148435	-0.5400661	0.59143445	0.99978775	-4.7928224
TBC1D8B	-0.0516785	2.9475168	-0.2946267	0.76944061	0.99978775	-4.7267061
SLC4A7	-0.051684	5.56667474	-0.2223938	0.82487001	0.99978775	-4.8596142
RPS6	-0.0516854	8.04865449	-0.5328254	0.59640207	0.99978775	-4.8129922
ENOPH1	-0.0517546	5.13243688	-0.379974	0.70549714	0.99978775	-4.8426432
ERLIN1	-0.0517699	4.12591634	-0.3908307	0.69750345	0.99978775	-4.7660527
RAN	-0.051776	7.63851419	-0.4738056	0.63760254	0.99978775	-4.8292491
ZC3H10	-0.0518251	1.67576051	-0.2418216	0.80986023	0.99978775	-4.6473249
PIP4K2B	-0.0518348	6.63270142	-0.6934923	0.49105674	0.99978775	-4.786692
CHD1L	-0.0519199	4.76184559	-0.3865617	0.70064267	0.99978775	-4.8205453
ITGB3	-0.0519534	7.33057829	-0.159719	0.87371515	0.99978775	-4.8837905
SUN2	-0.0519697	7.08001777	-0.5926377	0.55596713	0.99978775	-4.8106934
ENSCAFG00000019644	-0.052044	7.64993428	-0.3229398	0.74802318	0.99978775	-4.8547976
BANF1	-0.0521457	4.5162012	-0.4081284	0.68483869	0.99978775	-4.810637
TOPBP1	-0.0522303	6.23043684	-0.4014719	0.68970183	0.99978775	-4.8598633
SMN	-0.0522454	5.1569911	-0.5502092	0.58450873	0.99978775	-4.8038706
RALA	-0.0522535	5.67713765	-0.3896496	0.69837143	0.99978775	-4.8513878
ENSCAFG00000007479	-0.0522812	6.2021243	-0.7217958	0.47362176	0.99978775	-4.7761859
CPT1C	-0.0522943	3.80474827	-0.1540857	0.87813281	0.99978775	-4.7234981
ATG9A	-0.0523374	5.85524114	-0.4799097	0.6332845	0.99978775	-4.838656
TSC2	-0.0523641	6.44352892	-0.5088268	0.61300488	0.99978775	-4.8374001
LMAN2	-0.0523668	7.14960831	-0.6000296	0.55106703	0.99978775	-4.8097211
YRDC	-0.0523995	4.48783679	-0.4489261	0.65533212	0.99978775	-4.792931
GNL3	-0.0524204	5.66530894	-0.4116536	0.6822687	0.99978775	-4.845453
DDX10	-0.0524228	4.71961301	-0.4889772	0.62689385	0.99978775	-4.788778
THOC2	-0.0524331	5.95947549	-0.4720785	0.63882658	0.99978775	-4.8415099
TMEM218	-0.0524566	3.84669712	-0.270286	0.78799963	0.99978775	-4.7827523
AGPAT5	-0.0525193	5.91006272	-0.63369	0.52903361	0.99978775	-4.8006312
ENSCAFG00000006238	-0.0525367	3.42508828	-0.3437439	0.73241141	0.99978775	-4.7346668
SYNE1	-0.0525805	7.16689863	-0.2864157	0.77568666	0.99978775	-4.8766344
CBR4	-0.0525822	1.34099887	-0.1802901	0.85761876	0.99978775	-4.6453497
KATNAL1	-0.0525897	2.39224359	-0.2022991	0.84046456	0.99978775	-4.6919919
CGGBP1	-0.0526491	4.61214894	-0.5319589	0.59699787	0.99978775	-4.7821856
CIPC	-0.052673	3.10540361	-0.3666278	0.71536977	0.99978775	-4.7003699

PIN1	-0.0526845	4.0680618	-0.4439326	0.65891525	0.99978775	-4.7673059
DDX55	-0.0527282	4.18146245	-0.4627096	0.64548418	0.99978775	-4.7668756
CSKMT	-0.0527373	2.68744415	-0.2569065	0.79825483	0.99978775	-4.7092602
FARP2	-0.0527636	3.87353387	-0.3783047	0.70672925	0.99978775	-4.7509047
ENSCAFG00000012457	-0.0528676	2.18607357	-0.2358651	0.81445482	0.99978775	-4.6812366
AGTPBP1	-0.0528787	4.10681222	-0.4793462	0.63368258	0.99978775	-4.755923
PARVA	-0.0529514	6.82703924	-0.5209053	0.60462236	0.99978775	-4.8345213
MRPL49	-0.052967	3.90817004	-0.4246437	0.67283117	0.99978775	-4.7665072
MAP3K12	-0.0529857	4.56235642	-0.3695376	0.71321306	0.99978775	-4.7946059
KCTD15	-0.0530475	4.65848396	-0.3688011	0.71375869	0.99978775	-4.838776
SHF	-0.0531202	1.52911749	-0.2483753	0.80481276	0.99978775	-4.7132761
DNAJC21	-0.0531421	3.87582774	-0.5126376	0.61035449	0.99978775	-4.7464594
GLI3	-0.0531451	5.11045926	-0.1415809	0.8879528	0.99978775	-4.8415824
WASHC4	-0.0531767	5.55753391	-0.4893002	0.62666675	0.99978775	-4.8273159
ENSCAFG00000011368	-0.0532554	3.20542114	-0.1592058	0.87411745	0.99978775	-4.7644241
GGN	-0.053259	2.66194882	-0.3024255	0.76352236	0.99978775	-4.7245598
CMPK1	-0.0532608	5.58153888	-0.4449765	0.65816546	0.99978775	-4.8392305
ZMYM1	-0.0533891	4.27566929	-0.4174761	0.67803213	0.99978775	-4.7779328
ENSCAFG00000014234	-0.0534606	0.0077134	-0.1781344	0.8593028	0.99978775	-4.6331245
OPHN1	-0.0535137	2.52692602	-0.2843196	0.7772836	0.99978775	-4.68728
KAZN	-0.0536343	1.74071269	-0.2300806	0.81892305	0.99978775	-4.6685945
COMMD9	-0.0536787	4.62655932	-0.5439483	0.5887791	0.99978775	-4.788455
PTPN4	-0.0536824	2.5161631	-0.2971949	0.76749015	0.99978775	-4.701181
NCBP1	-0.0537017	6.413071	-0.6307873	0.53091537	0.99978775	-4.8056418
WNK3	-0.0537039	4.78125389	-0.4884967	0.62723178	0.99978775	-4.7892814
THOC5	-0.0537151	6.56509756	-0.8062179	0.42375321	0.99978775	-4.7482659
RSRC2	-0.053786	5.44427993	-0.5228991	0.60324379	0.99978775	-4.8156977
ZNF609	-0.0537941	4.9720912	-0.4203872	0.6759179	0.99978775	-4.8076387
FPGT	-0.0538354	3.42217671	-0.3553166	0.72377559	0.99978775	-4.7400169
MINDY2	-0.0539356	2.77168284	-0.1945794	0.84647303	0.99978775	-4.6770688
L3MBTL4	-0.0539523	-0.2610959	-0.1367383	0.89176054	0.99978775	-4.6318634
PRRC2A	-0.0540237	8.62735788	-0.836092	0.40689143	0.99978775	-4.7120133
KIAA1143	-0.0540694	2.51036572	-0.3589259	0.72108962	0.99978775	-4.6811978
PPP2R5C	-0.0541479	6.87090708	-0.7855332	0.43567094	0.99978775	-4.7556155
NDUFB4	-0.0541479	4.61554657	-0.4849678	0.62971609	0.99978775	-4.8013735
DNAJC7	-0.0541697	4.98027909	-0.528024	0.5997069	0.99978775	-4.7939209
SSR3	-0.0542209	5.07958901	-0.3816113	0.70428943	0.99978775	-4.8342684
ETHE1	-0.0543231	5.03987361	-0.2497126	0.80378386	0.99978775	-4.8515762
CA11	-0.0543727	1.42293215	-0.0988892	0.92160277	0.99978775	-4.6288367
TINF2	-0.0543793	4.44705372	-0.4089388	0.68424755	0.99978775	-4.8043179
ARHGEF37	-0.0543975	1.81848531	-0.1897035	0.85027286	0.99978775	-4.7506838
EPS8	-0.0544468	8.30256924	-0.2553786	0.79942824	0.99978775	-4.8564647
TBL3	-0.0544559	5.65364242	-0.6356654	0.52775505	0.99978775	-4.7997761
AASS	-0.0544863	-2.3218381	-0.0921225	0.92695122	0.99978775	-4.6082602
RALGPS1	-0.0545004	2.02011896	-0.3059375	0.76086177	0.99978775	-4.6790454
ODF2L	-0.054509	1.33904291	-0.1991208	0.84293723	0.99978775	-4.6549295
SLC44A1	-0.0545221	5.74263896	-0.21973	0.82693334	0.99978775	-4.8752793
ZC3H3	-0.0545651	4.32260341	-0.4502172	0.654407	0.99978775	-4.7722509
MTFMT	-0.0546233	2.80009463	-0.2759182	0.78369382	0.99978775	-4.6934721
PPP4R3B	-0.0546324	6.19524458	-0.723663	0.47248402	0.99978775	-4.775959

CDKL5	-0.0546557	1.48062851	-0.2477331	0.80530703	0.99978775	-4.6482517
ADD3	-0.0546877	6.16854452	-0.1614008	0.87239707	0.99978775	-4.8620796
C4H5orf51	-0.05469	6.7787239	-0.5000178	0.61915143	0.99978775	-4.8376396
OAS1	-0.0547128	3.75831191	-0.3981233	0.69215325	0.99978775	-4.7715043
NUFIP1	-0.0547297	3.72244504	-0.3728481	0.71076218	0.99978775	-4.7612829
FAM91A1	-0.0547542	4.9999408	-0.4709013	0.63966147	0.99978775	-4.8148957
GID4	-0.0547727	4.58554693	-0.4597414	0.64759956	0.99978775	-4.7764094
CDK5RAP3	-0.0548013	5.06248003	-0.5283325	0.59949432	0.99978775	-4.8048895
CDH5	-0.054837	0.5517619	-0.0186842	0.98516387	0.99978775	-4.6141447
SERTAD1	-0.0548396	4.01550364	-0.28927	0.77351374	0.99978775	-4.7792961
REPS1	-0.0548425	4.61919233	-0.5652961	0.57427965	0.99978775	-4.7680557
SRP9	-0.0549123	3.90443609	-0.4179962	0.6776542	0.99978775	-4.7721509
IGSF8	-0.0549463	5.18659918	-0.2658873	0.7913671	0.99978775	-4.8547983
PGP	-0.054984	3.57412234	-0.3261874	0.74557894	0.99978775	-4.7471073
DGKQ	-0.0549846	2.90786256	-0.2675082	0.79012578	0.99978775	-4.7040087
KIF16B	-0.0551265	6.28533673	-0.4836479	0.63064641	0.99978775	-4.8419843
RAD21	-0.0551327	7.30420961	-0.3970821	0.69291618	0.99978775	-4.8557061
TMEM42	-0.0551804	0.11846557	-0.1383982	0.89045502	0.99978775	-4.6244399
CMSS1	-0.0552086	3.05903806	-0.2565092	0.79855995	0.99978775	-4.7277005
POLR2E	-0.0552163	5.07575481	-0.4996993	0.61937418	0.99978775	-4.8221083
CRTC3	-0.0553417	5.47220995	-0.4977402	0.62074511	0.99978775	-4.8233621
TMEM185B	-0.0553719	4.25751482	-0.4185661	0.67724021	0.99978775	-4.7852046
ENSCAFG00000002360	-0.0553773	2.58706407	-0.3070027	0.76005544	0.99978775	-4.681448
CHD4	-0.0554222	8.0447834	-0.6672954	0.50750523	0.99978775	-4.7807813
LZIC	-0.0555134	5.31614624	-0.5579979	0.57921702	0.99978775	-4.8041466
ARL4D	-0.055514	2.43365085	-0.1388466	0.89010247	0.99978775	-4.6673226
EHBP1L1	-0.0555697	7.86209658	-0.3156916	0.75348785	0.99978775	-4.8581806
NDST2	-0.0557438	4.73049659	-0.3074437	0.75972162	0.99978775	-4.8213853
DPH5	-0.0557502	4.32524472	-0.4370065	0.6638984	0.99978775	-4.7839272
SIK2	-0.0559375	4.25131	-0.4291009	0.66960509	0.99978775	-4.7727274
TMEM214	-0.0560652	7.67718783	-0.5139162	0.60946637	0.99978775	-4.8228077
FZD1	-0.0560838	2.72469032	-0.2064106	0.83726842	0.99978775	-4.8048842
PPM1A	-0.0561124	5.09230581	-0.6753175	0.50243691	0.99978775	-4.770248
TAF6	-0.0561287	5.47427736	-0.7816061	0.43795585	0.99978775	-4.747902
CDC26	-0.0561414	2.56177606	-0.3628017	0.71820923	0.99978775	-4.6840964
XPC	-0.0562898	4.51372865	-0.4342348	0.66589694	0.99978775	-4.783261
SHMT1	-0.0563883	1.39539234	-0.1215069	0.90375328	0.99978775	-4.6513968
DDHD2	-0.0564828	5.22366071	-0.5921215	0.55631009	0.99978775	-4.7836716
EPN2	-0.0564867	4.7143847	-0.2544296	0.80015732	0.99978775	-4.7922349
GLT8D2	-0.0564996	4.70541704	-0.1373103	0.89131063	0.99978775	-4.810606
CRIPT	-0.0565703	2.67596727	-0.3581255	0.72168493	0.99978775	-4.6890876
SWI5	-0.0565791	3.59086345	-0.4343415	0.66581994	0.99978775	-4.7271816
ENSCAFG00000002230	-0.0565871	1.24871086	-0.0789908	0.93734016	0.99978775	-4.6286178
NUDT14	-0.0567425	1.80817802	-0.2110633	0.8336548	0.99978775	-4.6606153
OTULIN	-0.0567557	3.86564053	-0.5535822	0.58221425	0.99978775	-4.7408582
ZNF432	-0.0567687	2.05863324	-0.1669707	0.86803438	0.99978775	-4.6635559
MCCC1	-0.0568204	4.76616308	-0.4182209	0.67749096	0.99978775	-4.8076382
IREB2	-0.0568296	5.92769881	-0.6782272	0.50060541	0.99978775	-4.7897038
PSMB5	-0.0568373	6.18110702	-0.5703434	0.57087712	0.99978775	-4.8222232
SRSF3	-0.0568397	5.82501238	-0.4424403	0.65998757	0.99978775	-4.8490196

ENSCAFG00000009894	-0.0568456	1.03312166	-0.163889	0.8704477	0.99978775	-4.6559031
SLC11A1	-0.0568777	1.04819114	-0.1530579	0.87893923	0.99978775	-4.6398973
TMEM98	-0.0568791	4.59167402	-0.367499	0.71472377	0.99978775	-4.8286363
TUBB4B	-0.057027	7.86506786	-0.3338125	0.73985042	0.99978775	-4.8475874
CHP1	-0.0570301	5.23916683	-0.5279237	0.59977604	0.99978775	-4.8032922
EXOC5	-0.0570342	5.72388323	-0.4575808	0.64914123	0.99978775	-4.8393417
AP1S1	-0.0570593	4.62789444	-0.6148562	0.54130485	0.99978775	-4.76313
ABCD1	-0.0570711	3.96358385	-0.4334187	0.66648583	0.99978775	-4.7742452
DXO	-0.0571133	4.10855667	-0.5195729	0.6055445	0.99978775	-4.7561122
ATXN7L2	-0.0571787	2.45933614	-0.2499257	0.80361991	0.99978775	-4.6707447
NSD3	-0.0572814	5.78418854	-0.6463966	0.52083762	0.99978775	-4.7923864
COX5B	-0.057292	5.55981593	-0.5842688	0.5615411	0.99978775	-4.8075129
POLR3F	-0.0574225	3.35545354	-0.4455617	0.65774539	0.99978775	-4.7440978
ZNF423	-0.0574236	3.0811471	-0.1550041	0.87741234	0.99978775	-4.7333008
RFC3	-0.0574435	2.66177747	-0.1991602	0.84290655	0.99978775	-4.7387873
FAM102B	-0.0574912	4.42169315	-0.2659028	0.79135523	0.99978775	-4.8115437
NANS	-0.0575418	5.23882942	-0.4403448	0.66149461	0.99978775	-4.8304623
ASL	-0.0575979	4.09338672	-0.3915988	0.69693919	0.99978775	-4.7707826
OLFML1	-0.0577509	0.48674733	-0.1604694	0.87312701	0.99978775	-4.7119975
SCAP	-0.0577546	6.09421157	-0.4812223	0.63235761	0.99978775	-4.8419723
PAQR6	-0.0578427	1.21924244	-0.0992678	0.92130364	0.99978775	-4.6360749
PRKAR1A	-0.0579499	7.39008282	-0.7454301	0.45933524	0.99978775	-4.7641505
XPOT	-0.0580049	8.34740268	-0.5564856	0.58024269	0.99978775	-4.7915205
ENSCAFG00000026428	-0.0580073	-0.0840418	-0.1608417	0.87283525	0.99978775	-4.6216672
ATAD3A	-0.0580493	5.28586916	-0.4088797	0.68429063	0.99978775	-4.8356914
NRG2	-0.0580731	-1.1359316	-0.085139	0.93247466	0.99978775	-4.6139357
ANKRD39	-0.0580808	3.25338967	-0.3755053	0.70879724	0.99978775	-4.7233353
THAP11	-0.0581089	3.38426449	-0.3924891	0.69628545	0.99978775	-4.7413729
THBS3	-0.0581661	7.4731549	-0.23489	0.81520759	0.99978775	-4.8710515
ERI2	-0.0582578	3.32902321	-0.4183488	0.677398	0.99978775	-4.726419
RPAP1	-0.058302	5.28487556	-0.667754	0.50721472	0.99978775	-4.7778159
PIBF1	-0.058329	3.86887635	-0.4561432	0.65016784	0.99978775	-4.745669
ENSCAFG00000010867	-0.0583622	5.58822349	-0.850742	0.39877517	0.99978775	-4.7214294
CDC5L	-0.0583903	6.44615637	-0.7094948	0.48115587	0.99978775	-4.7815984
INTS7	-0.0584868	4.56783417	-0.3624866	0.71844321	0.99978775	-4.8081009
ZNF143	-0.0584939	3.2588663	-0.5160408	0.607992	0.99978775	-4.7009843
ENSCAFG00000009917	-0.0585153	4.04687434	-0.4879563	0.62761199	0.99978775	-4.7671432
GMPPB	-0.0585181	4.73637727	-0.4214113	0.67517468	0.99978775	-4.8185193
ENSCAFG00000013040	-0.0585654	4.24007992	-0.3648046	0.71672232	0.99978775	-4.7816225
HSPA2	-0.0585692	3.16340862	-0.1517405	0.87997302	0.99978775	-4.7273334
ENSCAFG00000010854	-0.0585706	0.09227171	-0.1677291	0.86744064	0.99978775	-4.6240293
PXN	-0.0585903	8.15478685	-0.3705335	0.7124754	0.99978775	-4.8426956
E2F4	-0.0589376	4.29938514	-0.453116	0.65233191	0.99978775	-4.7722079
STRAP	-0.0589782	7.21180916	-0.5706438	0.57067492	0.99978775	-4.8168984
LRWD1	-0.0590255	4.32041622	-0.5079048	0.61364686	0.99978775	-4.7749441
NCKIPSD	-0.0590282	3.44669536	-0.3682997	0.71413028	0.99978775	-4.719779
MRPL39	-0.0590323	5.01749524	-0.5893173	0.55817532	0.99978775	-4.787081
ENPEP	-0.0590539	1.93058688	-0.2128841	0.83224163	0.99978775	-4.8219986
FAM207A	-0.0591071	2.32029373	-0.3105394	0.75738002	0.99978775	-4.68066
ZNF235	-0.0592042	2.11633998	-0.2792372	0.78115958	0.99978775	-4.6641513

SETD2	-0.0592285	6.36041439	-0.6812988	0.49867591	0.99978775	-4.7900206
KLC2	-0.0592608	4.14770392	-0.5812083	0.56358643	0.99978775	-4.768381
ZMIZ1	-0.0592633	6.25374722	-0.4080429	0.68490113	0.99978775	-4.856677
PRPSAP2	-0.0593716	2.54216104	-0.2483398	0.80484007	0.99978775	-4.6820464
ARHGEF19	-0.0593947	1.3026585	-0.1596202	0.87379265	0.99978775	-4.6444354
ITPKB	-0.0594303	3.42511365	-0.220128	0.82662496	0.99978775	-4.7720194
HAUS6	-0.0594304	4.39704899	-0.4335703	0.66637637	0.99978775	-4.7958571
SSBP4	-0.0595281	4.88668871	-0.3752229	0.70900595	0.99978775	-4.8087461
FNIP1	-0.0595532	4.94459979	-0.5000815	0.61910686	0.99978775	-4.8034665
PSMD13	-0.0597112	6.61060357	-0.7110056	0.48022692	0.99978775	-4.7810796
FBXL6	-0.0597132	3.74625921	-0.4445523	0.6584701	0.99978775	-4.7448118
SYMPK	-0.0597322	5.89879488	-0.7372462	0.46425396	0.99978775	-4.7682325
PTK2	-0.0598369	6.8038169	-0.4599425	0.64745619	0.99978775	-4.8445351
SHPRH	-0.0598812	4.72830045	-0.5262505	0.60092974	0.99978775	-4.7869147
C19H4orf33	-0.059935	3.95503232	-0.4641273	0.6444749	0.99978775	-4.7585244
ABHD10	-0.0599856	3.14590787	-0.3002864	0.76514425	0.99978775	-4.7263777
DDX27	-0.0599869	5.78607079	-0.5323405	0.59673549	0.99978775	-4.8248468
TMEM167A	-0.0600032	3.5274749	-0.403802	0.68799796	0.99978775	-4.7356484
TATDN3	-0.0600148	3.30472945	-0.3893694	0.69857742	0.99978775	-4.7216704
PHAX	-0.0600194	3.29340783	-0.4081505	0.6848226	0.99978775	-4.7249024
ENSCAFG00000032146	-0.0600564	4.62632201	-0.4444569	0.65853865	0.99978775	-4.7994969
CLIC1	-0.0601015	7.10338485	-0.4586573	0.64837294	0.99978775	-4.8425693
AIFM2	-0.0601032	5.09920153	-0.4057853	0.68654905	0.99978775	-4.833585
ITPK1	-0.060105	4.58218485	-0.5639659	0.57517806	0.99978775	-4.7710283
GPX8	-0.0601394	5.89287907	-0.408233	0.68476238	0.99978775	-4.8566414
TRIM37	-0.0601803	5.37351576	-0.4851883	0.62956074	0.99978775	-4.8258295
SMARCAD1	-0.0601843	4.47740032	-0.3856733	0.70129657	0.99978775	-4.7898038
ENSCAFG00000016140	-0.0602015	5.13030719	-0.5402722	0.59129339	0.99978775	-4.8058537
COMMD4	-0.0602199	4.40055782	-0.575946	0.56711186	0.99978775	-4.774276
NDUFA13	-0.0602232	4.79355268	-0.5149732	0.60873263	0.99978775	-4.7919301
COBLL1	-0.0602367	6.83886529	-0.2417826	0.80989027	0.99978775	-4.8738193
TET3	-0.0602399	3.53232645	-0.2618409	0.79446844	0.99978775	-4.7268245
OGFRL1	-0.0602507	4.01559428	-0.4278355	0.67052037	0.99978775	-4.7516794
SHC1	-0.0603202	8.24841364	-0.4710002	0.6395913	0.99978775	-4.8298694
ZNF771	-0.0603385	2.02279361	-0.2707439	0.78764936	0.99978775	-4.6691198
MRPS10	-0.0603595	3.2414652	-0.3233851	0.74768786	0.99978775	-4.7242678
IQCE	-0.0603705	4.19914692	-0.5946221	0.55464953	0.99978775	-4.7460223
RABEP2	-0.0604334	4.53473115	-0.5275049	0.60006474	0.99978775	-4.7873147
DENND2C	-0.0605501	4.56542282	-0.3584011	0.72147993	0.99978775	-4.8236396
TCF7L2	-0.0606119	5.06138942	-0.2829995	0.77828977	0.99978775	-4.8384387
USF1	-0.0608044	4.65557075	-0.7254456	0.47139923	0.99978775	-4.7364115
ZNF581	-0.0608217	0.30337018	-0.1811508	0.85694657	0.99978775	-4.6286147
IRF3	-0.060837	6.0756129	-0.3210909	0.74941589	0.99978775	-4.8607006
TRDMT1	-0.0608451	2.93376803	-0.3587698	0.72120569	0.99978775	-4.717599
ZWILCH	-0.0609054	3.50035111	-0.2811726	0.77968295	0.99978775	-4.7673984
TFE3	-0.0609063	6.50459651	-0.5028717	0.61715707	0.99978775	-4.838802
SCAF11	-0.0609318	6.73920228	-0.5412162	0.59064722	0.99978775	-4.8290874
SLC7A6	-0.0609511	3.83137304	-0.365806	0.71597927	0.99978775	-4.7736398
SMARCA5	-0.0609687	7.23445739	-0.4854323	0.62938885	0.99978775	-4.8370598
TMEM260	-0.0610107	5.04780914	-0.4353229	0.66511202	0.99978775	-4.8124082

RPS8	-0.0610713	9.08164692	-0.660147	0.51204471	0.99978775	-4.7562837
RPRD2	-0.0612435	4.97777483	-0.5823571	0.56281827	0.99978775	-4.7780711
TARBP1	-0.061252	4.44778479	-0.3972462	0.6927959	0.99978775	-4.795675
NCAPH2	-0.0613113	5.77320293	-0.5091912	0.6127512	0.99978775	-4.8315244
TADA2B	-0.0613148	2.38079917	-0.4125893	0.68158719	0.99978775	-4.6763723
AKT2	-0.0613275	5.28615221	-0.4463578	0.657174	0.99978775	-4.8256786
ENSCAFG00000018717	-0.0613362	5.62216824	-0.5517593	0.5834537	0.99978775	-4.81837
PLRG1	-0.0613606	5.35089985	-0.8324965	0.40889876	0.99978775	-4.7249412
MAPK10	-0.061366	-1.2028343	-0.0660773	0.94756721	0.99978775	-4.608613
UTP15	-0.0613689	4.33097734	-0.5244158	0.60219605	0.99978775	-4.7744652
XPO4	-0.0613733	4.31720728	-0.4876721	0.62781194	0.99978775	-4.7708969
CRNKL1	-0.0615474	5.24773366	-0.6137731	0.54201496	0.99978775	-4.7845021
TBC1D7	-0.0615479	4.64335564	-0.5802981	0.56419541	0.99978775	-4.7765787
THNSL1	-0.0615873	3.67289184	-0.3852239	0.70162749	0.99978775	-4.754856
ENSCAFG00000013185	-0.0616673	3.24480633	-0.2833216	0.77804425	0.99978775	-4.7370527
GNG11	-0.0616844	3.55030932	-0.1774145	0.85986536	0.99978775	-4.8232463
ZNF532	-0.0617215	5.70244743	-0.390606	0.69766854	0.99978775	-4.8373416
RPL7A	-0.0617697	9.65991161	-0.5708302	0.57054948	0.99978775	-4.7674838
COPG2	-0.0617997	4.59861522	-0.7159433	0.47719792	0.99978775	-4.7334874
TNKS	-0.0618112	4.86462998	-0.5978127	0.55253435	0.99978775	-4.7779835
ENSCAFG00000006508	-0.0618422	3.9477721	-0.4408933	0.66110001	0.99978775	-4.7535497
ARFIP2	-0.0618668	4.45913925	-0.5714105	0.57015901	0.99978775	-4.7626388
USF3	-0.0619945	4.99764106	-0.5731966	0.56895809	0.99978775	-4.7856619
TSPO	-0.0620083	5.70871741	-0.347698	0.7294568	0.99978775	-4.8665145
OAT	-0.0620293	6.09762402	-0.468239	0.6415514	0.99978775	-4.8388249
SCN3A	-0.0620456	0.71326076	-0.088602	0.92973529	0.99978775	-4.658291
DCTN1	-0.0620496	7.94628318	-0.8980943	0.37323457	0.99978775	-4.7013238
SEC16A	-0.0620759	7.38438859	-0.7883057	0.43406205	0.99978775	-4.7467734
POLR2A	-0.0620847	7.33078357	-0.7468637	0.45847672	0.99978775	-4.763287
REXO2	-0.0620987	5.66017811	-0.4445163	0.65849596	0.99978775	-4.8397208
RPL11	-0.0621323	8.02448102	-0.6031387	0.54901256	0.99978775	-4.7964623
MED17	-0.0621763	5.25176333	-0.7743917	0.44217184	0.99978775	-4.7476634
TSSC4	-0.0622282	3.98242255	-0.5884385	0.55876044	0.99978775	-4.7538824
ENSCAFG00000019817	-0.0622334	5.82694266	-0.6923677	0.49175673	0.99978775	-4.7814296
ENSCAFG00000030348	-0.0622548	1.65060729	-0.3756335	0.70870248	0.99978775	-4.6477866
UBXN8	-0.0622868	4.20450563	-0.6391169	0.52552493	0.99978775	-4.7288069
ENSCAFG00000017143	-0.0623272	9.17185137	-0.5932195	0.55558067	0.99978775	-4.763216
ZFPL1	-0.0624356	5.59706778	-0.6274413	0.53308879	0.99978775	-4.7993935
NEPRO	-0.0624372	3.87854085	-0.6144895	0.5415452	0.99978775	-4.7204415
AGAP3	-0.0624405	4.56169113	-0.3913523	0.69712026	0.99978775	-4.7822422
PPP1R15B	-0.0624533	6.30257087	-0.6754895	0.50232851	0.99978775	-4.7923195
KIAA2026	-0.0625122	4.31578679	-0.5289921	0.59903989	0.99978775	-4.7491488
NT5C	-0.0625535	4.12400491	-0.4022965	0.68909866	0.99978775	-4.7929238
RNF219	-0.0625561	4.70263011	-0.4763935	0.63577025	0.99978775	-4.8034174
TRAM1L1	-0.0626263	2.57869805	-0.3112912	0.75681164	0.99978775	-4.6955189
ACAP2	-0.0627722	6.04083014	-0.6813785	0.49862591	0.99978775	-4.7877286
SETX	-0.0627908	5.95201212	-0.6457438	0.52125705	0.99978775	-4.7920105
FAM20C	-0.0627918	7.81801694	-0.3527383	0.72569651	0.99978775	-4.8510231
TAPBPL	-0.0627993	2.42126308	-0.2253499	0.82258177	0.99978775	-4.6839589
SHARPIN	-0.0628052	5.43847539	-0.5133253	0.60987673	0.99978775	-4.8266101

FASTKD2	-0.0628266	4.04172717	-0.5047202	0.61586681	0.99978775	-4.7611986
HNRNPAB	-0.0628359	5.96193744	-0.49175	0.62494536	0.99978775	-4.8392102
RNPS1	-0.0628645	6.07710643	-0.6015336	0.55007274	0.99978775	-4.8128087
STK33	-0.0628805	2.31350186	-0.3855788	0.70136614	0.99978775	-4.6839326
SRRD	-0.0629015	2.60698866	-0.4070272	0.6856423	0.99978775	-4.6790206
ENSCAFG00000028760	-0.0629048	4.15723492	-0.4431972	0.65944358	0.99978775	-4.7802979
CDC42EP1	-0.0629173	5.14345844	-0.2935291	0.77027472	0.99978775	-4.8681895
NFATC2IP	-0.0629283	4.97603993	-0.7195782	0.47497506	0.99978775	-4.756911
G6PC3	-0.0630009	4.78179424	-0.5281547	0.59961683	0.99978775	-4.7924735
SPAG9	-0.0630263	6.82195226	-0.5780235	0.56571878	0.99978775	-4.8192542
EIF3A	-0.0630434	9.08256673	-0.6626979	0.51042227	0.99978775	-4.7547314
DNAJB14	-0.063051	3.12751949	-0.3921069	0.69656608	0.99978775	-4.7209242
GTF2IRD1	-0.063063	2.91187499	-0.3777926	0.70710741	0.99978775	-4.737296
IFT52	-0.0631942	4.15109769	-0.4622183	0.6458341	0.99978775	-4.7672006
BLOC1S4	-0.0631943	2.63172754	-0.2866035	0.77554364	0.99978775	-4.7005241
BAG6	-0.0632142	7.76126174	-0.7944806	0.43049156	0.99978775	-4.7398569
RPL23	-0.0633539	7.71134339	-0.4886146	0.62714884	0.99978775	-4.8309071
IFT81	-0.0633626	3.38073875	-0.3185248	0.75135029	0.99978775	-4.7283359
TRRAP	-0.063415	6.35395015	-0.6284969	0.53240262	0.99978775	-4.8056976
TNPO3	-0.0634365	6.83250484	-0.8456084	0.40160776	0.99978775	-4.7316934
MED20	-0.0634367	2.55333937	-0.4758464	0.63615741	0.99978775	-4.6692233
OXSR1	-0.0634582	6.73778408	-0.7750923	0.44176138	0.99978775	-4.7585616
SLC38A2	-0.0634814	8.91012585	-0.4973267	0.62103461	0.99978775	-4.7983786
SPOP	-0.0634861	5.97422167	-0.56588	0.57388558	0.99978775	-4.8182335
UTP18	-0.0634989	4.6874609	-0.6236155	0.53557957	0.99978775	-4.768846
NPC2	-0.0635225	6.6891091	-0.2827615	0.77847121	0.99978775	-4.8782844
AKAP8L	-0.0635969	4.79628163	-0.672827	0.50400741	0.99978775	-4.7490447
ENSCAFG00000005319	-0.0636725	0.22724766	-0.1925957	0.84801853	0.99978775	-4.6700259
NDUFB11	-0.0637708	4.41772913	-0.5212999	0.60434942	0.99978775	-4.7716577
RRAGC	-0.0638285	6.78313409	-0.5896926	0.55792551	0.99978775	-4.8150143
DDX50	-0.0639026	5.9582824	-0.6712852	0.50498105	0.99978775	-4.7897783
CDK2AP2	-0.063945	3.44044772	-0.4663471	0.64289587	0.99978775	-4.7260921
ENSCAFG00000029044	-0.0639891	3.87604029	-0.5056779	0.61519887	0.99978775	-4.7512059
EXOSC1	-0.0640249	3.00226287	-0.5735128	0.56874561	0.99978775	-4.6963735
C5H1orf123	-0.0640605	3.56857811	-0.4315094	0.66786443	0.99978775	-4.7345071
KAT5	-0.0641495	4.88290664	-0.6628113	0.51035025	0.99978775	-4.7600465
ENSCAFG00000022269	-0.0641873	1.03777044	-0.2417892	0.80988523	0.99978775	-4.6453962
ZNF318	-0.0642055	5.48850876	-0.5981225	0.55232917	0.99978775	-4.8020126
PTAR1	-0.0642221	3.14484738	-0.2857883	0.77616453	0.99978775	-4.7045743
GAS8	-0.0642505	2.77798814	-0.3822444	0.70382265	0.99978775	-4.6838146
UBTD1	-0.0643088	3.95813535	-0.3056221	0.76110062	0.99978775	-4.7866879
ZSWIM4	-0.0643504	4.77554241	-0.262258	0.79414859	0.99978775	-4.8242972
THRAP3	-0.0644219	7.84511564	-0.753917	0.4542663	0.99978775	-4.7538725
THRA	-0.0644294	3.64739781	-0.3599947	0.72029489	0.99978775	-4.7400043
TM2D3	-0.0644534	3.1339937	-0.3588656	0.72113442	0.99978775	-4.7193217
PSMB1	-0.064454	6.57716184	-0.687491	0.49479863	0.99978775	-4.7884002
PRCP	-0.0644637	4.76956828	-0.336517	0.73782212	0.99978775	-4.8233538
C3H5orf30	-0.0644813	2.57796853	-0.3767645	0.70786674	0.99978775	-4.6950619
ETV1	-0.0644949	5.26085838	-0.3766501	0.70795129	0.99978775	-4.8178532
ACBD4	-0.0645925	2.65175038	-0.2721295	0.78658952	0.99978775	-4.6893604

CHFR	-0.0646141	5.5505291	-0.642676	0.52323052	0.99978775	-4.7975671
GPC2	-0.064652	-1.106233	-0.1641634	0.87023277	0.99978775	-4.6249883
CTPS1	-0.0647049	5.31266088	-0.3527897	0.72565819	0.99978775	-4.851723
ENSCAFG00000010861	-0.0647197	2.20200227	-0.3289089	0.74353264	0.99978775	-4.6834606
SKAP2	-0.0647352	5.82826563	-0.4951504	0.62255946	0.99978775	-4.8300916
NBEA	-0.0647368	4.12654322	-0.3948855	0.69452672	0.99978775	-4.7984452
ORAI2	-0.0648048	3.21417971	-0.3046664	0.76182439	0.99978775	-4.7299557
STAM	-0.064814	5.59066038	-0.6856985	0.4959193	0.99978775	-4.780192
KMT5A	-0.0648146	5.31720958	-0.8332696	0.40846663	0.99978775	-4.7246041
RUVBL1	-0.0648856	5.9629281	-0.4809376	0.63255859	0.99978775	-4.8418336
SNRNP25	-0.0649159	3.72382755	-0.5980529	0.55237523	0.99978775	-4.7256662
RELL1	-0.0649269	4.729813	-0.4340608	0.66602246	0.99978775	-4.8286073
TTLL5	-0.0649271	4.49099867	-0.6041569	0.54834058	0.99978775	-4.7622066
NDUFAF3	-0.0649681	3.83043318	-0.5985469	0.55204819	0.99978775	-4.7273615
ENSCAFG00000002743	-0.0649861	0.03079799	-0.1656811	0.86904415	0.99978775	-4.6287575
SEPHS1	-0.0650013	6.43563289	-0.6377811	0.52638741	0.99978775	-4.8034846
SLC10A7	-0.0650216	3.69060803	-0.5120452	0.61076614	0.99978775	-4.728564
SERPINB9	-0.0650737	4.9684067	-0.2775881	0.78241848	0.99978775	-4.8424552
C1H9orf64	-0.0651044	5.54924407	-0.8879179	0.37863372	0.99978775	-4.707224
ENSCAFG00000003564	-0.0651066	4.85222134	-0.4404029	0.66145282	0.99978775	-4.8087399
RYBP	-0.0651106	3.07941527	-0.5073104	0.61406099	0.99978775	-4.6986778
PDE8A	-0.0651107	6.33132054	-0.6972868	0.48869889	0.99978775	-4.7854807
ITGB1BP1	-0.0651211	3.84563411	-0.5027877	0.61721567	0.99978775	-4.7547418
BCL9L	-0.0651871	6.66548341	-0.3900311	0.69809105	0.99978775	-4.8611357
GPN2	-0.06526	3.05398829	-0.3927735	0.69607665	0.99978775	-4.7118308
MSX2	-0.0652766	1.4575407	-0.2107485	0.83389917	0.99978775	-4.666644
FGGY	-0.0652886	2.6912644	-0.3603149	0.72005685	0.99978775	-4.7002739
EZR	-0.0653172	8.40784569	-0.3734304	0.71033141	0.99978775	-4.8355551
OGG1	-0.0653808	1.23238783	-0.277554	0.78244449	0.99978775	-4.643816
ZNF691	-0.0654956	1.5740074	-0.301286	0.76438623	0.99978775	-4.6481238
SIRT3	-0.0654989	3.84184501	-0.6199818	0.53795081	0.99978775	-4.7246824
LYPLA1	-0.0655301	3.93411456	-0.5792293	0.56491094	0.99978775	-4.744288
ENSCAFG00000004287	-0.0655314	-1.6828654	-0.187086	0.85231417	0.99978775	-4.6197324
LRRK2	-0.0655347	7.17560262	-0.4758561	0.63615054	0.99978775	-4.8385909
ENSCAFG00000018518	-0.0655354	0.25117409	-0.1849832	0.8539548	0.99978775	-4.6324261
APOL6	-0.0655385	3.31543066	-0.3089888	0.75855261	0.99978775	-4.708055
GSE1	-0.0655396	4.54151861	-0.4579767	0.64885863	0.99978775	-4.7802643
CYB561	-0.0655448	5.2001592	-0.4821152	0.63172744	0.99978775	-4.8215579
PIH1D1	-0.0655776	3.91487019	-0.4029492	0.68862143	0.99978775	-4.7521137
AKAP3	-0.0656183	-0.7157493	-0.1313269	0.89601854	0.99978775	-4.6155219
KCTD17	-0.0656291	3.10074502	-0.4299221	0.66901135	0.99978775	-4.7079909
ASXL1	-0.0656445	6.24040501	-0.7366652	0.46460431	0.99978775	-4.7711261
HSD11B1	-0.0658181	-0.0919274	-0.0248643	0.98025745	0.99978775	-4.6072304
DNAH8	-0.0658181	-2.5811498	-0.0958224	0.92402636	0.99978775	-4.6071259
SPDL1	-0.0658213	4.39171476	-0.2718711	0.78678713	0.99978775	-4.8224963
LIN52	-0.0658536	2.37321157	-0.3763534	0.70817046	0.99978775	-4.6795097
RPL3	-0.0658747	9.33848412	-0.665681	0.50852849	0.99978775	-4.7515042
MBOAT7	-0.0659353	5.09940607	-0.4779456	0.63467248	0.99978775	-4.825262
MICU1	-0.0659465	5.54412292	-0.8236206	0.41388002	0.99978775	-4.7333299
OPLAH	-0.0659635	4.71935589	-0.2858933	0.77608461	0.99978775	-4.819974

EPB41	-0.0660052	4.53959018	-0.3494124	0.72817703	0.99978775	-4.7830919
CDC42SE1	-0.0660319	3.95140749	-0.4124912	0.68165858	0.99978775	-4.7614099
PLEKHM2	-0.0661642	7.47244001	-0.3854357	0.70147155	0.99978775	-4.8416098
ARPC4	-0.0661656	5.9366223	-0.6893658	0.49362802	0.99978775	-4.7859136
QRSL1	-0.0661739	4.04480685	-0.5793307	0.56484303	0.99978775	-4.7487791
SIK3	-0.0662134	5.93265934	-0.7434932	0.46049667	0.99978775	-4.7686749
ENSCAFG00000007675	-0.0662548	6.05116025	-0.7507282	0.45616708	0.99978775	-4.7660287
MAN2C1	-0.0663442	5.45362219	-0.7061131	0.48323877	0.99978775	-4.7723605
SDR39U1	-0.0663694	3.31352423	-0.4988753	0.61995059	0.99978775	-4.7329828
FBRSL1	-0.0664783	4.01208634	-0.4523648	0.65286939	0.99978775	-4.7659548
CCDC158	-0.0664825	1.74690757	-0.2781615	0.78198065	0.99978775	-4.6530888
DIS3L2	-0.0664897	3.52937098	-0.6405599	0.52459403	0.99978775	-4.7000936
ELF4	-0.0665373	5.72711381	-0.4036012	0.68814479	0.99978775	-4.8556309
ENSCAFG00000032012	-0.0665575	3.79706863	-0.3465882	0.73028564	0.99978775	-4.7568456
WNT5B	-0.0666327	3.48875626	-0.1630089	0.87113714	0.99978775	-4.7481097
NME1	-0.0666348	7.35580287	-0.6302075	0.53129162	0.99978775	-4.7994551
ZGPAT	-0.0666734	4.74194756	-0.5770041	0.5664021	0.99978775	-4.7854154
FAIM	-0.0667204	3.05155098	-0.461238	0.64653262	0.99978775	-4.7068003
SLC12A6	-0.0667308	5.08016818	-0.4784232	0.63433488	0.99978775	-4.8046868
MAP3K5	-0.0667382	3.49505676	-0.110801	0.91219656	0.99978775	-4.6712504
MSH6	-0.0667522	5.29991648	-0.5879483	0.55908699	0.99978775	-4.8043306
MLLT6	-0.0667546	4.65573042	-0.3171406	0.75239434	0.99978775	-4.8076946
GPRC5B	-0.0667798	4.22066034	-0.1853307	0.85368363	0.99978775	-4.7705252
KAT6B	-0.0667989	5.11425401	-0.5592009	0.57840176	0.99978775	-4.7874223
GTF2E2	-0.066835	5.54979947	-0.8645881	0.39119714	0.99978775	-4.7174829
ARHGAP17	-0.0669177	5.70064012	-0.6624873	0.51055611	0.99978775	-4.7875667
HAUS3	-0.0669332	4.5602354	-0.4014749	0.6896996	0.99978775	-4.79881
DCAF1	-0.0669485	5.29132694	-0.8088145	0.42227109	0.99978775	-4.7300915
MEAF6	-0.0669655	3.60114492	-0.6412637	0.52414035	0.99978775	-4.7035721
RGP1	-0.0670061	4.05339221	-0.7439374	0.46023018	0.99978775	-4.7116197
ZNF394	-0.067013	3.56121625	-0.5739173	0.56847387	0.99978775	-4.7049647
MTA2	-0.0670242	6.60097312	-0.7455292	0.45927592	0.99978775	-4.7690996
ABHD14A	-0.0670851	3.73415757	-0.3926034	0.69620153	0.99978775	-4.744224
DHX38	-0.0673014	5.12390791	-0.88619	0.37955541	0.99978775	-4.7016403
TANK	-0.0673033	4.68855076	-0.4075445	0.68526476	0.99978775	-4.8075493
ENSCAFG00000026010	-0.0673824	1.43315976	-0.2166408	0.8293277	0.99978775	-4.6534891
CFAP20	-0.0673968	3.89700364	-0.5451114	0.58798467	0.99978775	-4.7300006
FHOD1	-0.0675371	5.18013351	-0.360157	0.72017427	0.99978775	-4.8256752
BRWD3	-0.0676848	3.98169419	-0.5446865	0.58827484	0.99978775	-4.747761
TMED9	-0.0676951	6.51390885	-0.7182182	0.47580606	0.99978775	-4.7782348
ZKSCAN2	-0.067761	4.61052842	-0.5851527	0.56095112	0.99978775	-4.7682434
TFDP1	-0.0678413	5.78605069	-0.8920929	0.37641268	0.99978775	-4.7125704
CCDC12	-0.0680208	2.22884565	-0.3707456	0.71231838	0.99978775	-4.6671296
YBEY	-0.0680528	3.10984951	-0.5317671	0.59712976	0.99978775	-4.6968854
SRSF7	-0.0680541	4.72471607	-0.4601517	0.64730697	0.99978775	-4.8132707
CAMKK1	-0.0681097	4.28881074	-0.4324182	0.66720807	0.99978775	-4.7955589
ZNF697	-0.0681586	3.19528694	-0.3354675	0.73860893	0.99978775	-4.7288727
MEX3D	-0.0681799	3.6603844	-0.3852571	0.70160303	0.99978775	-4.7524392
SVBP	-0.0681887	2.55531377	-0.460207	0.64726753	0.99978775	-4.6764168
FAM178B	-0.0683372	-1.4799205	-0.1235155	0.90217047	0.99978775	-4.6123115

ENPP4	-0.0683829	3.06905706	-0.2498978	0.80364139	0.99978775	-4.7060509
ENSCAFG00000015774	-0.0683882	-0.8866356	-0.2044439	0.83879692	0.99978775	-4.6246944
ARMC5	-0.0684919	3.52600222	-0.503733	0.61655571	0.99978775	-4.724462
CEP57L1	-0.0684959	2.78514206	-0.3762167	0.70827148	0.99978775	-4.6953561
KDM4A	-0.0685363	7.25275251	-1.1337397	0.26205294	0.99978775	-4.6033305
PCBP4	-0.0685455	5.45335818	-0.5014655	0.61813935	0.99978775	-4.8238083
DLGAP4	-0.0686818	6.73065004	-0.8217997	0.41490646	0.99978775	-4.7418593
ZNF789	-0.0686965	0.36279594	-0.2135235	0.83174547	0.99978775	-4.6324507
RBBP5	-0.0687427	4.74321049	-0.7698238	0.44485359	0.99978775	-4.7172334
MFSD14B	-0.0688828	5.31395084	-0.857599	0.39501103	0.99978775	-4.7120887
MFSD1	-0.0689059	5.04980863	-0.6746834	0.50283653	0.99978775	-4.7689396
WRN	-0.0689234	4.82212729	-0.5206892	0.60477189	0.99978775	-4.7973687
CTTN	-0.0689545	8.47982679	-0.7935202	0.43104571	0.99978775	-4.7299067
ENSCAFG00000014664	-0.0689814	0.70456689	-0.2858954	0.776083	0.99978775	-4.6522309
GOLPH3	-0.069027	4.98713743	-0.8438291	0.40259245	0.99978775	-4.7154466
RAD54L2	-0.0690352	4.69053383	-0.6684879	0.50675005	0.99978775	-4.748848
PTBP1	-0.069044	8.10506742	-0.5674209	0.57284607	0.99978775	-4.7971429
LTA4H	-0.0690678	6.46518822	-0.8834662	0.3810111	0.99978775	-4.7187794
GBGT1	-0.0690776	5.8314676	-0.6510202	0.51787206	0.99978775	-4.7920926
ENSCAFG00000031564	-0.0691198	6.72467317	-0.4318705	0.66760361	0.99978775	-4.8487346
PSME1	-0.0692522	4.74987768	-0.3017269	0.76405195	0.99978775	-4.8235647
TOP2B	-0.0693104	6.96378321	-0.6137952	0.54200048	0.99978775	-4.809181
MRPL43	-0.069384	3.09956643	-0.4392904	0.66225351	0.99978775	-4.7075397
SNRPB	-0.0694214	5.53376062	-0.5936039	0.55532537	0.99978775	-4.8051232
ENSCAFG00000017325	-0.069423	5.56010432	-0.4057206	0.6865963	0.99978775	-4.8404883
NXF1	-0.0694233	6.14107541	-1.0802233	0.28497889	0.99978775	-4.6314809
GSTO1	-0.0694251	5.39642436	-0.6192029	0.53845981	0.99978775	-4.7927098
EI24	-0.0694799	5.94227236	-0.7582595	0.45168526	0.99978775	-4.7625729
TARS2	-0.0695013	4.71041666	-0.6617483	0.51102593	0.99978775	-4.7635282
AKNA	-0.0695212	2.99505333	-0.4157458	0.67929006	0.99978775	-4.7152237
ERCC6	-0.0695489	4.09839327	-0.4946701	0.62289627	0.99978775	-4.7428559
DBI	-0.0696053	5.1145561	-0.5457105	0.58757564	0.99978775	-4.8041549
PHF1	-0.0697464	4.17713669	-0.4201185	0.6761129	0.99978775	-4.7768066
BTRC	-0.0697933	4.75823859	-0.4962625	0.6217801	0.99978775	-4.7877615
AP1AR	-0.0698531	2.86480177	-0.405684	0.686623	0.99978775	-4.7023338
PATL1	-0.0698807	7.1070075	-0.2945055	0.76953271	0.99978775	-4.8752747
DNHD1	-0.0699347	-0.7256076	-0.1360703	0.89228595	0.99978775	-4.615343
SYNJ2	-0.0699616	4.09263887	-0.4074589	0.68532724	0.99978775	-4.8042588
ZMYM6	-0.0700883	5.3247538	-0.6893601	0.49363157	0.99978775	-4.7669388
APEH	-0.0700948	5.55134985	-0.697188	0.48876025	0.99978775	-4.7714836
AP4B1	-0.0701295	4.22757706	-0.7428229	0.460899	0.99978775	-4.7189066
DECR1	-0.0702375	3.63776684	-0.2667752	0.79068703	0.99978775	-4.7621438
NUP107	-0.0702615	4.56747098	-0.4802002	0.63307934	0.99978775	-4.7877939
ASB3	-0.070391	5.22122265	-0.8363839	0.40672874	0.99978775	-4.7198773
NLGN2	-0.0704136	2.85040171	-0.3009472	0.76464307	0.99978775	-4.6833037
CKAP2	-0.0705508	4.62321665	-0.3735128	0.71027047	0.99978775	-4.8393263
ENSCAFG00000011681	-0.070629	0.27743801	-0.1711964	0.86472727	0.99978775	-4.6310718
HEXIM1	-0.0706711	5.27299097	-0.5021637	0.6176515	0.99978775	-4.8202135
PRRT4	-0.070767	1.32501503	-0.2700786	0.78815834	0.99978775	-4.665345
GPR153	-0.0710417	3.95916961	-0.5893676	0.55814184	0.99978775	-4.7512673

METTL22	-0.0710499	4.12746849	-0.5695852	0.57138763	0.99978775	-4.7574805
ENSCAFG00000017763	-0.0711007	3.65938683	-0.4141403	0.68045806	0.99978775	-4.7487755
HSD17B4	-0.0711194	6.15555406	-1.1197236	0.26792676	0.99978775	-4.6122258
KDM5C	-0.0711667	6.74384385	-1.2817882	0.20554734	0.99978775	-4.5264016
CMTM6	-0.0711996	2.58241434	-0.4104404	0.68315277	0.99978775	-4.676188
SLC9A9	-0.0712524	4.25074613	-0.278278	0.78189176	0.99978775	-4.7622355
ZBTB44	-0.0712956	4.43962125	-0.5129281	0.61015264	0.99978775	-4.7698221
TMEM263	-0.0713008	4.3829487	-0.4868406	0.62839713	0.99978775	-4.7807825
ATG2B	-0.0713264	5.78419035	-0.8157265	0.41834115	0.99978775	-4.7364832
ZNF768	-0.0713273	4.97689033	-0.6459004	0.52115641	0.99978775	-4.7713445
SLC16A7	-0.0713849	-1.3801964	-0.084904	0.93266054	0.99978775	-4.6103946
ENSCAFG00000013883	-0.0714531	4.96131001	-0.7809445	0.43834147	0.99978775	-4.7279666
ZNF7	-0.0715236	5.59521603	-0.719887	0.47478649	0.99978775	-4.7640696
YEATS4	-0.0715602	3.36497535	-0.5860189	0.56037321	0.99978775	-4.7037849
STAT1	-0.0715654	7.092521	-0.9018786	0.37123934	0.99978775	-4.7088914
SNCG	-0.071575	-2.4693619	-0.0950904	0.92460498	0.99978775	-4.6072344
PPIL2	-0.071793	4.72656416	-0.9259049	0.35873104	0.99978775	-4.6737203
KDR	-0.0718008	3.3146084	-0.1182895	0.90628961	0.99978775	-4.7262393
DOCK10	-0.0718794	6.12969012	-0.2534198	0.80093334	0.99978775	-4.8754709
LIN7B	-0.0718852	0.79795794	-0.1993033	0.84279521	0.99978775	-4.6335305
RCN3	-0.0718999	6.60524034	-0.2978691	0.76697833	0.99978775	-4.8763808
DESI1	-0.0719531	3.98125196	-0.4312319	0.66806484	0.99978775	-4.7751656
MCCC2	-0.0719792	6.21562116	-0.7446291	0.45981539	0.99978775	-4.7693614
RHOC	-0.0719903	6.32145762	-0.7314206	0.46777362	0.99978775	-4.7736927
TFPT	-0.072023	2.61384984	-0.5993903	0.55148995	0.99978775	-4.6694014
TUBB6	-0.0720659	7.95998083	-0.3854291	0.70147636	0.99978775	-4.8330269
ENSCAFG00000007808	-0.072085	3.98272938	-0.6972402	0.48872786	0.99978775	-4.7090847
AKAP8	-0.0721499	4.59781006	-0.7060588	0.48327231	0.99978775	-4.7389581
C20H3orf67	-0.0721827	4.69905276	-0.5730476	0.56905819	0.99978775	-4.7654593
CYC1	-0.0721944	5.94543938	-0.5630141	0.57582131	0.99978775	-4.8218905
EIF4A3	-0.0722611	6.3694956	-0.7602355	0.45051365	0.99978775	-4.7645384
PAM	-0.0723149	7.53365053	-0.379688	0.70570823	0.99978775	-4.8628859
ASPA	-0.0723642	5.16885631	-0.1043501	0.91728907	0.99978775	-4.7518693
KNOP1	-0.0724141	3.3554475	-0.5435115	0.58907759	0.99978775	-4.7131707
ENSCAFG00000009798	-0.0724237	3.09188314	-0.3659686	0.71585867	0.99978775	-4.6925982
ENSCAFG00000003222	-0.0724863	4.66987362	-0.6875871	0.49473858	0.99978775	-4.7514591
FBXW4	-0.0724885	2.91211742	-0.3900471	0.69807929	0.99978775	-4.6937762
RNASEL	-0.0725013	3.60465068	-0.2888444	0.77383763	0.99978775	-4.7060685
SAE1	-0.072531	6.35181528	-0.6196336	0.53817836	0.99978775	-4.8087989
PHF14	-0.072535	4.67393159	-0.5189844	0.60595193	0.99978775	-4.7867111
ENSCAFG00000023283	-0.0726714	4.86655658	-0.6649454	0.50899517	0.99978775	-4.762221
ARGLU1	-0.0727023	5.48945517	-0.7223361	0.47329239	0.99978775	-4.766527
FREM1	-0.0728246	1.85327127	-0.1068154	0.91534245	0.99978775	-4.6767466
IRF9	-0.0729244	3.44866411	-0.3850492	0.70175612	0.99978775	-4.7281112
COPRS	-0.072974	4.5493309	-0.4809822	0.63252712	0.99978775	-4.8178917
ZMYND19	-0.0730275	2.98997842	-0.4872356	0.62811911	0.99978775	-4.7022428
MLLT3	-0.0730496	3.35865038	-0.2932673	0.77047365	0.99978775	-4.7215889
NDUFAF1	-0.0730506	3.88785711	-0.704299	0.48435824	0.99978775	-4.7098486
TMEM231	-0.073129	3.70017704	-0.4194401	0.67660542	0.99978775	-4.7407219
CNPY4	-0.0731471	3.73881769	-0.499957	0.6191939	0.99978775	-4.7288248

NDUFAF4	-0.0732667	4.71859151	-0.5024533	0.61744922	0.99978775	-4.794453
TULP3	-0.0732878	5.94244158	-0.6653502	0.50873835	0.99978775	-4.7891364
DYM	-0.0733102	7.62507249	-0.6292939	0.53188488	0.99978775	-4.7924916
ENSCAFG00000004842	-0.0733325	4.47616806	-0.4803688	0.63296022	0.99978775	-4.7860656
ZFP30	-0.0733935	1.9605296	-0.2871155	0.77515375	0.99978775	-4.6698675
ELK1	-0.0734488	4.08062778	-0.5231332	0.603082	0.99978775	-4.7584113
ARMC9	-0.073466	3.53836929	-0.3445446	0.7318128	0.99978775	-4.729321
C18H11orf49	-0.0734754	3.69239039	-0.4495397	0.65489236	0.99978775	-4.7526463
METTL17	-0.0735011	3.59659921	-0.4913054	0.6252576	0.99978775	-4.7251936
PDHA1	-0.0735075	7.57271292	-0.7840596	0.43652749	0.99978775	-4.7488098
TFCP2	-0.0735331	5.22340943	-0.7898413	0.43317249	0.99978775	-4.731815
NACA	-0.0736061	7.56950308	-0.5932982	0.55552836	0.99978775	-4.8092052
SIGLEC1	-0.0736174	5.34084357	-0.621517	0.5369483	0.99978775	-4.7884542
AAMDC	-0.0736944	1.60008337	-0.2932045	0.77052139	0.99978775	-4.6507156
MECP2	-0.0737023	4.46129052	-0.8574916	0.39506976	0.99978775	-4.6930917
MACROD1	-0.0737574	2.00891196	-0.2903007	0.77272953	0.99978775	-4.662114
ENSCAFG00000019210	-0.0738009	3.7685408	-0.462429	0.64568407	0.99978775	-4.7771059
RAD17	-0.0738015	5.15893752	-0.8342684	0.4079088	0.99978775	-4.7192356
AP5M1	-0.0738035	4.49197771	-0.6201232	0.53785847	0.99978775	-4.7605437
MRPS15	-0.0738189	5.18233157	-0.7980608	0.4284294	0.99978775	-4.7340968
NUDT18	-0.0738452	2.7018124	-0.5193732	0.60568271	0.99978775	-4.6828468
TAF11	-0.0738605	4.0099911	-0.6000374	0.5510619	0.99978775	-4.7440647
LIN37	-0.0738796	2.13718215	-0.4682102	0.64157186	0.99978775	-4.6560247
PMS2	-0.0739065	3.91730116	-0.7489947	0.45720227	0.99978775	-4.693378
ENSCAFG00000029758	-0.0739544	3.40010543	-0.4856317	0.62924838	0.99978775	-4.7359055
GPAT4	-0.0739812	6.76449319	-0.8966077	0.37402023	0.99978775	-4.7126943
AUTS2	-0.07401	5.43011041	-0.3461338	0.73062511	0.99978775	-4.8567128
POLR3B	-0.0740391	5.04527939	-0.5849938	0.56105713	0.99978775	-4.7975236
ENSCAFG00000025882	-0.0740437	-1.1800188	-0.1394573	0.88962224	0.99978775	-4.6136401
VPS37C	-0.0740926	3.07096756	-0.5701222	0.57102604	0.99978775	-4.6918888
SLC4A4	-0.0742052	1.62371928	-0.2482527	0.80490711	0.99978775	-4.6652032
SETD1B	-0.0742374	4.63753753	-0.6722004	0.50440297	0.99978775	-4.7495233
CHUK	-0.0742409	3.96630504	-0.6365697	0.52717028	0.99978775	-4.7224568
TMEM19	-0.0742956	4.66446693	-0.8150067	0.41874939	0.99978775	-4.7076356
ENSCAFG00000032355	-0.0743349	1.53755049	-0.1371566	0.89143155	0.99978775	-4.6464275
RHPN1	-0.0743379	2.81697249	-0.3536165	0.72504205	0.99978775	-4.6930763
TMEM159	-0.0744204	2.22433116	-0.2799305	0.7806305	0.99978775	-4.6733275
EIF2B2	-0.0745077	4.43392788	-0.2736784	0.78540536	0.99978775	-4.7943948
TTC4	-0.0745531	5.33365966	-0.9694423	0.33676845	0.99978775	-4.6725527
ENSCAFG00000009583	-0.0745894	4.8894343	-0.5365423	0.59384963	0.99978775	-4.8000904
ENSA	-0.0747156	5.08854999	-0.7054771	0.48363108	0.99978775	-4.7558234
ENSCAFG00000009311	-0.0747212	1.71776931	-0.3336883	0.73994359	0.99978775	-4.6535324
C27H12orf57	-0.0747846	4.82828565	-0.5442873	0.58854752	0.99978775	-4.7907549
MAP3K1	-0.0748973	3.80503201	-0.3011149	0.76451593	0.99978775	-4.7472859
TGFB2	-0.074912	6.70713497	-0.178392	0.85910151	0.99978775	-4.8896307
ENSCAFG00000030682	-0.0750755	3.99664844	-0.2009004	0.84155256	0.99978775	-4.811198
PTGS2	-0.0750916	5.03281494	-0.1259007	0.90029133	0.99978775	-4.7963466
CDK5RAP1	-0.0750931	4.21353155	-0.6986077	0.48787959	0.99978775	-4.7212046
PCYT1A	-0.0751966	5.22985475	-0.7984756	0.42819087	0.99978775	-4.7304404
LPCAT1	-0.0752695	4.9952826	-0.6114236	0.54355705	0.99978775	-4.7843195

RPL36AL	-0.0753747	6.04181798	-0.8018522	0.42625209	0.99978775	-4.7471303
ADGRB2	-0.0753891	2.21923231	-0.3104212	0.75746936	0.99978775	-4.7494037
RIOX1	-0.0755433	3.5566636	-0.5350601	0.59486681	0.99978775	-4.7184148
FAM117B	-0.0756117	4.50612753	-0.3084496	0.75896051	0.99978775	-4.8079823
COL5A2	-0.0756118	13.3695441	-0.325955	0.74575374	0.99978775	-4.7162858
GOPC	-0.0756198	4.32041142	-0.7616541	0.44967356	0.99978775	-4.7092459
DHRS4	-0.0756838	5.27844814	-0.5106105	0.61176362	0.99978775	-4.8143126
PABPC4	-0.0757182	7.14345513	-0.6624672	0.5105689	0.99978775	-4.7935298
ROBO1	-0.0757275	7.66896332	-0.2206146	0.82624798	0.99978775	-4.860079
PPP5C	-0.0757312	5.16226654	-0.7818918	0.43778935	0.99978775	-4.7349438
CCDC22	-0.0757488	3.61800354	-0.6145983	0.54147391	0.99978775	-4.7174519
SLC22A5	-0.0757877	0.50736775	-0.2455209	0.8070101	0.99978775	-4.6278513
ZDHHC18	-0.0757901	1.84746502	-0.2322936	0.81721287	0.99978775	-4.6498945
WDR7	-0.0757967	5.61770307	-1.0347435	0.30553032	0.99978775	-4.6492393
BORA	-0.0758285	3.26494777	-0.514048	0.60937486	0.99978775	-4.7148756
TRMT13	-0.0758311	1.73623005	-0.3850672	0.70174288	0.99978775	-4.6472624
PVR	-0.0758611	4.87234995	-0.3960368	0.6936824	0.99978775	-4.8374695
NICN1	-0.0759262	4.03481088	-0.5209784	0.6045718	0.99978775	-4.7442167
ADGRA2	-0.0760067	6.28867137	-0.2900484	0.77292146	0.99978775	-4.876462
CISH	-0.0760381	4.64124122	-0.3919727	0.69666458	0.99978775	-4.8066097
ENSCAFG00000006478	-0.0760833	6.12531961	-0.3976984	0.6924646	0.99978775	-4.855726
MSH3	-0.0761213	5.14342405	-0.6019204	0.54981715	0.99978775	-4.7953681
MDN1	-0.0761646	5.82082755	-0.4573994	0.64927077	0.99978775	-4.8402142
PLPP5	-0.0761706	3.02399152	-0.525552	0.60141175	0.99978775	-4.6972915
DPAGT1	-0.0762944	4.75590067	-0.8635039	0.39178725	0.99978775	-4.7051264
AMD1	-0.0763894	4.98451483	-0.5731169	0.56901161	0.99978775	-4.7961815
ALKBH3	-0.0764457	4.18833814	-1.0414993	0.3024151	0.99978775	-4.622384
NOL6	-0.0764482	4.79063095	-0.4914419	0.62516171	0.99978775	-4.8033481
ZNF250	-0.0764514	3.22985942	-0.4073523	0.68540507	0.99978775	-4.7121002
PSMB6	-0.0765548	6.00473737	-0.710239	0.48069816	0.99978775	-4.780674
ENSCAFG00000002945	-0.0765646	2.57832851	-0.3464988	0.73035245	0.99978775	-4.6888611
UFD1	-0.0765717	5.11789707	-0.792027	0.43190819	0.99978775	-4.7328614
ST3GAL2	-0.0765836	4.55134817	-0.710307	0.48065635	0.99978775	-4.7371396
RNPEP	-0.0766229	6.22548804	-0.6882728	0.49431028	0.99978775	-4.7873187
HNRNPH1	-0.0766525	7.58431323	-0.4904446	0.62586233	0.99978775	-4.8271703
MAP7D3	-0.0767012	6.55742657	-0.7442475	0.46004418	0.99978775	-4.7699821
LPIN3	-0.0767302	4.39663435	-0.3943903	0.69489002	0.99978775	-4.7638075
TSGA10	-0.0767441	-0.6956902	-0.1484915	0.88252361	0.99978775	-4.6184535
MGST1	-0.0767798	0.3798117	-0.0367614	0.97081462	0.99978775	-4.6082014
PRPF38B	-0.0767872	5.3651576	-0.7102348	0.48070075	0.99978775	-4.7649425
SETDB2	-0.0768329	4.35245085	-0.5135038	0.60975272	0.99978775	-4.775043
ENSCAFG00000005494	-0.0768383	1.4741341	-0.2986905	0.76635501	0.99978775	-4.6699734
ACIN1	-0.0768599	6.95831685	-1.0627768	0.29274608	0.99978775	-4.6396961
ERGIC1	-0.0769995	7.05811808	-0.5076385	0.6138324	0.99978775	-4.836201
PODXL	-0.0770048	1.1863166	-0.0677089	0.94627452	0.99978775	-4.6469886
ENSCAFG00000016006	-0.0770226	5.43645498	-0.8566424	0.39553477	0.99978775	-4.7211007
TPD52L1	-0.0772107	0.27292045	-0.115268	0.90867229	0.99978775	-4.6281332
CCDC59	-0.0772587	3.27863499	-0.6288091	0.53219978	0.99978775	-4.6920092
ACTL6A	-0.0773039	4.55322055	-0.8566857	0.39551106	0.99978775	-4.694114
ENSCAFG00000014120	-0.0773535	4.3244863	-0.6095356	0.54479783	0.99978775	-4.7621946

YBX3	-0.0773896	7.07778623	-0.5460076	0.5873729	0.99978775	-4.828065
EEFSEC	-0.0774232	3.62118438	-0.9419935	0.35050937	0.99978775	-4.6499149
DCHS1	-0.0775158	5.2954497	-0.2674883	0.79014098	0.99978775	-4.8751434
TRPV3	-0.0776202	-1.5856284	-0.1131043	0.91037914	0.99978775	-4.6079509
ENSCAFG00000017072	-0.0776283	2.86192946	-0.3760135	0.70842161	0.99978775	-4.6917338
PLCD1	-0.0777619	7.32062318	-0.5965793	0.5533515	0.99978775	-4.8110525
DIS3	-0.0778068	4.76539229	-0.5196944	0.60546034	0.99978775	-4.7917535
NUDT6	-0.0779177	1.43787126	-0.2237253	0.82383915	0.99978775	-4.6405735
SMIM4	-0.0779193	2.3915021	-0.3362664	0.73800999	0.99978775	-4.6763225
UBQLN4	-0.0779624	4.03152541	-0.645154	0.52163616	0.99978775	-4.737061
ZNF397	-0.0779823	2.29820661	-0.3904706	0.69776808	0.99978775	-4.6612266
ERBB2	-0.0780659	5.96768952	-0.7063885	0.48306896	0.99978775	-4.7821992
MFHAS1	-0.0780731	6.67909324	-0.6133489	0.54229324	0.99978775	-4.8097478
BMP1	-0.0781333	7.66059912	-0.4312844	0.66802695	0.99978775	-4.8418552
SLC7A11	-0.0781456	3.81055033	-0.3059733	0.76083469	0.99978775	-4.7899626
AAGAB	-0.0781751	4.42386374	-0.6496018	0.51878086	0.99978775	-4.7441644
MAVS	-0.0782535	5.35475761	-0.5936135	0.55531897	0.99978775	-4.8004502
MMP15	-0.0782788	2.08183415	-0.2798325	0.78070528	0.99978775	-4.66862
ENSCAFG00000030317	-0.0782874	0.48330478	-0.1899899	0.85004957	0.99978775	-4.6247906
DHX35	-0.0784357	2.22118217	-0.3793471	0.70595979	0.99978775	-4.671547
TRIM26	-0.0784836	3.45839191	-0.3957066	0.69392455	0.99978775	-4.7347691
RFNG	-0.0785159	5.78695329	-0.5405059	0.59113337	0.99978775	-4.8251626
ENSCAFG00000014844	-0.0785846	-0.6529729	-0.1639442	0.87040442	0.99978775	-4.6215152
ENSCAFG00000014560	-0.0785915	2.36756135	-0.4517165	0.65333336	0.99978775	-4.6795011
TYW3	-0.0786037	1.99516156	-0.4638306	0.64468606	0.99978775	-4.6570841
MAP2K3	-0.078687	6.43463629	-0.8698473	0.38834251	0.99978775	-4.7236363
GAB1	-0.0787113	5.89837716	-0.4693456	0.64076553	0.99978775	-4.8438151
IPO4	-0.0787189	6.16756516	-0.7133618	0.47878021	0.99978775	-4.7798594
NUDCD2	-0.0787265	2.81969468	-0.4265212	0.67147153	0.99978775	-4.686005
ZFAND5	-0.0787919	5.70316613	-0.3874445	0.69999303	0.99978775	-4.842016
KIAA0825	-0.078845	2.37641646	-0.3644839	0.71696033	0.99978775	-4.6708376
RPS19	-0.0789261	9.11983009	-0.6866429	0.49532867	0.99978775	-4.7503188
LAMA2	-0.0789398	9.11359841	-0.1488293	0.88225841	0.99978775	-4.8536129
IRF2BP2	-0.0789629	5.66272314	-0.5208382	0.60466877	0.99978775	-4.8240535
EIF4B	-0.0789794	8.85412773	-0.6923552	0.49176457	0.99978775	-4.75624
TXNRD2	-0.0790666	3.12490098	-0.5902863	0.55753041	0.99978775	-4.6892173
ENSCAFG00000030159	-0.079219	1.77093386	-0.3452944	0.73125232	0.99978775	-4.6511914
EPHA2	-0.0792421	6.94462431	-0.3910949	0.69730936	0.99978775	-4.8589502
CC2D2A	-0.0792768	4.73801867	-0.4526993	0.65263003	0.99978775	-4.8033309
RBM26	-0.0793676	5.12187952	-0.7418004	0.46151308	0.99978775	-4.7492899
MAATS1	-0.0793892	-0.5607924	-0.2013453	0.84120647	0.99978775	-4.618683
NBN	-0.0794543	6.29754901	-0.7585234	0.45152868	0.99978775	-4.7645823
CDKN2AIP	-0.0794806	3.88624051	-0.5975755	0.5526914	0.99978775	-4.7236202
CRYZ	-0.0795203	1.84812086	-0.2542472	0.80029753	0.99978775	-4.6564426
MAP2K7	-0.07954	5.00790756	-0.7159276	0.47720759	0.99978775	-4.7410907
HSPH1	-0.0796656	6.3858396	-0.4375054	0.66353893	0.99978775	-4.8528798
S100A13	-0.0796938	2.76442657	-0.5596454	0.57810069	0.99978775	-4.6863066
TMEM206	-0.07974	2.53940598	-0.3429262	0.73302294	0.99978775	-4.6869761
FOXJ2	-0.0797434	4.75061112	-0.7934894	0.4310635	0.99978775	-4.7255307
CDC14B	-0.0797635	2.8931499	-0.3089237	0.75860186	0.99978775	-4.6959669

DHRS13	-0.0797986	6.60914804	-0.5418724	0.59019831	0.99978775	-4.8291901
LCN2	-0.0799071	0.54460346	-0.0731499	0.94196478	0.99978775	-4.6166709
UPF1	-0.0799597	5.71834357	-0.9979535	0.32287822	0.99978775	-4.6648197
BEND7	-0.0799706	3.02924587	-0.4170398	0.67834925	0.99978775	-4.7282207
E2F6	-0.0800037	3.80623	-0.5667426	0.57330352	0.99978775	-4.7315763
LRSAM1	-0.0800079	4.48189233	-0.7840064	0.43655841	0.99978775	-4.71961
ENSCAFG00000004548	-0.0800302	1.19823327	-0.3396069	0.7355071	0.99978775	-4.6367479
CLSTN3	-0.0800925	-0.5273745	-0.1984545	0.84345574	0.99978775	-4.6307167
ZSCAN22	-0.0801054	3.75939283	-0.7454656	0.45931399	0.99978775	-4.6935848
SNRPA1	-0.0801536	4.21169148	-0.4233828	0.67374497	0.99978775	-4.7837735
ZNF598	-0.0801813	5.16141021	-0.6997654	0.48716219	0.99978775	-4.7602361
PPIH	-0.0802227	3.54863036	-0.519027	0.60592243	0.99978775	-4.7263499
TMLHE	-0.080254	3.66411363	-0.5904416	0.55742714	0.99978775	-4.7279029
CROT	-0.0802847	5.21865746	-0.5676538	0.57268906	0.99978775	-4.792824
ENSCAFG00000014568	-0.0803217	1.30395308	-0.3894113	0.69854665	0.99978775	-4.6432127
UBP1	-0.0803629	6.69260251	-0.9577975	0.34255378	0.99978775	-4.687814
HNRNPUL1	-0.0804126	8.2363349	-0.9468517	0.34805105	0.99978775	-4.6778834
NEDD1	-0.0804646	5.64027739	-0.744057	0.46015843	0.99978775	-4.7635306
ADK	-0.0805222	4.92500666	-0.4675487	0.64204179	0.99978775	-4.8107415
C7	-0.0805294	-2.2244404	-0.1021838	0.919	0.99978775	-4.6091336
INTS2	-0.0805905	4.7564203	-0.8322156	0.40905584	0.99978775	-4.705878
ZCCHC7	-0.0805929	3.71249439	-0.5903866	0.55746369	0.99978775	-4.7147165
ELAC2	-0.0806056	4.74167209	-0.4337947	0.66621448	0.99978775	-4.7929035
MIOS	-0.080663	4.92661122	-0.7969906	0.4290452	0.99978775	-4.7246415
FAM114A2	-0.0806731	6.06701985	-0.6023669	0.54952221	0.99978775	-4.8115338
ENSCAFG00000003457	-0.0807222	-0.520032	-0.1655771	0.86912556	0.99978775	-4.6310922
CCNL1	-0.0807567	5.06713212	-0.4773405	0.63510037	0.99978775	-4.8022296
FUBP1	-0.0807807	6.46578176	-0.8579593	0.39481385	0.99978775	-4.728635
ENSCAFG00000015098	-0.0807915	4.27765093	-0.7985298	0.4281597	0.99978775	-4.7050501
RPRD1A	-0.0808126	3.57909658	-0.5438465	0.58884868	0.99978775	-4.7098365
APH1A	-0.0808288	5.23556939	-0.7213119	0.4739169	0.99978775	-4.7556367
RABEPK	-0.080924	3.98098573	-0.6731193	0.50382294	0.99978775	-4.7211324
HSD17B8	-0.0809637	3.92260731	-0.4788457	0.63403622	0.99978775	-4.7541665
PUF60	-0.08097	6.67158339	-1.1102313	0.27195724	0.99978775	-4.617217
INTS10	-0.0809714	5.53862595	-0.8195398	0.41618255	0.99978775	-4.7309367
ZBED4	-0.0809806	4.82490886	-0.7174504	0.47627556	0.99978775	-4.749557
TMEM14A	-0.0810141	1.55684516	-0.4359024	0.66469423	0.99978775	-4.6606149
DNAJA4	-0.0810637	2.46951998	-0.3153999	0.75370799	0.99978775	-4.7345606
GLI2	-0.0810777	4.48332999	-0.2184614	0.82791642	0.99978775	-4.8021168
ENSCAFG00000011635	-0.0810832	2.52632903	-0.4124931	0.68165723	0.99978775	-4.6771723
WTAP	-0.0811539	4.83203231	-0.8477555	0.40042152	0.99978775	-4.7023108
CYTH2	-0.0811926	4.45657453	-0.6127433	0.54269062	0.99978775	-4.7598584
AP1M1	-0.0812036	5.81392722	-0.8071367	0.42322842	0.99978775	-4.7429428
CNTLN	-0.0812208	4.59344941	-0.5666712	0.57335168	0.99978775	-4.7732734
ENSCAFG00000012763	-0.0812451	6.07722068	-0.583466	0.56207727	0.99978775	-4.816936
ABCA2	-0.0812714	5.38144236	-0.5286123	0.59930154	0.99978775	-4.806343
MPHOSPH10	-0.0813435	4.79479008	-0.6164359	0.54027003	0.99978775	-4.7699368
ZDHHC8	-0.0814256	5.37201892	-0.508764	0.61304861	0.99978775	-4.8138312
ERG28	-0.0814932	3.89073991	-0.3671806	0.71495985	0.99978775	-4.7430366
RAB34	-0.0815326	6.24042566	-0.2617332	0.79455099	0.99978775	-4.8800624

GGCX	-0.081547	6.24645092	-0.8212527	0.41521513	0.99978775	-4.7422716
TAF1A	-0.0815587	2.6755924	-0.3603407	0.72003767	0.99978775	-4.6677758
NRCAM	-0.0815638	0.61208347	-0.12071	0.90438144	0.99978775	-4.6256804
CBX5	-0.0815804	4.79094085	-0.5395228	0.59180651	0.99978775	-4.8003334
EPS8L2	-0.0816403	-0.4819632	-0.1771047	0.86010743	0.99978775	-4.628388
TFDP2	-0.0816715	3.07424772	-0.4292646	0.66948671	0.99978775	-4.6722817
THBD	-0.0816869	4.7067387	-0.337929	0.73676391	0.99978775	-4.8301606
LONRF1	-0.0817162	3.55841893	-0.6094477	0.54485564	0.99978775	-4.7224382
MCAM	-0.0817255	3.05517692	-0.1676515	0.86750138	0.99978775	-4.7666223
BCAT2	-0.0817986	5.0965718	-0.5265109	0.60075013	0.99978775	-4.7946009
DNMT3A	-0.0818008	2.64252318	-0.4606686	0.64693844	0.99978775	-4.6895014
ACSL4	-0.0818038	7.19389533	-0.5619943	0.57651088	0.99978775	-4.8209913
COPS6	-0.0818314	5.49200929	-0.9989931	0.32237908	0.99978775	-4.6629014
SLC4A10	-0.0818595	0.20621082	-0.1656182	0.86909339	0.99978775	-4.6668127
RGS10	-0.0818604	0.33766123	-0.1728922	0.86340079	0.99978775	-4.6266725
IMP3	-0.0818898	3.16863287	-0.4774029	0.63505623	0.99978775	-4.7221239
ADPRH	-0.0820234	2.95468619	-0.6689035	0.50648702	0.99978775	-4.6720351
UMAD1	-0.0820686	0.28515492	-0.2478027	0.80525343	0.99978775	-4.6301806
SSSCA1	-0.0821253	3.45563634	-0.5629202	0.57588477	0.99978775	-4.7126037
SLC9A5	-0.0821317	3.008812	-0.3943774	0.6948995	0.99978775	-4.6878805
NOTCH4	-0.0821327	-1.8277868	-0.0749678	0.94052518	0.99978775	-4.6076723
HNRNPL	-0.0822739	7.88525077	-0.9875185	0.32791677	0.99978775	-4.6659502
KRCC1	-0.0823775	4.40370599	-0.9145174	0.36462509	0.99978775	-4.6730361
UNK	-0.0824022	4.48593504	-0.6312003	0.53064739	0.99978775	-4.7576239
ENSCAFG00000015055	-0.0824134	4.06786138	-0.7547953	0.45374357	0.99978775	-4.6994682
SETBP1	-0.082438	1.89086488	-0.2255838	0.82240077	0.99978775	-4.685775
POLDIP2	-0.0825072	5.55307723	-0.7742863	0.4422336	0.99978775	-4.7520926
TTC14	-0.0827888	6.3700382	-0.5355362	0.59454003	0.99978775	-4.8309916
SFRP5	-0.0828502	-2.0883914	-0.1529099	0.87905535	0.99978775	-4.6114557
ADIPOR2	-0.0828563	6.3243382	-0.9540984	0.34440511	0.99978775	-4.6881383
GGACT	-0.082901	0.95949267	-0.3092234	0.75837514	0.99978775	-4.6364016
DENND4A	-0.0829322	5.57213221	-0.6359551	0.52756769	0.99978775	-4.7900469
PPWD1	-0.0829461	4.63395668	-0.7767314	0.44080193	0.99978775	-4.7174775
MRPL44	-0.0829587	3.90624404	-0.7939998	0.43076895	0.99978775	-4.6920101
ENSCAFG00000014968	-0.082999	6.6784405	-0.741431	0.46173505	0.99978775	-4.7708556
MECR	-0.0830147	4.23801339	-0.7575855	0.45208532	0.99978775	-4.7168058
UMPS	-0.0831017	5.29362568	-0.8570884	0.39529056	0.99978775	-4.7144851
TMEM120A	-0.0831619	4.7319856	-0.7714594	0.44389224	0.99978775	-4.7220271
PEX2	-0.0832929	4.28112093	-0.7093541	0.48124246	0.99978775	-4.7250893
MCM3AP	-0.0832997	6.73766037	-1.3970368	0.16827813	0.99978775	-4.4589896
UBE3C	-0.0833195	5.94940266	-1.2937123	0.20142615	0.99978775	-4.5192368
ENSCAFG00000029900	-0.0833343	0.92961476	-0.3321328	0.74111102	0.99978775	-4.6410029
PIGF	-0.0835305	2.3073507	-0.5149483	0.60874991	0.99978775	-4.6660218
PRPF3	-0.0835712	5.28665532	-0.8417645	0.40373685	0.99978775	-4.719677
SPG21	-0.0835885	5.05873716	-0.4846074	0.62997004	0.99978775	-4.8172213
RBCK1	-0.0835905	6.24449991	-0.7080609	0.48203846	0.99978775	-4.7810512
C30H15orf41	-0.0836036	4.24035512	-0.7904299	0.4328318	0.99978775	-4.7034484
CCDC9B	-0.0836252	5.19844418	-0.4342828	0.66586225	0.99978775	-4.8219015
SND1	-0.0836566	8.48999145	-0.8408881	0.40422325	0.99978775	-4.7137442
MCM9	-0.0836832	3.92998775	-0.8935667	0.37563065	0.99978775	-4.6693637

SMARCA4	-0.0836914	7.11001732	-0.7348456	0.46570251	0.99978775	-4.7677587
CAVIN1	-0.0837806	10.0113288	-0.8514205	0.39840171	0.99978775	-4.6901815
PRR7	-0.0838124	-0.1135051	-0.2039939	0.83914678	0.99978775	-4.6280256
ARHGDIB	-0.0838273	2.50667046	-0.0911151	0.92774778	0.99978775	-4.6590599
SGO2	-0.0838604	4.14636091	-0.3851635	0.70167192	0.99978775	-4.8163796
RPL28	-0.0838647	8.56224119	-0.6058286	0.54723825	0.99978775	-4.782601
SRSF4	-0.0838975	4.78889874	-0.8393812	0.40506041	0.99978775	-4.7067961
MTMR4	-0.0839736	5.8077587	-0.6576006	0.51366703	0.99978775	-4.7897457
FAM155B	-0.084024	1.45320341	-0.2970563	0.76759537	0.99978775	-4.6844176
RIPOR1	-0.084053	7.26897633	-1.202684	0.23449322	0.99978775	-4.5685638
RNF170	-0.0841219	2.83389846	-0.4797964	0.63336456	0.99978775	-4.6927376
C29H8orf37	-0.0841681	1.71861554	-0.3236396	0.74749626	0.99978775	-4.6446113
ENTPD5	-0.0842943	2.83012686	-0.3789695	0.70623844	0.99978775	-4.7053409
GALNT16	-0.0843801	5.49571427	-0.2839356	0.77757623	0.99978775	-4.8617644
SP1	-0.0843963	6.80312632	-0.8773273	0.38430494	0.99978775	-4.7208826
SH3BP4	-0.0844579	5.30967679	-0.6572014	0.51392158	0.99978775	-4.7956889
DMXL2	-0.0845988	4.71931529	-0.6601735	0.51202788	0.99978775	-4.7517531
DTX2	-0.0846059	4.69470417	-0.5202216	0.60509545	0.99978775	-4.7783024
MYDGF	-0.084728	5.24161506	-0.6477212	0.51998712	0.99978775	-4.7830385
ANKHD1	-0.0847518	7.02528847	-0.8196522	0.41611905	0.99978775	-4.7421556
PPHLN1	-0.0847629	4.35528655	-0.7782728	0.43990082	0.99978775	-4.7092078
PRELID1	-0.0848406	6.68232502	-0.7704386	0.44449211	0.99978775	-4.7605833
VAMP5	-0.0848415	1.72094188	-0.2319317	0.81749249	0.99978775	-4.6443802
TBC1D5	-0.0848918	6.86941182	-0.5984761	0.55209505	0.99978775	-4.8123813
CTTNBP2	-0.0849258	4.15702054	-0.4309908	0.66823906	0.99978775	-4.7681812
ENSCAFG00000008104	-0.0849422	-2.0219504	-0.1010907	0.91986344	0.99978775	-4.6077617
TGFBR3	-0.0849676	7.19898778	-0.156314	0.87638489	0.99978775	-4.8797002
OTUD1	-0.0849858	2.6706301	-0.4488319	0.65539964	0.99978775	-4.7028003
SF1	-0.0849898	7.40278439	-1.2635996	0.21195454	0.99978775	-4.535513
SF3B4	-0.0851535	5.45350889	-0.7933371	0.43115141	0.99978775	-4.7457312
PPP1R9A	-0.0851856	-1.3959218	-0.1404761	0.88882126	0.99978775	-4.6137253
TBCB	-0.0852046	5.67032931	-0.7257308	0.47122584	0.99978775	-4.7687541
BCR	-0.0852178	6.18552652	-0.442226	0.66014166	0.99978775	-4.8497932
DDX20	-0.0852637	5.44793732	-0.814919	0.41879914	0.99978775	-4.7337228
HDLBP	-0.0852712	9.89877006	-1.1175464	0.26884745	0.99978775	-4.5940971
SBNO2	-0.0853622	5.72774215	-0.6416741	0.52387585	0.99978775	-4.7974107
FIBP	-0.0853775	5.87048119	-0.7172026	0.47642719	0.99978775	-4.7762248
CHST11	-0.0853995	4.5217534	-0.1971444	0.84447557	0.99978775	-4.8349511
ALDH3A2	-0.0854033	4.67637236	-0.6118764	0.54325969	0.99978775	-4.7693182
SOX13	-0.0854157	1.37717036	-0.1479239	0.88296937	0.99978775	-4.6544821
HYLS1	-0.0854687	2.65658766	-0.6553976	0.51507273	0.99978775	-4.6696198
SMARCAL1	-0.0854718	4.85239588	-0.9721877	0.33541398	0.99978775	-4.6606397
LNPEP	-0.0855098	3.71130552	-0.4763952	0.63576908	0.99978775	-4.7448515
PPP4C	-0.0855101	5.77931843	-0.8410709	0.40412176	0.99978775	-4.7315868
NOP53	-0.0855141	6.90806113	-0.5262319	0.60094259	0.99978775	-4.8322337
CYP27A1	-0.0855453	3.067818	-0.0797556	0.93673479	0.99978775	-4.6718972
BROX	-0.0857367	5.90345593	-0.9343192	0.35441567	0.99978775	-4.6945738
PAGR1	-0.0857919	0.99948917	-0.4452321	0.657982	0.99978775	-4.6318684
DCAF6	-0.0858118	5.29066229	-0.7855057	0.43568691	0.99978775	-4.7317586
CDYL	-0.0858275	5.28900241	-0.5408864	0.59087297	0.99978775	-4.8053844

METTL23	-0.0858902	1.74709074	-0.3759063	0.70850088	0.99978775	-4.6573825
LRRC17	-0.0858922	0.85013018	-0.1693944	0.86613725	0.99978775	-4.6822292
ENSCAFG00000028808	-0.085937	2.38143253	-0.5750471	0.56771512	0.99978775	-4.6832622
CLUH	-0.0860111	6.31141211	-0.6854257	0.49609002	0.99978775	-4.7882597
ENSCAFG00000032308	-0.0860224	4.14190413	-0.6471036	0.5203836	0.99978775	-4.7368913
PRICKLE1	-0.0860534	5.48559743	-0.269852	0.78833171	0.99978775	-4.8763617
APPL2	-0.0860663	5.26196638	-0.5967558	0.55323454	0.99978775	-4.8066631
COPE	-0.0860923	6.53666122	-0.8930692	0.37589451	0.99978775	-4.7146077
EFTUD2	-0.0861673	6.40740427	-0.7985379	0.42815504	0.99978775	-4.7510822
NUP88	-0.0862076	5.34478672	-0.9962846	0.32368051	0.99978775	-4.6623582
SNRNP70	-0.0862176	6.38574508	-0.900856	0.37177778	0.99978775	-4.7117387
EIF2AK4	-0.0862263	5.77476782	-1.034778	0.30551434	0.99978775	-4.6478656
DOHH	-0.086273	3.28789209	-0.5654983	0.57414316	0.99978775	-4.7189123
NPRL3	-0.0863631	3.96252966	-0.4705414	0.63991683	0.99978775	-4.7466647
ZBTB22	-0.0865426	3.15924233	-0.5460722	0.5873288	0.99978775	-4.6961742
BCCIP	-0.0865648	4.06088483	-0.7135433	0.47866886	0.99978775	-4.7117005
ZER1	-0.0866014	5.2237418	-0.666128	0.50824511	0.99978775	-4.7728655
NDUFV3	-0.0866545	5.71207773	-1.2134288	0.23039551	0.99978775	-4.5625353
USP30	-0.0866568	5.03605913	-1.0956312	0.27823957	0.99978775	-4.6129253
FAM198A	-0.0866649	0.17007251	-0.1055434	0.91634679	0.99978775	-4.621241
EXOC8	-0.0867002	3.4896498	-0.6308043	0.53090431	0.99978775	-4.6978381
ZC3HAV1	-0.0867183	3.71620878	-0.2908913	0.77228026	0.99978775	-4.7221406
RPA1	-0.0868006	6.47489143	-0.7219739	0.47351315	0.99978775	-4.7773123
SCRN1	-0.0868476	5.96827891	-0.330633	0.74223731	0.99978775	-4.8717619
SMG9	-0.0869523	5.13007602	-0.6248515	0.53477421	0.99978775	-4.7839412
PTPN22	-0.0870128	0.82344559	-0.2069473	0.83685133	0.99978775	-4.6666561
PPID	-0.0872394	5.57389175	-0.6409854	0.52431973	0.99978775	-4.7919634
LETM1	-0.0873641	6.25913505	-0.8421262	0.4035362	0.99978775	-4.7346651
HOXB7	-0.0874004	1.30859471	-0.1423285	0.88736516	0.99978775	-4.6573734
POLR2F	-0.087402	4.03505938	-0.6158461	0.54065626	0.99978775	-4.7382836
KLF11	-0.0874288	3.34272284	-0.3063781	0.76052819	0.99978775	-4.7224441
PPRC1	-0.0874409	5.95416433	-0.8697296	0.38840623	0.99978775	-4.7231568
ENSCAFG00000028127	-0.0874583	3.08465746	-0.3669246	0.71514968	0.99978775	-4.7263303
DZIP1	-0.0875129	7.83882059	-0.5386856	0.59238005	0.99978775	-4.8130735
SRSF9	-0.0875453	5.38515	-1.1716859	0.24661175	0.99978775	-4.5823018
TMEM8A	-0.0875505	5.39093732	-0.5888266	0.55850199	0.99978775	-4.8023365
DENND3	-0.0875524	4.53713584	-0.4197479	0.67638195	0.99978775	-4.8058495
ARNTL2	-0.0875589	5.87554305	-0.5027753	0.61722437	0.99978775	-4.8322596
ATPAF1	-0.0876099	3.39552805	-0.5221986	0.603728	0.99978775	-4.7073972
GIPC1	-0.0876248	5.25452954	-0.7196974	0.47490226	0.99978775	-4.7575528
MCRS1	-0.0877569	5.33042482	-0.9672528	0.3378513	0.99978775	-4.6734635
SLC12A2	-0.0877617	5.24914372	-0.607197	0.54633671	0.99978775	-4.7984408
PLEKHA5	-0.0878065	4.82621606	-0.642593	0.52328394	0.99978775	-4.7606075
CFDP1	-0.0880558	5.88925492	-0.9138285	0.36498367	0.99978775	-4.7036718
DOCK6	-0.0880798	6.22158705	-0.4792393	0.63375811	0.99978775	-4.8395666
TIAL1	-0.0881266	5.46988032	-0.8140548	0.41928958	0.99978775	-4.7352875
ENSCAFG00000011389	-0.0881466	6.96162839	-0.6910902	0.49255262	0.99978775	-4.7861881
WDHD1	-0.0881482	3.96898951	-0.4275736	0.67070983	0.99978775	-4.7887635
ENSCAFG00000000136	-0.0881822	9.0687853	-1.0625538	0.29284627	0.99978775	-4.6224969
MX2	-0.0882686	-0.9177766	-0.1692399	0.86625816	0.99978775	-4.6212767

TRIP6	-0.0882929	5.00696966	-0.8888136	0.37815657	0.99978775	-4.7011895
ENSCAFG00000032192	-0.0883188	4.58445275	-0.3513568	0.72672653	0.99978775	-4.8122708
ZNF865	-0.0883505	3.32141622	-0.5580905	0.57915422	0.99978775	-4.7070299
ATP5MC1	-0.0883678	4.97847755	-0.5875622	0.55934426	0.99978775	-4.7934343
RPA3	-0.0883914	1.14910398	-0.4340437	0.66603482	0.99978775	-4.6392385
PTPN23	-0.0884292	6.51592003	-1.1033488	0.27490616	0.99978775	-4.6207867
PCBP1	-0.0884413	6.51081298	-1.011798	0.31627392	0.99978775	-4.6640272
FKBP4	-0.0884627	5.85803371	-0.7153106	0.47758547	0.99978775	-4.7778976
PLEKHO2	-0.0884885	6.58751577	-0.7549639	0.45364328	0.99978775	-4.7659321
MRGPRF	-0.0885429	4.76354217	-0.255256	0.79952247	0.99978775	-4.872877
CTDP1	-0.0886216	4.6892026	-0.9928363	0.32534254	0.99978775	-4.6497374
LIAS	-0.0887443	2.71282771	-0.6640824	0.50954292	0.99978775	-4.65857
FRA10AC1	-0.0887462	3.93433337	-0.6386415	0.52583182	0.99978775	-4.7236359
RIF1	-0.0887609	6.0617686	-0.7532195	0.45468168	0.99978775	-4.765153
VAC14	-0.0888115	5.70465993	-0.9709324	0.33603285	0.99978775	-4.678777
METTL18	-0.088819	2.59059149	-0.5375509	0.59315783	0.99978775	-4.6673589
CSNK1G2	-0.0888673	5.85737127	-0.9955681	0.3240254	0.99978775	-4.6672484
RAB11FIP5	-0.0888725	6.77570599	-0.7881843	0.43413244	0.99978775	-4.7542044
ABHD6	-0.0888736	1.72695873	-0.319185	0.75085248	0.99978775	-4.6532853
RBM14	-0.0889039	5.22307944	-0.6679025	0.50712067	0.99978775	-4.7827671
CEBPG	-0.0889345	3.70191907	-0.6571017	0.51398522	0.99978775	-4.7274794
SPR	-0.0889546	5.14235092	-0.7639052	0.44834245	0.99978775	-4.7483454
OTUD7B	-0.0891298	4.51664466	-0.910366	0.36678924	0.99978775	-4.6697389
ENSCAFG00000001588	-0.0891739	5.35788908	-0.6480463	0.51977848	0.99978775	-4.7886135
FAM120A	-0.0891896	8.1961598	-0.9274561	0.35793296	0.99978775	-4.6845653
ENSCAFG00000019111	-0.0892124	4.11525802	-0.7658747	0.44717971	0.99978775	-4.7043458
ISY1	-0.0892825	5.3667869	-0.9156738	0.36402374	0.99978775	-4.695313
ALG6	-0.0892885	4.12899872	-0.6752447	0.50248275	0.99978775	-4.7168637
EFL1	-0.0893187	5.14432543	-1.0054967	0.31926845	0.99978775	-4.6527369
ISOC1	-0.0893203	4.63016225	-0.4232535	0.67383869	0.99978775	-4.8047267
SMARCC1	-0.0893816	7.45643471	-0.9000574	0.37219866	0.99978775	-4.706766
CERCAM	-0.0894659	7.13398859	-0.3149445	0.75405182	0.99978775	-4.873448
ENSCAFG00000023152	-0.089485	-0.6366087	-0.1699868	0.8656737	0.99978775	-4.6177583
PHLDB1	-0.0894942	8.06624691	-0.724919	0.47171957	0.99978775	-4.7599308
CD83	-0.0895007	-0.3026577	-0.1696286	0.86595399	0.99978775	-4.6266954
JMJD8	-0.0895358	4.94950451	-0.7194438	0.47505714	0.99978775	-4.7580043
DRG2	-0.0895399	5.46906608	-1.2293354	0.22442586	0.99978775	-4.552781
SLC11A2	-0.0895408	3.78673059	-0.6746491	0.50285811	0.99978775	-4.6985456
RBM22	-0.0895607	4.7843584	-0.9736535	0.3346923	0.99978775	-4.6597968
NOC2L	-0.089608	5.61496038	-0.8904279	0.37729745	0.99978775	-4.7114354
GEMIN8	-0.0896099	3.03033918	-0.7051497	0.48383312	0.99978775	-4.6735093
ENSCAFG00000013864	-0.0896235	3.39640841	-0.5685444	0.57208879	0.99978775	-4.7085209
DHX16	-0.0897322	5.58565227	-1.1535935	0.25389025	0.99978775	-4.5925804
CEP76	-0.0897496	3.24101638	-0.5885486	0.55868715	0.99978775	-4.7076206
CASKIN2	-0.0897881	4.09499792	-0.4883513	0.62733408	0.99978775	-4.7644151
RPS21	-0.0898007	7.41555487	-0.5937561	0.5552243	0.99978775	-4.8083687
ABHD16A	-0.0898341	5.41426704	-0.7889127	0.43371032	0.99978775	-4.7361398
NUCKS1	-0.0898453	6.31102739	-0.8189587	0.41651106	0.99978775	-4.7433198
MANF	-0.089895	5.38990367	-0.5188592	0.60603866	0.99978775	-4.8197271
ENSCAFG00000013253	-0.0899891	4.28171512	-0.8308572	0.40981597	0.99978775	-4.6913085

NUDT19	-0.0900351	3.24892633	-0.6430727	0.52297512	0.99978775	-4.6799446
DCLRE1A	-0.0900496	4.82441085	-0.9316695	0.35577095	0.99978775	-4.6773347
IMMP1L	-0.0901204	3.19489386	-0.9263288	0.35851284	0.99978775	-4.634971
RSL1D1	-0.0901267	5.64788876	-0.6071759	0.54635064	0.99978775	-4.8015894
CNOT10	-0.0903324	5.87346147	-0.9099409	0.36701132	0.99978775	-4.7070201
SCN1B	-0.090341	2.83947512	-0.2917132	0.77165514	0.99978775	-4.6892933
SELENOO	-0.0903809	4.17238291	-0.699994	0.48702059	0.99978775	-4.7282779
TOX2	-0.0903904	-1.3008599	-0.0914091	0.9275153	0.99978775	-4.6091398
ROMO1	-0.0904064	-0.3700941	-0.2892154	0.77355528	0.99978775	-4.6242647
ZHX3	-0.0904112	4.53392391	-0.3217075	0.74895133	0.99978775	-4.7682998
GATAD2A	-0.0904357	6.97641534	-1.0846712	0.28302185	0.99978775	-4.6287922
PLD2	-0.090496	5.06139331	-0.5983734	0.55216304	0.99978775	-4.7949106
MYLIP	-0.090669	2.51372229	-0.351532	0.72659583	0.99978775	-4.715319
VTI1B	-0.0906735	0.47158995	-0.3875274	0.69993208	0.99978775	-4.6333756
NDUFB6	-0.0907058	3.78794842	-0.5476381	0.58626058	0.99978775	-4.7408068
FAM208B	-0.0907218	6.60799072	-0.8825465	0.38150344	0.99978775	-4.7190029
PPP2R1B	-0.0907788	5.88046316	-0.7566394	0.45264723	0.99978775	-4.7617847
ENSCAFG00000014638	-0.090845	6.9636302	-1.5440411	0.12858365	0.99978775	-4.3659288
PARP3	-0.0908609	6.09373736	-0.7232024	0.47276452	0.99978775	-4.7768721
ARL10	-0.0908893	1.53203891	-0.4605767	0.64700397	0.99978775	-4.6524211
CDH23	-0.0909197	-2.1360186	-0.1282966	0.89840432	0.99978775	-4.6070371
HMGN4	-0.0909907	2.97146802	-0.6050248	0.54776817	0.99978775	-4.6739352
TEDC1	-0.0910336	3.17959911	-0.5963662	0.55349277	0.99978775	-4.6939713
TDRD7	-0.0910439	2.82115454	-0.2696546	0.78848279	0.99978775	-4.7280768
MBD3	-0.0910857	6.1283969	-1.0766253	0.28656891	0.99978775	-4.6336205
ZFP69B	-0.0910898	0.46051078	-0.2937905	0.77007605	0.99978775	-4.6308843
MTHFD1	-0.0911369	7.19809497	-0.8586326	0.39444552	0.99978775	-4.7261715
CEP170B	-0.091223	5.30594047	-0.7853096	0.43580085	0.99978775	-4.747951
MRM1	-0.0912363	1.14859754	-0.3847716	0.70196057	0.99978775	-4.6374018
ENSCAFG00000024573	-0.0912642	0.01990416	-0.2341029	0.8158154	0.99978775	-4.6284775
STARD6	-0.0912726	2.17698391	-0.3249079	0.74654161	0.99978775	-4.6622826
NUP188	-0.0913722	6.5796527	-0.8367923	0.40650118	0.99978775	-4.7366264
LEMD2	-0.091489	4.35434104	-0.7625597	0.44913777	0.99978775	-4.7127725
ZNF606	-0.0915132	2.77985179	-0.4493932	0.65499738	0.99978775	-4.6842274
HAUS1	-0.0915152	2.28338053	-0.4459772	0.65744715	0.99978775	-4.676886
PKNOX1	-0.0915184	3.12645794	-0.63864	0.52583274	0.99978775	-4.6761617
BEND3	-0.0915395	3.20151303	-0.620617	0.53753593	0.99978775	-4.7021089
NUP155	-0.091542	5.40088765	-0.6073093	0.5462628	0.99978775	-4.8022282
ARMCX5	-0.0915899	1.12112233	-0.3729499	0.71068689	0.99978775	-4.6311237
ENSCAFG00000031288	-0.0916427	5.51633425	-0.4459265	0.65748352	0.99978775	-4.838908
PANK2	-0.0916899	2.99451202	-0.649465	0.51886857	0.99978775	-4.6739188
ZFP37	-0.0916961	0.96168748	-0.2684388	0.78941326	0.99978775	-4.6469651
NOL10	-0.0917097	6.0415373	-0.7796178	0.43911542	0.99978775	-4.755603
SFMBT2	-0.0917503	2.57480551	-0.372913	0.71071418	0.99978775	-4.7105826
RAD51C	-0.091781	2.61753714	-0.6414624	0.5240123	0.99978775	-4.6659659
AP3M2	-0.0918295	1.2069978	-0.2701228	0.78812447	0.99978775	-4.6374077
CMTR1	-0.0918618	5.52121365	-1.4473986	0.15372898	0.99978775	-4.4299492
DRAM2	-0.0918879	3.32879449	-0.6260434	0.53399818	0.99978775	-4.6913387
AP5S1	-0.09199	2.14051981	-0.5115299	0.61112435	0.99978775	-4.6636699
ENSCAFG00000005160	-0.0920199	4.67104121	-0.5445444	0.58837189	0.99978775	-4.7821604

SH3PXD2B	-0.0920279	6.40176296	-0.5883393	0.55882653	0.99978775	-4.8169716
ZNF341	-0.092061	1.98800224	-0.4566751	0.64978792	0.99978775	-4.6495132
MMP14	-0.092101	9.57996585	-0.4250398	0.67254422	0.99978775	-4.7845329
ENSCAFG00000014246	-0.0922207	3.75485901	-0.6969785	0.48889023	0.99978775	-4.7031728
SMARCD2	-0.092235	5.83424612	-0.4906541	0.62571516	0.99978775	-4.8304312
MANBA	-0.0922699	6.44678438	-0.690985	0.49261818	0.99978775	-4.7873321
TFPI2	-0.0923264	4.87605859	-0.1745058	0.86213902	0.99978775	-4.871041
RALGPS2	-0.0923424	1.87796718	-0.3438976	0.73229652	0.99978775	-4.7401488
ZNHIT2	-0.0923825	2.74143881	-0.5272764	0.60022224	0.99978775	-4.6941656
CHST7	-0.0924106	-1.5762672	-0.1035447	0.91792509	0.99978775	-4.6075834
SLBP	-0.0924333	3.42741686	-0.5406088	0.59106291	0.99978775	-4.7144962
PXDN	-0.0924507	7.5787346	-0.3869178	0.70038056	0.99978775	-4.8340769
SP2	-0.0924579	4.35540375	-0.7119046	0.47967462	0.99978775	-4.730089
ENSCAFG00000017667	-0.0924824	4.13855844	-0.6703659	0.50556202	0.99978775	-4.7214911
LSM14B	-0.0925151	4.81217489	-0.9668051	0.33807297	0.99978775	-4.6617551
GMIP	-0.0925161	4.48106656	-0.7708362	0.44425837	0.99978775	-4.7193241
TRAPPC8	-0.0925517	5.87390424	-1.1420836	0.25859998	0.99978775	-4.5991894
MTMR3	-0.0925907	5.30362805	-1.0480839	0.29939981	0.99978775	-4.6397385
NCKAP5L	-0.0926037	5.88637395	-0.7995984	0.42754562	0.99978775	-4.745355
PLEKHO1	-0.0926783	4.59562019	-0.4102781	0.68327105	0.99978775	-4.8178211
CSTF1	-0.092734	4.41362304	-0.9093951	0.36729656	0.99978775	-4.6732241
ENSCAFG00000031490	-0.0927465	14.3903636	-0.432416	0.66720963	0.99978775	-4.6974818
ANKRD54	-0.0927776	4.31333623	-0.7834046	0.43690856	0.99978775	-4.7091189
GLS2	-0.0928658	-0.7293362	-0.200007	0.84224759	0.99978775	-4.6174023
COQ8A	-0.0931523	3.24556251	-0.3000812	0.76529991	0.99978775	-4.6964297
ENSCAFG00000015686	-0.0931777	7.63235479	-1.3278629	0.1899664	0.99978775	-4.4998028
HIST1H2AC	-0.0931849	0.30377041	-0.374397	0.70961653	0.99978775	-4.6349993
ENSCAFG00000031969	-0.0932154	-2.4163279	-0.1536197	0.87849842	0.99978775	-4.607004
LRRC32	-0.0932207	3.19170531	-0.2023128	0.84045394	0.99978775	-4.7879864
SMCO2	-0.0932955	-2.3419129	-0.1050899	0.91670486	0.99978775	-4.6069391
PRDM1	-0.0932955	-2.3633501	-0.1406863	0.88865605	0.99978775	-4.6068338
ZBTB16	-0.0932955	1.13167081	-0.0507401	0.95972511	0.99978775	-4.607041
ENSCAFG00000003424	-0.0933279	0.55339742	-0.2982118	0.76671824	0.99978775	-4.640933
IP6K1	-0.093345	4.88900852	-0.8420924	0.40355496	0.99978775	-4.7041154
SMC6	-0.0934	6.30195703	-0.5484233	0.58572534	0.99978775	-4.8278001
CXXC1	-0.0934229	5.16709846	-0.9414222	0.35079918	0.99978775	-4.6826199
GZF1	-0.0934496	4.23680407	-0.8301264	0.41022531	0.99978775	-4.6943491
APEX1	-0.0934683	7.0286347	-0.8417177	0.40376284	0.99978775	-4.7310936
SLC38A7	-0.093601	5.14826032	-0.7579908	0.45184474	0.99978775	-4.7491854
PARD3	-0.0936333	6.89704344	-0.7983547	0.42826039	0.99978775	-4.7509448
NUP133	-0.0936613	5.1711746	-1.0552207	0.29615502	0.99978775	-4.6304506
RNFT1	-0.0936709	4.11818627	-0.6416139	0.52391465	0.99978775	-4.7428975
ENSCAFG00000008385	-0.0936733	2.67662873	-0.5200891	0.60518718	0.99978775	-4.6703311
ENSCAFG00000016940	-0.0937726	4.77229166	-1.3640019	0.17838481	0.99978775	-4.4826409
MELTF	-0.093779	-0.1935801	-0.2032738	0.83970663	0.99978775	-4.6220016
B4GALT7	-0.0938	5.14850711	-0.7896677	0.43327301	0.99978775	-4.7392664
NCDN	-0.0938066	5.14953242	-0.7497625	0.4567436	0.99978775	-4.7481873
TBC1D22B	-0.0938076	2.9422708	-0.6439846	0.52238824	0.99978775	-4.6788559
GRASP	-0.0938268	2.41715802	-0.3456103	0.73101625	0.99978775	-4.6799019
CENPV	-0.0938542	3.5203652	-0.5109273	0.61154332	0.99978775	-4.7190068

B3GAT3	-0.0939061	5.38449404	-0.7360626	0.46496784	0.99978775	-4.7624034
KIN	-0.0940423	3.48991148	-0.7558938	0.45309032	0.99978775	-4.6763208
SLC25A13	-0.0940493	3.90963024	-0.588802	0.55851839	0.99978775	-4.7511542
SNED1	-0.0940544	2.58473137	-0.1494724	0.88175344	0.99978775	-4.6703438
DDAH2	-0.0940777	3.9700773	-0.5260202	0.60108869	0.99978775	-4.7546966
MYO9B	-0.0940796	8.20458465	-0.67195	0.50456113	0.99978775	-4.7624266
VEGFB	-0.0941524	4.99204313	-0.519426	0.60564618	0.99978775	-4.8168012
CHERP	-0.0941537	5.57107129	-1.0552691	0.2961331	0.99978775	-4.6390127
ATG101	-0.0942331	5.347599	-0.9161408	0.36378106	0.99978775	-4.6984961
NDUFS8	-0.0942545	5.35656304	-0.4506393	0.65410469	0.99978775	-4.829465
AAMP	-0.0942806	5.49009833	-0.9215501	0.36097773	0.99978775	-4.6919961
KIAA1109	-0.0943041	4.92125844	-0.6826372	0.49783651	0.99978775	-4.7587433
ARHGEF6	-0.0943758	4.59295802	-0.3967076	0.69319064	0.99978775	-4.8323993
ZNF837	-0.0943852	1.22647517	-0.3956863	0.69393941	0.99978775	-4.6385694
THOC1	-0.094588	3.45382931	-0.6613768	0.51126223	0.99978775	-4.6990441
ZBTB40	-0.094626	4.61624652	-0.6659816	0.50833788	0.99978775	-4.7481796
SLC39A13	-0.0946788	4.80830273	-0.5161862	0.60789115	0.99978775	-4.7960457
ARRDC1	-0.0948381	4.04490099	-0.5024203	0.61747228	0.99978775	-4.7492738
ENSCAFG00000024864	-0.0948451	2.80564648	-0.2462983	0.80641153	0.99978775	-4.7107678
PDHX	-0.0948781	4.06448298	-0.8261723	0.41244422	0.99978775	-4.6822085
GPATCH4	-0.0949115	5.85892296	-0.7246709	0.47187051	0.99978775	-4.7733487
ODC1	-0.0950786	6.55919895	-0.4675419	0.64204664	0.99978775	-4.846521
GCN1	-0.0951303	7.14726194	-0.9539417	0.34448371	0.99978775	-4.6858287
PRIMPOL	-0.0951884	3.97543061	-0.6080799	0.54575547	0.99978775	-4.7265596
FAM135A	-0.0952215	5.15027763	-0.6055564	0.54741768	0.99978775	-4.789096
TSEN2	-0.0952282	3.45097427	-0.5594138	0.57825757	0.99978775	-4.7107825
ZNF75D	-0.0952507	1.60574277	-0.4549984	0.65098585	0.99978775	-4.6458825
MTHFD1L	-0.0952675	7.06860025	-0.5770049	0.56640154	0.99978775	-4.8152343
RABGAP1	-0.0952861	6.21782085	-0.9391249	0.35196622	0.99978775	-4.6951709
MUM1	-0.0953014	4.05495852	-0.7311191	0.46795618	0.99978775	-4.7089
DISP1	-0.0953087	5.30253876	-0.6926969	0.49155182	0.99978775	-4.7719754
SURF2	-0.0953412	3.33541054	-0.6988016	0.48775944	0.99978775	-4.673994
TEN1	-0.0954088	2.66491904	-0.6263921	0.53377124	0.99978775	-4.6766954
TRMT61A	-0.0954959	4.37296273	-0.603869	0.54853054	0.99978775	-4.7685293
ZFYVE19	-0.0955128	2.50114479	-0.6157904	0.54069274	0.99978775	-4.6655031
GTPBP2	-0.0955476	4.92527285	-0.6514745	0.51758117	0.99978775	-4.7778983
GRINA	-0.0955601	6.44132044	-0.5801287	0.56430882	0.99978775	-4.8195608
ENSCAFG00000000496	-0.0955877	3.03319345	-0.7025918	0.48541305	0.99978775	-4.67176
CHKB	-0.0956006	3.33068491	-0.5831622	0.56228021	0.99978775	-4.6991954
LIFR	-0.0956146	4.07103139	-0.3459395	0.73077029	0.99978775	-4.7809121
LYSMD4	-0.0956932	4.54252149	-0.8868248	0.37921665	0.99978775	-4.6825151
POMT1	-0.0957134	5.02958669	-0.9373114	0.35288927	0.99978775	-4.6844691
BTK	-0.0957814	2.06227302	-0.4153554	0.67957395	0.99978775	-4.7174531
GLB1L	-0.0957891	4.67394407	-0.4420687	0.66025472	0.99978775	-4.7979695
PCDH12	-0.09584	-2.0374583	-0.0883321	0.92994874	0.99978775	-4.6074025
DESI2	-0.0958457	6.24351207	-1.1847928	0.24143368	0.99978775	-4.5790209
FAM151B	-0.095886	4.21916188	-0.9194152	0.36208243	0.99978775	-4.6584968
ALDH1L2	-0.0959903	7.96374324	-0.5622687	0.57632526	0.99978775	-4.8032662
ZNF613	-0.0959972	-0.0762538	-0.2772829	0.78265151	0.99978775	-4.6188522
NCL	-0.0960009	8.98919283	-0.6289618	0.53210057	0.99978775	-4.7675537

ENSCAFG00000013668	-0.0960158	5.63848091	-0.3098363	0.75791166	0.99978775	-4.8658568
OAS2	-0.0960461	6.04142535	-0.7234261	0.47262827	0.99978775	-4.7753307
RABGGTA	-0.096073	4.28120618	-0.7034906	0.48485754	0.99978775	-4.7276739
GPR173	-0.0961588	0.55128257	-0.23816	0.81268387	0.99978775	-4.6294084
RNF168	-0.0961589	5.55460078	-1.1244351	0.26594197	0.99978775	-4.6051777
RITA1	-0.0963516	4.70694518	-0.8048433	0.42453904	0.99978775	-4.7170986
STUB1	-0.096404	5.59937513	-1.1219309	0.26699562	0.99978775	-4.6059335
TRIM28	-0.0964142	7.40767371	-1.0281697	0.30858258	0.99978775	-4.6507034
NCOA5	-0.0964194	4.62064674	-1.1428502	0.25828438	0.99978775	-4.5882204
PPP1R37	-0.0964431	5.25800053	-0.9340052	0.35457609	0.99978775	-4.6846334
HINT3	-0.0964759	1.14259404	-0.2688844	0.78907224	0.99978775	-4.6362893
TRIM11	-0.0965463	3.31730323	-0.6660389	0.50830158	0.99978775	-4.6882751
ZXDC	-0.0967964	4.70632244	-1.0565401	0.2955578	0.99978775	-4.6252304
VPS35L	-0.0969292	5.53710994	-1.0357371	0.30507076	0.99978775	-4.6479409
EIF4G1	-0.0969471	9.33560172	-1.0827971	0.28384529	0.99978775	-4.6114227
ENSCAFG00000011367	-0.0969556	4.12875217	-0.6395978	0.52521461	0.99978775	-4.7373758
PPP4R3A	-0.0969771	5.93463922	-0.8752486	0.38542433	0.99978775	-4.719189
POLE3	-0.0970826	5.50040218	-0.9276768	0.35781949	0.99978775	-4.6934554
CCDC91	-0.0970998	5.62491108	-0.8217209	0.41495092	0.99978775	-4.7355316
SCAF1	-0.0971149	6.37954269	-1.1451503	0.25733908	0.99978775	-4.599876
S100A6	-0.0971334	7.90896856	-0.4914426	0.62516126	0.99978775	-4.8324155
DPEP1	-0.097292	-1.9351777	-0.1003599	0.92044078	0.99978775	-4.6069508
FGL1	-0.097292	-1.6448821	-0.0698632	0.94456788	0.99978775	-4.6070132
NAA30	-0.0972938	2.54755886	-0.587811	0.55917851	0.99978775	-4.6709348
DOP1B	-0.0973794	3.9804485	-0.2532834	0.80103814	0.99978775	-4.8246157
IBTK	-0.0974203	6.68432745	-0.7864997	0.43510971	0.99978775	-4.7553903
ENSCAFG00000013076	-0.0974316	4.11111875	-0.8488649	0.39980947	0.99978775	-4.6843547
ENSCAFG00000012274	-0.0974619	3.36072564	-0.6809639	0.49888613	0.99978775	-4.6785315
CBX1	-0.097493	3.69328215	-0.8301464	0.41021407	0.99978775	-4.6708318
USPL1	-0.0976232	3.492265	-0.630053	0.53139195	0.99978775	-4.6996277
B3GALNT1	-0.0976311	3.65908047	-0.499457	0.61954364	0.99978775	-4.7324368
PEX16	-0.0976592	3.23010235	-0.7274996	0.4701511	0.99978775	-4.6747019
DNAAF2	-0.0978255	4.49268074	-0.7533067	0.45462976	0.99978775	-4.7317054
HMGB1	-0.0978524	7.43143909	-0.5881149	0.55897601	0.99978775	-4.8045487
TM6SF1	-0.0978661	-1.3951329	-0.1692945	0.86621548	0.99978775	-4.6132127
ELOVL1	-0.0978736	5.25754825	-0.7955543	0.4298725	0.99978775	-4.7343654
TMEM254	-0.0979078	3.04397197	-0.5790704	0.56501739	0.99978775	-4.6875204
GID8	-0.097944	3.92723483	-0.8671242	0.38981896	0.99978775	-4.66603
TP73	-0.0979811	0.2963893	-0.2882811	0.77426639	0.99978775	-4.6484036
FZD5	-0.0979852	0.20525826	-0.2346797	0.81536994	0.99978775	-4.6279828
NGRN	-0.0980472	4.39829522	-0.9634196	0.33975254	0.99978775	-4.6500953
SRA1	-0.0981085	4.89748985	-0.6294094	0.53180985	0.99978775	-4.7667003
ENSCAFG00000017861	-0.0981194	6.05771898	-1.0299641	0.30774737	0.99978775	-4.6547289
CRY1	-0.098175	4.15741717	-0.9922435	0.32562884	0.99978775	-4.6333247
ENTR1	-0.098182	4.06115078	-0.8602337	0.39357058	0.99978775	-4.6761236
C4H1orf131	-0.0982	3.91976181	-0.9330611	0.35505876	0.99978775	-4.6475608
ENSCAFG00000030005	-0.0982453	6.33494527	-0.7651174	0.44762657	0.99978775	-4.7628716
ENSCAFG00000031829	-0.0983267	4.80831918	-1.2390737	0.22082767	0.99978775	-4.5449181
PKN1	-0.0983353	6.5798841	-0.8470438	0.40081451	0.99978775	-4.7328888
GNL1	-0.0983456	5.15842454	-1.0677762	0.29050553	0.99978775	-4.6278245

CSRNP1	-0.0984407	4.69872805	-0.5657249	0.5739902	0.99978775	-4.8002389
INTS1	-0.0984696	6.43262224	-0.8156067	0.41840911	0.99978775	-4.7447847
VDR	-0.0985147	3.98226463	-0.2649558	0.79208074	0.99978775	-4.7684233
WASF1	-0.0985767	1.26684319	-0.2695305	0.78857775	0.99978775	-4.6378906
RSRC1	-0.0986706	4.64562636	-0.8262812	0.41238298	0.99978775	-4.7126259
HM13	-0.098746	7.52996544	-0.7866756	0.43500756	0.99978775	-4.748305
MT-ND6	-0.098752	5.89530935	-0.7442608	0.46003619	0.99978775	-4.7644659
SNX33	-0.0987741	6.53793795	-0.4678917	0.64179807	0.99978775	-4.8459635
AP3M1	-0.0987803	4.91133825	-0.9121615	0.36585225	0.99978775	-4.6812546
ADH5	-0.0988521	2.052532	-0.3688726	0.71370575	0.99978775	-4.6609335
INTS5	-0.0989001	5.01295375	-1.009536	0.3173467	0.99978775	-4.6493448
ANAPC7	-0.0989767	4.72689974	-1.0751523	0.28722159	0.99978775	-4.6199415
CLCF1	-0.0990711	3.08873904	-0.4357443	0.66480822	0.99978775	-4.7080571
RRNAD1	-0.0991732	4.79496152	-1.109616	0.27221996	0.99978775	-4.6050921
RHBDF1	-0.0992138	6.42016933	-0.9043131	0.36995937	0.99978775	-4.7103396
DBN1	-0.0992205	7.36409768	-0.8459105	0.40144073	0.99978775	-4.7262714
ENSCAFG00000032015	-0.0992224	5.35682137	-1.1873697	0.24042496	0.99978775	-4.5728608
ENSCAFG00000004882	-0.0992259	5.57002483	-0.8469371	0.40087345	0.99978775	-4.7249351
KIF2A	-0.0992339	6.43358776	-0.885746	0.37979243	0.99978775	-4.7178589
MIF4GD	-0.0993874	4.05600774	-0.514473	0.60907981	0.99978775	-4.7572016
LNX1	-0.0993935	-0.6960893	-0.1915544	0.84882996	0.99978775	-4.6224244
GPANK1	-0.0994354	2.64764115	-0.629857	0.53151918	0.99978775	-4.6587349
SNRNP40	-0.0994385	4.63506824	-0.7648683	0.44777363	0.99978775	-4.7268022
PLA2G4E	-0.0994545	-1.9393248	-0.1513247	0.88029944	0.99978775	-4.6093946
COQ6	-0.099544	4.25701281	-0.9764406	0.33332292	0.99978775	-4.6501469
DYRK3	-0.0995633	2.07787107	-0.4796592	0.63346144	0.99978775	-4.650413
SLC30A1	-0.09967	6.65113742	-0.5579876	0.57922398	0.99978775	-4.8253784
ENSCAFG00000017103	-0.0997438	6.11179498	-0.9197833	0.36189183	0.99978775	-4.7008803
SELL	-0.0997457	-2.9833131	-0.217092	0.8289779	0.99978775	-4.6065053
RNF144A	-0.0997483	3.20360759	-0.3453475	0.73121265	0.99978775	-4.707302
LRRC8A	-0.0998946	6.86392086	-0.7916699	0.43211459	0.99978775	-4.7529658
ANXA11	-0.0999109	7.76381979	-0.8051252	0.42437784	0.99978775	-4.7411869
RASSF4	-0.1000762	2.95696596	-0.3577301	0.7219791	0.99978775	-4.7448114
FOSL1	-0.1000938	4.94975264	-0.4175031	0.67801248	0.99978775	-4.8330679
NVL	-0.1000952	4.90872747	-0.9497134	0.34660827	0.99978775	-4.6735343
SEPT8	-0.1001069	6.54168827	-0.9282491	0.35752539	0.99978775	-4.7004492
LAMTOR1	-0.1001422	4.65193479	-0.6608574	0.51159259	0.99978775	-4.7650495
PNPT1	-0.1001515	4.8731292	-0.8608897	0.39321246	0.99978775	-4.7027038
PPEF1	-0.1001787	-1.2161665	-0.2005044	0.84186065	0.99978775	-4.6147821
QTRT1	-0.1001978	3.306272	-0.5890049	0.55838325	0.99978775	-4.7019151
NHEJ1	-0.1002141	3.02815187	-0.6340528	0.52879868	0.99978775	-4.6796858
BICDL1	-0.1002253	-1.6639379	-0.1626344	0.87143051	0.99978775	-4.6082412
MUL1	-0.1002897	5.20695648	-1.3641612	0.17833499	0.99978775	-4.4797322
ABTB1	-0.1003062	3.15133945	-0.3410977	0.73439102	0.99978775	-4.7082037
TAGLN2	-0.1003447	9.41789977	-0.6155831	0.54082852	0.99978775	-4.7538511
PHACTR1	-0.1003785	2.82280078	-0.2028441	0.84004075	0.99978775	-4.6957775
ENSCAFG00000031862	-0.1004718	2.4447902	-0.5990494	0.55171555	0.99978775	-4.6543524
LOXL3	-0.1004792	7.40096144	-0.3165904	0.75280951	0.99978775	-4.8726888
MED31	-0.1004884	2.41205817	-0.6493071	0.51896978	0.99978775	-4.6536554
SENP1	-0.1005138	3.96224506	-0.771989	0.44358123	0.99978775	-4.6935549

SRSF11	-0.1005353	6.50147372	-1.0752258	0.287189	0.99978775	-4.6344607
SLC25A32	-0.1005787	4.28298863	-0.8257798	0.41266485	0.99978775	-4.6892531
KLHL5	-0.1005828	6.51513014	-0.5847942	0.56119039	0.99978775	-4.8155731
RNF19B	-0.1007073	4.33046541	-0.6852137	0.49622266	0.99978775	-4.7364947
ZFAT	-0.100724	3.20973232	-0.79538	0.42997292	0.99978775	-4.6580522
RPL37	-0.1007317	2.22482638	-0.3108883	0.75711625	0.99978775	-4.6505292
ERCC8	-0.100749	2.86951484	-0.6489979	0.51916806	0.99978775	-4.6795174
IQSEC1	-0.1007566	4.89267905	-0.7138169	0.47850105	0.99978775	-4.7509373
RPS23	-0.1007661	7.95870082	-0.7778173	0.44016699	0.99978775	-4.7420207
PCBD2	-0.1008109	2.44362246	-0.7219563	0.47352388	0.99978775	-4.6560473
KAT8	-0.1008688	4.09366248	-0.7761316	0.4411529	0.99978775	-4.6976411
LUC7L2	-0.1009716	5.84755127	-1.3066977	0.19700902	0.99978775	-4.5118898
RAB32	-0.1010344	5.34508787	-0.6251927	0.53455197	0.99978775	-4.7979973
CLIP3	-0.1010394	5.79738972	-0.7363908	0.46476983	0.99978775	-4.7631815
TBCCD1	-0.1011012	3.96578603	-0.826067	0.41250342	0.99978775	-4.6774176
PLCD3	-0.1012681	4.81784769	-0.5835976	0.56198937	0.99978775	-4.7773007
CFAP69	-0.1013544	0.21864858	-0.0819069	0.93503217	0.99978775	-4.6110502
G2E3	-0.1013615	4.61190761	-0.6130472	0.54249115	0.99978775	-4.7705378
TAF15	-0.1013974	6.42519299	-1.1392915	0.25975183	0.99978775	-4.6022709
BFSP1	-0.1015325	3.05466557	-0.4856152	0.62926	0.99978775	-4.7087043
HARS2	-0.1015341	3.33665781	-0.7011336	0.48631503	0.99978775	-4.6740848
GNB3	-0.1015867	1.76277897	-0.3593514	0.72077318	0.99978775	-4.6814341
DPP7	-0.1016971	6.05777395	-0.4970232	0.6212472	0.99978775	-4.8334839
STEAP2	-0.1017102	4.49254694	-0.2082709	0.83582309	0.99978775	-4.7319926
CREBRF	-0.1017115	3.25697378	-0.2894259	0.77339505	0.99978775	-4.6917338
IKBKE	-0.1017683	5.75451119	-0.7927598	0.4314848	0.99978775	-4.744868
TUBB2A	-0.1018043	5.09528515	-0.3122644	0.75607619	0.99978775	-4.8249231
ASIC3	-0.1018272	0.19008905	-0.2957598	0.76857991	0.99978775	-4.6234889
CWC27	-0.101846	4.64931967	-0.9990149	0.32236864	0.99978775	-4.6427717
TUBGCP2	-0.1018468	7.0653204	-1.386797	0.1713625	0.99978775	-4.4652976
TCF12	-0.1019053	6.70043183	-0.6227741	0.53612814	0.99978775	-4.8079066
ENSCAFG00000003386	-0.1019541	5.7731446	-1.1458022	0.25707162	0.99978775	-4.5973922
METTL7A	-0.1019681	3.41928974	-0.1331866	0.89455484	0.99978775	-4.7019445
DNASE1L1	-0.102023	5.32758487	-0.7408573	0.46207995	0.99978775	-4.752543
CCDC113	-0.1021122	4.60976602	-0.4185266	0.67726891	0.99978775	-4.809364
RAB4A	-0.1021485	3.62244643	-1.0519253	0.29765029	0.99978775	-4.6048119
NOS1AP	-0.1021746	-1.3894006	-0.1995254	0.84262235	0.99978775	-4.6155344
D2HGDH	-0.1023295	4.03227668	-0.4937024	0.623575	0.99978775	-4.7624329
CBLC	-0.1024062	3.03361988	-0.4252301	0.67240643	0.99978775	-4.7434219
ENSCAFG00000029381	-0.1024717	4.5446769	-0.6951751	0.49001033	0.99978775	-4.7361215
ENSCAFG00000021718	-0.10248	0.67462483	-0.2487919	0.80449221	0.99978775	-4.6336029
COX5A	-0.1025017	0.80379051	-0.2606534	0.7953792	0.99978775	-4.63539
TIMM8B	-0.102529	2.43226304	-0.4892614	0.62669404	0.99978775	-4.6773293
GOLM1	-0.1025395	6.25731834	-0.4055444	0.68672492	0.99978775	-4.8570531
HSPB8	-0.1026624	6.36305177	-0.482863	0.63119991	0.99978775	-4.8432963
CPSF6	-0.1026846	5.38794178	-1.0857142	0.2825643	0.99978775	-4.6233222
GPS2	-0.1027974	4.4387714	-0.8158268	0.4182843	0.99978775	-4.7069467
RWDD2B	-0.102824	2.49217268	-0.4773146	0.63511872	0.99978775	-4.6706343
ABCB9	-0.1028433	3.80364342	-0.6086308	0.54539294	0.99978775	-4.7076611
C9H17orf80	-0.1028779	2.73417779	-0.711706	0.47979663	0.99978775	-4.6560484

SBF1	-0.1028929	6.23811072	-1.1633219	0.24995765	0.99978775	-4.5902111
SDK1	-0.1028935	1.11329973	-0.4517864	0.65328334	0.99978775	-4.6979569
WDR43	-0.1029386	5.87861537	-0.8312417	0.40960073	0.99978775	-4.7346797
ENSCAFG00000026367	-0.1029423	-0.7053524	-0.231759	0.81762591	0.99978775	-4.6169551
TRAPPC4	-0.1029566	3.22482734	-0.8389206	0.40531652	0.99978775	-4.6540455
CCDC89	-0.1029583	0.03560701	-0.3077055	0.75952356	0.99978775	-4.6246449
DHX15	-0.1030195	7.00006559	-0.9475179	0.34771484	0.99978775	-4.6903737
TNRC6B	-0.1031413	5.69188262	-1.1272669	0.26475407	0.99978775	-4.6053498
PHYKPL	-0.1031434	3.57785323	-0.6697096	0.50597705	0.99978775	-4.6989453
MRPL51	-0.1034226	4.73324255	-0.4761641	0.63593262	0.99978775	-4.8008633
COTL1	-0.1034794	6.03462324	-0.3438645	0.73232128	0.99978775	-4.8612127
STAG1	-0.1034891	5.58030262	-0.8780055	0.38394017	0.99978775	-4.716313
CERK	-0.1035272	7.28511296	-0.5909916	0.5570613	0.99978775	-4.8114513
CLCN6	-0.1035456	5.49387051	-0.7672874	0.44634676	0.99978775	-4.7549481
GDNF	-0.1035778	5.40804963	-0.2529318	0.8013084	0.99978775	-4.8785608
ENSCAFG00000022725	-0.1036738	-0.9310643	-0.2499021	0.80363804	0.99978775	-4.6102184
TXNDC16	-0.1037298	3.45156728	-0.3462005	0.73057531	0.99978775	-4.6936528
ZBTB12	-0.1037298	2.32749709	-0.5408298	0.5909117	0.99978775	-4.6660362
CCDC93	-0.1037453	5.52726015	-0.9359726	0.35357171	0.99978775	-4.6898133
KLHL4	-0.1037724	-2.4574168	-0.1325412	0.89506281	0.99978775	-4.6074498
SLC25A1	-0.1037887	5.85777614	-1.2174739	0.22886652	0.99978775	-4.5610114
HTATIP2	-0.1038304	3.84036823	-0.7041247	0.48446588	0.99978775	-4.6949429
DTD2	-0.1038673	3.32194654	-0.4828943	0.63117783	0.99978775	-4.7145867
ARPC1B	-0.1038713	7.91554864	-0.6411536	0.52421131	0.99978775	-4.7952934
ORC3	-0.103888	4.76861833	-0.9606993	0.34110601	0.99978775	-4.6612756
GLT1D1	-0.1039961	4.0236172	-0.430209	0.66880403	0.99978775	-4.8145818
ENSCAFG00000011131	-0.1039984	3.17525547	-0.6636328	0.50982842	0.99978775	-4.6829742
PIGU	-0.1040005	4.72521436	-0.7916698	0.43211467	0.99978775	-4.7253345
ENSCAFG00000011658	-0.1040373	-0.4401498	-0.2824197	0.77873187	0.99978775	-4.62939
SLC25A37	-0.1040552	5.41901168	-0.65431	0.51576751	0.99978775	-4.7858655
POLL	-0.1042261	3.33765181	-0.7463819	0.45876514	0.99978775	-4.6660576
BOP1	-0.104274	5.89856558	-0.795859	0.42969691	0.99978775	-4.7490098
TAZ	-0.1043395	4.91680383	-0.7819463	0.43775761	0.99978775	-4.7368521
PFDN6	-0.104365	3.31362776	-0.7814202	0.4380642	0.99978775	-4.6659061
HYAL2	-0.1043785	4.55375508	-0.3646991	0.71680062	0.99978775	-4.8021564
OASL2	-0.1043801	3.7554549	-0.8929273	0.3759698	0.99978775	-4.6557807
POT1	-0.1043921	5.15495777	-1.1687714	0.24777396	0.99978775	-4.5794595
NABP2	-0.1044105	6.8390907	-1.0378454	0.30409725	0.99978775	-4.6509082
ENSCAFG00000029004	-0.1045083	4.902639	-0.689984	0.49324233	0.99978775	-4.7568426
SNUPN	-0.1045772	4.22857968	-0.5817148	0.56324772	0.99978775	-4.7387383
EMC6	-0.1046478	3.79617732	-0.5654704	0.57416204	0.99978775	-4.728844
FYCO1	-0.1046688	6.25079509	-0.5868652	0.55980885	0.99978775	-4.8172899
ENSCAFG00000010311	-0.1046877	2.44606754	-0.5942656	0.55488608	0.99978775	-4.6561093
ADD1	-0.1047018	7.7233389	-1.4164803	0.16253971	0.99978775	-4.448763
C21H11orf54	-0.1048088	4.26728235	-0.8519428	0.39811438	0.99978775	-4.6707437
PFKFB2	-0.1048333	2.73297198	-0.6691043	0.50635996	0.99978775	-4.6561343
UBN1	-0.1049044	5.0974746	-0.9021244	0.37110994	0.99978775	-4.6939131
SART3	-0.1049616	5.09856603	-1.1499728	0.25536514	0.99978775	-4.588688
CCM2L	-0.1049694	-2.2582964	-0.1285513	0.89820375	0.99978775	-4.6076211
ARFGAP2	-0.1049775	5.13110491	-1.4138562	0.16330519	0.99978775	-4.4524852

SUMO1	-0.1049788	4.03500832	-1.1320542	0.26275441	0.99978775	-4.5842929
VSTM4	-0.1049868	3.23946697	-0.6466007	0.52070652	0.99978775	-4.7064118
ENPP1	-0.105	5.79410957	-0.3990851	0.69144882	0.99978775	-4.844415
NUBP2	-0.1050463	4.4088075	-0.8841926	0.38062258	0.99978775	-4.6787471
IRAK1	-0.1050821	6.33368023	-0.7841315	0.43648569	0.99978775	-4.7544852
LMO2	-0.1051042	2.07954193	-0.0916822	0.92729936	0.99978775	-4.6339393
ENSCAFG00000017697	-0.1051524	2.77348314	-0.3121952	0.75612846	0.99978775	-4.6804549
M6PR	-0.1052378	6.51994088	-1.1448282	0.2574713	0.99978775	-4.6000368
FASTKD1	-0.1052496	3.50368305	-0.628743	0.53224272	0.99978775	-4.6993345
RRP1	-0.1052502	4.94327025	-0.9580451	0.3424301	0.99978775	-4.6699246
CX3CL1	-0.1053097	4.57752648	-0.1787721	0.85880455	0.99978775	-4.7253
TRA2B	-0.1053134	5.28917455	-0.8995276	0.37247807	0.99978775	-4.7042218
THOC3	-0.1053412	4.20448008	-0.7074799	0.48239631	0.99978775	-4.7180534
ENSCAFG00000016929	-0.1053737	5.92225057	-1.3562556	0.18082069	0.99978775	-4.4834917
ENSCAFG00000032208	-0.1054012	5.06544947	-1.0458339	0.30042781	0.99978775	-4.6362658
GON7	-0.1054928	3.50967747	-0.6773949	0.50112891	0.99978775	-4.6887408
SWAP70	-0.1055127	6.46249574	-1.1981627	0.23623327	0.99978775	-4.5722709
CST6	-0.105584	-1.4152273	-0.1408868	0.88849843	0.99978775	-4.6110853
COIL	-0.1055919	3.95545698	-0.9491196	0.34690731	0.99978775	-4.6425938
DNM1	-0.1055927	1.43972328	-0.0427264	0.9660817	0.99978775	-4.6195157
GRHPR	-0.1056137	3.65692973	-0.9304323	0.3564049	0.99978775	-4.645797
TRMO	-0.1056142	2.92637721	-0.5430028	0.58942533	0.99978775	-4.6860265
MSRB2	-0.1056558	2.27096647	-0.5246574	0.60202921	0.99978775	-4.6540125
ABHD15	-0.1056726	2.23639479	-0.4157803	0.67926495	0.99978775	-4.6660034
TWNK	-0.1056817	1.74102922	-0.4418205	0.6604332	0.99978775	-4.6386435
SMAD3	-0.1057112	5.55659356	-0.5373778	0.59327657	0.99978775	-4.8206905
THADA	-0.1057483	4.48730866	-1.0589529	0.29446782	0.99978775	-4.6196093
PIDD1	-0.105779	4.29876738	-0.600443	0.55079363	0.99978775	-4.7446165
PRKD2	-0.105784	4.49160849	-0.6648475	0.50905725	0.99978775	-4.7422728
PSME2	-0.1058	4.72809883	-0.9410317	0.35099736	0.99978775	-4.6687817
BAZ1B	-0.1058538	6.09290799	-1.2108828	0.23136172	0.99978775	-4.5644668
PTGFRN	-0.1058951	6.28706513	-0.3188109	0.75113454	0.99978775	-4.8717312
RTTN	-0.1059673	3.32014306	-0.534058	0.59555507	0.99978775	-4.7230782
EIF3B	-0.1060737	7.84451107	-1.0099212	0.31716381	0.99978775	-4.6571428
STK10	-0.1060948	4.79264554	-0.6490978	0.51910399	0.99978775	-4.764059
LAMA4	-0.1061029	10.0593272	-0.5832459	0.56222433	0.99978775	-4.7581396
FLT4	-0.1062074	2.64417272	-0.1932643	0.84749754	0.99978775	-4.7081078
TENT4A	-0.1062441	5.84659044	-1.3237547	0.19131825	0.99978775	-4.5022481
EFCAB7	-0.1062724	2.01678798	-0.412744	0.68147447	0.99978775	-4.6489845
MKKS	-0.1063149	5.9907705	-0.96617	0.33838763	0.99978775	-4.6829194
INO80E	-0.1063236	3.83747973	-0.9426468	0.35017811	0.99978775	-4.6518798
ENSCAFG00000004543	-0.1063246	-0.0654039	-0.2683144	0.78950851	0.99978775	-4.6249269
BORCS8	-0.1063373	2.98793712	-0.6985842	0.48789415	0.99978775	-4.6831758
MGST3	-0.106348	3.95432601	-0.5085591	0.61319127	0.99978775	-4.7300235
ALMS1	-0.1063753	4.85969076	-0.777993	0.44006434	0.99978775	-4.7340569
ENSCAFG00000002561	-0.1063757	4.22465332	-1.1701901	0.24720775	0.99978775	-4.5726754
WDR19	-0.1064232	4.63203735	-0.6520023	0.51724334	0.99978775	-4.7442236
MNAT1	-0.1064802	4.05168506	-0.6758592	0.50209567	0.99978775	-4.723377
ARMCX6	-0.1065872	2.69254428	-0.7665726	0.44676806	0.99978775	-4.6528118
FAM13C	-0.10668	4.10129814	-0.6626128	0.51047639	0.99978775	-4.7365159

ARL6IP4	-0.1066865	4.55917395	-1.0355448	0.30515968	0.99978775	-4.630065
BCL2L2	-0.1067283	2.7537509	-0.6049403	0.54782386	0.99978775	-4.6606881
CCDC157	-0.1067289	2.06625233	-0.448172	0.65587269	0.99978775	-4.6524359
STK16	-0.1067927	4.77961447	-0.9835319	0.32985548	0.99978775	-4.6540896
ISPD	-0.1068263	4.10314021	-0.8865076	0.37938588	0.99978775	-4.6695857
ENSCAFG00000028476	-0.1068384	5.82537392	-0.9914645	0.32600532	0.99978775	-4.6694167
DDX39B	-0.106853	6.65474438	-1.2157876	0.229503	0.99978775	-4.5627703
ENSCAFG00000019472	-0.1068959	9.5032056	-0.7506974	0.45618546	0.99978775	-4.723443
KITLG	-0.1069451	7.74868341	-0.4729417	0.63821466	0.99978775	-4.829502
THG1L	-0.1069838	2.26238732	-0.5828895	0.56246243	0.99978775	-4.6523703
GTPBP1	-0.1069895	4.51347371	-1.0392218	0.30346286	0.99978775	-4.628368
CKAP5	-0.1070687	6.91783263	-1.159464	0.2515119	0.99978775	-4.5915313
CHCHD3	-0.1070743	5.6200915	-0.9179657	0.36283375	0.99978775	-4.6976923
GREM2	-0.1072054	3.70099984	-0.2549654	0.79974572	0.99978775	-4.7547858
TLNRD1	-0.10723	2.93965212	-0.5980476	0.55237875	0.99978775	-4.700619
KAT14	-0.1072369	5.73815943	-1.3947865	0.16895226	0.99978775	-4.4607857
ZCRB1	-0.1072762	5.21675964	-1.0346367	0.30557974	0.99978775	-4.64241
OAS3	-0.1073066	3.07672524	-0.6650336	0.50893922	0.99978775	-4.6988113
LARS	-0.1073689	6.9370501	-1.060999	0.29354568	0.99978775	-4.639601
SERPINE2	-0.1073811	8.85253751	-0.3179678	0.75177037	0.99978775	-4.8402761
ZNF227	-0.1074353	2.34667254	-0.4325663	0.66710113	0.99978775	-4.6700857
TMEM189	-0.1074615	6.70861106	-0.7085626	0.48172955	0.99978775	-4.7818226
LURAP1L	-0.1075434	1.3231444	-0.3076928	0.75953312	0.99978775	-4.6785307
CSGALNACT1	-0.1075993	6.79909972	-0.381495	0.70437519	0.99978775	-4.8633425
ENSCAFG00000029341	-0.1076145	4.36708826	-0.7581104	0.45177376	0.99978775	-4.706312
CUL9	-0.1076791	5.91276861	-0.7858402	0.43549262	0.99978775	-4.7520972
PTPN5	-0.1076825	-1.8184413	-0.2089711	0.83527926	0.99978775	-4.6122059
GSN	-0.1076849	10.8260509	-0.2614116	0.79479761	0.99978775	-4.8270627
CDKN2AIPNL	-0.1077342	0.58851627	-0.3469947	0.72998205	0.99978775	-4.6291313
CDC34	-0.1078106	5.67133829	-1.0889403	0.28115228	0.99978775	-4.6242783
TP53I13	-0.1078224	3.82931036	-0.668478	0.50675634	0.99978775	-4.7194448
NF2	-0.1078866	7.08040868	-1.2884845	0.20322528	0.99978775	-4.5219564
DNAJC16	-0.107967	3.48297927	-0.8620701	0.39256854	0.99978775	-4.6534563
XRCC2	-0.1079797	0.06649105	-0.3008921	0.76468488	0.99978775	-4.6241255
AEN	-0.1079847	6.16656266	-0.9681316	0.3374164	0.99978775	-4.6828463
ENSCAFG00000009766	-0.1080268	0.34370467	-0.3224668	0.7483794	0.99978775	-4.6262
STK17B	-0.1080415	4.88821312	-0.5465899	0.58697554	0.99978775	-4.7903472
MMP27	-0.1080709	-3.0291078	-0.2093502	0.83498491	0.99978775	-4.6065449
XRCC1	-0.1081052	4.7184082	-0.8660494	0.39040266	0.99978775	-4.6922523
PPP1R9B	-0.1081956	8.08723843	-0.841079	0.40411726	0.99978775	-4.7208945
SLC35C1	-0.1082324	4.85148293	-0.8634485	0.39181744	0.99978775	-4.704894
OGDHL	-0.1082357	3.6811384	-0.6431409	0.52293116	0.99978775	-4.7030642
BLOC1S6	-0.1083082	1.9747654	-0.5516945	0.58349781	0.99978775	-4.646501
ENSCAFG00000012222	-0.1083655	0.81829617	-0.4496539	0.65481052	0.99978775	-4.6362953
PCDH9	-0.1083704	0.1342254	-0.3101021	0.75771066	0.99978775	-4.6499189
SEMA3D	-0.1083896	6.21127443	-0.3243835	0.74693629	0.99978775	-4.8681385
ZNF35	-0.1088488	1.66866288	-0.5745699	0.56803551	0.99978775	-4.6357879
SNAPIN	-0.1088649	2.34225431	-0.4881195	0.62749714	0.99978775	-4.6687726
TMEM175	-0.1088686	4.72900559	-0.8672416	0.38975523	0.99978775	-4.6979485
MRPL20	-0.1089102	4.72347502	-0.8909407	0.37702482	0.99978775	-4.6915287

ELP1	-0.109043	5.74094693	-1.1779633	0.24412186	0.99978775	-4.5812655
ENSCAFG00000031030	-0.1090867	1.93506987	-0.3729431	0.71069191	0.99978775	-4.6458371
PFDN2	-0.1091728	4.02284718	-0.9338712	0.35464455	0.99978775	-4.6570174
GEMIN5	-0.1092068	6.15789887	-0.9401996	0.35141995	0.99978775	-4.6951101
ZC3H12A	-0.1092128	3.29089907	-0.4471689	0.6565921	0.99978775	-4.7163212
POLH	-0.1092561	4.0426142	-0.6203326	0.53772166	0.99978775	-4.7418885
SLC29A1	-0.1092563	9.60385031	-1.0942222	0.27885116	0.99978775	-4.6057485
ENSCAFG00000029671	-0.1094511	2.16109095	-0.618933	0.53863623	0.99978775	-4.6453892
ENSCAFG00000031645	-0.1094765	0.51568937	-0.2360435	0.81431714	0.99978775	-4.6274907
AURKAIP1	-0.1095343	4.52579057	-0.8673219	0.38971161	0.99978775	-4.6953209
PAPSS1	-0.1095426	6.73062488	-0.9678277	0.33756673	0.99978775	-4.6832889
DENND4B	-0.1095706	5.10936863	-0.6523193	0.51704049	0.99978775	-4.7722061
NFXL1	-0.1097457	3.96530151	-0.9289122	0.35718483	0.99978775	-4.6464305
ZNF583	-0.1097815	1.50132265	-0.5315439	0.59728333	0.99978775	-4.6363787
TMEM71	-0.109831	0.89358349	-0.3886254	0.69912447	0.99978775	-4.6538231
PCID2	-0.1098536	5.33432554	-1.2681466	0.21033904	0.99978775	-4.5313964
ZNF518A	-0.1098756	4.03863424	-0.8780846	0.38389763	0.99978775	-4.6671403
UBE2D1	-0.1099789	4.41003785	-1.0476573	0.29959455	0.99978775	-4.624209
ZNF205	-0.1100431	3.76931422	-0.8190222	0.41647513	0.99978775	-4.6718365
ICE1	-0.1100488	5.60303359	-0.6872063	0.49497656	0.99978775	-4.7685892
ENSCAFG00000030875	-0.1100636	4.02331336	-0.4094814	0.68385187	0.99978775	-4.8066573
CP	-0.1100847	-1.0602812	-0.09222	0.92687415	0.99978775	-4.611102
ENSCAFG00000029095	-0.1101186	0.08938255	-0.3060522	0.76077498	0.99978775	-4.622269
TSTD2	-0.1101716	3.95957409	-0.9471403	0.34790539	0.99978775	-4.6502903
ENSCAFG00000016171	-0.1101724	2.28410284	-0.5090642	0.6128396	0.99978775	-4.6533062
ENSCAFG00000000576	-0.1102094	1.57615102	-0.3962047	0.69355936	0.99978775	-4.6454841
IL17RC	-0.1102217	4.69760074	-0.5481195	0.58593243	0.99978775	-4.7742468
GPCPD1	-0.1103458	5.37322149	-0.7306049	0.46826761	0.99978775	-4.7538152
MANEAL	-0.110385	1.93128968	-0.316829	0.75262945	0.99978775	-4.6638634
HEPH	-0.1103984	-2.3338748	-0.1240743	0.90173017	0.99978775	-4.6070819
BRF1	-0.1104066	4.1668225	-0.9871915	0.32807552	0.99978775	-4.6373253
KIAA1841	-0.1104306	2.40191045	-0.5624365	0.57621179	0.99978775	-4.672525
KPNA4	-0.1105236	6.27820046	-0.6830238	0.49759413	0.99978775	-4.7895287
RIC8A	-0.1105468	4.51199086	-1.1051063	0.27415103	0.99978775	-4.6023809
INTS12	-0.1105599	3.44956965	-0.5549202	0.58130527	0.99978775	-4.7015006
SLTM	-0.1105735	5.89040648	-0.9565442	0.34318032	0.99978775	-4.6854138
PYURF	-0.1105834	5.43161217	-1.0447725	0.3009136	0.99978775	-4.6424521
SGPL1	-0.1107141	6.0470342	-0.7770124	0.44063757	0.99978775	-4.7587085
METTL25	-0.1107235	1.67945455	-0.4444645	0.65853313	0.99978775	-4.6436879
WDR73	-0.1107466	4.27580039	-0.9656672	0.33863686	0.99978775	-4.6499574
AHDC1	-0.1107556	5.35727926	-0.4939754	0.62338345	0.99978775	-4.8165386
ENSCAFG00000004800	-0.1107778	1.1914738	-0.5233697	0.60291859	0.99978775	-4.6359016
MRPS25	-0.1109016	4.27721685	-0.8861284	0.37958826	0.99978775	-4.675283
C6H16orf91	-0.1109863	1.0259863	-0.4093844	0.68392263	0.99978775	-4.6394742
KIAA0930	-0.1110247	4.93982049	-0.5846717	0.56127211	0.99978775	-4.7835491
SPPL2B	-0.1110513	4.32123964	-1.0788704	0.28557605	0.99978775	-4.6084862
COMMD5	-0.1110792	3.17168086	-0.6227065	0.53617225	0.99978775	-4.7002145
PEPD	-0.1111472	5.06182212	-0.82788	0.41148502	0.99978775	-4.7252137
KIF7	-0.1112051	3.85782984	-0.6449878	0.521743	0.99978775	-4.7189745
QTRT2	-0.1112214	3.40673562	-0.649258	0.51900127	0.99978775	-4.69413

CCDC102A	-0.1112481	5.30413819	-0.8987216	0.37290337	0.99978775	-4.7004226
CTTNBP2NL	-0.111308	5.00190032	-0.766459	0.44683509	0.99978775	-4.7280253
CABIN1	-0.1113556	5.81731464	-1.0527221	0.29728828	0.99978775	-4.6440116
SRSF6	-0.1114238	6.71837264	-1.3124288	0.19508287	0.99978775	-4.509006
TAMM41	-0.1114249	3.2187606	-0.6470038	0.52044767	0.99978775	-4.6820363
P3H1	-0.111434	6.86336484	-1.1931516	0.23817277	0.99978775	-4.5743352
MRPL4	-0.1114898	4.51988342	-0.8190276	0.4164721	0.99978775	-4.7077636
NOP58	-0.1115165	5.78750861	-0.6904628	0.4929437	0.99978775	-4.7826179
ZNF140	-0.1116281	1.58702861	-0.3909844	0.69739053	0.99978775	-4.643841
STX10	-0.111821	3.57011815	-0.6848561	0.49644646	0.99978775	-4.6889694
HSD17B7	-0.1118303	3.50363801	-0.5479845	0.5860244	0.99978775	-4.6949952
ATF6B	-0.1118949	5.91396339	-1.1954478	0.23728262	0.99978775	-4.572497
HSF1	-0.1119976	5.44352986	-1.0555219	0.2960186	0.99978775	-4.6372995
TRMT112	-0.1121743	5.31928987	-1.0928989	0.27942645	0.99978775	-4.6184231
CSNK1G1	-0.1122568	3.46264406	-0.9392068	0.35192456	0.99978775	-4.6294034
EEF1AKMT1	-0.1122815	2.81966758	-0.8749417	0.38558976	0.99978775	-4.6314754
RTL6	-0.1122939	3.65175178	-0.8932569	0.37579492	0.99978775	-4.6548022
TMEM25	-0.1122997	2.49136945	-0.3996446	0.69103914	0.99978775	-4.6730084
NFKBID	-0.1123105	-0.4865799	-0.3019248	0.76390187	0.99978775	-4.6165017
MFSD12	-0.1124962	4.77957413	-0.5829801	0.56240192	0.99978775	-4.7682791
ENSCAFG00000013365	-0.1125005	2.96252888	-1.0359499	0.30497242	0.99978775	-4.6010848
ENSCAFG00000028725	-0.1125567	2.03670276	-0.5346016	0.59518171	0.99978775	-4.6467702
FAM185A	-0.1126081	2.05578372	-0.4626162	0.64555068	0.99978775	-4.6549989
CCAR1	-0.1126282	6.40653835	-1.1054295	0.27401231	0.99978775	-4.619755
SWT1	-0.1126289	3.09730485	-0.5761128	0.56699996	0.99978775	-4.6907886
ENSCAFG00000006639	-0.1126476	5.61298189	-0.8240865	0.41361764	0.99978775	-4.7349562
UVSSA	-0.1127362	4.87009461	-0.7021268	0.4857006	0.99978775	-4.742684
SLC46A3	-0.1128455	3.70830219	-0.3201095	0.7501555	0.99978775	-4.7701994
NNT	-0.1128852	7.64444366	-1.1664671	0.24869564	0.99978775	-4.583682
C20H19orf44	-0.1131126	4.44422765	-0.31544	0.75367777	0.99978775	-4.7576005
IPP	-0.1131935	3.54157308	-0.6625774	0.51049891	0.99978775	-4.6961037
ZNF639	-0.1132486	4.12465672	-1.1367625	0.26079826	0.99978775	-4.5836191
PAK1IP1	-0.1132876	4.25858653	-0.9667372	0.33810663	0.99978775	-4.6483754
STRN	-0.1133669	5.0176039	-1.0294802	0.30797244	0.99978775	-4.6435375
KLHL12	-0.1134047	3.99198357	-1.0723325	0.28847397	0.99978775	-4.6071665
POP5	-0.1134262	2.92702153	-0.784833	0.43607782	0.99978775	-4.6543568
TBC1D19	-0.1134503	4.39940864	-1.0182711	0.31321754	0.99978775	-4.6295997
SCRN3	-0.1134721	4.38733466	-0.9872453	0.3280494	0.99978775	-4.6414225
EHMT1	-0.1135598	5.95335443	-1.5544282	0.1260888	0.99978775	-4.359583
RING1	-0.1135766	5.53780792	-1.1536588	0.25386372	0.99978775	-4.5926372
R3HDM1	-0.1135907	5.77936303	-1.2207006	0.22765219	0.99978775	-4.5590028
DAPK1	-0.1136368	5.08406055	-0.5433516	0.58918686	0.99978775	-4.8083217
ANKRD42	-0.1136417	3.81156902	-0.7250441	0.47164346	0.99978775	-4.6965791
GGA2	-0.1136588	4.25110894	-0.8383662	0.40562487	0.99978775	-4.6881197
TMEM39B	-0.1136991	2.50908148	-0.6043282	0.5482276	0.99978775	-4.651317
FMO5	-0.1137162	2.56488215	-0.3359077	0.73827891	0.99978775	-4.6663906
ENSCAFG00000006193	-0.1137311	4.70550839	-0.995454	0.32408035	0.99978775	-4.6484691
NOG	-0.1137997	1.26480178	-0.5165276	0.60765437	0.99978775	-4.6790362
EPN1	-0.1138279	6.64859795	-1.1071612	0.27326993	0.99978775	-4.618654
NUDC	-0.1140669	6.22647039	-0.992227	0.32563677	0.99978775	-4.6724077

FBH1	-0.1142952	5.49512445	-1.1543309	0.25359064	0.99978775	-4.5917367
AKT1	-0.1143484	8.10198925	-1.2059546	0.23324035	0.99978775	-4.5628004
ZBTB38	-0.1143906	6.22868304	-0.8632285	0.39193726	0.99978775	-4.726647
GCLM	-0.1144524	3.96708453	-0.710849	0.48032318	0.99978775	-4.721898
HOXB9	-0.1144827	-2.0162674	-0.2156485	0.83009717	0.99978775	-4.6129889
ENSCAFG00000018825	-0.1145103	7.77089186	-0.1772487	0.85999496	0.99978775	-4.8536365
KLHL41	-0.1145366	2.11211608	-0.5954277	0.55411506	0.99978775	-4.6532531
AMOTL1	-0.1145604	5.17935543	-0.9142266	0.36477642	0.99978775	-4.6859455
NPHP4	-0.1146309	4.36199028	-0.6552525	0.51516544	0.99978775	-4.7362027
C10H2orf42	-0.1146576	5.44422378	-1.3378254	0.18671818	0.99978775	-4.494204
CCDC125	-0.1146594	3.6804955	-0.7702618	0.444596	0.99978775	-4.6793158
TRMT10A	-0.1146662	4.23380294	-0.8857729	0.37977807	0.99978775	-4.6706274
TCF20	-0.1147016	5.80644603	-1.4757655	0.14597802	0.99978775	-4.4118756
S1PR1	-0.1147402	3.61059256	-0.1363557	0.8920615	0.99978775	-4.713481
PCNX4	-0.1147849	3.85352152	-0.6068767	0.54654768	0.99978775	-4.7205515
ENSCAFG00000023888	-0.1148167	0.35379144	-0.3224863	0.74836472	0.99978775	-4.6226113
ARMCX1	-0.1148722	5.07905601	-0.8030621	0.42555869	0.99978775	-4.7260909
TULP4	-0.1148775	4.82750398	-0.7069828	0.48270265	0.99978775	-4.738605
MRPL3	-0.1148946	5.08184977	-1.2292793	0.22444672	0.99978775	-4.5507232
U2AF2	-0.1149217	6.69666348	-1.2762627	0.20747828	0.99978775	-4.5295267
PLEK2	-0.1149299	0.63209732	-0.2665986	0.79082233	0.99978775	-4.6341666
BICRA	-0.1150515	3.99297077	-0.5523987	0.58301882	0.99978775	-4.7293387
BRI3	-0.1150758	3.15115814	-0.6382629	0.52607629	0.99978775	-4.6923375
SZT2	-0.1150805	5.20383365	-1.1281605	0.26438001	0.99978775	-4.599553
PPP1R16B	-0.115094	2.28464075	-0.209961	0.83451056	0.99978775	-4.666435
FBXO6	-0.1151223	4.65745343	-0.7553527	0.453412	0.99978775	-4.7322528
MPP6	-0.1151465	3.17532067	-0.3244994	0.74684908	0.99978775	-4.7273131
ANKRD27	-0.1151517	5.0209543	-1.2146631	0.22992817	0.99978775	-4.5566443
ARHGEF40	-0.1152691	7.7021462	-0.89263	0.37612758	0.99978775	-4.7046594
SMG5	-0.1152762	6.00100808	-1.2110834	0.23128549	0.99978775	-4.5650849
CDK16	-0.1153721	6.4694118	-1.2747392	0.20801307	0.99978775	-4.5304841
SEPT11	-0.115403	5.08517736	-0.7086898	0.48165125	0.99978775	-4.7498124
ATF4	-0.1154875	8.70471116	-1.6023794	0.11507044	0.99978775	-4.3426734
ENSCAFG00000007340	-0.115492	5.26485585	-1.2853531	0.20430866	0.99978775	-4.5224653
ANAPC11	-0.1156768	4.32284893	-0.8309832	0.40974543	0.99978775	-4.6879282
AP4M1	-0.1157112	3.57925974	-0.738582	0.46344909	0.99978775	-4.6768806
MRPL38	-0.1157258	4.28972992	-1.1381748	0.2602135	0.99978775	-4.5858958
KLC4	-0.1157792	4.40886912	-0.9356875	0.35371713	0.99978775	-4.6572695
HEPHL1	-0.115806	-2.1137427	-0.2233761	0.82410946	0.99978775	-4.6144729
SSH1	-0.1158075	4.56619593	-0.7865052	0.4351065	0.99978775	-4.7083507
ACTG2	-0.1158894	8.67138471	-0.1306209	0.89657433	0.99978775	-4.8479581
NSUN6	-0.1160645	3.81630476	-1.04317	0.30164806	0.99978775	-4.6136928
NAT10	-0.1161042	5.30327607	-1.3876172	0.17111383	0.99978775	-4.4663406
CREBZF	-0.1161184	2.64375403	-0.6468818	0.52052599	0.99978775	-4.6678129
CHAC1	-0.1162468	4.82859664	-0.3135883	0.75507596	0.99978775	-4.8392962
PKIB	-0.1164009	-0.6971211	-0.0856245	0.93209056	0.99978775	-4.6093716
IGF2R	-0.1165758	7.60879364	-0.8974282	0.37358643	0.99978775	-4.7028528
HDAC6	-0.1166577	4.97074231	-0.8159701	0.41820306	0.99978775	-4.716238
PIKFYVE	-0.1166673	4.86161622	-1.1132727	0.27066121	0.99978775	-4.6007375
EXOSC2	-0.1166746	3.87504616	-0.9136073	0.36509883	0.99978775	-4.6514359

LMLN	-0.1166776	5.19761026	-0.9493192	0.34680677	0.99978775	-4.678701
CUEDC1	-0.116684	5.36059098	-0.8017031	0.42633759	0.99978775	-4.73973
SMARCB1	-0.1167422	5.58373276	-1.3281648	0.18986734	0.99978775	-4.499645
NCAM2	-0.1167976	-1.3502151	-0.1287622	0.8980377	0.99978775	-4.6077056
PHIP	-0.116871	5.01130959	-0.8199924	0.41592681	0.99978775	-4.7144983
WDR3	-0.1168815	6.44575613	-0.9587256	0.34209031	0.99978775	-4.6873295
FAM78A	-0.1169289	-0.0303127	-0.2091595	0.83513298	0.99978775	-4.6221569
FGF23	-0.1169588	-3.0296274	-0.2374506	0.81323117	0.99978775	-4.606596
NENF	-0.1170868	4.64466395	-0.796252	0.42947052	0.99978775	-4.7047597
EHHADH	-0.1171051	4.19670756	-0.6905921	0.4928631	0.99978775	-4.7121958
ENSCAFG00000032541	-0.1171065	-2.5665156	-0.2155113	0.83020355	0.99978775	-4.6073789
CXXC5	-0.11719	5.36532302	-0.7462411	0.45884945	0.99978775	-4.7543846
NUP205	-0.1172516	6.43530858	-1.0077545	0.31819329	0.99978775	-4.6657586
ENSCAFG00000019232	-0.1172593	6.03512606	-2.0508653	0.04528465	0.99978775	-3.9870283
DCPS	-0.1172652	4.23402031	-0.9222649	0.36060831	0.99978775	-4.6690098
ATF7IP2	-0.1172703	-2.1109788	-0.1461595	0.88435512	0.99978775	-4.6074182
COA6	-0.1173773	1.16314336	-0.5037799	0.61652298	0.99978775	-4.6324951
KCTD2	-0.1174593	2.63443822	-0.6849845	0.49636609	0.99978775	-4.6588777
LIPT1	-0.1175602	3.1243151	-0.6915606	0.49225945	0.99978775	-4.6700075
ATG4C	-0.1177429	4.11083333	-1.0117938	0.31627589	0.99978775	-4.625616
WDFY2	-0.1177721	4.6791598	-0.6864388	0.49545632	0.99978775	-4.744099
ATR	-0.1178498	5.1984676	-0.8380042	0.40582635	0.99978775	-4.7183344
TMEM131	-0.117951	7.23204359	-1.1713062	0.24676293	0.99978775	-4.5837075
ACCS	-0.1180285	4.4633628	-0.8336443	0.40825731	0.99978775	-4.7020539
METTL1	-0.1180903	2.70601222	-0.6057118	0.54731521	0.99978775	-4.696976
TMEM161A	-0.1181053	4.91453005	-0.7630311	0.44885906	0.99978775	-4.7305274
SARNP	-0.1181188	4.88791968	-0.7955972	0.42984775	0.99978775	-4.7259059
THOC6	-0.118167	4.24897285	-0.8252969	0.41293644	0.99978775	-4.7074837
GPR1	-0.1181682	-1.6763171	-0.0956854	0.92413465	0.99978775	-4.6069618
PEX11G	-0.1181791	0.71323495	-0.356998	0.72252386	0.99978775	-4.6331827
LRRC7	-0.1182281	-0.8968235	-0.2118808	0.83302022	0.99978775	-4.648386
LMOD2	-0.1182337	0.07734732	-0.2423898	0.80942229	0.99978775	-4.6200874
PAG1	-0.1182409	-1.0117599	-0.2425717	0.80928214	0.99978775	-4.6198428
TEX10	-0.1182969	4.623447	-1.0592312	0.2943423	0.99978775	-4.6231124
FGD1	-0.1183267	4.36055667	-1.0960171	0.2780722	0.99978775	-4.6032461
RRM1	-0.1183495	6.20348913	-0.5881825	0.558931	0.99978775	-4.8174258
MARK3	-0.1183708	5.78988392	-1.2656459	0.21122638	0.99978775	-4.5345544
SLC6A9	-0.1184441	5.72383691	-0.6350988	0.52812158	0.99978775	-4.8036476
TMEM62	-0.118519	2.69646236	-0.7697153	0.44491741	0.99978775	-4.645344
STK11IP	-0.1185568	3.22661217	-0.7520251	0.45539349	0.99978775	-4.6623508
ZNF24	-0.1185691	3.96038125	-1.0215569	0.31167379	0.99978775	-4.6213094
ENSCAFG00000001985	-0.1185737	0.84478127	-0.3358701	0.7383071	0.99978775	-4.6322367
ENSCAFG00000031486	-0.1185883	4.63959544	-0.6522429	0.51708938	0.99978775	-4.7439932
ACSS2	-0.1186278	6.85737746	-0.8919977	0.37646323	0.99978775	-4.7152553
STAP2	-0.1186434	-0.5157274	-0.2594697	0.79628734	0.99978775	-4.6164763
FEN1	-0.1187073	3.3508986	-0.5472228	0.58654381	0.99978775	-4.7381203
TAB1	-0.1187663	4.72712077	-1.1381622	0.26021872	0.99978775	-4.5890935
RSC1A1	-0.1187792	3.88636221	-0.7494744	0.45691567	0.99978775	-4.69949
NPHP1	-0.1188447	2.47660231	-0.566695	0.57333563	0.99978775	-4.6566889
CHMP7	-0.1189107	4.01653801	-1.2360161	0.22195279	0.99978775	-4.5445738

UGDH	-0.119019	8.06225367	-0.3406146	0.73475265	0.99978775	-4.8371955
NOC4L	-0.1191188	4.97301739	-0.709236	0.48131509	0.99978775	-4.7651669
ALKAL1	-0.1191625	-2.9395916	-0.246712	0.806093	0.99978775	-4.6075317
OTUB1	-0.1192199	3.15067353	-0.9684884	0.33723992	0.99978775	-4.6230932
FAM50A	-0.1192293	5.04449506	-1.254267	0.21529922	0.99978775	-4.5382527
BCL2L13	-0.1192315	5.04522854	-1.1829764	0.24214652	0.99978775	-4.5732764
IDNK	-0.1192752	2.42373995	-0.4197872	0.67635341	0.99978775	-4.6800452
CCDC97	-0.1192886	4.78272161	-1.0487059	0.29911605	0.99978775	-4.6281111
TANC2	-0.1193048	4.95236702	-0.4589246	0.6481822	0.99978775	-4.830612
PPT2	-0.1193342	2.68837625	-0.4736533	0.63771041	0.99978775	-4.6710058
TMEM74	-0.1193846	0.26288949	-0.3753893	0.70888298	0.99978775	-4.6463234
FNBP4	-0.1193897	5.00343248	-1.0582565	0.29478216	0.99978775	-4.6244935
GALNT17	-0.1193948	1.434169	-0.2106808	0.83395174	0.99978775	-4.6744702
ESS2	-0.1195638	3.79394311	-0.9362711	0.35341946	0.99978775	-4.6391369
RBM17	-0.1195738	5.29010223	-1.2910069	0.20235569	0.99978775	-4.5197443
PACSIN2	-0.1196573	5.85656787	-0.9622794	0.34031938	0.99978775	-4.6820605
C1QTNF1	-0.1197468	2.77507676	-0.3475174	0.72959164	0.99978775	-4.7146716
PINK1	-0.11979	3.94766504	-0.9040437	0.37010088	0.99978775	-4.6514897
PABPN1	-0.1198661	6.34223829	-1.9774291	0.05325264	0.99978775	-4.0444484
ENSCAFG00000029785	-0.1198682	2.04612171	-0.6054562	0.54748374	0.99978775	-4.6430306
TSC22D1	-0.1198874	7.12326368	-0.6560555	0.51465271	0.99978775	-4.7961197
CAPN10	-0.1199434	4.06361687	-0.9365967	0.35325344	0.99978775	-4.6572985
PLSCR3	-0.1199514	4.50683844	-0.8739544	0.38612231	0.99978775	-4.6824294
DNAJC27	-0.1199608	-0.2525896	-0.2902356	0.77277901	0.99978775	-4.6201827
ANKRD13D	-0.1200518	3.9210572	-0.9425867	0.35020859	0.99978775	-4.6463241
JRKL	-0.1200785	3.66857381	-0.9333435	0.35491432	0.99978775	-4.6356385
PLEKHN1	-0.1201263	-0.0045588	-0.3558522	0.72337684	0.99978775	-4.6235811
ENSCAFG00000009353	-0.1201397	-0.7382751	-0.3229151	0.74804179	0.99978775	-4.6212567
POU3F3	-0.1201802	-2.9081363	-0.2195504	0.82707249	0.99978775	-4.6066887
FAM192A	-0.1202393	4.21785878	-1.3872643	0.17122079	0.99978775	-4.4777232
RALBP1	-0.1203376	5.86062721	-1.5044105	0.13846671	0.99978775	-4.3934917
NUPR1	-0.1203431	6.01802043	-0.5467439	0.5868705	0.99978775	-4.8225666
LUC7L	-0.1203694	4.33634339	-0.7495265	0.45688454	0.99978775	-4.7141019
PLA2G7	-0.1204519	7.42563784	-0.3372343	0.73728448	0.99978775	-4.8456188
HNRNPR	-0.1204727	6.78419236	-0.9292536	0.35700958	0.99978775	-4.6990951
EARS2	-0.1205884	2.69612434	-0.7363921	0.46476903	0.99978775	-4.647169
RSPH6A	-0.1206076	0.86096635	-0.4203494	0.67594529	0.99978775	-4.6371645
GOT1	-0.1206281	5.1674917	-0.9496541	0.34663814	0.99978775	-4.6783406
FBRS	-0.1206499	5.1349323	-1.0277475	0.3087793	0.99978775	-4.6417354
TBL1XR1	-0.1206652	3.93037342	-0.8323398	0.40898639	0.99978775	-4.6736613
ENSCAFG00000016314	-0.1206982	1.0476502	-0.3081601	0.75917955	0.99978775	-4.6438513
SPTLC3	-0.1207149	-0.3519401	-0.173051	0.86327661	0.99978775	-4.6197522
ENSCAFG00000030732	-0.1207615	2.79885525	-0.7954692	0.42992155	0.99978775	-4.6428495
NRIP1	-0.1207635	3.97491422	-0.6854244	0.49609082	0.99978775	-4.7028293
BCL7C	-0.1208339	3.87711044	-0.7583591	0.45162613	0.99978775	-4.7043178
ENSCAFG00000007045	-0.1209767	4.22264213	-0.2363024	0.81411728	0.99978775	-4.8104574
DPP3	-0.1209965	6.05617164	-1.3180067	0.19322189	0.99978775	-4.5056677
TNFRSF1A	-0.1210462	6.12463935	-0.9596284	0.34163985	0.99978775	-4.6852368
MLST8	-0.1211537	2.85509461	-0.6782445	0.50059453	0.99978775	-4.6706213
SAT2	-0.1211647	2.90749522	-0.5070886	0.61421553	0.99978775	-4.6818429

ALOX15B	-0.1212162	4.0368592	-0.2616563	0.79460997	0.99978775	-4.7360889
NSMCE4A	-0.1212705	3.7189942	-0.7445677	0.45985218	0.99978775	-4.6897968
SFXN3	-0.1212825	5.16160013	-0.964841	0.3390467	0.99978775	-4.6746347
KTI12	-0.1213609	3.01723477	-0.9637857	0.33957064	0.99978775	-4.6216708
PRR12	-0.1213747	5.05116211	-0.8905059	0.377256	0.99978775	-4.6945026
XPO1	-0.1213778	6.69266382	-1.0142349	0.31512095	0.99978775	-4.6627208
GALNT5	-0.1214022	0.1978423	-0.1947224	0.84636165	0.99978775	-4.6325959
RRP7	-0.1215209	3.08968734	-0.7115245	0.47990813	0.99978775	-4.6726999
BAD	-0.1215275	2.48700801	-0.7194546	0.47505051	0.99978775	-4.6499028
TFAP2B	-0.1215717	-0.9788351	-0.243906	0.80825401	0.99978775	-4.6147261
KIAA1328	-0.1215943	3.36496647	-0.8977896	0.37339552	0.99978775	-4.6367208
SIRT7	-0.1216152	3.99821996	-0.9353682	0.35388005	0.99978775	-4.6480567
TMEM43	-0.121619	7.16114335	-0.9408208	0.35110445	0.99978775	-4.6926053
ENSCAFG00000032029	-0.1216911	2.62495842	-0.3920602	0.69660036	0.99978775	-4.6819438
GADD45GIP1	-0.1217195	3.29934773	-0.7505532	0.45627149	0.99978775	-4.687069
RHOBTB1	-0.1217322	3.58762614	-0.9251499	0.35911991	0.99978775	-4.6583375
MAU2	-0.1218158	4.90935165	-0.980876	0.33115131	0.99978775	-4.6529532
ZNF624	-0.1218626	1.36141325	-0.5424376	0.58981173	0.99978775	-4.632378
KMT2D	-0.1219129	5.41372365	-0.8166361	0.41782566	0.99978775	-4.7264413
PRMT5	-0.1219669	5.71375479	-1.1251472	0.26564289	0.99978775	-4.6077364
MPND	-0.1220097	2.85507897	-0.7759571	0.44125499	0.99978775	-4.658
DNPEP	-0.1220814	4.18797389	-0.7365279	0.46468717	0.99978775	-4.7083883
SLC27A4	-0.1220996	4.85639595	-1.1384605	0.26009532	0.99978775	-4.5944339
MFSD11	-0.1221005	5.92514803	-1.1870244	0.24055995	0.99978775	-4.577251
MLX	-0.1221033	4.53336028	-1.4185455	0.16193924	0.99978775	-4.4557668
ENSCAFG00000010687	-0.122119	4.88353896	-0.8261734	0.4124436	0.99978775	-4.7198578
SNX9	-0.1221206	6.19232886	-0.9727839	0.33512034	0.99978775	-4.6802316
EXOSC7	-0.1222562	3.66903722	-0.6246814	0.53488497	0.99978775	-4.698806
RIDA	-0.1222697	3.59937637	-0.7995569	0.42756946	0.99978775	-4.6681099
CYP2J2	-0.1223254	0.3510108	-0.3638611	0.71742257	0.99978775	-4.6933812
NOLC1	-0.1223683	6.00189351	-0.9134324	0.36518994	0.99978775	-4.7055238
GGA1	-0.1223709	4.27230841	-1.2939568	0.20134229	0.99978775	-4.5194845
ENSCAFG00000016095	-0.1223743	7.81679451	-1.1257437	0.26539255	0.99978775	-4.6046276
PGGHG	-0.1223765	4.20865598	-0.6482442	0.51965149	0.99978775	-4.7191641
PLLP	-0.1224131	-2.4774415	-0.2181497	0.82815798	0.99978775	-4.6084975
ENSCAFG00000016132	-0.1224421	6.39988308	-1.1953149	0.23733405	0.99978775	-4.5732562
GMPR2	-0.1225506	4.35995011	-1.2666182	0.21088105	0.99978775	-4.5311745
DMAC1	-0.1225814	4.26379266	-1.1945295	0.23763831	0.99978775	-4.5624271
LMBR1L	-0.1226099	3.79259561	-0.8571532	0.39525507	0.99978775	-4.6573188
ACAT1	-0.1226245	5.92276185	-1.1568822	0.25255591	0.99978775	-4.5915466
SLC27A1	-0.1226739	5.22527666	-0.5687276	0.57196533	0.99978775	-4.7699416
ANGEL1	-0.1227679	2.20178658	-0.5233876	0.60290623	0.99978775	-4.6453923
RAD18	-0.1228402	2.05614048	-0.5107582	0.6116609	0.99978775	-4.6656491
RFX2	-0.1230258	2.99407379	-0.5366365	0.59378499	0.99978775	-4.67814
CLIP2	-0.1231387	7.52901455	-0.800698	0.42691423	0.99978775	-4.745414
RTN4IP1	-0.1231403	3.45866393	-1.0787786	0.28561658	0.99978775	-4.5969184
EVA1B	-0.1231922	3.21045129	-0.4471602	0.65659833	0.99978775	-4.71287
RPS9	-0.1232234	8.36228488	-1.0593184	0.29430296	0.99978775	-4.6315941
PLAGL2	-0.1233343	4.38222654	-0.920014	0.36177238	0.99978775	-4.6584296
TMEM88	-0.1233556	2.32206913	-0.2724606	0.78633634	0.99978775	-4.6622675

STK32C	-0.1234144	-0.3141814	-0.2775988	0.7824103	0.99978775	-4.6208406
PCCB	-0.1234381	4.74964975	-0.8278068	0.41152612	0.99978775	-4.7051574
CABYR	-0.1234988	3.09914938	-0.7182914	0.47576133	0.99978775	-4.6883178
VRK3	-0.1236272	3.78007053	-0.582668	0.56261049	0.99978775	-4.7197872
PPM1M	-0.1236309	4.00793104	-0.4417447	0.66048767	0.99978775	-4.7447781
ENSCAFG00000016148	-0.1236891	4.26728082	-1.314271	0.19446676	0.99978775	-4.5098919
MAP2K2	-0.1237709	6.488166	-1.3788433	0.17378815	0.99978775	-4.4698918
AP3D1	-0.123793	7.60843712	-2.2350357	0.02969276	0.99978775	-3.8471893
ENSCAFG00000016475	-0.1239028	2.33621149	-0.2462627	0.80643891	0.99978775	-4.6582702
ENSCAFG00000031244	-0.1241017	2.88008364	-0.713382	0.47876783	0.99978775	-4.6640657
SCLY	-0.1241018	4.35298424	-1.02229	0.31133006	0.99978775	-4.6320864
GNA13	-0.1241712	4.61222363	-1.5033652	0.13873532	0.99978775	-4.4107283
MTX1	-0.1241957	4.70554833	-1.0138841	0.31528674	0.99978775	-4.6449566
CNST	-0.1242121	2.55947806	-0.6212682	0.5371107	0.99978775	-4.6696712
TNS2	-0.1242286	7.37713145	-0.5551507	0.58114872	0.99978775	-4.8221934
COQ8B	-0.1242478	3.90136945	-0.9847493	0.32926266	0.99978775	-4.6356051
GEMIN4	-0.1242664	4.61034519	-0.9367422	0.35317928	0.99978775	-4.6660783
ZNF830	-0.1242959	2.70010104	-0.7236493	0.47249233	0.99978775	-4.6465436
WDR91	-0.1243024	4.26723946	-0.6482009	0.51967928	0.99978775	-4.7215896
ENSCAFG00000025748	-0.1243568	-0.6460874	-0.2314504	0.81786439	0.99978775	-4.6172633
TMEM179B	-0.1244344	4.21806859	-0.7680941	0.44587152	0.99978775	-4.705695
IMP4	-0.1244514	5.42616318	-0.9205147	0.36151324	0.99978775	-4.6958238
DLG5	-0.1246706	6.23220138	-1.4631961	0.14937367	0.99978775	-4.4177076
ENSCAFG00000002166	-0.1246762	2.63320015	-0.7270272	0.47043801	0.99978775	-4.6609529
KLHDC3	-0.1247842	5.08327937	-1.516194	0.13546714	0.99978775	-4.3922633
SMAD5	-0.1248208	5.17175277	-1.0430718	0.30169312	0.99978775	-4.6373278
STK25	-0.1248332	5.96891316	-1.4408726	0.1555569	0.99978775	-4.4320041
CISD3	-0.1249119	2.94498163	-1.1335859	0.26211691	0.99978775	-4.5795274
DIDO1	-0.12492	5.22306521	-2.0495419	0.04541856	0.99978775	-4.0179492
ENSCAFG00000030445	-0.1250612	2.25471604	-0.4931618	0.62395426	0.99978775	-4.6568292
AGK	-0.125068	4.72541263	-1.3543845	0.18141287	0.99978775	-4.4869974
HIVEP1	-0.125135	3.9756984	-0.55751	0.57954781	0.99978775	-4.7543244
ALYREF	-0.125171	4.99102436	-0.8078	0.42284982	0.99978775	-4.7264456
VPS51	-0.1251783	5.17228583	-0.8321116	0.40911405	0.99978775	-4.7190107
SNX3	-0.1251972	5.42186772	-1.2765105	0.20739139	0.99978775	-4.5272611
HNRNPF	-0.1252729	7.52104398	-1.2506982	0.21658851	0.99978775	-4.5424737
C15H4orf46	-0.1252998	1.05477083	-0.4024415	0.68899261	0.99978775	-4.636345
SPESP1	-0.1253072	2.60394607	-0.5721163	0.56968432	0.99978775	-4.6577739
SH3RF3	-0.1253182	4.60906705	-0.5516743	0.58351152	0.99978775	-4.7673281
RCC1L	-0.1254455	3.76274754	-1.1149403	0.26995246	0.99978775	-4.589629
ENSCAFG00000006577	-0.1254592	4.26404855	-0.5382855	0.59265425	0.99978775	-4.7502757
TRPV4	-0.1254707	4.50188671	-1.0530034	0.29716051	0.99978775	-4.6282047
RRAS2	-0.125473	5.08903379	-1.0933324	0.2792379	0.99978775	-4.61663
RABAC1	-0.1255442	7.07590219	-1.0380991	0.30398026	0.99978775	-4.650375
PAQR8	-0.1255881	1.09858696	-0.4578895	0.64892088	0.99978775	-4.6468622
RBL1	-0.1256293	4.06811837	-0.7754228	0.44156785	0.99978775	-4.7043892
AP2A1	-0.1256377	7.64657397	-0.8848525	0.38026976	0.99978775	-4.713475
FZR1	-0.1256636	4.80078869	-1.192356	0.23848175	0.99978775	-4.5669611
FTSJ3	-0.1256901	5.09564065	-0.8693336	0.38862076	0.99978775	-4.7087014
MOCS3	-0.1257437	1.77695041	-0.730553	0.4682991	0.99978775	-4.6270901

RSRP1	-0.1257994	2.19123228	-0.5221514	0.60376062	0.99978775	-4.6742731
ASB15	-0.1258289	-0.1909579	-0.1905771	0.84959179	0.99978775	-4.6241325
CAPN15	-0.1258346	5.02328771	-0.8043005	0.42484964	0.99978775	-4.7216528
ENSCAFG00000000686	-0.1258501	5.65542926	-0.4280516	0.67036397	0.99978775	-4.8534619
BTAF1	-0.1260514	5.29767166	-1.0712405	0.28895994	0.99978775	-4.6264001
IMPDH2	-0.1261849	7.4668371	-0.7314183	0.46777499	0.99978775	-4.7687783
MON1B	-0.1262124	3.61840419	-0.7271347	0.4703727	0.99978775	-4.6903529
RELA	-0.12624	6.41762738	-1.1520219	0.2545297	0.99978775	-4.596345
MIPOL1	-0.1262839	4.79098773	-0.8204495	0.41566861	0.99978775	-4.7114148
MTBP	-0.1263111	2.96692246	-0.8277593	0.41155278	0.99978775	-4.6443868
TLE2	-0.1263316	0.99925067	-0.0945124	0.9250618	0.99978775	-4.6156904
ZNF687	-0.1263932	4.44376359	-0.9239444	0.35974138	0.99978775	-4.6694869
PRMT9	-0.1264431	3.81547626	-1.0240306	0.31051496	0.99978775	-4.6150628
METTL15	-0.1265089	3.72837152	-0.8549153	0.39648158	0.99978775	-4.6718092
ZDHHC3	-0.1265256	5.87327177	-0.9086128	0.36770567	0.99978775	-4.7039076
ENSCAFG00000010507	-0.1265314	-0.5734331	-0.3422995	0.73349174	0.99978775	-4.6197885
YJU2	-0.1265451	2.90370802	-0.6976387	0.48848059	0.99978775	-4.6573808
RNF14	-0.1265945	5.10742287	-1.2981966	0.19989243	0.99978775	-4.5158242
CRK	-0.1267042	4.88769663	-1.4851539	0.14348161	0.99978775	-4.4153882
CACNA1H	-0.1267168	-2.1279442	-0.2061929	0.83743753	0.99978775	-4.6090126
AMT	-0.1267176	4.56121723	-0.892931	0.37596782	0.99978775	-4.6763333
STIP1	-0.1267325	6.29914522	-1.1375117	0.26048792	0.99978775	-4.603578
SLC4A3	-0.1267612	1.76095567	-0.4696012	0.64058405	0.99978775	-4.6637314
ENSCAFG00000029731	-0.1268145	0.06838708	-0.3953134	0.69421286	0.99978775	-4.6366835
YTHDC1	-0.1269722	5.44947024	-1.0901707	0.28061509	0.99978775	-4.6198732
EXD3	-0.127065	0.28644135	-0.243123	0.80885724	0.99978775	-4.6270556
ZSWIM1	-0.1271341	2.41803559	-0.7398291	0.46269834	0.99978775	-4.645232
CNP	-0.1272359	6.01262652	-1.343383	0.18492459	0.99978775	-4.4909773
PHETA2	-0.1272617	3.16505193	-0.5910536	0.55702007	0.99978775	-4.7054647
SMCHD1	-0.1273132	5.03027608	-0.879353	0.3832161	0.99978775	-4.69867
DEXI	-0.1273632	2.36419335	-0.4245887	0.67287108	0.99978775	-4.6612986
ENSCAFG00000014991	-0.1274678	2.59101256	-0.6557248	0.51486382	0.99978775	-4.6507093
FAM237B	-0.1274731	-1.3168054	-0.1984707	0.84344314	0.99978775	-4.6071593
MRPL24	-0.127497	3.91475979	-0.7732627	0.44283378	0.99978775	-4.697233
PURG	-0.1275057	0.76305078	-0.4131168	0.68120305	0.99978775	-4.628753
MRPL11	-0.1275468	4.41962878	-1.1889113	0.23982296	0.99978775	-4.5654789
TET2	-0.1276332	4.92455052	-0.8865331	0.37937226	0.99978775	-4.6937062
B4GAT1	-0.1276485	3.95279417	-0.7291314	0.4691608	0.99978775	-4.6931393
UBL7	-0.1278135	5.39836345	-1.1822124	0.24244683	0.99978775	-4.5758006
POLR1A	-0.1278291	5.61253594	-1.2346513	0.22245637	0.99978775	-4.5512556
SKA3	-0.1278419	2.52196811	-0.4167542	0.67855684	0.99978775	-4.7029226
CBX4	-0.1279782	4.3806969	-0.9939452	0.32480746	0.99978775	-4.6407789
SV2A	-0.1279966	2.00522708	-0.5067359	0.61446128	0.99978775	-4.6545493
CCDC28B	-0.1281134	1.94186538	-0.5188932	0.60601513	0.99978775	-4.6549904
TCFL5	-0.1282611	4.30296309	-0.8969525	0.37383789	0.99978775	-4.6735675
PPP6R2	-0.1283129	5.77452353	-1.4805994	0.14468843	0.99978775	-4.4082896
SLC39A1	-0.128488	6.53597266	-1.638896	0.10721305	0.99978775	-4.3004899
CCDC62	-0.1284881	2.40907176	-0.6981773	0.4881465	0.99978775	-4.6494356
ENSCAFG00000009085	-0.1285022	-0.3584398	-0.3724525	0.71105488	0.99978775	-4.6188632
MRRF	-0.1285954	4.56069209	-0.9911692	0.32614808	0.99978775	-4.647376

SDHAF1	-0.1287896	1.81879451	-0.5739765	0.56843409	0.99978775	-4.6417635
TMEM140	-0.1288363	0.02941825	-0.2585223	0.79701445	0.99978775	-4.6187628
ENSCAFG00000029520	-0.1288519	0.71433465	-0.3614383	0.719222	0.99978775	-4.6298355
MAGI3	-0.128859	3.48990802	-0.679975	0.49950697	0.99978775	-4.6877092
ENSCAFG00000011780	-0.1288978	3.07196184	-0.8748859	0.38561985	0.99978775	-4.6344998
RASSF7	-0.1289848	0.22549307	-0.2960702	0.76834412	0.99978775	-4.6265619
DECR2	-0.1290088	2.12885758	-0.6388824	0.52567629	0.99978775	-4.6409607
PAFAH1B3	-0.1290542	4.97435435	-0.6394077	0.52533728	0.99978775	-4.7736159
ENSCAFG00000030398	-0.1290723	-0.4578446	-0.2938548	0.77002719	0.99978775	-4.6226665
KLHL15	-0.1290784	2.90653985	-0.7024996	0.48547007	0.99978775	-4.6549892
ETV4	-0.1291212	4.22302006	-0.3589638	0.72106146	0.99978775	-4.7234242
ADGRG1	-0.129131	1.33603691	-0.1900269	0.85002072	0.99978775	-4.6664565
BMF	-0.1291467	2.62863826	-0.3845235	0.70214325	0.99978775	-4.7039106
CHORDC1	-0.1291913	5.66883703	-0.9867164	0.32830625	0.99978775	-4.6733709
RUVBL2	-0.1293639	5.25972829	-1.0692959	0.28982685	0.99978775	-4.6285544
PCGF2	-0.1294093	4.93334159	-1.0167745	0.31392238	0.99978775	-4.6438819
EDEM2	-0.1294527	5.83862672	-0.9266602	0.35834228	0.99978775	-4.690527
HOMER3	-0.1295459	4.36738407	-0.559569	0.57815244	0.99978775	-4.7730663
NUP37	-0.1295504	3.16047385	-0.7801022	0.43883275	0.99978775	-4.660318
ENSCAFG00000002541	-0.1295582	6.55113914	-1.3298106	0.18932802	0.99978775	-4.4989551
CD9	-0.1295917	7.3309108	-0.5421233	0.59002671	0.99978775	-4.8280034
KIF20B	-0.1296147	4.75641835	-0.5773957	0.56613953	0.99978775	-4.793005
NINL	-0.1297103	3.34408815	-0.7772501	0.4404986	0.99978775	-4.6648771
PGM5	-0.12972	2.52017323	-0.4734874	0.63782797	0.99978775	-4.8031955
SREK1IP1	-0.1297342	2.66235847	-0.742193	0.46127726	0.99978775	-4.6379159
DGKG	-0.1297414	0.05748936	-0.3513219	0.72675255	0.99978775	-4.626836
GATAD2B	-0.1297775	4.50657105	-1.2291318	0.22450152	0.99978775	-4.5473882
ENSCAFG00000016869	-0.1298618	2.696443	-0.6437711	0.52252561	0.99978775	-4.6547743
ZNF827	-0.1300176	4.75725648	-1.177183	0.24443035	0.99978775	-4.5753212
STAMBP	-0.1300195	3.07973046	-1.0778677	0.28601917	0.99978775	-4.59291
SLC25A6	-0.130022	9.15525183	-0.9956466	0.32398758	0.99978775	-4.647558
ENSCAFG00000032656	-0.1300533	2.05324432	-0.4115764	0.68232494	0.99978775	-4.6457014
BRAT1	-0.1300591	4.71749603	-0.9790589	0.33203986	0.99978775	-4.650841
ZMAT2	-0.1300601	3.55081975	-0.7621649	0.4493713	0.99978775	-4.6773518
REXO4	-0.130218	5.75921675	-1.2610964	0.21284784	0.99978775	-4.5373863
RBPJ	-0.1302424	4.98744254	-0.8746862	0.38572754	0.99978775	-4.681426
CCDC84	-0.1303058	1.52154833	-0.5880706	0.55900553	0.99978775	-4.6314101
ENSCAFG00000009957	-0.1303382	0.76722704	-0.4962247	0.62180652	0.99978775	-4.642871
CCS	-0.130339	3.37139028	-0.4283968	0.67011431	0.99978775	-4.7011543
PSMB8	-0.1303505	0.09930866	-0.2832652	0.77808723	0.99978775	-4.6246101
PRPF6	-0.1303891	6.95304542	-1.5105684	0.13689266	0.99978775	-4.3875503
ENSCAFG00000012020	-0.1304263	5.85666021	-1.8002244	0.07757002	0.99978775	-4.1863382
MMAB	-0.1305411	2.2300642	-0.8001284	0.42724123	0.99978775	-4.6298019
ENSCAFG00000000491	-0.1305491	0.16884211	-0.4407846	0.66117823	0.99978775	-4.631291
ENSCAFG00000001499	-0.1305617	2.10571335	-0.7946199	0.43041119	0.99978775	-4.635358
CWF19L1	-0.1306433	3.11533184	-0.8214159	0.415123	0.99978775	-4.653146
ENSCAFG00000028519	-0.1306804	0.56901414	-0.4461821	0.65730004	0.99978775	-4.6246196
MEI4	-0.1307665	-0.731203	-0.3044735	0.76197047	0.99978775	-4.6178714
SLC1A4	-0.1307978	6.4191015	-0.7615362	0.44974336	0.99978775	-4.7636219
XRCC5	-0.1308081	5.19955919	-1.329709	0.18936127	0.99978775	-4.4988223

UBN2	-0.1308664	2.76713601	-0.7581905	0.4517262	0.99978775	-4.6452434
FGF2	-0.1309004	2.32105953	-0.5008816	0.61854747	0.99978775	-4.6525794
ENSCAFG00000023983	-0.1309119	0.51043744	-0.3946581	0.69469354	0.99978775	-4.6256474
L3MBTL3	-0.1309611	3.87052133	-0.7927575	0.43148613	0.99978775	-4.6928664
MRPL46	-0.130972	3.387607	-1.0919946	0.27982005	0.99978775	-4.5929817
UQCC2	-0.1309907	3.6454523	-1.0175044	0.31357849	0.99978775	-4.6177183
INTS4	-0.1311052	4.75843911	-1.176834	0.24456846	0.99978775	-4.5744566
RP9	-0.1311279	1.45919803	-0.6543502	0.51574181	0.99978775	-4.6274293
ZNF383	-0.131138	2.03924893	-0.507026	0.61425911	0.99978775	-4.6497855
ZNF462	-0.1311644	5.86087395	-1.1181877	0.26857601	0.99978775	-4.6096733
ABCC10	-0.131169	4.8426603	-1.134428	0.26176686	0.99978775	-4.5985225
ARFGEF2	-0.1312202	6.01187395	-1.4934848	0.1412947	0.99978775	-4.3996007
SNAP23	-0.1312709	3.49543635	-0.8725723	0.38686851	0.99978775	-4.643948
NPEPL1	-0.1313174	4.92648769	-1.29815	0.19990833	0.99978775	-4.5156652
SLC5A6	-0.1313261	5.55885027	-0.7045646	0.48419423	0.99978775	-4.7736197
AARS	-0.1313694	8.02468129	-0.9707248	0.33613526	0.99978775	-4.6664525
SNCAIP	-0.1314091	1.58818427	-0.6445664	0.52201399	0.99978775	-4.7054934
MRPL2	-0.1314267	4.16561702	-1.0818701	0.28425322	0.99978775	-4.6037383
PLCB3	-0.1314583	7.02007897	-1.6656987	0.10172859	0.99978775	-4.2824676
CRTC2	-0.1314805	4.55174521	-0.9118598	0.36600957	0.99978775	-4.6698215
WDR18	-0.131554	2.96496226	-0.8337709	0.40818657	0.99978775	-4.646797
TC2N	-0.1316128	-2.0404074	-0.1731457	0.86320256	0.99978775	-4.6095064
POC1B	-0.131747	3.12956739	-0.9017163	0.37132476	0.99978775	-4.6293981
MRPS18A	-0.1318203	3.50010517	-1.0537444	0.29682422	0.99978775	-4.6045653
LDLRAD3	-0.1318356	3.02716943	-0.4858313	0.62910783	0.99978775	-4.6894999
CHIC1	-0.1319355	1.21516608	-0.5206103	0.60482647	0.99978775	-4.6312062
MAP3K21	-0.1319709	2.17385093	-0.4584012	0.64855566	0.99978775	-4.6506804
ABL1	-0.1320592	7.69835129	-1.1198943	0.26785464	0.99978775	-4.6100062
ZNRF3	-0.1320738	2.02047371	-0.5111305	0.61140202	0.99978775	-4.6537634
UBXN1	-0.132213	5.29606862	-1.2725516	0.20878273	0.99978775	-4.5291409
WRNIP1	-0.1322182	5.18810282	-1.6145712	0.1123968	0.99978775	-4.3357151
UBE2J2	-0.1322871	4.98875993	-1.679374	0.09902009	0.99978775	-4.295175
UQCC1	-0.1323555	3.01767841	-0.8603751	0.39349335	0.99978775	-4.6351452
HNRNPH3	-0.1323914	5.89449177	-1.025853	0.30966314	0.99978775	-4.6560942
ENSCAFG00000006533	-0.132427	0.89235657	-0.3987865	0.69166747	0.99978775	-4.6342628
ATP5F1D	-0.1324325	6.1528558	-1.0784741	0.28575114	0.99978775	-4.6320655
NECAB3	-0.1324551	3.71759278	-0.3792975	0.70599635	0.99978775	-4.7259032
ZNF829	-0.1324666	2.59851339	-0.6555079	0.51500234	0.99978775	-4.6602306
TNKS1BP1	-0.1324872	7.7644093	-1.3183358	0.19311252	0.99978775	-4.5051247
HDGFL2	-0.1325475	5.1720761	-1.3542469	0.18145644	0.99978775	-4.4859149
TOMM34	-0.1325758	3.47101126	-0.9249266	0.35923494	0.99978775	-4.6395529
RPS19BP1	-0.132576	4.98873195	-1.3565441	0.18072952	0.99978775	-4.4844371
RANBP1	-0.1326293	5.95441716	-0.7819988	0.43772706	0.99978775	-4.755701
SERPINB6	-0.1326509	7.03981694	-0.8650665	0.39093695	0.99978775	-4.7214901
PPIE	-0.1327139	4.44067685	-1.3279544	0.18993635	0.99978775	-4.5034283
ZFPM2	-0.1328297	5.56432564	-0.458428	0.64853652	0.99978775	-4.8358434
LLPH	-0.1329115	4.16179789	-1.0518243	0.29769618	0.99978775	-4.6142567
BPHL	-0.1330177	2.50299168	-0.2936115	0.77021206	0.99978775	-4.670283
SORCS2	-0.1330369	-1.3961597	-0.2302036	0.81882795	0.99978775	-4.6191446
DET1	-0.1331863	4.14450989	-1.0521559	0.29754547	0.99978775	-4.6176783

CCDC130	-0.1332744	3.02354505	-1.0276685	0.30881611	0.99978775	-4.6036108
MPST	-0.1332987	4.72635398	-0.8206447	0.41555838	0.99978775	-4.7129833
HSPA9	-0.1333038	8.06120897	-1.2538712	0.21544193	0.99978775	-4.5387281
ENSCAFG00000011598	-0.1334075	5.13427795	-0.6029327	0.54914859	0.99978775	-4.7950498
HABP4	-0.1334181	3.18596411	-0.7659533	0.44713331	0.99978775	-4.6616341
SPRED2	-0.1334333	4.98356029	-0.6174436	0.53961042	0.99978775	-4.7495854
PLEKHM3	-0.1334692	4.3710216	-1.1169646	0.26909388	0.99978775	-4.5945579
RCL1	-0.1334751	5.23068372	-0.5366655	0.59376509	0.99978775	-4.7960441
SLC25A15	-0.1335457	3.38877518	-1.1207093	0.26751067	0.99978775	-4.5872321
MYO19	-0.1335789	4.22627267	-1.1872853	0.24045794	0.99978775	-4.5685855
ENSCAFG00000024456	-0.1335846	0.65442082	-0.5163944	0.60774676	0.99978775	-4.6251946
EFCAB2	-0.133614	-0.006052	-0.4286396	0.66993871	0.99978775	-4.6248093
AGPS	-0.133615	4.70095525	-1.2241504	0.22635914	0.99978775	-4.5510446
AATF	-0.1336322	6.33146581	-1.06036	0.29383344	0.99978775	-4.641522
QSER1	-0.1336543	6.57499638	-1.1753643	0.24515054	0.99978775	-4.5842343
HEATR3	-0.1336622	5.42103241	-1.0930443	0.27936317	0.99978775	-4.6213377
SERBP1	-0.1338022	7.76941589	-1.4937043	0.14123745	0.99978775	-4.4021865
ZBTB34	-0.1338047	1.93353474	-0.6301568	0.53132456	0.99978775	-4.6358228
RPS12	-0.1338132	8.19672879	-1.1488732	0.25581426	0.99978775	-4.5911886
HERPUD1	-0.1338323	6.92578749	-1.2241376	0.22636392	0.99978775	-4.558281
ZSCAN29	-0.133913	3.51213691	-0.8724074	0.3869576	0.99978775	-4.64905
FARP1	-0.1339206	6.60315428	-0.514078	0.60935406	0.99978775	-4.8256057
DYNC1LI1	-0.1339266	4.81176378	-1.1487614	0.25585996	0.99978775	-4.5858809
CFAP45	-0.1339717	0.79232939	-0.316327	0.75300825	0.99978775	-4.636212
ENSCAFG00000015181	-0.1339873	4.61491903	-0.8789088	0.38345471	0.99978775	-4.690806
YTHDC2	-0.1340815	5.2529279	-1.4133792	0.16344464	0.99978775	-4.4510395
RNF185	-0.1342567	2.72766387	-1.0243511	0.31036503	0.99978775	-4.6025424
TOR2A	-0.1344083	3.26884113	-1.1479354	0.25619777	0.99978775	-4.5750777
AFAP1L1	-0.1344418	-1.7989179	-0.1203163	0.90469172	0.99978775	-4.6082162
UPF3B	-0.1344651	3.40549805	-0.7752709	0.44165676	0.99978775	-4.6732263
CALR	-0.1344862	9.53753792	-0.9515784	0.34567015	0.99978775	-4.6591225
ASTE1	-0.1345341	1.78015652	-0.5637973	0.57529197	0.99978775	-4.6430335
TOP3A	-0.1346232	4.76182781	-1.2146973	0.22991524	0.99978775	-4.5564462
CYB561D1	-0.1346601	0.87924733	-0.4131485	0.68117999	0.99978775	-4.632651
PTCHD1	-0.1346646	-1.7336028	-0.1760115	0.86096189	0.99978775	-4.6133093
POMT2	-0.1346795	5.09809976	-1.5450949	0.12832875	0.99978775	-4.3737659
ENSCAFG00000026308	-0.1347183	0.76155217	-0.4487737	0.65544136	0.99978775	-4.6359885
ENSCAFG00000030295	-0.1347276	0.38040256	-0.462197	0.64584927	0.99978775	-4.6236551
CAVIN3	-0.1347539	6.05958869	-0.728031	0.46982848	0.99978775	-4.7741817
TSC1	-0.1349037	4.99554828	-1.3238403	0.19128999	0.99978775	-4.5022909
APH1B	-0.1349688	2.67378178	-0.5482748	0.58582654	0.99978775	-4.6624856
GCHFR	-0.1350183	1.34889335	-0.4969894	0.62127087	0.99978775	-4.6394332
CLIC5	-0.1350458	-0.607578	-0.1642089	0.8701971	0.99978775	-4.6151576
TBCEL	-0.135076	4.39178681	-1.0711519	0.28899944	0.99978775	-4.6127254
NCOR1	-0.1351351	7.31357413	-1.8340187	0.07232009	0.99978775	-4.1625005
ENSCAFG00000012643	-0.135192	3.49357345	-0.351512	0.72661077	0.99978775	-4.7624352
UFM1	-0.1352188	2.63751277	-0.7593996	0.45100902	0.99978775	-4.6457996
COX7A1	-0.13522	2.39647811	-0.5738478	0.56852049	0.99978775	-4.6538989
MRPL32	-0.1353072	3.71129151	-1.3116946	0.19532883	0.99978775	-4.51508
CNTRL	-0.1353283	4.81780879	-1.2989959	0.19961996	0.99978775	-4.5152801

BLVRB	-0.1353323	5.05722693	-0.7917876	0.43204657	0.99978775	-4.7309863
CYB5R4	-0.1353669	3.7552852	-1.314902	0.19425608	0.99978775	-4.5149047
PC	-0.1353887	4.95503679	-0.6097898	0.54463067	0.99978775	-4.7834402
ENSCAFG00000024514	-0.1354468	1.88256093	-0.3321941	0.74106504	0.99978775	-4.6461819
ENSCAFG00000028483	-0.1355983	3.29614335	-0.5324825	0.59663779	0.99978775	-4.7044229
ENSCAFG00000006580	-0.1356045	7.34804727	-0.8287287	0.41100878	0.99978775	-4.7347304
CDC42EP2	-0.1358007	2.45475495	-0.3013256	0.76435614	0.99978775	-4.66826
DPH7	-0.1359009	4.25157403	-0.9432415	0.3498768	0.99978775	-4.6494374
SMPD4	-0.1359872	5.86723948	-1.6457441	0.10578935	0.99978775	-4.2970239
FANCE	-0.1360008	3.99293062	-0.9938359	0.32486016	0.99978775	-4.6263181
ENSCAFG00000029533	-0.1360181	-1.6073569	-0.3330231	0.74044276	0.99978775	-4.614185
GTF2F1	-0.1360253	5.76709256	-1.5049832	0.13831972	0.99978775	-4.3926829
CANT1	-0.1360494	4.46269318	-1.1164015	0.26933252	0.99978775	-4.5944156
DNLZ	-0.1360606	1.26161827	-0.5008602	0.61856243	0.99978775	-4.6340682
NOL7	-0.1361588	4.25022403	-1.0537239	0.29683352	0.99978775	-4.6194013
MOK	-0.1362186	1.70957435	-0.5063026	0.61476333	0.99978775	-4.6361715
SLF2	-0.1362241	3.31467468	-0.7399936	0.46259938	0.99978775	-4.6566876
DNAJC25	-0.1362611	4.83811047	-0.8951859	0.3747726	0.99978775	-4.6874486
ERF	-0.1362962	5.26572899	-0.8467545	0.40097433	0.99978775	-4.7177539
NDUFS2	-0.1363437	6.48854166	-1.2425575	0.21955083	0.99978775	-4.5483346
CASP6	-0.1363474	4.04661041	-1.3078836	0.19660926	0.99978775	-4.5144455
GTPBP4	-0.1363703	6.32153224	-1.0884267	0.28137676	0.99978775	-4.6278347
TOMM70	-0.1364305	5.67005693	-1.340769	0.18576656	0.99978775	-4.4925476
EXOSC3	-0.1365634	2.02765653	-0.7263087	0.47087454	0.99978775	-4.6331424
ATAD2	-0.1365682	5.9572499	-0.5316304	0.59722383	0.99978775	-4.8318679
C9H17orf58	-0.1366526	0.17248399	-0.3203492	0.74997483	0.99978775	-4.6228726
ACSF2	-0.1367659	2.40917381	-0.3806816	0.70497514	0.99978775	-4.6482783
ENSCAFG00000023441	-0.1367689	7.19527865	-0.8731941	0.38653269	0.99978775	-4.7199668
TTC5	-0.1367847	3.45160779	-1.1173351	0.26893694	0.99978775	-4.5846885
PTPRS	-0.1368378	6.83704815	-0.7961114	0.42955149	0.99978775	-4.7516263
TRIP10	-0.1369178	5.22688799	-0.7351028	0.46554717	0.99978775	-4.7396762
MDC1	-0.136939	6.89505483	-2.0064278	0.04997258	0.99978775	-4.022262
BET1L	-0.136975	3.65996882	-1.2381924	0.22115153	0.99978775	-4.5443064
CIAO2A	-0.1369793	4.1497717	-1.2222529	0.2270697	0.99978775	-4.5500166
TRAPPC6B	-0.1371265	2.64254972	-0.8716279	0.38737898	0.99978775	-4.6251189
ENSCAFG00000030681	-0.1371434	-0.7426994	-0.4225791	0.67432766	0.99978775	-4.6221216
PPM1L	-0.1371877	0.58717707	-0.3793016	0.70599336	0.99978775	-4.6290174
KALRN	-0.1372358	1.207602	-0.3673376	0.71484347	0.99978775	-4.6540448
ENSCAFG00000010704	-0.1372735	3.93845468	-0.7298071	0.46875111	0.99978775	-4.6887609
TUFM	-0.1373997	6.1474339	-1.1209653	0.26740264	0.99978775	-4.6112808
IFFO1	-0.1375082	3.4094141	-0.8859698	0.37967295	0.99978775	-4.6460832
CDK2	-0.1376112	2.3208061	-0.5761186	0.56699601	0.99978775	-4.6784352
TTLL4	-0.1376167	4.01482153	-0.642274	0.52348939	0.99978775	-4.7242203
CHCHD2	-0.137685	6.36486916	-1.4517841	0.15251005	0.99978775	-4.4249343
MRI1	-0.1377622	3.56064632	-0.6574385	0.51377038	0.99978775	-4.6987259
ASMTL	-0.1379039	3.75001549	-0.7089015	0.48152095	0.99978775	-4.6981101
NOVA1	-0.1379624	2.86735394	-0.9664422	0.33825274	0.99978775	-4.6183852
UNG	-0.1380556	2.13670798	-0.6388292	0.52571066	0.99978775	-4.6505797
PPP1R12C	-0.1382398	5.56970692	-1.3052721	0.19749033	0.99978775	-4.5124735
BRD4	-0.1383602	6.37254859	-2.1090081	0.03973011	0.99978775	-3.9329803

AFG3L2	-0.1384277	5.68972715	-1.2823352	0.2053569	0.99978775	-4.5247404
MIPEP	-0.138453	5.07998619	-1.2467323	0.21802796	0.99978775	-4.5416963
DLAT	-0.1384625	5.2911637	-1.2182603	0.22857014	0.99978775	-4.557588
NAP1L1	-0.1385459	7.56507618	-0.9500076	0.34646016	0.99978775	-4.6871765
OSGIN1	-0.1385568	3.22477252	-0.4810252	0.63249675	0.99978775	-4.6990814
TERF1	-0.1385869	3.30861837	-0.7266477	0.47066855	0.99978775	-4.6854538
TRIM44	-0.1387127	2.91334372	-1.2040639	0.23396402	0.99978775	-4.5581177
MRM2	-0.1387427	2.4419378	-0.9512351	0.34584269	0.99978775	-4.6100483
RAB9A	-0.138749	5.21634415	-1.4969433	0.14039461	0.99978775	-4.4033406
DPCD	-0.1387877	3.26370333	-0.5807922	0.56386481	0.99978775	-4.6973147
TMC7	-0.1388501	1.72387577	-0.3369205	0.73751965	0.99978775	-4.6490317
TACO1	-0.1388629	2.62865685	-1.114985	0.26993349	0.99978775	-4.5826431
C11H9orf43	-0.1388761	0.09419882	-0.3903635	0.6978468	0.99978775	-4.619919
MRPS26	-0.1389	2.59555469	-0.7976653	0.42865692	0.99978775	-4.6408101
TMEM131L	-0.1389048	5.23125617	-1.0425489	0.30193307	0.99978775	-4.6350892
CPTP	-0.1389986	4.34361098	-0.7496987	0.45678173	0.99978775	-4.7062811
ENSCAFG00000028220	-0.1390572	2.61126173	-0.5479065	0.58607758	0.99978775	-4.7129224
TP53INP1	-0.1390575	1.45983865	-0.3495982	0.72803839	0.99978775	-4.6288872
DUSP1	-0.1390654	8.23874217	-0.7672964	0.44634144	0.99978775	-4.7460126
ENSCAFG00000011993	-0.1391598	5.14980297	-1.2487161	0.21730704	0.99978775	-4.5412061
JMJD4	-0.1392243	3.82599854	-1.1010622	0.27589087	0.99978775	-4.59466
RTN2	-0.1394088	3.24123007	-0.6236707	0.53554355	0.99978775	-4.6744879
ZSCAN12	-0.1395357	3.01466975	-1.0814163	0.28445308	0.99978775	-4.5921522
TLCD1	-0.139553	1.73629031	-0.4724459	0.63856606	0.99978775	-4.6427271
PLIN4	-0.1395999	1.1002831	-0.4751473	0.63665233	0.99978775	-4.6335278
FAM129B	-0.1396299	8.34580213	-0.999361	0.32220257	0.99978775	-4.6619888
SP110	-0.1396397	4.57940951	-1.1154202	0.26974875	0.99978775	-4.5943085
ZFP1	-0.1396606	2.4766289	-1.0081777	0.31799202	0.99978775	-4.6016356
PIAS4	-0.1396726	4.21691917	-1.4045103	0.16605419	0.99978775	-4.468527
ANKRD13B	-0.1396911	2.85331612	-0.5549524	0.58128338	0.99978775	-4.6776657
TFAM	-0.1397886	2.40967983	-0.8232812	0.41407122	0.99978775	-4.6338224
POLR2I	-0.1398532	3.43429407	-1.1432874	0.25810449	0.99978775	-4.5769931
RASSF1	-0.1398877	2.74342989	-0.764975	0.44771065	0.99978775	-4.656561
PPP6R1	-0.1399978	6.25698387	-1.8099338	0.07602994	0.99978775	-4.1756233
MFSD8	-0.1401958	1.86131728	-0.8945178	0.37512647	0.99978775	-4.612975
ENSCAFG00000025953	-0.1402099	-1.1678195	-0.2514888	0.80241779	0.99978775	-4.6105643
KRBA1	-0.1402475	3.05201597	-0.7474571	0.45812168	0.99978775	-4.6588936
ATAD2B	-0.1404867	3.47463606	-0.8687022	0.3889629	0.99978775	-4.6542026
DYRK1B	-0.1405043	4.39097802	-0.8323451	0.40898345	0.99978775	-4.6876397
ABCA8	-0.1406227	7.07689942	-0.4654589	0.64352748	0.99978775	-4.8459409
ENSCAFG00000013154	-0.1406253	3.8494681	-1.3818051	0.17288182	0.99978775	-4.4835232
BCL2L1	-0.1406296	4.94934218	-1.0128357	0.3157826	0.99978775	-4.6365004
ASH2L	-0.1406481	4.07953141	-1.2278804	0.22496712	0.99978775	-4.5478318
SYTL1	-0.1408562	1.92344458	-0.3158967	0.75333301	0.99978775	-4.6578472
ZNF787	-0.1408803	3.12553585	-0.9661842	0.3383806	0.99978775	-4.6251309
ATG4A	-0.1409823	3.28358898	-0.9359912	0.3535622	0.99978775	-4.6295554
CTR9	-0.1410106	6.13370038	-1.7935756	0.07863965	0.99978775	-4.1903985
TAF9B	-0.1411199	2.97810428	-0.5410134	0.59078601	0.99978775	-4.7206587
HCFC1	-0.1411638	6.81474872	-1.5998637	0.11562846	0.99978775	-4.3287342
C1QTNF5	-0.1411903	2.64161305	-0.7285068	0.46953971	0.99978775	-4.6937936

MGRN1	-0.1412823	6.75696414	-1.3048571	0.19763062	0.99978775	-4.5133953
FMNL3	-0.1412877	6.67188475	-0.6053351	0.54756357	0.99978775	-4.8127626
TACC2	-0.1412988	6.37765432	-0.9789047	0.33211531	0.99978775	-4.6780198
TSPAN5	-0.1413467	5.7323286	-0.5694781	0.57145978	0.99978775	-4.7847883
ZNF48	-0.1413769	4.11249961	-1.1571081	0.25246444	0.99978775	-4.575827
POR	-0.1413781	5.39499835	-1.3855522	0.17174038	0.99978775	-4.4679679
XPA	-0.1413812	3.13979999	-0.7100391	0.4808211	0.99978775	-4.6644036
ENSCAFG00000032037	-0.1414054	1.52820531	-0.4682805	0.64152192	0.99978775	-4.6411603
CDCA7L	-0.1414379	5.06504798	-0.7837681	0.43669703	0.99978775	-4.7394579
RRP1B	-0.1414637	5.82425623	-1.4918354	0.14172556	0.99978775	-4.4013726
PHF2	-0.1415584	5.45215834	-1.6272304	0.10967422	0.99978775	-4.3166622
NFKB1	-0.1415801	5.65335798	-1.4569167	0.15109313	0.99978775	-4.4244562
ANAPC2	-0.1416591	5.04005647	-0.9655409	0.3386995	0.99978775	-4.6662036
MAN1A1	-0.1418187	6.75534846	-0.4902278	0.62601468	0.99978775	-4.8396838
GPAM	-0.141834	6.57932408	-0.7501508	0.45651174	0.99978775	-4.7680501
TMEM102	-0.1418965	2.03994121	-0.7538297	0.45431829	0.99978775	-4.6342708
BCOR	-0.1419536	5.20508477	-1.2519405	0.21613904	0.99978775	-4.541791
SPNS1	-0.1420087	5.53629228	-1.1925656	0.23840032	0.99978775	-4.573811
ZNF484	-0.1420645	1.26686016	-0.4555844	0.65056707	0.99978775	-4.6335865
MCL1	-0.1421401	7.72291207	-1.2999786	0.19928539	0.99978775	-4.5155421
PHF8	-0.1422019	4.06889848	-1.418325	0.16200325	0.99978775	-4.4687829
GTPBP8	-0.1422096	2.39012665	-0.8558975	0.39594297	0.99978775	-4.6256689
GAR1	-0.1423352	3.65564796	-0.990121	0.32665526	0.99978775	-4.6265718
ENSCAFG00000013514	-0.1424337	3.23647809	-1.0853693	0.28271553	0.99978775	-4.5922882
SEPT6	-0.1424845	7.30007186	-0.6007818	0.55056965	0.99978775	-4.8073856
C15H1orf122	-0.1425471	1.03434309	-0.3961764	0.69358007	0.99978775	-4.6355432
MCC	-0.1425785	4.62101885	-0.8046467	0.4246515	0.99978775	-4.7311949
ZSWIM7	-0.1426559	0.20689944	-0.4234952	0.67366349	0.99978775	-4.6295233
GARS	-0.1427192	8.81519476	-1.038394	0.30384431	0.99978775	-4.6317843
TRA2A	-0.1427287	4.24939902	-1.1679401	0.24810618	0.99978775	-4.5724019
MYCBPAP	-0.1427372	0.65953867	-0.3338467	0.73982476	0.99978775	-4.6253455
HDDC3	-0.1427581	3.79932604	-1.0643007	0.29206186	0.99978775	-4.6053944
BRD9	-0.1427769	5.48698772	-1.4183964	0.16198253	0.99978775	-4.4478723
DOCK9	-0.1428261	6.18850057	-0.8695287	0.38851507	0.99978775	-4.707065
CTDNEP1	-0.1428299	5.72982597	-2.0385517	0.04654389	0.99978775	-4.007703
UPF3A	-0.1428303	2.49164589	-0.8612538	0.39301377	0.99978775	-4.6228992
KLF15	-0.1428522	1.47998159	-0.2941764	0.76978275	0.99978775	-4.63902
ABCD4	-0.1430766	3.29469198	-0.8197195	0.416081	0.99978775	-4.6555489
AIF1L	-0.1430931	-0.4667838	-0.2901624	0.7728347	0.99978775	-4.6226291
WDR90	-0.1431769	5.78363491	-1.1095452	0.27225021	0.99978775	-4.6126224
TRAPPC5	-0.1432177	3.55801831	-1.120513	0.2675935	0.99978775	-4.5859774
KIF5A	-0.143331	3.31950803	-0.8720172	0.38716851	0.99978775	-4.6554887
EPHX2	-0.1433319	-0.3764546	-0.2943911	0.76961966	0.99978775	-4.6194
HECTD3	-0.1433321	5.58984213	-1.5364933	0.13042118	0.99978775	-4.3725312
CDON	-0.143348	4.8674569	-0.3187562	0.75117575	0.99978775	-4.873488
POU6F1	-0.1433719	4.04189979	-1.0189451	0.31290045	0.99978775	-4.6326735
SRBD1	-0.1433821	4.29324093	-1.2441049	0.21898546	0.99978775	-4.5411578
PDAP1	-0.1435381	5.29803498	-1.1005733	0.27610174	0.99978775	-4.6162774
GGH	-0.1435685	5.86750691	-1.1593437	0.25156047	0.99978775	-4.5895223
SH3BP1	-0.1435736	4.54593116	-0.4005238	0.69039557	0.99978775	-4.7687233

EMCN	-0.1435898	1.34591164	-0.0612914	0.95135978	0.99978775	-4.6185387
ALAD	-0.1436023	1.98764204	-0.6157526	0.54071753	0.99978775	-4.6507155
SEPT9	-0.143644	8.10153288	-1.2869049	0.20377126	0.99978775	-4.5215916
SF3B2	-0.1437855	7.47905754	-1.74629	0.08660805	0.99978775	-4.2311426
CIZ1	-0.1437976	4.84097434	-1.0907089	0.28038033	0.99978775	-4.6113337
SPSB2	-0.1438496	1.07106025	-0.4465708	0.65702112	0.99978775	-4.6343849
MME	-0.1439022	2.97990586	-0.391304	0.69715578	0.99978775	-4.8049337
TCF3	-0.1439437	4.89238672	-1.2612741	0.21278435	0.99978775	-4.5344873
C7H1orf53	-0.143988	-0.080018	-0.4011435	0.68994208	0.99978775	-4.6205455
RPS6KA1	-0.1440499	1.65073347	-0.5186198	0.60620447	0.99978775	-4.6483963
SCAPER	-0.1440927	4.22945509	-1.2340311	0.22268551	0.99978775	-4.5454098
ENSCAFG00000008682	-0.1441239	1.75970756	-0.550209	0.58450887	0.99978775	-4.6445765
POMGNT2	-0.1441489	3.87580658	-0.778602	0.43970852	0.99978775	-4.6873571
DCAF15	-0.1441501	4.02188809	-1.2514248	0.21632554	0.99978775	-4.5379472
ENSCAFG00000002074	-0.1441913	1.63032561	-0.6127153	0.54270898	0.99978775	-4.6345857
RPTOR	-0.1442088	5.74833815	-1.6936454	0.09625695	0.99978775	-4.2658722
MT2A	-0.1442676	4.20496631	-0.1556144	0.87693358	0.99978775	-4.6675786
FBN1	-0.1442751	10.8263102	-0.5669897	0.57313689	0.99978775	-4.7372933
PLXNA1	-0.144421	6.99414898	-1.2279036	0.22495849	0.99978775	-4.5558176
PGM1	-0.1444723	6.68238521	-1.4100991	0.16440604	0.99978775	-4.4508674
GINS3	-0.1444795	3.57876426	-1.0318503	0.30687109	0.99978775	-4.6071372
ENSCAFG00000020261	-0.1444931	3.55907237	-1.0654467	0.29154803	0.99978775	-4.6005213
TTLL1	-0.1445323	3.72889184	-1.0035273	0.32020827	0.99978775	-4.6259842
IMMT	-0.1445555	6.68226978	-1.838501	0.07164677	0.99978775	-4.153492
CCDC85B	-0.1445627	2.61248266	-0.5232169	0.60302416	0.99978775	-4.6662835
ECSIT	-0.1446461	3.65593083	-1.0419581	0.30220435	0.99978775	-4.6133546
MIA3	-0.1446976	7.54277217	-1.9328706	0.05865261	0.99978775	-4.0933577
SEZ6L	-0.1448156	-1.6984945	-0.111469	0.91166941	0.99978775	-4.606953
HACD3	-0.1448346	3.99476857	-1.0233489	0.31083401	0.99978775	-4.6242801
YIF1B	-0.1448457	5.59016538	-0.6590596	0.51273714	0.99978775	-4.7833285
ARHGAP25	-0.1448535	-2.2240503	-0.2178892	0.82835989	0.99978775	-4.6075994
AMMECR1	-0.1448676	3.14174272	-1.0058054	0.31912132	0.99978775	-4.6098952
PCOLCE2	-0.1449048	3.69848304	-0.2301249	0.8188888	0.99978775	-4.7180348
TBC1D9	-0.1449075	4.55491746	-0.5453065	0.58785144	0.99978775	-4.8241853
WIPF1	-0.1449211	5.83996814	-0.9851012	0.32909141	0.99978775	-4.6736569
OCIAD2	-0.1450362	4.61771842	-0.3912075	0.69722667	0.99978775	-4.8117462
HDHD5	-0.1450788	5.52272048	-1.2876384	0.20351758	0.99978775	-4.5222513
VPS26C	-0.1451867	4.2911058	-1.4141803	0.16321049	0.99978775	-4.464235
NOL8	-0.1451958	5.70749829	-1.4326034	0.15789741	0.99978775	-4.438183
RALGAPA2	-0.1452524	3.87698932	-0.582414	0.56278022	0.99978775	-4.7317526
PTDSS2	-0.1452682	4.91061791	-1.2004558	0.23534959	0.99978775	-4.5649919
L2HGDH	-0.1452683	2.14098542	-0.765793	0.44722791	0.99978775	-4.629888
PANX2	-0.1453626	3.33132735	-0.7270906	0.47039946	0.99978775	-4.6757985
TNRC18	-0.1453694	6.82802781	-1.639099	0.10717062	0.99978775	-4.3007029
ANKRD45	-0.145376	-0.5159195	-0.2472995	0.80564072	0.99978775	-4.6122771
ARPIN	-0.1453908	4.58255326	-1.4633537	0.1493307	0.99978775	-4.4316069
PCED1A	-0.1453944	4.33394389	-0.6524975	0.51692648	0.99978775	-4.7346014
MDGA1	-0.145432	2.50688425	-0.2146918	0.83083915	0.99978775	-4.6678619
GEMIN6	-0.1456388	4.22801981	-1.1238256	0.26619815	0.99978775	-4.5865574
METTL3	-0.1457558	4.73252098	-1.4755216	0.14604332	0.99978775	-4.4209496

HIRA	-0.1457808	5.36495289	-1.5633589	0.12397492	0.99978775	-4.3595315
ENSCAFG00000016252	-0.1458084	-1.2909956	-0.3009085	0.76467246	0.99978775	-4.6158371
ENSCAFG00000013858	-0.1458192	-0.517585	-0.3706063	0.71242151	0.99978775	-4.6189342
ADARB1	-0.1458443	4.85370066	-0.6831493	0.49751548	0.99978775	-4.7646241
ATG7	-0.1458681	4.46338849	-1.1088002	0.27256856	0.99978775	-4.5963692
CTNS	-0.1459325	5.67997369	-0.3983927	0.6919559	0.99978775	-4.8130262
RCE1	-0.1459447	2.79057873	-0.8075668	0.42298287	0.99978775	-4.6473217
RNF7	-0.1459701	4.2574542	-1.2761292	0.20752508	0.99978775	-4.5270822
UBL4A	-0.1459809	2.66428026	-0.9657447	0.33859846	0.99978775	-4.6147361
CNTF	-0.1460342	-0.3575102	-0.3336637	0.73996205	0.99978775	-4.6170176
EMP3	-0.1460483	6.90693016	-0.7116491	0.47983158	0.99978775	-4.7804742
AGBL5	-0.1460895	3.59017661	-1.1066706	0.27348008	0.99978775	-4.5899618
BACE2	-0.1461553	4.06418202	-0.2829482	0.7783289	0.99978775	-4.7630243
FBXO30	-0.1461758	3.92353329	-1.2709768	0.20933815	0.99978775	-4.5302014
MLXIP	-0.1462435	5.67692433	-1.3499063	0.18283615	0.99978775	-4.4876946
TP53	-0.1462978	6.45994328	-1.1828244	0.24220625	0.99978775	-4.5803703
HDAC8	-0.1463062	2.57539767	-1.0198722	0.31246465	0.99978775	-4.602139
CDK5RAP2	-0.1463404	5.65903876	-1.5249441	0.13327335	0.99978775	-4.380235
L3MBTL2	-0.146373	5.17481073	-1.3429793	0.18505445	0.99978775	-4.4914692
HSBP1	-0.146392	4.42665521	-1.1898688	0.23944962	0.99978775	-4.5641239
KCTD6	-0.1464285	0.71586757	-0.3927413	0.69610026	0.99978775	-4.6264018
HSPA12B	-0.1465173	-2.3268282	-0.2307912	0.81837379	0.99978775	-4.6076862
ZFP2	-0.1466071	2.02045723	-0.603993	0.54844877	0.99978775	-4.6382301
THYN1	-0.1466109	4.53374736	-1.2505584	0.21663913	0.99978775	-4.5387535
KCNG1	-0.1466692	-0.5006424	-0.2842058	0.77737031	0.99978775	-4.6267889
BAG2	-0.1467021	4.28807026	-0.6606379	0.51173225	0.99978775	-4.7419963
CREB1	-0.1467558	2.83698745	-0.8375268	0.40609207	0.99978775	-4.6409012
THAP4	-0.146862	4.29919039	-1.4698238	0.14757553	0.99978775	-4.4346287
EIF5A	-0.1468638	7.46219816	-1.0040986	0.31993542	0.99978775	-4.6621698
GTF3C5	-0.1468734	4.94390118	-1.4291797	0.15887444	0.99978775	-4.4478986
ENSCAFG00000014151	-0.1469397	3.04187981	-0.8782258	0.38382177	0.99978775	-4.6410309
GHR	-0.1469421	5.15575636	-0.6399117	0.52501208	0.99978775	-4.7753295
LRPPRC	-0.1469739	7.25749583	-1.2902993	0.20259935	0.99978775	-4.5212652
LOXL1	-0.1470568	8.18591154	-0.6119574	0.54320649	0.99978775	-4.7761422
BCORL1	-0.1471134	4.05314821	-1.0692756	0.28983589	0.99978775	-4.6046818
PNPO	-0.1471213	3.41181666	-0.5697442	0.57128053	0.99978775	-4.6855603
AFTPH	-0.1471254	4.73738163	-1.2452316	0.21857447	0.99978775	-4.5410708
ZBTB2	-0.1471506	5.15959894	-1.2196689	0.22803995	0.99978775	-4.5560963
NQO1	-0.1472114	8.45451983	-0.7766232	0.44086524	0.99978775	-4.7427863
MTX3	-0.1472443	0.80079858	-0.5180158	0.60662285	0.99978775	-4.6226707
SOGA1	-0.1472502	5.28072767	-1.4614951	0.14983792	0.99978775	-4.422282
PRKCZ	-0.1472722	0.00653376	-0.3014367	0.76427197	0.99978775	-4.6208768
ENSCAFG00000016181	-0.1474577	3.85066849	-1.0200079	0.31240088	0.99978775	-4.6208731
EFNA5	-0.1474842	-1.0502482	-0.2277275	0.82074239	0.99978775	-4.6172016
SRSF1	-0.147534	5.44132339	-1.4042388	0.1661346	0.99978775	-4.455635
JAG1	-0.1475429	4.67424778	-0.4066904	0.68588815	0.99978775	-4.8229534
MCM6	-0.147545	6.72201187	-0.7118965	0.47967964	0.99978775	-4.7759503
TEP1	-0.1475697	5.7308841	-0.9932281	0.32515341	0.99978775	-4.6701175
CAPN5	-0.1475706	3.1587222	-0.4975135	0.62090382	0.99978775	-4.697706
STX2	-0.1476128	4.86478575	-1.4649271	0.14890239	0.99978775	-4.4264433

PACSIN1	-0.1476337	4.58070358	-0.7941626	0.43067497	0.99978775	-4.7118799
SLC1A5	-0.1479245	7.29937115	-1.0969261	0.27767828	0.99978775	-4.6184524
ACSL3	-0.1479723	6.83385787	-0.9819039	0.33064942	0.99978775	-4.6772967
ENSCAFG00000009420	-0.1480039	3.3081265	-0.928626	0.3573318	0.99978775	-4.6279604
SLC25A38	-0.1482047	4.11515577	-1.0190437	0.31285409	0.99978775	-4.6316265
ENSCAFG00000029457	-0.1482711	3.40914135	-0.8472133	0.40072091	0.99978775	-4.6581754
C8orf33	-0.1483239	1.95032875	-0.7183016	0.4757551	0.99978775	-4.6341112
ZNF599	-0.1483941	2.32377947	-0.7631313	0.44879983	0.99978775	-4.6315151
CLASP2	-0.1483953	5.33059702	-1.5139578	0.13603238	0.99978775	-4.3929578
NUP93	-0.1484792	5.29558751	-1.1846844	0.24147616	0.99978775	-4.5731146
RPRD1B	-0.1485321	3.67984916	-0.9416305	0.35069348	0.99978775	-4.6323701
AAAS	-0.1485843	5.10608982	-1.1854478	0.24117697	0.99978775	-4.5740775
ENSCAFG00000030220	-0.1486561	5.27758465	-2.188074	0.03313581	0.99978775	-3.9010515
SPG11	-0.1486701	5.53494314	-1.6477402	0.10537728	0.99978775	-4.3019748
MZF1	-0.1488217	3.03803499	-0.9079967	0.36802806	0.99978775	-4.6280537
ENSCAFG00000010865	-0.1488456	7.69833011	-1.0153688	0.31458544	0.99978775	-4.6537904
CYP1B1	-0.1489516	8.40251439	-0.4356268	0.6648929	0.99978775	-4.8252371
HCCS	-0.1490589	3.65649288	-1.5651199	0.12356145	0.99978775	-4.4148451
LRRC49	-0.1491481	4.75765299	-0.6692052	0.50629614	0.99978775	-4.7532966
AHNAK	-0.1491544	10.7390471	-0.9037708	0.37024421	0.99978775	-4.6640853
UTP20	-0.1491955	5.85260295	-1.2081798	0.23239073	0.99978775	-4.5660949
PSIP1	-0.1493051	6.03380028	-0.9748088	0.3341242	0.99978775	-4.6771448
CHCHD1	-0.1493781	3.00875719	-1.1030885	0.27501815	0.99978775	-4.5875826
KLHL20	-0.1493962	5.22345576	-1.2721997	0.20890676	0.99978775	-4.5291951
CYCS	-0.1496521	6.07640143	-0.9188029	0.3623997	0.99978775	-4.7038714
CPOX	-0.149671	4.50645994	-1.0252242	0.30995687	0.99978775	-4.6268917
ISCA2	-0.1496864	1.83721814	-0.6969539	0.48890555	0.99978775	-4.6311096
FADD	-0.1497709	2.88502023	-0.8760093	0.38501443	0.99978775	-4.6319369
CHST1	-0.1497803	2.79943828	-0.2905067	0.77257279	0.99978775	-4.6880188
ENSCAFG00000019479	-0.1499258	2.76239379	-0.988823	0.32728402	0.99978775	-4.60906
NDUFV1	-0.1499975	6.01374764	-1.3509994	0.18248794	0.99978775	-4.4865226
PLCG1	-0.1500551	6.30405126	-1.1960374	0.23705444	0.99978775	-4.5727028
TPCN2	-0.1500597	4.23069407	-0.6297955	0.53155915	0.99978775	-4.7341895
LRRK1	-0.1503284	5.27756796	-1.0688615	0.29002073	0.99978775	-4.6292354
SNRPA	-0.1503326	3.8298988	-0.9653484	0.33879498	0.99978775	-4.6454692
PGD	-0.1505223	8.63174073	-1.2734129	0.20847945	0.99978775	-4.5284959
TBC1D24	-0.1505955	3.22553569	-0.8415228	0.40387093	0.99978775	-4.6467813
CCSAP	-0.1506382	2.55095519	-0.7870084	0.43481445	0.99978775	-4.649583
NEDD4	-0.1506928	6.63006997	-1.157035	0.25249403	0.99978775	-4.5935695
SPRY2	-0.1507685	3.84603218	-0.4059281	0.68644476	0.99978775	-4.6960062
ZC3H18	-0.1507776	5.84133518	-1.8596834	0.06853591	0.99978775	-4.1427797
MRM3	-0.1507833	3.63807582	-1.3655319	0.17790671	0.99978775	-4.500656
PLTP	-0.1508032	0.54696458	-0.4432293	0.65942052	0.99978775	-4.6358389
ZNF514	-0.1508131	3.11682593	-0.8087318	0.42231828	0.99978775	-4.6494147
ISOC2	-0.1509302	4.05397688	-0.9562218	0.34334157	0.99978775	-4.6394607
LIG3	-0.1510115	5.3160103	-1.8144885	0.07531636	0.99978775	-4.1853324
TLE3	-0.15104	4.61637108	-0.6245575	0.53496573	0.99978775	-4.7437555
PAFAH2	-0.1512105	3.9577275	-1.0426083	0.30190578	0.99978775	-4.6107029
TMUB1	-0.151305	3.87886115	-1.2848199	0.20449357	0.99978775	-4.5249934
SSBP1	-0.1513474	2.45324363	-0.999773	0.32200499	0.99978775	-4.6049673

PTPN13	-0.1515724	6.7373035	-0.5461764	0.58725768	0.99978775	-4.8277516
ARRDC4	-0.1515733	2.71036158	-0.5114037	0.61121208	0.99978775	-4.6450094
ENSCAFG00000028982	-0.1516206	2.74731918	-0.508478	0.61324767	0.99978775	-4.7274402
APBA3	-0.1516606	2.94539894	-0.7474127	0.45814823	0.99978775	-4.6535003
ENSCAFG00000029455	-0.1516743	2.21753115	-0.5810589	0.56368636	0.99978775	-4.680201
MTRR	-0.1517556	5.14798683	-1.6319096	0.10868155	0.99978775	-4.3218148
ENSCAFG00000003095	-0.1518123	1.75584658	-0.7415734	0.46164947	0.99978775	-4.630695
DUS1L	-0.1519979	5.31502963	-1.2167004	0.22915831	0.99978775	-4.5588187
TNIK	-0.1520046	5.58238035	-0.4352469	0.66516687	0.99978775	-4.84967
PES1	-0.1520111	5.61192903	-1.5378534	0.13008851	0.99978775	-4.374566
MALL	-0.1520843	-2.0247891	-0.1497781	0.88151342	0.99978775	-4.6072511
URB2	-0.1522042	6.0559803	-1.7721568	0.0821697	0.99978775	-4.2056413
PAM16	-0.152229	3.73326685	-1.2715646	0.20913072	0.99978775	-4.5313235
CUL7	-0.1522469	6.52739065	-0.9767213	0.33318519	0.99978775	-4.6790316
PCNX3	-0.152305	6.40210247	-1.6842953	0.09806	0.99978775	-4.2681592
MRPL37	-0.1524161	5.34416643	-1.0674225	0.29066365	0.99978775	-4.6328106
TCEA1	-0.1524608	6.22992952	-1.5261402	0.13297568	0.99978775	-4.3770622
ENSCAFG00000000554	-0.152479	3.69863072	-1.1353711	0.26137526	0.99978775	-4.5796678
SNX7	-0.1525921	4.85308354	-1.3149654	0.19423489	0.99978775	-4.5068563
LGALS9	-0.1525985	0.06511081	-0.2336733	0.81614712	0.99978775	-4.6239393
PGAM5	-0.1528111	4.6619143	-1.2926142	0.20180303	0.99978775	-4.5184867
TEAD4	-0.1528336	4.28238889	-0.856773	0.39546328	0.99978775	-4.6826133
ING2	-0.1529507	2.69993007	-0.911312	0.36629538	0.99978775	-4.6197672
ENSCAFG00000011417	-0.1529611	2.37548666	-0.1899318	0.85009481	0.99978775	-4.7043821
NIPAL3	-0.1530248	3.61698099	-0.8674845	0.38962341	0.99978775	-4.6522651
ENSCAFG00000029663	-0.1530403	1.25273632	-0.7253055	0.47148444	0.99978775	-4.6208327
RNF220	-0.1530553	5.04061623	-1.3390044	0.18633661	0.99978775	-4.4939301
ZFP36L2	-0.1530602	6.61114318	-0.5441831	0.5886187	0.99978775	-4.8201601
MRPS35	-0.1531006	4.31575666	-1.2183402	0.22854004	0.99978775	-4.5522813
SYDE1	-0.1531224	5.06026752	-1.0413878	0.30246634	0.99978775	-4.6412262
RECQL5	-0.1531834	3.25134292	-1.0182249	0.31323927	0.99978775	-4.6071639
PELP1	-0.153188	5.56461709	-1.2703598	0.20955602	0.99978775	-4.5317328
NAP1L3	-0.153215	-0.7475436	-0.3818553	0.70410952	0.99978775	-4.6196747
ENSCAFG00000031218	-0.1532301	5.17711324	-1.0051991	0.31941033	0.99978775	-4.6498556
BCAR1	-0.1532968	7.27655091	-0.8669294	0.38992469	0.99978775	-4.7203166
GALNT12	-0.1533341	2.3549773	-0.4382486	0.66300364	0.99978775	-4.6761316
ILF3	-0.1533987	7.29681159	-1.5474361	0.12776392	0.99978775	-4.3660547
UBE4B	-0.1534025	6.36299781	-1.3502696	0.18272036	0.99978775	-4.4869452
GIPC2	-0.1534324	0.01250285	-0.2665611	0.79085104	0.99978775	-4.6166405
ARL6IP6	-0.1534993	4.11698489	-1.2473239	0.21781276	0.99978775	-4.5396827
MPHOSPH9	-0.1535031	5.57201924	-1.6050346	0.1144838	0.99978775	-4.3312185
HTATSF1	-0.1535634	5.95734353	-1.7707212	0.08241096	0.99978775	-4.2075714
HNRNPA3	-0.1535703	6.34580802	-1.1400341	0.25944512	0.99978775	-4.6024463
ADD2	-0.1536119	4.20498401	-0.6776067	0.50099569	0.99978775	-4.7691046
SART1	-0.1536755	5.48634371	-1.5248078	0.13330731	0.99978775	-4.3817655
COMTD1	-0.1537912	2.0554519	-0.4883074	0.62736498	0.99978775	-4.6471671
DYNLL1	-0.153792	5.02428257	-1.0049787	0.31951547	0.99978775	-4.64946
RILPL2	-0.1538424	3.35154793	-0.6076964	0.54600791	0.99978775	-4.6770827
ENSCAFG00000018563	-0.153863	2.51740419	-1.2455344	0.21846411	0.99978775	-4.5556034
ALG13	-0.1538694	3.09129316	-0.9197826	0.36189218	0.99978775	-4.6325371

KRT23	-0.1538969	-2.7918558	-0.2460113	0.80663248	0.99978775	-4.606565
ENSCAFG00000026245	-0.1539048	0.03560899	-0.4712023	0.63944797	0.99978775	-4.624144
ENSCAFG00000011075	-0.1539098	5.19831784	-1.391189	0.17003426	0.99978775	-4.4647425
GRIPAP1	-0.1539353	5.04867297	-1.7722715	0.08215045	0.99978775	-4.2348689
RNF40	-0.1539993	5.65414696	-1.7367793	0.08828924	0.99978775	-4.2397329
KLHL34	-0.1540333	0.88923509	-0.3435011	0.73259298	0.99978775	-4.6256227
LDLR	-0.1540632	8.88852787	-1.1250308	0.26569179	0.99978775	-4.5972871
CREG1	-0.1541699	7.03483437	-0.7084114	0.48182262	0.99978775	-4.7804708
CISD2	-0.1542172	2.84003715	-0.938776	0.35214368	0.99978775	-4.6241651
LIN9	-0.1542658	2.89810133	-0.6477574	0.51996387	0.99978775	-4.6764896
TMEM183A	-0.1542791	5.82705261	-1.815552	0.07515056	0.99978775	-4.1774858
ALG2	-0.1542837	3.95686517	-1.1594064	0.25153518	0.99978775	-4.5744071
HNRNPA0	-0.1543279	5.06927099	-1.1748836	0.24534111	0.99978775	-4.5768896
WDR4	-0.1544331	3.64883225	-1.1453152	0.25727138	0.99978775	-4.5783029
SIRT6	-0.1544549	2.84351026	-0.8817422	0.38193431	0.99978775	-4.6293358
SAP30	-0.1544684	3.16378786	-1.139032	0.25985903	0.99978775	-4.5771723
MAGEF1	-0.1545478	2.40073783	-0.7706645	0.44435927	0.99978775	-4.6508382
EPOP	-0.1546193	0.34611096	-0.3969657	0.69300151	0.99978775	-4.6216708
MTMR9	-0.1548202	5.05895667	-1.3572159	0.18051733	0.99978775	-4.4853351
PPARD	-0.1548398	6.55585612	-0.9867852	0.32827279	0.99978775	-4.6751754
SGF29	-0.1548816	2.84788137	-0.8243689	0.41345864	0.99978775	-4.6386532
CDCP1	-0.1549583	6.37226587	-0.3901391	0.69801167	0.99978775	-4.8617299
SPIN1	-0.1550034	6.48234647	-2.00101	0.05057181	0.99978775	-4.0245856
LRRC47	-0.1550652	5.84407812	-1.9209038	0.06018018	0.99978775	-4.0943713
TTC9C	-0.1550853	3.0606914	-1.1811512	0.24286437	0.99978775	-4.5655412
CCDC173	-0.1551724	-0.1587695	-0.3241381	0.74712097	0.99978775	-4.6177578
NFATC3	-0.1552263	4.89668229	-1.2600556	0.2132201	0.99978775	-4.5346226
CEP44	-0.1553671	2.82118434	-1.0040557	0.31995594	0.99978775	-4.607621
RNASEH2B	-0.1554116	4.94594885	-0.9713769	0.33581364	0.99978775	-4.6573537
ENSCAFG00000030829	-0.1554203	3.16686095	-0.8135153	0.41959599	0.99978775	-4.6486818
SLC2A4	-0.1554451	-0.8127179	-0.304367	0.76205115	0.99978775	-4.6175106
LOXL2	-0.1555503	9.36606187	-0.3470069	0.72997291	0.99978775	-4.7855983
SLC9B1	-0.1555568	1.42563308	-0.388544	0.6991843	0.99978775	-4.6409299
SMAGP	-0.1556552	2.2133011	-0.8619287	0.39264561	0.99978775	-4.626436
ENSCAFG00000030662	-0.1557692	5.64453218	-0.4288947	0.66975416	0.99978775	-4.8479853
C2CD5	-0.1558578	5.14310509	-1.122718	0.26666411	0.99978775	-4.6001642
ENSCAFG00000010185	-0.1559572	1.75548056	-0.7347123	0.46578302	0.99978775	-4.6296651
ZC3H4	-0.1559578	5.06435651	-1.7992718	0.07772252	0.99978775	-4.2095839
CYP51A1	-0.1559888	6.3890704	-0.9734707	0.33478227	0.99978775	-4.6783195
MRE11	-0.1560044	5.11800105	-1.1464703	0.2567977	0.99978775	-4.5910182
NUPL2	-0.1560323	3.98087615	-1.2516585	0.21624101	0.99978775	-4.5381523
ARF6	-0.1560411	5.41003468	-1.0942587	0.27883532	0.99978775	-4.6159521
CNTROB	-0.1560736	3.25595704	-1.1092628	0.27237084	0.99978775	-4.5856441
ENSCAFG00000028613	-0.1560751	5.08716099	-1.4736131	0.14655514	0.99978775	-4.4193639
RBM8A	-0.1561107	4.43922537	-1.4821946	0.14426484	0.99978775	-4.4247506
CTDSP1	-0.1561169	5.73537308	-0.8857793	0.37977468	0.99978775	-4.7106543
DNAAF4	-0.1561847	2.04223163	-0.5883559	0.55881545	0.99978775	-4.6513955
RNH1	-0.1561902	7.4367759	-1.1715361	0.24667138	0.99978775	-4.5834211
HUS1	-0.1562355	5.708614	-1.1671763	0.24841174	0.99978775	-4.586369
OFD1	-0.1562364	4.96566204	-1.1712358	0.24679098	0.99978775	-4.5761913

WDR59	-0.156258	4.25718223	-1.6614761	0.10257708	0.99978775	-4.3464905
GNAZ	-0.1562765	1.81513835	-0.3865357	0.70066177	0.99978775	-4.649543
RABL6	-0.1562831	6.59186917	-2.1390258	0.03710244	0.99978775	-3.9078951
ENSCAFG00000013986	-0.1562923	1.96719908	-0.4857201	0.62918615	0.99978775	-4.6365549
BLMH	-0.1563007	5.29510628	-1.9598752	0.05532675	0.99978775	-4.0787529
EPHA4	-0.1563371	1.74139116	-0.3277982	0.74436758	0.99978775	-4.6453711
BPNT1	-0.1564666	4.59991123	-0.8214678	0.41509372	0.99978775	-4.696167
ADGRV1	-0.156524	-0.4099286	-0.3527598	0.7256805	0.99978775	-4.6187922
ENSCAFG00000021568	-0.1565769	-1.0921401	-0.3535824	0.72506741	0.99978775	-4.6115515
ENSCAFG00000005812	-0.1566772	4.9378976	-1.3418934	0.18540405	0.99978775	-4.4934431
PPP1CB	-0.1567293	6.29172353	-1.9654202	0.0546642	0.99978775	-4.0529714
WDR20	-0.156763	3.36518144	-1.2189161	0.22832317	0.99978775	-4.5542215
PML	-0.1568907	5.39424613	-0.8409815	0.4041714	0.99978775	-4.7219698
ENSCAFG00000000640	-0.1570941	1.34482532	-0.4099674	0.68349755	0.99978775	-4.6338193
ENSCAFG00000000172	-0.1571246	3.57079613	-1.188608	0.23994132	0.99978775	-4.5625263
PPP1R3C	-0.1572289	2.04397474	-0.3168912	0.75258255	0.99978775	-4.6454358
MOCS1	-0.1572837	2.73251574	-0.780474	0.43861585	0.99978775	-4.65504
ENSCAFG00000019388	-0.1573692	2.72490972	-0.8359448	0.40697352	0.99978775	-4.632554
DAAM1	-0.1573894	5.40201975	-0.7473373	0.45819331	0.99978775	-4.7628013
NR4A1	-0.1575177	5.23508488	-0.3861489	0.70094649	0.99978775	-4.8501766
ENSCAFG00000016809	-0.1575399	3.84733671	-0.8838202	0.38082175	0.99978775	-4.65406
COL4A6	-0.1575888	-2.5797783	-0.2587311	0.79685416	0.99978775	-4.6091749
ENSCAFG00000014154	-0.1576075	0.47915416	-0.1350276	0.89310627	0.99978775	-4.6146334
RALY	-0.1576408	6.47440167	-2.0398982	0.04640473	0.99978775	-3.9916144
TSPYL1	-0.1577461	4.04806072	-2.1843867	0.03342048	0.99978775	-4.0330327
ARF5	-0.1577543	3.77117248	-1.0006876	0.32156665	0.99978775	-4.6248372
LMNB2	-0.1578145	4.07867914	-0.9444754	0.34925208	0.99978775	-4.6534418
HAT1	-0.1578163	4.85393586	-1.0969302	0.2776765	0.99978775	-4.6121808
MEDAG	-0.1578298	7.57469783	-0.3869396	0.70036453	0.99978775	-4.8527077
ENSCAFG00000008074	-0.1580063	-0.712953	-0.3692585	0.71341984	0.99978775	-4.6172182
DDHD1	-0.1580535	4.00999807	-0.7491192	0.45712788	0.99978775	-4.691673
DHX37	-0.1581937	5.92694481	-1.5681583	0.1228507	0.99978775	-4.3507148
NUMA1	-0.1581955	8.19390911	-1.958389	0.05550549	0.99978775	-4.0818042
EEF1B2	-0.1583281	8.02690746	-1.3556516	0.18101166	0.99978775	-4.4840808
FAM110A	-0.1583303	1.17376923	-0.4823351	0.63157233	0.99978775	-4.6284148
REXO5	-0.158382	3.46974603	-0.5709837	0.57044618	0.99978775	-4.7328684
ENSCAFG00000017211	-0.1583982	5.54311769	-1.851138	0.06977687	0.99978775	-4.1515058
ZFYVE1	-0.1584033	5.45768519	-1.5351773	0.13074368	0.99978775	-4.3776558
AXL	-0.1584654	8.43908994	-0.4218733	0.67483953	0.99978775	-4.8138604
ADAT1	-0.1585096	3.14470249	-0.9954437	0.32408531	0.99978775	-4.6160318
HSPB6	-0.158531	6.00751686	-0.5180461	0.6066019	0.99978775	-4.8317764
ENSCAFG00000005629	-0.1585379	2.54596453	-0.9166506	0.36351625	0.99978775	-4.6204281
ZFP64	-0.158573	4.74293888	-1.4869236	0.14301484	0.99978775	-4.4154566
GSDMD	-0.1586711	4.75010134	-0.9897761	0.32682227	0.99978775	-4.647192
MPHOSPH6	-0.1587595	3.24738554	-1.0126287	0.31588058	0.99978775	-4.6142762
MRPL52	-0.158827	3.45668521	-1.08691	0.28204034	0.99978775	-4.594936
CBY1	-0.1588385	2.66292295	-0.8898039	0.37762939	0.99978775	-4.6311321
GABRE	-0.1588732	-0.972141	-0.2236772	0.82387638	0.99978775	-4.6161117
ZNF740	-0.1589297	4.75753177	-1.8146789	0.07528666	0.99978775	-4.2248623
PLPP3	-0.158937	6.10851328	-0.3389222	0.73601987	0.99978775	-4.8247541

AK2	-0.1589698	2.99433755	-0.9839431	0.32965516	0.99978775	-4.6135417
LONP1	-0.1589762	6.36832786	-1.7055169	0.0940071	0.99978775	-4.2528721
PLD1	-0.1589834	7.10603949	-0.5223123	0.60364937	0.99978775	-4.8321544
DVL2	-0.1590043	4.63838118	-1.170072	0.24725486	0.99978775	-4.5759455
FAM120B	-0.1590072	5.36597299	-1.7281248	0.0898425	0.99978775	-4.2571104
ESYT1	-0.1590183	8.51296574	-1.0652346	0.2916431	0.99978775	-4.6243792
EHBP1	-0.1590873	6.04479139	-0.7848275	0.43608104	0.99978775	-4.7437437
ENSCAFG00000029415	-0.1591183	-0.4298036	-0.3139011	0.75483974	0.99978775	-4.6257599
TIFA	-0.159454	2.00685155	-0.5124172	0.61050761	0.99978775	-4.6505682
UQCRC1	-0.1594705	6.49956292	-1.5627685	0.12411378	0.99978775	-4.3526848
SLIT3	-0.1594912	7.69396959	-0.4299223	0.66901124	0.99978775	-4.8514046
CC2D1B	-0.1595274	5.91965872	-1.5672407	0.123065	0.99978775	-4.352372
CARD19	-0.1595654	3.08677829	-0.7906633	0.43269678	0.99978775	-4.6641237
FXN	-0.159577	2.82761449	-0.766371	0.44688696	0.99978775	-4.6463483
MUM1L1	-0.1596467	2.6775386	-0.7633717	0.44865767	0.99978775	-4.648935
VARS2	-0.1596693	6.39545936	-1.7840946	0.08018626	0.99978775	-4.1948962
RASL12	-0.1596903	-0.322066	-0.2725281	0.78628472	0.99978775	-4.6195128
ENSCAFG00000004640	-0.1597659	-0.307969	-0.5606368	0.57742937	0.99978775	-4.621751
ENSCAFG00000024665	-0.1598827	-0.0526219	-0.5262668	0.6009185	0.99978775	-4.6198963
UVRAG	-0.1598963	4.69000336	-1.4305321	0.15848796	0.99978775	-4.4505402
MPP1	-0.1599843	4.91408053	-1.094345	0.27879781	0.99978775	-4.6120377
CIAO3	-0.1600355	3.26170428	-1.3219765	0.19190564	0.99978775	-4.5223093
DNAJB1	-0.1600538	5.70191629	-1.5648233	0.12363101	0.99978775	-4.3574322
ENSCAFG00000000042	-0.1600722	0.75410544	-0.5397349	0.59166125	0.99978775	-4.623326
STXBP4	-0.1602137	3.46716852	-0.9436924	0.34964838	0.99978775	-4.632382
BST2	-0.1602178	0.48735289	-0.2674812	0.79014645	0.99978775	-4.6324515
MAP3K14	-0.1602208	2.61733822	-0.4637689	0.64472994	0.99978775	-4.6711667
TIMELESS	-0.1602746	3.49432417	-0.8253083	0.41293005	0.99978775	-4.6790875
COQ4	-0.1602777	4.81054347	-1.9340752	0.05850068	0.99978775	-4.1218383
POLRMT	-0.1603739	4.91603986	-1.2246636	0.22616724	0.99978775	-4.5516429
C12H6orf203	-0.160452	3.88859527	-1.1555775	0.25308468	0.99978775	-4.5733167
RIPOR3	-0.1605469	-0.2372848	-0.1413816	0.88810946	0.99978775	-4.6156197
MRPL34	-0.1606724	2.1769909	-0.9057558	0.36920219	0.99978775	-4.6151459
CALML6	-0.160733	-1.5972411	-0.2381289	0.81270784	0.99978775	-4.6129136
NECTIN1	-0.1607868	4.64674901	-0.7495045	0.4568977	0.99978775	-4.7392512
TTC39C	-0.1608305	4.38537179	-0.6745286	0.50293409	0.99978775	-4.7346648
SLC25A5	-0.1608965	6.79937018	-1.2269976	0.22529601	0.99978775	-4.5566721
HARBI1	-0.160905	1.48888272	-0.5274254	0.60011951	0.99978775	-4.6319234
PYGM	-0.1610572	0.10652529	-0.3714035	0.71183131	0.99978775	-4.6206931
PUS7	-0.1610788	4.8350015	-0.8462341	0.40126183	0.99978775	-4.7069969
MATK	-0.161169	-2.4078318	-0.2913805	0.77190819	0.99978775	-4.6066969
KHDRBS1	-0.1612052	5.6736118	-1.2538854	0.21543679	0.99978775	-4.5408624
RGS16	-0.1612474	-2.3622269	-0.2511516	0.80267703	0.99978775	-4.6075516
EDC4	-0.1612672	6.25393693	-2.0068744	0.04992345	0.99978775	-4.0196016
ZBTB10	-0.1614211	4.23855517	-1.2222419	0.2270738	0.99978775	-4.5507033
PRIM2	-0.1614466	3.61706384	-0.9741507	0.33444774	0.99978775	-4.6345395
ENSCAFG00000002290	-0.1614773	-0.3290737	-0.4133215	0.681054	0.99978775	-4.6205835
GALK1	-0.1614976	3.98977356	-1.0330322	0.30632287	0.99978775	-4.6251994
HPS1	-0.1615869	4.93286614	-1.3997584	0.1674656	0.99978775	-4.4614037
RER1	-0.1616111	3.79921306	-1.1763792	0.24474845	0.99978775	-4.566393

PPOX	-0.1616667	4.0901926	-1.0408749	0.3027021	0.99978775	-4.6194741
TBCC	-0.1616919	5.1762559	-1.0543099	0.29656776	0.99978775	-4.6339151
KIAA0753	-0.1616959	4.68796061	-1.5628083	0.12410441	0.99978775	-4.3771085
EDIL3	-0.1617054	7.58666188	-0.4869563	0.62831569	0.99978775	-4.8403877
MCOLN3	-0.1617715	-1.3054759	-0.4551989	0.6508426	0.99978775	-4.6196248
WDR5	-0.1618022	6.05080329	-1.5927916	0.11720895	0.99978775	-4.3332588
NCLN	-0.1619705	6.79772482	-1.1296183	0.26377053	0.99978775	-4.6067905
SOX12	-0.1620661	2.15742621	-0.9083219	0.36785784	0.99978775	-4.617783
NOP2	-0.1621013	5.2773313	-1.1098047	0.27213935	0.99978775	-4.6120477
TNMD	-0.1621368	-2.9499742	-0.3685556	0.71394066	0.99978775	-4.6059291
ENSCAFG00000030659	-0.1621768	3.80484719	-1.499107	0.1398338	0.99978775	-4.4379856
LRRC74A	-0.1622712	-1.0385427	-0.243661	0.80844273	0.99978775	-4.6114085
USP28	-0.1622968	4.42426144	-1.172637	0.24623335	0.99978775	-4.5707638
RRP9	-0.1623457	4.34918685	-1.2797815	0.20624702	0.99978775	-4.5249658
DGKD	-0.1624121	4.55454942	-1.1525788	0.25430297	0.99978775	-4.5830757
SLC38A1	-0.1626595	6.32412222	-0.9163243	0.36368574	0.99978775	-4.7050768
CCND2	-0.1627902	6.49480035	-0.4439134	0.65892899	0.99978775	-4.8125187
HEMK1	-0.1628766	2.61001782	-1.2249727	0.22605172	0.99978775	-4.5579605
SEZ6	-0.1630276	-0.5743356	-0.2845134	0.77713591	0.99978775	-4.6127776
IRF2BP1	-0.1630548	3.50166936	-1.1652797	0.24917155	0.99978775	-4.5700626
ENSCAFG00000015065	-0.1630924	5.36738407	-1.5697575	0.12247793	0.99978775	-4.3522691
BANK1	-0.1631318	4.05684151	-0.6948356	0.49022131	0.99978775	-4.6970118
SLC26A11	-0.163206	3.60181909	-0.6742911	0.50308384	0.99978775	-4.7070462
ELMSAN1	-0.1632319	5.0188536	-1.4217594	0.16100817	0.99978775	-4.4509862
BRMS1	-0.1633298	3.32926691	-1.3320059	0.18861043	0.99978775	-4.5144045
IPO13	-0.1634288	6.06806469	-1.7188375	0.09153446	0.99978775	-4.2441605
GIGYF1	-0.1634483	4.30301906	-0.9815378	0.33082808	0.99978775	-4.6352632
EEF1D	-0.1634896	8.12532472	-1.1878631	0.24023214	0.99978775	-4.5733196
ENSCAFG00000014771	-0.1635018	2.83678608	-1.0746379	0.28744978	0.99978775	-4.5919093
IGSF3	-0.1635701	3.26842036	-0.4199996	0.67619923	0.99978775	-4.7584477
ENSCAFG00000013366	-0.1635846	5.31537517	-1.2044076	0.23383236	0.99978775	-4.5646372
SMG6	-0.1637922	5.82351151	-0.8563075	0.39571831	0.99978775	-4.7249902
ANGEL2	-0.1638005	4.25775607	-1.6657048	0.10172737	0.99978775	-4.326828
PTPDC1	-0.1639039	0.96290027	-0.6904991	0.49292109	0.99978775	-4.6274241
TJP2	-0.1640044	5.80628996	-0.702992	0.48516567	0.99978775	-4.7770715
ENSCAFG00000024406	-0.1640579	2.45528032	-0.8210804	0.41531238	0.99978775	-4.6350671
SHISA2	-0.1641509	-1.3991736	-0.2741932	0.78501185	0.99978775	-4.6143188
RECQL	-0.1642286	4.27366875	-0.7370777	0.4643556	0.99978775	-4.7098233
PTGS1	-0.164232	-1.4919182	-0.1689298	0.8665009	0.99978775	-4.6082097
MRPS28	-0.1642635	3.31112342	-1.0663571	0.2911403	0.99978775	-4.6009443
BAK1	-0.1642639	4.39033863	-1.4128475	0.16360018	0.99978775	-4.4614986
DAZAP1	-0.1642833	5.48528904	-1.1805737	0.24309183	0.99978775	-4.5794241
ENSCAFG00000005346	-0.1642868	3.8441156	-1.7770351	0.08135429	0.99978775	-4.3026833
PHOSPHO2	-0.1643929	2.98439495	-1.0767127	0.28653021	0.99978775	-4.5921906
ODF2	-0.1644965	5.41558612	-1.5805563	0.11998449	0.99978775	-4.3471377
EXOC3L2	-0.1645015	-2.6020725	-0.249708	0.80378741	0.99978775	-4.6068102
TPCN1	-0.1645286	4.19781482	-0.8431406	0.40297384	0.99978775	-4.6769185
SMUG1	-0.1645411	4.7079469	-1.5512465	0.12684886	0.99978775	-4.3805682
ADAR	-0.1646851	6.70774219	-1.4073234	0.165223	0.99978775	-4.4525805
ENSCAFG00000017933	-0.1647245	2.44042383	-0.772349	0.44336991	0.99978775	-4.6382112

FYTTD1	-0.1647773	1.84469691	-0.9968519	0.32340762	0.99978775	-4.6020567
PCOLCE	-0.1648264	8.34794461	-0.4175163	0.67800293	0.99978775	-4.8286428
KCNU1	-0.1648286	-0.9977661	-0.2208884	0.8260359	0.99978775	-4.6133002
ENSCAFG00000004091	-0.1648687	-0.6243392	-0.4576312	0.64910524	0.99978775	-4.6211334
GNG4	-0.1649499	0.84632029	-0.3994756	0.69116285	0.99978775	-4.6381055
TRAP1	-0.1650949	6.2240982	-1.5412381	0.1292636	0.99978775	-4.3676596
NKAIN1	-0.1651139	4.39941029	-0.8971305	0.3737438	0.99978775	-4.687412
TNFRSF13C	-0.1651653	0.03279303	-0.5546301	0.58150231	0.99978775	-4.6169082
LYVE1	-0.1652116	3.31228287	-0.3101288	0.75769042	0.99978775	-4.7513477
NAV1	-0.1653585	7.57201324	-1.2072742	0.23273623	0.99978775	-4.5643429
PLEKHH3	-0.1654155	2.5590272	-0.4525911	0.65270748	0.99978775	-4.6613881
CNOT3	-0.1654217	5.08685115	-1.7066673	0.09379141	0.99978775	-4.2732834
AHR	-0.1654827	5.1065552	-0.5530803	0.58255537	0.99978775	-4.7962093
TICRR	-0.1654886	2.77150277	-0.5901835	0.5575988	0.99978775	-4.6919011
C27H12orf10	-0.1655293	5.34451275	-1.4029151	0.16652698	0.99978775	-4.4567651
ATPAF2	-0.1655324	3.5308841	-1.2580229	0.21394849	0.99978775	-4.5388221
ZW10	-0.1656784	5.56834276	-1.303934	0.19794296	0.99978775	-4.5132469
TYMS	-0.1657635	3.96056554	-0.7408995	0.46205457	0.99978775	-4.7255527
BMS1	-0.1657691	6.42194637	-1.4699923	0.14753004	0.99978775	-4.4134178
ASB1	-0.1658188	2.74433474	-1.0065859	0.31874946	0.99978775	-4.6059749
SVIL	-0.1659128	4.59260243	-0.3831347	0.70316649	0.99978775	-4.79636
BCAS4	-0.166039	2.85991351	-0.3733007	0.71042734	0.99978775	-4.7176764
IWS1	-0.1660555	6.21603352	-1.6299582	0.10909464	0.99978775	-4.3076719
CYB5RL	-0.1662301	2.89959705	-1.2051074	0.23356441	0.99978775	-4.5610347
ORC6	-0.1663773	1.71838711	-0.5218185	0.60399075	0.99978775	-4.6577939
SF3A1	-0.1664006	7.32229415	-2.4164118	0.01918417	0.99978775	-3.6691787
NTRK3	-0.1664012	-1.7530522	-0.3033155	0.76284787	0.99978775	-4.6130741
POLA1	-0.1664282	4.61957797	-0.8383087	0.40565688	0.99978775	-4.7118226
MRPL28	-0.166432	4.69856666	-1.0912588	0.28014058	0.99978775	-4.6087526
FRMD6	-0.1664368	5.87527874	-1.1459934	0.2569932	0.99978775	-4.597083
MKS1	-0.1664848	1.96927553	-0.7404601	0.46231879	0.99978775	-4.6291742
HPS3	-0.1664957	4.09963559	-1.0063359	0.31886856	0.99978775	-4.6320019
MICALL2	-0.1665699	4.38406553	-0.9295215	0.35687206	0.99978775	-4.6747304
ENSCAFG00000029845	-0.1666039	2.8547518	-1.134185	0.26186783	0.99978775	-4.5782935
ACSL1	-0.1666282	6.15351725	-1.6242772	0.11030451	0.99978775	-4.3105879
ENSCAFG00000018189	-0.1666373	1.49724717	-0.622552	0.536273	0.99978775	-4.6342223
SLC2A11	-0.1666884	0.94788379	-0.4271913	0.67098649	0.99978775	-4.6254602
TXNL4A	-0.1667686	3.85896869	-1.4318657	0.15810753	0.99978775	-4.464729
ENSCAFG00000028195	-0.1668901	2.43394856	-0.5265688	0.60071024	0.99978775	-4.6593669
TDP1	-0.1670848	2.29091591	-0.8980151	0.37327637	0.99978775	-4.6177791
CADM1	-0.1671287	4.8276592	-0.5650973	0.5744139	0.99978775	-4.7814936
GPKOW	-0.1672227	4.20541668	-1.6173278	0.11179933	0.99978775	-4.3570902
FTL	-0.1672704	8.42419227	-0.8788854	0.38346724	0.99978775	-4.7068546
KHSRP	-0.1674181	6.27489711	-2.0444507	0.0459369	0.99978775	-3.9886942
IRAK3	-0.1674361	2.43540964	-0.4659224	0.6431978	0.99978775	-4.6568801
DENND6B	-0.1675031	4.70907788	-1.2861492	0.20403284	0.99978775	-4.5221269
HOXB2	-0.1675262	1.25571454	-0.4415627	0.66061857	0.99978775	-4.6362489
EFR3B	-0.167616	1.89140291	-0.8262357	0.41240858	0.99978775	-4.6337083
HECA	-0.1676933	3.72501266	-1.2775306	0.20703399	0.99978775	-4.5325351
ADAM33	-0.1677657	5.36852499	-0.4933984	0.62378827	0.99978775	-4.8298602

ENSCAFG00000031148	-0.1678417	1.96130306	-0.691321	0.49240879	0.99978775	-4.6360357
ICE2	-0.1678508	4.55255433	-1.2369759	0.22159915	0.99978775	-4.5447408
DCLRE1B	-0.1678849	3.9454339	-0.8943785	0.3752003	0.99978775	-4.6767464
TDRKH	-0.1679836	3.33086605	-0.9849098	0.32918455	0.99978775	-4.6184877
CENPQ	-0.1680879	2.42184379	-0.6266663	0.53359288	0.99978775	-4.6761826
ENTPD6	-0.1681982	4.29402075	-1.2841125	0.2047391	0.99978775	-4.5232247
PRPF40B	-0.1682348	3.47208833	-1.3907978	0.17015225	0.99978775	-4.488855
PYCR1	-0.1682901	6.3802591	-1.0198921	0.31245529	0.99978775	-4.660337
LARS2	-0.1684554	3.78347005	-1.4200431	0.16150486	0.99978775	-4.4701731
MPG	-0.1684659	3.48010674	-0.9991069	0.32232448	0.99978775	-4.6194929
MAP3K4	-0.1684696	3.76508546	-1.3843061	0.17211934	0.99978775	-4.4889467
ATXN2L	-0.168613	7.31093369	-2.8230779	0.00670437	0.99978775	-3.2754845
EXD2	-0.1687907	2.17234215	-0.8497428	0.39932552	0.99978775	-4.6241039
PECR	-0.1689044	1.79689795	-0.621411	0.53701749	0.99978775	-4.6320268
ULK3	-0.1689051	3.99819241	-1.0490534	0.29895758	0.99978775	-4.6079911
IRX2	-0.1689768	-0.4466286	-0.3093676	0.75826607	0.99978775	-4.6239892
ENSCAFG00000013651	-0.1690385	2.18260929	-0.2025371	0.84027953	0.99978775	-4.6668263
PPP2R5B	-0.1691852	3.51256525	-0.9521453	0.34538529	0.99978775	-4.6269135
PAIP2B	-0.1692392	1.25119514	-0.5937545	0.55522538	0.99978775	-4.6235268
FANCB	-0.1696277	1.01577972	-0.5320927	0.59690585	0.99978775	-4.6346509
RBM6	-0.1696857	4.94295736	-1.9437227	0.05729607	0.99978775	-4.1246064
ENSCAFG00000002179	-0.1697088	4.57275424	-1.5719593	0.12196619	0.99978775	-4.3684684
GLRX	-0.1697375	3.48972467	-0.8196118	0.41614187	0.99978775	-4.6614635
PPIB	-0.1702146	9.09239507	-1.037695	0.30416665	0.99978775	-4.6349422
RRP8	-0.1702762	3.36237376	-0.8269563	0.41200366	0.99978775	-4.6622571
ZNF408	-0.1703508	2.77464694	-1.0570428	0.29533048	0.99978775	-4.5944465
ENSCAFG00000028877	-0.1703637	3.10597231	-1.0898294	0.28076402	0.99978775	-4.5890999
ULK4	-0.1704472	3.79704349	-0.8411267	0.4040908	0.99978775	-4.6612305
XYLT2	-0.1704481	4.10606609	-1.3959653	0.16859886	0.99978775	-4.4752898
TRMT1	-0.1706055	3.71992266	-1.4774753	0.14552083	0.99978775	-4.4504695
OSBPL5	-0.1706534	5.89449559	-1.0473676	0.29972682	0.99978775	-4.6423014
ENSCAFG00000005550	-0.1707324	5.42208027	-1.9362548	0.05822666	0.99978775	-4.0970589
WDR48	-0.1707897	5.02418416	-1.3563134	0.18080243	0.99978775	-4.4857097
ZNHIT1	-0.1708645	3.43693575	-1.1590539	0.25167753	0.99978775	-4.5715699
MOB1A	-0.1708653	5.20674025	-1.9863582	0.05222348	0.99978775	-4.06634
UBE2N	-0.1708712	-1.0445684	-0.3706959	0.71235517	0.99978775	-4.6117892
ENSCAFG00000004229	-0.1708753	1.06332555	-0.7154132	0.47752263	0.99978775	-4.6257547
INO80C	-0.1708768	3.47483481	-1.3115923	0.1953631	0.99978775	-4.5167895
CEP83	-0.1709211	4.12152935	-1.1067459	0.27344783	0.99978775	-4.5968445
FAXC	-0.1709872	2.12685327	-0.6700167	0.5057828	0.99978775	-4.6349784
RBM33	-0.1711092	4.72428717	-2.0776422	0.04264796	0.99978775	-4.0456799
GBA2	-0.1714012	4.61235138	-1.3174161	0.19341831	0.99978775	-4.5064123
WDR81	-0.171474	5.169403	-1.2500196	0.21683431	0.99978775	-4.5411133
ZNF510	-0.1715207	2.50211581	-0.8900547	0.37749596	0.99978775	-4.6189931
ENSCAFG00000010209	-0.1715278	-2.0638862	-0.1628259	0.87128045	0.99978775	-4.6071445
C5H1orf174	-0.171603	3.54433317	-1.5391771	0.12976541	0.99978775	-4.4362445
CTCF	-0.1716279	5.33796559	-1.6575578	0.10336958	0.99978775	-4.2957895
ZNRF1	-0.1716352	1.6826319	-0.5928465	0.55582841	0.99978775	-4.640424
OCLN	-0.171758	-2.5492486	-0.2639107	0.79288162	0.99978775	-4.6069744
MOB1B	-0.1718773	1.65217092	-0.5632002	0.57569549	0.99978775	-4.6366207

KMT2B	-0.1718831	6.29536562	-2.0463432	0.04574363	0.99978775	-3.987329
ENSCAFG00000010224	-0.1719506	6.10247741	-1.7338677	0.0888093	0.99978775	-4.2343208
TXNDC12	-0.171963	6.42547302	-1.8020004	0.07728638	0.99978775	-4.1823199
GALC	-0.1720288	3.10238605	-1.1311776	0.26311975	0.99978775	-4.5789949
EID1	-0.172116	6.07067155	-1.5728473	0.12176029	0.99978775	-4.3471201
PPM1G	-0.1721403	6.25186631	-1.5886642	0.11813939	0.99978775	-4.3352579
FAM53B	-0.1722462	6.36738259	-1.043278	0.30159853	0.99978775	-4.6491508
NOTCH1	-0.1723041	4.59637677	-0.445414	0.65785138	0.99978775	-4.7775245
DDX54	-0.1723716	6.46146471	-1.7181341	0.09166367	0.99978775	-4.2437283
CLPP	-0.1727121	4.15621449	-1.6179231	0.11167063	0.99978775	-4.3580145
WDR24	-0.1727964	4.22703357	-1.3668414	0.17749823	0.99978775	-4.4868592
FZD9	-0.1728497	-0.3078046	-0.427552	0.67072546	0.99978775	-4.6261786
ENSCAFG00000030475	-0.1729928	3.74628665	-1.0354506	0.30520322	0.99978775	-4.6104617
PHB2	-0.1730484	6.23060654	-1.3061146	0.19720578	0.99978775	-4.5126131
UBE3D	-0.1731127	3.15280917	-1.0983871	0.27704597	0.99978775	-4.5882147
IFT22	-0.1732112	2.47328816	-0.8914719	0.37674254	0.99978775	-4.6244432
ARHGAP32	-0.1734377	3.77339615	-1.0816948	0.28433043	0.99978775	-4.6054842
HNRNPA2B1	-0.1734462	8.37843248	-1.1888391	0.23985113	0.99978775	-4.5698755
THAP7	-0.1735941	2.91908509	-1.1977062	0.23640947	0.99978775	-4.5607512
EPB41L3	-0.1736608	3.64463161	-0.6564536	0.51439864	0.99978775	-4.7844021
ENSCAFG00000008773	-0.1736827	2.73650794	-0.9079266	0.36806474	0.99978775	-4.624978
C12H6orf120	-0.1737317	4.07134663	-0.8348614	0.40757779	0.99978775	-4.6822873
PHTF1	-0.1738549	5.16469866	-1.6562207	0.10364117	0.99978775	-4.3050568
CDK4	-0.1739445	5.91930233	-1.2960286	0.20063281	0.99978775	-4.5183069
TRIM59	-0.173998	3.54244611	-1.1208413	0.26745495	0.99978775	-4.5851645
TECPR1	-0.1741273	4.62099683	-1.8379787	0.07172496	0.99978775	-4.2048608
ENSCAFG00000016847	-0.1742051	2.78541808	-1.036433	0.30474923	0.99978775	-4.601643
SLC26A6	-0.1742681	4.50851899	-1.0499707	0.2985396	0.99978775	-4.6160381
SLC25A39	-0.1742764	6.34961064	-1.2407477	0.22021346	0.99978775	-4.5492688
ZNF358	-0.1743413	5.53191319	-1.1132013	0.27069162	0.99978775	-4.612792
MYPOP	-0.1743705	0.97193296	-0.6051513	0.54768475	0.99978775	-4.6283129
TCF19	-0.1744083	2.12130167	-0.6311042	0.53070979	0.99978775	-4.6601601
C9H17orf53	-0.1744543	1.05696346	-0.4838795	0.63048315	0.99978775	-4.6390912
LRFN4	-0.1744986	3.62595923	-0.6049571	0.54781281	0.99978775	-4.7245118
ZNF354C	-0.1745389	-1.0399656	-0.4787201	0.63412499	0.99978775	-4.617773
VANGL1	-0.1746278	3.82295599	-1.3875639	0.17112999	0.99978775	-4.4836532
RNPEPL1	-0.1747523	6.18062128	-1.5005143	0.13947001	0.99978775	-4.3937604
RAB43	-0.1749097	2.63554534	-1.0160097	0.31428301	0.99978775	-4.6026184
TMEM177	-0.1749575	1.87758042	-0.9615177	0.34069845	0.99978775	-4.6052916
POLR3H	-0.1749867	3.66275756	-1.3025094	0.19842568	0.99978775	-4.521478
NUDT8	-0.1750115	2.73861966	-0.8507576	0.3987666	0.99978775	-4.6352749
PARP15	-0.175245	3.64601509	-0.632115	0.53005421	0.99978775	-4.7371947
ADGRG4	-0.1754084	-2.6493299	-0.3092074	0.75838728	0.99978775	-4.60597
NEK11	-0.1755039	0.30233712	-0.3814288	0.70442405	0.99978775	-4.6203862
VAV2	-0.1755164	5.76339546	-1.2177253	0.22877175	0.99978775	-4.5612417
OXCT1	-0.1755173	4.10668755	-0.4298295	0.66907829	0.99978775	-4.7565322
ADPRHL2	-0.1755372	3.68608333	-0.986932	0.32820153	0.99978775	-4.6258475
TSPAN9	-0.1757533	5.49823104	-0.8296192	0.41050952	0.99978775	-4.7191962
RPL22L1	-0.1758131	4.46264683	-0.9141823	0.36479951	0.99978775	-4.6717471
RAB8B	-0.1758823	4.9448576	-0.8712424	0.38758743	0.99978775	-4.6849527

ENSCAFG00000011833	-0.1759332	5.31495896	-1.7493057	0.08608055	0.99978775	-4.2330776
ENSCAFG00000023585	-0.1760811	-2.1665897	-0.2060669	0.83753546	0.99978775	-4.610578
ATP7B	-0.176124	3.32386763	-1.3225163	0.19172718	0.99978775	-4.5156648
ENSCAFG00000006187	-0.1761533	5.58787777	-1.4015648	0.16692799	0.99978775	-4.4569555
CEP135	-0.1762355	3.86063782	-0.8969154	0.37385752	0.99978775	-4.6574685
LRRC59	-0.1763143	6.41094659	-0.805272	0.42429387	0.99978775	-4.7471968
CARS	-0.1763899	6.95764824	-1.3174444	0.19340888	0.99978775	-4.5059185
ERP29	-0.176427	5.31232757	-1.4286039	0.15903922	0.99978775	-4.4431277
NUP160	-0.1765004	4.61462713	-1.2713719	0.20919868	0.99978775	-4.5288102
BIN1	-0.1765123	5.70252407	-0.9699055	0.33653969	0.99978775	-4.6779974
RDH5	-0.1766132	2.02118535	-0.7746899	0.44199709	0.99978775	-4.6254021
IL17RE	-0.1766148	0.08236917	-0.4789168	0.63398599	0.99978775	-4.6197136
PTMA	-0.176806	8.30298408	-0.977105	0.332997	0.99978775	-4.6648453
CCDC191	-0.1769128	3.05448247	-0.8120625	0.42042165	0.99978775	-4.6433096
TFEB	-0.1769246	3.74245024	-1.1279073	0.26448593	0.99978775	-4.5820398
PEX26	-0.1769797	3.45672439	-1.1471827	0.25650585	0.99978775	-4.57519
ACSS1	-0.1772119	5.74014382	-0.9172708	0.36319429	0.99978775	-4.7025466
WDR54	-0.1773609	2.5821635	-0.9011062	0.371646	0.99978775	-4.6178219
PDLIM4	-0.1774459	5.29347597	-0.5665777	0.5734148	0.99978775	-4.774169
EXO1	-0.177453	2.1216952	-0.4747697	0.63691965	0.99978775	-4.7102583
ABLIM3	-0.1774722	3.02423546	-0.2538116	0.80063222	0.99978775	-4.7218605
CCDC115	-0.1776562	4.60439053	-1.5258367	0.13305115	0.99978775	-4.3985346
TAL2	-0.1776762	0.08347129	-0.521855	0.60396556	0.99978775	-4.6211578
DCLK1	-0.1777822	5.41378668	-0.8252426	0.41296699	0.99978775	-4.7404993
ARHGEF15	-0.1778761	-1.2707203	-0.1596209	0.8737921	0.99978775	-4.6104459
TMCO4	-0.1779377	0.93600803	-0.4222212	0.67458721	0.99978775	-4.6242827
ENSCAFG00000002956	-0.1779638	1.29028409	-0.4169893	0.6783859	0.99978775	-4.6354583
H2AFY2	-0.1779712	1.23744875	-0.7989535	0.42791615	0.99978775	-4.6553684
ANKS3	-0.1780071	4.03896026	-1.7269069	0.09006291	0.99978775	-4.3018651
PHC1	-0.1780752	3.83954244	-1.1019107	0.27552517	0.99978775	-4.5962801
TARBP2	-0.1781795	4.8521096	-1.3750828	0.17494414	0.99978775	-4.4747117
ENSCAFG00000003519	-0.1781812	3.29360458	-1.3710238	0.17619852	0.99978775	-4.5010158
NIPSNAP1	-0.1781987	3.00124855	-1.023861	0.31059433	0.99978775	-4.6048922
DCAKD	-0.1782047	4.00763085	-1.1631953	0.25000856	0.99978775	-4.574872
EBNA1BP2	-0.1782994	5.72308947	-0.9463657	0.34829647	0.99978775	-4.6882612
GUK1	-0.1784528	4.81818222	-1.7261025	0.09020871	0.99978775	-4.2648911
FSD1L	-0.1787985	2.19957869	-0.7289346	0.46928015	0.99978775	-4.6356505
VWDE	-0.17881	0.55991528	-0.3085274	0.75890166	0.99978775	-4.625508
MMS22L	-0.1788865	3.54450897	-1.0032752	0.32032869	0.99978775	-4.6278108
GPX7	-0.1789669	2.71166915	-0.8501384	0.39910758	0.99978775	-4.6665764
ARAP1	-0.1792182	5.90368755	-1.0323704	0.30662978	0.99978775	-4.648627
ZNF692	-0.179228	3.29279427	-1.0576259	0.29506695	0.99978775	-4.5981887
PXK	-0.1793036	5.41278337	-1.2247664	0.22612881	0.99978775	-4.5543112
ATP5S	-0.1795298	2.06798431	-1.217797	0.22874472	0.99978775	-4.5645102
RPS6KL1	-0.1795892	-2.2329549	-0.3108549	0.75714149	0.99978775	-4.6067665
PPM1J	-0.1796594	-0.5956036	-0.5005014	0.61881321	0.99978775	-4.6124886
GALNT18	-0.1796724	-2.0490124	-0.2634954	0.79319994	0.99978775	-4.6084374
MAFK	-0.1797144	2.60546168	-0.9891983	0.32710217	0.99978775	-4.6056474
ENSCAFG00000010918	-0.1797763	6.18354067	-2.3037971	0.0252223	0.99978775	-3.7635097
ENSCAFG00000017632	-0.1798509	1.83564511	-0.5207927	0.60470028	0.99978775	-4.65259

ENSCAFG00000012580	-0.1800007	2.37790763	-0.8954657	0.37462446	0.99978775	-4.6192731
CPNE1	-0.1800281	7.01283699	-1.8537301	0.06939846	0.99978775	-4.1446104
ACAP3	-0.1800541	4.30639452	-1.4891101	0.14243977	0.99978775	-4.4258724
RAB24	-0.180082	4.16695546	-1.5179509	0.13502437	0.99978775	-4.4200266
ENSCAFG00000024887	-0.1802712	-0.0787079	-0.4239376	0.67334283	0.99978775	-4.6203171
IL4R	-0.1803019	4.85229738	-0.9504906	0.3462171	0.99978775	-4.6653707
SCRN2	-0.1803534	1.65904306	-0.702335	0.4855718	0.99978775	-4.6324063
STK36	-0.180378	4.51063402	-1.5285076	0.13238807	0.99978775	-4.4014045
CDH18	-0.1804013	-0.178652	-0.2772742	0.78265812	0.99978775	-4.6199379
ACBD6	-0.1805016	4.55290184	-1.5809132	0.11990279	0.99978775	-4.3678771
ENSCAFG00000029336	-0.1806245	1.89926387	-0.5850273	0.56103478	0.99978775	-4.6431547
ENSCAFG00000032253	-0.1806746	-0.8415835	-0.4085005	0.68456729	0.99978775	-4.6150347
MAD1L1	-0.1809458	3.91974685	-1.4177953	0.16215717	0.99978775	-4.4688078
MS4A13	-0.1811235	-0.6086769	-0.3418644	0.73381728	0.99978775	-4.6179374
IL20RB	-0.1811712	0.11548293	-0.5662726	0.57362063	0.99978775	-4.6273754
CBFB	-0.1812225	3.35197441	-1.2324992	0.22325217	0.99978775	-4.5483147
DOK1	-0.1813273	5.65991394	-1.0213794	0.31175701	0.99978775	-4.6521704
TMA16	-0.1813872	3.65344734	-1.3796748	0.17353335	0.99978775	-4.4958533
SLC9A3R2	-0.1814121	4.72609612	-0.7997484	0.42745941	0.99978775	-4.7020533
ILVBL	-0.1815002	5.31331043	-1.4126672	0.16365294	0.99978775	-4.4530459
CCHCR1	-0.1815326	4.40775378	-1.2420862	0.21972326	0.99978775	-4.5420244
ENSCAFG00000007115	-0.1815468	-0.6631117	-0.4383179	0.66295368	0.99978775	-4.6175162
NLN	-0.1816091	3.27475692	-1.3820809	0.17279762	0.99978775	-4.5006987
ANKRD66	-0.1817671	-1.0990603	-0.2773654	0.78258851	0.99978775	-4.6147357
ENSCAFG00000005880	-0.1817832	2.24587706	-1.0911844	0.28017302	0.99978775	-4.5872462
MTG2	-0.1818075	3.09086948	-1.2334084	0.22291572	0.99978775	-4.5507158
KLKB1	-0.1818418	-2.0396507	-0.2631023	0.79350129	0.99978775	-4.6083766
KBTBD3	-0.1819366	3.23561015	-1.2047814	0.23368919	0.99978775	-4.559346
MOV10	-0.18198	4.69072411	-0.9299742	0.35663984	0.99978775	-4.6803352
ENSCAFG00000031454	-0.1820271	1.77145541	-0.649441	0.51888399	0.99978775	-4.6401371
ENSCAFG00000031661	-0.1820517	0.95957923	-0.5271873	0.60028367	0.99978775	-4.6229999
GFOD1	-0.1821225	2.80602276	-0.6863535	0.49550965	0.99978775	-4.6716364
ZBTB8OS	-0.1821358	2.67183204	-1.2967057	0.20040136	0.99978775	-4.5423053
CCDC34	-0.1821686	5.00316657	-1.6221498	0.11076039	0.99978775	-4.3382671
DOCK11	-0.182258	4.53612826	-0.5751288	0.56766031	0.99978775	-4.7599341
HS1BP3	-0.1823577	5.81040316	-1.2407089	0.22022769	0.99978775	-4.5483792
SRGN	-0.1824115	1.57428248	-0.3180702	0.7516931	0.99978775	-4.6417223
TMEM92	-0.1824597	-1.0318032	-0.366315	0.71560177	0.99978775	-4.6148662
KCTD16	-0.1825031	1.88900067	-0.5682165	0.57230972	0.99978775	-4.6607902
CEP192	-0.1825099	5.55459168	-1.7649075	0.083394	0.99978775	-4.2221978
PCCA	-0.1825759	3.85840974	-1.2378543	0.22127586	0.99978775	-4.5446611
COL27A1	-0.1826053	3.92957974	-0.632201	0.52999847	0.99978775	-4.7518842
IL15RA	-0.1826547	3.0605132	-0.6689168	0.50647859	0.99978775	-4.6715366
ENSCAFG00000003353	-0.182745	1.11478812	-0.8834941	0.38099619	0.99978775	-4.6072532
IL16	-0.1827597	2.69554059	-0.354749	0.72419834	0.99978775	-4.7036744
DEPTOR	-0.1828124	1.61451455	-0.5322349	0.59680809	0.99978775	-4.6601289
DPP9	-0.1828771	5.79447664	-1.5523296	0.12658972	0.99978775	-4.3619627
ENSCAFG00000010233	-0.1830413	4.3309968	-1.5784051	0.12047792	0.99978775	-4.3799677
EVC2	-0.1830774	6.37490032	-0.7182934	0.47576011	0.99978775	-4.778315
TFAP4	-0.1832387	1.8003859	-0.5297901	0.5984903	0.99978775	-4.6381431

ENSCAFG00000030272	-0.1832478	-0.7358798	-0.5850979	0.56098769	0.99978775	-4.6196791
SPICE1	-0.1832674	4.33712744	-1.4077146	0.16510769	0.99978775	-4.4648541
ISG20L2	-0.1834084	4.9238536	-1.8627234	0.06809896	0.99978775	-4.1629697
BTBD8	-0.1834488	1.11971012	-0.6367115	0.52707862	0.99978775	-4.6230616
GRWD1	-0.1835342	2.81753422	-1.104569	0.27438174	0.99978775	-4.5861785
ZNF879	-0.1835558	3.16848951	-1.3636554	0.17849326	0.99978775	-4.5133549
KCNIP2	-0.1835637	-0.2582298	-0.4147538	0.68001163	0.99978775	-4.6142155
HNRNPU	-0.1835671	8.19341685	-1.9213481	0.06012287	0.99978775	-4.1219816
C15H1orf109	-0.1836438	4.39468016	-1.8009729	0.07745037	0.99978775	-4.2363916
FLCN	-0.183972	5.16484571	-1.0253633	0.30989188	0.99978775	-4.6498595
RANGAP1	-0.1840347	4.42110247	-0.9969608	0.32335527	0.99978775	-4.6484909
ABHD11	-0.1840356	2.08807581	-1.0708264	0.28914439	0.99978775	-4.5914335
NFATC4	-0.1840684	5.16498227	-1.0113845	0.31646984	0.99978775	-4.6512238
RELT	-0.184151	0.63867708	-0.4867282	0.62847627	0.99978775	-4.6336786
RBM15B	-0.1842032	4.82083403	-1.7352609	0.08856013	0.99978775	-4.262077
COX10	-0.1842226	4.67538781	-1.7226009	0.0908457	0.99978775	-4.2765169
MAML1	-0.1843693	5.72918095	-1.7225393	0.09085695	0.99978775	-4.2466183
KLF6	-0.1845317	6.70204524	-1.1839642	0.24175865	0.99978775	-4.579776
TP53I11	-0.1845969	-1.0290795	-0.3081408	0.75919415	0.99978775	-4.6148833
SRM	-0.1847042	6.01292147	-0.824342	0.41347377	0.99978775	-4.7412134
NAXE	-0.1847974	4.36242615	-1.3236779	0.1913436	0.99978775	-4.5049382
ELK4	-0.1849943	4.18903325	-1.6500731	0.10489734	0.99978775	-4.366238
PDIA5	-0.185006	5.25449298	-1.0078213	0.31816154	0.99978775	-4.6477812
SOCS3	-0.1850105	2.54128476	-0.6133191	0.54231278	0.99978775	-4.6780661
POLD3	-0.1850388	2.66447904	-0.9726635	0.33517962	0.99978775	-4.6149309
ENSCAFG00000030459	-0.1851173	4.51388362	-1.2794769	0.20635338	0.99978775	-4.525723
SLC19A1	-0.185173	4.97116282	-1.0503686	0.29835839	0.99978775	-4.635053
PPFIA4	-0.1851955	4.34898576	-0.696596	0.48912771	0.99978775	-4.7214238
NME7	-0.1852536	2.31510035	-1.0346975	0.30555159	0.99978775	-4.5966262
ORAI3	-0.1853019	5.92387106	-1.3165257	0.19371469	0.99978775	-4.5063846
NCAPD3	-0.1853886	4.61922918	-0.907498	0.36828912	0.99978775	-4.6862554
RETSAT	-0.1854461	4.4478135	-1.0136068	0.31541782	0.99978775	-4.6349773
PPP1CA	-0.1854733	7.08712697	-1.9287725	0.05917196	0.99978775	-4.0870562
ZNF618	-0.1855182	2.78855624	-1.1238232	0.26619914	0.99978775	-4.5807391
CLUAP1	-0.1855565	2.33281967	-0.9367176	0.35319183	0.99978775	-4.61037
GRK7	-0.1856408	-0.9490629	-0.3780899	0.70688784	0.99978775	-4.615943
SCG3	-0.1858165	1.75401889	-0.4603199	0.64718705	0.99978775	-4.6352679
ENSCAFG00000031537	-0.1859391	2.89665255	-0.6384493	0.52595588	0.99978775	-4.683003
CTBP1	-0.1859412	5.96520311	-1.8775381	0.06600317	0.99978775	-4.1257502
RBM12B	-0.1859711	3.29558221	-1.4874388	0.14287916	0.99978775	-4.4651228
MTA1	-0.1860157	5.76255916	-2.1386327	0.03713584	0.99978775	-3.922968
ARL4C	-0.1860631	4.75913527	-0.4690851	0.64095051	0.99978775	-4.8166953
ENSCAFG00000029777	-0.1861298	4.78749028	-1.8297055	0.07297302	0.99978775	-4.209831
ZNF496	-0.1861619	5.37109412	-2.1628314	0.03512803	0.99978775	-3.9140342
PLSCR4	-0.1862851	2.96270194	-0.5954969	0.55406917	0.99978775	-4.6725417
GMEB2	-0.1863442	3.65400746	-1.4432089	0.15490055	0.99978775	-4.4676145
NOP56	-0.1863722	6.72578782	-1.4455116	0.15425576	0.99978775	-4.4289
FASTK	-0.1867698	5.38041058	-1.8309527	0.07278371	0.99978775	-4.1735299
NET1	-0.1867844	3.06291912	-0.5805217	0.56404581	0.99978775	-4.6793924
VCPKMT	-0.1867963	0.30663006	-0.6020724	0.54971674	0.99978775	-4.614799

HDGF	-0.1868305	7.57444346	-1.5090211	0.13728684	0.99978775	-4.3920275
RFXAP	-0.186925	3.80834488	-1.1668325	0.24854933	0.99978775	-4.5737884
CAMKMT	-0.1869867	1.86797384	-0.9021542	0.37109428	0.99978775	-4.6136871
C31H21orf91	-0.1870211	2.18774827	-1.0238885	0.31058144	0.99978775	-4.5977105
TIGD2	-0.1870382	2.33028428	-0.9338171	0.35467221	0.99978775	-4.6128056
TMEM68	-0.1870669	4.15923756	-1.3264741	0.19042258	0.99978775	-4.5072113
SDE2	-0.1870824	5.05046814	-1.9461824	0.05699237	0.99978775	-4.1040706
ZSWIM3	-0.1871479	1.99672001	-0.8951179	0.37480859	0.99978775	-4.6112329
FAM193A	-0.1873335	4.65278265	-2.0885854	0.04160944	0.99978775	-4.0547173
CYP2R1	-0.1873825	2.73428925	-0.6825104	0.49791601	0.99978775	-4.6803701
SOX18	-0.1877126	-1.6460676	-0.1633738	0.87085124	0.99978775	-4.6078663
C1QBP	-0.1877288	5.60776592	-1.4952161	0.14084355	0.99978775	-4.4004356
ENSCAFG00000011354	-0.1877378	4.16512699	-1.2148727	0.2298489	0.99978775	-4.5535818
OSGIN2	-0.187802	0.89686687	-0.3319579	0.74124233	0.99978775	-4.6349505
TEX28	-0.1878324	-0.6917002	-0.2887421	0.77391545	0.99978775	-4.6184219
SAFB	-0.1879044	5.39427099	-1.755385	0.0850253	0.99978775	-4.2313525
LLGL2	-0.1880645	0.14564568	-0.4015813	0.68962178	0.99978775	-4.6250886
GPX1	-0.1881518	7.9554538	-1.0886634	0.28127328	0.99978775	-4.6210863
FBXW9	-0.1883886	2.039829	-1.0333924	0.30615594	0.99978775	-4.5966152
PEX10	-0.1884051	0.93438006	-0.6901133	0.49316171	0.99978775	-4.6202554
INCA1	-0.1884074	-1.0295694	-0.3936209	0.69545457	0.99978775	-4.6100152
ACTN2	-0.1886395	0.61094167	-0.441634	0.66056731	0.99978775	-4.6696547
SFSWAP	-0.1887176	4.81500482	-2.20115	0.03214345	0.99978775	-3.9229143
ADAMTS10	-0.1887863	4.52230952	-1.1356749	0.26124919	0.99978775	-4.5927806
ENSCAFG00000008287	-0.1888103	0.28386217	-0.7143756	0.47815844	0.99978775	-4.617517
EML3	-0.1890183	5.28753292	-1.3564116	0.18077138	0.99978775	-4.4852396
SARS	-0.1890187	7.37338095	-1.5560419	0.12570472	0.99978775	-4.362056
NAA40	-0.1890284	2.8546713	-1.5038141	0.13861991	0.99978775	-4.4761235
ENSCAFG00000012367	-0.189149	3.74197089	-1.2427745	0.21947149	0.99978775	-4.5430605
SCAMP4	-0.1892316	6.52778896	-1.9259542	0.0595314	0.99978775	-4.0848377
ENSCAFG00000014021	-0.1892395	3.07190572	-1.173815	0.24576522	0.99978775	-4.5686567
GCC1	-0.1895041	3.7354749	-1.7363987	0.08835707	0.99978775	-4.3439196
ENSCAFG00000029571	-0.1895575	-0.565874	-0.5190961	0.60587458	0.99978775	-4.6152568
FGFR3	-0.1896498	1.4249508	-0.4686173	0.64128269	0.99978775	-4.6360442
HOXB6	-0.1896856	1.52336972	-0.5575985	0.57948784	0.99978775	-4.6344783
ENSCAFG00000014647	-0.1897445	3.5289512	-1.1256498	0.26543197	0.99978775	-4.5807059
BCL3	-0.1897517	2.57064395	-0.8389767	0.40528531	0.99978775	-4.646682
ENSCAFG00000032245	-0.1897701	2.6524941	-1.2190086	0.22828834	0.99978775	-4.5591346
ENSCAFG00000006726	-0.1898188	5.0392092	-1.4344723	0.15736604	0.99978775	-4.443807
ENSCAFG00000003953	-0.1899244	4.2485713	-1.4738115	0.14650187	0.99978775	-4.4440445
ATP11A	-0.1899737	6.73362186	-1.6301082	0.10906285	0.99978775	-4.3067829
DNAH5	-0.1899868	1.97461399	-0.5386761	0.59238661	0.99978775	-4.6557558
NAT9	-0.1899979	2.98099701	-1.3664573	0.17761797	0.99978775	-4.5154348
KHK	-0.1900196	0.46789199	-0.4285575	0.66999806	0.99978775	-4.6186942
ENSCAFG00000007135	-0.1900696	3.79474914	-1.3679453	0.17715449	0.99978775	-4.495558
ENSCAFG00000000061	-0.1900765	3.502437	-1.3337985	0.18802601	0.99978775	-4.5146948
PLAUR	-0.190119	6.8222721	-0.4072636	0.68546975	0.99978775	-4.8569113
ENSCAFG00000005722	-0.1901309	1.82813059	-0.5690581	0.57174265	0.99978775	-4.6343648
NHSL1	-0.1902086	6.40118963	-0.4892238	0.62672048	0.99978775	-4.8415446
KCTD5	-0.1903811	2.66705964	-0.9045141	0.36985381	0.99978775	-4.6187878

C6	-0.1903966	0.52368939	-0.2238639	0.82373181	0.99978775	-4.7072333
MNT	-0.1904068	5.1794015	-1.2849524	0.20444762	0.99978775	-4.5226256
SQSTM1	-0.1904991	7.32504682	-1.2613787	0.21274696	0.99978775	-4.5371907
PBX1	-0.1905853	5.08202869	-0.6743508	0.5030462	0.99978775	-4.7904656
NIPA1	-0.1906378	2.55365491	-0.7935885	0.43100631	0.99978775	-4.6394932
COL16A1	-0.1908379	8.13382579	-0.6624307	0.51059211	0.99978775	-4.7540821
GLIS2	-0.1909714	4.83042127	-0.9389693	0.35204532	0.99978775	-4.6817894
HGFAC	-0.1909758	0.00381717	-0.5854427	0.56075758	0.99978775	-4.6189028
WASHC1	-0.1911241	4.85558292	-1.4202293	0.16145092	0.99978775	-4.4512474
ACSF3	-0.1911813	2.98958263	-1.0886759	0.28126783	0.99978775	-4.5884888
EMG1	-0.191211	3.89005504	-1.1783373	0.2439741	0.99978775	-4.5670474
VRK1	-0.1912264	3.16773849	-0.8533382	0.39734737	0.99978775	-4.6462772
GHDC	-0.1912525	3.96978575	-1.1043981	0.27445515	0.99978775	-4.5900101
KAT7	-0.1914571	3.48047533	-1.5078674	0.13758131	0.99978775	-4.4458972
ENSCAFG00000028991	-0.1915678	1.93448642	-1.0096399	0.31729735	0.99978775	-4.5992942
RHBDF2	-0.1915727	3.42101488	-0.8551583	0.39634829	0.99978775	-4.6514099
TTC38	-0.1916955	3.39310162	-0.9062037	0.36896729	0.99978775	-4.6426235
PSEN2	-0.1917287	2.07083477	-0.8232693	0.41407794	0.99978775	-4.6268334
ARHGAP6	-0.1917683	2.22379388	-0.2527385	0.801457	0.99978775	-4.6500896
TUT1	-0.1918824	4.87921205	-1.6998316	0.09507909	0.99978775	-4.2905391
CEP131	-0.1918976	5.14617252	-1.3665214	0.177598	0.99978775	-4.4794915
ENSCAFG00000009016	-0.191941	2.64526613	-1.1805732	0.24309203	0.99978775	-4.5665181
ENSCAFG00000004935	-0.1919565	-1.2367366	-0.1752145	0.86158493	0.99978775	-4.6067676
ENSCAFG00000000072	-0.1919888	5.80487646	-1.6165057	0.11197724	0.99978775	-4.3224715
SRRT	-0.1920556	6.6099321	-2.0147508	0.04906394	0.99978775	-4.0128991
LDOC1	-0.1921973	3.57093315	-0.8948529	0.37494897	0.99978775	-4.6468116
TAF4B	-0.1922013	2.90756418	-0.8137328	0.41947244	0.99978775	-4.6315536
ZNF311	-0.1923357	3.39794992	-1.6467	0.10559184	0.99978775	-4.4040059
RAB3IP	-0.1923427	4.08984349	-1.0672907	0.29072261	0.99978775	-4.6188631
HNRNPM	-0.1923674	7.25719877	-1.4760009	0.145915	0.99978775	-4.4113284
CPSF1	-0.1924289	6.58253149	-1.8295465	0.0729972	0.99978775	-4.1603796
UCP3	-0.192537	3.99303719	-1.7952531	0.07836862	0.99978775	-4.2676747
ENSCAFG00000030140	-0.1926252	11.414126	-0.9407784	0.35112598	0.99978775	-4.6433377
FJX1	-0.1926652	1.20991045	-0.1279694	0.89866201	0.99978775	-4.6167693
MIB2	-0.1926856	3.52059578	-1.122884	0.26659425	0.99978775	-4.5842569
CARM1	-0.1927336	5.55461529	-1.7249408	0.09041963	0.99978775	-4.249753
EIF2AK2	-0.1928539	3.15769721	-1.0593573	0.29428543	0.99978775	-4.5971614
VARS	-0.1928809	7.43137476	-1.6388991	0.1072124	0.99978775	-4.3074326
RNASEH2A	-0.1928819	3.34998755	-1.299827	0.19933698	0.99978775	-4.5258632
DDX23	-0.1929121	5.87764325	-2.0241271	0.04805744	0.99978775	-4.0095292
TMEM164	-0.1929631	4.44394377	-0.909578	0.36720096	0.99978775	-4.6611521
CHRAC1	-0.1929992	2.84451355	-1.3953966	0.16876928	0.99978775	-4.5187046
RAI1	-0.1930556	5.29270093	-1.429827	0.15868936	0.99978775	-4.4417117
PAX8	-0.1931318	0.02939457	-0.6030485	0.54907212	0.99978775	-4.6242954
ZNF446	-0.193257	2.15406201	-1.1097638	0.27215685	0.99978775	-4.584428
NELFB	-0.1934059	5.68491185	-2.5778883	0.0127841	0.99978775	-3.5309911
LIG1	-0.1934705	5.16860233	-1.2205638	0.22770357	0.99978775	-4.5561672
PMM2	-0.1934723	4.42048258	-1.9009443	0.06280352	0.99978775	-4.1734099
DCK	-0.1935292	3.13742056	-0.5242392	0.60231804	0.99978775	-4.7286896
RCBTB1	-0.1935879	3.59243054	-1.0798162	0.28515851	0.99978775	-4.594097

GABPB2	-0.1936215	2.97623484	-1.3372452	0.18690621	0.99978775	-4.5252904
CYB561A3	-0.1936986	3.19849166	-1.2883137	0.20328425	0.99978775	-4.5296312
PTGES2	-0.1937162	2.47042785	-0.9572104	0.34284719	0.99978775	-4.608203
ZNF215	-0.1937869	2.71273966	-1.185694	0.24108053	0.99978775	-4.5692796
SNAI2	-0.1938211	6.39763582	-0.6580462	0.51338291	0.99978775	-4.7967863
ZADH2	-0.1942262	4.15503075	-1.3287903	0.18966223	0.99978775	-4.5063534
SETD9	-0.1943026	1.47266617	-1.0562056	0.29570914	0.99978775	-4.5920814
RAB11FIP3	-0.1943054	5.41403281	-1.6611889	0.102635	0.99978775	-4.2993394
GALE	-0.1943387	3.9802772	-0.8759382	0.38505273	0.99978775	-4.6754385
CCDC117	-0.1945321	4.20155764	-1.5743787	0.12140584	0.99978775	-4.3851519
RNF157	-0.19455	5.54250915	-0.8370388	0.40636384	0.99978775	-4.7270209
ENSCAFG00000028464	-0.1946137	1.31988583	-0.6716987	0.50471982	0.99978775	-4.6196075
PLPPR5	-0.1946298	1.31189924	-0.5265272	0.6007389	0.99978775	-4.6752525
RAD52	-0.1946971	2.37751295	-0.9771306	0.33298444	0.99978775	-4.6067676
PCDHGA1	-0.1947051	1.10511604	-0.5557211	0.58076147	0.99978775	-4.6314449
EHMT2	-0.194792	6.00903886	-1.4675577	0.1481884	0.99978775	-4.4154035
ELMO3	-0.1949012	4.54376332	-1.4879449	0.142746	0.99978775	-4.4239082
UTP3	-0.1949725	5.25376463	-2.0238113	0.04809105	0.99978775	-4.0424861
ZFP36	-0.1951201	6.6222716	-0.6360797	0.5274871	0.99978775	-4.8018031
TRMT44	-0.1951942	3.16765413	-1.6870169	0.09753234	0.99978775	-4.3953245
USP45	-0.1954471	1.8227534	-0.6691142	0.50635372	0.99978775	-4.6394366
DUSP18	-0.1954599	2.88091881	-0.7212197	0.47397309	0.99978775	-4.6574759
ENSCAFG00000013974	-0.1954621	0.83522094	-0.8692334	0.38867504	0.99978775	-4.613114
KHNYN	-0.1954627	4.63936463	-1.8391641	0.07154761	0.99978775	-4.2122173
EZH2	-0.1955208	3.75326582	-1.048119	0.29938381	0.99978775	-4.6168104
ADGRF3	-0.1957081	-0.1773496	-0.4440858	0.65880519	0.99978775	-4.6156651
ARL4A	-0.1957832	3.13455525	-0.4722794	0.63868414	0.99978775	-4.6601205
GALK2	-0.1959868	5.13069772	-1.657966	0.10328679	0.99978775	-4.3089242
PALMD	-0.1964329	-2.5305246	-0.2582645	0.79721229	0.99978775	-4.6065128
LLGL1	-0.1964688	5.2676831	-1.5339982	0.1310332	0.99978775	-4.3852917
TCOF1	-0.196511	4.57416295	-1.5175982	0.13511317	0.99978775	-4.4008477
MAST3	-0.1965578	3.88180308	-1.2134202	0.23039877	0.99978775	-4.5554893
HSF2BP	-0.1965826	3.1742414	-1.7968977	0.07810368	0.99978775	-4.3563949
CILP2	-0.1966573	2.55725369	-0.4648773	0.6439412	0.99978775	-4.6740556
TLX2	-0.1967666	3.77803496	-0.8035613	0.42527277	0.99978775	-4.7001483
RASGRP2	-0.1968038	2.38187692	-0.3449998	0.73147251	0.99978775	-4.6517999
GPD1L	-0.1968172	3.61365663	-0.773446	0.44272626	0.99978775	-4.6598363
TAF4	-0.1970383	3.75535066	-1.5262391	0.13295108	0.99978775	-4.4332252
GBP5	-0.1970985	1.09374249	-0.2879289	0.77453443	0.99978775	-4.6269682
SMC3	-0.1971249	6.30974763	-1.4321079	0.15803853	0.99978775	-4.4372582
SMIM12	-0.1971821	2.80077484	-1.1897456	0.23949761	0.99978775	-4.5644845
NADSYN1	-0.1972323	3.99788713	-1.461963	0.1497101	0.99978775	-4.4525289
ASF1A	-0.1972899	2.65511186	-1.0238113	0.31061759	0.99978775	-4.6012138
LHFPL2	-0.1973566	3.40849533	-1.0867445	0.28211281	0.99978775	-4.5933506
MIIP	-0.1973826	2.60695332	-1.0751123	0.28723934	0.99978775	-4.5917036
PPP1R18	-0.197526	6.82356872	-2.1662257	0.03485416	0.99978775	-3.885239
INHBB	-0.197558	1.41216517	-0.5633778	0.57557547	0.99978775	-4.6839203
ATXN7L3B	-0.1976477	-0.5901109	-0.4263773	0.67157571	0.99978775	-4.6135283
ZBTB20	-0.1977522	2.68185624	-0.6343132	0.52863006	0.99978775	-4.6452769
ZNF638	-0.1977843	5.70608058	-1.6956935	0.09586567	0.99978775	-4.2687299

RFC5	-0.1979648	4.04430785	-1.0029325	0.32049248	0.99978775	-4.6390473
SERPINB8	-0.198449	1.65203138	-0.7834093	0.43690581	0.99978775	-4.6775434
ENSCAFG00000003595	-0.1985573	6.40657931	-1.4992699	0.13979166	0.99978775	-4.3945457
PBX2	-0.1985819	3.77778813	-1.2328759	0.22311271	0.99978775	-4.5473167
NSMAF	-0.1986344	5.36802865	-2.228481	0.03015351	0.99978775	-3.865414
SLC3A2	-0.1988063	6.31776527	-1.507367	0.1377092	0.99978775	-4.3892836
ENSCAFG00000018497	-0.1988183	4.26427716	-1.1356022	0.26127935	0.99978775	-4.5866002
FOXRED1	-0.1988364	5.09532846	-1.7776741	0.08124798	0.99978775	-4.2187761
DIMT1	-0.1989769	4.61996711	-1.5710039	0.12218803	0.99978775	-4.3780618
RABL3	-0.1990546	2.42585986	-1.3440069	0.18472406	0.99978775	-4.5385227
SMC4	-0.1990818	6.16743097	-0.8048832	0.42451621	0.99978775	-4.7487044
DOT1L	-0.1993966	4.70060373	-1.4875897	0.14283945	0.99978775	-4.4123179
DENND1A	-0.1994514	4.61841964	-1.7239189	0.09060551	0.99978775	-4.2815652
CLIP1	-0.1995318	7.26922504	-1.9627829	0.05497847	0.99978775	-4.0669028
PSAT1	-0.1996159	6.82075534	-0.6241449	0.53523452	0.99978775	-4.7998846
AK1	-0.1996786	4.52227704	-1.1954155	0.23729513	0.99978775	-4.5621107
CLCA2	-0.1996946	-1.5005782	-0.2909696	0.77222066	0.99978775	-4.6085333
SAFB2	-0.1997196	4.90124386	-2.0243955	0.0480289	0.99978775	-4.0684414
C15H1orf216	-0.1997507	-0.0849684	-0.364991	0.716584	0.99978775	-4.6192621
NDUFS7	-0.1998692	4.4649986	-1.1862817	0.24085048	0.99978775	-4.5658053
ENSCAFG00000032636	-0.199921	2.04502158	-1.0956188	0.27824495	0.99978775	-4.5867686
ENSCAFG00000004189	-0.1999386	-0.1660509	-0.5888555	0.55848274	0.99978775	-4.6159628
ENSCAFG00000032445	-0.2000147	1.47559336	-0.6660463	0.50829685	0.99978775	-4.705451
EHD2	-0.2001641	8.47995369	-1.8970243	0.06333001	0.99978775	-4.1472142
SNX21	-0.2002129	4.16668755	-1.3025049	0.19842719	0.99978775	-4.5145175
SERF2	-0.2002993	0.68209013	-0.6862612	0.49556738	0.99978775	-4.6191947
ID4	-0.2003124	-2.1498309	-0.4155297	0.67944724	0.99978775	-4.6095387
CAMK2A	-0.2003974	3.07311306	-1.0530675	0.29713145	0.99978775	-4.6404643
MYBBP1A	-0.2005389	7.41515193	-1.3886356	0.17080549	0.99978775	-4.4647064
ANKRD33B	-0.200552	1.44864302	-0.6664683	0.50802937	0.99978775	-4.6325468
BCLAF3	-0.2005614	2.57531511	-0.9726635	0.33517961	0.99978775	-4.6150828
PDSS2	-0.2005783	2.57155707	-1.5424613	0.12896654	0.99978775	-4.4906547
ENSCAFG00000031225	-0.200595	-0.2525833	-0.4489751	0.65529702	0.99978775	-4.6206908
ENSCAFG00000018296	-0.2008065	2.34372372	-1.1736269	0.24583992	0.99978775	-4.572243
RAB20	-0.2008143	1.83939033	-0.4280754	0.67034682	0.99978775	-4.6320394
NR5A2	-0.2008777	-2.5764496	-0.3425032	0.73333934	0.99978775	-4.6059177
TMEM120B	-0.2009176	2.77318876	-1.3473715	0.18364551	0.99978775	-4.5273532
PHF11	-0.2009179	3.26271535	-1.6647122	0.1019263	0.99978775	-4.3975078
ELMO1	-0.2009205	5.88398721	-0.7855618	0.43565433	0.99978775	-4.75537
LGALS1	-0.2009475	8.62081572	-1.0005291	0.32164259	0.99978775	-4.6511698
LEMD3	-0.2011274	3.60202763	-0.9206461	0.36144522	0.99978775	-4.6318753
TRMT10B	-0.2011561	4.00062353	-1.9811656	0.05281987	0.99978775	-4.1807404
BEX4	-0.2011755	2.08549309	-0.8944898	0.37514134	0.99978775	-4.6127036
HMGN2	-0.2015002	4.9759265	-0.949584	0.34667343	0.99978775	-4.6789322
SNX31	-0.2015823	-0.5411017	-0.2563698	0.79866695	0.99978775	-4.6185083
ARHGEF2	-0.2016339	8.13644697	-1.3143937	0.19442579	0.99978775	-4.5071781
NUDT3	-0.2017114	4.75475092	-2.1961827	0.0325173	0.99978775	-3.9618292
APEX2	-0.2019323	3.03643828	-1.2467543	0.21801992	0.99978775	-4.5476955
FOXN3	-0.2019608	-0.3931148	-0.4599502	0.64745067	0.99978775	-4.6137465
PTK2B	-0.2021125	4.43495429	-0.7522389	0.45526601	0.99978775	-4.6652816

GLE1	-0.2022311	4.44375067	-1.4619028	0.14972653	0.99978775	-4.4371512
EMC9	-0.2022658	2.01082709	-0.6503902	0.51827565	0.99978775	-4.6375353
NEIL1	-0.2022759	0.46304159	-0.3759978	0.70843326	0.99978775	-4.6207487
ENSCAFG00000004331	-0.2022852	2.18518626	-0.5772596	0.56623079	0.99978775	-4.6462361
SIPA1L3	-0.2022931	6.70917796	-1.3750103	0.17496651	0.99978775	-4.4724328
MBD6	-0.2023228	4.95840866	-1.3269207	0.19027578	0.99978775	-4.5004408
RNF26	-0.2025894	4.05451454	-1.4010586	0.16707851	0.99978775	-4.4740486
FAM219A	-0.2026929	1.86701972	-1.0000584	0.3218682	0.99978775	-4.5991611
ENSCAFG00000003909	-0.2027472	2.81752233	-1.1647218	0.24939538	0.99978775	-4.5709379
ENSCAFG00000030307	-0.2028004	3.27769642	-0.7340939	0.46615662	0.99978775	-4.7566535
RNF114	-0.2028427	4.88400876	-1.5037519	0.13863589	0.99978775	-4.4022363
VAT1	-0.2028678	6.65317273	-1.0835626	0.28350876	0.99978775	-4.6302756
PTRH1	-0.2029301	2.49588595	-1.0602598	0.29387858	0.99978775	-4.5934273
NCKAP1L	-0.2029573	-1.633106	-0.3804617	0.70513734	0.99978775	-4.6116708
SLC2A13	-0.2035688	2.71124949	-0.99485	0.32437129	0.99978775	-4.6106703
EID2	-0.2035693	1.52721668	-0.8634817	0.39179935	0.99978775	-4.6110718
STAC2	-0.2037963	1.27909241	-0.7037164	0.48471805	0.99978775	-4.6491104
XRCC4	-0.2038986	2.12925569	-1.0669383	0.29088024	0.99978775	-4.5908487
TYK2	-0.203976	6.02403465	-1.791805	0.07892658	0.99978775	-4.1913789
ENSCAFG00000031105	-0.2040725	-0.1944861	-0.4853761	0.62942848	0.99978775	-4.6191781
TRIM45	-0.2043573	2.12375555	-0.7753401	0.44161624	0.99978775	-4.6345503
WNK2	-0.204392	0.65309868	-0.390049	0.69807787	0.99978775	-4.6353194
MB21D2	-0.2044475	3.67941728	-1.1180438	0.2686369	0.99978775	-4.585259
PIGV	-0.2045035	2.93610351	-1.1617322	0.25059726	0.99978775	-4.5721451
ENSCAFG00000015189	-0.2046063	4.75270152	-1.5989719	0.11582681	0.99978775	-4.3548198
ENSCAFG00000019620	-0.2046447	5.5273945	-1.1516266	0.2546907	0.99978775	-4.5936171
WDR35	-0.2048727	4.87671767	-1.5713232	0.12211386	0.99978775	-4.3635968
HMGN1	-0.2049737	4.94007954	-1.4838373	0.14382966	0.99978775	-4.4108886
PRPSAP1	-0.2050398	5.41448295	-1.467213	0.14828181	0.99978775	-4.4189383
FUNDC1	-0.2050972	2.55167032	-0.9362276	0.35344165	0.99978775	-4.6119997
SMC1A	-0.2051335	5.84408411	-1.2056832	0.23334413	0.99978775	-4.5679124
ABCG4	-0.205147	0.2264876	-0.5780409	0.56570712	0.99978775	-4.6173866
SERPINH1	-0.2051706	9.93055425	-1.4011136	0.16706214	0.99978775	-4.4720906
DFFA	-0.2051964	3.51919729	-1.5891199	0.11803637	0.99978775	-4.4161831
INPP5B	-0.2059435	5.8029148	-2.2752099	0.02700255	0.99978775	-3.8016243
ENSCAFG00000005635	-0.2059558	-0.9155572	-0.396514	0.69333259	0.99978775	-4.6119623
EIF4EBP1	-0.2059756	4.67559152	-1.4526123	0.15228071	0.99978775	-4.4353803
MLLT1	-0.206001	6.37274875	-2.0010878	0.05056317	0.99978775	-4.0248099
CDC25C	-0.2060078	0.32981913	-0.6061219	0.54704494	0.99978775	-4.6242965
RBM10	-0.2061059	6.17966168	-2.3059743	0.0250911	0.99978775	-3.7610942
ENSCAFG00000019285	-0.206398	1.95136898	-1.3829776	0.17252402	0.99978775	-4.5336946
MRPL27	-0.2064482	3.60669007	-1.245141	0.2186075	0.99978775	-4.5425609
ENSCAFG00000024501	-0.2064721	3.94118052	-1.5116312	0.13662243	0.99978775	-4.4310718
C33H3orf38	-0.2065169	4.42053407	-2.2654582	0.02763488	0.99978775	-3.9386884
KLHL22	-0.2065816	4.17560532	-1.7999031	0.07762144	0.99978775	-4.273321
ZNF283	-0.2066306	1.36713309	-0.8309798	0.40974733	0.99978775	-4.6121324
ABCA12	-0.2067883	0.86292964	-0.4983291	0.62033286	0.99978775	-4.6247762
COL9A2	-0.2068524	1.71075269	-0.5197428	0.60542684	0.99978775	-4.6544495
RCC2	-0.2068784	5.92140073	-2.1567409	0.03562419	0.99978775	-3.896952
LUC7L3	-0.2068798	5.08677062	-1.8661286	0.06761232	0.99978775	-4.1618517

ATF1	-0.2070246	4.28319902	-2.2511704	0.02858506	0.99978775	-3.9657428
ENSCAFG00000028878	-0.2071397	2.64944009	-0.3060072	0.76080901	0.99978775	-4.6844025
PLEKHG3	-0.2073059	4.09005854	-0.825517	0.41281267	0.99978775	-4.7011649
ENSCAFG00000015889	-0.2074188	1.58898209	-1.0083136	0.31792743	0.99978775	-4.599045
FAM173B	-0.2074965	2.72539232	-1.3552714	0.18113197	0.99978775	-4.5252019
CALHM2	-0.2075483	3.52570427	-0.7168763	0.4766268	0.99978775	-4.6651528
ACACA	-0.2075787	6.8027735	-1.5207384	0.13432421	0.99978775	-4.3808951
STK40	-0.2075888	5.13356358	-2.0976653	0.04076454	0.99978775	-4.0014551
HSD17B14	-0.2076083	2.51436314	-0.5037892	0.61651651	0.99978775	-4.6606475
ERICH6	-0.207655	2.99944087	-0.8609878	0.39315889	0.99978775	-4.6358318
KHDC4	-0.2076572	3.67975148	-1.5593353	0.12492374	0.99978775	-4.4129537
TENT5B	-0.2080145	3.62354915	-0.6502392	0.51837239	0.99978775	-4.6875833
EMILIN1	-0.2081184	7.41248396	-0.829757	0.4104323	0.99978775	-4.7384731
OLR1	-0.2081295	-0.218807	-0.3318617	0.74131458	0.99978775	-4.6337605
NUDT16L1	-0.2081832	0.99358845	-0.6034526	0.54880535	0.99978775	-4.6292349
FLT3LG	-0.2082957	2.76028738	-0.7167317	0.4767153	0.99978775	-4.6556521
ENSCAFG00000013638	-0.2083763	1.79220285	-0.9234645	0.35998892	0.99978775	-4.6087765
SH3GL1	-0.2084714	6.39517208	-1.5165817	0.13536934	0.99978775	-4.3837748
TMOD3	-0.208485	6.43781242	-1.5753586	0.12117948	0.99978775	-4.3458764
SURF1	-0.2084967	3.85217455	-1.5213472	0.1341717	0.99978775	-4.4289762
TRAF7	-0.2084988	5.92596894	-1.5257644	0.13306913	0.99978775	-4.379368
CXCL8	-0.2085128	0.69913413	-0.1610232	0.87269302	0.99978775	-4.6175566
KIAA1147	-0.208614	-1.5593615	-0.2689081	0.7890541	0.99978775	-4.6093902
NUSAP1	-0.2087175	2.97437187	-0.6853184	0.49615715	0.99978775	-4.6840919
HSPBP1	-0.2089031	4.97596671	-1.963807	0.05485625	0.99978775	-4.0967295
JAKMIP1	-0.2089962	-1.4825824	-0.2801359	0.78047379	0.99978775	-4.6065814
ABCF3	-0.2090045	5.01112141	-2.0117214	0.04939301	0.99978775	-4.0638683
KDM6B	-0.209275	4.30741445	-1.2280609	0.22489994	0.99978775	-4.5482044
FAM19A4	-0.209282	-0.936743	-0.3431412	0.73286215	0.99978775	-4.6102229
ANKRD35	-0.2092907	-0.1913761	-0.5514125	0.58368965	0.99978775	-4.6113121
PTRHD1	-0.2092978	1.6765098	-0.6883791	0.49424394	0.99978775	-4.6280892
ENSCAFG00000018271	-0.2096587	0.79322048	-0.5074748	0.61394644	0.99978775	-4.6302159
HMGCR	-0.2096695	6.97980541	-1.0557753	0.29590387	0.99978775	-4.643637
POLR3E	-0.2097264	4.49279694	-2.1112283	0.03953035	0.99978775	-4.039017
ENSCAFG00000010021	-0.2098089	0.51786913	-0.6419164	0.52371977	0.99978775	-4.618146
TOMM40L	-0.2098388	3.40960449	-1.7448779	0.08685596	0.99978775	-4.3568853
SIGIRR	-0.2098568	2.62384739	-0.6787941	0.50024896	0.99978775	-4.6421375
MDM4	-0.2099041	3.92109204	-1.3210569	0.19220996	0.99978775	-4.5114885
CD2BP2	-0.2099504	5.92976766	-2.3253827	0.02394811	0.99978775	-3.7500446
CLCN5	-0.2099725	2.03347069	-1.2446895	0.21877214	0.99978775	-4.5595052
TCF15	-0.2099845	-0.9391241	-0.3926161	0.69619218	0.99978775	-4.6133998
AXIN2	-0.2099954	1.29670051	-0.4056824	0.68662417	0.99978775	-4.6299878
YPEL2	-0.2100043	1.9691284	-0.3976848	0.69247453	0.99978775	-4.6343559
PMAIP1	-0.2100049	-1.876937	-0.3781928	0.70681187	0.99978775	-4.6091313
ENSCAFG00000023425	-0.2103443	0.9244874	-0.6233561	0.53574868	0.99978775	-4.6267944
KRI1	-0.2103557	3.95096217	-1.1945857	0.23761652	0.99978775	-4.5604066
ENSCAFG00000024183	-0.2105757	5.07165647	-1.6763531	0.09961326	0.99978775	-4.2970167
RTN1	-0.2106362	-0.0989145	-0.4566055	0.64983768	0.99978775	-4.6263876
TLE1	-0.2107166	5.96561085	-0.9383638	0.35235342	0.99978775	-4.6957557
SNRPF	-0.210767	4.09972969	-1.2746247	0.20805327	0.99978775	-4.5282191

OSBPL10	-0.2107939	3.98130098	-0.9602684	0.34132075	0.99978775	-4.6652087
ZNF790	-0.2108096	1.21008116	-0.7299104	0.46868848	0.99978775	-4.6179949
SGSM3	-0.2108325	4.72028804	-1.6296974	0.10914994	0.99978775	-4.3377228
ZNF521	-0.2110633	5.3170181	-0.7817913	0.43784791	0.99978775	-4.7385739
UCKL1	-0.2110655	4.38258798	-1.163598	0.24984669	0.99978775	-4.5724619
HID1	-0.2110951	-0.1018076	-0.3414361	0.73413779	0.99978775	-4.6160729
TBL2	-0.2113189	3.97031387	-2.0052768	0.05009936	0.99978775	-4.1707243
TRIOBP	-0.2113664	6.37358525	-1.69741	0.09553875	0.99978775	-4.2607865
PXMP4	-0.2113726	2.98066796	-1.060689	0.29368527	0.99978775	-4.5956343
SLC16A2	-0.2114162	-0.8244123	-0.3406991	0.73468936	0.99978775	-4.614823
FBXL19	-0.2114472	3.38655536	-1.6006508	0.11545364	0.99978775	-4.4148879
PGS1	-0.2114818	4.93756711	-2.165509	0.03491184	0.99978775	-3.9278358
SNRPD1	-0.2115101	4.2935639	-1.6405821	0.10686107	0.99978775	-4.3441897
MMP16	-0.2115716	-1.5975644	-0.6575674	0.51368822	0.99978775	-4.6107141
ELK3	-0.2116006	7.12782594	-1.1336921	0.26207274	0.99978775	-4.6046065
ENSCAFG00000031286	-0.2117467	5.0567364	-1.458779	0.15058158	0.99978775	-4.4256071
KBTBD4	-0.212029	3.05297583	-1.5390673	0.12979218	0.99978775	-4.4624428
WWOX	-0.2122657	2.79017295	-1.1107372	0.27174138	0.99978775	-4.5837749
TIMM21	-0.2123355	3.57136593	-1.6495237	0.10501019	0.99978775	-4.3846063
ENSCAFG00000004704	-0.2124521	1.90739496	-0.869958	0.38828256	0.99978775	-4.6130935
AMPD3	-0.2125319	4.64411408	-0.4996229	0.61942759	0.99978775	-4.7305827
TIMM44	-0.2126592	4.16265287	-1.8405156	0.07134586	0.99978775	-4.2375524
NCOA7	-0.2127313	4.22365725	-0.9501622	0.34638237	0.99978775	-4.6441923
ENSCAFG00000029403	-0.2127378	6.57649021	-2.5160264	0.01496248	0.99978775	-3.5601305
ENSCAFG00000032728	-0.2127496	5.99870463	-1.5322964	0.13145194	0.99978775	-4.3734908
ENSCAFG00000024219	-0.2127567	4.99218211	-1.7905701	0.0791272	0.99978775	-4.2279027
PACSIN3	-0.2129672	3.73647082	-1.0517138	0.29774641	0.99978775	-4.6125158
ENSCAFG00000006021	-0.2130591	4.68247439	-1.7639121	0.08356331	0.99978775	-4.2661186
RAB23	-0.21314	3.99534081	-1.3726478	0.17569584	0.99978775	-4.4842075
RPA2	-0.2132285	3.09895619	-1.0980247	0.27720273	0.99978775	-4.5902966
NAB2	-0.2133074	5.04006786	-1.1084797	0.2727056	0.99978775	-4.6051192
CTNNBL1	-0.2133184	5.05960054	-1.9987806	0.05082018	0.99978775	-4.0824901
ENSCAFG00000005585	-0.213514	2.6637389	-1.445878	0.15415338	0.99978775	-4.4911773
ENSCAFG00000000399	-0.213538	3.36474782	-0.8670976	0.3898334	0.99978775	-4.6324547
NT5DC2	-0.2135423	6.15164898	-0.8700022	0.38825861	0.99978775	-4.7191586
ENSCAFG00000028596	-0.2135654	-1.1470597	-0.4584311	0.64853431	0.99978775	-4.6142059
AKAP12	-0.2136451	8.2607104	-0.8352751	0.40734699	0.99978775	-4.7167591
ZSCAN30	-0.2137221	1.24760478	-0.7671876	0.44640556	0.99978775	-4.6161786
ENSCAFG00000029170	-0.2139913	-2.3456163	-0.2757854	0.78379523	0.99978775	-4.6074116
TGDS	-0.2143459	3.99355265	-1.5280487	0.1325018	0.99978775	-4.4217872
DAXX	-0.2143814	4.58116178	-1.6923198	0.09651091	0.99978775	-4.2969104
CSTF2T	-0.214509	3.59200113	-1.3810138	0.1731236	0.99978775	-4.4903734
ZNF407	-0.2146393	4.36952713	-2.3679708	0.02160138	0.99978775	-3.8718364
RHPN2	-0.2146602	1.1058866	-0.5583341	0.57898914	0.99978775	-4.6316978
MUS81	-0.2147448	4.84640011	-2.5281996	0.01450892	0.99978775	-3.6522362
SLC45A4	-0.2150763	3.22464812	-1.4677067	0.14814806	0.99978775	-4.4750327
LMF2	-0.2152519	5.8149493	-1.4900809	0.14218504	0.99978775	-4.4012666
G6PD	-0.2153729	7.04589372	-1.4236953	0.16044937	0.99978775	-4.4429141
PDLIM1	-0.2155872	7.00579487	-1.1484447	0.25598943	0.99978775	-4.5968161
WDR41	-0.2156333	3.33600076	-1.7083737	0.09347221	0.99978775	-4.3712549

PHRF1	-0.2157796	6.03124955	-2.3268487	0.02386369	0.99978775	-3.7470872
NDOR1	-0.2158303	4.51781417	-1.7572917	0.08469654	0.99978775	-4.2601384
SNX15	-0.2158341	3.91336168	-2.0057534	0.05004684	0.99978775	-4.1729609
ENSCAFG00000004676	-0.2158458	2.9781164	-0.7620536	0.44943718	0.99978775	-4.6523861
ENSCAFG00000007610	-0.2158575	2.00537825	-1.2167455	0.22914129	0.99978775	-4.5673892
SETD1A	-0.2158746	4.73581267	-1.8828738	0.06526186	0.99978775	-4.1799335
DCAF11	-0.2159947	4.65467928	-1.9212793	0.06013173	0.99978775	-4.1541585
GATAD1	-0.2160455	4.21493284	-1.2707323	0.20942446	0.99978775	-4.5302114
FRMD8	-0.216064	5.12442612	-1.2113826	0.23117181	0.99978775	-4.5562757
TRMT1L	-0.2161009	4.58563204	-1.2737694	0.20835401	0.99978775	-4.5277973
COA7	-0.216139	2.97722032	-1.3178016	0.19329008	0.99978775	-4.5280653
SELE	-0.2163401	-1.4001075	-0.1567195	0.87606688	0.99978775	-4.6078106
ENSCAFG00000031771	-0.2163475	3.28064046	-1.408031	0.16501443	0.99978775	-4.4884693
SLC7A5	-0.2166474	7.66991262	-0.6689392	0.50646444	0.99978775	-4.7721236
SAC3D1	-0.2167318	2.12162158	-0.9860308	0.32863934	0.99978775	-4.603021
TIRAP	-0.2167328	1.74424389	-1.023373	0.31082274	0.99978775	-4.5968441
TTC7A	-0.2167669	7.42416354	-1.2324182	0.22328215	0.99978775	-4.5534712
MRGBP	-0.2167833	1.31989611	-1.0865053	0.28221758	0.99978775	-4.5885908
TBC1D2	-0.2168176	3.63448248	-1.1231644	0.26647626	0.99978775	-4.584255
PLEKHF1	-0.2169985	2.50211671	-0.7741942	0.44228758	0.99978775	-4.6487586
IL27RA	-0.2170501	5.0323357	-1.2543346	0.21527484	0.99978775	-4.5372471
RAB30	-0.2170804	2.40918583	-0.7567075	0.45260675	0.99978775	-4.6378078
FBXL15	-0.217128	1.35219597	-0.7848763	0.43605262	0.99978775	-4.6183189
SLC10A6	-0.2171781	0.55953506	-0.4305127	0.66858449	0.99978775	-4.6199995
TADA2A	-0.217535	4.97766711	-1.9800084	0.05295357	0.99978775	-4.0861333
ENSCAFG00000011119	-0.217707	-0.3686976	-0.595439	0.55410758	0.99978775	-4.6143577
MNDA	-0.2177634	6.3334411	-1.5301875	0.13197235	0.99978775	-4.3756119
DHX57	-0.2181287	4.24726347	-1.854914	0.0692262	0.99978775	-4.2393067
ENSCAFG00000013888	-0.2182242	4.92103528	-1.3711358	0.17616382	0.99978775	-4.4781758
DDX28	-0.2182582	3.31571162	-1.532226	0.13146928	0.99978775	-4.4458725
FGD4	-0.2184696	0.57045451	-0.3926642	0.69615689	0.99978775	-4.6187225
PNKD	-0.2185331	2.23751611	-0.973094	0.33496763	0.99978775	-4.6085239
ENSCAFG00000001208	-0.2186414	2.6582642	-0.9833903	0.32992452	0.99978775	-4.6079906
LTBP2	-0.2187788	10.4774857	-0.5425146	0.58975905	0.99978775	-4.7584846
GFPT2	-0.2188852	6.99008966	-0.5689011	0.57184842	0.99978775	-4.8211992
ENSCAFG00000020060	-0.2189233	2.44855589	-0.7870553	0.43478721	0.99978775	-4.6471125
MYC	-0.2190287	6.81914157	-1.3748695	0.17500989	0.99978775	-4.4722841
ENSCAFG00000002375	-0.2199541	-0.5133666	-0.5518442	0.58339598	0.99978775	-4.6153912
XYLT1	-0.2199795	2.39285299	-0.8093156	0.42198544	0.99978775	-4.6574717
PLK3	-0.2199923	4.77913558	-1.2807949	0.20589346	0.99978775	-4.5251942
NQO2	-0.2200435	3.43946395	-1.2795749	0.20631916	0.99978775	-4.5361074
GLRX5	-0.220155	2.49181616	-0.843598	0.40272045	0.99978775	-4.6214887
CDC25A	-0.2202164	2.7397374	-0.8751521	0.38547632	0.99978775	-4.6310734
MVD	-0.2203466	6.36873157	-1.4251261	0.16003732	0.99978775	-4.4418758
ZNF300	-0.2203648	2.95975854	-1.2640559	0.21179201	0.99978775	-4.5451467
ANP32A	-0.2204288	5.3825945	-1.4174257	0.16226462	0.99978775	-4.4498081
NIPAL2	-0.220593	3.41552677	-0.8486779	0.39991258	0.99978775	-4.7038114
PFAS	-0.2206294	5.7787013	-1.575661	0.12110971	0.99978775	-4.3458466
IFT122	-0.2208358	4.74081199	-2.0274163	0.04770861	0.99978775	-4.0792203
SIRT1	-0.2208514	4.08643618	-1.6682071	0.10122729	0.99978775	-4.3389118

ACER2	-0.2208552	4.97649405	-1.4103598	0.16432947	0.99978775	-4.4528016
TPPP3	-0.2208598	1.601602	-0.4454515	0.65782447	0.99978775	-4.6275434
SCUBE2	-0.2208799	2.76706016	-0.4441904	0.65873001	0.99978775	-4.6979378
MCPH1	-0.2209271	4.43258248	-2.0203187	0.04846408	0.99978775	-4.1238932
RIN1	-0.2211092	4.03641926	-1.0940892	0.27890895	0.99978775	-4.5933897
TUBE1	-0.221263	3.32833981	-1.3258994	0.19061161	0.99978775	-4.5270004
LRRC42	-0.2213023	3.69337691	-1.4821824	0.14426807	0.99978775	-4.4572171
MED16	-0.2215101	5.66752824	-1.5347512	0.13084825	0.99978775	-4.3718081
DYNLRB2	-0.2217129	0.77832115	-0.6286243	0.53231981	0.99978775	-4.6208752
ZNF322	-0.2219881	3.47536184	-1.6823986	0.0984291	0.99978775	-4.3783054
PYCR3	-0.221995	3.30162686	-1.3444346	0.1845867	0.99978775	-4.5174994
ENSCAFG00000022297	-0.2220647	6.0897769	-0.9510239	0.34594891	0.99978775	-4.6885555
ENSCAFG00000030979	-0.2220653	3.68106466	-1.1980475	0.23627773	0.99978775	-4.5590802
STIL	-0.2221867	2.23134393	-0.6929989	0.49136378	0.99978775	-4.6614527
SPSB3	-0.2222121	3.18292412	-1.2579193	0.21398567	0.99978775	-4.5480193
PLEKHH2	-0.2222268	3.30649613	-0.7309184	0.46807772	0.99978775	-4.6684133
P2RX2	-0.2222495	-1.3051823	-0.4154973	0.67947075	0.99978775	-4.6079479
NOP9	-0.2224495	4.87843088	-2.3197747	0.02427351	0.99978775	-3.8378048
COL5A1	-0.2225083	11.5584985	-0.5392177	0.59201553	0.99978775	-4.710489
ENSCAFG00000031506	-0.2225316	4.05889283	-1.346682	0.18386614	0.99978775	-4.500024
TMEM17	-0.2226091	2.78747873	-1.1494992	0.2555585	0.99978775	-4.5758743
HERC6	-0.2226774	3.15119223	-0.9635032	0.33971097	0.99978775	-4.6189078
BPI	-0.2226897	-1.5353168	-0.4054125	0.6868213	0.99978775	-4.6066537
DEAF1	-0.2227153	3.74729563	-1.1963779	0.23692273	0.99978775	-4.5597561
PKD1L1	-0.2231069	-1.4272776	-0.472952	0.63820733	0.99978775	-4.6147464
FAM160B2	-0.2231356	4.90267507	-1.5385199	0.12992574	0.99978775	-4.3843983
HIST1H1C	-0.223185	1.28620681	-0.8860997	0.3796036	0.99978775	-4.6134581
CYP7B1	-0.2232727	3.49168013	-0.7712917	0.44399077	0.99978775	-4.6954823
SYNE2	-0.2236009	3.43358945	-0.5617236	0.57669395	0.99978775	-4.7278406
DENND5B	-0.2237057	4.08929491	-0.9902465	0.32659453	0.99978775	-4.6414137
SLX4	-0.2237243	4.36465289	-2.092831	0.04121251	0.99978775	-4.0455792
SSRP1	-0.2238004	7.02968645	-1.8052923	0.07676294	0.99978775	-4.1826228
RHOB	-0.2238071	5.88194845	-0.9157126	0.36400358	0.99978775	-4.7042069
EMC8	-0.2239571	3.77724706	-1.9664557	0.05454123	0.99978775	-4.2032763
SUV39H1	-0.224329	2.69182648	-0.815934	0.41822353	0.99978775	-4.6495423
CACTIN	-0.2243427	4.28968898	-1.8910476	0.06413995	0.99978775	-4.2038101
ENSCAFG00000012747	-0.2243762	2.99967914	-1.1587227	0.25181135	0.99978775	-4.5718587
MRNIP	-0.2246405	0.63322046	-1.0429715	0.30173913	0.99978775	-4.5933387
PTGR1	-0.2247672	8.54628055	-1.2535514	0.21555726	0.99978775	-4.5395529
BICRAL	-0.2248162	4.64213756	-1.3991291	0.16765322	0.99978775	-4.4674994
ENSCAFG00000001866	-0.2249613	3.63300784	-0.197503	0.84419644	0.99978775	-4.6496693
LGALS3	-0.225006	9.40339579	-0.6664474	0.5080426	0.99978775	-4.7491436
ATL1	-0.2250763	0.86145682	-0.6838917	0.49705033	0.99978775	-4.6172694
RILP	-0.225105	0.54575663	-0.3795336	0.70582211	0.99978775	-4.6177281
ENSCAFG00000003969	-0.2255571	2.79978301	-1.3869477	0.1713168	0.99978775	-4.5170992
PAQR4	-0.2258196	2.3752637	-0.7576723	0.45203377	0.99978775	-4.6351288
MEX3B	-0.2258916	2.50863593	-1.1572807	0.25239457	0.99978775	-4.5727953
MYO18A	-0.2261687	6.22697217	-1.4174505	0.16225739	0.99978775	-4.4463748
HOOK1	-0.2263295	2.46021771	-1.0482254	0.29933523	0.99978775	-4.5951417
ENSCAFG00000016965	-0.2264444	1.59214989	-0.4857962	0.62913256	0.99978775	-4.6382123

ZNF319	-0.2264585	2.26061003	-0.8406028	0.40438165	0.99978775	-4.6243624
SRXN1	-0.2264841	2.66390456	-0.9709989	0.33600006	0.99978775	-4.611451
CUTC	-0.2265108	1.61957512	-1.122006	0.26696397	0.99978775	-4.5840922
OSCP1	-0.2266212	2.65348967	-0.8631777	0.39196493	0.99978775	-4.6270115
ENSCAFG00000002828	-0.2267502	5.2495218	-2.2587431	0.02807791	0.99978775	-3.8447354
DTX3L	-0.2268104	3.23234668	-0.6218665	0.53672022	0.99978775	-4.6734412
RBM39	-0.2272493	6.81669319	-1.9685332	0.05429524	0.99978775	-4.051347
RRBP1	-0.2272733	9.36355574	-1.6949854	0.0960008	0.99978775	-4.3020947
H2AFX	-0.2273335	1.93201588	-0.7847174	0.43614503	0.99978775	-4.6374268
SLIT2	-0.2273979	5.52233809	-0.3277223	0.7444246	0.99978775	-4.8273037
ENSCAFG00000013515	-0.2275066	5.52837117	-1.5762722	0.12096875	0.99978775	-4.3505168
TIGD5	-0.2275148	3.09768196	-1.1573716	0.25235776	0.99978775	-4.5720561
IRF2BPL	-0.2275413	6.16361924	-1.2867652	0.20381957	0.99978775	-4.5235717
ENSCAFG00000000033	-0.2277505	1.1691067	-1.130623	0.2633511	0.99978775	-4.5832819
CDK20	-0.2277852	3.46365313	-1.2466768	0.21804812	0.99978775	-4.542929
TNFRSF6B	-0.2278862	1.53363529	-0.5954806	0.55408	0.99978775	-4.6284028
OGFOD1	-0.2280609	4.35279397	-1.8062338	0.07661377	0.99978775	-4.2526297
HMG20B	-0.2280729	4.64440084	-1.7718518	0.08222091	0.99978775	-4.258578
SPOCK1	-0.2281929	-1.661037	-0.2714985	0.78707211	0.99978775	-4.6068406
SH3BP5	-0.2282946	5.55220422	-0.9870774	0.32813092	0.99978775	-4.6657105
INF2	-0.2283723	6.58757229	-1.4565123	0.15120439	0.99978775	-4.4219408
DHCR7	-0.2283881	6.28825722	-1.1567243	0.25261986	0.99978775	-4.5928408
ENSCAFG00000029903	-0.2285616	-0.6455266	-0.5244014	0.60220602	0.99978775	-4.614287
ENSCAFG00000015869	-0.2287539	-0.2629923	-0.6181778	0.53913011	0.99978775	-4.6130765
NEMP1	-0.2290097	5.33692198	-1.6531047	0.10427633	0.99978775	-4.3084077
RAI14	-0.2292658	7.73395509	-0.9825503	0.33033402	0.99978775	-4.6711886
IL33	-0.2293468	2.14652763	-0.1354937	0.89273962	0.99978775	-4.6207203
SHROOM1	-0.2293782	3.1614383	-1.3321537	0.18856221	0.99978775	-4.5113099
MRPL15	-0.2295354	4.07916943	-1.6387381	0.10724606	0.99978775	-4.3573741
PLEKHA8	-0.2296025	4.02247834	-1.5864695	0.11863655	0.99978775	-4.3853831
ANXA5	-0.229704	8.83048103	-1.9689335	0.05424796	0.99978775	-4.0876935
CDK10	-0.2299244	3.29392553	-1.6349145	0.10804795	0.99978775	-4.4141666
ENSCAFG00000001559	-0.2299644	-1.1634435	-0.6543271	0.51575658	0.99978775	-4.6171117
MAZ	-0.2303409	4.65251962	-1.5840451	0.11918773	0.99978775	-4.3527906
CDR2L	-0.2304942	6.98076363	-1.6019702	0.11516105	0.99978775	-4.3267519
JUNB	-0.2305505	6.92248632	-0.8288014	0.41096805	0.99978775	-4.7396462
LEO1	-0.2306329	4.39299471	-1.5486047	0.1274827	0.99978775	-4.3912554
AVPR1A	-0.2307569	0.66512271	-0.0655525	0.94798301	0.99978775	-4.6076286
TMEM143	-0.230908	2.95690361	-1.3429891	0.1850513	0.99978775	-4.5309774
P2RY11	-0.2309206	4.69370618	-1.9606739	0.05523088	0.99978775	-4.1176898
ANO8	-0.2309222	3.9380031	-1.3627852	0.17876575	0.99978775	-4.4993917
ENSCAFG00000012933	-0.2310471	2.03702332	-0.894046	0.3753765	0.99978775	-4.6148647
GADD45A	-0.231169	5.76250786	-1.2285748	0.22470869	0.99978775	-4.5552006
ENSCAFG00000019360	-0.2313249	4.7510099	-1.6205962	0.11109426	0.99978775	-4.3366577
L3HYPDH	-0.2314996	3.12557439	-1.016359	0.31411828	0.99978775	-4.6052845
JUND	-0.2317753	5.48230974	-0.9542617	0.34432328	0.99978775	-4.6808128
TYW5	-0.2318828	3.10885566	-1.2416164	0.21989524	0.99978775	-4.5476325
SETMAR	-0.2319711	1.52937572	-0.6578303	0.51352058	0.99978775	-4.623806
WDPCP	-0.2322192	0.42928544	-0.7255154	0.47135681	0.99978775	-4.6154347
NSD1	-0.2322916	5.47260594	-1.8729448	0.06664705	0.99978775	-4.1317527

LNX2	-0.2323015	4.25869073	-1.5537131	0.12625932	0.99978775	-4.3869761
SCARB1	-0.2324324	6.46553959	-1.1990239	0.23590111	0.99978775	-4.5708657
RARG	-0.2326464	4.13471623	-0.9040382	0.37010378	0.99978775	-4.678188
ANKRD11	-0.2327855	7.61971844	-2.452057	0.01756383	0.99978775	-3.6574521
POP1	-0.2329156	3.92462477	-1.3444971	0.18456665	0.99978775	-4.4972349
C1QTNF2	-0.2329287	3.25477773	-0.9447751	0.34910046	0.99978775	-4.6331981
SDCCAG8	-0.2330121	3.31866193	-1.415574	0.16280377	0.99978775	-4.4906444
SREK1	-0.23324	5.54894551	-1.8071152	0.07647435	0.99978775	-4.1954305
ENSCAFG00000024028	-0.2332915	2.2814571	-0.7667953	0.44663681	0.99978775	-4.6263823
KLHL42	-0.2333019	4.71737915	-2.1694159	0.03459847	0.99978775	-3.9699839
ENSCAFG00000012703	-0.2333117	1.12600768	-0.7615166	0.44975498	0.99978775	-4.6142819
BBS9	-0.2333308	3.82396481	-1.6183734	0.11157337	0.99978775	-4.3940059
TPD52L2	-0.2333463	5.32933363	-2.6340471	0.01106085	0.99978775	-3.5053619
ATP23	-0.2337032	2.4115919	-1.1460528	0.25696886	0.99978775	-4.5773968
PCNT	-0.2337187	6.33412402	-3.15399	0.00266354	0.99978775	-2.8957385
ENSCAFG00000030118	-0.2338096	2.52777006	-1.225248	0.22594886	0.99978775	-4.558578
ENSCAFG00000002619	-0.2338739	1.02860724	-0.9067165	0.36869853	0.99978775	-4.6042149
MMAA	-0.2341033	2.18077311	-1.3099398	0.19591764	0.99978775	-4.5503943
MON1A	-0.2342077	3.84826701	-1.8301324	0.07290818	0.99978775	-4.2794498
RARRES2	-0.2343401	1.80844145	-0.1916491	0.84875615	0.99978775	-4.6188363
TTI1	-0.2345564	3.86710618	-2.5965247	0.01218685	0.99978775	-3.8403821
EMP1	-0.2345711	10.3635437	-1.4007018	0.16718466	0.99978775	-4.4700212
KLK4	-0.2347304	-0.6484782	-0.4564159	0.64997304	0.99978775	-4.6132065
CLEC3B	-0.234743	0.98037806	-0.1400751	0.88913656	0.99978775	-4.6241529
SENP8	-0.2347519	1.63744905	-1.1159657	0.26951728	0.99978775	-4.5846404
TMEM8B	-0.2347774	1.94848635	-1.1596108	0.25145263	0.99978775	-4.5776001
ENSCAFG00000030810	-0.2348311	6.03494983	-1.1222383	0.26686609	0.99978775	-4.6071112
TSHZ1	-0.2350571	5.17338793	-1.1327409	0.26246847	0.99978775	-4.5984531
CENPJ	-0.2351397	3.59562333	-0.9787937	0.33216965	0.99978775	-4.6325593
SECISBP2	-0.2351544	4.94117641	-2.8905257	0.00558066	0.99978775	-3.3306096
SPIDR	-0.235735	3.65448489	-1.6093383	0.11353811	0.99978775	-4.4021823
PROCR	-0.2358393	3.19028218	-0.4018784	0.68940449	0.99978775	-4.6455827
STX18	-0.2361109	4.14383231	-1.696975	0.09562152	0.99978775	-4.3291688
PPP1R14A	-0.2361227	-1.1816139	-0.4844944	0.63004968	0.99978775	-4.6099698
TNIP2	-0.2364619	2.48626962	-1.611327	0.11310328	0.99978775	-4.4669479
B3GALT6	-0.2365671	1.63177968	-0.8932166	0.37581632	0.99978775	-4.6106026
NOP14	-0.2366261	6.13058916	-2.0820942	0.04222277	0.99978775	-3.9576133
RIT1	-0.2366594	3.56839861	-1.6172486	0.11181646	0.99978775	-4.4017713
RFX5	-0.2367026	3.23379465	-1.2580592	0.21393544	0.99978775	-4.5469883
TXNRD3	-0.2367034	4.3116265	-1.0030729	0.32042537	0.99978775	-4.6244172
VWF	-0.2367574	0.51666074	-0.0939411	0.92551343	0.99978775	-4.6172964
NDUFAF8	-0.236817	-0.0267353	-0.5434742	0.5891031	0.99978775	-4.6153398
CREB3L1	-0.2368744	7.54885651	-0.9385209	0.35227347	0.99978775	-4.6804345
TNFAIP2	-0.2369173	3.46094956	-0.4717318	0.63907239	0.99978775	-4.6924877
MAP3K6	-0.2369291	3.29964783	-1.4592217	0.15046017	0.99978775	-4.4626871
PHKA2	-0.2369621	3.66594016	-0.9147971	0.36447962	0.99978775	-4.6443407
ENSCAFG00000018145	-0.2369703	2.48296972	-0.8907297	0.377137	0.99978775	-4.6227226
RPS6KA4	-0.2370176	5.05835719	-2.0297393	0.04746358	0.99978775	-4.0330618
CSK	-0.2374023	5.46248635	-2.8247945	0.00667335	0.99978775	-3.3136106
HAGHL	-0.2375946	2.67456549	-1.2393736	0.22071755	0.99978775	-4.5552901

RHBDL3	-0.2376845	-1.7665823	-0.3933501	0.69565333	0.99978775	-4.605841
SOCS2	-0.2378372	2.37326511	-0.9633095	0.33980724	0.99978775	-4.6211554
CREB3L4	-0.2379269	0.35306927	-0.5934565	0.55542324	0.99978775	-4.6198517
BAIAP2	-0.2380561	4.70224385	-1.192668	0.23836057	0.99978775	-4.5628495
WNT2	-0.2380911	2.93770673	-0.3785438	0.70655274	0.99978775	-4.6893066
FLVCR1	-0.2381196	4.0680137	-1.1983785	0.23614999	0.99978775	-4.5593093
SLC20A1	-0.2381723	8.74847637	-0.927456	0.35793298	0.99978775	-4.684825
PMVK	-0.2383946	2.28787704	-1.1926635	0.2383623	0.99978775	-4.5715938
GYPC	-0.2384809	-0.1576448	-0.249846	0.8036812	0.99978775	-4.6171302
RND1	-0.2384897	0.04799534	-0.5773785	0.5661511	0.99978775	-4.6149869
COL6A1	-0.2385094	11.1176766	-0.640497	0.5246346	0.99978775	-4.7199506
ENSCAFG00000031903	-0.2385875	3.58363564	-2.054065	0.04496232	0.99978775	-4.2001098
ABL2	-0.2386873	4.23949364	-1.8465133	0.07045636	0.99978775	-4.2246264
FAM122B	-0.2386987	3.4899919	-1.7263603	0.09016197	0.99978775	-4.3276677
STK11	-0.2387326	5.78537416	-2.0975168	0.04077823	0.99978775	-3.9566346
PDE7A	-0.2389195	0.29011626	-0.5854102	0.56077925	0.99978775	-4.6146685
RNF215	-0.2390529	2.72362009	-1.236257	0.221864	0.99978775	-4.5570366
MSH2	-0.2392298	4.10503201	-1.1572314	0.25241452	0.99978775	-4.5773415
ENSCAFG00000000687	-0.2392624	-0.5051318	-0.5008087	0.61859844	0.99978775	-4.6091013
CROCC	-0.2392842	2.23475877	-1.0989229	0.27681436	0.99978775	-4.5858614
SNX29	-0.2393744	4.57081467	-1.7871902	0.0796785	0.99978775	-4.2293643
ZNF689	-0.2395476	1.84359435	-0.9997453	0.3220183	0.99978775	-4.5991485
PI15	-0.2395869	2.47770477	-0.5647682	0.57463613	0.99978775	-4.6953153
BHLHE40	-0.2397419	8.36933735	-0.9231458	0.36015345	0.99978775	-4.6802934
CPLX1	-0.2398272	1.17597479	-0.9959324	0.32385002	0.99978775	-4.5981946
MRPL41	-0.2399029	2.81678068	-1.2293312	0.22442741	0.99978775	-4.552495
ACTR5	-0.2399836	2.39374421	-1.1662959	0.24876425	0.99978775	-4.5750724
SDC1	-0.2407125	5.55387874	-0.8282919	0.41125386	0.99978775	-4.739997
CELSR1	-0.2408285	-0.7619613	-0.3234345	0.74765066	0.99978775	-4.6149331
CRADD	-0.2408382	2.08295576	-0.6864616	0.49544207	0.99978775	-4.6296853
IKZF2	-0.2409091	0.92646313	-0.4187914	0.67707656	0.99978775	-4.6219184
C1QTNF6	-0.2409263	5.34535882	-1.1317842	0.26286689	0.99978775	-4.6058433
GRAMD4	-0.2409452	4.01828102	-1.2737277	0.20836867	0.99978775	-4.5313362
MOGS	-0.2411118	4.04462435	-1.8487518	0.07012677	0.99978775	-4.2377841
TMEM268	-0.241132	3.95271305	-1.6406922	0.10683812	0.99978775	-4.3525337
LRRTM2	-0.2411676	2.26471713	-0.2769137	0.78293348	0.99978775	-4.6307402
ENSCAFG00000003752	-0.2414666	-0.2958707	-0.8346454	0.40769831	0.99978775	-4.6310893
ENSCAFG00000030667	-0.2415896	0.82178004	-0.7393323	0.46299733	0.99978775	-4.612615
EIF1B	-0.2417006	3.16950216	-1.9371003	0.05812066	0.99978775	-4.2925486
ENSCAFG00000009910	-0.2418585	7.08545099	-1.6248246	0.11018747	0.99978775	-4.3119859
IFIT2	-0.2418637	-2.3913403	-0.4169558	0.67841029	0.99978775	-4.6061157
ARL5B	-0.2420318	2.04574887	-1.0315051	0.30703131	0.99978775	-4.5957597
MMP19	-0.2424666	4.8685653	-0.8118055	0.42056778	0.99978775	-4.6853069
FBL	-0.2425022	6.33485077	-1.3644577	0.17824228	0.99978775	-4.478522
GLS	-0.2425729	6.889966	-1.7285345	0.08976848	0.99978775	-4.2401237
LPAR6	-0.2426246	-0.9731942	-0.4148469	0.67994387	0.99978775	-4.6095688
EFCAB6	-0.2426577	-0.4510961	-0.4358774	0.66471226	0.99978775	-4.6152357
TNFRSF14	-0.2428696	2.02291801	-0.7007559	0.48654881	0.99978775	-4.629242
ENSCAFG00000002216	-0.2432146	4.12647439	-1.8694465	0.06714099	0.99978775	-4.2318919
THAP2	-0.2432783	1.12950803	-0.871968	0.38719512	0.99978775	-4.6063807

HAUS7	-0.2433006	2.2996031	-0.9222628	0.36060944	0.99978775	-4.6229133
PLEKHG1	-0.2434004	1.98880093	-0.6020841	0.54970902	0.99978775	-4.6661136
ZNF502	-0.2436373	2.2870573	-1.4389062	0.156111	0.99978775	-4.5227204
SLCO2B1	-0.243721	-2.8782709	-0.4237317	0.67349211	0.99978775	-4.605298
FLAD1	-0.2439037	5.20611688	-1.8177448	0.07480967	0.99978775	-4.2024857
SLC6A17	-0.2441753	5.1451757	-1.5760321	0.12102412	0.99978775	-4.3748354
ZNF146	-0.2442887	4.18652626	-1.8551837	0.069187	0.99978775	-4.2346061
OTUD3	-0.2445721	4.03978476	-1.2750479	0.2079046	0.99978775	-4.5293429
ARHGEF10	-0.2445933	5.96519942	-2.0661715	0.04376067	0.99978775	-3.9788756
GCDH	-0.2446737	2.90574607	-1.1875212	0.24036574	0.99978775	-4.5659723
MFSD3	-0.2447903	3.39315642	-0.8496186	0.39939398	0.99978775	-4.6516467
CTH	-0.2448894	3.35477546	-0.8786271	0.38360604	0.99978775	-4.6511736
TARDBP	-0.2450736	4.83382894	-2.985979	0.00428662	0.99978775	-3.227036
INTS3	-0.2451514	6.14907871	-2.7682364	0.00776853	0.99978775	-3.3180574
ECE2	-0.2453275	2.51285392	-1.1444294	0.25763508	0.99978775	-4.5768884
FEM1C	-0.2456775	4.07290305	-1.2178711	0.22871679	0.99978775	-4.5520138
NECTIN3	-0.2458131	5.09894505	-1.1178742	0.26870868	0.99978775	-4.6082913
PIGO	-0.2460306	5.82884089	-2.2533092	0.028441	0.99978775	-3.8232608
ENSCAFG00000009915	-0.2460602	0.12473576	-0.8373491	0.40619102	0.99978775	-4.6055974
RBP1	-0.2460953	-2.3496512	-0.3799152	0.70554054	0.99978775	-4.6060728
DNAJC30	-0.2462149	1.56536441	-0.7219962	0.47349957	0.99978775	-4.6204043
FSCN1	-0.2462152	8.00763656	-1.170289	0.2471683	0.99978775	-4.5821895
ENSCAFG00000032688	-0.2463125	1.46564125	-1.0248193	0.31014612	0.99978775	-4.5951837
NDC1	-0.2464082	5.44486356	-1.4718152	0.14703857	0.99978775	-4.4144664
SH2B3	-0.2465032	6.54982222	-1.5704039	0.12232751	0.99978775	-4.3484166
USP1	-0.2465513	5.57433051	-1.2988837	0.19965821	0.99978775	-4.5164393
IFT172	-0.2467941	4.67108569	-1.3796966	0.17352666	0.99978775	-4.479762
ITGA10	-0.2468415	3.50549859	-0.2907305	0.77240256	0.99978775	-4.645482
PDGFRB	-0.2469161	8.54252208	-0.6359541	0.52756828	0.99978775	-4.78009
DHX34	-0.2469648	4.06896085	-1.7298993	0.0895222	0.99978775	-4.3204808
ENSCAFG00000013990	-0.2473099	-0.646353	-0.7664487	0.44684115	0.99978775	-4.6072538
MAN1C1	-0.2473576	3.08858153	-0.4365895	0.66419893	0.99978775	-4.6783271
ENSCAFG00000030283	-0.2475282	-0.664724	-0.5023976	0.61748817	0.99978775	-4.6099183
COLEC12	-0.2476911	0.39624789	-0.1413512	0.88813335	0.99978775	-4.6139344
TRUB2	-0.247949	3.79231853	-1.7722232	0.08215855	0.99978775	-4.2997194
ENSCAFG00000032152	-0.2481253	5.64112667	-1.4333979	0.15767136	0.99978775	-4.4386092
ENDOG	-0.2484029	1.11372064	-0.8437549	0.4026335	0.99978775	-4.6064008
ENSCAFG00000028627	-0.2484065	2.55177132	-1.0379343	0.30405625	0.99978775	-4.5963418
IQCK	-0.2486143	0.87987494	-0.7618081	0.44958242	0.99978775	-4.6149857
VPS9D1	-0.248633	3.0812108	-1.2936908	0.2014335	0.99978775	-4.5373139
ADCK1	-0.2486483	1.6152187	-1.2759404	0.20759133	0.99978775	-4.5588446
ENSCAFG00000018000	-0.2487548	4.16515703	-2.14038	0.03698757	0.99978775	-4.064389
GATB	-0.2488291	2.20116697	-1.5466376	0.12795634	0.99978775	-4.4923617
FADS1	-0.2488387	7.71206618	-1.0778485	0.28602764	0.99978775	-4.6290493
APRT	-0.2489426	3.8357184	-1.2618137	0.21259159	0.99978775	-4.5364237
ARHGAP24	-0.2490756	2.07704339	-0.3970496	0.69293998	0.99978775	-4.6567244
CHCHD10	-0.2491663	3.10037194	-0.7511845	0.45589483	0.99978775	-4.6699297
ENSCAFG00000028871	-0.249337	-1.0674847	-0.3211837	0.74934599	0.99978775	-4.6147363
NHLRC1	-0.2493892	0.37140767	-0.6866872	0.49530101	0.99978775	-4.6144349
PLEK	-0.2494697	-1.0092032	-0.3957642	0.69388229	0.99978775	-4.6094113

PRR5	-0.2496109	1.29637247	-0.517017	0.6073151	0.99978775	-4.6257488
DNAAF3	-0.2496471	1.42319735	-0.6083511	0.54557699	0.99978775	-4.6227291
LRRN4CL	-0.2496572	4.57835753	-0.3645158	0.71693666	0.99978775	-4.7443668
PPARG	-0.2496649	-2.2013405	-0.2977076	0.76710092	0.99978775	-4.6067094
KLF3	-0.2498328	6.00996465	-1.5304919	0.13189712	0.99978775	-4.3760041
GLIPR2	-0.2498501	6.86134228	-1.1547306	0.25342831	0.99978775	-4.5948298
QSOX2	-0.2499174	5.10489371	-2.0843684	0.042007	0.99978775	-4.0067199
ZNF584	-0.2499235	1.28094336	-0.8757265	0.38516677	0.99978775	-4.6069246
PIM1	-0.2505268	4.07124167	-1.4318136	0.15812237	0.99978775	-4.4402296
ITGB3BP	-0.2505569	0.62661735	-0.792548	0.43160714	0.99978775	-4.6110381
ENSCAFG00000017892	-0.2505691	6.51728383	-1.3195482	0.19270999	0.99978775	-4.5049197
MCM8	-0.2507508	3.01826467	-1.5015182	0.13921095	0.99978775	-4.4762615
GIPC3	-0.2507711	-0.8297826	-0.4729139	0.63823434	0.99978775	-4.6131973
ENSCAFG00000016098	-0.2507834	0.25550451	-0.542166	0.58999748	0.99978775	-4.6224973
MAML3	-0.2508609	-1.3549592	-0.4649323	0.64390208	0.99978775	-4.6063327
POLR1D	-0.2508795	3.96809113	-1.9379166	0.05801846	0.99978775	-4.2174238
NEFH	-0.2509875	-1.4460486	-0.3862098	0.70090161	0.99978775	-4.6075128
DVL1	-0.2509901	5.17216115	-2.4040926	0.01977468	0.99978775	-3.7205069
SLC25A43	-0.2510903	3.20374486	-0.9729285	0.33504912	0.99978775	-4.6127402
ENSCAFG00000030092	-0.2513587	-1.4610266	-0.5008434	0.61857413	0.99978775	-4.609964
ENSCAFG00000014799	-0.2513862	0.98324054	-0.2594843	0.79627612	0.99978775	-4.625122
ENSCAFG00000031808	-0.2514725	4.44028941	-1.1021483	0.27542284	0.99978775	-4.6004254
CYP1A1	-0.2515006	0.29385167	-0.5738396	0.56852604	0.99978775	-4.645772
CTSD	-0.2515297	8.49077369	-1.2039827	0.23399514	0.99978775	-4.5632321
ZNF862	-0.2520847	3.99413742	-1.9585044	0.05549159	0.99978775	-4.1955663
KIAA1549	-0.2521531	2.57052292	-1.1132485	0.27067149	0.99978775	-4.5830632
TGM1	-0.2522563	0.1342025	-0.7378424	0.46389464	0.99978775	-4.6084491
ENSCAFG00000011124	-0.2523875	-0.5009396	-0.5583348	0.57898866	0.99978775	-4.6129663
HIRIP3	-0.2525452	4.07486725	-1.6467781	0.10557572	0.99978775	-4.3587209
MTRF1L	-0.2525541	2.09149144	-1.284282	0.20468023	0.99978775	-4.5520423
ARHGAP26	-0.2526223	0.46059635	-0.5780758	0.56568371	0.99978775	-4.6153455
TSC22D3	-0.2526306	6.01667717	-1.5462419	0.12805177	0.99978775	-4.3771756
ENSCAFG00000023746	-0.2526464	3.01167154	-1.5823518	0.11957391	0.99978775	-4.4515731
KIFC2	-0.2527073	4.26423307	-1.3841136	0.17217792	0.99978775	-4.4880711
SLC25A33	-0.2528731	3.32256943	-1.3955276	0.16873001	0.99978775	-4.4957175
ZFP14	-0.2530577	2.44111205	-1.1766081	0.24465786	0.99978775	-4.5694809
ENSCAFG00000002078	-0.2530673	1.27751136	-0.2870456	0.77520698	0.99978775	-4.6324131
PTGDR	-0.2531578	3.69936316	-0.4577294	0.64903513	0.99978775	-4.6770393
SPATA5L1	-0.2532791	3.04046907	-1.5757678	0.12108506	0.99978775	-4.4500991
ENSCAFG00000004177	-0.253488	2.90223577	-1.8298841	0.07294589	0.99978775	-4.3775692
LRRC45	-0.2535709	4.32144673	-1.5669532	0.12313222	0.99978775	-4.390242
SLC25A42	-0.2536887	1.44986483	-0.9118791	0.36599952	0.99978775	-4.6055969
KLHDC9	-0.2537026	-0.7516143	-0.5523694	0.58303877	0.99978775	-4.6112091
PDK4	-0.2537899	3.11916294	-0.1393965	0.88967009	0.99978775	-4.6153115
AKAP6	-0.2538693	-1.0559573	-0.3632162	0.71790136	0.99978775	-4.610401
PRKRIP1	-0.2541237	3.03462094	-2.3065765	0.02505491	0.99978775	-4.1495049
RUFY1	-0.2542683	4.25975656	-1.6442859	0.10609119	0.99978775	-4.3441349
IRAK1BP1	-0.2545315	1.135397	-0.9104614	0.36673939	0.99978775	-4.6037071
BCAT1	-0.2545519	6.03790505	-1.4113514	0.16403847	0.99978775	-4.4507884
PCDH18	-0.2546322	6.20310367	-0.8852347	0.38006553	0.99978775	-4.7178966

CRYL1	-0.2547701	4.01815836	-1.1289984	0.26402957	0.99978775	-4.5851197
PDGFA	-0.2548794	5.43523877	-0.8081609	0.42264387	0.99978775	-4.7465133
LRP1B	-0.2549167	-1.8349279	-0.4784188	0.63433798	0.99978775	-4.6065617
ST6GALNAC2	-0.2549328	-1.6169704	-0.3480499	0.72919407	0.99978775	-4.6076689
PDCD2L	-0.2550909	3.87722264	-2.6136647	0.01166013	0.99978775	-3.7545118
WRAP53	-0.2552527	3.87846704	-1.7479107	0.08632422	0.99978775	-4.312608
PPM1F	-0.2552659	3.89124871	-0.7367628	0.46454547	0.99978775	-4.6896525
DEK	-0.2553886	3.80652642	-1.3800439	0.17342034	0.99978775	-4.4894597
ENSCAFG00000006763	-0.2554341	-2.3306515	-0.3076652	0.759554	0.99978775	-4.6060243
ENSCAFG00000009225	-0.255464	2.02838789	-1.3426753	0.18515226	0.99978775	-4.5328171
MPPED2	-0.2556095	-1.6177844	-0.5155851	0.60830811	0.99978775	-4.6118288
AACS	-0.2556873	5.60679649	-1.4733324	0.14663055	0.99978775	-4.4144857
CCDC77	-0.2557455	1.45797895	-0.99579	0.32391858	0.99978775	-4.5989736
TP53I3	-0.2559556	1.37126911	-0.9923987	0.32555386	0.99978775	-4.5980683
MLLT11	-0.2560335	2.00360011	-0.4769225	0.63539605	0.99978775	-4.6527336
FIGNL1	-0.2561086	2.45716355	-1.5653528	0.12350686	0.99978775	-4.4588911
FAM118B	-0.2562225	3.26529172	-1.525528	0.13312797	0.99978775	-4.4508805
NIF3L1	-0.2562504	3.3068121	-1.3954776	0.16874499	0.99978775	-4.4962413
ZFHX4	-0.2562995	4.41599922	-0.5498557	0.58474941	0.99978775	-4.7966815
SAMM50	-0.2563449	6.35115118	-2.1550738	0.03576106	0.99978775	-3.8959129
DGKZ	-0.2564452	6.23339404	-2.1590236	0.03543751	0.99978775	-3.8903037
NLRX1	-0.2566586	4.7211342	-2.2618881	0.02786963	0.99978775	-3.8918084
USHBP1	-0.2568423	-2.5091245	-0.3689033	0.71368302	0.99978775	-4.6055237
NFE2L3	-0.2571222	-0.1178528	-0.4553222	0.65075444	0.99978775	-4.6164278
PRR14	-0.257151	4.78926676	-2.2366454	0.02958057	0.99978775	-3.9021437
RNF5	-0.2572361	3.67495683	-1.926054	0.05951864	0.99978775	-4.2350476
GJC1	-0.2573276	1.9274373	-0.9456423	0.34866196	0.99978775	-4.6129407
GRAMD2B	-0.2575177	5.08865301	-1.4111631	0.1640937	0.99978775	-4.4563598
DNAJC17	-0.2576016	1.94325935	-1.3641191	0.17834817	0.99978775	-4.5408213
RFC2	-0.257721	3.78584673	-1.4646733	0.14897142	0.99978775	-4.4461853
IDH1	-0.2577678	4.96179142	-1.2757081	0.20767285	0.99978775	-4.526733
GUF1	-0.2577682	2.80798012	-1.7648336	0.08340657	0.99978775	-4.3878405
BTG1	-0.2577845	4.2023154	-1.0419748	0.30219667	0.99978775	-4.6092975
TRPM4	-0.2580437	3.20372798	-1.262712	0.212271	0.99978775	-4.5424777
CUBN	-0.2580987	4.03999697	-0.6993397	0.4874259	0.99978775	-4.6951318
LRTOMT	-0.2583408	1.68342952	-1.0773345	0.28625498	0.99978775	-4.5895254
ALKBH2	-0.2583415	1.1087255	-1.1538584	0.2537826	0.99978775	-4.583881
HELZ2	-0.2584608	3.81092917	-0.9810999	0.33104193	0.99978775	-4.6255261
SEL1L3	-0.2584711	0.72357999	-0.6716099	0.5047759	0.99978775	-4.6369141
ZNF76	-0.2585883	4.05733037	-1.9402105	0.05773214	0.99978775	-4.185447
SLC27A3	-0.2588997	3.1030235	-1.0856989	0.282571	0.99978775	-4.5901578
NSD2	-0.2589161	6.02834709	-1.2921595	0.20195927	0.99978775	-4.5205923
SLC35C2	-0.2589226	4.80496307	-1.8536055	0.0694166	0.99978775	-4.1834297
CFAP299	-0.2589413	1.05453903	-0.7210368	0.47408469	0.99978775	-4.6451974
OIP5	-0.2591254	-0.9170596	-0.5454205	0.5877736	0.99978775	-4.6185009
UHRF2	-0.2591642	4.24869982	-1.6229992	0.1105782	0.99978775	-4.3806866
RUNX1T1	-0.2592099	5.3013215	-0.9239415	0.35974284	0.99978775	-4.6930851
SGSH	-0.2592594	5.26703999	-0.775244	0.44167251	0.99978775	-4.7378436
INPP5E	-0.2593908	3.30271725	-1.1720095	0.24648294	0.99978775	-4.5681959
MTFR2	-0.259623	3.52720247	-1.3702069	0.17645182	0.99978775	-4.4867752

ENSCAFG00000032163	-0.2599006	0.16558932	-0.7055086	0.48361164	0.99978775	-4.6207502
DDIT3	-0.260248	4.3224317	-1.4325615	0.15790934	0.99978775	-4.4484903
CAPG	-0.2607996	9.34559939	-1.1720514	0.24646627	0.99978775	-4.5729141
FOXO4	-0.260891	3.04777079	-1.2151775	0.22973361	0.99978775	-4.560197
TRIB2	-0.2609904	4.96691331	-0.6205546	0.53757668	0.99978775	-4.6961621
ENSCAFG00000031693	-0.2612004	1.7049538	-0.8877836	0.37870532	0.99978775	-4.6090534
USP27X	-0.2618467	0.70103479	-0.9031643	0.37056302	0.99978775	-4.6030834
ENSCAFG00000031730	-0.261935	2.58880692	-1.2592933	0.21349305	0.99978775	-4.5505091
TAF5	-0.2620195	1.66829808	-1.0891495	0.28106092	0.99978775	-4.5882943
IRF1	-0.2624187	5.05051633	-1.3236696	0.19134632	0.99978775	-4.5038301
FBF1	-0.2624915	2.76174151	-1.1560186	0.25290581	0.99978775	-4.5741278
ACLY	-0.2624968	8.7160456	-1.4195416	0.1616502	0.99978775	-4.4491334
LMNA	-0.2625555	9.85301017	-1.8324194	0.07256162	0.99978775	-4.2320737
PGGT1B	-0.2627193	2.65076316	-1.4807969	0.14463591	0.99978775	-4.4879858
ARAP3	-0.2628855	5.38190667	-1.0571214	0.29529495	0.99978775	-4.6345791
CAD	-0.2631113	7.47998122	-2.2542236	0.02837961	0.99978775	-3.8289262
SPHK2	-0.2631859	4.19748494	-1.901139	0.06277747	0.99978775	-4.1752084
SAMD1	-0.2635013	3.7915128	-1.4572353	0.15100551	0.99978775	-4.4616632
FAM199X	-0.2636663	1.85832009	-1.0134253	0.31550365	0.99978775	-4.5970717
SFXN4	-0.2637379	2.37843753	-1.1677659	0.24817583	0.99978775	-4.572764
MID2	-0.2639659	4.64839114	-1.1072497	0.27323201	0.99978775	-4.5913967
PASK	-0.2640692	2.27971751	-1.392865	0.16952952	0.99978775	-4.5250907
ENSCAFG00000032623	-0.2644419	2.14065415	-1.2972203	0.20022558	0.99978775	-4.5510014
PIWIL4	-0.2644485	0.9314054	-1.1178301	0.26872734	0.99978775	-4.583451
GALNT15	-0.2644604	1.80698786	-0.0744949	0.94089968	0.99978775	-4.61374
PCGF1	-0.264462	3.43622999	-1.2778484	0.20692277	0.99978775	-4.5345341
SNX8	-0.2645596	5.84675219	-2.3750262	0.02123313	0.99978775	-3.699579
C1H19orf47	-0.2645986	3.2635935	-2.4998208	0.01558622	0.99978775	-4.0241876
HOXA13	-0.2648394	-1.4962939	-0.4075263	0.68527805	0.99978775	-4.6162168
LRRC46	-0.2653485	2.09669157	-0.9678904	0.33753572	0.99978775	-4.6027679
MAP3K20	-0.2654035	4.6670574	-1.3281679	0.1898663	0.99978775	-4.5022494
VSIG10	-0.2657889	5.33715598	-2.1068057	0.03992915	0.99978775	-3.9432533
ENSCAFG00000016577	-0.2658503	3.02977079	-1.3538664	0.18157707	0.99978775	-4.4980833
VWA5A	-0.2659802	0.02830419	-0.2907724	0.77237067	0.99978775	-4.6161452
CTBP2	-0.2660908	5.15002544	-2.2538162	0.02840695	0.99978775	-3.8887829
RWDD3	-0.2663928	2.22131611	-1.432411	0.1579522	0.99978775	-4.5183388
SLC25A19	-0.2665187	3.49959702	-1.347922	0.1834695	0.99978775	-4.506167
INPP1	-0.2666873	5.00992151	-2.3577035	0.02214748	0.99978775	-3.8008527
XYLB	-0.2668976	1.95047434	-1.165175	0.24921355	0.99978775	-4.5753161
MVK	-0.2668977	4.48886867	-1.3867855	0.17136598	0.99978775	-4.4744791
SLC35E4	-0.2669251	2.7616628	-1.1602607	0.25119037	0.99978775	-4.573014
ZNF23	-0.2670031	3.62838081	-2.1232127	0.03846708	0.99978775	-4.1293434
ENSCAFG00000008716	-0.2670895	5.53598873	-1.260459	0.21307575	0.99978775	-4.5380031
ENSCAFG00000030366	-0.2673084	4.0586319	-1.8175981	0.07483243	0.99978775	-4.2685034
LTBP4	-0.2675315	5.5577178	-0.5175716	0.60693069	0.99978775	-4.8068213
COL1A2	-0.267777	13.6859788	-0.802051	0.42613812	0.99978775	-4.6475992
KAT2A	-0.2679128	4.81763988	-2.2928114	0.02589368	0.99978775	-3.8707124
TMEM97	-0.2680508	3.71999808	-0.861093	0.3931015	0.99978775	-4.6441064
HINT2	-0.2681479	2.56579915	-1.5655609	0.12345809	0.99978775	-4.4793735
GATA3	-0.2682092	0.78112251	-0.6088659	0.54523831	0.99978775	-4.6359849

ITGA2B	-0.2682462	-0.8548918	-0.6153521	0.54097986	0.99978775	-4.6083039
C15H12orf73	-0.2683245	1.5897415	-1.1658023	0.24896203	0.99978775	-4.5781648
CARD10	-0.268576	3.59451803	-0.6224725	0.53632484	0.99978775	-4.6824976
SIX1	-0.2687981	4.02359944	-0.5790021	0.56506312	0.99978775	-4.7283017
PCTP	-0.2690955	1.82624233	-1.4768132	0.14569774	0.99978775	-4.5218794
TMEM156	-0.2693551	-0.9392814	-0.3682302	0.71418182	0.99978775	-4.6106782
ENSCAFG00000013221	-0.2694803	-1.3678109	-0.5280948	0.59965811	0.99978775	-4.6125532
ERMARD	-0.2698998	3.27644348	-1.6916208	0.09664503	0.99978775	-4.3825736
ENSCAFG00000013321	-0.2699343	1.91628789	-0.9404027	0.35131677	0.99978775	-4.6047804
JADE2	-0.270128	4.8216867	-0.6385	0.52592317	0.99978775	-4.719914
ACP6	-0.2701473	2.57223597	-1.2870115	0.20373435	0.99978775	-4.5506058
LPIN1	-0.2701737	3.23148603	-0.8073395	0.42311261	0.99978775	-4.6318427
ENSCAFG00000028442	-0.270399	0.19390985	-0.5044775	0.61603613	0.99978775	-4.6281359
GIT1	-0.2705941	4.91416704	-2.2820503	0.0265667	0.99978775	-3.8741552
NDST3	-0.2708304	-0.6920199	-0.556049	0.58053894	0.99978775	-4.6193599
USP35	-0.270886	2.18265548	-1.3968531	0.1683331	0.99978775	-4.5318209
FAM189A2	-0.270975	-0.0680034	-0.3738159	0.71004632	0.99978775	-4.6196034
C20H19orf38	-0.2711298	-1.7348549	-0.4985087	0.62020718	0.99978775	-4.6049877
GTPBP6	-0.2717932	2.28131562	-1.6751197	0.09985627	0.99978775	-4.4646328
HAUS8	-0.271931	2.52165623	-1.3613921	0.17920267	0.99978775	-4.5126008
INTS13	-0.2720444	5.00273481	-1.5354577	0.13067491	0.99978775	-4.3900286
SMOX	-0.2724528	3.11546181	-1.0656915	0.29143839	0.99978775	-4.5913724
PTER	-0.2725878	1.66041977	-0.7653878	0.44746695	0.99978775	-4.6169631
ENSCAFG00000031865	-0.2726329	-0.1052852	-0.234286	0.81567397	0.99978775	-4.6132848
FDFT1	-0.2729303	6.33989262	-1.3880066	0.17099588	0.99978775	-4.4649599
ZCCHC8	-0.2730215	4.18111519	-1.9054295	0.06220569	0.99978775	-4.2046157
ZNF236	-0.2730975	3.4485869	-1.8168408	0.07495005	0.99978775	-4.3467301
ABCA7	-0.273141	0.40041002	-0.6401378	0.52486626	0.99978775	-4.6191127
CEBPB	-0.2732139	3.12674277	-0.9809057	0.33113682	0.99978775	-4.6208752
TRAIP	-0.2732471	2.62379817	-1.1958396	0.23713096	0.99978775	-4.5624864
APBA1	-0.2733406	0.0298347	-0.4960485	0.62192999	0.99978775	-4.6151871
CLBA1	-0.2736542	1.04247176	-0.9270686	0.35813217	0.99978775	-4.6024474
FARSA	-0.2736745	4.94810072	-2.0379453	0.04660667	0.99978775	-4.0409529
CDCA4	-0.2737766	2.40060869	-1.3193937	0.19276125	0.99978775	-4.5345637
GREM1	-0.2738504	7.33920337	-0.4736631	0.63770346	0.99978775	-4.8263814
MTHFR	-0.2739762	3.37944356	-1.1875543	0.24035279	0.99978775	-4.5636617
BTG2	-0.2741232	3.29584726	-0.9647957	0.33906917	0.99978775	-4.6276796
DLL4	-0.2742396	-1.7701092	-0.2230851	0.82433478	0.99978775	-4.606608
PDP2	-0.2743385	4.4004688	-1.9039502	0.06240233	0.99978775	-4.182754
SCRIB	-0.2743615	6.57003458	-2.5701857	0.01303864	0.99978775	-3.5110105
ABCC1	-0.2747646	4.65313114	-1.027519	0.30888579	0.99978775	-4.6311693
CCDC171	-0.2747677	0.4855636	-0.7726271	0.44320668	0.99978775	-4.6087631
RASGRP3	-0.2747845	-0.5413941	-0.2153679	0.83031476	0.99978775	-4.6128358
ENSCAFG00000008862	-0.2750573	-2.1077991	-0.4068762	0.6857525	0.99978775	-4.6081923
SLC25A14	-0.2750777	2.41410269	-1.1084127	0.27273428	0.99978775	-4.5840605
LYAR	-0.2750832	3.52572536	-1.2996953	0.1993818	0.99978775	-4.5265909
TSPAN15	-0.2751919	-2.482014	-0.3845266	0.70214099	0.99978775	-4.6054181
AGAP1	-0.2752634	4.25025002	-1.9786721	0.05310833	0.99978775	-4.1531811
ENO3	-0.2753814	2.8615036	-1.6660724	0.10165379	0.99978775	-4.4131326
ENSCAFG00000032330	-0.2753984	2.00210015	-1.2411461	0.22006748	0.99978775	-4.5634461

FAM161A	-0.2760366	2.7814026	-2.0167648	0.04884623	0.99978775	-4.2889194
HRAS	-0.2764853	3.56359545	-1.7920584	0.07888546	0.99978775	-4.326375
ANKS1B	-0.2765152	1.5528949	-0.5874561	0.55941494	0.99978775	-4.6243483
ARRB2	-0.2766403	2.83594653	-1.4903583	0.14211231	0.99978775	-4.4995386
SLAIN1	-0.2766733	-0.0936654	-0.37725	0.70750813	0.99978775	-4.6102931
CBR3	-0.2766976	1.93825823	-0.5904134	0.55744584	0.99978775	-4.6325904
ZNF385A	-0.2768324	4.81395569	-0.685515	0.4960341	0.99978775	-4.7371193
C1H19orf12	-0.27702	2.12837685	-1.3266552	0.19036306	0.99978775	-4.5463062
PLS1	-0.277158	-0.0726909	-0.278132	0.78200321	0.99978775	-4.6116939
CCDC137	-0.2771719	3.06569953	-1.5551235	0.12592319	0.99978775	-4.4551584
DNAL1	-0.2772834	1.78355314	-1.0272099	0.30902991	0.99978775	-4.595264
STPG2	-0.2773553	-0.6247397	-0.5779562	0.56576383	0.99978775	-4.6103739
ETS1	-0.2774489	5.95281965	-1.2526126	0.21589618	0.99978775	-4.5421877
IL34	-0.2775247	4.80372675	-0.9341658	0.35449404	0.99978775	-4.6734471
LRRC73	-0.2777464	0.4460068	-0.8471208	0.40077197	0.99978775	-4.6066023
P2RX1	-0.2778793	-2.214232	-0.4649079	0.64391941	0.99978775	-4.6058459
YARS	-0.2779725	6.60574575	-2.144245	0.03666144	0.99978775	-3.904146
IRS1	-0.2780948	3.40958874	-0.800108	0.4272529	0.99978775	-4.6712789
DPH2	-0.2787005	2.61364681	-1.3964225	0.16846195	0.99978775	-4.5234906
JUN	-0.2791988	5.69964719	-1.1425446	0.25841014	0.99978775	-4.5984962
ANAPC1	-0.2794097	5.74950509	-2.2297773	0.03006189	0.99978775	-3.8401816
STAT5A	-0.2794165	4.4667562	-2.2451042	0.02899716	0.99978775	-3.9593416
ASS1	-0.2796106	0.59859318	-0.4896615	0.62641278	0.99978775	-4.6186574
PCBP3	-0.2796948	3.59288259	-0.8999316	0.37226499	0.99978775	-4.6471297
ZNF699	-0.2796993	1.86866728	-1.0005512	0.32163202	0.99978775	-4.5979581
C20H19orf66	-0.279752	3.49459051	-1.8509674	0.06980184	0.99978775	-4.3151937
FAM107B	-0.2798645	2.66807502	-1.2169058	0.22908081	0.99978775	-4.5577265
TLN2	-0.2799605	7.20698043	-0.8268194	0.41208059	0.99978775	-4.7404603
SMARCD1	-0.2800717	4.3423337	-1.5999308	0.11561355	0.99978775	-4.3673351
ENSCAFG00000004288	-0.2801284	1.19069528	-0.8729321	0.3866742	0.99978775	-4.6055599
LAMB1	-0.2804196	9.29820033	-1.9701529	0.05410411	0.99978775	-4.0920013
ENSCAFG00000015158	-0.2804294	0.7533447	-0.8775596	0.38417996	0.99978775	-4.6049162
NPM3	-0.2804746	5.39806351	-1.7790672	0.08101662	0.99978775	-4.2118978
KYAT1	-0.280633	2.76103187	-0.9626733	0.3401235	0.99978775	-4.6074805
MAD2L2	-0.2806856	3.52546679	-1.6672958	0.10140918	0.99978775	-4.3749687
ANP32B	-0.2809302	6.37006518	-1.8963502	0.06342092	0.99978775	-4.1087267
CCDC28A	-0.2810422	3.360279	-1.2069631	0.23285499	0.99978775	-4.5589641
ZNF684	-0.281047	1.48049562	-0.9239875	0.35971913	0.99978775	-4.6047176
CIC	-0.2811636	6.86086681	-1.7816714	0.0805856	0.99978775	-4.1990259
ACADL	-0.2812732	3.07347066	-1.6568155	0.10352029	0.99978775	-4.3934423
CLNS1A	-0.2813774	2.73645959	-1.5741723	0.12145358	0.99978775	-4.4677949
HLCS	-0.281437	2.99079218	-1.8387539	0.07160894	0.99978775	-4.3767375
ART4	-0.2816821	-1.4311944	-0.2970081	0.76763193	0.99978775	-4.6077474
GBP6	-0.2817291	0.45181596	-0.3489611	0.72851381	0.99978775	-4.6162645
ZNF550	-0.2817513	0.40760288	-0.8746372	0.38575397	0.99978775	-4.6035532
CEP85L	-0.2818259	1.48352751	-0.9888823	0.32725527	0.99978775	-4.5986788
WARS2	-0.2818525	3.4963183	-1.4011866	0.16704044	0.99978775	-4.492225
ENSCAFG00000017940	-0.2821625	-2.3330853	-0.4696972	0.64051596	0.99978775	-4.6052647
NDST1	-0.2822905	6.00836967	-2.1815964	0.03363732	0.99978775	-3.8732985
ENSCAFG00000017277	-0.2824133	3.99244556	-1.2576323	0.21408865	0.99978775	-4.5469535

PNKP	-0.2824767	4.03101161	-2.1526992	0.03595683	0.99978775	-4.0787269
NAT14	-0.2833155	1.4874961	-0.8781534	0.38386069	0.99978775	-4.609524
WNT11	-0.2836097	-2.0321218	-0.3673833	0.71480958	0.99978775	-4.6057886
ZBTB49	-0.2837079	2.54841366	-1.7167045	0.09192674	0.99978775	-4.4445246
CYB5D2	-0.2837392	2.50944375	-1.3860618	0.17158562	0.99978775	-4.5272068
GMNN	-0.2838632	2.96312441	-1.3053264	0.19747199	0.99978775	-4.5235679
ENSCAFG00000019220	-0.284049	3.76017281	-1.2791615	0.20646359	0.99978775	-4.5280488
PCNA	-0.2841113	5.09915568	-1.456249	0.15127686	0.99978775	-4.4265768
NEURL2	-0.284184	0.02754444	-0.8766755	0.38465573	0.99978775	-4.6030317
JDP2	-0.284212	3.57380733	-2.0500639	0.0453657	0.99978775	-4.1557475
BAHCC1	-0.284488	4.17551752	-1.5677432	0.12294762	0.99978775	-4.385102
STAMBPL1	-0.2845028	4.32484457	-2.7233057	0.00875415	0.99978775	-3.5637217
ENSCAFG00000007622	-0.284596	2.34946298	-1.6382002	0.10735856	0.99978775	-4.4641019
ANGPTL2	-0.2848734	6.41508378	-1.0259961	0.30959633	0.99978775	-4.6566767
ENSCAFG00000007461	-0.2852921	0.61967385	-0.9379783	0.35254962	0.99978775	-4.6019475
POGZ	-0.2854069	4.79202032	-2.3371416	0.02327842	0.99978775	-3.8377184
C20H3orf14	-0.285419	0.04479291	-0.7984372	0.42821294	0.99978775	-4.6076059
ANKRD2	-0.2855773	0.46077058	-0.5242836	0.60228733	0.99978775	-4.6203583
ENSCAFG00000030711	-0.2857797	0.29026772	-0.8662678	0.39028397	0.99978775	-4.6036423
ARHGAP39	-0.2858692	3.24921098	-1.4447569	0.15446687	0.99978775	-4.4904776
CEP164	-0.2859885	5.65834274	-2.8982817	0.0054633	0.99978775	-3.2108545
PALB2	-0.2862246	3.31488552	-1.565966	0.12336321	0.99978775	-4.4374977
CSTF3	-0.2865068	4.35207574	-2.0477856	0.04559679	0.99978775	-4.1209397
RPUSD1	-0.2865738	4.12486845	-1.669897	0.1008907	0.99978775	-4.3455747
GEN1	-0.2865752	2.88736082	-0.9222981	0.36059118	0.99978775	-4.6334734
PUS10	-0.2866453	2.94624191	-1.5714558	0.12208305	0.99978775	-4.454942
C18H7orf57	-0.2875471	4.06609155	-1.3959601	0.16860043	0.99978775	-4.4778325
GPR137	-0.2877742	4.22730793	-1.9110727	0.0614604	0.99978775	-4.1846931
ARHGEF5	-0.2878019	1.20386934	-0.6880625	0.49444163	0.99978775	-4.6249557
TSPOAP1	-0.2880805	2.68275072	-0.6111396	0.54374357	0.99978775	-4.6794324
STK32B	-0.2881461	-0.5236849	-0.5222665	0.60368107	0.99978775	-4.6117379
ACAT2	-0.2883268	6.06883676	-1.340526	0.18584499	0.99978775	-4.4926269
HGH1	-0.28843	3.10581297	-1.7524523	0.08553301	0.99978775	-4.3716175
EVA1A	-0.2886562	1.99728069	-0.6365308	0.5271954	0.99978775	-4.6576617
SH3D21	-0.2887529	3.98663472	-1.1227383	0.26665557	0.99978775	-4.5852286
FIBIN	-0.2887585	3.84015194	-0.5537845	0.58207674	0.99978775	-4.7326532
ENSCAFG00000030144	-0.2889005	2.80795962	-1.0124168	0.31598086	0.99978775	-4.5997499
PER1	-0.2889207	5.40506273	-1.2557625	0.21476062	0.99978775	-4.5359394
C1H19orf54	-0.2889282	1.57395636	-1.5001383	0.13956714	0.99978775	-4.5290634
ENSCAFG00000003676	-0.2889908	2.92305277	-1.5706321	0.12227446	0.99978775	-4.4414735
TBPL1	-0.2889964	-0.198727	-0.8496735	0.39936371	0.99978775	-4.6033006
H3F3B	-0.289228	6.83722073	-1.9640313	0.05482952	0.99978775	-4.0560421
DDX39A	-0.2893247	4.66539723	-1.421813	0.16099267	0.99978775	-4.4513857
HOMER2	-0.2893266	-0.1846912	-0.8130117	0.41988207	0.99978775	-4.6156083
ABHD14B	-0.2896132	3.0993317	-1.3319752	0.18862046	0.99978775	-4.5245799
EVC	-0.2898278	6.1477358	-1.019567	0.31260806	0.99978775	-4.6604126
MRC2	-0.2898306	8.26843982	-1.3202563	0.19247516	0.99978775	-4.5043357
RAB36	-0.2900942	-0.2155975	-0.8334867	0.40834534	0.99978775	-4.6043399
CHAF1B	-0.2901736	2.593173	-1.1082162	0.27281834	0.99978775	-4.5853383
NUDT13	-0.2904261	1.72777917	-1.0918319	0.27989091	0.99978775	-4.5879272

RNF32	-0.2904559	0.46004271	-0.8250224	0.41309084	0.99978775	-4.6070127
DSC3	-0.2905658	-1.0925912	-0.5201471	0.60514699	0.99978775	-4.6092148
TSHZ2	-0.2906121	5.0680453	-1.3592853	0.17986498	0.99978775	-4.4865235
ZNF527	-0.2906192	1.99061911	-1.3629009	0.17872949	0.99978775	-4.5457487
MFAP2	-0.2908204	3.17876196	-0.4245065	0.67293059	0.99978775	-4.694517
SLC17A9	-0.2910127	3.78110255	-1.1103634	0.27190087	0.99978775	-4.586968
CCKBR	-0.2910178	-0.837193	-0.2886042	0.77402043	0.99978775	-4.6122071
HYKK	-0.2911422	1.17784774	-0.5611131	0.57710706	0.99978775	-4.6234976
HDAC10	-0.2911452	4.16302583	-1.5707981	0.12223586	0.99978775	-4.3883793
KIF18A	-0.2911635	3.26292163	-1.3326657	0.18839517	0.99978775	-4.5124818
ASPM	-0.2912761	4.36273533	-0.8716248	0.38738063	0.99978775	-4.7176172
STX4	-0.2917242	5.04430556	-2.6808097	0.00979086	0.99978775	-3.4996819
ENSCAFG00000020269	-0.291946	0.07607593	-0.6907209	0.49278284	0.99978775	-4.6088136
USE1	-0.2925125	2.80194771	-0.996065	0.32378622	0.99978775	-4.6041993
OCA2	-0.2928408	0.34380605	-0.7446782	0.45978593	0.99978775	-4.6193583
KIAA1614	-0.2928427	2.63060429	-1.3393354	0.18622957	0.99978775	-4.5350917
KLF7	-0.2931245	3.38191198	-1.6985307	0.0953258	0.99978775	-4.3889585
SSNA1	-0.2932123	2.46417963	-1.7931545	0.07870781	0.99978775	-4.4198121
ZNF84	-0.293351	3.2039445	-1.3791144	0.17370504	0.99978775	-4.5118051
SORD	-0.2934158	2.24808665	-1.0779706	0.28597365	0.99978775	-4.5902673
ABCB8	-0.2936956	4.75817506	-2.1084052	0.03978452	0.99978775	-4.0125208
LSM10	-0.2937396	3.68002524	-1.7303182	0.08944672	0.99978775	-4.3387132
ADPRM	-0.293778	2.52312893	-1.2837898	0.20485115	0.99978775	-4.5510453
PLEC	-0.2939329	10.5069828	-1.5312864	0.13170096	0.99978775	-4.4180585
ENSCAFG00000030302	-0.2941272	0.44468448	-0.6644404	0.50931561	0.99978775	-4.6123862
DUSP22	-0.2945233	1.89637211	-0.7406368	0.46221248	0.99978775	-4.633489
GRK5	-0.2945591	5.57965498	-1.6622987	0.10241133	0.99978775	-4.3035789
UBA7	-0.2948709	4.10198301	-0.9918251	0.32583097	0.99978775	-4.6260122
PLAU	-0.2950853	5.38987808	-1.1023435	0.27533877	0.99978775	-4.6151079
ZBTB46	-0.2952722	-1.7733035	-0.4715965	0.63916836	0.99978775	-4.6057917
ENSCAFG00000023300	-0.2956251	-0.3579022	-0.7576139	0.45206844	0.99978775	-4.6073967
PXYLP1	-0.2957183	3.42330662	-1.5657026	0.1234249	0.99978775	-4.4145233
RFC4	-0.2959175	3.99638211	-1.3711445	0.17616112	0.99978775	-4.4826307
STARD4	-0.2961393	3.22321042	-2.2985801	0.02553918	0.99978775	-4.1669749
ZNF674	-0.2962381	1.29179212	-1.0665953	0.29103371	0.99978775	-4.5912604
CCDC112	-0.2963587	3.82970664	-2.0031145	0.05033831	0.99978775	-4.2103896
SPOUT1	-0.2964726	3.5176118	-2.075187	0.04288403	0.99978775	-4.1923573
SOCS1	-0.2965564	-2.0868792	-0.4966563	0.62150421	0.99978775	-4.6057335
HSPA1L	-0.2965871	1.03166094	-0.8441228	0.40242979	0.99978775	-4.6109275
FLNB	-0.2966798	9.71457116	-1.4140474	0.16324931	0.99978775	-4.4585409
TEPSIN	-0.2968568	2.82277938	-2.1218904	0.03858315	0.99978775	-4.2697978
DBF4	-0.2973084	3.55613337	-1.3793705	0.17362657	0.99978775	-4.4872007
SLC2A3	-0.2977693	3.81296539	-0.510856	0.61159291	0.99978775	-4.6810298
VSIG10L	-0.2979501	1.19873241	-0.887502	0.37885546	0.99978775	-4.6047767
BARD1	-0.2979884	2.99029557	-1.1236665	0.26626506	0.99978775	-4.5832672
BBS7	-0.2980305	3.7215546	-2.0964179	0.04087972	0.99978775	-4.1657547
LRR1	-0.2981089	1.79812432	-1.1983208	0.23617226	0.99978775	-4.5700085
ZNF333	-0.2982014	3.42751409	-1.3642208	0.17831634	0.99978775	-4.5090043
TET1	-0.2984048	0.83787146	-0.8692588	0.38866127	0.99978775	-4.6110141
PAMR1	-0.2985817	3.66191292	-0.1776512	0.85968036	0.99978775	-4.6293321

NATD1	-0.2987786	0.61314119	-0.891322	0.37682219	0.99978775	-4.6040223
GPR19	-0.2990807	-0.483806	-0.6654456	0.50867779	0.99978775	-4.6101565
POLD1	-0.2992004	3.6013827	-1.5707796	0.12224017	0.99978775	-4.399291
MSMO1	-0.2993305	2.01070052	-0.5703274	0.57088793	0.99978775	-4.6275648
ARG2	-0.2994719	2.57272484	-0.77618	0.44112457	0.99978775	-4.6486485
SLC38A6	-0.2996481	2.83186175	-1.2811114	0.2057831	0.99978775	-4.5400377
MFSD4A	-0.3001033	-1.4696641	-0.3029325	0.76313804	0.99978775	-4.607803
ENSCAFG00000028851	-0.3003069	0.85196326	-0.9144208	0.36467535	0.99978775	-4.60205
TTYH3	-0.3005225	4.17010335	-1.3196408	0.19267924	0.99978775	-4.5126027
WDR89	-0.3006206	1.45855929	-1.2282014	0.22484765	0.99978775	-4.5734036
CDH10	-0.3007538	-1.3334104	-0.5520059	0.58328594	0.99978775	-4.6129757
PRKAR2B	-0.3008921	3.67621397	-0.8631749	0.39196643	0.99978775	-4.6628107
ENSCAFG00000011616	-0.3009947	1.11316494	-0.9071106	0.36849206	0.99978775	-4.6044939
ZNF71	-0.3010735	1.20907216	-0.9365603	0.35327199	0.99978775	-4.6025962
HMGB3	-0.3011565	3.53902155	-1.0850018	0.28287675	0.99978775	-4.5997105
NTHL1	-0.3015679	1.52386688	-1.2861889	0.20401909	0.99978775	-4.5642693
ST3GAL6	-0.301574	3.41539742	-1.1967968	0.23676079	0.99978775	-4.5595269
ANPEP	-0.3019819	7.57278366	-0.5542327	0.58177221	0.99978775	-4.8123799
REEP4	-0.3021272	3.16752338	-1.4597811	0.15030687	0.99978775	-4.4703223
RCC1	-0.3022092	4.12948656	-1.5721459	0.1219229	0.99978775	-4.3832865
TBC1D32	-0.3023565	3.0645833	-1.9928597	0.05148494	0.99978775	-4.2781791
ZNF366	-0.302656	2.51422823	-0.4953186	0.62244157	0.99978775	-4.6371336
TSC22D2	-0.3028233	4.91023911	-2.3598377	0.02203296	0.99978775	-3.7498786
EEF2K	-0.3029013	6.83063567	-1.1992608	0.23580977	0.99978775	-4.5715612
MED22	-0.3029221	3.18359825	-1.639249	0.10713928	0.99978775	-4.4091809
ACAP1	-0.3030089	2.49281919	-1.0215964	0.31165523	0.99978775	-4.5971174
P4HA3	-0.3030999	5.60830146	-1.1368015	0.26078209	0.99978775	-4.6024757
SLC7A6OS	-0.3034213	2.54888704	-1.6904032	0.09687906	0.99978775	-4.4435338
CHKA	-0.3034733	3.06376001	-1.8288999	0.07309553	0.99978775	-4.3662529
HIC1	-0.3041559	4.85285793	-1.4265602	0.15962518	0.99978775	-4.445086
NFIL3	-0.304226	4.46863541	-1.5363666	0.1304522	0.99978775	-4.3999434
PHF19	-0.3042407	4.45594713	-1.347886	0.18348101	0.99978775	-4.4889492
FAM161B	-0.3043585	1.79063061	-1.7133454	0.09254735	0.99978775	-4.4599077
FOXRED2	-0.3047754	0.70501313	-0.5143582	0.60915951	0.99978775	-4.6193704
ADGRG6	-0.3048073	6.47187663	-0.6387937	0.52573352	0.99978775	-4.8018389
FAM69C	-0.3051685	-2.0451935	-0.7252038	0.47154632	0.99978775	-4.6061516
ENSCAFG00000004589	-0.3052221	5.85542512	-1.7133521	0.09254611	0.99978775	-4.2538399
ENSCAFG00000015742	-0.3056768	-0.8851693	-0.597815	0.55253281	0.99978775	-4.6084685
C4H5orf34	-0.3058484	-1.0330073	-0.6094531	0.54485206	0.99978775	-4.6126764
IGFBP3	-0.3060121	-2.4323249	-0.3969844	0.69298777	0.99978775	-4.6053002
ENSCAFG00000002517	-0.3061419	0.26076318	-1.2580192	0.21394981	0.99978775	-4.5739543
ZSCAN25	-0.3062683	2.43939824	-1.4471457	0.15379948	0.99978775	-4.516357
METTL4	-0.3063896	3.36677298	-1.8613473	0.06829645	0.99978775	-4.2825251
MEI1	-0.306718	0.75925777	-0.903874	0.37019002	0.99978775	-4.6078068
CSDC2	-0.3069514	0.26196036	-0.6864013	0.49547973	0.99978775	-4.6127707
ENSCAFG00000022718	-0.3069918	-1.3140875	-0.5295861	0.59863079	0.99978775	-4.6063117
TM7SF2	-0.3072425	3.89378528	-0.7612315	0.44992374	0.99978775	-4.6608808
RPUSD3	-0.3073791	3.41721889	-2.0112557	0.04944376	0.99978775	-4.2428503
KCNQ5	-0.3075839	1.21350709	-0.3530614	0.72545569	0.99978775	-4.6407747
PARP16	-0.3076505	2.1152211	-1.0412877	0.30251233	0.99978775	-4.5940276

ENSCAFG00000028156	-0.3077109	-0.0811985	-0.7409005	0.46205395	0.99978775	-4.6088851
MED11	-0.3078828	1.95589432	-1.2855435	0.20424268	0.99978775	-4.557111
ENSCAFG00000017707	-0.3078887	-0.3620258	-1.0955376	0.27828015	0.99978775	-4.5901374
HSPA12A	-0.3091457	1.01786688	-0.6256596	0.53424804	0.99978775	-4.6235279
CCNQ	-0.3093186	1.86611944	-1.2861988	0.20401566	0.99978775	-4.5587648
HOXB5	-0.3095692	0.1205839	-0.4300976	0.66888455	0.99978775	-4.6206973
ENSCAFG00000013404	-0.3097979	4.38239494	-1.8653339	0.06772562	0.99978775	-4.2082461
FAM83G	-0.3101644	-0.4715043	-0.6808292	0.49897066	0.99978775	-4.6149628
ENSCAFG00000032235	-0.3102409	-0.9155718	-0.8399755	0.40473012	0.99978775	-4.6046894
ENSCAFG00000017087	-0.3102452	1.20159489	-1.5929232	0.11717939	0.99978775	-4.5266048
OAF	-0.3102641	6.60125865	-1.1112251	0.27153327	0.99978775	-4.6127894
NDNF	-0.3103398	-2.5511	-0.4550733	0.65093235	0.99978775	-4.6054322
ROM1	-0.3107128	1.04459776	-0.9651275	0.33890454	0.99978775	-4.5991291
SUSD2	-0.3108848	2.47036203	-0.4142928	0.68034704	0.99978775	-4.6758709
CBX8	-0.3109008	1.96879726	-1.0140583	0.31520441	0.99978775	-4.597645
NABP1	-0.3116565	3.70900577	-1.8267519	0.073423	0.99978775	-4.3227465
CCDC57	-0.3117095	1.16961645	-1.0442874	0.30113582	0.99978775	-4.5932254
TAF12	-0.3117125	1.3722429	-1.1365183	0.26089947	0.99978775	-4.5838835
TCTA	-0.3117772	2.76427071	-1.4482774	0.15348411	0.99978775	-4.5008737
SNAPC5	-0.3119425	0.21643192	-0.806345	0.42368058	0.99978775	-4.6087107
EBF3	-0.3122164	1.57656284	-0.3557082	0.72348403	0.99978775	-4.6373083
NR2C2AP	-0.3125237	1.55982909	-1.7769687	0.08136535	0.99978775	-4.4775131
GPR157	-0.3127112	1.36185443	-0.9268808	0.35822877	0.99978775	-4.6057672
FASN	-0.3127896	9.04505082	-1.918852	0.06044547	0.99978775	-4.1433186
CCL17	-0.3128419	-0.9513943	-0.4845448	0.63001418	0.99978775	-4.6094718
ENSCAFG00000024837	-0.3128485	0.15900886	-0.9227797	0.36034243	0.99978775	-4.6017392
MRPS34	-0.313126	2.8720261	-1.0128979	0.31575317	0.99978775	-4.6066917
SESN2	-0.3131398	5.92767666	-1.499511	0.13972928	0.99978775	-4.3948251
NAALAD2	-0.3133119	-0.226347	-0.5053463	0.61543007	0.99978775	-4.6172187
BCL6B	-0.3133579	-1.4139101	-0.2429735	0.8089725	0.99978775	-4.6072721
TIMM8A	-0.3137894	2.18313255	-1.4048022	0.1659678	0.99978775	-4.529855
LAYN	-0.3139145	2.40919253	-1.2797603	0.20625443	0.99978775	-4.5431596
FAM102A	-0.3140015	3.56187113	-1.1429822	0.25823005	0.99978775	-4.5768195
HIST2H2BF	-0.3140942	1.15426969	-1.5052127	0.13826083	0.99978775	-4.5284221
BCKDHB	-0.3143449	3.75156889	-1.9660347	0.0545912	0.99978775	-4.2036499
MCRIP1	-0.3144954	3.45744512	-1.6997927	0.09508647	0.99978775	-4.367728
FAAP100	-0.3145271	3.8469488	-1.8691221	0.06718694	0.99978775	-4.2581705
OLFM1	-0.3147615	-0.1840374	-0.5325837	0.59656823	0.99978775	-4.6190589
MASTL	-0.3150882	2.53255405	-0.9382437	0.35241452	0.99978775	-4.6215252
NEIL2	-0.31527	2.78439198	-0.9055059	0.36933328	0.99978775	-4.6140042
TRIM14	-0.3152899	1.63027179	-0.8002999	0.42714272	0.99978775	-4.6206944
LENG1	-0.315326	2.36994291	-1.5071947	0.13775326	0.99978775	-4.5120368
CCDC170	-0.3159752	-0.6194424	-0.3615766	0.71911919	0.99978775	-4.610325
MUTYH	-0.3163786	4.61364217	-2.422146	0.01891475	0.99978775	-3.7609693
BHLHE41	-0.3164904	3.6732107	-1.2874299	0.20358967	0.99978775	-4.5278853
COL6A2	-0.3167277	9.9184793	-0.9639269	0.3395005	0.99978775	-4.6533182
CA4	-0.3167537	-0.9359724	-0.2996733	0.76560933	0.99978775	-4.6075726
CIP2A	-0.317158	3.92750156	-1.1952243	0.23736914	0.99978775	-4.561548
ENSCAFG00000031973	-0.3172241	3.88438731	-1.1730521	0.24606831	0.99978775	-4.5676506
SPHK1	-0.3172603	4.83652505	-0.8938614	0.37547435	0.99978775	-4.6788914

ENSCAFG00000010596	-0.3174528	3.27882817	-1.1330906	0.26232292	0.99978775	-4.5789496
AOC2	-0.3177901	-0.4896079	-0.7450284	0.45957599	0.99978775	-4.6052269
SOX6	-0.318243	-0.7868932	-0.6749618	0.50266104	0.99978775	-4.6164727
HOOK2	-0.3182748	1.78725122	-1.2916848	0.20212249	0.99978775	-4.5600606
ANGPT4	-0.3182764	2.05296357	-0.2498648	0.80366677	0.99978775	-4.630052
PSMG3	-0.3183067	1.42123356	-1.5174478	0.13515103	0.99978775	-4.5253599
CACNB1	-0.31928	1.01962349	-1.6282885	0.10944911	0.99978775	-4.5161788
CHAF1A	-0.3195592	3.7155544	-1.1044072	0.27445123	0.99978775	-4.59757
DSCAML1	-0.3196691	-0.8507673	-0.202513	0.84029826	0.99978775	-4.6069066
PAK1	-0.3197358	4.48367227	-1.1162987	0.26937608	0.99978775	-4.60316
SLC35F2	-0.3200097	3.39974139	-0.9610298	0.34094142	0.99978775	-4.6367445
CAV1	-0.3200706	6.86145557	-1.3974418	0.16815703	0.99978775	-4.4586856
RBM20	-0.3201092	-0.0248797	-0.5056899	0.61519045	0.99978775	-4.6207189
SLC6A6	-0.3205097	7.02594217	-0.9628113	0.34005488	0.99978775	-4.6839048
CENPL	-0.3206315	3.14311832	-1.2094764	0.23189671	0.99978775	-4.5556801
B4GALNT3	-0.3207519	1.26309035	-2.628791	0.01121264	0.99978775	-3.5559821
ADCY4	-0.3209205	3.60501672	-1.0610079	0.29354168	0.99978775	-4.6018492
ESRRA	-0.3213505	3.77095873	-1.8738779	0.06651582	0.99978775	-4.276377
PCK2	-0.3214778	7.35566834	-1.9795781	0.05300337	0.99978775	-4.0604989
GPT	-0.3215086	1.5385227	-1.4563739	0.15124248	0.99978775	-4.5425206
DPF1	-0.3216123	0.42344352	-0.5624531	0.57620055	0.99978775	-4.6132501
ENSCAFG00000017118	-0.3216973	2.50466483	-1.484881	0.14355369	0.99978775	-4.5036758
TCEA2	-0.3219001	2.54525449	-0.8600556	0.39366782	0.99978775	-4.6179082
CDC14A	-0.32199	3.23368171	-1.2378363	0.2212825	0.99978775	-4.5537574
PRAG1	-0.322012	1.12555844	-0.428931	0.66972797	0.99978775	-4.6282213
SH3BGRL3	-0.3221813	3.98614635	-0.7048059	0.48404531	0.99978775	-4.6694024
MCM7	-0.3225106	5.86995376	-1.4556381	0.15144513	0.99978775	-4.422657
KDM1B	-0.3225329	6.02199676	-1.3561382	0.1808578	0.99978775	-4.4837142
THOP1	-0.3225823	5.72690142	-2.7369987	0.00844222	0.99978775	-3.3518255
C19H2orf76	-0.3230744	1.84611287	-1.1506531	0.25508754	0.99978775	-4.57919
HMMR	-0.323112	3.07851368	-1.2559617	0.21468896	0.99978775	-4.5393555
ENSCAFG00000014028	-0.3232454	-1.6932979	-0.6628029	0.51035557	0.99978775	-4.6071824
AKR1E2	-0.3237684	1.95880029	-1.0723234	0.288478	0.99978775	-4.5901652
KCNN4	-0.3238175	4.53507336	-1.8847477	0.06500319	0.99978775	-4.2520052
TIMP4	-0.3240531	-1.8056745	-0.558425	0.5789275	0.99978775	-4.6039486
VSIR	-0.3241509	3.69288468	-0.8118487	0.4205432	0.99978775	-4.6612965
SWSAP1	-0.3241567	1.68960247	-1.230319	0.22406046	0.99978775	-4.5732447
PECAM1	-0.3243713	1.09794492	-0.0959631	0.92391516	0.99978775	-4.6148223
ENSCAFG00000019368	-0.3245027	2.793517	-1.7266025	0.09011806	0.99978775	-4.4348148
ZNF180	-0.3245123	1.1114463	-1.2774989	0.20704509	0.99978775	-4.5700537
PPA2	-0.3246648	3.37787364	-2.3962317	0.02015997	0.99978775	-4.051306
GTPBP3	-0.3246784	2.8014865	-1.998882	0.05080886	0.99978775	-4.3313451
AHRR	-0.3246904	2.4993674	-1.1658471	0.24894407	0.99978775	-4.5705048
CCDC78	-0.325054	1.810528	-1.0104857	0.31689598	0.99978775	-4.596602
XIRP1	-0.3251375	-0.9267881	-0.7210843	0.47405572	0.99978775	-4.6136207
SREBF2	-0.3253059	7.74476374	-1.8997849	0.06295886	0.99978775	-4.1160562
CEP68	-0.3258556	3.09242194	-1.4471441	0.15379994	0.99978775	-4.49657
RIN2	-0.3267161	5.31203648	-1.3186541	0.19300676	0.99978775	-4.5069593
ENSCAFG00000023364	-0.3269949	-1.3523497	-0.5657318	0.57398555	0.99978775	-4.6072865
OLFML2A	-0.3270894	4.32933087	-0.4267201	0.67132754	0.99978775	-4.7378489

EGLN3	-0.3271456	3.27780823	-0.4801129	0.63314095	0.99978775	-4.6284288
ENSCAFG00000011263	-0.3274315	-0.3176185	-0.8801176	0.38280558	0.99978775	-4.6031068
THAP3	-0.3274322	1.4715559	-1.7711089	0.08234574	0.99978775	-4.4875802
RRS1	-0.3274951	3.04392437	-1.8542282	0.06932594	0.99978775	-4.3427562
TCAP	-0.3279331	0.08738283	-0.8457477	0.40153075	0.99978775	-4.6046857
SLC43A2	-0.3282377	0.8479031	-0.606124	0.5470436	0.99978775	-4.6142841
ENSCAFG00000015864	-0.3284736	1.4129465	-1.1232361	0.26644606	0.99978775	-4.5843455
TIGD4	-0.3285009	0.75250704	-0.9960839	0.32377708	0.99978775	-4.5963938
ENSCAFG00000001540	-0.3288871	3.86866872	-1.8291115	0.07306334	0.99978775	-4.2931091
IER5	-0.3289512	1.61917127	-1.1328177	0.26243648	0.99978775	-4.5830814
KIAA0513	-0.3291016	2.2796016	-1.722856	0.09079917	0.99978775	-4.4303748
PALM	-0.3292963	2.11801266	-0.4007396	0.69023763	0.99978775	-4.6404688
POLI	-0.3296351	2.98393988	-2.128589	0.03799826	0.99978775	-4.2550118
CD34	-0.3296533	1.01024607	-0.1523323	0.87950861	0.99978775	-4.6175136
ENSCAFG00000030184	-0.329806	2.94909404	-1.3217072	0.19199472	0.99978775	-4.5347887
CSPG4	-0.3299272	6.50680467	-0.7809971	0.43831081	0.99978775	-4.7550455
ENSCAFG00000027901	-0.3300748	-0.0607055	-0.9359663	0.35357489	0.99978775	-4.5999409
MNS1	-0.3301286	2.64455599	-1.8245534	0.07375947	0.99978775	-4.3883448
ENSCAFG00000007577	-0.3307122	0.01105121	-0.8113445	0.42083005	0.99978775	-4.6079097
DNAL4	-0.3307677	1.29291832	-1.0880502	0.2815414	0.99978775	-4.5893154
UBE2S	-0.330968	5.03546516	-1.4841453	0.14374816	0.99978775	-4.4072578
RGS17	-0.3310605	0.29636278	-0.5019139	0.61782605	0.99978775	-4.614873
ELOF1	-0.3311847	1.38269808	-1.1071305	0.27328305	0.99978775	-4.5858361
TELO2	-0.3312891	3.95394023	-2.3529873	0.02240245	0.99978775	-3.9870027
LYRM2	-0.3314792	1.00390381	-1.0933608	0.27922554	0.99978775	-4.5883102
ENSCAFG00000004578	-0.3316922	1.88826711	-1.5449377	0.12836676	0.99978775	-4.4868954
TTC39B	-0.3317087	2.882168	-1.3225468	0.19171711	0.99978775	-4.5258239
KLF5	-0.3318263	4.25173949	-1.5979324	0.11605836	0.99978775	-4.3588437
FBLN1	-0.3318649	4.25980957	-0.3261134	0.74563463	0.99978775	-4.7083136
SELP	-0.3320751	-2.0639863	-0.3668981	0.71516929	0.99978775	-4.606239
IER2	-0.3323308	5.43331018	-1.116388	0.26933822	0.99978775	-4.6061049
FAR2	-0.3324079	4.18706364	-1.1563324	0.25277864	0.99978775	-4.5780538
KCNH3	-0.3325187	0.56140193	-0.6983014	0.48806951	0.99978775	-4.6118192
KIF26B	-0.332582	2.41047575	-0.7385553	0.46346516	0.99978775	-4.6362407
ENSCAFG00000007873	-0.3327566	-2.2240514	-0.5727331	0.56926963	0.99978775	-4.6072272
SNAI1	-0.3330699	3.63105143	-1.0218548	0.31153407	0.99978775	-4.6084846
ENSCAFG00000011673	-0.3331285	0.2671794	-1.3297828	0.18933711	0.99978775	-4.5691183
KCNJ12	-0.333207	-1.6970313	-0.6388525	0.52569559	0.99978775	-4.6054207
FAM189A1	-0.3334271	3.69354073	-1.5465256	0.12798335	0.99978775	-4.3723869
ENSCAFG00000024449	-0.3337278	-0.5182005	-0.8124997	0.42017307	0.99978775	-4.6031328
ENSCAFG00000019739	-0.3342617	-0.3908717	-0.7585611	0.4515063	0.99978775	-4.6060586
PAAF1	-0.3344249	2.0818657	-1.2891759	0.20298664	0.99978775	-4.5578456
GABBR2	-0.3344411	-0.6231428	-0.5834915	0.5620602	0.99978775	-4.6123786
FADS2	-0.3345768	8.64331684	-1.5561506	0.12567889	0.99978775	-4.3683649
ATG14	-0.3347495	2.5050139	-1.5710007	0.12218878	0.99978775	-4.4857726
FAM110D	-0.3349421	-2.4868743	-0.4849571	0.62972363	0.99978775	-4.6045523
MRPS30	-0.33504	4.20360806	-2.6516291	0.01056666	0.99978775	-3.6999936
SMTNL2	-0.3352333	-0.7003132	-0.572175	0.56964484	0.99978775	-4.6093137
MIGA2	-0.3352538	3.48443993	-1.7245886	0.09048366	0.99978775	-4.3842608
SAYSD1	-0.3352654	1.6396012	-1.3350917	0.18760527	0.99978775	-4.5553613

GSTT4	-0.3353414	-1.5357785	-0.6703347	0.50558178	0.99978775	-4.60389
CES2	-0.3355112	0.3924751	-0.5742743	0.56823402	0.99978775	-4.6122392
ZNF470	-0.3355931	0.16017798	-0.8648353	0.39106268	0.99978775	-4.6049153
ZWINT	-0.3357912	2.3638377	-1.0371709	0.30440851	0.99978775	-4.6021362
NFKBIL1	-0.336034	3.44990975	-1.7795668	0.08093377	0.99978775	-4.3383505
DLA-DMA	-0.3362811	0.99374401	-0.8852465	0.38005927	0.99978775	-4.6059197
ZNRD1	-0.336386	2.53457927	-1.9478266	0.05679013	0.99978775	-4.3657826
CENPN	-0.336558	2.07443513	-0.986876	0.32822872	0.99978775	-4.6124642
SLC4A11	-0.3365651	0.25435433	-0.580656	0.5639559	0.99978775	-4.6166193
SPATA2L	-0.3365774	1.97337438	-1.4003391	0.16729262	0.99978775	-4.5395721
DCAF4	-0.3369197	3.20349947	-2.3073485	0.0250086	0.99978775	-4.1010604
WNT5A	-0.3370043	2.8090384	-0.4148862	0.67991531	0.99978775	-4.6748206
PAQR5	-0.3372706	-2.4098398	-0.5439512	0.58877711	0.99978775	-4.6038758
IGSF9	-0.3374266	2.48179915	-1.7371548	0.08822236	0.99978775	-4.3648494
SYNGR1	-0.3374454	0.87866775	-0.7203781	0.47448664	0.99978775	-4.6190316
GINS4	-0.3374791	3.2500334	-1.2416542	0.21988139	0.99978775	-4.5441235
ENSCAFG00000013452	-0.3375051	-1.9572234	-0.5696316	0.57135637	0.99978775	-4.6052755
IRX5	-0.3375479	0.72761158	-0.4681287	0.64162968	0.99978775	-4.6243903
DGAT1	-0.3377297	3.3634403	-2.0765948	0.04274853	0.99978775	-4.2043812
PCYT2	-0.3377869	4.68615595	-2.2813158	0.0266132	0.99978775	-3.9114126
METTL27	-0.3378758	0.75296813	-1.2234205	0.22663225	0.99978775	-4.5722778
HPCAL1	-0.3380222	5.69268715	-1.0197339	0.31252962	0.99978775	-4.6571272
CCNB3	-0.3382379	0.59648511	-0.6332209	0.52933752	0.99978775	-4.6304142
ENSCAFG00000029124	-0.3382398	0.16078262	-1.1034006	0.27488391	0.99978775	-4.5889333
NAV2	-0.3382496	4.24946627	-1.0083126	0.3179279	0.99978775	-4.6485866
COX11	-0.338276	1.84815412	-1.7402545	0.08767182	0.99978775	-4.4757802
MTMR11	-0.3383077	3.77060708	-1.1052003	0.27411065	0.99978775	-4.5868148
GDPD5	-0.3385045	3.08412649	-1.2982311	0.19988068	0.99978775	-4.5328464
RBP4	-0.3385097	5.04063148	-1.0384893	0.30380038	0.99978775	-4.651649
DGKE	-0.3385725	1.04431124	-1.0912766	0.28013281	0.99978775	-4.5895168
ENSCAFG00000005848	-0.3386872	1.11106235	-1.1041157	0.27457649	0.99978775	-4.5882421
R3HCC1L	-0.3387862	2.67970642	-1.6110736	0.11315861	0.99978775	-4.4701052
MAFG	-0.3391722	1.65605907	-1.6934259	0.09629896	0.99978775	-4.4906379
ENSCAFG00000030449	-0.3392852	1.19021085	-1.6514551	0.10461387	0.99978775	-4.5110935
TSPAN14	-0.3394221	3.44272764	-1.043419	0.30153385	0.99978775	-4.6063579
FBXO48	-0.3399648	0.49905563	-0.6898266	0.4933405	0.99978775	-4.6134019
ALOX15	-0.3400078	0.96896776	-0.3043115	0.76209323	0.99978775	-4.6180526
CASP14	-0.3400957	1.99309286	-0.4603755	0.64714743	0.99978775	-4.6409669
BDH1	-0.3401752	1.62351902	-0.8001523	0.42722746	0.99978775	-4.6200262
ALDH5A1	-0.3403729	2.25908882	-0.9241035	0.35965928	0.99978775	-4.6126629
COL1A1	-0.3404663	13.7530172	-0.8787536	0.3835381	0.99978775	-4.6314306
SCAI	-0.3404695	1.14632621	-1.1745626	0.24546845	0.99978775	-4.5812337
NEK10	-0.3406111	2.25840699	-1.4189511	0.1618215	0.99978775	-4.5323934
LIPC	-0.3410966	0.27595524	-0.6901086	0.49316463	0.99978775	-4.6199575
C2CD2	-0.3411253	4.26676628	-2.1898106	0.03300248	0.99978775	-4.0291107
PRXL2A	-0.3411469	3.6270672	-1.5906096	0.11770011	0.99978775	-4.4119658
IL7R	-0.3412936	-1.6317383	-0.3156942	0.7534859	0.99978775	-4.6076869
SIX4	-0.341894	3.82710122	-0.9098692	0.36704879	0.99978775	-4.654216
AADAT	-0.3419114	0.04163067	-0.6522017	0.51711573	0.99978775	-4.6225414
ENSCAFG00000019542	-0.3422183	3.60576003	-2.0902186	0.04145636	0.99978775	-4.1950277

ENSCAFG00000000081	-0.3422777	-0.6936317	-0.8625943	0.3922828	0.99978775	-4.6019955
HIST1H1A	-0.3423372	-1.6158942	-0.7336294	0.46643737	0.99978775	-4.6061399
MOB3A	-0.3423708	3.84966453	-1.7035558	0.09437575	0.99978775	-4.3362085
ENSCAFG00000030257	-0.3424412	2.18764113	-2.121182	0.03864546	0.99978775	-4.3432443
TPPP	-0.3424445	-1.2745072	-0.6620257	0.51084956	0.99978775	-4.6065381
FHL3	-0.3429255	6.54081902	-1.7811798	0.08066681	0.99978775	-4.1973673
GPR160	-0.3431425	2.71742588	-1.5429947	0.12883715	0.99978775	-4.4276902
NSDHL	-0.3433605	5.02241458	-1.4991021	0.13983506	0.99978775	-4.4159371
COL8A2	-0.3436592	1.2560848	-0.9935686	0.32498912	0.99978775	-4.6008728
TMEM53	-0.3437808	2.14291266	-1.4889659	0.14247764	0.99978775	-4.5168711
CACNA1A	-0.3438732	2.01340326	-0.6903244	0.49303001	0.99978775	-4.6284381
ENSCAFG00000023943	-0.3441206	-0.1381762	-0.7478031	0.45791471	0.99978775	-4.6055717
PROM1	-0.3443649	-1.0387402	-0.4172858	0.67817046	0.99978775	-4.6157385
MAMSTR	-0.3446904	-0.6195647	-0.7551903	0.45350862	0.99978775	-4.6044263
RPS6KA2	-0.345799	6.90589128	-1.3402932	0.18592014	0.99978775	-4.4928103
LIMK1	-0.3458377	2.66645716	-1.7152763	0.09219018	0.99978775	-4.4374449
TMPO	-0.3461215	4.63408801	-1.4030691	0.16648128	0.99978775	-4.4592737
TTK	-0.346211	1.89257687	-0.818218	0.41693002	0.99978775	-4.651239
CCDC18	-0.3465039	3.47986932	-1.317233	0.19347923	0.99978775	-4.5270462
ZNF367	-0.3467403	2.23656997	-1.1892971	0.23967245	0.99978775	-4.5711606
SLC25A21	-0.3467972	0.76476598	-1.1752689	0.24518834	0.99978775	-4.5822637
GRAMD1C	-0.3468816	-0.1419079	-0.8791139	0.3833445	0.99978775	-4.6023996
ENSCAFG00000012184	-0.3469347	1.76549518	-1.1861254	0.24091164	0.99978775	-4.5757957
FAM117A	-0.3471808	2.58730862	-1.4381072	0.15633658	0.99978775	-4.5207613
ADAMTS2	-0.347319	6.80620218	-0.8116751	0.42064195	0.99978775	-4.7451951
FIGN	-0.347994	-0.2843656	-0.7347603	0.46575403	0.99978775	-4.6081622
LRP4	-0.3481016	3.60103632	-0.906286	0.36892414	0.99978775	-4.6408517
SLC22A15	-0.3481911	-0.5006164	-0.6844321	0.49671194	0.99978775	-4.6087493
PGBD1	-0.3482515	0.99209484	-1.2777632	0.20695257	0.99978775	-4.569366
EFHD2	-0.3483783	3.94220624	-2.3185673	0.02434408	0.99978775	-3.9223765
UQCC3	-0.3486312	1.04185201	-1.2162786	0.22931757	0.99978775	-4.5750565
GPRASP1	-0.3487193	1.53197588	-1.0536425	0.29687048	0.99978775	-4.5923757
ENSCAFG00000026794	-0.3487376	2.8437105	-0.8892902	0.37790278	0.99978775	-4.6455381
RPGRIP1L	-0.3489297	2.71146958	-1.7476972	0.08636157	0.99978775	-4.4197829
RRAGD	-0.3493245	3.16824735	-1.3828952	0.17254915	0.99978775	-4.4825126
ATF5	-0.3493718	5.29103268	-1.1968914	0.23672421	0.99978775	-4.5715199
SHOX2	-0.3495021	4.93774611	-0.8447764	0.40206802	0.99978775	-4.7255173
TRAF3IP1	-0.3495042	2.34177813	-1.4753925	0.1460779	0.99978775	-4.5279402
ENSCAFG00000000340	-0.3496044	0.02691359	-0.9558112	0.34354709	0.99978775	-4.5984249
FGF18	-0.3499189	-1.0959812	-0.6591227	0.51269698	0.99978775	-4.6065853
LAPTM5	-0.3509312	-0.6170092	-0.1900414	0.85000943	0.99978775	-4.6112614
PAN2	-0.3511573	3.47544466	-2.0973605	0.04079266	0.99978775	-4.2116622
FAH	-0.3511794	2.38817825	-0.8254125	0.41287141	0.99978775	-4.6273742
H2AFZ	-0.3513558	5.47021635	-1.8058798	0.07666983	0.99978775	-4.1862199
MSX1	-0.3514469	3.98124877	-0.9624413	0.34023886	0.99978775	-4.6333691
ENSCAFG00000025983	-0.3518125	5.20012778	-1.5045847	0.13842198	0.99978775	-4.39299
ENSCAFG00000018380	-0.3519247	1.59687593	-1.8548148	0.06924062	0.99978775	-4.4603575
ENSCAFG00000030495	-0.3520533	3.62246759	-2.7620239	0.00789844	0.99978775	-3.6978981
TMEM253	-0.3520543	0.21883729	-0.7718761	0.44364754	0.99978775	-4.6058989
ENSCAFG00000017016	-0.3520577	0.09363467	-0.6361019	0.52747276	0.99978775	-4.6243366

CEP128	-0.3524164	2.60882473	-1.2553891	0.21489502	0.99978775	-4.548469
ENSCAFG00000005454	-0.3528119	1.44890297	-1.1538378	0.25379096	0.99978775	-4.581576
DFFB	-0.3530577	2.20855043	-1.6200832	0.11120468	0.99978775	-4.495795
LRP3	-0.3534628	-0.1340837	-0.8292195	0.41073357	0.99978775	-4.6113398
OSGEP	-0.3537105	4.59742565	-2.1855643	0.03332933	0.99978775	-3.9693515
ZBTB45	-0.3538866	1.36696438	-1.1287468	0.26413478	0.99978775	-4.5837855
ENSCAFG00000009800	-0.354158	-1.1385418	-0.6696316	0.5060264	0.99978775	-4.6061753
ENSCAFG00000010539	-0.3541808	2.95771728	-1.658731	0.10313177	0.99978775	-4.393809
IRX3	-0.3541841	2.74080169	-0.6965329	0.48916691	0.99978775	-4.6561222
ELL3	-0.3542197	-0.1556579	-0.8843543	0.38053609	0.99978775	-4.6023778
HYI	-0.3543378	2.00972401	-1.1171628	0.26900991	0.99978775	-4.5836364
TMEM35A	-0.3545566	-0.6168435	-0.8815924	0.38201461	0.99978775	-4.6001093
GNPDA1	-0.354789	4.53548929	-1.8961408	0.06344918	0.99978775	-4.1702198
ENSCAFG00000010945	-0.3548696	1.08039383	-1.2163352	0.22929618	0.99978775	-4.5764617
FXYD6	-0.3555446	-0.3943601	-0.2427586	0.80913811	0.99978775	-4.6104576
ENSCAFG00000023462	-0.3560967	0.77807568	-1.2306431	0.22394018	0.99978775	-4.5731262
RGMA	-0.3562802	2.49150395	-0.6299571	0.53145421	0.99978775	-4.6372812
ENSCAFG00000002332	-0.3565859	-1.6233724	-0.7750225	0.44180224	0.99978775	-4.6036455
DCP2	-0.3569324	1.31176844	-1.1533553	0.2539871	0.99978775	-4.5829449
MBOAT2	-0.3570922	0.38748535	-0.6555946	0.51494695	0.99978775	-4.6131275
PRX	-0.3571106	1.62518902	-1.3219497	0.19191449	0.99978775	-4.5588553
TWF2	-0.357589	4.19496994	-2.5575716	0.01346545	0.99978775	-3.8025679
LURAP1	-0.3583622	1.66194673	-1.0212418	0.31182158	0.99978775	-4.5954737
FBXO4	-0.3584596	2.80932037	-1.7786192	0.08109095	0.99978775	-4.3995703
PKD1L3	-0.3586922	0.91622821	-1.001529	0.32116376	0.99978775	-4.5965435
TTLL12	-0.3587208	6.35841965	-2.1248763	0.03832148	0.99978775	-3.9206846
ENSCAFG00000023074	-0.3590336	1.24757997	-0.5318751	0.59705548	0.99978775	-4.6378412
PRR5L	-0.3593068	-1.5849708	-0.4041085	0.68777402	0.99978775	-4.6059821
ST8SIA5	-0.3597568	-1.2527317	-0.6240444	0.53530001	0.99978775	-4.6091854
ENSCAFG00000009315	-0.3598018	1.0121828	-1.2724498	0.20881861	0.99978775	-4.5690629
IFIT3	-0.3598044	0.28629187	-0.6365875	0.52715879	0.99978775	-4.6185235
ENSCAFG00000032467	-0.3600964	2.00617597	-1.1712388	0.24678979	0.99978775	-4.5756575
TMEM267	-0.3602179	2.90762925	-1.8711665	0.06689774	0.99978775	-4.3479623
FBP2	-0.3602485	-2.1260203	-0.649522	0.51883203	0.99978775	-4.6052038
OASL	-0.3606972	0.21181098	-0.6272098	0.53323934	0.99978775	-4.6130569
DUSP4	-0.3613478	4.73022148	-1.1942405	0.23775033	0.99978775	-4.5620204
ITPR3	-0.3613599	6.49561707	-1.4104551	0.16430149	0.99978775	-4.4526524
TMEM132B	-0.3614935	0.44643222	-0.5483861	0.58575064	0.99978775	-4.6191267
ENSCAFG00000026614	-0.3615648	0.65771663	-1.0892709	0.28100788	0.99978775	-4.5898239
ZNF668	-0.3616856	2.77043901	-2.04711	0.04566551	0.99978775	-4.3174841
IGFBP6	-0.3618953	6.97734273	-0.7430571	0.46075838	0.99978775	-4.7666454
EHD3	-0.361943	2.30714182	-1.0117886	0.31627838	0.99978775	-4.5986704
TIMP3	-0.361999	9.77982555	-1.0708946	0.28911404	0.99978775	-4.6220942
UBE2T	-0.36239	2.26249694	-1.3791485	0.17369461	0.99978775	-4.5166282
PEG10	-0.3623947	3.84245025	-0.819401	0.41626099	0.99978775	-4.6762066
MXRA5	-0.3624292	2.82567345	-0.8653018	0.390809	0.99978775	-4.7256614
ZSWIM9	-0.3627116	0.25690412	-1.0026377	0.32063341	0.99978775	-4.5957656
ZNF672	-0.3630163	0.4515771	-1.142789	0.25830956	0.99978775	-4.5852537
PSD4	-0.3636497	4.0933111	-1.5320306	0.13151745	0.99978775	-4.3868746
ACKR2	-0.3637268	-0.7423362	-0.4870722	0.62823414	0.99978775	-4.6100566

IMPA2	-0.3640572	1.24690804	-1.228977	0.22455909	0.99978775	-4.5745699
TRAF1	-0.3642905	0.8646167	-0.8009327	0.42677955	0.99978775	-4.613423
HPDL	-0.364485	1.51461938	-1.1841921	0.24166923	0.99978775	-4.5767548
ENSCAFG00000019431	-0.3648571	2.12074194	-1.5733036	0.12165459	0.99978775	-4.4949038
TGFBI	-0.3648942	1.75145622	-0.8192282	0.41635869	0.99978775	-4.7370663
CEP290	-0.3650382	2.84041969	-1.6361283	0.10779285	0.99978775	-4.4436945
ACSL5	-0.3650648	5.04620938	-1.0107142	0.31678761	0.99978775	-4.6282984
PSMB10	-0.3653405	3.57105999	-2.0240044	0.0480705	0.99978775	-4.2248711
PUS1	-0.3657286	4.16265203	-3.4942072	0.00097441	0.99968241	-3.0827985
FAM76B	-0.3658266	1.82603738	-1.3670218	0.17744204	0.99978775	-4.5497747
AGFG2	-0.3660214	4.89588601	-1.8924476	0.06394944	0.99978775	-4.1853513
IFI44L	-0.3661875	0.16575205	-0.6443008	0.52218483	0.99978775	-4.6100079
ENSCAFG00000014370	-0.3662316	2.82611662	-1.2110019	0.23131644	0.99978775	-4.560706
FAM129C	-0.3663223	-2.1445261	-0.6219451	0.53666892	0.99978775	-4.602895
ENSCAFG00000029003	-0.3663751	-0.88705	-0.9358718	0.35362308	0.99978775	-4.5991402
ENSCAFG00000015313	-0.3665039	0.41193495	-0.6614744	0.51120009	0.99978775	-4.610024
ENSCAFG00000028034	-0.3666977	1.30886287	-1.2707235	0.20942759	0.99978775	-4.5686177
OPTC	-0.3668135	-0.8244774	-0.7536028	0.45445341	0.99978775	-4.6067191
ENSCAFG00000020713	-0.3669046	-1.3069798	-0.7862137	0.4352757	0.99978775	-4.6024368
APOL5	-0.3671797	4.66342223	-0.9626238	0.34014813	0.99978775	-4.6394639
MCAT	-0.3672048	4.70992116	-2.255478	0.02829559	0.99978775	-3.9044669
SMPDL3A	-0.3678614	-1.4209703	-0.2727216	0.78613682	0.99978775	-4.606768
LIMA1	-0.368014	7.37349867	-2.5129193	0.01508029	0.99978775	-3.5783142
ENSCAFG00000013770	-0.3680694	0.10392772	-0.4156699	0.67934521	0.99978775	-4.6167965
PPP1R35	-0.3683275	2.74036666	-1.8265584	0.07345257	0.99978775	-4.3690454
RAB3IL1	-0.3689516	6.57747389	-1.1403344	0.25932116	0.99978775	-4.601953
ENSCAFG00000028833	-0.3690863	3.25503124	-1.480385	0.14474544	0.99978775	-4.4687584
CDC42EP5	-0.3693864	3.0778215	-1.5896531	0.11791591	0.99978775	-4.4224327
GPAT3	-0.369613	4.15873801	-1.2973944	0.20016613	0.99978775	-4.5294976
DTL	-0.3697952	3.0417621	-1.2195549	0.22808283	0.99978775	-4.5512784
TBX3	-0.3699614	2.56654654	-0.6701387	0.5057057	0.99978775	-4.6517453
KIAA1324L	-0.3701974	-1.2099941	-0.6138249	0.54198101	0.99978775	-4.6072521
USP21	-0.3709857	3.22606155	-2.1002219	0.04052936	0.99978775	-4.2300796
PTGDS	-0.3712046	1.05816204	-0.437538	0.66351543	0.99978775	-4.6238652
ENSCAFG00000020257	-0.3718446	1.29342049	-1.5158061	0.13556506	0.99978775	-4.53735
KIF24	-0.3718817	1.90713511	-1.0938259	0.27902337	0.99978775	-4.5867816
PHLDA1	-0.3726794	3.01445128	-0.9011814	0.37160642	0.99978775	-4.6202867
FDPS	-0.3734583	6.92196888	-1.9506336	0.05644627	0.99978775	-4.0654007
IAH1	-0.3736294	3.34480863	-1.4618715	0.14973509	0.99978775	-4.4784393
ENSCAFG00000017868	-0.373658	1.70154647	-1.9890592	0.05191556	0.99978775	-4.4029548
ANKRD6	-0.373928	-0.4706389	-0.7558728	0.4531028	0.99978775	-4.612836
ENSCAFG00000020100	-0.374676	-0.2491652	-0.9280963	0.35760386	0.99978775	-4.6007633
CACNA2D2	-0.3751142	4.41896862	-0.9662364	0.33835474	0.99978775	-4.6410355
S100A16	-0.3761374	5.70695536	-1.4889072	0.14249307	0.99978775	-4.4065784
ENSCAFG00000019869	-0.3761528	4.06738722	-1.216292	0.22931249	0.99978775	-4.5540124
ENSCAFG00000019510	-0.3765073	4.07963673	-1.1536316	0.25387476	0.99978775	-4.5781093
PNMA8A	-0.3765924	0.53958342	-0.9967463	0.32345842	0.99978775	-4.5964702
ST6GAL1	-0.3766824	0.44953505	-0.2795124	0.78094954	0.99978775	-4.6169078
RNF151	-0.3769629	0.03139209	-1.0293192	0.30804735	0.99978775	-4.594291
CPT1B	-0.3773903	2.4644837	-1.4997888	0.13965746	0.99978775	-4.5052944

CENPW	-0.3776972	0.84673751	-1.2308425	0.22386618	0.99978775	-4.5681592
ENSCAFG00000021239	-0.3777642	1.19526637	-0.8684211	0.38911535	0.99978775	-4.6083592
SARDH	-0.3779397	2.44653295	-1.0054306	0.31929998	0.99978775	-4.603327
DEF6	-0.3780154	3.70920014	-1.5027761	0.13888687	0.99978775	-4.4547152
ITPKC	-0.3780755	2.39413889	-1.9772559	0.05327276	0.99978775	-4.4062251
KIF26A	-0.3781331	-2.2972604	-0.5489658	0.58535561	0.99978775	-4.6036179
SLC6A4	-0.3790775	-2.5916995	-0.4210438	0.67544138	0.99978775	-4.6050996
ENSCAFG00000029798	-0.3792225	4.44381308	-1.6206596	0.11108062	0.99978775	-4.3476604
PREX2	-0.3792835	4.40379786	-0.7099763	0.48085971	0.99978775	-4.7447221
TRIM7	-0.3793844	3.12768123	-1.3721814	0.1758401	0.99978775	-4.5174432
RBM38	-0.3802746	2.85485275	-1.5145315	0.13588718	0.99978775	-4.4912745
N6AMT1	-0.380652	1.13875603	-1.4462504	0.15404934	0.99978775	-4.5512607
RFFL	-0.3809892	2.83821586	-2.0831867	0.042119	0.99978775	-4.2981122
TMEM132C	-0.3814898	0.11037489	-1.055914	0.29584108	0.99978775	-4.5946542
EXOSC5	-0.3820101	2.64933704	-2.2256334	0.03035563	0.99978775	-4.2980869
AGTR1	-0.3825868	-0.7211992	-0.5922532	0.55622258	0.99978775	-4.6205457
FBXO45	-0.3826786	0.96717141	-1.2856079	0.20422035	0.99978775	-4.5711457
ENSCAFG00000019779	-0.3826979	0.91177441	-1.4870356	0.14298532	0.99978775	-4.5408466
GPC1	-0.383306	6.78070459	-2.0119849	0.0493643	0.99978775	-4.0184558
MAPK13	-0.3833916	0.96106511	-0.8084224	0.42249471	0.99978775	-4.6130141
EDNRA	-0.3835299	2.63387083	-0.2836437	0.77779876	0.99978775	-4.6408255
ANXA9	-0.3844415	1.42854183	-1.1555276	0.2531049	0.99978775	-4.5793662
MDFI	-0.3849859	-1.6437441	-0.6308759	0.53085788	0.99978775	-4.6062212
GARNL3	-0.3855285	3.10990116	-1.4444331	0.15455749	0.99978775	-4.4866577
ENSCAFG00000014684	-0.3855982	5.73566695	-2.114176	0.03926647	0.99978775	-3.9448181
FAM19A5	-0.3859389	-0.2877282	-0.8458364	0.40148168	0.99978775	-4.6038002
PDPN	-0.3868548	7.63650921	-0.6273429	0.53315277	0.99978775	-4.805826
TLR4	-0.3869066	-1.3982327	-0.3678245	0.71448249	0.99978775	-4.6067178
STX3	-0.3873777	1.11816982	-1.12236	0.26681486	0.99978775	-4.5847211
NBEAL2	-0.3874207	3.16651316	-0.9283815	0.35745737	0.99978775	-4.6218177
WDR34	-0.3877047	3.20719055	-1.4461479	0.15407797	0.99978775	-4.4706592
SSC5D	-0.3877376	5.66639084	-0.9740225	0.3345108	0.99978775	-4.6783525
IGF1	-0.3882528	-2.2762051	-0.6155753	0.54083366	0.99978775	-4.6034478
CHCHD4	-0.3883085	1.60155164	-1.8262788	0.0734953	0.99978775	-4.4713507
VASN	-0.3884743	6.27938838	-0.81245	0.42020133	0.99978775	-4.7456491
ARID3B	-0.3889623	1.3232676	-1.2099859	0.23170279	0.99978775	-4.5782606
LIMD1	-0.3891637	5.06317433	-2.0426932	0.04611701	0.99978775	-4.0291154
SH3BP2	-0.3898111	5.12437883	-2.1547572	0.03578711	0.99978775	-3.9702927
ENSCAFG00000001339	-0.389963	-0.9378118	-0.8189411	0.41652102	0.99978775	-4.6012237
LYRM7	-0.3901778	-0.2116795	-0.9392693	0.35189276	0.99978775	-4.5992716
ENSCAFG00000025886	-0.3905033	1.30814676	-1.7207034	0.09119243	0.99978775	-4.4960359
ABLIM2	-0.3908914	-0.0955441	-0.3491533	0.72837039	0.99978775	-4.6131655
HAPLN4	-0.3908998	-0.1164107	-0.6928383	0.49146375	0.99978775	-4.6060984
IQCC	-0.3911886	1.71314229	-1.3125513	0.19504187	0.99978775	-4.5568269
ENSCAFG00000031436	-0.3915775	0.96593086	-1.3796534	0.1735399	0.99978775	-4.5640276
PTPRQ	-0.3916621	-1.9368187	-0.6942579	0.49058049	0.99978775	-4.6052814
CD248	-0.3920098	7.72235696	-0.9006216	0.37190128	0.99978775	-4.7114591
TTC26	-0.3920884	3.177673	-1.7629466	0.08372778	0.99978775	-4.4033305
ENSCAFG00000011847	-0.3920934	1.10823595	-1.0379982	0.30402677	0.99978775	-4.5939258
PLEKHA4	-0.3924855	5.29610619	-1.6227302	0.11063587	0.99978775	-4.319928

NNAT	-0.392716	-1.525471	-0.6820568	0.49820041	0.99978775	-4.6037374
LRRC75A	-0.3929251	2.8986669	-0.9891264	0.32713697	0.99978775	-4.6118597
DDB2	-0.3931715	2.44130916	-1.6410496	0.10676364	0.99978775	-4.412537
SPC24	-0.3934641	2.34282854	-1.3566264	0.1807035	0.99978775	-4.5083678
TNFSF9	-0.3935733	0.37276461	-0.5714311	0.5701452	0.99978775	-4.6122943
DNAH10	-0.3935929	-0.3739359	-0.7617981	0.44958834	0.99978775	-4.6059655
NWD1	-0.3936103	-0.7219904	-0.7058211	0.48341891	0.99978775	-4.6054451
TPBG	-0.3937356	4.7705371	-1.4021685	0.16674862	0.99978775	-4.4606021
GJB3	-0.3940268	4.14150571	-1.1417859	0.25872262	0.99978775	-4.5879907
RDH13	-0.394057	2.36860389	-1.6565818	0.10356776	0.99978775	-4.4630811
CCDC181	-0.3941022	2.23472318	-1.6626355	0.10234353	0.99978775	-4.4871275
MAPK8IP2	-0.3942904	1.91794745	-1.4053039	0.16581939	0.99978775	-4.5322939
ENSCAFG00000030356	-0.3950722	0.54292895	-0.9650212	0.33895729	0.99978775	-4.5985743
RPP40	-0.3951904	2.76976982	-1.8207228	0.07434878	0.99978775	-4.3714736
TIMM10B	-0.3961294	1.98779244	-1.5870256	0.11851043	0.99978775	-4.4943116
FHOD3	-0.397255	1.61845531	-1.0498283	0.29860447	0.99978775	-4.6080974
PDE3A	-0.3972676	4.79122342	-1.2489081	0.21723737	0.99978775	-4.5390859
ENSCAFG00000007015	-0.3976623	0.43413495	-1.1378212	0.26035983	0.99978775	-4.5870422
NCAPD2	-0.3978588	5.69309494	-1.8994665	0.06300157	0.99978775	-4.1106173
MTR	-0.3980627	4.12768787	-2.8448657	0.00632032	0.99978775	-3.5781902
WEE1	-0.3981549	3.29092861	-2.2249346	0.03040541	0.99978775	-4.1607317
MAP4K2	-0.3983525	2.84536346	-1.4878415	0.1427732	0.99978775	-4.4946984
AARS2	-0.3984041	3.0209945	-2.8179272	0.00679826	0.99978775	-3.8820614
INCENP	-0.3984279	5.4923989	-2.1981611	0.03236795	0.99978775	-3.8647431
TRIB3	-0.3985335	5.55981559	-2.4525183	0.01754369	0.99978775	-3.6233939
ENSCAFG00000019300	-0.3987373	-1.4531836	-0.5159734	0.60803871	0.99978775	-4.6043455
LY6E	-0.3988604	-1.097823	-0.7392017	0.46307597	0.99978775	-4.6086439
MAFB	-0.3991222	-0.6736826	-0.8638083	0.39162153	0.99978775	-4.6038529
S100A2	-0.3992418	-0.0537232	-1.26314	0.21211838	0.99978775	-4.5813716
ENSCAFG00000029083	-0.3993268	0.35945969	-0.8901997	0.37741881	0.99978775	-4.6071344
ENSCAFG00000022715	-0.3994298	-0.9656539	-0.8475486	0.40053577	0.99978775	-4.6012667
TRIP13	-0.3996738	4.26660096	-1.4803846	0.14474555	0.99978775	-4.4216568
CD3G	-0.3997125	-0.9491827	-0.4347205	0.6655465	0.99978775	-4.6103435
SCN4B	-0.4000854	2.65257022	-0.7686083	0.44556875	0.99978775	-4.6434536
ANLN	-0.4001733	6.43922139	-1.0127777	0.31581005	0.99978775	-4.6600424
BORCS6	-0.4007357	0.30768162	-0.9395632	0.35174337	0.99978775	-4.5998739
FANCM	-0.401012	1.70260788	-0.9622267	0.3403456	0.99978775	-4.6116673
DTNA	-0.4015807	1.45070899	-0.7062611	0.48314752	0.99978775	-4.6349346
ANK3	-0.4017025	2.81723627	-1.0426577	0.30188312	0.99978775	-4.6071427
STIM1	-0.4017327	4.7352015	-1.3239604	0.19125037	0.99978775	-4.5120919
ING5	-0.4019101	1.16051023	-1.4807142	0.14465792	0.99978775	-4.5512456
SH2D3C	-0.4020088	-0.8468748	-0.3005931	0.76491163	0.99978775	-4.6084102
SYNE3	-0.4022401	4.57829124	-0.8389825	0.40528209	0.99978775	-4.6707719
SERINC5	-0.4029719	3.10932616	-1.4501088	0.1529748	0.99978775	-4.507134
PINX1	-0.403078	2.80628116	-1.7850707	0.08002586	0.99978775	-4.4016701
MCM4	-0.4031759	5.99669172	-1.4206234	0.16133679	0.99978775	-4.4444417
CXCR4	-0.4032908	-0.6287164	-0.2191546	0.82737917	0.99978775	-4.6096544
POLA2	-0.4037875	4.0099608	-1.2804404	0.20601708	0.99978775	-4.5244559
GLI4	-0.4040943	2.04365185	-1.3753071	0.17487505	0.99978775	-4.5437596
SLC19A2	-0.4046999	3.67272177	-1.3018544	0.19864792	0.99978775	-4.5263215

IL1R1	-0.4048483	5.30297048	-1.0786317	0.2856815	0.99978775	-4.5990595
RCSD1	-0.4049621	-1.6367331	-0.5513662	0.58372119	0.99978775	-4.6037397
SIPA1	-0.405086	4.4931708	-1.9802071	0.0529306	0.99978775	-4.1610026
COX19	-0.4054197	0.84900105	-1.505584	0.13816564	0.99978775	-4.5480176
DLEC1	-0.4057321	1.65941047	-1.2467997	0.21800344	0.99978775	-4.5732232
CHDH	-0.405758	1.22267281	-1.3898714	0.17043191	0.99978775	-4.5216299
BAMBI	-0.4062744	0.23022716	-0.6347348	0.52835716	0.99978775	-4.6240349
THSD4	-0.4069986	-1.3361152	-0.5811723	0.56361052	0.99978775	-4.6071404
NDE1	-0.4073096	2.79091867	-1.7266113	0.09011647	0.99978775	-4.4267326
EGR3	-0.4075686	0.8842015	-0.479957	0.63325105	0.99978775	-4.6143212
SLC9A3R1	-0.4077578	2.57988681	-1.333711	0.1880545	0.99978775	-4.5357617
ARRB1	-0.4081361	0.506759	-0.3614012	0.71924954	0.99978775	-4.6130127
ENSCAFG00000000069	-0.4084615	2.11942306	-1.6615319	0.10256583	0.99978775	-4.488404
CD74	-0.408723	-1.5569588	-0.4656276	0.64340749	0.99978775	-4.6059135
ITGB7	-0.4087573	0.05408684	-0.7948932	0.43025362	0.99978775	-4.6047205
ENSCAFG00000031848	-0.4088415	2.52012118	-0.7179343	0.47597965	0.99978775	-4.6305083
MSC	-0.4090195	4.90473729	-0.9701745	0.33640688	0.99978775	-4.6756895
ENSCAFG00000029063	-0.409142	2.28554565	-2.1883556	0.03311416	0.99978775	-4.3376615
ENSCAFG00000008941	-0.4099364	1.65466795	-0.8586511	0.39443539	0.99978775	-4.6160831
ENSCAFG00000004696	-0.4107738	2.51982163	-2.1616186	0.03522635	0.99978775	-4.339768
ZBED2	-0.4112829	0.79096934	-0.8063474	0.4236792	0.99978775	-4.6193405
CENPP	-0.4114913	3.0393783	-1.4324157	0.15795085	0.99978775	-4.4959871
ENSCAFG00000018374	-0.4116704	-0.7133856	-0.7713097	0.44398018	0.99978775	-4.6036264
ENSCAFG00000016467	-0.4118727	2.56776953	-1.3490055	0.18312345	0.99978775	-4.5071542
INSR	-0.4121415	3.85954007	-1.462437	0.14958072	0.99978775	-4.4828366
APOE	-0.412226	7.0498555	-0.6506676	0.51809793	0.99978775	-4.7990439
GALNT14	-0.4122591	-2.6463336	-0.7271028	0.47039209	0.99978775	-4.6010232
KPTN	-0.4123255	1.58824389	-1.6735058	0.10017499	0.99978775	-4.5046888
PTK7	-0.4126857	6.75670385	-1.2609837	0.21288815	0.99978775	-4.5375471
ATP8B4	-0.4127693	1.30708631	-0.5475673	0.58630889	0.99978775	-4.6306031
ENSCAFG00000021068	-0.4138984	-1.3274356	-1.0089678	0.31761655	0.99978775	-4.5951462
ABCB4	-0.4140833	1.64702361	-1.2387981	0.22092891	0.99978775	-4.5552367
NSUN5	-0.4141921	3.76350133	-3.5095187	0.00093014	0.99968241	-3.1360882
TCHP	-0.4143179	3.89102145	-2.4972607	0.01568688	0.99978775	-3.8618505
CELSR2	-0.4144064	1.40786486	-0.9336418	0.35476182	0.99978775	-4.6013713
CENPK	-0.4144576	1.07875038	-1.0292254	0.30809098	0.99978775	-4.59527
THUMPD2	-0.4155585	1.797351	-1.6048737	0.1145193	0.99978775	-4.5064868
CEP250	-0.4159007	3.39118133	-2.2376865	0.02950821	0.99978775	-4.1258401
TIMM29	-0.4159449	1.55057002	-1.6249204	0.11016699	0.99978775	-4.5221515
SEC31B	-0.4162513	0.71788501	-1.2200105	0.2279115	0.99978775	-4.5777635
ENSCAFG00000030154	-0.4168655	-0.9060881	-1.2000582	0.23550262	0.99978775	-4.5844473
MYORG	-0.4171565	2.39208981	-1.4285202	0.15906318	0.99978775	-4.5334598
PTGFR	-0.4172444	1.11022633	-1.3908513	0.17013612	0.99978775	-4.537908
ENSCAFG00000032647	-0.4181654	0.24291198	-1.0772091	0.28631047	0.99978775	-4.5918358
ACTL7A	-0.4187031	0.74862725	-1.1776052	0.24426341	0.99978775	-4.5832801
CALML4	-0.4188019	-0.6755144	-0.7963498	0.42941418	0.99978775	-4.6034365
ZNF524	-0.418894	1.88341803	-1.3668831	0.17748525	0.99978775	-4.5451137
ALG14	-0.4196509	1.53280483	-1.4815212	0.14444352	0.99978775	-4.5282201
ENKD1	-0.4203737	2.40293519	-2.3040098	0.02520946	0.99978775	-4.3116499
HIC2	-0.4212347	2.0950312	-1.7091262	0.09333174	0.99978775	-4.5029039

PLPP7	-0.4212929	1.06625127	-1.1862137	0.24087708	0.99978775	-4.5810429
ENSCAFG00000032625	-0.4216155	4.16682466	-0.8400394	0.40469463	0.99978775	-4.6966463
OLFML3	-0.4216559	5.38709119	-1.0353202	0.30526352	0.99978775	-4.6507261
PKN3	-0.4224243	4.00451283	-1.2626601	0.21228951	0.99978775	-4.5347129
COLEC11	-0.422495	-0.8019878	-0.5151455	0.60861308	0.99978775	-4.6130439
ALX1	-0.4225249	-2.3297662	-0.7377301	0.46396232	0.99978775	-4.6020034
CPZ	-0.4226798	-0.7117012	-1.2734931	0.20845122	0.99978775	-4.5760706
FMO3	-0.4227127	1.08588132	-0.5505127	0.58430205	0.99978775	-4.619876
TBX15	-0.4229301	3.31365775	-0.6964947	0.48919061	0.99978775	-4.6867294
PPARGC1B	-0.4230566	2.20688743	-0.9733616	0.33483594	0.99978775	-4.6018903
HCK	-0.4231241	-2.4934683	-0.8108228	0.42112697	0.99978775	-4.5999668
TBC1D10C	-0.4231512	-1.4674525	-0.6830477	0.49757919	0.99978775	-4.6033002
DDX11	-0.4242766	3.95363771	-2.3945045	0.02024552	0.99978775	-3.9409072
ENSCAFG00000008243	-0.4246702	2.80890498	-2.2407682	0.02929493	0.99978775	-4.2541527
MDM1	-0.424717	2.12930601	-1.994775	0.05126908	0.99978775	-4.4033194
CENPH	-0.4260505	2.7969691	-1.4182485	0.16202547	0.99978775	-4.4790766
RTN4RL1	-0.4263865	-2.1085276	-0.6050649	0.54774174	0.99978775	-4.6032041
LDHD	-0.4264599	1.85208125	-0.9047802	0.3697141	0.99978775	-4.6067943
PPP1R3F	-0.4273549	0.55738223	-1.2942	0.2012589	0.99978775	-4.5710013
ISLR	-0.4274861	2.10660697	-0.6061633	0.54701769	0.99978775	-4.6336189
EMB	-0.4277374	-2.0526989	-0.6197053	0.53813149	0.99978775	-4.6034743
RAVER2	-0.4277502	2.01466901	-0.9166724	0.36350495	0.99978775	-4.6058198
UCP2	-0.4285751	5.63954679	-0.9524	0.34525736	0.99978775	-4.6832003
HBEGF	-0.4288189	1.80092799	-0.8007805	0.42686688	0.99978775	-4.619011
MAD2L1BP	-0.4289155	2.8384634	-1.4671721	0.14829289	0.99978775	-4.4968572
BMPER	-0.4289716	-1.1978604	-0.8485426	0.39998725	0.99978775	-4.6027461
PPP1R15A	-0.429048	5.97397542	-2.0876857	0.04169399	0.99978775	-3.9698417
TRIM35	-0.4292691	2.72520993	-2.1142232	0.03926225	0.99978775	-4.2895899
SMIM8	-0.4293436	1.60037085	-1.7279671	0.08987102	0.99978775	-4.4954185
CDKL4	-0.4294005	-1.1072158	-0.6011502	0.55032613	0.99978775	-4.6056147
ISL1	-0.429643	-2.0328845	-0.6311722	0.53066564	0.99978775	-4.6039908
CYP26B1	-0.4298682	2.13742116	-0.7483559	0.45758408	0.99978775	-4.64446
EGFL7	-0.4298708	1.05429051	-0.4095618	0.68379322	0.99978775	-4.6215632
RNF150	-0.4301819	0.6368432	-0.7209546	0.47413487	0.99978775	-4.6120313
MRC1	-0.4303039	-0.4994329	-0.7987953	0.42800708	0.99978775	-4.6020431
FAM193B	-0.4304611	4.02610014	-2.3390061	0.02317378	0.99978775	-4.0309185
ENSCAFG00000018572	-0.4305199	6.50556048	-1.7518497	0.08563765	0.99978775	-4.2253421
SCD	-0.4310185	9.55027775	-1.9354328	0.05832987	0.99978775	-4.139561
TJP3	-0.4312047	-2.1658848	-0.7283185	0.46965399	0.99978775	-4.6013117
ENSCAFG00000023349	-0.4317587	2.17599004	-0.8778245	0.38403752	0.99978775	-4.6153679
CCL27	-0.4319105	-0.4599433	-1.2472201	0.2178505	0.99978775	-4.5837336
TNFAIP8	-0.4320599	0.5325183	-0.9047586	0.36972546	0.99978775	-4.6038141
ENSCAFG00000010570	-0.4326477	-0.8869214	-1.0300087	0.30772664	0.99978775	-4.5943232
ERRFI1	-0.4328463	5.82247194	-1.2146748	0.22992376	0.99978775	-4.552966
ENSCAFG00000032034	-0.4336735	-0.2537344	-1.128821	0.26410373	0.99978775	-4.5894821
EVA1C	-0.4337718	1.94510241	-0.5942257	0.55491256	0.99978775	-4.6104284
ENSCAFG00000024295	-0.4339286	-0.5777194	-0.896824	0.37390585	0.99978775	-4.6007895
HR	-0.4343612	5.86634712	-1.0880509	0.28154109	0.99978775	-4.6115838
TRIM25	-0.4346641	6.14548369	-2.6287815	0.01121291	0.99978775	-3.4571837
TMC6	-0.4350843	0.19819133	-0.7917245	0.43208306	0.99978775	-4.6047164

ENSCAFG00000026287	-0.4352496	0.10750605	-1.0978632	0.27727261	0.99978775	-4.5903294
ENSCAFG00000013278	-0.4353836	1.84057376	-1.5332414	0.13121929	0.99978775	-4.4841948
ADCK2	-0.4354914	3.18986568	-1.7046785	0.09416456	0.99978775	-4.4214129
AURKA	-0.4363682	2.59319552	-1.1892844	0.23967744	0.99978775	-4.5647144
AFF2	-0.4364626	-1.0199488	-0.7747434	0.44196579	0.99978775	-4.6030278
KLHL31	-0.4367597	0.11922372	-1.0170959	0.31377095	0.99978775	-4.595514
OSR2	-0.4367742	2.52982543	-0.6507547	0.51804213	0.99978775	-4.6331633
ECT2	-0.4374421	4.32306595	-1.202304	0.23463911	0.99978775	-4.5664059
ARRDC3	-0.4382894	6.2911321	-1.8376796	0.07176976	0.99978775	-4.1692379
CCDC149	-0.4383831	-0.1865643	-0.9943856	0.32459508	0.99978775	-4.5970094
C28H10orf71	-0.4384121	-1.9679268	-1.0480129	0.29943221	0.99978775	-4.5934731
PCSK4	-0.4397703	-0.3545024	-0.9817248	0.33073684	0.99978775	-4.5961308
CIART	-0.4397758	2.13589389	-1.5884762	0.11818191	0.99978775	-4.4923768
PCLO	-0.4401009	0.42674306	-0.9004098	0.37201291	0.99978775	-4.6014017
ENSCAFG00000026225	-0.4409904	-0.1989667	-1.5013048	0.13926599	0.99978775	-4.5619637
VEPH1	-0.4411997	1.06533883	-0.4954643	0.62233947	0.99978775	-4.615427
ALDH16A1	-0.4418541	4.39194838	-2.7446543	0.00827232	0.99978775	-3.5337377
ENSCAFG00000032099	-0.4420284	-0.2449551	-0.7968121	0.42914796	0.99978775	-4.6042324
ENSCAFG00000025074	-0.4424197	2.30436944	-2.1820946	0.03359852	0.99978775	-4.3376445
SMC2	-0.4429639	4.16502832	-1.3060667	0.19722194	0.99978775	-4.5118546
DSCC1	-0.4430181	1.74838873	-1.7232449	0.09072826	0.99978775	-4.4675841
TREX2	-0.4432282	1.92726163	-1.6571115	0.10346016	0.99978775	-4.4518976
TTC9	-0.4432449	-0.4134229	-0.856315	0.39571419	0.99978775	-4.6027436
LRRC27	-0.4432646	0.51058875	-1.1724805	0.24629556	0.99978775	-4.5851417
MKNK2	-0.4438762	5.53869072	-3.133982	0.00282095	0.99978775	-2.9577683
STON2	-0.4441159	3.9316105	-0.7401157	0.46252591	0.99978775	-4.7110696
STS	-0.4445051	1.56429635	-1.4257732	0.15985125	0.99978775	-4.5406883
ENSCAFG00000031575	-0.4461862	-1.4549271	-0.8800131	0.38286168	0.99978775	-4.5997645
SLAMF9	-0.4465494	-0.4937764	-0.6376774	0.52645443	0.99978775	-4.607943
IGSF11	-0.4465911	-1.425066	-0.8369598	0.40640786	0.99978775	-4.6024365
PXMP2	-0.4469314	1.15178028	-1.9897236	0.05184006	0.99978775	-4.44359
ADAMTS6	-0.4472293	4.75646485	-1.1947338	0.23755914	0.99978775	-4.5629745
ENSCAFG00000023004	-0.4475208	2.01226251	-0.8352618	0.40735439	0.99978775	-4.623721
ENSCAFG00000014043	-0.4475843	-0.5886494	-1.0507171	0.29819977	0.99978775	-4.5927281
PRSS35	-0.4481644	0.78791706	-0.9693471	0.3368155	0.99978775	-4.5998175
PDE3B	-0.4484182	2.86626419	-1.0762619	0.28672981	0.99978775	-4.5926071
ISYNA1	-0.4491617	3.96806836	-1.5200102	0.13450685	0.99978775	-4.4509525
ST8SIA1	-0.4496905	-1.5436863	-0.6731075	0.50383042	0.99978775	-4.6037478
CCNE2	-0.4497351	0.2678163	-0.9639525	0.33948776	0.99978775	-4.5995589
ABTB2	-0.4500852	1.21927963	-0.7314915	0.46773067	0.99978775	-4.6118961
CAND2	-0.4501694	0.13199035	-1.3390787	0.18631257	0.99978775	-4.5569346
SHLD1	-0.4501892	1.15215572	-1.65361	0.10417312	0.99978775	-4.5198716
TOMM6	-0.4505495	2.56733788	-2.5648069	0.01321911	0.99978775	-4.1412078
MIS18BP1	-0.4508472	3.50378794	-1.7129137	0.09262736	0.99978775	-4.338728
ARHGEF39	-0.4508532	2.95600224	-1.3787678	0.1738113	0.99978775	-4.4924982
TUBD1	-0.4510639	1.59337451	-1.81172	0.07574942	0.99978775	-4.478305
ROGDI	-0.4512581	2.02869481	-1.4368086	0.15670376	0.99978775	-4.5342596
CARHSP1	-0.4514002	1.77419223	-1.1665089	0.24867891	0.99978775	-4.5804363
ENSCAFG00000000747	-0.4518012	0.15447047	-1.6064898	0.11416333	0.99978775	-4.5479215
NES	-0.4518347	7.89437553	-1.4054701	0.16577025	0.99978775	-4.454619

CYP2S1	-0.4521149	-0.8588856	-0.7016718	0.48598198	0.99978775	-4.6097988
NCAPG2	-0.4521434	4.65959816	-1.8768699	0.06609652	0.99978775	-4.1556267
JAG2	-0.4521943	1.85035907	-0.9750014	0.33402958	0.99978775	-4.6003423
PLXND1	-0.4528901	6.42182977	-1.1744412	0.24551664	0.99978775	-4.5839663
BRIP1	-0.4542939	1.77014548	-1.4186267	0.16191566	0.99978775	-4.528591
ABI3	-0.4553334	-2.5224818	-0.725657	0.47127067	0.99978775	-4.6010657
CLEC11A	-0.4554655	3.23185799	-0.8956663	0.37451826	0.99978775	-4.6370071
FOS	-0.4555229	6.70273312	-0.9395995	0.35172488	0.99978775	-4.6953302
CCDC65	-0.4556932	-0.5177533	-1.1575218	0.25229697	0.99978775	-4.5887495
ADAMTS7	-0.4561067	2.98035825	-0.7089743	0.48147611	0.99978775	-4.673446
DSN1	-0.4561332	2.89534636	-1.3214539	0.19207854	0.99978775	-4.5163995
BANP	-0.456711	2.36000263	-1.5908922	0.11763641	0.99978775	-4.4813085
ADAMTSL1	-0.456891	6.44255466	-1.3631406	0.17865443	0.99978775	-4.4795601
PIK3CD	-0.457168	2.1679155	-1.2812128	0.20574778	0.99978775	-4.5545304
ATF3	-0.4578665	4.34452016	-1.4014587	0.16695953	0.99978775	-4.4642639
MEGF6	-0.4581287	3.38518092	-2.044642	0.04591732	0.99978775	-3.9893297
SH2D4A	-0.458651	2.98402202	-1.1253495	0.265558	0.99978775	-4.5804783
MCM3	-0.4593873	4.91320548	-1.2012299	0.23505183	0.99978775	-4.5684495
A4GALT	-0.4595561	2.33759476	-0.7353985	0.46536863	0.99978775	-4.6241001
PRSS53	-0.4603649	0.35785794	-0.8022667	0.42601443	0.99978775	-4.6150118
SLCO1C1	-0.4605021	-1.0648391	-0.7961125	0.42955089	0.99978775	-4.6019776
LINGO2	-0.4619909	-2.1276468	-0.9004628	0.371985	0.99978775	-4.5991217
FOLH1	-0.4620105	4.09061513	-1.2206971	0.2276535	0.99978775	-4.5509016
E2F7	-0.4624844	3.51484851	-1.6746456	0.09994982	0.99978775	-4.3239535
TMEM80	-0.4628148	0.38561138	-1.212731	0.23066006	0.99978775	-4.5813038
FRAS1	-0.4629199	3.64898311	-0.4066587	0.68591134	0.99978775	-4.6664913
KIF27	-0.4629236	1.93344844	-1.3972678	0.16820905	0.99978775	-4.5448307
SLC2A9	-0.463567	0.29703675	-0.5877871	0.55919441	0.99978775	-4.6132526
ENSCAFG00000028262	-0.4636072	-0.4371639	-0.9829041	0.33016152	0.99978775	-4.5965467
ANO3	-0.4638371	-2.8476093	-0.7990763	0.42784557	0.99978775	-4.599585
ZBTB3	-0.4640009	0.52779702	-1.3138462	0.1946087	0.99978775	-4.5730112
GPR156	-0.4642012	-0.3326749	-1.0294892	0.30796825	0.99978775	-4.5942758
ADAMTS12	-0.4644272	4.62111284	-1.1509201	0.25497865	0.99978775	-4.5748541
TTYH2	-0.4646786	4.19851406	-1.6498122	0.10495092	0.99978775	-4.3298768
EGFL8	-0.4647706	-0.3349693	-1.0872489	0.28189199	0.99978775	-4.5914805
FOXM1	-0.4653736	3.00310077	-1.6170027	0.11186966	0.99978775	-4.3721234
TCF7	-0.4660514	-2.1715833	-0.7845215	0.43625887	0.99978775	-4.601329
TBC1D2B	-0.4663951	4.10772919	-1.0909489	0.28027567	0.99978775	-4.5925433
ENSCAFG00000025410	-0.4664783	-1.7122828	-0.6690083	0.50642073	0.99978775	-4.6041545
ENSCAFG00000022659	-0.4667957	-1.0249716	-0.9142174	0.36478123	0.99978775	-4.5985233
RNF122	-0.4667973	-1.2701202	-1.2627483	0.21225804	0.99978775	-4.5806958
ORC1	-0.4668785	1.92365252	-1.2056918	0.23334083	0.99978775	-4.5595029
MEF2B	-0.4669885	-0.4209331	-0.9701326	0.33642759	0.99978775	-4.59675
PFKFB4	-0.4670455	2.08876544	-1.6055464	0.11437101	0.99978775	-4.5295698
DHTKD1	-0.4672591	1.89953257	-1.73026	0.0894572	0.99978775	-4.4321639
AGAP2	-0.4679174	-0.2863162	-0.9533853	0.34476277	0.99978775	-4.597852
CRACR2B	-0.4681119	-0.5427277	-0.663635	0.50982701	0.99978775	-4.6061459
NEU3	-0.4683488	0.68900582	-1.3327704	0.18836102	0.99978775	-4.5718146
APOLD1	-0.4684916	-1.1742918	-0.8081342	0.42265909	0.99978775	-4.6009493
IL11	-0.4686077	0.01190317	-0.9616041	0.34065545	0.99978775	-4.5978312

NR6A1	-0.4697308	0.24370878	-1.2383871	0.22107997	0.99978775	-4.5809792
ENSCAFG00000023205	-0.4701304	3.14103484	-2.3180306	0.0243755	0.99978775	-4.1092756
PALM3	-0.4705179	2.3939057	-0.9855881	0.3288546	0.99978775	-4.6072322
ENSCAFG00000031933	-0.4707436	3.1470828	-1.6509197	0.1047236	0.99978775	-4.4132647
ENSCAFG00000007079	-0.4718377	0.51524526	-1.6697779	0.1009144	0.99978775	-4.5221719
ENSCAFG00000000142	-0.4718785	1.81774148	-1.9571777	0.05565154	0.99978775	-4.3831277
PCDH19	-0.4722879	-0.7758202	-0.7095796	0.48110372	0.99978775	-4.6032313
CDKN2C	-0.4725065	2.91043433	-1.5447088	0.1284221	0.99978775	-4.4284797
BLM	-0.4730537	2.77052977	-1.4473919	0.15373084	0.99978775	-4.4894652
DNA2	-0.4736397	1.51190913	-1.1631633	0.25002141	0.99978775	-4.5740981
ZDHHC14	-0.4741228	-1.7078186	-0.7991579	0.42779867	0.99978775	-4.6009092
TMEM150C	-0.474203	-0.2243974	-0.6870452	0.49507723	0.99978775	-4.6063787
ZNF572	-0.4743473	0.48619098	-1.3790103	0.17373694	0.99978775	-4.5663339
ENSCAFG00000019344	-0.4747782	1.87605508	-1.3229812	0.19157359	0.99978775	-4.5619172
ZCCHC9	-0.4747882	1.01848694	-2.0033906	0.05030774	0.99978775	-4.4784232
HMGB2	-0.4760003	4.7107839	-1.680575	0.09878509	0.99978775	-4.3030335
ENSCAFG00000018512	-0.4776914	0.23120401	-1.5078318	0.13759042	0.99978775	-4.5517645
CPNE2	-0.4778963	2.99530825	-1.700912	0.09487461	0.99978775	-4.4336686
ENSCAFG00000000564	-0.4782025	1.43130544	-1.5586298	0.12509073	0.99978775	-4.5330326
TMEM141	-0.4783125	0.91929501	-1.3017667	0.19867769	0.99978775	-4.571631
TMEM67	-0.4789353	3.48268166	-1.3652648	0.1779901	0.99978775	-4.5139297
LBX2	-0.4789843	0.38543423	-1.3155175	0.19405071	0.99978775	-4.5721092
SEC16B	-0.4793153	0.98127865	-1.8794591	0.06573546	0.99978775	-4.4433232
APBB3	-0.4794559	1.67809756	-0.6788043	0.50024258	0.99978775	-4.6169417
SAMHD1	-0.4794851	2.76521889	-1.3279254	0.18994588	0.99978775	-4.5449913
STARD5	-0.4797898	1.42972869	-1.9354946	0.0583221	0.99978775	-4.4652463
ENSCAFG00000030021	-0.4801697	-1.4372332	-0.6506042	0.51813853	0.99978775	-4.60375
KCTD21	-0.4803262	-0.2636377	-1.0200076	0.31240104	0.99978775	-4.5947435
ENGASE	-0.4806882	0.82393245	-1.3459776	0.18409176	0.99978775	-4.5665197
SLC39A8	-0.4808402	0.09820864	-1.1192273	0.26813644	0.99978775	-4.5876303
PROCA1	-0.4808833	1.40728418	-1.4469553	0.15385261	0.99978775	-4.5476915
ADGRG2	-0.4809549	2.97843728	-0.7209546	0.47413483	0.99978775	-4.6486576
NYX	-0.4821968	-1.7372675	-0.7691382	0.44525692	0.99978775	-4.6012596
LRRC71	-0.4828665	0.85008832	-1.914519	0.061009	0.99978775	-4.4960276
FANCD2	-0.4829851	3.67492864	-1.5225402	0.1338732	0.99978775	-4.4009553
THY1	-0.483138	7.56655599	-1.0686306	0.29012383	0.99978775	-4.6318573
ENSCAFG00000024792	-0.483221	-2.0008497	-0.6598677	0.5122225	0.99978775	-4.6028545
DLX3	-0.4835819	-1.5065369	-0.6134594	0.54222077	0.99978775	-4.60372
PIF1	-0.4836911	-0.2912591	-1.2696286	0.20981449	0.99978775	-4.5790214
TCEANC	-0.4839671	1.02966074	-1.5220429	0.13399757	0.99978775	-4.5446741
IGF2BP1	-0.4840287	0.18218211	-1.1431105	0.25817725	0.99978775	-4.5830182
PROSER2	-0.4846465	-0.692787	-0.9516305	0.34564392	0.99978775	-4.5993894
ENSCAFG00000001830	-0.4858692	-1.2045764	-1.2962437	0.20055929	0.99978775	-4.5787715
ENSCAFG00000022282	-0.4859002	-0.4130013	-1.0562183	0.29570338	0.99978775	-4.5930677
CASP12	-0.4859881	-0.8963088	-0.6577056	0.51360009	0.99978775	-4.6040439
CNTNAP4	-0.4861948	-0.278338	-0.4872479	0.62811048	0.99978775	-4.6096185
ENSCAFG00000023972	-0.4866894	1.23213753	-1.3668992	0.17748024	0.99978775	-4.5480486
IGFLR1	-0.4867409	0.03759584	-1.3320285	0.18860305	0.99978775	-4.5758766
ENSCAFG00000022575	-0.4868201	-0.45051	-0.9805053	0.33133247	0.99978775	-4.5974014
SPTBN4	-0.4871223	-2.195512	-0.8028075	0.42570454	0.99978775	-4.5998323

BRCA1	-0.487771	3.55443054	-1.58017	0.12007298	0.99978775	-4.3914365
TMEM201	-0.4878679	2.78644408	-2.6430257	0.0108059	0.99978775	-4.0722253
ENSCAFG00000025619	-0.4881379	0.23598284	-1.3599622	0.17965198	0.99978775	-4.5645226
ENSCAFG00000029897	-0.488381	-2.3062978	-0.6231235	0.53590033	0.99978775	-4.603635
NLRC5	-0.488872	1.1482854	-1.0665988	0.29103214	0.99978775	-4.5909928
PLPP4	-0.4891341	-1.3373715	-1.2915826	0.20215761	0.99978775	-4.575963
XKR5	-0.4897962	-1.4552389	-0.9994971	0.32213732	0.99978775	-4.5961548
NOVA2	-0.4900952	-2.0632027	-0.7389163	0.46324778	0.99978775	-4.600949
ELOVL6	-0.4902478	3.07797467	-1.6649209	0.10188446	0.99978775	-4.4238301
ENSCAFG00000023760	-0.4909432	3.64064604	-1.9768666	0.05331805	0.99978775	-4.2216183
TEX15	-0.4911963	0.01087401	-1.2403066	0.22037517	0.99978775	-4.5806708
ENSCAFG00000002412	-0.4914968	1.03019135	-1.5999135	0.11561741	0.99978775	-4.5373799
SQLE	-0.4916697	7.17496977	-1.8309453	0.07278484	0.99978775	-4.1599422
CATSPER2	-0.4929202	-0.4282717	-1.3760268	0.1746534	0.99978775	-4.5744951
GALNT6	-0.4933156	2.52314551	-0.550593	0.5842474	0.99978775	-4.6328676
GJA4	-0.4938826	-2.6067085	-0.8035255	0.42529325	0.99978775	-4.6000594
ENSCAFG00000010825	-0.4938957	0.48755319	-1.4762322	0.14585312	0.99978775	-4.5443002
DNAJC9	-0.4941366	1.3524649	-2.066633	0.04371542	0.99978775	-4.4056926
CKAP2L	-0.4941816	3.12352555	-1.307332	0.19679512	0.99978775	-4.5140188
ADAMTS14	-0.4943879	2.93861095	-1.6700009	0.10087004	0.99978775	-4.3191441
SFMBT1	-0.4951714	2.75510127	-1.8755213	0.06628523	0.99978775	-4.3899598
FBXO5	-0.4952138	2.31375465	-1.6022421	0.11510083	0.99978775	-4.4473669
SASS6	-0.4959773	1.94085396	-1.0904202	0.28050622	0.99978775	-4.5882983
SNN	-0.4967476	-0.5317783	-1.1467168	0.25669669	0.99978775	-4.5892992
PLPPR2	-0.4968869	-1.225495	-0.8501166	0.39911958	0.99978775	-4.6007238
CDC42EP4	-0.4976683	3.66432697	-1.8587955	0.06866397	0.99978775	-4.3240889
ENSCAFG00000031011	-0.4976981	-1.1706022	-0.9638362	0.33954554	0.99978775	-4.5969715
FAM228B	-0.4980137	-0.3198945	-1.2565109	0.21449149	0.99978775	-4.5841008
PLXNA4	-0.4984804	1.56029544	-0.9815158	0.33083887	0.99978775	-4.6009954
CXCL12	-0.4986943	4.27505792	-0.6188611	0.53868327	0.99978775	-4.658461
ENSCAFG00000029818	-0.4988847	-0.9504914	-0.988657	0.32736449	0.99978775	-4.5959169
AUNIP	-0.4990568	-0.9242294	-0.9051855	0.36950138	0.99978775	-4.600331
KDM8	-0.4991953	0.14508997	-1.6783044	0.09922978	0.99978775	-4.542099
ENSCAFG00000031912	-0.4992539	-1.8670267	-0.7250378	0.47164727	0.99978775	-4.601095
TMEM203	-0.4995224	2.09822256	-1.9612192	0.05516553	0.99978775	-4.410694
DIAPH3	-0.4999346	4.93910765	-1.1656555	0.24902088	0.99978775	-4.5879929
ENSCAFG00000016594	-0.5012155	2.68337566	-1.5553988	0.12585767	0.99978775	-4.4733955
RGS9	-0.5012397	-0.3765027	-0.9580193	0.34244297	0.99978775	-4.5985982
IGLON5	-0.5012901	-2.0988856	-1.0211591	0.31186038	0.99978775	-4.5946845
SREBF1	-0.5022142	6.73460128	-3.2323761	0.00212297	0.99968241	-2.8159639
TUB	-0.5026615	-0.1611449	-0.9378751	0.35260215	0.99978775	-4.6000086
TRIL	-0.502939	0.83766574	-0.9582726	0.34231646	0.99978775	-4.6009605
ST8SIA2	-0.5038414	-0.4129297	-0.8966708	0.37398681	0.99978775	-4.6041995
ARHGAP22	-0.5038627	2.62253166	-1.4528503	0.15221487	0.99978775	-4.5300385
MSMP	-0.5039339	-2.4494956	-0.6650858	0.50890605	0.99978775	-4.6022735
DDX25	-0.5043597	-1.2477927	-0.9157443	0.36398711	0.99978775	-4.598314
PRDM15	-0.5045914	1.83525427	-1.6522416	0.10445283	0.99978775	-4.5052705
CEP152	-0.5047721	3.01141926	-1.9824524	0.05267154	0.99978775	-4.2811433
ACOT11	-0.5052352	2.14407091	-0.911294	0.36630474	0.99978775	-4.6170255
CLEC2L	-0.5056146	-1.1130781	-0.7151537	0.47768162	0.99978775	-4.603769

KCNAB2	-0.5064279	1.01173612	-1.5378929	0.13007886	0.99978775	-4.542936
SEMA4D	-0.5066518	-1.6498309	-1.1692795	0.24757108	0.99978775	-4.5881837
SDR16C5	-0.5070293	1.85268669	-1.1425769	0.25839686	0.99978775	-4.5831623
LHFPL4	-0.5071772	0.95886411	-1.1412587	0.25893991	0.99978775	-4.5808523
RNASEH2C	-0.5081141	2.18163965	-2.0169386	0.04882747	0.99978775	-4.395821
COX16	-0.5084201	0.63221471	-1.5062322	0.13799955	0.99978775	-4.5565802
GJB5	-0.5086539	2.89866811	-1.0889852	0.2811327	0.99978775	-4.5885173
PARP2	-0.5088765	3.52144334	-2.2465004	0.02890184	0.99978775	-4.0702542
ANK1	-0.5091375	0.37929854	-1.0394727	0.30334731	0.99978775	-4.5936332
TMEM121	-0.509177	1.95793715	-1.5617205	0.12436057	0.99978775	-4.5074944
CPEB3	-0.5094588	-0.646982	-1.0532992	0.29702625	0.99978775	-4.5932853
KCNJ2	-0.5097695	-1.2443892	-0.7811085	0.43824585	0.99978775	-4.6026246
C10H22orf23	-0.51037	-0.6652833	-1.1774857	0.24431066	0.99978775	-4.5879537
BEND6	-0.510468	3.52078309	-2.1710522	0.03446797	0.99978775	-4.0270775
DUOX1	-0.5108909	-1.9408292	-0.9444022	0.3492891	0.99978775	-4.5979583
ENSCAFG00000007307	-0.511327	1.48190794	-0.25878	0.7968166	0.99978775	-4.6187347
EMP2	-0.5124877	3.39608123	-1.6750886	0.0998624	0.99978775	-4.4217416
FOXF1	-0.512664	4.39287181	-0.9473485	0.34780031	0.99978775	-4.6510094
FAM83D	-0.5127872	3.21287378	-1.8052711	0.0767663	0.99978775	-4.3194194
FGFR1OP	-0.5133791	2.98112523	-2.3089934	0.02491016	0.99978775	-4.1908966
AZIN2	-0.5134765	1.13332538	-1.3854454	0.17177285	0.99978775	-4.5603846
NLGN3	-0.5143106	0.54178632	-1.1400504	0.25943836	0.99978775	-4.5868961
HDAC11	-0.5147938	-1.1154196	-0.8991311	0.37268724	0.99978775	-4.5990311
ENSCAFG00000004905	-0.5152251	-0.441071	-0.2934199	0.77035769	0.99978775	-4.6108607
BBOF1	-0.5154771	1.09070584	-1.6515439	0.10459566	0.99978775	-4.5260787
DUSP2	-0.5157915	-0.9687508	-0.8956798	0.37451111	0.99978775	-4.5982885
NT5E	-0.5163251	-0.2708938	-0.6424745	0.52336024	0.99978775	-4.6085238
FCHO1	-0.5164168	-1.396738	-1.0241568	0.31045593	0.99978775	-4.5945669
TEX14	-0.5171161	0.44938086	-1.3153049	0.19412161	0.99978775	-4.5755919
NEIL3	-0.5173028	-1.3035454	-1.1335309	0.26213978	0.99978775	-4.5883115
HOXC4	-0.5174004	2.00132154	-1.0928097	0.27946526	0.99978775	-4.5873413
ENSCAFG00000030774	-0.5175976	0.28895006	-1.785173	0.08000907	0.99978775	-4.5253528
CCDC61	-0.5177777	2.29834612	-1.7537522	0.08530766	0.99978775	-4.4583516
ENSCAFG00000023653	-0.5180182	-0.1133055	-1.3900635	0.17037389	0.99978775	-4.5702746
BLOC1S3	-0.5181454	-0.1772615	-1.2612747	0.21278414	0.99978775	-4.5790398
LGR4	-0.5181954	4.57077728	-1.3324827	0.18845486	0.99978775	-4.5161225
TNXB	-0.5186681	3.58182137	-0.7413841	0.46176327	0.99978775	-4.6662579
IRF5	-0.5189506	1.14201043	-1.4486131	0.15339064	0.99978775	-4.5568716
CDKN3	-0.5191644	-0.3713124	-1.0725204	0.28839039	0.99978775	-4.5908211
RAB11FIP4	-0.5191834	-1.0306189	-0.9870613	0.32813873	0.99978775	-4.5960593
GRHL1	-0.5199109	0.43955718	-1.3392757	0.18624887	0.99978775	-4.5759471
ZNF385D	-0.5199177	1.55874568	-1.0317059	0.30693812	0.99978775	-4.5977788
P2RY1	-0.5207068	-2.0123882	-0.8324317	0.40893503	0.99978775	-4.5994826
VWA1	-0.521422	1.62638892	-1.177115	0.24445726	0.99978775	-4.5821021
PRELID3A	-0.5228111	0.79704572	-1.6550464	0.10388016	0.99978775	-4.5381251
ENSCAFG00000029649	-0.522847	1.4420256	-2.100893	0.04046783	0.99978775	-4.4404824
EFS	-0.52289	4.46684408	-0.9142349	0.36477211	0.99978775	-4.6672656
RIIAD1	-0.5229157	0.33793943	-1.7372288	0.08820917	0.99978775	-4.5404788
FLT1	-0.522924	4.35648471	-0.8777831	0.38405977	0.99978775	-4.7026042
ENSCAFG00000009806	-0.5235718	-1.2390727	-0.4105868	0.68304601	0.99978775	-4.6056206

HAUS5	-0.5240949	3.01574352	-2.7740628	0.0076485	0.99978775	-3.89509
CENPO	-0.5250588	1.64749823	-1.9260007	0.05952546	0.99978775	-4.4071972
PARPBP	-0.5257872	1.92461551	-1.4749442	0.14619802	0.99978775	-4.4909662
ZNF185	-0.5262653	2.0054001	-1.4791241	0.14508106	0.99978775	-4.4805373
PLBD1	-0.5276021	-1.8710217	-0.7078078	0.48219433	0.99978775	-4.6017875
NGEF	-0.5276534	3.89880296	-1.8923687	0.06396017	0.99978775	-4.1656678
RNF138	-0.5278315	2.05402275	-1.895646	0.06351602	0.99978775	-4.4421735
ZC3H8	-0.5280925	1.28583762	-1.790555	0.07912966	0.99978775	-4.5010194
FDXR	-0.5288255	1.67198772	-2.3849997	0.02072217	0.99978775	-4.3582107
ENSCAFG00000025209	-0.5290002	0.52129526	-1.5186352	0.13485223	0.99978775	-4.5589222
CACHD1	-0.5293047	2.11033734	-1.6786025	0.09917131	0.99978775	-4.4853849
PHYHD1	-0.5293705	1.98485788	-2.2294265	0.03008666	0.99978775	-4.4132675
DNAH11	-0.5296258	0.00731149	-1.1761018	0.24485833	0.99978775	-4.5870688
YOD1	-0.5304492	1.13102188	-1.6615087	0.1025705	0.99978775	-4.5290554
SFRP1	-0.5312686	-2.4520505	-0.74035	0.46238501	0.99978775	-4.6013151
TMEM200B	-0.5328544	1.28918133	-1.3489604	0.18313785	0.99978775	-4.5581812
ILDR2	-0.5329918	-0.0961148	-0.5674314	0.57283897	0.99978775	-4.6111807
ENSCAFG00000005299	-0.5338197	-0.1286251	-1.1395486	0.25964561	0.99978775	-4.58595
HCN3	-0.5339184	-0.1529598	-1.5556395	0.12580041	0.99978775	-4.5562299
RAD51AP1	-0.5345963	1.23678465	-1.3828646	0.17255848	0.99978775	-4.5346345
TST	-0.5346341	0.45522117	-1.370782	0.17627347	0.99978775	-4.5683613
LETM2	-0.534646	-0.5552253	-1.554491	0.12607384	0.99978775	-4.56585
POPDC3	-0.5351334	-0.786792	-1.0384935	0.30379841	0.99978775	-4.5936944
KLF9	-0.5357359	2.11429284	-0.6373594	0.52665987	0.99978775	-4.6186726
TNFSF13B	-0.5365012	1.33551063	-0.9226233	0.36042323	0.99978775	-4.6011571
AMER1	-0.5365671	2.2404141	-1.9020827	0.06265133	0.99978775	-4.4460903
PLEKHA7	-0.5369703	-1.4651832	-0.6704706	0.50549587	0.99978775	-4.6035736
GCAT	-0.5370971	2.84292069	-1.3302586	0.18918139	0.99978775	-4.5362404
ADGRF5	-0.5371391	-0.9591881	-0.329859	0.74281871	0.99978775	-4.6080478
ABHD12B	-0.5372123	0.30402178	-0.8118368	0.42054998	0.99978775	-4.6063887
HOXA4	-0.5393924	-0.691941	-1.0778686	0.28601879	0.99978775	-4.5919563
ENSCAFG00000029256	-0.5404685	1.90712784	-1.5499549	0.12715843	0.99978775	-4.5152696
GPER1	-0.5404951	0.62614261	-0.8217965	0.41490826	0.99978775	-4.6092511
RARB	-0.540807	-0.6693702	-0.8309138	0.40978433	0.99978775	-4.6028391
ENSCAFG00000011327	-0.5409242	-0.9080865	-1.1582016	0.252022	0.99978775	-4.5872897
RNF213	-0.5410885	3.4394205	-1.1160039	0.26950111	0.99978775	-4.5826887
ZNF554	-0.5419249	-0.2634637	-1.4508533	0.15276814	0.99978775	-4.5709793
ALPL	-0.5420077	-0.5895369	-0.8523541	0.39788822	0.99978775	-4.600331
CKS2	-0.5424438	2.99395449	-2.0535076	0.04501833	0.99978775	-4.2531487
TFPI	-0.5426483	6.67418327	-1.7945233	0.07848644	0.99978775	-4.2233408
KIAA1549L	-0.5448826	-0.632862	-1.0167109	0.31395236	0.99978775	-4.5950856
RASIP1	-0.5467642	1.20333546	-0.5565551	0.58019552	0.99978775	-4.6208255
ENSCAFG00000002706	-0.5468744	1.20012678	-1.5447751	0.12840606	0.99978775	-4.5340552
CACNB2	-0.5477474	-0.9166937	-1.3098148	0.19595961	0.99978775	-4.574346
TMEM145	-0.5480484	-1.0297323	-1.2546547	0.2151595	0.99978775	-4.5860229
ENSCAFG00000029636	-0.54805	0.53893998	-1.5818047	0.1196989	0.99978775	-4.5491574
NUDT1	-0.5482441	1.72406525	-1.803178	0.07709879	0.99978775	-4.4673006
NPR1	-0.5494429	-0.2306568	-0.5843019	0.56151899	0.99978775	-4.6096061
TMOD4	-0.5507074	-1.8608049	-0.8825127	0.38152156	0.99978775	-4.5986447
C30H15orf39	-0.551041	3.10220431	-2.0602432	0.04434556	0.99978775	-4.2789792

CASP8	-0.5513452	2.43150553	-1.6897984	0.09699547	0.99978775	-4.4791961
CHTF18	-0.5513668	2.62367941	-1.6349151	0.10804781	0.99978775	-4.4352942
ZCCHC3	-0.5526021	0.76258438	-1.5211323	0.13422553	0.99978775	-4.5527831
NREP	-0.5532301	1.342136	-1.7414437	0.08746136	0.99978775	-4.4030494
STMN3	-0.5533389	-1.3478152	-0.8909422	0.37702405	0.99978775	-4.5983397
GAREM1	-0.5537209	1.40394802	-0.8842277	0.3806038	0.99978775	-4.6043657
ENSCAFG00000032225	-0.5539204	-0.1106271	-1.4516118	0.15255781	0.99978775	-4.56804
RTKN	-0.5552256	0.61379184	-1.1140292	0.27033953	0.99978775	-4.5892545
ESRP1	-0.555351	-0.8761479	-1.1676756	0.24821196	0.99978775	-4.5836374
S100A4	-0.5555381	7.50032601	-1.7071342	0.093704	0.99978775	-4.2523102
TRIM66	-0.5556557	-0.0067919	-1.2229784	0.22679782	0.99978775	-4.5822749
METTL21A	-0.5566647	1.009242	-1.5741056	0.121469	0.99978775	-4.5420007
CPXM2	-0.5578232	1.43544655	-0.7002971	0.48683286	0.99978775	-4.6272235
ENSCAFG00000002800	-0.5605971	1.91184413	-1.659586	0.10295874	0.99978775	-4.5064308
POLE	-0.5608344	4.39838281	-1.5138272	0.13606544	0.99978775	-4.3947676
HOXA1	-0.5614687	1.12250579	-1.1775924	0.24426846	0.99978775	-4.5816478
CCDC69	-0.5618974	-2.4326739	-1.1416909	0.25876178	0.99978775	-4.591046
DGAT2	-0.5619546	0.50952168	-0.9881718	0.32759981	0.99978775	-4.5962191
FOXF2	-0.562997	2.05839304	-0.3101922	0.75764253	0.99978775	-4.6234115
OTUB2	-0.5639154	-0.7083066	-1.3540357	0.18152342	0.99978775	-4.5797959
TMEM54	-0.5640579	-0.7746729	-1.2220172	0.22715808	0.99978775	-4.5850498
ENSCAFG00000031494	-0.5642841	0.02596232	-0.936014	0.35355058	0.99978775	-4.6002596
WDR76	-0.5657419	3.41672795	-1.9585836	0.05548205	0.99978775	-4.2303675
EYA1	-0.5659615	-1.7940801	-0.6964406	0.48922421	0.99978775	-4.6046321
HOXB8	-0.5663621	-2.0823089	-0.9356187	0.35375223	0.99978775	-4.5972167
FAM217B	-0.5670838	-0.3135057	-1.4624786	0.14956934	0.99978775	-4.5672803
TWIST2	-0.5674269	2.03349663	-1.3347234	0.18772503	0.99978775	-4.5454389
GUCY1A1	-0.5691451	-2.2865639	-0.7791361	0.43939662	0.99978775	-4.6005045
NHS	-0.5692068	4.41131855	-1.5734655	0.1216171	0.99978775	-4.3755141
ENSCAFG00000011656	-0.5693024	5.40231159	-1.2447576	0.21874732	0.99978775	-4.5420756
CHEK2	-0.5698343	3.20353203	-3.0361423	0.00372452	0.99978775	-3.8199947
S100A5	-0.5714376	-0.8476991	-0.8295652	0.41053976	0.99978775	-4.6006787
ENSCAFG00000016149	-0.5714555	5.6951819	-0.9842965	0.32948308	0.99978775	-4.6748544
SNPH	-0.5721043	0.90487444	-1.5851783	0.11892984	0.99978775	-4.5380927
SCARA3	-0.5725221	7.70125713	-1.7152618	0.09219286	0.99978775	-4.2511543
LMNB1	-0.5725661	5.31693115	-1.8075165	0.07641094	0.99978775	-4.1839843
SMOC1	-0.5730542	3.12559624	-0.9620017	0.34045757	0.99978775	-4.6369024
CLSPN	-0.573728	2.20153996	-1.3154784	0.19406377	0.99978775	-4.5233349
MROH8	-0.5744685	0.22270899	-1.6808827	0.09872495	0.99978775	-4.5398103
MTCL1	-0.5745133	5.6371378	-3.199374	0.00233658	0.99968241	-2.8786853
AVPI1	-0.5756531	1.52954691	-1.7898927	0.07923744	0.99978775	-4.4647628
PPFIBP2	-0.5757105	0.20900941	-1.2135888	0.23033492	0.99978775	-4.5765762
ITGA7	-0.5773411	4.53744476	-0.7297199	0.46880398	0.99978775	-4.6452897
PDZRN4	-0.5776546	-1.0770693	-0.8852528	0.38005586	0.99978775	-4.6004665
TRIB1	-0.5781879	4.722287	-1.8933848	0.06382218	0.99978775	-4.1826659
ABAT	-0.5783166	2.39213997	-1.6408371	0.10680793	0.99978775	-4.4909179
SNRPG	-0.5784253	-1.2439366	-1.1511771	0.2548739	0.99978775	-4.5891404
DRC3	-0.5788347	-0.0272647	-1.4795868	0.14495782	0.99978775	-4.5655552
POC1A	-0.5789365	2.79407029	-2.0283309	0.04761201	0.99978775	-4.2639762
ENSCAFG00000030498	-0.5799239	-0.7040241	-1.579569	0.12021074	0.99978775	-4.5583919

PARD6G	-0.5801774	-0.7047458	-1.0489492	0.2990051	0.99978775	-4.5933844
ENSCAFG00000006797	-0.5808663	1.23348267	-2.4120285	0.01939244	0.99978775	-4.3682441
FAM180A	-0.581313	2.53721955	-0.4569668	0.64957963	0.99978775	-4.6408672
KBTBD7	-0.5823016	0.87115383	-1.8853493	0.06492034	0.99978775	-4.5096166
KCNQ2	-0.5825083	-0.8031661	-0.8191028	0.41642961	0.99978775	-4.6004156
SYNE4	-0.5839237	0.40874615	-1.5796416	0.1201941	0.99978775	-4.5528858
ADAMTSL2	-0.5839353	-2.3316856	-0.980354	0.33140638	0.99978775	-4.595843
SERP2	-0.5842754	0.49961334	-1.9283351	0.05922762	0.99978775	-4.4989886
C8H14orf93	-0.5845289	1.95192934	-2.1143644	0.03924966	0.99978775	-4.4007463
FANCA	-0.5866253	1.67519394	-1.730107	0.08948478	0.99978775	-4.4427966
NOD2	-0.5877909	0.41216427	-1.173078	0.24605801	0.99978775	-4.5860117
ENSCAFG00000006595	-0.587965	-1.8850211	-1.1214319	0.26720592	0.99978775	-4.590322
RRH	-0.5898293	-0.6623784	-1.3536893	0.18163326	0.99978775	-4.5779254
DOK7	-0.5909041	-0.9174661	-1.2117752	0.23102271	0.99978775	-4.5844237
ADORA2A	-0.5920277	0.32939106	-1.6178732	0.11168142	0.99978775	-4.5324922
EEPD1	-0.5933423	1.14638788	-1.2121581	0.23087738	0.99978775	-4.5785251
SOD3	-0.5940773	-0.3353125	-0.9470445	0.34795373	0.99978775	-4.5992372
ENSCAFG00000029677	-0.5944167	-1.832291	-0.7465144	0.45868583	0.99978775	-4.6015972
CBX2	-0.5944305	1.37371141	-2.3720399	0.02138831	0.99978775	-4.3808046
BIRC5	-0.595079	3.11767438	-1.7747187	0.08174063	0.99978775	-4.3025782
CFAP46	-0.5957744	-0.9568705	-1.1451765	0.25732833	0.99978775	-4.588878
TMEM150B	-0.5975615	-0.4509708	-1.4550178	0.15161614	0.99978775	-4.5703866
FBN2	-0.5993888	1.29245355	-1.5826506	0.11950568	0.99978775	-4.3542721
HLA-DRB1	-0.5997107	-1.7038769	-0.7362616	0.4648478	0.99978775	-4.6013261
SLC16A12	-0.599804	-1.396313	-0.8119095	0.42050864	0.99978775	-4.6044799
DZANK1	-0.6000875	1.63840336	-2.3984104	0.02005251	0.99978775	-4.3674714
FBXO10	-0.6001517	2.01715603	-1.2883144	0.20328401	0.99978775	-4.563908
MTFP1	-0.6001755	-0.2088735	-1.3601064	0.17960663	0.99978775	-4.5753991
WDFY4	-0.6001848	1.05492284	-0.8253361	0.41291439	0.99978775	-4.6041713
EGR1	-0.6007816	8.71392041	-1.7243841	0.09052084	0.99978775	-4.259276
TRAPPC6A	-0.6012973	0.05916388	-1.3899359	0.17041243	0.99978775	-4.5687435
ARSE	-0.6013371	1.27706586	-0.9014861	0.37144595	0.99978775	-4.6045962
NPDC1	-0.6017464	1.41999711	-0.8389761	0.40528565	0.99978775	-4.6076571
MELK	-0.6043104	3.060461	-1.8390242	0.07156852	0.99978775	-4.2750029
RANGRF	-0.6054628	1.61888673	-2.9103287	0.00528554	0.99978775	-4.1873307
FLVCR2	-0.6073703	-1.4584546	-0.988508	0.32743672	0.99978775	-4.5956778
CDC6	-0.6075939	4.02300777	-1.5050019	0.13831492	0.99978775	-4.4078701
CMKLR1	-0.6078863	1.04951238	-0.5709853	0.57044511	0.99978775	-4.6169581
TAL1	-0.6080246	-0.7777109	-1.071369	0.28890276	0.99978775	-4.5922605
FMNL1	-0.6086499	1.18193586	-0.8703544	0.38806793	0.99978775	-4.6092636
EFCAB1	-0.6087641	0.57227358	-1.5956276	0.11657308	0.99978775	-4.5487467
LSS	-0.6087686	6.98265932	-2.4994624	0.01560028	0.99978775	-3.5793049
RIT2	-0.6090729	-1.421733	-0.845928	0.40143107	0.99978775	-4.6007104
CAVIN4	-0.6098358	-0.3856723	-1.6260763	0.10992018	0.99978775	-4.5330241
SGSM1	-0.6099611	0.15800155	-0.9876846	0.32783617	0.99978775	-4.5960483
PAPLN	-0.6113393	-0.5342001	-0.7654747	0.44741568	0.99978775	-4.6034265
PTP4A3	-0.6113553	-0.514082	-1.2668444	0.21080077	0.99978775	-4.582213
PDXP	-0.6119179	1.17331256	-2.0824194	0.04219185	0.99978775	-4.4698659
ENSCAFG00000027581	-0.61291	-0.8788866	-1.172497	0.246289	0.99978775	-4.5886913
XPNPEP2	-0.6139872	-0.3689654	-0.6889712	0.49387423	0.99978775	-4.6049887

NDC80	-0.6146952	2.93254923	-1.8567767	0.06895591	0.99978775	-4.2759488
PPCDC	-0.6150961	2.61384016	-3.6275803	0.00064769	0.99968241	-3.7502
MTUS2	-0.6151063	1.34522117	-1.8223216	0.07410234	0.99978775	-4.3873938
TMEM250	-0.6154734	0.73670186	-1.7740807	0.08184731	0.99978775	-4.5221656
CENPE	-0.6156903	4.56911856	-1.6725667	0.10036084	0.99978775	-4.295453
KCNIP1	-0.6157747	0.7562579	-1.1759665	0.24491192	0.99978775	-4.579498
ENSCAFG00000006785	-0.616684	2.26448087	-2.5792288	0.01274027	0.99978775	-4.2366543
CEACAM20	-0.6168239	-1.0735287	-0.9741555	0.33444537	0.99978775	-4.5965958
ENSCAFG00000008380	-0.6173336	0.48750394	-1.7209741	0.09114291	0.99978775	-4.5279498
ATP8A1	-0.6184799	-0.85103	-1.1344558	0.2617553	0.99978775	-4.5893342
CFAP410	-0.6187436	2.82541018	-2.0115886	0.04940747	0.99978775	-4.3461511
CPED1	-0.6188929	2.03083168	-0.4553956	0.65070201	0.99978775	-4.6237963
ENSCAFG00000025524	-0.6190722	6.18427384	-2.4735562	0.01664724	0.99978775	-3.649954
RFX1	-0.6190762	2.61903277	-2.5180182	0.0148874	0.99978775	-4.1805928
ENSCAFG00000026748	-0.6193625	-0.5165165	-1.3391464	0.18629066	0.99978775	-4.5796019
TCP11	-0.6231856	0.66529014	-2.2745654	0.02704394	0.99978775	-4.437722
HELLS	-0.6235707	2.56580799	-1.6106923	0.11324191	0.99978775	-4.4153452
CDK1	-0.6245668	3.6134057	-1.7875403	0.07962126	0.99978775	-4.2697353
TRIM47	-0.6247404	3.55974823	-1.1502968	0.25523293	0.99978775	-4.5740674
FRMD3	-0.6262703	1.2521285	-1.1138463	0.27041727	0.99978775	-4.5848945
F2RL2	-0.6284386	1.19259364	-0.2889246	0.77377657	0.99978775	-4.6122603
COL5A3	-0.62851	4.37166208	-0.5547738	0.58140467	0.99978775	-4.6511322
ASPHD2	-0.6295363	-0.5465675	-1.2013195	0.23501735	0.99978775	-4.5858008
C24H20orf96	-0.6303552	-0.5582792	-1.323749	0.19132012	0.99978775	-4.579033
PARP14	-0.631992	3.00550425	-1.6519698	0.10450845	0.99978775	-4.4464124
PRC1	-0.633343	4.31818703	-1.5025958	0.1389333	0.99978775	-4.3975145
PTPRR	-0.6355189	0.10055388	-1.2367565	0.22167995	0.99978775	-4.5778805
ENSCAFG00000000626	-0.6357073	1.44776662	-1.6863905	0.09765357	0.99978775	-4.5169976
ENSCAFG00000030031	-0.6361302	-0.4712561	-0.7633094	0.44869451	0.99978775	-4.6027102
ACOT6	-0.6363252	-0.9733958	-1.182859	0.24219266	0.99978775	-4.5881827
HS3ST3B1	-0.6364193	3.20587196	-0.4131329	0.68119133	0.99978775	-4.6231913
IBA57	-0.6390856	1.32737505	-1.7007305	0.09490894	0.99978775	-4.5181862
CENPI	-0.6403236	1.1635249	-1.7748151	0.08172451	0.99978775	-4.4779189
TRIAP1	-0.6403427	0.26063186	-1.6058434	0.1143056	0.99978775	-4.5536502
FNDC1	-0.6408035	4.30871776	-1.6249861	0.11015295	0.99978775	-4.3112299
SIX2	-0.6420364	-0.3102929	-1.0734129	0.28799366	0.99978775	-4.5917057
ENSCAFG00000013158	-0.6424301	-0.1303482	-1.2991472	0.19956842	0.99978775	-4.5733305
ABCB1	-0.6428273	0.94649627	-0.4852857	0.6294921	0.99978775	-4.617916
ENSCAFG00000025482	-0.6431587	-0.810576	-1.4949004	0.14092574	0.99978775	-4.5710497
FANCI	-0.6441648	3.43696579	-1.9242694	0.05974717	0.99978775	-4.2054016
MCM2	-0.644597	5.00333041	-1.7369669	0.08825581	0.99978775	-4.2396273
SALL1	-0.6448664	-1.3452963	-1.0811111	0.28458752	0.99978775	-4.5920658
ENSCAFG00000020954	-0.6449099	0.87843127	-1.8460115	0.07053041	0.99978775	-4.4910796
GEMIN7	-0.6462588	1.85863347	-2.8836663	0.00568639	0.99978775	-4.1798596
ENSCAFG00000030758	-0.6469165	-0.9027192	-1.3271986	0.19018449	0.99978775	-4.580403
CHRNB2	-0.6470184	-0.03963	-1.7225974	0.09084634	0.99978775	-4.5489135
RNF125	-0.6476074	0.66246088	-1.5288001	0.13231561	0.99978775	-4.5413804
STRIP2	-0.6480038	-1.1111366	-1.269077	0.21000963	0.99978775	-4.5817882
TRIM46	-0.6482504	-0.5239129	-1.2568423	0.21437237	0.99978775	-4.5846049
PTPRU	-0.6482829	3.50213017	-0.756178	0.45292136	0.99978775	-4.636086

FAM49A	-0.6492061	1.09243382	-0.8487084	0.39989576	0.99978775	-4.6095687
ACP5	-0.6495027	0.26352158	-1.075644	0.28700362	0.99978775	-4.5911292
PDE7B	-0.650121	-0.0105961	-0.8858904	0.37971532	0.99978775	-4.6029682
TMEM117	-0.6511139	3.67350418	-1.7726107	0.08209355	0.99978775	-4.40536
FANK1	-0.6521411	0.86889436	-0.98207	0.33056836	0.99978775	-4.5977253
ENSCAFG00000031198	-0.6527054	0.83989264	-1.962197	0.05504849	0.99978775	-4.5031311
CEP72	-0.6535245	0.21347802	-1.2201374	0.22786379	0.99978775	-4.5791505
TMEM246	-0.6538082	0.47337196	-1.4655361	0.14873684	0.99978775	-4.5574395
CENPU	-0.6544588	0.4220799	-1.358366	0.18015455	0.99978775	-4.5521889
ACE	-0.6544952	-0.1074593	-0.3820008	0.70400226	0.99978775	-4.609407
ETFBKMT	-0.6548981	0.21089308	-1.4230877	0.16062458	0.99978775	-4.5672317
SHC4	-0.6549951	1.42863083	-1.7418712	0.08738581	0.99978775	-4.5231588
ENSCAFG00000010830	-0.6550786	-0.2615955	-1.3688837	0.17686267	0.99978775	-4.575208
SLC22A13	-0.6550806	-0.6866664	-1.8071016	0.07647649	0.99978775	-4.5257096
FOSB	-0.6551847	4.77456875	-1.0070915	0.31850877	0.99978775	-4.6308027
CAPS	-0.6552925	0.78142679	-1.7251008	0.09039055	0.99978775	-4.469117
ENSCAFG00000032172	-0.6555913	-0.7320529	-1.6672991	0.10140853	0.99978775	-4.5504003
ZNF792	-0.6585288	1.71342681	-1.8590617	0.06862555	0.99978775	-4.4780593
CLIC6	-0.6589675	0.33171477	-0.4886156	0.62714815	0.99978775	-4.6163991
MCM10	-0.659044	3.64727217	-1.7577031	0.08462576	0.99978775	-4.2730989
ENSCAFG00000011251	-0.6590526	-1.5893632	-0.7096166	0.48108093	0.99978775	-4.6030776
LGALSL	-0.6607006	-0.3973356	-1.4690092	0.14779562	0.99978775	-4.5567638
DNPH1	-0.6608856	2.02931578	-2.6894539	0.00957128	0.99978775	-4.1571633
KIAA1958	-0.6614975	-0.6189739	-1.5225147	0.13387957	0.99978775	-4.5652057
LEF1	-0.6618267	-0.9388119	-0.8311032	0.40967828	0.99978775	-4.6006225
MYRFL	-0.6623265	0.20525505	-1.317712	0.19331988	0.99978775	-4.5566435
KIAA0408	-0.6623881	-1.4407712	-0.8625276	0.39231916	0.99978775	-4.6004813
TPX2	-0.6636505	4.52303022	-1.6415224	0.10666518	0.99978775	-4.3130113
ENSCAFG00000003715	-0.66437	-2.3241724	-0.9478866	0.34752882	0.99978775	-4.5965685
DLA-DMB	-0.6643992	0.37976087	-1.3936105	0.16930536	0.99978775	-4.5659635
ENSCAFG00000007435	-0.6644644	2.7250538	-1.5801999	0.12006613	0.99978775	-4.439113
PKP3	-0.6660404	-1.1761199	-1.0409227	0.30268011	0.99978775	-4.5939288
RIMBP2	-0.6664592	-1.6592253	-1.0037083	0.32012182	0.99978775	-4.595188
KIF4A	-0.6672059	2.85983707	-1.6007835	0.11542419	0.99978775	-4.3909442
PSMC3IP	-0.6673629	-0.7197002	-1.5633463	0.12397788	0.99978775	-4.5574092
HJURP	-0.6686659	1.77239066	-1.4087921	0.16479032	0.99978775	-4.5000081
GPR34	-0.6687027	1.36953868	-0.7897384	0.43323207	0.99978775	-4.6154081
USP2	-0.669166	0.94220372	-1.870793	0.06695051	0.99978775	-4.4819907
ENSCAFG00000005269	-0.6705753	-0.4681269	-1.7958722	0.0782688	0.99978775	-4.546078
ENSCAFG00000008236	-0.6714338	4.28066974	-1.8071773	0.07646453	0.99978775	-4.2135918
TRIM9	-0.6720959	-1.0409808	-0.9375614	0.35276193	0.99978775	-4.5971444
UNC5A	-0.6731022	2.36465863	-1.9785405	0.0531236	0.99978775	-4.4390967
FCMR	-0.6732089	-0.1713453	-1.8123414	0.07565203	0.99978775	-4.5427971
SOAT2	-0.6751572	0.64634703	-1.6484957	0.10522164	0.99978775	-4.5381985
TBX4	-0.6759479	-2.7479095	-1.2127908	0.23063737	0.99978775	-4.5892179
EN1	-0.6759671	1.57758585	-1.0934642	0.27918061	0.99978775	-4.5879434
RACGAP1	-0.6760856	5.38590242	-1.7326236	0.08903229	0.99978775	-4.2360415
IL4I1	-0.6766006	-0.8471784	-1.424876	0.16010928	0.99978775	-4.5762356
SLC16A14	-0.6777206	-2.9896149	-1.5715081	0.12207093	0.99978775	-4.5777562
CPM	-0.6787423	-1.83967	-1.1772649	0.24439798	0.99978775	-4.5899502

ASB9	-0.6792061	-1.3434072	-1.2706672	0.20944744	0.99978775	-4.5833927
GATM	-0.6806249	-0.6922685	-1.3344626	0.18780984	0.99978775	-4.5567592
PRR11	-0.6840476	1.77020153	-1.4795038	0.14497991	0.99978775	-4.4979533
DDIAS	-0.6843432	0.47004729	-1.5567226	0.12554298	0.99978775	-4.5356506
KLF4	-0.6846617	6.09052916	-2.0690905	0.04347514	0.99978775	-3.9828453
EGFLAM	-0.6848056	1.8312268	-0.8473629	0.40063827	0.99978775	-4.6151374
CCDC114	-0.6851708	0.10887197	-1.54181	0.12912464	0.99978775	-4.5613572
RAD51	-0.6854872	2.08094161	-1.4472816	0.1537616	0.99978775	-4.4848503
LYSMD2	-0.6859829	-1.4167079	-1.2609675	0.21289392	0.99978775	-4.5854696
C28H10orf90	-0.6887269	3.16323875	-1.3925287	0.1696307	0.99978775	-4.5091446
COL14A1	-0.6891635	3.45767758	-1.0902026	0.28060117	0.99978775	-4.5958243
GNGT2	-0.6910153	-2.2544939	-0.8947453	0.37500594	0.99978775	-4.597805
HASPIN	-0.6938328	1.76436867	-1.6927359	0.09643113	0.99978775	-4.4484916
ANGPT2	-0.6945625	-1.002193	-0.5769667	0.56642716	0.99978775	-4.6060145
GAS2L3	-0.6950652	1.28590047	-1.7963955	0.0781845	0.99978775	-4.4598395
ENSCAFG00000026443	-0.6974277	-0.3493823	-1.4911086	0.14191576	0.99978775	-4.5697572
LFNG	-0.6983175	0.516915	-0.8468087	0.40094435	0.99978775	-4.6038509
ENSCAFG00000017554	-0.6991649	-1.5227278	-1.4287559	0.15899571	0.99978775	-4.5744498
CDCA5	-0.6992882	1.1686823	-1.4364455	0.15680654	0.99978775	-4.5255318
CEP126	-0.7010666	0.18688115	-1.4163256	0.16258476	0.99978775	-4.5717315
ENSCAFG00000030329	-0.7016075	1.39079646	-1.8919348	0.06401916	0.99978775	-4.3447908
PDGFD	-0.7028415	4.13214087	-0.6939448	0.49077525	0.99978775	-4.6549831
IFT27	-0.7031691	1.78164167	-2.8639888	0.00600013	0.99978775	-4.2490021
LRIG1	-0.7032092	5.59631253	-1.9680945	0.05434711	0.99978775	-4.0754486
ENSCAFG00000022714	-0.7033754	1.37868889	-1.675102	0.09985976	0.99978775	-4.5352307
KIAA1211	-0.7056855	-1.4279154	-1.3411867	0.18563183	0.99978775	-4.5805161
ADCY1	-0.7060593	-0.1189364	-1.6378273	0.10743662	0.99978775	-4.5531872
GAP43	-0.7068003	4.30948182	-1.4279962	0.15921329	0.99978775	-4.4595081
KCNMA1	-0.7076461	-0.8445589	-1.5511497	0.12687204	0.99978775	-4.5496876
TK1	-0.7078629	4.22638765	-1.7722728	0.08215023	0.99978775	-4.2363255
ESM1	-0.7079586	4.50133142	-0.8511669	0.39854125	0.99978775	-4.6234971
KCND1	-0.7081369	0.52767203	-0.7177799	0.47607409	0.99978775	-4.6033741
MCF2L	-0.7100795	0.39862411	-1.4208629	0.16126746	0.99978775	-4.56964
SCD5	-0.7109658	0.27585708	-1.5393662	0.12971929	0.99978775	-4.5515305
NPTX1	-0.7114021	5.30481201	-0.7008769	0.48647388	0.99978775	-4.6876637
WSCD1	-0.7114376	-2.0059804	-0.6524592	0.51695096	0.99978775	-4.602311
PKHD1L1	-0.7114469	-2.0161209	-0.928593	0.3573487	0.99978775	-4.5974844
ENSCAFG00000023994	-0.7118707	-0.6147012	-1.418006	0.16209592	0.99978775	-4.5695093
FAM78B	-0.7140379	-0.9136366	-1.3527763	0.18192302	0.99978775	-4.5808508
DPT	-0.7151719	2.39260891	-0.7630459	0.44885027	0.99978775	-4.6336896
LRRC8D	-0.7152263	2.41706017	-1.5652262	0.12353655	0.99978775	-4.5048937
SPNS3	-0.7162737	-0.2886098	-1.5016925	0.139166	0.99978775	-4.556462
TNNI1	-0.7164857	-0.9330709	-1.5995296	0.11570275	0.99978775	-4.5582538
FAM71E1	-0.71692	-0.8141914	-1.6335969	0.10832539	0.99978775	-4.5664177
MMRN2	-0.7172424	-0.3544173	-0.3426473	0.73323155	0.99978775	-4.6085394
ENSCAFG00000029292	-0.7175328	1.54039865	-0.6549905	0.51533276	0.99978775	-4.6146132
PELI2	-0.7184205	0.56511266	-1.6246455	0.11022575	0.99978775	-4.5264527
GBP1	-0.7184836	-1.4157779	-0.8116065	0.42068099	0.99978775	-4.5994609
CCDC150	-0.7186799	1.79541714	-1.6749077	0.09989809	0.99978775	-4.4597529
ENSCAFG00000025725	-0.7196155	-1.4668539	-1.3598435	0.1796893	0.99978775	-4.5810345

SLC22A4	-0.7205741	-0.6680487	-1.5416181	0.12917127	0.99978775	-4.5691979
CYP4F8	-0.7215547	2.0086532	-2.2834287	0.02647963	0.99978775	-4.2547342
ADGRL4	-0.7216672	-0.7017578	-0.3902922	0.69789919	0.99978775	-4.6084052
CCDC142	-0.7230928	1.24937118	-2.5525057	0.01364041	0.99978775	-4.3926803
CORO2A	-0.7245585	2.4669925	-2.310932	0.0247946	0.99978775	-4.3343296
MYCT1	-0.7250411	-0.2444229	-0.4543316	0.65146256	0.99978775	-4.6074814
TRIM65	-0.7250746	2.27666882	-1.4839152	0.14380905	0.99978775	-4.5362443
HMGA1	-0.7266945	7.73834716	-2.0541776	0.04495102	0.99978775	-3.9828974
PCYT1B	-0.7274649	0.3179664	-1.525378	0.13316529	0.99978775	-4.5668561
MXD3	-0.7282845	-0.8864779	-1.7605544	0.08413647	0.99978775	-4.5483025
LRIG3	-0.728536	1.70668332	-1.8524477	0.06958546	0.99978775	-4.378161
IQCG	-0.7292359	0.81382929	-2.3347453	0.02341352	0.99978775	-4.4530225
KANK4	-0.7296693	4.06878424	-1.38038	0.17331746	0.99978775	-4.4717163
KCNK5	-0.7304116	-0.7554383	-1.0736782	0.28787583	0.99978775	-4.5924842
WISP1	-0.7333317	4.63252454	-1.2630821	0.21213899	0.99978775	-4.5361835
ENSCAFG00000008439	-0.7336302	0.2751852	-2.1697216	0.03457406	0.99978775	-4.4907683
ALDH3B1	-0.7365686	-0.031419	-0.8512918	0.39847252	0.99978775	-4.6017642
PLEKHG4	-0.73709	2.95872513	-1.8824107	0.06532592	0.99978775	-4.3552573
INSIG1	-0.7382947	7.89348401	-1.9309501	0.0588955	0.99978775	-4.0833772
FAM81A	-0.7434136	2.9539037	-2.0829997	0.04213674	0.99978775	-4.1816305
ENSCAFG00000007173	-0.7438538	-0.9313717	-2.3825609	0.02084609	0.99978775	-4.4927905
RSPO3	-0.7439779	-1.6413206	-1.4921372	0.14164667	0.99978775	-4.5680593
PRRG2	-0.7460304	-1.0223714	-1.5785632	0.1204416	0.99978775	-4.5701597
CLDN6	-0.7481715	0.07832792	-1.396725	0.1683714	0.99978775	-4.5714362
FAT2	-0.7486032	-1.4050915	-1.3123373	0.19511351	0.99978775	-4.5804736
KBTBD8	-0.7488415	0.95471031	-2.297848	0.02558393	0.99978775	-4.4187858
HDHD3	-0.7492599	-0.0918569	-2.1597783	0.03537599	0.99978775	-4.5039597
MAP2K6	-0.7498738	-0.9931269	-0.8684032	0.38912501	0.99978775	-4.5994499
ARSI	-0.7510449	1.72018033	-3.1010868	0.00309887	0.99978775	-2.9796341
FHDC1	-0.7548744	0.06721632	-1.1905919	0.23916793	0.99978775	-4.584563
PLK2	-0.7550852	3.78230173	-1.1620744	0.2504595	0.99978775	-4.5708745
PLA2G3	-0.7560926	2.91327538	-1.2602443	0.21315258	0.99978775	-4.5582755
P3H2	-0.7566674	-0.2430172	-1.2246032	0.22618982	0.99978775	-4.5836676
KIFC1	-0.7568656	3.95171078	-1.9561753	0.05577264	0.99978775	-4.1236628
CEP55	-0.7572217	1.88216827	-1.4875969	0.14283756	0.99978775	-4.4820574
NKX2-3	-0.7575894	-2.8575074	-1.223038	0.22677548	0.99978775	-4.5890778
ERCC6L	-0.7582867	0.91111004	-1.5237021	0.133583	0.99978775	-4.5093642
IL1RL1	-0.7584146	4.22272228	-0.5260978	0.60103515	0.99978775	-4.6552895
MGLL	-0.7592724	-1.7462477	-1.0903802	0.28052369	0.99978775	-4.5925134
CDCA2	-0.7595251	2.27881887	-1.7523691	0.08554745	0.99978775	-4.3914862
IFI35	-0.7596298	1.00233649	-1.5651467	0.12355517	0.99978775	-4.5485686
ITGA4	-0.7609807	-1.307274	-1.1472459	0.25647996	0.99978775	-4.5863461
SH3BGRL2	-0.7616668	-1.3153317	-1.2745931	0.20806438	0.99978775	-4.5842999
PTGES	-0.7622864	1.48248599	-1.1686398	0.24782653	0.99978775	-4.5797842
RNF152	-0.7634039	-0.2496215	-1.7922474	0.0788548	0.99978775	-4.5394193
SPC25	-0.7641082	1.72872669	-1.7136663	0.09248791	0.99978775	-4.4493379
IGFBP5	-0.7657218	1.19583724	-1.2947367	0.20107499	0.99978775	-4.5188141
HOXC6	-0.7660161	1.73759728	-1.4190525	0.16179207	0.99978775	-4.5416454
EPB41L4B	-0.7661234	0.33760605	-1.2918268	0.20207366	0.99978775	-4.5782835
ADRA2A	-0.767002	0.26681124	-1.296315	0.20053489	0.99978775	-4.5785355

IL12A	-0.7673495	1.1271824	-1.7109941	0.09298381	0.99978775	-4.5330561
ADAMTS17	-0.769615	2.5771749	-1.5542636	0.12612803	0.99978775	-4.4275399
TNN	-0.7697605	-2.4204493	-1.4440262	0.15467145	0.99978775	-4.5813005
MBOAT1	-0.7700676	1.69923953	-1.4338164	0.15755237	0.99978775	-4.5435498
ENSCAFG00000004198	-0.7718968	0.0379329	-2.5213214	0.01476364	0.99978775	-4.4388348
SYT4	-0.772687	0.87557215	-1.1267905	0.26495366	0.99978775	-4.582163
PLK1	-0.7735977	3.96393411	-1.8105537	0.07593249	0.99978775	-4.207311
KIF23	-0.7743333	3.88064763	-1.6526257	0.10437425	0.99978775	-4.3075652
SPON1	-0.7743713	-0.6861866	-0.8744993	0.38582831	0.99978775	-4.6053551
SULF2	-0.774981	5.42183034	-1.1186883	0.26836429	0.99978775	-4.6092607
CARD9	-0.7770745	-0.0555542	-1.4671788	0.14829107	0.99978775	-4.5681358
FKBP5	-0.7791045	5.69999514	-1.7582816	0.08452629	0.99978775	-4.3725073
MTSS1	-0.7798314	-0.0689433	-0.8750153	0.38555009	0.99978775	-4.5991949
GAS1	-0.7815789	2.01075041	-1.1297696	0.26370734	0.99978775	-4.5839538
DHCR24	-0.7816002	5.45802816	-2.3252022	0.02395852	0.99978775	-3.8688499
RAD54B	-0.7823952	0.7577782	-2.2410665	0.02927436	0.99978775	-4.4289941
ADORA1	-0.7825403	-1.9026081	-2.4304836	0.01852909	0.99978775	-4.4896791
LOXL4	-0.7840085	3.08826733	-2.5802644	0.0127065	0.99978775	-3.5442063
LAMC2	-0.784109	2.76545382	-0.9486321	0.34715296	0.99978775	-4.6013455
ENSCAFG00000010412	-0.784173	1.46810782	-1.7704715	0.08245297	0.99978775	-4.4319976
RAB39B	-0.7848903	1.37488715	-3.0822511	0.00326936	0.99978775	-4.2599205
PAX1	-0.7867154	-0.2208942	-1.2547321	0.21513162	0.99978775	-4.5749904
ENSCAFG00000008627	-0.7869522	2.34784355	-2.1314421	0.03775151	0.99978775	-4.3799489
FZD10	-0.7879684	-1.7155915	-1.3401685	0.18596039	0.99978775	-4.5835576
RADIL	-0.7883526	2.67757457	-2.34611	0.02277895	0.99978775	-4.204382
LSP1	-0.7892747	7.26409735	-1.3642385	0.17831081	0.99978775	-4.479002
MGMT	-0.7895423	-0.0711427	-1.5662934	0.12328656	0.99978775	-4.5634155
SGO1	-0.7906729	1.79582218	-1.8281416	0.073211	0.99978775	-4.386671
DACH1	-0.7909685	-1.7107025	-0.8337169	0.40821677	0.99978775	-4.5992757
HOXA7	-0.7917007	0.84443617	-1.2990508	0.19960128	0.99978775	-4.5690063
PRSS12	-0.7927281	0.50212146	-1.946748	0.05692273	0.99978775	-4.4623563
NCAPH	-0.7953132	3.05574958	-1.8200808	0.07444793	0.99978775	-4.2685139
LHX8	-0.7956599	-2.5231878	-1.1298768	0.26366259	0.99978775	-4.5916805
PTGER2	-0.7960302	0.38549787	-1.0592654	0.29432685	0.99978775	-4.5930439
NUF2	-0.7977414	2.145945	-1.7334538	0.08888342	0.99978775	-4.3546392
PRAP1	-0.7982485	-1.2040318	-0.9227406	0.36036263	0.99978775	-4.5976153
KIF2C	-0.7991085	2.6161793	-2.8289763	0.00659832	0.99978775	-3.8319375
MCM5	-0.8016748	3.84631382	-1.8107239	0.07590575	0.99978775	-4.1986242
C3	-0.8031096	1.67458846	-0.2937319	0.77012054	0.99978775	-4.6142558
CCNF	-0.8043936	2.2004665	-1.8121053	0.07568902	0.99978775	-4.3405809
CNRIP1	-0.8054423	3.46186628	-1.4544886	0.15176215	0.99978775	-4.495718
ENSCAFG00000032230	-0.8056194	0.56700199	-2.4308557	0.01851204	0.99978775	-4.4462702
FAM131C	-0.8058686	0.36134282	-1.5210577	0.13424421	0.99978775	-4.561176
TMEM187	-0.8061793	0.87510893	-2.4409643	0.01805434	0.99978775	-4.4300771
DHDH	-0.8070243	-0.5144266	-0.9950563	0.32427189	0.99978775	-4.595559
CIT	-0.8087239	3.27813893	-1.9335137	0.05857145	0.99978775	-4.1780225
SYNM	-0.8104661	1.61053057	-1.6401189	0.10695766	0.99978775	-4.5193262
ENSCAFG00000009113	-0.8106712	1.92251091	-0.378243	0.70677479	0.99978775	-4.6206005
PIMREG	-0.8110289	1.58845818	-1.9733457	0.05372905	0.99978775	-4.2941125
IL1R2	-0.812448	-1.0473804	-1.1906959	0.23912744	0.99978775	-4.5893402

UHRF1	-0.8131369	3.76952247	-1.7758526	0.08155131	0.99978775	-4.2437149
HSD17B1	-0.81558	0.35239858	-1.9470111	0.05689035	0.99978775	-4.5283571
RHBG	-0.8156897	1.07519731	-2.5084422	0.01525152	0.99978775	-4.4111038
CDC45	-0.8163486	1.36577256	-1.5942957	0.11687137	0.99978775	-4.4742226
WDR62	-0.8169982	2.73324683	-2.5703658	0.01303263	0.99978775	-3.9636382
ENSCAFG00000029335	-0.8172243	-0.9878321	-1.8884808	0.06449048	0.99978775	-4.5352933
GTSE1	-0.8183487	2.41712049	-1.9363543	0.05821418	0.99978775	-4.227028
RASGEF1B	-0.8200323	1.28053752	-0.9322953	0.35545057	0.99978775	-4.6001664
BGLAP	-0.822753	0.83859313	-1.0999154	0.27638564	0.99978775	-4.590207
HAS2	-0.8252662	1.76904726	-0.9789502	0.33209304	0.99978775	-4.6016395
KNSTRN	-0.8255247	0.59597314	-1.7774449	0.08128609	0.99978775	-4.4952017
DEPDC1B	-0.8260824	1.35768853	-1.9120674	0.06132982	0.99978775	-4.38664
CD70	-0.8269266	-0.7705579	-1.2057527	0.23331756	0.99978775	-4.5881478
CDT1	-0.8317904	2.52163037	-1.9083912	0.0618136	0.99978775	-4.3149696
BDKRB2	-0.8318122	4.26340923	-1.5695798	0.12251932	0.99978775	-4.3724106
NOTUM	-0.8332762	-1.7115986	-1.3657518	0.17783805	0.99978775	-4.5809581
RELN	-0.8346428	-0.0345753	-0.5048638	0.61576662	0.99978775	-4.6102338
WFDC1	-0.8349229	4.40282081	-1.1843368	0.24161247	0.99978775	-4.5647435
ENSCAFG00000016482	-0.83538	0.88552958	-1.8115804	0.07577132	0.99978775	-4.4632536
PTN	-0.8356018	0.78759304	-1.631953	0.10867238	0.99978775	-4.4455373
KCNF1	-0.8360521	0.82684841	-1.269307	0.20992825	0.99978775	-4.5743858
KNTC1	-0.8392405	3.41163548	-2.0125441	0.04930345	0.99978775	-4.1237785
KAZALD1	-0.8396349	2.26901159	-0.7725296	0.44326387	0.99978775	-4.614959
TMEM51	-0.8402626	0.36111115	-2.2175887	0.03093313	0.99978775	-4.4216752
STAB1	-0.8412583	0.33084218	-0.4963271	0.62173481	0.99978775	-4.6112407
ANOS1	-0.8415991	0.6971804	-1.9788191	0.05309129	0.99978775	-4.2721077
ENSCAFG00000017264	-0.8442328	2.46290096	-2.3217852	0.02415639	0.99978775	-4.0744528
RHOU	-0.8443791	0.66196481	-1.4467244	0.15391702	0.99978775	-4.5598944
ANKLE1	-0.8464584	1.50716755	-2.183233	0.03350999	0.99978775	-4.3638885
ENSCAFG00000007078	-0.8473078	-0.8260503	-2.0296501	0.04747296	0.99978775	-4.5332056
RAD54L	-0.8500774	1.77278521	-1.9226596	0.05995394	0.99978775	-4.3841441
KIF14	-0.8503478	1.92821224	-1.7141975	0.0923896	0.99978775	-4.3852477
FAM167A	-0.8506062	-1.7383792	-1.3749986	0.1749701	0.99978775	-4.5829439
TFAP2C	-0.853147	0.39830847	-0.900854	0.37177888	0.99978775	-4.5984912
RECQL4	-0.8533219	1.09553026	-1.9060141	0.06212813	0.99978775	-4.4519742
RHEBL1	-0.8560456	0.05174752	-2.5622266	0.01330649	0.99978775	-4.4746684
ENSCAFG00000029779	-0.856084	-0.2847138	-1.5929975	0.1171627	0.99978775	-4.5610412
PLVAP	-0.8563326	-0.9724757	-0.4666209	0.6427012	0.99978775	-4.6064321
PKMYT1	-0.8584689	1.46784496	-1.886496	0.06476265	0.99978775	-4.3400941
CTRL	-0.8587328	-0.2868209	-2.3886616	0.02053735	0.99978775	-4.4828527
PLK4	-0.8606159	2.17253853	-1.7496763	0.08601592	0.99978775	-4.3707131
EPHB1	-0.8618386	-1.3389676	-1.1265665	0.26504752	0.99978775	-4.5905955
TNFAIP8L3	-0.862911	0.26342232	-1.2600094	0.21323662	0.99978775	-4.5717075
ENSCAFG00000029032	-0.8655328	-1.4858243	-1.7542884	0.08521484	0.99978775	-4.554894
IGSF9B	-0.8664576	1.12439566	-1.4813958	0.14447683	0.99978775	-4.5635369
E2F8	-0.8665167	1.58622104	-1.8771756	0.0660538	0.99978775	-4.3394102
HHAT	-0.8679975	-0.7251539	-1.8325203	0.07254636	0.99978775	-4.5369139
PADI2	-0.8692806	-0.5401628	-1.1675917	0.24824551	0.99978775	-4.5880143
GNG7	-0.8726205	-0.2089582	-2.3975439	0.02009519	0.99978775	-4.4817301
HOXA9	-0.872672	0.13193816	-0.7846792	0.4361672	0.99978775	-4.607343

HEXIM2	-0.8729002	-0.1871007	-1.9566958	0.05570973	0.99978775	-4.5398177
ATP6AP1L	-0.8730772	-0.7183946	-1.6545499	0.10398134	0.99978775	-4.5388799
HOXA6	-0.8731686	-0.7051624	-1.2075342	0.23263698	0.99978775	-4.5860319
HOXA2	-0.8743949	-0.7891397	-1.1745491	0.24547384	0.99978775	-4.5883149
TOP2A	-0.8748219	5.05662049	-1.6136705	0.11259259	0.99978775	-4.3205547
C1QL1	-0.8765118	0.3669359	-0.9644065	0.33926236	0.99978775	-4.5973622
DLGAP5	-0.8780203	3.98761	-1.650528	0.10480395	0.99978775	-4.3121774
TBC1D30	-0.8791871	-1.3274785	-1.3573487	0.18047541	0.99978775	-4.5833403
LIPG	-0.8801808	3.94878975	-1.7106019	0.09305678	0.99978775	-4.387751
GLI1	-0.8840216	1.78248665	-1.9511787	0.0563797	0.99978775	-4.4348037
CENPM	-0.8851448	1.0826575	-2.2228248	0.03055616	0.99978775	-4.3423179
CRLF1	-0.8870898	5.06065829	-0.8546473	0.39662863	0.99978775	-4.628897
NCAPG	-0.888784	3.94267794	-1.7905216	0.0791351	0.99978775	-4.2164213
GRIA3	-0.8902505	1.93233345	-1.3422174	0.18529967	0.99978775	-4.5378046
ENSCAFG00000015941	-0.8914659	-0.1909041	-2.3269015	0.02386065	0.99978775	-4.4761486
BRINP2	-0.89199	-1.25678	-1.3753614	0.17485831	0.99978775	-4.5790689
GPRIN1	-0.8932405	1.15757503	-2.2521882	0.02851643	0.99978775	-4.476648
ZNF296	-0.8956755	1.67353703	-2.6232363	0.01137511	0.99978775	-4.323586
KCTD12	-0.897295	0.13628565	-1.1038592	0.2746867	0.99978775	-4.5906178
TMEFF2	-0.8998172	4.23503089	-1.3822411	0.1727487	0.99978775	-4.5303252
ASTN1	-0.9010269	0.15382447	-0.4901999	0.62603435	0.99978775	-4.6088861
BUB1	-0.9012354	2.87869941	-2.0145755	0.04908294	0.99978775	-4.1551534
ENSCAFG00000007705	-0.9028237	2.22689234	-1.7882555	0.07950439	0.99978775	-4.3143437
TNFAIP6	-0.905347	3.15515391	-0.880916	0.38237727	0.99978775	-4.6233493
ALPK2	-0.905739	3.54207641	-2.2940032	0.02582008	0.99978775	-3.909181
STMN1	-0.9072128	3.70774434	-1.8462735	0.07049174	0.99978775	-4.2055375
SYT7	-0.9089865	0.22014342	-1.3876926	0.17109099	0.99978775	-4.574373
CDCA3	-0.9095703	2.94442187	-2.1237593	0.03841918	0.99978775	-4.0764706
KIF11	-0.9105082	3.96598798	-1.7531779	0.08540715	0.99978775	-4.24848
PDE4B	-0.911156	3.01280613	-0.83175	0.40931632	0.99978775	-4.6135108
ZIC2	-0.9123738	-1.415745	-1.5131621	0.13623395	0.99978775	-4.5770606
MDH1B	-0.9161257	0.29545006	-2.0635444	0.04401903	0.99978775	-4.5075645
FGF7	-0.916724	2.38912617	-0.426529	0.67146587	0.99978775	-4.6197085
CA2	-0.9174803	-1.0671979	-1.0682502	0.29029372	0.99978775	-4.5926376
TENM3	-0.9178044	0.84911758	-2.4339366	0.01837145	0.99978775	-3.911495
TMEM63C	-0.918226	-0.9664915	-1.8210098	0.07430449	0.99978775	-4.5453616
AIPL1	-0.9198757	-0.4834242	-2.0422682	0.04616066	0.99978775	-4.4673911
UBE2C	-0.9215286	3.06218269	-1.7688902	0.08271951	0.99978775	-4.305192
COL7A1	-0.9250899	3.00165812	-1.749024	0.08612971	0.99978775	-4.4417854
RRM2	-0.9253855	3.84028169	-1.8638396	0.06793912	0.99978775	-4.1686086
CDC20	-0.9281113	4.1247562	-1.8144458	0.07532303	0.99978775	-4.2135126
IL22RA1	-0.933925	-2.0218124	-2.0382533	0.04657477	0.99978775	-4.5333697
MND1	-0.9350618	-0.2296693	-1.6989348	0.09524912	0.99978775	-4.5314987
TONSL	-0.9369285	2.25002464	-2.2945199	0.02578823	0.99978775	-4.2641534
ENSCAFG00000005785	-0.9379379	0.65212639	-2.2032823	0.03198414	0.99978775	-4.4899841
CSMD2	-0.9397788	1.73883934	-1.2631035	0.21213138	0.99978775	-4.5700607
CDCA8	-0.9401891	3.49285204	-2.0219339	0.04829126	0.99978775	-4.1547191
HOXA10	-0.9406637	-0.8876733	-1.0081642	0.31799847	0.99978775	-4.5952495
TOX	-0.9408827	2.00502152	-1.8389729	0.0715762	0.99978775	-4.4330798
TRPT1	-0.9418681	1.03215508	-2.3214198	0.02417764	0.99978775	-4.4290596

ENSCAFG00000031334	-0.9425034	-1.0329811	-1.4492391	0.15321649	0.99978775	-4.5800866
ENSCAFG00000000451	-0.9438618	-0.3934548	-2.0020687	0.05045423	0.99978775	-4.4259875
RCOR2	-0.944706	1.03906829	-2.1752548	0.03413476	0.99978775	-4.4714695
HIST1H2BB	-0.9473664	-0.8726181	-1.9640584	0.05482628	0.99978775	-4.5176377
SHE	-0.9474585	-0.8213408	-0.6123775	0.54293065	0.99978775	-4.6044422
CNNM1	-0.9484776	-0.4639612	-1.6898909	0.09697766	0.99978775	-4.5534014
CENPA	-0.9500799	-0.1372319	-1.935432	0.05832997	0.99978775	-4.4892878
DEPDC1	-0.9503254	1.74539171	-2.0189338	0.04861269	0.99978775	-4.2877053
KNL1	-0.9518146	3.85372243	-1.8418924	0.07114085	0.99978775	-4.1833596
TSPAN7	-0.9527823	1.19491108	-1.9191089	0.0604122	0.99978775	-4.4877283
IGSF10	-0.9600227	-2.1986623	-2.3321161	0.02356256	0.99978775	-4.5189795
MKI67	-0.962334	5.08001107	-1.6614267	0.10258703	0.99978775	-4.286303
STXBP6	-0.9625906	6.35119308	-1.6043067	0.11464439	0.99978775	-4.3255733
HECW1	-0.9642029	0.03110437	-0.9254995	0.3589398	0.99978775	-4.5981646
PHYHIPL	-0.9646683	1.18183442	-0.581047	0.56369432	0.99978775	-4.610136
ENPP5	-0.9661334	-0.7060167	-1.5506003	0.12700366	0.99978775	-4.5713744
HAND2	-0.969962	-0.2698417	-1.4164562	0.16254672	0.99978775	-4.5556702
SYBU	-0.9720422	0.57076436	-1.2003286	0.23539853	0.99978775	-4.586838
KIF15	-0.9765523	2.66993304	-1.956115	0.05577994	0.99978775	-4.2637893
MAB21L1	-0.9882324	-1.6584775	-1.1485181	0.25595942	0.99978775	-4.5903115
NOS2	-0.9888386	-1.5621625	-1.9417276	0.05754343	0.99978775	-4.5504968
PPL	-0.9906166	7.1138253	-1.7618508	0.08391478	0.99978775	-4.2595498
DLA-79	-0.9907481	-0.0965412	-1.0303937	0.30754764	0.99978775	-4.5942791
MYBL2	-0.9927485	4.34721564	-1.794771	0.07844645	0.99978775	-4.1980957
GRID2	-0.9958442	-0.7281409	-1.6369383	0.1076229	0.99978775	-4.5524519
CCNB2	-1.0008146	2.08877982	-2.1358739	0.03737101	0.99978775	-4.1650869
ENSCAFG00000030825	-1.0011326	-0.9131369	-2.0686906	0.04351417	0.99978775	-4.5241156
ENSCAFG00000010422	-1.002623	-1.7810131	-1.5534648	0.12631856	0.99978775	-4.5734181
SLC30A3	-1.0056178	-0.9838493	-1.8249682	0.07369587	0.99978775	-4.5445944
ELN	-1.0090261	6.99509032	-1.7030817	0.09446505	0.99978775	-4.2711617
ESCO2	-1.0112363	2.72533096	-1.996726	0.05105002	0.99978775	-4.1853505
TSNAXIP1	-1.0116794	-0.0645357	-2.4722011	0.0167037	0.99978775	-4.4801277
ENSCAFG00000022040	-1.0137755	-0.9405767	-2.122104	0.03856439	0.99978775	-4.5377336
BUB1B	-1.0150497	3.27301826	-2.1228216	0.03850138	0.99978775	-4.0464685
CCNA2	-1.0168192	2.95979731	-1.8428737	0.07099502	0.99978775	-4.2379484
PPP2R2B	-1.0181122	-1.1224114	-1.513778	0.1360779	0.99978775	-4.5771534
WT1	-1.0182744	-1.5915956	-1.9076111	0.06191667	0.99978775	-4.5377129
ENSCAFG00000018724	-1.0287616	2.72844051	-2.0573977	0.0446287	0.99978775	-4.1368139
HIST2H2AC	-1.0288779	0.27900842	-2.9604286	0.00460244	0.99978775	-4.3564456
KIF18B	-1.0301011	0.99062424	-1.7910735	0.07904537	0.99978775	-4.4560381
TNFSF18	-1.0311567	-0.6892093	-0.9313181	0.35595092	0.99978775	-4.5970849
RAB38	-1.0324559	-1.1311008	-1.6333527	0.10837688	0.99978775	-4.5673039
TACC3	-1.0325846	3.43882406	-2.1761116	0.03406718	0.99978775	-4.0391407
FAM180B	-1.034724	-1.6502539	-1.3381296	0.18661968	0.99978775	-4.584155
TMOD2	-1.0356266	-1.252721	-1.6059552	0.11428099	0.99978775	-4.569899
CHL1	-1.0416636	0.37227892	-1.8581646	0.06875509	0.99978775	-4.481701
BCL2A1	-1.0425409	1.93752457	-1.2365811	0.22174459	0.99978775	-4.5810124
AURKB	-1.0515709	2.36099139	-2.2987074	0.0255314	0.99978775	-4.1684188
DHRS3	-1.0531803	2.40472693	-1.6079743	0.11383716	0.99978775	-4.4822966
KIF22	-1.0567011	2.93932997	-2.3223165	0.02412553	0.99978775	-4.0613355

EGR2	-1.0569805	4.36018588	-2.0180336	0.0487095	0.99978775	-4.2759902
ENSCAFG00000009779	-1.0607866	-0.8546883	-2.6430733	0.01080457	0.99978775	-4.4798235
ENSCAFG00000012593	-1.0615233	2.8561408	-2.0183495	0.04867551	0.99978775	-4.1619236
NEK2	-1.0638389	1.72857142	-2.2471625	0.02885674	0.99978775	-4.2057595
NCKAP5	-1.0694527	1.7065621	-1.0299502	0.30775382	0.99978775	-4.5942488
C5H1orf159	-1.0708749	1.10641664	-3.3738065	0.00139946	0.99968241	-4.2015826
GPR68	-1.0739827	0.32467405	-1.4363142	0.15684372	0.99978775	-4.5639598
PIH1D2	-1.0780575	-0.5361791	-2.8294866	0.00658922	0.99978775	-4.4713943
KLHL13	-1.0796672	-1.6280735	-2.944091	0.00481561	0.99978775	-4.431101
BAIAP2L1	-1.0829581	-1.461397	-1.5783972	0.12047973	0.99978775	-4.567362
GNG2	-1.0837695	3.75337496	-1.2172749	0.22894156	0.99978775	-4.56033
WNT2B	-1.0854716	-1.9075177	-2.2157477	0.03106665	0.99978775	-4.53184
ENSCAFG00000025115	-1.0919003	2.35604266	-1.3780082	0.17404437	0.99978775	-4.5555788
SHCBP1	-1.0921714	2.77692621	-2.1104934	0.03959637	0.99978775	-4.087288
RASD2	-1.0956221	0.89714886	-0.3810553	0.7046995	0.99978775	-4.6088561
IQGAP3	-1.096392	2.54814344	-2.2779716	0.02682582	0.99978775	-4.0198228
HEY1	-1.1001373	1.96485765	-2.426229	0.018725	0.99978775	-4.1900447
GAS7	-1.1018366	1.44053771	-0.5600698	0.57781329	0.99978775	-4.6113575
CXHXorf57	-1.1020053	-1.5967137	-1.7202486	0.09127571	0.99978775	-4.5643215
OSBPL3	-1.1043507	2.34663038	-1.1106432	0.27178147	0.99978775	-4.5906418
ESPL1	-1.1054652	3.09332204	-2.3881205	0.02056457	0.99978775	-3.9405046
ACAN	-1.1075875	-2.2068472	-2.2806814	0.02665342	0.99978775	-4.5354521
CDK15	-1.1085629	-1.4424823	-2.124775	0.03833033	0.99978775	-4.5204833
FABP3	-1.1138296	6.52977842	-2.0673854	0.04364173	0.99978775	-3.9970115
BDKRB1	-1.1274712	-0.4471751	-1.9497354	0.0565561	0.99978775	-4.529682
ENSCAFG00000030486	-1.1279713	2.07220023	-2.2026589	0.03203064	0.99978775	-4.2021924
SOX9	-1.1362658	2.67022967	-1.3242988	0.19113878	0.99978775	-4.5563456
ERAP2	-1.1507807	-0.7630399	-1.8078259	0.07636209	0.99978775	-4.5493693
ENSCAFG00000008369	-1.1546664	1.26067942	-1.9422512	0.05747842	0.99978775	-4.3821949
CHST9	-1.1569987	-1.7713271	-2.3462077	0.02277356	0.99978775	-4.5124416
MIS18A	-1.1575275	0.05472122	-2.4513366	0.01759531	0.99978775	-4.4333624
CDC25B	-1.1608806	4.92963499	-1.8139833	0.07539523	0.99978775	-4.226519
CORO1A	-1.1714194	0.39280816	-2.9955708	0.00417337	0.99978775	-4.370915
HTRA3	-1.183208	2.89396758	-2.5288869	0.01448369	0.99978775	-4.1149146
TROAP	-1.1867411	1.76990755	-2.3223454	0.02412385	0.99978775	-4.1828757
ENSCAFG00000007550	-1.1897273	-0.7308348	-2.1958542	0.03254216	0.99978775	-4.5282584
CCBE1	-1.1906112	-2.0993781	-1.541298	0.12924905	0.99978775	-4.5765934
TIAM1	-1.1911747	1.78488371	-0.9391247	0.35196629	0.99978775	-4.6001341
GDF15	-1.1960294	-1.9539139	-1.768146	0.0828452	0.99978775	-4.567078
HAPLN1	-1.2021239	-0.0968556	-1.3717257	0.17598113	0.99978775	-4.5707317
HOXA3	-1.2096772	1.08788004	-1.6659871	0.10167086	0.99978775	-4.5277895
P2RY2	-1.2124505	-1.927151	-2.2720906	0.0272034	0.99978775	-4.5413684
VIT	-1.2257566	1.58375932	-0.7702054	0.44462919	0.99978775	-4.6110743
PSTPIP1	-1.2429139	-1.0953914	-2.2895617	0.02609531	0.99978775	-4.5219533
TMEM236	-1.2457036	-0.4864429	-1.9023044	0.06262172	0.99978775	-4.5512769
MTURN	-1.2526482	-0.2879536	-2.6738074	0.00997211	0.99978775	-4.4627353
ENSCAFG00000001604	-1.2593492	0.56530101	-3.2895443	0.0017959	0.99968241	-4.3827138
MASP1	-1.26295	-2.3637125	-1.9467547	0.0569219	0.99978775	-4.5560564
SEMA3A	-1.2678859	-0.8700701	-2.298714	0.025531	0.99978775	-4.508756
CAPN3	-1.2699622	-0.2450089	-2.6398065	0.01089669	0.99978775	-4.47235

LRRN2	-1.2873456	-0.367953	-2.2321555	0.02989444	0.99978775	-4.4868216
CPLX2	-1.3090306	0.26205595	-2.9275707	0.00504047	0.99978775	-4.2934358
KIF20A	-1.3107603	3.64193971	-2.3496444	0.02258476	0.99978775	-3.8483201
PLB1	-1.3152944	-0.846441	-2.1558655	0.035696	0.99978775	-4.4904137
NRXN2	-1.3168416	-1.4583275	-2.536555	0.01420486	0.99978775	-4.4985211
KIF21B	-1.3390614	0.28507584	-2.1437592	0.03670229	0.99978775	-4.5025879
SEPT4	-1.3402201	1.17156772	-1.6123044	0.11289005	0.99978775	-4.553044
ITGA11	-1.3531356	5.0079142	-1.7268604	0.09007132	0.99978775	-4.2691774
PRR16	-1.36403	0.92222824	-1.0940603	0.27892152	0.99978775	-4.5909448
GUCY1B1	-1.3648703	0.5145515	-1.4903368	0.14211794	0.99978775	-4.5602783
ENSCAFG00000030087	-1.3882541	2.24417987	-2.2092743	0.03154017	0.99978775	-4.1991541
CRABP2	-1.3899256	3.5940927	-1.7056955	0.09397359	0.99978775	-4.403104
FBP1	-1.4075594	-1.2220774	-1.8800117	0.06565862	0.99978775	-4.5477794
CCL7	-1.4110418	0.85058472	-1.2973417	0.20018414	0.99978775	-4.5830809
TNK1	-1.4134498	-0.6240887	-2.0385762	0.04654135	0.99978775	-4.5341974
HOXA5	-1.4177449	2.08668437	-2.1307186	0.03781396	0.99978775	-4.4148028
ACKR3	-1.4226236	1.83695606	-0.7124465	0.47934192	0.99978775	-4.6120786
CA12	-1.4620041	0.46009075	-1.2751718	0.20786111	0.99978775	-4.5787073
CSF1R	-1.4625195	-0.8433924	-2.0112319	0.04944635	0.99978775	-4.5457708
ALDH1A3	-1.4689544	1.32837458	-1.5859067	0.11876433	0.99978775	-4.5233292
COL18A1	-1.4801369	4.74625314	-1.5422066	0.12902834	0.99978775	-4.5080248
SPSB4	-1.501314	-0.1978061	-3.2212474	0.00219286	0.99968241	-4.2802231
CRISPLD2	-1.5086825	4.38373398	-0.9538427	0.34453337	0.99978775	-4.6038345
CCL14	-1.5135946	1.91605552	-0.6265939	0.53363998	0.99978775	-4.6060597
CADM3	-1.5209614	-0.4243994	-1.6622947	0.10241214	0.99978775	-4.5574979
HS3ST2	-1.5295886	-0.8438936	-2.6272117	0.01125861	0.99978775	-4.4241301
ZNF804B	-1.5358326	-0.1984831	-2.4954797	0.01575726	0.99978775	-4.4229127
KCNJ8	-1.5452676	-1.9241512	-3.3137223	0.00167243	0.99968241	-4.439592
LMX1B	-1.5525965	-0.9875692	-1.6878372	0.09737374	0.99978775	-4.5577397
SYT9	-1.5583774	-1.5751443	-2.7911414	0.00730643	0.99978775	-4.4720974
PITX1	-1.5621919	-1.145302	-1.9284332	0.05921513	0.99978775	-4.5475879
ROR2	-1.5847211	4.3605026	-2.5332988	0.01432266	0.99978775	-3.7302291
COL6A3	-1.6138894	8.31029764	-2.7422975	0.00832429	0.99978775	-3.3614468
SH3GL3	-1.6404122	-0.5208578	-4.0108819	0.00019246	0.99968241	-4.2326269
GPC3	-1.6427119	-0.7738471	-2.2402009	0.02933409	0.99978775	-4.5259202
ANO2	-1.656001	-0.578664	-1.4739984	0.14645171	0.99978775	-4.5743689
NETO1	-1.661697	-2.0465051	-2.1932468	0.03274006	0.99978775	-4.5394688
EMX2	-1.6641913	0.61535807	-2.1786465	0.03386791	0.99978775	-4.4589126
HOXD8	-1.6666932	1.36661568	-1.8431669	0.07095149	0.99978775	-4.5053643
ENSCAFG00000013249	-1.6773827	0.52073242	-2.0696253	0.043423	0.99978775	-4.5190018
ABI3BP	-1.7081887	2.47393901	-1.1646672	0.24941728	0.99978775	-4.5782861
COMP	-1.7228443	0.650462	-2.0428808	0.04609776	0.99978775	-4.5173784
IQGAP2	-1.7274838	-0.3742561	-3.3442223	0.00152811	0.99968241	-4.3607372
HOXA11	-1.7609044	-1.3653077	-1.8708692	0.06693973	0.99978775	-4.5518491
CRTAC1	-1.7623096	-0.4720131	-3.6440525	0.00061551	0.99968241	-4.1983023
GREB1	-1.7709306	-0.7346542	-2.3158313	0.02450467	0.99978775	-4.4734457
SPINK5	-1.8961936	-1.0728539	-2.8413739	0.00638047	0.99978775	-4.4191754
HGF	-1.9445464	3.70924098	-2.1289733	0.03796495	0.99978775	-4.4061644
SV2C	-1.9747145	-0.1672486	-2.2821253	0.02656196	0.99978775	-4.509118
POSTN	-1.9907367	3.94390444	-1.1971948	0.236607	0.99978775	-4.566714

DMRT2	-2.1383927	-0.8655674	-2.4050226	0.01972954	0.99978775	-4.4797926
FGF5	-2.3031995	-1.0781096	-3.8772692	0.00029567	0.99968241	-4.2647812
