# Supplementary material for: Locational memory of macrovessel vascular cells is transcriptionally imprinted
Source: Sci Rep. 2023 Aug 10;13:13028. doi: 10.1038/s41598-023-38880-6 (PMC10415317; doi:10.1038/s41598-023-38880-6)
Supplement: Supplementary file 12 — Supplementary Table 3. [file 41598_2023_38880_MOESM12_ESM.pdf]

Supplemental Table 3. Statistical analyses of the tube formation assay parameters.

Generalized linear mixed model fit by maximum likelihood (Laplace Approximation) [glmerMod]
Family: poisson (log)
Formula: Parameter ~ 1 + (1 | Donor:Vessel) + (1 | Batch) + (1 | Donor) + Type

N_{Donor}	5
N_{Vessel}	9
N_{Batch}	5
Observations	39

				Random effects		
**Parameter**	**Incidence Rate Ratio**	**Confidence interval**	**P.Value**	**τ_{00} Donor:Vessel**	**τ_{00} Batch**	**τ_{00} Donor**
Total network length	0.53	0.36 – 0.78	0.001	0.11	0.02	0.00
Total segments length	0.37	0.20 – 0.68	0.001	0.25	0.08	0.00
Number of master junctions	0.49	0.31 – 0.77	0.002	0.12	0.10	0.02
Total master segments length	0.42	0.24 – 0.73	0.002	0.21	0.04	0.00
Total branching length	0.50	0.33 – 0.78	0.002	0.13	0.02	0.00
Number of meshes	0.27	0.11 – 0.64	0.003	0.39	0.30	0.00
Number of master segments	0.50	0.30 – 0.81	0.01	0.15	0.06	0.03
Total meshes area	0.14	0.03 – 0.56	0.01	1.43	0.10	0.00
Number of segments	0.50	0.27 – 0.92	0.03	0.27	0.00	0.00
Number of junctions	0.59	0.32 – 1.08	0.09	0.27	0.01	0.00
Total branches length	0.71	0.47 – 1.08	0.11	0.12	0.04	0.00
Number of nodes	0.60	0.31 – 1.16	0.13	0.31	0.03	0.00
Number of peaces	0.68	0.37 – 1.27	0.23	0.27	0.04	0.00
Branching interval	0.47	0.13 – 1.74	0.26	1.00	1.00	1.00
Mean mesh size	0.66	0.18 – 2.43	0.53	1.00	1.00	1.00
Number of isolated segments	1.10	0.72 – 1.70	0.66	0.08	0.13	0.07
Number of branches	0.86	0.38 – 1.94	0.72	0.42	0.22	0.00
Total isolated branches length	0.95	0.62 – 1.44	0.80	0.09	0.12	0.11
Number of extremities	0.92	0.48 – 1.78	0.80	0.27	0.18	0.00
Mesh index	0.92	0.25 – 3.40	0.90	1.00	1.00	1.00
