# Supplementary material for: Locational memory of macrovessel vascular cells is transcriptionally imprinted
Source: Sci Rep. 2023 Aug 10;13:13028. doi: 10.1038/s41598-023-38880-6 (PMC10415317; doi:10.1038/s41598-023-38880-6)
Supplement: Supplementary file 14 — Supplementary Table 5. [file 41598_2023_38880_MOESM14_ESM.pdf]

Supplemental Table 5. Top 5 differentially expressed genes per vessel and cell type. Genes are annotated in symbols when available. For each gene, log2-fold-change (LogFC), average log2-expression (AveExpr), moderated t-statistic (t), raw P value (p), adjusted P value (Adj.p), and log-odds that the gene is differentially expressed (B) were calculated.

ECs

Vessel	Genes	LogFC	AveExpr	t	p	Adj. p	B
**ACOR**	HTR4	5.8	-2.54	15.35	4.15E-18	5.23E-14	28.87
**ACOR**	C7	6.12	-2.22	13.4	3.61E-16	2.27E-12	25.06
**ACOR**	NFASC	5.43	-0.41	12.08	9.47E-15	3.98E-11	22.91
**ACOR**	CNTNAP4	4.85	-0.28	11.67	2.73E-14	8.58E-11	22.24
**ACOR**	SH3TC2	4.92	-1.09	10.08	2.05E-12	5.16E-09	17.84
**VCAV**	ENSCAFG00000023188	2.35	1.26	8.53	1.88E-10	2.37E-06	13.58
**VCAV**	PLVAP	7.71	-0.97	8.12	6.53E-10	3.02E-06	12.6
**VCAV**	ENTPD1	5.48	-1.67	8.09	7.19E-10	3.02E-06	12.49
**VCAV**	IL3RA	6.1	-2.5	7.73	2.22E-09	5.79E-06	11.34
**VCAV**	GNGT2	5.02	-2.25	7.68	2.56E-09	5.79E-06	11.1
**VPOR**	ENSCAFG00000017317	2.17	3.35	10.59	4.94E-13	6.22E-09	19.42
**VPOR**	ENSCAFG00000015445	1.97	2.67	8.54	1.87E-10	1.18E-06	13.71
**VPOR**	HAND2	4.43	-0.27	8.01	9.40E-10	3.95E-06	11.3
**VPOR**	EYA4	4.02	1.99	7.72	2.25E-09	7.06E-06	11.06
**VPOR**	ENSCAFG00000004690	1.48	3.67	7.65	2.80E-09	7.06E-06	11.19
**APUL**	NR1H4	2.62	0.68	8.4	2.83E-10	3.57E-06	13.17
**APUL**	TBX5	3.44	-0.89	7.64	2.86E-09	1.80E-05	9.77
**APUL**	HOXA2	2.54	-0.79	6.6	7.75E-08	2.91E-04	7.02
**APUL**	TCF21	3.25	-0.17	6.54	9.25E-08	2.91E-04	7.03
**APUL**	HOXB3	1.59	2.64	6.4	1.47E-07	3.70E-04	7.37
**VPUL**	TBX5	4.39	-0.89	10.97	1.77E-13	2.22E-09	16.44
**VPUL**	HOXA3	2.32	1.09	8.04	8.48E-10	5.34E-06	11.01
**VPUL**	TCF21	3.72	-0.17	7.89	1.35E-09	5.69E-06	9.82
**VPUL**	NR1H4	2.34	0.68	7.32	7.79E-09	2.45E-05	9.86
**VPUL**	EYA4	3.25	1.99	6.25	2.33E-07	5.86E-04	6
**AORT**	ENSCAFG00000015899	2.34	0.71	7.57	3.60E-09	4.53E-05	9.73
**AORT**	HOXC10	3.6	-0.04	7.17	1.26E-08	5.36E-05	9.08
**AORT**	ENSCAFG00000023188	2.1	1.26	7.17	1.28E-08	5.36E-05	8.95
**AORT**	ENSCAFG00000017317	1.59	3.35	6.95	2.55E-08	8.02E-05	9.02
**AORT**	WISP1	4.06	4.63	6.78	4.36E-08	1.10E-04	8.52
**AFEM**	HTR2A	4.62	-1.07	10.39	8.63E-13	5.64E-09	17.48
**AFEM**	TBX15	2.75	3.31	10.38	8.95E-13	5.64E-09	18.97
**AFEM**	MT3	5.04	-1.14	8.99	4.86E-11	2.04E-07	14.95
**AFEM**	HOXA9	3.75	0.13	8.78	9.08E-11	2.86E-07	13.97
**AFEM**	ADRA2A	2.92	0.27	8.59	1.59E-10	4.00E-07	13.65
**VFEM**	HOXC10	4.55	-0.04	11.44	4.99E-14	6.29E-10	19.02

**VFEM**	TBX15	2.07	3.31	7.81	1.69E-09	1.07E-05	11.59
**VFEM**	EYA1	4.21	-1.79	7.54	3.97E-09	1.67E-05	8.69
**VFEM**	FBXO10	1.8	2.02	6.37	1.60E-07	4.91E-04	7.28
**VFEM**	HOXA9	2.82	0.13	6.31	1.95E-07	4.91E-04	6.56
**VSAP**	GPR1	5.85	-1.68	15.01	8.73E-18	1.10E-13	29.17
**VSAP**	TBX15	3.09	3.31	11.92	1.43E-14	9.02E-11	23.07
**VSAP**	LMX1B	4.76	-0.99	11.53	3.98E-14	1.67E-10	20.92
**VSAP**	DSCAML1	4.8	-0.85	11.13	1.15E-13	3.61E-10	20.94
**VSAP**	HOXA11	5.11	-1.37	10.66	4.16E-13	1.05E-09	18.79

VSMCs

Vessel	Genes	LogFC	AveExpr	t	p	Adj. p	B
**ACOR**	SYNPO2L	4.62	0.53	17.91	2.11E-20	2.66E-16	36.22
**ACOR**	COLGALT2	5.33	-2.34	16.37	4.68E-19	2.95E-15	31.86
**ACOR**	EXPH5	5.98	-0.95	15.72	1.88E-18	7.89E-15	31.63
**ACOR**	C15orf48	4.8	-2.75	15.5	3.00E-18	9.44E-15	29.38
**ACOR**	SORBS2	4.38	4.44	15.24	5.33E-18	1.34E-14	30.9
**VCAV**	WT1	3.04	-1.59	7.32	7.76E-09	9.78E-05	8.35
**VCAV**	MYRF	1.95	3.55	6.72	5.25E-08	3.31E-04	8.36
**VCAV**	ZNF385B	2.16	-0.15	6.49	1.08E-07	4.54E-04	7.48
**VCAV**	HOXC6	2.1	1.74	6.16	3.13E-07	9.85E-04	6.42
**VCAV**	SLITRK5	3.39	-1.04	6.06	4.23E-07	1.07E-03	5.36
**VPOR**	NKX2-3	5.36	-2.86	15.15	6.42E-18	8.09E-14	21.92
**VPOR**	TRPM2	5.14	-2.31	9.04	4.18E-11	2.63E-07	12.35
**VPOR**	CHL1	2.93	0.37	8.56	1.72E-10	7.22E-07	13.52
**VPOR**	ALKAL1	3.65	-2.94	8.35	3.32E-10	1.05E-06	9.35
**VPOR**	HAND2	2.8	-0.27	7.61	3.22E-09	8.11E-06	10.6
**APUL**	HOXA3	3.04	1.09	12.01	1.14E-14	1.44E-10	15.44
**APUL**	TBX5	3.38	-0.89	7.66	2.75E-09	1.73E-05	5.47
**APUL**	MBNL3	2.28	-2.24	5.53	2.31E-06	7.35E-03	0.38
**APUL**	HOXA2	2.55	-0.79	5.53	2.33E-06	7.35E-03	0.83
**APUL**	HOXB5	2.38	0.12	5.39	3.65E-06	9.21E-03	1.96
**VPUL**	HOXA3	3.48	1.09	13.55	2.51E-16	3.17E-12	21.97
**VPUL**	TBX5	3.88	-0.89	8.6	1.57E-10	9.87E-07	10.05
**VPUL**	MBNL3	3.15	-2.24	8.07	7.73E-10	3.24E-06	7.17
**VPUL**	GATA5	1.89	1.18	7.49	4.65E-09	1.46E-05	10.51
**VPUL**	MYRF	1.99	3.55	6.94	2.61E-08	6.58E-05	8.96
**AORT**	HOXC10	2.91	-0.04	6.69	5.81E-08	4.81E-04	6.77
**AORT**	CARM1	2.11	-0.94	6.6	7.64E-08	4.81E-04	7.17
**AORT**	EYA4	1.58	1.99	6.3	2.01E-07	8.43E-04	7.05
**AORT**	ENSCAFG00000028801	2.34	-1.03	5.82	9.27E-07	2.92E-03	3.31
**AORT**	MXRA5	-3.12	2.83	-5.59	1.95E-06	4.91E-03	4.8
**AFEM**	HOXC10	4.16	-0.04	11.25	8.44E-14	1.06E-09	19.09

**AFEM**	LMX1B	3.29	-0.99	7.43	5.66E-09	3.57E-05	9.04
**AFEM**	TLR2	1.93	0.23	6.33	1.83E-07	6.17E-04	6.85
**AFEM**	TBX15	1.67	3.31	6.31	1.96E-07	6.17E-04	7.11
**AFEM**	EYA1	3.62	-1.79	6.13	3.45E-07	7.75E-04	5.21
**VFEM**	HOXC10	3.78	-0.04	10.17	1.63E-12	2.05E-08	15.93
**VFEM**	EYA1	4.27	-1.79	8.99	4.88E-11	3.08E-07	11.92
**VFEM**	LMX1B	3.43	-0.99	7.97	1.05E-09	4.40E-06	9.79
**VFEM**	GATM	2.64	-0.69	7.68	2.53E-09	7.96E-06	10.76
**VFEM**	HOXA10	3.03	-0.89	7.39	6.30E-09	1.59E-05	8.27
**VSAP**	HOXA11	5.16	-1.37	12.12	8.59E-15	1.08E-10	21.38
**VSAP**	LMX1B	4.37	-0.99	11.5	4.26E-14	2.68E-10	20.05
**VSAP**	TBX15	2.25	3.31	9.25	2.26E-11	9.49E-08	15.85
**VSAP**	LHX8	4.72	-2.52	8.97	5.19E-11	1.50E-07	12.95
**VSAP**	HOXA10	3.43	-0.89	8.92	5.96E-11	1.50E-07	13.66
