# Supplementary material for: Locational memory of macrovessel vascular cells is transcriptionally imprinted
Source: Sci Rep. 2023 Aug 10;13:13028. doi: 10.1038/s41598-023-38880-6 (PMC10415317; doi:10.1038/s41598-023-38880-6)
Supplement: Supplementary file 16 — Supplementary Table 7. [file 41598_2023_38880_MOESM16_ESM.pdf]

Supplemental Table 7. KEGG pathways for contrasts of all sample groups.

ID	Description	GeneRatio	BgRatio	p value	p.adjust	q value	geneID	Count	vessel
cfa04151	PI3K-Akt signaling pathway	37/357	354/7854	1.5014E-06	0.00010473	7.6652E-05	609293/403617/403714/612582/403811/403851/490280/403985/442986/483273/399544/442939/482154/481464/403417/403784/403466/479444/475108/478232/475266/100855806/607616/482932/477374/607922/403608/611773/487421/403872/100856563/607513/479460/403788/403897/475034/491587 IL3RA, GNGT2, TEK, IL7R, KIT, IL2RG, PPP2R5A, IL6, PDGFB, RELN, VWF, LAMC2, KDR, FLT4, TLR4, NOS3, COL4A5, PPP2R1B, PTK2, MTOR, CREB5, GNG11, ANGPT2, VEGFC, PPP2R2A, CREB1, PPP2CA, FGF23, EPHA2, NRAS, ITGA6, GNG4, PDGFD, ITGB3, NR4A1, LAMA4, PPP2R2D	37	VCAV_EC-up
cfa05416	Viral myocarditis	5/407	51/7854	0.1227398	0.76328292	0.73371129	609265/609307/476623/474836/611066 SGCA, CD55, DAG1, DLA88, SGCB	5	ACOR_VSMC-up
cfa05168	Herpes simplex virus 1 infection	35/407	359/7854	0.0002063	0.05941352	0.05711168	100856077/403416/414288/482905/102155260/100687500/484217/100855560/477402/484542/484559/100687233/100687206/106558995/100686614/100685774/100685146/403522/607899/100855518/100684862/474836/484933/480688/100855741/106558983/100683215/100687631/403421/484934/100855887/102156745/102152692/100686024/111089961 IFNGR1, BCL2, ZNF331, TLR3, ZNF550, ZNF782, ZNF484, DDX58, , HKR1, , ZNF641, ZNF398, , ZNF175, ZNF79, ZNF432, CCL5, IRF9, ZNF853, ZNF74, DLA88, ZNF791, TSC1, B2M, , ZFP90, ZNF461, ZNF252, , ZNF226, MAP3K7, ZNF529, ZNF425, ZNF454	35	ACOR_VSMC-up
cfa04141	Protein processing in endoplasmic reticulum	33/279	160/7854	7.5115E-17	1.968E-14	1.6683E-14	483369/478279/480726/476694/481295/403948/490691/404019/478523/479406/475668/476547/479410/480295/609416/403908/480409/476186/403951/478826/483104/482715/474593/611781/479441/607439/489300/480438/478464/480361/484922/476516/607653 P4HB, , HSPA5, CALR, CKAP4, TRAM1, ERO1A, HSP90B1, SEC24D, HSPA8, , EDEM1, HYOU1, SEC23A, , CANX, SEL1L, LMAN1, SSR1, DNAJC10, MOGS, PDIA4, ERLEC1, BAG1, CRYAB, DDIT3, STT3A, HSP90AA1, MAPK10, EIF2S1, RAD23A, RPN1, SEC13	33	VSAP_EC-down
cfa04010	MAPK signaling pathway	33/357	288/7854	7.3901E-07	9.5115E-05	6.9618E-05	403714/611329/403811/483732/442986/482154/479155/481464/485189/488148/403486/480481/612821/607616/489943/488126/482932/478175/484713/483225/479406/612814/100856359/480632/486683/485692/611773/487421/403872/491768/479460/403897/487514 TEK, RASGRP3, KIT, MAP3K11, PDGFB, KDR, MEF2C, FLT4, ARRB1, MECOM, PPP3CA, MAP3K3, MAPK13, ANGPT2, TAOK2, IL1RAP, VEGFC, STMN1, FLNB, RAP1A, HSPA8, PAK2, RAPGEF2, TAOK1, DUSP16, RASA2, FGF23, EPHA2, NRAS, RPS6KA3, PDGFD, NR4A1, PLA2G4E	33	VCAV_EC-up
cfa03040	Spliceosome	33/388	132/7854	3.2308E-15	2.9293E-13	2.7434E-13	476725/476015/475922/100856058/100687048/608074/477148/477624/102152026/403687/478422/478663/611914/100855638/483177/607948/474489/479743/483721/607232/480100/488856/474839/612188/479669/478014/478052/485806/475326/474921/606899/607245/477538 HNRNPM, SF3B2, EIF4A3, SRSF2, U2AF2, HNRNPA3, SNRPB2, DDX23, , SRSF3, U2AF1, TRA2B, SNRNP40, HNRNPK, SF3B4, ALYREF, PHF5A, , SART1, PPIE, HNRNPU, DHX15, DDX39B, SF3A2, DHX38, RBM17, TCERG1, SNRPB, , CDC5L, PPIH, PUF60, SF3A1	33	ACOR_VSMC-down

cfa04015	Rap1 signaling pathway	29/357	209/7854	6.0826E-08	1.6971E-05	1.2421E-05	403714/611329/403811/482407/442986/482154/481464/491286/607269/483881/487188/486591/480271/478227/612821/483730/607616/482932/483225/484427/100856359/485874/611773/487421/403872/609521/479460/609091/403788 TEK, RASGRP3, KIT, DOCK4, PDGFB, KDR, FLT4, RAPGEF1, CALML6, RALB, ARAP3, RAPGEF3, ADCY4, GNAI1, MAPK13, SIPA1, ANGPT2, VEGFC, RAP1A, PRKD2, RAPGEF2, PLCG1, FGF23, EPHA2, NRAS, , PDGFD, PRKD1, ITGB3	29	VCAV_EC-up
cfa04014	Ras signaling pathway	28/357	225/7854	1.0227E-06	9.5115E-05	6.9618E-05	403617/403714/611329/403811/442986/482154/481464/607269/483881/490548/481145/100855806/607616/489287/482932/483225/612814/489692/485692/485874/611773/487421/403872/607513/479460/487514/481739/479473 GNGT2, TEK, RASGRP3, KIT, PDGFB, KDR, FLT4, CALML6, RALB, RALBP1, TBK1, GNG11, ANGPT2, ETS1, VEGFC, RAP1A, PAK2, PLCG2, RASA2, PLCG1, FGF23, EPHA2, NRAS, GNG4, PDGFD, PLA2G4E, RGL2, PLD2	28	VCAV_EC-up
cfa05165	Human papillomavirus infection	27/357	328/7854	0.00193478	0.02076166	0.01519623	490280/480676/483273/399544/442939/403466/479444/488473/475108/478232/481726/475266/481145/482905/610303/477714/485769/442942/477374/607922/403608/403872/100856563/403788/479010/475034/491587 PPP2R5A, NOTCH1, RELN, VWF, LAMC2, COL4A5, PPP2R1B, CASP8, PTK2, MTOR, NOTCH4, CREB5, TBK1, TLR3, MFNG, CHD4, JAG1, PTGS2, PPP2R2A, CREB1, PPP2CA, NRAS, ITGA6, ITGB3, UBE3A, LAMA4, PPP2R2D	27	VCAV_EC-up
cfa05166	Human T-cell leukemia virus 1 infection	25/388	209/7854	3.346E-05	0.00075843	0.00071029	480129/476694/479307/489287/492093/477857/100499482/477563/475750/475359/481131/478324/488828/403872/607879/489717/483724/476070/100855704/483845/474681/100855774/608350/480272/489644 FDPS, CALR, , ETS1, SLC25A5, BUB3, RAN, RANBP1, ANAPC1, CDKN2C, CDK4, CCNB2, MSX1, NRAS, ESPL1, VAC14, FOSL1, , CDK2, CCNA2, VDAC1, CDC20, BUB1B, NFATC4, PIK3CD	25	ACOR_VSMC-down
cfa04151	PI3K-Akt signaling pathway	24/441	354/7854	0.19346946	0.66536335	0.5989803	100682877/612271/476179/403915/483776/479460/486148/492265/491290/491278/491313/442938/485672/478127/474800/487235/403924/612305/484648/476626/483755/608847/611939/489038 TNXB, IL6R, PHLPP1, FGF7, CHRM1, PDGFD, IRS1, MTCP1, LAMC3, RXRA, PKN3, FLT3LG, PPP2R3A, RBL2, LPAR1, PIK3R1, MYC, CRTC2, RAF1, LAMB2, PPP2R5B, CCND3, MAP2K2, DDIT4	24	VSAP_EC-up
cfa04510	Focal adhesion	24/279	198/7854	1.1705E-07	1.0223E-05	8.666E-06	403802/480369/403936/489208/607885/484713/608287/476836/475411/477956/485856/484087/482736/491597/484526/477032/488437/479252/100856393/478575/403788/607207/478464/612814 VEGFA, ACTN1, VASP, ITGA2, PAK1, FLNB, ITGA10, ILK, PPP1R12A, ITGB1, MYL9, THBS2, ZYX, PDGFA, ACTN4, CTNNB1, ITGAV, VCL, TLN1, GSK3B, ITGB3, , MAPK10, PAK2	24	VSAP_EC-down
cfa05163	Human cytomegalovirus infection	24/357	220/7854	5.6645E-05	0.00160623	0.00117566	403617/403850/483900/403985/606815/607269/403486/485930/488473/475108/478232/480271/475266/481145/478227/100855806/612821/479409/442942/607922/102152063/403872/607513/403788 GNGT2, CXCL8, CXCR4, IL6, TMEM173, CALML6, PPP3CA, NFATC2, CASP8, PTK2, MTOR, ADCY4, CREB5, TBK1, GNAI1, GNG11, MAPK13, ARHGEF12, PTGS2, CREB1, RIPK1, NRAS, GNG4, ITGB3	24	VCAV_EC-up

cfa05225	Hepatocellular carcinoma	23/441	165/7854	4.4414E-05	0.00329774	0.00296872	482294/485183/490903/486894/480477/483626/476006/477683/485149/609050/478813/479912/487235/403924/476005/484648/490099/476335/442987/611939/477039/476713/610935 FZD1, WNT11, AXIN2, TCF7L2, SMARCD2, DDB2, , MGST1, FZD4, MGST3, NFE2L2, GSTM4, PIK3R1, MYC, GSTP1, RAF1, AXIN1, SMARCA2, HMOX1, MAP2K2, TGFBR2, KEAP1, NQO1	23	VSAP_EC-up
cfa05167	Kaposi sarcoma-associated herpesvirus infection	23/357	178/7854	5.1236E-06	0.0002859	0.00020926	403617/403850/403985/442986/607269/403486/485930/488473/478232/481145/100855806/612821/100856077/607616/482905/403764/442942/489692/607922/485874/403872/607513/480348 GNGT2, CXCL8, IL6, PDGFB, CALML6, PPP3CA, NFATC2, CASP8, MTOR, TBK1, GNG11, MAPK13, IFNGR1, ANGPT2, TLR3, CD86, PTGS2, PLCG2, CREB1, PLCG1, NRAS, GNG4, HIF1A	23	VCAV_EC-up
cfa04714	Thermogenesis	22/441	227/7854	0.00847825	0.11990668	0.10794364	476754/476215/478634/480477/478880/608530/612448/478981/476631/479887/611480/486295/475978/476503/403607/476040/478807/476335/474695/475631/608597/476004 NDUFS7, MAP3K5, SDHA, SMARCD2, NDUFS1, , RPS6KA2, NDUFS2, NDUFAF3, NDUFB10, , PRKAB1, NDUFS3, UQCRFS1, LIPE, COX8A, , SMARCA2, KDM3B, ACSL1, COA1, NDUFV1	22	VSAP_EC-up
cfa04151	PI3K-Akt signaling pathway	22/279	354/7854	0.00739842	0.08076606	0.06846782	403802/477374/487421/403647/404019/489208/608287/477956/487870/484087/491597/475590/488437/480438/478575/484955/403788/491369/403897/474890/608458/100687866 VEGFA, PPP2R2A, EPHA2, SGK1, HSP90B1, ITGA2, ITGA10, ITGB1, EIF4E, THBS2, PDGFA, EIF4EBP1, ITGAV, HSP90AA1, GSK3B, CDC37, ITGB3, CREB3L2, NR4A1, CDKN1A, EFNA5, YWHAH	22	VSAP_EC-down
cfa05170	Human immunodeficiency virus 1 infection	22/357	200/7854	0.00010209	0.00237366	0.00173737	403617/483900/606815/403417/607269/403486/485930/488473/475108/478232/487437/481145/478227/100855806/612821/612814/489692/485874/102152063/403872/607513/607683 GNGT2, CXCR4, TMEM173, TLR4, CALML6, PPP3CA, NFATC2, CASP8, PTK2, MTOR, TNFRSF1B, TBK1, GNAI1, GNG11, MAPK13, PAK2, PLCG2, PLCG1, RIPK1, NRAS, GNG4, CUL4A	22	VCAV_EC-up
cfa04151	PI3K-Akt signaling pathway	22/241	354/7854	0.00123353	0.04951748	0.04451843	442985/608977/403656/403824/610359/403668/489223/609956/100856491/449021/482666/480173/611795/608847/490942/403651/403857/486148/403582/611782/481954/475590 PDGFRB, ITGB5, ANGPT1, COL1A2, THBS3, COL6A1, OSMR, , COL6A2, AKT2, PDGFC, LAMA3, CSF1, CCND3, G6PC3, COL1A1, FGF2, IRS1, COL6A3, CCND2, FOXO3, EIF4EBP1	22	VCAV_EC-down
cfa03030	DNA replication	22/388	36/7854	2.0553E-20	5.5903E-18	5.2355E-18	476131/477871/612550/479737/100855690/481839/480869/484622/476025/486223/489798/477499/477308/100856421/100686967/478667/476063/477166/610047/474950/610886/483096 MCM6, MCM4, POLE3, MCM7, RPA1, MCM3, POLA1, MCM2, POLA2, POLE, RFC2, RFC5, RFC3, RPA2, LIG1, RFC4, FEN1, PCNA, POLD1, PRIM2, RNASEH2C,	22	ACOR_VSMC-down
cfa04114	Oocyte meiosis	22/388	117/7854	4.5386E-08	3.0785E-06	2.8831E-06	480928/479444/479307/490050/610343/612020/100856079/475750/481806/489971/478324/607879/487443/477237/476070/486886/100855704/608420/100682599/100855774/485940/481746 SMC1A, PPP2R1B, , PKMYT1, SGO1, FBXO5, CDK1, ANAPC1, PPP2R5D, PLK1, CCNB2, ESPL1, MAD2L2, YWHAB, , SMC3, CDK2, , BUB1, CDC20, AURKA, ITPR3	22	ACOR_VSMC-down

cfa05165	Human papillomavirus infection	20/441	328/7854	0.38357854	0.85964878	0.77388194	482294/100682877/485183/490903/486894/485149/491290/485672/478127/485116/487235/484648/476626/483755/490099/485158/476412/608847/611939/611262 FZD1, TNXB, WNT11, AXIN2, TCF7L2, FZD4, LAMC3, PPP2R3A, RBL2, MAML2, PIK3R1, RAF1, LAMB2, PPP2R5B, AXIN1, DLG2, IRF3, CCND3, MAP2K2, TRADD	20 VSAP_EC-up
cfa05165	Human papillomavirus infection	20/279	328/7854	0.01269321	0.11467658	0.09721478	403802/477374/479975/477714/489208/403874/403567/488036/608287/442942/477956/484087/475590/477032/488437/478575/403788/491369/487309/474890 VEGFA, PPP2R2A, PRKACB, CHD4, ITGA2, , CASP3, DLG1, ITGA10, PTGS2, ITGB1, THBS2, EIF4EBP1, CTNNB1, ITGAV, GSK3B, ITGB3, CREB3L2, HDAC1, CDKN1A	20 VSAP_EC-down
cfa04022	cGMP-PKG signaling pathway	20/357	165/7854	5.4256E-05	0.00160623	0.00117566	403862/490455/609574/403480/479155/403784/607269/403486/490755/482663/485930/480271/475266/477098/478227/553102/607922/403558/488584/479797 EDNRB, NPR1, PDE2A, ATP2B1, MEF2C, NOS3, CALML6, PPP3CA, SLC8A3, GUCY1A1, NFATC2, ADCY4, CREB5, ATP1B3, GNAI1, GUCY1B1, CREB1, PPP1CB, RGS2, ATP2A1	20 VCAV_EC-up
cfa04510	Focal adhesion	20/357	198/7854	0.00064601	0.00901187	0.00659613	442986/483273/399544/442939/482154/481464/491286/403466/486934/475108/475294/482932/484713/483225/612814/403558/100856563/479460/403788/475034 PDGFB, RELN, VWF, LAMC2, KDR, FLT4, RAPGEF1, COL4A5, DOCK1, PTK2, CAV2, VEGFC, FLNB, RAP1A, PAK2, PPP1CB, ITGA6, PDGFD, ITGB3, LAMA4	20 VCAV_EC-up
cfa04144	Endocytosis	20/357	235/7854	0.00501483	0.03959584	0.02898167	403851/483900/475659/100856769/403957/487188/485189/608079/475294/474657/479406/612505/100856747/608795/477520/612856/490341/476331/479473/477199 IL2RG, CXCR4, ASAP2, WWP1, RAB11A, ARAP3, ARRB1, CYTH1, CAV2, SNX2, HSPA8, SH3GLB1, , EHD4, GIT2, ARPC5L, DNM3, PIP5K1B, PLD2, ITCH	20 VCAV_EC-up
cfa04218	Cellular senescence	20/388	151/7854	4.88E-05	0.00102105	0.00095624	489287/492093/100856079/486743/481131/478324/486308/478454/403872/611881/100855704/483845/608420/474681/483696/484780/481746/449494/480272/489644 ETS1, SLC25A5, CDK1, FOXM1, CDK4, CCNB2, TRPV4, LIN54, NRAS, LIN9, CDK2, CCNA2, , VDAC1, RAD9A, CDC25A, ITPR3, CCNB3, NFATC4, PIK3CD	20 ACOR_VSMC-down
cfa04066	HIF-1 signaling pathway	19/279	112/7854	1.1179E-08	1.4644E-06	1.2414E-06	403802/403476/476882/480964/487139/479597/100856683/480348/487870/482437/612212/475590/480622/403703/477952/610764/474890/489050/476828 VEGFA, SERPINE1, , PGK1, PFKFB3, ENO1, ENO2, HIF1A, EIF4E, SLC2A1, MKNK2, EIF4EBP1, ALDOC, TFRC, CUL2, CAMK2D, CDKN1A, CAMK2G,	19 VSAP_EC-down
cfa05205	Proteoglycans in cancer	19/279	202/7854	9.627E-05	0.00360325	0.00305459	403802/479975/489208/403567/607885/477488/484713/475411/491924/480348/477956/477032/488437/403939/403788/485893/610764/474890/489050 VEGFA, PRKACB, ITGA2, CASP3, PAK1, PTPN11, FLNB, PPP1R12A, MSN, HIF1A, ITGB1, CTNNB1, ITGAV, CD44, ITGB3, SDC4, CAMK2D, CDKN1A, CAMK2G	19 VSAP_EC-down
cfa04530	Tight junction	18/279	170/7854	3.1083E-05	0.00162874	0.00138073	403498/481280/477374/480369/403936/479975/488036/491924/477956/485856/484526/403702/474680/479327/607207/478464/481396/100688336 ARHGEF2, MYH9, PPP2R2A, ACTN1, VASP, PRKACB, DLG1, MSN, ITGB1, MYL9, ACTN4, ACTR3, HSPA4, SYNPO, , MAPK10, ACTR2, MYH14	18 VSAP_EC-down

cfa04360	Axon guidance	18/279	179/7854	6.1878E-05	0.00270202	0.00229058	487421/607885/477488/476836/477956/485856/610879/487635/476022/478575/607207/489307/612814/610764/608458/477955/489050/476828 EPHA2, PAK1, PTPN11, ILK, ITGB1, MYL9, RGS3, NEO1, CFL1, GSK3B, , ROBO3, PAK2, CAMK2D, EFNA5, NRP1, CAMK2G,	18	VSAP_EC-down
cfa04810	Regulation of actin cytoskeleton	18/279	211/7854	0.00049206	0.01611501	0.01366118	480369/489208/607885/608287/475411/491924/477956/485856/476022/491597/484526/488437/479252/403788/607207/100856747/612814/100683370 ACTN1, ITGA2, PAK1, ITGA10, PPP1R12A, MSN, ITGB1, MYL9, CFL1, PDGFA, ACTN4, ITGAV, VCL, ITGB3, , , PAK2,	18	VSAP_EC-down
cfa04261	Adrenergic signaling in cardiomyocytes	18/357	147/7854	0.00011196	0.00240277	0.00175867	490280/403480/607269/479444/486591/490755/480271/403887/475266/477098/478227/612821/477374/607922/403608/403558/479797/491587 PPP2R5A, ATP2B1, CALML6, PPP2R1B, RAPGEF3, SLC8A3, ADCY4, CREM, CREB5, ATP1B3, GNAI1, MAPK13, PPP2R2A, CREB1, PPP2CA, PPP1CB, ATP2A1, PPP2R2D	18	VCAV_EC-up
cfa04072	Phospholipase D signaling pathway	18/357	148/7854	0.00012226	0.00243651	0.00178337	403811/403850/442986/476932/483881/486591/608079/478232/480271/482270/489692/485874/490341/403872/479460/476331/487514/479473 KIT, CXCL8, PDGFB, DGKH, RALB, RAPGEF3, CYTH1, MTOR, ADCY4, GRM8, PLCG2, PLCG1, DNM3, NRAS, PDGFD, PIP5K1B, PLA2G4E, PLD2	18	VCAV_EC-up
cfa04360	Axon guidance	18/357	179/7854	0.00125423	0.0152144	0.01113598	485671/483900/475431/403486/485930/475108/489830/478227/479409/478949/612814/485677/609483/489692/485874/487421/403872/484763 EPHB1, CXCR4, NTN4, PPP3CA, NFATC2, PTK2, EPHB4, GNAI1, ARHGEF12, , PAK2, NCK1, FES, PLCG2, PLCG1, EPHA2, NRAS, SEMA3F	18	VCAV_EC-up
cfa04810	Regulation of actin cytoskeleton	18/357	211/7854	0.0073983	0.0480029	0.03513511	483900/442986/485552/486934/475108/478828/479409/479177/612814/100856747/403558/611773/612856/403872/100856563/479460/476331/403788 CXCR4, PDGFB, ARHGEF7, DOCK1, PTK2, NCKAP1, ARHGEF12, IQGAP2, PAK2, , PPP1CB, FGF23, ARPC5L, NRAS, ITGA6, PDGFD, PIP5K1B, ITGB3	18	VCAV_EC-up
cfa04510	Focal adhesion	18/241	198/7854	3.4369E-05	0.00965757	0.00868259	442985/608977/403824/610359/478331/403668/609956/100856491/485864/449021/482666/480173/608847/403651/403582/611782/490439/481271 PDGFRB, ITGB5, COL1A2, THBS3, TLN2, COL6A1, , COL6A2, SRC, AKT2, PDGFC, LAMA3, CCND3, COL1A1, COL6A3, CCND2, SHC1, RAC2	18	VCAV_EC-down
cfa04151	PI3K-Akt signaling pathway	18/407	354/7854	0.56936905	0.90219582	0.86724232	491749/611795/488087/403416/403656/489571/482666/403402/415125/611782/611641/480349/474890/480173/611934/611993/480688/489038 VEGFD, CSF1, GNB4, BCL2, ANGPT1, PRKAA2, PDGFC, NGF, FGFR2, CCND2, FGF18, PPP2R5E, CDKN1A, LAMA3, CREB3, GYS1, TSC1, DDIT4	18	ACOR_VSMC-up
cfa04932	Non-alcoholic fatty liver disease (NAFLD)	17/441	145/7854	0.00303392	0.06436254	0.05794111	612271/476754/476215/478634/486148/478880/491278/608530/487235/478981/479887/611480/486295/475978/476503/476040/476004 IL6R, NDUFS7, MAP3K5, SDHA, IRS1, NDUFS1, RXRA, , PIK3R1, NDUFS2, NDUFB10, , PRKAB1, NDUFS3, UQCRFS1, COX8A, NDUFV1	17	VSAP_EC-up
cfa05010	Alzheimer disease	17/441	168/7854	0.0130504	0.15503871	0.13957056	481746/476754/478634/478880/476833/608530/403626/478981/479887/611480/475978/476503/476040/478807/479248/476004/479458 ITPR3, NDUFS7, SDHA, NDUFS1, APBB1, , LPL, NDUFS2, NDUFB10, , NDUFS3, UQCRFS1, COX8A, , PPP3CB, NDUFV1, CASP12	17	VSAP_EC-up

cfa05016	Huntington disease	17/441	257/7854	0.27564482	0.74424101	0.66998836	476754/476215/478634/476258/478880/608530/483634/483756/478981/479887/611480/475978/476503/476040/478807/476004/477437 NDUFS7, MAP3K5, SDHA, SOD2, NDUFS1, , AMBRA1, ATG2A, NDUFS2, NDUFB10, , NDUFS3, UQCRFS1, COX8A, , NDUFV1, ULK1	17	VSAP_EC-up
cfa04010	MAPK signaling pathway	17/441	288/7854	0.45169158	0.89991478	0.81013062	490926/403915/476215/479460/612101/442938/403924/612448/485189/485790/477024/481245/484648/479248/611939/477039/611262 MAP3K14, FGF7, MAP3K5, PDGFD, RASGRP2, FLT3LG, MYC, RPS6KA2, ARRB1, CDC25B, MYD88, TAB1, RAF1, PPP3CB, MAP2K2, TGFBR2, TRADD	17	VSAP_EC-up
cfa04728	Dopaminergic synapse	17/357	132/7854	9.2216E-05	0.00233893	0.00171195	403617/490280/607269/485189/403486/479444/475266/478227/100855806/612821/488355/477374/607922/403608/403558/607513/491587 GNGT2, PPP2R5A, CALML6, ARRB1, PPP3CA, PPP2R1B, CREB5, GNAI1, GNG11, MAPK13, KCNJ3, PPP2R2A, CREB1, PPP2CA, PPP1CB, GNG4, PPP2R2D	17	VCAV_EC-up
cfa04921	Oxytocin signaling pathway	17/357	151/7854	0.00047442	0.0069665	0.00509904	490455/479155/403784/607269/403486/482663/485930/480271/478227/488355/403717/553102/442942/403558/403872/488584/487514 NPR1, MEF2C, NOS3, CALML6, PPP3CA, GUCY1A1, NFATC2, ADCY4, GNAI1, KCNJ3, KCNJ2, GUCY1B1, PTGS2, PPP1CB, NRAS, RGS2, PLA2G4E	17	VCAV_EC-up
cfa05202	Transcriptional misregulation in cancer	17/357	172/7854	0.00204741	0.02115656	0.01548528	403850/403985/489286/403606/479155/475456/482978/475108/479221/608175/403641/487819/487760/403764/479077/608366/477966 CXCL8, IL6, FLI1, PPARG, MEF2C, SMAD1, , PTK2, JMJD1C, HHEX, ETV4, BMP2K, ERG, CD86, NSD2, ARNT2, ZEB1	17	VCAV_EC-up
cfa04024	cAMP signaling pathway	17/357	211/7854	0.01548456	0.07640893	0.05592655	490455/403480/487221/607269/403424/487188/479540/486591/480271/475266/477098/478227/483225/607922/403558/479797/479473 NPR1, ATP2B1, PDE4D, CALML6, EDN1, ARAP3, PDE4B, RAPGEF3, ADCY4, CREB5, ATP1B3, GNAI1, RAP1A, CREB1, PPP1CB, ATP2A1, PLD2	17	VCAV_EC-up
cfa05132	Salmonella infection	17/357	212/7854	0.01615816	0.07640893	0.05592655	403850/403985/482252/403417/608079/488473/612821/478828/484713/479497/490710/100856747/474541/100855591/612856/102152063/111089957 CXCL8, IL6, PODXL, TLR4, CYTH1, CASP8, MAPK13, NCKAP1, FLNB, MYH10, EXOC5, , GCC2, , ARPC5L, RIPK1, TLR6	17	VCAV_EC-up
cfa05165	Human papillomavirus infection	17/241	328/7854	0.02375891	0.24158651	0.21719709	483148/442985/608977/403824/610359/483376/403668/609956/100856491/449021/480173/608847/403651/479877/403582/611782/475590 NOTCH2, PDGFRB, ITGB5, COL1A2, THBS3, RFNG, COL6A1, , COL6A2, AKT2, LAMA3, CCND3, COL1A1, ATP6V0C, COL6A3, CCND2, EIF4EBP1	17	VCAV_EC-down
cfa04140	Autophagy - animal	17/407	136/7854	0.00062471	0.0899579	0.08647269	403416/489571/488471/476548/612919/484142/486077/607408/481015/489525/490477/481037/485205/480688/489038/102156745/490768 BCL2, PRKAA2, CFLAR, ITPR1, GABARAPL1, DAPK1, CTSB, BNIP3, ATG4A, ULK2, PIK3C3, LAMP2, ATG16L2, TSC1, DDIT4, MAP3K7, ZFYVE1	17	ACOR_VSMC-up
cfa04144	Endocytosis	17/407	235/7854	0.10204494	0.71680351	0.68903262	489553/476331/415125/607044/485693/481628/477520/474741/611619/482044/479353/475356/474836/480605/479233/403941/475213 DNAJC6, PIP5K1B, FGFR2, RNF41, GRK7, TGFBR1, GIT2, , RAB11B, ASAP1, DAB2, EPS15, DLA88, AP2B1, VPS26A, RAB5C, WASL	17	ACOR_VSMC-up

cfa04010	MAPK signaling pathway	17/407	288/7854	0.32375326	0.83250838	0.80025476	491749/611795/487085/403656/478061/482666/403402/415125/475734/611641/486563/481628/612101/102156745/480618/489532/608125 VEGFD, CSF1, MAP3K8, ANGPT1, MAP3K1, PDGFC, NGF, FGFR2, MAP4K3, FGF18, CACNB3, TGFBR1, RASGRP2, MAP3K7, NF1, MAPK7, CRKL	17	ACOR_VSMC-up
cfa03013	RNA transport	17/388	158/7854	0.00198442	0.03175069	0.02973555	479880/475922/479766/100684187/100688577/481234/100499482/607867/607948/484370/478119/491307/474839/480242/483618/474470/100855650 RNPS1, EIF4A3, EIF3B, NDC1, EIF4A1, RANGAP1, RAN, AAAS, ALYREF, NUP62, NUP93, NUP188, DDX39B, PRMT5, NUP160, NUP50, GEMIN7	17	ACOR_VSMC-down
cfa05203	Viral carcinogenesis	17/388	189/7854	0.01203183	0.13636078	0.12770631	474827/100856079/477563/481131/100855638/403872/477237/403955/489717/611089/100855704/483845/100855774/100688636/482339/477714/489644 MRPS18B, CDK1, RANBP1, CDK4, HNRNPK, NRAS, YWHAB, RAC1, VAC14, HIST1H2BB, CDK2, CCNA2, CDC20, PSMC1, HDAC9, CHD4, PIK3CD	17	ACOR_VSMC-down
cfa01200	Carbon metabolism	16/441	116/7854	0.00071515	0.0358674	0.03228892	486834/100856446/478634/490779/481088/403978/478230/477517/488110/477205/483800/483704/477002/478889/479066/477177 GOT1, DLST, SDHA, ALDH6A1, G6PD, DLD, MTHFR, ACADS, EHHADH, ACSS2, TKFC, PC, ACSS1, IDH1, IDH3A,	16	VSAP_EC-up
cfa04390	Hippo signaling pathway	16/441	153/7854	0.01175675	0.1487619	0.13391998	482294/485183/490903/442858/486894/480319/485149/403924/490688/100856566/490099/485158/403609/608847/477039/477343 FZD1, WNT11, AXIN2, CDH1, TCF7L2, SAV1, FZD4, MYC, FRMD6, LIMD1, AXIN1, DLG2, PPP1CA, CCND3, TGFBR2, LATS2	16	VSAP_EC-up
cfa04144	Endocytosis	16/441	235/7854	0.24644393	0.70505074	0.63470809	491319/484849/476674/489533/475444/475424/485054/477292/487599/611619/485189/100856651/488409/483069/477039/477834 DNM1, EPS15L1, MVB12A, EPN2, , EEA1, PIP5K1C, VPS36, SPG21, RAB11B, ARRB1, RAB11FIP5, WIPF1, PSD4, TGFBR2, RAB11FIP2	16	VSAP_EC-up
cfa05132	Salmonella infection	16/279	212/7854	0.00354118	0.04883104	0.04139554	481280/404019/403567/607885/490710/484713/485856/609233/477032/478680/480438/478585/607207/100856747/478464/100688336 MYH9, HSP90B1, CASP3, PAK1, EXOC5, FLNB, MYL9, DYNLL1, CTNNB1, KPNA4, HSP90AA1, KPNA1, , , MAPK10, MYH14	16	VSAP_EC-down
cfa04010	MAPK signaling pathway	16/279	288/7854	0.05055696	0.25472931	0.21594168	403802/487421/479975/479406/403567/607885/484713/481628/491768/612212/491597/607439/478464/612814/403897/608458 VEGFA, EPHA2, PRKACB, HSPA8, CASP3, PAK1, FLNB, TGFBR1, RPS6KA3, MKNK2, PDGFA, DDIT3, MAPK10, PAK2, NR4A1, EFNA5	16	VSAP_EC-down
cfa04668	TNF signaling pathway	16/357	108/7854	2.6305E-05	0.00122317	0.00089528	403999/403985/484006/403424/488473/487437/475266/488471/612821/482932/488472/485769/442942/607922/102152063/477199 SELE, IL6, TNFAIP3, EDN1, CASP8, TNFRSF1B, CREB5, CFLAR, MAPK13, VEGFC, CASP10, JAG1, PTGS2, CREB1, RIPK1, ITCH	16	VCAV_EC-up
cfa04926	Relaxin signaling pathway	16/357	127/7854	0.00019185	0.00356849	0.00261191	403617/403862/403784/403466/403424/485189/480271/475266/478227/100855806/612821/482932/607922/403822/403872/607513 GNGT2, EDNRB, NOS3, COL4A5, EDN1, ARRB1, ADCY4, CREB5, GNAI1, GNG11, MAPK13, VEGFC, CREB1, NOS2, NRAS, GNG4	16	VCAV_EC-up
cfa05152	Tuberculosis	16/357	171/7854	0.00472768	0.03885933	0.0284426	403985/403417/607269/403486/488473/612821/100856077/479409/488472/484657/607922/490183/403822/111089957/482620/474897 IL6, TLR4, CALML6, PPP3CA, CASP8, MAPK13, IFNGR1, ARHGEF12, CASP10, VHL, CREB1, BCL10, NOS2, TLR6, APAF1, NFYA	16	VCAV_EC-up

cfa04020	Calcium signaling pathway	16/357	192/7854	0.01382777	0.07419132	0.0543034	403862/483900/448778/403480/403784/607269/403486/490755/480271/490383/489692/485874/403822/489042/448781/479797 EDNRB, CXCR4, P2RX7, ATP2B1, NOS3, CALML6, PPP3CA, SLC8A3, ADCY4, ITPKB, PLCG2, PLCG1, NOS2, MCU, P2RX2, ATP2A1	16 VCAV_EC-up
cfa04360	Axon guidance	16/407	179/7854	0.02308368	0.36933889	0.3550297	478622/489692/611745/492244/610764/490270/477955/479239/478227/486093/477623/100855988/476828/489132/483699/607997 SEMA5A, PLCG2, EFNB2, L1CAM, CAMK2D, PLXNA2, NRP1, UNC5B, GNAI1, FZD3, RND1, SEMA6D, , SLIT3, SSH3, LRRC4	16 ACOR_VSMC-up
cfa04914	Progesterone-mediated oocyte maturation	16/388	89/7854	5.9125E-06	0.00016082	0.00015061	490050/100856079/475750/489971/489938/478324/487443/476070/100855704/483845/608420/100682599/484780/485940/449494/489644 PKMYT1, CDK1, ANAPC1, PLK1, KIF22, CCNB2, MAD2L2, , CDK2, CCNA2, , BUB1, CDC25A, AURKA, CCNB3, PIK3CD	16 ACOR_VSMC-down
cfa04146	Peroxisome	15/441	82/7854	4.2153E-05	0.00329774	0.00296872	484000/477023/487007/476258/480254/478001/483028/610008/491304/488110/486309/478889/608697/475631/481805 PEX7, ACAA1, PEX2, SOD2, DHRS4, , XDH, PXMP4, CRAT, EHHADH, MVK, IDH1, HACL1, ACSL1, PEX6	15 VSAP_EC-up
cfa05226	Gastric cancer	15/441	149/7854	0.01984333	0.19644896	0.17684932	482294/485183/490903/442858/403915/486894/483626/485149/491278/487235/403924/484648/490099/611939/477039 FZD1, WNT11, AXIN2, CDH1, FGF7, TCF7L2, DDB2, FZD4, RXRA, PIK3R1, MYC, RAF1, AXIN1, MAP2K2, TGFBR2	15 VSAP_EC-up
cfa04360	Axon guidance	15/441	179/7854	0.07771052	0.41125246	0.37022196	595148/491292/482251/478622/484736/487235/489132/480272/484137/486107/475431/484648/483191/479248/485677 SLIT2, ABL1, PLXNA4, SEMA5A, SEMA3G, PIK3R1, SLIT3, NFATC4, PTCH1, DPYSL2, NTN4, RAF1, SEMA6C, PPP3CB, NCK1	15 VSAP_EC-up
cfa05163	Human cytomegalovirus infection	15/279	220/7854	0.01159201	0.1084681	0.09195166	403802/478279/476694/479975/403567/442942/475590/403928/477032/488437/478575/403788/480416/491369/474890 VEGFA, , CALR, PRKACB, CASP3, PTGS2, EIF4EBP1, GNAQ, CTNNB1, ITGAV, GSK3B, ITGB3, CALM1, CREB3L2, CDKN1A	15 VSAP_EC-down
cfa05142	Chagas disease (American trypanosomiasis)	15/357	103/7854	5.7571E-05	0.00160623	0.00117566	403850/403985/610668/403417/479444/488473/488471/478227/612821/100856077/477374/403608/403822/111089957/491587 CXCL8, IL6, ACE, TLR4, PPP2R1B, CASP8, CFLAR, GNAI1, MAPK13, IFNGR1, PPP2R2A, PPP2CA, NOS2, TLR6, PPP2R2D	15 VCAV_EC-up
cfa05135	Yersinia infection	15/357	117/7854	0.00024947	0.00435016	0.00318405	403850/403985/487245/485552/403417/485930/486934/475108/481145/612821/479409/477520/485874/491768/476331 CXCL8, IL6, ARHGEF28, ARHGEF7, TLR4, NFATC2, DOCK1, PTK2, TBK1, MAPK13, ARHGEF12, GIT2, PLCG1, RPS6KA3, PIP5K1B	15 VCAV_EC-up
cfa05416	Viral myocarditis	4/388	51/7854	0.24351845	0.99972301	0.9362731	403980/100683675/475258/403955 CAV1, BID, CYCS, RAC1	4 ACOR_VSMC-down
cfa05205	Proteoglycans in cancer	15/357	202/7854	0.0415736	0.14145163	0.10353373	100684969/482154/403417/475108/478232/475294/612821/479409/484713/489692/403558/485874/403872/480348/403788 HCLS1, KDR, TLR4, PTK2, MTOR, CAV2, MAPK13, ARHGEF12, FLNB, PLCG2, PPP1CB, PLCG1, NRAS, HIF1A, ITGB3	15 VCAV_EC-up
cfa04142	Lysosome	15/407	118/7854	0.00108073	0.10374981	0.09973026	475042/479915/478435/403400/475678/486077/474969/484681/485334/611983/481037/476010/479053/483352/475983 LAPTM4B, SORT1, SCARB2, CTSS, LAPTM4A, CTSB, SLC17A5, SUMF1, SMPD1, CTSZ, LAMP2, CTSF, AP3B2, GAA, ACP2	15 ACOR_VSMC-up

cfa04371	Apelin signaling pathway	15/407	135/7854	0.00412011	0.14832401	0.14257754	488087/489571/479127/477625/476548/612919/403607/478227/609934/481628/490477/488526/100686627/403928/475738 GNB4, PRKAA2, PPARGC1A, ADCY6, ITPR1, GABARAPL1, LIPE, GNAI1, PRKCE, TGFBR1, PIK3C3, PRKAG3, RYR3, GNAQ, SLC8A1	15 ACOR_VSMC-up
cfa04015	Rap1 signaling pathway	15/407	209/7854	0.12503523	0.76328292	0.73371129	491749/488969/611795/403656/482666/403402/415125/611641/477625/480433/478227/480379/612101/403928/608125 VEGFD, SIPA1L2, CSF1, ANGPT1, PDGFC, NGF, FGFR2, FGF18, ADCY6, EVL, GNAI1, SIPA1L1, RASGRP2, GNAQ, CRKL	15 ACOR_VSMC-up
cfa04060	Cytokine-cytokine receptor interaction	15/407	270/7854	0.42849797	0.88820767	0.85379612	100856077/403616/611795/403402/490695/403545/606826/491935/100174780/483880/489632/481628/403522/100855848/611438 IFNGR1, LEP, CSF1, NGF, BMP4, IL6ST, GDF11, EDA, TNFSF10, INHBB, TNFRSF25, TGFBR1, CCL5, TNFRSF11B, IL18R1	15 ACOR_VSMC-up
cfa05169	Epstein-Barr virus infection	15/388	193/7854	0.05440149	0.46241271	0.43306453	492247/608738/491018/476694/479671/475934/475980/481131/100683675/475258/403955/100855704/483845/100688636/489644 IRAK1, NEDD4, PSMD3, CALR, PSMD7, PSMD13, , CDK4, BID, CYCS, RAC1, CDK2, CCNA2, PSMC1, PIK3CD	15 ACOR_VSMC-down
cfa05170	Human immunodeficiency virus 1 infection	15/388	200/7854	0.06964719	0.54125816	0.50690586	492247/476694/607885/100856079/100683675/478324/403872/475258/403955/479733/608420/481746/449494/480272/489644 IRAK1, CALR, PAK1, CDK1, BID, CCNB2, NRAS, CYCS, RAC1, GNB2, , ITPR3, CCNB3, NFATC4, PIK3CD	15 ACOR_VSMC-down
cfa05205	Proteoglycans in cancer	15/388	202/7854	0.07447853	0.55088328	0.5159201	484713/482986/100856354/607885/403980/403872/403788/403955/479050/484056/489208/486185/479215/481746/489644 FLNB, SDC1, , PAK1, CAV1, NRAS, ITGB3, RAC1, IQGAP1, EZR, ITGA2, TWIST2, ANK3, ITPR3, PIK3CD	15 ACOR_VSMC-down
cfa04151	PI3K-Akt signaling pathway	15/388	354/7854	0.76881056	0.99972301	0.9362731	479444/481806/481131/491313/403437/403872/403788/477237/403955/479733/100855704/608459/475882/489208/489644 PPP2R1B, PPP2R5D, CDK4, PKN3, BRCA1, NRAS, ITGB3, YWHAB, RAC1, GNB2, CDK2, FGF5, LAMB1, ITGA2, PIK3CD	15 ACOR_VSMC-down
cfa04152	AMPK signaling pathway	14/441	121/7854	0.00766947	0.11389162	0.10252869	485884/100685468/479812/486148/485672/486263/611619/487235/476744/486295/612305/403607/483755/477437 HNF4A, ACACB, EEF2K, IRS1, PPP2R3A, CAMKK2, RAB11B, PIK3R1, EEF2, PRKAB1, CRTC2, LIPE, PPP2R5B, ULK1	14 VSAP_EC-up
cfa05012	Parkinson disease	14/441	138/7854	0.02255117	0.20930307	0.18842098	476754/478634/478880/608530/478981/479887/611480/608118/475978/476503/476040/478807/475782/476004 NDUFS7, SDHA, NDUFS1, , NDUFS2, NDUFB10, , UBA7, NDUFS3, UQCRFS1, COX8A, , HTRA2, NDUFV1	14 VSAP_EC-up
cfa04218	Cellular senescence	14/441	151/7854	0.04392147	0.32611692	0.29358036	481746/478127/490748/487235/403924/480272/611243/100856710/484648/403609/479248/608847/611939/477039 ITPR3, RBL2, ZFP36L1, PIK3R1, MYC, NFATC4, E2F4, ZFP36L2, RAF1, PPP1CA, PPP3CB, CCND3, MAP2K2, TGFBR2	14 VSAP_EC-up
cfa05161	Hepatitis B	14/441	164/7854	0.07666637	0.41125246	0.37022196	483626/490968/100142679/487235/403924/480272/477024/481245/484648/485789/476412/611939/477039/479458 DDB2, STAT5A, STAT6, PIK3R1, MYC, NFATC4, MYD88, TAB1, RAF1, MAVS, IRF3, MAP2K2, TGFBR2, CASP12	14 VSAP_EC-up
cfa05202	Transcriptional misregulation in cancer	14/441	172/7854	0.1031408	0.48420266	0.43589394	100683897/482373/482372/486180/488770/483626/489398/491278/403924/477966/491872/100855805/486073/477039 HOXA11, HOXA10, HOXA9, PER2, BCL2A1, DDB2, ZBTB16, RXRA, MYC, ZEB1, TFE3, CDK9, HPGD, TGFBR2	14 VSAP_EC-up

cfa05203	Viral carcinogenesis	14/279	189/7854	0.00728869	0.08076606	0.06846782	480369/479975/477714/403874/403567/488036/484526/475204/477886/476881/491369/487309/474890/100687866 ACTN1, PRKACB, CHD4, , CASP3, DLG1, ACTN4, SND1, LYN, GTF2H1, CREB3L2, HDAC1, CDKN1A, YWHAH	14	VSAP_EC-down
cfa04371	Apelin signaling pathway	14/357	135/7854	0.00320161	0.02881446	0.02109038	403617/479155/403784/607269/483497/490755/478232/480271/478227/100855806/485769/403822/403872/607513 GNGT2, MEF2C, NOS3, CALML6, APLNR, SLC8A3, MTOR, ADCY4, GNAI1, GNG11, JAG1, NOS2, NRAS, GNG4	14	VCAV_EC-up
cfa05160	Hepatitis C	14/357	157/7854	0.01194514	0.06801417	0.04978211	403844/479444/488473/481145/486241/488471/482905/477490/477374/403608/102152063/482620/403872/491587 OCLN, PPP2R1B, CASP8, TBK1, SCARB1, CFLAR, TLR3, OAS3, PPP2R2A, PPP2CA, RIPK1, APAF1, NRAS, PPP2R2D	14	VCAV_EC-up
cfa04530	Tight junction	14/357	170/7854	0.02249735	0.09713048	0.07109343	100684969/481022/403844/479444/403752/483225/479497/100856359/477374/403608/609521/403498/403854/491587 HCLS1, AMOT, OCLN, PPP2R1B, TJP1, RAP1A, MYH10, RAPGEF2, PPP2R2A, PPP2CA, , ARHGEF2, TJP2, PPP2R2D	14	VCAV_EC-up
cfa05206	MicroRNAs in cancer	14/357	281/7854	0.40080461	0.6577911	0.48146189	480676/442986/478232/481726/481772/478175/442942/489692/485874/403872/607313/403788/477966/489880 NOTCH1, PDGFB, MTOR, NOTCH4, PIM1, STMN1, PTGS2, PLCG2, PLCG1, NRAS, BMF, ITGB3, ZEB1, FSCN1	14	VCAV_EC-up
cfa05205	Proteoglycans in cancer	14/241	202/7854	0.00363546	0.1004926	0.09034735	608977/403904/482599/485893/403824/403733/609956/485864/449021/403651/403857/477945/610764/486185 ITGB5, DCN, LUM, SDC4, COL1A2, MMP2, , SRC, AKT2, COL1A1, FGF2, SDC2, CAMK2D, TWIST2	14	VCAV_EC-down
cfa04152	AMPK signaling pathway	14/407	121/7854	0.0038123	0.14832401	0.14257754	403616/489571/479127/403849/480349/403607/489688/611619/611934/611993/488526/480688/102156745/487797 LEP, PRKAA2, PPARGC1A, PFKM, PPP2R5E, LIPE, MLYCD, RAB11B, CREB3, GYS1, PRKAG3, TSC1, MAP3K7, PFKL	14	ACOR_VSMC-up
cfa04921	Oxytocin signaling pathway	14/407	151/7854	0.02451187	0.37154836	0.35715358	489571/477625/476548/610764/486563/474890/481253/478227/488584/488526/490230/100686627/403928/489532 PRKAA2, ADCY6, ITPR1, CAMK2D, CACNB3, CDKN1A, KCNJ4, GNAI1, RGS2, PRKAG3, PPP1R12B, RYR3, GNAQ, MAPK7	14	ACOR_VSMC-up
cfa05169	Epstein-Barr virus infection	14/407	193/7854	0.127026	0.76328292	0.73371129	489692/403416/489228/611782/100855667/100855560/479180/486814/474890/607899/485604/474836/100855741/102156745 PLCG2, BCL2, SKP2, CCND2, ISG15, DDX58, POLK, BLNK, CDKN1A, IRF9, ENTPD3, DLA88, B2M, MAP3K7	14	ACOR_VSMC-up
cfa04014	Ras signaling pathway	14/407	225/7854	0.27726618	0.82260001	0.79073027	491749/489692/611795/488087/403656/482666/403402/415125/611641/612101/476045/478514/403941/480618 VEGFD, PLCG2, CSF1, GNB4, ANGPT1, PDGFC, NGF, FGFR2, FGF18, RASGRP2, PLA2G16, CFI, RAB5C, NF1	14	ACOR_VSMC-up
cfa03460	Fanconi anemia pathway	14/388	49/7854	5.659E-08	3.0785E-06	2.8831E-06	479549/100855690/608713/489537/607154/403568/100856370/484659/100856421/403437/474915/100687493/608504/608527 USP1, RPA1, FANCA, TOP3A, UBE2T, RAD51, FANCI, FANCD2, RPA2, BRCA1, POLH, FANCB, ATRIP, PALB2	14	ACOR_VSMC-down
cfa04360	Axon guidance	13/131	179/7854	8.0671E-06	0.00187964	0.00172382	100856339/100855988/487930/100856151/492067/487979/608458/492244/485864/607997/489497/489307/477955 , SEMA6D, EPHA3, RGMA, PAK3, BOC, EFNA5, L1CAM, SRC, LRRC4, NTN1, ROBO3, NRP1	13	ACOR_EC-up
cfa00280	Valine, leucine and isoleucine degradation	13/441	48/7854	1.4638E-06	0.00043474	0.00039136	477023/484394/490779/403978/484488/479856/100856316/474978/477517/488110/479610/481486/478991 ACAA1, BCAT2, ALDH6A1, DLD, , ABAT, IVD, BCKDHB, ACADS, EHHADH, , ALDH7A1, ALDH9A1	13	VSAP_EC-up

cfa04210	Apoptosis	13/441	131/7854	0.03200667	0.2569184	0.23128575	490926/481746/476215/403400/488770/489623/487235/484648/476010/475782/611939/479458/611262 MAP3K14, ITPR3, MAP3K5, CTSS, BCL2A1, DFFB, PIK3R1, RAF1, CTSF, HTRA2, MAP2K2, CASP12, TRADD	13	VSAP_EC-up
cfa05416	Viral myocarditis	3/357	51/7854	0.41095525	0.65826592	0.48180942	488473/606758/403764 CASP8, DMD, CD86	3	VCAV_EC-up
cfa05224	Breast cancer	13/441	142/7854	0.05536731	0.35748022	0.32181456	482294/485183/490903/403915/486894/100688936/483626/485149/487235/403924/484648/490099/611939 FZD1, WNT11, AXIN2, FGF7, TCF7L2, DLL1, DDB2, FZD4, PIK3R1, MYC, RAF1, AXIN1, MAP2K2	13	VSAP_EC-up
cfa04150	mTOR signaling pathway	13/441	152/7854	0.08484578	0.43238824	0.38924902	482294/485183/486148/485149/487235/612448/609909/475482/484648/477437/611939/611588/489038 FZD1, WNT11, IRS1, FZD4, PIK3R1, RPS6KA2, CASTOR1, FNIP2, RAF1, ULK1, MAP2K2, NPRL3, DDIT4	13	VSAP_EC-up
cfa05205	Proteoglycans in cancer	13/441	202/7854	0.34598981	0.8287014	0.74602217	482294/486185/485183/481746/608707/485149/487235/487731/403924/484137/484648/403609/611939 FZD1, TWIST2, WNT11, ITPR3, HPSE, FZD4, PIK3R1, TIAM1, MYC, PTCH1, RAF1, PPP1CA, MAP2K2	13	VSAP_EC-up
cfa05166	Human T-cell leukemia virus 1 infection	13/441	209/7854	0.39235603	0.85964878	0.77388194	490926/491236/488828/490968/487235/403924/480272/612305/481679/479248/608847/611939/477039 MAP3K14, ANAPC2, MSX1, STAT5A, PIK3R1, MYC, NFATC4, CRTC2, CDC26, PPP3CB, CCND3, MAP2K2, TGFBR2	13	VSAP_EC-up
cfa01230	Biosynthesis of amino acids	13/279	74/7854	1.6262E-06	0.00010651	9.0295E-05	475240/607458/483372/476318/403874/480964/477711/477786/479597/100856683/477781/480622/486633 ASNS, SHMT2, PYCR1, PSAT1, , PGK1, TPI1, PGAM1, ENO1, ENO2, ALDH18A1, ALDOC, BCAT1	13	VSAP_EC-down
cfa05416	Viral myocarditis	2/279	51/7854	0.54574129	0.79879452	0.67716209	403567/606758 CASP3, DMD	2	VSAP_EC-down
cfa05016	Huntington disease	13/279	257/7854	0.1262301	0.4066908	0.344764	403567/100855651/607407/474830/478701/403928/479074/482163/478464/606983/480578/491369/487309 CASP3, KIF5B, NDUFA4L2, TUBB, TUBB2A, GNAQ, HTT, REST, MAPK10, DNAH5, CLTC, CREB3L2, HDAC1	13	VSAP_EC-down
cfa04925	Aldosterone synthesis and secretion	13/357	94/7854	0.00030245	0.0049638	0.00363319	490455/609574/403480/607269/480271/475266/477098/486241/484427/607922/478754/609091/403897 NPR1, PDE2A, ATP2B1, CALML6, ADCY4, CREB5, ATP1B3, SCARB1, PRKD2, CREB1, NR4A2, PRKD1, NR4A1	13	VCAV_EC-up
cfa04928	Parathyroid hormone synthesis, secretion and action	13/357	107/7854	0.00106745	0.01353725	0.00990842	487221/479155/487134/485189/479540/480271/475266/478227/485849/607922/611773/478754/479473 PDE4D, MEF2C, GATA3, ARRB1, PDE4B, ADCY4, CREB5, GNAI1, MMP24, CREB1, FGF23, NR4A2, PLD2	13	VCAV_EC-up
cfa04670	Leukocyte transendothelial migration	13/357	113/7854	0.0017702	0.02045734	0.0149735	483900/403844/489775/489309/486591/475108/478227/612821/483730/483225/489692/474698/485874 CXCR4, OCLN, CDH5, ESAM, RAPGEF3, PTK2, GNAI1, MAPK13, SIPA1, RAP1A, PLCG2, CTNNA1, PLCG1	13	VCAV_EC-up
cfa05416	Viral myocarditis	44106	51/7854	0.00179955	0.01834863	0.00804764	612079/606758 SGCD, DMD	2	AORT_EC-up
cfa04621	NOD-like receptor signaling pathway	13/357	158/7854	0.02746881	0.1083193	0.07928294	490170/403850/403985/484006/448778/606815/403417/488473/481145/612821/477490/102152063/489042 GBP5, CXCL8, IL6, TNFAIP3, P2RX7, TMEM173, TLR4, CASP8, TBK1, MAPK13, OAS3, RIPK1, MCU	13	VCAV_EC-up
cfa05161	Hepatitis B	13/357	164/7854	0.03571133	0.1293956	0.09470948	403850/403985/403417/485930/488473/475266/481145/612821/482905/488472/607922/482620/403872 CXCL8, IL6, TLR4, NFATC2, CASP8, CREB5, TBK1, MAPK13, TLR3, CASP10, CREB1, APAF1, NRAS	13	VCAV_EC-up
cfa05416	Viral myocarditis	34700	51/7854	0.46347416	0.83791788	0.83274402	403469 CD40	1	AFEM_EC-up
cfa03010	Ribosome	13/241	139/7854	0.00032482	0.02816767	0.02532399	102155201/403722/474877/476753/403683/479612/476463/611398/403688/488727/612291/476672/609159 , UBA52, RPS10, RPS15, RPL23, RPL13, RPS16, RPL27A, RPL27, MRPS11, RPS27, , RPLP0	13	VCAV_EC-down

cfa04668	TNF signaling pathway	13/407	108/7854	0.00370792	0.14832401	0.14257754	491749/488472/611795/487085/488471/491356/403982/403522/403424/611934/487286/102156745/611438 VEGFD, CASP10, CSF1, MAP3K8, CFLAR, DAB2IP, VCAM1, CCL5, EDN1, CREB3, NOD2, MAP3K7, IL18R1	13	ACOR_VSMC-up
cfa04066	HIF-1 signaling pathway	13/407	112/7854	0.0050752	0.16240647	0.15611441	489692/100856077/403416/403656/477072/403849/610764/488971/474890/476828/403424/480622/487797 PLCG2, IFNGR1, BCL2, ANGPT1, , PFKM, CAMK2D, EGLN1, CDKN1A, , EDN1, ALDOC, PFKL	13	ACOR_VSMC-up
cfa04068	FoxO signaling pathway	13/407	127/7854	0.01413781	0.30996841	0.2979594	489228/489571/611782/478442/481563/608099/612919/607408/474890/100174780/481628/488526/403474 SKP2, PRKAA2, CCND2, CCNG2, CDKN2B, FBXO25, GABARAPL1, BNIP3, CDKN1A, TNFSF10, TGFBR1, PRKAG3, CAT	13	ACOR_VSMC-up
cfa04020	Calcium signaling pathway	13/407	192/7854	0.19602301	0.80256807	0.77147442	403962/489692/403881/403813/476548/403812/610764/484156/448778/403652/100686627/403928/475738 ADRA1B, PLCG2, HTR2B, HRH1, ITPR1, HRH2, CAMK2D, GNA14, P2RX7, PDE1A, RYR3, GNAQ, SLC8A1	13	ACOR_VSMC-up
cfa04714	Thermogenesis	13/407	227/7854	0.39572337	0.87388357	0.84002697	489571/487441/479127/474670/477625/403607/475541/474444/484774/611934/488526/480688/607407 PRKAA2, NPPB, PPARGC1A, ACSL6, ADCY6, LIPE, SMARCD3, FRS2, SLC25A20, CREB3, PRKAG3, TSC1, NDUFA4L2	13	ACOR_VSMC-up
cfa03420	Nucleotide excision repair	13/388	43/7854	8.0765E-08	3.6613E-06	3.429E-06	612550/100855690/486223/489798/477499/477308/100856421/100686967/478667/477166/484922/610047/483096 POLE3, RPA1, POLE, RFC2, RFC5, RFC3, RPA2, LIG1, RFC4, PCNA, RAD23A, POLD1,	13	ACOR_VSMC-down
cfa04810	Regulation of actin cytoskeleton	13/388	211/7854	0.24374942	0.99972301	0.9362731	100856354/607885/485474/403872/403788/403955/479050/479177/608459/484056/479744/489208/489644 , PAK1, DIAPH3, NRAS, ITGB3, RAC1, IQGAP1, IQGAP2, FGF5, EZR, ARPC1B, ITGA2, PIK3CD	13	ACOR_VSMC-down
cfa05206	MicroRNAs in cancer	13/388	281/7854	0.63803693	0.99972301	0.9362731	489880/476715/478355/475511/478175/100855638/403437/403872/403788/484056/612071/484780/489644 FSCN1, , KIF23, EZH2, STMN1, HNRNPK, BRCA1, NRAS, ITGB3, EZR, CDCA5, CDC25A, PIK3CD	13	ACOR_VSMC-down
cfa05165	Human papillomavirus infection	13/388	328/7854	0.83223353	0.99972301	0.9362731	479444/403420/481806/481131/403872/403788/100855704/483845/475882/489208/100688636/477714/489644 PPP2R1B, HEY1, PPP2R5D, CDK4, NRAS, ITGB3, CDK2, CCNA2, LAMB1, ITGA2, PSMC1, CHD4, PIK3CD	13	ACOR_VSMC-down
cfa00190	Oxidative phosphorylation	12/441	131/7854	0.0637333	0.39434981	0.35500568	476754/478634/478880/608530/478981/479887/611480/475978/476503/476040/478807/476004 NDUFS7, SDHA, NDUFS1, , NDUFS2, NDUFB10, , NDUFS3, UQCRFS1, COX8A, , NDUFV1	12	VSAP_EC-up
cfa04910	Insulin signaling pathway	12/441	134/7854	0.07299554	0.41125246	0.37022196	476726/100685468/486148/480857/487235/490006/486295/403607/484648/474831/403609/611939 TRIP10, ACACB, IRS1, PHKA2, PIK3R1, SOCS1, PRKAB1, LIPE, RAF1, FLOT1, PPP1CA, MAP2K2	12	VSAP_EC-up
cfa04723	Retrograde endocannabinoid signaling	12/441	149/7854	0.13177234	0.55744768	0.50183133	100685330/492220/481746/476754/478880/482510/478981/479887/611480/479756/475978/476004 NAPEPLD, GABRE, ITPR3, NDUFS7, NDUFS1, FAAH, NDUFS2, NDUFB10, , DAGLB, NDUFS3, NDUFV1	12	VSAP_EC-up
cfa04310	Wnt signaling pathway	12/441	151/7854	0.1411627	0.58229615	0.52420068	482294/485183/490903/486894/485149/403924/480272/612240/490099/483133/479248/608847 FZD1, WNT11, AXIN2, TCF7L2, FZD4, MYC, NFATC4, CTBP1, AXIN1, VANGL1, PPP3CB, CCND3	12	VSAP_EC-up
cfa04024	cAMP signaling pathway	12/441	211/7854	0.5249945	0.92381513	0.83164644	483776/485523/487235/487731/484137/481627/403607/484648/476487/486591/403609/611939 CHRM1, ABCC4, PIK3R1, TIAM1, PTCH1, GABBR2, LIPE, RAF1, FXYD1, RAPGEF3, PPP1CA, MAP2K2	12	VSAP_EC-up

cfa04810	Regulation of actin cytoskeleton	12/441	211/7854	0.5249945	0.92381513	0.83164644	610290/403915/483776/479460/485054/474800/487235/487731/491034/484648/403609/611939 ARHGEF6, FGF7, CHRM1, PDGFD, PIP5K1C, LPAR1, PIK3R1, TIAM1, PIP4K2B, RAF1, PPP1CA, MAP2K2	12	VSAP_EC-up
cfa05132	Salmonella infection	12/441	212/7854	0.53174902	0.92381513	0.83164644	477331/480649/486894/487657/403924/486615/477024/481245/484648/489374/611939/611262 WASF3, RILP, TCF7L2, SNX33, MYC, FGD4, MYD88, TAB1, RAF1, VPS11, MAP2K2, TRADD	12	VSAP_EC-up
cfa05416	Viral myocarditis	42370	51/7854	0.09907646	0.28484483	0.25420935	403980 CAV1	1	VSAP_VSMC-up
cfa04015	Rap1 signaling pathway	12/279	209/7854	0.06857317	0.3346501	0.28369293	403802/480379/487421/403936/477956/491597/403928/477032/100856393/403788/480416/608458 VEGFA, SIPA1L1, EPHA2, VASP, ITGB1, PDGFA, GNAQ, CTNNB1, TLN1, ITGB3, CALM1, EFNA5	12	VSAP_EC-down
cfa04933	AGE-RAGE signaling pathway in diabetic complications	12/357	100/7854	0.0018331	0.02045734	0.0149735	403999/403850/403985/403784/403466/403424/481772/612821/482932/489692/485874/403872 SELE, CXCL8, IL6, NOS3, COL4A5, EDN1, PIM1, MAPK13, VEGFC, PLCG2, PLCG1, NRAS	12	VCAV_EC-up
cfa04659	Th17 cell differentiation	12/357	102/7854	0.00217347	0.02132038	0.01560518	403851/403985/487134/403486/485930/478232/612821/100856077/488126/485874/475251/480348 IL2RG, IL6, GATA3, PPP3CA, NFATC2, MTOR, MAPK13, IFNGR1, IL1RAP, PLCG1, AHR, HIF1A	12	VCAV_EC-up
cfa04066	HIF-1 signaling pathway	12/357	112/7854	0.00473555	0.03885933	0.0284426	403714/403985/403417/403784/403424/478232/100856077/607616/489692/485874/403822/480348 TEK, IL6, TLR4, NOS3, EDN1, MTOR, IFNGR1, ANGPT2, PLCG2, PLCG1, NOS2, HIF1A	12	VCAV_EC-up
cfa04724	Glutamatergic synapse	12/357	112/7854	0.00473555	0.03885933	0.0284426	403617/484813/403486/477633/480271/478227/100855806/488355/482270/607513/487514/479473 GNGT2, HOMER3, PPP3CA, SLC38A1, ADCY4, GNAI1, GNG11, KCNJ3, GRM8, GNG4, PLA2G4E, PLD2	12	VCAV_EC-up
cfa04114	Oocyte meiosis	12/357	117/7854	0.00671149	0.04681264	0.03426392	490280/607269/403486/479444/480271/486876/612821/403608/403558/488821/491768/612020 PPP2R5A, CALML6, PPP3CA, PPP2R1B, ADCY4, SLK, MAPK13, PPP2CA, PPP1CB, CPEB2, RPS6KA3, FBXO5	12	VCAV_EC-up
cfa04722	Neurotrophin signaling pathway	12/357	118/7854	0.00717522	0.04766397	0.03488704	611999/491286/607269/480481/612821/483225/484657/489692/485874/403872/491768/486666 MATK, RAPGEF1, CALML6, MAP3K3, MAPK13, RAP1A, VHL, PLCG2, PLCG1, NRAS, RPS6KA3, ARHGDIB	12	VCAV_EC-up
cfa04152	AMPK signaling pathway	12/357	121/7854	0.00871917	0.05405885	0.03956769	490280/403606/479444/478232/475266/485442/477374/607922/403608/477732/479115/491587 PPP2R5A, PPARG, PPP2R1B, MTOR, CREB5, CAB39L, PPP2R2A, CREB1, PPP2CA, ADIPOR2, TBC1D1, PPP2R2D	12	VCAV_EC-up
cfa00230	Purine metabolism	12/357	130/7854	0.01493816	0.07640893	0.05592655	486810/490455/609574/487221/479540/480098/482663/483996/480271/479116/553102/482188 ENTPD1, NPR1, PDE2A, PDE4D, PDE4B, ADSS, GUCY1A1, PDE7B, ADCY4, PGM2, GUCY1B1, DCK	12	VCAV_EC-up
cfa04962	Vasopressin-regulated water reabsorption	6/407	43/7854	0.02237705	0.36933889	0.3550297	477625/611619/478797/611934/403941/475236 ADCY6, RAB11B, DYNC1I2, CREB3, RAB5C, DYNC1I1	6	ACOR_VSMC-up
cfa04514	Cell adhesion molecules (CAMs)	12/357	139/7854	0.02406215	0.10019912	0.07333948	403994/403999/415130/480080/403844/489775/489309/610435/611060/475881/403764/100856563 SELP, SELE, CD34, SELL, OCLN, CDH5, ESAM, CADM3, NECTIN2, NRCAM, CD86, ITGA6	12	VCAV_EC-up
cfa04062	Chemokine signaling pathway	12/357	174/7854	0.09803562	0.26815625	0.19627358	403617/403850/483900/485189/475108/480271/478227/100855806/612913/483225/403872/607513 GNGT2, CXCL8, CXCR4, ARRB1, PTK2, ADCY4, GNAI1, GNG11, ELMO1, RAP1A, NRAS, GNG4	12	VCAV_EC-up
cfa04714	Thermogenesis	12/357	227/7854	0.33674279	0.60225153	0.4408104	490455/403606/478232/480271/475266/612821/476710/607922/486548/403872/491768/475763 NPR1, PPARG, MTOR, ADCY4, CREB5, MAPK13, SMARCA4, CREB1, SMARCD1, NRAS, RPS6KA3, KDM3A	12	VCAV_EC-up

cfa04015	Rap1 signaling pathway	12/241	209/7854	0.02710309	0.24158651	0.21719709	442985/403656/477625/478331/609956/485864/449021/482666/611795/403410/403857/481271 PDGFRB, ANGPT1, ADCY6, TLN2, , SRC, AKT2, PDGFC, CSF1, ADORA2B, FGF2, RAC2	12	VCAV_EC-down
cfa05016	Huntington disease	12/241	257/7854	0.09693646	0.41271433	0.37104866	611480/612644/478983/491061/486347/476040/489104/486762/475994/442961/478553/100686835 , , SDHC, ATP5MC1, UQCR10, COX8A, CLTB, TUBA8, AP2A2, GPX1, IFT57,	12	VCAV_EC-down
cfa04210	Apoptosis	12/407	131/7854	0.03864222	0.46370665	0.4457414	488472/403416/403484/403402/403400/488471/476548/491356/486077/100174780/611983/476010 CASP10, BCL2, SPTAN1, NGF, CTSS, CFLAR, ITPR1, DAB2IP, CTSB, TNFSF10, CTSZ, CTSF	12	ACOR_VSMC-up
cfa05226	Gastric cancer	12/407	149/7854	0.08521671	0.64585296	0.62083088	403416/475818/415125/611641/481563/479180/485149/474890/486093/481628/480522/100306933 BCL2, , FGFR2, FGF18, CDKN2B, POLK, FZD4, CDKN1A, FZD3, TGFBR1, JUP, LRP5	12	ACOR_VSMC-up
cfa04022	cGMP-PKG signaling pathway	12/407	165/7854	0.14766989	0.76328292	0.73371129	403962/486079/487441/403825/477625/476548/478227/609934/488584/611934/403928/475738 ADRA1B, GATA4, NPPB, PDE5A, ADCY6, ITPR1, GNAI1, PRKCE, RGS2, CREB3, GNAQ, SLC8A1	12	ACOR_VSMC-up
cfa05167	Kaposi sarcoma-associated herpesvirus infection	12/407	178/7854	0.21221343	0.80256807	0.77147442	489692/100856077/488087/487977/482905/476548/403545/612919/474890/490477/607899/474836 PLCG2, IFNGR1, GNB4, , TLR3, ITPR1, IL6ST, GABARAPL1, CDKN1A, PIK3C3, IRF9, DLA88	12	ACOR_VSMC-up
cfa05132	Salmonella infection	12/407	212/7854	0.41886997	0.88820767	0.85379612	403416/477331/611857/100174780/607701/490477/609544/478797/102156745/403941/475213/475236 BCL2, WASF3, ARHGEF26, TNFSF10, BRK1, PIK3C3, TIRAP, DYNC1I2, MAP3K7, RAB5C, WASL, DYNC1I1	12	ACOR_VSMC-up
cfa05163	Human cytomegalovirus infection	12/407	220/7854	0.47029065	0.88820767	0.85379612	488087/477625/476548/474890/478227/403522/611934/474836/480688/100855741/403928/608125 GNB4, ADCY6, ITPR1, CDKN1A, GNAI1, CCL5, CREB3, DLA88, TSC1, B2M, GNAQ, CRKL	12	ACOR_VSMC-up
cfa04530	Tight junction	12/388	170/7854	0.13489995	0.82883085	0.77622704	608738/479444/477535/481131/477166/481054/403728/403955/484056/403498/485122/487746 NEDD4, PPP2R1B, NF2, CDK4, PCNA, RAP2C, YBX3, RAC1, EZR, ARHGEF2, AMOTL1, RUNX1	12	ACOR_VSMC-down
cfa05202	Transcriptional misregulation in cancer	12/388	172/7854	0.14322525	0.82883085	0.77622704	479077/488669/100856381/475359/609786/483845/482373/486545/482372/490720/475916/487746 NSD2, PBX1, FUS, CDKN2C, EWSR1, CCNA2, HOXA10, ATF1, HOXA9, SIX4, H3F3B, RUNX1	12	ACOR_VSMC-down
cfa05163	Human cytomegalovirus infection	12/388	220/7854	0.40426793	0.99972301	0.9362731	476694/100856354/481131/100683675/403872/403788/475258/403955/479733/481746/480272/489644 CALR, , CDK4, BID, NRAS, ITGB3, CYCS, RAC1, GNB2, ITPR3, NFATC4, PIK3CD	12	ACOR_VSMC-down
cfa04072	Phospholipase D signaling pathway	11/441	148/7854	0.20895747	0.68198206	0.61394097	491319/479981/479460/485054/479557/474800/487235/403836/484648/486591/611939 DNM1, PTGFR, PDGFD, PIP5K1C, PLPP3, LPAR1, PIK3R1, AGTR1, RAF1, RAPGEF3, MAP2K2	11	VSAP_EC-up
cfa04921	Oxytocin signaling pathway	11/441	151/7854	0.22763631	0.70424983	0.63398709	481746/479812/486263/480272/476744/486295/484648/484665/403609/479248/611939 ITPR3, EEF2K, CAMKK2, NFATC4, EEF2, PRKAB1, RAF1, CAMK1, PPP1CA, PPP3CB, MAP2K2	11	VSAP_EC-up
cfa04621	NOD-like receptor signaling pathway	11/441	158/7854	0.27356676	0.74424101	0.66998836	481746/487439/485818/477024/481245/489370/480259/489664/485789/476412/479458 ITPR3, MFN2, RBCK1, MYD88, TAB1, NLRX1, , CYBA, MAVS, IRF3, CASP12	11	VSAP_EC-up
cfa05164	Influenza A	11/441	161/7854	0.29410107	0.75621155	0.68076461	611619/487235/477024/476822/489370/484648/485789/476412/608847/611939/611262 RAB11B, PIK3R1, MYD88, NUP98, NLRX1, RAF1, MAVS, IRF3, CCND3, MAP2K2, TRADD	11	VSAP_EC-up
cfa05169	Epstein-Barr virus infection	11/441	193/7854	0.52464906	0.92381513	0.83164644	490926/483626/487235/485604/403924/477024/481245/485789/476412/608847/611262 MAP3K14, DDB2, PIK3R1, ENTPD3, MYC, MYD88, TAB1, MAVS, IRF3, CCND3, TRADD	11	VSAP_EC-up

cfa04922	Glucagon signaling pathway	11/279	101/7854	0.00086585	0.025206	0.02136788	476882/479975/403874/477786/482437/403928/476411/480416/491369/610764/489050 , PRKACB, , PGAM1, SLC2A1, GNAQ, PRMT1, CALM1, CREB3L2, CAMK2D, CAMK2G	11	VSAP_EC-down
cfa04145	Phagosome	11/279	138/7854	0.00988142	0.09957433	0.08441215	476694/489208/403908/477956/474830/478701/484087/486241/488437/403703/403788 CALR, ITGA2, CANX, ITGB1, TUBB, TUBB2A, THBS2, SCARB1, ITGAV, TFRC, ITGB3	11	VSAP_EC-down
cfa04261	Adrenergic signaling in cardiomyocytes	11/279	147/7854	0.01537062	0.12584695	0.10668424	477374/481598/479975/609879/403480/403928/480416/491369/480137/610764/489050 PPP2R2A, TPM2, PRKACB, TPM4, ATP2B1, GNAQ, CALM1, CREB3L2, TPM3, CAMK2D, CAMK2G	11	VSAP_EC-down
cfa03013	RNA transport	11/279	158/7854	0.02487619	0.16683866	0.14143414	477197/487870/477447/474427/474839/475590/491042/486663/480442/480361/607653 EIF2S2, EIF4E, EIF2B1, XPOT, DDX39B, EIF4EBP1, KPNB1, STRAP, EIF5, EIF2S1, SEC13	11	VSAP_EC-down
cfa05166	Human T-cell leukemia virus 1 infection	11/279	209/7854	0.12449645	0.4066908	0.344764	476694/479975/488036/481628/403908/482437/100856393/478464/491369/474890/477955 CALR, PRKACB, DLG1, TGFBR1, CANX, SLC2A1, TLN1, MAPK10, CREB3L2, CDKN1A, NRP1	11	VSAP_EC-down
cfa04014	Ras signaling pathway	11/279	225/7854	0.17679241	0.47367346	0.4015472	403802/487421/479975/607885/477488/477762/491597/478464/480416/612814/608458 VEGFA, EPHA2, PRKACB, PAK1, PTPN11, HTR7, PDGFA, MAPK10, CALM1, PAK2, EFNA5	11	VSAP_EC-down
cfa05206	MicroRNAs in cancer	11/279	281/7854	0.41452121	0.73226635	0.62076416	403802/403567/489880/442942/488575/491597/403939/403788/487309/474890/608458 VEGFA, CASP3, FSCN1, PTGS2, SLC45A3, PDGFA, CD44, ITGB3, HDAC1, CDKN1A, EFNA5	11	VSAP_EC-down
cfa04370	VEGF signaling pathway	11/357	57/7854	4.1121E-05	0.00160623	0.00117566	482154/403784/403486/485930/475108/612821/442942/489692/485874/403872/487514 KDR, NOS3, PPP3CA, NFATC2, PTK2, MAPK13, PTGS2, PLCG2, PLCG1, NRAS, PLA2G4E	11	VCAV_EC-up
cfa05235	PD-L1 expression and PD-1 checkpoint pathway in cancer	11/357	89/7854	0.0022161	0.02132038	0.01560518	403417/403486/485930/478232/480481/483048/612821/100856077/485874/403872/480348 TLR4, PPP3CA, NFATC2, MTOR, MAP3K3, EML4, MAPK13, IFNGR1, PLCG1, NRAS, HIF1A	11	VCAV_EC-up
cfa05032	Morphine addiction	11/357	92/7854	0.00288941	0.02687152	0.01966827	403617/609574/487221/485189/479540/483996/480271/478227/100855806/488355/607513 GNGT2, PDE2A, PDE4D, ARRB1, PDE4B, PDE7B, ADCY4, GNAI1, GNG11, KCNJ3, GNG4	11	VCAV_EC-up
cfa04625	C-type lectin receptor signaling pathway	11/357	99/7854	0.00510914	0.03959584	0.02898167	403985/607269/403486/485930/488473/612821/479409/442942/489692/490183/403872 IL6, CALML6, PPP3CA, NFATC2, CASP8, MAPK13, ARHGEF12, PTGS2, PLCG2, BCL10, NRAS	11	VCAV_EC-up
cfa04919	Thyroid hormone signaling pathway	11/357	116/7854	0.01610621	0.07640893	0.05592655	480676/485494/478232/481726/477098/489692/485874/403872/480348/403788/479797 NOTCH1, TBC1D4, MTOR, NOTCH4, ATP1B3, PLCG2, PLCG1, NRAS, HIF1A, ITGB3, ATP2A1	11	VCAV_EC-up
cfa04071	Sphingolipid signaling pathway	11/357	122/7854	0.02262896	0.09713048	0.07109343	490280/403784/479444/480403/478227/612821/477374/403608/403872/479473/491587 PPP2R5A, NOS3, PPP2R1B, SPTLC2, GNAI1, MAPK13, PPP2R2A, PPP2CA, NRAS, PLD2, PPP2R2D	11	VCAV_EC-up
cfa04962	Vasopressin-regulated water reabsorption	4/357	43/7854	0.12987638	0.31883099	0.23336431	403957/475266/607922/486666 RAB11A, CREB5, CREB1, ARHGDIB	4	VCAV_EC-up
cfa04218	Cellular senescence	11/357	151/7854	0.08223238	0.24620394	0.18020586	403850/403985/607269/403486/485930/478232/612821/489287/403558/403872/489042 CXCL8, IL6, CALML6, PPP3CA, NFATC2, MTOR, MAPK13, ETS1, PPP1CB, NRAS, MCU	11	VCAV_EC-up
cfa04217	Necroptosis	11/357	160/7854	0.11171587	0.29129651	0.2132108	484006/403417/488473/488471/100856077/482905/477348/403170/102152063/100855593/487514 TNFAIP3, TLR4, CASP8, CFLAR, IFNGR1, TLR3, PARP4, HMGB1, RIPK1, HIST2H2AC, PLA2G4E	11	VCAV_EC-up

cfa05164	Influenza A	11/357	161/7854	0.11532562	0.29519127	0.21606153	403850/403985/403417/403957/488473/481145/100856077/482905/477490/487314/482620 CXCL8, IL6, TLR4, RAB11A, CASP8, TBK1, IFNGR1, TLR3, OAS3, KPNA6, APAF1	11	VCAV_EC-up
cfa05169	Epstein-Barr virus infection	11/357	193/7854	0.26221047	0.51654618	0.37807945	486810/403985/484006/488473/481145/612821/477490/489692/102152063/482620/480465 ENTPD1, IL6, TNFAIP3, CASP8, TBK1, MAPK13, OAS3, PLCG2, RIPK1, APAF1, PSMD12	11	VCAV_EC-up
cfa05206	MicroRNAs in cancer	11/241	281/7854	0.24490262	0.59576107	0.53561587	483148/442985/403477/609956/485548/483038/486148/483909/611782/490439/100686499 NOTCH2, PDGFRB, RECK, , IRS2, CYP1B1, IRS1, ZEB2, CCND2, SHC1, GLS2	11	VCAV_EC-down
cfa04750	Inflammatory mediator regulation of TRP channels	11/407	100/7854	0.01405697	0.30996841	0.2979594	489692/403881/403402/403813/477625/476548/488940/610764/610195/609934/403928 PLCG2, HTR2B, NGF, HRH1, ADCY6, ITPR1, F2RL1, CAMK2D, CYP2J2, PRKCE, GNAQ	11	ACOR_VSMC-up
cfa04922	Glucagon signaling pathway	11/407	101/7854	0.01506791	0.30996841	0.2979594	489571/479127/403849/476548/610764/611078/611934/611993/488526/403928/487797 PRKAA2, PPARGC1A, PFKM, ITPR1, CAMK2D, PYGM, CREB3, GYS1, PRKAG3, GNAQ, PFKL	11	ACOR_VSMC-up
cfa04722	Neurotrophin signaling pathway	11/407	118/7854	0.04176582	0.48114226	0.46250152	489692/403416/479915/478061/403402/610764/481150/474444/403408/489532/608125 PLCG2, BCL2, SORT1, MAP3K1, NGF, CAMK2D, IRAK3, FRS2, PSEN1, MAPK7, CRKL	11	ACOR_VSMC-up
cfa04962	Vasopressin-regulated water reabsorption	3/131	43/7854	0.03447448	0.33468971	0.3069438	475236/486666/403556 DYNC1I1, ARHGDIB, PRKACA	3	ACOR_EC-up
cfa04261	Adrenergic signaling in cardiomyocytes	11/407	147/7854	0.14030095	0.76328292	0.73371129	403532/403962/403416/477625/610764/480349/486563/478227/611934/403928/475738 TNNT2, ADRA1B, BCL2, ADCY6, CAMK2D, PPP2R5E, CACNB3, GNAI1, CREB3, GNAQ, SLC8A1	11	ACOR_VSMC-up
cfa04310	Wnt signaling pathway	11/407	151/7854	0.15951562	0.78597746	0.75552658	490231/611782/486339/485149/610764/484699/486093/403408/100856496/102156745/100306933 LGR6, CCND2, ZNRF3, FZD4, CAMK2D, PRICKLE2, FZD3, PSEN1, SIAH1, MAP3K7, LRP5	11	ACOR_VSMC-up
cfa04934	Cushing syndrome	11/407	152/7854	0.16451728	0.78968296	0.75908852	481563/477625/476548/485149/610764/488171/474890/478227/486093/611934/403928 CDKN2B, ADCY6, ITPR1, FZD4, CAMK2D, NCEH1, CDKN1A, GNAI1, FZD3, CREB3, GNAQ	11	ACOR_VSMC-up
cfa04217	Necroptosis	11/407	160/7854	0.20719124	0.80256807	0.77147442	100856077/403416/482905/488471/610764/100174780/485334/611078/474741/607899/490969 IFNGR1, BCL2, TLR3, CFLAR, CAMK2D, TNFSF10, SMPD1, PYGM, , IRF9, STAT5B	11	ACOR_VSMC-up
cfa05161	Hepatitis B	11/407	164/7854	0.23015112	0.80256807	0.77147442	488472/403416/478061/482905/100855560/474890/481628/609544/611934/102156745/490969 CASP10, BCL2, MAP3K1, TLR3, DDX58, CDKN1A, TGFBR1, TIRAP, CREB3, MAP3K7, STAT5B	11	ACOR_VSMC-up
cfa05152	Tuberculosis	11/407	171/7854	0.2725259	0.82260001	0.79073027	488834/488472/100856077/403416/403400/610764/490477/481037/609544/487286/403941 TLR1, CASP10, IFNGR1, BCL2, CTSS, CAMK2D, PIK3C3, LAMP2, TIRAP, NOD2, RAB5C	11	ACOR_VSMC-up
cfa05205	Proteoglycans in cancer	11/407	202/7854	0.47749745	0.88820767	0.85379612	489692/476548/485149/610764/474890/486093/474444/479446/490230/477818/477945 PLCG2, ITPR1, FZD4, CAMK2D, CDKN1A, FZD3, FRS2, RDX, PPP1R12B, PDCD4, SDC2	11	ACOR_VSMC-up
cfa05166	Human T-cell leukemia virus 1 infection	11/407	209/7854	0.52372215	0.88929092	0.8548374	478061/611782/481563/477625/474890/477955/481628/611934/474836/100855741/490969 MAP3K1, CCND2, CDKN2B, ADCY6, CDKN1A, NRP1, TGFBR1, CREB3, DLA88, B2M, STAT5B	11	ACOR_VSMC-up
cfa04810	Regulation of actin cytoskeleton	11/407	211/7854	0.53670863	0.89195014	0.85739359	476331/482666/415125/611641/607701/483699/479446/490230/475213/608414/608125 PIP5K1B, PDGFC, FGFR2, FGF18, BRK1, SSH3, RDX, PPP1R12B, WASL, , CRKL	11	ACOR_VSMC-up
cfa03410	Base excision repair	11/388	34/7854	3.7982E-07	1.4759E-05	1.3822E-05	403170/612550/491145/486223/100686967/476063/477166/475639/610047/482558/483096 HMGB1, POLE3, LIG3, POLE, LIG1, FEN1, PCNA, NEIL3, POLD1, APEX1,	11	ACOR_VSMC-down

cfa04962	Vasopressin-regulated water reabsorption	3/441	43/7854	0.43742422	0.88982872	0.80105084	611619/479491/489919 RAB11B, , STX4	3	VSAP_EC-up
cfa05161	Hepatitis B	11/388	164/7854	0.18727706	0.94332149	0.88345124	492247/442936/477166/100683675/403872/477237/475258/100855704/483845/480272/489644 IRAK1, , PCNA, BID, NRAS, YWHAB, CYCS, CDK2, CCNA2, NFATC4, PIK3CD	11	ACOR_VSMC-down
cfa04024	cAMP signaling pathway	10/131	211/7854	0.00269477	0.1255761	0.1151658	489198/403464/100856339/403966/100683695/486808/488402/479473/403556/610836 HTR4, SOX9, , ATP1B1, PDE4A, PLCE1, RAPGEF4, PLD2, PRKACA, PDE3A	10	ACOR_EC-up
cfa00640	Propanoate metabolism	10/441	34/7854	1.0892E-05	0.00161751	0.00145613	100685468/490779/403978/484488/479856/474978/477517/488110/477205/477002 ACACB, ALDH6A1, DLD, , ABAT, BCKDHB, ACADS, EHHADH, ACSS2, ACSS1	10	VSAP_EC-up
cfa04722	Neurotrophin signaling pathway	10/441	118/7854	0.12578916	0.54144031	0.48742101	491292/476215/481150/486148/612792/479915/487235/612448/484648/611939 ABL1, MAP3K5, IRAK3, IRS1, SH2B1, SORT1, PIK3R1, RPS6KA2, RAF1, MAP2K2	10	VSAP_EC-up
cfa00230	Purine metabolism	10/441	130/7854	0.1940171	0.66536335	0.5989803	484169/476342/403781/483028/485604/477809/488744/483309/474746/475393 GDA, , PDE6D, XDH, ENTPD3, NT5C2, HDDC3, NT5C, NUDT2, PNP	10	VSAP_EC-up
cfa04550	Signaling pathways regulating pluripotency of stem cells	10/441	135/7854	0.22646485	0.70424983	0.63398709	482294/485183/490903/481657/485149/487235/403924/484648/490099/611939 FZD1, WNT11, AXIN2, KLF4, FZD4, PIK3R1, MYC, RAF1, AXIN1, MAP2K2	10	VSAP_EC-up
cfa04140	Autophagy - animal	10/441	136/7854	0.23319655	0.70505074	0.63470809	486148/486263/483634/487235/483756/484648/102154798/477437/611939/489038 IRS1, CAMKK2, AMBRA1, PIK3R1, ATG2A, RAF1, ATG9A, ULK1, MAP2K2, DDIT4	10	VSAP_EC-up
cfa04120	Ubiquitin mediated proteolysis	10/441	139/7854	0.25382736	0.71119554	0.64023982	491236/483626/474161/100856557/490006/608118/479036/481679/611303/476713 ANAPC2, DDB2, NHLRC1, FBXO4, SOCS1, UBA7, DET1, CDC26, RNFT2, KEAP1	10	VSAP_EC-up
cfa04934	Cushing syndrome	10/441	152/7854	0.34904425	0.82932914	0.74658728	482294/485183/490903/481746/486894/485149/403836/488171/490099/611939 FZD1, WNT11, AXIN2, ITPR3, TCF7L2, FZD4, AGTR1, NCEH1, AXIN1, MAP2K2	10	VSAP_EC-up
cfa04925	Aldosterone synthesis and secretion	10/279	94/7854	0.00176589	0.03304746	0.02801533	479975/403480/486241/403928/478754/480416/491369/610764/403897/489050 PRKACB, ATP2B1, SCARB1, GNAQ, NR4A2, CALM1, CREB3L2, CAMK2D, NR4A1, CAMK2G	10	VSAP_EC-down
cfa04962	Vasopressin-regulated water reabsorption	3/279	43/7854	0.19551081	0.51223831	0.43423979	479975/609233/491369 PRKACB, DYNLL1, CREB3L2	3	VSAP_EC-down
cfa04670	Leukocyte transendothelial migration	10/279	113/7854	0.00672948	0.080142	0.06793878	480369/403936/477488/491924/477956/485856/484526/477032/479252/607207 ACTN1, VASP, PTPN11, MSN, ITGB1, MYL9, ACTN4, CTNNB1, VCL,	10	VSAP_EC-down
cfa04728	Dopaminergic synapse	10/279	132/7854	0.01884258	0.1473612	0.12492251	477374/479975/100855651/403928/478575/478464/480416/491369/610764/489050 PPP2R2A, PRKACB, KIF5B, GNAQ, GSK3B, MAPK10, CALM1, CREB3L2, CAMK2D, CAMK2G	10	VSAP_EC-down
cfa04921	Oxytocin signaling pathway	10/279	151/7854	0.04232094	0.24104538	0.2043414	479975/475411/442942/485856/488584/403928/480416/610764/474890/489050 PRKACB, PPP1R12A, PTGS2, MYL9, RGS2, GNAQ, CALM1, CAMK2D, CDKN1A, CAMK2G	10	VSAP_EC-down
cfa04934	Cushing syndrome	10/279	152/7854	0.04394397	0.24366258	0.20656008	479975/486241/403928/477032/478575/491369/610764/403897/474890/489050 PRKACB, SCARB1, GNAQ, CTNNB1, GSK3B, CREB3L2, CAMK2D, NR4A1, CDKN1A, CAMK2G	10	VSAP_EC-down
cfa04022	cGMP-PKG signaling pathway	10/279	165/7854	0.06897368	0.3346501	0.28369293	403936/403480/609616/475411/489796/485856/488584/403928/480416/491369 VASP, ATP2B1, PRKG1, PPP1R12A, GTF2IRD1, MYL9, RGS2, GNAQ, CALM1, CREB3L2	10	VSAP_EC-down
cfa05167	Kaposi sarcoma-associated herpesvirus infection	10/279	178/7854	0.10162037	0.38701439	0.32808371	403802/403567/442942/480348/477032/477886/478575/478464/480416/474890 VEGFA, CASP3, PTGS2, HIF1A, CTNNB1, LYN, GSK3B, MAPK10, CALM1, CDKN1A	10	VSAP_EC-down

cfa05169	Epstein-Barr virus infection	10/279	193/7854	0.14876704	0.43794343	0.37125779	478279/476694/403567/477886/403939/478464/484700/487309/492019/474890 , CALR, CASP3, LYN, CD44, MAPK10, PSMD6, HDAC1, BTK, CDKN1A	10 VSAP_EC-down
cfa04024	cAMP signaling pathway	10/279	211/7854	0.2174817	0.53252528	0.45143766	479975/607885/403480/475411/485856/478464/480416/491369/610764/489050 PRKACB, PAK1, ATP2B1, PPP1R12A, MYL9, MAPK10, CALM1, CREB3L2, CAMK2D, CAMK2G	10 VSAP_EC-down
cfa04924	Renin secretion	10/357	69/7854	0.00102681	0.01353725	0.00990842	490455/610668/607269/403424/403486/482663/478227/403717/553102/607922 NPR1, ACE, CALML6, EDN1, PPP3CA, GUCY1A1, GNAI1, KCNJ2, GUCY1B1, CREB1	10 VCAV_EC-up
cfa04658	Th1 and Th2 cell differentiation	10/357	87/7854	0.00586575	0.0442309	0.03237425	403851/480676/607575/487134/403486/485930/612821/100856077/485769/485874 IL2RG, NOTCH1, DLL4, GATA3, PPP3CA, NFATC2, MAPK13, IFNGR1, JAG1, PLCG1	10 VCAV_EC-up
cfa04064	NF-kappa B signaling pathway	10/357	93/7854	0.00932277	0.0565446	0.0413871	403850/484006/403417/488471/486753/442942/489692/490183/485874/102152063 CXCL8, TNFAIP3, TLR4, CFLAR, ERC1, PTGS2, PLCG2, BCL10, PLCG1, RIPK1	10 VCAV_EC-up
cfa05231	Choline metabolism in cancer	10/357	96/7854	0.01154992	0.06713392	0.04913783	442986/476932/478232/485874/403872/479460/476331/480348/487514/479473 PDGFB, DGKH, MTOR, PLCG1, NRAS, PDGFD, PIP5K1B, HIF1A, PLA2G4E, PLD2	10 VCAV_EC-up
cfa04070	Phosphatidylinositol signaling system	10/357	97/7854	0.0123752	0.0690536	0.05054291	100688270/476932/607269/485399/490383/606918/489692/485874/477925/476331 INPP5D, DGKH, CALML6, PIK3C2A, ITPKB, MTMR2, PLCG2, PLCG1, IMPA1, PIP5K1B	10 VCAV_EC-up
cfa04713	Circadian entrainment	10/357	98/7854	0.01324418	0.07245346	0.0530314	403617/607269/482663/480271/478227/100855806/488355/553102/607922/607513 GNGT2, CALML6, GUCY1A1, ADCY4, GNAI1, GNG11, KCNJ3, GUCY1B1, CREB1, GNG4	10 VCAV_EC-up
cfa04620	Toll-like receptor signaling pathway	10/357	101/7854	0.01612692	0.07640893	0.05592655	403850/403985/403417/488473/481145/612821/482905/403764/102152063/111089957 CXCL8, IL6, TLR4, CASP8, TBK1, MAPK13, TLR3, CD86, RIPK1, TLR6	10 VCAV_EC-up
cfa05146	Amoebiasis	10/357	102/7854	0.01718423	0.07990668	0.05848669	403850/403985/442939/403417/403466/480364/475108/488192/403822/475034 CXCL8, IL6, LAMC2, TLR4, COL4A5, ARG2, PTK2, SERPINB9, NOS2, LAMA4	10 VCAV_EC-up
cfa05145	Toxoplasmosis	10/357	109/7854	0.02606895	0.10540924	0.07715296	442939/403417/488473/478227/612821/100856077/479406/403822/100856563/475034 LAMC2, TLR4, CASP8, GNAI1, MAPK13, IFNGR1, HSPA8, NOS2, ITGA6, LAMA4	10 VCAV_EC-up
cfa04726	Serotonergic synapse	10/357	110/7854	0.02756513	0.1083193	0.07928294	403617/403544/486591/478227/100855806/488355/442942/403872/607513/487514 GNGT2, PTGS1, RAPGEF3, GNAI1, GNG11, KCNJ3, PTGS2, NRAS, GNG4, PLA2G4E	10 VCAV_EC-up
cfa04725	Cholinergic synapse	10/357	113/7854	0.03241995	0.1206022	0.08827326	403617/480271/475266/478227/100855806/488355/403717/607922/403872/607513 GNGT2, ADCY4, CREB5, GNAI1, GNG11, KCNJ3, KCNJ2, CREB1, NRAS, GNG4	10 VCAV_EC-up
cfa04935	Growth hormone synthesis, secretion and action	10/357	116/7854	0.03784673	0.13366125	0.09783167	475108/478232/480271/475266/478227/612821/489692/607922/485874/403872 PTK2, MTOR, ADCY4, CREB5, GNAI1, MAPK13, PLCG2, CREB1, PLCG1, NRAS	10 VCAV_EC-up
cfa04210	Apoptosis	10/357	131/7854	0.07436868	0.22800947	0.16688865	609293/403458/488473/488471/488472/477348/100502558/102152063/482620/403872 IL3RA, CTSC, CASP8, CFLAR, CASP10, PARP4, PMAIP1, RIPK1, APAF1, NRAS	10 VCAV_EC-up
cfa05162	Measles	10/357	136/7854	0.09023231	0.25737195	0.18838015	403851/403985/484006/403417/488473/481145/609365/479406/477490/482620 IL2RG, IL6, TNFAIP3, TLR4, CASP8, TBK1, , HSPA8, OAS3, APAF1	10 VCAV_EC-up
cfa05034	Alcoholism	10/357	171/7854	0.25039632	0.50993119	0.3732377	403617/607269/475266/478227/100855806/607922/403558/403872/607513/100855593 GNGT2, CALML6, CREB5, GNAI1, GNG11, CREB1, PPP1CB, NRAS, GNG4, HIST2H2AC	10 VCAV_EC-up

cfa05166	Human T-cell leukemia virus 1 infection	10/357	209/7854	0.48049512	0.71307521	0.52192639	403851/403985/403486/485930/480271/480481/475266/489287/607922/403872 IL2RG, IL6, PPP3CA, NFATC2, ADCY4, MAP3K3, CREB5, ETS1, CREB1, NRAS	10 VCAV_EC-up
cfa04142	Lysosome	10/241	118/7854	0.0032818	0.1004926	0.09034735	474457/490965/479915/489104/479877/492194/476697/487633/475020/475678 ARSA, NAGLU, SORT1, CLTB, ATP6V0C, IDS, DNASE2, , CD164, LAPTM4A	10 VCAV_EC-down
cfa04152	AMPK signaling pathway	10/241	121/7854	0.00393387	0.1004926	0.09034735	609956/610130/449021/479812/485548/490942/486148/476744/481954/475590 , AKT1S1, AKT2, EEF2K, IRS2, G6PC3, IRS1, EEF2, FOXO3, EIF4EBP1	10 VCAV_EC-down
cfa04141	Protein processing in endoplasmic reticulum	10/241	160/7854	0.02520946	0.24158651	0.21719709	481082/403950/482715/404012/483745/477124/404018/484941/477223/404006 SSR4, SSR2, PDIA4, DDOST, CAPN1, SSR3, SEC61B, PRKCSH, RPN2, SEC61A1	10 VCAV_EC-down
cfa04360	Axon guidance	10/241	179/7854	0.04855826	0.34112175	0.30668372	611862/609956/485864/475930/100688869/610764/489132/487979/481271/100856151 RHOD, , SRC, SEMA3C, PLXNA1, CAMK2D, SLIT3, BOC, RAC2, RGMA	10 VCAV_EC-down
cfa04014	Ras signaling pathway	10/241	225/7854	0.15360887	0.50190805	0.45123781	442985/403656/609956/449021/482666/611795/489819/403857/490439/481271 PDGFRB, ANGPT1, , AKT2, PDGFC, CSF1, , FGF2, SHC1, RAC2	10 VCAV_EC-down
cfa04714	Thermogenesis	10/241	227/7854	0.15975279	0.51052385	0.45898379	477625/608597/610130/611480/612644/478983/491061/486347/612448/476040 ADCY6, COA1, AKT1S1, , , SDHC, ATP5MC1, UQCR10, RPS6KA2, COX8A	10 VCAV_EC-down
cfa04115	p53 signaling pathway	10/407	69/7854	0.00273011	0.14832401	0.14257754	403416/611782/478442/474890/485118/476218/100856496/481956/476177/100855542 BCL2, CCND2, CCNG2, CDKN1A, SESN3, PERP, SIAH1, SESN1, SERPINB5, ZMAT3	10 ACOR_VSMC-up
cfa04962	Vasopressin-regulated water reabsorption	44197	43/7854	0.10902294	0.34415573	0.29640206	489919 STX4	1 ACOR_EC-down
cfa04725	Cholinergic synapse	10/407	113/7854	0.06759707	0.62799859	0.60366824	488087/403416/489828/477625/476548/610764/481253/478227/611934/403928 GNB4, BCL2, ACHE, ADCY6, ITPR1, CAMK2D, KCNJ4, GNAI1, CREB3, GNAQ	10 ACOR_VSMC-up
cfa04218	Cellular senescence	10/407	151/7854	0.25656302	0.82260001	0.79073027	486079/475947/611782/481563/476548/482772/474890/481628/474836/480688 GATA4, HIPK3, CCND2, CDKN2B, ITPR1, HIPK2, CDKN1A, TGFBR1, DLA88, TSC1	10 ACOR_VSMC-up
cfa04150	mTOR signaling pathway	10/407	152/7854	0.26304512	0.82260001	0.79073027	489228/489571/609909/485149/489525/486093/480688/489038/487210/100306933 SKP2, PRKAA2, CASTOR1, FZD4, ULK2, FZD3, TSC1, DDIT4, SLC38A9, LRP5	10 ACOR_VSMC-up
cfa04621	NOD-like receptor signaling pathway	10/407	158/7854	0.30296466	0.82260001	0.79073027	403416/478274/476548/612919/486077/448778/403522/607899/487286/102156745 BCL2, TP53BP1, ITPR1, GABARAPL1, CTSB, P2RX7, CCL5, IRF9, NOD2, MAP3K7	10 ACOR_VSMC-up
cfa04530	Tight junction	10/407	170/7854	0.38644085	0.87388357	0.84002697	486079/485669/478061/489571/609934/479446/488526/489271/474708/481944 GATA4, AMOTL2, MAP3K1, PRKAA2, PRKCE, RDX, PRKAG3, JAM3, MPDZ, BVES	10 ACOR_VSMC-up
cfa05203	Viral carcinogenesis	10/407	189/7854	0.51994567	0.88929092	0.8548374	489228/611782/481563/403545/477402/474890/607899/611934/474836/490969 SKP2, CCND2, CDKN2B, IL6ST, , CDKN1A, IRF9, CREB3, DLA88, STAT5B	10 ACOR_VSMC-up
cfa05170	Human immunodeficiency virus 1 infection	10/407	200/7854	0.59318327	0.90408536	0.86905866	489692/488087/403416/476548/478227/474836/100855741/403928/102156745/608125 PLCG2, GNB4, BCL2, ITPR1, GNAI1, DLA88, B2M, GNAQ, MAP3K7, CRKL	10 ACOR_VSMC-up
cfa03440	Homologous recombination	10/388	41/7854	2.1795E-05	0.00053892	0.00050472	100855690/489537/475368/403568/100856421/477070/403437/480175/610047/608527 RPA1, TOP3A, RAD54L, RAD51, RPA2, TOPBP1, BRCA1, RBBP8, POLD1, PALB2	10 ACOR_VSMC-down
cfa04115	p53 signaling pathway	10/388	69/7854	0.00192185	0.03175069	0.02973555	100856079/481131/100683675/478324/608855/482963/475258/100855704/489620/608420 CDK1, CDK4, BID, CCNB2, GTSE1, RRM2, CYCS, CDK2, TP73,	10 ACOR_VSMC-down

cfa03008	Ribosome biogenesis in eukaryotes	10/388	79/7854	0.00525779	0.07150598	0.06696767	612871/100499482/478021/476460/487790/492263/100856234/477708/479820/489733 NOP56, RAN, GTPBP4, FBL, , , NOP58, EMG1, REXO5, NOB1	10	ACOR_VSMC-down
cfa03320	PPAR signaling pathway	10/388	80/7854	0.00575244	0.07450784	0.06977902	483792/486839/478927/481018/480872/479344/491317/481756/475631/478895 FADS2, SCD, ACSL3, ACSL4, , , SLC27A4, PPARD, ACSL1, ACADL	10	ACOR_VSMC-down
cfa04068	FoxO signaling pathway	10/388	127/7854	0.09691288	0.67590519	0.63300719	476411/489971/475380/478324/403872/476087/100855704/608420/449494/489644 PRMT1, PLK1, PLK3, CCNB2, NRAS, PLK4, CDK2, , CCNB3, PIK3CD	10	ACOR_VSMC-down
cfa04210	Apoptosis	10/388	131/7854	0.11290047	0.73116496	0.68475975	480124/474663/442936/482737/100683675/403872/475258/481048/481746/489644 LMNA, LMNB1, , CASP2, BID, NRAS, CYCS, AIFM1, ITPR3, PIK3CD	10	ACOR_VSMC-down
cfa04714	Thermogenesis	10/388	227/7854	0.69151343	0.99972301	0.9362731	478927/481018/476640/403872/486407/475121/475631/480149/478807/476756 ACSL3, ACSL4, SMARCC1, NRAS, SMARCB1, CYC1, ACSL1, ATP5F1A, , ATP5F1D	10	ACOR_VSMC-down
cfa04962	Vasopressin-regulated water reabsorption	44105	43/7854	0.0534497	0.17686461	0.16548735	611619 RAB11B	1	VPOR_EC-down
cfa00480	Glutathione metabolism	9/441	52/7854	0.00217113	0.04960195	0.04465318	476006/477683/609050/479912/481088/476005/403913/478889/475596 , MGST1, MGST3, GSTM4, G6PD, GSTP1, ANPEP, IDH1,	9	VSAP_EC-up
cfa00980	Metabolism of xenobiotics by cytochrome P450	9/441	52/7854	0.00217113	0.04960195	0.04465318	449023/403786/487748/476003/476006/477683/609050/479912/476005 HSD11B1, DHDH, CBR3, ALDH3B1, , MGST1, MGST3, GSTM4, GSTP1	9	VSAP_EC-up
cfa05213	Endometrial cancer	9/441	59/7854	0.00524116	0.09728903	0.08758254	490903/442858/486894/483626/487235/403924/484648/490099/611939 AXIN2, CDH1, TCF7L2, DDB2, PIK3R1, MYC, RAF1, AXIN1, MAP2K2	9	VSAP_EC-up
cfa05221	Acute myeloid leukemia	9/441	67/7854	0.01202116	0.1487619	0.13391998	486180/486894/488770/489398/490968/487235/403924/484648/611939 PER2, TCF7L2, BCL2A1, ZBTB16, STAT5A, PIK3R1, MYC, RAF1, MAP2K2	9	VSAP_EC-up
cfa01230	Biosynthesis of amino acids	9/441	74/7854	0.02204768	0.20930307	0.18842098	486834/484394/403443/480112/483704/478889/479066/477177/480693 GOT1, BCAT2, GLUL, , PC, IDH1, IDH3A, , ASS1	9	VSAP_EC-up
cfa05220	Chronic myeloid leukemia	9/441	76/7854	0.02577492	0.21992842	0.19798625	491292/483626/490968/487235/403924/484648/612240/611939/477039 ABL1, DDB2, STAT5A, PIK3R1, MYC, RAF1, CTBP1, MAP2K2, TGFBR2	9	VSAP_EC-up
cfa05210	Colorectal cancer	9/441	85/7854	0.04809698	0.33577344	0.30227345	490903/486894/483626/487235/403924/484648/490099/611939/477039 AXIN2, TCF7L2, DDB2, PIK3R1, MYC, RAF1, AXIN1, MAP2K2, TGFBR2	9	VSAP_EC-up
cfa04931	Insulin resistance	9/441	109/7854	0.15806233	0.61400684	0.55274761	489190/100685468/486148/487235/474666/612448/486295/612305/403609 PPARGC1B, ACACB, IRS1, PIK3R1, SLC27A6, RPS6KA2, PRKAB1, CRTC2, PPP1CA	9	VSAP_EC-up
cfa04142	Lysosome	9/441	118/7854	0.21727613	0.69388184	0.62465351	403400/479915/100856090/475678/479053/489052/474457/476010/490019 CTSS, SORT1, CTNS, LAPTM4A, AP3B2, AP3M1, ARSA, CTSF, NAGPA	9	VSAP_EC-up
cfa04071	Sphingolipid signaling pathway	9/441	122/7854	0.24604905	0.70505074	0.63470809	100856377/476215/485008/485672/487235/484648/483755/611939/611262 SMPD2, MAP3K5, CERS4, PPP2R3A, PIK3R1, RAF1, PPP2R5B, MAP2K2, TRADD	9	VSAP_EC-up
cfa04962	Vasopressin-regulated water reabsorption	42736	43/7854	0.08919347	0.32364194	0.30122906	482994 ADCY3	1	VFEM_EC-up
cfa05160	Hepatitis C	9/441	157/7854	0.52403445	0.92381513	0.83164644	608207/491278/487235/403924/484648/485789/476412/611939/611262 CLDN1, RXRA, PIK3R1, MYC, RAF1, MAVS, IRF3, MAP2K2, TRADD	9	VSAP_EC-up
cfa03013	RNA transport	9/441	158/7854	0.5318348	0.92381513	0.83164644	476840/481101/475445/476822/487655/612971/608665/492002/476197 EIF3F, PYM1, NUP37, NUP98, SNUPN, EIF3G, EIF3D, PABPC5, ELAC1	9	VSAP_EC-up
NA	NA	NA	NA	NA	NA	NA	NA NA	NA	ACOR_EC-down
NA	NA	NA	NA	NA	NA	NA	NA NA	NA	ACOR_EC-down
NA	NA	NA	NA	NA	NA	NA	NA NA	NA	ACOR_EC-down
NA	NA	NA	NA	NA	NA	NA	NA NA	NA	ACOR_EC-down
NA	NA	NA	NA	NA	NA	NA	NA NA	NA	ACOR_EC-down
NA	NA	NA	NA	NA	NA	NA	NA NA	NA	ACOR_EC-down
NA	NA	NA	NA	NA	NA	NA	NA NA	NA	ACOR_EC-down

NA	NA	NA	NA	NA	NA	NA	NA	NA	NA	ACOR_EC-down
NA	NA	NA	NA	NA	NA	NA	NA	NA	NA	ACOR_EC-down
NA	NA	NA	NA	NA	NA	NA	NA	NA	NA	ACOR_EC-down
NA	NA	NA	NA	NA	NA	NA	NA	NA	NA	ACOR_EC-down
NA	NA	NA	NA	NA	NA	NA	NA	NA	NA	ACOR_EC-down
NA	NA	NA	NA	NA	NA	NA	NA	NA	NA	ACOR_EC-down
NA	NA	NA	NA	NA	NA	NA	NA	NA	NA	ACOR_EC-down
NA	NA	NA	NA	NA	NA	NA	NA	NA	NA	ACOR_EC-down
NA	NA	NA	NA	NA	NA	NA	NA	NA	NA	ACOR_EC-down
NA	NA	NA	NA	NA	NA	NA	NA	NA	NA	ACOR_EC-down
NA	NA	NA	NA	NA	NA	NA	NA	NA	NA	ACOR_EC-down
NA	NA	NA	NA	NA	NA	NA	NA	NA	NA	ACOR_EC-down
NA	NA	NA	NA	NA	NA	NA	NA	NA	NA	ACOR_EC-down
NA	NA	NA	NA	NA	NA	NA	NA	NA	NA	ACOR_EC-down
NA	NA	NA	NA	NA	NA	NA	NA	NA	NA	ACOR_EC-down
NA	NA	NA	NA	NA	NA	NA	NA	NA	NA	ACOR_EC-down
NA	NA	NA	NA	NA	NA	NA	NA	NA	NA	ACOR_EC-down
NA	NA	NA	NA	NA	NA	NA	NA	NA	NA	ACOR_EC-down
NA	NA	NA	NA	NA	NA	NA	NA	NA	NA	ACOR_EC-down
NA	NA	NA	NA	NA	NA	NA	NA	NA	NA	ACOR_EC-down
NA	NA	NA	NA	NA	NA	NA	NA	NA	NA	ACOR_EC-down
NA	NA	NA	NA	NA	NA	NA	NA	NA	NA	ACOR_EC-down
NA	NA	NA	NA	NA	NA	NA	NA	NA	NA	ACOR_EC-down
NA	NA	NA	NA	NA	NA	NA	NA	NA	NA	ACOR_EC-down
NA	NA	NA	NA	NA	NA	NA	NA	NA	NA	ACOR_EC-down
NA	NA	NA	NA	NA	NA	NA	NA	NA	NA	ACOR_EC-down
NA	NA	NA	NA	NA	NA	NA	NA	NA	NA	ACOR_EC-down
NA	NA	NA	NA	NA	NA	NA	NA	NA	NA	ACOR_EC-down
NA	NA	NA	NA	NA	NA	NA	NA	NA	NA	ACOR_EC-down
NA	NA	NA	NA	NA	NA	NA	NA	NA	NA	ACOR_EC-down
NA	NA	NA	NA	NA	NA	NA	NA	NA	NA	ACOR_EC-down
NA	NA	NA	NA	NA	NA	NA	NA	NA	NA	ACOR_EC-down
NA	NA	NA	NA	NA	NA	NA	NA	NA	NA	ACOR_EC-down
NA	NA	NA	NA	NA	NA	NA	NA	NA	NA	ACOR_EC-down
NA	NA	NA	NA	NA	NA	NA	NA	NA	NA	ACOR_EC-down
NA	NA	NA	NA	NA	NA	NA	NA	NA	NA	ACOR_EC-down
NA	NA	NA	NA	NA	NA	NA	NA	NA	NA	ACOR_EC-down
NA	NA	NA	NA	NA	NA	NA	NA	NA	NA	ACOR_EC-down
NA	NA	NA	NA	NA	NA	NA	NA	NA	NA	ACOR_EC-down
NA	NA	NA	NA	NA	NA	NA	NA	NA	NA	ACOR_EC-down
NA	NA	NA	NA	NA	NA	NA	NA	NA	NA	ACOR_EC-down
NA	NA	NA	NA	NA	NA	NA	NA	NA	NA	ACOR_EC-down
NA	NA	NA	NA	NA	NA	NA	NA	NA	NA	ACOR_EC-down
NA	NA	NA	NA	NA	NA	NA	NA	NA	NA	ACOR_EC-down
NA	NA	NA	NA	NA	NA	NA	NA	NA	NA	ACOR_EC-down
NA	NA	NA	NA	NA	NA	NA	NA	NA	NA	ACOR_EC-down
NA	NA	NA	NA	NA	NA	NA	NA	NA	NA	ACOR_EC-down
NA	NA	NA	NA	NA	NA	NA	NA	NA	NA	ACOR_EC-down
NA	NA	NA	NA	NA	NA	NA	NA	NA	NA	ACOR_EC-down
NA	NA	NA	NA	NA	NA	NA	NA	NA	NA	ACOR_EC-down
NA	NA	NA	NA	NA	NA	NA	NA	NA	NA	ACOR_EC-down
NA	NA	NA	NA	NA	NA	NA	NA	NA	NA	ACOR_EC-down
NA	NA	NA	NA	NA	NA	NA	NA	NA	NA	ACOR_EC-down
NA	NA	NA	NA	NA	NA	NA	NA	NA	NA	ACOR_EC-down
NA	NA	NA	NA	NA	NA	NA	NA	NA	NA	ACOR_EC-down
NA	NA	NA	NA	NA	NA	NA	NA	NA	NA	ACOR_EC-down
NA	NA	NA	NA	NA	NA	NA	NA	NA	NA	ACOR_EC-down
NA	NA	NA	NA	NA	NA	NA	NA	NA	NA	ACOR_EC-down
NA	NA	NA	NA	NA	NA	NA	NA	NA	NA	ACOR_EC-down
NA	NA	NA	NA	NA	NA	NA	NA	NA	NA	ACOR_EC-down
NA	NA	NA	NA	NA	NA	NA	NA	NA	NA	ACOR_EC-down
NA	NA	NA	NA	NA	NA	NA	NA	NA	NA	ACOR_EC-down
NA	NA	NA	NA	NA	NA	NA	NA	NA	NA	ACOR_EC-down
NA	NA	NA	NA	NA	NA	NA	NA	NA	NA	ACOR_EC-down
NA	NA	NA	NA	NA	NA	NA	NA	NA	NA	ACOR_EC-down
NA	NA	NA	NA	NA	NA	NA	NA	NA	NA	ACOR_EC-down
NA	NA	NA	NA	NA	NA	NA	NA	NA	NA	ACOR_EC-down
NA	NA	NA	NA	NA	NA	NA	NA	NA	NA	ACOR_EC-down
NA	NA	NA	NA	NA	NA	NA	NA	NA	NA	ACOR_EC-down
NA	NA	NA	NA	NA	NA	NA	NA	NA	NA	ACOR_EC-down
NA	NA	NA	NA	NA	NA	NA	NA	NA	NA	ACOR_EC-down
NA	NA	NA	NA	NA	NA	NA	NA	NA	NA	ACOR_EC-down
NA	NA	NA	NA	NA	NA	NA	NA	NA	NA	ACOR_EC-down
NA	NA	NA	NA	NA	NA	NA	NA	NA	NA	ACOR_EC-down

NA	NA	NA	NA	NA	NA	NA	NA	NA	NA	ACOR_EC-down
NA	NA	NA	NA	NA	NA	NA	NA	NA	NA	ACOR_EC-down
NA	NA	NA	NA	NA	NA	NA	NA	NA	NA	ACOR_EC-down
NA	NA	NA	NA	NA	NA	NA	NA	NA	NA	ACOR_EC-down
NA	NA	NA	NA	NA	NA	NA	NA	NA	NA	ACOR_EC-down
NA	NA	NA	NA	NA	NA	NA	NA	NA	NA	ACOR_EC-down
NA	NA	NA	NA	NA	NA	NA	NA	NA	NA	ACOR_EC-down
NA	NA	NA	NA	NA	NA	NA	NA	NA	NA	ACOR_EC-down
NA	NA	NA	NA	NA	NA	NA	NA	NA	NA	ACOR_EC-down
NA	NA	NA	NA	NA	NA	NA	NA	NA	NA	ACOR_EC-down
NA	NA	NA	NA	NA	NA	NA	NA	NA	NA	ACOR_EC-down
NA	NA	NA	NA	NA	NA	NA	NA	NA	NA	ACOR_EC-down
NA	NA	NA	NA	NA	NA	NA	NA	NA	NA	ACOR_EC-down
NA	NA	NA	NA	NA	NA	NA	NA	NA	NA	ACOR_EC-down
NA	NA	NA	NA	NA	NA	NA	NA	NA	NA	ACOR_EC-down
NA	NA	NA	NA	NA	NA	NA	NA	NA	NA	ACOR_EC-down
NA	NA	NA	NA	NA	NA	NA	NA	NA	NA	ACOR_EC-down
NA	NA	NA	NA	NA	NA	NA	NA	NA	NA	ACOR_EC-down
NA	NA	NA	NA	NA	NA	NA	NA	NA	NA	ACOR_EC-down
NA	NA	NA	NA	NA	NA	NA	NA	NA	NA	ACOR_EC-down
NA	NA	NA	NA	NA	NA	NA	NA	NA	NA	ACOR_EC-down
NA	NA	NA	NA	NA	NA	NA	NA	NA	NA	ACOR_EC-down
NA	NA	NA	NA	NA	NA	NA	NA	NA	NA	ACOR_EC-down
NA	NA	NA	NA	NA	NA	NA	NA	NA	NA	ACOR_EC-down
NA	NA	NA	NA	NA	NA	NA	NA	NA	NA	ACOR_EC-down
NA	NA	NA	NA	NA	NA	NA	NA	NA	NA	ACOR_EC-down
NA	NA	NA	NA	NA	NA	NA	NA	NA	NA	ACOR_EC-down
NA	NA	NA	NA	NA	NA	NA	NA	NA	NA	ACOR_EC-down
NA	NA	NA	NA	NA	NA	NA	NA	NA	NA	ACOR_EC-down
NA	NA	NA	NA	NA	NA	NA	NA	NA	NA	ACOR_EC-down
NA	NA	NA	NA	NA	NA	NA	NA	NA	NA	ACOR_EC-down
NA	NA	NA	NA	NA	NA	NA	NA	NA	NA	ACOR_EC-down
NA	NA	NA	NA	NA	NA	NA	NA	NA	NA	ACOR_EC-down
NA	NA	NA	NA	NA	NA	NA	NA	NA	NA	ACOR_EC-down
NA	NA	NA	NA	NA	NA	NA	NA	NA	NA	ACOR_EC-down
NA	NA	NA	NA	NA	NA	NA	NA	NA	NA	ACOR_EC-down
NA	NA	NA	NA	NA	NA	NA	NA	NA	NA	ACOR_EC-down
NA	NA	NA	NA	NA	NA	NA	NA	NA	NA	ACOR_EC-down
NA	NA	NA	NA	NA	NA	NA	NA	NA	NA	ACOR_EC-down
NA	NA	NA	NA	NA	NA	NA	NA	NA	NA	ACOR_EC-down
NA	NA	NA	NA	NA	NA	NA	NA	NA	NA	ACOR_EC-down
NA	NA	NA	NA	NA	NA	NA	NA	NA	NA	ACOR_EC-down
NA	NA	NA	NA	NA	NA	NA	NA	NA	NA	ACOR_EC-down
NA	NA	NA	NA	NA	NA	NA	NA	NA	NA	ACOR_EC-down
NA	NA	NA	NA	NA	NA	NA	NA	NA	NA	ACOR_EC-down
NA	NA	NA	NA	NA	NA	NA	NA	NA	NA	ACOR_EC-down
NA	NA	NA	NA	NA	NA	NA	NA	NA	NA	ACOR_EC-down
NA	NA	NA	NA	NA	NA	NA	NA	NA	NA	ACOR_EC-down
NA	NA	NA	NA	NA	NA	NA	NA	NA	NA	ACOR_EC-down
NA	NA	NA	NA	NA	NA	NA	NA	NA	NA	ACOR_EC-down
NA	NA	NA	NA	NA	NA	NA	NA	NA	NA	ACOR_EC-down
NA	NA	NA	NA	NA	NA	NA	NA	NA	NA	ACOR_EC-down
NA	NA	NA	NA	NA	NA	NA	NA	NA	NA	ACOR_EC-down
cfa04217	Necroptosis	9/441	160/7854	0.54729754	0.92381513	0.83164644	403443/490968/100142679/485818/486669/480259/477042/608743/611262	GLUL, STAT5A, STAT6, RBCK1, H2AFJ, , FTL, SPATA2L, TRADD	9	VSAP_EC-up
cfa04630	JAK-STAT signaling pathway	9/441	160/7854	0.54729754	0.92381513	0.83164644	612271/490968/100142679/484757/487235/403924/490006/484648/608847	IL6R, STAT5A, STAT6, CISH, PIK3R1, MYC, SOCS1, RAF1, CCND3	9	VSAP_EC-up
cfa05167	Kaposi sarcoma-associated herpesvirus infection	9/441	178/7854	0.67566623	0.99864063	0.89900663	481746/487235/403924/480272/484648/479248/476412/611939/611262	ITPR3, PIK3R1, MYC, NFATC4, RAF1, PPP3CB, IRF3, MAP2K2, TRADD	9	VSAP_EC-up
cfa04270	Vascular smooth muscle contraction	12/357	133/7854	0.01762346	0.08060564	0.05899828	480515/490455/490722/488438/607269/403424/482663/480271/479409/553102/403558/487514	, NPR1, PRKCH, CALCRL, CALML6, EDN1, GUCY1A1, ADCY4, ARHGEF12, GUCY1B1, PPP1CB, PLA2G4E	12	VCAV_EC-up
cfa04270	Vascular smooth muscle contraction	11/407	133/7854	0.08366203	0.64585296	0.62083088	403962/487441/484429/477625/476548/609934/403424/490230/478514/403928/403817	ADRA1B, NPPB, PTGIR, ADCY6, ITPR1, PRKCE, EDN1, PPP1R12B, CFI, GNAQ, ADM	11	ACOR_VSMC-up

cfa04931	Insulin resistance	9/279	109/7854	0.01520328	0.12584695	0.10668424	477488/485819/482437/491768/477766/485494/478575/478464/491369 PTPN11, TRIB3, SLC2A1, RPS6KA3, PPP1R3C, TBC1D4, GSK3B, MAPK10, CREB3L2	9	VSAP_EC-down
cfa01200	Carbon metabolism	9/279	116/7854	0.02196995	0.15557097	0.13188218	607458/476318/403874/480964/477711/477786/479597/100856683/480622 SHMT2, PSAT1, , PGK1, TPI1, PGAM1, ENO1, ENO2, ALDOC	9	VSAP_EC-down
cfa04919	Thyroid hormone signaling pathway	9/279	116/7854	0.02196995	0.15557097	0.13188218	479975/480348/482437/477032/488437/485494/478575/403788/487309 PRKACB, HIF1A, SLC2A1, CTNNB1, ITGAV, TBC1D4, GSK3B, ITGB3, HDAC1	9	VSAP_EC-down
cfa04722	Neurotrophin signaling pathway	9/279	118/7854	0.02424775	0.16683866	0.14143414	477488/491768/480923/478575/478464/100683018/480416/610764/489050 PTPN11, RPS6KA3, MAGED1, GSK3B, MAPK10, SH2B3, CALM1, CAMK2D, CAMK2G	9	VSAP_EC-down
cfa04910	Insulin signaling pathway	9/279	134/7854	0.04877531	0.25472931	0.21594168	479975/480459/487870/612212/475590/477766/478575/478464/480416 PRKACB, PRKAR1A, EIF4E, MKNK2, EIF4EBP1, PPP1R3C, GSK3B, MAPK10, CALM1	9	VSAP_EC-down
cfa05160	Hepatitis C	9/279	157/7854	0.10627808	0.38701439	0.32808371	477374/403567/486241/477032/478575/480361/474890/100687866/488471 PPP2R2A, CASP3, SCARB1, CTNNB1, GSK3B, EIF2S1, CDKN1A, YWHAH, CFLAR	9	VSAP_EC-down
cfa05202	Transcriptional misregulation in cancer	9/279	172/7854	0.1583437	0.44134096	0.37413798	486685/482840/488575/403641/491597/607439/487309/490720/474890 ETV6, , SLC45A3, ETV4, PDGFA, DDIT3, HDAC1, SIX4, CDKN1A	9	VSAP_EC-down
cfa04020	Calcium signaling pathway	9/279	192/7854	0.24313601	0.56373129	0.47789193	479975/403480/477762/403928/403652/403846/480416/610764/489050 PRKACB, ATP2B1, HTR7, GNAQ, PDE1A, ASPH, CALM1, CAMK2D, CAMK2G	9	VSAP_EC-down
cfa05170	Human immunodeficiency virus 1 infection	9/279	200/7854	0.28085069	0.61044809	0.51749517	478279/476694/403567/607885/476022/403928/478464/480416/612814 , CALR, CASP3, PAK1, CFL1, GNAQ, MAPK10, CALM1, PAK2	9	VSAP_EC-down
cfa04144	Endocytosis	9/279	235/7854	0.45743614	0.73430489	0.62249229	479406/481628/100855651/476150/403703/612343/612505/100856747/480578 HSPA8, TGFBR1, KIF5B, STAM2, TFRC, CBLC, SH3GLB1, , CLTC	9	VSAP_EC-down
cfa05100	Bacterial invasion of epithelial cells	9/357	75/7854	0.00663181	0.04681264	0.03426392	100684969/486934/475108/475294/612913/100856747/474698/612856/490341 HCLS1, DOCK1, PTK2, CAV2, ELMO1, , CTNNA1, ARPC5L, DNM3	9	VCAV_EC-up
cfa04666	Fc gamma R-mediated phagocytosis	9/357	90/7854	0.02063715	0.09139307	0.06689401	100688270/475659/100856747/489692/485874/612856/476331/487514/479473 INPP5D, ASAP2, , PLCG2, PLCG1, ARPC5L, PIP5K1B, PLA2G4E, PLD2	9	VCAV_EC-up
cfa01522	Endocrine resistance	9/357	93/7854	0.02498529	0.10251317	0.07503322	480676/607575/475108/478232/480271/481726/612821/485769/403872 NOTCH1, DLL4, PTK2, MTOR, ADCY4, NOTCH4, MAPK13, JAG1, NRAS	9	VCAV_EC-up
cfa04750	Inflammatory mediator regulation of TRP channels	9/357	100/7854	0.03758353	0.13366125	0.09783167	490722/607269/480271/612821/488126/489692/403558/485874/487514 PRKCH, CALML6, ADCY4, MAPK13, IL1RAP, PLCG2, PPP1CB, PLCG1, PLA2G4E	9	VCAV_EC-up
cfa05224	Breast cancer	9/357	142/7854	0.19703834	0.42615269	0.31191708	403811/480676/481464/607575/478232/481726/485769/611773/403872 KIT, NOTCH1, FLT4, DLL4, MTOR, NOTCH4, JAG1, FGF23, NRAS	9	VCAV_EC-up
cfa04934	Cushing syndrome	9/357	152/7854	0.25435121	0.5142318	0.37638547	480271/475266/486241/478227/483225/607922/475251/488568/403897 ADCY4, CREB5, SCARB1, GNAI1, RAP1A, CREB1, AHR, RBBP5, NR4A1	9	VCAV_EC-up
cfa04390	Hippo signaling pathway	9/357	153/7854	0.26037047	0.51654618	0.37807945	481022/475456/479444/477162/477374/403608/474698/403558/491587 AMOT, SMAD1, PPP2R1B, BMP2, PPP2R2A, PPP2CA, CTNNA1, PPP1CB, PPP2R2D	9	VCAV_EC-up
cfa04630	JAK-STAT signaling pathway	9/357	160/7854	0.30366098	0.57244197	0.41899167	609293/612582/403851/403985/442986/478232/481772/100856077/403623 IL3RA, IL7R, IL2RG, IL6, PDGFB, MTOR, PIM1, IFNGR1, IL13RA1	9	VCAV_EC-up
cfa01521	EGFR tyrosine kinase inhibitor resistance	9/241	80/7854	0.00072086	0.03376005	0.0303518	442985/609956/485864/449021/482666/403857/490439/481954/475590 PDGFRB, , SRC, AKT2, PDGFC, FGF2, SHC1, FOXO3, EIF4EBP1	9	VCAV_EC-down

cfa04270	Vascular smooth muscle contraction	8/279	133/7854	0.10059896	0.38701439	0.32808371	479975/609616/475411/485856/475792/477587/403928/480416 PRKACB, PRKG1, PPP1R12A, MYL9, ACTG2, ACTA2, GNAQ, CALM1	8	VSAP_EC-down
cfa04926	Relaxin signaling pathway	9/241	127/7854	0.01594426	0.23580714	0.21200118	477625/403824/403733/478835/609956/485864/449021/403651/490439 ADCY6, COL1A2, MMP2, COL3A1, , SRC, AKT2, COL1A1, SHC1	9	VCAV_EC-down
cfa04145	Phagosome	9/241	138/7854	0.02586951	0.24158651	0.21719709	608977/610359/479877/486762/489664/488576/404018/404006/475816 ITGB5, THBS3, ATP6V0C, TUBA8, CYBA, RAB7B, SEC61B, SEC61A1, SEC22B	9	VCAV_EC-down
cfa04932	Non-alcoholic fatty liver disease (NAFLD)	9/241	145/7854	0.03410271	0.27379603	0.24615489	609956/449021/611480/612644/485548/478983/486347/476040/486148 , AKT2, , , IRS2, SDHC, UQCR10, COX8A, IRS1	9	VCAV_EC-down
cfa05010	Alzheimer disease	9/241	168/7854	0.07334927	0.38682027	0.34776874	481124/611480/612644/478983/491061/483745/486347/476040/476833 LRP1, , , SDHC, ATP5MC1, CAPN1, UQCR10, COX8A, APBB1	9	VCAV_EC-down
cfa04010	MAPK signaling pathway	9/241	288/7854	0.5265277	0.84753502	0.76197193	442985/403656/449021/482666/486563/611795/612448/403857/481271 PDGFRB, ANGPT1, AKT2, PDGFC, CACNB3, CSF1, RPS6KA2, FGF2, RAC2	9	VCAV_EC-down
cfa04080	Neuroactive ligand-receptor interaction	34943	327/7854	0.01704126	0.5083977	0.50525852	403616/100686684/403858/403599/403963/481142/478047/485056/403817 LEP, NPFFR2, CHRM2, THRB, HTR1D, AVPR1A, NR3C1, TBXA2R, ADM	9	AFEM_EC-up
cfa05412	Arrhythmogenic right ventricular cardiomyopathy (ARVC)	9/407	77/7854	0.01750667	0.33612816	0.32310565	479191/403418/609265/486563/497091/476623/480522/611066/475738 ACTN2, GJA1, SGCA, CACNB3, DES, DAG1, JUP, SGCB, SLC8A1	9	ACOR_VSMC-up
cfa04211	Longevity regulating pathway	9/407	88/7854	0.03784587	0.46370665	0.4457414	489571/479127/477625/485118/481956/611934/488526/480688/403474 PRKAA2, PPARGC1A, ADCY6, SESN3, SESN1, CREB3, PRKAG3, TSC1, CAT	9	ACOR_VSMC-up
cfa04540	Gap junction	9/407	88/7854	0.03784587	0.46370665	0.4457414	403881/482666/100855815/403418/477625/476548/478227/403928/489532 HTR2B, PDGFC, TUBB4A, GJA1, ADCY6, ITPR1, GNAI1, GNAQ, MAPK7	9	ACOR_VSMC-up
cfa04064	NF-kappa B signaling pathway	9/407	93/7854	0.05097917	0.56469236	0.54281466	489692/403416/100855560/488471/486814/491935/403982/609544/102156745 PLCG2, BCL2, DDX58, CFLAR, BLNK, EDA, VCAM1, TIRAP, MAP3K7	9	ACOR_VSMC-up
cfa00564	Glycerophospholipid metabolism	9/407	95/7854	0.05697874	0.60777321	0.58422644	479680/610214/489828/100856443/607530/476045/478514/484993/484502 LCAT, CHPT1, ACHE, PLPP2, CRLS1, PLA2G16, CFI, PNPLA6, PLD3	9	ACOR_VSMC-up
cfa04070	Phosphatidylinositol signaling system	9/407	97/7854	0.06341729	0.62573034	0.60148786	476331/489692/478276/476548/483196/490477/480647/608414/485399 PIP5K1B, PLCG2, PPIP5K1, ITPR1, PI4KB, PIK3C3, INPP5K, , PIK3C2A	9	ACOR_VSMC-up
cfa04726	Serotonergic synapse	9/407	110/7854	0.11618387	0.76328292	0.73371129	403451/488087/403881/403450/476548/610195/478227/403407/403928 MAOB, GNB4, HTR2B, MAOA, ITPR1, CYP2J2, GNAI1, APP, GNAQ	9	ACOR_VSMC-up
cfa04935	Growth hormone synthesis, secretion and action	9/407	116/7854	0.14679601	0.76328292	0.73371129	489692/478061/477625/476548/478227/611934/403928/490969/608125 PLCG2, MAP3K1, ADCY6, ITPR1, GNAI1, CREB3, GNAQ, STAT5B, CRKL	9	ACOR_VSMC-up
cfa04380	Osteoclast differentiation	9/407	125/7854	0.19938872	0.80256807	0.77147442	489692/100856077/611795/486814/481628/608412/607899/100855848/102156745 PLCG2, IFNGR1, CSF1, BLNK, TGFBR1, FOSL2, IRF9, TNFRSF11B, MAP3K7	9	ACOR_VSMC-up
cfa04926	Relaxin signaling pathway	9/407	127/7854	0.21204399	0.80256807	0.77147442	100855494/491749/488087/489428/477625/478227/481628/403424/611934 RXFP1, VEGFD, GNB4, MMP1, ADCY6, GNAI1, TGFBR1, EDN1, CREB3	9	ACOR_VSMC-up
cfa00230	Purine metabolism	9/407	130/7854	0.23160582	0.80256807	0.77147442	403825/477236/477625/481823/403652/489554/485604/477809/611022 PDE5A, ADA, ADCY6, ENPP4, PDE1A, , ENTPD3, NT5C2, PDE9A	9	ACOR_VSMC-up
cfa04728	Dopaminergic synapse	9/407	132/7854	0.24500305	0.81104457	0.77962252	403451/488087/403450/476548/610764/480349/478227/611934/403928 MAOB, GNB4, MAOA, ITPR1, CAMK2D, PPP2R5E, GNAI1, CREB3, GNAQ	9	ACOR_VSMC-up
cfa04270	Vascular smooth muscle contraction	7/441	133/7854	0.62500913	0.97336925	0.87625657	481746/481256/403836/403817/484648/403609/611939 ITPR3, PLA2G6, AGTR1, ADM, RAF1, PPP1CA, MAP2K2	7	VSAP_EC-up

cfa04145	Phagosome	9/407	138/7854	0.28666472	0.82260001	0.79073027	100855815/403400/490477/481037/403729/478797/474836/403941/475236 TUBB4A, CTSS, PIK3C3, LAMP2, ATP6V0E1, DYNC1I2, DLA88, RAB5C, DYNC1I1	9	ACOR_VSMC-up
cfa04390	Hippo signaling pathway	9/407	153/7854	0.39700256	0.87388357	0.84002697	490695/611782/477343/479297/485149/486093/481628/479465/610713 BMP4, CCND2, LATS2, WWC1, FZD4, FZD3, TGFBR1, YAP1, TEAD1	9	ACOR_VSMC-up
cfa04141	Protein processing in endoplasmic reticulum	9/407	160/7854	0.44945399	0.88820767	0.85379612	403416/487898/488956/474419/485628/481509/478116/100683583/482113 BCL2, SEC24B, ERO1B, OS9, STT3B, SAR1B, HERPUD1, TXNDC5, WFS1	9	ACOR_VSMC-up
cfa05225	Hepatocellular carcinoma	9/407	165/7854	0.48656522	0.88820767	0.85379612	489692/479180/485149/474890/477558/475541/486093/481628/100306933 PLCG2, POLK, FZD4, CDKN1A, , SMARCD3, FZD3, TGFBR1, LRP5	9	ACOR_VSMC-up
cfa05202	Transcriptional misregulation in cancer	9/407	172/7854	0.53739752	0.89195014	0.85739359	481556/611782/487501/479180/474890/486564/448804/480522/486073 MLLT3, CCND2, SPINT1, POLK, CDKN1A, CCNT1, FUT8, JUP, HPGD	9	ACOR_VSMC-up
cfa03430	Mismatch repair	9/388	23/7854	7.0429E-07	2.3946E-05	2.2426E-05	100855690/489798/477499/477308/100856421/100686967/478667/477166/610047 RPA1, RFC2, RFC5, RFC3, RPA2, LIG1, RFC4, PCNA, POLD1	9	ACOR_VSMC-down
cfa00480	Glutathione metabolism	9/388	52/7854	0.00088647	0.01607469	0.01505447	481088/476823/403913/442961/482963/477813/475596/609843/478236 G6PD, RRM1, ANPEP, GPX1, RRM2, GSTO1, , TXNDC12, PGD	9	ACOR_VSMC-down
cfa03015	mRNA surveillance pathway	9/388	90/7854	0.03302707	0.32083441	0.30047186	479880/475922/479444/100683271/481806/476066/607948/474839/100856093 RNPS1, EIF4A3, PPP2R1B, DAZAP1, PPP2R5D, CPSF7, ALYREF, DDX39B, WDR33	9	ACOR_VSMC-down
cfa01200	Carbon metabolism	9/388	116/7854	0.11919931	0.75400497	0.70615016	481088/476588/475775/481011/476574/480858/484063/478236/489406 G6PD, TKT, SUCLG1, PRPS1, , PDHA1, ACAT2, PGD, DLAT	9	ACOR_VSMC-down
cfa04152	AMPK signaling pathway	9/388	121/7854	0.14349938	0.82883085	0.77622704	486839/483378/403475/479182/479444/481806/477732/483845/489644 SCD, FASN, SREBF1, HMGCR, PPP2R1B, PPP2R5D, ADIPOR2, CCNA2, PIK3CD	9	ACOR_VSMC-down
cfa04071	Sphingolipid signaling pathway	9/388	122/7854	0.1486418	0.82883085	0.77622704	479444/483329/481806/100683675/403872/484401/403955/489032/489644 PPP2R1B, SPHK1, PPP2R5D, BID, NRAS, SPHK2, RAC1, SGPL1, PIK3CD	9	ACOR_VSMC-down
cfa04120	Ubiquitin mediated proteolysis	9/388	139/7854	0.24859922	0.99972301	0.9362731	403671/608738/475750/403437/606935/609531/100855774/485898/476490 UBE2S, NEDD4, ANAPC1, BRCA1, UBE2J2, UBE2K, CDC20, UBE2C, UBA2	9	ACOR_VSMC-down
cfa05160	Hepatitis C	9/388	157/7854	0.37211341	0.99972301	0.9362731	479444/100302056/481131/100683675/403872/477237/475258/100855704/489644 PPP2R1B, EIF2AK1, CDK4, BID, NRAS, YWHAB, CYCS, CDK2, PIK3CD	9	ACOR_VSMC-down
cfa05164	Influenza A	9/388	161/7854	0.40064043	0.99972301	0.9362731	476452/480129/492093/481131/100683675/480469/475258/474681/489644 HNRNPUL1, FDPS, SLC25A5, CDK4, BID, , CYCS, VDAC1, PIK3CD	9	ACOR_VSMC-down
cfa05034	Alcoholism	9/388	171/7854	0.47186253	0.99972301	0.9362731	442966/403872/479733/611089/478799/100855593/489372/482339/475916 SLC29A1, NRAS, GNB2, HIST1H2BB, HAT1, HIST2H2AC, H2AFX, HDAC9, H3F3B	9	ACOR_VSMC-down
cfa05167	Kaposi sarcoma-associated herpesvirus infection	9/388	178/7854	0.52072973	0.99972301	0.9362731	481131/100683675/403872/475258/403955/479733/481746/480272/489644 CDK4, BID, NRAS, CYCS, RAC1, GNB2, ITPR3, NFATC4, PIK3CD	9	ACOR_VSMC-down
cfa04140	Autophagy - animal	8/131	136/7854	0.00190615	0.11103303	0.10182835	608254/485205/100856339/403416/610064/403556/494005/478564 PRAP1, ATG16L2, , BCL2, TP53INP2, PRKACA, PRKCD,	8	ACOR_EC-up
cfa04014	Ras signaling pathway	8/131	225/7854	0.03419475	0.33468971	0.3069438	100856339/486808/492067/478514/608458/479473/403556/442938 , PLCE1, PAK3, CFI, EFNA5, PLD2, PRKACA, FLT3LG	8	ACOR_EC-up
cfa05168	Herpes simplex virus 1 infection	8/131	359/7854	0.24979142	0.60001444	0.55027301	100856339/403416/100855518/485789/485864/481740/106557467/476728 , BCL2, ZNF853, MAVS, SRC, TAPBP, ZNF229, C3	8	ACOR_EC-up
cfa00620	Pyruvate metabolism	8/441	37/7854	0.00084546	0.0358674	0.03228892	100685468/610390/403978/477205/483704/477002/481486/478991 ACACB, LDHD, DLD, ACSS2, PC, ACSS1, ALDH7A1, ALDH9A1	8	VSAP_EC-up

cfa05216	Thyroid cancer	8/441	38/7854	0.00101933	0.0358674	0.03228892	442858/486894/483626/491278/403924/403494/611939/477750 CDH1, TCF7L2, DDB2, RXRA, MYC, RET, MAP2K2, NCOA4	8 VSAP_EC-up
cfa04270	Vascular smooth muscle contraction	5/131	133/7854	0.07117784	0.36854302	0.33799067	484429/478514/553102/403556/494005 PTGIR, CFI, GUCY1B1, PRKACA, PRKCD	5 ACOR_EC-up
cfa05204	Chemical carcinogenesis	8/441	55/7854	0.01102833	0.1487619	0.13391998	449023/476003/403892/476006/477683/609050/479912/476005 HSD11B1, ALDH3B1, SULT1A1, , MGST1, MGST3, GSTM4, GSTP1	8 VSAP_EC-up
cfa00310	Lysine degradation	8/441	58/7854	0.01500599	0.17141454	0.15431257	100856446/475133/403978/476696/488110/476549/481486/478991 DLST, HYKK, DLD, GCDH, EHHADH, SETMAR, ALDH7A1, ALDH9A1	8 VSAP_EC-up
cfa00561	Glycerolipid metabolism	8/441	59/7854	0.01653772	0.175418	0.15791661	478320/485876/479557/403626/483800/481486/478991/475585 LIPC, LPIN3, PLPP3, LPL, TKFC, ALDH7A1, ALDH9A1, PLPP5	8 VSAP_EC-up
cfa05217	Basal cell carcinoma	8/441	63/7854	0.02379966	0.21419692	0.19282658	482294/485183/490903/486894/483626/485149/484137/490099 FZD1, WNT11, AXIN2, TCF7L2, DDB2, FZD4, PTCH1, AXIN1	8 VSAP_EC-up
cfa04137	Mitophagy - animal	8/441	64/7854	0.02591749	0.21992842	0.19798625	483634/487439/484372/491872/102154798/479893/477437/484010 AMBRA1, MFN2, TBC1D17, TFE3, ATG9A, RHOT2, ULK1, CITED2	8 VSAP_EC-up
cfa03320	PPAR signaling pathway	8/441	80/7854	0.07892724	0.41125246	0.37022196	476724/477023/610489/491278/403626/474666/488110/475631 ANGPTL4, ACAA1, CYP27A1, RXRA, LPL, SLC27A6, EHHADH, ACSL1	8 VSAP_EC-up
cfa00564	Glycerophospholipid metabolism	8/441	95/7854	0.16353552	0.61481778	0.55347764	478238/480663/485876/479557/481256/612975/479680/475585 LPCAT4, PNPLA7, LPIN3, PLPP3, PLA2G6, TAZ, LCAT, PLPP5	8 VSAP_EC-up
cfa05231	Choline metabolism in cancer	8/441	96/7854	0.17029499	0.62441498	0.56211733	477331/479460/485054/479557/474792/487235/484648/611939 WASF3, PDGFD, PIP5K1C, PLPP3, SLC44A1, PIK3R1, RAF1, MAP2K2	8 VSAP_EC-up
cfa04070	Phosphatidylinositol signaling system	8/441	97/7854	0.17717917	0.62645494	0.56395376	481746/607443/485054/478276/487235/491034/492211/486443 ITPR3, INPP5E, PIP5K1C, PPIP5K1, PIK3R1, PIP4K2B, MTMR1, PI4KA	8 VSAP_EC-up
cfa05215	Prostate cancer	8/441	97/7854	0.17717917	0.62645494	0.56395376	486894/479460/476006/487235/477966/476005/484648/611939 TCF7L2, PDGFD, , PIK3R1, ZEB1, GSTP1, RAF1, MAP2K2	8 VSAP_EC-up
cfa04935	Growth hormone synthesis, secretion and action	8/441	116/7854	0.32645523	0.80885538	0.72815618	490069/481746/486148/490968/487235/490006/484648/611939 IGFALS, ITPR3, IRS1, STAT5A, PIK3R1, SOCS1, RAF1, MAP2K2	8 VSAP_EC-up
cfa04114	Oocyte meiosis	8/441	117/7854	0.33498051	0.80885538	0.72815618	481746/491236/612448/483755/481679/490622/403609/479248 ITPR3, ANAPC2, RPS6KA2, PPP2R5B, CDC26, REC8, PPP1CA, PPP3CB	8 VSAP_EC-up
cfa05135	Yersinia infection	8/441	117/7854	0.33498051	0.80885538	0.72815618	485054/487235/612448/477024/481245/488409/476412/611939 PIP5K1C, PIK3R1, RPS6KA2, MYD88, TAB1, WIPF1, IRF3, MAP2K2	8 VSAP_EC-up
cfa04380	Osteoclast differentiation	8/441	125/7854	0.40406621	0.86594207	0.77954735	490926/487235/490006/481245/609452/489664/479248/477039 MAP3K14, PIK3R1, SOCS1, TAB1, , CYBA, PPP3CB, TGFBR2	8 VSAP_EC-up
cfa04068	FoxO signaling pathway	8/441	127/7854	0.42142185	0.87526076	0.78793632	486148/476258/478127/487235/486295/484648/611939/477039 IRS1, SOD2, RBL2, PIK3R1, PRKAB1, RAF1, MAP2K2, TGFBR2	8 VSAP_EC-up
cfa00010	Glycolysis / Gluconeogenesis	8/279	62/7854	0.00146482	0.02952173	0.02502646	476882/403874/480964/477711/477786/479597/100856683/480622 , , PGK1, TPI1, PGAM1, ENO1, ENO2, ALDOC	8 VSAP_EC-down
cfa05211	Renal cell carcinoma	8/279	67/7854	0.00242529	0.03971411	0.03366685	403802/607885/477488/480348/482437/477952/612814/474890 VEGFA, PAK1, PTPN11, HIF1A, SLC2A1, CUL2, PAK2, CDKN1A	8 VSAP_EC-down
cfa04520	Adherens junction	8/279	70/7854	0.0032033	0.04662583	0.03952611	480369/486940/481628/403917/484526/477032/479252/476948 ACTN1, SNAI2, TGFBR1, YES1, ACTN4, CTNNB1, VCL, LMO7	8 VSAP_EC-down
cfa04012	ErbB signaling pathway	8/279	85/7854	0.01035327	0.10046508	0.08516727	607885/475590/478575/478464/612814/610764/474890/489050 PAK1, EIF4EBP1, GSK3B, MAPK10, PAK2, CAMK2D, CDKN1A, CAMK2G	8 VSAP_EC-down

cfa05215	Prostate cancer	8/279	97/7854	0.02164766	0.15557097	0.13188218	404019/482840/491597/477032/480438/478575/491369/474890 HSP90B1, , PDGFA, CTNNB1, HSP90AA1, GSK3B, CREB3L2, CDKN1A	8 VSAP_EC-down
cfa04916	Melanogenesis	8/279	100/7854	0.02547155	0.16683866	0.14143414	479975/403928/477032/478575/480416/491369/610764/489050 PRKACB, GNAQ, CTNNB1, GSK3B, CALM1, CREB3L2, CAMK2D, CAMK2G	8 VSAP_EC-down
cfa04068	FoxO signaling pathway	8/279	127/7854	0.08212014	0.3573146	0.30290631	475380/403647/481628/607408/478464/476411/474890/488933 PLK3, SGK1, TGFBR1, BNIP3, MAPK10, PRMT1, CDKN1A, HOMER1	8 VSAP_EC-down
cfa03040	Spliceosome	8/279	132/7854	0.09736835	0.38701439	0.32808371	479406/477148/476152/100855927/474839/479743/475805/607102 HSPA8, SNRPB2, PRPF40A, PRPF3, DDX39B, , BCAS2, SNRPA1	8 VSAP_EC-down
cfa04270	Vascular smooth muscle contraction	34759	133/7854	0.21737171	0.81029448	0.80529119	481142/403817/612579 AVPR1A, ADM, PPP1R14A	3 AFEM_EC-up
cfa04140	Autophagy - animal	8/279	136/7854	0.11064826	0.38701439	0.32808371	479975/111097413/480348/607408/612505/478464/480361/488471 PRKACB, VMP1, HIF1A, BNIP3, SH3GLB1, MAPK10, EIF2S1, CFLAR	8 VSAP_EC-down
cfa04310	Wnt signaling pathway	8/279	151/7854	0.16855783	0.46009869	0.39003947	100856007/479975/477032/478575/490714/478464/610764/489050 PRICKLE1, PRKACB, CTNNB1, GSK3B, DAAM1, MAPK10, CAMK2D, CAMK2G	8 VSAP_EC-down
cfa04390	Hippo signaling pathway	8/279	153/7854	0.17717557	0.47367346	0.4015472	477374/403476/486940/488036/481628/477032/478575/100687866 PPP2R2A, SERPINE1, SNAI2, DLG1, TGFBR1, CTNNB1, GSK3B, YWHAH	8 VSAP_EC-down
cfa05211	Renal cell carcinoma	8/357	67/7854	0.01054842	0.06261723	0.04583189	442986/491286/489287/483225/612814/403872/480348/608366 PDGFB, RAPGEF1, ETS1, RAP1A, PAK2, NRAS, HIF1A, ARNT2	8 VCAV_EC-up
cfa05133	Pertussis	8/357	71/7854	0.01471926	0.07640893	0.05592655	403850/403985/403417/607269/489673/478227/612821/403822 CXCL8, IL6, TLR4, CALML6, IRF8, GNAI1, MAPK13, NOS2	8 VCAV_EC-up
cfa00562	Inositol phosphate metabolism	8/357	72/7854	0.01592642	0.07640893	0.05592655	100688270/485399/490383/606918/489692/485874/477925/476331 INPP5D, PIK3C2A, ITPKB, MTMR2, PLCG2, PLCG1, IMPA1, PIP5K1B	8 VCAV_EC-up
cfa05412	Arrhythmogenic right ventricular cardiomyopathy (ARVC)	8/357	77/7854	0.02305336	0.09745283	0.07132937	403860/490755/490500/606758/474698/100856563/403788/479797 DSC2, SLC8A3, DSG2, DMD, CTNNA1, ITGA6, ITGB3, ATP2A1	8 VCAV_EC-up
cfa01521	EGFR tyrosine kinase inhibitor resistance	8/357	80/7854	0.02827236	0.1095554	0.08018769	403985/442986/482154/478232/489692/485874/403872/479460 IL6, PDGFB, KDR, MTOR, PLCG2, PLCG1, NRAS, PDGFD	8 VCAV_EC-up
cfa04657	IL-17 signaling pathway	8/357	86/7854	0.04104781	0.14145163	0.10353373	403850/403985/484006/478311/488473/481145/612821/442942 CXCL8, IL6, TNFAIP3, MAPK6, CASP8, TBK1, MAPK13, PTGS2	8 VCAV_EC-up
cfa04540	Gap junction	8/357	88/7854	0.04604056	0.15292044	0.11192819	442986/482663/480271/403752/478227/553102/403872/479460 PDGFB, GUCY1A1, ADCY4, TJP1, GNAI1, GUCY1B1, NRAS, PDGFD	8 VCAV_EC-up
cfa04270	Vascular smooth muscle contraction	42767	133/7854	0.03278359	0.32364194	0.30122906	481142/482994 AVPR1A, ADCY3	2 VFEM_EC-up
cfa05222	Small cell lung cancer	8/357	92/7854	0.0571804	0.18128785	0.13269135	442939/403466/475108/442942/403822/482620/100856563/475034 LAMC2, COL4A5, PTK2, PTGS2, NOS2, APAF1, ITGA6, LAMA4	8 VCAV_EC-up
cfa05215	Prostate cancer	8/357	97/7854	0.07332779	0.22731614	0.16638118	442986/478232/475266/487760/607922/403872/479460/477966 PDGFB, MTOR, CREB5, ERG, CREB1, NRAS, PDGFD, ZEB1	8 VCAV_EC-up
cfa04916	Melanogenesis	8/357	100/7854	0.08421899	0.24733787	0.18103583	403862/403811/607269/403424/480271/478227/607922/403872 EDNRB, KIT, CALML6, EDN1, ADCY4, GNAI1, CREB1, NRAS	8 VCAV_EC-up
cfa04660	T cell receptor signaling pathway	8/357	102/7854	0.09198117	0.25737195	0.18838015	403486/485930/612821/612814/485677/490183/485874/403872 PPP3CA, NFATC2, MAPK13, PAK2, NCK1, BCL10, PLCG1, NRAS	8 VCAV_EC-up
cfa04931	Insulin resistance	8/357	109/7854	0.1222566	0.31008719	0.2269644	403985/485494/403784/478232/475266/607922/403558/491768 IL6, TBC1D4, NOS3, MTOR, CREB5, CREB1, PPP1CB, RPS6KA3	8 VCAV_EC-up

cfa04380	Osteoclast differentiation	8/357	125/7854	0.20803402	0.44647302	0.32679029	403606/403486/485930/612821/100856077/489692/607922/403788 PPARG, PPP3CA, NFATC2, MAPK13, IFNGR1, PLCG2, CREB1, ITGB3	8	VCAV_EC-up
cfa04140	Autophagy - animal	8/357	136/7854	0.27756395	0.54154085	0.39637399	478232/481145/488471/612505/403608/403170/403872/480348 MTOR, TBK1, CFLAR, SH3GLB1, PPP2CA, HMGB1, NRAS, HIF1A	8	VCAV_EC-up
cfa04723	Retrograde endocannabinoid signaling	8/357	149/7854	0.36623987	0.62687683	0.45883458	403617/480271/478227/100855806/612821/488355/442942/607513 GNGT2, ADCY4, GNAI1, GNG11, MAPK13, KCNJ3, PTGS2, GNG4	8	VCAV_EC-up
cfa04213	Longevity regulating pathway - multiple species	8/241	59/7854	0.00040096	0.02816767	0.02532399	477625/609956/610130/449021/485548/486148/476815/481954 ADCY6, , AKT1S1, AKT2, IRS2, IRS1, CLPB, FOXO3	8	VCAV_EC-down
cfa04211	Longevity regulating pathway	8/241	88/7854	0.00542868	0.10896145	0.09796123	477625/609956/610130/449021/485548/486148/481954/475590 ADCY6, , AKT1S1, AKT2, IRS2, IRS1, FOXO3, EIF4EBP1	8	VCAV_EC-down
cfa04974	Protein digestion and absorption	8/241	91/7854	0.00663738	0.12434019	0.11178739	403824/607775/478835/403668/100856491/403651/403582/483777 COL1A2, ELN, COL3A1, COL6A1, COL6A2, COL1A1, COL6A3, SLC3A2	8	VCAV_EC-down
cfa04722	Neurotrophin signaling pathway	8/241	118/7854	0.02837137	0.24158651	0.21719709	609956/449021/479915/612448/486148/610764/490439/481954 , AKT2, SORT1, RPS6KA2, IRS1, CAMK2D, SHC1, FOXO3	8	VCAV_EC-down
cfa04270	Vascular smooth muscle contraction	42736	133/7854	0.25221729	0.31249863	0.27046665	606992 MYL6B	1	APUL_EC-up
cfa04068	FoxO signaling pathway	8/241	127/7854	0.0412468	0.32195419	0.28945123	609956/479479/449021/485548/490942/486148/611782/481954 , GABARAP, AKT2, IRS2, G6PC3, IRS1, CCND2, FOXO3	8	VCAV_EC-down
cfa04218	Cellular senescence	8/241	151/7854	0.09267571	0.40842728	0.36719441	609956/449021/608847/483745/490748/611782/481954/475590 , AKT2, CCND3, CAPN1, ZFP36L1, CCND2, FOXO3, EIF4EBP1	8	VCAV_EC-down
cfa04810	Regulation of actin cytoskeleton	8/241	211/7854	0.32116811	0.69421722	0.62413235	442985/608977/609956/485864/482666/403857/474417/481271 PDGFRB, ITGB5, , SRC, PDGFC, FGF2, PIP4K2C, RAC2	8	VCAV_EC-down
cfa04144	Endocytosis	8/241	235/7854	0.43357553	0.79541394	0.71511274	476816/485864/488409/489104/475994/489597/608485/100856651 FOLR2, SRC, WIPF1, CLTB, AP2A2, ACAP3, RAB31, RAB11FIP5	8	VCAV_EC-down
cfa04611	Platelet activation	15/357	121/7854	0.00036164	0.00560542	0.00410282	399544/403784/403544/482663/478268/480271/478227/612821/479409/483225/553102/489692/403558/403788/487514 VWF, NOS3, PTGS1, GUCY1A1, SNAP23, ADCY4, GNAI1, MAPK13, ARHGEF12, RAP1A, GUCY1B1, PLCG2, PPP1CB, ITGB3, PLA2G4E	15	VCAV_EC-up
cfa04666	Fc gamma R-mediated phagocytosis	8/407	90/7854	0.09387054	0.6931978	0.66634145	476331/489692/477331/100856443/612998/609934/482044/608125 PIP5K1B, PLCG2, WASF3, PLPP2, MARCKSL1, PRKCE, ASAP1, CRKL	8	ACOR_VSMC-up
cfa04611	Platelet activation	12/279	121/7854	0.00117902	0.02574197	0.02182224	403936/479975/489208/609616/475411/477956/403928/477886/100856393/403788/607207/492019 VASP, PRKACB, ITGA2, PRKG1, PPP1R12A, ITGB1, GNAQ, LYN, TLN1, ITGB3, , BTK	12	VSAP_EC-down
cfa04916	Melanogenesis	8/407	100/7854	0.14624699	0.76328292	0.73371129	477625/485149/610764/478227/486093/403424/611934/403928 ADCY6, FZD4, CAMK2D, GNAI1, FZD3, EDN1, CREB3, GNAQ	8	ACOR_VSMC-up
cfa04933	AGE-RAGE signaling pathway in diabetic complications	8/407	100/7854	0.14624699	0.76328292	0.73371129	491749/489692/403416/403982/609934/481628/403424/490969 VEGFD, PLCG2, BCL2, VCAM1, PRKCE, TGFBR1, EDN1, STAT5B	8	ACOR_VSMC-up
cfa04928	Parathyroid hormone synthesis, secretion and action	8/407	107/7854	0.1898534	0.80256807	0.77147442	403416/477625/476548/474890/478227/611934/403928/100306933 BCL2, ADCY6, ITPR1, CDKN1A, GNAI1, CREB3, GNAQ, LRP5	8	ACOR_VSMC-up
cfa04910	Insulin signaling pathway	8/407	134/7854	0.39253289	0.87388357	0.84002697	489571/479127/403607/611078/611993/488526/480688/608125 PRKAA2, PPARGC1A, LIPE, PYGM, GYS1, PRKAG3, TSC1, CRKL	8	ACOR_VSMC-up
cfa04550	Signaling pathways regulating pluripotency of stem cells	8/407	135/7854	0.40052997	0.87388357	0.84002697	486794/490695/415125/403545/485149/483880/486093/403547 PCGF5, BMP4, FGFR2, IL6ST, FZD4, INHBB, FZD3, ID3	8	ACOR_VSMC-up

cfa04514	Cell adhesion molecules (CAMs)	8/407	139/7854	0.43249944	0.88820767	0.85379612	488565/484186/492244/403982/607997/474836/489271/477945 NFASC, CD274, L1CAM, VCAM1, LRRC4, DLA88, JAM3, SDC2	8 ACOR_VSMC-up
cfa04630	JAK-STAT signaling pathway	8/407	160/7854	0.5932902	0.90408536	0.86905866	100856077/403616/403416/611782/403545/474890/607899/490969 IFNGR1, LEP, BCL2, CCND2, IL6ST, CDKN1A, IRF9, STAT5B	8 ACOR_VSMC-up
cfa05164	Influenza A	8/407	161/7854	0.60042103	0.90773643	0.87256828	100856077/403745/482905/100855560/100174780/403522/607899/611619 IFNGR1, MX1, TLR3, DDX58, TNFSF10, CCL5, IRF9, RAB11B	8 ACOR_VSMC-up
cfa00900	Terpenoid backbone biosynthesis	8/388	21/7854	3.8083E-06	0.0001151	0.00010779	479182/480129/479344/489663/100687634/484063/484102/607944 HMGCR, FDPS, , MVD, , ACAT2, NUS1, PDSS1	8 ACOR_VSMC-down
cfa00240	Pyrimidine metabolism	8/388	52/7854	0.00367343	0.052588	0.04925037	483009/476823/100855947/607417/482963/482188/609526/100687720 CAD, RRM1, TK1, TYMS, RRM2, DCK, DUT, CTPS1	8 ACOR_VSMC-down
cfa01524	Platinum drug resistance	8/388	72/7854	0.0249189	0.25103488	0.23510232	442936/100683675/403437/474915/475258/477813/480525/489644 , BID, BRCA1, POLH, CYCS, GSTO1, TOP2A, PIK3CD	8 ACOR_VSMC-down
cfa03018	RNA degradation	8/388	78/7854	0.03790565	0.35552884	0.33296432	487647/474697/488057/489751/485491/609536/478233/611350 EDC3, HSPA9, TENT4A, EDC4, DIS3, DCPS, EXOSC10, EXOSC5	8 ACOR_VSMC-down
cfa00230	Purine metabolism	8/388	130/7854	0.3129313	0.99972301	0.9362731	476823/489490/610836/482963/482188/481011/488752/477123 RRM1, PFAS, PDE3A, RRM2, DCK, PRPS1, PDE8A, GMPS	8 ACOR_VSMC-down
cfa04611	Platelet activation	8/241	121/7854	0.03230212	0.26696755	0.24001578	477625/403824/478331/478835/609956/485864/449021/403651 ADCY6, COL1A2, TLN2, COL3A1, , SRC, AKT2, COL1A1	8 VCAV_EC-down
cfa05162	Measles	8/388	136/7854	0.35789315	0.99972301	0.9362731	492247/100302056/481131/100683675/475258/100855704/489620/489644 IRAK1, EIF2AK1, CDK4, BID, CYCS, CDK2, TP73, PIK3CD	8 ACOR_VSMC-down
cfa05012	Parkinson disease	8/388	138/7854	0.37305553	0.99972301	0.9362731	492093/606935/475258/474681/475121/480149/478807/476756 SLC25A5, UBE2J2, CYCS, VDAC1, CYC1, ATP5F1A, , ATP5F1D	8 ACOR_VSMC-down
cfa04141	Protein processing in endoplasmic reticulum	8/388	160/7854	0.53811501	0.99972301	0.9362731	476694/403809/100302056/483007/403948/606935/484922/476275 CALR, RRBP1, EIF2AK1, PREB, TRAM1, UBE2J2, RAD23A, MAN1A1	8 ACOR_VSMC-down
cfa04217	Necroptosis	8/388	160/7854	0.53811501	0.99972301	0.9362731	403170/492093/486221/100683675/474681/481048/100855593/489372 HMGB1, SLC25A5, PGAM5, BID, VDAC1, AIFM1, HIST2H2AC, H2AFX	8 ACOR_VSMC-down
cfa05152	Tuberculosis	8/388	171/7854	0.61446917	0.99972301	0.9362731	492247/474697/483329/475380/100683675/484401/475258/489949 IRAK1, HSPA9, SPHK1, PLK3, BID, SPHK2, CYCS, CORO1A	8 ACOR_VSMC-down
cfa04510	Focal adhesion	8/388	198/7854	0.76959924	0.99972301	0.9362731	484713/607885/403980/403788/403955/475882/489208/489644 FLNB, PAK1, CAV1, ITGB3, RAC1, LAMB1, ITGA2, PIK3CD	8 ACOR_VSMC-down
cfa04015	Rap1 signaling pathway	8/388	209/7854	0.81807348	0.99972301	0.9362731	612583/403872/403788/403955/608459/483036/483730/489644 SIPA1L3, NRAS, ITGB3, RAC1, FGF5, PRKD3, SIPA1, PIK3CD	8 ACOR_VSMC-down
cfa04921	Oxytocin signaling pathway	7/131	151/7854	0.01297182	0.25186945	0.23098938	100855763/609157/403756/553102/484665/485864/403556 , GNAO1, CD38, GUCY1B1, CAMK1, SRC, PRKACA	7 ACOR_EC-up
cfa04020	Calcium signaling pathway	7/131	192/7854	0.04126992	0.36641541	0.33603943	489198/403652/486808/403756/489784/484665/403556 HTR4, PDE1A, PLCE1, CD38, PHKG1, CAMK1, PRKACA	7 ACOR_EC-up
cfa04060	Cytokine-cytokine receptor interaction	7/131	270/7854	0.16433377	0.52441163	0.48093771	484943/403810/481588/486042/489632/606826/483880 EPOR, IL33, IL11RA, TNFRSF19, TNFRSF25, GDF11, INHBB	7 ACOR_EC-up
cfa00020	Citrate cycle (TCA cycle)	7/441	29/7854	0.00088924	0.0358674	0.03228892	100856446/478634/403978/483704/478889/479066/477177 DLST, SDHA, DLD, PC, IDH1, IDH3A,	7 VSAP_EC-up
cfa00410	beta-Alanine metabolism	7/441	30/7854	0.0011047	0.0358674	0.03228892	476003/490779/479856/477517/488110/481486/478991 ALDH3B1, ALDH6A1, ABAT, ACADS, EHHADH, ALDH7A1, ALDH9A1	7 VSAP_EC-up
cfa00760	Nicotinate and nicotinamide metabolism	7/441	37/7854	0.00397699	0.07874438	0.07088808	488820/489589/609412/477809/483674/483309/475393 BST1, NADK, NAPRT, NT5C2, NADSYN1, NT5C, PNP	7 VSAP_EC-up

cfa00071	Fatty acid degradation	7/441	41/7854	0.00715895	0.11190575	0.10074095	477023/476696/477517/488110/481486/478991/475631 ACAA1, GCDH, ACADS, EHHADH, ALDH7A1, ALDH9A1, ACSL1	7	VSAP_EC-up
cfa00380	Tryptophan metabolism	7/441	41/7854	0.00715895	0.11190575	0.10074095	100856446/403978/476696/488110/481486/478991/482846 DLST, DLD, GCDH, EHHADH, ALDH7A1, ALDH9A1, IDO2	7	VSAP_EC-up
cfa00982	Drug metabolism - cytochrome P450	7/441	49/7854	0.01863283	0.19082591	0.17178728	476003/476006/477683/609050/475819/479912/476005 ALDH3B1, , MGST1, MGST3, FMO5, GSTM4, GSTP1	7	VSAP_EC-up
cfa00983	Drug metabolism - other enzymes	7/441	63/7854	0.06181817	0.39063824	0.3516644	476006/477683/609050/479912/483028/476005/489769 , MGST1, MGST3, GSTM4, XDH, GSTP1, CES2	7	VSAP_EC-up
cfa04929	GnRH secretion	7/441	65/7854	0.07082093	0.41125246	0.37022196	481746/487235/485189/481627/484464/484648/611939 ITPR3, PIK3R1, ARRB1, GABBR2, KCNN4, RAF1, MAP2K2	7	VSAP_EC-up
cfa04720	Long-term potentiation	7/441	66/7854	0.07560002	0.41125246	0.37022196	481746/612448/484648/486591/403609/479248/611939 ITPR3, RPS6KA2, RAF1, RAPGEF3, PPP1CA, PPP3CB, MAP2K2	7	VSAP_EC-up
cfa04920	Adipocytokine signaling pathway	7/441	70/7854	0.09655853	0.47012924	0.42322462	100685468/486148/491278/486263/486295/475631/611262 ACACB, IRS1, RXRA, CAMKK2, PRKAB1, ACSL1, TRADD	7	VSAP_EC-up
cfa01524	Platinum drug resistance	7/441	72/7854	0.10812701	0.48657156	0.4380265	476215/476006/477683/609050/479912/487235/476005 MAP3K5, , MGST1, MGST3, GSTM4, PIK3R1, GSTP1	7	VSAP_EC-up
cfa05218	Melanoma	7/441	73/7854	0.11417727	0.49868604	0.44893232	442858/403915/479460/483626/487235/484648/611939 CDH1, FGF7, PDGFD, DDB2, PIK3R1, RAF1, MAP2K2	7	VSAP_EC-up
cfa04012	ErbB signaling pathway	7/441	85/7854	0.19938498	0.66536335	0.5989803	491292/490968/487235/403924/484648/485677/611939 ABL1, STAT5A, PIK3R1, MYC, RAF1, NCK1, MAP2K2	7	VSAP_EC-up
cfa04211	Longevity regulating pathway	7/441	88/7854	0.22381905	0.70424983	0.63398709	481956/486148/476258/486263/487235/486295/477437 SESN1, IRS1, SOD2, CAMKK2, PIK3R1, PRKAB1, ULK1	7	VSAP_EC-up
cfa05235	PD-L1 expression and PD-1 checkpoint pathway in cancer	7/441	89/7854	0.23218907	0.70505074	0.63470809	484186/487235/483754/477024/484648/479248/611939 CD274, PIK3R1, BATF2, MYD88, RAF1, PPP3CB, MAP2K2	7	VSAP_EC-up
cfa04919	Thyroid hormone signaling pathway	7/441	116/7854	0.47845662	0.92381513	0.83164644	403599/491278/487235/403924/484648/486989/611939 THRB, RXRA, PIK3R1, MYC, RAF1, NCOA2, MAP2K2	7	VSAP_EC-up
cfa04611	Platelet activation	7/407	121/7854	0.43843286	0.88820767	0.85379612	489692/484429/477625/476548/478227/612101/403928 PLCG2, PTGIR, ADCY6, ITPR1, GNAI1, RASGRP2, GNAQ	7	ACOR_VSMC-up
cfa04371	Apelin signaling pathway	7/441	135/7854	0.64071275	0.99110253	0.8922206	481746/442858/403836/486295/403607/484648/611939 ITPR3, CDH1, AGTR1, PRKAB1, LIPE, RAF1, MAP2K2	7	VSAP_EC-up
cfa05162	Measles	7/441	136/7854	0.64842103	0.9926858	0.89364591	490968/487235/477024/485789/476412/608847/611262 STAT5A, PIK3R1, MYD88, MAVS, IRF3, CCND3, TRADD	7	VSAP_EC-up
cfa04612	Antigen processing and presentation	7/279	54/7854	0.00280335	0.04320457	0.03662581	478279/480726/476694/479406/403908/474680/480438 , HSPA5, CALR, HSPA8, CANX, HSPA4, HSP90AA1	7	VSAP_EC-down
cfa00970	Aminoacyl-tRNA biosynthesis	7/279	66/7854	0.00859738	0.09010056	0.07638095	475998/490124/100856659/479656/478148/479370/607631 CARS, SARS, GARS, AARS, YARS, TARS, NARS	7	VSAP_EC-down
cfa04918	Thyroid hormone synthesis	7/279	72/7854	0.01359405	0.11872133	0.10064363	480726/479975/404019/403908/482715/403928/491369 HSPA5, PRKACB, HSP90B1, CANX, PDIA4, GNAQ, CREB3L2	7	VSAP_EC-down
cfa05412	Arrhythmogenic right ventricular cardiomyopathy (ARVC)	7/279	77/7854	0.01912321	0.1473612	0.12492251	489208/608287/477956/606758/477032/488437/403788 ITGA2, ITGA10, ITGB1, DMD, CTNNB1, ITGAV, ITGB3	7	VSAP_EC-down
cfa04657	IL-17 signaling pathway	7/279	86/7854	0.03272787	0.19054894	0.16153406	478311/404019/403567/442942/480438/478575/478464 MAPK6, HSP90B1, CASP3, PTGS2, HSP90AA1, GSK3B, MAPK10	7	VSAP_EC-down
cfa05142	Chagas disease (American trypanosomiasis)	7/279	103/7854	0.07327682	0.34283086	0.29062801	477374/476694/403476/481628/403928/478464/488471 PPP2R2A, CALR, SERPINE1, TGFBR1, GNAQ, MAPK10, CFLAR	7	VSAP_EC-down
cfa05135	Yersinia infection	7/279	117/7854	0.12223297	0.4066908	0.344764	477956/491768/482366/403702/478575/478464/481396 ITGB1, RPS6KA3, SKAP2, ACTR3, GSK3B, MAPK10, ACTR2	7	VSAP_EC-down
cfa04611	Platelet activation	5/131	121/7854	0.05137804	0.36641541	0.33603943	100856339/484429/553102/485864/403556 , PTGIR, GUCY1B1, SRC, PRKACA	5	ACOR_EC-up
cfa04371	Apelin signaling pathway	7/279	135/7854	0.20398316	0.51886979	0.43986149	403476/479975/482840/481628/477587/403928/480416 SERPINE1, PRKACB, , TGFBR1, ACTA2, GNAQ, CALM1	7	VSAP_EC-down
cfa04150	mTOR signaling pathway	7/279	152/7854	0.29554401	0.62195513	0.52725003	403647/487335/487870/491768/475590/478575/607653 SGK1, SESN2, EIF4E, RPS6KA3, EIF4EBP1, GSK3B, SEC13	7	VSAP_EC-down

cfa04630	JAK-STAT signaling pathway	7/279	160/7854	0.34150897	0.67274699	0.57030782	474581/477488/403623/490563/476150/491597/474890 SOCS5, PTPN11, IL13RA1, PTPN2, STAM2, PDGFA, CDKN1A	7	VSAP_EC-down
cfa05010	Alzheimer disease	7/279	168/7854	0.38830591	0.71904705	0.60955776	403567/607407/475662/403928/476438/478575/480416 CASP3, NDUFA4L2, ADAM17, GNAQ, APOE, GSK3B, CALM1	7	VSAP_EC-down
cfa05144	Malaria	7/357	49/7854	0.00624431	0.04584635	0.03355666	100856110/403994/403999/403850/403985/403417/609898 ACKR1, SELP, SELE, CXCL8, IL6, TLR4, GYPC	7	VCAV_EC-up
cfa05134	Legionellosis	7/357	50/7854	0.00698509	0.04753266	0.03479093	403850/403985/403417/488473/102155289/479406/482620 CXCL8, IL6, TLR4, CASP8, , HSPA8, APAF1	7	VCAV_EC-up
cfa04730	Long-term depression	7/357	60/7854	0.01837041	0.08266684	0.06050695	479444/482663/478227/553102/403608/403872/487514 PPP2R1B, GUCY1A1, GNAI1, GUCY1B1, PPP2CA, NRAS, PLA2G4E	7	VCAV_EC-up
cfa04720	Long-term potentiation	7/357	66/7854	0.02944373	0.11253151	0.08236602	607269/403486/486591/483225/403558/403872/491768 CALML6, PPP3CA, RAPGEF3, RAP1A, PPP1CB, NRAS, RPS6KA3	7	VCAV_EC-up
cfa04622	RIG-I-like receptor signaling pathway	7/357	67/7854	0.03164818	0.1193222	0.08733638	403850/606815/488473/481145/612821/488472/102152063 CXCL8, TMEM173, CASP8, TBK1, MAPK13, CASP10, RIPK1	7	VCAV_EC-up
cfa04662	B cell receptor signaling pathway	7/357	72/7854	0.04432396	0.1489926	0.10905326	611329/100688270/403486/485930/489692/490183/403872 RASGRP3, INPP5D, PPP3CA, NFATC2, PLCG2, BCL10, NRAS	7	VCAV_EC-up
cfa04970	Salivary secretion	7/357	78/7854	0.06334833	0.19858634	0.14535277	474442/403480/607269/482663/480271/477098/553102 LYZ, ATP2B1, CALML6, GUCY1A1, ADCY4, ATP1B3, GUCY1B1	7	VCAV_EC-up
cfa04012	ErbB signaling pathway	7/357	85/7854	0.09097885	0.25737195	0.18838015	475108/478232/612814/485677/489692/485874/403872 PTK2, MTOR, PAK2, NCK1, PLCG2, PLCG1, NRAS	7	VCAV_EC-up
cfa04211	Longevity regulating pathway	7/357	88/7854	0.10460331	0.28061851	0.20539517	403606/478232/480271/475266/607922/477732/403872 PPARG, MTOR, ADCY4, CREB5, CREB1, ADIPOR2, NRAS	7	VCAV_EC-up
cfa04611	Platelet activation	5/388	121/7854	0.7207419	0.99972301	0.9362731	100856354/403788/489208/481746/489644 , ITGB3, ITGA2, ITPR3, PIK3CD	5	ACOR_VSMC-down
cfa04912	GnRH signaling pathway	7/357	89/7854	0.10937727	0.2883378	0.21104521	607269/480271/480481/612821/403872/487514/479473 CALML6, ADCY4, MAP3K3, MAPK13, NRAS, PLA2G4E, PLD2	7	VCAV_EC-up
cfa03015	mRNA surveillance pathway	7/357	90/7854	0.11426559	0.2951861	0.21605773	490280/479444/477374/403608/403558/487130/491587 PPP2R5A, PPP2R1B, PPP2R2A, PPP2CA, PPP1CB, UPF2, PPP2R2D	7	VCAV_EC-up
cfa04640	Hematopoietic cell lineage	7/357	93/7854	0.12960307	0.31883099	0.23336431	609293/612582/403811/403985/415130/100856563/403788 IL3RA, IL7R, KIT, IL6, CD34, ITGA6, ITGB3	7	VCAV_EC-up
cfa04972	Pancreatic secretion	7/357	94/7854	0.13493459	0.32736304	0.23960925	403480/403957/480271/477098/483225/476708/479797 ATP2B1, RAB11A, ADCY4, ATP1B3, RAP1A, RAB3D, ATP2A1	7	VCAV_EC-up
cfa04922	Glucagon signaling pathway	7/357	101/7854	0.17512928	0.3954409	0.28943797	607269/403486/475266/487787/612974/607922/474603 CALML6, PPP3CA, CREB5, , PPP4R3A, CREB1, PPP4R3B	7	VCAV_EC-up
cfa04120	Ubiquitin mediated proteolysis	7/357	139/7854	0.44622546	0.67499155	0.49405154	100856769/474993/477392/607683/608578/479010/477199 WWP1, UBE2J1, CUL3, CUL4A, UBE2D1, UBE3A, ITCH	7	VCAV_EC-up
cfa00280	Valine, leucine and isoleucine degradation	7/241	48/7854	0.00059019	0.0331687	0.02982015	484394/477023/484488/607926/479610/474978/489421 BCAT2, ACAA1, , , , BCKDHB, ACAT1	7	VCAV_EC-down
cfa01522	Endocrine resistance	7/241	93/7854	0.02380502	0.24158651	0.21719709	483148/477625/403733/609956/485864/449021/490439 NOTCH2, ADCY6, MMP2, , SRC, AKT2, SHC1	7	VCAV_EC-down
cfa05231	Choline metabolism in cancer	7/241	96/7854	0.02774796	0.24158651	0.21719709	442985/609956/449021/482666/607962/481271/475590 PDGFRB, , AKT2, PDGFC, PLPP1, RAC2, EIF4EBP1	7	VCAV_EC-down
cfa04931	Insulin resistance	7/241	109/7854	0.04990064	0.34200197	0.30747508	609956/449021/485548/474653/612448/490942/486148 , AKT2, IRS2, GFPT2, RPS6KA2, G6PC3, IRS1	7	VCAV_EC-down
cfa05135	Yersinia infection	7/241	117/7854	0.06790787	0.38682027	0.34776874	609956/485864/449021/488409/612448/475549/481271 , SRC, AKT2, WIPF1, RPS6KA2, ACTR3B, RAC2	7	VCAV_EC-down
cfa00190	Oxidative phosphorylation	7/241	131/7854	0.10770647	0.43236456	0.38871509	611480/612644/478983/491061/486347/476040/479877 , , SDHC, ATP5MC1, UQCR10, COX8A, ATP6V0C	7	VCAV_EC-down
cfa04910	Insulin signaling pathway	7/241	134/7854	0.11757952	0.45260061	0.40690821	609956/449021/485548/490942/486148/490439/475590 , AKT2, IRS2, G6PC3, IRS1, SHC1, EIF4EBP1	7	VCAV_EC-down
cfa04611	Platelet activation	44197	121/7854	0.27853152	0.38235335	0.32929954	477886 LYN	1	ACOR_EC-down

cfa04140	Autophagy - animal	7/241	136/7854	0.12441526	0.47244173	0.42474627	609956/479479/610130/449021/485548/486148/488576 , GABARAP, AKT1S1, AKT2, IRS2, IRS1, RAB7B	7 VCAV_EC-down
cfa05012	Parkinson disease	7/241	138/7854	0.13144979	0.47881756	0.43047842	611480/612644/478983/491061/486347/476040/479595 , , SDHC, ATP5MC1, UQCR10, COX8A, PARK7	7 VCAV_EC-down
cfa04072	Phospholipase D signaling pathway	7/241	148/7854	0.16945174	0.51199935	0.46031034	442985/477625/609956/449021/482666/607962/490439 PDGFRB, ADCY6, , AKT2, PDGFC, PLPP1, SHC1	7 VCAV_EC-down
cfa04921	Oxytocin signaling pathway	7/241	151/7854	0.18171038	0.51619497	0.46408238	477625/485864/479812/486563/476744/610764/606992 ADCY6, SRC, EEF2K, CACNB3, EEF2, CAMK2D, MYL6B	7 VCAV_EC-down
cfa04150	mTOR signaling pathway	7/241	152/7854	0.18587803	0.52095886	0.46836534	609956/610130/449021/612448/486148/483777/475590 , AKT1S1, AKT2, RPS6KA2, IRS1, SLC3A2, EIF4EBP1	7 VCAV_EC-down
cfa04630	JAK-STAT signaling pathway	7/241	160/7854	0.22057199	0.56978807	0.51226498	442985/492162/489223/609956/449021/608847/611782 PDGFRB, FHL1, OSMR, , AKT2, CCND3, CCND2	7 VCAV_EC-down
cfa04062	Chemokine signaling pathway	7/241	174/7854	0.2860629	0.65352581	0.58754896	477625/609956/485864/449021/490439/481271/481954 ADCY6, , SRC, AKT2, SHC1, RAC2, FOXO3	7 VCAV_EC-down
cfa04024	cAMP signaling pathway	7/241	211/7854	0.47159253	0.8129908	0.73091512	477625/476487/609956/449021/483244/610764/481271 ADCY6, FXYD1, , AKT2, GLI3, CAMK2D, RAC2	7 VCAV_EC-down
cfa05165	Human papillomavirus infection	34881	328/7854	0.10155447	0.81029448	0.80529119	486137/490903/482528/100682877/490937/476665/481689 ATP6V1B2, AXIN2, ATP6V0B, TNXB, FZD2, COMP, TNC	7 AFEM_EC-up
cfa04924	Renin secretion	7/407	69/7854	0.06518024	0.62573034	0.60148786	477625/476548/486077/403652/478227/403424/403928 ADCY6, ITPR1, CTSB, PDE1A, GNAI1, EDN1, GNAQ	7 ACOR_VSMC-up
cfa00562	Inositol phosphate metabolism	7/407	72/7854	0.07820227	0.64585296	0.62083088	476331/489692/483196/490477/480647/608414/485399 PIP5K1B, PLCG2, PI4KB, PIK3C3, INPP5K, , PIK3C2A	7 ACOR_VSMC-up
cfa04971	Gastric acid secretion	7/407	72/7854	0.07820227	0.64585296	0.62083088	100855695/477625/476548/403812/610764/478227/403928 SLC26A7, ADCY6, ITPR1, HRH2, CAMK2D, GNAI1, GNAQ	7 ACOR_VSMC-up
cfa04350	TGF-beta signaling pathway	7/407	92/7854	0.19897306	0.80256807	0.77147442	488560/487612/490695/481563/483880/481628/403547 FMOD, SMAD6, BMP4, CDKN2B, INHBB, TGFBR1, ID3	7 ACOR_VSMC-up
cfa04925	Aldosterone synthesis and secretion	7/407	94/7854	0.21384117	0.80256807	0.77147442	477625/476548/610764/403607/609934/611934/403928 ADCY6, ITPR1, CAMK2D, LIPE, PRKCE, CREB3, GNAQ	7 ACOR_VSMC-up
cfa05017	Spinocerebellar ataxia	7/407	96/7854	0.22910205	0.80256807	0.77147442	486270/476548/478328/489525/490477/474467/403928 ATXN2, ITPR1, RORA, ULK2, PIK3C3, ATXN10, GNAQ	7 ACOR_VSMC-up
cfa05231	Choline metabolism in cancer	7/407	96/7854	0.22910205	0.80256807	0.77147442	476331/482666/610214/477331/100856443/484951/480688 PIP5K1B, PDGFC, CHPT1, WASF3, PLPP2, SLC44A2, TSC1	7 ACOR_VSMC-up
cfa05215	Prostate cancer	7/407	97/7854	0.23686905	0.80256807	0.77147442	403416/482666/415125/487501/403588/474890/611934 BCL2, PDGFC, FGFR2, SPINT1, AR, CDKN1A, CREB3	7 ACOR_VSMC-up
cfa04713	Circadian entrainment	7/407	98/7854	0.24472124	0.81104457	0.77962252	488087/477625/476548/610764/478227/100686627/403928 GNB4, ADCY6, ITPR1, CAMK2D, GNAI1, RYR3, GNAQ	7 ACOR_VSMC-up
cfa05142	Chagas disease (American trypanosomiasis)	7/407	103/7854	0.28510257	0.82260001	0.79073027	100856077/488471/484156/478227/481628/403522/403928 IFNGR1, CFLAR, GNA14, GNAI1, TGFBR1, CCL5, GNAQ	7 ACOR_VSMC-up
cfa04931	Insulin resistance	7/407	109/7854	0.33542164	0.85487994	0.82175959	489571/479127/611078/609934/611934/611993/488526 PRKAA2, PPARGC1A, PYGM, PRKCE, CREB3, GYS1, PRKAG3	7 ACOR_VSMC-up
cfa04611	Platelet activation	42736	121/7854	0.2321912	0.32364194	0.30122906	482994 ADCY3	1 VFEM_EC-up
cfa05162	Measles	7/407	136/7854	0.56282149	0.90174277	0.86680683	403416/611782/403745/100855560/607899/102156745/490969 BCL2, CCND2, MX1, DDX58, IRF9, MAP3K7, STAT5B	7 ACOR_VSMC-up
cfa04120	Ubiquitin mediated proteolysis	7/407	139/7854	0.58625329	0.90408536	0.86905866	491737/489228/478061/475718/607816/611303/100856496 MID1, SKP2, MAP3K1, BIRC6, UBE2H, RNFT2, SIAH1	7 ACOR_VSMC-up
cfa00310	Lysine degradation	7/388	58/7854	0.0232977	0.2437298	0.22826088	479077/484834/475511/491868/486059/484063/477995 NSD2, COLGALT1, EZH2, SUV39H1, AADAT, ACAT2,	7 ACOR_VSMC-down
cfa05222	Small cell lung cancer	7/388	92/7854	0.16872143	0.88254287	0.82653009	481131/100686141/475258/100855704/475882/489208/489644 CDK4, CKS2, CYCS, CDK2, LAMB1, ITGA2, PIK3CD	7 ACOR_VSMC-down
cfa04932	Non-alcoholic fatty liver disease (NAFLD)	7/388	145/7854	0.58016215	0.99972301	0.9362731	403475/477732/100683675/475258/403955/475121/489644 SREBF1, ADIPOR2, BID, CYCS, RAC1, CYC1, PIK3CD	7 ACOR_VSMC-down

cfa04934	Cushing syndrome	7/388	152/7854	0.63030929	0.99972301	0.9362731	489447/488669/475359/481131/100855704/488752/481746 AIPL1, PBX1, CDKN2C, CDK4, CDK2, PDE8A, ITPR3	7	ACOR_VSMC-down
cfa05010	Alzheimer disease	7/388	168/7854	0.73118839	0.99972301	0.9362731	100683675/475258/475121/480149/478807/481746/476756 BID, CYCS, CYC1, ATP5F1A, , ITPR3, ATP5F1D	7	ACOR_VSMC-down
cfa04924	Renin secretion	6/131	69/7854	0.00097365	0.07562038	0.06935143	403732/403652/553102/403589/403556/610836 AQP1, PDE1A, GUCY1B1, PTGER4, PRKACA, PDE3A	6	ACOR_EC-up
cfa00564	Glycerophospholipid metabolism	6/131	95/7854	0.00496632	0.16530761	0.15160354	607962/478514/610214/479473/491249/484502 PLPP1, CFI, CHPT1, PLD2, AGPAT2, PLD3	6	ACOR_EC-up
cfa04919	Thyroid hormone signaling pathway	6/131	116/7854	0.01278653	0.25186945	0.23098938	486079/100856339/403966/486808/485864/403556 GATA4, , ATP1B1, PLCE1, SRC, PRKACA	6	ACOR_EC-up
cfa04611	Platelet activation	34700	121/7854	0.77325851	0.87603338	0.87062417	485056 TBXA2R	1	AFEM_EC-up
cfa04611	Platelet activation	42370	121/7854	0.22015221	0.34920695	0.31164922	608643 STIM1	1	VSAP_VSMC-up
cfa04514	Cell adhesion molecules (CAMs)	6/131	139/7854	0.02846567	0.31583342	0.28965071	488565/611010/490960/492244/607997/403982 NFASC, CDH3, CNTNAP1, L1CAM, LRRC4, VCAM1	6	ACOR_EC-up
cfa04015	Rap1 signaling pathway	6/131	209/7854	0.13613173	0.50347132	0.46173336	100856339/609157/486808/608458/488402/485864 , GNAO1, PLCE1, EFNA5, RAPGEF4, SRC	6	ACOR_EC-up
cfa05132	Salmonella infection	6/131	212/7854	0.14278677	0.50408057	0.4622921	100688336/475236/492067/403416/100856310/611857 MYH14, DYNC1I1, PAK3, BCL2, VPS16, ARHGEF26	6	ACOR_EC-up
cfa05163	Human cytomegalovirus infection	6/131	220/7854	0.16125097	0.52441163	0.48093771	100856339/609157/403589/485864/481740/403556 , GNAO1, PTGER4, SRC, TAPBP, PRKACA	6	ACOR_EC-up
cfa04010	MAPK signaling pathway	6/131	288/7854	0.3486121	0.68154985	0.6250491	485409/480488/608458/481183/403556/442938 PTPN5, MAPT, EFNA5, MAPK8IP2, PRKACA, FLT3LG	6	ACOR_EC-up
cfa04080	Neuroactive ligand-receptor interaction	6/131	327/7854	0.46547305	0.72788738	0.66754524	489198/403779/484429/487329/403589/476728 HTR4, PTH1R, PTGIR, OPRD1, PTGER4, C3	6	ACOR_EC-up
cfa00565	Ether lipid metabolism	6/441	43/7854	0.03163874	0.2569184	0.23128575	482026/478238/478174/479557/481256/476449 ENPP2, LPCAT4, PAFAH2, PLPP3, PLA2G6, PAFAH1B3	6	VSAP_EC-up
cfa00330	Arginine and proline metabolism	6/441	47/7854	0.04626871	0.33516603	0.30172664	486834/480112/478144/481486/478991/490715 GOT1, , AZIN2, ALDH7A1, ALDH9A1, L3HYPDH	6	VSAP_EC-up
cfa00520	Amino sugar and nucleotide sugar metabolism	6/441	48/7854	0.05048523	0.34077532	0.30677629	403694/612532/100533995/474479/474486/478043 GALK1, RENBP, FPGT, CYB5R3, PMM1, GNPDA1	6	VSAP_EC-up
cfa04978	Mineral absorption	8/441	51/7854	0.00700969	0.11190575	0.10074095	100686073/486588/403768/488106/477042/475222/482291/442987 , VDR, MT2A, CLCN2, FTL, , STEAP2, HMOX1	8	VSAP_EC-up
cfa00010	Glycolysis / Gluconeogenesis	6/441	62/7854	0.1332619	0.55744768	0.50183133	476003/403978/477205/477002/481486/478991 ALDH3B1, DLD, ACSS2, ACSS1, ALDH7A1, ALDH9A1	6	VSAP_EC-up
cfa05230	Central carbon metabolism in cancer	6/441	66/7854	0.16447335	0.61481778	0.55347764	487235/481088/403924/484648/403494/611939 PIK3R1, G6PD, MYC, RAF1, RET, MAP2K2	6	VSAP_EC-up
cfa05223	Non-small cell lung cancer	6/441	67/7854	0.17272673	0.62560778	0.56319112	483626/491278/490968/487235/484648/611939 DDB2, RXRA, STAT5A, PIK3R1, RAF1, MAP2K2	6	VSAP_EC-up
cfa04917	Prolactin signaling pathway	6/441	70/7854	0.19846341	0.66536335	0.5989803	490968/484757/487235/490006/484648/611939 STAT5A, CISH, PIK3R1, SOCS1, RAF1, MAP2K2	6	VSAP_EC-up
cfa00562	Inositol phosphate metabolism	6/441	72/7854	0.21636409	0.69388184	0.62465351	607443/485054/490779/491034/492211/486443 INPP5E, PIP5K1C, ALDH6A1, PIP4K2B, MTMR1, PI4KA	6	VSAP_EC-up
cfa01521	EGFR tyrosine kinase inhibitor resistance	6/441	80/7854	0.29259838	0.75621155	0.68076461	612271/479460/487235/607745/484648/611939 IL6R, PDGFD, PIK3R1, GAS6, RAF1, MAP2K2	6	VSAP_EC-up
cfa04914	Progesterone-mediated oocyte maturation	6/441	89/7854	0.38344513	0.85964878	0.77388194	491236/487235/612448/485790/484648/481679 ANAPC2, PIK3R1, RPS6KA2, CDC25B, RAF1, CDC26	6	VSAP_EC-up
cfa03015	mRNA surveillance pathway	6/441	90/7854	0.39364388	0.85964878	0.77388194	485672/481101/484740/483755/403609/492002 PPP2R3A, PYM1, WDR82, PPP2R5B, PPP1CA, PABPC5	6	VSAP_EC-up
cfa04666	Fc gamma R-mediated phagocytosis	6/441	90/7854	0.39364388	0.85964878	0.77388194	477331/485054/479557/487235/481256/484648 WASF3, PIP5K1C, PLPP3, PIK3R1, PLA2G6, RAF1	6	VSAP_EC-up
cfa05222	Small cell lung cancer	6/441	92/7854	0.41401944	0.86594207	0.77954735	483626/491290/491278/487235/403924/476626 DDB2, LAMC3, RXRA, PIK3R1, MYC, LAMB2	6	VSAP_EC-up
cfa05017	Spinocerebellar ataxia	6/441	96/7854	0.45450241	0.89991478	0.81013062	481746/476215/483634/487235/483756/477437 ITPR3, MAP3K5, AMBRA1, PIK3R1, ATG2A, ULK1	6	VSAP_EC-up
cfa04625	C-type lectin receptor signaling pathway	6/441	99/7854	0.48444156	0.92381513	0.83164644	490926/481746/487235/480272/484648/479248 MAP3K14, ITPR3, PIK3R1, NFATC4, RAF1, PPP3CB	6	VSAP_EC-up

cfa04916	Melanogenesis	6/441	100/7854	0.49431136	0.92381513	0.83164644	482294/485183/486894/485149/484648/611939 FZD1, WNT11, TCF7L2, FZD4, RAF1, MAP2K2	6	VSAP_EC-up
cfa04922	Glucagon signaling pathway	6/441	101/7854	0.50411778	0.92381513	0.83164644	481746/100685468/480857/486295/612305/479248 ITPR3, ACACB, PHKA2, PRKAB1, CRTC2, PPP3CB	6	VSAP_EC-up
cfa04659	Th17 cell differentiation	6/441	102/7854	0.51385608	0.92381513	0.83164644	612271/491278/490968/100142679/479248/477039 IL6R, RXRA, STAT5A, STAT6, PPP3CB, TGFBR2	6	VSAP_EC-up
cfa04660	T cell receptor signaling pathway	6/441	102/7854	0.51385608	0.92381513	0.83164644	490926/487235/484648/479248/485677/611939 MAP3K14, PIK3R1, RAF1, PPP3CB, NCK1, MAP2K2	6	VSAP_EC-up
cfa04928	Parathyroid hormone synthesis, secretion and action	6/441	107/7854	0.56137427	0.93144223	0.83851259	481746/486588/491278/485189/484648/403762 ITPR3, VDR, RXRA, ARRB1, RAF1, BGLAP	6	VSAP_EC-up
cfa04668	TNF signaling pathway	6/441	108/7854	0.57061581	0.94151609	0.84758138	490926/476215/487235/481245/607954/611262 MAP3K14, MAP3K5, PIK3R1, TAB1, BAG4, TRADD	6	VSAP_EC-up
cfa04720	Long-term potentiation	6/279	66/7854	0.02913937	0.18177416	0.15409541	479975/491768/403928/480416/610764/489050 PRKACB, RPS6KA3, GNAQ, CALM1, CAMK2D, CAMK2G	6	VSAP_EC-down
cfa05230	Central carbon metabolism in cancer	6/279	66/7854	0.02913937	0.18177416	0.15409541	476882/403874/477786/480348/482437/476828 , , PGAM1, HIF1A, SLC2A1,	6	VSAP_EC-down
cfa05031	Amphetamine addiction	6/279	67/7854	0.03108907	0.1894264	0.16058244	479975/480416/491369/610764/487309/489050 PRKACB, CALM1, CREB3L2, CAMK2D, HDAC1, CAMK2G	6	VSAP_EC-down
cfa05100	Bacterial invasion of epithelial cells	6/279	75/7854	0.04976496	0.25472931	0.21594168	476836/477956/477032/479252/100856747/480578 ILK, ITGB1, CTNNB1, VCL, , CLTC	6	VSAP_EC-down
cfa04911	Insulin secretion	6/279	85/7854	0.08112099	0.3573146	0.30290631	479975/482437/403928/491369/610764/489050 PRKACB, SLC2A1, GNAQ, CREB3L2, CAMK2D, CAMK2G	6	VSAP_EC-down
cfa05210	Colorectal cancer	6/279	85/7854	0.08112099	0.3573146	0.30290631	403567/481628/477032/478575/478464/474890 CASP3, TGFBR1, CTNNB1, GSK3B, MAPK10, CDKN1A	6	VSAP_EC-down
cfa04540	Gap junction	6/279	88/7854	0.09227472	0.38374567	0.32531272	479975/609616/474830/478701/491597/403928 PRKACB, PRKG1, TUBB, TUBB2A, PDGFA, GNAQ	6	VSAP_EC-down
cfa04912	GnRH signaling pathway	6/279	89/7854	0.09616831	0.38701439	0.32808371	479975/403928/478464/480416/610764/489050 PRKACB, GNAQ, MAPK10, CALM1, CAMK2D, CAMK2G	6	VSAP_EC-down
cfa04666	Fc gamma R-mediated phagocytosis	6/279	90/7854	0.10014861	0.38701439	0.32808371	403936/607885/476022/477886/100856747/612998 VASP, PAK1, CFL1, LYN, , MARCKSL1	6	VSAP_EC-down
cfa05222	Small cell lung cancer	6/279	92/7854	0.10836621	0.38701439	0.32808371	489208/403567/442942/477956/488437/474890 ITGA2, CASP3, PTGS2, ITGB1, ITGAV, CDKN1A	6	VSAP_EC-down
cfa04713	Circadian entrainment	6/279	98/7854	0.13500559	0.42616224	0.36127052	479975/609616/403928/480416/610764/489050 PRKACB, PRKG1, GNAQ, CALM1, CAMK2D, CAMK2G	6	VSAP_EC-down
cfa04625	C-type lectin receptor signaling pathway	6/279	99/7854	0.13972343	0.431399	0.36570988	475380/607885/477488/442942/478464/480416 PLK3, PAK1, PTPN11, PTGS2, MAPK10, CALM1	6	VSAP_EC-down
cfa04750	Inflammatory mediator regulation of TRP channels	6/279	100/7854	0.14451729	0.431399	0.36570988	479975/403928/478464/480416/610764/489050 PRKACB, GNAQ, MAPK10, CALM1, CAMK2D, CAMK2G	6	VSAP_EC-down
cfa05146	Amoebiasis	6/279	102/7854	0.15432752	0.44134096	0.37413798	480369/479975/403567/484526/403928/479252 ACTN1, PRKACB, CASP3, ACTN4, GNAQ, VCL	6	VSAP_EC-down
cfa04114	Oocyte meiosis	6/279	117/7854	0.23613907	0.55239675	0.4682833	479975/491768/480416/610764/100687866/489050 PRKACB, RPS6KA3, CALM1, CAMK2D, YWHAH, CAMK2G	6	VSAP_EC-down
cfa04926	Relaxin signaling pathway	6/279	127/7854	0.29673432	0.62195513	0.52725003	403802/479975/481628/477587/478464/491369 VEGFA, PRKACB, TGFBR1, ACTA2, MAPK10, CREB3L2	6	VSAP_EC-down
cfa00230	Purine metabolism	6/279	130/7854	0.31551022	0.64580999	0.54747251	100856150/403874/486117/478504/403652/490204 ENPP1, , ENTPD4, PAPSS1, PDE1A, AK5	6	VSAP_EC-down
cfa05162	Measles	6/279	136/7854	0.35356131	0.68617084	0.58168762	479406/403567/491924/478575/478464/480361 HSPA8, CASP3, MSN, GSK3B, MAPK10, EIF2S1	6	VSAP_EC-down
cfa04514	Cell adhesion molecules (CAMs)	6/279	139/7854	0.37273624	0.70990446	0.60180732	477956/476439/487635/487323/488437/485893 ITGB1, PVR, NEO1, SDC3, ITGAV, SDC4	6	VSAP_EC-down
cfa04932	Non-alcoholic fatty liver disease (NAFLD)	6/279	145/7854	0.41114522	0.73226635	0.62076416	403567/607407/607439/478575/478464/480361 CASP3, NDUFA4L2, DDIT3, GSK3B, MAPK10, EIF2S1	6	VSAP_EC-down
cfa04218	Cellular senescence	6/279	151/7854	0.44933513	0.73430489	0.62249229	403476/481628/475590/482772/480416/474890 SERPINE1, TGFBR1, EIF4EBP1, HIPK2, CALM1, CDKN1A	6	VSAP_EC-down
cfa05225	Hepatocellular carcinoma	6/279	165/7854	0.53582835	0.79879452	0.67716209	477558/476710/481628/477032/478575/474890 , SMARCA4, TGFBR1, CTNNB1, GSK3B, CDKN1A	6	VSAP_EC-down

cfa05152	Tuberculosis	6/279	171/7854	0.5711458	0.82674144	0.70085353	475380/403567/478464/480416/610764/489050 PLK3, CASP3, MAPK10, CALM1, CAMK2D, CAMK2G	6	VSAP_EC-down
cfa04923	Regulation of lipolysis in adipocytes	6/357	54/7854	0.03478066	0.12768162	0.09345495	490455/403544/480271/478227/442942/608476 NPR1, PTGS1, ADCY4, GNAI1, PTGS2,	6	VCAV_EC-up
cfa00310	Lysine degradation	6/357	58/7854	0.04694847	0.15410144	0.1127926	491177/486059/489094/479077/485441/475584 PIPOX, AADAT, NSD1, NSD2, SETDB2, NSD3	6	VCAV_EC-up
cfa04664	Fc epsilon RI signaling pathway	6/357	67/7854	0.08295043	0.24620394	0.18020586	100688270/612821/489692/485874/403872/487514 INPP5D, MAPK13, PLCG2, PLCG1, NRAS, PLA2G4E	6	VCAV_EC-up
cfa05140	Leishmaniasis	6/357	70/7854	0.09760866	0.26815625	0.19627358	403417/612821/100856077/102155289/442942/403822 TLR4, MAPK13, IFNGR1, , PTGS2, NOS2	6	VCAV_EC-up
cfa05214	Glioma	6/357	75/7854	0.12486501	0.3138499	0.22971847	442986/607269/478232/489692/485874/403872 PDGFB, CALML6, MTOR, PLCG2, PLCG1, NRAS	6	VCAV_EC-up
cfa04610	Complement and coagulation cascades	6/357	80/7854	0.15541728	0.36746967	0.26896479	403875/399544/475624/403921/609365/485848 F8, VWF, KLKB1, TFPI, , PROCR	6	VCAV_EC-up
cfa04914	Progesterone-mediated oocyte maturation	6/357	89/7854	0.21751915	0.45975639	0.33651289	489121/480271/478227/612821/488821/491768 STK10, ADCY4, GNAI1, MAPK13, CPEB2, RPS6KA3	6	VCAV_EC-up
cfa04727	GABAergic synapse	6/357	91/7854	0.23235046	0.48019096	0.35146971	403617/477633/480271/478227/100855806/607513 GNGT2, SLC38A1, ADCY4, GNAI1, GNG11, GNG4	6	VCAV_EC-up
cfa04978	Mineral absorption	4/241	51/7854	0.07042259	0.38682027	0.34776874	478796/403768/486588/403713 CYBRD1, MT2A, VDR, ATOX1	4	VCAV_EC-down
cfa04650	Natural killer cell mediated cytotoxicity	6/357	103/7854	0.32663985	0.59176959	0.43313828	403486/485930/100856077/489692/485874/403872 PPP3CA, NFATC2, IFNGR1, PLCG2, PLCG1, NRAS	6	VCAV_EC-up
cfa04068	FoxO signaling pathway	6/357	127/7854	0.52116167	0.74950571	0.54859124	478063/612582/403985/484813/612821/403872 PLK2, IL7R, IL6, HOMER3, MAPK13, NRAS	6	VCAV_EC-up
cfa03040	Spliceosome	6/357	132/7854	0.55939765	0.7843691	0.57410905	479406/607232/490529/478052/477103/474807 HSPA8, PPIE, THOC1, TCERG1, U2SURP, PRPF4	6	VCAV_EC-up
cfa00480	Glutathione metabolism	6/241	52/7854	0.0048998	0.10591097	0.0952187	476005/403913/476006/479912/442961/479081 GSTP1, ANPEP, , GSTM4, GPX1, LAP3	6	VCAV_EC-down
cfa04917	Prolactin signaling pathway	6/241	70/7854	0.02003191	0.24158651	0.21719709	609956/485864/449021/611782/490439/481954 , SRC, AKT2, CCND2, SHC1, FOXO3	6	VCAV_EC-down
cfa04012	ErbB signaling pathway	6/241	85/7854	0.04594868	0.33131754	0.2978693	609956/485864/449021/610764/490439/475590 , SRC, AKT2, CAMK2D, SHC1, EIF4EBP1	6	VCAV_EC-down
cfa04350	TGF-beta signaling pathway	6/241	92/7854	0.06299411	0.38481182	0.34596306	486006/403904/478293/489077/487475/100856151 SMAD9, DCN, FBN1, BMPR1A, GREM1, RGMA	6	VCAV_EC-down
cfa05215	Prostate cancer	6/241	97/7854	0.0771775	0.38682027	0.34776874	476005/442985/609956/449021/476006/482666 GSTP1, PDGFRB, , AKT2, , PDGFC	6	VCAV_EC-down
cfa04933	AGE-RAGE signaling pathway in diabetic complications	6/241	100/7854	0.08648717	0.4045956	0.36374955	403824/403733/478835/609956/449021/403651 COL1A2, MMP2, COL3A1, , AKT2, COL1A1	6	VCAV_EC-down
cfa05146	Amoebiasis	6/241	102/7854	0.09302258	0.40842728	0.36719441	403824/478835/609956/480173/403651/488576 COL1A2, COL3A1, , LAMA3, COL1A1, RAB7B	6	VCAV_EC-down
cfa01200	Carbon metabolism	6/241	116/7854	0.14578592	0.4861441	0.43706531	485151/489642/478983/610090/477205/489421 ME3, H6PD, SDHC, PGLS, ACSS2, ACAT1	6	VCAV_EC-down
cfa04935	Growth hormone synthesis, secretion and action	6/241	116/7854	0.14578592	0.4861441	0.43706531	477625/609956/449021/485548/486148/490439 ADCY6, , AKT2, IRS2, IRS1, SHC1	6	VCAV_EC-down
cfa04621	NOD-like receptor signaling pathway	6/241	158/7854	0.35666052	0.73692358	0.66252728	489522/479479/612601/489664/485789/474519 TRPV2, GABARAP, ANTXR1, CYBA, MAVS, TXN2	6	VCAV_EC-down
cfa05225	Hepatocellular carcinoma	6/241	165/7854	0.39531164	0.78213515	0.70317451	476005/609956/449021/476006/479912/490439 GSTP1, , AKT2, , GSTM4, SHC1	6	VCAV_EC-down
cfa05202	Transcriptional misregulation in cancer	6/241	172/7854	0.43389281	0.79541394	0.71511274	491872/487044/611782/448804/489398/475223 TFE3, RUNX1T1, CCND2, FUT8, ZBTB16, CDK14	6	VCAV_EC-down
cfa05169	Epstein-Barr virus infection	6/241	193/7854	0.54592114	0.84753502	0.76197193	609956/449021/485604/608847/485789/611782 , AKT2, ENTPD3, CCND3, MAVS, CCND2	6	VCAV_EC-down
cfa05166	Human T-cell leukemia virus 1 infection	6/241	209/7854	0.62428977	0.8815348	0.79253925	477625/478331/609956/449021/608847/611782 ADCY6, TLN2, , AKT2, CCND3, CCND2	6	VCAV_EC-down
cfa04142	Lysosome	34851	118/7854	0.00296754	0.32190301	0.31991537	490004/403400/474969/482528/611983/479915 , CTSS, SLC17A5, ATP6V0B, CTSZ, SORT1	6	AFEM_EC-up

cfa04145	Phagosome	34851	138/7854	0.00638353	0.32190301	0.31991537	403400/486137/607342/404007/482528/476665 CTSS, ATP6V1B2, COLEC12, RAB7A, ATP6V0B, COMP	6	AFEM_EC-up
cfa04151	PI3K-Akt signaling pathway	34851	354/7854	0.25673739	0.81029448	0.80529119	609626/403858/482666/100682877/476665/481689 ANGPT4, CHRM2, PDGFC, TNXB, COMP, TNC	6	AFEM_EC-up
cfa04978	Mineral absorption	3/131	51/7854	0.05289902	0.36641541	0.33603943	478837/403966/477072 SLC40A1, ATP1B1,	3	ACOR_EC-up
cfa00330	Arginine and proline metabolism	6/407	47/7854	0.03318628	0.45512609	0.43749328	403451/403450/485085/479242/479081/478144 MAOB, MAOA, GAMT, P4HA1, LAP3, AZIN2	6	ACOR_VSMC-up
cfa04970	Salivary secretion	6/407	78/7854	0.21654419	0.80256807	0.77147442	403962/477625/476548/607874/100686627/403928 ADRA1B, ADCY6, ITPR1, CST3, RYR3, GNAQ	6	ACOR_VSMC-up
cfa03320	PPAR signaling pathway	6/407	80/7854	0.23350208	0.80256807	0.77147442	476724/489428/403626/474670/479564/100856329 ANGPTL4, MMP1, LPL, ACSL6, SCP2, PLIN2	6	ACOR_VSMC-up
cfa04978	Mineral absorption	3/357	51/7854	0.41095525	0.65826592	0.48180942	403480/490755/477098 ATP2B1, SLC8A3, ATP1B3	3	VCAV_EC-up
cfa04912	GnRH signaling pathway	6/407	89/7854	0.31402283	0.82260001	0.79073027	478061/477625/476548/610764/403928/489532 MAP3K1, ADCY6, ITPR1, CAMK2D, GNAQ, MAPK7	6	ACOR_VSMC-up
cfa05222	Small cell lung cancer	6/407	92/7854	0.34187995	0.86027195	0.8269427	403416/489228/481563/479180/474890/480173 BCL2, SKP2, CDKN2B, POLK, CDKN1A, LAMA3	6	ACOR_VSMC-up
cfa04620	Toll-like receptor signaling pathway	6/407	101/7854	0.42627797	0.88820767	0.85379612	488834/487085/482905/403522/609544/102156745 TLR1, MAP3K8, TLR3, CCL5, TIRAP, MAP3K7	6	ACOR_VSMC-up
cfa04724	Glutamatergic synapse	6/407	112/7854	0.52680016	0.88929092	0.8548374	488087/403751/477625/476548/478227/403928 GNB4, SLC1A1, ADCY6, ITPR1, GNAI1, GNAQ	6	ACOR_VSMC-up
cfa04114	Oocyte meiosis	6/407	117/7854	0.57013763	0.90219582	0.86724232	403588/477625/476548/610764/480349/479287 AR, ADCY6, ITPR1, CAMK2D, PPP2R5E, CPEB4	6	ACOR_VSMC-up
cfa04071	Sphingolipid signaling pathway	6/407	122/7854	0.61145965	0.90773643	0.87256828	403416/480349/485334/478227/609934/403928 BCL2, PPP2R5E, SMPD1, GNAI1, PRKCE, GNAQ	6	ACOR_VSMC-up
cfa00983	Drug metabolism - other enzymes	6/388	63/7854	0.08961546	0.64145801	0.60074628	476823/100855947/482963/477813/609526/477123 RRM1, TK1, RRM2, GSTO1, DUT, GMPS	6	ACOR_VSMC-down
cfa05100	Bacterial invasion of epithelial cells	6/388	75/7854	0.16493862	0.88254287	0.82653009	403980/487443/612892/403955/479744/489644 CAV1, MAD2L2, SEPT6, RAC1, ARPC1B, PIK3CD	6	ACOR_VSMC-down
cfa04666	Fc gamma R-mediated phagocytosis	6/388	90/7854	0.28483997	0.99972301	0.9362731	483329/607885/484401/403955/479744/489644 SPHK1, PAK1, SPHK2, RAC1, ARPC1B, PIK3CD	6	ACOR_VSMC-down
cfa04625	C-type lectin receptor signaling pathway	6/388	99/7854	0.36416525	0.99972301	0.9362731	607885/475380/403872/481746/480272/489644 PAK1, PLK3, NRAS, ITPR3, NFATC4, PIK3CD	6	ACOR_VSMC-down
cfa04919	Thyroid hormone signaling pathway	6/388	116/7854	0.51410213	0.99972301	0.9362731	476769/403872/403788/477098/485094/489644 MED17, NRAS, ITGB3, ATP1B3, MED16, PIK3CD	6	ACOR_VSMC-down
cfa04722	Neurotrophin signaling pathway	6/388	118/7854	0.53101303	0.99972301	0.9362731	492247/403872/475924/403955/489620/489644 IRAK1, NRAS, ARHGDIA, RAC1, TP73, PIK3CD	6	ACOR_VSMC-down
cfa04145	Phagosome	6/388	138/7854	0.68371247	0.99972301	0.9362731	476694/403703/403788/403955/489949/489208 CALR, TFRC, ITGB3, RAC1, CORO1A, ITGA2	6	ACOR_VSMC-down
cfa05224	Breast cancer	6/388	142/7854	0.7100269	0.99972301	0.9362731	403420/481131/403437/403872/608459/489644 HEY1, CDK4, BRCA1, NRAS, FGF5, PIK3CD	6	ACOR_VSMC-down
cfa04390	Hippo signaling pathway	6/388	153/7854	0.77450593	0.99972301	0.9362731	486940/479444/442936/477535/477237/489620 SNAI2, PPP2R1B, , NF2, YWHAB, TP73	6	ACOR_VSMC-down
cfa04022	cGMP-PKG signaling pathway	6/388	165/7854	0.83194172	0.99972301	0.9362731	492093/610836/477098/474681/481746/480272 SLC25A5, PDE3A, ATP1B3, VDAC1, ITPR3, NFATC4	6	ACOR_VSMC-down
cfa05225	Hepatocellular carcinoma	6/388	165/7854	0.83194172	0.99972301	0.9362731	481131/476640/403872/477813/486407/489644 CDK4, SMARCC1, NRAS, GSTO1, SMARCB1, PIK3CD	6	ACOR_VSMC-down
cfa00565	Ether lipid metabolism	5/131	43/7854	0.00069234	0.07562038	0.06935143	607962/478514/610214/479473/484502 PLPP1, CFI, CHPT1, PLD2, PLD3	5	ACOR_EC-up
cfa01524	Platinum drug resistance	5/131	72/7854	0.00685319	0.19959928	0.18305243	474938/100856339/477558/403416/476903 , , , BCL2, ATP7B	5	ACOR_EC-up
cfa05100	Bacterial invasion of epithelial cells	5/131	75/7854	0.00812631	0.21038126	0.19294057	100856339/477251/485864/489008/611857 , ELMO2, SRC, , ARHGEF26	5	ACOR_EC-up
cfa04970	Salivary secretion	5/131	78/7854	0.00955519	0.22263596	0.20417935	403966/403756/553102/607874/403556 ATP1B1, CD38, GUCY1B1, CST3, PRKACA	5	ACOR_EC-up
cfa05032	Morphine addiction	5/131	92/7854	0.01852575	0.29765577	0.27297999	403652/609157/100683695/403556/610836 PDE1A, GNAO1, PDE4A, PRKACA, PDE3A	5	ACOR_EC-up

cfa04640	Hematopoietic cell lineage	5/131	93/7854	0.0193241	0.29765577	0.27297999	475945/484943/403756/481588/442938 CD59, EPOR, CD38, IL11RA, FLT3LG	5 ACOR_EC-up
cfa04750	Inflammatory mediator regulation of TRP channels	5/131	100/7854	0.02554985	0.29765577	0.27297999	100856339/403589/485864/403556/494005 , PTGER4, SRC, PRKACA, PRKCD	5 ACOR_EC-up
cfa04933	AGE-RAGE signaling pathway in diabetic complications	5/131	100/7854	0.02554985	0.29765577	0.27297999	100856339/486808/403416/403982/494005 , PLCE1, BCL2, VCAM1, PRKCD	5 ACOR_EC-up
cfa04928	Parathyroid hormone synthesis, secretion and action	5/131	107/7854	0.03293681	0.33468971	0.3069438	403779/100683695/403416/479473/403556 PTH1R, PDE4A, BCL2, PLD2, PRKACA	5 ACOR_EC-up
cfa04978	Mineral absorption	3/407	51/7854	0.49701759	0.88820767	0.85379612	477072/478796/475738 , CYBRD1, SLC8A1	3 ACOR_VSMC-up
cfa00230	Purine metabolism	5/131	130/7854	0.06588192	0.36641541	0.33603943	403652/100683695/553102/480429/610836 PDE1A, PDE4A, GUCY1B1, AK7, PDE3A	5 ACOR_EC-up
cfa04210	Apoptosis	5/131	131/7854	0.06762173	0.36641541	0.33603943	480571/100856339/488472/403416/611983 SEPT4, , CASP10, BCL2, CTSZ	5 ACOR_EC-up
cfa04978	Mineral absorption	34731	51/7854	0.12627509	0.81029448	0.80529119	403768/100686073 MT2A,	2 AFEM_EC-up
cfa04072	Phospholipase D signaling pathway	5/131	148/7854	0.101037	0.45272346	0.41519252	100856339/607962/488402/479473/491249 , PLPP1, RAPGEF4, PLD2, AGPAT2	5 ACOR_EC-up
cfa04621	NOD-like receptor signaling pathway	5/131	158/7854	0.12393044	0.48996541	0.44934709	100855763/487286/403416/485789/494005 , NOD2, BCL2, MAVS, PRKCD	5 ACOR_EC-up
cfa04630	JAK-STAT signaling pathway	5/131	160/7854	0.12877848	0.49189156	0.45111357	100856339/484943/481588/403416/478350 , EPOR, IL11RA, BCL2, PIAS1	5 ACOR_EC-up
cfa05161	Hepatitis B	5/131	164/7854	0.13873129	0.50408057	0.4622921	100856339/488472/403416/485789/485864 , CASP10, BCL2, MAVS, SRC	5 ACOR_EC-up
cfa04022	cGMP-PKG signaling pathway	5/131	165/7854	0.14127173	0.50408057	0.4622921	486079/403966/487329/553102/610836 GATA4, ATP1B1, OPRD1, GUCY1B1, PDE3A	5 ACOR_EC-up
cfa05152	Tuberculosis	5/131	171/7854	0.15693579	0.52441163	0.48093771	487286/488472/403416/485864/476728 NOD2, CASP10, BCL2, SRC, C3	5 ACOR_EC-up
cfa04062	Chemokine signaling pathway	5/131	174/7854	0.16502809	0.52441163	0.48093771	100856339/485693/485864/403556/494005 , GRK7, SRC, PRKACA, PRKCD	5 ACOR_EC-up
cfa05170	Human immunodeficiency virus 1 infection	5/131	200/7854	0.24129589	0.60001444	0.55027301	100856339/609157/492067/403416/481740 , GNAO1, PAK3, BCL2, TAPBP	5 ACOR_EC-up
cfa05205	Proteoglycans in cancer	5/131	202/7854	0.24753927	0.60001444	0.55027301	100856339/100855544/486808/485864/403556 , FZD5, PLCE1, SRC, PRKACA	5 ACOR_EC-up
cfa04144	Endocytosis	5/131	235/7854	0.35502698	0.68154985	0.6250491	611239/485693/479473/485864/485842 SH3GL2, GRK7, PLD2, SRC, CHMP4B	5 ACOR_EC-up
cfa01210	2-Oxocarboxylic acid metabolism	5/441	17/7854	0.0019222	0.04960195	0.04465318	486834/484394/478889/479066/477177 GOT1, BCAT2, IDH1, IDH3A,	5 VSAP_EC-up
cfa00860	Porphyrin and chlorophyll metabolism	5/441	33/7854	0.03528802	0.27580374	0.24828691	478980/609051/610360/487948/442987 PPOX, FXN, FECH, CPOX, HMOX1	5 VSAP_EC-up
cfa00250	Alanine, aspartate and glutamate metabolism	5/441	37/7854	0.05407847	0.3569179	0.32130833	486834/403443/476775/479856/480693 GOT1, GLUL, FOLH1, ABAT, ASS1	5 VSAP_EC-up
cfa04216	Ferroptosis	5/441	45/7854	0.10597028	0.48420266	0.43589394	483885/477042/475631/442987/477750 STEAP3, FTL, ACSL1, HMOX1, NCOA4	5 VSAP_EC-up
cfa00270	Cysteine and methionine metabolism	5/441	50/7854	0.14737275	0.59958501	0.53976464	486834/484394/476715/474515/611790 GOT1, BCAT2, , MPST, AHCYL1	5 VSAP_EC-up
cfa04330	Notch signaling pathway	5/441	51/7854	0.15637989	0.61400684	0.55274761	100688936/479723/480381/485116/612240 DLL1, DTX2, NUMB, MAML2, CTBP1	5 VSAP_EC-up
cfa00240	Pyrimidine metabolism	5/441	52/7854	0.16560748	0.61481778	0.55347764	485604/477809/483309/474746/475393 ENTPD3, NT5C2, NT5C, NUDT2, PNP	5 VSAP_EC-up
cfa04622	RIG-I-like receptor signaling pathway	5/441	67/7854	0.32312181	0.80885538	0.72815618	489370/483800/485789/476412/611262 NLRX1, TKFC, MAVS, IRF3, TRADD	5 VSAP_EC-up
cfa05212	Pancreatic cancer	5/441	75/7854	0.41365528	0.86594207	0.77954735	610290/483626/487235/484648/477039 ARHGEF6, DDB2, PIK3R1, RAF1, TGFBR2	5 VSAP_EC-up
cfa05214	Glioma	5/441	75/7854	0.41365528	0.86594207	0.77954735	483626/487235/484648/484665/611939 DDB2, PIK3R1, RAF1, CAMK1, MAP2K2	5 VSAP_EC-up
cfa04970	Salivary secretion	5/441	78/7854	0.44732287	0.89991478	0.81013062	488820/481746/607874/484464/479491 BST1, ITPR3, CST3, KCNN4,	5 VSAP_EC-up
cfa04658	Th1 and Th2 cell differentiation	5/441	87/7854	0.54455715	0.92381513	0.83164644	100688936/490968/100142679/485116/479248 DLL1, STAT5A, STAT6, MAML2, PPP3CB	5 VSAP_EC-up
cfa04540	Gap junction	5/441	88/7854	0.55487697	0.93106475	0.83817276	481746/479460/474800/484648/611939 ITPR3, PDGFD, LPAR1, RAF1, MAP2K2	5 VSAP_EC-up
cfa04974	Protein digestion and absorption	5/441	91/7854	0.58512674	0.95484968	0.85958469	492124/485197/475085/482315/484464 XPNPEP2, KCNE3, COL14A1, COL28A1, KCNN4	5 VSAP_EC-up
cfa01522	Endocrine resistance	5/441	93/7854	0.60466376	0.96551148	0.86918276	100688936/488390/487235/484648/611939 DLL1, ABCB11, PIK3R1, RAF1, MAP2K2	5 VSAP_EC-up

cfa04064	NF-kappa B signaling pathway	5/441	93/7854	0.60466376	0.96551148	0.86918276	490926/488770/477024/481245/611262 MAP3K14, BCL2A1, MYD88, TAB1, TRADD	5	VSAP_EC-up
cfa04925	Aldosterone synthesis and secretion	5/441	94/7854	0.61423351	0.97035825	0.87354597	481746/403836/479756/403607/484665 ITPR3, AGTR1, DAGLB, LIPE, CAMK1	5	VSAP_EC-up
cfa04750	Inflammatory mediator regulation of TRP channels	5/441	100/7854	0.66871271	0.99864063	0.89900663	481746/488940/487235/481256/403609 ITPR3, F2RL1, PIK3R1, PLA2G6, PPP1CA	5	VSAP_EC-up
cfa04620	Toll-like receptor signaling pathway	5/441	101/7854	0.67728537	0.99864063	0.89900663	487235/477024/481245/476412/611939 PIK3R1, MYD88, TAB1, IRF3, MAP2K2	5	VSAP_EC-up
cfa03060	Protein export	5/279	23/7854	0.00108444	0.02574197	0.02182224	480726/403953/609416/489293/403944 HSPA5, SRP54, , SRPRA, SRP72	5	VSAP_EC-down
cfa04927	Cortisol synthesis and secretion	5/279	63/7854	0.07230659	0.34283086	0.29062801	479975/486241/403928/491369/403897 PRKACB, SCARB1, GNAQ, CREB3L2, NR4A1	5	VSAP_EC-down
cfa05133	Pertussis	5/279	71/7854	0.10722597	0.38701439	0.32808371	403567/477956/476022/478464/480416 CASP3, ITGB1, CFL1, MAPK10, CALM1	5	VSAP_EC-down
cfa04971	Gastric acid secretion	5/279	72/7854	0.11208555	0.38701439	0.32808371	479975/403928/480416/610764/489050 PRKACB, GNAQ, CALM1, CAMK2D, CAMK2G	5	VSAP_EC-down
cfa05214	Glioma	5/279	75/7854	0.12728491	0.4066908	0.344764	491597/480416/610764/474890/489050 PDGFA, CALM1, CAMK2D, CDKN1A, CAMK2G	5	VSAP_EC-down
cfa04970	Salivary secretion	5/279	78/7854	0.14337358	0.431399	0.36570988	479975/403480/609616/403928/480416 PRKACB, ATP2B1, PRKG1, GNAQ, CALM1	5	VSAP_EC-down
cfa01521	EGFR tyrosine kinase inhibitor resistance	5/279	80/7854	0.15456458	0.44134096	0.37413798	403802/487870/491597/475590/478575 VEGFA, EIF4E, PDGFA, EIF4EBP1, GSK3B	5	VSAP_EC-down
cfa04211	Longevity regulating pathway	5/279	88/7854	0.20263079	0.51886979	0.43986149	479975/487335/487870/475590/491369 PRKACB, SESN2, EIF4E, EIF4EBP1, CREB3L2	5	VSAP_EC-down
cfa04933	AGE-RAGE signaling pathway in diabetic complications	5/279	100/7854	0.28229259	0.61044809	0.51749517	403802/403476/403567/481628/478464 VEGFA, SERPINE1, CASP3, TGFBR1, MAPK10	5	VSAP_EC-down
cfa04660	T cell receptor signaling pathway	5/279	102/7854	0.29616777	0.62195513	0.52725003	607885/488036/478575/478464/612814 PAK1, DLG1, GSK3B, MAPK10, PAK2	5	VSAP_EC-down
cfa04928	Parathyroid hormone synthesis, secretion and action	5/279	107/7854	0.33129907	0.66259814	0.56170433	479975/403928/478754/491369/474890 PRKACB, GNAQ, NR4A2, CREB3L2, CDKN1A	5	VSAP_EC-down
cfa04668	TNF signaling pathway	5/279	108/7854	0.33838013	0.67163329	0.5693637	403567/442942/478464/491369/488471 CASP3, PTGS2, MAPK10, CREB3L2, CFLAR	5	VSAP_EC-down
cfa04726	Serotonergic synapse	5/279	110/7854	0.35257644	0.68617084	0.58168762	479975/403567/442942/477762/403928 PRKACB, CASP3, PTGS2, HTR7, GNAQ	5	VSAP_EC-down
cfa04725	Cholinergic synapse	5/279	113/7854	0.37391914	0.70990446	0.60180732	479975/403928/491369/610764/489050 PRKACB, GNAQ, CREB3L2, CAMK2D, CAMK2G	5	VSAP_EC-down
cfa04935	Growth hormone synthesis, secretion and action	5/279	116/7854	0.39526225	0.72002051	0.610383	479975/403928/478575/478464/491369 PRKACB, GNAQ, GSK3B, MAPK10, CREB3L2	5	VSAP_EC-down
cfa04380	Osteoclast differentiation	5/279	125/7854	0.45869656	0.73430489	0.62249229	474544/481628/403788/478464/492019 FHL2, TGFBR1, ITGB3, MAPK10, BTK	5	VSAP_EC-down
cfa04210	Apoptosis	5/279	131/7854	0.49995909	0.77508451	0.6570624	403567/607439/478464/480361/488471 CASP3, DDIT3, MAPK10, EIF2S1, CFLAR	5	VSAP_EC-down
cfa04723	Retrograde endocannabinoid signaling	5/279	149/7854	0.61519413	0.84772198	0.71863937	479975/442942/607407/403928/478464 PRKACB, PTGS2, NDUFA4L2, GNAQ, MAPK10	5	VSAP_EC-down
cfa04330	Notch signaling pathway	5/357	51/7854	0.08056808	0.24433145	0.17883532	480676/607575/481726/610303/485769 NOTCH1, DLL4, NOTCH4, MFNG, JAG1	5	VCAV_EC-up
cfa04961	Endocrine and other factor-regulated calcium reabsorption	5/357	52/7854	0.08599569	0.24992499	0.18292944	403480/403957/490755/477098/490341 ATP2B1, RAB11A, SLC8A3, ATP1B3, DNM3	5	VCAV_EC-up
cfa05014	Amyotrophic lateral sclerosis (ALS)	5/357	56/7854	0.10954769	0.2883378	0.21104521	403486/487437/612821/482620/100856109 PPP3CA, TNFRSF1B, MAPK13, APAF1, ALS2	5	VCAV_EC-up
cfa05321	Inflammatory bowel disease (IBD)	5/357	61/7854	0.14287255	0.34363311	0.25151791	403851/403985/403417/487134/100856077 IL2RG, IL6, TLR4, GATA3, IFNGR1	5	VCAV_EC-up
cfa04927	Cortisol synthesis and secretion	5/357	63/7854	0.15730281	0.36880238	0.26994024	480271/475266/486241/607922/403897 ADCY4, CREB5, SCARB1, CREB1, NR4A1	5	VCAV_EC-up
cfa05230	Central carbon metabolism in cancer	5/357	66/7854	0.18000356	0.3954409	0.28943797	403811/478232/403494/403872/480348 KIT, MTOR, RET, NRAS, HIF1A	5	VCAV_EC-up
cfa05031	Amphetamine addiction	5/357	67/7854	0.18783043	0.40941164	0.29966368	607269/403486/475266/607922/403558 CALML6, PPP3CA, CREB5, CREB1, PPP1CB	5	VCAV_EC-up

cfa04520	Adherens junction	5/357	70/7854	0.21201216	0.45153734	0.33049705	481162/403917/611060/403752/474698<br>PTPRB, YES1, NECTIN2, TJP1, CTNNA1	5	VCAV_EC-up
cfa04971	Gastric acid secretion	5/357	72/7854	0.2286596	0.47872221	0.35039468	607269/480271/477098/478227/403717<br>CALML6, ADCY4, ATP1B3, GNAI1, KCNJ2	5	VCAV_EC-up
cfa04061	Viral protein interaction with cytokine and cytokine receptor	5/357	83/7854	0.32549478	0.59176959	0.43313828	403851/403850/483900/403985/487437<br>IL2RG, CXCL8, CXCR4, IL6, TNFRSF1B	5	VCAV_EC-up
cfa05323	Rheumatoid arthritis	5/357	83/7854	0.32549478	0.59176959	0.43313828	403714/403850/403985/403417/403764<br>TEK, CXCL8, IL6, TLR4, CD86	5	VCAV_EC-up
cfa04911	Insulin secretion	5/357	85/7854	0.34368169	0.61074644	0.44702814	475066/480271/475266/477098/607922<br>RIMS2, ADCY4, CREB5, ATP1B3, CREB1	5	VCAV_EC-up
cfa04974	Protein digestion and absorption	5/357	91/7854	0.39847921	0.6577911	0.48146189	476788/403466/490755/477098/481780<br>PRCP, COL4A5, SLC8A3, ATP1B3, KCNK5	5	VCAV_EC-up
cfa04350	TGF-beta signaling pathway	5/357	92/7854	0.40759391	0.65826592	0.48180942	475456/479444/477162/487628/403608<br>SMAD1, PPP2R1B, BMP2, THSD4, PPP2CA	5	VCAV_EC-up
cfa05017	Spinocerebellar ataxia	5/357	96/7854	0.44383056	0.67499155	0.49405154	483273/480229/478232/474897/479797<br>RELN, , MTOR, NFYA, ATP2A1	5	VCAV_EC-up
cfa00513	Various types of N-glycan biosynthesis	5/241	40/7854	0.00714553	0.12549329	0.11282408	404012/481579/448804/487633/477223<br>DDOST, B4GALT1, FUT8, , RPN2	5	VCAV_EC-down
cfa04340	Hedgehog signaling pathway	5/241	47/7854	0.01401413	0.23164538	0.20825957	611166/611156/483244/611782/487979<br>EVC, EVC2, GLI3, CCND2, BOC	5	VCAV_EC-down
NA	NA	NA	NA	NA	NA	NA	NA NA	NA	AORT_EC-up
NA	NA	NA	NA	NA	NA	NA	NA NA	NA	AORT_EC-up
NA	NA	NA	NA	NA	NA	NA	NA NA	NA	AORT_EC-up
NA	NA	NA	NA	NA	NA	NA	NA NA	NA	AORT_EC-up
NA	NA	NA	NA	NA	NA	NA	NA NA	NA	AORT_EC-up
NA	NA	NA	NA	NA	NA	NA	NA NA	NA	AORT_EC-up
NA	NA	NA	NA	NA	NA	NA	NA NA	NA	AORT_EC-up
NA	NA	NA	NA	NA	NA	NA	NA NA	NA	AORT_EC-up
NA	NA	NA	NA	NA	NA	NA	NA NA	NA	AORT_EC-up
NA	NA	NA	NA	NA	NA	NA	NA NA	NA	AORT_EC-up
NA	NA	NA	NA	NA	NA	NA	NA NA	NA	AORT_EC-up
NA	NA	NA	NA	NA	NA	NA	NA NA	NA	AORT_EC-up
NA	NA	NA	NA	NA	NA	NA	NA NA	NA	AORT_EC-up
NA	NA	NA	NA	NA	NA	NA	NA NA	NA	AORT_EC-up
NA	NA	NA	NA	NA	NA	NA	NA NA	NA	AORT_EC-up
NA	NA	NA	NA	NA	NA	NA	NA NA	NA	AORT_EC-up
NA	NA	NA	NA	NA	NA	NA	NA NA	NA	AORT_EC-up
NA	NA	NA	NA	NA	NA	NA	NA NA	NA	AORT_EC-up
NA	NA	NA	NA	NA	NA	NA	NA NA	NA	AORT_EC-up
NA	NA	NA	NA	NA	NA	NA	NA NA	NA	AORT_EC-up
NA	NA	NA	NA	NA	NA	NA	NA NA	NA	AORT_EC-up
NA	NA	NA	NA	NA	NA	NA	NA NA	NA	AORT_EC-up
NA	NA	NA	NA	NA	NA	NA	NA NA	NA	AORT_EC-up
NA	NA	NA	NA	NA	NA	NA	NA NA	NA	AORT_EC-up
NA	NA	NA	NA	NA	NA	NA	NA NA	NA	AORT_EC-up
NA	NA	NA	NA	NA	NA	NA	NA NA	NA	AORT_EC-up
NA	NA	NA	NA	NA	NA	NA	NA NA	NA	AORT_EC-up
NA	NA	NA	NA	NA	NA	NA	NA NA	NA	AORT_EC-up
NA	NA	NA	NA	NA	NA	NA	NA NA	NA	AORT_EC-up
NA	NA	NA	NA	NA	NA	NA	NA NA	NA	AORT_EC-up
NA	NA	NA	NA	NA	NA	NA	NA NA	NA	AORT_EC-up
NA	NA	NA	NA	NA	NA	NA	NA NA	NA	AORT_EC-up
NA	NA	NA	NA	NA	NA	NA	NA NA	NA	AORT_EC-up
NA	NA	NA	NA	NA	NA	NA	NA NA	NA	AORT_EC-up
NA	NA	NA	NA	NA	NA	NA	NA NA	NA	AORT_EC-up
NA	NA	NA	NA	NA	NA	NA	NA NA	NA	AORT_EC-up
NA	NA	NA	NA	NA	NA	NA	NA NA	NA	AORT_EC-up
NA	NA	NA	NA	NA	NA	NA	NA NA	NA	AORT_EC-up
NA	NA	NA	NA	NA	NA	NA	NA NA	NA	AORT_EC-up
NA	NA	NA	NA	NA	NA	NA	NA NA	NA	AORT_EC-up
NA	NA	NA	NA	NA	NA	NA	NA NA	NA	AORT_EC-up
NA	NA	NA	NA	NA	NA	NA	NA NA	NA	AORT_EC-up
NA	NA	NA	NA	NA	NA	NA	NA NA	NA	AORT_EC-up
NA	NA	NA	NA	NA	NA	NA	NA NA	NA	AORT_EC-up
NA	NA	NA	NA	NA	NA	NA	NA NA	NA	AORT_EC-up
NA	NA	NA	NA	NA	NA	NA	NA NA	NA	AORT_EC-up
NA	NA	NA	NA	NA	NA	NA	NA NA	NA	AORT_EC-up
NA	NA	NA	NA	NA	NA	NA	NA NA	NA	AORT_EC-up
NA	NA	NA	NA	NA	NA	NA	NA NA	NA	AORT_EC-up
NA	NA	NA	NA	NA	NA	NA	NA NA	NA	AORT_EC-up
NA	NA	NA	NA	NA	NA	NA	NA NA	NA	AORT_EC-up
NA	NA	NA	NA	NA	NA	NA	NA NA	NA	AORT_EC-up
NA	NA	NA	NA	NA	NA	NA	NA NA	NA	AORT_EC-up

NA	NA	NA	NA	NA	NA	NA	NA	NA	NA	AORT_EC-up
NA	NA	NA	NA	NA	NA	NA	NA	NA	NA	AORT_EC-up
NA	NA	NA	NA	NA	NA	NA	NA	NA	NA	AORT_EC-up
NA	NA	NA	NA	NA	NA	NA	NA	NA	NA	AORT_EC-up
NA	NA	NA	NA	NA	NA	NA	NA	NA	NA	AORT_EC-up
NA	NA	NA	NA	NA	NA	NA	NA	NA	NA	AORT_EC-up
NA	NA	NA	NA	NA	NA	NA	NA	NA	NA	AORT_EC-up
NA	NA	NA	NA	NA	NA	NA	NA	NA	NA	AORT_EC-up
NA	NA	NA	NA	NA	NA	NA	NA	NA	NA	AORT_EC-up
NA	NA	NA	NA	NA	NA	NA	NA	NA	NA	AORT_EC-up
NA	NA	NA	NA	NA	NA	NA	NA	NA	NA	AORT_EC-up
NA	NA	NA	NA	NA	NA	NA	NA	NA	NA	AORT_EC-up
NA	NA	NA	NA	NA	NA	NA	NA	NA	NA	AORT_EC-up
NA	NA	NA	NA	NA	NA	NA	NA	NA	NA	AORT_EC-up
NA	NA	NA	NA	NA	NA	NA	NA	NA	NA	AORT_EC-up
NA	NA	NA	NA	NA	NA	NA	NA	NA	NA	AORT_EC-up
NA	NA	NA	NA	NA	NA	NA	NA	NA	NA	AORT_EC-up
NA	NA	NA	NA	NA	NA	NA	NA	NA	NA	AORT_EC-up
NA	NA	NA	NA	NA	NA	NA	NA	NA	NA	AORT_EC-up
NA	NA	NA	NA	NA	NA	NA	NA	NA	NA	AORT_EC-up
NA	NA	NA	NA	NA	NA	NA	NA	NA	NA	AORT_EC-up
NA	NA	NA	NA	NA	NA	NA	NA	NA	NA	AORT_EC-up
NA	NA	NA	NA	NA	NA	NA	NA	NA	NA	AORT_EC-up
NA	NA	NA	NA	NA	NA	NA	NA	NA	NA	AORT_EC-up
NA	NA	NA	NA	NA	NA	NA	NA	NA	NA	AORT_EC-up
NA	NA	NA	NA	NA	NA	NA	NA	NA	NA	AORT_EC-up
NA	NA	NA	NA	NA	NA	NA	NA	NA	NA	AORT_EC-up
NA	NA	NA	NA	NA	NA	NA	NA	NA	NA	AORT_EC-up
NA	NA	NA	NA	NA	NA	NA	NA	NA	NA	AORT_EC-up
NA	NA	NA	NA	NA	NA	NA	NA	NA	NA	AORT_EC-up
NA	NA	NA	NA	NA	NA	NA	NA	NA	NA	AORT_EC-up
NA	NA	NA	NA	NA	NA	NA	NA	NA	NA	AORT_EC-up
NA	NA	NA	NA	NA	NA	NA	NA	NA	NA	AORT_EC-up
NA	NA	NA	NA	NA	NA	NA	NA	NA	NA	AORT_EC-up
NA	NA	NA	NA	NA	NA	NA	NA	NA	NA	AORT_EC-up
NA	NA	NA	NA	NA	NA	NA	NA	NA	NA	AORT_EC-up
NA	NA	NA	NA	NA	NA	NA	NA	NA	NA	AORT_EC-up
NA	NA	NA	NA	NA	NA	NA	NA	NA	NA	AORT_EC-up
NA	NA	NA	NA	NA	NA	NA	NA	NA	NA	AORT_EC-up
NA	NA	NA	NA	NA	NA	NA	NA	NA	NA	AORT_EC-up
NA	NA	NA	NA	NA	NA	NA	NA	NA	NA	AORT_EC-up
NA	NA	NA	NA	NA	NA	NA	NA	NA	NA	AORT_EC-up
NA	NA	NA	NA	NA	NA	NA	NA	NA	NA	AORT_EC-up
NA	NA	NA	NA	NA	NA	NA	NA	NA	NA	AORT_EC-up
NA	NA	NA	NA	NA	NA	NA	NA	NA	NA	AORT_EC-up
NA	NA	NA	NA	NA	NA	NA	NA	NA	NA	AORT_EC-up
NA	NA	NA	NA	NA	NA	NA	NA	NA	NA	AORT_EC-up
NA	NA	NA	NA	NA	NA	NA	NA	NA	NA	AORT_EC-up
NA	NA	NA	NA	NA	NA	NA	NA	NA	NA	AORT_EC-up
NA	NA	NA	NA	NA	NA	NA	NA	NA	NA	AORT_EC-up
NA	NA	NA	NA	NA	NA	NA	NA	NA	NA	AORT_EC-up
NA	NA	NA	NA	NA	NA	NA	NA	NA	NA	AORT_EC-up
NA	NA	NA	NA	NA	NA	NA	NA	NA	NA	AORT_EC-up
NA	NA	NA	NA	NA	NA	NA	NA	NA	NA	AORT_EC-up
NA	NA	NA	NA	NA	NA	NA	NA	NA	NA	AORT_EC-up
NA	NA	NA	NA	NA	NA	NA	NA	NA	NA	AORT_EC-up
NA	NA	NA	NA	NA	NA	NA	NA	NA	NA	AORT_EC-up
NA	NA	NA	NA	NA	NA	NA	NA	NA	NA	AORT_EC-up
NA	NA	NA	NA	NA	NA	NA	NA	NA	NA	AORT_EC-up
NA	NA	NA	NA	NA	NA	NA	NA	NA	NA	AORT_EC-up
NA	NA	NA	NA	NA	NA	NA	NA	NA	NA	AORT_EC-up
NA	NA	NA	NA	NA	NA	NA	NA	NA	NA	AORT_EC-up
NA	NA	NA	NA	NA	NA	NA	NA	NA	NA	AORT_EC-up
NA	NA	NA	NA	NA	NA	NA	NA	NA	NA	AORT_EC-up
NA	NA	NA	NA	NA	NA	NA	NA	NA	NA	AORT_EC-up
NA	NA	NA	NA	NA	NA	NA	NA	NA	NA	AORT_EC-up
NA	NA	NA	NA	NA	NA	NA	NA	NA	NA	AORT_EC-up
NA	NA	NA	NA	NA	NA	NA	NA	NA	NA	AORT_EC-up
NA	NA	NA	NA	NA	NA	NA	NA	NA	NA	AORT_EC-up
NA	NA	NA	NA	NA	NA	NA	NA	NA	NA	AORT_EC-up
NA	NA	NA	NA	NA	NA	NA	NA	NA	NA	AORT_EC-up
NA	NA	NA	NA	NA	NA	NA	NA	NA	NA	AORT_EC-up
NA	NA	NA	NA	NA	NA	NA	NA	NA	NA	AORT_EC-up
NA	NA	NA	NA	NA	NA	NA	NA	NA	NA	AORT_EC-up

NA	NA	NA	NA	NA	NA	NA	NA	NA	NA	AORT_EC-up
NA	NA	NA	NA	NA	NA	NA	NA	NA	NA	AORT_EC-up
NA	NA	NA	NA	NA	NA	NA	NA	NA	NA	AORT_EC-up
NA	NA	NA	NA	NA	NA	NA	NA	NA	NA	AORT_EC-up
NA	NA	NA	NA	NA	NA	NA	NA	NA	NA	AORT_EC-up
NA	NA	NA	NA	NA	NA	NA	NA	NA	NA	AORT_EC-up
NA	NA	NA	NA	NA	NA	NA	NA	NA	NA	AORT_EC-up
NA	NA	NA	NA	NA	NA	NA	NA	NA	NA	AORT_EC-up
NA	NA	NA	NA	NA	NA	NA	NA	NA	NA	AORT_EC-up
NA	NA	NA	NA	NA	NA	NA	NA	NA	NA	AORT_EC-up
NA	NA	NA	NA	NA	NA	NA	NA	NA	NA	AORT_EC-up
NA	NA	NA	NA	NA	NA	NA	NA	NA	NA	AORT_EC-up
NA	NA	NA	NA	NA	NA	NA	NA	NA	NA	AORT_EC-up
NA	NA	NA	NA	NA	NA	NA	NA	NA	NA	AORT_EC-up
NA	NA	NA	NA	NA	NA	NA	NA	NA	NA	AORT_EC-up
NA	NA	NA	NA	NA	NA	NA	NA	NA	NA	AORT_EC-up
NA	NA	NA	NA	NA	NA	NA	NA	NA	NA	AORT_EC-up
NA	NA	NA	NA	NA	NA	NA	NA	NA	NA	AORT_EC-up
NA	NA	NA	NA	NA	NA	NA	NA	NA	NA	AORT_EC-up
NA	NA	NA	NA	NA	NA	NA	NA	NA	NA	AORT_EC-up
NA	NA	NA	NA	NA	NA	NA	NA	NA	NA	AORT_EC-up
NA	NA	NA	NA	NA	NA	NA	NA	NA	NA	AORT_EC-up
NA	NA	NA	NA	NA	NA	NA	NA	NA	NA	AORT_EC-up
NA	NA	NA	NA	NA	NA	NA	NA	NA	NA	AORT_EC-up
NA	NA	NA	NA	NA	NA	NA	NA	NA	NA	AORT_EC-up
NA	NA	NA	NA	NA	NA	NA	NA	NA	NA	AORT_EC-up
NA	NA	NA	NA	NA	NA	NA	NA	NA	NA	AORT_EC-up
NA	NA	NA	NA	NA	NA	NA	NA	NA	NA	AORT_EC-up
NA	NA	NA	NA	NA	NA	NA	NA	NA	NA	AORT_EC-up
NA	NA	NA	NA	NA	NA	NA	NA	NA	NA	AORT_EC-up
NA	NA	NA	NA	NA	NA	NA	NA	NA	NA	AORT_EC-up
NA	NA	NA	NA	NA	NA	NA	NA	NA	NA	AORT_EC-up
NA	NA	NA	NA	NA	NA	NA	NA	NA	NA	AORT_EC-up
NA	NA	NA	NA	NA	NA	NA	NA	NA	NA	AORT_EC-up
NA	NA	NA	NA	NA	NA	NA	NA	NA	NA	AORT_EC-up
NA	NA	NA	NA	NA	NA	NA	NA	NA	NA	AORT_EC-up
NA	NA	NA	NA	NA	NA	NA	NA	NA	NA	AORT_EC-up
NA	NA	NA	NA	NA	NA	NA	NA	NA	NA	AORT_EC-up
NA	NA	NA	NA	NA	NA	NA	NA	NA	NA	AORT_EC-up
NA	NA	NA	NA	NA	NA	NA	NA	NA	NA	AORT_EC-up
NA	NA	NA	NA	NA	NA	NA	NA	NA	NA	AORT_EC-up
NA	NA	NA	NA	NA	NA	NA	NA	NA	NA	AORT_EC-up
NA	NA	NA	NA	NA	NA	NA	NA	NA	NA	AORT_EC-up
NA	NA	NA	NA	NA	NA	NA	NA	NA	NA	AORT_EC-up
NA	NA	NA	NA	NA	NA	NA	NA	NA	NA	AORT_EC-up
NA	NA	NA	NA	NA	NA	NA	NA	NA	NA	AORT_EC-up
NA	NA	NA	NA	NA	NA	NA	NA	NA	NA	AORT_EC-up
NA	NA	NA	NA	NA	NA	NA	NA	NA	NA	AORT_EC-up
NA	NA	NA	NA	NA	NA	NA	NA	NA	NA	AORT_EC-up
NA	NA	NA	NA	NA	NA	NA	NA	NA	NA	AORT_EC-up
NA	NA	NA	NA	NA	NA	NA	NA	NA	NA	AORT_EC-up
NA	NA	NA	NA	NA	NA	NA	NA	NA	NA	AORT_EC-up
NA	NA	NA	NA	NA	NA	NA	NA	NA	NA	AORT_EC-up
NA	NA	NA	NA	NA	NA	NA	NA	NA	NA	AORT_EC-up
NA	NA	NA	NA	NA	NA	NA	NA	NA	NA	AORT_EC-up
NA	NA	NA	NA	NA	NA	NA	NA	NA	NA	AORT_EC-up
NA	NA	NA	NA	NA	NA	NA	NA	NA	NA	AORT_EC-up
NA	NA	NA	NA	NA	NA	NA	NA	NA	NA	AORT_EC-up
NA	NA	NA	NA	NA	NA	NA	NA	NA	NA	AORT_EC-up
NA	NA	NA	NA	NA	NA	NA	NA	NA	NA	AORT_EC-up
NA	NA	NA	NA	NA	NA	NA	NA	NA	NA	AORT_EC-up
NA	NA	NA	NA	NA	NA	NA	NA	NA	NA	AORT_EC-up
NA	NA	NA	NA	NA	NA	NA	NA	NA	NA	AORT_EC-up
cfa04978	Mineral absorption	2/388	51/7854	0.72544179	0.99972301	0.9362731	100286799/477098	SLC30A1, ATP1B3	2	ACOR_VSMC-down
NA	NA	NA	NA	NA	NA	NA	NA	NA	NA	AORT_EC-down
NA	NA	NA	NA	NA	NA	NA	NA	NA	NA	AORT_EC-down
NA	NA	NA	NA	NA	NA	NA	NA	NA	NA	AORT_EC-down
NA	NA	NA	NA	NA	NA	NA	NA	NA	NA	AORT_EC-down
NA	NA	NA	NA	NA	NA	NA	NA	NA	NA	AORT_EC-down
NA	NA	NA	NA	NA	NA	NA	NA	NA	NA	AORT_EC-down
NA	NA	NA	NA	NA	NA	NA	NA	NA	NA	AORT_EC-down
NA	NA	NA	NA	NA	NA	NA	NA	NA	NA	AORT_EC-down
NA	NA	NA	NA	NA	NA	NA	NA	NA	NA	AORT_EC-down

NA	NA	NA	NA	NA	NA	NA	NA	NA	NA	AORT_EC-down
NA	NA	NA	NA	NA	NA	NA	NA	NA	NA	AORT_EC-down
NA	NA	NA	NA	NA	NA	NA	NA	NA	NA	AORT_EC-down
NA	NA	NA	NA	NA	NA	NA	NA	NA	NA	AORT_EC-down
NA	NA	NA	NA	NA	NA	NA	NA	NA	NA	AORT_EC-down
NA	NA	NA	NA	NA	NA	NA	NA	NA	NA	AORT_EC-down
NA	NA	NA	NA	NA	NA	NA	NA	NA	NA	AORT_EC-down
NA	NA	NA	NA	NA	NA	NA	NA	NA	NA	AORT_EC-down
NA	NA	NA	NA	NA	NA	NA	NA	NA	NA	AORT_EC-down
NA	NA	NA	NA	NA	NA	NA	NA	NA	NA	AORT_EC-down
NA	NA	NA	NA	NA	NA	NA	NA	NA	NA	AORT_EC-down
NA	NA	NA	NA	NA	NA	NA	NA	NA	NA	AORT_EC-down
NA	NA	NA	NA	NA	NA	NA	NA	NA	NA	AORT_EC-down
NA	NA	NA	NA	NA	NA	NA	NA	NA	NA	AORT_EC-down
NA	NA	NA	NA	NA	NA	NA	NA	NA	NA	AORT_EC-down
NA	NA	NA	NA	NA	NA	NA	NA	NA	NA	AORT_EC-down
NA	NA	NA	NA	NA	NA	NA	NA	NA	NA	AORT_EC-down
NA	NA	NA	NA	NA	NA	NA	NA	NA	NA	AORT_EC-down
NA	NA	NA	NA	NA	NA	NA	NA	NA	NA	AORT_EC-down
NA	NA	NA	NA	NA	NA	NA	NA	NA	NA	AORT_EC-down
NA	NA	NA	NA	NA	NA	NA	NA	NA	NA	AORT_EC-down
NA	NA	NA	NA	NA	NA	NA	NA	NA	NA	AORT_EC-down
NA	NA	NA	NA	NA	NA	NA	NA	NA	NA	AORT_EC-down
NA	NA	NA	NA	NA	NA	NA	NA	NA	NA	AORT_EC-down
NA	NA	NA	NA	NA	NA	NA	NA	NA	NA	AORT_EC-down
NA	NA	NA	NA	NA	NA	NA	NA	NA	NA	AORT_EC-down
NA	NA	NA	NA	NA	NA	NA	NA	NA	NA	AORT_EC-down
NA	NA	NA	NA	NA	NA	NA	NA	NA	NA	AORT_EC-down
NA	NA	NA	NA	NA	NA	NA	NA	NA	NA	AORT_EC-down
NA	NA	NA	NA	NA	NA	NA	NA	NA	NA	AORT_EC-down
NA	NA	NA	NA	NA	NA	NA	NA	NA	NA	AORT_EC-down
NA	NA	NA	NA	NA	NA	NA	NA	NA	NA	AORT_EC-down
NA	NA	NA	NA	NA	NA	NA	NA	NA	NA	AORT_EC-down
NA	NA	NA	NA	NA	NA	NA	NA	NA	NA	AORT_EC-down
NA	NA	NA	NA	NA	NA	NA	NA	NA	NA	AORT_EC-down
NA	NA	NA	NA	NA	NA	NA	NA	NA	NA	AORT_EC-down
NA	NA	NA	NA	NA	NA	NA	NA	NA	NA	AORT_EC-down
NA	NA	NA	NA	NA	NA	NA	NA	NA	NA	AORT_EC-down
NA	NA	NA	NA	NA	NA	NA	NA	NA	NA	AORT_EC-down
NA	NA	NA	NA	NA	NA	NA	NA	NA	NA	AORT_EC-down
NA	NA	NA	NA	NA	NA	NA	NA	NA	NA	AORT_EC-down
NA	NA	NA	NA	NA	NA	NA	NA	NA	NA	AORT_EC-down
NA	NA	NA	NA	NA	NA	NA	NA	NA	NA	AORT_EC-down
NA	NA	NA	NA	NA	NA	NA	NA	NA	NA	AORT_EC-down
NA	NA	NA	NA	NA	NA	NA	NA	NA	NA	AORT_EC-down
NA	NA	NA	NA	NA	NA	NA	NA	NA	NA	AORT_EC-down
NA	NA	NA	NA	NA	NA	NA	NA	NA	NA	AORT_EC-down
NA	NA	NA	NA	NA	NA	NA	NA	NA	NA	AORT_EC-down
NA	NA	NA	NA	NA	NA	NA	NA	NA	NA	AORT_EC-down
NA	NA	NA	NA	NA	NA	NA	NA	NA	NA	AORT_EC-down
NA	NA	NA	NA	NA	NA	NA	NA	NA	NA	AORT_EC-down
NA	NA	NA	NA	NA	NA	NA	NA	NA	NA	AORT_EC-down
NA	NA	NA	NA	NA	NA	NA	NA	NA	NA	AORT_EC-down
NA	NA	NA	NA	NA	NA	NA	NA	NA	NA	AORT_EC-down
NA	NA	NA	NA	NA	NA	NA	NA	NA	NA	AORT_EC-down
NA	NA	NA	NA	NA	NA	NA	NA	NA	NA	AORT_EC-down
NA	NA	NA	NA	NA	NA	NA	NA	NA	NA	AORT_EC-down
NA	NA	NA	NA	NA	NA	NA	NA	NA	NA	AORT_EC-down
NA	NA	NA	NA	NA	NA	NA	NA	NA	NA	AORT_EC-down
NA	NA	NA	NA	NA	NA	NA	NA	NA	NA	AORT_EC-down
NA	NA	NA	NA	NA	NA	NA	NA	NA	NA	AORT_EC-down
NA	NA	NA	NA	NA	NA	NA	NA	NA	NA	AORT_EC-down
NA	NA	NA	NA	NA	NA	NA	NA	NA	NA	AORT_EC-down
NA	NA	NA	NA	NA	NA	NA	NA	NA	NA	AORT_EC-down
NA	NA	NA	NA	NA	NA	NA	NA	NA	NA	AORT_EC-down
NA	NA	NA	NA	NA	NA	NA	NA	NA	NA	AORT_EC-down
NA	NA	NA	NA	NA	NA	NA	NA	NA	NA	AORT_EC-down
NA	NA	NA	NA	NA	NA	NA	NA	NA	NA	AORT_EC-down
NA	NA	NA	NA	NA	NA	NA	NA	NA	NA	AORT_EC-down
NA	NA	NA	NA	NA	NA	NA	NA	NA	NA	AORT_EC-down
NA	NA	NA	NA	NA	NA	NA	NA	NA	NA	AORT_EC-down
NA	NA	NA	NA	NA	NA	NA	NA	NA	NA	AORT_EC-down
NA	NA	NA	NA	NA	NA	NA	NA	NA	NA	AORT_EC-down
NA	NA	NA	NA	NA	NA	NA	NA	NA	NA	AORT_EC-down

NA	NA	NA	NA	NA	NA	NA	NA	NA	NA	AORT_EC-down
NA	NA	NA	NA	NA	NA	NA	NA	NA	NA	AORT_EC-down
NA	NA	NA	NA	NA	NA	NA	NA	NA	NA	AORT_EC-down
NA	NA	NA	NA	NA	NA	NA	NA	NA	NA	AORT_EC-down
NA	NA	NA	NA	NA	NA	NA	NA	NA	NA	AORT_EC-down
NA	NA	NA	NA	NA	NA	NA	NA	NA	NA	AORT_EC-down
NA	NA	NA	NA	NA	NA	NA	NA	NA	NA	AORT_EC-down
NA	NA	NA	NA	NA	NA	NA	NA	NA	NA	AORT_EC-down
NA	NA	NA	NA	NA	NA	NA	NA	NA	NA	AORT_EC-down
NA	NA	NA	NA	NA	NA	NA	NA	NA	NA	AORT_EC-down
NA	NA	NA	NA	NA	NA	NA	NA	NA	NA	AORT_EC-down
NA	NA	NA	NA	NA	NA	NA	NA	NA	NA	AORT_EC-down
NA	NA	NA	NA	NA	NA	NA	NA	NA	NA	AORT_EC-down
NA	NA	NA	NA	NA	NA	NA	NA	NA	NA	AORT_EC-down
NA	NA	NA	NA	NA	NA	NA	NA	NA	NA	AORT_EC-down
NA	NA	NA	NA	NA	NA	NA	NA	NA	NA	AORT_EC-down
NA	NA	NA	NA	NA	NA	NA	NA	NA	NA	AORT_EC-down
NA	NA	NA	NA	NA	NA	NA	NA	NA	NA	AORT_EC-down
NA	NA	NA	NA	NA	NA	NA	NA	NA	NA	AORT_EC-down
NA	NA	NA	NA	NA	NA	NA	NA	NA	NA	AORT_EC-down
NA	NA	NA	NA	NA	NA	NA	NA	NA	NA	AORT_EC-down
NA	NA	NA	NA	NA	NA	NA	NA	NA	NA	AORT_EC-down
NA	NA	NA	NA	NA	NA	NA	NA	NA	NA	AORT_EC-down
NA	NA	NA	NA	NA	NA	NA	NA	NA	NA	AORT_EC-down
NA	NA	NA	NA	NA	NA	NA	NA	NA	NA	AORT_EC-down
NA	NA	NA	NA	NA	NA	NA	NA	NA	NA	AORT_EC-down
NA	NA	NA	NA	NA	NA	NA	NA	NA	NA	AORT_EC-down
NA	NA	NA	NA	NA	NA	NA	NA	NA	NA	AORT_EC-down
NA	NA	NA	NA	NA	NA	NA	NA	NA	NA	AORT_EC-down
NA	NA	NA	NA	NA	NA	NA	NA	NA	NA	AORT_EC-down
NA	NA	NA	NA	NA	NA	NA	NA	NA	NA	AORT_EC-down
NA	NA	NA	NA	NA	NA	NA	NA	NA	NA	AORT_EC-down
NA	NA	NA	NA	NA	NA	NA	NA	NA	NA	AORT_EC-down
NA	NA	NA	NA	NA	NA	NA	NA	NA	NA	AORT_EC-down
NA	NA	NA	NA	NA	NA	NA	NA	NA	NA	AORT_EC-down
NA	NA	NA	NA	NA	NA	NA	NA	NA	NA	AORT_EC-down
NA	NA	NA	NA	NA	NA	NA	NA	NA	NA	AORT_EC-down
NA	NA	NA	NA	NA	NA	NA	NA	NA	NA	AORT_EC-down
NA	NA	NA	NA	NA	NA	NA	NA	NA	NA	AORT_EC-down
NA	NA	NA	NA	NA	NA	NA	NA	NA	NA	AORT_EC-down
NA	NA	NA	NA	NA	NA	NA	NA	NA	NA	AORT_EC-down
NA	NA	NA	NA	NA	NA	NA	NA	NA	NA	AORT_EC-down
NA	NA	NA	NA	NA	NA	NA	NA	NA	NA	AORT_EC-down
NA	NA	NA	NA	NA	NA	NA	NA	NA	NA	AORT_EC-down
NA	NA	NA	NA	NA	NA	NA	NA	NA	NA	AORT_EC-down
NA	NA	NA	NA	NA	NA	NA	NA	NA	NA	AORT_EC-down
NA	NA	NA	NA	NA	NA	NA	NA	NA	NA	AORT_EC-down
NA	NA	NA	NA	NA	NA	NA	NA	NA	NA	AORT_EC-down
NA	NA	NA	NA	NA	NA	NA	NA	NA	NA	AORT_EC-down
NA	NA	NA	NA	NA	NA	NA	NA	NA	NA	AORT_EC-down
NA	NA	NA	NA	NA	NA	NA	NA	NA	NA	AORT_EC-down
NA	NA	NA	NA	NA	NA	NA	NA	NA	NA	AORT_EC-down
NA	NA	NA	NA	NA	NA	NA	NA	NA	NA	AORT_EC-down
NA	NA	NA	NA	NA	NA	NA	NA	NA	NA	AORT_EC-down
NA	NA	NA	NA	NA	NA	NA	NA	NA	NA	AORT_EC-down
NA	NA	NA	NA	NA	NA	NA	NA	NA	NA	AORT_EC-down
NA	NA	NA	NA	NA	NA	NA	NA	NA	NA	AORT_EC-down
NA	NA	NA	NA	NA	NA	NA	NA	NA	NA	AORT_EC-down
NA	NA	NA	NA	NA	NA	NA	NA	NA	NA	AORT_EC-down
NA	NA	NA	NA	NA	NA	NA	NA	NA	NA	AORT_EC-down
NA	NA	NA	NA	NA	NA	NA	NA	NA	NA	AORT_EC-down
NA	NA	NA	NA	NA	NA	NA	NA	NA	NA	AORT_EC-down
NA	NA	NA	NA	NA	NA	NA	NA	NA	NA	AORT_EC-down
NA	NA	NA	NA	NA	NA	NA	NA	NA	NA	AORT_EC-down
NA	NA	NA	NA	NA	NA	NA	NA	NA	NA	AORT_EC-down
NA	NA	NA	NA	NA	NA	NA	NA	NA	NA	AORT_EC-down
NA	NA	NA	NA	NA	NA	NA	NA	NA	NA	AORT_EC-down
NA	NA	NA	NA	NA	NA	NA	NA	NA	NA	AORT_EC-down
NA	NA	NA	NA	NA	NA	NA	NA	NA	NA	AORT_EC-down
NA	NA	NA	NA	NA	NA	NA	NA	NA	NA	AORT_EC-down
NA	NA	NA	NA	NA	NA	NA	NA	NA	NA	AORT_EC-down
NA	NA	NA	NA	NA	NA	NA	NA	NA	NA	AORT_EC-down
NA	NA	NA	NA	NA	NA	NA	NA	NA	NA	AORT_EC-down
NA	NA	NA	NA	NA	NA	NA	NA	NA	NA	AORT_EC-down

NA	NA	NA	NA	NA	NA	NA	NA NA	NA	AORT_EC-down
NA	NA	NA	NA	NA	NA	NA	NA NA	NA	AORT_EC-down
NA	NA	NA	NA	NA	NA	NA	NA NA	NA	AORT_EC-down
NA	NA	NA	NA	NA	NA	NA	NA NA	NA	AORT_EC-down
NA	NA	NA	NA	NA	NA	NA	NA NA	NA	AORT_EC-down
NA	NA	NA	NA	NA	NA	NA	NA NA	NA	AORT_EC-down
NA	NA	NA	NA	NA	NA	NA	NA NA	NA	AORT_EC-down
NA	NA	NA	NA	NA	NA	NA	NA NA	NA	AORT_EC-down
NA	NA	NA	NA	NA	NA	NA	NA NA	NA	AORT_EC-down
NA	NA	NA	NA	NA	NA	NA	NA NA	NA	AORT_EC-down
NA	NA	NA	NA	NA	NA	NA	NA NA	NA	AORT_EC-down
NA	NA	NA	NA	NA	NA	NA	NA NA	NA	AORT_EC-down
NA	NA	NA	NA	NA	NA	NA	NA NA	NA	AORT_EC-down
NA	NA	NA	NA	NA	NA	NA	NA NA	NA	AORT_EC-down
NA	NA	NA	NA	NA	NA	NA	NA NA	NA	AORT_EC-down
NA	NA	NA	NA	NA	NA	NA	NA NA	NA	AORT_EC-down
NA	NA	NA	NA	NA	NA	NA	NA NA	NA	AORT_EC-down
NA	NA	NA	NA	NA	NA	NA	NA NA	NA	AORT_EC-down
NA	NA	NA	NA	NA	NA	NA	NA NA	NA	AORT_EC-down
NA	NA	NA	NA	NA	NA	NA	NA NA	NA	AORT_EC-down
NA	NA	NA	NA	NA	NA	NA	NA NA	NA	AORT_EC-down
NA	NA	NA	NA	NA	NA	NA	NA NA	NA	AORT_EC-down
NA	NA	NA	NA	NA	NA	NA	NA NA	NA	AORT_EC-down
NA	NA	NA	NA	NA	NA	NA	NA NA	NA	AORT_EC-down
NA	NA	NA	NA	NA	NA	NA	NA NA	NA	AORT_EC-down
NA	NA	NA	NA	NA	NA	NA	NA NA	NA	AORT_EC-down
NA	NA	NA	NA	NA	NA	NA	NA NA	NA	AORT_EC-down
NA	NA	NA	NA	NA	NA	NA	NA NA	NA	AORT_EC-down
NA	NA	NA	NA	NA	NA	NA	NA NA	NA	AORT_EC-down
NA	NA	NA	NA	NA	NA	NA	NA NA	NA	AORT_EC-down
NA	NA	NA	NA	NA	NA	NA	NA NA	NA	AORT_EC-down
NA	NA	NA	NA	NA	NA	NA	NA NA	NA	AORT_EC-down
NA	NA	NA	NA	NA	NA	NA	NA NA	NA	AORT_EC-down
NA	NA	NA	NA	NA	NA	NA	NA NA	NA	AORT_EC-down
NA	NA	NA	NA	NA	NA	NA	NA NA	NA	AORT_EC-down
NA	NA	NA	NA	NA	NA	NA	NA NA	NA	AORT_EC-down
NA	NA	NA	NA	NA	NA	NA	NA NA	NA	AORT_EC-down
NA	NA	NA	NA	NA	NA	NA	NA NA	NA	AORT_EC-down
NA	NA	NA	NA	NA	NA	NA	NA NA	NA	AORT_EC-down
NA	NA	NA	NA	NA	NA	NA	NA NA	NA	AORT_EC-down
NA	NA	NA	NA	NA	NA	NA	NA NA	NA	AORT_EC-down
NA	NA	NA	NA	NA	NA	NA	NA NA	NA	AORT_EC-down
cfa00980	Metabolism of xenobiotics by cytochrome P450	5/241	52/7854	0.02100027	0.24158651	0.21719709	476005/476006/480113/479912/483038 GSTP1, , EPHX1, GSTM4, CYP1B1	5	VCAV_EC-down
cfa04923	Regulation of lipolysis in adipocytes	5/241	54/7854	0.02433262	0.24158651	0.21719709	477625/609956/449021/485548/486148 ADCY6, , AKT2, IRS2, IRS1	5	VCAV_EC-down
cfa05204	Chemical carcinogenesis	5/241	55/7854	0.0261193	0.24158651	0.21719709	476005/476006/480113/479912/483038 GSTP1, , EPHX1, GSTM4, CYP1B1	5	VCAV_EC-down
cfa04137	Mitophagy - animal	5/241	64/7854	0.04598357	0.33131754	0.2978693	479479/485864/491872/488576/481954 GABARAP, SRC, TFE3, RAB7B, FOXO3	5	VCAV_EC-down
cfa05221	Acute myeloid leukemia	5/241	67/7854	0.05416573	0.36239455	0.32580893	609956/449021/487044/489398/475590 , AKT2, RUNX1T1, ZBTB16, EIF4EBP1	5	VCAV_EC-down
cfa01524	Platinum drug resistance	5/241	72/7854	0.0695602	0.38682027	0.34776874	476005/609956/449021/476006/479912 GSTP1, , AKT2, , GSTM4	5	VCAV_EC-down
cfa05218	Melanoma	5/241	73/7854	0.07290114	0.38682027	0.34776874	442985/609956/449021/482666/403857 PDGFRB, , AKT2, PDGFC, FGF2	5	VCAV_EC-down
cfa05100	Bacterial invasion of epithelial cells	5/241	75/7854	0.07984191	0.38682027	0.34776874	609956/485864/489104/482639/490439 , SRC, CLTB, ARHGAP10, SHC1	5	VCAV_EC-down
cfa05214	Glioma	5/241	75/7854	0.07984191	0.38682027	0.34776874	442985/609956/449021/610764/490439 PDGFRB, , AKT2, CAMK2D, SHC1	5	VCAV_EC-down
cfa04540	Gap junction	5/241	88/7854	0.13297223	0.47881756	0.43047842	442985/477625/485864/482666/486762 PDGFRB, ADCY6, SRC, PDGFC, TUBA8	5	VCAV_EC-down
cfa04914	Progesterone-mediated oocyte maturation	5/241	89/7854	0.13759627	0.48330691	0.43451455	477625/609956/449021/612448/100683974 ADCY6, , AKT2, RPS6KA2,	5	VCAV_EC-down
cfa04727	GABAergic synapse	5/241	91/7854	0.14705427	0.4861441	0.43706531	477625/479479/485864/478858/100686499 ADCY6, GABARAP, SRC, PLCL1, GLS2	5	VCAV_EC-down
cfa04750	Inflammatory mediator regulation of TRP channels	5/241	100/7854	0.19277757	0.52592716	0.47283206	477625/489522/609956/485864/610764 ADCY6, TRPV2, , SRC, CAMK2D	5	VCAV_EC-down
cfa04066	HIF-1 signaling pathway	5/241	112/7854	0.26017909	0.62487455	0.5617902	403656/609956/449021/610764/475590 ANGPT1, , AKT2, CAMK2D, EIF4EBP1	5	VCAV_EC-down
cfa04670	Leukocyte transendothelial migration	5/241	113/7854	0.26604421	0.62962523	0.56606127	403733/609956/489365/489664/481271 MMP2, , THY1, CYBA, RAC2	5	VCAV_EC-down

cfa04919	Thyroid hormone signaling pathway	5/241	116/7854	0.28381166	0.65352581	0.58754896	483148/609956/485864/449021/403599 NOTCH2, , SRC, AKT2, THRB	5	VCAV_EC-down
cfa00230	Purine metabolism	5/241	130/7854	0.36890083	0.75116762	0.67533332	477625/477809/485604/479253/479913 ADCY6, NT5C2, ENTPD3, ADK, AMPD2	5	VCAV_EC-down
cfa04550	Signaling pathways regulating pluripotency of stem cells	5/241	135/7854	0.39958389	0.78213515	0.70317451	486006/489077/609956/449021/403857 SMAD9, BMPR1A, , AKT2, FGF2	5	VCAV_EC-down
cfa05162	Measles	5/241	136/7854	0.40570835	0.78213515	0.70317451	609956/449021/608847/485789/611782 , AKT2, CCND3, MAVS, CCND2	5	VCAV_EC-down
cfa05224	Breast cancer	5/241	142/7854	0.44225363	0.79541394	0.71511274	483148/609956/449021/403857/490439 NOTCH2, , AKT2, FGF2, SHC1	5	VCAV_EC-down
cfa04310	Wnt signaling pathway	5/241	151/7854	0.49597456	0.82102262	0.73813609	484699/608847/611782/610764/481271 PRICKLE2, CCND3, CCND2, CAMK2D, RAC2	5	VCAV_EC-down
cfa03013	RNA transport	5/241	158/7854	0.53639899	0.84753502	0.76197193	487655/476840/478152/481101/475590 SNUPN, EIF3F, EIF3I, PYM1, EIF4EBP1	5	VCAV_EC-down
cfa04530	Tight junction	5/241	170/7854	0.60201773	0.8750588	0.78671703	485864/489898/486762/606992/475549 SRC, MICALL2, TUBA8, MYL6B, ACTR3B	5	VCAV_EC-down
cfa05152	Tuberculosis	5/241	171/7854	0.60724721	0.8750588	0.78671703	485864/449021/486588/479877/610764 SRC, AKT2, VDR, ATP6V0C, CAMK2D	5	VCAV_EC-down
cfa05202	Transcriptional misregulation in cancer	42856	172/7854	2.3757E-05	0.00225696	0.00210066	477910/482372/482373/490719/488770 EYA1, HOXA9, HOXA10, SIX1, BCL2A1	5	VFEM_EC-up
cfa04310	Wnt signaling pathway	34820	151/7854	0.03581315	0.64105544	0.63709715	612941/484699/490231/490903/490937 CAMK2A, PRICKLE2, LGR6, AXIN2, FZD2	5	AFEM_EC-up
cfa04020	Calcium signaling pathway	34820	192/7854	0.08269331	0.81029448	0.80529119	403858/481142/612941/485056/608401 CHRM2, AVPR1A, CAMK2A, TBXA2R, CASQ1	5	AFEM_EC-up
cfa05016	Huntington disease	34820	257/7854	0.19969949	0.81029448	0.80529119	610370/479127/612614/476258/403559 DNAL1, PPARGC1A, COX7A1, SOD2, SOD1	5	AFEM_EC-up
cfa04060	Cytokine-cytokine receptor interaction	34820	270/7854	0.22798516	0.81029448	0.80529119	403616/403469/100856510/403522/4865 37 LEP, CD40, ACKR4, CCL5, ACVR1B	5	AFEM_EC-up
cfa00563	Glycosylphosphatidylinositol (GPI)-anchor biosynthesis	5/407	25/7854	0.00819211	0.2359328	0.22679213	491748/476182/491170/479136/477244 , PIGN, PIGS, PIGG, PIGT	5	ACOR_VSMC-up
cfa04392	Hippo signaling pathway - multiple species	5/407	28/7854	0.01334337	0.30996841	0.2979594	485781/477343/479297/479465/610713 RASSF2, LATS2, WWC1, YAP1, TEAD1	5	ACOR_VSMC-up
cfa00500	Starch and sucrose metabolism	5/407	33/7854	0.02609966	0.37583516	0.3612743	491717/611078/611614/611993/483352 GYG2, PYGM, GYG1, GYS1, GAA	5	ACOR_VSMC-up
cfa00565	Ether lipid metabolism	5/407	43/7854	0.06980624	0.62825613	0.6039158	610214/100856443/476045/478514/4845 02 CHPT1, PLPP2, PLA2G16, CFI, PLD3	5	ACOR_VSMC-up
cfa00600	Sphingolipid metabolism	5/407	48/7854	0.10111284	0.71680351	0.68903262	481666/100856443/609655/485334/4851 95 UGCG, PLPP2, KDSR, SMPD1, NEU3	5	ACOR_VSMC-up
cfa00982	Drug metabolism - cytochrome P450	5/407	49/7854	0.1080947	0.7412208	0.71250391	403451/403450/490345/477558/403603 MAOB, MAOA, FMO4, , FMO3	5	ACOR_VSMC-up
cfa04978	Mineral absorption	44197	51/7854	0.12801527	0.34415573	0.29640206	482291 STEAP2	1	ACOR_EC-down
cfa04978	Mineral absorption	44105	51/7854	0.06310521	0.08414028	0.03690363	102155200	1	AORT_EC-up
cfa04927	Cortisol synthesis and secretion	5/407	63/7854	0.22678375	0.80256807	0.77147442	477625/476548/488171/611934/403928 ADCY6, ITPR1, NCEH1, CREB3, GNAQ	5	ACOR_VSMC-up
cfa05217	Basal cell carcinoma	5/407	63/7854	0.22678375	0.80256807	0.77147442	490695/479180/485149/474890/486093 BMP4, POLK, FZD4, CDKN1A, FZD3	5	ACOR_VSMC-up
cfa04137	Mitophagy - animal	5/407	64/7854	0.23644604	0.80256807	0.77147442	480613/480509/484010/612919/607408 RHOT1, NBR1, CITED2, GABARAPL1, BNIP3	5	ACOR_VSMC-up
cfa04622	RIG-I-like receptor signaling pathway	5/407	67/7854	0.26607111	0.82260001	0.79073027	488472/478061/100855667/100855560/1 02156745 CASP10, MAP3K1, ISG15, DDX58, MAP3K7	5	ACOR_VSMC-up
cfa05223	Non-small cell lung cancer	5/407	67/7854	0.26607111	0.82260001	0.79073027	489692/483048/479180/474890/490969 PLCG2, EML4, POLK, CDKN1A, STAT5B	5	ACOR_VSMC-up
cfa04920	Adipocytokine signaling pathway	5/407	70/7854	0.29646746	0.82260001	0.79073027	403616/489571/479127/474670/488526 LEP, PRKAA2, PPARGC1A, ACSL6, PRKAG3	5	ACOR_VSMC-up
cfa04918	Thyroid hormone synthesis	5/407	72/7854	0.31704375	0.82260001	0.79073027	478781/477625/476548/611934/403928 LRP2, ADCY6, ITPR1, CREB3, GNAQ	5	ACOR_VSMC-up
cfa05220	Chronic myeloid leukemia	5/407	76/7854	0.35864202	0.86074085	0.82739343	479180/474890/481628/490969/608125 POLK, CDKN1A, TGFBR1, STAT5B, CRKL	5	ACOR_VSMC-up
cfa01521	EGFR tyrosine kinase inhibitor resistance	5/407	80/7854	0.4004109	0.87388357	0.84002697	489692/403416/482666/415125/480618 PLCG2, BCL2, PDGFC, FGFR2, NF1	5	ACOR_VSMC-up
cfa04610	Complement and coagulation cascades	5/407	80/7854	0.4004109	0.87388357	0.84002697	478952/475945/609307/486714/489221 , CD59, CD55, C1S, C7	5	ACOR_VSMC-up
cfa04061	Viral protein interaction with cytokine and cytokine receptor	5/407	83/7854	0.43156664	0.88820767	0.85379612	611795/403545/100174780/403522/6114 38 CSF1, IL6ST, TNFSF10, CCL5, IL18R1	5	ACOR_VSMC-up

cfa05323	Rheumatoid arthritis	5/407	83/7854	0.43156664	0.88820767	0.85379612	611795/403656/489428/403522/403729 CSF1, ANGPT1, MMP1, CCL5, ATP6V0E1	5	ACOR_VSMC-up
cfa04012	ErbB signaling pathway	5/407	85/7854	0.45215564	0.88820767	0.85379612	489692/610764/474890/490969/608125 PLCG2, CAMK2D, CDKN1A, STAT5B, CRKL	5	ACOR_VSMC-up
cfa04911	Insulin secretion	5/407	85/7854	0.45215564	0.88820767	0.85379612	477625/610764/484826/611934/403928 ADCY6, CAMK2D, RAB3A, CREB3, GNAQ	5	ACOR_VSMC-up
cfa04727	GABAergic synapse	5/407	91/7854	0.51253821	0.88929092	0.8548374	478858/488087/477625/612919/478227 PLCL1, GNB4, ADCY6, GABARAPL1, GNAI1	5	ACOR_VSMC-up
cfa04659	Th17 cell differentiation	5/407	102/7854	0.61514517	0.90773643	0.87256828	100856077/403545/478328/481628/4909 69 IFNGR1, IL6ST, RORA, TGFBR1, STAT5B	5	ACOR_VSMC-up
cfa00100	Steroid biosynthesis	5/388	20/7854	0.00240023	0.0362702	0.03396822	608021/491611/489573/477362/475225 SQLE, LSS, DHCR24, FDFT1, CYP51A1	5	ACOR_VSMC-down
cfa01040	Biosynthesis of unsaturated fatty acids	5/388	26/7854	0.00798071	0.09867057	0.0924082	483792/486839/483793/100855761/4879 00 FADS2, SCD, FADS1, ELOVL1, ELOVL6	5	ACOR_VSMC-down
cfa00020	Citrate cycle (TCA cycle)	5/388	29/7854	0.01276429	0.13887544	0.13006137	607852/475775/476574/480858/489406 ACLY, SUCLG1, , PDHA1, DLAT	5	ACOR_VSMC-down
cfa00071	Fatty acid degradation	5/388	41/7854	0.04988586	0.45229848	0.42359223	478927/481018/475631/484063/478895 ACSL3, ACSL4, ACSL1, ACAT2, ACADL	5	ACOR_VSMC-down
cfa04216	Ferroptosis	5/388	45/7854	0.06946521	0.54125816	0.50690586	478927/481018/403703/475631/477596 ACSL3, ACSL4, TFRC, ACSL1, PCBP2	5	ACOR_VSMC-down
cfa03050	Proteasome	5/388	46/7854	0.07493633	0.55088328	0.5159201	491018/479671/475934/475980/1006886 36 PSMD3, PSMD7, PSMD13, , PSMC1	5	ACOR_VSMC-down
cfa04370	VEGF signaling pathway	5/388	57/7854	0.14931144	0.82883085	0.77622704	483329/403872/484401/403955/489644 SPHK1, NRAS, SPHK2, RAC1, PIK3CD	5	ACOR_VSMC-down
cfa00970	Aminoacyl-tRNA biosynthesis	5/388	66/7854	0.22615808	0.99972301	0.9362731	481716/476695/474833/100856659/6098 03 VARS, FARSA, VARS2, GARS, RARS	5	ACOR_VSMC-down
cfa05230	Central carbon metabolism in cancer	5/388	66/7854	0.22615808	0.99972301	0.9362731	481088/403872/476574/480858/489644 G6PD, NRAS, , PDHA1, PIK3CD	5	ACOR_VSMC-down
cfa05211	Renal cell carcinoma	5/388	67/7854	0.23534897	0.99972301	0.9362731	489287/607885/403872/403955/489644 ETS1, PAK1, NRAS, RAC1, PIK3CD	5	ACOR_VSMC-down
cfa05221	Acute myeloid leukemia	5/388	67/7854	0.23534897	0.99972301	0.9362731	403872/481756/483845/487746/489644 NRAS, PPARD, CCNA2, RUNX1, PIK3CD	5	ACOR_VSMC-down
cfa01230	Biosynthesis of amino acids	5/388	74/7854	0.30213481	0.99972301	0.9362731	486633/480364/476588/481011/479991 BCAT1, ARG2, TKT, PRPS1, CTH	5	ACOR_VSMC-down
cfa05210	Colorectal cancer	5/388	85/7854	0.41111943	0.99972301	0.9362731	442936/403872/475258/403955/489644 , NRAS, CYCS, RAC1, PIK3CD	5	ACOR_VSMC-down
cfa04978	Mineral absorption	12420	51/7854	0.19905848	0.52353656	0.51664792	102155200	1	VPOR_EC-up
cfa04650	Natural killer cell mediated cytotoxicity	5/388	103/7854	0.58084738	0.99972301	0.9362731	607885/100683675/403872/403955/4896 44 PAK1, BID, NRAS, RAC1, PIK3CD	5	ACOR_VSMC-down
cfa04066	HIF-1 signaling pathway	5/388	112/7854	0.65528828	0.99972301	0.9362731	612212/403703/476574/480858/489644 MKNK2, TFRC, , PDHA1, PIK3CD	5	ACOR_VSMC-down
cfa05322	Systemic lupus erythematosus	5/388	113/7854	0.66301808	0.99972301	0.9362731	611089/485806/100855593/489372/4759 16 HIST1H2BB, SNRPB, HIST2H2AC, H2AFX, H3F3B	5	ACOR_VSMC-down
cfa04978	Mineral absorption	44013	51/7854	0.04459529	0.1193917	0.07937398	490755 SLC8A3	1	VCAV_VSMC-up
cfa04380	Osteoclast differentiation	5/388	125/7854	0.74682793	0.99972301	0.9362731	403788/403955/483724/609452/489644 ITGB3, RAC1, FOSL1, , PIK3CD	5	ACOR_VSMC-down
cfa04910	Insulin signaling pathway	5/388	134/7854	0.79884855	0.99972301	0.9362731	483378/403475/612212/403872/489644 FASN, SREBF1, MKNK2, NRAS, PIK3CD	5	ACOR_VSMC-down
cfa04371	Apelin signaling pathway	5/388	135/7854	0.80407389	0.99972301	0.9362731	483329/403872/484401/479733/481746 SPHK1, NRAS, SPHK2, GNB2, ITPR3	5	ACOR_VSMC-down
cfa04976	Bile secretion	4/131	66/7854	0.02405032	0.29765577	0.27297999	612928/403732/403966/403556 NR1H4, AQP1, ATP1B1, PRKACA	4	ACOR_EC-up
cfa01521	EGFR tyrosine kinase inhibitor resistance	4/131	80/7854	0.04425818	0.36641541	0.33603943	100856339/607745/403416/485864 , GAS6, BCL2, SRC	4	ACOR_EC-up
cfa04610	Complement and coagulation cascades	4/131	80/7854	0.04425818	0.36641541	0.33603943	489221/475945/442971/476728 C7, CD59, CLU, C3	4	ACOR_EC-up
cfa04540	Gap junction	4/131	88/7854	0.05904934	0.36641541	0.33603943	553102/485864/403556/100855815 GUCY1B1, SRC, PRKACA, TUBB4A	4	ACOR_EC-up
cfa04912	GnRH signaling pathway	4/131	89/7854	0.06106285	0.36641541	0.33603943	479473/485864/403556/494005 PLD2, SRC, PRKACA, PRKCD	4	ACOR_EC-up
cfa04666	Fc gamma R-mediated phagocytosis	4/131	90/7854	0.06311251	0.36641541	0.33603943	100856339/607962/479473/494005 , PLPP1, PLD2, PRKCD	4	ACOR_EC-up
cfa04727	GABAergic synapse	4/131	91/7854	0.06519816	0.36641541	0.33603943	478858/609157/485864/403556 PLCL1, GNAO1, SRC, PRKACA	4	ACOR_EC-up
cfa04350	TGF-beta signaling pathway	4/131	92/7854	0.06731964	0.36641541	0.33603943	488560/100856151/487612/483880 FMOD, RGMA, SMAD6, INHBB	4	ACOR_EC-up
cfa01522	Endocrine resistance	4/131	93/7854	0.06947674	0.36791092	0.33741096	100856339/403416/485864/403556 , BCL2, SRC, PRKACA	4	ACOR_EC-up

cfa05231	Choline metabolism in cancer	4/131	96/7854	0.07615975	0.38576571	0.35378558	100856339/607962/610214/479473 , PLPP1, CHPT1, PLD2	4	ACOR_EC-up
cfa04668	TNF signaling pathway	4/131	108/7854	0.10594571	0.45713613	0.41923937	100856339/487286/488472/403982 , NOD2, CASP10, VCAM1	4	ACOR_EC-up
cfa04066	HIF-1 signaling pathway	4/131	112/7854	0.11689898	0.48935098	0.44878359	100856339/477072/403416/480622 , , BCL2, ALDOC	4	ACOR_EC-up
cfa04670	Leukocyte transendothelial migration	4/131	113/7854	0.11971247	0.48935098	0.44878359	100856339/488402/489008/403982 , RAPGEF4, , VCAM1	4	ACOR_EC-up
cfa04725	Cholinergic synapse	4/131	113/7854	0.11971247	0.48935098	0.44878359	100856339/609157/403416/403556 , GNAO1, BCL2, PRKACA	4	ACOR_EC-up
cfa04722	Neurotrophin signaling pathway	4/131	118/7854	0.13421178	0.50347132	0.46173336	100856339/403416/486666/494005 , BCL2, ARHGDIB, PRKCD	4	ACOR_EC-up
cfa04071	Sphingolipid signaling pathway	4/131	122/7854	0.14630532	0.50879313	0.46661398	100856339/487329/403416/479473 , OPRD1, BCL2, PLD2	4	ACOR_EC-up
NA	NA	NA	NA	NA	NA	NA	NA NA	NA	APUL_EC-up
NA	NA	NA	NA	NA	NA	NA	NA NA	NA	APUL_EC-up
NA	NA	NA	NA	NA	NA	NA	NA NA	NA	APUL_EC-up
NA	NA	NA	NA	NA	NA	NA	NA NA	NA	APUL_EC-up
NA	NA	NA	NA	NA	NA	NA	NA NA	NA	APUL_EC-up
NA	NA	NA	NA	NA	NA	NA	NA NA	NA	APUL_EC-up
NA	NA	NA	NA	NA	NA	NA	NA NA	NA	APUL_EC-up
NA	NA	NA	NA	NA	NA	NA	NA NA	NA	APUL_EC-up
NA	NA	NA	NA	NA	NA	NA	NA NA	NA	APUL_EC-up
NA	NA	NA	NA	NA	NA	NA	NA NA	NA	APUL_EC-up
NA	NA	NA	NA	NA	NA	NA	NA NA	NA	APUL_EC-up
NA	NA	NA	NA	NA	NA	NA	NA NA	NA	APUL_EC-up
NA	NA	NA	NA	NA	NA	NA	NA NA	NA	APUL_EC-up
NA	NA	NA	NA	NA	NA	NA	NA NA	NA	APUL_EC-up
NA	NA	NA	NA	NA	NA	NA	NA NA	NA	APUL_EC-up
NA	NA	NA	NA	NA	NA	NA	NA NA	NA	APUL_EC-up
NA	NA	NA	NA	NA	NA	NA	NA NA	NA	APUL_EC-up
NA	NA	NA	NA	NA	NA	NA	NA NA	NA	APUL_EC-up
NA	NA	NA	NA	NA	NA	NA	NA NA	NA	APUL_EC-up
NA	NA	NA	NA	NA	NA	NA	NA NA	NA	APUL_EC-up
NA	NA	NA	NA	NA	NA	NA	NA NA	NA	APUL_EC-up
NA	NA	NA	NA	NA	NA	NA	NA NA	NA	APUL_EC-up
NA	NA	NA	NA	NA	NA	NA	NA NA	NA	APUL_EC-up
NA	NA	NA	NA	NA	NA	NA	NA NA	NA	APUL_EC-up
NA	NA	NA	NA	NA	NA	NA	NA NA	NA	APUL_EC-up
NA	NA	NA	NA	NA	NA	NA	NA NA	NA	APUL_EC-up
NA	NA	NA	NA	NA	NA	NA	NA NA	NA	APUL_EC-up
NA	NA	NA	NA	NA	NA	NA	NA NA	NA	APUL_EC-up
NA	NA	NA	NA	NA	NA	NA	NA NA	NA	APUL_EC-up
NA	NA	NA	NA	NA	NA	NA	NA NA	NA	APUL_EC-up
NA	NA	NA	NA	NA	NA	NA	NA NA	NA	APUL_EC-up
NA	NA	NA	NA	NA	NA	NA	NA NA	NA	APUL_EC-up
NA	NA	NA	NA	NA	NA	NA	NA NA	NA	APUL_EC-up
NA	NA	NA	NA	NA	NA	NA	NA NA	NA	APUL_EC-up
NA	NA	NA	NA	NA	NA	NA	NA NA	NA	APUL_EC-up
NA	NA	NA	NA	NA	NA	NA	NA NA	NA	APUL_EC-up
NA	NA	NA	NA	NA	NA	NA	NA NA	NA	APUL_EC-up
NA	NA	NA	NA	NA	NA	NA	NA NA	NA	APUL_EC-up
NA	NA	NA	NA	NA	NA	NA	NA NA	NA	APUL_EC-up
NA	NA	NA	NA	NA	NA	NA	NA NA	NA	APUL_EC-up
NA	NA	NA	NA	NA	NA	NA	NA NA	NA	APUL_EC-up
NA	NA	NA	NA	NA	NA	NA	NA NA	NA	APUL_EC-up
NA	NA	NA	NA	NA	NA	NA	NA NA	NA	APUL_EC-up
NA	NA	NA	NA	NA	NA	NA	NA NA	NA	APUL_EC-up
NA	NA	NA	NA	NA	NA	NA	NA NA	NA	APUL_EC-up
NA	NA	NA	NA	NA	NA	NA	NA NA	NA	APUL_EC-up
NA	NA	NA	NA	NA	NA	NA	NA NA	NA	APUL_EC-up
NA	NA	NA	NA	NA	NA	NA	NA NA	NA	APUL_EC-up
NA	NA	NA	NA	NA	NA	NA	NA NA	NA	APUL_EC-up
NA	NA	NA	NA	NA	NA	NA	NA NA	NA	APUL_EC-up
NA	NA	NA	NA	NA	NA	NA	NA NA	NA	APUL_EC-up
NA	NA	NA	NA	NA	NA	NA	NA NA	NA	APUL_EC-up
NA	NA	NA	NA	NA	NA	NA	NA NA	NA	APUL_EC-up
NA	NA	NA	NA	NA	NA	NA	NA NA	NA	APUL_EC-up
NA	NA	NA	NA	NA	NA	NA	NA NA	NA	APUL_EC-up
NA	NA	NA	NA	NA	NA	NA	NA NA	NA	APUL_EC-up
NA	NA	NA	NA	NA	NA	NA	NA NA	NA	APUL_EC-up
NA	NA	NA	NA	NA	NA	NA	NA NA	NA	APUL_EC-up
NA	NA	NA	NA	NA	NA	NA	NA NA	NA	APUL_EC-up
NA	NA	NA	NA	NA	NA	NA	NA NA	NA	APUL_EC-up
NA	NA	NA	NA	NA	NA	NA	NA NA	NA	APUL_EC-up

NA	NA	NA	NA	NA	NA	NA	NA NA	NA	APUL_EC-up
NA	NA	NA	NA	NA	NA	NA	NA NA	NA	APUL_EC-up
NA	NA	NA	NA	NA	NA	NA	NA NA	NA	APUL_EC-up
NA	NA	NA	NA	NA	NA	NA	NA NA	NA	APUL_EC-up
NA	NA	NA	NA	NA	NA	NA	NA NA	NA	APUL_EC-up
NA	NA	NA	NA	NA	NA	NA	NA NA	NA	APUL_EC-up
NA	NA	NA	NA	NA	NA	NA	NA NA	NA	APUL_EC-up
NA	NA	NA	NA	NA	NA	NA	NA NA	NA	APUL_EC-up
NA	NA	NA	NA	NA	NA	NA	NA NA	NA	APUL_EC-up
NA	NA	NA	NA	NA	NA	NA	NA NA	NA	APUL_EC-up
NA	NA	NA	NA	NA	NA	NA	NA NA	NA	APUL_EC-up
NA	NA	NA	NA	NA	NA	NA	NA NA	NA	APUL_EC-up
NA	NA	NA	NA	NA	NA	NA	NA NA	NA	APUL_EC-up
NA	NA	NA	NA	NA	NA	NA	NA NA	NA	APUL_EC-up
NA	NA	NA	NA	NA	NA	NA	NA NA	NA	APUL_EC-up
NA	NA	NA	NA	NA	NA	NA	NA NA	NA	APUL_EC-up
NA	NA	NA	NA	NA	NA	NA	NA NA	NA	APUL_EC-up
NA	NA	NA	NA	NA	NA	NA	NA NA	NA	APUL_EC-up
NA	NA	NA	NA	NA	NA	NA	NA NA	NA	APUL_EC-up
NA	NA	NA	NA	NA	NA	NA	NA NA	NA	APUL_EC-up
NA	NA	NA	NA	NA	NA	NA	NA NA	NA	APUL_EC-up
NA	NA	NA	NA	NA	NA	NA	NA NA	NA	APUL_EC-up
NA	NA	NA	NA	NA	NA	NA	NA NA	NA	APUL_EC-up
NA	NA	NA	NA	NA	NA	NA	NA NA	NA	APUL_EC-up
NA	NA	NA	NA	NA	NA	NA	NA NA	NA	APUL_EC-up
NA	NA	NA	NA	NA	NA	NA	NA NA	NA	APUL_EC-up
NA	NA	NA	NA	NA	NA	NA	NA NA	NA	APUL_EC-up
NA	NA	NA	NA	NA	NA	NA	NA NA	NA	APUL_EC-up
NA	NA	NA	NA	NA	NA	NA	NA NA	NA	APUL_EC-up
NA	NA	NA	NA	NA	NA	NA	NA NA	NA	APUL_EC-up
NA	NA	NA	NA	NA	NA	NA	NA NA	NA	APUL_EC-up
NA	NA	NA	NA	NA	NA	NA	NA NA	NA	APUL_EC-up
NA	NA	NA	NA	NA	NA	NA	NA NA	NA	APUL_EC-up
NA	NA	NA	NA	NA	NA	NA	NA NA	NA	APUL_EC-up
NA	NA	NA	NA	NA	NA	NA	NA NA	NA	APUL_EC-up
NA	NA	NA	NA	NA	NA	NA	NA NA	NA	APUL_EC-up
NA	NA	NA	NA	NA	NA	NA	NA NA	NA	APUL_EC-up
NA	NA	NA	NA	NA	NA	NA	NA NA	NA	APUL_EC-up
NA	NA	NA	NA	NA	NA	NA	NA NA	NA	APUL_EC-up
NA	NA	NA	NA	NA	NA	NA	NA NA	NA	APUL_EC-up
NA	NA	NA	NA	NA	NA	NA	NA NA	NA	APUL_EC-up
NA	NA	NA	NA	NA	NA	NA	NA NA	NA	APUL_EC-up
NA	NA	NA	NA	NA	NA	NA	NA NA	NA	APUL_EC-up
NA	NA	NA	NA	NA	NA	NA	NA NA	NA	APUL_EC-up
NA	NA	NA	NA	NA	NA	NA	NA NA	NA	APUL_EC-up
NA	NA	NA	NA	NA	NA	NA	NA NA	NA	APUL_EC-up
NA	NA	NA	NA	NA	NA	NA	NA NA	NA	APUL_EC-up
NA	NA	NA	NA	NA	NA	NA	NA NA	NA	APUL_EC-up
NA	NA	NA	NA	NA	NA	NA	NA NA	NA	APUL_EC-up
NA	NA	NA	NA	NA	NA	NA	NA NA	NA	APUL_EC-up
cfa04926	Relaxin signaling pathway	4/131	127/7854	0.16199577	0.52441163	0.48093771	100856339/609157/485864/403556 , GNAO1, SRC, PRKACA	4	ACOR_EC-up
cfa04261	Adrenergic signaling in cardiomyocytes	4/131	147/7854	0.23005394	0.60001444	0.55027301	403966/403416/488402/403556 ATP1B1, BCL2, RAPGEF4, PRKACA	4	ACOR_EC-up
cfa05226	Gastric cancer	4/131	149/7854	0.23723541	0.60001444	0.55027301	100856339/100855544/403416/489008 , FZD5, BCL2,	4	ACOR_EC-up
cfa05160	Hepatitis C	4/131	157/7854	0.2664614	0.61470798	0.56374845	100856339/478350/485789/611606 , PIAS1, MAVS, CD81	4	ACOR_EC-up
cfa05225	Hepatocellular carcinoma	4/131	165/7854	0.29631652	0.66386297	0.60882848	474938/100856339/100855544/477558 , , FZD5,	4	ACOR_EC-up
cfa04530	Tight junction	4/131	170/7854	0.31519711	0.68000857	0.6236356	486079/100688336/485864/403556 GATA4, MYH14, SRC, PRKACA	4	ACOR_EC-up
cfa05167	Kaposi sarcoma-associated herpesvirus infection	4/131	178/7854	0.34561009	0.68154985	0.6250491	100856339/485864/478564/476728 , SRC, , C3	4	ACOR_EC-up
cfa05203	Viral carcinogenesis	4/131	189/7854	0.38751704	0.70025305	0.6422018	100856339/485864/403556/476728 , SRC, PRKACA, C3	4	ACOR_EC-up
cfa05169	Epstein-Barr virus infection	4/131	193/7854	0.40269937	0.70755799	0.64890116	100856339/403416/485789/481740 , BCL2, MAVS, TAPBP	4	ACOR_EC-up
cfa04510	Focal adhesion	4/131	198/7854	0.42158557	0.71180752	0.6527984	100856339/492067/403416/485864 , PAK3, BCL2, SRC	4	ACOR_EC-up
cfa00650	Butanoate metabolism	4/441	25/7854	0.04861366	0.33577344	0.30227345	608888/479856/477517/488110 ACSM3, ABAT, ACADS, EHHADH	4	VSAP_EC-up
cfa04392	Hippo signaling pathway - multiple species	4/441	28/7854	0.06906853	0.41125246	0.37022196	480319/490688/100856566/477343 SAV1, FRMD6, LIMD1, LATS2	4	VSAP_EC-up

cfa00630	Glyoxylate and dicarboxylate metabolism	4/441	29/7854	0.07672926	0.41125246	0.37022196	403443/403978/477205/477002 GLUL, DLD, ACSS2, ACSS1	4	VSAP_EC-up
cfa00051	Fructose and mannose metabolism	4/441	32/7854	0.10210646	0.48420266	0.43589394	100170647/483800/100533995/474486 KHK, TKFC, FPGT, PMM1	4	VSAP_EC-up
cfa05219	Bladder cancer	4/441	41/7854	0.19645091	0.66536335	0.5989803	442858/403924/484648/611939 CDH1, MYC, RAF1, MAP2K2	4	VSAP_EC-up
cfa04340	Hedgehog signaling pathway	4/441	47/7854	0.26985031	0.74208835	0.66805047	489296/607496/485189/484137 CDON, GAS1, ARRB1, PTCH1	4	VSAP_EC-up
cfa00600	Sphingolipid metabolism	4/441	48/7854	0.28255866	0.74928501	0.67452913	100856377/485008/479557/474457 SMPD2, CERS4, PLPP3, ARSA	4	VSAP_EC-up
cfa00830	Retinol metabolism	4/441	49/7854	0.29535535	0.75621155	0.68076461	477888/480254/483083/488391 SDR16C5, DHRS4, RETSAT, DHRS9	4	VSAP_EC-up
cfa05014	Amyotrophic lateral sclerosis (ALS)	4/441	56/7854	0.38603738	0.85964878	0.77388194	476215/442988/479248/479458 MAP3K5, CCS, PPP3CB, CASP12	4	VSAP_EC-up
cfa04370	VEGF signaling pathway	4/441	57/7854	0.39896941	0.86491908	0.77862643	487235/484648/479248/611939 PIK3R1, RAF1, PPP3CB, MAP2K2	4	VSAP_EC-up
cfa04213	Longevity regulating pathway - multiple species	4/441	59/7854	0.42468137	0.87590533	0.78851658	486148/476258/487235/486295 IRS1, SOD2, PIK3R1, PRKAB1	4	VSAP_EC-up
cfa00590	Arachidonic acid metabolism	4/441	61/7854	0.45011209	0.89991478	0.81013062	487748/609101/481256/480698 CBR3, PTGES2, PLA2G6, PTGES	4	VSAP_EC-up
cfa00970	Aminoacyl-tRNA biosynthesis	4/441	66/7854	0.51191827	0.92381513	0.83164644	483145/488204/476628/476652 WARS2, FARS2, QARS, LARS2	4	VSAP_EC-up
cfa04976	Bile secretion	4/441	66/7854	0.51191827	0.92381513	0.83164644	485523/488390/491278/488171 ABCC4, ABCB11, RXRA, NCEH1	4	VSAP_EC-up
cfa05211	Renal cell carcinoma	4/441	67/7854	0.52390534	0.92381513	0.83164644	487235/491872/484648/611939 PIK3R1, TFE3, RAF1, MAP2K2	4	VSAP_EC-up
cfa04115	p53 signaling pathway	4/441	69/7854	0.547446	0.92381513	0.83164644	481956/483626/483885/608847 SESN1, DDB2, STEAP3, CCND3	4	VSAP_EC-up
cfa04520	Adherens junction	4/441	70/7854	0.55898719	0.93144223	0.83851259	477331/442858/486894/477039 WASF3, CDH1, TCF7L2, TGFBR2	4	VSAP_EC-up
cfa04662	B cell receptor signaling pathway	4/441	72/7854	0.5815841	0.95431203	0.85910068	487235/484648/479248/611939 PIK3R1, RAF1, PPP3CB, MAP2K2	4	VSAP_EC-up
cfa05100	Bacterial invasion of epithelial cells	4/441	75/7854	0.61419886	0.97035825	0.87354597	491319/442858/487235/482639 DNM1, CDH1, PIK3R1, ARHGAP10	4	VSAP_EC-up
cfa03018	RNA degradation	4/441	78/7854	0.64519401	0.9926858	0.89364591	100684605/481233/476654/492002 BTG1, TOB2, EXOSC7, PABPC5	4	VSAP_EC-up
cfa05216	Thyroid cancer	4/279	38/7854	0.04464047	0.24366258	0.20656008	488993/477032/480137/474890 CCDC6, CTNNB1, TPM3, CDKN1A	4	VSAP_EC-down
cfa00330	Arginine and proline metabolism	4/279	47/7854	0.08455536	0.3573146	0.30290631	474672/483372/479242/477781 P4HA2, PYCR1, P4HA1, ALDH18A1	4	VSAP_EC-down
cfa00514	Other types of O-glycan biosynthesis	4/279	47/7854	0.08455536	0.3573146	0.30290631	480161/100855959/479730/100682763 GALNT1, POFUT2, PLOD3, GALNT2	4	VSAP_EC-down
cfa04340	Hedgehog signaling pathway	4/279	47/7854	0.08455536	0.3573146	0.30290631	479975/476134/478575/480548 PRKACB, SPOPL, GSK3B, SPOP	4	VSAP_EC-down
cfa04961	Endocrine and other factor-regulated calcium reabsorption	4/279	52/7854	0.11226372	0.38701439	0.32808371	479975/403480/403928/480578 PRKACB, ATP2B1, GNAQ, CLTC	4	VSAP_EC-down
cfa04923	Regulation of lipolysis in adipocytes	4/279	54/7854	0.12434407	0.4066908	0.344764	479975/609616/442942/485570 PRKACB, PRKG1, PTGS2, ABHD5	4	VSAP_EC-down
cfa04213	Longevity regulating pathway - multiple species	4/279	59/7854	0.1567702	0.44134096	0.37413798	479975/479406/479441/487309 PRKACB, HSPA8, CRYAB, HDAC1	4	VSAP_EC-down
cfa05213	Endometrial cancer	4/279	59/7854	0.1567702	0.44134096	0.37413798	476836/477032/478575/474890 ILK, CTNNB1, GSK3B, CDKN1A	4	VSAP_EC-down
cfa04976	Bile secretion	4/279	66/7854	0.2066685	0.52064564	0.44136693	612928/479975/482437/486241 NR1H4, PRKACB, SLC2A1, SCARB1	4	VSAP_EC-down
cfa04115	p53 signaling pathway	4/279	69/7854	0.22932434	0.54128807	0.45886614	403476/403567/487335/474890 SERPINE1, CASP3, SESN2, CDKN1A	4	VSAP_EC-down
cfa04924	Renin secretion	4/279	69/7854	0.22932434	0.54128807	0.45886614	479975/403928/403652/480416 PRKACB, GNAQ, PDE1A, CALM1	4	VSAP_EC-down
cfa05212	Pancreatic cancer	4/279	75/7854	0.2762701	0.61044809	0.51749517	403802/481628/478464/474890 VEGFA, TGFBR1, MAPK10, CDKN1A	4	VSAP_EC-down
cfa05220	Chronic myeloid leukemia	4/279	76/7854	0.28425446	0.61044809	0.51749517	477488/481628/487309/474890 PTPN11, TGFBR1, HDAC1, CDKN1A	4	VSAP_EC-down
cfa05410	Hypertrophic cardiomyopathy (HCM)	10/407	91/7854	0.01886611	0.33958992	0.3264333	403532/489571/609265/486563/497091/476623/403424/488526/611066/475738 TNNT2, PRKAA2, SGCA, CACNB3, DES, DAG1, EDN1, PRKAG3, SGCB, SLC8A1	10	ACOR_VSMC-up
cfa04914	Progesterone-mediated oocyte maturation	4/279	89/7854	0.38971252	0.71904705	0.60955776	479975/491768/480438/478464 PRKACB, RPS6KA3, HSP90AA1, MAPK10	4	VSAP_EC-down
cfa04350	TGF-beta signaling pathway	4/279	92/7854	0.41398396	0.73226635	0.62076416	475720/481628/487635/483245 LTBP1, TGFBR1, NEO1, INHBA	4	VSAP_EC-down
cfa04064	NF-kappa B signaling pathway	4/279	93/7854	0.42203137	0.73226635	0.62076416	442942/477886/492019/488471 PTGS2, LYN, BTK, CFLAR	4	VSAP_EC-down

cfa04640	Hematopoietic cell lineage	4/279	93/7854	0.42203137	0.73226635	0.62076416	489208/403703/403939/403788 ITGA2, TFRC, CD44, ITGB3	4	VSAP_EC-down
cfa05231	Choline metabolism in cancer	4/279	96/7854	0.4460041	0.73430489	0.62249229	480348/491597/475590/478464 HIF1A, PDGFA, EIF4EBP1, MAPK10	4	VSAP_EC-down
cfa04659	Th17 cell differentiation	4/279	102/7854	0.49295466	0.76877454	0.65171325	481628/480348/480438/478464 TGFBR1, HIF1A, HSP90AA1, MAPK10	4	VSAP_EC-down
cfa05145	Toxoplasmosis	4/279	109/7854	0.54552011	0.79879452	0.67716209	479406/403567/477956/478464 HSPA8, CASP3, ITGB1, MAPK10	4	VSAP_EC-down
cfa04724	Glutamatergic synapse	4/279	112/7854	0.56717647	0.82555686	0.69984933	479975/477633/403928/488933 PRKACB, SLC38A1, GNAQ, HOMER1	4	VSAP_EC-down
cfa04142	Lysosome	4/279	118/7854	0.60872674	0.84772198	0.71863937	486117/607494/480578/482897 ENTPD4, AP3S1, CLTC, ASAH1	4	VSAP_EC-down
cfa04152	AMPK signaling pathway	4/279	121/7854	0.62856712	0.84772198	0.71863937	477374/487139/475590/491369 PPP2R2A, PFKFB3, EIF4EBP1, CREB3L2	4	VSAP_EC-down
cfa04071	Sphingolipid signaling pathway	4/279	122/7854	0.63503695	0.84772198	0.71863937	477374/403928/478464/482897 PPP2R2A, GNAQ, MAPK10, ASAH1	4	VSAP_EC-down
cfa05410	Hypertrophic cardiomyopathy (HCM)	9/279	91/7854	0.00484811	0.06048592	0.05127573	481598/489208/609879/608287/477956/606758/488437/403788/480137 TPM2, ITGA2, TPM4, ITGA10, ITGB1, DMD, ITGAV, ITGB3, TPM3	9	VSAP_EC-down
cfa05216	Thyroid cancer	4/357	38/7854	0.09224801	0.25737195	0.18838015	403606/403494/480045/403872 PPARG, RET, TPR, NRAS	4	VCAV_EC-up
cfa05410	Hypertrophic cardiomyopathy (HCM)	8/357	91/7854	0.05424902	0.173971	0.12733588	403985/610668/403424/490755/606758/100856563/403788/479797 IL6, ACE, EDN1, SLC8A3, DMD, ITGA6, ITGB3, ATP2A1	8	VCAV_EC-up
cfa04913	Ovarian steroidogenesis	4/357	46/7854	0.15504683	0.36746967	0.26896479	480271/486241/442942/487514 ADCY4, SCARB1, PTGS2, PLA2G4E	4	VCAV_EC-up
cfa00330	Arginine and proline metabolism	4/357	47/7854	0.16381677	0.37772628	0.27647198	403784/475666/480364/403822 NOS3, ODC1, ARG2, NOS2	4	VCAV_EC-up
cfa04340	Hedgehog signaling pathway	4/357	47/7854	0.16381677	0.37772628	0.27647198	481481/485189/477392/489296 CSNK1G3, ARRB1, CUL3, CDON	4	VCAV_EC-up
cfa05410	Hypertrophic cardiomyopathy (HCM)	4/388	91/7854	0.664815	0.99972301	0.9362731	480124/403788/489208/480137 LMNA, ITGB3, ITGA2, TPM3	4	ACOR_VSMC-down
cfa04623	Cytosolic DNA-sensing pathway	4/357	57/7854	0.25951722	0.51654618	0.37807945	403985/606815/481145/102152063 IL6, TMEM173, TBK1, RIPK1	4	VCAV_EC-up
cfa04213	Longevity regulating pathway - multiple species	4/357	59/7854	0.2799024	0.54231091	0.39693762	478232/480271/479406/403872 MTOR, ADCY4, HSPA8, NRAS	4	VCAV_EC-up
cfa00590	Arachidonic acid metabolism	4/357	61/7854	0.30052747	0.57038887	0.41748893	403544/612760/442942/487514 PTGS1, PTGES3, PTGS2, PLA2G4E	4	VCAV_EC-up
cfa04137	Mitophagy - animal	4/357	64/7854	0.33176077	0.59716938	0.43709059	481145/474451/403872/480348 TBK1, TBC1D15, NRAS, HIF1A	4	VCAV_EC-up
cfa04976	Bile secretion	4/357	66/7854	0.3526824	0.6188578	0.45296515	480271/477098/486241/478472 ADCY4, ATP1B3, SCARB1, ABCG2	4	VCAV_EC-up
cfa05221	Acute myeloid leukemia	4/357	67/7854	0.36314919	0.62542361	0.45777091	403811/478232/481772/403872 KIT, MTOR, PIM1, NRAS	4	VCAV_EC-up
cfa05223	Non-small cell lung cancer	4/357	67/7854	0.36314919	0.62542361	0.45777091	483048/489692/485874/403872 EML4, PLCG2, PLCG1, NRAS	4	VCAV_EC-up
cfa01524	Platinum drug resistance	4/357	72/7854	0.41525018	0.65826592	0.48180942	488473/477044/100502558/482620 CASP8, TOP2B, PMAIP1, APAF1	4	VCAV_EC-up
cfa04918	Thyroid hormone synthesis	4/357	72/7854	0.41525018	0.65826592	0.48180942	480271/475266/477098/607922 ADCY4, CREB5, ATP1B3, CREB1	4	VCAV_EC-up
cfa05218	Melanoma	4/357	73/7854	0.42557637	0.66705509	0.48824255	442986/611773/403872/479460 PDGFB, FGF23, NRAS, PDGFD	4	VCAV_EC-up
cfa05212	Pancreatic cancer	4/357	75/7854	0.44608497	0.67499155	0.49405154	483881/478232/490548/479473 RALB, MTOR, RALBP1, PLD2	4	VCAV_EC-up
cfa03018	RNA degradation	4/357	78/7854	0.47640548	0.71078679	0.52025141	481463/486024/482895/489086 CNOT6, PAN3, CNOT7, DCP2	4	VCAV_EC-up
cfa05210	Colorectal cancer	4/357	85/7854	0.54442614	0.77718781	0.5688528	483881/478232/100502558/403872 RALB, MTOR, PMAIP1, NRAS	4	VCAV_EC-up
cfa03060	Protein export	4/241	23/7854	0.0048348	0.10591097	0.0952187	476592/404003/404018/404006 SPCS1, SEC11C, SEC61B, SEC61A1	4	VCAV_EC-down
cfa00640	Propanoate metabolism	4/241	34/7854	0.01947657	0.24158651	0.21719709	484488/477205/474978/489421 , ACSS2, BCKDHB, ACAT1	4	VCAV_EC-down
cfa00520	Amino sugar and nucleotide sugar metabolism	4/241	48/7854	0.05879722	0.38423298	0.34544265	474479/606823/474653/487633 CYB5R3, CYB5R1, GFPT2,	4	VCAV_EC-down
cfa00982	Drug metabolism - cytochrome P450	4/241	49/7854	0.06254939	0.38481182	0.34596306	476005/403450/476006/479912 GSTP1, MAOA, , GSTM4	4	VCAV_EC-down
cfa05144	Malaria	4/241	49/7854	0.06254939	0.38481182	0.34596306	481124/610359/477945/611606 LRP1, THBS3, SDC2, CD81	4	VCAV_EC-down
cfa00510	N-Glycan biosynthesis	4/241	50/7854	0.06642491	0.38682027	0.34776874	404012/481579/448804/477223 DDOST, B4GALT1, FUT8, RPN2	4	VCAV_EC-down
cfa05410	Hypertrophic cardiomyopathy (HCM)	3/241	91/7854	0.53274686	0.84753502	0.76197193	608977/492249/486563 ITGB5, EMD, CACNB3	3	VCAV_EC-down
cfa04961	Endocrine and other factor-regulated calcium reabsorption	4/241	52/7854	0.07454112	0.38682027	0.34776874	477625/486588/489104/475994 ADCY6, VDR, CLTB, AP2A2	4	VCAV_EC-down

cfa04370	VEGF signaling pathway	4/241	57/7854	0.09689098	0.41271433	0.37104866	609956/485864/449021/481271 , SRC, AKT2, RAC2	4	VCAV_EC-down
cfa05410	Hypertrophic cardiomyopathy (HCM)	44106	91/7854	0.00562466	0.01834863	0.00804764	612079/606758 SGCD, DMD	2	AORT_EC-up
cfa05230	Central carbon metabolism in cancer	4/241	66/7854	0.14386419	0.4861441	0.43706531	442985/609956/449021/100686499 PDGFRB, , AKT2, GLS2	4	VCAV_EC-down
cfa04115	p53 signaling pathway	4/241	69/7854	0.16120333	0.51052385	0.45898379	608847/100855542/611782/479236 CCND3, ZMAT3, CCND2, AIFM2	4	VCAV_EC-down
cfa04920	Adipocytokine signaling pathway	4/241	70/7854	0.1671466	0.51052385	0.45898379	449021/485548/490942/486148 AKT2, IRS2, G6PC3, IRS1	4	VCAV_EC-down
cfa04662	B cell receptor signaling pathway	4/241	72/7854	0.17926143	0.51619497	0.46408238	609956/449021/611606/481271 , AKT2, CD81, RAC2	4	VCAV_EC-down
cfa05412	Arrhythmogenic right ventricular cardiomyopathy (ARVC)	4/241	77/7854	0.21074529	0.55867384	0.50227279	608977/492249/486563/480169 ITGB5, EMD, CACNB3, CDH2	4	VCAV_EC-down
cfa05410	Hypertrophic cardiomyopathy (HCM)	44013	91/7854	0.07836872	0.1193917	0.07937398	490755 SLC8A3	1	VCAV_VSMC-up
cfa04146	Peroxisome	4/241	82/7854	0.24364708	0.59576107	0.53561587	477023/479564/486710/611056 ACAA1, SCP2, PEX5, MPV17	4	VCAV_EC-down
cfa04912	GnRH signaling pathway	4/241	89/7854	0.29140517	0.66036172	0.59369474	477625/403733/485864/610764 ADCY6, MMP2, SRC, CAMK2D	4	VCAV_EC-down
cfa04666	Fc gamma R-mediated phagocytosis	4/241	90/7854	0.29834072	0.67066995	0.60296231	609956/449021/607962/481271 , AKT2, PLPP1, RAC2	4	VCAV_EC-down
cfa00564	Glycerophospholipid metabolism	4/241	95/7854	0.33326902	0.70498356	0.63381178	486890/479680/607962/485626 GPAM, LCAT, PLPP1, GPD1L	4	VCAV_EC-down
cfa05418	Fluid shear stress and atherosclerosis	13/441	135/7854	0.03947377	0.30060794	0.2706164	476215/476006/477683/609050/478813/479912/487235/476005/489664/480693/442987/476713/610935 MAP3K5, , MGST1, MGST3, NFE2L2, GSTM4, PIK3R1, GSTP1, CYBA, ASS1, HMOX1, KEAP1, NQO1	13	VSAP_EC-up
cfa04725	Cholinergic synapse	4/241	113/7854	0.45858903	0.80539699	0.72408795	477625/609956/449021/610764 ADCY6, , AKT2, CAMK2D	4	VCAV_EC-down
cfa04114	Oocyte meiosis	4/241	117/7854	0.48555222	0.81424413	0.73204192	477625/612448/100683974/610764 ADCY6, RPS6KA2, , CAMK2D	4	VCAV_EC-down
cfa04071	Sphingolipid signaling pathway	4/241	122/7854	0.5184765	0.84753502	0.76197193	609956/449021/100856377/481271 , AKT2, SMPD2, RAC2	4	VCAV_EC-down
cfa04380	Osteoclast differentiation	4/241	125/7854	0.53775546	0.84753502	0.76197193	609956/449021/611795/489664 , AKT2, CSF1, CYBA	4	VCAV_EC-down
cfa04210	Apoptosis	4/241	131/7854	0.57511262	0.87354944	0.78536005	609956/449021/483745/486762 , AKT2, CAPN1, TUBA8	4	VCAV_EC-down
cfa04514	Cell adhesion molecules (CAMs)	4/241	139/7854	0.62217827	0.8815348	0.79253925	485893/477945/488922/480169 SDC4, SDC2, VCAN, CDH2	4	VCAV_EC-down
cfa04213	Longevity regulating pathway - multiple species	34790	59/7854	0.00550163	0.32190301	0.31991537	485548/479441/476258/403559 IRS2, CRYAB, SOD2, SOD1	4	AFEM_EC-up
cfa04920	Adipocytokine signaling pathway	34790	70/7854	0.01002331	0.35883458	0.3566189	403616/485548/100685468/479127 LEP, IRS2, ACACB, PPARGC1A	4	AFEM_EC-up
cfa00564	Glycerophospholipid metabolism	34790	95/7854	0.02773365	0.62054046	0.61670884	483023/486890/476045/491249 LCLAT1, GPAM, PLA2G16, AGPAT2	4	AFEM_EC-up
cfa04152	AMPK signaling pathway	34790	121/7854	0.05858153	0.81029448	0.80529119	403616/485548/100685468/479127 LEP, IRS2, ACACB, PPARGC1A	4	AFEM_EC-up
cfa04140	Autophagy - animal	34790	136/7854	0.08224867	0.81029448	0.80529119	485548/608254/404007/111097413 IRS2, PRAP1, RAB7A, VMP1	4	AFEM_EC-up
cfa05152	Tuberculosis	34790	171/7854	0.15240212	0.81029448	0.80529119	403400/612941/404007/482528 CTSS, CAMK2A, RAB7A, ATP6V0B	4	AFEM_EC-up
cfa04510	Focal adhesion	34790	198/7854	0.21777575	0.81029448	0.80529119	482666/100682877/476665/481689 PDGFC, TNXB, COMP, TNC	4	AFEM_EC-up
cfa05205	Proteoglycans in cancer	34790	202/7854	0.22806819	0.81029448	0.80529119	486185/612941/490937/611362 TWIST2, CAMK2A, FZD2, HSPB2	4	AFEM_EC-up
cfa04024	cAMP signaling pathway	34790	211/7854	0.25166355	0.81029448	0.80529119	403858/403963/612941/476487 CHRM2, HTR1D, CAMK2A, FXYD1	4	AFEM_EC-up
cfa05206	MicroRNAs in cancer	34790	281/7854	0.44347612	0.83791788	0.83274402	485548/403477/100682877/481689 IRS2, RECK, TNXB, TNC	4	AFEM_EC-up
cfa00051	Fructose and mannose metabolism	4/407	32/7854	0.08140479	0.64585296	0.62083088	480185/403849/480622/487797 ENOSF1, PFKM, ALDOC, PFKL	4	ACOR_VSMC-up
cfa04136	Autophagy - other	4/407	32/7854	0.08140479	0.64585296	0.62083088	612919/481015/489525/490477 GABARAPL1, ATG4A, ULK2, PIK3C3	4	ACOR_VSMC-up
cfa00260	Glycine, serine and threonine metabolism	4/407	40/7854	0.15106641	0.76328292	0.73371129	403451/403450/485085/491277 MAOB, MAOA, GAMT, SARDH	4	ACOR_VSMC-up
cfa00380	Tryptophan metabolism	4/407	41/7854	0.16101622	0.78597746	0.75552658	403451/478506/403450/403474 MAOB, HADH, MAOA, CAT	4	ACOR_VSMC-up
cfa05030	Cocaine addiction	4/407	48/7854	0.23634964	0.80256807	0.77147442	403451/403450/478227/611934 MAOB, MAOA, GNAI1, CREB3	4	ACOR_VSMC-up
cfa04961	Endocrine and other factor-regulated calcium reabsorption	4/407	52/7854	0.28259427	0.82260001	0.79073027	477625/480605/403928/475738 ADCY6, AP2B1, GNAQ, SLC8A1	4	ACOR_VSMC-up
cfa04612	Antigen processing and presentation	4/407	54/7854	0.30622195	0.82260001	0.79073027	403400/486077/474836/100855741 CTSS, CTSB, DLA88, B2M	4	ACOR_VSMC-up

cfa04923	Regulation of lipolysis in adipocytes	4/407	54/7854	0.30622195	0.82260001	0.79073027	477625/403607/478227/476045 ADCY6, LIPE, GNAI1, PLA2G16	4	ACOR_VSMC-up
cfa04213	Longevity regulating pathway - multiple species	4/407	59/7854	0.36592967	0.87097311	0.83722927	489571/477625/488526/403474 PRKAA2, ADCY6, PRKAG3, CAT	4	ACOR_VSMC-up
cfa05321	Inflammatory bowel disease (IBD)	4/407	61/7854	0.38981826	0.87388357	0.84002697	100856077/478328/487286/611438 IFNGR1, RORA, NOD2, IL18R1	4	ACOR_VSMC-up
cfa05230	Central carbon metabolism in cancer	4/407	66/7854	0.44881775	0.88820767	0.85379612	415125/403849/476828/487797 FGFR2, PFKM, , PFKL	4	ACOR_VSMC-up
cfa05031	Amphetamine addiction	4/407	67/7854	0.46042071	0.88820767	0.85379612	403451/403450/610764/611934 MAOB, MAOA, CAMK2D, CREB3	4	ACOR_VSMC-up
cfa04520	Adherens junction	4/407	70/7854	0.49469218	0.88820767	0.85379612	477331/481628/102156745/475213 WASF3, TGFBR1, MAP3K7, WASL	4	ACOR_VSMC-up
cfa01524	Platinum drug resistance	4/407	72/7854	0.5170238	0.88929092	0.8548374	403416/474890/477558/406185 BCL2, CDKN1A, , ATP7A	4	ACOR_VSMC-up
cfa04662	B cell receptor signaling pathway	4/407	72/7854	0.5170238	0.88929092	0.8548374	489692/478491/486814/611606 PLCG2, DAPP1, BLNK, CD81	4	ACOR_VSMC-up
cfa05218	Melanoma	4/407	73/7854	0.52801649	0.88929092	0.8548374	482666/611641/479180/474890 PDGFC, FGF18, POLK, CDKN1A	4	ACOR_VSMC-up
cfa01230	Biosynthesis of amino acids	4/407	74/7854	0.53888654	0.89195014	0.85739359	479301/403849/480622/487797 MAT2B, PFKM, ALDOC, PFKL	4	ACOR_VSMC-up
cfa05214	Glioma	4/407	75/7854	0.54962905	0.90029392	0.86541411	489692/479180/610764/474890 PLCG2, POLK, CAMK2D, CDKN1A	4	ACOR_VSMC-up
cfa03018	RNA degradation	4/407	78/7854	0.58104747	0.90408536	0.86905866	403849/477734/482708/487797 PFKM, DCP1B, CNOT4, PFKL	4	ACOR_VSMC-up
cfa04721	Synaptic vesicle cycle	4/407	78/7854	0.58104747	0.90408536	0.86905866	403751/484826/403729/480605 SLC1A1, RAB3A, ATP6V0E1, AP2B1	4	ACOR_VSMC-up
cfa04146	Peroxisome	4/407	82/7854	0.62091693	0.90773643	0.87256828	474670/479564/489688/403474 ACSL6, SCP2, MLYCD, CAT	4	ACOR_VSMC-up
cfa00061	Fatty acid biosynthesis	4/388	18/7854	0.01033295	0.12219833	0.11444271	478927/483378/481018/475631 ACSL3, FASN, ACSL4, ACSL1	4	ACOR_VSMC-down
cfa00030	Pentose phosphate pathway	4/388	29/7854	0.05246914	0.4603744	0.43115559	481088/476588/481011/478236 G6PD, TKT, PRPS1, PGD	4	ACOR_VSMC-down
cfa00620	Pyruvate metabolism	4/388	37/7854	0.10781487	0.73116496	0.68475975	476574/480858/484063/489406 , PDHA1, ACAT2, DLAT	4	ACOR_VSMC-down
cfa00520	Amino sugar and nucleotide sugar metabolism	4/388	48/7854	0.21145709	0.99166085	0.92872262	100856660/479107/100855555/490013 GMPPB, UGDH, GALE, PMM2	4	ACOR_VSMC-down
cfa00270	Cysteine and methionine metabolism	4/388	50/7854	0.23270441	0.99972301	0.9362731	486633/476715/481960/479991 BCAT1, , AMD1, CTH	4	ACOR_VSMC-down
cfa05418	Fluid shear stress and atherosclerosis	13/279	135/7854	0.00098025	0.02568257	0.02177188	403498/403802/404019/482840/477558/491597/477032/488437/480438/403788/485893/478464/480416 ARHGEF2, VEGFA, HSP90B1, , , PDGFA, CTNNB1, ITGAV, HSP90AA1, ITGB3, SDC4, MAPK10, CALM1	13	VSAP_EC-down
cfa05014	Amyotrophic lateral sclerosis (ALS)	4/388	56/7854	0.29900406	0.99972301	0.9362731	100683675/442961/475258/403955 BID, GPX1, CYCS, RAC1	4	ACOR_VSMC-down
cfa04920	Adipocytokine signaling pathway	4/388	70/7854	0.45721663	0.99972301	0.9362731	478927/481018/477732/475631 ACSL3, ACSL4, ADIPOR2, ACSL1	4	ACOR_VSMC-down
cfa04918	Thyroid hormone synthesis	4/388	72/7854	0.4791237	0.99972301	0.9362731	442961/477098/475596/481746 GPX1, ATP1B3, , ITPR3	4	ACOR_VSMC-down
cfa05218	Melanoma	4/388	73/7854	0.48994914	0.99972301	0.9362731	481131/403872/608459/489644 CDK4, NRAS, FGF5, PIK3CD	4	ACOR_VSMC-down
cfa05212	Pancreatic cancer	4/388	75/7854	0.51131502	0.99972301	0.9362731	403568/481131/403955/489644 RAD51, CDK4, RAC1, PIK3CD	4	ACOR_VSMC-down
cfa05220	Chronic myeloid leukemia	4/388	76/7854	0.52184492	0.99972301	0.9362731	481131/403872/487746/489644 CDK4, NRAS, RUNX1, PIK3CD	4	ACOR_VSMC-down
cfa04146	Peroxisome	4/388	82/7854	0.58260098	0.99972301	0.9362731	478927/481018/475631/475375 ACSL3, ACSL4, ACSL1, PRDX1	4	ACOR_VSMC-down
cfa05418	Fluid shear stress and atherosclerosis	13/357	135/7854	0.00823446	0.05221397	0.03821736	403999/442986/482154/479155/403784/489775/607269/403424/475108/475294/612821/403788/403498 SELE, PDGFB, KDR, MEF2C, NOS3, CDH5, CALML6, EDN1, PTK2, CAV2, MAPK13, ITGB3, ARHGEF2	13	VCAV_EC-up
cfa01522	Endocrine resistance	4/388	93/7854	0.68150846	0.99972301	0.9362731	475359/481131/403872/489644 CDKN2C, CDK4, NRAS, PIK3CD	4	ACOR_VSMC-down
cfa04640	Hematopoietic cell lineage	4/388	93/7854	0.68150846	0.99972301	0.9362731	403913/403703/403788/489208 ANPEP, TFRC, ITGB3, ITGA2	4	ACOR_VSMC-down
cfa04925	Aldosterone synthesis and secretion	4/388	94/7854	0.68963459	0.99972301	0.9362731	477098/486545/483036/481746 ATP1B3, ATF1, PRKD3, ITPR3	4	ACOR_VSMC-down
cfa05418	Fluid shear stress and atherosclerosis	13/241	135/7854	0.00024337	0.02816767	0.02532399	476005/485893/403733/489077/609956/485864/449021/476006/479912/477945/489664/474519/481271 GSTP1, SDC4, MMP2, BMPR1A, , SRC, AKT2, , GSTM4, SDC2, CYBA, TXN2, RAC2	13	VCAV_EC-down

cfa04933	AGE-RAGE signaling pathway in diabetic complications	4/388	100/7854	0.73529649	0.99972301	0.9362731	481131/403872/403955/489644 CDK4, NRAS, RAC1, PIK3CD	4	ACOR_VSMC-down
cfa04922	Glucagon signaling pathway	4/388	101/7854	0.74239367	0.99972301	0.9362731	476411/476574/480858/481746 PRMT1, , PDHA1, ITPR3	4	ACOR_VSMC-down
cfa04660	T cell receptor signaling pathway	4/388	102/7854	0.74934583	0.99972301	0.9362731	607885/481131/403872/489644 PAK1, CDK4, NRAS, PIK3CD	4	ACOR_VSMC-down
cfa05146	Amoebiasis	4/388	102/7854	0.74934583	0.99972301	0.9362731	480364/475375/475882/489644 ARG2, PRDX1, LAMB1, PIK3CD	4	ACOR_VSMC-down
cfa05142	Chagas disease (American trypanosomiasis)	4/388	103/7854	0.75615385	0.99972301	0.9362731	492247/479444/476694/489644 IRAK1, PPP2R1B, CALR, PIK3CD	4	ACOR_VSMC-down
cfa04670	Leukocyte transendothelial migration	4/388	113/7854	0.81656783	0.99972301	0.9362731	403955/484056/483730/489644 RAC1, EZR, SIPA1, PIK3CD	4	ACOR_VSMC-down
cfa04725	Cholinergic synapse	4/388	113/7854	0.81656783	0.99972301	0.9362731	403872/479733/481746/489644 NRAS, GNB2, ITPR3, PIK3CD	4	ACOR_VSMC-down
cfa05135	Yersinia infection	4/388	117/7854	0.83703991	0.99972301	0.9362731	492247/100856354/403955/489644 IRAK1, , RAC1, PIK3CD	4	ACOR_VSMC-down
cfa05202	Transcriptional misregulation in cancer	42461	172/7854	0.00032901	0.01513467	0.01350691	100683897/482373/482372/480879 HOXA11, HOXA10, HOXA9, TSPAN7	4	VSAP_VSMC-up
cfa05202	Transcriptional misregulation in cancer	44016	172/7854	7.386E-06	8.1246E-05	3.8874E-05	477910/482373/482372/100683897 EYA1, HOXA10, HOXA9, HOXA11	4	VFEM_VSMC-up
cfa04964	Proximal tubule bicarbonate reclamation	3/131	21/7854	0.00484149	0.16530761	0.15160354	480591/403732/403966 CA4, AQP1, ATP1B1	3	ACOR_EC-up
cfa04975	Fat digestion and absorption	3/131	38/7854	0.02501682	0.29765577	0.27297999	607962/478514/491249 PLPP1, CFI, AGPAT2	3	ACOR_EC-up
cfa05418	Fluid shear stress and atherosclerosis	9/407	135/7854	0.26557918	0.82260001	0.79073027	403416/489571/490695/477558/403982/403424/102156745/489532/477945 BCL2, PRKAA2, BMP4, , VCAM1, EDN1, MAP3K7, MAPK7, SDC2	9	ACOR_VSMC-up
cfa04340	Hedgehog signaling pathway	3/131	47/7854	0.04319026	0.36641541	0.33603943	403416/487979/403556 BCL2, BOC, PRKACA	3	ACOR_EC-up
cfa00982	Drug metabolism - cytochrome P450	3/131	49/7854	0.04792262	0.36641541	0.33603943	474938/477558/403603 , , FMO3	3	ACOR_EC-up
cfa05418	Fluid shear stress and atherosclerosis	8/388	135/7854	0.35033717	0.99972301	0.9362731	482986/403980/486308/403788/403955/477813/403498/489644 SDC1, CAV1, TRPV4, ITGB3, RAC1, GSTO1, ARHGEF2, PIK3CD	8	ACOR_VSMC-down
cfa00480	Glutathione metabolism	3/131	52/7854	0.05547722	0.36641541	0.33603943	474938/486400/477558 , ,	3	ACOR_EC-up
cfa00980	Metabolism of xenobiotics by cytochrome P450	3/131	52/7854	0.05547722	0.36641541	0.33603943	474938/477558/484491 , , CYP2S1	3	ACOR_EC-up
cfa04961	Endocrine and other factor-regulated calcium reabsorption	3/131	52/7854	0.05547722	0.36641541	0.33603943	403779/403966/403556 PTH1R, ATP1B1, PRKACA	3	ACOR_EC-up
cfa04730	Long-term depression	3/131	60/7854	0.07817126	0.38752986	0.35540349	609157/553102/607445 GNAO1, GUCY1B1, GNAZ	3	ACOR_EC-up
cfa00590	Arachidonic acid metabolism	3/131	61/7854	0.08125508	0.39442571	0.36172766	486400/403740/478514 , PTGDS, CFI	3	ACOR_EC-up
cfa00983	Drug metabolism - other enzymes	3/131	63/7854	0.08757836	0.41644403	0.38192066	474938/477558/489769 , , CES2	3	ACOR_EC-up
cfa04012	ErbB signaling pathway	3/131	85/7854	0.16880203	0.52441163	0.48093771	100856339/492067/485864 , PAK3, SRC	3	ACOR_EC-up
cfa04911	Insulin secretion	3/131	85/7854	0.16880203	0.52441163	0.48093771	403966/488402/403556 ATP1B1, RAPGEF4, PRKACA	3	ACOR_EC-up
cfa04925	Aldosterone synthesis and secretion	3/131	94/7854	0.20672638	0.59465738	0.54536005	403966/484665/403556 ATP1B1, CAMK1, PRKACA	3	ACOR_EC-up
cfa04972	Pancreatic secretion	3/131	94/7854	0.20672638	0.59465738	0.54536005	403966/403756/478514 ATP1B1, CD38, CFI	3	ACOR_EC-up
cfa04713	Circadian entrainment	3/131	98/7854	0.22416932	0.60001444	0.55027301	609157/553102/403556 GNAO1, GUCY1B1, PRKACA	3	ACOR_EC-up
cfa04625	C-type lectin receptor signaling pathway	3/131	99/7854	0.22857601	0.60001444	0.55027301	100856339/485864/494005 , SRC, PRKCD	3	ACOR_EC-up
cfa04916	Melanogenesis	3/131	100/7854	0.23299931	0.60001444	0.55027301	100855544/609157/403556 FZD5, GNAO1, PRKACA	3	ACOR_EC-up
cfa05142	Chagas disease (American trypanosomiasis)	3/131	103/7854	0.24636041	0.60001444	0.55027301	100856339/609157/476728 , GNAO1, C3	3	ACOR_EC-up
cfa04726	Serotonergic synapse	3/131	110/7854	0.27796156	0.63495141	0.5823137	489198/609157/403556 HTR4, GNAO1, PRKACA	3	ACOR_EC-up
cfa04724	Glutamatergic synapse	3/131	112/7854	0.28707103	0.64939369	0.5955587	609157/479473/403556 GNAO1, PLD2, PRKACA	3	ACOR_EC-up
cfa04728	Dopaminergic synapse	3/131	132/7854	0.37860787	0.70012408	0.64208353	491697/609157/403556 CALY, GNAO1, PRKACA	3	ACOR_EC-up
cfa04910	Insulin signaling pathway	3/131	134/7854	0.38769375	0.70025305	0.6422018	100856339/489784/403556 , PHKG1, PRKACA	3	ACOR_EC-up

cfa04550	Signaling pathways regulating pluripotency of stem cells	3/131	135/7854	0.3922255	0.70298878	0.64471074	100856339/100855544/483880 , FZD5, INHBB	3	ACOR_EC-up
cfa05162	Measles	3/131	136/7854	0.39674913	0.70566829	0.64716812	100856339/403416/485789 , BCL2, MAVS	3	ACOR_EC-up
cfa04145	Phagosome	3/131	138/7854	0.40577001	0.70755799	0.64890116	475236/100855815/476728 DYNC1I1, TUBB4A, C3	3	ACOR_EC-up
cfa04310	Wnt signaling pathway	3/131	151/7854	0.46331538	0.72788738	0.66754524	480903/100855544/403556 PORCN, FZD5, PRKACA	3	ACOR_EC-up
cfa04141	Protein processing in endoplasmic reticulum	3/131	160/7854	0.50173906	0.73408049	0.67322493	477209/403416/483007 EDEM2, BCL2, PREB	3	ACOR_EC-up
cfa04217	Necroptosis	3/131	160/7854	0.50173906	0.73408049	0.67322493	403810/403416/485842 IL33, BCL2, CHMP4B	3	ACOR_EC-up
cfa05164	Influenza A	3/131	161/7854	0.50592415	0.73408049	0.67322493	100856339/403810/485789 , IL33, MAVS	3	ACOR_EC-up
cfa05034	Alcoholism	3/131	171/7854	0.54675287	0.74756244	0.68558922	609157/610744/403556 GNAO1, PKIA, PRKACA	3	ACOR_EC-up
cfa05166	Human T-cell leukemia virus 1 infection	3/131	209/7854	0.68259033	0.80099827	0.7345952	100856339/403556/477955 , PRKACA, NRP1	3	ACOR_EC-up
cfa04810	Regulation of actin cytoskeleton	3/131	211/7854	0.68883716	0.80099827	0.7345952	100856339/492067/485864 , PAK3, SRC	3	ACOR_EC-up
cfa00920	Sulfur metabolism	3/441	2174929	0.01569023	0.17259249	0.155373	475847/481103/474515 SELENBP1, SUOX, MPST	3	VSAP_EC-up
cfa00061	Fatty acid biosynthesis	3/441	18/7854	0.07661987	0.41125246	0.37022196	100685468/607895/475631 ACACB, HSD17B8, ACSL1	3	VSAP_EC-up
cfa00220	Arginine biosynthesis	3/441	19/7854	0.08735116	0.43238824	0.38924902	486834/403443/480693 GOT1, GLUL, ASS1	3	VSAP_EC-up
cfa00532	Glycosaminoglycan biosynthesis - chondroitin sulfate / dermatan sulfate	3/441	19/7854	0.08735116	0.43238824	0.38924902	608435/476271/491852 CHST14, DSE, CHST7	3	VSAP_EC-up
cfa00340	Histidine metabolism	3/441	21/7854	0.11055249	0.49006104	0.44116784	476003/481486/478991 ALDH3B1, ALDH7A1, ALDH9A1	3	VSAP_EC-up
cfa04710	Circadian rhythm	3/441	30/7854	0.23597479	0.70505074	0.63470809	486180/474552/486295 PER2, NPAS2, PRKAB1	3	VSAP_EC-up
cfa05020	Prion diseases	3/441	33/7854	0.28223324	0.74928501	0.67452913	487705/611939/479458 NCAM2, MAP2K2, CASP12	3	VSAP_EC-up
cfa04130	SNARE interactions in vesicular transport	3/441	36/7854	0.32918561	0.80885538	0.72815618	609734/479491/489919 USE1, , STX4	3	VSAP_EC-up
cfa05143	African trypanosomiasis	3/441	38/7854	0.3604949	0.84304712	0.75893662	488940/477024/482846 F2RL1, MYD88, IDO2	3	VSAP_EC-up
cfa00260	Glycine, serine and threonine metabolism	3/441	40/7854	0.39158068	0.85964878	0.77388194	484723/403978/481486 CHDH, DLD, ALDH7A1	3	VSAP_EC-up
cfa05418	Fluid shear stress and atherosclerosis	6/131	135/7854	0.02511472	0.29765577	0.27297999	474938/100856339/477558/403416/485864/403982 , , , BCL2, SRC, VCAM1	6	ACOR_EC-up
cfa04930	Type II diabetes mellitus	3/441	46/7854	0.481908	0.92381513	0.83164644	486148/487235/490006 IRS1, PIK3R1, SOCS1	3	VSAP_EC-up
cfa02010	ABC transporters	3/441	48/7854	0.51062033	0.92381513	0.83164644	485523/488390/480456 ABCC4, ABCB11, ABCA9	3	VSAP_EC-up
cfa04923	Regulation of lipolysis in adipocytes	3/441	54/7854	0.59134772	0.95972826	0.86397653	486148/487235/403607 IRS1, PIK3R1, LIPE	3	VSAP_EC-up
cfa04730	Long-term depression	3/441	60/7854	0.66297612	0.99864063	0.89900663	481746/484648/611939 ITPR3, RAF1, MAP2K2	3	VSAP_EC-up
cfa00670	One carbon pool by folate	3/279	20/7854	0.03232005	0.19054894	0.16153406	607458/474534/483107 SHMT2, ALDH1L2,	3	VSAP_EC-down
cfa00051	Fructose and mannose metabolism	3/279	32/7854	0.10342187	0.38701439	0.32808371	487139/477711/480622 PFKFB3, TPI1, ALDOC	3	VSAP_EC-down
cfa00620	Pyruvate metabolism	3/279	37/7854	0.14287367	0.431399	0.36570988	476882/403874/474894 , , GLO1	3	VSAP_EC-down
cfa00260	Glycine, serine and threonine metabolism	3/279	40/7854	0.16858578	0.46009869	0.39003947	607458/476318/477786 SHMT2, PSAT1, PGAM1	3	VSAP_EC-down
cfa05418	Fluid shear stress and atherosclerosis	42795	135/7854	0.00283087	0.10246963	0.08868717	478464/474938/477558 MAPK10, ,	3	APUL_EC-up
cfa04216	Ferroptosis	3/279	45/7854	0.21400947	0.52896679	0.44842103	486124/403703/483821 SLC39A14, TFRC, SLC7A11	3	VSAP_EC-down
cfa04913	Ovarian steroidogenesis	3/279	46/7854	0.2233961	0.53783066	0.45593519	479975/442942/486241 PRKACB, PTGS2, SCARB1	3	VSAP_EC-down
cfa00270	Cysteine and methionine metabolism	3/279	50/7854	0.2616555	0.58592941	0.49670995	476882/476318/486633 , PSAT1, BCAT1	3	VSAP_EC-down
cfa00510	N-Glycan biosynthesis	3/279	50/7854	0.2616555	0.58592941	0.49670995	483104/489300/476516 MOGS, STT3A, RPN1	3	VSAP_EC-down
cfa05134	Legionellosis	3/279	50/7854	0.2616555	0.58592941	0.49670995	479406/403567/607408 HSPA8, CASP3, BNIP3	3	VSAP_EC-down
cfa00240	Pyrimidine metabolism	3/279	52/7854	0.2810975	0.61044809	0.51749517	100856150/486117/610291 ENPP1, ENTPD4, CMPK1	3	VSAP_EC-down
cfa04730	Long-term depression	3/279	60/7854	0.35955833	0.69267855	0.5872044	609616/403928/477886 PRKG1, GNAQ, LYN	3	VSAP_EC-down
cfa05217	Basal cell carcinoma	3/279	63/7854	0.38880333	0.71904705	0.60955776	477032/478575/474890 CTNNB1, GSK3B, CDKN1A	3	VSAP_EC-down
cfa04137	Mitophagy - animal	3/279	64/7854	0.39848463	0.72002051	0.610383	480348/607408/478464 HIF1A, BNIP3, MAPK10	3	VSAP_EC-down

cfa04664	Fc epsilon RI signaling pathway	3/279	67/7854	0.4272596	0.73430489	0.62249229	477886/478464/492019 LYN, MAPK10, BTK	3	VSAP_EC-down
cfa04917	Prolactin signaling pathway	3/279	70/7854	0.45554071	0.73430489	0.62249229	474581/478575/478464 SOCS5, GSK3B, MAPK10	3	VSAP_EC-down
cfa04920	Adipocytokine signaling pathway	3/279	70/7854	0.45554071	0.73430489	0.62249229	477488/482437/478464 PTPN11, SLC2A1, MAPK10	3	VSAP_EC-down
cfa05140	Leishmaniasis	3/279	70/7854	0.45554071	0.73430489	0.62249229	442942/477956/612998 PTGS2, ITGB1, MARCKSL1	3	VSAP_EC-down
cfa01524	Platinum drug resistance	3/279	72/7854	0.47406916	0.74822963	0.63429672	403567/477558/474890 CASP3, , CDKN1A	3	VSAP_EC-down
cfa04662	B cell receptor signaling pathway	3/279	72/7854	0.47406916	0.74822963	0.63429672	477886/478575/492019 LYN, GSK3B, BTK	3	VSAP_EC-down
cfa03018	RNA degradation	3/279	78/7854	0.52781296	0.79475284	0.67373584	479597/100856683/482895 ENO1, ENO2, CNOT7	3	VSAP_EC-down
cfa04721	Synaptic vesicle cycle	3/279	78/7854	0.52781296	0.79475284	0.67373584	100683511/482526/480578 UNC13B, SLC6A9, CLTC	3	VSAP_EC-down
cfa04610	Complement and coagulation cascades	3/279	80/7854	0.54504485	0.79879452	0.67716209	403476/482840/477699 SERPINE1, , A2M	3	VSAP_EC-down
cfa05235	PD-L1 expression and PD-1 checkpoint pathway in cancer	3/279	89/7854	0.61785166	0.84772198	0.71863937	477488/480348/483048 PTPN11, HIF1A, EML4	3	VSAP_EC-down
cfa03015	mRNA surveillance pathway	3/279	90/7854	0.6254389	0.84772198	0.71863937	477374/474839/479846 PPP2R2A, DDX39B, GSPT1	3	VSAP_EC-down
cfa04727	GABAergic synapse	3/279	91/7854	0.63292274	0.84772198	0.71863937	479975/477633/478858 PRKACB, SLC38A1, PLCL1	3	VSAP_EC-down
cfa04974	Protein digestion and absorption	3/279	91/7854	0.63292274	0.84772198	0.71863937	474778/476788/481881 COL15A1, PRCP, COL12A1	3	VSAP_EC-down
cfa01522	Endocrine resistance	3/279	93/7854	0.64757841	0.84772198	0.71863937	479975/478464/474890 PRKACB, MAPK10, CDKN1A	3	VSAP_EC-down
cfa05418	Fluid shear stress and atherosclerosis	12451	135/7854	0.11529741	0.52353656	0.51664792	403498/476140 ARHGEF2, ACVR2A	2	VPOR_EC-up
cfa04020	Calcium signaling pathway	42795	192/7854	0.00759528	0.10246963	0.08868717	489198/477762/403652 HTR4, HTR7, PDE1A	3	APUL_EC-up
cfa05132	Salmonella infection	42795	212/7854	0.00996901	0.10312167	0.0892515	478464/611857/606992 MAPK10, ARHGEF26, MYL6B	3	APUL_EC-up
cfa04014	Ras signaling pathway	42795	225/7854	0.01172148	0.10549336	0.09130419	478464/477762/484147 MAPK10, HTR7, NTRK2	3	APUL_EC-up
cfa00220	Arginine biosynthesis	3/357	19/7854	0.05257069	0.17054911	0.12483126	403784/480364/403822 NOS3, ARG2, NOS2	3	VCAV_EC-up
cfa04215	Apoptosis - multiple species	3/357	32/7854	0.17630195	0.3954409	0.28943797	488473/100502558/482620 CASP8, PMAIP1, APAF1	3	VCAV_EC-up
cfa05143	African trypanosomiasis	3/357	38/7854	0.24802604	0.50881812	0.372423	403999/403985/475034 SELE, IL6, LAMA4	3	VCAV_EC-up
cfa04672	Intestinal immune network for IgA production	3/357	43/7854	0.31061338	0.57822417	0.42322388	483900/403985/403764 CXCR4, IL6, CD86	3	VCAV_EC-up
cfa03050	Proteasome	3/357	46/7854	0.34848096	0.61535562	0.45040177	474594/474867/480465 PSME4, PSMB9, PSMD12	3	VCAV_EC-up
cfa00514	Other types of O-glycan biosynthesis	3/357	47/7854	0.3610717	0.62542361	0.45777091	478668/485390/610303 ST6GAL1, GALNT18, MFNG	3	VCAV_EC-up
cfa00520	Amino sugar and nucleotide sugar metabolism	3/357	48/7854	0.37362574	0.63176716	0.462414	479116/490313/100855555 PGM2, NPL, GALE	3	VCAV_EC-up
cfa05030	Cocaine addiction	3/357	48/7854	0.37362574	0.63176716	0.462414	475266/478227/607922 CREB5, GNAI1, CREB1	3	VCAV_EC-up
cfa00510	N-Glycan biosynthesis	3/357	50/7854	0.39857815	0.6577911	0.48146189	478668/474562/609002 ST6GAL1, MGAT4A,	3	VCAV_EC-up
cfa05418	Fluid shear stress and atherosclerosis	41671	135/7854	0.02331359	0.3227867	0.32089906	477558/403498 , ARHGEF2	2	VPUL_EC-up
cfa05418	Fluid shear stress and atherosclerosis	44105	135/7854	0.1592674	0.215011	0.20117988	476005 GSTP1	1	VPOR_EC-down
cfa00240	Pyrimidine metabolism	3/357	52/7854	0.4232531	0.66705509	0.48824255	486810/487393/482188 ENTPD1, CDA, DCK	3	VCAV_EC-up
cfa04612	Antigen processing and presentation	3/357	54/7854	0.44757505	0.67499155	0.49405154	479406/607922/474897 HSPA8, CREB1, NFYA	3	VCAV_EC-up
cfa00532	Glycosaminoglycan biosynthesis - chondroitin sulfate / dermatan sulfate	3/241	19/7854	0.01922387	0.24158651	0.21719709	476271/488539/476238 DSE, OBSL1, UST	3	VCAV_EC-down
cfa00531	Glycosaminoglycan degradation	3/241	20/7854	0.02211458	0.24158651	0.21719709	490965/492194/487633 NAGLU, IDS,	3	VCAV_EC-down
cfa00620	Pyruvate metabolism	3/241	37/7854	0.10353499	0.42358973	0.38082613	485151/477205/489421 ME3, ACSS2, ACAT1	3	VCAV_EC-down
cfa00071	Fatty acid degradation	3/241	41/7854	0.13024625	0.47881756	0.43047842	477023/607926/489421 ACAA1, , ACAT1	3	VCAV_EC-down
cfa00380	Tryptophan metabolism	3/241	41/7854	0.13024625	0.47881756	0.43047842	403450/483038/489421 MAOA, CYP1B1, ACAT1	3	VCAV_EC-down

cfa04973	Carbohydrate digestion and absorption	3/241	43/7854	0.14442292	0.4861441	0.43706531	609956/449021/490942 , AKT2, G6PC3	3	VCAV_EC-down
cfa04930	Type II diabetes mellitus	3/241	46/7854	0.16657489	0.51052385	0.45898379	609956/485548/486148 , IRS2, IRS1	3	VCAV_EC-down
cfa00330	Arginine and proline metabolism	3/241	47/7854	0.17417072	0.51517865	0.46316867	403450/100855552/479081 MAOA, , LAP3	3	VCAV_EC-down
cfa00514	Other types of O-glycan biosynthesis	3/241	47/7854	0.17417072	0.51517865	0.46316867	483376/609881/481579 RFNG, POFUT1, B4GALT1	3	VCAV_EC-down
cfa00600	Sphingolipid metabolism	3/241	48/7854	0.18186228	0.51619497	0.46408238	474457/607962/100856377 ARSA, PLPP1, SMPD2	3	VCAV_EC-down
cfa04330	Notch signaling pathway	3/241	51/7854	0.20545145	0.54982722	0.49431929	483148/483376/612491 NOTCH2, RFNG, NUMBL	3	VCAV_EC-down
cfa05213	Endometrial cancer	3/241	59/7854	0.27111976	0.62962523	0.56606127	609956/449021/481954 , AKT2, FOXO3	3	VCAV_EC-down
cfa00983	Drug metabolism - other enzymes	3/241	63/7854	0.30482155	0.67980044	0.61117103	476005/476006/479912 GSTP1, , GSTM4	3	VCAV_EC-down
cfa04664	Fc epsilon RI signaling pathway	3/241	67/7854	0.33869317	0.70498356	0.63381178	609956/449021/481271 , AKT2, RAC2	3	VCAV_EC-down
cfa05211	Renal cell carcinoma	3/241	67/7854	0.33869317	0.70498356	0.63381178	609956/449021/491872 , AKT2, TFE3	3	VCAV_EC-down
cfa05223	Non-small cell lung cancer	3/241	67/7854	0.33869317	0.70498356	0.63381178	609956/449021/481954 , AKT2, FOXO3	3	VCAV_EC-down
cfa04918	Thyroid hormone synthesis	3/241	72/7854	0.38086801	0.76995619	0.69222508	477625/482715/442961 ADCY6, PDIA4, GPX1	3	VCAV_EC-down
cfa05212	Pancreatic cancer	3/241	75/7854	0.40591058	0.78213515	0.70317451	609956/449021/481271 , AKT2, RAC2	3	VCAV_EC-down
cfa05220	Chronic myeloid leukemia	3/241	76/7854	0.41419498	0.78213515	0.70317451	609956/449021/490439 , AKT2, SHC1	3	VCAV_EC-down
cfa04721	Synaptic vesicle cycle	3/241	78/7854	0.43065171	0.79541394	0.71511274	489104/479877/475994 CLTB, ATP6V0C, AP2A2	3	VCAV_EC-down
cfa04970	Salivary secretion	3/241	78/7854	0.43065171	0.79541394	0.71511274	607874/477625/488820 CST3, ADCY6, BST1	3	VCAV_EC-down
cfa03320	PPAR signaling pathway	3/241	80/7854	0.44694119	0.79541394	0.71511274	477023/485151/479564 ACAA1, ME3, SCP2	3	VCAV_EC-down
cfa05323	Rheumatoid arthritis	3/241	83/7854	0.47101911	0.8129908	0.73091512	403656/611795/479877 ANGPT1, CSF1, ATP6V0C	3	VCAV_EC-down
cfa05210	Colorectal cancer	3/241	85/7854	0.48680788	0.81424413	0.73204192	609956/449021/481271 , AKT2, RAC2	3	VCAV_EC-down
cfa05418	Fluid shear stress and atherosclerosis	42736	135/7854	0.2555068	0.32364194	0.30122906	480454 MAP2K6	1	VFEM_EC-up
cfa05222	Small cell lung cancer	3/241	92/7854	0.54017868	0.84753502	0.76197193	609956/449021/480173 , AKT2, LAMA3	3	VCAV_EC-down
cfa04625	C-type lectin receptor signaling pathway	3/241	99/7854	0.59024422	0.8750588	0.78671703	609956/485864/449021 , SRC, AKT2	3	VCAV_EC-down
cfa04922	Glucagon signaling pathway	3/241	101/7854	0.6038925	0.8750588	0.78671703	449021/490942/610764 AKT2, G6PC3, CAMK2D	3	VCAV_EC-down
cfa04650	Natural killer cell mediated cytotoxicity	3/241	103/7854	0.61723908	0.88042732	0.79154357	609956/490439/481271 , SHC1, RAC2	3	VCAV_EC-down
cfa00230	Purine metabolism	12479	130/7854	0.01824455	0.52353656	0.51664792	490204/475272/481988 AK5, PDE1C, RRM2B	3	VPOR_EC-up
cfa03013	RNA transport	12479	158/7854	0.03030902	0.52353656	0.51664792	474427/102155289/480442 XPOT, , EIF5	3	VPOR_EC-up
cfa04015	Rap1 signaling pathway	42795	209/7854	0.00958827	0.32364194	0.30122906	480454/487731/482994 MAP2K6, TIAM1, ADCY3	3	VFEM_EC-up
cfa05020	Prion diseases	34759	33/7854	0.00719336	0.32190301	0.31991537	487705/403522/403559 NCAM2, CCL5, SOD1	3	AFEM_EC-up
cfa00561	Glycerolipid metabolism	34759	59/7854	0.03424514	0.64105544	0.63709715	483023/486890/491249 LCLAT1, GPAM, AGPAT2	3	AFEM_EC-up
cfa05323	Rheumatoid arthritis	34759	83/7854	0.07891695	0.81029448	0.80529119	486137/482528/403522 ATP6V1B2, ATP6V0B, CCL5	3	AFEM_EC-up
cfa04211	Longevity regulating pathway	34759	88/7854	0.09036311	0.81029448	0.80529119	485548/479127/476258 IRS2, PPARGC1A, SOD2	3	AFEM_EC-up
cfa05418	Fluid shear stress and atherosclerosis	44166	135/7854	0.18795922	0.2588504	0.24219921	477746 MAPK8	1	AFEM_EC-down
cfa04350	TGF-beta signaling pathway	34759	92/7854	0.09999147	0.81029448	0.80529119	487475/490367/486537 GREM1, GREM2, ACVR1B	3	AFEM_EC-up
cfa04922	Glucagon signaling pathway	34759	101/7854	0.12306614	0.81029448	0.80529119	612941/100685468/479127 CAMK2A, ACACB, PPARGC1A	3	AFEM_EC-up
cfa04931	Insulin resistance	34759	109/7854	0.14504624	0.81029448	0.80529119	485548/100685468/479127 IRS2, ACACB, PPARGC1A	3	AFEM_EC-up
cfa00190	Oxidative phosphorylation	34759	131/7854	0.21105778	0.81029448	0.80529119	486137/482528/612614 ATP6V1B2, ATP6V0B, COX7A1	3	AFEM_EC-up
cfa05418	Fluid shear stress and atherosclerosis	42370	135/7854	0.24245908	0.36210801	0.32316276	403980 CAV1	1	VSAP_VSMC-up
cfa04910	Insulin signaling pathway	34759	134/7854	0.22054388	0.81029448	0.80529119	485548/100685468/479127 IRS2, ACACB, PPARGC1A	3	AFEM_EC-up
cfa04550	Signaling pathways regulating pluripotency of stem cells	34759	135/7854	0.22372574	0.81029448	0.80529119	490903/490937/486537 AXIN2, FZD2, ACVR1B	3	AFEM_EC-up
cfa05224	Breast cancer	34759	142/7854	0.24624226	0.81029448	0.80529119	100688936/490903/490937 DLL1, AXIN2, FZD2	3	AFEM_EC-up
cfa04932	Non-alcoholic fatty liver disease (NAFLD)	34759	145/7854	0.25600487	0.81029448	0.80529119	403616/485548/612614 LEP, IRS2, COX7A1	3	AFEM_EC-up

cfa04072	Phospholipase D signaling pathway	34759	148/7854	0.26582282	0.81029448	0.80529119	481142/482666/491249 AVPR1A, PDGFC, AGPAT2	3	AFEM_EC-up
cfa04934	Cushing syndrome	34759	152/7854	0.27898531	0.81866182	0.81360687	612941/490903/490937 CAMK2A, AXIN2, FZD2	3	AFEM_EC-up
cfa05202	Transcriptional misregulation in cancer	34759	172/7854	0.34541861	0.83791788	0.83274402	482372/482373/403469 HOXA9, HOXA10, CD40	3	AFEM_EC-up
cfa04810	Regulation of actin cytoskeleton	34759	211/7854	0.47223525	0.83791788	0.83274402	403858/482666/483856 CHRM2, PDGFC, ARHGEF4	3	AFEM_EC-up
cfa04014	Ras signaling pathway	34759	225/7854	0.5152364	0.86267127	0.85734458	609626/476045/482666 ANGPT4, PLA2G16, PDGFC	3	AFEM_EC-up
cfa00062	Fatty acid elongation	3/407	26/7854	0.14942104	0.76328292	0.73371129	478506/607926/608997 HADH, , ELOVL7	3	ACOR_VSMC-up
cfa00591	Linoleic acid metabolism	3/407	26/7854	0.14942104	0.76328292	0.73371129	610195/476045/478514 CYP2J2, PLA2G16, CFI	3	ACOR_VSMC-up
cfa04977	Vitamin digestion and absorption	3/407	26/7854	0.14942104	0.76328292	0.73371129	100856550/486355/482514 LMBRD1, TCN2,	3	ACOR_VSMC-up
cfa00030	Pentose phosphate pathway	3/407	29/7854	0.18815249	0.80256807	0.77147442	403849/480622/487797 PFKM, ALDOC, PFKL	3	ACOR_VSMC-up
cfa00052	Galactose metabolism	3/407	30/7854	0.2015944	0.80256807	0.77147442	403849/483352/487797 PFKM, GAA, PFKL	3	ACOR_VSMC-up
cfa04710	Circadian rhythm	3/407	30/7854	0.2015944	0.80256807	0.77147442	489571/478328/488526 PRKAA2, RORA, PRKAG3	3	ACOR_VSMC-up
cfa00760	Nicotinate and nicotinamide metabolism	3/407	37/7854	0.30021302	0.82260001	0.79073027	484162/477809/100856724 NMRK1, NT5C2, NMNAT1	3	ACOR_VSMC-up
cfa04975	Fat digestion and absorption	3/407	38/7854	0.31463833	0.82260001	0.79073027	481651/100856443/478514 ABCA1, PLPP2, CFI	3	ACOR_VSMC-up
cfa05143	African trypanosomiasis	3/407	38/7854	0.31463833	0.82260001	0.79073027	488940/403982/403928 F2RL1, VCAM1, GNAQ	3	ACOR_VSMC-up
cfa00513	Various types of N-glycan biosynthesis	3/407	40/7854	0.34351137	0.86027195	0.8269427	485628/448804/488745 STT3B, FUT8, MAN2A2	3	ACOR_VSMC-up
cfa00071	Fatty acid degradation	3/407	41/7854	0.35791888	0.86074085	0.82739343	478506/474670/607926 HADH, ACSL6,	3	ACOR_VSMC-up
cfa05219	Bladder cancer	3/407	41/7854	0.35791888	0.86074085	0.82739343	489428/484142/474890 MMP1, DAPK1, CDKN1A	3	ACOR_VSMC-up
cfa04216	Ferroptosis	3/407	45/7854	0.41493641	0.88820767	0.85379612	477072/474670/479619 , ACSL6,	3	ACOR_VSMC-up
cfa00514	Other types of O-glycan biosynthesis	3/407	47/7854	0.44285915	0.88820767	0.85379612	478774/489165/494221 GALNT3, GALNT10, EOGT	3	ACOR_VSMC-up
cfa04340	Hedgehog signaling pathway	3/407	47/7854	0.44285915	0.88820767	0.85379612	403416/478781/611782 BCL2, LRP2, CCND2	3	ACOR_VSMC-up
cfa00280	Valine, leucine and isoleucine degradation	3/407	48/7854	0.45662645	0.88820767	0.85379612	479610/478506/607926 , HADH,	3	ACOR_VSMC-up
cfa02010	ABC transporters	3/407	48/7854	0.45662645	0.88820767	0.85379612	486638/481651/444852 ABCC9, ABCA1, ABCA4	3	ACOR_VSMC-up
cfa03460	Fanconi anemia pathway	3/407	49/7854	0.47024882	0.88820767	0.85379612	479180/477027/479017 POLK, WDR48, FAN1	3	ACOR_VSMC-up
cfa05144	Malaria	3/407	49/7854	0.47024882	0.88820767	0.85379612	403982/611606/477945 VCAM1, CD81, SDC2	3	ACOR_VSMC-up
cfa00270	Cysteine and methionine metabolism	3/407	50/7854	0.48371575	0.88820767	0.85379612	479301/611790/475197 MAT2B, AHCYL1, AHCYL2	3	ACOR_VSMC-up
cfa00510	N-Glycan biosynthesis	3/407	50/7854	0.48371575	0.88820767	0.85379612	485628/448804/488745 STT3B, FUT8, MAN2A2	3	ACOR_VSMC-up
cfa04330	Notch signaling pathway	3/407	51/7854	0.49701759	0.88820767	0.85379612	483461/480381/403408 DTX4, NUMB, PSEN1	3	ACOR_VSMC-up
cfa05418	Fluid shear stress and atherosclerosis	43891	135/7854	0.05069115	0.06878116	0.02413374	477558	1	VSAP_VSMC-down
cfa05014	Amyotrophic lateral sclerosis (ALS)	3/407	56/7854	0.56077051	0.90174277	0.86680683	403416/477618/403474 BCL2, PRPH, CAT	3	ACOR_VSMC-up
cfa04730	Long-term depression	3/407	60/7854	0.6081078	0.90773643	0.87256828	476548/478227/403928 ITPR1, GNAI1, GNAQ	3	ACOR_VSMC-up
cfa00590	Arachidonic acid metabolism	3/407	61/7854	0.61939864	0.90773643	0.87256828	610195/476045/478514 CYP2J2, PLA2G16, CFI	3	ACOR_VSMC-up
cfa00010	Glycolysis / Gluconeogenesis	3/407	62/7854	0.63046704	0.91668454	0.88116972	403849/480622/487797 PFKM, ALDOC, PFKL	3	ACOR_VSMC-up
cfa04614	Renin-angiotensin system	3/388	24/7854	0.11258557	0.73116496	0.68475975	476748/403913/476788 THOP1, ANPEP, PRCP	3	ACOR_VSMC-down
cfa00052	Galactose metabolism	3/388	30/7854	0.18300092	0.93917454	0.87956749	607537/481579/100855555 AKR1B1, B4GALT1, GALE	3	ACOR_VSMC-down
cfa00051	Fructose and mannose metabolism	3/388	32/7854	0.20861999	0.99166085	0.92872262	100856660/607537/490013 GMPPB, AKR1B1, PMM2	3	ACOR_VSMC-down
cfa04215	Apoptosis - multiple species	3/388	32/7854	0.20861999	0.99166085	0.92872262	442936/100683675/475258 , BID, CYCS	3	ACOR_VSMC-down
cfa00330	Arginine and proline metabolism	3/388	47/7854	0.41210772	0.99972301	0.9362731	481960/609381/480364 AMD1, P4HA3, ARG2	3	ACOR_VSMC-down
cfa00280	Valine, leucine and isoleucine degradation	3/388	48/7854	0.42548443	0.99972301	0.9362731	486633/479344/484063 BCAT1, , ACAT2	3	ACOR_VSMC-down
cfa00600	Sphingolipid metabolism	3/388	48/7854	0.42548443	0.99972301	0.9362731	483329/484401/489032 SPHK1, SPHK2, SGPL1	3	ACOR_VSMC-down

cfa00561	Glycerolipid metabolism	3/388	59/7854	0.56354409	0.99972301	0.9362731	480872/607537/478320 , AKR1B1, LIPC	3	ACOR_VSMC-down
cfa04730	Long-term depression	3/388	60/7854	0.57507835	0.99972301	0.9362731	479444/403872/481746 PPP2R1B, NRAS, ITPR3	3	ACOR_VSMC-down
cfa00010	Glycolysis / Gluconeogenesis	3/388	62/7854	0.59756865	0.99972301	0.9362731	476574/480858/489406 , PDHA1, DLAT	3	ACOR_VSMC-down
cfa04927	Cortisol synthesis and secretion	3/388	63/7854	0.60851879	0.99972301	0.9362731	488669/488752/481746 PBX1, PDE8A, ITPR3	3	ACOR_VSMC-down
cfa04929	GnRH secretion	3/388	65/7854	0.62981776	0.99972301	0.9362731	403872/481746/489644 NRAS, ITPR3, PIK3CD	3	ACOR_VSMC-down
cfa04664	Fc epsilon RI signaling pathway	3/388	67/7854	0.65030479	0.99972301	0.9362731	403872/403955/489644 NRAS, RAC1, PIK3CD	3	ACOR_VSMC-down
cfa05223	Non-small cell lung cancer	3/388	67/7854	0.65030479	0.99972301	0.9362731	481131/403872/489644 CDK4, NRAS, PIK3CD	3	ACOR_VSMC-down
cfa04520	Adherens junction	3/388	70/7854	0.6794994	0.99972301	0.9362731	486940/403955/479050 SNAI2, RAC1, IQGAP1	3	ACOR_VSMC-down
cfa04662	B cell receptor signaling pathway	3/388	72/7854	0.69793823	0.99972301	0.9362731	403872/403955/489644 NRAS, RAC1, PIK3CD	3	ACOR_VSMC-down
cfa04971	Gastric acid secretion	3/388	72/7854	0.69793823	0.99972301	0.9362731	477098/484056/481746 ATP1B3, EZR, ITPR3	3	ACOR_VSMC-down
cfa05214	Glioma	3/388	75/7854	0.72407383	0.99972301	0.9362731	481131/403872/489644 CDK4, NRAS, PIK3CD	3	ACOR_VSMC-down
cfa05412	Arrhythmogenic right ventricular cardiomyopathy (ARVC)	3/388	77/7854	0.74049751	0.99972301	0.9362731	480124/403788/489208 LMNA, ITGB3, ITGA2	3	ACOR_VSMC-down
cfa04970	Salivary secretion	3/388	78/7854	0.74841442	0.99972301	0.9362731	486551/477098/481746 , ATP1B3, ITPR3	3	ACOR_VSMC-down
cfa05418	Fluid shear stress and atherosclerosis	44013	135/7854	0.11433254	0.14482122	0.09628004	477558	1	VCAV_VSMC-up
cfa04012	ErbB signaling pathway	3/388	85/7854	0.79853373	0.99972301	0.9362731	607885/403872/489644 PAK1, NRAS, PIK3CD	3	ACOR_VSMC-down
cfa04211	Longevity regulating pathway	3/388	88/7854	0.81731721	0.99972301	0.9362731	477732/403872/489644 ADIPOR2, NRAS, PIK3CD	3	ACOR_VSMC-down
cfa04540	Gap junction	3/388	88/7854	0.81731721	0.99972301	0.9362731	100856079/403872/481746 CDK1, NRAS, ITPR3	3	ACOR_VSMC-down
cfa04912	GnRH signaling pathway	3/388	89/7854	0.82323977	0.99972301	0.9362731	403823/403872/481746 MMP14, NRAS, ITPR3	3	ACOR_VSMC-down
cfa04974	Protein digestion and absorption	3/388	91/7854	0.83459523	0.99972301	0.9362731	476788/477098/607775 PRCP, ATP1B3, ELN	3	ACOR_VSMC-down
cfa05032	Morphine addiction	3/388	92/7854	0.84003433	0.99972301	0.9362731	610836/479733/488752 PDE3A, GNB2, PDE8A	3	ACOR_VSMC-down
cfa04972	Pancreatic secretion	3/388	94/7854	0.85045097	0.99972301	0.9362731	403955/477098/481746 RAC1, ATP1B3, ITPR3	3	ACOR_VSMC-down
cfa05017	Spinocerebellar ataxia	3/388	96/7854	0.86027321	0.99972301	0.9362731	612871/481746/489644 NOP56, ITPR3, PIK3CD	3	ACOR_VSMC-down
cfa05231	Choline metabolism in cancer	3/388	96/7854	0.86027321	0.99972301	0.9362731	403872/403955/489644 NRAS, RAC1, PIK3CD	3	ACOR_VSMC-down
cfa05215	Prostate cancer	3/388	97/7854	0.86496934	0.99972301	0.9362731	403872/100855704/489644 NRAS, CDK2, PIK3CD	3	ACOR_VSMC-down
cfa05205	Proteoglycans in cancer	42430	202/7854	0.00732981	0.11239042	0.10030267	448807/479215/403980 TLR2, ANK3, CAV1	3	VSAP_VSMC-up
cfa04151	PI3K-Akt signaling pathway	42430	354/7854	0.03284928	0.22413514	0.20002907	476665/448807/608459 COMP, TLR2, FGF5	3	VSAP_VSMC-up
cfa05202	Transcriptional misregulation in cancer	43924	172/7854	4.0631E-05	0.00085325	0.00059877	477910/482372/482373 EYA1, HOXA9, HOXA10	3	AFEM_VSMC-up
cfa00563	Glycosylphosphatidylinositol (GPI)-anchor biosynthesis	2/131	25/7854	0.06453594	0.36641541	0.33603943	477244/491170 PIGT, PIGS	2	ACOR_EC-up
cfa04215	Apoptosis - multiple species	2/131	32/7854	0.09904873	0.45251676	0.41500295	480571/403416 SEPT4, BCL2	2	ACOR_EC-up
cfa05020	Prion diseases	2/131	33/7854	0.10433058	0.45713613	0.41923937	489221/403556 C7, PRKACA	2	ACOR_EC-up
cfa00760	Nicotinate and nicotinamide metabolism	2/131	37/7854	0.12617135	0.48996541	0.44934709	610576/403756 NMNAT2, CD38	2	ACOR_EC-up
cfa04960	Aldosterone-regulated sodium reabsorption	2/131	37/7854	0.12617135	0.48996541	0.44934709	100856339/403966 , ATP1B1	2	ACOR_EC-up
cfa04973	Carbohydrate digestion and absorption	2/131	43/7854	0.16067211	0.52441163	0.48093771	100856339/403966 , ATP1B1	2	ACOR_EC-up
cfa04216	Ferroptosis	2/131	45/7854	0.17252999	0.52894063	0.48509125	478837/477072 SLC40A1,	2	ACOR_EC-up
cfa04930	Type II diabetes mellitus	2/131	46/7854	0.1785124	0.54017389	0.49539326	100856339/494005 , PRKCD	2	ACOR_EC-up
cfa05144	Malaria	2/131	49/7854	0.19664035	0.57996459	0.5318853	611606/403982 CD81, VCAM1	2	ACOR_EC-up
cfa04923	Regulation of lipolysis in adipocytes	2/131	54/7854	0.22730336	0.60001444	0.55027301	100856339/403556 , PRKACA	2	ACOR_EC-up
cfa05204	Chemical carcinogenesis	2/131	55/7854	0.23348221	0.60001444	0.55027301	474938/477558 ,	2	ACOR_EC-up

cfa04370	VEGF signaling pathway	2/131	57/7854	0.24586885	0.60001444	0.55027301	100856339/485864 , SRC	2 ACOR_EC-up
cfa04623	Cytosolic DNA-sensing pathway	2/131	57/7854	0.24586885	0.60001444	0.55027301	403810/485789 IL33, MAVS	2 ACOR_EC-up
cfa00561	Glycerolipid metabolism	2/131	59/7854	0.25828075	0.60179416	0.55190519	607962/491249 PLPP1, AGPAT2	2 ACOR_EC-up
cfa04213	Longevity regulating pathway - multiple species	2/131	59/7854	0.25828075	0.60179416	0.55190519	100856339/403556 , PRKACA	2 ACOR_EC-up
cfa05213	Endometrial cancer	2/131	59/7854	0.25828075	0.60179416	0.55190519	100856339/489008 ,	2 ACOR_EC-up
cfa04622	RIG-I-like receptor signaling pathway	2/131	67/7854	0.30789308	0.6742616	0.61836505	488472/485789 CASP10, MAVS	2 ACOR_EC-up
cfa05211	Renal cell carcinoma	2/131	67/7854	0.30789308	0.6742616	0.61836505	100856339/492067 , PAK3	2 ACOR_EC-up
cfa04520	Adherens junction	2/131	70/7854	0.3263695	0.68154985	0.6250491	485864/489008 SRC,	2 ACOR_EC-up
cfa04917	Prolactin signaling pathway	2/131	70/7854	0.3263695	0.68154985	0.6250491	100856339/485864 , SRC	2 ACOR_EC-up
cfa04662	B cell receptor signaling pathway	2/131	72/7854	0.33861802	0.68154985	0.6250491	100856339/611606 , CD81	2 ACOR_EC-up
cfa04918	Thyroid hormone synthesis	2/131	72/7854	0.33861802	0.68154985	0.6250491	403966/403556 ATP1B1, PRKACA	2 ACOR_EC-up
cfa04971	Gastric acid secretion	2/131	72/7854	0.33861802	0.68154985	0.6250491	403966/403556 ATP1B1, PRKACA	2 ACOR_EC-up
cfa05212	Pancreatic cancer	2/131	75/7854	0.35686301	0.68154985	0.6250491	100856339/479473 , PLD2	2 ACOR_EC-up
cfa05214	Glioma	2/131	75/7854	0.35686301	0.68154985	0.6250491	100856339/484665 , CAMK1	2 ACOR_EC-up
cfa04742	Taste transduction	2/131	77/7854	0.36892832	0.69322821	0.63575933	403652/403556 PDE1A, PRKACA	2 ACOR_EC-up
cfa05412	Arrhythmogenic right ventricular cardiomyopathy (ARVC)	2/131	77/7854	0.36892832	0.69322821	0.63575933	479191/489008 ACTN2,	2 ACOR_EC-up
cfa05210	Colorectal cancer	2/131	85/7854	0.41624027	0.70895463	0.65018202	100856339/403416 , BCL2	2 ACOR_EC-up
cfa04211	Longevity regulating pathway	2/131	88/7854	0.43353277	0.72071968	0.66097174	100856339/403556 , PRKACA	2 ACOR_EC-up
cfa04914	Progesterone-mediated oocyte maturation	2/131	89/7854	0.43923689	0.72071968	0.66097174	100856339/403556 , PRKACA	2 ACOR_EC-up
cfa04974	Protein digestion and absorption	2/131	91/7854	0.45055151	0.72788738	0.66754524	403966/481734 ATP1B1, COL11A2	2 ACOR_EC-up
cfa05222	Small cell lung cancer	2/131	92/7854	0.45616093	0.72788738	0.66754524	100856339/403416 , BCL2	2 ACOR_EC-up
cfa04064	NF-kappa B signaling pathway	2/131	93/7854	0.46173774	0.72788738	0.66754524	403416/403982 BCL2, VCAM1	2 ACOR_EC-up
cfa04070	Phosphatidylinositol signaling system	2/131	97/7854	0.48370972	0.73408049	0.67322493	100856339/486808 , PLCE1	2 ACOR_EC-up
cfa05215	Prostate cancer	2/131	97/7854	0.48370972	0.73408049	0.67322493	100856339/403416 , BCL2	2 ACOR_EC-up
cfa04922	Glucagon signaling pathway	2/131	101/7854	0.50512605	0.73408049	0.67322493	489784/403556 PHKG1, PRKACA	2 ACOR_EC-up
cfa04660	T cell receptor signaling pathway	2/131	102/7854	0.51039072	0.73408049	0.67322493	100856339/492067 , PAK3	2 ACOR_EC-up
cfa05146	Amoebiasis	2/131	102/7854	0.51039072	0.73408049	0.67322493	100856339/403556 , PRKACA	2 ACOR_EC-up
cfa04931	Insulin resistance	2/131	109/7854	0.54621236	0.74756244	0.68558922	100856339/494005 , PRKCD	2 ACOR_EC-up
cfa05145	Toxoplasmosis	2/131	109/7854	0.54621236	0.74756244	0.68558922	609157/403416 GNAO1, BCL2	2 ACOR_EC-up
cfa05322	Systemic lupus erythematosus	2/131	113/7854	0.56585469	0.75339511	0.69093836	489221/476728 C7, C3	2 ACOR_EC-up
cfa04935	Growth hormone synthesis, secretion and action	2/131	116/7854	0.58018509	0.76374647	0.70043159	100856339/403556 , PRKACA	2 ACOR_EC-up
cfa05135	Yersinia infection	2/131	117/7854	0.58488501	0.76560791	0.70213872	100856339/485864 , SRC	2 ACOR_EC-up
cfa04142	Lysosome	2/131	118/7854	0.58954641	0.7673984	0.70378078	611983/477456 CTSZ, ABCB9	2 ACOR_EC-up
cfa04120	Ubiquitin mediated proteolysis	2/131	139/7854	0.67857228	0.80099827	0.7345952	478350/607816 PIAS1, UBE2H	2 ACOR_EC-up
cfa05224	Breast cancer	2/131	142/7854	0.68993218	0.80099827	0.7345952	100856339/100855544 , FZD5	2 ACOR_EC-up
cfa00061	Fatty acid biosynthesis	44228	18/7854	0.00101532	0.06701139	0.05771316	481018/478927 ACSL4, ACSL3	2 ACOR_EC-down
cfa00071	Fatty acid degradation	44228	41/7854	0.00524382	0.13838834	0.11918613	481018/478927 ACSL4, ACSL3	2 ACOR_EC-down
cfa04216	Ferroptosis	44228	45/7854	0.00629038	0.13838834	0.11918613	481018/478927 ACSL4, ACSL3	2 ACOR_EC-down
cfa01212	Fatty acid metabolism	11/388	58/7854	0.00010346	0.00201013	0.00188255	483792/486839/478927/483793/483378/481018/100855761/487900/475631/484063/478895 FADS2, SCD, ACSL3, FADS1, FASN, ACSL4, ELOVL1, ELOVL6, ACSL1, ACAT2, ACADL	11 ACOR_VSMC-down
cfa04730	Long-term depression	44228	60/7854	0.01097881	0.14492033	0.12481176	477886/442951 LYN, IGF1R	2 ACOR_EC-down
cfa04920	Adipocytokine signaling pathway	44228	70/7854	0.01474132	0.16215447	0.13965457	481018/478927 ACSL4, ACSL3	2 ACOR_EC-down
cfa03320	PPAR signaling pathway	44228	80/7854	0.01898241	0.16405787	0.14129386	481018/478927 ACSL4, ACSL3	2 ACOR_EC-down
cfa04146	Peroxisome	44228	82/7854	0.0198858	0.16405787	0.14129386	481018/478927 ACSL4, ACSL3	2 ACOR_EC-down
cfa00230	Purine metabolism	44228	130/7854	0.04650701	0.27309434	0.23520086	474499/486463 TNRC6B, PAPSS2	2 ACOR_EC-down
cfa04530	Tight junction	44228	170/7854	0.07477532	0.34415573	0.29640206	403728/608738 YBX3, NEDD4	2 ACOR_EC-down
cfa05169	Epstein-Barr virus infection	44228	193/7854	0.0930033	0.34415573	0.29640206	477886/608738 LYN, NEDD4	2 ACOR_EC-down
cfa04015	Rap1 signaling pathway	44228	209/7854	0.1063896	0.34415573	0.29640206	442951/612583 IGF1R, SIPA1L3	2 ACOR_EC-down
cfa04714	Thermogenesis	44228	227/7854	0.12204893	0.34415573	0.29640206	481018/478927 ACSL4, ACSL3	2 ACOR_EC-down
cfa04144	Endocytosis	44228	235/7854	0.1291907	0.34415573	0.29640206	442951/608738 IGF1R, NEDD4	2 ACOR_EC-down

cfa04151	PI3K-Akt signaling pathway	44228	354/7854	0.2440011	0.36600165	0.31521673	442951/476179 IGF1R, PHLPP1	2	ACOR_EC-down
cfa00604	Glycosphingolipid biosynthesis - ganglio series	2/441	15/7854	0.2045923	0.67515459	0.60779467	481737/491324 B3GALT4, ST6GALNAC6	2	VSAP_EC-up
cfa00053	Ascorbate and aldarate metabolism	2/441	17/7854	0.24688646	0.70505074	0.63470809	481486/478991 ALDH7A1, ALDH9A1	2	VSAP_EC-up
cfa00360	Phenylalanine metabolism	2/441	17/7854	0.24688646	0.70505074	0.63470809	476003/486834 ALDH3B1, GOT1	2	VSAP_EC-up
cfa00910	Nitrogen metabolism	2/441	17/7854	0.24688646	0.70505074	0.63470809	403443/100856271 GLUL, CA5B	2	VSAP_EC-up
cfa00120	Primary bile acid biosynthesis	2/441	18/7854	0.26816978	0.74208835	0.66805047	610489/489917 CYP27A1, HSD3B7	2	VSAP_EC-up
cfa00670	One carbon pool by folate	2/441	20/7854	0.31063901	0.78854519	0.70987233	610157/478230 , MTHFR	2	VSAP_EC-up
cfa00592	alpha-Linolenic acid metabolism	2/441	22/7854	0.35257615	0.83107236	0.74815658	477023/481256 ACAA1, PLA2G6	2	VSAP_EC-up
cfa04614	Renin-angiotensin system	2/441	24/7854	0.39359191	0.85964878	0.77388194	403836/403913 AGTR1, ANPEP	2	VSAP_EC-up
cfa00790	Folate biosynthesis	2/441	25/7854	0.41365792	0.86594207	0.77954735	490482/609680 MOCOS, PCBD1	2	VSAP_EC-up
cfa01040	Biosynthesis of unsaturated fatty acids	2/441	26/7854	0.43338796	0.88769809	0.79913278	477023/611337 ACAA1, HACD4	2	VSAP_EC-up
cfa00512	Mucin type O-glycan biosynthesis	2/441	31/7854	0.52623365	0.92381513	0.83164644	477056/482809 GALNT15, GALNT11	2	VSAP_EC-up
cfa01523	Antifolate resistance	2/441	31/7854	0.52623365	0.92381513	0.83164644	485523/478230 ABCC4, MTHFR	2	VSAP_EC-up
cfa00350	Tyrosine metabolism	2/441	32/7854	0.54353616	0.92381513	0.83164644	476003/486834 ALDH3B1, GOT1	2	VSAP_EC-up
cfa04136	Autophagy - other	2/441	32/7854	0.54353616	0.92381513	0.83164644	483756/102154798 ATG2A, ATG9A	2	VSAP_EC-up
cfa04960	Aldosterone-regulated sodium reabsorption	2/441	37/7854	0.6233174	0.97336925	0.87625657	486148/487235 IRS1, PIK3R1	2	VSAP_EC-up
cfa00920	Sulfur metabolism	2/279	2174929	0.04687166	0.2506199	0.21245801	478289/478504 , PAPSS1	2	VSAP_EC-down
cfa00770	Pantothenate and CoA biosynthesis	2/279	17/7854	0.12058558	0.4066908	0.344764	100856150/486633 ENPP1, BCAT1	2	VSAP_EC-down
cfa00532	Glycosaminoglycan biosynthesis - chondroitin sulfate / dermatan sulfate	2/279	19/7854	0.14489737	0.431399	0.36570988	488704/476238 CHSY1, UST	2	VSAP_EC-down
cfa00534	Glycosaminoglycan biosynthesis - heparan sulfate / heparin	2/279	24/7854	0.20908387	0.52171404	0.44227265	479963/482024 , EXT1	2	VSAP_EC-down
cfa00512	Mucin type O-glycan biosynthesis	2/279	31/7854	0.30210627	0.62818923	0.53253487	480161/100682763 GALNT1, GALNT2	2	VSAP_EC-down
cfa04215	Apoptosis - multiple species	2/279	32/7854	0.3153519	0.64580999	0.54747251	403567/478464 CASP3, MAPK10	2	VSAP_EC-down
cfa05020	Prion diseases	2/279	33/7854	0.32854061	0.66213569	0.56131229	480726/479975 HSPA5, PRKACB	2	VSAP_EC-down
cfa00760	Nicotinate and nicotinamide metabolism	2/279	37/7854	0.38048756	0.71717799	0.60797331	100856150/610576 ENPP1, NMNAT2	2	VSAP_EC-down
cfa00513	Various types of N-glycan biosynthesis	2/279	40/7854	0.41832099	0.73226635	0.62076416	489300/476516 STT3A, RPN1	2	VSAP_EC-down
cfa05219	Bladder cancer	2/279	41/7854	0.43067393	0.73430489	0.62249229	403802/474890 VEGFA, CDKN1A	2	VSAP_EC-down
cfa03022	Basal transcription factors	2/279	42/7854	0.44288678	0.73430489	0.62249229	489796/476881 GTF2IRD1, GTF2H1	2	VSAP_EC-down
cfa03420	Nucleotide excision repair	2/279	43/7854	0.4549539	0.73430489	0.62249229	476881/484922 GTF2H1, RAD23A	2	VSAP_EC-down
cfa04930	Type II diabetes mellitus	2/279	46/7854	0.49023278	0.76877454	0.65171325	403874/478464 , MAPK10	2	VSAP_EC-down
cfa00600	Sphingolipid metabolism	2/279	48/7854	0.51294412	0.78134511	0.6623697	481666/482897 UGCG, ASAH1	2	VSAP_EC-down
cfa01212	Fatty acid metabolism	6/441	58/7854	0.10520638	0.48420266	0.43589394	477023/607895/611337/477517/488110/475631 ACAA1, HSD17B8, HACD4, ACADS, EHHADH, ACSL1	6	VSAP_EC-up
cfa05030	Cocaine addiction	2/279	48/7854	0.51294412	0.78134511	0.6623697	479975/491369 PRKACB, CREB3L2	2	VSAP_EC-down
cfa04330	Notch signaling pathway	2/279	51/7854	0.54574129	0.79879452	0.67716209	475662/487309 ADAM17, HDAC1	2	VSAP_EC-down
cfa01212	Fatty acid metabolism	5/407	58/7854	0.18046583	0.80256807	0.77147442	478506/474670/479564/607926/608997 HADH, ACSL6, SCP2, , ELOVL7	5	ACOR_VSMC-up
cfa05204	Chemical carcinogenesis	2/279	55/7854	0.58702707	0.84044313	0.71246886	477558/442942 , PTGS2	2	VSAP_EC-down
cfa04370	VEGF signaling pathway	2/279	57/7854	0.60660699	0.84772198	0.71863937	403802/442942 VEGFA, PTGS2	2	VSAP_EC-down
cfa00310	Lysine degradation	2/279	58/7854	0.61613054	0.84772198	0.71863937	485702/479730 PLOD2, PLOD3	2	VSAP_EC-down
NA	NA	NA	NA	NA	NA	NA	NA NA	NA	VPOR_EC-up
NA	NA	NA	NA	NA	NA	NA	NA NA	NA	VPOR_EC-up
NA	NA	NA	NA	NA	NA	NA	NA NA	NA	VPOR_EC-up
NA	NA	NA	NA	NA	NA	NA	NA NA	NA	VPOR_EC-up
NA	NA	NA	NA	NA	NA	NA	NA NA	NA	VPOR_EC-up
NA	NA	NA	NA	NA	NA	NA	NA NA	NA	VPOR_EC-up
NA	NA	NA	NA	NA	NA	NA	NA NA	NA	VPOR_EC-up
NA	NA	NA	NA	NA	NA	NA	NA NA	NA	VPOR_EC-up
NA	NA	NA	NA	NA	NA	NA	NA NA	NA	VPOR_EC-up

NA	NA	NA	NA	NA	NA	NA	NA	NA	NA	VPOR_EC-up
NA	NA	NA	NA	NA	NA	NA	NA	NA	NA	VPOR_EC-up
NA	NA	NA	NA	NA	NA	NA	NA	NA	NA	VPOR_EC-up
NA	NA	NA	NA	NA	NA	NA	NA	NA	NA	VPOR_EC-up
NA	NA	NA	NA	NA	NA	NA	NA	NA	NA	VPOR_EC-up
NA	NA	NA	NA	NA	NA	NA	NA	NA	NA	VPOR_EC-up
NA	NA	NA	NA	NA	NA	NA	NA	NA	NA	VPOR_EC-up
NA	NA	NA	NA	NA	NA	NA	NA	NA	NA	VPOR_EC-up
NA	NA	NA	NA	NA	NA	NA	NA	NA	NA	VPOR_EC-up
NA	NA	NA	NA	NA	NA	NA	NA	NA	NA	VPOR_EC-up
NA	NA	NA	NA	NA	NA	NA	NA	NA	NA	VPOR_EC-up
NA	NA	NA	NA	NA	NA	NA	NA	NA	NA	VPOR_EC-up
NA	NA	NA	NA	NA	NA	NA	NA	NA	NA	VPOR_EC-up
NA	NA	NA	NA	NA	NA	NA	NA	NA	NA	VPOR_EC-up
NA	NA	NA	NA	NA	NA	NA	NA	NA	NA	VPOR_EC-up
NA	NA	NA	NA	NA	NA	NA	NA	NA	NA	VPOR_EC-up
NA	NA	NA	NA	NA	NA	NA	NA	NA	NA	VPOR_EC-up
NA	NA	NA	NA	NA	NA	NA	NA	NA	NA	VPOR_EC-up
NA	NA	NA	NA	NA	NA	NA	NA	NA	NA	VPOR_EC-up
NA	NA	NA	NA	NA	NA	NA	NA	NA	NA	VPOR_EC-up
NA	NA	NA	NA	NA	NA	NA	NA	NA	NA	VPOR_EC-up
NA	NA	NA	NA	NA	NA	NA	NA	NA	NA	VPOR_EC-up
NA	NA	NA	NA	NA	NA	NA	NA	NA	NA	VPOR_EC-up
NA	NA	NA	NA	NA	NA	NA	NA	NA	NA	VPOR_EC-up
NA	NA	NA	NA	NA	NA	NA	NA	NA	NA	VPOR_EC-up
NA	NA	NA	NA	NA	NA	NA	NA	NA	NA	VPOR_EC-up
NA	NA	NA	NA	NA	NA	NA	NA	NA	NA	VPOR_EC-up
NA	NA	NA	NA	NA	NA	NA	NA	NA	NA	VPOR_EC-up
NA	NA	NA	NA	NA	NA	NA	NA	NA	NA	VPOR_EC-up
NA	NA	NA	NA	NA	NA	NA	NA	NA	NA	VPOR_EC-up
NA	NA	NA	NA	NA	NA	NA	NA	NA	NA	VPOR_EC-up
NA	NA	NA	NA	NA	NA	NA	NA	NA	NA	VPOR_EC-up
NA	NA	NA	NA	NA	NA	NA	NA	NA	NA	VPOR_EC-up
NA	NA	NA	NA	NA	NA	NA	NA	NA	NA	VPOR_EC-up
NA	NA	NA	NA	NA	NA	NA	NA	NA	NA	VPOR_EC-up
NA	NA	NA	NA	NA	NA	NA	NA	NA	NA	VPOR_EC-up
NA	NA	NA	NA	NA	NA	NA	NA	NA	NA	VPOR_EC-up
NA	NA	NA	NA	NA	NA	NA	NA	NA	NA	VPOR_EC-up
NA	NA	NA	NA	NA	NA	NA	NA	NA	NA	VPOR_EC-up
NA	NA	NA	NA	NA	NA	NA	NA	NA	NA	VPOR_EC-up
NA	NA	NA	NA	NA	NA	NA	NA	NA	NA	VPOR_EC-up
NA	NA	NA	NA	NA	NA	NA	NA	NA	NA	VPOR_EC-up
NA	NA	NA	NA	NA	NA	NA	NA	NA	NA	VPOR_EC-up
NA	NA	NA	NA	NA	NA	NA	NA	NA	NA	VPOR_EC-up
NA	NA	NA	NA	NA	NA	NA	NA	NA	NA	VPOR_EC-up
NA	NA	NA	NA	NA	NA	NA	NA	NA	NA	VPOR_EC-up
NA	NA	NA	NA	NA	NA	NA	NA	NA	NA	VPOR_EC-up
NA	NA	NA	NA	NA	NA	NA	NA	NA	NA	VPOR_EC-up
NA	NA	NA	NA	NA	NA	NA	NA	NA	NA	VPOR_EC-up
NA	NA	NA	NA	NA	NA	NA	NA	NA	NA	VPOR_EC-up
NA	NA	NA	NA	NA	NA	NA	NA	NA	NA	VPOR_EC-up
NA	NA	NA	NA	NA	NA	NA	NA	NA	NA	VPOR_EC-up
NA	NA	NA	NA	NA	NA	NA	NA	NA	NA	VPOR_EC-up
NA	NA	NA	NA	NA	NA	NA	NA	NA	NA	VPOR_EC-up
NA	NA	NA	NA	NA	NA	NA	NA	NA	NA	VPOR_EC-up
NA	NA	NA	NA	NA	NA	NA	NA	NA	NA	VPOR_EC-up
NA	NA	NA	NA	NA	NA	NA	NA	NA	NA	VPOR_EC-up
NA	NA	NA	NA	NA	NA	NA	NA	NA	NA	VPOR_EC-up
NA	NA	NA	NA	NA	NA	NA	NA	NA	NA	VPOR_EC-up
NA	NA	NA	NA	NA	NA	NA	NA	NA	NA	VPOR_EC-up
NA	NA	NA	NA	NA	NA	NA	NA	NA	NA	VPOR_EC-up
NA	NA	NA	NA	NA	NA	NA	NA	NA	NA	VPOR_EC-up
NA	NA	NA	NA	NA	NA	NA	NA	NA	NA	VPOR_EC-up
NA	NA	NA	NA	NA	NA	NA	NA	NA	NA	VPOR_EC-up
NA	NA	NA	NA	NA	NA	NA	NA	NA	NA	VPOR_EC-up
NA	NA	NA	NA	NA	NA	NA	NA	NA	NA	VPOR_EC-up
NA	NA	NA	NA	NA	NA	NA	NA	NA	NA	VPOR_EC-up
NA	NA	NA	NA	NA	NA	NA	NA	NA	NA	VPOR_EC-up
NA	NA	NA	NA	NA	NA	NA	NA	NA	NA	VPOR_EC-up
NA	NA	NA	NA	NA	NA	NA	NA	NA	NA	VPOR_EC-up
NA	NA	NA	NA	NA	NA	NA	NA	NA	NA	VPOR_EC-up
NA	NA	NA	NA	NA	NA	NA	NA	NA	NA	VPOR_EC-up
NA	NA	NA	NA	NA	NA	NA	NA	NA	NA	VPOR_EC-up
NA	NA	NA	NA	NA	NA	NA	NA	NA	NA	VPOR_EC-up

NA	NA	NA	NA	NA	NA	NA	NA NA	NA	VPOR_EC-up
NA	NA	NA	NA	NA	NA	NA	NA NA	NA	VPOR_EC-up
NA	NA	NA	NA	NA	NA	NA	NA NA	NA	VPOR_EC-up
NA	NA	NA	NA	NA	NA	NA	NA NA	NA	VPOR_EC-up
NA	NA	NA	NA	NA	NA	NA	NA NA	NA	VPOR_EC-up
NA	NA	NA	NA	NA	NA	NA	NA NA	NA	VPOR_EC-up
NA	NA	NA	NA	NA	NA	NA	NA NA	NA	VPOR_EC-up
NA	NA	NA	NA	NA	NA	NA	NA NA	NA	VPOR_EC-up
NA	NA	NA	NA	NA	NA	NA	NA NA	NA	VPOR_EC-up
NA	NA	NA	NA	NA	NA	NA	NA NA	NA	VPOR_EC-up
NA	NA	NA	NA	NA	NA	NA	NA NA	NA	VPOR_EC-up
NA	NA	NA	NA	NA	NA	NA	NA NA	NA	VPOR_EC-up
NA	NA	NA	NA	NA	NA	NA	NA NA	NA	VPOR_EC-up
NA	NA	NA	NA	NA	NA	NA	NA NA	NA	VPOR_EC-up
NA	NA	NA	NA	NA	NA	NA	NA NA	NA	VPOR_EC-up
NA	NA	NA	NA	NA	NA	NA	NA NA	NA	VPOR_EC-up
NA	NA	NA	NA	NA	NA	NA	NA NA	NA	VPOR_EC-up
NA	NA	NA	NA	NA	NA	NA	NA NA	NA	VPOR_EC-up
NA	NA	NA	NA	NA	NA	NA	NA NA	NA	VPOR_EC-up
NA	NA	NA	NA	NA	NA	NA	NA NA	NA	VPOR_EC-up
NA	NA	NA	NA	NA	NA	NA	NA NA	NA	VPOR_EC-up
NA	NA	NA	NA	NA	NA	NA	NA NA	NA	VPOR_EC-up
NA	NA	NA	NA	NA	NA	NA	NA NA	NA	VPOR_EC-up
NA	NA	NA	NA	NA	NA	NA	NA NA	NA	VPOR_EC-up
NA	NA	NA	NA	NA	NA	NA	NA NA	NA	VPOR_EC-up
NA	NA	NA	NA	NA	NA	NA	NA NA	NA	VPOR_EC-up
NA	NA	NA	NA	NA	NA	NA	NA NA	NA	VPOR_EC-up
NA	NA	NA	NA	NA	NA	NA	NA NA	NA	VPOR_EC-up
NA	NA	NA	NA	NA	NA	NA	NA NA	NA	VPOR_EC-up
NA	NA	NA	NA	NA	NA	NA	NA NA	NA	VPOR_EC-up
NA	NA	NA	NA	NA	NA	NA	NA NA	NA	VPOR_EC-up
NA	NA	NA	NA	NA	NA	NA	NA NA	NA	VPOR_EC-up
NA	NA	NA	NA	NA	NA	NA	NA NA	NA	VPOR_EC-up
NA	NA	NA	NA	NA	NA	NA	NA NA	NA	VPOR_EC-up
NA	NA	NA	NA	NA	NA	NA	NA NA	NA	VPOR_EC-up
NA	NA	NA	NA	NA	NA	NA	NA NA	NA	VPOR_EC-up
NA	NA	NA	NA	NA	NA	NA	NA NA	NA	VPOR_EC-up
NA	NA	NA	NA	NA	NA	NA	NA NA	NA	VPOR_EC-up
NA	NA	NA	NA	NA	NA	NA	NA NA	NA	VPOR_EC-up
NA	NA	NA	NA	NA	NA	NA	NA NA	NA	VPOR_EC-up
NA	NA	NA	NA	NA	NA	NA	NA NA	NA	VPOR_EC-up
NA	NA	NA	NA	NA	NA	NA	NA NA	NA	VPOR_EC-up
NA	NA	NA	NA	NA	NA	NA	NA NA	NA	VPOR_EC-up
NA	NA	NA	NA	NA	NA	NA	NA NA	NA	VPOR_EC-up
NA	NA	NA	NA	NA	NA	NA	NA NA	NA	VPOR_EC-up
NA	NA	NA	NA	NA	NA	NA	NA NA	NA	VPOR_EC-up
NA	NA	NA	NA	NA	NA	NA	NA NA	NA	VPOR_EC-up
NA	NA	NA	NA	NA	NA	NA	NA NA	NA	VPOR_EC-up
NA	NA	NA	NA	NA	NA	NA	NA NA	NA	VPOR_EC-up
NA	NA	NA	NA	NA	NA	NA	NA NA	NA	VPOR_EC-up
NA	NA	NA	NA	NA	NA	NA	NA NA	NA	VPOR_EC-up
NA	NA	NA	NA	NA	NA	NA	NA NA	NA	VPOR_EC-up
NA	NA	NA	NA	NA	NA	NA	NA NA	NA	VPOR_EC-up
cfa00590	Arachidonic acid metabolism	2/279	61/7854	0.64364007	0.84772198	0.71863937	442942/100856661 PTGS2, PTGIS	2	VSAP_EC-down
cfa01212	Fatty acid metabolism	4/241	58/7854	0.10169963	0.42358973	0.38082613	477023/479564/607926/489421 ACAA1, SCP2, , ACAT1	4	VCAV_EC-down
cfa05412	Arrhythmogenic right ventricular cardiomyopathy (ARVC)	44106	77/7854	0.00405743	0.01834863	0.00804764	612079/606758 SGCD, DMD	2	AORT_EC-up
cfa01212	Fatty acid metabolism	44228	58/7854	0.01028605	0.14492033	0.12481176	481018/478927 ACSL4, ACSL3	2	ACOR_EC-down
cfa01212	Fatty acid metabolism	1/131	58/7854	0.62437032	0.79933124	0.73306637	607926	1	ACOR_EC-up
cfa04310	Wnt signaling pathway	44106	151/7854	0.01493334	0.03584002	0.01571931	478502/481783 DKK2, DAAM2	2	AORT_EC-up
cfa04010	MAPK signaling pathway	44106	288/7854	0.049645	0.0838079	0.03675785	480632/611653 TAOK1, DUSP2	2	AORT_EC-up
cfa00982	Drug metabolism - cytochrome P450	42767	49/7854	0.00488513	0.10246963	0.08868717	474938/477558 ,	2	APUL_EC-up
cfa00480	Glutathione metabolism	42767	52/7854	0.00548729	0.10246963	0.08868717	474938/477558 ,	2	APUL_EC-up
cfa00980	Metabolism of xenobiotics by cytochrome P450	42767	52/7854	0.00548729	0.10246963	0.08868717	474938/477558 ,	2	APUL_EC-up
cfa05204	Chemical carcinogenesis	42767	55/7854	0.00612189	0.10246963	0.08868717	474938/477558 ,	2	APUL_EC-up
cfa00983	Drug metabolism - other enzymes	42767	63/7854	0.00796986	0.10246963	0.08868717	474938/477558 ,	2	APUL_EC-up

cfa01524	Platinum drug resistance	42767	72/7854	0.01031217	0.10312167	0.0892515	474938/477558 ,	2	APUL_EC-up
cfa04657	IL-17 signaling pathway	42767	86/7854	0.01448666	0.11852719	0.10258494	478464/489428 MAPK10, MMP1	2	APUL_EC-up
cfa04726	Serotonergic synapse	42767	110/7854	0.02305016	0.17287622	0.14962387	489198/477762 HTR4, HTR7	2	APUL_EC-up
cfa04722	Neurotrophin signaling pathway	42767	118/7854	0.02627418	0.1818982	0.15743236	478464/484147 MAPK10, NTRK2	2	APUL_EC-up
cfa04926	Relaxin signaling pathway	42767	127/7854	0.03010899	0.1935578	0.16752371	478464/489428 MAPK10, MMP1	2	APUL_EC-up
cfa04310	Wnt signaling pathway	42767	151/7854	0.04134615	0.24807689	0.21470982	478464/481148 MAPK10, WIF1	2	APUL_EC-up
cfa05225	Hepatocellular carcinoma	42767	165/7854	0.04853246	0.27108113	0.23461992	474938/477558 ,	2	APUL_EC-up
cfa04530	Tight junction	42767	170/7854	0.05120421	0.27108113	0.23461992	478464/606992 MAPK10, MYL6B	2	APUL_EC-up
cfa04024	cAMP signaling pathway	42767	211/7854	0.07500526	0.31221218	0.27021873	489198/478464 HTR4, MAPK10	2	APUL_EC-up
cfa05016	Huntington disease	42767	257/7854	0.10513496	0.31221218	0.27021873	478464/100855815 MAPK10, TUBB4A	2	APUL_EC-up
cfa04010	MAPK signaling pathway	42767	288/7854	0.12707153	0.31221218	0.27021873	478464/484147 MAPK10, NTRK2	2	APUL_EC-up
cfa04080	Neuroactive ligand-receptor interaction	42767	327/7854	0.15612528	0.31221218	0.27021873	489198/477762 HTR4, HTR7	2	APUL_EC-up
cfa00770	Pantothenate and CoA biosynthesis	2/357	17/7854	0.17933964	0.3954409	0.28943797	489133/486633 PANK3, BCAT1	2	VCAV_EC-up
cfa01210	2-Oxocarboxylic acid metabolism	2/357	17/7854	0.17933964	0.3954409	0.28943797	486059/486633 AADAT, BCAT1	2	VCAV_EC-up
cfa00100	Steroid biosynthesis	2/357	20/7854	0.22992393	0.47872221	0.35039468	100856363/490391 LIPA, LBR	2	VCAV_EC-up
NA	NA	NA	NA	NA	NA	NA	NA NA	NA	VPOR_EC-down
NA	NA	NA	NA	NA	NA	NA	NA NA	NA	VPOR_EC-down
NA	NA	NA	NA	NA	NA	NA	NA NA	NA	VPOR_EC-down
NA	NA	NA	NA	NA	NA	NA	NA NA	NA	VPOR_EC-down
NA	NA	NA	NA	NA	NA	NA	NA NA	NA	VPOR_EC-down
NA	NA	NA	NA	NA	NA	NA	NA NA	NA	VPOR_EC-down
NA	NA	NA	NA	NA	NA	NA	NA NA	NA	VPOR_EC-down
NA	NA	NA	NA	NA	NA	NA	NA NA	NA	VPOR_EC-down
NA	NA	NA	NA	NA	NA	NA	NA NA	NA	VPOR_EC-down
NA	NA	NA	NA	NA	NA	NA	NA NA	NA	VPOR_EC-down
NA	NA	NA	NA	NA	NA	NA	NA NA	NA	VPOR_EC-down
NA	NA	NA	NA	NA	NA	NA	NA NA	NA	VPOR_EC-down
NA	NA	NA	NA	NA	NA	NA	NA NA	NA	VPOR_EC-down
NA	NA	NA	NA	NA	NA	NA	NA NA	NA	VPOR_EC-down
NA	NA	NA	NA	NA	NA	NA	NA NA	NA	VPOR_EC-down
NA	NA	NA	NA	NA	NA	NA	NA NA	NA	VPOR_EC-down
NA	NA	NA	NA	NA	NA	NA	NA NA	NA	VPOR_EC-down
NA	NA	NA	NA	NA	NA	NA	NA NA	NA	VPOR_EC-down
NA	NA	NA	NA	NA	NA	NA	NA NA	NA	VPOR_EC-down
NA	NA	NA	NA	NA	NA	NA	NA NA	NA	VPOR_EC-down
NA	NA	NA	NA	NA	NA	NA	NA NA	NA	VPOR_EC-down
NA	NA	NA	NA	NA	NA	NA	NA NA	NA	VPOR_EC-down
NA	NA	NA	NA	NA	NA	NA	NA NA	NA	VPOR_EC-down
NA	NA	NA	NA	NA	NA	NA	NA NA	NA	VPOR_EC-down
NA	NA	NA	NA	NA	NA	NA	NA NA	NA	VPOR_EC-down
NA	NA	NA	NA	NA	NA	NA	NA NA	NA	VPOR_EC-down
NA	NA	NA	NA	NA	NA	NA	NA NA	NA	VPOR_EC-down
NA	NA	NA	NA	NA	NA	NA	NA NA	NA	VPOR_EC-down
NA	NA	NA	NA	NA	NA	NA	NA NA	NA	VPOR_EC-down
NA	NA	NA	NA	NA	NA	NA	NA NA	NA	VPOR_EC-down
NA	NA	NA	NA	NA	NA	NA	NA NA	NA	VPOR_EC-down
NA	NA	NA	NA	NA	NA	NA	NA NA	NA	VPOR_EC-down
NA	NA	NA	NA	NA	NA	NA	NA NA	NA	VPOR_EC-down
NA	NA	NA	NA	NA	NA	NA	NA NA	NA	VPOR_EC-down
NA	NA	NA	NA	NA	NA	NA	NA NA	NA	VPOR_EC-down
NA	NA	NA	NA	NA	NA	NA	NA NA	NA	VPOR_EC-down
NA	NA	NA	NA	NA	NA	NA	NA NA	NA	VPOR_EC-down
NA	NA	NA	NA	NA	NA	NA	NA NA	NA	VPOR_EC-down
NA	NA	NA	NA	NA	NA	NA	NA NA	NA	VPOR_EC-down
NA	NA	NA	NA	NA	NA	NA	NA NA	NA	VPOR_EC-down
NA	NA	NA	NA	NA	NA	NA	NA NA	NA	VPOR_EC-down
NA	NA	NA	NA	NA	NA	NA	NA NA	NA	VPOR_EC-down
NA	NA	NA	NA	NA	NA	NA	NA NA	NA	VPOR_EC-down
NA	NA	NA	NA	NA	NA	NA	NA NA	NA	VPOR_EC-down
NA	NA	NA	NA	NA	NA	NA	NA NA	NA	VPOR_EC-down
NA	NA	NA	NA	NA	NA	NA	NA NA	NA	VPOR_EC-down
NA	NA	NA	NA	NA	NA	NA	NA NA	NA	VPOR_EC-down
NA	NA	NA	NA	NA	NA	NA	NA NA	NA	VPOR_EC-down
NA	NA	NA	NA	NA	NA	NA	NA NA	NA	VPOR_EC-down
NA	NA	NA	NA	NA	NA	NA	NA NA	NA	VPOR_EC-down
NA	NA	NA	NA	NA	NA	NA	NA NA	NA	VPOR_EC-down
NA	NA	NA	NA	NA	NA	NA	NA NA	NA	VPOR_EC-down

NA	NA	NA	NA	NA	NA	NA	NA	NA	NA	NA	VPOR_EC-down
NA	NA	NA	NA	NA	NA	NA	NA	NA	NA	NA	VPOR_EC-down
NA	NA	NA	NA	NA	NA	NA	NA	NA	NA	NA	VPOR_EC-down
NA	NA	NA	NA	NA	NA	NA	NA	NA	NA	NA	VPOR_EC-down
NA	NA	NA	NA	NA	NA	NA	NA	NA	NA	NA	VPOR_EC-down
NA	NA	NA	NA	NA	NA	NA	NA	NA	NA	NA	VPOR_EC-down
NA	NA	NA	NA	NA	NA	NA	NA	NA	NA	NA	VPOR_EC-down
NA	NA	NA	NA	NA	NA	NA	NA	NA	NA	NA	VPOR_EC-down
NA	NA	NA	NA	NA	NA	NA	NA	NA	NA	NA	VPOR_EC-down
NA	NA	NA	NA	NA	NA	NA	NA	NA	NA	NA	VPOR_EC-down
NA	NA	NA	NA	NA	NA	NA	NA	NA	NA	NA	VPOR_EC-down
NA	NA	NA	NA	NA	NA	NA	NA	NA	NA	NA	VPOR_EC-down
NA	NA	NA	NA	NA	NA	NA	NA	NA	NA	NA	VPOR_EC-down
NA	NA	NA	NA	NA	NA	NA	NA	NA	NA	NA	VPOR_EC-down
NA	NA	NA	NA	NA	NA	NA	NA	NA	NA	NA	VPOR_EC-down
NA	NA	NA	NA	NA	NA	NA	NA	NA	NA	NA	VPOR_EC-down
NA	NA	NA	NA	NA	NA	NA	NA	NA	NA	NA	VPOR_EC-down
NA	NA	NA	NA	NA	NA	NA	NA	NA	NA	NA	VPOR_EC-down
NA	NA	NA	NA	NA	NA	NA	NA	NA	NA	NA	VPOR_EC-down
NA	NA	NA	NA	NA	NA	NA	NA	NA	NA	NA	VPOR_EC-down
NA	NA	NA	NA	NA	NA	NA	NA	NA	NA	NA	VPOR_EC-down
NA	NA	NA	NA	NA	NA	NA	NA	NA	NA	NA	VPOR_EC-down
NA	NA	NA	NA	NA	NA	NA	NA	NA	NA	NA	VPOR_EC-down
NA	NA	NA	NA	NA	NA	NA	NA	NA	NA	NA	VPOR_EC-down
NA	NA	NA	NA	NA	NA	NA	NA	NA	NA	NA	VPOR_EC-down
NA	NA	NA	NA	NA	NA	NA	NA	NA	NA	NA	VPOR_EC-down
NA	NA	NA	NA	NA	NA	NA	NA	NA	NA	NA	VPOR_EC-down
NA	NA	NA	NA	NA	NA	NA	NA	NA	NA	NA	VPOR_EC-down
NA	NA	NA	NA	NA	NA	NA	NA	NA	NA	NA	VPOR_EC-down
NA	NA	NA	NA	NA	NA	NA	NA	NA	NA	NA	VPOR_EC-down
NA	NA	NA	NA	NA	NA	NA	NA	NA	NA	NA	VPOR_EC-down
NA	NA	NA	NA	NA	NA	NA	NA	NA	NA	NA	VPOR_EC-down
NA	NA	NA	NA	NA	NA	NA	NA	NA	NA	NA	VPOR_EC-down
NA	NA	NA	NA	NA	NA	NA	NA	NA	NA	NA	VPOR_EC-down
NA	NA	NA	NA	NA	NA	NA	NA	NA	NA	NA	VPOR_EC-down
NA	NA	NA	NA	NA	NA	NA	NA	NA	NA	NA	VPOR_EC-down
NA	NA	NA	NA	NA	NA	NA	NA	NA	NA	NA	VPOR_EC-down
NA	NA	NA	NA	NA	NA	NA	NA	NA	NA	NA	VPOR_EC-down
NA	NA	NA	NA	NA	NA	NA	NA	NA	NA	NA	VPOR_EC-down
NA	NA	NA	NA	NA	NA	NA	NA	NA	NA	NA	VPOR_EC-down
NA	NA	NA	NA	NA	NA	NA	NA	NA	NA	NA	VPOR_EC-down
NA	NA	NA	NA	NA	NA	NA	NA	NA	NA	NA	VPOR_EC-down
NA	NA	NA	NA	NA	NA	NA	NA	NA	NA	NA	VPOR_EC-down
NA	NA	NA	NA	NA	NA	NA	NA	NA	NA	NA	VPOR_EC-down
NA	NA	NA	NA	NA	NA	NA	NA	NA	NA	NA	VPOR_EC-down
NA	NA	NA	NA	NA	NA	NA	NA	NA	NA	NA	VPOR_EC-down
NA	NA	NA	NA	NA	NA	NA	NA	NA	NA	NA	VPOR_EC-down
NA	NA	NA	NA	NA	NA	NA	NA	NA	NA	NA	VPOR_EC-down
NA	NA	NA	NA	NA	NA	NA	NA	NA	NA	NA	VPOR_EC-down
NA	NA	NA	NA	NA	NA	NA	NA	NA	NA	NA	VPOR_EC-down
NA	NA	NA	NA	NA	NA	NA	NA	NA	NA	NA	VPOR_EC-down
NA	NA	NA	NA	NA	NA	NA	NA	NA	NA	NA	VPOR_EC-down
NA	NA	NA	NA	NA	NA	NA	NA	NA	NA	NA	VPOR_EC-down
NA	NA	NA	NA	NA	NA	NA	NA	NA	NA	NA	VPOR_EC-down
NA	NA	NA	NA	NA	NA	NA	NA	NA	NA	NA	VPOR_EC-down
NA	NA	NA	NA	NA	NA	NA	NA	NA	NA	NA	VPOR_EC-down
NA	NA	NA	NA	NA	NA	NA	NA	NA	NA	NA	VPOR_EC-down
NA	NA	NA	NA	NA	NA	NA	NA	NA	NA	NA	VPOR_EC-down
NA	NA	NA	NA	NA	NA	NA	NA	NA	NA	NA	VPOR_EC-down
NA	NA	NA	NA	NA	NA	NA	NA	NA	NA	NA	VPOR_EC-down
NA	NA	NA	NA	NA	NA	NA	NA	NA	NA	NA	VPOR_EC-down
NA	NA	NA	NA	NA	NA	NA	NA	NA	NA	NA	VPOR_EC-down
NA	NA	NA	NA	NA	NA	NA	NA	NA	NA	NA	VPOR_EC-down
NA	NA	NA	NA	NA	NA	NA	NA	NA	NA	NA	VPOR_EC-down
NA	NA	NA	NA	NA	NA	NA	NA	NA	NA	NA	VPOR_EC-down
NA	NA	NA	NA	NA	NA	NA	NA	NA	NA	NA	VPOR_EC-down
NA	NA	NA	NA	NA	NA	NA	NA	NA	NA	NA	VPOR_EC-down
NA	NA	NA	NA	NA	NA	NA	NA	NA	NA	NA	VPOR_EC-down
NA	NA	NA	NA	NA	NA	NA	NA	NA	NA	NA	VPOR_EC-down
NA	NA	NA	NA	NA	NA	NA	NA	NA	NA	NA	VPOR_EC-down
NA	NA	NA	NA	NA	NA	NA	NA	NA	NA	NA	VPOR_EC-down
NA	NA	NA	NA	NA	NA	NA	NA	NA	NA	NA	VPOR_EC-down
NA	NA	NA	NA	NA	NA	NA	NA	NA	NA	NA	VPOR_EC-down
NA	NA	NA	NA	NA	NA	NA	NA	NA	NA	NA	VPOR_EC-down

NA	NA	NA	NA	NA	NA	NA	NA	NA	NA	VPOR_EC-down
NA	NA	NA	NA	NA	NA	NA	NA	NA	NA	VPOR_EC-down
NA	NA	NA	NA	NA	NA	NA	NA	NA	NA	VPOR_EC-down
NA	NA	NA	NA	NA	NA	NA	NA	NA	NA	VPOR_EC-down
NA	NA	NA	NA	NA	NA	NA	NA	NA	NA	VPOR_EC-down
NA	NA	NA	NA	NA	NA	NA	NA	NA	NA	VPOR_EC-down
NA	NA	NA	NA	NA	NA	NA	NA	NA	NA	VPOR_EC-down
NA	NA	NA	NA	NA	NA	NA	NA	NA	NA	VPOR_EC-down
NA	NA	NA	NA	NA	NA	NA	NA	NA	NA	VPOR_EC-down
NA	NA	NA	NA	NA	NA	NA	NA	NA	NA	VPOR_EC-down
NA	NA	NA	NA	NA	NA	NA	NA	NA	NA	VPOR_EC-down
NA	NA	NA	NA	NA	NA	NA	NA	NA	NA	VPOR_EC-down
NA	NA	NA	NA	NA	NA	NA	NA	NA	NA	VPOR_EC-down
NA	NA	NA	NA	NA	NA	NA	NA	NA	NA	VPOR_EC-down
NA	NA	NA	NA	NA	NA	NA	NA	NA	NA	VPOR_EC-down
NA	NA	NA	NA	NA	NA	NA	NA	NA	NA	VPOR_EC-down
NA	NA	NA	NA	NA	NA	NA	NA	NA	NA	VPOR_EC-down
NA	NA	NA	NA	NA	NA	NA	NA	NA	NA	VPOR_EC-down
NA	NA	NA	NA	NA	NA	NA	NA	NA	NA	VPOR_EC-down
NA	NA	NA	NA	NA	NA	NA	NA	NA	NA	VPOR_EC-down
NA	NA	NA	NA	NA	NA	NA	NA	NA	NA	VPOR_EC-down
NA	NA	NA	NA	NA	NA	NA	NA	NA	NA	VPOR_EC-down
NA	NA	NA	NA	NA	NA	NA	NA	NA	NA	VPOR_EC-down
NA	NA	NA	NA	NA	NA	NA	NA	NA	NA	VPOR_EC-down
NA	NA	NA	NA	NA	NA	NA	NA	NA	NA	VPOR_EC-down
NA	NA	NA	NA	NA	NA	NA	NA	NA	NA	VPOR_EC-down
NA	NA	NA	NA	NA	NA	NA	NA	NA	NA	VPOR_EC-down
NA	NA	NA	NA	NA	NA	NA	NA	NA	NA	VPOR_EC-down
NA	NA	NA	NA	NA	NA	NA	NA	NA	NA	VPOR_EC-down
NA	NA	NA	NA	NA	NA	NA	NA	NA	NA	VPOR_EC-down
NA	NA	NA	NA	NA	NA	NA	NA	NA	NA	VPOR_EC-down
NA	NA	NA	NA	NA	NA	NA	NA	NA	NA	VPOR_EC-down
NA	NA	NA	NA	NA	NA	NA	NA	NA	NA	VPOR_EC-down
NA	NA	NA	NA	NA	NA	NA	NA	NA	NA	VPOR_EC-down
NA	NA	NA	NA	NA	NA	NA	NA	NA	NA	VPOR_EC-down
NA	NA	NA	NA	NA	NA	NA	NA	NA	NA	VPOR_EC-down
NA	NA	NA	NA	NA	NA	NA	NA	NA	NA	VPOR_EC-down
NA	NA	NA	NA	NA	NA	NA	NA	NA	NA	VPOR_EC-down
NA	NA	NA	NA	NA	NA	NA	NA	NA	NA	VPOR_EC-down
NA	NA	NA	NA	NA	NA	NA	NA	NA	NA	VPOR_EC-down
NA	NA	NA	NA	NA	NA	NA	NA	NA	NA	VPOR_EC-down
NA	NA	NA	NA	NA	NA	NA	NA	NA	NA	VPOR_EC-down
NA	NA	NA	NA	NA	NA	NA	NA	NA	NA	VPOR_EC-down
NA	NA	NA	NA	NA	NA	NA	NA	NA	NA	VPOR_EC-down
NA	NA	NA	NA	NA	NA	NA	NA	NA	NA	VPOR_EC-down
NA	NA	NA	NA	NA	NA	NA	NA	NA	NA	VPOR_EC-down
NA	NA	NA	NA	NA	NA	NA	NA	NA	NA	VPOR_EC-down
cfa04614	Renin-angiotensin system	2/357	24/7854	0.29838976	0.57021057	0.41735843	476788/610668	PRCP, ACE	2	VCAV_EC-up
cfa04950	Maturity onset diabetes of the young	2/357	24/7854	0.29838976	0.57021057	0.41735843	490252/608175	NR5A2, HHEX	2	VCAV_EC-up
cfa05332	Graft-versus-host disease	2/357	25/7854	0.31542723	0.58280926	0.42657987	403985/403764	IL6, CD86	2	VCAV_EC-up
cfa00601	Glycosphingolipid biosynthesis - lacto and neolacto series	2/357	29/7854	0.38237613	0.64266831	0.47039296	102154559/478535	B3GNT5, ST3GAL6	2	VCAV_EC-up
cfa00052	Galactose metabolism	2/357	30/7854	0.39869911	0.6577911	0.48146189	479116/100855555	PGM2, GALE	2	VCAV_EC-up
cfa01523	Antifolate resistance	2/357	31/7854	0.41481879	0.65826592	0.48180942	403985/478472	IL6, ABCG2	2	VCAV_EC-up
cfa04136	Autophagy - other	2/357	32/7854	0.43071991	0.67134556	0.4913829	478232/403608	MTOR, PPP2CA	2	VCAV_EC-up
cfa00860	Porphyrin and chlorophyll metabolism	2/357	33/7854	0.44638903	0.67499155	0.49405154	442963/475867	CP, BLVRA	2	VCAV_EC-up
cfa05020	Prion diseases	2/357	33/7854	0.44638903	0.67499155	0.49405154	403985/480676	IL6, NOTCH1	2	VCAV_EC-up
cfa03410	Base excision repair	2/357	34/7854	0.46181434	0.69272151	0.50702876	477348/403170	PARP4, HMGB1	2	VCAV_EC-up
cfa04130	SNARE interactions in vesicular transport	2/357	36/7854	0.49189404	0.71852585	0.52591592	478268/484019	SNAP23, STX11	2	VCAV_EC-up
cfa04940	Type I diabetes mellitus	2/357	36/7854	0.49189404	0.71852585	0.52591592	475242/403764	ICA1, CD86	2	VCAV_EC-up
cfa05340	Primary immunodeficiency	2/357	36/7854	0.49189404	0.71852585	0.52591592	612582/403851	IL7R, IL2RG	2	VCAV_EC-up
cfa04975	Fat digestion and absorption	2/357	38/7854	0.52089356	0.74950571	0.54859124	486241/481651	SCARB1, ABCA1	2	VCAV_EC-up
cfa00513	Various types of N-glycan biosynthesis	2/357	40/7854	0.54876702	0.77718781	0.5688528	474562/609002	MGAT4A,	2	VCAV_EC-up
cfa03440	Homologous recombination	2/357	41/7854	0.56227175	0.7843691	0.57410905	100685609/477070	BLM, TOPBP1	2	VCAV_EC-up

cfa05219	Bladder cancer	2/357	41/7854	0.56227175	0.7843691	0.57410905	403850/403872 CXCL8, NRAS	2	VCAV_EC-up
cfa00130	Ubiquinone and other terpenoid-quinone biosynthesis	2/241	2174960	0.04296853	0.32632853	0.29338396	479775/475769 VKORC1, GGCX	2	VCAV_EC-down
cfa00533	Glycosaminoglycan biosynthesis - keratan sulfate	2/241	15/7854	0.07574019	0.38682027	0.34776874	481579/448804 B4GALT1, FUT8	2	VCAV_EC-down
cfa00604	Glycosphingolipid biosynthesis - ganglio series	2/241	15/7854	0.07574019	0.38682027	0.34776874	485724/487633 SLC33A1,	2	VCAV_EC-down
cfa00603	Glycosphingolipid biosynthesis - globo and isoglobo series	2/241	16/7854	0.08486223	0.40417437	0.36337085	481222/487633 A4GALT,	2	VCAV_EC-down
cfa00120	Primary bile acid biosynthesis	2/241	18/7854	0.10401314	0.42358973	0.38082613	489917/479564 HSD3B7, SCP2	2	VCAV_EC-down
cfa00340	Histidine metabolism	2/241	21/7854	0.13461419	0.47881756	0.43047842	403450/476133 MAOA, HNMT	2	VCAV_EC-down
cfa00515	Mannose type O-glycan biosynthesis	2/241	24/7854	0.16690636	0.51052385	0.45898379	481579/474555 B4GALT1, CHST10	2	VCAV_EC-down
cfa04614	Renin-angiotensin system	2/241	24/7854	0.16690636	0.51052385	0.45898379	403913/100682978 ANPEP, ATP6AP2	2	VCAV_EC-down
cfa00563	Glycosylphosphatidylinositol (GPI)-anchor biosynthesis	2/241	25/7854	0.17794995	0.51619497	0.46408238	477244/491170 PIGT, PIGS	2	VCAV_EC-down
cfa01040	Biosynthesis of unsaturated fatty acids	2/241	26/7854	0.18910251	0.52095886	0.46836534	477023/479564 ACAA1, SCP2	2	VCAV_EC-down
cfa04977	Vitamin digestion and absorption	2/241	26/7854	0.18910251	0.52095886	0.46836534	477059/491606 BTD, SLC19A1	2	VCAV_EC-down
cfa00030	Pentose phosphate pathway	2/241	29/7854	0.22304871	0.56978807	0.51226498	489642/610090 H6PD, PGLS	2	VCAV_EC-down
cfa00601	Glycosphingolipid biosynthesis - lacto and neolacto series	2/241	29/7854	0.22304871	0.56978807	0.51226498	481579/481222 B4GALT1, A4GALT	2	VCAV_EC-down
cfa00630	Glyoxylate and dicarboxylate metabolism	2/241	29/7854	0.22304871	0.56978807	0.51226498	477205/489421 ACSS2, ACAT1	2	VCAV_EC-down
cfa00052	Galactose metabolism	2/241	30/7854	0.23447636	0.59358431	0.53365886	490942/481579 G6PC3, B4GALT1	2	VCAV_EC-down
cfa01523	Antifolate resistance	2/241	31/7854	0.24593695	0.59576107	0.53561587	476816/491606 FOLR2, SLC19A1	2	VCAV_EC-down
cfa00860	Porphyrin and chlorophyll metabolism	2/241	33/7854	0.2689068	0.62962523	0.56606127	492116/610360 UROS, FECH	2	VCAV_EC-down
cfa00250	Alanine, aspartate and glutamate metabolism	2/241	37/7854	0.31472999	0.68557464	0.61636229	474653/100686499 GFPT2, GLS2	2	VCAV_EC-down
cfa00760	Nicotinate and nicotinamide metabolism	2/241	37/7854	0.31472999	0.68557464	0.61636229	488820/477809 BST1, NT5C2	2	VCAV_EC-down
cfa04960	Aldosterone-regulated sodium reabsorption	2/241	37/7854	0.31472999	0.68557464	0.61636229	609956/486148 , IRS1	2	VCAV_EC-down
cfa05219	Bladder cancer	2/241	41/7854	0.3598985	0.73818598	0.66366224	403733/485864 MMP2, SRC	2	VCAV_EC-down
cfa04216	Ferroptosis	2/241	45/7854	0.40393727	0.78213515	0.70317451	485783/483777 PRNP, SLC3A2	2	VCAV_EC-down
cfa04913	Ovarian steroidogenesis	2/241	46/7854	0.41472647	0.78213515	0.70317451	477625/483038 ADCY6, CYP1B1	2	VCAV_EC-down
cfa00830	Retinol metabolism	2/241	49/7854	0.44649077	0.79541394	0.71511274	483083/486998 RETSAT, RDH10	2	VCAV_EC-down
cfa00270	Cysteine and methionine metabolism	2/241	50/7854	0.45686502	0.80539699	0.72408795	484394/611790 BCAT2, AHCYL1	2	VCAV_EC-down
cfa00240	Pyrimidine metabolism	2/241	52/7854	0.47727322	0.81376158	0.73160809	477809/485604 NT5C2, ENTPD3	2	VCAV_EC-down
cfa00561	Glycerolipid metabolism	2/241	59/7854	0.54488145	0.84753502	0.76197193	486890/607962 GPAM, PLPP1	2	VCAV_EC-down
cfa00010	Glycolysis / Gluconeogenesis	2/241	62/7854	0.57194187	0.8734547	0.78527488	490942/477205 G6PC3, ACSS2	2	VCAV_EC-down
cfa04929	GnRH secretion	2/241	65/7854	0.59782059	0.8750588	0.78671703	609956/449021 , AKT2	2	VCAV_EC-down
cfa04720	Long-term potentiation	2/241	66/7854	0.60618296	0.8750588	0.78671703	612448/610764 RPS6KA2, CAMK2D	2	VCAV_EC-down
cfa04976	Bile secretion	2/241	66/7854	0.60618296	0.8750588	0.78671703	477625/480113 ADCY6, EPHX1	2	VCAV_EC-down
cfa05031	Amphetamine addiction	2/241	67/7854	0.61441342	0.88042732	0.79154357	403450/610764 MAOA, CAMK2D	2	VCAV_EC-down
cfa04216	Ferroptosis	12451	45/7854	0.01603103	0.52353656	0.51664792	483821/102155200 SLC7A11,	2	VPOR_EC-up
cfa03018	RNA degradation	12451	78/7854	0.04451198	0.52353656	0.51664792	612549/612778 C1D, CNOT10	2	VPOR_EC-up
cfa04910	Insulin signaling pathway	12451	134/7854	0.11388786	0.52353656	0.51664792	612212/476299 MKNK2, FBP1	2	VPOR_EC-up
cfa04550	Signaling pathways regulating pluripotency of stem cells	12451	135/7854	0.11529741	0.52353656	0.51664792	612954/476140 ISL1, ACVR2A	2	VPOR_EC-up
cfa04915	Estrogen signaling pathway	11/357	134/7854	0.04114659	0.14145163	0.10353373	491005/491004/403784/607269/480271/475266/478227/488355/479406/607922/403872 KRT23, KRT39, NOS3, CALML6, ADCY4, CREB5, GNAI1, KCNJ3, HSPA8, CREB1, NRAS	11	VCAV_EC-up
cfa03010	Ribosome	12451	139/7854	0.12098131	0.52353656	0.51664792	478313/490672 RSL24D1, RPL10L	2	VPOR_EC-up
cfa04360	Axon guidance	12451	179/7854	0.18106539	0.52353656	0.51664792	483386/475929 SEMA3A, SEMA3D	2	VPOR_EC-up

cfa04020	Calcium signaling pathway	12451	192/7854	0.20151539	0.52353656	0.51664792	477762/475272 HTR7, PDE1C	2	VPOR_EC-up
cfa05132	Salmonella infection	12451	212/7854	0.2334892	0.52353656	0.51664792	474541/100856747 GCC2,	2	VPOR_EC-up
cfa04080	Neuroactive ligand-receptor interaction	12451	327/7854	0.41684714	0.54445341	0.53728955	486704/477762 C3AR1, HTR7	2	VPOR_EC-up
cfa04912	GnRH signaling pathway	42767	89/7854	0.01546225	0.32364194	0.30122906	480454/482994 MAP2K6, ADCY3	2	VFEM_EC-up
cfa04750	Inflammatory mediator regulation of TRP channels	42767	100/7854	0.01927461	0.32364194	0.30122906	480454/482994 MAP2K6, ADCY3	2	VFEM_EC-up
cfa04935	Growth hormone synthesis, secretion and action	42767	116/7854	0.02545161	0.32364194	0.30122906	480454/482994 MAP2K6, ADCY3	2	VFEM_EC-up
cfa04915	Estrogen signaling pathway	7/279	134/7854	0.19896681	0.51613172	0.43754035	479975/404019/479406/403928/480438/480416/491369 PRKACB, HSP90B1, HSPA8, GNAQ, HSP90AA1, CALM1, CREB3L2	7	VSAP_EC-down
cfa04072	Phospholipase D signaling pathway	42767	148/7854	0.03986474	0.32364194	0.30122906	481142/482994 AVPR1A, ADCY3	2	VFEM_EC-up
cfa04723	Retrograde endocannabinoid signaling	42767	149/7854	0.04035619	0.32364194	0.30122906	611480/482994 , ADCY3	2	VFEM_EC-up
cfa05010	Alzheimer disease	42767	168/7854	0.05012905	0.32364194	0.30122906	477120/611480 MME,	2	VFEM_EC-up
cfa04530	Tight junction	42767	170/7854	0.05120421	0.32364194	0.30122906	403728/487731 YBX3, TIAM1	2	VFEM_EC-up
cfa04062	Chemokine signaling pathway	42767	174/7854	0.05338001	0.32364194	0.30122906	487731/482994 TIAM1, ADCY3	2	VFEM_EC-up
cfa04020	Calcium signaling pathway	42767	192/7854	0.06357499	0.32364194	0.30122906	481142/482994 AVPR1A, ADCY3	2	VFEM_EC-up
cfa04024	cAMP signaling pathway	42767	211/7854	0.07500526	0.32364194	0.30122906	487731/482994 TIAM1, ADCY3	2	VFEM_EC-up
cfa05163	Human cytomegalovirus infection	42767	220/7854	0.08064072	0.32364194	0.30122906	480454/482994 MAP2K6, ADCY3	2	VFEM_EC-up
cfa04714	Thermogenesis	42767	227/7854	0.08511608	0.32364194	0.30122906	611480/482994 , ADCY3	2	VFEM_EC-up
cfa04915	Estrogen signaling pathway	7/241	134/7854	0.11757952	0.45260061	0.40690821	477625/403733/609956/485864/449021/481759/490439 ADCY6, MMP2, , SRC, AKT2, FKBP5, SHC1	7	VCAV_EC-down
cfa04360	Axon guidance	41671	179/7854	0.0392714	0.3227867	0.32089906	608458/487635 EFNA5, NEO1	2	VPUL_EC-up
cfa04014	Ras signaling pathway	41671	225/7854	0.05929617	0.3227867	0.32089906	608458/477762 EFNA5, HTR7	2	VPUL_EC-up
cfa04080	Neuroactive ligand-receptor interaction	41671	327/7854	0.11316596	0.3227867	0.32089906	477762/403797 HTR7, PTGER2	2	VPUL_EC-up
cfa00532	Glycosaminoglycan biosynthesis - chondroitin sulfate / dermatan sulfate	34731	19/7854	0.02165811	0.55382877	0.55040907	494008/486786 XYLT1, CSGALNACT2	2	AFEM_EC-up
cfa05144	Malaria	34731	49/7854	0.11824704	0.81029448	0.80529119	403469/476665 CD40, COMP	2	AFEM_EC-up
cfa04330	Notch signaling pathway	34731	51/7854	0.12627509	0.81029448	0.80529119	100688936/475662 DLL1, ADAM17	2	AFEM_EC-up
cfa04915	Estrogen signaling pathway	6/131	134/7854	0.0243203	0.29765577	0.27297999	100856339/609157/403416/485864/403556/494005 , GNAO1, BCL2, SRC, PRKACA, PRKCD	6	ACOR_EC-up
cfa00980	Metabolism of xenobiotics by cytochrome P450	34731	52/7854	0.13033721	0.81029448	0.80529119	449023/476003 HSD11B1, ALDH3B1	2	AFEM_EC-up
cfa04612	Antigen processing and presentation	34731	54/7854	0.13855098	0.81029448	0.80529119	403400/476669 CTSS, IFI30	2	AFEM_EC-up
cfa04923	Regulation of lipolysis in adipocytes	34731	54/7854	0.13855098	0.81029448	0.80529119	485548/476045 IRS2, PLA2G16	2	AFEM_EC-up
cfa05204	Chemical carcinogenesis	34731	55/7854	0.14270004	0.81029448	0.80529119	449023/476003 HSD11B1, ALDH3B1	2	AFEM_EC-up
cfa05014	Amyotrophic lateral sclerosis (ALS)	34731	56/7854	0.14687555	0.81029448	0.80529119	442940/403559 NEFH, SOD1	2	AFEM_EC-up
cfa05217	Basal cell carcinoma	34731	63/7854	0.17674456	0.81029448	0.80529119	490903/490937 AXIN2, FZD2	2	AFEM_EC-up
cfa04721	Synaptic vesicle cycle	34731	78/7854	0.24319414	0.81029448	0.80529119	486137/482528 ATP6V1B2, ATP6V0B	2	AFEM_EC-up
cfa04610	Complement and coagulation cascades	34731	80/7854	0.25218106	0.81029448	0.80529119	100685858/490153 F5, F3	2	AFEM_EC-up
cfa04146	Peroxisome	34731	82/7854	0.26117777	0.81029448	0.80529119	476258/403559 SOD2, SOD1	2	AFEM_EC-up
cfa04061	Viral protein interaction with cytokine and cytokine receptor	34731	83/7854	0.26567804	0.81029448	0.80529119	100856510/403522 ACKR4, CCL5	2	AFEM_EC-up
cfa04064	NF-kappa B signaling pathway	34731	93/7854	0.31059697	0.83791788	0.83274402	403469/611649 CD40, CYLD	2	AFEM_EC-up
cfa04916	Melanogenesis	34731	100/7854	0.34174536	0.83791788	0.83274402	612941/490937 CAMK2A, FZD2	2	AFEM_EC-up
cfa04620	Toll-like receptor signaling pathway	34731	101/7854	0.34616312	0.83791788	0.83274402	403469/403522 CD40, CCL5	2	AFEM_EC-up
cfa04066	HIF-1 signaling pathway	34731	112/7854	0.39405511	0.83791788	0.83274402	609626/612941 ANGPT4, CAMK2A	2	AFEM_EC-up
cfa04725	Cholinergic synapse	34731	113/7854	0.3983355	0.83791788	0.83274402	403858/612941 CHRM2, CAMK2A	2	AFEM_EC-up
NA	NA	NA	NA	NA	NA	NA	NA NA	NA	VFEM_EC-up
NA	NA	NA	NA	NA	NA	NA	NA NA	NA	VFEM_EC-up
NA	NA	NA	NA	NA	NA	NA	NA NA	NA	VFEM_EC-up

NA	NA	NA	NA	NA	NA	NA	NA	NA	NA	VFEM_EC-up
NA	NA	NA	NA	NA	NA	NA	NA	NA	NA	VFEM_EC-up
NA	NA	NA	NA	NA	NA	NA	NA	NA	NA	VFEM_EC-up
NA	NA	NA	NA	NA	NA	NA	NA	NA	NA	VFEM_EC-up
NA	NA	NA	NA	NA	NA	NA	NA	NA	NA	VFEM_EC-up
NA	NA	NA	NA	NA	NA	NA	NA	NA	NA	VFEM_EC-up
NA	NA	NA	NA	NA	NA	NA	NA	NA	NA	VFEM_EC-up
NA	NA	NA	NA	NA	NA	NA	NA	NA	NA	VFEM_EC-up
NA	NA	NA	NA	NA	NA	NA	NA	NA	NA	VFEM_EC-up
NA	NA	NA	NA	NA	NA	NA	NA	NA	NA	VFEM_EC-up
NA	NA	NA	NA	NA	NA	NA	NA	NA	NA	VFEM_EC-up
NA	NA	NA	NA	NA	NA	NA	NA	NA	NA	VFEM_EC-up
NA	NA	NA	NA	NA	NA	NA	NA	NA	NA	VFEM_EC-up
NA	NA	NA	NA	NA	NA	NA	NA	NA	NA	VFEM_EC-up
NA	NA	NA	NA	NA	NA	NA	NA	NA	NA	VFEM_EC-up
NA	NA	NA	NA	NA	NA	NA	NA	NA	NA	VFEM_EC-up
NA	NA	NA	NA	NA	NA	NA	NA	NA	NA	VFEM_EC-up
NA	NA	NA	NA	NA	NA	NA	NA	NA	NA	VFEM_EC-up
NA	NA	NA	NA	NA	NA	NA	NA	NA	NA	VFEM_EC-up
NA	NA	NA	NA	NA	NA	NA	NA	NA	NA	VFEM_EC-up
NA	NA	NA	NA	NA	NA	NA	NA	NA	NA	VFEM_EC-up
NA	NA	NA	NA	NA	NA	NA	NA	NA	NA	VFEM_EC-up
NA	NA	NA	NA	NA	NA	NA	NA	NA	NA	VFEM_EC-up
NA	NA	NA	NA	NA	NA	NA	NA	NA	NA	VFEM_EC-up
NA	NA	NA	NA	NA	NA	NA	NA	NA	NA	VFEM_EC-up
NA	NA	NA	NA	NA	NA	NA	NA	NA	NA	VFEM_EC-up
NA	NA	NA	NA	NA	NA	NA	NA	NA	NA	VFEM_EC-up
NA	NA	NA	NA	NA	NA	NA	NA	NA	NA	VFEM_EC-up
NA	NA	NA	NA	NA	NA	NA	NA	NA	NA	VFEM_EC-up
NA	NA	NA	NA	NA	NA	NA	NA	NA	NA	VFEM_EC-up
NA	NA	NA	NA	NA	NA	NA	NA	NA	NA	VFEM_EC-up
NA	NA	NA	NA	NA	NA	NA	NA	NA	NA	VFEM_EC-up
NA	NA	NA	NA	NA	NA	NA	NA	NA	NA	VFEM_EC-up
NA	NA	NA	NA	NA	NA	NA	NA	NA	NA	VFEM_EC-up
NA	NA	NA	NA	NA	NA	NA	NA	NA	NA	VFEM_EC-up
NA	NA	NA	NA	NA	NA	NA	NA	NA	NA	VFEM_EC-up
NA	NA	NA	NA	NA	NA	NA	NA	NA	NA	VFEM_EC-up
NA	NA	NA	NA	NA	NA	NA	NA	NA	NA	VFEM_EC-up
NA	NA	NA	NA	NA	NA	NA	NA	NA	NA	VFEM_EC-up
NA	NA	NA	NA	NA	NA	NA	NA	NA	NA	VFEM_EC-up
NA	NA	NA	NA	NA	NA	NA	NA	NA	NA	VFEM_EC-up
NA	NA	NA	NA	NA	NA	NA	NA	NA	NA	VFEM_EC-up
NA	NA	NA	NA	NA	NA	NA	NA	NA	NA	VFEM_EC-up
NA	NA	NA	NA	NA	NA	NA	NA	NA	NA	VFEM_EC-up
NA	NA	NA	NA	NA	NA	NA	NA	NA	NA	VFEM_EC-up
NA	NA	NA	NA	NA	NA	NA	NA	NA	NA	VFEM_EC-up
NA	NA	NA	NA	NA	NA	NA	NA	NA	NA	VFEM_EC-up
NA	NA	NA	NA	NA	NA	NA	NA	NA	NA	VFEM_EC-up
NA	NA	NA	NA	NA	NA	NA	NA	NA	NA	VFEM_EC-up
NA	NA	NA	NA	NA	NA	NA	NA	NA	NA	VFEM_EC-up
NA	NA	NA	NA	NA	NA	NA	NA	NA	NA	VFEM_EC-up
NA	NA	NA	NA	NA	NA	NA	NA	NA	NA	VFEM_EC-up
NA	NA	NA	NA	NA	NA	NA	NA	NA	NA	VFEM_EC-up
NA	NA	NA	NA	NA	NA	NA	NA	NA	NA	VFEM_EC-up
NA	NA	NA	NA	NA	NA	NA	NA	NA	NA	VFEM_EC-up
NA	NA	NA	NA	NA	NA	NA	NA	NA	NA	VFEM_EC-up
NA	NA	NA	NA	NA	NA	NA	NA	NA	NA	VFEM_EC-up
NA	NA	NA	NA	NA	NA	NA	NA	NA	NA	VFEM_EC-up
NA	NA	NA	NA	NA	NA	NA	NA	NA	NA	VFEM_EC-up
NA	NA	NA	NA	NA	NA	NA	NA	NA	NA	VFEM_EC-up
NA	NA	NA	NA	NA	NA	NA	NA	NA	NA	VFEM_EC-up
NA	NA	NA	NA	NA	NA	NA	NA	NA	NA	VFEM_EC-up
NA	NA	NA	NA	NA	NA	NA	NA	NA	NA	VFEM_EC-up
NA	NA	NA	NA	NA	NA	NA	NA	NA	NA	VFEM_EC-up
NA	NA	NA	NA	NA	NA	NA	NA	NA	NA	VFEM_EC-up
NA	NA	NA	NA	NA	NA	NA	NA	NA	NA	VFEM_EC-up
NA	NA	NA	NA	NA	NA	NA	NA	NA	NA	VFEM_EC-up
NA	NA	NA	NA	NA	NA	NA	NA	NA	NA	VFEM_EC-up
NA	NA	NA	NA	NA	NA	NA	NA	NA	NA	VFEM_EC-up
NA	NA	NA	NA	NA	NA	NA	NA	NA	NA	VFEM_EC-up
NA	NA	NA	NA	NA	NA	NA	NA	NA	NA	VFEM_EC-up
NA	NA	NA	NA	NA	NA	NA	NA	NA	NA	VFEM_EC-up
NA	NA	NA	NA	NA	NA	NA	NA	NA	NA	VFEM_EC-up
NA	NA	NA	NA	NA	NA	NA	NA	NA	NA	VFEM_EC-up

NA	NA	NA	NA	NA	NA	NA	NA	NA	NA	VFEM_EC-up
NA	NA	NA	NA	NA	NA	NA	NA	NA	NA	VFEM_EC-up
NA	NA	NA	NA	NA	NA	NA	NA	NA	NA	VFEM_EC-up
NA	NA	NA	NA	NA	NA	NA	NA	NA	NA	VFEM_EC-up
NA	NA	NA	NA	NA	NA	NA	NA	NA	NA	VFEM_EC-up
NA	NA	NA	NA	NA	NA	NA	NA	NA	NA	VFEM_EC-up
NA	NA	NA	NA	NA	NA	NA	NA	NA	NA	VFEM_EC-up
NA	NA	NA	NA	NA	NA	NA	NA	NA	NA	VFEM_EC-up
NA	NA	NA	NA	NA	NA	NA	NA	NA	NA	VFEM_EC-up
NA	NA	NA	NA	NA	NA	NA	NA	NA	NA	VFEM_EC-up
NA	NA	NA	NA	NA	NA	NA	NA	NA	NA	VFEM_EC-up
NA	NA	NA	NA	NA	NA	NA	NA	NA	NA	VFEM_EC-up
NA	NA	NA	NA	NA	NA	NA	NA	NA	NA	VFEM_EC-up
NA	NA	NA	NA	NA	NA	NA	NA	NA	NA	VFEM_EC-up
NA	NA	NA	NA	NA	NA	NA	NA	NA	NA	VFEM_EC-up
NA	NA	NA	NA	NA	NA	NA	NA	NA	NA	VFEM_EC-up
NA	NA	NA	NA	NA	NA	NA	NA	NA	NA	VFEM_EC-up
NA	NA	NA	NA	NA	NA	NA	NA	NA	NA	VFEM_EC-up
NA	NA	NA	NA	NA	NA	NA	NA	NA	NA	VFEM_EC-up
NA	NA	NA	NA	NA	NA	NA	NA	NA	NA	VFEM_EC-up
NA	NA	NA	NA	NA	NA	NA	NA	NA	NA	VFEM_EC-up
NA	NA	NA	NA	NA	NA	NA	NA	NA	NA	VFEM_EC-up
NA	NA	NA	NA	NA	NA	NA	NA	NA	NA	VFEM_EC-up
NA	NA	NA	NA	NA	NA	NA	NA	NA	NA	VFEM_EC-up
NA	NA	NA	NA	NA	NA	NA	NA	NA	NA	VFEM_EC-up
NA	NA	NA	NA	NA	NA	NA	NA	NA	NA	VFEM_EC-up
NA	NA	NA	NA	NA	NA	NA	NA	NA	NA	VFEM_EC-up
NA	NA	NA	NA	NA	NA	NA	NA	NA	NA	VFEM_EC-up
cfa04114	Oocyte meiosis	34731	117/7854	0.41531748	0.83791788	0.83274402	612941/479287	CAMK2A, CPEB4	2	AFEM_EC-up
NA	NA	NA	NA	NA	NA	NA	NA	NA	NA	VFEM_EC-down
NA	NA	NA	NA	NA	NA	NA	NA	NA	NA	VFEM_EC-down
NA	NA	NA	NA	NA	NA	NA	NA	NA	NA	VFEM_EC-down
NA	NA	NA	NA	NA	NA	NA	NA	NA	NA	VFEM_EC-down
NA	NA	NA	NA	NA	NA	NA	NA	NA	NA	VFEM_EC-down
NA	NA	NA	NA	NA	NA	NA	NA	NA	NA	VFEM_EC-down
NA	NA	NA	NA	NA	NA	NA	NA	NA	NA	VFEM_EC-down
NA	NA	NA	NA	NA	NA	NA	NA	NA	NA	VFEM_EC-down
NA	NA	NA	NA	NA	NA	NA	NA	NA	NA	VFEM_EC-down
NA	NA	NA	NA	NA	NA	NA	NA	NA	NA	VFEM_EC-down
NA	NA	NA	NA	NA	NA	NA	NA	NA	NA	VFEM_EC-down
NA	NA	NA	NA	NA	NA	NA	NA	NA	NA	VFEM_EC-down
NA	NA	NA	NA	NA	NA	NA	NA	NA	NA	VFEM_EC-down
NA	NA	NA	NA	NA	NA	NA	NA	NA	NA	VFEM_EC-down
NA	NA	NA	NA	NA	NA	NA	NA	NA	NA	VFEM_EC-down
NA	NA	NA	NA	NA	NA	NA	NA	NA	NA	VFEM_EC-down
NA	NA	NA	NA	NA	NA	NA	NA	NA	NA	VFEM_EC-down
NA	NA	NA	NA	NA	NA	NA	NA	NA	NA	VFEM_EC-down
NA	NA	NA	NA	NA	NA	NA	NA	NA	NA	VFEM_EC-down
NA	NA	NA	NA	NA	NA	NA	NA	NA	NA	VFEM_EC-down
NA	NA	NA	NA	NA	NA	NA	NA	NA	NA	VFEM_EC-down
NA	NA	NA	NA	NA	NA	NA	NA	NA	NA	VFEM_EC-down
NA	NA	NA	NA	NA	NA	NA	NA	NA	NA	VFEM_EC-down
NA	NA	NA	NA	NA	NA	NA	NA	NA	NA	VFEM_EC-down
NA	NA	NA	NA	NA	NA	NA	NA	NA	NA	VFEM_EC-down
NA	NA	NA	NA	NA	NA	NA	NA	NA	NA	VFEM_EC-down
NA	NA	NA	NA	NA	NA	NA	NA	NA	NA	VFEM_EC-down
NA	NA	NA	NA	NA	NA	NA	NA	NA	NA	VFEM_EC-down
NA	NA	NA	NA	NA	NA	NA	NA	NA	NA	VFEM_EC-down
NA	NA	NA	NA	NA	NA	NA	NA	NA	NA	VFEM_EC-down
NA	NA	NA	NA	NA	NA	NA	NA	NA	NA	VFEM_EC-down
NA	NA	NA	NA	NA	NA	NA	NA	NA	NA	VFEM_EC-down
NA	NA	NA	NA	NA	NA	NA	NA	NA	NA	VFEM_EC-down
NA	NA	NA	NA	NA	NA	NA	NA	NA	NA	VFEM_EC-down
NA	NA	NA	NA	NA	NA	NA	NA	NA	NA	VFEM_EC-down
NA	NA	NA	NA	NA	NA	NA	NA	NA	NA	VFEM_EC-down
NA	NA	NA	NA	NA	NA	NA	NA	NA	NA	VFEM_EC-down
NA	NA	NA	NA	NA	NA	NA	NA	NA	NA	VFEM_EC-down
NA	NA	NA	NA	NA	NA	NA	NA	NA	NA	VFEM_EC-down
NA	NA	NA	NA	NA	NA	NA	NA	NA	NA	VFEM_EC-down
NA	NA	NA	NA	NA	NA	NA	NA	NA	NA	VFEM_EC-down
NA	NA	NA	NA	NA	NA	NA	NA	NA	NA	VFEM_EC-down
NA	NA	NA	NA	NA	NA	NA	NA	NA	NA	VFEM_EC-down
NA	NA	NA	NA	NA	NA	NA	NA	NA	NA	VFEM_EC-down
NA	NA	NA	NA	NA	NA	NA	NA	NA	NA	VFEM_EC-down

NA	NA	NA	NA	NA	NA	NA	NA	NA	NA	VFEM_EC-down
NA	NA	NA	NA	NA	NA	NA	NA	NA	NA	VFEM_EC-down
NA	NA	NA	NA	NA	NA	NA	NA	NA	NA	VFEM_EC-down
NA	NA	NA	NA	NA	NA	NA	NA	NA	NA	VFEM_EC-down
NA	NA	NA	NA	NA	NA	NA	NA	NA	NA	VFEM_EC-down
NA	NA	NA	NA	NA	NA	NA	NA	NA	NA	VFEM_EC-down
NA	NA	NA	NA	NA	NA	NA	NA	NA	NA	VFEM_EC-down
NA	NA	NA	NA	NA	NA	NA	NA	NA	NA	VFEM_EC-down
NA	NA	NA	NA	NA	NA	NA	NA	NA	NA	VFEM_EC-down
NA	NA	NA	NA	NA	NA	NA	NA	NA	NA	VFEM_EC-down
NA	NA	NA	NA	NA	NA	NA	NA	NA	NA	VFEM_EC-down
NA	NA	NA	NA	NA	NA	NA	NA	NA	NA	VFEM_EC-down
NA	NA	NA	NA	NA	NA	NA	NA	NA	NA	VFEM_EC-down
NA	NA	NA	NA	NA	NA	NA	NA	NA	NA	VFEM_EC-down
NA	NA	NA	NA	NA	NA	NA	NA	NA	NA	VFEM_EC-down
NA	NA	NA	NA	NA	NA	NA	NA	NA	NA	VFEM_EC-down
NA	NA	NA	NA	NA	NA	NA	NA	NA	NA	VFEM_EC-down
NA	NA	NA	NA	NA	NA	NA	NA	NA	NA	VFEM_EC-down
NA	NA	NA	NA	NA	NA	NA	NA	NA	NA	VFEM_EC-down
NA	NA	NA	NA	NA	NA	NA	NA	NA	NA	VFEM_EC-down
NA	NA	NA	NA	NA	NA	NA	NA	NA	NA	VFEM_EC-down
NA	NA	NA	NA	NA	NA	NA	NA	NA	NA	VFEM_EC-down
NA	NA	NA	NA	NA	NA	NA	NA	NA	NA	VFEM_EC-down
NA	NA	NA	NA	NA	NA	NA	NA	NA	NA	VFEM_EC-down
NA	NA	NA	NA	NA	NA	NA	NA	NA	NA	VFEM_EC-down
NA	NA	NA	NA	NA	NA	NA	NA	NA	NA	VFEM_EC-down
NA	NA	NA	NA	NA	NA	NA	NA	NA	NA	VFEM_EC-down
NA	NA	NA	NA	NA	NA	NA	NA	NA	NA	VFEM_EC-down
NA	NA	NA	NA	NA	NA	NA	NA	NA	NA	VFEM_EC-down
NA	NA	NA	NA	NA	NA	NA	NA	NA	NA	VFEM_EC-down
NA	NA	NA	NA	NA	NA	NA	NA	NA	NA	VFEM_EC-down
NA	NA	NA	NA	NA	NA	NA	NA	NA	NA	VFEM_EC-down
NA	NA	NA	NA	NA	NA	NA	NA	NA	NA	VFEM_EC-down
NA	NA	NA	NA	NA	NA	NA	NA	NA	NA	VFEM_EC-down
NA	NA	NA	NA	NA	NA	NA	NA	NA	NA	VFEM_EC-down
NA	NA	NA	NA	NA	NA	NA	NA	NA	NA	VFEM_EC-down
NA	NA	NA	NA	NA	NA	NA	NA	NA	NA	VFEM_EC-down
NA	NA	NA	NA	NA	NA	NA	NA	NA	NA	VFEM_EC-down
NA	NA	NA	NA	NA	NA	NA	NA	NA	NA	VFEM_EC-down
NA	NA	NA	NA	NA	NA	NA	NA	NA	NA	VFEM_EC-down
NA	NA	NA	NA	NA	NA	NA	NA	NA	NA	VFEM_EC-down
NA	NA	NA	NA	NA	NA	NA	NA	NA	NA	VFEM_EC-down
NA	NA	NA	NA	NA	NA	NA	NA	NA	NA	VFEM_EC-down
NA	NA	NA	NA	NA	NA	NA	NA	NA	NA	VFEM_EC-down
NA	NA	NA	NA	NA	NA	NA	NA	NA	NA	VFEM_EC-down
NA	NA	NA	NA	NA	NA	NA	NA	NA	NA	VFEM_EC-down
NA	NA	NA	NA	NA	NA	NA	NA	NA	NA	VFEM_EC-down
NA	NA	NA	NA	NA	NA	NA	NA	NA	NA	VFEM_EC-down
NA	NA	NA	NA	NA	NA	NA	NA	NA	NA	VFEM_EC-down
NA	NA	NA	NA	NA	NA	NA	NA	NA	NA	VFEM_EC-down
NA	NA	NA	NA	NA	NA	NA	NA	NA	NA	VFEM_EC-down
NA	NA	NA	NA	NA	NA	NA	NA	NA	NA	VFEM_EC-down
NA	NA	NA	NA	NA	NA	NA	NA	NA	NA	VFEM_EC-down
NA	NA	NA	NA	NA	NA	NA	NA	NA	NA	VFEM_EC-down
NA	NA	NA	NA	NA	NA	NA	NA	NA	NA	VFEM_EC-down
NA	NA	NA	NA	NA	NA	NA	NA	NA	NA	VFEM_EC-down
NA	NA	NA	NA	NA	NA	NA	NA	NA	NA	VFEM_EC-down
NA	NA	NA	NA	NA	NA	NA	NA	NA	NA	VFEM_EC-down
NA	NA	NA	NA	NA	NA	NA	NA	NA	NA	VFEM_EC-down
NA	NA	NA	NA	NA	NA	NA	NA	NA	NA	VFEM_EC-down
NA	NA	NA	NA	NA	NA	NA	NA	NA	NA	VFEM_EC-down
NA	NA	NA	NA	NA	NA	NA	NA	NA	NA	VFEM_EC-down
NA	NA	NA	NA	NA	NA	NA	NA	NA	NA	VFEM_EC-down
NA	NA	NA	NA	NA	NA	NA	NA	NA	NA	VFEM_EC-down
NA	NA	NA	NA	NA	NA	NA	NA	NA	NA	VFEM_EC-down
NA	NA	NA	NA	NA	NA	NA	NA	NA	NA	VFEM_EC-down
NA	NA	NA	NA	NA	NA	NA	NA	NA	NA	VFEM_EC-down
NA	NA	NA	NA	NA	NA	NA	NA	NA	NA	VFEM_EC-down
NA	NA	NA	NA	NA	NA	NA	NA	NA	NA	VFEM_EC-down
NA	NA	NA	NA	NA	NA	NA	NA	NA	NA	VFEM_EC-down
NA	NA	NA	NA	NA	NA	NA	NA	NA	NA	VFEM_EC-down
NA	NA	NA	NA	NA	NA	NA	NA	NA	NA	VFEM_EC-down
NA	NA	NA	NA	NA	NA	NA	NA	NA	NA	VFEM_EC-down
NA	NA	NA	NA	NA	NA	NA	NA	NA	NA	VFEM_EC-down

NA	NA	NA	NA	NA	NA	NA	NA	NA	NA	VFEM_EC-down
NA	NA	NA	NA	NA	NA	NA	NA	NA	NA	VFEM_EC-down
NA	NA	NA	NA	NA	NA	NA	NA	NA	NA	VFEM_EC-down
NA	NA	NA	NA	NA	NA	NA	NA	NA	NA	VFEM_EC-down
NA	NA	NA	NA	NA	NA	NA	NA	NA	NA	VFEM_EC-down
NA	NA	NA	NA	NA	NA	NA	NA	NA	NA	VFEM_EC-down
NA	NA	NA	NA	NA	NA	NA	NA	NA	NA	VFEM_EC-down
NA	NA	NA	NA	NA	NA	NA	NA	NA	NA	VFEM_EC-down
NA	NA	NA	NA	NA	NA	NA	NA	NA	NA	VFEM_EC-down
NA	NA	NA	NA	NA	NA	NA	NA	NA	NA	VFEM_EC-down
NA	NA	NA	NA	NA	NA	NA	NA	NA	NA	VFEM_EC-down
NA	NA	NA	NA	NA	NA	NA	NA	NA	NA	VFEM_EC-down
NA	NA	NA	NA	NA	NA	NA	NA	NA	NA	VFEM_EC-down
NA	NA	NA	NA	NA	NA	NA	NA	NA	NA	VFEM_EC-down
NA	NA	NA	NA	NA	NA	NA	NA	NA	NA	VFEM_EC-down
NA	NA	NA	NA	NA	NA	NA	NA	NA	NA	VFEM_EC-down
NA	NA	NA	NA	NA	NA	NA	NA	NA	NA	VFEM_EC-down
NA	NA	NA	NA	NA	NA	NA	NA	NA	NA	VFEM_EC-down
NA	NA	NA	NA	NA	NA	NA	NA	NA	NA	VFEM_EC-down
NA	NA	NA	NA	NA	NA	NA	NA	NA	NA	VFEM_EC-down
NA	NA	NA	NA	NA	NA	NA	NA	NA	NA	VFEM_EC-down
NA	NA	NA	NA	NA	NA	NA	NA	NA	NA	VFEM_EC-down
NA	NA	NA	NA	NA	NA	NA	NA	NA	NA	VFEM_EC-down
NA	NA	NA	NA	NA	NA	NA	NA	NA	NA	VFEM_EC-down
NA	NA	NA	NA	NA	NA	NA	NA	NA	NA	VFEM_EC-down
NA	NA	NA	NA	NA	NA	NA	NA	NA	NA	VFEM_EC-down
NA	NA	NA	NA	NA	NA	NA	NA	NA	NA	VFEM_EC-down
NA	NA	NA	NA	NA	NA	NA	NA	NA	NA	VFEM_EC-down
NA	NA	NA	NA	NA	NA	NA	NA	NA	NA	VFEM_EC-down
NA	NA	NA	NA	NA	NA	NA	NA	NA	NA	VFEM_EC-down
NA	NA	NA	NA	NA	NA	NA	NA	NA	NA	VFEM_EC-down
NA	NA	NA	NA	NA	NA	NA	NA	NA	NA	VFEM_EC-down
NA	NA	NA	NA	NA	NA	NA	NA	NA	NA	VFEM_EC-down
NA	NA	NA	NA	NA	NA	NA	NA	NA	NA	VFEM_EC-down
NA	NA	NA	NA	NA	NA	NA	NA	NA	NA	VFEM_EC-down
NA	NA	NA	NA	NA	NA	NA	NA	NA	NA	VFEM_EC-down
NA	NA	NA	NA	NA	NA	NA	NA	NA	NA	VFEM_EC-down
NA	NA	NA	NA	NA	NA	NA	NA	NA	NA	VFEM_EC-down
NA	NA	NA	NA	NA	NA	NA	NA	NA	NA	VFEM_EC-down
NA	NA	NA	NA	NA	NA	NA	NA	NA	NA	VFEM_EC-down
NA	NA	NA	NA	NA	NA	NA	NA	NA	NA	VFEM_EC-down
NA	NA	NA	NA	NA	NA	NA	NA	NA	NA	VFEM_EC-down
NA	NA	NA	NA	NA	NA	NA	NA	NA	NA	VFEM_EC-down
NA	NA	NA	NA	NA	NA	NA	NA	NA	NA	VFEM_EC-down
NA	NA	NA	NA	NA	NA	NA	NA	NA	NA	VFEM_EC-down
NA	NA	NA	NA	NA	NA	NA	NA	NA	NA	VFEM_EC-down
NA	NA	NA	NA	NA	NA	NA	NA	NA	NA	VFEM_EC-down
NA	NA	NA	NA	NA	NA	NA	NA	NA	NA	VFEM_EC-down
NA	NA	NA	NA	NA	NA	NA	NA	NA	NA	VFEM_EC-down
NA	NA	NA	NA	NA	NA	NA	NA	NA	NA	VFEM_EC-down
NA	NA	NA	NA	NA	NA	NA	NA	NA	NA	VFEM_EC-down
NA	NA	NA	NA	NA	NA	NA	NA	NA	NA	VFEM_EC-down
NA	NA	NA	NA	NA	NA	NA	NA	NA	NA	VFEM_EC-down
NA	NA	NA	NA	NA	NA	NA	NA	NA	NA	VFEM_EC-down
NA	NA	NA	NA	NA	NA	NA	NA	NA	NA	VFEM_EC-down
NA	NA	NA	NA	NA	NA	NA	NA	NA	NA	VFEM_EC-down
NA	NA	NA	NA	NA	NA	NA	NA	NA	NA	VFEM_EC-down
NA	NA	NA	NA	NA	NA	NA	NA	NA	NA	VFEM_EC-down
NA	NA	NA	NA	NA	NA	NA	NA	NA	NA	VFEM_EC-down
NA	NA	NA	NA	NA	NA	NA	NA	NA	NA	VFEM_EC-down
NA	NA	NA	NA	NA	NA	NA	NA	NA	NA	VFEM_EC-down
NA	NA	NA	NA	NA	NA	NA	NA	NA	NA	VFEM_EC-down
NA	NA	NA	NA	NA	NA	NA	NA	NA	NA	VFEM_EC-down
NA	NA	NA	NA	NA	NA	NA	NA	NA	NA	VFEM_EC-down
NA	NA	NA	NA	NA	NA	NA	NA	NA	NA	VFEM_EC-down
NA	NA	NA	NA	NA	NA	NA	NA	NA	NA	VFEM_EC-down
NA	NA	NA	NA	NA	NA	NA	NA	NA	NA	VFEM_EC-down
NA	NA	NA	NA	NA	NA	NA	NA	NA	NA	VFEM_EC-down
NA	NA	NA	NA	NA	NA	NA	NA	NA	NA	VFEM_EC-down
NA	NA	NA	NA	NA	NA	NA	NA	NA	NA	VFEM_EC-down
NA	NA	NA	NA	NA	NA	NA	NA	NA	NA	VFEM_EC-down
NA	NA	NA	NA	NA	NA	NA	NA	NA	NA	VFEM_EC-down
NA	NA	NA	NA	NA	NA	NA	NA	NA	NA	VFEM_EC-down
NA	NA	NA	NA	NA	NA	NA	NA	NA	NA	VFEM_EC-down

NA	NA	NA	NA	NA	NA	NA	NA	NA	NA	VFEM_EC-down
NA	NA	NA	NA	NA	NA	NA	NA	NA	NA	VFEM_EC-down
NA	NA	NA	NA	NA	NA	NA	NA	NA	NA	VFEM_EC-down
NA	NA	NA	NA	NA	NA	NA	NA	NA	NA	VFEM_EC-down
NA	NA	NA	NA	NA	NA	NA	NA	NA	NA	VFEM_EC-down
NA	NA	NA	NA	NA	NA	NA	NA	NA	NA	VFEM_EC-down
cfa04722	Neurotrophin signaling pathway	34731	118/7854	0.41952667	0.83791788	0.83274402	612941/479915	CAMK2A, SORT1	2	AFEM_EC-up
cfa04380	Osteoclast differentiation	34731	125/7854	0.44855576	0.83791788	0.83274402	611649/608412	CYLD, FOSL2	2	AFEM_EC-up
cfa04068	FoxO signaling pathway	34731	127/7854	0.45670308	0.83791788	0.83274402	485548/476258	IRS2, SOD2	2	AFEM_EC-up
cfa04210	Apoptosis	34731	131/7854	0.47279165	0.83791788	0.83274402	403400/611983	CTSS, CTSZ	2	AFEM_EC-up
cfa04514	Cell adhesion molecules (CAMs)	34731	139/7854	0.5041076	0.86267127	0.85734458	487705/403469	NCAM2, CD40	2	AFEM_EC-up
cfa05226	Gastric cancer	34731	149/7854	0.54155288	0.86267127	0.85734458	490903/490937	AXIN2, FZD2	2	AFEM_EC-up
cfa04150	mTOR signaling pathway	34731	152/7854	0.55240526	0.86267127	0.85734458	486137/490937	ATP6V1B2, FZD2	2	AFEM_EC-up
cfa04390	Hippo signaling pathway	34731	153/7854	0.55598299	0.86267127	0.85734458	490903/490937	AXIN2, FZD2	2	AFEM_EC-up
cfa04141	Protein processing in endoplasmic reticulum	34731	160/7854	0.58046614	0.86577607	0.8604302	478178/479441	MAN1C1, CRYAB	2	AFEM_EC-up
cfa04217	Necroptosis	34731	160/7854	0.58046614	0.86577607	0.8604302	612941/611649	CAMK2A, CYLD	2	AFEM_EC-up
cfa05225	Hepatocellular carcinoma	34731	165/7854	0.59734915	0.86577607	0.8604302	490903/490937	AXIN2, FZD2	2	AFEM_EC-up
cfa05010	Alzheimer disease	34731	168/7854	0.60723599	0.86577607	0.8604302	612614/475662	COX7A1, ADAM17	2	AFEM_EC-up
cfa04530	Tight junction	34731	170/7854	0.61372594	0.86577607	0.8604302	403852/481944	TJP3, BVES	2	AFEM_EC-up
cfa04062	Chemokine signaling pathway	34731	174/7854	0.62646303	0.86577607	0.8604302	485828/403522	HCK, CCL5	2	AFEM_EC-up
cfa04360	Axon guidance	34731	179/7854	0.64193055	0.86577607	0.8604302	482251/612941	PLXNA4, CAMK2A	2	AFEM_EC-up
cfa04015	Rap1 signaling pathway	34731	209/7854	0.72441065	0.8660572	0.8607096	609626/482666	ANGPT4, PDGFC	2	AFEM_EC-up
cfa04714	Thermogenesis	34731	227/7854	0.76586993	0.87318928	0.86779764	479127/612614	PPARGC1A, COX7A1	2	AFEM_EC-up
cfa04010	MAPK signaling pathway	34731	288/7854	0.86886075	0.91485926	0.90921032	609626/482666	ANGPT4, PDGFC	2	AFEM_EC-up
cfa00020	Citrate cycle (TCA cycle)	44167	29/7854	0.00084921	0.06878597	0.06436115	476562/474403	SUCLG2, CS	2	AFEM_EC-down
cfa01200	Carbon metabolism	44167	116/7854	0.01295835	0.2588504	0.24219921	476562/474403	SUCLG2, CS	2	AFEM_EC-down
cfa04910	Insulin signaling pathway	44167	134/7854	0.01704988	0.2588504	0.24219921	477746/612212	MAPK8, MKNK2	2	AFEM_EC-down
cfa04932	Non-alcoholic fatty liver disease (NAFLD)	44167	145/7854	0.01978998	0.2588504	0.24219921	608244/477746	NDUFA10, MAPK8	2	AFEM_EC-down
cfa04723	Retrograde endocannabinoid signaling	44167	149/7854	0.02083005	0.2588504	0.24219921	608244/477746	NDUFA10, MAPK8	2	AFEM_EC-down
cfa05016	Huntington disease	44167	257/7854	0.05671538	0.2588504	0.24219921	608244/477746	NDUFA10, MAPK8	2	AFEM_EC-down
cfa04010	MAPK signaling pathway	44167	288/7854	0.06940971	0.2588504	0.24219921	477746/612212	MAPK8, MKNK2	2	AFEM_EC-down
cfa00740	Riboflavin metabolism	2/407	2174868	0.06095276	0.62573034	0.60148786	484159/475983	RFK, ACP2	2	ACOR_VSMC-up
cfa00533	Glycosaminoglycan biosynthesis - keratan sulfate	2/407	15/7854	0.18072536	0.80256807	0.77147442	486158/448804	B3GNT7, FUT8	2	ACOR_VSMC-up
cfa00360	Phenylalanine metabolism	2/407	17/7854	0.21921617	0.80256807	0.77147442	403451/403450	MAOB, MAOA	2	ACOR_VSMC-up
cfa00340	Histidine metabolism	2/407	21/7854	0.29747129	0.82260001	0.79073027	403451/403450	MAOB, MAOA	2	ACOR_VSMC-up
cfa00900	Terpenoid backbone biosynthesis	2/407	21/7854	0.29747129	0.82260001	0.79073027	479198/481418	GGPS1, PCYOX1	2	ACOR_VSMC-up
cfa00592	alpha-Linolenic acid metabolism	2/407	22/7854	0.31695457	0.82260001	0.79073027	476045/478514	PLA2G16, CFI	2	ACOR_VSMC-up
cfa00515	Mannose type O-glycan biosynthesis	2/407	24/7854	0.355499	0.86074085	0.82739343	479196/482834	B3GALNT2, POMK	2	ACOR_VSMC-up
cfa04614	Renin-angiotensin system	2/407	24/7854	0.355499	0.86074085	0.82739343	478081/100682978	NLN, ATP6AP2	2	ACOR_VSMC-up
cfa00790	Folate biosynthesis	2/407	25/7854	0.37448647	0.87388357	0.84002697	609680/611326	PCBD1, PTS	2	ACOR_VSMC-up
cfa01040	Biosynthesis of unsaturated fatty acids	2/407	26/7854	0.39324192	0.87388357	0.84002697	479564/608997	SCP2, ELOVL7	2	ACOR_VSMC-up
cfa04966	Collecting duct acid secretion	2/407	26/7854	0.39324192	0.87388357	0.84002697	488069/403729	SLC12A7, ATP6V0E1	2	ACOR_VSMC-up
NA	NA	NA	NA	NA	NA	NA	NA	NA	NA	VPUL_EC-up
NA	NA	NA	NA	NA	NA	NA	NA	NA	NA	VPUL_EC-up
NA	NA	NA	NA	NA	NA	NA	NA	NA	NA	VPUL_EC-up
NA	NA	NA	NA	NA	NA	NA	NA	NA	NA	VPUL_EC-up
NA	NA	NA	NA	NA	NA	NA	NA	NA	NA	VPUL_EC-up
NA	NA	NA	NA	NA	NA	NA	NA	NA	NA	VPUL_EC-up
NA	NA	NA	NA	NA	NA	NA	NA	NA	NA	VPUL_EC-up
NA	NA	NA	NA	NA	NA	NA	NA	NA	NA	VPUL_EC-up
NA	NA	NA	NA	NA	NA	NA	NA	NA	NA	VPUL_EC-up
NA	NA	NA	NA	NA	NA	NA	NA	NA	NA	VPUL_EC-up
NA	NA	NA	NA	NA	NA	NA	NA	NA	NA	VPUL_EC-up
NA	NA	NA	NA	NA	NA	NA	NA	NA	NA	VPUL_EC-up
NA	NA	NA	NA	NA	NA	NA	NA	NA	NA	VPUL_EC-up

NA	NA	NA	NA	NA	NA	NA	NA	NA	NA	VPUL_EC-up
NA	NA	NA	NA	NA	NA	NA	NA	NA	NA	VPUL_EC-up
NA	NA	NA	NA	NA	NA	NA	NA	NA	NA	VPUL_EC-up
NA	NA	NA	NA	NA	NA	NA	NA	NA	NA	VPUL_EC-up
NA	NA	NA	NA	NA	NA	NA	NA	NA	NA	VPUL_EC-up
NA	NA	NA	NA	NA	NA	NA	NA	NA	NA	VPUL_EC-up
NA	NA	NA	NA	NA	NA	NA	NA	NA	NA	VPUL_EC-up
NA	NA	NA	NA	NA	NA	NA	NA	NA	NA	VPUL_EC-up
NA	NA	NA	NA	NA	NA	NA	NA	NA	NA	VPUL_EC-up
NA	NA	NA	NA	NA	NA	NA	NA	NA	NA	VPUL_EC-up
NA	NA	NA	NA	NA	NA	NA	NA	NA	NA	VPUL_EC-up
NA	NA	NA	NA	NA	NA	NA	NA	NA	NA	VPUL_EC-up
NA	NA	NA	NA	NA	NA	NA	NA	NA	NA	VPUL_EC-up
NA	NA	NA	NA	NA	NA	NA	NA	NA	NA	VPUL_EC-up
NA	NA	NA	NA	NA	NA	NA	NA	NA	NA	VPUL_EC-up
NA	NA	NA	NA	NA	NA	NA	NA	NA	NA	VPUL_EC-up
NA	NA	NA	NA	NA	NA	NA	NA	NA	NA	VPUL_EC-up
NA	NA	NA	NA	NA	NA	NA	NA	NA	NA	VPUL_EC-up
NA	NA	NA	NA	NA	NA	NA	NA	NA	NA	VPUL_EC-up
NA	NA	NA	NA	NA	NA	NA	NA	NA	NA	VPUL_EC-up
NA	NA	NA	NA	NA	NA	NA	NA	NA	NA	VPUL_EC-up
NA	NA	NA	NA	NA	NA	NA	NA	NA	NA	VPUL_EC-up
NA	NA	NA	NA	NA	NA	NA	NA	NA	NA	VPUL_EC-up
NA	NA	NA	NA	NA	NA	NA	NA	NA	NA	VPUL_EC-up
NA	NA	NA	NA	NA	NA	NA	NA	NA	NA	VPUL_EC-up
NA	NA	NA	NA	NA	NA	NA	NA	NA	NA	VPUL_EC-up
NA	NA	NA	NA	NA	NA	NA	NA	NA	NA	VPUL_EC-up
NA	NA	NA	NA	NA	NA	NA	NA	NA	NA	VPUL_EC-up
NA	NA	NA	NA	NA	NA	NA	NA	NA	NA	VPUL_EC-up
NA	NA	NA	NA	NA	NA	NA	NA	NA	NA	VPUL_EC-up
NA	NA	NA	NA	NA	NA	NA	NA	NA	NA	VPUL_EC-up
NA	NA	NA	NA	NA	NA	NA	NA	NA	NA	VPUL_EC-up
NA	NA	NA	NA	NA	NA	NA	NA	NA	NA	VPUL_EC-up
NA	NA	NA	NA	NA	NA	NA	NA	NA	NA	VPUL_EC-up
NA	NA	NA	NA	NA	NA	NA	NA	NA	NA	VPUL_EC-up
NA	NA	NA	NA	NA	NA	NA	NA	NA	NA	VPUL_EC-up
NA	NA	NA	NA	NA	NA	NA	NA	NA	NA	VPUL_EC-up
NA	NA	NA	NA	NA	NA	NA	NA	NA	NA	VPUL_EC-up
NA	NA	NA	NA	NA	NA	NA	NA	NA	NA	VPUL_EC-up
NA	NA	NA	NA	NA	NA	NA	NA	NA	NA	VPUL_EC-up
NA	NA	NA	NA	NA	NA	NA	NA	NA	NA	VPUL_EC-up
NA	NA	NA	NA	NA	NA	NA	NA	NA	NA	VPUL_EC-up
NA	NA	NA	NA	NA	NA	NA	NA	NA	NA	VPUL_EC-up
NA	NA	NA	NA	NA	NA	NA	NA	NA	NA	VPUL_EC-up
NA	NA	NA	NA	NA	NA	NA	NA	NA	NA	VPUL_EC-up
NA	NA	NA	NA	NA	NA	NA	NA	NA	NA	VPUL_EC-up
NA	NA	NA	NA	NA	NA	NA	NA	NA	NA	VPUL_EC-up
NA	NA	NA	NA	NA	NA	NA	NA	NA	NA	VPUL_EC-up
NA	NA	NA	NA	NA	NA	NA	NA	NA	NA	VPUL_EC-up
NA	NA	NA	NA	NA	NA	NA	NA	NA	NA	VPUL_EC-up
NA	NA	NA	NA	NA	NA	NA	NA	NA	NA	VPUL_EC-up
NA	NA	NA	NA	NA	NA	NA	NA	NA	NA	VPUL_EC-up
NA	NA	NA	NA	NA	NA	NA	NA	NA	NA	VPUL_EC-up
NA	NA	NA	NA	NA	NA	NA	NA	NA	NA	VPUL_EC-up
NA	NA	NA	NA	NA	NA	NA	NA	NA	NA	VPUL_EC-up
NA	NA	NA	NA	NA	NA	NA	NA	NA	NA	VPUL_EC-up
NA	NA	NA	NA	NA	NA	NA	NA	NA	NA	VPUL_EC-up
NA	NA	NA	NA	NA	NA	NA	NA	NA	NA	VPUL_EC-up
NA	NA	NA	NA	NA	NA	NA	NA	NA	NA	VPUL_EC-up
NA	NA	NA	NA	NA	NA	NA	NA	NA	NA	VPUL_EC-up
NA	NA	NA	NA	NA	NA	NA	NA	NA	NA	VPUL_EC-up
NA	NA	NA	NA	NA	NA	NA	NA	NA	NA	VPUL_EC-up
NA	NA	NA	NA	NA	NA	NA	NA	NA	NA	VPUL_EC-up
NA	NA	NA	NA	NA	NA	NA	NA	NA	NA	VPUL_EC-up
NA	NA	NA	NA	NA	NA	NA	NA	NA	NA	VPUL_EC-up
NA	NA	NA	NA	NA	NA	NA	NA	NA	NA	VPUL_EC-up
NA	NA	NA	NA	NA	NA	NA	NA	NA	NA	VPUL_EC-up
NA	NA	NA	NA	NA	NA	NA	NA	NA	NA	VPUL_EC-up
NA	NA	NA	NA	NA	NA	NA	NA	NA	NA	VPUL_EC-up
NA	NA	NA	NA	NA	NA	NA	NA	NA	NA	VPUL_EC-up
NA	NA	NA	NA	NA	NA	NA	NA	NA	NA	VPUL_EC-up
NA	NA	NA	NA	NA	NA	NA	NA	NA	NA	VPUL_EC-up
NA	NA	NA	NA	NA	NA	NA	NA	NA	NA	VPUL_EC-up
NA	NA	NA	NA	NA	NA	NA	NA	NA	NA	VPUL_EC-up

NA	NA	NA	NA	NA	NA	NA	NA	NA	NA	VPUL_EC-up
NA	NA	NA	NA	NA	NA	NA	NA	NA	NA	VPUL_EC-up
NA	NA	NA	NA	NA	NA	NA	NA	NA	NA	VPUL_EC-up
NA	NA	NA	NA	NA	NA	NA	NA	NA	NA	VPUL_EC-up
NA	NA	NA	NA	NA	NA	NA	NA	NA	NA	VPUL_EC-up
NA	NA	NA	NA	NA	NA	NA	NA	NA	NA	VPUL_EC-up
NA	NA	NA	NA	NA	NA	NA	NA	NA	NA	VPUL_EC-up
NA	NA	NA	NA	NA	NA	NA	NA	NA	NA	VPUL_EC-up
NA	NA	NA	NA	NA	NA	NA	NA	NA	NA	VPUL_EC-up
NA	NA	NA	NA	NA	NA	NA	NA	NA	NA	VPUL_EC-up
NA	NA	NA	NA	NA	NA	NA	NA	NA	NA	VPUL_EC-up
NA	NA	NA	NA	NA	NA	NA	NA	NA	NA	VPUL_EC-up
NA	NA	NA	NA	NA	NA	NA	NA	NA	NA	VPUL_EC-up
NA	NA	NA	NA	NA	NA	NA	NA	NA	NA	VPUL_EC-up
NA	NA	NA	NA	NA	NA	NA	NA	NA	NA	VPUL_EC-up
NA	NA	NA	NA	NA	NA	NA	NA	NA	NA	VPUL_EC-up
NA	NA	NA	NA	NA	NA	NA	NA	NA	NA	VPUL_EC-up
NA	NA	NA	NA	NA	NA	NA	NA	NA	NA	VPUL_EC-up
NA	NA	NA	NA	NA	NA	NA	NA	NA	NA	VPUL_EC-up
NA	NA	NA	NA	NA	NA	NA	NA	NA	NA	VPUL_EC-up
NA	NA	NA	NA	NA	NA	NA	NA	NA	NA	VPUL_EC-up
NA	NA	NA	NA	NA	NA	NA	NA	NA	NA	VPUL_EC-up
NA	NA	NA	NA	NA	NA	NA	NA	NA	NA	VPUL_EC-up
NA	NA	NA	NA	NA	NA	NA	NA	NA	NA	VPUL_EC-up
NA	NA	NA	NA	NA	NA	NA	NA	NA	NA	VPUL_EC-up
NA	NA	NA	NA	NA	NA	NA	NA	NA	NA	VPUL_EC-up
NA	NA	NA	NA	NA	NA	NA	NA	NA	NA	VPUL_EC-up
NA	NA	NA	NA	NA	NA	NA	NA	NA	NA	VPUL_EC-up
NA	NA	NA	NA	NA	NA	NA	NA	NA	NA	VPUL_EC-up
NA	NA	NA	NA	NA	NA	NA	NA	NA	NA	VPUL_EC-up
NA	NA	NA	NA	NA	NA	NA	NA	NA	NA	VPUL_EC-up
NA	NA	NA	NA	NA	NA	NA	NA	NA	NA	VPUL_EC-up
NA	NA	NA	NA	NA	NA	NA	NA	NA	NA	VPUL_EC-up
NA	NA	NA	NA	NA	NA	NA	NA	NA	NA	VPUL_EC-up
NA	NA	NA	NA	NA	NA	NA	NA	NA	NA	VPUL_EC-up
NA	NA	NA	NA	NA	NA	NA	NA	NA	NA	VPUL_EC-up
NA	NA	NA	NA	NA	NA	NA	NA	NA	NA	VPUL_EC-up
NA	NA	NA	NA	NA	NA	NA	NA	NA	NA	VPUL_EC-up
NA	NA	NA	NA	NA	NA	NA	NA	NA	NA	VPUL_EC-up
NA	NA	NA	NA	NA	NA	NA	NA	NA	NA	VPUL_EC-up
NA	NA	NA	NA	NA	NA	NA	NA	NA	NA	VPUL_EC-up
NA	NA	NA	NA	NA	NA	NA	NA	NA	NA	VPUL_EC-up
NA	NA	NA	NA	NA	NA	NA	NA	NA	NA	VPUL_EC-up
NA	NA	NA	NA	NA	NA	NA	NA	NA	NA	VPUL_EC-up
NA	NA	NA	NA	NA	NA	NA	NA	NA	NA	VPUL_EC-up
NA	NA	NA	NA	NA	NA	NA	NA	NA	NA	VPUL_EC-up
NA	NA	NA	NA	NA	NA	NA	NA	NA	NA	VPUL_EC-up
NA	NA	NA	NA	NA	NA	NA	NA	NA	NA	VPUL_EC-up
NA	NA	NA	NA	NA	NA	NA	NA	NA	NA	VPUL_EC-up
NA	NA	NA	NA	NA	NA	NA	NA	NA	NA	VPUL_EC-up
NA	NA	NA	NA	NA	NA	NA	NA	NA	NA	VPUL_EC-up
NA	NA	NA	NA	NA	NA	NA	NA	NA	NA	VPUL_EC-up
NA	NA	NA	NA	NA	NA	NA	NA	NA	NA	VPUL_EC-up
NA	NA	NA	NA	NA	NA	NA	NA	NA	NA	VPUL_EC-up
NA	NA	NA	NA	NA	NA	NA	NA	NA	NA	VPUL_EC-up
NA	NA	NA	NA	NA	NA	NA	NA	NA	NA	VPUL_EC-up
NA	NA	NA	NA	NA	NA	NA	NA	NA	NA	VPUL_EC-up
NA	NA	NA	NA	NA	NA	NA	NA	NA	NA	VPUL_EC-up
NA	NA	NA	NA	NA	NA	NA	NA	NA	NA	VPUL_EC-up
NA	NA	NA	NA	NA	NA	NA	NA	NA	NA	VPUL_EC-up
NA	NA	NA	NA	NA	NA	NA	NA	NA	NA	VPUL_EC-up
NA	NA	NA	NA	NA	NA	NA	NA	NA	NA	VPUL_EC-up
NA	NA	NA	NA	NA	NA	NA	NA	NA	NA	VPUL_EC-up
NA	NA	NA	NA	NA	NA	NA	NA	NA	NA	VPUL_EC-up
NA	NA	NA	NA	NA	NA	NA	NA	NA	NA	VPUL_EC-up
NA	NA	NA	NA	NA	NA	NA	NA	NA	NA	VPUL_EC-up
NA	NA	NA	NA	NA	NA	NA	NA	NA	NA	VPUL_EC-up
NA	NA	NA	NA	NA	NA	NA	NA	NA	NA	VPUL_EC-up
NA	NA	NA	NA	NA	NA	NA	NA	NA	NA	VPUL_EC-up
NA	NA	NA	NA	NA	NA	NA	NA	NA	NA	VPUL_EC-up
NA	NA	NA	NA	NA	NA	NA	NA	NA	NA	VPUL_EC-up
NA	NA	NA	NA	NA	NA	NA	NA	NA	NA	VPUL_EC-up
NA	NA	NA	NA	NA	NA	NA	NA	NA	NA	VPUL_EC-up
NA	NA	NA	NA	NA	NA	NA	NA	NA	NA	VPUL_EC-up

NA	NA	NA	NA	NA	NA	NA	NA	NA	NA	VPUL_EC-up
NA	NA	NA	NA	NA	NA	NA	NA	NA	NA	VPUL_EC-up
NA	NA	NA	NA	NA	NA	NA	NA	NA	NA	VPUL_EC-up
cfa00512	Mucin type O-glycan biosynthesis	2/407	31/7854	0.48270946	0.88820767	0.85379612	478774/489165	GALNT3, GALNT10	2	ACOR_VSMC-up
cfa00350	Tyrosine metabolism	2/407	32/7854	0.49961681	0.88820767	0.85379612	403451/403450	MAOB, MAOA	2	ACOR_VSMC-up
cfa04215	Apoptosis - multiple species	2/407	32/7854	0.49961681	0.88820767	0.85379612	403416/475718	BCL2, BIRC6	2	ACOR_VSMC-up
cfa05020	Prion diseases	2/407	33/7854	0.51616592	0.88929092	0.8548374	489221/403522	C7, CCL5	2	ACOR_VSMC-up
cfa04940	Type I diabetes mellitus	2/407	36/7854	0.56358923	0.90174277	0.86680683	475492/474836	CPE, DLA88	2	ACOR_VSMC-up
cfa05340	Primary immunodeficiency	2/407	36/7854	0.56358923	0.90174277	0.86680683	477236/486814	ADA, BLNK	2	ACOR_VSMC-up
cfa00250	Alanine, aspartate and glutamate metabolism	2/407	37/7854	0.5786393	0.90408536	0.86905866	478540/486698	NIT2, RIMKLB	2	ACOR_VSMC-up
cfa05216	Thyroid cancer	2/407	38/7854	0.59330602	0.90408536	0.86905866	479180/474890	POLK, CDKN1A	2	ACOR_VSMC-up
cfa03440	Homologous recombination	2/407	41/7854	0.63500375	0.91668454	0.88116972	475711/611847	BABAM2,	2	ACOR_VSMC-up
cfa00072	Synthesis and degradation of ketone bodies	2/388	2174899	0.06961648	0.54125816	0.50690586	479344/484063	, ACAT2	2	ACOR_VSMC-down
cfa00533	Glycosaminoglycan biosynthesis - keratan sulfate	2/388	15/7854	0.16762747	0.88254287	0.82653009	474611/481579	B3GNT2, B4GALT1	2	ACOR_VSMC-down
cfa01210	2-Oxocarboxylic acid metabolism	2/388	17/7854	0.20392219	0.99166085	0.92872262	486633/486059	BCAT1, AADAT	2	ACOR_VSMC-down
cfa00670	One carbon pool by folate	2/388	20/7854	0.25961598	0.99972301	0.9362731	480352/607417	MTHFD1, TYMS	2	ACOR_VSMC-down
cfa04964	Proximal tubule bicarbonate reclamation	2/388	21/7854	0.27827989	0.99972301	0.9362731	100855592/477098	, ATP1B3	2	ACOR_VSMC-down
cfa00040	Pentose and glucuronate interconversions	2/388	22/7854	0.29691127	0.99972301	0.9362731	607537/479107	AKR1B1, UGDH	2	ACOR_VSMC-down
cfa00534	Glycosaminoglycan biosynthesis - heparan sulfate / heparin	2/388	24/7854	0.33391174	0.99972301	0.9362731	494008/489975	XYLT1, HS3ST2	2	ACOR_VSMC-down
cfa00650	Butanoate metabolism	2/388	25/7854	0.35220872	0.99972301	0.9362731	479344/484063	, ACAT2	2	ACOR_VSMC-down
cfa00790	Folate biosynthesis	2/388	25/7854	0.35220872	0.99972301	0.9362731	607537/100856436	AKR1B1, GGH	2	ACOR_VSMC-down
cfa00062	Fatty acid elongation	2/388	26/7854	0.37032838	0.99972301	0.9362731	100855761/487900	ELOVL1, ELOVL6	2	ACOR_VSMC-down
cfa04977	Vitamin digestion and absorption	2/388	26/7854	0.37032838	0.99972301	0.9362731	475698/476775	SLC5A6, FOLH1	2	ACOR_VSMC-down
cfa03020	RNA polymerase	2/388	28/7854	0.40593038	0.99972301	0.9362731	477143/474912	POLR3F, POLR1C	2	ACOR_VSMC-down
cfa04392	Hippo signaling pathway - multiple species	2/388	28/7854	0.40593038	0.99972301	0.9362731	607885/477535	PAK1, NF2	2	ACOR_VSMC-down
cfa00601	Glycosphingolipid biosynthesis - lacto and neolacto series	2/388	29/7854	0.42336754	0.99972301	0.9362731	474611/481579	B3GNT2, B4GALT1	2	ACOR_VSMC-down
cfa01523	Antifolate resistance	2/388	31/7854	0.45742142	0.99972301	0.9362731	607417/100856436	TYMS, GGH	2	ACOR_VSMC-down
cfa00640	Propanoate metabolism	2/388	34/7854	0.50624218	0.99972301	0.9362731	475775/484063	SUCLG1, ACAT2	2	ACOR_VSMC-down
cfa00250	Alanine, aspartate and glutamate metabolism	2/388	37/7854	0.5521207	0.99972301	0.9362731	483009/476775	CAD, FOLH1	2	ACOR_VSMC-down
cfa04960	Aldosterone-regulated sodium reabsorption	2/388	37/7854	0.5521207	0.99972301	0.9362731	477098/489644	ATP1B3, PIK3CD	2	ACOR_VSMC-down
cfa05216	Thyroid cancer	2/388	38/7854	0.56673196	0.99972301	0.9362731	403872/480137	NRAS, TPM3	2	ACOR_VSMC-down
cfa00513	Various types of N-glycan biosynthesis	2/388	40/7854	0.59491437	0.99972301	0.9362731	481579/476275	B4GALT1, MAN1A1	2	ACOR_VSMC-down
cfa00380	Tryptophan metabolism	2/388	41/7854	0.60848345	0.99972301	0.9362731	486059/484063	AADAT, ACAT2	2	ACOR_VSMC-down
cfa05219	Bladder cancer	2/388	41/7854	0.60848345	0.99972301	0.9362731	481131/403872	CDK4, NRAS	2	ACOR_VSMC-down
cfa04973	Carbohydrate digestion and absorption	2/388	43/7854	0.63457903	0.99972301	0.9362731	477098/489644	ATP1B3, PIK3CD	2	ACOR_VSMC-down
cfa00514	Other types of O-glycan biosynthesis	2/388	47/7854	0.68265685	0.99972301	0.9362731	484834/481579	COLGALT1, B4GALT1	2	ACOR_VSMC-down
cfa04915	Estrogen signaling pathway	42736	134/7854	0.25386375	0.32364194	0.30122906	482994	ADCY3	1	VFEM_EC-up
cfa00510	N-Glycan biosynthesis	2/388	50/7854	0.71522566	0.99972301	0.9362731	481579/476275	B4GALT1, MAN1A1	2	ACOR_VSMC-down

cfa04915	Estrogen signaling pathway	44013	134/7854	0.11352875	0.1508831	0.12145389	403640 ESR1		1	AORT_VSMC-up
cfa04923	Regulation of lipolysis in adipocytes	2/388	54/7854	0.75424629	0.99972301	0.9362731	485570/489644 ABHD5, PIK3CD		2	ACOR_VSMC-down
cfa04623	Cytosolic DNA-sensing pathway	2/388	57/7854	0.78041059	0.99972301	0.9362731	477143/474912 POLR3F, POLR1C		2	ACOR_VSMC-down
cfa04213	Longevity regulating pathway - multiple species	2/388	59/7854	0.79647028	0.99972301	0.9362731	403872/489644 NRAS, PIK3CD		2	ACOR_VSMC-down
cfa05213	Endometrial cancer	2/388	59/7854	0.79647028	0.99972301	0.9362731	403872/489644 NRAS, PIK3CD		2	ACOR_VSMC-down
cfa04137	Mitophagy - animal	2/388	64/7854	0.83215514	0.99972301	0.9362731	486221/403872 PGAM5, NRAS		2	ACOR_VSMC-down
cfa04720	Long-term potentiation	2/388	66/7854	0.84477903	0.99972301	0.9362731	403872/481746 NRAS, ITPR3		2	ACOR_VSMC-down
cfa04976	Bile secretion	2/388	66/7854	0.84477903	0.99972301	0.9362731	479182/477098 HMGCR, ATP1B3		2	ACOR_VSMC-down
cfa04924	Renin secretion	2/388	69/7854	0.86210528	0.99972301	0.9362731	610836/481746 PDE3A, ITPR3		2	ACOR_VSMC-down
cfa05144	Malaria	42401	49/7854	0.00432758	0.09953442	0.08882934	476665/448807 COMP, TLR2		2	VSAP_VSMC-up
cfa04610	Complement and coagulation cascades	42401	80/7854	0.01120903	0.1289039	0.11504009	488121/100685858 MASP1, F5		2	VSAP_VSMC-up
cfa04145	Phagosome	42401	138/7854	0.03130954	0.22413514	0.20002907	476665/448807 COMP, TLR2		2	VSAP_VSMC-up
cfa04510	Focal adhesion	42401	198/7854	0.0601903	0.28232016	0.25195621	476665/403980 COMP, CAV1		2	VSAP_VSMC-up
cfa05225	Hepatocellular carcinoma	43892	165/7854	0.00129799	0.0389396	0.01366302	481628/477558 TGFBR1,		2	VSAP_VSMC-down
cfa05224	Breast cancer	44014	142/7854	0.00642256	0.1508831	0.12145389	607180/403640 WNT7A, ESR1		2	AORT_VSMC-up
cfa05205	Proteoglycans in cancer	44014	202/7854	0.01269421	0.1508831	0.12145389	607180/403640 WNT7A, ESR1		2	AORT_VSMC-up
cfa00980	Metabolism of xenobiotics by cytochrome P450	44014	52/7854	0.00088397	0.01878532	0.01248886	100686778/477558 CYP1A1,		2	VCAV_VSMC-up
cfa05204	Chemical carcinogenesis	44014	55/7854	0.0009887	0.01878532	0.01248886	100686778/477558 CYP1A1,		2	VCAV_VSMC-up
cfa04151	PI3K-Akt signaling pathway	44106	354/7854	0.07175802	0.25090823	0.25090823	608459/403466 FGF5, COL4A5		2	VPOR_VSMC-up
cfa00780	Biotin metabolism	1/131	2174715	0.04921439	0.36641541	0.33603943	477059 BTD		1	ACOR_EC-up
cfa00440	Phosphonate and phosphinate metabolism	1/131	2174807	0.09602431	0.44747328	0.41037758	610214 CHPT1		1	ACOR_EC-up
cfa00430	Taurine and hypotaurine metabolism	1/131	13/7854	0.19654131	0.57996459	0.5318853		486400	1	ACOR_EC-up
cfa00730	Thiamine metabolism	1/131	14/7854	0.20996473	0.59660711	0.54714815	480429 AK7		1	ACOR_EC-up
cfa00533	Glycosaminoglycan biosynthesis - keratan sulfate	1/131	15/7854	0.22316558	0.60001444	0.55027301	486158 B3GNT7		1	ACOR_EC-up
cfa00910	Nitrogen metabolism	1/131	17/7854	0.24891411	0.60001444	0.55027301	480591 CA4		1	ACOR_EC-up
cfa00592	alpha-Linolenic acid metabolism	1/131	22/7854	0.30963945	0.6742616	0.61836505	478514 CFI		1	ACOR_EC-up
cfa03430	Mismatch repair	1/131	23/7854	0.32118659	0.68154985	0.6250491		611847	1	ACOR_EC-up
cfa00062	Fatty acid elongation	1/131	26/7854	0.35469046	0.68154985	0.6250491		607926	1	ACOR_EC-up
cfa00591	Linoleic acid metabolism	1/131	26/7854	0.35469046	0.68154985	0.6250491	478514 CFI		1	ACOR_EC-up
cfa04977	Vitamin digestion and absorption	1/131	26/7854	0.35469046	0.68154985	0.6250491	477059 BTD		1	ACOR_EC-up
cfa04744	Phototransduction	1/131	28/7854	0.37610935	0.70012408	0.64208353	485693 GRK7		1	ACOR_EC-up
cfa00030	Pentose phosphate pathway	1/131	29/7854	0.3865527	0.70025305	0.6422018	480622 ALDOC		1	ACOR_EC-up
cfa00512	Mucin type O-glycan biosynthesis	1/131	31/7854	0.40692176	0.70755799	0.64890116	488359 GALNT5		1	ACOR_EC-up
cfa00051	Fructose and mannose metabolism	1/131	32/7854	0.41685315	0.70895463	0.65018202	480622 ALDOC		1	ACOR_EC-up
cfa04136	Autophagy - other	1/131	32/7854	0.41685315	0.70895463	0.65018202		478564	1	ACOR_EC-up
cfa00640	Propanoate metabolism	1/131	34/7854	0.43622348	0.72071968	0.66097174	475414 ACSS3		1	ACOR_EC-up
cfa03410	Base excision repair	1/131	34/7854	0.43622348	0.72071968	0.66097174		611847	1	ACOR_EC-up
cfa03030	DNA replication	1/131	36/7854	0.45495513	0.72788738	0.66754524		611847	1	ACOR_EC-up
cfa00250	Alanine, aspartate and glutamate metabolism	1/131	37/7854	0.46408801	0.72788738	0.66754524	478540 NIT2		1	ACOR_EC-up
cfa05143	African trypanosomiasis	1/131	38/7854	0.47306901	0.73408049	0.67322493	403982 VCAM1		1	ACOR_EC-up
cfa00260	Glycine, serine and threonine metabolism	1/131	40/7854	0.49058535	0.73408049	0.67322493	491277 SARDH		1	ACOR_EC-up
cfa00071	Fatty acid degradation	1/131	41/7854	0.49912557	0.73408049	0.67322493		607926	1	ACOR_EC-up
cfa03440	Homologous recombination	1/131	41/7854	0.49912557	0.73408049	0.67322493		611847	1	ACOR_EC-up
cfa05219	Bladder cancer	1/131	41/7854	0.49912557	0.73408049	0.67322493	485864 SRC		1	ACOR_EC-up

cfa03420	Nucleotide excision repair	1/131	43/7854	0.51578207	0.73728357	0.67616248	611847	1	ACOR_EC-up
cfa04913	Ovarian steroidogenesis	1/131	46/7854	0.53974155	0.74756244	0.68558922	403556 PRKACA	1	ACOR_EC-up
cfa00330	Arginine and proline metabolism	1/131	47/7854	0.54746361	0.74756244	0.68558922	478144 AZIN2	1	ACOR_EC-up
cfa00514	Other types of O-glycan biosynthesis	1/131	47/7854	0.54746361	0.74756244	0.68558922	488359 GALNT5	1	ACOR_EC-up
cfa00280	Valine, leucine and isoleucine degradation	1/131	48/7854	0.55505709	0.74756244	0.68558922	607926	1	ACOR_EC-up
cfa00600	Sphingolipid metabolism	1/131	48/7854	0.55505709	0.74756244	0.68558922	607962 PLPP1	1	ACOR_EC-up
cfa02010	ABC transporters	1/131	48/7854	0.55505709	0.74756244	0.68558922	477456 ABCB9	1	ACOR_EC-up
cfa05030	Cocaine addiction	1/131	48/7854	0.55505709	0.74756244	0.68558922	403556 PRKACA	1	ACOR_EC-up
cfa00830	Retinol metabolism	1/131	49/7854	0.5625241	0.75326504	0.69081907	484491 CYP2S1	1	ACOR_EC-up
cfa05134	Legionellosis	1/131	50/7854	0.56986675	0.75442587	0.69188367	476728 C3	1	ACOR_EC-up
cfa04612	Antigen processing and presentation	1/131	54/7854	0.59803424	0.77412209	0.70994708	481740 TAPBP	1	ACOR_EC-up
cfa05014	Amyotrophic lateral sclerosis (ALS)	1/131	56/7854	0.61142363	0.78708125	0.72183191	403416 BCL2	1	ACOR_EC-up
cfa04512	ECM-receptor interaction	9/279	88/7854	0.00387551	0.05076914	0.04303853	489208/608287/477956/484087/479574/488437/403939/403788/485893 ITGA2, ITGA10, ITGB1, THBS2, AGRN, ITGAV, CD44, ITGB3, SDC4	9	VSAP_EC-down
cfa05321	Inflammatory bowel disease (IBD)	1/131	61/7854	0.64299194	0.80099827	0.7345952	487286 NOD2	1	ACOR_EC-up
cfa00010	Glycolysis / Gluconeogenesis	1/131	62/7854	0.64899323	0.80099827	0.7345952	480622 ALDOC	1	ACOR_EC-up
cfa04927	Cortisol synthesis and secretion	1/131	63/7854	0.65489439	0.80099827	0.7345952	403556 PRKACA	1	ACOR_EC-up
cfa05217	Basal cell carcinoma	1/131	63/7854	0.65489439	0.80099827	0.7345952	100855544 FZD5	1	ACOR_EC-up
cfa04137	Mitophagy - animal	1/131	64/7854	0.66069709	0.80099827	0.7345952	485864 SRC	1	ACOR_EC-up
cfa04929	GnRH secretion	1/131	65/7854	0.66640296	0.80099827	0.7345952	100856339	1	ACOR_EC-up
cfa00970	Aminoacyl-tRNA biosynthesis	1/131	66/7854	0.67201359	0.80099827	0.7345952	474833 VARS2	1	ACOR_EC-up
cfa04720	Long-term potentiation	1/131	66/7854	0.67201359	0.80099827	0.7345952	403556 PRKACA	1	ACOR_EC-up
cfa05230	Central carbon metabolism in cancer	1/131	66/7854	0.67201359	0.80099827	0.7345952	100856339	1	ACOR_EC-up
cfa04664	Fc epsilon RI signaling pathway	1/131	67/7854	0.67753057	0.80099827	0.7345952	100856339	1	ACOR_EC-up
cfa05031	Amphetamine addiction	1/131	67/7854	0.67753057	0.80099827	0.7345952	403556 PRKACA	1	ACOR_EC-up
cfa05221	Acute myeloid leukemia	1/131	67/7854	0.67753057	0.80099827	0.7345952	100856339	1	ACOR_EC-up
cfa05223	Non-small cell lung cancer	1/131	67/7854	0.67753057	0.80099827	0.7345952	100856339	1	ACOR_EC-up
cfa04115	p53 signaling pathway	1/131	69/7854	0.68828974	0.80099827	0.7345952	403416 BCL2	1	ACOR_EC-up
cfa00920	Sulfur metabolism	44197	2174929	0.0264335	0.1938457	0.16694845	486463 PAPSS2	1	ACOR_EC-down
cfa00450	Selenocompound metabolism	44197	16/7854	0.04197275	0.27309434	0.23520086	486463 PAPSS2	1	ACOR_EC-down
cfa00532	Glycosaminoglycan biosynthesis - chondroitin sulfate / dermatan sulfate	44197	19/7854	0.04965352	0.27309434	0.23520086	482831 CSGALNACT1	1	ACOR_EC-down
cfa04614	Renin-angiotensin system	44197	24/7854	0.06232462	0.31641731	0.27251251	403913 ANPEP	1	ACOR_EC-down
cfa04130	SNARE interactions in vesicular transport	44197	36/7854	0.09208208	0.34415573	0.29640206	489919 STX4	1	ACOR_EC-down
cfa00250	Alanine, aspartate and glutamate metabolism	44197	37/7854	0.09452085	0.34415573	0.29640206	474499 TNRC6B	1	ACOR_EC-down
cfa04512	ECM-receptor interaction	9/241	88/7854	0.00143627	0.05044899	0.0453559	608977/485893/403824/610359/403668/100856491/480173/403651/403582 ITGB5, SDC4, COL1A2, THBS3, COL6A1, COL6A2, LAMA3, COL1A1, COL6A3	9	VCAV_EC-down
cfa04913	Ovarian steroidogenesis	44197	46/7854	0.11619077	0.34415573	0.29640206	442951 IGF1R	1	ACOR_EC-down
cfa03460	Fanconi anemia pathway	44197	49/7854	0.12330367	0.34415573	0.29640206	608504 ATRIP	1	ACOR_EC-down
cfa04512	ECM-receptor interaction	7/357	88/7854	0.10460331	0.28061851	0.20539517	483273/399544/442939/403466/100856563/403788/475034 RELN, VWF, LAMC2, COL4A5, ITGA6, ITGB3, LAMA4	7	VCAV_EC-up
cfa00480	Glutathione metabolism	44197	52/7854	0.13036202	0.34415573	0.29640206	403913 ANPEP	1	ACOR_EC-down
cfa00561	Glycerolipid metabolism	44197	59/7854	0.14662163	0.35295632	0.30398152	483023 LCLAT1	1	ACOR_EC-down
cfa04213	Longevity regulating pathway - multiple species	44197	59/7854	0.14662163	0.35295632	0.30398152	442951 IGF1R	1	ACOR_EC-down
cfa04664	Fc epsilon RI signaling pathway	44197	67/7854	0.1648495	0.35295632	0.30398152	477886 LYN	1	ACOR_EC-down
cfa04520	Adherens junction	44197	70/7854	0.17158886	0.35295632	0.30398152	442951 IGF1R	1	ACOR_EC-down

cfa04662	B cell receptor signaling pathway	44197	72/7854	0.17605297	0.35295632	0.30398152	477886 LYN	1	ACOR_EC-down
cfa04918	Thyroid hormone synthesis	44197	72/7854	0.17605297	0.35295632	0.30398152	100684320 TTF2	1	ACOR_EC-down
cfa05218	Melanoma	44197	73/7854	0.17827642	0.35295632	0.30398152	442951 IGF1R	1	ACOR_EC-down
cfa05214	Glioma	44197	75/7854	0.18270618	0.35295632	0.30398152	442951 IGF1R	1	ACOR_EC-down
cfa01521	EGFR tyrosine kinase inhibitor resistance	44197	80/7854	0.19368132	0.35295632	0.30398152	442951 IGF1R	1	ACOR_EC-down
cfa04211	Longevity regulating pathway	44197	88/7854	0.21095011	0.35295632	0.30398152	442951 IGF1R	1	ACOR_EC-down
cfa04914	Progesterone-mediated oocyte maturation	44197	89/7854	0.21308378	0.35295632	0.30398152	442951 IGF1R	1	ACOR_EC-down
cfa04666	Fc gamma R-mediated phagocytosis	44197	90/7854	0.21521195	0.35295632	0.30398152	477886 LYN	1	ACOR_EC-down
cfa04974	Protein digestion and absorption	44197	91/7854	0.21733464	0.35295632	0.30398152	607775 ELN	1	ACOR_EC-down
cfa01522	Endocrine resistance	44197	93/7854	0.22156362	0.35295632	0.30398152	442951 IGF1R	1	ACOR_EC-down
cfa04064	NF-kappa B signaling pathway	44197	93/7854	0.22156362	0.35295632	0.30398152	477886 LYN	1	ACOR_EC-down
cfa04640	Hematopoietic cell lineage	44197	93/7854	0.22156362	0.35295632	0.30398152	403913 ANPEP	1	ACOR_EC-down
cfa00564	Glycerophospholipid metabolism	44197	95/7854	0.22577084	0.35295632	0.30398152	483023 LCLAT1	1	ACOR_EC-down
cfa05215	Prostate cancer	44197	97/7854	0.22995639	0.35295632	0.30398152	442951 IGF1R	1	ACOR_EC-down
cfa04066	HIF-1 signaling pathway	44197	112/7854	0.2606682	0.38231336	0.32926509	442951 IGF1R	1	ACOR_EC-down
cfa04114	Oocyte meiosis	44197	117/7854	0.27064361	0.38235335	0.32929954	442951 IGF1R	1	ACOR_EC-down
cfa04152	AMPK signaling pathway	44197	121/7854	0.27853152	0.38235335	0.32929954	442951 IGF1R	1	ACOR_EC-down
cfa04512	ECM-receptor interaction	6/407	88/7854	0.30481766	0.82260001	0.79073027	478552/487891/486002/479574/476623/480173 CD47, NPNT, FREM2, AGRN, DAG1, LAMA3	6	ACOR_VSMC-up
cfa04068	FoxO signaling pathway	44197	127/7854	0.29021122	0.38235335	0.32929954	442951 IGF1R	1	ACOR_EC-down
cfa04910	Insulin signaling pathway	44197	134/7854	0.30360995	0.38235335	0.32929954	100685147 PRKAR2A	1	ACOR_EC-down
cfa04550	Signaling pathways regulating pluripotency of stem cells	44197	135/7854	0.30550427	0.38235335	0.32929954	442951 IGF1R	1	ACOR_EC-down
cfa04140	Autophagy - animal	44197	136/7854	0.30739369	0.38235335	0.32929954	442951 IGF1R	1	ACOR_EC-down
cfa03010	Ribosome	44197	139/7854	0.31303261	0.38235335	0.32929954	477029 RPSA	1	ACOR_EC-down
cfa04120	Ubiquitin mediated proteolysis	44197	139/7854	0.31303261	0.38235335	0.32929954	608738 NEDD4	1	ACOR_EC-down
cfa05224	Breast cancer	44197	142/7854	0.31862779	0.38235335	0.32929954	442951 IGF1R	1	ACOR_EC-down
cfa04150	mTOR signaling pathway	44197	152/7854	0.33696662	0.39713923	0.34203379	442951 IGF1R	1	ACOR_EC-down
cfa05225	Hepatocellular carcinoma	44197	165/7854	0.36010515	0.41696386	0.35910763	442951 IGF1R	1	ACOR_EC-down
cfa05202	Transcriptional misregulation in cancer	44197	172/7854	0.37224372	0.42024258	0.36193141	442951 IGF1R	1	ACOR_EC-down
cfa04062	Chemokine signaling pathway	44197	174/7854	0.3756714	0.42024258	0.36193141	477886 LYN	1	ACOR_EC-down
cfa05167	Kaposi sarcoma-associated herpesvirus infection	44197	178/7854	0.38247336	0.42072069	0.36234318	477886 LYN	1	ACOR_EC-down
cfa05203	Viral carcinogenesis	44197	189/7854	0.40081674	0.43367057	0.37349618	477886 LYN	1	ACOR_EC-down
cfa04510	Focal adhesion	44197	198/7854	0.41543779	0.44191335	0.38059523	442951 IGF1R	1	ACOR_EC-down
cfa05205	Proteoglycans in cancer	44197	202/7854	0.42182638	0.44191335	0.38059523	442951 IGF1R	1	ACOR_EC-down
cfa04014	Ras signaling pathway	44197	225/7854	0.45728978	0.47158009	0.40614553	442951 IGF1R	1	ACOR_EC-down
cfa04060	Cytokine-cytokine receptor interaction	44197	270/7854	0.52077239	0.52878427	0.45541229	477603 ACVRL1	1	ACOR_EC-down
cfa04010	MAPK signaling pathway	44197	288/7854	0.54412955	0.54412955	0.46862832	442951 IGF1R	1	ACOR_EC-down
cfa00232	Caffeine metabolism	1/441	2174715	0.15918696	0.61400684	0.55274761	483028 XDH	1	VSAP_EC-up
cfa00290	Valine, leucine and isoleucine biosynthesis	1/441	2174715	0.15918696	0.61400684	0.55274761	484394 BCAT2	1	VSAP_EC-up
cfa00400	Phenylalanine, tyrosine and tryptophan biosynthesis	1/441	2174807	0.29308166	0.75621155	0.68076461	486834 GOT1	1	VSAP_EC-up
cfa00750	Vitamin B6 metabolism	1/441	2174837	0.33280528	0.80885538	0.72815618	480540 PNPO	1	VSAP_EC-up
cfa04122	Sulfur relay system	1/441	2174868	0.3703015	0.8592152	0.77349162	474515 MPST	1	VSAP_EC-up
cfa00130	Ubiquinone and other terpenoid-quinone biosynthesis	1/441	2174960	0.47063766	0.92381513	0.83164644	610935 NQO1	1	VSAP_EC-up
cfa00603	Glycosphingolipid biosynthesis - globo and isoglobo series	1/441	16/7854	0.60367235	0.96551148	0.86918276	481222 A4GALT	1	VSAP_EC-up
cfa00770	Pantothenate and CoA biosynthesis	1/441	17/7854	0.62597147	0.97336925	0.87625657	484394 BCAT2	1	VSAP_EC-up

cfa00100	Steroid biosynthesis	1/441	20/7854	0.6856337	0.99864063	0.89900663	490325 SOAT1	1	VSAP_EC-up
cfa00531	Glycosaminoglycan degradation	1/441	20/7854	0.6856337	0.99864063	0.89900663	608707 HPSE	1	VSAP_EC-up
cfa00290	Valine, leucine and isoleucine biosynthesis	1/279	2174715	0.10284164	0.38701439	0.32808371	486633 BCAT1	1	VSAP_EC-down
cfa00440	Phosphonate and phosphinate metabolism	1/279	2174807	0.19514087	0.51223831	0.43423979	100302747 SELENOI	1	VSAP_EC-down
cfa00750	Vitamin B6 metabolism	1/279	2174837	0.22375398	0.53783066	0.45593519	476318 PSAT1	1	VSAP_EC-down
cfa00740	Riboflavin metabolism	1/279	2174868	0.2513534	0.57767184	0.48970976	100856150 ENPP1	1	VSAP_EC-down
cfa00730	Thiamine metabolism	1/279	14/7854	0.39758079	0.72002051	0.610383	490204 AK5	1	VSAP_EC-down
cfa00450	Selenocompound metabolism	1/279	16/7854	0.43969678	0.73430489	0.62249229	478504 PAPSS1	1	VSAP_EC-down
cfa01210	2-Oxocarboxylic acid metabolism	1/279	17/7854	0.45964123	0.73430489	0.62249229	486633 BCAT1	1	VSAP_EC-down
cfa04614	Renin-angiotensin system	1/279	24/7854	0.58078336	0.83607276	0.70876397	476788 PRCP	1	VSAP_EC-down
cfa04977	Vitamin digestion and absorption	1/279	26/7854	0.61012815	0.84772198	0.71863937	486241 SCARB1	1	VSAP_EC-down
cfa04392	Hippo signaling pathway - multiple species	1/279	28/7854	0.63742568	0.84772198	0.71863937	607885 PAK1	1	VSAP_EC-down
cfa04744	Phototransduction	1/279	28/7854	0.63742568	0.84772198	0.71863937	480416 CALM1	1	VSAP_EC-down
cfa00030	Pentose phosphate pathway	1/279	29/7854	0.6503516	0.84772198	0.71863937	480622 ALDOC	1	VSAP_EC-down
NA	NA	NA	NA	NA	NA	NA	NA NA	NA	AFEM_EC-up
NA	NA	NA	NA	NA	NA	NA	NA NA	NA	AFEM_EC-up
NA	NA	NA	NA	NA	NA	NA	NA NA	NA	AFEM_EC-up
NA	NA	NA	NA	NA	NA	NA	NA NA	NA	AFEM_EC-up
NA	NA	NA	NA	NA	NA	NA	NA NA	NA	AFEM_EC-up
NA	NA	NA	NA	NA	NA	NA	NA NA	NA	AFEM_EC-up
NA	NA	NA	NA	NA	NA	NA	NA NA	NA	AFEM_EC-up
NA	NA	NA	NA	NA	NA	NA	NA NA	NA	AFEM_EC-up
NA	NA	NA	NA	NA	NA	NA	NA NA	NA	AFEM_EC-up
NA	NA	NA	NA	NA	NA	NA	NA NA	NA	AFEM_EC-up
NA	NA	NA	NA	NA	NA	NA	NA NA	NA	AFEM_EC-up
NA	NA	NA	NA	NA	NA	NA	NA NA	NA	AFEM_EC-up
NA	NA	NA	NA	NA	NA	NA	NA NA	NA	AFEM_EC-up
NA	NA	NA	NA	NA	NA	NA	NA NA	NA	AFEM_EC-up
NA	NA	NA	NA	NA	NA	NA	NA NA	NA	AFEM_EC-up
NA	NA	NA	NA	NA	NA	NA	NA NA	NA	AFEM_EC-up
NA	NA	NA	NA	NA	NA	NA	NA NA	NA	AFEM_EC-up
NA	NA	NA	NA	NA	NA	NA	NA NA	NA	AFEM_EC-up
NA	NA	NA	NA	NA	NA	NA	NA NA	NA	AFEM_EC-up
NA	NA	NA	NA	NA	NA	NA	NA NA	NA	AFEM_EC-up
NA	NA	NA	NA	NA	NA	NA	NA NA	NA	AFEM_EC-up
cfa04614	Renin-angiotensin system	44105	24/7854	0.03015793	0.06031586	0.02645433	488896 LNPEP	1	AORT_EC-up
cfa04216	Ferroptosis	44105	45/7854	0.05587193	0.0838079	0.03675785	102155200	1	AORT_EC-up
cfa04512	ECM-receptor interaction	5/388	88/7854	0.44070436	0.99972301	0.9362731	482986/611838/403788/475882/489208 SDC1, HMMR, ITGB3, LAMB1, ITGA2	5	ACOR_VSMC-down
cfa04064	NF-kappa B signaling pathway	44105	93/7854	0.11235763	0.13482916	0.0591356	486753 ERC1	1	AORT_EC-up
cfa04217	Necroptosis	44105	160/7854	0.18611871	0.19873108	0.08716275	102155200	1	AORT_EC-up
cfa05202	Transcriptional misregulation in cancer	44105	172/7854	0.19873108	0.19873108	0.08716275	482372 HOXA9	1	AORT_EC-up
cfa04970	Salivary secretion	43831	78/7854	0.00993125	0.00993125	NA	607874 CST3	1	AORT_EC-down
cfa00512	Mucin type O-glycan biosynthesis	42736	31/7854	0.0650863	0.31221218	0.27021873	489790 GALNT17	1	APUL_EC-up
cfa04215	Apoptosis - multiple species	42736	32/7854	0.06711794	0.31221218	0.27021873	478464 MAPK10	1	APUL_EC-up
cfa05219	Bladder cancer	42736	41/7854	0.08521666	0.31221218	0.27021873	489428 MMP1	1	APUL_EC-up
cfa04930	Type II diabetes mellitus	42736	46/7854	0.09512818	0.31221218	0.27021873	478464 MAPK10	1	APUL_EC-up
cfa00514	Other types of O-glycan biosynthesis	42736	47/7854	0.09709831	0.31221218	0.27021873	489790 GALNT17	1	APUL_EC-up
cfa04137	Mitophagy - animal	42736	64/7854	0.12997915	0.31221218	0.27021873	478464 MAPK10	1	APUL_EC-up
cfa04976	Bile secretion	42736	66/7854	0.13377251	0.31221218	0.27021873	612928 NR1H4	1	APUL_EC-up
cfa04622	RIG-I-like receptor signaling pathway	42736	67/7854	0.13566336	0.31221218	0.27021873	478464 MAPK10	1	APUL_EC-up
cfa04664	Fc epsilon RI signaling pathway	42736	67/7854	0.13566336	0.31221218	0.27021873	478464 MAPK10	1	APUL_EC-up
cfa04924	Renin secretion	42736	69/7854	0.13943339	0.31221218	0.27021873	403652 PDE1A	1	APUL_EC-up
cfa04917	Prolactin signaling pathway	42736	70/7854	0.1413126	0.31221218	0.27021873	478464 MAPK10	1	APUL_EC-up

cfa04920	Adipocytokine signaling pathway	42736	70/7854	0.1413126	0.31221218	0.27021873	478464 MAPK10	1	APUL_EC-up
cfa05133	Pertussis	42736	71/7854	0.14318794	0.31221218	0.27021873	478464 MAPK10	1	APUL_EC-up
cfa05100	Bacterial invasion of epithelial cells	42736	75/7854	0.15065084	0.31221218	0.27021873	611857 ARHGEF26	1	APUL_EC-up
cfa05212	Pancreatic cancer	42736	75/7854	0.15065084	0.31221218	0.27021873	478464 MAPK10	1	APUL_EC-up
cfa04742	Taste transduction	42736	77/7854	0.15435931	0.31221218	0.27021873	403652 PDE1A	1	APUL_EC-up
cfa03320	PPAR signaling pathway	42736	80/7854	0.15989345	0.31221218	0.27021873	489428 MMP1	1	APUL_EC-up
cfa05323	Rheumatoid arthritis	42736	83/7854	0.16539349	0.31221218	0.27021873	489428 MMP1	1	APUL_EC-up
cfa04012	ErbB signaling pathway	42736	85/7854	0.16904133	0.31221218	0.27021873	478464 MAPK10	1	APUL_EC-up
cfa05210	Colorectal cancer	42736	85/7854	0.16904133	0.31221218	0.27021873	478464 MAPK10	1	APUL_EC-up
cfa04658	Th1 and Th2 cell differentiation	42736	87/7854	0.17267417	0.31221218	0.27021873	478464 MAPK10	1	APUL_EC-up
cfa04512	ECM-receptor interaction	34759	88/7854	0.09036311	0.81029448	0.80529119	100682877/476665/481689 TNXB, COMP, TNC	3	AFEM_EC-up
cfa04540	Gap junction	42736	88/7854	0.17448497	0.31221218	0.27021873	100855815 TUBB4A	1	APUL_EC-up
cfa04912	GnRH signaling pathway	42736	89/7854	0.17629205	0.31221218	0.27021873	478464 MAPK10	1	APUL_EC-up
cfa04914	Progesterone-mediated oocyte maturation	42736	89/7854	0.17629205	0.31221218	0.27021873	478464 MAPK10	1	APUL_EC-up
cfa04727	GABAergic synapse	42736	91/7854	0.17989504	0.31221218	0.27021873	478858 PLCL1	1	APUL_EC-up
cfa05032	Morphine addiction	42736	92/7854	0.18169097	0.31221218	0.27021873	403652 PDE1A	1	APUL_EC-up
cfa01522	Endocrine resistance	42736	93/7854	0.18348319	0.31221218	0.27021873	478464 MAPK10	1	APUL_EC-up
cfa05017	Spinocerebellar ataxia	42736	96/7854	0.18883773	0.31221218	0.27021873	478464 MAPK10	1	APUL_EC-up
cfa05231	Choline metabolism in cancer	42736	96/7854	0.18883773	0.31221218	0.27021873	478464 MAPK10	1	APUL_EC-up
cfa04625	C-type lectin receptor signaling pathway	42736	99/7854	0.1941592	0.31221218	0.27021873	478464 MAPK10	1	APUL_EC-up
cfa04750	Inflammatory mediator regulation of TRP channels	42736	100/7854	0.19592572	0.31221218	0.27021873	478464 MAPK10	1	APUL_EC-up
cfa04933	AGE-RAGE signaling pathway in diabetic complications	42736	100/7854	0.19592572	0.31221218	0.27021873	478464 MAPK10	1	APUL_EC-up
cfa04620	Toll-like receptor signaling pathway	42736	101/7854	0.19768858	0.31221218	0.27021873	478464 MAPK10	1	APUL_EC-up
cfa04659	Th17 cell differentiation	42736	102/7854	0.19944781	0.31221218	0.27021873	478464 MAPK10	1	APUL_EC-up
cfa04660	T cell receptor signaling pathway	42736	102/7854	0.19944781	0.31221218	0.27021873	478464 MAPK10	1	APUL_EC-up
cfa05142	Chagas disease (American trypanosomiasis)	42736	103/7854	0.20120341	0.31221218	0.27021873	478464 MAPK10	1	APUL_EC-up
cfa04668	TNF signaling pathway	42736	108/7854	0.20992717	0.31228691	0.27028341	478464 MAPK10	1	APUL_EC-up
cfa04931	Insulin resistance	42736	109/7854	0.21166113	0.31228691	0.27028341	478464 MAPK10	1	APUL_EC-up
cfa05145	Toxoplasmosis	42736	109/7854	0.21166113	0.31228691	0.27028341	478464 MAPK10	1	APUL_EC-up
cfa04935	Growth hormone synthesis, secretion and action	42736	116/7854	0.22369892	0.31249863	0.27046665	478464 MAPK10	1	APUL_EC-up
cfa05135	Yersinia infection	42736	117/7854	0.22540442	0.31249863	0.27046665	478464 MAPK10	1	APUL_EC-up
cfa04071	Sphingolipid signaling pathway	42736	122/7854	0.23387912	0.31249863	0.27046665	478464 MAPK10	1	APUL_EC-up
cfa04380	Osteoclast differentiation	42736	125/7854	0.23892198	0.31249863	0.27046665	478464 MAPK10	1	APUL_EC-up
cfa04068	FoxO signaling pathway	42736	127/7854	0.24226651	0.31249863	0.27046665	478464 MAPK10	1	APUL_EC-up
cfa00230	Purine metabolism	42736	130/7854	0.24725738	0.31249863	0.27046665	403652 PDE1A	1	APUL_EC-up
cfa04210	Apoptosis	42736	131/7854	0.24891411	0.31249863	0.27046665	478464 MAPK10	1	APUL_EC-up
cfa04728	Dopaminergic synapse	42736	132/7854	0.25056741	0.31249863	0.27046665	478464 MAPK10	1	APUL_EC-up
cfa04512	ECM-receptor interaction	42736	88/7854	0.17448497	0.31221218	0.27021873	487891 NPNT	1	APUL_EC-up
cfa04910	Insulin signaling pathway	42736	134/7854	0.25386375	0.31249863	0.27046665	478464 MAPK10	1	APUL_EC-up
cfa04550	Signaling pathways regulating pluripotency of stem cells	42736	135/7854	0.2555068	0.31249863	0.27046665	612954 ISL1	1	APUL_EC-up
cfa04140	Autophagy - animal	42736	136/7854	0.25714644	0.31249863	0.27046665	478464 MAPK10	1	APUL_EC-up
cfa05162	Measles	42736	136/7854	0.25714644	0.31249863	0.27046665	478464 MAPK10	1	APUL_EC-up
cfa04145	Phagosome	42736	138/7854	0.26041553	0.31249863	0.27046665	100855815 TUBB4A	1	APUL_EC-up
cfa04932	Non-alcoholic fatty liver disease (NAFLD)	42736	145/7854	0.27175103	0.32181043	0.27852599	478464 MAPK10	1	APUL_EC-up
cfa04723	Retrograde endocannabinoid signaling	42736	149/7854	0.27815484	0.32461942	0.28095716	478464 MAPK10	1	APUL_EC-up
cfa04921	Oxytocin signaling pathway	42736	151/7854	0.28133683	0.32461942	0.28095716	606992 MYL6B	1	APUL_EC-up

cfa04621	NOD-like receptor signaling pathway	42736	158/7854	0.29237015	0.32832569	0.28416493	478464 MAPK10	1	APUL_EC-up
cfa04141	Protein processing in endoplasmic reticulum	42736	160/7854	0.29549312	0.32832569	0.28416493	478464 MAPK10	1	APUL_EC-up
cfa04217	Necroptosis	42736	160/7854	0.29549312	0.32832569	0.28416493	478464 MAPK10	1	APUL_EC-up
cfa05161	Hepatitis B	42736	164/7854	0.30170019	0.33113436	0.28659582	478464 MAPK10	1	APUL_EC-up
cfa05034	Alcoholism	42736	171/7854	0.31243889	0.33475596	0.2897303	484147 NTRK2	1	APUL_EC-up
cfa05152	Tuberculosis	42736	171/7854	0.31243889	0.33475596	0.2897303	478464 MAPK10	1	APUL_EC-up
cfa05167	Kaposi sarcoma-associated herpesvirus infection	42736	178/7854	0.32302203	0.34202332	0.29602018	478464 MAPK10	1	APUL_EC-up
cfa05169	Epstein-Barr virus infection	42736	193/7854	0.3451861	0.36124127	0.31265326	478464 MAPK10	1	APUL_EC-up
cfa04510	Focal adhesion	42736	198/7854	0.35242107	0.3633688	0.31449464	478464 MAPK10	1	APUL_EC-up
cfa05170	Human immunodeficiency virus 1 infection	42736	200/7854	0.35529394	0.3633688	0.31449464	478464 MAPK10	1	APUL_EC-up
cfa05166	Human T-cell leukemia virus 1 infection	42736	209/7854	0.36807404	0.3722097	0.32214641	478464 MAPK10	1	APUL_EC-up
cfa04151	PI3K-Akt signaling pathway	42736	354/7854	0.54381525	0.54381525	0.47067051	484147 NTRK2	1	APUL_EC-up
cfa00290	Valine, leucine and isoleucine biosynthesis	1/357	2174715	0.13027503	0.31883099	0.23336431	486633 BCAT1	1	VCAV_EC-up
cfa00740	Riboflavin metabolism	1/357	2174868	0.31087321	0.57822417	0.42322388	484159 RFK	1	VCAV_EC-up
cfa00533	Glycosaminoglycan biosynthesis - keratan sulfate	1/357	15/7854	0.50263825	0.73039621	0.53460428	483642 CHST1	1	VCAV_EC-up
cfa00360	Phenylalanine metabolism	1/357	17/7854	0.54691069	0.77718781	0.5688528	100856346 ALDH1A3	1	VCAV_EC-up
cfa00290	Valine, leucine and isoleucine biosynthesis	1/241	2174715	0.0892702	0.4045956	0.36374955	484394 BCAT2	1	VCAV_EC-down
NA	NA	NA	NA	NA	NA	NA	NA NA	NA	AFEM_EC-down
NA	NA	NA	NA	NA	NA	NA	NA NA	NA	AFEM_EC-down
NA	NA	NA	NA	NA	NA	NA	NA NA	NA	AFEM_EC-down
NA	NA	NA	NA	NA	NA	NA	NA NA	NA	AFEM_EC-down
NA	NA	NA	NA	NA	NA	NA	NA NA	NA	AFEM_EC-down
NA	NA	NA	NA	NA	NA	NA	NA NA	NA	AFEM_EC-down
NA	NA	NA	NA	NA	NA	NA	NA NA	NA	AFEM_EC-down
NA	NA	NA	NA	NA	NA	NA	NA NA	NA	AFEM_EC-down
NA	NA	NA	NA	NA	NA	NA	NA NA	NA	AFEM_EC-down
NA	NA	NA	NA	NA	NA	NA	NA NA	NA	AFEM_EC-down
NA	NA	NA	NA	NA	NA	NA	NA NA	NA	AFEM_EC-down
NA	NA	NA	NA	NA	NA	NA	NA NA	NA	AFEM_EC-down
NA	NA	NA	NA	NA	NA	NA	NA NA	NA	AFEM_EC-down
NA	NA	NA	NA	NA	NA	NA	NA NA	NA	AFEM_EC-down
NA	NA	NA	NA	NA	NA	NA	NA NA	NA	AFEM_EC-down
NA	NA	NA	NA	NA	NA	NA	NA NA	NA	AFEM_EC-down
NA	NA	NA	NA	NA	NA	NA	NA NA	NA	AFEM_EC-down
NA	NA	NA	NA	NA	NA	NA	NA NA	NA	AFEM_EC-down
NA	NA	NA	NA	NA	NA	NA	NA NA	NA	AFEM_EC-down
NA	NA	NA	NA	NA	NA	NA	NA NA	NA	AFEM_EC-down
NA	NA	NA	NA	NA	NA	NA	NA NA	NA	AFEM_EC-down
NA	NA	NA	NA	NA	NA	NA	NA NA	NA	AFEM_EC-down
NA	NA	NA	NA	NA	NA	NA	NA NA	NA	AFEM_EC-down
NA	NA	NA	NA	NA	NA	NA	NA NA	NA	AFEM_EC-down
NA	NA	NA	NA	NA	NA	NA	NA NA	NA	AFEM_EC-down
NA	NA	NA	NA	NA	NA	NA	NA NA	NA	AFEM_EC-down
NA	NA	NA	NA	NA	NA	NA	NA NA	NA	AFEM_EC-down
NA	NA	NA	NA	NA	NA	NA	NA NA	NA	AFEM_EC-down
NA	NA	NA	NA	NA	NA	NA	NA NA	NA	AFEM_EC-down
NA	NA	NA	NA	NA	NA	NA	NA NA	NA	AFEM_EC-down
NA	NA	NA	NA	NA	NA	NA	NA NA	NA	AFEM_EC-down
NA	NA	NA	NA	NA	NA	NA	NA NA	NA	AFEM_EC-down
NA	NA	NA	NA	NA	NA	NA	NA NA	NA	AFEM_EC-down
NA	NA	NA	NA	NA	NA	NA	NA NA	NA	AFEM_EC-down
NA	NA	NA	NA	NA	NA	NA	NA NA	NA	AFEM_EC-down
NA	NA	NA	NA	NA	NA	NA	NA NA	NA	AFEM_EC-down
NA	NA	NA	NA	NA	NA	NA	NA NA	NA	AFEM_EC-down
NA	NA	NA	NA	NA	NA	NA	NA NA	NA	AFEM_EC-down
NA	NA	NA	NA	NA	NA	NA	NA NA	NA	AFEM_EC-down
NA	NA	NA	NA	NA	NA	NA	NA NA	NA	AFEM_EC-down
NA	NA	NA	NA	NA	NA	NA	NA NA	NA	AFEM_EC-down
NA	NA	NA	NA	NA	NA	NA	NA NA	NA	AFEM_EC-down
NA	NA	NA	NA	NA	NA	NA	NA NA	NA	AFEM_EC-down

NA	NA	NA	NA	NA	NA	NA	NA	NA	NA	AFEM_EC-down
NA	NA	NA	NA	NA	NA	NA	NA	NA	NA	AFEM_EC-down
NA	NA	NA	NA	NA	NA	NA	NA	NA	NA	AFEM_EC-down
NA	NA	NA	NA	NA	NA	NA	NA	NA	NA	AFEM_EC-down
NA	NA	NA	NA	NA	NA	NA	NA	NA	NA	AFEM_EC-down
NA	NA	NA	NA	NA	NA	NA	NA	NA	NA	AFEM_EC-down
NA	NA	NA	NA	NA	NA	NA	NA	NA	NA	AFEM_EC-down
NA	NA	NA	NA	NA	NA	NA	NA	NA	NA	AFEM_EC-down
NA	NA	NA	NA	NA	NA	NA	NA	NA	NA	AFEM_EC-down
NA	NA	NA	NA	NA	NA	NA	NA	NA	NA	AFEM_EC-down
NA	NA	NA	NA	NA	NA	NA	NA	NA	NA	AFEM_EC-down
NA	NA	NA	NA	NA	NA	NA	NA	NA	NA	AFEM_EC-down
NA	NA	NA	NA	NA	NA	NA	NA	NA	NA	AFEM_EC-down
NA	NA	NA	NA	NA	NA	NA	NA	NA	NA	AFEM_EC-down
NA	NA	NA	NA	NA	NA	NA	NA	NA	NA	AFEM_EC-down
NA	NA	NA	NA	NA	NA	NA	NA	NA	NA	AFEM_EC-down
NA	NA	NA	NA	NA	NA	NA	NA	NA	NA	AFEM_EC-down
NA	NA	NA	NA	NA	NA	NA	NA	NA	NA	AFEM_EC-down
NA	NA	NA	NA	NA	NA	NA	NA	NA	NA	AFEM_EC-down
NA	NA	NA	NA	NA	NA	NA	NA	NA	NA	AFEM_EC-down
NA	NA	NA	NA	NA	NA	NA	NA	NA	NA	AFEM_EC-down
NA	NA	NA	NA	NA	NA	NA	NA	NA	NA	AFEM_EC-down
NA	NA	NA	NA	NA	NA	NA	NA	NA	NA	AFEM_EC-down
NA	NA	NA	NA	NA	NA	NA	NA	NA	NA	AFEM_EC-down
NA	NA	NA	NA	NA	NA	NA	NA	NA	NA	AFEM_EC-down
NA	NA	NA	NA	NA	NA	NA	NA	NA	NA	AFEM_EC-down
NA	NA	NA	NA	NA	NA	NA	NA	NA	NA	AFEM_EC-down
NA	NA	NA	NA	NA	NA	NA	NA	NA	NA	AFEM_EC-down
NA	NA	NA	NA	NA	NA	NA	NA	NA	NA	AFEM_EC-down
NA	NA	NA	NA	NA	NA	NA	NA	NA	NA	AFEM_EC-down
NA	NA	NA	NA	NA	NA	NA	NA	NA	NA	AFEM_EC-down
NA	NA	NA	NA	NA	NA	NA	NA	NA	NA	AFEM_EC-down
NA	NA	NA	NA	NA	NA	NA	NA	NA	NA	AFEM_EC-down
NA	NA	NA	NA	NA	NA	NA	NA	NA	NA	AFEM_EC-down
NA	NA	NA	NA	NA	NA	NA	NA	NA	NA	AFEM_EC-down
NA	NA	NA	NA	NA	NA	NA	NA	NA	NA	AFEM_EC-down
NA	NA	NA	NA	NA	NA	NA	NA	NA	NA	AFEM_EC-down
NA	NA	NA	NA	NA	NA	NA	NA	NA	NA	AFEM_EC-down
NA	NA	NA	NA	NA	NA	NA	NA	NA	NA	AFEM_EC-down
NA	NA	NA	NA	NA	NA	NA	NA	NA	NA	AFEM_EC-down
NA	NA	NA	NA	NA	NA	NA	NA	NA	NA	AFEM_EC-down
NA	NA	NA	NA	NA	NA	NA	NA	NA	NA	AFEM_EC-down
NA	NA	NA	NA	NA	NA	NA	NA	NA	NA	AFEM_EC-down
NA	NA	NA	NA	NA	NA	NA	NA	NA	NA	AFEM_EC-down
NA	NA	NA	NA	NA	NA	NA	NA	NA	NA	AFEM_EC-down
NA	NA	NA	NA	NA	NA	NA	NA	NA	NA	AFEM_EC-down
NA	NA	NA	NA	NA	NA	NA	NA	NA	NA	AFEM_EC-down
NA	NA	NA	NA	NA	NA	NA	NA	NA	NA	AFEM_EC-down
NA	NA	NA	NA	NA	NA	NA	NA	NA	NA	AFEM_EC-down
NA	NA	NA	NA	NA	NA	NA	NA	NA	NA	AFEM_EC-down
NA	NA	NA	NA	NA	NA	NA	NA	NA	NA	AFEM_EC-down
NA	NA	NA	NA	NA	NA	NA	NA	NA	NA	AFEM_EC-down
NA	NA	NA	NA	NA	NA	NA	NA	NA	NA	AFEM_EC-down
NA	NA	NA	NA	NA	NA	NA	NA	NA	NA	AFEM_EC-down
NA	NA	NA	NA	NA	NA	NA	NA	NA	NA	AFEM_EC-down
NA	NA	NA	NA	NA	NA	NA	NA	NA	NA	AFEM_EC-down
NA	NA	NA	NA	NA	NA	NA	NA	NA	NA	AFEM_EC-down
NA	NA	NA	NA	NA	NA	NA	NA	NA	NA	AFEM_EC-down
NA	NA	NA	NA	NA	NA	NA	NA	NA	NA	AFEM_EC-down
NA	NA	NA	NA	NA	NA	NA	NA	NA	NA	AFEM_EC-down
NA	NA	NA	NA	NA	NA	NA	NA	NA	NA	AFEM_EC-down
NA	NA	NA	NA	NA	NA	NA	NA	NA	NA	AFEM_EC-down
NA	NA	NA	NA	NA	NA	NA	NA	NA	NA	AFEM_EC-down
NA	NA	NA	NA	NA	NA	NA	NA	NA	NA	AFEM_EC-down
NA	NA	NA	NA	NA	NA	NA	NA	NA	NA	AFEM_EC-down
NA	NA	NA	NA	NA	NA	NA	NA	NA	NA	AFEM_EC-down
NA	NA	NA	NA	NA	NA	NA	NA	NA	NA	AFEM_EC-down
NA	NA	NA	NA	NA	NA	NA	NA	NA	NA	AFEM_EC-down
NA	NA	NA	NA	NA	NA	NA	NA	NA	NA	AFEM_EC-down
NA	NA	NA	NA	NA	NA	NA	NA	NA	NA	AFEM_EC-down
NA	NA	NA	NA	NA	NA	NA	NA	NA	NA	AFEM_EC-down
NA	NA	NA	NA	NA	NA	NA	NA	NA	NA	AFEM_EC-down
NA	NA	NA	NA	NA	NA	NA	NA	NA	NA	AFEM_EC-down
NA	NA	NA	NA	NA	NA	NA	NA	NA	NA	AFEM_EC-down

NA	NA	NA	NA	NA	NA	NA	NA NA	NA	AFEM_EC-down
NA	NA	NA	NA	NA	NA	NA	NA NA	NA	AFEM_EC-down
cfa00780	Biotin metabolism	1/241	2174715	0.0892702	0.4045956	0.36374955	477059 BTD	1	VCAV_EC-down
cfa00471	D-Glutamine and D-glutamate metabolism	1/241	2174746	0.11722663	0.45260061	0.40690821	100686499 GLS2	1	VCAV_EC-down
cfa00750	Vitamin B6 metabolism	1/241	2174837	0.1960709	0.52976849	0.47628559	487788 PDXK	1	VCAV_EC-down
cfa00072	Synthesis and degradation of ketone bodies	1/241	2174899	0.24469679	0.59576107	0.53561587	489421 ACAT1	1	VCAV_EC-down
cfa00920	Sulfur metabolism	1/241	2174929	0.26789986	0.62962523	0.56606127	481103 SUOX	1	VCAV_EC-down
cfa00360	Phenylalanine metabolism	1/241	17/7854	0.41160977	0.78213515	0.70317451	403450 MAOA	1	VCAV_EC-down
cfa00770	Pantothenate and CoA biosynthesis	1/241	17/7854	0.41160977	0.78213515	0.70317451	484394 BCAT2	1	VCAV_EC-down
cfa01210	2-Oxocarboxylic acid metabolism	1/241	17/7854	0.41160977	0.78213515	0.70317451	484394 BCAT2	1	VCAV_EC-down
cfa00220	Arginine biosynthesis	1/241	19/7854	0.44724343	0.79541394	0.71511274	100686499 GLS2	1	VCAV_EC-down
cfa00511	Other glycan degradation	1/241	19/7854	0.44724343	0.79541394	0.71511274	487633	1	VCAV_EC-down
cfa00100	Steroid biosynthesis	1/241	20/7854	0.46424589	0.81026768	0.72846692	490325 SOAT1	1	VCAV_EC-down
cfa00900	Terpenoid backbone biosynthesis	1/241	21/7854	0.48072748	0.81376158	0.73160809	489421 ACAT1	1	VCAV_EC-down
cfa04964	Proximal tubule bicarbonate reclamation	1/241	21/7854	0.48072748	0.81376158	0.73160809	100686499 GLS2	1	VCAV_EC-down
cfa00592	alpha-Linolenic acid metabolism	1/241	22/7854	0.49670408	0.82102262	0.73813609	477023 ACAA1	1	VCAV_EC-down
cfa00534	Glycosaminoglycan biosynthesis - heparan sulfate / heparin	1/241	24/7854	0.52720347	0.84753502	0.76197193	489516 HS3ST3B1	1	VCAV_EC-down
cfa00650	Butanoate metabolism	1/241	25/7854	0.5417557	0.84753502	0.76197193	489421 ACAT1	1	VCAV_EC-down
cfa00790	Folate biosynthesis	1/241	25/7854	0.5417557	0.84753502	0.76197193	479082 QDPR	1	VCAV_EC-down
cfa00062	Fatty acid elongation	1/241	26/7854	0.55586183	0.85353647	0.7673675	607926	1	VCAV_EC-down
cfa04966	Collecting duct acid secretion	1/241	26/7854	0.55586183	0.85353647	0.7673675	479877 ATP6V0C	1	VCAV_EC-down
cfa03020	RNA polymerase	1/241	28/7854	0.58278985	0.8750588	0.78671703	100686835	1	VCAV_EC-down
cfa04392	Hippo signaling pathway - multiple species	1/241	28/7854	0.58278985	0.8750588	0.78671703	610713 TEAD1	1	VCAV_EC-down
cfa00020	Citrate cycle (TCA cycle)	1/241	29/7854	0.59563774	0.8750588	0.78671703	478983 SDHC	1	VCAV_EC-down
cfa00350	Tyrosine metabolism	1/241	32/7854	0.63186488	0.88166467	0.792656	403450 MAOA	1	VCAV_EC-down
cfa00730	Thiamine metabolism	12420	14/7854	0.05897734	0.52353656	0.51664792	490204 AK5	1	VPOR_EC-up
cfa03060	Protein export	12420	23/7854	0.09509341	0.52353656	0.51664792	403953 SRP54	1	VPOR_EC-up
cfa00030	Pentose phosphate pathway	12420	29/7854	0.11841949	0.52353656	0.51664792	476299 FBP1	1	VPOR_EC-up
cfa04710	Circadian rhythm	12420	30/7854	0.12225	0.52353656	0.51664792	478328 RORA	1	VPOR_EC-up
cfa00051	Fructose and mannose metabolism	12420	32/7854	0.12986261	0.52353656	0.51664792	476299 FBP1	1	VPOR_EC-up
cfa04340	Hedgehog signaling pathway	12420	47/7854	0.18494988	0.52353656	0.51664792	477392 CUL3	1	VPOR_EC-up
cfa00520	Amino sugar and nucleotide sugar metabolism	12420	48/7854	0.18849948	0.52353656	0.51664792	480325 GNPNAT1	1	VPOR_EC-up
cfa05134	Legionellosis	12420	50/7854	0.19555372	0.52353656	0.51664792	102155289	1	VPOR_EC-up
cfa04512	ECM-receptor interaction	12420	88/7854	0.31881024	0.52906168	0.52210035	488943 SV2C	1	VPOR_EC-up
cfa00240	Pyrimidine metabolism	12420	52/7854	0.20254842	0.52353656	0.51664792	481988 RRM2B	1	VPOR_EC-up
cfa00480	Glutathione metabolism	12420	52/7854	0.20254842	0.52353656	0.51664792	481988 RRM2B	1	VPOR_EC-up
cfa04923	Regulation of lipolysis in adipocytes	12420	54/7854	0.20948408	0.52353656	0.51664792	485570 ABHD5	1	VPOR_EC-up
cfa00561	Glycerolipid metabolism	12420	59/7854	0.22656807	0.52353656	0.51664792	480872	1	VPOR_EC-up
cfa05321	Inflammatory bowel disease (IBD)	12420	61/7854	0.23330085	0.52353656	0.51664792	478328 RORA	1	VPOR_EC-up
cfa00010	Glycolysis / Gluconeogenesis	12420	62/7854	0.23664587	0.52353656	0.51664792	476299 FBP1	1	VPOR_EC-up
cfa00983	Drug metabolism - other enzymes	12420	63/7854	0.23997673	0.52353656	0.51664792	481988 RRM2B	1	VPOR_EC-up
cfa00970	Aminoacyl-tRNA biosynthesis	12420	66/7854	0.24988488	0.52353656	0.51664792	100856659 GARS	1	VPOR_EC-up
cfa04115	p53 signaling pathway	12420	69/7854	0.25966762	0.52353656	0.51664792	481988 RRM2B	1	VPOR_EC-up
cfa04924	Renin secretion	12420	69/7854	0.25966762	0.52353656	0.51664792	475272 PDE1C	1	VPOR_EC-up
cfa05140	Leishmaniasis	12420	70/7854	0.26290093	0.52353656	0.51664792	102155289	1	VPOR_EC-up
cfa05100	Bacterial invasion of epithelial cells	12420	75/7854	0.27886298	0.52353656	0.51664792	100856747	1	VPOR_EC-up
cfa04742	Taste transduction	12420	77/7854	0.28515341	0.52353656	0.51664792	475272 PDE1C	1	VPOR_EC-up

cfa05150	Staphylococcus aureus infection	12420	78/7854	0.28827862	0.52353656	0.51664792	486704 C3AR1	1	VPOR_EC-up
cfa03320	PPAR signaling pathway	12420	80/7854	0.29448932	0.52353656	0.51664792	480872	1	VPOR_EC-up
cfa04610	Complement and coagulation cascades	12420	80/7854	0.29448932	0.52353656	0.51664792	486704 C3AR1	1	VPOR_EC-up
cfa04512	ECM-receptor interaction	42370	88/7854	0.16511139	0.31976033	0.28536963	476665 COMP	1	VSAP_VSMC-up
cfa04666	Fc gamma R-mediated phagocytosis	12420	90/7854	0.32476214	0.52906168	0.52210035	100856747	1	VPOR_EC-up
cfa04350	TGF-beta signaling pathway	12420	92/7854	0.33066355	0.52906168	0.52210035	476140 ACVR2A	1	VPOR_EC-up
cfa05032	Morphine addiction	12420	92/7854	0.33066355	0.52906168	0.52210035	475272 PDE1C	1	VPOR_EC-up
cfa05017	Spinocerebellar ataxia	12420	96/7854	0.34231657	0.53434782	0.52731693	478328 RORA	1	VPOR_EC-up
cfa04922	Glucagon signaling pathway	12420	101/7854	0.35660619	0.53496219	0.52792322	476299 FBP1	1	VPOR_EC-up
cfa04659	Th17 cell differentiation	12420	102/7854	0.35942772	0.53496219	0.52792322	478328 RORA	1	VPOR_EC-up
cfa04931	Insulin resistance	12420	109/7854	0.37884495	0.53842172	0.53133723	477802 OGA	1	VPOR_EC-up
cfa04726	Serotonergic synapse	12420	110/7854	0.38157177	0.53842172	0.53133723	477762 HTR7	1	VPOR_EC-up
cfa04066	HIF-1 signaling pathway	12420	112/7854	0.38699061	0.53842172	0.53133723	612212 MKNK2	1	VPOR_EC-up
cfa01200	Carbon metabolism	12420	116/7854	0.39769039	0.54153584	0.53441037	476299 FBP1	1	VPOR_EC-up
cfa04152	AMPK signaling pathway	12420	121/7854	0.41081041	0.54445341	0.53728955	476299 FBP1	1	VPOR_EC-up
cfa04140	Autophagy - animal	12420	136/7854	0.44852779	0.56096597	0.55358484	610498 WDR41	1	VPOR_EC-up
cfa05162	Measles	12420	136/7854	0.44852779	0.56096597	0.55358484	477490 OAS3	1	VPOR_EC-up
cfa04120	Ubiquitin mediated proteolysis	12420	139/7854	0.45578485	0.56096597	0.55358484	477392 CUL3	1	VPOR_EC-up
cfa05160	Hepatitis C	12420	157/7854	0.49741969	0.57856335	0.57095068	477490 OAS3	1	VPOR_EC-up
cfa04621	NOD-like receptor signaling pathway	12420	158/7854	0.49963974	0.57856335	0.57095068	477490 OAS3	1	VPOR_EC-up
cfa04217	Necroptosis	12420	160/7854	0.50405132	0.57856335	0.57095068	102155200	1	VPOR_EC-up
cfa05164	Influenza A	12420	161/7854	0.50624293	0.57856335	0.57095068	477490 OAS3	1	VPOR_EC-up
cfa04530	Tight junction	12420	170/7854	0.52554904	0.59009015	0.58232581	403498 ARHGEF2	1	VPOR_EC-up
cfa05169	Epstein-Barr virus infection	12420	193/7854	0.57161775	0.63075062	0.62245127	477490 OAS3	1	VPOR_EC-up
cfa04810	Regulation of actin cytoskeleton	12420	211/7854	0.60461348	0.65585191	0.64722228	100856747	1	VPOR_EC-up
cfa04014	Ras signaling pathway	12420	225/7854	0.62855812	0.670462	0.66164013	477762 HTR7	1	VPOR_EC-up
cfa04144	Endocytosis	12420	235/7854	0.6447934	0.67650455	0.66760318	100856747	1	VPOR_EC-up
cfa04060	Cytokine-cytokine receptor interaction	12420	270/7854	0.69636982	0.71883336	0.70937503	476140 ACVR2A	1	VPOR_EC-up
cfa04010	MAPK signaling pathway	12420	288/7854	0.71998538	0.73141372	0.72178985	612212 MKNK2	1	VPOR_EC-up
cfa05168	Herpes simplex virus 1 infection	12420	359/7854	0.7969257	0.7969257	0.78643984	477490 OAS3	1	VPOR_EC-up
cfa00603	Glycosphingolipid biosynthesis - globo and isoglobo series	44105	16/7854	0.02019751	0.17686461	0.16548735	403833 GBGT1	1	VPOR_EC-down
cfa00120	Primary bile acid biosynthesis	44105	18/7854	0.02269621	0.17686461	0.16548735	489917 HSD3B7	1	VPOR_EC-down
cfa00513	Various types of N-glycan biosynthesis	44105	40/7854	0.04980585	0.17686461	0.16548735	481458 MGAT4B	1	VPOR_EC-down
cfa04512	ECM-receptor interaction	44105	88/7854	0.10661908	0.25090823	0.25090823	403466 COL4A5	1	VPOR_VSMC-up
cfa00982	Drug metabolism - cytochrome P450	44105	49/7854	0.06069968	0.17686461	0.16548735	476005 GSTP1	1	VPOR_EC-down
cfa00510	N-Glycan biosynthesis	44105	50/7854	0.06190314	0.17686461	0.16548735	481458 MGAT4B	1	VPOR_EC-down
cfa00480	Glutathione metabolism	44105	52/7854	0.0643059	0.17686461	0.16548735	476005 GSTP1	1	VPOR_EC-down
cfa00980	Metabolism of xenobiotics by cytochrome P450	44105	52/7854	0.0643059	0.17686461	0.16548735	476005 GSTP1	1	VPOR_EC-down
cfa05204	Chemical carcinogenesis	44105	55/7854	0.06789965	0.17686461	0.16548735	476005 GSTP1	1	VPOR_EC-down
cfa04213	Longevity regulating pathway - multiple species	44105	59/7854	0.07267199	0.17686461	0.16548735	476258 SOD2	1	VPOR_EC-down
cfa00983	Drug metabolism - other enzymes	44105	63/7854	0.07742233	0.17686461	0.16548735	476005 GSTP1	1	VPOR_EC-down
cfa04137	Mitophagy - animal	44105	64/7854	0.07860649	0.17686461	0.16548735	484010 CITED2	1	VPOR_EC-down
cfa01524	Platinum drug resistance	44105	72/7854	0.08803063	0.17943976	0.16789685	476005 GSTP1	1	VPOR_EC-down
cfa04970	Salivary secretion	44105	78/7854	0.0950417	0.17943976	0.16789685	607874 CST3	1	VPOR_EC-down
cfa04146	Peroxisome	44105	82/7854	0.09968875	0.17943976	0.16789685	476258 SOD2	1	VPOR_EC-down
cfa04211	Longevity regulating pathway	44105	88/7854	0.10661908	0.1799197	0.16834591	476258 SOD2	1	VPOR_EC-down
cfa05215	Prostate cancer	44105	97/7854	0.11692457	0.18570372	0.17375787	476005 GSTP1	1	VPOR_EC-down
cfa04152	AMPK signaling pathway	44105	121/7854	0.14388488	0.21387999	0.20012163	611619 RAB11B	1	VPOR_EC-down
cfa04068	FoxO signaling pathway	44105	127/7854	0.15050814	0.21387999	0.20012163	476258 SOD2	1	VPOR_EC-down

cfa05414	Dilated cardiomyopathy (DCM)	10/279	95/7854	0.00191324	0.03341792	0.02832937	481598/479975/489208/609879/608287/477956/606758/488437/403788/480137 TPM2, PRKACB, ITGA2, TPM4, ITGA10, ITGB1, DMD, ITGAV, ITGB3, TPM3	10	VSAP_EC-down
cfa05164	Influenza A	44105	161/7854	0.18717653	0.23175763	0.21684925	611619 RAB11B	1	VPOR_EC-down
cfa05225	Hepatocellular carcinoma	44105	165/7854	0.19139542	0.23175763	0.21684925	476005 GSTP1	1	VPOR_EC-down
cfa05034	Alcoholism	44105	171/7854	0.1976868	0.23175763	0.21684925	486589 HDAC7	1	VPOR_EC-down
cfa04360	Axon guidance	44105	179/7854	0.20600679	0.23175763	0.21684925	490270 PLXNA2	1	VPOR_EC-down
cfa05203	Viral carcinogenesis	44105	189/7854	0.21629753	0.23360133	0.21857434	486589 HDAC7	1	VPOR_EC-down
cfa04144	Endocytosis	44105	235/7854	0.26210604	0.27218704	0.25467794	611619 RAB11B	1	VPOR_EC-down
cfa05016	Huntington disease	44105	257/7854	0.2831504	0.2831504	0.26493605	476258 SOD2	1	VPOR_EC-down
cfa00910	Nitrogen metabolism	42736	17/7854	0.03620246	0.32364194	0.30122906	611933 CA9	1	VFEM_EC-up
cfa00532	Glycosaminoglycan biosynthesis - chondroitin sulfate / dermatan sulfate	42736	19/7854	0.04037953	0.32364194	0.30122906	491852 CHST7	1	VFEM_EC-up
cfa04964	Proximal tubule bicarbonate reclamation	42736	21/7854	0.04453955	0.32364194	0.30122906	480255 PCK2	1	VFEM_EC-up
cfa04614	Renin-angiotensin system	42736	24/7854	0.05074777	0.32364194	0.30122906	477120 MME	1	VFEM_EC-up
cfa00020	Citrate cycle (TCA cycle)	42736	29/7854	0.06101052	0.32364194	0.30122906	480255 PCK2	1	VFEM_EC-up
cfa00620	Pyruvate metabolism	42736	37/7854	0.07721397	0.32364194	0.30122906	480255 PCK2	1	VFEM_EC-up
cfa05414	Dilated cardiomyopathy (DCM)	8/407	95/7854	0.11850612	0.76328292	0.73371129	403532/609265/477625/486563/497091/476623/611066/475738 TNNT2, SGCA, ADCY6, CACNB3, DES, DAG1, SGCB, SLC8A1	8	ACOR_VSMC-up
cfa04913	Ovarian steroidogenesis	42736	46/7854	0.09512818	0.32364194	0.30122906	482994 ADCY3	1	VFEM_EC-up
cfa00830	Retinol metabolism	42736	49/7854	0.10102648	0.32364194	0.30122906	483120 CYP26B1	1	VFEM_EC-up
cfa04923	Regulation of lipolysis in adipocytes	42736	54/7854	0.11077664	0.32364194	0.30122906	482994 ADCY3	1	VFEM_EC-up
cfa05014	Amyotrophic lateral sclerosis (ALS)	42736	56/7854	0.11464877	0.32364194	0.30122906	480454 MAP2K6	1	VFEM_EC-up
cfa04213	Longevity regulating pathway - multiple species	42736	59/7854	0.12042721	0.32364194	0.30122906	482994 ADCY3	1	VFEM_EC-up
cfa00010	Glycolysis / Gluconeogenesis	42736	62/7854	0.12617015	0.32364194	0.30122906	480255 PCK2	1	VFEM_EC-up
cfa04927	Cortisol synthesis and secretion	42736	63/7854	0.12807661	0.32364194	0.30122906	482994 ADCY3	1	VFEM_EC-up
cfa04976	Bile secretion	42736	66/7854	0.13377251	0.32364194	0.30122906	482994 ADCY3	1	VFEM_EC-up
cfa04664	Fc epsilon RI signaling pathway	42736	67/7854	0.13566336	0.32364194	0.30122906	480454 MAP2K6	1	VFEM_EC-up
cfa05221	Acute myeloid leukemia	42736	67/7854	0.13566336	0.32364194	0.30122906	488770 BCL2A1	1	VFEM_EC-up
cfa04920	Adipocytokine signaling pathway	42736	70/7854	0.1413126	0.32364194	0.30122906	480255 PCK2	1	VFEM_EC-up
cfa04918	Thyroid hormone synthesis	42736	72/7854	0.14505943	0.32364194	0.30122906	482994 ADCY3	1	VFEM_EC-up
cfa04971	Gastric acid secretion	42736	72/7854	0.14505943	0.32364194	0.30122906	482994 ADCY3	1	VFEM_EC-up
cfa04970	Salivary secretion	42736	78/7854	0.15620782	0.32364194	0.30122906	482994 ADCY3	1	VFEM_EC-up
cfa03320	PPAR signaling pathway	42736	80/7854	0.15989345	0.32364194	0.30122906	480255 PCK2	1	VFEM_EC-up
cfa04911	Insulin secretion	42736	85/7854	0.16904133	0.32364194	0.30122906	482994 ADCY3	1	VFEM_EC-up
cfa04211	Longevity regulating pathway	42736	88/7854	0.17448497	0.32364194	0.30122906	482994 ADCY3	1	VFEM_EC-up
cfa04540	Gap junction	42736	88/7854	0.17448497	0.32364194	0.30122906	482994 ADCY3	1	VFEM_EC-up
cfa04914	Progesterone-mediated oocyte maturation	42736	89/7854	0.17629205	0.32364194	0.30122906	482994 ADCY3	1	VFEM_EC-up
cfa05235	PD-L1 expression and PD-1 checkpoint pathway in cancer	42736	89/7854	0.17629205	0.32364194	0.30122906	480454 MAP2K6	1	VFEM_EC-up
cfa04727	GABAergic synapse	42736	91/7854	0.17989504	0.32364194	0.30122906	482994 ADCY3	1	VFEM_EC-up
cfa04974	Protein digestion and absorption	42736	91/7854	0.17989504	0.32364194	0.30122906	477120 MME	1	VFEM_EC-up
cfa05032	Morphine addiction	42736	92/7854	0.18169097	0.32364194	0.30122906	482994 ADCY3	1	VFEM_EC-up
cfa01522	Endocrine resistance	42736	93/7854	0.18348319	0.32364194	0.30122906	482994 ADCY3	1	VFEM_EC-up
cfa04064	NF-kappa B signaling pathway	42736	93/7854	0.18348319	0.32364194	0.30122906	488770 BCL2A1	1	VFEM_EC-up
cfa04640	Hematopoietic cell lineage	42736	93/7854	0.18348319	0.32364194	0.30122906	477120 MME	1	VFEM_EC-up
cfa04925	Aldosterone synthesis and secretion	42736	94/7854	0.18527172	0.32364194	0.30122906	482994 ADCY3	1	VFEM_EC-up
cfa04972	Pancreatic secretion	42736	94/7854	0.18527172	0.32364194	0.30122906	482994 ADCY3	1	VFEM_EC-up

cfa05414	Dilated cardiomyopathy (DCM)	6/357	95/7854	0.26290164	0.51654618	0.37807945	490755/480271/606758/100856563/403788/479797 SLC8A3, ADCY4, DMD, ITGA6, ITGB3, ATP2A1	6	VCAV_EC-up
cfa04713	Circadian entrainment	42736	98/7854	0.19238904	0.32364194	0.30122906	482994 ADCY3	1	VFEM_EC-up
cfa04916	Melanogenesis	42736	100/7854	0.19592572	0.32364194	0.30122906	482994 ADCY3	1	VFEM_EC-up
cfa04620	Toll-like receptor signaling pathway	42736	101/7854	0.19768858	0.32364194	0.30122906	480454 MAP2K6	1	VFEM_EC-up
cfa04922	Glucagon signaling pathway	42736	101/7854	0.19768858	0.32364194	0.30122906	480255 PCK2	1	VFEM_EC-up
cfa04928	Parathyroid hormone synthesis, secretion and action	42736	107/7854	0.20818962	0.32364194	0.30122906	482994 ADCY3	1	VFEM_EC-up
cfa04668	TNF signaling pathway	42736	108/7854	0.20992717	0.32364194	0.30122906	480454 MAP2K6	1	VFEM_EC-up
cfa04931	Insulin resistance	42736	109/7854	0.21166113	0.32364194	0.30122906	480255 PCK2	1	VFEM_EC-up
cfa05145	Toxoplasmosis	42736	109/7854	0.21166113	0.32364194	0.30122906	480454 MAP2K6	1	VFEM_EC-up
cfa04724	Glutamatergic synapse	42736	112/7854	0.21684154	0.32364194	0.30122906	482994 ADCY3	1	VFEM_EC-up
cfa04725	Cholinergic synapse	42736	113/7854	0.21856121	0.32364194	0.30122906	482994 ADCY3	1	VFEM_EC-up
cfa04114	Oocyte meiosis	42736	117/7854	0.22540442	0.32364194	0.30122906	482994 ADCY3	1	VFEM_EC-up
cfa05135	Yersinia infection	42736	117/7854	0.22540442	0.32364194	0.30122906	480454 MAP2K6	1	VFEM_EC-up
cfa04152	AMPK signaling pathway	42736	121/7854	0.2321912	0.32364194	0.30122906	480255 PCK2	1	VFEM_EC-up
cfa05414	Dilated cardiomyopathy (DCM)	4/241	95/7854	0.33326902	0.70498356	0.63381178	608977/477625/492249/486563 ITGB5, ADCY6, EMD, CACNB3	4	VCAV_EC-down
cfa04380	Osteoclast differentiation	42736	125/7854	0.23892198	0.32364194	0.30122906	480454 MAP2K6	1	VFEM_EC-up
cfa04068	FoxO signaling pathway	42736	127/7854	0.24226651	0.32364194	0.30122906	480255 PCK2	1	VFEM_EC-up
cfa04926	Relaxin signaling pathway	42736	127/7854	0.24226651	0.32364194	0.30122906	482994 ADCY3	1	VFEM_EC-up
cfa00230	Purine metabolism	42736	130/7854	0.24725738	0.32364194	0.30122906	482994 ADCY3	1	VFEM_EC-up
cfa00190	Oxidative phosphorylation	42736	131/7854	0.24891411	0.32364194	0.30122906	611480	1	VFEM_EC-up
cfa04210	Apoptosis	42736	131/7854	0.24891411	0.32364194	0.30122906	488770 BCL2A1	1	VFEM_EC-up
cfa04910	Insulin signaling pathway	42736	134/7854	0.25386375	0.32364194	0.30122906	480255 PCK2	1	VFEM_EC-up
cfa05414	Dilated cardiomyopathy (DCM)	4/388	95/7854	0.69761324	0.99972301	0.9362731	480124/403788/489208/480137 LMNA, ITGB3, ITGA2, TPM3	4	ACOR_VSMC-down
cfa04371	Apelin signaling pathway	42736	135/7854	0.2555068	0.32364194	0.30122906	482994 ADCY3	1	VFEM_EC-up
cfa05414	Dilated cardiomyopathy (DCM)	44106	95/7854	0.00611621	0.01834863	0.00804764	612079/606758 SGCD, DMD	2	AORT_EC-up
cfa05012	Parkinson disease	42736	138/7854	0.26041553	0.32551941	0.30297651	611480	1	VFEM_EC-up
cfa04932	Non-alcoholic fatty liver disease (NAFLD)	42736	145/7854	0.27175103	0.33182312	0.30884368	611480	1	VFEM_EC-up
cfa04261	Adrenergic signaling in cardiomyocytes	42736	147/7854	0.27495959	0.33182312	0.30884368	482994 ADCY3	1	VFEM_EC-up
cfa04218	Cellular senescence	42736	151/7854	0.28133683	0.33182312	0.30884368	480454 MAP2K6	1	VFEM_EC-up
cfa04921	Oxytocin signaling pathway	42736	151/7854	0.28133683	0.33182312	0.30884368	482994 ADCY3	1	VFEM_EC-up
cfa04934	Cushing syndrome	42736	152/7854	0.28292287	0.33182312	0.30884368	482994 ADCY3	1	VFEM_EC-up
cfa05161	Hepatitis B	42736	164/7854	0.30170019	0.34708639	0.32304994	480454 MAP2K6	1	VFEM_EC-up
cfa04022	cGMP-PKG signaling pathway	42736	165/7854	0.3032439	0.34708639	0.32304994	482994 ADCY3	1	VFEM_EC-up
cfa05167	Kaposi sarcoma-associated herpesvirus infection	42736	178/7854	0.32302203	0.36532253	0.34002318	480454 MAP2K6	1	VFEM_EC-up
cfa05169	Epstein-Barr virus infection	42736	193/7854	0.3451861	0.38579623	0.35907904	480454 MAP2K6	1	VFEM_EC-up
cfa05170	Human immunodeficiency virus 1 infection	42736	200/7854	0.35529394	0.39108859	0.36400489	480454 MAP2K6	1	VFEM_EC-up
cfa05205	Proteoglycans in cancer	42736	202/7854	0.35815481	0.39108859	0.36400489	487731 TIAM1	1	VFEM_EC-up
cfa05166	Human T-cell leukemia virus 1 infection	42736	209/7854	0.36807404	0.39296309	0.36574958	482994 ADCY3	1	VFEM_EC-up
cfa04810	Regulation of actin cytoskeleton	42736	211/7854	0.3708815	0.39296309	0.36574958	487731 TIAM1	1	VFEM_EC-up
cfa05132	Salmonella infection	42736	212/7854	0.37228082	0.39296309	0.36574958	480454 MAP2K6	1	VFEM_EC-up
cfa04014	Ras signaling pathway	42736	225/7854	0.39020747	0.40735945	0.37914896	487731 TIAM1	1	VFEM_EC-up
cfa05016	Huntington disease	42736	257/7854	0.43230361	0.44640046	0.4154863	611480	1	VFEM_EC-up
cfa04010	MAPK signaling pathway	42736	288/7854	0.47046361	0.4805811	0.44729986	480454 MAP2K6	1	VFEM_EC-up
cfa04080	Neuroactive ligand-receptor interaction	42736	327/7854	0.51504644	0.52052566	0.48447818	481142 AVPR1A	1	VFEM_EC-up
cfa04151	PI3K-Akt signaling pathway	42736	354/7854	0.54381525	0.54381525	0.50615491	480255 PCK2	1	VFEM_EC-up
cfa04727	GABAergic synapse	43831	91/7854	0.01158645	0.01158645	NA	478858 PLCL1	1	VFEM_EC-down
cfa03050	Proteasome	41640	46/7854	0.07900976	0.3227867	0.32089906	489749 PSMB10	1	VPUL_EC-up

cfa00982	Drug metabolism - cytochrome P450	41640	49/7854	0.08395561	0.3227867	0.32089906		477558	1	VPUL_EC-up
cfa00480	Glutathione metabolism	41640	52/7854	0.08887679	0.3227867	0.32089906		477558	1	VPUL_EC-up
cfa00980	Metabolism of xenobiotics by cytochrome P450	41640	52/7854	0.08887679	0.3227867	0.32089906		477558	1	VPUL_EC-up
cfa05204	Chemical carcinogenesis	41640	55/7854	0.09377341	0.3227867	0.32089906		477558	1	VPUL_EC-up
cfa00983	Drug metabolism - other enzymes	41640	63/7854	0.10671186	0.3227867	0.32089906		477558	1	VPUL_EC-up
cfa04976	Bile secretion	41640	66/7854	0.1115194	0.3227867	0.32089906	612928 NR1H4		1	VPUL_EC-up
cfa04924	Renin secretion	41640	69/7854	0.1163029	0.3227867	0.32089906	403797 PTGER2		1	VPUL_EC-up
cfa01524	Platinum drug resistance	41640	72/7854	0.12106248	0.3227867	0.32089906		477558	1	VPUL_EC-up
cfa03018	RNA degradation	41640	78/7854	0.13051031	0.3227867	0.32089906	487817 CNOT6L		1	VPUL_EC-up
cfa04061	Viral protein interaction with cytokine and cytokine receptor	41640	83/7854	0.13831137	0.3227867	0.32089906	445452 CCL24		1	VPUL_EC-up
cfa04350	TGF-beta signaling pathway	41640	92/7854	0.15218974	0.3227867	0.32089906	487635 NEO1		1	VPUL_EC-up
cfa04925	Aldosterone synthesis and secretion	41640	94/7854	0.1552455	0.3227867	0.32089906	478754 NR4A2		1	VPUL_EC-up
cfa04750	Inflammatory mediator regulation of TRP channels	41640	100/7854	0.16435152	0.3227867	0.32089906	403797 PTGER2		1	VPUL_EC-up
cfa04928	Parathyroid hormone synthesis, secretion and action	41640	107/7854	0.17485999	0.3227867	0.32089906	478754 NR4A2		1	VPUL_EC-up
cfa04726	Serotonergic synapse	41640	110/7854	0.17932594	0.3227867	0.32089906	477762 HTR7		1	VPUL_EC-up
cfa04145	Phagosome	41640	138/7854	0.21993928	0.3463915	0.34436582	487114 MRC1		1	VPUL_EC-up
cfa04514	Cell adhesion molecules (CAMs)	41640	139/7854	0.22135463	0.3463915	0.34436582	487635 NEO1		1	VPUL_EC-up
cfa04310	Wnt signaling pathway	41640	151/7854	0.23815388	0.3463915	0.34436582	481148 WIF1		1	VPUL_EC-up
cfa05225	Hepatocellular carcinoma	41640	165/7854	0.25732742	0.3463915	0.34436582		477558	1	VPUL_EC-up
cfa04530	Tight junction	41640	170/7854	0.26406582	0.3463915	0.34436582	403498 ARHGEF2		1	VPUL_EC-up
cfa05152	Tuberculosis	41640	171/7854	0.26540667	0.3463915	0.34436582	487114 MRC1		1	VPUL_EC-up
cfa05202	Transcriptional misregulation in cancer	41640	172/7854	0.26674525	0.3463915	0.34436582	403641 ETV4		1	VPUL_EC-up
cfa04062	Chemokine signaling pathway	41640	174/7854	0.26941561	0.3463915	0.34436582	445452 CCL24		1	VPUL_EC-up
cfa04020	Calcium signaling pathway	41640	192/7854	0.29304582	0.36378101	0.36165364	477762 HTR7		1	VPUL_EC-up
cfa04015	Rap1 signaling pathway	41640	209/7854	0.31470971	0.36838158	0.3662273	608458 EFNA5		1	VPUL_EC-up
cfa04024	cAMP signaling pathway	41640	211/7854	0.31721747	0.36838158	0.3662273	403797 PTGER2		1	VPUL_EC-up
cfa05163	Human cytomegalovirus infection	41640	220/7854	0.32839729	0.36944695	0.36728644	403797 PTGER2		1	VPUL_EC-up
cfa04060	Cytokine-cytokine receptor interaction	41640	270/7854	0.38747385	0.41917648	0.41672515	445452 CCL24		1	VPUL_EC-up
cfa05206	MicroRNAs in cancer	41640	281/7854	0.39980564	0.41917648	0.41672515	608458 EFNA5		1	VPUL_EC-up
cfa04010	MAPK signaling pathway	41640	288/7854	0.40753269	0.41917648	0.41672515	608458 EFNA5		1	VPUL_EC-up
cfa04151	PI3K-Akt signaling pathway	41640	354/7854	0.47598063	0.47598063	0.47319711	608458 EFNA5		1	VPUL_EC-up
cfa00920	Sulfur metabolism	34700	2174929	0.11464375	0.81029448	0.80529119	486463 PAPSS2		1	AFEM_EC-up
cfa00130	Ubiquinone and other terpenoid-quinone biosynthesis	34700	2174960	0.12536645	0.81029448	0.80529119	475769 GGCX		1	AFEM_EC-up
cfa00430	Taurine and hypotaurine metabolism	34700	13/7854	0.14642783	0.81029448	0.80529119	474637 CDO1		1	AFEM_EC-up
cfa00450	Selenocompound metabolism	34700	16/7854	0.17708245	0.81029448	0.80529119	486463 PAPSS2		1	AFEM_EC-up
cfa00603	Glycosphingolipid biosynthesis - globo and isoglobo series	34700	16/7854	0.17708245	0.81029448	0.80529119	481222 A4GALT		1	AFEM_EC-up
cfa00360	Phenylalanine metabolism	34700	17/7854	0.18705657	0.81029448	0.80529119	476003 ALDH3B1		1	AFEM_EC-up
cfa00061	Fatty acid biosynthesis	34700	18/7854	0.19691106	0.81029448	0.80529119	100685468 ACACB		1	AFEM_EC-up
cfa00340	Histidine metabolism	34700	21/7854	0.22577084	0.81029448	0.80529119	476003 ALDH3B1		1	AFEM_EC-up
cfa00592	alpha-Linolenic acid metabolism	34700	22/7854	0.23516082	0.81029448	0.80529119	476045 PLA2G16		1	AFEM_EC-up
cfa00534	Glycosaminoglycan biosynthesis - heparan sulfate / heparin	34700	24/7854	0.25360404	0.81029448	0.80529119	494008 XYLT1		1	AFEM_EC-up
cfa05310	Asthma	34700	25/7854	0.26265993	0.81029448	0.80529119	403469 CD40		1	AFEM_EC-up

cfa00591	Linoleic acid metabolism	34700	26/7854	0.27160709	0.81029448	0.80529119	476045 PLA2G16	1	AFEM_EC-up
cfa04966	Collecting duct acid secretion	34700	26/7854	0.27160709	0.81029448	0.80529119	486137 ATP6V1B2	1	AFEM_EC-up
cfa00601	Glycosphingolipid biosynthesis - lacto and neolacto series	34700	29/7854	0.29780902	0.83791788	0.83274402	481222 A4GALT	1	AFEM_EC-up
cfa00410	beta-Alanine metabolism	34700	30/7854	0.30633402	0.83791788	0.83274402	476003 ALDH3B1	1	AFEM_EC-up
cfa04710	Circadian rhythm	34700	30/7854	0.30633402	0.83791788	0.83274402	483641 CRY2	1	AFEM_EC-up
cfa05330	Allograft rejection	34700	30/7854	0.30633402	0.83791788	0.83274402	403469 CD40	1	AFEM_EC-up
cfa00512	Mucin type O-glycan biosynthesis	34700	31/7854	0.3147566	0.83791788	0.83274402	488946 GCNT4	1	AFEM_EC-up
cfa00350	Tyrosine metabolism	34700	32/7854	0.32307798	0.83791788	0.83274402	476003 ALDH3B1	1	AFEM_EC-up
cfa00640	Propanoate metabolism	34700	34/7854	0.33942192	0.83791788	0.83274402	100685468 ACACB	1	AFEM_EC-up
cfa05340	Primary immunodeficiency	34700	36/7854	0.35537529	0.83791788	0.83274402	403469 CD40	1	AFEM_EC-up
cfa00620	Pyruvate metabolism	34700	37/7854	0.36320841	0.83791788	0.83274402	100685468 ACACB	1	AFEM_EC-up
cfa04975	Fat digestion and absorption	34700	38/7854	0.37094734	0.83791788	0.83274402	491249 AGPAT2	1	AFEM_EC-up
cfa00513	Various types of N-glycan biosynthesis	34700	40/7854	0.38614709	0.83791788	0.83274402	478178 MAN1C1	1	AFEM_EC-up
cfa03440	Homologous recombination	34700	41/7854	0.3936101	0.83791788	0.83274402	475711 BABAM2	1	AFEM_EC-up
cfa00565	Ether lipid metabolism	34700	43/7854	0.40826784	0.83791788	0.83274402	476045 PLA2G16	1	AFEM_EC-up
cfa04672	Intestinal immune network for IgA production	34700	43/7854	0.40826784	0.83791788	0.83274402	403469 CD40	1	AFEM_EC-up
cfa04216	Ferroptosis	34700	45/7854	0.42257491	0.83791788	0.83274402	486124 SLC39A14	1	AFEM_EC-up
cfa05320	Autoimmune thyroid disease	34700	45/7854	0.42257491	0.83791788	0.83274402	403469 CD40	1	AFEM_EC-up
cfa04930	Type II diabetes mellitus	34700	46/7854	0.42959955	0.83791788	0.83274402	485548 IRS2	1	AFEM_EC-up
cfa00330	Arginine and proline metabolism	34700	47/7854	0.43653962	0.83791788	0.83274402	479081 LAP3	1	AFEM_EC-up
cfa04340	Hedgehog signaling pathway	34700	47/7854	0.43653962	0.83791788	0.83274402	607496 GAS1	1	AFEM_EC-up
cfa00140	Steroid hormone biosynthesis	34700	48/7854	0.44339612	0.83791788	0.83274402	449023 HSD11B1	1	AFEM_EC-up
cfa02010	ABC transporters	34700	48/7854	0.44339612	0.83791788	0.83274402	482284 ABCB4	1	AFEM_EC-up
cfa05414	Dilated cardiomyopathy (DCM)	42736	95/7854	0.18705657	0.32364194	0.30122906	482994 ADCY3	1	VFEM_EC-up
cfa00982	Drug metabolism - cytochrome P450	34700	49/7854	0.45017006	0.83791788	0.83274402	476003 ALDH3B1	1	AFEM_EC-up
cfa00270	Cysteine and methionine metabolism	34700	50/7854	0.45686242	0.83791788	0.83274402	474637 CDO1	1	AFEM_EC-up
cfa00510	N-Glycan biosynthesis	34700	50/7854	0.45686242	0.83791788	0.83274402	478178 MAN1C1	1	AFEM_EC-up
cfa05414	Dilated cardiomyopathy (DCM)	44013	95/7854	0.08168906	0.1193917	0.07937398	490755 SLC8A3	1	VCAV_VSMC-up
cfa00480	Glutathione metabolism	34700	52/7854	0.47000626	0.83791788	0.83274402	479081 LAP3	1	AFEM_EC-up
cfa04623	Cytosolic DNA-sensing pathway	34700	57/7854	0.50150485	0.86267127	0.85734458	403522 CCL5	1	AFEM_EC-up
cfa05213	Endometrial cancer	34700	59/7854	0.51357911	0.86267127	0.85734458	490903 AXIN2	1	AFEM_EC-up
cfa00590	Arachidonic acid metabolism	34700	61/7854	0.52536393	0.86267127	0.85734458	476045 PLA2G16	1	AFEM_EC-up
cfa00010	Glycolysis / Gluconeogenesis	34700	62/7854	0.53114995	0.86267127	0.85734458	476003 ALDH3B1	1	AFEM_EC-up
cfa04137	Mitophagy - animal	34700	64/7854	0.54251341	0.86267127	0.85734458	404007 RAB7A	1	AFEM_EC-up
cfa04720	Long-term potentiation	34700	66/7854	0.55360429	0.86267127	0.85734458	612941 CAMK2A	1	AFEM_EC-up
cfa04976	Bile secretion	34700	66/7854	0.55360429	0.86267127	0.85734458	482284 ABCB4	1	AFEM_EC-up
cfa04622	RIG-I-like receptor signaling pathway	34700	67/7854	0.55904954	0.86267127	0.85734458	611649 CYLD	1	AFEM_EC-up
cfa05031	Amphetamine addiction	34700	67/7854	0.55904954	0.86267127	0.85734458	612941 CAMK2A	1	AFEM_EC-up
cfa05211	Renal cell carcinoma	34700	67/7854	0.55904954	0.86267127	0.85734458	474578 EPAS1	1	AFEM_EC-up
cfa04520	Adherens junction	34700	70/7854	0.57499403	0.86577607	0.8604302	481162 PTPRB	1	AFEM_EC-up
cfa04971	Gastric acid secretion	34700	72/7854	0.58530537	0.86577607	0.8604302	612941 CAMK2A	1	AFEM_EC-up
cfa05218	Melanoma	34700	73/7854	0.59036782	0.86577607	0.8604302	482666 PDGFC	1	AFEM_EC-up
cfa05214	Glioma	34700	75/7854	0.60030998	0.86577607	0.8604302	612941 CAMK2A	1	AFEM_EC-up
cfa04742	Taste transduction	34700	77/7854	0.61001331	0.86577607	0.8604302	403963 HTR1D	1	AFEM_EC-up
cfa01521	EGFR tyrosine kinase inhibitor resistance	34700	80/7854	0.62413289	0.86577607	0.8604302	482666 PDGFC	1	AFEM_EC-up
cfa04979	Cholesterol metabolism	9/441	48/7854	0.00120766	0.0358674	0.03228892	478320/476724/610489/488390/403626/479915/488171/479680/490325 LIPC, ANGPTL4, CYP27A1, ABCB11, LPL, SORT1, NCEH1, LCAT, SOAT1	9	VSAP_EC-up
cfa04012	ErbB signaling pathway	34700	85/7854	0.64654993	0.86577607	0.8604302	612941 CAMK2A	1	AFEM_EC-up

cfa04911	Insulin secretion	34700	85/7854	0.64654993	0.86577607	0.8604302	612941 CAMK2A	1	AFEM_EC-up
cfa05210	Colorectal cancer	34700	85/7854	0.64654993	0.86577607	0.8604302	490903 AXIN2	1	AFEM_EC-up
cfa04658	Th1 and Th2 cell differentiation	34700	87/7854	0.65514167	0.86577607	0.8604302	100688936 DLL1	1	AFEM_EC-up
cfa04540	Gap junction	34700	88/7854	0.65935971	0.86577607	0.8604302	482666 PDGFC	1	AFEM_EC-up
cfa04912	GnRH signaling pathway	34700	89/7854	0.6635267	0.86577607	0.8604302	612941 CAMK2A	1	AFEM_EC-up
cfa04914	Progesterone-mediated oocyte maturation	34700	89/7854	0.6635267	0.86577607	0.8604302	479287 CPEB4	1	AFEM_EC-up
cfa04666	Fc gamma R-mediated phagocytosis	34700	90/7854	0.66764324	0.86577607	0.8604302	485828 HCK	1	AFEM_EC-up
cfa01522	Endocrine resistance	34700	93/7854	0.67969622	0.86577607	0.8604302	100688936 DLL1	1	AFEM_EC-up
cfa04925	Aldosterone synthesis and secretion	34700	94/7854	0.68361696	0.86577607	0.8604302	612941 CAMK2A	1	AFEM_EC-up
cfa05231	Choline metabolism in cancer	34700	96/7854	0.69131653	0.86577607	0.8604302	482666 PDGFC	1	AFEM_EC-up
cfa05215	Prostate cancer	34700	97/7854	0.69509649	0.86577607	0.8604302	482666 PDGFC	1	AFEM_EC-up
cfa04713	Circadian entrainment	34700	98/7854	0.69883064	0.86577607	0.8604302	612941 CAMK2A	1	AFEM_EC-up
cfa04625	C-type lectin receptor signaling pathway	34700	99/7854	0.70251954	0.86577607	0.8604302	611649 CYLD	1	AFEM_EC-up
cfa04750	Inflammatory mediator regulation of TRP channels	34700	100/7854	0.70616372	0.86577607	0.8604302	612941 CAMK2A	1	AFEM_EC-up
cfa04933	AGE-RAGE signaling pathway in diabetic complications	34700	100/7854	0.70616372	0.86577607	0.8604302	490153 F3	1	AFEM_EC-up
cfa05146	Amoebiasis	34700	102/7854	0.71332009	0.8660572	0.8607096	404007 RAB7A	1	AFEM_EC-up
cfa05142	Chagas disease (American trypanosomiasis)	34700	103/7854	0.71683332	0.8660572	0.8607096	403522 CCL5	1	AFEM_EC-up
cfa04928	Parathyroid hormone synthesis, secretion and action	34700	107/7854	0.73046527	0.8660572	0.8607096	403987 PTHLH	1	AFEM_EC-up
cfa04668	TNF signaling pathway	34700	108/7854	0.73377053	0.8660572	0.8607096	403522 CCL5	1	AFEM_EC-up
cfa05145	Toxoplasmosis	34700	109/7854	0.73703567	0.8660572	0.8607096	403469 CD40	1	AFEM_EC-up
cfa04726	Serotonergic synapse	34700	110/7854	0.74026118	0.8660572	0.8607096	403963 HTR1D	1	AFEM_EC-up
cfa05322	Systemic lupus erythematosus	34700	113/7854	0.74970469	0.87068392	0.86530775	403469 CD40	1	AFEM_EC-up
cfa04919	Thyroid hormone signaling pathway	34700	116/7854	0.75880833	0.87068392	0.86530775	403599 THRB	1	AFEM_EC-up
cfa04935	Growth hormone synthesis, secretion and action	34700	116/7854	0.75880833	0.87068392	0.86530775	485548 IRS2	1	AFEM_EC-up
cfa04979	Cholesterol metabolism	8/407	48/7854	0.00294715	0.14832401	0.14257754	478781/479915/476724/479680/481651/403626/488171/479072 LRP2, SORT1, ANGPTL4, LCAT, ABCA1, LPL, NCEH1, LRPAP1	8	ACOR_VSMC-up
cfa00230	Purine metabolism	34700	130/7854	0.79714271	0.89735201	0.89181117	486463 PAPSS2	1	AFEM_EC-up
cfa04728	Dopaminergic synapse	34700	132/7854	0.80210236	0.89735201	0.89181117	612941 CAMK2A	1	AFEM_EC-up
cfa04371	Apelin signaling pathway	34700	135/7854	0.80931771	0.89980043	0.89424447	479127 PPARGC1A	1	AFEM_EC-up
cfa05012	Parkinson disease	34700	138/7854	0.81627265	0.90193089	0.89636178	612614 COX7A1	1	AFEM_EC-up
cfa04261	Adrenergic signaling in cardiomyocytes	34700	147/7854	0.83566646	0.91485926	0.90921032	612941 CAMK2A	1	AFEM_EC-up
cfa04723	Retrograde endocannabinoid signaling	34700	149/7854	0.83969305	0.91485926	0.90921032	100685330 NAPEPLD	1	AFEM_EC-up
cfa04921	Oxytocin signaling pathway	34700	151/7854	0.84362199	0.91485926	0.90921032	612941 CAMK2A	1	AFEM_EC-up
cfa04621	NOD-like receptor signaling pathway	34700	158/7854	0.85663765	0.91485926	0.90921032	403522 CCL5	1	AFEM_EC-up
cfa04630	JAK-STAT signaling pathway	34700	160/7854	0.86015538	0.91485926	0.90921032	403616 LEP	1	AFEM_EC-up
cfa05164	Influenza A	34700	161/7854	0.86188208	0.91485926	0.90921032	403522 CCL5	1	AFEM_EC-up
cfa04022	cGMP-PKG signaling pathway	34700	165/7854	0.86858044	0.91485926	0.90921032	485548 IRS2	1	AFEM_EC-up
cfa05034	Alcoholism	34700	171/7854	0.87802978	0.9191072	0.91343203	482339 HDAC9	1	AFEM_EC-up
cfa05167	Kaposi sarcoma-associated herpesvirus infection	34700	178/7854	0.88820706	0.92435503	0.91864745	485828 HCK	1	AFEM_EC-up
cfa05203	Viral carcinogenesis	34700	189/7854	0.90252749	0.93334262	0.92757955	482339 HDAC9	1	AFEM_EC-up
cfa05169	Epstein-Barr virus infection	34700	193/7854	0.9072716	0.93334262	0.92757955	403469 CD40	1	AFEM_EC-up
cfa05166	Human T-cell leukemia virus 1 infection	34700	209/7854	0.92406863	0.94266346	0.93684283	403469 CD40	1	AFEM_EC-up

cfa05132	Salmonella infection	34700	212/7854	0.92686463	0.94266346	0.93684283	404007 RAB7A	1	AFEM_EC-up
cfa05163	Human cytomegalovirus infection	34700	220/7854	0.93383231	0.94438409	0.93855284	403522 CCL5	1	AFEM_EC-up
cfa04144	Endocytosis	34700	235/7854	0.94517301	0.95048297	0.94461406	404007 RAB7A	1	AFEM_EC-up
cfa05168	Herpes simplex virus 1 infection	34700	359/7854	0.98857789	0.98857789	0.98247376	403522 CCL5	1	AFEM_EC-up
cfa01210	2-Oxocarboxylic acid metabolism	44166	17/7854	0.02568481	0.2588504	0.24219921	474403 CS	1	AFEM_EC-down
cfa00534	Glycosaminoglycan biosynthesis - heparan sulfate / heparin	44166	24/7854	0.03608401	0.2588504	0.24219921	479925 EXTL2	1	AFEM_EC-down
cfa00630	Glyoxylate and dicarboxylate metabolism	44166	29/7854	0.04344961	0.2588504	0.24219921	474403 CS	1	AFEM_EC-down
cfa04215	Apoptosis - multiple species	44166	32/7854	0.04784417	0.2588504	0.24219921	477746 MAPK8	1	AFEM_EC-down
cfa00640	Propanoate metabolism	44166	34/7854	0.05076359	0.2588504	0.24219921	476562 SUCLG2	1	AFEM_EC-down
cfa04930	Type II diabetes mellitus	44166	46/7854	0.06810846	0.2588504	0.24219921	477746 MAPK8	1	AFEM_EC-down
cfa00561	Glycerolipid metabolism	44166	59/7854	0.08657053	0.2588504	0.24219921	100684122 AGPAT5	1	AFEM_EC-down
cfa04137	Mitophagy - animal	44166	64/7854	0.09358159	0.2588504	0.24219921	477746 MAPK8	1	AFEM_EC-down
cfa00970	Aminoacyl-tRNA biosynthesis	44166	66/7854	0.09637218	0.2588504	0.24219921	476132 DARS	1	AFEM_EC-down
cfa04622	RIG-I-like receptor signaling pathway	44166	67/7854	0.09776452	0.2588504	0.24219921	477746 MAPK8	1	AFEM_EC-down
cfa04664	Fc epsilon RI signaling pathway	44166	67/7854	0.09776452	0.2588504	0.24219921	477746 MAPK8	1	AFEM_EC-down
cfa04917	Prolactin signaling pathway	44166	70/7854	0.10192975	0.2588504	0.24219921	477746 MAPK8	1	AFEM_EC-down
cfa04920	Adipocytokine signaling pathway	44166	70/7854	0.10192975	0.2588504	0.24219921	477746 MAPK8	1	AFEM_EC-down
cfa05133	Pertussis	44166	71/7854	0.10331423	0.2588504	0.24219921	477746 MAPK8	1	AFEM_EC-down
cfa01230	Biosynthesis of amino acids	44166	74/7854	0.10745596	0.2588504	0.24219921	474403 CS	1	AFEM_EC-down
cfa05212	Pancreatic cancer	44166	75/7854	0.10883264	0.2588504	0.24219921	477746 MAPK8	1	AFEM_EC-down
cfa04742	Taste transduction	44166	77/7854	0.11158015	0.2588504	0.24219921	100855710 SCN9A	1	AFEM_EC-down
cfa04012	ErbB signaling pathway	44166	85/7854	0.12249273	0.2588504	0.24219921	477746 MAPK8	1	AFEM_EC-down
cfa05210	Colorectal cancer	44166	85/7854	0.12249273	0.2588504	0.24219921	477746 MAPK8	1	AFEM_EC-down
cfa04657	IL-17 signaling pathway	44166	86/7854	0.12384812	0.2588504	0.24219921	477746 MAPK8	1	AFEM_EC-down
cfa04658	Th1 and Th2 cell differentiation	44166	87/7854	0.1252016	0.2588504	0.24219921	477746 MAPK8	1	AFEM_EC-down
cfa04912	GnRH signaling pathway	44166	89/7854	0.12790281	0.2588504	0.24219921	477746 MAPK8	1	AFEM_EC-down
cfa04914	Progesterone-mediated oocyte maturation	44166	89/7854	0.12790281	0.2588504	0.24219921	477746 MAPK8	1	AFEM_EC-down
cfa01522	Endocrine resistance	44166	93/7854	0.13328231	0.2588504	0.24219921	477746 MAPK8	1	AFEM_EC-down
cfa00564	Glycerophospholipid metabolism	44166	95/7854	0.13596063	0.2588504	0.24219921	100684122 AGPAT5	1	AFEM_EC-down
cfa05017	Spinocerebellar ataxia	44166	96/7854	0.13729695	0.2588504	0.24219921	477746 MAPK8	1	AFEM_EC-down
cfa05231	Choline metabolism in cancer	44166	96/7854	0.13729695	0.2588504	0.24219921	477746 MAPK8	1	AFEM_EC-down
cfa04625	C-type lectin receptor signaling pathway	44166	99/7854	0.14129453	0.2588504	0.24219921	477746 MAPK8	1	AFEM_EC-down
cfa04750	Inflammatory mediator regulation of TRP channels	44166	100/7854	0.14262329	0.2588504	0.24219921	477746 MAPK8	1	AFEM_EC-down
cfa04933	AGE-RAGE signaling pathway in diabetic complications	44166	100/7854	0.14262329	0.2588504	0.24219921	477746 MAPK8	1	AFEM_EC-down
cfa04620	Toll-like receptor signaling pathway	44166	101/7854	0.14395015	0.2588504	0.24219921	477746 MAPK8	1	AFEM_EC-down
cfa04659	Th17 cell differentiation	44166	102/7854	0.14527514	0.2588504	0.24219921	477746 MAPK8	1	AFEM_EC-down
cfa04660	T cell receptor signaling pathway	44166	102/7854	0.14527514	0.2588504	0.24219921	477746 MAPK8	1	AFEM_EC-down
cfa05142	Chagas disease (American trypanosomiasis)	44166	103/7854	0.14659824	0.2588504	0.24219921	477746 MAPK8	1	AFEM_EC-down
cfa04668	TNF signaling pathway	44166	108/7854	0.15318564	0.2588504	0.24219921	477746 MAPK8	1	AFEM_EC-down
cfa04931	Insulin resistance	44166	109/7854	0.15449751	0.2588504	0.24219921	477746 MAPK8	1	AFEM_EC-down
cfa05145	Toxoplasmosis	44166	109/7854	0.15449751	0.2588504	0.24219921	477746 MAPK8	1	AFEM_EC-down
cfa04066	HIF-1 signaling pathway	44166	112/7854	0.15842196	0.2588504	0.24219921	612212 MKNK2	1	AFEM_EC-down
cfa04935	Growth hormone synthesis, secretion and action	44166	116/7854	0.16362859	0.2588504	0.24219921	477746 MAPK8	1	AFEM_EC-down
cfa05135	Yersinia infection	44166	117/7854	0.16492563	0.2588504	0.24219921	477746 MAPK8	1	AFEM_EC-down

cfa04722	Neurotrophin signaling pathway	44166	118/7854	0.16622082	0.2588504	0.24219921	477746 MAPK8	1	AFEM_EC-down
cfa04071	Sphingolipid signaling pathway	44166	122/7854	0.17138319	0.2588504	0.24219921	477746 MAPK8	1	AFEM_EC-down
cfa04380	Osteoclast differentiation	44166	125/7854	0.17523572	0.2588504	0.24219921	477746 MAPK8	1	AFEM_EC-down
cfa04068	FoxO signaling pathway	44166	127/7854	0.17779495	0.2588504	0.24219921	477746 MAPK8	1	AFEM_EC-down
cfa04926	Relaxin signaling pathway	44166	127/7854	0.17779495	0.2588504	0.24219921	477746 MAPK8	1	AFEM_EC-down
cfa00190	Oxidative phosphorylation	44166	131/7854	0.18289157	0.2588504	0.24219921	608244 NDUFA10	1	AFEM_EC-down
cfa04210	Apoptosis	44166	131/7854	0.18289157	0.2588504	0.24219921	477746 MAPK8	1	AFEM_EC-down
cfa03040	Spliceosome	44166	132/7854	0.1841612	0.2588504	0.24219921	403667 SRSF6	1	AFEM_EC-down
cfa04728	Dopaminergic synapse	44166	132/7854	0.1841612	0.2588504	0.24219921	477746 MAPK8	1	AFEM_EC-down
cfa04979	Cholesterol metabolism	5/241	48/7854	0.0152635	0.23580714	0.21200118	481124/479680/479915/479072/490325 LRP1, LCAT, SORT1, LRPAP1, SOAT1	5	VCAV_EC-down
cfa04140	Autophagy - animal	44166	136/7854	0.18922163	0.2588504	0.24219921	477746 MAPK8	1	AFEM_EC-down
cfa05162	Measles	44166	136/7854	0.18922163	0.2588504	0.24219921	477746 MAPK8	1	AFEM_EC-down
cfa05012	Parkinson disease	44166	138/7854	0.19174104	0.2588504	0.24219921	608244 NDUFA10	1	AFEM_EC-down
cfa04072	Phospholipase D signaling pathway	44166	148/7854	0.20423079	0.2711917	0.25374662	100684122 AGPAT5	1	AFEM_EC-down
cfa04310	Wnt signaling pathway	44166	151/7854	0.20794307	0.27166756	0.25419186	477746 MAPK8	1	AFEM_EC-down
cfa04621	NOD-like receptor signaling pathway	44166	158/7854	0.2165434	0.27268675	0.25514549	477746 MAPK8	1	AFEM_EC-down
cfa04141	Protein processing in endoplasmic reticulum	44166	160/7854	0.21898486	0.27268675	0.25514549	477746 MAPK8	1	AFEM_EC-down
cfa04217	Necroptosis	44166	160/7854	0.21898486	0.27268675	0.25514549	477746 MAPK8	1	AFEM_EC-down
cfa05161	Hepatitis B	44166	164/7854	0.22384688	0.27268675	0.25514549	477746 MAPK8	1	AFEM_EC-down
cfa05010	Alzheimer disease	44166	168/7854	0.22868114	0.27268675	0.25514549	608244 NDUFA10	1	AFEM_EC-down
cfa04530	Tight junction	44166	170/7854	0.23108791	0.27268675	0.25514549	477746 MAPK8	1	AFEM_EC-down
cfa05152	Tuberculosis	44166	171/7854	0.23228871	0.27268675	0.25514549	477746 MAPK8	1	AFEM_EC-down
cfa05167	Kaposi sarcoma-associated herpesvirus infection	44166	178/7854	0.2406463	0.27846214	0.26054937	477746 MAPK8	1	AFEM_EC-down
cfa05169	Epstein-Barr virus infection	44166	193/7854	0.25827528	0.29465208	0.27569785	477746 MAPK8	1	AFEM_EC-down
cfa04510	Focal adhesion	44166	198/7854	0.26406771	0.29556462	0.27655169	477746 MAPK8	1	AFEM_EC-down
cfa05170	Human immunodeficiency virus 1 infection	44166	200/7854	0.26637305	0.29556462	0.27655169	477746 MAPK8	1	AFEM_EC-down
cfa05166	Human T-cell leukemia virus 1 infection	44166	209/7854	0.27666539	0.29849209	0.27929084	477746 MAPK8	1	AFEM_EC-down
cfa04024	cAMP signaling pathway	44166	211/7854	0.27893453	0.29849209	0.27929084	477746 MAPK8	1	AFEM_EC-down
cfa05132	Salmonella infection	44166	212/7854	0.28006665	0.29849209	0.27929084	477746 MAPK8	1	AFEM_EC-down
cfa04014	Ras signaling pathway	44166	225/7854	0.29463668	0.30827154	0.2884412	477746 MAPK8	1	AFEM_EC-down
cfa04714	Thermogenesis	44166	227/7854	0.29685407	0.30827154	0.2884412	608244 NDUFA10	1	AFEM_EC-down
cfa04144	Endocytosis	44166	235/7854	0.30565991	0.31339813	0.29323802	490648 SNX6	1	AFEM_EC-down
cfa05206	MicroRNAs in cancer	44166	281/7854	0.35436188	0.3587914	0.33571125	489880 FSCN1	1	AFEM_EC-down
cfa05168	Herpes simplex virus 1 infection	44166	359/7854	0.42984374	0.42984374	0.40219297	403667 SRSF6	1	AFEM_EC-down
cfa00785	Lipoic acid metabolism	1/407	2174715	0.14756298	0.76328292	0.73371129	485196 LIPT2	1	ACOR_VSMC-up
cfa00440	Phosphonate and phosphinate metabolism	1/407	2174807	0.27339666	0.82260001	0.79073027	610214 CHPT1	1	ACOR_VSMC-up
cfa00730	Thiamine metabolism	1/407	14/7854	0.52554933	0.88929092	0.8548374	489554	1	ACOR_VSMC-up
cfa00604	Glycosphingolipid biosynthesis - ganglio series	1/407	15/7854	0.55017961	0.90029392	0.86541411	612022 ST3GAL5	1	ACOR_VSMC-up
cfa00061	Fatty acid biosynthesis	1/407	18/7854	0.61667677	0.90773643	0.87256828	474670 ACSL6	1	ACOR_VSMC-up
cfa00120	Primary bile acid biosynthesis	1/407	18/7854	0.61667677	0.90773643	0.87256828	479564 SCP2	1	ACOR_VSMC-up
cfa00511	Other glycan degradation	1/407	19/7854	0.63658649	0.91668454	0.88116972	485195 NEU3	1	ACOR_VSMC-up
cfa00290	Valine, leucine and isoleucine biosynthesis	1/388	2174715	0.14102081	0.82883085	0.77622704	486633 BCAT1	1	ACOR_VSMC-down
cfa00440	Phosphonate and phosphinate metabolism	1/388	2174807	0.26219872	0.99972301	0.9362731	607608 PCYT2	1	ACOR_VSMC-down
cfa00750	Vitamin B6 metabolism	1/388	2174837	0.29867513	0.99972301	0.9362731	100688635 PDXP	1	ACOR_VSMC-down
cfa03450	Non-homologous end-joining	1/388	13/7854	0.48270562	0.99972301	0.9362731	476063 FEN1	1	ACOR_VSMC-down
cfa00450	Selenocompound metabolism	1/388	16/7854	0.55576977	0.99972301	0.9362731	479991 CTH	1	ACOR_VSMC-down

cfa00053	Ascorbate and aldarate metabolism	1/388	17/7854	0.57776024	0.99972301	0.9362731	479107 UGDH	1	ACOR_VSMC-down
cfa00770	Pantothenate and CoA biosynthesis	1/388	17/7854	0.57776024	0.99972301	0.9362731	486633 BCAT1	1	ACOR_VSMC-down
cfa00220	Arginine biosynthesis	1/388	19/7854	0.61853694	0.99972301	0.9362731	480364 ARG2	1	ACOR_VSMC-down
cfa00511	Other glycan degradation	1/388	19/7854	0.61853694	0.99972301	0.9362731	487883 MANBA	1	ACOR_VSMC-down
cfa00532	Glycosaminoglycan biosynthesis - chondroitin sulfate / dermatan sulfate	1/388	19/7854	0.61853694	0.99972301	0.9362731	494008 XYLT1	1	ACOR_VSMC-down
cfa00592	alpha-Linolenic acid metabolism	1/388	22/7854	0.67245507	0.99972301	0.9362731	483792 FADS2	1	ACOR_VSMC-down
cfa00515	Mannose type O-glycan biosynthesis	1/388	24/7854	0.70410654	0.99972301	0.9362731	481579 B4GALT1	1	ACOR_VSMC-down
cfa04744	Phototransduction	1/388	28/7854	0.75854866	0.99972301	0.9362731	490902 RGS9	1	ACOR_VSMC-down
cfa00630	Glyoxylate and dicarboxylate metabolism	1/388	29/7854	0.77051941	0.99972301	0.9362731	484063 ACAT2	1	ACOR_VSMC-down
cfa00760	Nicotinate and nicotinamide metabolism	1/388	37/7854	0.84725313	0.99972301	0.9362731	479342 NNT	1	ACOR_VSMC-down
cfa05143	African trypanosomiasis	1/388	38/7854	0.85483479	0.99972301	0.9362731	476748 THOP1	1	ACOR_VSMC-down
cfa00910	Nitrogen metabolism	42370	17/7854	0.03410752	0.22413514	0.20002907	478333 CA12	1	VSAP_VSMC-up
cfa04977	Vitamin digestion and absorption	42370	26/7854	0.05171987	0.28232016	0.25195621	476775 FOLH1	1	VSAP_VSMC-up
cfa00512	Mucin type O-glycan biosynthesis	42370	31/7854	0.06137395	0.28232016	0.25195621	489790 GALNT17	1	VSAP_VSMC-up
cfa00250	Alanine, aspartate and glutamate metabolism	42370	37/7854	0.07283722	0.28484483	0.25420935	476775 FOLH1	1	VSAP_VSMC-up
cfa00260	Glycine, serine and threonine metabolism	42370	40/7854	0.0785195	0.28484483	0.25420935	414733 GATM	1	VSAP_VSMC-up
cfa00330	Arginine and proline metabolism	42370	47/7854	0.09165147	0.28484483	0.25420935	414733 GATM	1	VSAP_VSMC-up
cfa00514	Other types of O-glycan biosynthesis	42370	47/7854	0.09165147	0.28484483	0.25420935	489790 GALNT17	1	VSAP_VSMC-up
cfa05134	Legionellosis	42370	50/7854	0.09722557	0.28484483	0.25420935	448807 TLR2	1	VSAP_VSMC-up
cfa04979	Cholesterol metabolism	4/357	48/7854	0.17276108	0.39508478	0.28917731	486241/481651/488230/100856363 SCARB1, ABCA1, MYLIP, LIPA	4	VCAV_EC-up
cfa05321	Inflammatory bowel disease (IBD)	42370	61/7854	0.11739078	0.31764564	0.28348238	448807 TLR2	1	VSAP_VSMC-up
cfa05140	Leishmaniasis	42370	70/7854	0.13357468	0.31976033	0.28536963	448807 TLR2	1	VSAP_VSMC-up
cfa05218	Melanoma	42370	73/7854	0.1389072	0.31976033	0.28536963	608459 FGF5	1	VSAP_VSMC-up
cfa05100	Bacterial invasion of epithelial cells	42370	75/7854	0.1424451	0.31976033	0.28536963	403980 CAV1	1	VSAP_VSMC-up
cfa05150	Staphylococcus aureus infection	42370	78/7854	0.14772641	0.31976033	0.28536963	488121 MASP1	1	VSAP_VSMC-up
cfa05323	Rheumatoid arthritis	42370	83/7854	0.15646088	0.31976033	0.28536963	448807 TLR2	1	VSAP_VSMC-up
cfa04979	Cholesterol metabolism	2/279	48/7854	0.51294412	0.78134511	0.6623697	486241/476438 SCARB1, APOE	2	VSAP_EC-down
cfa05235	PD-L1 expression and PD-1 checkpoint pathway in cancer	42370	89/7854	0.16683147	0.31976033	0.28536963	448807 TLR2	1	VSAP_VSMC-up
cfa04620	Toll-like receptor signaling pathway	42370	101/7854	0.18721508	0.32466894	0.28975032	448807 TLR2	1	VSAP_VSMC-up
cfa05146	Amoebiasis	42370	102/7854	0.18889244	0.32466894	0.28975032	448807 TLR2	1	VSAP_VSMC-up
cfa05142	Chagas disease (American trypanosomiasis)	42370	103/7854	0.19056655	0.32466894	0.28975032	448807 TLR2	1	VSAP_VSMC-up
cfa05145	Toxoplasmosis	42370	109/7854	0.2005434	0.32946415	0.29402979	448807 TLR2	1	VSAP_VSMC-up
cfa04979	Cholesterol metabolism	2/388	48/7854	0.69383826	0.99972301	0.9362731	474681/478320 VDAC1, LIPC	2	ACOR_VSMC-down
cfa04979	Cholesterol metabolism	34700	48/7854	0.44339612	0.83791788	0.83274402	479915 SORT1	1	AFEM_EC-up
cfa05162	Measles	42370	136/7854	0.24402931	0.36210801	0.32316276	448807 TLR2	1	VSAP_VSMC-up
cfa05224	Breast cancer	42370	142/7854	0.25338684	0.36424358	0.32506864	608459 FGF5	1	VSAP_VSMC-up
cfa05226	Gastric cancer	42370	149/7854	0.26416668	0.36823234	0.32862841	608459 FGF5	1	VSAP_VSMC-up
cfa05161	Hepatitis B	42370	164/7854	0.28677692	0.38799231	0.34626316	448807 TLR2	1	VSAP_VSMC-up
cfa05152	Tuberculosis	42370	171/7854	0.29710396	0.39047949	0.34848284	448807 TLR2	1	VSAP_VSMC-up
cfa04020	Calcium signaling pathway	42370	192/7854	0.3272503	0.39810246	0.35528595	608643 STIM1	1	VSAP_VSMC-up
cfa05169	Epstein-Barr virus infection	42370	193/7854	0.32865516	0.39810246	0.35528595	448807 TLR2	1	VSAP_VSMC-up

cfa05170	Human immunodeficiency virus 1 infection	42370	200/7854	0.3384124	0.39810246	0.35528595	448807	TLR2	1	VSAP_VSMC-up
NA	NA	NA	NA	NA	NA	NA	NA	NA	NA	VSAP_VSMC-up
NA	NA	NA	NA	NA	NA	NA	NA	NA	NA	VSAP_VSMC-up
NA	NA	NA	NA	NA	NA	NA	NA	NA	NA	VSAP_VSMC-up
NA	NA	NA	NA	NA	NA	NA	NA	NA	NA	VSAP_VSMC-up
NA	NA	NA	NA	NA	NA	NA	NA	NA	NA	VSAP_VSMC-up
NA	NA	NA	NA	NA	NA	NA	NA	NA	NA	VSAP_VSMC-up
NA	NA	NA	NA	NA	NA	NA	NA	NA	NA	VSAP_VSMC-up
NA	NA	NA	NA	NA	NA	NA	NA	NA	NA	VSAP_VSMC-up
NA	NA	NA	NA	NA	NA	NA	NA	NA	NA	VSAP_VSMC-up
NA	NA	NA	NA	NA	NA	NA	NA	NA	NA	VSAP_VSMC-up
NA	NA	NA	NA	NA	NA	NA	NA	NA	NA	VSAP_VSMC-up
NA	NA	NA	NA	NA	NA	NA	NA	NA	NA	VSAP_VSMC-up
NA	NA	NA	NA	NA	NA	NA	NA	NA	NA	VSAP_VSMC-up
NA	NA	NA	NA	NA	NA	NA	NA	NA	NA	VSAP_VSMC-up
NA	NA	NA	NA	NA	NA	NA	NA	NA	NA	VSAP_VSMC-up
NA	NA	NA	NA	NA	NA	NA	NA	NA	NA	VSAP_VSMC-up
NA	NA	NA	NA	NA	NA	NA	NA	NA	NA	VSAP_VSMC-up
NA	NA	NA	NA	NA	NA	NA	NA	NA	NA	VSAP_VSMC-up
NA	NA	NA	NA	NA	NA	NA	NA	NA	NA	VSAP_VSMC-up
NA	NA	NA	NA	NA	NA	NA	NA	NA	NA	VSAP_VSMC-up
NA	NA	NA	NA	NA	NA	NA	NA	NA	NA	VSAP_VSMC-up
NA	NA	NA	NA	NA	NA	NA	NA	NA	NA	VSAP_VSMC-up
NA	NA	NA	NA	NA	NA	NA	NA	NA	NA	VSAP_VSMC-up
NA	NA	NA	NA	NA	NA	NA	NA	NA	NA	VSAP_VSMC-up
NA	NA	NA	NA	NA	NA	NA	NA	NA	NA	VSAP_VSMC-up
NA	NA	NA	NA	NA	NA	NA	NA	NA	NA	VSAP_VSMC-up
NA	NA	NA	NA	NA	NA	NA	NA	NA	NA	VSAP_VSMC-up
NA	NA	NA	NA	NA	NA	NA	NA	NA	NA	VSAP_VSMC-up
NA	NA	NA	NA	NA	NA	NA	NA	NA	NA	VSAP_VSMC-up
NA	NA	NA	NA	NA	NA	NA	NA	NA	NA	VSAP_VSMC-up
NA	NA	NA	NA	NA	NA	NA	NA	NA	NA	VSAP_VSMC-up
NA	NA	NA	NA	NA	NA	NA	NA	NA	NA	VSAP_VSMC-up
NA	NA	NA	NA	NA	NA	NA	NA	NA	NA	VSAP_VSMC-up
NA	NA	NA	NA	NA	NA	NA	NA	NA	NA	VSAP_VSMC-up
NA	NA	NA	NA	NA	NA	NA	NA	NA	NA	VSAP_VSMC-up
NA	NA	NA	NA	NA	NA	NA	NA	NA	NA	VSAP_VSMC-up
NA	NA	NA	NA	NA	NA	NA	NA	NA	NA	VSAP_VSMC-up
NA	NA	NA	NA	NA	NA	NA	NA	NA	NA	VSAP_VSMC-up
NA	NA	NA	NA	NA	NA	NA	NA	NA	NA	VSAP_VSMC-up
NA	NA	NA	NA	NA	NA	NA	NA	NA	NA	VSAP_VSMC-up
NA	NA	NA	NA	NA	NA	NA	NA	NA	NA	VSAP_VSMC-up
NA	NA	NA	NA	NA	NA	NA	NA	NA	NA	VSAP_VSMC-up
NA	NA	NA	NA	NA	NA	NA	NA	NA	NA	VSAP_VSMC-up
NA	NA	NA	NA	NA	NA	NA	NA	NA	NA	VSAP_VSMC-up
NA	NA	NA	NA	NA	NA	NA	NA	NA	NA	VSAP_VSMC-up
NA	NA	NA	NA	NA	NA	NA	NA	NA	NA	VSAP_VSMC-up
NA	NA	NA	NA	NA	NA	NA	NA	NA	NA	VSAP_VSMC-up
NA	NA	NA	NA	NA	NA	NA	NA	NA	NA	VSAP_VSMC-up
NA	NA	NA	NA	NA	NA	NA	NA	NA	NA	VSAP_VSMC-up
NA	NA	NA	NA	NA	NA	NA	NA	NA	NA	VSAP_VSMC-up
NA	NA	NA	NA	NA	NA	NA	NA	NA	NA	VSAP_VSMC-up
NA	NA	NA	NA	NA	NA	NA	NA	NA	NA	VSAP_VSMC-up
NA	NA	NA	NA	NA	NA	NA	NA	NA	NA	VSAP_VSMC-up
NA	NA	NA	NA	NA	NA	NA	NA	NA	NA	VSAP_VSMC-up
NA	NA	NA	NA	NA	NA	NA	NA	NA	NA	VSAP_VSMC-up
NA	NA	NA	NA	NA	NA	NA	NA	NA	NA	VSAP_VSMC-up
NA	NA	NA	NA	NA	NA	NA	NA	NA	NA	VSAP_VSMC-up
NA	NA	NA	NA	NA	NA	NA	NA	NA	NA	VSAP_VSMC-up
NA	NA	NA	NA	NA	NA	NA	NA	NA	NA	VSAP_VSMC-up
NA	NA	NA	NA	NA	NA	NA	NA	NA	NA	VSAP_VSMC-up
NA	NA	NA	NA	NA	NA	NA	NA	NA	NA	VSAP_VSMC-up
NA	NA	NA	NA	NA	NA	NA	NA	NA	NA	VSAP_VSMC-up
NA	NA	NA	NA	NA	NA	NA	NA	NA	NA	VSAP_VSMC-up
NA	NA	NA	NA	NA	NA	NA	NA	NA	NA	VSAP_VSMC-up
NA	NA	NA	NA	NA	NA	NA	NA	NA	NA	VSAP_VSMC-up
NA	NA	NA	NA	NA	NA	NA	NA	NA	NA	VSAP_VSMC-up
NA	NA	NA	NA	NA	NA	NA	NA	NA	NA	VSAP_VSMC-up
NA	NA	NA	NA	NA	NA	NA	NA	NA	NA	VSAP_VSMC-up
NA	NA	NA	NA	NA	NA	NA	NA	NA	NA	VSAP_VSMC-up
NA	NA	NA	NA	NA	NA	NA	NA	NA	NA	VSAP_VSMC-up
NA	NA	NA	NA	NA	NA	NA	NA	NA	NA	VSAP_VSMC-up

NA	NA	NA	NA	NA	NA	NA	NA	NA	NA	NA	VSAP_VSMC-up
NA	NA	NA	NA	NA	NA	NA	NA	NA	NA	NA	VSAP_VSMC-up
NA	NA	NA	NA	NA	NA	NA	NA	NA	NA	NA	VSAP_VSMC-up
NA	NA	NA	NA	NA	NA	NA	NA	NA	NA	NA	VSAP_VSMC-up
NA	NA	NA	NA	NA	NA	NA	NA	NA	NA	NA	VSAP_VSMC-up
NA	NA	NA	NA	NA	NA	NA	NA	NA	NA	NA	VSAP_VSMC-up
NA	NA	NA	NA	NA	NA	NA	NA	NA	NA	NA	VSAP_VSMC-up
NA	NA	NA	NA	NA	NA	NA	NA	NA	NA	NA	VSAP_VSMC-up
NA	NA	NA	NA	NA	NA	NA	NA	NA	NA	NA	VSAP_VSMC-up
NA	NA	NA	NA	NA	NA	NA	NA	NA	NA	NA	VSAP_VSMC-up
NA	NA	NA	NA	NA	NA	NA	NA	NA	NA	NA	VSAP_VSMC-up
NA	NA	NA	NA	NA	NA	NA	NA	NA	NA	NA	VSAP_VSMC-up
NA	NA	NA	NA	NA	NA	NA	NA	NA	NA	NA	VSAP_VSMC-up
NA	NA	NA	NA	NA	NA	NA	NA	NA	NA	NA	VSAP_VSMC-up
NA	NA	NA	NA	NA	NA	NA	NA	NA	NA	NA	VSAP_VSMC-up
NA	NA	NA	NA	NA	NA	NA	NA	NA	NA	NA	VSAP_VSMC-up
NA	NA	NA	NA	NA	NA	NA	NA	NA	NA	NA	VSAP_VSMC-up
NA	NA	NA	NA	NA	NA	NA	NA	NA	NA	NA	VSAP_VSMC-up
NA	NA	NA	NA	NA	NA	NA	NA	NA	NA	NA	VSAP_VSMC-up
NA	NA	NA	NA	NA	NA	NA	NA	NA	NA	NA	VSAP_VSMC-up
NA	NA	NA	NA	NA	NA	NA	NA	NA	NA	NA	VSAP_VSMC-up
NA	NA	NA	NA	NA	NA	NA	NA	NA	NA	NA	VSAP_VSMC-up
NA	NA	NA	NA	NA	NA	NA	NA	NA	NA	NA	VSAP_VSMC-up
NA	NA	NA	NA	NA	NA	NA	NA	NA	NA	NA	VSAP_VSMC-up
NA	NA	NA	NA	NA	NA	NA	NA	NA	NA	NA	VSAP_VSMC-up
NA	NA	NA	NA	NA	NA	NA	NA	NA	NA	NA	VSAP_VSMC-up
NA	NA	NA	NA	NA	NA	NA	NA	NA	NA	NA	VSAP_VSMC-up
NA	NA	NA	NA	NA	NA	NA	NA	NA	NA	NA	VSAP_VSMC-up
NA	NA	NA	NA	NA	NA	NA	NA	NA	NA	NA	VSAP_VSMC-up
NA	NA	NA	NA	NA	NA	NA	NA	NA	NA	NA	VSAP_VSMC-up
NA	NA	NA	NA	NA	NA	NA	NA	NA	NA	NA	VSAP_VSMC-up
NA	NA	NA	NA	NA	NA	NA	NA	NA	NA	NA	VSAP_VSMC-up
NA	NA	NA	NA	NA	NA	NA	NA	NA	NA	NA	VSAP_VSMC-up
NA	NA	NA	NA	NA	NA	NA	NA	NA	NA	NA	VSAP_VSMC-up
NA	NA	NA	NA	NA	NA	NA	NA	NA	NA	NA	VSAP_VSMC-up
NA	NA	NA	NA	NA	NA	NA	NA	NA	NA	NA	VSAP_VSMC-up
NA	NA	NA	NA	NA	NA	NA	NA	NA	NA	NA	VSAP_VSMC-up
NA	NA	NA	NA	NA	NA	NA	NA	NA	NA	NA	VSAP_VSMC-up
NA	NA	NA	NA	NA	NA	NA	NA	NA	NA	NA	VSAP_VSMC-up
NA	NA	NA	NA	NA	NA	NA	NA	NA	NA	NA	VSAP_VSMC-up
NA	NA	NA	NA	NA	NA	NA	NA	NA	NA	NA	VSAP_VSMC-up
NA	NA	NA	NA	NA	NA	NA	NA	NA	NA	NA	VSAP_VSMC-up
NA	NA	NA	NA	NA	NA	NA	NA	NA	NA	NA	VSAP_VSMC-up
NA	NA	NA	NA	NA	NA	NA	NA	NA	NA	NA	VSAP_VSMC-up
NA	NA	NA	NA	NA	NA	NA	NA	NA	NA	NA	VSAP_VSMC-up
NA	NA	NA	NA	NA	NA	NA	NA	NA	NA	NA	VSAP_VSMC-up
NA	NA	NA	NA	NA	NA	NA	NA	NA	NA	NA	VSAP_VSMC-up
NA	NA	NA	NA	NA	NA	NA	NA	NA	NA	NA	VSAP_VSMC-up
NA	NA	NA	NA	NA	NA	NA	NA	NA	NA	NA	VSAP_VSMC-up
NA	NA	NA	NA	NA	NA	NA	NA	NA	NA	NA	VSAP_VSMC-up
NA	NA	NA	NA	NA	NA	NA	NA	NA	NA	NA	VSAP_VSMC-up
NA	NA	NA	NA	NA	NA	NA	NA	NA	NA	NA	VSAP_VSMC-up
NA	NA	NA	NA	NA	NA	NA	NA	NA	NA	NA	VSAP_VSMC-up
NA	NA	NA	NA	NA	NA	NA	NA	NA	NA	NA	VSAP_VSMC-up
NA	NA	NA	NA	NA	NA	NA	NA	NA	NA	NA	VSAP_VSMC-up
NA	NA	NA	NA	NA	NA	NA	NA	NA	NA	NA	VSAP_VSMC-up
NA	NA	NA	NA	NA	NA	NA	NA	NA	NA	NA	VSAP_VSMC-up
NA	NA	NA	NA	NA	NA	NA	NA	NA	NA	NA	VSAP_VSMC-up
NA	NA	NA	NA	NA	NA	NA	NA	NA	NA	NA	VSAP_VSMC-up
NA	NA	NA	NA	NA	NA	NA	NA	NA	NA	NA	VSAP_VSMC-up
NA	NA	NA	NA	NA	NA	NA	NA	NA	NA	NA	VSAP_VSMC-up
NA	NA	NA	NA	NA	NA	NA	NA	NA	NA	NA	VSAP_VSMC-up
NA	NA	NA	NA	NA	NA	NA	NA	NA	NA	NA	VSAP_VSMC-up
NA	NA	NA	NA	NA	NA	NA	NA	NA	NA	NA	VSAP_VSMC-up
NA	NA	NA	NA	NA	NA	NA	NA	NA	NA	NA	VSAP_VSMC-up
NA	NA	NA	NA	NA	NA	NA	NA	NA	NA	NA	VSAP_VSMC-up
NA	NA	NA	NA	NA	NA	NA	NA	NA	NA	NA	VSAP_VSMC-up
NA	NA	NA	NA	NA	NA	NA	NA	NA	NA	NA	VSAP_VSMC-up
NA	NA	NA	NA	NA	NA	NA	NA	NA	NA	NA	VSAP_VSMC-up
NA	NA	NA	NA	NA	NA	NA	NA	NA	NA	NA	VSAP_VSMC-up
NA	NA	NA	NA	NA	NA	NA	NA	NA	NA	NA	VSAP_VSMC-up
NA	NA	NA	NA	NA	NA	NA	NA	NA	NA	NA	VSAP_VSMC-up
NA	NA	NA	NA	NA	NA	NA	NA	NA	NA	NA	VSAP_VSMC-up
NA	NA	NA	NA	NA	NA	NA	NA	NA	NA	NA	VSAP_VSMC-up

NA	NA	NA	NA	NA	NA	NA	NA NA	NA	VSAP_VSMC-up
NA	NA	NA	NA	NA	NA	NA	NA NA	NA	VSAP_VSMC-up
NA	NA	NA	NA	NA	NA	NA	NA NA	NA	VSAP_VSMC-up
NA	NA	NA	NA	NA	NA	NA	NA NA	NA	VSAP_VSMC-up
NA	NA	NA	NA	NA	NA	NA	NA NA	NA	VSAP_VSMC-up
NA	NA	NA	NA	NA	NA	NA	NA NA	NA	VSAP_VSMC-up
NA	NA	NA	NA	NA	NA	NA	NA NA	NA	VSAP_VSMC-up
NA	NA	NA	NA	NA	NA	NA	NA NA	NA	VSAP_VSMC-up
NA	NA	NA	NA	NA	NA	NA	NA NA	NA	VSAP_VSMC-up
cfa04015	Rap1 signaling pathway	42370	209/7854	0.35076214	0.39810246	0.35528595	608459 FGF5	1	VSAP_VSMC-up
cfa04810	Regulation of actin cytoskeleton	42370	211/7854	0.35347701	0.39810246	0.35528595	608459 FGF5	1	VSAP_VSMC-up
cfa05132	Salmonella infection	42370	212/7854	0.35483046	0.39810246	0.35528595	448807 TLR2	1	VSAP_VSMC-up
cfa04014	Ras signaling pathway	42370	225/7854	0.37218523	0.40763145	0.36379008	608459 FGF5	1	VSAP_VSMC-up
cfa04144	Endocytosis	42370	235/7854	0.3852362	0.41211314	0.36778976	403980 CAV1	1	VSAP_VSMC-up
cfa04010	MAPK signaling pathway	42370	288/7854	0.45026407	0.47073062	0.42010284	608459 FGF5	1	VSAP_VSMC-up
cfa05165	Human papillomavirus infection	42370	328/7854	0.49500917	0.50600937	0.45158731	476665 COMP	1	VSAP_VSMC-up
cfa05168	Herpes simplex virus 1 infection	42370	359/7854	0.52731308	0.52731308	0.47059978	448807 TLR2	1	VSAP_VSMC-up
cfa00730	Thiamine metabolism	43891	14/7854	0.00533875	0.06878116	0.02413374	490204 AK5	1	VSAP_VSMC-down
cfa00982	Drug metabolism - cytochrome P450	43891	49/7854	0.0186024	0.06878116	0.02413374	477558	1	VSAP_VSMC-down
cfa00480	Glutathione metabolism	43891	52/7854	0.01973377	0.06878116	0.02413374	477558	1	VSAP_VSMC-down
cfa00980	Metabolism of xenobiotics by cytochrome P450	43891	52/7854	0.01973377	0.06878116	0.02413374	477558	1	VSAP_VSMC-down
cfa05204	Chemical carcinogenesis	43891	55/7854	0.02086427	0.06878116	0.02413374	477558	1	VSAP_VSMC-down
cfa00983	Drug metabolism - other enzymes	43891	63/7854	0.02387467	0.06878116	0.02413374	477558	1	VSAP_VSMC-down
cfa04520	Adherens junction	43891	70/7854	0.02650371	0.06878116	0.02413374	481628 TGFBR1	1	VSAP_VSMC-down
cfa01524	Platinum drug resistance	43891	72/7854	0.027254	0.06878116	0.02413374	477558	1	VSAP_VSMC-down
cfa05212	Pancreatic cancer	43891	75/7854	0.02837871	0.06878116	0.02413374	481628 TGFBR1	1	VSAP_VSMC-down
cfa05220	Chronic myeloid leukemia	43891	76/7854	0.02875342	0.06878116	0.02413374	481628 TGFBR1	1	VSAP_VSMC-down
cfa05210	Colorectal cancer	43891	85/7854	0.03212147	0.06878116	0.02413374	481628 TGFBR1	1	VSAP_VSMC-down
cfa04350	TGF-beta signaling pathway	43891	92/7854	0.03473567	0.06878116	0.02413374	481628 TGFBR1	1	VSAP_VSMC-down
cfa04933	AGE-RAGE signaling pathway in diabetic complications	43891	100/7854	0.03771756	0.06878116	0.02413374	481628 TGFBR1	1	VSAP_VSMC-down
cfa04659	Th17 cell differentiation	43891	102/7854	0.03846208	0.06878116	0.02413374	481628 TGFBR1	1	VSAP_VSMC-down
cfa05142	Chagas disease (American trypanosomiasis)	43891	103/7854	0.03883419	0.06878116	0.02413374	481628 TGFBR1	1	VSAP_VSMC-down
cfa04380	Osteoclast differentiation	43891	125/7854	0.04699639	0.06878116	0.02413374	481628 TGFBR1	1	VSAP_VSMC-down
cfa04068	FoxO signaling pathway	43891	127/7854	0.0477361	0.06878116	0.02413374	481628 TGFBR1	1	VSAP_VSMC-down
cfa04926	Relaxin signaling pathway	43891	127/7854	0.0477361	0.06878116	0.02413374	481628 TGFBR1	1	VSAP_VSMC-down
cfa00230	Purine metabolism	43891	130/7854	0.04884496	0.06878116	0.02413374	490204 AK5	1	VSAP_VSMC-down
cfa04371	Apelin signaling pathway	43891	135/7854	0.05069115	0.06878116	0.02413374	481628 TGFBR1	1	VSAP_VSMC-down
cfa04110	Cell cycle	36/388	123/7854	5.9262E-19	8.0596E-17	7.5481E-17	476131/477871/479737/481839/480928/479307/490050/484622/477857/100856079/475750/475359/489971/481131/477166/478324/482492/607879/487443/480527/477237/100855917/475219/476070/486886/100855704/483845/485674/608420/100682599/100855774/481895/608350/486421/484780/449494 MCM6, MCM4, MCM7, MCM3, SMC1A, , PKMYT1, MCM2, BUB3, CDK1, ANAPC1, CDKN2C, PLK1, CDK4, PCNA, CCNB2, ORC6, ESPL1, MAD2L2, CDC6, YWHAB, ORC1, DBF4, , SMC3, CDK2, CCNA2, STAG1, , BUB1, CDC20, TTK, BUB1B, CDC45, CDC25A, CCNB3	36	ACOR_VSMC-down
cfa05226	Gastric cancer	43891	149/7854	0.05584776	0.06878116	0.02413374	481628 TGFBR1	1	VSAP_VSMC-down
NA	NA	NA	NA	NA	NA	NA	NA NA	NA	VSAP_VSMC-down
NA	NA	NA	NA	NA	NA	NA	NA NA	NA	VSAP_VSMC-down
NA	NA	NA	NA	NA	NA	NA	NA NA	NA	VSAP_VSMC-down
NA	NA	NA	NA	NA	NA	NA	NA NA	NA	VSAP_VSMC-down
NA	NA	NA	NA	NA	NA	NA	NA NA	NA	VSAP_VSMC-down

NA	NA	NA	NA	NA	NA	NA	NA	NA	NA	VSAP_VSMC-down
NA	NA	NA	NA	NA	NA	NA	NA	NA	NA	VSAP_VSMC-down
NA	NA	NA	NA	NA	NA	NA	NA	NA	NA	VSAP_VSMC-down
NA	NA	NA	NA	NA	NA	NA	NA	NA	NA	VSAP_VSMC-down
NA	NA	NA	NA	NA	NA	NA	NA	NA	NA	VSAP_VSMC-down
NA	NA	NA	NA	NA	NA	NA	NA	NA	NA	VSAP_VSMC-down
NA	NA	NA	NA	NA	NA	NA	NA	NA	NA	VSAP_VSMC-down
NA	NA	NA	NA	NA	NA	NA	NA	NA	NA	VSAP_VSMC-down
NA	NA	NA	NA	NA	NA	NA	NA	NA	NA	VSAP_VSMC-down
NA	NA	NA	NA	NA	NA	NA	NA	NA	NA	VSAP_VSMC-down
NA	NA	NA	NA	NA	NA	NA	NA	NA	NA	VSAP_VSMC-down
NA	NA	NA	NA	NA	NA	NA	NA	NA	NA	VSAP_VSMC-down
NA	NA	NA	NA	NA	NA	NA	NA	NA	NA	VSAP_VSMC-down
NA	NA	NA	NA	NA	NA	NA	NA	NA	NA	VSAP_VSMC-down
NA	NA	NA	NA	NA	NA	NA	NA	NA	NA	VSAP_VSMC-down
NA	NA	NA	NA	NA	NA	NA	NA	NA	NA	VSAP_VSMC-down
NA	NA	NA	NA	NA	NA	NA	NA	NA	NA	VSAP_VSMC-down
NA	NA	NA	NA	NA	NA	NA	NA	NA	NA	VSAP_VSMC-down
NA	NA	NA	NA	NA	NA	NA	NA	NA	NA	VSAP_VSMC-down
NA	NA	NA	NA	NA	NA	NA	NA	NA	NA	VSAP_VSMC-down
NA	NA	NA	NA	NA	NA	NA	NA	NA	NA	VSAP_VSMC-down
NA	NA	NA	NA	NA	NA	NA	NA	NA	NA	VSAP_VSMC-down
NA	NA	NA	NA	NA	NA	NA	NA	NA	NA	VSAP_VSMC-down
NA	NA	NA	NA	NA	NA	NA	NA	NA	NA	VSAP_VSMC-down
NA	NA	NA	NA	NA	NA	NA	NA	NA	NA	VSAP_VSMC-down
NA	NA	NA	NA	NA	NA	NA	NA	NA	NA	VSAP_VSMC-down
NA	NA	NA	NA	NA	NA	NA	NA	NA	NA	VSAP_VSMC-down
NA	NA	NA	NA	NA	NA	NA	NA	NA	NA	VSAP_VSMC-down
NA	NA	NA	NA	NA	NA	NA	NA	NA	NA	VSAP_VSMC-down
NA	NA	NA	NA	NA	NA	NA	NA	NA	NA	VSAP_VSMC-down
NA	NA	NA	NA	NA	NA	NA	NA	NA	NA	VSAP_VSMC-down
NA	NA	NA	NA	NA	NA	NA	NA	NA	NA	VSAP_VSMC-down
NA	NA	NA	NA	NA	NA	NA	NA	NA	NA	VSAP_VSMC-down
NA	NA	NA	NA	NA	NA	NA	NA	NA	NA	VSAP_VSMC-down
NA	NA	NA	NA	NA	NA	NA	NA	NA	NA	VSAP_VSMC-down
NA	NA	NA	NA	NA	NA	NA	NA	NA	NA	VSAP_VSMC-down
NA	NA	NA	NA	NA	NA	NA	NA	NA	NA	VSAP_VSMC-down
NA	NA	NA	NA	NA	NA	NA	NA	NA	NA	VSAP_VSMC-down
NA	NA	NA	NA	NA	NA	NA	NA	NA	NA	VSAP_VSMC-down
NA	NA	NA	NA	NA	NA	NA	NA	NA	NA	VSAP_VSMC-down
NA	NA	NA	NA	NA	NA	NA	NA	NA	NA	VSAP_VSMC-down
NA	NA	NA	NA	NA	NA	NA	NA	NA	NA	VSAP_VSMC-down
NA	NA	NA	NA	NA	NA	NA	NA	NA	NA	VSAP_VSMC-down
NA	NA	NA	NA	NA	NA	NA	NA	NA	NA	VSAP_VSMC-down
NA	NA	NA	NA	NA	NA	NA	NA	NA	NA	VSAP_VSMC-down
NA	NA	NA	NA	NA	NA	NA	NA	NA	NA	VSAP_VSMC-down
NA	NA	NA	NA	NA	NA	NA	NA	NA	NA	VSAP_VSMC-down
NA	NA	NA	NA	NA	NA	NA	NA	NA	NA	VSAP_VSMC-down
NA	NA	NA	NA	NA	NA	NA	NA	NA	NA	VSAP_VSMC-down
NA	NA	NA	NA	NA	NA	NA	NA	NA	NA	VSAP_VSMC-down
NA	NA	NA	NA	NA	NA	NA	NA	NA	NA	VSAP_VSMC-down
NA	NA	NA	NA	NA	NA	NA	NA	NA	NA	VSAP_VSMC-down
NA	NA	NA	NA	NA	NA	NA	NA	NA	NA	VSAP_VSMC-down
NA	NA	NA	NA	NA	NA	NA	NA	NA	NA	VSAP_VSMC-down
NA	NA	NA	NA	NA	NA	NA	NA	NA	NA	VSAP_VSMC-down
NA	NA	NA	NA	NA	NA	NA	NA	NA	NA	VSAP_VSMC-down
NA	NA	NA	NA	NA	NA	NA	NA	NA	NA	VSAP_VSMC-down
NA	NA	NA	NA	NA	NA	NA	NA	NA	NA	VSAP_VSMC-down
NA	NA	NA	NA	NA	NA	NA	NA	NA	NA	VSAP_VSMC-down
NA	NA	NA	NA	NA	NA	NA	NA	NA	NA	VSAP_VSMC-down
NA	NA	NA	NA	NA	NA	NA	NA	NA	NA	VSAP_VSMC-down
NA	NA	NA	NA	NA	NA	NA	NA	NA	NA	VSAP_VSMC-down
NA	NA	NA	NA	NA	NA	NA	NA	NA	NA	VSAP_VSMC-down
NA	NA	NA	NA	NA	NA	NA	NA	NA	NA	VSAP_VSMC-down
NA	NA	NA	NA	NA	NA	NA	NA	NA	NA	VSAP_VSMC-down
NA	NA	NA	NA	NA	NA	NA	NA	NA	NA	VSAP_VSMC-down
NA	NA	NA	NA	NA	NA	NA	NA	NA	NA	VSAP_VSMC-down
NA	NA	NA	NA	NA	NA	NA	NA	NA	NA	VSAP_VSMC-down
NA	NA	NA	NA	NA	NA	NA	NA	NA	NA	VSAP_VSMC-down
NA	NA	NA	NA	NA	NA	NA	NA	NA	NA	VSAP_VSMC-down
NA	NA	NA	NA	NA	NA	NA	NA	NA	NA	VSAP_VSMC-down
NA	NA	NA	NA	NA	NA	NA	NA	NA	NA	VSAP_VSMC-down
NA	NA	NA	NA	NA	NA	NA	NA	NA	NA	VSAP_VSMC-down
NA	NA	NA	NA	NA	NA	NA	NA	NA	NA	VSAP_VSMC-down

NA	NA	NA	NA	NA	NA	NA	NA	NA	NA	VSAP_VSMC-down
NA	NA	NA	NA	NA	NA	NA	NA	NA	NA	VSAP_VSMC-down
NA	NA	NA	NA	NA	NA	NA	NA	NA	NA	VSAP_VSMC-down
NA	NA	NA	NA	NA	NA	NA	NA	NA	NA	VSAP_VSMC-down
NA	NA	NA	NA	NA	NA	NA	NA	NA	NA	VSAP_VSMC-down
NA	NA	NA	NA	NA	NA	NA	NA	NA	NA	VSAP_VSMC-down
NA	NA	NA	NA	NA	NA	NA	NA	NA	NA	VSAP_VSMC-down
NA	NA	NA	NA	NA	NA	NA	NA	NA	NA	VSAP_VSMC-down
NA	NA	NA	NA	NA	NA	NA	NA	NA	NA	VSAP_VSMC-down
NA	NA	NA	NA	NA	NA	NA	NA	NA	NA	VSAP_VSMC-down
NA	NA	NA	NA	NA	NA	NA	NA	NA	NA	VSAP_VSMC-down
NA	NA	NA	NA	NA	NA	NA	NA	NA	NA	VSAP_VSMC-down
NA	NA	NA	NA	NA	NA	NA	NA	NA	NA	VSAP_VSMC-down
NA	NA	NA	NA	NA	NA	NA	NA	NA	NA	VSAP_VSMC-down
NA	NA	NA	NA	NA	NA	NA	NA	NA	NA	VSAP_VSMC-down
NA	NA	NA	NA	NA	NA	NA	NA	NA	NA	VSAP_VSMC-down
NA	NA	NA	NA	NA	NA	NA	NA	NA	NA	VSAP_VSMC-down
NA	NA	NA	NA	NA	NA	NA	NA	NA	NA	VSAP_VSMC-down
NA	NA	NA	NA	NA	NA	NA	NA	NA	NA	VSAP_VSMC-down
NA	NA	NA	NA	NA	NA	NA	NA	NA	NA	VSAP_VSMC-down
NA	NA	NA	NA	NA	NA	NA	NA	NA	NA	VSAP_VSMC-down
NA	NA	NA	NA	NA	NA	NA	NA	NA	NA	VSAP_VSMC-down
NA	NA	NA	NA	NA	NA	NA	NA	NA	NA	VSAP_VSMC-down
NA	NA	NA	NA	NA	NA	NA	NA	NA	NA	VSAP_VSMC-down
NA	NA	NA	NA	NA	NA	NA	NA	NA	NA	VSAP_VSMC-down
NA	NA	NA	NA	NA	NA	NA	NA	NA	NA	VSAP_VSMC-down
NA	NA	NA	NA	NA	NA	NA	NA	NA	NA	VSAP_VSMC-down
NA	NA	NA	NA	NA	NA	NA	NA	NA	NA	VSAP_VSMC-down
NA	NA	NA	NA	NA	NA	NA	NA	NA	NA	VSAP_VSMC-down
NA	NA	NA	NA	NA	NA	NA	NA	NA	NA	VSAP_VSMC-down
NA	NA	NA	NA	NA	NA	NA	NA	NA	NA	VSAP_VSMC-down
NA	NA	NA	NA	NA	NA	NA	NA	NA	NA	VSAP_VSMC-down
NA	NA	NA	NA	NA	NA	NA	NA	NA	NA	VSAP_VSMC-down
NA	NA	NA	NA	NA	NA	NA	NA	NA	NA	VSAP_VSMC-down
NA	NA	NA	NA	NA	NA	NA	NA	NA	NA	VSAP_VSMC-down
NA	NA	NA	NA	NA	NA	NA	NA	NA	NA	VSAP_VSMC-down
NA	NA	NA	NA	NA	NA	NA	NA	NA	NA	VSAP_VSMC-down
NA	NA	NA	NA	NA	NA	NA	NA	NA	NA	VSAP_VSMC-down
NA	NA	NA	NA	NA	NA	NA	NA	NA	NA	VSAP_VSMC-down
NA	NA	NA	NA	NA	NA	NA	NA	NA	NA	VSAP_VSMC-down
NA	NA	NA	NA	NA	NA	NA	NA	NA	NA	VSAP_VSMC-down
NA	NA	NA	NA	NA	NA	NA	NA	NA	NA	VSAP_VSMC-down
NA	NA	NA	NA	NA	NA	NA	NA	NA	NA	VSAP_VSMC-down
NA	NA	NA	NA	NA	NA	NA	NA	NA	NA	VSAP_VSMC-down
NA	NA	NA	NA	NA	NA	NA	NA	NA	NA	VSAP_VSMC-down
NA	NA	NA	NA	NA	NA	NA	NA	NA	NA	VSAP_VSMC-down
NA	NA	NA	NA	NA	NA	NA	NA	NA	NA	VSAP_VSMC-down
NA	NA	NA	NA	NA	NA	NA	NA	NA	NA	VSAP_VSMC-down
NA	NA	NA	NA	NA	NA	NA	NA	NA	NA	VSAP_VSMC-down
NA	NA	NA	NA	NA	NA	NA	NA	NA	NA	VSAP_VSMC-down
NA	NA	NA	NA	NA	NA	NA	NA	NA	NA	VSAP_VSMC-down
NA	NA	NA	NA	NA	NA	NA	NA	NA	NA	VSAP_VSMC-down
NA	NA	NA	NA	NA	NA	NA	NA	NA	NA	VSAP_VSMC-down
NA	NA	NA	NA	NA	NA	NA	NA	NA	NA	VSAP_VSMC-down
NA	NA	NA	NA	NA	NA	NA	NA	NA	NA	VSAP_VSMC-down
NA	NA	NA	NA	NA	NA	NA	NA	NA	NA	VSAP_VSMC-down
NA	NA	NA	NA	NA	NA	NA	NA	NA	NA	VSAP_VSMC-down
NA	NA	NA	NA	NA	NA	NA	NA	NA	NA	VSAP_VSMC-down
NA	NA	NA	NA	NA	NA	NA	NA	NA	NA	VSAP_VSMC-down
NA	NA	NA	NA	NA	NA	NA	NA	NA	NA	VSAP_VSMC-down
NA	NA	NA	NA	NA	NA	NA	NA	NA	NA	VSAP_VSMC-down
NA	NA	NA	NA	NA	NA	NA	NA	NA	NA	VSAP_VSMC-down
NA	NA	NA	NA	NA	NA	NA	NA	NA	NA	VSAP_VSMC-down
NA	NA	NA	NA	NA	NA	NA	NA	NA	NA	VSAP_VSMC-down
NA	NA	NA	NA	NA	NA	NA	NA	NA	NA	VSAP_VSMC-down
NA	NA	NA	NA	NA	NA	NA	NA	NA	NA	VSAP_VSMC-down
NA	NA	NA	NA	NA	NA	NA	NA	NA	NA	VSAP_VSMC-down
NA	NA	NA	NA	NA	NA	NA	NA	NA	NA	VSAP_VSMC-down
NA	NA	NA	NA	NA	NA	NA	NA	NA	NA	VSAP_VSMC-down
NA	NA	NA	NA	NA	NA	NA	NA	NA	NA	VSAP_VSMC-down
NA	NA	NA	NA	NA	NA	NA	NA	NA	NA	VSAP_VSMC-down
NA	NA	NA	NA	NA	NA	NA	NA	NA	NA	VSAP_VSMC-down
NA	NA	NA	NA	NA	NA	NA	NA	NA	NA	VSAP_VSMC-down
NA	NA	NA	NA	NA	NA	NA	NA	NA	NA	VSAP_VSMC-down

NA	NA	NA	NA	NA	NA	NA	NA NA	NA	VSAP_VSMC-down
NA	NA	NA	NA	NA	NA	NA	NA NA	NA	VSAP_VSMC-down
NA	NA	NA	NA	NA	NA	NA	NA NA	NA	VSAP_VSMC-down
NA	NA	NA	NA	NA	NA	NA	NA NA	NA	VSAP_VSMC-down
NA	NA	NA	NA	NA	NA	NA	NA NA	NA	VSAP_VSMC-down
NA	NA	NA	NA	NA	NA	NA	NA NA	NA	VSAP_VSMC-down
NA	NA	NA	NA	NA	NA	NA	NA NA	NA	VSAP_VSMC-down
NA	NA	NA	NA	NA	NA	NA	NA NA	NA	VSAP_VSMC-down
NA	NA	NA	NA	NA	NA	NA	NA NA	NA	VSAP_VSMC-down
NA	NA	NA	NA	NA	NA	NA	NA NA	NA	VSAP_VSMC-down
NA	NA	NA	NA	NA	NA	NA	NA NA	NA	VSAP_VSMC-down
NA	NA	NA	NA	NA	NA	NA	NA NA	NA	VSAP_VSMC-down
NA	NA	NA	NA	NA	NA	NA	NA NA	NA	VSAP_VSMC-down
NA	NA	NA	NA	NA	NA	NA	NA NA	NA	VSAP_VSMC-down
NA	NA	NA	NA	NA	NA	NA	NA NA	NA	VSAP_VSMC-down
NA	NA	NA	NA	NA	NA	NA	NA NA	NA	VSAP_VSMC-down
NA	NA	NA	NA	NA	NA	NA	NA NA	NA	VSAP_VSMC-down
cfa04218	Cellular senescence	43891	151/7854	0.05658289	0.06878116	0.02413374	481628 TGFBR1	1	VSAP_VSMC-down
cfa04390	Hippo signaling pathway	43891	153/7854	0.05731763	0.06878116	0.02413374	481628 TGFBR1	1	VSAP_VSMC-down
cfa05161	Hepatitis B	43891	164/7854	0.06135193	0.07079069	0.02483884	481628 TGFBR1	1	VSAP_VSMC-down
cfa05166	Human T-cell leukemia virus 1 infection	43891	209/7854	0.07773603	0.08637337	0.03030644	481628 TGFBR1	1	VSAP_VSMC-down
cfa04144	Endocytosis	43891	235/7854	0.08711491	0.09333741	0.03274997	481628 TGFBR1	1	VSAP_VSMC-down
cfa04060	Cytokine-cytokine receptor interaction	43891	270/7854	0.09963962	0.10307547	0.03616683	481628 TGFBR1	1	VSAP_VSMC-down
cfa04010	MAPK signaling pathway	43891	288/7854	0.10603605	0.10603605	0.03720563	481628 TGFBR1	1	VSAP_VSMC-down
cfa00220	Arginine biosynthesis	44013	19/7854	0.01681802	0.1508831	0.12145389	480364 ARG2	1	AORT_VSMC-up
cfa00534	Glycosaminoglycan biosynthesis - heparan sulfate / heparin	44013	24/7854	0.0212033	0.1508831	0.12145389	489975 HS3ST2	1	AORT_VSMC-up
cfa00330	Arginine and proline metabolism	44013	47/7854	0.04116036	0.1508831	0.12145389	480364 ARG2	1	AORT_VSMC-up
cfa00600	Sphingolipid metabolism	44013	48/7854	0.04202008	0.1508831	0.12145389	483329 SPHK1	1	AORT_VSMC-up
cfa04961	Endocrine and other factor-regulated calcium reabsorption	44013	52/7854	0.04545238	0.1508831	0.12145389	403640 ESR1	1	AORT_VSMC-up
cfa04370	VEGF signaling pathway	44013	57/7854	0.04972792	0.1508831	0.12145389	483329 SPHK1	1	AORT_VSMC-up
cfa05217	Basal cell carcinoma	44013	63/7854	0.0548369	0.1508831	0.12145389	607180 WNT7A	1	AORT_VSMC-up
cfa04917	Prolactin signaling pathway	44013	70/7854	0.0607676	0.1508831	0.12145389	403640 ESR1	1	AORT_VSMC-up
cfa01230	Biosynthesis of amino acids	44013	74/7854	0.06414223	0.1508831	0.12145389	480364 ARG2	1	AORT_VSMC-up
cfa04721	Synaptic vesicle cycle	44013	78/7854	0.06750646	0.1508831	0.12145389	403751 SLC1A1	1	AORT_VSMC-up
cfa04666	Fc gamma R-mediated phagocytosis	44013	90/7854	0.07753703	0.1508831	0.12145389	483329 SPHK1	1	AORT_VSMC-up
cfa04974	Protein digestion and absorption	44013	91/7854	0.07836872	0.1508831	0.12145389	403751 SLC1A1	1	AORT_VSMC-up
cfa01522	Endocrine resistance	44013	93/7854	0.08003017	0.1508831	0.12145389	403640 ESR1	1	AORT_VSMC-up
cfa04916	Melanogenesis	44013	100/7854	0.08582506	0.1508831	0.12145389	607180 WNT7A	1	AORT_VSMC-up
cfa05146	Amoebiasis	44013	102/7854	0.08747498	0.1508831	0.12145389	480364 ARG2	1	AORT_VSMC-up
cfa04724	Glutamatergic synapse	44013	112/7854	0.09568636	0.1508831	0.12145389	403751 SLC1A1	1	AORT_VSMC-up
cfa04919	Thyroid hormone signaling pathway	44013	116/7854	0.09895313	0.1508831	0.12145389	403640 ESR1	1	AORT_VSMC-up
cfa04071	Sphingolipid signaling pathway	44013	122/7854	0.10383432	0.1508831	0.12145389	483329 SPHK1	1	AORT_VSMC-up
cfa04110	Cell cycle	9/441	123/7854	0.25344078	0.71119554	0.64023982	491236/491292/478127/479926/403924/485790/611243/481679/608847 ANAPC2, ABL1, RBL2, CDC14A, MYC, CDC25B, E2F4, CDC26, CCND3	9	VSAP_EC-up
cfa04371	Apelin signaling pathway	44013	135/7854	0.11433254	0.1508831	0.12145389	483329 SPHK1	1	AORT_VSMC-up
cfa04550	Signaling pathways regulating pluripotency of stem cells	44013	135/7854	0.11433254	0.1508831	0.12145389	607180 WNT7A	1	AORT_VSMC-up
cfa04072	Phospholipase D signaling pathway	44013	148/7854	0.12472517	0.1508831	0.12145389	483329 SPHK1	1	AORT_VSMC-up
cfa05226	Gastric cancer	44013	149/7854	0.12552026	0.1508831	0.12145389	607180 WNT7A	1	AORT_VSMC-up
cfa04310	Wnt signaling pathway	44013	151/7854	0.12710857	0.1508831	0.12145389	607180 WNT7A	1	AORT_VSMC-up
cfa04150	mTOR signaling pathway	44013	152/7854	0.1279018	0.1508831	0.12145389	607180 WNT7A	1	AORT_VSMC-up
cfa04934	Cushing syndrome	44013	152/7854	0.1279018	0.1508831	0.12145389	607180 WNT7A	1	AORT_VSMC-up
cfa04390	Hippo signaling pathway	44013	153/7854	0.12869441	0.1508831	0.12145389	607180 WNT7A	1	AORT_VSMC-up
NA	NA	NA	NA	NA	NA	NA	NA NA	NA	AORT_VSMC-up
NA	NA	NA	NA	NA	NA	NA	NA NA	NA	AORT_VSMC-up

NA	NA	NA	NA	NA	NA	NA	NA	NA	NA	AORT_VSMC-up
NA	NA	NA	NA	NA	NA	NA	NA	NA	NA	AORT_VSMC-up
NA	NA	NA	NA	NA	NA	NA	NA	NA	NA	AORT_VSMC-up
NA	NA	NA	NA	NA	NA	NA	NA	NA	NA	AORT_VSMC-up
NA	NA	NA	NA	NA	NA	NA	NA	NA	NA	AORT_VSMC-up
NA	NA	NA	NA	NA	NA	NA	NA	NA	NA	AORT_VSMC-up
NA	NA	NA	NA	NA	NA	NA	NA	NA	NA	AORT_VSMC-up
NA	NA	NA	NA	NA	NA	NA	NA	NA	NA	AORT_VSMC-up
NA	NA	NA	NA	NA	NA	NA	NA	NA	NA	AORT_VSMC-up
NA	NA	NA	NA	NA	NA	NA	NA	NA	NA	AORT_VSMC-up
NA	NA	NA	NA	NA	NA	NA	NA	NA	NA	AORT_VSMC-up
NA	NA	NA	NA	NA	NA	NA	NA	NA	NA	AORT_VSMC-up
NA	NA	NA	NA	NA	NA	NA	NA	NA	NA	AORT_VSMC-up
NA	NA	NA	NA	NA	NA	NA	NA	NA	NA	AORT_VSMC-up
NA	NA	NA	NA	NA	NA	NA	NA	NA	NA	AORT_VSMC-up
NA	NA	NA	NA	NA	NA	NA	NA	NA	NA	AORT_VSMC-up
NA	NA	NA	NA	NA	NA	NA	NA	NA	NA	AORT_VSMC-up
NA	NA	NA	NA	NA	NA	NA	NA	NA	NA	AORT_VSMC-up
NA	NA	NA	NA	NA	NA	NA	NA	NA	NA	AORT_VSMC-up
NA	NA	NA	NA	NA	NA	NA	NA	NA	NA	AORT_VSMC-up
NA	NA	NA	NA	NA	NA	NA	NA	NA	NA	AORT_VSMC-up
NA	NA	NA	NA	NA	NA	NA	NA	NA	NA	AORT_VSMC-up
NA	NA	NA	NA	NA	NA	NA	NA	NA	NA	AORT_VSMC-up
NA	NA	NA	NA	NA	NA	NA	NA	NA	NA	AORT_VSMC-up
NA	NA	NA	NA	NA	NA	NA	NA	NA	NA	AORT_VSMC-up
NA	NA	NA	NA	NA	NA	NA	NA	NA	NA	AORT_VSMC-up
NA	NA	NA	NA	NA	NA	NA	NA	NA	NA	AORT_VSMC-up
NA	NA	NA	NA	NA	NA	NA	NA	NA	NA	AORT_VSMC-up
NA	NA	NA	NA	NA	NA	NA	NA	NA	NA	AORT_VSMC-up
NA	NA	NA	NA	NA	NA	NA	NA	NA	NA	AORT_VSMC-up
NA	NA	NA	NA	NA	NA	NA	NA	NA	NA	AORT_VSMC-up
NA	NA	NA	NA	NA	NA	NA	NA	NA	NA	AORT_VSMC-up
NA	NA	NA	NA	NA	NA	NA	NA	NA	NA	AORT_VSMC-up
NA	NA	NA	NA	NA	NA	NA	NA	NA	NA	AORT_VSMC-up
NA	NA	NA	NA	NA	NA	NA	NA	NA	NA	AORT_VSMC-up
NA	NA	NA	NA	NA	NA	NA	NA	NA	NA	AORT_VSMC-up
NA	NA	NA	NA	NA	NA	NA	NA	NA	NA	AORT_VSMC-up
NA	NA	NA	NA	NA	NA	NA	NA	NA	NA	AORT_VSMC-up
NA	NA	NA	NA	NA	NA	NA	NA	NA	NA	AORT_VSMC-up
NA	NA	NA	NA	NA	NA	NA	NA	NA	NA	AORT_VSMC-up
NA	NA	NA	NA	NA	NA	NA	NA	NA	NA	AORT_VSMC-up
NA	NA	NA	NA	NA	NA	NA	NA	NA	NA	AORT_VSMC-up
NA	NA	NA	NA	NA	NA	NA	NA	NA	NA	AORT_VSMC-up
NA	NA	NA	NA	NA	NA	NA	NA	NA	NA	AORT_VSMC-up
NA	NA	NA	NA	NA	NA	NA	NA	NA	NA	AORT_VSMC-up
NA	NA	NA	NA	NA	NA	NA	NA	NA	NA	AORT_VSMC-up
NA	NA	NA	NA	NA	NA	NA	NA	NA	NA	AORT_VSMC-up
NA	NA	NA	NA	NA	NA	NA	NA	NA	NA	AORT_VSMC-up
NA	NA	NA	NA	NA	NA	NA	NA	NA	NA	AORT_VSMC-up
NA	NA	NA	NA	NA	NA	NA	NA	NA	NA	AORT_VSMC-up
NA	NA	NA	NA	NA	NA	NA	NA	NA	NA	AORT_VSMC-up
NA	NA	NA	NA	NA	NA	NA	NA	NA	NA	AORT_VSMC-up
NA	NA	NA	NA	NA	NA	NA	NA	NA	NA	AORT_VSMC-up
NA	NA	NA	NA	NA	NA	NA	NA	NA	NA	AORT_VSMC-up
NA	NA	NA	NA	NA	NA	NA	NA	NA	NA	AORT_VSMC-up
NA	NA	NA	NA	NA	NA	NA	NA	NA	NA	AORT_VSMC-up
NA	NA	NA	NA	NA	NA	NA	NA	NA	NA	AORT_VSMC-up
NA	NA	NA	NA	NA	NA	NA	NA	NA	NA	AORT_VSMC-up
NA	NA	NA	NA	NA	NA	NA	NA	NA	NA	AORT_VSMC-up
NA	NA	NA	NA	NA	NA	NA	NA	NA	NA	AORT_VSMC-up
NA	NA	NA	NA	NA	NA	NA	NA	NA	NA	AORT_VSMC-up
NA	NA	NA	NA	NA	NA	NA	NA	NA	NA	AORT_VSMC-up
NA	NA	NA	NA	NA	NA	NA	NA	NA	NA	AORT_VSMC-up
NA	NA	NA	NA	NA	NA	NA	NA	NA	NA	AORT_VSMC-up
NA	NA	NA	NA	NA	NA	NA	NA	NA	NA	AORT_VSMC-up
NA	NA	NA	NA	NA	NA	NA	NA	NA	NA	AORT_VSMC-up
NA	NA	NA	NA	NA	NA	NA	NA	NA	NA	AORT_VSMC-up
NA	NA	NA	NA	NA	NA	NA	NA	NA	NA	AORT_VSMC-up
NA	NA	NA	NA	NA	NA	NA	NA	NA	NA	AORT_VSMC-up
NA	NA	NA	NA	NA	NA	NA	NA	NA	NA	AORT_VSMC-up
NA	NA	NA	NA	NA	NA	NA	NA	NA	NA	AORT_VSMC-up
NA	NA	NA	NA	NA	NA	NA	NA	NA	NA	AORT_VSMC-up
NA	NA	NA	NA	NA	NA	NA	NA	NA	NA	AORT_VSMC-up
NA	NA	NA	NA	NA	NA	NA	NA	NA	NA	AORT_VSMC-up

NA	NA	NA	NA	NA	NA	NA	NA	NA	NA	NA	AORT_VSMC-up
NA	NA	NA	NA	NA	NA	NA	NA	NA	NA	NA	AORT_VSMC-up
NA	NA	NA	NA	NA	NA	NA	NA	NA	NA	NA	AORT_VSMC-up
NA	NA	NA	NA	NA	NA	NA	NA	NA	NA	NA	AORT_VSMC-up
NA	NA	NA	NA	NA	NA	NA	NA	NA	NA	NA	AORT_VSMC-up
NA	NA	NA	NA	NA	NA	NA	NA	NA	NA	NA	AORT_VSMC-up
NA	NA	NA	NA	NA	NA	NA	NA	NA	NA	NA	AORT_VSMC-up
NA	NA	NA	NA	NA	NA	NA	NA	NA	NA	NA	AORT_VSMC-up
NA	NA	NA	NA	NA	NA	NA	NA	NA	NA	NA	AORT_VSMC-up
NA	NA	NA	NA	NA	NA	NA	NA	NA	NA	NA	AORT_VSMC-up
NA	NA	NA	NA	NA	NA	NA	NA	NA	NA	NA	AORT_VSMC-up
NA	NA	NA	NA	NA	NA	NA	NA	NA	NA	NA	AORT_VSMC-up
NA	NA	NA	NA	NA	NA	NA	NA	NA	NA	NA	AORT_VSMC-up
NA	NA	NA	NA	NA	NA	NA	NA	NA	NA	NA	AORT_VSMC-up
NA	NA	NA	NA	NA	NA	NA	NA	NA	NA	NA	AORT_VSMC-up
NA	NA	NA	NA	NA	NA	NA	NA	NA	NA	NA	AORT_VSMC-up
NA	NA	NA	NA	NA	NA	NA	NA	NA	NA	NA	AORT_VSMC-up
NA	NA	NA	NA	NA	NA	NA	NA	NA	NA	NA	AORT_VSMC-up
NA	NA	NA	NA	NA	NA	NA	NA	NA	NA	NA	AORT_VSMC-up
NA	NA	NA	NA	NA	NA	NA	NA	NA	NA	NA	AORT_VSMC-up
NA	NA	NA	NA	NA	NA	NA	NA	NA	NA	NA	AORT_VSMC-up
NA	NA	NA	NA	NA	NA	NA	NA	NA	NA	NA	AORT_VSMC-up
NA	NA	NA	NA	NA	NA	NA	NA	NA	NA	NA	AORT_VSMC-up
NA	NA	NA	NA	NA	NA	NA	NA	NA	NA	NA	AORT_VSMC-up
NA	NA	NA	NA	NA	NA	NA	NA	NA	NA	NA	AORT_VSMC-up
NA	NA	NA	NA	NA	NA	NA	NA	NA	NA	NA	AORT_VSMC-up
NA	NA	NA	NA	NA	NA	NA	NA	NA	NA	NA	AORT_VSMC-up
NA	NA	NA	NA	NA	NA	NA	NA	NA	NA	NA	AORT_VSMC-up
NA	NA	NA	NA	NA	NA	NA	NA	NA	NA	NA	AORT_VSMC-up
NA	NA	NA	NA	NA	NA	NA	NA	NA	NA	NA	AORT_VSMC-up
NA	NA	NA	NA	NA	NA	NA	NA	NA	NA	NA	AORT_VSMC-up
NA	NA	NA	NA	NA	NA	NA	NA	NA	NA	NA	AORT_VSMC-up
NA	NA	NA	NA	NA	NA	NA	NA	NA	NA	NA	AORT_VSMC-up
NA	NA	NA	NA	NA	NA	NA	NA	NA	NA	NA	AORT_VSMC-up
NA	NA	NA	NA	NA	NA	NA	NA	NA	NA	NA	AORT_VSMC-up
NA	NA	NA	NA	NA	NA	NA	NA	NA	NA	NA	AORT_VSMC-up
NA	NA	NA	NA	NA	NA	NA	NA	NA	NA	NA	AORT_VSMC-up
NA	NA	NA	NA	NA	NA	NA	NA	NA	NA	NA	AORT_VSMC-up
NA	NA	NA	NA	NA	NA	NA	NA	NA	NA	NA	AORT_VSMC-up
NA	NA	NA	NA	NA	NA	NA	NA	NA	NA	NA	AORT_VSMC-up
NA	NA	NA	NA	NA	NA	NA	NA	NA	NA	NA	AORT_VSMC-up
NA	NA	NA	NA	NA	NA	NA	NA	NA	NA	NA	AORT_VSMC-up
NA	NA	NA	NA	NA	NA	NA	NA	NA	NA	NA	AORT_VSMC-up
NA	NA	NA	NA	NA	NA	NA	NA	NA	NA	NA	AORT_VSMC-up
NA	NA	NA	NA	NA	NA	NA	NA	NA	NA	NA	AORT_VSMC-up
NA	NA	NA	NA	NA	NA	NA	NA	NA	NA	NA	AORT_VSMC-up
NA	NA	NA	NA	NA	NA	NA	NA	NA	NA	NA	AORT_VSMC-up
NA	NA	NA	NA	NA	NA	NA	NA	NA	NA	NA	AORT_VSMC-up
NA	NA	NA	NA	NA	NA	NA	NA	NA	NA	NA	AORT_VSMC-up
NA	NA	NA	NA	NA	NA	NA	NA	NA	NA	NA	AORT_VSMC-up
NA	NA	NA	NA	NA	NA	NA	NA	NA	NA	NA	AORT_VSMC-up
NA	NA	NA	NA	NA	NA	NA	NA	NA	NA	NA	AORT_VSMC-up
NA	NA	NA	NA	NA	NA	NA	NA	NA	NA	NA	AORT_VSMC-up
NA	NA	NA	NA	NA	NA	NA	NA	NA	NA	NA	AORT_VSMC-up
NA	NA	NA	NA	NA	NA	NA	NA	NA	NA	NA	AORT_VSMC-up
NA	NA	NA	NA	NA	NA	NA	NA	NA	NA	NA	AORT_VSMC-up
NA	NA	NA	NA	NA	NA	NA	NA	NA	NA	NA	AORT_VSMC-up
NA	NA	NA	NA	NA	NA	NA	NA	NA	NA	NA	AORT_VSMC-up
NA	NA	NA	NA	NA	NA	NA	NA	NA	NA	NA	AORT_VSMC-up
NA	NA	NA	NA	NA	NA	NA	NA	NA	NA	NA	AORT_VSMC-up
NA	NA	NA	NA	NA	NA	NA	NA	NA	NA	NA	AORT_VSMC-up
NA	NA	NA	NA	NA	NA	NA	NA	NA	NA	NA	AORT_VSMC-up
NA	NA	NA	NA	NA	NA	NA	NA	NA	NA	NA	AORT_VSMC-up
NA	NA	NA	NA	NA	NA	NA	NA	NA	NA	NA	AORT_VSMC-up
NA	NA	NA	NA	NA	NA	NA	NA	NA	NA	NA	AORT_VSMC-up
NA	NA	NA	NA	NA	NA	NA	NA	NA	NA	NA	AORT_VSMC-up
NA	NA	NA	NA	NA	NA	NA	NA	NA	NA	NA	AORT_VSMC-up
NA	NA	NA	NA	NA	NA	NA	NA	NA	NA	NA	AORT_VSMC-up
NA	NA	NA	NA	NA	NA	NA	NA	NA	NA	NA	AORT_VSMC-up
NA	NA	NA	NA	NA	NA	NA	NA	NA	NA	NA	AORT_VSMC-up
NA	NA	NA	NA	NA	NA	NA	NA	NA	NA	NA	AORT_VSMC-up
NA	NA	NA	NA	NA	NA	NA	NA	NA	NA	NA	AORT_VSMC-up
NA	NA	NA	NA	NA	NA	NA	NA	NA	NA	NA	AORT_VSMC-up
NA	NA	NA	NA	NA	NA	NA	NA	NA	NA	NA	AORT_VSMC-up

NA	NA	NA	NA	NA	NA	NA	NA NA	NA	AORT_VSMC-up
NA	NA	NA	NA	NA	NA	NA	NA NA	NA	AORT_VSMC-up
NA	NA	NA	NA	NA	NA	NA	NA NA	NA	AORT_VSMC-up
NA	NA	NA	NA	NA	NA	NA	NA NA	NA	AORT_VSMC-up
NA	NA	NA	NA	NA	NA	NA	NA NA	NA	AORT_VSMC-up
NA	NA	NA	NA	NA	NA	NA	NA NA	NA	AORT_VSMC-up
NA	NA	NA	NA	NA	NA	NA	NA NA	NA	AORT_VSMC-up
NA	NA	NA	NA	NA	NA	NA	NA NA	NA	AORT_VSMC-up
NA	NA	NA	NA	NA	NA	NA	NA NA	NA	AORT_VSMC-up
NA	NA	NA	NA	NA	NA	NA	NA NA	NA	AORT_VSMC-up
NA	NA	NA	NA	NA	NA	NA	NA NA	NA	AORT_VSMC-up
NA	NA	NA	NA	NA	NA	NA	NA NA	NA	AORT_VSMC-up
NA	NA	NA	NA	NA	NA	NA	NA NA	NA	AORT_VSMC-up
NA	NA	NA	NA	NA	NA	NA	NA NA	NA	AORT_VSMC-up
NA	NA	NA	NA	NA	NA	NA	NA NA	NA	AORT_VSMC-up
NA	NA	NA	NA	NA	NA	NA	NA NA	NA	AORT_VSMC-up
cfa05225	Hepatocellular carcinoma	44013	165/7854	0.13815768	0.15261445	0.12284755	607180 WNT7A	1	AORT_VSMC-up
cfa05152	Tuberculosis	44013	171/7854	0.14285619	0.15261445	0.12284755	483329 SPHK1	1	AORT_VSMC-up
cfa05202	Transcriptional misregulation in cancer	44013	172/7854	0.14363713	0.15261445	0.12284755	482372 HOXA9	1	AORT_VSMC-up
cfa04020	Calcium signaling pathway	44013	192/7854	0.15912849	0.16395056	0.13197259	483329 SPHK1	1	AORT_VSMC-up
cfa05165	Human papillomavirus infection	44013	328/7854	0.25824141	0.25824141	0.20787235	607180 WNT7A	1	AORT_VSMC-up
cfa00910	Nitrogen metabolism	44013	17/7854	0.0150592	0.1193917	0.07937398	477928 CA2	1	VCAV_VSMC-up
cfa04964	Proximal tubule bicarbonate reclamation	44013	21/7854	0.01857415	0.1193917	0.07937398	477928 CA2	1	VCAV_VSMC-up
cfa04966	Collecting duct acid secretion	44013	26/7854	0.02295272	0.1193917	0.07937398	477928 CA2	1	VCAV_VSMC-up
cfa05020	Prion diseases	44013	33/7854	0.02905457	0.1193917	0.07937398	479348 C6	1	VCAV_VSMC-up
cfa00380	Tryptophan metabolism	44013	41/7854	0.0359881	0.1193917	0.07937398	100686778 CYP1A1	1	VCAV_VSMC-up
cfa04913	Ovarian steroidogenesis	44013	46/7854	0.04029997	0.1193917	0.07937398	100686778 CYP1A1	1	VCAV_VSMC-up
cfa00140	Steroid hormone biosynthesis	44013	48/7854	0.04202008	0.1193917	0.07937398	100686778 CYP1A1	1	VCAV_VSMC-up
cfa00830	Retinol metabolism	44013	49/7854	0.04287915	0.1193917	0.07937398	100686778 CYP1A1	1	VCAV_VSMC-up
cfa00982	Drug metabolism - cytochrome P450	44013	49/7854	0.04287915	0.1193917	0.07937398	477558	1	VCAV_VSMC-up
cfa04110	Cell cycle	4/279	123/7854	0.64143398	0.84772198	0.71863937	478575/487309/474890/100687866 GSK3B, HDAC1, CDKN1A, YWHAH	4	VSAP_EC-down
cfa00480	Glutathione metabolism	44013	52/7854	0.04545238	0.1193917	0.07937398	477558	1	VCAV_VSMC-up
cfa04961	Endocrine and other factor-regulated calcium reabsorption	44013	52/7854	0.04545238	0.1193917	0.07937398	490755 SLC8A3	1	VCAV_VSMC-up
cfa00983	Drug metabolism - other enzymes	44013	63/7854	0.0548369	0.1193917	0.07937398	477558	1	VCAV_VSMC-up
cfa04976	Bile secretion	44013	66/7854	0.05738255	0.1193917	0.07937398	477928 CA2	1	VCAV_VSMC-up
cfa01524	Platinum drug resistance	44013	72/7854	0.06245622	0.1193917	0.07937398	477558	1	VCAV_VSMC-up
cfa04971	Gastric acid secretion	44013	72/7854	0.06245622	0.1193917	0.07937398	477928 CA2	1	VCAV_VSMC-up
cfa05412	Arrhythmogenic right ventricular cardiomyopathy (ARVC)	44013	77/7854	0.06666638	0.1193917	0.07937398	490755 SLC8A3	1	VCAV_VSMC-up
cfa04610	Complement and coagulation cascades	44013	80/7854	0.06918469	0.1193917	0.07937398	479348 C6	1	VCAV_VSMC-up
cfa04260	Cardiac muscle contraction	4/279	81/7854	0.32460687	0.65927908	0.55889066	481598/609879/403846/480137 TPM2, TPM4, ASPH, TPM3	4	VSAP_EC-down
cfa04666	Fc gamma R-mediated phagocytosis	44013	90/7854	0.07753703	0.1193917	0.07937398	489800 LIMK1	1	VCAV_VSMC-up
cfa04974	Protein digestion and absorption	44013	91/7854	0.07836872	0.1193917	0.07937398	490755 SLC8A3	1	VCAV_VSMC-up
cfa04260	Cardiac muscle contraction	4/241	81/7854	0.23697076	0.59454272	0.53452052	612644/486563/486347/476040 , CACNB3, UQCR10, COX8A	4	VCAV_EC-down
cfa04972	Pancreatic secretion	44013	94/7854	0.08085994	0.1193917	0.07937398	477928 CA2	1	VCAV_VSMC-up
cfa04260	Cardiac muscle contraction	3/388	81/7854	0.77101034	0.99972301	0.9362731	477098/475121/480137 ATP1B3, CYC1, TPM3	3	ACOR_VSMC-down
cfa05322	Systemic lupus erythematosus	44013	113/7854	0.096504	0.13539976	0.09001646	479348 C6	1	VCAV_VSMC-up
cfa05135	Yersinia infection	44013	117/7854	0.09976824	0.13539976	0.09001646	489800 LIMK1	1	VCAV_VSMC-up
cfa04371	Apelin signaling pathway	44013	135/7854	0.11433254	0.14482122	0.09628004	490755 SLC8A3	1	VCAV_VSMC-up
cfa04260	Cardiac muscle contraction	34700	81/7854	0.62872607	0.86577607	0.8604302	612614 COX7A1	1	AFEM_EC-up

cfa04261	Adrenergic signaling in cardiomyocytes	44013	147/7854	0.12392947	0.15191354	0.10099515	490755 SLC8A3		1	VCAV_VSMC-up
cfa04022	cGMP-PKG signaling pathway	44013	165/7854	0.13815768	0.15909066	0.10576665	490755 SLC8A3		1	VCAV_VSMC-up
cfa05225	Hepatocellular carcinoma	44013	165/7854	0.13815768	0.15909066	0.10576665		477558	1	VCAV_VSMC-up
cfa05202	Transcriptional misregulation in cancer	44013	172/7854	0.14363713	0.16053562	0.10672728	609253 WT1		1	VCAV_VSMC-up
cfa04360	Axon guidance	44013	179/7854	0.1490867	0.16186556	0.10761145	489800 LIMK1		1	VCAV_VSMC-up
NA	NA	NA	NA	NA	NA	NA	NA NA		NA	VCAV_VSMC-up
NA	NA	NA	NA	NA	NA	NA	NA NA		NA	VCAV_VSMC-up
NA	NA	NA	NA	NA	NA	NA	NA NA		NA	VCAV_VSMC-up
NA	NA	NA	NA	NA	NA	NA	NA NA		NA	VCAV_VSMC-up
NA	NA	NA	NA	NA	NA	NA	NA NA		NA	VCAV_VSMC-up
NA	NA	NA	NA	NA	NA	NA	NA NA		NA	VCAV_VSMC-up
NA	NA	NA	NA	NA	NA	NA	NA NA		NA	VCAV_VSMC-up
NA	NA	NA	NA	NA	NA	NA	NA NA		NA	VCAV_VSMC-up
NA	NA	NA	NA	NA	NA	NA	NA NA		NA	VCAV_VSMC-up
NA	NA	NA	NA	NA	NA	NA	NA NA		NA	VCAV_VSMC-up
NA	NA	NA	NA	NA	NA	NA	NA NA		NA	VCAV_VSMC-up
NA	NA	NA	NA	NA	NA	NA	NA NA		NA	VCAV_VSMC-up
NA	NA	NA	NA	NA	NA	NA	NA NA		NA	VCAV_VSMC-up
NA	NA	NA	NA	NA	NA	NA	NA NA		NA	VCAV_VSMC-up
NA	NA	NA	NA	NA	NA	NA	NA NA		NA	VCAV_VSMC-up
NA	NA	NA	NA	NA	NA	NA	NA NA		NA	VCAV_VSMC-up
NA	NA	NA	NA	NA	NA	NA	NA NA		NA	VCAV_VSMC-up
NA	NA	NA	NA	NA	NA	NA	NA NA		NA	VCAV_VSMC-up
NA	NA	NA	NA	NA	NA	NA	NA NA		NA	VCAV_VSMC-up
NA	NA	NA	NA	NA	NA	NA	NA NA		NA	VCAV_VSMC-up
NA	NA	NA	NA	NA	NA	NA	NA NA		NA	VCAV_VSMC-up
NA	NA	NA	NA	NA	NA	NA	NA NA		NA	VCAV_VSMC-up
NA	NA	NA	NA	NA	NA	NA	NA NA		NA	VCAV_VSMC-up
NA	NA	NA	NA	NA	NA	NA	NA NA		NA	VCAV_VSMC-up
NA	NA	NA	NA	NA	NA	NA	NA NA		NA	VCAV_VSMC-up
NA	NA	NA	NA	NA	NA	NA	NA NA		NA	VCAV_VSMC-up
NA	NA	NA	NA	NA	NA	NA	NA NA		NA	VCAV_VSMC-up
NA	NA	NA	NA	NA	NA	NA	NA NA		NA	VCAV_VSMC-up
NA	NA	NA	NA	NA	NA	NA	NA NA		NA	VCAV_VSMC-up
NA	NA	NA	NA	NA	NA	NA	NA NA		NA	VCAV_VSMC-up
NA	NA	NA	NA	NA	NA	NA	NA NA		NA	VCAV_VSMC-up
NA	NA	NA	NA	NA	NA	NA	NA NA		NA	VCAV_VSMC-up
NA	NA	NA	NA	NA	NA	NA	NA NA		NA	VCAV_VSMC-up
NA	NA	NA	NA	NA	NA	NA	NA NA		NA	VCAV_VSMC-up
NA	NA	NA	NA	NA	NA	NA	NA NA		NA	VCAV_VSMC-up
NA	NA	NA	NA	NA	NA	NA	NA NA		NA	VCAV_VSMC-up
NA	NA	NA	NA	NA	NA	NA	NA NA		NA	VCAV_VSMC-up
NA	NA	NA	NA	NA	NA	NA	NA NA		NA	VCAV_VSMC-up
NA	NA	NA	NA	NA	NA	NA	NA NA		NA	VCAV_VSMC-up
NA	NA	NA	NA	NA	NA	NA	NA NA		NA	VCAV_VSMC-up
NA	NA	NA	NA	NA	NA	NA	NA NA		NA	VCAV_VSMC-up
NA	NA	NA	NA	NA	NA	NA	NA NA		NA	VCAV_VSMC-up
NA	NA	NA	NA	NA	NA	NA	NA NA		NA	VCAV_VSMC-up
NA	NA	NA	NA	NA	NA	NA	NA NA		NA	VCAV_VSMC-up
NA	NA	NA	NA	NA	NA	NA	NA NA		NA	VCAV_VSMC-up
NA	NA	NA	NA	NA	NA	NA	NA NA		NA	VCAV_VSMC-up
NA	NA	NA	NA	NA	NA	NA	NA NA		NA	VCAV_VSMC-up
NA	NA	NA	NA	NA	NA	NA	NA NA		NA	VCAV_VSMC-up
NA	NA	NA	NA	NA	NA	NA	NA NA		NA	VCAV_VSMC-up
NA	NA	NA	NA	NA	NA	NA	NA NA		NA	VCAV_VSMC-up
NA	NA	NA	NA	NA	NA	NA	NA NA		NA	VCAV_VSMC-up
NA	NA	NA	NA	NA	NA	NA	NA NA		NA	VCAV_VSMC-up
NA	NA	NA	NA	NA	NA	NA	NA NA		NA	VCAV_VSMC-up
NA	NA	NA	NA	NA	NA	NA	NA NA		NA	VCAV_VSMC-up
NA	NA	NA	NA	NA	NA	NA	NA NA		NA	VCAV_VSMC-up
NA	NA	NA	NA	NA	NA	NA	NA NA		NA	VCAV_VSMC-up
NA	NA	NA	NA	NA	NA	NA	NA NA		NA	VCAV_VSMC-up
NA	NA	NA	NA	NA	NA	NA	NA NA		NA	VCAV_VSMC-up
NA	NA	NA	NA	NA	NA	NA	NA NA		NA	VCAV_VSMC-up
NA	NA	NA	NA	NA	NA	NA	NA NA		NA	VCAV_VSMC-up
NA	NA	NA	NA	NA	NA	NA	NA NA		NA	VCAV_VSMC-up
NA	NA	NA	NA	NA	NA	NA	NA NA		NA	VCAV_VSMC-up
NA	NA	NA	NA	NA	NA	NA	NA NA		NA	VCAV_VSMC-up
NA	NA	NA	NA	NA	NA	NA	NA NA		NA	VCAV_VSMC-up
NA	NA	NA	NA	NA	NA	NA	NA NA		NA	VCAV_VSMC-up

NA	NA	NA	NA	NA	NA	NA	NA	NA	NA	VCAV_VSMC-up
NA	NA	NA	NA	NA	NA	NA	NA	NA	NA	VCAV_VSMC-up
NA	NA	NA	NA	NA	NA	NA	NA	NA	NA	VCAV_VSMC-up
NA	NA	NA	NA	NA	NA	NA	NA	NA	NA	VCAV_VSMC-up
NA	NA	NA	NA	NA	NA	NA	NA	NA	NA	VCAV_VSMC-up
NA	NA	NA	NA	NA	NA	NA	NA	NA	NA	VCAV_VSMC-up
NA	NA	NA	NA	NA	NA	NA	NA	NA	NA	VCAV_VSMC-up
NA	NA	NA	NA	NA	NA	NA	NA	NA	NA	VCAV_VSMC-up
NA	NA	NA	NA	NA	NA	NA	NA	NA	NA	VCAV_VSMC-up
NA	NA	NA	NA	NA	NA	NA	NA	NA	NA	VCAV_VSMC-up
NA	NA	NA	NA	NA	NA	NA	NA	NA	NA	VCAV_VSMC-up
NA	NA	NA	NA	NA	NA	NA	NA	NA	NA	VCAV_VSMC-up
NA	NA	NA	NA	NA	NA	NA	NA	NA	NA	VCAV_VSMC-up
NA	NA	NA	NA	NA	NA	NA	NA	NA	NA	VCAV_VSMC-up
NA	NA	NA	NA	NA	NA	NA	NA	NA	NA	VCAV_VSMC-up
NA	NA	NA	NA	NA	NA	NA	NA	NA	NA	VCAV_VSMC-up
NA	NA	NA	NA	NA	NA	NA	NA	NA	NA	VCAV_VSMC-up
NA	NA	NA	NA	NA	NA	NA	NA	NA	NA	VCAV_VSMC-up
NA	NA	NA	NA	NA	NA	NA	NA	NA	NA	VCAV_VSMC-up
NA	NA	NA	NA	NA	NA	NA	NA	NA	NA	VCAV_VSMC-up
NA	NA	NA	NA	NA	NA	NA	NA	NA	NA	VCAV_VSMC-up
NA	NA	NA	NA	NA	NA	NA	NA	NA	NA	VCAV_VSMC-up
NA	NA	NA	NA	NA	NA	NA	NA	NA	NA	VCAV_VSMC-up
NA	NA	NA	NA	NA	NA	NA	NA	NA	NA	VCAV_VSMC-up
NA	NA	NA	NA	NA	NA	NA	NA	NA	NA	VCAV_VSMC-up
NA	NA	NA	NA	NA	NA	NA	NA	NA	NA	VCAV_VSMC-up
NA	NA	NA	NA	NA	NA	NA	NA	NA	NA	VCAV_VSMC-up
NA	NA	NA	NA	NA	NA	NA	NA	NA	NA	VCAV_VSMC-up
NA	NA	NA	NA	NA	NA	NA	NA	NA	NA	VCAV_VSMC-up
NA	NA	NA	NA	NA	NA	NA	NA	NA	NA	VCAV_VSMC-up
NA	NA	NA	NA	NA	NA	NA	NA	NA	NA	VCAV_VSMC-up
NA	NA	NA	NA	NA	NA	NA	NA	NA	NA	VCAV_VSMC-up
NA	NA	NA	NA	NA	NA	NA	NA	NA	NA	VCAV_VSMC-up
NA	NA	NA	NA	NA	NA	NA	NA	NA	NA	VCAV_VSMC-up
NA	NA	NA	NA	NA	NA	NA	NA	NA	NA	VCAV_VSMC-up
NA	NA	NA	NA	NA	NA	NA	NA	NA	NA	VCAV_VSMC-up
NA	NA	NA	NA	NA	NA	NA	NA	NA	NA	VCAV_VSMC-up
NA	NA	NA	NA	NA	NA	NA	NA	NA	NA	VCAV_VSMC-up
NA	NA	NA	NA	NA	NA	NA	NA	NA	NA	VCAV_VSMC-up
NA	NA	NA	NA	NA	NA	NA	NA	NA	NA	VCAV_VSMC-up
NA	NA	NA	NA	NA	NA	NA	NA	NA	NA	VCAV_VSMC-up
NA	NA	NA	NA	NA	NA	NA	NA	NA	NA	VCAV_VSMC-up
NA	NA	NA	NA	NA	NA	NA	NA	NA	NA	VCAV_VSMC-up
NA	NA	NA	NA	NA	NA	NA	NA	NA	NA	VCAV_VSMC-up
NA	NA	NA	NA	NA	NA	NA	NA	NA	NA	VCAV_VSMC-up
NA	NA	NA	NA	NA	NA	NA	NA	NA	NA	VCAV_VSMC-up
NA	NA	NA	NA	NA	NA	NA	NA	NA	NA	VCAV_VSMC-up
NA	NA	NA	NA	NA	NA	NA	NA	NA	NA	VCAV_VSMC-up
NA	NA	NA	NA	NA	NA	NA	NA	NA	NA	VCAV_VSMC-up
NA	NA	NA	NA	NA	NA	NA	NA	NA	NA	VCAV_VSMC-up
NA	NA	NA	NA	NA	NA	NA	NA	NA	NA	VCAV_VSMC-up
NA	NA	NA	NA	NA	NA	NA	NA	NA	NA	VCAV_VSMC-up
NA	NA	NA	NA	NA	NA	NA	NA	NA	NA	VCAV_VSMC-up
NA	NA	NA	NA	NA	NA	NA	NA	NA	NA	VCAV_VSMC-up
NA	NA	NA	NA	NA	NA	NA	NA	NA	NA	VCAV_VSMC-up
NA	NA	NA	NA	NA	NA	NA	NA	NA	NA	VCAV_VSMC-up
NA	NA	NA	NA	NA	NA	NA	NA	NA	NA	VCAV_VSMC-up
NA	NA	NA	NA	NA	NA	NA	NA	NA	NA	VCAV_VSMC-up
NA	NA	NA	NA	NA	NA	NA	NA	NA	NA	VCAV_VSMC-up
NA	NA	NA	NA	NA	NA	NA	NA	NA	NA	VCAV_VSMC-up
NA	NA	NA	NA	NA	NA	NA	NA	NA	NA	VCAV_VSMC-up
NA	NA	NA	NA	NA	NA	NA	NA	NA	NA	VCAV_VSMC-up
NA	NA	NA	NA	NA	NA	NA	NA	NA	NA	VCAV_VSMC-up
NA	NA	NA	NA	NA	NA	NA	NA	NA	NA	VCAV_VSMC-up
NA	NA	NA	NA	NA	NA	NA	NA	NA	NA	VCAV_VSMC-up
NA	NA	NA	NA	NA	NA	NA	NA	NA	NA	VCAV_VSMC-up
NA	NA	NA	NA	NA	NA	NA	NA	NA	NA	VCAV_VSMC-up
NA	NA	NA	NA	NA	NA	NA	NA	NA	NA	VCAV_VSMC-up
NA	NA	NA	NA	NA	NA	NA	NA	NA	NA	VCAV_VSMC-up
NA	NA	NA	NA	NA	NA	NA	NA	NA	NA	VCAV_VSMC-up
NA	NA	NA	NA	NA	NA	NA	NA	NA	NA	VCAV_VSMC-up
NA	NA	NA	NA	NA	NA	NA	NA	NA	NA	VCAV_VSMC-up
NA	NA	NA	NA	NA	NA	NA	NA	NA	NA	VCAV_VSMC-up
NA	NA	NA	NA	NA	NA	NA	NA	NA	NA	VCAV_VSMC-up

NA	NA	NA	NA	NA	NA	NA	NA NA	NA	VCAV_VSMC-up
NA	NA	NA	NA	NA	NA	NA	NA NA	NA	VCAV_VSMC-up
NA	NA	NA	NA	NA	NA	NA	NA NA	NA	VCAV_VSMC-up
NA	NA	NA	NA	NA	NA	NA	NA NA	NA	VCAV_VSMC-up
NA	NA	NA	NA	NA	NA	NA	NA NA	NA	VCAV_VSMC-up
NA	NA	NA	NA	NA	NA	NA	NA NA	NA	VCAV_VSMC-up
NA	NA	NA	NA	NA	NA	NA	NA NA	NA	VCAV_VSMC-up
NA	NA	NA	NA	NA	NA	NA	NA NA	NA	VCAV_VSMC-up
NA	NA	NA	NA	NA	NA	NA	NA NA	NA	VCAV_VSMC-up
NA	NA	NA	NA	NA	NA	NA	NA NA	NA	VCAV_VSMC-up
NA	NA	NA	NA	NA	NA	NA	NA NA	NA	VCAV_VSMC-up
NA	NA	NA	NA	NA	NA	NA	NA NA	NA	VCAV_VSMC-up
NA	NA	NA	NA	NA	NA	NA	NA NA	NA	VCAV_VSMC-up
NA	NA	NA	NA	NA	NA	NA	NA NA	NA	VCAV_VSMC-up
NA	NA	NA	NA	NA	NA	NA	NA NA	NA	VCAV_VSMC-up
NA	NA	NA	NA	NA	NA	NA	NA NA	NA	VCAV_VSMC-up
NA	NA	NA	NA	NA	NA	NA	NA NA	NA	VCAV_VSMC-up
NA	NA	NA	NA	NA	NA	NA	NA NA	NA	VCAV_VSMC-up
NA	NA	NA	NA	NA	NA	NA	NA NA	NA	VCAV_VSMC-up
NA	NA	NA	NA	NA	NA	NA	NA NA	NA	VCAV_VSMC-up
NA	NA	NA	NA	NA	NA	NA	NA NA	NA	VCAV_VSMC-up
NA	NA	NA	NA	NA	NA	NA	NA NA	NA	VCAV_VSMC-up
NA	NA	NA	NA	NA	NA	NA	NA NA	NA	VCAV_VSMC-up
cfa04020	Calcium signaling pathway	44013	192/7854	0.15912849	0.16796896	0.11166911	490755 SLC8A3	1	VCAV_VSMC-up
cfa05170	Human immunodeficiency virus 1 infection	44013	200/7854	0.16525742	0.16972384	0.11283579	489800 LIMK1	1	VCAV_VSMC-up
cfa04810	Regulation of actin cytoskeleton	44013	211/7854	0.17362216	0.17362216	0.11542747	489800 LIMK1	1	VCAV_VSMC-up
cfa00830	Retinol metabolism	44105	49/7854	0.06069968	0.25090823	0.25090823	483120 CYP26B1	1	VPOR_VSMC-up
cfa04929	GnRH secretion	44105	65/7854	0.07978928	0.25090823	0.25090823	486004 TRPC4	1	VPOR_VSMC-up
cfa05218	Melanoma	44105	73/7854	0.08920252	0.25090823	0.25090823	608459 FGF5	1	VPOR_VSMC-up
cfa05150	Staphylococcus aureus infection	44105	78/7854	0.0950417	0.25090823	0.25090823	486704 C3AR1	1	VPOR_VSMC-up
cfa03320	PPAR signaling pathway	44105	80/7854	0.09736792	0.25090823	0.25090823	486694 OLR1	1	VPOR_VSMC-up
cfa04610	Complement and coagulation cascades	44105	80/7854	0.09736792	0.25090823	0.25090823	486704 C3AR1	1	VPOR_VSMC-up
cfa04260	Cardiac muscle contraction	44013	81/7854	0.07002283	0.1193917	0.07937398	490755 SLC8A3	1	VCAV_VSMC-up
cfa04974	Protein digestion and absorption	44105	91/7854	0.1100662	0.25090823	0.25090823	403466 COL4A5	1	VPOR_VSMC-up
cfa04350	TGF-beta signaling pathway	44105	92/7854	0.11121258	0.25090823	0.25090823	487081 BAMBI	1	VPOR_VSMC-up
cfa05222	Small cell lung cancer	44105	92/7854	0.11121258	0.25090823	0.25090823	403466 COL4A5	1	VPOR_VSMC-up
cfa04933	AGE-RAGE signaling pathway in diabetic complications	44105	100/7854	0.12033588	0.25090823	0.25090823	403466 COL4A5	1	VPOR_VSMC-up
cfa05146	Amoebiasis	44105	102/7854	0.12260349	0.25090823	0.25090823	403466 COL4A5	1	VPOR_VSMC-up
cfa04926	Relaxin signaling pathway	44105	127/7854	0.15050814	0.25090823	0.25090823	403466 COL4A5	1	VPOR_VSMC-up
cfa00230	Purine metabolism	44105	130/7854	0.15380244	0.25090823	0.25090823	100855769 KIAA1211	1	VPOR_VSMC-up
cfa04550	Signaling pathways regulating pluripotency of stem cells	44105	135/7854	0.1592674	0.25090823	0.25090823	612954 ISL1	1	VPOR_VSMC-up
cfa04145	Phagosome	44105	138/7854	0.16253111	0.25090823	0.25090823	486694 OLR1	1	VPOR_VSMC-up
cfa05224	Breast cancer	44105	142/7854	0.16686499	0.25090823	0.25090823	608459 FGF5	1	VPOR_VSMC-up
cfa05226	Gastric cancer	44105	149/7854	0.17440073	0.25090823	0.25090823	608459 FGF5	1	VPOR_VSMC-up
cfa04310	Wnt signaling pathway	44105	151/7854	0.1765425	0.25090823	0.25090823	487081 BAMBI	1	VPOR_VSMC-up
cfa04921	Oxytocin signaling pathway	44105	151/7854	0.1765425	0.25090823	0.25090823	100855763	1	VPOR_VSMC-up
cfa04621	NOD-like receptor signaling pathway	44105	158/7854	0.18399937	0.25090823	0.25090823	100855763	1	VPOR_VSMC-up
cfa04360	Axon guidance	44105	179/7854	0.20600679	0.2687045	0.2687045	486004 TRPC4	1	VPOR_VSMC-up
cfa04510	Focal adhesion	44105	198/7854	0.22545639	0.27521058	0.27521058	403466 COL4A5	1	VPOR_VSMC-up
cfa04015	Rap1 signaling pathway	44105	209/7854	0.23651968	0.27521058	0.27521058	608459 FGF5	1	VPOR_VSMC-up
cfa04810	Regulation of actin cytoskeleton	44105	211/7854	0.23851584	0.27521058	0.27521058	608459 FGF5	1	VPOR_VSMC-up
cfa04014	Ras signaling pathway	44105	225/7854	0.25235793	0.2803977	0.2803977	608459 FGF5	1	VPOR_VSMC-up
cfa04010	MAPK signaling pathway	44105	288/7854	0.31188725	0.33416491	0.33416491	608459 FGF5	1	VPOR_VSMC-up
NA	NA	NA	NA	NA	NA	NA	NA NA	NA	VPOR_VSMC-up
NA	NA	NA	NA	NA	NA	NA	NA NA	NA	VPOR_VSMC-up
NA	NA	NA	NA	NA	NA	NA	NA NA	NA	VPOR_VSMC-up
NA	NA	NA	NA	NA	NA	NA	NA NA	NA	VPOR_VSMC-up

NA	NA	NA	NA	NA	NA	NA	NA	NA	NA	NA	VPOR_VSMC-up
NA	NA	NA	NA	NA	NA	NA	NA	NA	NA	NA	VPOR_VSMC-up
NA	NA	NA	NA	NA	NA	NA	NA	NA	NA	NA	VPOR_VSMC-up
NA	NA	NA	NA	NA	NA	NA	NA	NA	NA	NA	VPOR_VSMC-up
NA	NA	NA	NA	NA	NA	NA	NA	NA	NA	NA	VPOR_VSMC-up
NA	NA	NA	NA	NA	NA	NA	NA	NA	NA	NA	VPOR_VSMC-up
NA	NA	NA	NA	NA	NA	NA	NA	NA	NA	NA	VPOR_VSMC-up
NA	NA	NA	NA	NA	NA	NA	NA	NA	NA	NA	VPOR_VSMC-up
NA	NA	NA	NA	NA	NA	NA	NA	NA	NA	NA	VPOR_VSMC-up
NA	NA	NA	NA	NA	NA	NA	NA	NA	NA	NA	VPOR_VSMC-up
NA	NA	NA	NA	NA	NA	NA	NA	NA	NA	NA	VPOR_VSMC-up
NA	NA	NA	NA	NA	NA	NA	NA	NA	NA	NA	VPOR_VSMC-up
NA	NA	NA	NA	NA	NA	NA	NA	NA	NA	NA	VPOR_VSMC-up
NA	NA	NA	NA	NA	NA	NA	NA	NA	NA	NA	VPOR_VSMC-up
NA	NA	NA	NA	NA	NA	NA	NA	NA	NA	NA	VPOR_VSMC-up
NA	NA	NA	NA	NA	NA	NA	NA	NA	NA	NA	VPOR_VSMC-up
NA	NA	NA	NA	NA	NA	NA	NA	NA	NA	NA	VPOR_VSMC-up
NA	NA	NA	NA	NA	NA	NA	NA	NA	NA	NA	VPOR_VSMC-up
NA	NA	NA	NA	NA	NA	NA	NA	NA	NA	NA	VPOR_VSMC-up
NA	NA	NA	NA	NA	NA	NA	NA	NA	NA	NA	VPOR_VSMC-up
NA	NA	NA	NA	NA	NA	NA	NA	NA	NA	NA	VPOR_VSMC-up
NA	NA	NA	NA	NA	NA	NA	NA	NA	NA	NA	VPOR_VSMC-up
NA	NA	NA	NA	NA	NA	NA	NA	NA	NA	NA	VPOR_VSMC-up
NA	NA	NA	NA	NA	NA	NA	NA	NA	NA	NA	VPOR_VSMC-up
NA	NA	NA	NA	NA	NA	NA	NA	NA	NA	NA	VPOR_VSMC-up
NA	NA	NA	NA	NA	NA	NA	NA	NA	NA	NA	VPOR_VSMC-up
NA	NA	NA	NA	NA	NA	NA	NA	NA	NA	NA	VPOR_VSMC-up
NA	NA	NA	NA	NA	NA	NA	NA	NA	NA	NA	VPOR_VSMC-up
NA	NA	NA	NA	NA	NA	NA	NA	NA	NA	NA	VPOR_VSMC-up
NA	NA	NA	NA	NA	NA	NA	NA	NA	NA	NA	VPOR_VSMC-up
NA	NA	NA	NA	NA	NA	NA	NA	NA	NA	NA	VPOR_VSMC-up
NA	NA	NA	NA	NA	NA	NA	NA	NA	NA	NA	VPOR_VSMC-up
NA	NA	NA	NA	NA	NA	NA	NA	NA	NA	NA	VPOR_VSMC-up
NA	NA	NA	NA	NA	NA	NA	NA	NA	NA	NA	VPOR_VSMC-up
NA	NA	NA	NA	NA	NA	NA	NA	NA	NA	NA	VPOR_VSMC-up
NA	NA	NA	NA	NA	NA	NA	NA	NA	NA	NA	VPOR_VSMC-up
NA	NA	NA	NA	NA	NA	NA	NA	NA	NA	NA	VPOR_VSMC-up
NA	NA	NA	NA	NA	NA	NA	NA	NA	NA	NA	VPOR_VSMC-up
NA	NA	NA	NA	NA	NA	NA	NA	NA	NA	NA	VPOR_VSMC-up
NA	NA	NA	NA	NA	NA	NA	NA	NA	NA	NA	VPOR_VSMC-up
NA	NA	NA	NA	NA	NA	NA	NA	NA	NA	NA	VPOR_VSMC-up
NA	NA	NA	NA	NA	NA	NA	NA	NA	NA	NA	VPOR_VSMC-up
NA	NA	NA	NA	NA	NA	NA	NA	NA	NA	NA	VPOR_VSMC-up
NA	NA	NA	NA	NA	NA	NA	NA	NA	NA	NA	VPOR_VSMC-up
NA	NA	NA	NA	NA	NA	NA	NA	NA	NA	NA	VPOR_VSMC-up
NA	NA	NA	NA	NA	NA	NA	NA	NA	NA	NA	VPOR_VSMC-up
NA	NA	NA	NA	NA	NA	NA	NA	NA	NA	NA	VPOR_VSMC-up
NA	NA	NA	NA	NA	NA	NA	NA	NA	NA	NA	VPOR_VSMC-up
NA	NA	NA	NA	NA	NA	NA	NA	NA	NA	NA	VPOR_VSMC-up
NA	NA	NA	NA	NA	NA	NA	NA	NA	NA	NA	VPOR_VSMC-up
NA	NA	NA	NA	NA	NA	NA	NA	NA	NA	NA	VPOR_VSMC-up
NA	NA	NA	NA	NA	NA	NA	NA	NA	NA	NA	VPOR_VSMC-up
NA	NA	NA	NA	NA	NA	NA	NA	NA	NA	NA	VPOR_VSMC-up
NA	NA	NA	NA	NA	NA	NA	NA	NA	NA	NA	VPOR_VSMC-up
NA	NA	NA	NA	NA	NA	NA	NA	NA	NA	NA	VPOR_VSMC-up
NA	NA	NA	NA	NA	NA	NA	NA	NA	NA	NA	VPOR_VSMC-up
NA	NA	NA	NA	NA	NA	NA	NA	NA	NA	NA	VPOR_VSMC-up
NA	NA	NA	NA	NA	NA	NA	NA	NA	NA	NA	VPOR_VSMC-up
NA	NA	NA	NA	NA	NA	NA	NA	NA	NA	NA	VPOR_VSMC-up
NA	NA	NA	NA	NA	NA	NA	NA	NA	NA	NA	VPOR_VSMC-up
NA	NA	NA	NA	NA	NA	NA	NA	NA	NA	NA	VPOR_VSMC-up
NA	NA	NA	NA	NA	NA	NA	NA	NA	NA	NA	VPOR_VSMC-up
NA	NA	NA	NA	NA	NA	NA	NA	NA	NA	NA	VPOR_VSMC-up
NA	NA	NA	NA	NA	NA	NA	NA	NA	NA	NA	VPOR_VSMC-up
NA	NA	NA	NA	NA	NA	NA	NA	NA	NA	NA	VPOR_VSMC-up
NA	NA	NA	NA	NA	NA	NA	NA	NA	NA	NA	VPOR_VSMC-up
NA	NA	NA	NA	NA	NA	NA	NA	NA	NA	NA	VPOR_VSMC-up
NA	NA	NA	NA	NA	NA	NA	NA	NA	NA	NA	VPOR_VSMC-up
NA	NA	NA	NA	NA	NA	NA	NA	NA	NA	NA	VPOR_VSMC-up
NA	NA	NA	NA	NA	NA	NA	NA	NA	NA	NA	VPOR_VSMC-up
NA	NA	NA	NA	NA	NA	NA	NA	NA	NA	NA	VPOR_VSMC-up
NA	NA	NA	NA	NA	NA	NA	NA	NA	NA	NA	VPOR_VSMC-up
NA	NA	NA	NA	NA	NA	NA	NA	NA	NA	NA	VPOR_VSMC-up
NA	NA	NA	NA	NA	NA	NA	NA	NA	NA	NA	VPOR_VSMC-up

NA	NA	NA	NA	NA	NA	NA	NA	NA	NA	VPOR_VSMC-up
NA	NA	NA	NA	NA	NA	NA	NA	NA	NA	VPOR_VSMC-up
NA	NA	NA	NA	NA	NA	NA	NA	NA	NA	VPOR_VSMC-up
NA	NA	NA	NA	NA	NA	NA	NA	NA	NA	VPOR_VSMC-up
NA	NA	NA	NA	NA	NA	NA	NA	NA	NA	VPOR_VSMC-up
NA	NA	NA	NA	NA	NA	NA	NA	NA	NA	VPOR_VSMC-up
NA	NA	NA	NA	NA	NA	NA	NA	NA	NA	VPOR_VSMC-up
NA	NA	NA	NA	NA	NA	NA	NA	NA	NA	VPOR_VSMC-up
NA	NA	NA	NA	NA	NA	NA	NA	NA	NA	VPOR_VSMC-up
NA	NA	NA	NA	NA	NA	NA	NA	NA	NA	VPOR_VSMC-up
NA	NA	NA	NA	NA	NA	NA	NA	NA	NA	VPOR_VSMC-up
NA	NA	NA	NA	NA	NA	NA	NA	NA	NA	VPOR_VSMC-up
NA	NA	NA	NA	NA	NA	NA	NA	NA	NA	VPOR_VSMC-up
NA	NA	NA	NA	NA	NA	NA	NA	NA	NA	VPOR_VSMC-up
NA	NA	NA	NA	NA	NA	NA	NA	NA	NA	VPOR_VSMC-up
NA	NA	NA	NA	NA	NA	NA	NA	NA	NA	VPOR_VSMC-up
NA	NA	NA	NA	NA	NA	NA	NA	NA	NA	VPOR_VSMC-up
NA	NA	NA	NA	NA	NA	NA	NA	NA	NA	VPOR_VSMC-up
NA	NA	NA	NA	NA	NA	NA	NA	NA	NA	VPOR_VSMC-up
NA	NA	NA	NA	NA	NA	NA	NA	NA	NA	VPOR_VSMC-up
NA	NA	NA	NA	NA	NA	NA	NA	NA	NA	VPOR_VSMC-up
NA	NA	NA	NA	NA	NA	NA	NA	NA	NA	VPOR_VSMC-up
NA	NA	NA	NA	NA	NA	NA	NA	NA	NA	VPOR_VSMC-up
NA	NA	NA	NA	NA	NA	NA	NA	NA	NA	VPOR_VSMC-up
NA	NA	NA	NA	NA	NA	NA	NA	NA	NA	VPOR_VSMC-up
NA	NA	NA	NA	NA	NA	NA	NA	NA	NA	VPOR_VSMC-up
NA	NA	NA	NA	NA	NA	NA	NA	NA	NA	VPOR_VSMC-up
NA	NA	NA	NA	NA	NA	NA	NA	NA	NA	VPOR_VSMC-up
NA	NA	NA	NA	NA	NA	NA	NA	NA	NA	VPOR_VSMC-up
NA	NA	NA	NA	NA	NA	NA	NA	NA	NA	VPOR_VSMC-up
NA	NA	NA	NA	NA	NA	NA	NA	NA	NA	VPOR_VSMC-up
NA	NA	NA	NA	NA	NA	NA	NA	NA	NA	VPOR_VSMC-up
NA	NA	NA	NA	NA	NA	NA	NA	NA	NA	VPOR_VSMC-up
NA	NA	NA	NA	NA	NA	NA	NA	NA	NA	VPOR_VSMC-up
NA	NA	NA	NA	NA	NA	NA	NA	NA	NA	VPOR_VSMC-up
NA	NA	NA	NA	NA	NA	NA	NA	NA	NA	VPOR_VSMC-up
NA	NA	NA	NA	NA	NA	NA	NA	NA	NA	VPOR_VSMC-up
NA	NA	NA	NA	NA	NA	NA	NA	NA	NA	VPOR_VSMC-up
NA	NA	NA	NA	NA	NA	NA	NA	NA	NA	VPOR_VSMC-up
NA	NA	NA	NA	NA	NA	NA	NA	NA	NA	VPOR_VSMC-up
NA	NA	NA	NA	NA	NA	NA	NA	NA	NA	VPOR_VSMC-up
NA	NA	NA	NA	NA	NA	NA	NA	NA	NA	VPOR_VSMC-up
NA	NA	NA	NA	NA	NA	NA	NA	NA	NA	VPOR_VSMC-up
NA	NA	NA	NA	NA	NA	NA	NA	NA	NA	VPOR_VSMC-up
NA	NA	NA	NA	NA	NA	NA	NA	NA	NA	VPOR_VSMC-up
NA	NA	NA	NA	NA	NA	NA	NA	NA	NA	VPOR_VSMC-up
NA	NA	NA	NA	NA	NA	NA	NA	NA	NA	VPOR_VSMC-up
NA	NA	NA	NA	NA	NA	NA	NA	NA	NA	VPOR_VSMC-up
NA	NA	NA	NA	NA	NA	NA	NA	NA	NA	VPOR_VSMC-up
NA	NA	NA	NA	NA	NA	NA	NA	NA	NA	VPOR_VSMC-up
NA	NA	NA	NA	NA	NA	NA	NA	NA	NA	VPOR_VSMC-up
NA	NA	NA	NA	NA	NA	NA	NA	NA	NA	VPOR_VSMC-up
NA	NA	NA	NA	NA	NA	NA	NA	NA	NA	VPOR_VSMC-up
NA	NA	NA	NA	NA	NA	NA	NA	NA	NA	VPOR_VSMC-up
NA	NA	NA	NA	NA	NA	NA	NA	NA	NA	VPOR_VSMC-up
NA	NA	NA	NA	NA	NA	NA	NA	NA	NA	VPOR_VSMC-up
NA	NA	NA	NA	NA	NA	NA	NA	NA	NA	VPOR_VSMC-up
NA	NA	NA	NA	NA	NA	NA	NA	NA	NA	VPOR_VSMC-up
NA	NA	NA	NA	NA	NA	NA	NA	NA	NA	VPOR_VSMC-up
NA	NA	NA	NA	NA	NA	NA	NA	NA	NA	VPOR_VSMC-up
NA	NA	NA	NA	NA	NA	NA	NA	NA	NA	VPOR_VSMC-up
NA	NA	NA	NA	NA	NA	NA	NA	NA	NA	VPOR_VSMC-up
NA	NA	NA	NA	NA	NA	NA	NA	NA	NA	VPOR_VSMC-up
NA	NA	NA	NA	NA	NA	NA	NA	NA	NA	VPOR_VSMC-up
NA	NA	NA	NA	NA	NA	NA	NA	NA	NA	VPOR_VSMC-up
NA	NA	NA	NA	NA	NA	NA	NA	NA	NA	VPOR_VSMC-up
NA	NA	NA	NA	NA	NA	NA	NA	NA	NA	VPOR_VSMC-up
NA	NA	NA	NA	NA	NA	NA	NA	NA	NA	VPOR_VSMC-up
NA	NA	NA	NA	NA	NA	NA	NA	NA	NA	VPOR_VSMC-up
NA	NA	NA	NA	NA	NA	NA	NA	NA	NA	VPOR_VSMC-up
NA	NA	NA	NA	NA	NA	NA	NA	NA	NA	VPOR_VSMC-up
NA	NA	NA	NA	NA	NA	NA	NA	NA	NA	VPOR_VSMC-up
NA	NA	NA	NA	NA	NA	NA	NA	NA	NA	VPOR_VSMC-up
NA	NA	NA	NA	NA	NA	NA	NA	NA	NA	VPOR_VSMC-up

NA	NA	NA	NA	NA	NA	NA	NA NA	NA	VPOR_VSMC-up
NA	NA	NA	NA	NA	NA	NA	NA NA	NA	VPOR_VSMC-up
NA	NA	NA	NA	NA	NA	NA	NA NA	NA	VPOR_VSMC-up
NA	NA	NA	NA	NA	NA	NA	NA NA	NA	VPOR_VSMC-up
NA	NA	NA	NA	NA	NA	NA	NA NA	NA	VPOR_VSMC-up
NA	NA	NA	NA	NA	NA	NA	NA NA	NA	VPOR_VSMC-up
NA	NA	NA	NA	NA	NA	NA	NA NA	NA	VPOR_VSMC-up
NA	NA	NA	NA	NA	NA	NA	NA NA	NA	VPOR_VSMC-up
NA	NA	NA	NA	NA	NA	NA	NA NA	NA	VPOR_VSMC-up
NA	NA	NA	NA	NA	NA	NA	NA NA	NA	VPOR_VSMC-up
NA	NA	NA	NA	NA	NA	NA	NA NA	NA	VPOR_VSMC-up
NA	NA	NA	NA	NA	NA	NA	NA NA	NA	VPOR_VSMC-up
NA	NA	NA	NA	NA	NA	NA	NA NA	NA	VPOR_VSMC-up
NA	NA	NA	NA	NA	NA	NA	NA NA	NA	VPOR_VSMC-up
NA	NA	NA	NA	NA	NA	NA	NA NA	NA	VPOR_VSMC-up
NA	NA	NA	NA	NA	NA	NA	NA NA	NA	VPOR_VSMC-up
NA	NA	NA	NA	NA	NA	NA	NA NA	NA	VPOR_VSMC-up
NA	NA	NA	NA	NA	NA	NA	NA NA	NA	VPOR_VSMC-up
cfa04080	Neuroactive ligand-receptor interaction	44105	327/7854	0.34656559	0.34743371	0.34743371	486704 C3AR1	1	VPOR_VSMC-up
cfa05165	Human papillomavirus infection	44105	328/7854	0.34743371	0.34743371	0.34743371	403466 COL4A5	1	VPOR_VSMC-up
cfa00534	Glycosaminoglycan biosynthesis - heparan sulfate / heparin	44013	24/7854	0.0212033	0.07546065	0.03610557	489975 HS3ST2	1	VFEM_VSMC-up
cfa00030	Pentose phosphate pathway	44013	29/7854	0.02557181	0.07546065	0.03610557	476299 FBP1	1	VFEM_VSMC-up
cfa00051	Fructose and mannose metabolism	44013	32/7854	0.02818488	0.07546065	0.03610557	476299 FBP1	1	VFEM_VSMC-up
cfa00260	Glycine, serine and threonine metabolism	44013	40/7854	0.03512374	0.07546065	0.03610557	414733 GATM	1	VFEM_VSMC-up
cfa00330	Arginine and proline metabolism	44013	47/7854	0.04116036	0.07546065	0.03610557	414733 GATM	1	VFEM_VSMC-up
cfa00010	Glycolysis / Gluconeogenesis	44013	62/7854	0.05398705	0.08483679	0.04059176	476299 FBP1	1	VFEM_VSMC-up
cfa04922	Glucagon signaling pathway	44013	101/7854	0.08665034	0.11332461	0.0542223	476299 FBP1	1	VFEM_VSMC-up
cfa01200	Carbon metabolism	44013	116/7854	0.09895313	0.11332461	0.0542223	476299 FBP1	1	VFEM_VSMC-up
cfa04152	AMPK signaling pathway	44013	121/7854	0.10302237	0.11332461	0.0542223	476299 FBP1	1	VFEM_VSMC-up
NA	NA	NA	NA	NA	NA	NA	NA NA	NA	VFEM_VSMC-up
NA	NA	NA	NA	NA	NA	NA	NA NA	NA	VFEM_VSMC-up
NA	NA	NA	NA	NA	NA	NA	NA NA	NA	VFEM_VSMC-up
NA	NA	NA	NA	NA	NA	NA	NA NA	NA	VFEM_VSMC-up
NA	NA	NA	NA	NA	NA	NA	NA NA	NA	VFEM_VSMC-up
NA	NA	NA	NA	NA	NA	NA	NA NA	NA	VFEM_VSMC-up
NA	NA	NA	NA	NA	NA	NA	NA NA	NA	VFEM_VSMC-up
NA	NA	NA	NA	NA	NA	NA	NA NA	NA	VFEM_VSMC-up
NA	NA	NA	NA	NA	NA	NA	NA NA	NA	VFEM_VSMC-up
NA	NA	NA	NA	NA	NA	NA	NA NA	NA	VFEM_VSMC-up
NA	NA	NA	NA	NA	NA	NA	NA NA	NA	VFEM_VSMC-up
NA	NA	NA	NA	NA	NA	NA	NA NA	NA	VFEM_VSMC-up
NA	NA	NA	NA	NA	NA	NA	NA NA	NA	VFEM_VSMC-up
NA	NA	NA	NA	NA	NA	NA	NA NA	NA	VFEM_VSMC-up
NA	NA	NA	NA	NA	NA	NA	NA NA	NA	VFEM_VSMC-up
NA	NA	NA	NA	NA	NA	NA	NA NA	NA	VFEM_VSMC-up
NA	NA	NA	NA	NA	NA	NA	NA NA	NA	VFEM_VSMC-up
NA	NA	NA	NA	NA	NA	NA	NA NA	NA	VFEM_VSMC-up
NA	NA	NA	NA	NA	NA	NA	NA NA	NA	VFEM_VSMC-up
NA	NA	NA	NA	NA	NA	NA	NA NA	NA	VFEM_VSMC-up
NA	NA	NA	NA	NA	NA	NA	NA NA	NA	VFEM_VSMC-up
NA	NA	NA	NA	NA	NA	NA	NA NA	NA	VFEM_VSMC-up
NA	NA	NA	NA	NA	NA	NA	NA NA	NA	VFEM_VSMC-up
NA	NA	NA	NA	NA	NA	NA	NA NA	NA	VFEM_VSMC-up
NA	NA	NA	NA	NA	NA	NA	NA NA	NA	VFEM_VSMC-up
NA	NA	NA	NA	NA	NA	NA	NA NA	NA	VFEM_VSMC-up
NA	NA	NA	NA	NA	NA	NA	NA NA	NA	VFEM_VSMC-up
NA	NA	NA	NA	NA	NA	NA	NA NA	NA	VFEM_VSMC-up
NA	NA	NA	NA	NA	NA	NA	NA NA	NA	VFEM_VSMC-up
NA	NA	NA	NA	NA	NA	NA	NA NA	NA	VFEM_VSMC-up
NA	NA	NA	NA	NA	NA	NA	NA NA	NA	VFEM_VSMC-up
NA	NA	NA	NA	NA	NA	NA	NA NA	NA	VFEM_VSMC-up
NA	NA	NA	NA	NA	NA	NA	NA NA	NA	VFEM_VSMC-up
NA	NA	NA	NA	NA	NA	NA	NA NA	NA	VFEM_VSMC-up
NA	NA	NA	NA	NA	NA	NA	NA NA	NA	VFEM_VSMC-up

NA	NA	NA	NA	NA	NA	NA	NA	NA	NA	VFEM_VSMC-up
NA	NA	NA	NA	NA	NA	NA	NA	NA	NA	VFEM_VSMC-up
NA	NA	NA	NA	NA	NA	NA	NA	NA	NA	VFEM_VSMC-up
NA	NA	NA	NA	NA	NA	NA	NA	NA	NA	VFEM_VSMC-up
NA	NA	NA	NA	NA	NA	NA	NA	NA	NA	VFEM_VSMC-up
NA	NA	NA	NA	NA	NA	NA	NA	NA	NA	VFEM_VSMC-up
NA	NA	NA	NA	NA	NA	NA	NA	NA	NA	VFEM_VSMC-up
NA	NA	NA	NA	NA	NA	NA	NA	NA	NA	VFEM_VSMC-up
NA	NA	NA	NA	NA	NA	NA	NA	NA	NA	VFEM_VSMC-up
NA	NA	NA	NA	NA	NA	NA	NA	NA	NA	VFEM_VSMC-up
NA	NA	NA	NA	NA	NA	NA	NA	NA	NA	VFEM_VSMC-up
NA	NA	NA	NA	NA	NA	NA	NA	NA	NA	VFEM_VSMC-up
NA	NA	NA	NA	NA	NA	NA	NA	NA	NA	VFEM_VSMC-up
NA	NA	NA	NA	NA	NA	NA	NA	NA	NA	VFEM_VSMC-up
NA	NA	NA	NA	NA	NA	NA	NA	NA	NA	VFEM_VSMC-up
NA	NA	NA	NA	NA	NA	NA	NA	NA	NA	VFEM_VSMC-up
NA	NA	NA	NA	NA	NA	NA	NA	NA	NA	VFEM_VSMC-up
NA	NA	NA	NA	NA	NA	NA	NA	NA	NA	VFEM_VSMC-up
NA	NA	NA	NA	NA	NA	NA	NA	NA	NA	VFEM_VSMC-up
NA	NA	NA	NA	NA	NA	NA	NA	NA	NA	VFEM_VSMC-up
NA	NA	NA	NA	NA	NA	NA	NA	NA	NA	VFEM_VSMC-up
NA	NA	NA	NA	NA	NA	NA	NA	NA	NA	VFEM_VSMC-up
NA	NA	NA	NA	NA	NA	NA	NA	NA	NA	VFEM_VSMC-up
NA	NA	NA	NA	NA	NA	NA	NA	NA	NA	VFEM_VSMC-up
NA	NA	NA	NA	NA	NA	NA	NA	NA	NA	VFEM_VSMC-up
NA	NA	NA	NA	NA	NA	NA	NA	NA	NA	VFEM_VSMC-up
NA	NA	NA	NA	NA	NA	NA	NA	NA	NA	VFEM_VSMC-up
NA	NA	NA	NA	NA	NA	NA	NA	NA	NA	VFEM_VSMC-up
NA	NA	NA	NA	NA	NA	NA	NA	NA	NA	VFEM_VSMC-up
NA	NA	NA	NA	NA	NA	NA	NA	NA	NA	VFEM_VSMC-up
NA	NA	NA	NA	NA	NA	NA	NA	NA	NA	VFEM_VSMC-up
NA	NA	NA	NA	NA	NA	NA	NA	NA	NA	VFEM_VSMC-up
NA	NA	NA	NA	NA	NA	NA	NA	NA	NA	VFEM_VSMC-up
NA	NA	NA	NA	NA	NA	NA	NA	NA	NA	VFEM_VSMC-up
NA	NA	NA	NA	NA	NA	NA	NA	NA	NA	VFEM_VSMC-up
NA	NA	NA	NA	NA	NA	NA	NA	NA	NA	VFEM_VSMC-up
NA	NA	NA	NA	NA	NA	NA	NA	NA	NA	VFEM_VSMC-up
NA	NA	NA	NA	NA	NA	NA	NA	NA	NA	VFEM_VSMC-up
NA	NA	NA	NA	NA	NA	NA	NA	NA	NA	VFEM_VSMC-up
NA	NA	NA	NA	NA	NA	NA	NA	NA	NA	VFEM_VSMC-up
NA	NA	NA	NA	NA	NA	NA	NA	NA	NA	VFEM_VSMC-up
NA	NA	NA	NA	NA	NA	NA	NA	NA	NA	VFEM_VSMC-up
NA	NA	NA	NA	NA	NA	NA	NA	NA	NA	VFEM_VSMC-up
NA	NA	NA	NA	NA	NA	NA	NA	NA	NA	VFEM_VSMC-up
NA	NA	NA	NA	NA	NA	NA	NA	NA	NA	VFEM_VSMC-up
NA	NA	NA	NA	NA	NA	NA	NA	NA	NA	VFEM_VSMC-up
NA	NA	NA	NA	NA	NA	NA	NA	NA	NA	VFEM_VSMC-up
NA	NA	NA	NA	NA	NA	NA	NA	NA	NA	VFEM_VSMC-up
NA	NA	NA	NA	NA	NA	NA	NA	NA	NA	VFEM_VSMC-up
NA	NA	NA	NA	NA	NA	NA	NA	NA	NA	VFEM_VSMC-up
NA	NA	NA	NA	NA	NA	NA	NA	NA	NA	VFEM_VSMC-up
NA	NA	NA	NA	NA	NA	NA	NA	NA	NA	VFEM_VSMC-up
NA	NA	NA	NA	NA	NA	NA	NA	NA	NA	VFEM_VSMC-up
NA	NA	NA	NA	NA	NA	NA	NA	NA	NA	VFEM_VSMC-up
NA	NA	NA	NA	NA	NA	NA	NA	NA	NA	VFEM_VSMC-up
NA	NA	NA	NA	NA	NA	NA	NA	NA	NA	VFEM_VSMC-up
NA	NA	NA	NA	NA	NA	NA	NA	NA	NA	VFEM_VSMC-up
NA	NA	NA	NA	NA	NA	NA	NA	NA	NA	VFEM_VSMC-up
NA	NA	NA	NA	NA	NA	NA	NA	NA	NA	VFEM_VSMC-up
NA	NA	NA	NA	NA	NA	NA	NA	NA	NA	VFEM_VSMC-up
NA	NA	NA	NA	NA	NA	NA	NA	NA	NA	VFEM_VSMC-up
NA	NA	NA	NA	NA	NA	NA	NA	NA	NA	VFEM_VSMC-up
NA	NA	NA	NA	NA	NA	NA	NA	NA	NA	VFEM_VSMC-up
NA	NA	NA	NA	NA	NA	NA	NA	NA	NA	VFEM_VSMC-up
NA	NA	NA	NA	NA	NA	NA	NA	NA	NA	VFEM_VSMC-up
NA	NA	NA	NA	NA	NA	NA	NA	NA	NA	VFEM_VSMC-up
NA	NA	NA	NA	NA	NA	NA	NA	NA	NA	VFEM_VSMC-up
NA	NA	NA	NA	NA	NA	NA	NA	NA	NA	VFEM_VSMC-up
NA	NA	NA	NA	NA	NA	NA	NA	NA	NA	VFEM_VSMC-up
NA	NA	NA	NA	NA	NA	NA	NA	NA	NA	VFEM_VSMC-up
NA	NA	NA	NA	NA	NA	NA	NA	NA	NA	VFEM_VSMC-up
NA	NA	NA	NA	NA	NA	NA	NA	NA	NA	VFEM_VSMC-up
NA	NA	NA	NA	NA	NA	NA	NA	NA	NA	VFEM_VSMC-up
NA	NA	NA	NA	NA	NA	NA	NA	NA	NA	VFEM_VSMC-up

NA	NA	NA	NA	NA	NA	NA	NA NA	NA	VFEM_VSMC-up
NA	NA	NA	NA	NA	NA	NA	NA NA	NA	VFEM_VSMC-up
NA	NA	NA	NA	NA	NA	NA	NA NA	NA	VFEM_VSMC-up
NA	NA	NA	NA	NA	NA	NA	NA NA	NA	VFEM_VSMC-up
NA	NA	NA	NA	NA	NA	NA	NA NA	NA	VFEM_VSMC-up
NA	NA	NA	NA	NA	NA	NA	NA NA	NA	VFEM_VSMC-up
NA	NA	NA	NA	NA	NA	NA	NA NA	NA	VFEM_VSMC-up
NA	NA	NA	NA	NA	NA	NA	NA NA	NA	VFEM_VSMC-up
NA	NA	NA	NA	NA	NA	NA	NA NA	NA	VFEM_VSMC-up
NA	NA	NA	NA	NA	NA	NA	NA NA	NA	VFEM_VSMC-up
NA	NA	NA	NA	NA	NA	NA	NA NA	NA	VFEM_VSMC-up
NA	NA	NA	NA	NA	NA	NA	NA NA	NA	VFEM_VSMC-up
NA	NA	NA	NA	NA	NA	NA	NA NA	NA	VFEM_VSMC-up
NA	NA	NA	NA	NA	NA	NA	NA NA	NA	VFEM_VSMC-up
NA	NA	NA	NA	NA	NA	NA	NA NA	NA	VFEM_VSMC-up
NA	NA	NA	NA	NA	NA	NA	NA NA	NA	VFEM_VSMC-up
NA	NA	NA	NA	NA	NA	NA	NA NA	NA	VFEM_VSMC-up
NA	NA	NA	NA	NA	NA	NA	NA NA	NA	VFEM_VSMC-up
NA	NA	NA	NA	NA	NA	NA	NA NA	NA	VFEM_VSMC-up
NA	NA	NA	NA	NA	NA	NA	NA NA	NA	VFEM_VSMC-up
NA	NA	NA	NA	NA	NA	NA	NA NA	NA	VFEM_VSMC-up
NA	NA	NA	NA	NA	NA	NA	NA NA	NA	VFEM_VSMC-up
NA	NA	NA	NA	NA	NA	NA	NA NA	NA	VFEM_VSMC-up
NA	NA	NA	NA	NA	NA	NA	NA NA	NA	VFEM_VSMC-up
NA	NA	NA	NA	NA	NA	NA	NA NA	NA	VFEM_VSMC-up
NA	NA	NA	NA	NA	NA	NA	NA NA	NA	VFEM_VSMC-up
NA	NA	NA	NA	NA	NA	NA	NA NA	NA	VFEM_VSMC-up
NA	NA	NA	NA	NA	NA	NA	NA NA	NA	VFEM_VSMC-up
NA	NA	NA	NA	NA	NA	NA	NA NA	NA	VFEM_VSMC-up
NA	NA	NA	NA	NA	NA	NA	NA NA	NA	VFEM_VSMC-up
NA	NA	NA	NA	NA	NA	NA	NA NA	NA	VFEM_VSMC-up
NA	NA	NA	NA	NA	NA	NA	NA NA	NA	VFEM_VSMC-up
NA	NA	NA	NA	NA	NA	NA	NA NA	NA	VFEM_VSMC-up
NA	NA	NA	NA	NA	NA	NA	NA NA	NA	VFEM_VSMC-up
NA	NA	NA	NA	NA	NA	NA	NA NA	NA	VFEM_VSMC-up
NA	NA	NA	NA	NA	NA	NA	NA NA	NA	VFEM_VSMC-up
NA	NA	NA	NA	NA	NA	NA	NA NA	NA	VFEM_VSMC-up
NA	NA	NA	NA	NA	NA	NA	NA NA	NA	VFEM_VSMC-up
NA	NA	NA	NA	NA	NA	NA	NA NA	NA	VFEM_VSMC-up
NA	NA	NA	NA	NA	NA	NA	NA NA	NA	VFEM_VSMC-up
NA	NA	NA	NA	NA	NA	NA	NA NA	NA	VFEM_VSMC-up
NA	NA	NA	NA	NA	NA	NA	NA NA	NA	VFEM_VSMC-up
NA	NA	NA	NA	NA	NA	NA	NA NA	NA	VFEM_VSMC-up
NA	NA	NA	NA	NA	NA	NA	NA NA	NA	VFEM_VSMC-up
NA	NA	NA	NA	NA	NA	NA	NA NA	NA	VFEM_VSMC-up
NA	NA	NA	NA	NA	NA	NA	NA NA	NA	VFEM_VSMC-up
NA	NA	NA	NA	NA	NA	NA	NA NA	NA	VFEM_VSMC-up
NA	NA	NA	NA	NA	NA	NA	NA NA	NA	VFEM_VSMC-up
NA	NA	NA	NA	NA	NA	NA	NA NA	NA	VFEM_VSMC-up
NA	NA	NA	NA	NA	NA	NA	NA NA	NA	VFEM_VSMC-up
NA	NA	NA	NA	NA	NA	NA	NA NA	NA	VFEM_VSMC-up
NA	NA	NA	NA	NA	NA	NA	NA NA	NA	VFEM_VSMC-up
NA	NA	NA	NA	NA	NA	NA	NA NA	NA	VFEM_VSMC-up
NA	NA	NA	NA	NA	NA	NA	NA NA	NA	VFEM_VSMC-up
NA	NA	NA	NA	NA	NA	NA	NA NA	NA	VFEM_VSMC-up
NA	NA	NA	NA	NA	NA	NA	NA NA	NA	VFEM_VSMC-up
NA	NA	NA	NA	NA	NA	NA	NA NA	NA	VFEM_VSMC-up
NA	NA	NA	NA	NA	NA	NA	NA NA	NA	VFEM_VSMC-up
NA	NA	NA	NA	NA	NA	NA	NA NA	NA	VFEM_VSMC-up
NA	NA	NA	NA	NA	NA	NA	NA NA	NA	VFEM_VSMC-up
NA	NA	NA	NA	NA	NA	NA	NA NA	NA	VFEM_VSMC-up
NA	NA	NA	NA	NA	NA	NA	NA NA	NA	VFEM_VSMC-up
NA	NA	NA	NA	NA	NA	NA	NA NA	NA	VFEM_VSMC-up
NA	NA	NA	NA	NA	NA	NA	NA NA	NA	VFEM_VSMC-up
NA	NA	NA	NA	NA	NA	NA	NA NA	NA	VFEM_VSMC-up
NA	NA	NA	NA	NA	NA	NA	NA NA	NA	VFEM_VSMC-up
NA	NA	NA	NA	NA	NA	NA	NA NA	NA	VFEM_VSMC-up
NA	NA	NA	NA	NA	NA	NA	NA NA	NA	VFEM_VSMC-up
NA	NA	NA	NA	NA	NA	NA	NA NA	NA	VFEM_VSMC-up
NA	NA	NA	NA	NA	NA	NA	NA NA	NA	VFEM_VSMC-up
NA	NA	NA	NA	NA	NA	NA	NA NA	NA	VFEM_VSMC-up
NA	NA	NA	NA	NA	NA	NA	NA NA	NA	VFEM_VSMC-up
NA	NA	NA	NA	NA	NA	NA	NA NA	NA	VFEM_VSMC-up
NA	NA	NA	NA	NA	NA	NA	NA NA	NA	VFEM_VSMC-up

NA	NA	NA	NA	NA	NA	NA	NA NA	NA	VFEM_VSMC-up
NA	NA	NA	NA	NA	NA	NA	NA NA	NA	VFEM_VSMC-up
NA	NA	NA	NA	NA	NA	NA	NA NA	NA	VFEM_VSMC-up
NA	NA	NA	NA	NA	NA	NA	NA NA	NA	VFEM_VSMC-up
NA	NA	NA	NA	NA	NA	NA	NA NA	NA	VFEM_VSMC-up
NA	NA	NA	NA	NA	NA	NA	NA NA	NA	VFEM_VSMC-up
cfa04910	Insulin signaling pathway	44013	134/7854	0.11352875	0.11352875	0.05431997	476299 FBP1	1	VFEM_VSMC-up
cfa05221	Acute myeloid leukemia	43831	67/7854	0.00853069	0.01960784	NA	480522 JUP	1	VFEM_VSMC-down
cfa05412	Arrhythmogenic right ventricular cardiomyopathy (ARVC)	43831	77/7854	0.00980392	0.01960784	NA	480522 JUP	1	VFEM_VSMC-down
cfa05226	Gastric cancer	43831	149/7854	0.01897122	0.02189967	NA	480522 JUP	1	VFEM_VSMC-down
NA	NA	NA	NA	NA	NA	NA	NA NA	NA	VFEM_VSMC-down
NA	NA	NA	NA	NA	NA	NA	NA NA	NA	VFEM_VSMC-down
NA	NA	NA	NA	NA	NA	NA	NA NA	NA	VFEM_VSMC-down
NA	NA	NA	NA	NA	NA	NA	NA NA	NA	VFEM_VSMC-down
NA	NA	NA	NA	NA	NA	NA	NA NA	NA	VFEM_VSMC-down
NA	NA	NA	NA	NA	NA	NA	NA NA	NA	VFEM_VSMC-down
NA	NA	NA	NA	NA	NA	NA	NA NA	NA	VFEM_VSMC-down
NA	NA	NA	NA	NA	NA	NA	NA NA	NA	VFEM_VSMC-down
NA	NA	NA	NA	NA	NA	NA	NA NA	NA	VFEM_VSMC-down
NA	NA	NA	NA	NA	NA	NA	NA NA	NA	VFEM_VSMC-down
NA	NA	NA	NA	NA	NA	NA	NA NA	NA	VFEM_VSMC-down
NA	NA	NA	NA	NA	NA	NA	NA NA	NA	VFEM_VSMC-down
NA	NA	NA	NA	NA	NA	NA	NA NA	NA	VFEM_VSMC-down
NA	NA	NA	NA	NA	NA	NA	NA NA	NA	VFEM_VSMC-down
NA	NA	NA	NA	NA	NA	NA	NA NA	NA	VFEM_VSMC-down
NA	NA	NA	NA	NA	NA	NA	NA NA	NA	VFEM_VSMC-down
NA	NA	NA	NA	NA	NA	NA	NA NA	NA	VFEM_VSMC-down
NA	NA	NA	NA	NA	NA	NA	NA NA	NA	VFEM_VSMC-down
NA	NA	NA	NA	NA	NA	NA	NA NA	NA	VFEM_VSMC-down
NA	NA	NA	NA	NA	NA	NA	NA NA	NA	VFEM_VSMC-down
NA	NA	NA	NA	NA	NA	NA	NA NA	NA	VFEM_VSMC-down
NA	NA	NA	NA	NA	NA	NA	NA NA	NA	VFEM_VSMC-down
NA	NA	NA	NA	NA	NA	NA	NA NA	NA	VFEM_VSMC-down
NA	NA	NA	NA	NA	NA	NA	NA NA	NA	VFEM_VSMC-down
NA	NA	NA	NA	NA	NA	NA	NA NA	NA	VFEM_VSMC-down
NA	NA	NA	NA	NA	NA	NA	NA NA	NA	VFEM_VSMC-down
NA	NA	NA	NA	NA	NA	NA	NA NA	NA	VFEM_VSMC-down
NA	NA	NA	NA	NA	NA	NA	NA NA	NA	VFEM_VSMC-down
NA	NA	NA	NA	NA	NA	NA	NA NA	NA	VFEM_VSMC-down

NA	NA	NA	NA	NA	NA	NA	NA	NA	NA	VFEM_VSMC-down
NA	NA	NA	NA	NA	NA	NA	NA	NA	NA	VFEM_VSMC-down
NA	NA	NA	NA	NA	NA	NA	NA	NA	NA	VFEM_VSMC-down
NA	NA	NA	NA	NA	NA	NA	NA	NA	NA	VFEM_VSMC-down
NA	NA	NA	NA	NA	NA	NA	NA	NA	NA	VFEM_VSMC-down
NA	NA	NA	NA	NA	NA	NA	NA	NA	NA	VFEM_VSMC-down
NA	NA	NA	NA	NA	NA	NA	NA	NA	NA	VFEM_VSMC-down
NA	NA	NA	NA	NA	NA	NA	NA	NA	NA	VFEM_VSMC-down
NA	NA	NA	NA	NA	NA	NA	NA	NA	NA	VFEM_VSMC-down
NA	NA	NA	NA	NA	NA	NA	NA	NA	NA	VFEM_VSMC-down
NA	NA	NA	NA	NA	NA	NA	NA	NA	NA	VFEM_VSMC-down
NA	NA	NA	NA	NA	NA	NA	NA	NA	NA	VFEM_VSMC-down
NA	NA	NA	NA	NA	NA	NA	NA	NA	NA	VFEM_VSMC-down
NA	NA	NA	NA	NA	NA	NA	NA	NA	NA	VFEM_VSMC-down
NA	NA	NA	NA	NA	NA	NA	NA	NA	NA	VFEM_VSMC-down
NA	NA	NA	NA	NA	NA	NA	NA	NA	NA	VFEM_VSMC-down
NA	NA	NA	NA	NA	NA	NA	NA	NA	NA	VFEM_VSMC-down
NA	NA	NA	NA	NA	NA	NA	NA	NA	NA	VFEM_VSMC-down
NA	NA	NA	NA	NA	NA	NA	NA	NA	NA	VFEM_VSMC-down
NA	NA	NA	NA	NA	NA	NA	NA	NA	NA	VFEM_VSMC-down
NA	NA	NA	NA	NA	NA	NA	NA	NA	NA	VFEM_VSMC-down
NA	NA	NA	NA	NA	NA	NA	NA	NA	NA	VFEM_VSMC-down
NA	NA	NA	NA	NA	NA	NA	NA	NA	NA	VFEM_VSMC-down
NA	NA	NA	NA	NA	NA	NA	NA	NA	NA	VFEM_VSMC-down
NA	NA	NA	NA	NA	NA	NA	NA	NA	NA	VFEM_VSMC-down
NA	NA	NA	NA	NA	NA	NA	NA	NA	NA	VFEM_VSMC-down
NA	NA	NA	NA	NA	NA	NA	NA	NA	NA	VFEM_VSMC-down
NA	NA	NA	NA	NA	NA	NA	NA	NA	NA	VFEM_VSMC-down
NA	NA	NA	NA	NA	NA	NA	NA	NA	NA	VFEM_VSMC-down
NA	NA	NA	NA	NA	NA	NA	NA	NA	NA	VFEM_VSMC-down
NA	NA	NA	NA	NA	NA	NA	NA	NA	NA	VFEM_VSMC-down
NA	NA	NA	NA	NA	NA	NA	NA	NA	NA	VFEM_VSMC-down
NA	NA	NA	NA	NA	NA	NA	NA	NA	NA	VFEM_VSMC-down
NA	NA	NA	NA	NA	NA	NA	NA	NA	NA	VFEM_VSMC-down
NA	NA	NA	NA	NA	NA	NA	NA	NA	NA	VFEM_VSMC-down
NA	NA	NA	NA	NA	NA	NA	NA	NA	NA	VFEM_VSMC-down
NA	NA	NA	NA	NA	NA	NA	NA	NA	NA	VFEM_VSMC-down

NA	NA	NA	NA	NA	NA	NA	NA	NA	NA	VFEM_VSMC-down
NA	NA	NA	NA	NA	NA	NA	NA	NA	NA	VFEM_VSMC-down
NA	NA	NA	NA	NA	NA	NA	NA	NA	NA	VFEM_VSMC-down
NA	NA	NA	NA	NA	NA	NA	NA	NA	NA	VFEM_VSMC-down
NA	NA	NA	NA	NA	NA	NA	NA	NA	NA	VFEM_VSMC-down
NA	NA	NA	NA	NA	NA	NA	NA	NA	NA	VFEM_VSMC-down
NA	NA	NA	NA	NA	NA	NA	NA	NA	NA	VFEM_VSMC-down
NA	NA	NA	NA	NA	NA	NA	NA	NA	NA	VFEM_VSMC-down
NA	NA	NA	NA	NA	NA	NA	NA	NA	NA	VFEM_VSMC-down
NA	NA	NA	NA	NA	NA	NA	NA	NA	NA	VFEM_VSMC-down
NA	NA	NA	NA	NA	NA	NA	NA	NA	NA	VFEM_VSMC-down
NA	NA	NA	NA	NA	NA	NA	NA	NA	NA	VFEM_VSMC-down
NA	NA	NA	NA	NA	NA	NA	NA	NA	NA	VFEM_VSMC-down
NA	NA	NA	NA	NA	NA	NA	NA	NA	NA	VFEM_VSMC-down
NA	NA	NA	NA	NA	NA	NA	NA	NA	NA	VFEM_VSMC-down
NA	NA	NA	NA	NA	NA	NA	NA	NA	NA	VFEM_VSMC-down
NA	NA	NA	NA	NA	NA	NA	NA	NA	NA	VFEM_VSMC-down
NA	NA	NA	NA	NA	NA	NA	NA	NA	NA	VFEM_VSMC-down
NA	NA	NA	NA	NA	NA	NA	NA	NA	NA	VFEM_VSMC-down
NA	NA	NA	NA	NA	NA	NA	NA	NA	NA	VFEM_VSMC-down
NA	NA	NA	NA	NA	NA	NA	NA	NA	NA	VFEM_VSMC-down
NA	NA	NA	NA	NA	NA	NA	NA	NA	NA	VFEM_VSMC-down
NA	NA	NA	NA	NA	NA	NA	NA	NA	NA	VFEM_VSMC-down
NA	NA	NA	NA	NA	NA	NA	NA	NA	NA	VFEM_VSMC-down
NA	NA	NA	NA	NA	NA	NA	NA	NA	NA	VFEM_VSMC-down
NA	NA	NA	NA	NA	NA	NA	NA	NA	NA	VFEM_VSMC-down
NA	NA	NA	NA	NA	NA	NA	NA	NA	NA	VFEM_VSMC-down
NA	NA	NA	NA	NA	NA	NA	NA	NA	NA	VFEM_VSMC-down
NA	NA	NA	NA	NA	NA	NA	NA	NA	NA	VFEM_VSMC-down
NA	NA	NA	NA	NA	NA	NA	NA	NA	NA	VFEM_VSMC-down
NA	NA	NA	NA	NA	NA	NA	NA	NA	NA	VFEM_VSMC-down
NA	NA	NA	NA	NA	NA	NA	NA	NA	NA	VFEM_VSMC-down
NA	NA	NA	NA	NA	NA	NA	NA	NA	NA	VFEM_VSMC-down
NA	NA	NA	NA	NA	NA	NA	NA	NA	NA	VFEM_VSMC-down
NA	NA	NA	NA	NA	NA	NA	NA	NA	NA	VFEM_VSMC-down
NA	NA	NA	NA	NA	NA	NA	NA	NA	NA	VFEM_VSMC-down
NA	NA	NA	NA	NA	NA	NA	NA	NA	NA	VFEM_VSMC-down

NA	NA	NA	NA	NA	NA	NA	NA	NA	NA	VFEM_VSMC-down
NA	NA	NA	NA	NA	NA	NA	NA	NA	NA	VFEM_VSMC-down
NA	NA	NA	NA	NA	NA	NA	NA	NA	NA	VFEM_VSMC-down
NA	NA	NA	NA	NA	NA	NA	NA	NA	NA	VFEM_VSMC-down
NA	NA	NA	NA	NA	NA	NA	NA	NA	NA	VFEM_VSMC-down
NA	NA	NA	NA	NA	NA	NA	NA	NA	NA	VFEM_VSMC-down
NA	NA	NA	NA	NA	NA	NA	NA	NA	NA	VFEM_VSMC-down
NA	NA	NA	NA	NA	NA	NA	NA	NA	NA	VFEM_VSMC-down
NA	NA	NA	NA	NA	NA	NA	NA	NA	NA	VFEM_VSMC-down
NA	NA	NA	NA	NA	NA	NA	NA	NA	NA	VFEM_VSMC-down
NA	NA	NA	NA	NA	NA	NA	NA	NA	NA	VFEM_VSMC-down
NA	NA	NA	NA	NA	NA	NA	NA	NA	NA	VFEM_VSMC-down
NA	NA	NA	NA	NA	NA	NA	NA	NA	NA	VFEM_VSMC-down
NA	NA	NA	NA	NA	NA	NA	NA	NA	NA	VFEM_VSMC-down
NA	NA	NA	NA	NA	NA	NA	NA	NA	NA	VFEM_VSMC-down
NA	NA	NA	NA	NA	NA	NA	NA	NA	NA	VFEM_VSMC-down
NA	NA	NA	NA	NA	NA	NA	NA	NA	NA	VFEM_VSMC-down
NA	NA	NA	NA	NA	NA	NA	NA	NA	NA	VFEM_VSMC-down
NA	NA	NA	NA	NA	NA	NA	NA	NA	NA	VFEM_VSMC-down
NA	NA	NA	NA	NA	NA	NA	NA	NA	NA	VFEM_VSMC-down
NA	NA	NA	NA	NA	NA	NA	NA	NA	NA	VFEM_VSMC-down
NA	NA	NA	NA	NA	NA	NA	NA	NA	NA	VFEM_VSMC-down
NA	NA	NA	NA	NA	NA	NA	NA	NA	NA	VFEM_VSMC-down
NA	NA	NA	NA	NA	NA	NA	NA	NA	NA	VFEM_VSMC-down
NA	NA	NA	NA	NA	NA	NA	NA	NA	NA	VFEM_VSMC-down
NA	NA	NA	NA	NA	NA	NA	NA	NA	NA	VFEM_VSMC-down
NA	NA	NA	NA	NA	NA	NA	NA	NA	NA	VFEM_VSMC-down
NA	NA	NA	NA	NA	NA	NA	NA	NA	NA	VFEM_VSMC-down
NA	NA	NA	NA	NA	NA	NA	NA	NA	NA	VFEM_VSMC-down
NA	NA	NA	NA	NA	NA	NA	NA	NA	NA	VFEM_VSMC-down
NA	NA	NA	NA	NA	NA	NA	NA	NA	NA	VFEM_VSMC-down
NA	NA	NA	NA	NA	NA	NA	NA	NA	NA	VFEM_VSMC-down
NA	NA	NA	NA	NA	NA	NA	NA	NA	NA	VFEM_VSMC-down
NA	NA	NA	NA	NA	NA	NA	NA	NA	NA	VFEM_VSMC-down
NA	NA	NA	NA	NA	NA	NA	NA	NA	NA	VFEM_VSMC-down
NA	NA	NA	NA	NA	NA	NA	NA	NA	NA	VFEM_VSMC-down
NA	NA	NA	NA	NA	NA	NA	NA	NA	NA	VFEM_VSMC-down

NA	NA	NA	NA	NA	NA	NA	NA	NA	NA	VFEM_VSMC-down
NA	NA	NA	NA	NA	NA	NA	NA	NA	NA	VFEM_VSMC-down
NA	NA	NA	NA	NA	NA	NA	NA	NA	NA	VFEM_VSMC-down
NA	NA	NA	NA	NA	NA	NA	NA	NA	NA	VFEM_VSMC-down
NA	NA	NA	NA	NA	NA	NA	NA	NA	NA	VFEM_VSMC-down
NA	NA	NA	NA	NA	NA	NA	NA	NA	NA	VFEM_VSMC-down
NA	NA	NA	NA	NA	NA	NA	NA	NA	NA	VFEM_VSMC-down
NA	NA	NA	NA	NA	NA	NA	NA	NA	NA	VFEM_VSMC-down
NA	NA	NA	NA	NA	NA	NA	NA	NA	NA	VFEM_VSMC-down
NA	NA	NA	NA	NA	NA	NA	NA	NA	NA	VFEM_VSMC-down
NA	NA	NA	NA	NA	NA	NA	NA	NA	NA	VFEM_VSMC-down
NA	NA	NA	NA	NA	NA	NA	NA	NA	NA	VFEM_VSMC-down
NA	NA	NA	NA	NA	NA	NA	NA	NA	NA	VFEM_VSMC-down
NA	NA	NA	NA	NA	NA	NA	NA	NA	NA	VFEM_VSMC-down
NA	NA	NA	NA	NA	NA	NA	NA	NA	NA	VFEM_VSMC-down
NA	NA	NA	NA	NA	NA	NA	NA	NA	NA	VFEM_VSMC-down
NA	NA	NA	NA	NA	NA	NA	NA	NA	NA	VFEM_VSMC-down
NA	NA	NA	NA	NA	NA	NA	NA	NA	NA	VFEM_VSMC-down
NA	NA	NA	NA	NA	NA	NA	NA	NA	NA	VFEM_VSMC-down
NA	NA	NA	NA	NA	NA	NA	NA	NA	NA	VFEM_VSMC-down
NA	NA	NA	NA	NA	NA	NA	NA	NA	NA	VFEM_VSMC-down
NA	NA	NA	NA	NA	NA	NA	NA	NA	NA	VFEM_VSMC-down
NA	NA	NA	NA	NA	NA	NA	NA	NA	NA	VFEM_VSMC-down
NA	NA	NA	NA	NA	NA	NA	NA	NA	NA	VFEM_VSMC-down
NA	NA	NA	NA	NA	NA	NA	NA	NA	NA	VFEM_VSMC-down
NA	NA	NA	NA	NA	NA	NA	NA	NA	NA	VFEM_VSMC-down
NA	NA	NA	NA	NA	NA	NA	NA	NA	NA	VFEM_VSMC-down
NA	NA	NA	NA	NA	NA	NA	NA	NA	NA	VFEM_VSMC-down
NA	NA	NA	NA	NA	NA	NA	NA	NA	NA	VFEM_VSMC-down
NA	NA	NA	NA	NA	NA	NA	NA	NA	NA	VFEM_VSMC-down
NA	NA	NA	NA	NA	NA	NA	NA	NA	NA	VFEM_VSMC-down
NA	NA	NA	NA	NA	NA	NA	NA	NA	NA	VFEM_VSMC-down
NA	NA	NA	NA	NA	NA	NA	NA	NA	NA	VFEM_VSMC-down
NA	NA	NA	NA	NA	NA	NA	NA	NA	NA	VFEM_VSMC-down
NA	NA	NA	NA	NA	NA	NA	NA	NA	NA	VFEM_VSMC-down
NA	NA	NA	NA	NA	NA	NA	NA	NA	NA	VFEM_VSMC-down
NA	NA	NA	NA	NA	NA	NA	NA	NA	NA	VFEM_VSMC-down

NA	NA	NA	NA	NA	NA	NA	NA	NA	NA	VFEM_VSMC-down
NA	NA	NA	NA	NA	NA	NA	NA	NA	NA	VFEM_VSMC-down
NA	NA	NA	NA	NA	NA	NA	NA	NA	NA	VFEM_VSMC-down
NA	NA	NA	NA	NA	NA	NA	NA	NA	NA	VFEM_VSMC-down
NA	NA	NA	NA	NA	NA	NA	NA	NA	NA	VFEM_VSMC-down
NA	NA	NA	NA	NA	NA	NA	NA	NA	NA	VFEM_VSMC-down
NA	NA	NA	NA	NA	NA	NA	NA	NA	NA	VFEM_VSMC-down
NA	NA	NA	NA	NA	NA	NA	NA	NA	NA	VFEM_VSMC-down
NA	NA	NA	NA	NA	NA	NA	NA	NA	NA	VFEM_VSMC-down
NA	NA	NA	NA	NA	NA	NA	NA	NA	NA	VFEM_VSMC-down
NA	NA	NA	NA	NA	NA	NA	NA	NA	NA	VFEM_VSMC-down
NA	NA	NA	NA	NA	NA	NA	NA	NA	NA	VFEM_VSMC-down
NA	NA	NA	NA	NA	NA	NA	NA	NA	NA	VFEM_VSMC-down
NA	NA	NA	NA	NA	NA	NA	NA	NA	NA	VFEM_VSMC-down
NA	NA	NA	NA	NA	NA	NA	NA	NA	NA	VFEM_VSMC-down
NA	NA	NA	NA	NA	NA	NA	NA	NA	NA	VFEM_VSMC-down
NA	NA	NA	NA	NA	NA	NA	NA	NA	NA	VFEM_VSMC-down
NA	NA	NA	NA	NA	NA	NA	NA	NA	NA	VFEM_VSMC-down
NA	NA	NA	NA	NA	NA	NA	NA	NA	NA	VFEM_VSMC-down
cfa05202	Transcriptional misregulation in cancer	43831	172/7854	0.02189967	0.02189967	NA	480522	JUP	1	VFEM_VSMC-down
cfa04061	Viral protein interaction with cytokine and cytokine receptor	43862	83/7854	0.02102538	0.06008704	0.01581238	612279	IL22RA1	1	VPUL_VSMC-up
cfa04630	JAK-STAT signaling pathway	43862	160/7854	0.0403311	0.06008704	0.01581238	612279	IL22RA1	1	VPUL_VSMC-up
cfa04360	Axon guidance	43862	179/7854	0.04506528	0.06008704	0.01581238	595148	SLIT2	1	VPUL_VSMC-up
NA	NA	NA	NA	NA	NA	NA	NA	NA	NA	VPUL_VSMC-up
NA	NA	NA	NA	NA	NA	NA	NA	NA	NA	VPUL_VSMC-up
NA	NA	NA	NA	NA	NA	NA	NA	NA	NA	VPUL_VSMC-up
NA	NA	NA	NA	NA	NA	NA	NA	NA	NA	VPUL_VSMC-up
NA	NA	NA	NA	NA	NA	NA	NA	NA	NA	VPUL_VSMC-up
NA	NA	NA	NA	NA	NA	NA	NA	NA	NA	VPUL_VSMC-up
NA	NA	NA	NA	NA	NA	NA	NA	NA	NA	VPUL_VSMC-up
NA	NA	NA	NA	NA	NA	NA	NA	NA	NA	VPUL_VSMC-up
NA	NA	NA	NA	NA	NA	NA	NA	NA	NA	VPUL_VSMC-up
NA	NA	NA	NA	NA	NA	NA	NA	NA	NA	VPUL_VSMC-up
NA	NA	NA	NA	NA	NA	NA	NA	NA	NA	VPUL_VSMC-up
NA	NA	NA	NA	NA	NA	NA	NA	NA	NA	VPUL_VSMC-up
NA	NA	NA	NA	NA	NA	NA	NA	NA	NA	VPUL_VSMC-up
NA	NA	NA	NA	NA	NA	NA	NA	NA	NA	VPUL_VSMC-up
NA	NA	NA	NA	NA	NA	NA	NA	NA	NA	VPUL_VSMC-up
NA	NA	NA	NA	NA	NA	NA	NA	NA	NA	VPUL_VSMC-up
NA	NA	NA	NA	NA	NA	NA	NA	NA	NA	VPUL_VSMC-up
NA	NA	NA	NA	NA	NA	NA	NA	NA	NA	VPUL_VSMC-up
NA	NA	NA	NA	NA	NA	NA	NA	NA	NA	VPUL_VSMC-up
NA	NA	NA	NA	NA	NA	NA	NA	NA	NA	VPUL_VSMC-up
NA	NA	NA	NA	NA	NA	NA	NA	NA	NA	VPUL_VSMC-up
NA	NA	NA	NA	NA	NA	NA	NA	NA	NA	VPUL_VSMC-up
NA	NA	NA	NA	NA	NA	NA	NA	NA	NA	VPUL_VSMC-up
NA	NA	NA	NA	NA	NA	NA	NA	NA	NA	VPUL_VSMC-up
NA	NA	NA	NA	NA	NA	NA	NA	NA	NA	VPUL_VSMC-up
NA	NA	NA	NA	NA	NA	NA	NA	NA	NA	VPUL_VSMC-up
NA	NA	NA	NA	NA	NA	NA	NA	NA	NA	VPUL_VSMC-up

NA	NA	NA	NA	NA	NA	NA	NA	NA	NA	VPUL_VSMC-up
NA	NA	NA	NA	NA	NA	NA	NA	NA	NA	VPUL_VSMC-up
NA	NA	NA	NA	NA	NA	NA	NA	NA	NA	VPUL_VSMC-up
NA	NA	NA	NA	NA	NA	NA	NA	NA	NA	VPUL_VSMC-up
NA	NA	NA	NA	NA	NA	NA	NA	NA	NA	VPUL_VSMC-up
NA	NA	NA	NA	NA	NA	NA	NA	NA	NA	VPUL_VSMC-up
NA	NA	NA	NA	NA	NA	NA	NA	NA	NA	VPUL_VSMC-up
NA	NA	NA	NA	NA	NA	NA	NA	NA	NA	VPUL_VSMC-up
NA	NA	NA	NA	NA	NA	NA	NA	NA	NA	VPUL_VSMC-up
NA	NA	NA	NA	NA	NA	NA	NA	NA	NA	VPUL_VSMC-up
NA	NA	NA	NA	NA	NA	NA	NA	NA	NA	VPUL_VSMC-up
NA	NA	NA	NA	NA	NA	NA	NA	NA	NA	VPUL_VSMC-up
NA	NA	NA	NA	NA	NA	NA	NA	NA	NA	VPUL_VSMC-up
NA	NA	NA	NA	NA	NA	NA	NA	NA	NA	VPUL_VSMC-up
NA	NA	NA	NA	NA	NA	NA	NA	NA	NA	VPUL_VSMC-up
NA	NA	NA	NA	NA	NA	NA	NA	NA	NA	VPUL_VSMC-up
NA	NA	NA	NA	NA	NA	NA	NA	NA	NA	VPUL_VSMC-up
NA	NA	NA	NA	NA	NA	NA	NA	NA	NA	VPUL_VSMC-up
NA	NA	NA	NA	NA	NA	NA	NA	NA	NA	VPUL_VSMC-up
NA	NA	NA	NA	NA	NA	NA	NA	NA	NA	VPUL_VSMC-up
NA	NA	NA	NA	NA	NA	NA	NA	NA	NA	VPUL_VSMC-up
NA	NA	NA	NA	NA	NA	NA	NA	NA	NA	VPUL_VSMC-up
NA	NA	NA	NA	NA	NA	NA	NA	NA	NA	VPUL_VSMC-up
NA	NA	NA	NA	NA	NA	NA	NA	NA	NA	VPUL_VSMC-up
NA	NA	NA	NA	NA	NA	NA	NA	NA	NA	VPUL_VSMC-up
NA	NA	NA	NA	NA	NA	NA	NA	NA	NA	VPUL_VSMC-up
NA	NA	NA	NA	NA	NA	NA	NA	NA	NA	VPUL_VSMC-up
NA	NA	NA	NA	NA	NA	NA	NA	NA	NA	VPUL_VSMC-up
NA	NA	NA	NA	NA	NA	NA	NA	NA	NA	VPUL_VSMC-up
NA	NA	NA	NA	NA	NA	NA	NA	NA	NA	VPUL_VSMC-up
NA	NA	NA	NA	NA	NA	NA	NA	NA	NA	VPUL_VSMC-up
NA	NA	NA	NA	NA	NA	NA	NA	NA	NA	VPUL_VSMC-up
NA	NA	NA	NA	NA	NA	NA	NA	NA	NA	VPUL_VSMC-up
NA	NA	NA	NA	NA	NA	NA	NA	NA	NA	VPUL_VSMC-up
NA	NA	NA	NA	NA	NA	NA	NA	NA	NA	VPUL_VSMC-up
NA	NA	NA	NA	NA	NA	NA	NA	NA	NA	VPUL_VSMC-up
NA	NA	NA	NA	NA	NA	NA	NA	NA	NA	VPUL_VSMC-up
NA	NA	NA	NA	NA	NA	NA	NA	NA	NA	VPUL_VSMC-up
NA	NA	NA	NA	NA	NA	NA	NA	NA	NA	VPUL_VSMC-up
NA	NA	NA	NA	NA	NA	NA	NA	NA	NA	VPUL_VSMC-up
NA	NA	NA	NA	NA	NA	NA	NA	NA	NA	VPUL_VSMC-up
NA	NA	NA	NA	NA	NA	NA	NA	NA	NA	VPUL_VSMC-up
NA	NA	NA	NA	NA	NA	NA	NA	NA	NA	VPUL_VSMC-up
NA	NA	NA	NA	NA	NA	NA	NA	NA	NA	VPUL_VSMC-up
NA	NA	NA	NA	NA	NA	NA	NA	NA	NA	VPUL_VSMC-up
NA	NA	NA	NA	NA	NA	NA	NA	NA	NA	VPUL_VSMC-up
NA	NA	NA	NA	NA	NA	NA	NA	NA	NA	VPUL_VSMC-up
NA	NA	NA	NA	NA	NA	NA	NA	NA	NA	VPUL_VSMC-up
NA	NA	NA	NA	NA	NA	NA	NA	NA	NA	VPUL_VSMC-up
NA	NA	NA	NA	NA	NA	NA	NA	NA	NA	VPUL_VSMC-up
NA	NA	NA	NA	NA	NA	NA	NA	NA	NA	VPUL_VSMC-up
NA	NA	NA	NA	NA	NA	NA	NA	NA	NA	VPUL_VSMC-up
NA	NA	NA	NA	NA	NA	NA	NA	NA	NA	VPUL_VSMC-up
NA	NA	NA	NA	NA	NA	NA	NA	NA	NA	VPUL_VSMC-up
NA	NA	NA	NA	NA	NA	NA	NA	NA	NA	VPUL_VSMC-up
NA	NA	NA	NA	NA	NA	NA	NA	NA	NA	VPUL_VSMC-up
NA	NA	NA	NA	NA	NA	NA	NA	NA	NA	VPUL_VSMC-up
NA	NA	NA	NA	NA	NA	NA	NA	NA	NA	VPUL_VSMC-up
NA	NA	NA	NA	NA	NA	NA	NA	NA	NA	VPUL_VSMC-up
NA	NA	NA	NA	NA	NA	NA	NA	NA	NA	VPUL_VSMC-up
NA	NA	NA	NA	NA	NA	NA	NA	NA	NA	VPUL_VSMC-up
NA	NA	NA	NA	NA	NA	NA	NA	NA	NA	VPUL_VSMC-up
NA	NA	NA	NA	NA	NA	NA	NA	NA	NA	VPUL_VSMC-up
NA	NA	NA	NA	NA	NA	NA	NA	NA	NA	VPUL_VSMC-up
NA	NA	NA	NA	NA	NA	NA	NA	NA	NA	VPUL_VSMC-up
NA	NA	NA	NA	NA	NA	NA	NA	NA	NA	VPUL_VSMC-up
NA	NA	NA	NA	NA	NA	NA	NA	NA	NA	VPUL_VSMC-up
NA	NA	NA	NA	NA	NA	NA	NA	NA	NA	VPUL_VSMC-up
NA	NA	NA	NA	NA	NA	NA	NA	NA	NA	VPUL_VSMC-up
NA	NA	NA	NA	NA	NA	NA	NA	NA	NA	VPUL_VSMC-up
NA	NA	NA	NA	NA	NA	NA	NA	NA	NA	VPUL_VSMC-up
NA	NA	NA	NA	NA	NA	NA	NA	NA	NA	VPUL_VSMC-up
NA	NA	NA	NA	NA	NA	NA	NA	NA	NA	VPUL_VSMC-up
NA	NA	NA	NA	NA	NA	NA	NA	NA	NA	VPUL_VSMC-up

NA	NA	NA	NA	NA	NA	NA	NA	NA	NA	VPUL_VSMC-up
NA	NA	NA	NA	NA	NA	NA	NA	NA	NA	VPUL_VSMC-up
NA	NA	NA	NA	NA	NA	NA	NA	NA	NA	VPUL_VSMC-up
NA	NA	NA	NA	NA	NA	NA	NA	NA	NA	VPUL_VSMC-up
NA	NA	NA	NA	NA	NA	NA	NA	NA	NA	VPUL_VSMC-up
NA	NA	NA	NA	NA	NA	NA	NA	NA	NA	VPUL_VSMC-up
NA	NA	NA	NA	NA	NA	NA	NA	NA	NA	VPUL_VSMC-up
NA	NA	NA	NA	NA	NA	NA	NA	NA	NA	VPUL_VSMC-up
NA	NA	NA	NA	NA	NA	NA	NA	NA	NA	VPUL_VSMC-up
NA	NA	NA	NA	NA	NA	NA	NA	NA	NA	VPUL_VSMC-up
NA	NA	NA	NA	NA	NA	NA	NA	NA	NA	VPUL_VSMC-up
NA	NA	NA	NA	NA	NA	NA	NA	NA	NA	VPUL_VSMC-up
NA	NA	NA	NA	NA	NA	NA	NA	NA	NA	VPUL_VSMC-up
NA	NA	NA	NA	NA	NA	NA	NA	NA	NA	VPUL_VSMC-up
NA	NA	NA	NA	NA	NA	NA	NA	NA	NA	VPUL_VSMC-up
NA	NA	NA	NA	NA	NA	NA	NA	NA	NA	VPUL_VSMC-up
NA	NA	NA	NA	NA	NA	NA	NA	NA	NA	VPUL_VSMC-up
NA	NA	NA	NA	NA	NA	NA	NA	NA	NA	VPUL_VSMC-up
NA	NA	NA	NA	NA	NA	NA	NA	NA	NA	VPUL_VSMC-up
NA	NA	NA	NA	NA	NA	NA	NA	NA	NA	VPUL_VSMC-up
NA	NA	NA	NA	NA	NA	NA	NA	NA	NA	VPUL_VSMC-up
NA	NA	NA	NA	NA	NA	NA	NA	NA	NA	VPUL_VSMC-up
NA	NA	NA	NA	NA	NA	NA	NA	NA	NA	VPUL_VSMC-up
NA	NA	NA	NA	NA	NA	NA	NA	NA	NA	VPUL_VSMC-up
NA	NA	NA	NA	NA	NA	NA	NA	NA	NA	VPUL_VSMC-up
NA	NA	NA	NA	NA	NA	NA	NA	NA	NA	VPUL_VSMC-up
NA	NA	NA	NA	NA	NA	NA	NA	NA	NA	VPUL_VSMC-up
NA	NA	NA	NA	NA	NA	NA	NA	NA	NA	VPUL_VSMC-up
NA	NA	NA	NA	NA	NA	NA	NA	NA	NA	VPUL_VSMC-up
NA	NA	NA	NA	NA	NA	NA	NA	NA	NA	VPUL_VSMC-up
NA	NA	NA	NA	NA	NA	NA	NA	NA	NA	VPUL_VSMC-up
NA	NA	NA	NA	NA	NA	NA	NA	NA	NA	VPUL_VSMC-up
NA	NA	NA	NA	NA	NA	NA	NA	NA	NA	VPUL_VSMC-up
NA	NA	NA	NA	NA	NA	NA	NA	NA	NA	VPUL_VSMC-up
NA	NA	NA	NA	NA	NA	NA	NA	NA	NA	VPUL_VSMC-up
NA	NA	NA	NA	NA	NA	NA	NA	NA	NA	VPUL_VSMC-up
NA	NA	NA	NA	NA	NA	NA	NA	NA	NA	VPUL_VSMC-up
NA	NA	NA	NA	NA	NA	NA	NA	NA	NA	VPUL_VSMC-up
NA	NA	NA	NA	NA	NA	NA	NA	NA	NA	VPUL_VSMC-up
NA	NA	NA	NA	NA	NA	NA	NA	NA	NA	VPUL_VSMC-up
NA	NA	NA	NA	NA	NA	NA	NA	NA	NA	VPUL_VSMC-up
NA	NA	NA	NA	NA	NA	NA	NA	NA	NA	VPUL_VSMC-up
NA	NA	NA	NA	NA	NA	NA	NA	NA	NA	VPUL_VSMC-up
NA	NA	NA	NA	NA	NA	NA	NA	NA	NA	VPUL_VSMC-up
NA	NA	NA	NA	NA	NA	NA	NA	NA	NA	VPUL_VSMC-up
NA	NA	NA	NA	NA	NA	NA	NA	NA	NA	VPUL_VSMC-up
NA	NA	NA	NA	NA	NA	NA	NA	NA	NA	VPUL_VSMC-up
NA	NA	NA	NA	NA	NA	NA	NA	NA	NA	VPUL_VSMC-up
NA	NA	NA	NA	NA	NA	NA	NA	NA	NA	VPUL_VSMC-up
NA	NA	NA	NA	NA	NA	NA	NA	NA	NA	VPUL_VSMC-up
NA	NA	NA	NA	NA	NA	NA	NA	NA	NA	VPUL_VSMC-up
NA	NA	NA	NA	NA	NA	NA	NA	NA	NA	VPUL_VSMC-up
NA	NA	NA	NA	NA	NA	NA	NA	NA	NA	VPUL_VSMC-up
NA	NA	NA	NA	NA	NA	NA	NA	NA	NA	VPUL_VSMC-up
NA	NA	NA	NA	NA	NA	NA	NA	NA	NA	VPUL_VSMC-up
NA	NA	NA	NA	NA	NA	NA	NA	NA	NA	VPUL_VSMC-up
NA	NA	NA	NA	NA	NA	NA	NA	NA	NA	VPUL_VSMC-up
NA	NA	NA	NA	NA	NA	NA	NA	NA	NA	VPUL_VSMC-up
NA	NA	NA	NA	NA	NA	NA	NA	NA	NA	VPUL_VSMC-up
NA	NA	NA	NA	NA	NA	NA	NA	NA	NA	VPUL_VSMC-up
NA	NA	NA	NA	NA	NA	NA	NA	NA	NA	VPUL_VSMC-up
NA	NA	NA	NA	NA	NA	NA	NA	NA	NA	VPUL_VSMC-up
NA	NA	NA	NA	NA	NA	NA	NA	NA	NA	VPUL_VSMC-up
NA	NA	NA	NA	NA	NA	NA	NA	NA	NA	VPUL_VSMC-up
NA	NA	NA	NA	NA	NA	NA	NA	NA	NA	VPUL_VSMC-up
NA	NA	NA	NA	NA	NA	NA	NA	NA	NA	VPUL_VSMC-up
NA	NA	NA	NA	NA	NA	NA	NA	NA	NA	VPUL_VSMC-up
NA	NA	NA	NA	NA	NA	NA	NA	NA	NA	VPUL_VSMC-up
NA	NA	NA	NA	NA	NA	NA	NA	NA	NA	VPUL_VSMC-up
NA	NA	NA	NA	NA	NA	NA	NA	NA	NA	VPUL_VSMC-up
NA	NA	NA	NA	NA	NA	NA	NA	NA	NA	VPUL_VSMC-up
NA	NA	NA	NA	NA	NA	NA	NA	NA	NA	VPUL_VSMC-up
NA	NA	NA	NA	NA	NA	NA	NA	NA	NA	VPUL_VSMC-up
NA	NA	NA	NA	NA	NA	NA	NA	NA	NA	VPUL_VSMC-up

NA	NA	NA	NA	NA	NA	NA	NA	NA	NA	VPUL_VSMC-up
NA	NA	NA	NA	NA	NA	NA	NA	NA	NA	VPUL_VSMC-up
NA	NA	NA	NA	NA	NA	NA	NA	NA	NA	VPUL_VSMC-up
NA	NA	NA	NA	NA	NA	NA	NA	NA	NA	VPUL_VSMC-up
NA	NA	NA	NA	NA	NA	NA	NA	NA	NA	VPUL_VSMC-up
NA	NA	NA	NA	NA	NA	NA	NA	NA	NA	VPUL_VSMC-up
NA	NA	NA	NA	NA	NA	NA	NA	NA	NA	VPUL_VSMC-up
NA	NA	NA	NA	NA	NA	NA	NA	NA	NA	VPUL_VSMC-up
NA	NA	NA	NA	NA	NA	NA	NA	NA	NA	VPUL_VSMC-up
NA	NA	NA	NA	NA	NA	NA	NA	NA	NA	VPUL_VSMC-up
NA	NA	NA	NA	NA	NA	NA	NA	NA	NA	VPUL_VSMC-up
NA	NA	NA	NA	NA	NA	NA	NA	NA	NA	VPUL_VSMC-up
NA	NA	NA	NA	NA	NA	NA	NA	NA	NA	VPUL_VSMC-up
NA	NA	NA	NA	NA	NA	NA	NA	NA	NA	VPUL_VSMC-up
NA	NA	NA	NA	NA	NA	NA	NA	NA	NA	VPUL_VSMC-up
NA	NA	NA	NA	NA	NA	NA	NA	NA	NA	VPUL_VSMC-up
NA	NA	NA	NA	NA	NA	NA	NA	NA	NA	VPUL_VSMC-up
NA	NA	NA	NA	NA	NA	NA	NA	NA	NA	VPUL_VSMC-up
NA	NA	NA	NA	NA	NA	NA	NA	NA	NA	VPUL_VSMC-up
NA	NA	NA	NA	NA	NA	NA	NA	NA	NA	VPUL_VSMC-up
cfa04060	Cytokine-cytokine receptor interaction	43862	270/7854	0.0675772	0.0675772	0.01778347	612279	IL22RA1	1	VPUL_VSMC-up
cfa05144	Malaria	43922	49/7854	0.02472755	0.10381312	0.07285131	448807	TLR2	1	AFEM_VSMC-up
cfa05134	Legionellosis	43922	50/7854	0.02522736	0.10381312	0.07285131	448807	TLR2	1	AFEM_VSMC-up
cfa05321	Inflammatory bowel disease (IBD)	43922	61/7854	0.03071271	0.10381312	0.07285131	448807	TLR2	1	AFEM_VSMC-up
cfa05140	Leishmaniasis	43922	70/7854	0.03518347	0.10381312	0.07285131	448807	TLR2	1	AFEM_VSMC-up
cfa05323	Rheumatoid arthritis	43922	83/7854	0.04161391	0.10381312	0.07285131	448807	TLR2	1	AFEM_VSMC-up
cfa05235	PD-L1 expression and PD-1 checkpoint pathway in cancer	43922	89/7854	0.04457094	0.10381312	0.07285131	448807	TLR2	1	AFEM_VSMC-up
cfa04620	Toll-like receptor signaling pathway	43922	101/7854	0.05046446	0.10381312	0.07285131	448807	TLR2	1	AFEM_VSMC-up
cfa05146	Amoebiasis	43922	102/7854	0.05095435	0.10381312	0.07285131	448807	TLR2	1	AFEM_VSMC-up
cfa05142	Chagas disease (American trypanosomiasis)	43922	103/7854	0.05144406	0.10381312	0.07285131	448807	TLR2	1	AFEM_VSMC-up
cfa05145	Toxoplasmosis	43922	109/7854	0.0543783	0.10381312	0.07285131	448807	TLR2	1	AFEM_VSMC-up
cfa05162	Measles	43922	136/7854	0.06749824	0.1105967	0.07761172	448807	TLR2	1	AFEM_VSMC-up
cfa04145	Phagosome	43922	138/7854	0.06846462	0.1105967	0.07761172	448807	TLR2	1	AFEM_VSMC-up
cfa05161	Hepatitis B	43922	164/7854	0.08095941	0.11461136	0.08042902	448807	TLR2	1	AFEM_VSMC-up
cfa05152	Tuberculosis	43922	171/7854	0.0843018	0.11461136	0.08042902	448807	TLR2	1	AFEM_VSMC-up
cfa05169	Epstein-Barr virus infection	43922	193/7854	0.09474715	0.11461136	0.08042902	448807	TLR2	1	AFEM_VSMC-up
cfa05170	Human immunodeficiency virus 1 infection	43922	200/7854	0.09805185	0.11461136	0.08042902	448807	TLR2	1	AFEM_VSMC-up
cfa05205	Proteoglycans in cancer	43922	202/7854	0.09899439	0.11461136	0.08042902	448807	TLR2	1	AFEM_VSMC-up
cfa05132	Salmonella infection	43922	212/7854	0.10369599	0.11461136	0.08042902	448807	TLR2	1	AFEM_VSMC-up
cfa04151	PI3K-Akt signaling pathway	43922	354/7854	0.16849321	0.17070879	0.11979564	448807	TLR2	1	AFEM_VSMC-up
cfa05168	Herpes simplex virus 1 infection	43922	359/7854	0.17070879	0.17070879	0.11979564	448807	TLR2	1	AFEM_VSMC-up
