# Supplementary material for: Locational memory of macrovessel vascular cells is transcriptionally imprinted
Source: Sci Rep. 2023 Aug 10;13:13028. doi: 10.1038/s41598-023-38880-6 (PMC10415317; doi:10.1038/s41598-023-38880-6)
Supplement: Supplementary file 17 — Supplementary Table 8. [file 41598_2023_38880_MOESM17_ESM.pdf]

Supplemental Table 8. Module membership values of the genes in the endothelial cell dataset. Module membership value (MM) and the corresponding p value (p.val) for all genes in the endothelial cell WGCNA dataset. Ensembl IDs (Gene) and their gene names (Gene_sym) are provided. Each module has a color and a numeric identifier (Module_num).

Gene	Gene_sym	Module	Module_num	darkgrey_MM	darkgrey_p.val	turquoise_MM	turquoise_p.val	cyan_MM	cyan_p.val	paleturquoise_	paleturquoise_p.val	darkgreen_MN	darkgreen_p.val	violet_MM	violet_p.val	darkolivegreer	darkolivegreen_p.val	darkmagenta_	darkmagenta_p.val	magenta_MM	magenta_p.val	grey_MM	grey_p.val
ENSCAFG0000000139	HNRNPK	darkgrey	EC_M8	0.94	4.2E-13	0.13	5.2E-01	-0.47	1.4E-02	-0.07	7.3E-01	-0.23	2.5E-01	0.23	2.6E-01	-0.41	3.7E-02	-0.18	3.7E-01	-0.44	2.5E-02	-0.29	1.5E-01
ENSCAFG0000000589	CKAP2	darkgrey	EC_M8	0.94	1.8E-12	0.24	2.3E-01	-0.48	1.2E-02	0.01	9.7E-01	-0.23	2.5E-01	0.06	7.8E-01	-0.26	2.0E-01	-0.15	4.7E-01	-0.23	2.5E-01	-0.24	2.4E-01
ENSCAFG0000001083	CENPE	darkgrey	EC_M8	0.93	3.6E-12	0.16	4.4E-01	-0.44	2.5E-02	-0.07	7.5E-01	-0.24	2.5E-01	0.16	4.5E-01	-0.18	3.9E-01	-0.10	6.4E-01	-0.19	3.4E-01	-0.24	2.5E-01
ENSCAFG0000000809	HELLS	darkgrey	EC_M8	0.93	5.5E-12	0.22	2.8E-01	-0.47	1.5E-02	-0.03	8.8E-01	-0.23	2.5E-01	0.20	3.2E-01	-0.33	1.0E-01	-0.13	5.2E-01	-0.30	1.4E-01	-0.24	2.3E-01
ENSCAFG0000001106	SGO2	darkgrey	EC_M8	0.93	8.4E-12	0.17	4.1E-01	-0.56	3.2E-03	-0.09	6.7E-01	-0.11	5.9E-01	0.19	3.6E-01	-0.20	3.3E-01	0.01	9.7E-01	-0.18	3.8E-01	-0.37	6.1E-02
ENSCAFG0000001853	SERBP1	darkgrey	EC_M8	0.92	1.6E-11	0.12	5.7E-01	-0.43	2.9E-02	-0.06	7.6E-01	-0.23	2.6E-01	0.13	5.3E-01	-0.37	6.4E-02	-0.09	6.5E-01	-0.33	9.8E-02	-0.26	1.9E-01
ENSCAFG0000001429	SMC4	darkgrey	EC_M8	0.92	1.9E-11	0.35	8.4E-02	-0.36	7.3E-02	-0.03	9.0E-01	-0.41	3.5E-02	0.15	4.6E-01	-0.17	4.0E-01	-0.17	4.0E-01	-0.13	5.2E-01	-0.05	8.0E-01
ENSCAFG0000000739	CENPJ	darkgrey	EC_M8	0.92	2.1E-11	0.08	6.9E-01	-0.47	1.4E-02	-0.15	4.7E-01	-0.18	3.7E-01	0.22	2.8E-01	-0.25	2.3E-01	-0.11	5.9E-01	-0.30	1.4E-01	-0.31	1.2E-01
ENSCAFG0000001006	NOLC1	darkgrey	EC_M8	0.92	2.7E-11	0.16	4.4E-01	-0.41	3.9E-02	-0.15	4.7E-01	-0.27	1.9E-01	0.23	2.6E-01	-0.27	1.8E-01	-0.16	4.5E-01	-0.37	6.4E-02	-0.23	2.6E-01
ENSCAFG0000001852	HNRNPM	darkgrey	EC_M8	0.92	2.9E-11	0.28	1.6E-01	-0.43	2.8E-02	-0.11	5.8E-01	-0.28	1.7E-01	0.16	4.3E-01	-0.13	5.4E-01	-0.11	5.8E-01	-0.31	1.3E-01	-0.21	3.1E-01
ENSCAFG0000000572	GINS4	darkgrey	EC_M8	0.92	3.5E-11	0.31	1.2E-01	-0.52	7.1E-03	-0.08	6.9E-01	-0.20	3.3E-01	0.18	3.8E-01	-0.28	1.6E-01	-0.16	4.4E-01	-0.30	1.4E-01	-0.30	1.4E-01
ENSCAFG0000001408	MIS18BP1	darkgrey	EC_M8	0.92	6.0E-11	0.10	6.3E-01	-0.30	1.4E-01	0.01	9.7E-01	-0.39	5.1E-02	0.13	5.3E-01	-0.23	2.5E-01	-0.17	4.2E-01	-0.19	3.5E-01	-0.10	6.3E-01
ENSCAFG0000000033	HNRNPH1	darkgrey	EC_M8	0.91	7.0E-11	0.40	4.5E-02	-0.49	1.1E-02	0.05	7.9E-01	-0.28	1.7E-01	0.13	5.3E-01	-0.17	4.1E-01	-0.06	7.8E-01	-0.22	2.9E-01	-0.21	3.0E-01
ENSCAFG0000000232	NOL8	darkgrey	EC_M8	0.91	7.5E-11	0.35	7.6E-02	-0.51	7.3E-03	-0.11	5.9E-01	-0.26	2.1E-01	0.04	8.4E-01	-0.41	3.8E-02	-0.07	7.5E-01	-0.37	6.2E-02	-0.26	2.0E-01
ENSCAFG0000001016	NUCKS1	darkgrey	EC_M8	0.91	9.0E-11	0.09	6.6E-01	-0.51	7.4E-03	-0.03	8.9E-01	-0.16	4.4E-01	0.19	3.4E-01	-0.08	6.9E-01	-0.01	9.8E-01	-0.13	5.4E-01	-0.31	1.3E-01
ENSCAFG0000000725	UBA2	darkgrey	EC_M8	0.91	9.8E-11	0.10	6.1E-01	-0.53	5.2E-03	-0.01	9.5E-01	-0.14	4.8E-01	0.23	2.5E-01	-0.18	3.7E-01	-0.17	4.0E-01	-0.06	7.6E-01	-0.34	8.5E-02
ENSCAFG0000000059	LMNB1	darkgrey	EC_M8	0.91	1.1E-10	0.31	1.3E-01	-0.56	3.1E-03	0.00	9.8E-01	-0.14	4.9E-01	0.15	4.6E-01	-0.22	2.7E-01	-0.15	4.6E-01	-0.26	2.0E-01	-0.35	8.3E-02
ENSCAFG0000001750	ENSCAFG0000001750	darkgrey	EC_M8	0.91	1.1E-10	0.27	1.8E-01	-0.53	5.3E-03	-0.05	8.1E-01	-0.21	3.1E-01	0.16	4.5E-01	-0.29	1.6E-01	-0.13	5.2E-01	-0.42	3.5E-02	-0.29	1.4E-01
ENSCAFG0000002907	PTGES3	darkgrey	EC_M8	0.91	1.1E-10	0.37	6.1E-02	-0.61	1.1E-03	0.08	6.8E-01	-0.17	4.1E-01	0.08	6.8E-01	-0.15	4.5E-01	-0.20	3.2E-01	-0.36	7.1E-02	-0.30	1.4E-01
ENSCAFG0000000681	MTREX	darkgrey	EC_M8	0.91	1.4E-10	0.05	8.1E-01	-0.37	6.6E-02	0.02	9.2E-01	-0.31	1.3E-01	0.17	4.1E-01	-0.16	4.3E-01	-0.31	1.3E-01	-0.42	3.2E-02	-0.19	3.5E-01
ENSCAFG0000001753	ILF2	darkgrey	EC_M8	0.91	1.4E-10	0.47	1.6E-02	-0.46	1.9E-02	-0.01	9.6E-01	-0.32	1.1E-01	0.08	7.0E-01	-0.29	1.5E-01	-0.24	2.4E-01	-0.25	2.1E-01	-0.15	4.7E-01
ENSCAFG0000000645	ENSCAFG0000000645	darkgrey	EC_M8	0.91	1.7E-10	0.32	1.1E-01	-0.69	1.1E-04	-0.09	6.7E-01	-0.06	7.7E-01	0.16	4.4E-01	-0.25	2.3E-01	-0.14	4.8E-01	-0.32	1.1E-01	-0.46	1.9E-02
ENSCAFG0000000659	MCM4	darkgrey	EC_M8	0.91	2.0E-10	0.14	5.0E-01	-0.28	1.7E-01	-0.02	9.2E-01	-0.40	4.3E-02	0.13	5.1E-01	-0.42	3.1E-02	-0.16	4.4E-01	-0.20	3.2E-01	-0.08	6.8E-01
ENSCAFG0000000129	HSPA9	darkgrey	EC_M8	0.90	2.2E-10	-0.03	8.9E-01	-0.58	1.7E-03	-0.07	7.3E-01	-0.01	9.5E-01	0.32	1.1E-01	-0.25	2.1E-01	-0.12	5.7E-01	-0.34	8.5E-02	-0.48	1.4E-02
ENSCAFG0000001270	BUB3	darkgrey	EC_M8	0.90	2.3E-10	0.28	1.6E-01	-0.52	6.9E-03	0.10	6.3E-01	-0.20	3.3E-01	0.21	3.1E-01	-0.21	3.0E-01	-0.25	2.1E-01	-0.03	8.9E-01	-0.25	2.1E-01
ENSCAFG0000000567	RIF1	darkgrey	EC_M8	0.90	2.4E-10	0.19	3.5E-01	-0.49	1.1E-02	0.00	9.9E-01	-0.22	2.8E-01	0.08	7.1E-01	-0.30	1.3E-01	-0.24	2.3E-01	-0.42	3.1E-02	-0.26	2.0E-01
ENSCAFG0000001411	KIF15	darkgrey	EC_M8	0.90	2.4E-10	0.16	4.2E-01	-0.31	1.2E-01	0.03	9.0E-01	-0.36	7.2E-02	0.13	5.3E-01	-0.36	6.8E-02	-0.15	4.7E-01	-0.21	3.1E-01	-0.09	6.5E-01
ENSCAFG0000001320	DHX9	darkgrey	EC_M8	0.90	3.2E-10	0.17	4.2E-01	-0.65	3.4E-04	-0.06	7.9E-01	0.02	9.1E-01	0.28	1.6E-01	-0.41	3.9E-02	-0.12	5.5E-01	-0.24	2.5E-01	-0.49	1.1E-02
ENSCAFG0000001839	NDC80	darkgrey	EC_M8	0.90	3.9E-10	0.13	5.2E-01	-0.47	1.5E-02	-0.02	9.2E-01	-0.16	4.3E-01	0.23	2.6E-01	-0.26	1.9E-01	-0.20	3.3E-01	-0.18	3.8E-01	-0.31	1.2E-01
ENSCAFG0000003272	ENSCAFG0000003272	darkgrey	EC_M8	0.90	4.1E-10	0.15	4.6E-01	-0.50	9.9E-03	0.17	4.0E-01	-0.19	3.4E-01	0.25	2.2E-01	-0.37	6.5E-02	-0.21	3.0E-01	-0.35	8.0E-02	-0.30	1.4E-01
ENSCAFG0000001090	CPSF2	darkgrey	EC_M8	0.90	4.2E-10	0.35	7.8E-02	-0.63	5.9E-04	-0.06	7.8E-01	-0.09	6.5E-01	0.08	6.9E-01	-0.18	3.7E-01	-0.07	7.3E-01	-0.18	3.7E-01	-0.36	7.1E-02
ENSCAFG0000000516	DARS	darkgrey	EC_M8	0.90	4.4E-10	0.09	6.7E-01	-0.51	8.2E-03	0.09	6.5E-01	-0.14	5.0E-01	0.20	3.2E-01	-0.38	5.4E-02	-0.14	4.8E-01	-0.33	9.7E-02	-0.34	8.5E-02
ENSCAFG0000001849	SUZ12	darkgrey	EC_M8	0.90	4.6E-10	0.32	1.2E-01	-0.42	3.1E-02	0.15	4.7E-01	-0.36	6.9E-02	0.18	3.8E-01	-0.12	5.7E-01	-0.29	1.5E-01	-0.25	2.2E-01	-0.11	5.8E-01
ENSCAFG0000003090	TRIM59	darkgrey	EC_M8	0.90	5.8E-10	0.27	1.8E-01	-0.30	1.4E-01	0.11	5.9E-01	-0.49	1.0E-02	0.09	6.6E-01	-0.30	1.3E-01	-0.14	5.1E-01	-0.34	9.1E-02	-0.01	9.5E-01
ENSCAFG0000000749	KIF11	darkgrey	EC_M8	0.90	6.1E-10	0.21	2.9E-01	-0.30	1.3E-01	0.01	9.5E-01	-0.39	4.9E-02	0.23	2.6E-01	-0.20	3.3E-01	-0.20	3.3E-01	-0.20	3.3E-01	-0.08	7.0E-01
ENSCAFG0000000159	HAUS6	darkgrey	EC_M8	0.89	7.1E-10	0.29	1.5E-01	-0.57	2.4E-03	-0.09	6.7E-01	-0.16	4.3E-01	0.09	6.4E-01	-0.15	4.6E-01	-0.09	6.6E-01	-0.26	1.9E-01	-0.33	9.6E-02
ENSCAFG0000001148	BCAT1	darkgrey	EC_M8	0.89	7.2E-10	0.30	1.4E-01	-0.49	1.1E-02	0.06	7.6E-01	-0.24	2.3E-01	0.05	8.2E-01	-0.34	9.2E-02	-0.19	3.6E-01	-0.19	3.6E-01	-0.25	2.1E-01
ENSCAFG0000003048	ENSCAFG0000003048	darkgrey	EC_M8	0.89	9.2E-10	0.25	2.2E-01	-0.37	6.5E-02	0.00	9.8E-01	-0.31	1.2E-01	0.15	4.5E-01	-0.32	1.2E-01	-0.34	8.5E-02	-0.26	1.9E-01	-0.14	5.1E-01
ENSCAFG0000000266	SMC2	darkgrey	EC_M8	0.89	9.3E-10	0.27	1.8E-01	-0.44	2.5E-02	-0.10	6.1E-01	-0.24	2.5E-01	0.11	6.0E-01	-0.29	1.5E-01	-0.13	5.3E-01	-0.18	3.8E-01	-0.21	3.1E-01
ENSCAFG0000001583	HNRNPU	darkgrey	EC_M8	0.89	9.7E-10	0.29	1.5E-01	-0.21	3.1E-01	0.06	7.6E-01	-0.57	2.2E-03	0.13	5.3E-01	-0.25	2.2E-01	-0.13	5.2E-01	-0.34	8.5E-02	0.08	6.8E-01
ENSCAFG0000001848	ATAD5	darkgrey	EC_M8	0.89	1.1E-09	0.21	3.0E-01	-0.35	8.3E-02	0.08	6.9E-01	-0.35	8.4E-02	0.09	6.5E-01	-0.20	3.3E-01	-0.26	2.0E-01	-0.25	2.2E-01	-0.12	5.6E-01
ENSCAFG0000000219	MCM3	darkgrey	EC_M8	0.89	1.2E-09	0.19	3.6E-01	-0.47	1.6E-02	-0.13	5.4E-01	-0.17	4.2E-01	0.13	5.3E-01	-0.36	7.3E-02	-0.07	7.4E-01	-0.12	5.7E-01	-0.30	1.4E-01
ENSCAFG0000001759	CENPI	darkgrey	EC_M8	0.89	1.2E-09	0.26	2.0E-01	-0.39	4.7E-02	0.09	6.8E-01	-0.32	1.1E-01	0.24	2.4E-01	-0.18	3.8E-01	-0.12	5.4E-01	-0.15	4.6E-01	-0.15	4.7E-01
ENSCAFG0000001330	BAZ1A	darkgrey	EC_M8	0.89	1.9E-09	0.51	7.4E-03	-0.49	1.1E-02	-0.13	5.4E-01	-0.34	9.1E-02	0.09	6.6E-01	-0.19	3.6E-01	-0.22	2.8E-01	-0.25	2.1E-01	-0.13	5.3E-01
ENSCAFG0000001683	KIF4A	darkgrey	EC_M8	0.88	2.0E-09	0.21	2.9E-01	-0.40	4.1E-02	-0.10	6.2E-01	-0.25	2.1E-01	0.21	3.0E-01	-0.25	2.2E-01	-0.09	6.6E-01	-0.10	6.4E-01	-0.20	3.2E-01
ENSCAFG0000000953	NUSAP1	darkgrey	EC_M8	0.88	2.0E-09	0.15	4.6E-01	-0.37	6.1E-02	-0.03	8.9E-01	-0.27	1.8E-01	0.15	4.8E-01	-0.24	2.5E-01	-0.16	4.5E-01	-0.06	7.7E-01	-0.17	4.1E-01
ENSCAFG0000000134	MATR3	darkgrey	EC_M8	0.88	2.1E-09	0.17	4.1E-01	-0.33	9.7E-02	0.05	8.1E-01	-0.42	3.4E-02	0.19	3.6E-01	-0.24	2.3E-01	-0.06	7.8E-01	-0.22	2.8E-01	-0.07	7.4E-01
ENSCAFG0000001325	MKI67	darkgrey	EC_M8	0.88	2.2E-09	0.26	1.9E-01	-0.31	1.3E-01	0.00	9.9E-01	-0.38	5.2E-02	0.18	3.7E-01	-0.29	1.5E-01	-0.27	1.8E-01	-0.19	3.4E-01	-0.08	7.1E-01
ENSCAFG0000000251	TWISTNB	darkgrey	EC_M8	0.88	2.4E-09	0.17	4.2E-01	-0.59	1.6E-03	0.02	9.2E-01	-0.08	7.0E-01	0.17	3.9E-01	-0.16	4.3E-01	-0.23	2.6E-01	-0.27	1.8E-01	-0.39	4.6E-02
ENSCAFG0000000894	HNRNPDL	darkgrey	EC_M8	0.88	3.0E-09	0.18	3.7E-01	-0.29	1.5E-01	0.00	9.9E-01	-0.40	4.5E-02	0.21	3.0E-01	-0.25	2.1E-01	-0.08	7.0E-01	-0.13	5.3E-01	-0.07	7.5E-01
ENSCAFG0000000788	CEP55	darkgrey	EC_M8	0.88	3.1E-09	0.05	8.2E-01	-0.44	2.6E-02	-0.04	8.5E-01	-0.15	4.7E-01	0.28	1.6E-01	-0.33	9.7E-02	-0.14	4.8E-01	-0.18	3.9E-01	-0.32	1.2E-01
ENSCAFG0000000863	USP16	darkgrey	EC_M8	0.88	3.3E-09	0.01	9.6E-01	-0.27	1.8E-01	0.06	7.6E-01	-0.41	3.7E-02	0.15	4.7E-01	-0.34	8.7E-02	-0.23	2.6E-01	-0.29	1.5E-01	-0.05	8.2E-01
ENSCAFG0000000184	DBF4	darkgrey	EC_M8	0.88	3.4E-09	0.20	3.3E-01	-0.54	4.3E-03	0.12	5.7E-01	-0.16	4.4E-01	0.21	3.0E-01	-0.09	6.8E-01	0.04	8.6E-01	-0.20	3.3E-01	-0.30	1.4E-01
ENSCAFG0000000525	WDR43	darkgrey	EC_M8	0.88	3.4E-09	-0.08	7.1E-01	-0.52	6.4E-03	-0.11	5.8E-01	-0.03	8.9E-01	0.21	3.1E-01	-0.25	2.2E-01	-0.23	2.6E-01	-0.27	1.8E-01	-0.44	2.6E-02
ENSCAFG0000002896	SLC25A32	darkgrey	EC_M8	0.88	4.2E-09	0.15	4.6E-01	-0.46	1.9E-02	0.06	7.7E-01	-0.19	3.6E-01	0.15	4.6E-01	-0.29	1.5E-01	-0.32	1.1E-01	-0.24	2.4E-01	-0.23	2.6E-01
ENSCAFG0000000288	HNRNPA2B1	darkgrey	EC_M8	0.88	4.4E-09	0.29	1.5E-01	-0.29	1.5E-01	-0.07	7.3E-01	-0.46	1.9E-02	0.05	8.3E-01	-0.02	9.3E-01	-0.03	8.8E-01	-0.19	3.4E-01	-0.01	9.5E-01
ENSCAFG0000003008	ENSCAFG0000003008	darkgrey	EC_M8	0.88	4.6E-09	0.26	1.9E-01	-0.35	8.4E-02	-0.03	8.9E-01	-0.33	1.0E-01	0.22	2.7E-01	-0.19	3.6E-01	-0.21	3.0E-01	-0.18	3.8E-01	-0.13	5.4E-01
ENSCAFG0000001545	ECT2	darkgrey	EC_M8	0.88	5.0E-09	0.15	4.6E-01	-0.46	1.8E-02	-0.11	5.8E-01	-0.15	4.7E-01	0.20	3.2E-01	-0.27	1.8E-01	-0.05	8.1E-01	-0.13	5.3E-01	-0.29	1.5E-01
ENSCAFG0000001103	NCL	darkgrey	EC_M8	0.87	5.1E-09	-0.11	5.9E-01	-0.51	7.9E-03	0.00	9.9E-01	-0.04	8.6E-01	0.20	3.3E-01	-0.35	7.9E-02	0.04	8.4E-01	-0.24	2.5E-01	-0.44	2.4E-02
ENSCAFG0000001435	NMD3	darkgrey	EC_M8	0.87	5.2E-09	0.00	9.9E-01	-0.44	2.5E-02	0.02	9.0E-01	-0.20	3.4E-01	0.23	2.6E-01	-0.16	4.3E-01	-0.24	2.3E-01	-0.38	5.7E-02	-0.27	1.9E-01
ENSCAFG0000000808	TNPO1	darkgrey	EC_M8	0.87	5.3E-09	0.20	3.2E-01	-0.65	3.7E-04	0.02	9.3E-01	0.00	1.0E+00	0.10	6.1E-01	-0.35	7.6E-02	-0.18	3.7E-01	-0.24	2.4E-01	-0.48	1.3E-02
ENSCAFG0000000359	ENSCAFG0000000359	darkgrey	EC_M8	0.87	5.8E-09	0.30	1.4E-01	-0.34	8.6E-02	0.13	5.1E-01	-0.38	5.3E-02	0.19	3.4E-01	-0.19	3.5E-01	-0.36	6.8E-02	-0.14	5.1E-01	-0.07	7.2E-01
ENSCAFG0000002002	SASS6	darkgrey	EC_M8	0.87	5.8E-09	0.34	9.3E-02	-0.38	5.6E-02	-0.11	5.8E-01	-0.33	9.9E-02	0.12	5.4E-01	-0.22	2.8E-01	-0.17	4.0E-01	-0.11	5.9E-01	-0.12	5.6E-01
ENSCAFG0000001609	TOP2A	darkgrey	EC_M8	0.87	5.9E-09	0.21	3.0E-01	-0.24	2.4E-01	-0.06	7.6E-01	-0.42	3.1E-02	0.15	4.6E-01	-0.29	1.5E-01	-0.15	4.7E-01	-0.19	3.5E-01	-0.03	8.8E-01
ENSCAFG0000001457	CDC45	darkgrey	EC_M8	0.87	6.0E-09	0.28	1.6E-01	-0.45	2.1E-02	-0.15	4.7E-01	-0.22	2.7E-01	0.22	2.7E-01	-0.31	1.2E-01	-0.05	8.0E-01	-0.13	5.3E-01	-0.25	2.2E-01
ENSCAFG0000000920	KNL1	darkgrey	EC_M8	0.87	6.8E-09	0.20	3.3E-01	-0.26	2.0E-01	-0.03	8.8E-01	-0.40	4.3E-02	0.18	3.7E-01	-0.30	1.3E-01	-0.16	4.4E-01	-0.17	3.9E-01	-0.06	7.7E-01
ENSCAFG0000000706	BUB1	darkgrey	EC_M8	0.87	7.0E-09	0.20	3.3E-01	-0.27	1.8E-01	-0.08	6.9E-01	-0.39	4.7E-02	0.11	5.9E-01	-0.35	7.6E-02	-0.13	5.2E-01	-0.09	6.5E-01	-0.07	7.4E-01
ENSCAFG0000000256	TEX10	darkgrey	EC_M8	0.87	7.6E-09	0.12	5.6E-01	-0.38	5.3E-02	-0.07	7.2E-01	-0.25	2.1E-01	0.14	5.0E-01	-0.31	1.2E-01	-0.21	3.1E-01	-0.07	7.4E-01	-0.21	3.0E-01
ENSCAFG0000000371	NAA15	darkgrey	EC_M8	0.87	7.7E-09	0.10	6.2E-01	-0.76	6.6E-06	-0.19	3.6E-01	0.17	4.0E-01	0.23	2.6E-01	-0.27	1.8E-01	-0.14	4.8E-01	-0.18	3.7E-01	-0.63	5.3E-04
ENSCAFG0000000878	GMPS	darkgrey	EC_M8	0.87	7.8E-09	0.10	6.1E-01	-0.50	1.0E-02	-0.04	8.4E-01	-0.14	5.0E-01	0.13	5.3E-01	-0.17	4.0E-01	-0.16	4.2E-01	-0.36	6.7E-02	-0.34	9.2E-02
ENSCAFG0000001325	NUF2	darkgrey	EC_M8	0.87	7.9E-09	0.16	4.2E-01	-0.35	7.9E-02	-0.08	7.1E-01	-0.26	2.0E-01	0.16	4.3E-01	-0.31	1.2E-01	-0.13	5.4E-01	-0.11	6.0E-01	-0.17	4.1E-01
ENSCAFG0000000054	FBXO5	darkgrey	EC_M8	0.87	7.9E-09	0.35	7.5E-02	-0.35	7.8E-02	0.07	7.5E-01	-0.35	7.5E-02	0.18	3.8E-01	-0.28	1.6E-01	-0.25	2.2E-01	-0.24	2.4E-01	-0.08	6.8E-01
ENSCAFG0000001660	CCNB2	darkgrey	EC_M8	0.87	8.6E-09	0.17	4.2E-01	-0.27	1.8E-01	0.00	9.8E-01	-0.36	6.9E-02	0.23	2.7E-01	-0.27	1.8E-01	-0.22	2.7E-01	-0.21	3.0E-01	-0.08	6.9E-01
ENSCAFG0000000735	LSM14A	darkgrey	EC_M8	0.87	8.8E-09	0.38	5.2E-02	-0.58	2.1E-03	-0.09	6.7E-01	-0.18	3.8E-01	0.33	1.0E-01	-0.31	1.2E-01	-0.04	8.6E-01	-0.32	1.2E-01	-0.31	1.2E-01
ENSCAFG0000003016	COMMD2	darkgrey	EC_M8	0.87	1.0E-08	-0.01	9.7E-01	-0.42	3.2E-02	0.26	2.0E-01	-0.20	3.4E-01	0.36	7.4E-02	-0.20	3.2E-01	-0.37	6.0E-02	-0.31	1.2E-01	-0.26	2.0E-01
ENSCAFG0000000837	ESCO2	darkgrey	EC_M8	0.87	1.1E-08	0.26	1.9E-01	-0.32	1.1E-01	-0.05	8.2E-01	-0.34	9.4E-02	0.16	4.3E-01	-0.29	1.5E-01	-0.10	6.1E-01	-0.09	6.7E-01	-0.10	6.2E-01
ENSCAFG0000000829	NUP160	darkgrey	EC_M8	0.86	1.4E-08	0.08	7.1E-01	-0.28	1.7E-01	0.11	6.0E-01	-0.37	5.9E-02	0.00	1.0E+00	-0.20	3.3E-01	-0.10	6.4E-01	-0.36	6.8E-02	-0.10	6.4E-01
ENSCAFG0000001624	SMC1A	darkgrey	EC_M8	0.86	1.4E-08	0.22	2.9E-01	-0.21	2.9E-01	-0.02	9.2E-01	-0.46	1.7E-02	0.11	5.8E-01	-0.14	4.9E-01	-0.14	4.9E-01	-0.37	6.4E-02	0.01	9.5E-01
ENSCAFG0000001114	KIF14	darkgrey	EC_M8	0.86	1.5E-08	0.13	5.1E-01	-0.32	1.1E-01	-0.03	9.0E-01	-0.29	1.5E-01	0.28	1.7E-01	-0.30	1.4E-01	-0.19	3.5E-01	-0.26	2.0E-01	-0.16	4.4E-01
ENSCAFG0000001214	PRC1	darkgrey	EC_M8	0.86	1.5E-08	0.16	4.5E-01	-0.29	1.4E-01	-0.09	6.7E-01	-0.33	9.6E-02	0.19	3.6E-01	-0.22	2.8E-01	-0.21	3.1E-01	-0.15	4.7E-01	-0.11	6.0E-01
ENSCAFG0000000372	SMC6	darkgrey	EC_M8	0.86	1.5E-08	0.26	1.9E-01	-0.12	5.6E-01	0.11	5.9E-01	-0.66	2.3E-04	0.06	7.7E-01	-0.20	3.3E-01	-0.20	3.3E-01	-0.31	1.2E-01	0.20	3.4E-01
ENSCAFG0000000629	CUL4A	darkgrey	EC_M8	0.86	1.6E-08	0.45	2.0E-02	-0.55	3.8E-03	0.04	8.4E-01	-0.22	2.8E-01	0.09	6.5E-01	-0.36	7.5E-02	-0.17	4.1E-01	0.00	9.8E-01	-0.21	2.9E-01
ENSCAFG0000000782	BDP1	darkgrey	EC_M8	0.86	1.6E-08	0.44	2.4E-02	-0.65	3.7E-04	-0.03	8.7E-01	-0.12	5.5E-01	0.07	7.2E-01	-0.26	1.9E-01	-0.18	3.7E-01	-0.27	1.9E-01	-0.33	9.8E-02
ENSCAFG0000001140	ASPM	darkgrey	EC_M8	0.86	1.6E-08	0.18	3.8E-01	-0.27	1.8E-01	-0.01	9.4E-01	-0.36	7.2E-02	0.18	3.8E-01	-0.26	2.0E-01	-0.18	3.7E-01	-0.17	4.0E-01	-0.07	7.4E-01
ENSCAFG0000000024	BCLAF1	darkgrey	EC_M8	0.86	1.7E-08	0.05	8.0E-01	-0.66	2.4E-04	-0.11	5.8E-01	0.05	7.9E-01	0.32	1.1E-01	-0.30	1.3E-01	0.01	9.5E-01	-0.29	1.6E-01	-0.53	5.3E-03
ENSCAFG0000000701	TGS1	darkgrey	EC_M8	0.86	1.7E-08	0.05	8.1E-01	-0.49	1.0E-02	-0.01	9.6E-01	-0.17	4.0E-01	0.36	6.7E-02	-0.27	1.8E-01	0.07	7.3E-01	-0.25	2.1E-01	-0.31	1.2E-01
ENSCAFG0000000652	TMPO	darkgrey	EC_M8	0.86	1.7E-08	0.19	3.6E-01	-0.16	4.4E-01	0.04	8.6E-01	-0.54	4.0E-03	0.19	3.4E-01	-0.23	2.7E-01	-0.32	1.1E-01	-0.37	6.3E-02	0.09	6.6E-01
ENSCAFG0000000759	ABCE1	darkgrey	EC_M8	0.86	1.8E-08	0.16	4.4E-01	-0.70	6.3E-05	-0.13	5.1E-01	0.13	5.2E-01	0.20	3.3E-01	-0.27	1.8E-01	-0.03	8.8E-01	-0.31	1.2E-01	-0.58	2.0E-03
ENSCAFG0000001646	ENSCAFG0000001646	darkgrey	EC_M8	0.86	1.8E-08	0.26	2.0E-01	-0.26	2.0E-01	-0.04	8.4E-01	-0.43	3.0E-02	0.09	6.8E-01	-0.16	4.2E-01	-0.07	7.2E-01	-0.13	5.1E-01	-0.01	9.7E-01
ENSCAFG0000000340	RRM2	darkgrey	EC_M8	0.86	1.8E-08	0.21	3.0E-01	-0.31	1.2E-01	-0.01	9.8E-01	-0.32	1.1E-01	0.24	2.5E-01	-0.30	1.4E-01	-0.17	4.1E-01	-0.20	3.2E-01	-0.13	5.3E-01
ENSCAFG0000001753	KIF23	darkgrey	EC_M8	0.86	1.8E-08	0.26	1.9E-01	-0.40	4.2E-02	-0.07	7.4E-01	-0.23	2.5E-01	0.15	4.7E-01	-0.20	3.2E-01	-0.19	3.6E-01	-0.16	4.3E-01	-0.21	3.1E-01
ENSCAFG0000000403	STIL	darkgrey	EC_M8	0.86	2.1E-08	0.24	2.3E-01	-0.32	1.1E-01	-0.23	2.5E-01	-0.37	6.0E-02	-0.10	6.3E-01	-0.27	1.9E-01	-0.19	3.6E-01	-0.22	2.8E-01	-0.07	7.5E-01
ENSCAFG0000000932	WDR75	darkgrey	EC_M8	0.86	2.1E-08	0.15	4.8E-01	-0.76	5.9E-06	0.04	8.5E-01	0.12	5.4E-01	0.30	1.4E-01	-0.20	3.3E-01	-0.07	7.5E-01	-0.12	5.6E-01	-0.59	1.4E-03
ENSCAFG0000001259	ENSCAFG0000001259	darkgrey	EC_M8	0.86	2.3E-08	0.17	4.0E-01	-0.34	9.2E-02	-0.04	8.3E-01	-0.28	1.6E-01	0.17	4.1E-01	-0.33	1.0E-01	-0.07	7.5E-01	-0.13	5.2E-01	-0.16	4.3E-01
ENSCAFG0000000797	U2SURP	darkgrey	EC_M8	0.86	2.5E-08	0.36	7.2E-02	-0.56	3.2E-03	0.05	8.2E-01	-0.22	2.9E-01	0.18	3.7E-01	-0.23	2.6E-01	0.03	9.0E-01	-0.06	7.8E-01	-0.25	2.2E-01
ENSCAFG0000000293	DCK	darkgrey	EC_M8	0.86	2.5E-08	0.45	2.0E-02	-0.52	6.7E-03	-0.06	7.7E-01	-0.22	2.8E-01	0.09	6.6E-01	-0.22	2.8E-01	-0.07	7.2E-01	-0.04	8.5E-01	-0.22	2.9E-01
ENSCAFG0000000717	KIF20B	darkgrey	EC_M8	0.86	2.6E-08	-0.08	7.0E-01	-0.22	2.8E-01	-0.01	9.4E-01	-0.38	5.9E-02	0.14	5.1E-01	-0.17	4.2E-01	-0.08	6.9E-01	-0.29	1.5E-01	-0.07	7.3E-01
ENSCAFG0000001857	THOC2	darkgrey	EC_M8	0.86	2.6E-08	0.27	1.9E-01	-0.27	1.9E-01	-0.10	6.2E-01	-0.49	1.1E-02	0.13	5.4E-01	-0.30	1.4E-01	-0.03	8.8E-01	-0.26	1.9E-01	0.02	9.1E-01
ENSCAFG0000001077	KHDRBS1	darkgrey	EC_M8	0.86	2.6E-08	0.35	7.8E-02	-0.29	1.5E-01	-0.03	8.7E-01	-0.44	2.5E-02	0.13	5.3E-01	-0.22	2.9E-01	-0.25	2.2E-01	-0.29	1.6E-01	-0.02	9.2E-01
ENSCAFG0000000966	MDM4	darkgrey	EC_M8	0.85	2.7E-08	0.14	5.0E-01	-0.44	2.4E-02	0.15	4.7E-01	-0.27	1.8E-01	0.05	7.9E-01	-0.22	2.9E-01	-0.30	1.4E-01	-0.26	2.1E-01	-0.20	3.4E-01
ENSCAFG0000001498	WDHD1	darkgrey	EC_M8	0.85	2.7E-08	0.13	5.3E-01	-0.51	7.1E-03	0.12	5.7E-01	-0.09	6.7E-01	0.27	1.8E-01	-0.24	2.5E-01	-0.08	6.8E-01	-0.23	2.6E-01	-0.36	6.9E-02
ENSCAFG0000000120	KIF20A	darkgrey	EC_M8	0.85	2.7E-08	0.22	2.8E-01	-0.32	1.1E-01	-0.03	8.9E-01	-0.31	1.3E-01	0.16	4.2E-01	-0.31	1.3E-01	-0.20	3.2E-01	-0.14	5.1E-01	-0.13	5.3E-01
ENSCAFG0000001330	HNRNPR	darkgrey	EC_M8	0.85	2.8E-08	0.19	3.5E-01	-0.07	7.2E-01	0.14	5.1E-01	-0.68	1.3E-04	0.09	6.5E-01	-0.17	4.1E-01	-0.17	4.0E-01	-0.22	2.8E-01	0.21	3.0E-01
ENSCAFG0000000423	DNAJC2	darkgrey	EC_M8	0.85	2.8E-08	0.05	8.1E-01	-0.61	1.0E-03	0.26	2.0E-01	-0.03	8.9E-01	0.31	1.2E-01	-0.31	1.2E-01	-0.07	7.5E-01	-0.23	2.6E-01	-0.44	2.5E-02
ENSCAFG0000000104	DDX46	darkgrey	EC_M8	0.85	2.8E-08	0.25	2.1E-01	-0.59	1.6E-03	-0.09	6.5E-01	-0.04	8.6E-01	0.23	2.6E-01	-0.12	5.4E-01	-0.06	7.7E-01	-0.14	4.9E-01	-0.38	5.4E-02
ENSCAFG0000000012	TIMELESS	darkgrey	EC_M8	0.85	2.9E-08	0.56	3.0E-03	-0.43	2.7E-02	-0.05	8.0E-01	-0.35	7.7E-02	0.00	9.9E-01	-0.22	2.8E-01	-0.13	5.2E-01	-0.24	2.3E-01	-0.11	5.9E-01
ENSCAFG0000001398	CDCA5	darkgrey	EC_M8	0.85	3.2E-08	0.00	9.9E-01	-0.43	2.8E-02	-0.10	6.2E-01	-0.12	5.7E-01	0.14	5.0E-01	-0.20	3.3E-01	-0.06	7.5E-01	-0.10	6.3E-01	-0.32	1.1E-01
ENSCAFG0000001502	DLGAP5	darkgrey	EC_M8	0.85	3.2E-08	0.04	8.6E-01	-0.29	1.5E-01	0.04	8.3E-01	-0.30	1.3E-01	0.10	6.4E-01	-0.25	2.2E-01	-0.16	4.4E-01	-0.24	2.5E-01	-0.15	4.7E-01
ENSCAFG0000000696	UTP20	darkgrey	EC_M8	0.85	3.8E-08	-0.03	8.9E-01	-0.41	3.7E-02	0.02	9.3E-01	-0.18	3.7E-01	0.04	8.5E-01	-0.37	6.4E-02	-0.06	7.9E-01	-0.30	1.3E-01	-0.28	1.6E-01
ENSCAFG0000000071	NUDCD1	darkgrey	EC_M8	0.85	3.8E-08	0.14	4.8E-01	-0.52	6.1E-03	0.04	8.3E-01	-0.13	5.2E-01	0.13	5.4E-01	-0.12	5.7E-01	-0.14	5.1E-01	-0.48	1.2E-02	-0.33	9.7E-02
ENSCAFG0000000786	ATR	darkgrey	EC_M8	0.85	3.9E-08	-0.05	8.1E-01	-0.57	2.3E-03	-0.07	7.5E-01	-0.02	9.2E-01	0.22	2.9E-01	-0.30	1.4E-01	0.10	6.4E-01	-0.22	2.9E-01	-0.45	2.0E-02
ENSCAFG0000000284	TTK	darkgrey	EC_M8	0.85	4.0E-08	0.26	2.0E-01	-0.31	1.2E-01	-0.07	7.3E-01	-0.33	1.0E-01	0.10	6.3E-01	-0.22	2.8E-01	-0.11	5.8E-01	-0.14	5.0E-01	-0.10	6.3E-01

ENSCAFG0000001983	PSMA5	darkgrey	EC_M8	0.85	4.2E-08	0.28	1.6E-01	-0.41	3.6E-02	-0.04	8.6E-01	-0.24	2.5E-01	0.17	4.1E-01	-0.26	1.9E-01	-0.25	2.2E-01	-0.21	3.0E-01	-0.21	3.0E-01
ENSCAFG0000001205	EIF3A	darkgrey	EC_M8	0.85	4.3E-08	-0.06	7.7E-01	-0.67	1.9E-04	-0.06	7.9E-01	0.12	5.6E-01	0.19	3.5E-01	-0.42	3.1E-02	-0.01	9.6E-01	-0.32	1.1E-01	-0.59	1.6E-03
ENSCAFG0000001376	CCAR1	darkgrey	EC_M8	0.85	4.5E-08	0.31	1.2E-01	-0.45	2.2E-02	0.21	3.1E-01	-0.32	1.1E-01	0.18	3.7E-01	-0.39	5.0E-02	-0.18	3.7E-01	-0.37	6.5E-02	-0.15	4.6E-01
ENSCAFG0000000722	XPO4	darkgrey	EC_M8	0.85	4.7E-08	0.24	2.4E-01	-0.64	3.8E-04	-0.06	7.6E-01	-0.02	9.3E-01	0.11	6.0E-01	-0.38	5.6E-02	-0.16	4.4E-01	-0.20	3.2E-01	-0.41	3.7E-02
ENSCAFG0000001872	ENSCAFG0000001872	darkgrey	EC_M8	0.85	4.7E-08	0.29	1.6E-01	-0.31	1.3E-01	-0.06	7.7E-01	-0.37	6.6E-02	0.20	3.2E-01	-0.18	3.8E-01	-0.15	4.8E-01	-0.09	6.7E-01	-0.08	6.8E-01
ENSCAFG0000000580	SGO1	darkgrey	EC_M8	0.85	4.7E-08	0.09	6.7E-01	-0.29	1.5E-01	0.07	7.4E-01	-0.30	1.4E-01	0.25	2.2E-01	-0.28	1.7E-01	-0.07	7.2E-01	-0.17	4.1E-01	-0.14	5.0E-01
ENSCAFG0000000338	THRAP3	darkgrey	EC_M8	0.85	4.8E-08	0.25	2.2E-01	-0.54	4.1E-03	-0.03	8.7E-01	-0.13	5.2E-01	0.32	1.1E-01	-0.29	1.5E-01	-0.08	7.1E-01	-0.14	5.0E-01	-0.32	1.1E-01
ENSCAFG0000001667	NCAPG	darkgrey	EC_M8	0.85	4.9E-08	0.15	4.6E-01	-0.21	3.1E-01	-0.05	8.2E-01	-0.42	3.1E-02	0.16	4.5E-01	-0.30	1.4E-01	-0.12	5.6E-01	-0.17	4.1E-01	-0.01	9.5E-01
ENSCAFG0000000370	SHCBP1	darkgrey	EC_M8	0.85	5.6E-08	0.15	4.7E-01	-0.36	7.4E-02	0.06	7.8E-01	-0.23	2.5E-01	0.24	2.5E-01	-0.30	1.4E-01	-0.14	5.0E-01	-0.15	4.5E-01	-0.19	3.6E-01
ENSCAFG0000001244	NOP58	darkgrey	EC_M8	0.84	6.0E-08	-0.14	5.1E-01	-0.43	2.8E-02	0.00	9.9E-01	-0.09	6.7E-01	0.22	2.9E-01	-0.34	9.1E-02	-0.11	5.8E-01	-0.32	1.2E-01	-0.39	4.8E-02
ENSCAFG0000001379	DDX21	darkgrey	EC_M8	0.84	6.1E-08	-0.21	3.1E-01	-0.45	2.0E-02	0.07	7.4E-01	-0.06	7.8E-01	0.23	2.6E-01	-0.20	3.2E-01	-0.08	7.0E-01	-0.39	4.9E-02	-0.41	3.8E-02
ENSCAFG0000000930	TOMM7C	darkgrey	EC_M8	0.84	6.2E-08	-0.04	8.5E-01	-0.28	1.6E-01	0.17	4.2E-01	-0.38	5.9E-02	0.19	3.6E-01	-0.31	1.2E-01	-0.23	2.6E-01	-0.21	3.0E-01	-0.10	6.2E-01
ENSCAFG0000000099	ATAD2	darkgrey	EC_M8	0.84	6.9E-08	0.17	4.1E-01	-0.76	7.8E-06	-0.05	8.0E-01	0.16	4.4E-01	0.22	2.9E-01	-0.10	6.4E-01	-0.08	7.1E-01	-0.29	1.4E-01	-0.61	8.4E-04
ENSCAFG0000002042	DEPDC1	darkgrey	EC_M8	0.84	7.0E-08	0.19	3.4E-01	-0.39	5.1E-02	-0.12	5.5E-01	-0.22	2.7E-01	0.18	3.8E-01	-0.29	1.5E-01	-0.24	2.3E-01	-0.18	3.7E-01	-0.19	3.4E-01
ENSCAFG0000001500	TACC3	darkgrey	EC_M8	0.84	7.4E-08	0.17	4.1E-01	-0.36	7.2E-02	-0.08	7.0E-01	-0.24	2.5E-01	0.19	3.5E-01	-0.22	2.8E-01	-0.12	5.4E-01	-0.10	6.3E-01	-0.19	3.5E-01
ENSCAFG0000000226	FAM120A	darkgrey	EC_M8	0.84	8.6E-08	0.00	1.0E+00	-0.24	2.4E-01	0.15	4.8E-01	-0.38	5.3E-02	0.09	6.7E-01	-0.60	1.1E-03	-0.10	6.2E-01	-0.27	1.8E-01	-0.09	6.5E-01
ENSCAFG0000001290	CDK1	darkgrey	EC_M8	0.84	8.9E-08	0.18	3.8E-01	-0.23	2.6E-01	0.00	9.8E-01	-0.39	4.8E-02	0.11	5.9E-01	-0.21	3.1E-01	-0.24	2.3E-01	-0.27	1.9E-01	-0.02	9.4E-01
ENSCAFG0000000626	DNAJC13	darkgrey	EC_M8	0.84	9.2E-08	0.29	1.6E-01	-0.54	4.8E-03	-0.08	6.9E-01	-0.15	4.6E-01	0.07	7.5E-01	-0.40	4.5E-02	-0.19	3.5E-01	-0.39	4.8E-02	-0.28	1.7E-01
ENSCAFG0000000518	FANCD2	darkgrey	EC_M8	0.84	1.0E-07	0.26	1.9E-01	-0.50	1.0E-02	-0.12	5.4E-01	-0.11	5.9E-01	0.13	5.3E-01	-0.17	4.1E-01	-0.06	7.8E-01	-0.10	6.4E-01	-0.31	1.2E-01
ENSCAFG0000001488	HAUS3	darkgrey	EC_M8	0.84	1.0E-07	0.36	6.8E-02	-0.65	3.3E-04	0.14	5.1E-01	-0.09	6.6E-01	0.07	7.3E-01	-0.22	2.8E-01	-0.01	9.6E-01	-0.28	1.6E-01	-0.38	5.6E-02
ENSCAFG0000000887	BUB1B	darkgrey	EC_M8	0.84	1.1E-07	0.24	2.3E-01	-0.27	1.9E-01	0.00	9.9E-01	-0.38	5.6E-02	0.17	4.0E-01	-0.22	2.8E-01	-0.21	2.9E-01	-0.10	6.4E-01	-0.05	8.0E-01
ENSCAFG0000000136	SRPK1	darkgrey	EC_M8	0.83	1.2E-07	0.33	1.0E-01	-0.62	7.6E-04	-0.12	5.7E-01	-0.06	7.6E-01	0.21	3.1E-01	-0.43	2.9E-02	-0.23	2.6E-01	-0.26	2.1E-01	-0.41	3.7E-02
ENSCAFG0000000650	TCERG1	darkgrey	EC_M8	0.83	1.2E-07	0.50	1.0E-02	-0.71	5.1E-05	-0.05	8.2E-01	0.01	9.7E-01	0.20	3.4E-01	-0.16	4.3E-01	-0.03	8.7E-01	-0.12	5.7E-01	-0.44	2.3E-02
ENSCAFG0000002504	HNRNPD	darkgrey	EC_M8	0.83	1.3E-07	0.29	1.5E-01	-0.26	2.1E-01	-0.01	9.5E-01	-0.44	2.6E-02	0.04	8.4E-01	-0.08	6.9E-01	-0.01	9.5E-01	-0.14	4.8E-01	-0.01	9.8E-01
ENSCAFG0000001240	UMPS	darkgrey	EC_M8	0.83	1.3E-07	0.12	5.6E-01	-0.53	5.5E-03	0.03	9.0E-01	-0.08	6.9E-01	0.23	2.5E-01	-0.38	5.7E-02	-0.08	6.8E-01	-0.01	9.5E-01	-0.35	8.0E-02
ENSCAFG0000000943	E2F8	darkgrey	EC_M8	0.83	1.3E-07	0.29	1.5E-01	-0.35	7.8E-02	-0.08	6.8E-01	-0.27	1.8E-01	0.16	4.2E-01	-0.16	4.3E-01	-0.10	6.3E-01	-0.12	5.6E-01	-0.15	4.8E-01
ENSCAFG0000001792	REXO5	darkgrey	EC_M8	0.83	1.3E-07	0.14	5.0E-01	-0.46	1.9E-02	-0.13	5.2E-01	-0.10	6.3E-01	0.04	8.5E-01	-0.24	2.3E-01	-0.15	4.6E-01	-0.15	4.7E-01	-0.32	1.1E-01
ENSCAFG0000000896	CDCA2	darkgrey	EC_M8	0.83	1.4E-07	0.13	5.1E-01	-0.23	2.6E-01	-0.14	5.1E-01	-0.38	5.6E-02	0.12	5.6E-01	-0.19	3.5E-01	-0.05	8.0E-01	-0.19	3.6E-01	-0.05	8.2E-01
ENSCAFG0000003180	ZNF146	darkgrey	EC_M8	0.83	1.4E-07	0.42	3.3E-02	-0.34	9.0E-02	0.02	9.4E-01	-0.45	2.0E-02	0.14	4.8E-01	-0.15	4.5E-01	-0.10	6.3E-01	-0.17	4.1E-01	-0.03	8.9E-01
ENSCAFG0000001213	NEK2	darkgrey	EC_M8	0.83	1.5E-07	0.13	5.1E-01	-0.29	1.5E-01	-0.02	9.2E-01	-0.29	1.5E-01	0.21	2.9E-01	-0.31	1.3E-01	-0.35	7.9E-02	-0.18	3.7E-01	-0.13	5.3E-01
ENSCAFG0000002042	SRSF11	darkgrey	EC_M8	0.83	1.5E-07	0.22	2.8E-01	-0.25	2.3E-01	0.25	2.2E-01	-0.49	1.1E-02	0.19	3.5E-01	-0.31	1.2E-01	-0.07	7.5E-01	-0.22	2.8E-01	0.03	8.8E-01
ENSCAFG0000000823	ENSCAFG0000000823	darkgrey	EC_M8	0.83	1.5E-07	0.11	5.9E-01	-0.46	1.8E-02	-0.03	8.9E-01	-0.10	6.2E-01	0.17	3.9E-01	-0.17	4.0E-01	-0.10	6.4E-01	-0.11	5.9E-01	-0.32	1.1E-01
ENSCAFG0000001495	NSD2	darkgrey	EC_M8	0.83	1.7E-07	0.52	6.9E-03	-0.49	1.2E-02	-0.11	6.1E-01	-0.25	2.2E-01	0.10	6.3E-01	-0.02	9.4E-01	-0.06	7.7E-01	-0.22	2.9E-01	-0.15	4.5E-01
ENSCAFG0000001725	DENND4A	darkgrey	EC_M8	0.83	1.7E-07	0.44	2.5E-02	-0.64	4.7E-04	0.05	8.0E-01	-0.11	5.8E-01	0.00	9.8E-01	-0.32	1.1E-01	-0.02	9.4E-01	-0.05	7.9E-01	-0.34	9.0E-02
ENSCAFG0000001707	NONO	darkgrey	EC_M8	0.83	1.8E-07	0.19	3.4E-01	-0.28	1.7E-01	0.00	1.0E+00	-0.39	5.2E-02	0.11	5.9E-01	-0.02	9.0E-01	-0.07	7.3E-01	-0.39	4.9E-02	-0.08	6.8E-01
ENSCAFG0000000713	DEPDC1B	darkgrey	EC_M8	0.83	1.9E-07	0.26	2.0E-01	-0.35	8.1E-02	-0.21	2.9E-01	-0.29	1.5E-01	0.30	1.3E-01	-0.10	6.3E-01	-0.11	5.8E-01	-0.14	4.8E-01	-0.15	4.7E-01
ENSCAFG0000002888	SIKE1	darkgrey	EC_M8	0.82	2.2E-07	0.11	5.9E-01	-0.64	4.2E-04	-0.01	9.4E-01	0.01	9.5E-01	0.27	1.8E-01	-0.32	1.2E-01	0.01	9.5E-01	-0.15	4.7E-01	-0.45	2.3E-02
ENSCAFG0000001893	C4H5orf22	darkgrey	EC_M8	0.82	2.2E-07	0.32	1.2E-01	-0.72	3.7E-05	0.09	6.6E-01	0.05	7.9E-01	0.14	5.0E-01	-0.33	1.0E-01	-0.06	7.6E-01	-0.12	5.7E-01	-0.49	1.0E-02
ENSCAFG0000001189	HJURP	darkgrey	EC_M8	0.82	2.2E-07	0.29	1.5E-01	-0.26	2.0E-01	-0.09	6.5E-01	-0.38	5.6E-02	0.15	4.5E-01	-0.26	2.0E-01	-0.21	3.1E-01	-0.12	5.5E-01	-0.04	8.3E-01
ENSCAFG0000000783	KNTC1	darkgrey	EC_M8	0.82	2.3E-07	0.20	3.2E-01	-0.44	2.5E-02	-0.09	6.5E-01	-0.13	5.1E-01	0.28	1.6E-01	-0.17	4.0E-01	-0.19	3.6E-01	-0.17	4.0E-01	-0.29	1.6E-01
ENSCAFG0000000553	GTPBP4	darkgrey	EC_M8	0.82	2.3E-07	-0.08	6.9E-01	-0.09	6.6E-01	0.04	8.6E-01	-0.53	5.9E-03	0.10	6.3E-01	-0.12	5.6E-01	0.01	9.6E-01	-0.32	1.1E-01	0.07	7.4E-01
ENSCAFG0000001465	CDCA3	darkgrey	EC_M8	0.82	2.3E-07	0.30	1.4E-01	-0.36	6.7E-02	-0.15	4.8E-01	-0.27	1.9E-01	0.13	5.2E-01	-0.28	1.7E-01	-0.04	8.4E-01	-0.10	6.3E-01	-0.16	4.5E-01
ENSCAFG0000001661	IQGAP3	darkgrey	EC_M8	0.82	2.4E-07	0.22	2.7E-01	-0.23	2.6E-01	0.00	1.0E+00	-0.38	5.4E-02	0.12	5.6E-01	-0.25	2.1E-01	-0.22	2.8E-01	-0.28	1.7E-01	-0.05	8.1E-01
ENSCAFG0000001686	FUS	darkgrey	EC_M8	0.82	2.4E-07	0.15	4.7E-01	-0.33	9.4E-02	-0.07	7.2E-01	-0.24	2.4E-01	0.21	3.0E-01	-0.01	9.6E-01	-0.13	5.2E-01	-0.19	3.4E-01	-0.18	3.8E-01
ENSCAFG0000001025	CIP2A	darkgrey	EC_M8	0.82	2.5E-07	0.08	7.1E-01	-0.58	1.8E-03	-0.14	5.0E-01	0.04	8.3E-01	0.23	2.7E-01	-0.21	3.1E-01	-0.05	8.0E-01	-0.04	8.5E-01	-0.48	1.4E-02
ENSCAFG0000000374	ORC1	darkgrey	EC_M8	0.82	2.5E-07	0.33	9.7E-02	-0.50	9.4E-03	-0.19	3.5E-01	-0.11	6.0E-01	0.17	4.1E-01	-0.10	6.4E-01	-0.17	4.2E-01	-0.15	4.7E-01	-0.32	1.2E-01
ENSCAFG0000000659	HMGB1	darkgrey	EC_M8	0.82	2.6E-07	0.44	2.5E-02	-0.22	2.9E-01	0.03	8.7E-01	-0.56	2.7E-03	0.06	7.8E-01	-0.09	6.5E-01	-0.23	2.6E-01	-0.24	2.5E-01	0.15	4.6E-01
ENSCAFG0000000770	ENSCAFG0000000770	darkgrey	EC_M8	0.82	2.6E-07	0.19	3.6E-01	-0.35	7.6E-02	-0.12	5.7E-01	-0.23	2.5E-01	0.15	4.6E-01	-0.27	1.9E-01	-0.17	4.1E-01	-0.12	5.7E-01	-0.19	3.5E-01
ENSCAFG0000003077	PALB2	darkgrey	EC_M8	0.82	2.6E-07	0.04	8.3E-01	-0.19	3.6E-01	-0.27	1.8E-01	-0.41	3.5E-02	0.19	3.6E-01	-0.25	2.2E-01	0.08	7.0E-01	-0.16	4.5E-01	-0.02	9.0E-01
ENSCAFG0000001663	EIF4A1	darkgrey	EC_M8	0.82	2.7E-07	-0.10	6.2E-01	-0.36	6.7E-02	-0.03	8.7E-01	-0.16	4.4E-01	0.25	2.1E-01	-0.22	2.8E-01	-0.01	9.4E-01	-0.38	5.8E-02	-0.32	1.2E-01
ENSCAFG0000001460	BRCA1	darkgrey	EC_M8	0.82	2.7E-07	0.15	4.7E-01	-0.47	1.7E-02	-0.17	4.1E-01	-0.12	5.7E-01	0.04	8.6E-01	-0.18	3.8E-01	-0.13	5.2E-01	-0.05	8.1E-01	-0.30	1.3E-01
ENSCAFG0000002353	SRRT	darkgrey	EC_M8	0.82	2.7E-07	0.15	4.8E-01	-0.28	1.7E-01	-0.14	4.9E-01	-0.35	7.8E-02	0.25	2.2E-01	-0.32	1.1E-01	-0.10	6.3E-01	-0.16	4.2E-01	-0.10	6.3E-01
ENSCAFG0000000221	PPAT	darkgrey	EC_M8	0.82	2.7E-07	-0.09	6.7E-01	-0.46	1.7E-02	-0.03	8.9E-01	-0.04	8.3E-01	0.31	1.2E-01	-0.31	1.2E-01	-0.12	5.7E-01	0.01	9.7E-01	-0.37	6.5E-02
ENSCAFG0000000405	MCM2	darkgrey	EC_M8	0.82	2.7E-07	0.11	6.1E-01	-0.22	2.7E-01	-0.11	5.8E-01	-0.36	7.0E-02	0.05	8.2E-01	-0.24	2.3E-01	-0.07	7.3E-01	-0.13	5.3E-01	-0.08	6.8E-01
ENSCAFG0000000374	GEN1	darkgrey	EC_M8	0.82	2.8E-07	0.31	1.3E-01	-0.22	2.7E-01	-0.01	9.6E-01	-0.47	1.5E-02	-0.04	8.5E-01	-0.12	5.7E-01	-0.20	3.3E-01	-0.14	5.0E-01	0.04	8.5E-01
ENSCAFG0000000711	BMS1	darkgrey	EC_M8	0.82	2.9E-07	-0.14	4.9E-01	-0.58	2.0E-03	-0.09	6.5E-01	0.08	6.9E-01	0.38	5.8E-02	-0.27	1.9E-01	-0.05	7.9E-01	-0.29	1.5E-01	-0.53	4.9E-03
ENSCAFG0000000286	IBTK	darkgrey	EC_M8	0.82	3.0E-07	-0.03	8.7E-01	-0.51	8.0E-03	-0.14	4.9E-01	-0.07	7.5E-01	0.26	2.1E-01	-0.26	2.0E-01	0.00	1.0E+00	-0.35	7.6E-02	-0.40	4.1E-02
ENSCAFG0000001646	SEPSECS	darkgrey	EC_M8	0.82	3.0E-07	0.03	9.0E-01	-0.42	3.3E-02	-0.05	8.2E-01	-0.15	4.5E-01	0.28	1.7E-01	-0.46	1.9E-02	-0.02	9.2E-01	-0.41	4.0E-02	-0.30	1.4E-01
ENSCAFG0000002025	NOB1	darkgrey	EC_M8	0.82	3.1E-07	0.08	7.1E-01	-0.24	2.3E-01	0.00	9.8E-01	-0.36	6.9E-02	0.23	2.6E-01	-0.16	4.4E-01	-0.08	6.9E-01	-0.39	4.7E-02	-0.10	6.4E-01
ENSCAFG0000001340	PSMA6	darkgrey	EC_M8	0.82	3.2E-07	0.44	2.4E-02	-0.57	2.4E-03	0.11	5.9E-01	-0.13	5.1E-01	-0.07	7.4E-01	-0.25	2.2E-01	-0.22	2.9E-01	-0.17	4.1E-01	-0.26	1.9E-01
ENSCAFG0000001174	ANP32E	darkgrey	EC_M8	0.82	3.3E-07	0.57	2.6E-03	-0.70	6.0E-05	-0.09	6.7E-01	-0.06	7.6E-01	0.22	2.9E-01	-0.12	5.7E-01	-0.19	3.6E-01	-0.08	7.0E-01	-0.35	7.8E-02
ENSCAFG0000003184	NUP35	darkgrey	EC_M8	0.82	3.4E-07	0.33	1.0E-01	-0.34	8.7E-02	-0.26	2.0E-01	-0.32	1.1E-01	0.09	6.5E-01	-0.30	1.3E-01	-0.04	8.5E-01	-0.14	4.9E-01	-0.11	6.0E-01
ENSCAFG0000001575	EXO1	darkgrey	EC_M8	0.82	3.5E-07	0.02	9.3E-01	-0.46	1.9E-02	-0.07	7.2E-01	-0.07	7.5E-01	0.13	5.4E-01	-0.39	4.9E-02	-0.04	8.5E-01	-0.18	3.9E-01	-0.38	5.5E-02
ENSCAFG0000000922	RAD51	darkgrey	EC_M8	0.82	3.6E-07	0.21	3.0E-01	-0.29	1.5E-01	0.00	9.8E-01	-0.32	1.1E-01	0.14	5.0E-01	-0.41	3.9E-02	-0.06	7.7E-01	-0.21	2.9E-01	-0.11	5.9E-01
ENSCAFG0000000122	CDC25C	darkgrey	EC_M8	0.82	3.6E-07	0.15	4.7E-01	-0.48	1.3E-02	0.04	8.4E-01	-0.09	6.6E-01	0.16	4.5E-01	-0.28	1.7E-01	-0.27	1.8E-01	-0.19	3.5E-01	-0.36	7.4E-02
ENSCAFG0000000738	WDR36	darkgrey	EC_M8	0.82	3.9E-07	0.12	5.7E-01	-0.50	9.3E-03	-0.12	5.5E-01	-0.04	8.4E-01	0.08	7.0E-01	-0.43	3.0E-02	-0.20	3.3E-01	-0.06	7.7E-01	-0.36	7.2E-02
ENSCAFG0000000142	PPIL1	darkgrey	EC_M8	0.82	3.9E-07	0.00	9.9E-01	-0.25	2.2E-01	0.02	9.1E-01	-0.34	8.4E-02	0.13	5.4E-01	-0.49	1.1E-02	0.00	9.8E-01	-0.07	7.2E-01	-0.07	7.5E-01
ENSCAFG0000003207	EIF1AX	darkgrey	EC_M8	0.82	3.9E-07	-0.11	5.8E-01	-0.56	2.7E-03	0.00	9.9E-01	0.02	9.2E-01	0.27	1.7E-01	-0.23	2.7E-01	-0.14	5.0E-01	-0.24	2.4E-01	-0.44	2.6E-02
ENSCAFG0000000170	IFT74	darkgrey	EC_M8	0.81	4.0E-07	-0.03	8.8E-01	-0.35	8.2E-02	0.05	8.1E-01	-0.21	2.9E-01	0.01	9.7E-01	-0.45	2.1E-02	0.01	9.6E-01	-0.15	4.5E-01	-0.21	3.1E-01
ENSCAFG0000000492	PPM1G	darkgrey	EC_M8	0.81	4.0E-07	0.01	9.7E-01	-0.62	6.7E-04	-0.16	4.4E-01	0.11	6.0E-01	0.31	1.2E-01	-0.16	4.4E-01	-0.11	5.8E-01	-0.45	2.1E-02	-0.55	3.7E-03
ENSCAFG0000001478	CEP152	darkgrey	EC_M8	0.81	4.1E-07	0.12	5.4E-01	-0.27	1.9E-01	0.13	5.3E-01	-0.38	5.7E-02	-0.04	8.3E-01	-0.07	7.4E-01	-0.24	2.4E-01	-0.31	1.2E-01	-0.04	8.3E-01
ENSCAFG0000001070	CCDC150	darkgrey	EC_M8	0.81	4.2E-07	0.37	6.1E-02	-0.50	9.7E-03	-0.14	5.1E-01	-0.18	3.9E-01	0.19	3.5E-01	-0.16	4.3E-01	0.03	8.9E-01	-0.09	6.7E-01	-0.27	1.9E-01
ENSCAFG0000000305	XPO1	darkgrey	EC_M8	0.81	4.2E-07	0.27	1.9E-01	-0.22	2.9E-01	0.01	9.5E-01	-0.52	5.9E-03	0.00	9.8E-01	-0.38	5.8E-02	-0.02	9.1E-01	-0.31	1.2E-01	0.08	6.8E-01
ENSCAFG0000003190	RNF219	darkgrey	EC_M8	0.81	4.6E-07	0.21	3.1E-01	-0.33	1.0E-01	0.04	8.3E-01	-0.39	4.8E-02	0.23	2.6E-01	-0.26	2.0E-01	0.02	9.3E-01	-0.31	1.3E-01	-0.07	7.2E-01
ENSCAFG0000001185	SPC25	darkgrey	EC_M8	0.81	4.8E-07	0.13	5.2E-01	-0.37	6.4E-02	-0.13	5.3E-01	-0.19	3.5E-01	-0.01	9.5E-01	-0.20	3.3E-01	-0.08	6.8E-01	-0.12	5.6E-01	-0.21	3.1E-01
ENSCAFG0000000621	POLE	darkgrey	EC_M8	0.81	4.8E-07	0.23	2.6E-01	-0.37	6.4E-02	-0.14	5.0E-01	-0.22	2.8E-01	0.07	7.4E-01	-0.32	1.1E-01	-0.07	7.5E-01	-0.15	4.7E-01	-0.21	3.1E-01
ENSCAFG0000000665	NOP56	darkgrey	EC_M8	0.81	4.8E-07	-0.03	8.8E-01	-0.14	4.8E-01	0.02	9.2E-01	-0.42	3.3E-02	0.18	3.9E-01	-0.37	6.0E-02	-0.11	5.8E-01	-0.07	7.5E-01	-0.02	9.3E-01
ENSCAFG0000000387	PLK4	darkgrey	EC_M8	0.81	4.9E-07	0.26	2.0E-01	-0.29	1.5E-01	-0.04	8.5E-01	-0.32	1.1E-01	0.15	4.7E-01	-0.32	1.1E-01	-0.11	5.8E-01	-0.20	3.2E-01	-0.09	6.5E-01
ENSCAFG0000000157	CEP78	darkgrey	EC_M8	0.81	5.0E-07	-0.04	8.6E-01	-0.27	1.9E-01	0.05	8.0E-01	-0.27	1.8E-01	0.26	2.0E-01	-0.28	1.6E-01	-0.16	4.4E-01	-0.32	1.1E-01	-0.16	4.4E-01
ENSCAFG0000000490	DIAPH3	darkgrey	EC_M8	0.81	5.0E-07	0.09	6.5E-01	-0.37	6.2E-02	-0.14	4.8E-01	-0.17	3.9E-01	0.03	9.0E-01	-0.41	3.6E-02	0.00	9.9E-01	-0.14	4.9E-01	-0.24	2.5E-01
ENSCAFG0000000302	SYNCRIP	darkgrey	EC_M8	0.81	5.1E-07	0.09	6.6E-01	-0.78	2.1E-06	-0.15	4.7E-01	0.27	1.8E-01	0.23	2.6E-01	-0.28	1.7E-01	-0.05	8.1E-01	-0.33	1.0E-01	-0.70	7.0E-05
ENSCAFG0000001896	RBMX	darkgrey	EC_M8	0.81	5.1E-07	0.25	2.3E-01	-0.16	4.3E-01	-0.11	5.9E-01	-0.53	5.6E-03	0.21	3.2E-01	-0.08	6.8E-01	-0.04	8.4E-01	-0.27	1.8E-01	0.08	7.0E-01
ENSCAFG0000000427	RAD54L	darkgrey	EC_M8	0.81	5.2E-07	0.05	8.0E-01	-0.43	2.9E-02	-0.22	2.8E-01	-0.09	6.5E-01	0.16	4.4E-01	-0.14	4.8E-01	0.02	9.0E-01	-0.07	7.5E-01	-0.31	1.2E-01
ENSCAFG0000000847	MRPL39	darkgrey	EC_M8	0.81	5.2E-07	-0.02	9.4E-01	-0.60	1.1E-03	0.09	6.7E-01	0.06	7.9E-01	0.27	1.8E-01	-0.33	9.5E-02	-0.23	2.5E-01	-0.34	9.3E-02	-0.51	7.3E-03
ENSCAFG0000001886	RSL1D1	darkgrey	EC_M8	0.81	5.2E-07	-0.08	6.9E-01	-0.33	1.0E-01	0.01	9.6E-01	-0.18	3.9E-01	0.22	2.8E-01	-0.25	2.2E-01	-0.06	7.9E-01	-0.27	1.9E-01	-0.28	1.7E-01
ENSCAFG0000002369	ERCC6L	darkgrey	EC_M8	0.81	5.5E-07	0.22	2.9E-01	-0.38	5.4E-02	-0.26	2.0E-01	-0.20	3.2E-01	0.22	2.8E-01	-0.22	2.7E-01	-0.24	2.5E-01	-0.14	5.0E-01	-0.22	2.8E-01
ENSCAFG0000001258	G2E3	darkgrey	EC_M8	0.81	5.5E-07	0.38	5.3E-02	-0.83	1.1E-07	-0.09	6.6E-01	0.16	4.5E-01	0.14	4.9E-01	-0.16	4.2E-01	-0.09	6.6E-01	-0.23	2.7E-01	-0.58	2.1E-03
ENSCAFG0000000404	HDAC2	darkgrey	EC_M8	0.81	5.5E-07	0.55	3.4E-03	-0.53	4.9E-03	-0.03	8.8E-01	-0.22	2.7E-01	0.16	4.4E-01	-0.23	2.5E-01	-0.03	9.0E-01	-0.32	1.1E-01	-0.20	3.2E-01
ENSCAFG0000001602	SMARCE1	darkgrey	EC_M8	0.81	5.6E-07	0.37	5.9E-02	-0.22	2.9E-01	-0.23	2.5E-01	-0.53	5.7E-03	0.12	5.8E-01	-0.36	6.9E-02	-0.02	9.1E-01	-0.25	2.1E-01	0.08	7.1E-01
ENSCAFG0000001217	INTS7	darkgrey	EC_M8	0.81	6.0E-07	0.27	1.9E-01	-0.34	8.7E-02	0.02	9.1E-01	-0.35	8.2E-02	0.08	6.9E-01	-0.39	4.7E-02	-0.09	6.6E-01	-0.61	9.5E-04	-0.12	5.5E-01
ENSCAFG0000000538	FNTA	darkgrey	EC_M8	0.81	6.0E-07	0.15	4.8E-01	-0.63	5.6E-04	0.00	9.9E-01	0.05	8.0E-01	0.16	4.3E-01	-0.44	2.4E-02	-0.24	2.4E-01	-0.43	2.7E-02	-0.47	1.4E-02
ENSCAFG0000000246	ANP32B	darkgrey	EC_M8	0.81	6.1E-07	-0.10	6.4E-01	-0.12	5.5E-01	0.08	7.1E-01	-0.46	1.9E-02	0.15	4.7E-01	-0.28	1.7E-01	0.04	8.4E-01	-0.26	2.0E-01	0.01	9.5E-01
ENSCAFG0000001352	POLA1	darkgrey	EC_M8	0.81	6.1E-07	-0.06	7.6E-01	-0.15	4.5E-01	-0.02	9.2E-01	-0.38	5.7E-02	-0.01	9.4E-01	-0.14	5.1E-01	-0.02	9.1E-01	-0.15	4.5E-01	-0.01	9.4E-01
ENSCAFG0000000557	SUPT16H	darkgrey	EC_M8	0.81	6.2E-07	0.23	2.5E-01	-0.66	2.4E-04	-0.08	6.9E-01	0.02	9.1E-01	0.21	3.0E-01	-0.13	5.4E-01	0.02	9.4E-01	-0.26	2.1E-01	-0.45	2.3E-02
ENSCAFG0000000359	ZMYM1	grey	EC_M10	0.81	6.3E-07	0.10	6.2E-01	-0.24	2.4E-01	0.09	6.6E-01	-0.43	2.9E-02	0.07	7.2E-01	-0.22	2.7E-01	-0.18	3.9E-01	-0.45	2.0E-02	-0.01	9.6E-01
ENSCAFG0000001937	HASPIN	darkgrey	EC_M8	0.81	6.8E-07	0.05	8.2E-01	-0.31	1.3E-01	-0.12	5.5E-01	-0.23	2.6E-01	0.05	7.9E-01	-0.17	4.1E-01	0.07	7.2E-01	-0.07	7.4E-01	-0.19	3.6E-01
ENSCAFG0000002933	AURKA	darkgrey	EC_M8	0.81	7.0E-07	0.08	6.9E-01	-0.46	1.9E-02	-0.10	6.4E-01	-0.07	7.2E-01	0.15	4.7E-01	-0.31	1.2E-01	0.08	7.0E-01	-0.05	8.2E-01	-0.34	9.3E-02
ENSCAFG0000000207	ENSCAFG0000000207	darkgrey	EC_M8	0.80	7.4E-07	0.09	6.5E-01	-0.41	3.5E-02	0.01	9.5E-01	-0.22	2.7E-01	0.31	1.2E-01	-0.34	9.0E-02	-0.04	8.3E-01	-0.14	5.0E-01	-0.23	2.7E-01
ENSCAFG0000000752	MPHOSPH9	darkgrey	EC_M8	0.80	7.4E-07	0.36	7.1E-02	-0.45	2.3E-02	0.09	6.8E-01	-0.26	2.0E-01	0.04	8.3E-01	-0.47	1.5E-02	-0.33	9.5E-02	-0.04	8.3E-01	-0.17	4.0E-01
ENSCAFG0000000845	ENSCAFG0000000845	darkgrey	EC_M8	0.80	7.5E-07	0.28	1.6E-01	-0.82	3.4E-07	0.06	7.7E-01	0.18	3.8E-01	0.22	2.8E-01	-0.28	1.7E-01	-0.13	5.1E-01	-0.32	1.1E-01	-0.61	9.5E-04
ENSCAFG0000000188	SMC5	darkgrey	EC_M8	0.80	7.6E-07	0.37	6.1E-02	-0.64	4.8E-04	0.04	8.5E-01	-0.11	5.9E-01	0.19	3.4E-01	-0.11	6.1E-01	-0.19	3.4E-01	-0.21	2.9E-01	-0.32	1.1E-01
ENSCAFG0000000570	TOP2B	darkgrey	EC_M8	0.80	7.9E-07	0.50	9.8E-03	-0.35	8.4E-02	0.04	8.4E-01	-0.47	1.5E-02	0.05	8.2E-01	-0.14	4.9E-01	-0.13	5.4E-01	-0.26	2.0E-01	0.04	8.6E-01
ENSCAFG0000000724	CKAP2L	darkgrey	EC_M8	0.80	8.1E-07	0.08	7.1E-01	-0.53	5.7E-03	-0.16	4.4E-01	0.00	1.0E+00	0.22	2.9E-01	0.07	7.2E-01	0.02	9.3E-01	-0.12	5.5E-01	-0.42	3.4E-02
ENSCAFG0000000775	GK5	darkgrey	EC_M8	0.80	8.2E-07	0.46	1.8E-02	-0.48	1.3E-02	0.07	7.2E-01	-0.28	1.7E-01	-0.05	8.2E-01	-0.13	5.3E-01	-0.15	4.6E-01	-0.37	6.1E-02	-0.14	4.9E-01
ENSCAFG0000001101	ENSCAFG0000001101	darkgrey	EC_M8	0.80	8.6E-07	0.00	1.0E+00	-0.71	5.8E-05	0.01	9.5E-01	0.18	3.8E-01	0.23	2.5E-01	-0.31	1.2E-01	-0.03	8.8E-01	-0.24	2.3E-01	-0.59	1.4E-03
ENSCAFG0000000627	RFC3	darkgrey	EC_M8	0.80	8.9E-07	0.11	6.0E-01	-0.49	1.1E-02	-0.04	8.4E-01	-0.08	7.0E-01	0.21	3.0E-01	0.03	8.9E-01	0.18	3.8E-01	-0.15	4.7E-01	-0.34	9.4E-02
ENSCAFG0000000706	NUP58	darkgrey	EC_M8	0.80	9.0E-07	0.18	3.7E-01	-0.77	3.5E-06	-0.05	8.2E-01	0.22	2.9E-01	0.21	3.1E-01	-0.26	2.0E-01	-0.20	3.3E-01	-0.15	4.6E-01	-0.64	4.0E-04
ENSCAFG0000000710	ANAPC1	darkgrey	EC_M8	0.80	9.0E-07	0.31	1.2E-01	-0.33	9.5E-02	-0.09	6.7E-01	-0.30	1.4E-01	-0.05	8.0E-01	-0.28	1.6E-01	-0.08	7.2E-01	-0.13	5.3E-01	-0.11	6.1E-01
ENSCAFG0000000413	CCNA2	darkgrey	EC_M8	0.80	9.0E-07	0.17	4.1E-01	-0.25	2.1E-01	-0.03	8.7E-01	-0.33	1.0E-01	0.25	2.1E-01	-0.21	3.0E-01	-0.16	4.2E-01	-0.12	5.5E-01	-0.08	6.9E-01
ENSCAFG0000000170	MCM5	darkgrey	EC_M8	0.80	9.1E-07	0.22	2.8E-01	-0.31	1.2E-01	-0.06	7.7E-01	-0.28	1.7E-01	0.14	4.9E-01	-0.18	3.7E-01	-0.07	7.4E-01	-0.04	8.4E-01	-0.14	5.0E-01
ENSCAFG0000000933	MYBL2	darkgrey	EC_M8	0.80	9.3E-07	0.26	2.0E-01	-0.37	6.0E-02	-0.09	6.5E-01	-0.24	2.4E-01	0.23	2.6E-01	-0.20	3.3E-01	-0.12	5.7E-01	-0.01	9.6E-01	-0.19	3.6E-01
ENSCAFG0000001606	RFC1	darkgrey	EC_M8	0.80	9.5E-07	-0.07	7.2E-01	-0.29	1.5E-01	-0.04	8.5E-01	-0.26	2.0E-01	0.13	5.3E-01	-0.25	2.3E-01	-0.22	2.8E-01	-0.22	2.8E-01	-0.14	5.0E-01
ENSCAFG0000001091	HEATR1	darkgrey	EC_M8	0.80	9.6E-07	-0.09	6.8E-01	-0.56	2.7E-03	0.00	9.9E-01	0.07	7.4E-01	0.19	3.5E-01	-0.35	7.7E-02	-0.08	7.0E-01	-0.32	1.1E-01	-0.48	1.3E-02
ENSCAFG0000000938	PRPF4B	darkgrey	EC_M8	0.80	9.6E-07	0.30	1.4E-01	-0.42	3.5E-02	0.10	6.4E-01	-0.32	1.1E-01	0.05	8.1E-01	-0.16	4.4E-01	-0.10	6.3E-01	-0.19	3.4E-01	-0.08	6.8E-01
ENSCAFG0000000905	TOP1	darkgrey	EC_M8	0.80	9.8E-07	0.49	1.2E-02	-0.42	3.5E-02	0.06	7.8E-01	-0.37	5.9E-02	0.07	7.4E-01	-0.29	1.5E-01	-0.04	8.4E-01	0.00	9.9E-01	-0.07	7.4E-01
ENSCAFG0000001534	RAD51AP1	darkgrey	EC_M8	0.80	9.9E-07	0.17	4.1E-01	-0.38	5.4E-02	-0.12	5.6E-01	-0.17	4.1E-01	0.06	7.7E-01	-0.11	6.1E-01	-0.23	2.6E-01	-0.21	3.0E-01	-0.22	2.8E-01

ENSCAFG0000001699	SPDL1	darkgrey	EC_M8	0.80	1.0E-06	-0.16	4.4E-01	-0.22	2.9E-01	-0.12	5.6E-01	-0.28	1.7E-01	0.22	2.7E-01	-0.39	5.1E-02	-0.10	6.3E-01	-0.19	3.7E-01	-0.16	4.3E-01
ENSCAFG0000000439	YME1L1	darkgrey	EC_M8	0.80	1.0E-06	0.21	3.1E-01	-0.38	5.7E-02	0.06	7.8E-01	-0.34	8.8E-02	0.17	4.0E-01	-0.05	8.0E-01	-0.03	8.9E-01	-0.17	4.0E-01	-0.09	6.5E-01
ENSCAFG0000001035	MPHOSPH10	grey	EC_M10	0.80	1.0E-06	-0.02	9.4E-01	-0.24	2.4E-01	0.10	6.1E-01	-0.40	4.0E-02	0.28	1.6E-01	-0.13	5.3E-01	-0.19	3.6E-01	-0.44	2.6E-02	-0.05	8.0E-01
ENSCAFG0000000398	MTPAP	grey	EC_M10	0.80	1.1E-06	0.02	9.1E-01	-0.16	4.2E-01	0.20	3.3E-01	-0.51	7.5E-03	0.22	2.9E-01	-0.24	2.3E-01	-0.09	6.6E-01	-0.36	7.4E-02	0.06	7.7E-01
ENSCAFG0000002320	WTAP	darkgrey	EC_M8	0.80	1.1E-06	0.40	4.1E-02	-0.49	1.0E-02	0.06	7.8E-01	-0.26	2.0E-01	-0.07	7.3E-01	-0.39	4.7E-02	-0.03	8.9E-01	-0.22	2.9E-01	-0.17	4.0E-01
ENSCAFG0000000368	DDX1	darkgrey	EC_M8	0.80	1.1E-06	0.24	2.4E-01	-0.67	1.8E-04	-0.18	3.8E-01	0.09	6.6E-01	0.22	2.7E-01	-0.13	5.2E-01	-0.14	4.9E-01	-0.39	5.1E-02	-0.49	1.2E-02
ENSCAFG0000001241	SSB	darkgrey	EC_M8	0.80	1.1E-06	-0.08	6.8E-01	-0.66	2.4E-04	-0.12	5.6E-01	0.16	4.4E-01	0.24	2.3E-01	-0.31	1.2E-01	0.13	5.2E-01	-0.12	5.5E-01	-0.59	1.5E-03
ENSCAFG0000001371	TPR	darkgrey	EC_M8	0.80	1.1E-06	0.38	5.7E-02	-0.54	4.3E-03	0.12	5.5E-01	-0.18	3.9E-01	0.03	8.7E-01	-0.39	4.9E-02	-0.23	2.5E-01	-0.35	8.3E-02	-0.24	2.5E-01
ENSCAFG0000001680	ENSCAFG0000001680	darkgrey	EC_M8	0.80	1.2E-06	0.37	6.1E-02	-0.62	6.9E-04	0.16	4.4E-01	-0.09	6.6E-01	0.03	8.7E-01	-0.21	3.0E-01	-0.05	8.2E-01	-0.24	2.4E-01	-0.34	9.4E-02
ENSCAFG0000000343	NOL10	darkgrey	EC_M8	0.80	1.2E-06	-0.07	7.4E-01	-0.62	7.5E-04	-0.10	6.4E-01	0.13	5.3E-01	0.18	3.7E-01	-0.17	4.0E-01	0.05	7.9E-01	-0.37	6.3E-02	-0.57	2.3E-03
ENSCAFG0000002012	DNTTIP2	darkgrey	EC_M8	0.80	1.2E-06	0.29	1.5E-01	-0.83	1.3E-07	-0.04	8.6E-01	0.19	3.5E-01	0.29	1.4E-01	-0.13	5.2E-01	-0.11	5.8E-01	-0.24	2.5E-01	-0.62	7.4E-04
ENSCAFG0000001170	ENSCAFG0000001170	darkgrey	EC_M8	0.80	1.2E-06	0.11	6.1E-01	-0.76	8.2E-06	0.02	9.0E-01	0.20	3.2E-01	0.25	2.1E-01	-0.23	2.7E-01	0.00	9.9E-01	-0.05	8.2E-01	-0.63	5.8E-04
ENSCAFG0000000010	ENSCAFG0000000010	darkgrey	EC_M8	0.79	1.2E-06	-0.03	8.8E-01	-0.47	1.4E-02	-0.15	4.7E-01	0.01	9.6E-01	0.27	1.8E-01	-0.24	2.4E-01	0.01	9.6E-01	-0.18	3.7E-01	-0.44	2.4E-02
ENSCAFG0000001567	LYAR	darkgrey	EC_M8	0.79	1.2E-06	0.19	3.7E-01	-0.13	5.1E-01	0.09	6.5E-01	-0.57	2.4E-03	0.03	8.7E-01	-0.24	2.4E-01	-0.07	7.3E-01	-0.28	1.7E-01	0.11	5.8E-01
ENSCAFG0000000707	TPX2	darkgrey	EC_M8	0.79	1.2E-06	0.13	5.2E-01	-0.29	1.5E-01	-0.06	7.5E-01	-0.27	1.8E-01	0.12	5.5E-01	-0.12	5.5E-01	-0.02	9.3E-01	-0.10	6.4E-01	-0.14	5.0E-01
ENSCAFG0000001769	ILF3	darkgrey	EC_M8	0.79	1.3E-06	0.11	6.1E-01	-0.38	5.4E-02	-0.07	7.4E-01	-0.14	4.9E-01	0.15	4.7E-01	-0.16	4.5E-01	-0.08	7.1E-01	-0.43	2.8E-02	-0.29	1.5E-01
ENSCAFG0000001406	FANCM	darkgrey	EC_M8	0.79	1.4E-06	0.26	2.0E-01	-0.52	5.9E-03	-0.31	1.2E-01	-0.07	7.4E-01	0.24	2.4E-01	-0.29	1.6E-01	-0.17	4.1E-01	-0.11	5.9E-01	-0.35	7.8E-02
ENSCAFG0000000650	NEDD1	darkgrey	EC_M8	0.79	1.4E-06	0.35	8.3E-02	-0.60	1.2E-03	-0.01	9.6E-01	-0.10	6.4E-01	0.11	5.9E-01	-0.38	5.6E-02	0.00	1.0E+00	-0.42	3.1E-02	-0.35	7.6E-02
ENSCAFG0000001574	DNAJC7	darkgrey	EC_M8	0.79	1.4E-06	0.18	3.8E-01	-0.10	6.2E-01	0.35	8.4E-02	-0.59	1.4E-03	0.13	5.1E-01	-0.28	1.7E-01	-0.21	3.1E-01	-0.38	5.3E-02	0.17	4.0E-01
ENSCAFG0000001016	PRDM10	darkgrey	EC_M8	0.79	1.5E-06	0.16	4.3E-01	-0.25	2.2E-01	0.11	5.8E-01	-0.39	4.8E-02	0.28	1.7E-01	-0.30	1.4E-01	0.01	9.7E-01	-0.32	1.1E-01	-0.03	8.7E-01
ENSCAFG0000001981	FANCA	darkgrey	EC_M8	0.79	1.5E-06	0.35	8.3E-02	-0.17	4.2E-01	0.06	7.7E-01	-0.50	9.9E-03	0.04	8.5E-01	-0.24	2.4E-01	-0.07	7.3E-01	-0.16	4.2E-01	0.09	6.8E-01
ENSCAFG0000001893	UHRF1	darkgrey	EC_M8	0.79	1.5E-06	0.24	2.5E-01	-0.37	6.0E-02	-0.09	6.5E-01	-0.20	3.3E-01	0.19	3.6E-01	-0.18	3.7E-01	-0.23	2.5E-01	-0.09	6.7E-01	-0.21	3.1E-01
ENSCAFG0000000656	NCAPH	darkgrey	EC_M8	0.79	1.5E-06	0.36	6.9E-02	-0.38	5.5E-02	-0.17	4.0E-01	-0.22	2.7E-01	0.15	4.6E-01	-0.27	1.9E-01	-0.13	5.3E-01	-0.04	8.3E-01	-0.17	4.0E-01
ENSCAFG0000001011	CIT	darkgrey	EC_M8	0.79	1.7E-06	0.10	6.2E-01	-0.18	3.7E-01	-0.03	8.7E-01	-0.37	6.6E-02	0.16	4.4E-01	-0.16	4.4E-01	-0.29	1.5E-01	-0.24	2.4E-01	-0.04	8.6E-01
ENSCAFG0000001885	GSPT1	darkgrey	EC_M8	0.79	1.7E-06	0.13	5.4E-01	-0.85	2.9E-08	-0.13	5.4E-01	0.34	9.3E-02	0.27	1.9E-01	-0.32	1.1E-01	-0.08	6.9E-01	-0.15	4.6E-01	-0.74	1.3E-05
ENSCAFG0000000098	KIFC1	darkgrey	EC_M8	0.79	1.8E-06	0.08	7.0E-01	-0.18	3.9E-01	-0.06	7.6E-01	-0.37	6.1E-02	0.13	5.4E-01	-0.23	2.5E-01	-0.04	8.3E-01	-0.09	6.5E-01	-0.04	8.6E-01
ENSCAFG0000001494	PRRC2C	darkgrey	EC_M8	0.79	1.8E-06	0.43	2.7E-02	-0.58	1.9E-03	-0.07	7.2E-01	-0.13	5.3E-01	-0.01	9.8E-01	-0.31	1.3E-01	0.03	9.0E-01	-0.26	2.0E-01	-0.30	1.4E-01
ENSCAFG0000001474	MCM7	darkgrey	EC_M8	0.79	1.8E-06	0.19	3.5E-01	-0.20	3.2E-01	-0.05	7.9E-01	-0.40	4.6E-02	0.10	6.4E-01	-0.15	4.6E-01	-0.15	4.5E-01	-0.09	6.7E-01	-0.02	9.2E-01
ENSCAFG0000003186	ZNF518A	darkgrey	EC_M8	0.79	1.8E-06	0.44	2.4E-02	-0.52	7.0E-03	-0.03	8.9E-01	-0.24	2.4E-01	0.08	6.9E-01	-0.26	2.1E-01	-0.01	9.5E-01	-0.18	3.9E-01	-0.18	3.9E-01
ENSCAFG0000000661	SUCLG2	darkgrey	EC_M8	0.79	2.0E-06	0.14	5.1E-01	-0.20	3.3E-01	0.06	7.8E-01	-0.45	2.2E-02	-0.02	9.1E-01	-0.61	9.2E-04	-0.03	9.0E-01	-0.13	5.2E-01	0.02	9.0E-01
ENSCAFG0000002962	SRSF7	darkgrey	EC_M8	0.79	2.0E-06	0.26	2.0E-01	-0.41	3.6E-02	-0.27	1.8E-01	-0.15	4.6E-01	0.14	5.0E-01	-0.09	6.6E-01	0.01	9.6E-01	-0.14	5.0E-01	-0.25	2.2E-01
ENSCAFG0000000594	RRM1	darkgrey	EC_M8	0.79	2.0E-06	0.28	1.6E-01	-0.37	6.1E-02	-0.07	7.2E-01	-0.22	2.9E-01	0.09	6.6E-01	-0.09	6.6E-01	-0.19	3.5E-01	-0.41	3.9E-02	-0.17	3.9E-01
ENSCAFG0000001647	NSD1	darkgrey	EC_M8	0.78	2.1E-06	0.40	4.3E-02	-0.19	3.5E-01	0.06	7.6E-01	-0.57	2.6E-03	-0.03	8.8E-01	-0.30	1.3E-01	-0.25	2.2E-01	-0.39	5.0E-02	0.11	5.8E-01
ENSCAFG0000001562	LEO1	darkgrey	EC_M8	0.78	2.1E-06	0.23	2.5E-01	-0.74	1.7E-05	-0.21	3.1E-01	0.14	5.0E-01	0.25	2.1E-01	-0.13	5.3E-01	0.09	6.6E-01	-0.26	1.9E-01	-0.56	2.7E-03
ENSCAFG0000000635	METAP2	darkgrey	EC_M8	0.78	2.1E-06	0.20	3.2E-01	-0.50	1.0E-02	0.07	7.2E-01	-0.14	5.0E-01	0.21	3.1E-01	-0.08	6.8E-01	-0.11	6.0E-01	-0.52	5.9E-03	-0.25	2.2E-01
ENSCAFG0000000344	CUL1	darkgrey	EC_M8	0.78	2.2E-06	0.15	4.6E-01	-0.27	1.7E-01	0.10	6.1E-01	-0.38	5.4E-02	0.14	4.9E-01	-0.50	1.0E-02	-0.40	4.4E-02	-0.28	1.7E-01	-0.06	7.6E-01
ENSCAFG0000000043	CCT2	darkgrey	EC_M8	0.78	2.3E-06	0.01	9.6E-01	-0.79	2.0E-06	-0.10	6.2E-01	0.32	1.1E-01	0.37	6.2E-02	-0.18	3.9E-01	-0.14	4.9E-01	-0.28	1.6E-01	-0.74	1.7E-05
ENSCAFG0000001387	PNN	darkgrey	EC_M8	0.78	2.3E-06	0.37	6.0E-02	-0.43	2.8E-02	0.09	6.6E-01	-0.23	2.6E-01	0.07	7.5E-01	-0.28	1.7E-01	0.12	5.7E-01	-0.03	9.0E-01	-0.16	4.2E-01
ENSCAFG0000000518	XRN2	darkgrey	EC_M8	0.78	2.4E-06	0.38	5.4E-02	-0.57	2.6E-03	-0.13	5.4E-01	-0.09	6.6E-01	0.21	3.0E-01	-0.50	8.8E-03	-0.20	3.2E-01	0.07	7.3E-01	-0.32	1.1E-01
ENSCAFG0000000750	SBNO1	darkgrey	EC_M8	0.78	2.5E-06	0.22	2.9E-01	-0.74	1.7E-05	-0.18	3.7E-01	0.12	5.5E-01	0.33	1.0E-01	-0.11	5.9E-01	-0.18	3.9E-01	-0.36	7.2E-02	-0.56	2.7E-03
ENSCAFG0000000469	ENSCAFG0000000469	darkgrey	EC_M8	0.78	2.5E-06	0.23	2.5E-01	-0.73	2.7E-05	0.16	4.5E-01	0.09	6.6E-01	0.19	3.6E-01	-0.29	1.6E-01	-0.14	4.9E-01	-0.26	2.0E-01	-0.50	1.0E-02
ENSCAFG0000001531	AP4E1	darkgrey	EC_M8	0.78	2.6E-06	0.41	3.5E-02	-0.77	3.7E-06	0.05	8.0E-01	0.10	6.2E-01	0.19	3.5E-01	-0.35	8.3E-02	-0.05	8.0E-01	-0.20	3.2E-01	-0.52	6.9E-03
ENSCAFG0000001566	EIF2AK1	darkgrey	EC_M8	0.78	2.6E-06	-0.18	3.9E-01	-0.47	1.4E-02	0.01	9.7E-01	0.03	9.0E-01	0.10	6.4E-01	-0.33	1.0E-01	-0.10	6.2E-01	-0.28	1.6E-01	-0.45	2.2E-02
ENSCAFG0000000039	NUP43	darkgrey	EC_M8	0.78	2.6E-06	-0.04	8.6E-01	-0.27	1.9E-01	0.04	8.4E-01	-0.24	2.4E-01	0.02	9.4E-01	-0.55	3.8E-03	0.06	7.6E-01	-0.22	2.7E-01	-0.19	3.5E-01
ENSCAFG0000000736	EIF3M	grey	EC_M10	0.78	2.7E-06	-0.01	9.5E-01	-0.26	2.1E-01	0.15	4.7E-01	-0.38	5.7E-02	0.19	3.5E-01	-0.29	1.5E-01	-0.16	4.3E-01	-0.48	1.3E-02	-0.09	6.5E-01
ENSCAFG0000000308	CCT4	darkgrey	EC_M8	0.78	2.7E-06	-0.05	8.0E-01	-0.60	1.3E-03	-0.05	8.0E-01	0.14	4.8E-01	0.27	1.8E-01	-0.25	2.1E-01	-0.16	4.3E-01	-0.35	7.7E-02	-0.56	2.9E-03
ENSCAFG0000001503	NOP2	darkgrey	EC_M8	0.78	2.7E-06	-0.06	7.8E-01	-0.30	1.4E-01	-0.15	4.6E-01	-0.20	3.3E-01	0.19	3.6E-01	-0.39	4.8E-02	0.02	9.4E-01	-0.26	2.0E-01	-0.24	2.4E-01
ENSCAFG0000000223	PAICS	darkgrey	EC_M8	0.78	2.8E-06	0.03	9.0E-01	-0.19	3.6E-01	-0.10	6.4E-01	-0.35	8.1E-02	0.19	3.4E-01	-0.33	9.6E-02	-0.09	6.8E-01	-0.13	5.2E-01	-0.05	8.0E-01
ENSCAFG0000001162	FANCI	darkgrey	EC_M8	0.78	2.8E-06	0.18	3.8E-01	-0.41	3.9E-02	-0.23	2.5E-01	-0.12	5.7E-01	0.07	7.4E-01	-0.36	7.1E-02	-0.05	8.1E-01	-0.09	6.5E-01	-0.26	2.1E-01
ENSCAFG0000001031	METAP1	darkgrey	EC_M8	0.78	2.8E-06	-0.06	7.6E-01	-0.07	7.5E-01	0.00	1.0E+00	-0.51	7.7E-03	0.15	4.7E-01	-0.38	5.5E-02	-0.12	5.5E-01	-0.33	9.7E-02	0.09	6.6E-01
ENSCAFG0000000556	IPO5	darkgrey	EC_M8	0.78	2.8E-06	-0.20	3.3E-01	-0.18	3.9E-01	0.15	4.7E-01	-0.34	8.8E-02	0.08	6.9E-01	-0.34	8.7E-02	-0.13	5.2E-01	-0.38	5.8E-02	-0.12	5.7E-01
ENSCAFG0000002022	PKN2	darkgrey	EC_M8	0.78	2.8E-06	0.32	1.1E-01	-0.29	1.5E-01	0.14	5.0E-01	-0.48	1.3E-02	0.05	8.0E-01	-0.40	4.4E-02	-0.10	6.3E-01	-0.25	2.3E-01	0.04	8.3E-01
ENSCAFG0000000703	ESPL1	darkgrey	EC_M8	0.78	3.0E-06	0.13	5.4E-01	-0.19	3.4E-01	-0.14	5.1E-01	-0.36	7.4E-02	0.15	4.8E-01	-0.09	6.6E-01	-0.12	5.6E-01	-0.22	2.8E-01	-0.05	8.2E-01
ENSCAFG0000000773	CENPU	darkgrey	EC_M8	0.78	3.0E-06	0.23	2.6E-01	-0.37	6.6E-02	-0.04	8.5E-01	-0.19	3.4E-01	0.10	6.2E-01	-0.21	3.1E-01	-0.11	5.8E-01	-0.07	7.2E-01	-0.22	2.7E-01
ENSCAFG0000001473	NOP14	darkgrey	EC_M8	0.78	3.0E-06	0.01	9.6E-01	-0.37	6.6E-02	-0.11	5.9E-01	-0.13	5.4E-01	0.19	3.6E-01	-0.12	5.6E-01	-0.10	6.2E-01	-0.34	9.2E-02	-0.27	1.8E-01
ENSCAFG0000000887	MIS18A	darkgrey	EC_M8	0.78	3.2E-06	0.34	8.5E-02	-0.23	2.6E-01	0.05	8.3E-01	-0.42	3.2E-02	0.07	7.4E-01	-0.17	3.9E-01	-0.17	4.0E-01	0.01	9.5E-01	0.02	9.1E-01
ENSCAFG0000000725	IPO7	darkgrey	EC_M8	0.78	3.2E-06	0.07	7.3E-01	-0.60	1.3E-03	-0.05	8.1E-01	0.04	8.5E-01	0.27	1.8E-01	-0.22	2.9E-01	-0.29	1.5E-01	-0.54	4.6E-03	-0.47	1.5E-02
ENSCAFG0000001879	BRIX1	darkgrey	EC_M8	0.77	3.4E-06	0.07	7.3E-01	-0.63	5.0E-04	0.09	6.8E-01	0.08	7.1E-01	0.14	5.0E-01	-0.28	1.7E-01	-0.05	8.1E-01	-0.42	3.1E-02	-0.48	1.3E-02
ENSCAFG0000002316	HMGN2	darkgrey	EC_M8	0.77	3.4E-06	-0.11	6.1E-01	0.01	9.8E-01	0.07	7.3E-01	-0.57	2.5E-03	0.11	6.0E-01	-0.37	6.0E-02	-0.05	8.0E-01	-0.08	7.1E-01	0.17	4.2E-01
ENSCAFG0000000078	GTSE1	darkgrey	EC_M8	0.77	3.4E-06	0.18	3.9E-01	-0.27	1.8E-01	0.13	5.3E-01	-0.28	1.6E-01	0.21	2.9E-01	-0.22	2.7E-01	-0.17	4.1E-01	-0.13	5.1E-01	-0.12	5.6E-01
ENSCAFG0000000498	TSEN2	darkgrey	EC_M8	0.77	3.5E-06	-0.04	8.4E-01	-0.14	4.9E-01	0.10	6.2E-01	-0.45	2.1E-02	0.00	9.9E-01	-0.09	6.7E-01	-0.09	6.5E-01	-0.49	1.1E-02	0.01	9.7E-01
ENSCAFG0000001150	GAR1	darkgrey	EC_M8	0.77	3.5E-06	-0.15	4.8E-01	-0.56	3.1E-03	-0.07	7.3E-01	0.08	6.9E-01	0.29	1.5E-01	-0.16	4.3E-01	-0.14	5.0E-01	-0.36	7.4E-02	-0.52	6.0E-03
ENSCAFG0000000353	CLSPN	darkgrey	EC_M8	0.77	3.7E-06	0.36	7.2E-02	-0.34	8.7E-02	-0.12	5.5E-01	-0.27	1.9E-01	0.25	2.2E-01	-0.23	2.6E-01	-0.18	3.7E-01	0.00	9.9E-01	-0.12	5.7E-01
ENSCAFG0000002376	ENSCAFG0000002376	darkgrey	EC_M8	0.77	3.8E-06	0.29	1.5E-01	-0.22	2.7E-01	0.18	3.8E-01	-0.48	1.3E-02	-0.05	8.1E-01	-0.10	6.3E-01	-0.23	2.5E-01	-0.23	2.6E-01	0.11	6.0E-01
ENSCAFG0000001452	CENPL	darkgrey	EC_M8	0.77	3.8E-06	-0.01	9.7E-01	-0.52	6.9E-03	-0.13	5.1E-01	0.06	7.8E-01	0.07	7.2E-01	-0.35	8.3E-02	-0.03	8.9E-01	-0.01	9.4E-01	-0.43	2.8E-02
ENSCAFG0000001610	CDC6	darkgrey	EC_M8	0.77	3.8E-06	0.01	9.7E-01	-0.69	1.1E-04	-0.14	5.1E-01	0.22	2.9E-01	0.27	1.8E-01	-0.34	8.9E-02	-0.13	5.2E-01	-0.27	1.8E-01	-0.65	3.6E-04
ENSCAFG0000000219	CKS2	darkgrey	EC_M8	0.77	3.8E-06	0.19	3.6E-01	-0.46	1.7E-02	-0.14	4.9E-01	-0.14	5.0E-01	0.11	5.8E-01	-0.19	3.6E-01	0.17	4.0E-01	-0.09	6.4E-01	-0.30	1.4E-01
ENSCAFG0000000697	TCEA1	darkgrey	EC_M8	0.77	3.8E-06	0.02	9.4E-01	-0.62	8.0E-04	0.22	2.8E-01	0.05	8.1E-01	0.18	3.9E-01	-0.28	1.6E-01	-0.04	8.5E-01	-0.33	1.0E-01	-0.49	1.2E-02
ENSCAFG0000001907	CHAF1A	darkgrey	EC_M8	0.77	4.0E-06	0.04	8.5E-01	-0.18	3.7E-01	0.00	1.0E+00	-0.34	9.2E-02	0.15	4.6E-01	-0.40	4.4E-02	-0.08	7.0E-01	-0.12	5.6E-01	-0.07	7.4E-01
ENSCAFG0000001226	DTL	darkgrey	EC_M8	0.77	4.0E-06	0.06	7.6E-01	-0.30	1.4E-01	-0.11	5.9E-01	-0.19	3.5E-01	0.10	6.4E-01	-0.26	2.0E-01	-0.08	6.9E-01	-0.32	1.1E-01	-0.20	3.2E-01
ENSCAFG0000000644	TOPBP1	darkgrey	EC_M8	0.77	4.0E-06	0.41	3.6E-02	-0.53	5.3E-03	-0.13	5.2E-01	-0.09	6.7E-01	0.11	5.8E-01	-0.35	7.9E-02	-0.17	4.1E-01	0.02	9.4E-01	-0.28	1.7E-01
ENSCAFG0000000655	PRKDC	darkgrey	EC_M8	0.77	4.0E-06	-0.05	7.9E-01	-0.14	4.8E-01	-0.03	8.8E-01	-0.44	2.4E-02	-0.13	5.4E-01	-0.37	6.1E-02	-0.14	5.1E-01	-0.28	1.7E-01	0.02	9.3E-01
ENSCAFG0000002015	GLMN	darkgrey	EC_M8	0.77	4.0E-06	0.23	2.6E-01	-0.54	4.7E-03	0.03	9.0E-01	-0.09	6.6E-01	0.19	3.4E-01	-0.22	2.7E-01	-0.30	1.4E-01	-0.16	4.3E-01	-0.30	1.4E-01
ENSCAFG0000000979	NCAPD3	darkgrey	EC_M8	0.77	4.1E-06	0.34	9.3E-02	-0.48	1.4E-02	-0.25	2.2E-01	-0.11	5.9E-01	0.10	6.1E-01	-0.12	5.6E-01	-0.02	9.4E-01	0.01	9.5E-01	-0.28	1.7E-01
ENSCAFG0000001820	RIOK3	darkgrey	EC_M8	0.77	4.1E-06	0.47	1.6E-02	-0.47	1.6E-02	0.08	7.1E-01	-0.25	2.1E-01	0.04	8.6E-01	-0.35	8.2E-02	-0.22	2.8E-01	-0.42	3.1E-02	-0.14	4.8E-01
ENSCAFG0000001512	NCAPD2	darkgrey	EC_M8	0.77	4.2E-06	0.01	9.4E-01	-0.01	9.5E-01	0.09	6.6E-01	-0.53	5.4E-03	0.15	4.6E-01	-0.30	1.4E-01	-0.26	2.0E-01	-0.17	4.0E-01	0.14	5.0E-01
ENSCAFG0000000731	QSER1	darkgrey	EC_M8	0.77	4.2E-06	0.47	1.6E-02	-0.40	4.0E-02	0.02	9.1E-01	-0.36	7.2E-02	0.00	1.0E+00	-0.36	7.3E-02	-0.11	6.0E-01	-0.18	3.9E-01	-0.09	6.7E-01
ENSCAFG0000001518	ENSCAFG0000001518	darkgrey	EC_M8	0.77	4.3E-06	-0.06	7.6E-01	-0.34	9.3E-02	-0.06	7.6E-01	-0.12	5.5E-01	0.16	4.2E-01	-0.40	4.4E-02	-0.11	5.9E-01	-0.17	4.2E-01	-0.27	1.8E-01
ENSCAFG0000001595	PDS5A	darkgrey	EC_M8	0.77	4.3E-06	0.40	4.3E-02	-0.24	2.4E-01	0.15	4.7E-01	-0.53	5.0E-03	-0.04	8.5E-01	-0.18	3.8E-01	-0.19	3.5E-01	-0.28	1.7E-01	0.11	6.0E-01
ENSCAFG0000001367	POLA2	darkgrey	EC_M8	0.77	4.3E-06	0.27	1.7E-01	-0.28	1.6E-01	-0.17	4.1E-01	-0.29	1.5E-01	0.06	7.8E-01	-0.21	3.1E-01	-0.08	6.9E-01	-0.10	6.1E-01	-0.08	6.9E-01
ENSCAFG0000000758	PTCD3	darkgrey	EC_M8	0.77	4.4E-06	-0.01	9.8E-01	0.07	7.3E-01	0.17	4.1E-01	-0.68	1.4E-04	0.00	9.8E-01	-0.46	1.7E-02	-0.12	5.5E-01	-0.29	1.5E-01	0.25	2.2E-01
ENSCAFG0000000767	CHD1	darkgrey	EC_M8	0.77	4.4E-06	0.48	1.2E-02	-0.58	1.7E-03	0.03	8.7E-01	-0.17	4.1E-01	0.13	5.2E-01	-0.13	5.3E-01	-0.18	3.7E-01	-0.29	1.6E-01	-0.24	2.4E-01
ENSCAFG0000000530	BIRC5	darkgrey	EC_M8	0.77	4.6E-06	0.19	3.5E-01	-0.32	1.1E-01	-0.06	7.8E-01	-0.23	2.7E-01	0.15	4.7E-01	-0.31	1.2E-01	-0.06	7.8E-01	-0.05	8.2E-01	-0.16	4.3E-01
ENSCAFG0000001352	CTDSPL2	darkgrey	EC_M8	0.77	4.7E-06	0.50	9.5E-03	-0.34	8.5E-02	0.09	6.6E-01	-0.45	2.2E-02	0.08	7.0E-01	-0.11	6.0E-01	-0.26	1.9E-01	-0.31	1.2E-01	0.04	8.6E-01
ENSCAFG0000000785	SAP30	darkgrey	EC_M8	0.77	4.7E-06	0.23	2.7E-01	-0.09	6.6E-01	0.21	3.1E-01	-0.57	2.2E-03	-0.01	9.7E-01	-0.40	4.3E-02	-0.19	3.5E-01	-0.44	2.4E-02	0.16	4.3E-01
ENSCAFG0000000645	PSMD13	darkgrey	EC_M8	0.77	4.7E-06	0.30	1.3E-01	-0.82	2.5E-07	-0.07	7.4E-01	0.23	2.5E-01	0.24	2.3E-01	-0.15	4.7E-01	0.17	4.2E-01	-0.09	6.8E-01	-0.65	3.5E-04
ENSCAFG0000001217	TIAL1	darkgrey	EC_M8	0.77	4.8E-06	0.25	2.1E-01	-0.72	3.4E-05	-0.10	6.1E-01	0.08	7.0E-01	0.31	1.2E-01	-0.06	7.7E-01	-0.09	6.7E-01	-0.35	7.8E-02	-0.49	1.1E-02
ENSCAFG0000000334	TIA1	darkgrey	EC_M8	0.77	4.8E-06	0.19	3.4E-01	-0.22	2.8E-01	0.11	5.9E-01	-0.46	1.7E-02	0.39	4.9E-02	-0.20	3.3E-01	-0.18	3.9E-01	-0.40	4.1E-02	0.02	9.1E-01
ENSCAFG0000001669	ICE2	darkgrey	EC_M8	0.77	5.0E-06	0.27	1.8E-01	-0.07	7.3E-01	-0.11	5.8E-01	-0.65	3.1E-04	-0.12	5.7E-01	-0.26	1.9E-01	-0.23	2.7E-01	-0.35	8.1E-02	0.22	2.9E-01
ENSCAFG0000000797	TERF1	darkgrey	EC_M8	0.77	5.0E-06	0.21	3.1E-01	-0.69	9.5E-05	-0.08	6.9E-01	0.11	6.0E-01	0.22	2.8E-01	-0.42	3.1E-02	0.10	6.2E-01	-0.20	3.2E-01	-0.53	5.0E-03
ENSCAFG0000000771	CENPH	darkgrey	EC_M8	0.76	5.3E-06	0.07	7.4E-01	-0.40	4.2E-02	-0.07	7.4E-01	-0.07	7.3E-01	0.17	4.0E-01	-0.34	8.8E-02	-0.01	9.6E-01	-0.23	2.6E-01	-0.33	9.7E-02
ENSCAFG0000000608	CEBPZ	darkgrey	EC_M8	0.76	5.3E-06	-0.13	5.2E-01	-0.51	7.7E-03	-0.06	7.6E-01	0.03	8.7E-01	0.27	1.8E-01	-0.07	7.4E-01	-0.03	8.8E-01	-0.38	5.3E-02	-0.43	3.0E-02
ENSCAFG0000000501	DIS3	darkgrey	EC_M8	0.76	5.3E-06	0.11	5.9E-01	-0.72	3.3E-05	-0.02	9.3E-01	0.24	2.4E-01	0.28	1.7E-01	-0.33	9.9E-02	-0.19	3.5E-01	-0.39	4.8E-02	-0.62	7.2E-04
ENSCAFG0000001358	IVNS1ABP	darkgrey	EC_M8	0.76	5.4E-06	0.44	2.5E-02	-0.46	1.9E-02	-0.09	6.6E-01	-0.28	1.6E-01	0.10	6.3E-01	-0.29	1.5E-01	-0.08	6.9E-01	0.18	3.7E-01	-0.13	5.3E-01
ENSCAFG0000000834	RACGAP1	darkgrey	EC_M8	0.76	5.6E-06	0.12	5.6E-01	-0.27	1.9E-01	-0.09	6.6E-01	-0.26	1.9E-01	0.06	7.6E-01	-0.22	2.7E-01	0.13	5.3E-01	-0.08	6.9E-01	-0.14	5.1E-01
ENSCAFG0000001352	AGPS	darkgrey	EC_M8	0.76	5.6E-06	0.33	1.0E-01	-0.44	2.6E-02	0.07	7.2E-01	-0.21	3.1E-01	-0.15	4.7E-01	-0.24	2.5E-01	-0.23	2.5E-01	-0.10	6.2E-01	-0.18	3.7E-01
ENSCAFG0000000525	CDC20	darkgrey	EC_M8	0.76	5.6E-06	0.03	8.9E-01	-0.26	2.0E-01	0.00	9.8E-01	-0.24	2.5E-01	0.12	5.7E-01	-0.21	2.9E-01	0.01	9.7E-01	-0.05	8.2E-01	-0.16	4.4E-01
ENSCAFG0000000751	ARFGEF1	darkgrey	EC_M8	0.76	5.7E-06	0.44	2.6E-02	-0.51	7.6E-03	0.00	9.9E-01	-0.25	2.1E-01	-0.04	8.6E-01	-0.33	1.0E-01	0.09	6.5E-01	-0.17	4.1E-01	-0.18	3.8E-01
ENSCAFG0000000746	IDE	darkgrey	EC_M8	0.76	5.7E-06	-0.02	9.4E-01	-0.62	7.7E-04	0.05	7.9E-01	0.08	6.8E-01	0.27	1.9E-01	-0.17	4.0E-01	-0.01	9.7E-01	-0.42	3.4E-02	-0.53	5.0E-03
ENSCAFG0000001159	ENSCAFG0000001159	darkgrey	EC_M8	0.76	5.9E-06	0.19	3.6E-01	-0.54	4.6E-03	-0.11	6.0E-01	0.05	8.2E-01	0.06	7.6E-01	-0.31	1.2E-01	-0.06	7.6E-01	-0.19	3.5E-01	-0.40	4.2E-02
ENSCAFG0000000022	YTHDC2	darkgrey	EC_M8	0.76	5.9E-06	0.26	2.0E-01	-0.39	4.9E-02	0.11	6.0E-01	-0.32	1.1E-01	0.02	9.2E-01	-0.36	7.1E-02	-0.17	4.1E-01	-0.21	3.0E-01	-0.11	6.0E-01
ENSCAFG0000001596	WAPL	darkgrey	EC_M8	0.76	5.9E-06	0.26	2.0E-01	-0.47	1.5E-02	0.10	6.2E-01	-0.21	2.9E-01	-0.07	7.4E-01	-0.40	4.3E-02	-0.07	7.4E-01	-0.11	6.0E-01	-0.17	4.1E-01
ENSCAFG0000000310	MDN1	darkgrey	EC_M8	0.76	6.2E-06	-0.14	5.0E-01	-0.34	9.3E-02	-0.08	7.2E-01	-0.14	4.9E-01	0.06	7.9E-01	-0.24	2.5E-01	-0.09	6.5E-01	-0.26	1.9E-01	-0.27	1.8E-01
ENSCAFG0000000203	PUM3	darkgrey	EC_M8	0.76	6.2E-06	-0.01	9.7E-01	-0.67	1.9E-04	0.23	2.6E-01	0.15	4.8E-01	0.23	2.6E-01	-0.30	1.4E-01	-0.13	5.4E-01	-0.08	7.0E-01	-0.58	1.8E-03
ENSCAFG0000001719	HMMR	darkgrey	EC_M8	0.76	6.2E-06	0.16	4.3E-01	-0.23	2.5E-01	0.00	9.9E-01	-0.32	1.1E-01	0.18	3.9E-01	-0.21	3.0E-01	-0.26	2.0E-01	-0.05	7.9E-01	-0.05	7.9E-01
ENSCAFG0000000309	COMMD1	darkgrey	EC_M8	0.76	6.7E-06	0.20	3.2E-01	-0.11	5.9E-01	0.13	5.1E-01	-0.56	3.0E-03	0.17	4.1E-01	-0.46	1.7E-02	-0.28	1.6E-01	-0.21	3.1E-01	0.14	4.9E-01
ENSCAFG0000000595	TPP2	darkgrey	EC_M8	0.76	6.9E-06	-0.01	9.7E-01	-0.71	5.4E-05	0.03	8.7E-01	0.19	3.5E-01	0.16	4.5E-01	-0.25	2.2E-01	0.01	9.7E-01	-0.34	8.6E-02	-0.60	1.1E-03
ENSCAFG0000001610	LIN9	darkgrey	EC_M8	0.76	6.9E-06	0.41	3.7E-02	-0.45	2.2E-02	-0.33	1.1E-01	-0.17	4.2E-01	0.10	6.2E-01	-0.20	3.4E-01	-0.04	8.6E-01	-0.22	2.8E-01	-0.22	2.8E-01
ENSCAFG0000000341	EZH2	darkgrey	EC_M8	0.76	7.0E-06	0.58	1.7E-03	-0.35	8.3E-02	-0.03	8.7E-01	-0.36	7.3E-02	-0.06	7.7E-01	-0.08	6.9E-01	-0.27	1.8E-01	-0.21	3.0E-01	-0.03	8.8E-01
ENSCAFG0000000787	ENSCAFG0000000787	darkgrey	EC_M8	0.76	7.1E-06	0.09	6.7E-01	-0.22	2.8E-01	0.32	1.1E-01	-0.38	5.8E-02	0.10	6.3E-01	-0.30	1.3E-01	-0.31	1.3E-01	-0.55	3.8E-03	-0.02	9.2E-01
ENSCAFG0000000825	UTP15	darkgrey	EC_M8	0.76	7.3E-06	0.08	7.0E-01	-0.59	1.5E-03	0.00	9.9E-01	0.09	6.6E-01	0.20	3.3E-01	-0.43	2.9E-02	-0.24	2.3E-01	-0.25	2.1E-01	-0.49	1.1E-02
ENSCAFG0000002037	ZZZ3	darkgrey	EC_M8	0.76	7.4E-06	0.08	7.0E-01	-0.48	1.3E-02	0.05	8.2E-01	-0.14	4.8E-01	0.24	2.3E-01	-0.51	8.4E-03	-0.13	5.2E-01	-0.29	1.4E-01	-0.29	1.5E-01
ENSCAFG0000000879	NAA25	darkgrey	EC_M8	0.76	7.4E-06	0.11	6.0E-01	-0.63	5.1E-04	-0.04	8.4E-01	0.15	4.5E-01	0.26	1.9E-01	-0.15	4.7E-01	-0.20	3.3E-01	-0.31	1.2E-01	-0.54	4.5E-03
ENSCAFG0000000729	PARPBP	darkgrey	EC_M8	0.76	7.4E-06	-0.09	6.7E-01	-0.24	2.4E-01	-0.06	7.8E-01	-0.21	3.1E-01	0.24	2.3E-01	-0.33	9.8E-02	-0.32	1.2E-01	-0.23	2.7E-01	-0.17	4.2E-01
ENSCAFG0000000434	SETDB2	darkgrey	EC_M8	0.76	7.5E-06	0.27	1.8E-01	-0.71	5.7E-05	0.08	6.8E-01	0.03	8.7E-01	0.26	2.1E-01	-0.11	5.8E-01	0.06	7.8E-01	-0.14	4.9E-01	-0.45	2.0E-02
ENSCAFG0000000735	BTAF1	darkgrey	EC_M8	0.76	7.5E-06	0.09	6.7E-01	-0.59	1.5E-03	0.05	8.2E-01	0.03	8.9E-01	0.38	5.6E-02	-0.29	1.6E-01	-0.37	6.4E-02	-0.59	1.7E-03	-0.47	1.5E-02

ENSCAFG0000000861	DSN1	darkgrey	EC_M8	0.76	7.7E-06	0.16	4.3E-01	-0.62	7.1E-04	-0.01	9.5E-01	0.15	4.6E-01	0.27	1.8E-01	-0.24	2.4E-01	-0.05	8.1E-01	-0.09	6.6E-01	-0.53	5.7E-03
ENSCAFG0000001289	HAT1	darkgrey	EC_M8	0.76	8.0E-06	0.05	7.9E-01	-0.62	7.6E-04	-0.20	3.2E-01	0.13	5.1E-01	0.25	2.1E-01	-0.18	3.8E-01	-0.12	5.7E-01	-0.04	8.5E-01	-0.50	9.8E-03
ENSCAFG0000000031	ENSCAFG0000000031	darkgrey	EC_M8	0.76	8.2E-06	-0.23	2.5E-01	-0.61	9.9E-04	-0.04	8.4E-01	0.19	3.5E-01	0.41	3.6E-02	-0.33	9.9E-02	0.09	6.8E-01	-0.19	3.4E-01	-0.60	1.2E-03
ENSCAFG0000000286	PPP4R3B	darkgrey	EC_M8	0.75	8.3E-06	0.38	5.4E-02	-0.25	2.2E-01	0.20	3.4E-01	-0.50	9.0E-03	0.09	6.5E-01	-0.20	3.3E-01	-0.28	1.6E-01	-0.25	2.1E-01	0.10	6.1E-01
ENSCAFG0000001682	CCT3	darkgrey	EC_M8	0.75	8.5E-06	-0.15	4.5E-01	-0.59	1.6E-03	-0.12	5.7E-01	0.21	3.1E-01	0.23	2.7E-01	-0.29	1.5E-01	-0.05	7.9E-01	-0.26	2.1E-01	-0.60	1.3E-03
ENSCAFG0000001726	ENSCAFG0000001726	darkgrey	EC_M8	0.75	8.6E-06	0.09	6.5E-01	-0.26	2.0E-01	-0.17	4.2E-01	-0.24	2.3E-01	0.13	5.4E-01	-0.31	1.3E-01	-0.01	9.6E-01	-0.20	3.2E-01	-0.16	4.5E-01
ENSCAFG0000000944	TRIM33	darkgrey	EC_M8	0.75	8.6E-06	0.10	6.2E-01	-0.18	3.8E-01	0.04	8.4E-01	-0.44	2.4E-02	0.04	8.3E-01	-0.40	4.1E-02	-0.15	4.8E-01	-0.31	1.2E-01	0.02	9.4E-01
ENSCAFG0000000233	MELK	darkgrey	EC_M8	0.75	8.8E-06	0.18	3.8E-01	-0.30	1.4E-01	-0.18	3.8E-01	-0.23	2.6E-01	0.10	6.4E-01	-0.28	1.7E-01	-0.17	3.9E-01	-0.22	2.7E-01	-0.16	4.4E-01
ENSCAFG0000000368	ORC6	darkgrey	EC_M8	0.75	8.8E-06	-0.01	9.5E-01	-0.43	2.8E-02	-0.04	8.3E-01	-0.04	8.5E-01	0.35	7.9E-02	-0.20	3.4E-01	-0.23	2.7E-01	-0.17	4.1E-01	-0.37	6.2E-02
ENSCAFG0000000495	DDX18	darkgrey	EC_M8	0.75	9.0E-06	0.20	3.3E-01	-0.70	7.0E-05	-0.29	1.4E-01	0.21	3.1E-01	0.22	2.8E-01	-0.36	7.4E-02	-0.05	8.1E-01	-0.07	7.3E-01	-0.58	1.9E-03
ENSCAFG0000000571	E2F7	darkgrey	EC_M8	0.75	9.0E-06	-0.04	8.4E-01	-0.21	3.0E-01	0.10	6.2E-01	-0.27	1.8E-01	0.20	3.2E-01	-0.38	5.5E-02	-0.05	8.0E-01	-0.31	1.2E-01	-0.14	4.9E-01
ENSCAFG0000001773	BRIP1	darkgrey	EC_M8	0.75	9.1E-06	0.03	8.9E-01	-0.09	6.5E-01	0.13	5.2E-01	-0.45	2.0E-02	0.05	8.1E-01	-0.44	2.6E-02	0.00	9.9E-01	-0.18	3.8E-01	0.07	7.5E-01
ENSCAFG0000001784	G3BP1	darkgrey	EC_M8	0.75	9.2E-06	0.04	8.5E-01	-0.49	1.1E-02	-0.04	8.3E-01	0.04	8.6E-01	0.15	4.6E-01	-0.28	1.6E-01	0.09	6.6E-01	-0.06	7.7E-01	-0.40	4.1E-02
ENSCAFG0000001057	U2AF1	darkgrey	EC_M8	0.75	9.2E-06	0.42	3.4E-02	-0.41	3.7E-02	-0.17	4.1E-01	-0.21	3.0E-01	0.25	2.1E-01	-0.04	8.6E-01	-0.32	1.2E-01	-0.32	1.1E-01	-0.19	3.4E-01
ENSCAFG0000001282	EDRF1	darkgrey	EC_M8	0.75	9.3E-06	0.14	5.1E-01	-0.64	4.4E-04	0.06	7.8E-01	0.02	9.1E-01	0.32	1.1E-01	-0.26	2.0E-01	-0.14	4.9E-01	-0.28	1.7E-01	-0.44	2.3E-02
ENSCAFG0000001734	THG1L	darkgrey	EC_M8	0.75	1.0E-05	0.16	4.4E-01	-0.46	1.9E-02	0.37	6.1E-02	-0.11	6.0E-01	0.25	2.2E-01	-0.42	3.1E-02	-0.07	7.5E-01	-0.24	2.4E-01	-0.31	1.2E-01
ENSCAFG0000002039	RABGGTB	darkgrey	EC_M8	0.75	1.0E-05	0.25	2.2E-01	-0.35	7.6E-02	-0.06	7.8E-01	-0.30	1.4E-01	0.14	5.0E-01	-0.13	5.4E-01	-0.01	9.5E-01	-0.38	5.6E-02	-0.12	5.7E-01
ENSCAFG0000001495	RPL22L1	darkgrey	EC_M8	0.75	1.0E-05	-0.23	2.5E-01	-0.30	1.4E-01	0.00	1.0E+00	-0.17	4.1E-01	0.25	2.3E-01	-0.13	5.2E-01	0.00	9.9E-01	-0.22	2.8E-01	-0.27	1.9E-01
ENSCAFG0000000252	LRPPRC	darkgrey	EC_M8	0.75	1.0E-05	-0.30	1.4E-01	-0.09	6.5E-01	0.02	9.0E-01	-0.37	6.3E-02	0.10	6.4E-01	-0.29	1.5E-01	-0.12	5.5E-01	-0.38	5.3E-02	-0.03	8.7E-01
ENSCAFG0000000587	ALYREF	darkgrey	EC_M8	0.75	1.0E-05	0.15	4.7E-01	-0.47	1.6E-02	-0.04	8.6E-01	-0.02	9.2E-01	0.12	5.6E-01	-0.18	3.7E-01	-0.11	6.1E-01	-0.29	1.5E-01	-0.36	7.4E-02
ENSCAFG0000000795	APPL1	grey	EC_M10	0.75	1.1E-05	0.05	8.0E-01	-0.51	7.2E-03	0.11	5.8E-01	-0.02	9.2E-01	-0.04	8.6E-01	-0.48	1.2E-02	-0.11	6.1E-01	-0.25	2.2E-01	-0.38	5.7E-02
ENSCAFG0000001578	CEP170	darkgrey	EC_M8	0.75	1.1E-05	0.19	3.5E-01	-0.52	6.3E-03	-0.08	7.0E-01	-0.12	5.6E-01	0.24	2.3E-01	-0.27	1.8E-01	-0.04	8.3E-01	-0.11	5.8E-01	-0.30	1.4E-01
ENSCAFG0000002001	CENPN	darkgrey	EC_M8	0.75	1.1E-05	0.19	3.6E-01	-0.38	5.6E-02	-0.19	3.6E-01	-0.12	5.5E-01	0.03	8.7E-01	-0.16	4.2E-01	-0.13	5.3E-01	-0.23	2.5E-01	-0.27	1.8E-01
ENSCAFG0000001196	TICRR	darkgrey	EC_M8	0.75	1.1E-05	0.11	5.9E-01	-0.14	5.0E-01	0.08	6.9E-01	-0.40	4.2E-02	0.08	6.9E-01	-0.16	4.4E-01	-0.21	3.1E-01	-0.19	3.4E-01	0.04	8.6E-01
ENSCAFG0000000815	GCFC2	darkgrey	EC_M8	0.75	1.1E-05	0.27	1.8E-01	-0.25	2.1E-01	-0.02	9.4E-01	-0.45	2.3E-02	0.11	5.8E-01	-0.41	3.7E-02	-0.08	7.1E-01	-0.07	7.3E-01	0.03	9.0E-01
ENSCAFG0000000836	ENSCAFG0000000836	darkgrey	EC_M8	0.75	1.2E-05	0.24	2.5E-01	-0.27	1.9E-01	-0.18	3.9E-01	-0.28	1.6E-01	0.00	1.0E+00	-0.28	1.7E-01	-0.09	6.7E-01	-0.12	5.5E-01	-0.09	6.7E-01
ENSCAFG0000000218	CEP135	grey	EC_M10	0.75	1.2E-05	-0.05	8.3E-01	-0.26	1.9E-01	-0.05	8.0E-01	-0.28	1.6E-01	0.07	7.2E-01	-0.24	2.4E-01	-0.36	7.1E-02	-0.53	5.5E-03	-0.10	6.2E-01
ENSCAFG0000001215	NUP133	darkgrey	EC_M8	0.75	1.2E-05	0.47	1.4E-02	-0.37	6.3E-02	0.03	8.7E-01	-0.34	9.2E-02	-0.02	9.1E-01	-0.26	2.0E-01	-0.24	2.5E-01	-0.16	4.4E-01	-0.03	8.8E-01
ENSCAFG0000001150	NOL11	darkgrey	EC_M8	0.75	1.2E-05	0.15	4.6E-01	-0.44	2.3E-02	-0.08	7.1E-01	-0.05	8.0E-01	0.34	9.1E-02	-0.34	8.9E-02	0.08	7.0E-01	-0.28	1.7E-01	-0.35	8.4E-02
ENSCAFG0000000189	XPO5	darkgrey	EC_M8	0.75	1.2E-05	0.17	3.9E-01	-0.14	5.0E-01	0.01	9.8E-01	-0.48	1.3E-02	-0.21	3.1E-01	-0.15	4.6E-01	-0.11	5.8E-01	-0.22	2.8E-01	0.06	7.8E-01
ENSCAFG0000000046	POP1	darkgrey	EC_M8	0.75	1.2E-05	-0.06	7.9E-01	-0.40	4.5E-02	0.00	9.8E-01	-0.05	8.0E-01	0.23	2.6E-01	-0.40	4.5E-02	-0.11	5.8E-01	-0.07	7.2E-01	-0.35	8.0E-02
ENSCAFG0000001071	KCTD3	darkgrey	EC_M8	0.75	1.2E-05	0.40	4.4E-02	-0.56	3.0E-03	-0.06	7.5E-01	-0.09	6.8E-01	0.21	3.1E-01	-0.11	6.1E-01	-0.11	5.9E-01	-0.27	1.8E-01	-0.31	1.2E-01
ENSCAFG0000000641	WRN	grey	EC_M10	0.75	1.3E-05	-0.18	3.7E-01	-0.11	5.9E-01	0.09	6.5E-01	-0.41	3.9E-02	0.03	9.0E-01	-0.10	6.2E-01	-0.08	7.1E-01	-0.30	1.4E-01	0.03	8.8E-01
ENSCAFG0000002005	ODF2	darkgrey	EC_M8	0.75	1.3E-05	0.30	1.4E-01	-0.40	4.4E-02	0.07	7.5E-01	-0.21	3.0E-01	0.13	5.4E-01	-0.29	1.6E-01	-0.04	8.5E-01	-0.35	8.3E-02	-0.19	3.6E-01
ENSCAFG0000001832	THOC1	darkgrey	EC_M8	0.74	1.3E-05	0.52	6.6E-03	-0.17	4.1E-01	0.09	6.8E-01	-0.62	8.2E-04	-0.04	8.6E-01	-0.19	3.6E-01	-0.13	5.3E-01	-0.18	3.9E-01	0.23	2.7E-01
ENSCAFG0000002371	SARNP	darkgrey	EC_M8	0.74	1.3E-05	0.16	4.3E-01	-0.81	5.6E-07	0.05	8.1E-01	0.25	2.2E-01	0.37	6.3E-02	0.02	9.1E-01	-0.18	3.9E-01	-0.21	2.9E-01	-0.67	1.9E-04
ENSCAFG0000003032	ENSCAFG0000003032	darkgrey	EC_M8	0.74	1.3E-05	0.04	8.4E-01	-0.14	5.0E-01	-0.09	6.7E-01	-0.38	5.6E-02	0.12	5.7E-01	-0.26	1.9E-01	-0.09	6.7E-01	-0.09	6.5E-01	-0.01	9.6E-01
ENSCAFG0000001371	DNA2	darkgrey	EC_M8	0.74	1.3E-05	0.07	7.2E-01	-0.47	1.4E-02	0.01	9.6E-01	0.03	8.9E-01	0.39	5.2E-02	-0.18	3.9E-01	-0.17	4.1E-01	-0.19	3.4E-01	-0.39	4.7E-02
ENSCAFG0000001564	MAPK6	darkgrey	EC_M8	0.74	1.4E-05	0.46	1.8E-02	-0.87	1.1E-08	-0.02	9.0E-01	0.21	3.0E-01	0.17	4.0E-01	-0.03	8.7E-01	-0.12	5.6E-01	-0.22	2.9E-01	-0.63	6.3E-04
ENSCAFG0000000038	MED23	grey	EC_M10	0.74	1.4E-05	0.49	1.2E-02	-0.39	4.7E-02	-0.05	8.2E-01	-0.33	9.9E-02	0.10	6.3E-01	-0.31	1.3E-01	-0.32	1.1E-01	-0.34	8.5E-02	-0.07	7.4E-01
ENSCAFG0000000454	TRDMT1	darkgrey	EC_M8	0.74	1.4E-05	-0.10	6.3E-01	-0.34	9.0E-02	-0.05	7.9E-01	-0.12	5.6E-01	0.36	7.4E-02	-0.30	1.4E-01	-0.36	7.5E-02	-0.08	7.0E-01	-0.27	1.9E-01
ENSCAFG0000002919	STMN1	darkgrey	EC_M8	0.74	1.4E-05	0.55	3.5E-03	-0.30	1.4E-01	-0.12	5.5E-01	-0.36	7.2E-02	0.05	8.1E-01	-0.19	3.6E-01	-0.31	1.2E-01	-0.15	4.7E-01	-0.02	9.2E-01
ENSCAFG0000001657	LAS1L	darkgrey	EC_M8	0.74	1.5E-05	0.16	4.3E-01	-0.30	1.4E-01	-0.01	9.8E-01	-0.28	1.7E-01	0.22	2.8E-01	-0.31	1.3E-01	-0.27	1.8E-01	0.00	9.8E-01	-0.11	5.9E-01
ENSCAFG0000001083	RRP15	darkgrey	EC_M8	0.74	1.5E-05	0.00	1.0E+00	-0.60	1.2E-03	0.07	7.3E-01	0.08	7.1E-01	0.25	2.2E-01	-0.25	2.2E-01	0.03	8.7E-01	-0.26	2.1E-01	-0.49	1.1E-02
ENSCAFG0000000798	SUCLG1	darkgrey	EC_M8	0.74	1.5E-05	-0.27	1.8E-01	-0.37	6.1E-02	0.02	9.3E-01	-0.05	8.0E-01	0.13	5.2E-01	-0.10	6.3E-01	0.10	6.4E-01	-0.35	7.9E-02	-0.33	1.0E-01
ENSCAFG0000000533	NKTR	darkgrey	EC_M8	0.74	1.5E-05	0.46	1.8E-02	-0.56	2.9E-03	0.19	3.5E-01	-0.17	4.0E-01	0.16	4.2E-01	-0.28	1.7E-01	0.08	6.8E-01	-0.13	5.3E-01	-0.25	2.1E-01
ENSCAFG0000001008	CCT5	darkgrey	EC_M8	0.74	1.5E-05	-0.15	4.6E-01	-0.65	3.2E-04	-0.02	9.2E-01	0.28	1.7E-01	0.24	2.4E-01	-0.33	9.7E-02	-0.03	8.9E-01	-0.32	1.1E-01	-0.68	1.5E-04
ENSCAFG0000000199	CDC5L	grey	EC_M10	0.74	1.5E-05	-0.10	6.1E-01	-0.13	5.3E-01	0.02	9.3E-01	-0.40	4.4E-02	0.07	7.3E-01	-0.24	2.4E-01	-0.01	9.7E-01	-0.49	1.0E-02	0.02	9.3E-01
ENSCAFG0000000296	CYB5R4	darkgrey	EC_M8	0.74	1.5E-05	0.27	1.9E-01	-0.46	2.0E-02	0.04	8.6E-01	-0.20	3.3E-01	0.19	3.4E-01	-0.43	2.8E-02	-0.29	1.6E-01	-0.14	4.9E-01	-0.21	2.9E-01
ENSCAFG0000001503	DCUN1D5	darkgrey	EC_M8	0.74	1.5E-05	0.35	8.1E-02	-0.75	1.2E-05	0.00	9.9E-01	0.17	4.1E-01	0.42	3.3E-02	-0.25	2.3E-01	-0.17	4.0E-01	-0.18	3.7E-01	-0.57	2.3E-03
ENSCAFG0000003047	ABCF1	darkgrey	EC_M8	0.74	1.5E-05	0.12	5.7E-01	-0.47	1.4E-02	0.06	7.7E-01	-0.01	9.6E-01	0.16	4.4E-01	-0.27	1.8E-01	-0.23	2.6E-01	-0.23	2.6E-01	-0.36	6.8E-02
ENSCAFG0000001216	EWSR1	darkgrey	EC_M8	0.74	1.5E-05	0.43	3.0E-02	-0.20	3.2E-01	0.01	9.6E-01	-0.51	7.3E-03	0.11	5.9E-01	0.00	9.8E-01	-0.13	5.2E-01	-0.12	5.4E-01	0.09	6.5E-01
ENSCAFG0000001887	HPRT1	darkgrey	EC_M8	0.74	1.6E-05	0.48	1.3E-02	-0.55	3.6E-03	-0.18	3.7E-01	-0.14	5.0E-01	-0.07	7.3E-01	-0.17	4.1E-01	0.31	1.3E-01	-0.03	8.8E-01	-0.25	2.2E-01
ENSCAFG0000000410	CEP57	darkgrey	EC_M8	0.74	1.6E-05	0.40	4.5E-02	-0.40	4.1E-02	0.21	3.0E-01	-0.33	1.0E-01	0.11	6.1E-01	-0.09	6.5E-01	-0.17	3.9E-01	-0.29	1.5E-01	-0.10	6.2E-01
ENSCAFG0000000522	FAM208B	grey	EC_M10	0.74	1.6E-05	0.28	1.7E-01	-0.72	3.1E-05	-0.09	6.5E-01	0.13	5.3E-01	0.19	3.6E-01	-0.23	2.6E-01	-0.19	3.6E-01	-0.42	3.2E-02	-0.56	3.2E-03
ENSCAFG0000000323	ETAA1	darkgrey	EC_M8	0.74	1.7E-05	0.31	1.2E-01	-0.20	3.3E-01	0.22	2.9E-01	-0.55	3.4E-03	0.13	5.3E-01	-0.20	3.3E-01	-0.27	1.8E-01	-0.30	1.3E-01	0.13	5.3E-01
ENSCAFG0000000322	ENSCAFG0000000322	darkgrey	EC_M8	0.74	1.7E-05	0.26	2.0E-01	-0.66	2.2E-04	-0.13	5.2E-01	0.13	5.2E-01	0.36	7.5E-02	0.02	9.1E-01	-0.08	6.9E-01	-0.41	3.8E-02	-0.53	5.8E-03
ENSCAFG0000000859	LRRCC1	darkgrey	EC_M8	0.74	1.7E-05	0.40	4.6E-02	-0.73	2.3E-05	-0.05	8.0E-01	0.06	7.6E-01	0.12	5.7E-01	-0.22	2.8E-01	-0.15	4.8E-01	-0.34	8.9E-02	-0.47	1.6E-02
ENSCAFG0000001797	PRPS1	darkgrey	EC_M8	0.74	1.7E-05	0.03	8.9E-01	-0.45	2.1E-02	-0.05	8.1E-01	0.01	9.6E-01	0.31	1.2E-01	-0.15	4.5E-01	-0.32	1.1E-01	-0.29	1.5E-01	-0.39	4.8E-02
ENSCAFG0000002960	FOXN2	darkgrey	EC_M8	0.74	1.7E-05	0.56	2.9E-03	-0.45	2.1E-02	0.04	8.6E-01	-0.33	9.7E-02	0.12	5.7E-01	-0.23	2.6E-01	-0.15	4.8E-01	-0.22	2.9E-01	-0.05	8.0E-01
ENSCAFG0000003053	LLPH	darkgrey	EC_M8	0.74	1.7E-05	0.22	2.8E-01	-0.61	9.6E-04	-0.17	4.0E-01	0.02	9.3E-01	0.26	1.9E-01	-0.22	2.9E-01	0.13	5.3E-01	-0.36	7.2E-02	-0.44	2.6E-02
ENSCAFG0000000475	MCM10	darkgrey	EC_M8	0.74	1.7E-05	0.07	7.5E-01	-0.60	1.2E-03	-0.29	1.5E-01	0.16	4.3E-01	0.13	5.4E-01	-0.13	5.2E-01	0.03	9.0E-01	-0.09	6.4E-01	-0.56	3.2E-03
ENSCAFG0000000861	TROAP	darkgrey	EC_M8	0.74	1.7E-05	0.22	2.8E-01	-0.18	3.7E-01	-0.04	8.6E-01	-0.34	8.5E-02	0.05	8.0E-01	-0.18	3.7E-01	-0.07	7.3E-01	-0.07	7.4E-01	0.00	9.9E-01
ENSCAFG0000000593	STRN	darkgrey	EC_M8	0.74	1.8E-05	0.51	7.3E-03	-0.65	2.8E-04	-0.04	8.5E-01	-0.03	9.0E-01	0.04	8.4E-01	-0.13	5.4E-01	-0.16	4.3E-01	-0.28	1.6E-01	-0.36	7.0E-02
ENSCAFG0000000276	FSD1L	darkgrey	EC_M8	0.74	1.8E-05	0.38	5.4E-02	-0.46	1.9E-02	0.05	8.1E-01	-0.18	3.9E-01	0.04	8.5E-01	-0.09	6.7E-01	-0.02	9.1E-01	-0.41	3.8E-02	-0.20	3.3E-01
ENSCAFG0000001868	NUP155	darkgrey	EC_M8	0.74	1.8E-05	-0.19	3.5E-01	-0.61	1.0E-03	0.01	9.7E-01	0.22	2.9E-01	0.37	6.3E-02	-0.05	8.2E-01	-0.13	5.2E-01	-0.32	1.1E-01	-0.61	9.0E-04
ENSCAFG0000001776	ENSCAFG0000001776	darkgrey	EC_M8	0.74	1.8E-05	0.33	1.0E-01	-0.55	4.0E-03	0.24	2.4E-01	-0.03	8.7E-01	0.09	6.5E-01	-0.30	1.3E-01	-0.07	7.2E-01	-0.47	1.6E-02	-0.37	6.4E-02
ENSCAFG0000003097	ENSCAFG0000003097	darkgrey	EC_M8	0.74	1.8E-05	0.33	9.7E-02	-0.19	3.5E-01	0.14	5.0E-01	-0.50	9.0E-03	0.14	5.0E-01	-0.26	2.0E-01	-0.43	2.7E-02	-0.33	9.6E-02	0.12	5.6E-01
ENSCAFG0000000643	LARS	darkgrey	EC_M8	0.74	1.9E-05	-0.31	1.3E-01	-0.24	2.3E-01	0.08	7.1E-01	-0.19	3.6E-01	0.23	2.5E-01	-0.43	2.9E-02	-0.14	5.0E-01	-0.36	7.1E-02	-0.25	2.1E-01
ENSCAFG0000002021	DHODH	darkgrey	EC_M8	0.74	1.9E-05	-0.14	5.1E-01	-0.21	2.9E-01	-0.20	3.4E-01	-0.22	2.7E-01	0.01	9.5E-01	-0.27	1.8E-01	-0.06	7.7E-01	-0.20	3.2E-01	-0.18	3.8E-01
ENSCAFG0000001890	MYSM1	darkgrey	EC_M8	0.73	1.9E-05	0.22	2.8E-01	-0.50	9.7E-03	0.19	3.4E-01	-0.14	4.9E-01	0.24	2.4E-01	-0.31	1.3E-01	-0.01	9.7E-01	-0.11	5.9E-01	-0.24	2.4E-01
ENSCAFG0000000909	KNSTRN	darkgrey	EC_M8	0.73	2.0E-05	0.32	1.2E-01	-0.20	3.3E-01	-0.03	8.7E-01	-0.40	4.1E-02	-0.15	4.8E-01	-0.15	4.7E-01	-0.11	5.9E-01	-0.05	8.2E-01	0.03	8.8E-01
ENSCAFG0000002941	ENSCAFG0000002941	darkgrey	EC_M8	0.73	2.0E-05	0.08	6.8E-01	-0.48	1.3E-02	0.11	6.0E-01	-0.02	9.1E-01	0.19	3.6E-01	-0.27	1.8E-01	-0.36	7.4E-02	-0.20	3.2E-01	-0.37	5.9E-02
ENSCAFG0000000671	DHX29	darkgrey	EC_M8	0.73	2.0E-05	-0.25	2.3E-01	-0.37	6.4E-02	-0.01	9.6E-01	-0.07	7.2E-01	0.29	1.5E-01	-0.44	2.5E-02	0.03	8.7E-01	-0.07	7.2E-01	-0.33	1.0E-01
ENSCAFG0000001937	NCBP3	darkgrey	EC_M8	0.73	2.0E-05	0.09	6.8E-01	-0.58	2.1E-03	-0.01	9.8E-01	0.04	8.6E-01	0.26	1.9E-01	-0.35	7.6E-02	-0.17	4.0E-01	-0.03	8.8E-01	-0.38	5.8E-02
ENSCAFG0000000149	TMEM209	darkgrey	EC_M8	0.73	2.1E-05	-0.17	4.0E-01	-0.20	3.2E-01	-0.03	8.7E-01	-0.26	1.9E-01	0.14	4.9E-01	-0.48	1.4E-02	0.02	9.3E-01	-0.44	2.6E-02	-0.15	4.7E-01
ENSCAFG0000001960	NOL9	darkgrey	EC_M8	0.73	2.1E-05	0.13	5.2E-01	-0.23	2.6E-01	-0.12	5.5E-01	-0.30	1.4E-01	0.01	9.7E-01	-0.29	1.5E-01	-0.05	7.9E-01	-0.31	1.3E-01	-0.10	6.2E-01
ENSCAFG0000001061	TIMM17A	darkgrey	EC_M8	0.73	2.1E-05	-0.08	7.1E-01	-0.62	7.1E-04	-0.05	8.2E-01	0.18	3.9E-01	0.19	3.6E-01	-0.37	5.9E-02	0.04	8.6E-01	-0.41	4.0E-02	-0.58	1.9E-03
ENSCAFG0000002997	OTUD6B	darkgrey	EC_M8	0.73	2.1E-05	0.01	9.8E-01	-0.76	7.0E-06	-0.07	7.3E-01	0.26	2.0E-01	0.29	1.5E-01	-0.33	9.7E-02	-0.01	9.4E-01	-0.25	2.1E-01	-0.65	3.0E-04
ENSCAFG0000001237	PPIG	darkgrey	EC_M8	0.73	2.1E-05	0.38	5.5E-02	-0.61	8.6E-04	0.02	9.4E-01	-0.07	7.2E-01	0.17	4.1E-01	-0.23	2.6E-01	0.12	5.6E-01	-0.13	5.3E-01	-0.30	1.3E-01
ENSCAFG0000001888	CEP192	grey	EC_M10	0.73	2.2E-05	0.02	9.1E-01	0.05	7.9E-01	0.07	7.2E-01	-0.66	2.6E-04	-0.14	5.1E-01	-0.34	9.4E-02	-0.20	3.3E-01	-0.26	2.1E-01	0.28	1.6E-01
ENSCAFG0000001937	CCNF	darkgrey	EC_M8	0.73	2.2E-05	0.11	6.0E-01	-0.33	9.9E-02	-0.12	5.5E-01	-0.13	5.3E-01	0.19	3.5E-01	-0.17	4.2E-01	-0.37	6.6E-02	-0.18	3.8E-01	-0.25	2.1E-01
ENSCAFG0000001678	GPATCH4	darkgrey	EC_M8	0.73	2.2E-05	-0.21	3.0E-01	-0.15	4.7E-01	0.02	9.2E-01	-0.29	1.5E-01	0.13	5.1E-01	-0.33	1.0E-01	-0.02	9.2E-01	-0.39	4.9E-02	-0.14	5.1E-01
ENSCAFG0000001554	POLR3A	darkgrey	EC_M8	0.73	2.3E-05	0.21	3.0E-01	-0.80	1.1E-06	-0.13	5.2E-01	0.29	1.5E-01	0.22	2.7E-01	-0.27	1.8E-01	-0.18	3.7E-01	-0.24	2.3E-01	-0.66	2.1E-04
ENSCAFG0000001277	FTSJ3	darkgrey	EC_M8	0.73	2.3E-05	-0.39	4.8E-02	-0.12	5.5E-01	0.17	4.1E-01	-0.28	1.7E-01	0.15	4.5E-01	-0.13	5.2E-01	-0.06	7.8E-01	-0.18	3.7E-01	-0.12	5.6E-01
ENSCAFG0000002973	BRMS1L	darkgrey	EC_M8	0.73	2.3E-05	0.03	8.8E-01	-0.31	1.3E-01	0.08	6.8E-01	-0.24	2.5E-01	0.27	1.8E-01	-0.17	4.2E-01	-0.08	7.0E-01	-0.14	4.9E-01	-0.13	5.1E-01
ENSCAFG0000000582	PRPF40A	darkgrey	EC_M8	0.73	2.4E-05	0.25	2.2E-01	-0.91	1.9E-10	-0.08	7.1E-01	0.36	6.9E-02	0.24	2.5E-01	-0.28	1.7E-01	0.05	8.1E-01	-0.13	5.4E-01	-0.74	1.8E-05
ENSCAFG0000001475	TFPI	darkgrey	EC_M8	0.73	2.4E-05	0.52	7.0E-03	-0.47	1.6E-02	-0.12	5.6E-01	-0.27	1.8E-01	0.09	6.7E-01	-0.13	5.2E-01	-0.08	7.1E-01	-0.25	2.3E-01	-0.17	4.2E-01
ENSCAFG0000001650	DHX15	darkgrey	EC_M8	0.73	2.4E-05	0.30	1.3E-01	-0.81	7.0E-07	-0.20	3.3E-01	0.30	1.4E-01	0.32	1.2E-01	-0.08	6.9E-01	-0.15	4.7E-01	-0.34	9.0E-02	-0.68	1.3E-04
ENSCAFG0000000423	ENSCAFG0000000423	grey	EC_M10	0.73	2.4E-05	-0.08	6.9E-01	-0.12	5.5E-01	0.14	5.0E-01	-0.43	2.9E-02	0.17	4.0E-01	-0.05	8.0E-01	-0.20	3.3E-01	-0.59	1.5E-03	0.02	9.4E-01
ENSCAFG0000001334	TRA2B	darkgrey	EC_M8	0.73	2.5E-05	0.16	4.5E-01	-0.32	1.1E-01	-0.25	2.2E-01	-0.22	2.8E-01	0.15	4.7E-01	-0.16	4.3E-01	-0.06	7.9E-01	-0.32	1.1E-01	-0.20	3.3E-01
ENSCAFG0000001021	KIF18A	darkgrey	EC_M8	0.73	2.5E-05	-0.08	7.1E-01	-0.10	6.2E-01	-0.02	9.2E-01	-0.41	3.7E-02	0.16	4.3E-01	-0.04	8.5E-01	-0.11	5.8E-01	-0.29	1.4E-01	0.00	9.8E-01
ENSCAFG0000000609	KITLG	darkgrey	EC_M8	0.73	2.5E-05	0.38	5.6E-02	-0.62	7.4E-04	0.08	6.9E-01	0.00	9.9E-01	0.13	5.3E-01	-0.08	6.9E-01	-0.13	5.4E-01	-0.22	2.8E-01	-0.42	3.3E-02
ENSCAFG0000000451	NASP	darkgrey	EC_M8	0.73	2.5E-05	0.58	2.1E-03	-0.70	6.8E-05	-0.02	9.1E-01	0.01	9.7E-01	0.20	3.4E-01	0.01	9.4E-01	-0.02	9.3E-01	-0.23	2.5E-01	-0.39	4.6E-02
ENSCAFG0000002447	ENSCAFG0000002447	darkgrey	EC_M8	0.73	2.6E-05	0.15	4.8E-01	-0.42	3.3E-02	0.12	5.6E-01	-0.16	4.4E-01	0.10	6.2E-01	-0.41	3.8E-02	-0.08	6.9E-01	-0.30	1.4E-01	-0.21	3.0E-01
ENSCAFG0000000932	RIPK1	darkgrey	EC_M8	0.73	2.6E-05	0.42	3.3E-02	-0.53	5.6E-03	-0.28	1.6E-01	-0.09	6.7E-01	0.10	6.2E-01	-0.36	6.8E-02	0.02	9.2E-01	-0.23	2.6E-01	-0.31	1.2E-01
ENSCAFG0000001014	CCT6A	darkgrey	EC_M8	0.73	2.6E-05	0.14	5.0E-01	-0.84	1.1E-07	0.00	9.9E-01	0.35	8.3E-02	0.11	5.9E-01	-0.22	2.8E-01	0.04	8.3E-01	-0.12	5.7E-01	-0.70	6.2E-05
ENSCAFG0000001361	HNRNPH3	darkgrey	EC_M8	0.73	2.7E-05	0.35	7.5E-02	-0.46	1.8E-02	-0.11	6.1E-01	-0.18	3.9E-01	0.08	6.8E-01	0.25	2.2E-01	0.10	6.2E-01	-0.04	8.6E-01	-0.19	3.5E-01
ENSCAFG0000000651	EML4	turquoise	EC_M6	0.73	2.7E-05	0.60	1.2E-03	-0.68	1.4E-04	-0.02	9.1E-01	-0.02	9.2E-01	0.01	9.5E-01	-0.27	1.8E-01	-0.09	6.7E-01	-0.19	3.5E-01	-0.37	6.0E-02
ENSCAFG0000000566	ENSCAFG0000000566	darkgrey	EC_M8	0.73	2.7E-05	0.14	5.1E-01	-0.47	1.5E-02	0.23	2.6E-01	-0.16	4.5E-01	0.12	5.6E-01	-0.28	1.7E-01	-0.19	3.4E-01	-0.11	5.9E-01	-0.21	3.0E-01
ENSCAFG0000000323	NUP205	darkgrey	EC_M8	0.73	2.8E-05	0.18	3.9E-01	-0.71	5.3E-05	-0.08	6.8E-01	0.24	2.4E-01	0.29	1.5E-01	-0.32	1.1E-01	-0.28	1.6E-01	-0.22	2.9E-01	-0.64	4.0E-04
ENSCAFG0000000002	ZNF407	grey	EC_M10	0.72	2.9E-05	0.51	7.8E-03	-0.34	8.7E-02	0.19	3.5E-01	-0.38	5.5E-02	0.15	4.8E-01	-0.20	3.2E-01	-0.24	2.3E-01	-0.32	1.2E-01	0.01	9.7E-01
ENSCAFG0000000657	ENSCAFG0000000657	darkgrey	EC_M8	0.72	3.0E-05	0.40	4.3E-02	-0.79	1.6E-06	-0.12	5.5E-01	0.21	3.0E-01	0.10	6.2E-01	-0.39	4.8E-02	0.06	7.8E-01	-0.16	4.4E-01	-0.61	9.9E-04
ENSCAFG0000000265	MSH2	darkgrey	EC_M8	0.72	3.0E-05	0.02	9.3E-01	-0.03	8.7E-01	0.03	8.8E-01	-0.48	1.3E-02	-0.08	7.0E-01	-0.44	2.5E-02	-0.21	2.9E-01	-0.23	2.5E-01	0.14	4.9E-01
ENSCAFG0000001725	ATRX	darkgrey	EC_M8	0.72	3.0E-05	0.28	1.7E-01	-0.66	2.6E-04	-0.10	6.3E-01	0.05	8.1E-01	0.11	6.1E-01	-0.39	5.2E-02	-0.06	7.7E-01	-0.23	2.6E-01	-0.42	3.4E-02
ENSCAFG0000001821	EIF5	darkgrey	EC_M8	0.72	3.0E-05	0.21	3.1E-01	-0.86	1.4E-08	0.01	9.8E-01	0.36	6.8E-02	0.21	3.0E-01	-0.22	2.7E-01	-0.05	8.3E-01	-0.22	2.8E-01	-0.72	2.9E-05
ENSCAFG0000001962	ENSCAFG0000001962	darkgrey	EC_M8	0.72	3.2E-05	-0.26	2.0E-01	-0.20	3.2E-01	-0.14	5.0E-01	-0.19	3.5E-01	0.18	3.8E-01	-0.23	2.6E-01	-0.05	8.1E-01	-0.18	3.7E-01	-0.21	3.0E-01
ENSCAFG0000000638	BRCA2	darkgrey	EC_M8	0.72	3.2E-05	-0.08	7.1E-01	-0.09	6.5E-01	0.13	5.3E-01	-0.40	4.0E-02	0.05	8.3E-01	-0.02	9.2E-01	0.00	9.8E-01	-0.18	3.8E-01	0.04	8.3E-01
ENSCAFG0000003269	TFB2M	darkgrey	EC_M8	0.72	3.2E-05	-0.13	5.4E-01	-0.69	1.1E-04	-0.02	9.2E-01	0.29	1.5E-01	0.28	1.6E-01	-0.31	1.2E-01	-0.04	8.5E-01	-0.21	2.9E-01	-0.68	1.3E-04
ENSCAFG0000000869	PPID	darkgrey	EC_M8	0.72	3.2E-05	-0.28	1.7E-01	-0.59	1.5E-03	0.05	8.0E-01	0.20	3.2E-01	0.28	1.7E-01	-0.28	1.7E-01	-0.11	5.8E-01	-0.25	2.2E-01	-0.61	8.9E-04
ENSCAFG0000001645	ENSCAFG0000001645	darkgrey	EC_M8	0.72	3.2E-05	0.06	7.6E-01	-0.64	4.2E-04	0.04	8.3E-01	0.09	6.5E-01	0.20	3.3E-01	-0.21	3.0E-01	-0.02	9.0E-01	-0.41	3.8E-02	-0.48	1.4E-02
ENSCAFG0000001416	CWC22	darkgrey	EC_M8	0.72	3.2E-05	0.17	4.0E-01	-0.43	2.7E-02	-0.01	9.5E-01	-0.18	3.8E-01	0.25	2.3E-01	-0.40	4.1E-02	0.00	9.8E-01	0.05	8.2E-01	-0.18	3.7E-01
ENSCAFG0000000128	FANCC	darkgrey	EC_M8	0.72	3.2E-05	0.28	1.6E-01	-0.49	1.2E-02	-0.07	7.2E-01	-0.08	7.0E-01	-0.07	7.2E-01	-0.29	1.5E-01	0.14	4.9E-01	-0.18	3.8E-01	-0.31	1.3E-01
ENSCAFG0000000655	MAPK8	darkgrey	EC_M8	0.72	3.3E-05	0.21	3.0E-01	-0.71	4.3E-05	0.02	9.2E-01	0.17	4.1E-01	0.20	3.3E-01	-0.42	3.2E-02	-0.13	5.4E-01	-0.04	8.6E-01	-0.54	4.2E-03
ENSCAFG0000000403	PUS7	darkgrey	EC_M8	0.72	3.3E-05	-0.31	1.3E-01	-0.29	1.5E-01	-0.10	6.3E-01	-0.07	7.2E-01	0.11	5.9E-01	-0.35	8.1E-02	0.03	8.8E-01	-0.24	2.3E-01	-0.33	1.0E-01
ENSCAFG0000000299	ANKRD17	grey	EC_M10	0.72	3.3E-05	0.18	3.9E-01	-0.66	2.4E-04	-0.05	8.0E-01	0.14	5.0E-01	0.16	4.3E-01	-0.38	5.5E-02	-0.04	8.3E-01	-0.49	1.0E-02	-0.54	4.4E-03

ENSCAFG0000000413	EXOSC9	darkgrey	EC_M8	0.72	3.4E-05	-0.12	5.6E-01	-0.58	2.0E-03	-0.21	3.0E-01	0.13	5.2E-01	0.26	2.0E-01	-0.05	8.2E-01	-0.06	7.6E-01	-0.54	4.8E-03	-0.53	5.5E-03
ENSCAFG0000001011	UBE3A	darkgrey	EC_M8	0.72	3.5E-05	0.39	4.7E-02	-0.82	3.7E-07	-0.16	4.3E-01	0.20	3.3E-01	0.25	2.2E-01	-0.22	2.9E-01	-0.02	9.1E-01	-0.25	2.2E-01	-0.58	2.1E-03
ENSCAFG0000000584	ARL6IP6	darkgrey	EC_M8	0.72	3.5E-05	0.36	7.3E-02	-0.43	2.7E-02	-0.04	8.6E-01	-0.25	2.3E-01	0.28	1.7E-01	-0.15	4.5E-01	-0.19	3.5E-01	-0.35	7.7E-02	-0.14	4.8E-01
ENSCAFG0000000831	ENSCAFG0000000831	darkgrey	EC_M8	0.72	3.5E-05	0.24	2.4E-01	-0.80	9.1E-07	-0.06	7.8E-01	0.28	1.7E-01	0.36	6.7E-02	-0.04	8.3E-01	-0.01	9.7E-01	-0.10	6.3E-01	-0.67	1.8E-04
ENSCAFG0000000962	CWF19L1	darkgrey	EC_M8	0.72	3.5E-05	0.54	4.2E-03	-0.28	1.6E-01	-0.06	7.7E-01	-0.42	3.1E-02	-0.09	6.7E-01	-0.08	7.1E-01	0.02	9.3E-01	-0.07	7.3E-01	0.05	8.0E-01
ENSCAFG0000000645	ELK3	darkgrey	EC_M8	0.72	3.6E-05	0.30	1.4E-01	-0.30	1.4E-01	0.10	6.1E-01	-0.31	1.2E-01	0.07	7.3E-01	-0.10	6.4E-01	-0.39	4.8E-02	-0.44	2.5E-02	-0.10	6.4E-01
ENSCAFG0000000904	TENT2	darkgrey	EC_M8	0.72	3.6E-05	0.55	3.3E-03	-0.50	9.9E-03	0.10	6.3E-01	-0.24	2.3E-01	0.16	4.5E-01	-0.32	1.1E-01	-0.12	5.7E-01	-0.15	4.5E-01	-0.13	5.1E-01
ENSCAFG0000001076	SMC3	darkgrey	EC_M8	0.72	3.6E-05	0.10	6.4E-01	0.06	7.6E-01	0.20	3.4E-01	-0.73	2.7E-05	0.13	5.3E-01	-0.15	4.7E-01	-0.14	5.0E-01	-0.29	1.5E-01	0.34	8.5E-02
ENSCAFG0000003021	DTD2	darkgrey	EC_M8	0.72	3.6E-05	-0.28	1.6E-01	-0.16	4.2E-01	-0.09	6.7E-01	-0.24	2.4E-01	0.18	3.9E-01	-0.31	1.2E-01	-0.16	4.4E-01	-0.33	1.0E-01	-0.17	4.0E-01
ENSCAFG0000001025	NAP1L4	darkgrey	EC_M8	0.72	3.6E-05	0.05	8.3E-01	-0.61	8.5E-04	-0.24	2.4E-01	0.21	3.1E-01	0.15	4.7E-01	-0.12	5.7E-01	0.02	9.2E-01	-0.34	9.3E-02	-0.55	3.5E-03
ENSCAFG0000001772	INTS2	darkgrey	EC_M8	0.72	3.6E-05	0.08	7.0E-01	-0.23	2.6E-01	-0.05	8.1E-01	-0.35	7.6E-02	0.28	1.6E-01	-0.02	9.1E-01	-0.20	3.3E-01	-0.53	5.8E-03	-0.07	7.4E-01
ENSCAFG0000001760	MRPL22	darkgrey	EC_M8	0.72	3.6E-05	-0.12	5.6E-01	-0.40	4.3E-02	0.11	5.8E-01	-0.06	7.8E-01	0.20	3.3E-01	-0.21	2.9E-01	0.03	8.8E-01	-0.03	8.9E-01	-0.31	1.2E-01
ENSCAFG0000001881	AFG3L2	darkgrey	EC_M8	0.72	3.7E-05	-0.30	1.3E-01	-0.29	1.5E-01	-0.16	4.2E-01	-0.10	6.3E-01	0.15	4.8E-01	-0.22	2.8E-01	0.02	9.3E-01	-0.21	2.9E-01	-0.26	2.0E-01
ENSCAFG0000001705	RARS	darkgrey	EC_M8	0.72	3.8E-05	-0.11	6.0E-01	-0.71	5.3E-05	0.03	9.0E-01	0.32	1.1E-01	0.27	1.9E-01	-0.24	2.5E-01	-0.02	9.4E-01	-0.24	2.3E-01	-0.70	6.3E-05
ENSCAFG0000003207	ENSCAFG0000003207	darkgrey	EC_M8	0.72	3.8E-05	0.33	1.0E-01	-0.67	2.1E-04	-0.15	4.6E-01	0.07	7.3E-01	0.16	4.4E-01	-0.14	4.8E-01	-0.18	3.7E-01	-0.22	2.9E-01	-0.43	2.7E-02
ENSCAFG0000001887	ZC3H7A	darkgrey	EC_M8	0.72	3.9E-05	0.37	6.2E-02	-0.56	2.8E-03	0.06	7.8E-01	-0.10	6.2E-01	0.36	7.2E-02	-0.35	7.7E-02	-0.22	2.8E-01	-0.04	8.5E-01	-0.30	1.3E-01
ENSCAFG0000000380	NRDC	darkgrey	EC_M8	0.72	3.9E-05	0.42	3.3E-02	-0.79	1.3E-06	-0.12	5.7E-01	0.21	3.1E-01	0.12	5.7E-01	-0.41	3.6E-02	-0.14	4.9E-01	-0.20	3.4E-01	-0.57	2.1E-03
ENSCAFG0000001893	ENSCAFG0000001893	darkgrey	EC_M8	0.72	3.9E-05	-0.03	8.7E-01	-0.65	2.9E-04	0.00	9.9E-01	0.23	2.7E-01	0.09	6.8E-01	-0.48	1.4E-02	-0.01	9.6E-01	-0.28	1.7E-01	-0.60	1.1E-03
ENSCAFG0000000777	PRMT9	grey	EC_M10	0.72	4.0E-05	0.14	4.8E-01	-0.13	5.2E-01	0.31	1.2E-01	-0.50	8.7E-03	-0.25	2.1E-01	-0.31	1.3E-01	0.00	9.9E-01	-0.20	3.3E-01	0.15	4.7E-01
ENSCAFG0000001086	ENSCAFG0000001086	darkgrey	EC_M8	0.72	4.0E-05	-0.25	2.1E-01	-0.64	4.4E-04	-0.05	7.9E-01	0.29	1.5E-01	0.27	1.8E-01	-0.20	3.3E-01	-0.06	7.8E-01	-0.27	1.7E-01	-0.68	1.1E-04
ENSCAFG0000000421	MED17	darkgrey	EC_M8	0.72	4.0E-05	0.21	3.1E-01	-0.31	1.2E-01	0.24	2.5E-01	-0.29	1.5E-01	0.30	1.4E-01	-0.46	1.7E-02	-0.32	1.1E-01	-0.30	1.4E-01	-0.09	6.7E-01
ENSCAFG0000000743	FER	darkgrey	EC_M8	0.72	4.0E-05	0.38	5.8E-02	-0.69	1.0E-04	0.19	3.6E-01	0.03	8.7E-01	0.09	6.8E-01	-0.47	1.6E-02	0.00	1.0E+00	-0.13	5.3E-01	-0.42	3.3E-02
ENSCAFG0000000953	NRAS	darkgrey	EC_M8	0.71	4.1E-05	0.44	2.4E-02	-0.06	7.7E-01	0.14	5.1E-01	-0.67	2.0E-04	-0.14	5.1E-01	-0.23	2.7E-01	-0.19	3.7E-01	-0.33	9.9E-02	0.28	1.7E-01
ENSCAFG0000002976	PRPF38B	darkgrey	EC_M8	0.71	4.1E-05	0.47	1.4E-02	-0.32	1.1E-01	-0.10	6.3E-01	-0.41	4.0E-02	0.17	4.1E-01	-0.27	1.8E-01	0.05	8.2E-01	-0.28	1.7E-01	0.03	8.9E-01
ENSCAFG0000001395	KIF18B	darkgrey	EC_M8	0.71	4.2E-05	0.16	4.3E-01	-0.09	6.5E-01	-0.09	6.8E-01	-0.43	2.9E-02	0.11	5.8E-01	-0.24	2.3E-01	-0.11	5.8E-01	-0.05	8.0E-01	0.05	7.9E-01
ENSCAFG0000000856	AGPAT5	darkgrey	EC_M8	0.71	4.2E-05	0.35	8.1E-02	-0.40	4.3E-02	-0.09	6.5E-01	-0.21	3.1E-01	0.21	3.0E-01	-0.50	8.8E-03	-0.25	2.3E-01	-0.19	3.6E-01	-0.13	5.3E-01
ENSCAFG0000000141	SRSF3	darkgrey	EC_M8	0.71	4.3E-05	0.23	2.6E-01	-0.31	1.2E-01	-0.20	3.3E-01	-0.18	3.9E-01	-0.01	9.6E-01	-0.07	7.4E-01	-0.14	5.0E-01	-0.29	1.6E-01	-0.15	4.5E-01
ENSCAFG0000000899	NBN	darkgrey	EC_M8	0.71	4.3E-05	-0.19	3.6E-01	-0.40	4.1E-02	0.05	8.0E-01	-0.07	7.3E-01	0.29	1.5E-01	-0.46	1.9E-02	-0.01	9.6E-01	-0.09	6.6E-01	-0.33	9.4E-02
ENSCAFG0000001419	DGCR8	darkgrey	EC_M8	0.71	4.4E-05	0.43	2.9E-02	-0.44	2.5E-02	-0.04	8.4E-01	-0.27	1.8E-01	0.44	2.3E-02	-0.06	7.8E-01	-0.17	4.1E-01	-0.17	4.0E-01	-0.14	4.8E-01
ENSCAFG0000000974	UBE2C	darkgrey	EC_M8	0.71	4.5E-05	-0.06	7.7E-01	-0.22	2.8E-01	-0.10	6.1E-01	-0.21	3.0E-01	0.10	6.2E-01	-0.25	2.2E-01	0.02	9.1E-01	-0.03	8.7E-01	-0.17	4.1E-01
ENSCAFG0000001933	PKMYT1	darkgrey	EC_M8	0.71	4.5E-05	0.13	5.3E-01	-0.28	1.7E-01	-0.01	9.4E-01	-0.19	3.4E-01	0.04	8.4E-01	-0.29	1.5E-01	-0.11	6.1E-01	-0.01	9.6E-01	-0.16	4.4E-01
ENSCAFG0000003098	TSNAX-DISC1	darkgrey	EC_M8	0.71	4.5E-05	0.27	1.9E-01	-0.74	1.7E-05	-0.08	7.0E-01	0.13	5.2E-01	0.19	3.5E-01	-0.29	1.5E-01	0.03	8.9E-01	-0.12	5.5E-01	-0.51	8.1E-03
ENSCAFG0000001726	SPAG9	darkgrey	EC_M8	0.71	4.5E-05	0.51	7.7E-03	-0.27	1.8E-01	0.08	7.1E-01	-0.49	1.2E-02	-0.13	5.1E-01	-0.17	4.1E-01	-0.16	4.4E-01	-0.41	3.5E-02	0.08	6.8E-01
ENSCAFG0000001114	SNRNP40	darkgrey	EC_M8	0.71	4.6E-05	-0.11	6.0E-01	-0.37	6.0E-02	0.09	6.5E-01	-0.05	8.2E-01	0.18	3.9E-01	-0.24	2.4E-01	0.01	9.6E-01	-0.17	4.0E-01	-0.33	1.0E-01
ENSCAFG0000000546	SNX5	grey	EC_M10	0.71	4.6E-05	-0.03	8.8E-01	-0.13	5.3E-01	0.20	3.3E-01	-0.46	1.9E-02	0.13	5.3E-01	-0.13	5.3E-01	-0.04	8.5E-01	-0.04	8.5E-01	0.08	7.1E-01
ENSCAFG0000001641	EIF3B	darkgrey	EC_M8	0.71	4.6E-05	-0.12	5.5E-01	-0.55	3.7E-03	-0.12	5.6E-01	0.20	3.3E-01	0.23	2.7E-01	-0.33	1.0E-01	0.05	8.2E-01	-0.21	3.1E-01	-0.57	2.1E-03
ENSCAFG0000000498	BORA	darkgrey	EC_M8	0.71	4.6E-05	0.00	9.8E-01	-0.42	3.4E-02	-0.30	1.3E-01	-0.10	6.2E-01	0.01	9.8E-01	-0.23	2.5E-01	0.10	6.4E-01	-0.11	6.0E-01	-0.31	1.2E-01
ENSCAFG0000000833	TMEM161B	darkgrey	EC_M8	0.71	4.6E-05	0.29	1.5E-01	-0.41	4.0E-02	0.27	1.8E-01	-0.17	4.1E-01	0.10	6.3E-01	-0.15	4.8E-01	-0.20	3.2E-01	-0.14	4.9E-01	-0.15	4.7E-01
ENSCAFG0000000282	MTIF2	darkgrey	EC_M8	0.71	4.6E-05	0.05	7.9E-01	-0.41	3.7E-02	-0.10	6.3E-01	-0.08	6.8E-01	-0.04	8.3E-01	-0.26	2.0E-01	-0.23	2.5E-01	-0.09	6.5E-01	-0.25	2.1E-01
ENSCAFG0000001880	USP1	grey	EC_M10	0.71	4.7E-05	0.03	8.8E-01	-0.03	8.8E-01	0.21	3.1E-01	-0.50	9.9E-03	0.11	5.9E-01	-0.26	2.0E-01	-0.34	8.6E-02	-0.49	1.1E-02	0.13	5.1E-01
ENSCAFG0000000905	CKAP5	darkgrey	EC_M8	0.71	4.7E-05	-0.19	3.6E-01	-0.46	1.8E-02	0.16	4.4E-01	0.05	8.0E-01	0.15	4.8E-01	-0.27	1.7E-01	-0.09	6.7E-01	-0.31	1.2E-01	-0.40	4.1E-02
ENSCAFG0000000878	MSH3	darkgrey	EC_M8	0.71	4.9E-05	-0.25	2.1E-01	-0.43	2.8E-02	-0.02	9.3E-01	0.05	8.1E-01	0.01	9.7E-01	-0.41	3.5E-02	-0.14	4.8E-01	-0.32	1.1E-01	-0.45	2.2E-02
ENSCAFG0000000825	RBM12	darkgrey	EC_M8	0.71	4.9E-05	0.38	5.8E-02	-0.02	9.2E-01	0.11	5.9E-01	-0.62	7.5E-04	-0.09	6.6E-01	-0.39	4.9E-02	-0.16	4.4E-01	0.01	9.6E-01	0.26	2.1E-01
ENSCAFG0000000935	ASNSD1	darkgrey	EC_M8	0.71	4.9E-05	0.35	7.6E-02	-0.50	9.9E-03	0.00	9.9E-01	-0.12	5.6E-01	0.20	3.2E-01	-0.14	4.9E-01	0.01	9.7E-01	-0.27	1.9E-01	-0.22	2.8E-01
ENSCAFG0000001096	MRPS35	grey	EC_M10	0.71	5.0E-05	0.08	7.0E-01	-0.54	4.2E-03	0.12	5.6E-01	0.01	9.6E-01	0.26	2.0E-01	-0.37	6.2E-02	-0.09	6.7E-01	-0.35	7.7E-02	-0.44	2.4E-02
ENSCAFG0000000440	NINL	grey	EC_M10	0.71	5.0E-05	0.65	3.3E-04	-0.45	2.1E-02	-0.25	2.1E-01	-0.26	2.1E-01	0.08	7.0E-01	-0.12	5.6E-01	-0.11	6.1E-01	-0.14	4.9E-01	-0.11	5.9E-01
ENSCAFG0000001863	RICTOR	darkgrey	EC_M8	0.71	5.0E-05	0.42	3.2E-02	-0.30	1.4E-01	0.17	4.2E-01	-0.40	4.1E-02	0.01	9.8E-01	-0.30	1.3E-01	-0.37	6.1E-02	-0.33	9.6E-02	0.03	8.8E-01
ENSCAFG0000001731	ZWILCH	darkgrey	EC_M8	0.71	5.1E-05	0.02	9.3E-01	-0.73	2.5E-05	-0.21	3.1E-01	0.32	1.1E-01	0.14	4.9E-01	-0.25	2.1E-01	0.11	6.1E-01	-0.09	6.6E-01	-0.70	6.7E-05
ENSCAFG0000000044	MDC1	darkgrey	EC_M8	0.71	5.2E-05	0.27	1.8E-01	-0.54	4.8E-03	-0.05	8.1E-01	-0.05	8.2E-01	0.15	4.6E-01	-0.53	5.4E-03	0.18	3.9E-01	-0.15	4.7E-01	-0.34	8.5E-02
ENSCAFG0000000380	SCLT1	darkgrey	EC_M8	0.71	5.2E-05	0.13	5.4E-01	-0.53	5.0E-03	0.09	6.5E-01	0.02	9.4E-01	0.12	5.7E-01	-0.54	4.5E-03	0.03	8.9E-01	-0.04	8.6E-01	-0.38	5.2E-02
ENSCAFG0000000659	CBX5	darkgrey	EC_M8	0.71	5.3E-05	0.14	5.1E-01	-0.76	7.1E-06	-0.16	4.4E-01	0.24	2.4E-01	0.19	3.5E-01	-0.14	4.9E-01	0.05	7.9E-01	0.00	9.8E-01	-0.63	5.2E-04
ENSCAFG0000000842	CNOT1	darkgrey	EC_M8	0.71	5.3E-05	0.39	5.0E-02	-0.33	1.0E-01	0.05	8.2E-01	-0.35	7.6E-02	0.01	9.7E-01	-0.04	8.3E-01	-0.16	4.3E-01	-0.45	2.0E-02	-0.04	8.4E-01
ENSCAFG0000000826	MND1	darkgrey	EC_M8	0.71	5.6E-05	0.10	6.2E-01	-0.27	1.9E-01	-0.31	1.3E-01	-0.23	2.6E-01	0.25	2.2E-01	-0.10	6.2E-01	-0.27	1.9E-01	-0.20	3.2E-01	-0.15	4.6E-01
ENSCAFG0000000202	GCC2	darkgrey	EC_M8	0.71	5.6E-05	0.20	3.3E-01	-0.75	8.6E-06	0.07	7.2E-01	0.16	4.2E-01	0.26	2.0E-01	-0.28	1.7E-01	-0.01	9.8E-01	-0.29	1.6E-01	-0.55	3.4E-03
ENSCAFG0000002017	CMTR2	darkgrey	EC_M8	0.71	5.6E-05	0.04	8.6E-01	-0.24	2.3E-01	0.21	3.0E-01	-0.26	2.0E-01	0.35	8.0E-02	-0.22	2.9E-01	-0.04	8.7E-01	-0.38	5.6E-02	-0.10	6.4E-01
ENSCAFG0000000456	PRDX1	darkgrey	EC_M8	0.71	5.7E-05	-0.14	5.1E-01	-0.06	7.7E-01	-0.03	8.9E-01	-0.36	6.8E-02	0.12	5.5E-01	-0.27	1.9E-01	-0.15	4.5E-01	-0.23	2.7E-01	0.01	9.6E-01
ENSCAFG0000000929	IQGAP2	turquoise	EC_M6	0.71	5.7E-05	0.62	6.4E-04	-0.60	1.2E-03	0.00	1.0E+00	-0.08	7.1E-01	-0.01	9.8E-01	-0.15	4.7E-01	-0.31	1.2E-01	-0.18	3.9E-01	-0.31	1.2E-01
ENSCAFG0000000520	RBM17	darkgrey	EC_M8	0.70	5.8E-05	-0.02	9.1E-01	-0.37	5.9E-02	0.16	4.3E-01	-0.11	6.0E-01	0.24	2.3E-01	-0.14	4.9E-01	0.06	7.6E-01	-0.08	6.9E-01	-0.26	2.0E-01
ENSCAFG0000002039	PDP2	darkgrey	EC_M8	0.70	5.9E-05	0.01	9.6E-01	-0.05	8.1E-01	0.12	5.6E-01	-0.50	9.4E-03	-0.12	5.5E-01	-0.31	1.2E-01	-0.02	9.2E-01	-0.37	6.0E-02	0.09	6.6E-01
ENSCAFG0000000088	DSCC1	darkgrey	EC_M8	0.70	5.9E-05	0.21	2.9E-01	-0.30	1.4E-01	0.00	9.9E-01	-0.22	2.9E-01	0.06	7.8E-01	-0.39	4.6E-02	-0.25	2.1E-01	0.00	1.0E+00	-0.12	5.7E-01
ENSCAFG0000000514	MCM6	darkgrey	EC_M8	0.70	6.0E-05	0.20	3.4E-01	-0.63	5.9E-04	-0.16	4.4E-01	0.20	3.3E-01	0.17	3.9E-01	-0.16	4.3E-01	-0.29	1.6E-01	-0.27	1.8E-01	-0.51	7.8E-03
ENSCAFG0000001234	FASTKD1	darkgrey	EC_M8	0.70	6.1E-05	0.23	2.5E-01	-0.54	4.2E-03	-0.01	9.7E-01	-0.04	8.3E-01	0.21	2.9E-01	-0.21	3.0E-01	-0.19	3.6E-01	-0.30	1.4E-01	-0.36	7.4E-02
ENSCAFG0000000676	API5	darkgrey	EC_M8	0.70	6.2E-05	0.30	1.3E-01	-0.31	1.2E-01	0.12	5.4E-01	-0.27	1.8E-01	0.04	8.6E-01	-0.37	6.1E-02	-0.14	5.1E-01	-0.11	6.0E-01	-0.05	8.2E-01
ENSCAFG0000001320	HDGFL3	darkgrey	EC_M8	0.70	6.2E-05	0.47	1.5E-02	-0.41	3.8E-02	-0.08	6.8E-01	-0.28	1.7E-01	0.15	4.6E-01	-0.23	2.5E-01	-0.14	5.1E-01	0.15	4.7E-01	-0.09	6.8E-01
ENSCAFG0000000162	TONSL	darkgrey	EC_M8	0.70	6.2E-05	0.10	6.2E-01	-0.17	4.1E-01	0.10	6.1E-01	-0.35	7.6E-02	0.10	6.1E-01	0.07	7.5E-01	-0.29	1.6E-01	-0.18	3.7E-01	-0.01	9.5E-01
ENSCAFG0000000524	RBM26	darkgrey	EC_M8	0.70	6.3E-05	0.04	8.5E-01	-0.28	1.6E-01	0.03	8.9E-01	-0.29	1.5E-01	0.16	4.3E-01	-0.21	3.2E-01	-0.11	5.9E-01	-0.23	2.6E-01	-0.14	4.9E-01
ENSCAFG0000001124	PRMT5	darkgrey	EC_M8	0.70	6.3E-05	-0.15	4.6E-01	-0.17	4.0E-01	-0.01	9.7E-01	-0.23	2.5E-01	0.13	5.1E-01	-0.37	6.5E-02	0.08	6.8E-01	-0.24	2.5E-01	-0.16	4.3E-01
ENSCAFG0000002951	ZNF829	darkgrey	EC_M8	0.70	6.3E-05	0.39	4.7E-02	-0.11	5.8E-01	0.12	5.6E-01	-0.57	2.5E-03	0.07	7.5E-01	-0.37	6.2E-02	0.07	7.2E-01	0.03	8.9E-01	0.23	2.5E-01
ENSCAFG0000000565	HNRNPL	darkgrey	EC_M8	0.70	6.5E-05	0.06	7.6E-01	-0.14	4.9E-01	0.06	7.9E-01	-0.38	5.7E-02	0.07	7.2E-01	-0.25	2.2E-01	-0.17	4.2E-01	-0.45	2.0E-02	-0.04	8.4E-01
ENSCAFG0000001291	CDC25A	darkgrey	EC_M8	0.70	6.6E-05	-0.17	4.1E-01	-0.43	3.0E-02	-0.07	7.4E-01	0.07	7.2E-01	0.15	4.7E-01	-0.26	1.9E-01	-0.15	4.7E-01	-0.34	9.4E-02	-0.42	3.4E-02
ENSCAFG0000000906	COPS4	grey	EC_M10	0.70	6.7E-05	-0.24	2.3E-01	-0.18	3.8E-01	0.03	8.9E-01	-0.30	1.4E-01	0.19	3.4E-01	-0.17	4.1E-01	-0.01	9.7E-01	-0.16	4.4E-01	-0.06	7.9E-01
ENSCAFG0000001765	PLK1	darkgrey	EC_M8	0.70	6.7E-05	0.09	6.7E-01	-0.16	4.4E-01	-0.04	8.4E-01	-0.31	1.2E-01	0.21	3.1E-01	-0.17	4.1E-01	-0.11	5.9E-01	-0.01	9.6E-01	-0.03	8.8E-01
ENSCAFG0000001302	HEATR5A	darkgrey	EC_M8	0.70	6.8E-05	0.27	1.9E-01	-0.74	1.6E-05	0.03	8.7E-01	0.21	3.1E-01	0.13	5.2E-01	-0.25	2.1E-01	-0.36	7.0E-02	-0.28	1.7E-01	-0.59	1.4E-03
ENSCAFG0000000972	PUS7L	darkgrey	EC_M8	0.70	7.1E-05	-0.06	7.7E-01	-0.17	4.0E-01	0.19	3.5E-01	-0.37	6.7E-02	0.21	3.1E-01	-0.38	5.5E-02	-0.10	6.3E-01	-0.16	4.3E-01	-0.03	8.9E-01
ENSCAFG0000000095	TBC1D32	darkgrey	EC_M8	0.70	7.2E-05	0.00	9.9E-01	-0.35	8.3E-02	-0.22	2.8E-01	-0.18	3.8E-01	-0.01	9.5E-01	-0.48	1.3E-02	-0.09	6.5E-01	-0.12	5.6E-01	-0.20	3.2E-01
ENSCAFG0000000416	MRE11	grey	EC_M10	0.70	7.3E-05	-0.18	3.8E-01	-0.01	9.6E-01	0.16	4.2E-01	-0.52	7.1E-03	-0.17	4.1E-01	-0.30	1.3E-01	0.02	9.1E-01	-0.11	5.9E-01	0.13	5.2E-01
ENSCAFG0000000289	PNPT1	darkgrey	EC_M8	0.70	7.5E-05	-0.15	4.7E-01	-0.14	5.0E-01	0.08	7.1E-01	-0.30	1.4E-01	-0.01	9.5E-01	-0.47	1.5E-02	0.17	4.1E-01	0.02	9.1E-01	-0.03	8.7E-01
ENSCAFG0000001417	LRR1	darkgrey	EC_M8	0.70	7.5E-05	0.29	1.6E-01	-0.33	1.0E-01	0.09	6.7E-01	-0.21	3.0E-01	0.00	1.0E+00	-0.09	6.7E-01	-0.15	4.8E-01	0.00	1.0E+00	-0.14	4.9E-01
ENSCAFG0000001367	DDX20	darkgrey	EC_M8	0.70	7.6E-05	-0.11	5.9E-01	-0.56	2.8E-03	-0.17	4.0E-01	0.19	3.5E-01	0.08	6.8E-01	-0.32	1.1E-01	0.11	5.9E-01	-0.16	4.4E-01	-0.54	4.5E-03
ENSCAFG0000000641	PPP4R2	darkgrey	EC_M8	0.70	7.8E-05	0.31	1.2E-01	-0.82	3.8E-07	0.07	7.2E-01	0.20	3.3E-01	0.25	2.2E-01	-0.15	4.8E-01	-0.11	5.8E-01	-0.21	3.0E-01	-0.58	1.8E-03
ENSCAFG0000001999	DPH5	turquoise	EC_M6	0.70	7.9E-05	0.51	7.6E-03	-0.61	1.0E-03	-0.04	8.4E-01	-0.04	8.5E-01	0.19	3.5E-01	-0.42	3.2E-02	-0.06	7.7E-01	-0.32	1.1E-01	-0.38	5.5E-02
ENSCAFG0000000328	GNL2	darkgrey	EC_M8	0.70	8.0E-05	-0.17	4.1E-01	-0.64	4.4E-04	0.03	8.9E-01	0.28	1.6E-01	0.33	1.0E-01	-0.18	3.7E-01	0.04	8.4E-01	-0.24	2.3E-01	-0.67	1.7E-04
ENSCAFG0000001372	ZW10	turquoise	EC_M6	0.70	8.0E-05	0.56	3.1E-03	-0.63	5.1E-04	-0.05	8.1E-01	0.05	8.3E-01	0.12	5.6E-01	-0.19	3.5E-01	-0.29	1.5E-01	-0.42	3.5E-02	-0.38	5.6E-02
ENSCAFG0000000289	ENSCAFG0000000289	darkgrey	EC_M8	0.70	8.1E-05	0.47	1.4E-02	-0.17	3.9E-01	0.24	2.4E-01	-0.58	1.7E-03	0.03	8.7E-01	-0.15	4.7E-01	-0.10	6.3E-01	-0.26	1.9E-01	0.21	3.0E-01
ENSCAFG0000001309	BCLAF3	darkgrey	EC_M8	0.69	8.2E-05	0.04	8.3E-01	-0.16	4.4E-01	0.14	4.9E-01	-0.40	4.0E-02	-0.03	9.0E-01	-0.35	7.5E-02	-0.30	1.3E-01	-0.37	6.3E-02	0.02	9.2E-01
ENSCAFG0000000299	ZMPSTE24	darkgrey	EC_M8	0.69	8.3E-05	-0.16	4.3E-01	-0.71	5.1E-05	-0.10	6.3E-01	0.36	7.0E-02	0.28	1.7E-01	-0.24	2.4E-01	-0.06	7.6E-01	-0.14	5.1E-01	-0.71	5.0E-05
ENSCAFG0000000726	CSTF3	grey	EC_M10	0.69	8.3E-05	0.00	1.0E+00	-0.38	5.4E-02	0.06	7.7E-01	-0.17	4.2E-01	0.10	6.3E-01	-0.27	1.8E-01	-0.08	6.8E-01	-0.46	1.9E-02	-0.27	1.9E-01
ENSCAFG0000001721	ABCB7	grey	EC_M10	0.69	8.4E-05	-0.07	7.2E-01	-0.19	3.5E-01	0.16	4.2E-01	-0.37	6.0E-02	0.16	4.3E-01	-0.32	1.1E-01	-0.05	8.0E-01	-0.16	4.2E-01	-0.02	9.1E-01
ENSCAFG0000000227	EIF5B	darkgreen	EC_M4	0.69	8.4E-05	0.15	4.6E-01	-0.87	8.8E-09	-0.21	3.0E-01	0.41	3.7E-02	0.25	2.1E-01	-0.12	5.6E-01	-0.02	9.3E-01	-0.33	1.0E-01	-0.77	4.9E-06
ENSCAFG0000001061	H2AFZ	darkgrey	EC_M8	0.69	8.4E-05	0.34	8.9E-02	-0.48	1.3E-02	-0.08	7.0E-01	-0.04	8.5E-01	0.17	4.0E-01	-0.04	8.6E-01	-0.34	9.4E-02	-0.09	6.5E-01	-0.33	1.0E-01
ENSCAFG0000000992	RBMS1	darkgrey	EC_M8	0.69	8.6E-05	0.26	2.0E-01	-0.54	4.2E-03	-0.14	5.0E-01	-0.01	9.7E-01	0.14	5.0E-01	-0.04	8.4E-01	0.02	9.2E-01	-0.03	9.0E-01	-0.40	4.4E-02
ENSCAFG0000001672	EXOSC10	darkgrey	EC_M8	0.69	8.7E-05	0.34	9.2E-02	-0.08	7.0E-01	0.05	8.0E-01	-0.56	3.1E-03	0.05	8.1E-01	-0.11	6.1E-01	-0.13	5.4E-01	-0.48	1.3E-02	0.16	4.3E-01
ENSCAFG0000000761	POLR1A	darkgrey	EC_M8	0.69	8.8E-05	-0.24	2.5E-01	-0.11	5.8E-01	-0.11	6.0E-01	-0.26	1.9E-01	0.11	5.8E-01	-0.25	2.2E-01	-0.15	4.7E-01	-0.32	1.1E-01	-0.13	5.4E-01
ENSCAFG0000000098	VDAC1	darkgrey	EC_M8	0.69	8.8E-05	0.15	4.7E-01	-0.28	1.7E-01	-0.09	6.5E-01	-0.20	3.3E-01	-0.03	8.9E-01	-0.43	2.8E-02	-0.04	8.5E-01	-0.11	6.1E-01	-0.11	5.9E-01
ENSCAFG0000000035	XPOT	darkgrey	EC_M8	0.69	8.8E-05	-0.05	8.1E-01	-0.72	3.8E-05	0.14	4.9E-01	0.28	1.7E-01	0.35	7.8E-02	-0.25	2.1E-01	-0.12	5.6E-01	-0.28	1.6E-01	-0.69	1.1E-04
ENSCAFG0000001599	CCNB3	darkgrey	EC_M8	0.69	8.8E-05	0.24	2.4E-01	-0.23	2.6E-01	0.05	8.3E-01	-0.28	1.7E-01	0.14	5.0E-01	-0.26	1.9E-01	-0.07	7.2E-01	-0.04	8.4E-01	-0.04	8.4E-01
ENSCAFG0000001082	SF3B1	darkgrey	EC_M8	0.69	8.8E-05	0.44	2.3E-02	-0.12	5.6E-01	0.23	2.7E-01	-0.64	4.5E-04	-0.04	8.6E-01	-0.14	5.0E-01	-0.08	6.8E-01	-0.21	3.1E-01	0.26	2.0E-01
ENSCAFG0000000282	YTHDC1	darkgrey	EC_M8	0.69	8.9E-05	0.59	1.6E-03	-0.47	1.5E-02	0.02	9.2E-01	-0.28	1.7E-01	0.21	3.1E-01	0.04	8.5E-01	-0.11	5.9E-01	-0.17	4.2E-01	-0.09	6.7E-01
ENSCAFG0000002318	ENSCAFG0000002318	darkgrey	EC_M8	0.69	8.9E-05	0.30	1.3E-01	-0.47	1.6E-02	0.25	2.3E-01	-0.16	4.4E-01	0.02	9.2E-01	-0.28	1.6E-01	-0.28	1.6E-01	-0.28	1.7E-01	-0.19	3.5E-01
ENSCAFG0000000540	HOOK3	darkgrey	EC_M8	0.69	8.9E-05	0.27	1.8E-01	-0.83	1.1E-07	0.10	6.2E-01	0.25	2.2E-01	0.23	2.5E-01	-0.12	5.5E-01	-0.17	4.0E-01	-0.18	3.7E-01	-0.63	6.0E-04
ENSCAFG0000000340	CAPZA2	darkgrey	EC_M8	0.69	9.0E-05	0.03	8.9E-01	-0.78	2.8E-06	0.07	7.3E-01	0.33	1.0E-01	0.17	4.0E-01	-0.36	6.9E-02	-0.10	6.3E-01	-0.41	3.7E-02	-0.69	1.0E-04
ENSCAFG0000001057	UCHL5	darkgreen	EC_M4	0.69	9.1E-05	0.11	5.8E-01	-0.86	1.3E-08	-0.10	6.3E-01	0.43	3.0E-02	0.23	2.6E-01	-0.17	4.1E-01	0.00	9.8E-01	-0.19	3.6E-01	-0.78	2.6E-06
ENSCAFG0000002953	ENSCAFG0000002953	darkgrey	EC_M8	0.69	9.1E-05	0.06	7.9E-01	-0.39	4.7E-02	0.08	7.0E-01	-0.18	3.8E-01	0.21	3.0E-01	-0.28	1.7E-01	-0.26	2.0E-01	-0.29	1.6E-01	-0.21	3.0E-01
ENSCAFG0000000266	MSH6	grey	EC_M10	0.69	9.2E-05	0.58	2.1E-03	-0.30	1.4E-01	0.08	7.0E-01	-0.39	5.0E-02	0.07	7.3E-01	-0.10	6.2E-01	-0.06	7.7E-01	-0.25	2.3E-01	0.04	8.4E-01
ENSCAFG0000001635	DDX39A	darkgrey	EC_M8	0.69	9.2E-05	0.12	5.4E-01	-0.40	4.2E-02	-0.17	4.0E-01	-0.02	9.1E-01	0.11	5.9E-01	-0.22	2.8E-01	0.01	9.8E-01	-0.18	3.8E-01	-0.32	1.1E-01
ENSCAFG0000000242	BZW2	darkgrey	EC_M8	0.69	9.3E-05	-0.13	5.1E-01	-0.49	1.1E-02	-0.11	5.8E-01	0.10	6.3E-01	0.10	6.3E-01	-0.31	1.2E-01	0.03	8.7E-01	0.11	5.8E-01	-0.43	2.8E-02
ENSCAFG0000000774	RIOK2	darkgreen	EC_M4	0.69	9.4E-05	0.16	4.3E-01	-0.84	7.1E-08	-0.16	4.3E-01	0.41	3.6E-02	0.27	1.8E-01	-0.32	1.1E-01	-0.04	8.4E-01	-0.30	1.4E-01	-0.75	8.5E-06
ENSCAFG0000001044	GMNN	darkgrey	EC_M8	0.69	9.6E-05	0.17	4.0E-01	-0.06	7.7E-01	-0.03	8.8E-01	-0.48	1.3E-02	0.13	5.3E-01	-0.25	2.2E-01	-0.41	3.8E-02	-0.20	3.2E-01	0.15	4.7E-01
ENSCAFG0000001145	PSMD12	darkgrey	EC_M8	0.69	9.6E-05	0.33	9.6E-02	-0.87	9.7E-09	-0.16	4.4E-01	0.35	8.3E-02	0.31	1.2E-01	-0.19	3.6E-01	-0.08	7.0E-01	-0.16	4.4E-01	-0.68	1.3E-04
ENSCAFG0000000958	ENSCAFG0000000958	darkgrey	EC_M8	0.69	9.6E-05	-0.13	5.1E-01	-0.63	5.1E-04	-0.04	8.5E-01	0.25	2.2E-01	0.16	4.3E-01	-0.57	2.5E-03	-0.01	9.7E-01	-0.13	5.1E-01	-0.61	9.0E-04
ENSCAFG0000001083	TRIP13	darkgrey	EC_M8	0.69	9.9E-05	-0.06	7.8E-01	-0.29	1.5E-01	-0.25	2.2E-01	-0.09	6.6E-01	0.18	3.7E-01	-0.29	1.5E-01	-0.07	7.2E-01	-0.05	7.9E-01	-0.24	2.4E-01
ENSCAFG0000000343	UFL1	darkgrey	EC_M8	0.69	9.9E-05	0.07	7.3E-01	-0.47	1.4E-02	0.23	2.7E-01	-0.11	5.8E-01	0.17	4.1E-01	-0.39	4.8E-02	0.05	7.9E-01	-0.21	2.9E-01	-0.29	1.5E-01
ENSCAFG0000000163	MRPS10	darkgrey	EC_M8	0.69	1.0E-04	-0.18	3.8E-01	-0.54	4.8E-03	0.08	6.9E-01	0.15	4.7E-01	0.21	3.0E-01	-0.50	9.5E-03	-0.21	3.1E-01	-0.08	6.9E-01	-0.51	7.5E-03
ENSCAFG0000001667	KPNB1	darkgrey	EC_M8	0.69	1.0E-04	-0.01	9.7E-01	-0.79	2.0E-06	-0.18	3.9E-01	0.41	3.5E-02	0.29	1.5E-01	-0.28	1.6E-01	0.12	5.4E-01	-0.08	6.9E-01	-0.78	2.6E-06
ENSCAFG0000000244	NCBP1	darkgrey	EC_M8	0.69	1.0E-04	0.13	5.4E-01	-0.64	4.2E-04	-0.26	1.9E-01	0.21	3.0E-01	0.21	3.0E-01	-0.52	6.9E-03	-0.07	7.3E-01	0.03	8.7E-01	-0.56	2.7E-03
ENSCAFG0000000830	AQR	darkgrey	EC_M8	0.69	1.0E-04	0.11	5.9E-01	-0.10	6.3E-01	0.25	2.1E-01	-0.52	6.1E-03	0.05	8.0E-01	-0.40	4.5E-02	-0.39	4.9E-02	-0.44	2.5E-02	0.14	5.0E-01

ENSCAFG0000000423	CEP295	darkgrey	EC_M8	0.69	1.1E-04	0.32	1.1E-01	-0.03	8.9E-01	-0.06	7.7E-01	-0.68	1.4E-04	-0.21	3.1E-01	-0.29	1.6E-01	-0.12	5.7E-01	-0.35	7.8E-02	0.30	1.4E-01
ENSCAFG0000000507	R3HDM1	darkgrey	EC_M8	0.69	1.1E-04	0.18	3.8E-01	-0.30	1.4E-01	-0.13	5.4E-01	-0.28	1.7E-01	0.13	5.3E-01	-0.54	4.3E-03	-0.04	8.5E-01	-0.24	2.4E-01	-0.16	4.4E-01
ENSCAFG0000002002	TRMT13	grey	EC_M10	0.69	1.1E-04	0.11	5.9E-01	-0.15	4.7E-01	0.09	6.7E-01	-0.46	1.9E-02	0.22	2.9E-01	-0.28	1.7E-01	-0.10	6.3E-01	-0.39	5.0E-02	0.09	6.8E-01
ENSCAFG0000001899	USP7	turquoise	EC_M6	0.69	1.1E-04	0.70	8.0E-05	-0.50	9.8E-03	-0.02	9.1E-01	-0.19	3.6E-01	0.05	7.9E-01	-0.28	1.7E-01	-0.15	4.6E-01	-0.19	3.6E-01	-0.15	4.7E-01
ENSCAFG0000001942	DOT1L	turquoise	EC_M6	0.69	1.1E-04	0.45	2.1E-02	-0.64	4.1E-04	-0.03	8.7E-01	0.04	8.6E-01	0.02	9.1E-01	-0.28	1.6E-01	-0.03	8.9E-01	-0.32	1.1E-01	-0.42	3.3E-02
ENSCAFG0000000968	PAK1IP1	darkgrey	EC_M8	0.69	1.1E-04	-0.31	1.2E-01	-0.21	3.0E-01	-0.08	7.1E-01	-0.14	5.1E-01	0.25	2.3E-01	-0.23	2.5E-01	-0.10	6.4E-01	-0.26	2.0E-01	-0.23	2.5E-01
ENSCAFG0000001557	FKBP4	darkgrey	EC_M8	0.69	1.1E-04	-0.16	4.5E-01	-0.28	1.7E-01	-0.03	8.7E-01	-0.09	6.6E-01	0.25	2.1E-01	-0.11	5.8E-01	0.01	9.5E-01	-0.15	4.5E-01	-0.25	2.2E-01
ENSCAFG0000000632	MAK16	darkgrey	EC_M8	0.69	1.1E-04	-0.30	1.4E-01	-0.55	3.3E-03	-0.08	6.9E-01	0.23	2.6E-01	0.29	1.5E-01	-0.13	5.2E-01	0.11	6.0E-01	-0.17	4.1E-01	-0.60	1.3E-03
ENSCAFG0000000662	WDR62	darkgrey	EC_M8	0.68	1.2E-04	-0.07	7.2E-01	-0.19	3.4E-01	-0.05	8.2E-01	-0.22	2.8E-01	0.15	4.6E-01	-0.37	5.9E-02	-0.21	3.0E-01	-0.15	4.5E-01	-0.16	4.2E-01
ENSCAFG0000000513	RPL14	grey	EC_M10	0.68	1.2E-04	-0.09	6.5E-01	-0.19	3.5E-01	0.23	2.6E-01	-0.33	1.0E-01	0.24	2.4E-01	0.11	5.9E-01	-0.21	3.1E-01	-0.55	3.4E-03	-0.07	7.5E-01
ENSCAFG0000001874	AIFM1	darkgrey	EC_M8	0.68	1.2E-04	0.18	3.7E-01	-0.13	5.3E-01	0.06	7.8E-01	-0.46	1.9E-02	-0.12	5.5E-01	-0.15	4.6E-01	0.17	4.0E-01	0.08	7.0E-01	0.14	5.1E-01
ENSCAFG0000002468	ENSCAFG0000002468	darkgrey	EC_M8	0.68	1.2E-04	0.17	4.1E-01	-0.41	3.7E-02	0.33	9.7E-02	-0.18	3.9E-01	0.06	7.7E-01	-0.33	1.0E-01	-0.26	2.0E-01	-0.48	1.4E-02	-0.18	3.8E-01
ENSCAFG0000000451	ZC3H13	darkgrey	EC_M8	0.68	1.2E-04	0.27	1.9E-01	-0.45	2.2E-02	0.06	7.8E-01	-0.18	3.7E-01	-0.07	7.5E-01	-0.36	6.9E-02	0.14	5.0E-01	-0.15	4.8E-01	-0.18	3.7E-01
ENSCAFG0000002011	ABCD3	darkgrey	EC_M8	0.68	1.2E-04	0.07	7.2E-01	-0.75	1.3E-05	-0.13	5.3E-01	0.27	1.9E-01	0.10	6.2E-01	-0.31	1.2E-01	0.15	4.6E-01	-0.22	2.7E-01	-0.61	9.6E-04
ENSCAFG0000001814	ENSCAFG0000001814	darkgrey	EC_M8	0.68	1.2E-04	0.20	3.2E-01	-0.43	3.0E-02	0.23	2.6E-01	-0.18	3.9E-01	0.10	6.2E-01	0.09	6.5E-01	0.03	8.7E-01	-0.11	6.0E-01	-0.21	3.0E-01
ENSCAFG0000001345	FASTKD2	darkgrey	EC_M8	0.68	1.2E-04	-0.10	6.4E-01	-0.14	5.0E-01	-0.13	5.3E-01	-0.32	1.1E-01	0.05	7.9E-01	-0.52	6.6E-03	-0.34	9.2E-02	-0.03	8.9E-01	-0.03	9.0E-01
ENSCAFG0000003178	CASP6	darkgrey	EC_M8	0.68	1.3E-04	0.16	4.4E-01	-0.01	9.5E-01	0.08	7.1E-01	-0.59	1.6E-03	0.03	8.7E-01	-0.04	8.3E-01	0.04	8.4E-01	-0.19	3.6E-01	0.21	3.0E-01
ENSCAFG0000000653	HSPH1	darkgreen	EC_M4	0.68	1.3E-04	0.51	8.2E-03	-0.79	1.2E-06	-0.29	1.5E-01	0.20	3.2E-01	0.14	4.8E-01	-0.15	4.5E-01	-0.07	7.2E-01	-0.34	9.3E-02	-0.55	3.4E-03
ENSCAFG0000000342	ENSCAFG0000000342	darkgrey	EC_M8	0.68	1.3E-04	0.25	2.1E-01	-0.41	3.5E-02	0.13	5.3E-01	-0.23	2.7E-01	0.09	6.5E-01	-0.05	8.1E-01	-0.15	4.6E-01	-0.24	2.3E-01	-0.15	4.7E-01
ENSCAFG0000001571	ENSCAFG0000001571	darkgrey	EC_M8	0.68	1.3E-04	-0.17	4.1E-01	-0.37	6.4E-02	-0.15	4.8E-01	0.00	9.9E-01	0.36	7.5E-02	-0.16	4.3E-01	0.02	9.1E-01	-0.10	6.3E-01	-0.38	5.8E-02
ENSCAFG0000001758	PRR11	darkgrey	EC_M8	0.68	1.3E-04	0.02	9.2E-01	-0.06	7.8E-01	0.13	5.3E-01	-0.41	3.6E-02	0.12	5.7E-01	-0.26	2.0E-01	-0.23	2.6E-01	-0.19	3.7E-01	0.06	7.8E-01
ENSCAFG0000001537	KIAA0586	darkgrey	EC_M8	0.68	1.3E-04	-0.28	1.6E-01	-0.38	5.9E-02	-0.06	7.8E-01	0.02	9.3E-01	0.15	4.6E-01	-0.47	1.5E-02	0.04	8.3E-01	-0.36	7.0E-02	-0.41	3.7E-02
ENSCAFG0000001621	MRPL44	darkgrey	EC_M8	0.68	1.3E-04	-0.02	9.1E-01	-0.16	4.2E-01	-0.02	9.2E-01	-0.38	5.9E-02	0.23	2.6E-01	-0.05	8.1E-01	-0.12	5.7E-01	-0.10	6.3E-01	0.02	9.3E-01
ENSCAFG0000000538	PARP2	darkgrey	EC_M8	0.68	1.3E-04	0.55	3.6E-03	-0.09	6.7E-01	0.17	4.1E-01	-0.59	1.6E-03	-0.03	8.9E-01	-0.31	1.2E-01	-0.28	1.6E-01	-0.28	1.7E-01	0.24	2.3E-01
ENSCAFG0000001908	NDC1	darkgrey	EC_M8	0.68	1.3E-04	-0.20	3.3E-01	-0.31	1.3E-01	-0.17	4.1E-01	-0.07	7.5E-01	0.21	3.1E-01	-0.11	6.0E-01	-0.05	8.0E-01	-0.13	5.1E-01	-0.32	1.1E-01
ENSCAFG0000000879	DUSP11	grey	EC_M10	0.68	1.3E-04	0.48	1.2E-02	-0.35	8.1E-02	0.18	3.7E-01	-0.36	7.0E-02	0.26	1.9E-01	-0.40	4.3E-02	-0.16	4.5E-01	-0.03	8.8E-01	-0.02	9.2E-01
ENSCAFG0000000391	BCAP29	darkgrey	EC_M8	0.68	1.3E-04	-0.42	3.3E-02	-0.12	5.5E-01	0.08	7.0E-01	-0.21	3.0E-01	0.20	3.4E-01	-0.15	4.7E-01	-0.05	8.2E-01	-0.27	1.9E-01	-0.15	4.7E-01
ENSCAFG0000001116	PAPSS1	darkgrey	EC_M8	0.68	1.4E-04	-0.21	3.0E-01	-0.51	7.7E-03	0.08	6.9E-01	0.12	5.5E-01	0.24	2.5E-01	-0.17	4.1E-01	0.03	8.9E-01	-0.14	5.0E-01	-0.50	8.8E-03
ENSCAFG0000001544	ENSCAFG0000001544	darkgrey	EC_M8	0.68	1.4E-04	0.19	3.6E-01	-0.58	1.8E-03	0.37	6.5E-02	0.02	9.3E-01	0.22	2.8E-01	-0.26	2.0E-01	-0.28	1.7E-01	-0.26	2.0E-01	-0.36	6.8E-02
ENSCAFG0000001041	ENSCAFG0000001041	darkgrey	EC_M8	0.68	1.4E-04	0.04	8.6E-01	-0.09	6.4E-01	-0.17	4.0E-01	-0.37	6.1E-02	0.12	5.6E-01	-0.12	5.6E-01	-0.14	5.0E-01	-0.17	4.0E-01	0.00	9.9E-01
ENSCAFG0000000831	FNBP4	darkgrey	EC_M8	0.68	1.4E-04	0.47	1.4E-02	-0.10	6.4E-01	0.04	8.4E-01	-0.62	6.8E-04	0.08	6.9E-01	-0.09	6.5E-01	0.02	9.1E-01	0.02	9.4E-01	0.27	1.8E-01
ENSCAFG0000000429	CHORDC1	darkgrey	EC_M8	0.68	1.4E-04	0.03	8.8E-01	-0.61	9.7E-04	-0.20	3.2E-01	0.21	3.1E-01	0.19	3.5E-01	-0.48	1.3E-02	0.04	8.5E-01	-0.42	3.3E-02	-0.59	1.5E-03
ENSCAFG0000001798	THUMPD1	darkgrey	EC_M8	0.68	1.4E-04	0.03	8.8E-01	-0.35	8.3E-02	-0.05	7.9E-01	-0.14	5.1E-01	0.20	3.3E-01	-0.18	3.8E-01	-0.14	4.8E-01	-0.21	3.1E-01	-0.19	3.6E-01
ENSCAFG0000003228	ZNF420	darkgrey	EC_M8	0.68	1.4E-04	0.17	4.1E-01	-0.54	4.1E-03	-0.07	7.4E-01	-0.04	8.5E-01	0.11	6.0E-01	-0.04	8.3E-01	-0.12	5.5E-01	0.01	9.6E-01	-0.33	1.0E-01
ENSCAFG0000000019	RPS12	grey	EC_M10	0.68	1.4E-04	-0.17	4.2E-01	-0.05	8.0E-01	0.25	2.2E-01	-0.44	2.6E-02	0.15	4.6E-01	-0.19	3.4E-01	-0.10	6.4E-01	-0.61	9.5E-04	0.01	9.6E-01
ENSCAFG0000000188	GTPBP10	grey	EC_M10	0.68	1.4E-04	-0.05	8.2E-01	-0.41	4.0E-02	0.16	4.5E-01	-0.11	5.9E-01	0.21	3.1E-01	-0.36	7.1E-02	-0.18	3.9E-01	-0.24	2.4E-01	-0.26	2.0E-01
ENSCAFG0000000808	FAM208A	darkgrey	EC_M8	0.68	1.5E-04	0.20	3.2E-01	-0.39	4.7E-02	0.17	4.1E-01	-0.23	2.6E-01	0.05	8.0E-01	-0.62	6.8E-04	-0.02	9.3E-01	-0.15	4.6E-01	-0.16	4.3E-01
ENSCAFG0000003203	CCDC58	darkgrey	EC_M8	0.68	1.5E-04	0.08	7.1E-01	-0.31	1.3E-01	-0.07	7.4E-01	-0.25	2.2E-01	0.09	6.5E-01	-0.03	9.0E-01	-0.14	4.8E-01	0.00	9.9E-01	-0.14	4.9E-01
ENSCAFG0000000851	GABPA	darkgrey	EC_M8	0.68	1.5E-04	0.29	1.5E-01	-0.08	6.9E-01	0.08	6.9E-01	-0.62	6.6E-04	0.03	8.7E-01	-0.45	2.2E-02	-0.09	6.7E-01	-0.06	7.8E-01	0.25	2.2E-01
ENSCAFG0000001722	LUC7L3	darkgrey	EC_M8	0.68	1.5E-04	0.23	2.6E-01	-0.41	3.9E-02	0.21	3.0E-01	-0.15	4.7E-01	0.06	7.6E-01	-0.26	2.0E-01	0.07	7.3E-01	0.13	5.3E-01	-0.20	3.3E-01
ENSCAFG0000001418	PPP2R1B	grey	EC_M10	0.68	1.5E-04	0.65	3.6E-04	-0.44	2.6E-02	-0.09	6.8E-01	-0.29	1.5E-01	-0.10	6.3E-01	-0.14	5.1E-01	-0.20	3.4E-01	-0.33	1.0E-01	-0.04	8.3E-01
ENSCAFG0000000928	SLC38A1	turquoise	EC_M6	0.68	1.5E-04	0.54	4.4E-03	-0.76	7.3E-06	-0.04	8.6E-01	0.17	4.1E-01	0.06	7.5E-01	-0.23	2.7E-01	-0.25	2.1E-01	-0.31	1.3E-01	-0.55	3.7E-03
ENSCAFG0000000057	SCAF8	darkgrey	EC_M8	0.68	1.5E-04	0.31	1.2E-01	-0.06	7.8E-01	-0.01	9.5E-01	-0.63	5.6E-04	0.06	7.6E-01	-0.42	3.1E-02	-0.15	4.7E-01	-0.14	5.0E-01	0.25	2.1E-01
ENSCAFG0000000471	NIFK	darkgrey	EC_M8	0.68	1.5E-04	-0.05	7.9E-01	-0.75	9.7E-06	-0.14	4.9E-01	0.39	4.8E-02	0.33	1.0E-01	-0.23	2.5E-01	-0.11	5.8E-01	-0.29	1.6E-01	-0.78	2.9E-06
ENSCAFG0000000841	ZBTB10	darkgrey	EC_M8	0.68	1.5E-04	0.27	1.8E-01	-0.73	2.6E-05	-0.01	9.5E-01	0.18	3.8E-01	0.40	4.3E-02	-0.12	5.6E-01	-0.44	2.3E-02	-0.21	3.0E-01	-0.55	3.8E-03
ENSCAFG0000001757	PPP4R3A	darkgrey	EC_M8	0.68	1.5E-04	0.53	5.0E-03	-0.03	8.7E-01	0.06	7.7E-01	-0.72	3.8E-05	-0.06	7.6E-01	-0.02	9.4E-01	-0.24	2.4E-01	-0.26	2.0E-01	0.36	7.2E-02
ENSCAFG0000000524	NET1	turquoise	EC_M6	0.68	1.5E-04	0.64	4.5E-04	-0.38	5.3E-02	-0.09	6.6E-01	-0.33	1.0E-01	-0.11	5.9E-01	-0.32	1.1E-01	-0.11	6.0E-01	0.14	5.0E-01	-0.03	8.8E-01
ENSCAFG0000000957	ZBED6	darkgrey	EC_M8	0.68	1.5E-04	0.23	2.5E-01	-0.62	7.5E-04	0.07	7.3E-01	0.06	7.9E-01	0.02	9.4E-01	-0.27	1.8E-01	-0.02	9.2E-01	-0.40	4.4E-02	-0.42	3.5E-02
ENSCAFG0000001386	ENSCAFG0000001386	darkgrey	EC_M8	0.68	1.5E-04	0.05	8.2E-01	-0.58	2.0E-03	-0.02	9.1E-01	0.12	5.6E-01	0.33	1.0E-01	-0.14	4.9E-01	-0.30	1.3E-01	-0.14	4.9E-01	-0.50	9.0E-03
ENSCAFG0000000659	ANKS1B	darkgrey	EC_M8	0.68	1.5E-04	0.19	3.6E-01	-0.57	2.5E-03	-0.25	2.2E-01	0.08	6.9E-01	0.25	2.2E-01	-0.37	6.5E-02	0.11	5.8E-01	0.14	5.1E-01	-0.42	3.1E-02
ENSCAFG0000000827	HLTF	darkgrey	EC_M8	0.68	1.5E-04	0.18	3.8E-01	-0.84	8.4E-08	-0.13	5.2E-01	0.34	9.2E-02	0.25	2.2E-01	-0.15	4.6E-01	-0.09	6.5E-01	0.10	6.2E-01	-0.71	4.8E-05
ENSCAFG0000001802	TRAPPC8	grey	EC_M10	0.68	1.5E-04	0.28	1.6E-01	-0.06	7.7E-01	0.15	4.7E-01	-0.59	1.4E-03	0.12	5.7E-01	-0.38	5.7E-02	-0.25	2.3E-01	-0.18	3.9E-01	0.23	2.6E-01
ENSCAFG0000000102	FAM91A1	turquoise	EC_M6	0.67	1.6E-04	0.51	7.6E-03	-0.74	1.5E-05	-0.08	6.9E-01	0.11	5.8E-01	0.12	5.7E-01	-0.09	6.5E-01	0.02	9.3E-01	0.21	3.0E-01	-0.46	1.8E-02
ENSCAFG0000001658	SLTM	darkgrey	EC_M8	0.67	1.6E-04	0.55	3.4E-03	-0.24	2.4E-01	0.03	8.9E-01	-0.53	5.1E-03	-0.06	7.7E-01	-0.19	3.6E-01	-0.10	6.1E-01	-0.19	3.6E-01	0.18	3.8E-01
ENSCAFG0000001471	PSME3	darkgrey	EC_M8	0.67	1.6E-04	-0.14	5.0E-01	-0.50	9.5E-03	-0.16	4.4E-01	0.16	4.4E-01	0.29	1.5E-01	-0.40	4.1E-02	-0.11	6.0E-01	-0.10	6.2E-01	-0.50	9.4E-03
ENSCAFG0000001096	ENSCAFG0000001096	darkgrey	EC_M8	0.67	1.6E-04	-0.20	3.3E-01	-0.60	1.1E-03	0.07	7.3E-01	0.23	2.5E-01	0.39	5.2E-02	-0.26	2.0E-01	0.02	9.3E-01	-0.17	3.9E-01	-0.56	2.8E-03
ENSCAFG0000000590	ZMAT2	darkgrey	EC_M8	0.67	1.6E-04	0.03	8.8E-01	-0.53	5.3E-03	-0.01	9.7E-01	0.06	7.6E-01	0.04	8.4E-01	-0.08	6.8E-01	0.33	9.6E-02	-0.29	1.5E-01	-0.41	3.6E-02
ENSCAFG0000000041	NUP107	darkgrey	EC_M8	0.67	1.6E-04	0.39	5.0E-02	-0.39	4.8E-02	-0.10	6.1E-01	-0.15	4.8E-01	-0.03	8.7E-01	-0.18	3.9E-01	0.09	6.7E-01	-0.04	8.5E-01	-0.18	3.8E-01
ENSCAFG0000001869	NIPBL	darkgrey	EC_M8	0.67	1.6E-04	0.30	1.4E-01	-0.35	7.9E-02	0.16	4.4E-01	-0.33	1.0E-01	0.08	6.9E-01	-0.27	1.9E-01	-0.14	5.0E-01	-0.28	1.6E-01	-0.06	7.5E-01
ENSCAFG0000000718	SKA3	darkgrey	EC_M8	0.67	1.6E-04	-0.14	4.9E-01	0.00	1.0E+00	-0.12	5.7E-01	-0.42	3.4E-02	0.09	6.7E-01	-0.13	5.2E-01	0.03	8.7E-01	0.04	8.4E-01	0.09	6.5E-01
ENSCAFG0000000169	PLAA	darkgreen	EC_M4	0.67	1.6E-04	0.14	5.1E-01	-0.87	7.8E-09	-0.04	8.6E-01	0.44	2.5E-02	0.26	2.1E-01	-0.27	1.8E-01	-0.14	5.0E-01	-0.27	1.9E-01	-0.78	2.3E-06
ENSCAFG0000000769	RNF7	grey	EC_M10	0.67	1.7E-04	-0.07	7.4E-01	-0.35	7.9E-02	-0.15	4.8E-01	-0.15	4.6E-01	0.37	6.2E-02	0.07	7.5E-01	-0.18	3.8E-01	-0.43	2.9E-02	-0.22	2.9E-01
ENSCAFG0000000930	SCAF11	darkgrey	EC_M8	0.67	1.7E-04	0.59	1.5E-03	-0.23	2.5E-01	0.16	4.5E-01	-0.55	3.5E-03	-0.08	7.0E-01	-0.15	4.7E-01	-0.12	5.6E-01	-0.14	5.0E-01	0.18	3.8E-01
ENSCAFG0000002037	USP33	darkgrey	EC_M8	0.67	1.7E-04	0.50	9.6E-03	-0.71	4.7E-05	0.07	7.4E-01	0.04	8.5E-01	0.22	2.8E-01	-0.33	9.9E-02	-0.22	2.9E-01	-0.13	5.4E-01	-0.39	4.6E-02
ENSCAFG0000003183	PSMD7	darkgrey	EC_M8	0.67	1.8E-04	-0.05	8.1E-01	-0.77	4.4E-06	-0.14	4.9E-01	0.38	5.5E-02	0.31	1.2E-01	-0.11	5.8E-01	0.00	9.9E-01	-0.28	1.7E-01	-0.72	3.3E-05
ENSCAFG0000001648	ENSCAFG0000001648	darkgrey	EC_M8	0.67	1.8E-04	0.03	9.0E-01	-0.12	5.5E-01	-0.05	8.1E-01	-0.33	1.0E-01	0.01	9.6E-01	0.00	9.9E-01	-0.11	6.1E-01	-0.27	1.8E-01	-0.04	8.3E-01
ENSCAFG0000000569	NAP1L1	darkgrey	EC_M8	0.67	1.8E-04	0.16	4.3E-01	0.17	4.0E-01	0.14	5.0E-01	-0.83	1.3E-07	-0.04	8.4E-01	-0.26	1.9E-01	-0.12	5.5E-01	-0.29	1.5E-01	0.45	2.2E-02
ENSCAFG0000000086	RAD50	grey	EC_M10	0.67	1.8E-04	-0.13	5.1E-01	-0.34	8.7E-02	0.13	5.3E-01	-0.15	4.5E-01	0.10	6.2E-01	-0.41	3.7E-02	-0.01	9.5E-01	-0.25	2.2E-01	-0.25	2.2E-01
ENSCAFG0000000190	KIF24	darkgrey	EC_M8	0.67	1.8E-04	-0.09	6.7E-01	-0.19	3.6E-01	-0.07	7.2E-01	-0.18	3.9E-01	0.07	7.2E-01	-0.31	1.3E-01	-0.07	7.3E-01	-0.09	6.7E-01	-0.15	4.7E-01
ENSCAFG0000000632	DDX51	darkgrey	EC_M8	0.67	1.8E-04	0.35	7.7E-02	-0.21	3.1E-01	-0.03	8.7E-01	-0.38	5.5E-02	0.22	2.7E-01	-0.30	1.4E-01	0.00	9.8E-01	-0.32	1.1E-01	0.04	8.6E-01
ENSCAFG0000001066	ENSCAFG0000001066	turquoise	EC_M6	0.67	1.8E-04	0.64	4.4E-04	-0.68	1.3E-04	-0.13	5.3E-01	0.01	9.8E-01	0.16	4.3E-01	-0.19	3.4E-01	-0.17	4.1E-01	-0.26	2.0E-01	-0.37	6.3E-02
ENSCAFG0000001089	ENSCAFG0000001089	darkgrey	EC_M8	0.67	1.9E-04	0.38	5.3E-02	-0.16	4.3E-01	-0.03	8.8E-01	-0.55	3.6E-03	-0.10	6.2E-01	-0.24	2.4E-01	-0.01	9.5E-01	0.03	8.9E-01	0.20	3.2E-01
ENSCAFG0000000655	APAF1	darkgrey	EC_M8	0.67	1.9E-04	0.61	1.0E-03	-0.40	4.0E-02	-0.03	8.7E-01	-0.31	1.2E-01	0.22	2.8E-01	-0.15	4.5E-01	-0.06	7.9E-01	-0.31	1.2E-01	-0.06	7.6E-01
ENSCAFG0000001094	CHD1L	darkgrey	EC_M8	0.67	1.9E-04	-0.07	7.2E-01	-0.69	8.8E-05	-0.14	4.9E-01	0.35	8.1E-02	0.26	2.1E-01	-0.32	1.1E-01	0.02	9.1E-01	-0.05	8.1E-01	-0.70	8.0E-05
ENSCAFG0000001764	MYO9A	darkgrey	EC_M8	0.67	1.9E-04	0.51	8.2E-03	-0.44	2.5E-02	-0.05	8.1E-01	-0.26	2.0E-01	-0.02	9.3E-01	-0.17	4.0E-01	-0.11	6.0E-01	-0.47	1.5E-02	-0.11	5.8E-01
ENSCAFG0000001857	XIAP	darkgrey	EC_M8	0.67	1.9E-04	0.36	7.1E-02	-0.69	1.1E-04	0.21	3.1E-01	0.11	6.0E-01	0.06	7.6E-01	-0.44	2.3E-02	-0.09	6.7E-01	-0.18	3.7E-01	-0.45	2.2E-02
ENSCAFG0000000457	ENSCAFG0000000457	darkgrey	EC_M8	0.67	1.9E-04	0.30	1.3E-01	-0.28	1.7E-01	-0.01	9.5E-01	-0.30	1.3E-01	0.16	4.5E-01	0.03	8.7E-01	0.09	6.7E-01	0.13	5.2E-01	-0.05	8.1E-01
ENSCAFG0000003203	ENSCAFG0000003203	darkgrey	EC_M8	0.67	2.0E-04	0.07	7.3E-01	0.04	8.6E-01	0.03	9.0E-01	-0.56	2.7E-03	0.16	4.2E-01	-0.31	1.2E-01	-0.05	8.3E-01	-0.28	1.7E-01	0.23	2.5E-01
ENSCAFG0000000409	ENSCAFG0000000409	darkgrey	EC_M8	0.67	2.0E-04	0.26	2.0E-01	-0.58	2.0E-03	0.27	1.9E-01	0.00	1.0E+00	0.24	2.3E-01	-0.34	8.7E-02	-0.12	5.5E-01	-0.38	5.5E-02	-0.36	6.7E-02
ENSCAFG0000000756	IMMT	grey	EC_M10	0.67	2.0E-04	0.01	9.5E-01	-0.01	9.8E-01	-0.01	9.5E-01	-0.54	4.6E-03	-0.06	7.8E-01	-0.31	1.2E-01	-0.26	2.1E-01	-0.23	2.6E-01	0.17	3.9E-01
ENSCAFG0000000213	ENSCAFG0000000213	darkgrey	EC_M8	0.67	2.0E-04	0.03	8.8E-01	-0.73	2.1E-05	0.13	5.1E-01	0.28	1.7E-01	0.14	5.1E-01	-0.33	1.0E-01	-0.06	7.8E-01	-0.19	3.4E-01	-0.61	9.2E-04
ENSCAFG0000001281	DDX42	turquoise	EC_M6	0.67	2.0E-04	0.62	8.1E-04	-0.64	4.1E-04	-0.05	8.0E-01	-0.06	7.7E-01	0.25	2.3E-01	-0.17	4.2E-01	-0.18	3.7E-01	0.08	6.9E-01	-0.29	1.5E-01
ENSCAFG0000001770	MED13	darkgrey	EC_M8	0.67	2.0E-04	0.01	9.5E-01	-0.54	4.2E-03	0.14	4.9E-01	0.01	9.8E-01	0.28	1.6E-01	-0.24	2.4E-01	-0.16	4.2E-01	-0.34	9.4E-02	-0.40	4.0E-02
ENSCAFG0000001900	BLMH	darkgrey	EC_M8	0.67	2.0E-04	0.16	4.2E-01	-0.20	3.2E-01	0.11	5.8E-01	-0.34	8.9E-02	0.24	2.3E-01	-0.25	2.2E-01	-0.19	3.5E-01	-0.07	7.3E-01	0.00	1.0E+00
ENSCAFG0000001768	ENSCAFG0000001768	grey	EC_M10	0.67	2.0E-04	-0.21	2.9E-01	-0.15	4.6E-01	0.21	2.9E-01	-0.34	8.8E-02	0.17	4.0E-01	-0.06	7.6E-01	0.02	9.2E-01	-0.44	2.3E-02	-0.06	7.7E-01
ENSCAFG0000000637	ENSCAFG0000000637	darkgrey	EC_M8	0.67	2.0E-04	0.20	3.4E-01	-0.62	8.1E-04	0.21	3.1E-01	0.05	8.1E-01	0.00	9.8E-01	-0.32	1.1E-01	-0.14	4.8E-01	-0.19	3.4E-01	-0.38	5.4E-02
ENSCAFG0000000271	SENP6	darkgrey	EC_M8	0.67	2.1E-04	0.46	1.9E-02	-0.67	2.0E-04	0.07	7.4E-01	0.01	9.8E-01	0.21	3.1E-01	-0.20	3.2E-01	-0.05	8.0E-01	-0.27	1.8E-01	-0.36	7.3E-02
ENSCAFG0000002932	FAM133B	darkgrey	EC_M8	0.67	2.1E-04	0.33	9.8E-02	-0.52	6.1E-03	0.02	9.3E-01	-0.08	6.8E-01	0.24	2.4E-01	-0.07	7.3E-01	-0.33	1.0E-01	-0.28	1.7E-01	-0.26	1.9E-01
ENSCAFG0000001056	SNRPA1	darkgrey	EC_M8	0.67	2.1E-04	0.29	1.5E-01	-0.78	2.8E-06	-0.16	4.2E-01	0.31	1.2E-01	0.15	4.6E-01	-0.07	7.5E-01	0.09	6.7E-01	-0.08	6.8E-01	-0.64	4.6E-04
ENSCAFG0000001811	TCOF1	grey	EC_M10	0.66	2.1E-04	0.06	7.7E-01	0.16	4.3E-01	0.21	3.1E-01	-0.75	9.1E-06	-0.07	7.4E-01	-0.16	4.2E-01	-0.16	4.5E-01	-0.33	9.9E-02	0.35	7.9E-02
ENSCAFG0000001251	WDR12	darkgrey	EC_M8	0.66	2.1E-04	-0.12	5.6E-01	-0.44	2.4E-02	-0.01	9.7E-01	0.08	7.0E-01	0.15	4.6E-01	-0.09	6.7E-01	-0.58	2.0E-03	-0.35	7.7E-02	-0.43	2.9E-02
ENSCAFG0000001002	SMARCAD1	darkgrey	EC_M8	0.66	2.1E-04	0.27	1.9E-01	-0.14	4.9E-01	0.16	4.3E-01	-0.57	2.6E-03	-0.04	8.3E-01	-0.19	3.6E-01	-0.06	7.7E-01	-0.15	4.8E-01	0.18	3.9E-01
ENSCAFG0000000390	FAF1	grey	EC_M10	0.66	2.1E-04	-0.11	6.0E-01	-0.17	4.2E-01	0.13	5.4E-01	-0.26	2.1E-01	0.04	8.4E-01	-0.36	6.7E-02	-0.29	1.6E-01	-0.41	3.9E-02	-0.07	7.5E-01
ENSCAFG0000000792	FRA10AC1	grey	EC_M10	0.66	2.2E-04	-0.04	8.5E-01	-0.31	1.2E-01	0.16	4.3E-01	-0.22	2.8E-01	0.26	2.0E-01	-0.35	7.9E-02	-0.19	3.4E-01	-0.04	8.4E-01	-0.14	5.1E-01
ENSCAFG0000000969	ENSCAFG0000000969	grey	EC_M10	0.66	2.2E-04	0.09	6.5E-01	-0.19	3.4E-01	0.03	8.7E-01	-0.39	4.6E-02	-0.12	5.6E-01	-0.08	7.1E-01	-0.15	4.5E-01	-0.04	8.3E-01	0.07	7.2E-01
ENSCAFG0000003082	ENSCAFG0000003082	darkgrey	EC_M8	0.66	2.2E-04	-0.42	3.3E-02	-0.13	5.1E-01	0.15	4.8E-01	-0.21	3.1E-01	0.10	6.4E-01	-0.44	2.4E-02	0.06	7.9E-01	-0.11	6.0E-01	-0.14	4.8E-01
ENSCAFG0000000055	CSNK1G3	darkgrey	EC_M8	0.66	2.2E-04	0.58	1.9E-03	-0.74	1.4E-05	-0.03	8.8E-01	0.08	7.0E-01	0.13	5.2E-01	-0.29	1.5E-01	-0.01	9.5E-01	-0.13	5.2E-01	-0.41	3.7E-02
ENSCAFG0000002418	ENSCAFG0000002418	darkgrey	EC_M8	0.66	2.2E-04	0.45	2.0E-02	0.04	8.4E-01	0.12	5.6E-01	-0.76	6.0E-06	-0.21	3.0E-01	-0.18	3.7E-01	-0.12	5.5E-01	-0.37	6.4E-02	0.40	4.2E-02
ENSCAFG0000000374	PRPF38A	grey	EC_M10	0.66	2.2E-04	-0.07	7.3E-01	-0.04	8.4E-01	0.15	4.5E-01	-0.49	1.1E-02	0.08	6.8E-01	-0.21	3.0E-01	-0.38	5.3E-02	-0.47	1.5E-02	0.09	6.6E-01
ENSCAFG0000001041	PDCD11	darkgrey	EC_M8	0.66	2.2E-04	-0.06	7.7E-01	-0.76	6.0E-06	-0.26	2.0E-01	0.42	3.4E-02	0.27	1.7E-01	-0.17	4.1E-01	0.08	7.0E-01	-0.10	6.4E-01	-0.78	3.2E-06
ENSCAFG0000001644	PRELID1	darkgrey	EC_M8	0.66	2.2E-04	0.53	5.6E-03	-0.43	2.9E-02	-0.14	4.8E-01	-0.14	5.0E-01	0.03	8.7E-01	-0.11	6.1E-01	-0.03	8.9E-01	-0.27	1.9E-01	-0.22	2.7E-01
ENSCAFG0000000125	TNRC6B	darkgrey	EC_M8	0.66	2.3E-04	-0.31	1.2E-01	-0.27	1.8E-01	-0.02	9.3E-01	-0.06	7.7E-01	0.23	2.5E-01	-0.28	1.6E-01	-0.28	1.6E-01	-0.52	5.9E-03	-0.30	1.3E-01
ENSCAFG0000002071	ENSCAFG0000002071	darkgrey	EC_M8	0.66	2.3E-04	0.13	5.4E-01	-0.17	4.0E-01	-0.01	9.6E-01	-0.37	6.5E-02	-0.07	7.2E-01	-0.37	6.5E-02	-0.08	7.1E-01	-0.01	9.8E-01	0.02	9.2E-01
ENSCAFG0000002628	ENSCAFG0000002628	grey	EC_M10	0.66	2.3E-04	0.02	9.4E-01	-0.23	2.7E-01	-0.01	9.8E-01	-0.32	1.1E-01	0.39	4.8E-02	-0.23	2.7E-01	0.05	8.2E-01	-0.23	2.6E-01	-0.08	6.8E-01
ENSCAFG0000000737	PARP4	darkgrey	EC_M8	0.66	2.3E-04	0.36	6.9E-02	-0.32	1.1E-01	-0.11	5.9E-01	-0.35	8.0E-02	0.10	6.4E-01	-0.26	2.1E-01	-0.06	7.7E-01	-0.28	1.7E-01	-0.02	9.3E-01
ENSCAFG0000001086	ENSCAFG0000001086	darkgrey	EC_M8	0.66	2.3E-04	0.25	2.2E-01	-0.60	1.1E-03	0.11	5.8E-01	0.05	8.3E-01	0.14	4.9E-01	-0.26	1.9E-01	-0.31	1.2E-01	-0.25	2.1E-01	-0.37	6.3E-02
ENSCAFG0000001952	DAZAP1	darkgrey	EC_M8	0.66	2.4E-04	0.17	4.0E-01	-0.18	3.7E-01	-0.05	8.0E-01	-0.29	1.5E-01	0.05	8.1E-01	-0.12	5.7E-01	-0.12	5.4E-01	-0.05	8.2E-01	-0.05	7.9E-01
ENSCAFG0000001236	FLVCR1	darkgrey	EC_M8	0.66	2.4E-04	0.16	4.2E-01	-0.09	6.5E-01	0.10	6.4E-01	-0.44	2.3E-02	0.21	3.0E-01	-0.14	5.1E-01	-0.33	9.5E-02	-0.49	1.2E-02	0.08	6.9E-01
ENSCAFG0000000355	ASCC3	darkgrey	EC_M8	0.66	2.4E-04	-0.23	2.6E-01	-0.68	1.5E-04	-0.07	7.2E-01	0.37	6.7E-02	0.27	1.9E-01	-0.21	2.9E-01	-0.28	1.7E-01	-0.14	4.9E-01	-0.71	5.0E-05
ENSCAFG0000000406	NT5DC1	darkgrey	EC_M8	0.66	2.4E-04	0.29	1.5E-01	-0.20	3.3E-01	-0.31	1.2E-01	-0.36	6.7E-02	-0.10	6.4E-01	-0.20	3.2E-01	0.06	7.7E-01	-0.11	6.0E-01	0.04	8.6E-01
ENSCAFG0000000744	TDG	darkgrey	EC_M8	0.66	2.4E-04	0.29	1.5E-01	-0.56	3.2E-03	-0.31	1.2E-01	-0.06	7.6E-01	0.28	1.7E-01	-0.11	6.1E-01	0.06	7.6E-01	-0.15	4.7E-01	-0.34	9.4E-02
ENSCAFG0000001126	CSE1L	darkgrey	EC_M8	0.66	2.4E-04	0.10	6.3E-01	-0.67	2.0E-04	-0.21	2.9E-01	0.25	2.1E-01	0.15	4.6E-01	-0.52	6.3E-03	0.01	9.5E-01	0.11	5.9E-01	-0.58	1.7E-03
ENSCAFG0000000430	AMMECR1L	turquoise	EC_M6	0.66	2.4E-04	0.61	9.6E-04	-0.45	2.0E-02	0.05	7.9E-01	-0.24	2.4E-01	0.01	9.6E-01	-0.17	4.2E-01	-0.10	6.1E-01	-0.02	9.1E-01	-0.11	5.8E-01
ENSCAFG0000001091	RBM6	darkgrey	EC_M8	0.66	2.4E-04	0.47	1.5E-02	-0.10	6.2E-01	0.25	2.2E-01	-0.62	8.1E-04	-0.09	6.6E-01	-0.12	5.5E-01	-0.24	2.5E-01	-0.04	8.6E-01	0.27	1.8E-01
ENSCAFG0000002008	PTBP2	grey	EC_M10	0.66	2.4E-04	0.21	2.9E-01	-0.58	1.8E-03	0.02	9.1E-01	0.06	7.7E-01	0.40	4.4E-02	-0.02	9.3E-01	-0.19	3.6E-01	0.14	4.8E-01	-0.39	5.2E-02
ENSCAFG0000003271	ENSCAFG0000003271	darkgreen	EC_M4	0.66	2.4E-04	0.11	5.8E-01	-0.73	2.5E-05	-0.16	4.2E-01	0.29	1.4E-01	0.28	1.6E-01	-0.21	2.9E-01	-0.14	4.8E-01	-0.52	7.1E-03	-0.63	5.7E-04

ENSCAFG0000002042	ANKRD13C	darkgrey	EC_M8	0.66	2.5E-04	0.01	9.6E-01	-0.47	1.5E-02	0.17	4.0E-01	-0.04	8.3E-01	0.16	4.3E-01	-0.07	7.3E-01	-0.16	4.3E-01	-0.20	3.3E-01	-0.28	1.7E-01
ENSCAFG0000000477	KIF2C	darkgrey	EC_M8	0.66	2.5E-04	0.27	1.8E-01	-0.49	1.1E-02	-0.13	5.4E-01	0.04	8.4E-01	0.24	2.4E-01	-0.10	6.1E-01	-0.06	7.8E-01	-0.12	5.4E-01	-0.35	7.8E-02
ENSCAFG0000001238	BLM	darkgrey	EC_M8	0.66	2.5E-04	0.23	2.6E-01	-0.26	2.0E-01	-0.11	5.9E-01	-0.29	1.5E-01	0.25	2.2E-01	-0.02	9.1E-01	-0.15	4.5E-01	-0.12	5.6E-01	-0.10	6.2E-01
ENSCAFG0000001638	EIF2S1	darkgreen	EC_M4	0.66	2.5E-04	0.09	6.6E-01	-0.87	6.4E-09	-0.22	2.9E-01	0.48	1.3E-02	0.24	2.4E-01	-0.19	3.6E-01	0.11	6.1E-01	-0.18	3.9E-01	-0.82	2.3E-07
ENSCAFG0000001720	RLIM	darkgrey	EC_M8	0.66	2.5E-04	0.14	4.8E-01	-0.76	5.7E-06	0.01	9.5E-01	0.27	1.8E-01	0.19	3.5E-01	-0.17	4.0E-01	-0.07	7.2E-01	-0.18	3.7E-01	-0.58	1.7E-03
ENSCAFG0000000558	ESF1	grey	EC_M10	0.66	2.5E-04	-0.06	7.7E-01	-0.16	4.3E-01	0.15	4.7E-01	-0.40	4.5E-02	-0.07	7.4E-01	-0.25	2.2E-01	0.00	9.9E-01	0.05	8.2E-01	0.06	7.8E-01
ENSCAFG0000000949	INO80	darkgrey	EC_M8	0.66	2.5E-04	0.31	1.2E-01	-0.47	1.4E-02	-0.16	4.5E-01	-0.10	6.3E-01	0.12	5.5E-01	-0.09	6.8E-01	-0.07	7.2E-01	-0.20	3.2E-01	-0.27	1.9E-01
ENSCAFG0000000836	ENSCAFG0000000836	grey	EC_M10	0.66	2.5E-04	0.27	1.9E-01	-0.51	7.2E-03	0.30	1.3E-01	0.01	9.5E-01	0.41	4.0E-02	-0.16	4.4E-01	-0.17	4.0E-01	-0.27	1.9E-01	-0.36	6.8E-02
ENSCAFG0000000717	POLR1B	darkgrey	EC_M8	0.66	2.5E-04	-0.39	4.7E-02	-0.12	5.5E-01	0.00	9.9E-01	-0.18	3.8E-01	0.06	7.9E-01	-0.32	1.1E-01	0.07	7.5E-01	-0.24	2.3E-01	-0.18	3.7E-01
ENSCAFG0000000965	PPP1R15B	grey	EC_M10	0.66	2.6E-04	0.35	8.1E-02	-0.34	9.3E-02	0.20	3.3E-01	-0.28	1.7E-01	0.20	3.2E-01	-0.06	7.6E-01	-0.42	3.0E-02	-0.49	1.1E-02	-0.13	5.4E-01
ENSCAFG0000002307	POC1A	darkgrey	EC_M8	0.66	2.6E-04	0.18	3.7E-01	-0.12	5.7E-01	0.08	6.8E-01	-0.38	5.3E-02	0.07	7.3E-01	-0.28	1.6E-01	-0.16	4.3E-01	0.00	1.0E+00	0.04	8.5E-01
ENSCAFG0000000438	MASTL	darkgrey	EC_M8	0.66	2.6E-04	0.41	4.0E-02	-0.60	1.2E-03	-0.19	3.6E-01	0.07	7.4E-01	0.11	6.1E-01	-0.02	9.3E-01	0.08	7.1E-01	-0.15	4.6E-01	-0.41	3.8E-02
ENSCAFG0000000176	IREB2	turquoise	EC_M6	0.66	2.6E-04	0.58	1.8E-03	-0.71	4.4E-05	-0.33	1.0E-01	0.08	6.9E-01	0.16	4.5E-01	-0.24	2.3E-01	-0.04	8.3E-01	-0.03	9.0E-01	-0.45	2.0E-02
ENSCAFG0000000833	ENSCAFG0000000833	darkgrey	EC_M8	0.66	2.6E-04	0.20	3.3E-01	-0.67	1.8E-04	-0.03	8.7E-01	0.13	5.4E-01	0.19	3.4E-01	0.02	9.2E-01	0.01	9.4E-01	-0.23	2.6E-01	-0.50	9.4E-03
ENSCAFG0000000860	SH2B3	darkgrey	EC_M8	0.66	2.6E-04	0.51	8.3E-03	-0.63	5.4E-04	-0.21	3.0E-01	0.08	6.8E-01	0.10	6.1E-01	-0.04	8.6E-01	-0.16	4.4E-01	-0.12	5.6E-01	-0.41	3.6E-02
ENSCAFG0000000008	CDK2	darkgrey	EC_M8	0.66	2.6E-04	0.52	6.3E-03	-0.37	6.4E-02	0.09	6.6E-01	-0.25	2.1E-01	-0.07	7.3E-01	0.09	6.5E-01	-0.23	2.6E-01	-0.13	5.1E-01	-0.10	6.3E-01
ENSCAFG0000001474	ANXA7	grey	EC_M10	0.66	2.6E-04	-0.02	9.4E-01	-0.07	7.2E-01	0.07	7.5E-01	-0.45	2.2E-02	-0.01	9.8E-01	-0.09	6.7E-01	-0.21	3.1E-01	-0.50	9.4E-03	0.10	6.2E-01
ENSCAFG0000000245	ZNF451	darkgrey	EC_M8	0.66	2.7E-04	0.16	4.5E-01	-0.29	1.5E-01	0.16	4.5E-01	-0.32	1.1E-01	0.30	1.3E-01	-0.38	5.8E-02	-0.10	6.4E-01	-0.29	1.5E-01	-0.04	8.4E-01
ENSCAFG0000000429	RNASEH2B	darkgrey	EC_M8	0.66	2.7E-04	-0.21	3.0E-01	-0.09	6.7E-01	0.09	6.7E-01	-0.32	1.1E-01	-0.07	7.4E-01	-0.27	1.9E-01	0.01	9.6E-01	-0.03	8.9E-01	-0.05	8.1E-01
ENSCAFG0000000280	PHIP	darkgrey	EC_M8	0.66	2.7E-04	0.16	4.3E-01	0.13	5.4E-01	0.15	4.5E-01	-0.78	2.1E-06	0.11	5.8E-01	-0.29	1.5E-01	-0.20	3.3E-01	-0.14	4.9E-01	0.41	3.6E-02
ENSCAFG0000002326	PRIM2	darkgrey	EC_M8	0.66	2.7E-04	-0.02	9.3E-01	-0.32	1.1E-01	0.04	8.6E-01	-0.06	7.8E-01	0.00	9.9E-01	-0.53	5.8E-03	-0.21	3.1E-01	-0.27	1.9E-01	-0.30	1.3E-01
ENSCAFG0000001763	LARP1	darkgrey	EC_M8	0.66	2.7E-04	0.02	9.3E-01	-0.21	3.0E-01	0.04	8.4E-01	-0.23	2.5E-01	0.37	6.1E-02	-0.49	1.0E-02	-0.11	6.0E-01	-0.35	7.8E-02	-0.15	4.5E-01
ENSCAFG0000000724	IPO11	darkgrey	EC_M8	0.66	2.8E-04	0.45	2.1E-02	-0.60	1.3E-03	-0.14	5.1E-01	0.10	6.2E-01	0.09	6.7E-01	-0.31	1.3E-01	-0.13	5.4E-01	-0.21	2.9E-01	-0.43	2.9E-02
ENSCAFG0000000139	RMI1	darkgrey	EC_M8	0.66	2.8E-04	0.10	6.4E-01	-0.05	8.2E-01	0.14	5.0E-01	-0.55	3.8E-03	0.24	2.5E-01	-0.14	4.9E-01	-0.31	1.2E-01	-0.23	2.6E-01	0.20	3.4E-01
ENSCAFG0000000303	ENSCAFG0000000303	darkgrey	EC_M8	0.66	2.8E-04	-0.23	2.6E-01	-0.34	9.2E-02	-0.03	8.7E-01	-0.04	8.4E-01	0.10	6.4E-01	-0.37	6.6E-02	0.13	5.4E-01	-0.05	8.2E-01	-0.30	1.4E-01
ENSCAFG0000001784	ENSCAFG0000001784	darkgrey	EC_M8	0.66	2.8E-04	0.20	3.2E-01	-0.38	5.8E-02	0.25	2.2E-01	-0.21	3.0E-01	-0.08	7.1E-01	-0.27	1.9E-01	-0.40	4.2E-02	-0.41	4.0E-02	-0.12	5.4E-01
ENSCAFG0000001842	UPF3B	darkgrey	EC_M8	0.65	2.9E-04	-0.04	8.5E-01	-0.73	1.9E-05	-0.01	9.5E-01	0.33	9.8E-02	0.15	4.5E-01	-0.31	1.2E-01	0.10	6.3E-01	-0.33	9.8E-02	-0.70	7.7E-05
ENSCAFG0000001591	RAB27A	turquoise	EC_M6	0.65	2.9E-04	0.66	2.6E-04	-0.50	9.3E-03	0.06	7.8E-01	-0.19	3.5E-01	0.04	8.3E-01	-0.18	3.8E-01	-0.26	2.0E-01	0.01	9.5E-01	-0.17	4.0E-01
ENSCAFG0000000974	NIPA2	darkgrey	EC_M8	0.65	2.9E-04	-0.18	3.8E-01	-0.64	4.1E-04	-0.07	7.4E-01	0.35	8.1E-02	0.22	2.8E-01	-0.34	9.4E-02	0.01	9.4E-01	-0.26	2.0E-01	-0.68	1.4E-04
ENSCAFG0000000208	CD2AP	turquoise	EC_M6	0.65	2.9E-04	0.63	6.3E-04	-0.66	2.4E-04	0.05	7.9E-01	-0.02	9.3E-01	0.09	6.7E-01	-0.40	4.3E-02	-0.04	8.3E-01	-0.26	1.9E-01	-0.35	7.9E-02
ENSCAFG0000000759	ZNF507	darkgrey	EC_M8	0.65	2.9E-04	0.28	1.7E-01	0.01	9.7E-01	0.30	1.3E-01	-0.70	6.5E-05	-0.05	8.2E-01	-0.14	4.9E-01	-0.15	4.6E-01	-0.21	3.1E-01	0.33	9.9E-02
ENSCAFG0000000650	CHAMP1	grey	EC_M10	0.65	2.9E-04	0.34	8.7E-02	-0.14	5.0E-01	-0.03	8.7E-01	-0.51	7.5E-03	-0.05	8.1E-01	-0.17	4.1E-01	0.28	1.7E-01	-0.29	1.4E-01	0.14	4.9E-01
ENSCAFG0000000767	ENSCAFG0000000767	darkgrey	EC_M8	0.65	2.9E-04	0.60	1.1E-03	-0.41	3.6E-02	-0.12	5.5E-01	-0.31	1.2E-01	0.17	4.2E-01	0.10	6.4E-01	-0.10	6.2E-01	-0.16	4.4E-01	-0.03	8.8E-01
ENSCAFG0000000352	CCNC	darkgrey	EC_M8	0.65	2.9E-04	0.01	9.5E-01	-0.46	1.8E-02	-0.21	3.0E-01	0.02	9.2E-01	0.29	1.5E-01	0.13	5.4E-01	-0.13	5.2E-01	-0.31	1.2E-01	-0.38	5.2E-02
ENSCAFG0000001508	MED28	darkgrey	EC_M8	0.65	2.9E-04	0.09	6.6E-01	-0.41	3.7E-02	-0.12	5.7E-01	0.00	1.0E+00	0.13	5.2E-01	-0.07	7.3E-01	-0.17	4.1E-01	-0.24	2.4E-01	-0.39	5.0E-02
ENSCAFG0000000033	MON2	grey	EC_M10	0.65	2.9E-04	-0.20	3.3E-01	-0.28	1.7E-01	0.03	8.9E-01	-0.16	4.4E-01	0.16	4.3E-01	-0.08	7.0E-01	-0.32	1.2E-01	-0.27	1.8E-01	-0.19	3.6E-01
ENSCAFG0000002036	MIGA1	darkgrey	EC_M8	0.65	3.0E-04	-0.18	3.8E-01	-0.52	6.2E-03	0.20	3.4E-01	0.08	6.8E-01	0.18	3.8E-01	-0.32	1.2E-01	-0.08	6.9E-01	0.03	9.0E-01	-0.46	1.7E-02
ENSCAFG0000000014	C1H18orf54	darkgrey	EC_M8	0.65	3.0E-04	0.38	5.3E-02	-0.39	5.1E-02	0.14	4.8E-01	-0.29	1.5E-01	0.00	9.9E-01	-0.03	9.0E-01	-0.20	3.4E-01	-0.14	5.0E-01	-0.07	7.3E-01
ENSCAFG0000000908	PBRM1	grey	EC_M10	0.65	3.0E-04	-0.10	6.2E-01	0.05	8.1E-01	0.04	8.5E-01	-0.53	5.6E-03	-0.08	6.9E-01	-0.40	4.4E-02	-0.14	4.8E-01	-0.43	2.8E-02	0.21	3.1E-01
ENSCAFG0000001046	MTR	grey	EC_M10	0.65	3.0E-04	0.23	2.6E-01	-0.03	9.0E-01	-0.07	7.5E-01	-0.58	1.7E-03	-0.25	2.3E-01	-0.25	2.2E-01	0.01	9.8E-01	-0.28	1.7E-01	0.25	2.2E-01
ENSCAFG0000003042	ENSCAFG0000003042	darkgrey	EC_M8	0.65	3.1E-04	0.34	9.1E-02	-0.78	3.2E-06	0.05	7.9E-01	0.21	3.0E-01	0.22	2.9E-01	-0.25	2.1E-01	-0.21	3.0E-01	-0.24	2.3E-01	-0.55	3.4E-03
ENSCAFG0000000899	CFAP298	darkgrey	EC_M8	0.65	3.1E-04	-0.16	4.4E-01	-0.45	2.0E-02	0.02	9.3E-01	0.04	8.4E-01	0.38	5.7E-02	-0.03	8.9E-01	0.02	9.4E-01	-0.25	2.1E-01	-0.41	3.5E-02
ENSCAFG0000000433	PDSS1	darkgrey	EC_M8	0.65	3.1E-04	0.03	8.7E-01	-0.63	5.2E-04	-0.10	6.2E-01	0.29	1.5E-01	0.13	5.3E-01	-0.03	8.9E-01	-0.21	2.9E-01	-0.35	7.9E-02	-0.63	6.0E-04
ENSCAFG0000001108	CAMSAP2	darkgrey	EC_M8	0.65	3.1E-04	0.29	1.5E-01	-0.54	4.5E-03	0.03	8.9E-01	-0.06	7.6E-01	0.13	5.1E-01	-0.17	4.1E-01	-0.19	3.6E-01	-0.50	9.9E-03	-0.29	1.5E-01
ENSCAFG0000000386	CDC40	darkgrey	EC_M8	0.65	3.1E-04	0.05	8.1E-01	-0.41	3.5E-02	0.15	4.7E-01	-0.13	5.2E-01	0.18	3.7E-01	0.02	9.3E-01	0.01	9.7E-01	0.04	8.5E-01	-0.21	2.9E-01
ENSCAFG0000000272	PSME4	darkgreen	EC_M4	0.65	3.1E-04	0.44	2.3E-02	-0.87	1.0E-08	-0.09	6.5E-01	0.28	1.7E-01	0.20	3.4E-01	-0.25	2.3E-01	-0.17	4.1E-01	-0.28	1.7E-01	-0.64	4.4E-04
ENSCAFG0000000459	PTER	grey	EC_M10	0.65	3.1E-04	0.28	1.7E-01	-0.26	2.0E-01	-0.05	8.1E-01	-0.35	7.7E-02	0.10	6.2E-01	-0.28	1.7E-01	-0.09	6.8E-01	-0.10	6.2E-01	0.01	9.8E-01
ENSCAFG0000001175	RAB9A	darkgrey	EC_M8	0.65	3.1E-04	0.21	3.0E-01	-0.53	5.4E-03	-0.04	8.4E-01	-0.05	8.0E-01	0.32	1.1E-01	-0.19	3.6E-01	-0.12	5.6E-01	-0.29	1.6E-01	-0.37	6.4E-02
ENSCAFG0000000775	GALNT6	darkgrey	EC_M8	0.65	3.2E-04	-0.30	1.4E-01	-0.30	1.4E-01	-0.05	8.0E-01	-0.07	7.3E-01	0.12	5.6E-01	-0.31	1.2E-01	-0.01	9.5E-01	-0.37	6.6E-02	-0.34	8.5E-02
ENSCAFG0000002015	SF3B3	darkgrey	EC_M8	0.65	3.2E-04	0.18	3.8E-01	-0.12	5.5E-01	-0.21	3.0E-01	-0.35	8.0E-02	0.14	4.8E-01	-0.14	4.9E-01	-0.14	5.1E-01	0.02	9.4E-01	0.00	9.8E-01
ENSCAFG0000001206	ORC2	darkgrey	EC_M8	0.65	3.2E-04	0.39	4.9E-02	-0.38	5.5E-02	0.08	6.9E-01	-0.27	1.8E-01	0.06	7.6E-01	-0.20	3.2E-01	0.18	3.9E-01	0.22	2.9E-01	-0.08	7.0E-01
ENSCAFG0000000362	USP38	grey	EC_M10	0.65	3.2E-04	0.46	1.7E-02	-0.24	2.4E-01	0.26	2.0E-01	-0.40	4.1E-02	-0.13	5.2E-01	-0.48	1.3E-02	-0.27	1.8E-01	-0.24	2.4E-01	0.07	7.3E-01
ENSCAFG0000002857	NXT2	grey	EC_M10	0.65	3.3E-04	-0.01	9.7E-01	-0.14	4.8E-01	0.12	5.5E-01	-0.38	5.6E-02	0.05	8.1E-01	-0.27	1.9E-01	-0.13	5.4E-01	-0.34	9.1E-02	0.06	7.9E-01
ENSCAFG0000000061	UBR5	darkgrey	EC_M8	0.65	3.3E-04	0.34	9.4E-02	-0.50	8.8E-03	0.01	9.6E-01	-0.13	5.2E-01	0.15	4.6E-01	-0.11	5.9E-01	-0.18	3.8E-01	-0.23	2.6E-01	-0.23	2.5E-01
ENSCAFG0000001763	C7H18orf25	turquoise	EC_M6	0.65	3.3E-04	0.54	4.7E-03	-0.71	5.3E-05	-0.14	4.8E-01	0.10	6.1E-01	0.17	4.0E-01	-0.16	4.3E-01	-0.16	4.3E-01	-0.14	4.8E-01	-0.44	2.4E-02
ENSCAFG0000000300	USP34	darkgrey	EC_M8	0.65	3.3E-04	0.23	2.5E-01	-0.13	5.3E-01	0.06	7.8E-01	-0.49	1.1E-02	-0.08	7.0E-01	-0.30	1.3E-01	-0.29	1.5E-01	-0.28	1.6E-01	0.12	5.4E-01
ENSCAFG0000000666	SLC7A1	darkgrey	EC_M8	0.65	3.4E-04	0.28	1.7E-01	-0.66	2.4E-04	0.01	9.6E-01	0.21	2.9E-01	0.17	4.0E-01	-0.30	1.4E-01	-0.25	2.2E-01	-0.44	2.6E-02	-0.58	2.0E-03
ENSCAFG0000001196	RFC2	darkgrey	EC_M8	0.65	3.4E-04	-0.04	8.5E-01	-0.05	8.0E-01	0.13	5.2E-01	-0.34	8.6E-02	-0.08	7.0E-01	-0.17	4.2E-01	-0.24	2.3E-01	-0.37	6.3E-02	0.02	9.2E-01
ENSCAFG0000002036	FUBP1	turquoise	EC_M6	0.65	3.4E-04	0.62	7.5E-04	-0.66	2.6E-04	-0.04	8.6E-01	0.04	8.3E-01	0.11	5.8E-01	-0.15	4.6E-01	-0.14	4.9E-01	-0.05	8.2E-01	-0.38	5.9E-02
ENSCAFG0000002561	ENSCAFG0000002561	darkgrey	EC_M8	0.65	3.4E-04	-0.17	4.0E-01	-0.26	2.0E-01	-0.03	8.8E-01	-0.08	6.8E-01	0.09	6.5E-01	-0.28	1.6E-01	-0.20	3.4E-01	-0.08	6.9E-01	-0.24	2.5E-01
ENSCAFG0000001051	SLK	darkgrey	EC_M8	0.65	3.4E-04	0.59	1.4E-03	-0.41	4.0E-02	-0.06	7.7E-01	-0.31	1.3E-01	-0.07	7.2E-01	-0.23	2.6E-01	0.09	6.5E-01	0.13	5.4E-01	-0.01	9.7E-01
ENSCAFG0000000752	SMARCA5	darkgrey	EC_M8	0.65	3.4E-04	0.47	1.4E-02	-0.22	2.9E-01	0.08	6.8E-01	-0.53	5.5E-03	0.02	9.2E-01	-0.22	2.7E-01	0.01	9.4E-01	0.08	7.1E-01	0.17	4.1E-01
ENSCAFG0000001112	HAUS2	darkgrey	EC_M8	0.65	3.5E-04	0.19	3.6E-01	-0.74	1.4E-05	-0.10	6.4E-01	0.23	2.6E-01	0.18	3.8E-01	-0.32	1.1E-01	-0.12	5.6E-01	-0.13	5.3E-01	-0.61	1.0E-03
ENSCAFG0000000408	CENPO	darkgrey	EC_M8	0.65	3.5E-04	0.20	3.4E-01	-0.31	1.3E-01	-0.16	4.4E-01	-0.12	5.6E-01	0.28	1.6E-01	0.02	9.3E-01	-0.19	3.6E-01	-0.06	7.6E-01	-0.18	3.8E-01
ENSCAFG0000001301	PDE8A	darkgrey	EC_M8	0.65	3.5E-04	0.11	6.1E-01	-0.57	2.5E-03	-0.24	2.4E-01	0.09	6.7E-01	0.41	3.6E-02	-0.12	5.6E-01	-0.26	2.0E-01	-0.43	2.9E-02	-0.50	9.6E-03
ENSCAFG0000000828	POLR3G	darkgrey	EC_M8	0.65	3.5E-04	-0.08	7.1E-01	-0.42	3.5E-02	-0.15	4.6E-01	0.01	9.6E-01	0.03	8.7E-01	-0.30	1.4E-01	0.21	3.0E-01	-0.02	9.2E-01	-0.37	6.0E-02
ENSCAFG0000000177	ANXA1	grey	EC_M10	0.65	3.5E-04	0.22	2.9E-01	-0.53	5.6E-03	-0.11	5.9E-01	0.02	9.2E-01	0.07	7.2E-01	-0.27	1.8E-01	-0.02	9.3E-01	-0.28	1.7E-01	-0.38	5.6E-02
ENSCAFG0000002430	HIST1H1A	darkgrey	EC_M8	0.65	3.5E-04	0.42	3.3E-02	-0.35	7.9E-02	-0.17	4.1E-01	-0.22	2.9E-01	0.10	6.3E-01	-0.18	3.9E-01	-0.19	3.5E-01	0.05	7.9E-01	-0.08	7.1E-01
ENSCAFG0000001823	MIB1	darkgrey	EC_M8	0.65	3.6E-04	0.54	4.4E-03	-0.76	5.6E-06	-0.04	8.5E-01	0.11	5.8E-01	0.10	6.4E-01	-0.08	7.1E-01	-0.15	4.7E-01	-0.22	2.9E-01	-0.47	1.5E-02
ENSCAFG0000002019	ZNF326	darkgrey	EC_M8	0.65	3.6E-04	0.37	5.9E-02	-0.83	1.9E-07	-0.06	7.7E-01	0.29	1.6E-01	0.21	3.0E-01	-0.22	2.8E-01	0.00	9.9E-01	0.07	7.3E-01	-0.61	8.3E-04
ENSCAFG0000001761	GEMIN5	darkgrey	EC_M8	0.65	3.7E-04	-0.27	1.8E-01	-0.57	2.2E-03	-0.08	7.0E-01	0.29	1.5E-01	0.25	2.2E-01	-0.30	1.4E-01	-0.08	7.0E-01	-0.41	3.9E-02	-0.65	3.0E-04
ENSCAFG0000000864	FAM98B	darkgreen	EC_M4	0.65	3.7E-04	0.10	6.4E-01	-0.73	2.3E-05	-0.14	4.8E-01	0.33	9.5E-02	0.15	4.7E-01	-0.37	6.3E-02	-0.05	8.2E-01	-0.30	1.3E-01	-0.69	8.7E-05
ENSCAFG0000001924	RPA1	darkgrey	EC_M8	0.65	3.7E-04	-0.13	5.3E-01	-0.67	1.6E-04	-0.01	9.6E-01	0.31	1.3E-01	0.20	3.4E-01	-0.13	5.1E-01	-0.21	3.0E-01	-0.36	7.3E-02	-0.68	1.2E-04
ENSCAFG0000000710	CAPRIN1	darkgrey	EC_M8	0.65	3.7E-04	0.14	5.1E-01	-0.35	7.7E-02	-0.06	7.6E-01	-0.12	5.7E-01	0.08	7.0E-01	-0.23	2.6E-01	0.33	1.0E-01	0.12	5.7E-01	-0.22	2.7E-01
ENSCAFG0000001175	RCC1	darkgrey	EC_M8	0.65	3.7E-04	-0.11	5.8E-01	-0.22	2.7E-01	-0.16	4.3E-01	-0.12	5.7E-01	0.20	3.2E-01	-0.16	4.2E-01	-0.09	6.7E-01	-0.02	9.4E-01	-0.21	3.1E-01
ENSCAFG0000001967	PMPCA	darkgrey	EC_M8	0.64	3.8E-04	-0.15	4.6E-01	-0.19	3.5E-01	0.05	8.1E-01	-0.15	4.6E-01	0.24	2.3E-01	-0.42	3.4E-02	-0.01	9.7E-01	-0.09	6.8E-01	-0.20	3.3E-01
ENSCAFG0000000016	MEX3C	darkgrey	EC_M8	0.64	3.8E-04	0.10	6.3E-01	-0.46	1.8E-02	-0.17	4.0E-01	-0.01	9.4E-01	0.20	3.2E-01	-0.61	8.6E-04	-0.03	8.8E-01	-0.30	1.4E-01	-0.36	7.3E-02
ENSCAFG0000000212	CENPQ	darkgrey	EC_M8	0.64	3.8E-04	0.06	7.7E-01	-0.11	6.0E-01	0.22	2.9E-01	-0.32	1.1E-01	0.00	1.0E+00	-0.36	6.7E-02	0.07	7.3E-01	-0.01	9.8E-01	0.03	8.9E-01
ENSCAFG0000000266	FBXO11	darkgrey	EC_M8	0.64	3.8E-04	0.38	5.3E-02	-0.46	1.8E-02	0.08	7.1E-01	-0.23	2.5E-01	0.05	8.2E-01	-0.18	3.9E-01	0.04	8.6E-01	-0.15	4.6E-01	-0.12	5.6E-01
ENSCAFG0000001806	CCP110	grey	EC_M10	0.64	3.8E-04	0.08	6.8E-01	-0.38	5.8E-02	0.09	6.8E-01	-0.13	5.2E-01	-0.08	6.9E-01	-0.06	7.8E-01	-0.25	2.2E-01	-0.36	7.4E-02	-0.23	2.6E-01
ENSCAFG0000000357	ENSCAFG0000000357	darkgrey	EC_M8	0.64	3.9E-04	0.29	1.4E-01	-0.79	1.9E-06	-0.10	6.1E-01	0.29	1.5E-01	0.21	3.1E-01	-0.15	4.5E-01	-0.09	6.8E-01	0.09	6.8E-01	-0.63	6.0E-04
ENSCAFG0000003154	SFR1	darkgrey	EC_M8	0.64	3.9E-04	0.15	4.6E-01	-0.08	6.9E-01	-0.01	9.8E-01	-0.52	6.7E-03	0.09	6.6E-01	-0.20	3.4E-01	-0.25	2.1E-01	-0.35	8.0E-02	0.16	4.5E-01
ENSCAFG0000000400	ENSCAFG0000000400	darkgrey	EC_M8	0.64	3.9E-04	0.14	5.0E-01	-0.56	2.7E-03	0.21	3.1E-01	0.04	8.6E-01	0.15	4.6E-01	-0.41	3.5E-02	-0.05	8.0E-01	-0.23	2.6E-01	-0.38	5.8E-02
ENSCAFG0000000688	GAS2L3	darkgrey	EC_M8	0.64	4.0E-04	0.45	2.2E-02	-0.02	9.2E-01	-0.08	7.1E-01	-0.56	2.8E-03	-0.01	9.8E-01	-0.18	3.7E-01	-0.15	4.6E-01	-0.18	3.9E-01	0.25	2.1E-01
ENSCAFG0000000320	ENSCAFG0000000320	darkgrey	EC_M8	0.64	4.0E-04	0.07	7.2E-01	-0.06	7.7E-01	-0.17	4.0E-01	-0.45	2.2E-02	0.23	2.5E-01	-0.24	2.3E-01	-0.11	5.8E-01	-0.06	7.9E-01	0.11	5.9E-01
ENSCAFG0000000832	RBM39	darkgrey	EC_M8	0.64	4.0E-04	0.31	1.2E-01	-0.29	1.5E-01	0.15	4.6E-01	-0.41	3.6E-02	0.11	6.1E-01	-0.17	4.1E-01	0.03	9.0E-01	0.00	1.0E+00	0.03	8.9E-01
ENSCAFG0000000172	ENSCAFG0000000172	darkgrey	EC_M8	0.64	4.1E-04	0.33	9.7E-02	-0.34	9.0E-02	-0.12	5.7E-01	-0.14	4.9E-01	0.05	8.0E-01	0.01	9.6E-01	-0.13	5.1E-01	-0.29	1.5E-01	-0.18	3.9E-01
ENSCAFG0000001275	AUNIP	darkgrey	EC_M8	0.64	4.1E-04	0.11	5.9E-01	-0.51	7.5E-03	-0.03	8.8E-01	0.12	5.7E-01	0.35	7.6E-02	-0.22	2.9E-01	-0.24	2.5E-01	-0.05	8.2E-01	-0.44	2.5E-02
ENSCAFG0000001379	ENSCAFG0000001379	darkgrey	EC_M8	0.64	4.1E-04	0.38	5.4E-02	-0.77	5.1E-06	-0.11	6.0E-01	0.20	3.4E-01	0.20	3.2E-01	0.01	9.8E-01	0.16	4.4E-01	-0.13	5.2E-01	-0.56	3.2E-03
ENSCAFG0000002854	ZFP30	darkgrey	EC_M8	0.64	4.2E-04	0.12	5.6E-01	-0.35	7.9E-02	0.08	7.1E-01	-0.22	2.8E-01	0.03	8.9E-01	-0.07	7.3E-01	0.31	1.3E-01	-0.14	5.0E-01	-0.14	5.1E-01
ENSCAFG0000001803	HSP90AA1	darkgreen	EC_M4	0.64	4.2E-04	0.16	4.3E-01	-0.79	1.4E-06	-0.09	6.7E-01	0.40	4.5E-02	0.32	1.1E-01	-0.07	7.4E-01	0.01	9.6E-01	-0.06	7.6E-01	-0.71	4.7E-05
ENSCAFG0000000379	C19H4orf33	darkgrey	EC_M8	0.64	4.2E-04	0.36	7.5E-02	-0.27	1.9E-01	0.05	8.0E-01	-0.40	4.2E-02	0.13	5.3E-01	-0.41	3.9E-02	-0.44	2.6E-02	-0.12	5.7E-01	0.06	7.8E-01
ENSCAFG0000001540	BOD1L1	darkgrey	EC_M8	0.64	4.2E-04	0.17	4.0E-01	-0.50	9.8E-03	0.07	7.3E-01	-0.06	7.7E-01	0.09	6.5E-01	-0.27	1.8E-01	-0.20	3.3E-01	-0.49	1.0E-02	-0.29	1.5E-01
ENSCAFG0000002346	ENSCAFG0000002346	grey	EC_M10	0.64	4.2E-04	0.11	5.9E-01	-0.52	6.3E-03	0.26	2.0E-01	-0.01	9.7E-01	0.12	5.7E-01	-0.11	5.9E-01	-0.03	8.8E-01	-0.02	9.1E-01	-0.34	9.3E-02
ENSCAFG0000002271	ENSCAFG0000002271	darkgrey	EC_M8	0.64	4.2E-04	0.10	6.2E-01	-0.22	2.8E-01	0.24	2.3E-01	-0.39	5.2E-02	0.02	9.3E-01	-0.24	2.3E-01	-0.12	5.5E-01	-0.36	7.0E-02	0.05	8.1E-01
ENSCAFG0000000038	KATNA1	darkgrey	EC_M8	0.64	4.3E-04	0.02	9.3E-01	-0.02	9.2E-01	0.08	7.0E-01	-0.49	1.1E-02	0.11	5.8E-01	-0.26	2.0E-01	-0.21	3.0E-01	-0.14	5.0E-01	0.15	4.5E-01
ENSCAFG0000000778	XRN1	darkgrey	EC_M8	0.64	4.3E-04	0.03	9.0E-01	-0.21	3.0E-01	0.20	3.2E-01	-0.37	6.1E-02	0.09	6.8E-01	-0.23	2.6E-01	-0.10	6.3E-01	-0.33	1.0E-01	0.01	9.5E-01
ENSCAFG0000000046	CNOT6	turquoise	EC_M6	0.64	4.3E-04	0.57	2.5E-03	-0.46	1.7E-02	0.07	7.3E-01	-0.17	4.0E-01	0.03	8.9E-01	-0.20	3.2E-01	-0.19	3.5E-01	-0.03	8.9E-01	-0.14	5.0E-01
ENSCAFG0000003006	HMGN4	darkgrey	EC_M8	0.64	4.3E-04	0.35	8.1E-02	-0.55	3.4E-03	-0.09	6.8E-01	-0.02	9.4E-01	0.01	9.7E-01	-0.19	3.4E-01	0.23	2.6E-01	-0.24	2.4E-01	-0.31	1.2E-01
ENSCAFG0000002029	TANGO6	grey	EC_M10	0.64	4.3E-04	0.16	4.3E-01	-0.35	8.3E-02	-0.17	4.0E-01	-0.16	4.4E-01	-0.04	8.5E-01	-0.30	1.4E-01	0.04	8.3E-01	-0.25	2.1E-01	-0.19	3.5E-01
ENSCAFG0000000094	MRPL13	darkgrey	EC_M8	0.64	4.4E-04	0.10	6.1E-01	-0.68	1.3E-04	0.31	1.2E-01	0.26	2.0E-01	0.26	1.9E-01	-0.24	2.5E-01	-0.07	7.2E-01	-0.08	6.8E-01	-0.59	1.6E-03
ENSCAFG0000002514	ENSCAFG0000002514	darkgrey	EC_M8	0.64	4.4E-04	0.15	4.5E-01	-0.42	3.2E-02	0.08	6.8E-01	-0.09	6.5E-01	0.13	5.2E-01	-0.17	4.1E-01	-0.10	6.2E-01	-0.33	9.5E-02	-0.23	2.6E-01
ENSCAFG0000000185	GUF1	grey	EC_M10	0.64	4.4E-04	0.26	2.0E-01	-0.03	8.7E-01	0.14	4.9E-01	-0.56	3.1E-03	0.12	5.5E-01	-0.25	2.1E-01	-0.31	1.2E-01	-0.27	1.9E-01	0.22	2.7E-01
ENSCAFG0000003234	ENSCAFG0000003234	darkgrey	EC_M8	0.64	4.4E-04	0.07	7.4E-01	-0.60	1.1E-03	0.33	1.0E-01	0.10	6.3E-01	0.25	2.1E-01	-0.31	1.2E-01	-0.19	3.5E-01	-0.31	1.3E-01	-0.46	1.9E-02
ENSCAFG0000000155	PSIP1	darkgrey	EC_M8	0.64	4.4E-04	0.08	7.1E-01	0.15	4.6E-01	0.25	2.2E-01	-0.77	5.0E-06	-0.01	9.6E-01	-0.24	2.5E-01	-0.08	6.9E-01	-0.31	1.3E-01	0.41	3.5E-02
ENSCAFG0000001406	EFTUD2	darkgrey	EC_M8	0.64	4.5E-04	-0.07	7.4E-01	-0.15	4.6E-01	-0.26	2.0E-01	-0.27	1.8E-01	0.20	3.3E-01	-0.09	6.7E-01	-0.05	8.2E-01	-0.28	1.6E-01	-0.12	5.7E-01
ENSCAFG0000000781	SMN	darkgrey	EC_M8	0.64	4.5E-04	-0.07	7.3E-01	-0.15	4.6E-01	-0.01	9.8E-01	-0.27	1.9E-01	0.14	4.9E-01	-0.26	2.0E-01	0.02	9.1E-01	-0.20	3.3E-01	-0.11	5.9E-01
ENSCAFG0000003001	IMPA1	darkgrey	EC_M8	0.64	4.5E-04	0.26	2.0E-01	-0.89	9.8E-10	-0.11	5.9E-01	0.41	3.8E-02	0.20	3.3E-01	-0.24	2.4E-01	-0.09	6.4E-01	-0.12	5.7E-01	-0.72	3.1E-05
ENSCAFG0000000039	CAND1	darkgreen	EC_M4	0.64	4.6E-04	0.36	7.0E-02	-0.84	7.7E-08	-0.08	7.0E-01	0.35	8.3E-02	0.26	2.0E-01	-0.07	7.3E-01	-0.01	9.7E-01	-0.29	1.4E-01	-0.68	1.3E-04
ENSCAFG0000000611	EXOSC8	darkgrey	EC_M8	0.64	4.6E-04	-0.06	7.8E-01	0.05	8.0E-01	0.11	5.9E-01	-0.51	8.0E-03	0.04	8.5E-01	-0.06	7.6E-01	-0.20	3.2E-01	-0.05	8.1E-01	0.17	4.1E-01
ENSCAFG0000002904	ACSL3	grey	EC_M10	0.64	4.6E-04	-0.03	9.0E-01	-0.25	2.1E-01	0.32	1.1E-01	-0.22	2.8E-01	0.08	7.0E-01	-0.31	1.3E-01	-0.18	3.8E-01	-0.53	5.4E-03	-0.15	4.5E-01
ENSCAFG0000001129	TBCE	darkgrey	EC_M8	0.64	4.6E-04	-0.31	1.3E-01	-0.43	2.7E-02	0.15	4.5E-01	0.11	5.8E-01	0.14	4.9E-01	-0.52	7.0E-03	-0.21	3.0E-01	-0.17	3.9E-01	-0.46	1.8E-02
ENSCAFG0000001643	MRM2	grey	EC_M10	0.64	4.6E-04	-0.08	7.0E-01	-0.32	1.1E-01	0.18	3.8E-01	-0.15	4.7E-01	-0.19	3.6E-01	-0.26	2.1E-01	0.21	2.9E-01	-0.22	2.9E-01	-0.22	2.9E-01
ENSCAFG0000000272	CDCA7L	darkgrey	EC_M8	0.64	4.6E-04	-0.29	1.6E-01	0.03	8.8E-01	0.08	7.0E-01	-0.40	4.0E-02	-0.03	8.7E-01	-0.13	5.1E-01	-0.09	6.6E-01	-0.11	6.0E-01	0.04	8.4E-01
ENSCAFG0000001838	PSMD11	darkgreen	EC_M4	0.64	4.7E-04	0.11	6.1E-01	-0.84	8.0E-08	-0.24	2.4E-01	0.46	1.9E-02	0.36	7.3E-02	-0.02	9.2E-01	-0.02	9.4E-01	-0.21	3.0E-01	-0.78	3.0E-06
ENSCAFG0000000504	TBC1D4	turquoise	EC_M6	0.64	4.7E-04	0.72	3.1E-05	-0.45	2.1E-02	0.02	9.3E-01	-0.25	2.2E-01	-0.12	5.6E-01	-0.29	1.5E-01	0.00	9.9E-01	0.19	3.6E-01	-0.08	6.9E-01

ENSCAFG0000000373	C12H6orf203	grey	EC_M10	0.64	4.7E-04	-0.19	3.4E-01	0.00	9.8E-01	0.01	9.5E-01	-0.43	2.6E-02	0.02	9.2E-01	-0.29	1.5E-01	-0.12	5.7E-01	-0.54	4.1E-03	0.08	7.0E-01
ENSCAFG0000000805	MTMR9	darkgrey	EC_M8	0.64	4.8E-04	0.29	1.6E-01	-0.13	5.2E-01	0.13	5.3E-01	-0.49	1.1E-02	-0.02	9.4E-01	-0.29	1.5E-01	-0.20	3.2E-01	-0.25	2.1E-01	0.18	3.8E-01
ENSCAFG0000000161	DENND4C	darkgrey	EC_M8	0.64	4.8E-04	0.03	8.8E-01	-0.63	6.0E-04	0.05	7.9E-01	0.13	5.3E-01	0.03	9.0E-01	-0.17	4.1E-01	-0.12	5.4E-01	-0.34	9.0E-02	-0.48	1.3E-02
ENSCAFG0000000779	PDE12	darkgrey	EC_M8	0.64	4.8E-04	-0.11	5.9E-01	-0.55	3.5E-03	0.01	9.7E-01	0.19	3.4E-01	0.09	6.7E-01	-0.48	1.2E-02	-0.06	7.7E-01	-0.20	3.4E-01	-0.52	6.8E-03
ENSCAFG0000002443	GEMIN4	darkgrey	EC_M8	0.64	4.8E-04	-0.24	2.4E-01	0.11	5.9E-01	-0.03	9.0E-01	-0.53	5.0E-03	0.05	8.2E-01	-0.25	2.3E-01	-0.02	9.3E-01	-0.13	5.4E-01	0.16	4.2E-01
ENSCAFG0000000529	TK1	darkgrey	EC_M8	0.64	4.9E-04	-0.10	6.3E-01	-0.08	7.0E-01	-0.03	8.8E-01	-0.29	1.5E-01	-0.09	6.8E-01	-0.14	5.1E-01	-0.17	4.2E-01	0.05	8.0E-01	-0.02	9.1E-01
ENSCAFG0000000198	SLC29A1	grey	EC_M10	0.64	4.9E-04	-0.14	5.0E-01	0.15	4.7E-01	0.11	6.0E-01	-0.60	1.3E-03	0.08	7.0E-01	-0.14	5.1E-01	-0.21	3.0E-01	-0.48	1.4E-02	0.24	2.4E-01
ENSCAFG0000000345	MMS22L	darkgrey	EC_M8	0.63	4.9E-04	0.05	8.1E-01	-0.01	9.8E-01	-0.02	9.2E-01	-0.47	1.5E-02	0.24	2.3E-01	-0.02	9.2E-01	-0.32	1.1E-01	-0.32	1.1E-01	0.12	5.4E-01
ENSCAFG0000000184	NFX1	turquoise	EC_M6	0.63	4.9E-04	0.62	7.3E-04	-0.49	1.1E-02	0.10	6.3E-01	-0.20	3.4E-01	0.03	8.8E-01	-0.23	2.7E-01	-0.19	3.6E-01	-0.32	1.1E-01	-0.16	4.4E-01
ENSCAFG0000001085	PSMD1	darkgreen	EC_M4	0.63	5.0E-04	0.03	8.9E-01	-0.84	7.9E-08	-0.20	3.3E-01	0.49	1.2E-02	0.24	2.4E-01	-0.26	2.0E-01	-0.09	6.7E-01	-0.20	3.3E-01	-0.80	8.7E-07
ENSCAFG0000000611	PRKD3	darkgrey	EC_M8	0.63	5.0E-04	0.37	6.3E-02	-0.06	7.8E-01	-0.02	9.3E-01	-0.59	1.5E-03	0.04	8.6E-01	-0.25	2.2E-01	-0.48	1.4E-02	-0.40	4.5E-02	0.26	2.1E-01
ENSCAFG0000000533	EBNA1BP2	darkgrey	EC_M8	0.63	5.0E-04	-0.22	2.9E-01	-0.53	5.0E-03	-0.13	5.1E-01	0.21	2.9E-01	0.15	4.8E-01	-0.09	6.7E-01	0.19	3.6E-01	-0.36	7.0E-02	-0.57	2.3E-03
ENSCAFG0000001372	CDC27	darkgrey	EC_M8	0.63	5.0E-04	-0.11	5.8E-01	-0.71	4.8E-05	-0.10	6.2E-01	0.38	5.2E-02	0.32	1.1E-01	-0.52	6.0E-03	0.02	9.2E-01	-0.19	3.5E-01	-0.72	3.4E-05
ENSCAFG0000000359	HACE1	grey	EC_M10	0.63	5.0E-04	0.40	4.2E-02	-0.29	1.5E-01	-0.04	8.3E-01	-0.33	9.8E-02	0.19	3.5E-01	-0.22	2.7E-01	-0.37	6.6E-02	-0.38	5.4E-02	0.01	9.7E-01
ENSCAFG0000002922	ZNF770	darkgrey	EC_M8	0.63	5.1E-04	0.40	4.1E-02	-0.44	2.5E-02	0.11	5.9E-01	-0.23	2.5E-01	0.17	4.0E-01	0.03	8.7E-01	0.02	9.3E-01	-0.15	4.6E-01	-0.13	5.4E-01
ENSCAFG0000001199	UTP25	grey	EC_M10	0.63	5.1E-04	0.01	9.7E-01	0.10	6.2E-01	0.11	5.9E-01	-0.62	6.8E-04	-0.09	6.5E-01	-0.09	6.7E-01	0.06	7.8E-01	-0.36	7.4E-02	0.27	1.8E-01
ENSCAFG0000001404	PRPF39	darkgrey	EC_M8	0.63	5.1E-04	0.43	2.7E-02	-0.16	4.5E-01	0.25	2.1E-01	-0.56	3.1E-03	0.02	9.2E-01	-0.19	3.7E-01	-0.07	7.4E-01	-0.10	6.3E-01	0.22	2.9E-01
ENSCAFG0000002978	SAAL1	darkgrey	EC_M8	0.63	5.1E-04	-0.04	8.4E-01	-0.22	2.8E-01	-0.15	4.7E-01	-0.19	3.5E-01	-0.07	7.5E-01	-0.35	8.3E-02	-0.03	8.9E-01	0.00	1.0E+00	-0.14	4.8E-01
ENSCAFG0000000256	FOXJ3	darkgrey	EC_M8	0.63	5.2E-04	0.25	2.2E-01	-0.39	4.8E-02	0.03	8.8E-01	-0.20	3.3E-01	0.11	5.9E-01	-0.17	4.1E-01	-0.12	5.7E-01	-0.23	2.5E-01	-0.21	2.9E-01
ENSCAFG0000000294	ENSCAFG0000000294	grey	EC_M10	0.63	5.2E-04	-0.13	5.4E-01	-0.08	7.0E-01	0.16	4.3E-01	-0.43	2.7E-02	0.16	4.5E-01	-0.08	6.8E-01	0.13	5.4E-01	-0.45	2.1E-02	0.05	8.2E-01
ENSCAFG0000001349	HNRNPA3	darkgrey	EC_M8	0.63	5.2E-04	0.15	4.8E-01	-0.05	8.1E-01	-0.21	2.9E-01	-0.43	2.7E-02	0.00	9.9E-01	-0.17	4.0E-01	-0.01	9.6E-01	-0.07	7.3E-01	0.09	6.5E-01
ENSCAFG0000002983	CENPW	darkgrey	EC_M8	0.63	5.2E-04	-0.07	7.2E-01	-0.25	2.1E-01	-0.15	4.7E-01	-0.10	6.1E-01	0.04	8.4E-01	-0.10	6.3E-01	-0.16	4.4E-01	-0.02	9.4E-01	-0.23	2.6E-01
ENSCAFG0000001723	HACD3	darkgrey	EC_M8	0.63	5.3E-04	-0.25	2.2E-01	-0.38	5.8E-02	-0.12	5.7E-01	0.09	6.6E-01	0.15	4.8E-01	-0.39	4.8E-02	-0.16	4.3E-01	-0.14	5.0E-01	-0.40	4.3E-02
ENSCAFG0000000877	ENSCAFG0000000877	darkgrey	EC_M8	0.63	5.3E-04	0.30	1.3E-01	-0.42	3.2E-02	0.13	5.3E-01	-0.19	3.6E-01	-0.02	9.2E-01	-0.09	6.8E-01	-0.28	1.7E-01	-0.43	2.9E-02	-0.16	4.2E-01
ENSCAFG0000001422	NEMF	grey	EC_M10	0.63	5.3E-04	-0.01	9.6E-01	-0.26	1.9E-01	0.02	9.3E-01	-0.24	2.4E-01	0.25	2.2E-01	-0.32	1.1E-01	-0.14	4.9E-01	-0.57	2.4E-03	-0.11	5.8E-01
ENSCAFG0000001722	SPTLC2	darkgreen	EC_M4	0.63	5.4E-04	0.56	3.2E-03	-0.79	1.8E-06	-0.23	2.5E-01	0.19	3.4E-01	-0.03	9.0E-01	-0.17	3.9E-01	-0.05	8.1E-01	-0.26	2.0E-01	-0.52	6.2E-03
ENSCAFG0000003131	ENSCAFG0000003131	grey	EC_M10	0.63	5.4E-04	0.13	5.3E-01	-0.39	5.0E-02	0.23	2.5E-01	-0.18	3.9E-01	0.19	3.5E-01	-0.23	2.5E-01	0.05	8.0E-01	0.07	7.4E-01	-0.19	3.5E-01
ENSCAFG0000001270	OSBPL11	darkgrey	EC_M8	0.63	5.4E-04	0.14	5.0E-01	-0.72	3.7E-05	-0.07	7.4E-01	0.26	2.0E-01	0.22	2.8E-01	-0.27	1.9E-01	-0.35	8.3E-02	0.09	6.6E-01	-0.59	1.4E-03
ENSCAFG0000001368	RFC4	darkgrey	EC_M8	0.63	5.4E-04	0.04	8.3E-01	-0.27	1.9E-01	-0.19	3.6E-01	-0.10	6.4E-01	-0.01	9.8E-01	-0.18	3.8E-01	-0.19	3.5E-01	-0.19	3.6E-01	-0.23	2.5E-01
ENSCAFG0000000917	RAD54B	darkgreen	EC_M4	0.63	5.4E-04	0.24	2.3E-01	-0.78	3.2E-06	0.07	7.3E-01	0.32	1.1E-01	0.33	9.5E-02	-0.23	2.7E-01	0.05	8.2E-01	-0.16	4.5E-01	-0.69	8.3E-05
ENSCAFG0000001196	PSME2	grey	EC_M10	0.63	5.4E-04	0.20	3.3E-01	-0.17	4.1E-01	0.13	5.4E-01	-0.41	4.0E-02	-0.10	6.3E-01	-0.05	8.0E-01	0.08	7.1E-01	-0.30	1.4E-01	0.02	9.2E-01
ENSCAFG0000001331	SIRT1	grey	EC_M10	0.63	5.5E-04	-0.10	6.2E-01	-0.17	4.0E-01	0.33	1.0E-01	-0.32	1.1E-01	0.18	3.9E-01	-0.31	1.2E-01	-0.22	2.9E-01	-0.20	3.4E-01	-0.01	9.6E-01
ENSCAFG0000000416	PMPCB	darkgrey	EC_M8	0.63	5.5E-04	-0.03	8.7E-01	-0.13	5.3E-01	-0.08	7.1E-01	-0.33	1.0E-01	-0.11	6.0E-01	-0.43	2.8E-02	-0.04	8.4E-01	-0.03	8.8E-01	0.01	9.4E-01
ENSCAFG0000001357	EIF3J	darkgreen	EC_M4	0.63	5.5E-04	-0.02	9.1E-01	-0.81	6.2E-07	-0.09	6.5E-01	0.44	2.5E-02	0.27	1.8E-01	-0.28	1.7E-01	-0.03	9.0E-01	-0.21	3.0E-01	-0.77	3.4E-06
ENSCAFG0000000700	TMEM68	darkgrey	EC_M8	0.63	5.6E-04	0.09	6.6E-01	-0.78	2.6E-06	-0.03	8.9E-01	0.33	1.0E-01	0.39	4.7E-02	-0.09	6.7E-01	-0.11	6.0E-01	-0.39	5.1E-02	-0.70	7.8E-05
ENSCAFG0000001554	SUV39H1	darkgrey	EC_M8	0.63	5.6E-04	-0.15	4.6E-01	-0.21	2.9E-01	-0.09	6.8E-01	-0.11	5.9E-01	0.15	4.7E-01	-0.35	7.8E-02	0.06	7.7E-01	-0.10	6.3E-01	-0.20	3.2E-01
ENSCAFG0000001750	ANP32A	darkgrey	EC_M8	0.63	5.6E-04	0.27	1.9E-01	-0.02	9.2E-01	0.05	8.1E-01	-0.62	8.0E-04	-0.08	6.8E-01	-0.08	7.1E-01	0.15	4.6E-01	-0.02	9.3E-01	0.28	1.6E-01
ENSCAFG0000001731	ENSCAFG0000001731	darkgrey	EC_M8	0.63	5.6E-04	0.10	6.4E-01	-0.60	1.2E-03	0.33	1.0E-01	0.10	6.4E-01	0.22	2.7E-01	-0.14	5.1E-01	-0.23	2.6E-01	-0.42	3.1E-02	-0.43	2.6E-02
ENSCAFG0000001127	ZNF639	grey	EC_M10	0.63	5.7E-04	0.04	8.4E-01	0.03	8.7E-01	0.04	8.5E-01	-0.56	2.7E-03	0.06	7.9E-01	-0.11	5.9E-01	-0.09	6.8E-01	-0.48	1.3E-02	0.25	2.2E-01
ENSCAFG0000001560	RPS24	grey	EC_M10	0.63	5.7E-04	0.03	8.9E-01	-0.28	1.7E-01	0.17	4.1E-01	-0.24	2.4E-01	0.18	3.8E-01	0.07	7.5E-01	0.01	9.6E-01	-0.46	1.8E-02	-0.17	4.2E-01
ENSCAFG0000001339	SETD2	darkgrey	EC_M8	0.63	5.7E-04	0.43	2.9E-02	-0.24	2.4E-01	0.13	5.2E-01	-0.49	1.2E-02	-0.07	7.5E-01	-0.16	4.4E-01	-0.14	4.8E-01	-0.28	1.7E-01	0.12	5.6E-01
ENSCAFG0000001739	ZNF711	darkgrey	EC_M8	0.63	5.7E-04	0.49	1.1E-02	-0.18	3.9E-01	0.13	5.1E-01	-0.49	1.1E-02	-0.12	5.5E-01	-0.04	8.3E-01	-0.24	2.4E-01	-0.33	9.5E-02	0.16	4.3E-01
ENSCAFG0000003238	DENR	darkgreen	EC_M4	0.63	5.7E-04	-0.11	5.9E-01	-0.80	7.5E-07	0.00	9.9E-01	0.46	1.7E-02	0.23	2.5E-01	-0.30	1.3E-01	-0.07	7.3E-01	-0.26	1.9E-01	-0.82	2.8E-07
ENSCAFG0000001879	DNAJC21	darkgrey	EC_M8	0.63	5.7E-04	0.24	2.4E-01	-0.81	6.5E-07	0.00	9.9E-01	0.31	1.2E-01	0.24	2.3E-01	-0.15	4.5E-01	-0.14	4.9E-01	-0.13	5.4E-01	-0.63	5.5E-04
ENSCAFG0000002317	RBM8A	darkgrey	EC_M8	0.63	5.8E-04	0.19	3.6E-01	-0.63	5.9E-04	-0.02	9.3E-01	0.17	4.1E-01	0.31	1.3E-01	-0.11	6.0E-01	0.11	6.1E-01	0.09	6.5E-01	-0.47	1.5E-02
ENSCAFG0000000521	NCAPG2	darkgrey	EC_M8	0.63	5.8E-04	0.00	9.8E-01	0.05	8.2E-01	-0.02	9.3E-01	-0.46	1.7E-02	0.04	8.4E-01	-0.17	4.0E-01	-0.41	3.9E-02	-0.19	3.4E-01	0.17	4.0E-01
ENSCAFG0000000203	FIP1L1	grey	EC_M10	0.63	5.9E-04	0.17	4.1E-01	-0.38	5.5E-02	0.12	5.6E-01	-0.18	3.9E-01	0.15	4.7E-01	-0.22	2.9E-01	0.03	8.9E-01	0.11	5.8E-01	-0.20	3.4E-01
ENSCAFG0000000884	CPNE3	darkgrey	EC_M8	0.63	5.9E-04	0.27	1.8E-01	-0.55	3.8E-03	0.09	6.6E-01	-0.02	9.2E-01	-0.06	7.5E-01	-0.40	4.6E-02	0.23	2.6E-01	0.08	7.0E-01	-0.33	1.0E-01
ENSCAFG0000000798	ENSCAFG0000000798	darkgrey	EC_M8	0.63	5.9E-04	0.12	5.8E-01	-0.28	1.7E-01	0.09	6.6E-01	-0.25	2.1E-01	0.04	8.4E-01	-0.32	1.1E-01	-0.30	1.4E-01	-0.48	1.3E-02	-0.07	7.5E-01
ENSCAFG0000000633	CACNA2D1	darkgrey	EC_M8	0.63	5.9E-04	0.32	1.1E-01	-0.63	6.1E-04	-0.01	9.5E-01	0.04	8.4E-01	0.02	9.2E-01	-0.35	7.8E-02	-0.07	7.2E-01	-0.32	1.1E-01	-0.41	4.0E-02
ENSCAFG0000002871	HMGB3	darkgrey	EC_M8	0.63	6.0E-04	0.33	1.0E-01	-0.37	6.2E-02	-0.08	7.0E-01	-0.07	7.3E-01	0.15	4.6E-01	-0.10	6.2E-01	-0.05	8.2E-01	-0.04	8.4E-01	-0.22	2.8E-01
ENSCAFG0000000050	DDX39B	darkgrey	EC_M8	0.63	6.0E-04	0.23	2.5E-01	-0.42	3.5E-02	0.09	6.5E-01	-0.04	8.5E-01	0.20	3.4E-01	-0.11	5.9E-01	-0.27	1.9E-01	0.09	6.7E-01	-0.27	1.8E-01
ENSCAFG0000000548	POLD3	grey	EC_M10	0.63	6.1E-04	-0.06	7.5E-01	0.03	8.8E-01	0.05	8.2E-01	-0.55	3.8E-03	-0.05	8.1E-01	-0.12	5.7E-01	0.00	9.9E-01	-0.12	5.7E-01	0.22	2.9E-01
ENSCAFG0000000041	MTHFD1L	darkgrey	EC_M8	0.63	6.1E-04	0.23	2.7E-01	-0.38	5.6E-02	-0.05	8.0E-01	-0.09	6.8E-01	-0.06	7.8E-01	-0.46	1.8E-02	-0.03	8.8E-01	-0.48	1.4E-02	-0.27	1.9E-01
ENSCAFG0000001055	TROVE2	darkgreen	EC_M4	0.63	6.1E-04	0.50	9.0E-03	-0.86	2.3E-08	-0.05	8.2E-01	0.28	1.7E-01	0.20	3.2E-01	-0.08	6.9E-01	-0.07	7.3E-01	-0.08	6.8E-01	-0.62	7.6E-04
ENSCAFG0000001412	ENSCAFG0000001412	grey	EC_M10	0.63	6.1E-04	0.16	4.4E-01	-0.56	3.2E-03	-0.28	1.7E-01	0.08	6.8E-01	0.02	9.4E-01	-0.29	1.4E-01	-0.26	2.0E-01	-0.29	1.5E-01	-0.43	3.0E-02
ENSCAFG0000001775	UQCRC2	darkgrey	EC_M8	0.63	6.2E-04	0.11	5.8E-01	-0.17	4.1E-01	-0.05	8.1E-01	-0.29	1.6E-01	0.11	6.1E-01	-0.30	1.3E-01	-0.19	3.4E-01	-0.30	1.3E-01	0.00	9.9E-01
ENSCAFG0000001609	ENSCAFG0000001609	darkgrey	EC_M8	0.63	6.2E-04	-0.29	1.5E-01	-0.59	1.6E-03	0.03	8.7E-01	0.32	1.1E-01	0.23	2.6E-01	-0.42	3.2E-02	0.03	8.8E-01	-0.21	3.0E-01	-0.66	2.2E-04
ENSCAFG0000000016	ME2	darkgrey	EC_M8	0.63	6.3E-04	-0.07	7.4E-01	-0.15	4.7E-01	-0.12	5.6E-01	-0.26	2.1E-01	-0.01	9.6E-01	-0.53	5.5E-03	0.06	7.6E-01	0.14	5.1E-01	-0.04	8.4E-01
ENSCAFG0000001437	ENSCAFG0000001437	darkgrey	EC_M8	0.63	6.3E-04	0.14	4.9E-01	-0.44	2.5E-02	0.19	3.6E-01	-0.10	6.2E-01	0.03	9.0E-01	-0.17	4.1E-01	-0.25	2.1E-01	-0.39	5.1E-02	-0.22	2.7E-01
ENSCAFG0000002601	ENSCAFG0000002601	grey	EC_M10	0.63	6.3E-04	0.15	4.7E-01	-0.42	3.4E-02	0.03	8.7E-01	-0.14	4.9E-01	0.19	3.5E-01	0.23	2.5E-01	-0.15	4.8E-01	-0.20	3.4E-01	-0.23	2.6E-01
ENSCAFG0000000751	PPIP5K2	grey	EC_M10	0.63	6.3E-04	-0.34	9.3E-02	-0.40	4.4E-02	0.09	6.5E-01	0.02	9.3E-01	0.25	2.1E-01	-0.05	8.0E-01	-0.11	5.9E-01	-0.31	1.2E-01	-0.41	3.8E-02
ENSCAFG0000001963	DNAJC11	darkgrey	EC_M8	0.63	6.3E-04	-0.13	5.4E-01	-0.59	1.5E-03	-0.15	4.7E-01	0.32	1.1E-01	0.13	5.3E-01	-0.35	7.8E-02	0.12	5.7E-01	-0.22	2.8E-01	-0.61	9.2E-04
ENSCAFG0000002041	CTH	darkgrey	EC_M8	0.63	6.4E-04	-0.50	8.9E-03	-0.15	4.5E-01	0.03	8.8E-01	-0.12	5.7E-01	0.06	7.9E-01	-0.23	2.6E-01	-0.07	7.3E-01	-0.42	3.3E-02	-0.25	2.2E-01
ENSCAFG0000001069	NAA50	darkgrey	EC_M8	0.62	6.4E-04	-0.18	3.7E-01	-0.50	1.0E-02	-0.20	3.2E-01	0.16	4.3E-01	0.27	1.9E-01	0.00	9.9E-01	-0.14	4.9E-01	0.00	9.9E-01	-0.51	7.5E-03
ENSCAFG0000001342	EIF2S3	grey	EC_M10	0.62	6.4E-04	-0.04	8.4E-01	-0.22	2.8E-01	0.20	3.3E-01	-0.27	1.8E-01	0.10	6.3E-01	-0.16	4.4E-01	-0.13	5.1E-01	-0.52	6.9E-03	-0.09	6.6E-01
ENSCAFG0000001581	ADSS	turquoise	EC_M6	0.62	6.6E-04	0.74	1.8E-05	-0.52	6.0E-03	-0.07	7.2E-01	-0.12	5.7E-01	-0.13	5.1E-01	-0.25	2.1E-01	-0.01	9.5E-01	-0.11	5.8E-01	-0.17	4.1E-01
ENSCAFG0000000445	ENSCAFG0000000445	grey	EC_M10	0.62	6.6E-04	0.30	1.4E-01	-0.53	5.6E-03	-0.16	4.2E-01	-0.04	8.6E-01	0.24	2.4E-01	-0.21	3.0E-01	-0.20	3.4E-01	-0.22	2.9E-01	-0.27	1.8E-01
ENSCAFG0000001397	KLHL28	darkgrey	EC_M8	0.62	6.7E-04	-0.06	7.8E-01	-0.51	8.3E-03	0.18	3.9E-01	0.02	9.2E-01	0.33	9.5E-02	-0.07	7.2E-01	-0.33	1.0E-01	-0.32	1.1E-01	-0.36	7.0E-02
ENSCAFG0000002517	ENSCAFG0000002517	darkgrey	EC_M8	0.62	6.7E-04	0.21	3.1E-01	-0.34	9.4E-02	0.35	7.8E-02	-0.24	2.5E-01	0.04	8.5E-01	-0.14	4.9E-01	-0.29	1.6E-01	-0.43	2.9E-02	-0.09	6.7E-01
ENSCAFG0000003239	SCAMP1	darkgrey	EC_M8	0.62	6.8E-04	0.54	4.4E-03	-0.75	1.2E-05	0.09	6.6E-01	0.14	5.0E-01	0.16	4.3E-01	-0.18	3.8E-01	-0.22	2.8E-01	-0.17	4.1E-01	-0.45	2.2E-02
ENSCAFG0000000817	LARP4	darkgrey	EC_M8	0.62	6.9E-04	0.02	9.3E-01	-0.77	4.8E-06	-0.03	9.0E-01	0.36	7.1E-02	0.29	1.6E-01	-0.14	5.1E-01	0.01	9.7E-01	-0.12	5.5E-01	-0.68	1.2E-04
ENSCAFG0000001296	SEPT2	darkgrey	EC_M8	0.62	6.9E-04	0.44	2.3E-02	-0.36	7.1E-02	0.14	5.0E-01	-0.31	1.3E-01	0.08	6.9E-01	-0.29	1.5E-01	-0.08	6.9E-01	-0.11	6.0E-01	-0.03	8.9E-01
ENSCAFG0000001165	ENSCAFG0000001165	darkgrey	EC_M8	0.62	7.0E-04	0.25	2.1E-01	-0.72	3.5E-05	0.10	6.4E-01	0.21	3.0E-01	0.01	9.6E-01	-0.18	3.9E-01	-0.09	6.6E-01	-0.28	1.6E-01	-0.53	5.3E-03
ENSCAFG0000000885	PPP2R2A	darkgreen	EC_M4	0.62	7.0E-04	0.39	4.6E-02	-0.94	1.3E-12	-0.09	6.6E-01	0.44	2.6E-02	0.22	2.8E-01	-0.16	4.5E-01	-0.02	9.2E-01	-0.02	9.2E-01	-0.79	1.7E-06
ENSCAFG0000000028	ENSCAFG0000000028	darkgrey	EC_M8	0.62	7.0E-04	0.23	2.7E-01	-0.50	9.2E-03	0.26	2.0E-01	-0.06	7.6E-01	0.04	8.5E-01	-0.37	6.3E-02	-0.26	2.1E-01	-0.35	8.0E-02	-0.28	1.7E-01
ENSCAFG0000000305	SNX30	darkgrey	EC_M8	0.62	7.0E-04	-0.09	6.7E-01	-0.53	5.1E-03	0.15	4.7E-01	0.15	4.7E-01	0.02	9.3E-01	-0.12	5.6E-01	-0.23	2.5E-01	-0.24	2.4E-01	-0.48	1.4E-02
ENSCAFG0000001664	ENSCAFG0000001664	darkgrey	EC_M8	0.62	7.1E-04	0.15	4.7E-01	-0.39	5.0E-02	0.09	6.8E-01	-0.11	5.8E-01	-0.01	9.8E-01	-0.12	5.5E-01	-0.21	3.1E-01	-0.18	3.8E-01	-0.16	4.2E-01
ENSCAFG0000000062	CLIC1	darkgrey	EC_M8	0.62	7.1E-04	0.48	1.4E-02	-0.22	2.7E-01	0.03	8.9E-01	-0.36	7.1E-02	-0.01	9.6E-01	-0.20	3.3E-01	0.10	6.1E-01	-0.10	6.2E-01	0.01	9.5E-01
ENSCAFG0000000647	RRP8	darkgrey	EC_M8	0.62	7.1E-04	-0.07	7.2E-01	-0.21	3.0E-01	0.13	5.4E-01	-0.16	4.5E-01	0.16	4.4E-01	-0.23	2.5E-01	0.13	5.2E-01	0.00	1.0E+00	-0.17	4.0E-01
ENSCAFG0000001187	BZW1	darkgreen	EC_M4	0.62	7.2E-04	0.47	1.6E-02	-0.93	5.2E-12	-0.11	6.1E-01	0.42	3.3E-02	0.18	3.9E-01	-0.22	2.7E-01	-0.03	8.8E-01	-0.25	2.2E-01	-0.75	9.4E-06
ENSCAFG0000002450	ENSCAFG0000002450	grey	EC_M10	0.62	7.2E-04	-0.10	6.4E-01	-0.48	1.2E-02	0.08	6.8E-01	0.07	7.4E-01	0.12	5.6E-01	-0.24	2.4E-01	-0.29	1.5E-01	-0.37	6.1E-02	-0.42	3.3E-02
ENSCAFG0000000996	PSMD14	darkgreen	EC_M4	0.62	7.2E-04	0.11	6.0E-01	-0.82	3.1E-07	-0.15	4.6E-01	0.47	1.7E-02	0.10	6.3E-01	-0.27	1.7E-01	0.03	8.8E-01	-0.07	7.4E-01	-0.77	3.7E-06
ENSCAFG0000001552	ZWINT	darkgrey	EC_M8	0.62	7.2E-04	0.03	8.9E-01	-0.19	3.6E-01	0.00	9.9E-01	-0.24	2.3E-01	-0.19	3.6E-01	-0.26	2.0E-01	0.03	8.8E-01	-0.23	2.7E-01	-0.09	6.5E-01
ENSCAFG0000000133	TUT7	darkgrey	EC_M8	0.62	7.2E-04	0.66	2.5E-04	-0.34	9.2E-02	0.07	7.2E-01	-0.40	4.4E-02	-0.03	8.8E-01	-0.18	3.8E-01	-0.10	6.3E-01	-0.15	4.6E-01	0.06	7.6E-01
ENSCAFG0000000185	POLR3B	darkgrey	EC_M8	0.62	7.3E-04	-0.30	1.4E-01	-0.45	2.2E-02	0.07	7.4E-01	0.16	4.2E-01	0.30	1.4E-01	-0.38	5.8E-02	0.02	9.1E-01	-0.14	5.0E-01	-0.53	5.9E-03
ENSCAFG0000001426	COP1	darkgrey	EC_M8	0.62	7.3E-04	0.27	1.8E-01	-0.62	7.0E-04	0.03	8.9E-01	0.10	6.3E-01	0.16	4.2E-01	-0.18	3.9E-01	0.00	9.9E-01	-0.20	3.2E-01	-0.42	3.1E-02
ENSCAFG0000001596	UBE2K	darkgrey	EC_M8	0.62	7.4E-04	0.17	4.1E-01	-0.78	2.1E-06	-0.19	3.6E-01	0.41	3.8E-02	0.34	9.0E-02	0.06	7.6E-01	-0.01	9.4E-01	-0.32	1.1E-01	-0.75	1.0E-05
ENSCAFG0000000859	NUP54	darkgrey	EC_M8	0.62	7.4E-04	0.07	7.4E-01	-0.19	3.6E-01	-0.09	6.6E-01	-0.29	1.6E-01	0.20	3.4E-01	-0.20	3.4E-01	-0.01	9.4E-01	-0.47	1.6E-02	-0.09	6.6E-01
ENSCAFG0000002849	ENSCAFG0000002849	grey	EC_M10	0.62	7.4E-04	0.35	7.6E-02	-0.14	4.9E-01	0.15	4.8E-01	-0.48	1.3E-02	0.14	5.0E-01	-0.35	8.3E-02	-0.03	8.7E-01	0.10	6.2E-01	0.18	3.9E-01
ENSCAFG0000001847	TEFM	darkgrey	EC_M8	0.62	7.5E-04	-0.18	3.9E-01	-0.10	6.3E-01	0.04	8.5E-01	-0.31	1.2E-01	0.20	3.4E-01	-0.30	1.4E-01	0.14	5.0E-01	-0.15	4.5E-01	-0.02	9.2E-01
ENSCAFG0000002488	ENSCAFG0000002488	grey	EC_M10	0.62	7.6E-04	-0.07	7.5E-01	-0.35	7.5E-02	0.17	4.1E-01	-0.08	7.0E-01	0.05	8.0E-01	-0.24	2.4E-01	0.12	5.5E-01	-0.04	8.6E-01	-0.25	2.1E-01
ENSCAFG0000000755	MRPL35	darkgrey	EC_M8	0.62	7.6E-04	0.26	2.0E-01	-0.56	2.8E-03	0.02	9.2E-01	0.11	5.9E-01	0.17	4.0E-01	-0.39	4.7E-02	-0.41	3.6E-02	-0.22	2.8E-01	-0.42	3.1E-02
ENSCAFG0000001713	CLPX	darkgrey	EC_M8	0.62	7.6E-04	0.12	5.5E-01	-0.01	9.8E-01	0.20	3.2E-01	-0.59	1.4E-03	-0.12	5.5E-01	-0.18	3.8E-01	-0.06	7.9E-01	-0.19	3.5E-01	0.29	1.4E-01
ENSCAFG0000001274	OAT	darkgrey	EC_M8	0.62	7.7E-04	-0.02	9.1E-01	-0.16	4.2E-01	-0.14	4.9E-01	-0.26	2.0E-01	-0.10	6.3E-01	-0.19	3.6E-01	0.20	3.4E-01	-0.13	5.2E-01	-0.03	8.9E-01
ENSCAFG0000003120	ENSCAFG0000003120	darkgrey	EC_M8	0.62	7.8E-04	0.48	1.3E-02	-0.43	2.8E-02	0.12	5.7E-01	-0.22	2.9E-01	0.16	4.5E-01	-0.20	3.3E-01	-0.07	7.4E-01	-0.25	2.1E-01	-0.11	6.0E-01
ENSCAFG0000001903	LIPG	darkgrey	EC_M8	0.62	7.8E-04	0.17	4.1E-01	-0.01	9.5E-01	0.01	9.4E-01	-0.55	3.5E-03	0.22	2.8E-01	-0.13	5.2E-01	-0.29	1.5E-01	-0.57	2.3E-03	0.18	3.8E-01
ENSCAFG0000000738	STAG1	grey	EC_M10	0.62	7.8E-04	0.29	1.6E-01	-0.15	4.6E-01	-0.08	7.1E-01	-0.42	3.4E-02	0.20	3.2E-01	-0.12	5.5E-01	-0.18	3.7E-01	-0.23	2.6E-01	0.12	5.7E-01
ENSCAFG0000001026	RAP1GDS1	darkgreen	EC_M4	0.62	7.8E-04	0.16	4.5E-01	-0.73	2.4E-05	-0.08	6.9E-01	0.28	1.7E-01	-0.02	9.2E-01	-0.35	7.9E-02	-0.05	8.2E-01	-0.32	1.2E-01	-0.62	6.9E-04
ENSCAFG0000001866	LIFR	darkgrey	EC_M8	0.62	7.8E-04	0.35	8.4E-02	-0.03	9.0E-01	0.40	4.5E-02	-0.62	7.6E-04	-0.06	7.6E-01	-0.25	2.2E-01	-0.15	4.7E-01	-0.01	9.5E-01	0.32	1.1E-01
ENSCAFG0000001392	ABL2	darkgrey	EC_M8	0.62	7.8E-04	0.13	5.2E-01	-0.67	2.0E-04	0.00	9.9E-01	0.19	3.6E-01	-0.03	8.8E-01	-0.12	5.6E-01	-0.04	8.5E-01	0.08	6.9E-01	-0.51	7.3E-03
ENSCAFG0000000670	ZNF214	darkgrey	EC_M8	0.62	7.8E-04	-0.12	5.7E-01	0.00	9.8E-01	-0.09	6.6E-01	-0.46	1.8E-02	0.18	3.7E-01	-0.29	1.5E-01	0.31	1.3E-01	0.02	9.3E-01	0.14	5.0E-01
ENSCAFG0000001858	STAG2	darkgrey	EC_M8	0.62	7.9E-04	0.27	1.8E-01	0.13	5.3E-01	0.17	4.1E-01	-0.80	7.6E-07	-0.08	7.2E-01	-0.03	9.0E-01	-0.06	7.7E-01	-0.18	3.9E-01	0.46	1.9E-02
ENSCAFG0000000644	LTA4H	darkgrey	EC_M8	0.62	7.9E-04	-0.34	8.5E-02	-0.34	9.1E-02	0.19	3.6E-01	0.02	9.4E-01	0.18	3.7E-01	-0.41	3.8E-02	-0.07	7.3E-01	-0.44	2.5E-02	-0.39	4.8E-02
ENSCAFG0000002303	ENSCAFG0000002303	darkgrey	EC_M8	0.62	8.0E-04	0.27	1.8E-01	-0.47	1.6E-02	0.35	7.9E-02	-0.08	6.9E-01	-0.06	7.8E-01	-0.26	2.0E-01	-0.10	6.3E-01	-0.48	1.4E-02	-0.22	2.7E-01
ENSCAFG0000003244	ENSCAFG0000003244	grey	EC_M10	0.62	8.0E-04	0.04	8.5E-01	-0.14	4.9E-01	0.13	5.3E-01	-0.38	5.5E-02	0.20	3.2E-01	-0.33	1.0E-01	-0.11	5.8E-01	-0.11	6.0E-01	0.05	8.2E-01
ENSCAFG0000000085	NUP50	darkgreen	EC_M4	0.62	8.0E-04	0.20	3.3E-01	-0.76	5.8E-06	-0.12	5.7E-01	0.36	7.4E-02	0.30	1.3E-01	-0.25	2.1E-01	-0.09	6.6E-01	-0.33	1.0E-01	-0.71	5.0E-05
ENSCAFG0000001136	ACTL6A	grey	EC_M10	0.62	8.0E-04	0.47	1.5E-02	-0.23	2.7E-01	0.19	3.5E-01	-0.39	5.0E-02	-0.15	4.6E-01	-0.17	3.9E-01	-0.01	9.5E-01	-0.21	3.1E-01	0.09	6.7E-01
ENSCAFG0000000693	ENSCAFG0000000693	darkgrey	EC_M8	0.62	8.0E-04	0.08	7.1E-01	-0.51	8.0E-03	0.20	3.4E-01	0.00	1.0E+00	0.18	3.7E-01	-0.24	2.5E-01	-0.18	3.8E-01	-0.16	4.3E-01	-0.30	1.4E-01
ENSCAFG0000001730	GTF2A1	darkgrey	EC_M8	0.62	8.0E-04	0.03	8.7E-01	-0.18	3.9E-01	0.20	3.3E-01	-0.39	5.0E-02	0.27	1.7E-01	-0.12	5.4E-01	-0.14	4.9E-01	-0.41	3.9E-02	0.05	8.2E-01
ENSCAFG0000001994	EXOSC2	darkgrey	EC_M8	0.62	8.0E-04	-0.07	7.4E-01	-0.11	6.0E-01	0.07	7.3E-01	-0.32	1.1E-01	0.06	7.8E-01	-0.12	5.5E-01	0.21	3.1E-01	-0.30	1.3E-01	-0.05	8.0E-01
ENSCAFG0000000624	NR2C1	darkgrey	EC_M8	0.62	8.1E-04	0.21	2.9E-01	-0.64	4.7E-04	0.01	9.8E-01	0.08	7.1E-01	0.19	3.6E-01	-0.15	4.5E-01	-0.16	4.5E-01	-0.06	7.8E-01	-0.42	3.4E-02
ENSCAFG0000001018	DEK	darkgrey	EC_M8	0.62	8.1E-04	0.29	1.6E-01	0.01	9.5E-01	0.16	4.3E-01	-0.63	5.4E-04	0.06	7.6E-01	0.00	9.9E-01	0.00	9.9E-01	-0.25	2.2E-01	0.30	1.4E-01
ENSCAFG0000000134	NAA35	darkgrey	EC_M8	0.61	8.3E-04	0.00	9.9E-01	-0.09	6.7E-01	-0.13	5.2E-01	-0.34	8.4E-02	-0.09	6.7E-01	-0.13	5.4E-01	0.03	8.7E-01	-0.22	2.8E-01	0.05	8.0E-01
ENSCAFG0000000233	ENSCAFG0000000233	darkgrey	EC_M8	0.61	8.3E-04	0.22	2.9E-01	-0.48	1.3E-02	-0.18	3.9E-01	-0.01	9.7E-01	0.38	5.9E-02	-0.20	3.2E-01	-0.11	6.1E-01	-0.28	1.7E-01	-0.32	1.1E-01
ENSCAFG0000000104	XRCC6	darkgrey	EC_M8	0.61	8.3E-04	0.14	4.8E-01	-0.62	8.0E-04	0.14	5.0E-01	0.18	3.8E-01	0.09	6.6E-01	-0.35	8.0E-02	-0.09	6.5E-01	-0.05	7.9E-01	-0.47	1.4E-02

ENSCAFG0000001184	C4H1orf131	darkgrey	EC_M8	0.61	8.4E-04	0.19	3.5E-01	-0.14	4.9E-01	0.10	6.1E-01	-0.38	5.5E-02	0.12	5.6E-01	0.04	8.3E-01	-0.06	7.6E-01	-0.11	6.1E-01	0.08	6.9E-01
ENSCAFG0000003009	C1D	darkgrey	EC_M8	0.61	8.4E-04	0.05	8.2E-01	-0.67	2.1E-04	0.11	5.8E-01	0.20	3.2E-01	0.38	5.3E-02	-0.19	3.5E-01	-0.21	3.1E-01	-0.14	5.0E-01	-0.53	5.7E-03
ENSCAFG0000002018	CDC7	grey	EC_M10	0.61	8.5E-04	-0.09	6.6E-01	-0.37	6.4E-02	-0.02	9.1E-01	-0.06	7.9E-01	0.17	4.2E-01	0.19	3.4E-01	0.21	3.0E-01	-0.44	2.3E-02	-0.30	1.4E-01
ENSCAFG0000001763	TIMM8A	darkgrey	EC_M8	0.61	8.5E-04	-0.18	3.7E-01	-0.24	2.4E-01	-0.16	4.4E-01	-0.11	6.0E-01	0.22	2.8E-01	-0.26	2.1E-01	-0.16	4.5E-01	-0.45	2.1E-02	-0.27	1.9E-01
ENSCAFG0000000207	ARHGEF39	darkgrey	EC_M8	0.61	8.6E-04	0.21	2.9E-01	-0.22	2.8E-01	-0.16	4.4E-01	-0.20	3.2E-01	0.09	6.7E-01	-0.13	5.2E-01	-0.15	4.7E-01	0.06	7.9E-01	-0.10	6.3E-01
ENSCAFG0000001153	TARBP1	darkgrey	EC_M8	0.61	8.6E-04	0.54	4.3E-03	-0.03	9.0E-01	0.08	7.0E-01	-0.66	2.3E-04	-0.07	7.5E-01	-0.03	8.9E-01	-0.19	3.5E-01	-0.21	3.0E-01	0.35	8.2E-02
ENSCAFG0000002584	ENSCAFG0000002584	grey	EC_M10	0.61	8.6E-04	0.17	4.0E-01	-0.15	4.7E-01	0.24	2.3E-01	-0.46	1.9E-02	0.31	1.3E-01	-0.21	3.1E-01	-0.03	8.9E-01	-0.16	4.2E-01	0.08	7.1E-01
ENSCAFG0000000483	ENSCAFG0000000483	darkgrey	EC_M8	0.61	8.7E-04	0.24	2.4E-01	-0.60	1.3E-03	-0.24	2.3E-01	0.20	3.3E-01	0.22	2.8E-01	-0.09	6.7E-01	-0.01	9.6E-01	-0.10	6.1E-01	-0.53	5.1E-03
ENSCAFG0000001854	MIER1	darkgrey	EC_M8	0.61	8.7E-04	0.34	8.9E-02	-0.18	3.9E-01	0.02	9.1E-01	-0.51	8.1E-03	0.08	6.9E-01	0.05	8.2E-01	-0.14	4.8E-01	-0.29	1.5E-01	0.15	4.6E-01
ENSCAFG0000001090	PHF5A	darkgrey	EC_M8	0.61	8.7E-04	0.34	9.1E-02	-0.69	8.7E-05	-0.14	4.9E-01	0.21	2.9E-01	0.18	3.7E-01	-0.04	8.5E-01	-0.27	1.8E-01	-0.11	5.9E-01	-0.58	1.9E-03
ENSCAFG0000000786	TRAM1	darkgreen	EC_M4	0.61	8.7E-04	0.22	2.8E-01	-0.91	1.3E-10	-0.01	9.5E-01	0.44	2.3E-02	0.37	5.9E-02	-0.22	2.9E-01	-0.14	4.9E-01	-0.21	3.0E-01	-0.78	2.3E-06
ENSCAFG0000000956	RIOK1	darkgrey	EC_M8	0.61	8.7E-04	-0.15	4.6E-01	-0.54	4.0E-03	-0.23	2.7E-01	0.18	3.8E-01	0.12	5.5E-01	-0.16	4.5E-01	0.13	5.3E-01	0.04	8.3E-01	-0.50	8.9E-03
ENSCAFG0000001695	KIF22	darkgrey	EC_M8	0.61	8.8E-04	-0.12	5.6E-01	-0.04	8.5E-01	-0.04	8.3E-01	-0.28	1.6E-01	0.12	5.5E-01	-0.12	5.5E-01	0.00	9.9E-01	-0.10	6.2E-01	-0.03	8.8E-01
ENSCAFG0000000445	CD3EAP	grey	EC_M10	0.61	9.1E-04	-0.11	6.1E-01	-0.08	6.9E-01	0.01	9.7E-01	-0.34	8.8E-02	0.07	7.4E-01	0.04	8.4E-01	-0.06	7.6E-01	-0.41	4.0E-02	-0.04	8.6E-01
ENSCAFG0000000984	NOL7	grey	EC_M10	0.61	9.2E-04	0.07	7.5E-01	-0.20	3.2E-01	0.19	3.5E-01	-0.26	1.9E-01	0.08	7.1E-01	-0.09	6.7E-01	-0.40	4.4E-02	-0.45	2.2E-02	-0.04	8.6E-01
ENSCAFG0000000873	MOB1A	darkgrey	EC_M8	0.61	9.2E-04	0.05	7.9E-01	-0.05	8.0E-01	-0.04	8.4E-01	-0.41	3.9E-02	0.06	7.8E-01	-0.19	3.4E-01	-0.32	1.2E-01	-0.31	1.3E-01	0.08	6.9E-01
ENSCAFG0000001885	CEP76	darkgrey	EC_M8	0.61	9.2E-04	-0.10	6.2E-01	-0.31	1.2E-01	-0.09	6.8E-01	-0.03	9.0E-01	0.40	4.6E-02	0.13	5.2E-01	-0.07	7.3E-01	-0.18	3.8E-01	-0.31	1.2E-01
ENSCAFG0000000015	NEMP1	grey	EC_M10	0.61	9.2E-04	0.01	9.5E-01	-0.41	3.8E-02	0.15	4.6E-01	-0.10	6.3E-01	-0.01	9.7E-01	-0.26	2.0E-01	0.08	6.9E-01	-0.08	7.0E-01	-0.29	1.5E-01
ENSCAFG0000000098	TBC1D31	grey	EC_M10	0.61	9.3E-04	0.17	4.0E-01	-0.20	3.4E-01	0.07	7.3E-01	-0.34	8.6E-02	0.12	5.6E-01	-0.06	7.7E-01	-0.22	2.8E-01	-0.38	5.4E-02	0.05	8.1E-01
ENSCAFG0000000216	ENSCAFG0000000216	darkgrey	EC_M8	0.61	9.4E-04	0.02	9.2E-01	-0.41	4.0E-02	0.28	1.6E-01	-0.07	7.2E-01	0.35	8.0E-02	-0.22	2.7E-01	-0.23	2.6E-01	-0.36	7.4E-02	-0.28	1.6E-01
ENSCAFG0000003180	ENSCAFG0000003180	grey	EC_M10	0.61	9.4E-04	0.51	7.2E-03	-0.20	3.4E-01	0.01	9.6E-01	-0.45	2.0E-02	0.05	8.0E-01	-0.37	6.0E-02	0.00	9.9E-01	-0.19	3.5E-01	0.08	7.0E-01
ENSCAFG0000001886	PHF6	darkgrey	EC_M8	0.61	9.5E-04	0.38	5.3E-02	-0.64	4.4E-04	0.07	7.3E-01	0.07	7.3E-01	0.07	7.3E-01	-0.31	1.2E-01	-0.10	6.2E-01	-0.32	1.1E-01	-0.41	3.8E-02
ENSCAFG0000000797	KATNBL1	darkgrey	EC_M8	0.61	9.5E-04	0.03	9.0E-01	-0.63	5.8E-04	-0.07	7.4E-01	0.26	1.9E-01	0.13	5.1E-01	-0.09	6.7E-01	0.04	8.4E-01	-0.16	4.4E-01	-0.58	1.8E-03
ENSCAFG0000001588	INCENP	darkgrey	EC_M8	0.61	9.5E-04	0.28	1.7E-01	-0.25	2.2E-01	-0.20	3.2E-01	-0.21	3.1E-01	0.04	8.6E-01	0.03	8.9E-01	-0.14	5.0E-01	0.00	9.8E-01	-0.09	6.6E-01
ENSCAFG0000001588	ENSCAFG0000001588	darkgrey	EC_M8	0.61	9.5E-04	0.19	3.6E-01	-0.17	4.0E-01	0.05	8.2E-01	-0.33	1.1E-01	0.33	1.0E-01	-0.08	6.9E-01	-0.44	2.3E-02	-0.29	1.4E-01	-0.02	9.4E-01
ENSCAFG0000000003	RTTN	darkgrey	EC_M8	0.61	9.7E-04	-0.06	7.9E-01	-0.15	4.7E-01	0.05	8.2E-01	-0.28	1.7E-01	-0.06	7.9E-01	-0.01	9.5E-01	-0.52	6.5E-03	-0.19	3.4E-01	-0.05	8.0E-01
ENSCAFG0000002271	ENSCAFG0000002271	darkgrey	EC_M8	0.61	9.7E-04	0.05	8.1E-01	-0.25	2.3E-01	-0.09	6.5E-01	-0.25	2.2E-01	0.26	2.0E-01	-0.11	6.1E-01	-0.05	8.0E-01	-0.45	2.1E-02	-0.10	6.4E-01
ENSCAFG0000000767	SLC30A5	darkgrey	EC_M8	0.61	9.7E-04	0.07	7.3E-01	-0.74	1.3E-05	-0.03	8.7E-01	0.31	1.2E-01	0.30	1.4E-01	-0.28	1.6E-01	-0.09	6.7E-01	-0.31	1.3E-01	-0.64	4.9E-04
ENSCAFG0000001492	CHD4	turquoise	EC_M6	0.61	9.8E-04	0.50	9.4E-03	-0.68	1.5E-04	-0.02	9.4E-01	0.05	7.9E-01	-0.02	9.2E-01	0.13	5.4E-01	-0.10	6.2E-01	-0.06	7.9E-01	-0.39	4.8E-02
ENSCAFG0000001743	SRSF1	darkgrey	EC_M8	0.61	9.9E-04	0.00	9.9E-01	-0.17	4.0E-01	-0.03	8.7E-01	-0.21	3.1E-01	0.16	4.2E-01	-0.14	5.0E-01	0.10	6.3E-01	-0.03	8.9E-01	-0.13	5.3E-01
ENSCAFG0000000291	RUFY3	darkgrey	EC_M8	0.61	9.9E-04	0.06	7.6E-01	-0.75	1.0E-05	0.01	9.5E-01	0.32	1.2E-01	0.32	1.1E-01	0.01	9.5E-01	-0.17	4.2E-01	-0.22	2.8E-01	-0.65	3.7E-04
ENSCAFG0000001075	QTRT2	darkgrey	EC_M8	0.61	9.9E-04	-0.11	5.8E-01	-0.32	1.1E-01	0.07	7.4E-01	-0.05	8.0E-01	0.23	2.6E-01	-0.17	4.1E-01	-0.07	7.5E-01	0.20	3.4E-01	-0.24	2.3E-01
ENSCAFG0000000552	ORC4	darkgrey	EC_M8	0.61	9.9E-04	0.06	7.8E-01	-0.63	5.1E-04	-0.12	5.5E-01	0.24	2.4E-01	0.39	4.7E-02	-0.26	2.1E-01	-0.20	3.2E-01	-0.25	2.1E-01	-0.58	1.9E-03
ENSCAFG0000003073	ENSCAFG0000003073	darkgrey	EC_M8	0.61	1.0E-03	0.30	1.3E-01	-0.74	1.5E-05	0.01	9.7E-01	0.25	2.2E-01	0.18	3.7E-01	-0.13	5.4E-01	-0.02	9.2E-01	-0.13	5.1E-01	-0.55	3.6E-03
ENSCAFG0000000337	ITGB1BP1	darkgrey	EC_M8	0.61	1.0E-03	0.28	1.7E-01	-0.42	3.3E-02	0.17	4.2E-01	-0.05	8.2E-01	0.03	8.9E-01	-0.13	5.2E-01	-0.14	4.9E-01	-0.21	3.1E-01	-0.23	2.6E-01
ENSCAFG0000001211	VPS33B	darkgrey	EC_M8	0.61	1.0E-03	0.08	7.0E-01	-0.47	1.6E-02	-0.06	7.8E-01	0.03	9.0E-01	0.09	6.6E-01	-0.36	6.7E-02	-0.14	5.0E-01	-0.17	4.1E-01	-0.35	7.8E-02
ENSCAFG0000001181	DGKD	darkgrey	EC_M8	0.61	1.0E-03	0.36	7.4E-02	-0.41	4.0E-02	-0.13	5.3E-01	-0.09	6.6E-01	0.03	8.7E-01	0.07	7.4E-01	-0.26	2.1E-01	-0.04	8.4E-01	-0.20	3.3E-01
ENSCAFG0000001765	FAM114A2	darkgreen	EC_M4	0.61	1.0E-03	0.14	4.9E-01	-0.90	6.0E-10	-0.15	4.5E-01	0.53	5.3E-03	0.26	2.0E-01	-0.09	6.6E-01	0.12	5.7E-01	-0.24	2.3E-01	-0.86	2.0E-08
ENSCAFG0000000745	DDX55	darkgrey	EC_M8	0.61	1.0E-03	0.32	1.2E-01	-0.11	5.9E-01	0.14	5.1E-01	-0.49	1.0E-02	-0.09	6.6E-01	-0.01	9.4E-01	-0.06	7.8E-01	-0.03	8.7E-01	0.19	3.6E-01
ENSCAFG0000002062	ENSCAFG0000002062	grey	EC_M10	0.61	1.1E-03	0.15	4.5E-01	-0.20	3.3E-01	0.07	7.2E-01	-0.35	7.7E-02	0.10	6.3E-01	-0.27	1.9E-01	-0.35	7.7E-02	-0.02	9.2E-01	0.01	9.6E-01
ENSCAFG0000002454	GBP5	turquoise	EC_M6	0.60	1.1E-03	0.75	1.2E-05	-0.71	4.6E-05	-0.08	7.2E-01	0.10	6.2E-01	-0.02	9.4E-01	-0.08	7.2E-01	-0.17	4.1E-01	-0.09	6.7E-01	-0.42	3.1E-02
ENSCAFG0000000220	AASDH	grey	EC_M10	0.60	1.1E-03	-0.31	1.3E-01	-0.40	4.1E-02	0.03	8.7E-01	0.04	8.4E-01	0.43	3.0E-02	-0.17	4.1E-01	-0.05	8.0E-01	-0.10	6.4E-01	-0.35	7.7E-02
ENSCAFG0000000675	SCYL2	darkgrey	EC_M8	0.60	1.1E-03	0.22	2.8E-01	-0.82	3.5E-07	-0.11	5.8E-01	0.31	1.2E-01	0.32	1.1E-01	-0.26	2.0E-01	-0.01	9.4E-01	-0.20	3.2E-01	-0.65	3.6E-04
ENSCAFG0000000554	RAD18	darkgrey	EC_M8	0.60	1.1E-03	0.00	9.8E-01	-0.35	8.1E-02	-0.48	1.2E-02	-0.06	7.7E-01	-0.15	4.6E-01	-0.25	2.3E-01	0.21	3.0E-01	-0.24	2.4E-01	-0.28	1.7E-01
ENSCAFG0000002912	ENSCAFG0000002912	darkgrey	EC_M8	0.60	1.1E-03	0.36	7.3E-02	-0.54	4.4E-03	0.27	1.8E-01	-0.08	6.9E-01	0.05	8.0E-01	-0.19	3.4E-01	-0.04	8.4E-01	-0.13	5.1E-01	-0.22	2.7E-01
ENSCAFG0000001825	MARK3	turquoise	EC_M6	0.60	1.1E-03	0.51	7.8E-03	-0.64	3.8E-04	-0.06	7.7E-01	0.01	9.5E-01	0.26	2.0E-01	-0.43	2.8E-02	0.06	7.8E-01	-0.09	6.5E-01	-0.36	7.1E-02
ENSCAFG0000000327	CDCA8	darkgrey	EC_M8	0.60	1.1E-03	-0.02	9.4E-01	-0.35	7.9E-02	-0.14	4.9E-01	0.03	8.9E-01	0.10	6.3E-01	-0.12	5.5E-01	0.05	7.9E-01	0.02	9.0E-01	-0.32	1.1E-01
ENSCAFG0000001531	TIPRL	darkgrey	EC_M8	0.60	1.1E-03	-0.03	9.0E-01	-0.63	5.3E-04	0.16	4.3E-01	0.18	3.8E-01	0.32	1.1E-01	-0.15	4.5E-01	-0.13	5.2E-01	-0.23	2.6E-01	-0.51	7.5E-03
ENSCAFG0000000475	MRPS7	darkgrey	EC_M8	0.60	1.1E-03	0.23	2.6E-01	-0.71	5.5E-05	-0.20	3.3E-01	0.33	1.0E-01	0.14	5.0E-01	-0.29	1.6E-01	0.06	7.7E-01	-0.16	4.2E-01	-0.65	2.8E-04
ENSCAFG0000000715	ENSCAFG0000000715	darkgrey	EC_M8	0.60	1.1E-03	0.43	3.0E-02	0.01	9.7E-01	0.13	5.4E-01	-0.71	4.2E-05	-0.04	8.5E-01	-0.19	3.5E-01	-0.20	3.3E-01	-0.19	3.6E-01	0.39	5.0E-02
ENSCAFG0000001256	PSMD4	darkgrey	EC_M8	0.60	1.1E-03	0.39	4.9E-02	-0.41	3.5E-02	0.16	4.5E-01	-0.08	7.1E-01	0.10	6.2E-01	-0.25	2.2E-01	-0.08	7.0E-01	-0.39	5.0E-02	-0.24	2.4E-01
ENSCAFG0000000983	PRMT3	grey	EC_M10	0.60	1.1E-03	-0.28	1.6E-01	0.12	5.8E-01	0.26	1.9E-01	-0.53	5.3E-03	0.05	8.1E-01	-0.41	4.0E-02	-0.22	2.8E-01	-0.12	5.6E-01	0.21	3.1E-01
ENSCAFG0000000707	ENSCAFG0000000707	grey	EC_M10	0.60	1.1E-03	0.04	8.5E-01	-0.26	2.0E-01	0.20	3.3E-01	-0.21	3.0E-01	-0.03	8.7E-01	-0.04	8.4E-01	-0.47	1.6E-02	-0.44	2.6E-02	-0.11	5.9E-01
ENSCAFG0000001888	NUFIP2	darkgrey	EC_M8	0.60	1.1E-03	0.03	8.8E-01	-0.55	3.3E-03	-0.07	7.2E-01	0.06	7.7E-01	0.17	4.1E-01	-0.22	2.8E-01	0.11	5.8E-01	-0.08	7.1E-01	-0.41	3.9E-02
ENSCAFG0000001597	NEDD4	darkgrey	EC_M8	0.60	1.1E-03	0.09	6.4E-01	0.04	8.6E-01	0.17	4.0E-01	-0.60	1.1E-03	-0.03	8.7E-01	-0.12	5.7E-01	-0.27	1.9E-01	-0.60	1.1E-03	0.25	2.2E-01
ENSCAFG0000001030	EIF4E	darkgreen	EC_M4	0.60	1.1E-03	-0.01	9.5E-01	-0.90	5.3E-10	-0.14	4.9E-01	0.58	2.0E-03	0.28	1.7E-01	-0.17	4.2E-01	0.03	9.0E-01	-0.14	4.8E-01	-0.89	9.4E-10
ENSCAFG0000003045	MBLAC2	grey	EC_M10	0.60	1.1E-03	0.04	8.3E-01	-0.21	3.0E-01	0.03	9.0E-01	-0.28	1.7E-01	-0.08	7.0E-01	-0.31	1.2E-01	-0.09	6.8E-01	-0.12	5.6E-01	0.01	9.7E-01
ENSCAFG0000000566	ZMYND11	grey	EC_M10	0.60	1.1E-03	0.00	9.8E-01	-0.02	9.0E-01	0.24	2.3E-01	-0.51	7.2E-03	0.04	8.3E-01	-0.33	1.0E-01	-0.03	8.7E-01	0.03	8.8E-01	0.19	3.4E-01
ENSCAFG0000000226	REV1	darkgrey	EC_M8	0.60	1.1E-03	0.28	1.7E-01	0.04	8.3E-01	0.10	6.4E-01	-0.68	1.3E-04	-0.17	4.2E-01	-0.01	9.8E-01	-0.03	8.9E-01	0.02	9.3E-01	0.35	7.8E-02
ENSCAFG0000000893	ENSCAFG0000000893	darkgrey	EC_M8	0.60	1.1E-03	0.18	3.7E-01	-0.50	9.0E-03	0.22	2.7E-01	0.04	8.6E-01	0.04	8.5E-01	-0.19	3.6E-01	-0.04	8.3E-01	-0.40	4.1E-02	-0.35	7.9E-02
ENSCAFG0000000688	CNOT7	darkgreen	EC_M4	0.60	1.1E-03	0.42	3.5E-02	-0.90	5.5E-10	0.02	9.4E-01	0.37	6.1E-02	0.32	1.1E-01	-0.23	2.7E-01	-0.21	3.0E-01	-0.09	6.6E-01	-0.71	4.8E-05
ENSCAFG0000003121	ENSCAFG0000003121	grey	EC_M10	0.60	1.1E-03	0.02	9.3E-01	-0.11	5.9E-01	0.18	3.9E-01	-0.43	2.7E-02	-0.27	1.7E-01	-0.14	4.9E-01	0.02	9.1E-01	0.03	8.9E-01	0.09	6.7E-01
ENSCAFG0000001872	ATG4C	grey	EC_M10	0.60	1.1E-03	0.04	8.3E-01	-0.30	1.4E-01	0.30	1.4E-01	-0.21	3.1E-01	0.18	3.7E-01	-0.32	1.2E-01	-0.24	2.4E-01	-0.43	2.6E-02	-0.13	5.2E-01
ENSCAFG0000001417	RANBP1	grey	EC_M10	0.60	1.2E-03	-0.07	7.2E-01	-0.50	1.0E-02	-0.15	4.6E-01	0.23	2.6E-01	0.30	1.4E-01	-0.21	3.1E-01	-0.08	7.0E-01	-0.13	5.3E-01	-0.54	4.4E-03
ENSCAFG0000000087	TAF2	darkgreen	EC_M4	0.60	1.2E-03	0.03	8.8E-01	-0.80	8.5E-07	-0.15	4.6E-01	0.48	1.4E-02	0.23	2.5E-01	-0.41	3.6E-02	-0.02	9.4E-01	-0.12	5.5E-01	-0.79	1.8E-06
ENSCAFG0000000704	HNRNPF	grey	EC_M10	0.60	1.2E-03	0.02	9.1E-01	0.29	1.6E-01	0.16	4.4E-01	-0.82	2.6E-07	0.08	7.1E-01	-0.14	4.9E-01	-0.12	5.6E-01	-0.46	1.7E-02	0.48	1.4E-02
ENSCAFG0000002925	ENSCAFG0000002925	grey	EC_M10	0.60	1.2E-03	0.15	4.7E-01	-0.08	7.0E-01	0.11	6.0E-01	-0.49	1.0E-02	-0.06	7.5E-01	-0.29	1.5E-01	-0.13	5.2E-01	0.01	9.6E-01	0.19	3.6E-01
ENSCAFG0000001803	KNOP1	darkgrey	EC_M8	0.60	1.2E-03	0.25	2.2E-01	-0.28	1.7E-01	0.19	3.4E-01	-0.34	8.6E-02	0.18	3.7E-01	-0.16	4.4E-01	0.17	4.0E-01	-0.31	1.2E-01	-0.02	9.1E-01
ENSCAFG0000001033	NSUN2	darkgrey	EC_M8	0.60	1.2E-03	-0.09	6.5E-01	-0.67	2.0E-04	-0.15	4.7E-01	0.41	3.8E-02	0.25	2.3E-01	-0.23	2.6E-01	-0.06	7.7E-01	-0.19	3.4E-01	-0.73	2.6E-05
ENSCAFG0000000654	ENSCAFG0000000654	grey	EC_M10	0.60	1.2E-03	-0.23	2.5E-01	0.25	2.2E-01	0.04	8.3E-01	-0.66	2.6E-04	-0.03	8.7E-01	-0.32	1.1E-01	-0.19	3.6E-01	-0.35	7.7E-02	0.33	1.0E-01
ENSCAFG0000000161	VPS13A	grey	EC_M10	0.60	1.2E-03	0.03	8.7E-01	-0.52	6.9E-03	0.10	6.3E-01	0.08	7.0E-01	-0.15	4.6E-01	-0.31	1.2E-01	-0.14	5.1E-01	-0.25	2.2E-01	-0.39	5.0E-02
ENSCAFG0000000889	URB1	darkgrey	EC_M8	0.60	1.2E-03	0.19	3.5E-01	0.01	9.6E-01	-0.15	4.6E-01	-0.49	1.0E-02	-0.17	3.9E-01	-0.48	1.4E-02	-0.22	2.8E-01	-0.29	1.4E-01	0.19	3.6E-01
ENSCAFG0000002542	DDX23	grey	EC_M10	0.60	1.2E-03	0.49	1.1E-02	-0.31	1.3E-01	0.07	7.5E-01	-0.29	1.5E-01	0.13	5.1E-01	0.22	2.7E-01	-0.08	7.0E-01	-0.39	5.1E-02	-0.04	8.5E-01
ENSCAFG0000002904	ENSCAFG0000002904	turquoise	EC_M6	0.60	1.2E-03	0.51	7.7E-03	-0.56	2.7E-03	-0.28	1.6E-01	0.05	7.9E-01	-0.06	7.5E-01	-0.19	3.5E-01	0.30	1.4E-01	-0.05	8.0E-01	-0.35	8.2E-02
ENSCAFG0000000483	UPF2	darkgrey	EC_M8	0.60	1.2E-03	0.42	3.1E-02	-0.81	6.3E-07	-0.06	7.6E-01	0.26	2.0E-01	0.19	3.6E-01	-0.17	4.0E-01	0.12	5.7E-01	0.07	7.2E-01	-0.57	2.6E-03
ENSCAFG0000000485	ENSCAFG0000000485	grey	EC_M10	0.60	1.2E-03	0.33	1.0E-01	-0.45	2.0E-02	0.39	4.6E-02	-0.11	5.8E-01	0.09	6.6E-01	0.09	6.8E-01	-0.35	7.7E-02	-0.21	3.0E-01	-0.23	2.6E-01
ENSCAFG0000002948	GEMIN6	darkgrey	EC_M8	0.60	1.2E-03	-0.35	8.4E-02	0.10	6.4E-01	0.04	8.3E-01	-0.42	3.4E-02	-0.02	9.2E-01	-0.17	4.0E-01	0.00	9.9E-01	-0.41	3.9E-02	0.09	6.8E-01
ENSCAFG0000001335	SLC16A1	darkgrey	EC_M8	0.60	1.2E-03	-0.25	2.1E-01	-0.37	6.1E-02	-0.15	4.7E-01	0.02	9.1E-01	0.21	3.0E-01	-0.11	5.8E-01	0.11	5.9E-01	-0.43	2.8E-02	-0.35	8.0E-02
ENSCAFG0000001700	AURKB	darkgrey	EC_M8	0.60	1.2E-03	0.13	5.1E-01	0.16	4.2E-01	0.06	7.6E-01	-0.65	3.6E-04	0.10	6.1E-01	-0.05	8.2E-01	-0.22	2.8E-01	0.03	9.0E-01	0.37	6.4E-02
ENSCAFG0000000409	MTMR2	turquoise	EC_M6	0.60	1.2E-03	0.78	2.6E-06	-0.49	1.0E-02	-0.04	8.3E-01	-0.19	3.6E-01	-0.10	6.3E-01	-0.26	1.9E-01	-0.11	5.8E-01	0.14	5.0E-01	-0.10	6.2E-01
ENSCAFG0000000051	SRFBP1	darkgrey	EC_M8	0.60	1.2E-03	0.20	3.3E-01	-0.53	5.4E-03	0.14	4.8E-01	0.02	9.4E-01	0.08	6.8E-01	-0.11	5.8E-01	0.07	7.2E-01	-0.18	3.8E-01	-0.31	1.2E-01
ENSCAFG0000000879	TMA16	darkgrey	EC_M8	0.60	1.2E-03	-0.20	3.2E-01	-0.39	4.7E-02	-0.36	7.2E-02	0.05	8.2E-01	0.19	3.6E-01	-0.12	5.5E-01	0.14	5.0E-01	-0.17	4.1E-01	-0.38	5.5E-02
ENSCAFG0000000785	RSRC2	darkgrey	EC_M8	0.60	1.3E-03	0.41	4.0E-02	-0.45	2.2E-02	0.07	7.4E-01	-0.22	2.9E-01	0.18	3.7E-01	-0.05	8.2E-01	-0.08	7.0E-01	-0.20	3.4E-01	-0.11	5.8E-01
ENSCAFG0000000049	ENSCAFG0000000049	grey	EC_M10	0.60	1.3E-03	0.18	3.8E-01	-0.32	1.1E-01	0.22	2.8E-01	-0.19	3.5E-01	0.06	7.7E-01	-0.30	1.4E-01	-0.55	3.6E-03	-0.36	6.8E-02	-0.14	4.9E-01
ENSCAFG0000000562	CHD8	darkgrey	EC_M8	0.60	1.3E-03	0.62	7.2E-04	-0.37	6.2E-02	0.02	9.3E-01	-0.34	8.5E-02	0.01	9.5E-01	-0.10	6.3E-01	-0.01	9.4E-01	-0.14	4.8E-01	0.02	9.2E-01
ENSCAFG0000000614	TUBGCP3	darkgrey	EC_M8	0.60	1.3E-03	0.05	8.1E-01	-0.25	2.2E-01	0.01	9.6E-01	-0.16	4.4E-01	0.07	7.3E-01	-0.40	4.5E-02	-0.15	4.7E-01	-0.05	7.9E-01	-0.15	4.5E-01
ENSCAFG0000000884	KLHL2	darkgreen	EC_M4	0.60	1.3E-03	0.52	6.7E-03	-0.78	2.6E-06	0.01	9.6E-01	0.23	2.6E-01	0.20	3.3E-01	-0.27	1.7E-01	0.03	8.9E-01	-0.42	3.5E-02	-0.57	2.4E-03
ENSCAFG0000000959	PCNP	darkgrey	EC_M8	0.60	1.3E-03	0.25	2.1E-01	-0.21	3.1E-01	0.27	1.9E-01	-0.43	3.0E-02	0.08	7.1E-01	-0.11	5.9E-01	-0.16	4.2E-01	0.22	2.7E-01	0.12	5.7E-01
ENSCAFG0000000272	ASB3	darkgrey	EC_M8	0.60	1.3E-03	0.03	9.0E-01	-0.63	5.7E-04	0.00	9.9E-01	0.26	2.1E-01	0.35	8.4E-02	-0.27	1.8E-01	-0.33	9.8E-02	-0.11	6.0E-01	-0.57	2.4E-03
ENSCAFG0000001546	PIMREG	darkgrey	EC_M8	0.60	1.3E-03	0.38	5.2E-02	-0.11	5.9E-01	-0.13	5.4E-01	-0.37	6.2E-02	0.09	6.5E-01	-0.26	1.9E-01	-0.05	7.9E-01	-0.14	4.9E-01	0.05	7.9E-01
ENSCAFG0000000273	CTPS1	darkgrey	EC_M8	0.60	1.3E-03	-0.14	4.9E-01	-0.39	4.9E-02	-0.19	3.6E-01	0.08	7.1E-01	0.21	3.1E-01	-0.31	1.2E-01	0.10	6.4E-01	0.01	9.5E-01	-0.41	3.8E-02
ENSCAFG0000000785	HMGB2	grey	EC_M10	0.60	1.3E-03	-0.08	7.1E-01	0.28	1.7E-01	0.25	2.2E-01	-0.77	4.0E-06	0.03	8.8E-01	-0.18	3.7E-01	-0.12	5.7E-01	-0.29	1.5E-01	0.46	1.8E-02
ENSCAFG0000001807	ZNF317	grey	EC_M10	0.60	1.3E-03	0.55	3.3E-03	-0.33	1.0E-01	0.10	6.2E-01	-0.27	1.9E-01	0.08	6.8E-01	-0.10	6.1E-01	0.12	5.7E-01	-0.24	2.3E-01	-0.05	8.1E-01
ENSCAFG0000000470	HSPA14	grey	EC_M10	0.60	1.3E-03	0.27	1.7E-01	-0.67	2.0E-04	-0.11	6.0E-01	0.23	2.7E-01	0.36	7.2E-02	0.05	8.1E-01	-0.01	9.7E-01	-0.13	5.3E-01	-0.55	3.9E-03
ENSCAFG0000001461	CUL5	darkgrey	EC_M8	0.60	1.3E-03	0.03	8.8E-01	-0.61	8.7E-04	-0.01	9.4E-01	0.16	4.5E-01	0.21	3.1E-01	-0.31	1.2E-01	0.00	1.0E+00	-0.24	2.3E-01	-0.46	1.8E-02
ENSCAFG0000000756	SREK1	darkgrey	EC_M8	0.60	1.3E-03	0.22	2.8E-01	-0.19	3.5E-01	0.21	3.1E-01	-0.42	3.4E-02	0.02	9.1E-01	-0.06	7.7E-01	0.12	5.7E-01	-0.18	3.9E-01	0.11	6.0E-01
ENSCAFG0000000153	ENSCAFG0000000153	darkgrey	EC_M8	0.60	1.3E-03	0.30	1.4E-01	-0.58	2.1E-03	0.11	6.0E-01	0.03	8.8E-01	0.14	5.0E-01	-0.09	6.7E-01	-0.23	2.5E-01	-0.30	1.3E-01	-0.32	1.1E-01
ENSCAFG0000000183	SMU1	darkgrey	EC_M8	0.60	1.3E-03	-0.07	7.3E-01	-0.60	1.1E-03	-0.08	7.1E-01	0.21	3.1E-01	0.25	2.2E-01	-0.22	2.9E-01	-0.07	7.4E-01	-0.21	2.9E-01	-0.54	4.5E-03
ENSCAFG0000000808	PINX1	darkgreen	EC_M4	0.60	1.3E-03	0.38	5.5E-02	-0.76	7.7E-06	-0.12	5.7E-01	0.29	1.5E-01	0.16	4.4E-01	-0.05	8.2E-01	-0.06	7.9E-01	-0.36	7.3E-02	-0.61	9.2E-04
ENSCAFG0000002896	LRRIQ1	darkgrey	EC_M8	0.60	1.3E-03	-0.04	8.4E-01	-0.17	4.0E-01	-0.33	1.0E-01	-0.27	1.8E-01	0.00	9.9E-01	-0.01	9.5E-01	0.00	9.9E-01	0.02	9.3E-01	-0.04	8.5E-01
ENSCAFG0000000341	ODC1	turquoise	EC_M6	0.60	1.3E-03	0.78	3.2E-06	-0.50	8.9E-03	-0.17	4.0E-01	-0.11	5.8E-01	0.03	8.9E-01	-0.30	1.3E-01	-0.16	4.3E-01	-0.31	1.3E-01	-0.20	3.3E-01
ENSCAFG0000001186	FANCB	darkgrey	EC_M8	0.59	1.4E-03	0.19	3.5E-01	-0.28	1.6E-01	-0.12	5.5E-01	-0.17	4.2E-01	-0.36	6.8E-02	-0.02	9.3E-01	0.17	4.0E-01	0.07	7.2E-01	-0.12	5.6E-01
ENSCAFG0000001730	RPL4	grey	EC_M10	0.59	1.4E-03	-0.22	2.9E-01	0.11	5.8E-01	0.27	1.9E-01	-0.55	3.4E-03	0.11	5.8E-01	-0.06	7.5E-01	-0.18	3.7E-01	-0.60	1.3E-03	0.19	3.5E-01
ENSCAFG0000000093	ASF1A	turquoise	EC_M6	0.59	1.4E-03	0.55	3.7E-03	-0.10	6.3E-01	0.01	9.7E-01	-0.53	5.2E-03	-0.12	5.6E-01	-0.26	2.0E-01	-0.15	4.8E-01	-0.06	7.8E-01	0.26	1.9E-01
ENSCAFG0000000752	KDM3A	turquoise	EC_M6	0.59	1.4E-03	0.64	4.7E-04	-0.67	2.0E-04	0.01	9.5E-01	-0.01	9.6E-01	0.17	4.0E-01	-0.08	7.0E-01	-0.03	8.9E-01	0.11	6.0E-01	-0.32	1.1E-01
ENSCAFG0000002015	EVI5	darkgrey	EC_M8	0.59	1.4E-03	0.57	2.1E-03	-0.33	9.7E-02	0.16	4.3E-01	-0.36	7.0E-02	0.09	6.5E-01	-0.36	6.7E-02	-0.15	4.6E-01	-0.18	3.7E-01	0.04	8.4E-01
ENSCAFG0000000131	CHCHD3	darkgrey	EC_M8	0.59	1.4E-03	-0.33	1.0E-01	-0.31	1.2E-01	-0.10	6.2E-01	0.05	8.1E-01	0.20	3.3E-01	-0.03	9.0E-01	0.05	7.9E-01	-0.47	1.6E-02	-0.40	4.6E-02
ENSCAFG0000000422	ENSCAFG0000000422	grey	EC_M10	0.59	1.4E-03	0.14	4.8E-01	-0.23	2.6E-01	0.07	7.3E-01	-0.26	2.0E-01	0.08	6.9E-01	-0.53	5.8E-03	-0.13	5.3E-01	-0.18	3.8E-01	-0.04	8.3E-01
ENSCAFG0000002020	SCAI	darkgrey	EC_M8	0.59	1.4E-03	0.48	1.2E-02	-0.44	2.4E-02	-0.07	7.5E-01	-0.16	4.3E-01	-0.03	8.8E-01	0.02	9.4E-01	0.02	9.2E-01	-0.08	6.9E-01	-0.14	4.9E-01
ENSCAFG0000000706	MSL2	grey	EC_M10	0.59	1.4E-03	0.33	9.5E-02	-0.13	5.4E-01	0.10	6.4E-01	-0.47	1.6E-02	0.26	2.0E-01	-0.22	2.8E-01	-0.14	5.0E-01	-0.35	8.1E-02	0.15	4.7E-01
ENSCAFG0000001171	ZBED9	darkgrey	EC_M8	0.59	1.4E-03	0.20	3.2E-01	-0.22	2.8E-01	0.15	4.8E-01	-0.34	9.2E-02	0.05	8.1E-01	-0.23	2.6E-01	0.05	7.9E-01	-0.51	7.5E-03	0.03	8.9E-01
ENSCAFG0000000750	DBR1	grey	EC_M10	0.59	1.4E-03	0.17	4.2E-01	-0.23	2.6E-01	-0.18	3.9E-01	-0.27	1.9E-01	0.02	9.2E-01	-0.15	4.8E-01	-0.11	6.0E-01	-0.51	7.1E-03	-0.05	8.1E-01
ENSCAFG0000000032	USP15	darkgrey	EC_M8	0.59	1.4E-03	0.14	5.1E-01	-0.51	8.4E-03	-0.07	7.2E-01	0.06	7.6E-01	0.10	6.2E-01	-0.41	3.6E-02	-0.11	5.9E-01	-0.50	9.3E-03	-0.35	7.8E-02
ENSCAFG0000002507	ENSCAFG0000002507	darkgrey	EC_M8	0.59	1.4E-03	0.24	2.4E-01	-0.05	7.9E-01	0.08	6.9E-01	-0.57	2.4E-03	-0.05	8.2E-01	-0.24	2.4E-01	-0.19	3.6E-01	-0.18	3.7E-01	0.26	2.0E-01
ENSCAFG0000001753	SPC24	darkgrey	EC_M8	0.59	1.4E-03	0.01	9.6E-01	-0.06	7.6E-01	0.07	7.5E-01	-0.32	1.1E-01	-0.05	8.1E-01	-0.24	2.3E-01	0.01	9.6E-01	0.13	5.2E-01	0.01	9.4E-01
ENSCAFG0000001497	LETM1	darkgrey	EC_M8	0.59	1.4E-03	-0.15	4.5E-01	-0.28	1.7E-01	-0.27	1.9E-01	0.01	9.8E-01	0.15	4.7E-01	-0.22	2.8E-01	-0.04	8.3E-01	-0.31	1.2E-01	-0.29	1.5E-01

ENSCAFG0000000777	RAD17	grey	EC_M10	0.59	1.4E-03	0.47	1.6E-02	-0.72	3.8E-05	0.10	6.4E-01	0.16	4.5E-01	0.20	3.2E-01	-0.13	5.3E-01	-0.10	6.4E-01	-0.35	7.9E-02	-0.46	1.8E-02
ENSCAFG0000000809	CYP2C18	grey	EC_M10	0.59	1.4E-03	0.36	7.4E-02	-0.60	1.2E-03	0.01	9.5E-01	0.11	5.8E-01	0.26	2.0E-01	-0.39	4.9E-02	-0.07	7.2E-01	-0.02	9.3E-01	-0.43	2.7E-02
ENSCAFG0000000222	PDCL3	darkgreen	EC_M4	0.59	1.5E-03	0.02	9.4E-01	-0.74	1.5E-05	-0.16	4.5E-01	0.38	5.6E-02	0.14	4.9E-01	-0.39	5.1E-02	0.00	9.9E-01	-0.27	1.8E-01	-0.71	4.9E-05
ENSCAFG0000002880	ENSCAFG0000002880	darkgrey	EC_M8	0.59	1.5E-03	0.09	6.6E-01	-0.26	2.1E-01	0.28	1.7E-01	-0.26	2.0E-01	0.04	8.4E-01	-0.09	6.6E-01	-0.23	2.5E-01	-0.33	1.0E-01	-0.06	7.7E-01
ENSCAFG0000001292	WBP11	darkgrey	EC_M8	0.59	1.5E-03	-0.14	5.1E-01	-0.21	3.0E-01	-0.03	8.9E-01	-0.15	4.5E-01	0.24	2.4E-01	-0.22	2.8E-01	0.11	5.9E-01	0.07	7.4E-01	-0.17	4.2E-01
ENSCAFG0000002360	ZNF622	grey	EC_M10	0.59	1.5E-03	0.52	5.9E-03	-0.29	1.5E-01	-0.12	5.7E-01	-0.31	1.3E-01	-0.16	4.4E-01	-0.17	4.0E-01	-0.18	3.9E-01	-0.45	2.0E-02	0.01	9.7E-01
ENSCAFG0000003230	ENSCAFG0000003230	turquoise	EC_M6	0.59	1.5E-03	0.45	2.2E-02	-0.62	7.4E-04	-0.08	7.0E-01	0.06	7.8E-01	0.05	8.1E-01	-0.15	4.8E-01	0.10	6.4E-01	-0.01	9.8E-01	-0.33	9.5E-02
ENSCAFG0000000445	CENPA	darkgrey	EC_M8	0.59	1.5E-03	0.32	1.1E-01	-0.24	2.4E-01	-0.25	2.2E-01	-0.20	3.3E-01	0.02	9.1E-01	-0.23	2.5E-01	-0.24	2.3E-01	-0.10	6.2E-01	-0.10	6.4E-01
ENSCAFG0000000670	ZNF215	grey	EC_M10	0.59	1.5E-03	-0.24	2.4E-01	-0.11	5.8E-01	0.29	1.6E-01	-0.28	1.6E-01	0.20	3.2E-01	-0.33	9.5E-02	-0.20	3.4E-01	-0.30	1.4E-01	-0.05	8.0E-01
ENSCAFG0000000270	ENSCAFG0000000270	darkgrey	EC_M8	0.59	1.5E-03	0.23	2.7E-01	-0.77	3.7E-06	-0.22	2.8E-01	0.30	1.4E-01	0.27	1.9E-01	-0.21	3.0E-01	0.06	7.8E-01	0.11	6.0E-01	-0.60	1.1E-03
ENSCAFG0000001578	RAB8A	darkgrey	EC_M8	0.59	1.5E-03	0.04	8.3E-01	-0.54	4.8E-03	-0.16	4.3E-01	0.17	3.9E-01	0.03	8.7E-01	-0.30	1.4E-01	0.05	8.2E-01	-0.45	2.1E-02	-0.49	1.1E-02
ENSCAFG0000001268	CEP350	grey	EC_M10	0.59	1.5E-03	0.13	5.3E-01	-0.56	2.8E-03	-0.07	7.3E-01	0.05	7.9E-01	0.08	6.9E-01	-0.02	9.2E-01	-0.18	3.8E-01	-0.38	5.6E-02	-0.39	4.9E-02
ENSCAFG0000000521	ULK4	darkgrey	EC_M8	0.59	1.5E-03	0.01	9.6E-01	-0.68	1.3E-04	-0.12	5.5E-01	0.33	1.0E-01	0.19	3.5E-01	-0.44	2.4E-02	-0.22	2.8E-01	-0.20	3.2E-01	-0.67	1.6E-04
ENSCAFG0000000467	CNOT10	darkgrey	EC_M8	0.59	1.5E-03	-0.10	6.3E-01	-0.55	3.8E-03	0.13	5.2E-01	0.16	4.3E-01	0.42	3.1E-02	-0.33	9.9E-02	-0.30	1.3E-01	-0.32	1.1E-01	-0.53	5.2E-03
ENSCAFG0000001351	BROX	darkgrey	EC_M8	0.59	1.5E-03	0.32	1.2E-01	-0.60	1.2E-03	0.01	9.6E-01	0.06	7.7E-01	0.12	5.7E-01	-0.21	3.0E-01	-0.07	7.2E-01	-0.43	2.8E-02	-0.36	7.3E-02
ENSCAFG0000001815	SMG1	grey	EC_M10	0.59	1.6E-03	0.18	3.8E-01	-0.52	6.1E-03	-0.06	7.9E-01	0.06	7.7E-01	0.07	7.4E-01	-0.10	6.4E-01	-0.21	3.0E-01	-0.24	2.4E-01	-0.36	7.2E-02
ENSCAFG0000001348	KLF7	grey	EC_M10	0.59	1.6E-03	0.63	5.8E-04	-0.34	8.5E-02	0.02	9.3E-01	-0.29	1.5E-01	0.00	9.8E-01	-0.21	3.1E-01	-0.22	2.9E-01	-0.40	4.2E-02	-0.01	9.8E-01
ENSCAFG0000000029	VTA1	grey	EC_M10	0.59	1.6E-03	0.39	4.6E-02	-0.50	9.2E-03	0.06	7.9E-01	-0.07	7.5E-01	0.19	3.5E-01	-0.49	1.2E-02	-0.06	7.7E-01	-0.45	2.0E-02	-0.25	2.1E-01
ENSCAFG0000000868	CCT8	darkgreen	EC_M4	0.59	1.6E-03	0.05	7.9E-01	-0.85	5.1E-08	-0.16	4.3E-01	0.56	3.0E-03	0.26	2.0E-01	-0.10	6.4E-01	-0.07	7.2E-01	-0.22	2.9E-01	-0.85	4.0E-08
ENSCAFG0000002448	ENSCAFG0000002448	darkgrey	EC_M8	0.59	1.6E-03	0.13	5.2E-01	-0.43	2.8E-02	0.29	1.5E-01	-0.10	6.1E-01	0.07	7.4E-01	-0.23	2.5E-01	-0.21	3.1E-01	-0.22	2.9E-01	-0.21	3.1E-01
ENSCAFG0000000785	ZCCHC8	darkgrey	EC_M8	0.59	1.6E-03	0.18	3.7E-01	0.15	4.8E-01	0.18	3.7E-01	-0.75	9.6E-06	0.05	7.9E-01	0.01	9.4E-01	-0.31	1.2E-01	-0.39	4.7E-02	0.42	3.4E-02
ENSCAFG0000000243	TSTD2	turquoise	EC_M6	0.59	1.6E-03	0.56	2.8E-03	-0.78	2.1E-06	-0.24	2.3E-01	0.28	1.6E-01	0.08	7.0E-01	-0.32	1.1E-01	-0.19	3.4E-01	-0.09	6.8E-01	-0.57	2.3E-03
ENSCAFG0000000730	CWC27	grey	EC_M10	0.59	1.6E-03	0.29	1.5E-01	-0.50	9.2E-03	-0.14	5.0E-01	0.03	8.9E-01	0.27	1.9E-01	-0.21	3.0E-01	0.15	4.8E-01	0.01	9.6E-01	-0.31	1.2E-01
ENSCAFG0000001742	MRPS23	darkgrey	EC_M8	0.59	1.6E-03	0.02	9.3E-01	-0.29	1.5E-01	-0.32	1.1E-01	-0.05	8.1E-01	0.38	5.7E-02	-0.24	2.5E-01	-0.19	3.5E-01	-0.13	5.4E-01	-0.25	2.2E-01
ENSCAFG0000000967	CHAF1B	darkgrey	EC_M8	0.59	1.6E-03	0.27	1.8E-01	-0.07	7.4E-01	-0.16	4.4E-01	-0.40	4.6E-02	0.17	4.2E-01	0.03	8.7E-01	-0.02	9.1E-01	-0.12	5.6E-01	0.14	5.1E-01
ENSCAFG0000001003	BRWD1	darkgrey	EC_M8	0.59	1.6E-03	0.76	7.2E-06	-0.35	8.2E-02	0.06	7.9E-01	-0.37	6.5E-02	-0.08	6.9E-01	-0.11	6.0E-01	-0.12	5.7E-01	-0.22	2.7E-01	0.07	7.4E-01
ENSCAFG0000000139	KCTD20	grey	EC_M10	0.59	1.6E-03	0.32	1.1E-01	0.02	9.2E-01	0.20	3.3E-01	-0.59	1.4E-03	-0.01	9.6E-01	-0.48	1.3E-02	-0.02	9.2E-01	-0.24	2.4E-01	0.29	1.5E-01
ENSCAFG0000003170	ENSCAFG0000003170	turquoise	EC_M6	0.59	1.7E-03	0.50	9.2E-03	-0.69	1.0E-04	-0.10	6.2E-01	0.18	3.7E-01	-0.10	6.4E-01	-0.11	5.8E-01	0.12	5.5E-01	-0.06	7.5E-01	-0.48	1.4E-02
ENSCAFG0000002332	RASSF9	turquoise	EC_M6	0.59	1.7E-03	0.49	1.2E-02	-0.53	5.1E-03	-0.27	1.8E-01	-0.04	8.4E-01	0.01	9.6E-01	-0.14	5.5E-01	0.12	5.5E-01	-0.04	8.3E-01	-0.27	1.8E-01
ENSCAFG0000001656	ADAM10	grey	EC_M10	0.59	1.7E-03	0.53	5.4E-03	-0.64	4.0E-04	-0.23	2.6E-01	0.12	5.7E-01	0.09	6.5E-01	-0.29	1.5E-01	-0.16	4.4E-01	-0.40	4.2E-02	-0.39	4.8E-02
ENSCAFG0000002967	NAF1	darkgrey	EC_M8	0.59	1.7E-03	-0.37	6.4E-02	-0.25	2.2E-01	0.00	9.8E-01	0.00	9.9E-01	0.22	2.9E-01	-0.24	2.4E-01	0.01	9.7E-01	-0.12	5.7E-01	-0.29	1.5E-01
ENSCAFG0000001050	ENSCAFG0000001050	darkgrey	EC_M8	0.59	1.7E-03	0.00	9.9E-01	-0.35	8.1E-02	0.13	5.4E-01	-0.11	5.9E-01	0.27	1.9E-01	-0.26	2.0E-01	-0.01	9.5E-01	-0.42	3.5E-02	-0.21	3.0E-01
ENSCAFG0000001620	RSC1A1	turquoise	EC_M6	0.59	1.7E-03	0.65	3.3E-04	-0.57	2.3E-03	-0.18	3.8E-01	-0.02	9.1E-01	0.12	5.7E-01	-0.09	6.7E-01	-0.23	2.7E-01	-0.24	2.3E-01	-0.27	1.8E-01
ENSCAFG0000001380	ENSCAFG0000001380	grey	EC_M10	0.59	1.7E-03	0.10	6.2E-01	-0.34	9.1E-02	0.26	2.1E-01	-0.17	4.1E-01	0.15	4.5E-01	-0.21	3.0E-01	0.06	7.8E-01	-0.63	4.9E-04	-0.19	3.5E-01
ENSCAFG0000002447	ENSCAFG0000002447	darkgrey	EC_M8	0.59	1.7E-03	-0.25	2.1E-01	-0.10	6.3E-01	0.24	2.3E-01	-0.24	2.3E-01	0.28	1.7E-01	-0.22	2.7E-01	-0.15	4.7E-01	-0.29	1.5E-01	-0.07	7.2E-01
ENSCAFG0000002964	ENSCAFG0000002964	darkgrey	EC_M8	0.58	1.7E-03	0.09	6.8E-01	0.22	2.8E-01	0.27	1.9E-01	-0.75	1.0E-05	-0.11	5.9E-01	-0.15	4.8E-01	0.02	9.3E-01	-0.19	3.4E-01	0.45	2.0E-02
ENSCAFG0000000734	ENSCAFG0000000734	grey	EC_M10	0.58	1.7E-03	-0.22	2.8E-01	0.04	8.3E-01	0.22	2.9E-01	-0.39	4.9E-02	0.07	7.2E-01	-0.48	1.4E-02	-0.03	8.8E-01	-0.42	3.3E-02	0.09	6.7E-01
ENSCAFG0000001779	MORF4L2	darkgreen	EC_M4	0.58	1.7E-03	0.27	1.9E-01	-0.91	8.0E-11	-0.12	5.5E-01	0.50	9.6E-03	0.35	8.2E-02	-0.10	6.3E-01	-0.04	8.5E-01	-0.30	1.4E-01	-0.84	1.0E-07
ENSCAFG0000001564	FOXM1	darkgrey	EC_M8	0.58	1.7E-03	0.10	6.1E-01	-0.36	6.9E-02	-0.16	4.5E-01	0.04	8.5E-01	0.17	4.1E-01	-0.04	8.4E-01	-0.15	4.6E-01	-0.31	1.2E-01	-0.31	1.2E-01
ENSCAFG0000000303	PPIE	turquoise	EC_M6	0.58	1.7E-03	0.52	6.3E-03	-0.56	2.8E-03	0.17	4.2E-01	0.00	9.9E-01	-0.14	5.0E-01	-0.17	4.1E-01	-0.32	1.1E-01	-0.39	5.0E-02	-0.32	1.1E-01
ENSCAFG0000001543	ANKLE1	darkgrey	EC_M8	0.58	1.7E-03	0.06	7.6E-01	0.10	6.3E-01	0.13	5.2E-01	-0.50	8.6E-03	-0.04	8.6E-01	-0.27	1.8E-01	-0.21	2.9E-01	-0.12	5.7E-01	0.23	2.5E-01
ENSCAFG0000000606	TMTC3	darkgreen	EC_M4	0.58	1.7E-03	0.18	3.8E-01	-0.83	1.3E-07	-0.06	7.8E-01	0.39	4.8E-02	0.22	2.7E-01	-0.25	2.2E-01	-0.04	8.6E-01	-0.28	1.7E-01	-0.72	3.9E-05
ENSCAFG0000000460	TOE1	grey	EC_M10	0.58	1.7E-03	0.32	1.1E-01	-0.63	5.7E-04	-0.12	5.5E-01	0.13	5.3E-01	0.25	2.1E-01	-0.19	3.6E-01	-0.13	5.3E-01	-0.03	9.0E-01	-0.47	1.4E-02
ENSCAFG0000001467	STIP1	darkgreen	EC_M4	0.58	1.8E-03	0.04	8.6E-01	-0.81	5.8E-07	-0.30	1.4E-01	0.51	8.2E-03	0.34	9.1E-02	-0.10	6.2E-01	0.15	4.7E-01	-0.20	3.3E-01	-0.82	3.4E-07
ENSCAFG0000000293	ENSCAFG0000000293	darkgrey	EC_M8	0.58	1.8E-03	0.29	1.5E-01	-0.52	6.4E-03	0.29	1.6E-01	-0.02	9.3E-01	0.18	3.7E-01	0.04	8.3E-01	-0.26	2.0E-01	-0.24	2.4E-01	-0.25	2.1E-01
ENSCAFG0000001088	TRIP11	darkgrey	EC_M8	0.58	1.8E-03	0.36	6.8E-02	-0.58	1.8E-03	0.03	8.8E-01	-0.02	9.3E-01	0.04	8.4E-01	-0.02	9.1E-01	-0.14	4.9E-01	-0.23	2.6E-01	-0.31	1.3E-01
ENSCAFG0000000938	INTS8	grey	EC_M10	0.58	1.8E-03	0.26	1.9E-01	-0.36	7.0E-02	-0.30	1.4E-01	-0.18	3.9E-01	-0.14	4.9E-01	0.00	1.0E+00	0.11	6.0E-01	-0.15	4.7E-01	-0.16	4.5E-01
ENSCAFG0000001800	PSMD10	darkgrey	EC_M8	0.58	1.8E-03	0.10	6.1E-01	-0.68	1.3E-04	-0.18	3.7E-01	0.27	1.9E-01	0.25	2.1E-01	-0.12	5.4E-01	-0.08	7.1E-01	0.05	8.2E-01	-0.59	1.5E-03
ENSCAFG0000000588	ENSCAFG0000000588	darkgrey	EC_M8	0.58	1.8E-03	0.12	5.6E-01	-0.42	3.4E-02	0.00	1.0E+00	0.02	9.3E-01	0.02	9.3E-01	-0.17	4.1E-01	-0.22	2.7E-01	-0.37	6.1E-02	-0.37	6.2E-02
ENSCAFG0000000189	COMMD8	grey	EC_M10	0.58	1.8E-03	-0.45	2.0E-02	-0.15	4.6E-01	0.18	3.9E-01	-0.15	4.8E-01	0.22	2.9E-01	-0.48	1.4E-02	-0.06	7.8E-01	-0.31	1.2E-01	-0.18	3.8E-01
ENSCAFG0000001590	RSL24D1	grey	EC_M10	0.58	1.8E-03	-0.17	4.0E-01	-0.64	4.9E-04	0.22	2.7E-01	0.30	1.3E-01	0.42	3.3E-02	0.02	9.1E-01	-0.15	4.6E-01	-0.17	4.2E-01	-0.61	8.7E-04
ENSCAFG0000001299	UBXN7	grey	EC_M10	0.58	1.8E-03	0.25	2.2E-01	-0.29	1.4E-01	0.19	3.4E-01	-0.26	2.0E-01	0.20	3.3E-01	-0.12	5.7E-01	-0.27	1.9E-01	0.04	8.5E-01	-0.04	8.4E-01
ENSCAFG0000000803	NOC3L	darkgrey	EC_M8	0.58	1.8E-03	-0.20	3.3E-01	-0.66	2.4E-04	-0.24	2.3E-01	0.39	5.1E-02	0.32	1.2E-01	-0.28	1.6E-01	0.09	6.7E-01	-0.11	6.0E-01	-0.72	3.9E-05
ENSCAFG0000001067	ENSCAFG0000001067	grey	EC_M10	0.58	1.8E-03	0.07	7.3E-01	-0.38	5.9E-02	-0.03	8.8E-01	-0.11	5.8E-01	-0.05	8.2E-01	-0.11	5.9E-01	0.07	7.3E-01	-0.07	7.4E-01	-0.19	3.4E-01
ENSCAFG0000001367	RUFY2	grey	EC_M10	0.58	1.8E-03	-0.20	3.2E-01	-0.25	2.2E-01	0.15	4.6E-01	-0.17	4.1E-01	0.06	7.7E-01	-0.39	4.9E-02	-0.21	3.1E-01	-0.14	4.8E-01	-0.12	5.6E-01
ENSCAFG0000001889	TAOK1	darkgrey	EC_M8	0.58	1.9E-03	0.37	6.2E-02	-0.47	1.5E-02	-0.05	8.0E-01	-0.12	5.7E-01	0.06	7.7E-01	-0.13	5.3E-01	-0.10	6.3E-01	-0.23	2.7E-01	-0.17	4.1E-01
ENSCAFG0000001870	RALBP1	grey	EC_M10	0.58	1.9E-03	0.37	6.3E-02	-0.36	6.7E-02	0.11	6.0E-01	-0.16	4.3E-01	0.04	8.3E-01	-0.47	1.5E-02	-0.03	8.7E-01	-0.11	5.9E-01	-0.10	6.1E-01
ENSCAFG0000001826	TOP3A	darkgrey	EC_M8	0.58	1.9E-03	0.19	3.6E-01	0.14	4.8E-01	0.11	6.1E-01	-0.68	1.4E-04	0.00	9.8E-01	-0.12	5.5E-01	-0.04	8.5E-01	-0.09	6.7E-01	0.38	5.6E-02
ENSCAFG0000001420	ENSCAFG0000001420	darkgrey	EC_M8	0.58	1.9E-03	0.18	3.7E-01	-0.71	5.2E-05	0.03	8.9E-01	0.26	2.0E-01	0.15	4.6E-01	-0.22	2.9E-01	-0.09	6.8E-01	-0.31	1.2E-01	-0.56	3.0E-03
ENSCAFG0000000143	PLEC	darkgrey	EC_M8	0.58	1.9E-03	0.28	1.7E-01	0.10	6.4E-01	0.00	9.9E-01	-0.64	4.4E-04	-0.09	6.7E-01	-0.37	6.6E-02	-0.14	4.8E-01	-0.07	7.3E-01	0.33	9.7E-02
ENSCAFG0000001834	TYMS	darkgrey	EC_M8	0.58	1.9E-03	0.15	4.7E-01	-0.28	1.6E-01	-0.14	4.8E-01	-0.07	7.3E-01	0.10	6.3E-01	0.08	7.0E-01	-0.08	7.0E-01	-0.01	9.5E-01	-0.17	3.9E-01
ENSCAFG0000001092	ACSL5	darkgrey	EC_M8	0.58	1.9E-03	0.09	6.7E-01	0.05	8.1E-01	0.07	7.3E-01	-0.47	1.5E-02	0.00	9.9E-01	-0.23	2.6E-01	-0.13	5.1E-01	-0.44	2.4E-02	0.21	3.1E-01
ENSCAFG0000000089	KIF3A	darkgrey	EC_M8	0.58	1.9E-03	-0.08	7.1E-01	-0.65	2.9E-04	-0.09	6.6E-01	0.33	9.6E-02	0.19	3.6E-01	-0.46	1.7E-02	-0.25	2.2E-01	-0.28	1.6E-01	-0.60	1.1E-03
ENSCAFG0000001407	ENSCAFG0000001407	darkgrey	EC_M8	0.58	1.9E-03	0.31	1.2E-01	-0.43	3.0E-02	0.13	5.2E-01	-0.10	6.4E-01	-0.07	7.3E-01	-0.09	6.7E-01	-0.33	9.6E-02	-0.10	6.2E-01	-0.19	3.5E-01
ENSCAFG0000001377	DDX50	darkgrey	EC_M8	0.58	1.9E-03	0.10	6.3E-01	-0.14	5.1E-01	0.16	4.4E-01	-0.46	1.8E-02	0.05	8.1E-01	-0.18	3.7E-01	0.10	6.4E-01	-0.28	1.7E-01	0.11	6.0E-01
ENSCAFG0000001706	RPL26	grey	EC_M10	0.58	1.9E-03	0.28	1.6E-01	-0.04	8.5E-01	0.02	9.4E-01	-0.56	3.2E-03	-0.06	7.8E-01	-0.04	8.3E-01	-0.24	2.5E-01	-0.36	7.4E-02	0.23	2.6E-01
ENSCAFG0000001793	ERI2	grey	EC_M10	0.58	1.9E-03	0.16	4.2E-01	-0.20	3.3E-01	0.00	9.9E-01	-0.34	9.3E-02	0.24	2.3E-01	-0.16	4.4E-01	0.02	9.1E-01	-0.16	4.3E-01	0.06	7.6E-01
ENSCAFG0000000339	TAF1B	darkgrey	EC_M8	0.58	1.9E-03	-0.27	1.8E-01	-0.53	5.1E-03	-0.01	9.8E-01	0.25	2.2E-01	0.26	2.0E-01	-0.42	3.1E-02	0.03	8.7E-01	-0.05	8.0E-01	-0.54	4.6E-03
ENSCAFG0000001052	CDC73	darkgreen	EC_M4	0.58	1.9E-03	0.35	8.0E-02	-0.82	4.0E-07	-0.30	1.3E-01	0.35	8.1E-02	0.32	1.1E-01	-0.16	4.3E-01	-0.12	5.7E-01	-0.21	2.9E-01	-0.66	2.4E-04
ENSCAFG0000001023	CUL3	darkgrey	EC_M8	0.58	1.9E-03	0.28	1.6E-01	-0.76	7.0E-06	0.00	9.8E-01	0.28	1.7E-01	0.26	1.9E-01	-0.21	3.0E-01	-0.19	3.6E-01	-0.43	2.7E-02	-0.57	2.3E-03
ENSCAFG0000000572	ENSCAFG0000000572	grey	EC_M10	0.58	2.0E-03	-0.14	4.8E-01	-0.36	7.1E-02	0.21	3.0E-01	-0.06	7.7E-01	0.16	4.5E-01	-0.20	3.4E-01	-0.21	3.1E-01	-0.19	3.5E-01	-0.25	2.2E-01
ENSCAFG0000000131	CTNNA1	darkgreen	EC_M4	0.58	2.0E-03	0.39	5.0E-02	-0.87	6.4E-09	-0.22	2.9E-01	0.42	3.2E-02	0.08	6.9E-01	-0.33	1.0E-01	0.08	7.0E-01	-0.28	1.7E-01	-0.72	2.9E-05
ENSCAFG0000001851	ENSCAFG0000001851	grey	EC_M10	0.58	2.0E-03	0.15	4.6E-01	-0.42	3.2E-02	-0.07	7.2E-01	-0.05	8.0E-01	-0.17	4.1E-01	-0.39	5.1E-02	0.05	8.1E-01	-0.51	8.4E-03	-0.29	1.5E-01
ENSCAFG0000000255	SRBD1	grey	EC_M10	0.58	2.0E-03	-0.16	4.4E-01	0.04	8.5E-01	0.02	9.3E-01	-0.49	1.0E-02	-0.01	9.6E-01	-0.32	1.1E-01	-0.05	8.1E-01	0.05	8.1E-01	0.16	4.3E-01
ENSCAFG0000000730	TNKS2	darkgrey	EC_M8	0.58	2.0E-03	0.58	1.8E-03	-0.53	5.6E-03	0.16	4.4E-01	-0.13	5.2E-01	0.18	3.9E-01	-0.18	3.8E-01	-0.21	3.0E-01	-0.15	4.6E-01	-0.18	3.7E-01
ENSCAFG0000001583	RAD52	darkgrey	EC_M8	0.58	2.0E-03	0.21	3.1E-01	0.20	3.2E-01	0.14	5.0E-01	-0.79	1.9E-06	0.03	9.0E-01	-0.04	8.4E-01	-0.18	6.3E-01	-0.18	3.7E-01	0.47	1.6E-02
ENSCAFG0000000654	UBA3	darkgreen	EC_M4	0.58	2.0E-03	-0.09	6.6E-01	-0.82	2.5E-07	-0.02	9.2E-01	0.50	9.3E-03	0.21	3.1E-01	-0.24	2.3E-01	-0.01	9.4E-01	-0.13	5.1E-01	-0.83	2.1E-07
ENSCAFG0000001748	LCMT1	darkgrey	EC_M8	0.58	2.0E-03	-0.21	3.0E-01	-0.10	6.2E-01	-0.01	9.7E-01	-0.24	2.4E-01	-0.04	8.6E-01	-0.44	2.6E-02	-0.15	4.8E-01	-0.44	2.5E-02	-0.11	6.0E-01
ENSCAFG0000001122	FGFR1OP2	darkgrey	EC_M8	0.58	2.0E-03	0.50	8.5E-03	-0.26	2.0E-01	0.18	3.7E-01	-0.36	6.7E-02	-0.04	8.4E-01	-0.12	5.4E-01	-0.11	5.9E-01	-0.16	4.3E-01	0.08	6.9E-01
ENSCAFG0000000869	RBL1	darkgrey	EC_M8	0.58	2.0E-03	0.30	1.4E-01	-0.27	1.8E-01	-0.08	7.1E-01	-0.22	2.8E-01	0.04	8.4E-01	-0.04	8.4E-01	-0.08	6.9E-01	0.12	5.7E-01	-0.05	8.3E-01
ENSCAFG0000001534	DCAF6	darkgrey	EC_M8	0.58	2.0E-03	0.05	7.9E-01	0.17	4.0E-01	0.14	4.9E-01	-0.72	3.2E-05	-0.07	7.3E-01	-0.38	5.6E-02	-0.17	4.1E-01	-0.38	5.8E-02	0.40	4.4E-02
ENSCAFG0000000940	CCNE2	darkgrey	EC_M8	0.58	2.1E-03	0.36	6.9E-02	-0.09	6.5E-01	0.10	6.2E-01	-0.42	3.1E-02	0.03	8.9E-01	-0.03	8.8E-01	-0.16	4.3E-01	-0.04	8.5E-01	0.15	4.8E-01
ENSCAFG0000001756	RBBP6	darkgrey	EC_M8	0.58	2.1E-03	0.38	5.3E-02	-0.24	2.4E-01	0.22	2.8E-01	-0.42	3.3E-02	0.10	6.4E-01	-0.01	9.5E-01	-0.02	9.0E-01	-0.27	1.8E-01	0.08	7.0E-01
ENSCAFG0000000719	KIF2A	darkgreen	EC_M4	0.58	2.1E-03	0.15	4.6E-01	-0.86	2.6E-08	-0.12	5.4E-01	0.47	1.5E-02	0.29	1.5E-01	-0.15	4.7E-01	-0.19	3.5E-01	-0.33	9.5E-02	-0.78	3.1E-06
ENSCAFG0000001324	PRR14L	grey	EC_M10	0.58	2.1E-03	0.41	3.7E-02	-0.49	1.0E-02	0.10	6.3E-01	-0.05	8.0E-01	0.09	6.5E-01	-0.40	4.4E-02	-0.15	4.7E-01	-0.39	4.6E-02	-0.25	2.1E-01
ENSCAFG0000001842	SMCHD1	darkgrey	EC_M8	0.57	2.1E-03	0.24	2.4E-01	0.24	2.5E-01	0.18	3.7E-01	-0.86	2.5E-08	-0.03	8.7E-01	-0.26	2.1E-01	-0.26	2.0E-01	-0.26	2.0E-01	0.55	3.6E-03
ENSCAFG0000003035	ENSCAFG0000003035	grey	EC_M10	0.57	2.1E-03	0.32	1.1E-01	-0.12	5.7E-01	0.24	2.3E-01	-0.46	1.9E-02	-0.08	6.9E-01	-0.22	2.8E-01	0.10	6.3E-01	-0.16	4.3E-01	0.13	5.4E-01
ENSCAFG0000000756	CEP70	grey	EC_M10	0.57	2.1E-03	-0.09	6.8E-01	-0.04	8.3E-01	-0.05	8.0E-01	-0.38	5.7E-02	0.14	4.9E-01	-0.37	6.3E-02	-0.35	7.8E-02	-0.18	3.8E-01	0.08	6.9E-01
ENSCAFG0000000308	PABPC4	darkgrey	EC_M8	0.57	2.1E-03	-0.16	4.5E-01	-0.34	9.2E-02	-0.05	8.1E-01	0.04	8.3E-01	0.13	5.3E-01	-0.47	1.6E-02	0.10	6.2E-01	-0.02	9.3E-01	-0.39	4.9E-02
ENSCAFG0000000995	ENSCAFG0000000995	darkgrey	EC_M8	0.57	2.1E-03	0.19	3.5E-01	-0.40	4.1E-02	0.40	4.4E-02	-0.14	4.9E-01	-0.02	9.4E-01	-0.14	5.0E-01	-0.08	7.0E-01	-0.20	3.3E-01	-0.18	3.9E-01
ENSCAFG0000001052	RBBP4	grey	EC_M10	0.57	2.2E-03	0.22	2.9E-01	-0.50	8.7E-03	0.05	8.0E-01	0.05	8.0E-01	0.35	8.4E-02	-0.08	6.8E-01	-0.38	5.7E-02	-0.10	6.4E-01	-0.32	1.1E-01
ENSCAFG0000003153	ENSCAFG0000003153	grey	EC_M10	0.57	2.2E-03	0.20	3.3E-01	-0.45	2.2E-02	0.22	2.8E-01	-0.06	7.7E-01	0.33	9.5E-02	-0.26	2.0E-01	-0.25	2.3E-01	-0.32	1.1E-01	-0.28	1.6E-01
ENSCAFG0000000364	ENSCAFG0000000364	darkgrey	EC_M8	0.57	2.2E-03	0.12	5.8E-01	-0.41	3.6E-02	0.26	1.9E-01	-0.10	6.2E-01	0.29	1.5E-01	-0.26	2.1E-01	-0.33	9.5E-02	-0.31	1.2E-01	-0.22	2.9E-01
ENSCAFG0000001554	TBL1XR1	darkgrey	EC_M8	0.57	2.2E-03	0.43	2.8E-02	0.05	8.2E-01	0.25	2.1E-01	-0.72	3.9E-05	-0.14	5.1E-01	-0.20	3.3E-01	0.06	7.8E-01	-0.11	6.0E-01	0.42	3.3E-02
ENSCAFG0000000941	CDYL	turquoise	EC_M6	0.57	2.2E-03	0.63	5.4E-04	-0.65	3.1E-04	0.00	9.9E-01	0.04	8.5E-01	0.18	3.7E-01	-0.13	5.4E-01	-0.31	1.3E-01	-0.22	2.7E-01	-0.38	5.6E-02
ENSCAFG0000002852	TRMT10B	grey	EC_M10	0.57	2.2E-03	0.08	6.8E-01	-0.19	3.6E-01	0.17	4.1E-01	-0.32	1.2E-01	0.06	7.6E-01	-0.47	1.6E-02	-0.32	1.1E-01	-0.37	5.9E-02	-0.03	8.8E-01
ENSCAFG0000000054	MTRF1L	grey	EC_M10	0.57	2.2E-03	-0.06	7.7E-01	-0.56	2.9E-03	0.35	8.1E-02	0.17	4.1E-01	0.37	6.3E-02	-0.25	2.2E-01	-0.01	9.4E-01	-0.36	6.7E-02	-0.51	7.2E-03
ENSCAFG0000001980	GAS8	darkgrey	EC_M8	0.57	2.2E-03	-0.32	1.1E-01	0.12	5.5E-01	0.12	5.6E-01	-0.51	7.6E-03	0.22	2.7E-01	-0.13	5.3E-01	-0.12	5.6E-01	-0.19	3.5E-01	0.17	4.1E-01
ENSCAFG0000001677	VPS13C	grey	EC_M10	0.57	2.2E-03	-0.07	7.2E-01	0.07	7.3E-01	0.15	4.6E-01	-0.54	4.4E-03	-0.20	3.2E-01	-0.21	2.9E-01	-0.21	3.0E-01	-0.42	3.4E-02	0.23	2.6E-01
ENSCAFG0000000311	CASP8AP2	darkgrey	EC_M8	0.57	2.3E-03	0.53	5.5E-03	-0.49	1.2E-02	0.06	7.6E-01	-0.14	4.9E-01	0.09	6.5E-01	0.04	8.4E-01	-0.25	2.2E-01	-0.21	3.1E-01	-0.18	3.9E-01
ENSCAFG0000002135	ENSCAFG0000002135	grey	EC_M10	0.57	2.3E-03	0.23	2.6E-01	-0.34	9.1E-02	0.13	5.3E-01	-0.19	3.6E-01	-0.07	7.2E-01	-0.13	5.2E-01	-0.15	4.6E-01	-0.14	4.9E-01	-0.13	5.2E-01
ENSCAFG0000000452	COG3	darkgrey	EC_M8	0.57	2.3E-03	0.22	2.9E-01	-0.48	1.3E-02	0.37	6.0E-02	-0.08	6.8E-01	0.11	6.0E-01	-0.20	3.2E-01	-0.23	2.7E-01	-0.25	2.2E-01	-0.26	2.0E-01
ENSCAFG0000000406	LUC7L2	darkgrey	EC_M8	0.57	2.3E-03	0.16	4.4E-01	-0.29	1.4E-01	0.21	3.0E-01	-0.28	1.7E-01	0.04	8.6E-01	-0.25	2.2E-01	0.10	6.3E-01	-0.01	9.6E-01	-0.05	8.1E-01
ENSCAFG0000000227	PHF14	grey	EC_M10	0.57	2.3E-03	0.57	2.5E-03	-0.65	3.1E-04	-0.11	6.0E-01	0.01	9.6E-01	0.14	4.9E-01	-0.21	3.1E-01	0.06	7.7E-01	-0.29	1.5E-01	-0.35	8.0E-02
ENSCAFG0000000366	ENSCAFG0000000366	darkgrey	EC_M8	0.57	2.3E-03	0.12	5.7E-01	-0.27	1.8E-01	0.05	8.2E-01	-0.21	3.0E-01	0.00	9.9E-01	0.00	9.9E-01	-0.43	2.8E-02	-0.38	5.5E-02	-0.11	5.9E-01
ENSCAFG0000002404	ENSCAFG0000002404	grey	EC_M10	0.57	2.3E-03	0.18	3.7E-01	-0.34	8.7E-02	-0.18	3.7E-01	-0.14	4.9E-01	-0.26	2.0E-01	-0.20	3.3E-01	-0.21	3.1E-01	-0.18	3.7E-01	-0.13	5.1E-01
ENSCAFG0000003176	NDUFAF4	darkgreen	EC_M4	0.57	2.3E-03	-0.12	5.6E-01	-0.66	2.8E-04	-0.10	6.3E-01	0.37	6.5E-02	0.18	3.9E-01	-0.21	3.1E-01	0.15	4.6E-01	-0.33	1.0E-01	-0.68	1.2E-04
ENSCAFG0000000176	PWP1	darkgrey	EC_M8	0.57	2.3E-03	-0.30	1.3E-01	-0.54	4.1E-03	-0.16	4.4E-01	0.36	7.3E-02	0.30	1.4E-01	-0.23	2.6E-01	-0.18	3.8E-01	-0.14	5.1E-01	-0.65	3.5E-04
ENSCAFG0000001223	ENSCAFG0000001223	darkgreen	EC_M4	0.57	2.3E-03	0.18	3.8E-01	-0.85	5.3E-08	-0.14	5.1E-01	0.49	1.1E-02	0.12	5.6E-01	0.03	8.8E-01	0.18	3.7E-01	-0.20	3.2E-01	-0.79	1.3E-06
ENSCAFG0000001676	NES	darkgreen	EC_M4	0.57	2.3E-03	0.41	4.0E-02	-0.79	1.5E-06	-0.21	3.0E-01	0.33	1.0E-01	0.16	4.3E-01	0.03	9.0E-01	0.05	8.2E-01	0.17	4.0E-01	-0.63	6.3E-04
ENSCAFG0000000201	ENSCAFG0000000201	darkgreen	EC_M4	0.57	2.3E-03	0.33	1.0E-01	-0.86	1.9E-08	-0.03	8.9E-01	0.36	7.3E-02	0.44	2.4E-02	-0.21	3.0E-01	-0.20	3.3E-01	-0.08	7.0E-01	-0.69	1.0E-04
ENSCAFG0000000806	ENSCAFG0000000806	grey	EC_M10	0.57	2.3E-03	0.30	1.4E-01	-0.28	1.7E-01	0.23	2.6E-01	-0.31	1.3E-01	0.06	7.6E-01	-0.50	9.9E-03	-0.26	1.9E-01	-0.28	1.7E-01	-0.04	8.3E-01
ENSCAFG0000001056	NEPRO	grey	EC_M10	0.57	2.3E-03	-0.14	5.0E-01	-0.39	4.7E-02	-0.05	8.1E-01	-0.01	9.5E-01	0.20	3.2E-01	-0.31	1.2E-01	0.11	5.8E-01	0.08	7.0E-01	-0.31	1.3E-01
ENSCAFG0000000317	DPY19L1	darkgrey	EC_M8	0.57	2.4E-03	0.31	1.3E-01	0.00	9.9E-01	0.00	1.0E+00	-0.60	1.2E-03	-0.14	4.9E-01	-0.12	5.5E-01	0.02	9.2E-01	-0.19	3.5E-01	0.29	1.4E-01
ENSCAFG0000001778	PAPOLA	turquoise	EC_M6	0.57	2.4E-03	0.58	2.0E-03	-0.54	4.4E-03	-0.16	4.2E-01	-0.01	9.5E-01	0.07	7.3E-01	-0.42	3.3E-02	0.16	4.3E-01	0.03	8.9E-01	-0.26	1.9E-01
ENSCAFG0000001105	TRAIP	darkgrey	EC_M8	0.57	2.4E-03	0.03	8.9E-01	-0.20	3.4E-01	0.03	8.7E-01	-0.11	6.0E-01	0.11	6.0E-01	-0.19	3.6E-01	0.04	8.4E-01	-0.02	9.1E-01	-0.15	4.7E-01

ENSCAFG0000001875	ZNF280C	darkgrey	EC_M8	0.57	2.4E-03	0.12	5.7E-01	-0.63	5.8E-04	-0.27	1.8E-01	0.23	2.5E-01	0.02	9.2E-01	-0.39	4.9E-02	0.04	8.3E-01	-0.21	3.1E-01	-0.52	6.8E-03
ENSCAFG0000000658	ENSCAFG0000000658	grey	EC_M10	0.57	2.4E-03	0.09	6.8E-01	0.32	1.1E-01	0.07	7.3E-01	-0.82	3.1E-07	-0.03	8.7E-01	-0.01	9.4E-01	-0.18	3.8E-01	-0.43	3.0E-02	0.50	8.7E-03
ENSCAFG0000000456	NUFIP1	darkgreen	EC_M4	0.57	2.4E-03	-0.27	1.8E-01	-0.67	2.0E-04	-0.16	4.4E-01	0.45	2.1E-02	0.39	4.6E-02	-0.25	2.2E-01	0.05	8.0E-01	-0.22	2.9E-01	-0.75	1.2E-05
ENSCAFG0000000503	PIBF1	grey	EC_M10	0.57	2.4E-03	-0.32	1.1E-01	-0.05	8.0E-01	0.25	2.2E-01	-0.32	1.1E-01	0.17	4.1E-01	-0.46	1.9E-02	0.01	9.7E-01	-0.37	6.1E-02	-0.04	8.4E-01
ENSCAFG0000000748	CEP89	darkgrey	EC_M8	0.57	2.4E-03	-0.28	1.7E-01	-0.52	7.1E-03	-0.14	5.1E-01	0.29	1.5E-01	0.27	1.8E-01	0.01	9.8E-01	-0.03	8.9E-01	-0.25	2.1E-01	-0.63	4.9E-04
ENSCAFG0000000618	ATL2	grey	EC_M10	0.57	2.4E-03	0.19	3.6E-01	-0.31	1.3E-01	0.17	4.1E-01	-0.24	2.3E-01	0.38	5.5E-02	-0.14	4.9E-01	-0.36	7.3E-02	-0.10	6.4E-01	-0.07	7.3E-01
ENSCAFG0000001432	DDX10	grey	EC_M10	0.57	2.4E-03	0.03	8.9E-01	0.04	8.4E-01	0.15	4.7E-01	-0.54	4.0E-03	-0.20	3.3E-01	-0.13	5.3E-01	-0.13	5.3E-01	-0.60	1.3E-03	0.22	2.9E-01
ENSCAFG0000000013	TXNL1	darkgreen	EC_M4	0.57	2.4E-03	0.28	1.6E-01	-0.71	4.3E-05	-0.23	2.6E-01	0.33	9.8E-02	0.22	2.9E-01	-0.28	1.6E-01	-0.13	5.3E-01	-0.26	2.1E-01	-0.64	4.2E-04
ENSCAFG0000000922	WDR41	grey	EC_M10	0.57	2.5E-03	0.02	9.2E-01	-0.26	2.0E-01	0.20	3.2E-01	-0.13	5.2E-01	0.23	2.6E-01	-0.40	4.3E-02	-0.29	1.5E-01	-0.31	1.3E-01	-0.16	4.3E-01
ENSCAFG0000000360	CNTRL	darkgrey	EC_M8	0.57	2.5E-03	0.01	9.7E-01	0.13	5.2E-01	0.12	5.5E-01	-0.66	2.4E-04	0.02	9.3E-01	-0.06	7.6E-01	-0.08	7.1E-01	0.00	9.8E-01	0.37	6.5E-02
ENSCAFG0000001232	PYROXD1	darkgreen	EC_M4	0.57	2.5E-03	-0.13	5.2E-01	-0.74	1.6E-05	-0.13	5.2E-01	0.45	2.0E-02	0.25	2.1E-01	-0.20	3.2E-01	0.03	9.0E-01	-0.18	3.8E-01	-0.74	1.7E-05
ENSCAFG0000000612	ATP2B1	darkgreen	EC_M4	0.57	2.5E-03	0.52	6.4E-03	-0.82	3.8E-07	-0.09	6.5E-01	0.33	1.0E-01	0.17	4.1E-01	-0.14	4.8E-01	-0.11	5.9E-01	-0.34	8.7E-02	-0.62	6.6E-04
ENSCAFG0000003224	ENSCAFG0000003224	darkgrey	EC_M8	0.57	2.5E-03	-0.06	7.5E-01	-0.65	2.9E-04	-0.02	9.4E-01	0.32	1.1E-01	0.25	2.2E-01	-0.40	4.1E-02	-0.03	8.9E-01	-0.04	8.4E-01	-0.65	3.2E-04
ENSCAFG0000000878	WWP1	grey	EC_M10	0.57	2.5E-03	0.63	5.7E-04	-0.62	6.6E-04	-0.16	4.5E-01	-0.02	9.2E-01	0.06	7.7E-01	-0.14	5.0E-01	-0.04	8.6E-01	-0.05	8.3E-01	-0.27	1.7E-01
ENSCAFG0000001970	QSOX2	grey	EC_M10	0.57	2.5E-03	-0.20	3.2E-01	0.19	3.4E-01	0.06	7.9E-01	-0.57	2.5E-03	0.09	6.8E-01	-0.11	5.8E-01	-0.16	4.3E-01	-0.43	2.7E-02	0.25	2.2E-01
ENSCAFG0000000253	STX17	darkgrey	EC_M8	0.57	2.5E-03	0.08	6.9E-01	-0.45	2.0E-02	0.01	9.5E-01	0.00	9.9E-01	0.18	3.8E-01	-0.05	7.9E-01	-0.08	7.2E-01	-0.27	1.8E-01	-0.29	1.6E-01
ENSCAFG0000000529	ENSCAFG0000000529	darkgrey	EC_M8	0.57	2.5E-03	0.05	8.2E-01	-0.27	1.9E-01	0.23	2.5E-01	-0.25	2.1E-01	0.22	2.8E-01	-0.14	5.0E-01	-0.29	1.6E-01	-0.29	1.6E-01	-0.05	8.0E-01
ENSCAFG0000001734	STXBP4	darkgrey	EC_M8	0.57	2.5E-03	-0.37	5.9E-02	-0.34	8.9E-02	0.09	6.5E-01	0.03	8.9E-01	0.32	1.1E-01	-0.33	9.7E-02	0.09	6.8E-01	-0.06	7.8E-01	-0.37	6.4E-02
ENSCAFG0000000350	MRPL32	darkgrey	EC_M8	0.57	2.5E-03	-0.03	8.9E-01	-0.48	1.4E-02	-0.10	6.4E-01	0.18	3.7E-01	0.01	9.5E-01	-0.42	3.1E-02	-0.08	7.1E-01	0.04	8.5E-01	-0.48	1.3E-02
ENSCAFG0000000432	WDR33	darkgrey	EC_M8	0.57	2.5E-03	0.31	1.3E-01	0.12	5.7E-01	-0.14	5.0E-01	-0.70	6.3E-05	0.05	8.0E-01	0.01	9.5E-01	-0.03	8.7E-01	-0.37	6.5E-02	0.40	4.1E-02
ENSCAFG0000000073	TCP1	darkgreen	EC_M4	0.57	2.5E-03	0.05	8.0E-01	-0.83	1.2E-07	-0.18	3.9E-01	0.56	3.1E-03	0.22	2.7E-01	-0.16	4.5E-01	-0.13	5.4E-01	-0.17	4.1E-01	-0.84	8.8E-08
ENSCAFG0000000413	CWC15	darkgrey	EC_M8	0.57	2.5E-03	0.25	2.2E-01	-0.48	1.2E-02	0.02	9.2E-01	0.03	8.9E-01	0.16	4.3E-01	0.15	4.6E-01	-0.02	9.3E-01	0.02	9.2E-01	-0.34	8.6E-02
ENSCAFG0000000622	DHX57	grey	EC_M10	0.57	2.5E-03	0.14	5.0E-01	-0.52	6.2E-03	-0.13	5.3E-01	0.06	7.7E-01	-0.14	4.9E-01	-0.49	1.1E-02	-0.03	9.0E-01	-0.19	3.6E-01	-0.38	5.6E-02
ENSCAFG0000000686	THOC7	grey	EC_M10	0.57	2.5E-03	-0.20	3.3E-01	-0.33	9.6E-02	-0.25	2.1E-01	-0.01	9.6E-01	0.14	4.8E-01	-0.09	6.5E-01	0.02	9.3E-01	-0.35	8.0E-02	-0.31	1.2E-01
ENSCAFG0000001064	RRP1	darkgrey	EC_M8	0.57	2.5E-03	0.38	5.5E-02	-0.09	6.5E-01	0.00	1.0E+00	-0.43	2.8E-02	-0.18	3.7E-01	0.07	7.3E-01	-0.07	7.2E-01	-0.28	1.6E-01	0.13	5.2E-01
ENSCAFG0000001691	NPM1	darkgrey	EC_M8	0.57	2.6E-03	-0.41	3.8E-02	-0.01	9.5E-01	0.04	8.3E-01	-0.23	2.6E-01	0.17	3.9E-01	-0.15	4.6E-01	-0.07	7.2E-01	-0.50	9.0E-03	-0.11	5.9E-01
ENSCAFG0000001171	LARP7	darkgreen	EC_M4	0.57	2.6E-03	-0.05	7.9E-01	-0.74	1.8E-05	-0.10	6.3E-01	0.42	3.1E-02	0.30	1.3E-01	-0.34	8.7E-02	-0.17	4.0E-01	-0.15	4.5E-01	-0.71	5.8E-05
ENSCAFG0000001055	CHEK1	darkgrey	EC_M8	0.57	2.6E-03	0.24	2.4E-01	0.12	5.5E-01	-0.15	4.7E-01	-0.65	3.6E-04	-0.02	9.3E-01	0.13	5.1E-01	-0.08	7.1E-01	-0.08	6.8E-01	0.35	7.8E-02
ENSCAFG0000000767	RASA2	darkgrey	EC_M8	0.57	2.6E-03	0.37	5.9E-02	-0.76	8.1E-06	-0.08	6.9E-01	0.27	1.8E-01	0.24	2.4E-01	-0.28	1.7E-01	0.08	6.8E-01	-0.20	3.4E-01	-0.59	1.6E-03
ENSCAFG0000001732	ENSCAFG0000001732	darkgrey	EC_M8	0.57	2.6E-03	-0.15	4.7E-01	-0.41	3.5E-02	0.22	2.9E-01	0.00	9.9E-01	0.18	3.8E-01	0.06	7.7E-01	0.07	7.4E-01	-0.38	5.3E-02	-0.34	8.5E-02
ENSCAFG0000001588	ENSCAFG0000001588	darkgrey	EC_M8	0.57	2.6E-03	0.30	1.3E-01	-0.49	1.1E-02	0.19	3.6E-01	-0.03	9.0E-01	0.25	2.2E-01	-0.29	1.5E-01	-0.28	1.7E-01	-0.09	6.6E-01	-0.25	2.1E-01
ENSCAFG0000000396	ANKRD50	turquoise	EC_M6	0.57	2.6E-03	0.57	2.4E-03	-0.39	4.9E-02	0.12	5.6E-01	-0.21	3.1E-01	-0.13	5.1E-01	-0.33	1.0E-01	0.16	4.5E-01	0.22	2.7E-01	-0.09	6.4E-01
ENSCAFG0000003162	FAM200A	grey	EC_M10	0.57	2.6E-03	-0.04	8.6E-01	-0.44	2.4E-02	0.30	1.4E-01	0.04	8.5E-01	0.24	2.5E-01	-0.30	1.4E-01	0.11	6.0E-01	-0.14	4.9E-01	-0.34	9.2E-02
ENSCAFG0000000252	ENSCAFG0000000252	darkgrey	EC_M8	0.57	2.6E-03	0.27	1.8E-01	-0.32	1.1E-01	0.44	2.5E-02	-0.23	2.7E-01	0.07	7.4E-01	-0.10	6.3E-01	-0.49	1.1E-02	-0.27	1.9E-01	-0.04	8.3E-01
ENSCAFG0000001088	CEP72	darkgrey	EC_M8	0.57	2.6E-03	0.30	1.3E-01	-0.34	9.1E-02	-0.18	3.9E-01	-0.09	6.6E-01	0.00	1.0E+00	-0.34	8.9E-02	0.02	9.3E-01	-0.24	2.5E-01	-0.21	3.1E-01
ENSCAFG0000001315	GALE	turquoise	EC_M6	0.57	2.6E-03	0.57	2.3E-03	-0.74	1.8E-05	-0.05	8.2E-01	0.24	2.3E-01	0.10	6.2E-01	-0.07	7.4E-01	-0.08	7.0E-01	-0.20	3.3E-01	-0.54	4.5E-03
ENSCAFG0000000829	GFM2	grey	EC_M10	0.56	2.6E-03	0.01	9.6E-01	-0.14	5.1E-01	0.00	1.0E+00	-0.30	1.3E-01	-0.10	6.3E-01	-0.51	8.3E-03	0.25	2.3E-01	0.03	8.7E-01	0.03	8.7E-01
ENSCAFG0000002457	ENSCAFG0000002457	grey	EC_M10	0.56	2.6E-03	-0.01	9.6E-01	-0.07	7.3E-01	0.20	3.3E-01	-0.40	4.3E-02	0.09	6.5E-01	-0.19	3.6E-01	0.06	7.6E-01	-0.28	1.7E-01	0.12	5.5E-01
ENSCAFG0000002901	CACYBP	darkgreen	EC_M4	0.56	2.6E-03	-0.10	6.3E-01	-0.68	1.4E-04	-0.06	7.6E-01	0.34	8.6E-02	0.28	1.7E-01	-0.11	6.1E-01	0.24	2.5E-01	-0.22	2.8E-01	-0.65	3.2E-04
ENSCAFG0000000402	RINT1	darkgrey	EC_M8	0.56	2.7E-03	-0.16	4.4E-01	-0.72	3.5E-05	-0.04	8.3E-01	0.49	1.1E-02	0.21	3.0E-01	-0.41	3.6E-02	-0.03	8.8E-01	-0.05	8.1E-01	-0.78	2.8E-06
ENSCAFG0000000358	ZMYM4	darkgrey	EC_M8	0.56	2.7E-03	0.29	1.5E-01	-0.57	2.1E-03	0.09	6.5E-01	0.01	9.5E-01	0.16	4.5E-01	-0.33	1.0E-01	-0.15	4.8E-01	-0.23	2.5E-01	-0.38	5.9E-02
ENSCAFG0000001637	ENSCAFG0000001637	grey	EC_M10	0.56	2.7E-03	0.38	5.4E-02	-0.33	9.7E-02	0.28	1.7E-01	-0.22	2.7E-01	0.05	7.9E-01	-0.19	3.4E-01	-0.05	7.9E-01	-0.06	7.7E-01	-0.02	9.2E-01
ENSCAFG0000003128	ZNF134	grey	EC_M10	0.56	2.7E-03	-0.04	8.4E-01	-0.09	6.7E-01	0.37	6.4E-02	-0.41	3.9E-02	0.07	7.3E-01	-0.14	4.9E-01	0.15	4.5E-01	0.01	9.4E-01	0.10	6.2E-01
ENSCAFG0000000138	UHRF2	darkgrey	EC_M8	0.56	2.7E-03	0.50	1.0E-02	-0.42	3.5E-02	0.04	8.3E-01	-0.21	3.0E-01	0.06	7.6E-01	-0.25	2.1E-01	-0.13	5.2E-01	-0.33	9.7E-02	-0.09	6.5E-01
ENSCAFG0000001039	TAF5	grey	EC_M10	0.56	2.7E-03	0.02	9.1E-01	-0.15	4.7E-01	0.02	9.3E-01	-0.32	1.1E-01	0.09	6.5E-01	0.13	5.1E-01	0.02	9.4E-01	-0.40	4.4E-02	-0.01	9.7E-01
ENSCAFG0000001793	EDC3	grey	EC_M10	0.56	2.7E-03	0.31	1.3E-01	-0.73	2.0E-05	-0.30	1.3E-01	0.32	1.1E-01	0.26	2.0E-01	-0.07	7.2E-01	-0.44	2.4E-02	-0.24	2.4E-01	-0.60	1.2E-03
ENSCAFG0000001704	PANK3	grey	EC_M10	0.56	2.7E-03	0.74	1.3E-05	-0.42	3.1E-02	-0.07	7.4E-01	-0.25	2.2E-01	0.09	6.5E-01	-0.09	6.5E-01	-0.26	2.0E-01	-0.35	8.1E-02	-0.05	8.0E-01
ENSCAFG0000001589	ENSCAFG0000001589	darkgrey	EC_M8	0.56	2.8E-03	0.18	3.9E-01	-0.47	1.5E-02	0.22	2.7E-01	-0.04	8.4E-01	0.10	6.1E-01	-0.18	3.7E-01	-0.43	2.8E-02	-0.26	2.1E-01	-0.25	2.1E-01
ENSCAFG0000002218	ENSCAFG0000002218	grey	EC_M10	0.56	2.8E-03	0.24	2.3E-01	-0.40	4.3E-02	0.11	6.0E-01	-0.10	6.2E-01	0.08	7.0E-01	-0.19	3.6E-01	0.11	5.9E-01	-0.24	2.3E-01	-0.23	2.6E-01
ENSCAFG0000000844	ANAPC7	darkgrey	EC_M8	0.56	2.8E-03	-0.19	3.5E-01	-0.57	2.5E-03	0.04	8.5E-01	0.28	1.7E-01	0.37	6.3E-02	-0.17	4.0E-01	-0.09	6.7E-01	-0.09	6.7E-01	-0.58	1.9E-03
ENSCAFG0000000650	ENSCAFG0000000650	darkgrey	EC_M8	0.56	2.8E-03	-0.35	7.9E-02	-0.13	5.1E-01	0.09	6.8E-01	-0.12	5.6E-01	0.18	3.7E-01	-0.23	2.5E-01	0.04	8.5E-01	-0.40	4.5E-02	-0.18	3.7E-01
ENSCAFG0000001201	MCM3AP	darkgrey	EC_M8	0.56	2.8E-03	-0.31	1.3E-01	-0.07	7.3E-01	0.14	4.8E-01	-0.27	1.8E-01	0.14	4.9E-01	-0.20	3.4E-01	-0.26	2.0E-01	-0.17	4.0E-01	-0.02	9.4E-01
ENSCAFG0000001235	NSL1	darkgrey	EC_M8	0.56	2.8E-03	0.30	1.3E-01	-0.40	4.6E-02	-0.17	4.2E-01	-0.09	6.5E-01	0.25	2.1E-01	0.00	1.0E+00	0.33	1.0E-01	0.00	9.9E-01	-0.21	3.1E-01
ENSCAFG0000002958	ENSCAFG0000002958	grey	EC_M10	0.56	2.8E-03	-0.06	7.7E-01	-0.29	1.6E-01	0.28	1.7E-01	-0.11	6.1E-01	-0.18	3.8E-01	-0.21	3.1E-01	-0.12	5.6E-01	-0.17	4.2E-01	-0.21	3.1E-01
ENSCAFG0000003190	IMP4	darkgrey	EC_M8	0.56	2.8E-03	-0.38	5.7E-02	-0.05	8.1E-01	-0.02	9.4E-01	-0.24	2.4E-01	0.22	2.8E-01	-0.02	9.1E-01	-0.09	6.7E-01	-0.45	2.0E-02	-0.12	5.5E-01
ENSCAFG0000000012	NARS	darkgrey	EC_M8	0.56	2.9E-03	-0.22	2.8E-01	-0.76	6.8E-06	-0.03	8.8E-01	0.55	3.7E-03	0.36	7.2E-02	-0.22	2.8E-01	-0.03	8.9E-01	-0.22	2.7E-01	-0.86	2.3E-08
ENSCAFG0000000924	VIRMA	darkgrey	EC_M8	0.56	2.9E-03	0.08	6.9E-01	-0.47	1.4E-02	0.20	3.4E-01	0.00	9.8E-01	0.27	1.8E-01	-0.14	5.1E-01	-0.17	4.2E-01	-0.41	3.8E-02	-0.36	6.7E-02
ENSCAFG0000000946	MARCH7	grey	EC_M10	0.56	2.9E-03	0.35	7.7E-02	-0.52	6.0E-03	0.21	3.1E-01	-0.03	9.0E-01	0.23	2.6E-01	-0.31	1.2E-01	-0.07	7.3E-01	-0.14	5.0E-01	-0.28	1.7E-01
ENSCAFG0000002413	FASTKD5	darkgrey	EC_M8	0.56	2.9E-03	0.15	4.6E-01	0.19	3.6E-01	0.07	7.5E-01	-0.68	1.4E-04	-0.01	9.6E-01	-0.22	2.9E-01	-0.21	3.0E-01	-0.02	9.1E-01	0.41	3.9E-02
ENSCAFG0000000289	DOP1A	darkgrey	EC_M8	0.56	2.9E-03	-0.18	3.9E-01	0.06	7.6E-01	0.09	6.5E-01	-0.54	4.4E-03	0.15	4.5E-01	-0.25	2.3E-01	-0.26	1.9E-01	-0.23	2.6E-01	0.23	2.6E-01
ENSCAFG0000000819	ENSCAFG0000000819	grey	EC_M10	0.56	2.9E-03	0.34	8.7E-02	-0.43	2.7E-02	-0.29	1.5E-01	-0.05	8.1E-01	0.32	1.1E-01	-0.17	4.2E-01	-0.18	3.7E-01	-0.13	5.1E-01	-0.22	2.8E-01
ENSCAFG0000001072	SMNDC1	grey	EC_M10	0.56	2.9E-03	0.36	6.7E-02	-0.41	3.5E-02	0.06	7.7E-01	-0.15	4.8E-01	0.35	8.3E-02	0.03	8.7E-01	-0.34	8.7E-02	-0.37	6.5E-02	-0.17	4.0E-01
ENSCAFG0000001684	ENSCAFG0000001684	darkgrey	EC_M8	0.56	2.9E-03	0.05	8.0E-01	0.20	3.4E-01	0.08	7.0E-01	-0.75	1.2E-05	0.16	4.2E-01	-0.04	8.6E-01	-0.24	2.4E-01	-0.34	8.9E-02	0.42	3.3E-02
ENSCAFG0000002746	ENSCAFG0000002746	grey	EC_M10	0.56	2.9E-03	0.39	5.1E-02	-0.50	8.8E-03	0.08	7.0E-01	-0.08	7.0E-01	0.20	3.2E-01	-0.22	2.8E-01	-0.31	1.3E-01	-0.36	7.0E-02	-0.29	1.6E-01
ENSCAFG0000000076	MAP3K4	darkgrey	EC_M8	0.56	3.0E-03	0.36	7.2E-02	0.06	7.5E-01	0.11	5.9E-01	-0.66	2.3E-04	-0.20	3.4E-01	-0.34	9.1E-02	-0.13	5.3E-01	-0.29	1.5E-01	0.36	7.2E-02
ENSCAFG0000000549	AZI2	grey	EC_M10	0.56	3.0E-03	0.03	8.9E-01	-0.58	1.9E-03	0.15	4.6E-01	0.16	4.3E-01	0.36	6.8E-02	-0.34	8.7E-02	0.10	6.2E-01	-0.07	7.4E-01	-0.49	1.0E-02
ENSCAFG0000002374	ENSCAFG0000002374	darkgrey	EC_M8	0.56	3.0E-03	0.28	1.7E-01	0.04	8.4E-01	0.10	6.3E-01	-0.64	4.7E-04	0.26	1.9E-01	-0.28	1.7E-01	-0.14	5.0E-01	-0.11	5.8E-01	0.32	1.2E-01
ENSCAFG0000000966	MGA	darkgrey	EC_M8	0.56	3.0E-03	0.23	2.5E-01	0.03	8.7E-01	0.05	7.9E-01	-0.62	8.1E-04	-0.23	2.6E-01	-0.12	5.6E-01	-0.14	5.0E-01	-0.29	1.5E-01	0.32	1.2E-01
ENSCAFG0000000445	MED4	grey	EC_M10	0.56	3.0E-03	-0.19	3.6E-01	-0.20	3.4E-01	-0.02	9.4E-01	-0.21	3.1E-01	0.31	1.3E-01	-0.18	3.7E-01	-0.09	6.7E-01	-0.20	3.3E-01	-0.14	4.8E-01
ENSCAFG0000002035	CTCF	darkgrey	EC_M8	0.56	3.0E-03	0.45	2.2E-02	0.20	3.4E-01	0.18	3.8E-01	-0.86	2.5E-08	-0.15	4.8E-01	-0.12	5.6E-01	-0.14	4.9E-01	-0.16	4.5E-01	0.55	3.3E-03
ENSCAFG0000000156	RECQL4	darkgrey	EC_M8	0.56	3.0E-03	0.22	2.8E-01	-0.27	1.7E-01	-0.10	6.2E-01	-0.11	6.0E-01	0.12	5.6E-01	-0.05	8.2E-01	0.01	9.7E-01	-0.11	6.0E-01	-0.16	4.2E-01
ENSCAFG0000001703	ENSCAFG0000001703	darkgrey	EC_M8	0.56	3.1E-03	0.29	1.5E-01	-0.43	2.7E-02	0.11	5.9E-01	-0.06	7.6E-01	0.32	1.1E-01	-0.31	1.3E-01	-0.11	5.9E-01	-0.27	1.9E-01	-0.20	3.3E-01
ENSCAFG0000003210	ENSCAFG0000003210	darkgrey	EC_M8	0.56	3.1E-03	0.02	9.3E-01	-0.24	2.3E-01	-0.13	5.3E-01	-0.06	7.6E-01	-0.12	5.7E-01	-0.07	7.4E-01	-0.08	7.1E-01	-0.09	6.6E-01	-0.20	3.3E-01
ENSCAFG0000001426	UBTF	darkgrey	EC_M8	0.56	3.1E-03	-0.16	4.4E-01	0.04	8.3E-01	-0.17	4.0E-01	-0.40	4.3E-02	0.13	5.2E-01	-0.38	5.6E-02	0.02	9.1E-01	0.06	7.6E-01	0.09	6.7E-01
ENSCAFG0000001048	YARS	darkgrey	EC_M8	0.56	3.1E-03	-0.07	7.5E-01	-0.48	1.4E-02	0.13	5.4E-01	0.14	4.9E-01	0.17	4.0E-01	-0.32	1.1E-01	-0.17	4.0E-01	-0.14	5.0E-01	-0.50	8.9E-03
ENSCAFG0000000278	TRA2A	grey	EC_M10	0.56	3.1E-03	0.26	2.1E-01	-0.32	1.1E-01	0.16	4.4E-01	-0.22	2.7E-01	-0.06	7.8E-01	-0.41	3.5E-02	0.11	5.9E-01	-0.37	6.1E-02	-0.14	4.9E-01
ENSCAFG0000000565	METTL3	darkgrey	EC_M8	0.56	3.1E-03	0.20	3.2E-01	0.01	9.7E-01	0.08	7.1E-01	-0.48	1.4E-02	0.04	8.5E-01	-0.03	8.8E-01	-0.42	3.2E-02	-0.18	3.8E-01	0.16	4.2E-01
ENSCAFG0000001902	ATP11C	darkgreen	EC_M4	0.56	3.1E-03	0.48	1.3E-02	-0.74	1.3E-05	-0.01	9.5E-01	0.24	2.3E-01	0.25	2.2E-01	-0.21	2.9E-01	-0.10	6.4E-01	-0.35	8.0E-02	-0.55	3.7E-03
ENSCAFG0000000291	UTP3	grey	EC_M10	0.56	3.1E-03	0.06	7.8E-01	-0.04	8.6E-01	0.09	6.5E-01	-0.50	9.0E-03	0.12	5.7E-01	0.04	8.5E-01	0.05	7.9E-01	-0.46	1.8E-02	0.18	3.8E-01
ENSCAFG0000001942	MKNK2	grey	EC_M10	0.56	3.1E-03	0.02	9.4E-01	-0.30	1.4E-01	0.33	9.5E-02	-0.12	5.5E-01	0.26	1.9E-01	-0.34	9.0E-02	-0.16	4.2E-01	-0.19	3.5E-01	-0.23	2.6E-01
ENSCAFG0000001303	PDHA1	grey	EC_M10	0.56	3.2E-03	-0.41	3.6E-02	0.00	9.9E-01	-0.11	6.1E-01	-0.25	2.1E-01	0.09	6.7E-01	-0.37	6.6E-02	0.05	8.3E-01	-0.06	7.6E-01	-0.03	8.8E-01
ENSCAFG0000003222	ENSCAFG0000003222	darkgreen	EC_M4	0.56	3.2E-03	0.31	1.2E-01	-0.87	7.4E-09	-0.21	3.1E-01	0.47	1.5E-02	0.28	1.6E-01	-0.10	6.3E-01	0.03	8.9E-01	-0.20	3.4E-01	-0.76	6.4E-06
ENSCAFG0000001126	ENSCAFG0000001126	darkgrey	EC_M8	0.56	3.2E-03	0.10	6.2E-01	-0.66	2.2E-04	0.08	7.1E-01	0.26	2.0E-01	0.38	5.2E-02	-0.08	7.1E-01	-0.04	8.6E-01	-0.46	1.7E-02	-0.56	2.8E-03
ENSCAFG0000001037	ENSCAFG0000001037	darkgrey	EC_M8	0.56	3.2E-03	0.18	3.8E-01	-0.44	2.6E-02	0.37	6.2E-02	-0.03	8.8E-01	-0.04	8.6E-01	0.00	1.0E+00	-0.12	5.7E-01	-0.34	8.7E-02	-0.23	2.5E-01
ENSCAFG0000000095	MTBP	grey	EC_M10	0.56	3.2E-03	-0.04	8.6E-01	-0.24	2.4E-01	0.01	9.8E-01	-0.17	4.1E-01	0.27	1.8E-01	0.09	6.5E-01	-0.30	1.4E-01	-0.46	1.7E-02	-0.13	5.4E-01
ENSCAFG0000003018	ZNF383	grey	EC_M10	0.56	3.2E-03	0.35	8.2E-02	-0.47	1.5E-02	0.23	2.7E-01	-0.08	7.0E-01	-0.07	7.4E-01	-0.17	4.2E-01	0.04	8.4E-01	0.01	9.7E-01	-0.19	3.4E-01
ENSCAFG0000001819	ALG13	grey	EC_M10	0.56	3.2E-03	0.10	6.3E-01	-0.67	1.7E-04	0.00	9.9E-01	0.27	1.8E-01	0.46	1.9E-02	-0.08	6.8E-01	-0.10	6.3E-01	-0.03	9.0E-01	-0.62	7.6E-04
ENSCAFG0000001095	IARS2	darkgrey	EC_M8	0.56	3.2E-03	-0.23	2.6E-01	-0.54	4.3E-03	-0.12	5.5E-01	0.32	1.2E-01	0.09	6.6E-01	-0.30	1.4E-01	-0.14	5.1E-01	-0.24	2.3E-01	-0.57	2.4E-03
ENSCAFG0000000045	TBC1D15	darkgrey	EC_M8	0.56	3.2E-03	0.38	5.4E-02	-0.13	5.2E-01	0.13	5.4E-01	-0.50	8.9E-03	-0.02	9.2E-01	-0.07	7.3E-01	-0.12	5.5E-01	-0.39	4.9E-02	0.20	3.2E-01
ENSCAFG0000000229	POLR2B	turquoise	EC_M6	0.56	3.3E-03	0.41	3.6E-02	-0.57	2.3E-03	-0.15	4.5E-01	0.07	7.4E-01	0.27	1.9E-01	0.10	6.4E-01	0.02	9.4E-01	0.05	8.3E-01	-0.31	1.2E-01
ENSCAFG0000000395	ENSCAFG0000000395	grey	EC_M10	0.55	3.3E-03	-0.10	6.2E-01	-0.56	3.1E-03	0.23	2.6E-01	0.24	2.4E-01	0.15	4.5E-01	-0.16	4.4E-01	-0.35	8.2E-02	-0.42	3.1E-02	-0.54	4.1E-03
ENSCAFG0000000595	MCM8	grey	EC_M10	0.55	3.3E-03	0.33	9.8E-02	-0.69	8.9E-05	0.00	9.9E-01	0.24	2.5E-01	0.10	6.1E-01	0.04	8.5E-01	0.19	3.6E-01	-0.01	9.4E-01	-0.53	5.8E-03
ENSCAFG0000000991	ENSCAFG0000000991	grey	EC_M10	0.55	3.3E-03	0.29	1.5E-01	-0.09	6.6E-01	0.18	3.7E-01	-0.47	1.6E-02	-0.42	3.3E-02	-0.11	5.8E-01	0.05	8.1E-01	-0.49	1.1E-02	0.16	4.4E-01
ENSCAFG0000001672	ENSCAFG0000001672	darkgreen	EC_M4	0.55	3.3E-03	-0.08	7.0E-01	-0.86	2.5E-08	-0.17	4.0E-01	0.58	1.8E-03	0.41	3.7E-02	-0.18	3.7E-01	0.11	5.8E-01	-0.23	2.6E-01	-0.88	2.3E-09
ENSCAFG0000000486	MBD4	darkgrey	EC_M8	0.55	3.3E-03	0.45	2.2E-02	-0.23	2.6E-01	0.19	3.6E-01	-0.40	4.2E-02	0.24	2.5E-01	-0.05	8.1E-01	-0.31	1.2E-01	-0.16	4.4E-01	0.09	6.7E-01
ENSCAFG0000000109	FAM49B	darkgreen	EC_M4	0.55	3.3E-03	0.42	3.4E-02	-0.87	9.3E-09	-0.06	7.7E-01	0.42	3.2E-02	0.21	2.9E-01	-0.05	8.2E-01	-0.23	2.5E-01	-0.12	5.5E-01	-0.71	4.3E-05
ENSCAFG0000000068	ENSCAFG0000000068	grey	EC_M10	0.55	3.3E-03	0.25	2.2E-01	-0.56	3.1E-03	0.12	5.7E-01	0.06	7.9E-01	0.15	4.7E-01	-0.13	5.2E-01	0.06	7.6E-01	-0.18	3.8E-01	-0.38	5.3E-02
ENSCAFG0000001481	CNOT9	darkgrey	EC_M8	0.55	3.3E-03	0.28	1.7E-01	-0.54	4.5E-03	-0.20	3.4E-01	0.16	4.2E-01	0.13	5.3E-01	-0.29	1.5E-01	-0.09	6.5E-01	-0.18	3.7E-01	-0.45	2.1E-02
ENSCAFG0000001138	DDX27	darkgrey	EC_M8	0.55	3.4E-03	-0.30	1.3E-01	0.13	5.4E-01	0.10	6.4E-01	-0.44	2.4E-02	0.21	3.0E-01	-0.23	2.6E-01	0.04	8.3E-01	0.15	4.8E-01	0.15	4.6E-01
ENSCAFG0000000786	ENSCAFG0000000786	darkgrey	EC_M8	0.55	3.4E-03	0.07	7.5E-01	-0.56	3.2E-03	0.32	1.1E-01	0.14	4.9E-01	0.18	3.8E-01	-0.30	1.3E-01	-0.23	2.7E-01	-0.13	5.2E-01	-0.43	2.7E-02
ENSCAFG0000001675	TARDBP	grey	EC_M10	0.55	3.4E-03	0.39	5.1E-02	-0.63	5.0E-04	-0.10	6.3E-01	0.24	2.3E-01	0.06	7.7E-01	-0.34	8.9E-02	0.25	2.2E-01	-0.10	6.3E-01	-0.52	6.7E-03
ENSCAFG0000002019	ENSCAFG0000002019	darkgrey	EC_M8	0.55	3.4E-03	-0.11	5.9E-01	-0.17	4.1E-01	0.06	7.7E-01	-0.17	4.2E-01	0.08	6.9E-01	-0.38	5.4E-02	-0.12	5.6E-01	-0.20	3.3E-01	-0.12	5.5E-01
ENSCAFG0000000587	FAM98A	darkgreen	EC_M4	0.55	3.4E-03	0.05	8.0E-01	-0.84	6.6E-08	-0.15	4.5E-01	0.51	8.4E-03	0.21	2.9E-01	-0.26	2.0E-01	0.05	7.9E-01	-0.21	2.9E-01	-0.82	2.6E-07
ENSCAFG0000000947	BCAS2	darkgrey	EC_M8	0.55	3.4E-03	0.26	1.9E-01	-0.72	2.9E-05	0.11	5.9E-01	0.30	1.3E-01	0.21	3.1E-01	-0.35	7.7E-02	-0.14	4.8E-01	-0.12	5.8E-01	-0.57	2.6E-03
ENSCAFG0000001313	SMARCC1	darkgrey	EC_M8	0.55	3.5E-03	-0.05	8.2E-01	-0.76	6.6E-06	-0.11	6.0E-01	0.51	8.0E-03	0.21	3.0E-01	-0.35	7.7E-02	-0.17	4.0E-01	-0.30	1.4E-01	-0.81	4.2E-07
ENSCAFG0000001088	PPA2	grey	EC_M10	0.55	3.5E-03	0.08	6.8E-01	0.09	6.6E-01	0.11	5.8E-01	-0.56	3.2E-03	-0.11	5.8E-01	-0.37	6.5E-02	-0.19	3.5E-01	-0.24	2.3E-01	0.27	1.9E-01
ENSCAFG0000000817	MRPL19	turquoise	EC_M6	0.55	3.5E-03	0.30	1.4E-01	-0.56	2.8E-03	-0.15	4.6E-01	0.06	7.8E-01	0.03	8.9E-01	-0.03	8.7E-01	0.05	8.3E-01	-0.06	7.7E-01	-0.33	1.0E-01
ENSCAFG0000001758	SMARCA4	darkgreen	EC_M4	0.55	3.5E-03	0.53	5.8E-03	-0.85	3.5E-08	-0.19	3.5E-01	0.38	5.6E-02	0.04	8.4E-01	-0.10	6.2E-01	0.09	6.7E-01	0.09	6.5E-01	-0.66	2.3E-04
ENSCAFG0000002499	TMOD3	darkgrey	EC_M8	0.55	3.5E-03	0.54	4.8E-03	0.07	7.5E-01	0.02	9.2E-01	-0.75	1.2E-05	-0.17	4.1E-01	-0.24	2.3E-01	-0.16	4.5E-01	0.02	9.0E-01	0.48	1.3E-02
ENSCAFG0000001817	TAF15	darkgrey	EC_M8	0.55	3.5E-03	0.02	9.2E-01	-0.45	2.2E-02	-0.03	8.8E-01	0.05	8.0E-01	-0.01	9.7E-01	-0.52	6.3E-03	0.18	3.8E-01	-0.02	9.4E-01	-0.39	4.6E-02
ENSCAFG0000000282	LCA5	grey	EC_M10	0.55	3.5E-03	0.41	3.6E-02	-0.64	4.3E-04	-0.03	8.8E-01	0.07	7.2E-01	0.30	1.4E-01	-0.01	9.5E-01	-0.01	9.6E-01	-0.12	5.4E-01	-0.38	5.4E-02
ENSCAFG0000000309	ENSCAFG0000000309	grey	EC_M10	0.55	3.5E-03	0.28	1.7E-01	-0.70	6.7E-05	-0.07	7.5E-01	0.28	1.6E-01	0.18	3.8E-01	-0.12	5.6E-01	-0.04	8.6E-01	-0.27	1.8E-01	-0.53	4.9E-03
ENSCAFG0000000285	CYCS	darkgreen	EC_M4	0.55	3.5E-03	-0.05	8.3E-01	-0.74	1.3E-05	-0.19	3.5E-01	0.52	6.6E-03	0.29	1.5E-01	-0.19	3.6E-01	0.01	9.4E-01	-0.15	4.8E-01	-0.78	2.5E-06
ENSCAFG0000001158	FXR1	darkgrey	EC_M8	0.55	3.5E-03	0.23	2.7E-01	-0.41	3.9E-02	0.11	5.9E-01	-0.15	4.7E-01	0.11	6.0E-01	-0.17	4.2E-01	-0.05	8.0E-01	-0.44	2.5E-02	-0.19	3.6E-01
ENSCAFG0000000653	COPZ1	grey	EC_M10	0.55	3.5E-03	0.16	4.3E-01	-0.26	2.0E-01	-0.27	1.7E-01	-0.21	3.1E-01	-0.20	3.3E-01	-0.13	5.2E-01	-0.11	6.0E-01	-0.35	7.8E-02	-0.14	5.0E-01
ENSCAFG0000000408	CCDC82	grey	EC_M10	0.55	3.6E-03	0.08	7.0E-01	-0.34	8.9E-02	-0.07	7.4E-01	-0.14	4.9E-01	0.22	2.8E-01	-0.14	4.9E-01	-0.09	6.6E-01	-0.22	2.9E-01	-0.14	5.0E-01

ENSCAFG0000001244	AEBP2	grey	EC_M10	0.55	3.6E-03	-0.34	8.9E-02	-0.57	2.4E-03	0.02	9.3E-01	0.29	1.5E-01	0.41	3.9E-02	-0.04	8.4E-01	-0.13	5.2E-01	-0.31	1.2E-01	-0.62	8.0E-04
ENSCAFG0000003061	MZT1	darkgreen	EC_M4	0.55	3.6E-03	0.20	3.2E-01	-0.93	6.8E-12	0.05	8.2E-01	0.52	6.5E-03	0.28	1.7E-01	-0.15	4.7E-01	-0.16	4.2E-01	-0.16	4.4E-01	-0.82	3.8E-07
ENSCAFG0000000356	ENSCAFG0000000356	darkgrey	EC_M8	0.55	3.6E-03	0.38	5.8E-02	-0.59	1.4E-03	0.16	4.2E-01	0.06	7.8E-01	0.20	3.4E-01	-0.27	1.8E-01	0.00	1.0E+00	-0.23	2.6E-01	-0.34	9.0E-02
ENSCAFG0000000024	MTFR2	darkgrey	EC_M8	0.55	3.6E-03	-0.31	1.2E-01	-0.10	6.4E-01	-0.09	6.8E-01	-0.13	5.2E-01	-0.03	9.0E-01	-0.35	8.2E-02	-0.01	9.8E-01	0.09	6.7E-01	-0.14	5.1E-01
ENSCAFG0000001170	CEP95	darkgrey	EC_M8	0.55	3.6E-03	-0.07	7.5E-01	0.16	4.4E-01	0.14	5.0E-01	-0.65	3.2E-04	0.08	7.1E-01	-0.20	3.4E-01	0.03	9.0E-01	-0.20	3.3E-01	0.33	9.7E-02
ENSCAFG0000001739	ENSCAFG0000001739	darkgrey	EC_M8	0.55	3.6E-03	0.02	9.3E-01	-0.20	3.2E-01	-0.12	5.7E-01	-0.10	6.3E-01	-0.11	6.0E-01	-0.10	6.2E-01	-0.08	7.0E-01	-0.10	6.3E-01	-0.16	4.2E-01
ENSCAFG0000001874	ENSCAFG0000001874	darkgrey	EC_M8	0.55	3.7E-03	0.19	3.5E-01	-0.56	2.9E-03	0.19	3.6E-01	0.09	6.7E-01	0.26	1.9E-01	-0.34	9.1E-02	-0.20	3.2E-01	-0.26	2.0E-01	-0.36	6.9E-02
ENSCAFG0000000193	AKAP9	darkgrey	EC_M8	0.55	3.7E-03	0.58	2.0E-03	-0.56	3.0E-03	0.02	9.2E-01	-0.09	6.8E-01	-0.05	7.9E-01	0.04	8.6E-01	-0.17	4.1E-01	-0.22	2.8E-01	-0.21	2.9E-01
ENSCAFG0000001092	KMT5B	grey	EC_M10	0.55	3.7E-03	0.47	1.5E-02	-0.38	5.4E-02	0.11	6.1E-01	-0.20	3.2E-01	0.05	8.1E-01	-0.20	3.2E-01	-0.23	2.6E-01	0.00	9.8E-01	-0.07	7.3E-01
ENSCAFG0000000426	HADHA	grey	EC_M10	0.55	3.7E-03	-0.10	6.4E-01	-0.35	7.7E-02	0.06	7.5E-01	-0.05	8.1E-01	0.06	7.8E-01	-0.14	4.9E-01	0.13	5.3E-01	0.38	5.7E-02	-0.22	2.8E-01
ENSCAFG0000001210	CBL	grey	EC_M10	0.55	3.7E-03	0.58	1.7E-03	-0.36	7.0E-02	-0.02	9.2E-01	-0.25	2.3E-01	-0.21	3.1E-01	-0.04	8.5E-01	-0.14	5.0E-01	-0.24	2.4E-01	-0.04	8.4E-01
ENSCAFG0000001381	ENSCAFG0000001381	grey	EC_M10	0.55	3.7E-03	-0.18	3.9E-01	-0.34	8.9E-02	0.12	5.6E-01	0.02	9.0E-01	0.08	7.0E-01	-0.25	2.1E-01	0.17	4.0E-01	-0.05	7.9E-01	-0.34	8.9E-02
ENSCAFG0000000527	THAP12	grey	EC_M10	0.55	3.7E-03	-0.25	2.2E-01	0.11	6.0E-01	-0.07	7.5E-01	-0.48	1.3E-02	-0.03	8.8E-01	-0.27	1.8E-01	-0.03	8.8E-01	-0.40	4.2E-02	0.18	3.9E-01
ENSCAFG0000001502	ENSCAFG0000001502	darkgrey	EC_M8	0.55	3.7E-03	0.16	4.3E-01	-0.51	8.0E-03	0.03	9.0E-01	0.03	8.9E-01	0.03	9.0E-01	-0.24	2.4E-01	-0.27	1.9E-01	-0.41	3.7E-02	-0.32	1.1E-01
ENSCAFG0000000612	MRPL3	grey	EC_M10	0.55	3.7E-03	-0.22	2.7E-01	-0.07	7.2E-01	0.29	1.4E-01	-0.34	9.3E-02	0.08	7.1E-01	-0.36	7.4E-02	-0.16	4.4E-01	-0.24	2.3E-01	0.02	9.4E-01
ENSCAFG0000000529	ENSCAFG0000000529	grey	EC_M10	0.55	3.7E-03	0.41	3.7E-02	-0.49	1.1E-02	0.19	3.5E-01	-0.01	9.5E-01	-0.27	1.8E-01	-0.24	2.3E-01	-0.24	2.3E-01	-0.02	9.4E-01	-0.29	1.5E-01
ENSCAFG0000001528	ENSCAFG0000001528	turquoise	EC_M6	0.55	3.7E-03	0.46	1.8E-02	-0.67	2.0E-04	-0.24	2.3E-01	0.22	2.8E-01	0.23	2.7E-01	-0.53	5.2E-03	-0.04	8.6E-01	-0.34	9.4E-02	-0.51	7.2E-03
ENSCAFG0000000391	RPF2	grey	EC_M10	0.55	3.7E-03	-0.52	6.3E-03	-0.19	3.5E-01	0.21	3.0E-01	-0.06	7.6E-01	0.28	1.7E-01	-0.18	3.8E-01	0.00	9.9E-01	-0.28	1.7E-01	-0.27	1.9E-01
ENSCAFG0000002271	ENSCAFG0000002271	grey	EC_M10	0.55	3.8E-03	-0.06	7.7E-01	-0.11	5.8E-01	0.29	1.5E-01	-0.35	7.5E-02	0.13	5.1E-01	-0.36	6.9E-02	-0.01	9.5E-01	-0.35	8.3E-02	0.05	7.9E-01
ENSCAFG0000000233	ENSCAFG0000000233	darkgrey	EC_M8	0.55	3.8E-03	-0.06	7.8E-01	-0.15	4.5E-01	0.18	3.7E-01	-0.16	4.3E-01	0.07	7.5E-01	-0.13	5.1E-01	0.18	3.8E-01	0.14	4.8E-01	-0.09	6.4E-01
ENSCAFG0000002597	ENSCAFG0000002597	grey	EC_M10	0.55	3.8E-03	0.00	9.8E-01	-0.16	4.3E-01	0.02	9.3E-01	-0.34	8.7E-02	0.19	3.4E-01	-0.22	2.9E-01	-0.13	5.4E-01	-0.09	6.6E-01	0.02	9.2E-01
ENSCAFG0000000042	YEATS4	grey	EC_M10	0.55	3.8E-03	0.20	3.3E-01	-0.05	8.0E-01	0.01	9.6E-01	-0.46	1.7E-02	0.15	4.7E-01	-0.36	7.1E-02	-0.10	6.2E-01	-0.13	5.2E-01	0.19	3.5E-01
ENSCAFG0000001400	MYEF2	turquoise	EC_M6	0.55	3.8E-03	0.64	4.5E-04	-0.79	1.6E-06	-0.24	2.3E-01	0.25	2.1E-01	0.11	6.0E-01	0.07	7.4E-01	0.02	9.1E-01	0.10	6.1E-01	-0.53	5.7E-03
ENSCAFG0000000914	RBM19	grey	EC_M10	0.55	3.8E-03	-0.18	3.7E-01	-0.35	7.9E-02	-0.03	9.0E-01	0.14	5.1E-01	0.15	4.6E-01	-0.48	1.3E-02	-0.10	6.4E-01	-0.35	7.6E-02	-0.43	2.7E-02
ENSCAFG0000002483	KIF21B	grey	EC_M10	0.55	3.8E-03	0.63	5.5E-04	-0.47	1.5E-02	-0.12	5.7E-01	-0.13	5.4E-01	0.06	7.9E-01	-0.13	5.3E-01	-0.28	1.7E-01	-0.39	4.8E-02	-0.18	3.9E-01
ENSCAFG0000000675	POLR1D	grey	EC_M10	0.55	3.8E-03	-0.06	7.7E-01	0.25	2.2E-01	-0.05	8.2E-01	-0.71	5.1E-05	0.12	5.6E-01	-0.04	8.3E-01	0.05	8.0E-01	-0.42	3.5E-02	0.39	4.7E-02
ENSCAFG0000000976	PPHLN1	grey	EC_M10	0.55	3.8E-03	0.27	1.9E-01	-0.47	1.6E-02	0.30	1.4E-01	0.01	9.6E-01	0.33	9.8E-02	-0.28	1.7E-01	-0.39	5.0E-02	-0.15	4.7E-01	-0.27	1.8E-01
ENSCAFG0000001052	DNM1L	darkgreen	EC_M4	0.55	3.8E-03	-0.02	9.4E-01	-0.78	2.9E-06	-0.14	5.1E-01	0.52	6.7E-03	0.27	1.9E-01	-0.39	4.7E-02	-0.03	8.8E-01	-0.16	4.2E-01	-0.79	1.4E-06
ENSCAFG0000000322	PRPF4	grey	EC_M10	0.55	3.8E-03	0.27	1.8E-01	-0.60	1.3E-03	-0.21	3.1E-01	0.21	3.0E-01	0.21	2.9E-01	0.19	3.4E-01	0.01	9.5E-01	-0.33	9.9E-02	-0.47	1.4E-02
ENSCAFG0000001922	PRPF8	grey	EC_M10	0.55	3.9E-03	0.12	5.7E-01	-0.40	4.4E-02	-0.19	3.4E-01	0.05	8.2E-01	0.17	4.2E-01	-0.03	8.9E-01	-0.15	4.7E-01	-0.40	4.2E-02	-0.33	1.0E-01
ENSCAFG0000002421	USP4	grey	EC_M10	0.55	3.9E-03	0.36	7.4E-02	-0.22	2.8E-01	0.05	8.2E-01	-0.23	2.6E-01	0.14	4.8E-01	-0.23	2.5E-01	-0.26	1.9E-01	-0.63	5.2E-04	-0.04	8.3E-01
ENSCAFG0000001171	DDX5	darkgrey	EC_M8	0.55	3.9E-03	0.59	1.6E-03	-0.28	1.7E-01	0.03	8.9E-01	-0.40	4.4E-02	-0.03	9.0E-01	-0.18	3.8E-01	0.04	8.6E-01	0.21	3.1E-01	0.12	5.6E-01
ENSCAFG0000000223	MIOS	darkgrey	EC_M8	0.55	3.9E-03	-0.27	1.8E-01	0.13	5.4E-01	0.12	5.5E-01	-0.54	4.1E-03	0.17	4.0E-01	-0.16	4.4E-01	-0.07	7.2E-01	-0.38	5.3E-02	0.25	2.2E-01
ENSCAFG0000001209	FAM126B	grey	EC_M10	0.55	4.0E-03	0.48	1.3E-02	-0.34	9.1E-02	-0.05	7.9E-01	-0.22	2.9E-01	-0.07	7.2E-01	0.21	3.0E-01	-0.17	4.2E-01	-0.07	7.2E-01	-0.03	8.7E-01
ENSCAFG0000000852	SDAD1	darkgreen	EC_M4	0.55	4.0E-03	-0.13	5.2E-01	-0.77	4.5E-06	-0.13	5.2E-01	0.52	7.1E-03	0.39	5.1E-02	-0.27	1.9E-01	0.05	8.1E-01	-0.05	8.2E-01	-0.82	2.7E-07
ENSCAFG0000002274	ENSCAFG0000002274	darkgrey	EC_M8	0.54	4.0E-03	-0.11	6.1E-01	-0.65	3.6E-04	-0.02	9.2E-01	0.34	8.7E-02	0.23	2.5E-01	-0.10	6.4E-01	0.15	4.7E-01	-0.19	3.5E-01	-0.65	3.0E-04
ENSCAFG0000001814	SS18	darkgreen	EC_M4	0.54	4.0E-03	-0.15	4.7E-01	-0.69	9.4E-05	-0.06	7.7E-01	0.40	4.6E-02	0.39	4.8E-02	-0.39	4.9E-02	0.07	7.4E-01	0.02	9.4E-01	-0.70	7.6E-05
ENSCAFG0000001055	ZBTB8OS	darkgrey	EC_M8	0.54	4.0E-03	0.00	9.9E-01	-0.28	1.6E-01	0.07	7.3E-01	-0.10	6.4E-01	0.20	3.4E-01	-0.23	2.5E-01	0.01	9.7E-01	-0.18	3.7E-01	-0.18	3.8E-01
ENSCAFG0000002228	ENSCAFG0000002228	grey	EC_M10	0.54	4.0E-03	0.06	7.6E-01	0.09	6.6E-01	0.09	6.7E-01	-0.59	1.5E-03	-0.14	5.1E-01	-0.08	6.9E-01	0.06	7.8E-01	-0.32	1.1E-01	0.29	1.4E-01
ENSCAFG0000002638	ENSCAFG0000002638	grey	EC_M10	0.54	4.1E-03	-0.09	6.6E-01	0.01	9.6E-01	0.30	1.3E-01	-0.41	3.6E-02	0.20	3.2E-01	-0.12	5.5E-01	0.07	7.3E-01	-0.39	5.1E-02	0.11	6.0E-01
ENSCAFG0000001814	ENSCAFG0000001814	darkgrey	EC_M8	0.54	4.1E-03	0.24	2.4E-01	-0.74	1.9E-05	-0.02	9.0E-01	0.31	1.3E-01	0.00	9.8E-01	-0.14	4.9E-01	-0.15	4.7E-01	-0.20	3.3E-01	-0.56	3.0E-03
ENSCAFG0000003160	GRPEL2	darkgreen	EC_M4	0.54	4.1E-03	0.38	5.5E-02	-0.76	5.6E-06	-0.20	3.4E-01	0.31	1.3E-01	0.29	1.5E-01	-0.08	6.8E-01	-0.01	9.8E-01	-0.34	9.0E-02	-0.61	8.8E-04
ENSCAFG0000001040	TJP1	darkgreen	EC_M4	0.54	4.1E-03	0.61	8.9E-04	-0.81	6.1E-07	-0.15	4.5E-01	0.26	1.9E-01	0.04	8.6E-01	-0.21	3.1E-01	-0.12	5.6E-01	-0.37	6.6E-02	-0.59	1.7E-03
ENSCAFG0000002394	ENSCAFG0000002394	grey	EC_M10	0.54	4.1E-03	0.16	4.2E-01	-0.27	1.9E-01	0.44	2.4E-02	-0.24	2.3E-01	0.11	5.8E-01	-0.24	2.4E-01	-0.32	1.2E-01	-0.20	3.3E-01	-0.03	9.0E-01
ENSCAFG0000002042	LRRC40	grey	EC_M10	0.54	4.1E-03	-0.01	9.4E-01	-0.23	2.5E-01	0.26	2.0E-01	-0.21	2.9E-01	0.23	2.5E-01	-0.37	6.3E-02	-0.15	4.6E-01	0.20	3.3E-01	-0.06	7.6E-01
ENSCAFG0000001316	SP3	darkgrey	EC_M8	0.54	4.1E-03	0.18	3.8E-01	0.16	4.2E-01	0.22	2.7E-01	-0.76	5.5E-06	0.00	9.8E-01	-0.12	5.5E-01	-0.18	3.8E-01	-0.22	2.8E-01	0.48	1.4E-02
ENSCAFG0000000609	SUPT20H	darkgrey	EC_M8	0.54	4.1E-03	0.01	9.5E-01	0.10	6.2E-01	0.13	5.4E-01	-0.57	2.3E-03	-0.04	8.3E-01	-0.26	2.1E-01	-0.22	2.9E-01	-0.04	8.4E-01	0.25	2.1E-01
ENSCAFG0000001315	ENSCAFG0000001315	darkgrey	EC_M8	0.54	4.1E-03	0.01	9.5E-01	-0.01	9.4E-01	-0.37	6.4E-02	-0.35	8.3E-02	0.08	6.9E-01	-0.28	1.6E-01	-0.16	4.2E-01	-0.07	7.4E-01	0.07	7.4E-01
ENSCAFG0000000885	BMP2K	turquoise	EC_M6	0.54	4.2E-03	0.84	9.3E-08	-0.57	2.4E-03	0.02	9.3E-01	-0.09	6.5E-01	-0.03	8.8E-01	-0.11	5.8E-01	0.08	7.1E-01	-0.09	6.6E-01	-0.21	3.1E-01
ENSCAFG0000001197	PDZD8	grey	EC_M10	0.54	4.2E-03	0.67	1.6E-04	-0.37	6.2E-02	-0.15	4.5E-01	-0.26	2.0E-01	0.10	6.3E-01	-0.28	1.6E-01	-0.34	9.4E-02	-0.17	4.0E-01	-0.03	8.9E-01
ENSCAFG0000000948	ENTPD7	grey	EC_M10	0.54	4.2E-03	0.11	6.0E-01	-0.66	2.2E-04	0.05	7.9E-01	0.31	1.2E-01	0.22	2.9E-01	-0.06	7.9E-01	-0.37	6.2E-02	-0.38	5.7E-02	-0.62	6.6E-04
ENSCAFG0000001087	TET2	darkgrey	EC_M8	0.54	4.2E-03	0.65	3.4E-04	-0.15	4.6E-01	0.18	3.7E-01	-0.56	3.2E-03	0.01	9.6E-01	-0.31	1.3E-01	-0.03	8.9E-01	-0.08	7.0E-01	0.24	2.4E-01
ENSCAFG0000001986	ENSCAFG0000001986	grey	EC_M10	0.54	4.2E-03	-0.05	8.1E-01	-0.27	1.8E-01	0.18	3.8E-01	-0.14	5.0E-01	-0.01	9.5E-01	-0.08	7.1E-01	-0.53	5.2E-03	-0.15	4.7E-01	-0.14	5.0E-01
ENSCAFG0000001732	UTP18	darkgrey	EC_M8	0.54	4.2E-03	-0.21	3.1E-01	-0.40	4.4E-02	0.10	6.2E-01	0.15	4.8E-01	0.39	4.8E-02	-0.44	2.5E-02	0.12	5.7E-01	-0.05	7.9E-01	-0.44	2.6E-02
ENSCAFG0000000195	SEPT10	grey	EC_M10	0.54	4.2E-03	-0.07	7.3E-01	-0.18	3.8E-01	-0.06	7.8E-01	-0.23	2.5E-01	0.01	9.6E-01	-0.23	2.6E-01	-0.20	3.2E-01	-0.52	7.1E-03	-0.07	7.4E-01
ENSCAFG0000003220	ENSCAFG0000003220	darkgrey	EC_M8	0.54	4.3E-03	0.12	5.6E-01	-0.22	2.8E-01	0.20	3.3E-01	-0.23	2.5E-01	0.14	4.9E-01	-0.38	5.3E-02	-0.36	7.4E-02	-0.08	6.9E-01	-0.04	8.5E-01
ENSCAFG0000000595	SLC25A15	grey	EC_M10	0.54	4.3E-03	0.20	3.3E-01	0.04	8.6E-01	0.30	1.4E-01	-0.48	1.4E-02	-0.08	7.0E-01	-0.34	9.1E-02	-0.28	1.7E-01	-0.53	5.3E-03	0.20	3.4E-01
ENSCAFG0000000725	IFT88	grey	EC_M10	0.54	4.3E-03	-0.32	1.2E-01	-0.07	7.3E-01	0.19	3.4E-01	-0.28	1.6E-01	0.30	1.3E-01	-0.45	2.0E-02	-0.23	2.7E-01	-0.15	4.7E-01	-0.03	8.7E-01
ENSCAFG0000002383	ENSCAFG0000002383	turquoise	EC_M6	0.54	4.3E-03	0.49	1.1E-02	-0.72	3.5E-05	-0.31	1.2E-01	0.26	1.9E-01	0.12	5.6E-01	-0.10	6.4E-01	0.12	5.6E-01	-0.07	7.2E-01	-0.56	3.0E-03
ENSCAFG0000002012	GCLM	grey	EC_M10	0.54	4.3E-03	0.33	9.9E-02	-0.02	9.1E-01	0.11	5.9E-01	-0.52	6.3E-03	0.19	3.6E-01	-0.09	6.6E-01	-0.22	2.9E-01	-0.06	7.6E-01	0.27	1.8E-01
ENSCAFG0000000941	ZDHHC13	turquoise	EC_M6	0.54	4.3E-03	0.51	7.8E-03	-0.09	6.6E-01	0.03	8.9E-01	-0.48	1.3E-02	-0.08	7.0E-01	-0.21	3.1E-01	0.06	7.9E-01	-0.28	1.7E-01	0.21	2.9E-01
ENSCAFG0000000936	PKP4	darkgrey	EC_M8	0.54	4.3E-03	0.23	2.5E-01	-0.27	1.8E-01	-0.33	1.0E-01	-0.15	4.8E-01	0.07	7.5E-01	-0.41	3.9E-02	0.12	5.6E-01	0.00	9.9E-01	-0.12	5.5E-01
ENSCAFG0000000308	RNGTT	grey	EC_M10	0.54	4.3E-03	-0.27	1.9E-01	-0.52	7.0E-03	-0.04	8.5E-01	0.22	2.8E-01	0.14	4.9E-01	-0.09	6.6E-01	-0.20	3.3E-01	-0.29	1.5E-01	-0.50	8.7E-03
ENSCAFG0000000552	SNRPB2	darkgrey	EC_M8	0.54	4.3E-03	0.21	3.1E-01	-0.35	8.0E-02	0.03	8.8E-01	-0.12	5.7E-01	0.32	1.1E-01	-0.09	6.7E-01	-0.07	7.3E-01	0.19	3.5E-01	-0.22	2.7E-01
ENSCAFG0000002480	ENSCAFG0000002480	grey	EC_M10	0.54	4.3E-03	0.01	9.7E-01	-0.23	2.5E-01	0.28	1.7E-01	-0.16	4.4E-01	0.08	7.1E-01	-0.08	7.0E-01	0.03	8.9E-01	-0.64	4.5E-04	-0.14	4.9E-01
ENSCAFG0000000095	PFDN6	grey	EC_M10	0.54	4.4E-03	-0.23	2.7E-01	-0.28	1.6E-01	0.22	2.9E-01	-0.05	8.1E-01	0.14	4.8E-01	-0.12	5.6E-01	-0.04	8.3E-01	-0.33	9.6E-02	-0.29	1.6E-01
ENSCAFG0000000274	ENSCAFG0000000274	grey	EC_M10	0.54	4.4E-03	0.31	1.2E-01	-0.33	9.9E-02	0.31	1.2E-01	-0.17	4.1E-01	0.18	3.8E-01	0.02	9.1E-01	-0.20	3.3E-01	-0.27	1.9E-01	-0.10	6.3E-01
ENSCAFG0000001751	PSMC1	darkgreen	EC_M4	0.54	4.4E-03	0.08	6.8E-01	-0.83	1.3E-07	-0.23	2.7E-01	0.56	3.0E-03	0.24	2.3E-01	-0.08	7.1E-01	-0.06	7.7E-01	-0.31	1.3E-01	-0.83	1.8E-07
ENSCAFG0000003195	ENSCAFG0000003195	grey	EC_M10	0.54	4.5E-03	0.42	3.0E-02	-0.52	6.3E-03	-0.01	9.7E-01	0.03	9.0E-01	-0.02	9.1E-01	-0.01	9.5E-01	-0.14	4.9E-01	-0.31	1.2E-01	-0.27	1.9E-01
ENSCAFG0000000510	RPSA	grey	EC_M10	0.54	4.5E-03	-0.31	1.2E-01	-0.18	3.7E-01	0.20	3.4E-01	-0.11	6.1E-01	0.25	2.2E-01	0.11	5.8E-01	-0.09	6.5E-01	-0.67	2.1E-04	-0.24	2.5E-01
ENSCAFG0000001125	POLR3C	grey	EC_M10	0.54	4.5E-03	-0.11	5.9E-01	0.21	3.1E-01	0.27	1.8E-01	-0.62	7.0E-04	0.25	2.2E-01	-0.32	1.2E-01	-0.24	2.5E-01	-0.20	3.3E-01	0.31	1.2E-01
ENSCAFG0000001543	ENSCAFG0000001543	grey	EC_M10	0.54	4.5E-03	0.14	4.8E-01	-0.21	3.1E-01	0.17	4.2E-01	-0.29	1.4E-01	-0.20	3.4E-01	-0.20	3.3E-01	-0.03	8.8E-01	-0.39	4.7E-02	0.02	9.2E-01
ENSCAFG0000001880	SAFB	darkgrey	EC_M8	0.54	4.5E-03	0.46	1.8E-02	-0.08	6.8E-01	0.09	6.4E-01	-0.51	7.6E-03	0.19	3.6E-01	0.18	3.8E-01	-0.02	9.1E-01	-0.29	1.5E-01	0.23	2.6E-01
ENSCAFG0000001308	JMJD1C	grey	EC_M10	0.54	4.5E-03	0.60	1.2E-03	-0.44	2.4E-02	0.05	8.2E-01	-0.16	4.3E-01	0.15	4.7E-01	-0.15	4.8E-01	-0.32	1.1E-01	-0.35	7.6E-02	-0.12	5.7E-01
ENSCAFG0000002498	ZSCAN23	grey	EC_M10	0.54	4.5E-03	0.13	5.3E-01	-0.33	9.9E-02	-0.15	4.7E-01	-0.12	5.7E-01	0.04	8.6E-01	-0.10	6.3E-01	-0.10	6.1E-01	-0.13	5.2E-01	-0.16	4.3E-01
ENSCAFG0000003259	PTMA	grey	EC_M10	0.54	4.5E-03	0.17	4.0E-01	0.34	9.0E-02	0.06	7.5E-01	-0.84	9.7E-08	-0.02	9.2E-01	-0.19	3.5E-01	-0.13	5.3E-01	-0.13	5.1E-01	0.56	2.9E-03
ENSCAFG0000001837	ENSCAFG0000001837	darkgrey	EC_M8	0.54	4.6E-03	-0.04	8.6E-01	-0.40	4.3E-02	0.47	1.7E-02	-0.01	9.7E-01	0.18	3.8E-01	0.01	9.7E-01	-0.21	3.1E-01	-0.23	2.6E-01	-0.26	2.0E-01
ENSCAFG0000002536	INTS6	darkgrey	EC_M8	0.54	4.6E-03	0.52	6.1E-03	-0.36	7.4E-02	0.13	5.4E-01	-0.30	1.4E-01	-0.20	3.2E-01	-0.14	5.0E-01	-0.21	2.9E-01	-0.10	6.4E-01	0.01	9.5E-01
ENSCAFG0000001286	CCDC47	darkgrey	EC_M8	0.54	4.6E-03	0.26	2.0E-01	-0.53	5.4E-03	0.09	6.6E-01	0.06	7.8E-01	0.31	1.2E-01	-0.01	9.5E-01	-0.16	4.4E-01	-0.49	1.1E-02	-0.32	1.1E-01
ENSCAFG0000001571	FAM220A	darkgrey	EC_M8	0.54	4.6E-03	-0.13	5.3E-01	-0.53	5.1E-03	-0.27	1.8E-01	0.24	2.5E-01	0.19	3.5E-01	-0.20	3.3E-01	0.19	3.4E-01	-0.42	3.3E-02	-0.54	4.2E-03
ENSCAFG0000000228	TXNDC9	darkgreen	EC_M4	0.54	4.6E-03	0.37	6.7E-02	-0.88	4.1E-09	-0.13	5.4E-01	0.43	3.0E-02	0.17	4.0E-01	-0.19	3.5E-01	-0.07	7.5E-01	-0.12	5.5E-01	-0.71	4.3E-05
ENSCAFG0000000447	IWS1	darkgreen	EC_M4	0.54	4.6E-03	0.60	1.3E-03	-0.80	8.5E-07	-0.15	4.8E-01	0.22	2.8E-01	0.09	6.5E-01	-0.06	7.6E-01	0.05	8.1E-01	-0.02	9.1E-01	-0.51	7.9E-03
ENSCAFG0000000879	CTNNBL1	grey	EC_M10	0.54	4.6E-03	-0.11	5.8E-01	-0.29	1.5E-01	-0.09	6.5E-01	-0.05	8.0E-01	0.04	8.5E-01	0.11	5.8E-01	0.14	4.8E-01	0.37	6.3E-02	-0.17	3.9E-01
ENSCAFG0000000794	SSRP1	grey	EC_M10	0.54	4.6E-03	-0.17	3.9E-01	0.36	7.0E-02	0.15	4.6E-01	-0.73	2.3E-05	-0.06	7.6E-01	-0.21	3.0E-01	-0.11	5.9E-01	-0.22	2.8E-01	0.43	2.7E-02
ENSCAFG0000000542	POLR3F	darkgrey	EC_M8	0.54	4.7E-03	-0.08	7.0E-01	-0.54	4.4E-03	-0.07	7.4E-01	0.24	2.3E-01	0.61	1.0E-03	-0.22	2.8E-01	-0.10	6.4E-01	0.14	4.8E-01	-0.53	5.2E-03
ENSCAFG0000000678	LNX2	darkgrey	EC_M8	0.54	4.7E-03	0.74	1.8E-05	-0.23	2.5E-01	0.05	8.0E-01	-0.48	1.3E-02	-0.02	9.2E-01	-0.04	8.5E-01	-0.23	2.7E-01	-0.26	2.1E-01	0.18	3.8E-01
ENSCAFG0000001397	ENSCAFG0000001397	darkgrey	EC_M8	0.54	4.7E-03	0.17	4.2E-01	-0.19	3.6E-01	0.20	3.3E-01	-0.32	1.1E-01	0.06	7.5E-01	-0.13	5.2E-01	-0.60	1.3E-03	-0.45	2.2E-02	0.04	8.5E-01
ENSCAFG0000001338	ACOT9	grey	EC_M10	0.54	4.7E-03	0.35	8.2E-02	-0.22	2.9E-01	-0.12	5.5E-01	-0.27	1.8E-01	-0.21	3.0E-01	-0.22	2.7E-01	-0.09	6.8E-01	-0.04	8.5E-01	0.04	8.5E-01
ENSCAFG0000002966	HNRNPLL	grey	EC_M10	0.54	4.7E-03	-0.10	6.3E-01	-0.39	4.7E-02	0.19	3.6E-01	0.03	8.9E-01	0.19	3.6E-01	-0.54	4.8E-03	-0.14	5.1E-01	-0.28	1.6E-01	-0.35	7.8E-02
ENSCAFG0000001821	RBBP8	darkgreen	EC_M4	0.54	4.7E-03	0.08	6.9E-01	-0.84	1.0E-07	0.03	8.9E-01	0.51	8.3E-03	0.22	2.8E-01	-0.21	2.9E-01	-0.07	7.3E-01	-0.03	8.8E-01	-0.79	1.3E-06
ENSCAFG0000000460	PCF11	darkgrey	EC_M8	0.54	4.7E-03	0.01	9.6E-01	0.21	3.0E-01	0.22	2.7E-01	-0.74	1.3E-05	0.04	8.6E-01	0.04	8.5E-01	-0.07	7.3E-01	-0.27	1.9E-01	0.45	2.0E-02
ENSCAFG0000000396	ENSCAFG0000000396	darkgrey	EC_M8	0.54	4.7E-03	-0.13	5.3E-01	-0.38	5.4E-02	-0.29	1.4E-01	0.14	5.1E-01	-0.01	9.7E-01	-0.09	6.5E-01	0.03	8.9E-01	-0.29	1.5E-01	-0.40	4.5E-02
ENSCAFG0000002578	ENSCAFG0000002578	grey	EC_M10	0.54	4.7E-03	0.13	5.3E-01	-0.05	8.1E-01	0.33	1.0E-01	-0.49	1.1E-02	0.00	1.0E+00	-0.07	7.3E-01	-0.13	5.1E-01	-0.07	7.5E-01	0.21	3.1E-01
ENSCAFG0000000147	ENSCAFG0000000147	grey	EC_M10	0.54	4.7E-03	0.07	7.5E-01	-0.44	2.4E-02	0.28	1.7E-01	0.01	9.8E-01	-0.15	4.7E-01	-0.16	4.3E-01	-0.02	9.2E-01	-0.15	4.5E-01	-0.28	1.7E-01
ENSCAFG0000000307	RARS2	grey	EC_M10	0.54	4.7E-03	-0.15	4.6E-01	-0.21	3.0E-01	0.18	3.9E-01	-0.11	6.1E-01	0.06	7.7E-01	-0.57	2.2E-03	-0.12	5.7E-01	0.06	7.7E-01	-0.13	5.4E-01
ENSCAFG0000000991	WDR3	darkgrey	EC_M8	0.54	4.8E-03	-0.49	1.0E-02	-0.44	2.3E-02	-0.10	6.2E-01	0.32	1.1E-01	0.25	2.2E-01	-0.23	2.5E-01	0.01	9.8E-01	-0.27	1.8E-01	-0.60	1.1E-03
ENSCAFG0000001345	TSEN15	darkgrey	EC_M8	0.54	4.8E-03	-0.08	7.0E-01	-0.07	7.4E-01	0.04	8.5E-01	-0.27	1.8E-01	0.01	9.7E-01	-0.58	2.0E-03	-0.18	3.7E-01	0.02	9.3E-01	0.01	9.5E-01
ENSCAFG0000000186	NOL6	grey	EC_M10	0.54	4.8E-03	0.07	7.4E-01	-0.43	3.0E-02	-0.15	4.7E-01	0.15	4.5E-01	0.16	4.5E-01	-0.22	2.9E-01	0.09	6.5E-01	-0.09	6.8E-01	-0.43	2.8E-02
ENSCAFG0000000826	HK2	darkgrey	EC_M8	0.54	4.8E-03	0.06	7.8E-01	-0.63	5.9E-04	-0.09	6.6E-01	0.32	1.1E-01	0.25	2.1E-01	0.09	6.5E-01	-0.20	3.2E-01	-0.15	4.5E-01	-0.58	1.8E-03
ENSCAFG0000000667	ERI1	darkgreen	EC_M4	0.54	4.8E-03	-0.07	7.2E-01	-0.67	1.7E-04	-0.17	4.0E-01	0.38	5.8E-02	0.09	6.4E-01	-0.07	7.4E-01	-0.16	4.4E-01	-0.35	8.1E-02	-0.66	2.5E-04
ENSCAFG0000001969	PTBP1	darkgrey	EC_M8	0.54	4.8E-03	0.27	1.8E-01	-0.30	1.4E-01	-0.09	6.7E-01	-0.06	7.7E-01	0.22	2.9E-01	-0.31	1.2E-01	-0.23	2.5E-01	-0.31	1.2E-01	-0.23	2.6E-01
ENSCAFG0000001439	XRCC5	darkgrey	EC_M8	0.54	4.8E-03	-0.34	9.0E-02	-0.31	1.2E-01	0.17	4.2E-01	0.11	6.1E-01	0.18	3.8E-01	-0.50	9.1E-03	0.00	9.8E-01	-0.21	3.1E-01	-0.39	5.1E-02
ENSCAFG0000001264	NBEAL1	grey	EC_M10	0.54	4.9E-03	0.21	3.0E-01	-0.53	5.0E-03	-0.02	9.1E-01	0.04	8.5E-01	-0.19	3.6E-01	0.02	9.1E-01	-0.13	5.4E-01	-0.14	4.9E-01	-0.32	1.1E-01
ENSCAFG0000001428	C9H17orf53	darkgrey	EC_M8	0.53	4.9E-03	0.21	3.1E-01	-0.12	5.6E-01	-0.11	6.0E-01	-0.30	1.4E-01	-0.26	2.0E-01	-0.24	2.4E-01	0.05	8.0E-01	0.10	6.1E-01	0.01	9.7E-01
ENSCAFG0000001919	ENSCAFG0000001919	grey	EC_M10	0.53	4.9E-03	0.22	2.8E-01	-0.56	3.1E-03	0.14	5.0E-01	0.08	6.8E-01	0.04	8.5E-01	-0.11	5.8E-01	-0.10	6.2E-01	-0.43	2.7E-02	-0.39	5.0E-02
ENSCAFG0000000489	RSF1	darkgrey	EC_M8	0.53	4.9E-03	0.47	1.6E-02	-0.35	8.1E-02	-0.06	7.8E-01	-0.24	2.4E-01	-0.02	9.4E-01	-0.12	5.7E-01	-0.01	9.7E-01	-0.36	6.7E-02	-0.03	8.7E-01
ENSCAFG0000000673	PAN3	darkgrey	EC_M8	0.53	5.0E-03	0.45	2.0E-02	-0.11	6.1E-01	0.07	7.2E-01	-0.53	5.1E-03	-0.30	1.4E-01	-0.20	3.3E-01	-0.14	4.9E-01	-0.08	7.0E-01	0.24	2.3E-01
ENSCAFG0000000498	ACTR3	darkgreen	EC_M4	0.53	5.0E-03	0.09	6.5E-01	-0.92	5.1E-11	-0.17	3.9E-01	0.63	6.1E-04	0.29	1.5E-01	-0.13	5.1E-01	-0.01	9.7E-01	-0.11	6.0E-01	-0.91	1.5E-10
ENSCAFG0000000249	PHF3	darkgrey	EC_M8	0.53	5.0E-03	0.34	9.1E-02	0.01	9.5E-01	0.10	6.1E-01	-0.64	4.1E-04	0.01	9.5E-01	-0.21	3.0E-01	-0.14	5.0E-01	-0.33	1.0E-01	0.35	7.9E-02
ENSCAFG0000000014	NACA	grey	EC_M10	0.53	5.0E-03	-0.18	3.8E-01	0.09	6.7E-01	0.27	1.8E-01	-0.53	5.2E-03	0.11	6.0E-01	0.05	7.9E-01	-0.05	8.1E-01	-0.55	3.8E-03	0.20	3.3E-01
ENSCAFG0000000823	HMBOX1	turquoise	EC_M6	0.53	5.0E-03	0.58	1.8E-03	-0.65	3.1E-04	-0.01	9.4E-01	0.11	5.8E-01	0.17	4.2E-01	-0.16	4.4E-01	-0.22	2.9E-01	-0.33	9.5E-02	-0.40	4.6E-02
ENSCAFG0000000901	ENSCAFG0000000901	grey	EC_M10	0.53	5.1E-03	-0.15	4.6E-01	-0.05	8.2E-01	0.16	4.4E-01	-0.35	8.4E-02	0.18	3.7E-01	-0.04	8.6E-01	-0.20	3.2E-01	-0.08	6.9E-01	0.07	7.2E-01
ENSCAFG0000001587	ERC1	darkgreen	EC_M4	0.53	5.1E-03	0.34	8.9E-02	-0.80	9.4E-07	-0.16	4.3E-01	0.35	8.2E-02	0.02	9.1E-01	-0.10	6.2E-01	-0.03	8.7E-01	-0.06	7.9E-01	-0.59	1.5E-03
ENSCAFG0000002361	ENSCAFG0000002361	grey	EC_M10	0.53	5.1E-03	0.33	1.0E-01	-0.50	9.9E-03	0.11	5.8E-01	0.04	8.6E-01	-0.03	8.8E-01	-0.39	4.6E-02	-0.36	6.9E-02	-0.07	7.3E-01	-0.27	1.8E-01
ENSCAFG0000001408	GJC1	darkgrey	EC_M8	0.53	5.1E-03	-0.04	8.4E-01	-0.26	1.9E-01	-0.14	4.9E-01	-0.10	6.1E-01	0.28	1.7E-01	-0.10	6.3E-01	-0.04	8.3E-01	0.26	2.0E-01	-0.21	3.0E-01
ENSCAFG0000000397	ENSCAFG0000000397	darkgrey	EC_M8	0.53	5.1E-03	0.21	3.1E-01	-0.26	2.0E-01	-0.20	3.2E-01	-0.20	3.2E-01	-0.20	3.2E-01	-0.15	4.7E-01	-0.15	4.7E-01	-0.21	3.1E-01	-0.06	7.8E-01

ENSCAFG0000001703	EME1	darkgrey	EC_M8	0.53	5.1E-03	0.12	5.7E-01	0.06	7.7E-01	-0.07	7.2E-01	-0.53	5.7E-03	0.02	9.2E-01	-0.09	6.5E-01	-0.10	6.4E-01	0.07	7.3E-01	0.26	2.0E-01
ENSCAFG0000000774	SLC10A7	grey	EC_M10	0.53	5.1E-03	-0.10	6.4E-01	-0.18	3.7E-01	0.28	1.6E-01	-0.25	2.2E-01	0.23	2.7E-01	-0.29	1.4E-01	-0.34	9.3E-02	-0.55	3.4E-03	-0.10	6.3E-01
ENSCAFG0000001449	ZC3H15	darkgreen	EC_M4	0.53	5.1E-03	0.22	2.7E-01	-0.92	1.8E-11	-0.09	6.6E-01	0.53	5.6E-03	0.23	2.7E-01	-0.16	4.4E-01	0.11	6.0E-01	-0.03	8.8E-01	-0.81	5.5E-07
ENSCAFG0000000966	SCD	grey	EC_M10	0.53	5.1E-03	0.11	5.8E-01	-0.18	3.9E-01	-0.01	9.8E-01	-0.27	1.9E-01	-0.08	6.9E-01	-0.25	2.3E-01	-0.15	4.7E-01	-0.41	3.6E-02	-0.07	7.5E-01
ENSCAFG0000002925	ENSCAFG0000002925	grey	EC_M10	0.53	5.1E-03	0.07	7.4E-01	-0.50	8.7E-03	0.00	9.9E-01	0.10	6.2E-01	-0.02	9.4E-01	-0.20	3.2E-01	-0.38	5.6E-02	-0.30	1.4E-01	-0.42	3.3E-02
ENSCAFG0000001130	MFN1	grey	EC_M10	0.53	5.2E-03	-0.15	4.7E-01	-0.26	2.0E-01	0.03	8.7E-01	-0.13	5.3E-01	-0.13	5.1E-01	-0.27	1.9E-01	0.26	1.9E-01	-0.10	6.2E-01	-0.17	4.0E-01
ENSCAFG0000002020	RABEPK	darkgrey	EC_M8	0.53	5.2E-03	0.01	9.5E-01	0.14	4.9E-01	0.16	4.5E-01	-0.55	3.5E-03	-0.16	4.5E-01	-0.40	4.5E-02	-0.29	1.4E-01	-0.23	2.5E-01	0.24	2.4E-01
ENSCAFG0000001570	DDX11	darkgrey	EC_M8	0.53	5.2E-03	0.51	7.7E-03	-0.02	9.4E-01	0.12	5.6E-01	-0.62	7.4E-04	-0.17	4.0E-01	-0.02	9.3E-01	0.07	7.4E-01	-0.29	1.6E-01	0.34	9.2E-02
ENSCAFG0000001303	SF3B2	grey	EC_M10	0.53	5.2E-03	-0.01	9.6E-01	0.10	6.4E-01	-0.04	8.4E-01	-0.49	1.0E-02	0.05	8.3E-01	0.25	2.1E-01	-0.03	8.9E-01	-0.39	4.9E-02	0.22	2.8E-01
ENSCAFG0000001422	ENSCAFG0000001422	darkgrey	EC_M8	0.53	5.2E-03	0.10	6.4E-01	-0.20	3.2E-01	0.20	3.4E-01	-0.24	2.3E-01	-0.12	5.6E-01	-0.44	2.3E-02	-0.12	5.5E-01	-0.40	4.6E-02	-0.03	8.9E-01
ENSCAFG0000001635	MPP5	turquoise	EC_M6	0.53	5.2E-03	0.56	3.2E-03	-0.70	7.3E-05	-0.24	2.3E-01	0.18	3.9E-01	0.13	5.4E-01	0.01	9.7E-01	0.05	7.9E-01	-0.18	3.7E-01	-0.43	2.7E-02
ENSCAFG0000002011	ARHGAP29	darkgreen	EC_M4	0.53	5.2E-03	0.47	1.5E-02	-0.80	1.2E-06	-0.14	5.0E-01	0.27	1.8E-01	0.25	2.2E-01	-0.36	6.7E-02	-0.05	8.2E-01	-0.34	8.9E-02	-0.60	1.3E-03
ENSCAFG0000003261	ZSCAN16	darkgrey	EC_M8	0.53	5.2E-03	0.48	1.3E-02	-0.29	1.5E-01	0.06	7.6E-01	-0.32	1.1E-01	-0.11	6.0E-01	-0.12	5.6E-01	-0.14	5.0E-01	-0.22	2.9E-01	0.04	8.4E-01
ENSCAFG0000001702	PFAS	darkgrey	EC_M8	0.53	5.3E-03	-0.20	3.2E-01	0.20	3.3E-01	0.11	6.1E-01	-0.50	8.7E-03	0.06	7.6E-01	-0.18	3.9E-01	-0.25	2.2E-01	-0.06	7.7E-01	0.22	2.8E-01
ENSCAFG0000001030	ETS1	turquoise	EC_M6	0.53	5.4E-03	0.80	9.7E-07	-0.18	3.7E-01	-0.17	4.0E-01	-0.47	1.5E-02	-0.18	3.8E-01	0.01	9.7E-01	-0.11	5.9E-01	0.02	9.3E-01	0.22	2.8E-01
ENSCAFG0000001657	ENSCAFG0000001657	darkgrey	EC_M8	0.53	5.4E-03	-0.11	6.0E-01	-0.54	4.2E-03	0.18	3.8E-01	0.20	3.3E-01	0.15	4.7E-01	-0.29	1.5E-01	-0.09	6.5E-01	-0.21	3.0E-01	-0.48	1.4E-02
ENSCAFG0000001191	CHEK2	grey	EC_M10	0.53	5.4E-03	0.02	9.3E-01	-0.06	7.8E-01	0.02	9.1E-01	-0.45	2.1E-02	0.18	3.7E-01	-0.03	9.0E-01	-0.17	4.1E-01	-0.15	4.8E-01	0.13	5.1E-01
ENSCAFG0000000389	AGK	grey	EC_M10	0.53	5.4E-03	0.26	1.9E-01	-0.32	1.2E-01	0.00	9.9E-01	-0.12	5.5E-01	0.24	2.5E-01	-0.48	1.4E-02	-0.13	5.4E-01	-0.14	5.0E-01	-0.13	5.1E-01
ENSCAFG0000001452	DARS2	darkgrey	EC_M8	0.53	5.4E-03	0.03	8.9E-01	0.06	7.8E-01	0.02	9.4E-01	-0.43	2.7E-02	0.03	8.9E-01	-0.37	6.2E-02	-0.19	3.4E-01	-0.01	9.5E-01	0.16	4.2E-01
ENSCAFG0000001939	EHMT1	darkgrey	EC_M8	0.53	5.4E-03	0.60	1.2E-03	0.00	9.9E-01	0.19	3.4E-01	-0.66	2.2E-04	0.03	9.0E-01	-0.18	3.7E-01	-0.03	8.8E-01	-0.14	5.0E-01	0.36	6.9E-02
ENSCAFG0000002027	ZNHIT6	darkgreen	EC_M4	0.53	5.4E-03	0.31	1.2E-01	-0.76	5.6E-06	-0.36	7.5E-02	0.40	4.2E-02	0.16	4.3E-01	-0.12	5.5E-01	0.01	9.5E-01	-0.28	1.7E-01	-0.67	2.0E-04
ENSCAFG0000000762	OTUD4	darkgrey	EC_M8	0.53	5.5E-03	0.59	1.5E-03	0.00	9.9E-01	-0.02	9.2E-01	-0.64	3.9E-04	-0.17	4.0E-01	-0.23	2.7E-01	-0.24	2.3E-01	-0.34	9.0E-02	0.36	7.4E-02
ENSCAFG0000000958	CHUK	darkgrey	EC_M8	0.53	5.5E-03	0.19	3.6E-01	-0.63	6.0E-04	-0.06	7.5E-01	0.22	2.7E-01	0.23	2.6E-01	-0.29	1.5E-01	-0.12	5.5E-01	-0.18	3.7E-01	-0.47	1.5E-02
ENSCAFG0000001649	RPL19	grey	EC_M10	0.53	5.5E-03	-0.11	5.9E-01	-0.10	6.3E-01	0.23	2.5E-01	-0.32	1.1E-01	0.13	5.3E-01	0.09	6.8E-01	-0.09	6.6E-01	-0.66	2.2E-04	0.01	9.5E-01
ENSCAFG0000001720	DPP8	darkgrey	EC_M8	0.53	5.5E-03	0.45	2.0E-02	-0.73	2.0E-05	0.03	8.8E-01	0.19	3.4E-01	0.15	4.6E-01	-0.33	9.9E-02	0.03	8.9E-01	-0.02	9.1E-01	-0.47	1.6E-02
ENSCAFG0000001720	VIPAS39	grey	EC_M10	0.53	5.5E-03	0.10	6.4E-01	-0.59	1.6E-03	-0.18	3.8E-01	0.21	3.1E-01	0.48	1.4E-02	-0.18	3.8E-01	0.06	7.6E-01	-0.03	8.9E-01	-0.52	6.5E-03
ENSCAFG0000001081	PTP4A2	darkgrey	EC_M8	0.53	5.5E-03	-0.24	2.3E-01	-0.53	5.7E-03	0.12	5.6E-01	0.27	1.9E-01	0.19	3.5E-01	-0.51	8.1E-03	0.08	7.1E-01	-0.32	1.1E-01	-0.58	1.7E-03
ENSCAFG0000001571	RAC1	turquoise	EC_M6	0.53	5.7E-03	0.45	2.2E-02	-0.62	7.4E-04	-0.27	1.8E-01	0.21	3.0E-01	0.16	4.3E-01	-0.24	2.3E-01	-0.17	4.2E-01	-0.29	1.5E-01	-0.47	1.6E-02
ENSCAFG0000000635	PDS5B	grey	EC_M10	0.53	5.7E-03	0.16	4.3E-01	-0.56	3.2E-03	-0.02	9.2E-01	0.07	7.4E-01	0.40	4.1E-02	-0.10	6.4E-01	-0.12	5.7E-01	-0.14	4.9E-01	-0.42	3.4E-02
ENSCAFG0000001017	KDM1B	grey	EC_M10	0.53	5.8E-03	-0.32	1.1E-01	0.00	9.9E-01	0.06	7.7E-01	-0.33	1.0E-01	0.05	8.1E-01	-0.31	1.2E-01	0.05	8.0E-01	0.09	6.7E-01	0.01	9.5E-01
ENSCAFG0000001814	ETFA	darkgrey	EC_M8	0.53	5.9E-03	-0.10	6.4E-01	-0.08	7.0E-01	0.03	8.9E-01	-0.25	2.2E-01	0.06	7.5E-01	-0.42	3.3E-02	0.05	8.1E-01	0.29	1.5E-01	0.00	1.0E+00
ENSCAFG0000000866	SEPT11	turquoise	EC_M6	0.52	5.9E-03	0.65	3.5E-04	-0.42	3.2E-02	0.01	9.4E-01	-0.15	4.6E-01	-0.10	6.3E-01	-0.48	1.4E-02	-0.10	6.2E-01	0.04	8.6E-01	-0.11	5.8E-01
ENSCAFG0000000433	ISY1	darkgrey	EC_M8	0.52	5.9E-03	-0.35	7.5E-02	-0.55	3.4E-03	0.12	5.7E-01	0.35	7.9E-02	0.41	3.6E-02	-0.19	3.5E-01	0.03	8.9E-01	-0.23	2.6E-01	-0.65	3.0E-04
ENSCAFG0000003017	RNF8	darkgrey	EC_M8	0.52	6.0E-03	-0.21	3.0E-01	-0.64	4.3E-04	-0.04	8.6E-01	0.44	2.6E-02	0.27	1.8E-01	-0.10	6.4E-01	-0.09	6.6E-01	-0.10	6.3E-01	-0.68	1.5E-04
ENSCAFG0000001622	DDI2	turquoise	EC_M6	0.52	6.0E-03	0.71	4.6E-05	-0.48	1.3E-02	-0.09	6.5E-01	-0.08	7.0E-01	-0.13	5.3E-01	-0.05	8.1E-01	-0.02	9.3E-01	-0.12	5.7E-01	-0.16	4.2E-01
ENSCAFG0000000586	IK	grey	EC_M10	0.52	6.0E-03	0.20	3.4E-01	-0.20	3.2E-01	0.14	5.1E-01	-0.28	1.6E-01	0.13	5.3E-01	-0.07	7.4E-01	-0.17	4.1E-01	-0.13	5.3E-01	0.07	7.2E-01
ENSCAFG0000000776	TAF9	darkgrey	EC_M8	0.52	6.0E-03	0.06	7.8E-01	-0.37	6.6E-02	0.07	7.4E-01	0.06	7.6E-01	0.09	6.7E-01	-0.57	2.3E-03	-0.08	7.1E-01	-0.31	1.2E-01	-0.36	6.9E-02
ENSCAFG0000001693	RANBP17	grey	EC_M10	0.52	6.0E-03	0.35	8.4E-02	-0.63	5.1E-04	-0.10	6.1E-01	0.21	3.0E-01	0.20	3.4E-01	-0.42	3.2E-02	-0.27	1.9E-01	-0.02	9.1E-01	-0.50	8.6E-03
ENSCAFG0000001546	ZNF518B	darkgrey	EC_M8	0.52	6.0E-03	0.64	4.3E-04	-0.19	3.5E-01	0.06	7.6E-01	-0.50	9.0E-03	-0.09	6.4E-01	-0.15	4.6E-01	0.06	7.6E-01	-0.05	8.0E-01	0.21	3.1E-01
ENSCAFG0000000349	USP45	darkgrey	EC_M8	0.52	6.1E-03	-0.16	4.3E-01	-0.44	2.3E-02	0.01	9.5E-01	0.18	3.8E-01	0.07	7.2E-01	-0.38	5.4E-02	0.05	8.1E-01	-0.12	5.5E-01	-0.50	1.0E-02
ENSCAFG0000000529	ENSCAFG0000000529	darkgrey	EC_M8	0.52	6.1E-03	0.10	6.1E-01	-0.20	3.2E-01	0.07	7.3E-01	-0.17	4.0E-01	-0.02	9.3E-01	-0.37	6.5E-02	-0.06	7.8E-01	0.05	8.1E-01	-0.08	7.0E-01
ENSCAFG0000000709	ENSCAFG0000000709	grey	EC_M10	0.52	6.1E-03	0.28	1.7E-01	-0.51	8.1E-03	-0.17	4.1E-01	0.07	7.2E-01	0.62	7.0E-04	-0.09	6.6E-01	-0.13	5.1E-01	-0.10	6.3E-01	-0.34	9.2E-02
ENSCAFG0000002540	ZNF684	darkgrey	EC_M8	0.52	6.1E-03	-0.04	8.3E-01	-0.52	6.6E-03	-0.02	9.2E-01	0.20	3.3E-01	0.34	9.4E-02	-0.41	3.5E-02	0.14	5.0E-01	-0.01	9.7E-01	-0.48	1.4E-02
ENSCAFG0000001166	ETNK1	darkgreen	EC_M4	0.52	6.2E-03	0.31	1.3E-01	-0.70	6.2E-05	0.06	7.5E-01	0.27	1.9E-01	0.12	5.6E-01	0.06	7.8E-01	-0.23	2.6E-01	-0.35	8.2E-02	-0.56	3.1E-03
ENSCAFG0000000792	TTC37	darkgrey	EC_M8	0.52	6.2E-03	0.05	7.9E-01	-0.70	6.6E-05	0.14	5.0E-01	0.31	1.3E-01	0.16	4.5E-01	-0.12	5.6E-01	-0.14	4.8E-01	-0.32	1.1E-01	-0.61	1.0E-03
ENSCAFG0000000759	CFAP97	grey	EC_M10	0.52	6.2E-03	0.15	4.6E-01	-0.45	2.2E-02	0.10	6.2E-01	-0.02	9.1E-01	0.07	7.5E-01	-0.36	6.9E-02	-0.03	8.9E-01	-0.40	4.1E-02	-0.26	2.0E-01
ENSCAFG0000001470	MYNN	grey	EC_M10	0.52	6.3E-03	0.34	8.5E-02	-0.57	2.2E-03	0.00	1.0E+00	0.05	8.2E-01	0.34	8.7E-02	0.09	6.7E-01	-0.10	6.3E-01	-0.10	6.4E-01	-0.34	9.1E-02
ENSCAFG0000000773	ATP1B3	darkgreen	EC_M4	0.52	6.3E-03	0.44	2.5E-02	-0.79	1.7E-06	-0.23	2.5E-01	0.37	6.2E-02	0.09	6.7E-01	-0.11	5.9E-01	-0.08	7.1E-01	-0.44	2.4E-02	-0.63	6.1E-04
ENSCAFG0000002345	HAVCR1	turquoise	EC_M6	0.52	6.3E-03	0.66	2.8E-04	-0.67	1.6E-04	-0.01	9.6E-01	0.08	7.1E-01	0.29	1.5E-01	-0.08	6.9E-01	0.02	9.3E-01	0.09	6.6E-01	-0.35	7.6E-02
ENSCAFG0000000503	CCNT2	darkgrey	EC_M8	0.52	6.3E-03	0.40	4.2E-02	-0.17	4.0E-01	0.29	1.5E-01	-0.44	2.4E-02	0.05	8.0E-01	-0.36	7.1E-02	-0.15	4.7E-01	0.02	9.0E-01	0.16	4.3E-01
ENSCAFG0000001011	ENSCAFG0000001011	darkgrey	EC_M8	0.52	6.3E-03	-0.12	5.6E-01	-0.45	2.0E-02	-0.29	1.5E-01	0.17	4.0E-01	-0.02	9.3E-01	-0.17	3.9E-01	0.16	4.4E-01	-0.42	3.5E-02	-0.48	1.3E-02
ENSCAFG0000001897	NSRP1	darkgrey	EC_M8	0.52	6.4E-03	0.14	4.9E-01	-0.64	4.2E-04	0.01	9.5E-01	0.18	3.7E-01	0.26	1.9E-01	0.03	8.9E-01	0.16	4.3E-01	-0.22	2.9E-01	-0.48	1.2E-02
ENSCAFG0000000503	KIN	grey	EC_M10	0.52	6.4E-03	0.12	5.5E-01	-0.51	7.4E-03	-0.05	8.1E-01	0.01	9.5E-01	0.38	5.4E-02	-0.06	7.6E-01	-0.09	6.8E-01	-0.05	8.2E-01	-0.37	6.0E-02
ENSCAFG0000000989	RFC5	darkgrey	EC_M8	0.52	6.4E-03	-0.12	5.8E-01	-0.02	9.3E-01	0.03	8.7E-01	-0.25	2.2E-01	0.11	5.9E-01	-0.26	2.1E-01	-0.09	6.6E-01	0.10	6.3E-01	0.03	8.9E-01
ENSCAFG0000000249	PTP4A1	darkgreen	EC_M4	0.52	6.4E-03	0.26	2.0E-01	-0.85	4.9E-08	0.06	7.7E-01	0.42	3.4E-02	0.30	1.3E-01	-0.22	2.8E-01	-0.04	8.6E-01	-0.23	2.6E-01	-0.71	4.6E-05
ENSCAFG0000000713	ENSCAFG0000000713	darkgrey	EC_M8	0.52	6.5E-03	0.26	1.9E-01	-0.13	5.3E-01	0.12	5.6E-01	-0.43	2.8E-02	0.19	3.6E-01	-0.27	1.9E-01	-0.05	8.2E-01	-0.12	5.5E-01	0.17	4.2E-01
ENSCAFG0000000462	DDIAS	darkgrey	EC_M8	0.52	6.5E-03	-0.10	6.4E-01	-0.62	6.6E-04	-0.09	6.7E-01	0.38	5.2E-02	0.27	1.8E-01	0.01	9.6E-01	-0.25	2.1E-01	-0.19	3.6E-01	-0.65	2.8E-04
ENSCAFG0000001456	PDCD10	darkgreen	EC_M4	0.52	6.5E-03	0.34	8.9E-02	-0.90	5.0E-10	-0.21	3.1E-01	0.46	1.7E-02	0.28	1.6E-01	-0.12	5.7E-01	0.02	9.3E-01	-0.14	4.9E-01	-0.74	1.3E-05
ENSCAFG0000003045	LZIC	darkgrey	EC_M8	0.52	6.5E-03	0.16	4.3E-01	0.22	2.8E-01	0.22	2.9E-01	-0.74	1.7E-05	-0.12	5.5E-01	-0.32	1.2E-01	-0.06	7.8E-01	-0.23	2.6E-01	0.49	1.0E-02
ENSCAFG0000000596	MRPS31	darkgreen	EC_M4	0.52	6.5E-03	-0.15	4.7E-01	-0.69	9.2E-05	0.07	7.5E-01	0.45	2.0E-02	0.14	5.0E-01	-0.25	2.1E-01	-0.09	6.7E-01	-0.28	1.7E-01	-0.74	1.5E-05
ENSCAFG0000001762	DHX40	darkgrey	EC_M8	0.52	6.5E-03	0.30	1.4E-01	-0.43	2.8E-02	0.06	7.9E-01	-0.12	5.5E-01	0.19	3.5E-01	-0.27	1.8E-01	-0.14	4.8E-01	-0.27	1.7E-01	-0.17	4.0E-01
ENSCAFG0000002624	ENSCAFG0000002624	grey	EC_M10	0.52	6.5E-03	0.31	1.2E-01	-0.25	2.2E-01	-0.26	1.9E-01	-0.23	2.5E-01	0.24	2.4E-01	-0.06	7.7E-01	0.01	9.5E-01	-0.05	8.1E-01	-0.05	8.2E-01
ENSCAFG0000000100	CENPM	darkgrey	EC_M8	0.52	6.5E-03	-0.08	7.1E-01	0.10	6.2E-01	-0.22	2.9E-01	-0.44	2.3E-02	-0.14	5.0E-01	0.15	4.7E-01	-0.16	4.3E-01	0.00	9.9E-01	0.19	3.5E-01
ENSCAFG0000001468	ENSCAFG0000001468	darkgrey	EC_M8	0.52	6.5E-03	0.19	3.6E-01	-0.70	7.3E-05	-0.30	1.4E-01	0.28	1.6E-01	0.17	4.0E-01	-0.02	9.1E-01	-0.17	4.1E-01	-0.07	7.3E-01	-0.57	2.4E-03
ENSCAFG0000001611	ENSCAFG0000001611	darkgrey	EC_M8	0.52	6.5E-03	-0.12	5.7E-01	-0.36	7.3E-02	0.41	3.9E-02	-0.02	9.2E-01	0.07	7.5E-01	-0.20	3.2E-01	-0.24	2.3E-01	-0.23	2.6E-01	-0.28	1.6E-01
ENSCAFG0000000900	GNL3	darkgreen	EC_M4	0.52	6.6E-03	-0.13	5.4E-01	-0.74	1.8E-05	-0.08	7.1E-01	0.50	9.4E-03	0.26	2.0E-01	-0.05	8.2E-01	-0.03	8.7E-01	-0.33	1.0E-01	-0.78	2.8E-06
ENSCAFG0000000939	PMS1	grey	EC_M10	0.52	6.6E-03	-0.45	2.0E-02	-0.16	4.3E-01	0.35	7.6E-02	-0.09	6.7E-01	0.13	5.4E-01	-0.30	1.4E-01	-0.11	6.0E-01	-0.12	5.4E-01	-0.16	4.5E-01
ENSCAFG0000001402	ENSCAFG0000001402	darkgrey	EC_M8	0.52	6.6E-03	0.21	3.0E-01	-0.11	6.0E-01	0.14	5.0E-01	-0.45	2.1E-02	-0.24	2.3E-01	-0.35	8.4E-02	-0.15	4.6E-01	-0.28	1.7E-01	0.16	4.3E-01
ENSCAFG0000001539	VDAC2	darkgreen	EC_M4	0.52	6.6E-03	0.22	2.7E-01	-0.89	1.9E-09	-0.32	1.2E-01	0.59	1.4E-03	0.26	1.9E-01	-0.14	5.0E-01	-0.07	7.2E-01	-0.12	5.6E-01	-0.85	4.3E-08
ENSCAFG0000001893	MAP7D3	grey	EC_M10	0.52	6.6E-03	0.26	2.0E-01	0.06	7.8E-01	0.10	6.3E-01	-0.60	1.3E-03	-0.13	5.4E-01	-0.02	9.4E-01	-0.40	4.3E-02	-0.03	9.0E-01	0.35	7.7E-02
ENSCAFG0000000935	POC5	darkgrey	EC_M8	0.52	6.6E-03	0.01	9.6E-01	-0.58	1.8E-03	0.03	9.0E-01	0.28	1.7E-01	0.21	3.0E-01	-0.47	1.6E-02	-0.16	4.5E-01	-0.01	9.7E-01	-0.53	5.0E-03
ENSCAFG0000001411	ENSCAFG0000001411	darkgrey	EC_M8	0.52	6.6E-03	-0.38	5.6E-02	-0.02	9.4E-01	0.00	1.0E+00	-0.21	2.9E-01	0.00	1.0E+00	-0.46	1.9E-02	-0.18	3.8E-01	-0.01	9.8E-01	-0.06	7.5E-01
ENSCAFG0000001135	SART3	grey	EC_M10	0.52	6.7E-03	0.34	9.0E-02	-0.24	2.5E-01	0.08	6.9E-01	-0.26	2.1E-01	0.27	1.9E-01	0.22	2.9E-01	-0.08	6.9E-01	-0.45	2.1E-02	-0.04	8.6E-01
ENSCAFG0000003173	ENSCAFG0000003173	grey	EC_M10	0.52	6.7E-03	0.51	7.7E-03	-0.43	2.7E-02	-0.17	4.2E-01	-0.03	8.9E-01	0.24	2.4E-01	-0.12	5.6E-01	0.21	3.1E-01	-0.15	4.7E-01	-0.21	2.9E-01
ENSCAFG0000000602	UFM1	darkgreen	EC_M4	0.52	6.7E-03	-0.17	4.0E-01	-0.66	2.4E-04	-0.20	3.4E-01	0.42	3.3E-02	0.26	2.0E-01	-0.22	2.8E-01	-0.16	4.5E-01	-0.23	2.5E-01	-0.73	2.2E-05
ENSCAFG0000001871	ENSCAFG0000001871	darkgrey	EC_M8	0.52	6.7E-03	-0.49	1.1E-02	0.00	1.0E+00	0.08	7.0E-01	-0.26	2.0E-01	0.21	3.0E-01	-0.36	7.3E-02	0.14	5.1E-01	-0.41	3.8E-02	-0.07	7.3E-01
ENSCAFG0000000671	ACTR6	darkgrey	EC_M8	0.52	6.8E-03	-0.37	6.2E-02	-0.47	1.5E-02	0.21	3.0E-01	0.22	2.8E-01	0.18	3.8E-01	-0.18	3.7E-01	-0.25	2.1E-01	-0.18	3.9E-01	-0.51	8.0E-03
ENSCAFG0000001886	PTPN2	darkgreen	EC_M4	0.52	6.8E-03	0.05	8.1E-01	-0.74	1.3E-05	-0.06	7.7E-01	0.34	8.7E-02	0.38	5.9E-02	-0.07	7.2E-01	-0.01	9.7E-01	-0.11	6.0E-01	-0.66	2.3E-04
ENSCAFG0000002304	ENSCAFG0000002304	darkgrey	EC_M8	0.52	6.8E-03	0.42	3.4E-02	-0.37	6.1E-02	-0.17	4.1E-01	-0.06	7.7E-01	-0.18	3.9E-01	0.31	1.3E-01	-0.08	6.9E-01	-0.11	6.1E-01	-0.16	4.3E-01
ENSCAFG0000000472	PDCD6IP	darkgreen	EC_M4	0.52	6.8E-03	0.19	3.4E-01	-0.82	2.4E-07	-0.17	4.1E-01	0.45	2.1E-02	0.06	7.8E-01	-0.34	8.9E-02	0.11	6.0E-01	-0.27	1.9E-01	-0.72	3.1E-05
ENSCAFG0000003089	ZFP62	darkgrey	EC_M8	0.52	6.8E-03	0.16	4.5E-01	0.12	5.6E-01	0.23	2.6E-01	-0.65	2.9E-04	0.03	8.9E-01	-0.38	5.6E-02	0.04	8.3E-01	-0.01	9.5E-01	0.38	5.7E-02
ENSCAFG0000001402	LANCL1	darkgrey	EC_M8	0.52	6.9E-03	-0.04	8.4E-01	-0.61	9.7E-04	-0.23	2.6E-01	0.36	7.5E-02	0.24	2.4E-01	-0.34	8.5E-02	-0.09	6.6E-01	-0.19	3.4E-01	-0.59	1.6E-03
ENSCAFG0000000882	ALMS1	darkgrey	EC_M8	0.52	6.9E-03	0.08	6.8E-01	0.24	2.4E-01	0.06	7.8E-01	-0.76	5.5E-06	0.06	7.8E-01	-0.10	6.4E-01	-0.16	4.3E-01	-0.14	4.9E-01	0.46	1.7E-02
ENSCAFG0000000308	GARS	darkgreen	EC_M4	0.52	6.9E-03	-0.01	9.5E-01	-0.76	6.5E-06	0.02	9.4E-01	0.49	1.1E-02	0.31	1.3E-01	-0.25	2.2E-01	-0.15	4.6E-01	-0.36	6.9E-02	-0.81	5.8E-07
ENSCAFG0000001110	GNA13	darkgrey	EC_M8	0.52	6.9E-03	0.16	4.3E-01	-0.18	3.9E-01	0.13	5.3E-01	-0.31	1.3E-01	0.12	5.6E-01	-0.17	4.2E-01	-0.07	7.3E-01	-0.36	6.8E-02	0.05	7.9E-01
ENSCAFG0000000801	UQCC1	grey	EC_M10	0.52	7.0E-03	-0.05	8.1E-01	-0.32	1.1E-01	0.10	6.2E-01	-0.10	6.4E-01	0.23	2.5E-01	-0.32	1.2E-01	-0.06	7.6E-01	0.37	6.1E-02	-0.21	3.1E-01
ENSCAFG0000000563	KAT6A	darkgrey	EC_M8	0.52	7.0E-03	0.25	2.1E-01	-0.07	7.2E-01	0.20	3.3E-01	-0.50	8.5E-03	0.09	6.7E-01	-0.06	7.6E-01	-0.06	7.5E-01	-0.37	6.5E-02	0.21	3.0E-01
ENSCAFG0000001269	KMT2A	grey	EC_M10	0.52	7.1E-03	0.07	7.3E-01	-0.31	1.2E-01	0.02	9.3E-01	-0.12	5.7E-01	0.01	9.6E-01	-0.25	2.2E-01	-0.30	1.4E-01	-0.29	1.5E-01	-0.17	4.0E-01
ENSCAFG0000000283	ENSCAFG0000000283	grey	EC_M10	0.52	7.1E-03	-0.20	3.3E-01	-0.15	4.8E-01	0.22	2.7E-01	-0.19	3.5E-01	-0.10	6.2E-01	-0.40	4.3E-02	-0.26	2.0E-01	-0.60	1.1E-03	-0.10	6.2E-01
ENSCAFG0000001580	MTA2	grey	EC_M10	0.52	7.1E-03	0.65	3.2E-04	-0.05	8.1E-01	-0.10	6.2E-01	-0.57	2.3E-03	-0.09	6.5E-01	0.02	9.3E-01	0.07	7.2E-01	0.04	8.4E-01	0.29	1.5E-01
ENSCAFG0000001857	ZNF131	grey	EC_M10	0.51	7.1E-03	0.15	4.7E-01	-0.37	6.2E-02	-0.45	2.2E-02	-0.07	7.5E-01	0.08	6.8E-01	-0.17	4.0E-01	0.14	5.0E-01	-0.05	7.9E-01	-0.19	3.5E-01
ENSCAFG0000002978	ENSCAFG0000002978	grey	EC_M10	0.51	7.1E-03	0.06	7.8E-01	-0.42	3.4E-02	0.34	9.0E-02	0.03	8.7E-01	0.23	2.5E-01	-0.31	1.2E-01	-0.09	6.5E-01	-0.40	4.1E-02	-0.34	9.4E-02
ENSCAFG0000000928	SRSF6	grey	EC_M10	0.51	7.3E-03	0.10	6.3E-01	0.16	4.3E-01	0.30	1.3E-01	-0.64	4.1E-04	0.03	8.8E-01	-0.52	6.2E-03	-0.13	5.3E-01	0.05	8.0E-01	0.35	8.1E-02
ENSCAFG0000001500	HP1BP3	darkgrey	EC_M8	0.51	7.3E-03	0.45	2.0E-02	-0.13	5.2E-01	0.22	2.7E-01	-0.50	9.6E-03	-0.12	5.5E-01	-0.11	5.9E-01	-0.25	2.2E-01	-0.09	6.7E-01	0.23	2.5E-01
ENSCAFG0000000643	CDV3	darkgreen	EC_M4	0.51	7.3E-03	0.12	5.7E-01	-0.90	4.6E-10	-0.11	5.8E-01	0.61	9.0E-04	0.25	2.2E-01	-0.18	3.7E-01	-0.10	6.3E-01	-0.20	3.2E-01	-0.88	2.3E-09
ENSCAFG0000000554	UCP3	grey	EC_M10	0.51	7.3E-03	-0.10	6.3E-01	-0.28	1.7E-01	0.08	7.1E-01	-0.11	5.8E-01	0.17	4.0E-01	-0.27	1.8E-01	-0.30	1.3E-01	-0.14	5.0E-01	-0.17	4.2E-01
ENSCAFG0000000391	GTF3C6	grey	EC_M10	0.51	7.3E-03	0.18	3.9E-01	-0.30	1.3E-01	0.15	4.6E-01	-0.13	5.3E-01	-0.04	8.6E-01	-0.58	1.8E-03	0.11	5.9E-01	0.03	8.7E-01	-0.19	3.6E-01
ENSCAFG0000000497	OXSR1	grey	EC_M10	0.51	7.4E-03	-0.12	5.6E-01	-0.15	4.8E-01	-0.15	4.7E-01	-0.24	2.4E-01	0.26	1.9E-01	0.07	7.4E-01	-0.32	1.1E-01	-0.37	6.4E-02	-0.07	7.2E-01
ENSCAFG0000000774	CDK7	grey	EC_M10	0.51	7.4E-03	0.24	2.5E-01	-0.53	4.9E-03	0.09	6.8E-01	0.10	6.4E-01	0.22	2.9E-01	-0.16	4.3E-01	0.30	1.4E-01	-0.05	8.1E-01	-0.35	8.1E-02
ENSCAFG0000000902	FAM83D	darkgrey	EC_M8	0.51	7.4E-03	0.01	9.4E-01	0.26	1.9E-01	-0.02	9.4E-01	-0.65	3.2E-04	0.03	8.7E-01	-0.28	1.6E-01	-0.15	4.6E-01	0.04	8.4E-01	0.41	3.8E-02
ENSCAFG0000002029	ENSCAFG0000002029	grey	EC_M10	0.51	7.5E-03	-0.30	1.3E-01	-0.32	1.1E-01	-0.03	8.7E-01	0.17	4.0E-01	0.13	5.4E-01	-0.35	8.3E-02	-0.20	3.3E-01	-0.25	2.3E-01	-0.45	2.3E-02
ENSCAFG0000000345	ENSCAFG0000000345	grey	EC_M10	0.51	7.5E-03	-0.06	7.8E-01	-0.48	1.4E-02	-0.08	6.9E-01	0.15	4.7E-01	0.05	8.0E-01	-0.14	5.0E-01	-0.13	5.3E-01	-0.09	6.5E-01	-0.38	5.3E-02
ENSCAFG0000001873	ELF4	turquoise	EC_M6	0.51	7.5E-03	0.75	1.1E-05	-0.02	9.1E-01	-0.03	8.9E-01	-0.64	4.4E-04	-0.15	4.6E-01	-0.14	5.0E-01	-0.11	5.9E-01	-0.28	1.7E-01	0.36	6.9E-02
ENSCAFG0000001170	MNDA	grey	EC_M10	0.51	7.6E-03	0.08	7.0E-01	0.22	2.9E-01	-0.02	9.1E-01	-0.67	1.6E-04	-0.01	9.7E-01	-0.17	4.1E-01	-0.06	7.8E-01	-0.35	8.0E-02	0.38	5.8E-02
ENSCAFG0000000268	PPP1R21	grey	EC_M10	0.51	7.6E-03	-0.31	1.3E-01	0.10	6.2E-01	0.13	5.1E-01	-0.43	2.7E-02	-0.03	9.0E-01	-0.31	1.2E-01	-0.15	4.6E-01	-0.46	1.8E-02	0.14	5.0E-01
ENSCAFG0000001443	RC3H1	grey	EC_M10	0.51	7.6E-03	0.14	4.9E-01	-0.48	1.3E-02	-0.05	8.0E-01	0.05	8.1E-01	0.04	8.3E-01	-0.04	8.4E-01	-0.26	2.1E-01	-0.35	7.6E-02	-0.34	9.2E-02
ENSCAFG0000000541	FBL	grey	EC_M10	0.51	7.6E-03	-0.19	3.5E-01	0.27	1.9E-01	0.05	8.2E-01	-0.57	2.2E-03	0.05	8.2E-01	0.04	8.5E-01	-0.05	8.3E-01	-0.26	2.0E-01	0.27	1.8E-01
ENSCAFG0000000731	RRS1	darkgrey	EC_M8	0.51	7.7E-03	-0.24	2.4E-01	-0.01	9.6E-01	-0.10	6.4E-01	-0.25	2.2E-01	0.08	7.0E-01	0.03	8.7E-01	-0.06	7.6E-01	0.07	7.4E-01	-0.02	9.3E-01
ENSCAFG0000003235	UBE2D1	grey	EC_M10	0.51	7.7E-03	0.37	6.3E-02	-0.54	4.4E-03	0.13	5.4E-01	0.02	9.2E-01	0.35	7.9E-02	-0.36	7.4E-02	-0.20	3.2E-01	-0.31	1.3E-01	-0.33	1.0E-01
ENSCAFG0000000112	ENSCAFG0000000112	darkgrey	EC_M8	0.51	7.7E-03	0.34	8.9E-02	-0.49	1.1E-02	0.22	2.7E-01	-0.02	9.1E-01	0.22	2.7E-01	-0.25	2.2E-01	-0.15	4.7E-01	-0.43	3.0E-02	-0.26	2.0E-01
ENSCAFG0000001445	ATM	grey	EC_M10	0.51	7.7E-03	0.17	4.1E-01	-0.48	1.4E-02	-0.14	4.9E-01	0.07	7.3E-01	-0.20	3.2E-01	-0.17	4.1E-01	0.01	9.6E-01	-0.15	4.6E-01	-0.38	5.3E-02
ENSCAFG0000003101	ENSCAFG0000003101	grey	EC_M10	0.51	7.7E-03	0.14	4.9E-01	-0.27	1.9E-01	0.17	4.2E-01	-0.20	3.2E-01	0.17	3.9E-01	-0.15	4.5E-01	0.00	9.9E-01	-0.01	9.7E-01	-0.06	7.8E-01
ENSCAFG0000000858	SPATS2	turquoise	EC_M6	0.51	7.7E-03	0.59	1.5E-03	-0.62	6.7E-04	-0.10	6.1E-01	0.07	7.2E-01	0.04	8.4E-01	-0.25	2.3E-01	0.17	4.0E-01	0.21	2.9E-01	-0.35	7.8E-02
ENSCAFG0000000698	MRPL15	darkgrey	EC_M8	0.51	7.7E-03	-0.01	9.6E-01	-0.06	7.8E-01	0.14	5.0E-01	-0.24	2.3E-01	0.12	5.6E-01	-0.25	2.3E-01	-0.46	1.8E-02	-0.23	2.6E-01	-0.03	8.8E-01
ENSCAFG0000001997	ENSCAFG0000001997	darkgrey	EC_M8	0.51	7.7E-03	0.18	3.7E-01	-0.40	4.4E-02	0.32	1.1E-01	-0.06	7.8E-01	0.07	7.5E-01	-0.01	9.7E-01	-0.24	2.3E-01	-0.33	1.0E-01	-0.19	3.5E-01
ENSCAFG0000001011	TMEFF2	grey	EC_M10	0.51	7.8E-03	0.30	1.3E-01	0.27	1.8E-01	0.06	7.6E-01	-0.81	5.9E-07	-0.25	2.1E-01	-0.42	3.1E-02	0.02	9.4E-01	-0.12	5.6E-01	0.53	5.3E-03

ENSCAFG0000001138	MRPL47	grey	EC_M10	0.51	7.8E-03	-0.13	5.3E-01	-0.47	1.5E-02	0.02	9.1E-01	0.14	5.0E-01	0.04	8.5E-01	-0.18	3.9E-01	-0.10	6.4E-01	-0.10	6.2E-01	-0.40	4.1E-02
ENSCAFG0000002508	PAXBP1	darkgrey	EC_M8	0.51	7.8E-03	0.46	1.7E-02	-0.13	5.3E-01	0.19	3.4E-01	-0.52	6.1E-03	-0.02	9.3E-01	0.06	7.8E-01	-0.11	6.1E-01	-0.11	5.8E-01	0.24	2.4E-01
ENSCAFG0000003240	LIN52	grey	EC_M10	0.51	7.8E-03	0.09	6.7E-01	-0.19	3.5E-01	-0.06	7.6E-01	-0.20	3.2E-01	0.09	6.7E-01	-0.19	3.5E-01	-0.12	5.6E-01	-0.43	2.8E-02	-0.07	7.4E-01
ENSCAFG0000001939	RNPS1	darkgrey	EC_M8	0.51	7.9E-03	0.23	2.7E-01	0.07	7.4E-01	-0.29	1.5E-01	-0.47	1.6E-02	-0.13	5.4E-01	-0.17	3.9E-01	-0.01	9.7E-01	-0.01	9.4E-01	0.20	3.3E-01
ENSCAFG0000001999	SLC30A7	darkgreen	EC_M4	0.51	7.9E-03	-0.13	5.2E-01	-0.69	8.8E-05	-0.03	9.0E-01	0.46	1.7E-02	0.37	6.4E-02	-0.34	8.9E-02	-0.21	3.0E-01	-0.14	5.0E-01	-0.77	3.7E-06
ENSCAFG0000001244	DDX6	darkgrey	EC_M8	0.51	7.9E-03	0.30	1.3E-01	-0.47	1.6E-02	0.09	6.7E-01	-0.01	9.6E-01	-0.04	8.5E-01	-0.29	1.4E-01	0.03	8.9E-01	-0.15	4.6E-01	-0.21	2.9E-01
ENSCAFG0000000775	CASP3	grey	EC_M10	0.51	7.9E-03	0.20	3.2E-01	-0.50	9.1E-03	0.02	9.2E-01	0.08	6.9E-01	0.05	8.1E-01	-0.20	3.4E-01	-0.13	5.3E-01	0.28	1.7E-01	-0.34	8.7E-02
ENSCAFG0000001713	OGT	darkgrey	EC_M8	0.51	7.9E-03	0.23	2.7E-01	0.09	6.5E-01	0.33	1.0E-01	-0.69	1.1E-04	-0.06	7.8E-01	-0.12	5.5E-01	-0.10	6.4E-01	-0.27	1.8E-01	0.36	6.9E-02
ENSCAFG0000000860	LTN1	darkgrey	EC_M8	0.51	8.0E-03	-0.07	7.2E-01	-0.48	1.3E-02	-0.19	3.6E-01	0.19	3.6E-01	-0.05	8.1E-01	-0.43	2.9E-02	-0.04	8.5E-01	-0.34	9.3E-02	-0.43	2.8E-02
ENSCAFG0000000999	ENSCAFG0000000999	darkgrey	EC_M8	0.51	8.0E-03	0.22	2.7E-01	-0.28	1.6E-01	0.08	7.1E-01	-0.21	3.0E-01	0.01	9.7E-01	-0.13	5.2E-01	-0.32	1.1E-01	-0.38	5.4E-02	-0.06	7.6E-01
ENSCAFG0000003154	PIK3C2A	darkgreen	EC_M4	0.51	8.0E-03	0.74	1.6E-05	-0.78	2.1E-06	-0.08	6.9E-01	0.22	2.9E-01	0.05	8.0E-01	0.04	8.6E-01	-0.11	5.9E-01	-0.08	7.0E-01	-0.49	1.1E-02
ENSCAFG0000002033	ADGRL2	turquoise	EC_M6	0.51	8.0E-03	0.68	1.5E-04	-0.42	3.3E-02	-0.02	9.3E-01	-0.16	4.3E-01	-0.18	3.9E-01	-0.26	2.0E-01	-0.06	7.8E-01	0.17	4.1E-01	-0.10	6.1E-01
ENSCAFG0000000096	HSF2	darkgrey	EC_M8	0.51	8.1E-03	-0.01	9.8E-01	-0.50	8.5E-03	0.09	6.5E-01	0.13	5.3E-01	0.19	3.4E-01	-0.08	7.1E-01	-0.22	2.8E-01	-0.27	1.8E-01	-0.37	6.4E-02
ENSCAFG0000001823	IL13RA2	grey	EC_M10	0.51	8.1E-03	-0.11	6.1E-01	-0.26	2.1E-01	-0.21	3.0E-01	-0.05	8.2E-01	0.08	7.0E-01	-0.17	4.0E-01	0.11	6.1E-01	-0.54	4.2E-03	-0.26	2.0E-01
ENSCAFG0000000747	NUDT12	grey	EC_M10	0.51	8.1E-03	-0.23	2.7E-01	-0.20	3.3E-01	0.27	1.9E-01	-0.22	2.8E-01	0.05	7.9E-01	-0.10	6.4E-01	0.08	7.1E-01	0.01	9.4E-01	-0.06	7.8E-01
ENSCAFG0000000871	MRPL1	darkgreen	EC_M4	0.51	8.2E-03	-0.06	7.6E-01	-0.71	5.4E-05	0.25	2.1E-01	0.44	2.6E-02	0.11	6.0E-01	-0.13	5.1E-01	-0.08	7.1E-01	-0.02	9.4E-01	-0.71	4.8E-05
ENSCAFG0000000003	ENSCAFG0000000003	grey	EC_M10	0.51	8.2E-03	-0.13	5.1E-01	-0.04	8.5E-01	0.18	3.8E-01	-0.26	2.0E-01	-0.04	8.6E-01	-0.51	8.3E-03	-0.07	7.3E-01	-0.17	4.0E-01	0.05	8.1E-01
ENSCAFG0000000577	UBE2E1	turquoise	EC_M6	0.51	8.2E-03	0.52	6.1E-03	-0.63	5.7E-04	-0.13	5.1E-01	0.11	6.0E-01	0.06	7.6E-01	-0.04	8.4E-01	-0.04	8.3E-01	-0.21	3.1E-01	-0.42	3.1E-02
ENSCAFG0000001520	SNAP29	turquoise	EC_M6	0.51	8.2E-03	0.47	1.5E-02	-0.48	1.2E-02	-0.32	1.1E-01	0.01	9.5E-01	-0.05	8.0E-01	-0.52	5.9E-03	0.18	3.7E-01	0.02	9.1E-01	-0.26	2.0E-01
ENSCAFG0000000904	DHX35	grey	EC_M10	0.51	8.2E-03	0.29	1.4E-01	-0.01	9.6E-01	-0.07	7.4E-01	-0.55	3.6E-03	0.09	6.5E-01	-0.17	4.0E-01	-0.28	1.6E-01	0.08	7.0E-01	0.26	2.0E-01
ENSCAFG0000001451	MCU	turquoise	EC_M6	0.51	8.3E-03	0.52	5.9E-03	-0.16	4.3E-01	-0.15	4.5E-01	-0.36	6.9E-02	-0.19	3.6E-01	-0.36	7.4E-02	-0.38	5.7E-02	-0.20	3.4E-01	0.13	5.4E-01
ENSCAFG0000001510	GABPB1	darkgreen	EC_M4	0.51	8.3E-03	0.68	1.2E-04	-0.81	6.8E-07	-0.06	7.7E-01	0.30	1.4E-01	0.07	7.3E-01	-0.11	6.0E-01	-0.07	7.2E-01	-0.08	7.0E-01	-0.55	3.3E-03
ENSCAFG0000000718	ENSCAFG0000000718	grey	EC_M10	0.51	8.3E-03	0.23	2.5E-01	-0.32	1.1E-01	0.35	8.2E-02	-0.10	6.4E-01	0.22	2.8E-01	0.03	9.0E-01	-0.16	4.4E-01	-0.26	2.0E-01	-0.16	4.4E-01
ENSCAFG0000002949	ZNF260	turquoise	EC_M6	0.51	8.3E-03	0.58	1.9E-03	-0.67	1.9E-04	0.08	7.1E-01	0.11	6.0E-01	0.08	7.0E-01	-0.17	4.0E-01	0.04	8.5E-01	0.10	6.1E-01	-0.38	5.7E-02
ENSCAFG0000000910	HELQ	grey	EC_M10	0.51	8.3E-03	-0.07	7.4E-01	-0.24	2.5E-01	0.22	2.8E-01	-0.13	5.2E-01	-0.16	4.4E-01	-0.40	4.5E-02	0.30	1.4E-01	0.27	1.8E-01	-0.10	6.1E-01
ENSCAFG0000001828	NLE1	darkgrey	EC_M8	0.51	8.3E-03	-0.24	2.3E-01	-0.02	9.4E-01	-0.20	3.2E-01	-0.22	2.8E-01	0.14	4.9E-01	0.03	8.7E-01	0.05	8.0E-01	-0.39	4.8E-02	-0.06	7.7E-01
ENSCAFG0000001921	ENSCAFG0000001921	darkgrey	EC_M8	0.51	8.4E-03	0.23	2.5E-01	0.15	4.6E-01	0.03	8.7E-01	-0.57	2.4E-03	0.02	9.2E-01	0.07	7.3E-01	-0.25	2.1E-01	0.02	9.4E-01	0.34	9.1E-02
ENSCAFG0000001461	HIRA	grey	EC_M10	0.51	8.4E-03	0.11	6.0E-01	0.29	1.5E-01	0.04	8.4E-01	-0.72	2.9E-05	0.00	9.9E-01	-0.26	2.0E-01	-0.29	1.6E-01	-0.02	9.4E-01	0.45	2.1E-02
ENSCAFG0000001851	MRPS30	grey	EC_M10	0.51	8.4E-03	-0.19	3.6E-01	0.21	3.0E-01	0.08	7.1E-01	-0.59	1.5E-03	-0.11	5.8E-01	-0.39	5.0E-02	-0.02	9.4E-01	-0.20	3.3E-01	0.30	1.4E-01
ENSCAFG0000000194	CYP51A1	grey	EC_M10	0.51	8.4E-03	0.07	7.3E-01	-0.34	8.9E-02	-0.07	7.2E-01	-0.05	8.2E-01	-0.02	9.4E-01	-0.01	9.6E-01	-0.09	6.4E-01	-0.62	7.9E-04	-0.27	1.9E-01
ENSCAFG0000001373	MIPOL1	grey	EC_M10	0.51	8.4E-03	-0.19	3.4E-01	-0.13	5.2E-01	0.14	4.9E-01	-0.22	2.9E-01	0.03	8.9E-01	-0.31	1.3E-01	0.00	9.8E-01	-0.33	1.0E-01	-0.09	6.7E-01
ENSCAFG0000002031	RPF1	darkgrey	EC_M8	0.51	8.4E-03	-0.35	7.8E-02	-0.28	1.6E-01	0.35	7.8E-02	0.10	6.2E-01	0.18	3.9E-01	0.00	9.9E-01	-0.35	7.7E-02	-0.43	3.0E-02	-0.35	8.3E-02
ENSCAFG0000001925	ATAD3A	grey	EC_M10	0.51	8.5E-03	-0.30	1.3E-01	0.13	5.3E-01	-0.04	8.5E-01	-0.35	7.6E-02	-0.06	7.8E-01	-0.26	1.9E-01	-0.03	8.8E-01	-0.44	2.6E-02	0.07	7.2E-01
ENSCAFG0000001046	ENSCAFG0000001046	darkgrey	EC_M8	0.51	8.5E-03	-0.36	7.2E-02	0.01	9.7E-01	-0.14	5.0E-01	-0.23	2.6E-01	0.22	2.9E-01	-0.13	5.2E-01	-0.16	4.4E-01	-0.06	7.6E-01	-0.04	8.6E-01
ENSCAFG0000000965	TWF1	darkgreen	EC_M4	0.50	8.5E-03	0.00	1.0E+00	-0.88	4.0E-09	-0.01	9.6E-01	0.57	2.2E-03	0.32	1.1E-01	-0.15	4.6E-01	-0.02	9.2E-01	-0.25	2.1E-01	-0.86	2.1E-08
ENSCAFG0000000715	ZC3H8	darkgrey	EC_M8	0.50	8.6E-03	-0.25	2.2E-01	-0.57	2.5E-03	-0.13	5.2E-01	0.32	1.1E-01	0.30	1.4E-01	-0.11	5.8E-01	0.12	5.6E-01	0.04	8.6E-01	-0.61	9.0E-04
ENSCAFG0000001041	C35H6orf62	grey	EC_M10	0.50	8.6E-03	0.26	2.0E-01	-0.43	2.7E-02	0.16	4.4E-01	-0.05	8.2E-01	0.08	6.9E-01	-0.40	4.2E-02	-0.08	6.9E-01	0.23	2.5E-01	-0.24	2.4E-01
ENSCAFG0000002933	ENSCAFG0000002933	darkgrey	EC_M8	0.50	8.6E-03	0.11	5.9E-01	-0.08	7.1E-01	-0.03	9.0E-01	-0.28	1.6E-01	-0.02	9.1E-01	-0.03	8.8E-01	0.13	5.4E-01	0.00	9.9E-01	0.03	8.7E-01
ENSCAFG0000000917	GTF2H1	darkgreen	EC_M4	0.50	8.7E-03	-0.24	2.3E-01	-0.67	1.8E-04	-0.04	8.5E-01	0.42	3.4E-02	0.29	1.6E-01	-0.19	3.6E-01	0.20	3.4E-01	0.05	8.2E-01	-0.72	3.4E-05
ENSCAFG0000001426	BARD1	grey	EC_M10	0.50	8.7E-03	0.09	6.7E-01	0.25	2.2E-01	0.10	6.2E-01	-0.68	1.4E-04	-0.04	8.3E-01	-0.16	4.2E-01	-0.24	2.5E-01	-0.35	7.9E-02	0.41	3.9E-02
ENSCAFG0000000905	LIN54	grey	EC_M10	0.50	8.7E-03	0.22	2.8E-01	-0.01	9.5E-01	0.15	4.5E-01	-0.44	2.5E-02	-0.31	1.2E-01	-0.33	9.8E-02	-0.21	3.0E-01	-0.35	7.8E-02	0.22	2.7E-01
ENSCAFG0000001669	NOP16	darkgrey	EC_M8	0.50	8.7E-03	-0.16	4.3E-01	-0.37	6.0E-02	0.00	9.9E-01	0.14	4.9E-01	0.10	6.2E-01	-0.24	2.3E-01	0.43	3.0E-02	-0.10	6.4E-01	-0.44	2.5E-02
ENSCAFG0000001825	ENSCAFG0000001825	darkgrey	EC_M8	0.50	8.7E-03	0.18	3.9E-01	-0.33	9.6E-02	0.34	8.6E-02	-0.14	4.9E-01	0.13	5.3E-01	-0.06	7.7E-01	-0.26	2.0E-01	-0.35	7.7E-02	-0.11	6.0E-01
ENSCAFG0000001143	SCN9A	grey	EC_M10	0.50	8.8E-03	-0.01	9.7E-01	-0.14	4.9E-01	0.32	1.1E-01	-0.30	1.4E-01	-0.16	4.4E-01	-0.22	2.7E-01	0.20	3.2E-01	-0.18	3.9E-01	-0.02	9.2E-01
ENSCAFG0000001824	SNRPD1	grey	EC_M10	0.50	8.8E-03	-0.04	8.3E-01	0.26	1.9E-01	0.17	4.2E-01	-0.69	1.0E-04	-0.10	6.2E-01	0.08	7.1E-01	0.02	9.4E-01	-0.39	4.7E-02	0.40	4.3E-02
ENSCAFG0000000877	FAM192A	turquoise	EC_M6	0.50	8.8E-03	0.40	4.1E-02	-0.26	2.0E-01	0.09	6.5E-01	-0.20	3.2E-01	0.24	2.3E-01	-0.13	5.3E-01	0.03	8.7E-01	-0.47	1.6E-02	-0.07	7.4E-01
ENSCAFG0000000911	ABRAXAS1	darkgrey	EC_M8	0.50	8.9E-03	-0.26	2.0E-01	-0.36	7.0E-02	-0.09	6.7E-01	0.10	6.2E-01	0.02	9.2E-01	-0.18	3.7E-01	0.17	4.2E-01	-0.09	6.6E-01	-0.39	4.9E-02
ENSCAFG0000000054	CEP120	grey	EC_M10	0.50	8.9E-03	0.72	3.5E-05	-0.61	9.4E-04	-0.01	9.5E-01	-0.02	9.4E-01	0.03	8.8E-01	-0.09	6.7E-01	-0.07	7.2E-01	-0.13	5.4E-01	-0.25	2.2E-01
ENSCAFG0000002453	ENSCAFG0000002453	grey	EC_M10	0.50	8.9E-03	-0.40	4.5E-02	-0.04	8.4E-01	0.03	8.9E-01	-0.20	3.3E-01	-0.02	9.4E-01	-0.23	2.6E-01	-0.09	6.8E-01	-0.45	2.0E-02	-0.11	6.1E-01
ENSCAFG0000000393	ENSCAFG0000000393	darkgreen	EC_M4	0.50	8.9E-03	0.54	4.1E-03	-0.75	1.0E-05	-0.01	9.6E-01	0.21	3.1E-01	0.25	2.3E-01	-0.28	1.6E-01	-0.24	2.3E-01	-0.02	9.3E-01	-0.51	7.8E-03
ENSCAFG0000000275	CENPC	darkgreen	EC_M4	0.50	8.9E-03	0.45	2.0E-02	-0.67	2.0E-04	0.04	8.3E-01	0.17	4.1E-01	0.18	3.8E-01	-0.13	5.2E-01	-0.12	5.7E-01	-0.25	2.2E-01	-0.42	3.5E-02
ENSCAFG0000003027	ENSCAFG0000003027	darkgrey	EC_M8	0.50	9.0E-03	0.18	3.8E-01	-0.70	7.9E-05	0.09	6.8E-01	0.30	1.3E-01	0.13	5.3E-01	-0.32	1.1E-01	0.08	7.0E-01	-0.32	1.1E-01	-0.58	2.1E-03
ENSCAFG0000001800	ENSCAFG0000001800	grey	EC_M10	0.50	9.0E-03	0.04	8.6E-01	-0.16	4.5E-01	0.26	2.0E-01	-0.30	1.4E-01	-0.07	7.3E-01	0.14	5.0E-01	-0.06	7.7E-01	-0.40	4.4E-02	-0.02	9.3E-01
ENSCAFG0000001667	BNIP2	turquoise	EC_M6	0.50	9.0E-03	0.63	6.4E-04	-0.59	1.5E-03	-0.15	4.5E-01	0.07	7.5E-01	-0.06	7.5E-01	-0.02	9.3E-01	-0.03	8.7E-01	-0.23	2.5E-01	-0.29	1.5E-01
ENSCAFG0000001921	CALML6	turquoise	EC_M6	0.50	9.0E-03	0.53	5.2E-03	-0.49	1.0E-02	-0.12	5.5E-01	-0.04	8.6E-01	0.14	5.1E-01	0.10	6.2E-01	-0.19	3.5E-01	-0.35	7.7E-02	-0.27	1.9E-01
ENSCAFG0000000482	ALG8	darkgrey	EC_M8	0.50	9.0E-03	-0.28	1.7E-01	-0.51	7.6E-03	-0.12	5.4E-01	0.36	6.8E-02	0.30	1.3E-01	0.00	9.9E-01	-0.13	5.4E-01	-0.23	2.5E-01	-0.63	5.3E-04
ENSCAFG0000002156	ENSCAFG0000002156	darkgrey	EC_M8	0.50	9.1E-03	0.40	4.4E-02	-0.02	9.1E-01	0.10	6.2E-01	-0.53	5.4E-03	0.09	6.6E-01	0.06	7.9E-01	-0.17	4.0E-01	-0.31	1.2E-01	0.25	2.2E-01
ENSCAFG0000000699	ZNF664	darkgrey	EC_M8	0.50	9.1E-03	0.55	3.9E-03	-0.37	6.1E-02	0.08	7.0E-01	-0.25	2.2E-01	-0.11	6.1E-01	-0.30	1.4E-01	0.03	8.9E-01	-0.06	7.8E-01	-0.02	9.3E-01
ENSCAFG0000001166	HSPA8	darkgreen	EC_M4	0.50	9.1E-03	0.34	8.9E-02	-0.86	1.3E-08	-0.23	2.6E-01	0.49	1.2E-02	0.09	6.7E-01	-0.05	8.0E-01	0.10	6.4E-01	-0.19	3.4E-01	-0.76	6.7E-06
ENSCAFG0000001731	TPM3	grey	EC_M10	0.50	9.1E-03	0.47	1.5E-02	-0.68	1.2E-04	-0.31	1.3E-01	0.29	1.5E-01	0.04	8.5E-01	-0.35	7.9E-02	0.12	5.7E-01	-0.16	4.3E-01	-0.57	2.5E-03
ENSCAFG0000003252	ENSCAFG0000003252	grey	EC_M10	0.50	9.1E-03	0.42	3.3E-02	-0.59	1.6E-03	0.12	5.7E-01	0.07	7.5E-01	0.10	6.1E-01	-0.16	4.4E-01	-0.29	1.5E-01	-0.18	3.9E-01	-0.34	9.1E-02
ENSCAFG0000001776	APPBP2	darkgrey	EC_M8	0.50	9.2E-03	0.33	1.0E-01	-0.07	7.5E-01	0.10	6.2E-01	-0.51	7.7E-03	-0.10	6.4E-01	-0.24	2.3E-01	-0.02	9.4E-01	-0.14	5.1E-01	0.26	1.9E-01
ENSCAFG0000000304	ZNF292	darkgrey	EC_M8	0.50	9.2E-03	0.61	9.8E-04	-0.41	3.8E-02	0.01	9.4E-01	-0.22	2.7E-01	0.04	8.3E-01	0.02	9.3E-01	0.00	9.9E-01	-0.09	6.6E-01	-0.04	8.3E-01
ENSCAFG0000001193	EYA3	grey	EC_M10	0.50	9.2E-03	-0.22	2.8E-01	-0.28	1.6E-01	0.05	8.2E-01	-0.03	8.7E-01	0.06	7.7E-01	-0.20	3.3E-01	-0.15	4.6E-01	-0.45	2.0E-02	-0.25	2.2E-01
ENSCAFG0000001142	KRAS	darkgreen	EC_M4	0.50	9.2E-03	0.32	1.1E-01	-0.70	7.9E-05	0.00	9.9E-01	0.23	2.6E-01	0.21	3.1E-01	-0.32	1.1E-01	0.09	6.7E-01	-0.06	7.6E-01	-0.55	3.6E-03
ENSCAFG0000001411	MORF4L1	darkgreen	EC_M4	0.50	9.3E-03	0.40	4.2E-02	-0.84	9.5E-08	-0.09	6.7E-01	0.43	2.8E-02	0.40	4.3E-02	-0.02	9.3E-01	-0.11	5.8E-01	-0.24	2.3E-01	-0.69	1.1E-04
ENSCAFG0000001327	E2F2	grey	EC_M10	0.50	9.3E-03	0.45	2.0E-02	-0.25	2.3E-01	-0.15	4.7E-01	-0.25	2.3E-01	-0.16	4.4E-01	-0.14	4.8E-01	-0.18	3.9E-01	-0.06	7.9E-01	0.01	9.6E-01
ENSCAFG0000001632	ENSCAFG0000001632	darkgrey	EC_M8	0.50	9.3E-03	0.25	2.1E-01	-0.41	3.9E-02	0.30	1.4E-01	-0.09	6.5E-01	0.16	4.3E-01	-0.30	1.3E-01	-0.22	2.9E-01	-0.29	1.5E-01	-0.15	4.6E-01
ENSCAFG0000000970	ENSCAFG0000000970	grey	EC_M10	0.50	9.3E-03	0.41	3.8E-02	-0.43	2.6E-02	-0.23	2.5E-01	-0.08	6.9E-01	-0.03	8.9E-01	0.04	8.5E-01	0.02	9.3E-01	-0.05	8.2E-01	-0.17	4.0E-01
ENSCAFG0000001374	BLOC1S6	grey	EC_M10	0.50	9.4E-03	0.27	1.8E-01	-0.54	4.1E-03	-0.29	1.4E-01	0.09	6.5E-01	0.01	9.7E-01	-0.40	4.5E-02	0.15	4.7E-01	-0.34	9.0E-02	-0.38	5.4E-02
ENSCAFG0000001935	PDPK1	grey	EC_M10	0.50	9.4E-03	-0.03	9.0E-01	-0.53	5.2E-03	0.00	9.9E-01	0.17	4.0E-01	0.46	1.8E-02	-0.17	4.0E-01	-0.24	2.4E-01	-0.48	1.2E-02	-0.47	1.4E-02
ENSCAFG0000000437	SYMPK	darkgrey	EC_M8	0.50	9.4E-03	0.47	1.6E-02	-0.37	6.1E-02	-0.30	1.4E-01	-0.07	7.2E-01	0.05	8.2E-01	-0.02	9.4E-01	-0.31	1.2E-01	-0.36	6.9E-02	-0.21	3.0E-01
ENSCAFG0000000359	C5	turquoise	EC_M6	0.50	9.4E-03	0.52	6.1E-03	-0.30	1.4E-01	-0.14	4.9E-01	-0.24	2.4E-01	-0.15	4.5E-01	-0.17	4.1E-01	-0.17	4.0E-01	-0.15	4.5E-01	-0.01	9.6E-01
ENSCAFG0000001991	STXBP3	darkgrey	EC_M8	0.50	9.4E-03	0.34	8.6E-02	-0.28	1.6E-01	0.10	6.2E-01	-0.29	1.5E-01	-0.09	6.7E-01	-0.40	4.2E-02	0.04	8.5E-01	-0.06	7.8E-01	0.03	8.9E-01
ENSCAFG0000003058	ZNF782	darkgrey	EC_M8	0.50	9.5E-03	0.43	2.7E-02	-0.08	6.8E-01	0.05	8.2E-01	-0.49	1.1E-02	-0.06	7.7E-01	-0.35	8.0E-02	0.00	1.0E+00	-0.06	7.5E-01	0.23	2.7E-01
ENSCAFG0000000392	COG5	grey	EC_M10	0.50	9.5E-03	0.21	3.0E-01	-0.51	7.2E-03	-0.08	6.8E-01	0.16	4.2E-01	0.01	9.4E-01	-0.55	3.9E-03	0.02	9.3E-01	-0.15	4.6E-01	-0.40	4.1E-02
ENSCAFG0000002462	ZCCHC7	darkgrey	EC_M8	0.50	9.5E-03	0.19	3.6E-01	-0.23	2.6E-01	0.12	5.6E-01	-0.31	1.3E-01	0.18	3.7E-01	-0.11	5.9E-01	0.30	1.4E-01	-0.23	2.7E-01	0.00	9.9E-01
ENSCAFG0000003259	MRPL50	grey	EC_M10	0.50	9.6E-03	-0.40	4.4E-02	-0.15	4.7E-01	0.08	7.1E-01	-0.10	6.2E-01	0.39	4.7E-02	-0.26	1.9E-01	0.06	7.8E-01	-0.08	7.1E-01	-0.15	4.6E-01
ENSCAFG0000001250	ENSCAFG0000001250	grey	EC_M10	0.50	9.6E-03	0.22	2.8E-01	-0.65	3.1E-04	0.06	7.8E-01	0.24	2.4E-01	-0.08	7.0E-01	-0.01	9.6E-01	-0.30	1.3E-01	-0.28	1.7E-01	-0.52	6.3E-03
ENSCAFG0000001314	BAZ1B	darkgrey	EC_M8	0.50	9.7E-03	0.50	1.0E-02	0.02	9.4E-01	0.13	5.3E-01	-0.65	2.9E-04	-0.01	9.8E-01	-0.27	1.8E-01	-0.02	9.1E-01	-0.28	1.7E-01	0.36	6.9E-02
ENSCAFG0000001764	CLTC	darkgreen	EC_M4	0.50	9.7E-03	0.10	6.4E-01	-0.88	3.6E-09	-0.11	6.1E-01	0.60	1.3E-03	0.28	1.7E-01	0.02	9.3E-01	-0.07	7.4E-01	-0.32	1.1E-01	-0.87	8.3E-09
ENSCAFG0000003003	ERP29	darkgrey	EC_M8	0.50	9.7E-03	-0.28	1.7E-01	-0.49	1.1E-02	0.01	9.7E-01	0.34	8.9E-02	0.30	1.4E-01	-0.27	1.8E-01	0.12	5.6E-01	-0.36	7.3E-02	-0.60	1.2E-03
ENSCAFG0000000977	TTC3	darkgreen	EC_M4	0.50	9.7E-03	0.51	7.2E-03	-0.69	9.2E-05	0.07	7.5E-01	0.16	4.3E-01	-0.06	7.8E-01	-0.23	2.7E-01	-0.18	3.9E-01	-0.04	8.5E-01	-0.42	3.1E-02
ENSCAFG0000000088	SAMM50	grey	EC_M10	0.50	9.7E-03	0.20	3.2E-01	0.25	2.2E-01	-0.02	9.1E-01	-0.71	4.4E-05	-0.15	4.6E-01	-0.33	1.0E-01	-0.23	2.7E-01	-0.50	9.1E-03	0.43	2.8E-02
ENSCAFG0000000356	CDK5RAP2	grey	EC_M10	0.50	9.7E-03	-0.33	9.9E-02	-0.35	8.4E-02	-0.15	4.7E-01	0.21	3.0E-01	0.06	7.7E-01	-0.30	1.4E-01	0.14	4.9E-01	-0.18	3.8E-01	-0.49	1.2E-02
ENSCAFG0000001794	TBC1D8B	turquoise	EC_M6	0.50	9.7E-03	0.49	1.0E-02	-0.62	7.5E-04	-0.05	7.9E-01	0.12	5.7E-01	0.06	7.7E-01	-0.04	8.5E-01	-0.05	8.2E-01	0.04	8.4E-01	-0.40	4.1E-02
ENSCAFG0000001643	ANAPC4	darkgreen	EC_M4	0.50	9.7E-03	0.01	9.5E-01	-0.70	6.7E-05	-0.05	8.1E-01	0.41	4.0E-02	0.27	1.9E-01	-0.29	1.5E-01	-0.19	3.5E-01	-0.01	9.6E-01	-0.68	1.5E-04
ENSCAFG0000001340	ENSCAFG0000001340	darkgrey	EC_M8	0.50	9.9E-03	0.12	5.7E-01	0.09	6.6E-01	0.16	4.5E-01	-0.61	9.8E-04	0.07	7.4E-01	-0.34	9.4E-02	0.03	8.7E-01	-0.01	9.5E-01	0.37	6.4E-02
ENSCAFG0000002886	ENSCAFG0000002886	turquoise	EC_M6	0.50	9.9E-03	0.58	1.7E-03	-0.34	8.5E-02	0.29	1.4E-01	-0.23	2.5E-01	0.09	6.7E-01	-0.41	3.7E-02	0.07	7.3E-01	0.02	9.2E-01	-0.05	8.0E-01
ENSCAFG0000001262	TXLNG	darkgrey	EC_M8	0.50	9.9E-03	0.06	7.7E-01	0.05	8.2E-01	0.26	2.1E-01	-0.57	2.4E-03	-0.04	8.4E-01	0.05	8.1E-01	-0.10	6.2E-01	-0.01	9.5E-01	0.31	1.2E-01
ENSCAFG0000001301	RNF168	grey	EC_M10	0.50	9.9E-03	0.32	1.1E-01	-0.62	7.5E-04	-0.04	8.6E-01	0.11	5.8E-01	0.31	1.2E-01	0.02	9.1E-01	-0.08	7.0E-01	0.19	3.5E-01	-0.40	4.4E-02
ENSCAFG0000000760	FLNB	darkgreen	EC_M4	0.50	9.9E-03	0.37	6.6E-02	-0.88	3.6E-09	-0.07	7.4E-01	0.48	1.2E-02	0.17	4.2E-01	-0.29	1.5E-01	-0.23	2.7E-01	-0.17	4.0E-01	-0.77	3.4E-06
ENSCAFG0000000400	SYPL1	darkgrey	EC_M8	0.50	1.0E-02	0.22	2.7E-01	-0.57	2.2E-03	0.07	7.2E-01	0.17	4.1E-01	0.25	2.3E-01	-0.21	3.0E-01	-0.14	5.1E-01	-0.26	2.0E-01	-0.38	5.3E-02
ENSCAFG0000001283	ENSCAFG0000001283	darkgrey	EC_M8	0.50	1.0E-02	-0.01	9.6E-01	-0.40	4.5E-02	0.07	7.4E-01	0.00	9.9E-01	0.02	9.2E-01	-0.10	6.3E-01	-0.07	7.3E-01	-0.35	8.0E-02	-0.27	1.8E-01
ENSCAFG0000000362	CASP2	darkgrey	EC_M8	0.50	1.0E-02	0.34	9.2E-02	-0.37	6.3E-02	-0.23	2.6E-01	0.02	9.1E-01	0.28	1.6E-01	-0.34	8.6E-02	0.04	8.5E-01	-0.12	5.7E-01	-0.24	2.3E-01
ENSCAFG0000003054	FEN1	darkgrey	EC_M8	0.50	1.0E-02	0.15	4.8E-01	-0.67	1.9E-04	-0.29	1.5E-01	0.42	3.4E-02	0.20	3.3E-01	-0.15	4.8E-01	0.25	2.2E-01	-0.11	6.1E-01	-0.68	1.4E-04
ENSCAFG0000001608	AKAP8	grey	EC_M10	0.50	1.0E-02	0.11	5.8E-01	-0.06	7.7E-01	-0.09	6.6E-01	-0.40	4.4E-02	0.20	3.3E-01	0.09	6.7E-01	0.13	5.2E-01	-0.19	3.6E-01	0.12	5.7E-01
ENSCAFG0000000177	ENSCAFG0000000177	grey	EC_M10	0.50	1.0E-02	0.40	4.4E-02	-0.69	8.4E-05	0.08	7.0E-01	0.25	2.2E-01	0.17	4.0E-01	-0.26	2.0E-01	0.06	7.7E-01	-0.14	4.9E-01	-0.50	8.9E-03
ENSCAFG0000000443	RB1	darkgreen	EC_M4	0.50	1.0E-02	0.26	2.0E-01	-0.76	6.1E-06	0.18	3.8E-01	0.33	9.8E-02	0.26	2.0E-01	-0.23	2.7E-01	-0.20	3.2E-01	-0.07	7.3E-01	-0.61	1.0E-03
ENSCAFG0000002935	TOR1AIP2	darkgreen	EC_M4	0.50	1.0E-02	-0.15	4.6E-01	-0.64	4.1E-04	-0.11	6.0E-01	0.36	7.3E-02	0.28	1.7E-01	-0.06	7.9E-01	0.14	5.0E-01	-0.40	4.2E-02	-0.67	2.0E-04
ENSCAFG0000000742	PJA2	darkgreen	EC_M4	0.50	1.0E-02	0.48	1.2E-02	-0.88	2.9E-09	-0.06	7.7E-01	0.38	5.4E-02	0.25	2.3E-01	-0.15	4.6E-01	-0.12	5.7E-01	-0.10	6.4E-01	-0.65	3.5E-04
ENSCAFG0000003215	ENSCAFG0000003215	grey	EC_M10	0.50	1.0E-02	0.32	1.1E-01	-0.39	4.9E-02	-0.14	5.1E-01	-0.04	8.4E-01	-0.07	7.3E-01	-0.07	7.4E-01	0.06	7.5E-01	-0.42	3.4E-02	-0.21	3.1E-01
ENSCAFG0000002437	RPP38	grey	EC_M10	0.50	1.0E-02	0.16	4.5E-01	-0.23	2.6E-01	0.11	5.9E-01	-0.22	2.7E-01	-0.11	5.8E-01	-0.12	5.5E-01	0.20	3.3E-01	-0.40	4.3E-02	-0.07	7.5E-01
ENSCAFG0000000634	ENSCAFG0000000634	grey	EC_M10	0.50	1.0E-02	0.34	8.8E-02	-0.26	2.0E-01	0.04	8.4E-01	-0.26	2.0E-01	-0.42	3.4E-02	0.04	8.3E-01	0.08	7.0E-01	-0.03	9.0E-01	0.01	9.5E-01
ENSCAFG0000003258	ENSCAFG0000003258	turquoise	EC_M6	0.50	1.0E-02	0.59	1.4E-03	-0.27	1.8E-01	0.01	9.6E-01	-0.33	9.6E-02	0.15	4.6E-01	-0.20	3.3E-01	-0.28	1.7E-01	-0.09	6.7E-01	0.08	6.8E-01
ENSCAFG0000000093	AFF4	darkgreen	EC_M4	0.49	1.0E-02	0.32	1.1E-01	-0.80	1.1E-06	-0.13	5.2E-01	0.36	7.1E-02	0.12	5.6E-01	-0.06	7.9E-01	-0.08	6.9E-01	-0.28	1.7E-01	-0.63	5.5E-04
ENSCAFG0000001770	DICER1	darkgreen	EC_M4	0.49	1.0E-02	0.34	9.1E-02	-0.84	5.8E-08	-0.15	4.6E-01	0.47	1.5E-02	0.21	3.0E-01	-0.30	1.3E-01	-0.17	4.0E-01	-0.29	1.5E-01	-0.72	3.4E-05
ENSCAFG0000000763	CTR9	grey	EC_M10	0.49	1.0E-02	-0.51	8.2E-03	-0.03	8.8E-01	0.15	4.7E-01	-0.22	2.9E-01	0.23	2.7E-01	-0.31	1.2E-01	-0.16	4.2E-01	-0.36	7.3E-02	-0.06	7.6E-01
ENSCAFG0000000865	ENSCAFG0000000865	darkgrey	EC_M8	0.49	1.0E-02	0.21	3.0E-01	-0.30	1.4E-01	0.31	1.2E-01	-0.18	3.9E-01	0.02	9.3E-01	-0.15	4.5E-01	-0.33	1.0E-01	-0.20	3.2E-01	-0.06	7.7E-01
ENSCAFG0000001623	DCP2	darkgrey	EC_M8	0.49	1.0E-02	0.59	1.6E-03	-0.37	6.6E-02	0.02	9.4E-01	-0.26	2.0E-01	-0.03	8.9E-01	-0.23	2.6E-01	-0.24	2.3E-01	-0.24	2.5E-01	-0.04	8.4E-01
ENSCAFG0000001222	RECQL	darkgreen	EC_M4	0.49	1.0E-02	0.15	4.7E-01	-0.84	6.8E-08	-0.20	3.3E-01	0.51	8.3E-03	0.34	9.2E-02	0.08	7.1E-01	-0.02	9.2E-01	-0.11	5.9E-01	-0.80	1.2E-06
ENSCAFG0000001079	SLC9B1	darkgrey	EC_M8	0.49	1.0E-02	-0.46	1.8E-02	-0.09	6.7E-01	0.11	5.8E-01	-0.13	5.3E-01	0.08	7.1E-01	-0.11	5.8E-01	0.25	2.3E-01	-0.11	6.0E-01	-0.18	3.8E-01
ENSCAFG0000003103	E2F3	turquoise	EC_M6	0.49	1.0E-02	0.33	1.0E-01	-0.57	2.3E-03	-0.25	2.1E-01	0.18	3.8E-01	0.04	8.5E-01	-0.13	5.2E-01	0.15	4.7E-01	0.02	9.1E-01	-0.42	3.1E-02
ENSCAFG0000001516	KTN1	darkgreen	EC_M4	0.49	1.0E-02	0.42	3.1E-02	-0.97	1.3E-15	-0.16	4.5E-01	0.55	3.3E-03	0.18	3.9E-01	-0.11	6.0E-01	-0.05	8.2E-01	-0.16	4.4E-01	-0.81	4.4E-07
ENSCAFG0000002332	METTL18	darkgrey	EC_M8	0.49	1.0E-02	0.58	1.9E-03	-0.21	3.1E-01	0.31	1.2E-01	-0.40	4.4E-02	-0.06	7.6E-01	-0.16	4.4E-01	-0.37	6.6E-02	-0.18	3.8E-01	0.14	4.8E-01
ENSCAFG0000001988	GPSM2	grey	EC_M10	0.49	1.0E-02	-0.10	6.3E-01	-0.31	1.3E-01	0.15	4.7E-01	0.02	9.3E-01	-0.01	9.7E-01	-0.50	9.4E-03	-0.09	6.6E-01	-0.32	1.1E-01	-0.26	2.0E-01

ENSCAFG0000000037	PPIL4	darkgrey	EC_M8	0.49	1.0E-02	0.01	9.7E-01	0.19	3.4E-01	0.27	1.9E-01	-0.71	5.0E-05	-0.01	9.5E-01	-0.05	8.1E-01	-0.04	8.6E-01	-0.03	8.7E-01	0.43	2.8E-02
ENSCAFG0000003166	ENSCAFG0000003166	grey	EC_M10	0.49	1.0E-02	0.41	3.9E-02	-0.02	9.3E-01	0.07	7.4E-01	-0.55	3.3E-03	-0.08	7.1E-01	-0.10	6.4E-01	-0.34	8.9E-02	-0.32	1.1E-01	0.30	1.4E-01
ENSCAFG0000001138	ZBTB41	darkgrey	EC_M8	0.49	1.0E-02	0.04	8.4E-01	-0.47	1.4E-02	0.20	3.3E-01	0.02	9.3E-01	0.07	7.2E-01	-0.14	5.1E-01	-0.21	3.0E-01	-0.27	1.7E-01	-0.32	1.1E-01
ENSCAFG0000000926	SLC38A2	darkgrey	EC_M8	0.49	1.1E-02	0.27	1.9E-01	-0.08	7.1E-01	0.40	4.3E-02	-0.48	1.2E-02	0.01	9.7E-01	-0.22	2.8E-01	-0.33	1.0E-01	-0.40	4.4E-02	0.21	3.1E-01
ENSCAFG0000001399	NKAPD1	grey	EC_M10	0.49	1.1E-02	-0.03	8.7E-01	-0.25	2.2E-01	0.12	5.6E-01	-0.19	3.6E-01	0.19	3.4E-01	-0.54	4.7E-03	0.11	6.1E-01	-0.11	6.1E-01	-0.10	6.3E-01
ENSCAFG0000000811	CCDC66	darkgrey	EC_M8	0.49	1.1E-02	0.13	5.4E-01	-0.52	6.0E-03	-0.14	4.9E-01	0.07	7.4E-01	0.11	6.0E-01	-0.07	7.3E-01	0.10	6.2E-01	-0.18	3.9E-01	-0.33	9.8E-02
ENSCAFG0000003182	ENSCAFG0000003182	darkgrey	EC_M8	0.49	1.1E-02	0.13	5.1E-01	-0.63	5.2E-04	0.03	9.0E-01	0.35	7.9E-02	0.14	4.9E-01	-0.37	6.4E-02	-0.09	6.4E-01	0.01	9.8E-01	-0.61	9.8E-04
ENSCAFG0000001878	RRN3	grey	EC_M10	0.49	1.1E-02	-0.12	5.5E-01	-0.31	1.3E-01	-0.23	2.7E-01	0.03	8.8E-01	0.25	2.2E-01	-0.17	4.2E-01	0.10	6.3E-01	-0.04	8.5E-01	-0.23	2.5E-01
ENSCAFG0000001414	RPL10L	darkgrey	EC_M8	0.49	1.1E-02	0.06	7.8E-01	-0.40	4.4E-02	0.24	2.4E-01	-0.04	8.5E-01	0.18	3.7E-01	-0.12	5.5E-01	-0.11	6.1E-01	-0.39	4.6E-02	-0.26	2.0E-01
ENSCAFG0000003193	ENSCAFG0000003193	grey	EC_M10	0.49	1.1E-02	-0.11	5.8E-01	-0.07	7.4E-01	0.36	7.3E-02	-0.34	8.9E-02	0.25	2.1E-01	-0.17	4.0E-01	-0.32	1.1E-01	-0.09	6.6E-01	0.05	8.2E-01
ENSCAFG0000002898	ENSCAFG0000002898	grey	EC_M10	0.49	1.1E-02	0.32	1.1E-01	-0.54	4.6E-03	0.05	8.1E-01	0.08	6.8E-01	-0.04	8.6E-01	0.09	6.5E-01	0.34	9.4E-02	-0.03	8.7E-01	-0.36	6.7E-02
ENSCAFG0000003212	C1QTNF6	grey	EC_M10	0.49	1.1E-02	0.39	4.7E-02	-0.59	1.7E-03	0.06	7.7E-01	0.15	4.5E-01	0.27	1.7E-01	0.07	7.5E-01	-0.17	4.2E-01	-0.06	7.8E-01	-0.46	1.7E-02
ENSCAFG0000001113	MSANTD2	grey	EC_M10	0.49	1.1E-02	0.27	1.8E-01	-0.47	1.6E-02	-0.13	5.4E-01	0.02	9.3E-01	-0.08	7.1E-01	-0.20	3.3E-01	-0.08	7.1E-01	0.16	4.3E-01	-0.31	1.2E-01
ENSCAFG0000000490	CLNS1A	darkgrey	EC_M8	0.49	1.1E-02	-0.35	8.4E-02	-0.22	2.9E-01	-0.31	1.2E-01	0.00	9.9E-01	0.21	3.0E-01	0.06	7.6E-01	-0.05	8.1E-01	-0.11	5.8E-01	-0.31	1.2E-01
ENSCAFG0000002861	ENSCAFG0000002861	grey	EC_M10	0.49	1.1E-02	0.36	6.9E-02	-0.10	6.4E-01	0.01	9.7E-01	-0.42	3.3E-02	0.02	9.2E-01	0.11	5.9E-01	-0.32	1.1E-01	-0.48	1.4E-02	0.13	5.2E-01
ENSCAFG0000001278	BRMS1	grey	EC_M10	0.49	1.1E-02	0.11	6.0E-01	-0.20	3.2E-01	-0.02	9.1E-01	-0.28	1.6E-01	-0.08	6.8E-01	-0.31	1.3E-01	-0.13	5.2E-01	-0.29	1.5E-01	-0.01	9.7E-01
ENSCAFG0000000090	ZUP1	grey	EC_M10	0.49	1.1E-02	0.30	1.4E-01	-0.50	9.1E-03	-0.23	2.6E-01	0.00	9.8E-01	0.21	3.1E-01	-0.08	7.0E-01	-0.06	7.7E-01	-0.18	3.7E-01	-0.31	1.2E-01
ENSCAFG0000000505	ZRANB3	grey	EC_M10	0.49	1.1E-02	0.08	7.0E-01	-0.29	1.6E-01	0.07	7.3E-01	-0.14	4.9E-01	-0.06	7.8E-01	-0.40	4.2E-02	-0.01	9.6E-01	-0.32	1.1E-01	-0.15	4.7E-01
ENSCAFG0000000952	NUDT9	grey	EC_M10	0.49	1.1E-02	0.18	3.9E-01	-0.48	1.3E-02	0.06	7.6E-01	0.08	6.8E-01	0.04	8.4E-01	-0.58	1.9E-03	0.07	7.2E-01	0.08	7.0E-01	-0.30	1.3E-01
ENSCAFG0000000154	TNPO3	darkgrey	EC_M8	0.49	1.1E-02	-0.17	4.2E-01	-0.51	7.4E-03	-0.14	5.1E-01	0.34	9.1E-02	0.35	8.2E-02	-0.39	5.1E-02	-0.33	1.0E-01	-0.17	4.0E-01	-0.62	8.0E-04
ENSCAFG0000001524	TRRAP	grey	EC_M10	0.49	1.1E-02	0.36	7.3E-02	-0.26	2.0E-01	-0.13	5.2E-01	-0.23	2.5E-01	-0.12	5.7E-01	-0.11	6.0E-01	-0.09	6.7E-01	-0.31	1.2E-01	-0.08	7.2E-01
ENSCAFG0000001068	DENND5B	grey	EC_M10	0.49	1.1E-02	0.07	7.4E-01	-0.60	1.3E-03	-0.12	5.5E-01	0.23	2.5E-01	0.21	3.0E-01	-0.25	2.1E-01	-0.08	6.8E-01	-0.38	5.6E-02	-0.57	2.4E-03
ENSCAFG0000000411	FAM76B	darkgrey	EC_M8	0.49	1.1E-02	0.49	1.1E-02	0.00	9.8E-01	0.09	6.6E-01	-0.61	8.6E-04	0.03	8.9E-01	-0.15	4.7E-01	-0.17	4.1E-01	0.01	9.5E-01	0.35	8.1E-02
ENSCAFG0000000030	PEX3	grey	EC_M10	0.49	1.1E-02	0.29	1.6E-01	-0.37	6.0E-02	-0.11	5.9E-01	-0.04	8.5E-01	0.04	8.6E-01	-0.13	5.4E-01	-0.43	2.8E-02	-0.17	4.2E-01	-0.16	4.5E-01
ENSCAFG0000000170	CARNMT1	darkgrey	EC_M8	0.49	1.1E-02	-0.33	9.6E-02	-0.22	2.8E-01	0.09	6.8E-01	0.05	8.1E-01	-0.04	8.5E-01	-0.23	2.6E-01	0.05	7.9E-01	-0.08	7.0E-01	-0.32	1.1E-01
ENSCAFG0000000993	OGA	darkgrey	EC_M8	0.49	1.1E-02	0.08	6.8E-01	-0.59	1.6E-03	0.23	2.7E-01	0.22	2.7E-01	0.23	2.6E-01	-0.31	1.3E-01	0.00	9.8E-01	-0.34	8.8E-02	-0.47	1.6E-02
ENSCAFG0000000697	PLK2	turquoise	EC_M6	0.49	1.1E-02	0.85	5.5E-08	-0.47	1.6E-02	-0.10	6.2E-01	-0.15	4.5E-01	-0.05	8.0E-01	-0.18	3.7E-01	-0.01	9.4E-01	-0.14	5.1E-01	-0.12	5.5E-01
ENSCAFG0000001786	AATF	darkgrey	EC_M8	0.49	1.1E-02	-0.41	3.9E-02	-0.51	7.6E-03	-0.05	8.1E-01	0.39	4.6E-02	0.36	6.9E-02	-0.23	2.5E-01	-0.02	9.2E-01	-0.19	3.5E-01	-0.65	3.2E-04
ENSCAFG0000000695	DHX37	darkgrey	EC_M8	0.49	1.1E-02	-0.02	9.4E-01	-0.15	4.6E-01	0.05	8.1E-01	-0.12	5.5E-01	0.02	9.3E-01	-0.36	7.3E-02	-0.23	2.6E-01	-0.26	2.1E-01	-0.13	5.2E-01
ENSCAFG0000001010	BMPR1B	darkgrey	EC_M8	0.49	1.1E-02	-0.13	5.1E-01	-0.29	1.5E-01	-0.24	2.4E-01	0.04	8.6E-01	0.10	6.3E-01	0.00	1.0E+00	0.10	6.2E-01	0.18	3.8E-01	-0.25	2.2E-01
ENSCAFG0000002424	LRRC8D	turquoise	EC_M6	0.49	1.1E-02	0.78	3.1E-06	-0.36	7.4E-02	0.08	7.1E-01	-0.25	2.3E-01	-0.08	7.0E-01	-0.33	1.0E-01	-0.08	6.8E-01	0.21	3.1E-01	0.00	9.9E-01
ENSCAFG0000001621	MICAL3	turquoise	EC_M6	0.49	1.1E-02	0.74	1.6E-05	-0.49	1.2E-02	-0.21	3.0E-01	-0.13	5.3E-01	0.00	9.9E-01	-0.04	8.3E-01	-0.23	2.6E-01	0.18	3.9E-01	-0.11	5.9E-01
ENSCAFG0000002115	ENSCAFG0000002115	grey	EC_M10	0.49	1.1E-02	0.02	9.3E-01	-0.13	5.4E-01	0.09	6.7E-01	-0.27	1.9E-01	0.40	4.2E-02	-0.12	5.7E-01	-0.35	8.0E-02	-0.47	1.7E-02	-0.01	9.6E-01
ENSCAFG0000001506	ENSCAFG0000001506	darkgreen	EC_M4	0.49	1.1E-02	0.25	2.1E-01	-0.75	9.1E-06	-0.11	5.8E-01	0.41	3.5E-02	0.37	6.1E-02	-0.18	3.8E-01	0.00	9.9E-01	-0.10	6.2E-01	-0.72	2.8E-05
ENSCAFG0000001141	RPL36AL	grey	EC_M10	0.49	1.1E-02	-0.15	4.8E-01	-0.14	4.8E-01	0.29	1.6E-01	-0.23	2.6E-01	0.11	6.1E-01	-0.16	4.5E-01	-0.06	7.6E-01	-0.50	9.8E-03	-0.04	8.5E-01
ENSCAFG0000001478	USP37	grey	EC_M10	0.49	1.1E-02	0.53	5.6E-03	-0.42	3.4E-02	0.12	5.5E-01	-0.17	4.0E-01	0.11	6.0E-01	0.09	6.7E-01	0.02	9.3E-01	-0.17	4.1E-01	-0.09	6.5E-01
ENSCAFG0000000953	OIP5	darkgrey	EC_M8	0.49	1.1E-02	0.00	1.0E+00	0.04	8.5E-01	-0.01	9.5E-01	-0.33	1.0E-01	0.13	5.2E-01	-0.16	4.4E-01	-0.21	3.1E-01	-0.11	5.8E-01	0.13	5.2E-01
ENSCAFG0000000581	ENSCAFG0000000581	darkgrey	EC_M8	0.49	1.1E-02	-0.36	7.3E-02	-0.28	1.6E-01	0.06	7.6E-01	0.11	5.8E-01	0.27	1.7E-01	-0.33	9.9E-02	-0.05	8.1E-01	-0.27	1.8E-01	-0.39	5.1E-02
ENSCAFG0000001228	ENSCAFG0000001228	grey	EC_M10	0.49	1.1E-02	0.06	7.8E-01	-0.66	2.8E-04	-0.08	7.1E-01	0.30	1.3E-01	0.23	2.6E-01	0.22	2.8E-01	0.05	8.0E-01	-0.29	1.6E-01	-0.61	8.8E-04
ENSCAFG0000000704	MRPS5	darkgrey	EC_M8	0.49	1.1E-02	-0.33	1.0E-01	-0.35	8.1E-02	-0.16	4.4E-01	0.19	3.6E-01	0.18	3.9E-01	-0.08	7.0E-01	0.06	7.7E-01	-0.22	2.9E-01	-0.42	3.1E-02
ENSCAFG0000002008	ZFP1	grey	EC_M10	0.49	1.1E-02	-0.39	4.8E-02	0.10	6.3E-01	0.36	7.5E-02	-0.41	3.9E-02	0.30	1.4E-01	-0.31	1.3E-01	-0.17	4.2E-01	-0.13	5.2E-01	0.14	5.1E-01
ENSCAFG0000001814	TADA2A	grey	EC_M10	0.49	1.1E-02	-0.23	2.5E-01	0.14	5.1E-01	0.38	5.4E-02	-0.52	7.1E-03	-0.14	5.1E-01	-0.42	3.4E-02	-0.19	3.5E-01	-0.21	3.1E-01	0.23	2.7E-01
ENSCAFG0000002270	ENSCAFG0000002270	darkgrey	EC_M8	0.49	1.1E-02	0.02	9.2E-01	0.01	9.7E-01	0.25	2.2E-01	-0.51	8.3E-03	-0.05	8.1E-01	-0.28	1.6E-01	-0.11	5.8E-01	-0.30	1.4E-01	0.25	2.1E-01
ENSCAFG0000002273	ND4L	cyan	EC_M2	0.49	1.1E-02	-0.25	2.1E-01	0.20	3.3E-01	0.21	3.0E-01	-0.58	2.0E-03	0.08	7.1E-01	-0.26	2.0E-01	-0.02	9.1E-01	-0.34	9.2E-02	0.29	1.5E-01
ENSCAFG0000001699	USP3	darkgrey	EC_M8	0.49	1.1E-02	0.38	5.7E-02	-0.47	1.5E-02	0.12	5.6E-01	-0.08	7.0E-01	0.24	2.4E-01	0.00	9.9E-01	-0.41	3.8E-02	-0.33	1.0E-01	-0.21	3.0E-01
ENSCAFG0000001642	ARG2	turquoise	EC_M6	0.49	1.1E-02	0.61	1.0E-03	-0.62	6.8E-04	-0.36	7.0E-02	0.13	5.2E-01	0.28	1.7E-01	0.02	9.3E-01	-0.28	1.6E-01	-0.20	3.3E-01	-0.37	6.1E-02
ENSCAFG0000000371	SNRNP70	grey	EC_M10	0.49	1.2E-02	0.32	1.1E-01	0.08	7.1E-01	0.15	4.6E-01	-0.64	4.5E-04	-0.12	5.6E-01	-0.05	8.1E-01	0.00	1.0E+00	0.03	8.8E-01	0.35	7.9E-02
ENSCAFG0000000749	EIF2S2	darkgreen	EC_M4	0.49	1.2E-02	0.04	8.6E-01	-0.83	1.3E-07	-0.02	9.2E-01	0.58	1.8E-03	0.31	1.2E-01	-0.28	1.7E-01	0.02	9.3E-01	0.03	8.9E-01	-0.87	9.4E-09
ENSCAFG0000001655	EXD2	grey	EC_M10	0.49	1.2E-02	0.14	4.9E-01	-0.58	1.8E-03	-0.14	5.0E-01	0.22	2.7E-01	-0.25	2.2E-01	-0.26	1.9E-01	-0.19	3.4E-01	-0.18	3.7E-01	-0.52	6.4E-03
ENSCAFG0000001143	ENSCAFG0000001143	grey	EC_M10	0.49	1.2E-02	0.37	6.4E-02	-0.19	3.4E-01	0.28	1.7E-01	-0.26	1.9E-01	0.25	2.1E-01	-0.16	4.4E-01	-0.20	3.4E-01	-0.15	4.5E-01	0.02	9.2E-01
ENSCAFG0000000251	ENSCAFG0000000251	darkgrey	EC_M8	0.49	1.2E-02	0.15	4.5E-01	0.12	5.5E-01	0.15	4.7E-01	-0.62	6.9E-04	0.23	2.7E-01	-0.20	3.4E-01	-0.25	2.1E-01	-0.34	8.7E-02	0.35	8.3E-02
ENSCAFG0000000563	ENSCAFG0000000563	darkgreen	EC_M4	0.49	1.2E-02	-0.05	8.0E-01	-0.84	1.0E-07	-0.21	3.1E-01	0.62	7.6E-04	0.25	2.1E-01	-0.11	5.9E-01	-0.08	7.1E-01	-0.32	1.2E-01	-0.88	2.5E-09
ENSCAFG0000000036	TBC1D30	turquoise	EC_M6	0.49	1.2E-02	0.76	6.3E-06	-0.59	1.5E-03	-0.11	5.9E-01	0.04	8.3E-01	0.16	4.4E-01	-0.27	1.8E-01	-0.08	6.9E-01	-0.10	6.2E-01	-0.31	1.3E-01
ENSCAFG0000000684	ENSCAFG0000000684	darkgreen	EC_M4	0.49	1.2E-02	0.44	2.3E-02	-0.78	2.5E-06	0.02	9.2E-01	0.28	1.7E-01	0.11	5.9E-01	-0.20	3.3E-01	-0.07	7.4E-01	-0.13	5.4E-01	-0.53	5.7E-03
ENSCAFG0000001462	TMX1	grey	EC_M10	0.49	1.2E-02	0.39	5.1E-02	-0.40	4.3E-02	-0.03	8.7E-01	-0.01	9.7E-01	-0.21	3.0E-01	0.21	3.2E-01	-0.20	3.3E-01	-0.39	4.8E-02	-0.23	2.5E-01
ENSCAFG0000001306	PAK2	turquoise	EC_M6	0.49	1.2E-02	0.65	3.0E-04	-0.78	3.2E-06	-0.09	6.7E-01	0.24	2.4E-01	0.11	6.0E-01	-0.14	5.0E-01	-0.19	3.6E-01	0.28	1.7E-01	-0.48	1.3E-02
ENSCAFG0000001986	CDT1	darkgrey	EC_M8	0.49	1.2E-02	0.34	9.2E-02	-0.26	2.0E-01	-0.21	2.9E-01	-0.06	7.7E-01	0.07	7.4E-01	-0.11	5.8E-01	-0.09	6.7E-01	-0.17	4.2E-01	-0.18	3.8E-01
ENSCAFG0000001231	TFAM	grey	EC_M10	0.49	1.2E-02	0.51	8.4E-03	-0.25	2.3E-01	0.02	9.2E-01	-0.33	1.0E-01	0.02	9.2E-01	-0.16	4.2E-01	0.14	5.1E-01	0.04	8.4E-01	0.06	7.6E-01
ENSCAFG0000001014	SLC39A10	darkgreen	EC_M4	0.49	1.2E-02	0.31	1.2E-01	-0.74	1.3E-05	-0.28	1.6E-01	0.34	9.4E-02	-0.03	9.0E-01	-0.15	4.6E-01	-0.03	8.8E-01	0.08	7.0E-01	-0.57	2.2E-03
ENSCAFG0000000280	HMGN3	grey	EC_M10	0.49	1.2E-02	0.15	4.6E-01	-0.05	8.1E-01	-0.10	6.4E-01	-0.37	6.5E-02	-0.37	6.3E-02	-0.40	4.0E-02	0.10	6.2E-01	-0.14	5.0E-01	0.09	6.6E-01
ENSCAFG0000003049	ENSCAFG0000003049	grey	EC_M10	0.49	1.2E-02	0.24	2.5E-01	-0.67	1.7E-04	-0.09	6.6E-01	0.32	1.1E-01	0.42	3.2E-02	-0.04	8.6E-01	-0.02	9.4E-01	-0.17	4.0E-01	-0.57	2.4E-03
ENSCAFG0000000121	SLC35D2	darkgrey	EC_M8	0.49	1.2E-02	0.07	7.5E-01	-0.07	7.5E-01	-0.06	7.9E-01	-0.28	1.6E-01	0.00	9.9E-01	-0.40	4.5E-02	-0.21	3.1E-01	-0.33	1.0E-01	0.05	8.1E-01
ENSCAFG0000002694	ENSCAFG0000002694	darkgrey	EC_M8	0.49	1.2E-02	-0.05	8.2E-01	-0.55	3.3E-03	0.20	3.4E-01	0.32	1.1E-01	0.15	4.7E-01	-0.21	3.1E-01	-0.19	3.4E-01	-0.21	3.0E-01	-0.60	1.1E-03
ENSCAFG0000000011	CS	grey	EC_M10	0.49	1.2E-02	-0.40	4.2E-02	0.27	1.8E-01	0.19	3.5E-01	-0.51	7.6E-03	-0.01	9.5E-01	-0.44	2.4E-02	-0.05	8.1E-01	-0.23	2.5E-01	0.24	2.4E-01
ENSCAFG0000002887	RAB6A	darkgreen	EC_M4	0.48	1.2E-02	0.58	2.0E-03	-0.91	1.6E-10	-0.10	6.4E-01	0.41	3.8E-02	0.21	3.0E-01	-0.13	5.4E-01	0.04	8.6E-01	-0.04	8.3E-01	-0.69	9.4E-05
ENSCAFG0000000337	CPSF3	grey	EC_M10	0.48	1.2E-02	-0.22	2.9E-01	-0.23	2.6E-01	0.19	3.6E-01	-0.08	7.1E-01	-0.01	9.7E-01	0.04	8.4E-01	-0.16	4.3E-01	-0.22	2.7E-01	-0.17	4.1E-01
ENSCAFG0000001843	ENSCAFG0000001843	grey	EC_M10	0.48	1.2E-02	-0.24	2.4E-01	-0.20	3.4E-01	0.26	2.0E-01	-0.15	4.7E-01	0.13	5.4E-01	0.06	7.5E-01	-0.15	4.7E-01	-0.12	5.5E-01	-0.13	5.1E-01
ENSCAFG0000001901	PMM2	grey	EC_M10	0.48	1.2E-02	0.37	6.0E-02	-0.42	3.2E-02	-0.17	4.0E-01	0.01	9.5E-01	-0.09	6.6E-01	-0.17	3.9E-01	0.09	6.8E-01	-0.19	3.6E-01	-0.31	1.2E-01
ENSCAFG0000000640	CD36	turquoise	EC_M6	0.48	1.2E-02	0.43	2.9E-02	-0.44	2.6E-02	-0.19	3.5E-01	-0.06	7.9E-01	0.50	8.8E-03	-0.17	4.0E-01	0.19	3.5E-01	-0.11	6.1E-01	-0.18	3.7E-01
ENSCAFG0000000414	MLLT10	darkgrey	EC_M8	0.48	1.2E-02	0.41	3.6E-02	0.06	7.7E-01	0.23	2.5E-01	-0.67	1.8E-04	-0.17	4.0E-01	-0.27	1.8E-01	-0.08	7.1E-01	0.08	7.1E-01	0.42	3.4E-02
ENSCAFG0000000845	CCNJ	darkgreen	EC_M4	0.48	1.2E-02	0.61	8.5E-04	-0.79	1.5E-06	-0.02	9.3E-01	0.25	2.2E-01	0.24	2.4E-01	-0.08	7.0E-01	-0.04	8.6E-01	-0.21	3.1E-01	-0.53	5.0E-03
ENSCAFG0000001824	ESCO1	darkgrey	EC_M8	0.48	1.2E-02	0.69	9.6E-05	-0.36	7.0E-02	-0.01	9.7E-01	-0.28	1.7E-01	-0.01	9.6E-01	0.00	9.8E-01	-0.22	2.7E-01	-0.09	6.7E-01	0.03	8.7E-01
ENSCAFG0000000281	ELP1	grey	EC_M10	0.48	1.2E-02	-0.44	2.4E-02	-0.48	1.3E-02	-0.13	5.2E-01	0.35	7.7E-02	0.09	6.8E-01	-0.29	1.5E-01	0.11	6.1E-01	-0.13	5.3E-01	-0.62	7.1E-04
ENSCAFG0000001078	ENSCAFG0000001078	grey	EC_M10	0.48	1.2E-02	-0.07	7.5E-01	-0.28	1.7E-01	0.27	1.8E-01	-0.05	8.0E-01	0.01	9.5E-01	-0.27	1.9E-01	-0.22	2.8E-01	0.06	7.6E-01	-0.21	3.0E-01
ENSCAFG0000002025	ODF2L	grey	EC_M10	0.48	1.2E-02	-0.15	4.6E-01	-0.41	3.7E-02	-0.07	7.4E-01	0.12	5.5E-01	-0.03	9.0E-01	-0.45	2.1E-02	-0.14	5.0E-01	-0.03	8.7E-01	-0.39	5.0E-02
ENSCAFG0000003239	CCDC117	darkgrey	EC_M8	0.48	1.2E-02	0.51	7.4E-03	-0.09	6.8E-01	0.08	7.1E-01	-0.53	5.3E-03	0.04	8.4E-01	-0.08	7.1E-01	-0.32	1.1E-01	-0.40	4.3E-02	0.26	2.0E-01
ENSCAFG0000002988	SLC25A36	darkgrey	EC_M8	0.48	1.2E-02	0.35	7.8E-02	-0.40	4.3E-02	0.02	9.1E-01	-0.16	4.3E-01	0.10	6.3E-01	-0.08	7.1E-01	0.00	1.0E+00	-0.32	1.2E-01	-0.15	4.7E-01
ENSCAFG0000000556	EPC2	darkgrey	EC_M8	0.48	1.2E-02	0.10	6.2E-01	0.17	4.0E-01	0.21	3.1E-01	-0.71	4.9E-05	0.16	4.4E-01	-0.21	3.0E-01	-0.25	2.1E-01	-0.15	4.6E-01	0.46	1.8E-02
ENSCAFG0000001728	TIPIN	darkgrey	EC_M8	0.48	1.2E-02	0.08	6.9E-01	-0.58	1.8E-03	-0.40	4.5E-02	0.24	2.4E-01	0.23	2.7E-01	-0.31	1.2E-01	0.24	2.3E-01	0.01	9.8E-01	-0.53	5.5E-03
ENSCAFG0000002813	ENSCAFG0000002813	grey	EC_M10	0.48	1.2E-02	0.05	8.1E-01	-0.03	9.0E-01	0.31	1.2E-01	-0.42	3.5E-02	0.40	4.3E-02	-0.05	8.0E-01	-0.29	1.5E-01	-0.37	6.3E-02	0.14	5.0E-01
ENSCAFG0000000378	BTF3L4	darkgrey	EC_M8	0.48	1.2E-02	0.29	1.5E-01	-0.34	9.1E-02	0.21	3.1E-01	-0.16	4.3E-01	0.24	2.3E-01	0.11	5.9E-01	-0.18	3.7E-01	-0.26	1.9E-01	-0.11	5.9E-01
ENSCAFG0000001038	POP5	grey	EC_M10	0.48	1.2E-02	-0.29	1.5E-01	0.06	7.6E-01	0.34	8.5E-02	-0.31	1.2E-01	-0.04	8.4E-01	-0.23	2.7E-01	0.11	6.0E-01	-0.15	4.6E-01	0.08	6.8E-01
ENSCAFG0000001111	COPS7B	darkgrey	EC_M8	0.48	1.2E-02	0.33	1.0E-01	-0.40	4.4E-02	-0.18	3.9E-01	0.01	9.7E-01	0.02	9.1E-01	-0.16	4.4E-01	-0.25	2.2E-01	-0.19	3.5E-01	-0.25	2.3E-01
ENSCAFG0000002169	ENSCAFG0000002169	grey	EC_M10	0.48	1.2E-02	0.29	1.5E-01	0.07	7.2E-01	0.15	4.6E-01	-0.51	8.0E-03	-0.22	2.9E-01	-0.25	2.2E-01	-0.17	4.2E-01	-0.23	2.5E-01	0.24	2.4E-01
ENSCAFG0000002039	ENSCAFG0000002039	grey	EC_M10	0.48	1.2E-02	0.24	2.3E-01	-0.42	3.1E-02	0.25	2.2E-01	0.02	9.3E-01	0.32	1.2E-01	-0.21	2.9E-01	-0.27	1.8E-01	0.03	8.9E-01	-0.25	2.2E-01
ENSCAFG0000000142	COPG2	grey	EC_M10	0.48	1.3E-02	-0.10	6.4E-01	-0.06	7.8E-01	0.22	2.9E-01	-0.29	1.5E-01	-0.41	3.9E-02	-0.24	2.4E-01	0.00	9.9E-01	-0.15	4.7E-01	0.03	8.8E-01
ENSCAFG0000001984	ANKRD11	darkgreen	EC_M4	0.48	1.3E-02	0.33	1.0E-01	-0.67	1.7E-04	0.00	9.9E-01	0.23	2.5E-01	0.31	1.2E-01	-0.11	5.8E-01	-0.16	4.2E-01	-0.39	5.0E-02	-0.54	4.2E-03
ENSCAFG0000001073	KPNA6	darkgreen	EC_M4	0.48	1.3E-02	0.42	3.2E-02	-0.77	4.8E-06	-0.20	3.2E-01	0.36	7.4E-02	0.24	2.5E-01	-0.24	2.3E-01	-0.11	5.9E-01	-0.31	1.3E-01	-0.61	1.0E-03
ENSCAFG0000000476	SLC25A19	grey	EC_M10	0.48	1.3E-02	0.01	9.7E-01	-0.57	2.3E-03	-0.19	3.5E-01	0.38	5.5E-02	0.24	2.4E-01	-0.16	4.2E-01	-0.10	6.3E-01	-0.09	6.7E-01	-0.61	9.4E-04
ENSCAFG0000001568	USP42	turquoise	EC_M6	0.48	1.3E-02	0.43	2.7E-02	-0.05	8.1E-01	0.01	9.7E-01	-0.47	1.5E-02	-0.14	5.0E-01	0.01	9.7E-01	-0.13	5.4E-01	-0.29	1.5E-01	0.21	3.1E-01
ENSCAFG0000000700	ENSCAFG0000000700	grey	EC_M10	0.48	1.3E-02	-0.07	7.3E-01	-0.21	3.1E-01	-0.16	4.4E-01	-0.14	5.0E-01	-0.19	3.6E-01	-0.18	3.8E-01	-0.14	4.8E-01	-0.04	8.4E-01	-0.11	6.1E-01
ENSCAFG0000000224	SRP72	darkgreen	EC_M4	0.48	1.3E-02	-0.18	3.7E-01	-0.78	3.0E-06	-0.06	7.7E-01	0.59	1.4E-03	0.39	5.1E-02	-0.23	2.5E-01	-0.01	9.7E-01	-0.28	1.6E-01	-0.88	3.1E-09
ENSCAFG0000001710	ENSCAFG0000001710	darkgrey	EC_M8	0.48	1.3E-02	0.71	5.2E-05	-0.10	6.4E-01	0.10	6.3E-01	-0.57	2.5E-03	-0.18	3.7E-01	-0.05	8.0E-01	-0.28	1.6E-01	-0.17	4.1E-01	0.33	9.9E-02
ENSCAFG0000003178	ENSCAFG0000003178	grey	EC_M10	0.48	1.3E-02	0.13	5.4E-01	-0.48	1.3E-02	-0.08	7.0E-01	0.15	4.5E-01	0.63	5.5E-04	-0.12	5.5E-01	-0.10	6.2E-01	-0.16	4.2E-01	-0.41	3.8E-02
ENSCAFG0000003227	CINP	darkgrey	EC_M8	0.48	1.3E-02	-0.39	4.7E-02	0.03	8.8E-01	0.00	9.9E-01	-0.25	2.3E-01	0.23	2.6E-01	-0.05	8.0E-01	-0.19	3.5E-01	-0.16	4.3E-01	-0.05	8.3E-01
ENSCAFG0000001000	PSMG1	darkgreen	EC_M4	0.48	1.3E-02	0.03	8.9E-01	-0.71	5.3E-05	-0.03	8.8E-01	0.49	1.1E-02	0.22	2.9E-01	-0.30	1.3E-01	-0.07	7.2E-01	-0.35	7.8E-02	-0.75	9.6E-06
ENSCAFG0000000878	RSPRY1	turquoise	EC_M6	0.48	1.3E-02	0.50	8.6E-03	-0.49	1.1E-02	0.10	6.4E-01	0.04	8.5E-01	-0.05	8.2E-01	-0.49	1.2E-02	-0.16	4.4E-01	-0.03	8.8E-01	-0.27	1.9E-01
ENSCAFG0000002781	ENSCAFG0000002781	darkgrey	EC_M8	0.48	1.3E-02	0.03	8.8E-01	0.06	7.8E-01	0.19	3.5E-01	-0.53	5.2E-03	-0.07	7.4E-01	-0.32	1.1E-01	-0.04	8.3E-01	-0.31	1.3E-01	0.25	2.3E-01
ENSCAFG0000001081	PPP6R3	darkgreen	EC_M4	0.48	1.3E-02	0.51	7.2E-03	-0.75	9.0E-06	-0.17	4.2E-01	0.28	1.7E-01	0.13	5.2E-01	-0.20	3.4E-01	-0.01	9.7E-01	0.05	8.2E-01	-0.51	7.5E-03
ENSCAFG0000000044	CNOT2	grey	EC_M10	0.48	1.3E-02	0.14	5.0E-01	-0.40	4.1E-02	0.06	7.8E-01	0.02	9.0E-01	0.23	2.7E-01	-0.42	3.2E-02	-0.29	1.5E-01	-0.50	8.5E-03	-0.33	1.0E-01
ENSCAFG0000001908	FMR1	darkgrey	EC_M8	0.48	1.3E-02	0.14	5.0E-01	0.10	6.3E-01	0.41	3.9E-02	-0.62	7.3E-04	-0.06	7.6E-01	-0.24	2.4E-01	-0.17	4.0E-01	-0.34	8.9E-02	0.36	7.5E-02
ENSCAFG0000000054	FBXO43	darkgrey	EC_M8	0.48	1.3E-02	0.03	8.9E-01	-0.22	2.7E-01	-0.11	5.8E-01	-0.09	6.5E-01	-0.13	5.2E-01	-0.11	6.0E-01	-0.10	6.3E-01	-0.16	4.2E-01	-0.13	5.3E-01
ENSCAFG0000000412	RUVBL1	grey	EC_M10	0.48	1.3E-02	-0.44	2.4E-02	-0.40	4.1E-02	-0.16	4.3E-01	0.32	1.1E-01	0.18	3.7E-01	-0.27	1.7E-01	-0.02	9.4E-01	-0.07	7.3E-01	-0.58	2.1E-03
ENSCAFG0000000392	ATAD2B	darkgrey	EC_M8	0.48	1.3E-02	0.50	9.7E-03	0.09	6.6E-01	0.08	7.0E-01	-0.70	7.4E-05	0.03	9.0E-01	-0.37	6.5E-02	-0.21	2.9E-01	-0.06	7.6E-01	0.45	2.0E-02
ENSCAFG0000000922	ENSCAFG0000000922	darkgrey	EC_M8	0.48	1.3E-02	0.15	4.5E-01	-0.40	4.5E-02	0.21	3.0E-01	-0.06	7.6E-01	0.26	1.9E-01	-0.06	7.7E-01	-0.04	8.3E-01	-0.28	1.7E-01	-0.18	3.8E-01
ENSCAFG0000000119	AGO2	grey	EC_M10	0.48	1.3E-02	0.08	7.1E-01	-0.65	2.9E-04	0.01	9.8E-01	0.34	8.6E-02	0.19	3.5E-01	0.01	9.8E-01	-0.50	8.8E-03	-0.21	3.1E-01	-0.62	7.3E-04
ENSCAFG0000000480	GOLGA4	grey	EC_M10	0.48	1.3E-02	0.47	1.5E-02	-0.62	6.9E-04	0.00	9.8E-01	0.06	7.7E-01	0.04	8.6E-01	-0.01	9.4E-01	-0.22	2.8E-01	-0.14	5.0E-01	-0.33	9.9E-02
ENSCAFG0000000340	CAV2	turquoise	EC_M6	0.48	1.3E-02	0.71	4.3E-05	-0.63	5.4E-04	-0.05	7.9E-01	0.11	5.9E-01	-0.11	5.9E-01	-0.20	3.3E-01	0.15	4.5E-01	-0.11	5.8E-01	-0.35	7.7E-02
ENSCAFG0000003148	RWDD1	grey	EC_M10	0.48	1.3E-02	-0.28	1.6E-01	-0.25	2.2E-01	0.32	1.1E-01	-0.01	9.6E-01	0.14	5.0E-01	0.09	6.6E-01	-0.01	9.6E-01	-0.38	5.3E-02	-0.24	2.4E-01
ENSCAFG0000002483	ENSCAFG0000002483	grey	EC_M10	0.48	1.3E-02	-0.10	6.1E-01	-0.45	2.0E-02	0.22	2.8E-01	0.12	5.4E-01	0.09	6.7E-01	0.09	6.8E-01	-0.30	1.3E-01	-0.01	9.6E-01	-0.41	3.9E-02
ENSCAFG0000001878	RPL23A	cyan	EC_M2	0.48	1.3E-02	-0.25	2.2E-01	0.24	2.5E-01	0.07	7.2E-01	-0.57	2.3E-03	0.03	9.0E-01	0.06	7.7E-01	-0.05	8.0E-01	-0.38	5.4E-02	0.26	2.0E-01
ENSCAFG0000001349	RNF2	turquoise	EC_M6	0.48	1.3E-02	0.65	2.9E-04	-0.60	1.1E-03	-0.13	5.4E-01	0.04	8.6E-01	-0.08	7.1E-01	-0.19	3.6E-01	0.03	8.8E-01	0.36	7.3E-02	-0.27	1.8E-01
ENSCAFG0000000969	CHD9	darkgrey	EC_M8	0.48	1.3E-02	0.35	8.1E-02	-0.14	5.0E-01	0.17	4.0E-01	-0.45	2.2E-02	0.04	8.5E-01	-0.15	4.7E-01	-0.19	3.5E-01	-0.12	5.6E-01	0.16	4.2E-01
ENSCAFG0000002383	GPRIN1	darkgrey	EC_M8	0.48	1.4E-02	0.31	1.2E-01	-0.26	2.0E-01	-0.20	3.3E-01	-0.15	4.8E-01	-0.05	8.1E-01	-0.27	1.8E-01	-0.03	8.8E-01	-0.24	2.4E-01	-0.10	6.2E-01
ENSCAFG0000001125	INTS13	darkgrey	EC_M8	0.48	1.4E-02	-0.30	1.3E-01	-0.66	2.2E-04	-0.02	9.2E-01	0.51	8.2E-03	0.39	5.2E-02	-0.26	2.1E-01	0.15	4.6E-01	-0.18	3.8E-01	-0.80	9.0E-07
ENSCAFG0000002476	ENSCAFG0000002476	darkgrey	EC_M8	0.48	1.4E-02	0.11	5.9E-01	-0.61	8.5E-04	0.04	8.3E-01	0.21	2.9E-01	0.05	8.0E-01	-0.24	2.3E-01	0.01	9.6E-01	-0.13	5.4E-01	-0.45	2.1E-02

ENSCAFG0000000093	CEP85L	grey	EC_M1C	0.48	1.4E-02	0.63	6.4E-04	-0.28	1.7E-01	-0.10	6.4E-01	-0.30	1.3E-01	0.10	6.4E-01	-0.25	2.1E-01	-0.04	8.3E-01	-0.19	3.5E-01	0.07	7.2E-01
ENSCAFG0000000761	ST8SIA4	darkgreen	EC_M4	0.48	1.4E-02	0.61	8.6E-04	-0.83	1.3E-07	-0.06	7.7E-01	0.37	5.9E-02	0.02	9.3E-01	-0.21	3.1E-01	-0.02	9.4E-01	-0.11	5.9E-01	-0.64	4.1E-04
ENSCAFG0000000363	ZNF330	grey	EC_M1C	0.48	1.4E-02	-0.17	4.0E-01	-0.52	6.2E-03	0.17	3.9E-01	0.22	2.8E-01	0.20	3.4E-01	0.16	4.3E-01	-0.03	8.9E-01	-0.42	3.2E-02	-0.48	1.3E-02
ENSCAFG0000001791	ZNF397	grey	EC_M1C	0.48	1.4E-02	0.28	1.7E-01	-0.15	4.6E-01	0.37	6.1E-02	-0.34	8.5E-02	-0.28	1.7E-01	-0.11	6.1E-01	-0.01	9.8E-01	-0.05	8.2E-01	0.12	5.6E-01
ENSCAFG0000001722	ENSCAFG0000001722	grey	EC_M1C	0.48	1.4E-02	-0.07	7.5E-01	0.18	3.7E-01	0.08	7.1E-01	-0.57	2.2E-03	0.12	5.6E-01	-0.28	1.7E-01	0.03	8.7E-01	-0.10	6.2E-01	0.31	1.2E-01
ENSCAFG0000000984	TTF2	grey	EC_M1C	0.48	1.4E-02	0.05	7.9E-01	-0.14	4.9E-01	-0.02	9.2E-01	-0.16	4.4E-01	-0.13	5.1E-01	-0.17	4.2E-01	-0.22	2.7E-01	-0.63	5.2E-04	-0.06	7.7E-01
ENSCAFG0000000521	KIZ	grey	EC_M1C	0.48	1.4E-02	-0.17	4.2E-01	0.09	6.7E-01	0.24	2.3E-01	-0.47	1.7E-02	0.11	5.9E-01	-0.46	1.7E-02	0.03	8.9E-01	0.18	3.8E-01	0.19	3.5E-01
ENSCAFG0000001554	C1QBP	grey	EC_M1C	0.48	1.4E-02	-0.38	5.8E-02	-0.23	2.6E-01	-0.10	6.2E-01	0.10	6.4E-01	0.02	9.1E-01	-0.31	1.3E-01	0.15	4.5E-01	0.15	4.8E-01	-0.33	9.8E-02
ENSCAFG0000001884	PRELID3A	grey	EC_M1C	0.48	1.4E-02	-0.35	8.0E-02	-0.24	2.4E-01	-0.28	1.7E-01	-0.01	9.5E-01	0.17	4.1E-01	-0.03	8.9E-01	0.06	7.7E-01	-0.03	8.8E-01	-0.28	1.6E-01
ENSCAFG0000001555	HAUS8	grey	EC_M1C	0.48	1.4E-02	0.33	9.7E-02	-0.61	9.8E-04	0.14	4.9E-01	0.22	2.9E-01	0.15	4.6E-01	-0.25	2.1E-01	0.18	3.9E-01	0.07	7.5E-01	-0.50	9.2E-03
ENSCAFG0000002601	ENSCAFG0000002601	grey	EC_M1C	0.48	1.4E-02	0.25	2.3E-01	-0.10	6.4E-01	-0.03	8.9E-01	-0.34	8.6E-02	0.00	9.9E-01	0.12	5.5E-01	-0.26	2.0E-01	0.07	7.3E-01	0.11	5.9E-01
ENSCAFG0000000602	CEP290	darkgrey	EC_M8	0.48	1.4E-02	0.55	3.7E-03	-0.06	7.6E-01	0.17	4.1E-01	-0.55	3.7E-03	-0.17	4.0E-01	0.03	9.0E-01	-0.10	6.3E-01	-0.15	4.6E-01	0.31	1.2E-01
ENSCAFG0000002014	ENSCAFG0000002014	grey	EC_M1C	0.48	1.4E-02	0.23	2.5E-01	-0.68	1.1E-04	0.23	2.5E-01	0.30	1.4E-01	0.24	2.4E-01	-0.30	1.4E-01	-0.01	9.7E-01	0.00	9.8E-01	-0.54	4.6E-03
ENSCAFG0000001671	SFXN1	darkgreen	EC_M4	0.48	1.4E-02	0.00	9.9E-01	-0.80	1.0E-06	-0.18	3.9E-01	0.55	3.4E-03	0.32	1.1E-01	-0.09	6.6E-01	-0.04	8.4E-01	-0.41	3.9E-02	-0.84	9.3E-08
ENSCAFG0000001523	EXOC5	darkgreen	EC_M4	0.48	1.4E-02	0.40	4.5E-02	-0.92	4.1E-11	-0.12	5.7E-01	0.51	8.3E-03	0.13	5.2E-01	-0.24	2.5E-01	0.02	9.3E-01	-0.08	6.9E-01	-0.79	1.5E-06
ENSCAFG0000003076	IKZF5	darkgrey	EC_M8	0.48	1.4E-02	0.45	2.0E-02	-0.27	1.9E-01	0.09	6.7E-01	-0.31	1.3E-01	0.28	1.7E-01	-0.11	5.9E-01	-0.03	9.0E-01	-0.21	2.9E-01	0.04	8.5E-01
ENSCAFG0000001707	TTLL5	turquoise	EC_M6	0.48	1.4E-02	0.41	3.6E-02	-0.43	2.7E-02	-0.15	4.8E-01	-0.02	9.0E-01	-0.01	9.7E-01	-0.44	2.5E-02	-0.11	5.8E-01	-0.26	2.0E-01	-0.25	2.2E-01
ENSCAFG0000001901	CPD	grey	EC_M1C	0.48	1.4E-02	-0.32	1.1E-01	-0.18	3.8E-01	0.27	1.8E-01	-0.09	6.7E-01	-0.02	9.4E-01	-0.16	4.3E-01	-0.06	7.7E-01	-0.58	2.1E-03	-0.17	4.0E-01
ENSCAFG0000000979	GXYLT1	darkgrey	EC_M8	0.48	1.4E-02	0.05	8.2E-01	-0.60	1.1E-03	-0.43	3.0E-02	0.33	9.5E-02	0.03	8.7E-01	-0.46	1.9E-02	-0.10	6.4E-01	0.05	8.0E-01	-0.55	3.6E-03
ENSCAFG0000001570	FH	darkgrey	EC_M8	0.48	1.4E-02	-0.55	3.6E-03	-0.06	7.8E-01	-0.03	8.8E-01	-0.06	7.7E-01	0.09	6.6E-01	-0.29	1.5E-01	0.10	6.2E-01	-0.23	2.6E-01	-0.18	3.7E-01
ENSCAFG0000003189	TENT5A	grey	EC_M1C	0.48	1.4E-02	-0.01	9.4E-01	0.10	6.2E-01	0.30	1.3E-01	-0.52	6.1E-03	-0.06	7.7E-01	-0.21	3.1E-01	0.04	8.3E-01	0.31	1.2E-01	0.26	2.0E-01
ENSCAFG0000000948	MTDH	darkgreen	EC_M4	0.47	1.4E-02	0.06	7.6E-01	-0.83	1.8E-07	0.09	6.7E-01	0.54	4.2E-03	0.46	1.7E-02	-0.05	8.0E-01	-0.17	4.0E-01	-0.30	1.4E-01	-0.80	1.0E-06
ENSCAFG0000001245	CCDC6	darkgreen	EC_M4	0.47	1.4E-02	0.22	2.8E-01	-0.79	2.0E-06	0.07	7.2E-01	0.45	2.1E-02	0.23	2.6E-01	-0.45	2.0E-02	-0.09	6.7E-01	-0.16	4.4E-01	-0.75	1.2E-05
ENSCAFG0000000198	RBM48	grey	EC_M1C	0.47	1.4E-02	0.27	1.9E-01	-0.45	2.0E-02	-0.15	4.8E-01	-0.01	9.6E-01	0.40	4.3E-02	-0.16	4.2E-01	-0.24	2.3E-01	-0.41	3.7E-02	-0.26	2.0E-01
ENSCAFG0000002023	SH3GLB1	grey	EC_M1C	0.47	1.4E-02	0.39	4.6E-02	-0.67	1.8E-04	0.07	7.3E-01	0.20	3.3E-01	-0.01	9.8E-01	-0.27	1.9E-01	0.03	8.9E-01	-0.14	4.9E-01	-0.48	1.2E-02
ENSCAFG0000001424	ENSCAFG0000001424	darkgreen	EC_M4	0.47	1.4E-02	-0.03	8.7E-01	-0.80	1.1E-06	-0.22	2.8E-01	0.59	1.4E-03	0.28	1.6E-01	-0.29	1.4E-01	0.03	8.7E-01	-0.01	9.7E-01	-0.82	2.4E-07
ENSCAFG0000001563	MNAT1	darkgreen	EC_M4	0.47	1.4E-02	0.08	7.1E-01	-0.66	2.2E-04	-0.11	6.0E-01	0.35	7.6E-02	0.16	4.4E-01	-0.34	9.0E-02	-0.20	3.2E-01	-0.25	2.1E-01	-0.63	5.3E-04
ENSCAFG0000001267	STRAP	darkgreen	EC_M4	0.47	1.4E-02	0.00	1.0E+0C	-0.89	1.7E-09	-0.02	9.0E-01	0.65	2.9E-04	0.25	2.2E-01	-0.20	3.3E-01	0.05	8.0E-01	-0.27	1.8E-01	-0.91	1.4E-10
ENSCAFG0000001213	ABCB10	grey	EC_M1C	0.47	1.4E-02	-0.19	3.6E-01	0.31	1.2E-01	0.01	9.6E-01	-0.66	2.7E-04	-0.08	7.2E-01	-0.16	4.4E-01	-0.22	2.9E-01	-0.14	5.1E-01	0.42	3.5E-02
ENSCAFG0000001462	ENSCAFG0000001462	darkgrey	EC_M8	0.47	1.4E-02	0.29	1.6E-01	-0.54	4.7E-03	0.22	2.9E-01	0.10	6.2E-01	0.23	2.5E-01	-0.16	4.3E-01	-0.39	5.2E-02	-0.22	2.9E-01	-0.35	8.1E-02
ENSCAFG0000000306	ENSCAFG0000000306	darkgrey	EC_M8	0.47	1.5E-02	0.13	5.3E-01	-0.13	5.3E-01	0.31	1.2E-01	-0.28	1.7E-01	0.29	1.5E-01	-0.37	6.5E-02	-0.31	1.3E-01	-0.31	1.2E-01	0.04	8.3E-01
ENSCAFG0000002330	ENSCAFG0000002330	turquoise	EC_M6	0.47	1.5E-02	0.49	1.1E-02	-0.58	2.0E-03	0.06	7.6E-01	0.13	5.2E-01	0.26	2.1E-01	-0.17	4.2E-01	-0.16	4.4E-01	-0.06	7.7E-01	-0.41	3.5E-02
ENSCAFG0000001530	SPPL2A	darkgrey	EC_M8	0.47	1.5E-02	0.48	1.4E-02	-0.53	5.3E-03	0.18	3.8E-01	0.00	1.0E+0C	-0.01	9.7E-01	-0.13	5.4E-01	-0.14	4.9E-01	-0.24	2.3E-01	-0.21	3.1E-01
ENSCAFG0000001291	HECTD1	grey	EC_M1C	0.47	1.5E-02	0.35	7.6E-02	-0.50	9.9E-03	-0.06	7.8E-01	0.05	8.2E-01	-0.11	6.1E-01	-0.23	2.6E-01	-0.19	3.4E-01	-0.42	3.1E-02	-0.28	1.6E-01
ENSCAFG0000000518	MRPL33	grey	EC_M1C	0.47	1.5E-02	0.04	8.6E-01	-0.50	8.6E-03	0.02	9.4E-01	0.17	4.0E-01	0.09	6.8E-01	-0.35	8.2E-02	-0.24	2.5E-01	-0.03	8.8E-01	-0.38	5.3E-02
ENSCAFG0000000149	ENSCAFG0000000149	grey	EC_M1C	0.47	1.5E-02	-0.12	5.5E-01	-0.30	1.4E-01	-0.16	4.3E-01	0.05	8.1E-01	0.14	4.9E-01	0.11	6.1E-01	0.15	4.5E-01	-0.22	2.9E-01	-0.32	1.1E-01
ENSCAFG0000003266	SPCS2	grey	EC_M1C	0.47	1.5E-02	-0.21	3.0E-01	-0.40	4.3E-02	0.19	3.4E-01	0.17	4.2E-01	0.41	3.7E-02	-0.10	6.3E-01	-0.07	7.3E-01	-0.45	2.2E-02	-0.47	1.5E-02
ENSCAFG0000001062	RRP1B	darkgrey	EC_M8	0.47	1.5E-02	-0.11	5.9E-01	-0.20	3.2E-01	-0.26	2.0E-01	-0.01	9.7E-01	0.00	9.9E-01	-0.22	2.9E-01	0.17	4.1E-01	0.15	4.5E-01	-0.25	2.2E-01
ENSCAFG0000001020	GCN1	darkgrey	EC_M8	0.47	1.5E-02	-0.20	3.2E-01	-0.42	3.3E-02	-0.35	7.6E-02	0.24	2.3E-01	0.10	6.2E-01	-0.08	7.1E-01	0.02	9.4E-01	-0.48	1.3E-02	-0.51	7.4E-03
ENSCAFG0000001486	ENSCAFG0000001486	grey	EC_M1C	0.47	1.5E-02	0.25	2.2E-01	-0.55	3.3E-03	-0.11	5.8E-01	0.15	4.8E-01	-0.08	6.9E-01	-0.26	2.0E-01	-0.32	1.1E-01	0.03	9.0E-01	-0.37	6.5E-02
ENSCAFG0000000776	CCDC125	darkgrey	EC_M8	0.47	1.5E-02	0.66	2.7E-04	-0.13	5.4E-01	0.00	9.9E-01	-0.49	1.1E-02	-0.04	8.3E-01	-0.23	2.7E-01	-0.31	1.2E-01	-0.25	2.2E-01	0.28	1.7E-01
ENSCAFG0000001834	TIMM44	grey	EC_M1C	0.47	1.5E-02	0.01	9.7E-01	0.22	2.7E-01	-0.07	7.4E-01	-0.58	1.8E-03	-0.18	3.7E-01	0.05	8.2E-01	-0.23	2.6E-01	-0.26	1.9E-01	0.34	9.2E-02
ENSCAFG0000002534	SPCS3	darkgreen	EC_M4	0.47	1.5E-02	0.13	5.4E-01	-0.90	5.0E-10	0.00	9.9E-01	0.60	1.1E-03	0.31	1.3E-01	-0.01	9.6E-01	-0.09	6.5E-01	-0.06	7.8E-01	-0.89	1.9E-09
ENSCAFG0000003207	TIGD7	darkgrey	EC_M8	0.47	1.5E-02	-0.57	2.5E-03	-0.08	6.9E-01	-0.05	8.1E-01	-0.04	8.4E-01	0.05	8.1E-01	-0.24	2.4E-01	0.19	3.6E-01	-0.28	1.7E-01	-0.20	3.3E-01
ENSCAFG0000000649	CDC16	darkgrey	EC_M8	0.47	1.5E-02	-0.28	1.7E-01	-0.58	2.0E-03	-0.11	6.1E-01	0.42	3.4E-02	0.42	3.2E-02	-0.26	2.0E-01	0.02	9.2E-01	0.11	5.9E-01	-0.68	1.5E-04
ENSCAFG0000001254	ATRIP	darkgrey	EC_M8	0.47	1.5E-02	0.17	3.9E-01	0.16	4.4E-01	0.13	5.4E-01	-0.60	1.2E-03	-0.06	7.7E-01	-0.24	2.4E-01	-0.27	1.8E-01	-0.61	8.3E-04	0.34	8.8E-02
ENSCAFG0000002961	ENSCAFG0000002961	grey	EC_M1C	0.47	1.5E-02	0.10	6.1E-01	-0.26	2.0E-01	0.34	8.5E-02	-0.18	3.9E-01	0.00	9.9E-01	-0.09	6.7E-01	-0.16	4.4E-01	-0.07	7.5E-01	-0.06	7.6E-01
ENSCAFG0000001291	DHX32	turquoise	EC_M6	0.47	1.5E-02	0.64	4.4E-04	-0.34	9.0E-02	-0.06	7.6E-01	-0.14	5.0E-01	-0.03	9.0E-01	0.10	6.4E-01	-0.17	4.0E-01	-0.30	1.3E-01	-0.09	6.7E-01
ENSCAFG0000000591	METTL25	grey	EC_M1C	0.47	1.5E-02	0.35	7.8E-02	-0.43	2.8E-02	-0.42	3.2E-02	-0.04	8.6E-01	0.24	2.4E-01	-0.18	3.9E-01	-0.15	4.8E-01	-0.23	2.6E-01	-0.20	3.2E-01
ENSCAFG0000002882	UBE2V2	darkgreen	EC_M4	0.47	1.5E-02	0.08	6.9E-01	-0.92	3.3E-11	-0.21	3.1E-01	0.64	4.4E-04	0.28	1.7E-01	-0.15	4.6E-01	0.07	7.3E-01	-0.14	4.9E-01	-0.90	5.5E-10
ENSCAFG0000003112	ENSCAFG0000003112	darkgrey	EC_M8	0.47	1.5E-02	-0.48	1.4E-02	-0.24	2.5E-01	-0.20	3.2E-01	0.14	4.8E-01	0.09	6.5E-01	-0.02	9.3E-01	0.07	7.5E-01	-0.33	1.0E-01	-0.39	5.0E-02
ENSCAFG0000001772	ATG2B	grey	EC_M1C	0.47	1.6E-02	-0.07	7.5E-01	0.32	1.1E-01	0.24	2.4E-01	-0.74	1.8E-05	-0.02	9.3E-01	-0.34	8.6E-02	-0.25	2.2E-01	-0.19	3.5E-01	0.51	7.3E-03
ENSCAFG0000000093	NUS1	darkgreen	EC_M4	0.47	1.6E-02	0.15	4.5E-01	-0.81	4.3E-07	-0.13	5.3E-01	0.51	8.4E-03	0.43	2.7E-02	-0.23	2.7E-01	-0.05	8.0E-01	-0.22	2.7E-01	-0.80	1.1E-06
ENSCAFG0000001760	BTBD7	grey	EC_M1C	0.47	1.6E-02	0.61	9.0E-04	-0.27	1.8E-01	-0.28	1.7E-01	-0.28	1.7E-01	0.04	8.3E-01	-0.21	3.0E-01	-0.19	3.4E-01	-0.19	3.5E-01	0.06	7.6E-01
ENSCAFG0000002007	CFDP1	darkgreen	EC_M4	0.47	1.6E-02	-0.25	2.3E-01	-0.61	9.9E-04	0.03	8.9E-01	0.38	5.5E-02	0.12	5.7E-01	-0.01	9.5E-01	0.11	6.1E-01	-0.30	1.3E-01	-0.67	1.7E-04
ENSCAFG0000001934	SRRM2	darkgrey	EC_M8	0.47	1.6E-02	0.54	4.6E-03	-0.20	3.3E-01	0.25	2.2E-01	-0.42	3.5E-02	-0.05	8.1E-01	-0.26	2.0E-01	-0.06	7.8E-01	-0.07	7.4E-01	0.15	4.6E-01
ENSCAFG0000000963	ABCG2	grey	EC_M1C	0.47	1.6E-02	0.53	5.4E-03	-0.28	1.7E-01	0.31	1.2E-01	-0.27	1.7E-01	-0.04	8.5E-01	-0.14	4.9E-01	0.07	7.3E-01	-0.13	5.1E-01	0.03	8.8E-01
ENSCAFG0000000257	MSANTD3-TMEFF1	turquoise	EC_M6	0.47	1.6E-02	0.64	3.8E-04	-0.52	6.8E-03	-0.10	6.4E-01	-0.06	7.8E-01	0.16	4.5E-01	0.00	9.9E-01	-0.20	3.2E-01	0.20	3.2E-01	-0.19	3.6E-01
ENSCAFG0000001739	COIL	darkgrey	EC_M8	0.47	1.6E-02	0.22	2.8E-01	-0.31	1.3E-01	0.34	9.4E-02	-0.17	3.9E-01	-0.08	6.9E-01	-0.17	4.0E-01	-0.26	2.0E-01	-0.38	5.3E-02	-0.07	7.3E-01
ENSCAFG0000000035	TBK1	grey	EC_M1C	0.47	1.6E-02	0.68	1.2E-04	-0.51	7.5E-03	-0.01	9.5E-01	-0.10	6.3E-01	0.11	5.8E-01	-0.11	6.1E-01	-0.04	8.3E-01	-0.33	1.0E-01	-0.15	4.7E-01
ENSCAFG0000000498	ACOX1	darkgrey	EC_M8	0.47	1.6E-02	0.58	2.1E-03	0.00	9.9E-01	0.15	4.7E-01	-0.65	3.4E-04	-0.16	4.3E-01	0.02	9.2E-01	-0.24	2.4E-01	-0.16	4.4E-01	0.39	5.0E-02
ENSCAFG0000000056	ENSCAFG0000000056	grey	EC_M1C	0.47	1.6E-02	0.15	4.5E-01	-0.29	1.5E-01	0.06	7.6E-01	-0.12	5.7E-01	0.00	9.9E-01	-0.09	6.6E-01	-0.03	8.7E-01	-0.35	8.1E-02	-0.10	6.1E-01
ENSCAFG0000000424	ARHGAP21	turquoise	EC_M6	0.47	1.6E-02	0.62	6.6E-04	-0.14	5.1E-01	0.13	5.3E-01	-0.49	1.1E-02	-0.19	3.6E-01	-0.45	2.2E-02	-0.07	7.4E-01	0.16	4.4E-01	0.26	2.0E-01
ENSCAFG0000001547	AIPL1	darkgrey	EC_M8	0.47	1.6E-02	0.38	5.3E-02	0.12	5.4E-01	-0.22	2.7E-01	-0.57	2.5E-03	-0.19	3.5E-01	-0.19	3.5E-01	-0.16	4.4E-01	-0.21	3.0E-01	0.34	8.6E-02
ENSCAFG0000001466	ENSCAFG0000001466	darkgrey	EC_M8	0.47	1.6E-02	0.31	1.2E-01	-0.63	5.2E-04	0.17	4.1E-01	0.18	3.7E-01	0.36	7.3E-02	-0.25	2.2E-01	-0.14	4.9E-01	-0.06	7.7E-01	-0.43	2.6E-02
ENSCAFG0000000630	NOC4L	grey	EC_M1C	0.47	1.6E-02	-0.08	7.1E-01	-0.45	2.1E-02	-0.17	4.0E-01	0.28	1.6E-01	0.18	3.8E-01	-0.23	2.6E-01	0.05	8.2E-01	0.06	7.7E-01	-0.53	4.9E-03
ENSCAFG0000001011	NIPSNAP2	grey	EC_M1C	0.47	1.6E-02	-0.02	9.1E-01	-0.33	1.0E-01	-0.18	3.9E-01	0.00	1.0E+0C	-0.07	7.3E-01	-0.24	2.3E-01	0.28	1.6E-01	0.06	7.8E-01	-0.22	2.8E-01
ENSCAFG0000000543	ABCC4	grey	EC_M1C	0.47	1.6E-02	0.11	5.9E-01	0.09	6.5E-01	0.02	9.2E-01	-0.51	7.2E-03	0.01	9.5E-01	-0.29	1.5E-01	-0.17	4.1E-01	-0.57	2.2E-03	0.26	1.9E-01
ENSCAFG0000000368	VPS35	darkgreen	EC_M4	0.47	1.7E-02	-0.03	9.0E-01	-0.82	2.8E-07	-0.08	7.0E-01	0.60	1.3E-03	0.24	2.3E-01	-0.33	1.0E-01	-0.15	4.5E-01	-0.29	1.4E-01	-0.83	1.5E-07
ENSCAFG0000002905	ENSCAFG0000002905	grey	EC_M1C	0.47	1.7E-02	-0.07	7.2E-01	-0.27	1.8E-01	0.20	3.3E-01	-0.05	8.0E-01	-0.17	4.1E-01	-0.35	8.3E-02	0.28	1.7E-01	0.08	6.9E-01	-0.17	4.2E-01
ENSCAFG0000001468	HTT	darkgreen	EC_M4	0.47	1.7E-02	0.22	2.9E-01	-0.75	1.3E-05	-0.28	1.6E-01	0.46	1.8E-02	0.12	5.5E-01	-0.12	5.5E-01	-0.07	7.4E-01	0.00	1.0E+0C	-0.72	3.8E-05
ENSCAFG0000001785	ENSCAFG0000001785	grey	EC_M1C	0.47	1.7E-02	-0.11	6.0E-01	-0.30	1.3E-01	0.38	5.6E-02	0.02	9.1E-01	0.41	4.0E-02	0.00	1.0E+0C	-0.19	3.6E-01	-0.36	7.1E-02	-0.30	1.4E-01
ENSCAFG0000001601	ENSCAFG0000001601	turquoise	EC_M6	0.47	1.7E-02	0.59	1.5E-03	-0.46	1.9E-02	-0.12	5.7E-01	-0.02	9.3E-01	0.44	2.5E-02	-0.14	5.0E-01	-0.15	4.8E-01	-0.22	2.8E-01	-0.24	2.3E-01
ENSCAFG0000002901	NREP	grey	EC_M1C	0.47	1.7E-02	0.32	1.1E-01	-0.70	6.4E-05	-0.17	3.9E-01	0.31	1.2E-01	0.52	6.4E-03	0.01	9.5E-01	-0.04	8.5E-01	-0.21	3.0E-01	-0.59	1.7E-03
ENSCAFG0000000835	ZC2HC1A	darkgreen	EC_M4	0.47	1.7E-02	0.08	7.0E-01	-0.88	3.1E-09	-0.25	2.2E-01	0.59	1.6E-03	0.16	4.2E-01	-0.12	5.7E-01	0.11	6.0E-01	-0.08	7.2E-01	-0.83	1.2E-07
ENSCAFG0000001822	ENSCAFG0000001822	darkgrey	EC_M8	0.47	1.7E-02	0.02	9.3E-01	-0.24	2.3E-01	0.15	4.8E-01	-0.13	5.3E-01	0.03	8.8E-01	-0.21	3.1E-01	-0.51	7.1E-03	-0.43	3.0E-02	-0.10	6.4E-01
ENSCAFG0000000059	PHAX	grey	EC_M1C	0.46	1.7E-02	0.01	9.7E-01	-0.29	1.5E-01	-0.18	3.7E-01	-0.11	5.8E-01	0.02	9.2E-01	-0.17	4.0E-01	0.39	4.7E-02	0.12	5.6E-01	-0.17	4.1E-01
ENSCAFG0000000694	GPBP1	darkgrey	EC_M8	0.46	1.7E-02	0.39	5.2E-02	0.01	9.5E-01	0.10	6.3E-01	-0.59	1.4E-03	-0.19	3.4E-01	-0.03	8.7E-01	-0.23	2.5E-01	-0.23	2.6E-01	0.36	7.2E-02
ENSCAFG0000002934	ENSCAFG0000002934	grey	EC_M1C	0.46	1.7E-02	-0.06	7.8E-01	-0.25	2.2E-01	0.21	3.1E-01	-0.04	8.4E-01	0.09	6.5E-01	0.03	8.7E-01	0.22	2.9E-01	-0.07	7.4E-01	-0.21	3.0E-01
ENSCAFG0000001592	N4BP2	darkgrey	EC_M8	0.46	1.7E-02	0.38	5.3E-02	-0.11	6.0E-01	0.20	3.2E-01	-0.49	1.0E-02	0.04	8.5E-01	0.01	9.8E-01	-0.29	1.6E-01	-0.30	1.4E-01	0.20	3.2E-01
ENSCAFG0000000689	ENSCAFG0000000689	grey	EC_M1C	0.46	1.7E-02	0.14	4.8E-01	-0.21	3.0E-01	0.37	6.7E-02	-0.19	3.6E-01	0.06	7.6E-01	-0.16	4.2E-01	-0.01	9.5E-01	-0.48	1.4E-02	-0.04	8.5E-01
ENSCAFG0000001958	FAM50A	grey	EC_M1C	0.46	1.7E-02	0.14	4.9E-01	-0.34	8.5E-02	-0.10	6.3E-01	-0.03	9.0E-01	0.05	8.1E-01	-0.13	5.1E-01	0.24	2.5E-01	-0.33	1.0E-01	-0.29	1.5E-01
ENSCAFG0000003017	RFK	darkgrey	EC_M8	0.46	1.7E-02	0.35	7.7E-02	-0.70	7.5E-05	0.10	6.4E-01	0.23	2.6E-01	0.18	3.7E-01	-0.15	4.8E-01	0.27	1.9E-01	-0.22	2.9E-01	-0.48	1.3E-02
ENSCAFG0000000281	MPP6	grey	EC_M1C	0.46	1.7E-02	-0.37	6.2E-02	-0.21	3.0E-01	0.21	3.1E-01	0.01	9.5E-01	0.20	3.2E-01	-0.14	4.9E-01	0.17	3.9E-01	-0.01	9.6E-01	-0.31	1.3E-01
ENSCAFG0000001174	MCCC1	darkgrey	EC_M8	0.46	1.7E-02	-0.02	9.1E-01	0.21	3.0E-01	0.12	5.7E-01	-0.65	3.5E-04	-0.12	5.5E-01	-0.52	5.9E-03	0.06	7.8E-01	0.21	3.1E-01	0.41	3.6E-02
ENSCAFG0000000073	EBAG9	darkgrey	EC_M8	0.46	1.7E-02	0.32	1.1E-01	0.02	9.0E-01	0.06	7.5E-01	-0.55	3.6E-03	0.03	8.9E-01	-0.34	9.2E-02	0.16	4.5E-01	-0.18	3.7E-01	0.33	9.8E-02
ENSCAFG0000003188	ZNF200	darkgrey	EC_M8	0.46	1.7E-02	0.21	3.0E-01	0.01	9.6E-01	0.03	8.7E-01	-0.51	7.2E-03	-0.12	5.5E-01	-0.34	8.9E-02	-0.09	6.5E-01	-0.21	3.1E-01	0.25	2.1E-01
ENSCAFG0000000499	PPARG	turquoise	EC_M6	0.46	1.7E-02	0.87	7.7E-09	-0.44	2.3E-02	-0.06	7.7E-01	-0.16	4.4E-01	-0.07	7.4E-01	-0.18	3.7E-01	-0.19	3.6E-01	-0.25	2.2E-01	-0.10	6.3E-01
ENSCAFG0000001888	ZFR	darkgreen	EC_M4	0.46	1.7E-02	0.18	3.9E-01	-0.89	8.1E-10	-0.08	7.0E-01	0.58	1.9E-03	0.18	3.8E-01	-0.12	5.5E-01	0.11	5.9E-01	-0.11	6.0E-01	-0.81	4.1E-07
ENSCAFG0000000181	DNAJA1	grey	EC_M1C	0.46	1.7E-02	0.10	6.2E-01	-0.39	4.9E-02	-0.25	2.2E-01	0.03	8.7E-01	0.00	9.9E-01	0.13	5.2E-01	-0.02	9.2E-01	-0.50	9.9E-03	-0.28	1.7E-01
ENSCAFG0000000276	UBA6	darkgreen	EC_M4	0.46	1.7E-02	0.14	4.9E-01	-0.92	4.9E-11	-0.15	4.5E-01	0.65	3.5E-04	0.28	1.6E-01	-0.37	6.7E-02	-0.04	8.5E-01	0.01	9.8E-01	-0.89	9.7E-10
ENSCAFG0000001009	ENSCAFG0000001009	darkgrey	EC_M8	0.46	1.7E-02	0.01	9.6E-01	-0.34	8.8E-02	0.17	4.1E-01	-0.03	8.8E-01	-0.19	3.6E-01	-0.04	8.6E-01	-0.07	7.4E-01	-0.02	9.2E-01	-0.17	4.0E-01
ENSCAFG0000000063	SLC12A2	darkgreen	EC_M4	0.46	1.7E-02	0.00	9.8E-01	-0.83	1.1E-07	-0.10	6.2E-01	0.61	9.8E-04	0.24	2.4E-01	-0.17	4.0E-01	-0.08	7.0E-01	-0.32	1.1E-01	-0.86	2.0E-08
ENSCAFG0000002923	SKA2	darkgreen	EC_M4	0.46	1.8E-02	-0.04	8.6E-01	-0.74	1.7E-05	0.00	9.9E-01	0.50	9.0E-03	0.03	8.7E-01	-0.47	1.6E-02	-0.03	8.7E-01	0.03	8.8E-01	-0.73	2.7E-05
ENSCAFG0000001824	SLC26A2	grey	EC_M1C	0.46	1.8E-02	0.07	7.2E-01	-0.03	8.9E-01	-0.32	1.1E-01	-0.32	1.1E-01	0.09	6.6E-01	-0.07	7.3E-01	-0.24	2.5E-01	-0.21	3.0E-01	0.08	7.1E-01
ENSCAFG0000000536	INTS10	darkgrey	EC_M8	0.46	1.8E-02	-0.03	8.8E-01	0.31	1.2E-01	0.24	2.4E-01	-0.76	7.6E-06	-0.05	8.2E-01	-0.37	6.1E-02	-0.22	2.8E-01	-0.12	5.4E-01	0.52	6.4E-03
ENSCAFG0000001641	CDK12	darkgreen	EC_M4	0.46	1.8E-02	0.37	6.1E-02	-0.82	2.6E-07	-0.22	2.9E-01	0.45	2.0E-02	0.36	6.9E-02	-0.17	4.2E-01	0.06	7.7E-01	-0.02	9.2E-01	-0.70	6.6E-05
ENSCAFG0000002917	NFYB	grey	EC_M1C	0.46	1.8E-02	0.22	2.8E-01	-0.16	4.3E-01	0.02	9.1E-01	-0.34	8.8E-02	0.24	2.3E-01	0.02	9.1E-01	-0.27	1.8E-01	-0.11	6.0E-01	0.11	6.0E-01
ENSCAFG0000001400	TOGARAM1	grey	EC_M1C	0.46	1.8E-02	-0.06	7.7E-01	-0.16	4.4E-01	0.22	2.8E-01	-0.20	3.3E-01	-0.31	1.3E-01	-0.26	1.9E-01	-0.17	4.0E-01	-0.57	2.3E-03	-0.07	7.3E-01
ENSCAFG0000002388	ZNF227	grey	EC_M1C	0.46	1.8E-02	0.11	6.0E-01	-0.30	1.3E-01	0.30	1.3E-01	-0.13	5.3E-01	-0.07	7.2E-01	-0.50	8.9E-03	0.00	9.8E-01	0.00	9.8E-01	-0.13	5.2E-01
ENSCAFG0000001646	MAD1L1	darkgrey	EC_M8	0.46	1.8E-02	0.02	9.1E-01	0.08	7.1E-01	-0.18	3.9E-01	-0.37	6.3E-02	0.20	3.3E-01	-0.07	7.4E-01	-0.03	8.9E-01	-0.06	7.8E-01	0.17	4.1E-01
ENSCAFG0000001665	FAM81A	grey	EC_M1C	0.46	1.8E-02	-0.20	3.4E-01	0.19	3.5E-01	0.05	8.1E-01	-0.50	9.8E-03	-0.04	8.4E-01	-0.27	1.8E-01	-0.10	6.3E-01	-0.20	3.2E-01	0.26	2.0E-01
ENSCAFG0000001863	ENSCAFG0000001863	grey	EC_M1C	0.46	1.8E-02	-0.18	3.7E-01	-0.20	3.2E-01	-0.06	7.9E-01	-0.06	7.7E-01	0.09	6.8E-01	-0.15	4.7E-01	-0.26	2.0E-01	-0.27	1.9E-01	-0.24	2.4E-01
ENSCAFG0000000087	PARVG	turquoise	EC_M6	0.46	1.8E-02	0.82	3.6E-07	-0.44	2.6E-02	-0.08	7.1E-01	-0.12	5.4E-01	-0.13	5.2E-01	-0.13	5.3E-01	-0.09	6.6E-01	-0.15	4.6E-01	-0.10	6.4E-01
ENSCAFG0000000206	ADGRF1	turquoise	EC_M6	0.46	1.8E-02	0.82	3.6E-07	-0.44	2.6E-02	-0.08	7.1E-01	-0.12	5.4E-01	-0.13	5.2E-01	-0.13	5.3E-01	-0.09	6.6E-01	-0.15	4.6E-01	-0.10	6.4E-01
ENSCAFG0000001096	HCRTR1	turquoise	EC_M6	0.46	1.8E-02	0.82	3.6E-07	-0.44	2.6E-02	-0.08	7.1E-01	-0.12	5.4E-01	-0.13	5.2E-01	-0.13	5.3E-01	-0.09	6.6E-01	-0.15	4.6E-01	-0.10	6.4E-01
ENSCAFG0000000994	STMND1	darkgrey	EC_M8	0.46	1.8E-02	0.17	4.2E-01	-0.30	1.4E-01	-0.08	7.2E-01	0.02	9.2E-01	-0.08	7.1E-01	-0.05	8.2E-01	-0.05	8.2E-01	0.06	7.6E-01	-0.23	2.6E-01
ENSCAFG0000000821	USP25	darkgrey	EC_M8	0.46	1.8E-02	0.37	6.6E-02	0.10	6.4E-01	0.04	8.5E-01	-0.65	3.2E-04	-0.04	8.3E-01	-0.27	1.8E-01	-0.05	8.0E-01	-0.08	6.9E-01	0.43	2.8E-02
ENSCAFG0000000639	SNRPF	darkgrey	EC_M8	0.46	1.8E-02	-0.11	5.9E-01	-0.24	2.3E-01	-0.22	2.8E-01	0.04	8.5E-01	0.27	1.8E-01	-0.19	3.5E-01	0.02	9.2E-01	0.01	9.6E-01	-0.30	1.3E-01
ENSCAFG0000000121	PTK2	turquoise	EC_M6	0.46	1.8E-02	0.73	2.1E-05	-0.67	1.6E-04	-0.17	4.1E-01	0.16	4.3E-01	0.14	5.1E-01	-0.23	2.6E-01	-0.16	4.3E-01	-0.18	3.8E-01	-0.43	2.8E-02
ENSCAFG0000000486	USP6NL	turquoise	EC_M6	0.46	1.8E-02	0.74	1.5E-05	-0.59	1.7E-03	-0.26	1.9E-01	0.01	9.8E-01	-0.05	7.9E-01	0.00	9.8E-01	0.20	3.2E-01	0.05	8.2E-01	-0.28	1.7E-01
ENSCAFG0000001939	RER1	darkgrey	EC_M8	0.46	1.8E-02	0.14	5.1E-01	-0.74	1.3E-05	-0.14	4.8E-01	0.42	3.1E-02	0.24	2.4E-01	-0.08	7.2E-01	0.00	1.0E+0C	-0.22	2.9E-01	-0.64	4.6E-04
ENSCAFG0000000365	NBAS	grey	EC_M1C	0.46	1.8E-02	0.14	4.9E-01	-0.33	9.9E-02	0.16	4.5E-01	0.00	1.0E+0C	-0.06	7.7E-01	-0.54	4.2E-03	-0.19	3.5E-01	-0.44	2.3E-02	-0.26	2.0E-01
ENSCAFG0000000324	ANLN	darkgrey	EC_M8	0.46	1.8E-02	-0.04	8.6E-01	-0.50	9.5E-03	-0.09	6.6E-01	0.30	1.4E-01	0.13	5.4E-01	-0.08	7.1E-01	0.05	8.0E-01	0.22	2.9E-01	-0.48	1.3E-02
ENSCAFG0000001050	UBE2T	darkgrey	EC_M8	0.46	1.8E-02	-0.01	9.7E-01	-0.30	1.4E-01	-0.06	7.8E-01	0.09	6.7E-01	0.18	3.7E-01	0.04	8.4E-01	-0.07	7.3E-01	0.19	3.6E-01	-0.31	1.3E-01
ENSCAFG0000000921	ENSCAFG0000000921	darkgreen	EC_M4	0.46	1.8E-02	0.25	2.2E-01	-0.79	1.4E-06	-0.26	2.0E-01	0.48	1.2E-02	0.15	4.6E-01	-0.04	8.6E-01	0.14	4.9E-01	0.23	2.5E-01	-0.72	2.9E-05

ENSCAFG0000000279	CCDC88A	grey	EC_M10	0.46	1.8E-02	-0.64	3.9E-04	-0.05	8.1E-01	0.13	5.2E-01	-0.10	6.2E-01	0.07	7.2E-01	-0.29	1.5E-01	0.09	6.6E-01	-0.06	7.8E-01	-0.16	4.3E-01
ENSCAFG0000001143	ARID4B	darkgrey	EC_M8	0.46	1.8E-02	0.59	1.7E-03	-0.25	2.1E-01	0.20	3.4E-01	-0.40	4.4E-02	-0.08	7.0E-01	-0.18	3.7E-01	-0.03	8.7E-01	-0.08	7.0E-01	0.15	4.6E-01
ENSCAFG0000001423	ENSCAFG0000001423	darkgrey	EC_M8	0.46	1.8E-02	0.14	5.0E-01	-0.35	8.3E-02	0.54	4.5E-03	-0.06	7.6E-01	0.10	6.4E-01	-0.17	4.1E-01	-0.46	1.7E-02	-0.24	2.5E-01	-0.16	4.5E-01
ENSCAFG0000000403	CMPK1	darkgreen	EC_M4	0.46	1.8E-02	0.09	6.6E-01	-0.88	5.0E-09	-0.15	4.6E-01	0.62	7.6E-04	0.26	2.0E-01	-0.26	2.0E-01	0.15	4.6E-01	-0.19	3.4E-01	-0.87	6.0E-09
ENSCAFG0000001731	MBTD1	darkgrey	EC_M8	0.46	1.8E-02	0.67	2.0E-04	-0.10	6.4E-01	0.15	4.8E-01	-0.52	6.0E-03	-0.09	6.5E-01	0.02	9.1E-01	-0.24	2.3E-01	-0.12	5.8E-01	0.29	1.4E-01
ENSCAFG0000000205	RFX3	darkgrey	EC_M8	0.46	1.8E-02	0.53	5.3E-03	0.00	9.9E-01	0.19	3.5E-01	-0.58	1.8E-03	-0.07	7.5E-01	-0.09	6.6E-01	-0.23	2.5E-01	-0.05	8.0E-01	0.34	8.9E-02
ENSCAFG0000000657	ENSCAFG0000000657	grey	EC_M10	0.46	1.8E-02	0.15	4.6E-01	-0.48	1.3E-02	0.26	2.0E-01	0.09	6.6E-01	0.19	3.4E-01	-0.12	5.7E-01	0.10	6.4E-01	-0.30	1.3E-01	-0.36	7.1E-02
ENSCAFG0000002473	TRIM24	turquoise	EC_M6	0.46	1.8E-02	0.72	3.5E-05	-0.54	4.7E-03	-0.39	4.6E-02	-0.02	9.4E-01	0.00	1.0E+00	-0.21	3.1E-01	-0.26	1.9E-01	0.01	9.5E-01	-0.21	3.1E-01
ENSCAFG0000000737	ZNF143	darkgrey	EC_M8	0.46	1.8E-02	0.43	2.7E-02	-0.08	7.0E-01	-0.13	5.1E-01	-0.45	2.0E-02	0.25	2.3E-01	0.00	9.8E-01	0.07	7.5E-01	-0.28	1.6E-01	0.20	3.2E-01
ENSCAFG0000000026	RPA3	darkgrey	EC_M8	0.46	1.8E-02	-0.11	5.9E-01	-0.21	3.0E-01	0.00	9.9E-01	-0.04	8.5E-01	0.26	2.0E-01	-0.36	6.8E-02	-0.24	2.4E-01	0.04	8.4E-01	-0.16	4.5E-01
ENSCAFG0000001342	ACAP2	grey	EC_M10	0.46	1.9E-02	-0.27	1.8E-01	-0.39	4.9E-02	0.14	5.0E-01	0.10	6.3E-01	0.20	3.2E-01	-0.07	7.3E-01	-0.02	9.1E-01	0.02	9.3E-01	-0.38	5.3E-02
ENSCAFG0000000425	UGGT1	grey	EC_M10	0.46	1.9E-02	-0.43	2.9E-02	-0.50	1.0E-02	0.04	8.6E-01	0.33	9.9E-02	0.22	2.8E-01	-0.17	4.0E-01	-0.01	9.7E-01	-0.52	6.9E-03	-0.61	9.6E-04
ENSCAFG0000001729	CEP128	grey	EC_M10	0.46	1.9E-02	-0.12	5.7E-01	0.05	8.1E-01	0.16	4.3E-01	-0.37	6.2E-02	-0.09	6.5E-01	0.03	9.0E-01	0.35	7.7E-02	-0.12	5.7E-01	0.12	5.7E-01
ENSCAFG0000002847	ENSCAFG0000002847	darkgrey	EC_M8	0.46	1.9E-02	-0.25	2.1E-01	-0.36	6.7E-02	-0.02	9.2E-01	0.19	3.5E-01	0.08	7.1E-01	-0.02	9.4E-01	0.12	5.7E-01	-0.08	6.9E-01	-0.46	1.9E-02
ENSCAFG0000001210	PRKAR2A	grey	EC_M10	0.46	1.9E-02	-0.32	1.1E-01	0.01	9.7E-01	-0.03	9.0E-01	-0.21	3.1E-01	0.03	8.9E-01	-0.11	6.0E-01	-0.37	6.1E-02	-0.54	4.8E-03	-0.02	9.4E-01
ENSCAFG0000001526	MYBBP1A	grey	EC_M10	0.46	1.9E-02	-0.23	2.7E-01	-0.11	5.9E-01	-0.08	6.9E-01	-0.03	8.8E-01	0.04	8.5E-01	-0.24	2.5E-01	0.04	8.3E-01	-0.16	4.5E-01	-0.21	3.0E-01
ENSCAFG0000001764	ATP5F1A	grey	EC_M10	0.46	1.9E-02	-0.33	1.0E-01	0.22	2.9E-01	-0.06	7.9E-01	-0.42	3.3E-02	-0.02	9.2E-01	-0.17	3.9E-01	-0.16	4.2E-01	-0.28	1.6E-01	0.22	2.8E-01
ENSCAFG0000001497	ENSCAFG0000001497	darkgrey	EC_M8	0.46	1.9E-02	0.40	4.5E-02	-0.27	1.9E-01	0.28	1.6E-01	-0.25	2.1E-01	-0.10	6.3E-01	-0.31	1.2E-01	-0.30	1.4E-01	-0.31	1.2E-01	0.03	8.8E-01
ENSCAFG0000000179	SLC30A9	darkgreen	EC_M4	0.46	1.9E-02	-0.04	8.5E-01	-0.89	1.4E-09	-0.21	3.1E-01	0.68	1.4E-04	0.40	4.2E-02	-0.11	6.0E-01	-0.06	7.8E-01	-0.09	6.5E-01	-0.90	3.2E-10
ENSCAFG0000002085	ENSCAFG0000002085	grey	EC_M10	0.46	1.9E-02	0.14	4.8E-01	-0.19	3.6E-01	0.06	7.8E-01	-0.18	3.8E-01	0.13	5.3E-01	-0.46	1.8E-02	-0.07	7.2E-01	-0.22	2.9E-01	-0.12	5.6E-01
ENSCAFG0000000663	TNKS	grey	EC_M10	0.46	1.9E-02	0.07	7.3E-01	-0.48	1.4E-02	0.01	9.8E-01	0.09	6.5E-01	-0.08	6.9E-01	-0.18	3.8E-01	0.11	6.0E-01	-0.36	6.8E-02	-0.37	6.5E-02
ENSCAFG0000003163	ENSCAFG0000003163	grey	EC_M10	0.46	1.9E-02	0.05	8.0E-01	-0.29	1.5E-01	0.49	1.2E-02	-0.06	7.6E-01	-0.13	5.3E-01	-0.12	5.4E-01	-0.12	5.4E-01	-0.13	5.2E-01	-0.17	4.1E-01
ENSCAFG0000002019	ZNF644	darkgreen	EC_M4	0.46	1.9E-02	0.22	2.8E-01	-0.75	1.2E-05	-0.02	9.1E-01	0.38	5.3E-02	0.32	1.1E-01	0.09	6.7E-01	-0.24	2.3E-01	-0.11	5.9E-01	-0.59	1.4E-03
ENSCAFG0000000550	ENSCAFG0000000550	darkgrey	EC_M8	0.46	1.9E-02	0.41	3.6E-02	-0.37	6.1E-02	0.03	8.9E-01	-0.04	8.5E-01	-0.10	6.4E-01	-0.31	1.3E-01	0.21	3.1E-01	0.23	2.5E-01	-0.21	3.0E-01
ENSCAFG0000000551	EOMES	grey	EC_M10	0.46	1.9E-02	0.09	6.5E-01	-0.48	1.2E-02	-0.05	8.3E-01	0.15	4.5E-01	-0.13	5.2E-01	-0.08	7.0E-01	-0.12	5.7E-01	0.33	9.6E-02	-0.39	4.9E-02
ENSCAFG0000002333	ENSCAFG0000002333	grey	EC_M10	0.46	1.9E-02	-0.11	6.0E-01	-0.36	6.8E-02	-0.24	2.3E-01	0.11	5.8E-01	0.39	4.8E-02	-0.19	3.5E-01	0.19	3.4E-01	-0.11	6.0E-01	-0.31	1.2E-01
ENSCAFG0000001604	ENSCAFG0000001604	turquoise	EC_M6	0.46	1.9E-02	0.45	2.0E-02	-0.24	2.4E-01	-0.16	4.4E-01	-0.26	2.0E-01	-0.24	2.4E-01	-0.23	2.6E-01	-0.19	3.5E-01	-0.03	8.9E-01	0.02	9.1E-01
ENSCAFG0000001134	ITPR2	grey	EC_M10	0.46	1.9E-02	0.31	1.3E-01	-0.44	2.5E-02	-0.03	8.7E-01	0.05	8.2E-01	-0.08	6.8E-01	-0.15	4.5E-01	-0.14	5.0E-01	-0.62	7.1E-04	-0.31	1.3E-01
ENSCAFG0000000586	TMTC4	darkgreen	EC_M4	0.46	1.9E-02	0.56	2.8E-03	-0.65	3.3E-04	-0.05	8.2E-01	0.18	3.8E-01	0.13	5.1E-01	-0.05	8.1E-01	0.10	6.2E-01	-0.43	2.8E-02	-0.44	2.3E-02
ENSCAFG0000000867	DHX36	darkgreen	EC_M4	0.46	1.9E-02	0.36	7.3E-02	-0.78	2.8E-06	-0.10	6.1E-01	0.42	3.1E-02	0.23	2.6E-01	0.00	9.8E-01	-0.04	8.6E-01	-0.01	9.6E-01	-0.65	3.0E-04
ENSCAFG0000000333	TMEM168	darkgrey	EC_M8	0.46	1.9E-02	0.36	7.4E-02	0.03	8.8E-01	0.02	9.1E-01	-0.57	2.2E-03	-0.06	7.5E-01	-0.15	4.6E-01	-0.13	5.3E-01	0.38	5.7E-02	0.38	5.9E-02
ENSCAFG0000001327	DLG1	turquoise	EC_M6	0.46	1.9E-02	0.53	5.1E-03	-0.67	1.9E-04	0.05	7.9E-01	0.16	4.4E-01	0.16	4.4E-01	-0.13	5.4E-01	-0.15	4.5E-01	0.29	1.5E-01	-0.42	3.3E-02
ENSCAFG0000000128	ETF1	darkgreen	EC_M4	0.46	1.9E-02	-0.03	8.9E-01	-0.80	9.5E-07	-0.19	3.6E-01	0.55	3.9E-03	0.41	3.7E-02	-0.06	7.9E-01	-0.09	6.5E-01	-0.34	8.8E-02	-0.79	1.7E-06
ENSCAFG0000003154	PRELID2	grey	EC_M10	0.46	1.9E-02	0.35	7.8E-02	-0.08	6.9E-01	0.08	6.9E-01	-0.47	1.6E-02	0.00	9.8E-01	-0.07	7.4E-01	0.12	5.5E-01	-0.28	1.7E-01	0.16	4.2E-01
ENSCAFG0000001150	RBM34	darkgreen	EC_M4	0.46	1.9E-02	0.18	3.7E-01	-0.84	1.0E-07	-0.16	4.5E-01	0.49	1.1E-02	0.25	2.2E-01	-0.08	7.1E-01	-0.25	2.2E-01	-0.13	5.3E-01	-0.72	3.8E-05
ENSCAFG0000003184	ENSCAFG0000003184	darkgrey	EC_M8	0.46	1.9E-02	0.33	1.0E-01	0.18	3.8E-01	0.19	3.6E-01	-0.73	2.3E-05	-0.16	4.5E-01	-0.15	4.8E-01	-0.10	6.4E-01	-0.23	2.5E-01	0.48	1.2E-02
ENSCAFG0000001828	CSNK1A1	grey	EC_M10	0.46	1.9E-02	-0.19	3.5E-01	-0.19	3.5E-01	0.34	8.9E-02	-0.07	7.2E-01	0.12	5.5E-01	-0.12	5.7E-01	0.02	9.0E-01	-0.18	3.8E-01	-0.16	4.5E-01
ENSCAFG0000002954	C7H18orf21	darkgrey	EC_M8	0.45	2.0E-02	-0.27	1.8E-01	-0.59	1.7E-03	-0.07	7.5E-01	0.47	1.7E-02	0.41	3.7E-02	-0.09	6.7E-01	-0.22	2.8E-01	-0.32	1.1E-01	-0.72	2.9E-05
ENSCAFG0000000583	PCCA	grey	EC_M10	0.45	2.0E-02	0.07	7.2E-01	0.19	3.5E-01	0.15	4.7E-01	-0.56	2.8E-03	-0.25	2.3E-01	-0.41	3.7E-02	0.04	8.6E-01	0.16	4.4E-01	0.39	4.8E-02
ENSCAFG0000001401	DLAT	grey	EC_M10	0.45	2.0E-02	-0.22	2.9E-01	0.20	3.4E-01	0.06	7.6E-01	-0.46	1.9E-02	-0.14	5.1E-01	-0.33	9.5E-02	-0.22	2.8E-01	-0.33	9.5E-02	0.20	3.2E-01
ENSCAFG0000000332	IFRD1	darkgreen	EC_M4	0.45	2.0E-02	0.69	8.2E-05	-0.82	2.4E-07	-0.22	2.8E-01	0.30	1.3E-01	0.18	3.7E-01	-0.09	6.5E-01	-0.14	5.1E-01	-0.15	4.7E-01	-0.57	2.4E-03
ENSCAFG0000000277	ENSCAFG0000000277	darkgrey	EC_M8	0.45	2.0E-02	-0.14	4.8E-01	-0.43	2.8E-02	0.15	4.6E-01	0.12	5.6E-01	0.09	6.6E-01	-0.26	2.0E-01	-0.24	2.3E-01	-0.25	2.1E-01	-0.36	7.5E-02
ENSCAFG0000000721	DIMT1	darkgrey	EC_M8	0.45	2.0E-02	-0.47	1.6E-02	-0.01	9.5E-01	-0.11	6.0E-01	-0.10	6.1E-01	0.07	7.3E-01	-0.34	8.6E-02	0.08	7.1E-01	-0.20	3.4E-01	-0.14	4.8E-01
ENSCAFG0000000667	RAN	grey	EC_M10	0.45	2.0E-02	-0.24	2.5E-01	-0.43	3.0E-02	-0.18	3.8E-01	0.34	9.1E-02	0.26	2.0E-01	-0.16	4.3E-01	0.07	7.5E-01	-0.23	2.5E-01	-0.58	1.9E-03
ENSCAFG0000000418	SAE1	darkgrey	EC_M8	0.45	2.0E-02	-0.54	4.2E-03	-0.15	4.5E-01	-0.08	6.8E-01	0.09	6.6E-01	0.14	5.0E-01	-0.31	1.3E-01	-0.02	9.3E-01	-0.29	1.5E-01	-0.34	9.2E-02
ENSCAFG0000000384	ABHD18	darkgreen	EC_M4	0.45	2.0E-02	0.47	1.6E-02	-0.76	5.8E-06	-0.04	8.5E-01	0.30	1.4E-01	0.32	1.1E-01	0.08	6.9E-01	-0.03	8.7E-01	0.02	9.4E-01	-0.57	2.4E-03
ENSCAFG0000000922	RPAP3	darkgrey	EC_M8	0.45	2.0E-02	0.02	9.1E-01	-0.57	2.4E-03	0.04	8.3E-01	0.24	2.3E-01	0.22	2.7E-01	-0.37	6.4E-02	-0.15	4.5E-01	-0.13	5.3E-01	-0.44	2.5E-02
ENSCAFG0000000169	OSTF1	turquoise	EC_M6	0.45	2.0E-02	0.85	5.2E-08	-0.27	1.9E-01	-0.11	6.0E-01	-0.36	6.7E-02	-0.14	5.1E-01	-0.10	6.3E-01	-0.06	7.7E-01	-0.13	5.2E-01	0.14	5.1E-01
ENSCAFG0000000167	MTAP	darkgreen	EC_M4	0.45	2.0E-02	-0.07	7.2E-01	-0.59	1.7E-03	-0.01	9.5E-01	0.36	7.0E-02	0.08	7.1E-01	-0.59	1.6E-03	0.01	9.5E-01	-0.30	1.3E-01	-0.61	8.3E-04
ENSCAFG0000001489	CDKN3	darkgrey	EC_M8	0.45	2.0E-02	0.13	5.4E-01	-0.04	8.4E-01	-0.04	8.5E-01	-0.26	2.0E-01	-0.23	2.6E-01	-0.16	4.3E-01	-0.19	3.6E-01	0.14	5.1E-01	0.07	7.4E-01
ENSCAFG0000000908	NUP93	darkgrey	EC_M8	0.45	2.0E-02	-0.18	3.7E-01	0.12	5.5E-01	-0.21	3.1E-01	-0.36	7.0E-02	0.06	7.6E-01	-0.12	5.5E-01	-0.06	7.6E-01	-0.20	3.3E-01	0.11	5.9E-01
ENSCAFG0000002939	HAS2	darkgreen	EC_M4	0.45	2.0E-02	-0.11	5.9E-01	-0.65	3.4E-04	-0.13	5.1E-01	0.44	2.4E-02	0.47	1.4E-02	-0.20	3.2E-01	0.09	6.7E-01	-0.19	3.6E-01	-0.74	1.5E-05
ENSCAFG0000000883	ZFYVE16	grey	EC_M10	0.45	2.0E-02	0.58	2.1E-03	-0.68	1.5E-04	-0.09	6.7E-01	0.13	5.2E-01	0.04	8.5E-01	-0.01	9.4E-01	0.01	9.8E-01	-0.16	4.4E-01	-0.38	5.3E-02
ENSCAFG0000000649	ENSCAFG0000000649	grey	EC_M10	0.45	2.1E-02	0.16	4.2E-01	-0.23	2.6E-01	0.10	6.4E-01	-0.10	6.2E-01	-0.07	7.3E-01	-0.13	5.1E-01	-0.04	8.5E-01	-0.32	1.1E-01	-0.10	6.1E-01
ENSCAFG0000001775	ENSCAFG0000001775	grey	EC_M10	0.45	2.1E-02	-0.04	8.4E-01	-0.56	3.2E-03	-0.13	5.3E-01	0.29	1.6E-01	0.47	1.5E-02	-0.16	4.3E-01	-0.15	4.8E-01	-0.22	2.8E-01	-0.53	5.1E-03
ENSCAFG0000000158	TREML2	grey	EC_M10	0.45	2.1E-02	-0.24	2.3E-01	-0.38	5.6E-02	-0.14	4.8E-01	0.13	5.2E-01	0.30	1.4E-01	-0.13	5.3E-01	0.24	2.3E-01	-0.09	6.7E-01	-0.35	7.8E-02
ENSCAFG0000000598	ENSCAFG0000000598	darkgrey	EC_M8	0.45	2.1E-02	0.18	3.9E-01	-0.65	3.4E-04	-0.17	3.9E-01	0.31	1.2E-01	0.02	9.0E-01	-0.30	1.3E-01	0.00	9.9E-01	-0.26	2.0E-01	-0.53	5.5E-03
ENSCAFG0000001435	NCKAP1	darkgreen	EC_M4	0.45	2.1E-02	0.52	6.0E-03	-0.75	1.2E-05	0.06	7.8E-01	0.27	1.9E-01	0.09	6.7E-01	-0.13	5.3E-01	-0.19	3.5E-01	-0.30	1.4E-01	-0.50	1.0E-02
ENSCAFG0000001465	ENSCAFG0000001465	darkgrey	EC_M8	0.45	2.1E-02	0.20	3.2E-01	-0.38	5.6E-02	0.39	5.0E-02	-0.04	8.4E-01	-0.14	5.0E-01	-0.02	9.1E-01	-0.41	3.5E-02	-0.29	1.5E-01	-0.17	4.1E-01
ENSCAFG0000000042	DHX16	turquoise	EC_M6	0.45	2.1E-02	0.57	2.3E-03	-0.23	2.5E-01	0.15	4.7E-01	-0.30	1.3E-01	0.40	4.5E-02	-0.02	9.1E-01	-0.14	4.9E-01	-0.08	6.9E-01	0.04	8.3E-01
ENSCAFG0000000094	ARFGAP3	grey	EC_M10	0.45	2.1E-02	0.11	5.8E-01	-0.52	6.8E-03	-0.14	4.9E-01	0.11	5.9E-01	0.33	9.7E-02	0.14	5.0E-01	-0.21	2.9E-01	-0.32	1.1E-01	-0.39	4.7E-02
ENSCAFG0000001778	BHLHB9	grey	EC_M10	0.45	2.1E-02	0.22	2.8E-01	-0.07	7.3E-01	-0.03	9.0E-01	-0.38	5.4E-02	-0.04	8.6E-01	0.03	8.7E-01	0.01	9.4E-01	-0.56	2.7E-03	0.16	4.2E-01
ENSCAFG0000000123	UHRF1BP1	darkgrey	EC_M8	0.45	2.1E-02	0.28	1.7E-01	-0.19	3.5E-01	-0.33	1.0E-01	-0.12	5.5E-01	-0.17	4.1E-01	0.13	5.3E-01	0.09	6.6E-01	-0.03	8.8E-01	-0.06	7.8E-01
ENSCAFG0000002577	ENSCAFG0000002577	grey	EC_M10	0.45	2.1E-02	-0.06	7.6E-01	0.13	5.4E-01	-0.09	6.8E-01	-0.50	9.8E-03	0.36	7.0E-02	0.02	9.2E-01	-0.34	9.2E-02	-0.42	3.4E-02	0.23	2.6E-01
ENSCAFG0000001679	BNIP1	grey	EC_M10	0.45	2.1E-02	-0.12	5.7E-01	-0.61	1.0E-03	0.18	3.9E-01	0.36	7.2E-02	0.14	4.9E-01	-0.22	2.8E-01	0.01	9.5E-01	-0.16	4.5E-01	-0.67	1.9E-04
ENSCAFG0000001553	ENSCAFG0000001553	grey	EC_M10	0.45	2.1E-02	0.02	9.0E-01	-0.29	1.6E-01	0.18	3.8E-01	-0.10	6.2E-01	-0.05	8.0E-01	-0.22	2.8E-01	0.07	7.3E-01	-0.52	6.0E-03	-0.14	5.0E-01
ENSCAFG0000001204	RAE1	darkgrey	EC_M8	0.45	2.1E-02	0.15	4.6E-01	-0.15	4.6E-01	-0.01	9.8E-01	-0.18	3.7E-01	-0.08	7.0E-01	-0.23	2.6E-01	-0.22	2.8E-01	0.32	1.1E-01	-0.03	8.9E-01
ENSCAFG0000001462	MECOM	turquoise	EC_M6	0.45	2.1E-02	0.69	9.1E-05	-0.67	1.7E-04	-0.18	3.8E-01	0.17	4.0E-01	0.07	7.4E-01	-0.32	1.2E-01	-0.01	9.5E-01	0.17	3.9E-01	-0.41	3.8E-02
ENSCAFG0000001663	HEPH	turquoise	EC_M6	0.45	2.1E-02	0.83	1.8E-07	-0.37	6.3E-02	0.08	7.1E-01	-0.20	3.2E-01	-0.16	4.3E-01	-0.16	4.5E-01	-0.17	3.9E-01	-0.20	3.3E-01	-0.04	8.3E-01
ENSCAFG0000000339	ENSCAFG0000000339	grey	EC_M10	0.45	2.1E-02	-0.23	2.5E-01	-0.21	3.1E-01	0.28	1.6E-01	-0.13	5.3E-01	0.33	1.0E-01	-0.19	3.6E-01	-0.23	2.6E-01	-0.17	4.0E-01	-0.11	5.8E-01
ENSCAFG0000001215	EIF2B5	darkgrey	EC_M8	0.45	2.1E-02	-0.07	7.3E-01	0.15	4.6E-01	0.19	3.6E-01	-0.53	5.2E-03	0.16	4.5E-01	-0.12	5.8E-01	0.18	3.8E-01	-0.35	7.6E-02	0.30	1.4E-01
ENSCAFG0000001482	USP48	darkgreen	EC_M4	0.45	2.1E-02	0.34	8.5E-02	-0.79	1.7E-06	-0.03	8.9E-01	0.35	7.8E-02	0.23	2.5E-01	-0.24	2.3E-01	-0.14	5.1E-01	-0.13	5.3E-01	-0.63	5.3E-04
ENSCAFG0000000603	PCNA	grey	EC_M10	0.45	2.1E-02	0.08	7.0E-01	0.08	6.8E-01	0.10	6.4E-01	-0.42	3.2E-02	0.13	5.4E-01	-0.12	5.5E-01	-0.57	2.4E-03	0.05	7.9E-01	0.23	2.7E-01
ENSCAFG0000002356	DMD	darkgreen	EC_M4	0.45	2.1E-02	0.28	1.7E-01	-0.82	3.6E-07	-0.14	5.1E-01	0.51	7.1E-03	0.03	8.9E-01	-0.05	8.2E-01	-0.05	7.9E-01	0.01	9.8E-01	-0.73	2.7E-05
ENSCAFG0000001433	NR2C2AP	darkgrey	EC_M8	0.45	2.1E-02	-0.20	3.3E-01	-0.32	1.1E-01	0.04	8.6E-01	0.14	5.1E-01	0.13	5.3E-01	-0.05	8.0E-01	0.16	4.4E-01	-0.19	3.4E-01	-0.34	8.7E-02
ENSCAFG0000000948	RPGRIP1L	grey	EC_M10	0.45	2.2E-02	-0.51	8.3E-03	0.07	7.2E-01	0.21	3.0E-01	-0.27	1.8E-01	0.33	1.0E-01	-0.18	3.9E-01	0.03	9.0E-01	-0.19	3.6E-01	0.01	9.5E-01
ENSCAFG0000003149	ENSCAFG0000003149	grey	EC_M10	0.45	2.2E-02	0.22	2.7E-01	-0.31	1.3E-01	0.25	2.2E-01	-0.09	6.6E-01	-0.11	6.0E-01	0.08	6.8E-01	0.03	8.8E-01	-0.58	2.0E-03	-0.15	4.8E-01
ENSCAFG0000001776	KRI1	grey	EC_M10	0.45	2.2E-02	0.23	2.5E-01	0.04	8.6E-01	0.09	6.5E-01	-0.46	1.9E-02	0.12	5.6E-01	0.02	9.4E-01	-0.15	4.6E-01	-0.32	1.1E-01	0.25	2.2E-01
ENSCAFG0000000734	ZMYM2	darkgrey	EC_M8	0.45	2.2E-02	0.59	1.4E-03	-0.16	4.4E-01	0.03	8.7E-01	-0.48	1.3E-02	0.02	9.1E-01	-0.27	1.8E-01	0.01	9.5E-01	-0.01	9.5E-01	0.22	2.9E-01
ENSCAFG0000002354	ENSCAFG0000002354	grey	EC_M10	0.45	2.2E-02	-0.02	9.3E-01	-0.20	3.4E-01	0.00	9.9E-01	-0.10	6.4E-01	0.09	6.5E-01	-0.10	6.2E-01	0.07	7.5E-01	-0.64	4.5E-04	-0.12	5.7E-01
ENSCAFG0000001885	TARS	darkgreen	EC_M4	0.45	2.2E-02	-0.03	8.8E-01	-0.86	2.5E-08	-0.04	8.3E-01	0.66	2.7E-04	0.38	5.4E-02	-0.19	3.5E-01	-0.07	7.3E-01	-0.23	2.6E-01	-0.92	1.5E-11
ENSCAFG0000003105	MANEA	darkgrey	EC_M8	0.45	2.2E-02	0.17	4.2E-01	-0.50	8.6E-03	0.24	2.4E-01	0.09	6.7E-01	0.14	4.9E-01	-0.31	1.2E-01	-0.21	3.1E-01	-0.09	6.5E-01	-0.32	1.1E-01
ENSCAFG0000000617	SPART	grey	EC_M10	0.45	2.2E-02	0.54	4.5E-03	-0.27	1.8E-01	0.04	8.5E-01	-0.25	2.1E-01	0.16	4.5E-01	-0.19	3.6E-01	-0.33	9.8E-02	-0.31	1.2E-01	0.03	8.7E-01
ENSCAFG0000001203	SPO11	darkgrey	EC_M8	0.45	2.2E-02	0.13	5.2E-01	-0.21	3.0E-01	-0.05	8.0E-01	-0.05	8.0E-01	-0.12	5.4E-01	-0.09	6.8E-01	-0.04	8.4E-01	-0.15	4.5E-01	-0.15	4.7E-01
ENSCAFG0000002107	ENSCAFG0000002107	darkgrey	EC_M8	0.45	2.2E-02	-0.14	5.1E-01	-0.20	3.2E-01	-0.40	4.3E-02	-0.03	8.7E-01	0.20	3.4E-01	0.01	9.5E-01	-0.02	9.3E-01	-0.33	9.7E-02	-0.20	3.2E-01
ENSCAFG0000001473	CALCRL	turquoise	EC_M6	0.45	2.2E-02	0.85	3.3E-08	-0.57	2.6E-03	-0.14	5.1E-01	0.01	9.6E-01	-0.15	4.6E-01	-0.08	7.0E-01	-0.13	5.2E-01	0.14	4.8E-01	-0.23	2.5E-01
ENSCAFG0000003100	ZNF354A	darkgrey	EC_M8	0.45	2.2E-02	0.48	1.3E-02	-0.05	8.1E-01	0.15	4.8E-01	-0.57	2.5E-03	0.01	9.7E-01	-0.29	1.5E-01	0.01	9.8E-01	-0.04	8.6E-01	0.34	9.0E-02
ENSCAFG0000003037	ENSCAFG0000003037	turquoise	EC_M6	0.45	2.2E-02	0.64	4.8E-04	-0.36	6.7E-02	0.05	8.1E-01	-0.19	3.5E-01	-0.09	6.5E-01	-0.19	3.5E-01	-0.26	2.0E-01	-0.16	4.5E-01	-0.02	9.1E-01
ENSCAFG0000000307	ENSCAFG0000000307	grey	EC_M10	0.45	2.2E-02	0.02	9.1E-01	-0.55	3.4E-03	0.33	1.0E-01	0.22	2.8E-01	0.10	6.2E-01	-0.09	6.5E-01	-0.31	1.2E-01	-0.43	2.7E-02	-0.49	1.1E-02
ENSCAFG0000001399	ENSCAFG0000001399	grey	EC_M10	0.45	2.2E-02	0.04	8.3E-01	-0.19	3.5E-01	0.21	3.1E-01	-0.20	3.3E-01	0.30	1.3E-01	0.08	7.1E-01	-0.49	1.1E-02	-0.32	1.2E-01	-0.06	7.8E-01
ENSCAFG0000000321	SLC31A1	darkgreen	EC_M4	0.45	2.2E-02	0.45	2.1E-02	-0.66	2.4E-04	-0.18	3.7E-01	0.26	1.9E-01	0.25	2.2E-01	0.23	2.6E-01	-0.11	6.0E-01	-0.26	2.0E-01	-0.51	8.2E-03
ENSCAFG0000002306	RANBP2	darkgreen	EC_M4	0.45	2.2E-02	0.37	6.0E-02	-0.80	1.1E-06	-0.14	4.8E-01	0.39	4.7E-02	0.10	6.2E-01	0.05	8.1E-01	-0.04	8.5E-01	-0.33	9.4E-02	-0.63	6.1E-04
ENSCAFG0000000024	HNRNPAB	grey	EC_M10	0.45	2.2E-02	0.00	9.9E-01	-0.57	2.5E-03	-0.27	1.9E-01	0.42	3.4E-02	0.32	1.1E-01	-0.12	5.5E-01	-0.12	5.6E-01	-0.09	6.6E-01	-0.66	2.6E-04
ENSCAFG0000001278	XPR1	darkgrey	EC_M8	0.45	2.2E-02	0.12	5.6E-01	0.13	5.2E-01	0.17	4.0E-01	-0.67	1.9E-04	0.02	9.4E-01	-0.16	4.4E-01	-0.29	1.5E-01	-0.06	7.6E-01	0.40	4.1E-02
ENSCAFG0000001552	TEAD4	turquoise	EC_M6	0.45	2.2E-02	0.54	4.6E-03	-0.42	3.2E-02	0.03	8.9E-01	-0.02	9.2E-01	0.04	8.3E-01	-0.02	9.1E-01	-0.26	2.0E-01	-0.17	4.1E-01	-0.22	2.8E-01
ENSCAFG0000001769	HNRNPH2	grey	EC_M10	0.45	2.2E-02	0.20	3.3E-01	-0.41	4.0E-02	-0.02	9.4E-01	-0.05	8.1E-01	0.09	6.7E-01	0.19	3.6E-01	0.07	7.3E-01	-0.22	2.8E-01	-0.20	3.3E-01
ENSCAFG0000001134	ENSCAFG0000001134	darkgrey	EC_M8	0.45	2.2E-02	0.20	3.3E-01	-0.57	2.4E-03	0.18	3.8E-01	0.18	3.7E-01	0.07	7.4E-01	-0.21	3.0E-01	-0.14	5.0E-01	-0.09	6.5E-01	-0.38	5.4E-02
ENSCAFG0000000149	KLHDC10	darkgreen	EC_M4	0.45	2.2E-02	-0.26	1.9E-01	-0.67	1.6E-04	0.12	5.7E-01	0.46	1.9E-02	0.14	5.0E-01	-0.24	2.4E-01	-0.04	8.4E-01	-0.13	5.3E-01	-0.73	2.6E-05
ENSCAFG0000000235	TRIM28	darkgrey	EC_M8	0.45	2.3E-02	0.22	2.8E-01	0.07	7.4E-01	-0.03	8.9E-01	-0.41	4.0E-02	0.01	9.7E-01	-0.02	9.2E-01	-0.27	1.8E-01	-0.29	1.5E-01	0.16	4.3E-01
ENSCAFG0000002559	HUS1	grey	EC_M10	0.45	2.3E-02	-0.44	2.3E-02	-0.34	8.9E-02	-0.05	8.3E-01	0.23	2.5E-01	0.20	3.2E-01	-0.07	7.5E-01	-0.01	9.7E-01	-0.11	6.0E-01	-0.52	6.4E-03
ENSCAFG0000001198	SPP2	grey	EC_M10	0.44	2.3E-02	-0.13	5.3E-01	-0.33	9.5E-02	-0.18	3.9E-01	0.08	6.9E-01	0.31	1.2E-01	-0.19	3.5E-01	0.19	3.5E-01	-0.05	8.0E-01	-0.37	6.2E-02
ENSCAFG0000001917	ZNF185	grey	EC_M10	0.44	2.3E-02	0.37	5.9E-02	-0.57	2.2E-03	0.07	7.3E-01	0.16	4.3E-01	0.25	2.1E-01	0.24	2.4E-01	-0.37	6.5E-02	-0.34	9.0E-02	-0.43	2.8E-02
ENSCAFG0000001725	RAB11A	darkgreen	EC_M4	0.44	2.3E-02	0.57	2.4E-03	-0.75	8.3E-06	-0.04	8.6E-01	0.29	1.6E-01	0.01	9.7E-01	-0.16	4.4E-01	0.02	9.2E-01	-0.04	8.6E-01	-0.49	1.2E-02
ENSCAFG0000002964	ENSCAFG0000002964	grey	EC_M10	0.44	2.3E-02	0.40	4.1E-02	-0.39	4.9E-02	0.15	4.5E-01	-0.13	5.1E-01	-0.24	2.4E-01	-0.23	2.7E-01	-0.23	2.5E-01	0.05	8.0E-01	-0.11	5.9E-01
ENSCAFG0000003186	ENSCAFG0000003186	darkgrey	EC_M8	0.44	2.3E-02	0.35	8.1E-02	-0.09	6.7E-01	0.00	9.9E-01	-0.47	1.5E-02	-0.31	1.3E-01	0.00	9.9E-01	-0.09	6.6E-01	0.11	5.8E-01	0.23	2.6E-01
ENSCAFG0000001368	RAP1A	darkgrey	EC_M8	0.44	2.3E-02	0.29	1.5E-01	-0.70	6.1E-05	-0.10	6.2E-01	0.31	1.2E-01	0.13	5.2E-01	-0.17	4.2E-01	-0.06	7.8E-01	-0.15	4.8E-01	-0.53	5.8E-03
ENSCAFG0000000976	ENSCAFG0000000976	darkgrey	EC_M8	0.44	2.3E-02	0.24	2.3E-01	-0.31	1.2E-01	0.08	7.1E-01	-0.16	4.4E-01	0.18	3.7E-01	-0.25	2.1E-01	-0.06	7.6E-01	-0.18	3.7E-01	-0.09	6.5E-01
ENSCAFG0000000887	ENSCAFG0000000887	darkgrey	EC_M8	0.44	2.3E-02	-0.20	3.3E-01	-0.51	7.5E-03	-0.01	9.6E-01	0.38	5.4E-02	0.11	5.9E-01	-0.33	9.6E-02	0.06	7.7E-01	-0.25	2.1E-01	-0.63	5.7E-04
ENSCAFG0000000986	EHD4	turquoise	EC_M6	0.44	2.3E-02	0.41	4.0E-02	-0.25	2.1E-01	0.07	7.5E-01	-0.19	3.6E-01	-0.09	6.7E-01	-0.24	2.4E-01	-0.08	7.0E-01	-0.64	4.3E-04	-0.04	8.5E-01
ENSCAFG0000000871	RAPGEF2	darkgreen	EC_M4	0.44	2.3E-02	0.66	2.1E-04	-0.76	7.3E-06	-0.20	3.3E-01	0.29	1.4E-01	0.01	9.7E-01	-0.14	5.0E-01	-0.06	7.7E-01	-0.14	4.8E-01	-0.51	7.6E-03
ENSCAFG0000000762	ENSCAFG0000000762	grey	EC_M10	0.44	2.3E-02	0.11	5.8E-01	0.22	2.8E-01	0.26	1.9E-01	-0.66	2.5E-04	-0.14	5.0E-01	-0.28	1.7E-01	-0.17	4.2E-01	-0.15	4.6E-01	0.40	4.2E-02
ENSCAFG0000001890	ENSCAFG0000001890	turquoise	EC_M6	0.44	2.3E-02	0.64	4.5E-04	-0.50	8.6E-03	-0.03	8.9E-01	0.01	9.5E-01	-0.16	4.4E-01	-0.14	4.9E-01	-0.12	5.4E-01	-0.22	2.9E-01	-0.27	1.8E-01
ENSCAFG0000000951	HMGCR	grey	EC_M10	0.44	2.3E-02	0.06	7.7E-01	-0.55	3.9E-03	-0.06	7.6E-01	0.25	2.2E-01	0.02	9.1E-01	0.02	9.1E-01	-0.14	5.1E-01	-0.56	2.7E-03	-0.52	6.7E-03
ENSCAFG0000002236	ENSCAFG0000002236	grey	EC_M10	0.44	2.3E-02	-0.19	3.6E-01	0.00	9.8E-01	0.01	9.7E-01	-0.35	7.9E-02	0.38	5.8E-02	-0.05	8.1E-01	-0.19	3.5E-01	-0.22	2.8E-01	0.11	6.0E-01
ENSCAFG0000002375	CCDC59	grey	EC_M10	0.44	2.3E-02	0.06	7.6E-01	-0.65	3.6E-04	0.20	3.4E-01	0.35	8.2E-02	0.45	2.0E-02	-0.09	6.7E-01	0.10	6.2E-01	-0.40	4.4E-02	-0.60	1.2E-03
ENSCAFG0000000094	HSPA4	darkgreen	EC_M4	0.44	2.3E-02	-0.13	5.3E-01	-0.85	5.5E-08	-0.21	2.9E-01	0.70	7.4E-05	0.23	2.6E-01	-0.10	6.2E-01	0.08	6.9E-01	-0.09	6.7E-01	-0.92	6.0E-11

ENSCAFG0000003104	UBE2D2	darkgreen	EC_M4	0.44	2.3E-02	0.35	7.7E-02	-0.82	2.6E-07	-0.24	2.5E-01	0.50	9.6E-03	0.14	4.9E-01	-0.25	2.1E-01	-0.15	4.7E-01	-0.29	1.5E-01	-0.73	2.0E-05
ENSCAFG0000001567	PTEN	darkgrey	EC_M8	0.44	2.4E-02	-0.08	7.0E-01	0.09	6.6E-01	0.30	1.3E-01	-0.49	1.1E-02	0.02	9.2E-01	-0.26	2.1E-01	0.03	8.8E-01	-0.31	1.2E-01	0.25	2.1E-01
ENSCAFG0000001841	NKRF	darkgreen	EC_M4	0.44	2.4E-02	-0.20	3.2E-01	-0.62	8.0E-04	-0.31	1.3E-01	0.45	2.0E-02	0.08	7.0E-01	0.10	6.2E-01	0.06	7.6E-01	-0.19	3.5E-01	-0.68	1.3E-04
ENSCAFG0000002249	ENSCAFG0000002249	grey	EC_M1C	0.44	2.4E-02	0.15	4.6E-01	-0.06	7.6E-01	0.26	2.1E-01	-0.37	6.4E-02	0.25	2.2E-01	-0.16	4.5E-01	0.23	2.6E-01	-0.09	6.5E-01	0.15	4.7E-01
ENSCAFG0000002273	ND5	cyan	EC_M2	0.44	2.4E-02	-0.29	1.5E-01	0.17	4.0E-01	0.20	3.2E-01	-0.49	1.1E-02	0.08	6.9E-01	-0.57	2.2E-03	-0.02	9.1E-01	-0.28	1.7E-01	0.22	2.8E-01
ENSCAFG0000001429	ZC3H12C	turquoise	EC_M6	0.44	2.4E-02	0.60	1.2E-03	-0.26	2.0E-01	-0.08	7.0E-01	-0.24	2.4E-01	-0.27	1.9E-01	0.08	6.8E-01	-0.23	2.6E-01	-0.15	4.5E-01	0.06	7.7E-01
ENSCAFG0000000561	EIF4A3	grey	EC_M1C	0.44	2.4E-02	0.24	2.3E-01	0.35	7.5E-02	-0.09	6.5E-01	-0.78	3.3E-06	-0.02	9.1E-01	-0.09	6.5E-01	-0.18	3.8E-01	-0.12	5.5E-01	0.55	3.3E-03
ENSCAFG0000001659	ENSCAFG0000001659	darkgrey	EC_M8	0.44	2.4E-02	-0.17	4.0E-01	-0.19	3.5E-01	-0.12	5.7E-01	0.00	9.9E-01	0.30	1.4E-01	-0.10	6.2E-01	-0.07	7.2E-01	-0.02	9.1E-01	-0.28	1.6E-01
ENSCAFG0000002495	ENSCAFG0000002495	grey	EC_M1C	0.44	2.4E-02	0.48	1.2E-02	-0.53	5.4E-03	-0.14	5.1E-01	0.12	5.6E-01	0.10	6.2E-01	-0.17	4.0E-01	0.24	2.3E-01	-0.09	6.6E-01	-0.34	9.3E-02
ENSCAFG0000001663	ENSCAFG0000001663	grey	EC_M1C	0.44	2.4E-02	0.49	1.1E-02	0.05	8.1E-01	0.30	1.4E-01	-0.57	2.4E-03	-0.30	1.4E-01	-0.23	2.5E-01	-0.16	4.5E-01	-0.34	9.0E-02	0.34	9.3E-02
ENSCAFG0000000390	AMD1	darkgrey	EC_M8	0.44	2.4E-02	-0.42	3.3E-02	-0.30	1.4E-01	-0.08	6.9E-01	0.16	4.4E-01	0.12	5.5E-01	-0.20	3.2E-01	0.04	8.4E-01	-0.19	3.4E-01	-0.41	3.5E-02
ENSCAFG0000003155	ENSCAFG0000003155	grey	EC_M1C	0.44	2.4E-02	0.26	1.9E-01	-0.42	3.0E-02	-0.15	4.8E-01	0.08	7.1E-01	0.13	5.1E-01	-0.18	3.7E-01	-0.24	2.4E-01	-0.22	2.8E-01	-0.31	1.3E-01
ENSCAFG0000001461	ECD	grey	EC_M1C	0.44	2.4E-02	-0.10	6.2E-01	-0.02	9.2E-01	0.07	7.2E-01	-0.36	6.9E-02	0.02	9.4E-01	-0.41	3.6E-02	-0.03	8.8E-01	0.05	8.1E-01	0.12	5.6E-01
ENSCAFG0000000972	SLF2	grey	EC_M1C	0.44	2.4E-02	0.40	4.1E-02	-0.32	1.1E-01	0.15	4.8E-01	-0.13	5.3E-01	-0.15	4.7E-01	-0.32	1.1E-01	-0.02	9.1E-01	-0.08	7.2E-01	-0.05	8.0E-01
ENSCAFG0000000768	MMAA	grey	EC_M1C	0.44	2.4E-02	0.39	5.1E-02	-0.15	4.6E-01	0.14	4.8E-01	-0.34	8.6E-02	-0.13	5.4E-01	-0.10	6.2E-01	-0.35	8.4E-02	0.01	9.5E-01	0.07	7.4E-01
ENSCAFG0000002588	ENSCAFG0000002588	darkgrey	EC_M8	0.44	2.4E-02	0.40	4.5E-02	0.03	8.7E-01	0.38	5.4E-02	-0.58	2.0E-03	-0.14	5.0E-01	-0.06	7.6E-01	-0.09	6.5E-01	0.00	9.8E-01	0.34	9.3E-02
ENSCAFG0000000645	CDK17	turquoise	EC_M6	0.44	2.4E-02	0.84	9.4E-08	-0.67	1.6E-04	-0.07	7.3E-01	0.11	6.0E-01	0.11	6.1E-01	-0.13	5.4E-01	-0.06	7.7E-01	-0.19	3.5E-01	-0.35	7.6E-02
ENSCAFG0000000659	USPL1	grey	EC_M1C	0.44	2.4E-02	0.30	1.3E-01	-0.47	1.6E-02	0.01	9.6E-01	0.06	7.6E-01	0.46	1.7E-02	0.08	6.8E-01	-0.19	3.6E-01	-0.30	1.3E-01	-0.27	1.8E-01
ENSCAFG0000001784	ELP2	darkgreen	EC_M4	0.44	2.4E-02	0.09	6.7E-01	-0.72	3.7E-05	-0.13	5.3E-01	0.50	8.7E-03	0.33	1.0E-01	-0.08	7.0E-01	-0.32	1.1E-01	-0.32	1.1E-01	-0.73	2.6E-05
ENSCAFG0000001386	PRRG1	turquoise	EC_M6	0.44	2.4E-02	0.59	1.7E-03	-0.48	1.4E-02	-0.04	8.5E-01	-0.06	7.9E-01	0.05	8.1E-01	0.17	4.0E-01	-0.15	4.8E-01	-0.20	3.2E-01	-0.20	3.4E-01
ENSCAFG0000002625	ENSCAFG0000002625	grey	EC_M1C	0.44	2.4E-02	-0.07	7.2E-01	-0.12	5.6E-01	0.09	6.7E-01	-0.25	2.2E-01	0.34	8.7E-02	-0.22	2.8E-01	0.11	6.1E-01	-0.01	9.6E-01	0.02	9.0E-01
ENSCAFG0000000487	PCDH17	darkgreen	EC_M4	0.44	2.4E-02	0.13	5.4E-01	-0.82	2.3E-07	-0.13	5.2E-01	0.56	2.7E-03	0.17	3.9E-01	-0.21	3.1E-01	0.04	8.5E-01	-0.10	6.1E-01	-0.83	1.2E-07
ENSCAFG0000002674	ENSCAFG0000002674	grey	EC_M1C	0.44	2.4E-02	0.19	3.5E-01	-0.08	6.9E-01	0.12	5.7E-01	-0.39	5.1E-02	-0.07	7.5E-01	0.21	3.0E-01	0.19	3.6E-01	-0.01	9.5E-01	0.13	5.3E-01
ENSCAFG0000000979	STAT1	turquoise	EC_M6	0.44	2.4E-02	0.51	7.4E-03	-0.41	3.7E-02	-0.02	9.3E-01	-0.02	9.3E-01	0.14	5.0E-01	-0.57	2.4E-03	-0.04	8.6E-01	0.10	6.2E-01	-0.21	3.1E-01
ENSCAFG0000001240	COL7A1	grey	EC_M1C	0.44	2.4E-02	0.00	1.0E+00	-0.52	6.9E-03	0.00	9.9E-01	0.16	4.4E-01	0.34	9.3E-02	0.08	7.0E-01	-0.04	8.6E-01	-0.18	3.9E-01	-0.45	2.1E-02
ENSCAFG0000001850	UTP6	darkgrey	EC_M8	0.44	2.4E-02	-0.33	1.0E-01	-0.66	2.2E-04	-0.08	7.0E-01	0.53	5.0E-03	0.35	7.8E-02	-0.04	8.5E-01	-0.06	7.8E-01	-0.14	5.1E-01	-0.80	1.2E-06
ENSCAFG0000000012	ONECUT2	darkgrey	EC_M8	0.44	2.4E-02	-0.24	2.4E-01	-0.17	4.1E-01	-0.18	3.7E-01	-0.01	9.4E-01	0.46	1.7E-02	-0.09	6.5E-01	-0.09	6.5E-01	-0.10	6.1E-01	-0.19	3.6E-01
ENSCAFG0000000645	PPP2CB	darkgreen	EC_M4	0.44	2.4E-02	0.48	1.4E-02	-0.84	7.4E-08	-0.25	2.2E-01	0.47	1.5E-02	0.18	3.7E-01	-0.10	6.2E-01	-0.31	1.3E-01	-0.06	7.8E-01	-0.69	1.1E-04
ENSCAFG0000001014	NUP153	grey	EC_M1C	0.44	2.4E-02	0.06	7.8E-01	0.31	1.3E-01	0.14	5.0E-01	-0.71	4.3E-05	0.02	9.2E-01	-0.26	1.9E-01	-0.31	1.2E-01	-0.21	3.0E-01	0.50	9.2E-03
ENSCAFG0000001181	GNPAT	grey	EC_M1C	0.44	2.4E-02	0.13	5.3E-01	-0.25	2.2E-01	-0.40	4.5E-02	-0.05	7.9E-01	-0.03	8.8E-01	-0.40	4.3E-02	0.11	5.9E-01	-0.23	2.5E-01	-0.15	4.6E-01
ENSCAFG0000001783	GGNBP2	grey	EC_M1C	0.44	2.5E-02	-0.25	2.2E-01	-0.43	3.0E-02	0.12	5.5E-01	0.14	4.9E-01	0.17	4.0E-01	0.01	9.7E-01	0.04	8.4E-01	-0.13	5.3E-01	-0.35	8.3E-02
ENSCAFG0000000063	VWA7	darkgrey	EC_M8	0.44	2.5E-02	-0.07	7.2E-01	-0.09	6.6E-01	0.12	5.5E-01	-0.20	3.4E-01	0.13	5.2E-01	-0.23	2.6E-01	-0.12	5.5E-01	0.05	8.0E-01	-0.07	7.4E-01
ENSCAFG0000001068	ENSCAFG0000001068	darkgrey	EC_M8	0.44	2.5E-02	0.02	9.3E-01	0.17	4.1E-01	-0.05	8.1E-01	-0.44	2.4E-02	0.02	9.3E-01	-0.44	2.4E-02	-0.09	6.5E-01	-0.13	5.4E-01	0.20	3.3E-01
ENSCAFG0000001229	NDST3	grey	EC_M1C	0.44	2.5E-02	-0.20	3.2E-01	-0.14	4.9E-01	0.19	3.5E-01	-0.17	4.1E-01	0.04	8.6E-01	-0.20	3.3E-01	0.16	4.3E-01	0.30	1.3E-01	-0.09	6.8E-01
ENSCAFG0000001579	WDR89	grey	EC_M1C	0.44	2.5E-02	-0.31	1.2E-01	-0.02	9.4E-01	0.35	7.8E-02	-0.27	1.7E-01	0.14	4.9E-01	-0.22	2.9E-01	-0.09	6.7E-01	-0.20	3.2E-01	0.01	9.6E-01
ENSCAFG0000000844	USO1	darkgreen	EC_M4	0.44	2.5E-02	0.15	4.5E-01	-0.92	3.3E-11	-0.08	7.1E-01	0.64	4.0E-04	0.28	1.7E-01	-0.18	3.9E-01	-0.09	6.6E-01	-0.27	1.8E-01	-0.89	7.1E-10
ENSCAFG0000001919	ANKS3	grey	EC_M1C	0.44	2.5E-02	0.07	7.2E-01	0.07	7.4E-01	0.19	3.6E-01	-0.47	1.4E-02	-0.24	2.4E-01	-0.15	4.5E-01	0.12	5.5E-01	0.08	6.9E-01	0.23	2.7E-01
ENSCAFG0000000437	ACBD5	grey	EC_M1C	0.44	2.5E-02	0.11	6.1E-01	0.04	8.3E-01	0.16	4.4E-01	-0.52	5.9E-03	-0.15	4.8E-01	-0.18	3.8E-01	0.19	3.5E-01	-0.21	3.0E-01	0.31	1.3E-01
ENSCAFG0000001161	GOLGB1	darkgreen	EC_M4	0.44	2.5E-02	0.35	8.4E-02	-0.75	8.9E-06	-0.08	6.8E-01	0.31	1.3E-01	0.14	4.9E-01	-0.10	6.4E-01	-0.06	7.7E-01	0.09	6.7E-01	-0.56	2.9E-03
ENSCAFG0000000663	SNAI2	grey	EC_M1C	0.44	2.5E-02	-0.17	4.0E-01	-0.58	1.7E-03	0.07	7.2E-01	0.33	1.0E-01	0.25	2.3E-01	-0.13	5.2E-01	0.07	7.3E-01	0.15	4.6E-01	-0.61	9.0E-04
ENSCAFG0000002891	ZSCAN21	grey	EC_M1C	0.44	2.5E-02	0.27	1.8E-01	-0.18	3.8E-01	-0.19	3.4E-01	-0.26	2.1E-01	0.08	6.8E-01	0.10	6.3E-01	0.08	7.0E-01	0.04	8.5E-01	0.01	9.6E-01
ENSCAFG0000001082	TTC23	darkgrey	EC_M8	0.44	2.5E-02	0.44	2.3E-02	0.00	1.0E+00	0.12	5.5E-01	-0.51	7.3E-03	-0.02	9.4E-01	0.03	8.9E-01	-0.13	5.3E-01	-0.65	3.2E-04	0.27	1.8E-01
ENSCAFG0000001449	ENSCAFG0000001449	grey	EC_M1C	0.44	2.5E-02	0.38	5.5E-02	-0.57	2.4E-03	0.06	7.8E-01	0.10	6.2E-01	-0.02	9.2E-01	0.11	5.8E-01	0.04	8.4E-01	-0.10	6.2E-01	-0.37	6.5E-02
ENSCAFG0000000824	ACTR8	darkgreen	EC_M4	0.44	2.5E-02	0.38	5.3E-02	-0.72	3.3E-05	-0.26	2.0E-01	0.39	4.8E-02	0.38	5.3E-02	-0.13	5.3E-01	-0.01	9.7E-01	-0.24	2.3E-01	-0.58	1.8E-03
ENSCAFG0000000441	ARL5B	grey	EC_M1C	0.44	2.5E-02	0.53	4.9E-03	-0.27	1.8E-01	0.10	6.2E-01	-0.28	1.7E-01	0.04	8.6E-01	0.15	4.8E-01	-0.13	5.4E-01	-0.18	3.7E-01	0.02	9.2E-01
ENSCAFG0000000370	CUL2	darkgreen	EC_M4	0.44	2.5E-02	0.29	1.5E-01	-0.91	2.1E-10	0.00	9.9E-01	0.58	2.0E-03	0.19	3.4E-01	-0.16	4.2E-01	-0.02	9.1E-01	-0.24	2.3E-01	-0.81	6.8E-07
ENSCAFG0000003148	ENSCAFG0000003148	darkgrey	EC_M8	0.44	2.5E-02	0.35	8.1E-02	-0.26	2.1E-01	0.29	1.5E-01	-0.21	3.0E-01	0.07	7.4E-01	-0.13	5.3E-01	-0.34	9.3E-02	-0.33	9.6E-02	-0.02	9.3E-01
ENSCAFG0000001687	ZNF410	turquoise	EC_M6	0.44	2.5E-02	0.60	1.2E-03	-0.54	4.2E-03	0.07	7.5E-01	0.02	9.3E-01	0.12	5.5E-01	0.17	4.0E-01	-0.35	8.0E-02	-0.24	2.4E-01	-0.24	2.4E-01
ENSCAFG0000002874	ENSCAFG0000002874	turquoise	EC_M6	0.44	2.5E-02	0.89	1.4E-09	-0.42	3.1E-02	-0.08	6.9E-01	-0.15	4.6E-01	-0.13	5.3E-01	-0.12	5.5E-01	-0.09	6.5E-01	-0.15	4.6E-01	-0.06	7.9E-01
ENSCAFG0000001796	ENSCAFG0000001796	darkgrey	EC_M8	0.44	2.5E-02	-0.10	6.2E-01	-0.12	5.5E-01	-0.13	5.3E-01	-0.09	6.6E-01	0.64	4.3E-04	-0.12	5.6E-01	-0.07	7.5E-01	-0.18	3.7E-01	-0.13	5.3E-01
ENSCAFG0000000384	RUVBL2	grey	EC_M1C	0.44	2.5E-02	-0.33	9.6E-02	0.23	2.6E-01	-0.05	8.2E-01	-0.42	3.4E-02	0.14	5.0E-01	-0.02	9.3E-01	0.09	6.6E-01	-0.09	6.6E-01	0.17	3.9E-01
ENSCAFG0000001060	ELF3	grey	EC_M1C	0.44	2.6E-02	0.18	3.7E-01	0.06	7.7E-01	0.08	7.0E-01	-0.47	1.6E-02	-0.07	7.5E-01	0.10	6.2E-01	0.12	5.7E-01	-0.02	9.3E-01	0.26	2.0E-01
ENSCAFG0000001248	ENSCAFG0000001248	darkgrey	EC_M8	0.44	2.6E-02	0.23	2.5E-01	-0.07	7.2E-01	-0.20	3.2E-01	-0.33	1.0E-01	-0.27	1.9E-01	-0.22	2.7E-01	-0.21	3.1E-01	-0.09	6.5E-01	0.05	8.0E-01
ENSCAFG0000000751	NLN	grey	EC_M1C	0.44	2.6E-02	0.46	1.8E-02	-0.19	3.6E-01	0.32	1.1E-01	-0.32	1.1E-01	0.06	7.7E-01	-0.12	5.6E-01	-0.18	3.9E-01	-0.10	6.4E-01	0.08	7.0E-01
ENSCAFG0000001485	COPS2	grey	EC_M1C	0.44	2.6E-02	-0.32	1.1E-01	-0.14	4.8E-01	0.30	1.4E-01	-0.13	5.1E-01	0.08	6.9E-01	-0.16	4.2E-01	0.07	7.5E-01	-0.37	6.6E-02	-0.10	6.2E-01
ENSCAFG0000001126	AJUBA	darkgrey	EC_M8	0.44	2.6E-02	0.46	1.9E-02	0.15	4.5E-01	0.21	3.0E-01	-0.73	2.1E-05	-0.17	4.2E-01	-0.12	5.5E-01	-0.12	5.7E-01	0.27	1.9E-01	0.51	7.4E-03
ENSCAFG0000000518	EDNRB	turquoise	EC_M6	0.44	2.6E-02	0.85	3.5E-08	-0.27	1.9E-01	-0.05	8.0E-01	-0.33	9.8E-02	-0.19	3.6E-01	-0.18	3.8E-01	-0.12	5.5E-01	-0.16	4.4E-01	0.07	7.3E-01
ENSCAFG0000001577	ENSCAFG0000001577	grey	EC_M1C	0.44	2.6E-02	0.23	2.6E-01	-0.23	2.5E-01	0.01	9.6E-01	-0.17	4.0E-01	0.21	3.0E-01	-0.24	2.4E-01	-0.34	8.7E-02	0.01	9.6E-01	-0.08	7.1E-01
ENSCAFG0000001100	CAMKV	grey	EC_M1C	0.44	2.6E-02	0.32	1.2E-01	-0.23	2.5E-01	-0.15	4.7E-01	-0.17	4.1E-01	-0.15	4.8E-01	-0.07	7.3E-01	-0.15	4.8E-01	0.03	8.9E-01	0.00	9.9E-01
ENSCAFG0000000220	SPIN1	grey	EC_M1C	0.44	2.6E-02	0.28	1.7E-01	-0.23	2.6E-01	0.15	4.8E-01	-0.16	4.3E-01	0.21	3.0E-01	-0.29	1.5E-01	-0.37	6.4E-02	-0.08	7.1E-01	-0.09	6.7E-01
ENSCAFG0000001197	PARL	grey	EC_M1C	0.44	2.6E-02	-0.36	7.0E-02	-0.52	6.3E-03	-0.16	4.5E-01	0.44	2.6E-02	0.21	3.1E-01	-0.40	4.5E-02	0.16	4.4E-01	-0.11	6.1E-01	-0.66	2.2E-04
ENSCAFG0000001432	PPM1L	grey	EC_M1C	0.44	2.6E-02	0.22	2.8E-01	-0.21	2.9E-01	-0.14	4.9E-01	-0.15	4.5E-01	0.33	9.7E-02	-0.22	2.8E-01	-0.36	7.0E-02	0.16	4.4E-01	-0.03	9.0E-01
ENSCAFG0000003275	NABP1	turquoise	EC_M6	0.44	2.6E-02	0.41	3.8E-02	-0.57	2.5E-03	-0.14	4.8E-01	0.12	5.5E-01	0.09	6.6E-01	0.03	8.8E-01	0.24	2.4E-01	-0.06	7.9E-01	-0.38	5.4E-02
ENSCAFG0000000615	ENSCAFG0000000615	darkgrey	EC_M8	0.44	2.6E-02	0.26	2.0E-01	-0.43	2.7E-02	0.27	1.9E-01	0.03	8.7E-01	0.11	5.8E-01	-0.24	2.4E-01	-0.25	2.1E-01	-0.19	3.6E-01	-0.23	2.5E-01
ENSCAFG0000000166	RBM28	darkgrey	EC_M8	0.44	2.6E-02	-0.19	3.4E-01	-0.66	2.4E-04	-0.37	6.2E-02	0.55	3.8E-03	0.34	8.7E-02	-0.01	9.4E-01	0.14	5.1E-01	0.00	1.0E+00	-0.77	5.0E-06
ENSCAFG0000002933	FOPNL	grey	EC_M1C	0.44	2.6E-02	-0.21	3.1E-01	-0.58	2.1E-03	-0.08	7.0E-01	0.32	1.2E-01	0.21	3.0E-01	-0.03	8.8E-01	0.18	3.9E-01	0.16	4.4E-01	-0.57	2.4E-03
ENSCAFG0000000049	ENSCAFG0000000049	grey	EC_M1C	0.44	2.6E-02	0.17	4.0E-01	-0.51	8.2E-03	-0.35	7.8E-02	0.24	2.4E-01	0.50	9.4E-03	-0.10	6.4E-01	-0.32	1.1E-01	-0.07	7.4E-01	-0.48	1.3E-02
ENSCAFG0000001873	KIAA0100	grey	EC_M1C	0.44	2.6E-02	0.37	5.9E-02	-0.11	6.0E-01	0.05	8.1E-01	-0.32	1.1E-01	-0.20	3.4E-01	-0.49	1.2E-02	-0.14	4.8E-01	-0.06	7.7E-01	0.15	4.7E-01
ENSCAFG0000000557	NDUFAF5	grey	EC_M1C	0.44	2.6E-02	-0.32	1.1E-01	-0.42	3.2E-02	0.05	8.3E-01	0.22	2.8E-01	0.19	3.6E-01	-0.13	5.4E-01	0.18	3.8E-01	0.08	6.8E-01	-0.46	1.8E-02
ENSCAFG0000000935	ENSCAFG0000000935	grey	EC_M1C	0.44	2.6E-02	0.29	1.5E-01	-0.54	4.5E-03	0.30	1.4E-01	0.16	4.5E-01	-0.06	7.9E-01	-0.02	9.1E-01	-0.07	7.3E-01	-0.26	2.0E-01	-0.38	5.4E-02
ENSCAFG0000001644	MED1	darkgreen	EC_M4	0.44	2.6E-02	0.23	2.7E-01	-0.74	1.7E-05	-0.14	5.0E-01	0.47	1.5E-02	0.29	1.5E-01	-0.28	1.7E-01	-0.22	2.8E-01	-0.32	1.1E-01	-0.69	1.0E-04
ENSCAFG0000001901	ENSCAFG0000001901	grey	EC_M1C	0.44	2.6E-02	0.25	2.2E-01	-0.35	7.6E-02	0.06	7.5E-01	-0.04	8.5E-01	0.08	6.9E-01	-0.29	1.6E-01	-0.10	6.4E-01	-0.09	6.6E-01	-0.16	4.5E-01
ENSCAFG0000000350	FAM3C	darkgrey	EC_M8	0.44	2.6E-02	0.76	5.8E-06	-0.25	2.1E-01	-0.01	9.5E-01	-0.38	5.9E-02	-0.17	4.1E-01	0.11	6.0E-01	-0.08	7.1E-01	-0.10	6.2E-01	0.15	4.8E-01
ENSCAFG0000001420	IL12A	darkgrey	EC_M8	0.44	2.6E-02	-0.12	5.5E-01	0.15	4.7E-01	0.26	2.0E-01	-0.57	2.6E-03	0.11	6.0E-01	-0.28	1.6E-01	-0.07	7.5E-01	-0.12	5.5E-01	0.32	1.2E-01
ENSCAFG0000001438	MAP4K5	darkgreen	EC_M4	0.44	2.6E-02	0.32	1.1E-01	-0.70	7.9E-05	-0.11	6.0E-01	0.31	1.3E-01	0.28	1.7E-01	-0.04	8.4E-01	-0.41	4.0E-02	-0.30	1.4E-01	-0.54	4.6E-03
ENSCAFG0000000292	GRSF1	grey	EC_M1C	0.43	2.6E-02	-0.08	6.8E-01	0.12	5.8E-01	0.26	2.1E-01	-0.48	1.4E-02	0.09	6.7E-01	-0.26	2.0E-01	-0.33	9.6E-02	0.17	4.2E-01	0.28	1.7E-01
ENSCAFG0000001929	METTL16	darkgrey	EC_M8	0.43	2.6E-02	-0.43	2.8E-02	-0.48	1.4E-02	-0.14	4.9E-01	0.43	3.0E-02	0.46	1.8E-02	-0.14	4.9E-01	-0.07	7.2E-01	-0.15	4.6E-01	-0.63	5.0E-04
ENSCAFG0000002987	ZNF180	grey	EC_M1C	0.43	2.7E-02	0.22	2.7E-01	-0.13	5.4E-01	-0.04	8.3E-01	-0.33	1.0E-01	-0.08	7.0E-01	-0.23	2.5E-01	-0.34	8.7E-02	-0.13	5.3E-01	0.06	7.6E-01
ENSCAFG0000001466	SLC35F2	darkgrey	EC_M8	0.43	2.7E-02	-0.41	3.6E-02	0.07	7.5E-01	-0.19	3.5E-01	-0.21	2.9E-01	-0.10	6.3E-01	0.09	6.5E-01	0.02	9.3E-01	-0.06	7.6E-01	0.01	9.8E-01
ENSCAFG0000000070	EIF3E	darkgrey	EC_M8	0.43	2.7E-02	-0.06	7.6E-01	0.32	1.2E-01	0.25	2.3E-01	-0.73	1.9E-05	-0.02	9.4E-01	-0.07	7.3E-01	-0.16	4.5E-01	-0.60	1.3E-03	0.47	1.5E-02
ENSCAFG0000000178	ZNF300	darkgrey	EC_M8	0.43	2.7E-02	0.46	1.7E-02	-0.20	3.3E-01	0.20	3.4E-01	-0.35	8.0E-02	-0.18	3.8E-01	-0.04	8.5E-01	0.26	2.0E-01	0.16	4.5E-01	0.15	4.6E-01
ENSCAFG0000000679	STARD7	turquoise	EC_M6	0.43	2.7E-02	0.63	5.9E-04	-0.08	7.0E-01	0.05	8.0E-01	-0.45	2.1E-02	-0.17	4.0E-01	-0.21	3.1E-01	-0.29	1.5E-01	-0.14	4.9E-01	0.28	1.6E-01
ENSCAFG0000000710	PDE4D	turquoise	EC_M6	0.43	2.7E-02	0.76	7.5E-06	-0.62	7.2E-04	-0.09	6.4E-01	0.10	6.1E-01	0.18	3.8E-01	-0.13	5.3E-01	0.01	9.7E-01	-0.01	9.5E-01	-0.34	8.8E-02
ENSCAFG0000003264	MRRF	turquoise	EC_M6	0.43	2.7E-02	0.74	1.7E-05	-0.53	4.9E-03	-0.06	7.6E-01	0.06	7.5E-01	0.09	6.8E-01	-0.31	1.3E-01	0.05	8.1E-01	-0.19	3.5E-01	-0.29	1.5E-01
ENSCAFG0000001696	CLK2	darkgrey	EC_M8	0.43	2.7E-02	0.56	2.7E-03	0.02	9.2E-01	0.18	3.7E-01	-0.61	9.1E-04	0.27	1.8E-01	-0.08	7.1E-01	-0.09	6.5E-01	-0.01	9.5E-01	0.37	6.0E-02
ENSCAFG0000001507	PDAP1	darkgreen	EC_M4	0.43	2.7E-02	0.08	7.0E-01	-0.80	8.1E-07	-0.09	6.5E-01	0.58	2.0E-03	0.29	1.4E-01	0.09	6.6E-01	0.17	4.0E-01	-0.17	4.0E-01	-0.83	1.1E-07
ENSCAFG0000001732	BRWD3	grey	EC_M1C	0.43	2.7E-02	0.29	1.5E-01	-0.40	4.4E-02	0.01	9.5E-01	-0.07	7.3E-01	0.11	5.8E-01	-0.11	5.8E-01	-0.08	7.0E-01	-0.27	1.9E-01	-0.17	4.0E-01
ENSCAFG0000001417	MFSD1	grey	EC_M1C	0.43	2.7E-02	0.10	6.4E-01	0.15	4.5E-01	0.07	7.4E-01	-0.58	1.7E-03	-0.01	9.8E-01	-0.45	2.0E-02	-0.05	8.1E-01	-0.19	3.6E-01	0.34	8.9E-02
ENSCAFG0000001877	LONP1	turquoise	EC_M6	0.43	2.7E-02	0.58	2.1E-03	-0.65	3.1E-04	-0.24	2.3E-01	0.26	1.9E-01	0.17	4.0E-01	0.07	7.4E-01	-0.07	7.3E-01	-0.03	8.8E-01	-0.50	9.2E-03
ENSCAFG0000000237	POLR1E	grey	EC_M1C	0.43	2.7E-02	-0.30	1.4E-01	0.23	2.7E-01	0.09	6.7E-01	-0.45	2.3E-02	0.12	5.5E-01	-0.26	2.1E-01	-0.22	2.9E-01	-0.34	9.2E-02	0.18	3.9E-01
ENSCAFG0000002848	ENSCAFG0000002848	grey	EC_M1C	0.43	2.7E-02	0.12	5.5E-01	-0.63	5.7E-04	0.24	2.5E-01	0.31	1.2E-01	0.34	8.5E-02	-0.02	9.4E-01	-0.27	1.9E-01	-0.30	1.4E-01	-0.54	4.0E-03
ENSCAFG0000000522	GDI2	darkgrey	EC_M8	0.43	2.7E-02	0.13	5.1E-01	-0.21	3.1E-01	-0.14	4.8E-01	0.00	9.9E-01	0.08	7.1E-01	-0.21	3.1E-01	-0.13	5.3E-01	-0.13	5.2E-01	-0.20	3.3E-01
ENSCAFG0000000717	ENSCAFG0000000717	grey	EC_M1C	0.43	2.7E-02	0.26	1.9E-01	-0.42	3.4E-02	0.20	3.2E-01	0.06	7.6E-01	-0.05	8.1E-01	-0.14	4.9E-01	-0.49	1.2E-02	-0.19	3.6E-01	-0.29	1.5E-01
ENSCAFG0000001769	USP31	turquoise	EC_M6	0.43	2.8E-02	0.82	2.3E-07	-0.40	4.1E-02	-0.09	6.8E-01	-0.15	4.8E-01	-0.11	6.1E-01	-0.17	3.9E-01	-0.13	5.3E-01	-0.39	4.7E-02	-0.08	6.9E-01
ENSCAFG0000001276	UBE4A	darkgreen	EC_M4	0.43	2.8E-02	0.16	4.4E-01	-0.81	5.7E-07	-0.19	3.4E-01	0.57	2.5E-03	0.20	3.3E-01	-0.25	2.2E-01	-0.01	9.6E-01	-0.33	1.0E-01	-0.79	1.7E-06
ENSCAFG0000001578	CCDC77	grey	EC_M1C	0.43	2.8E-02	-0.03	8.9E-01	-0.05	8.1E-01	0.05	8.0E-01	-0.33	9.9E-02	-0.14	5.0E-01	-0.13	5.3E-01	0.28	1.7E-01	0.14	4.9E-01	0.06	7.6E-01
ENSCAFG0000000246	THADA	grey	EC_M1C	0.43	2.8E-02	-0.01	9.5E-01	0.29	1.6E-01	0.24	2.3E-01	-0.62	7.1E-04	-0.23	2.5E-01	-0.23	2.5E-01	-0.19	3.5E-01	-0.50	9.9E-03	0.41	3.8E-02
ENSCAFG0000001883	RAP2C	grey	EC_M1C	0.43	2.8E-02	0.05	8.2E-01	-0.56	2.9E-03	0.08	6.9E-01	0.23	2.6E-01	0.12	5.6E-01	-0.08	7.1E-01	-0.20	3.2E-01	-0.25	2.1E-01	-0.52	6.0E-03
ENSCAFG0000001963	VBP1	grey	EC_M1C	0.43	2.8E-02	0.07	7.4E-01	-0.41	3.9E-02	0.08	6.9E-01	0.11	5.9E-01	-0.19	3.6E-01	-0.21	2.9E-01	0.41	3.8E-02	-0.05	7.9E-01	-0.33	9.5E-02
ENSCAFG0000000743	EIF2B1	darkgrey	EC_M8	0.43	2.8E-02	-0.23	2.6E-01	-0.66	2.4E-04	-0.01	9.6E-01	0.56	2.9E-03	0.19	3.6E-01	-0.24	2.4E-01	0.09	6.6E-01	-0.17	4.1E-01	-0.80	1.1E-06
ENSCAFG0000000716	CHD7	turquoise	EC_M6	0.43	2.8E-02	0.83	1.4E-07	-0.69	8.6E-05	-0.06	7.7E-01	0.17	4.1E-01	0.02	9.1E-01	-0.15	4.6E-01	-0.04	8.6E-01	0.07	7.4E-01	-0.40	4.0E-02
ENSCAFG0000000310	UBE2J1	darkgreen	EC_M4	0.43	2.8E-02	0.35	7.9E-02	-0.83	2.0E-07	-0.06	7.8E-01	0.44	2.3E-02	0.17	4.1E-01	-0.09	6.5E-01	-0.03	9.0E-01	-0.36	7.1E-02	-0.69	9.4E-05
ENSCAFG0000000585	ENSCAFG0000000585	grey	EC_M1C	0.43	2.8E-02	0.23	2.6E-01	-0.45	2.1E-02	0.06	7.8E-01	0.11	6.0E-01	0.21	3.0E-01	-0.28	1.6E-01	-0.24	2.3E-01	-0.24	2.4E-01	-0.31	1.2E-01
ENSCAFG0000001149	MRPL46	grey	EC_M1C	0.43	2.8E-02	-0.20	3.3E-01	0.17	3.9E-01	0.16	4.5E-01	-0.46	1.7E-02	0.06	7.7E-01	0.01	9.6E-01	-0.29	1.4E-01	-0.03	8.9E-01	0.29	1.6E-01
ENSCAFG0000002106	ENSCAFG0000002106	grey	EC_M1C	0.43	2.8E-02	0.42	3.2E-02	-0.13	5.4E-01	0.14	5.0E-01	-0.36	6.8E-02	-0.03	8.7E-01	-0.13	5.3E-01	-0.08	7.1E-01	-0.36	7.1E-02	0.12	5.5E-01
ENSCAFG0000001553	DHX33	darkgrey	EC_M8	0.43	2.8E-02	0.34	9.4E-02	-0.18	3.9E-01	-0.31	1.2E-01	-0.16	4.4E-01	0.09	6.7E-01	-0.23	2.6E-01	-0.07	7.2E-01	0.04	8.4E-01	-0.06	7.5E-01
ENSCAFG0000001838	METTL4	grey	EC_M1C	0.43	2.8E-02	-0.26	1.9E-01	-0.25	2.3E-01	0.07	7.5E-01	-0.02	9.4E-01	0.27	1.8E-01	-0.18	3.8E-01	-0.18	3.7E-01	-0.38	5.5E-02	-0.30	1.4E-01
ENSCAFG0000000180	TOPORS	darkgrey	EC_M8	0.43	2.8E-02	0.30	1.4E-01	-0.16	4.4E-01	0.02	9.2E-01	-0.38	5.8E-02	-0.05	8.0E-01	-0.02	9.3E-01	-0.03	8.7E-01	-0.37	6.4E-02	0.13	5.3E-01
ENSCAFG0000000319	ENSCAFG0000000319	grey	EC_M1C	0.43	2.8E-02	0.25	2.1E-01	-0.56	2.9E-03	0.35	8.2E-02	0.23	2.6E-01	0.16	4.4E-01	-0.11	5.9E-01	-0.36	7.1E-02	-0.08	7.1E-01	-0.40	4.1E-02
ENSCAFG0000001989	SETX	grey	EC_M1C	0.43	2.9E-02	0.27	1.8E-01	-0.55	3.3E-03	0.18	3.7E-01	0.18	3.9E-01	0.02	9.1E-01	-0.16	4.4E-01	-0.19	3.4E-01	-0.39	5.2E-02	-0.40	4.1E-02
ENSCAFG0000000825	ENSCAFG0000000825	grey	EC_M1C	0.43	2.9E-02	0.20	3.3E-01	-0.58	1.7E-03	0.15	4.5E-01	0.22	2.7E-01	0.20	3.3E-01	-0.14	4.8E-01	-0.23	2.5E-01	-0.37	6.0E-02	-0.46	1.9E-02
ENSCAFG0000001938	LMNB2	darkgrey	EC_M8	0.43	2.9E-02	-0.27	1.9E-01	0.11	6.0E-01	-0.17	4.1E-01	-0.29	1.5E-01	0.37	6.3E-02	-0.05	8.0E-01	-0.43	2.7E-02	-0.45	2.0E-02	0.05	8.2E-01
ENSCAFG0000001807	TRAF3	grey	EC_M1C	0.43	2.9E-02	0.01	9.7E-01	-0.58	1.9E-03	-0.12	5.5E-01	0.40	4.4E-02	0.26	1.9E-01	-0.01	9.5E-01	-0.09	6.5E-01	-0.18	3.9E-01	-0.59	1.4E-03
ENSCAFG0000000767	ENSCAFG0000000767	grey	EC_M1C	0.43	2.9E-02	0.08	6.9E-01	-0.44	2.3E-02	-0.17	4.0E-01	0.19	3.6E-01	0.24	2.3E-01	-0.13	5.3E-01	-0.16	4.3E-01	-0.03	9.0E-01	-0.40	4.5E-02
ENSCAFG0000000300	RLF	darkgrey	EC_M8	0.43	2.9E-02	0.43	3.0E-02	-0.40	4.4E-02	0.01	9.7E-01	-0.14	4.8E-01	0.22	2.9E-01	0.06	7.8E-01	-0.11	5.9E-01	-0.30	1.3E-01	-0.13	5.4E-01
ENSCAFG0000002265	ENSCAFG0000002265	grey	EC_M1C	0.43	2.9E-02	0.08	7.0E-01	0.05	8.1E-01	0.10	6.2E-01	-0.44	2.4E-02	-0.08	6.8E-01	0.07	7.2E-01	0.00	9.9E-01	-0.05	8.1E-01	0.23	2.7E-01

ENSCAFG0000001168	TRUB1	grey	EC_M10	0.43	2.9E-02	-0.13	5.2E-01	0.11	5.9E-01	0.17	4.0E-01	-0.47	1.7E-02	0.00	1.0E+00	-0.22	2.9E-01	-0.02	9.1E-01	-0.31	1.2E-01	0.23	2.7E-01
ENSCAFG0000001022	CDKAL1	grey	EC_M10	0.43	2.9E-02	-0.05	8.0E-01	-0.02	9.3E-01	0.01	9.5E-01	-0.30	1.4E-01	0.06	7.5E-01	-0.42	3.2E-02	-0.34	8.4E-02	-0.13	5.1E-01	0.04	8.3E-01
ENSCAFG0000000112	PHF20L1	darkgreen	EC_M4	0.43	2.9E-02	0.20	3.3E-01	-0.81	5.5E-07	-0.21	3.0E-01	0.46	1.7E-02	0.44	2.6E-02	-0.21	3.1E-01	-0.03	9.0E-01	-0.12	5.7E-01	-0.72	3.4E-05
ENSCAFG0000001128	OSTC	grey	EC_M10	0.43	2.9E-02	-0.10	6.2E-01	-0.56	2.8E-03	0.19	3.5E-01	0.29	1.5E-01	0.52	6.3E-03	-0.08	7.0E-01	-0.11	5.9E-01	-0.39	4.7E-02	-0.57	2.6E-03
ENSCAFG0000000571	ZDHHC17	darkgrey	EC_M8	0.43	2.9E-02	0.38	5.5E-02	-0.10	6.2E-01	0.15	4.7E-01	-0.48	1.4E-02	-0.18	3.8E-01	-0.21	2.9E-01	-0.10	6.4E-01	-0.24	2.4E-01	0.24	2.4E-01
ENSCAFG0000000693	HAUS5	darkgrey	EC_M8	0.43	2.9E-02	0.40	4.1E-02	0.11	6.1E-01	-0.06	7.7E-01	-0.55	3.6E-03	-0.06	7.6E-01	-0.13	5.2E-01	-0.34	8.9E-02	0.21	3.1E-01	0.38	5.8E-02
ENSCAFG0000000760	OSBP	darkgreen	EC_M4	0.43	2.9E-02	0.56	3.1E-03	-0.76	6.5E-06	-0.08	7.0E-01	0.27	1.8E-01	0.16	4.2E-01	0.02	9.3E-01	-0.18	3.9E-01	-0.20	3.2E-01	-0.54	4.4E-03
ENSCAFG0000001535	BAIAP2L1	turquoise	EC_M6	0.43	2.9E-02	0.85	5.5E-08	-0.40	4.5E-02	-0.15	4.5E-01	-0.15	4.7E-01	-0.03	8.9E-01	-0.17	4.0E-01	-0.06	7.8E-01	0.04	8.6E-01	-0.05	8.1E-01
ENSCAFG0000000951	YWHAB	grey	EC_M10	0.43	2.9E-02	0.31	1.2E-01	-0.80	7.4E-07	-0.25	2.1E-01	0.55	3.7E-03	0.13	5.2E-01	-0.26	2.0E-01	-0.12	5.6E-01	0.08	7.1E-01	-0.74	1.8E-05
ENSCAFG0000000529	ENSCAFG0000000529	grey	EC_M10	0.43	2.9E-02	0.06	7.6E-01	-0.07	7.3E-01	-0.11	6.0E-01	-0.26	2.0E-01	-0.19	3.6E-01	-0.19	3.5E-01	-0.16	4.2E-01	-0.20	3.2E-01	0.04	8.5E-01
ENSCAFG0000001155	BPTF	darkgrey	EC_M8	0.43	2.9E-02	0.40	4.3E-02	-0.25	2.3E-01	0.00	9.9E-01	-0.29	1.5E-01	-0.17	4.1E-01	-0.24	2.3E-01	-0.10	6.3E-01	-0.26	1.9E-01	0.04	8.6E-01
ENSCAFG0000000056	ZNF608	darkgrey	EC_M8	0.43	2.9E-02	0.21	3.1E-01	0.17	4.0E-01	0.20	3.4E-01	-0.67	1.6E-04	-0.23	2.6E-01	-0.01	9.5E-01	0.12	5.7E-01	0.05	8.0E-01	0.42	3.4E-02
ENSCAFG0000002367	ENSCAFG0000002367	grey	EC_M10	0.43	2.9E-02	-0.08	7.2E-01	-0.41	3.5E-02	0.07	7.4E-01	0.14	4.8E-01	0.14	4.8E-01	-0.04	8.4E-01	-0.01	9.6E-01	-0.35	8.4E-02	-0.34	8.5E-02
ENSCAFG0000003088	BMT2	grey	EC_M10	0.43	2.9E-02	0.37	6.0E-02	-0.42	3.3E-02	-0.07	7.4E-01	-0.08	7.0E-01	-0.10	6.4E-01	-0.32	1.1E-01	-0.01	9.8E-01	0.14	4.9E-01	-0.15	4.7E-01
ENSCAFG0000000424	ASXL2	grey	EC_M10	0.43	3.0E-02	0.33	1.0E-01	-0.11	5.9E-01	-0.04	8.4E-01	-0.38	5.7E-02	-0.29	1.4E-01	-0.20	3.2E-01	-0.18	3.9E-01	-0.49	1.2E-02	0.12	5.7E-01
ENSCAFG0000001067	ASB7	darkgrey	EC_M8	0.43	3.0E-02	0.55	3.7E-03	-0.08	7.1E-01	0.04	8.4E-01	-0.53	5.7E-03	-0.01	9.5E-01	-0.19	3.6E-01	-0.10	6.3E-01	0.04	8.4E-01	0.27	1.8E-01
ENSCAFG0000003034	C33H3orf52	turquoise	EC_M6	0.43	3.0E-02	0.63	6.2E-04	-0.42	3.1E-02	0.09	6.6E-01	-0.07	7.3E-01	-0.19	3.5E-01	-0.19	3.6E-01	-0.23	2.5E-01	0.08	7.1E-01	-0.12	5.6E-01
ENSCAFG0000000659	ENSCAFG0000000659	darkgrey	EC_M8	0.43	3.0E-02	0.05	8.0E-01	-0.17	3.9E-01	-0.08	6.9E-01	-0.03	8.8E-01	-0.13	5.3E-01	-0.05	7.9E-01	-0.07	7.2E-01	-0.14	5.0E-01	-0.16	4.2E-01
ENSCAFG0000001806	ACSL4	darkgreen	EC_M4	0.43	3.0E-02	-0.03	8.9E-01	-0.66	2.4E-04	0.09	6.8E-01	0.39	4.8E-02	0.15	4.5E-01	-0.09	6.4E-01	-0.29	1.5E-01	-0.57	2.6E-03	-0.66	2.1E-04
ENSCAFG0000000407	KIF5B	darkgreen	EC_M4	0.43	3.0E-02	0.26	2.0E-01	-0.96	2.4E-15	-0.19	3.6E-01	0.70	5.9E-05	0.24	2.4E-01	-0.15	4.7E-01	0.01	9.5E-01	-0.08	7.0E-01	-0.91	7.7E-11
ENSCAFG0000000888	CCNL1	darkgrey	EC_M8	0.43	3.0E-02	0.48	1.3E-02	0.11	5.8E-01	0.26	2.0E-01	-0.71	4.6E-05	-0.07	7.2E-01	0.06	7.6E-01	-0.12	5.6E-01	-0.21	3.0E-01	0.47	1.6E-02
ENSCAFG0000002826	ENSCAFG0000002826	grey	EC_M10	0.43	3.0E-02	0.13	5.3E-01	-0.21	3.1E-01	0.25	2.1E-01	-0.21	3.1E-01	0.29	1.6E-01	-0.26	2.0E-01	-0.53	5.4E-03	-0.15	4.6E-01	-0.02	9.3E-01
ENSCAFG0000002504	ENSCAFG0000002504	grey	EC_M10	0.43	3.0E-02	0.16	4.4E-01	-0.56	3.1E-03	-0.08	6.9E-01	0.26	2.0E-01	0.09	6.5E-01	-0.21	3.1E-01	-0.04	8.4E-01	-0.13	5.2E-01	-0.46	1.9E-02
ENSCAFG0000001734	GPR65	darkgreen	EC_M4	0.43	3.0E-02	-0.09	6.6E-01	-0.68	1.4E-04	-0.09	6.5E-01	0.47	1.6E-02	0.32	1.1E-01	-0.25	2.3E-01	-0.15	4.5E-01	-0.18	3.8E-01	-0.75	8.4E-06
ENSCAFG0000001764	HAUS1	grey	EC_M10	0.43	3.0E-02	0.36	6.7E-02	-0.11	5.9E-01	-0.12	5.6E-01	-0.31	1.2E-01	-0.52	6.4E-03	-0.09	6.8E-01	-0.22	2.9E-01	0.02	9.3E-01	0.09	6.5E-01
ENSCAFG0000000213	ERMP1	grey	EC_M10	0.43	3.0E-02	0.18	3.8E-01	-0.28	1.7E-01	-0.11	6.0E-01	-0.01	9.7E-01	-0.21	3.1E-01	-0.47	1.7E-02	0.04	8.5E-01	-0.19	3.6E-01	-0.15	4.6E-01
ENSCAFG0000001888	FAM122B	darkgreen	EC_M4	0.43	3.0E-02	0.06	7.7E-01	-0.77	3.6E-06	-0.20	3.3E-01	0.57	2.4E-03	0.36	7.0E-02	0.10	6.4E-01	0.02	9.0E-01	-0.07	7.4E-01	-0.76	6.0E-06
ENSCAFG0000000093	PACSIN2	turquoise	EC_M6	0.43	3.0E-02	0.75	1.1E-05	-0.72	3.2E-05	-0.23	2.6E-01	0.28	1.7E-01	-0.01	9.7E-01	0.01	9.8E-01	0.00	9.9E-01	0.00	9.9E-01	-0.48	1.4E-02
ENSCAFG0000001225	LTB4R	turquoise	EC_M6	0.43	3.0E-02	0.91	1.5E-10	-0.42	3.5E-02	-0.08	6.9E-01	-0.16	4.3E-01	-0.13	5.3E-01	-0.12	5.5E-01	-0.09	6.5E-01	-0.15	4.6E-01	-0.04	8.5E-01
ENSCAFG0000003220	TYW3	grey	EC_M10	0.43	3.0E-02	-0.05	8.2E-01	-0.13	5.4E-01	0.52	7.0E-03	-0.22	2.8E-01	0.03	9.0E-01	-0.07	7.3E-01	-0.19	3.4E-01	-0.32	1.1E-01	0.01	9.5E-01
ENSCAFG0000001182	PRPF3	darkgreen	EC_M4	0.43	3.0E-02	0.01	9.7E-01	-0.65	3.0E-04	0.03	8.7E-01	0.41	4.0E-02	0.32	1.2E-01	0.24	2.4E-01	-0.05	8.2E-01	-0.49	1.1E-02	-0.65	3.5E-04
ENSCAFG0000000904	SENP1	darkgrey	EC_M8	0.43	3.0E-02	0.42	3.3E-02	0.21	3.0E-01	0.28	1.7E-01	-0.78	2.3E-06	-0.19	3.5E-01	-0.17	3.9E-01	-0.19	3.5E-01	-0.17	4.0E-01	0.54	4.3E-03
ENSCAFG0000003253	RCHY1	darkgrey	EC_M8	0.43	3.0E-02	-0.05	7.9E-01	0.23	2.6E-01	0.22	2.8E-01	-0.64	4.8E-04	0.11	6.0E-01	-0.01	9.5E-01	-0.11	5.9E-01	0.04	8.4E-01	0.43	2.9E-02
ENSCAFG0000001288	SEC11A	darkgreen	EC_M4	0.42	3.0E-02	-0.19	3.6E-01	-0.59	1.5E-03	0.04	8.5E-01	0.39	4.7E-02	0.26	1.9E-01	0.00	9.9E-01	0.17	4.0E-01	-0.32	1.1E-01	-0.66	2.3E-04
ENSCAFG0000002521	FAM111B	darkgrey	EC_M8	0.42	3.0E-02	0.06	7.8E-01	0.18	3.8E-01	0.24	2.4E-01	-0.66	2.2E-04	-0.04	8.5E-01	0.04	8.5E-01	0.14	5.1E-01	-0.05	8.3E-01	0.41	3.7E-02
ENSCAFG0000001372	SLC25A16	darkgreen	EC_M4	0.42	3.0E-02	-0.17	4.0E-01	-0.75	9.2E-06	0.09	6.5E-01	0.59	1.6E-03	0.40	4.3E-02	0.00	9.9E-01	-0.16	4.2E-01	-0.28	1.7E-01	-0.82	3.4E-07
ENSCAFG0000001703	CIAO2A	darkgrey	EC_M8	0.42	3.0E-02	-0.08	6.9E-01	0.32	1.1E-01	0.09	6.5E-01	-0.74	1.6E-05	-0.03	8.8E-01	-0.04	8.5E-01	-0.06	7.6E-01	-0.39	4.6E-02	0.49	1.1E-02
ENSCAFG0000002218	ENSCAFG0000002218	grey	EC_M10	0.42	3.1E-02	0.02	9.3E-01	-0.15	4.6E-01	-0.16	4.3E-01	-0.15	4.7E-01	-0.10	6.2E-01	-0.10	6.3E-01	-0.07	7.3E-01	-0.06	7.7E-01	-0.07	7.3E-01
ENSCAFG0000000144	PIM1	turquoise	EC_M6	0.42	3.1E-02	0.51	8.4E-03	-0.40	4.5E-02	0.05	8.1E-01	-0.13	5.3E-01	0.14	5.0E-01	0.02	9.4E-01	-0.30	1.3E-01	-0.06	7.6E-01	-0.14	4.8E-01
ENSCAFG0000000893	SMYD5	darkgrey	EC_M8	0.42	3.1E-02	-0.14	5.0E-01	-0.45	2.3E-02	-0.02	9.2E-01	0.30	1.3E-01	0.41	3.6E-02	-0.23	2.6E-01	-0.03	8.8E-01	-0.05	7.9E-01	-0.54	4.7E-03
ENSCAFG0000001122	ENSCAFG0000001122	grey	EC_M10	0.42	3.1E-02	0.30	1.3E-01	-0.50	9.4E-03	-0.08	6.9E-01	0.15	4.5E-01	0.03	8.8E-01	-0.30	1.4E-01	-0.41	3.9E-02	-0.05	8.0E-01	-0.35	8.1E-02
ENSCAFG0000000549	THUMPD3	grey	EC_M10	0.42	3.1E-02	0.23	2.6E-01	-0.56	2.9E-03	-0.35	8.3E-02	0.19	3.5E-01	-0.19	3.6E-01	-0.11	5.9E-01	0.15	4.8E-01	0.07	7.5E-01	-0.43	3.0E-02
ENSCAFG0000000626	ENSCAFG0000000626	grey	EC_M10	0.42	3.1E-02	0.19	3.4E-01	-0.27	1.8E-01	0.24	2.5E-01	-0.13	5.4E-01	-0.19	3.6E-01	-0.12	5.5E-01	-0.23	2.6E-01	-0.18	3.8E-01	-0.11	6.0E-01
ENSCAFG0000000377	AFG1L	darkgrey	EC_M8	0.42	3.1E-02	0.53	5.8E-03	-0.13	5.3E-01	0.17	4.0E-01	-0.46	1.8E-02	-0.21	3.0E-01	-0.29	1.5E-01	-0.13	5.2E-01	-0.06	7.6E-01	0.21	3.0E-01
ENSCAFG0000000438	CTSC	turquoise	EC_M6	0.42	3.1E-02	0.89	1.1E-09	-0.41	3.7E-02	-0.07	7.5E-01	-0.17	4.1E-01	-0.03	9.0E-01	-0.13	5.4E-01	-0.19	3.6E-01	-0.09	6.6E-01	-0.08	6.9E-01
ENSCAFG0000002912	ENSCAFG0000002912	darkgrey	EC_M8	0.42	3.1E-02	0.32	1.1E-01	-0.52	6.8E-03	0.16	4.3E-01	0.12	5.7E-01	0.36	7.2E-02	-0.26	1.9E-01	-0.37	6.6E-02	-0.16	4.5E-01	-0.35	8.4E-02
ENSCAFG0000001436	GRPEL1	darkgrey	EC_M8	0.42	3.1E-02	-0.05	8.1E-01	-0.56	3.0E-03	-0.28	1.7E-01	0.37	6.4E-02	0.18	3.9E-01	0.08	7.0E-01	0.17	4.0E-01	-0.01	9.6E-01	-0.57	2.6E-03
ENSCAFG0000001068	SINHCAF	grey	EC_M10	0.42	3.1E-02	0.40	4.2E-02	-0.41	3.6E-02	-0.11	6.0E-01	0.05	8.0E-01	0.07	7.3E-01	-0.05	8.0E-01	-0.07	7.3E-01	-0.33	1.0E-01	-0.28	1.6E-01
ENSCAFG0000001896	ATF7IP2	turquoise	EC_M6	0.42	3.1E-02	0.83	2.1E-07	-0.31	1.2E-01	-0.19	3.6E-01	-0.24	2.3E-01	-0.19	3.6E-01	-0.14	4.9E-01	-0.20	3.3E-01	0.19	3.4E-01	0.06	7.9E-01
ENSCAFG0000002193	ENSCAFG0000002193	darkgrey	EC_M8	0.42	3.1E-02	0.00	9.9E-01	-0.35	8.4E-02	0.17	4.1E-01	-0.05	8.2E-01	0.08	6.9E-01	-0.13	5.3E-01	-0.02	9.2E-01	-0.36	6.9E-02	-0.23	2.7E-01
ENSCAFG0000002917	ENSCAFG0000002917	turquoise	EC_M6	0.42	3.1E-02	0.55	3.7E-03	-0.10	6.3E-01	-0.11	5.9E-01	-0.35	7.9E-02	-0.20	3.3E-01	-0.13	5.1E-01	-0.12	5.6E-01	-0.18	3.7E-01	0.17	4.1E-01
ENSCAFG0000001981	VAV2	grey	EC_M10	0.42	3.2E-02	0.22	2.8E-01	-0.58	2.0E-03	-0.15	4.8E-01	0.29	1.5E-01	0.16	4.4E-01	0.22	2.7E-01	-0.15	4.8E-01	-0.26	2.0E-01	-0.52	6.6E-03
ENSCAFG0000001313	FOSL1	darkgrey	EC_M8	0.42	3.2E-02	-0.08	6.9E-01	0.02	9.4E-01	-0.05	8.0E-01	-0.25	2.2E-01	0.01	9.8E-01	-0.02	9.1E-01	0.13	5.3E-01	-0.04	8.5E-01	0.03	8.7E-01
ENSCAFG0000001881	RAI14	darkgreen	EC_M4	0.42	3.2E-02	0.25	2.3E-01	-0.80	1.1E-06	0.05	7.9E-01	0.45	2.0E-02	0.27	1.8E-01	-0.15	4.8E-01	-0.06	7.8E-01	-0.14	4.8E-01	-0.74	1.4E-05
ENSCAFG0000001514	USP8	grey	EC_M10	0.42	3.2E-02	0.49	1.1E-02	-0.61	9.8E-04	-0.08	6.8E-01	0.12	5.6E-01	0.05	8.0E-01	-0.12	5.6E-01	-0.10	6.2E-01	-0.21	2.9E-01	-0.33	1.0E-01
ENSCAFG0000001585	JAKMIP1	darkgreen	EC_M4	0.42	3.2E-02	0.34	9.3E-02	-0.81	6.9E-07	-0.34	9.3E-02	0.48	1.3E-02	0.30	1.4E-01	-0.06	7.8E-01	0.07	7.5E-01	-0.02	9.1E-01	-0.70	6.8E-05
ENSCAFG0000000666	KBTBD8	grey	EC_M10	0.42	3.2E-02	0.02	9.4E-01	-0.49	1.1E-02	-0.11	6.0E-01	0.24	2.4E-01	0.31	1.2E-01	-0.09	6.7E-01	-0.21	3.0E-01	-0.34	8.9E-02	-0.50	1.0E-02
ENSCAFG0000002967	ENSCAFG0000002967	darkgrey	EC_M8	0.42	3.2E-02	0.06	7.6E-01	-0.42	3.4E-02	0.31	1.2E-01	0.03	9.0E-01	0.16	4.3E-01	0.02	9.3E-01	-0.03	8.8E-01	-0.19	3.7E-01	-0.32	1.1E-01
ENSCAFG0000002272	MT-ATP8	darkgrey	EC_M8	0.42	3.2E-02	-0.02	9.3E-01	0.07	7.4E-01	0.19	3.7E-01	-0.50	9.1E-03	0.04	8.4E-01	-0.37	6.5E-02	0.04	8.6E-01	-0.04	8.6E-01	0.24	2.3E-01
ENSCAFG0000002983	SMIM8	grey	EC_M10	0.42	3.2E-02	-0.41	3.8E-02	-0.18	3.8E-01	0.21	3.1E-01	-0.01	9.6E-01	0.12	5.7E-01	-0.14	4.8E-01	0.10	6.4E-01	-0.56	3.1E-03	-0.27	1.9E-01
ENSCAFG0000001132	ENSCAFG0000001132	grey	EC_M10	0.42	3.2E-02	0.39	5.1E-02	-0.22	2.9E-01	0.15	4.5E-01	-0.15	4.6E-01	0.07	7.2E-01	-0.27	1.8E-01	-0.20	3.3E-01	-0.12	5.7E-01	-0.06	7.7E-01
ENSCAFG0000001984	SARS	darkgreen	EC_M4	0.42	3.2E-02	0.09	6.7E-01	-0.83	2.0E-07	0.01	9.6E-01	0.60	1.2E-03	0.35	8.0E-02	-0.33	1.0E-01	-0.03	8.8E-01	-0.21	3.0E-01	-0.87	9.5E-09
ENSCAFG0000003006	MFAP3	turquoise	EC_M6	0.42	3.2E-02	0.84	9.6E-08	-0.42	3.1E-02	0.01	9.7E-01	-0.16	4.4E-01	-0.12	5.6E-01	-0.13	5.1E-01	-0.16	4.3E-01	-0.34	9.1E-02	-0.06	7.6E-01
ENSCAFG0000001100	TYW5	darkgreen	EC_M4	0.42	3.2E-02	0.07	7.3E-01	-0.84	9.1E-08	-0.05	8.1E-01	0.55	3.9E-03	0.35	7.6E-02	0.05	7.9E-01	0.01	9.6E-01	-0.13	5.2E-01	-0.80	1.0E-06
ENSCAFG0000000915	TRIO	grey	EC_M10	0.42	3.2E-02	0.50	9.3E-03	-0.51	8.2E-03	-0.17	4.2E-01	0.05	8.3E-01	-0.01	9.5E-01	-0.16	4.5E-01	-0.15	4.8E-01	-0.21	3.0E-01	-0.29	1.5E-01
ENSCAFG0000000678	MTIF3	grey	EC_M10	0.42	3.2E-02	-0.11	6.1E-01	0.00	9.9E-01	0.07	7.3E-01	-0.35	7.7E-02	-0.04	8.3E-01	-0.27	1.8E-01	-0.09	6.7E-01	-0.17	4.2E-01	0.09	6.6E-01
ENSCAFG0000001085	LSAMP	turquoise	EC_M6	0.42	3.2E-02	0.59	1.4E-03	-0.42	3.3E-02	-0.06	7.7E-01	-0.03	9.0E-01	-0.12	5.5E-01	-0.11	5.9E-01	-0.08	6.9E-01	-0.13	5.1E-01	-0.19	3.5E-01
ENSCAFG0000001339	ENSCAFG0000001339	turquoise	EC_M6	0.42	3.2E-02	0.43	3.0E-02	-0.23	2.6E-01	0.12	5.5E-01	-0.21	3.0E-01	-0.10	6.4E-01	-0.01	9.7E-01	-0.37	6.1E-02	-0.09	6.8E-01	0.03	8.9E-01
ENSCAFG0000001123	LYST	turquoise	EC_M6	0.42	3.2E-02	0.77	4.8E-06	-0.19	3.5E-01	-0.11	5.9E-01	-0.41	4.0E-02	-0.24	2.4E-01	-0.20	3.4E-01	-0.04	8.6E-01	0.06	7.9E-01	0.22	2.7E-01
ENSCAFG0000001683	CBX1	turquoise	EC_M6	0.42	3.2E-02	0.60	1.3E-03	-0.48	1.3E-02	-0.10	6.2E-01	-0.04	8.4E-01	0.14	5.0E-01	0.02	9.1E-01	0.27	1.8E-01	0.02	9.4E-01	-0.20	3.2E-01
ENSCAFG0000001420	DNAAF2	grey	EC_M10	0.42	3.2E-02	0.04	8.6E-01	0.17	4.1E-01	0.23	2.5E-01	-0.55	3.8E-03	-0.09	6.6E-01	-0.03	8.8E-01	-0.01	9.5E-01	0.40	4.2E-02	0.37	6.0E-02
ENSCAFG0000000881	ENSCAFG0000000881	grey	EC_M10	0.42	3.2E-02	0.04	8.6E-01	0.09	6.6E-01	0.42	3.2E-02	-0.43	3.0E-02	-0.10	6.1E-01	-0.05	8.1E-01	-0.28	1.7E-01	-0.44	2.6E-02	0.26	2.1E-01
ENSCAFG0000001581	ACLY	grey	EC_M10	0.42	3.3E-02	-0.16	4.4E-01	-0.09	6.7E-01	0.04	8.5E-01	-0.19	3.4E-01	-0.15	4.5E-01	-0.02	9.3E-01	0.05	8.0E-01	-0.62	8.1E-04	-0.08	6.9E-01
ENSCAFG0000001064	IPO9	grey	EC_M10	0.42	3.3E-02	0.52	6.1E-03	0.16	4.4E-01	-0.05	8.3E-01	-0.68	1.1E-04	-0.01	9.6E-01	-0.18	3.7E-01	-0.06	7.7E-01	-0.02	9.1E-01	0.46	1.8E-02
ENSCAFG0000001215	LPGAT1	grey	EC_M10	0.42	3.3E-02	-0.38	5.6E-02	-0.32	1.1E-01	-0.01	9.5E-01	0.10	6.3E-01	0.16	4.5E-01	-0.17	4.0E-01	0.15	4.8E-01	-0.58	1.9E-03	-0.39	4.8E-02
ENSCAFG0000001853	CUL4B	darkgrey	EC_M8	0.42	3.3E-02	-0.01	9.5E-01	0.14	5.0E-01	0.16	4.2E-01	-0.61	9.4E-04	0.03	8.8E-01	-0.17	4.0E-01	0.20	3.3E-01	0.15	4.6E-01	0.34	8.5E-02
ENSCAFG0000000545	KAT14	darkgrey	EC_M8	0.42	3.3E-02	0.16	4.2E-01	0.21	3.0E-01	0.03	8.8E-01	-0.69	1.0E-04	-0.09	6.6E-01	-0.19	3.5E-01	-0.35	7.7E-02	-0.04	8.3E-01	0.46	1.8E-02
ENSCAFG0000000926	AGGF1	darkgrey	EC_M8	0.42	3.3E-02	-0.09	6.5E-01	0.06	7.8E-01	0.08	7.0E-01	-0.43	2.9E-02	0.04	8.5E-01	-0.06	7.8E-01	-0.19	3.4E-01	-0.06	7.7E-01	0.25	2.2E-01
ENSCAFG0000001978	RBM15	grey	EC_M10	0.42	3.3E-02	0.24	2.3E-01	-0.44	2.6E-02	0.01	9.6E-01	0.10	6.2E-01	-0.22	2.9E-01	0.21	3.0E-01	-0.03	8.8E-01	-0.42	3.2E-02	-0.31	1.3E-01
ENSCAFG0000001808	DSC3	turquoise	EC_M6	0.42	3.3E-02	0.53	5.4E-03	-0.06	7.9E-01	0.18	3.7E-01	-0.42	3.3E-02	-0.12	5.5E-01	-0.35	8.4E-02	-0.14	4.8E-01	-0.20	3.2E-01	0.21	3.0E-01
ENSCAFG0000000181	APTX	darkgrey	EC_M8	0.42	3.3E-02	-0.16	4.4E-01	-0.29	1.6E-01	0.00	9.9E-01	0.14	4.9E-01	0.26	2.0E-01	-0.53	5.4E-03	-0.13	5.2E-01	0.06	7.6E-01	-0.32	1.1E-01
ENSCAFG0000000985	DNAH5	darkgreen	EC_M4	0.42	3.3E-02	0.00	9.9E-01	-0.68	1.3E-04	-0.30	1.4E-01	0.47	1.5E-02	-0.03	9.0E-01	-0.22	2.8E-01	0.21	2.9E-01	0.10	6.4E-01	-0.70	7.9E-05
ENSCAFG0000001802	COL4A5	turquoise	EC_M6	0.42	3.3E-02	0.92	4.2E-11	-0.31	1.2E-01	-0.03	8.9E-01	-0.28	1.7E-01	-0.16	4.5E-01	-0.14	4.8E-01	-0.15	4.6E-01	-0.19	3.5E-01	0.07	7.2E-01
ENSCAFG0000001328	GLRX3	darkgreen	EC_M4	0.42	3.3E-02	0.00	9.9E-01	-0.61	1.1E-03	-0.15	4.6E-01	0.44	2.5E-02	0.40	4.5E-02	-0.26	2.0E-01	-0.02	9.4E-01	-0.36	7.0E-02	-0.68	1.3E-04
ENSCAFG0000001333	RXRG	grey	EC_M10	0.42	3.3E-02	-0.16	4.2E-01	-0.16	4.3E-01	-0.19	3.6E-01	-0.10	6.4E-01	0.28	1.6E-01	-0.22	2.8E-01	-0.18	3.8E-01	-0.18	3.9E-01	-0.13	5.2E-01
ENSCAFG0000002363	ENSCAFG0000002363	grey	EC_M10	0.42	3.3E-02	0.31	1.3E-01	-0.07	7.5E-01	0.34	8.8E-02	-0.38	5.7E-02	0.42	3.3E-02	-0.23	2.6E-01	-0.28	1.7E-01	-0.03	8.8E-01	0.17	4.1E-01
ENSCAFG0000000977	NAV2	turquoise	EC_M6	0.42	3.3E-02	0.76	7.0E-06	-0.09	6.5E-01	-0.03	8.9E-01	-0.48	1.3E-02	-0.21	3.1E-01	0.06	7.5E-01	-0.08	7.1E-01	0.18	3.8E-01	0.28	1.7E-01
ENSCAFG0000001624	ENSCAFG0000001624	grey	EC_M10	0.42	3.3E-02	0.09	6.5E-01	-0.19	3.4E-01	-0.10	6.3E-01	-0.21	3.1E-01	0.24	2.3E-01	-0.30	1.3E-01	0.38	5.9E-02	-0.18	3.7E-01	-0.04	8.6E-01
ENSCAFG0000000701	NRIP3	turquoise	EC_M6	0.42	3.3E-02	0.76	6.0E-06	-0.53	5.3E-03	-0.15	4.7E-01	0.06	7.8E-01	-0.16	4.5E-01	0.03	9.0E-01	0.03	8.7E-01	-0.11	5.9E-01	-0.24	2.3E-01
ENSCAFG0000003113	ENSCAFG0000003113	grey	EC_M10	0.42	3.3E-02	0.26	2.0E-01	-0.23	2.5E-01	-0.17	4.2E-01	-0.12	5.4E-01	0.31	1.3E-01	-0.10	6.4E-01	-0.17	4.0E-01	-0.01	9.5E-01	-0.08	7.1E-01
ENSCAFG0000000391	DUS4L	grey	EC_M10	0.42	3.3E-02	0.12	5.6E-01	0.43	2.7E-02	0.16	4.3E-01	-0.88	3.1E-09	-0.01	9.8E-01	-0.08	7.1E-01	-0.14	5.0E-01	-0.11	6.1E-01	0.65	3.4E-04
ENSCAFG0000001015	ANO3	turquoise	EC_M6	0.42	3.3E-02	0.84	8.5E-08	-0.38	5.6E-02	0.06	7.8E-01	-0.18	3.8E-01	-0.15	4.7E-01	-0.13	5.2E-01	-0.11	5.9E-01	-0.08	6.9E-01	-0.02	9.1E-01
ENSCAFG0000000084	FGFR1OP	grey	EC_M10	0.42	3.3E-02	0.13	5.2E-01	-0.09	6.6E-01	0.45	2.0E-02	-0.29	1.5E-01	0.20	3.3E-01	-0.06	7.8E-01	-0.28	1.6E-01	-0.13	5.3E-01	0.07	7.2E-01
ENSCAFG0000001113	ENSCAFG0000001113	darkgreen	EC_M4	0.42	3.3E-02	0.12	5.5E-01	-0.77	3.8E-06	0.12	5.5E-01	0.56	3.2E-03	0.28	1.7E-01	-0.10	6.4E-01	0.00	9.9E-01	0.10	6.4E-01	-0.75	1.0E-05
ENSCAFG0000002024	RABGAP1	darkgrey	EC_M8	0.42	3.4E-02	0.37	6.4E-02	-0.23	2.5E-01	0.14	5.0E-01	-0.32	1.1E-01	-0.05	8.2E-01	-0.42	3.3E-02	-0.18	3.8E-01	-0.07	7.3E-01	0.08	6.8E-01
ENSCAFG0000000538	ENSCAFG0000000538	grey	EC_M10	0.42	3.4E-02	0.12	5.6E-01	-0.39	4.6E-02	0.11	5.8E-01	0.06	7.6E-01	-0.02	9.3E-01	-0.14	5.0E-01	-0.15	4.8E-01	-0.22	2.9E-01	-0.25	2.1E-01
ENSCAFG0000003220	OSGEP	grey	EC_M10	0.42	3.4E-02	-0.51	7.6E-03	0.30	1.4E-01	0.25	2.1E-01	-0.46	1.7E-02	0.13	5.3E-01	-0.14	5.1E-01	-0.06	7.7E-01	-0.19	3.5E-01	0.21	3.0E-01
ENSCAFG0000001619	AP1S3	turquoise	EC_M6	0.42	3.4E-02	0.88	3.3E-09	-0.39	4.9E-02	-0.09	6.5E-01	-0.18	3.9E-01	-0.16	4.2E-01	-0.07	7.5E-01	-0.13	5.3E-01	-0.23	2.6E-01	-0.03	8.9E-01
ENSCAFG0000001347	ENSCAFG0000001347	grey	EC_M10	0.42	3.4E-02	0.10	6.3E-01	-0.73	2.4E-05	-0.33	1.0E-01	0.54	4.2E-03	0.20	3.4E-01	-0.42	3.1E-02	0.06	7.6E-01	-0.01	9.5E-01	-0.75	9.4E-06
ENSCAFG0000002271	ENSCAFG0000002271	darkgrey	EC_M8	0.42	3.4E-02	0.22	2.9E-01	-0.18	3.9E-01	-0.23	2.7E-01	-0.11	6.0E-01	-0.18	3.8E-01	0.11	6.0E-01	-0.15	4.5E-01	-0.09	6.8E-01	-0.06	7.7E-01
ENSCAFG0000000841	C33H3orf38	grey	EC_M10	0.42	3.4E-02	0.20	3.3E-01	-0.49	1.1E-02	-0.23	2.6E-01	0.11	5.8E-01	0.46	1.9E-02	-0.27	1.9E-01	0.02	9.1E-01	0.15	4.7E-01	-0.34	8.9E-02
ENSCAFG0000001614	KLHL5	darkgreen	EC_M4	0.42	3.4E-02	0.29	1.5E-01	-0.85	4.9E-08	0.00	9.8E-01	0.51	7.2E-03	0.31	1.3E-01	-0.27	1.9E-01	-0.02	9.2E-01	-0.05	8.1E-01	-0.79	1.7E-06
ENSCAFG0000001856	C4H5orf34	grey	EC_M10	0.42	3.4E-02	0.18	3.9E-01	-0.42	3.3E-02	-0.20	3.2E-01	0.13	5.3E-01	0.36	7.2E-02	0.02	9.2E-01	-0.21	3.1E-01	0.11	6.0E-01	-0.34	9.1E-02
ENSCAFG0000000767	HPF1	grey	EC_M10	0.42	3.4E-02	-0.09	6.6E-01	-0.06	7.6E-01	0.29	1.4E-01	-0.26	1.9E-01	0.24	2.3E-01	-0.25	2.1E-01	0.15	4.7E-01	0.27	1.8E-01	0.05	8.3E-01
ENSCAFG0000001897	USP24	grey	EC_M10	0.42	3.4E-02	0.09	6.7E-01	-0.35	7.6E-02	-0.05	8.2E-01	0.02	9.1E-01	-0.26	2.0E-01	-0.19	3.6E-01	0.04	8.5E-01	-0.22	2.8E-01	-0.22	2.8E-01
ENSCAFG0000000903	NSUN3	grey	EC_M10	0.42	3.4E-02	0.18	3.8E-01	-0.44	2.6E-02	0.15	4.6E-01	0.02	9.2E-01	0.40	4.1E-02	-0.19	3.6E-01	-0.09	6.5E-01	0.03	9.0E-01	-0.28	1.6E-01
ENSCAFG0000002847	ZFP82	grey	EC_M10	0.42	3.4E-02	0.33	9.6E-02	-0.62	7.6E-04	0.02	9.1E-01	0.18	3.9E-01	0.24	2.4E-01	-0.25	2.1E-01	0.11	5.9E-01	0.05	8.0E-01	-0.43	3.0E-02
ENSCAFG0000000909	ENSCAFG0000000909	grey	EC_M10	0.42	3.4E-02	0.35	8.1E-02	-0.32	1.2E-01	0.25	2.2E-01	-0.10	6.4E-01	-0.19	3.6E-01	-0.22	2.7E-01	-0.17	4.1E-01	-0.22	2.9E-01	-0.11	5.8E-01
ENSCAFG0000002040	NAE1	darkgreen	EC_M4	0.42	3.4E-02	0.03	9.0E-01	-0.76	5.5E-06	0.02	9.2E-01	0.55	3.5E-03	0.16	4.2E-01	-0.45	2.0E-02	-0.09	6.7E-01	-0.03	9.0E-01	-0.74	1.6E-05
ENSCAFG0000001230	PPP2R5A	turquoise	EC_M6	0.42	3.5E-02	0.85	2.9E-08	-0.16	4.2E-01	0.02	9.4E-01	-0.46	1.7E-02	-0.27	1.8E-01	-0.10	6.1E-01	-0.13	5.3E-01	-0.15	4.7E-01	0.26	2.1E-01
ENSCAFG0000003271	ENSCAFG0000003271	darkgreen	EC_M4	0.42	3.5E-02	0.16	4.4E-01	-0.73	2.2E-05	-0.29	1.5E-01	0.46	1.8E-02	0.31	1.3E-01	-0.03	8.8E-01	-0.25	2.1E-01	-0.28	1.7E-01	-0.70	6.4E-05
ENSCAFG0000001914	UBN1	darkgrey	EC_M8	0.42	3.5E-02	0.45	2.0E-02	0.20	3.3E-01	0.16	4.3E-01	-0.75	1.1E-05	-0.16	4.5E-01	-0.11	6.1E-01	-0.41	3.9E-02	-0.19	3.6E-01	0.53	5.5E-03
ENSCAFG0000001911	YJU2	grey	EC_M10	0.42	3.5E-02	0.30	1.4E-01	-0.06	7.8E-01	0.35	8.2E-02	-0.43	3.0E-02	-0.19	3.6E-01	-0.07	7.4E-01	0.33	1.1E-01	-0.09	6.7E-01	0.18	3.8E-01
ENSCAFG0000002889	ENSCAFG0000002889	grey	EC_M10	0.42	3.5E-02	0.18	3.7E-01	-0.25	2.2E-01	-0.08	7.0E-01	-0.10	6.3E-01	-0.13	5.1E-01	-0.11	5.8E-01	-0.11	6.0E-01	-0.05	8.2E-01	-0.08	7.1E-01
ENSCAFG0000002487	ENSCAFG0000002487	grey	EC_M10	0.42	3.5E-02	0.31	1.3E-01	-0.19	3.5E-01	-0.13	5.4E-01	-0.23	2.6E-01	0.06	7.9E-01	-0.10	6.1E-01	0.34	9.4E-02	-0.26	2.1E-01	-0.01	9.5E-01
ENSCAFG0000000287	UBE3D	grey	EC_M10	0.42	3.5E-02	-0.37	6.2E-02	0.13	5.3E-01	0.07	7.3E-01	-0.33	1.0E-01	0.17	4.1E-01	-0.49	1.1E-02	-0.27	1.9E-01	-0.11	6.0E-01	0.09	6.6E-01

ENSCAFG0000003163	GNG4	turquoise	EC_M6	0.42	3.5E-02	0.73	2.1E-05	-0.26	1.9E-01	-0.03	8.7E-01	-0.28	1.6E-01	-0.04	8.5E-01	-0.15	4.6E-01	0.12	5.5E-01	0.05	8.2E-01	0.06	7.6E-01
ENSCAFG0000001929	PAFAH1B1	darkgreen	EC_M4	0.41	3.5E-02	0.11	6.0E-01	-0.89	1.5E-09	-0.14	4.9E-01	0.66	2.7E-04	0.26	2.0E-01	-0.05	8.1E-01	0.07	7.2E-01	-0.11	5.8E-01	-0.89	1.2E-09
ENSCAFG0000001886	MYO18A	turquoise	EC_M6	0.41	3.5E-02	0.72	3.7E-05	-0.11	5.9E-01	-0.03	8.7E-01	-0.45	2.3E-02	-0.30	1.3E-01	-0.20	3.2E-01	-0.05	8.0E-01	-0.05	8.1E-01	0.26	2.1E-01
ENSCAFG0000000906	EXOSC1	grey	EC_M10	0.41	3.5E-02	-0.11	6.1E-01	0.09	6.7E-01	-0.18	3.9E-01	-0.40	4.3E-02	0.11	5.9E-01	-0.23	2.6E-01	-0.39	5.2E-02	-0.04	8.3E-01	0.20	3.3E-01
ENSCAFG0000000053	ENSCAFG0000000053	grey	EC_M10	0.41	3.5E-02	0.16	4.5E-01	-0.08	6.9E-01	0.01	9.6E-01	-0.28	1.6E-01	0.04	8.3E-01	-0.34	8.7E-02	-0.06	7.7E-01	-0.36	6.8E-02	0.07	7.5E-01
ENSCAFG0000000700	GAPT	turquoise	EC_M6	0.41	3.5E-02	0.76	6.9E-06	-0.21	3.0E-01	-0.13	5.1E-01	-0.29	1.5E-01	-0.14	4.9E-01	-0.13	5.4E-01	-0.07	7.3E-01	-0.21	3.1E-01	0.07	7.3E-01
ENSCAFG0000002514	TMX2	darkgrey	EC_M8	0.41	3.5E-02	-0.31	1.3E-01	-0.57	2.3E-03	-0.26	2.0E-01	0.50	1.0E-02	0.29	1.5E-01	-0.21	3.1E-01	0.10	6.1E-01	-0.35	7.9E-02	-0.73	2.0E-05
ENSCAFG0000001806	HMG20A	turquoise	EC_M6	0.41	3.5E-02	0.71	4.5E-05	-0.30	1.4E-01	-0.12	5.5E-01	-0.28	1.7E-01	-0.28	1.7E-01	-0.18	3.9E-01	-0.11	6.1E-01	-0.25	2.3E-01	0.06	7.7E-01
ENSCAFG0000000191	POLH	darkgrey	EC_M8	0.41	3.5E-02	-0.21	3.1E-01	-0.48	1.2E-02	0.08	7.0E-01	0.36	7.1E-02	0.26	2.0E-01	-0.06	7.6E-01	-0.06	7.6E-01	0.07	7.5E-01	-0.59	1.6E-03
ENSCAFG0000000479	MTRF1	grey	EC_M10	0.41	3.5E-02	-0.37	6.6E-02	-0.13	5.2E-01	0.00	9.9E-01	-0.06	7.7E-01	0.03	9.0E-01	-0.48	1.4E-02	-0.09	6.5E-01	-0.03	9.0E-01	-0.20	3.4E-01
ENSCAFG0000001839	ARL15	turquoise	EC_M6	0.41	3.6E-02	0.66	2.2E-04	-0.33	1.0E-01	-0.08	7.0E-01	-0.19	3.6E-01	0.18	3.7E-01	-0.01	9.5E-01	-0.16	4.2E-01	0.00	9.9E-01	0.00	9.9E-01
ENSCAFG0000003097	GYPC	grey	EC_M10	0.41	3.6E-02	0.42	3.3E-02	0.01	9.5E-01	0.21	3.0E-01	-0.49	1.0E-02	-0.13	5.1E-01	-0.30	1.4E-01	-0.03	9.0E-01	-0.27	1.9E-01	0.25	2.2E-01
ENSCAFG0000000752	CBR4	cyan	EC_M2	0.41	3.6E-02	-0.25	2.2E-01	0.32	1.1E-01	0.13	5.3E-01	-0.66	2.5E-04	0.04	8.5E-01	-0.03	8.9E-01	-0.01	9.6E-01	-0.08	6.8E-01	0.44	2.5E-02
ENSCAFG0000001513	VAMP1	grey	EC_M10	0.41	3.6E-02	0.02	9.1E-01	-0.53	5.6E-03	-0.34	9.3E-02	0.29	1.6E-01	0.06	7.7E-01	-0.06	7.6E-01	0.21	2.9E-01	0.06	7.6E-01	-0.49	1.2E-02
ENSCAFG0000002474	TRIM68	grey	EC_M10	0.41	3.6E-02	0.25	2.3E-01	-0.07	7.3E-01	0.21	2.9E-01	-0.38	5.2E-02	0.04	8.3E-01	-0.22	2.7E-01	0.09	6.7E-01	0.04	8.5E-01	0.14	5.1E-01
ENSCAFG0000000545	ENSCAFG0000000545	darkgrey	EC_M8	0.41	3.6E-02	0.11	6.1E-01	-0.36	7.4E-02	0.18	3.8E-01	0.03	8.8E-01	-0.03	9.0E-01	-0.02	9.4E-01	-0.10	6.4E-01	-0.52	6.3E-03	-0.23	2.6E-01
ENSCAFG0000002921	TXNDC12	grey	EC_M10	0.41	3.6E-02	-0.05	8.0E-01	0.20	3.2E-01	0.21	3.1E-01	-0.59	1.6E-03	-0.28	1.6E-01	0.29	1.5E-01	0.10	6.1E-01	-0.12	5.7E-01	0.36	7.1E-02
ENSCAFG0000001078	STT3A	darkgrey	EC_M8	0.41	3.6E-02	-0.20	3.2E-01	-0.65	2.9E-04	-0.10	6.2E-01	0.56	3.1E-03	0.32	1.1E-01	-0.26	2.0E-01	-0.01	9.6E-01	-0.15	4.6E-01	-0.81	4.7E-07
ENSCAFG0000000962	ZNF248	darkgrey	EC_M8	0.41	3.6E-02	0.52	6.9E-03	-0.25	2.1E-01	-0.02	9.1E-01	-0.34	8.9E-02	0.10	6.1E-01	-0.13	5.2E-01	0.15	4.8E-01	0.11	6.1E-01	0.10	6.2E-01
ENSCAFG0000000073	ENSCAFG0000000073	darkgreen	EC_M4	0.41	3.6E-02	0.11	6.0E-01	-0.69	1.1E-04	0.07	7.5E-01	0.43	2.9E-02	0.24	2.4E-01	0.00	1.0E+00	-0.12	5.7E-01	-0.31	1.2E-01	-0.63	6.4E-04
ENSCAFG0000001968	LUC7L	grey	EC_M10	0.41	3.6E-02	0.39	5.2E-02	-0.29	1.5E-01	-0.02	9.2E-01	-0.18	3.9E-01	0.29	1.6E-01	0.04	8.6E-01	-0.12	5.5E-01	0.17	4.2E-01	-0.05	8.0E-01
ENSCAFG0000000295	ENSCAFG0000000295	grey	EC_M10	0.41	3.6E-02	0.32	1.1E-01	-0.71	4.8E-05	0.06	7.8E-01	0.35	8.3E-02	0.24	2.4E-01	-0.10	6.2E-01	0.01	9.6E-01	0.01	9.8E-01	-0.53	5.5E-03
ENSCAFG0000002853	KCNU1	grey	EC_M10	0.41	3.6E-02	0.13	5.2E-01	-0.60	1.2E-03	-0.29	1.6E-01	0.37	6.1E-02	0.31	1.2E-01	-0.09	6.6E-01	0.04	8.6E-01	0.14	4.8E-01	-0.56	2.7E-03
ENSCAFG0000000882	FAM151B	grey	EC_M10	0.41	3.6E-02	-0.33	9.7E-02	0.05	8.0E-01	-0.06	7.6E-01	-0.27	1.8E-01	-0.07	7.4E-01	-0.12	5.5E-01	-0.24	2.4E-01	-0.35	8.0E-02	0.08	7.0E-01
ENSCAFG0000000096	FSTL4	grey	EC_M10	0.41	3.6E-02	-0.04	8.5E-01	-0.31	1.2E-01	-0.20	3.4E-01	0.09	6.8E-01	0.10	6.2E-01	-0.17	4.0E-01	0.16	4.2E-01	0.01	9.5E-01	-0.28	1.6E-01
ENSCAFG0000002992	ENSCAFG0000002992	grey	EC_M10	0.41	3.7E-02	0.07	7.4E-01	-0.33	9.6E-02	0.29	1.5E-01	0.00	9.9E-01	0.34	8.7E-02	-0.22	2.9E-01	0.11	5.9E-01	-0.15	4.7E-01	-0.23	2.6E-01
ENSCAFG0000000766	USP39	grey	EC_M10	0.41	3.7E-02	0.10	6.2E-01	-0.42	3.5E-02	0.03	8.8E-01	0.22	2.7E-01	0.02	9.3E-01	-0.22	2.8E-01	0.08	7.1E-01	-0.21	3.0E-01	-0.45	2.1E-02
ENSCAFG0000002977	ENSCAFG0000002977	grey	EC_M10	0.41	3.7E-02	0.47	1.5E-02	-0.64	4.0E-04	0.18	3.9E-01	0.27	1.9E-01	0.24	2.3E-01	0.04	8.4E-01	-0.14	5.1E-01	-0.23	2.7E-01	-0.49	1.1E-02
ENSCAFG0000001143	ENSCAFG0000001143	darkgrey	EC_M8	0.41	3.7E-02	-0.12	5.5E-01	-0.54	4.3E-03	0.16	4.3E-01	0.38	5.3E-02	0.17	4.0E-01	-0.40	4.3E-02	-0.19	3.5E-01	-0.38	5.7E-02	-0.60	1.3E-03
ENSCAFG0000001068	LINS1	grey	EC_M10	0.41	3.7E-02	0.00	9.9E-01	-0.63	5.5E-04	0.24	2.3E-01	0.32	1.1E-01	0.35	7.6E-02	-0.11	5.8E-01	-0.01	9.8E-01	-0.05	8.1E-01	-0.53	5.7E-03
ENSCAFG0000000028	ABRACL	darkgreen	EC_M4	0.41	3.7E-02	0.36	7.0E-02	-0.91	1.3E-10	-0.03	8.7E-01	0.57	2.4E-03	0.13	5.4E-01	-0.03	8.9E-01	0.09	6.5E-01	0.00	1.0E+00	-0.80	8.0E-07
ENSCAFG0000000874	MAPKAPK5	darkgreen	EC_M4	0.41	3.7E-02	0.27	1.8E-01	-0.70	8.0E-05	0.04	8.6E-01	0.38	5.7E-02	0.17	4.2E-01	0.06	7.6E-01	-0.04	8.4E-01	-0.31	1.2E-01	-0.58	1.9E-03
ENSCAFG0000001177	C2CD5	darkgrey	EC_M8	0.41	3.7E-02	-0.06	7.7E-01	0.43	2.7E-02	0.30	1.4E-01	-0.85	4.8E-08	-0.17	4.0E-01	-0.13	5.1E-01	-0.08	6.8E-01	-0.21	3.0E-01	0.61	9.6E-04
ENSCAFG0000002642	ENSCAFG0000002642	grey	EC_M10	0.41	3.7E-02	0.28	1.6E-01	-0.10	6.1E-01	-0.12	5.6E-01	-0.32	1.1E-01	-0.03	8.8E-01	-0.02	9.3E-01	0.30	1.4E-01	0.17	3.9E-01	0.09	6.6E-01
ENSCAFG0000001290	ENSCAFG0000001290	grey	EC_M10	0.41	3.7E-02	-0.19	3.6E-01	-0.26	2.0E-01	-0.14	4.9E-01	0.00	9.9E-01	0.20	3.3E-01	-0.22	2.9E-01	-0.03	8.9E-01	-0.15	4.6E-01	-0.27	1.8E-01
ENSCAFG0000001341	MFAP1	grey	EC_M10	0.41	3.7E-02	0.12	5.5E-01	-0.21	3.0E-01	-0.21	3.0E-01	-0.14	4.8E-01	0.35	7.9E-02	-0.17	4.1E-01	0.04	8.3E-01	-0.39	5.1E-02	-0.06	7.6E-01
ENSCAFG0000001411	CABYR	grey	EC_M10	0.41	3.7E-02	-0.04	8.5E-01	0.01	9.7E-01	0.01	9.6E-01	-0.26	2.1E-01	0.14	5.0E-01	-0.68	1.3E-04	-0.22	2.9E-01	-0.31	1.2E-01	0.03	8.8E-01
ENSCAFG0000000011	PAN2	darkgrey	EC_M8	0.41	3.7E-02	0.64	4.6E-04	0.06	7.6E-01	0.10	6.4E-01	-0.68	1.3E-04	-0.05	8.1E-01	-0.13	5.4E-01	-0.03	8.7E-01	-0.17	4.1E-01	0.45	2.0E-02
ENSCAFG0000001018	DCAF1	turquoise	EC_M6	0.41	3.7E-02	0.44	2.3E-02	-0.40	4.1E-02	-0.07	7.4E-01	-0.01	9.5E-01	0.11	5.9E-01	0.29	1.4E-01	-0.19	3.6E-01	-0.35	8.4E-02	-0.21	2.9E-01
ENSCAFG0000001068	SP110	darkgrey	EC_M8	0.41	3.7E-02	0.12	5.5E-01	0.18	3.7E-01	0.14	5.0E-01	-0.60	1.2E-03	0.13	5.3E-01	-0.07	7.5E-01	-0.10	6.4E-01	-0.22	2.7E-01	0.37	6.1E-02
ENSCAFG0000001799	ENSCAFG0000001799	darkgreen	EC_M4	0.41	3.7E-02	0.32	1.1E-01	-0.73	2.1E-05	-0.16	4.4E-01	0.45	2.1E-02	0.06	7.8E-01	0.05	8.1E-01	-0.15	4.6E-01	-0.34	8.5E-02	-0.62	8.2E-04
ENSCAFG0000000648	ENSCAFG0000000648	grey	EC_M10	0.41	3.7E-02	0.03	8.9E-01	-0.44	2.5E-02	0.13	5.1E-01	0.16	4.3E-01	0.19	3.6E-01	-0.19	3.5E-01	-0.37	6.0E-02	-0.31	1.2E-01	-0.43	2.8E-02
ENSCAFG0000003004	RBM18	darkgreen	EC_M4	0.41	3.7E-02	0.68	1.5E-04	-0.86	2.1E-08	-0.13	5.2E-01	0.41	3.9E-02	0.18	3.7E-01	-0.02	9.4E-01	-0.10	6.4E-01	-0.14	5.1E-01	-0.63	5.9E-04
ENSCAFG0000000670	NFKBID	darkgrey	EC_M8	0.41	3.7E-02	0.20	3.4E-01	0.12	5.7E-01	0.30	1.4E-01	-0.60	1.2E-03	-0.18	3.7E-01	0.29	1.5E-01	-0.01	9.6E-01	-0.05	7.9E-01	0.38	5.6E-02
ENSCAFG0000000473	DGKH	darkgreen	EC_M4	0.41	3.7E-02	0.65	3.4E-04	-0.77	4.6E-06	-0.10	6.4E-01	0.34	9.0E-02	0.05	8.1E-01	-0.04	8.3E-01	-0.29	1.5E-01	-0.14	5.0E-01	-0.55	3.6E-03
ENSCAFG0000002620	ENSCAFG0000002620	grey	EC_M10	0.41	3.7E-02	0.07	7.5E-01	-0.34	8.7E-02	-0.11	6.1E-01	-0.02	9.2E-01	0.10	6.1E-01	-0.15	4.8E-01	-0.17	4.1E-01	0.07	7.3E-01	-0.18	3.7E-01
ENSCAFG0000000096	RRP7	darkgrey	EC_M8	0.41	3.7E-02	-0.38	5.7E-02	-0.29	1.5E-01	-0.07	7.3E-01	0.22	2.8E-01	0.26	2.1E-01	-0.21	3.0E-01	-0.08	7.0E-01	0.06	7.7E-01	-0.44	2.5E-02
ENSCAFG0000001294	TFRC	darkgreen	EC_M4	0.41	3.8E-02	0.51	8.4E-03	-0.84	7.8E-08	-0.18	3.9E-01	0.50	9.9E-03	0.22	2.8E-01	-0.12	5.5E-01	-0.20	3.2E-01	-0.05	8.2E-01	-0.69	8.3E-05
ENSCAFG0000002905	UCHL3	grey	EC_M10	0.41	3.8E-02	-0.50	9.9E-03	-0.51	8.3E-03	-0.06	7.6E-01	0.47	1.5E-02	0.16	4.4E-01	-0.28	1.6E-01	0.15	4.7E-01	-0.16	4.5E-01	-0.70	6.8E-05
ENSCAFG0000000667	SPINK5	darkgreen	EC_M4	0.41	3.8E-02	0.53	5.2E-03	-0.66	2.7E-04	-0.15	4.5E-01	0.26	2.1E-01	0.03	9.0E-01	-0.21	3.0E-01	-0.15	4.6E-01	-0.24	2.4E-01	-0.48	1.4E-02
ENSCAFG0000000648	GTF2E2	grey	EC_M10	0.41	3.8E-02	-0.13	5.1E-01	-0.37	6.4E-02	0.34	9.0E-02	0.06	7.6E-01	0.37	6.5E-02	-0.24	2.5E-01	-0.14	5.1E-01	-0.19	3.4E-01	-0.31	1.2E-01
ENSCAFG0000000298	PUS10	darkgrey	EC_M8	0.41	3.8E-02	0.22	2.9E-01	-0.19	3.6E-01	0.26	1.9E-01	-0.33	1.0E-01	-0.03	9.0E-01	-0.06	7.6E-01	0.00	9.8E-01	-0.09	6.6E-01	0.07	7.2E-01
ENSCAFG0000000877	ENSCAFG0000000877	grey	EC_M10	0.41	3.8E-02	-0.05	8.0E-01	-0.25	2.3E-01	-0.20	3.2E-01	0.00	9.9E-01	0.21	3.1E-01	-0.13	5.4E-01	-0.20	3.2E-01	-0.09	6.5E-01	-0.17	4.1E-01
ENSCAFG0000003068	ENSCAFG0000003068	grey	EC_M10	0.41	3.8E-02	0.06	7.9E-01	-0.63	5.2E-04	-0.21	2.9E-01	0.38	5.7E-02	0.14	5.0E-01	-0.01	9.7E-01	-0.06	7.8E-01	-0.25	2.2E-01	-0.60	1.2E-03
ENSCAFG0000001524	AP5M1	darkgreen	EC_M4	0.41	3.8E-02	0.54	4.1E-03	-0.73	2.5E-05	0.00	1.0E+00	0.32	1.1E-01	0.11	5.8E-01	-0.24	2.3E-01	-0.23	2.7E-01	-0.16	4.3E-01	-0.51	8.1E-03
ENSCAFG0000001455	ENSCAFG0000001455	grey	EC_M10	0.41	3.8E-02	0.23	2.6E-01	0.04	8.5E-01	-0.02	9.1E-01	-0.48	1.3E-02	-0.23	2.6E-01	-0.12	5.7E-01	-0.08	7.1E-01	0.17	4.1E-01	0.27	1.8E-01
ENSCAFG0000000469	DCLRE1C	darkgrey	EC_M8	0.41	3.8E-02	0.40	4.1E-02	0.12	5.8E-01	0.08	7.1E-01	-0.63	5.3E-04	-0.08	7.1E-01	-0.06	7.8E-01	0.23	2.6E-01	-0.07	7.2E-01	0.44	2.4E-02
ENSCAFG0000001992	EMC8	grey	EC_M10	0.41	3.8E-02	-0.18	3.7E-01	-0.18	3.8E-01	-0.20	3.4E-01	0.08	6.8E-01	0.18	3.7E-01	-0.22	2.8E-01	-0.16	4.3E-01	-0.30	1.4E-01	-0.32	1.1E-01
ENSCAFG0000000938	POLK	grey	EC_M10	0.41	3.8E-02	-0.18	3.7E-01	-0.38	5.9E-02	0.03	8.7E-01	0.09	6.7E-01	0.04	8.3E-01	-0.16	4.4E-01	0.18	3.8E-01	-0.26	2.1E-01	-0.32	1.2E-01
ENSCAFG0000000716	NDUFAF2	darkgrey	EC_M8	0.41	3.8E-02	-0.24	2.5E-01	-0.38	5.3E-02	-0.17	4.0E-01	0.25	2.2E-01	0.18	3.7E-01	-0.19	3.6E-01	0.05	7.9E-01	-0.23	2.6E-01	-0.51	8.2E-03
ENSCAFG0000000033	ENSCAFG0000000033	grey	EC_M10	0.41	3.8E-02	-0.33	9.9E-02	-0.37	6.6E-02	-0.14	5.1E-01	0.19	3.5E-01	0.31	1.2E-01	-0.29	1.5E-01	0.20	3.4E-01	-0.08	6.8E-01	-0.42	3.3E-02
ENSCAFG0000001575	RCC2	turquoise	EC_M6	0.41	3.8E-02	0.41	4.0E-02	-0.48	1.3E-02	0.03	8.9E-01	0.14	5.0E-01	0.16	4.5E-01	0.01	9.6E-01	-0.44	2.4E-02	-0.32	1.1E-01	-0.39	4.9E-02
ENSCAFG0000001163	NGDN	darkgrey	EC_M8	0.41	3.8E-02	0.42	3.4E-02	0.03	8.7E-01	0.08	6.9E-01	-0.58	2.1E-03	-0.19	3.6E-01	0.29	1.5E-01	0.00	1.0E+00	-0.11	5.9E-01	0.37	6.3E-02
ENSCAFG0000001059	TRIP12	darkgreen	EC_M4	0.41	3.8E-02	0.41	3.8E-02	-0.89	8.4E-10	-0.16	4.5E-01	0.55	3.8E-03	0.05	8.1E-01	-0.18	3.7E-01	-0.02	9.2E-01	-0.11	5.8E-01	-0.76	8.0E-06
ENSCAFG0000000803	DIP2B	darkgreen	EC_M4	0.41	3.8E-02	0.44	2.6E-02	-0.82	2.2E-07	-0.18	3.7E-01	0.45	2.3E-02	0.16	4.4E-01	-0.19	3.6E-01	-0.07	7.3E-01	-0.21	2.9E-01	-0.68	1.2E-04
ENSCAFG0000001975	ABO	grey	EC_M10	0.41	3.8E-02	0.49	1.2E-02	-0.33	1.1E-01	-0.14	5.0E-01	-0.06	7.8E-01	-0.17	4.2E-01	-0.13	5.4E-01	-0.15	4.6E-01	-0.05	8.2E-01	-0.12	5.7E-01
ENSCAFG0000000428	FAM185A	grey	EC_M10	0.41	3.9E-02	-0.17	4.2E-01	0.13	5.1E-01	0.14	5.0E-01	-0.41	3.9E-02	0.05	8.3E-01	-0.05	8.1E-01	-0.05	8.0E-01	-0.01	9.5E-01	0.21	3.0E-01
ENSCAFG0000000839	DPH6	grey	EC_M10	0.41	3.9E-02	0.06	7.8E-01	-0.54	4.7E-03	-0.12	5.6E-01	0.25	2.2E-01	0.07	7.3E-01	-0.33	9.5E-02	0.36	7.0E-02	0.04	8.5E-01	-0.50	9.2E-03
ENSCAFG0000001753	CHTOP	turquoise	EC_M6	0.41	3.9E-02	0.60	1.3E-03	-0.44	2.4E-02	0.11	5.8E-01	-0.01	9.6E-01	0.05	8.0E-01	0.04	8.6E-01	0.15	4.6E-01	-0.01	9.7E-01	-0.17	4.0E-01
ENSCAFG0000000462	PRCP	turquoise	EC_M6	0.41	3.9E-02	0.80	1.1E-06	-0.43	2.7E-02	0.03	8.7E-01	-0.09	6.7E-01	-0.10	6.2E-01	-0.02	9.3E-01	-0.28	1.7E-01	-0.23	2.7E-01	-0.15	4.6E-01
ENSCAFG0000001222	HINFP	grey	EC_M10	0.41	3.9E-02	-0.03	8.7E-01	-0.51	7.3E-03	0.04	8.3E-01	0.35	7.9E-02	0.29	1.5E-01	-0.18	3.8E-01	-0.28	1.7E-01	-0.09	6.8E-01	-0.57	2.5E-03
ENSCAFG0000000761	ENSCAFG0000000761	grey	EC_M10	0.41	3.9E-02	-0.22	2.7E-01	-0.46	1.8E-02	0.12	5.8E-01	0.27	1.9E-01	-0.09	6.7E-01	-0.13	5.4E-01	0.28	1.7E-01	0.04	8.5E-01	-0.48	1.3E-02
ENSCAFG0000001167	ENSCAFG0000001167	grey	EC_M10	0.41	3.9E-02	0.31	1.2E-01	-0.22	2.8E-01	0.17	4.1E-01	-0.16	4.2E-01	0.11	5.8E-01	0.09	6.6E-01	-0.23	2.6E-01	-0.48	1.3E-02	-0.06	7.8E-01
ENSCAFG0000003189	ENSCAFG0000003189	grey	EC_M10	0.41	3.9E-02	-0.10	6.3E-01	-0.05	8.1E-01	0.14	5.0E-01	-0.26	1.9E-01	0.13	5.1E-01	-0.22	2.9E-01	-0.28	1.7E-01	-0.10	6.2E-01	0.00	1.0E+00
ENSCAFG0000001498	SLC2A2	darkgrey	EC_M8	0.41	3.9E-02	-0.14	5.1E-01	-0.57	2.3E-03	0.10	6.1E-01	0.43	2.9E-02	0.24	2.5E-01	-0.13	5.4E-01	-0.15	4.7E-01	-0.05	8.1E-01	-0.64	4.8E-04
ENSCAFG0000003158	ZNF34	darkgrey	EC_M8	0.41	3.9E-02	0.39	5.1E-02	0.18	3.9E-01	0.27	1.9E-01	-0.71	4.9E-05	-0.11	6.1E-01	-0.35	8.2E-02	-0.08	6.9E-01	0.09	6.7E-01	0.48	1.2E-02
ENSCAFG0000001756	TRIM37	darkgrey	EC_M8	0.41	3.9E-02	-0.06	7.7E-01	0.31	1.2E-01	0.08	7.0E-01	-0.68	1.1E-04	-0.18	3.7E-01	-0.03	8.8E-01	-0.05	8.0E-01	0.04	8.3E-01	0.50	8.7E-03
ENSCAFG0000001217	SLC26A6	grey	EC_M10	0.41	3.9E-02	0.48	1.2E-02	-0.12	5.6E-01	-0.12	5.6E-01	-0.38	5.5E-02	0.05	8.2E-01	0.08	7.1E-01	0.13	5.3E-01	-0.19	3.6E-01	0.11	6.1E-01
ENSCAFG0000003202	ENSCAFG0000003202	grey	EC_M10	0.41	3.9E-02	-0.02	9.1E-01	-0.63	5.2E-04	0.06	7.7E-01	0.39	4.9E-02	0.27	1.8E-01	-0.29	1.5E-01	0.29	1.5E-01	0.04	8.3E-01	-0.61	9.7E-04
ENSCAFG0000000195	ENSCAFG0000000195	grey	EC_M10	0.41	3.9E-02	0.16	4.5E-01	0.11	6.0E-01	0.36	7.2E-02	-0.48	1.3E-02	0.16	4.5E-01	0.01	9.6E-01	-0.26	1.9E-01	-0.42	3.1E-02	0.26	2.1E-01
ENSCAFG0000000789	CDKN2AIP	darkgrey	EC_M8	0.41	3.9E-02	0.29	1.5E-01	-0.12	5.6E-01	0.09	6.7E-01	-0.39	4.8E-02	0.12	5.7E-01	0.02	9.3E-01	-0.20	3.3E-01	-0.31	1.3E-01	0.17	4.2E-01
ENSCAFG0000002505	ENSCAFG0000002505	grey	EC_M10	0.41	3.9E-02	0.31	1.2E-01	-0.59	1.6E-03	-0.11	5.9E-01	0.29	1.6E-01	0.22	2.8E-01	-0.13	5.2E-01	-0.16	4.3E-01	-0.31	1.2E-01	-0.53	5.2E-03
ENSCAFG0000000079	GPSM3	grey	EC_M10	0.41	3.9E-02	0.38	5.8E-02	-0.18	3.8E-01	-0.11	5.9E-01	-0.24	2.4E-01	-0.06	7.8E-01	0.26	2.0E-01	0.08	6.8E-01	0.11	6.0E-01	0.03	8.7E-01
ENSCAFG0000003274	UBL3	grey	EC_M10	0.41	3.9E-02	0.27	1.9E-01	-0.29	1.5E-01	0.26	1.9E-01	-0.17	4.0E-01	0.13	5.2E-01	-0.28	1.6E-01	-0.12	5.6E-01	-0.03	8.8E-01	-0.02	9.4E-01
ENSCAFG0000000479	NUDT5	grey	EC_M10	0.41	4.0E-02	-0.36	7.1E-02	0.06	7.6E-01	0.35	8.0E-02	-0.20	3.3E-01	-0.10	6.2E-01	-0.18	3.8E-01	0.09	6.6E-01	0.00	1.0E+00	0.01	9.7E-01
ENSCAFG0000001024	PXN	grey	EC_M10	0.41	4.0E-02	0.42	3.1E-02	-0.51	7.2E-03	-0.34	8.9E-02	0.20	3.2E-01	-0.08	7.1E-01	-0.50	9.5E-03	0.12	5.6E-01	-0.05	8.1E-01	-0.41	3.6E-02
ENSCAFG0000001016	TPMT	grey	EC_M10	0.41	4.0E-02	-0.57	2.6E-03	-0.01	9.7E-01	-0.02	9.4E-01	-0.08	6.9E-01	0.06	7.7E-01	-0.20	3.2E-01	0.22	2.7E-01	0.12	5.7E-01	-0.16	4.4E-01
ENSCAFG0000000539	DYNC1LI1	darkgreen	EC_M4	0.41	4.0E-02	0.01	9.5E-01	-0.84	7.9E-08	-0.12	5.4E-01	0.68	1.4E-04	0.26	2.0E-01	-0.12	5.7E-01	-0.07	7.3E-01	-0.22	2.9E-01	-0.87	9.7E-09
ENSCAFG0000000640	UBA5	darkgreen	EC_M4	0.41	4.0E-02	0.19	3.5E-01	-0.87	5.6E-09	-0.12	5.6E-01	0.64	4.2E-04	0.28	1.6E-01	-0.21	3.1E-01	-0.23	2.7E-01	-0.22	2.9E-01	-0.85	3.6E-08
ENSCAFG0000001245	UBR3	darkgrey	EC_M8	0.41	4.0E-02	0.12	5.7E-01	-0.45	2.0E-02	0.16	4.3E-01	0.12	5.5E-01	-0.03	8.9E-01	-0.21	3.1E-01	-0.02	9.2E-01	-0.21	3.0E-01	-0.27	1.8E-01
ENSCAFG0000001236	ALS2	darkgrey	EC_M8	0.41	4.0E-02	0.51	8.2E-03	-0.13	5.3E-01	0.21	3.0E-01	-0.45	2.0E-02	-0.28	1.7E-01	-0.19	3.6E-01	-0.06	7.9E-01	-0.05	8.0E-01	0.20	3.3E-01
ENSCAFG0000001144	USP13	turquoise	EC_M6	0.41	4.0E-02	0.75	1.2E-05	-0.38	5.5E-02	0.18	3.7E-01	-0.19	3.4E-01	-0.12	5.6E-01	0.09	6.5E-01	-0.06	7.8E-01	0.13	5.1E-01	-0.03	9.0E-01
ENSCAFG0000001237	VASH2	darkgreen	EC_M4	0.40	4.0E-02	0.16	4.3E-01	-0.90	5.7E-10	-0.23	2.5E-01	0.65	3.2E-04	0.20	3.4E-01	0.00	9.9E-01	0.14	5.0E-01	-0.02	9.4E-01	-0.88	2.1E-09
ENSCAFG0000000100	ENSCAFG0000000100	grey	EC_M10	0.40	4.0E-02	-0.35	7.9E-02	0.02	9.2E-01	0.28	1.7E-01	-0.26	2.0E-01	0.10	6.2E-01	-0.17	4.2E-01	0.01	9.5E-01	-0.52	6.9E-03	0.01	9.5E-01
ENSCAFG0000001455	NIN	grey	EC_M10	0.40	4.0E-02	0.23	2.6E-01	0.17	4.0E-01	0.11	6.0E-01	-0.62	7.9E-04	-0.06	7.7E-01	-0.14	4.9E-01	0.03	8.9E-01	-0.28	1.7E-01	0.45	2.0E-02
ENSCAFG0000000960	SLC35B3	grey	EC_M10	0.40	4.0E-02	0.20	3.3E-01	-0.53	5.3E-03	0.14	4.9E-01	0.13	5.3E-01	0.18	3.8E-01	0.01	9.7E-01	-0.08	7.1E-01	-0.15	4.8E-01	-0.38	5.9E-02
ENSCAFG0000003156	ZPR1	darkgreen	EC_M4	0.40	4.0E-02	0.01	9.7E-01	-0.85	4.9E-08	-0.13	5.3E-01	0.70	7.2E-05	0.21	3.1E-01	-0.17	3.9E-01	0.09	6.7E-01	-0.01	9.6E-01	-0.90	2.3E-10
ENSCAFG0000000302	CHN2	turquoise	EC_M6	0.40	4.0E-02	0.93	1.4E-11	-0.44	2.5E-02	-0.14	5.0E-01	-0.12	5.6E-01	-0.05	8.3E-01	-0.13	5.3E-01	-0.15	4.5E-01	-0.04	8.3E-01	-0.08	7.0E-01
ENSCAFG0000001838	SLC25A5	grey	EC_M10	0.40	4.1E-02	-0.46	1.9E-02	0.09	6.5E-01	-0.02	9.3E-01	-0.21	3.1E-01	0.08	7.0E-01	-0.19	3.4E-01	0.03	8.8E-01	-0.03	8.7E-01	-0.03	9.0E-01
ENSCAFG0000000300	CREB5	turquoise	EC_M6	0.40	4.1E-02	0.60	1.1E-03	-0.25	2.3E-01	-0.28	1.6E-01	-0.21	3.1E-01	0.05	8.2E-01	-0.29	1.5E-01	-0.17	4.2E-01	-0.01	9.8E-01	0.00	9.9E-01
ENSCAFG0000000681	RPL21	grey	EC_M10	0.40	4.1E-02	-0.37	6.1E-02	0.14	4.8E-01	0.37	6.6E-02	-0.42	3.2E-02	0.24	2.4E-01	0.00	9.9E-01	-0.09	6.5E-01	-0.36	6.7E-02	0.14	4.9E-01
ENSCAFG0000000485	INTS4	darkgreen	EC_M4	0.40	4.1E-02	0.59	1.5E-03	-0.77	4.7E-06	-0.24	2.4E-01	0.38	5.3E-02	0.00	9.9E-01	-0.27	1.8E-01	-0.08	6.9E-01	-0.18	3.7E-01	-0.58	1.7E-03
ENSCAFG0000002868	MRPS25	grey	EC_M10	0.40	4.1E-02	-0.14	4.9E-01	-0.37	6.3E-02	-0.02	9.2E-01	0.24	2.4E-01	0.29	1.5E-01	0.02	9.3E-01	0.24	2.4E-01	-0.23	2.5E-01	-0.48	1.3E-02
ENSCAFG0000001259	NDUFA10	grey	EC_M10	0.40	4.1E-02	-0.34	8.5E-02	0.19	3.4E-01	0.11	6.0E-01	-0.35	7.7E-02	-0.14	4.8E-01	-0.50	9.4E-03	-0.04	8.6E-01	-0.03	9.0E-01	0.18	3.8E-01
ENSCAFG0000002998	RNF139	darkgrey	EC_M8	0.40	4.1E-02	0.21	3.0E-01	0.18	3.8E-01	0.15	4.7E-01	-0.67	1.8E-04	0.11	6.1E-01	-0.18	3.7E-01	-0.24	2.3E-01	-0.19	3.7E-01	0.47	1.6E-02
ENSCAFG0000000661	KATNAL1	darkgreen	EC_M4	0.40	4.1E-02	-0.13	5.3E-01	-0.66	2.6E-04	-0.02	9.1E-01	0.47	1.5E-02	0.09	6.7E-01	-0.01	9.5E-01	-0.04	8.6E-01	-0.17	4.0E-01	-0.72	3.4E-05
ENSCAFG0000003150	ENSCAFG0000003150	grey	EC_M10	0.40	4.1E-02	0.31	1.3E-01	-0.64	4.2E-04	0.22	2.8E-01	0.22	2.8E-01	0.11	6.1E-01	-0.05	8.0E-01	0.00	9.8E-01	0.05	8.2E-01	-0.46	1.8E-02
ENSCAFG0000000711	PCM1	grey	EC_M10	0.40	4.1E-02	-0.09	6.8E-01	-0.13	5.1E-01	0.01	9.6E-01	-0.18	3.7E-01	-0.16	4.3E-01	-0.29	1.5E-01	-0.03	9.0E-01	-0.16	4.4E-01	-0.02	9.3E-01
ENSCAFG0000000710	ENSCAFG0000000710	grey	EC_M10	0.40	4.1E-02	0.27	1.9E-01	-0.25	2.1E-01	0.18	3.8E-01	-0.12	5.5E-01	-0.23	2.6E-01	-0.23	2.5E-01	-0.22	2.8E-01	-0.27	1.7E-01	-0.12	5.5E-01
ENSCAFG0000000593	ENSCAFG0000000593	grey	EC_M10	0.40	4.1E-02	0.21	3.1E-01	-0.26	2.1E-01	0.41	4.0E-02	-0.07	7.4E-01	-0.11	5.8E-01	-0.12	5.5E-01	-0.09	6.7E-01	-0.13	5.2E-01	-0.13	5.2E-01
ENSCAFG0000001633	ENSCAFG0000001633	grey	EC_M10	0.40	4.2E-02	-0.12	5.5E-01	-0.13	5.2E-01	-0.20	3.3E-01	-0.10	6.2E-01	-0.24	2.4E-01	-0.17	4.2E-01	0.32	1.1E-01	-0.03	8.8E-01	-0.13	5.2E-01
ENSCAFG0000001581	SYNE2	turquoise	EC_M6	0.40	4.2E-02	0.81	4.5E-07	-0.20	3.2E-01	-0.18	3.7E-01	-0.34	9.3E-02	-0.09	6.6E-01	-0.13	5.2E-01	-0.13	5.2E-01	-0.11	5.9E-01	0.14	5.0E-01
ENSCAFG0000001281	CDKL5	darkgrey	EC_M8	0.40	4.2E-02	0.36	7.3E-02	-0.03	8.7E-01	0.27	1.8E-01	-0.44	2.5E-02	-0.11	6.0E-01	-0.08	7.0E-01	-0.04	8.3E-01	-0.04	8.4E-01	0.24	2.3E-01
ENSCAFG0000000740	CDK5RAP1	darkgrey	EC_M8	0.40	4.2E-02	-0.05	8.0E-01	0.20	3.2E-01	-0.16	4.3E-01	-0.60	1.1E-03	-0.10	6.4E-01	-0.08	6.8E-01	-0.10	6.2E-01	0.14	5.0E-01	0.41	3.9E-02
ENSCAFG0000003075	ZCRB1	grey	EC_M10	0.40	4.2E-02	-0.19	3.5E-01	-0.20	3.2E-01	0.10	6.3E-01	-0.12	5.4E-01	0.10	6.3E-01	-0.16	4.3E-01	-0.01	9.7E-01	-0.07	7.2E-01	-0.14	5.0E-01
ENSCAFG0000001872	SKP2	grey	EC_M10	0.40	4.2E-02	-0.22	2.7E-01	0.37	6.0E-02	0.21	3.1E-01	-0.71	5.4E-05	-0.15	4.5E-01	-0.28	1.7E-01	0.16	4.4E-01	0.01	9.6E-01	0.46	1.9E-02

ENSCAFG0000000970	IRAK4	grey	EC_M10	0.40	4.2E-02	0.33	1.0E-01	-0.35	8.0E-02	-0.08	7.0E-01	-0.11	5.8E-01	0.02	9.4E-01	-0.14	4.8E-01	0.15	4.7E-01	-0.29	1.5E-01	-0.16	4.3E-01
ENSCAFG0000000034	ENSCAFG0000000034	grey	EC_M10	0.40	4.2E-02	-0.04	8.5E-01	0.05	8.0E-01	0.06	7.9E-01	-0.38	5.6E-02	0.26	1.9E-01	-0.06	7.8E-01	-0.36	7.2E-02	-0.17	4.0E-01	0.15	4.7E-01
ENSCAFG0000000246	SNX13	darkgreen	EC_M4	0.40	4.2E-02	0.41	3.7E-02	-0.74	1.5E-05	-0.10	6.4E-01	0.34	9.4E-02	0.29	1.4E-01	-0.07	7.4E-01	0.18	3.8E-01	-0.08	7.1E-01	-0.55	3.6E-03
ENSCAFG0000001820	TMEM241	grey	EC_M10	0.40	4.2E-02	-0.15	4.7E-01	0.24	2.3E-01	0.23	2.7E-01	-0.56	3.1E-03	-0.07	7.3E-01	0.00	9.9E-01	-0.27	1.8E-01	-0.39	4.6E-02	0.35	7.8E-02
ENSCAFG0000001609	ENSCAFG0000001609	grey	EC_M10	0.40	4.2E-02	-0.26	1.9E-01	0.16	4.3E-01	0.05	8.2E-01	-0.36	6.8E-02	-0.10	6.3E-01	-0.34	9.4E-02	0.04	8.3E-01	-0.05	8.0E-01	0.14	4.8E-01
ENSCAFG0000001094	CCDC91	grey	EC_M10	0.40	4.2E-02	0.22	2.8E-01	-0.55	3.6E-03	0.06	7.7E-01	0.16	4.3E-01	-0.01	9.6E-01	-0.16	4.3E-01	-0.01	9.7E-01	-0.31	1.2E-01	-0.42	3.2E-02
ENSCAFG0000002344	ENSCAFG0000002344	grey	EC_M10	0.40	4.2E-02	-0.25	2.1E-01	-0.11	6.1E-01	0.43	2.8E-02	-0.07	7.3E-01	0.40	4.0E-02	-0.10	6.4E-01	-0.15	4.7E-01	-0.21	3.1E-01	-0.12	5.4E-01
ENSCAFG0000000944	UQCRB	darkgreen	EC_M4	0.40	4.2E-02	-0.12	5.5E-01	-0.63	5.0E-04	-0.11	6.1E-01	0.44	2.5E-02	0.20	3.3E-01	-0.09	6.6E-01	0.26	1.9E-01	-0.19	3.6E-01	-0.68	1.5E-04
ENSCAFG0000001747	FEM1B	grey	EC_M10	0.40	4.2E-02	-0.10	6.4E-01	0.04	8.4E-01	0.33	1.0E-01	-0.37	5.9E-02	0.01	9.5E-01	0.26	2.0E-01	-0.12	5.5E-01	-0.39	5.0E-02	0.15	4.7E-01
ENSCAFG0000001846	COPS3	darkgrey	EC_M8	0.40	4.3E-02	-0.21	3.0E-01	-0.68	1.4E-04	0.08	7.0E-01	0.59	1.6E-03	0.35	7.6E-02	-0.44	2.4E-02	-0.08	7.1E-01	-0.14	5.0E-01	-0.79	1.4E-06
ENSCAFG0000000331	ENSCAFG0000000331	darkgrey	EC_M8	0.40	4.3E-02	0.14	5.0E-01	-0.28	1.6E-01	0.33	1.0E-01	-0.11	5.9E-01	0.06	7.5E-01	-0.13	5.2E-01	-0.30	1.4E-01	-0.09	6.5E-01	-0.07	7.3E-01
ENSCAFG0000000480	ENSCAFG0000000480	grey	EC_M10	0.40	4.3E-02	0.02	9.3E-01	-0.08	6.8E-01	0.47	1.5E-02	-0.26	1.9E-01	-0.01	9.7E-01	-0.22	2.8E-01	-0.08	6.8E-01	-0.35	7.9E-02	0.02	9.1E-01
ENSCAFG0000000065	ENSCAFG0000000065	grey	EC_M10	0.40	4.3E-02	0.34	8.5E-02	-0.17	4.1E-01	0.46	1.7E-02	-0.25	2.2E-01	-0.22	2.8E-01	-0.16	4.4E-01	-0.20	3.3E-01	-0.02	9.1E-01	0.02	9.1E-01
ENSCAFG0000000504	RAB3GAP1	darkgreen	EC_M4	0.40	4.3E-02	0.10	6.2E-01	-0.76	6.4E-06	-0.02	9.2E-01	0.53	5.3E-03	0.32	1.1E-01	-0.12	5.6E-01	-0.24	2.4E-01	-0.48	1.3E-02	-0.76	5.6E-06
ENSCAFG0000003167	ENSCAFG0000003167	darkgrey	EC_M8	0.40	4.3E-02	0.04	8.3E-01	0.28	1.7E-01	0.35	7.7E-02	-0.72	3.4E-05	-0.18	3.8E-01	-0.48	1.3E-02	0.02	9.3E-01	-0.14	5.1E-01	0.48	1.3E-02
ENSCAFG0000000854	MCRS1	darkgrey	EC_M8	0.40	4.3E-02	0.40	4.5E-02	-0.33	9.8E-02	-0.17	4.2E-01	0.03	8.8E-01	-0.08	7.1E-01	-0.33	9.6E-02	-0.10	6.1E-01	0.01	9.6E-01	-0.22	2.8E-01
ENSCAFG0000001975	ENSCAFG0000001975	grey	EC_M10	0.40	4.3E-02	0.26	1.9E-01	0.14	4.9E-01	0.05	8.2E-01	-0.59	1.7E-03	0.12	5.5E-01	-0.01	9.6E-01	-0.21	3.1E-01	-0.15	4.7E-01	0.39	4.8E-02
ENSCAFG0000002157	ENSCAFG0000002157	grey	EC_M10	0.40	4.4E-02	0.10	6.2E-01	-0.25	2.2E-01	0.12	5.6E-01	-0.13	5.3E-01	-0.28	1.7E-01	-0.31	1.3E-01	0.11	5.9E-01	-0.20	3.3E-01	-0.14	5.0E-01
ENSCAFG0000001927	ENSCAFG0000001927	darkgrey	EC_M8	0.40	4.4E-02	-0.23	2.6E-01	-0.48	1.3E-02	-0.31	1.2E-01	0.33	9.8E-02	0.06	7.7E-01	-0.24	2.5E-01	0.46	1.8E-02	-0.23	2.6E-01	-0.57	2.6E-03
ENSCAFG0000001999	PTPA	grey	EC_M10	0.40	4.4E-02	-0.15	4.5E-01	-0.39	5.1E-02	-0.17	4.1E-01	0.29	1.5E-01	-0.05	8.1E-01	-0.02	9.1E-01	0.09	6.6E-01	-0.04	8.5E-01	-0.47	1.5E-02
ENSCAFG0000003266	ENSCAFG0000003266	grey	EC_M10	0.40	4.4E-02	0.03	8.8E-01	-0.64	4.2E-04	0.21	2.9E-01	0.39	4.6E-02	0.16	4.3E-01	-0.10	6.1E-01	-0.07	7.3E-01	-0.51	7.7E-03	-0.62	7.9E-04
ENSCAFG0000000728	ENSCAFG0000000728	grey	EC_M10	0.40	4.4E-02	0.00	9.9E-01	-0.10	6.1E-01	0.44	2.5E-02	-0.21	3.1E-01	0.04	8.5E-01	-0.12	5.5E-01	-0.17	4.1E-01	-0.09	6.5E-01	0.01	9.7E-01
ENSCAFG0000002397	ENSCAFG0000002397	grey	EC_M10	0.40	4.4E-02	0.36	7.4E-02	-0.30	1.4E-01	0.02	9.4E-01	-0.09	6.5E-01	-0.02	9.1E-01	0.51	8.2E-03	-0.16	4.4E-01	0.08	7.1E-01	-0.10	6.3E-01
ENSCAFG0000000379	ENSCAFG0000000379	darkgreen	EC_M4	0.40	4.4E-02	-0.05	7.9E-01	-0.69	1.0E-04	-0.12	5.6E-01	0.47	1.6E-02	0.19	3.5E-01	-0.20	3.4E-01	-0.09	6.6E-01	-0.15	4.5E-01	-0.70	8.1E-05
ENSCAFG0000001854	NNT	grey	EC_M10	0.40	4.4E-02	0.16	4.5E-01	-0.40	4.3E-02	-0.06	7.8E-01	0.09	6.7E-01	-0.28	1.6E-01	-0.15	4.6E-01	-0.15	4.7E-01	-0.13	5.2E-01	-0.25	2.2E-01
ENSCAFG0000000306	SLC35A1	darkgreen	EC_M4	0.40	4.4E-02	0.43	2.7E-02	-0.82	3.4E-07	-0.12	5.5E-01	0.44	2.5E-02	0.27	1.8E-01	-0.18	3.8E-01	-0.10	6.1E-01	-0.32	1.1E-01	-0.71	5.5E-05
ENSCAFG0000001807	DCTN4	grey	EC_M10	0.40	4.4E-02	0.26	2.1E-01	0.03	8.9E-01	-0.03	8.7E-01	-0.45	2.0E-02	-0.04	8.3E-01	-0.16	4.5E-01	-0.26	1.9E-01	-0.53	5.0E-03	0.25	2.1E-01
ENSCAFG0000000270	PRPF31	grey	EC_M10	0.40	4.4E-02	0.09	6.6E-01	0.19	3.7E-01	0.38	5.8E-02	-0.54	4.8E-03	-0.04	8.3E-01	-0.16	4.5E-01	-0.16	4.3E-01	0.00	9.8E-01	0.32	1.1E-01
ENSCAFG0000003216	ZNF792	turquoise	EC_M6	0.40	4.4E-02	0.86	2.6E-08	-0.14	5.0E-01	0.05	8.1E-01	-0.48	1.4E-02	-0.01	9.7E-01	-0.01	9.4E-01	-0.20	3.2E-01	-0.09	6.5E-01	0.27	1.9E-01
ENSCAFG0000000994	HEATR3	darkgrey	EC_M8	0.40	4.4E-02	-0.36	7.5E-02	-0.37	6.1E-02	-0.07	7.4E-01	0.32	1.2E-01	0.19	3.5E-01	-0.46	1.7E-02	-0.24	2.3E-01	-0.14	5.0E-01	-0.53	5.6E-03
ENSCAFG0000003186	ENSCAFG0000003186	turquoise	EC_M6	0.40	4.4E-02	0.76	5.6E-06	-0.34	9.0E-02	-0.19	3.6E-01	-0.22	2.9E-01	-0.18	3.8E-01	-0.15	4.6E-01	0.22	2.9E-01	-0.08	7.1E-01	0.02	9.1E-01
ENSCAFG0000000402	TUBE1	grey	EC_M10	0.40	4.4E-02	0.03	8.9E-01	-0.11	5.8E-01	0.28	1.6E-01	-0.26	1.9E-01	-0.05	8.1E-01	-0.01	9.6E-01	0.07	7.4E-01	-0.19	3.4E-01	0.02	9.3E-01
ENSCAFG0000000482	DHTKD1	grey	EC_M10	0.40	4.4E-02	-0.29	1.5E-01	0.22	2.7E-01	0.03	9.0E-01	-0.46	1.9E-02	0.13	5.4E-01	-0.08	7.0E-01	-0.08	7.2E-01	-0.03	8.7E-01	0.29	1.5E-01
ENSCAFG0000001709	ZNF609	darkgrey	EC_M8	0.40	4.4E-02	0.59	1.5E-03	0.12	5.7E-01	0.10	6.4E-01	-0.71	4.8E-05	-0.13	5.3E-01	-0.09	6.6E-01	-0.18	3.8E-01	-0.24	2.4E-01	0.49	1.1E-02
ENSCAFG0000000501	GATA3	turquoise	EC_M6	0.40	4.4E-02	0.74	1.3E-05	-0.56	2.7E-03	-0.25	2.3E-01	0.07	7.3E-01	-0.13	5.3E-01	0.07	7.2E-01	-0.27	1.8E-01	-0.10	6.2E-01	-0.29	1.5E-01
ENSCAFG0000000800	ENSCAFG0000000800	paleturquoise	EC_M11	0.40	4.4E-02	0.07	7.5E-01	-0.34	9.4E-02	0.61	8.8E-04	0.03	8.8E-01	-0.15	4.8E-01	-0.14	5.0E-01	-0.12	5.7E-01	-0.19	3.5E-01	-0.26	2.1E-01
ENSCAFG0000003124	FAM111A	grey	EC_M10	0.40	4.4E-02	-0.39	5.1E-02	-0.20	3.4E-01	0.30	1.4E-01	0.06	7.7E-01	0.30	1.4E-01	-0.27	1.8E-01	-0.09	6.6E-01	-0.44	2.4E-02	-0.35	7.7E-02
ENSCAFG0000003229	ENSCAFG0000003229	grey	EC_M10	0.40	4.4E-02	0.07	7.4E-01	-0.17	4.1E-01	0.09	6.6E-01	-0.16	4.4E-01	0.17	4.1E-01	0.07	7.2E-01	-0.43	2.7E-02	-0.05	8.1E-01	-0.03	8.8E-01
ENSCAFG0000001142	DYRK3	turquoise	EC_M6	0.40	4.4E-02	0.60	1.1E-03	0.03	9.0E-01	-0.12	5.7E-01	-0.56	2.8E-03	-0.21	3.0E-01	-0.17	4.1E-01	-0.01	9.7E-01	-0.09	6.6E-01	0.35	8.2E-02
ENSCAFG0000000919	PHTF1	grey	EC_M10	0.40	4.4E-02	0.25	2.2E-01	0.08	7.1E-01	0.16	4.2E-01	-0.53	5.5E-03	-0.35	7.7E-02	-0.29	1.6E-01	-0.20	3.3E-01	-0.40	4.3E-02	0.30	1.3E-01
ENSCAFG0000000101	PPP2CA	turquoise	EC_M6	0.40	4.5E-02	0.60	1.2E-03	-0.69	8.8E-05	0.08	7.1E-01	0.31	1.2E-01	0.17	4.0E-01	-0.14	5.1E-01	-0.32	1.1E-01	-0.22	2.8E-01	-0.48	1.3E-02
ENSCAFG0000001197	RPA2	darkgrey	EC_M8	0.40	4.5E-02	0.31	1.3E-01	-0.14	4.9E-01	-0.21	3.1E-01	-0.15	4.7E-01	0.03	8.9E-01	0.05	8.0E-01	0.04	8.5E-01	-0.14	5.1E-01	-0.03	8.8E-01
ENSCAFG0000000780	STOX2	turquoise	EC_M6	0.40	4.5E-02	0.85	4.8E-08	-0.69	1.1E-04	-0.04	8.5E-01	0.17	4.2E-01	0.04	8.4E-01	-0.06	7.8E-01	-0.24	2.3E-01	-0.16	4.2E-01	-0.38	5.5E-02
ENSCAFG0000001902	GOSR1	grey	EC_M10	0.40	4.5E-02	0.40	4.1E-02	-0.16	4.5E-01	0.14	4.8E-01	-0.35	8.3E-02	-0.04	8.4E-01	-0.14	4.9E-01	0.05	8.2E-01	-0.12	5.5E-01	0.15	4.8E-01
ENSCAFG0000001456	ENSCAFG0000001456	grey	EC_M10	0.40	4.5E-02	0.29	1.5E-01	-0.58	2.1E-03	0.18	3.9E-01	0.19	3.4E-01	0.10	6.4E-01	-0.08	7.0E-01	-0.01	9.7E-01	-0.20	3.4E-01	-0.38	5.8E-02
ENSCAFG0000003120	ZCCHC10	grey	EC_M10	0.40	4.5E-02	0.38	5.6E-02	-0.05	8.2E-01	-0.15	4.6E-01	-0.43	3.0E-02	0.03	8.8E-01	-0.13	5.3E-01	-0.28	1.7E-01	-0.21	3.1E-01	0.21	2.9E-01
ENSCAFG0000001545	ENSCAFG0000001545	darkgreen	EC_M4	0.40	4.5E-02	0.44	2.3E-02	-0.67	2.1E-04	-0.13	5.3E-01	0.27	1.8E-01	-0.24	2.4E-01	-0.33	9.8E-02	-0.13	5.2E-01	-0.16	4.2E-01	-0.50	8.9E-03
ENSCAFG0000001343	WDR76	grey	EC_M10	0.40	4.5E-02	0.08	7.0E-01	0.36	7.1E-02	0.15	4.7E-01	-0.73	2.2E-05	0.00	9.8E-01	-0.01	9.4E-01	-0.43	2.9E-02	-0.09	6.7E-01	0.56	3.2E-03
ENSCAFG0000003235	ENSCAFG0000003235	darkgrey	EC_M8	0.40	4.5E-02	0.58	1.9E-03	0.01	9.5E-01	0.21	3.1E-01	-0.55	3.8E-03	-0.04	8.3E-01	-0.34	8.9E-02	-0.17	4.0E-01	-0.33	9.6E-02	0.34	8.7E-02
ENSCAFG0000000745	GPATCH1	turquoise	EC_M6	0.40	4.5E-02	0.62	7.8E-04	-0.24	2.4E-01	0.06	7.7E-01	-0.24	2.4E-01	-0.12	5.7E-01	0.13	5.3E-01	0.04	8.6E-01	-0.04	8.3E-01	0.07	7.2E-01
ENSCAFG0000002475	ENSCAFG0000002475	grey	EC_M10	0.40	4.5E-02	0.21	2.9E-01	-0.67	2.0E-04	-0.38	5.8E-02	0.35	8.3E-02	0.35	8.1E-02	-0.07	7.4E-01	0.15	4.7E-01	-0.02	9.1E-01	-0.61	9.5E-04
ENSCAFG0000001377	ENSCAFG0000001377	grey	EC_M10	0.40	4.6E-02	0.17	4.0E-01	-0.17	4.1E-01	0.42	3.4E-02	-0.22	2.9E-01	0.09	6.7E-01	-0.03	8.8E-01	-0.45	2.2E-02	-0.14	5.0E-01	0.01	9.8E-01
ENSCAFG0000003155	ENSCAFG0000003155	grey	EC_M10	0.40	4.6E-02	0.12	5.4E-01	-0.25	2.2E-01	0.15	4.7E-01	-0.07	7.3E-01	0.08	7.2E-01	-0.18	3.9E-01	0.00	1.0E+00	-0.37	6.1E-02	-0.14	4.9E-01
ENSCAFG0000000443	ENSCAFG0000000443	grey	EC_M10	0.40	4.6E-02	0.11	6.0E-01	-0.35	7.6E-02	0.07	7.3E-01	0.13	5.4E-01	0.26	2.0E-01	-0.01	9.7E-01	0.11	5.9E-01	-0.23	2.6E-01	-0.38	5.4E-02
ENSCAFG0000001053	ENSCAFG0000001053	darkgrey	EC_M8	0.40	4.6E-02	-0.19	3.5E-01	-0.57	2.4E-03	-0.07	7.4E-01	0.42	3.1E-02	0.20	3.3E-01	-0.15	4.7E-01	-0.14	4.9E-01	0.18	3.7E-01	-0.65	3.7E-04
ENSCAFG0000003245	ARPC5L	turquoise	EC_M6	0.40	4.6E-02	0.81	4.7E-07	-0.62	7.9E-04	-0.18	3.9E-01	0.16	4.3E-01	-0.08	6.9E-01	-0.14	5.0E-01	-0.15	4.6E-01	-0.05	8.0E-01	-0.33	9.7E-02
ENSCAFG0000001240	KHNYN	grey	EC_M10	0.39	4.6E-02	0.42	3.4E-02	0.16	4.3E-01	0.06	7.7E-01	-0.63	5.0E-04	-0.36	7.5E-02	0.09	6.7E-01	-0.06	7.6E-01	-0.36	7.2E-02	0.42	3.5E-02
ENSCAFG0000000437	ENTPD6	darkgreen	EC_M4	0.39	4.6E-02	-0.12	5.7E-01	-0.65	3.4E-04	-0.40	4.4E-02	0.50	8.8E-03	0.12	5.6E-01	-0.13	5.3E-01	0.31	1.3E-01	-0.17	4.0E-01	-0.75	1.1E-05
ENSCAFG0000000662	SFSWAP	grey	EC_M10	0.39	4.6E-02	0.36	7.2E-02	-0.17	4.1E-01	-0.29	1.4E-01	-0.27	1.8E-01	0.19	3.5E-01	-0.26	2.0E-01	-0.31	1.2E-01	-0.20	3.3E-01	0.06	7.8E-01
ENSCAFG0000000993	ENSCAFG0000000993	grey	EC_M10	0.39	4.6E-02	0.26	2.0E-01	-0.52	6.3E-03	0.16	4.3E-01	0.17	4.2E-01	0.15	4.7E-01	0.09	6.6E-01	-0.20	3.3E-01	-0.26	2.0E-01	-0.38	5.3E-02
ENSCAFG0000003084	ENSCAFG0000003084	grey	EC_M10	0.39	4.6E-02	0.32	1.1E-01	-0.48	1.4E-02	-0.19	3.4E-01	0.18	3.7E-01	0.11	6.0E-01	-0.10	6.4E-01	-0.13	5.2E-01	0.10	6.4E-01	-0.37	6.1E-02
ENSCAFG0000001766	PTRH2	grey	EC_M10	0.39	4.6E-02	-0.04	8.4E-01	-0.55	3.5E-03	-0.01	9.7E-01	0.33	1.0E-01	0.31	1.3E-01	0.11	5.8E-01	-0.11	5.8E-01	-0.30	1.4E-01	-0.58	1.7E-03
ENSCAFG0000000135	KIAA2026	darkgrey	EC_M8	0.39	4.6E-02	0.22	2.9E-01	0.20	3.2E-01	0.06	7.6E-01	-0.71	4.2E-05	-0.19	3.6E-01	-0.38	5.4E-02	-0.10	6.3E-01	-0.26	2.0E-01	0.48	1.3E-02
ENSCAFG0000000122	DENND3	turquoise	EC_M6	0.39	4.6E-02	0.84	1.1E-07	-0.46	1.7E-02	-0.03	8.9E-01	-0.07	7.5E-01	0.04	8.3E-01	0.01	9.8E-01	-0.16	4.5E-01	0.03	8.7E-01	-0.16	4.3E-01
ENSCAFG0000001841	C9H17orf75	grey	EC_M10	0.39	4.6E-02	0.04	8.3E-01	0.16	4.4E-01	0.08	6.8E-01	-0.47	1.5E-02	-0.07	7.3E-01	-0.02	9.2E-01	-0.19	3.5E-01	-0.57	2.5E-03	0.29	1.5E-01
ENSCAFG0000002914	BEND3	grey	EC_M10	0.39	4.6E-02	0.34	9.0E-02	-0.49	1.0E-02	-0.35	7.5E-02	0.22	2.8E-01	0.05	7.9E-01	0.09	6.5E-01	0.06	7.6E-01	0.15	4.5E-01	-0.41	4.0E-02
ENSCAFG0000002912	ENSCAFG0000002912	darkgreen	EC_M4	0.39	4.6E-02	0.29	1.6E-01	-0.71	4.6E-05	-0.15	4.7E-01	0.38	5.2E-02	0.05	8.1E-01	-0.19	3.6E-01	0.06	7.9E-01	-0.17	4.0E-01	-0.60	1.3E-03
ENSCAFG0000001367	EFL1	grey	EC_M10	0.39	4.7E-02	0.23	2.7E-01	0.04	8.3E-01	0.16	4.4E-01	-0.48	1.4E-02	-0.07	7.3E-01	-0.04	8.4E-01	-0.39	5.0E-02	-0.46	1.8E-02	0.25	2.1E-01
ENSCAFG0000002305	ENSCAFG0000002305	grey	EC_M10	0.39	4.7E-02	-0.14	5.1E-01	-0.30	1.4E-01	-0.09	6.7E-01	0.05	8.3E-01	0.39	5.0E-02	-0.13	5.3E-01	-0.17	4.0E-01	-0.09	6.6E-01	-0.26	2.0E-01
ENSCAFG0000000774	PRIMPOL	grey	EC_M10	0.39	4.7E-02	-0.37	6.0E-02	-0.28	1.6E-01	0.05	8.2E-01	0.12	5.5E-01	0.00	9.9E-01	-0.31	1.2E-01	0.27	1.8E-01	-0.11	5.8E-01	-0.37	6.4E-02
ENSCAFG0000000702	NAT10	grey	EC_M10	0.39	4.7E-02	-0.25	2.2E-01	0.32	1.1E-01	0.05	8.3E-01	-0.52	6.2E-03	-0.17	4.1E-01	0.06	7.7E-01	-0.02	9.3E-01	-0.30	1.4E-01	0.32	1.1E-01
ENSCAFG0000000109	ASAP1	turquoise	EC_M6	0.39	4.7E-02	0.60	1.3E-03	0.00	1.0E+00	-0.06	7.8E-01	-0.52	6.2E-03	-0.33	1.0E-01	-0.25	2.1E-01	0.09	6.8E-01	0.15	4.5E-01	0.31	1.2E-01
ENSCAFG0000003197	ZNF10	turquoise	EC_M6	0.39	4.7E-02	0.37	6.2E-02	-0.14	4.9E-01	0.06	7.7E-01	-0.25	2.1E-01	-0.17	3.9E-01	-0.17	4.2E-01	-0.08	6.8E-01	-0.15	4.6E-01	0.06	7.7E-01
ENSCAFG0000000185	ADAM22	grey	EC_M10	0.39	4.7E-02	0.35	7.5E-02	-0.31	1.2E-01	0.02	9.4E-01	-0.09	6.5E-01	-0.26	2.0E-01	-0.26	2.1E-01	-0.27	1.9E-01	0.26	2.0E-01	-0.06	7.5E-01
ENSCAFG0000001317	OLA1	grey	EC_M10	0.39	4.7E-02	-0.66	2.7E-04	-0.15	4.5E-01	0.12	5.6E-01	0.10	6.1E-01	0.07	7.3E-01	-0.22	2.7E-01	0.11	6.0E-01	-0.30	1.4E-01	-0.33	9.9E-02
ENSCAFG0000001563	SLC2A9	turquoise	EC_M6	0.39	4.7E-02	0.62	8.0E-04	-0.20	3.2E-01	-0.11	5.8E-01	-0.31	1.2E-01	-0.08	7.0E-01	-0.02	9.1E-01	0.12	5.5E-01	-0.15	4.5E-01	0.06	7.8E-01
ENSCAFG0000001982	GNAI3	darkgreen	EC_M4	0.39	4.7E-02	-0.05	7.9E-01	-0.85	4.2E-08	-0.16	4.2E-01	0.70	8.0E-05	0.28	1.6E-01	-0.18	3.9E-01	0.03	8.9E-01	-0.26	2.0E-01	-0.92	1.4E-11
ENSCAFG0000000808	GATB	grey	EC_M10	0.39	4.8E-02	0.02	9.3E-01	0.26	2.1E-01	0.16	4.3E-01	-0.64	4.5E-04	-0.15	4.5E-01	-0.23	2.5E-01	-0.22	2.9E-01	0.03	8.7E-01	0.44	2.4E-02
ENSCAFG0000000071	CYP21A2	grey	EC_M10	0.39	4.8E-02	0.28	1.7E-01	-0.50	9.4E-03	0.08	6.8E-01	0.22	2.9E-01	0.07	7.5E-01	0.09	6.6E-01	-0.27	1.8E-01	0.17	4.0E-01	-0.35	7.5E-02
ENSCAFG0000000799	RPS3A	cyan	EC_M2	0.39	4.8E-02	-0.16	4.3E-01	0.23	2.5E-01	0.17	4.0E-01	-0.58	2.0E-03	0.12	5.5E-01	0.16	4.4E-01	0.05	8.0E-01	-0.44	2.5E-02	0.30	1.4E-01
ENSCAFG0000001790	ENSCAFG0000001790	grey	EC_M10	0.39	4.8E-02	-0.07	7.4E-01	0.04	8.5E-01	0.42	3.4E-02	-0.40	4.4E-02	-0.18	3.9E-01	-0.18	3.8E-01	-0.24	2.4E-01	0.08	7.1E-01	0.17	3.9E-01
ENSCAFG0000000927	DCLRE1B	darkgrey	EC_M8	0.39	4.8E-02	-0.05	8.0E-01	-0.58	1.9E-03	-0.37	6.0E-02	0.47	1.6E-02	0.09	6.5E-01	-0.21	3.1E-01	0.07	7.3E-01	-0.11	6.0E-01	-0.68	1.2E-04
ENSCAFG0000000833	ENSCAFG0000000833	grey	EC_M10	0.39	4.8E-02	0.09	6.5E-01	-0.29	1.5E-01	-0.01	9.5E-01	0.06	7.6E-01	-0.14	4.9E-01	-0.10	6.2E-01	-0.09	6.6E-01	-0.22	2.9E-01	-0.29	1.5E-01
ENSCAFG0000000461	MUTYH	grey	EC_M10	0.39	4.8E-02	0.28	1.7E-01	-0.26	2.0E-01	0.12	5.6E-01	-0.18	3.7E-01	0.08	7.0E-01	0.01	9.5E-01	-0.08	7.0E-01	-0.20	3.4E-01	-0.12	5.6E-01
ENSCAFG0000000337	ASAP2	turquoise	EC_M6	0.39	4.8E-02	0.80	8.5E-07	-0.34	9.3E-02	-0.04	8.4E-01	-0.23	2.6E-01	-0.18	3.8E-01	-0.16	4.3E-01	-0.03	8.8E-01	0.29	1.5E-01	0.02	9.1E-01
ENSCAFG0000001142	ACIN1	darkgrey	EC_M8	0.39	4.8E-02	0.43	3.0E-02	0.18	3.7E-01	0.21	3.0E-01	-0.75	1.1E-05	0.08	7.0E-01	-0.14	4.9E-01	0.04	8.4E-01	-0.15	4.6E-01	0.51	7.6E-03
ENSCAFG0000002883	ENSCAFG0000002883	grey	EC_M10	0.39	4.8E-02	0.38	5.8E-02	-0.35	8.3E-02	-0.16	4.4E-01	0.01	9.7E-01	-0.16	4.4E-01	-0.07	7.3E-01	-0.17	4.0E-01	0.30	1.4E-01	-0.14	4.9E-01
ENSCAFG0000000476	CAD	darkgrey	EC_M8	0.39	4.8E-02	-0.14	5.0E-01	-0.10	6.3E-01	-0.15	4.6E-01	-0.09	6.7E-01	0.03	8.7E-01	0.05	7.9E-01	0.01	9.6E-01	0.13	5.2E-01	-0.16	4.2E-01
ENSCAFG0000001119	ENSCAFG0000001119	grey	EC_M10	0.39	4.8E-02	0.02	9.4E-01	-0.41	3.6E-02	-0.18	3.8E-01	0.19	3.6E-01	-0.15	4.8E-01	-0.11	6.1E-01	-0.01	9.5E-01	0.03	9.0E-01	-0.37	6.0E-02
ENSCAFG0000001688	ARHGEF2	darkgreen	EC_M4	0.39	4.8E-02	0.25	2.1E-01	-0.65	2.9E-04	0.16	4.4E-01	0.32	1.2E-01	0.24	2.3E-01	-0.16	4.2E-01	-0.02	9.4E-01	-0.03	8.8E-01	-0.60	1.1E-03
ENSCAFG0000000957	TUBGCP5	grey	EC_M10	0.39	4.8E-02	-0.43	3.0E-02	0.11	6.0E-01	0.15	4.6E-01	-0.27	1.8E-01	-0.02	9.2E-01	-0.47	1.5E-02	-0.08	6.8E-01	-0.21	3.0E-01	0.08	6.9E-01
ENSCAFG0000001422	IFT80	darkgrey	EC_M8	0.39	4.8E-02	-0.16	4.3E-01	0.39	4.9E-02	0.31	1.3E-01	-0.80	1.1E-06	-0.06	7.6E-01	-0.19	3.5E-01	-0.19	3.4E-01	-0.18	3.7E-01	0.55	3.7E-03
ENSCAFG0000001586	ENSCAFG0000001586	darkgrey	EC_M8	0.39	4.8E-02	-0.05	8.2E-01	-0.24	2.3E-01	0.34	9.1E-02	-0.09	6.5E-01	-0.07	7.2E-01	-0.03	8.7E-01	0.03	8.9E-01	-0.27	1.9E-01	-0.11	6.0E-01
ENSCAFG0000001063	KIAA1551	turquoise	EC_M6	0.39	4.8E-02	0.75	9.4E-06	-0.33	9.8E-02	-0.06	7.8E-01	-0.24	2.5E-01	-0.16	4.4E-01	-0.12	5.7E-01	0.11	5.9E-01	-0.17	4.2E-01	0.02	9.1E-01
ENSCAFG0000003263	ENSCAFG0000003263	grey	EC_M10	0.39	4.9E-02	0.17	4.1E-01	-0.46	1.8E-02	-0.19	3.4E-01	0.17	4.1E-01	0.20	3.3E-01	-0.26	2.0E-01	0.17	4.0E-01	-0.03	8.8E-01	-0.36	6.8E-02
ENSCAFG0000001291	METAP1D	grey	EC_M10	0.39	4.9E-02	-0.40	4.3E-02	0.13	5.3E-01	0.34	8.6E-02	-0.31	1.3E-01	0.10	6.1E-01	-0.39	5.0E-02	-0.39	4.8E-02	-0.33	9.5E-02	0.09	6.7E-01
ENSCAFG0000001428	DNAJC10	darkgreen	EC_M4	0.39	4.9E-02	0.14	5.0E-01	-0.86	2.3E-08	0.00	9.9E-01	0.61	9.3E-04	0.18	3.8E-01	-0.14	4.8E-01	0.12	5.5E-01	0.08	7.1E-01	-0.80	8.5E-07
ENSCAFG0000000696	SCARB1	turquoise	EC_M6	0.39	4.9E-02	0.77	5.0E-06	-0.44	2.6E-02	-0.15	4.7E-01	-0.05	7.9E-01	-0.19	3.4E-01	-0.15	4.7E-01	-0.04	8.3E-01	0.08	7.1E-01	-0.16	4.2E-01
ENSCAFG0000001069	USF3	grey	EC_M10	0.39	4.9E-02	0.09	6.7E-01	-0.17	4.1E-01	-0.03	8.9E-01	-0.14	4.8E-01	0.04	8.6E-01	-0.08	6.9E-01	-0.21	3.1E-01	0.06	7.5E-01	-0.05	7.9E-01
ENSCAFG0000001857	SGIP1	grey	EC_M10	0.39	4.9E-02	0.48	1.3E-02	-0.67	1.7E-04	-0.15	4.7E-01	0.36	7.1E-02	0.27	1.9E-01	0.25	2.1E-01	-0.12	5.5E-01	-0.18	3.9E-01	-0.54	4.4E-03
ENSCAFG0000001909	ENSCAFG0000001909	turquoise	EC_M6	0.39	4.9E-02	0.30	1.3E-01	-0.21	3.1E-01	0.38	5.6E-02	-0.23	2.6E-01	0.40	4.1E-02	-0.13	5.3E-01	-0.19	3.5E-01	-0.15	4.7E-01	0.06	7.8E-01
ENSCAFG0000001442	EMG1	grey	EC_M10	0.39	4.9E-02	-0.34	9.1E-02	0.34	8.7E-02	0.00	9.9E-01	-0.50	9.1E-03	0.00	1.0E+00	-0.30	1.4E-01	-0.02	9.2E-01	-0.14	5.1E-01	0.29	1.5E-01
ENSCAFG0000001183	PITPNB	darkgreen	EC_M4	0.39	4.9E-02	0.37	6.5E-02	-0.89	1.0E-09	-0.14	4.8E-01	0.63	5.3E-04	0.34	8.5E-02	-0.20	3.4E-01	-0.15	4.7E-01	-0.19	3.5E-01	-0.81	6.0E-07
ENSCAFG0000002891	ENSCAFG0000002891	grey	EC_M10	0.39	4.9E-02	-0.04	8.6E-01	-0.20	3.3E-01	-0.04	8.5E-01	-0.11	5.9E-01	0.13	5.2E-01	0.11	5.8E-01	-0.17	4.0E-01	-0.06	7.6E-01	-0.09	6.6E-01
ENSCAFG0000000775	USP47	grey	EC_M10	0.39	4.9E-02	-0.10	6.1E-01	-0.55	3.4E-03	0.02	9.4E-01	0.33	1.0E-01	0.24	2.4E-01	-0.02	9.2E-01	-0.23	2.6E-01	-0.55	3.4E-03	-0.54	4.7E-03
ENSCAFG0000001709	CALR	darkgreen	EC_M4	0.39	4.9E-02	-0.13	5.2E-01	-0.80	8.3E-07	-0.06	7.8E-01	0.70	6.6E-05	0.33	1.0E-01	-0.03	8.7E-01	0.03	8.8E-01	-0.13	5.2E-01	-0.92	5.0E-11
ENSCAFG0000000112	KLHL3	turquoise	EC_M6	0.39	4.9E-02	0.76	5.4E-06	-0.67	2.1E-04	-0.16	4.3E-01	0.16	4.3E-01	-0.01	9.4E-01	0.03	8.8E-01	0.21	3.0E-01	0.24	2.4E-01	-0.38	5.8E-02
ENSCAFG0000002966	ENSCAFG0000002966	grey	EC_M10	0.39	5.0E-02	-0.14	4.9E-01	-0.29	1.4E-01	-0.34	8.8E-02	0.04	8.4E-01	0.16	4.3E-01	-0.18	3.8E-01	0.14	5.1E-01	-0.14	5.0E-01	-0.26	2.0E-01
ENSCAFG0000002025	ENSCAFG0000002025	darkgreen	EC_M4	0.39	5.0E-02	0.64	3.8E-04	-0.68	1.5E-04	0.05	7.9E-01	0.22	2.9E-01	0.05	8.1E-01	-0.11	5.8E-01	-0.13	5.3E-01	-0.15	4.6E-01	-0.41	3.5E-02
ENSCAFG0000001396	ENSCAFG0000001396	turquoise	EC_M6	0.39	5.0E-02	0.53	5.5E-03	-0.32	1.1E-01	-0.15	4.7E-01	-0.16	4.4E-01	0.41	3.6E-02	-0.11	5.8E-01	-0.16	4.4E-01	-0.12	5.6E-01	-0.01	9.6E-01
ENSCAFG0000000561	SLC4A7	darkgreen	EC_M4	0.39	5.0E-02	0.53	4.9E-03	-0.85	2.9E-08	-0.18	3.8E-01	0.46	1.8E-02	0.07	7.4E-01	-0.05	8.1E-01	-0.08	7.0E-01	-0.26	1.9E-01	-0.69	1.0E-04
ENSCAFG0000001608	PARP1	grey	EC_M10	0.39	5.0E-02	-0.25	2.1E-01	0.26	2.0E-01	-0.07	7.2E-01	-0.46	1.8E-02	-0.22	2.7E-01	-0.17	4.1E-01	0.08	7.0E-01	0.18	3.7E-01	0.28	1.7E-01
ENSCAFG0000000090	KPNA5	grey	EC_M10	0.39	5.0E-02	0.35	8.4E-02	-0.57	2.3E-03	0.19	3.6E-01	0.15	4.7E-01	-0.17	4.1E-01	0.01	9.8E-01	-0.13	5.2E-01	-0.01	9.6E-01	-0.35	8.2E-02
ENSCAFG0000002761	ENSCAFG0000002761	grey	EC_M10	0.39	5.0E-02	0.18	3.7E-01	-0.14	5.1E-01	-0.26	2.0E-01	-0.27	1.8E-01	0.08	6.9E-01	-0.02	9.4E-01	0.17	4.1E-01	-0.18	3.8E-01	0.02	9.2E-01
ENSCAFG0000000104	TATDN1	darkgreen	EC_M4	0.39	5.0E-02	-0.26	2.0E-01	-0.57	2.1E-03	-0.25	2.3E-01	0.47	1.5E-02	0.46	1.7E-02	-0.04	8.4E-01	0.15	4.6E-01	-0.08	7.1E-01	-0.70	7.9E-05
ENSCAFG0000001812	CHST9	turquoise	EC_M6	0.39	5.0E-02	0.68	1.2E-04	-0.60	1.2E-03	-0.07	7.2E-01	0.10	6.3E-01	0.15	4.7E-01	-0.17	4.1E-01	-0.13	5.2E-01	0.01	9.4E-01	-0.31	1.2E-01

ENSCAFG0000003000	COA7	grey	EC_M10	0.39	5.0E-02	-0.27	1.9E-01	0.16	4.3E-01	0.41	3.8E-02	-0.37	6.7E-02	-0.11	5.8E-01	-0.34	8.7E-02	0.16	4.2E-01	-0.33	1.0E-01	0.18	3.8E-01
ENSCAFG0000001644	RDH11	grey	EC_M10	0.39	5.0E-02	0.19	3.4E-01	-0.61	8.4E-04	0.09	6.5E-01	0.34	8.6E-02	0.11	6.0E-01	-0.15	4.6E-01	0.05	8.3E-01	-0.22	2.8E-01	-0.58	1.8E-03
ENSCAFG0000000923	CMSS1	grey	EC_M10	0.39	5.0E-02	-0.56	3.1E-03	-0.06	7.6E-01	0.08	6.9E-01	-0.03	8.9E-01	0.20	3.3E-01	-0.01	9.6E-01	0.20	3.2E-01	-0.11	5.9E-01	-0.21	3.0E-01
ENSCAFG0000000789	CLIP1	grey	EC_M10	0.39	5.0E-02	0.00	9.8E-01	-0.56	2.8E-03	0.13	5.2E-01	0.27	1.8E-01	0.27	1.8E-01	-0.21	2.9E-01	-0.19	3.5E-01	-0.34	8.5E-02	-0.53	4.9E-03
ENSCAFG0000000951	GLS	darkgreen	EC_M4	0.39	5.1E-02	0.11	5.9E-01	-0.80	8.1E-07	-0.05	8.0E-01	0.54	4.4E-03	0.10	6.1E-01	-0.17	4.0E-01	-0.12	5.5E-01	-0.24	2.4E-01	-0.76	5.5E-06
ENSCAFG0000000222	C1GALT1	darkgrey	EC_M8	0.39	5.1E-02	0.39	4.7E-02	0.05	7.9E-01	0.05	8.0E-01	-0.62	7.6E-04	-0.05	8.1E-01	0.06	7.8E-01	-0.04	8.5E-01	0.20	3.3E-01	0.40	4.0E-02
ENSCAFG0000000548	SETD5	turquoise	EC_M6	0.39	5.1E-02	0.63	5.8E-04	-0.60	1.1E-03	0.03	8.9E-01	0.09	6.8E-01	0.19	3.5E-01	0.09	6.7E-01	-0.03	8.9E-01	-0.07	7.2E-01	-0.35	8.1E-02
ENSCAFG0000002867	ENSCAFG0000002867	grey	EC_M10	0.39	5.1E-02	-0.13	5.2E-01	-0.14	4.9E-01	-0.12	5.6E-01	0.00	9.9E-01	0.63	6.4E-04	-0.03	8.8E-01	-0.07	7.2E-01	-0.09	6.6E-01	-0.19	3.5E-01
ENSCAFG0000001664	ENSCAFG0000001664	turquoise	EC_M6	0.39	5.1E-02	0.39	4.6E-02	-0.31	1.3E-01	-0.15	4.6E-01	-0.13	5.2E-01	0.49	1.2E-02	-0.11	5.9E-01	-0.16	4.3E-01	-0.11	5.9E-01	-0.03	8.7E-01
ENSCAFG0000002921	RBAK	darkgrey	EC_M8	0.39	5.1E-02	0.22	2.8E-01	-0.18	3.9E-01	0.35	8.4E-02	-0.27	1.9E-01	0.20	3.2E-01	0.09	6.6E-01	-0.04	8.4E-01	-0.15	4.7E-01	0.09	6.6E-01
ENSCAFG0000001222	PRELID3B	darkgreen	EC_M4	0.39	5.1E-02	0.16	4.5E-01	-0.79	1.9E-06	-0.17	3.9E-01	0.55	3.7E-03	0.37	5.9E-02	0.01	9.5E-01	0.14	4.9E-01	0.34	9.3E-02	-0.74	1.4E-05
ENSCAFG0000000828	CETN3	darkgrey	EC_M8	0.39	5.1E-02	-0.10	6.1E-01	0.31	1.2E-01	0.25	2.2E-01	-0.72	3.0E-05	-0.05	8.0E-01	-0.36	6.9E-02	-0.01	9.6E-01	-0.07	7.3E-01	0.50	9.1E-03
ENSCAFG0000001168	ATP11B	grey	EC_M10	0.39	5.1E-02	0.40	4.4E-02	-0.56	3.0E-03	0.13	5.2E-01	0.14	5.0E-01	-0.15	4.7E-01	-0.12	5.7E-01	0.01	9.6E-01	0.04	8.4E-01	-0.32	1.1E-01
ENSCAFG0000000315	NT5C3A	grey	EC_M10	0.39	5.1E-02	0.15	4.6E-01	-0.17	4.1E-01	0.06	7.9E-01	-0.18	3.9E-01	-0.13	5.2E-01	-0.51	7.8E-03	-0.17	4.0E-01	-0.23	2.7E-01	-0.05	8.2E-01
ENSCAFG0000000289	ENSCAFG0000000289	grey	EC_M10	0.39	5.2E-02	0.05	8.0E-01	-0.33	9.6E-02	-0.15	4.6E-01	0.06	7.7E-01	-0.16	4.3E-01	-0.16	4.5E-01	0.52	6.7E-03	0.04	8.4E-01	-0.27	1.9E-01
ENSCAFG0000000869	CNOT6L	grey	EC_M10	0.39	5.2E-02	0.24	2.4E-01	-0.11	6.0E-01	0.12	5.5E-01	-0.34	9.3E-02	-0.02	9.3E-01	-0.19	3.5E-01	0.12	5.7E-01	-0.08	7.0E-01	0.08	7.0E-01
ENSCAFG0000000044	TUBB	grey	EC_M10	0.39	5.2E-02	0.00	9.8E-01	-0.59	1.5E-03	-0.23	2.5E-01	0.48	1.3E-02	0.22	2.9E-01	-0.18	3.7E-01	0.06	7.7E-01	-0.03	8.7E-01	-0.69	1.0E-04
ENSCAFG0000001163	IQCB1	grey	EC_M10	0.39	5.2E-02	0.18	3.7E-01	-0.11	5.8E-01	0.11	6.1E-01	-0.25	2.1E-01	0.12	5.6E-01	0.24	2.4E-01	-0.35	7.9E-02	0.11	5.9E-01	0.09	6.8E-01
ENSCAFG0000002938	TIGAR	darkgrey	EC_M8	0.39	5.2E-02	-0.32	1.1E-01	-0.46	1.8E-02	-0.05	8.2E-01	0.37	6.2E-02	0.16	4.4E-01	-0.64	4.7E-04	-0.07	7.4E-01	-0.32	1.1E-01	-0.56	2.7E-03
ENSCAFG0000002020	AP1G1	darkgreen	EC_M4	0.39	5.2E-02	0.23	2.7E-01	-0.81	4.3E-07	-0.23	2.6E-01	0.55	3.9E-03	0.16	4.3E-01	-0.19	3.6E-01	-0.04	8.6E-01	-0.34	8.7E-02	-0.76	6.9E-06
ENSCAFG0000001086	SCN3A	turquoise	EC_M6	0.39	5.2E-02	0.87	1.2E-08	-0.45	2.3E-02	-0.12	5.8E-01	-0.07	7.3E-01	-0.11	5.9E-01	-0.28	1.7E-01	-0.22	2.7E-01	-0.17	4.0E-01	-0.13	5.3E-01
ENSCAFG0000002379	E2F6	grey	EC_M10	0.39	5.2E-02	0.43	2.9E-02	-0.30	1.3E-01	0.07	7.4E-01	-0.14	4.9E-01	0.24	2.4E-01	0.41	3.6E-02	-0.18	3.7E-01	-0.17	4.2E-01	-0.06	7.9E-01
ENSCAFG0000001195	ENSCAFG0000001195	grey	EC_M10	0.39	5.2E-02	0.08	6.8E-01	0.04	8.6E-01	0.17	4.0E-01	-0.36	6.7E-02	-0.29	1.6E-01	0.04	8.6E-01	-0.25	2.3E-01	-0.33	1.0E-01	0.17	4.0E-01
ENSCAFG0000000077	ENSCAFG0000000077	grey	EC_M10	0.39	5.2E-02	-0.19	3.6E-01	-0.32	1.1E-01	-0.04	8.4E-01	0.14	5.1E-01	-0.13	5.1E-01	-0.07	7.4E-01	-0.08	7.1E-01	-0.16	4.4E-01	-0.34	8.6E-02
ENSCAFG0000001159	HCCS	grey	EC_M10	0.39	5.2E-02	0.12	5.5E-01	-0.37	6.4E-02	-0.07	7.3E-01	0.19	3.5E-01	-0.17	4.0E-01	-0.33	1.0E-01	0.18	3.8E-01	0.01	9.6E-01	-0.35	7.8E-02
ENSCAFG0000000478	CDC123	grey	EC_M10	0.39	5.2E-02	-0.60	1.2E-03	-0.22	2.9E-01	-0.05	8.2E-01	0.20	3.4E-01	0.25	2.2E-01	-0.38	5.3E-02	0.01	9.6E-01	-0.28	1.6E-01	-0.41	3.9E-02
ENSCAFG0000000173	ZNF800	darkgreen	EC_M4	0.38	5.2E-02	0.48	1.4E-02	-0.70	7.0E-05	-0.08	7.0E-01	0.29	1.5E-01	0.08	7.1E-01	-0.02	9.3E-01	0.12	5.7E-01	-0.27	1.8E-01	-0.50	9.1E-03
ENSCAFG0000002134	ENSCAFG0000002134	turquoise	EC_M6	0.38	5.2E-02	0.41	4.0E-02	-0.21	3.1E-01	-0.21	2.9E-01	-0.12	5.6E-01	0.33	1.0E-01	-0.20	3.3E-01	-0.19	3.6E-01	-0.28	1.6E-01	-0.10	6.2E-01
ENSCAFG0000000502	ENSCAFG0000000502	grey	EC_M10	0.38	5.2E-02	-0.31	1.2E-01	-0.14	5.1E-01	-0.01	9.4E-01	0.04	8.6E-01	0.13	5.3E-01	-0.25	2.2E-01	-0.10	6.4E-01	-0.30	1.3E-01	-0.21	3.0E-01
ENSCAFG0000003489	ENSCAFG0000003489	grey	EC_M10	0.38	5.2E-02	-0.14	4.9E-01	-0.25	2.2E-01	-0.12	5.4E-01	0.01	9.6E-01	0.41	3.8E-02	-0.10	6.4E-01	-0.10	6.2E-01	-0.05	8.2E-01	-0.22	2.8E-01
ENSCAFG0000002434	ENSCAFG0000002434	grey	EC_M10	0.38	5.2E-02	0.10	6.2E-01	-0.51	8.0E-03	0.21	3.0E-01	0.20	3.3E-01	0.37	6.4E-02	-0.16	4.3E-01	-0.17	4.1E-01	-0.21	3.0E-01	-0.39	4.9E-02
ENSCAFG0000000459	UBP1	grey	EC_M10	0.38	5.3E-02	0.19	3.4E-01	0.38	5.4E-02	-0.02	9.2E-01	-0.76	7.4E-06	-0.27	1.9E-01	-0.37	6.7E-02	0.03	8.9E-01	-0.17	4.1E-01	0.57	2.4E-03
ENSCAFG0000001210	SFXN4	grey	EC_M10	0.38	5.3E-02	-0.39	5.1E-02	0.22	2.8E-01	-0.07	7.3E-01	-0.35	8.1E-02	0.12	5.7E-01	-0.16	4.4E-01	-0.03	8.9E-01	-0.28	1.7E-01	0.11	6.0E-01
ENSCAFG0000000596	TEX30	grey	EC_M10	0.38	5.3E-02	0.15	4.7E-01	-0.35	7.7E-02	-0.04	8.6E-01	0.04	8.5E-01	0.39	4.9E-02	-0.01	9.5E-01	-0.45	2.2E-02	-0.24	2.4E-01	-0.28	1.6E-01
ENSCAFG0000000765	NEK1	grey	EC_M10	0.38	5.3E-02	0.56	2.8E-03	-0.64	4.3E-04	0.01	9.5E-01	0.14	4.9E-01	0.06	7.6E-01	0.09	6.6E-01	0.03	8.7E-01	0.09	6.8E-01	-0.38	5.7E-02
ENSCAFG0000000161	RPS6	cyan	EC_M2	0.38	5.3E-02	-0.27	1.9E-01	0.24	2.3E-01	0.25	2.1E-01	-0.52	6.1E-03	0.15	4.5E-01	0.14	5.1E-01	-0.17	4.2E-01	-0.63	5.9E-04	0.26	2.0E-01
ENSCAFG0000002911	ZNF354B	grey	EC_M10	0.38	5.3E-02	0.16	4.3E-01	-0.59	1.6E-03	0.07	7.4E-01	0.29	1.5E-01	0.35	7.6E-02	-0.13	5.1E-01	0.28	1.7E-01	-0.03	8.9E-01	-0.47	1.6E-02
ENSCAFG0000001159	TTC21B	grey	EC_M10	0.38	5.3E-02	0.01	9.6E-01	0.30	1.4E-01	0.24	2.4E-01	-0.67	1.6E-04	-0.25	2.2E-01	-0.21	2.9E-01	0.10	6.1E-01	0.23	2.7E-01	0.48	1.3E-02
ENSCAFG0000000065	TMEM181	grey	EC_M10	0.38	5.3E-02	0.25	2.3E-01	-0.69	9.8E-05	-0.05	8.1E-01	0.42	3.2E-02	0.15	4.6E-01	-0.43	2.7E-02	0.16	4.4E-01	-0.01	9.7E-01	-0.60	1.3E-03
ENSCAFG0000003037	ENSCAFG0000003037	grey	EC_M10	0.38	5.3E-02	-0.02	9.3E-01	-0.08	6.9E-01	0.36	7.4E-02	-0.19	3.5E-01	-0.21	3.1E-01	-0.17	4.1E-01	-0.23	2.5E-01	0.15	4.8E-01	0.01	9.4E-01
ENSCAFG0000000309	B3GNT2	grey	EC_M10	0.38	5.3E-02	-0.02	9.4E-01	-0.19	3.5E-01	0.12	5.7E-01	-0.13	5.2E-01	0.44	2.4E-02	-0.13	5.3E-01	-0.17	4.1E-01	0.16	4.4E-01	-0.07	7.4E-01
ENSCAFG0000002095	ENSCAFG0000002095	grey	EC_M10	0.38	5.3E-02	-0.29	1.4E-01	-0.08	7.0E-01	-0.09	6.6E-01	-0.15	4.6E-01	0.07	7.2E-01	-0.22	2.8E-01	0.00	1.0E+00	-0.27	1.9E-01	-0.11	5.8E-01
ENSCAFG0000001920	HMOX2	turquoise	EC_M6	0.38	5.3E-02	0.51	7.8E-03	-0.16	4.4E-01	-0.14	4.9E-01	-0.27	1.7E-01	-0.05	8.0E-01	-0.14	5.1E-01	0.03	8.7E-01	0.39	5.1E-02	0.11	5.9E-01
ENSCAFG0000000277	FKTN	grey	EC_M10	0.38	5.3E-02	0.20	3.2E-01	-0.13	5.4E-01	-0.03	8.7E-01	-0.26	2.0E-01	-0.18	3.7E-01	-0.22	2.7E-01	-0.13	5.4E-01	0.02	9.1E-01	0.08	6.9E-01
ENSCAFG0000000897	ENOPH1	darkgrey	EC_M8	0.38	5.3E-02	-0.19	3.5E-01	-0.61	1.0E-03	-0.08	7.1E-01	0.56	2.7E-03	0.19	3.4E-01	-0.06	7.5E-01	0.02	9.2E-01	-0.24	2.3E-01	-0.76	6.3E-06
ENSCAFG0000002246	ENSCAFG0000002246	darkgrey	EC_M8	0.38	5.3E-02	-0.03	8.7E-01	0.01	9.6E-01	0.17	4.2E-01	-0.25	2.2E-01	0.12	5.5E-01	-0.42	3.5E-02	-0.19	3.5E-01	-0.07	7.3E-01	0.07	7.3E-01
ENSCAFG0000002010	RFWD3	turquoise	EC_M6	0.38	5.3E-02	0.61	1.0E-03	-0.02	9.2E-01	0.15	4.6E-01	-0.48	1.4E-02	-0.22	2.8E-01	-0.09	6.6E-01	0.04	8.5E-01	-0.15	4.7E-01	0.31	1.2E-01
ENSCAFG0000003013	ENSCAFG0000003013	grey	EC_M10	0.38	5.3E-02	0.37	6.5E-02	-0.25	2.2E-01	0.20	3.4E-01	-0.21	3.1E-01	0.16	4.2E-01	0.06	7.8E-01	-0.23	2.6E-01	-0.06	7.8E-01	-0.02	9.2E-01
ENSCAFG0000001780	VRK1	grey	EC_M10	0.38	5.3E-02	0.43	2.7E-02	0.17	4.2E-01	-0.25	2.1E-01	-0.60	1.1E-03	-0.09	6.8E-01	0.04	8.5E-01	-0.28	1.6E-01	-0.12	5.7E-01	0.44	2.3E-02
ENSCAFG0000002848	BLOC1S5	darkgreen	EC_M4	0.38	5.3E-02	-0.06	7.8E-01	-0.80	7.5E-07	-0.03	9.0E-01	0.60	1.1E-03	0.26	2.1E-01	-0.18	3.9E-01	0.02	9.4E-01	-0.20	3.2E-01	-0.84	6.7E-08
ENSCAFG0000000686	MICU3	turquoise	EC_M6	0.38	5.4E-02	0.64	4.0E-04	-0.57	2.2E-03	-0.04	8.3E-01	0.12	5.7E-01	-0.15	4.5E-01	-0.30	1.3E-01	0.07	7.5E-01	0.13	5.2E-01	-0.30	1.3E-01
ENSCAFG0000000950	ENSCAFG0000000950	darkgrey	EC_M8	0.38	5.4E-02	0.27	1.8E-01	0.07	7.5E-01	0.54	4.1E-03	-0.49	1.0E-02	-0.13	5.2E-01	-0.14	5.0E-01	-0.22	2.8E-01	0.12	5.6E-01	0.33	9.6E-02
ENSCAFG0000000550	VDAC3	grey	EC_M10	0.38	5.4E-02	-0.25	2.1E-01	-0.37	6.0E-02	0.10	6.1E-01	0.25	2.2E-01	0.16	4.4E-01	-0.04	8.4E-01	-0.15	4.7E-01	-0.39	4.8E-02	-0.45	2.0E-02
ENSCAFG0000000471	ENSCAFG0000000471	grey	EC_M10	0.38	5.4E-02	0.07	7.4E-01	-0.10	6.2E-01	0.49	1.2E-02	-0.23	2.6E-01	-0.15	4.7E-01	-0.13	5.4E-01	-0.15	4.7E-01	-0.08	7.0E-01	0.07	7.3E-01
ENSCAFG0000000596	TRMT6	darkgreen	EC_M4	0.38	5.4E-02	-0.09	6.6E-01	-0.74	1.3E-05	-0.19	3.5E-01	0.56	3.0E-03	0.40	4.4E-02	-0.13	5.4E-01	0.07	7.3E-01	0.26	2.1E-01	-0.77	4.4E-06
ENSCAFG0000000515	UBE3C	grey	EC_M10	0.38	5.4E-02	0.23	2.5E-01	-0.46	1.8E-02	0.06	7.6E-01	0.11	6.0E-01	0.18	3.9E-01	-0.58	1.9E-03	-0.07	7.4E-01	-0.14	4.8E-01	-0.27	1.9E-01
ENSCAFG0000000197	VCP	darkgreen	EC_M4	0.38	5.4E-02	0.12	5.7E-01	-0.77	4.7E-06	-0.36	7.3E-02	0.61	8.5E-04	0.22	2.8E-01	-0.12	5.6E-01	-0.01	9.7E-01	-0.27	1.9E-01	-0.80	8.4E-07
ENSCAFG0000001382	SEC23A	darkgreen	EC_M4	0.38	5.4E-02	0.24	2.4E-01	-0.91	1.2E-10	-0.05	7.9E-01	0.63	6.3E-04	0.35	7.7E-02	-0.04	8.5E-01	-0.08	6.8E-01	-0.15	4.5E-01	-0.87	8.6E-09
ENSCAFG0000001186	PCK2	grey	EC_M10	0.38	5.5E-02	-0.29	1.5E-01	0.02	9.1E-01	0.29	1.5E-01	-0.22	2.7E-01	0.14	5.1E-01	-0.26	2.0E-01	-0.15	4.6E-01	-0.24	2.3E-01	-0.06	7.9E-01
ENSCAFG0000001523	TRPM7	darkgrey	EC_M8	0.38	5.5E-02	-0.10	6.2E-01	0.09	6.7E-01	0.31	1.2E-01	-0.45	2.1E-02	-0.06	7.6E-01	-0.28	1.7E-01	-0.10	6.4E-01	-0.08	7.1E-01	0.21	3.1E-01
ENSCAFG0000001341	MTX2	grey	EC_M10	0.38	5.5E-02	-0.17	4.1E-01	-0.30	1.3E-01	-0.23	2.6E-01	0.20	3.4E-01	0.03	9.0E-01	-0.15	4.7E-01	-0.21	3.1E-01	-0.26	2.0E-01	-0.31	1.3E-01
ENSCAFG0000001296	PCYT1A	grey	EC_M10	0.38	5.5E-02	0.24	2.3E-01	-0.62	8.1E-04	-0.25	2.2E-01	0.37	6.6E-02	-0.04	8.5E-01	-0.09	6.7E-01	0.06	7.7E-01	0.33	9.5E-02	-0.48	1.3E-02
ENSCAFG0000000769	LPXN	grey	EC_M10	0.38	5.5E-02	0.44	2.5E-02	-0.74	1.7E-05	-0.32	1.1E-01	0.44	2.4E-02	0.21	3.0E-01	-0.16	4.4E-01	-0.07	7.5E-01	-0.15	4.6E-01	-0.66	2.6E-04
ENSCAFG0000001479	RP2	darkgrey	EC_M8	0.38	5.5E-02	0.09	6.6E-01	0.17	4.1E-01	0.20	3.3E-01	-0.60	1.2E-03	0.18	3.8E-01	-0.27	1.9E-01	-0.10	6.1E-01	-0.25	2.1E-01	0.32	1.1E-01
ENSCAFG0000000489	DBI	grey	EC_M10	0.38	5.5E-02	-0.23	2.5E-01	0.32	1.1E-01	0.13	5.2E-01	-0.52	6.5E-03	-0.09	6.5E-01	-0.29	1.4E-01	-0.20	3.2E-01	-0.14	5.1E-01	0.36	7.3E-02
ENSCAFG0000000161	BYSL	grey	EC_M10	0.38	5.5E-02	-0.09	6.5E-01	-0.36	7.4E-02	-0.04	8.4E-01	0.24	2.4E-01	0.29	1.6E-01	-0.38	5.7E-02	0.01	9.5E-01	0.04	8.3E-01	-0.47	1.5E-02
ENSCAFG0000001151	GTF2I	darkgrey	EC_M8	0.38	5.5E-02	0.51	8.4E-03	0.21	3.1E-01	-0.10	6.4E-01	-0.76	5.8E-06	-0.15	4.5E-01	-0.12	5.6E-01	0.04	8.5E-01	0.03	8.8E-01	0.57	2.3E-03
ENSCAFG0000000800	EIF6	grey	EC_M10	0.38	5.5E-02	0.03	8.9E-01	-0.28	1.7E-01	-0.22	2.9E-01	0.14	5.1E-01	0.20	3.4E-01	-0.14	5.1E-01	-0.11	5.8E-01	0.12	5.7E-01	-0.32	1.2E-01
ENSCAFG0000001630	GPHN	grey	EC_M10	0.38	5.5E-02	0.11	6.1E-01	-0.52	6.0E-03	0.06	7.8E-01	0.29	1.6E-01	0.26	2.0E-01	0.17	4.1E-01	-0.30	1.3E-01	-0.03	9.0E-01	-0.48	1.3E-02
ENSCAFG0000001540	CRACR2A	turquoise	EC_M6	0.38	5.5E-02	0.88	3.0E-09	-0.38	5.8E-02	-0.08	6.9E-01	-0.19	3.6E-01	-0.14	5.1E-01	-0.15	4.8E-01	-0.11	5.8E-01	-0.17	4.1E-01	0.00	9.9E-01
ENSCAFG0000000314	ENSCAFG0000000314	grey	EC_M10	0.38	5.5E-02	0.11	6.1E-01	0.00	9.8E-01	-0.01	9.6E-01	-0.38	5.2E-02	-0.06	7.8E-01	0.22	2.9E-01	0.21	3.1E-01	-0.14	5.0E-01	0.13	5.4E-01
ENSCAFG0000001050	ENSCAFG0000001050	turquoise	EC_M6	0.38	5.6E-02	0.60	1.1E-03	-0.41	4.0E-02	-0.07	7.4E-01	-0.03	8.7E-01	-0.13	5.3E-01	-0.11	6.0E-01	-0.11	6.1E-01	-0.18	3.9E-01	-0.16	4.3E-01
ENSCAFG0000003269	ZNF597	grey	EC_M10	0.38	5.6E-02	-0.15	4.5E-01	-0.15	4.8E-01	-0.21	3.0E-01	-0.04	8.3E-01	0.08	7.0E-01	-0.26	2.0E-01	-0.08	7.1E-01	0.00	9.8E-01	-0.18	3.7E-01
ENSCAFG0000001624	LBR	turquoise	EC_M6	0.38	5.6E-02	0.74	1.6E-05	-0.12	5.4E-01	0.02	9.3E-01	-0.37	5.9E-02	-0.04	8.5E-01	0.17	4.1E-01	-0.07	7.4E-01	-0.17	4.1E-01	0.20	3.4E-01
ENSCAFG0000001593	DNAAF4	grey	EC_M10	0.38	5.6E-02	-0.55	3.8E-03	-0.32	1.1E-01	-0.13	5.4E-01	0.30	1.4E-01	0.09	6.5E-01	-0.22	2.9E-01	0.09	6.6E-01	-0.17	4.1E-01	-0.49	1.0E-02
ENSCAFG0000000523	ENSCAFG0000000523	darkgrey	EC_M8	0.38	5.6E-02	0.05	8.2E-01	-0.14	5.1E-01	-0.12	5.5E-01	-0.10	6.2E-01	0.46	1.9E-02	-0.10	6.3E-01	-0.14	5.1E-01	-0.14	4.9E-01	-0.09	6.5E-01
ENSCAFG0000000374	SLC7A11	darkgreen	EC_M4	0.38	5.6E-02	0.17	4.2E-01	-0.74	1.7E-05	0.04	8.6E-01	0.53	5.1E-03	0.32	1.1E-01	-0.07	7.3E-01	-0.31	1.2E-01	-0.12	5.6E-01	-0.74	1.3E-05
ENSCAFG0000000434	ENSCAFG0000000434	grey	EC_M10	0.38	5.6E-02	0.06	7.9E-01	-0.22	2.7E-01	-0.12	5.6E-01	-0.06	7.7E-01	-0.12	5.6E-01	-0.14	4.8E-01	-0.08	7.0E-01	-0.12	5.7E-01	-0.13	5.3E-01
ENSCAFG0000000589	HARS	grey	EC_M10	0.38	5.6E-02	-0.04	8.3E-01	-0.64	4.7E-04	-0.14	4.9E-01	0.55	3.6E-03	0.14	5.0E-01	-0.41	3.9E-02	0.07	7.5E-01	-0.11	5.9E-01	-0.71	5.0E-05
ENSCAFG0000000830	ENSCAFG0000000830	grey	EC_M10	0.38	5.6E-02	0.32	1.1E-01	-0.57	2.5E-03	0.05	8.2E-01	0.25	2.3E-01	0.08	6.9E-01	-0.21	3.0E-01	-0.03	8.7E-01	0.55	3.9E-03	-0.43	2.9E-02
ENSCAFG0000000866	ENSCAFG0000000866	grey	EC_M10	0.38	5.6E-02	0.27	1.8E-01	-0.39	5.2E-02	0.18	3.7E-01	-0.03	8.8E-01	0.31	1.2E-01	-0.23	2.7E-01	-0.31	1.2E-01	0.13	5.1E-01	-0.17	4.0E-01
ENSCAFG0000003114	ENSCAFG0000003114	darkgreen	EC_M4	0.38	5.6E-02	0.04	8.6E-01	-0.78	2.4E-06	-0.05	8.2E-01	0.58	2.0E-03	0.22	2.8E-01	-0.28	1.7E-01	-0.01	9.7E-01	-0.10	6.3E-01	-0.74	1.5E-05
ENSCAFG0000000561	KIF5C	grey	EC_M10	0.38	5.6E-02	0.06	7.8E-01	-0.61	8.7E-04	-0.38	5.9E-02	0.41	3.9E-02	0.16	4.2E-01	-0.03	8.8E-01	0.14	5.0E-01	0.35	7.6E-02	-0.59	1.6E-03
ENSCAFG0000000545	ENSCAFG0000000545	grey	EC_M10	0.38	5.6E-02	0.46	1.9E-02	0.06	7.5E-01	0.00	9.8E-01	-0.53	4.9E-03	-0.27	1.9E-01	-0.20	3.2E-01	0.10	6.3E-01	0.22	2.9E-01	0.35	8.4E-02
ENSCAFG0000000233	ENSCAFG0000000233	darkgrey	EC_M8	0.38	5.6E-02	-0.09	6.6E-01	-0.23	2.5E-01	0.38	5.6E-02	-0.07	7.5E-01	0.08	7.1E-01	-0.13	5.3E-01	-0.27	1.9E-01	-0.14	5.0E-01	-0.09	6.8E-01
ENSCAFG0000001750	TDP1	turquoise	EC_M6	0.38	5.6E-02	0.45	2.0E-02	0.05	8.0E-01	0.03	8.8E-01	-0.53	5.5E-03	0.09	6.5E-01	0.04	8.5E-01	-0.09	6.5E-01	-0.06	7.8E-01	0.31	1.2E-01
ENSCAFG0000000013	ENSCAFG0000000013	grey	EC_M10	0.38	5.6E-02	-0.38	5.7E-02	0.07	7.4E-01	-0.08	7.1E-01	-0.14	5.1E-01	-0.05	8.2E-01	-0.29	1.5E-01	-0.10	6.4E-01	-0.12	5.7E-01	-0.01	9.8E-01
ENSCAFG0000001752	GLCE	grey	EC_M10	0.38	5.6E-02	-0.37	6.2E-02	0.22	2.7E-01	0.21	3.1E-01	-0.42	3.1E-02	-0.07	7.3E-01	-0.22	2.7E-01	-0.03	9.0E-01	0.02	9.3E-01	0.23	2.7E-01
ENSCAFG0000003077	ENSCAFG0000003077	grey	EC_M10	0.38	5.6E-02	-0.05	8.2E-01	-0.27	1.8E-01	-0.20	3.4E-01	0.06	7.6E-01	0.29	1.5E-01	-0.41	4.0E-02	-0.03	8.9E-01	0.11	5.9E-01	-0.28	1.7E-01
ENSCAFG0000001432	THOC3	turquoise	EC_M6	0.38	5.6E-02	0.67	2.0E-04	-0.47	1.5E-02	-0.25	2.1E-01	0.02	9.4E-01	0.14	5.0E-01	0.24	2.5E-01	-0.27	1.7E-01	0.13	5.2E-01	-0.19	3.6E-01
ENSCAFG0000000337	ZNF350	darkgrey	EC_M8	0.38	5.7E-02	0.36	6.8E-02	0.08	7.0E-01	0.30	1.4E-01	-0.62	8.1E-04	-0.07	7.4E-01	-0.29	1.4E-01	-0.07	7.2E-01	-0.17	4.1E-01	0.42	3.3E-02
ENSCAFG0000001774	VWA3A	turquoise	EC_M6	0.38	5.7E-02	0.65	3.4E-04	-0.11	5.8E-01	0.24	2.3E-01	-0.40	4.6E-02	0.24	2.5E-01	-0.19	3.6E-01	-0.14	4.9E-01	-0.29	1.6E-01	0.18	3.8E-01
ENSCAFG0000000849	GINS3	grey	EC_M10	0.38	5.7E-02	0.37	6.4E-02	0.25	2.2E-01	-0.06	7.5E-01	-0.68	1.3E-04	-0.20	3.4E-01	-0.04	8.6E-01	0.03	8.7E-01	-0.46	1.9E-02	0.46	1.9E-02
ENSCAFG0000001751	ENSCAFG0000001751	grey	EC_M10	0.38	5.7E-02	0.25	2.2E-01	-0.52	5.9E-03	-0.22	2.8E-01	0.25	2.1E-01	0.21	3.0E-01	-0.13	5.4E-01	-0.15	4.6E-01	-0.05	8.0E-01	-0.37	6.3E-02
ENSCAFG0000001961	CHTF18	darkgrey	EC_M8	0.38	5.7E-02	-0.19	3.6E-01	0.03	8.7E-01	-0.23	2.6E-01	-0.16	4.4E-01	-0.09	6.5E-01	-0.14	5.0E-01	0.06	7.8E-01	-0.04	8.5E-01	-0.03	8.7E-01
ENSCAFG0000003133	MED21	grey	EC_M10	0.38	5.7E-02	0.00	9.8E-01	-0.21	3.1E-01	-0.08	7.0E-01	-0.08	7.0E-01	-0.10	6.3E-01	-0.58	2.0E-03	-0.24	2.3E-01	0.07	7.2E-01	-0.07	7.2E-01
ENSCAFG0000000305	PLEKHA8	darkgrey	EC_M8	0.38	5.7E-02	-0.11	5.9E-01	-0.18	3.8E-01	0.45	2.1E-02	-0.15	4.7E-01	-0.05	8.2E-01	-0.12	5.7E-01	-0.19	3.5E-01	-0.46	1.8E-02	-0.07	7.2E-01
ENSCAFG0000001948	TP73	darkgrey	EC_M8	0.38	5.7E-02	0.19	3.4E-01	-0.39	4.7E-02	-0.28	1.7E-01	0.14	5.0E-01	0.07	7.3E-01	0.12	5.5E-01	-0.27	1.9E-01	0.04	8.4E-01	-0.37	6.0E-02
ENSCAFG0000000039	ENSCAFG0000000039	grey	EC_M10	0.38	5.7E-02	0.22	2.9E-01	-0.60	1.1E-03	-0.14	5.0E-01	0.31	1.2E-01	-0.04	8.6E-01	0.30	1.4E-01	0.17	4.1E-01	-0.23	2.5E-01	-0.53	5.1E-03
ENSCAFG0000001803	VPS35L	grey	EC_M10	0.38	5.7E-02	0.07	7.4E-01	0.11	6.1E-01	-0.11	5.9E-01	-0.41	3.7E-02	-0.23	2.6E-01	-0.18	3.8E-01	-0.20	3.4E-01	-0.39	5.1E-02	0.20	3.4E-01
ENSCAFG0000000053	SNX2	turquoise	EC_M6	0.38	5.8E-02	0.62	7.0E-04	-0.39	4.9E-02	0.06	7.6E-01	-0.15	4.5E-01	-0.02	9.3E-01	-0.10	6.2E-01	-0.14	4.9E-01	0.01	9.4E-01	-0.01	9.5E-01
ENSCAFG0000003018	ENSCAFG0000003018	grey	EC_M10	0.38	5.8E-02	-0.24	2.4E-01	0.25	2.1E-01	-0.12	5.4E-01	-0.54	4.1E-03	-0.02	9.2E-01	-0.16	4.3E-01	0.04	8.6E-01	-0.35	8.2E-02	0.32	1.2E-01
ENSCAFG0000001747	ZKSCAN2	darkgrey	EC_M8	0.38	5.8E-02	0.51	7.7E-03	-0.11	5.8E-01	0.20	3.3E-01	-0.45	2.2E-02	-0.20	3.2E-01	-0.33	1.0E-01	0.14	5.0E-01	-0.08	7.0E-01	0.21	3.1E-01
ENSCAFG0000001569	KAT2A	grey	EC_M10	0.38	5.8E-02	-0.14	4.8E-01	0.35	8.1E-02	-0.09	6.4E-01	-0.58	2.0E-03	-0.10	6.1E-01	-0.21	2.9E-01	0.22	2.7E-01	-0.05	8.1E-01	0.38	5.5E-02
ENSCAFG0000000267	SP4	darkgrey	EC_M8	0.38	5.8E-02	0.68	1.2E-04	-0.03	8.8E-01	0.18	3.9E-01	-0.57	2.5E-03	-0.04	8.4E-01	-0.05	8.0E-01	-0.11	5.8E-01	-0.10	6.3E-01	0.39	5.0E-02
ENSCAFG0000000964	REEP4	grey	EC_M10	0.38	5.8E-02	-0.15	4.6E-01	-0.45	2.1E-02	-0.18	3.8E-01	0.39	4.7E-02	0.25	2.2E-01	-0.22	2.8E-01	0.07	7.4E-01	-0.05	8.0E-01	-0.58	1.9E-03
ENSCAFG0000001817	RPS15A	cyan	EC_M2	0.38	5.8E-02	-0.35	7.6E-02	0.19	3.5E-01	0.17	3.9E-01	-0.43	2.8E-02	0.15	4.7E-01	0.09	6.5E-01	0.09	6.6E-01	-0.55	3.4E-03	0.17	4.0E-01
ENSCAFG0000000962	ENSCAFG0000000962	grey	EC_M10	0.38	5.8E-02	0.27	1.8E-01	-0.15	4.6E-01	0.34	9.0E-02	-0.27	1.8E-01	-0.28	1.6E-01	-0.29	1.5E-01	-0.29	1.4E-01	-0.34	9.1E-02	0.04	8.5E-01
ENSCAFG0000000723	TMEM41B	darkgreen	EC_M4	0.38	5.8E-02	0.39	4.9E-02	-0.74	1.8E-05	0.10	6.3E-01	0.33	9.9E-02	0.21	3.0E-01	0.09	6.6E-01	-0.25	2.1E-01	-0.30	1.4E-01	-0.55	3.9E-03
ENSCAFG0000001812	ENSCAFG0000001812	grey	EC_M10	0.38	5.9E-02	0.03	9.0E-01	-0.15	4.7E-01	0.49	1.1E-02	-0.17	4.1E-01	-0.12	5.5E-01	-0.20	3.4E-01	-0.16	4.5E-01	-0.21	3.1E-01	-0.05	8.0E-01
ENSCAFG0000001735	XPO6	darkgrey	EC_M8	0.38	5.9E-02	0.20	3.4E-01	-0.25	2.2E-01	-0.12	5.6E-01	0.03	9.0E-01	0.06	7.6E-01	-0.47	1.6E-02	-0.04	8.6E-01	0.05	8.2E-01	-0.22	2.9E-01
ENSCAFG0000000000	ADNP2	grey	EC_M10	0.38	5.9E-02	0.25	2.2E-01	0.26	1.9E-01	0.17	4.1E-01	-0.70	7.1E-05	0.04	8.5E-01	-0.21	3.0E-01	0.00	9.8E-01	0.18	3.8E-01	0.50	9.9E-03
ENSCAFG0000000792	ATF1	cyan	EC_M2	0.38	5.9E-02	-0.37	6.1E-02	0.42	3.3E-02	0.13	5.2E-01	-0.69	1.1E-04	0.13	5.4E-01	-0.27	1.8E-01	-0.04	8.4E-01	-0.05	7.9E-01	0.46	1.9E-02
ENSCAFG0000001123	ZNF184	darkgrey	EC_M8	0.38	5.9E-02	0.46	1.7E-02	0.08	7.0E-01	0.00	9.8E-01	-0.64	4.0E-04	-0.10	6.1E-01	-0.07	7.4E-01	-0.01	9.7E-01	0.06	7.6E-01	0.44	2.4E-02
ENSCAFG0000000898	ENSCAFG0000000898	turquoise	EC_M6	0.38	5.9E-02	0.84	5.8E-08	-0.32	1.1E-01	-0.04	8.6E-01	-0.21	3.0E-01	0.01	9.8E-01	-0.10	6.1E-01	-0.09	6.6E-01	-0.10	6.4E-01	-0.01	9.5E-01
ENSCAFG0000000033	SHPRH	darkgrey	EC_M8	0.38	5.9E-02	0.16	4.5E-01	-0.25	2.2E-01	0.40	4.2E-02	-0.17	4.1E-01	-0.07	7.4E-01	-0.08	7.1E-01	0.05	8.2E-01	-0.29	1.5E-01	-0.04	8.5E-01

ENSCAFG0000003017	ENSCAFG0000003017	grey	EC_M10	0.37	5.9E-02	0.10	6.2E-01	-0.44	2.6E-02	0.02	9.0E-01	0.14	4.8E-01	-0.15	4.7E-01	-0.17	4.2E-01	-0.11	6.0E-01	-0.18	3.7E-01	-0.38	5.8E-02
ENSCAFG0000000196	SH3RF3	turquoise	EC_M6	0.37	5.9E-02	0.54	4.6E-03	-0.09	6.5E-01	0.04	8.5E-01	-0.37	6.5E-02	-0.25	2.2E-01	-0.21	3.0E-01	0.15	4.5E-01	0.50	8.8E-03	0.19	3.5E-01
ENSCAFG0000002001	NUP188	grey	EC_M10	0.37	5.9E-02	-0.30	1.3E-01	-0.26	2.0E-01	-0.06	7.7E-01	0.20	3.2E-01	0.20	3.3E-01	-0.36	7.1E-02	-0.05	8.1E-01	0.08	6.9E-01	-0.39	4.6E-02
ENSCAFG0000000490	KDM4A	grey	EC_M10	0.37	5.9E-02	0.10	6.3E-01	-0.64	4.6E-04	0.04	8.6E-01	0.41	3.8E-02	0.57	2.3E-03	-0.19	3.6E-01	-0.23	2.6E-01	-0.27	1.8E-01	-0.66	2.6E-04
ENSCAFG0000000513	ENSCAFG0000000513	grey	EC_M10	0.37	5.9E-02	0.16	4.4E-01	-0.46	1.8E-02	-0.26	2.0E-01	0.29	1.5E-01	0.02	9.4E-01	-0.37	6.3E-02	0.22	2.7E-01	0.00	9.8E-01	-0.45	2.1E-02
ENSCAFG0000001103	GOLGA5	darkgreen	EC_M4	0.37	6.0E-02	0.31	1.2E-01	-0.75	1.3E-05	0.06	7.8E-01	0.38	5.6E-02	0.31	1.3E-01	-0.08	6.9E-01	-0.11	5.8E-01	-0.40	4.3E-02	-0.62	7.5E-04
ENSCAFG0000000919	ST3GAL6	turquoise	EC_M6	0.37	6.0E-02	0.42	3.1E-02	-0.27	1.8E-01	0.14	4.9E-01	-0.15	4.7E-01	-0.06	7.9E-01	0.01	9.7E-01	-0.23	2.6E-01	-0.73	2.6E-05	-0.06	7.6E-01
ENSCAFG0000001287	BCCIP	grey	EC_M10	0.37	6.0E-02	-0.16	4.4E-01	-0.02	9.1E-01	0.28	1.6E-01	-0.13	5.1E-01	0.14	4.9E-01	-0.11	6.0E-01	-0.18	3.8E-01	-0.07	7.2E-01	-0.06	7.8E-01
ENSCAFG0000001977	LRIF1	darkgrey	EC_M8	0.37	6.0E-02	-0.01	9.6E-01	0.09	6.7E-01	0.29	1.5E-01	-0.51	8.0E-03	0.33	9.8E-02	-0.04	8.3E-01	-0.05	8.1E-01	-0.39	5.1E-02	0.27	1.9E-01
ENSCAFG0000003039	MRPS15	grey	EC_M10	0.37	6.0E-02	-0.18	3.9E-01	0.16	4.3E-01	-0.30	1.4E-01	-0.37	6.3E-02	0.16	4.3E-01	-0.15	4.7E-01	0.06	7.6E-01	0.03	8.7E-01	0.18	3.8E-01
ENSCAFG0000001471	CWF19L2	darkgrey	EC_M8	0.37	6.0E-02	0.15	4.7E-01	0.28	1.6E-01	0.37	6.0E-02	-0.74	1.3E-05	-0.30	1.3E-01	-0.27	1.9E-01	-0.04	8.3E-01	-0.04	8.3E-01	0.57	2.4E-03
ENSCAFG0000001004	HMGN1	grey	EC_M10	0.37	6.0E-02	-0.17	4.2E-01	-0.24	2.5E-01	-0.04	8.4E-01	0.06	7.9E-01	0.12	5.7E-01	-0.06	7.5E-01	-0.09	6.6E-01	0.04	8.3E-01	-0.32	1.2E-01
ENSCAFG0000000846	FMNL3	grey	EC_M10	0.37	6.0E-02	0.58	1.8E-03	-0.72	4.0E-05	-0.15	4.7E-01	0.32	1.1E-01	0.17	4.1E-01	0.09	6.7E-01	-0.12	5.7E-01	0.15	4.6E-01	-0.54	4.6E-03
ENSCAFG0000002890	CDKN2C	grey	EC_M10	0.37	6.0E-02	0.06	7.5E-01	0.36	7.0E-02	0.13	5.1E-01	-0.67	2.1E-04	-0.18	3.9E-01	-0.06	7.6E-01	-0.23	2.6E-01	-0.01	9.8E-01	0.52	6.2E-03
ENSCAFG0000002929	GGTA1P	grey	EC_M10	0.37	6.0E-02	0.22	2.7E-01	-0.08	6.8E-01	-0.13	5.3E-01	-0.32	1.2E-01	-0.12	5.6E-01	0.46	1.8E-02	0.21	2.9E-01	-0.03	9.0E-01	0.14	5.1E-01
ENSCAFG0000000087	ENSCAFG0000000087	paleturquoise	EC_M11	0.37	6.0E-02	0.07	7.3E-01	-0.20	3.3E-01	0.47	1.4E-02	-0.10	6.4E-01	-0.14	4.9E-01	-0.15	4.8E-01	-0.13	5.3E-01	-0.22	2.9E-01	-0.10	6.2E-01
ENSCAFG0000000754	ENSCAFG0000000754	turquoise	EC_M6	0.37	6.0E-02	0.56	3.2E-03	-0.44	2.3E-02	-0.13	5.2E-01	0.07	7.4E-01	-0.15	4.7E-01	-0.15	4.6E-01	-0.10	6.2E-01	-0.07	7.3E-01	-0.24	2.3E-01
ENSCAFG0000000006	VPS4B	grey	EC_M10	0.37	6.0E-02	0.24	2.3E-01	-0.60	1.2E-03	-0.02	9.2E-01	0.29	1.6E-01	-0.14	4.9E-01	-0.34	9.0E-02	-0.09	6.7E-01	-0.15	4.6E-01	-0.44	2.5E-02
ENSCAFG0000000111	ARHGAP18	turquoise	EC_M6	0.37	6.0E-02	0.69	9.7E-05	-0.19	3.6E-01	-0.01	9.7E-01	-0.38	5.4E-02	-0.26	2.0E-01	-0.15	4.7E-01	0.29	1.5E-01	0.20	3.3E-01	0.18	3.8E-01
ENSCAFG0000000924	SLC38A4	grey	EC_M10	0.37	6.0E-02	-0.05	8.1E-01	-0.11	5.9E-01	0.20	3.3E-01	-0.15	4.6E-01	-0.19	3.6E-01	-0.22	2.9E-01	0.14	4.9E-01	-0.17	3.9E-01	-0.05	8.2E-01
ENSCAFG0000000315	VPS54	turquoise	EC_M6	0.37	6.0E-02	0.66	2.2E-04	-0.60	1.1E-03	0.00	9.8E-01	0.14	5.1E-01	-0.08	6.9E-01	0.03	8.7E-01	-0.01	9.7E-01	-0.01	9.7E-01	-0.27	1.8E-01
ENSCAFG0000000135	AGTPBP1	turquoise	EC_M6	0.37	6.0E-02	0.64	4.3E-04	-0.41	3.7E-02	-0.06	7.7E-01	-0.11	6.1E-01	-0.02	9.3E-01	-0.25	2.2E-01	-0.14	5.1E-01	-0.14	4.8E-01	-0.12	5.6E-01
ENSCAFG0000000777	CGGBP1	darkgrey	EC_M8	0.37	6.1E-02	0.07	7.2E-01	-0.02	9.1E-01	0.21	3.0E-01	-0.41	3.8E-02	-0.01	9.6E-01	-0.38	5.4E-02	-0.22	2.9E-01	-0.34	9.1E-02	0.18	3.9E-01
ENSCAFG0000002273	MT-ND4	cyan	EC_M2	0.37	6.1E-02	-0.27	1.9E-01	0.34	8.7E-02	0.18	3.9E-01	-0.64	4.5E-04	0.04	8.4E-01	-0.54	4.4E-03	-0.03	8.7E-01	-0.24	2.4E-01	0.40	4.2E-02
ENSCAFG0000000383	LARP1B	darkgreen	EC_M4	0.37	6.1E-02	0.15	4.8E-01	-0.72	3.6E-05	0.07	7.5E-01	0.44	2.3E-02	0.08	7.1E-01	-0.12	5.6E-01	-0.15	4.8E-01	-0.31	1.2E-01	-0.64	4.9E-04
ENSCAFG0000000379	WDR35	darkgrey	EC_M8	0.37	6.1E-02	0.13	5.2E-01	0.40	4.0E-02	0.09	6.5E-01	-0.83	1.2E-07	-0.22	2.7E-01	-0.18	3.7E-01	-0.18	3.8E-01	-0.09	6.8E-01	0.66	2.5E-04
ENSCAFG0000000191	PIP5K1B	turquoise	EC_M6	0.37	6.1E-02	0.87	1.0E-08	-0.48	1.3E-02	-0.24	2.5E-01	-0.06	7.8E-01	0.01	9.6E-01	-0.05	7.9E-01	-0.23	2.6E-01	-0.05	7.9E-01	-0.13	5.3E-01
ENSCAFG0000001828	ROCK1	darkgreen	EC_M4	0.37	6.1E-02	0.17	4.0E-01	-0.91	9.4E-11	-0.18	3.7E-01	0.67	1.8E-04	0.30	1.4E-01	-0.20	3.3E-01	-0.04	8.6E-01	-0.11	6.0E-01	-0.87	7.4E-09
ENSCAFG0000000687	ENSCAFG0000000687	grey	EC_M10	0.37	6.2E-02	0.12	5.7E-01	-0.46	1.7E-02	-0.14	4.9E-01	0.22	2.9E-01	-0.16	4.5E-01	-0.13	5.1E-01	-0.02	9.1E-01	-0.05	8.0E-01	-0.40	4.6E-02
ENSCAFG0000000038	LATS1	darkgrey	EC_M8	0.37	6.2E-02	-0.21	2.9E-01	0.28	1.7E-01	0.20	3.3E-01	-0.63	6.1E-04	0.17	4.1E-01	-0.10	6.3E-01	-0.13	5.1E-01	-0.29	1.5E-01	0.39	4.8E-02
ENSCAFG0000001989	TTF1	grey	EC_M10	0.37	6.2E-02	0.14	4.8E-01	-0.54	4.3E-03	0.14	5.0E-01	0.19	3.5E-01	-0.11	5.9E-01	-0.27	1.8E-01	0.00	1.0E+00	-0.10	6.3E-01	-0.38	5.5E-02
ENSCAFG0000000160	ENSCAFG0000000160	darkgrey	EC_M8	0.37	6.2E-02	0.26	1.9E-01	-0.40	4.1E-02	0.31	1.3E-01	0.04	8.5E-01	0.21	3.0E-01	-0.08	7.0E-01	-0.47	1.5E-02	-0.04	8.4E-01	-0.20	3.4E-01
ENSCAFG0000002002	SLC35A3	darkgreen	EC_M4	0.37	6.2E-02	0.58	2.0E-03	-0.83	1.1E-07	-0.11	5.9E-01	0.45	2.2E-02	0.07	7.5E-01	0.01	9.6E-01	0.04	8.4E-01	-0.16	4.4E-01	-0.62	7.1E-04
ENSCAFG0000000034	CANX	darkgreen	EC_M4	0.37	6.2E-02	-0.21	3.1E-01	-0.71	4.9E-05	0.00	9.9E-01	0.62	7.6E-04	0.24	2.4E-01	-0.24	2.3E-01	0.16	4.2E-01	-0.28	1.6E-01	-0.81	5.4E-07
ENSCAFG0000001225	GOLT1B	darkgreen	EC_M4	0.37	6.2E-02	-0.03	8.9E-01	-0.87	9.2E-09	-0.03	9.0E-01	0.68	1.4E-04	0.38	5.7E-02	-0.01	9.6E-01	-0.08	6.9E-01	-0.13	5.1E-01	-0.90	6.0E-10
ENSCAFG0000002041	ZRANB2	darkgrey	EC_M8	0.37	6.2E-02	0.57	2.6E-03	-0.34	8.9E-02	0.16	4.2E-01	-0.21	3.0E-01	0.19	3.6E-01	-0.02	9.3E-01	-0.04	8.3E-01	-0.16	4.2E-01	-0.01	9.7E-01
ENSCAFG0000002941	ENSCAFG0000002941	grey	EC_M10	0.37	6.2E-02	0.11	6.0E-01	-0.58	1.7E-03	0.13	5.2E-01	0.30	1.4E-01	0.09	6.7E-01	0.15	4.8E-01	0.00	9.9E-01	-0.12	5.7E-01	-0.48	1.3E-02
ENSCAFG0000001766	DCTN5	darkgrey	EC_M8	0.37	6.2E-02	-0.07	7.2E-01	-0.31	1.2E-01	0.08	6.9E-01	0.15	4.6E-01	0.05	8.0E-01	-0.26	1.9E-01	0.18	3.8E-01	0.29	1.4E-01	-0.34	9.3E-02
ENSCAFG0000000605	NSD3	darkgrey	EC_M8	0.37	6.2E-02	0.27	1.8E-01	-0.19	3.6E-01	0.18	3.8E-01	-0.30	1.4E-01	0.15	4.8E-01	-0.27	1.8E-01	-0.14	4.8E-01	-0.42	3.4E-02	0.05	8.1E-01
ENSCAFG0000001293	ENSCAFG0000001293	grey	EC_M10	0.37	6.3E-02	0.11	5.8E-01	-0.34	9.1E-02	-0.18	3.9E-01	0.08	7.0E-01	0.30	1.4E-01	0.28	1.6E-01	0.07	7.2E-01	0.08	6.8E-01	-0.24	2.4E-01
ENSCAFG0000000372	NOCT	turquoise	EC_M6	0.37	6.3E-02	0.63	6.2E-04	-0.56	2.7E-03	-0.05	8.0E-01	0.23	2.7E-01	0.12	5.6E-01	-0.05	8.0E-01	-0.11	5.9E-01	-0.01	9.5E-01	-0.40	4.6E-02
ENSCAFG0000000263	RDH13	grey	EC_M10	0.37	6.3E-02	-0.10	6.4E-01	0.23	2.5E-01	-0.18	3.8E-01	-0.45	2.2E-02	-0.20	3.2E-01	-0.15	4.7E-01	-0.25	2.2E-01	-0.06	7.6E-01	0.29	1.5E-01
ENSCAFG0000001240	RPS6KA1	turquoise	EC_M6	0.37	6.3E-02	0.67	1.7E-04	-0.49	1.2E-02	-0.10	6.2E-01	0.01	9.7E-01	0.04	8.4E-01	-0.18	3.8E-01	-0.15	4.7E-01	0.29	1.5E-01	-0.21	3.0E-01
ENSCAFG0000001185	TBCEL	grey	EC_M10	0.37	6.3E-02	0.40	4.5E-02	-0.11	5.9E-01	0.18	3.8E-01	-0.41	3.7E-02	0.02	9.3E-01	-0.24	2.4E-01	-0.15	4.7E-01	0.09	6.5E-01	0.18	3.7E-01
ENSCAFG0000001876	PIEZO2	turquoise	EC_M6	0.37	6.3E-02	0.67	1.9E-04	-0.73	2.8E-05	-0.15	4.8E-01	0.33	9.6E-02	-0.03	8.9E-01	-0.21	3.0E-01	-0.18	3.7E-01	-0.15	4.7E-01	-0.53	4.9E-03
ENSCAFG0000003029	ENSCAFG0000003029	grey	EC_M10	0.37	6.3E-02	-0.07	7.2E-01	-0.14	5.0E-01	0.27	1.8E-01	-0.10	6.3E-01	-0.22	2.9E-01	-0.17	4.1E-01	-0.21	3.0E-01	-0.12	5.4E-01	-0.05	8.3E-01
ENSCAFG0000000316	BBS9	grey	EC_M10	0.37	6.3E-02	0.04	8.4E-01	0.03	8.8E-01	0.28	1.7E-01	-0.40	4.2E-02	-0.17	4.1E-01	-0.67	1.8E-04	-0.12	5.7E-01	-0.35	7.6E-02	0.17	4.2E-01
ENSCAFG0000001626	ENSCAFG0000001626	grey	EC_M10	0.37	6.3E-02	0.24	2.4E-01	-0.62	7.6E-04	-0.13	5.2E-01	0.25	2.3E-01	0.20	3.3E-01	-0.21	3.0E-01	-0.22	2.7E-01	-0.01	9.8E-01	-0.49	1.0E-02
ENSCAFG0000000783	TFCP2	darkgrey	EC_M8	0.37	6.3E-02	0.62	7.6E-04	0.15	4.6E-01	0.23	2.7E-01	-0.73	2.3E-05	-0.26	1.9E-01	0.02	9.1E-01	-0.18	3.8E-01	-0.17	4.1E-01	0.54	4.8E-03
ENSCAFG0000002911	ZNF510	grey	EC_M10	0.37	6.3E-02	0.31	1.2E-01	-0.17	4.0E-01	0.11	5.8E-01	-0.30	1.3E-01	-0.03	8.9E-01	-0.17	4.1E-01	-0.12	5.8E-01	0.09	6.6E-01	0.06	7.6E-01
ENSCAFG0000002916	ENSCAFG0000002916	grey	EC_M10	0.37	6.3E-02	-0.22	2.8E-01	0.34	9.1E-02	0.01	9.6E-01	-0.54	4.8E-03	-0.16	4.4E-01	-0.18	3.9E-01	0.07	7.3E-01	-0.30	1.3E-01	0.39	5.1E-02
ENSCAFG0000002493	TMEM273	turquoise	EC_M6	0.37	6.3E-02	0.95	3.5E-13	-0.37	6.0E-02	-0.09	6.8E-01	-0.19	3.5E-01	-0.12	5.7E-01	-0.11	6.0E-01	-0.09	6.6E-01	-0.14	4.8E-01	0.02	9.4E-01
ENSCAFG0000003175	ENSCAFG0000003175	grey	EC_M10	0.37	6.3E-02	0.00	9.8E-01	0.03	9.0E-01	-0.13	5.3E-01	-0.27	1.7E-01	-0.16	4.3E-01	-0.14	5.1E-01	-0.10	6.3E-01	-0.11	5.9E-01	0.15	4.8E-01
ENSCAFG0000000477	EPM2AIP1	grey	EC_M10	0.37	6.3E-02	0.09	6.7E-01	-0.14	5.0E-01	0.28	1.6E-01	-0.26	2.0E-01	-0.16	4.4E-01	0.03	8.8E-01	0.30	1.3E-01	-0.13	5.3E-01	0.02	9.2E-01
ENSCAFG0000000593	CRLS1	grey	EC_M10	0.37	6.3E-02	-0.02	9.3E-01	0.13	5.3E-01	-0.05	8.0E-01	-0.42	3.0E-02	0.06	7.7E-01	0.00	1.0E+00	0.04	8.5E-01	0.34	9.0E-02	0.25	2.1E-01
ENSCAFG0000001942	AP3D1	turquoise	EC_M6	0.37	6.3E-02	0.53	5.8E-03	-0.49	1.1E-02	-0.01	9.8E-01	0.10	6.1E-01	0.04	8.5E-01	-0.10	6.4E-01	-0.02	9.3E-01	-0.34	9.3E-02	-0.34	9.1E-02
ENSCAFG0000000735	ENSCAFG0000000735	grey	EC_M10	0.37	6.4E-02	0.19	3.6E-01	0.31	1.2E-01	0.10	6.4E-01	-0.68	1.3E-04	-0.01	9.8E-01	-0.06	7.6E-01	0.08	7.1E-01	0.07	7.2E-01	0.53	4.9E-03
ENSCAFG0000000443	CHCHD4	grey	EC_M10	0.37	6.4E-02	0.23	2.5E-01	-0.36	6.8E-02	-0.13	5.4E-01	-0.01	9.7E-01	0.18	3.9E-01	0.31	1.3E-01	0.22	2.7E-01	0.17	4.1E-01	-0.16	4.3E-01
ENSCAFG0000000575	NR1D2	turquoise	EC_M6	0.37	6.4E-02	0.59	1.7E-03	-0.23	2.7E-01	0.00	9.9E-01	-0.31	1.2E-01	-0.19	3.6E-01	-0.08	7.0E-01	-0.07	7.4E-01	0.13	5.4E-01	0.16	4.5E-01
ENSCAFG0000001545	ENSCAFG0000001545	grey	EC_M10	0.37	6.4E-02	0.34	9.3E-02	-0.30	1.3E-01	-0.13	5.3E-01	-0.01	9.8E-01	-0.05	8.3E-01	0.04	8.5E-01	-0.25	2.2E-01	0.05	8.1E-01	-0.12	5.5E-01
ENSCAFG0000001908	RBFOX1	darkgrey	EC_M8	0.37	6.4E-02	-0.02	9.4E-01	-0.12	5.5E-01	-0.10	6.4E-01	-0.08	6.8E-01	0.37	5.9E-02	-0.14	5.1E-01	0.00	1.0E+00	-0.18	3.8E-01	-0.12	5.5E-01
ENSCAFG0000000961	STK4	darkgrey	EC_M8	0.37	6.4E-02	0.35	7.5E-02	0.14	5.1E-01	-0.10	6.3E-01	-0.65	3.0E-04	-0.34	8.7E-02	-0.11	6.0E-01	-0.03	8.8E-01	0.24	2.4E-01	0.44	2.4E-02
ENSCAFG0000000187	GNPDA2	grey	EC_M10	0.37	6.4E-02	-0.05	8.1E-01	-0.06	7.7E-01	0.21	3.0E-01	-0.22	2.9E-01	0.14	4.9E-01	-0.41	3.9E-02	0.08	6.9E-01	-0.19	3.5E-01	0.04	8.3E-01
ENSCAFG0000001777	ARMCX5	grey	EC_M10	0.37	6.4E-02	-0.14	5.0E-01	0.04	8.5E-01	0.06	7.7E-01	-0.28	1.6E-01	-0.13	5.3E-01	-0.43	2.8E-02	-0.04	8.6E-01	-0.31	1.3E-01	0.08	7.0E-01
ENSCAFG0000000674	PRAG1	turquoise	EC_M6	0.37	6.4E-02	0.77	4.6E-06	-0.37	6.1E-02	-0.06	7.6E-01	-0.13	5.2E-01	-0.04	8.4E-01	-0.31	1.2E-01	-0.11	5.9E-01	0.23	2.6E-01	-0.09	6.7E-01
ENSCAFG0000000974	ADAMTS20	turquoise	EC_M6	0.37	6.4E-02	0.79	1.9E-06	-0.39	5.2E-02	-0.17	4.2E-01	-0.08	6.9E-01	0.05	8.2E-01	-0.09	6.7E-01	-0.16	4.2E-01	0.17	4.1E-01	-0.06	7.6E-01
ENSCAFG0000001525	NME7	grey	EC_M10	0.37	6.4E-02	-0.27	1.8E-01	0.29	1.4E-01	0.27	1.8E-01	-0.54	4.8E-03	0.15	4.5E-01	-0.33	9.8E-02	-0.18	3.7E-01	-0.40	4.1E-02	0.33	9.7E-02
ENSCAFG0000002637	ENSCAFG0000002637	grey	EC_M10	0.37	6.4E-02	0.32	1.2E-01	-0.14	5.0E-01	-0.14	5.1E-01	-0.23	2.7E-01	-0.23	2.6E-01	-0.26	1.9E-01	0.04	8.4E-01	-0.23	2.5E-01	0.02	9.1E-01
ENSCAFG0000000625	UNC5D	grey	EC_M10	0.37	6.4E-02	0.05	7.9E-01	-0.17	4.2E-01	-0.11	5.9E-01	-0.06	7.7E-01	-0.10	6.2E-01	-0.09	6.7E-01	-0.01	9.4E-01	0.06	7.7E-01	-0.13	5.3E-01
ENSCAFG0000003092	ATP5MD	grey	EC_M10	0.37	6.4E-02	-0.07	7.4E-01	-0.14	5.1E-01	0.44	2.5E-02	-0.08	6.9E-01	-0.18	3.8E-01	-0.18	3.7E-01	-0.15	4.5E-01	-0.28	1.6E-01	-0.11	5.9E-01
ENSCAFG0000000253	PPM1B	grey	EC_M10	0.37	6.4E-02	-0.06	7.6E-01	-0.18	3.7E-01	-0.02	9.2E-01	-0.10	6.4E-01	-0.08	7.0E-01	-0.15	4.7E-01	0.23	2.7E-01	-0.17	4.0E-01	-0.12	5.7E-01
ENSCAFG0000000765	SMAD1	turquoise	EC_M6	0.37	6.4E-02	0.89	7.4E-10	-0.19	3.4E-01	-0.02	9.4E-01	-0.40	4.5E-02	-0.09	6.5E-01	-0.33	9.6E-02	-0.12	5.6E-01	-0.07	7.4E-01	0.19	3.4E-01
ENSCAFG0000002009	SLC25A25	turquoise	EC_M6	0.37	6.5E-02	0.45	2.0E-02	-0.10	6.1E-01	-0.11	5.9E-01	-0.37	6.3E-02	-0.20	3.3E-01	0.29	1.4E-01	-0.06	7.6E-01	-0.04	8.6E-01	0.20	3.2E-01
ENSCAFG0000002021	RPL35	grey	EC_M10	0.37	6.5E-02	-0.56	3.0E-03	-0.14	4.9E-01	0.11	5.9E-01	0.08	6.9E-01	0.29	1.5E-01	-0.05	8.2E-01	-0.04	8.5E-01	-0.47	1.6E-02	-0.35	8.4E-02
ENSCAFG0000001330	ENSCAFG0000001330	grey	EC_M10	0.37	6.5E-02	-0.21	3.1E-01	-0.26	1.9E-01	0.49	1.2E-02	0.00	9.8E-01	-0.12	5.7E-01	-0.14	5.1E-01	-0.15	4.6E-01	-0.19	3.6E-01	-0.24	2.5E-01
ENSCAFG0000001612	SDE2	grey	EC_M10	0.37	6.5E-02	0.34	8.9E-02	-0.43	3.0E-02	0.05	8.0E-01	0.04	8.3E-01	-0.07	7.3E-01	0.44	2.5E-02	0.18	3.8E-01	-0.11	6.0E-01	-0.21	3.1E-01
ENSCAFG0000000957	ERLIN1	cyan	EC_M2	0.37	6.5E-02	0.00	9.8E-01	0.50	9.7E-03	0.28	1.7E-01	-0.86	1.9E-08	-0.24	2.5E-01	-0.17	4.2E-01	-0.08	7.0E-01	-0.07	7.4E-01	0.67	2.1E-04
ENSCAFG0000000156	CNTLN	grey	EC_M10	0.37	6.5E-02	-0.09	6.8E-01	0.18	3.8E-01	0.01	9.5E-01	-0.44	2.4E-02	-0.14	5.1E-01	-0.05	8.0E-01	-0.03	8.8E-01	-0.02	9.2E-01	0.32	1.2E-01
ENSCAFG0000000228	GCLC	grey	EC_M10	0.37	6.5E-02	0.16	4.4E-01	0.08	7.0E-01	0.11	5.9E-01	-0.41	3.8E-02	-0.13	5.1E-01	0.01	9.6E-01	0.10	6.3E-01	0.06	7.7E-01	0.27	1.8E-01
ENSCAFG0000000798	PTCD2	grey	EC_M10	0.37	6.5E-02	0.26	2.0E-01	-0.34	8.5E-02	-0.18	3.9E-01	0.07	7.2E-01	0.09	6.7E-01	-0.04	8.3E-01	-0.17	4.2E-01	-0.19	3.6E-01	-0.21	3.0E-01
ENSCAFG0000000865	TLDC2	grey	EC_M10	0.37	6.5E-02	0.12	5.4E-01	0.21	3.0E-01	-0.27	1.8E-01	-0.59	1.7E-03	-0.26	2.0E-01	-0.25	2.3E-01	-0.24	2.4E-01	0.06	7.8E-01	0.38	5.3E-02
ENSCAFG0000000984	TIGD2	grey	EC_M10	0.37	6.5E-02	-0.38	5.7E-02	-0.22	2.8E-01	0.16	4.3E-01	0.06	7.8E-01	0.05	8.1E-01	0.05	8.2E-01	-0.05	8.0E-01	-0.20	3.2E-01	-0.26	1.9E-01
ENSCAFG0000001094	POGLUT1	grey	EC_M10	0.37	6.5E-02	-0.14	4.8E-01	-0.25	2.2E-01	0.35	8.3E-02	-0.02	9.1E-01	0.06	7.6E-01	-0.55	3.6E-03	-0.19	3.4E-01	0.31	1.2E-01	-0.18	3.7E-01
ENSCAFG0000000465	ENSCAFG0000000465	darkgrey	EC_M8	0.37	6.5E-02	0.20	3.2E-01	-0.64	4.7E-04	0.27	1.8E-01	0.29	1.5E-01	0.05	8.1E-01	-0.37	6.3E-02	-0.10	6.4E-01	-0.23	2.7E-01	-0.50	9.0E-03
ENSCAFG0000000217	ENSCAFG0000000217	grey	EC_M10	0.37	6.6E-02	-0.12	5.7E-01	-0.22	2.9E-01	-0.30	1.3E-01	0.01	9.5E-01	0.24	2.4E-01	0.07	7.2E-01	-0.25	2.3E-01	-0.03	8.9E-01	-0.26	1.9E-01
ENSCAFG0000000762	PREX2	turquoise	EC_M6	0.37	6.6E-02	0.62	8.1E-04	-0.48	1.4E-02	0.19	3.6E-01	0.01	9.4E-01	0.02	9.0E-01	-0.20	3.3E-01	0.05	8.0E-01	-0.12	5.6E-01	-0.27	1.7E-01
ENSCAFG0000000980	ENSCAFG0000000980	darkgrey	EC_M8	0.37	6.6E-02	0.49	1.1E-02	0.18	3.8E-01	0.04	8.6E-01	-0.65	3.0E-04	-0.08	6.8E-01	-0.14	4.8E-01	-0.23	2.5E-01	-0.05	8.1E-01	0.50	9.7E-03
ENSCAFG0000000385	ENSCAFG0000000385	grey	EC_M10	0.37	6.6E-02	0.05	8.1E-01	-0.43	2.8E-02	-0.07	7.2E-01	0.19	3.5E-01	0.53	5.2E-03	-0.10	6.4E-01	-0.12	5.6E-01	-0.19	3.4E-01	-0.40	4.3E-02
ENSCAFG0000002981	ENSCAFG0000002981	grey	EC_M10	0.37	6.6E-02	0.05	8.1E-01	-0.43	2.8E-02	-0.07	7.2E-01	0.19	3.5E-01	0.53	5.2E-03	-0.10	6.4E-01	-0.12	5.6E-01	-0.19	3.4E-01	-0.40	4.3E-02
ENSCAFG0000000989	ENSCAFG0000000989	grey	EC_M10	0.37	6.6E-02	-0.02	9.3E-01	-0.08	6.9E-01	-0.23	2.6E-01	-0.17	4.0E-01	-0.22	2.9E-01	-0.17	4.1E-01	0.22	2.8E-01	-0.28	1.7E-01	-0.03	8.7E-01
ENSCAFG0000000254	ENSCAFG0000000254	grey	EC_M10	0.37	6.6E-02	-0.44	2.5E-02	-0.26	2.1E-01	-0.02	9.1E-01	0.16	4.4E-01	0.12	5.6E-01	0.18	3.7E-01	0.14	4.8E-01	-0.53	5.7E-03	-0.40	4.3E-02
ENSCAFG0000001446	ENSCAFG0000001446	grey	EC_M10	0.37	6.6E-02	-0.25	2.1E-01	-0.22	2.7E-01	0.24	2.4E-01	-0.03	8.8E-01	0.21	2.9E-01	-0.15	4.5E-01	0.11	5.8E-01	-0.14	5.0E-01	-0.16	4.4E-01
ENSCAFG0000000934	SPTY2D1	grey	EC_M10	0.37	6.6E-02	0.28	1.6E-01	-0.60	1.2E-03	-0.10	6.3E-01	0.23	2.7E-01	0.00	9.9E-01	0.24	2.4E-01	0.07	7.2E-01	-0.30	1.3E-01	-0.43	2.8E-02
ENSCAFG0000000622	CEP83	grey	EC_M10	0.37	6.6E-02	-0.13	5.3E-01	0.26	2.1E-01	0.27	1.9E-01	-0.59	1.6E-03	0.01	9.5E-01	-0.12	5.7E-01	-0.23	2.7E-01	0.04	8.5E-01	0.40	4.2E-02
ENSCAFG0000000019	EYA4	grey	EC_M10	0.37	6.6E-02	-0.27	1.9E-01	-0.40	4.5E-02	0.22	2.7E-01	0.22	2.7E-01	0.26	2.1E-01	-0.21	3.0E-01	-0.07	7.5E-01	-0.02	9.1E-01	-0.43	2.8E-02
ENSCAFG0000000035	STXBP5	grey	EC_M10	0.37	6.7E-02	-0.28	1.7E-01	-0.50	8.7E-03	0.06	7.8E-01	0.37	6.3E-02	0.18	3.9E-01	-0.13	5.2E-01	-0.09	6.7E-01	-0.34	9.0E-02	-0.59	1.4E-03
ENSCAFG0000002910	ENSCAFG0000002910	grey	EC_M10	0.37	6.7E-02	0.37	6.0E-02	-0.19	3.6E-01	-0.08	6.8E-01	-0.26	2.0E-01	-0.18	3.9E-01	-0.14	4.9E-01	-0.04	8.5E-01	-0.13	5.3E-01	0.07	7.3E-01
ENSCAFG0000003047	ENSCAFG0000003047	grey	EC_M10	0.37	6.7E-02	-0.10	6.2E-01	-0.23	2.5E-01	-0.12	5.4E-01	0.05	8.0E-01	-0.05	8.0E-01	0.00	9.8E-01	-0.06	7.8E-01	0.11	6.0E-01	-0.22	2.8E-01
ENSCAFG0000000298	KIAA1841	grey	EC_M10	0.36	6.7E-02	-0.07	7.2E-01	-0.65	2.8E-04	-0.15	4.7E-01	0.54	4.7E-03	0.20	3.2E-01	-0.29	1.5E-01	0.05	8.1E-01	0.20	3.2E-01	-0.68	1.4E-04
ENSCAFG0000000777	LNPEP	darkgreen	EC_M4	0.36	6.7E-02	-0.01	9.7E-01	-0.79	1.8E-06	-0.01	9.6E-01	0.61	9.7E-04	0.07	7.4E-01	-0.03	8.8E-01	-0.26	2.0E-01	-0.10	6.3E-01	-0.77	4.4E-06
ENSCAFG0000000483	ENSCAFG0000000483	grey	EC_M10	0.36	6.7E-02	0.08	6.9E-01	-0.25	2.2E-01	-0.11	5.9E-01	0.00	9.9E-01	-0.07	7.4E-01	-0.08	6.8E-01	-0.10	6.3E-01	-0.06	7.7E-01	-0.22	2.8E-01
ENSCAFG0000001270	ENSCAFG0000001270	grey	EC_M10	0.36	6.7E-02	-0.18	3.8E-01	-0.38	5.6E-02	-0.06	7.7E-01	0.14	5.0E-01	-0.10	6.3E-01	-0.12	5.7E-01	-0.12	5.8E-01	-0.15	4.5E-01	-0.38	5.7E-02
ENSCAFG0000001247	PLEKHA5	darkgrey	EC_M8	0.36	6.7E-02	0.24	2.4E-01	0.23	2.6E-01	0.19	3.5E-01	-0.70	6.4E-05	0.04	8.5E-01	-0.10	6.2E-01	-0.07	7.2E-01	-0.51	7.9E-03	0.47	1.5E-02
ENSCAFG0000001302	WDR53	grey	EC_M10	0.36	6.7E-02	0.32	1.1E-01	-0.19	3.5E-01	0.12	5.6E-01	-0.28	1.6E-01	-0.28	1.7E-01	-0.11	5.9E-01	0.25	2.2E-01	0.39	4.9E-02	0.08	6.8E-01
ENSCAFG0000001866	ENSCAFG0000001866	grey	EC_M10	0.36	6.7E-02	-0.03	8.9E-01	-0.49	1.1E-02	-0.05	8.0E-01	0.25	2.2E-01	-0.15	4.5E-01	-0.14	5.1E-01	-0.15	4.7E-01	-0.10	6.4E-01	-0.43	3.0E-02
ENSCAFG0000000593	VPS36	darkgrey	EC_M8	0.36	6.7E-02	0.19	3.5E-01	0.32	1.1E-01	0.04	8.6E-01	-0.73	2.6E-05	-0.32	1.1E-01	-0.18	3.9E-01	0.02	9.1E-01	-0.25	2.1E-01	0.57	2.4E-03
ENSCAFG0000001086	RBM5	darkgrey	EC_M8	0.36	6.8E-02	0.48	1.3E-02	0.10	6.1E-01	0.19	3.4E-01	-0.69	9.0E-05	-0.12	5.5E-01	-0.06	7.8E-01	0.03	8.7E-01	-0.05	8.0E-01	0.48	1.4E-02
ENSCAFG0000000229	ENSCAFG0000000229	grey	EC_M10	0.36	6.8E-02	0.03	8.7E-01	-0.61	1.0E-03	0.24	2.3E-01	0.37	6.7E-02	0.13	5.3E-01	0.05	8.2E-01	0.02	9.0E-01	-0.11	6.1E-01	-0.52	6.1E-03
ENSCAFG0000001059	GSTO1	grey	EC_M10	0.36	6.8E-02	-0.10	6.1E-01	-0.17	4.0E-01	-0.05	8.2E-01	0.00	1.0E+00	0.17	3.9E-01	-0.09	6.6E-01	-0.12	5.7E-01	-0.11	5.8E-01	-0.15	4.6E-01
ENSCAFG0000003195	ENSCAFG0000003195	cyan	EC_M2	0.36	6.8E-02	-0.20	3.3E-01	0.40	4.5E-02	0.16	4.3E-01	-0.69	1.1E-04	0.25	2.1E-01	-0.02	9.3E-01	-0.15	4.7E-01	-0.48	1.4E-02	0.44	2.3E-02
ENSCAFG0000001723	ENSCAFG0000001723	darkgreen	EC_M4	0.36	6.8E-02	0.17	4.2E-01	-0.85	2.7E-08	-0.04	8.5E-01	0.63	6.0E-04	0.33	9.8E-02	-0.22	2.8E-01	-0.28	1.6E-01	-0.27	1.9E-01	-0.83	1.6E-07
ENSCAFG0000002991	SENP7	darkgrey	EC_M8	0.36	6.8E-02	0.34	8.8E-02	0.16	4.3E-01	0.05	8.0E-01	-0.66	2.6E-04	-0.16	4.5E-01	0.04	8.4E-01	0.14	5.0E-01	0.35	8.3E-02	0.48	1.3E-02
ENSCAFG0000001053	TRMT10A	darkgrey	EC_M8	0.36	6.8E-02	-0.10	6.1E-01	0.24	2.3E-01	0.16	4.3E-01	-0.64	4.0E-04	-0.04	8.6E-01	-0.16	4.4E-01	-0.03	8.7E-01	0.14	4.8E-01	0.43	2.9E-02
ENSCAFG0000001833	INF2	turquoise	EC_M6	0.36	6.8E-02	0.45	2.1E-02	0.02	9.2E-01	-0.08	6.8E-01	-0.44	2.3E-02	-0.21	3.1E-01	-0.15	4.7E-01	0.00	9.8E-01	0.27	1.8E-01	0.24	2.3E-01
ENSCAFG0000003115	PAIP2	darkgrey	EC_M8	0.36	6.8E-02	0.42	3.4E-02	-0.13	5.2E-01	0.24	2.4E-01	-0.36	6.8E-02	-0.08	6.9E-01	-0.24	2.3E-01	-0.03	8.8E-01	-0.28	1.7E-01	0.16	4.2E-01
ENSCAFG0000001079	SPATA17	grey	EC_M10	0.36	6.8E-02	-0.32	1.1E-01	-0.23	2.6E-01	-0.15	4.6E-01	0.07	7.3E-01	0.28	1.6E-01	-0.11	5.9E-01	-0.10	6.2E-01	-0.22	2.8E-01	-0.32	1.1E-01

ENSCAFG0000000838	ENSCAFG0000000838	grey	EC_M10	0.36	6.8E-02	-0.02	9.3E-01	-0.52	6.6E-03	-0.09	6.6E-01	0.31	1.2E-01	0.43	3.0E-02	-0.18	3.8E-01	-0.16	4.4E-01	-0.25	2.3E-01	-0.53	5.9E-03
ENSCAFG0000000956	ATP2B4	darkgreen	EC_M4	0.36	6.8E-02	0.37	6.1E-02	-0.83	1.3E-07	-0.24	2.5E-01	0.54	4.9E-03	0.14	5.0E-01	-0.04	8.6E-01	0.08	7.1E-01	-0.10	6.3E-01	-0.74	1.6E-05
ENSCAFG0000000992	ENSCAFG0000000992	grey	EC_M10	0.36	6.8E-02	0.21	3.0E-01	-0.31	1.2E-01	-0.03	9.0E-01	0.01	9.7E-01	-0.12	5.7E-01	-0.15	4.7E-01	-0.09	6.7E-01	-0.13	5.4E-01	-0.20	3.2E-01
ENSCAFG0000000539	PITRM1	darkgrey	EC_M8	0.36	6.8E-02	-0.22	2.8E-01	-0.51	7.7E-03	-0.09	6.6E-01	0.37	6.2E-02	0.07	7.3E-01	-0.32	1.1E-01	0.01	9.6E-01	0.23	2.5E-01	-0.57	2.4E-03
ENSCAFG0000000900	ACTR5	turquoise	EC_M6	0.36	6.8E-02	0.65	3.5E-04	-0.30	1.3E-01	-0.06	7.6E-01	-0.15	4.6E-01	0.24	2.4E-01	-0.05	8.2E-01	-0.36	6.9E-02	0.14	5.1E-01	0.00	9.9E-01
ENSCAFG0000000082	RAD21	darkgrey	EC_M8	0.36	6.8E-02	0.39	5.2E-02	0.27	1.9E-01	-0.01	9.4E-01	-0.78	2.7E-06	-0.11	6.1E-01	0.10	6.3E-01	-0.09	6.5E-01	-0.08	6.8E-01	0.60	1.1E-03
ENSCAFG0000001099	CHD2	darkgrey	EC_M8	0.36	6.8E-02	0.42	3.5E-02	-0.18	3.8E-01	0.04	8.5E-01	-0.37	6.6E-02	-0.17	4.0E-01	0.01	9.5E-01	-0.07	7.3E-01	-0.19	3.4E-01	0.14	4.9E-01
ENSCAFG0000002485	KPNA4	darkgreen	EC_M4	0.36	6.9E-02	0.12	5.8E-01	-0.91	1.4E-10	-0.16	4.4E-01	0.74	1.9E-05	0.28	1.6E-01	-0.16	4.3E-01	-0.03	8.8E-01	-0.03	8.7E-01	-0.94	2.4E-12
ENSCAFG0000001398	P3H2	turquoise	EC_M6	0.36	6.9E-02	0.46	1.9E-02	-0.30	1.3E-01	-0.04	8.6E-01	-0.10	6.4E-01	0.21	2.9E-01	-0.17	4.1E-01	-0.40	4.1E-02	0.08	6.9E-01	-0.11	6.0E-01
ENSCAFG0000001070	ENSCAFG0000001070	grey	EC_M10	0.36	6.9E-02	-0.19	3.6E-01	-0.16	4.3E-01	0.40	4.1E-02	-0.01	9.4E-01	0.33	9.8E-02	-0.20	3.3E-01	-0.21	3.1E-01	-0.24	2.4E-01	-0.13	5.3E-01
ENSCAFG0000001062	RASSF1	turquoise	EC_M6	0.36	6.9E-02	0.49	1.1E-02	-0.48	1.4E-02	-0.06	7.7E-01	0.12	5.5E-01	0.22	2.9E-01	0.09	6.6E-01	0.09	6.6E-01	0.39	5.1E-02	-0.27	1.8E-01
ENSCAFG0000001999	S1PR1	turquoise	EC_M6	0.36	6.9E-02	0.70	7.9E-05	-0.38	5.8E-02	-0.08	6.9E-01	-0.11	5.8E-01	0.02	9.3E-01	-0.31	1.3E-01	0.14	5.0E-01	0.17	4.2E-01	-0.14	5.0E-01
ENSCAFG0000001351	CREB1	turquoise	EC_M6	0.36	6.9E-02	0.64	4.8E-04	-0.20	3.4E-01	-0.06	7.9E-01	-0.23	2.7E-01	-0.20	3.3E-01	0.01	9.8E-01	-0.28	1.7E-01	-0.39	5.1E-02	0.04	8.4E-01
ENSCAFG0000000861	ENSCAFG0000000861	turquoise	EC_M6	0.36	6.9E-02	0.54	4.2E-03	-0.42	3.1E-02	-0.11	5.8E-01	0.00	9.8E-01	0.68	1.4E-04	-0.10	6.2E-01	-0.12	5.7E-01	-0.18	3.8E-01	-0.18	3.7E-01
ENSCAFG0000001915	ATCAY	turquoise	EC_M6	0.36	6.9E-02	0.54	4.2E-03	-0.42	3.1E-02	-0.11	5.8E-01	0.00	9.8E-01	0.68	1.4E-04	-0.10	6.2E-01	-0.12	5.7E-01	-0.18	3.8E-01	-0.18	3.7E-01
ENSCAFG0000000104	NCOA7	turquoise	EC_M6	0.36	6.9E-02	0.77	4.8E-06	-0.05	8.0E-01	0.06	7.6E-01	-0.54	4.1E-03	-0.22	2.8E-01	0.05	8.2E-01	-0.03	8.9E-01	0.01	9.7E-01	0.36	7.0E-02
ENSCAFG0000000247	ENSCAFG0000000247	violet	EC_M7	0.36	6.9E-02	-0.11	5.9E-01	-0.31	1.2E-01	-0.11	5.9E-01	0.16	4.4E-01	0.82	3.3E-07	-0.03	8.8E-01	-0.08	7.0E-01	0.00	9.9E-01	-0.34	8.9E-02
ENSCAFG0000001027	CARS	darkgreen	EC_M4	0.36	6.9E-02	0.11	5.8E-01	-0.76	7.1E-06	0.02	9.1E-01	0.55	3.5E-03	0.30	1.3E-01	-0.11	5.9E-01	-0.10	6.1E-01	-0.04	8.4E-01	-0.80	1.0E-06
ENSCAFG0000001664	MTOR	turquoise	EC_M6	0.36	6.9E-02	0.78	2.6E-06	-0.42	3.4E-02	-0.22	2.8E-01	-0.04	8.5E-01	-0.02	9.3E-01	-0.31	1.3E-01	-0.17	4.0E-01	-0.19	3.4E-01	-0.12	5.5E-01
ENSCAFG0000000468	EIF2B3	darkgreen	EC_M4	0.36	7.0E-02	-0.20	3.3E-01	-0.71	4.1E-05	-0.08	6.8E-01	0.63	5.7E-04	0.21	2.9E-01	-0.32	1.1E-01	0.01	9.5E-01	-0.12	5.5E-01	-0.83	1.4E-07
ENSCAFG0000000460	BIN1	grey	EC_M10	0.36	7.0E-02	0.20	3.2E-01	-0.07	7.3E-01	0.05	8.2E-01	-0.27	1.8E-01	-0.23	2.5E-01	-0.24	2.4E-01	0.30	1.4E-01	-0.13	5.3E-01	0.07	7.4E-01
ENSCAFG0000000032	UTRN	grey	EC_M10	0.36	7.0E-02	-0.03	8.9E-01	-0.27	1.8E-01	0.03	8.9E-01	0.03	8.7E-01	-0.03	8.9E-01	-0.31	1.3E-01	-0.21	3.1E-01	-0.48	1.2E-02	-0.23	2.5E-01
ENSCAFG0000001269	DYNC1I2	darkgreen	EC_M4	0.36	7.0E-02	0.05	8.0E-01	-0.82	3.6E-07	-0.17	4.0E-01	0.63	5.0E-04	0.27	1.8E-01	-0.33	9.8E-02	0.13	5.3E-01	0.10	6.4E-01	-0.81	5.2E-07
ENSCAFG0000000399	ARHGAP42	turquoise	EC_M6	0.36	7.0E-02	0.53	5.3E-03	-0.49	1.1E-02	0.16	4.3E-01	0.05	8.2E-01	-0.11	6.0E-01	-0.04	8.6E-01	-0.27	1.8E-01	0.21	2.9E-01	-0.24	2.3E-01
ENSCAFG0000000155	CCDC171	darkgrey	EC_M8	0.36	7.0E-02	0.32	1.1E-01	0.37	6.3E-02	0.35	8.1E-02	-0.87	6.0E-09	-0.12	5.5E-01	-0.15	4.8E-01	-0.15	4.7E-01	-0.09	6.7E-01	0.66	2.5E-04
ENSCAFG0000002686	ENSCAFG0000002686	grey	EC_M10	0.36	7.0E-02	0.20	3.2E-01	-0.16	4.2E-01	-0.11	5.9E-01	-0.09	6.7E-01	0.61	8.8E-04	-0.08	6.8E-01	-0.09	6.5E-01	-0.14	4.9E-01	-0.10	6.2E-01
ENSCAFG0000001560	PPM1A	grey	EC_M10	0.36	7.0E-02	0.42	3.3E-02	-0.15	4.6E-01	0.07	7.4E-01	-0.32	1.2E-01	0.14	4.8E-01	-0.06	7.7E-01	-0.13	5.4E-01	-0.61	8.3E-04	0.09	6.7E-01
ENSCAFG0000000319	ACTR2	darkgreen	EC_M4	0.36	7.0E-02	-0.13	5.1E-01	-0.81	4.0E-07	-0.27	1.7E-01	0.74	1.8E-05	0.28	1.7E-01	-0.07	7.4E-01	0.03	8.7E-01	-0.01	9.6E-01	-0.92	2.2E-11
ENSCAFG0000002924	ABT1	darkgreen	EC_M4	0.36	7.0E-02	0.18	3.9E-01	-0.87	1.1E-08	-0.24	2.3E-01	0.68	1.4E-04	0.25	2.1E-01	0.07	7.5E-01	-0.03	8.9E-01	-0.06	7.8E-01	-0.85	3.9E-08
ENSCAFG0000001412	ENSCAFG0000001412	turquoise	EC_M6	0.36	7.0E-02	0.80	1.1E-06	-0.35	7.7E-02	-0.07	7.5E-01	-0.15	4.7E-01	-0.13	5.2E-01	0.05	8.0E-01	-0.08	7.1E-01	-0.22	2.9E-01	0.00	9.9E-01
ENSCAFG0000001009	ENSCAFG0000001009	darkgreen	EC_M4	0.36	7.0E-02	-0.19	3.5E-01	-0.68	1.5E-04	0.00	9.9E-01	0.58	1.7E-03	-0.01	9.5E-01	-0.30	1.4E-01	0.11	5.8E-01	-0.02	9.1E-01	-0.79	1.6E-06
ENSCAFG0000000325	RNASEH1	darkgreen	EC_M4	0.36	7.0E-02	-0.01	9.5E-01	-0.65	3.1E-04	-0.06	7.6E-01	0.50	8.6E-03	0.14	5.0E-01	0.14	5.0E-01	0.05	8.1E-01	0.00	9.8E-01	-0.66	2.2E-04
ENSCAFG0000001994	FUBP3	darkgreen	EC_M4	0.36	7.0E-02	0.09	6.5E-01	-0.70	6.6E-05	-0.34	8.9E-02	0.52	7.0E-03	0.16	4.3E-01	-0.12	5.6E-01	0.04	8.6E-01	-0.20	3.2E-01	-0.76	5.4E-06
ENSCAFG0000001321	ENSCAFG0000001321	grey	EC_M10	0.36	7.0E-02	-0.07	7.3E-01	-0.18	3.8E-01	0.46	1.9E-02	-0.12	5.5E-01	0.11	5.8E-01	-0.20	3.3E-01	-0.11	5.9E-01	-0.17	4.1E-01	-0.04	8.6E-01
ENSCAFG0000001538	SAMD8	turquoise	EC_M6	0.36	7.0E-02	0.69	1.1E-04	-0.54	4.3E-03	0.01	9.6E-01	0.07	7.4E-01	-0.12	5.7E-01	0.13	5.2E-01	-0.10	6.2E-01	-0.21	2.9E-01	-0.24	2.4E-01
ENSCAFG0000002916	ZNF569	grey	EC_M10	0.36	7.0E-02	0.29	1.6E-01	-0.11	5.8E-01	0.37	6.2E-02	-0.34	9.3E-02	0.08	6.9E-01	-0.21	3.0E-01	0.00	9.9E-01	-0.12	5.6E-01	0.12	5.6E-01
ENSCAFG0000000330	DOCK4	turquoise	EC_M6	0.36	7.0E-02	0.88	2.1E-09	-0.33	1.0E-01	-0.23	2.5E-01	-0.21	3.1E-01	-0.14	5.0E-01	-0.17	4.0E-01	0.12	5.5E-01	0.17	4.1E-01	0.02	9.1E-01
ENSCAFG0000000603	LIG4	darkgreen	EC_M4	0.36	7.0E-02	0.17	4.0E-01	-0.73	2.6E-05	0.06	7.7E-01	0.42	3.5E-02	0.12	5.5E-01	-0.22	2.9E-01	0.10	6.3E-01	-0.35	7.9E-02	-0.64	4.3E-04
ENSCAFG0000000726	ACOX2	grey	EC_M10	0.36	7.0E-02	0.23	2.6E-01	-0.13	5.2E-01	-0.18	3.7E-01	-0.23	2.6E-01	-0.16	4.5E-01	-0.10	6.2E-01	0.16	4.4E-01	0.03	8.9E-01	0.08	7.0E-01
ENSCAFG0000000372	QRSL1	grey	EC_M10	0.36	7.0E-02	-0.29	1.4E-01	-0.41	3.8E-02	0.04	8.4E-01	0.32	1.1E-01	-0.04	8.6E-01	-0.38	5.8E-02	-0.03	8.7E-01	-0.15	4.7E-01	-0.48	1.3E-02
ENSCAFG0000002930	ITGB3BP	darkgrey	EC_M8	0.36	7.1E-02	-0.06	7.8E-01	-0.05	8.2E-01	-0.32	1.1E-01	-0.15	4.7E-01	0.30	1.3E-01	-0.07	7.2E-01	-0.20	3.4E-01	0.15	4.6E-01	-0.05	8.0E-01
ENSCAFG0000001612	EIF5A	grey	EC_M10	0.36	7.1E-02	-0.17	4.2E-01	-0.63	6.1E-04	-0.21	3.0E-01	0.59	1.4E-03	0.22	2.8E-01	-0.12	5.7E-01	0.11	5.9E-01	-0.15	4.8E-01	-0.78	3.0E-06
ENSCAFG0000002942	NETO2	grey	EC_M10	0.36	7.1E-02	-0.25	2.2E-01	-0.14	5.1E-01	-0.03	8.7E-01	0.04	8.4E-01	-0.02	9.4E-01	-0.25	2.2E-01	0.02	9.4E-01	-0.58	1.9E-03	-0.22	2.7E-01
ENSCAFG0000000244	BEND6	grey	EC_M10	0.36	7.1E-02	0.05	8.1E-01	0.32	1.1E-01	0.45	2.1E-02	-0.72	3.0E-05	-0.23	2.6E-01	-0.12	5.6E-01	-0.23	2.6E-01	0.20	3.2E-01	0.55	3.3E-03
ENSCAFG0000000993	SUDS3	grey	EC_M10	0.36	7.1E-02	0.47	1.4E-02	-0.58	2.1E-03	-0.17	4.0E-01	0.21	2.9E-01	0.07	7.4E-01	-0.38	5.5E-02	0.02	9.0E-01	-0.19	3.6E-01	-0.38	5.7E-02
ENSCAFG0000001847	ZNF3	darkgrey	EC_M8	0.36	7.1E-02	0.48	1.3E-02	0.04	8.6E-01	0.16	4.5E-01	-0.59	1.6E-03	0.22	2.8E-01	-0.05	8.2E-01	-0.18	3.9E-01	0.18	3.8E-01	0.40	4.5E-02
ENSCAFG0000000484	RALB	turquoise	EC_M6	0.36	7.1E-02	0.90	4.1E-10	-0.34	9.1E-02	0.02	9.4E-01	-0.23	2.6E-01	0.04	8.4E-01	-0.07	7.2E-01	-0.20	3.3E-01	-0.01	9.6E-01	0.06	7.7E-01
ENSCAFG0000001090	FAR2	grey	EC_M10	0.36	7.1E-02	0.11	5.9E-01	-0.63	6.0E-04	-0.07	7.4E-01	0.42	3.1E-02	0.18	3.9E-01	-0.20	3.3E-01	-0.20	3.4E-01	-0.28	1.6E-01	-0.63	5.6E-04
ENSCAFG0000000297	CEP162	darkgrey	EC_M8	0.36	7.1E-02	-0.15	4.8E-01	0.16	4.3E-01	0.24	2.3E-01	-0.51	7.4E-03	-0.10	6.1E-01	-0.06	7.7E-01	-0.27	1.7E-01	-0.15	4.5E-01	0.29	1.5E-01
ENSCAFG0000001535	C27H12orf4	grey	EC_M10	0.36	7.1E-02	-0.42	3.2E-02	-0.16	4.2E-01	0.08	6.8E-01	-0.02	9.4E-01	0.35	7.9E-02	-0.44	2.4E-02	-0.08	7.0E-01	-0.12	5.6E-01	-0.23	2.6E-01
ENSCAFG0000001004	PPRC1	grey	EC_M10	0.36	7.1E-02	-0.09	6.6E-01	-0.15	4.8E-01	-0.23	2.7E-01	0.03	8.9E-01	0.30	1.4E-01	-0.06	7.5E-01	-0.13	5.3E-01	-0.12	5.5E-01	-0.25	2.1E-01
ENSCAFG0000002953	BLOC1S2	darkgreen	EC_M4	0.36	7.1E-02	-0.15	4.7E-01	-0.67	2.1E-04	0.07	7.2E-01	0.53	4.9E-03	0.28	1.6E-01	-0.20	3.2E-01	-0.06	7.6E-01	-0.27	1.8E-01	-0.74	1.5E-05
ENSCAFG0000000201	VPS50	grey	EC_M10	0.36	7.1E-02	-0.01	9.7E-01	-0.31	1.2E-01	0.11	5.8E-01	0.03	8.8E-01	0.09	6.6E-01	-0.25	2.1E-01	0.21	3.0E-01	-0.32	1.1E-01	-0.20	3.4E-01
ENSCAFG0000001518	CRKL	grey	EC_M10	0.36	7.1E-02	0.34	9.0E-02	-0.02	9.1E-01	-0.17	4.2E-01	-0.38	5.9E-02	-0.08	6.9E-01	-0.19	3.6E-01	-0.31	1.2E-01	-0.56	3.0E-03	0.17	4.1E-01
ENSCAFG0000000602	CAPN7	grey	EC_M10	0.36	7.1E-02	-0.22	2.9E-01	-0.22	2.7E-01	0.02	9.3E-01	-0.02	9.1E-01	0.31	1.2E-01	-0.35	7.9E-02	-0.21	2.9E-01	-0.05	8.2E-01	-0.19	3.6E-01
ENSCAFG0000001449	ENSCAFG0000001449	grey	EC_M10	0.36	7.2E-02	0.09	6.6E-01	-0.13	5.2E-01	0.21	3.0E-01	-0.20	3.3E-01	-0.16	4.4E-01	-0.03	8.7E-01	-0.31	1.3E-01	-0.12	5.7E-01	0.04	8.5E-01
ENSCAFG0000001738	DGKE	grey	EC_M10	0.36	7.2E-02	0.34	9.4E-02	-0.40	4.3E-02	-0.23	2.6E-01	0.06	7.8E-01	0.09	6.5E-01	0.14	5.1E-01	0.29	1.5E-01	0.03	9.0E-01	-0.25	2.1E-01
ENSCAFG0000002909	LYPD6	turquoise	EC_M6	0.36	7.2E-02	0.85	3.5E-08	-0.48	1.4E-02	-0.12	5.5E-01	-0.05	8.3E-01	-0.04	8.4E-01	-0.15	4.5E-01	0.13	5.4E-01	0.14	4.8E-01	-0.16	4.4E-01
ENSCAFG0000000364	STARD3NL	darkgreen	EC_M4	0.36	7.2E-02	0.47	1.6E-02	-0.76	6.7E-06	-0.20	3.2E-01	0.43	2.8E-02	0.15	4.5E-01	0.04	8.5E-01	-0.09	6.6E-01	-0.37	6.5E-02	-0.63	6.4E-04
ENSCAFG0000002864	ENSCAFG0000002864	grey	EC_M10	0.36	7.2E-02	0.24	2.4E-01	-0.13	5.2E-01	-0.29	1.6E-01	-0.19	3.4E-01	0.14	4.9E-01	-0.25	2.1E-01	0.23	2.6E-01	0.17	4.0E-01	0.02	9.2E-01
ENSCAFG0000001667	LCORL	grey	EC_M10	0.36	7.2E-02	-0.01	9.5E-01	-0.31	1.2E-01	-0.01	9.8E-01	0.07	7.3E-01	-0.02	9.1E-01	0.30	1.4E-01	-0.17	4.1E-01	-0.28	1.7E-01	-0.25	2.2E-01
ENSCAFG0000000216	SLC25A13	grey	EC_M10	0.36	7.2E-02	-0.11	5.8E-01	-0.63	5.8E-04	-0.26	2.0E-01	0.53	5.6E-03	0.24	2.4E-01	-0.29	1.5E-01	-0.10	6.1E-01	0.11	6.0E-01	-0.71	4.1E-05
ENSCAFG0000000875	PRKAG1	cyan	EC_M2	0.36	7.2E-02	-0.40	4.4E-02	0.39	4.7E-02	0.26	2.0E-01	-0.61	1.0E-03	-0.04	8.3E-01	-0.46	1.7E-02	-0.09	6.6E-01	-0.04	8.4E-01	0.43	2.9E-02
ENSCAFG0000000687	CDK8	darkgreen	EC_M4	0.36	7.2E-02	0.00	1.0E+00	-0.80	1.0E-06	-0.13	5.3E-01	0.62	8.2E-04	0.39	4.6E-02	-0.06	7.9E-01	0.20	3.2E-01	0.09	6.6E-01	-0.84	6.3E-08
ENSCAFG0000002971	ENSCAFG0000002971	grey	EC_M10	0.36	7.3E-02	-0.18	3.7E-01	-0.07	7.3E-01	0.05	8.1E-01	-0.17	4.0E-01	0.30	1.4E-01	-0.03	8.8E-01	0.27	1.8E-01	-0.47	1.6E-02	-0.09	6.7E-01
ENSCAFG0000001881	SAFB2	darkgrey	EC_M8	0.36	7.3E-02	0.38	5.5E-02	-0.04	8.6E-01	0.20	3.3E-01	-0.48	1.4E-02	-0.10	6.3E-01	0.02	9.1E-01	-0.23	2.6E-01	-0.12	5.7E-01	0.25	2.1E-01
ENSCAFG0000001730	NFATC2IP	grey	EC_M10	0.36	7.3E-02	-0.13	5.3E-01	0.33	9.5E-02	0.23	2.5E-01	-0.53	5.8E-03	-0.13	5.1E-01	-0.17	4.1E-01	0.11	6.0E-01	-0.36	6.7E-02	0.34	9.4E-02
ENSCAFG0000001349	NSF	grey	EC_M10	0.36	7.3E-02	0.18	3.9E-01	-0.64	4.5E-04	-0.09	6.7E-01	0.38	5.4E-02	0.23	2.6E-01	-0.26	2.0E-01	-0.22	2.8E-01	-0.29	1.5E-01	-0.56	2.8E-03
ENSCAFG0000002025	ENSCAFG0000002025	grey	EC_M10	0.36	7.3E-02	0.03	8.9E-01	-0.06	7.6E-01	0.21	3.1E-01	-0.30	1.4E-01	0.10	6.3E-01	-0.04	8.3E-01	0.05	8.0E-01	-0.16	4.3E-01	0.10	6.2E-01
ENSCAFG0000000809	NRIP1	grey	EC_M10	0.36	7.3E-02	0.21	3.0E-01	-0.13	5.2E-01	0.22	2.7E-01	-0.22	2.9E-01	0.15	4.7E-01	-0.16	4.3E-01	0.04	8.6E-01	-0.60	1.3E-03	0.00	9.8E-01
ENSCAFG0000000959	ZBTB11	darkgrey	EC_M8	0.36	7.3E-02	0.21	3.1E-01	-0.13	5.3E-01	0.26	2.0E-01	-0.33	1.0E-01	-0.04	8.5E-01	-0.02	9.3E-01	-0.11	6.0E-01	-0.11	5.9E-01	0.11	5.9E-01
ENSCAFG0000000894	RALGAPB	darkgreen	EC_M4	0.36	7.3E-02	0.36	6.9E-02	-0.77	4.7E-06	-0.20	3.2E-01	0.45	2.1E-02	0.21	3.0E-01	-0.09	6.6E-01	-0.18	3.9E-01	0.29	1.5E-01	-0.62	8.0E-04
ENSCAFG0000001855	ENSCAFG0000001855	grey	EC_M10	0.36	7.3E-02	0.00	9.8E-01	-0.35	7.8E-02	0.48	1.4E-02	0.11	5.9E-01	-0.15	4.7E-01	-0.13	5.3E-01	-0.11	5.9E-01	-0.10	6.4E-01	-0.27	1.7E-01
ENSCAFG0000001226	THOC5	grey	EC_M10	0.36	7.4E-02	0.05	8.2E-01	-0.59	1.6E-03	0.07	7.2E-01	0.34	8.8E-02	0.39	5.1E-02	-0.41	4.0E-02	0.05	8.2E-01	0.16	4.3E-01	-0.53	5.8E-03
ENSCAFG0000000433	SELENOI	darkgreen	EC_M4	0.36	7.4E-02	-0.04	8.4E-01	-0.85	3.3E-08	-0.10	6.3E-01	0.73	2.1E-05	0.26	2.0E-01	-0.16	4.2E-01	-0.09	6.5E-01	-0.20	3.4E-01	-0.95	2.0E-13
ENSCAFG0000002868	TBCA	darkgreen	EC_M4	0.36	7.4E-02	0.08	7.1E-01	-0.76	5.9E-06	-0.19	3.6E-01	0.56	2.7E-03	0.36	7.5E-02	0.05	8.0E-01	0.12	5.8E-01	0.05	8.2E-01	-0.73	2.7E-05
ENSCAFG0000000015	POLI	grey	EC_M10	0.36	7.4E-02	-0.26	2.1E-01	0.11	6.1E-01	0.13	5.4E-01	-0.39	4.9E-02	-0.36	7.3E-02	-0.15	4.7E-01	-0.20	3.3E-01	-0.32	1.1E-01	0.18	3.8E-01
ENSCAFG0000001085	PRKAB2	darkgreen	EC_M4	0.36	7.4E-02	-0.14	5.0E-01	-0.73	2.7E-05	-0.10	6.4E-01	0.61	9.4E-04	0.13	5.2E-01	0.02	9.1E-01	0.14	4.9E-01	-0.13	5.3E-01	-0.80	8.4E-07
ENSCAFG0000001528	PSMA3	grey	EC_M10	0.36	7.4E-02	-0.19	3.5E-01	-0.50	8.6E-03	-0.08	6.9E-01	0.42	3.5E-02	0.23	2.5E-01	0.11	5.9E-01	-0.13	5.2E-01	-0.28	1.7E-01	-0.61	1.0E-03
ENSCAFG0000001273	IQCG	grey	EC_M10	0.36	7.5E-02	-0.43	2.9E-02	0.27	1.7E-01	0.06	7.8E-01	-0.45	2.1E-02	0.14	5.0E-01	-0.38	5.6E-02	-0.04	8.6E-01	-0.02	9.2E-01	0.20	3.2E-01
ENSCAFG0000001025	SFXN2	cyan	EC_M2	0.36	7.5E-02	-0.46	1.8E-02	0.39	4.7E-02	0.22	2.9E-01	-0.57	2.6E-03	-0.07	7.3E-01	-0.31	1.3E-01	0.10	6.3E-01	-0.31	1.2E-01	0.34	9.3E-02
ENSCAFG0000000992	LRRK2	grey	EC_M10	0.36	7.5E-02	-0.06	7.8E-01	0.04	8.5E-01	0.28	1.7E-01	-0.35	7.7E-02	-0.36	7.3E-02	0.29	1.5E-01	-0.04	8.6E-01	0.06	7.9E-01	0.20	3.3E-01
ENSCAFG0000001110	SNAP23	turquoise	EC_M6	0.36	7.5E-02	0.68	1.4E-04	-0.56	3.2E-03	-0.05	8.2E-01	0.08	7.0E-01	-0.01	9.4E-01	-0.08	6.9E-01	0.18	3.7E-01	-0.15	4.6E-01	-0.27	1.9E-01
ENSCAFG0000001694	DAP3	grey	EC_M10	0.36	7.5E-02	-0.03	8.8E-01	-0.34	9.0E-02	-0.05	8.1E-01	0.19	3.6E-01	0.25	2.1E-01	-0.09	6.5E-01	-0.01	9.8E-01	-0.01	9.7E-01	-0.39	5.0E-02
ENSCAFG0000000357	ENSCAFG0000000357	grey	EC_M10	0.36	7.5E-02	0.15	4.6E-01	0.05	8.1E-01	-0.01	9.6E-01	-0.44	2.6E-02	-0.18	3.8E-01	0.15	4.7E-01	0.13	5.3E-01	-0.02	9.2E-01	0.29	1.5E-01
ENSCAFG0000000487	PTPN4	turquoise	EC_M6	0.36	7.5E-02	0.67	2.0E-04	-0.36	7.1E-02	0.02	9.2E-01	-0.12	5.7E-01	0.08	7.0E-01	-0.22	2.8E-01	-0.18	3.7E-01	-0.15	4.7E-01	-0.08	6.9E-01
ENSCAFG0000003231	ZNF470	grey	EC_M10	0.36	7.5E-02	0.10	6.3E-01	-0.36	7.4E-02	-0.43	2.6E-02	0.15	4.5E-01	-0.07	7.2E-01	0.15	4.8E-01	0.08	7.1E-01	-0.14	5.1E-01	-0.35	7.6E-02
ENSCAFG0000001813	ENSCAFG0000001813	grey	EC_M10	0.36	7.5E-02	0.30	1.4E-01	-0.31	1.3E-01	-0.18	3.8E-01	-0.06	7.6E-01	0.11	6.1E-01	0.04	8.6E-01	0.36	6.7E-02	-0.09	6.7E-01	-0.12	5.5E-01
ENSCAFG0000001426	GATAD2A	grey	EC_M10	0.35	7.5E-02	0.33	1.0E-01	-0.49	1.1E-02	-0.17	4.1E-01	0.24	2.4E-01	-0.02	9.3E-01	-0.49	1.1E-02	-0.04	8.7E-01	-0.06	7.7E-01	-0.43	2.7E-02
ENSCAFG0000001998	TOR1B	grey	EC_M10	0.35	7.5E-02	0.18	3.9E-01	-0.64	4.0E-04	0.00	9.9E-01	0.48	1.4E-02	-0.03	9.0E-01	-0.28	1.7E-01	0.06	7.6E-01	0.03	8.9E-01	-0.63	5.4E-04
ENSCAFG0000000234	HMGCLL1	grey	EC_M10	0.35	7.5E-02	0.38	5.7E-02	-0.55	3.3E-03	-0.17	3.9E-01	0.24	2.3E-01	0.35	7.8E-02	-0.19	3.5E-01	0.11	6.1E-01	-0.23	2.6E-01	-0.42	3.2E-02
ENSCAFG0000002667	ENSCAFG0000002667	grey	EC_M10	0.35	7.5E-02	0.26	2.0E-01	-0.12	5.6E-01	-0.19	3.6E-01	-0.24	2.4E-01	-0.20	3.4E-01	0.02	9.2E-01	-0.17	4.0E-01	-0.22	2.7E-01	-0.01	9.7E-01
ENSCAFG0000001730	CD19	turquoise	EC_M6	0.35	7.5E-02	0.63	5.1E-04	-0.19	3.6E-01	0.16	4.3E-01	-0.30	1.3E-01	-0.15	4.8E-01	0.03	8.9E-01	-0.19	3.6E-01	-0.29	1.5E-01	0.09	6.5E-01
ENSCAFG0000001178	ZFAND2A	grey	EC_M10	0.35	7.6E-02	0.38	5.4E-02	-0.17	3.9E-01	-0.04	8.5E-01	-0.18	3.7E-01	0.14	4.8E-01	0.29	1.5E-01	-0.31	1.3E-01	0.00	9.9E-01	0.08	7.1E-01
ENSCAFG0000000103	SEC24A	darkgreen	EC_M4	0.35	7.6E-02	0.29	1.4E-01	-0.73	2.8E-05	0.01	9.5E-01	0.38	5.6E-02	0.27	1.7E-01	-0.09	6.4E-01	-0.11	6.0E-01	-0.23	2.5E-01	-0.59	1.6E-03
ENSCAFG0000003199	CBLL1	turquoise	EC_M6	0.35	7.6E-02	0.43	2.8E-02	-0.32	1.1E-01	-0.18	3.8E-01	-0.05	8.2E-01	-0.43	2.9E-02	0.07	7.3E-01	0.23	2.5E-01	0.06	7.8E-01	-0.10	6.1E-01
ENSCAFG0000003082	ENSCAFG0000003082	grey	EC_M10	0.35	7.6E-02	0.01	9.7E-01	0.20	3.2E-01	0.28	1.7E-01	-0.46	1.7E-02	0.10	6.3E-01	-0.25	2.3E-01	-0.32	1.2E-01	-0.08	7.1E-01	0.31	1.2E-01
ENSCAFG0000000453	EXOC3L2	turquoise	EC_M6	0.35	7.6E-02	0.86	1.4E-08	-0.35	7.9E-02	-0.10	6.3E-01	-0.15	4.6E-01	-0.16	4.3E-01	-0.03	8.8E-01	-0.01	9.8E-01	-0.18	3.7E-01	-0.04	8.6E-01
ENSCAFG0000000908	PLCG1	turquoise	EC_M6	0.35	7.6E-02	0.73	2.5E-05	0.00	9.9E-01	0.15	4.6E-01	-0.58	1.9E-03	-0.13	5.4E-01	-0.18	3.7E-01	-0.35	7.9E-02	0.15	4.5E-01	0.39	4.8E-02
ENSCAFG0000002903	ENSCAFG0000002903	grey	EC_M10	0.35	7.6E-02	-0.31	1.2E-01	0.09	6.7E-01	0.25	2.2E-01	-0.27	1.8E-01	0.06	7.7E-01	-0.18	3.7E-01	-0.27	1.8E-01	-0.33	1.0E-01	0.06	7.7E-01
ENSCAFG0000001635	ENSCAFG0000001635	darkgreen	EC_M4	0.35	7.6E-02	-0.24	2.4E-01	-0.74	1.4E-05	-0.03	8.7E-01	0.65	3.2E-04	0.23	2.7E-01	-0.24	2.4E-01	0.18	3.7E-01	-0.10	6.3E-01	-0.85	3.2E-08
ENSCAFG0000000790	SLC11A2	grey	EC_M10	0.35	7.6E-02	0.06	7.6E-01	0.15	4.6E-01	0.06	7.8E-01	-0.52	6.8E-03	-0.19	3.4E-01	-0.12	5.5E-01	-0.16	4.4E-01	-0.18	3.8E-01	0.26	2.0E-01
ENSCAFG0000001425	DDX3X	turquoise	EC_M6	0.35	7.6E-02	0.48	1.4E-02	-0.43	2.9E-02	0.02	9.4E-01	-0.04	8.3E-01	0.12	5.6E-01	0.24	2.3E-01	-0.07	7.3E-01	0.18	3.9E-01	-0.12	5.5E-01
ENSCAFG0000001754	RPS6KA5	turquoise	EC_M6	0.35	7.6E-02	0.68	1.2E-04	-0.30	1.3E-01	-0.17	4.2E-01	-0.18	3.9E-01	-0.06	7.8E-01	0.00	9.9E-01	-0.28	1.7E-01	0.34	9.4E-02	0.04	8.5E-01
ENSCAFG0000000022	ALDH8A1	turquoise	EC_M6	0.35	7.6E-02	0.45	2.2E-02	-0.58	2.0E-03	-0.01	9.6E-01	0.14	4.8E-01	-0.08	7.2E-01	-0.07	7.4E-01	0.12	5.5E-01	-0.07	7.3E-01	-0.38	5.6E-02
ENSCAFG0000000839	HEY1	darkgreen	EC_M4	0.35	7.6E-02	0.09	6.7E-01	-0.70	7.3E-05	0.10	6.1E-01	0.46	1.9E-02	0.14	5.1E-01	-0.04	8.4E-01	-0.23	2.6E-01	-0.29	1.5E-01	-0.70	6.3E-05
ENSCAFG0000001319	ENSCAFG0000001319	grey	EC_M10	0.35	7.6E-02	0.14	5.0E-01	-0.45	2.1E-02	-0.08	7.0E-01	0.20	3.2E-01	0.56	2.7E-03	-0.08	6.9E-01	-0.11	6.1E-01	-0.15	4.5E-01	-0.42	3.4E-02
ENSCAFG0000000259	FAM135A	grey	EC_M10	0.35	7.6E-02	-0.20	3.2E-01	-0.33	9.9E-02	0.12	5.7E-01	0.09	6.5E-01	0.11	5.8E-01	-0.22	2.8E-01	-0.26	2.0E-01	-0.26	2.0E-01	-0.28	1.7E-01
ENSCAFG0000001330	INO80D	grey	EC_M10	0.35	7.7E-02	0.43	2.7E-02	-0.04	8.5E-01	0.03	8.7E-01	-0.42	3.4E-02	-0.03	8.9E-01	0.07	7.3E-01	-0.37	6.1E-02	-0.06	7.8E-01	0.26	2.0E-01
ENSCAFG0000001858	ENSCAFG0000001858	grey	EC_M10	0.35	7.7E-02	-0.11	5.9E-01	-0.32	1.1E-01	-0.06	7.9E-01	0.10	6.1E-01	-0.10	6.2E-01	-0.10	6.2E-01	-0.12	5.7E-01	-0.07	7.2E-01	-0.32	1.1E-01
ENSCAFG0000000052	ENSCAFG0000000052	grey	EC_M10	0.35	7.7E-02	0.18	3.8E-01	-0.09	6.6E-01	0.25	2.3E-01	-0.32	1.1E-01	0.06	7.6E-01	-0.29	1.5E-01	-0.09	6.6E-01	-0.46	1.8E-02	0.11	5.9E-01
ENSCAFG0000000690	RNF6	darkgreen	EC_M4	0.35	7.7E-02	0.33	9.7E-02	-0.84	8.1E-08	-0.03	8.8E-01	0.52	6.8E-03	0.33	1.0E-01	-0.14	5.0E-01	0.03	8.8E-01	-0.09	6.7E-01	-0.70	6.1E-05
ENSCAFG0000003168	ZNF391	grey	EC_M10	0.35	7.7E-02	-0.32	1.1E-01	-0.07	7.3E-01	0.16	4.3E-01	-0.18	3.9E-01	0.15	4.8E-01	-0.05	8.1E-01	-0.34	8.5E-02	-0.20	3.3E-01	-0.06	7.6E-01
ENSCAFG0000000254	PPIH	darkgrey	EC_M8	0.35	7.7E-02	-0.16	4.3E-01	0.20	3.3E-01	-0.04	8.6E-01	-0.40	4.4E-02	-0.21	3.1E-01	-0.05	8.2E-01	-0.16	4.4E-01	0.03	9.0E-01	0.20	3.3E-01
ENSCAFG0000001794	CSK	darkgrey	EC_M8	0.35	7.7E-02	0.01	9.8E-01	-0.16	4.3E-01	-0.20	3.2E-01	0.00	9.8E-01	-0.03	8.7E-01	0.01	9.7E-01	0.19	3.6E-01	0.23	2.6E-01	-0.16	4.2E-01
ENSCAFG0000000145	GCAT	grey	EC_M10	0.35	7.7E-02	-0.39	4.9E-02	0.40	4.4E-02	0.14	5.0E-01	-0.52	5.9E-03	-0.14	4.8E-01	-0.19	3.6E-01	-0.15	4.7E-01	-0.10	6.4E-01	0.34	8.9E-02

ENSCAFG0000000494	PCDH9	grey	EC_M10	0.35	7.8E-02	0.12	5.6E-01	-0.60	1.3E-03	-0.16	4.2E-01	0.37	6.1E-02	0.05	8.3E-01	-0.21	3.0E-01	0.07	7.2E-01	0.09	6.5E-01	-0.55	3.3E-03
ENSCAFG0000001195	ENSCAFG0000001195	grey	EC_M10	0.35	7.8E-02	0.00	9.9E-01	-0.32	1.1E-01	-0.07	7.5E-01	0.11	6.0E-01	-0.13	5.2E-01	-0.08	7.1E-01	-0.03	8.9E-01	-0.05	8.2E-01	-0.32	1.1E-01
ENSCAFG0000002013	FNBP1L	turquoise	EC_M6	0.35	7.8E-02	0.74	1.9E-05	-0.73	2.7E-05	0.00	1.0E+00	0.29	1.5E-01	0.14	5.0E-01	-0.10	6.2E-01	-0.27	1.8E-01	0.08	6.9E-01	-0.50	9.9E-03
ENSCAFG0000000750	HHEX	turquoise	EC_M6	0.35	7.8E-02	0.89	1.1E-09	-0.44	2.6E-02	-0.17	4.0E-01	-0.10	6.4E-01	-0.13	5.2E-01	-0.05	8.2E-01	-0.08	7.1E-01	0.09	6.6E-01	-0.11	5.8E-01
ENSCAFG0000002032	TTLL7	grey	EC_M10	0.35	7.8E-02	-0.07	7.5E-01	-0.52	6.2E-03	0.09	6.4E-01	0.33	1.0E-01	-0.14	5.0E-01	-0.11	5.9E-01	0.00	9.8E-01	0.39	5.0E-02	-0.50	8.5E-03
ENSCAFG0000001513	LDB2	turquoise	EC_M6	0.35	7.8E-02	0.62	6.6E-04	0.19	3.5E-01	0.08	7.1E-01	-0.74	1.3E-05	-0.27	1.8E-01	-0.28	1.7E-01	-0.06	7.8E-01	0.10	6.2E-01	0.57	2.2E-03
ENSCAFG0000001208	ENSCAFG0000001208	grey	EC_M10	0.35	7.8E-02	0.58	1.8E-03	0.09	6.8E-01	0.08	6.9E-01	-0.58	1.8E-03	-0.01	9.8E-01	-0.05	8.1E-01	-0.18	3.9E-01	0.26	2.0E-01	0.44	2.3E-02
ENSCAFG0000001222	MCMBP	grey	EC_M10	0.35	7.8E-02	0.31	1.3E-01	0.35	8.2E-02	0.03	8.7E-01	-0.76	6.7E-06	0.02	9.4E-01	-0.07	7.4E-01	-0.23	2.7E-01	-0.30	1.3E-01	0.57	2.6E-03
ENSCAFG0000000384	OSBPL9	turquoise	EC_M6	0.35	7.8E-02	0.75	8.4E-06	-0.80	9.2E-07	-0.10	6.2E-01	0.37	6.2E-02	0.04	8.6E-01	-0.21	3.1E-01	-0.01	9.4E-01	-0.06	7.8E-01	-0.55	3.4E-03
ENSCAFG0000000636	NPHP3	darkgrey	EC_M8	0.35	7.8E-02	0.48	1.3E-02	0.01	9.7E-01	0.38	5.3E-02	-0.54	4.1E-03	-0.19	3.6E-01	-0.23	2.6E-01	-0.37	6.1E-02	-0.22	2.8E-01	0.34	9.4E-02
ENSCAFG0000000321	SEPT7	grey	EC_M10	0.35	7.9E-02	0.15	4.6E-01	-0.68	1.1E-04	-0.19	3.6E-01	0.38	5.6E-02	0.07	7.2E-01	-0.21	3.0E-01	0.31	1.2E-01	0.06	7.7E-01	-0.57	2.2E-03
ENSCAFG0000001115	ENSCAFG0000001115	grey	EC_M10	0.35	7.9E-02	0.12	5.4E-01	-0.64	4.1E-04	0.00	9.8E-01	0.30	1.4E-01	0.19	3.4E-01	-0.03	8.7E-01	0.01	9.5E-01	-0.09	6.5E-01	-0.54	4.7E-03
ENSCAFG0000000681	USP12	grey	EC_M10	0.35	7.9E-02	0.32	1.1E-01	-0.75	1.1E-05	-0.07	7.2E-01	0.44	2.3E-02	0.27	1.8E-01	-0.11	6.0E-01	-0.14	5.1E-01	0.27	1.8E-01	-0.61	8.3E-04
ENSCAFG0000001423	ENSCAFG0000001423	grey	EC_M10	0.35	7.9E-02	-0.18	3.9E-01	-0.18	3.7E-01	0.27	1.8E-01	-0.03	8.9E-01	0.22	2.8E-01	-0.26	1.9E-01	0.10	6.3E-01	-0.18	3.9E-01	-0.15	4.5E-01
ENSCAFG0000000159	MDFI	turquoise	EC_M6	0.35	7.9E-02	0.83	1.8E-07	-0.31	1.2E-01	-0.20	3.2E-01	-0.19	3.5E-01	-0.04	8.4E-01	0.02	9.3E-01	0.01	9.5E-01	-0.21	3.0E-01	0.01	9.7E-01
ENSCAFG0000000979	HIVEP1	darkgrey	EC_M8	0.35	7.9E-02	0.64	4.0E-04	0.00	9.9E-01	0.14	4.9E-01	-0.56	2.9E-03	-0.26	2.0E-01	-0.09	6.7E-01	-0.25	2.2E-01	0.09	6.7E-01	0.38	5.4E-02
ENSCAFG0000002110	ENSCAFG0000002110	grey	EC_M10	0.35	7.9E-02	0.18	3.7E-01	-0.32	1.1E-01	-0.19	3.6E-01	-0.06	7.8E-01	0.22	2.8E-01	-0.19	3.5E-01	-0.19	3.5E-01	-0.22	2.9E-01	-0.17	4.0E-01
ENSCAFG0000000244	AHR	grey	EC_M10	0.35	7.9E-02	0.43	2.6E-02	-0.20	3.4E-01	0.37	6.2E-02	-0.27	1.8E-01	0.04	8.4E-01	-0.20	3.3E-01	-0.01	9.5E-01	0.30	1.4E-01	0.07	7.2E-01
ENSCAFG0000000655	TMF1	grey	EC_M10	0.35	7.9E-02	0.46	1.8E-02	-0.57	2.2E-03	0.06	7.5E-01	0.09	6.6E-01	0.13	5.4E-01	0.04	8.5E-01	0.08	6.9E-01	-0.17	4.1E-01	-0.30	1.3E-01
ENSCAFG0000002318	ENSCAFG0000002318	violet	EC_M7	0.35	7.9E-02	-0.11	5.9E-01	-0.33	1.0E-01	-0.11	6.1E-01	0.17	4.0E-01	0.89	9.8E-10	-0.04	8.4E-01	-0.08	6.8E-01	-0.04	8.6E-01	-0.35	7.6E-02
ENSCAFG0000001457	P4HA1	darkgreen	EC_M4	0.35	7.9E-02	0.16	4.3E-01	-0.85	3.8E-08	-0.12	5.5E-01	0.63	5.6E-04	0.28	1.6E-01	0.17	4.2E-01	0.15	4.6E-01	-0.01	9.8E-01	-0.85	5.1E-08
ENSCAFG0000001806	RCOR1	turquoise	EC_M6	0.35	8.0E-02	0.45	2.1E-02	-0.56	3.2E-03	-0.09	6.8E-01	0.16	4.5E-01	0.04	8.4E-01	0.17	4.2E-01	-0.12	5.4E-01	0.18	3.8E-01	-0.34	8.5E-02
ENSCAFG0000000058	ENSCAFG0000000058	darkgreen	EC_M4	0.35	8.0E-02	0.11	5.9E-01	-0.85	3.4E-08	-0.38	5.6E-02	0.71	5.5E-05	0.28	1.6E-01	-0.05	8.2E-01	-0.01	9.6E-01	-0.17	4.0E-01	-0.87	8.2E-09
ENSCAFG0000000147	ENSCAFG0000000147	turquoise	EC_M6	0.35	8.0E-02	0.57	2.1E-03	-0.08	7.0E-01	-0.16	4.4E-01	-0.38	5.8E-02	-0.18	3.7E-01	-0.16	4.3E-01	-0.21	3.0E-01	0.10	6.4E-01	0.22	2.7E-01
ENSCAFG0000000187	POLR1C	cyan	EC_M2	0.35	8.0E-02	-0.28	1.7E-01	0.18	3.8E-01	0.01	9.6E-01	-0.34	9.1E-02	0.11	5.9E-01	0.07	7.3E-01	-0.35	8.2E-02	-0.44	2.5E-02	0.13	5.4E-01
ENSCAFG0000000742	HSP90B1	darkgreen	EC_M4	0.35	8.0E-02	-0.09	6.6E-01	-0.85	5.4E-08	-0.15	4.6E-01	0.77	4.5E-06	0.27	1.9E-01	0.01	9.6E-01	0.18	3.9E-01	-0.09	6.5E-01	-0.95	5.9E-14
ENSCAFG0000001138	SHROOM2	darkgreen	EC_M4	0.35	8.0E-02	0.36	6.9E-02	-0.88	2.8E-09	-0.12	5.6E-01	0.61	8.7E-04	0.14	4.9E-01	-0.22	2.9E-01	0.03	9.0E-01	-0.07	7.4E-01	-0.83	2.0E-07
ENSCAFG0000001351	ENSCAFG0000001351	turquoise	EC_M6	0.35	8.1E-02	0.51	7.8E-03	-0.09	6.5E-01	-0.08	6.9E-01	-0.31	1.2E-01	0.16	4.4E-01	-0.07	7.3E-01	-0.11	5.9E-01	0.09	6.6E-01	0.16	4.3E-01
ENSCAFG0000002963	ENSCAFG0000002963	grey	EC_M10	0.35	8.1E-02	-0.17	3.9E-01	-0.62	7.3E-04	0.02	9.3E-01	0.42	3.3E-02	0.34	9.4E-02	0.00	9.9E-01	0.20	3.4E-01	-0.27	1.9E-01	-0.62	8.1E-04
ENSCAFG0000003013	GBA2	turquoise	EC_M6	0.35	8.1E-02	0.49	1.0E-02	-0.07	7.2E-01	-0.08	6.9E-01	-0.31	1.2E-01	-0.26	2.0E-01	-0.38	5.3E-02	-0.11	5.9E-01	0.30	1.3E-01	0.18	3.9E-01
ENSCAFG0000001836	YES1	turquoise	EC_M6	0.35	8.1E-02	0.74	1.4E-05	-0.78	2.1E-06	-0.05	8.1E-01	0.36	7.1E-02	-0.01	9.6E-01	-0.17	4.1E-01	0.05	8.1E-01	0.00	1.0E+00	-0.55	3.4E-03
ENSCAFG0000000034	SRGAP1	grey	EC_M10	0.35	8.1E-02	-0.21	3.1E-01	-0.58	2.0E-03	-0.20	3.4E-01	0.42	3.4E-02	0.21	3.0E-01	-0.03	8.8E-01	-0.07	7.2E-01	-0.31	1.3E-01	-0.61	9.7E-04
ENSCAFG0000000892	OAS3	turquoise	EC_M6	0.35	8.2E-02	0.52	6.6E-03	-0.48	1.4E-02	0.28	1.7E-01	0.01	9.5E-01	0.15	4.8E-01	0.03	8.9E-01	-0.18	3.8E-01	-0.04	8.6E-01	-0.25	2.2E-01
ENSCAFG0000000881	ENSCAFG0000000881	turquoise	EC_M6	0.35	8.2E-02	0.55	3.5E-03	-0.11	5.9E-01	-0.13	5.2E-01	-0.33	1.0E-01	-0.15	4.7E-01	-0.08	6.9E-01	-0.12	5.7E-01	-0.11	6.1E-01	0.21	3.1E-01
ENSCAFG0000000752	ITCH	turquoise	EC_M6	0.35	8.2E-02	0.61	1.0E-03	-0.50	8.6E-03	0.01	9.6E-01	-0.01	9.8E-01	0.08	6.9E-01	-0.03	8.7E-01	-0.03	8.9E-01	0.38	5.6E-02	-0.19	3.5E-01
ENSCAFG0000002907	ZNF614	darkgrey	EC_M8	0.35	8.2E-02	0.37	6.5E-02	0.16	4.5E-01	0.21	3.1E-01	-0.64	4.4E-04	-0.01	9.8E-01	-0.03	9.0E-01	-0.10	6.2E-01	-0.12	5.7E-01	0.48	1.2E-02
ENSCAFG0000000056	PABPC1	grey	EC_M10	0.35	8.2E-02	-0.14	4.9E-01	0.35	7.7E-02	0.11	6.0E-01	-0.61	8.3E-04	0.02	9.4E-01	-0.35	8.3E-02	-0.11	6.0E-01	-0.42	3.4E-02	0.38	5.4E-02
ENSCAFG0000002981	EPHX4	turquoise	EC_M6	0.35	8.2E-02	0.79	1.4E-06	-0.28	1.7E-01	-0.09	6.6E-01	-0.22	2.7E-01	-0.11	5.8E-01	-0.15	4.6E-01	-0.14	5.1E-01	-0.20	3.2E-01	0.02	9.2E-01
ENSCAFG0000001211	URB2	darkgrey	EC_M8	0.35	8.2E-02	-0.34	9.3E-02	-0.39	4.8E-02	-0.06	7.8E-01	0.37	6.1E-02	0.22	2.8E-01	-0.35	7.7E-02	-0.25	2.1E-01	-0.28	1.6E-01	-0.54	4.7E-03
ENSCAFG0000000847	GPN3	darkgrey	EC_M8	0.35	8.2E-02	-0.27	1.7E-01	-0.53	5.5E-03	-0.33	9.9E-02	0.51	7.5E-03	0.28	1.7E-01	0.01	9.6E-01	0.03	9.0E-01	-0.05	8.0E-01	-0.69	1.0E-04
ENSCAFG0000002893	ENSCAFG0000002893	grey	EC_M10	0.35	8.2E-02	-0.17	4.0E-01	-0.02	9.1E-01	0.26	2.1E-01	-0.23	2.5E-01	0.13	5.2E-01	0.12	5.6E-01	-0.26	2.0E-01	-0.29	1.5E-01	0.00	9.9E-01
ENSCAFG0000002113	ENSCAFG0000002113	grey	EC_M10	0.35	8.3E-02	-0.16	4.3E-01	-0.13	5.2E-01	-0.50	8.9E-03	-0.07	7.2E-01	0.14	5.1E-01	-0.18	3.8E-01	0.08	6.9E-01	0.03	8.9E-01	-0.14	4.9E-01
ENSCAFG0000000920	AP3B1	darkgreen	EC_M4	0.35	8.3E-02	0.14	4.9E-01	-0.80	1.2E-06	-0.10	6.1E-01	0.58	2.1E-03	0.30	1.4E-01	-0.41	3.5E-02	-0.13	5.3E-01	-0.26	2.0E-01	-0.76	5.4E-06
ENSCAFG0000002595	ENSCAFG0000002595	darkgrey	EC_M8	0.35	8.3E-02	-0.02	9.3E-01	0.39	4.8E-02	0.27	1.8E-01	-0.77	4.3E-06	0.05	8.0E-01	-0.04	8.3E-01	-0.21	2.9E-01	-0.16	4.4E-01	0.56	3.0E-03
ENSCAFG0000002525	FOCAD	grey	EC_M10	0.35	8.3E-02	-0.57	2.2E-03	-0.39	4.7E-02	-0.07	7.4E-01	0.42	3.1E-02	0.20	3.4E-01	-0.16	4.3E-01	0.03	9.0E-01	-0.16	4.2E-01	-0.62	7.7E-04
ENSCAFG0000000920	ENSCAFG0000000920	grey	EC_M10	0.35	8.3E-02	-0.07	7.5E-01	-0.33	1.0E-01	0.23	2.6E-01	0.12	5.6E-01	-0.18	3.7E-01	-0.21	2.9E-01	-0.22	2.8E-01	-0.08	6.8E-01	-0.27	1.8E-01
ENSCAFG0000001701	HERC1	grey	EC_M10	0.35	8.3E-02	0.33	1.0E-01	-0.52	6.4E-03	0.02	9.2E-01	0.18	3.9E-01	0.04	8.5E-01	-0.14	5.0E-01	0.06	7.9E-01	-0.07	7.5E-01	-0.34	8.7E-02
ENSCAFG0000001355	ENSCAFG0000001355	grey	EC_M10	0.35	8.3E-02	-0.03	8.9E-01	-0.34	9.1E-02	0.05	8.1E-01	0.13	5.4E-01	0.17	3.9E-01	0.18	3.8E-01	-0.37	6.4E-02	-0.14	4.9E-01	-0.31	1.2E-01
ENSCAFG0000000891	RSRC1	grey	EC_M10	0.35	8.3E-02	-0.36	6.8E-02	0.00	9.8E-01	0.08	7.1E-01	-0.11	5.8E-01	0.24	2.4E-01	-0.10	6.2E-01	0.05	8.0E-01	-0.54	4.0E-03	-0.06	7.5E-01
ENSCAFG0000001427	RDX	grey	EC_M10	0.35	8.3E-02	0.21	3.1E-01	-0.75	1.1E-05	-0.10	6.1E-01	0.48	1.3E-02	-0.01	9.8E-01	-0.22	2.9E-01	0.07	7.2E-01	0.23	2.7E-01	-0.63	6.2E-04
ENSCAFG0000001969	ENO1	darkgreen	EC_M4	0.35	8.4E-02	0.13	5.2E-01	-0.75	8.7E-06	-0.23	2.5E-01	0.61	8.3E-04	0.11	6.0E-01	0.14	5.0E-01	0.14	5.1E-01	-0.14	4.9E-01	-0.80	8.2E-07
ENSCAFG0000003025	RIDA	darkgrey	EC_M8	0.35	8.4E-02	-0.35	7.8E-02	-0.49	1.0E-02	-0.24	2.3E-01	0.43	2.8E-02	0.24	2.4E-01	-0.21	3.1E-01	0.05	8.1E-01	-0.03	8.8E-01	-0.61	8.3E-04
ENSCAFG0000000273	RAPGEF5	turquoise	EC_M6	0.35	8.4E-02	0.60	1.1E-03	-0.37	6.6E-02	-0.13	5.2E-01	-0.08	7.0E-01	-0.19	3.5E-01	-0.04	8.4E-01	-0.12	5.6E-01	0.12	5.5E-01	-0.07	7.3E-01
ENSCAFG0000002112	ENSCAFG0000002112	grey	EC_M10	0.35	8.4E-02	-0.08	6.8E-01	-0.50	9.1E-03	0.04	8.4E-01	0.29	1.5E-01	0.27	1.8E-01	0.13	5.3E-01	-0.05	8.3E-01	0.05	8.0E-01	-0.48	1.3E-02
ENSCAFG0000001903	ENSCAFG0000001903	turquoise	EC_M6	0.35	8.4E-02	0.58	1.9E-03	-0.45	2.2E-02	-0.15	4.7E-01	0.07	7.4E-01	-0.14	4.9E-01	-0.17	4.1E-01	-0.11	5.8E-01	-0.16	4.3E-01	-0.25	2.2E-01
ENSCAFG0000003050	ZNF133	grey	EC_M10	0.35	8.4E-02	0.01	9.5E-01	-0.03	8.7E-01	0.00	9.8E-01	-0.25	2.1E-01	0.09	6.7E-01	0.11	5.9E-01	-0.11	6.0E-01	0.36	6.8E-02	0.13	5.4E-01
ENSCAFG0000001157	HIST2H2AC	grey	EC_M10	0.35	8.4E-02	0.18	3.9E-01	0.05	7.9E-01	0.07	7.4E-01	-0.42	3.2E-02	0.04	8.6E-01	-0.20	3.2E-01	-0.11	6.1E-01	-0.19	3.6E-01	0.26	1.9E-01
ENSCAFG0000000909	LXN	grey	EC_M10	0.34	8.4E-02	-0.05	8.2E-01	-0.52	7.0E-03	-0.24	2.3E-01	0.32	1.1E-01	-0.08	7.1E-01	-0.17	4.1E-01	0.40	4.0E-02	-0.18	3.7E-01	-0.52	7.0E-03
ENSCAFG0000000784	ENSCAFG0000000784	turquoise	EC_M6	0.34	8.4E-02	0.41	3.6E-02	-0.23	2.6E-01	0.20	3.3E-01	-0.18	3.8E-01	0.22	2.8E-01	-0.41	3.8E-02	-0.17	4.1E-01	-0.63	5.8E-04	-0.06	7.7E-01
ENSCAFG0000001141	ENSCAFG0000001141	grey	EC_M10	0.34	8.5E-02	-0.26	1.9E-01	-0.35	7.6E-02	-0.28	1.7E-01	0.24	2.5E-01	0.09	6.5E-01	0.16	4.5E-01	0.22	2.7E-01	-0.31	1.3E-01	-0.45	2.0E-02
ENSCAFG0000000146	PTPRD	grey	EC_M10	0.34	8.5E-02	0.05	8.0E-01	-0.25	2.2E-01	-0.14	5.0E-01	0.02	9.1E-01	-0.12	5.7E-01	0.19	3.5E-01	-0.21	3.1E-01	0.07	7.3E-01	-0.14	5.0E-01
ENSCAFG0000000741	PPWD1	darkgrey	EC_M8	0.34	8.5E-02	-0.11	5.9E-01	0.21	2.9E-01	-0.02	9.2E-01	-0.57	2.3E-03	0.20	3.3E-01	0.03	8.7E-01	-0.04	8.6E-01	-0.26	1.9E-01	0.36	6.8E-02
ENSCAFG0000001676	SLC49A3	turquoise	EC_M6	0.34	8.5E-02	0.77	3.7E-06	-0.33	1.1E-01	0.04	8.4E-01	-0.13	5.3E-01	-0.20	3.4E-01	0.05	7.9E-01	-0.14	4.8E-01	-0.20	3.2E-01	-0.04	8.4E-01
ENSCAFG0000001685	HOXB7	turquoise	EC_M6	0.34	8.5E-02	0.62	6.9E-04	-0.46	1.9E-02	0.15	4.5E-01	0.03	8.9E-01	-0.09	6.7E-01	0.14	4.8E-01	-0.30	1.4E-01	-0.16	4.2E-01	-0.19	3.5E-01
ENSCAFG0000000509	SNRPA	darkgrey	EC_M8	0.34	8.5E-02	-0.01	9.8E-01	0.02	9.4E-01	-0.06	7.6E-01	-0.17	4.2E-01	-0.05	8.2E-01	-0.14	4.9E-01	0.11	5.9E-01	-0.06	7.7E-01	-0.02	9.4E-01
ENSCAFG0000001862	WSB1	darkgreen	EC_M4	0.34	8.5E-02	0.59	1.6E-03	-0.83	1.2E-07	-0.07	7.2E-01	0.42	3.5E-02	0.17	4.0E-01	-0.04	8.5E-01	0.09	6.5E-01	0.11	5.9E-01	-0.62	7.5E-04
ENSCAFG0000000173	HMGXB4	darkgrey	EC_M8	0.34	8.5E-02	0.37	6.4E-02	0.24	2.4E-01	0.19	3.5E-01	-0.67	1.7E-04	-0.31	1.2E-01	0.13	5.4E-01	-0.22	2.9E-01	-0.10	6.4E-01	0.53	5.4E-03
ENSCAFG0000001940	LSM7	grey	EC_M10	0.34	8.5E-02	0.01	9.6E-01	0.26	1.9E-01	0.29	1.5E-01	-0.53	5.8E-03	-0.05	8.1E-01	-0.06	7.8E-01	-0.23	2.6E-01	-0.21	3.1E-01	0.30	1.4E-01
ENSCAFG0000000514	VHL	turquoise	EC_M6	0.34	8.6E-02	0.69	1.1E-04	0.17	4.2E-01	-0.08	7.1E-01	-0.71	4.8E-05	-0.16	4.4E-01	-0.14	4.8E-01	0.10	6.3E-01	-0.04	8.6E-01	0.53	5.3E-03
ENSCAFG0000003187	ENSCAFG0000003187	turquoise	EC_M6	0.34	8.6E-02	0.50	9.7E-03	-0.46	1.8E-02	0.16	4.4E-01	0.04	8.4E-01	0.20	3.3E-01	-0.13	5.4E-01	-0.51	7.7E-03	0.05	8.2E-01	-0.20	3.2E-01
ENSCAFG0000000210	ENSCAFG0000000210	grey	EC_M10	0.34	8.6E-02	0.27	1.9E-01	-0.53	5.5E-03	-0.04	8.5E-01	0.16	4.5E-01	0.31	1.2E-01	-0.25	2.1E-01	-0.03	8.7E-01	0.03	8.8E-01	-0.36	7.3E-02
ENSCAFG0000002965	ENSCAFG0000002965	grey	EC_M10	0.34	8.6E-02	0.07	7.2E-01	-0.47	1.5E-02	0.32	1.1E-01	0.16	4.3E-01	0.33	1.0E-01	0.07	7.2E-01	-0.23	2.7E-01	-0.30	1.4E-01	-0.40	4.6E-02
ENSCAFG0000001855	ENSCAFG0000001855	grey	EC_M10	0.34	8.6E-02	-0.04	8.5E-01	-0.09	6.8E-01	0.32	1.1E-01	-0.24	2.3E-01	0.12	5.7E-01	-0.16	4.2E-01	-0.11	6.1E-01	-0.24	2.4E-01	0.08	7.0E-01
ENSCAFG0000000401	ITSN2	turquoise	EC_M6	0.34	8.6E-02	0.65	3.6E-04	-0.33	1.0E-01	0.03	8.8E-01	-0.18	3.7E-01	-0.32	1.1E-01	-0.16	4.3E-01	0.06	7.8E-01	-0.20	3.4E-01	0.02	9.1E-01
ENSCAFG0000001023	ADCY2	turquoise	EC_M6	0.34	8.6E-02	0.78	2.7E-06	-0.34	8.7E-02	-0.08	7.1E-01	-0.12	5.6E-01	-0.11	5.9E-01	-0.14	4.9E-01	-0.11	5.8E-01	-0.18	3.9E-01	-0.03	8.7E-01
ENSCAFG0000000943	BAZ2B	turquoise	EC_M6	0.34	8.6E-02	0.54	4.4E-03	0.03	8.9E-01	0.12	5.6E-01	-0.49	1.2E-02	-0.12	5.7E-01	-0.23	2.6E-01	-0.05	8.0E-01	-0.10	6.3E-01	0.34	9.3E-02
ENSCAFG0000002533	PTPN12	darkgreen	EC_M4	0.34	8.6E-02	0.67	1.8E-04	-0.84	6.4E-08	-0.21	3.1E-01	0.45	2.0E-02	0.19	3.4E-01	0.03	9.0E-01	0.02	9.3E-01	-0.16	4.3E-01	-0.64	4.5E-04
ENSCAFG0000000504	KLF12	grey	EC_M10	0.34	8.7E-02	-0.31	1.2E-01	-0.16	4.3E-01	0.24	2.5E-01	0.04	8.6E-01	0.03	8.8E-01	-0.39	4.7E-02	-0.12	5.5E-01	-0.12	5.6E-01	-0.24	2.4E-01
ENSCAFG0000000225	ICK	darkgrey	EC_M8	0.34	8.7E-02	0.12	5.6E-01	0.15	4.6E-01	0.12	5.5E-01	-0.60	1.3E-03	-0.04	8.3E-01	-0.33	1.1E-01	0.22	2.7E-01	0.30	1.4E-01	0.38	5.9E-02
ENSCAFG0000002716	ENSCAFG0000002716	grey	EC_M10	0.34	8.7E-02	-0.16	4.3E-01	-0.14	4.9E-01	-0.11	6.1E-01	-0.02	9.4E-01	-0.10	6.4E-01	-0.04	8.5E-01	-0.05	7.9E-01	-0.10	6.3E-01	-0.14	5.0E-01
ENSCAFG0000000926	AP4B1	turquoise	EC_M6	0.34	8.7E-02	0.46	1.7E-02	-0.16	4.3E-01	-0.13	5.2E-01	-0.28	1.7E-01	0.01	9.8E-01	-0.28	1.6E-01	0.25	2.1E-01	0.18	3.7E-01	0.10	6.2E-01
ENSCAFG0000001698	YLPM1	grey	EC_M10	0.34	8.7E-02	-0.18	3.8E-01	0.08	6.9E-01	-0.11	5.8E-01	-0.36	7.1E-02	0.00	9.9E-01	-0.26	2.0E-01	0.31	1.3E-01	-0.41	4.0E-02	0.11	6.1E-01
ENSCAFG0000000453	CLASP2	darkgrey	EC_M8	0.34	8.7E-02	0.11	5.8E-01	-0.10	6.4E-01	0.13	5.2E-01	-0.31	1.3E-01	-0.27	1.8E-01	-0.37	6.0E-02	-0.01	9.7E-01	-0.16	4.4E-01	0.12	5.5E-01
ENSCAFG0000001815	ZNF521	turquoise	EC_M6	0.34	8.7E-02	0.63	5.3E-04	0.22	2.7E-01	0.08	6.9E-01	-0.79	1.3E-06	-0.14	5.0E-01	-0.24	2.4E-01	-0.11	6.0E-01	-0.09	6.7E-01	0.59	1.4E-03
ENSCAFG0000002413	ENSCAFG0000002413	darkgrey	EC_M8	0.34	8.7E-02	-0.10	6.2E-01	-0.09	6.6E-01	-0.13	5.2E-01	-0.05	8.3E-01	-0.08	6.9E-01	-0.02	9.3E-01	-0.08	6.9E-01	-0.04	8.6E-01	-0.12	5.4E-01
ENSCAFG0000000121	HMGA1	grey	EC_M10	0.34	8.7E-02	-0.20	3.3E-01	0.21	3.0E-01	0.18	3.9E-01	-0.41	3.8E-02	-0.21	3.1E-01	-0.18	3.9E-01	0.03	8.8E-01	-0.41	3.7E-02	0.19	3.6E-01
ENSCAFG0000001145	LRMP	turquoise	EC_M6	0.34	8.7E-02	0.81	5.5E-07	-0.11	5.8E-01	-0.13	5.4E-01	-0.39	4.9E-02	-0.13	5.3E-01	-0.14	5.0E-01	-0.11	6.1E-01	-0.23	2.7E-01	0.24	2.3E-01
ENSCAFG0000000932	UEVLD	grey	EC_M10	0.34	8.7E-02	0.40	4.2E-02	-0.15	4.7E-01	-0.22	2.8E-01	-0.27	1.8E-01	-0.27	1.9E-01	-0.05	8.2E-01	0.02	9.2E-01	-0.08	7.0E-01	0.09	6.4E-01
ENSCAFG0000001219	LDHB	grey	EC_M10	0.34	8.8E-02	-0.61	8.5E-04	0.08	6.9E-01	0.13	5.3E-01	-0.11	6.0E-01	0.05	8.3E-01	-0.16	4.4E-01	-0.10	6.3E-01	-0.24	2.5E-01	-0.06	7.6E-01
ENSCAFG0000000467	HECTD3	grey	EC_M10	0.34	8.8E-02	0.27	1.8E-01	0.38	5.8E-02	0.13	5.3E-01	-0.79	2.0E-06	-0.13	5.2E-01	-0.20	3.2E-01	-0.03	8.9E-01	-0.03	8.7E-01	0.62	7.8E-04
ENSCAFG0000002307	ENSCAFG0000002307	grey	EC_M10	0.34	8.8E-02	-0.35	7.5E-02	-0.40	4.1E-02	-0.18	3.7E-01	0.36	7.3E-02	0.24	2.3E-01	0.08	7.0E-01	0.18	3.7E-01	-0.27	1.7E-01	-0.58	1.9E-03
ENSCAFG0000001735	ENSCAFG0000001735	turquoise	EC_M6	0.34	8.8E-02	0.57	2.3E-03	-0.39	5.2E-02	-0.06	7.9E-01	-0.08	7.0E-01	-0.12	5.8E-01	-0.14	5.1E-01	-0.12	5.6E-01	-0.21	3.1E-01	-0.13	5.3E-01
ENSCAFG0000003040	ENSCAFG0000003040	grey	EC_M10	0.34	8.8E-02	-0.10	6.3E-01	0.09	6.6E-01	-0.09	6.5E-01	-0.33	1.0E-01	-0.07	7.3E-01	-0.15	4.8E-01	0.22	2.9E-01	-0.26	2.1E-01	0.13	5.1E-01
ENSCAFG0000002931	ZNF582	grey	EC_M10	0.34	8.8E-02	0.18	3.8E-01	0.07	7.5E-01	0.07	7.2E-01	-0.41	3.6E-02	-0.19	3.4E-01	-0.64	4.1E-04	-0.11	5.9E-01	0.08	6.8E-01	0.24	2.5E-01
ENSCAFG0000001722	ALKBH1	grey	EC_M10	0.34	8.8E-02	0.37	6.0E-02	-0.16	4.4E-01	-0.06	7.6E-01	-0.28	1.7E-01	0.06	7.9E-01	-0.42	3.2E-02	-0.26	2.1E-01	-0.07	7.3E-01	0.08	6.8E-01
ENSCAFG0000001935	P2RX1	grey	EC_M10	0.34	8.8E-02	0.00	9.9E-01	-0.28	1.7E-01	-0.06	7.6E-01	0.07	7.2E-01	-0.05	8.2E-01	-0.02	9.2E-01	-0.21	3.0E-01	-0.06	7.6E-01	-0.19	3.6E-01
ENSCAFG0000001376	ENSCAFG0000001376	grey	EC_M10	0.34	8.8E-02	0.33	1.0E-01	-0.60	1.2E-03	-0.15	4.8E-01	0.28	1.7E-01	0.16	4.3E-01	0.07	7.2E-01	0.23	2.6E-01	-0.16	4.3E-01	-0.47	1.6E-02
ENSCAFG0000001721	UPRT	grey	EC_M10	0.34	8.8E-02	0.01	9.7E-01	-0.31	1.3E-01	-0.06	7.8E-01	0.09	6.7E-01	0.02	9.2E-01	-0.38	5.4E-02	0.15	4.8E-01	-0.03	9.0E-01	-0.27	1.8E-01
ENSCAFG0000000715	ERCC8	grey	EC_M10	0.34	8.8E-02	-0.29	1.6E-01	-0.31	1.2E-01	-0.32	1.1E-01	0.17	4.1E-01	-0.12	5.5E-01	-0.35	8.1E-02	-0.05	8.2E-01	0.01	9.6E-01	-0.35	8.3E-02
ENSCAFG0000001720	NUDCD2	grey	EC_M10	0.34	8.8E-02	-0.02	9.4E-01	-0.43	2.9E-02	0.20	3.4E-01	0.16	4.4E-01	0.05	8.2E-01	-0.38	5.4E-02	-0.21	3.1E-01	-0.23	2.6E-01	-0.38	5.8E-02
ENSCAFG0000001643	MXD3	grey	EC_M10	0.34	8.8E-02	0.25	2.2E-01	-0.07	7.2E-01	0.09	6.6E-01	-0.26	2.0E-01	-0.04	8.4E-01	-0.33	1.0E-01	-0.09	6.5E-01	0.26	2.1E-01	0.11	6.0E-01
ENSCAFG0000000023	PDE7B	turquoise	EC_M6	0.34	8.9E-02	0.85	3.8E-08	-0.34	9.4E-02	-0.21	3.0E-01	-0.15	4.7E-01	-0.21	3.1E-01	-0.08	6.9E-01	0.03	8.7E-01	0.22	2.8E-01	-0.03	8.9E-01
ENSCAFG0000000301	PTBP3	turquoise	EC_M6	0.34	8.9E-02	0.65	2.9E-04	-0.48	1.4E-02	-0.16	4.3E-01	0.05	8.0E-01	-0.09	6.8E-01	-0.25	2.3E-01	-0.01	9.4E-01	0.08	7.0E-01	-0.26	2.0E-01
ENSCAFG0000002592	ENSCAFG0000002592	grey	EC_M10	0.34	8.9E-02	0.20	3.3E-01	0.03	8.9E-01	-0.15	4.7E-01	-0.35	8.0E-02	0.12	5.7E-01	-0.19	3.6E-01	0.18	3.7E-01	0.07	7.3E-01	0.20	3.3E-01
ENSCAFG0000000532	TGDS	grey	EC_M10	0.34	8.9E-02	0.14	5.1E-01	-0.43	2.9E-02	0.15	4.7E-01	0.11	5.9E-01	-0.15	4.5E-01	0.08	6.9E-01	0.10	6.3E-01	-0.35	7.9E-02	-0.31	1.2E-01
ENSCAFG0000003018	ENSCAFG0000003018	grey	EC_M10	0.34	8.9E-02	-0.18	3.8E-01	-0.14	4.9E-01	-0.15	4.6E-01	0.06	7.7E-01	0.17	4.1E-01	-0.33	9.8E-02	0.09	6.5E-01	0.07	7.5E-01	-0.27	1.9E-01
ENSCAFG0000001952	SAPCD2	darkgrey	EC_M8	0.34	8.9E-02	0.32	1.2E-01	-0.11	5.9E-01	-0.19	3.6E-01	-0.17	4.1E-01	-0.13	5.2E-01	0.22	2.7E-01	-0.09	6.7E-01	0.20	3.2E-01	-0.01	9.7E-01
ENSCAFG0000002601	ENSCAFG0000002601	grey	EC_M10	0.34	8.9E-02	0.30	1.4E-01	-0.25	2.1E-01	-0.02	9.3E-01	-0.07	7.3E-01	0.04	8.3E-01	-0.22	2.8E-01	0.33	1.0E-01	0.09	6.8E-01	-0.15	4.7E-01
ENSCAFG0000000796	MRPS27	grey	EC_M10	0.34	8.9E-02	-0.15	4.6E-01	-0.39	5.2E-02	0.00	9.9E-01	0.34	9.3E-02	0.19	3.4E-01	-0.29	1.5E-01	0.03	8.7E-01	0.09	6.6E-01	-0.46	1.7E-02
ENSCAFG0000001542	KIAA0753	grey	EC_M10	0.34	8.9E-02	0.19	3.5E-01	0.29	1.5E-01	0.16	4.4E-01	-0.70	6.4E-05	0.02	9.1E-01	-0.16	4.4E-01	-0.05	8.0E-01	0.01	9.6E-01	0.51	8.2E-03
ENSCAFG0000001368	PIKFYVE	darkgrey	EC_M8	0.34	8.9E-02	0.42	3.4E-02	0.05	8.2E-01	0.16	4.4E-01	-0.57	2.6E-03	-0.14	4.8E-01	-0.18	3.7E-01	-0.41	3.5E-02	-0.17	4.2E-01	0.37	6.0E-02
ENSCAFG0000001255	PRKD1	turquoise	EC_M6	0.34	8.9E-02	0.77	3.9E-06	-0.52	6.9E-03	0.01	9.7E-01	0.10	6.4E-01	-0.01	9.7E-01	0.03	8.7E-01	0.09	6.7E-01	0.08	6.9E-01	-0.27	1.8E-01
ENSCAFG0000001504	CDA	turquoise	EC_M6	0.34	8.9E-02	0.88	2.4E-09	-0.39	4.7E-02	-0.14	5.0E-01	-0.10	6.2E-01	-0.16	4.5E-01	-0.15	4.7E-01	-0.09	6.8E-01	-0.16	4.3E-01	-0.07	7.4E-01
ENSCAFG0000001688	COQ6	grey	EC_M10	0.34	8.9E-02	-0.32	1.1E-01	0.22	2.9E-01	-0.01	9.5E-01	-0.35	8.1E-02	0.02	9.3E-01	-0.28	1.6E-01	-0.17	4.1E-01	0.11	6.0E-01	0.21	3.1E-01
ENSCAFG0000001383	SUPV3L1	grey	EC_M10	0.34	9.0E-02	-0.05	7.9E-01	-0.02	9.4E-01	-0.09	6.6E-01	-0.31	1.3E-01	-0.03	8.9E-01	0.22	2.7E-01	0.08	6.9E-01	-0.36	7.5E-02	0.11	5.9E-01
ENSCAFG0000002465	LRRC8B	grey	EC_M10	0.34	9.0E-02	0.54	4.2E-03	-0.56	3.1E-03	-0.10	6.4E-01	0.26	2.0E-01	-0.23	2.5E-01	-0.07	7.3E-01	0.09	6.5E-01	0.07	7.4E-01	-0.41	3.8E-02

ENSCAFG0000001064	PPP3CA	turquoise	EC_M6	0.34	9.0E-02	0.77	5.0E-06	-0.43	2.9E-02	-0.09	6.5E-01	-0.12	5.6E-01	0.11	5.9E-01	-0.07	7.2E-01	-0.02	9.3E-01	0.20	3.3E-01	-0.08	7.0E-01
ENSCAFG000000009	ERBB3	grey	EC_M10	0.34	9.0E-02	0.17	4.1E-01	-0.04	8.6E-01	0.25	2.1E-01	-0.30	1.3E-01	-0.26	2.0E-01	-0.34	8.9E-02	0.03	8.7E-01	-0.13	5.2E-01	0.11	6.0E-01
ENSCAFG0000000695	SP1	darkgrey	EC_M8	0.34	9.0E-02	0.13	5.1E-01	0.38	5.6E-02	0.23	2.6E-01	-0.84	9.4E-08	-0.10	6.3E-01	-0.16	4.5E-01	-0.02	9.3E-01	-0.05	8.2E-01	0.63	6.1E-04
ENSCAFG0000002021	IST1	darkgrey	EC_M8	0.34	9.0E-02	0.55	3.7E-03	-0.12	5.7E-01	-0.11	6.0E-01	-0.40	4.4E-02	-0.14	4.9E-01	-0.03	8.8E-01	0.10	6.3E-01	0.03	9.0E-01	0.22	2.9E-01
ENSCAFG0000000176	KLHDC3	grey	EC_M10	0.34	9.0E-02	0.12	5.5E-01	0.09	6.5E-01	0.00	9.9E-01	-0.42	3.3E-02	0.34	8.8E-02	-0.15	4.8E-01	-0.53	5.5E-03	-0.28	1.6E-01	0.23	2.7E-01
ENSCAFG0000001500	CDK16	turquoise	EC_M6	0.34	9.0E-02	0.59	1.4E-03	-0.57	2.4E-03	-0.22	2.8E-01	0.23	2.7E-01	0.06	7.5E-01	-0.19	3.6E-01	0.00	9.8E-01	-0.12	5.7E-01	-0.45	2.2E-02
ENSCAFG0000000753	EXOC6	turquoise	EC_M6	0.34	9.0E-02	0.77	4.2E-06	-0.33	9.6E-02	0.00	9.9E-01	-0.17	4.2E-01	-0.31	1.3E-01	-0.17	4.2E-01	0.03	8.8E-01	-0.04	8.3E-01	0.00	9.8E-01
ENSCAFG0000001507	HDC	grey	EC_M10	0.34	9.0E-02	0.06	7.6E-01	-0.43	2.8E-02	-0.05	8.2E-01	0.27	1.8E-01	-0.16	4.4E-01	-0.12	5.6E-01	-0.05	7.9E-01	-0.16	4.2E-01	-0.44	2.4E-02
ENSCAFG0000003263	ZNF283	grey	EC_M10	0.34	9.0E-02	0.22	2.8E-01	0.23	2.6E-01	0.19	3.6E-01	-0.62	7.3E-04	0.00	9.9E-01	-0.02	9.2E-01	-0.25	2.1E-01	0.11	5.8E-01	0.50	8.8E-03
ENSCAFG0000000371	ENSCAFG0000000371	turquoise	EC_M6	0.34	9.0E-02	0.59	1.4E-03	-0.31	1.3E-01	0.09	6.5E-01	-0.18	3.8E-01	0.10	6.1E-01	-0.22	2.7E-01	-0.05	7.9E-01	-0.18	3.9E-01	-0.03	8.7E-01
ENSCAFG0000000840	P2RX7	turquoise	EC_M6	0.34	9.0E-02	0.91	7.0E-11	-0.36	6.9E-02	0.13	5.4E-01	-0.20	3.4E-01	-0.14	4.8E-01	-0.11	5.9E-01	-0.18	3.9E-01	0.07	7.3E-01	0.02	9.3E-01
ENSCAFG0000001235	TATDN3	grey	EC_M10	0.34	9.1E-02	-0.08	6.9E-01	0.28	1.6E-01	0.20	3.3E-01	-0.63	5.2E-04	0.04	8.6E-01	-0.15	4.7E-01	-0.10	6.3E-01	0.09	6.8E-01	0.45	2.1E-02
ENSCAFG0000001324	SNX6	grey	EC_M10	0.34	9.1E-02	0.34	9.2E-02	-0.28	1.6E-01	0.23	2.7E-01	-0.06	7.8E-01	0.02	9.3E-01	-0.67	1.6E-04	-0.14	4.8E-01	-0.24	2.5E-01	-0.09	6.8E-01
ENSCAFG0000000095	RPS18	cyan	EC_M2	0.34	9.1E-02	-0.49	1.2E-02	0.31	1.2E-01	0.23	2.6E-01	-0.47	1.5E-02	0.14	4.8E-01	-0.07	7.5E-01	0.01	9.6E-01	-0.42	3.4E-02	0.23	2.7E-01
ENSCAFG0000000300	PPT1	turquoise	EC_M6	0.34	9.1E-02	0.61	9.0E-04	-0.30	1.4E-01	0.18	3.9E-01	-0.17	4.2E-01	-0.23	2.5E-01	-0.19	3.5E-01	-0.10	6.1E-01	0.03	8.8E-01	0.02	9.3E-01
ENSCAFG0000000851	PPP1CC	cyan	EC_M2	0.34	9.1E-02	-0.43	2.7E-02	0.41	3.8E-02	0.32	1.1E-01	-0.63	5.7E-04	0.13	5.2E-01	-0.04	8.5E-01	-0.24	2.4E-01	-0.12	5.7E-01	0.43	2.7E-02
ENSCAFG0000000017	ENSCAFG0000000017	grey	EC_M10	0.34	9.1E-02	0.10	6.1E-01	-0.03	9.0E-01	0.00	9.9E-01	-0.31	1.2E-01	-0.28	1.6E-01	-0.38	5.5E-02	0.28	1.7E-01	-0.07	7.5E-01	0.17	4.2E-01
ENSCAFG0000000470	TSN	grey	EC_M10	0.34	9.1E-02	0.12	5.7E-01	-0.16	4.2E-01	-0.03	8.7E-01	-0.04	8.5E-01	-0.11	6.1E-01	-0.27	1.8E-01	-0.17	4.1E-01	-0.22	2.7E-01	-0.11	5.9E-01
ENSCAFG0000000449	ERCC3	grey	EC_M10	0.34	9.1E-02	-0.11	5.9E-01	0.38	5.3E-02	0.05	8.2E-01	-0.64	4.2E-04	-0.14	5.0E-01	-0.18	3.8E-01	0.09	6.8E-01	0.20	3.4E-01	0.51	8.0E-03
ENSCAFG0000002157	ENSCAFG0000002157	turquoise	EC_M6	0.34	9.2E-02	0.37	6.4E-02	-0.14	4.9E-01	0.12	5.7E-01	-0.24	2.3E-01	0.10	6.2E-01	-0.01	9.6E-01	-0.30	1.4E-01	0.17	4.1E-01	0.04	8.3E-01
ENSCAFG0000000077	QKI	turquoise	EC_M6	0.34	9.2E-02	0.95	1.1E-13	-0.35	8.2E-02	-0.05	8.0E-01	-0.21	3.1E-01	-0.14	5.0E-01	-0.11	6.1E-01	-0.17	4.1E-01	0.01	9.6E-01	0.04	8.5E-01
ENSCAFG0000001373	TET1	turquoise	EC_M6	0.34	9.2E-02	0.45	2.1E-02	-0.47	1.6E-02	-0.08	7.1E-01	0.09	6.5E-01	0.17	4.1E-01	0.11	5.8E-01	-0.09	6.6E-01	0.47	1.6E-02	-0.25	2.2E-01
ENSCAFG0000000412	ENSCAFG0000000412	grey	EC_M10	0.34	9.2E-02	0.12	5.7E-01	-0.44	2.3E-02	-0.24	2.3E-01	0.19	3.6E-01	0.16	4.2E-01	-0.21	3.1E-01	-0.24	2.4E-01	-0.11	5.8E-01	-0.34	8.5E-02
ENSCAFG0000000533	CRNKL1	darkgrey	EC_M8	0.34	9.2E-02	0.27	1.8E-01	0.02	9.4E-01	0.19	3.5E-01	-0.50	9.2E-03	0.15	4.7E-01	-0.18	3.9E-01	-0.03	8.8E-01	0.15	4.7E-01	0.30	1.3E-01
ENSCAFG0000000476	VWA8	grey	EC_M10	0.34	9.2E-02	-0.25	2.1E-01	0.07	7.5E-01	0.11	6.0E-01	-0.25	2.3E-01	-0.07	7.5E-01	-0.27	1.8E-01	-0.20	3.3E-01	-0.52	6.8E-03	0.09	6.5E-01
ENSCAFG0000000846	ENSCAFG0000000846	grey	EC_M10	0.34	9.2E-02	0.05	7.9E-01	-0.14	5.0E-01	-0.09	6.5E-01	-0.11	5.9E-01	-0.17	4.1E-01	-0.11	5.8E-01	-0.14	5.0E-01	-0.12	5.7E-01	-0.05	8.2E-01
ENSCAFG0000000194	CBWD2	grey	EC_M10	0.34	9.2E-02	0.26	2.0E-01	-0.38	5.8E-02	-0.22	2.9E-01	0.11	6.0E-01	0.30	1.3E-01	-0.25	2.2E-01	-0.24	2.3E-01	-0.37	6.0E-02	-0.29	1.6E-01
ENSCAFG0000001879	BFAR	turquoise	EC_M6	0.34	9.2E-02	0.68	1.5E-04	-0.46	1.9E-02	0.02	9.2E-01	0.05	8.2E-01	0.09	6.5E-01	-0.40	4.3E-02	-0.33	9.7E-02	-0.16	4.3E-01	-0.21	3.1E-01
ENSCAFG0000000726	DNMT3B	turquoise	EC_M6	0.34	9.2E-02	0.61	9.4E-04	-0.06	7.9E-01	0.22	2.8E-01	-0.44	2.6E-02	-0.25	2.2E-01	-0.17	4.1E-01	-0.04	8.4E-01	0.02	9.4E-01	0.28	1.6E-01
ENSCAFG0000000740	TLR3	grey	EC_M10	0.34	9.2E-02	0.53	5.5E-03	-0.11	5.8E-01	0.34	9.0E-02	-0.36	6.9E-02	-0.01	9.5E-01	-0.34	8.6E-02	0.02	9.1E-01	-0.03	8.7E-01	0.17	4.2E-01
ENSCAFG0000000632	KL	grey	EC_M10	0.34	9.2E-02	-0.18	3.9E-01	-0.14	4.8E-01	-0.09	6.7E-01	0.04	8.5E-01	0.62	7.7E-04	-0.05	8.2E-01	-0.06	7.7E-01	-0.18	3.7E-01	-0.19	3.4E-01
ENSCAFG0000001078	GPATCH2	grey	EC_M10	0.34	9.3E-02	0.13	5.4E-01	-0.38	5.2E-02	-0.11	5.8E-01	0.05	8.2E-01	0.36	7.0E-02	0.08	6.8E-01	0.25	2.1E-01	-0.10	6.2E-01	-0.28	1.7E-01
ENSCAFG0000002541	ENSCAFG0000002541	grey	EC_M10	0.34	9.3E-02	0.34	8.7E-02	-0.37	6.0E-02	-0.09	6.6E-01	0.02	9.3E-01	-0.12	5.7E-01	-0.06	7.6E-01	-0.14	4.8E-01	0.25	2.3E-01	-0.21	3.0E-01
ENSCAFG0000001939	ZMYND19	grey	EC_M10	0.34	9.3E-02	0.23	2.5E-01	-0.41	3.7E-02	-0.08	7.1E-01	0.20	3.2E-01	0.06	7.6E-01	-0.05	8.2E-01	-0.14	5.0E-01	-0.08	6.9E-01	-0.36	6.7E-02
ENSCAFG0000001596	MTHFD1	grey	EC_M10	0.34	9.3E-02	-0.47	1.5E-02	-0.34	8.7E-02	0.03	9.0E-01	0.37	6.0E-02	0.17	4.1E-01	-0.09	6.5E-01	-0.02	9.2E-01	-0.32	1.1E-01	-0.56	2.8E-03
ENSCAFG0000001642	WNK3	grey	EC_M10	0.34	9.3E-02	0.26	2.0E-01	-0.51	8.1E-03	-0.02	9.2E-01	0.18	3.8E-01	-0.09	6.7E-01	0.20	3.4E-01	0.10	6.3E-01	-0.16	4.4E-01	-0.39	4.9E-02
ENSCAFG0000001112	RAB3GAP2	grey	EC_M10	0.34	9.3E-02	0.27	1.8E-01	-0.60	1.3E-03	-0.15	4.7E-01	0.31	1.2E-01	0.22	2.8E-01	0.00	1.0E+00	-0.21	3.1E-01	-0.10	6.1E-01	-0.45	2.0E-02
ENSCAFG0000001727	DIS3L	darkgrey	EC_M8	0.34	9.3E-02	-0.06	7.8E-01	0.16	4.4E-01	0.10	6.3E-01	-0.53	5.7E-03	0.10	6.2E-01	-0.23	2.7E-01	-0.11	6.0E-01	-0.29	1.5E-01	0.31	1.3E-01
ENSCAFG0000001992	NUP214	turquoise	EC_M6	0.34	9.3E-02	0.32	1.1E-01	0.05	8.0E-01	-0.09	6.6E-01	-0.40	4.1E-02	-0.12	5.7E-01	-0.21	3.0E-01	-0.45	2.2E-02	-0.33	9.9E-02	0.23	2.6E-01
ENSCAFG0000001313	TLK2	grey	EC_M10	0.34	9.3E-02	0.25	2.2E-01	-0.29	1.4E-01	0.17	4.1E-01	-0.08	6.8E-01	-0.12	5.5E-01	-0.06	7.5E-01	0.07	7.4E-01	-0.44	2.4E-02	-0.08	6.9E-01
ENSCAFG0000001991	MTHFSD	darkgreen	EC_M4	0.34	9.3E-02	0.08	6.8E-01	-0.67	1.7E-04	-0.05	8.0E-01	0.47	1.6E-02	0.26	2.0E-01	-0.04	8.5E-01	-0.11	5.9E-01	0.04	8.3E-01	-0.64	4.5E-04
ENSCAFG0000000830	MEF2C	turquoise	EC_M6	0.34	9.3E-02	0.82	3.0E-07	-0.38	5.7E-02	-0.13	5.2E-01	-0.10	6.4E-01	-0.14	5.0E-01	-0.20	3.2E-01	-0.31	1.2E-01	0.20	3.2E-01	-0.06	7.7E-01
ENSCAFG0000000140	MKLN1	darkgreen	EC_M4	0.34	9.4E-02	0.40	4.2E-02	-0.78	2.5E-06	-0.16	4.5E-01	0.44	2.4E-02	0.05	8.2E-01	0.05	8.2E-01	0.07	7.4E-01	0.00	9.9E-01	-0.62	6.9E-04
ENSCAFG0000001631	HUWE1	grey	EC_M10	0.34	9.4E-02	0.23	2.6E-01	-0.54	4.2E-03	-0.11	5.9E-01	0.28	1.6E-01	-0.01	9.8E-01	-0.22	2.8E-01	-0.18	3.8E-01	-0.39	4.7E-02	-0.49	1.1E-02
ENSCAFG0000000154	SNAPC3	grey	EC_M10	0.34	9.4E-02	-0.06	7.7E-01	-0.07	7.2E-01	-0.05	8.1E-01	-0.23	2.7E-01	0.10	6.2E-01	-0.35	7.7E-02	0.21	2.9E-01	0.30	1.4E-01	0.00	1.0E+00
ENSCAFG0000002031	PRKACB	grey	EC_M10	0.34	9.4E-02	0.19	3.5E-01	-0.74	1.8E-05	-0.02	9.3E-01	0.49	1.0E-02	0.07	7.3E-01	-0.08	6.9E-01	-0.03	8.9E-01	0.43	2.9E-02	-0.62	7.5E-04
ENSCAFG0000000623	ACPP	darkgreen	EC_M4	0.34	9.4E-02	0.46	1.8E-02	-0.87	1.2E-08	-0.22	2.8E-01	0.59	1.6E-03	0.18	3.7E-01	-0.13	5.3E-01	0.03	8.7E-01	-0.23	2.5E-01	-0.78	2.5E-06
ENSCAFG0000000986	ENSCAFG0000000986	turquoise	EC_M6	0.34	9.4E-02	0.70	6.6E-05	-0.25	2.1E-01	-0.11	5.9E-01	-0.17	4.0E-01	-0.07	7.2E-01	-0.03	8.7E-01	-0.07	7.5E-01	0.02	9.2E-01	0.03	8.7E-01
ENSCAFG0000002134	ENSCAFG0000002134	turquoise	EC_M6	0.34	9.4E-02	0.70	6.6E-05	-0.25	2.1E-01	-0.11	5.9E-01	-0.17	4.0E-01	-0.07	7.2E-01	-0.03	8.7E-01	-0.07	7.5E-01	0.02	9.2E-01	0.03	8.7E-01
ENSCAFG0000000470	ENSCAFG0000000470	grey	EC_M10	0.34	9.4E-02	-0.20	3.2E-01	-0.46	1.7E-02	0.32	1.2E-01	0.35	7.9E-02	0.36	7.3E-02	-0.01	9.6E-01	0.01	9.6E-01	-0.25	2.2E-01	-0.53	5.3E-03
ENSCAFG0000001868	GTF2F1	grey	EC_M10	0.34	9.4E-02	-0.11	5.8E-01	-0.57	2.2E-03	-0.04	8.5E-01	0.48	1.3E-02	0.20	3.2E-01	0.12	5.5E-01	0.09	6.7E-01	-0.10	6.3E-01	-0.68	1.4E-04
ENSCAFG0000000445	NUDT15	grey	EC_M10	0.34	9.4E-02	-0.21	3.0E-01	-0.34	8.6E-02	0.09	6.5E-01	0.19	3.4E-01	0.16	4.4E-01	-0.01	9.6E-01	-0.17	4.2E-01	-0.40	4.3E-02	-0.43	2.7E-02
ENSCAFG0000001430	ATIC	cyan	EC_M2	0.34	9.4E-02	-0.15	4.6E-01	0.44	2.4E-02	0.16	4.4E-01	-0.67	1.8E-04	-0.16	4.3E-01	0.01	9.8E-01	-0.06	7.9E-01	-0.38	5.3E-02	0.48	1.3E-02
ENSCAFG0000003225	NMNAT1	turquoise	EC_M6	0.34	9.4E-02	0.39	4.8E-02	-0.31	1.3E-01	-0.18	3.8E-01	-0.05	8.3E-01	0.18	3.9E-01	-0.34	9.4E-02	0.10	6.1E-01	-0.37	6.1E-02	-0.13	5.3E-01
ENSCAFG0000001267	SCFD1	darkgreen	EC_M4	0.34	9.4E-02	0.17	3.9E-01	-0.91	2.0E-10	-0.24	2.4E-01	0.68	1.2E-04	0.33	1.0E-01	-0.02	9.3E-01	0.01	9.6E-01	-0.11	5.8E-01	-0.88	3.1E-09
ENSCAFG0000001263	DPP3	darkgrey	EC_M8	0.34	9.4E-02	0.07	7.4E-01	-0.26	1.9E-01	-0.19	3.5E-01	0.10	6.2E-01	-0.14	5.0E-01	-0.11	5.9E-01	0.19	3.6E-01	-0.02	9.3E-01	-0.27	1.8E-01
ENSCAFG0000001345	ATP13A3	grey	EC_M10	0.33	9.4E-02	-0.24	2.3E-01	-0.38	5.6E-02	-0.13	5.1E-01	0.18	3.7E-01	0.15	4.6E-01	0.22	2.8E-01	0.02	9.3E-01	-0.09	6.7E-01	-0.38	5.5E-02
ENSCAFG0000001616	BID	turquoise	EC_M6	0.33	9.4E-02	0.53	4.9E-03	-0.42	3.4E-02	0.23	2.6E-01	0.00	9.8E-01	0.10	6.4E-01	-0.05	8.2E-01	-0.38	5.8E-02	-0.28	1.7E-01	-0.16	4.3E-01
ENSCAFG0000000735	SGK3	darkgrey	EC_M8	0.33	9.4E-02	0.33	9.9E-02	0.13	5.4E-01	0.19	3.4E-01	-0.62	8.0E-04	-0.34	8.5E-02	-0.17	4.1E-01	-0.05	8.2E-01	-0.15	4.6E-01	0.45	2.1E-02
ENSCAFG0000001585	ENSCAFG0000001585	grey	EC_M10	0.33	9.5E-02	-0.30	1.4E-01	-0.37	6.4E-02	-0.13	5.4E-01	0.23	2.7E-01	0.34	8.9E-02	0.27	1.9E-01	-0.14	5.1E-01	-0.27	1.9E-01	-0.43	2.7E-02
ENSCAFG0000001626	PSMD3	grey	EC_M10	0.33	9.5E-02	0.44	2.4E-02	-0.41	3.7E-02	-0.30	1.3E-01	0.17	4.0E-01	0.22	2.8E-01	-0.04	8.5E-01	-0.17	4.1E-01	-0.26	2.1E-01	-0.32	1.1E-01
ENSCAFG0000001515	C7H1orf112	grey	EC_M10	0.33	9.5E-02	-0.41	3.6E-02	-0.19	3.5E-01	-0.35	8.3E-02	0.18	3.9E-01	0.16	4.2E-01	-0.10	6.4E-01	-0.03	9.0E-01	0.10	6.2E-01	-0.34	8.8E-02
ENSCAFG0000003184	CGRRF1	grey	EC_M10	0.33	9.5E-02	0.20	3.2E-01	-0.42	3.2E-02	0.15	4.6E-01	0.04	8.6E-01	0.15	4.6E-01	-0.39	4.9E-02	-0.39	4.9E-02	0.21	2.9E-01	-0.22	2.7E-01
ENSCAFG0000000997	RBBP5	turquoise	EC_M6	0.33	9.5E-02	0.71	5.7E-05	-0.29	1.5E-01	-0.10	6.4E-01	-0.23	2.5E-01	-0.16	4.3E-01	-0.14	5.0E-01	0.17	4.1E-01	-0.21	3.0E-01	0.07	7.5E-01
ENSCAFG0000001543	ENSCAFG0000001543	grey	EC_M10	0.33	9.5E-02	-0.34	9.4E-02	0.21	2.9E-01	0.24	2.3E-01	-0.38	5.8E-02	0.05	8.2E-01	-0.18	3.7E-01	-0.26	2.0E-01	-0.68	1.4E-04	0.19	3.6E-01
ENSCAFG0000000821	BTF3	cyan	EC_M2	0.33	9.5E-02	-0.28	1.6E-01	0.24	2.4E-01	0.05	8.2E-01	-0.45	2.0E-02	0.03	8.9E-01	0.07	7.3E-01	0.02	9.4E-01	-0.54	4.7E-03	0.22	2.8E-01
ENSCAFG0000000614	ASH2L	turquoise	EC_M6	0.33	9.5E-02	0.39	5.0E-02	-0.20	3.3E-01	0.13	5.4E-01	-0.14	4.9E-01	0.31	1.2E-01	-0.13	5.3E-01	-0.02	9.4E-01	0.14	4.8E-01	0.00	1.0E+00
ENSCAFG0000000845	CELF1	darkgreen	EC_M4	0.33	9.5E-02	0.32	1.1E-01	-0.86	2.4E-08	-0.16	4.2E-01	0.64	4.2E-04	0.19	3.5E-01	-0.05	8.3E-01	-0.02	9.3E-01	0.00	9.8E-01	-0.83	1.6E-07
ENSCAFG0000000842	COPB1	darkgreen	EC_M4	0.33	9.5E-02	-0.02	9.2E-01	-0.84	7.8E-08	-0.08	7.0E-01	0.72	3.9E-05	0.33	1.0E-01	-0.19	3.5E-01	-0.07	7.2E-01	-0.32	1.1E-01	-0.89	8.0E-10
ENSCAFG0000001727	ENSCAFG0000001727	turquoise	EC_M6	0.33	9.6E-02	0.85	5.2E-08	-0.31	1.2E-01	-0.15	4.6E-01	-0.17	4.0E-01	0.09	6.6E-01	0.06	7.5E-01	-0.11	5.8E-01	-0.23	2.6E-01	-0.02	9.1E-01
ENSCAFG0000001241	NSMCE4A	grey	EC_M10	0.33	9.6E-02	0.34	9.3E-02	0.24	2.4E-01	-0.09	6.8E-01	-0.64	4.4E-04	-0.01	9.6E-01	0.08	7.1E-01	-0.09	6.6E-01	-0.13	5.4E-01	0.49	1.2E-02
ENSCAFG0000000652	FRMD4B	turquoise	EC_M6	0.33	9.6E-02	0.88	2.2E-09	-0.54	4.6E-03	-0.20	3.2E-01	0.08	7.0E-01	-0.20	3.3E-01	-0.08	6.8E-01	0.08	6.9E-01	0.06	7.8E-01	-0.25	2.3E-01
ENSCAFG0000001190	ASB9	turquoise	EC_M6	0.33	9.6E-02	0.68	1.3E-04	-0.30	1.3E-01	0.02	9.4E-01	-0.13	5.4E-01	-0.30	1.3E-01	-0.06	7.6E-01	-0.01	9.5E-01	0.01	9.6E-01	-0.05	7.9E-01
ENSCAFG0000001036	MCEE	grey	EC_M10	0.33	9.6E-02	-0.05	8.2E-01	0.18	3.9E-01	0.21	3.0E-01	-0.51	7.6E-03	-0.06	7.6E-01	-0.09	6.8E-01	-0.23	2.6E-01	0.06	7.7E-01	0.33	1.0E-01
ENSCAFG0000001203	LCMT2	grey	EC_M10	0.33	9.6E-02	0.15	4.7E-01	0.05	8.2E-01	-0.13	5.3E-01	-0.31	1.2E-01	0.07	7.2E-01	-0.26	2.1E-01	-0.22	2.8E-01	-0.18	3.8E-01	0.18	3.9E-01
ENSCAFG0000000981	SFXN3	grey	EC_M10	0.33	9.6E-02	-0.47	1.5E-02	0.36	6.7E-02	0.05	8.1E-01	-0.43	2.8E-02	0.06	7.7E-01	-0.26	2.1E-01	-0.09	6.7E-01	-0.32	1.1E-01	0.25	2.3E-01
ENSCAFG0000000375	ENSCAFG0000000375	grey	EC_M10	0.33	9.7E-02	-0.20	3.4E-01	-0.08	7.0E-01	0.55	3.9E-03	-0.07	7.3E-01	0.56	2.7E-03	-0.08	6.8E-01	-0.12	5.8E-01	-0.21	3.0E-01	-0.09	6.7E-01
ENSCAFG0000002660	ENSCAFG0000002660	grey	EC_M10	0.33	9.7E-02	0.16	4.3E-01	-0.18	3.8E-01	-0.13	5.4E-01	-0.17	4.0E-01	0.42	3.5E-02	-0.21	3.1E-01	-0.22	2.7E-01	-0.28	1.6E-01	-0.02	9.0E-01
ENSCAFG0000000366	PNPLA8	grey	EC_M10	0.33	9.7E-02	-0.01	9.6E-01	-0.53	5.6E-03	-0.11	6.0E-01	0.35	7.6E-02	0.09	6.5E-01	-0.02	9.4E-01	-0.15	4.5E-01	-0.24	2.3E-01	-0.50	9.0E-03
ENSCAFG0000001756	SMAD2	turquoise	EC_M6	0.33	9.7E-02	0.51	8.2E-03	-0.39	4.7E-02	-0.21	2.9E-01	0.03	8.9E-01	0.10	6.2E-01	-0.29	1.4E-01	-0.27	1.9E-01	-0.47	1.5E-02	-0.18	3.7E-01
ENSCAFG0000000701	ENSCAFG0000000701	grey	EC_M10	0.33	9.7E-02	-0.07	7.5E-01	-0.17	4.1E-01	-0.21	2.9E-01	-0.07	7.2E-01	-0.17	4.1E-01	-0.29	1.5E-01	-0.28	1.6E-01	-0.24	2.3E-01	-0.07	7.5E-01
ENSCAFG0000003056	PDCL	turquoise	EC_M6	0.33	9.7E-02	0.64	4.2E-04	-0.44	2.3E-02	0.19	3.5E-01	-0.02	9.3E-01	0.05	8.3E-01	-0.26	2.1E-01	-0.31	1.3E-01	0.15	4.5E-01	-0.12	5.6E-01
ENSCAFG0000001011	MTRR	grey	EC_M10	0.33	9.8E-02	-0.32	1.1E-01	-0.36	6.9E-02	-0.10	6.3E-01	0.26	2.0E-01	-0.04	8.5E-01	-0.16	4.3E-01	0.10	6.2E-01	-0.26	1.9E-01	-0.46	1.7E-02
ENSCAFG0000001333	ENSCAFG0000001333	turquoise	EC_M6	0.33	9.8E-02	0.63	5.1E-04	-0.30	1.4E-01	-0.13	5.4E-01	-0.17	4.1E-01	-0.09	6.8E-01	-0.08	6.8E-01	-0.10	6.1E-01	-0.13	5.4E-01	-0.01	9.8E-01
ENSCAFG0000001891	RMI2	darkgrey	EC_M8	0.33	9.8E-02	0.06	7.8E-01	-0.11	5.9E-01	-0.08	7.1E-01	-0.06	7.8E-01	-0.15	4.8E-01	-0.09	6.5E-01	0.29	1.6E-01	-0.08	7.0E-01	-0.11	6.1E-01
ENSCAFG0000000755	PDCD5	grey	EC_M10	0.33	9.8E-02	-0.51	7.4E-03	-0.16	4.3E-01	-0.20	3.3E-01	0.16	4.5E-01	0.20	3.2E-01	-0.23	2.5E-01	0.21	3.1E-01	-0.08	7.1E-01	-0.35	8.2E-02
ENSCAFG0000000831	ANAPC5	grey	EC_M10	0.33	9.8E-02	-0.35	7.8E-02	0.20	3.2E-01	0.30	1.4E-01	-0.33	1.0E-01	-0.07	7.4E-01	-0.43	3.0E-02	-0.19	3.6E-01	-0.13	5.2E-01	0.15	4.5E-01
ENSCAFG0000003230	ZNF473	grey	EC_M10	0.33	9.8E-02	0.17	4.2E-01	-0.17	4.0E-01	-0.13	5.4E-01	-0.14	5.0E-01	-0.22	2.8E-01	0.08	6.8E-01	0.28	1.7E-01	0.10	6.2E-01	-0.04	8.3E-01
ENSCAFG0000000644	TEX15	grey	EC_M10	0.33	9.8E-02	-0.09	6.6E-01	-0.61	9.4E-04	0.12	5.5E-01	0.44	2.4E-02	0.35	7.9E-02	-0.13	5.2E-01	-0.11	5.9E-01	0.02	9.3E-01	-0.63	6.4E-04
ENSCAFG0000001600	RPL9	cyan	EC_M2	0.33	9.8E-02	-0.26	2.1E-01	0.16	4.4E-01	0.14	5.0E-01	-0.39	4.9E-02	0.13	5.4E-01	0.19	3.5E-01	0.14	5.0E-01	-0.47	1.5E-02	0.14	5.0E-01
ENSCAFG0000002015	RPL5	cyan	EC_M2	0.33	9.8E-02	-0.29	1.6E-01	0.32	1.1E-01	0.17	4.2E-01	-0.54	4.4E-03	0.01	9.5E-01	0.11	5.8E-01	-0.07	7.4E-01	-0.61	1.0E-03	0.31	1.3E-01
ENSCAFG0000000652	ZNF382	grey	EC_M10	0.33	9.9E-02	0.34	9.0E-02	-0.26	2.0E-01	0.06	7.7E-01	-0.15	4.7E-01	-0.02	9.1E-01	0.07	7.2E-01	0.04	8.5E-01	-0.27	1.8E-01	-0.04	8.6E-01
ENSCAFG0000000902	ARL13B	grey	EC_M10	0.33	9.9E-02	-0.59	1.4E-03	-0.12	5.5E-01	0.29	1.5E-01	-0.01	9.5E-01	0.27	1.7E-01	-0.20	3.3E-01	-0.11	5.9E-01	0.01	9.7E-01	-0.20	3.3E-01
ENSCAFG0000000027	HEBP2	grey	EC_M10	0.33	9.9E-02	0.29	1.4E-01	-0.08	6.8E-01	0.15	4.5E-01	-0.26	2.1E-01	0.00	9.9E-01	0.07	7.4E-01	0.02	9.2E-01	-0.37	6.3E-02	0.04	8.4E-01
ENSCAFG0000001794	ENSCAFG0000001794	turquoise	EC_M6	0.33	9.9E-02	0.83	1.7E-07	-0.41	3.8E-02	-0.14	4.8E-01	-0.05	8.1E-01	0.01	9.5E-01	-0.17	4.2E-01	-0.15	4.8E-01	0.00	1.0E+00	-0.13	5.4E-01
ENSCAFG0000002616	ENSCAFG0000002616	turquoise	EC_M6	0.33	9.9E-02	0.70	7.4E-05	-0.23	2.5E-01	-0.03	8.8E-01	-0.21	3.0E-01	-0.15	4.7E-01	-0.14	5.0E-01	-0.06	7.8E-01	-0.16	4.4E-01	0.04	8.5E-01
ENSCAFG0000001327	DUSP16	turquoise	EC_M6	0.33	9.9E-02	0.66	2.8E-04	-0.37	6.5E-02	-0.18	3.7E-01	-0.09	6.6E-01	-0.02	9.3E-01	0.03	8.8E-01	0.05	8.0E-01	0.32	1.1E-01	-0.06	7.7E-01
ENSCAFG0000000175	POT1	darkgrey	EC_M8	0.33	9.9E-02	0.03	8.9E-01	0.31	1.2E-01	0.09	6.7E-01	-0.74	1.4E-05	0.04	8.6E-01	-0.24	2.5E-01	0.02	9.3E-01	-0.01	9.6E-01	0.54	4.5E-03
ENSCAFG0000000713	MIPEP	grey	EC_M10	0.33	9.9E-02	-0.45	2.0E-02	0.16	4.4E-01	0.00	1.0E+00	-0.31	1.3E-01	-0.14	4.9E-01	-0.36	6.8E-02	0.13	5.2E-01	-0.22	2.7E-01	0.12	5.6E-01
ENSCAFG0000003085	ENSCAFG0000003085	grey	EC_M10	0.33	9.9E-02	-0.06	7.6E-01	-0.45	2.2E-02	-0.17	4.0E-01	0.24	2.4E-01	0.16	4.3E-01	0.07	7.3E-01	-0.17	3.9E-01	-0.20	3.2E-01	-0.42	3.2E-02
ENSCAFG0000000437	ZNF786	grey	EC_M10	0.33	9.9E-02	-0.01	9.6E-01	0.36	6.8E-02	0.13	5.3E-01	-0.69	1.1E-04	0.12	5.5E-01	-0.04	8.5E-01	-0.21	3.0E-01	-0.12	5.6E-01	0.51	7.2E-03
ENSCAFG0000001854	MCTS1	grey	EC_M10	0.33	9.9E-02	-0.06	7.7E-01	0.17	4.2E-01	0.25	2.1E-01	-0.52	6.9E-03	0.08	7.0E-01	-0.14	5.0E-01	-0.03	9.0E-01	-0.38	5.9E-02	0.26	2.0E-01
ENSCAFG0000001655	ZC4H2	turquoise	EC_M6	0.33	9.9E-02	0.56	2.8E-03	-0.57	2.3E-03	-0.20	3.3E-01	0.20	3.3E-01	0.20	3.3E-01	-0.01	9.7E-01	0.06	7.7E-01	-0.31	1.3E-01	-0.40	4.4E-02
ENSCAFG0000002508	PHOSPHO2	grey	EC_M10	0.33	9.9E-02	-0.48	1.3E-02	-0.09	6.6E-01	0.19	3.4E-01	-0.01	9.7E-01	0.38	5.8E-02	0.03	8.9E-01	-0.28	1.6E-01	-0.26	2.0E-01	-0.20	3.4E-01
ENSCAFG0000000414	ENSCAFG0000000414	turquoise	EC_M6	0.33	9.9E-02	0.70	8.1E-05	-0.33	9.8E-02	0.04	8.5E-01	-0.17	4.2E-01	-0.24	2.4E-01	-0.21	3.1E-01	0.16	4.3E-01	-0.12	5.5E-01	0.01	9.5E-01
ENSCAFG0000002969	ENSCAFG0000002969	turquoise	EC_M6	0.33	9.9E-02	0.59	1.5E-03	-0.58	1.8E-03	-0.06	7.6E-01	0.16	4.3E-01	0.07	7.2E-01	-0.13	5.1E-01	-0.26	1.9E-01	0.32	1.1E-01	-0.31	1.2E-01
ENSCAFG0000000739	CENPK	grey	EC_M10	0.33	9.9E-02	-0.03	9.0E-01	-0.51	7.3E-03	-0.24	2.4E-01	0.41	3.8E-02	0.11	6.0E-01	-0.10	6.4E-01	-0.23	2.5E-01	-0.19	3.6E-01	-0.62	7.4E-04
ENSCAFG0000000949	DENND2C	grey	EC_M10	0.33	9.9E-02	-0.49	1.2E-02	-0.18	3.7E-01	-0.01	9.6E-01	0.18	3.9E-01	0.23	2.5E-01	0.01	9.6E-01	0.03	8.9E-01	0.00	1.0E+00	-0.38	5.7E-02
ENSCAFG0000001347	HERC4	grey	EC_M10	0.33	9.9E-02	-0.06	7.6E-01	-0.64	4.0E-04	-0.02	9.4E-01	0.43	2.6E-02	0.17	4.0E-01	-0.06	7.7E-01	-0.14	4.9E-01	-0.19	3.7E-01	-0.62	7.8E-04
ENSCAFG0000002476	ENSCAFG0000002476	grey	EC_M10	0.33	9.9E-02	0.42	3.4E-02	-0.29	1.4E-01	-0.06	7.6E-01	-0.03	9.0E-01	-0.15	4.6E-01	-0.18	3.9E-01	-0.06	7.6E-01	-0.10	6.1E-01	-0.14	4.9E-01
ENSCAFG0000000472	NUP85	darkgrey	EC_M8	0.33	9.9E-02	0.24	2.4E-01	-0.34	9.2E-02	-0.31	1.2E-01	0.16	4.2E-01	0.29	1.5E-01	-0.35	8.1E-02	-0.05	8.0E-01	0.06	7.6E-01	-0.29	1.5E-01
ENSCAFG0000001601	FSCN1	turquoise	EC_M6	0.33	9.9E-02	0.57	2.4E-03	-0.36	7.3E-02	-0.22	2.9E-01	0.00	9.9E-01	-0.13	5.2E-01	-0.25	2.1E-01	0.12	5.5E-01	0.33	9.6E-02	-0.17	4.1E-01
ENSCAFG0000002019	LRRC8C	turquoise	EC_M6	0.33	1.0E-01	0.68	1.4E-04	-0.57	2.2E-03	-0.31	1.3E-01	0.21	3.1E-01	-0.04	8.5E-01	-0.29	1.6E-01	-0.14	4.8E-01	-0.17	3.9E-01	-0.37	6.0E-02
ENSCAFG0000000399	BBS12	grey	EC_M10	0.33	1.0E-01	-0.57	2.6E-03	-0.02	9.1E-01	0.07	7.5E-01	-0.06	7.6E-01	0.04	8.4E-01	-0.34	8.7E-02	-0.21	3.0E-01	-0.13	5.2E-01	-0.13	5.4E-01
ENSCAFG0000001056	CFAP43	grey	EC_M10	0.33	1.0E-01	-0.17	3.9E-01	-0.22	2.7E-01	-0.16	4.3E-01	0.01	9.8E-01	0.37	5.9E-02	-0.11	5.8E-01	-0.17	4.0E-01	-0.09	6.5E-01	-0.16	4.5E-01
ENSCAFG0000000913	MMS19	cyan	EC_M2	0.33	1.0E-01	0.42	3.2E-02	0.40	4.1E-02	0.21	3.1E-01	-0.91	6.7E-11	-0.21	3.0E-01	-0.11	5.8E-01	-0.16	4.4E-01	-0.05	7.9E-01	0.71	5.5E-05

ENSCAFG0000003031	ENSCAFG0000003031	darkgreen	EC_M4	0.33	1.0E-01	0.05	8.1E-01	-0.76	5.4E-06	-0.09	6.7E-01	0.58	1.7E-03	0.19	3.4E-01	-0.27	1.8E-01	0.09	6.6E-01	-0.01	9.7E-01	-0.78	3.0E-06
ENSCAFG0000000644	RYBP	grey	EC_M10	0.33	1.0E-01	0.27	1.8E-01	-0.54	4.1E-03	-0.40	4.2E-02	0.19	3.6E-01	0.02	9.2E-01	0.02	9.3E-01	-0.02	9.1E-01	0.13	5.2E-01	-0.38	5.8E-02
ENSCAFG0000001120	RNF115	grey	EC_M10	0.33	1.0E-01	0.08	7.1E-01	0.00	9.8E-01	-0.06	7.9E-01	-0.23	2.6E-01	0.10	6.3E-01	-0.34	9.2E-02	-0.32	1.1E-01	-0.42	3.3E-02	0.03	8.9E-01
ENSCAFG0000000353	ENSCAFG0000000353	turquoise	EC_M6	0.33	1.0E-01	0.95	1.2E-13	-0.37	6.3E-02	-0.14	4.9E-01	-0.17	4.0E-01	-0.08	6.9E-01	-0.13	5.2E-01	-0.12	5.6E-01	0.05	8.2E-01	0.01	9.6E-01
ENSCAFG0000001065	STK17B	grey	EC_M10	0.33	1.0E-01	-0.44	2.3E-02	-0.25	2.2E-01	0.17	4.2E-01	0.10	6.3E-01	0.46	1.8E-02	0.19	3.6E-01	-0.21	3.0E-01	-0.06	7.8E-01	-0.32	1.1E-01
ENSCAFG0000002327	KRT39	turquoise	EC_M6	0.33	1.0E-01	0.91	1.7E-10	-0.20	3.2E-01	0.01	9.7E-01	-0.34	9.2E-02	-0.14	4.9E-01	-0.17	4.2E-01	-0.14	5.0E-01	-0.24	2.5E-01	0.17	4.2E-01
ENSCAFG0000002145	ENSCAFG0000002145	grey	EC_M10	0.33	1.0E-01	-0.04	8.3E-01	-0.11	5.9E-01	0.26	1.9E-01	-0.14	5.1E-01	-0.12	5.8E-01	-0.19	3.4E-01	0.32	1.1E-01	-0.07	7.3E-01	-0.07	7.5E-01
ENSCAFG0000000358	PRMT1	grey	EC_M10	0.33	1.0E-01	-0.12	5.5E-01	-0.51	8.2E-03	-0.20	3.2E-01	0.47	1.4E-02	0.22	2.7E-01	0.04	8.4E-01	0.07	7.3E-01	-0.02	9.1E-01	-0.66	2.6E-04
ENSCAFG0000003180	RPP30	grey	EC_M10	0.33	1.0E-01	-0.25	2.2E-01	-0.04	8.4E-01	0.38	5.6E-02	-0.18	3.7E-01	0.25	2.1E-01	-0.31	1.2E-01	0.09	6.8E-01	0.31	1.3E-01	-0.01	9.7E-01
ENSCAFG0000000552	P4HA3	grey	EC_M10	0.33	1.0E-01	-0.10	6.2E-01	-0.02	9.3E-01	0.28	1.6E-01	-0.23	2.5E-01	-0.08	7.0E-01	-0.35	7.7E-02	-0.05	7.9E-01	-0.14	5.0E-01	0.00	9.9E-01
ENSCAFG0000000780	OCLN	turquoise	EC_M6	0.33	1.0E-01	0.92	2.4E-11	-0.31	1.2E-01	-0.11	5.8E-01	-0.21	3.0E-01	-0.13	5.2E-01	-0.13	5.2E-01	-0.12	5.7E-01	-0.01	9.4E-01	0.04	8.3E-01
ENSCAFG0000001094	MCTP2	turquoise	EC_M6	0.33	1.0E-01	0.94	4.1E-13	-0.32	1.1E-01	-0.10	6.3E-01	-0.22	2.7E-01	-0.12	5.7E-01	-0.11	6.0E-01	-0.08	7.0E-01	-0.14	4.8E-01	0.06	7.6E-01
ENSCAFG0000000314	ENSCAFG0000000314	grey	EC_M10	0.33	1.0E-01	0.18	3.8E-01	-0.28	1.7E-01	-0.02	9.1E-01	0.00	1.0E+00	-0.11	5.8E-01	-0.15	4.6E-01	-0.09	6.6E-01	-0.13	5.3E-01	-0.18	3.7E-01
ENSCAFG0000003108	RGS8	grey	EC_M10	0.33	1.0E-01	0.18	3.8E-01	-0.28	1.7E-01	-0.02	9.1E-01	0.00	1.0E+00	-0.11	5.8E-01	-0.15	4.6E-01	-0.09	6.6E-01	-0.13	5.3E-01	-0.18	3.7E-01
ENSCAFG0000000225	NOA1	grey	EC_M10	0.33	1.0E-01	0.07	7.2E-01	-0.35	7.9E-02	-0.11	5.9E-01	0.17	3.9E-01	0.14	4.8E-01	-0.26	2.0E-01	0.30	1.4E-01	0.01	9.5E-01	-0.38	5.3E-02
ENSCAFG0000001257	ZNF148	turquoise	EC_M6	0.33	1.0E-01	0.34	9.2E-02	-0.33	1.0E-01	0.10	6.3E-01	-0.02	9.2E-01	0.08	7.0E-01	-0.15	4.6E-01	-0.12	5.7E-01	0.49	1.0E-02	-0.08	6.8E-01
ENSCAFG0000000142	CPNE5	grey	EC_M10	0.33	1.0E-01	0.26	1.9E-01	-0.22	2.7E-01	-0.04	8.6E-01	-0.05	8.0E-01	-0.06	7.8E-01	-0.09	6.6E-01	-0.06	7.8E-01	-0.11	5.9E-01	-0.13	5.3E-01
ENSCAFG0000001871	RANBP3L	grey	EC_M10	0.33	1.0E-01	-0.03	8.9E-01	-0.38	5.7E-02	-0.07	7.3E-01	0.16	4.3E-01	0.09	6.5E-01	-0.01	9.6E-01	-0.08	7.1E-01	0.32	1.1E-01	-0.27	1.8E-01
ENSCAFG0000000591	ENSCAFG0000000591	grey	EC_M10	0.33	1.0E-01	0.26	1.9E-01	-0.14	4.9E-01	0.40	4.2E-02	-0.17	4.2E-01	-0.02	9.3E-01	-0.33	9.5E-02	-0.09	6.7E-01	0.06	7.9E-01	0.04	8.4E-01
ENSCAFG0000000222	ZNF169	grey	EC_M10	0.33	1.0E-01	-0.29	1.4E-01	-0.24	2.5E-01	0.12	5.8E-01	0.06	7.6E-01	0.23	2.6E-01	-0.47	1.6E-02	0.04	8.4E-01	-0.11	5.8E-01	-0.33	1.0E-01
ENSCAFG0000000331	ZNF277	grey	EC_M10	0.33	1.0E-01	0.26	2.1E-01	0.15	4.7E-01	0.44	2.5E-02	-0.56	3.2E-03	-0.30	1.3E-01	-0.10	6.4E-01	0.10	6.2E-01	-0.28	1.7E-01	0.37	6.5E-02
ENSCAFG0000001082	ZNF106	darkgreen	EC_M4	0.33	1.0E-01	0.28	1.6E-01	-0.86	1.5E-08	-0.06	7.8E-01	0.62	7.9E-04	0.08	7.0E-01	-0.04	8.3E-01	-0.10	6.3E-01	-0.26	2.0E-01	-0.79	1.4E-06
ENSCAFG0000000410	EPC1	turquoise	EC_M6	0.33	1.0E-01	0.51	7.3E-03	-0.26	2.0E-01	-0.06	7.7E-01	-0.22	2.8E-01	0.11	6.1E-01	-0.04	8.3E-01	-0.01	9.7E-01	-0.15	4.8E-01	0.04	8.4E-01
ENSCAFG0000000496	ENSCAFG0000000496	grey	EC_M10	0.33	1.0E-01	-0.14	5.1E-01	-0.26	2.0E-01	0.16	4.2E-01	0.09	6.5E-01	-0.22	2.8E-01	-0.20	3.2E-01	0.17	4.2E-01	-0.08	7.0E-01	-0.21	3.1E-01
ENSCAFG0000001005	ENSCAFG0000001005	grey	EC_M10	0.33	1.0E-01	-0.16	4.3E-01	-0.17	4.1E-01	0.25	2.3E-01	0.06	7.6E-01	0.02	9.4E-01	-0.47	1.6E-02	0.08	7.1E-01	-0.15	4.6E-01	-0.21	3.0E-01
ENSCAFG0000001533	PLVAP	turquoise	EC_M6	0.33	1.0E-01	0.84	8.3E-08	-0.32	1.1E-01	-0.06	7.8E-01	-0.17	4.1E-01	-0.12	5.7E-01	-0.15	4.8E-01	-0.15	4.6E-01	-0.19	3.6E-01	-0.03	8.8E-01
ENSCAFG0000000576	ARL5A	grey	EC_M10	0.33	1.0E-01	-0.33	1.0E-01	-0.19	3.5E-01	-0.06	7.7E-01	0.10	6.4E-01	0.19	3.6E-01	-0.45	2.1E-02	0.00	1.0E+00	-0.56	3.2E-03	-0.31	1.3E-01
ENSCAFG0000001266	TOR1AIP1	turquoise	EC_M6	0.33	1.0E-01	0.48	1.2E-02	-0.36	6.9E-02	-0.04	8.4E-01	-0.10	6.2E-01	0.02	9.3E-01	-0.08	7.1E-01	0.20	3.4E-01	0.28	1.7E-01	-0.04	8.6E-01
ENSCAFG0000000081	EIF3H	cyan	EC_M2	0.33	1.0E-01	-0.42	3.4E-02	0.39	4.7E-02	0.25	2.3E-01	-0.60	1.3E-03	0.05	7.9E-01	-0.16	4.4E-01	-0.24	2.3E-01	-0.47	1.5E-02	0.36	6.9E-02
ENSCAFG0000000085	ENSCAFG0000000085	turquoise	EC_M6	0.33	1.0E-01	0.39	4.6E-02	-0.33	9.8E-02	-0.23	2.6E-01	0.03	8.9E-01	-0.30	1.3E-01	0.08	7.1E-01	-0.14	5.0E-01	-0.13	5.4E-01	-0.14	4.9E-01
ENSCAFG0000000890	LCOR	grey	EC_M10	0.33	1.0E-01	0.21	3.0E-01	-0.19	3.5E-01	0.28	1.7E-01	-0.14	5.1E-01	0.24	2.3E-01	-0.12	5.7E-01	-0.15	4.8E-01	-0.22	2.8E-01	-0.05	8.2E-01
ENSCAFG0000000691	STK33	grey	EC_M10	0.33	1.0E-01	0.16	4.5E-01	0.13	5.4E-01	0.02	9.4E-01	-0.53	4.9E-03	-0.04	8.3E-01	-0.12	5.4E-01	-0.02	9.0E-01	-0.02	9.2E-01	0.33	9.9E-02
ENSCAFG0000000841	RASA1	darkgreen	EC_M4	0.33	1.0E-01	0.05	8.2E-01	-0.66	2.6E-04	0.08	7.1E-01	0.45	2.0E-02	0.24	2.4E-01	-0.19	3.5E-01	-0.09	6.8E-01	-0.40	4.2E-02	-0.69	1.1E-04
ENSCAFG0000001111	FABP3	grey	EC_M10	0.33	1.0E-01	-0.46	1.9E-02	0.09	6.5E-01	0.17	4.0E-01	-0.24	2.4E-01	0.01	9.5E-01	-0.11	5.8E-01	-0.04	8.6E-01	-0.50	9.6E-03	0.00	9.9E-01
ENSCAFG0000000082	UTP23	grey	EC_M10	0.33	1.0E-01	0.10	6.1E-01	-0.24	2.4E-01	0.15	4.6E-01	-0.12	5.6E-01	0.06	7.6E-01	0.04	8.4E-01	0.00	9.9E-01	-0.17	4.0E-01	-0.10	6.4E-01
ENSCAFG0000000033	FBXO30	turquoise	EC_M6	0.33	1.0E-01	0.62	6.6E-04	-0.36	7.3E-02	0.14	5.1E-01	-0.16	4.3E-01	0.01	9.5E-01	0.13	5.3E-01	-0.07	7.4E-01	0.02	9.4E-01	-0.02	9.2E-01
ENSCAFG0000001482	GNPNAT1	grey	EC_M10	0.33	1.0E-01	-0.61	9.2E-04	-0.28	1.7E-01	0.06	7.6E-01	0.27	1.8E-01	0.38	5.6E-02	-0.23	2.6E-01	-0.14	5.1E-01	-0.36	7.1E-02	-0.51	7.4E-03
ENSCAFG0000000265	MTO1	darkgreen	EC_M4	0.33	1.0E-01	-0.16	4.4E-01	-0.61	1.0E-03	-0.05	8.1E-01	0.54	4.8E-03	0.29	1.6E-01	0.01	9.7E-01	0.03	9.0E-01	-0.09	6.6E-01	-0.70	5.8E-05
ENSCAFG0000000865	MADD	turquoise	EC_M6	0.33	1.0E-01	0.77	5.3E-06	-0.73	2.7E-05	-0.08	6.9E-01	0.30	1.4E-01	0.11	6.1E-01	-0.08	7.0E-01	-0.13	5.2E-01	0.08	6.9E-01	-0.46	1.7E-02
ENSCAFG0000002013	AARS	darkgreen	EC_M4	0.33	1.0E-01	0.00	1.0E+00	-0.85	3.7E-08	-0.09	6.6E-01	0.74	1.5E-05	0.40	4.2E-02	-0.14	4.9E-01	-0.05	8.1E-01	-0.13	5.2E-01	-0.94	7.3E-13
ENSCAFG0000000936	ANO6	turquoise	EC_M6	0.33	1.0E-01	0.61	1.0E-03	-0.35	7.9E-02	0.04	8.4E-01	-0.12	5.7E-01	-0.03	9.0E-01	-0.03	8.9E-01	-0.10	6.4E-01	0.37	6.0E-02	-0.02	9.4E-01
ENSCAFG0000003024	ZNF304	grey	EC_M10	0.33	1.0E-01	0.47	1.5E-02	-0.34	8.8E-02	0.11	5.9E-01	-0.04	8.5E-01	0.04	8.4E-01	-0.32	1.1E-01	-0.26	2.0E-01	0.02	9.2E-01	-0.07	7.2E-01
ENSCAFG0000000766	EIF4G2	darkgreen	EC_M4	0.33	1.0E-01	-0.03	8.9E-01	-0.89	1.7E-09	-0.01	9.7E-01	0.77	5.2E-06	0.27	1.8E-01	-0.23	2.6E-01	0.06	7.8E-01	-0.06	7.7E-01	-0.95	1.5E-13
ENSCAFG0000002861	HYLS1	grey	EC_M10	0.33	1.0E-01	0.21	3.0E-01	0.10	6.1E-01	-0.12	5.8E-01	-0.36	7.2E-02	-0.36	7.0E-02	-0.14	5.1E-01	-0.01	9.7E-01	-0.02	9.4E-01	0.24	2.3E-01
ENSCAFG0000000303	ENSCAFG0000000303	grey	EC_M10	0.33	1.0E-01	-0.17	4.0E-01	-0.13	5.4E-01	-0.12	5.7E-01	-0.04	8.7E-01	0.50	9.5E-03	-0.15	4.7E-01	-0.15	4.6E-01	-0.25	2.1E-01	-0.18	3.9E-01
ENSCAFG0000002975	ENSCAFG0000002975	turquoise	EC_M6	0.33	1.0E-01	0.48	1.3E-02	-0.13	5.2E-01	-0.10	6.1E-01	-0.20	3.3E-01	-0.14	4.9E-01	-0.08	6.8E-01	-0.12	5.7E-01	-0.23	2.6E-01	0.03	8.9E-01
ENSCAFG0000000345	ENSCAFG0000000345	darkgreen	EC_M4	0.33	1.0E-01	0.23	2.6E-01	-0.78	2.3E-06	-0.25	2.2E-01	0.59	1.7E-03	0.17	4.1E-01	-0.03	8.8E-01	-0.03	8.7E-01	-0.15	4.6E-01	-0.77	4.8E-06
ENSCAFG0000000833	ALDH18A1	darkgreen	EC_M4	0.33	1.0E-01	0.10	6.4E-01	-0.85	2.8E-08	-0.17	4.2E-01	0.73	2.3E-05	0.33	9.8E-02	-0.17	4.1E-01	-0.05	8.1E-01	0.00	9.9E-01	-0.91	9.9E-11
ENSCAFG0000002938	ENSCAFG0000002938	grey	EC_M10	0.33	1.0E-01	0.15	4.8E-01	-0.02	9.3E-01	0.22	2.8E-01	-0.31	1.3E-01	0.09	6.7E-01	-0.04	8.5E-01	-0.07	7.5E-01	0.24	2.4E-01	0.19	3.5E-01
ENSCAFG0000001617	TLR6	turquoise	EC_M6	0.33	1.0E-01	0.72	3.9E-05	-0.44	2.5E-02	-0.16	4.2E-01	0.03	8.7E-01	0.05	7.9E-01	-0.15	4.8E-01	-0.19	3.6E-01	-0.19	3.4E-01	-0.19	3.6E-01
ENSCAFG0000000502	SRP68	darkgreen	EC_M4	0.33	1.0E-01	0.08	6.9E-01	-0.82	2.9E-07	-0.24	2.4E-01	0.66	2.2E-04	0.32	1.1E-01	0.16	4.4E-01	0.05	8.2E-01	-0.27	1.8E-01	-0.86	2.4E-08
ENSCAFG0000000947	COL4A3BP	darkgrey	EC_M8	0.33	1.0E-01	0.37	6.6E-02	0.24	2.3E-01	0.14	5.1E-01	-0.72	3.2E-05	-0.26	2.0E-01	-0.06	7.8E-01	-0.10	6.3E-01	-0.16	4.3E-01	0.56	3.1E-03
ENSCAFG0000003110	ENSCAFG0000003110	grey	EC_M10	0.33	1.0E-01	-0.45	2.2E-02	0.00	9.9E-01	-0.12	5.6E-01	-0.08	6.8E-01	0.35	7.8E-02	-0.26	2.0E-01	-0.16	4.5E-01	-0.37	6.3E-02	-0.10	6.4E-01
ENSCAFG0000001895	ANKRD13B	grey	EC_M10	0.33	1.0E-01	-0.39	4.7E-02	0.42	3.3E-02	0.11	5.8E-01	-0.53	5.4E-03	-0.08	7.1E-01	-0.29	1.5E-01	-0.08	6.9E-01	-0.04	8.4E-01	0.37	6.5E-02
ENSCAFG0000000168	ZNF16	grey	EC_M10	0.33	1.0E-01	0.33	1.0E-01	-0.19	3.5E-01	0.19	3.6E-01	-0.27	1.8E-01	-0.06	7.9E-01	-0.28	1.6E-01	-0.04	8.5E-01	0.10	6.4E-01	0.07	7.3E-01
ENSCAFG0000003247	ENSCAFG0000003247	darkgreen	EC_M4	0.33	1.0E-01	0.44	2.5E-02	-0.74	1.8E-05	0.00	9.9E-01	0.39	4.8E-02	0.03	8.9E-01	0.14	5.1E-01	0.06	7.7E-01	-0.06	7.5E-01	-0.57	2.6E-03
ENSCAFG0000001872	NDE1	grey	EC_M10	0.33	1.0E-01	-0.04	8.6E-01	0.27	1.8E-01	0.01	9.6E-01	-0.58	1.8E-03	-0.09	6.6E-01	0.31	1.3E-01	0.04	8.3E-01	0.14	4.8E-01	0.37	6.1E-02
ENSCAFG0000000414	ORC5	darkgreen	EC_M4	0.33	1.0E-01	-0.24	2.3E-01	-0.67	1.9E-04	-0.02	9.3E-01	0.62	7.8E-04	0.32	1.1E-01	-0.20	3.2E-01	0.16	4.4E-01	-0.08	7.2E-01	-0.82	3.2E-07
ENSCAFG0000000472	JPT1	darkgrey	EC_M8	0.33	1.1E-01	-0.20	3.2E-01	-0.50	8.6E-03	-0.11	5.8E-01	0.48	1.2E-02	0.05	8.0E-01	-0.43	2.7E-02	0.19	3.4E-01	-0.12	5.6E-01	-0.63	5.0E-04
ENSCAFG0000002466	ENSCAFG0000002466	grey	EC_M10	0.33	1.1E-01	-0.08	6.9E-01	0.04	8.6E-01	0.14	5.1E-01	-0.26	2.0E-01	0.24	2.4E-01	-0.03	8.9E-01	0.18	3.9E-01	-0.20	3.2E-01	0.08	6.8E-01
ENSCAFG0000000115	FAM13B	grey	EC_M10	0.33	1.1E-01	-0.09	6.5E-01	-0.29	1.6E-01	0.07	7.5E-01	0.16	4.4E-01	0.13	5.4E-01	-0.36	7.4E-02	-0.06	7.6E-01	-0.15	4.5E-01	-0.27	1.9E-01
ENSCAFG0000001547	ENSCAFG0000001547	grey	EC_M10	0.32	1.1E-01	0.13	5.2E-01	-0.05	8.3E-01	-0.03	8.8E-01	-0.33	9.8E-02	0.00	9.9E-01	-0.05	7.9E-01	0.12	5.7E-01	0.12	5.7E-01	0.18	3.7E-01
ENSCAFG0000000577	BIRC6	grey	EC_M10	0.32	1.1E-01	0.16	4.3E-01	-0.56	3.0E-03	-0.15	4.6E-01	0.37	6.3E-02	-0.03	8.9E-01	-0.19	3.4E-01	-0.25	2.2E-01	-0.32	1.1E-01	-0.52	6.8E-03
ENSCAFG0000000533	CAPN14	grey	EC_M10	0.32	1.1E-01	-0.10	6.4E-01	-0.61	1.0E-03	-0.12	5.5E-01	0.44	2.4E-02	0.70	6.2E-05	-0.15	4.5E-01	-0.16	4.3E-01	-0.03	8.8E-01	-0.65	3.7E-04
ENSCAFG0000001783	MYO19	grey	EC_M10	0.32	1.1E-01	-0.36	6.8E-02	-0.05	7.9E-01	-0.18	3.8E-01	-0.02	9.4E-01	0.31	1.2E-01	-0.50	9.3E-03	-0.06	7.6E-01	-0.11	5.9E-01	-0.21	3.0E-01
ENSCAFG0000001154	ELOVL6	grey	EC_M10	0.32	1.1E-01	-0.58	1.7E-03	0.24	2.3E-01	0.00	9.9E-01	-0.31	1.2E-01	0.01	9.8E-01	-0.21	3.1E-01	-0.02	9.1E-01	-0.18	3.9E-01	0.12	5.8E-01
ENSCAFG0000000654	PPP2R2B	darkgreen	EC_M4	0.32	1.1E-01	0.06	7.8E-01	-0.67	1.7E-04	-0.36	7.2E-02	0.55	3.6E-03	0.21	3.1E-01	-0.14	5.1E-01	0.03	9.0E-01	-0.03	8.7E-01	-0.72	3.3E-05
ENSCAFG0000000933	OLFML3	cyan	EC_M2	0.32	1.1E-01	-0.53	5.4E-03	0.27	1.8E-01	0.24	2.3E-01	-0.40	4.3E-02	0.14	4.9E-01	0.01	9.8E-01	0.05	8.0E-01	0.02	9.2E-01	0.20	3.3E-01
ENSCAFG0000000560	SPTLC3	grey	EC_M10	0.32	1.1E-01	0.32	1.1E-01	0.07	7.3E-01	-0.38	5.3E-02	-0.43	2.8E-02	-0.30	1.4E-01	-0.03	8.8E-01	0.24	2.3E-01	0.18	3.9E-01	0.30	1.4E-01
ENSCAFG0000001096	ZDHHC6	turquoise	EC_M6	0.32	1.1E-01	0.62	7.6E-04	-0.60	1.3E-03	-0.08	6.9E-01	0.24	2.3E-01	0.26	2.1E-01	-0.16	4.5E-01	-0.41	3.7E-02	-0.33	1.0E-01	-0.40	4.1E-02
ENSCAFG0000000923	WRNIP1	grey	EC_M10	0.32	1.1E-01	0.27	1.8E-01	0.27	1.8E-01	-0.16	4.4E-01	-0.62	6.9E-04	-0.16	4.4E-01	-0.20	3.3E-01	-0.12	5.6E-01	-0.35	8.1E-02	0.48	1.3E-02
ENSCAFG0000002014	CCDC18	cyan	EC_M2	0.32	1.1E-01	0.21	3.1E-01	0.38	5.7E-02	0.24	2.4E-01	-0.83	1.2E-07	-0.24	2.4E-01	-0.09	6.6E-01	-0.09	6.7E-01	-0.14	4.8E-01	0.66	2.8E-04
ENSCAFG0000000373	ELF2	darkgrey	EC_M8	0.32	1.1E-01	0.43	2.8E-02	0.30	1.3E-01	0.09	6.6E-01	-0.79	1.9E-06	-0.09	6.6E-01	0.02	9.3E-01	0.00	9.8E-01	-0.20	3.2E-01	0.61	9.8E-04
ENSCAFG0000000227	ELOVL5	grey	EC_M10	0.32	1.1E-01	0.05	8.0E-01	-0.63	6.1E-04	-0.08	6.9E-01	0.48	1.4E-02	0.26	2.0E-01	-0.35	8.1E-02	0.06	7.8E-01	-0.06	7.6E-01	-0.67	2.1E-04
ENSCAFG0000001579	GPKOW	grey	EC_M10	0.32	1.1E-01	0.02	9.1E-01	-0.36	7.1E-02	0.06	7.7E-01	0.10	6.2E-01	0.00	9.8E-01	0.19	3.6E-01	0.26	2.0E-01	0.23	2.6E-01	-0.29	1.5E-01
ENSCAFG0000001163	FAM160B1	grey	EC_M10	0.32	1.1E-01	0.23	2.6E-01	-0.42	3.2E-02	0.00	1.0E+00	0.10	6.4E-01	0.16	4.4E-01	0.03	8.8E-01	0.02	9.2E-01	-0.22	2.9E-01	-0.25	2.3E-01
ENSCAFG0000002022	DHX38	grey	EC_M10	0.32	1.1E-01	0.29	1.6E-01	-0.01	9.7E-01	0.07	7.5E-01	-0.31	1.2E-01	-0.06	7.7E-01	0.07	7.3E-01	-0.20	3.2E-01	-0.10	6.4E-01	0.19	3.4E-01
ENSCAFG0000001465	DNAJC9	grey	EC_M10	0.32	1.1E-01	0.20	3.2E-01	-0.16	4.5E-01	-0.17	4.1E-01	-0.05	8.2E-01	0.13	5.4E-01	-0.21	2.9E-01	-0.13	5.2E-01	0.23	2.6E-01	-0.08	6.8E-01
ENSCAFG0000000622	TMCC3	turquoise	EC_M6	0.32	1.1E-01	0.86	1.2E-08	-0.48	1.3E-02	-0.15	4.7E-01	0.00	9.9E-01	0.02	9.2E-01	-0.02	9.2E-01	-0.18	3.7E-01	-0.05	7.9E-01	-0.16	4.3E-01
ENSCAFG0000003063	ENSCAFG0000003063	grey	EC_M10	0.32	1.1E-01	-0.05	8.1E-01	0.01	9.8E-01	0.35	7.8E-02	-0.24	2.3E-01	-0.15	4.6E-01	-0.12	5.5E-01	-0.16	4.4E-01	-0.07	7.3E-01	0.12	5.7E-01
ENSCAFG0000001339	ENSCAFG0000001339	grey	EC_M10	0.32	1.1E-01	-0.06	7.5E-01	0.08	7.1E-01	0.00	9.8E-01	-0.41	3.6E-02	0.12	5.5E-01	-0.30	1.3E-01	0.17	4.1E-01	-0.02	9.2E-01	0.16	4.3E-01
ENSCAFG0000001994	USP10	darkgreen	EC_M4	0.32	1.1E-01	0.13	5.4E-01	-0.69	8.6E-05	-0.06	7.8E-01	0.57	2.3E-03	0.30	1.4E-01	0.04	8.6E-01	-0.16	4.3E-01	-0.45	2.2E-02	-0.75	8.4E-06
ENSCAFG0000000872	TET3	darkgreen	EC_M4	0.32	1.1E-01	0.27	1.8E-01	-0.75	1.1E-05	-0.12	5.4E-01	0.46	1.8E-02	0.28	1.6E-01	0.05	8.2E-01	-0.36	7.0E-02	-0.09	6.8E-01	-0.68	1.4E-04
ENSCAFG0000000568	NGLY1	grey	EC_M10	0.32	1.1E-01	-0.18	3.8E-01	0.02	9.3E-01	-0.13	5.2E-01	-0.22	2.9E-01	-0.13	5.3E-01	0.14	4.9E-01	-0.17	4.0E-01	-0.47	1.5E-02	0.07	7.5E-01
ENSCAFG0000000445	SUCLA2	grey	EC_M10	0.32	1.1E-01	-0.30	1.4E-01	-0.55	3.5E-03	-0.01	9.5E-01	0.44	2.4E-02	0.18	3.8E-01	-0.21	3.1E-01	-0.09	6.7E-01	0.00	9.9E-01	-0.59	1.5E-03
ENSCAFG0000001762	CNOT8	turquoise	EC_M6	0.32	1.1E-01	0.55	3.4E-03	0.10	6.1E-01	0.12	5.7E-01	-0.57	2.3E-03	0.10	6.1E-01	-0.04	8.5E-01	-0.25	2.1E-01	-0.24	2.4E-01	0.39	5.0E-02
ENSCAFG0000000371	RTN4IP1	grey	EC_M10	0.32	1.1E-01	-0.22	2.9E-01	-0.14	5.0E-01	0.16	4.4E-01	-0.07	7.3E-01	0.49	1.0E-02	-0.04	8.5E-01	-0.37	6.5E-02	0.08	7.1E-01	-0.10	6.4E-01
ENSCAFG0000000273	IL6	turquoise	EC_M6	0.32	1.1E-01	0.93	8.9E-12	-0.44	2.4E-02	-0.08	7.0E-01	-0.07	7.5E-01	-0.06	7.7E-01	-0.03	9.0E-01	-0.09	6.5E-01	-0.15	4.5E-01	-0.11	5.8E-01
ENSCAFG0000001810	SCAPER	darkgreen	EC_M4	0.32	1.1E-01	0.19	3.6E-01	-0.74	1.8E-05	-0.31	1.2E-01	0.52	6.0E-03	0.28	1.7E-01	-0.10	6.2E-01	-0.01	9.5E-01	-0.15	4.8E-01	-0.67	1.7E-04
ENSCAFG0000001365	CTTNBP2NL	turquoise	EC_M6	0.32	1.1E-01	0.73	2.3E-05	-0.30	1.4E-01	-0.04	8.3E-01	-0.23	2.6E-01	0.01	9.8E-01	-0.31	1.2E-01	-0.16	4.3E-01	-0.20	3.3E-01	0.01	9.8E-01
ENSCAFG0000000190	TJP2	turquoise	EC_M6	0.32	1.1E-01	0.77	4.1E-06	-0.34	8.9E-02	-0.30	1.4E-01	-0.09	6.5E-01	-0.21	3.1E-01	-0.16	4.4E-01	-0.02	9.2E-01	0.29	1.5E-01	-0.05	8.2E-01
ENSCAFG0000000031	PHACTR2	turquoise	EC_M6	0.32	1.1E-01	0.87	7.4E-09	-0.27	1.8E-01	0.02	9.3E-01	-0.24	2.4E-01	-0.10	6.4E-01	-0.47	1.6E-02	-0.02	9.4E-01	-0.06	7.9E-01	0.07	7.3E-01
ENSCAFG0000001148	GIGYF2	grey	EC_M10	0.32	1.1E-01	-0.07	7.2E-01	0.12	5.5E-01	0.19	3.4E-01	-0.39	4.6E-02	-0.16	4.3E-01	-0.16	4.3E-01	-0.03	8.8E-01	-0.63	5.7E-04	0.20	3.2E-01
ENSCAFG0000000912	TMEM67	cyan	EC_M2	0.32	1.1E-01	-0.27	1.7E-01	0.29	1.5E-01	0.26	2.1E-01	-0.55	3.5E-03	0.02	9.2E-01	-0.43	2.7E-02	0.04	8.3E-01	-0.01	9.6E-01	0.37	6.1E-02
ENSCAFG0000000347	BLVRA	turquoise	EC_M6	0.32	1.1E-01	0.59	1.7E-03	-0.50	1.0E-02	0.30	1.4E-01	0.07	7.2E-01	-0.02	9.2E-01	-0.15	4.7E-01	-0.22	2.8E-01	0.11	6.0E-01	-0.25	2.1E-01
ENSCAFG0000000852	GPR87	grey	EC_M10	0.32	1.1E-01	-0.13	5.2E-01	-0.47	1.6E-02	-0.02	9.1E-01	0.30	1.4E-01	0.78	2.2E-06	-0.09	6.6E-01	-0.10	6.1E-01	-0.18	3.7E-01	-0.50	8.5E-03
ENSCAFG0000001025	KIF21A	grey	EC_M10	0.32	1.1E-01	0.56	3.0E-03	-0.61	9.5E-04	-0.17	4.0E-01	0.26	1.9E-01	-0.01	9.7E-01	-0.17	4.2E-01	-0.05	8.2E-01	0.27	1.8E-01	-0.41	3.6E-02
ENSCAFG0000000523	PPP1CB	turquoise	EC_M6	0.32	1.1E-01	0.66	2.5E-04	-0.60	1.3E-03	-0.16	4.3E-01	0.18	3.8E-01	0.08	6.9E-01	0.09	6.7E-01	-0.12	5.6E-01	-0.11	6.0E-01	-0.31	1.2E-01
ENSCAFG0000001236	SLCO1A2	grey	EC_M10	0.32	1.1E-01	-0.20	3.2E-01	-0.08	6.9E-01	-0.23	2.6E-01	-0.07	7.2E-01	-0.12	5.7E-01	-0.12	5.7E-01	0.06	7.6E-01	0.08	7.1E-01	-0.08	6.9E-01
ENSCAFG0000001560	TADA1	grey	EC_M10	0.32	1.1E-01	-0.04	8.3E-01	0.12	5.5E-01	0.35	8.0E-02	-0.36	7.2E-02	0.00	1.0E+00	0.05	8.0E-01	0.09	6.5E-01	-0.04	8.6E-01	0.25	2.1E-01
ENSCAFG0000001391	HK1	grey	EC_M10	0.32	1.1E-01	0.42	3.4E-02	-0.42	3.4E-02	-0.27	1.8E-01	0.15	4.7E-01	-0.14	5.0E-01	-0.10	6.4E-01	0.14	5.1E-01	-0.11	6.0E-01	-0.25	2.1E-01
ENSCAFG0000000341	ST7	grey	EC_M10	0.32	1.1E-01	0.30	1.3E-01	-0.41	3.6E-02	-0.23	2.6E-01	0.19	3.5E-01	-0.01	9.7E-01	-0.29	1.6E-01	-0.25	2.2E-01	0.40	4.5E-02	-0.27	1.8E-01
ENSCAFG0000001447	SERPINC1	grey	EC_M10	0.32	1.1E-01	-0.11	5.8E-01	-0.27	1.8E-01	-0.09	6.6E-01	0.11	5.9E-01	0.63	6.3E-04	-0.09	6.5E-01	-0.11	5.9E-01	-0.05	7.9E-01	-0.29	1.5E-01
ENSCAFG0000002583	ENSCAFG0000002583	grey	EC_M10	0.32	1.1E-01	0.27	1.9E-01	-0.23	2.6E-01	-0.19	3.4E-01	-0.05	8.1E-01	0.28	1.7E-01	-0.26	2.0E-01	-0.30	1.3E-01	-0.01	9.6E-01	-0.08	7.1E-01
ENSCAFG0000001353	CCNYL1	grey	EC_M10	0.32	1.1E-01	0.32	1.1E-01	-0.49	1.1E-02	-0.22	2.9E-01	0.20	3.3E-01	-0.10	6.1E-01	-0.16	4.4E-01	0.09	6.6E-01	0.58	2.1E-03	-0.34	9.4E-02
ENSCAFG0000000774	ENSCAFG0000000774	grey	EC_M10	0.32	1.1E-01	0.31	1.2E-01	-0.59	1.4E-03	0.11	6.1E-01	0.36	7.0E-02	0.27	1.8E-01	-0.24	2.4E-01	0.09	6.4E-01	-0.10	6.3E-01	-0.49	1.1E-02
ENSCAFG0000000193	PGM5	turquoise	EC_M6	0.32	1.1E-01	0.76	5.8E-06	-0.57	2.4E-03	-0.30	1.4E-01	0.16	4.3E-01	0.06	7.8E-01	-0.14	5.0E-01	-0.23	2.5E-01	0.05	8.1E-01	-0.30	1.3E-01
ENSCAFG0000001415	BRINP2	grey	EC_M10	0.32	1.1E-01	-0.12	5.6E-01	-0.23	2.5E-01	0.15	4.6E-01	-0.01	9.7E-01	0.11	6.0E-01	-0.03	8.8E-01	0.02	9.3E-01	-0.24	2.4E-01	-0.26	1.9E-01
ENSCAFG0000001045	KDM5B	darkgreen	EC_M4	0.32	1.1E-01	0.58	2.1E-03	-0.86	1.9E-08	-0.03	8.8E-01	0.48	1.3E-02	0.19	3.4E-01	-0.04	8.6E-01	-0.02	9.3E-01	-0.08	6.9E-01	-0.67	2.0E-04
ENSCAFG0000000509	ENSCAFG0000000509	turquoise	EC_M6	0.32	1.1E-01	0.65	3.0E-04	0.05	8.1E-01	0.04	8.5E-01	-0.54	4.2E-03	-0.20	3.3E-01	-0.09	6.6E-01	0.01	9.6E-01	0.12	5.5E-01	0.40	4.2E-02
ENSCAFG0000001164	AP1AR	turquoise	EC_M6	0.32	1.1E-01	0.63	5.1E-04	-0.60	1.1E-03	-0.16	4.3E-01	0.27	1.8E-01	0.11	5.9E-01	0.18	3.9E-01	-0.11	5.9E-01	-0.10	6.4E-01	-0.42	3.4E-02
ENSCAFG0000000515	GCKR	grey	EC_M10	0.32	1.1E-01	-0.09	6.6E-01	-0.11	6.1E-01	0.07	7.3E-01	-0.14	5.0E-01	-0.02	9.1E-01	-0.07	7.5E-01	0.25	2.2E-01	0.41	3.9E-02	-0.06	7.6E-01
ENSCAFG0000000656	ARHGAP22	darkgrey	EC_M8	0.32	1.1E-01	0.10	6.3E-01	0.36	7.0E-02	0.25	2.2E-01	-0.76	7.6E-06	0.05	8.0E-01	0.09	6.7E-01	-0.06	7.8E-01	-0.22	2.7E-01	0.56	2.9E-03
ENSCAFG0000001200	CACUL1	grey	EC_M10	0.32	1.1E-01	-0.04	8.3E-01	-0.41	3.5E-02	0.04	8.5E-01	0.21	3.0E-01	-0.05	8.0E-01	0.22	2.8E-01	-0.06	7.7E-01	-0.35	8.1E-02	-0.37	6.3E-02
ENSCAFG0000000419	ENSCAFG0000000419	grey	EC_M10	0.32	1.1E-01	-0.06	7.8E-01	-0.26	1.9E-01	0.35	8.4E-02	0.06	7.6E-01	0.19	3.5E-01	-0.13	5.4E-01	-0.22	2.8E-01	0.00	9.8E-01	-0.22	2.8E-01
ENSCAFG0000001050	WDR4	grey	EC_M10	0.32	1.1E-01	-0.29	1.6E-01	-0.32	1.1E-01	-0.01	9.7E-01	0.29	1.4E-01	0.09	6.8E-01	-0.04	8.6E-01	-0.04	8.5E-01	0.12	5.7E-01	-0.48	1.2E-02

ENSCAFG0000000305	ENSCAFG0000000305	grey	EC_M10	0.32	1.1E-01	0.05	8.2E-01	-0.38	5.3E-02	-0.06	7.9E-01	0.24	2.4E-01	0.63	5.2E-04	-0.12	5.5E-01	-0.09	6.5E-01	-0.10	6.1E-01	-0.39	4.6E-02
ENSCAFG0000000693	AACS	grey	EC_M10	0.32	1.1E-01	0.07	7.5E-01	0.32	1.1E-01	0.12	5.4E-01	-0.65	3.7E-04	-0.36	6.8E-02	-0.08	7.1E-01	0.07	7.4E-01	-0.35	8.2E-02	0.46	1.8E-02
ENSCAFG0000001938	PRKCZ	grey	EC_M10	0.32	1.1E-01	-0.24	2.3E-01	-0.36	7.5E-02	0.32	1.1E-01	0.30	1.4E-01	0.10	6.2E-01	-0.25	2.1E-01	0.09	6.5E-01	0.19	3.5E-01	-0.44	2.4E-02
ENSCAFG0000000455	GPALPP1	grey	EC_M10	0.32	1.1E-01	0.06	7.6E-01	-0.03	8.7E-01	-0.04	8.3E-01	-0.34	8.7E-02	-0.05	8.2E-01	-0.07	7.2E-01	0.27	1.9E-01	0.11	5.9E-01	0.20	3.2E-01
ENSCAFG0000000432	CNBP	grey	EC_M10	0.32	1.1E-01	-0.15	4.6E-01	0.20	3.4E-01	-0.01	9.4E-01	-0.36	7.1E-02	-0.02	9.2E-01	-0.06	7.6E-01	-0.27	1.8E-01	-0.37	6.0E-02	0.23	2.6E-01
ENSCAFG0000000616	ENSCAFG0000000616	turquoise	EC_M6	0.32	1.1E-01	0.65	2.9E-04	-0.35	7.5E-02	-0.12	5.6E-01	-0.07	7.2E-01	0.51	7.8E-03	-0.10	6.3E-01	-0.13	5.2E-01	-0.19	3.5E-01	-0.11	6.1E-01
ENSCAFG0000000295	SNAP91	turquoise	EC_M6	0.32	1.1E-01	0.49	1.1E-02	-0.40	4.5E-02	0.01	9.7E-01	0.00	9.9E-01	-0.07	7.4E-01	-0.33	9.5E-02	-0.13	5.4E-01	0.07	7.5E-01	-0.18	3.7E-01
ENSCAFG0000000717	CHCHD5	grey	EC_M10	0.32	1.1E-01	-0.34	8.6E-02	-0.12	5.5E-01	0.09	6.6E-01	0.10	6.4E-01	0.13	5.3E-01	0.04	8.5E-01	0.34	9.0E-02	-0.25	2.2E-01	-0.30	1.4E-01
ENSCAFG0000001741	ZC3H14	grey	EC_M10	0.32	1.1E-01	0.37	6.2E-02	-0.66	2.2E-04	0.15	4.8E-01	0.38	5.6E-02	0.19	3.6E-01	-0.53	5.7E-03	-0.28	1.6E-01	0.03	8.7E-01	-0.55	4.0E-03
ENSCAFG0000000939	MAPK10	grey	EC_M10	0.32	1.1E-01	-0.07	7.2E-01	-0.46	1.7E-02	-0.05	7.9E-01	0.37	6.0E-02	-0.05	8.2E-01	-0.24	2.4E-01	0.28	1.7E-01	0.18	3.8E-01	-0.55	3.8E-03
ENSCAFG0000002634	ENSCAFG0000002634	grey	EC_M10	0.32	1.1E-01	-0.03	9.0E-01	-0.14	5.1E-01	-0.30	1.3E-01	-0.14	5.0E-01	0.13	5.2E-01	0.01	9.6E-01	0.01	9.6E-01	-0.20	3.4E-01	-0.05	8.1E-01
ENSCAFG0000001114	RGS9	darkgrey	EC_M8	0.32	1.1E-01	0.26	2.0E-01	-0.14	4.9E-01	0.00	9.8E-01	-0.05	8.0E-01	-0.01	9.5E-01	-0.06	7.6E-01	0.05	8.0E-01	0.08	6.8E-01	-0.07	7.4E-01
ENSCAFG0000002039	ACADM	darkgrey	EC_M8	0.32	1.1E-01	0.01	9.7E-01	0.38	5.7E-02	0.27	1.8E-01	-0.75	1.2E-05	-0.10	6.2E-01	-0.30	1.4E-01	-0.07	7.4E-01	-0.05	8.0E-01	0.61	9.6E-04
ENSCAFG0000000896	KCTD9	grey	EC_M10	0.32	1.1E-01	-0.59	1.6E-03	-0.21	3.1E-01	0.11	5.9E-01	0.15	4.6E-01	0.14	5.0E-01	0.04	8.4E-01	-0.01	9.5E-01	-0.47	1.5E-02	-0.34	8.8E-02
ENSCAFG0000000598	TRNT1	darkgreen	EC_M4	0.32	1.1E-01	-0.09	6.8E-01	-0.60	1.3E-03	-0.22	2.7E-01	0.42	3.1E-02	0.10	6.3E-01	-0.22	2.8E-01	0.00	1.0E+00	-0.19	3.6E-01	-0.60	1.3E-03
ENSCAFG0000000850	ZFAND1	grey	EC_M10	0.32	1.1E-01	-0.45	2.0E-02	-0.06	7.6E-01	0.32	1.1E-01	0.01	9.7E-01	0.16	4.4E-01	-0.06	7.6E-01	-0.42	3.2E-02	-0.27	1.8E-01	-0.16	4.3E-01
ENSCAFG0000000788	CEP44	grey	EC_M10	0.32	1.1E-01	-0.05	8.2E-01	-0.03	9.0E-01	0.23	2.6E-01	-0.21	3.0E-01	-0.06	7.7E-01	-0.31	1.3E-01	-0.01	9.4E-01	0.00	1.0E+00	0.09	6.5E-01
ENSCAFG0000000313	ENSCAFG0000000313	grey	EC_M10	0.32	1.1E-01	0.31	1.3E-01	-0.01	9.6E-01	0.46	1.8E-02	-0.34	8.5E-02	-0.20	3.3E-01	-0.18	3.8E-01	0.19	3.6E-01	-0.06	7.9E-01	0.22	2.8E-01
ENSCAFG0000000084	ENSCAFG0000000084	violet	EC_M7	0.32	1.1E-01	-0.16	4.2E-01	-0.26	1.9E-01	-0.12	5.7E-01	0.08	7.0E-01	0.79	1.9E-06	-0.12	5.7E-01	-0.12	5.6E-01	-0.18	3.8E-01	-0.28	1.6E-01
ENSCAFG0000001809	TMC5	grey	EC_M10	0.32	1.1E-01	-0.13	5.3E-01	-0.42	3.2E-02	-0.02	9.2E-01	0.22	2.8E-01	-0.11	6.0E-01	-0.08	7.1E-01	-0.09	6.8E-01	-0.11	6.0E-01	-0.42	3.4E-02
ENSCAFG0000001535	ENSCAFG0000001535	grey	EC_M10	0.32	1.1E-01	0.33	9.8E-02	-0.40	4.6E-02	-0.10	6.1E-01	0.05	7.9E-01	0.27	1.9E-01	-0.13	5.2E-01	-0.13	5.1E-01	-0.29	1.5E-01	-0.27	1.9E-01
ENSCAFG0000002644	ENSCAFG0000002644	grey	EC_M10	0.32	1.1E-01	-0.13	5.3E-01	-0.22	2.9E-01	0.08	6.8E-01	0.04	8.4E-01	0.14	4.9E-01	-0.17	4.0E-01	-0.30	1.4E-01	0.16	4.2E-01	-0.22	2.7E-01
ENSCAFG0000001088	PPP2R2D	darkgrey	EC_M8	0.32	1.1E-01	0.51	7.2E-03	-0.02	9.2E-01	0.19	3.6E-01	-0.44	2.6E-02	0.06	7.7E-01	-0.12	5.7E-01	-0.07	7.4E-01	-0.22	2.8E-01	0.31	1.3E-01
ENSCAFG0000002005	PALMD	turquoise	EC_M6	0.32	1.2E-01	0.96	3.1E-14	-0.27	1.8E-01	-0.10	6.4E-01	-0.26	2.0E-01	-0.14	5.0E-01	-0.13	5.3E-01	-0.14	5.1E-01	0.00	9.9E-01	0.11	6.0E-01
ENSCAFG0000001086	ENSCAFG0000001086	grey	EC_M10	0.32	1.2E-01	0.09	6.5E-01	0.33	9.8E-02	0.25	2.1E-01	-0.69	1.1E-04	0.06	7.7E-01	-0.33	9.7E-02	-0.19	3.7E-01	-0.03	8.8E-01	0.53	5.7E-03
ENSCAFG0000000362	RAB14	grey	EC_M10	0.32	1.2E-01	0.17	4.1E-01	-0.52	6.9E-03	0.18	3.7E-01	0.30	1.3E-01	-0.06	7.6E-01	-0.14	5.0E-01	-0.11	6.0E-01	-0.05	8.2E-01	-0.42	3.3E-02
ENSCAFG0000001152	AP1G2	grey	EC_M10	0.32	1.2E-01	0.26	1.9E-01	0.39	5.0E-02	0.13	5.2E-01	-0.77	4.3E-06	-0.07	7.3E-01	-0.20	3.2E-01	-0.18	3.9E-01	-0.10	6.3E-01	0.59	1.5E-03
ENSCAFG0000000189	MAMDC2	turquoise	EC_M6	0.32	1.2E-01	0.88	3.0E-09	-0.36	6.8E-02	0.01	9.6E-01	-0.10	6.2E-01	-0.16	4.3E-01	-0.07	7.5E-01	-0.14	4.9E-01	-0.18	3.9E-01	-0.06	7.6E-01
ENSCAFG0000000088	ENSCAFG0000000088	grey	EC_M10	0.32	1.2E-01	0.30	1.4E-01	-0.54	4.2E-03	-0.33	1.0E-01	0.28	1.7E-01	-0.01	9.5E-01	0.13	5.4E-01	-0.20	3.4E-01	0.07	7.4E-01	-0.42	3.1E-02
ENSCAFG0000000765	SPSB4	grey	EC_M10	0.32	1.2E-01	0.24	2.3E-01	-0.40	4.5E-02	-0.21	3.0E-01	0.19	3.6E-01	0.13	5.4E-01	-0.19	3.5E-01	-0.04	8.6E-01	0.20	3.4E-01	-0.37	6.4E-02
ENSCAFG0000000871	ENSCAFG0000000871	darkgreen	EC_M4	0.32	1.2E-01	-0.10	6.4E-01	-0.76	5.6E-06	-0.01	9.5E-01	0.68	1.2E-04	0.33	1.0E-01	-0.11	6.1E-01	-0.05	8.2E-01	-0.11	5.9E-01	-0.90	6.5E-10
ENSCAFG0000000933	DPY19L4	darkgreen	EC_M4	0.32	1.2E-01	0.13	5.1E-01	-0.82	2.8E-07	0.03	8.9E-01	0.57	2.3E-03	0.17	4.1E-01	-0.11	6.0E-01	-0.03	8.9E-01	-0.17	4.2E-01	-0.75	1.2E-05
ENSCAFG0000000549	ENSCAFG0000000549	darkgrey	EC_M8	0.32	1.2E-01	-0.02	9.3E-01	-0.23	2.5E-01	0.35	8.4E-02	-0.05	8.2E-01	0.12	5.7E-01	-0.18	3.8E-01	-0.32	1.1E-01	-0.15	4.7E-01	-0.08	7.1E-01
ENSCAFG0000000835	DCHS2	grey	EC_M10	0.32	1.2E-01	-0.14	5.0E-01	0.03	8.7E-01	-0.09	6.8E-01	-0.09	6.6E-01	-0.06	7.6E-01	0.03	9.0E-01	-0.01	9.7E-01	0.03	8.7E-01	-0.02	9.4E-01
ENSCAFG0000002758	ENSCAFG0000002758	grey	EC_M10	0.32	1.2E-01	-0.14	5.0E-01	0.03	8.7E-01	-0.09	6.8E-01	-0.09	6.6E-01	-0.06	7.6E-01	0.03	9.0E-01	-0.01	9.7E-01	0.03	8.7E-01	-0.02	9.4E-01
ENSCAFG0000000562	JAG1	turquoise	EC_M6	0.32	1.2E-01	0.66	2.3E-04	-0.46	1.8E-02	-0.11	5.8E-01	0.04	8.3E-01	-0.08	6.9E-01	-0.22	2.7E-01	-0.12	5.6E-01	0.57	2.5E-03	-0.19	3.6E-01
ENSCAFG0000001172	EGFL6	grey	EC_M10	0.32	1.2E-01	-0.13	5.2E-01	-0.48	1.3E-02	-0.01	9.8E-01	0.31	1.2E-01	0.67	1.8E-04	-0.09	6.5E-01	-0.10	6.2E-01	-0.19	3.6E-01	-0.52	6.5E-03
ENSCAFG0000002448	ENSCAFG0000002448	grey	EC_M10	0.32	1.2E-01	-0.13	5.2E-01	-0.48	1.3E-02	-0.01	9.8E-01	0.31	1.2E-01	0.67	1.8E-04	-0.09	6.5E-01	-0.10	6.2E-01	-0.19	3.6E-01	-0.52	6.5E-03
ENSCAFG0000002967	ENSCAFG0000002967	grey	EC_M10	0.32	1.2E-01	-0.13	5.2E-01	-0.48	1.3E-02	-0.01	9.8E-01	0.31	1.2E-01	0.67	1.8E-04	-0.09	6.5E-01	-0.10	6.2E-01	-0.19	3.6E-01	-0.52	6.5E-03
ENSCAFG0000003169	ENSCAFG0000003169	grey	EC_M10	0.32	1.2E-01	-0.13	5.2E-01	-0.48	1.3E-02	-0.01	9.8E-01	0.31	1.2E-01	0.67	1.8E-04	-0.09	6.5E-01	-0.10	6.2E-01	-0.19	3.6E-01	-0.52	6.5E-03
ENSCAFG0000000930	ENSCAFG0000000930	grey	EC_M10	0.32	1.2E-01	0.08	7.0E-01	-0.27	1.8E-01	-0.13	5.3E-01	0.05	8.3E-01	0.50	9.2E-03	-0.15	4.8E-01	-0.18	3.9E-01	-0.25	2.2E-01	-0.28	1.7E-01
ENSCAFG0000001595	ENSCAFG0000001595	grey	EC_M10	0.31	1.2E-01	-0.17	4.1E-01	-0.20	3.4E-01	-0.19	3.5E-01	0.09	6.5E-01	0.34	9.3E-02	0.19	3.6E-01	-0.17	4.0E-01	-0.21	2.9E-01	-0.22	2.8E-01
ENSCAFG0000000640	SH3RF2	grey	EC_M10	0.31	1.2E-01	0.47	1.6E-02	-0.42	3.3E-02	-0.27	1.9E-01	0.09	6.7E-01	0.15	4.8E-01	0.00	9.9E-01	-0.03	8.9E-01	0.51	7.3E-03	-0.21	3.0E-01
ENSCAFG0000003031	ENSCAFG0000003031	grey	EC_M10	0.31	1.2E-01	0.19	3.4E-01	-0.58	2.1E-03	-0.23	2.6E-01	0.31	1.2E-01	-0.03	8.7E-01	0.03	8.9E-01	-0.25	2.1E-01	-0.08	7.0E-01	-0.50	8.9E-03
ENSCAFG0000001034	NECTIN3	grey	EC_M10	0.31	1.2E-01	-0.22	2.7E-01	-0.64	4.5E-04	0.12	5.6E-01	0.52	6.0E-03	0.49	1.1E-02	-0.12	5.7E-01	-0.04	8.5E-01	-0.02	9.1E-01	-0.75	9.8E-06
ENSCAFG0000000347	ENSCAFG0000000347	grey	EC_M10	0.31	1.2E-01	-0.12	5.7E-01	-0.16	4.3E-01	-0.15	4.5E-01	-0.02	9.4E-01	-0.08	7.1E-01	-0.04	8.6E-01	0.00	1.0E+00	0.08	7.0E-01	-0.15	4.7E-01
ENSCAFG0000000461	PLXND1	turquoise	EC_M6	0.31	1.2E-01	0.63	6.2E-04	0.08	6.9E-01	0.05	8.1E-01	-0.54	4.0E-03	-0.27	1.8E-01	-0.04	8.4E-01	-0.16	4.5E-01	0.21	3.1E-01	0.38	5.8E-02
ENSCAFG0000000345	SEC61G	grey	EC_M10	0.31	1.2E-01	0.17	4.0E-01	-0.43	2.8E-02	-0.06	7.8E-01	0.19	3.6E-01	0.40	4.5E-02	0.03	8.8E-01	-0.42	3.3E-02	-0.21	3.1E-01	-0.38	5.5E-02
ENSCAFG0000000906	ZNF638	darkgrey	EC_M8	0.31	1.2E-01	0.14	4.9E-01	0.16	4.4E-01	0.16	4.3E-01	-0.58	1.8E-03	-0.08	6.8E-01	-0.10	6.4E-01	0.01	9.6E-01	-0.26	2.0E-01	0.41	3.8E-02
ENSCAFG0000001073	NFKB1	darkgrey	EC_M8	0.31	1.2E-01	0.54	4.2E-03	0.18	3.8E-01	0.11	6.0E-01	-0.67	1.8E-04	-0.04	8.3E-01	0.05	8.1E-01	-0.13	5.3E-01	-0.18	3.7E-01	0.50	1.0E-02
ENSCAFG0000001111	ENSCAFG0000001111	grey	EC_M10	0.31	1.2E-01	-0.33	9.7E-02	-0.57	2.2E-03	-0.06	7.8E-01	0.52	5.9E-03	0.32	1.2E-01	-0.04	8.3E-01	0.25	2.1E-01	-0.05	8.1E-01	-0.69	9.2E-05
ENSCAFG0000000449	GRIP2	darkgrey	EC_M8	0.31	1.2E-01	0.05	8.2E-01	0.00	9.9E-01	-0.02	9.4E-01	-0.16	4.5E-01	-0.14	4.9E-01	-0.09	6.6E-01	-0.01	9.5E-01	-0.16	4.2E-01	0.01	9.5E-01
ENSCAFG0000000063	ENSCAFG0000000063	grey	EC_M10	0.31	1.2E-01	-0.25	2.1E-01	-0.18	3.7E-01	-0.13	5.2E-01	-0.03	8.7E-01	0.33	9.6E-02	-0.22	2.7E-01	-0.13	5.3E-01	-0.25	2.1E-01	-0.19	3.5E-01
ENSCAFG0000000110	RANGAP1	grey	EC_M10	0.31	1.2E-01	-0.16	4.2E-01	0.38	5.4E-02	0.06	7.8E-01	-0.52	6.5E-03	-0.03	9.0E-01	-0.11	6.0E-01	-0.07	7.3E-01	-0.01	9.4E-01	0.41	3.8E-02
ENSCAFG0000000446	ENSCAFG0000000446	grey	EC_M10	0.31	1.2E-01	0.20	3.3E-01	-0.43	2.9E-02	-0.05	8.2E-01	0.16	4.2E-01	0.30	1.4E-01	0.25	2.2E-01	-0.09	6.5E-01	-0.25	2.3E-01	-0.34	8.9E-02
ENSCAFG0000002932	ZNF484	grey	EC_M10	0.31	1.2E-01	0.13	5.1E-01	-0.09	6.6E-01	0.09	6.6E-01	-0.22	2.7E-01	0.10	6.2E-01	-0.02	9.1E-01	-0.22	2.7E-01	0.30	1.4E-01	0.16	4.3E-01
ENSCAFG0000002548	ENSCAFG0000002548	darkgreen	EC_M4	0.31	1.2E-01	0.32	1.1E-01	-0.76	7.8E-06	-0.04	8.5E-01	0.48	1.4E-02	-0.02	9.0E-01	0.02	9.3E-01	0.13	5.4E-01	-0.16	4.2E-01	-0.66	2.6E-04
ENSCAFG0000001890	MTMR12	turquoise	EC_M6	0.31	1.2E-01	0.71	4.4E-05	-0.44	2.4E-02	-0.02	9.1E-01	0.04	8.5E-01	-0.27	1.9E-01	-0.14	5.0E-01	-0.28	1.7E-01	-0.11	5.9E-01	-0.15	4.8E-01
ENSCAFG0000000955	IMPG2	turquoise	EC_M6	0.31	1.2E-01	0.47	1.6E-02	-0.12	5.6E-01	0.13	5.3E-01	-0.33	1.0E-01	0.15	4.6E-01	0.02	9.2E-01	0.08	7.1E-01	0.21	2.9E-01	0.20	3.2E-01
ENSCAFG0000000340	MET	grey	EC_M10	0.31	1.2E-01	-0.09	6.7E-01	0.29	1.5E-01	-0.13	5.2E-01	-0.52	6.6E-03	-0.36	7.3E-02	-0.15	4.8E-01	0.07	7.5E-01	-0.02	9.2E-01	0.39	4.9E-02
ENSCAFG0000000760	URI1	cyan	EC_M2	0.31	1.2E-01	-0.37	6.6E-02	0.38	5.5E-02	0.09	6.8E-01	-0.62	7.7E-04	-0.15	4.5E-01	-0.21	3.0E-01	-0.04	8.4E-01	0.07	7.5E-01	0.46	1.9E-02
ENSCAFG0000003248	ENSCAFG0000003248	darkgreen	EC_M4	0.31	1.2E-01	-0.05	8.0E-01	-0.70	7.8E-05	-0.09	6.6E-01	0.60	1.1E-03	0.11	6.0E-01	0.07	7.3E-01	0.05	8.2E-01	-0.07	7.3E-01	-0.78	2.4E-06
ENSCAFG0000001022	PHKB	grey	EC_M10	0.31	1.2E-01	-0.46	1.8E-02	0.00	1.0E+00	0.13	5.1E-01	-0.10	6.2E-01	-0.10	6.4E-01	-0.50	9.9E-03	0.32	1.1E-01	-0.10	6.3E-01	-0.07	7.2E-01
ENSCAFG0000000661	ENSCAFG0000000661	turquoise	EC_M6	0.31	1.2E-01	0.44	2.6E-02	-0.29	1.5E-01	0.25	2.3E-01	-0.11	6.1E-01	0.04	8.6E-01	0.03	8.9E-01	-0.26	2.1E-01	-0.25	2.2E-01	-0.07	7.2E-01
ENSCAFG0000000839	PLRG1	grey	EC_M10	0.31	1.2E-01	-0.28	1.7E-01	-0.30	1.4E-01	0.02	9.0E-01	0.22	2.9E-01	0.26	2.0E-01	-0.29	1.6E-01	0.25	2.1E-01	-0.13	5.3E-01	-0.36	6.9E-02
ENSCAFG0000001232	WDR11	grey	EC_M10	0.31	1.2E-01	-0.26	2.0E-01	-0.13	5.3E-01	0.13	5.3E-01	0.01	9.5E-01	0.06	7.5E-01	-0.52	6.4E-03	0.04	8.4E-01	-0.24	2.4E-01	-0.12	5.5E-01
ENSCAFG0000000632	ENSCAFG0000000632	grey	EC_M10	0.31	1.2E-01	0.09	6.5E-01	-0.44	2.3E-02	-0.12	5.5E-01	0.24	2.4E-01	-0.16	4.5E-01	-0.16	4.4E-01	-0.11	5.9E-01	-0.10	6.2E-01	-0.41	3.9E-02
ENSCAFG0000000647	THUMPD2	grey	EC_M10	0.31	1.2E-01	-0.08	7.1E-01	0.23	2.7E-01	0.21	3.0E-01	-0.43	2.7E-02	0.00	9.9E-01	-0.33	9.6E-02	-0.33	1.0E-01	-0.31	1.3E-01	0.26	2.0E-01
ENSCAFG0000002585	ENSCAFG0000002585	grey	EC_M10	0.31	1.2E-01	0.07	7.2E-01	-0.09	6.5E-01	-0.10	6.1E-01	-0.16	4.5E-01	-0.09	6.8E-01	-0.06	7.6E-01	-0.12	5.5E-01	0.32	1.2E-01	0.03	8.9E-01
ENSCAFG0000000177	ENSCAFG0000000177	turquoise	EC_M6	0.31	1.2E-01	0.44	2.6E-02	-0.29	1.5E-01	0.33	1.0E-01	-0.02	9.1E-01	-0.20	3.3E-01	-0.13	5.2E-01	-0.07	7.5E-01	-0.17	4.2E-01	-0.12	5.6E-01
ENSCAFG0000000978	PRICKLE1	grey	EC_M10	0.31	1.2E-01	0.35	8.1E-02	-0.61	1.0E-03	0.07	7.4E-01	0.32	1.1E-01	0.19	3.6E-01	-0.16	4.4E-01	0.10	6.4E-01	0.24	2.3E-01	-0.51	8.2E-03
ENSCAFG0000001244	PROX1	turquoise	EC_M6	0.31	1.2E-01	0.85	4.3E-08	-0.37	6.7E-02	-0.04	8.3E-01	-0.13	5.2E-01	0.05	8.2E-01	-0.02	9.2E-01	-0.21	3.0E-01	-0.13	5.3E-01	-0.03	8.8E-01
ENSCAFG0000001615	TMEM156	grey	EC_M10	0.31	1.2E-01	0.18	3.9E-01	-0.47	1.6E-02	0.05	8.1E-01	0.23	2.5E-01	0.46	1.7E-02	0.00	9.8E-01	-0.30	1.4E-01	-0.41	3.8E-02	-0.47	1.6E-02
ENSCAFG0000001208	TSSK4	grey	EC_M10	0.31	1.2E-01	0.14	5.0E-01	0.15	4.6E-01	-0.05	8.1E-01	-0.44	2.4E-02	0.20	3.2E-01	-0.32	1.1E-01	-0.36	6.8E-02	-0.12	5.5E-01	0.27	1.8E-01
ENSCAFG0000001756	RAD51C	grey	EC_M10	0.31	1.2E-01	0.25	2.2E-01	0.30	1.4E-01	0.15	4.5E-01	-0.69	9.5E-05	-0.23	2.6E-01	-0.24	2.4E-01	0.02	9.1E-01	0.03	8.8E-01	0.55	3.3E-03
ENSCAFG0000002960	DMAC1	grey	EC_M10	0.31	1.2E-01	-0.10	6.3E-01	0.08	6.8E-01	0.02	9.1E-01	-0.33	9.6E-02	0.39	5.0E-02	-0.14	5.1E-01	-0.28	1.6E-01	-0.29	1.5E-01	0.17	4.2E-01
ENSCAFG0000001083	TC2N	turquoise	EC_M6	0.31	1.2E-01	0.92	1.6E-11	-0.24	2.4E-01	0.04	8.6E-01	-0.29	1.4E-01	-0.14	4.9E-01	-0.08	6.9E-01	-0.01	9.8E-01	-0.20	3.4E-01	0.14	5.1E-01
ENSCAFG0000001242	GABPB2	cyan	EC_M2	0.31	1.2E-01	-0.38	5.8E-02	0.38	5.7E-02	0.18	3.7E-01	-0.59	1.7E-03	0.01	9.7E-01	-0.14	4.8E-01	0.04	8.4E-01	-0.39	4.7E-02	0.39	4.7E-02
ENSCAFG0000001135	COX19	grey	EC_M10	0.31	1.2E-01	-0.24	2.4E-01	-0.05	8.1E-01	0.27	1.8E-01	-0.18	3.9E-01	0.09	6.7E-01	-0.02	9.1E-01	-0.06	7.7E-01	0.12	5.7E-01	0.00	9.9E-01
ENSCAFG0000002917	ENSCAFG0000002917	turquoise	EC_M6	0.31	1.2E-01	0.87	1.0E-08	-0.21	3.1E-01	-0.11	5.9E-01	-0.28	1.6E-01	-0.11	5.9E-01	-0.13	5.4E-01	-0.14	4.9E-01	-0.22	2.7E-01	0.11	6.0E-01
ENSCAFG0000001371	ENSCAFG0000001371	grey	EC_M10	0.31	1.2E-01	-0.27	1.7E-01	-0.17	4.1E-01	0.33	9.9E-02	0.04	8.5E-01	0.21	2.9E-01	-0.06	7.7E-01	-0.12	5.6E-01	-0.13	5.4E-01	-0.16	4.2E-01
ENSCAFG0000000946	NKX2-3	grey	EC_M10	0.31	1.2E-01	-0.13	5.2E-01	-0.48	1.3E-02	0.00	1.0E+00	0.31	1.2E-01	0.63	5.8E-04	-0.09	6.5E-01	-0.10	6.3E-01	-0.19	3.6E-01	-0.52	6.3E-03
ENSCAFG0000000481	SLC5A6	grey	EC_M10	0.31	1.2E-01	0.01	9.6E-01	-0.07	7.3E-01	0.01	9.6E-01	-0.15	4.8E-01	-0.01	9.6E-01	-0.02	9.2E-01	0.08	7.1E-01	0.62	8.2E-04	-0.02	9.3E-01
ENSCAFG0000001188	DNAJC8	turquoise	EC_M6	0.31	1.2E-01	0.50	1.0E-02	-0.48	1.4E-02	-0.20	3.3E-01	0.13	5.4E-01	-0.09	6.8E-01	0.14	4.9E-01	0.01	9.7E-01	-0.18	3.9E-01	-0.26	2.0E-01
ENSCAFG0000001417	ASTN1	grey	EC_M10	0.31	1.2E-01	-0.15	4.8E-01	-0.40	4.2E-02	0.14	4.8E-01	0.21	3.0E-01	0.31	1.2E-01	-0.10	6.2E-01	0.06	7.5E-01	-0.29	1.5E-01	-0.48	1.3E-02
ENSCAFG0000001166	PRPS2	grey	EC_M10	0.31	1.2E-01	0.61	9.4E-04	0.17	4.1E-01	-0.17	4.1E-01	-0.60	1.2E-03	-0.14	5.0E-01	0.04	8.4E-01	-0.18	3.7E-01	0.09	6.5E-01	0.49	1.0E-02
ENSCAFG0000002647	ENSCAFG0000002647	grey	EC_M10	0.31	1.2E-01	0.20	3.4E-01	0.10	6.4E-01	0.29	1.5E-01	-0.46	1.9E-02	-0.31	1.2E-01	-0.34	8.8E-02	0.08	6.9E-01	-0.03	8.7E-01	0.26	2.0E-01
ENSCAFG0000000003	DOK6	grey	EC_M10	0.31	1.2E-01	0.03	9.0E-01	-0.49	1.1E-02	-0.24	2.5E-01	0.33	9.9E-02	0.01	9.5E-01	-0.17	4.1E-01	0.30	1.4E-01	0.47	1.6E-02	-0.46	1.8E-02
ENSCAFG0000001653	POLR2A	grey	EC_M10	0.31	1.2E-01	-0.15	4.7E-01	-0.31	1.2E-01	-0.11	5.9E-01	0.24	2.4E-01	0.07	7.3E-01	-0.40	4.3E-02	0.30	1.4E-01	0.02	9.2E-01	-0.45	2.1E-02
ENSCAFG0000001450	TBX1	turquoise	EC_M6	0.31	1.2E-01	0.86	1.5E-08	-0.24	2.3E-01	-0.14	4.9E-01	-0.26	1.9E-01	-0.13	5.4E-01	-0.14	4.9E-01	-0.16	4.2E-01	-0.21	3.1E-01	0.10	6.3E-01
ENSCAFG0000002789	ENSCAFG0000002789	grey	EC_M10	0.31	1.2E-01	0.24	2.5E-01	-0.24	2.4E-01	-0.11	5.9E-01	-0.04	8.6E-01	0.33	9.9E-02	-0.08	6.9E-01	-0.16	4.4E-01	0.18	3.8E-01	-0.11	5.8E-01
ENSCAFG0000001757	MED7	grey	EC_M10	0.31	1.2E-01	0.15	4.7E-01	-0.34	8.5E-02	-0.36	7.2E-02	0.11	5.9E-01	-0.40	4.2E-02	-0.22	2.8E-01	0.36	7.1E-02	0.06	7.6E-01	-0.27	1.8E-01
ENSCAFG0000002907	CCDC126	grey	EC_M10	0.31	1.2E-01	0.26	1.9E-01	-0.58	1.9E-03	0.10	6.2E-01	0.25	2.2E-01	0.11	5.8E-01	-0.08	7.1E-01	0.01	9.8E-01	-0.36	7.1E-02	-0.45	2.1E-02
ENSCAFG0000000103	ENSCAFG0000000103	grey	EC_M10	0.31	1.2E-01	0.05	8.2E-01	-0.30	1.3E-01	-0.30	1.4E-01	0.23	2.6E-01	0.01	9.5E-01	-0.24	2.3E-01	-0.07	7.3E-01	-0.30	1.4E-01	-0.37	6.1E-02
ENSCAFG0000001551	POU2F1	turquoise	EC_M6	0.31	1.2E-01	0.39	4.6E-02	-0.14	4.8E-01	0.10	6.2E-01	-0.21	3.1E-01	0.12	5.6E-01	-0.03	8.7E-01	-0.43	2.8E-02	-0.36	7.2E-02	0.05	8.1E-01
ENSCAFG0000001401	FKBP3	grey	EC_M10	0.31	1.2E-01	-0.21	2.9E-01	-0.55	3.4E-03	-0.25	2.1E-01	0.49	1.0E-02	0.21	3.0E-01	-0.27	1.8E-01	0.28	1.6E-01	-0.06	7.8E-01	-0.67	2.0E-04
ENSCAFG0000002629	ENSCAFG0000002629	grey	EC_M10	0.31	1.2E-01	-0.01	9.5E-01	-0.24	2.3E-01	-0.17	4.1E-01	0.01	9.7E-01	0.44	2.3E-02	-0.11	6.1E-01	0.13	5.2E-01	0.38	5.3E-02	-0.18	3.9E-01
ENSCAFG0000001799	ACACA	cyan	EC_M2	0.31	1.2E-01	-0.48	1.3E-02	0.23	2.5E-01	0.31	1.3E-01	-0.39	4.7E-02	-0.16	4.4E-01	-0.37	6.3E-02	-0.19	3.6E-01	-0.07	7.3E-01	0.20	3.2E-01
ENSCAFG0000001024	SLC4A10	grey	EC_M10	0.31	1.2E-01	0.07	7.3E-01	-0.62	7.1E-04	0.02	9.1E-01	0.44	2.3E-02	0.21	2.9E-01	-0.16	4.5E-01	-0.05	8.3E-01	0.00	9.8E-01	-0.66	2.5E-04
ENSCAFG0000000132	PYCR3	grey	EC_M10	0.31	1.2E-01	-0.39	5.1E-02	0.27	1.8E-01	0.17	4.2E-01	-0.35	7.6E-02	0.07	7.5E-01	0.12	5.7E-01	-0.08	7.1E-01	-0.01	9.7E-01	0.22	2.8E-01
ENSCAFG0000001605	MNS1	grey	EC_M10	0.31	1.2E-01	-0.32	1.1E-01	-0.02	9.3E-01	0.06	7.7E-01	-0.18	3.8E-01	-0.06	7.7E-01	0.16	4.4E-01	-0.01	9.5E-01	-0.24	2.3E-01	0.00	9.9E-01
ENSCAFG0000000182	CEMIP2	grey	EC_M10	0.31	1.2E-01	0.53	5.0E-03	-0.90	3.8E-10	-0.25	2.2E-01	0.61	8.8E-04	0.23	2.6E-01	-0.03	8.8E-01	-0.05	7.9E-01	0.15	4.7E-01	-0.78	3.0E-06
ENSCAFG0000001777	GPRASP1	grey	EC_M10	0.31	1.2E-01	-0.55	3.9E-03	0.14	4.9E-01	0.37	6.4E-02	-0.23	2.5E-01	0.41	3.8E-02	-0.33	1.0E-01	-0.20	3.3E-01	-0.26	1.9E-01	0.06	7.6E-01
ENSCAFG0000001823	LRCH2	grey	EC_M10	0.31	1.3E-01	-0.28	1.6E-01	-0.39	5.0E-02	0.24	2.4E-01	0.28	1.7E-01	0.25	2.2E-01	-0.16	4.4E-01	0.09	6.7E-01	-0.49	1.1E-02	-0.51	7.4E-03
ENSCAFG0000001667	MED6	darkgrey	EC_M8	0.31	1.3E-01	0.17	4.0E-01	0.25	2.2E-01	0.20	3.4E-01	-0.66	2.2E-04	-0.19	3.5E-01	-0.10	6.3E-01	-0.12	5.7E-01	-0.08	7.0E-01	0.53	5.4E-03
ENSCAFG0000000676	PARG	grey	EC_M10	0.31	1.3E-01	-0.01	9.6E-01	-0.32	1.1E-01	-0.25	2.2E-01	0.09	6.5E-01	0.19	3.4E-01	-0.31	1.2E-01	-0.22	2.7E-01	-0.21	3.1E-01	-0.25	2.3E-01
ENSCAFG0000002865	ENSCAFG0000002865	grey	EC_M10	0.31	1.3E-01	0.01	9.5E-01	-0.14	4.9E-01	0.18	3.9E-01	-0.14	5.0E-01	0.07	7.2E-01	-0.56	3.2E-03	-0.11	5.9E-01	0.32	1.2E-01	-0.05	8.2E-01
ENSCAFG0000000930	ESRP1	grey	EC_M10	0.31	1.3E-01	-0.05	8.0E-01	-0.29	1.6E-01	0.20	3.3E-01	0.10	6.2E-01	0.32	1.1E-01	-0.24	2.3E-01	0.21	3.1E-01	0.10	6.2E-01	-0.28	1.6E-01
ENSCAFG0000001828	ENSCAFG0000001828	turquoise	EC_M6	0.31	1.3E-01	0.57	2.5E-03	-0.35	7.9E-02	-0.03	8.9E-01	-0.03	8.7E-01	-0.01	9.5E-01	0.03	8.7E-01	-0.06	7.8E-01	-0.23	2.7E-01	-0.07	7.3E-01
ENSCAFG0000000870	TRIM35	grey	EC_M10	0.31	1.3E-01	-0.36	6.8E-02	0.01	9.5E-01	-0.37	6.4E-02	-0.06	7.8E-01	0.17	4.0E-01	0.10	6.1E-01	0.01	9.7E-01	-0.17	4.2E-01	-0.09	6.6E-01
ENSCAFG0000001503	SH2D5	grey	EC_M10	0.31	1.3E-01	0.28	1.7E-01	-0.37	6.0E-02	-0.14	5.0E-01	0.12	5.5E-01	-0.20	3.3E-01	-0.07	7.2E-01	-0.12	5.7E-01	-0.04	8.4E-01	-0.24	2.4E-01
ENSCAFG0000000001	ENSCAFG0000000001	turquoise	EC_M6	0.31	1.3E-01	0.38	5.6E-02	-0.19	3.6E-01	-0.07	7.4E-01	-0.14	4.8E-01	-0.14	5.1E-01	-0.10	6.3E-01	-0.03	8.7E-01	-0.20	3.2E-01	-0.04	8.6E-01
ENSCAFG0000001868	MTCL1	grey	EC_M10	0.31	1.3E-01	0.04	8.4E-01	-0.59	1.5E-03	-0.30	1.4E-01	0.50	1.0E-02	0.09	6.6E-01	0.03	8.7E-01	-0.07	7.3E-01	-0.22	2.9E-01	-0.64	4.9E-04
ENSCAFG0000001715	RPS4X	cyan	EC_M2	0.31	1.3E-01	-0.41	3.7E-02	0.34	8.5E-02	0.21	3.0E-01	-0.50	9.9E-03	0.09	6.8E-01	0.12	5.6E-01	-0.10	6.3E-01	-0.56	2.7E-03	0.27	1.7E-01
ENSCAFG0000001703	RANGRF	grey	EC_M10	0.31	1.3E-01	-0.26	2.0E-01	0.26	2.1E-01	0.03	8.9E-01	-0.35	8.3E-02	0.04	8.4E-01	-0.07	7.2E-01	-0.29	1.5E-01	-0.14	4.9E-01	0.19	3.5E-01
ENSCAFG0000001693	C6H16orf58	grey	EC_M10	0.31	1.3E-01	0.18	3.7E-01	-0.39	5.0E-02	0.12	5.6E-01	0.21	3.1E-01	0.02	9.4E-01	-0.25	2.1E-01	0.23	2.6E-01	0.16	4.4E-01	-0.33	9.6E-02

ENSCAFG0000001655	C9H17orf98	grey	EC_M10	0.31	1.3E-01	-0.32	1.1E-01	0.00	9.8E-01	0.03	8.8E-01	-0.19	3.6E-01	0.03	9.0E-01	0.10	6.2E-01	0.05	8.0E-01	-0.38	5.9E-02	0.00	1.0E+00
ENSCAFG0000001747	DIAPH2	darkgreen	EC_M4	0.31	1.3E-01	0.04	8.4E-01	-0.84	5.8E-08	-0.29	1.6E-01	0.70	6.3E-05	0.22	2.9E-01	-0.02	9.4E-01	0.00	9.8E-01	-0.26	2.0E-01	-0.87	9.9E-09
ENSCAFG0000001610	ENSCAFG0000001610	turquoise	EC_M6	0.31	1.3E-01	0.79	2.0E-06	-0.34	9.3E-02	-0.05	8.1E-01	-0.14	5.0E-01	-0.11	5.9E-01	-0.09	6.6E-01	-0.11	6.1E-01	0.35	8.0E-02	0.00	9.9E-01
ENSCAFG0000000918	KCNJ3	turquoise	EC_M6	0.31	1.3E-01	0.90	3.5E-10	-0.39	5.1E-02	-0.09	6.6E-01	-0.09	6.6E-01	-0.14	5.0E-01	-0.10	6.3E-01	-0.12	5.6E-01	0.21	3.1E-01	-0.05	7.9E-01
ENSCAFG0000000266	CD109	cyan	EC_M2	0.31	1.3E-01	0.14	5.1E-01	0.51	7.7E-03	0.23	2.6E-01	-0.93	3.8E-12	-0.24	2.3E-01	-0.16	4.4E-01	-0.08	7.0E-01	0.00	9.9E-01	0.75	1.2E-05
ENSCAFG0000001963	NOTCH1	turquoise	EC_M6	0.31	1.3E-01	0.90	5.6E-10	-0.21	3.0E-01	0.03	8.8E-01	-0.32	1.1E-01	-0.19	3.6E-01	-0.04	8.4E-01	-0.07	7.3E-01	0.13	5.2E-01	0.15	4.8E-01
ENSCAFG0000003208	CBX2	turquoise	EC_M6	0.31	1.3E-01	0.57	2.2E-03	-0.32	1.1E-01	-0.11	5.9E-01	-0.08	7.0E-01	-0.17	4.0E-01	0.03	8.9E-01	0.14	5.0E-01	0.17	4.2E-01	-0.09	6.7E-01
ENSCAFG0000001317	ELP6	grey	EC_M10	0.31	1.3E-01	-0.39	4.6E-02	-0.21	3.0E-01	-0.03	9.0E-01	0.16	4.4E-01	0.07	7.4E-01	0.02	9.4E-01	0.18	3.7E-01	0.04	8.4E-01	-0.34	9.2E-02
ENSCAFG0000001230	KLHL41	grey	EC_M10	0.31	1.3E-01	-0.36	7.3E-02	0.16	4.3E-01	0.19	3.6E-01	-0.36	7.2E-02	0.14	4.8E-01	-0.11	6.1E-01	-0.34	9.3E-02	0.01	9.6E-01	0.20	3.2E-01
ENSCAFG0000001490	POLN	grey	EC_M10	0.31	1.3E-01	0.02	9.2E-01	-0.37	6.4E-02	-0.04	8.6E-01	0.14	5.1E-01	-0.08	7.1E-01	-0.42	3.1E-02	0.01	9.5E-01	-0.34	8.6E-02	-0.32	1.1E-01
ENSCAFG0000002778	ENSCAFG0000002778	grey	EC_M10	0.31	1.3E-01	0.23	2.6E-01	-0.19	3.5E-01	0.00	1.0E+00	-0.05	7.9E-01	-0.12	5.7E-01	-0.12	5.7E-01	-0.03	8.8E-01	-0.11	6.0E-01	-0.11	6.0E-01
ENSCAFG0000000426	ENSCAFG0000000426	grey	EC_M10	0.31	1.3E-01	-0.17	4.0E-01	-0.15	4.6E-01	-0.12	5.5E-01	-0.01	9.8E-01	0.36	7.2E-02	-0.09	6.7E-01	-0.03	8.8E-01	-0.04	8.5E-01	-0.17	4.0E-01
ENSCAFG0000001681	CDK5RAP3	cyan	EC_M2	0.31	1.3E-01	-0.13	5.2E-01	0.51	7.7E-03	0.23	2.6E-01	-0.81	6.9E-07	-0.15	4.7E-01	-0.04	8.5E-01	-0.16	4.2E-01	-0.34	8.8E-02	0.62	6.8E-04
ENSCAFG0000001308	ING5	darkgrey	EC_M8	0.31	1.3E-01	-0.03	8.7E-01	0.32	1.1E-01	0.17	4.0E-01	-0.67	1.6E-04	-0.02	9.1E-01	-0.25	2.1E-01	-0.15	4.5E-01	-0.17	3.9E-01	0.48	1.2E-02
ENSCAFG0000000324	WDR92	grey	EC_M10	0.31	1.3E-01	0.11	5.8E-01	-0.45	2.2E-02	0.04	8.6E-01	0.19	3.6E-01	0.11	6.0E-01	0.02	9.3E-01	0.06	7.5E-01	-0.22	2.9E-01	-0.32	1.1E-01
ENSCAFG0000003040	ENSCAFG0000003040	grey	EC_M10	0.31	1.3E-01	0.27	1.8E-01	0.00	9.8E-01	-0.06	7.8E-01	-0.41	4.0E-02	0.08	7.1E-01	-0.20	3.2E-01	0.45	2.0E-02	0.27	1.8E-01	0.18	3.7E-01
ENSCAFG0000001099	CCDC15	grey	EC_M10	0.31	1.3E-01	0.24	2.4E-01	-0.54	4.0E-03	-0.02	9.4E-01	0.20	3.2E-01	0.18	3.7E-01	-0.17	4.1E-01	0.37	6.6E-02	0.32	1.2E-01	-0.41	3.5E-02
ENSCAFG0000000880	CCDC65	grey	EC_M10	0.31	1.3E-01	-0.10	6.4E-01	0.02	9.0E-01	0.31	1.2E-01	-0.24	2.4E-01	0.17	4.1E-01	-0.26	2.1E-01	-0.19	3.5E-01	0.18	3.9E-01	0.04	8.4E-01
ENSCAFG0000001049	ENSCAFG0000001049	grey	EC_M10	0.31	1.3E-01	0.21	3.1E-01	-0.08	6.9E-01	0.17	4.2E-01	-0.14	5.0E-01	0.17	4.2E-01	-0.24	2.5E-01	-0.19	3.6E-01	0.00	9.9E-01	0.03	8.9E-01
ENSCAFG0000000784	GBE1	grey	EC_M10	0.31	1.3E-01	0.23	2.7E-01	0.27	1.7E-01	0.06	7.5E-01	-0.67	2.1E-04	-0.20	3.3E-01	0.08	7.0E-01	0.11	5.9E-01	-0.05	8.0E-01	0.54	4.8E-03
ENSCAFG0000000236	ADGRL3	grey	EC_M10	0.31	1.3E-01	0.09	6.5E-01	-0.30	1.4E-01	0.12	5.6E-01	0.04	8.4E-01	0.07	7.2E-01	-0.18	3.8E-01	0.29	1.5E-01	0.46	1.8E-02	-0.24	2.4E-01
ENSCAFG0000001144	CASC1	grey	EC_M10	0.30	1.3E-01	-0.42	3.3E-02	-0.29	1.5E-01	0.33	9.8E-02	0.28	1.7E-01	0.29	1.5E-01	-0.18	3.8E-01	-0.16	4.3E-01	-0.20	3.3E-01	-0.44	2.6E-02
ENSCAFG0000000700	RAB28	grey	EC_M10	0.30	1.3E-01	0.06	7.8E-01	0.18	3.9E-01	0.15	4.5E-01	-0.47	1.4E-02	-0.31	1.3E-01	-0.15	4.7E-01	-0.30	1.4E-01	-0.23	2.5E-01	0.34	9.0E-02
ENSCAFG0000001152	TTC14	darkgreen	EC_M4	0.30	1.3E-01	-0.31	1.3E-01	-0.71	4.5E-05	0.05	8.3E-01	0.67	1.9E-04	0.35	7.9E-02	-0.20	3.3E-01	-0.05	8.3E-01	-0.22	2.9E-01	-0.86	1.6E-08
ENSCAFG0000000086	AFDN	turquoise	EC_M6	0.30	1.3E-01	0.76	7.3E-06	-0.26	1.9E-01	0.06	7.9E-01	-0.24	2.3E-01	-0.09	6.7E-01	-0.30	1.4E-01	0.09	6.5E-01	0.13	5.4E-01	0.06	7.8E-01
ENSCAFG0000002032	DUS2	turquoise	EC_M6	0.30	1.3E-01	0.67	1.7E-04	-0.08	6.8E-01	0.30	1.3E-01	-0.34	9.2E-02	-0.33	1.0E-01	-0.18	3.9E-01	-0.21	3.0E-01	-0.10	6.4E-01	0.19	3.5E-01
ENSCAFG0000000870	ENSCAFG0000000870	grey	EC_M10	0.30	1.3E-01	-0.18	3.8E-01	-0.35	8.1E-02	-0.06	7.7E-01	0.32	1.1E-01	0.08	6.9E-01	-0.01	9.7E-01	-0.05	8.0E-01	-0.29	1.5E-01	-0.48	1.4E-02
ENSCAFG0000000271	DNAH11	grey	EC_M10	0.30	1.3E-01	-0.28	1.7E-01	0.13	5.2E-01	-0.06	7.8E-01	-0.29	1.5E-01	0.17	4.1E-01	0.02	9.2E-01	-0.28	1.6E-01	0.10	6.1E-01	0.15	4.5E-01
ENSCAFG0000000960	ZC3H11A	turquoise	EC_M6	0.30	1.3E-01	0.61	9.9E-04	-0.22	2.7E-01	-0.04	8.6E-01	-0.27	1.8E-01	0.04	8.6E-01	0.18	3.9E-01	0.04	8.4E-01	0.05	8.2E-01	0.12	5.7E-01
ENSCAFG0000000766	CLCN3	turquoise	EC_M6	0.30	1.3E-01	0.57	2.2E-03	-0.55	3.7E-03	0.09	6.7E-01	0.12	5.8E-01	0.16	4.3E-01	-0.19	3.5E-01	0.00	9.9E-01	0.37	6.2E-02	-0.28	1.6E-01
ENSCAFG0000000925	CYTIP	turquoise	EC_M6	0.30	1.3E-01	0.93	7.0E-12	-0.22	2.8E-01	-0.10	6.3E-01	-0.29	1.5E-01	-0.12	5.5E-01	-0.11	5.9E-01	-0.11	5.9E-01	0.01	9.6E-01	0.13	5.1E-01
ENSCAFG0000000506	PAXIP1	darkgrey	EC_M8	0.30	1.3E-01	0.44	2.6E-02	0.16	4.4E-01	0.09	6.6E-01	-0.61	9.3E-04	-0.07	7.2E-01	0.20	3.3E-01	-0.13	5.4E-01	0.03	8.9E-01	0.45	2.1E-02
ENSCAFG0000000209	SRD5A3	turquoise	EC_M6	0.30	1.3E-01	0.52	6.8E-03	-0.13	5.2E-01	0.48	1.2E-02	-0.32	1.1E-01	-0.18	3.9E-01	-0.35	7.8E-02	-0.25	2.1E-01	-0.09	6.5E-01	0.15	4.5E-01
ENSCAFG0000001823	ENSCAFG0000001823	turquoise	EC_M6	0.30	1.3E-01	-0.15	4.6E-01	-0.09	6.7E-01	0.46	1.8E-02	-0.14	5.1E-01	0.49	1.1E-02	-0.10	6.1E-01	-0.17	4.0E-01	-0.10	6.2E-01	0.01	9.8E-01
ENSCAFG0000000602	SH3BP5	turquoise	EC_M6	0.30	1.3E-01	0.86	1.9E-08	-0.08	6.9E-01	0.06	7.5E-01	-0.46	1.7E-02	-0.22	2.8E-01	-0.07	7.4E-01	0.00	9.9E-01	0.05	7.9E-01	0.30	1.3E-01
ENSCAFG0000002496	ENSCAFG0000002496	turquoise	EC_M6	0.30	1.3E-01	0.41	3.7E-02	-0.23	2.5E-01	0.07	7.3E-01	-0.15	4.8E-01	0.09	6.7E-01	0.24	2.4E-01	-0.21	2.9E-01	-0.38	5.6E-02	-0.06	7.8E-01
ENSCAFG0000000848	PDE3B	grey	EC_M10	0.30	1.3E-01	-0.34	9.4E-02	-0.29	1.4E-01	-0.03	8.9E-01	0.25	2.2E-01	0.19	3.5E-01	-0.06	7.5E-01	0.36	7.1E-02	-0.13	5.3E-01	-0.41	3.6E-02
ENSCAFG0000001005	ANO5	turquoise	EC_M6	0.30	1.3E-01	0.63	6.1E-04	-0.17	4.1E-01	-0.01	9.8E-01	-0.27	1.8E-01	-0.16	4.3E-01	0.11	5.9E-01	-0.31	1.2E-01	-0.22	2.8E-01	0.11	6.0E-01
ENSCAFG0000002048	ENSCAFG0000002048	grey	EC_M10	0.30	1.3E-01	0.07	7.5E-01	-0.44	2.6E-02	0.02	9.2E-01	0.22	2.7E-01	-0.13	5.3E-01	-0.09	6.5E-01	-0.10	6.3E-01	-0.19	3.6E-01	-0.42	3.5E-02
ENSCAFG0000002470	ENSCAFG0000002470	grey	EC_M10	0.30	1.3E-01	-0.05	8.0E-01	-0.24	2.4E-01	0.37	6.5E-02	0.01	9.6E-01	-0.15	4.7E-01	-0.17	4.0E-01	-0.19	3.5E-01	-0.13	5.2E-01	-0.14	4.8E-01
ENSCAFG0000003016	ENSCAFG0000003016	darkgreen	EC_M4	0.30	1.3E-01	-0.08	7.1E-01	-0.73	2.1E-05	-0.01	9.6E-01	0.60	1.2E-03	0.20	3.3E-01	0.10	6.2E-01	0.12	5.4E-01	-0.25	2.2E-01	-0.80	1.0E-06
ENSCAFG0000001083	SHOC2	turquoise	EC_M6	0.30	1.3E-01	0.55	3.9E-03	-0.28	1.6E-01	0.00	9.9E-01	-0.19	3.6E-01	0.07	7.3E-01	-0.18	3.8E-01	-0.16	4.2E-01	-0.08	6.9E-01	0.05	8.0E-01
ENSCAFG0000001587	CNST	grey	EC_M10	0.30	1.3E-01	0.32	1.1E-01	-0.43	3.0E-02	0.13	5.3E-01	0.05	8.0E-01	0.51	7.8E-03	-0.08	7.1E-01	-0.13	5.3E-01	-0.33	1.0E-01	-0.27	1.8E-01
ENSCAFG0000000559	ENSCAFG0000000559	grey	EC_M10	0.30	1.3E-01	-0.26	2.1E-01	0.18	3.9E-01	0.27	1.8E-01	-0.37	6.3E-02	-0.15	4.7E-01	-0.17	4.0E-01	-0.12	5.5E-01	-0.22	2.8E-01	0.20	3.2E-01
ENSCAFG0000000994	TANK	darkgreen	EC_M4	0.30	1.3E-01	0.26	2.0E-01	-0.90	6.1E-10	-0.34	8.6E-02	0.70	7.5E-05	0.35	8.3E-02	-0.16	4.4E-01	-0.01	9.7E-01	0.02	9.3E-01	-0.87	9.6E-09
ENSCAFG0000001031	ENSCAFG0000001031	grey	EC_M10	0.30	1.3E-01	0.33	1.0E-01	-0.31	1.3E-01	-0.24	2.4E-01	0.04	8.6E-01	0.35	8.0E-02	0.09	6.6E-01	-0.11	5.9E-01	-0.17	4.1E-01	-0.21	3.1E-01
ENSCAFG0000000753	ERBIN	turquoise	EC_M6	0.30	1.3E-01	0.57	2.4E-03	-0.22	2.7E-01	0.12	5.5E-01	-0.28	1.7E-01	-0.06	7.7E-01	-0.15	4.5E-01	-0.02	9.1E-01	0.28	1.7E-01	0.11	6.1E-01
ENSCAFG0000000788	MCCC2	grey	EC_M10	0.30	1.3E-01	-0.62	6.7E-04	-0.28	1.6E-01	-0.06	7.6E-01	0.32	1.1E-01	0.13	5.3E-01	-0.35	7.7E-02	0.07	7.4E-01	0.09	6.6E-01	-0.50	1.0E-02
ENSCAFG0000000134	ENSCAFG0000000134	grey	EC_M10	0.30	1.3E-01	-0.23	2.5E-01	-0.16	4.5E-01	0.39	4.8E-02	0.05	8.1E-01	0.30	1.3E-01	-0.22	2.9E-01	0.30	1.4E-01	-0.09	6.5E-01	-0.21	3.1E-01
ENSCAFG0000001214	MAP3K6	turquoise	EC_M6	0.30	1.3E-01	0.47	1.6E-02	0.17	4.0E-01	0.16	4.4E-01	-0.60	1.3E-03	-0.16	4.4E-01	-0.17	4.1E-01	-0.09	6.7E-01	0.42	3.5E-02	0.44	2.3E-02
ENSCAFG0000000111	TMEM71	turquoise	EC_M6	0.30	1.3E-01	0.63	5.4E-04	-0.15	4.5E-01	-0.32	1.1E-01	-0.29	1.6E-01	0.17	4.0E-01	-0.08	7.1E-01	-0.09	6.6E-01	0.06	7.6E-01	0.15	4.7E-01
ENSCAFG0000000004	TMX3	grey	EC_M10	0.30	1.3E-01	0.18	3.8E-01	-0.61	9.2E-04	0.05	8.0E-01	0.34	8.6E-02	0.17	4.2E-01	0.00	9.8E-01	0.14	5.0E-01	-0.32	1.1E-01	-0.54	4.6E-03
ENSCAFG0000001044	FOXRED1	grey	EC_M10	0.30	1.3E-01	-0.48	1.3E-02	-0.23	2.6E-01	-0.06	7.8E-01	0.26	2.1E-01	0.48	1.4E-02	-0.12	5.6E-01	0.09	6.7E-01	0.09	6.7E-01	-0.42	3.4E-02
ENSCAFG0000002572	ENSCAFG0000002572	grey	EC_M10	0.30	1.3E-01	0.21	2.9E-01	-0.55	3.6E-03	-0.27	1.8E-01	0.39	5.1E-02	-0.09	6.5E-01	0.02	9.3E-01	0.13	5.3E-01	-0.26	2.0E-01	-0.52	6.6E-03
ENSCAFG0000002896	ENSCAFG0000002896	grey	EC_M10	0.30	1.3E-01	0.08	6.8E-01	0.08	6.9E-01	0.47	1.5E-02	-0.35	7.5E-02	-0.16	4.4E-01	-0.15	4.6E-01	-0.15	4.6E-01	-0.16	4.3E-01	0.22	2.9E-01
ENSCAFG0000000232	ENSCAFG0000000232	grey	EC_M10	0.30	1.3E-01	0.20	3.2E-01	-0.31	1.2E-01	-0.06	7.6E-01	0.08	7.1E-01	-0.11	5.8E-01	-0.12	5.6E-01	0.46	1.9E-02	0.00	9.9E-01	-0.23	2.6E-01
ENSCAFG0000002087	ENSCAFG0000002087	grey	EC_M10	0.30	1.3E-01	0.16	4.3E-01	0.02	9.3E-01	0.33	1.0E-01	-0.30	1.4E-01	-0.19	3.4E-01	-0.17	4.1E-01	-0.13	5.4E-01	-0.30	1.4E-01	0.11	5.8E-01
ENSCAFG0000003131	TMEM236	turquoise	EC_M6	0.30	1.3E-01	0.58	1.9E-03	-0.55	3.3E-03	-0.09	6.6E-01	0.16	4.4E-01	0.22	2.8E-01	0.08	6.9E-01	-0.05	8.2E-01	-0.15	4.8E-01	-0.38	5.4E-02
ENSCAFG0000001244	SLC16A9	turquoise	EC_M6	0.30	1.4E-01	0.80	1.1E-06	-0.26	2.0E-01	0.14	4.8E-01	-0.18	3.8E-01	0.06	7.8E-01	-0.14	4.9E-01	-0.18	3.7E-01	-0.27	1.8E-01	0.02	9.1E-01
ENSCAFG0000001496	ATP8B4	turquoise	EC_M6	0.30	1.4E-01	0.55	3.3E-03	-0.51	7.3E-03	-0.16	4.4E-01	0.20	3.3E-01	0.10	6.3E-01	0.24	2.4E-01	-0.17	4.0E-01	-0.33	1.0E-01	-0.35	8.1E-02
ENSCAFG0000001964	RAB39B	grey	EC_M10	0.30	1.4E-01	-0.35	8.0E-02	-0.01	9.6E-01	0.40	4.3E-02	-0.20	3.3E-01	0.20	3.4E-01	0.02	9.3E-01	0.07	7.2E-01	-0.09	6.6E-01	-0.01	9.5E-01
ENSCAFG0000001019	NFRKB	grey	EC_M10	0.30	1.4E-01	0.31	1.2E-01	-0.33	9.6E-02	0.01	9.5E-01	0.13	5.4E-01	0.33	9.7E-02	-0.23	2.7E-01	-0.05	8.2E-01	0.22	2.9E-01	-0.27	1.9E-01
ENSCAFG0000000630	MORN2	grey	EC_M10	0.30	1.4E-01	-0.65	3.5E-04	-0.05	8.0E-01	0.06	7.7E-01	0.03	9.0E-01	0.04	8.5E-01	-0.14	5.0E-01	0.09	6.5E-01	-0.02	9.2E-01	-0.21	2.9E-01
ENSCAFG0000001431	APEX2	darkgrey	EC_M8	0.30	1.4E-01	0.14	5.0E-01	0.20	3.2E-01	0.00	1.0E+00	-0.39	4.7E-02	-0.15	4.6E-01	-0.16	4.2E-01	-0.24	2.4E-01	-0.14	5.1E-01	0.30	1.3E-01
ENSCAFG0000001649	CCDC149	grey	EC_M10	0.30	1.4E-01	0.39	5.2E-02	0.18	3.9E-01	-0.09	6.6E-01	-0.54	4.7E-03	-0.24	2.5E-01	0.05	8.2E-01	-0.21	3.1E-01	0.10	6.2E-01	0.41	3.7E-02
ENSCAFG0000001613	CECR2	turquoise	EC_M6	0.30	1.4E-01	0.62	6.5E-04	-0.37	6.0E-02	-0.06	7.5E-01	-0.10	6.4E-01	-0.08	7.1E-01	-0.16	4.3E-01	0.18	3.9E-01	0.26	2.0E-01	-0.09	6.5E-01
ENSCAFG0000000714	SACS	darkgreen	EC_M4	0.30	1.4E-01	-0.28	1.7E-01	-0.65	3.7E-04	-0.13	5.2E-01	0.63	5.3E-04	0.24	2.3E-01	0.07	7.5E-01	-0.12	5.7E-01	-0.41	3.9E-02	-0.80	9.1E-07
ENSCAFG0000003137	UBXN2A	darkgrey	EC_M8	0.30	1.4E-01	0.11	5.8E-01	0.17	4.1E-01	0.18	3.7E-01	-0.52	6.6E-03	-0.13	5.2E-01	-0.42	3.4E-02	-0.12	5.6E-01	-0.27	1.8E-01	0.39	5.1E-02
ENSCAFG0000001765	ENSCAFG0000001765	darkgreen	EC_M4	0.30	1.4E-01	0.22	2.9E-01	-0.76	8.0E-06	-0.29	1.5E-01	0.60	1.2E-03	0.24	2.5E-01	0.30	1.3E-01	0.09	6.8E-01	-0.08	7.0E-01	-0.76	6.3E-06
ENSCAFG0000000748	SNRNP35	grey	EC_M10	0.30	1.4E-01	-0.07	7.4E-01	-0.40	4.1E-02	-0.07	7.3E-01	0.24	2.3E-01	0.11	6.1E-01	-0.04	8.6E-01	0.03	8.8E-01	-0.32	1.1E-01	-0.38	5.6E-02
ENSCAFG0000003076	MSMP	turquoise	EC_M6	0.30	1.4E-01	0.83	1.2E-07	-0.29	1.5E-01	-0.11	5.9E-01	-0.17	4.1E-01	-0.11	5.8E-01	-0.13	5.2E-01	-0.13	5.2E-01	-0.20	3.3E-01	-0.01	9.5E-01
ENSCAFG0000000478	CAMK1D	grey	EC_M10	0.30	1.4E-01	0.34	8.7E-02	-0.59	1.7E-03	-0.09	6.5E-01	0.32	1.1E-01	0.01	9.7E-01	-0.07	7.5E-01	-0.08	6.9E-01	0.22	2.7E-01	-0.44	2.5E-02
ENSCAFG0000000998	ZBTB44	cyan	EC_M2	0.30	1.4E-01	0.25	2.3E-01	0.40	4.2E-02	0.18	3.7E-01	-0.81	5.3E-07	-0.21	3.1E-01	-0.22	2.9E-01	-0.08	7.0E-01	-0.04	8.4E-01	0.66	2.3E-04
ENSCAFG0000000758	MAST4	darkgreen	EC_M4	0.30	1.4E-01	0.33	1.0E-01	-0.89	9.1E-10	-0.09	6.5E-01	0.66	2.4E-04	0.25	2.2E-01	-0.07	7.5E-01	-0.07	7.3E-01	-0.21	3.0E-01	-0.86	2.0E-08
ENSCAFG0000003005	ZNF234	grey	EC_M10	0.30	1.4E-01	0.37	6.1E-02	-0.23	2.6E-01	0.17	4.1E-01	-0.18	3.8E-01	-0.02	9.2E-01	-0.15	4.6E-01	0.30	1.3E-01	0.06	7.8E-01	0.02	9.1E-01
ENSCAFG0000003187	RNF125	turquoise	EC_M6	0.30	1.4E-01	0.39	5.1E-02	-0.14	5.0E-01	-0.01	9.7E-01	-0.17	4.0E-01	-0.17	4.1E-01	-0.08	6.9E-01	0.11	6.1E-01	0.32	1.1E-01	0.10	6.1E-01
ENSCAFG0000002914	C4H5orf51	cyan	EC_M2	0.30	1.4E-01	0.16	4.3E-01	0.33	1.0E-01	0.29	1.5E-01	-0.73	2.0E-05	-0.19	3.4E-01	-0.52	6.4E-03	0.07	7.2E-01	0.10	6.2E-01	0.56	2.6E-03
ENSCAFG0000002399	ADGRE3	turquoise	EC_M6	0.30	1.4E-01	0.78	2.2E-06	-0.30	1.3E-01	-0.15	4.8E-01	-0.18	3.7E-01	0.02	9.1E-01	-0.15	4.6E-01	-0.14	4.8E-01	-0.19	3.4E-01	0.01	9.7E-01
ENSCAFG0000002026	TERF2	turquoise	EC_M6	0.30	1.4E-01	0.41	3.7E-02	-0.06	7.9E-01	-0.27	1.8E-01	-0.31	1.3E-01	0.16	4.4E-01	-0.06	7.7E-01	0.18	3.7E-01	0.19	3.5E-01	0.14	4.9E-01
ENSCAFG0000001885	PSMG2	grey	EC_M10	0.30	1.4E-01	-0.22	2.7E-01	0.41	3.8E-02	0.15	4.5E-01	-0.62	7.5E-04	-0.21	3.1E-01	-0.35	7.8E-02	-0.12	5.6E-01	-0.08	7.1E-01	0.49	1.1E-02
ENSCAFG0000000346	COQ3	cyan	EC_M2	0.30	1.4E-01	-0.31	1.2E-01	0.28	1.6E-01	-0.01	9.6E-01	-0.52	6.0E-03	0.23	2.5E-01	0.14	4.9E-01	-0.01	9.7E-01	-0.28	1.7E-01	0.31	1.3E-01
ENSCAFG0000000066	DCAF13	turquoise	EC_M6	0.30	1.4E-01	0.53	5.5E-03	-0.55	4.0E-03	-0.23	2.7E-01	0.24	2.4E-01	-0.01	9.6E-01	-0.15	4.5E-01	-0.42	3.4E-02	-0.05	8.0E-01	-0.37	6.1E-02
ENSCAFG0000000298	REL	turquoise	EC_M6	0.30	1.4E-01	0.73	2.6E-05	-0.57	2.5E-03	-0.07	7.5E-01	0.12	5.5E-01	0.24	2.4E-01	-0.09	6.7E-01	-0.03	8.8E-01	0.04	8.6E-01	-0.31	1.2E-01
ENSCAFG0000001555	ENSCAFG0000001555	grey	EC_M10	0.30	1.4E-01	0.21	3.1E-01	0.01	9.7E-01	-0.12	5.6E-01	-0.32	1.1E-01	-0.24	2.3E-01	0.02	9.1E-01	-0.11	5.8E-01	-0.35	7.7E-02	0.13	5.3E-01
ENSCAFG0000000022	MARS	grey	EC_M10	0.30	1.4E-01	-0.10	6.1E-01	-0.51	7.4E-03	-0.14	4.8E-01	0.45	2.2E-02	0.32	1.1E-01	-0.10	6.2E-01	-0.13	5.2E-01	0.03	8.8E-01	-0.65	3.3E-04
ENSCAFG0000000337	FIGNL1	turquoise	EC_M6	0.30	1.4E-01	0.79	1.8E-06	-0.27	1.9E-01	-0.15	4.6E-01	-0.25	2.2E-01	-0.10	6.3E-01	-0.26	1.9E-01	0.16	4.3E-01	0.34	8.7E-02	0.09	6.8E-01
ENSCAFG0000000023	AHI1	darkgreen	EC_M4	0.30	1.4E-01	-0.03	8.9E-01	-0.82	3.6E-07	-0.06	7.6E-01	0.68	1.3E-04	0.16	4.2E-01	0.07	7.4E-01	0.10	6.1E-01	-0.05	8.0E-01	-0.83	1.5E-07
ENSCAFG0000001403	ENSCAFG0000001403	grey	EC_M10	0.30	1.4E-01	-0.19	3.6E-01	-0.14	5.1E-01	0.46	1.7E-02	-0.08	7.1E-01	0.50	9.8E-03	-0.17	3.9E-01	-0.18	3.9E-01	-0.23	2.7E-01	-0.12	5.7E-01
ENSCAFG0000001233	ATF3	darkgreen	EC_M4	0.30	1.4E-01	0.67	2.1E-04	-0.73	2.5E-05	-0.12	5.7E-01	0.33	1.0E-01	0.05	8.2E-01	-0.03	8.8E-01	-0.07	7.3E-01	-0.10	6.4E-01	-0.50	1.0E-02
ENSCAFG0000001685	HOXB5	turquoise	EC_M6	0.30	1.4E-01	0.43	2.7E-02	-0.51	7.5E-03	0.09	6.6E-01	0.21	3.1E-01	0.24	2.4E-01	-0.20	3.3E-01	-0.33	1.0E-01	-0.45	2.0E-02	-0.40	4.3E-02
ENSCAFG0000001736	NIPAL4	grey	EC_M10	0.30	1.4E-01	-0.10	6.1E-01	-0.02	9.1E-01	-0.12	5.5E-01	-0.07	7.3E-01	-0.08	7.1E-01	0.01	9.8E-01	-0.06	7.8E-01	0.04	8.4E-01	-0.08	6.9E-01
ENSCAFG0000001815	HEATR6	turquoise	EC_M6	0.30	1.4E-01	0.96	6.9E-15	-0.41	3.8E-02	-0.10	6.4E-01	-0.11	6.0E-01	-0.13	5.4E-01	-0.08	7.1E-01	-0.07	7.5E-01	-0.08	7.0E-01	-0.04	8.3E-01
ENSCAFG0000000222	ASNS	darkgreen	EC_M4	0.30	1.4E-01	-0.07	7.3E-01	-0.69	1.0E-04	0.09	6.6E-01	0.58	1.8E-03	0.23	2.6E-01	-0.20	3.3E-01	-0.01	9.6E-01	-0.12	5.6E-01	-0.81	5.8E-07
ENSCAFG0000000912	ENSCAFG0000000912	darkgreen	EC_M4	0.30	1.4E-01	-0.10	6.1E-01	-0.74	1.6E-05	-0.07	7.3E-01	0.63	6.4E-04	0.28	1.6E-01	-0.19	3.5E-01	-0.10	6.4E-01	-0.13	5.2E-01	-0.76	5.8E-06
ENSCAFG0000001691	GNGT2	turquoise	EC_M6	0.30	1.4E-01	0.93	6.9E-12	-0.29	1.5E-01	-0.11	6.0E-01	-0.21	3.0E-01	-0.15	4.7E-01	-0.14	4.8E-01	-0.14	5.1E-01	-0.10	6.4E-01	0.04	8.5E-01
ENSCAFG0000003260	DRG1	darkgrey	EC_M8	0.30	1.4E-01	-0.28	1.7E-01	-0.43	2.7E-02	0.00	1.0E+00	0.46	1.9E-02	0.18	3.8E-01	-0.38	5.9E-02	-0.02	9.1E-01	-0.27	1.9E-01	-0.63	4.9E-04
ENSCAFG0000000997	ENSCAFG0000000997	grey	EC_M10	0.30	1.4E-01	0.07	7.4E-01	-0.22	2.8E-01	-0.04	8.5E-01	0.02	9.0E-01	-0.13	5.2E-01	-0.09	6.8E-01	-0.13	5.2E-01	0.12	5.5E-01	-0.22	2.8E-01
ENSCAFG0000003020	ENSCAFG0000003020	grey	EC_M10	0.30	1.4E-01	0.25	2.1E-01	-0.15	4.5E-01	0.04	8.5E-01	-0.21	3.1E-01	-0.13	5.3E-01	-0.44	2.4E-02	-0.01	9.8E-01	0.27	1.9E-01	0.06	7.7E-01
ENSCAFG0000000076	CELSR1	turquoise	EC_M6	0.30	1.4E-01	0.90	4.0E-10	-0.52	6.4E-03	-0.18	3.7E-01	0.06	7.9E-01	0.01	9.7E-01	-0.14	5.1E-01	-0.11	6.1E-01	-0.17	4.1E-01	-0.22	2.7E-01
ENSCAFG0000001779	ENSCAFG0000001779	darkgreen	EC_M4	0.30	1.4E-01	0.30	1.4E-01	-0.64	4.0E-04	-0.10	6.3E-01	0.31	1.2E-01	0.12	5.7E-01	0.04	8.3E-01	0.20	3.4E-01	0.08	7.1E-01	-0.51	8.0E-03
ENSCAFG0000000529	SLITRK5	grey	EC_M10	0.30	1.4E-01	0.15	4.8E-01	-0.27	1.9E-01	-0.21	3.0E-01	0.09	6.5E-01	-0.15	4.5E-01	-0.14	5.1E-01	0.37	6.1E-02	0.10	6.4E-01	-0.25	2.2E-01
ENSCAFG0000001776	KIAA1328	turquoise	EC_M6	0.30	1.4E-01	0.58	1.7E-03	-0.16	4.2E-01	0.01	9.6E-01	-0.32	1.1E-01	0.04	8.3E-01	-0.23	2.6E-01	0.09	6.7E-01	0.09	6.6E-01	0.20	3.3E-01
ENSCAFG0000003030	ENSCAFG0000003030	grey	EC_M10	0.30	1.4E-01	-0.07	7.3E-01	0.07	7.5E-01	0.50	8.6E-03	-0.25	2.2E-01	-0.01	9.7E-01	-0.21	3.1E-01	-0.26	2.0E-01	-0.25	2.1E-01	0.07	7.3E-01
ENSCAFG0000001337	ENSCAFG0000001337	turquoise	EC_M6	0.30	1.4E-01	0.54	4.0E-03	-0.27	1.8E-01	-0.14	4.9E-01	-0.12	5.7E-01	-0.15	4.6E-01	-0.15	4.7E-01	0.45	2.2E-02	-0.03	8.9E-01	-0.04	8.4E-01
ENSCAFG0000000749	SGTB	grey	EC_M10	0.30	1.4E-01	0.13	5.2E-01	-0.61	1.0E-03	0.14	4.8E-01	0.39	4.7E-02	0.24	2.3E-01	-0.35	7.7E-02	-0.33	9.6E-02	-0.17	4.2E-01	-0.55	3.5E-03
ENSCAFG0000002835	ENSCAFG0000002835	grey	EC_M10	0.30	1.4E-01	0.41	4.0E-02	-0.31	1.2E-01	-0.15	4.5E-01	-0.06	7.8E-01	-0.18	3.7E-01	0.35	7.7E-02	-0.06	7.6E-01	-0.14	4.8E-01	-0.10	6.2E-01
ENSCAFG0000001115	STARD9	darkgrey	EC_M8	0.30	1.4E-01	0.39	4.6E-02	0.22	2.7E-01	0.15	4.6E-01	-0.68	1.4E-04	-0.28	1.7E-01	-0.19	3.5E-01	0.09	6.6E-01	-0.24	2.4E-01	0.51	8.3E-03
ENSCAFG0000001117	NR5A2	grey	EC_M10	0.30	1.4E-01	0.28	1.7E-01	-0.27	1.9E-01	-0.13	5.4E-01	-0.06	7.6E-01	-0.17	3.9E-01	-0.19	3.6E-01	-0.22	2.8E-01	0.06	7.6E-01	-0.09	6.5E-01
ENSCAFG0000001385	GEMIN2	grey	EC_M10	0.30	1.4E-01	0.24	2.3E-01	-0.21	3.1E-01	-0.13	5.3E-01	-0.16	4.3E-01	-0.07	7.3E-01	0.14	5.1E-01	0.22	2.9E-01	-0.10	6.2E-01	-0.03	8.9E-01
ENSCAFG0000001931	THOC6	grey	EC_M10	0.30	1.4E-01	-0.07	7.5E-01	0.48	1.2E-02	0.16	4.3E-01	-0.73	2.5E-05	0.06	7.6E-01	0.06	7.7E-01	-0.15	4.7E-01	-0.11	5.9E-01	0.56	2.9E-03
ENSCAFG0000001076	TCHP	grey	EC_M10	0.30	1.4E-01	-0.45	2.1E-02	-0.01	9.8E-01	0.09	6.7E-01	-0.14	5.0E-01	0.21	3.0E-01	0.02	9.3E-01	-0.23	2.6E-01	-0.09	6.4E-01	-0.07	7.2E-01
ENSCAFG0000001692	ABI3	turquoise	EC_M6	0.30	1.4E-01	0.84	7.5E-08	-0.27	1.8E-01	-0.10	6.1E-01	-0.20	3.2E-01	-0.13	5.2E-01	-0.13	5.1E-01	-0.17	4.1E-01	-0.13	5.3E-01	0.02	9.1E-01
ENSCAFG0000000755	ENSCAFG0000000755	grey	EC_M10	0.30	1.4E-01	-0.18	3.8E-01	0.14	4.9E-01	0.04	8.6E-01	-0.34	8.6E-02	-0.12	5.5E-01	-0.01	9.5E-01	0.06	7.8E-01	-0.18	3.7E-01	0.15	4.6E-01
ENSCAFG0000000762	ENSCAFG0000000762	grey	EC_M10	0.30	1.4E-01	0.34	9.3E-02	-0.31	1.3E-01	0.24	2.3E-01	-0.04	8.5E-01	-0.19	3.5E-01	-0.21	3.1E-01	-0.25	2.2E-01	0.02	9.1E-01	-0.12	5.5E-01
ENSCAFG0000000634	SOS1	grey	EC_M10	0.30	1.4E-01	0.40	4.1E-02	-0.47	1.6E-02	0.22	2.9E-01	0.06	7.6E-01	0.29	1.5E-01	-0.23	2.6E-01	-0.25	2.2E-01	-0.07	7.3E-01	-0.24	2.4E-01
ENSCAFG0000000579	STAM2	darkgreen	EC_M4	0.30	1.4E-01	0.20	3.4E-01	-0.87	6.3E-09	-0.18	3.9E-01	0.67	1.7E-04	0.43	2.7E-02	-0.06	7.7E-01	-0.12	5.5E-01	0.00	9.9E-01	-0.83	1.5E-07

ENSCAFG0000000803	SLF1	grey	EC_M10	0.30	1.4E-01	-0.31	1.2E-01	-0.34	9.3E-02	-0.03	8.8E-01	0.22	2.9E-01	-0.11	6.1E-01	0.16	4.3E-01	-0.23	2.5E-01	0.09	6.6E-01	-0.38	5.9E-02
ENSCAFG0000001471	REX1BD	grey	EC_M10	0.30	1.4E-01	-0.10	6.3E-01	0.20	3.3E-01	0.01	9.6E-01	-0.41	3.7E-02	0.04	8.4E-01	-0.15	4.6E-01	0.33	9.9E-02	0.00	9.9E-01	0.21	3.1E-01
ENSCAFG0000002967	ENSCAFG0000002967	turquoise	EC_M6	0.30	1.4E-01	0.94	1.2E-12	-0.31	1.3E-01	0.00	1.0E+00	-0.20	3.4E-01	-0.16	4.4E-01	-0.13	5.1E-01	-0.19	3.6E-01	0.04	8.4E-01	0.05	7.9E-01
ENSCAFG0000002999	DDAH1	grey	EC_M10	0.30	1.4E-01	0.51	7.4E-03	-0.67	2.1E-04	-0.32	1.2E-01	0.38	5.2E-02	0.19	3.7E-01	-0.21	3.0E-01	-0.15	4.6E-01	-0.23	2.5E-01	-0.55	3.9E-03
ENSCAFG0000000041	ENSCAFG0000000041	grey	EC_M10	0.30	1.4E-01	0.35	8.0E-02	-0.01	9.6E-01	-0.09	6.6E-01	-0.35	7.5E-02	0.09	6.7E-01	-0.16	4.2E-01	-0.20	3.2E-01	-0.06	7.6E-01	0.24	2.4E-01
ENSCAFG0000001942	HCFC1	turquoise	EC_M6	0.30	1.4E-01	0.44	2.3E-02	-0.03	8.8E-01	-0.16	4.3E-01	-0.27	1.8E-01	-0.08	7.1E-01	-0.06	7.8E-01	-0.29	1.6E-01	-0.23	2.5E-01	0.09	6.7E-01
ENSCAFG0000002523	ENTPD5	grey	EC_M10	0.30	1.4E-01	0.13	5.4E-01	-0.66	2.3E-04	0.03	8.8E-01	0.42	3.3E-02	0.19	3.6E-01	-0.49	1.1E-02	0.12	5.6E-01	-0.12	5.5E-01	-0.64	4.3E-04
ENSCAFG0000000774	MYOF	grey	EC_M10	0.30	1.4E-01	-0.42	3.2E-02	-0.01	9.8E-01	0.21	3.1E-01	-0.11	5.8E-01	0.15	4.5E-01	0.17	4.1E-01	-0.24	2.4E-01	-0.44	2.4E-02	-0.10	6.4E-01
ENSCAFG0000000890	ARFGAP2	grey	EC_M10	0.30	1.4E-01	0.18	3.8E-01	0.41	3.7E-02	0.16	4.4E-01	-0.76	8.1E-06	-0.20	3.3E-01	-0.12	5.6E-01	-0.03	8.8E-01	0.06	7.8E-01	0.63	5.6E-04
ENSCAFG0000002344	FBP2	grey	EC_M10	0.30	1.4E-01	0.04	8.3E-01	-0.27	1.8E-01	-0.11	5.9E-01	0.13	5.4E-01	-0.06	7.7E-01	0.00	9.9E-01	0.08	7.0E-01	0.15	4.5E-01	-0.25	2.1E-01
ENSCAFG0000001360	MIA3	grey	EC_M10	0.30	1.4E-01	0.17	3.9E-01	-0.18	3.7E-01	-0.03	8.9E-01	-0.19	3.4E-01	-0.01	9.6E-01	0.09	6.5E-01	-0.32	1.1E-01	-0.18	3.8E-01	0.00	9.9E-01
ENSCAFG0000001012	RAD54L2	turquoise	EC_M6	0.30	1.4E-01	0.82	3.8E-07	-0.51	7.3E-03	-0.21	3.1E-01	0.07	7.4E-01	-0.05	7.9E-01	0.21	3.1E-01	-0.23	2.6E-01	-0.05	7.9E-01	-0.22	2.9E-01
ENSCAFG0000001189	WDR5B	grey	EC_M10	0.29	1.4E-01	0.24	2.4E-01	0.03	8.8E-01	-0.06	7.8E-01	-0.38	5.8E-02	0.04	8.5E-01	0.01	9.7E-01	0.14	5.1E-01	0.21	3.0E-01	0.25	2.3E-01
ENSCAFG0000002963	ZNF613	grey	EC_M10	0.29	1.4E-01	-0.05	8.1E-01	0.37	6.3E-02	0.19	3.6E-01	-0.68	1.3E-04	0.26	2.0E-01	-0.23	2.5E-01	0.09	6.7E-01	0.01	9.5E-01	0.53	5.0E-03
ENSCAFG0000001792	ENSCAFG0000001792	grey	EC_M10	0.29	1.4E-01	0.09	6.8E-01	-0.33	1.0E-01	-0.11	6.0E-01	0.10	6.1E-01	-0.13	5.4E-01	0.40	4.5E-02	-0.05	8.0E-01	-0.08	6.8E-01	-0.25	2.3E-01
ENSCAFG0000001874	NAPG	darkgreen	EC_M4	0.29	1.4E-01	-0.12	5.5E-01	-0.66	2.2E-04	-0.30	1.4E-01	0.61	1.1E-03	0.42	3.4E-02	-0.04	8.6E-01	0.19	3.6E-01	-0.29	1.6E-01	-0.75	1.1E-05
ENSCAFG0000000312	ITPRID1	grey	EC_M10	0.29	1.4E-01	0.19	3.5E-01	-0.20	3.2E-01	-0.03	8.7E-01	-0.06	7.7E-01	-0.09	6.8E-01	-0.14	4.9E-01	-0.09	6.5E-01	-0.14	4.9E-01	-0.12	5.6E-01
ENSCAFG0000000464	ENSCAFG0000000464	grey	EC_M10	0.29	1.4E-01	-0.10	6.2E-01	0.17	4.0E-01	0.10	6.4E-01	-0.39	5.1E-02	-0.45	2.2E-02	-0.11	5.8E-01	-0.36	7.5E-02	-0.04	8.4E-01	0.24	2.4E-01
ENSCAFG0000001310	ATF7IP	grey	EC_M10	0.29	1.4E-01	0.39	4.6E-02	-0.34	8.8E-02	0.06	7.6E-01	-0.08	7.1E-01	0.20	3.2E-01	-0.07	7.4E-01	0.04	8.5E-01	-0.22	2.9E-01	-0.15	4.5E-01
ENSCAFG0000002868	ANKRD49	turquoise	EC_M6	0.29	1.4E-01	0.41	3.8E-02	-0.21	3.0E-01	0.18	3.8E-01	-0.23	2.6E-01	-0.01	9.8E-01	0.20	3.3E-01	-0.21	3.0E-01	-0.04	8.6E-01	0.08	6.9E-01
ENSCAFG0000000991	NPM3	cyan	EC_M2	0.29	1.4E-01	-0.46	1.8E-02	0.37	6.1E-02	0.00	1.0E+00	-0.45	2.2E-02	0.09	6.7E-01	-0.05	8.2E-01	-0.12	5.6E-01	-0.38	5.4E-02	0.24	2.4E-01
ENSCAFG0000000645	ARFIP2	grey	EC_M10	0.29	1.4E-01	0.22	2.8E-01	0.03	8.7E-01	-0.40	4.2E-02	-0.27	1.7E-01	0.05	8.1E-01	-0.08	7.1E-01	0.07	7.3E-01	0.16	4.3E-01	0.14	4.8E-01
ENSCAFG0000003117	DCTD	grey	EC_M10	0.29	1.4E-01	-0.14	4.9E-01	-0.43	2.9E-02	-0.05	7.9E-01	0.33	1.0E-01	0.36	7.4E-02	-0.18	3.9E-01	-0.21	3.1E-01	-0.26	2.0E-01	-0.54	4.4E-03
ENSCAFG0000001738	RAB13	darkgrey	EC_M8	0.29	1.4E-01	0.52	6.5E-03	0.09	6.7E-01	0.15	4.7E-01	-0.57	2.5E-03	-0.38	5.8E-02	0.16	4.3E-01	0.12	5.4E-01	0.15	4.6E-01	0.44	2.3E-02
ENSCAFG0000000016	TNFAIP8	turquoise	EC_M6	0.29	1.4E-01	0.74	1.3E-05	-0.35	7.9E-02	-0.17	4.0E-01	-0.05	8.1E-01	0.01	9.6E-01	0.11	6.1E-01	-0.34	8.8E-02	0.03	8.9E-01	-0.09	6.5E-01
ENSCAFG0000001707	CSNK1G1	grey	EC_M10	0.29	1.4E-01	-0.07	7.3E-01	-0.01	9.7E-01	0.08	7.0E-01	-0.30	1.3E-01	-0.22	2.8E-01	0.06	7.8E-01	0.11	5.9E-01	0.04	8.4E-01	0.10	6.3E-01
ENSCAFG0000000383	PUM2	turquoise	EC_M6	0.29	1.4E-01	0.65	3.7E-04	-0.49	1.2E-02	-0.15	4.6E-01	0.07	7.5E-01	0.19	3.6E-01	-0.02	9.1E-01	-0.35	7.6E-02	0.01	9.7E-01	-0.21	3.1E-01
ENSCAFG0000000191	ALDH1L2	darkgreen	EC_M4	0.29	1.5E-01	-0.25	2.2E-01	-0.61	1.0E-03	0.01	9.5E-01	0.55	3.3E-03	0.26	1.9E-01	-0.30	1.3E-01	0.03	8.8E-01	-0.07	7.2E-01	-0.77	4.3E-06
ENSCAFG0000000024	CLK4	darkgrey	EC_M8	0.29	1.5E-01	0.20	3.3E-01	0.19	3.5E-01	0.15	4.5E-01	-0.63	5.6E-04	0.18	3.8E-01	0.09	6.6E-01	-0.16	4.2E-01	0.09	6.8E-01	0.49	1.2E-02
ENSCAFG0000000182	C1H9orf85	cyan	EC_M2	0.29	1.5E-01	-0.45	2.1E-02	0.31	1.2E-01	0.17	4.0E-01	-0.47	1.6E-02	-0.02	9.3E-01	-0.09	6.5E-01	-0.04	8.3E-01	-0.56	2.9E-03	0.28	1.6E-01
ENSCAFG0000000984	SPTBN5	grey	EC_M10	0.29	1.5E-01	-0.22	2.9E-01	0.13	5.2E-01	0.11	6.1E-01	-0.31	1.2E-01	-0.30	1.3E-01	-0.24	2.5E-01	0.03	8.9E-01	-0.13	5.2E-01	0.21	3.0E-01
ENSCAFG0000000067	RIMS2	turquoise	EC_M6	0.29	1.5E-01	0.70	7.0E-05	-0.47	1.6E-02	-0.11	5.9E-01	0.09	6.5E-01	0.03	8.8E-01	-0.20	3.4E-01	-0.20	3.4E-01	-0.03	8.7E-01	-0.23	2.5E-01
ENSCAFG0000003115	ENSCAFG0000003115	grey	EC_M10	0.29	1.5E-01	-0.05	8.0E-01	-0.36	6.8E-02	-0.15	4.6E-01	0.15	4.7E-01	0.37	6.5E-02	0.17	4.2E-01	0.21	3.0E-01	-0.08	7.1E-01	-0.31	1.2E-01
ENSCAFG0000000483	TRIM54	grey	EC_M10	0.29	1.5E-01	-0.14	4.8E-01	-0.01	9.5E-01	0.29	1.6E-01	-0.18	3.8E-01	-0.17	4.0E-01	0.53	4.9E-03	-0.13	5.3E-01	-0.07	7.3E-01	0.07	7.2E-01
ENSCAFG0000000540	SEC23B	darkgreen	EC_M4	0.29	1.5E-01	-0.25	2.2E-01	-0.69	1.1E-04	-0.25	2.2E-01	0.66	2.7E-04	0.28	1.7E-01	-0.02	9.1E-01	0.13	5.2E-01	-0.09	6.7E-01	-0.86	1.7E-08
ENSCAFG0000001818	ENSCAFG0000001818	grey	EC_M10	0.29	1.5E-01	-0.11	6.1E-01	-0.11	6.0E-01	-0.09	6.6E-01	0.01	9.7E-01	0.52	6.0E-03	0.00	9.8E-01	-0.08	7.0E-01	0.35	8.0E-02	-0.12	5.5E-01
ENSCAFG0000001158	GRAMD1B	grey	EC_M10	0.29	1.5E-01	0.08	7.1E-01	-0.17	4.0E-01	-0.17	4.2E-01	-0.01	9.6E-01	0.14	5.1E-01	0.34	9.3E-02	0.23	2.7E-01	0.10	6.3E-01	-0.12	5.7E-01
ENSCAFG0000000324	ENSCAFG0000000324	grey	EC_M10	0.29	1.5E-01	-0.05	8.0E-01	-0.31	1.2E-01	0.17	4.1E-01	0.09	6.4E-01	0.24	2.4E-01	-0.30	1.4E-01	0.01	9.5E-01	-0.17	4.0E-01	-0.22	2.7E-01
ENSCAFG0000000415	DNMT3A	turquoise	EC_M6	0.29	1.5E-01	0.66	2.2E-04	-0.20	3.4E-01	0.14	4.8E-01	-0.28	1.6E-01	-0.41	3.7E-02	0.03	8.9E-01	0.07	7.4E-01	-0.07	7.4E-01	0.11	6.0E-01
ENSCAFG0000000354	ROCK2	darkgreen	EC_M4	0.29	1.5E-01	-0.12	5.7E-01	-0.78	2.3E-06	-0.07	7.4E-01	0.70	7.5E-05	0.21	3.1E-01	0.18	3.8E-01	-0.02	9.1E-01	-0.24	2.4E-01	-0.85	4.8E-08
ENSCAFG0000002923	ENSCAFG0000002923	grey	EC_M10	0.29	1.5E-01	0.03	9.0E-01	-0.14	5.0E-01	0.25	2.2E-01	0.00	9.9E-01	0.36	7.4E-02	0.11	6.0E-01	-0.39	5.0E-02	-0.15	4.6E-01	-0.12	5.6E-01
ENSCAFG0000002590	ENSCAFG0000002590	turquoise	EC_M6	0.29	1.5E-01	0.75	1.2E-05	-0.20	3.4E-01	-0.11	6.0E-01	-0.23	2.5E-01	-0.06	7.9E-01	-0.05	8.2E-01	-0.08	7.1E-01	-0.04	8.6E-01	0.10	6.4E-01
ENSCAFG0000003004	ENSCAFG0000003004	grey	EC_M10	0.29	1.5E-01	0.25	2.1E-01	-0.32	1.1E-01	-0.21	3.1E-01	0.06	7.7E-01	-0.25	2.2E-01	0.18	3.7E-01	0.24	2.4E-01	-0.19	3.5E-01	-0.22	2.7E-01
ENSCAFG0000001781	ZNF699	darkgrey	EC_M8	0.29	1.5E-01	-0.08	6.8E-01	0.33	9.8E-02	0.34	8.9E-02	-0.70	6.1E-05	-0.04	8.5E-01	-0.32	1.2E-01	-0.05	8.0E-01	-0.08	6.8E-01	0.49	1.0E-02
ENSCAFG0000001234	HMBS	darkgrey	EC_M8	0.29	1.5E-01	-0.30	1.4E-01	0.18	3.9E-01	-0.07	7.3E-01	-0.24	2.3E-01	0.23	2.7E-01	0.15	4.6E-01	-0.32	1.1E-01	0.03	9.0E-01	0.11	6.1E-01
ENSCAFG0000000061	TMEM242	grey	EC_M10	0.29	1.5E-01	0.22	2.7E-01	-0.01	9.6E-01	0.08	7.0E-01	-0.38	5.7E-02	-0.01	9.4E-01	0.04	8.4E-01	-0.19	3.5E-01	-0.29	1.5E-01	0.22	2.8E-01
ENSCAFG0000000957	SNRNP48	darkgreen	EC_M4	0.29	1.5E-01	0.27	1.8E-01	-0.80	9.6E-07	0.00	1.0E+00	0.52	7.0E-03	0.02	9.0E-01	-0.05	8.1E-01	0.14	4.9E-01	-0.13	5.2E-01	-0.67	1.8E-04
ENSCAFG0000001235	RBM44	darkgrey	EC_M8	0.29	1.5E-01	0.33	9.7E-02	0.21	3.1E-01	0.20	3.2E-01	-0.65	3.5E-04	0.10	6.4E-01	-0.27	1.9E-01	-0.08	6.8E-01	-0.08	7.1E-01	0.48	1.3E-02
ENSCAFG0000002325	ERBB4	grey	EC_M10	0.29	1.5E-01	0.12	5.6E-01	-0.33	1.0E-01	-0.16	4.3E-01	0.19	3.6E-01	-0.16	4.3E-01	-0.09	6.5E-01	-0.11	5.9E-01	0.09	6.5E-01	-0.27	1.8E-01
ENSCAFG0000001136	ENSCAFG0000001136	darkgrey	EC_M8	0.29	1.5E-01	0.40	4.3E-02	0.28	1.7E-01	0.31	1.3E-01	-0.75	9.6E-06	-0.13	5.3E-01	-0.19	3.6E-01	-0.11	5.9E-01	0.01	9.6E-01	0.61	1.0E-03
ENSCAFG0000001755	GPR68	turquoise	EC_M6	0.29	1.5E-01	0.57	2.5E-03	-0.60	1.1E-03	-0.11	5.9E-01	0.22	2.9E-01	0.40	4.3E-02	-0.01	9.4E-01	-0.17	4.0E-01	-0.07	7.5E-01	-0.43	2.8E-02
ENSCAFG0000002812	ENSCAFG0000002812	grey	EC_M10	0.29	1.5E-01	0.00	9.8E-01	0.08	6.9E-01	0.11	6.1E-01	-0.35	8.4E-02	0.25	2.1E-01	-0.27	1.8E-01	0.03	8.8E-01	0.20	3.4E-01	0.15	4.6E-01
ENSCAFG0000003273	ENSCAFG0000003273	grey	EC_M10	0.29	1.5E-01	-0.06	7.6E-01	-0.53	5.0E-03	0.04	8.4E-01	0.41	3.7E-02	0.34	9.2E-02	-0.22	2.8E-01	-0.18	3.9E-01	-0.13	5.3E-01	-0.54	4.4E-03
ENSCAFG0000000147	ENSCAFG0000000147	grey	EC_M10	0.29	1.5E-01	-0.06	7.8E-01	-0.26	2.0E-01	0.04	8.5E-01	0.01	9.5E-01	-0.24	2.3E-01	-0.18	3.8E-01	0.41	3.9E-02	0.22	2.9E-01	-0.15	4.7E-01
ENSCAFG0000002851	ENSCAFG0000002851	violet	EC_M7	0.29	1.5E-01	-0.16	4.3E-01	-0.21	3.1E-01	0.30	1.4E-01	0.04	8.6E-01	0.68	1.3E-04	-0.12	5.4E-01	-0.18	3.8E-01	-0.21	3.1E-01	-0.21	3.0E-01
ENSCAFG0000000057	ENSCAFG0000000057	grey	EC_M10	0.29	1.5E-01	0.47	1.5E-02	-0.23	2.6E-01	0.12	5.5E-01	-0.24	2.5E-01	0.20	3.4E-01	0.02	9.3E-01	-0.30	1.4E-01	0.11	5.8E-01	0.09	6.5E-01
ENSCAFG0000001787	GALNT1	darkgreen	EC_M4	0.29	1.5E-01	0.33	1.0E-01	-0.87	5.5E-09	-0.11	5.8E-01	0.62	7.6E-04	0.12	5.6E-01	-0.11	6.1E-01	0.03	9.0E-01	-0.04	8.4E-01	-0.78	2.8E-06
ENSCAFG0000000338	FOXP2	turquoise	EC_M6	0.29	1.5E-01	0.86	1.5E-08	-0.46	1.8E-02	-0.03	8.9E-01	0.02	9.4E-01	0.08	6.9E-01	-0.11	6.1E-01	0.02	9.3E-01	-0.13	5.3E-01	-0.17	4.1E-01
ENSCAFG0000001611	ACBD3	darkgreen	EC_M4	0.29	1.5E-01	0.32	1.1E-01	-0.70	6.5E-05	-0.09	6.5E-01	0.40	4.3E-02	0.25	2.3E-01	0.04	8.5E-01	-0.05	8.0E-01	-0.28	1.6E-01	-0.61	1.0E-03
ENSCAFG0000000843	C30H15orf41	grey	EC_M10	0.29	1.5E-01	0.24	2.3E-01	0.17	4.0E-01	0.23	2.5E-01	-0.57	2.2E-03	-0.27	1.9E-01	-0.43	2.7E-02	-0.06	7.7E-01	0.23	2.7E-01	0.40	4.6E-02
ENSCAFG0000003073	HIST2H2BF	grey	EC_M10	0.29	1.5E-01	0.22	2.8E-01	-0.43	2.8E-02	0.27	1.8E-01	0.24	2.3E-01	0.08	6.9E-01	-0.11	6.1E-01	-0.10	6.2E-01	-0.08	7.1E-01	-0.33	9.5E-02
ENSCAFG0000001373	SPATA5L1	grey	EC_M10	0.29	1.5E-01	0.19	3.5E-01	0.07	7.2E-01	0.00	1.0E+00	-0.33	1.0E-01	-0.08	6.9E-01	0.06	7.6E-01	-0.59	1.7E-03	-0.27	1.9E-01	0.19	3.5E-01
ENSCAFG0000000004	ENSCAFG0000000004	grey	EC_M10	0.29	1.5E-01	0.07	7.4E-01	-0.19	3.5E-01	-0.08	6.8E-01	-0.05	7.9E-01	-0.13	5.2E-01	-0.08	6.9E-01	-0.13	5.2E-01	-0.07	7.2E-01	-0.07	7.3E-01
ENSCAFG0000001074	PGAP1	grey	EC_M10	0.29	1.5E-01	0.26	1.9E-01	-0.46	1.9E-02	0.09	6.6E-01	0.17	4.2E-01	-0.11	6.0E-01	-0.22	2.7E-01	0.01	9.5E-01	-0.28	1.6E-01	-0.34	8.9E-02
ENSCAFG0000002041	NEGR1	grey	EC_M10	0.29	1.5E-01	-0.12	5.5E-01	-0.61	1.0E-03	0.04	8.6E-01	0.50	9.6E-03	0.30	1.4E-01	-0.10	6.2E-01	-0.23	2.7E-01	0.15	4.6E-01	-0.70	7.3E-05
ENSCAFG0000003205	FAM216A	grey	EC_M10	0.29	1.5E-01	0.15	4.5E-01	-0.36	6.9E-02	-0.24	2.3E-01	0.20	3.2E-01	-0.17	4.1E-01	-0.37	6.4E-02	-0.04	8.6E-01	0.01	9.8E-01	-0.29	1.5E-01
ENSCAFG0000001744	KLHL4	turquoise	EC_M6	0.29	1.5E-01	0.95	1.4E-13	-0.30	1.4E-01	-0.13	5.3E-01	-0.21	3.1E-01	-0.14	5.0E-01	-0.13	5.2E-01	-0.01	9.5E-01	-0.15	4.6E-01	0.06	7.7E-01
ENSCAFG0000000703	LYN	grey	EC_M10	0.29	1.5E-01	-0.15	4.8E-01	-0.25	2.2E-01	0.23	2.5E-01	0.06	7.7E-01	0.01	9.6E-01	0.17	4.1E-01	-0.19	3.5E-01	-0.65	3.0E-04	-0.26	2.0E-01
ENSCAFG0000000029	ENSCAFG0000000029	grey	EC_M10	0.29	1.5E-01	0.14	4.8E-01	-0.13	5.3E-01	0.27	1.8E-01	-0.15	4.8E-01	-0.22	2.9E-01	0.06	7.7E-01	-0.14	4.9E-01	-0.27	1.8E-01	-0.01	9.5E-01
ENSCAFG0000000713	TMEM87B	darkgreen	EC_M4	0.29	1.5E-01	0.01	9.6E-01	-0.79	1.6E-06	0.09	6.5E-01	0.64	3.8E-04	0.18	3.8E-01	0.06	7.9E-01	-0.16	4.4E-01	-0.15	4.7E-01	-0.83	1.4E-07
ENSCAFG0000001445	NDP	grey	EC_M10	0.29	1.5E-01	-0.16	4.3E-01	0.08	7.1E-01	-0.12	5.4E-01	-0.18	3.9E-01	-0.10	6.1E-01	-0.06	7.9E-01	-0.11	6.0E-01	0.04	8.3E-01	0.09	6.8E-01
ENSCAFG0000001305	TANC2	darkgreen	EC_M4	0.29	1.5E-01	0.41	3.9E-02	-0.67	1.6E-04	-0.04	8.4E-01	0.34	8.8E-02	0.24	2.4E-01	-0.09	6.5E-01	-0.18	3.7E-01	-0.38	5.3E-02	-0.58	2.0E-03
ENSCAFG0000000431	PPP5C	grey	EC_M10	0.29	1.5E-01	-0.26	1.9E-01	0.16	4.2E-01	0.05	8.0E-01	-0.19	3.4E-01	0.08	7.1E-01	-0.16	4.5E-01	-0.02	9.2E-01	-0.06	7.5E-01	0.05	8.1E-01
ENSCAFG0000002644	ENSCAFG0000002644	darkgrey	EC_M8	0.29	1.5E-01	0.11	6.0E-01	0.22	2.9E-01	-0.03	8.9E-01	-0.60	1.1E-03	-0.08	7.0E-01	-0.18	3.7E-01	0.13	5.2E-01	0.01	9.5E-01	0.43	2.7E-02
ENSCAFG0000001495	ENSCAFG0000001495	turquoise	EC_M6	0.29	1.5E-01	0.34	8.7E-02	-0.24	2.4E-01	-0.14	4.8E-01	-0.10	6.2E-01	0.55	3.9E-03	-0.16	4.2E-01	-0.17	4.1E-01	-0.25	2.2E-01	-0.07	7.4E-01
ENSCAFG0000001824	AP2B1	turquoise	EC_M6	0.29	1.5E-01	0.34	8.5E-02	-0.25	2.1E-01	-0.07	7.2E-01	-0.08	7.1E-01	-0.27	1.8E-01	-0.18	3.8E-01	0.10	6.4E-01	0.15	4.6E-01	-0.11	5.8E-01
ENSCAFG0000000445	ABHD5	darkgreen	EC_M4	0.29	1.5E-01	0.10	6.3E-01	-0.77	4.3E-06	0.07	7.3E-01	0.62	8.1E-04	0.30	1.4E-01	-0.10	6.4E-01	-0.19	3.5E-01	-0.40	4.4E-02	-0.78	3.1E-06
ENSCAFG0000003017	ENSCAFG0000003017	grey	EC_M10	0.29	1.5E-01	0.17	4.2E-01	0.04	8.5E-01	0.06	7.8E-01	-0.40	4.4E-02	-0.13	5.3E-01	-0.15	4.7E-01	0.34	9.4E-02	0.09	6.6E-01	0.24	2.5E-01
ENSCAFG0000001921	DNAJA3	grey	EC_M10	0.29	1.5E-01	-0.45	2.0E-02	-0.15	4.5E-01	0.06	7.8E-01	0.18	3.7E-01	0.48	1.3E-02	0.07	7.2E-01	-0.02	9.1E-01	0.00	9.9E-01	-0.33	9.5E-02
ENSCAFG0000002970	ENSCAFG0000002970	grey	EC_M10	0.29	1.5E-01	0.20	3.2E-01	-0.48	1.2E-02	0.07	7.2E-01	0.36	7.2E-02	0.11	6.0E-01	-0.38	5.4E-02	-0.31	1.3E-01	-0.04	8.3E-01	-0.48	1.4E-02
ENSCAFG0000001859	ARHGAP28	turquoise	EC_M6	0.29	1.5E-01	0.82	2.4E-07	-0.34	9.0E-02	-0.09	6.6E-01	-0.14	5.0E-01	-0.12	5.5E-01	-0.19	3.5E-01	0.11	5.9E-01	0.45	2.2E-02	-0.02	9.3E-01
ENSCAFG0000001798	DYNC1H1	grey	EC_M10	0.29	1.5E-01	0.28	1.7E-01	-0.73	2.0E-05	-0.23	2.7E-01	0.55	3.5E-03	0.05	8.2E-01	-0.23	2.5E-01	-0.08	7.1E-01	-0.26	2.0E-01	-0.69	1.0E-04
ENSCAFG0000000348	ING3	cyan	EC_M2	0.29	1.5E-01	0.20	3.2E-01	0.45	2.0E-02	0.07	7.5E-01	-0.84	6.7E-08	-0.33	9.5E-02	-0.05	7.9E-01	0.09	6.6E-01	-0.07	7.3E-01	0.71	4.7E-05
ENSCAFG0000000694	SLC7A2	grey	EC_M10	0.29	1.5E-01	-0.21	3.0E-01	-0.19	3.6E-01	0.28	1.6E-01	0.00	9.9E-01	0.05	8.2E-01	-0.27	1.9E-01	-0.11	5.9E-01	0.28	1.7E-01	-0.17	4.1E-01
ENSCAFG0000002867	ENSCAFG0000002867	grey	EC_M10	0.29	1.5E-01	0.08	7.0E-01	0.06	7.6E-01	-0.14	5.0E-01	-0.29	1.5E-01	-0.16	4.3E-01	-0.11	5.8E-01	-0.13	5.2E-01	0.43	2.9E-02	0.18	3.9E-01
ENSCAFG0000001458	KDM6A	cyan	EC_M2	0.29	1.5E-01	0.29	1.5E-01	0.26	1.9E-01	0.32	1.1E-01	-0.74	1.6E-05	-0.16	4.4E-01	-0.28	1.6E-01	-0.06	7.6E-01	-0.06	7.7E-01	0.58	2.0E-03
ENSCAFG0000001031	SRSF9	grey	EC_M10	0.29	1.5E-01	0.08	7.0E-01	-0.44	2.3E-02	-0.05	8.0E-01	0.38	5.8E-02	0.08	6.8E-01	-0.04	8.4E-01	-0.06	7.7E-01	-0.24	2.4E-01	-0.52	5.9E-03
ENSCAFG0000001327	SMG7	turquoise	EC_M6	0.29	1.5E-01	0.69	1.1E-04	-0.64	4.6E-04	-0.12	5.7E-01	0.20	3.3E-01	0.07	7.3E-01	-0.10	6.2E-01	0.20	3.2E-01	0.25	2.1E-01	-0.38	5.5E-02
ENSCAFG0000000356	ENSCAFG0000000356	cyan	EC_M2	0.29	1.5E-01	-0.30	1.4E-01	0.26	2.0E-01	0.33	9.5E-02	-0.48	1.4E-02	0.08	7.2E-01	0.05	8.1E-01	-0.08	6.9E-01	-0.22	2.7E-01	0.26	2.1E-01
ENSCAFG0000000031	PLAGL1	turquoise	EC_M6	0.29	1.5E-01	0.59	1.6E-03	-0.34	8.9E-02	0.09	6.7E-01	-0.11	5.9E-01	0.04	8.4E-01	0.07	7.4E-01	-0.11	5.9E-01	-0.05	8.0E-01	-0.12	5.7E-01
ENSCAFG0000000583	ENSCAFG0000000583	grey	EC_M10	0.29	1.5E-01	0.11	5.8E-01	-0.59	1.6E-03	-0.02	9.2E-01	0.43	2.9E-02	-0.16	4.4E-01	-0.13	5.3E-01	-0.05	8.1E-01	-0.10	6.2E-01	-0.58	2.1E-03
ENSCAFG0000000017	ENSCAFG0000000017	grey	EC_M10	0.29	1.5E-01	0.14	4.9E-01	-0.19	3.5E-01	0.07	7.3E-01	-0.09	6.5E-01	0.25	2.2E-01	0.09	6.5E-01	-0.17	4.2E-01	-0.52	6.6E-03	-0.05	8.2E-01
ENSCAFG0000000742	SWAP70	grey	EC_M10	0.29	1.5E-01	0.37	5.9E-02	0.16	4.5E-01	-0.03	9.0E-01	-0.56	2.8E-03	-0.11	5.9E-01	0.25	2.2E-01	-0.08	7.0E-01	-0.40	4.5E-02	0.38	5.5E-02
ENSCAFG0000001139	CDH24	turquoise	EC_M6	0.29	1.5E-01	0.80	9.5E-07	-0.20	3.4E-01	0.19	3.5E-01	-0.29	1.5E-01	0.00	9.9E-01	-0.17	4.1E-01	-0.23	2.5E-01	0.31	1.2E-01	0.15	4.7E-01
ENSCAFG0000001385	ENSCAFG0000001385	darkgrey	EC_M8	0.29	1.5E-01	-0.11	5.8E-01	-0.20	3.4E-01	0.20	3.4E-01	0.01	9.6E-01	0.36	7.0E-02	-0.03	8.7E-01	-0.25	2.1E-01	-0.14	5.0E-01	-0.12	5.5E-01
ENSCAFG0000000871	MME	grey	EC_M10	0.29	1.5E-01	-0.17	4.0E-01	0.05	8.3E-01	0.01	9.5E-01	-0.20	3.3E-01	0.27	1.8E-01	0.42	3.5E-02	-0.05	8.0E-01	-0.32	1.1E-01	0.06	7.9E-01
ENSCAFG0000002435	CENPP	grey	EC_M10	0.29	1.5E-01	-0.40	4.3E-02	0.19	3.6E-01	-0.20	3.3E-01	-0.23	2.6E-01	-0.04	8.3E-01	-0.44	2.3E-02	-0.20	3.3E-01	-0.43	2.7E-02	0.08	6.8E-01
ENSCAFG0000000183	ENSCAFG0000000183	turquoise	EC_M6	0.29	1.5E-01	0.40	4.2E-02	-0.04	8.3E-01	-0.20	3.3E-01	-0.35	7.6E-02	-0.21	3.0E-01	-0.22	2.7E-01	0.10	6.1E-01	0.07	7.2E-01	0.17	4.0E-01
ENSCAFG0000000117	SGSM3	turquoise	EC_M6	0.29	1.5E-01	0.58	1.7E-03	-0.41	3.5E-02	-0.16	4.2E-01	0.02	9.1E-01	0.01	9.6E-01	0.02	9.1E-01	-0.21	3.1E-01	-0.34	8.7E-02	-0.20	3.3E-01
ENSCAFG0000000627	FGD6	turquoise	EC_M6	0.29	1.5E-01	0.73	2.4E-05	-0.72	3.7E-05	-0.15	4.5E-01	0.31	1.3E-01	-0.02	9.4E-01	-0.20	3.3E-01	-0.02	9.3E-01	0.06	7.6E-01	-0.47	1.6E-02
ENSCAFG0000000013	ENSCAFG0000000013	grey	EC_M10	0.29	1.5E-01	-0.02	9.4E-01	-0.31	1.3E-01	-0.43	2.7E-02	0.18	3.8E-01	0.13	5.2E-01	-0.02	9.2E-01	0.08	6.8E-01	-0.03	8.7E-01	-0.33	9.7E-02
ENSCAFG0000003206	ENSCAFG0000003206	grey	EC_M10	0.29	1.5E-01	-0.06	7.6E-01	-0.61	9.2E-04	0.18	3.9E-01	0.52	6.8E-03	0.17	3.9E-01	-0.10	6.4E-01	0.24	2.5E-01	0.06	7.8E-01	-0.65	3.7E-04
ENSCAFG0000001836	LONRF3	grey	EC_M10	0.29	1.5E-01	0.36	7.0E-02	-0.02	9.3E-01	0.04	8.4E-01	-0.39	4.7E-02	-0.25	2.1E-01	-0.05	7.9E-01	0.19	3.5E-01	0.34	9.3E-02	0.25	2.3E-01
ENSCAFG0000000604	TRPC4	grey	EC_M10	0.29	1.5E-01	-0.02	9.1E-01	-0.59	1.5E-03	0.15	4.7E-01	0.47	1.5E-02	0.17	4.0E-01	-0.14	4.8E-01	0.05	8.2E-01	-0.14	5.0E-01	-0.65	3.3E-04
ENSCAFG0000000579	ZNF385D	grey	EC_M10	0.29	1.5E-01	-0.45	2.3E-02	-0.18	3.7E-01	-0.16	4.3E-01	0.17	4.2E-01	0.29	1.5E-01	0.15	4.8E-01	0.25	2.2E-01	-0.12	5.8E-01	-0.32	1.1E-01
ENSCAFG0000002025	NFAT5	turquoise	EC_M6	0.29	1.5E-01	0.64	3.9E-04	-0.30	1.4E-01	0.06	7.7E-01	-0.18	3.8E-01	-0.15	4.5E-01	-0.28	1.7E-01	-0.20	3.2E-01	-0.08	7.0E-01	0.04	8.5E-01
ENSCAFG0000000465	CBLC	grey	EC_M10	0.29	1.5E-01	0.01	9.8E-01	-0.66	2.7E-04	0.04	8.3E-01	0.53	5.0E-03	0.28	1.7E-01	0.01	9.7E-01	0.10	6.4E-01	0.16	4.3E-01	-0.72	3.4E-05
ENSCAFG0000000621	SEMA3A	grey	EC_M10	0.29	1.5E-01	0.10	6.3E-01	-0.18	3.9E-01	0.39	5.2E-02	-0.08	6.9E-01	0.41	3.8E-02	-0.31	1.2E-01	-0.02	9.3E-01	-0.16	4.3E-01	-0.12	5.7E-01
ENSCAFG0000000751	ELP4	grey	EC_M10	0.29	1.5E-01	-0.11	5.9E-01	-0.51	7.5E-03	-0.12	5.5E-01	0.41	4.0E-02	0.40	4.1E-02	-0.08	7.0E-01	-0.35	8.0E-02	-0.01	9.7E-01	-0.55	3.9E-03
ENSCAFG0000000208	ENSCAFG0000000208	grey	EC_M10	0.29	1.5E-01	-0.13	5.4E-01	0.03	8.8E-01	0.35	7.8E-02	-0.29	1.6E-01	-0.42	3.3E-02	0.14	4.8E-01	-0.08	6.8E-01	-0.45	2.2E-02	0.12	5.6E-01
ENSCAFG0000000780	ENSCAFG0000000780	grey	EC_M10	0.29	1.6E-01	-0.15	4.6E-01	-0.36	6.8E-02	0.13	5.3E-01	0.25	2.1E-01	-0.26	2.1E-01	-0.35	8.4E-02	0.20	3.4E-01	0.00	9.9E-01	-0.37	6.2E-02
ENSCAFG0000001112	DDX59	turquoise	EC_M6	0.29	1.6E-01	0.40	4.2E-02	-0.29	1.5E-01	-0.04	8.4E-01	-0.09	6.5E-01	0.20	3.3E-01	-0.15	4.6E-01	-0.22	2.7E-01	-0.22	2.8E-01	-0.07	7.5E-01
ENSCAFG0000001252	TLK1	grey	EC_M10	0.29	1.6E-01	0.36	6.9E-02	-0.30	1.3E-01	0.10	6.4E-01	-0.11	5.9E-01	0.09	6.5E-01	-0.19	3.4E-01	-0.16	4.4E-01	-0.01	9.6E-01	-0.07	7.4E-01
ENSCAFG0000002014	MTF2	turquoise	EC_M6	0.29	1.6E-01	0.59	1.4E-03	-0.06	7.6E-01	0.11	6.0E-01	-0.38	5.9E-02	-0.07	7.4E-01	-0.06	7.8E-01	-0.29	1.6E-01	0.01	9.6E-01	0.28	1.7E-01
ENSCAFG0000001054	MTTP	turquoise	EC_M6	0.29	1.6E-01	0.89	1.4E-09	-0.37	6.6E-02	-0.13	5.3E-01	-0.08	7.0E-01	-0.12	5.6E-01	-0.10	6.4E-01	-0.12	5.7E-01	0.17	4.0E-01	-0.05	8.1E-01
ENSCAFG0000003118	PFN4	grey	EC_M10	0.29	1.6E-01	-0.16	4.3E-01	-0.26	2.0E-01	-0.19	3.6E-01	0.17	4.0E-01	-0.23	2.6E-01	-0.26	2.0E-01	-0.22	2.7E-01	-0.05	8.1E-01	-0.33	1.0E-01

ENSCAFG0000000721	ENSCAFG0000000721	grey	EC_M10	0.29	1.6E-01	0.08	6.9E-01	-0.07	7.2E-01	0.52	6.7E-03	-0.16	4.4E-01	-0.16	4.5E-01	-0.17	4.0E-01	-0.20	3.3E-01	0.00	1.0E+00	-0.01	9.4E-01
ENSCAFG0000000569	CHMP6	grey	EC_M10	0.29	1.6E-01	0.25	2.2E-01	-0.39	4.9E-02	0.10	6.2E-01	0.25	2.2E-01	0.03	9.0E-01	-0.40	4.4E-02	-0.14	5.0E-01	0.06	7.8E-01	-0.32	1.1E-01
ENSCAFG0000003106	ENSCAFG0000003106	grey	EC_M10	0.29	1.6E-01	-0.19	3.6E-01	-0.22	2.8E-01	0.33	1.0E-01	0.10	6.2E-01	0.21	3.0E-01	-0.22	2.8E-01	-0.12	5.6E-01	-0.06	7.9E-01	-0.24	2.3E-01
ENSCAFG0000002374	GCNA	grey	EC_M10	0.29	1.6E-01	0.10	6.4E-01	-0.12	5.5E-01	0.01	9.4E-01	-0.08	7.1E-01	-0.08	7.1E-01	0.21	2.9E-01	0.37	6.4E-02	-0.02	9.3E-01	-0.08	7.0E-01
ENSCAFG0000000603	ZNF605	grey	EC_M10	0.29	1.6E-01	-0.15	4.7E-01	0.04	8.4E-01	0.09	6.6E-01	-0.28	1.7E-01	-0.34	9.3E-02	0.09	6.5E-01	0.04	8.5E-01	0.05	8.1E-01	0.13	5.3E-01
ENSCAFG0000000251	MACC1	grey	EC_M10	0.29	1.6E-01	0.19	3.6E-01	-0.10	6.1E-01	0.44	2.6E-02	-0.16	4.2E-01	-0.10	6.2E-01	-0.05	8.0E-01	-0.13	5.2E-01	0.22	2.8E-01	0.04	8.6E-01
ENSCAFG0000000839	NEIL3	grey	EC_M10	0.29	1.6E-01	0.27	1.8E-01	-0.10	6.4E-01	0.13	5.3E-01	-0.10	6.2E-01	-0.20	3.3E-01	-0.15	4.7E-01	-0.21	3.0E-01	-0.11	6.0E-01	0.02	9.4E-01
ENSCAFG0000001734	CLINT1	darkgreen	EC_M4	0.29	1.6E-01	0.07	7.5E-01	-0.87	7.3E-09	-0.13	5.2E-01	0.73	2.5E-05	0.37	6.0E-02	-0.14	4.8E-01	-0.05	8.3E-01	-0.15	4.6E-01	-0.91	8.5E-11
ENSCAFG0000001887	HOOK1	grey	EC_M10	0.29	1.6E-01	-0.20	3.4E-01	-0.26	2.1E-01	0.15	4.6E-01	0.05	8.2E-01	0.28	1.6E-01	0.31	1.3E-01	-0.09	6.6E-01	0.03	8.7E-01	-0.25	2.2E-01
ENSCAFG0000000713	MERTK	turquoise	EC_M6	0.29	1.6E-01	0.80	7.6E-07	-0.44	2.5E-02	-0.13	5.4E-01	-0.01	9.8E-01	-0.21	3.1E-01	-0.12	5.7E-01	-0.19	3.4E-01	-0.02	9.1E-01	-0.15	4.7E-01
ENSCAFG0000000030	HIVEP2	grey	EC_M10	0.29	1.6E-01	-0.09	6.5E-01	-0.18	3.7E-01	-0.13	5.2E-01	0.05	7.9E-01	0.06	7.8E-01	0.30	1.4E-01	-0.32	1.1E-01	-0.51	7.2E-03	-0.16	4.4E-01
ENSCAFG0000001192	CLK1	grey	EC_M10	0.29	1.6E-01	0.22	2.7E-01	0.17	4.1E-01	0.22	2.9E-01	-0.54	4.4E-03	0.19	3.4E-01	-0.06	7.6E-01	-0.33	9.8E-02	0.16	4.3E-01	0.40	4.3E-02
ENSCAFG0000001883	TUBB6	grey	EC_M10	0.29	1.6E-01	-0.36	6.8E-02	-0.43	3.0E-02	-0.15	4.7E-01	0.47	1.6E-02	0.22	2.8E-01	-0.23	2.6E-01	0.11	5.9E-01	-0.09	6.6E-01	-0.64	4.2E-04
ENSCAFG0000000379	CEP57L1	grey	EC_M10	0.28	1.6E-01	-0.01	9.6E-01	-0.10	6.3E-01	0.34	9.0E-02	-0.13	5.4E-01	0.00	9.9E-01	-0.38	5.8E-02	-0.17	4.0E-01	-0.03	8.9E-01	0.01	9.5E-01
ENSCAFG0000001076	MANBA	grey	EC_M10	0.28	1.6E-01	-0.19	3.6E-01	0.23	2.6E-01	0.29	1.6E-01	-0.51	8.3E-03	-0.15	4.6E-01	-0.01	9.5E-01	0.06	7.7E-01	-0.03	8.8E-01	0.31	1.2E-01
ENSCAFG0000000506	WDR48	darkgreen	EC_M4	0.28	1.6E-01	0.01	9.7E-01	-0.64	4.5E-04	-0.19	3.6E-01	0.52	6.7E-03	0.12	5.4E-01	-0.31	1.2E-01	0.16	4.3E-01	-0.20	3.2E-01	-0.65	3.0E-04
ENSCAFG0000001943	NTHL1	grey	EC_M10	0.28	1.6E-01	0.37	6.0E-02	0.35	8.4E-02	0.21	3.1E-01	-0.74	1.8E-05	-0.18	3.9E-01	-0.01	9.5E-01	-0.42	3.3E-02	-0.17	4.0E-01	0.57	2.4E-03
ENSCAFG0000003041	RGS16	turquoise	EC_M6	0.28	1.6E-01	0.85	2.7E-08	-0.34	9.1E-02	-0.11	6.1E-01	-0.10	6.4E-01	-0.14	4.8E-01	0.02	9.2E-01	-0.14	5.1E-01	-0.22	2.8E-01	-0.05	7.9E-01
ENSCAFG0000001511	ENSCAFG0000001511	grey	EC_M10	0.28	1.6E-01	0.06	7.8E-01	-0.18	3.7E-01	-0.23	2.6E-01	-0.11	5.9E-01	0.39	4.8E-02	0.07	7.4E-01	-0.23	2.6E-01	-0.27	1.9E-01	-0.09	6.6E-01
ENSCAFG0000002124	ENSCAFG0000002124	paleturquoise	EC_M11	0.28	1.6E-01	0.01	9.5E-01	-0.13	5.2E-01	0.73	2.6E-05	-0.09	6.6E-01	-0.16	4.5E-01	-0.14	4.8E-01	-0.10	6.4E-01	-0.19	3.4E-01	-0.08	7.0E-01
ENSCAFG0000000452	TMEM126B	grey	EC_M10	0.28	1.6E-01	-0.08	6.9E-01	-0.53	5.8E-03	0.03	9.0E-01	0.45	2.0E-02	0.37	6.6E-02	-0.41	3.9E-02	-0.34	8.9E-02	-0.25	2.2E-01	-0.59	1.6E-03
ENSCAFG0000000516	ENSCAFG0000000516	grey	EC_M10	0.28	1.6E-01	-0.20	3.3E-01	0.00	9.8E-01	-0.05	8.2E-01	-0.18	3.9E-01	0.29	1.5E-01	-0.09	6.6E-01	-0.20	3.2E-01	-0.45	2.1E-02	-0.02	9.1E-01
ENSCAFG0000000775	ENSCAFG0000000775	grey	EC_M10	0.28	1.6E-01	-0.26	1.9E-01	-0.19	3.5E-01	-0.10	6.2E-01	0.11	6.0E-01	0.51	8.1E-03	0.19	3.6E-01	-0.14	5.0E-01	-0.21	2.9E-01	-0.26	2.0E-01
ENSCAFG0000003066	ENSCAFG0000003066	grey	EC_M10	0.28	1.6E-01	0.09	6.6E-01	0.12	5.5E-01	0.05	8.2E-01	-0.46	1.8E-02	0.14	5.0E-01	0.07	7.4E-01	-0.35	8.1E-02	-0.14	4.8E-01	0.34	8.9E-02
ENSCAFG0000000022	HBS1L	darkgreen	EC_M4	0.28	1.6E-01	0.00	9.9E-01	-0.80	9.4E-07	-0.16	4.2E-01	0.72	3.7E-05	0.07	7.2E-01	-0.15	4.5E-01	0.19	3.5E-01	-0.11	5.8E-01	-0.86	2.2E-08
ENSCAFG0000003047	TGIF2	turquoise	EC_M6	0.28	1.6E-01	0.38	5.5E-02	0.02	9.1E-01	-0.34	8.7E-02	-0.39	4.9E-02	-0.02	9.2E-01	-0.11	5.8E-01	0.11	5.9E-01	0.33	1.0E-01	0.23	2.7E-01
ENSCAFG0000000605	EIF2AK2	grey	EC_M10	0.28	1.6E-01	0.33	9.6E-02	0.22	2.8E-01	0.17	4.1E-01	-0.65	3.3E-04	-0.21	3.0E-01	-0.11	5.9E-01	-0.07	7.2E-01	0.13	5.1E-01	0.51	7.7E-03
ENSCAFG0000001572	SNAPC1	grey	EC_M10	0.28	1.6E-01	0.43	3.0E-02	-0.61	8.5E-04	-0.28	1.6E-01	0.21	3.0E-01	0.19	3.6E-01	-0.07	7.3E-01	-0.01	9.5E-01	0.03	8.8E-01	-0.38	5.7E-02
ENSCAFG0000002529	ENSCAFG0000002529	grey	EC_M10	0.28	1.6E-01	0.13	5.2E-01	-0.03	8.9E-01	0.10	6.4E-01	-0.22	2.8E-01	0.22	2.8E-01	0.06	7.8E-01	-0.44	2.5E-02	-0.48	1.3E-02	0.09	6.6E-01
ENSCAFG0000001973	ATP5PB	grey	EC_M10	0.28	1.6E-01	-0.56	2.9E-03	-0.37	6.3E-02	-0.08	6.8E-01	0.47	1.6E-02	0.22	2.9E-01	-0.25	2.1E-01	0.01	9.4E-01	-0.07	7.3E-01	-0.60	1.1E-03
ENSCAFG0000000373	PARD3	cyan	EC_M2	0.28	1.6E-01	-0.18	3.7E-01	0.50	9.0E-03	0.15	4.7E-01	-0.80	8.7E-07	-0.18	3.8E-01	-0.10	6.1E-01	0.09	6.7E-01	-0.20	3.3E-01	0.62	6.7E-04
ENSCAFG0000001395	FSCB	grey	EC_M10	0.28	1.6E-01	0.03	8.7E-01	-0.34	9.1E-02	-0.08	7.1E-01	0.24	2.4E-01	0.60	1.2E-03	-0.11	5.9E-01	-0.12	5.4E-01	-0.20	3.2E-01	-0.38	5.3E-02
ENSCAFG0000002005	GLE1	grey	EC_M10	0.28	1.6E-01	-0.27	1.9E-01	0.23	2.7E-01	-0.21	3.1E-01	-0.34	8.6E-02	-0.08	7.1E-01	-0.16	4.3E-01	0.28	1.7E-01	-0.19	3.6E-01	0.17	4.0E-01
ENSCAFG0000001185	ENSCAFG0000001185	grey	EC_M10	0.28	1.6E-01	-0.07	7.4E-01	-0.29	1.5E-01	-0.17	3.9E-01	0.18	3.8E-01	0.42	3.5E-02	-0.24	2.5E-01	0.10	6.4E-01	0.04	8.5E-01	-0.32	1.2E-01
ENSCAFG0000000261	ENSCAFG0000000261	turquoise	EC_M6	0.28	1.6E-01	0.40	4.5E-02	-0.41	3.9E-02	0.08	7.0E-01	0.05	8.2E-01	0.24	2.3E-01	-0.09	6.7E-01	0.04	8.5E-01	-0.09	6.6E-01	-0.16	4.2E-01
ENSCAFG0000002302	ARRDC5	grey	EC_M10	0.28	1.6E-01	-0.35	8.4E-02	-0.48	1.3E-02	-0.04	8.6E-01	0.45	2.2E-02	0.18	3.8E-01	-0.14	4.8E-01	0.25	2.2E-01	0.00	1.0E+00	-0.59	1.5E-03
ENSCAFG0000003199	MSANTD4	grey	EC_M10	0.28	1.6E-01	0.22	2.7E-01	0.06	7.7E-01	-0.09	6.6E-01	-0.45	2.0E-02	0.11	6.0E-01	0.04	8.6E-01	0.04	8.3E-01	-0.01	9.5E-01	0.30	1.4E-01
ENSCAFG0000000549	CMC1	cyan	EC_M2	0.28	1.6E-01	-0.44	2.4E-02	0.40	4.3E-02	-0.04	8.4E-01	-0.55	3.8E-03	-0.04	8.4E-01	0.07	7.3E-01	0.08	7.1E-01	-0.02	9.3E-01	0.42	3.4E-02
ENSCAFG0000000715	ENSCAFG0000000715	grey	EC_M10	0.28	1.6E-01	0.24	2.3E-01	-0.28	1.6E-01	0.53	5.1E-03	0.00	9.8E-01	0.07	7.5E-01	0.02	9.3E-01	-0.01	9.6E-01	0.10	6.4E-01	-0.12	5.5E-01
ENSCAFG0000002368	ENSCAFG0000002368	violet	EC_M7	0.28	1.6E-01	-0.08	7.0E-01	-0.36	7.3E-02	-0.10	6.2E-01	0.17	4.1E-01	0.78	3.2E-06	-0.06	7.7E-01	-0.12	5.5E-01	0.23	2.6E-01	-0.32	1.1E-01
ENSCAFG0000001829	ENSCAFG0000001829	cyan	EC_M2	0.28	1.6E-01	-0.28	1.7E-01	0.51	8.1E-03	0.33	1.0E-01	-0.66	2.1E-04	0.00	9.9E-01	0.05	8.2E-01	-0.05	8.1E-01	-0.28	1.7E-01	0.49	1.1E-02
ENSCAFG0000001023	ENSCAFG0000001023	grey	EC_M10	0.28	1.6E-01	0.51	7.8E-03	-0.19	3.6E-01	0.05	8.2E-01	-0.30	1.3E-01	-0.01	9.5E-01	0.11	5.8E-01	-0.17	4.1E-01	-0.25	2.2E-01	0.08	7.0E-01
ENSCAFG0000003005	CCSAP	grey	EC_M10	0.28	1.6E-01	0.03	8.9E-01	-0.59	1.6E-03	-0.12	5.6E-01	0.50	9.5E-03	0.31	1.3E-01	-0.18	3.9E-01	-0.09	6.7E-01	0.10	6.3E-01	-0.62	7.0E-04
ENSCAFG0000001076	TESMIN	turquoise	EC_M6	0.28	1.6E-01	0.41	4.0E-02	-0.14	5.0E-01	-0.15	4.7E-01	-0.10	6.2E-01	-0.18	3.8E-01	-0.11	5.9E-01	-0.06	7.7E-01	-0.14	4.9E-01	0.00	9.9E-01
ENSCAFG0000001582	DESI2	turquoise	EC_M6	0.28	1.6E-01	0.70	6.8E-05	-0.40	4.1E-02	-0.02	9.1E-01	-0.02	9.3E-01	0.00	1.0E+00	-0.05	7.9E-01	-0.42	3.4E-02	0.08	6.9E-01	-0.14	5.0E-01
ENSCAFG0000000866	ENSCAFG0000000866	grey	EC_M10	0.28	1.6E-01	0.39	5.0E-02	-0.17	4.1E-01	0.31	1.3E-01	-0.18	3.7E-01	-0.13	5.1E-01	-0.07	7.4E-01	-0.16	4.4E-01	-0.23	2.6E-01	0.02	9.1E-01
ENSCAFG0000001936	ITGAE	grey	EC_M10	0.28	1.6E-01	-0.28	1.7E-01	0.02	9.1E-01	-0.21	3.0E-01	-0.12	5.7E-01	0.01	9.7E-01	0.26	2.0E-01	0.09	6.7E-01	-0.24	2.5E-01	0.00	9.8E-01
ENSCAFG0000000904	SYNJ1	grey	EC_M10	0.28	1.6E-01	0.25	2.2E-01	-0.18	3.8E-01	-0.12	5.6E-01	-0.16	4.4E-01	0.13	5.3E-01	-0.22	2.9E-01	-0.28	1.7E-01	-0.33	1.0E-01	-0.01	9.5E-01
ENSCAFG0000000077	TRMU	grey	EC_M10	0.28	1.6E-01	-0.26	2.0E-01	0.11	5.8E-01	-0.30	1.3E-01	-0.24	2.4E-01	-0.16	4.3E-01	-0.02	9.1E-01	0.27	1.9E-01	0.01	9.5E-01	0.04	8.3E-01
ENSCAFG0000001123	CCDC186	grey	EC_M10	0.28	1.6E-01	0.22	2.9E-01	-0.36	6.9E-02	0.13	5.4E-01	0.01	9.8E-01	0.20	3.2E-01	-0.17	4.1E-01	-0.14	4.8E-01	-0.28	1.7E-01	-0.15	4.7E-01
ENSCAFG0000000691	MTMR7	grey	EC_M10	0.28	1.6E-01	0.11	6.0E-01	-0.44	2.4E-02	0.22	2.8E-01	0.22	2.8E-01	0.16	4.4E-01	-0.04	8.3E-01	-0.30	1.4E-01	0.33	9.5E-02	-0.33	1.0E-01
ENSCAFG0000000611	ARHGEF7	turquoise	EC_M6	0.28	1.6E-01	0.86	1.2E-08	-0.51	8.2E-03	-0.28	1.7E-01	0.05	7.9E-01	-0.09	6.6E-01	-0.20	3.3E-01	0.04	8.4E-01	-0.04	8.4E-01	-0.18	3.7E-01
ENSCAFG0000000670	ENSCAFG0000000670	paleturquoise	EC_M11	0.28	1.6E-01	0.01	9.5E-01	-0.14	5.1E-01	0.50	8.8E-03	-0.06	7.5E-01	-0.17	4.0E-01	-0.12	5.6E-01	-0.10	6.2E-01	0.12	5.6E-01	-0.09	6.6E-01
ENSCAFG0000000832	CP	turquoise	EC_M6	0.28	1.6E-01	0.92	4.4E-11	-0.23	2.5E-01	-0.06	7.8E-01	-0.25	2.1E-01	-0.16	4.5E-01	-0.17	4.2E-01	-0.11	5.9E-01	0.00	1.0E+00	0.10	6.3E-01
ENSCAFG0000003062	TIMM9	grey	EC_M10	0.28	1.6E-01	-0.52	6.0E-03	-0.11	6.0E-01	0.15	4.5E-01	0.01	9.8E-01	0.31	1.3E-01	-0.24	2.5E-01	-0.12	5.4E-01	-0.21	3.0E-01	-0.23	2.7E-01
ENSCAFG0000001984	SPG7	grey	EC_M10	0.28	1.6E-01	0.19	3.5E-01	-0.61	8.7E-04	0.13	5.3E-01	0.41	3.7E-02	0.21	3.1E-01	-0.25	2.1E-01	-0.14	4.8E-01	-0.17	4.1E-01	-0.54	4.6E-03
ENSCAFG0000001463	ENSCAFG0000001463	grey	EC_M10	0.28	1.6E-01	0.25	2.3E-01	-0.53	5.2E-03	-0.12	5.5E-01	0.42	3.2E-02	-0.08	6.8E-01	-0.33	1.0E-01	-0.01	9.5E-01	-0.12	5.4E-01	-0.49	1.1E-02
ENSCAFG0000002854	ENSCAFG0000002854	grey	EC_M10	0.28	1.6E-01	0.17	4.0E-01	-0.09	6.5E-01	0.31	1.2E-01	-0.18	3.8E-01	0.15	4.7E-01	0.04	8.5E-01	0.09	6.8E-01	-0.33	1.0E-01	0.00	9.9E-01
ENSCAFG0000001858	ENSCAFG0000001858	grey	EC_M10	0.28	1.6E-01	-0.23	2.6E-01	-0.08	7.1E-01	0.23	2.6E-01	-0.11	6.0E-01	0.14	5.1E-01	0.22	2.7E-01	-0.26	2.0E-01	0.05	8.0E-01	-0.01	9.5E-01
ENSCAFG0000001813	COQ7	grey	EC_M10	0.28	1.6E-01	-0.31	1.2E-01	0.15	4.6E-01	0.14	5.1E-01	-0.19	3.6E-01	-0.21	3.1E-01	-0.05	8.0E-01	0.18	3.8E-01	-0.36	6.7E-02	0.05	7.9E-01
ENSCAFG0000001171	GMEB1	turquoise	EC_M6	0.28	1.6E-01	0.76	6.7E-06	-0.21	3.1E-01	0.05	8.1E-01	-0.31	1.2E-01	-0.21	3.0E-01	0.03	9.0E-01	-0.02	9.1E-01	-0.03	8.9E-01	0.15	4.5E-01
ENSCAFG0000000301	CPVL	turquoise	EC_M6	0.28	1.6E-01	0.72	3.7E-05	-0.32	1.1E-01	-0.11	5.9E-01	-0.11	5.9E-01	-0.13	5.1E-01	-0.15	4.7E-01	0.29	1.4E-01	-0.11	5.9E-01	-0.06	7.8E-01
ENSCAFG0000001595	ENSCAFG0000001595	turquoise	EC_M6	0.28	1.6E-01	0.42	3.4E-02	-0.21	3.0E-01	0.29	1.5E-01	-0.13	5.3E-01	0.01	9.8E-01	-0.24	2.4E-01	-0.23	2.6E-01	-0.56	3.1E-03	-0.01	9.7E-01
ENSCAFG0000003252	AHNAK	grey	EC_M10	0.28	1.6E-01	-0.27	1.9E-01	0.38	5.4E-02	0.08	6.9E-01	-0.56	3.1E-03	-0.28	1.6E-01	-0.31	1.3E-01	-0.03	8.7E-01	-0.36	7.1E-02	0.43	3.0E-02
ENSCAFG0000003231	ENSCAFG0000003231	turquoise	EC_M6	0.28	1.6E-01	0.90	5.2E-10	-0.18	3.8E-01	-0.03	8.7E-01	-0.31	1.3E-01	-0.06	7.7E-01	-0.23	2.7E-01	-0.17	4.0E-01	0.01	9.6E-01	0.16	4.3E-01
ENSCAFG0000000435	PHF11	cyan	EC_M2	0.28	1.6E-01	0.19	3.4E-01	0.39	4.9E-02	0.44	2.6E-02	-0.76	5.5E-06	-0.20	3.2E-01	-0.17	4.0E-01	-0.17	4.1E-01	-0.22	2.7E-01	0.59	1.7E-03
ENSCAFG0000000092	TTLL1	grey	EC_M10	0.28	1.7E-01	-0.30	1.4E-01	-0.36	7.3E-02	-0.22	2.9E-01	0.35	7.5E-02	0.13	5.4E-01	-0.42	3.1E-02	0.02	9.2E-01	0.09	6.7E-01	-0.51	7.5E-03
ENSCAFG0000000229	LIPT1	grey	EC_M10	0.28	1.7E-01	0.11	5.9E-01	0.28	1.7E-01	0.09	6.6E-01	-0.57	2.4E-03	-0.03	8.7E-01	-0.23	2.6E-01	-0.12	5.6E-01	-0.04	8.5E-01	0.42	3.1E-02
ENSCAFG0000001773	POLR3E	grey	EC_M10	0.28	1.7E-01	-0.17	4.2E-01	0.42	3.5E-02	0.17	4.2E-01	-0.57	2.2E-03	-0.16	4.5E-01	-0.10	6.2E-01	0.03	9.0E-01	-0.06	7.7E-01	0.46	1.9E-02
ENSCAFG0000001182	B3GNT5	turquoise	EC_M6	0.28	1.7E-01	0.83	1.2E-07	-0.53	5.4E-03	-0.01	9.4E-01	0.12	5.6E-01	0.09	6.5E-01	-0.18	3.9E-01	-0.21	3.0E-01	-0.21	3.0E-01	-0.27	1.8E-01
ENSCAFG0000000781	CCDC62	grey	EC_M10	0.28	1.7E-01	-0.41	3.8E-02	-0.40	4.2E-02	-0.20	3.3E-01	0.41	4.0E-02	0.05	8.0E-01	-0.21	3.0E-01	0.05	8.2E-01	-0.47	1.5E-02	-0.59	1.5E-03
ENSCAFG0000000474	RPS8	cyan	EC_M2	0.28	1.7E-01	-0.32	1.1E-01	0.25	2.2E-01	0.22	2.7E-01	-0.41	3.9E-02	0.17	4.2E-01	0.14	4.9E-01	-0.11	6.1E-01	-0.57	2.2E-03	0.18	3.8E-01
ENSCAFG0000002966	NUDT19	grey	EC_M10	0.28	1.7E-01	-0.37	6.3E-02	0.09	6.5E-01	0.05	8.3E-01	-0.17	4.0E-01	0.13	5.2E-01	-0.58	2.1E-03	0.17	4.1E-01	0.13	5.1E-01	0.02	9.1E-01
ENSCAFG0000000047	PSORS1C2	grey	EC_M10	0.28	1.7E-01	0.07	7.3E-01	-0.41	3.7E-02	0.10	6.2E-01	0.28	1.7E-01	-0.02	9.3E-01	-0.08	7.1E-01	0.17	4.1E-01	-0.29	1.5E-01	-0.39	5.2E-02
ENSCAFG0000001294	SRMS	turquoise	EC_M6	0.28	1.7E-01	0.79	1.5E-06	-0.20	3.4E-01	-0.09	6.6E-01	-0.27	1.9E-01	-0.11	6.0E-01	-0.11	6.1E-01	-0.10	6.3E-01	-0.18	3.8E-01	0.13	5.2E-01
ENSCAFG0000000685	SLC38A9	grey	EC_M10	0.28	1.7E-01	0.06	7.8E-01	-0.16	4.3E-01	0.30	1.4E-01	-0.15	4.5E-01	0.36	7.2E-02	-0.37	6.6E-02	-0.04	8.4E-01	-0.06	7.6E-01	-0.03	8.9E-01
ENSCAFG0000001208	COG2	grey	EC_M10	0.28	1.7E-01	0.11	5.9E-01	-0.59	1.6E-03	-0.14	5.1E-01	0.43	3.0E-02	0.04	8.4E-01	-0.27	1.9E-01	0.23	2.6E-01	-0.06	7.7E-01	-0.55	3.5E-03
ENSCAFG0000000578	ENSCAFG0000000578	grey	EC_M10	0.28	1.7E-01	-0.06	7.7E-01	-0.35	8.2E-02	0.27	1.8E-01	0.21	3.1E-01	0.17	4.0E-01	-0.23	2.7E-01	-0.18	3.7E-01	-0.24	2.4E-01	-0.37	6.0E-02
ENSCAFG0000001094	SLC37A2	grey	EC_M10	0.28	1.7E-01	-0.12	5.7E-01	-0.67	1.7E-04	-0.04	8.3E-01	0.56	3.2E-03	0.47	1.6E-02	0.02	9.1E-01	0.01	9.6E-01	0.19	3.6E-01	-0.74	1.4E-05
ENSCAFG0000000096	ENSCAFG0000000096	grey	EC_M10	0.28	1.7E-01	-0.10	6.4E-01	-0.32	1.1E-01	0.02	9.3E-01	0.20	3.2E-01	-0.08	7.1E-01	-0.03	9.0E-01	-0.05	8.1E-01	0.01	9.5E-01	-0.36	6.8E-02
ENSCAFG0000000458	CLASRP	grey	EC_M10	0.28	1.7E-01	0.24	2.4E-01	-0.21	3.0E-01	-0.14	5.1E-01	-0.08	7.1E-01	0.11	5.9E-01	0.26	2.0E-01	0.04	8.5E-01	0.21	2.9E-01	-0.06	7.6E-01
ENSCAFG0000001658	ENSCAFG0000001658	grey	EC_M10	0.28	1.7E-01	0.09	6.7E-01	-0.31	1.2E-01	0.27	1.8E-01	0.11	6.0E-01	-0.37	6.0E-02	0.01	9.8E-01	-0.34	8.5E-02	-0.11	6.0E-01	-0.22	2.9E-01
ENSCAFG0000001482	PRKCI	darkgreen	EC_M4	0.28	1.7E-01	-0.34	9.1E-02	-0.65	3.2E-04	-0.02	9.4E-01	0.61	8.5E-04	0.21	3.1E-01	-0.21	3.0E-01	0.14	4.8E-01	0.05	8.3E-01	-0.78	2.1E-06
ENSCAFG0000000988	SNCA	turquoise	EC_M6	0.28	1.7E-01	0.80	9.7E-07	-0.25	2.2E-01	-0.10	6.1E-01	-0.24	2.3E-01	-0.08	6.9E-01	-0.11	6.1E-01	-0.10	6.3E-01	-0.15	4.6E-01	0.10	6.3E-01
ENSCAFG0000001103	ENSCAFG0000001103	grey	EC_M10	0.28	1.7E-01	0.27	1.7E-01	-0.44	2.5E-02	-0.14	4.9E-01	0.23	2.5E-01	0.12	5.6E-01	-0.10	6.3E-01	0.04	8.5E-01	0.08	6.9E-01	-0.37	6.6E-02
ENSCAFG0000001602	RFX7	darkgrey	EC_M8	0.28	1.7E-01	0.50	9.9E-03	0.00	9.9E-01	0.18	3.8E-01	-0.50	8.8E-03	-0.32	1.2E-01	-0.12	5.5E-01	0.05	8.2E-01	-0.16	4.2E-01	0.34	8.6E-02
ENSCAFG0000001644	ZCCHC4	grey	EC_M10	0.28	1.7E-01	-0.21	2.9E-01	-0.14	5.1E-01	0.25	2.3E-01	-0.06	7.9E-01	0.24	2.4E-01	-0.43	2.8E-02	0.07	7.3E-01	0.05	7.9E-01	-0.10	6.3E-01
ENSCAFG0000000901	MTX3	turquoise	EC_M6	0.28	1.7E-01	0.54	4.5E-03	-0.38	5.6E-02	-0.01	9.5E-01	0.03	8.8E-01	-0.19	3.6E-01	-0.26	2.0E-01	0.00	1.0E+00	0.17	4.0E-01	-0.12	5.5E-01
ENSCAFG0000001426	ENY2	grey	EC_M10	0.28	1.7E-01	0.29	1.4E-01	-0.30	1.4E-01	-0.33	1.0E-01	0.05	8.3E-01	-0.05	8.0E-01	-0.26	2.0E-01	0.29	1.5E-01	-0.15	4.7E-01	-0.25	2.3E-01
ENSCAFG0000001087	EPRS	darkgreen	EC_M4	0.28	1.7E-01	-0.21	3.0E-01	-0.76	7.9E-06	-0.13	5.4E-01	0.74	1.4E-05	0.31	1.2E-01	-0.09	6.7E-01	-0.08	7.0E-01	-0.35	8.3E-02	-0.91	1.8E-10
ENSCAFG0000002172	ENSCAFG0000002172	grey	EC_M10	0.28	1.7E-01	0.06	7.8E-01	-0.12	5.6E-01	0.29	1.5E-01	-0.15	4.8E-01	-0.15	4.5E-01	0.10	6.4E-01	-0.17	4.0E-01	-0.15	4.7E-01	0.01	9.7E-01
ENSCAFG0000002963	ENSCAFG0000002963	violet	EC_M7	0.28	1.7E-01	-0.11	5.8E-01	-0.18	3.8E-01	-0.11	5.8E-01	0.00	9.8E-01	0.72	3.3E-05	-0.11	6.0E-01	-0.12	5.6E-01	-0.19	3.6E-01	-0.20	3.2E-01
ENSCAFG0000001067	HECW2	turquoise	EC_M6	0.28	1.7E-01	0.44	2.3E-02	-0.10	6.4E-01	0.04	8.5E-01	-0.34	9.2E-02	0.02	9.2E-01	0.01	9.6E-01	-0.07	7.3E-01	-0.38	5.4E-02	0.13	5.2E-01
ENSCAFG0000003065	ENSCAFG0000003065	grey	EC_M10	0.28	1.7E-01	-0.07	7.4E-01	-0.07	7.4E-01	0.17	4.1E-01	-0.08	7.0E-01	0.09	6.6E-01	-0.04	8.5E-01	0.25	2.1E-01	-0.28	1.6E-01	-0.05	8.0E-01
ENSCAFG0000001411	CCDC43	grey	EC_M10	0.28	1.7E-01	0.07	7.3E-01	-0.35	8.0E-02	0.05	8.1E-01	0.10	6.4E-01	0.10	6.1E-01	0.35	8.0E-02	0.14	4.9E-01	0.08	6.8E-01	-0.25	2.2E-01
ENSCAFG0000002872	ENSCAFG0000002872	grey	EC_M10	0.28	1.7E-01	0.14	5.1E-01	0.06	7.7E-01	-0.38	5.7E-02	-0.38	5.8E-02	-0.13	5.4E-01	-0.02	9.2E-01	-0.21	3.0E-01	0.00	1.0E+00	0.24	2.4E-01
ENSCAFG0000001263	DERA	grey	EC_M10	0.28	1.7E-01	0.20	3.2E-01	-0.13	5.2E-01	0.16	4.5E-01	-0.18	3.8E-01	0.01	9.6E-01	-0.13	5.3E-01	-0.09	6.8E-01	-0.45	2.2E-02	-0.02	9.2E-01
ENSCAFG0000001788	INO80C	darkgreen	EC_M4	0.28	1.7E-01	0.33	1.0E-01	-0.74	1.4E-05	-0.17	4.0E-01	0.56	2.9E-03	0.12	5.7E-01	-0.34	8.8E-02	0.05	8.3E-01	-0.07	7.5E-01	-0.68	1.4E-04
ENSCAFG0000000393	INTU	grey	EC_M10	0.28	1.7E-01	-0.18	3.7E-01	0.34	9.0E-02	0.25	2.2E-01	-0.60	1.3E-03	0.07	7.3E-01	-0.07	7.4E-01	-0.11	6.0E-01	0.04	8.3E-01	0.41	3.8E-02
ENSCAFG0000001692	ENSCAFG0000001692	darkgrey	EC_M8	0.28	1.7E-01	0.45	2.0E-02	0.22	2.9E-01	0.17	4.1E-01	-0.73	2.3E-05	-0.18	3.7E-01	0.07	7.4E-01	-0.07	7.2E-01	-0.10	6.4E-01	0.55	3.6E-03
ENSCAFG0000001033	TMEM183A	grey	EC_M10	0.28	1.7E-01	0.18	3.7E-01	0.14	4.9E-01	-0.07	7.3E-01	-0.40	4.3E-02	-0.12	5.7E-01	0.20	3.3E-01	-0.22	2.7E-01	-0.30	1.3E-01	0.24	2.5E-01
ENSCAFG0000001062	SPICE1	grey	EC_M10	0.28	1.7E-01	-0.31	1.2E-01	0.14	4.9E-01	0.37	6.4E-02	-0.38	5.7E-02	-0.02	9.1E-01	-0.13	5.3E-01	0.07	7.5E-01	0.14	4.9E-01	0.21	3.1E-01
ENSCAFG0000000175	PPP2R5D	grey	EC_M10	0.28	1.7E-01	0.26	2.0E-01	0.08	7.1E-01	-0.21	3.1E-01	-0.34	8.4E-02	0.23	2.6E-01	0.11	6.0E-01	0.13	5.4E-01	0.11	6.1E-01	0.21	3.0E-01
ENSCAFG0000000307	ENSCAFG0000000307	darkgreen	EC_M4	0.28	1.7E-01	-0.17	4.1E-01	-0.65	3.2E-04	-0.25	2.2E-01	0.62	6.6E-04	0.31	1.2E-01	-0.30	1.4E-01	-0.07	7.3E-01	-0.17	4.1E-01	-0.77	4.0E-06
ENSCAFG0000000910	STC1	turquoise	EC_M6	0.28	1.7E-01	0.80	9.3E-07	-0.18	3.7E-01	-0.07	7.4E-01	-0.28	1.6E-01	-0.15	4.7E-01	-0.18	3.7E-01	-0.17	4.1E-01	0.08	7.1E-01	0.11	6.1E-01
ENSCAFG0000003264	PPP1R2	turquoise	EC_M6	0.28	1.7E-01	0.62	6.9E-04	-0.55	3.4E-03	0.16	4.3E-01	0.12	5.7E-01	0.12	5.6E-01	-0.22	2.9E-01	-0.02	9.2E-01	0.24	2.4E-01	-0.24	2.4E-01
ENSCAFG0000002910	SMIM30	grey	EC_M10	0.28	1.7E-01	0.35	7.9E-02	-0.66	2.2E-04	-0.17	4.1E-01	0.44	2.3E-02	0.06	7.8E-01	-0.04	8.5E-01	0.10	6.1E-01	-0.33	1.0E-01	-0.55	3.4E-03
ENSCAFG0000000839	MRPS28	grey	EC_M10	0.28	1.7E-01	-0.41	3.9E-02	-0.26	2.0E-01	-0.32	1.1E-01	0.26	1.9E-01	0.07	7.4E-01	-0.40	4.2E-02	-0.01	9.5E-01	-0.11	6.0E-01	-0.40	4.3E-02
ENSCAFG0000000109	ENSCAFG0000000109	grey	EC_M10	0.28	1.7E-01	-0.44	2.4E-02	0.00	9.9E-01	0.27	1.9E-01	-0.03	8.7E-01	0.09	6.7E-01	-0.07	7.3E-01	-0.14	4.9E-01	-0.36	6.9E-02	-0.17	4.1E-01
ENSCAFG0000002028	BCL10	darkgreen	EC_M4	0.28	1.7E-01	0.32	1.2E-01	-0.80	9.5E-07	-0.04	8.3E-01	0.55	3.6E-03	0.22	2.9E-01	-0.32	1.1E-01	-0.02	9.2E-01	-0.16	4.5E-01	-0.70	7.8E-05
ENSCAFG0000002001	RTCA	grey	EC_M10	0.28	1.7E-01	0.17	4.0E-01	-0.65	3.6E-04	-0.12	5.6E-01	0.52	7.1E-03	0.13	5.2E-01	-0.12	5.6E-01	0.10	6.1E-01	-0.33	1.0E-01	-0.61	8.5E-04
ENSCAFG0000002748	ENSCAFG0000002748	grey	EC_M10	0.28	1.7E-01	-0.06	7.8E-01	-0.33	1.0E-01	-0.02	9.2E-01	0.23	2.6E-01	0.34	8.8E-02	-0.16	4.3E-01	-0.05	8.0E-01	-0.22	2.8E-01	-0.39	5.0E-02
ENSCAFG0000000042	CPSF6	grey	EC_M10	0.28	1.7E-01	-0.27	1.9E-01	0.24	2.4E-01	0.13	5.4E-01	-0.33	9.7E-02	0.20	3.3E-01	-0.39	4.7E-02	-0.14	5.0E-01	-0.36	7.1E-02	0.13	5.2E-01
ENSCAFG0000001994	SLC25A24	cyan	EC_M2	0.28	1.7E-01	-0.02	9.2E-01	0.41	3.6E-02	0.29	1.6E-01	-0.71	5.6E-05	-0.36	7.2E-02	-0.33	1.0E-01	-0.08	6.9E-01	-0.23	2.7E-01	0.57	2.4E-03
ENSCAFG0000000859	ANGPT2	turquoise	EC_M6	0.28	1.7E-01	0.96	1.5E-14	-0.27	1.9E-01	-0.11	6.0E-01	-0.23	2.7E-01	-0.17	4.1E-01	-0.13	5.1E-01	-0.19	3.6E-01	0.10	6.2E-01	0.10	6.3E-01
ENSCAFG0000003161	IFIT3	turquoise	EC_M6	0.28	1.7E-01	0.61	9.6E-04	-0.09	6.7E-01	0.04	8.4E-01	-0.34	9.1E-02	-0.30	1.3E-01	-0.28	1.6E-01	-0.04	8.6E-01	0.39	4.7E-02	0.21	3.0E-01

ENSCAFG0000000390	BRAF	grey	EC_M10	0.28	1.7E-01	0.05	8.2E-01	-0.45	2.0E-02	0.16	4.3E-01	0.20	3.2E-01	0.07	7.2E-01	-0.38	5.8E-02	-0.15	4.7E-01	0.20	3.4E-01	-0.36	6.9E-02
ENSCAFG0000000662	ERCC6	darkgrey	EC_M8	0.28	1.7E-01	-0.23	2.6E-01	0.31	1.3E-01	0.20	3.4E-01	-0.59	1.6E-03	0.06	7.6E-01	0.21	3.1E-01	-0.19	3.6E-01	-0.09	6.6E-01	0.42	3.2E-02
ENSCAFG0000000178	ENSCAFG0000000178	grey	EC_M10	0.28	1.7E-01	0.11	6.1E-01	-0.35	7.9E-02	0.36	7.3E-02	0.12	5.7E-01	0.28	1.7E-01	0.09	6.5E-01	-0.23	2.7E-01	-0.33	1.0E-01	-0.26	2.0E-01
ENSCAFG0000003100	SNX16	grey	EC_M10	0.28	1.7E-01	-0.09	6.6E-01	-0.39	5.1E-02	-0.05	8.1E-01	0.16	4.3E-01	0.23	2.5E-01	0.02	9.4E-01	0.02	9.2E-01	-0.28	1.6E-01	-0.35	7.8E-02
ENSCAFG0000003105	TMEM69	grey	EC_M10	0.28	1.7E-01	-0.24	2.4E-01	-0.16	4.4E-01	-0.24	2.4E-01	0.09	6.5E-01	0.28	1.6E-01	-0.49	1.1E-02	0.20	3.4E-01	0.23	2.5E-01	-0.24	2.5E-01
ENSCAFG0000000725	ARMC1	grey	EC_M10	0.28	1.7E-01	0.19	3.4E-01	-0.40	4.3E-02	-0.15	4.6E-01	0.17	4.1E-01	-0.02	9.3E-01	-0.11	5.9E-01	-0.39	5.0E-02	-0.43	3.0E-02	-0.27	1.8E-01
ENSCAFG0000002039	CDH16	grey	EC_M10	0.28	1.7E-01	-0.08	7.0E-01	-0.01	9.7E-01	-0.12	5.5E-01	-0.15	4.7E-01	-0.07	7.2E-01	-0.01	9.8E-01	-0.09	6.7E-01	0.17	4.0E-01	0.07	7.3E-01
ENSCAFG0000000074	ENSCAFG0000000074	grey	EC_M10	0.27	1.7E-01	0.02	9.1E-01	-0.06	7.5E-01	-0.12	5.6E-01	-0.16	4.4E-01	-0.17	3.9E-01	-0.16	4.5E-01	-0.12	5.7E-01	0.13	5.3E-01	-0.04	8.4E-01
ENSCAFG0000001086	BRD9	grey	EC_M10	0.27	1.7E-01	-0.03	8.9E-01	0.55	3.4E-03	0.09	6.7E-01	-0.83	1.4E-07	-0.07	7.3E-01	-0.19	3.6E-01	0.10	6.1E-01	0.12	5.6E-01	0.70	7.7E-05
ENSCAFG0000003092	ENSCAFG0000003092	turquoise	EC_M6	0.27	1.7E-01	0.53	5.1E-03	-0.49	1.0E-02	0.01	9.7E-01	0.11	5.8E-01	-0.05	8.2E-01	-0.21	3.1E-01	-0.13	5.2E-01	-0.07	7.3E-01	-0.27	1.8E-01
ENSCAFG0000000043	FRS2	grey	EC_M10	0.27	1.7E-01	-0.54	4.9E-03	-0.19	3.6E-01	0.06	7.7E-01	0.12	5.5E-01	0.00	9.8E-01	-0.22	2.7E-01	0.09	6.8E-01	-0.09	6.8E-01	-0.31	1.2E-01
ENSCAFG0000003046	ENSCAFG0000003046	turquoise	EC_M6	0.27	1.7E-01	0.40	4.4E-02	-0.15	4.6E-01	-0.17	4.1E-01	-0.19	3.5E-01	-0.14	5.0E-01	-0.08	7.0E-01	0.38	5.5E-02	0.13	5.3E-01	0.10	6.1E-01
ENSCAFG0000000667	ENSCAFG0000000667	grey	EC_M10	0.27	1.7E-01	0.37	6.5E-02	-0.33	9.8E-02	-0.03	9.0E-01	0.02	9.2E-01	0.43	2.8E-02	-0.13	5.2E-01	-0.12	5.7E-01	-0.24	2.3E-01	-0.22	2.9E-01
ENSCAFG0000002424	ENSCAFG0000002424	turquoise	EC_M6	0.27	1.8E-01	0.88	4.2E-09	-0.21	3.0E-01	-0.15	4.6E-01	-0.24	2.3E-01	-0.17	4.2E-01	-0.12	5.6E-01	0.07	7.5E-01	0.16	4.2E-01	0.12	5.5E-01
ENSCAFG0000001908	MYO10	darkgreen	EC_M4	0.27	1.8E-01	0.30	1.3E-01	-0.86	1.9E-08	-0.20	3.3E-01	0.67	2.0E-04	0.13	5.2E-01	-0.15	4.7E-01	0.01	9.7E-01	-0.03	9.0E-01	-0.82	2.4E-07
ENSCAFG0000000720	SLC20A1	darkgreen	EC_M4	0.27	1.8E-01	-0.18	3.8E-01	-0.72	3.0E-05	-0.13	5.2E-01	0.69	9.1E-05	0.23	2.5E-01	-0.33	9.6E-02	0.10	6.1E-01	-0.19	3.5E-01	-0.87	5.4E-09
ENSCAFG0000000196	ANKIB1	grey	EC_M10	0.27	1.8E-01	-0.26	2.0E-01	-0.10	6.3E-01	0.17	3.9E-01	-0.10	6.2E-01	0.12	5.7E-01	-0.29	1.5E-01	-0.11	6.0E-01	-0.30	1.4E-01	-0.08	6.8E-01
ENSCAFG0000002272	MT-CO1	cyan	EC_M2	0.27	1.8E-01	-0.34	8.6E-02	0.48	1.3E-02	0.18	3.8E-01	-0.66	2.4E-04	-0.02	9.1E-01	-0.33	1.0E-01	-0.12	5.5E-01	-0.36	6.8E-02	0.49	1.1E-02
ENSCAFG0000000988	MMRN1	turquoise	EC_M6	0.27	1.8E-01	0.89	7.0E-10	-0.24	2.4E-01	-0.06	7.8E-01	-0.22	2.7E-01	-0.09	6.7E-01	-0.14	4.9E-01	-0.08	7.0E-01	-0.22	2.7E-01	0.09	6.6E-01
ENSCAFG0000001335	PTCHD1	turquoise	EC_M6	0.27	1.8E-01	0.93	4.1E-12	-0.29	1.6E-01	-0.08	6.8E-01	-0.21	3.1E-01	-0.13	5.3E-01	-0.12	5.8E-01	-0.13	5.1E-01	-0.04	8.3E-01	0.04	8.4E-01
ENSCAFG0000001555	NUP88	turquoise	EC_M6	0.27	1.8E-01	0.60	1.2E-03	0.06	7.9E-01	-0.04	8.3E-01	-0.41	3.8E-02	-0.26	1.9E-01	-0.23	2.6E-01	-0.14	4.9E-01	0.05	8.1E-01	0.31	1.2E-01
ENSCAFG0000001599	LIAS	grey	EC_M10	0.27	1.8E-01	-0.30	1.3E-01	0.14	4.8E-01	0.36	7.4E-02	-0.28	1.6E-01	0.00	9.9E-01	0.12	5.7E-01	-0.36	7.3E-02	-0.47	1.5E-02	0.13	5.3E-01
ENSCAFG0000000280	ENSCAFG0000000280	turquoise	EC_M6	0.27	1.8E-01	0.38	5.5E-02	-0.28	1.7E-01	0.17	3.9E-01	-0.11	6.0E-01	0.17	3.9E-01	-0.06	7.8E-01	-0.05	8.2E-01	0.35	7.9E-02	-0.03	8.7E-01
ENSCAFG0000002027	LHX6	turquoise	EC_M6	0.27	1.8E-01	0.92	2.2E-11	-0.34	8.7E-02	-0.02	9.3E-01	-0.12	5.5E-01	-0.08	7.0E-01	-0.10	6.2E-01	-0.09	6.7E-01	-0.24	2.4E-01	-0.03	8.7E-01
ENSCAFG0000000469	MRPL58	grey	EC_M10	0.27	1.8E-01	-0.61	1.0E-03	-0.16	4.4E-01	0.13	5.4E-01	0.21	3.1E-01	0.28	1.6E-01	-0.17	4.1E-01	-0.06	7.7E-01	-0.47	1.6E-02	-0.36	7.1E-02
ENSCAFG0000000865	ENSCAFG0000000865	grey	EC_M10	0.27	1.8E-01	0.33	1.0E-01	-0.15	4.6E-01	0.30	1.4E-01	-0.18	3.8E-01	0.29	1.5E-01	-0.23	2.6E-01	-0.21	3.0E-01	-0.30	1.3E-01	0.02	9.2E-01
ENSCAFG0000000068	FBN2	darkgreen	EC_M4	0.27	1.8E-01	-0.05	8.2E-01	-0.35	7.6E-02	-0.21	3.1E-01	0.24	2.4E-01	0.15	4.5E-01	-0.19	3.6E-01	-0.10	6.2E-01	-0.12	5.7E-01	-0.36	6.8E-02
ENSCAFG0000001734	COX11	grey	EC_M10	0.27	1.8E-01	-0.05	7.9E-01	0.25	2.2E-01	0.57	2.2E-03	-0.54	4.3E-03	-0.18	3.8E-01	-0.14	5.0E-01	-0.25	2.2E-01	-0.15	4.7E-01	0.37	6.5E-02
ENSCAFG0000001089	COQ10B	darkgreen	EC_M4	0.27	1.8E-01	0.04	8.3E-01	-0.75	1.1E-05	-0.16	4.4E-01	0.68	1.5E-04	0.24	2.4E-01	0.03	9.0E-01	-0.19	3.4E-01	-0.01	9.5E-01	-0.77	4.8E-06
ENSCAFG0000000913	GPAT3	cyan	EC_M2	0.27	1.8E-01	-0.18	3.7E-01	0.43	3.0E-02	0.19	3.6E-01	-0.66	2.4E-04	-0.09	6.4E-01	-0.17	4.2E-01	0.07	7.4E-01	-0.29	1.5E-01	0.51	8.4E-03
ENSCAFG0000000619	ATP11A	turquoise	EC_M6	0.27	1.8E-01	0.52	6.1E-03	0.05	8.1E-01	0.09	6.5E-01	-0.47	1.6E-02	-0.13	5.2E-01	-0.60	1.2E-03	-0.11	5.9E-01	-0.23	2.5E-01	0.31	1.2E-01
ENSCAFG0000000570	OSBPL8	darkgreen	EC_M4	0.27	1.8E-01	0.04	8.4E-01	-0.78	2.9E-06	-0.15	4.6E-01	0.63	5.9E-04	0.30	1.3E-01	-0.04	8.5E-01	0.08	7.1E-01	-0.31	1.2E-01	-0.79	1.7E-06
ENSCAFG0000000113	XPNPEP3	grey	EC_M10	0.27	1.8E-01	-0.37	6.3E-02	-0.39	4.7E-02	-0.23	2.5E-01	0.34	8.6E-02	0.18	3.9E-01	-0.27	1.8E-01	0.12	5.4E-01	0.12	5.7E-01	-0.49	1.1E-02
ENSCAFG0000000261	TNNI3	grey	EC_M10	0.27	1.8E-01	-0.19	3.5E-01	0.06	7.8E-01	0.07	7.3E-01	-0.25	2.1E-01	-0.18	3.8E-01	0.08	6.8E-01	0.07	7.4E-01	0.03	8.9E-01	0.09	6.7E-01
ENSCAFG0000001063	COBLL1	grey	EC_M10	0.27	1.8E-01	0.14	4.9E-01	-0.35	8.0E-02	0.04	8.5E-01	0.18	3.7E-01	0.01	9.7E-01	0.18	3.9E-01	-0.62	8.3E-04	-0.62	6.4E-04	-0.32	1.1E-01
ENSCAFG0000000923	GPD2	grey	EC_M10	0.27	1.8E-01	0.02	9.2E-01	0.16	4.3E-01	0.03	9.0E-01	-0.44	2.4E-02	-0.33	9.9E-02	0.27	1.8E-01	0.28	1.7E-01	0.22	2.8E-01	0.32	1.1E-01
ENSCAFG0000002034	ADGRL4	turquoise	EC_M6	0.27	1.8E-01	0.89	1.2E-09	-0.26	2.0E-01	-0.04	8.5E-01	-0.21	3.1E-01	-0.12	5.5E-01	-0.17	4.1E-01	-0.12	5.6E-01	-0.08	7.0E-01	0.04	8.4E-01
ENSCAFG0000000577	PPP1R12A	darkgreen	EC_M4	0.27	1.8E-01	0.01	9.8E-01	-0.88	4.0E-09	-0.13	5.1E-01	0.76	8.1E-06	0.31	1.2E-01	0.00	9.9E-01	0.07	7.5E-01	0.15	4.7E-01	-0.91	1.5E-10
ENSCAFG0000001578	PPP2R5E	darkgreen	EC_M4	0.27	1.8E-01	-0.04	8.3E-01	-0.75	1.2E-05	0.02	9.3E-01	0.63	5.1E-04	0.06	7.6E-01	-0.27	1.8E-01	0.04	8.4E-01	-0.03	8.7E-01	-0.76	6.2E-06
ENSCAFG0000000325	PNO1	grey	EC_M10	0.27	1.8E-01	-0.31	1.2E-01	-0.02	9.2E-01	0.00	1.0E+00	-0.03	8.9E-01	0.14	5.1E-01	-0.19	3.6E-01	-0.36	7.0E-02	-0.40	4.4E-02	-0.14	4.9E-01
ENSCAFG0000003087	ENSCAFG0000003087	grey	EC_M10	0.27	1.8E-01	-0.31	1.3E-01	0.12	5.5E-01	0.12	5.7E-01	-0.25	2.2E-01	0.05	8.2E-01	0.11	5.9E-01	0.16	4.4E-01	0.00	9.8E-01	0.12	5.8E-01
ENSCAFG0000001763	THSD4	turquoise	EC_M6	0.27	1.8E-01	0.82	2.8E-07	-0.12	5.6E-01	0.01	9.6E-01	-0.39	5.2E-02	-0.18	3.8E-01	-0.20	3.3E-01	-0.17	3.9E-01	-0.01	9.6E-01	0.21	3.0E-01
ENSCAFG0000000300	DNAJC25	grey	EC_M10	0.27	1.8E-01	-0.01	9.6E-01	-0.63	5.5E-04	-0.14	5.0E-01	0.45	2.2E-02	0.42	3.2E-02	0.24	2.4E-01	-0.02	9.2E-01	-0.19	3.4E-01	-0.60	1.1E-03
ENSCAFG0000000402	KIAA1109	darkgrey	EC_M8	0.27	1.8E-01	0.39	4.6E-02	-0.05	8.1E-01	0.08	7.0E-01	-0.37	6.7E-02	-0.29	1.5E-01	-0.11	5.8E-01	-0.15	4.5E-01	-0.33	9.6E-02	0.22	2.8E-01
ENSCAFG0000001335	SIPA1	turquoise	EC_M6	0.27	1.8E-01	0.81	6.1E-07	-0.03	8.9E-01	-0.08	7.0E-01	-0.44	2.5E-02	-0.09	6.7E-01	0.10	6.1E-01	-0.18	3.9E-01	-0.12	5.7E-01	0.30	1.4E-01
ENSCAFG0000002973	ENSCAFG0000002973	grey	EC_M10	0.27	1.8E-01	0.34	9.0E-02	-0.38	5.7E-02	0.21	2.9E-01	0.06	7.8E-01	0.01	9.7E-01	0.07	7.2E-01	-0.34	9.3E-02	-0.19	3.6E-01	-0.20	3.2E-01
ENSCAFG0000002662	ENSCAFG0000002662	turquoise	EC_M6	0.27	1.8E-01	0.49	1.0E-02	-0.05	8.0E-01	-0.13	5.4E-01	-0.28	1.7E-01	-0.13	5.4E-01	-0.08	7.1E-01	0.01	9.8E-01	-0.14	5.0E-01	0.16	4.4E-01
ENSCAFG0000001204	IPO4	grey	EC_M10	0.27	1.8E-01	-0.33	1.0E-01	0.40	4.2E-02	0.11	6.1E-01	-0.48	1.2E-02	-0.27	1.9E-01	-0.26	2.0E-01	-0.12	5.6E-01	-0.41	3.6E-02	0.32	1.2E-01
ENSCAFG0000001469	ALKBH8	grey	EC_M10	0.27	1.8E-01	0.16	4.4E-01	-0.08	6.9E-01	-0.21	3.0E-01	-0.21	3.0E-01	-0.09	6.7E-01	-0.35	8.3E-02	0.25	2.1E-01	0.00	9.9E-01	0.07	7.2E-01
ENSCAFG0000000716	TTL	grey	EC_M10	0.27	1.8E-01	0.22	2.9E-01	-0.08	7.1E-01	-0.03	8.9E-01	-0.24	2.4E-01	0.28	1.7E-01	-0.07	7.5E-01	-0.30	1.3E-01	-0.21	3.0E-01	0.11	6.0E-01
ENSCAFG0000000230	ENSCAFG0000000230	grey	EC_M10	0.27	1.8E-01	0.20	3.2E-01	-0.04	8.3E-01	0.09	6.6E-01	-0.32	1.1E-01	-0.10	6.4E-01	-0.27	1.8E-01	0.14	4.8E-01	-0.23	2.6E-01	0.18	3.7E-01
ENSCAFG0000000397	SLC37A3	darkgreen	EC_M4	0.27	1.8E-01	0.49	1.2E-02	-0.83	2.1E-07	0.01	9.7E-01	0.51	7.8E-03	0.20	3.2E-01	-0.07	7.5E-01	-0.05	8.2E-01	-0.17	4.0E-01	-0.70	6.7E-05
ENSCAFG0000001791	P2RY11	grey	EC_M10	0.27	1.8E-01	0.13	5.3E-01	0.05	8.3E-01	-0.33	1.0E-01	-0.22	2.8E-01	0.02	9.2E-01	-0.14	4.9E-01	0.00	9.8E-01	-0.06	7.8E-01	0.07	7.2E-01
ENSCAFG0000001923	TFAP4	cyan	EC_M2	0.27	1.8E-01	-0.17	4.0E-01	0.56	2.7E-03	0.15	4.7E-01	-0.81	4.0E-07	-0.03	9.0E-01	0.06	7.6E-01	-0.13	5.3E-01	-0.22	2.8E-01	0.65	3.3E-04
ENSCAFG0000000688	CD44	grey	EC_M10	0.27	1.8E-01	-0.26	1.9E-01	-0.40	4.2E-02	-0.20	3.3E-01	0.38	5.5E-02	0.20	3.3E-01	0.08	6.8E-01	0.15	4.7E-01	0.16	4.3E-01	-0.56	2.8E-03
ENSCAFG0000002943	ZNF19	grey	EC_M10	0.27	1.8E-01	0.28	1.6E-01	-0.20	3.2E-01	0.11	5.9E-01	-0.17	4.0E-01	-0.09	6.6E-01	0.31	1.3E-01	0.08	7.0E-01	-0.07	7.3E-01	0.01	9.8E-01
ENSCAFG0000000383	DLD	grey	EC_M10	0.27	1.8E-01	-0.32	1.2E-01	0.32	1.1E-01	-0.02	9.2E-01	-0.45	2.2E-02	-0.08	7.1E-01	-0.23	2.5E-01	-0.06	7.6E-01	-0.16	4.3E-01	0.36	7.3E-02
ENSCAFG0000000670	FLT1	grey	EC_M10	0.27	1.8E-01	0.49	1.1E-02	-0.59	1.4E-03	-0.18	3.9E-01	0.32	1.1E-01	-0.08	6.8E-01	-0.10	6.3E-01	-0.11	5.9E-01	0.41	3.8E-02	-0.42	3.3E-02
ENSCAFG0000002807	ENSCAFG0000002807	grey	EC_M10	0.27	1.8E-01	0.36	6.7E-02	-0.01	9.4E-01	0.18	3.8E-01	-0.38	5.7E-02	-0.34	9.2E-02	0.11	5.8E-01	-0.01	9.5E-01	0.11	5.9E-01	0.22	2.8E-01
ENSCAFG0000000432	RSBN1L	cyan	EC_M2	0.27	1.8E-01	0.04	8.3E-01	0.40	4.3E-02	0.19	3.6E-01	-0.72	3.3E-05	-0.31	1.2E-01	-0.27	1.8E-01	-0.20	3.3E-01	0.10	6.4E-01	0.59	1.4E-03
ENSCAFG0000001200	ARL4C	grey	EC_M10	0.27	1.8E-01	-0.46	1.9E-02	-0.33	9.6E-02	0.03	8.7E-01	0.30	1.4E-01	0.22	2.9E-01	-0.19	3.5E-01	0.18	3.9E-01	-0.01	9.7E-01	-0.50	8.8E-03
ENSCAFG0000002677	ENSCAFG0000002677	turquoise	EC_M6	0.27	1.8E-01	0.55	3.8E-03	-0.13	5.2E-01	-0.12	5.5E-01	-0.30	1.4E-01	-0.13	5.4E-01	-0.08	6.8E-01	-0.13	5.2E-01	-0.04	8.6E-01	0.21	3.1E-01
ENSCAFG0000000527	ENSCAFG0000000527	turquoise	EC_M6	0.27	1.8E-01	0.50	9.1E-03	-0.38	5.6E-02	-0.20	3.2E-01	0.04	8.4E-01	0.33	1.0E-01	-0.22	2.8E-01	-0.24	2.4E-01	-0.02	9.2E-01	-0.17	4.2E-01
ENSCAFG0000001271	ARHGAP30	grey	EC_M10	0.27	1.8E-01	0.09	6.5E-01	-0.08	7.1E-01	-0.09	6.5E-01	-0.16	4.4E-01	-0.13	5.4E-01	-0.13	5.2E-01	-0.14	4.9E-01	-0.07	7.2E-01	0.06	7.6E-01
ENSCAFG0000000323	MEIS1	grey	EC_M10	0.27	1.8E-01	-0.40	4.4E-02	0.01	9.5E-01	0.39	4.9E-02	-0.16	4.4E-01	0.18	3.8E-01	-0.23	2.6E-01	-0.11	5.9E-01	0.18	3.8E-01	-0.01	9.6E-01
ENSCAFG0000002899	ENSCAFG0000002899	grey	EC_M10	0.27	1.8E-01	0.16	4.4E-01	-0.69	9.4E-05	0.23	2.5E-01	0.49	1.0E-02	-0.08	6.8E-01	0.00	1.0E+00	0.11	6.1E-01	-0.09	6.5E-01	-0.62	7.4E-04
ENSCAFG0000000411	PSMB1	grey	EC_M10	0.27	1.8E-01	-0.02	9.1E-01	-0.69	9.0E-05	-0.27	1.8E-01	0.67	1.7E-04	0.21	3.0E-01	-0.18	3.8E-01	0.11	6.1E-01	-0.07	7.4E-01	-0.79	1.2E-06
ENSCAFG0000001901	LRG1	turquoise	EC_M6	0.27	1.8E-01	0.87	6.0E-09	-0.25	2.2E-01	-0.12	5.7E-01	-0.23	2.5E-01	-0.13	5.3E-01	-0.13	5.4E-01	-0.15	4.7E-01	0.14	5.0E-01	0.11	5.9E-01
ENSCAFG0000000745	KRCC1	cyan	EC_M2	0.27	1.8E-01	-0.37	6.0E-02	0.27	1.8E-01	0.34	9.2E-02	-0.46	1.7E-02	0.09	6.5E-01	-0.42	3.1E-02	-0.19	3.6E-01	-0.27	1.7E-01	0.30	1.4E-01
ENSCAFG0000000294	FANCL	grey	EC_M10	0.27	1.8E-01	-0.17	4.1E-01	-0.41	3.6E-02	0.08	7.1E-01	0.33	9.7E-02	0.07	7.3E-01	-0.57	2.5E-03	0.12	5.7E-01	-0.14	5.0E-01	-0.50	1.0E-02
ENSCAFG0000003185	ZNF235	grey	EC_M10	0.27	1.8E-01	0.36	7.2E-02	0.18	3.7E-01	-0.13	5.1E-01	-0.58	1.9E-03	-0.21	2.9E-01	-0.22	2.8E-01	-0.14	5.0E-01	0.30	1.4E-01	0.45	2.2E-02
ENSCAFG0000001130	CORO1C	darkgreen	EC_M4	0.27	1.8E-01	0.17	4.2E-01	-0.84	9.2E-08	-0.22	2.9E-01	0.71	5.0E-05	0.14	5.0E-01	0.19	3.5E-01	0.11	6.0E-01	-0.06	7.6E-01	-0.85	3.0E-08
ENSCAFG0000000448	SNRK	turquoise	EC_M6	0.27	1.8E-01	0.90	2.3E-10	-0.06	7.5E-01	0.06	7.7E-01	-0.47	1.4E-02	-0.19	3.4E-01	-0.17	4.2E-01	-0.11	5.8E-01	-0.13	5.2E-01	0.33	9.7E-02
ENSCAFG0000000190	DCAF12	turquoise	EC_M6	0.27	1.8E-01	0.57	2.3E-03	-0.14	5.1E-01	-0.02	9.1E-01	-0.29	1.6E-01	-0.02	9.1E-01	-0.11	5.9E-01	0.12	5.7E-01	0.30	1.4E-01	0.16	4.3E-01
ENSCAFG0000000196	KRIT1	cyan	EC_M2	0.27	1.8E-01	-0.05	8.1E-01	0.38	5.5E-02	0.08	7.0E-01	-0.71	5.2E-05	-0.10	6.3E-01	-0.10	6.4E-01	-0.23	2.7E-01	-0.19	3.6E-01	0.54	4.2E-03
ENSCAFG0000003040	ZNF624	darkgrey	EC_M8	0.27	1.8E-01	0.58	1.7E-03	0.00	9.9E-01	-0.08	7.1E-01	-0.47	1.5E-02	-0.16	4.4E-01	-0.07	7.5E-01	0.08	6.8E-01	0.06	7.6E-01	0.34	9.0E-02
ENSCAFG0000000329	GFPT1	darkgreen	EC_M4	0.27	1.8E-01	-0.35	7.7E-02	-0.60	1.1E-03	-0.08	7.1E-01	0.58	1.7E-03	0.27	1.7E-01	0.07	7.4E-01	-0.09	6.5E-01	-0.18	3.9E-01	-0.78	3.3E-06
ENSCAFG0000001543	LMTK2	turquoise	EC_M6	0.27	1.8E-01	0.59	1.5E-03	-0.56	3.0E-03	-0.06	7.8E-01	0.13	5.3E-01	-0.02	9.2E-01	-0.30	1.3E-01	0.02	9.2E-01	0.26	1.9E-01	-0.28	1.6E-01
ENSCAFG0000003184	ENSCAFG0000003184	darkgrey	EC_M8	0.27	1.8E-01	0.43	2.9E-02	-0.02	9.3E-01	0.19	3.5E-01	-0.44	2.5E-02	-0.54	4.2E-03	0.01	9.8E-01	0.08	7.0E-01	-0.09	6.6E-01	0.28	1.7E-01
ENSCAFG0000000045	RAB21	darkgreen	EC_M4	0.27	1.8E-01	0.32	1.1E-01	-0.69	8.3E-05	0.00	1.0E+00	0.44	2.5E-02	0.22	2.9E-01	0.01	9.4E-01	-0.06	7.7E-01	-0.32	1.1E-01	-0.55	3.9E-03
ENSCAFG0000000259	ZNF189	darkgrey	EC_M8	0.27	1.9E-01	0.47	1.6E-02	0.12	5.5E-01	0.06	7.9E-01	-0.59	1.4E-03	0.08	6.9E-01	-0.12	5.5E-01	0.06	7.8E-01	-0.08	7.1E-01	0.46	1.9E-02
ENSCAFG0000000900	GFM1	grey	EC_M10	0.27	1.9E-01	-0.33	9.6E-02	-0.25	2.1E-01	-0.02	9.3E-01	0.22	2.9E-01	0.47	1.5E-02	-0.25	2.1E-01	-0.32	1.1E-01	0.10	6.3E-01	-0.34	9.2E-02
ENSCAFG0000000021	PGGT1B	grey	EC_M10	0.27	1.9E-01	0.09	6.8E-01	-0.29	1.5E-01	0.33	1.0E-01	0.13	5.2E-01	0.28	1.7E-01	0.09	6.4E-01	-0.05	8.2E-01	-0.05	8.1E-01	-0.25	2.2E-01
ENSCAFG0000000786	MICAL2	grey	EC_M10	0.27	1.9E-01	0.35	7.8E-02	-0.82	3.6E-07	-0.23	2.7E-01	0.60	1.2E-03	0.15	4.6E-01	-0.08	6.9E-01	-0.03	8.9E-01	0.26	2.0E-01	-0.76	6.6E-06
ENSCAFG0000001066	ENSCAFG0000001066	grey	EC_M10	0.27	1.9E-01	0.20	3.2E-01	-0.67	1.7E-04	-0.14	4.8E-01	0.54	4.8E-03	0.37	6.1E-02	0.02	9.1E-01	-0.27	1.8E-01	-0.01	9.8E-01	-0.67	1.6E-04
ENSCAFG0000000720	KIF3B	grey	EC_M10	0.27	1.9E-01	-0.33	9.7E-02	-0.41	3.6E-02	-0.05	8.1E-01	0.36	6.8E-02	0.05	8.1E-01	-0.18	3.7E-01	0.06	7.6E-01	0.42	3.1E-02	-0.46	1.7E-02
ENSCAFG0000000181	ATP8A1	turquoise	EC_M6	0.27	1.9E-01	0.91	9.4E-11	-0.27	1.8E-01	-0.01	9.6E-01	-0.23	2.6E-01	-0.12	5.5E-01	-0.14	5.1E-01	-0.09	6.7E-01	-0.13	5.3E-01	0.10	6.3E-01
ENSCAFG0000001016	MX2	turquoise	EC_M6	0.27	1.9E-01	0.93	5.4E-12	-0.27	1.9E-01	0.06	7.7E-01	-0.23	2.7E-01	-0.17	4.0E-01	-0.17	4.1E-01	-0.15	4.7E-01	-0.01	9.5E-01	0.08	6.9E-01
ENSCAFG0000001810	ENSCAFG0000001810	grey	EC_M10	0.27	1.9E-01	-0.09	6.5E-01	-0.25	2.2E-01	-0.10	6.4E-01	0.07	7.4E-01	-0.06	7.6E-01	-0.04	8.3E-01	-0.07	7.5E-01	-0.03	8.9E-01	-0.21	3.0E-01
ENSCAFG0000001119	PDGFA	grey	EC_M10	0.27	1.9E-01	0.46	1.7E-02	-0.70	6.4E-05	-0.21	3.1E-01	0.46	1.9E-02	0.09	6.7E-01	0.06	7.8E-01	-0.21	3.1E-01	0.39	5.0E-02	-0.59	1.6E-03
ENSCAFG0000000250	ABCG5	violet	EC_M7	0.27	1.9E-01	-0.10	6.3E-01	-0.32	1.1E-01	-0.08	7.0E-01	0.19	3.6E-01	0.98	9.9E-19	-0.07	7.5E-01	-0.09	6.7E-01	-0.13	5.2E-01	-0.34	8.6E-02
ENSCAFG0000000427	ENSCAFG0000000427	violet	EC_M7	0.27	1.9E-01	-0.10	6.3E-01	-0.32	1.1E-01	-0.08	7.0E-01	0.19	3.6E-01	0.98	9.9E-19	-0.07	7.5E-01	-0.09	6.7E-01	-0.13	5.2E-01	-0.34	8.6E-02
ENSCAFG0000000737	CAMK4	violet	EC_M7	0.27	1.9E-01	-0.10	6.3E-01	-0.32	1.1E-01	-0.08	7.0E-01	0.19	3.6E-01	0.98	9.9E-19	-0.07	7.5E-01	-0.09	6.7E-01	-0.13	5.2E-01	-0.34	8.6E-02
ENSCAFG0000001203	HHAT	turquoise	EC_M6	0.27	1.9E-01	0.56	3.2E-03	-0.58	1.9E-03	0.06	7.8E-01	0.30	1.4E-01	0.14	4.9E-01	-0.28	1.7E-01	-0.28	1.7E-01	-0.37	6.6E-02	-0.45	2.0E-02
ENSCAFG0000000206	SLC1A1	grey	EC_M10	0.27	1.9E-01	0.01	9.7E-01	-0.69	1.0E-04	-0.23	2.5E-01	0.62	7.5E-04	0.19	3.5E-01	-0.21	3.1E-01	0.17	4.2E-01	0.34	9.4E-02	-0.73	2.2E-05
ENSCAFG0000002977	ZNF606	grey	EC_M10	0.27	1.9E-01	0.06	7.9E-01	0.06	7.6E-01	0.29	1.5E-01	-0.37	6.5E-02	-0.28	1.7E-01	-0.32	1.1E-01	-0.01	9.8E-01	0.07	7.5E-01	0.21	3.1E-01
ENSCAFG0000000575	ENSCAFG0000000575	grey	EC_M10	0.27	1.9E-01	0.11	5.8E-01	0.11	5.8E-01	-0.29	1.5E-01	-0.41	4.0E-02	0.10	6.1E-01	0.00	9.8E-01	-0.26	2.1E-01	0.05	8.2E-01	0.28	1.6E-01
ENSCAFG0000000231	ARL4A	darkgrey	EC_M8	0.27	1.9E-01	0.19	3.4E-01	0.31	1.2E-01	0.10	6.2E-01	-0.67	1.9E-04	-0.07	7.2E-01	-0.21	3.0E-01	-0.11	5.8E-01	-0.66	2.3E-04	0.50	8.8E-03
ENSCAFG0000000918	ENSCAFG0000000918	grey	EC_M10	0.27	1.9E-01	0.20	3.3E-01	-0.23	2.6E-01	0.55	3.4E-03	-0.02	9.2E-01	-0.27	1.8E-01	-0.24	2.5E-01	-0.27	1.8E-01	-0.30	1.3E-01	-0.14	4.9E-01
ENSCAFG0000001158	ENPEP	grey	EC_M10	0.27	1.9E-01	0.07	7.5E-01	-0.58	1.9E-03	0.07	7.5E-01	0.46	1.9E-02	0.05	8.1E-01	-0.21	2.9E-01	0.02	9.3E-01	-0.18	3.9E-01	-0.64	4.3E-04
ENSCAFG0000001498	ENSCAFG0000001498	turquoise	EC_M6	0.27	1.9E-01	0.90	6.5E-10	-0.17	4.2E-01	-0.11	6.0E-01	-0.32	1.1E-01	-0.10	6.1E-01	-0.13	5.3E-01	-0.13	5.1E-01	-0.21	3.0E-01	0.19	3.4E-01
ENSCAFG0000001918	HAUS7	grey	EC_M10	0.27	1.9E-01	-0.10	6.1E-01	-0.60	1.2E-03	-0.21	3.0E-01	0.58	1.8E-03	0.14	5.1E-01	-0.14	4.8E-01	0.05	8.0E-01	0.24	2.4E-01	-0.70	6.8E-05
ENSCAFG0000000863	DCP1A	grey	EC_M10	0.27	1.9E-01	0.09	6.5E-01	-0.65	2.9E-04	-0.02	9.1E-01	0.45	2.0E-02	0.41	3.8E-02	0.05	8.1E-01	-0.03	8.7E-01	-0.27	1.8E-01	-0.62	7.9E-04
ENSCAFG0000000360	GJB4	grey	EC_M10	0.27	1.9E-01	-0.06	7.8E-01	-0.05	8.1E-01	0.43	2.8E-02	-0.10	6.2E-01	0.53	5.8E-03	-0.06	7.6E-01	-0.14	5.1E-01	-0.11	6.0E-01	-0.06	7.6E-01
ENSCAFG0000001081	NCOA3	grey	EC_M10	0.27	1.9E-01	0.23	2.5E-01	-0.70	7.2E-05	0.02	9.3E-01	0.44	2.3E-02	0.29	1.5E-01	0.02	9.4E-01	-0.12	5.6E-01	0.33	9.9E-02	-0.60	1.2E-03
ENSCAFG0000001867	WDR70	grey	EC_M10	0.27	1.9E-01	-0.21	2.9E-01	0.02	9.4E-01	0.26	2.0E-01	-0.20	3.4E-01	-0.02	9.1E-01	-0.17	4.0E-01	-0.14	5.0E-01	-0.43	2.6E-02	0.00	9.9E-01
ENSCAFG0000000046	LAPTM4B	turquoise	EC_M6	0.27	1.9E-01	0.88	2.9E-09	-0.17	4.1E-01	0.09	6.5E-01	-0.32	1.1E-01	-0.17	3.9E-01	-0.16	4.4E-01	-0.19	3.6E-01	-0.06	7.6E-01	0.20	3.3E-01
ENSCAFG0000002478	PKHD1L1	turquoise	EC_M6	0.27	1.9E-01	0.90	4.7E-10	-0.15	4.7E-01	-0.10	6.1E-01	-0.34	9.1E-02	-0.13	5.4E-01	-0.15	4.6E-01	-0.14	4.8E-01	-0.09	6.6E-01	0.20	3.2E-01
ENSCAFG0000000288	KLK14	grey	EC_M10	0.27	1.9E-01	-0.16	4.3E-01	-0.35	8.3E-02	-0.07	7.4E-01	0.18	3.7E-01	0.46	1.9E-02	-0.11	5.9E-01	-0.14	5.1E-01	-0.21	3.0E-01	-0.40	4.0E-02
ENSCAFG0000002450	ENSCAFG0000002450	grey	EC_M10	0.27	1.9E-01	-0.19	3.5E-01	-0.17	4.1E-01	-0.25	2.2E-01	0.02	9.2E-01	0.11	5.8E-01	0.00	1.0E+00	0.09	6.6E-01	-0.18	3.8E-01	-0.20	3.2E-01
ENSCAFG0000001918	MATK	turquoise	EC_M6	0.27	1.9E-01	0.76	7.9E-06	-0.38	5.3E-02	0.07	7.5E-01	0.01	9.6E-01	0.04	8.4E-01	-0.20	3.3E-01	-0.24	2.4E-01	0.02	9.2E-01	-0.14	4.8E-01
ENSCAFG0000001377	BCR	turquoise	EC_M6	0.27	1.9E-01	0.91	8.3E-11	-0.26	2.0E-01	-0.12	5.6E-01	-0.22	2.8E-01	-0.16	4.3E-01	-0.06	7.6E-01	-0.10	6.4E-01	0.19	3.5E-01	0.11	5.9E-01
ENSCAFG0000000711	ENSCAFG0000000711	turquoise	EC_M6	0.27	1.9E-01	0.81	4.0E-07	-0.18	3.9E-01	0.08	7.1E-01	-0.32	1.1E-01	-0.16	4.4E-01	-0.13	5.4E-01	0.04	8.5E-01	-0.05	8.0E-01	0.15	4.8E-01
ENSCAFG0000001544	PPM1F	turquoise	EC_M6	0.27	1.9E-01	0.91	1.3E-10	-0.43	2.9E-02	-0.16	4.5E-01	0.01	9.6E-01	-0.19	3.6E-01	-0.14	4.9E-01	-0.03	8.9E-01	0.08	7.0E-01	-0.13	5.3E-01
ENSCAFG0000002864	ENSCAFG0000002864	grey	EC_M10	0.27	1.9E-01	0.24	2.4E-01	-0.15	4.6E-01	0.17	4.1E-01	-0.14	4.8E-01	0.28	1.7E-01	0.20	3.2E-01	0.23	2.5E-01	-0.26	2.0E-01	0.00	9.9E-01
ENSCAFG0000003189	ENSCAFG0000003189	paleturquoise	EC_M11	0.27	1.9E-01	-0.14	4.9E-01	-0.16	4.2E-01	0.58	1.9E-03	0.00	1.0E+00	-0.12	5.6E-01	-0.11	5.8E-01	-0.13	5.3E-01	-0.09	6.5E-01	-0.16	4.4E-01

ENSCAFG0000001409	BCOR	turquoise	EC_M6	0.27	1.9E-01	0.78	2.9E-06	-0.33	9.5E-02	-0.03	8.8E-01	-0.11	6.1E-01	0.03	8.8E-01	-0.02	9.3E-01	-0.11	6.0E-01	-0.23	2.6E-01	-0.04	8.5E-01
ENSCAFG0000002242	ANKMY2	grey	EC_M10	0.27	1.9E-01	-0.16	4.3E-01	0.02	9.1E-01	0.31	1.3E-01	-0.22	2.8E-01	-0.19	3.5E-01	-0.05	8.2E-01	0.03	8.8E-01	0.15	4.7E-01	0.15	4.6E-01
ENSCAFG0000000986	ZNF37A	grey	EC_M10	0.27	1.9E-01	0.04	8.6E-01	-0.24	2.4E-01	0.01	9.7E-01	0.05	8.0E-01	0.08	7.0E-01	-0.35	7.7E-02	-0.04	8.6E-01	0.16	4.4E-01	-0.17	4.1E-01
ENSCAFG0000001983	WDR5	cyan	EC_M2	0.27	1.9E-01	-0.32	1.1E-01	0.46	1.7E-02	0.26	2.0E-01	-0.62	7.0E-04	0.01	9.5E-01	-0.04	8.3E-01	-0.26	2.0E-01	-0.36	7.5E-02	0.42	3.1E-02
ENSCAFG0000001568	ENSCAFG0000001568	grey	EC_M10	0.27	1.9E-01	0.21	3.1E-01	0.14	5.0E-01	-0.03	8.9E-01	-0.47	1.5E-02	-0.10	6.3E-01	0.53	5.8E-03	0.03	8.9E-01	-0.19	3.6E-01	0.34	9.0E-02
ENSCAFG0000000491	ENSCAFG0000000491	grey	EC_M10	0.27	1.9E-01	0.13	5.1E-01	-0.50	1.0E-02	0.44	2.5E-02	0.27	1.8E-01	0.01	9.6E-01	-0.42	3.3E-02	0.00	9.9E-01	-0.02	9.3E-01	-0.41	3.6E-02
ENSCAFG0000001835	IL13RA1	turquoise	EC_M6	0.27	1.9E-01	0.55	3.7E-03	-0.39	5.2E-02	0.22	2.9E-01	-0.04	8.4E-01	-0.05	8.2E-01	0.13	5.3E-01	-0.06	7.7E-01	0.10	6.3E-01	-0.14	5.0E-01
ENSCAFG0000002886	ENSCAFG0000002886	turquoise	EC_M6	0.27	1.9E-01	0.42	3.1E-02	-0.21	3.1E-01	-0.14	4.9E-01	-0.13	5.1E-01	0.59	1.6E-03	-0.14	4.9E-01	-0.11	6.1E-01	-0.20	3.3E-01	0.00	9.9E-01
ENSCAFG0000002933	ENSCAFG0000002933	grey	EC_M10	0.27	1.9E-01	-0.24	2.4E-01	-0.24	2.3E-01	0.02	9.3E-01	0.26	2.0E-01	0.18	3.7E-01	-0.25	2.2E-01	0.09	6.6E-01	0.12	5.7E-01	-0.33	1.0E-01
ENSCAFG0000001603	ZBTB1	grey	EC_M10	0.27	1.9E-01	0.29	1.5E-01	-0.45	2.2E-02	-0.06	7.8E-01	0.07	7.2E-01	-0.05	8.1E-01	0.12	5.6E-01	-0.09	6.7E-01	-0.04	8.6E-01	-0.23	2.5E-01
ENSCAFG0000000524	ELOVL1	grey	EC_M10	0.27	1.9E-01	0.01	9.8E-01	-0.28	1.7E-01	-0.03	8.7E-01	0.20	3.3E-01	0.08	6.9E-01	-0.20	3.3E-01	-0.21	3.1E-01	-0.11	6.1E-01	-0.35	7.7E-02
ENSCAFG0000001992	GINS2	grey	EC_M10	0.27	1.9E-01	0.22	2.9E-01	-0.24	2.5E-01	-0.15	4.6E-01	0.05	8.1E-01	0.39	4.7E-02	0.15	4.5E-01	0.02	9.4E-01	-0.07	7.4E-01	-0.18	3.9E-01
ENSCAFG0000000905	ENSCAFG0000000905	grey	EC_M10	0.26	1.9E-01	-0.25	2.1E-01	-0.06	7.5E-01	0.37	6.6E-02	-0.11	6.0E-01	0.17	4.0E-01	-0.17	4.0E-01	0.16	4.3E-01	-0.11	6.0E-01	-0.07	7.2E-01
ENSCAFG0000000872	BACH1	grey	EC_M10	0.26	1.9E-01	-0.02	9.0E-01	-0.60	1.1E-03	-0.14	4.8E-01	0.45	2.2E-02	0.12	5.5E-01	0.00	9.8E-01	-0.07	7.2E-01	-0.12	5.5E-01	-0.63	5.0E-04
ENSCAFG0000001692	PHB	grey	EC_M10	0.26	1.9E-01	-0.09	6.6E-01	-0.26	2.0E-01	-0.28	1.6E-01	0.27	1.8E-01	0.24	2.4E-01	-0.12	5.4E-01	-0.09	6.6E-01	-0.25	2.2E-01	-0.41	3.6E-02
ENSCAFG0000000386	ENSCAFG0000000386	grey	EC_M10	0.26	1.9E-01	0.35	8.0E-02	-0.28	1.6E-01	-0.28	1.6E-01	0.00	9.9E-01	-0.23	2.6E-01	-0.18	3.8E-01	-0.01	9.6E-01	0.18	3.8E-01	-0.13	5.3E-01
ENSCAFG0000000282	SH3BGRL2	turquoise	EC_M6	0.26	1.9E-01	0.63	6.3E-04	-0.49	1.0E-02	-0.25	2.3E-01	0.15	4.8E-01	-0.06	7.6E-01	-0.02	9.1E-01	0.40	4.4E-02	0.12	5.7E-01	-0.29	1.5E-01
ENSCAFG0000002602	ENSCAFG0000002602	turquoise	EC_M6	0.26	1.9E-01	0.53	5.3E-03	-0.19	3.6E-01	-0.12	5.7E-01	-0.18	3.9E-01	-0.19	3.6E-01	-0.15	4.6E-01	-0.10	6.1E-01	-0.08	6.9E-01	0.08	7.1E-01
ENSCAFG0000001529	ASMTL	grey	EC_M10	0.26	1.9E-01	-0.16	4.3E-01	0.30	1.4E-01	0.04	8.5E-01	-0.47	1.5E-02	0.06	7.9E-01	0.20	3.2E-01	0.07	7.4E-01	0.07	7.2E-01	0.34	8.4E-02
ENSCAFG0000001212	ENSCAFG0000001212	turquoise	EC_M6	0.26	1.9E-01	0.72	3.3E-05	-0.41	4.0E-02	0.20	3.2E-01	-0.10	6.2E-01	0.07	7.3E-01	-0.01	9.6E-01	-0.04	8.5E-01	0.24	2.3E-01	-0.05	8.2E-01
ENSCAFG0000001084	GAP43	grey	EC_M10	0.26	1.9E-01	-0.21	3.1E-01	-0.35	7.9E-02	0.02	9.3E-01	0.24	2.4E-01	0.26	1.9E-01	-0.38	5.3E-02	0.20	3.4E-01	-0.13	5.2E-01	-0.49	1.2E-02
ENSCAFG0000001921	C20H19orf71	violet	EC_M7	0.26	1.9E-01	-0.15	4.7E-01	-0.06	7.7E-01	-0.12	5.6E-01	-0.05	8.1E-01	0.70	8.0E-05	-0.05	8.2E-01	-0.10	6.2E-01	-0.06	7.6E-01	-0.07	7.4E-01
ENSCAFG0000000642	PUS1	grey	EC_M10	0.26	1.9E-01	0.15	4.7E-01	0.29	1.5E-01	0.09	6.6E-01	-0.51	8.4E-03	-0.05	8.0E-01	0.01	9.4E-01	-0.12	5.5E-01	-0.31	1.2E-01	0.35	8.0E-02
ENSCAFG0000001455	DHX8	turquoise	EC_M6	0.26	1.9E-01	0.49	1.1E-02	-0.41	3.7E-02	0.01	9.7E-01	0.05	8.0E-01	-0.01	9.6E-01	0.17	4.2E-01	0.19	3.4E-01	-0.33	1.0E-01	-0.18	3.9E-01
ENSCAFG0000000889	PTPN11	darkgreen	EC_M4	0.26	1.9E-01	0.10	6.2E-01	-0.88	3.6E-09	-0.21	3.1E-01	0.76	5.7E-06	0.23	2.7E-01	-0.08	7.1E-01	0.10	6.4E-01	0.23	2.5E-01	-0.90	3.6E-10
ENSCAFG0000000523	TRAK1	darkgreen	EC_M4	0.26	1.9E-01	0.05	8.2E-01	-0.82	2.8E-07	-0.21	3.1E-01	0.74	1.6E-05	0.23	2.6E-01	-0.04	8.3E-01	-0.08	7.1E-01	-0.23	2.7E-01	-0.91	1.6E-10
ENSCAFG0000001510	BIRC3	turquoise	EC_M6	0.26	1.9E-01	0.59	1.4E-03	-0.09	6.5E-01	-0.01	9.6E-01	-0.27	1.9E-01	-0.21	3.1E-01	-0.12	5.7E-01	-0.19	3.5E-01	0.27	1.8E-01	0.17	4.0E-01
ENSCAFG0000001112	ENSCAFG0000001112	darkgreen	EC_M4	0.26	1.9E-01	0.33	1.0E-01	-0.75	9.2E-06	-0.37	6.4E-02	0.53	5.6E-03	0.25	2.2E-01	0.01	9.6E-01	-0.41	3.9E-02	-0.10	6.3E-01	-0.67	1.8E-04
ENSCAFG0000001660	SMOC1	turquoise	EC_M6	0.26	1.9E-01	0.85	2.8E-08	-0.31	1.2E-01	-0.14	4.9E-01	-0.14	4.8E-01	-0.17	4.0E-01	-0.23	2.5E-01	0.12	5.7E-01	0.17	3.9E-01	-0.01	9.5E-01
ENSCAFG0000003081	ENSCAFG0000003081	grey	EC_M10	0.26	1.9E-01	-0.03	9.0E-01	-0.18	3.8E-01	0.08	6.8E-01	0.02	9.3E-01	-0.19	3.6E-01	-0.16	4.4E-01	0.03	8.7E-01	-0.53	5.4E-03	-0.17	4.0E-01
ENSCAFG0000000883	ENSCAFG0000000883	grey	EC_M10	0.26	1.9E-01	0.03	8.9E-01	-0.29	1.5E-01	-0.16	4.5E-01	0.19	3.7E-01	0.36	7.3E-02	-0.11	5.8E-01	-0.10	6.1E-01	-0.09	6.7E-01	-0.29	1.5E-01
ENSCAFG0000001481	JADE3	turquoise	EC_M6	0.26	1.9E-01	0.91	1.4E-10	-0.41	3.6E-02	-0.14	4.9E-01	-0.07	7.4E-01	-0.07	7.3E-01	-0.17	4.1E-01	-0.01	9.4E-01	0.13	5.3E-01	-0.07	7.3E-01
ENSCAFG0000000314	AVL9	turquoise	EC_M6	0.26	1.9E-01	0.66	2.1E-04	-0.13	5.1E-01	0.08	7.1E-01	-0.34	8.5E-02	-0.27	1.8E-01	-0.15	4.7E-01	-0.34	9.1E-02	-0.19	3.5E-01	0.22	2.9E-01
ENSCAFG0000001804	ENSCAFG0000001804	grey	EC_M10	0.26	1.9E-01	-0.23	2.5E-01	-0.22	2.7E-01	-0.09	6.8E-01	0.19	3.5E-01	0.52	6.5E-03	-0.11	6.0E-01	-0.08	7.0E-01	-0.16	4.5E-01	-0.32	1.2E-01
ENSCAFG0000002857	ZBED8	grey	EC_M10	0.26	2.0E-01	-0.42	3.4E-02	-0.10	6.2E-01	0.16	4.3E-01	0.04	8.4E-01	-0.12	5.7E-01	0.04	8.6E-01	-0.37	6.1E-02	-0.34	9.0E-02	-0.20	3.3E-01
ENSCAFG0000000389	KIAA1147	turquoise	EC_M6	0.26	2.0E-01	0.88	3.1E-09	-0.15	4.7E-01	0.03	8.7E-01	-0.36	7.1E-02	-0.21	3.1E-01	-0.20	3.4E-01	-0.14	4.9E-01	0.11	6.0E-01	0.24	2.4E-01
ENSCAFG0000001841	ENSCAFG0000001841	grey	EC_M10	0.26	2.0E-01	-0.10	6.2E-01	-0.08	7.0E-01	-0.12	5.5E-01	-0.03	9.0E-01	0.53	5.4E-03	-0.02	9.0E-01	-0.04	8.6E-01	0.39	4.9E-02	-0.09	6.6E-01
ENSCAFG0000002440	ENSCAFG0000002440	grey	EC_M10	0.26	2.0E-01	-0.06	7.8E-01	0.10	6.2E-01	0.07	7.2E-01	-0.40	4.0E-02	0.04	8.4E-01	0.01	9.5E-01	0.26	2.0E-01	-0.33	1.0E-01	0.23	2.6E-01
ENSCAFG0000002966	CHN1	darkgreen	EC_M4	0.26	2.0E-01	0.08	7.0E-01	-0.76	5.8E-06	-0.05	8.1E-01	0.68	1.4E-04	0.29	1.5E-01	-0.04	8.6E-01	-0.15	4.5E-01	-0.31	1.3E-01	-0.85	5.3E-08
ENSCAFG0000000790	RHOBTB3	grey	EC_M10	0.26	2.0E-01	-0.64	3.9E-04	-0.20	3.2E-01	-0.01	9.7E-01	0.23	2.6E-01	0.17	4.0E-01	0.11	6.1E-01	0.18	3.7E-01	-0.01	9.6E-01	-0.37	5.9E-02
ENSCAFG0000000105	SQLE	grey	EC_M10	0.26	2.0E-01	-0.05	8.2E-01	-0.05	8.3E-01	0.00	9.8E-01	-0.15	4.7E-01	-0.06	7.9E-01	0.04	8.6E-01	-0.16	4.5E-01	-0.69	9.3E-05	-0.03	8.9E-01
ENSCAFG0000003027	ZNF140	grey	EC_M10	0.26	2.0E-01	0.23	2.5E-01	0.15	4.8E-01	0.20	3.2E-01	-0.49	1.1E-02	0.09	6.7E-01	-0.05	8.2E-01	-0.21	3.1E-01	0.09	6.6E-01	0.38	5.4E-02
ENSCAFG0000001066	ANO1	turquoise	EC_M6	0.26	2.0E-01	0.69	9.7E-05	-0.28	1.7E-01	-0.07	7.5E-01	-0.11	5.9E-01	-0.11	5.9E-01	0.04	8.6E-01	-0.12	5.7E-01	0.24	2.5E-01	-0.03	8.7E-01
ENSCAFG0000000181	DMTF1	grey	EC_M10	0.26	2.0E-01	0.13	5.2E-01	-0.39	4.8E-02	0.15	4.5E-01	0.05	8.1E-01	0.34	8.7E-02	0.03	8.9E-01	-0.24	2.3E-01	0.06	7.7E-01	-0.23	2.7E-01
ENSCAFG0000000918	ANKRD2	turquoise	EC_M6	0.26	2.0E-01	0.69	1.1E-04	-0.45	2.1E-02	-0.08	7.1E-01	0.03	8.9E-01	0.12	5.7E-01	-0.01	9.6E-01	0.14	4.8E-01	0.10	6.3E-01	-0.18	3.8E-01
ENSCAFG0000001383	ENSCAFG0000001383	grey	EC_M10	0.26	2.0E-01	-0.13	5.2E-01	-0.10	6.2E-01	0.60	1.1E-03	-0.09	6.5E-01	-0.09	6.8E-01	-0.08	7.2E-01	-0.11	5.9E-01	-0.09	6.6E-01	-0.04	8.5E-01
ENSCAFG0000002597	ENSCAFG0000002597	grey	EC_M10	0.26	2.0E-01	-0.02	9.4E-01	-0.27	1.8E-01	-0.41	3.8E-02	0.11	5.9E-01	0.13	5.3E-01	-0.13	5.3E-01	0.11	6.0E-01	-0.19	3.6E-01	-0.22	2.7E-01
ENSCAFG0000000154	EIF3D	cyan	EC_M2	0.26	2.0E-01	-0.16	4.3E-01	0.53	5.6E-03	0.24	2.4E-01	-0.77	4.7E-06	-0.08	7.0E-01	-0.05	8.2E-01	-0.13	5.3E-01	-0.52	6.3E-03	0.59	1.4E-03
ENSCAFG0000001599	KRT23	turquoise	EC_M6	0.26	2.0E-01	0.93	4.6E-12	-0.33	1.0E-01	-0.07	7.4E-01	-0.15	4.6E-01	-0.11	5.9E-01	-0.13	5.4E-01	-0.10	6.3E-01	-0.16	4.5E-01	0.03	8.8E-01
ENSCAFG0000003190	ZNF790	grey	EC_M10	0.26	2.0E-01	0.17	4.1E-01	-0.20	3.2E-01	0.23	2.6E-01	-0.05	8.1E-01	0.08	7.0E-01	0.01	9.8E-01	0.13	5.2E-01	-0.04	8.3E-01	-0.06	7.9E-01
ENSCAFG0000001879	PARN	cyan	EC_M2	0.26	2.0E-01	0.22	2.8E-01	0.22	2.8E-01	0.34	9.4E-02	-0.61	9.9E-04	-0.22	2.7E-01	-0.46	1.9E-02	-0.05	8.2E-01	0.00	9.9E-01	0.45	2.1E-02
ENSCAFG0000001463	FRMD6	grey	EC_M10	0.26	2.0E-01	0.47	1.5E-02	0.31	1.2E-01	0.02	9.3E-01	-0.72	3.6E-05	-0.34	8.8E-02	-0.09	6.7E-01	-0.01	9.6E-01	-0.34	8.6E-02	0.59	1.4E-03
ENSCAFG0000001709	TRIP4	grey	EC_M10	0.26	2.0E-01	0.04	8.3E-01	-0.28	1.7E-01	-0.09	6.5E-01	0.06	7.8E-01	-0.28	1.7E-01	-0.23	2.7E-01	0.13	5.3E-01	-0.08	6.9E-01	-0.23	2.6E-01
ENSCAFG0000000038	GRIP1	grey	EC_M10	0.26	2.0E-01	0.01	9.5E-01	-0.25	2.1E-01	-0.01	9.5E-01	0.12	5.7E-01	0.25	2.1E-01	0.12	5.7E-01	0.07	7.2E-01	0.25	2.2E-01	-0.28	1.7E-01
ENSCAFG0000000508	RBM33	grey	EC_M10	0.26	2.0E-01	0.38	5.6E-02	0.11	6.0E-01	-0.01	9.7E-01	-0.52	7.0E-03	0.13	5.4E-01	-0.12	5.7E-01	-0.17	4.2E-01	-0.33	9.9E-02	0.36	6.7E-02
ENSCAFG0000001007	ENSCAFG0000001007	grey	EC_M10	0.26	2.0E-01	-0.15	4.7E-01	-0.11	6.0E-01	0.33	1.0E-01	-0.06	7.5E-01	0.34	8.5E-02	-0.11	6.0E-01	-0.21	3.0E-01	0.07	7.5E-01	-0.10	6.3E-01
ENSCAFG0000002021	NR6A1	grey	EC_M10	0.26	2.0E-01	0.19	3.5E-01	0.23	2.7E-01	0.31	1.3E-01	-0.57	2.3E-03	-0.46	1.9E-02	0.01	9.6E-01	-0.18	3.7E-01	-0.18	3.9E-01	0.43	2.7E-02
ENSCAFG0000001099	PRKAR1A	darkgreen	EC_M4	0.26	2.0E-01	0.12	5.6E-01	-0.78	2.6E-06	0.22	2.8E-01	0.62	6.7E-04	0.29	1.5E-01	-0.08	7.1E-01	-0.05	8.0E-01	-0.16	4.4E-01	-0.76	7.3E-06
ENSCAFG0000002023	STRBP	turquoise	EC_M6	0.26	2.0E-01	0.66	2.3E-04	-0.64	4.1E-04	-0.04	8.4E-01	0.32	1.1E-01	-0.07	7.5E-01	-0.12	5.6E-01	-0.16	4.3E-01	-0.20	3.2E-01	-0.43	2.7E-02
ENSCAFG0000000276	IGF2BP3	turquoise	EC_M6	0.26	2.0E-01	0.60	1.2E-03	-0.34	9.2E-02	-0.22	2.8E-01	-0.05	8.2E-01	-0.03	8.8E-01	-0.22	2.8E-01	-0.05	8.0E-01	0.50	9.2E-03	-0.09	6.5E-01
ENSCAFG0000001686	HOXB8	turquoise	EC_M6	0.26	2.0E-01	0.95	1.4E-13	-0.28	1.7E-01	0.07	7.5E-01	-0.22	2.9E-01	-0.15	4.8E-01	-0.12	5.6E-01	-0.04	8.6E-01	-0.08	6.9E-01	0.08	7.1E-01
ENSCAFG0000000419	ARHGAP35	grey	EC_M10	0.26	2.0E-01	0.35	8.3E-02	0.20	3.2E-01	0.16	4.4E-01	-0.62	6.6E-04	-0.20	3.4E-01	-0.12	5.6E-01	-0.22	2.7E-01	-0.29	1.5E-01	0.49	1.1E-02
ENSCAFG0000002894	ENSCAFG0000002894	grey	EC_M10	0.26	2.0E-01	0.21	3.1E-01	-0.53	4.9E-03	-0.13	5.1E-01	0.36	7.5E-02	0.29	1.5E-01	-0.04	8.5E-01	0.14	4.9E-01	0.17	4.0E-01	-0.49	1.1E-02
ENSCAFG0000000170	SND1	grey	EC_M10	0.26	2.0E-01	-0.51	8.0E-03	-0.44	2.6E-02	-0.04	8.6E-01	0.51	8.1E-03	0.31	1.2E-01	-0.11	5.9E-01	0.05	8.1E-01	-0.24	2.5E-01	-0.71	5.3E-05
ENSCAFG0000000794	ENSCAFG0000000794	grey	EC_M10	0.26	2.0E-01	0.24	2.4E-01	-0.51	8.5E-03	-0.05	8.0E-01	0.28	1.7E-01	-0.37	6.7E-02	0.02	9.0E-01	-0.02	9.3E-01	0.13	5.4E-01	-0.37	6.0E-02
ENSCAFG0000000447	ANO10	darkgreen	EC_M4	0.26	2.0E-01	0.00	9.8E-01	-0.74	1.9E-05	-0.15	4.7E-01	0.67	1.7E-04	0.27	1.8E-01	-0.37	6.4E-02	-0.10	6.3E-01	-0.14	4.9E-01	-0.80	8.3E-07
ENSCAFG0000001027	ENSCAFG0000001027	grey	EC_M10	0.26	2.0E-01	0.03	8.7E-01	-0.39	4.7E-02	0.13	5.3E-01	0.20	3.3E-01	0.17	4.1E-01	-0.10	6.4E-01	-0.04	8.5E-01	0.05	8.1E-01	-0.30	1.4E-01
ENSCAFG0000000830	C31H21orf91	grey	EC_M10	0.26	2.0E-01	-0.16	4.3E-01	0.32	1.2E-01	0.11	5.9E-01	-0.52	7.0E-03	-0.16	4.2E-01	-0.04	8.5E-01	-0.12	5.4E-01	-0.04	8.3E-01	0.39	4.8E-02
ENSCAFG0000001322	DDX47	grey	EC_M10	0.26	2.0E-01	-0.61	9.0E-04	-0.38	5.9E-02	-0.14	5.0E-01	0.48	1.4E-02	0.48	1.3E-02	-0.14	5.0E-01	-0.01	9.8E-01	-0.15	4.8E-01	-0.62	7.1E-04
ENSCAFG0000002969	ENSCAFG0000002969	grey	EC_M10	0.26	2.0E-01	0.30	1.3E-01	-0.26	2.0E-01	-0.01	9.6E-01	0.00	9.9E-01	-0.08	7.0E-01	-0.07	7.3E-01	-0.07	7.3E-01	0.37	6.2E-02	-0.12	5.7E-01
ENSCAFG0000000755	ENSCAFG0000000755	grey	EC_M10	0.26	2.0E-01	-0.14	4.8E-01	-0.41	3.9E-02	-0.05	8.1E-01	0.33	1.0E-01	-0.10	6.2E-01	-0.08	7.1E-01	-0.06	7.7E-01	0.00	9.8E-01	-0.48	1.4E-02
ENSCAFG0000002610	ENSCAFG0000002610	grey	EC_M10	0.26	2.0E-01	-0.19	3.6E-01	-0.15	4.5E-01	-0.04	8.6E-01	0.05	8.3E-01	0.63	5.9E-04	-0.09	6.4E-01	-0.10	6.3E-01	-0.15	4.7E-01	-0.20	3.2E-01
ENSCAFG0000001808	DSC2	turquoise	EC_M6	0.26	2.0E-01	0.93	9.1E-12	-0.20	3.3E-01	-0.11	5.8E-01	-0.30	1.3E-01	-0.11	5.9E-01	-0.13	5.4E-01	-0.15	4.7E-01	-0.10	6.3E-01	0.18	3.7E-01
ENSCAFG0000002037	FHOD1	grey	EC_M10	0.26	2.0E-01	0.29	1.6E-01	0.45	2.1E-02	0.03	8.9E-01	-0.78	2.8E-06	-0.37	6.6E-02	-0.06	7.6E-01	0.04	8.5E-01	0.14	5.0E-01	0.67	1.6E-04
ENSCAFG0000003049	ZNF7	grey	EC_M10	0.26	2.0E-01	0.27	1.9E-01	0.19	3.5E-01	0.21	2.9E-01	-0.55	3.5E-03	0.06	7.8E-01	0.35	8.3E-02	-0.10	6.2E-01	-0.23	2.5E-01	0.44	2.6E-02
ENSCAFG0000001920	ENSCAFG0000001920	turquoise	EC_M6	0.26	2.0E-01	0.88	2.5E-09	-0.23	2.5E-01	-0.09	6.7E-01	-0.24	2.4E-01	-0.14	4.9E-01	-0.13	5.1E-01	-0.12	5.5E-01	-0.01	9.6E-01	0.07	7.4E-01
ENSCAFG0000000924	DGKZ	turquoise	EC_M6	0.26	2.0E-01	0.68	1.3E-04	-0.35	8.3E-02	-0.06	7.6E-01	0.01	9.7E-01	-0.11	6.0E-01	-0.09	6.7E-01	-0.21	3.1E-01	0.39	5.0E-02	-0.13	5.4E-01
ENSCAFG0000000597	ENSCAFG0000000597	turquoise	EC_M6	0.26	2.0E-01	0.41	3.5E-02	-0.04	8.3E-01	-0.14	5.0E-01	-0.25	2.1E-01	-0.13	5.3E-01	-0.23	2.7E-01	-0.63	6.2E-04	-0.34	9.0E-02	0.11	6.0E-01
ENSCAFG0000001070	CCDC28B	turquoise	EC_M6	0.26	2.0E-01	0.50	1.0E-02	-0.23	2.6E-01	0.11	6.0E-01	-0.20	3.4E-01	0.07	7.2E-01	0.13	5.4E-01	-0.10	6.3E-01	0.11	6.1E-01	0.02	9.1E-01
ENSCAFG0000000863	ARHGEF26	grey	EC_M10	0.26	2.0E-01	-0.03	8.9E-01	-0.09	6.8E-01	0.02	9.4E-01	-0.10	6.4E-01	0.07	7.2E-01	-0.27	1.7E-01	0.22	2.8E-01	0.35	7.8E-02	-0.09	6.6E-01
ENSCAFG0000001285	RAB1B	grey	EC_M10	0.26	2.0E-01	0.40	4.5E-02	-0.58	2.0E-03	-0.20	3.3E-01	0.35	8.2E-02	0.02	9.2E-01	-0.48	1.4E-02	-0.07	7.2E-01	-0.13	5.3E-01	-0.44	2.3E-02
ENSCAFG0000001658	SRSF5	darkgrey	EC_M8	0.26	2.0E-01	0.19	3.4E-01	0.27	1.8E-01	0.29	1.5E-01	-0.70	8.1E-05	-0.05	8.1E-01	-0.12	5.5E-01	-0.15	4.8E-01	-0.17	4.1E-01	0.55	3.6E-03
ENSCAFG0000000014	ENSCAFG0000000014	grey	EC_M10	0.26	2.0E-01	-0.10	6.3E-01	0.25	2.2E-01	0.43	2.9E-02	-0.42	3.3E-02	0.01	9.6E-01	-0.15	4.6E-01	-0.52	6.0E-03	0.13	5.2E-01	0.33	9.5E-02
ENSCAFG0000000331	SNRNP27	darkgreen	EC_M4	0.26	2.0E-01	0.17	4.0E-01	-0.74	1.5E-05	-0.25	2.2E-01	0.61	9.6E-04	0.28	1.7E-01	0.04	8.5E-01	0.02	9.2E-01	-0.01	9.6E-01	-0.73	2.7E-05
ENSCAFG0000001105	ACP6	grey	EC_M10	0.26	2.0E-01	0.02	9.3E-01	0.19	3.4E-01	0.35	8.3E-02	-0.44	2.5E-02	-0.39	4.7E-02	-0.31	1.2E-01	-0.32	1.1E-01	0.05	7.9E-01	0.29	1.5E-01
ENSCAFG0000000610	ANKLE2	turquoise	EC_M6	0.26	2.0E-01	0.54	4.7E-03	-0.35	7.9E-02	-0.23	2.6E-01	0.02	9.3E-01	-0.28	1.7E-01	-0.20	3.3E-01	0.04	8.3E-01	0.13	5.2E-01	-0.12	5.6E-01
ENSCAFG0000000221	RPL31	cyan	EC_M2	0.26	2.0E-01	-0.38	5.4E-02	0.09	6.6E-01	0.14	4.9E-01	-0.16	4.5E-01	0.36	7.4E-02	0.09	6.7E-01	-0.29	1.6E-01	-0.53	5.4E-03	-0.02	9.3E-01
ENSCAFG0000003078	ENSCAFG0000003078	grey	EC_M10	0.26	2.0E-01	0.18	3.9E-01	-0.04	8.4E-01	0.51	7.9E-03	-0.19	3.6E-01	-0.08	7.1E-01	-0.10	6.1E-01	-0.12	5.4E-01	-0.17	4.0E-01	0.02	9.1E-01
ENSCAFG0000002604	ENSCAFG0000002604	grey	EC_M10	0.26	2.0E-01	0.01	9.7E-01	-0.17	4.0E-01	-0.13	5.1E-01	-0.01	9.7E-01	-0.11	5.8E-01	-0.06	7.6E-01	0.26	2.0E-01	0.36	7.3E-02	-0.10	6.2E-01
ENSCAFG0000000936	TFG	grey	EC_M10	0.26	2.0E-01	0.45	2.2E-02	-0.52	5.9E-03	-0.23	2.5E-01	0.25	2.2E-01	0.09	6.8E-01	-0.30	1.3E-01	0.15	4.6E-01	0.11	5.9E-01	-0.41	3.5E-02
ENSCAFG0000001645	GNL3L	grey	EC_M10	0.26	2.0E-01	-0.07	7.3E-01	-0.53	5.8E-03	-0.41	3.6E-02	0.46	1.7E-02	0.23	2.6E-01	-0.15	4.7E-01	0.25	2.2E-01	0.10	6.2E-01	-0.61	9.8E-04
ENSCAFG0000001059	DNAJB14	grey	EC_M10	0.26	2.0E-01	0.26	1.9E-01	-0.64	4.0E-04	0.17	4.1E-01	0.40	4.3E-02	0.17	4.0E-01	0.06	7.6E-01	-0.23	2.6E-01	-0.08	6.9E-01	-0.52	5.9E-03
ENSCAFG0000002861	TMPRSS11E	grey	EC_M10	0.26	2.0E-01	-0.14	5.0E-01	-0.15	4.7E-01	0.64	4.7E-04	-0.01	9.5E-01	0.67	1.7E-04	-0.09	6.6E-01	-0.13	5.3E-01	-0.17	4.2E-01	-0.13	5.3E-01
ENSCAFG0000002567	ENSCAFG0000002567	grey	EC_M10	0.26	2.0E-01	0.22	2.8E-01	0.02	9.1E-01	-0.16	4.4E-01	-0.32	1.1E-01	0.31	1.3E-01	-0.12	5.5E-01	-0.19	3.4E-01	-0.23	2.5E-01	0.19	3.5E-01
ENSCAFG0000000216	SPTLC1	grey	EC_M10	0.26	2.0E-01	0.03	9.0E-01	-0.51	7.6E-03	0.18	3.9E-01	0.39	4.9E-02	0.07	7.3E-01	-0.35	7.7E-02	0.01	9.6E-01	-0.45	2.1E-02	-0.52	6.9E-03
ENSCAFG0000002892	ENSCAFG0000002892	grey	EC_M10	0.26	2.0E-01	-0.01	9.7E-01	-0.12	5.7E-01	-0.15	4.6E-01	0.01	9.7E-01	-0.17	4.2E-01	-0.17	4.0E-01	-0.16	4.3E-01	-0.18	3.8E-01	-0.13	5.3E-01
ENSCAFG0000001669	RNF40	turquoise	EC_M6	0.26	2.0E-01	0.52	6.8E-03	0.23	2.6E-01	0.07	7.2E-01	-0.71	5.7E-05	-0.29	1.5E-01	-0.21	3.1E-01	-0.07	7.2E-01	-0.03	8.9E-01	0.58	2.0E-03
ENSCAFG0000001408	ADAMTS14	grey	EC_M10	0.26	2.0E-01	0.45	2.2E-02	-0.66	2.5E-04	-0.02	9.3E-01	0.35	7.5E-02	0.40	4.6E-02	-0.12	5.6E-01	-0.15	4.5E-01	0.25	2.2E-01	-0.55	4.0E-03
ENSCAFG0000001268	EIF4G1	grey	EC_M10	0.26	2.0E-01	-0.13	5.3E-01	-0.62	6.6E-04	-0.26	2.0E-01	0.64	3.9E-04	0.19	3.4E-01	-0.34	9.2E-02	0.14	4.8E-01	-0.08	6.9E-01	-0.78	3.2E-06
ENSCAFG0000000722	HTR7	grey	EC_M10	0.26	2.0E-01	-0.10	6.1E-01	-0.53	5.5E-03	0.11	6.1E-01	0.41	3.6E-02	0.32	1.1E-01	-0.24	2.4E-01	0.16	4.5E-01	0.13	5.4E-01	-0.59	1.5E-03
ENSCAFG0000000558	PAAF1	grey	EC_M10	0.26	2.0E-01	0.15	4.7E-01	-0.12	5.6E-01	-0.39	5.1E-02	-0.06	7.7E-01	0.17	4.1E-01	0.27	1.7E-01	-0.28	1.6E-01	-0.08	7.0E-01	-0.06	7.7E-01
ENSCAFG0000000286	NFE2L3	turquoise	EC_M6	0.26	2.0E-01	0.86	1.3E-08	-0.24	2.3E-01	-0.18	3.7E-01	-0.17	3.9E-01	-0.20	3.3E-01	-0.08	7.0E-01	-0.08	7.0E-01	-0.09	6.8E-01	0.08	6.8E-01
ENSCAFG0000000447	EED	darkgreen	EC_M4	0.26	2.0E-01	-0.41	4.0E-02	-0.62	7.2E-04	-0.15	4.7E-01	0.65	3.4E-04	0.29	1.5E-01	-0.05	8.0E-01	0.01	9.6E-01	-0.04	8.3E-01	-0.78	2.4E-06
ENSCAFG0000001751	NPR1	turquoise	EC_M6	0.26	2.0E-01	0.92	4.1E-11	-0.25	2.1E-01	0.05	8.1E-01	-0.24	2.3E-01	-0.16	4.4E-01	-0.06	7.6E-01	-0.24	2.3E-01	-0.04	8.3E-01	0.10	6.3E-01
ENSCAFG0000000258	PLPPR1	grey	EC_M10	0.26	2.0E-01	-0.06	7.7E-01	-0.34	9.1E-02	-0.18	3.7E-01	0.23	2.6E-01	0.26	2.0E-01	0.07	7.5E-01	-0.18	3.8E-01	-0.25	2.1E-01	-0.40	4.1E-02
ENSCAFG0000000752	ARMC8	grey	EC_M10	0.26	2.1E-01	0.18	3.7E-01	-0.26	2.0E-01	-0.03	8.7E-01	0.05	8.1E-01	0.28	1.6E-01	-0.16	4.5E-01	-0.41	3.6E-02	-0.08	7.1E-01	-0.17	4.1E-01
ENSCAFG0000000624	MCF2L	grey	EC_M10	0.26	2.1E-01	-0.13	5.3E-01	0.11	6.1E-01	-0.09	6.6E-01	-0.33	1.0E-01	-0.09	6.6E-01	0.22	2.8E-01	-0.01	9.7E-01	0.03	8.8E-01	0.21	3.1E-01
ENSCAFG0000001894	NUBP1	grey	EC_M10	0.26	2.1E-01	0.11	5.8E-01	-0.01	9.4E-01	-0.24	2.4E-01	-0.13	5.2E-01	-0.33	9.9E-02	0.00	9.8E-01	-0.05	7.9E-01	-0.02	9.4E-01	0.05	8.2E-01
ENSCAFG0000001118	ARFGEF2	grey	EC_M10	0.26	2.1E-01	0.23	2.6E-01	-0.05	8.0E-01	0.19	3.6E-01	-0.32	1.1E-01	-0.07	7.2E-01	0.41	3.9E-02	-0.23	2.6E-01	0.05	8.1E-01	0.19	3.6E-01
ENSCAFG0000000234	IARS	grey	EC_M10	0.26	2.1E-01	-0.46	1.8E-02	-0.33	1.0E-01	0.09	6.5E-01	0.38	5.5E-02	0.22	2.8E-01	-0.44	2.6E-02	-0.09	6.8E-01	-0.15	4.6E-01	-0.56	2.9E-03
ENSCAFG0000001124	LAPTM5	turquoise	EC_M6	0.26	2.1E-01	0.89	1.6E-09	-0.22	2.8E-01	-0.07	7.3E-01	-0.24	2.3E-01	-0.09	6.6E-01	-0.17	4.0E-01	-0.12	5.6E-01	-0.03	8.7E-01	0.10	6.4E-01
ENSCAFG0000002363	ZBTB33	turquoise	EC_M6	0.26	2.1E-01	0.62	7.6E-04	-0.05	8.0E-01	-0.06	7.6E-01	-0.43	2.8E-02	-0.33	9.8E-02	-0.23	2.6E-01	0.14	4.9E-01	0.09	6.8E-01	0.27	1.9E-01
ENSCAFG0000001162	NFATC2	turquoise	EC_M6	0.26	2.1E-01	0.93	4.1E-12	-0.20	3.3E-01	-0.06	7.6E-01	-0.30	1.3E-01	-0.03	8.8E-01	-0.10	6.4E-01	-0.11	6.0E-01	0.03	8.8E-01	0.17	4.1E-01
ENSCAFG0000001507	ATL3	grey	EC_M10	0.26	2.1E-01	-0.28	1.7E-01	-0.21	3.1E-01	0.05	8.0E-01	0.11	5.8E-01	-0.04	8.5E-01	-0.14	4.9E-01	0.15	4.6E-01	-0.45	2.1E-02	-0.22	2.7E-01
ENSCAFG0000000874	ATP6V0D2	darkolivegreen	EC_M5	0.26	2.1E-01	0.08	7.1E-01	-0.36	7.3E-02	-0.17	4.2E-01	0.21	3.0E-01	0.02	9.0E-01	0.47	1.4E-02	-0.06	7.6E-01	0.04	8.6E-01	-0.32	1.1E-01
ENSCAFG0000001626	RELL1	turquoise	EC_M6	0.26	2.1E-01	0.50	9.4E-03	0.11	5.9E-01	0.18	3.7E-01	-0.53	5.8E-03	-0.10	6.3E-01	-0.15	4.7E-01	-0.10	6.3E-01	-0.44	2.3E-02	0.38	5.5E-02

ENSCAFG0000000637	ENSCAFG0000000637	grey	EC_M10	0.26	2.1E-01	-0.27	1.8E-01	0.10	6.4E-01	0.16	4.4E-01	-0.33	9.5E-02	-0.11	6.1E-01	-0.16	4.3E-01	0.08	7.0E-01	-0.14	4.9E-01	0.19	3.6E-01
ENSCAFG0000003265	EXOSC5	cyan	EC_M2	0.26	2.1E-01	-0.15	4.7E-01	0.33	9.7E-02	0.13	5.3E-01	-0.48	1.2E-02	0.14	5.0E-01	-0.14	5.0E-01	-0.36	7.0E-02	-0.45	2.3E-02	0.30	1.4E-01
ENSCAFG0000001271	LMLN	grey	EC_M10	0.26	2.1E-01	-0.22	2.8E-01	-0.50	9.3E-03	-0.07	7.2E-01	0.37	6.3E-02	0.33	1.0E-01	0.13	5.3E-01	-0.29	1.6E-01	-0.12	5.5E-01	-0.56	2.8E-03
ENSCAFG0000001337	ETV6	turquoise	EC_M6	0.26	2.1E-01	0.55	3.6E-03	-0.59	1.6E-03	-0.04	8.6E-01	0.26	2.0E-01	0.19	3.6E-01	0.03	8.7E-01	0.08	7.1E-01	0.43	2.8E-02	-0.41	3.5E-02
ENSCAFG0000002357	MINDY3	darkgreen	EC_M4	0.26	2.1E-01	0.21	3.0E-01	-0.86	1.8E-08	0.01	9.8E-01	0.69	1.0E-04	0.11	6.0E-01	-0.06	7.6E-01	0.12	5.5E-01	0.02	9.2E-01	-0.84	9.9E-08
ENSCAFG0000001865	NLK	darkgreen	EC_M4	0.26	2.1E-01	0.48	1.4E-02	-0.85	3.6E-08	-0.28	1.7E-01	0.62	7.2E-04	0.10	6.2E-01	-0.11	6.0E-01	-0.15	4.7E-01	-0.05	8.0E-01	-0.73	2.0E-05
ENSCAFG0000001548	RBM3	grey	EC_M10	0.26	2.1E-01	-0.32	1.1E-01	-0.47	1.4E-02	-0.14	5.0E-01	0.55	3.5E-03	0.24	2.3E-01	-0.24	2.4E-01	0.08	6.9E-01	-0.10	6.2E-01	-0.70	6.6E-05
ENSCAFG0000002508	KLHL23	grey	EC_M10	0.26	2.1E-01	0.18	3.7E-01	-0.56	2.6E-03	-0.25	2.3E-01	0.40	4.6E-02	-0.13	5.2E-01	0.38	5.6E-02	-0.10	6.3E-01	0.10	6.3E-01	-0.49	1.2E-02
ENSCAFG0000003198	HIST1H2BB	grey	EC_M10	0.26	2.1E-01	0.32	1.1E-01	0.08	6.9E-01	0.22	2.9E-01	-0.36	6.8E-02	-0.25	2.1E-01	-0.18	3.7E-01	0.17	4.0E-01	0.20	3.3E-01	0.28	1.7E-01
ENSCAFG0000003113	TIMM8B	grey	EC_M10	0.26	2.1E-01	0.19	3.6E-01	-0.64	4.0E-04	-0.07	7.2E-01	0.50	8.5E-03	0.26	2.0E-01	0.24	2.4E-01	-0.21	3.1E-01	-0.22	2.9E-01	-0.66	2.2E-04
ENSCAFG0000000592	ENSCAFG0000000592	grey	EC_M10	0.25	2.1E-01	0.27	1.9E-01	-0.26	1.9E-01	0.04	8.4E-01	0.02	9.2E-01	-0.32	1.1E-01	0.19	3.6E-01	0.31	1.2E-01	-0.20	3.3E-01	-0.15	4.5E-01
ENSCAFG0000001341	MAP3K11	turquoise	EC_M6	0.25	2.1E-01	0.93	3.3E-12	-0.38	5.6E-02	-0.14	5.1E-01	-0.09	6.7E-01	-0.07	7.4E-01	-0.07	7.5E-01	-0.16	4.4E-01	0.09	6.8E-01	-0.06	7.7E-01
ENSCAFG0000001179	MCF2L2	grey	EC_M10	0.25	2.1E-01	0.20	3.3E-01	-0.09	6.7E-01	-0.10	6.1E-01	-0.18	3.8E-01	-0.13	5.2E-01	-0.10	6.2E-01	-0.07	7.2E-01	0.29	1.5E-01	0.06	7.8E-01
ENSCAFG0000000225	REST	darkgreen	EC_M4	0.25	2.1E-01	0.19	3.6E-01	-0.83	1.2E-07	-0.10	6.3E-01	0.61	9.8E-04	0.23	2.7E-01	-0.10	6.3E-01	-0.01	9.4E-01	0.06	7.8E-01	-0.76	8.1E-06
ENSCAFG0000003074	FDPS	grey	EC_M10	0.25	2.1E-01	0.04	8.6E-01	0.24	2.3E-01	0.11	5.9E-01	-0.49	1.1E-02	-0.25	2.1E-01	-0.14	4.8E-01	-0.10	6.4E-01	-0.45	2.2E-02	0.31	1.2E-01
ENSCAFG0000000169	UBR2	darkgrey	EC_M8	0.25	2.1E-01	0.19	3.6E-01	0.11	5.9E-01	0.19	3.6E-01	-0.52	6.0E-03	-0.10	6.3E-01	-0.13	5.4E-01	-0.14	5.1E-01	0.03	8.9E-01	0.38	5.5E-02
ENSCAFG0000001892	DAB1	grey	EC_M10	0.25	2.1E-01	-0.20	3.3E-01	-0.25	2.3E-01	-0.16	4.4E-01	0.09	6.7E-01	0.27	1.9E-01	-0.13	5.3E-01	-0.17	3.9E-01	-0.12	5.5E-01	-0.24	2.3E-01
ENSCAFG0000001872	ENSCAFG0000001872	grey	EC_M10	0.25	2.1E-01	0.33	1.0E-01	-0.16	4.3E-01	0.24	2.4E-01	-0.15	4.7E-01	0.17	4.1E-01	0.06	7.7E-01	-0.42	3.1E-02	-0.09	6.8E-01	0.05	8.2E-01
ENSCAFG0000003042	PDCD2	grey	EC_M10	0.25	2.1E-01	0.03	8.9E-01	-0.28	1.6E-01	0.17	4.0E-01	0.11	5.9E-01	0.38	5.9E-02	0.38	5.3E-02	-0.48	1.3E-02	-0.34	8.5E-02	-0.25	2.1E-01
ENSCAFG0000002006	TRUB2	grey	EC_M10	0.25	2.1E-01	0.16	4.3E-01	0.35	7.7E-02	0.26	2.0E-01	-0.67	1.6E-04	-0.18	3.7E-01	-0.27	1.9E-01	-0.16	4.4E-01	0.06	7.8E-01	0.57	2.6E-03
ENSCAFG0000001578	KDM5A	turquoise	EC_M6	0.25	2.1E-01	0.54	4.6E-03	-0.24	2.4E-01	0.03	8.8E-01	-0.19	3.5E-01	-0.03	8.7E-01	-0.15	4.7E-01	-0.09	6.7E-01	-0.12	5.4E-01	0.08	6.9E-01
ENSCAFG0000002033	ENSCAFG0000002033	grey	EC_M10	0.25	2.1E-01	-0.05	8.0E-01	-0.54	4.2E-03	-0.15	4.6E-01	0.48	1.3E-02	0.15	4.7E-01	-0.17	4.1E-01	-0.10	6.3E-01	-0.10	6.4E-01	-0.63	5.3E-04
ENSCAFG0000001241	RPS6KC1	grey	EC_M10	0.25	2.1E-01	-0.29	1.5E-01	-0.55	3.4E-03	-0.03	8.9E-01	0.47	1.5E-02	0.23	2.5E-01	-0.16	4.4E-01	-0.02	9.4E-01	-0.33	9.9E-02	-0.61	8.5E-04
ENSCAFG0000001272	NTSR1	grey	EC_M10	0.25	2.1E-01	-0.20	3.2E-01	-0.20	3.3E-01	-0.02	9.4E-01	0.15	4.7E-01	-0.11	5.9E-01	-0.01	9.6E-01	-0.07	7.3E-01	-0.10	6.1E-01	-0.28	1.6E-01
ENSCAFG0000000014	RDH16	turquoise	EC_M6	0.25	2.1E-01	0.94	4.3E-13	-0.30	1.4E-01	-0.09	6.5E-01	-0.19	3.6E-01	-0.12	5.5E-01	0.11	5.8E-01	-0.10	6.2E-01	0.04	8.4E-01	0.07	7.4E-01
ENSCAFG0000000255	U2AF2	grey	EC_M10	0.25	2.1E-01	-0.13	5.1E-01	-0.09	6.7E-01	-0.16	4.3E-01	0.10	6.4E-01	0.01	9.8E-01	-0.09	6.6E-01	0.09	6.6E-01	-0.06	7.7E-01	-0.21	3.0E-01
ENSCAFG0000000503	EXOG	grey	EC_M10	0.25	2.1E-01	0.17	4.1E-01	-0.08	7.1E-01	0.27	1.8E-01	-0.19	3.6E-01	-0.05	8.1E-01	-0.36	7.2E-02	0.08	6.9E-01	-0.16	4.2E-01	0.11	5.8E-01
ENSCAFG0000003190	ENSCAFG0000003190	turquoise	EC_M6	0.25	2.1E-01	0.54	4.3E-03	-0.35	7.6E-02	-0.30	1.4E-01	0.02	9.4E-01	0.09	6.5E-01	-0.08	7.0E-01	-0.06	7.7E-01	-0.14	5.1E-01	-0.12	5.6E-01
ENSCAFG0000001988	GTF3C4	cyan	EC_M2	0.25	2.1E-01	0.13	5.2E-01	0.47	1.6E-02	0.20	3.2E-01	-0.81	6.4E-07	-0.31	1.2E-01	-0.28	1.7E-01	-0.03	8.8E-01	0.01	9.6E-01	0.67	1.7E-04
ENSCAFG0000000608	GOLGA3	turquoise	EC_M6	0.25	2.1E-01	0.50	8.7E-03	-0.56	2.9E-03	0.00	9.8E-01	0.23	2.6E-01	0.11	6.0E-01	0.25	2.2E-01	-0.12	5.7E-01	-0.32	1.1E-01	-0.37	6.3E-02
ENSCAFG0000001221	AP1S2	darkgreen	EC_M4	0.25	2.1E-01	-0.45	2.1E-02	-0.35	7.6E-02	0.07	7.3E-01	0.35	8.2E-02	0.22	2.9E-01	0.07	7.3E-01	-0.20	3.3E-01	-0.48	1.3E-02	-0.54	4.8E-03
ENSCAFG0000000347	PNISR	cyan	EC_M2	0.25	2.1E-01	0.23	2.6E-01	0.36	7.1E-02	0.34	8.5E-02	-0.81	6.1E-07	-0.18	3.7E-01	-0.08	7.1E-01	0.01	9.4E-01	0.03	8.7E-01	0.66	2.2E-04
ENSCAFG0000003081	PCDHB15	grey	EC_M10	0.25	2.1E-01	-0.11	5.8E-01	-0.37	6.4E-02	-0.21	3.1E-01	0.24	2.3E-01	0.19	3.5E-01	0.15	4.6E-01	0.13	5.3E-01	0.19	3.5E-01	-0.41	3.9E-02
ENSCAFG0000000893	KANSL2	turquoise	EC_M6	0.25	2.1E-01	0.49	1.1E-02	-0.36	6.8E-02	0.22	2.8E-01	0.04	8.5E-01	0.20	3.3E-01	-0.15	4.7E-01	-0.20	3.2E-01	0.23	2.6E-01	-0.16	4.2E-01
ENSCAFG0000000873	TM9SF3	darkgreen	EC_M4	0.25	2.1E-01	0.05	8.3E-01	-0.78	2.1E-06	-0.14	5.0E-01	0.67	1.6E-04	0.31	1.3E-01	-0.21	3.1E-01	0.17	4.0E-01	-0.14	4.8E-01	-0.79	1.9E-06
ENSCAFG0000001648	LGI2	turquoise	EC_M6	0.25	2.1E-01	0.42	3.2E-02	-0.09	6.5E-01	-0.12	5.5E-01	-0.29	1.6E-01	-0.12	5.4E-01	-0.08	7.0E-01	-0.13	5.2E-01	-0.01	9.4E-01	0.21	3.0E-01
ENSCAFG0000001468	SUCO	darkgreen	EC_M4	0.25	2.1E-01	0.16	4.3E-01	-0.76	6.2E-06	0.08	6.9E-01	0.55	3.7E-03	0.34	9.0E-02	-0.09	6.7E-01	-0.02	9.1E-01	-0.32	1.1E-01	-0.70	6.3E-05
ENSCAFG0000002968	ENSCAFG0000002968	grey	EC_M10	0.25	2.1E-01	0.17	4.1E-01	-0.28	1.6E-01	-0.07	7.3E-01	0.09	6.7E-01	-0.11	5.8E-01	-0.12	5.7E-01	0.59	1.4E-03	0.03	8.9E-01	-0.21	2.9E-01
ENSCAFG0000000283	CTNNAL1	grey	EC_M10	0.25	2.1E-01	0.52	6.4E-03	-0.82	3.4E-07	-0.20	3.2E-01	0.56	2.9E-03	-0.04	8.3E-01	-0.14	4.9E-01	-0.01	9.7E-01	0.27	1.9E-01	-0.66	2.5E-04
ENSCAFG0000001193	ARHGEF12	turquoise	EC_M6	0.25	2.1E-01	0.76	8.2E-06	-0.30	1.4E-01	-0.05	8.3E-01	-0.21	3.0E-01	-0.13	5.4E-01	-0.08	7.0E-01	-0.11	5.8E-01	0.13	5.3E-01	0.06	7.6E-01
ENSCAFG0000002132	ENSCAFG0000002132	grey	EC_M10	0.25	2.1E-01	0.11	5.9E-01	-0.11	6.0E-01	-0.12	5.7E-01	-0.13	5.2E-01	-0.14	4.9E-01	-0.16	4.3E-01	0.27	1.8E-01	-0.03	8.7E-01	-0.01	9.7E-01
ENSCAFG0000000829	ENSCAFG0000000829	turquoise	EC_M6	0.25	2.1E-01	0.43	2.8E-02	-0.35	8.3E-02	0.28	1.7E-01	0.02	9.3E-01	0.35	7.7E-02	-0.14	5.0E-01	-0.16	4.3E-01	-0.21	3.0E-01	-0.17	4.0E-01
ENSCAFG0000001062	SORCS1	grey	EC_M10	0.25	2.1E-01	-0.17	4.1E-01	-0.42	3.1E-02	0.12	5.6E-01	0.34	8.8E-02	0.30	1.3E-01	-0.14	5.1E-01	-0.21	3.0E-01	-0.24	2.4E-01	-0.50	9.1E-03
ENSCAFG0000000992	TAOK3	darkgrey	EC_M8	0.25	2.1E-01	0.27	1.8E-01	0.17	3.9E-01	0.15	4.7E-01	-0.61	9.8E-04	-0.18	3.8E-01	0.04	8.4E-01	0.05	8.2E-01	-0.10	6.4E-01	0.45	2.0E-02
ENSCAFG0000002768	ENSCAFG0000002768	grey	EC_M10	0.25	2.1E-01	-0.13	5.2E-01	-0.16	4.5E-01	0.22	2.7E-01	-0.03	8.8E-01	0.23	2.5E-01	-0.24	2.3E-01	0.00	9.8E-01	-0.19	3.4E-01	-0.12	5.4E-01
ENSCAFG0000002272	ENSCAFG0000002272	grey	EC_M10	0.25	2.1E-01	0.09	6.6E-01	-0.18	3.7E-01	0.01	9.8E-01	0.06	7.8E-01	0.18	3.9E-01	0.21	3.0E-01	0.06	7.8E-01	0.07	7.2E-01	-0.16	4.3E-01
ENSCAFG0000001673	EFNB1	turquoise	EC_M6	0.25	2.1E-01	0.36	7.4E-02	-0.10	6.2E-01	-0.08	7.0E-01	-0.20	3.3E-01	0.05	8.2E-01	0.10	6.3E-01	0.08	7.0E-01	0.11	5.8E-01	0.00	1.0E+00
ENSCAFG0000002967	ENSCAFG0000002967	grey	EC_M10	0.25	2.1E-01	0.14	4.8E-01	-0.19	3.6E-01	0.42	3.1E-02	0.00	1.0E+00	0.17	4.2E-01	0.13	5.2E-01	-0.27	1.8E-01	-0.31	1.2E-01	-0.11	6.1E-01
ENSCAFG0000000705	MALL	turquoise	EC_M6	0.25	2.1E-01	0.85	4.7E-08	-0.16	4.2E-01	-0.03	8.7E-01	-0.29	1.5E-01	-0.14	5.0E-01	-0.14	4.9E-01	-0.09	6.5E-01	0.09	6.8E-01	0.14	5.0E-01
ENSCAFG0000000657	FAM19A4	grey	EC_M10	0.25	2.1E-01	0.12	5.4E-01	-0.30	1.4E-01	-0.34	9.2E-02	0.13	5.4E-01	-0.05	8.1E-01	0.08	7.0E-01	0.15	4.6E-01	0.49	1.2E-02	-0.22	2.8E-01
ENSCAFG0000001362	TAF1A	darkgrey	EC_M8	0.25	2.1E-01	-0.21	3.0E-01	0.34	8.8E-02	0.24	2.4E-01	-0.61	8.6E-04	-0.03	9.0E-01	0.08	6.9E-01	0.07	7.3E-01	-0.17	4.0E-01	0.47	1.6E-02
ENSCAFG0000000715	ENSCAFG0000000715	grey	EC_M10	0.25	2.1E-01	0.22	2.8E-01	-0.51	7.7E-03	-0.06	7.8E-01	0.32	1.1E-01	0.12	5.5E-01	-0.24	2.3E-01	-0.15	4.7E-01	-0.24	2.4E-01	-0.53	5.2E-03
ENSCAFG0000001721	TUFM	grey	EC_M10	0.25	2.1E-01	-0.34	8.9E-02	0.59	1.4E-03	0.11	5.8E-01	-0.71	4.6E-05	-0.16	4.4E-01	-0.23	2.6E-01	-0.15	4.6E-01	-0.18	3.7E-01	0.56	2.9E-03
ENSCAFG0000002432	ENSCAFG0000002432	turquoise	EC_M6	0.25	2.2E-01	0.48	1.3E-02	-0.40	4.0E-02	0.27	1.8E-01	0.02	9.1E-01	0.06	7.6E-01	-0.27	1.8E-01	-0.18	3.7E-01	-0.27	1.9E-01	-0.16	4.5E-01
ENSCAFG0000001059	ENSCAFG0000001059	turquoise	EC_M6	0.25	2.2E-01	0.79	1.6E-06	-0.39	4.8E-02	-0.14	4.9E-01	0.01	9.6E-01	0.13	5.3E-01	0.07	7.4E-01	-0.23	2.7E-01	0.01	9.6E-01	-0.16	4.4E-01
ENSCAFG0000001240	ATE1	grey	EC_M10	0.25	2.2E-01	-0.23	2.7E-01	-0.01	9.6E-01	0.05	8.2E-01	-0.12	5.6E-01	0.23	2.6E-01	-0.13	5.3E-01	-0.20	3.3E-01	-0.08	7.1E-01	0.00	9.8E-01
ENSCAFG0000001793	ARID3B	turquoise	EC_M6	0.25	2.2E-01	0.74	1.5E-05	-0.33	9.5E-02	-0.10	6.2E-01	-0.09	6.5E-01	-0.16	4.4E-01	0.02	9.2E-01	0.19	3.5E-01	-0.11	5.8E-01	-0.05	8.1E-01
ENSCAFG0000003083	ENSCAFG0000003083	grey	EC_M10	0.25	2.2E-01	-0.23	2.6E-01	-0.10	6.2E-01	-0.09	6.6E-01	-0.02	9.1E-01	0.40	4.1E-02	-0.08	6.8E-01	-0.11	5.9E-01	-0.23	2.6E-01	-0.17	4.0E-01
ENSCAFG0000003076	STARD4	grey	EC_M10	0.25	2.2E-01	0.23	2.5E-01	-0.50	9.0E-03	-0.27	1.9E-01	0.31	1.3E-01	0.20	3.3E-01	-0.25	2.2E-01	0.16	4.4E-01	-0.10	6.4E-01	-0.44	2.3E-02
ENSCAFG0000002541	ENSCAFG0000002541	turquoise	EC_M6	0.25	2.2E-01	0.87	7.8E-09	-0.22	2.9E-01	-0.02	9.3E-01	-0.28	1.7E-01	-0.13	5.1E-01	-0.03	8.7E-01	-0.17	4.2E-01	-0.16	4.3E-01	0.15	4.8E-01
ENSCAFG0000001139	ENSCAFG0000001139	grey	EC_M10	0.25	2.2E-01	-0.18	3.9E-01	-0.23	2.6E-01	-0.07	7.5E-01	0.06	7.8E-01	0.38	5.4E-02	-0.17	4.0E-01	-0.20	3.4E-01	-0.18	3.8E-01	-0.20	3.3E-01
ENSCAFG0000002519	CASP8	grey	EC_M10	0.25	2.2E-01	0.43	2.8E-02	0.21	3.0E-01	-0.03	8.8E-01	-0.61	9.9E-04	-0.33	1.0E-01	0.11	6.1E-01	-0.25	2.1E-01	-0.10	6.3E-01	0.48	1.2E-02
ENSCAFG0000002906	ENSCAFG0000002906	grey	EC_M10	0.25	2.2E-01	0.06	7.6E-01	0.13	5.3E-01	-0.28	1.7E-01	-0.28	1.6E-01	0.12	5.7E-01	-0.21	3.1E-01	0.12	5.5E-01	-0.21	3.1E-01	0.17	4.1E-01
ENSCAFG0000000851	CFAP20	grey	EC_M10	0.25	2.2E-01	0.41	3.7E-02	0.20	3.2E-01	-0.17	4.1E-01	-0.56	3.1E-03	-0.25	2.1E-01	-0.13	5.2E-01	-0.19	3.6E-01	0.02	9.1E-01	0.50	9.6E-03
ENSCAFG0000000554	KIF16B	grey	EC_M10	0.25	2.2E-01	-0.39	4.7E-02	-0.38	5.5E-02	0.16	4.4E-01	0.30	1.4E-01	0.24	2.4E-01	-0.06	7.8E-01	0.15	4.7E-01	0.38	5.6E-02	-0.46	1.7E-02
ENSCAFG0000001912	MTM1	turquoise	EC_M6	0.25	2.2E-01	0.77	3.6E-06	-0.54	4.0E-03	-0.15	4.7E-01	0.14	4.9E-01	0.03	8.9E-01	-0.08	7.0E-01	-0.14	5.1E-01	-0.04	8.3E-01	-0.23	2.6E-01
ENSCAFG0000000980	DYRK1A	turquoise	EC_M6	0.25	2.2E-01	0.47	1.5E-02	-0.13	5.1E-01	-0.03	8.7E-01	-0.29	1.6E-01	0.31	1.2E-01	-0.08	7.1E-01	-0.12	5.6E-01	-0.25	2.2E-01	0.16	4.4E-01
ENSCAFG0000001859	LAMA1	grey	EC_M10	0.25	2.2E-01	-0.15	4.7E-01	-0.24	2.3E-01	0.04	8.3E-01	0.15	4.5E-01	-0.12	5.7E-01	-0.06	7.8E-01	-0.05	8.2E-01	-0.17	4.0E-01	-0.30	1.3E-01
ENSCAFG0000002009	WDR59	grey	EC_M10	0.25	2.2E-01	0.14	4.9E-01	0.28	1.6E-01	0.05	7.9E-01	-0.63	6.3E-04	-0.30	1.3E-01	-0.02	9.0E-01	-0.14	5.0E-01	-0.18	3.8E-01	0.48	1.3E-02
ENSCAFG0000001089	EI24	grey	EC_M10	0.25	2.2E-01	0.27	1.8E-01	-0.40	4.5E-02	-0.04	8.4E-01	0.28	1.6E-01	0.23	2.7E-01	-0.31	1.3E-01	-0.30	1.3E-01	-0.09	6.6E-01	-0.39	4.8E-02
ENSCAFG0000001151	ENSCAFG0000001151	grey	EC_M10	0.25	2.2E-01	-0.10	6.2E-01	-0.28	1.7E-01	0.01	9.5E-01	0.14	4.8E-01	-0.10	6.2E-01	-0.09	6.6E-01	-0.09	6.7E-01	-0.04	8.3E-01	-0.30	1.3E-01
ENSCAFG0000000245	XPA	grey	EC_M10	0.25	2.2E-01	0.47	1.5E-02	-0.06	7.8E-01	0.38	5.4E-02	-0.36	7.0E-02	-0.24	2.5E-01	-0.31	1.2E-01	-0.18	3.9E-01	0.01	9.5E-01	0.26	2.0E-01
ENSCAFG0000001988	ZC3H18	grey	EC_M10	0.25	2.2E-01	0.24	2.4E-01	-0.29	1.5E-01	-0.45	2.1E-02	0.16	4.4E-01	-0.04	8.6E-01	0.00	9.9E-01	0.29	1.6E-01	-0.22	2.9E-01	-0.26	1.9E-01
ENSCAFG0000000719	ASPH	grey	EC_M10	0.25	2.2E-01	-0.36	6.7E-02	-0.25	2.2E-01	0.18	3.9E-01	0.19	3.5E-01	0.07	7.4E-01	0.05	8.2E-01	0.23	2.7E-01	-0.19	3.6E-01	-0.38	5.9E-02
ENSCAFG0000001170	YTHDF2	grey	EC_M10	0.25	2.2E-01	0.30	1.3E-01	-0.63	5.7E-04	-0.06	7.8E-01	0.43	2.9E-02	-0.03	8.9E-01	-0.22	2.7E-01	0.12	5.6E-01	-0.08	7.0E-01	-0.54	4.6E-03
ENSCAFG0000000525	NDFIP2	turquoise	EC_M6	0.25	2.2E-01	0.62	7.4E-04	-0.72	3.0E-05	-0.15	4.6E-01	0.44	2.5E-02	0.05	8.1E-01	0.00	1.0E+00	-0.21	3.1E-01	-0.13	5.2E-01	-0.54	4.7E-03
ENSCAFG0000001745	ENSCAFG0000001745	turquoise	EC_M6	0.25	2.2E-01	0.76	5.4E-06	-0.26	1.9E-01	-0.11	6.0E-01	-0.14	5.1E-01	-0.08	7.0E-01	-0.02	9.1E-01	-0.08	7.0E-01	-0.02	9.4E-01	0.02	9.3E-01
ENSCAFG0000001808	RBM22	grey	EC_M10	0.25	2.2E-01	0.50	8.7E-03	0.31	1.3E-01	0.20	3.2E-01	-0.75	1.3E-05	0.03	8.7E-01	-0.08	7.1E-01	0.02	9.1E-01	0.05	8.1E-01	0.61	8.3E-04
ENSCAFG0000001901	ENSCAFG0000001901	grey	EC_M10	0.25	2.2E-01	0.12	5.6E-01	-0.45	2.0E-02	-0.21	3.1E-01	0.24	2.4E-01	0.28	1.7E-01	-0.16	4.2E-01	0.29	1.5E-01	-0.14	4.8E-01	-0.44	2.3E-02
ENSCAFG0000001314	MAP3K20	grey	EC_M10	0.25	2.2E-01	-0.39	5.1E-02	-0.36	7.1E-02	0.06	7.5E-01	0.34	9.2E-02	0.04	8.7E-01	0.03	8.8E-01	0.02	9.3E-01	0.19	3.5E-01	-0.48	1.4E-02
ENSCAFG0000001148	PABPN1	turquoise	EC_M6	0.25	2.2E-01	0.48	1.4E-02	-0.16	4.4E-01	0.18	3.8E-01	-0.17	4.2E-01	0.38	5.6E-02	-0.19	3.6E-01	-0.16	4.4E-01	0.04	8.3E-01	0.05	8.1E-01
ENSCAFG0000001022	ENSCAFG0000001022	turquoise	EC_M6	0.25	2.2E-01	0.63	5.2E-04	-0.50	8.7E-03	-0.18	3.7E-01	0.11	5.8E-01	-0.02	9.3E-01	-0.19	3.5E-01	0.16	4.2E-01	-0.27	1.8E-01	-0.30	1.4E-01
ENSCAFG0000000981	IGSF3	turquoise	EC_M6	0.25	2.2E-01	0.75	1.0E-05	-0.57	2.3E-03	-0.05	7.9E-01	0.19	3.6E-01	0.19	3.4E-01	0.10	6.1E-01	0.00	9.9E-01	0.20	3.3E-01	-0.33	9.5E-02
ENSCAFG0000001459	TNFSF18	grey	EC_M10	0.25	2.2E-01	-0.10	6.3E-01	-0.48	1.2E-02	-0.15	4.7E-01	0.43	2.8E-02	0.29	1.5E-01	-0.25	2.2E-01	-0.16	4.2E-01	-0.15	4.6E-01	-0.59	1.5E-03
ENSCAFG0000001552	WDR74	grey	EC_M10	0.25	2.2E-01	-0.03	9.0E-01	-0.10	6.2E-01	-0.32	1.1E-01	0.02	9.4E-01	0.00	9.9E-01	0.29	1.5E-01	-0.14	4.9E-01	0.03	8.9E-01	-0.12	5.6E-01
ENSCAFG0000000830	SMARCD1	turquoise	EC_M6	0.25	2.2E-01	0.86	2.6E-08	-0.16	4.4E-01	-0.03	8.8E-01	-0.32	1.1E-01	-0.26	2.0E-01	-0.21	2.9E-01	0.02	9.1E-01	0.22	2.9E-01	0.23	2.7E-01
ENSCAFG0000001947	ARHGEF16	grey	EC_M10	0.25	2.2E-01	0.13	5.3E-01	-0.45	2.1E-02	-0.11	5.8E-01	0.37	6.6E-02	-0.12	5.6E-01	-0.08	7.0E-01	-0.02	9.3E-01	0.07	7.5E-01	-0.44	2.3E-02
ENSCAFG0000001291	HDLBP	darkgreen	EC_M4	0.25	2.2E-01	0.03	8.8E-01	-0.67	2.0E-04	-0.01	9.5E-01	0.52	6.6E-03	0.23	2.5E-01	-0.21	3.0E-01	0.03	8.7E-01	-0.21	3.1E-01	-0.73	1.9E-05
ENSCAFG0000001885	HS6ST2	grey	EC_M10	0.25	2.2E-01	0.31	1.2E-01	-0.15	4.8E-01	-0.18	3.9E-01	-0.11	5.9E-01	-0.23	2.6E-01	-0.20	3.2E-01	-0.20	3.2E-01	0.06	7.8E-01	-0.01	9.7E-01
ENSCAFG0000003225	ENSCAFG0000003225	turquoise	EC_M6	0.25	2.2E-01	0.88	4.4E-09	-0.22	2.8E-01	-0.09	6.6E-01	-0.24	2.4E-01	-0.10	6.4E-01	-0.07	7.2E-01	-0.09	6.8E-01	-0.15	4.8E-01	0.12	5.5E-01
ENSCAFG0000001482	SECISBP2L	turquoise	EC_M6	0.25	2.2E-01	0.76	8.1E-06	-0.15	4.7E-01	0.01	9.7E-01	-0.35	8.1E-02	-0.13	5.4E-01	-0.05	8.1E-01	-0.09	6.8E-01	0.02	9.4E-01	0.26	2.0E-01
ENSCAFG0000000023	ENSCAFG0000000023	grey	EC_M10	0.25	2.2E-01	0.00	9.9E-01	0.09	6.6E-01	-0.11	5.8E-01	-0.22	2.8E-01	-0.10	6.4E-01	-0.13	5.3E-01	-0.13	5.4E-01	-0.20	3.4E-01	0.04	8.4E-01
ENSCAFG0000000214	IL1R1	darkgrey	EC_M8	0.25	2.2E-01	-0.16	4.4E-01	0.33	9.5E-02	0.31	1.2E-01	-0.60	1.2E-03	-0.12	5.7E-01	-0.05	8.0E-01	0.02	9.1E-01	-0.09	6.8E-01	0.42	3.4E-02
ENSCAFG0000000673	ENSCAFG0000000673	grey	EC_M10	0.25	2.2E-01	-0.03	9.0E-01	-0.01	9.4E-01	0.24	2.5E-01	-0.23	2.6E-01	-0.19	3.5E-01	0.17	4.0E-01	-0.21	3.0E-01	-0.15	4.6E-01	0.11	5.9E-01
ENSCAFG0000000293	VRK2	grey	EC_M10	0.25	2.2E-01	-0.33	1.0E-01	-0.36	7.2E-02	-0.21	3.0E-01	0.34	9.2E-02	0.14	4.8E-01	0.16	4.2E-01	-0.09	6.8E-01	-0.20	3.2E-01	-0.47	1.4E-02
ENSCAFG0000000021	SGK1	grey	EC_M10	0.25	2.2E-01	0.37	6.4E-02	-0.60	1.1E-03	0.09	6.5E-01	0.31	1.3E-01	0.03	8.7E-01	0.02	9.1E-01	0.07	7.4E-01	-0.04	8.3E-01	-0.46	1.9E-02
ENSCAFG0000001019	CCDC34	grey	EC_M10	0.25	2.2E-01	-0.46	2.0E-02	0.42	3.3E-02	0.08	7.0E-01	-0.48	1.2E-02	-0.13	5.3E-01	-0.13	5.3E-01	0.04	8.3E-01	-0.01	9.6E-01	0.39	4.8E-02
ENSCAFG0000001692	ZNF652	grey	EC_M10	0.25	2.2E-01	0.19	3.5E-01	-0.04	8.5E-01	0.00	9.9E-01	-0.32	1.1E-01	-0.11	5.8E-01	0.17	4.2E-01	-0.02	9.3E-01	-0.09	6.6E-01	0.21	3.1E-01
ENSCAFG0000001344	HOXD10	turquoise	EC_M6	0.25	2.2E-01	0.70	6.8E-05	-0.15	4.6E-01	0.03	8.8E-01	-0.24	2.3E-01	-0.17	4.1E-01	0.02	9.2E-01	-0.15	4.8E-01	-0.20	3.2E-01	0.15	4.5E-01
ENSCAFG0000000220	KIAA1211	grey	EC_M10	0.25	2.2E-01	-0.12	5.5E-01	-0.37	6.7E-02	-0.23	2.6E-01	0.29	1.5E-01	0.46	1.8E-02	0.05	8.0E-01	-0.20	3.2E-01	-0.07	7.4E-01	-0.47	1.6E-02
ENSCAFG0000002004	AGL	darkgreen	EC_M4	0.25	2.2E-01	-0.36	7.0E-02	-0.58	2.1E-03	-0.15	4.8E-01	0.58	2.0E-03	0.24	2.3E-01	0.14	4.9E-01	0.08	6.9E-01	-0.12	5.5E-01	-0.72	3.7E-05
ENSCAFG0000000864	ATG10	grey	EC_M10	0.25	2.2E-01	-0.32	1.1E-01	0.29	1.5E-01	-0.03	8.7E-01	-0.39	4.7E-02	0.15	4.5E-01	-0.21	3.0E-01	-0.14	5.1E-01	-0.20	3.3E-01	0.26	2.0E-01
ENSCAFG0000003200	ENSCAFG0000003200	grey	EC_M10	0.25	2.2E-01	0.47	1.5E-02	-0.61	9.7E-04	0.10	6.4E-01	0.35	8.2E-02	0.29	1.5E-01	-0.28	1.7E-01	-0.09	6.6E-01	0.30	1.4E-01	-0.42	3.2E-02
ENSCAFG0000000413	EFR3B	grey	EC_M10	0.25	2.2E-01	0.15	4.7E-01	-0.62	8.0E-04	0.05	7.9E-01	0.51	8.1E-03	-0.04	8.6E-01	0.02	9.2E-01	0.09	6.8E-01	0.03	8.8E-01	-0.63	5.5E-04
ENSCAFG0000001193	IMPDH2	cyan	EC_M2	0.25	2.2E-01	-0.44	2.4E-02	0.53	5.1E-03	0.13	5.1E-01	-0.65	3.7E-04	-0.11	5.9E-01	0.00	9.9E-01	0.08	7.0E-01	-0.25	2.3E-01	0.46	1.9E-02
ENSCAFG0000002543	C3AR1	grey	EC_M10	0.25	2.2E-01	-0.17	4.2E-01	-0.46	1.7E-02	0.13	5.3E-01	0.33	9.6E-02	0.43	2.7E-02	-0.12	5.5E-01	-0.13	5.4E-01	-0.22	2.9E-01	-0.52	6.1E-03
ENSCAFG0000000887	TIPARP	turquoise	EC_M6	0.25	2.2E-01	0.76	6.1E-06	-0.40	4.3E-02	-0.05	8.2E-01	-0.03	8.9E-01	-0.09	6.5E-01	0.15	4.6E-01	0.00	9.9E-01	-0.18	3.9E-01	-0.08	7.0E-01
ENSCAFG0000000915	OGFOD1	grey	EC_M10	0.25	2.2E-01	-0.42	3.4E-02	-0.40	4.1E-02	-0.02	9.1E-01	0.44	2.4E-02	0.13	5.4E-01	-0.39	4.7E-02	0.17	4.1E-01	0.05	7.9E-01	-0.55	3.5E-03
ENSCAFG0000001314	NUBPL	grey	EC_M10	0.25	2.3E-01	-0.27	1.9E-01	0.09	6.5E-01	-0.12	5.8E-01	-0.25	2.3E-01	0.09	6.6E-01	0.07	7.4E-01	0.02	9.2E-01	-0.07	7.2E-01	0.10	6.1E-01
ENSCAFG0000002038	AK5	grey	EC_M10	0.25	2.3E-01	-0.18	3.7E-01	-0.63	5.5E-04	0.17	4.2E-01	0.54	4.2E-03	0.19	3.4E-01	-0.11	6.0E-01	0.14	4.9E-01	-0.01	9.8E-01	-0.74	1.3E-05
ENSCAFG0000001729	UBLCP1	grey	EC_M10	0.25	2.3E-01	-0.09	6.5E-01	-0.64	4.9E-04	0.20	3.4E-01	0.56	2.8E-03	0.30	1.4E-01	-0.24	2.3E-01	-0.06	7.7E-01	0.20	3.3E-01	-0.68	1.3E-04
ENSCAFG0000002083	ENSCAFG0000002083	grey	EC_M10	0.25	2.3E-01	-0.05	8.2E-01	-0.03	8.8E-01	0.01	9.5E-01	-0.25	2.2E-01	0.08	7.0E-01	0.19	3.5E-01	-0.06	7.8E-01	0.02	9.3E-01	0.11	5.9E-01
ENSCAFG0000001329	SKA1	grey	EC_M10	0.25	2.3E-01	-0.48	1.3E-02	-0.27	1.8E-01	-0.04	8.3E-01	0.28	1.6E-01	0.19	3.5E-01	-0.43	2.7E-02	0.21	3.1E-01	0.21	2.9E-01	-0.41	3.6E-02
ENSCAFG0000001183	ST8SIA1	grey	EC_M10	0.25	2.3E-01	-0.14	4.9E-01	-0.15	4.6E-01	0.34	8.5E-02	0.02	9.2E-01	0.14	5.1E-01	-0.27	1.8E-01	0.13	5.3E-01	-0.14	4.9E-01	-0.21	3.1E-01
ENSCAFG0000003097	ZNF789	turquoise	EC_M6	0.25	2.3E-01	0.56	2.9E-03	0.16	4.2E-01	0.00	9.9E-01	-0.54	4.5E-03	-0.07	7.3E-01	-0.02	9.3E-01	-0.21	3.1E-01	-0.05	8.2E-01	0.44	2.5E-02
ENSCAFG0000000663	JAKMIP2	grey	EC_M10	0.25	2.3E-01	0.10	6.1E-01	-0.60	1.1E-03	-0.21	3.1E-01	0.49	1.1E-02	0.27	1.8E-01	-0.08	7.0E-01	0.14	4.8E-01	-0.21	3.1E-01	-0.69	9.4E-05

ENSCAFG0000003036	ENSCAFG0000003036	grey	EC_M10	0.25	2.3E-01	0.37	6.3E-02	0.03	8.8E-01	-0.15	4.6E-01	-0.40	4.2E-02	-0.14	4.8E-01	-0.14	5.0E-01	-0.13	5.3E-01	-0.10	6.2E-01	0.30	1.4E-01
ENSCAFG0000001399	SACM1L	grey	EC_M10	0.25	2.3E-01	-0.01	9.5E-01	0.17	4.0E-01	0.15	4.6E-01	-0.37	6.5E-02	-0.26	1.9E-01	-0.07	7.5E-01	-0.25	2.1E-01	-0.20	3.3E-01	0.28	1.7E-01
ENSCAFG0000001901	SEMA6B	turquoise	EC_M6	0.25	2.3E-01	0.88	2.8E-09	-0.25	2.2E-01	-0.15	4.6E-01	-0.17	4.0E-01	-0.10	6.3E-01	-0.15	4.6E-01	-0.12	5.5E-01	-0.01	9.4E-01	0.04	8.6E-01
ENSCAFG0000002131	ENSCAFG0000002131	grey	EC_M10	0.25	2.3E-01	-0.22	2.7E-01	0.15	4.8E-01	-0.01	9.5E-01	-0.36	7.5E-02	0.06	7.5E-01	-0.27	1.7E-01	-0.34	9.2E-02	-0.06	7.8E-01	0.22	2.8E-01
ENSCAFG0000000669	STX2	grey	EC_M10	0.25	2.3E-01	0.35	7.7E-02	-0.60	1.1E-03	-0.26	1.9E-01	0.38	5.7E-02	0.18	3.8E-01	-0.21	2.9E-01	0.10	6.3E-01	0.08	6.9E-01	-0.50	9.5E-03
ENSCAFG0000001377	UNC80	grey	EC_M10	0.25	2.3E-01	-0.09	6.5E-01	-0.43	2.9E-02	-0.10	6.1E-01	0.36	7.5E-02	0.27	1.8E-01	-0.12	5.4E-01	-0.12	5.6E-01	-0.25	2.2E-01	-0.50	9.2E-03
ENSCAFG0000000326	FBXO48	darkgrey	EC_M8	0.25	2.3E-01	0.42	3.0E-02	0.09	6.6E-01	0.28	1.7E-01	-0.52	6.8E-03	0.06	7.7E-01	-0.06	7.7E-01	-0.05	7.9E-01	0.07	7.3E-01	0.37	6.2E-02
ENSCAFG0000000924	WDFY3	grey	EC_M10	0.25	2.3E-01	0.11	5.9E-01	-0.29	1.5E-01	-0.08	6.9E-01	0.02	9.2E-01	-0.04	8.4E-01	-0.19	3.5E-01	-0.19	3.5E-01	-0.29	1.6E-01	-0.15	4.6E-01
ENSCAFG0000000168	ENSCAFG0000000168	darkgreen	EC_M4	0.24	2.3E-01	0.05	8.2E-01	-0.68	1.1E-04	-0.03	8.7E-01	0.54	4.2E-03	0.14	5.1E-01	-0.32	1.1E-01	0.16	4.4E-01	-0.18	3.8E-01	-0.72	4.0E-05
ENSCAFG0000002204	ENSCAFG0000002204	grey	EC_M10	0.24	2.3E-01	0.05	8.2E-01	0.08	6.9E-01	0.42	3.4E-02	-0.38	5.5E-02	-0.24	2.3E-01	0.00	1.0E+00	0.01	9.7E-01	-0.05	8.2E-01	0.27	1.8E-01
ENSCAFG0000001068	SLC39A8	turquoise	EC_M6	0.24	2.3E-01	0.83	1.9E-07	-0.31	1.2E-01	-0.04	8.5E-01	-0.12	5.5E-01	-0.06	7.5E-01	-0.14	5.0E-01	-0.14	4.8E-01	-0.25	2.2E-01	-0.01	9.5E-01
ENSCAFG0000002499	ENSCAFG0000002499	grey	EC_M10	0.24	2.3E-01	0.37	6.5E-02	-0.55	3.9E-03	-0.22	2.7E-01	0.32	1.1E-01	0.21	2.9E-01	-0.10	6.2E-01	-0.15	4.6E-01	-0.06	7.6E-01	-0.41	3.7E-02
ENSCAFG0000001336	ENSCAFG0000001336	grey	EC_M10	0.24	2.3E-01	0.25	2.1E-01	-0.24	2.5E-01	0.32	1.1E-01	-0.03	8.9E-01	0.11	5.9E-01	-0.09	6.5E-01	-0.49	1.1E-02	-0.05	8.2E-01	-0.11	5.8E-01
ENSCAFG0000003233	ENSCAFG0000003233	grey	EC_M10	0.24	2.3E-01	0.18	3.9E-01	-0.12	5.5E-01	0.29	1.5E-01	-0.12	5.5E-01	0.21	3.0E-01	-0.31	1.2E-01	-0.03	8.7E-01	0.16	4.2E-01	-0.02	9.2E-01
ENSCAFG0000000565	MKKS	grey	EC_M10	0.24	2.3E-01	-0.31	1.2E-01	0.04	8.4E-01	-0.01	9.6E-01	-0.14	5.0E-01	0.19	3.6E-01	-0.24	2.3E-01	-0.23	2.6E-01	0.45	2.2E-02	0.05	8.3E-01
ENSCAFG0000000157	PSAT1	darkgreen	EC_M4	0.24	2.3E-01	-0.09	6.7E-01	-0.72	3.9E-05	0.07	7.4E-01	0.70	6.8E-05	0.24	2.3E-01	-0.20	3.2E-01	-0.11	5.9E-01	-0.21	2.9E-01	-0.87	1.1E-08
ENSCAFG0000003096	ENSCAFG0000003096	turquoise	EC_M6	0.24	2.3E-01	0.62	7.7E-04	-0.37	6.0E-02	-0.07	7.2E-01	0.01	9.4E-01	-0.12	5.5E-01	-0.07	7.5E-01	-0.12	5.7E-01	-0.17	4.0E-01	-0.16	4.2E-01
ENSCAFG0000001250	TP53BP1	darkgreen	EC_M4	0.24	2.3E-01	-0.32	1.1E-01	-0.65	3.7E-04	-0.09	6.7E-01	0.61	9.4E-04	0.15	4.7E-01	-0.03	8.7E-01	0.04	8.3E-01	0.04	8.4E-01	-0.76	6.1E-06
ENSCAFG0000000144	EIF3L	cyan	EC_M2	0.24	2.3E-01	-0.46	1.9E-02	0.44	2.4E-02	0.27	1.8E-01	-0.54	4.6E-03	0.07	7.2E-01	-0.18	3.7E-01	-0.06	7.6E-01	-0.46	1.8E-02	0.40	4.4E-02
ENSCAFG0000001098	VTI1A	grey	EC_M10	0.24	2.3E-01	0.13	5.3E-01	-0.51	7.5E-03	0.08	6.8E-01	0.34	9.2E-02	0.20	3.3E-01	-0.15	4.5E-01	-0.07	7.3E-01	-0.30	1.3E-01	-0.53	5.3E-03
ENSCAFG0000001735	SPATA7	cyan	EC_M2	0.24	2.3E-01	-0.20	3.4E-01	0.27	1.9E-01	0.23	2.6E-01	-0.50	9.3E-03	-0.13	5.2E-01	-0.37	6.3E-02	-0.07	7.5E-01	-0.13	5.3E-01	0.35	7.6E-02
ENSCAFG0000000952	AMPD1	turquoise	EC_M6	0.24	2.3E-01	0.63	5.4E-04	-0.15	4.7E-01	-0.13	5.3E-01	-0.19	3.7E-01	-0.12	5.5E-01	0.36	7.2E-02	-0.05	8.3E-01	-0.08	7.0E-01	0.11	6.0E-01
ENSCAFG0000002963	EEF1B2	cyan	EC_M2	0.24	2.3E-01	-0.48	1.3E-02	0.36	7.3E-02	0.18	3.8E-01	-0.45	2.0E-02	0.00	9.8E-01	-0.10	6.3E-01	0.05	8.1E-01	-0.56	2.8E-03	0.28	1.6E-01
ENSCAFG0000001165	TIFA	turquoise	EC_M6	0.24	2.3E-01	0.75	1.2E-05	-0.20	3.3E-01	-0.08	7.1E-01	-0.24	2.4E-01	0.02	9.3E-01	0.09	6.6E-01	-0.20	3.3E-01	-0.16	4.5E-01	0.11	6.1E-01
ENSCAFG0000001711	CORO1A	grey	EC_M10	0.24	2.3E-01	-0.04	8.6E-01	0.05	8.0E-01	-0.16	4.4E-01	-0.19	3.6E-01	0.38	5.7E-02	0.16	4.3E-01	-0.04	8.5E-01	0.15	4.7E-01	0.05	8.1E-01
ENSCAFG0000001419	IQCJ-SCHIP1	grey	EC_M10	0.24	2.3E-01	0.49	1.1E-02	-0.70	8.0E-05	-0.09	6.8E-01	0.45	2.1E-02	-0.09	6.7E-01	-0.38	5.7E-02	0.04	8.6E-01	0.20	3.2E-01	-0.56	3.1E-03
ENSCAFG0000001075	MAP2K6	grey	EC_M10	0.24	2.3E-01	0.34	8.9E-02	0.19	3.5E-01	0.03	8.7E-01	-0.45	2.1E-02	-0.22	2.8E-01	0.01	9.4E-01	0.05	8.0E-01	0.02	9.3E-01	0.36	7.2E-02
ENSCAFG0000001730	SNAPC5	grey	EC_M10	0.24	2.3E-01	0.04	8.4E-01	-0.33	9.8E-02	0.01	9.6E-01	0.17	4.1E-01	0.24	2.5E-01	-0.22	2.8E-01	0.04	8.4E-01	-0.22	2.9E-01	-0.32	1.2E-01
ENSCAFG0000000541	THAP1	grey	EC_M10	0.24	2.3E-01	0.13	5.4E-01	-0.14	5.0E-01	0.24	2.4E-01	-0.10	6.1E-01	0.42	3.4E-02	-0.01	9.5E-01	-0.12	5.8E-01	-0.19	3.6E-01	-0.08	7.1E-01
ENSCAFG0000001317	RPS6KA3	darkgreen	EC_M4	0.24	2.3E-01	0.41	3.8E-02	-0.87	9.6E-09	-0.14	5.1E-01	0.66	2.2E-04	0.18	3.9E-01	-0.03	8.7E-01	-0.05	8.1E-01	-0.28	1.7E-01	-0.82	3.8E-07
ENSCAFG0000000048	ENSCAFG0000000048	grey	EC_M10	0.24	2.3E-01	0.26	2.1E-01	-0.25	2.2E-01	-0.15	4.8E-01	0.01	9.7E-01	-0.18	3.8E-01	0.16	4.3E-01	0.39	5.0E-02	-0.06	7.9E-01	-0.15	4.5E-01
ENSCAFG0000000099	SKP1	grey	EC_M10	0.24	2.3E-01	-0.28	1.7E-01	-0.41	3.6E-02	0.05	8.2E-01	0.34	8.8E-02	0.18	3.9E-01	-0.14	5.1E-01	0.30	1.3E-01	0.26	2.1E-01	-0.42	3.1E-02
ENSCAFG0000000853	ENSCAFG0000000853	grey	EC_M10	0.24	2.3E-01	-0.12	5.7E-01	-0.18	3.8E-01	-0.14	5.0E-01	0.08	6.9E-01	-0.07	7.4E-01	0.37	6.2E-02	-0.04	8.6E-01	0.10	6.3E-01	-0.18	3.8E-01
ENSCAFG0000001456	TRMT44	grey	EC_M10	0.24	2.3E-01	0.20	3.2E-01	0.01	9.4E-01	0.18	3.8E-01	-0.41	3.9E-02	-0.08	7.0E-01	0.06	7.7E-01	0.15	4.5E-01	-0.02	9.1E-01	0.23	2.6E-01
ENSCAFG0000000306	CFAP206	grey	EC_M10	0.24	2.3E-01	0.00	9.8E-01	-0.50	1.0E-02	-0.20	3.3E-01	0.44	2.3E-02	-0.09	6.6E-01	0.02	9.2E-01	0.18	3.7E-01	-0.37	6.5E-02	-0.61	9.7E-04
ENSCAFG0000000892	MMP16	grey	EC_M10	0.24	2.3E-01	-0.25	2.2E-01	-0.32	1.1E-01	-0.15	4.7E-01	0.32	1.1E-01	0.35	7.6E-02	-0.13	5.1E-01	0.36	7.3E-02	-0.04	8.4E-01	-0.43	3.0E-02
ENSCAFG0000000675	CEP63	grey	EC_M10	0.24	2.3E-01	-0.42	3.3E-02	-0.15	4.8E-01	0.14	4.9E-01	0.10	6.4E-01	0.28	1.6E-01	-0.23	2.5E-01	0.16	4.5E-01	-0.49	1.2E-02	-0.27	1.8E-01
ENSCAFG0000000045	ZFC3H1	darkgrey	EC_M8	0.24	2.3E-01	0.43	2.7E-02	0.02	9.3E-01	0.15	4.5E-01	-0.51	8.1E-03	-0.18	3.8E-01	-0.22	2.8E-01	-0.02	9.1E-01	-0.10	6.4E-01	0.34	8.8E-02
ENSCAFG0000001990	KLHDC4	grey	EC_M10	0.24	2.3E-01	-0.38	5.4E-02	-0.36	7.4E-02	-0.01	9.6E-01	0.42	3.1E-02	0.23	2.6E-01	0.25	2.1E-01	-0.11	5.8E-01	-0.08	6.8E-01	-0.54	4.3E-03
ENSCAFG0000001336	PPP2R3C	grey	EC_M10	0.24	2.3E-01	0.31	1.2E-01	0.05	8.2E-01	0.25	2.1E-01	-0.43	3.0E-02	0.08	6.9E-01	-0.13	5.4E-01	-0.33	1.0E-01	0.01	9.6E-01	0.33	1.0E-01
ENSCAFG0000000888	TLL1	turquoise	EC_M6	0.24	2.3E-01	0.52	6.8E-03	-0.49	1.1E-02	-0.05	7.9E-01	0.15	4.6E-01	0.10	6.1E-01	-0.17	4.1E-01	-0.12	5.4E-01	-0.29	1.5E-01	-0.30	1.3E-01
ENSCAFG0000000110	PTPRK	grey	EC_M10	0.24	2.3E-01	0.56	3.0E-03	-0.65	3.5E-04	-0.08	7.2E-01	0.34	8.7E-02	-0.01	9.6E-01	-0.09	6.5E-01	0.13	5.2E-01	0.32	1.1E-01	-0.49	1.1E-02
ENSCAFG0000000682	PRR5L	turquoise	EC_M6	0.24	2.3E-01	0.90	4.6E-10	-0.31	1.3E-01	-0.16	4.5E-01	-0.13	5.2E-01	-0.05	8.1E-01	-0.04	8.6E-01	-0.21	3.0E-01	0.13	5.2E-01	0.00	9.8E-01
ENSCAFG0000001299	GRHL3	grey	EC_M10	0.24	2.3E-01	-0.13	5.4E-01	-0.06	7.6E-01	-0.11	5.9E-01	-0.03	9.0E-01	0.39	4.8E-02	-0.06	7.7E-01	-0.10	6.3E-01	-0.21	3.1E-01	-0.14	4.9E-01
ENSCAFG0000001548	SCG3	turquoise	EC_M6	0.24	2.3E-01	0.55	3.5E-03	-0.55	3.6E-03	-0.01	9.5E-01	0.26	1.9E-01	-0.01	9.6E-01	-0.35	8.1E-02	0.10	6.4E-01	0.37	6.2E-02	-0.38	5.2E-02
ENSCAFG0000001461	ARPC2	darkgreen	EC_M4	0.24	2.3E-01	-0.02	9.4E-01	-0.79	1.7E-06	-0.16	4.5E-01	0.73	2.2E-05	0.28	1.7E-01	-0.14	4.9E-01	0.20	3.2E-01	-0.01	9.7E-01	-0.88	2.5E-09
ENSCAFG0000001421	MAP4K2	grey	EC_M10	0.24	2.3E-01	0.16	4.5E-01	-0.15	4.6E-01	-0.10	6.4E-01	-0.08	6.8E-01	-0.27	1.9E-01	0.15	4.7E-01	0.28	1.6E-01	0.22	2.7E-01	-0.09	6.7E-01
ENSCAFG0000000766	ACSL1	grey	EC_M10	0.24	2.3E-01	-0.16	4.4E-01	0.31	1.2E-01	-0.02	9.1E-01	-0.50	9.5E-03	-0.31	1.3E-01	0.03	8.9E-01	-0.05	8.0E-01	-0.46	1.7E-02	0.35	7.5E-02
ENSCAFG0000003214	NRSN2	grey	EC_M10	0.24	2.3E-01	0.33	1.0E-01	-0.13	5.1E-01	-0.15	4.8E-01	-0.17	4.0E-01	0.12	5.6E-01	-0.09	6.8E-01	-0.19	3.6E-01	-0.21	2.9E-01	0.05	8.1E-01
ENSCAFG0000003160	TRMT10C	grey	EC_M10	0.24	2.3E-01	0.09	6.6E-01	-0.25	2.2E-01	-0.13	5.1E-01	-0.04	8.6E-01	-0.10	6.2E-01	-0.07	7.2E-01	-0.11	5.9E-01	0.35	8.1E-02	-0.10	6.2E-01
ENSCAFG0000000457	ANKRD42	grey	EC_M10	0.24	2.3E-01	-0.62	7.6E-04	-0.20	3.3E-01	-0.14	4.8E-01	0.20	3.2E-01	0.19	3.6E-01	-0.18	3.7E-01	0.17	4.1E-01	-0.23	2.6E-01	-0.40	4.3E-02
ENSCAFG0000000717	PCCB	grey	EC_M10	0.24	2.3E-01	-0.34	9.1E-02	0.53	5.7E-03	0.16	4.3E-01	-0.65	3.1E-04	-0.11	5.9E-01	-0.33	9.8E-02	-0.10	6.1E-01	-0.02	9.3E-01	0.54	4.6E-03
ENSCAFG0000001671	TP53	grey	EC_M10	0.24	2.3E-01	0.22	2.9E-01	0.44	2.3E-02	-0.07	7.2E-01	-0.74	1.4E-05	-0.09	6.6E-01	-0.10	6.3E-01	0.03	9.0E-01	0.05	8.3E-01	0.57	2.1E-03
ENSCAFG0000001954	TMEM204	turquoise	EC_M6	0.24	2.3E-01	0.84	1.0E-07	-0.11	6.1E-01	-0.20	3.4E-01	-0.36	7.2E-02	-0.16	4.2E-01	-0.04	8.5E-01	-0.17	4.1E-01	-0.10	6.3E-01	0.23	2.6E-01
ENSCAFG0000000637	EP400	grey	EC_M10	0.24	2.4E-01	0.40	4.2E-02	-0.51	7.3E-03	-0.28	1.6E-01	0.28	1.7E-01	-0.01	9.8E-01	0.07	7.2E-01	-0.16	4.3E-01	-0.30	1.3E-01	-0.40	4.1E-02
ENSCAFG0000002393	ARID4A	turquoise	EC_M6	0.24	2.4E-01	0.56	2.7E-03	-0.33	1.0E-01	-0.05	8.2E-01	-0.08	7.1E-01	0.10	6.1E-01	-0.25	2.1E-01	-0.05	8.2E-01	0.04	8.5E-01	0.00	1.0E+00
ENSCAFG0000001374	CUX1	grey	EC_M10	0.24	2.4E-01	-0.02	9.1E-01	-0.69	9.8E-05	-0.01	9.5E-01	0.55	3.6E-03	0.30	1.4E-01	0.16	4.4E-01	0.01	9.5E-01	0.08	7.0E-01	-0.68	1.3E-04
ENSCAFG0000000095	WDR46	grey	EC_M10	0.24	2.4E-01	-0.28	1.7E-01	-0.18	3.9E-01	-0.15	4.5E-01	0.19	3.5E-01	0.26	2.1E-01	-0.24	2.3E-01	-0.02	9.3E-01	-0.04	8.4E-01	-0.33	9.7E-02
ENSCAFG0000001771	ARIH1	darkgreen	EC_M4	0.24	2.4E-01	0.10	6.2E-01	-0.64	3.8E-04	0.07	7.5E-01	0.47	1.6E-02	0.22	2.8E-01	-0.33	1.0E-01	-0.16	4.5E-01	-0.39	4.8E-02	-0.61	1.0E-03
ENSCAFG0000002627	ENSCAFG0000002627	grey	EC_M10	0.24	2.4E-01	-0.04	8.5E-01	0.21	3.1E-01	0.00	9.9E-01	-0.42	3.5E-02	0.18	3.9E-01	0.11	6.0E-01	0.03	8.7E-01	-0.01	9.5E-01	0.35	7.5E-02
ENSCAFG0000002026	PTGS1	turquoise	EC_M6	0.24	2.4E-01	0.83	1.9E-07	-0.31	1.2E-01	-0.13	5.3E-01	-0.15	4.6E-01	-0.03	8.8E-01	-0.19	3.4E-01	-0.12	5.7E-01	0.06	7.7E-01	-0.02	9.4E-01
ENSCAFG0000002001	DBT	grey	EC_M10	0.24	2.4E-01	-0.49	1.1E-02	0.30	1.3E-01	0.03	8.9E-01	-0.33	9.5E-02	0.15	4.5E-01	0.04	8.5E-01	0.07	7.2E-01	-0.24	2.3E-01	0.22	2.9E-01
ENSCAFG0000001809	AMMECR1	grey	EC_M10	0.24	2.4E-01	0.11	6.0E-01	0.36	7.1E-02	0.08	6.9E-01	-0.61	8.3E-04	-0.22	2.7E-01	-0.16	4.2E-01	-0.04	8.5E-01	-0.05	8.1E-01	0.53	5.6E-03
ENSCAFG0000000190	MTERF1	darkgrey	EC_M8	0.24	2.4E-01	0.02	9.4E-01	0.23	2.5E-01	0.12	5.6E-01	-0.54	4.6E-03	-0.28	1.6E-01	-0.24	2.4E-01	0.10	6.1E-01	0.02	9.3E-01	0.41	3.7E-02
ENSCAFG0000001177	GPR146	turquoise	EC_M6	0.24	2.4E-01	0.92	2.2E-11	-0.11	5.9E-01	0.02	9.2E-01	-0.38	5.5E-02	-0.18	3.8E-01	-0.18	3.8E-01	-0.09	6.6E-01	-0.05	8.1E-01	0.27	1.8E-01
ENSCAFG0000000187	CKAP4	grey	EC_M10	0.24	2.4E-01	-0.23	2.7E-01	-0.71	5.5E-05	-0.07	7.2E-01	0.74	1.4E-05	0.26	1.9E-01	0.05	8.1E-01	0.17	4.0E-01	0.06	7.9E-01	-0.88	2.0E-09
ENSCAFG0000001059	RGS2	grey	EC_M10	0.24	2.4E-01	0.47	1.5E-02	-0.71	5.1E-05	-0.24	2.3E-01	0.43	2.8E-02	0.07	7.4E-01	0.03	9.0E-01	0.04	8.5E-01	0.40	4.3E-02	-0.57	2.1E-03
ENSCAFG0000001206	BMX	turquoise	EC_M6	0.24	2.4E-01	0.76	5.6E-06	-0.06	7.5E-01	0.06	7.6E-01	-0.41	3.6E-02	-0.09	6.6E-01	-0.14	4.9E-01	-0.26	1.9E-01	0.31	1.2E-01	0.29	1.4E-01
ENSCAFG0000000090	TTLL12	grey	EC_M10	0.24	2.4E-01	0.29	1.5E-01	0.39	5.1E-02	-0.07	7.2E-01	-0.66	2.7E-04	-0.22	2.8E-01	-0.03	9.0E-01	-0.08	7.1E-01	-0.12	5.5E-01	0.52	6.8E-03
ENSCAFG0000000431	GSAP	darkgreen	EC_M4	0.24	2.4E-01	0.56	3.0E-03	-0.72	3.1E-05	-0.07	7.2E-01	0.37	6.1E-02	0.19	3.5E-01	0.22	2.9E-01	-0.08	6.8E-01	0.04	8.5E-01	-0.53	5.7E-03
ENSCAFG0000000380	PPFIA3	grey	EC_M10	0.24	2.4E-01	0.00	9.9E-01	-0.39	5.0E-02	0.04	8.5E-01	0.24	2.5E-01	0.19	3.6E-01	0.22	2.7E-01	0.00	1.0E+00	-0.24	2.4E-01	-0.45	2.0E-02
ENSCAFG0000003071	HIST1H2AB	grey	EC_M10	0.24	2.4E-01	0.04	8.6E-01	0.11	5.8E-01	0.27	1.9E-01	-0.28	1.6E-01	-0.33	1.0E-01	-0.34	8.8E-02	-0.30	1.3E-01	-0.42	3.5E-02	0.15	4.5E-01
ENSCAFG0000002479	ENSCAFG0000002479	grey	EC_M10	0.24	2.4E-01	0.33	1.0E-01	0.01	9.5E-01	-0.23	2.6E-01	-0.26	2.0E-01	-0.21	3.0E-01	-0.11	5.8E-01	0.11	5.8E-01	-0.24	2.4E-01	0.16	4.5E-01
ENSCAFG0000000064	SERAC1	turquoise	EC_M6	0.24	2.4E-01	0.75	1.1E-05	-0.32	1.1E-01	0.04	8.3E-01	-0.13	5.4E-01	-0.13	5.3E-01	-0.04	8.5E-01	-0.39	4.9E-02	-0.08	6.9E-01	0.02	9.4E-01
ENSCAFG0000000404	LIG1	grey	EC_M10	0.24	2.4E-01	-0.29	1.5E-01	0.09	6.7E-01	0.09	6.6E-01	-0.21	3.1E-01	-0.03	8.9E-01	0.16	4.5E-01	0.33	9.9E-02	0.00	9.9E-01	0.03	8.7E-01
ENSCAFG0000000746	TRAPPC13	darkgreen	EC_M4	0.24	2.4E-01	0.13	5.1E-01	-0.80	9.2E-07	-0.27	1.8E-01	0.72	3.0E-05	0.21	3.1E-01	-0.31	1.2E-01	-0.11	5.8E-01	0.00	9.9E-01	-0.84	8.4E-08
ENSCAFG0000000558	ENSCAFG0000000558	grey	EC_M10	0.24	2.4E-01	-0.40	4.6E-02	0.11	6.1E-01	0.02	9.1E-01	-0.16	4.2E-01	0.31	1.2E-01	-0.29	1.5E-01	0.06	7.8E-01	-0.06	7.8E-01	0.01	9.4E-01
ENSCAFG0000000570	MEMO1	turquoise	EC_M6	0.24	2.4E-01	0.50	9.6E-03	-0.59	1.7E-03	0.03	8.9E-01	0.30	1.3E-01	-0.04	8.3E-01	-0.54	4.0E-03	-0.01	9.8E-01	-0.20	3.2E-01	-0.44	2.4E-02
ENSCAFG0000001691	RIT1	turquoise	EC_M6	0.24	2.4E-01	0.80	7.6E-07	-0.12	5.5E-01	-0.08	7.0E-01	-0.31	1.2E-01	-0.08	6.9E-01	-0.15	4.7E-01	-0.12	5.6E-01	0.08	7.1E-01	0.22	2.8E-01
ENSCAFG0000000643	HPX	grey	EC_M10	0.24	2.4E-01	0.39	4.7E-02	-0.41	3.8E-02	-0.10	6.1E-01	0.09	6.6E-01	-0.19	3.5E-01	0.45	2.0E-02	-0.14	4.9E-01	0.10	6.4E-01	-0.22	2.8E-01
ENSCAFG0000003116	GID4	darkgrey	EC_M8	0.24	2.4E-01	0.34	9.0E-02	0.27	1.8E-01	-0.09	6.8E-01	-0.67	2.1E-04	-0.48	1.4E-02	0.17	4.1E-01	0.19	3.6E-01	0.05	8.1E-01	0.55	3.4E-03
ENSCAFG0000001609	SNCG	turquoise	EC_M6	0.24	2.4E-01	0.90	2.2E-10	-0.18	3.7E-01	0.06	7.8E-01	-0.29	1.6E-01	-0.11	5.9E-01	-0.10	6.2E-01	-0.15	4.8E-01	0.01	9.8E-01	0.14	4.9E-01
ENSCAFG0000003073	ENSCAFG0000003073	grey	EC_M10	0.24	2.4E-01	0.37	6.6E-02	-0.06	7.6E-01	0.26	2.0E-01	-0.32	1.1E-01	-0.13	5.1E-01	-0.07	7.5E-01	0.20	3.2E-01	0.28	1.7E-01	0.21	3.0E-01
ENSCAFG0000002583	ENSCAFG0000002583	grey	EC_M10	0.24	2.4E-01	0.10	6.4E-01	-0.25	2.1E-01	-0.07	7.2E-01	0.17	4.0E-01	-0.13	5.3E-01	-0.08	6.9E-01	0.00	9.8E-01	0.06	7.6E-01	-0.26	2.0E-01
ENSCAFG0000000121	ENSCAFG0000000121	grey	EC_M10	0.24	2.4E-01	-0.09	6.7E-01	0.16	4.2E-01	0.23	2.6E-01	-0.41	4.0E-02	-0.25	2.2E-01	-0.23	2.7E-01	-0.07	7.5E-01	-0.62	7.4E-04	0.24	2.3E-01
ENSCAFG0000000072	ACAT2	grey	EC_M10	0.24	2.4E-01	-0.24	2.3E-01	0.10	6.4E-01	0.15	4.6E-01	-0.23	2.5E-01	-0.22	2.8E-01	-0.24	2.4E-01	-0.05	8.1E-01	-0.49	1.0E-02	0.07	7.3E-01
ENSCAFG0000001738	HDX	grey	EC_M10	0.24	2.4E-01	0.45	2.2E-02	-0.52	6.7E-03	-0.16	4.5E-01	0.26	2.1E-01	-0.04	8.4E-01	0.04	8.5E-01	-0.04	8.5E-01	-0.21	3.1E-01	-0.41	3.8E-02
ENSCAFG0000003050	ENSCAFG0000003050	grey	EC_M10	0.24	2.4E-01	-0.33	1.1E-01	-0.21	2.9E-01	-0.26	2.0E-01	0.20	3.2E-01	-0.09	6.6E-01	-0.12	5.5E-01	0.27	1.8E-01	-0.28	1.6E-01	-0.38	5.4E-02
ENSCAFG0000000000	TXNL4A	grey	EC_M10	0.24	2.4E-01	-0.54	4.1E-03	-0.13	5.2E-01	0.04	8.5E-01	0.23	2.5E-01	-0.08	7.1E-01	-0.09	6.7E-01	0.12	5.7E-01	-0.10	6.4E-01	-0.35	8.3E-02
ENSCAFG0000003212	ENSCAFG0000003212	grey	EC_M10	0.24	2.4E-01	-0.54	4.7E-03	-0.19	3.4E-01	0.17	4.0E-01	0.15	4.7E-01	0.20	3.3E-01	-0.02	9.0E-01	0.06	7.7E-01	0.17	3.9E-01	-0.28	1.7E-01
ENSCAFG0000002886	ZNF22	darkgrey	EC_M8	0.24	2.4E-01	0.62	7.3E-04	0.17	4.0E-01	0.13	5.3E-01	-0.66	2.6E-04	0.01	9.5E-01	0.02	9.2E-01	-0.14	5.0E-01	-0.01	9.6E-01	0.52	6.1E-03
ENSCAFG0000000299	TAX1BP1	darkgreen	EC_M4	0.24	2.4E-01	0.08	6.9E-01	-0.84	1.0E-07	-0.15	4.8E-01	0.72	3.7E-05	0.25	2.3E-01	-0.02	9.2E-01	0.10	6.1E-01	0.04	8.6E-01	-0.84	8.0E-08
ENSCAFG0000000439	TMEM135	grey	EC_M10	0.24	2.4E-01	-0.04	8.5E-01	-0.48	1.2E-02	-0.04	8.5E-01	0.32	1.1E-01	0.01	9.4E-01	-0.13	5.4E-01	-0.09	6.6E-01	0.03	8.8E-01	-0.47	1.6E-02
ENSCAFG0000000101	TRDN	turquoise	EC_M6	0.24	2.4E-01	0.72	3.0E-05	-0.33	9.8E-02	-0.13	5.1E-01	-0.04	8.4E-01	-0.16	4.5E-01	-0.16	4.4E-01	-0.13	5.2E-01	-0.19	3.5E-01	-0.10	6.1E-01
ENSCAFG0000003156	ENSCAFG0000003156	darkgreen	EC_M4	0.24	2.4E-01	-0.10	6.3E-01	-0.76	5.6E-06	-0.06	7.7E-01	0.73	2.7E-05	0.27	1.8E-01	-0.30	1.3E-01	-0.08	6.9E-01	-0.10	6.3E-01	-0.85	3.8E-08
ENSCAFG0000000104	DESI1	grey	EC_M10	0.24	2.4E-01	0.61	9.7E-04	-0.62	7.4E-04	-0.07	7.2E-01	0.35	7.7E-02	-0.07	7.5E-01	-0.06	7.6E-01	0.05	8.0E-01	-0.18	3.7E-01	-0.49	1.1E-02
ENSCAFG0000000867	LMBR1L	turquoise	EC_M6	0.24	2.4E-01	0.77	5.1E-06	-0.13	5.4E-01	0.02	9.2E-01	-0.37	6.5E-02	-0.19	3.6E-01	-0.11	6.0E-01	-0.09	6.7E-01	-0.14	5.0E-01	0.22	2.7E-01
ENSCAFG0000000314	KBTBD2	turquoise	EC_M6	0.24	2.4E-01	0.80	1.1E-06	-0.56	3.1E-03	-0.18	3.8E-01	0.17	4.0E-01	0.08	7.1E-01	0.00	9.9E-01	0.06	7.6E-01	0.15	4.7E-01	-0.25	2.1E-01
ENSCAFG0000000007	TNFRSF11A	turquoise	EC_M6	0.24	2.4E-01	0.87	6.3E-09	-0.34	9.1E-02	-0.19	3.6E-01	-0.12	5.5E-01	0.07	7.4E-01	-0.09	6.7E-01	-0.02	9.2E-01	-0.01	9.4E-01	0.02	9.3E-01
ENSCAFG0000000151	STRIP2	turquoise	EC_M6	0.24	2.4E-01	0.79	1.9E-06	-0.24	2.3E-01	-0.16	4.5E-01	-0.14	5.1E-01	-0.17	4.0E-01	-0.15	4.5E-01	-0.16	4.3E-01	-0.01	9.7E-01	0.01	9.6E-01
ENSCAFG0000002536	ACKR1	turquoise	EC_M6	0.24	2.4E-01	0.86	2.3E-08	-0.21	3.1E-01	-0.09	6.6E-01	-0.22	2.7E-01	-0.14	4.9E-01	-0.16	4.3E-01	-0.16	4.2E-01	-0.07	7.2E-01	0.09	6.6E-01
ENSCAFG0000001403	PPA1	grey	EC_M10	0.24	2.4E-01	0.14	4.8E-01	0.15	4.6E-01	0.19	3.5E-01	-0.39	5.1E-02	-0.24	2.4E-01	-0.45	2.1E-02	-0.28	1.7E-01	-0.01	9.7E-01	0.22	2.7E-01
ENSCAFG0000000407	ENSCAFG0000000407	grey	EC_M10	0.24	2.4E-01	0.38	5.9E-02	0.10	6.2E-01	-0.10	6.3E-01	-0.37	6.2E-02	-0.10	6.1E-01	-0.06	7.6E-01	-0.07	7.2E-01	-0.21	3.1E-01	0.30	1.4E-01
ENSCAFG0000000694	ENSCAFG0000000694	grey	EC_M10	0.24	2.4E-01	0.25	2.2E-01	-0.72	3.5E-05	-0.15	4.6E-01	0.57	2.5E-03	0.24	2.5E-01	-0.03	8.7E-01	-0.02	9.1E-01	0.47	1.6E-02	-0.67	1.8E-04
ENSCAFG0000001094	ENSCAFG0000001094	turquoise	EC_M6	0.24	2.4E-01	0.41	3.8E-02	-0.01	9.7E-01	-0.33	9.5E-02	-0.21	3.0E-01	0.06	7.7E-01	-0.07	7.4E-01	0.06	7.9E-01	0.04	8.4E-01	0.10	6.3E-01
ENSCAFG0000001550	LYSMD2	turquoise	EC_M6	0.24	2.4E-01	0.77	3.9E-06	-0.21	2.9E-01	0.03	8.7E-01	-0.25	2.2E-01	-0.03	8.8E-01	-0.26	2.0E-01	-0.01	9.8E-01	0.34	8.5E-02	0.15	4.8E-01
ENSCAFG0000000015	HSD17B4	grey	EC_M10	0.24	2.4E-01	-0.31	1.2E-01	0.06	7.9E-01	-0.08	6.9E-01	-0.16	4.4E-01	-0.16	4.2E-01	0.07	7.4E-01	0.11	6.0E-01	-0.12	5.7E-01	0.08	6.9E-01
ENSCAFG0000000265	EEF1A1	cyan	EC_M2	0.24	2.4E-01	-0.16	4.3E-01	0.51	7.3E-03	0.14	4.9E-01	-0.74	1.4E-05	-0.08	7.1E-01	0.09	6.6E-01	-0.13	5.4E-01	-0.52	6.9E-03	0.58	2.0E-03
ENSCAFG0000003126	CYYR1	turquoise	EC_M6	0.24	2.4E-01	0.91	7.2E-11	-0.27	1.8E-01	-0.03	8.7E-01	-0.19	3.6E-01	-0.11	5.8E-01	-0.13	5.3E-01	-0.15	4.5E-01	0.10	6.3E-01	0.06	7.8E-01
ENSCAFG0000001803	B4GALT6	turquoise	EC_M6	0.24	2.4E-01	0.93	5.0E-12	-0.25	2.2E-01	-0.13	5.4E-01	-0.24	2.4E-01	-0.15	4.6E-01	-0.15	4.5E-01	-0.19	3.5E-01	0.05	8.1E-01	0.14	4.9E-01
ENSCAFG0000001651	PEAR1	turquoise	EC_M6	0.24	2.4E-01	0.72	3.0E-05	-0.24	2.4E-01	-0.26	1.9E-01	-0.12	5.5E-01	-0.24	2.4E-01	0.03	9.0E-01	0.07	7.2E-01	0.38	5.7E-02	0.04	8.4E-01
ENSCAFG0000001069	LTO1	grey	EC_M10	0.24	2.4E-01	0.28	1.7E-01	-0.65	3.1E-04	-0.22	2.8E-01	0.48	1.2E-02	0.06	7.7E-01	-0.12	5.7E-01	0.08	7.1E-01	0.37	6.6E-02	-0.58	1.8E-03
ENSCAFG0000000425	RAB10	grey	EC_M10	0.24	2.4E-01	-0.18	3.8E-01	-0.64	4.7E-04	-0.28	1.6E-01	0.66	2.5E-04	0.22	2.8E-01	-0.33	1.0E-01	0.12	5.6E-01	0.09	6.5E-01	-0.77	3.7E-06
ENSCAFG0000000071	ZBED4	turquoise	EC_M6	0.24	2.4E-01	0.47	1.6E-02	-0.20	3.2E-01	-0.19	3.6E-01	-0.10	6.2E-01	-0.16	4.5E-01	-0.09	6.5E-01	-0.25	2.2E-01	-0.19	3.5E-01	-0.08	7.1E-01
ENSCAFG0000001382	VPS26A	grey	EC_M10	0.24	2.4E-01	0.35	7.7E-02	-0.08	7.1E-01	0.17	4.1E-01	-0.30	1.3E-01	-0.05	8.1E-01	-0.29	1.5E-01	-0.26	1.9E-01	-0.21	3.0E-01	0.18	3.9E-01
ENSCAFG0000000860	SOX6	grey	EC_M10	0.24	2.4E-01	-0.16	4.4E-01	-0.50	8.5E-03	-0.13	5.4E-01	0.45	2.2E-02	-0.22	2.9E-01	-0.14	5.1E-01	-0.20	3.3E-01	0.08	7.1E-01	-0.56	2.9E-03

ENSCAFG0000001979	UBE4B	cyan	EC_M2	0.24	2.4E-01	0.14	5.0E-01	0.55	3.4E-03	0.29	1.6E-01	-0.93	3.4E-12	-0.21	2.9E-01	-0.19	3.5E-01	-0.18	3.9E-01	-0.17	3.9E-01	0.78	2.9E-06
ENSCAFG0000000745	NCK1	darkgrey	EC_M8	0.24	2.4E-01	0.52	6.2E-03	0.28	1.6E-01	0.03	8.7E-01	-0.76	6.5E-06	-0.24	2.4E-01	-0.10	6.3E-01	-0.01	9.7E-01	-0.20	3.3E-01	0.63	6.3E-04
ENSCAFG0000002032	ENSCAFG0000002032	grey	EC_M10	0.24	2.4E-01	-0.15	4.5E-01	-0.07	7.3E-01	0.24	2.4E-01	-0.10	6.2E-01	-0.18	3.9E-01	-0.16	4.5E-01	-0.19	3.5E-01	0.25	2.2E-01	-0.01	9.6E-01
ENSCAFG0000001551	ENSCAFG0000001551	grey	EC_M10	0.24	2.4E-01	-0.24	2.5E-01	-0.11	6.1E-01	0.15	4.7E-01	0.01	9.6E-01	0.42	3.1E-02	0.15	4.7E-01	-0.18	3.8E-01	-0.15	4.5E-01	-0.12	5.7E-01
ENSCAFG0000001870	EFCAB7	cyan	EC_M2	0.24	2.5E-01	-0.33	9.6E-02	0.48	1.3E-02	0.23	2.6E-01	-0.69	1.0E-04	-0.05	8.1E-01	-0.09	6.6E-01	-0.23	2.5E-01	-0.19	3.5E-01	0.53	5.5E-03
ENSCAFG0000001454	TBC1D22E	turquoise	EC_M6	0.24	2.5E-01	0.84	1.1E-07	-0.50	8.9E-03	-0.06	7.6E-01	0.10	6.3E-01	0.03	8.8E-01	-0.19	3.6E-01	-0.14	4.9E-01	-0.11	6.0E-01	-0.22	2.9E-01
ENSCAFG0000000019	ENSCAFG0000000019	grey	EC_M10	0.24	2.5E-01	-0.01	9.5E-01	-0.01	9.5E-01	-0.16	4.2E-01	-0.21	3.1E-01	-0.15	4.5E-01	0.16	4.3E-01	-0.13	5.3E-01	-0.13	5.4E-01	0.10	6.3E-01
ENSCAFG0000001585	DYDC1	grey	EC_M10	0.24	2.5E-01	-0.15	4.6E-01	-0.27	1.8E-01	0.01	9.8E-01	0.12	5.7E-01	-0.07	7.2E-01	-0.11	5.8E-01	-0.09	6.6E-01	-0.17	4.0E-01	-0.30	1.3E-01
ENSCAFG0000001635	ENSCAFG0000001635	grey	EC_M10	0.24	2.5E-01	-0.15	4.6E-01	-0.27	1.8E-01	0.01	9.8E-01	0.12	5.7E-01	-0.07	7.2E-01	-0.11	5.8E-01	-0.09	6.6E-01	-0.17	4.0E-01	-0.30	1.3E-01
ENSCAFG0000001009	ZNF713	grey	EC_M10	0.24	2.5E-01	0.18	3.8E-01	-0.65	2.9E-04	-0.11	6.1E-01	0.44	2.4E-02	0.26	2.0E-01	-0.10	6.4E-01	-0.08	7.1E-01	0.00	9.9E-01	-0.59	1.5E-03
ENSCAFG0000001410	SMARCB1	grey	EC_M10	0.24	2.5E-01	0.32	1.1E-01	0.16	4.4E-01	0.03	9.0E-01	-0.43	3.0E-02	-0.09	6.6E-01	0.45	2.1E-02	-0.20	3.3E-01	-0.05	8.2E-01	0.31	1.3E-01
ENSCAFG0000002000	EXTL2	grey	EC_M10	0.24	2.5E-01	-0.18	3.8E-01	-0.22	2.8E-01	0.24	2.4E-01	0.06	7.5E-01	0.24	2.4E-01	-0.55	3.4E-03	0.08	6.8E-01	0.29	1.6E-01	-0.22	2.8E-01
ENSCAFG0000001039	AK2	grey	EC_M10	0.24	2.5E-01	-0.15	4.6E-01	0.14	4.9E-01	-0.31	1.2E-01	-0.25	2.2E-01	0.06	7.5E-01	0.09	6.6E-01	0.01	9.6E-01	-0.16	4.2E-01	0.10	6.2E-01
ENSCAFG0000000019	SEMA6A	turquoise	EC_M6	0.24	2.5E-01	0.78	3.3E-06	-0.28	1.7E-01	0.17	4.1E-01	-0.15	4.6E-01	0.00	9.9E-01	-0.22	2.7E-01	-0.03	8.9E-01	-0.24	2.3E-01	0.01	9.7E-01
ENSCAFG0000001364	RBM45	grey	EC_M10	0.24	2.5E-01	0.25	2.2E-01	-0.50	9.0E-03	-0.06	7.8E-01	0.30	1.3E-01	-0.05	8.0E-01	-0.58	1.9E-03	-0.06	7.8E-01	-0.26	2.0E-01	-0.40	4.6E-02
ENSCAFG0000003178	ZNF655	grey	EC_M10	0.24	2.5E-01	-0.50	9.5E-03	-0.48	1.2E-02	0.00	9.8E-01	0.54	4.7E-03	0.18	3.7E-01	-0.16	4.2E-01	0.32	1.1E-01	0.08	6.9E-01	-0.66	2.8E-04
ENSCAFG0000001150	ENSCAFG0000001150	grey	EC_M10	0.24	2.5E-01	0.19	3.6E-01	-0.08	6.9E-01	0.49	1.0E-02	-0.17	4.1E-01	-0.25	2.3E-01	-0.29	1.5E-01	-0.20	3.3E-01	-0.02	9.2E-01	0.03	8.9E-01
ENSCAFG0000001594	ENSCAFG0000001594	grey	EC_M10	0.24	2.5E-01	-0.48	1.2E-02	0.01	9.7E-01	0.16	4.4E-01	-0.06	7.8E-01	0.10	6.4E-01	0.27	1.9E-01	0.09	6.6E-01	0.13	5.2E-01	-0.06	7.5E-01
ENSCAFG0000001467	ENSCAFG0000001467	grey	EC_M10	0.24	2.5E-01	0.54	4.8E-03	-0.66	2.1E-04	-0.26	2.0E-01	0.40	4.1E-02	-0.20	3.2E-01	0.01	9.6E-01	0.10	6.4E-01	0.14	4.8E-01	-0.53	5.0E-03
ENSCAFG0000001066	SLC16A14	turquoise	EC_M6	0.24	2.5E-01	0.52	6.0E-03	-0.14	4.8E-01	-0.11	6.0E-01	-0.22	2.9E-01	-0.05	8.0E-01	-0.05	7.9E-01	-0.12	5.7E-01	0.45	2.2E-02	0.11	6.0E-01
ENSCAFG0000003182	ENSCAFG0000003182	darkgreen	EC_M4	0.24	2.5E-01	-0.07	7.3E-01	-0.76	5.5E-06	-0.03	8.8E-01	0.68	1.2E-04	0.12	5.5E-01	-0.14	4.9E-01	0.09	6.8E-01	-0.26	2.0E-01	-0.82	2.2E-07
ENSCAFG0000001835	DRG2	grey	EC_M10	0.24	2.5E-01	0.03	9.0E-01	-0.20	3.2E-01	0.28	1.7E-01	0.10	6.4E-01	0.21	3.0E-01	-0.06	7.7E-01	-0.22	2.9E-01	0.22	2.8E-01	-0.21	3.0E-01
ENSCAFG0000001102	ROBO4	turquoise	EC_M6	0.24	2.5E-01	0.82	3.5E-07	-0.44	2.6E-02	-0.12	5.6E-01	0.02	9.1E-01	0.06	7.7E-01	-0.17	4.0E-01	-0.06	7.8E-01	0.08	7.0E-01	-0.18	3.9E-01
ENSCAFG0000001493	PSMC3IP	grey	EC_M10	0.23	2.5E-01	0.09	6.7E-01	-0.25	2.1E-01	-0.04	8.5E-01	0.16	4.4E-01	-0.08	7.1E-01	-0.08	7.0E-01	-0.04	8.6E-01	-0.27	1.9E-01	-0.27	1.8E-01
ENSCAFG0000000933	ARID2	darkgrey	EC_M8	0.23	2.5E-01	0.49	1.2E-02	0.25	2.1E-01	0.14	4.8E-01	-0.74	1.9E-05	-0.13	5.1E-01	-0.23	2.6E-01	-0.08	7.2E-01	-0.03	9.0E-01	0.61	9.9E-04
ENSCAFG0000000400	GRWD1	grey	EC_M10	0.23	2.5E-01	-0.29	1.5E-01	0.21	3.1E-01	0.11	5.9E-01	-0.23	2.5E-01	0.10	6.2E-01	0.02	9.1E-01	0.23	2.6E-01	-0.06	7.9E-01	0.09	6.6E-01
ENSCAFG0000001818	ENSCAFG0000001818	grey	EC_M10	0.23	2.5E-01	-0.06	7.9E-01	0.10	6.3E-01	-0.06	7.8E-01	-0.26	2.0E-01	0.35	7.9E-02	0.11	5.8E-01	-0.08	7.1E-01	-0.09	6.8E-01	0.18	3.9E-01
ENSCAFG0000000019	WDR7	grey	EC_M10	0.23	2.5E-01	-0.04	8.5E-01	-0.47	1.4E-02	0.18	3.8E-01	0.32	1.1E-01	0.05	7.9E-01	-0.07	7.3E-01	-0.23	2.7E-01	-0.43	2.8E-02	-0.45	2.1E-02
ENSCAFG0000003101	ENSCAFG0000003101	grey	EC_M10	0.23	2.5E-01	0.59	1.7E-03	-0.64	3.9E-04	-0.13	5.2E-01	0.42	3.3E-02	0.00	9.9E-01	-0.06	7.7E-01	-0.15	4.8E-01	0.05	8.2E-01	-0.51	7.2E-03
ENSCAFG0000001241	FAM13C	grey	EC_M10	0.23	2.5E-01	0.02	9.4E-01	-0.32	1.1E-01	-0.05	8.2E-01	0.17	4.2E-01	0.01	9.6E-01	0.08	6.8E-01	0.19	3.6E-01	-0.26	2.1E-01	-0.31	1.3E-01
ENSCAFG0000002380	ENSCAFG0000002380	grey	EC_M10	0.23	2.5E-01	0.05	7.9E-01	0.17	4.2E-01	0.12	5.6E-01	-0.36	6.7E-02	-0.31	1.2E-01	0.14	5.0E-01	0.14	4.9E-01	0.05	8.0E-01	0.29	1.5E-01
ENSCAFG0000001918	PITPNA	darkgreen	EC_M4	0.23	2.5E-01	0.28	1.7E-01	-0.80	9.3E-07	0.01	9.8E-01	0.58	1.8E-03	0.09	6.5E-01	-0.01	9.8E-01	0.04	8.5E-01	0.13	5.3E-01	-0.71	5.3E-05
ENSCAFG0000002059	ENSCAFG0000002059	grey	EC_M10	0.23	2.5E-01	0.32	1.1E-01	0.17	4.0E-01	-0.06	7.8E-01	-0.52	6.3E-03	-0.35	7.9E-02	0.03	8.9E-01	-0.13	5.2E-01	0.16	4.2E-01	0.40	4.3E-02
ENSCAFG0000001408	ENSCAFG0000001408	grey	EC_M10	0.23	2.5E-01	-0.17	4.2E-01	-0.23	2.5E-01	-0.12	5.5E-01	0.18	3.8E-01	-0.15	4.8E-01	-0.10	6.2E-01	-0.06	7.6E-01	-0.10	6.3E-01	-0.27	1.9E-01
ENSCAFG0000000013	BAZ2A	turquoise	EC_M6	0.23	2.5E-01	0.79	1.6E-06	0.07	7.4E-01	0.05	8.1E-01	-0.57	2.6E-03	-0.16	4.2E-01	0.01	9.5E-01	-0.19	3.6E-01	0.22	2.8E-01	0.46	1.8E-02
ENSCAFG0000000225	ICA1	turquoise	EC_M6	0.23	2.5E-01	0.90	6.6E-10	-0.50	8.7E-03	-0.18	3.9E-01	0.09	6.7E-01	-0.20	3.2E-01	-0.19	3.5E-01	-0.03	8.7E-01	0.11	5.9E-01	-0.20	3.3E-01
ENSCAFG0000000970	SYCP1	grey	EC_M10	0.23	2.5E-01	-0.06	7.7E-01	-0.08	7.1E-01	-0.07	7.3E-01	-0.01	9.5E-01	-0.04	8.6E-01	-0.01	9.5E-01	-0.04	8.3E-01	0.13	5.2E-01	-0.09	6.6E-01
ENSCAFG0000001954	POLR2E	grey	EC_M10	0.23	2.5E-01	-0.35	7.6E-02	0.23	2.6E-01	0.00	9.9E-01	-0.20	3.4E-01	-0.10	6.4E-01	-0.11	6.0E-01	-0.02	9.0E-01	-0.06	7.8E-01	0.10	6.4E-01
ENSCAFG0000000913	ENTPD4	darkgreen	EC_M4	0.23	2.5E-01	0.41	3.8E-02	-0.86	1.3E-08	-0.08	7.0E-01	0.66	2.4E-04	0.05	8.3E-01	-0.16	4.5E-01	-0.04	8.5E-01	0.05	7.9E-01	-0.76	7.5E-06
ENSCAFG0000000954	NDUFAF1	grey	EC_M10	0.23	2.5E-01	-0.34	9.1E-02	-0.12	5.5E-01	0.17	4.1E-01	0.00	9.9E-01	0.00	9.9E-01	0.02	9.1E-01	-0.04	8.6E-01	-0.18	3.8E-01	-0.14	5.0E-01
ENSCAFG0000001416	MDGA2	darkgreen	EC_M4	0.23	2.5E-01	0.18	3.8E-01	-0.40	4.1E-02	-0.16	4.3E-01	0.34	9.3E-02	-0.15	4.8E-01	-0.06	7.8E-01	-0.09	6.8E-01	0.13	5.4E-01	-0.38	5.2E-02
ENSCAFG0000000944	ENSCAFG0000000944	grey	EC_M10	0.23	2.5E-01	-0.10	6.4E-01	-0.21	3.0E-01	-0.08	7.1E-01	0.03	8.7E-01	-0.09	6.6E-01	-0.11	6.0E-01	-0.10	6.2E-01	-0.08	7.1E-01	-0.17	3.9E-01
ENSCAFG0000001180	ICAM2	turquoise	EC_M6	0.23	2.5E-01	0.88	3.6E-09	-0.32	1.1E-01	-0.08	7.1E-01	-0.11	5.9E-01	-0.08	9.0E-01	-0.06	7.6E-01	-0.01	9.5E-01	0.15	4.7E-01	-0.04	8.5E-01
ENSCAFG0000000355	CADPS2	grey	EC_M10	0.23	2.5E-01	0.31	1.3E-01	-0.41	3.5E-02	-0.38	5.7E-02	0.18	3.9E-01	0.08	7.1E-01	0.14	5.1E-01	0.17	4.0E-01	-0.19	3.4E-01	-0.33	1.0E-01
ENSCAFG0000000280	TMPRSS11F	turquoise	EC_M6	0.23	2.5E-01	0.90	6.2E-10	-0.26	2.0E-01	-0.08	7.0E-01	-0.21	3.0E-01	-0.09	6.8E-01	-0.07	7.2E-01	-0.07	7.2E-01	-0.12	5.7E-01	0.11	6.0E-01
ENSCAFG0000000434	EFCC1	turquoise	EC_M6	0.23	2.5E-01	0.90	6.2E-10	-0.26	2.0E-01	-0.08	7.0E-01	-0.21	3.0E-01	-0.09	6.8E-01	-0.07	7.2E-01	-0.07	7.2E-01	-0.12	5.7E-01	0.11	6.0E-01
ENSCAFG0000001376	LTF	turquoise	EC_M6	0.23	2.5E-01	0.90	6.2E-10	-0.26	2.0E-01	-0.08	7.0E-01	-0.21	3.0E-01	-0.09	6.8E-01	-0.07	7.2E-01	-0.07	7.2E-01	-0.12	5.7E-01	0.11	6.0E-01
ENSCAFG0000001054	GLRX2	darkgreen	EC_M4	0.23	2.5E-01	-0.05	8.1E-01	-0.80	1.0E-06	-0.11	5.8E-01	0.78	2.3E-06	0.35	8.3E-02	-0.04	8.4E-01	-0.08	6.9E-01	-0.03	8.8E-01	-0.90	4.8E-10
ENSCAFG0000000739	SLC25A46	darkgreen	EC_M4	0.23	2.5E-01	0.14	4.9E-01	-0.86	2.3E-08	-0.14	4.9E-01	0.73	2.4E-05	0.14	5.0E-01	-0.17	4.1E-01	0.13	5.3E-01	0.18	3.9E-01	-0.82	2.9E-07
ENSCAFG0000000442	VASP	darkgreen	EC_M4	0.23	2.5E-01	0.06	7.6E-01	-0.73	2.5E-05	-0.20	3.2E-01	0.66	2.4E-04	0.22	2.9E-01	0.13	5.4E-01	0.03	8.7E-01	-0.05	7.9E-01	-0.83	2.1E-07
ENSCAFG0000000840	EIF2A	cyan	EC_M2	0.23	2.5E-01	-0.58	1.7E-03	0.27	1.9E-01	0.13	5.1E-01	-0.32	1.1E-01	0.12	5.5E-01	-0.06	7.8E-01	-0.01	9.5E-01	-0.28	1.7E-01	0.14	4.8E-01
ENSCAFG0000001751	MTMR4	cyan	EC_M2	0.23	2.5E-01	-0.02	9.1E-01	0.58	1.7E-03	0.25	2.1E-01	-0.88	2.3E-09	-0.32	1.2E-01	0.04	8.6E-01	-0.13	5.2E-01	-0.19	3.5E-01	0.75	1.3E-05
ENSCAFG0000000458	RBSN	grey	EC_M10	0.23	2.5E-01	0.53	5.0E-03	-0.61	1.1E-03	-0.08	6.8E-01	0.40	4.5E-02	-0.02	9.3E-01	-0.29	1.5E-01	0.18	3.9E-01	-0.05	8.1E-01	-0.48	1.4E-02
ENSCAFG0000001231	ARID1A	turquoise	EC_M6	0.23	2.5E-01	0.61	9.0E-04	-0.04	8.5E-01	0.07	7.5E-01	-0.37	6.2E-02	-0.22	2.8E-01	-0.18	3.7E-01	0.06	7.9E-01	0.23	2.6E-01	0.26	2.0E-01
ENSCAFG0000002074	ENSCAFG0000002074	grey	EC_M10	0.23	2.5E-01	-0.14	5.0E-01	-0.53	5.0E-03	-0.06	7.7E-01	0.44	2.3E-02	0.59	1.5E-03	-0.15	4.7E-01	-0.11	5.9E-01	-0.16	4.3E-01	-0.59	1.5E-03
ENSCAFG0000001830	PPP1R13B	turquoise	EC_M6	0.23	2.5E-01	0.74	1.8E-05	-0.72	3.6E-05	-0.29	1.6E-01	0.37	6.1E-02	0.09	6.8E-01	-0.14	5.1E-01	-0.03	9.0E-01	-0.14	4.9E-01	-0.53	5.5E-03
ENSCAFG0000000768	SLCO5A1	turquoise	EC_M6	0.23	2.5E-01	0.83	2.1E-07	-0.51	7.4E-03	-0.21	3.0E-01	0.12	5.7E-01	-0.05	8.0E-01	-0.23	2.6E-01	-0.22	2.7E-01	-0.04	8.4E-01	-0.26	2.1E-01
ENSCAFG0000001589	GHITM	darkgreen	EC_M4	0.23	2.5E-01	0.00	9.9E-01	-0.80	9.7E-07	-0.16	4.5E-01	0.75	8.7E-06	0.26	2.0E-01	-0.24	2.5E-01	-0.07	7.2E-01	-0.01	9.7E-01	-0.84	6.2E-08
ENSCAFG0000001172	POLG2	grey	EC_M10	0.23	2.5E-01	0.01	9.6E-01	0.19	3.6E-01	0.42	3.2E-02	-0.43	2.9E-02	0.31	1.2E-01	0.15	4.7E-01	-0.20	3.2E-01	-0.51	8.2E-03	0.29	1.5E-01
ENSCAFG0000000569	PDE2A	turquoise	EC_M6	0.23	2.6E-01	0.88	3.4E-09	-0.19	3.4E-01	-0.04	8.4E-01	-0.25	2.2E-01	-0.13	5.1E-01	-0.14	4.8E-01	-0.11	5.9E-01	-0.06	7.8E-01	0.11	5.8E-01
ENSCAFG0000002366	PRDM6	grey	EC_M10	0.23	2.6E-01	-0.07	7.5E-01	-0.19	3.4E-01	-0.20	3.2E-01	0.12	5.4E-01	-0.14	4.8E-01	-0.05	8.0E-01	-0.13	5.1E-01	0.18	3.9E-01	-0.16	4.3E-01
ENSCAFG0000002581	ENSCAFG0000002581	turquoise	EC_M6	0.23	2.6E-01	0.76	6.5E-06	-0.27	1.8E-01	-0.08	7.0E-01	-0.14	5.0E-01	-0.12	5.7E-01	0.26	1.9E-01	-0.09	6.7E-01	0.19	3.6E-01	0.04	8.6E-01
ENSCAFG0000001297	ENSCAFG0000001297	grey	EC_M10	0.23	2.6E-01	0.07	7.5E-01	0.04	8.6E-01	0.39	4.7E-02	-0.21	3.0E-01	-0.10	6.3E-01	-0.16	4.3E-01	-0.18	3.9E-01	-0.16	4.3E-01	0.07	7.2E-01
ENSCAFG0000000488	EIF2B4	grey	EC_M10	0.23	2.6E-01	0.00	9.9E-01	-0.01	9.6E-01	0.26	2.1E-01	-0.19	3.5E-01	0.26	2.0E-01	-0.13	5.2E-01	-0.23	2.6E-01	0.30	1.4E-01	0.05	8.0E-01
ENSCAFG0000002389	CEACAM20	grey	EC_M10	0.23	2.6E-01	-0.21	3.0E-01	-0.46	1.7E-02	-0.16	4.4E-01	0.40	4.2E-02	0.13	5.3E-01	-0.03	8.7E-01	0.32	1.1E-01	-0.03	8.9E-01	-0.57	2.5E-03
ENSCAFG0000001265	FAM110D	turquoise	EC_M6	0.23	2.6E-01	0.90	5.7E-10	-0.22	2.7E-01	-0.11	6.1E-01	-0.21	2.9E-01	-0.12	5.6E-01	-0.13	5.3E-01	-0.15	4.6E-01	-0.21	3.1E-01	0.07	7.2E-01
ENSCAFG0000000189	UBAP2	grey	EC_M10	0.23	2.6E-01	0.47	1.6E-02	-0.67	1.9E-04	-0.24	2.3E-01	0.47	1.5E-02	0.03	8.7E-01	-0.34	9.3E-02	-0.06	7.5E-01	-0.10	6.4E-01	-0.61	8.7E-04
ENSCAFG0000001457	PRDX6	grey	EC_M10	0.23	2.6E-01	-0.13	5.4E-01	-0.14	5.1E-01	-0.12	5.5E-01	0.14	4.9E-01	0.25	2.1E-01	-0.39	4.6E-02	-0.06	7.7E-01	-0.09	6.5E-01	-0.25	2.3E-01
ENSCAFG0000001076	IPO8	grey	EC_M10	0.23	2.6E-01	-0.48	1.4E-02	0.22	2.8E-01	0.06	7.8E-01	-0.27	1.7E-01	-0.07	7.5E-01	-0.02	9.3E-01	0.11	6.0E-01	-0.30	1.4E-01	0.19	3.5E-01
ENSCAFG0000001229	ADCY4	turquoise	EC_M6	0.23	2.6E-01	0.83	1.9E-07	0.06	7.6E-01	-0.04	8.6E-01	-0.53	5.2E-03	-0.22	2.8E-01	-0.09	6.7E-01	-0.10	6.3E-01	-0.16	4.5E-01	0.42	3.2E-02
ENSCAFG0000001438	ENSCAFG0000001438	grey	EC_M10	0.23	2.6E-01	-0.06	7.9E-01	-0.39	4.9E-02	0.06	7.6E-01	0.31	1.3E-01	0.05	8.1E-01	0.02	9.4E-01	0.02	9.4E-01	-0.15	4.5E-01	-0.40	4.5E-02
ENSCAFG0000001725	ENSCAFG0000001725	grey	EC_M10	0.23	2.6E-01	0.34	9.2E-02	-0.52	6.0E-03	-0.36	6.8E-02	0.32	1.1E-01	0.23	2.6E-01	0.14	5.0E-01	0.30	1.4E-01	0.12	5.5E-01	-0.42	3.5E-02
ENSCAFG0000000705	CHCHD7	grey	EC_M10	0.23	2.6E-01	-0.04	8.7E-01	-0.46	1.8E-02	-0.18	3.8E-01	0.35	7.6E-02	0.14	4.8E-01	-0.11	5.8E-01	0.43	2.9E-02	-0.10	6.2E-01	-0.53	5.1E-03
ENSCAFG0000000085	UNC93A	grey	EC_M10	0.23	2.6E-01	-0.22	2.8E-01	-0.17	4.2E-01	-0.06	7.8E-01	0.09	6.8E-01	-0.12	5.5E-01	-0.12	5.7E-01	0.45	2.0E-02	-0.11	6.1E-01	-0.23	2.5E-01
ENSCAFG0000001301	RTKN2	grey	EC_M10	0.23	2.6E-01	-0.17	4.2E-01	-0.19	3.5E-01	-0.20	3.2E-01	0.18	3.8E-01	0.31	1.2E-01	0.10	6.2E-01	-0.03	8.7E-01	0.02	9.2E-01	-0.26	2.0E-01
ENSCAFG0000001624	PGM2	turquoise	EC_M6	0.23	2.6E-01	0.86	2.3E-08	-0.45	2.0E-02	0.05	8.1E-01	0.03	8.7E-01	-0.15	4.8E-01	-0.24	2.4E-01	-0.08	7.0E-01	0.06	7.7E-01	-0.13	5.3E-01
ENSCAFG0000000426	THNSL1	turquoise	EC_M6	0.23	2.6E-01	0.46	1.8E-02	-0.13	5.1E-01	-0.13	5.2E-01	-0.18	3.9E-01	-0.44	2.4E-02	-0.25	2.1E-01	-0.12	5.5E-01	-0.19	3.5E-01	0.11	5.9E-01
ENSCAFG0000000567	LGALS4	grey	EC_M10	0.23	2.6E-01	0.04	8.3E-01	-0.16	4.3E-01	-0.07	7.4E-01	0.02	9.2E-01	-0.12	5.5E-01	0.31	1.2E-01	-0.05	8.1E-01	-0.10	6.2E-01	-0.15	4.7E-01
ENSCAFG0000000358	IQUB	grey	EC_M10	0.23	2.6E-01	0.39	4.6E-02	-0.42	3.2E-02	-0.08	6.9E-01	0.11	6.0E-01	-0.23	2.6E-01	-0.27	1.9E-01	0.18	3.9E-01	0.08	6.9E-01	-0.27	1.8E-01
ENSCAFG0000002603	ENSCAFG0000002603	grey	EC_M10	0.23	2.6E-01	-0.18	3.7E-01	0.18	3.7E-01	-0.12	5.7E-01	-0.22	2.7E-01	-0.05	8.2E-01	-0.06	7.6E-01	-0.08	7.0E-01	-0.13	5.3E-01	0.12	5.6E-01
ENSCAFG0000000527	ENSCAFG0000000527	grey	EC_M10	0.23	2.6E-01	-0.46	1.7E-02	-0.07	7.3E-01	0.05	8.1E-01	0.03	9.0E-01	0.19	3.6E-01	-0.03	8.8E-01	0.22	2.9E-01	-0.24	2.4E-01	-0.20	3.4E-01
ENSCAFG0000001333	NDUFS1	grey	EC_M10	0.23	2.6E-01	-0.42	3.4E-02	0.30	1.3E-01	-0.08	6.9E-01	-0.37	5.9E-02	-0.05	8.1E-01	-0.18	3.8E-01	0.01	9.5E-01	0.02	9.3E-01	0.32	1.2E-01
ENSCAFG0000000934	ZFYVE19	grey	EC_M10	0.23	2.6E-01	0.32	1.2E-01	0.07	7.5E-01	-0.11	6.0E-01	-0.35	8.4E-02	-0.42	3.3E-02	0.01	9.5E-01	0.41	3.7E-02	0.03	9.0E-01	0.22	2.7E-01
ENSCAFG0000001079	SBDS	darkgreen	EC_M4	0.23	2.6E-01	-0.11	5.8E-01	-0.57	2.2E-03	0.07	7.3E-01	0.49	1.2E-02	0.04	8.3E-01	-0.07	7.2E-01	0.18	3.7E-01	-0.34	8.6E-02	-0.61	9.4E-04
ENSCAFG0000001012	ENSCAFG0000001012	violet	EC_M7	0.23	2.6E-01	-0.19	3.4E-01	-0.03	8.9E-01	-0.12	5.5E-01	-0.11	5.9E-01	0.68	1.5E-04	-0.12	5.6E-01	-0.14	5.0E-01	-0.22	2.9E-01	-0.03	8.8E-01
ENSCAFG0000001668	ANXA2	grey	EC_M10	0.23	2.6E-01	-0.31	1.2E-01	-0.43	2.6E-02	-0.20	3.3E-01	0.50	9.9E-03	0.01	9.5E-01	-0.26	2.1E-01	0.32	1.2E-01	0.14	4.9E-01	-0.60	1.3E-03
ENSCAFG0000001630	ARAP2	grey	EC_M10	0.23	2.6E-01	-0.07	7.4E-01	-0.57	2.6E-03	-0.13	5.4E-01	0.48	1.4E-02	0.15	4.6E-01	-0.11	5.9E-01	0.27	1.9E-01	0.57	2.6E-03	-0.59	1.6E-03
ENSCAFG0000001708	WWC1	grey	EC_M10	0.23	2.6E-01	0.09	6.5E-01	-0.39	4.7E-02	-0.19	3.6E-01	0.25	2.1E-01	0.34	8.5E-02	0.13	5.3E-01	-0.18	3.9E-01	-0.07	7.4E-01	-0.42	3.5E-02
ENSCAFG0000000544	TIMM50	grey	EC_M10	0.23	2.6E-01	-0.37	6.5E-02	-0.06	7.7E-01	-0.23	2.5E-01	0.14	4.8E-01	-0.03	8.8E-01	-0.03	8.8E-01	-0.15	4.6E-01	-0.32	1.1E-01	-0.28	1.7E-01
ENSCAFG0000000183	ABCB1	grey	EC_M10	0.23	2.6E-01	-0.09	6.7E-01	-0.25	2.1E-01	-0.21	3.0E-01	0.12	5.5E-01	0.25	2.2E-01	0.15	4.7E-01	-0.18	3.7E-01	-0.12	5.6E-01	-0.28	1.6E-01
ENSCAFG0000000612	PGAM5	grey	EC_M10	0.23	2.6E-01	-0.35	8.3E-02	0.30	1.4E-01	0.02	9.4E-01	-0.28	1.6E-01	-0.08	7.1E-01	-0.34	8.5E-02	0.14	4.9E-01	0.04	8.5E-01	0.18	3.9E-01
ENSCAFG0000000951	COX15	grey	EC_M10	0.23	2.6E-01	-0.17	4.2E-01	-0.46	1.9E-02	-0.23	2.5E-01	0.48	1.3E-02	0.33	9.7E-02	-0.28	1.7E-01	0.02	9.3E-01	-0.15	4.7E-01	-0.60	1.1E-03
ENSCAFG0000000196	ENSCAFG0000000196	grey	EC_M10	0.23	2.6E-01	-0.23	2.6E-01	-0.22	2.7E-01	-0.13	5.2E-01	0.17	4.1E-01	-0.19	3.6E-01	-0.14	4.9E-01	-0.08	7.0E-01	-0.15	4.6E-01	-0.34	9.2E-02
ENSCAFG0000001935	PLEKHN1	grey	EC_M10	0.23	2.6E-01	-0.15	4.6E-01	0.53	5.3E-03	-0.09	6.7E-01	-0.72	3.1E-05	-0.02	9.2E-01	0.08	7.1E-01	-0.16	4.5E-01	-0.21	3.1E-01	0.59	1.4E-03
ENSCAFG0000002532	ENSCAFG0000002532	grey	EC_M10	0.23	2.6E-01	0.12	5.6E-01	0.08	7.0E-01	0.26	1.9E-01	-0.31	1.2E-01	0.11	6.0E-01	-0.32	1.1E-01	-0.57	2.3E-03	-0.24	2.4E-01	0.16	4.4E-01
ENSCAFG0000000760	SH3RF1	turquoise	EC_M6	0.23	2.6E-01	0.76	7.1E-06	-0.24	2.3E-01	-0.21	3.0E-01	-0.15	4.7E-01	-0.11	5.9E-01	-0.01	9.6E-01	0.14	5.1E-01	0.04	8.4E-01	0.00	9.9E-01
ENSCAFG0000002271	MT-ND1	cyan	EC_M2	0.23	2.6E-01	-0.42	3.3E-02	0.25	2.2E-01	0.22	2.9E-01	-0.34	9.1E-02	0.16	4.3E-01	-0.52	6.1E-03	0.08	6.9E-01	-0.04	8.3E-01	0.20	3.2E-01
ENSCAFG0000000745	RCN1	grey	EC_M10	0.23	2.6E-01	0.25	2.2E-01	0.00	1.0E+00	0.06	7.6E-01	-0.30	1.4E-01	0.20	3.2E-01	0.45	2.0E-02	0.13	5.3E-01	0.21	3.0E-01	0.14	4.8E-01
ENSCAFG0000002286	ENSCAFG0000002286	grey	EC_M10	0.23	2.6E-01	-0.30	1.4E-01	-0.26	2.0E-01	0.23	2.7E-01	0.16	4.4E-01	0.45	2.0E-02	0.08	7.0E-01	0.08	7.1E-01	-0.16	4.4E-01	-0.28	1.7E-01
ENSCAFG0000001496	DYNC2H1	cyan	EC_M2	0.23	2.6E-01	-0.55	3.7E-03	0.23	2.6E-01	0.27	1.8E-01	-0.31	1.2E-01	-0.06	7.9E-01	-0.13	5.4E-01	-0.12	5.8E-01	-0.16	4.3E-01	0.16	4.3E-01
ENSCAFG0000000965	TFAP2A	grey	EC_M10	0.23	2.6E-01	-0.14	5.0E-01	0.07	7.4E-01	-0.09	6.7E-01	-0.21	2.9E-01	-0.14	5.1E-01	-0.10	6.3E-01	-0.12	5.6E-01	-0.07	7.2E-01	0.14	5.0E-01
ENSCAFG0000001676	PCGF3	darkgreen	EC_M4	0.23	2.6E-01	0.46	1.9E-02	-0.77	4.3E-06	-0.12	5.7E-01	0.52	6.2E-03	0.15	4.5E-01	-0.03	8.7E-01	0.10	6.4E-01	-0.25	2.1E-01	-0.69	1.0E-04
ENSCAFG0000001423	WDR61	grey	EC_M10	0.23	2.6E-01	-0.14	4.9E-01	-0.49	1.0E-02	-0.08	6.8E-01	0.53	5.7E-03	0.25	2.1E-01	-0.12	5.4E-01	0.12	5.5E-01	-0.08	6.9E-01	-0.64	4.6E-04
ENSCAFG0000001143	UBR1	darkgrey	EC_M8	0.23	2.6E-01	0.06	7.6E-01	0.29	1.5E-01	0.19	3.6E-01	-0.61	9.5E-04	-0.27	1.8E-01	-0.32	1.1E-01	-0.10	6.2E-01	-0.28	1.7E-01	0.49	1.1E-02
ENSCAFG0000002542	ENSCAFG0000002542	turquoise	EC_M6	0.23	2.6E-01	0.20	3.3E-01	0.05	8.1E-01	0.47	1.6E-02	-0.36	7.2E-02	-0.14	5.1E-01	-0.16	4.4E-01	-0.09	6.7E-01	-0.16	4.5E-01	0.25	2.3E-01
ENSCAFG0000003039	ZNF558	darkgrey	EC_M8	0.23	2.6E-01	0.53	4.9E-03	0.13	5.2E-01	0.00	9.8E-01	-0.59	1.7E-03	-0.01	9.5E-01	0.07	7.5E-01	-0.04	8.5E-01	0.08	7.0E-01	0.49	1.1E-02
ENSCAFG0000000388	TXNRD3	cyan	EC_M2	0.23	2.6E-01	-0.43	2.9E-02	0.49	1.1E-02	0.15	4.6E-01	-0.60	1.3E-03	-0.19	3.5E-01	-0.20	3.4E-01	-0.11	5.9E-01	-0.06	7.8E-01	0.50	9.8E-03
ENSCAFG0000000578	ANAPC15	darkgreen	EC_M4	0.23	2.6E-01	-0.19	3.6E-01	-0.73	2.7E-05	-0.20	3.3E-01	0.74	1.7E-05	0.28	1.6E-01	-0.17	4.0E-01	0.28	1.7E-01	-0.07	7.3E-01	-0.86	1.7E-08
ENSCAFG0000001398	RALGPS2	grey	EC_M10	0.23	2.6E-01	0.09	6.8E-01	-0.62	8.0E-04	-0.29	1.5E-01	0.53	4.9E-03	0.02	9.1E-01	0.19	3.5E-01	0.24	2.3E-01	0.04	8.5E-01	-0.64	3.8E-04
ENSCAFG0000000429	CCDC146	grey	EC_M10	0.23	2.6E-01	0.14	4.9E-01	0.14	4.8E-01	0.33	9.6E-02	-0.39	5.2E-02	-0.11	5.8E-01	-0.12	5.5E-01	-0.27	1.8E-01	-0.41	3.7E-02	0.27	1.9E-01
ENSCAFG0000001272	CGN	turquoise	EC_M6	0.23	2.6E-01	0.55	3.3E-03	-0.09	6.6E-01	0.67	1.9E-04	-0.30	1.3E-01	-0.10	6.2E-01	-0.10	6.4E-01	-0.12	5.6E-01	-0.15	4.6E-01	0.20	3.3E-01
ENSCAFG0000003039	AP3S2	grey	EC_M10	0.23	2.6E-01	-0.39	4.8E-02	-0.41	3.8E-02	-0.23	2.7E-01	0.50	9.3E-03	0.37	6.6E-02	-0.18	3.8E-01	-0.13	5.3E-01	-0.42	3.4E-02	-0.64	4.5E-04
ENSCAFG0000002306	NDST1	grey	EC_M10	0.23	2.6E-01	0.28	1.6E-01	-0.31	1.3E-01	-0.16	4.4E-01	0.07	7.5E-01	0.20	3.3E-01	-0.17	3.9E-01	0.08	6.9E-01	-0.47	1.7E-02	-0.27	1.8E-01
ENSCAFG0000001120	NHLRC2	darkgreen	EC_M4	0.23	2.6E-01	0.19	3.6E-01	-0.83	1.1E-07	-0.14	5.0E-01	0.71	4.1E-05	0.30	1.4E-01	-0.10	6.2E-01	-0.15	4.8E-01	-0.04	8.4E-01	-0.81	5.4E-07
ENSCAFG0000002025	ENSCAFG0000002025	grey	EC_M10	0.23	2.6E-01	-0.20	3.2E-01	0.09	6.5E-01	-0.07	7.2E-01	-0.17	4.0E-01	0.39	5.0E-02	-0.12	5.5E-01	-0.10	6.1E-01	-0.16	4.3E-01	0.05	7.9E-01
ENSCAFG0000002138	ENSCAFG0000002138	grey	EC_M10	0.23	2.6E-01	0.12	5.7E-01	-0.02	9.1E-01	0.12	5.6E-01	-0.26	1.9E-01	-0.01	9.5E-01	-0.05	8.2E-01	0.16	4.4E-01	-0.02	9.1E-01	0.12	5.7E-01

ENSCAFG0000001671	SIMC1	grey	EC_M10	0.23	2.6E-01	0.12	5.6E-01	-0.02	9.1E-01	0.07	7.2E-01	-0.21	3.0E-01	0.08	6.9E-01	0.12	5.5E-01	-0.06	7.8E-01	0.05	7.9E-01	0.08	6.9E-01
ENSCAFG0000000099	TCF7	turquoise	EC_M6	0.23	2.6E-01	0.86	1.2E-08	-0.11	6.0E-01	-0.11	5.9E-01	-0.34	8.9E-02	-0.12	5.7E-01	-0.11	5.9E-01	0.06	7.9E-01	0.09	6.5E-01	0.22	2.8E-01
ENSCAFG0000001674	MRPL24	turquoise	EC_M6	0.23	2.6E-01	0.39	4.7E-02	-0.04	8.5E-01	0.18	3.8E-01	-0.25	2.2E-01	-0.07	7.2E-01	0.13	5.2E-01	-0.46	1.9E-02	0.02	9.1E-01	0.17	4.1E-01
ENSCAFG0000000191	NFXL1	grey	EC_M10	0.23	2.6E-01	-0.13	5.1E-01	0.10	6.3E-01	0.22	2.9E-01	-0.30	1.4E-01	0.06	7.6E-01	0.15	4.5E-01	-0.35	8.1E-02	-0.52	6.2E-03	0.13	5.2E-01
ENSCAFG0000001279	PES1	grey	EC_M10	0.23	2.6E-01	-0.36	7.3E-02	0.33	9.7E-02	0.11	5.8E-01	-0.33	1.0E-01	0.14	4.8E-01	0.01	9.8E-01	-0.05	8.0E-01	-0.30	1.3E-01	0.20	3.4E-01
ENSCAFG0000003081	MRPL51	darkgreen	EC_M4	0.23	2.6E-01	-0.44	2.6E-02	-0.50	9.2E-03	-0.08	6.9E-01	0.57	2.5E-03	0.37	6.5E-02	-0.10	6.2E-01	-0.16	4.3E-01	-0.16	4.4E-01	-0.73	2.2E-05
ENSCAFG0000000968	HLCS	grey	EC_M10	0.23	2.6E-01	-0.13	5.1E-01	0.22	2.8E-01	-0.08	6.9E-01	-0.35	8.0E-02	0.29	1.6E-01	0.06	7.7E-01	0.29	1.6E-01	-0.17	3.9E-01	0.25	2.1E-01
ENSCAFG0000001322	ELOA	darkgreen	EC_M4	0.23	2.6E-01	0.14	4.9E-01	-0.82	2.6E-07	-0.21	3.0E-01	0.73	2.1E-05	0.33	1.0E-01	-0.18	3.7E-01	0.05	8.3E-01	-0.17	4.0E-01	-0.85	5.6E-08
ENSCAFG0000000806	ARHGEF3	turquoise	EC_M6	0.23	2.6E-01	0.77	4.9E-06	0.11	6.1E-01	0.06	7.8E-01	-0.62	8.2E-04	-0.23	2.7E-01	-0.23	2.7E-01	-0.19	3.5E-01	0.05	8.0E-01	0.52	6.4E-03
ENSCAFG0000003167	POLR2M	grey	EC_M10	0.23	2.6E-01	-0.30	1.3E-01	0.13	5.2E-01	0.04	8.4E-01	-0.33	1.0E-01	0.15	4.6E-01	-0.14	5.1E-01	0.23	2.5E-01	0.04	8.3E-01	0.21	3.0E-01
ENSCAFG0000000956	CLIC6	turquoise	EC_M6	0.23	2.6E-01	0.79	1.2E-06	-0.26	2.0E-01	-0.11	6.0E-01	-0.16	4.2E-01	-0.01	9.4E-01	-0.11	5.8E-01	-0.19	3.5E-01	0.37	6.5E-02	0.04	8.6E-01
ENSCAFG0000000683	PSMD6	darkgreen	EC_M4	0.23	2.6E-01	-0.13	5.3E-01	-0.75	1.1E-05	-0.10	6.1E-01	0.77	3.4E-06	0.31	1.3E-01	-0.01	9.5E-01	-0.04	8.5E-01	-0.19	3.6E-01	-0.88	3.7E-09
ENSCAFG0000001194	RTF2	grey	EC_M10	0.23	2.6E-01	-0.10	6.4E-01	-0.65	3.0E-04	-0.01	9.8E-01	0.59	1.6E-03	0.33	1.0E-01	-0.20	3.3E-01	-0.26	2.0E-01	0.09	6.7E-01	-0.69	9.3E-05
ENSCAFG0000003051	ENSCAFG0000003051	grey	EC_M10	0.23	2.6E-01	-0.13	5.4E-01	-0.05	8.0E-01	0.36	7.4E-02	-0.10	6.3E-01	-0.17	4.2E-01	-0.22	2.9E-01	-0.19	3.4E-01	-0.25	2.1E-01	-0.06	7.6E-01
ENSCAFG0000001689	SNF8	grey	EC_M10	0.23	2.6E-01	0.26	2.0E-01	0.22	2.9E-01	-0.05	7.9E-01	-0.50	9.3E-03	0.06	7.5E-01	0.05	7.9E-01	0.02	9.3E-01	0.00	9.8E-01	0.36	7.1E-02
ENSCAFG0000001780	BCL11B	violet	EC_M7	0.23	2.6E-01	-0.06	7.8E-01	-0.26	2.0E-01	-0.09	6.8E-01	0.15	4.7E-01	0.90	4.2E-10	-0.07	7.5E-01	-0.11	6.0E-01	-0.15	4.6E-01	-0.30	1.4E-01
ENSCAFG0000003211	ENSCAFG0000003211	grey	EC_M10	0.23	2.6E-01	0.05	7.9E-01	-0.25	2.3E-01	-0.05	8.1E-01	0.16	4.3E-01	-0.15	4.7E-01	-0.10	6.1E-01	-0.11	5.9E-01	-0.16	4.3E-01	-0.28	1.7E-01
ENSCAFG0000001481	PPP3CB	grey	EC_M10	0.23	2.6E-01	-0.56	2.8E-03	0.47	1.7E-02	0.28	1.6E-01	-0.56	3.0E-03	-0.09	6.5E-01	-0.17	3.9E-01	-0.10	6.3E-01	-0.20	3.2E-01	0.43	2.7E-02
ENSCAFG0000001501	ENSCAFG0000001501	grey	EC_M10	0.23	2.7E-01	-0.01	9.6E-01	0.10	6.3E-01	0.23	2.5E-01	-0.34	9.3E-02	0.01	9.6E-01	-0.04	8.4E-01	-0.01	9.6E-01	-0.12	5.5E-01	0.24	2.3E-01
ENSCAFG0000002812	ENSCAFG0000002812	grey	EC_M10	0.23	2.7E-01	-0.04	8.5E-01	0.06	7.7E-01	-0.12	5.6E-01	-0.22	2.9E-01	-0.05	7.9E-01	-0.02	9.3E-01	-0.10	6.3E-01	0.11	6.0E-01	0.14	4.9E-01
ENSCAFG0000000377	NRP1	turquoise	EC_M6	0.23	2.7E-01	0.52	6.6E-03	-0.02	9.1E-01	0.18	3.7E-01	-0.38	5.2E-02	-0.08	7.0E-01	-0.06	7.7E-01	0.11	6.0E-01	0.56	3.2E-03	0.27	1.8E-01
ENSCAFG0000000972	CYFIP1	grey	EC_M10	0.23	2.7E-01	-0.21	2.9E-01	-0.44	2.3E-02	-0.17	4.1E-01	0.48	1.3E-02	0.00	9.8E-01	-0.22	2.9E-01	-0.02	9.3E-01	-0.20	3.3E-01	-0.58	1.7E-03
ENSCAFG0000000064	AZIN1	darkgreen	EC_M4	0.23	2.7E-01	-0.09	6.7E-01	-0.81	4.4E-07	-0.09	6.7E-01	0.81	4.5E-07	0.36	7.2E-02	0.02	9.4E-01	-0.12	5.7E-01	-0.05	8.1E-01	-0.93	9.1E-12
ENSCAFG0000001416	ENSCAFG0000001416	grey	EC_M10	0.23	2.7E-01	0.19	3.4E-01	-0.46	1.8E-02	-0.08	6.9E-01	0.34	9.3E-02	-0.13	5.4E-01	-0.11	5.9E-01	-0.02	9.1E-01	-0.03	8.7E-01	-0.42	3.3E-02
ENSCAFG0000002917	ENSCAFG0000002917	grey	EC_M10	0.23	2.7E-01	-0.26	1.9E-01	-0.10	6.4E-01	0.29	1.5E-01	0.00	1.0E+00	0.04	8.4E-01	-0.33	9.9E-02	-0.02	9.3E-01	-0.18	3.7E-01	-0.12	5.5E-01
ENSCAFG0000001842	ITGA2	darkgreen	EC_M4	0.23	2.7E-01	-0.02	9.3E-01	-0.81	6.6E-07	-0.17	4.1E-01	0.74	1.4E-05	0.11	5.8E-01	-0.11	6.0E-01	0.09	6.6E-01	0.07	7.4E-01	-0.86	2.0E-08
ENSCAFG0000001868	CPLANE1	grey	EC_M10	0.23	2.7E-01	-0.16	4.3E-01	0.00	9.9E-01	0.00	9.9E-01	-0.17	4.1E-01	-0.24	2.3E-01	-0.26	2.0E-01	-0.10	6.1E-01	-0.45	2.0E-02	0.05	8.2E-01
ENSCAFG0000002622	ENSCAFG0000002622	turquoise	EC_M6	0.23	2.7E-01	0.43	2.8E-02	-0.12	5.5E-01	0.08	7.1E-01	-0.20	3.2E-01	0.04	8.6E-01	0.11	5.8E-01	0.01	9.7E-01	0.39	4.9E-02	0.11	5.8E-01
ENSCAFG0000000121	HSD17B3	turquoise	EC_M6	0.23	2.7E-01	0.44	2.3E-02	-0.16	4.3E-01	0.48	1.4E-02	-0.21	3.1E-01	-0.17	4.0E-01	-0.17	4.0E-01	-0.13	5.2E-01	-0.23	2.6E-01	0.06	7.7E-01
ENSCAFG0000001755	S100A5	turquoise	EC_M6	0.23	2.7E-01	0.63	6.2E-04	0.15	4.7E-01	0.02	9.3E-01	-0.59	1.6E-03	-0.35	8.2E-02	0.18	3.8E-01	-0.04	8.5E-01	-0.01	9.7E-01	0.48	1.2E-02
ENSCAFG0000000261	SMAP1	grey	EC_M10	0.23	2.7E-01	-0.22	2.9E-01	-0.35	7.9E-02	0.38	5.8E-02	0.26	2.0E-01	0.19	3.5E-01	-0.38	5.9E-02	-0.31	1.2E-01	0.08	7.0E-01	-0.37	5.9E-02
ENSCAFG0000000751	IMMP1L	grey	EC_M10	0.23	2.7E-01	0.24	2.4E-01	-0.69	1.0E-04	-0.12	5.4E-01	0.55	3.4E-03	0.35	8.3E-02	0.07	7.4E-01	-0.04	8.3E-01	0.09	6.7E-01	-0.64	4.2E-04
ENSCAFG0000001656	CWC25	grey	EC_M10	0.23	2.7E-01	0.34	8.5E-02	0.10	6.3E-01	0.38	5.5E-02	-0.52	6.7E-03	-0.10	6.3E-01	0.02	9.2E-01	-0.19	3.4E-01	-0.27	1.8E-01	0.39	4.6E-02
ENSCAFG0000003102	CDC26	grey	EC_M10	0.23	2.7E-01	-0.16	4.3E-01	0.34	9.2E-02	0.30	1.3E-01	-0.53	5.5E-03	-0.04	8.4E-01	0.04	8.4E-01	-0.33	9.9E-02	-0.30	1.4E-01	0.41	3.8E-02
ENSCAFG0000001097	SATB2	darkgreen	EC_M4	0.23	2.7E-01	-0.26	1.9E-01	-0.58	2.1E-03	-0.28	1.6E-01	0.56	2.8E-03	0.06	7.8E-01	0.05	8.0E-01	0.23	2.7E-01	0.00	9.9E-01	-0.72	3.5E-05
ENSCAFG0000000983	CD101	cyan	EC_M2	0.23	2.7E-01	-0.40	4.0E-02	0.17	4.1E-01	0.20	3.4E-01	-0.23	2.6E-01	0.39	5.2E-02	0.11	5.8E-01	-0.18	3.7E-01	-0.30	1.3E-01	0.10	6.4E-01
ENSCAFG0000003005	ENSCAFG0000003005	grey	EC_M10	0.23	2.7E-01	0.10	6.2E-01	-0.38	5.8E-02	0.00	1.0E+00	0.21	3.0E-01	-0.14	4.8E-01	0.41	3.9E-02	-0.04	8.4E-01	-0.14	4.8E-01	-0.34	8.7E-02
ENSCAFG0000000002	TIMM21	grey	EC_M10	0.23	2.7E-01	-0.25	2.2E-01	-0.06	7.7E-01	-0.19	3.6E-01	-0.02	9.4E-01	0.38	5.8E-02	-0.27	1.8E-01	-0.13	5.3E-01	-0.34	9.4E-02	-0.14	5.1E-01
ENSCAFG0000001351	ENSCAFG0000001351	grey	EC_M10	0.23	2.7E-01	-0.55	3.9E-03	0.03	8.8E-01	-0.08	7.0E-01	0.10	6.4E-01	-0.03	8.9E-01	-0.36	7.5E-02	0.09	6.6E-01	-0.03	8.7E-01	-0.17	4.0E-01
ENSCAFG0000000721	ASXL1	grey	EC_M10	0.23	2.7E-01	0.38	5.7E-02	0.27	1.9E-01	0.15	4.6E-01	-0.66	2.1E-04	0.05	8.1E-01	-0.02	9.3E-01	-0.19	3.4E-01	0.19	3.4E-01	0.51	7.5E-03
ENSCAFG0000002599	ENSCAFG0000002599	grey	EC_M10	0.23	2.7E-01	0.18	3.7E-01	0.25	2.2E-01	0.19	3.6E-01	-0.50	9.4E-03	-0.29	1.6E-01	0.25	2.2E-01	-0.21	3.0E-01	0.08	7.0E-01	0.41	3.9E-02
ENSCAFG0000000371	ENSCAFG0000000371	turquoise	EC_M6	0.23	2.7E-01	0.89	1.5E-09	-0.42	3.0E-02	-0.11	5.8E-01	0.04	8.3E-01	-0.15	4.7E-01	-0.04	8.6E-01	-0.11	6.1E-01	-0.14	4.8E-01	-0.15	4.6E-01
ENSCAFG0000003007	ZNF623	grey	EC_M10	0.23	2.7E-01	0.22	2.7E-01	0.33	9.8E-02	0.30	1.4E-01	-0.67	1.9E-04	0.04	8.6E-01	0.00	1.0E+00	-0.34	9.3E-02	0.04	8.5E-01	0.57	2.5E-03
ENSCAFG0000001501	ENSCAFG0000001501	grey	EC_M10	0.22	2.7E-01	0.09	6.5E-01	-0.52	6.5E-03	0.32	1.1E-01	0.40	4.3E-02	0.22	2.9E-01	-0.19	3.5E-01	-0.12	5.5E-01	-0.09	6.6E-01	-0.53	4.9E-03
ENSCAFG0000001518	SELP	turquoise	EC_M6	0.22	2.7E-01	0.94	1.1E-12	-0.39	5.0E-02	-0.12	5.7E-01	-0.06	7.8E-01	-0.06	7.6E-01	-0.14	5.0E-01	-0.15	4.7E-01	0.14	5.0E-01	-0.05	8.0E-01
ENSCAFG0000002621	ENSCAFG0000002621	grey	EC_M10	0.22	2.7E-01	-0.37	6.0E-02	-0.04	8.4E-01	0.14	4.9E-01	-0.09	6.6E-01	0.02	9.4E-01	0.00	9.9E-01	-0.01	9.5E-01	-0.04	8.5E-01	-0.03	8.7E-01
ENSCAFG0000000393	AGBL4	turquoise	EC_M6	0.22	2.7E-01	0.92	3.7E-11	-0.18	3.7E-01	-0.11	6.0E-01	-0.29	1.6E-01	-0.15	4.8E-01	0.05	8.0E-01	-0.14	4.9E-01	0.03	8.8E-01	0.16	4.3E-01
ENSCAFG0000002311	NFIA	grey	EC_M10	0.22	2.7E-01	0.25	2.2E-01	0.20	3.3E-01	0.11	5.8E-01	-0.54	4.4E-03	-0.03	9.0E-01	-0.34	8.8E-02	-0.19	3.4E-01	0.05	8.2E-01	0.38	5.9E-02
ENSCAFG0000000318	CEP68	turquoise	EC_M6	0.22	2.7E-01	0.80	9.1E-07	0.08	7.1E-01	-0.07	7.2E-01	-0.53	5.0E-03	-0.31	1.2E-01	-0.11	5.8E-01	-0.20	3.3E-01	-0.08	7.1E-01	0.45	2.1E-02
ENSCAFG0000000888	FGF5	grey	EC_M10	0.22	2.7E-01	-0.16	4.3E-01	-0.10	6.4E-01	-0.08	6.8E-01	0.00	9.9E-01	0.43	3.0E-02	0.02	9.0E-01	-0.11	5.9E-01	-0.17	4.0E-01	-0.12	5.7E-01
ENSCAFG0000000351	TLR4	turquoise	EC_M6	0.22	2.7E-01	0.88	3.3E-09	-0.11	5.8E-01	-0.08	7.1E-01	-0.34	9.3E-02	-0.19	3.5E-01	0.01	9.6E-01	-0.16	4.2E-01	-0.12	5.6E-01	0.22	2.9E-01
ENSCAFG0000000550	CYTH1	turquoise	EC_M6	0.22	2.7E-01	0.75	1.2E-05	0.10	6.4E-01	0.12	5.5E-01	-0.58	2.0E-03	-0.26	2.0E-01	-0.05	8.3E-01	-0.21	3.2E-01	-0.21	3.0E-01	0.47	1.6E-02
ENSCAFG0000000011	ANKRD52	grey	EC_M10	0.22	2.7E-01	0.22	2.7E-01	-0.73	2.3E-05	-0.07	7.4E-01	0.64	4.1E-04	0.12	5.6E-01	-0.12	5.7E-01	-0.10	6.4E-01	0.21	3.1E-01	-0.71	5.1E-05
ENSCAFG0000002365	ENSCAFG0000002365	grey	EC_M10	0.22	2.7E-01	0.17	4.0E-01	0.26	2.1E-01	0.07	7.3E-01	-0.54	4.2E-03	0.02	9.1E-01	-0.24	2.4E-01	-0.35	8.1E-02	-0.15	4.7E-01	0.38	5.6E-02
ENSCAFG0000000570	TMEM17	turquoise	EC_M6	0.22	2.7E-01	0.91	1.8E-10	-0.29	1.6E-01	-0.16	4.5E-01	-0.16	4.3E-01	-0.02	9.0E-01	-0.15	4.5E-01	-0.05	8.1E-01	-0.13	5.4E-01	0.03	8.8E-01
ENSCAFG0000002483	ZNF654	darkgreen	EC_M4	0.22	2.7E-01	0.27	1.8E-01	-0.64	4.1E-04	-0.19	3.6E-01	0.43	2.9E-02	0.27	1.8E-01	0.08	7.0E-01	0.04	8.3E-01	-0.40	4.3E-02	-0.55	3.9E-03
ENSCAFG0000001887	ENSCAFG0000001887	grey	EC_M10	0.22	2.7E-01	-0.35	7.7E-02	-0.62	7.5E-04	-0.15	4.5E-01	0.65	2.9E-04	0.23	2.6E-01	-0.12	5.6E-01	0.36	7.0E-02	0.15	4.6E-01	-0.77	3.4E-06
ENSCAFG0000002358	ENSCAFG0000002358	grey	EC_M10	0.22	2.7E-01	0.00	9.9E-01	-0.18	3.8E-01	-0.07	7.4E-01	0.06	7.6E-01	-0.13	5.2E-01	-0.09	6.8E-01	-0.03	9.0E-01	-0.16	4.3E-01	-0.19	3.5E-01
ENSCAFG0000001795	RBM41	grey	EC_M10	0.22	2.7E-01	0.31	1.3E-01	-0.37	6.5E-02	0.12	5.5E-01	0.05	8.3E-01	-0.06	7.6E-01	-0.01	9.7E-01	-0.20	3.3E-01	-0.43	2.9E-02	-0.22	2.8E-01
ENSCAFG0000000204	GNG11	turquoise	EC_M6	0.22	2.7E-01	0.77	4.6E-06	-0.52	5.9E-03	-0.32	1.2E-01	0.17	4.1E-01	0.02	9.2E-01	-0.03	8.8E-01	0.02	9.4E-01	0.39	4.8E-02	-0.27	1.9E-01
ENSCAFG0000000716	ZSWIM6	darkgreen	EC_M4	0.22	2.7E-01	0.55	3.7E-03	-0.77	3.3E-06	-0.23	2.5E-01	0.47	1.5E-02	0.18	3.9E-01	0.05	8.1E-01	-0.10	6.4E-01	0.01	9.6E-01	-0.60	1.3E-03
ENSCAFG0000001550	DAAM1	darkgreen	EC_M4	0.22	2.7E-01	0.29	1.5E-01	-0.79	1.3E-06	-0.04	8.5E-01	0.57	2.5E-03	0.20	3.2E-01	-0.11	6.0E-01	0.16	4.5E-01	0.09	6.6E-01	-0.72	3.7E-05
ENSCAFG0000001722	ENSCAFG0000001722	grey	EC_M10	0.22	2.7E-01	0.19	3.4E-01	0.16	4.4E-01	0.07	7.5E-01	-0.51	7.2E-03	-0.01	9.6E-01	-0.13	5.1E-01	0.03	9.0E-01	-0.18	3.8E-01	0.37	6.3E-02
ENSCAFG0000002899	TTC1	darkgreen	EC_M4	0.22	2.7E-01	0.05	8.0E-01	-0.71	5.5E-05	0.02	9.3E-01	0.57	2.5E-03	0.14	4.8E-01	0.09	6.8E-01	-0.13	5.3E-01	-0.27	1.8E-01	-0.69	1.1E-04
ENSCAFG0000001495	ENSCAFG0000001495	grey	EC_M10	0.22	2.7E-01	0.14	5.1E-01	-0.15	4.6E-01	0.31	1.3E-01	-0.10	6.2E-01	0.14	4.8E-01	-0.23	2.6E-01	0.00	9.9E-01	-0.30	1.3E-01	-0.03	8.9E-01
ENSCAFG0000001754	S100A14	turquoise	EC_M6	0.22	2.7E-01	0.57	2.2E-03	0.15	4.7E-01	-0.17	4.0E-01	-0.55	3.8E-03	0.09	6.5E-01	-0.17	4.0E-01	-0.12	5.5E-01	-0.23	2.6E-01	0.45	2.2E-02
ENSCAFG0000001937	THOP1	grey	EC_M10	0.22	2.7E-01	-0.23	2.5E-01	-0.28	1.7E-01	-0.13	5.4E-01	0.37	6.4E-02	0.12	5.5E-01	-0.14	4.8E-01	-0.02	9.0E-01	-0.01	9.5E-01	-0.47	1.5E-02
ENSCAFG0000001479	ENSCAFG0000001479	turquoise	EC_M6	0.22	2.7E-01	0.87	7.2E-09	-0.12	5.5E-01	-0.11	5.8E-01	-0.33	9.6E-02	-0.16	4.2E-01	-0.14	4.9E-01	-0.17	4.0E-01	-0.11	5.8E-01	0.20	3.3E-01
ENSCAFG0000001877	PROCA1	grey	EC_M10	0.22	2.7E-01	0.04	8.4E-01	0.12	5.7E-01	0.11	5.8E-01	-0.33	9.9E-02	0.14	5.0E-01	-0.11	5.9E-01	-0.40	4.1E-02	0.05	8.1E-01	0.22	2.7E-01
ENSCAFG0000000697	EPHB1	turquoise	EC_M6	0.22	2.7E-01	0.86	1.5E-08	-0.16	4.3E-01	0.02	9.1E-01	-0.29	1.5E-01	-0.15	4.6E-01	-0.16	4.5E-01	-0.17	4.0E-01	-0.26	2.1E-01	0.14	4.8E-01
ENSCAFG0000000301	TRIL	grey	EC_M10	0.22	2.7E-01	0.27	1.8E-01	-0.27	1.9E-01	-0.12	5.7E-01	0.05	8.2E-01	0.07	7.3E-01	-0.07	7.2E-01	0.16	4.5E-01	-0.19	3.4E-01	-0.24	2.4E-01
ENSCAFG0000003203	ENSCAFG0000003203	grey	EC_M10	0.22	2.7E-01	-0.32	1.1E-01	0.17	3.9E-01	0.14	4.9E-01	-0.35	8.0E-02	-0.24	2.4E-01	-0.13	5.2E-01	0.10	6.2E-01	-0.01	9.8E-01	0.20	3.2E-01
ENSCAFG0000001789	ENSCAFG0000001789	grey	EC_M10	0.22	2.7E-01	-0.23	2.5E-01	0.61	8.3E-04	0.02	9.3E-01	-0.77	4.4E-06	-0.14	5.0E-01	-0.16	4.4E-01	-0.16	4.4E-01	-0.34	9.3E-02	0.68	1.5E-04
ENSCAFG0000001707	TRMT1	cyan	EC_M2	0.22	2.7E-01	-0.22	2.8E-01	0.55	3.8E-03	0.05	7.9E-01	-0.72	3.2E-05	-0.17	3.9E-01	0.03	8.7E-01	-0.17	4.1E-01	-0.35	8.4E-02	0.56	2.7E-03
ENSCAFG0000003002	ENSCAFG0000003002	grey	EC_M10	0.22	2.7E-01	-0.04	8.4E-01	-0.15	4.6E-01	-0.09	6.5E-01	-0.01	9.4E-01	-0.03	8.8E-01	-0.04	8.4E-01	-0.07	7.4E-01	-0.06	7.9E-01	-0.12	5.6E-01
ENSCAFG0000001215	ARSJ	grey	EC_M10	0.22	2.7E-01	-0.63	5.7E-04	-0.25	2.1E-01	0.03	8.7E-01	0.32	1.1E-01	0.28	1.6E-01	-0.07	7.2E-01	0.15	4.7E-01	-0.30	1.4E-01	-0.50	8.8E-03
ENSCAFG0000001153	PTPN1	grey	EC_M10	0.22	2.8E-01	0.08	7.0E-01	-0.23	2.7E-01	-0.21	2.9E-01	0.05	8.0E-01	-0.05	7.9E-01	0.28	1.6E-01	-0.03	8.7E-01	0.31	1.3E-01	-0.16	4.2E-01
ENSCAFG0000002995	PKIB	grey	EC_M10	0.22	2.8E-01	0.30	1.4E-01	-0.42	3.4E-02	-0.25	2.1E-01	0.21	3.1E-01	-0.26	2.0E-01	-0.07	7.5E-01	0.23	2.6E-01	0.59	1.5E-03	-0.29	1.5E-01
ENSCAFG0000001963	F8	turquoise	EC_M6	0.22	2.8E-01	0.91	1.2E-10	-0.23	2.5E-01	0.06	7.7E-01	-0.25	2.1E-01	-0.13	5.2E-01	-0.15	4.6E-01	-0.18	3.8E-01	0.09	6.7E-01	0.15	4.7E-01
ENSCAFG0000000697	LYPLA1	grey	EC_M10	0.22	2.8E-01	-0.26	2.0E-01	-0.17	4.1E-01	-0.22	2.9E-01	0.18	3.7E-01	0.13	5.2E-01	-0.11	5.9E-01	0.15	4.6E-01	-0.19	3.4E-01	-0.27	1.8E-01
ENSCAFG0000000677	ENSCAFG0000000677	grey	EC_M10	0.22	2.8E-01	-0.21	3.0E-01	-0.02	9.3E-01	-0.10	6.1E-01	-0.09	6.5E-01	-0.10	6.4E-01	-0.14	5.1E-01	-0.03	9.0E-01	-0.07	7.2E-01	-0.06	7.7E-01
ENSCAFG0000000495	PAK1	darkgreen	EC_M4	0.22	2.8E-01	-0.18	3.7E-01	-0.58	2.1E-03	-0.16	4.3E-01	0.56	2.8E-03	0.20	3.4E-01	0.01	9.8E-01	0.07	7.3E-01	0.11	5.8E-01	-0.73	2.5E-05
ENSCAFG0000001795	TNIP1	turquoise	EC_M6	0.22	2.8E-01	0.94	1.3E-12	-0.20	3.3E-01	-0.10	6.1E-01	-0.26	1.9E-01	-0.21	3.1E-01	-0.13	5.4E-01	-0.04	8.6E-01	0.08	7.1E-01	0.16	4.3E-01
ENSCAFG0000000288	KLK13	grey	EC_M10	0.22	2.8E-01	-0.05	8.3E-01	-0.25	2.2E-01	-0.16	4.3E-01	0.18	3.8E-01	-0.19	3.5E-01	-0.08	7.0E-01	0.09	6.7E-01	0.58	2.0E-03	-0.25	2.2E-01
ENSCAFG0000001281	JAML	grey	EC_M10	0.22	2.8E-01	0.32	1.2E-01	-0.07	7.3E-01	-0.19	3.5E-01	-0.20	3.3E-01	-0.15	4.7E-01	-0.07	7.4E-01	-0.19	3.6E-01	0.33	1.0E-01	0.13	5.1E-01
ENSCAFG0000001893	GIT1	turquoise	EC_M6	0.22	2.8E-01	0.72	4.0E-05	0.21	3.0E-01	0.09	6.7E-01	-0.67	1.6E-04	-0.35	8.3E-02	-0.13	5.2E-01	-0.02	9.3E-01	-0.01	9.6E-01	0.56	3.1E-03
ENSCAFG0000000988	ENSCAFG0000000988	grey	EC_M10	0.22	2.8E-01	-0.18	3.9E-01	0.03	8.7E-01	-0.21	3.1E-01	-0.18	3.8E-01	-0.13	5.2E-01	-0.08	6.9E-01	-0.12	5.5E-01	-0.14	4.8E-01	0.02	9.0E-01
ENSCAFG0000000120	CDC23	grey	EC_M10	0.22	2.8E-01	-0.02	9.0E-01	-0.33	1.0E-01	0.20	3.3E-01	0.29	1.6E-01	0.20	3.2E-01	-0.14	4.9E-01	0.09	6.5E-01	0.01	9.6E-01	-0.36	7.5E-02
ENSCAFG0000000171	TEK	turquoise	EC_M6	0.22	2.8E-01	0.89	1.4E-09	-0.22	2.9E-01	-0.07	7.2E-01	-0.23	2.6E-01	-0.18	3.8E-01	-0.16	4.5E-01	-0.06	7.7E-01	0.18	3.8E-01	0.10	6.4E-01
ENSCAFG0000000760	COPB2	darkgreen	EC_M4	0.22	2.8E-01	0.03	8.9E-01	-0.86	1.4E-08	-0.17	4.2E-01	0.79	2.0E-06	0.36	6.8E-02	-0.02	9.2E-01	0.05	8.2E-01	-0.14	5.1E-01	-0.93	1.3E-11
ENSCAFG0000000937	DBX2	grey	EC_M10	0.22	2.8E-01	-0.13	5.2E-01	-0.10	6.4E-01	-0.12	5.5E-01	0.00	9.8E-01	-0.06	7.9E-01	-0.05	8.0E-01	-0.10	6.4E-01	0.02	9.4E-01	-0.10	6.3E-01
ENSCAFG0000003142	PRPSAP1	grey	EC_M10	0.22	2.8E-01	-0.32	1.1E-01	0.33	1.0E-01	0.22	2.8E-01	-0.45	2.3E-02	-0.01	9.7E-01	0.05	8.1E-01	-0.41	4.0E-02	-0.28	1.7E-01	0.31	1.2E-01
ENSCAFG0000002834	ENSCAFG0000002834	cyan	EC_M2	0.22	2.8E-01	-0.29	1.6E-01	0.28	1.7E-01	0.04	8.5E-01	-0.41	3.7E-02	-0.16	4.2E-01	0.05	8.2E-01	-0.21	3.0E-01	-0.20	3.2E-01	0.27	1.8E-01
ENSCAFG0000001484	SKIL	darkgreen	EC_M4	0.22	2.8E-01	0.47	1.6E-02	-0.80	7.4E-07	-0.18	3.8E-01	0.54	4.6E-03	0.29	1.6E-01	0.04	8.6E-01	-0.03	9.0E-01	-0.17	4.0E-01	-0.69	1.1E-04
ENSCAFG0000003104	ZNF84	cyan	EC_M2	0.22	2.8E-01	0.29	1.5E-01	0.22	2.9E-01	0.27	1.8E-01	-0.65	3.4E-04	-0.28	1.6E-01	-0.19	3.6E-01	-0.08	7.1E-01	0.06	7.8E-01	0.51	8.4E-03
ENSCAFG0000000245	ENSCAFG0000000245	grey	EC_M10	0.22	2.8E-01	0.15	4.7E-01	-0.24	2.3E-01	0.38	5.5E-02	-0.03	8.8E-01	0.18	3.7E-01	0.03	8.8E-01	-0.04	8.3E-01	-0.44	2.6E-02	-0.10	6.2E-01
ENSCAFG0000000860	CTSO	turquoise	EC_M6	0.22	2.8E-01	0.44	2.6E-02	-0.12	5.5E-01	0.24	2.4E-01	-0.24	2.4E-01	0.03	8.9E-01	-0.09	6.6E-01	-0.26	2.0E-01	0.47	1.5E-02	0.15	4.6E-01
ENSCAFG0000001093	RFTN2	turquoise	EC_M6	0.22	2.8E-01	0.76	5.9E-06	-0.25	2.3E-01	0.08	7.1E-01	-0.19	3.5E-01	-0.12	5.7E-01	-0.03	8.9E-01	0.19	3.5E-01	0.14	5.0E-01	0.08	6.9E-01
ENSCAFG0000002994	ENSCAFG0000002994	grey	EC_M10	0.22	2.8E-01	-0.44	2.4E-02	-0.03	9.0E-01	0.08	7.1E-01	-0.04	8.6E-01	0.21	3.0E-01	0.04	8.3E-01	-0.17	4.1E-01	-0.24	2.4E-01	-0.14	4.8E-01
ENSCAFG0000000590	HARS2	turquoise	EC_M6	0.22	2.8E-01	0.40	4.6E-02	-0.13	5.3E-01	-0.10	6.4E-01	-0.20	3.2E-01	0.17	4.0E-01	0.00	1.0E+00	-0.13	5.2E-01	0.11	5.9E-01	0.15	4.7E-01
ENSCAFG0000002900	ENSCAFG0000002900	grey	EC_M10	0.22	2.8E-01	0.17	4.1E-01	-0.39	5.1E-02	0.36	7.1E-02	0.22	2.9E-01	0.31	1.2E-01	0.06	7.7E-01	-0.25	2.2E-01	0.04	8.4E-01	-0.32	1.1E-01
ENSCAFG0000002035	CARMIL2	turquoise	EC_M6	0.22	2.8E-01	0.57	2.1E-03	-0.31	1.2E-01	-0.20	3.2E-01	0.02	9.0E-01	0.09	6.5E-01	0.19	3.5E-01	-0.20	3.2E-01	0.01	9.6E-01	-0.12	5.5E-01
ENSCAFG0000000216	MAP4K4	grey	EC_M10	0.22	2.8E-01	0.02	9.2E-01	0.47	1.6E-02	0.16	4.2E-01	-0.72	3.2E-05	-0.09	6.8E-01	-0.02	9.1E-01	-0.18	3.7E-01	-0.36	7.3E-02	0.62	7.2E-04
ENSCAFG0000002347	RPS27A	cyan	EC_M2	0.22	2.8E-01	-0.40	4.3E-02	0.47	1.6E-02	0.06	7.6E-01	-0.54	4.1E-03	0.01	9.6E-01	0.02	9.2E-01	-0.01	9.6E-01	-0.41	3.7E-02	0.39	5.2E-02
ENSCAFG0000000000	PARD6G	turquoise	EC_M6	0.22	2.8E-01	0.85	4.6E-08	-0.33	1.0E-01	-0.27	1.8E-01	-0.03	8.8E-01	-0.09	6.5E-01	0.04	8.5E-01	-0.11	6.0E-01	-0.10	6.4E-01	-0.07	7.2E-01
ENSCAFG0000001835	ENSCAFG0000001835	grey	EC_M10	0.22	2.8E-01	-0.07	7.5E-01	-0.34	8.5E-02	-0.18	3.7E-01	0.35	8.0E-02	0.35	8.0E-02	-0.04	8.5E-01	0.05	7.9E-01	-0.46	1.7E-02	-0.48	1.3E-02
ENSCAFG0000002902	ZNF226	grey	EC_M10	0.22	2.8E-01	0.21	3.0E-01	0.03	8.9E-01	0.35	8.0E-02	-0.30	1.4E-01	0.01	9.6E-01	0.06	7.7E-01	0.20	3.3E-01	-0.08	7.0E-01	0.22	2.9E-01
ENSCAFG0000001618	KLF3	cyan	EC_M2	0.22	2.8E-01	0.37	6.6E-02	0.34	8.9E-02	0.25	2.1E-01	-0.73	2.1E-05	-0.10	6.4E-01	-0.44	2.6E-02	-0.13	5.4E-01	-0.30	1.4E-01	0.61	8.7E-04
ENSCAFG0000000638	TFDP1	grey	EC_M10	0.22	2.8E-01	-0.03	9.0E-01	-0.56	2.8E-03	-0.03	8.8E-01	0.58	1.7E-03	0.31	1.2E-01	-0.31	1.2E-01	-0.03	9.0E-01	-0.03	8.7E-01	-0.65	2.9E-04
ENSCAFG0000000014	TCF4	turquoise	EC_M6	0.22	2.8E-01	0.89	1.7E-09	-0.30	1.4E-01	-0.12	5.6E-01	-0.13	5.2E-01	-0.17	4.1E-01	0.07	7.4E-01	0.03	8.9E-01	-0.09	6.5E-01	0.02	9.4E-01
ENSCAFG0000000075	GRAMD4	turquoise	EC_M6	0.22	2.8E-01	0.82	2.9E-07	-0.11	5.8E-01	-0.11	5.9E-01	-0.32	1.1E-01	-0.14	4.9E-01	0.00	1.0E+00	-0.22	2.8E-01	-0.04	8.4E-01	0.23	2.7E-01
ENSCAFG0000002881	ZBED5	grey	EC_M10	0.22	2.8E-01	0.05	8.1E-01	-0.12	5.8E-01	0.28	1.7E-01	-0.22	2.8E-01	0.09	6.7E-01	-0.15	4.6E-01	-0.04	8.5E-01	0.11	5.9E-01	0.08	6.8E-01
ENSCAFG0000003257	ZFP69B	grey	EC_M10	0.22	2.8E-01	0.19	3.6E-01	-0.20	3.4E-01	-0.01	9.7E-01	-0.04	8.3E-01	0.11	6.0E-01	-0.02	9.3E-01	0.12	5.7E-01	-0.38	5.7E-02	-0.09	6.6E-01
ENSCAFG0000001273	LRCH3	grey	EC_M10	0.22	2.8E-01	0.18	3.9E-01	-0.53	5.2E-03	-0.06	7.7E-01	0.27	1.8E-01	0.11	5.8E-01	0.00	9.8E-01	-0.04	8.5E-01	0.21	3.1E-01	-0.39	5.1E-02
ENSCAFG0000002992	SPDYA	grey	EC_M10	0.22	2.8E-01	0.20	3.2E-01	-0.51	7.5E-03	-0.22	2.8E-01	0.41	3.5E-02	0.18	3.7E-01	-0.21	3.0E-01	-0.24	2.4E-01	-0.15	4.8E-01	-0.46	1.8E-02
ENSCAFG0000003149	ENSCAFG0000003149	grey	EC_M10	0.22	2.8E-01	0.10	6.4E-01	-0.08	7.0E-01	0.22	2.8E-01	-0.18	3.7E-01	0.00	1.0E+00	0.05	8.0E-01	-0.18	3.8E-01	-0.09	6.6E-01	0.10	6.3E-01

ENSCAFG0000002867	ZNF23	grey	EC_M10	0.22	2.8E-01	-0.04	8.6E-01	0.17	4.2E-01	0.15	4.6E-01	-0.40	4.4E-02	0.01	9.7E-01	-0.12	5.6E-01	-0.07	7.4E-01	0.00	1.0E+00	0.27	1.9E-01
ENSCAFG0000001630	EFHD2	grey	EC_M10	0.22	2.8E-01	0.04	8.5E-01	0.50	8.6E-03	0.18	3.7E-01	-0.71	5.4E-05	-0.39	5.2E-02	-0.07	7.3E-01	0.07	7.2E-01	-0.43	2.7E-02	0.61	1.0E-03
ENSCAFG0000003067	ENSCAFG0000003067	grey	EC_M10	0.22	2.8E-01	0.34	9.0E-02	-0.60	1.2E-03	0.01	9.5E-01	0.45	2.2E-02	0.25	2.2E-01	-0.22	2.7E-01	0.35	8.1E-02	0.05	8.0E-01	-0.57	2.3E-03
ENSCAFG0000001813	TAF4B	grey	EC_M10	0.22	2.8E-01	-0.55	3.8E-03	0.43	2.8E-02	0.13	5.4E-01	-0.44	2.5E-02	-0.01	9.4E-01	-0.17	4.1E-01	0.06	7.6E-01	-0.22	2.8E-01	0.32	1.1E-01
ENSCAFG0000000532	GALNT14	grey	EC_M10	0.22	2.8E-01	-0.15	4.5E-01	-0.48	1.2E-02	0.08	6.9E-01	0.41	3.6E-02	0.39	5.2E-02	-0.16	4.3E-01	-0.16	4.4E-01	-0.12	5.5E-01	-0.54	4.2E-03
ENSCAFG0000000425	PRTFDC1	turquoise	EC_M6	0.22	2.8E-01	0.53	4.9E-03	-0.18	3.9E-01	-0.02	9.1E-01	-0.18	3.9E-01	-0.03	9.0E-01	-0.50	9.7E-03	-0.02	9.1E-01	-0.03	8.9E-01	0.10	6.3E-01
ENSCAFG0000001815	FBXO22	darkgreen	EC_M4	0.22	2.8E-01	-0.14	5.1E-01	-0.61	1.0E-03	-0.02	9.4E-01	0.59	1.5E-03	0.22	2.8E-01	-0.10	6.1E-01	0.05	8.0E-01	-0.01	9.6E-01	-0.70	7.6E-05
ENSCAFG0000001328	SMS	darkgreen	EC_M4	0.22	2.8E-01	-0.40	4.4E-02	-0.49	1.0E-02	0.18	3.7E-01	0.56	2.8E-03	0.25	2.1E-01	-0.18	3.7E-01	-0.14	5.1E-01	-0.40	4.0E-02	-0.70	6.4E-05
ENSCAFG0000000916	ENSCAFG0000000916	grey	EC_M10	0.22	2.8E-01	-0.16	4.3E-01	-0.25	2.2E-01	0.26	1.9E-01	0.12	5.5E-01	0.30	1.3E-01	-0.15	4.7E-01	-0.14	4.9E-01	-0.15	4.7E-01	-0.26	2.0E-01
ENSCAFG0000000486	DPH2	grey	EC_M10	0.22	2.8E-01	0.29	1.5E-01	0.00	1.0E+00	0.20	3.2E-01	-0.24	2.3E-01	-0.15	4.6E-01	0.16	4.2E-01	-0.19	3.6E-01	0.06	7.9E-01	0.09	6.7E-01
ENSCAFG0000002450	MPHOSPH6	grey	EC_M10	0.22	2.8E-01	0.24	2.3E-01	-0.60	1.1E-03	-0.29	1.5E-01	0.44	2.3E-02	0.38	5.7E-02	0.22	2.8E-01	-0.42	3.1E-02	-0.19	3.4E-01	-0.56	3.2E-03
ENSCAFG0000001636	NVL	grey	EC_M10	0.22	2.8E-01	-0.63	5.2E-04	0.40	4.2E-02	0.19	3.5E-01	-0.39	4.7E-02	0.10	6.4E-01	-0.17	4.1E-01	-0.01	9.4E-01	-0.06	7.6E-01	0.27	1.8E-01
ENSCAFG0000001954	JPT2	grey	EC_M10	0.22	2.8E-01	-0.25	2.1E-01	-0.36	7.4E-02	-0.34	8.6E-02	0.41	3.7E-02	0.11	5.9E-01	-0.10	6.2E-01	-0.13	5.2E-01	0.02	9.1E-01	-0.51	8.1E-03
ENSCAFG0000000194	LRRD1	grey	EC_M10	0.22	2.8E-01	0.13	5.4E-01	-0.49	1.1E-02	-0.10	6.2E-01	0.33	9.6E-02	0.31	1.3E-01	0.25	2.2E-01	-0.15	4.8E-01	-0.25	2.1E-01	-0.44	2.4E-02
ENSCAFG0000001733	ENSCAFG0000001733	grey	EC_M10	0.22	2.8E-01	0.13	5.4E-01	-0.12	5.5E-01	0.19	3.5E-01	-0.13	5.2E-01	0.07	7.3E-01	-0.02	9.2E-01	0.11	5.9E-01	-0.24	2.5E-01	-0.01	9.7E-01
ENSCAFG0000000047	CCHCR1	grey	EC_M10	0.22	2.8E-01	0.24	2.4E-01	0.27	1.8E-01	0.20	3.2E-01	-0.60	1.3E-03	-0.15	4.7E-01	0.06	7.8E-01	0.22	2.8E-01	-0.01	9.7E-01	0.48	1.3E-02
ENSCAFG0000001455	TPI1	darkgreen	EC_M4	0.22	2.8E-01	0.33	9.5E-02	-0.77	3.4E-06	-0.33	9.5E-02	0.64	4.6E-04	0.07	7.2E-01	0.18	3.8E-01	0.20	3.3E-01	0.05	8.1E-01	-0.75	9.5E-06
ENSCAFG0000000401	ATXN7L1	cyan	EC_M2	0.22	2.8E-01	-0.03	8.9E-01	0.51	7.2E-03	0.21	3.0E-01	-0.82	3.4E-07	-0.07	7.5E-01	-0.31	1.2E-01	0.02	9.1E-01	0.10	6.2E-01	0.68	1.5E-04
ENSCAFG0000001703	LRRC59	grey	EC_M10	0.22	2.8E-01	0.01	9.8E-01	-0.69	1.0E-04	-0.20	3.3E-01	0.69	9.2E-05	0.27	1.9E-01	0.05	8.1E-01	-0.10	6.3E-01	-0.05	8.1E-01	-0.82	2.6E-07
ENSCAFG0000000261	PRKCE	grey	EC_M10	0.22	2.8E-01	0.23	2.5E-01	-0.45	2.0E-02	0.14	5.0E-01	0.17	4.2E-01	0.23	2.6E-01	-0.34	9.4E-02	0.13	5.4E-01	0.06	7.8E-01	-0.35	8.2E-02
ENSCAFG0000000754	ENSCAFG0000000754	turquoise	EC_M6	0.22	2.8E-01	0.43	2.8E-02	-0.31	1.2E-01	0.51	7.6E-03	0.03	8.7E-01	-0.16	4.2E-01	-0.18	3.9E-01	-0.13	5.4E-01	-0.22	2.9E-01	-0.15	4.7E-01
ENSCAFG0000002534	RELN	turquoise	EC_M6	0.22	2.8E-01	0.92	4.3E-11	-0.22	2.8E-01	-0.08	7.1E-01	-0.24	2.4E-01	-0.10	6.4E-01	-0.16	4.3E-01	-0.09	6.5E-01	0.28	1.7E-01	0.13	5.1E-01
ENSCAFG0000001078	ENSCAFG0000001078	grey	EC_M10	0.22	2.8E-01	0.25	2.1E-01	-0.45	2.2E-02	0.23	2.7E-01	0.21	3.0E-01	0.03	8.8E-01	-0.29	1.5E-01	-0.29	1.5E-01	-0.01	9.5E-01	-0.35	8.4E-02
ENSCAFG0000000153	TSPAN33	violet	EC_M7	0.22	2.8E-01	-0.05	8.2E-01	-0.21	3.1E-01	-0.08	7.0E-01	0.08	7.0E-01	0.84	8.4E-08	-0.06	7.7E-01	-0.09	6.7E-01	-0.14	4.9E-01	-0.23	2.7E-01
ENSCAFG0000001359	ENSCAFG0000001359	violet	EC_M7	0.22	2.8E-01	-0.05	8.2E-01	-0.21	3.1E-01	-0.08	7.0E-01	0.08	7.0E-01	0.84	8.4E-08	-0.06	7.7E-01	-0.09	6.7E-01	-0.14	4.9E-01	-0.23	2.7E-01
ENSCAFG0000000715	RAB2A	darkgreen	EC_M4	0.22	2.8E-01	0.27	1.8E-01	-0.82	3.2E-07	-0.10	6.1E-01	0.63	5.6E-04	0.44	2.4E-02	0.08	6.9E-01	-0.05	8.1E-01	-0.15	4.5E-01	-0.77	4.4E-06
ENSCAFG0000000257	HIVEP3	darkgreen	EC_M4	0.22	2.8E-01	-0.14	5.0E-01	-0.71	4.2E-05	-0.08	6.8E-01	0.73	2.0E-05	0.25	2.1E-01	0.07	7.4E-01	0.01	9.6E-01	-0.30	1.4E-01	-0.89	1.6E-09
ENSCAFG0000001766	BTK	grey	EC_M10	0.22	2.8E-01	-0.12	5.7E-01	-0.56	2.9E-03	0.06	7.7E-01	0.53	5.6E-03	0.39	4.6E-02	-0.13	5.2E-01	0.05	8.0E-01	0.08	7.1E-01	-0.69	9.9E-05
ENSCAFG0000000772	SLC4A8	grey	EC_M10	0.22	2.8E-01	0.22	2.9E-01	-0.50	9.0E-03	-0.27	1.8E-01	0.37	6.6E-02	0.10	6.4E-01	-0.15	4.6E-01	-0.14	5.0E-01	-0.12	5.5E-01	-0.46	1.8E-02
ENSCAFG0000000711	CCM2L	turquoise	EC_M6	0.22	2.8E-01	0.94	2.0E-12	-0.27	1.8E-01	-0.11	6.0E-01	-0.18	3.8E-01	-0.04	8.6E-01	-0.13	5.3E-01	-0.17	4.1E-01	0.04	8.3E-01	0.05	8.0E-01
ENSCAFG0000003011	DDIT3	grey	EC_M10	0.22	2.8E-01	0.27	1.9E-01	-0.70	5.9E-05	0.18	3.9E-01	0.50	1.0E-02	0.21	3.0E-01	-0.13	5.3E-01	-0.01	9.5E-01	-0.08	7.0E-01	-0.61	8.3E-04
ENSCAFG0000001097	TIMMDC1	grey	EC_M10	0.22	2.9E-01	-0.28	1.6E-01	-0.35	7.6E-02	-0.14	5.0E-01	0.38	5.9E-02	0.15	4.7E-01	-0.38	5.9E-02	0.05	8.1E-01	0.35	8.3E-02	-0.48	1.2E-02
ENSCAFG0000000664	ENSCAFG0000000664	grey	EC_M10	0.22	2.9E-01	0.17	4.0E-01	-0.14	4.9E-01	-0.04	8.5E-01	-0.03	8.7E-01	-0.08	6.8E-01	-0.13	5.2E-01	-0.09	6.5E-01	-0.14	4.9E-01	-0.07	7.3E-01
ENSCAFG0000001677	ENSCAFG0000001677	grey	EC_M10	0.22	2.9E-01	0.17	4.0E-01	-0.14	4.9E-01	-0.04	8.5E-01	-0.03	8.7E-01	-0.08	6.8E-01	-0.13	5.2E-01	-0.09	6.5E-01	-0.14	4.9E-01	-0.07	7.3E-01
ENSCAFG0000000568	ENSCAFG0000000568	grey	EC_M10	0.22	2.9E-01	0.39	4.6E-02	-0.58	1.7E-03	-0.22	2.8E-01	0.34	9.3E-02	0.37	6.5E-02	-0.24	2.5E-01	-0.26	2.0E-01	0.05	8.2E-01	-0.44	2.4E-02
ENSCAFG0000000924	ENSCAFG0000000924	grey	EC_M10	0.22	2.9E-01	-0.16	4.2E-01	0.24	2.4E-01	0.24	2.4E-01	-0.42	3.3E-02	0.02	9.0E-01	0.07	7.4E-01	0.06	7.6E-01	-0.06	7.7E-01	0.30	1.4E-01
ENSCAFG0000001316	NPL	turquoise	EC_M6	0.22	2.9E-01	0.75	8.8E-06	-0.02	9.1E-01	-0.03	8.7E-01	-0.40	4.2E-02	0.01	9.7E-01	-0.11	6.0E-01	-0.44	2.4E-02	-0.16	4.4E-01	0.31	1.3E-01
ENSCAFG0000001182	TAGLN2	darkgreen	EC_M4	0.22	2.9E-01	0.11	5.9E-01	-0.59	1.4E-03	-0.06	7.8E-01	0.48	1.3E-02	0.09	6.5E-01	-0.05	8.2E-01	0.09	6.7E-01	0.03	8.8E-01	-0.65	2.8E-04
ENSCAFG0000002031	SLC7A6OS	grey	EC_M10	0.22	2.9E-01	0.11	5.9E-01	-0.21	3.0E-01	0.22	2.8E-01	0.04	8.3E-01	0.01	9.7E-01	0.07	7.2E-01	0.04	8.3E-01	-0.17	4.1E-01	-0.10	6.3E-01
ENSCAFG0000001625	MCC	turquoise	EC_M6	0.22	2.9E-01	0.46	1.8E-02	-0.45	2.2E-02	0.15	4.5E-01	0.15	4.7E-01	0.21	3.1E-01	-0.09	6.8E-01	-0.15	4.7E-01	-0.25	2.2E-01	-0.34	9.2E-02
ENSCAFG0000000997	ENSCAFG0000000997	grey	EC_M10	0.22	2.9E-01	-0.02	9.3E-01	-0.34	8.6E-02	0.26	2.0E-01	0.10	6.1E-01	0.24	2.4E-01	-0.03	8.7E-01	-0.11	5.9E-01	0.13	5.4E-01	-0.24	2.3E-01
ENSCAFG0000001801	SIN3A	cyan	EC_M2	0.22	2.9E-01	0.37	6.6E-02	0.42	3.2E-02	0.07	7.4E-01	-0.82	2.6E-07	-0.03	8.8E-01	-0.02	9.4E-01	-0.06	7.9E-01	0.17	3.9E-01	0.70	6.6E-05
ENSCAFG0000001885	SEZ6	grey	EC_M10	0.22	2.9E-01	-0.12	5.7E-01	-0.07	7.2E-01	0.04	8.5E-01	-0.04	8.4E-01	0.16	4.3E-01	0.36	6.8E-02	-0.43	3.0E-02	0.28	1.6E-01	-0.07	7.2E-01
ENSCAFG0000000114	AKAP7	darkgrey	EC_M8	0.22	2.9E-01	-0.19	3.6E-01	0.31	1.2E-01	0.37	6.5E-02	-0.55	3.9E-03	-0.33	9.7E-02	-0.03	9.0E-01	0.09	6.7E-01	-0.11	6.0E-01	0.44	2.6E-02
ENSCAFG0000001460	FERMT3	grey	EC_M10	0.22	2.9E-01	0.32	1.1E-01	-0.08	7.1E-01	-0.12	5.7E-01	-0.22	2.9E-01	-0.04	8.3E-01	-0.12	5.6E-01	-0.12	5.5E-01	-0.18	3.9E-01	0.05	7.9E-01
ENSCAFG0000003111	EFCAB11	grey	EC_M10	0.22	2.9E-01	-0.07	7.4E-01	0.28	1.6E-01	0.06	7.9E-01	-0.40	4.1E-02	-0.01	9.6E-01	-0.10	6.3E-01	0.00	1.0E+00	0.10	6.1E-01	0.27	1.9E-01
ENSCAFG0000001657	SEPT1	grey	EC_M10	0.22	2.9E-01	-0.17	3.9E-01	0.19	3.5E-01	-0.12	5.6E-01	-0.20	3.2E-01	-0.06	7.5E-01	-0.03	8.9E-01	-0.01	9.6E-01	0.02	9.1E-01	0.15	4.7E-01
ENSCAFG0000000761	CCNE1	grey	EC_M10	0.22	2.9E-01	-0.11	5.9E-01	-0.08	7.1E-01	-0.35	8.3E-02	0.03	8.9E-01	0.18	3.9E-01	-0.22	2.7E-01	-0.12	5.7E-01	0.12	5.5E-01	-0.13	5.1E-01
ENSCAFG0000000863	CCDC158	grey	EC_M10	0.22	2.9E-01	-0.02	9.3E-01	-0.46	1.7E-02	0.05	8.1E-01	0.32	1.1E-01	-0.02	9.4E-01	0.01	9.5E-01	0.25	2.2E-01	-0.18	3.8E-01	-0.41	3.6E-02
ENSCAFG0000000766	ENSCAFG0000000766	grey	EC_M10	0.22	2.9E-01	-0.20	3.2E-01	-0.06	7.8E-01	0.07	7.4E-01	-0.06	7.6E-01	-0.10	6.3E-01	-0.24	2.4E-01	-0.02	9.1E-01	0.05	8.2E-01	-0.12	5.7E-01
ENSCAFG0000002780	ENSCAFG0000002780	grey	EC_M10	0.22	2.9E-01	0.28	1.6E-01	-0.14	5.1E-01	0.02	9.2E-01	-0.22	2.8E-01	0.05	8.2E-01	-0.02	9.4E-01	0.08	6.9E-01	0.05	7.9E-01	0.12	5.5E-01
ENSCAFG0000000619	SPEF1	turquoise	EC_M6	0.22	2.9E-01	0.40	4.5E-02	-0.22	2.8E-01	-0.07	7.5E-01	-0.09	6.6E-01	0.17	4.1E-01	-0.03	8.8E-01	0.08	6.9E-01	0.34	8.5E-02	0.01	9.6E-01
ENSCAFG0000000568	ENDOV	grey	EC_M10	0.22	2.9E-01	0.10	6.1E-01	-0.38	5.8E-02	-0.36	6.7E-02	0.22	2.8E-01	0.31	1.2E-01	-0.03	8.7E-01	-0.15	4.7E-01	-0.19	3.5E-01	-0.33	9.6E-02
ENSCAFG0000003121	CXHXorf36	turquoise	EC_M6	0.22	2.9E-01	0.88	2.7E-09	-0.23	2.5E-01	-0.13	5.3E-01	-0.19	3.5E-01	-0.08	7.0E-01	-0.15	4.6E-01	-0.19	3.6E-01	-0.19	3.5E-01	0.06	7.8E-01
ENSCAFG0000001274	DIDO1	grey	EC_M10	0.22	2.9E-01	0.17	4.0E-01	0.05	8.0E-01	0.20	3.4E-01	-0.38	5.3E-02	0.00	9.9E-01	0.07	7.4E-01	-0.15	4.5E-01	0.25	2.2E-01	0.23	2.5E-01
ENSCAFG0000000540	BRPF1	cyan	EC_M2	0.22	2.9E-01	0.29	1.5E-01	0.28	1.6E-01	0.45	2.1E-02	-0.66	2.7E-04	-0.06	7.9E-01	0.14	4.8E-01	-0.21	2.9E-01	0.09	6.7E-01	0.56	2.7E-03
ENSCAFG0000000304	TRIT1	darkgreen	EC_M4	0.22	2.9E-01	-0.24	2.4E-01	-0.62	8.1E-04	0.06	7.6E-01	0.66	2.5E-04	0.40	4.1E-02	-0.09	6.7E-01	-0.26	1.9E-01	-0.31	1.3E-01	-0.79	1.8E-06
ENSCAFG0000000459	CCDC90B	grey	EC_M10	0.22	2.9E-01	-0.54	4.7E-03	0.11	6.0E-01	0.20	3.4E-01	-0.13	5.2E-01	-0.21	3.0E-01	-0.45	2.0E-02	0.15	4.8E-01	-0.19	3.6E-01	0.00	9.8E-01
ENSCAFG0000001801	CENPV	grey	EC_M10	0.22	2.9E-01	-0.59	1.7E-03	0.38	5.4E-02	-0.08	7.0E-01	-0.36	7.0E-02	-0.08	7.0E-01	0.04	8.4E-01	0.09	6.7E-01	-0.07	7.2E-01	0.21	3.1E-01
ENSCAFG0000001348	TMEM120A	grey	EC_M10	0.22	2.9E-01	0.37	6.3E-02	0.22	2.9E-01	-0.02	9.4E-01	-0.48	1.2E-02	-0.21	3.1E-01	0.01	9.6E-01	0.09	6.7E-01	0.04	8.6E-01	0.36	7.3E-02
ENSCAFG0000003220	OGFRL1	grey	EC_M10	0.21	2.9E-01	0.16	4.5E-01	-0.07	7.5E-01	0.27	1.9E-01	-0.20	3.3E-01	0.11	5.8E-01	-0.03	8.7E-01	-0.22	2.8E-01	-0.13	5.1E-01	0.13	5.4E-01
ENSCAFG0000000966	MORC3	darkgrey	EC_M8	0.21	2.9E-01	0.14	4.8E-01	0.40	4.0E-02	0.20	3.4E-01	-0.77	4.3E-06	-0.20	3.2E-01	-0.10	6.2E-01	-0.18	3.7E-01	-0.22	2.8E-01	0.67	1.9E-04
ENSCAFG0000000478	IFT122	cyan	EC_M2	0.21	2.9E-01	0.11	6.0E-01	0.46	1.9E-02	0.20	3.2E-01	-0.80	7.6E-07	-0.11	5.8E-01	-0.10	6.1E-01	-0.16	4.4E-01	0.17	4.2E-01	0.70	7.9E-05
ENSCAFG0000001272	RPL35A	cyan	EC_M2	0.21	2.9E-01	-0.28	1.7E-01	0.38	5.5E-02	0.15	4.7E-01	-0.54	4.4E-03	0.16	4.2E-01	0.25	2.1E-01	-0.12	5.7E-01	-0.23	2.6E-01	0.35	7.5E-02
ENSCAFG0000001985	TAF13	grey	EC_M10	0.21	2.9E-01	-0.04	8.3E-01	-0.11	5.9E-01	-0.05	7.9E-01	0.01	9.5E-01	0.29	1.5E-01	-0.22	2.8E-01	0.06	7.7E-01	0.02	9.2E-01	-0.14	5.0E-01
ENSCAFG0000000827	ARHGEF28	turquoise	EC_M6	0.21	2.9E-01	0.93	9.9E-12	-0.43	2.8E-02	-0.15	4.7E-01	-0.01	9.7E-01	-0.09	6.6E-01	0.03	8.8E-01	-0.08	6.9E-01	-0.11	6.0E-01	-0.13	5.3E-01
ENSCAFG0000000454	ENSCAFG0000000454	grey	EC_M10	0.21	2.9E-01	-0.25	2.1E-01	0.37	6.0E-02	-0.33	1.1E-01	-0.51	8.3E-03	0.14	5.0E-01	-0.02	9.2E-01	-0.12	5.5E-01	-0.18	3.7E-01	0.38	5.9E-02
ENSCAFG0000000789	PLS1	grey	EC_M10	0.21	2.9E-01	-0.14	5.0E-01	-0.24	2.5E-01	0.04	8.6E-01	0.10	6.2E-01	-0.19	3.6E-01	-0.23	2.6E-01	0.52	6.6E-03	0.27	1.8E-01	-0.23	2.7E-01
ENSCAFG0000001112	GSK3B	darkgreen	EC_M4	0.21	2.9E-01	0.22	2.7E-01	-0.82	3.4E-07	0.02	9.2E-01	0.63	5.4E-04	0.31	1.2E-01	-0.06	7.8E-01	-0.13	5.1E-01	0.04	8.6E-01	-0.77	3.8E-06
ENSCAFG0000000932	DNAJC17	grey	EC_M10	0.21	2.9E-01	-0.47	1.6E-02	0.10	6.1E-01	-0.06	7.6E-01	-0.11	5.8E-01	0.20	3.2E-01	0.14	4.9E-01	0.02	9.3E-01	-0.19	3.5E-01	-0.02	9.1E-01
ENSCAFG0000001635	STIM2	darkgreen	EC_M4	0.21	2.9E-01	0.40	4.1E-02	-0.72	2.9E-05	-0.02	9.2E-01	0.51	7.8E-03	0.07	7.2E-01	-0.22	2.9E-01	0.14	5.0E-01	-0.24	2.4E-01	-0.66	2.8E-04
ENSCAFG0000001367	MBIP	grey	EC_M10	0.21	2.9E-01	-0.26	1.9E-01	-0.59	1.4E-03	-0.14	5.1E-01	0.58	1.9E-03	0.45	2.1E-02	0.17	4.0E-01	0.10	6.4E-01	-0.11	6.1E-01	-0.72	3.8E-05
ENSCAFG0000002302	BIRC2	grey	EC_M10	0.21	2.9E-01	-0.15	4.6E-01	0.26	2.0E-01	0.06	7.8E-01	-0.37	6.3E-02	0.01	9.6E-01	-0.19	3.6E-01	-0.30	1.3E-01	-0.32	1.1E-01	0.30	1.4E-01
ENSCAFG0000002964	FXN	grey	EC_M10	0.21	2.9E-01	0.14	5.1E-01	0.32	1.1E-01	0.05	8.2E-01	-0.52	6.9E-03	-0.05	8.0E-01	-0.48	1.3E-02	-0.01	9.6E-01	-0.03	8.7E-01	0.44	2.5E-02
ENSCAFG0000002429	ENSCAFG0000002429	grey	EC_M10	0.21	2.9E-01	0.38	5.2E-02	0.12	5.7E-01	0.12	5.5E-01	-0.46	1.8E-02	-0.33	9.5E-02	0.00	1.0E+00	-0.06	7.6E-01	0.11	5.9E-01	0.39	4.7E-02
ENSCAFG0000000302	CXCL8	turquoise	EC_M6	0.21	2.9E-01	0.90	4.2E-10	-0.44	2.4E-02	-0.19	3.6E-01	0.05	8.2E-01	-0.07	7.5E-01	-0.03	8.7E-01	-0.18	3.9E-01	0.25	2.2E-01	-0.14	5.0E-01
ENSCAFG0000000626	PCID2	grey	EC_M10	0.21	2.9E-01	0.36	7.5E-02	-0.04	8.3E-01	-0.26	2.0E-01	-0.25	2.1E-01	0.11	6.1E-01	-0.03	9.0E-01	0.20	3.2E-01	0.15	4.8E-01	0.17	4.0E-01
ENSCAFG0000001649	ENSCAFG0000001649	grey	EC_M10	0.21	2.9E-01	0.25	2.2E-01	-0.31	1.2E-01	0.06	7.7E-01	0.05	8.0E-01	-0.15	4.5E-01	-0.16	4.3E-01	0.21	3.0E-01	0.15	4.6E-01	-0.11	5.8E-01
ENSCAFG0000001385	ACADL	cyan	EC_M2	0.21	2.9E-01	-0.52	6.2E-03	0.22	2.8E-01	0.29	1.5E-01	-0.31	1.2E-01	0.09	6.7E-01	0.07	7.4E-01	0.02	9.3E-01	0.04	8.3E-01	0.20	3.4E-01
ENSCAFG0000001203	ENSCAFG0000001203	grey	EC_M10	0.21	2.9E-01	-0.20	3.2E-01	0.15	4.6E-01	-0.21	3.1E-01	-0.28	1.7E-01	-0.14	5.1E-01	0.31	1.2E-01	-0.09	6.6E-01	-0.04	8.3E-01	0.22	2.9E-01
ENSCAFG0000002632	ENSCAFG0000002632	grey	EC_M10	0.21	2.9E-01	0.19	3.5E-01	0.17	4.0E-01	0.01	9.6E-01	-0.46	1.8E-02	-0.28	1.7E-01	0.14	5.0E-01	-0.30	1.3E-01	0.00	9.9E-01	0.36	6.9E-02
ENSCAFG0000001300	ITGA6	turquoise	EC_M6	0.21	2.9E-01	0.63	5.0E-04	-0.23	2.6E-01	-0.30	1.4E-01	-0.12	5.7E-01	-0.22	2.8E-01	-0.34	9.4E-02	0.09	6.7E-01	0.26	2.0E-01	0.04	8.5E-01
ENSCAFG0000000623	ENSCAFG0000000623	grey	EC_M10	0.21	3.0E-01	0.33	1.0E-01	0.01	9.6E-01	0.07	7.5E-01	-0.34	8.7E-02	0.24	2.5E-01	-0.03	8.7E-01	0.14	5.0E-01	-0.15	4.6E-01	0.22	2.9E-01
ENSCAFG0000002031	SLC7A6	grey	EC_M10	0.21	3.0E-01	-0.34	9.2E-02	0.14	5.1E-01	-0.18	3.7E-01	-0.15	4.6E-01	0.37	5.9E-02	0.26	1.9E-01	-0.12	5.5E-01	-0.08	7.1E-01	0.02	9.4E-01
ENSCAFG0000001602	SHROOM4	turquoise	EC_M6	0.21	3.0E-01	0.86	2.0E-08	-0.53	5.0E-03	-0.08	6.9E-01	0.12	5.7E-01	0.02	9.1E-01	-0.12	5.5E-01	-0.11	5.8E-01	0.17	4.1E-01	-0.25	2.3E-01
ENSCAFG0000002271	ENSCAFG0000002271	grey	EC_M10	0.21	3.0E-01	-0.05	8.1E-01	-0.17	4.1E-01	-0.07	7.2E-01	0.04	8.3E-01	0.20	3.3E-01	-0.47	1.5E-02	-0.03	8.7E-01	0.12	5.5E-01	-0.12	5.6E-01
ENSCAFG0000000022	KCNN2	grey	EC_M10	0.21	3.0E-01	-0.04	8.6E-01	0.05	8.2E-01	-0.12	5.5E-01	-0.18	3.7E-01	-0.08	7.0E-01	-0.02	9.2E-01	-0.12	5.7E-01	0.10	6.1E-01	0.11	6.0E-01
ENSCAFG0000001175	ATG16L1	grey	EC_M10	0.21	3.0E-01	0.03	8.8E-01	-0.64	4.7E-04	-0.04	8.3E-01	0.58	1.7E-03	0.23	2.6E-01	0.16	4.4E-01	0.07	7.3E-01	-0.02	9.3E-01	-0.65	3.5E-04
ENSCAFG0000000583	RNF121	darkgreen	EC_M4	0.21	3.0E-01	-0.08	7.0E-01	-0.66	2.7E-04	-0.07	7.4E-01	0.62	7.8E-04	0.43	3.0E-02	0.17	4.2E-01	-0.07	7.2E-01	-0.32	1.1E-01	-0.75	9.6E-06
ENSCAFG0000001690	FGF18	turquoise	EC_M6	0.21	3.0E-01	0.43	2.7E-02	-0.11	6.0E-01	-0.35	8.2E-02	-0.13	5.3E-01	0.04	8.6E-01	-0.37	6.0E-02	-0.11	6.0E-01	-0.38	5.4E-02	-0.01	9.5E-01
ENSCAFG0000002967	ENSCAFG0000002967	grey	EC_M10	0.21	3.0E-01	-0.06	7.8E-01	0.13	5.1E-01	-0.06	7.7E-01	-0.33	1.0E-01	0.16	4.2E-01	-0.53	5.7E-03	-0.26	2.0E-01	0.06	7.5E-01	0.17	4.0E-01
ENSCAFG0000002754	ENSCAFG0000002754	grey	EC_M10	0.21	3.0E-01	0.11	5.8E-01	0.04	8.6E-01	-0.05	8.0E-01	-0.23	2.7E-01	-0.07	7.3E-01	-0.10	6.4E-01	-0.08	6.9E-01	-0.15	4.7E-01	0.14	5.0E-01
ENSCAFG0000001212	ENSCAFG0000001212	grey	EC_M10	0.21	3.0E-01	0.34	9.0E-02	-0.31	1.3E-01	-0.24	2.3E-01	0.07	7.3E-01	-0.21	3.1E-01	0.14	4.9E-01	-0.11	5.9E-01	-0.14	4.9E-01	-0.16	4.5E-01
ENSCAFG0000002886	GPX8	darkgreen	EC_M4	0.21	3.0E-01	-0.56	2.8E-03	-0.42	3.2E-02	0.02	9.2E-01	0.52	6.3E-03	0.39	4.6E-02	0.04	8.4E-01	-0.11	5.9E-01	-0.34	8.7E-02	-0.68	1.4E-04
ENSCAFG0000001276	ACBD6	cyan	EC_M2	0.21	3.0E-01	-0.46	1.8E-02	0.17	4.0E-01	0.05	7.9E-01	-0.14	4.9E-01	0.06	7.6E-01	0.32	1.1E-01	-0.27	1.8E-01	-0.53	5.0E-03	0.01	9.5E-01
ENSCAFG0000000073	ENSCAFG0000000073	grey	EC_M10	0.21	3.0E-01	-0.19	3.4E-01	-0.30	1.3E-01	0.28	1.6E-01	0.24	2.4E-01	0.21	3.1E-01	-0.15	4.5E-01	0.20	3.3E-01	-0.03	8.9E-01	-0.34	8.6E-02
ENSCAFG0000000362	INPP4B	turquoise	EC_M6	0.21	3.0E-01	0.61	8.8E-04	-0.32	1.1E-01	-0.17	3.9E-01	-0.01	9.5E-01	0.00	9.8E-01	0.49	1.2E-02	-0.17	4.0E-01	0.01	9.4E-01	-0.09	6.5E-01
ENSCAFG0000000784	TIMM10	grey	EC_M10	0.21	3.0E-01	-0.23	2.5E-01	-0.36	7.2E-02	0.04	8.5E-01	0.44	2.3E-02	0.44	2.5E-02	0.10	6.3E-01	-0.12	5.4E-01	0.00	9.8E-01	-0.52	5.9E-03
ENSCAFG0000000960	CEP97	turquoise	EC_M6	0.21	3.0E-01	0.45	2.1E-02	-0.13	5.2E-01	0.03	9.0E-01	-0.29	1.5E-01	0.16	4.2E-01	-0.10	6.3E-01	0.02	9.2E-01	0.46	1.9E-02	0.19	3.5E-01
ENSCAFG0000000624	GNPDA1	cyan	EC_M2	0.21	3.0E-01	0.27	1.7E-01	0.46	1.8E-02	0.14	5.0E-01	-0.81	5.0E-07	-0.26	2.0E-01	-0.28	1.6E-01	-0.09	6.5E-01	-0.30	1.3E-01	0.68	1.3E-04
ENSCAFG0000000810	ENSCAFG0000000810	grey	EC_M10	0.21	3.0E-01	-0.17	4.2E-01	-0.54	4.7E-03	-0.10	6.4E-01	0.49	1.2E-02	0.42	3.0E-02	-0.14	5.0E-01	-0.09	6.7E-01	-0.14	5.0E-01	-0.62	8.0E-04
ENSCAFG0000000563	FCHSD2	turquoise	EC_M6	0.21	3.0E-01	0.59	1.7E-03	-0.45	2.3E-02	-0.18	3.8E-01	0.15	4.8E-01	-0.18	3.7E-01	-0.15	4.7E-01	0.22	2.9E-01	0.39	4.9E-02	-0.20	3.4E-01
ENSCAFG0000001608	HDHD5	grey	EC_M10	0.21	3.0E-01	-0.33	9.8E-02	-0.27	1.8E-01	-0.06	7.8E-01	0.35	8.1E-02	0.01	9.5E-01	-0.26	2.0E-01	0.02	9.1E-01	-0.19	3.5E-01	-0.49	1.1E-02
ENSCAFG0000002520	CA2	turquoise	EC_M6	0.21	3.0E-01	0.43	2.9E-02	-0.25	2.1E-01	-0.14	4.9E-01	-0.03	8.8E-01	-0.04	8.6E-01	-0.14	5.0E-01	0.14	5.0E-01	0.21	3.0E-01	-0.12	5.4E-01
ENSCAFG0000000741	CSPP1	turquoise	EC_M6	0.21	3.0E-01	0.36	7.4E-02	-0.36	6.8E-02	0.18	3.8E-01	0.04	8.6E-01	-0.06	7.6E-01	0.11	6.1E-01	0.06	7.8E-01	0.32	1.1E-01	-0.17	4.2E-01
ENSCAFG0000001240	ILKAP	cyan	EC_M2	0.21	3.0E-01	-0.02	9.4E-01	0.54	4.6E-03	0.13	5.4E-01	-0.80	7.4E-07	-0.09	6.5E-01	0.01	9.7E-01	0.01	9.8E-01	-0.21	2.9E-01	0.69	8.5E-05
ENSCAFG0000002274	MT-CYB	cyan	EC_M2	0.21	3.0E-01	-0.31	1.3E-01	0.32	1.1E-01	0.29	1.5E-01	-0.51	7.8E-03	0.05	8.2E-01	-0.49	1.1E-02	0.04	8.6E-01	-0.10	6.2E-01	0.34	9.2E-02
ENSCAFG0000000528	SPTBN4	turquoise	EC_M6	0.21	3.0E-01	0.84	1.1E-07	-0.18	3.7E-01	0.01	9.5E-01	-0.24	2.5E-01	-0.20	3.3E-01	0.14	5.0E-01	-0.15	4.6E-01	0.23	2.5E-01	0.14	5.0E-01
ENSCAFG0000001705	HIRIP3	grey	EC_M10	0.21	3.0E-01	-0.11	5.8E-01	0.47	1.6E-02	0.14	5.0E-01	-0.64	4.3E-04	-0.05	8.0E-01	-0.03	8.8E-01	0.17	4.2E-01	0.06	7.6E-01	0.58	2.1E-03
ENSCAFG0000001597	UGDH	darkgreen	EC_M4	0.21	3.0E-01	-0.34	8.8E-02	-0.67	1.9E-04	-0.09	6.5E-01	0.72	3.9E-05	0.47	1.4E-02	-0.04	8.4E-01	-0.11	5.9E-01	-0.09	6.5E-01	-0.85	2.8E-08
ENSCAFG0000001205	PDIA5	turquoise	EC_M6	0.21	3.0E-01	0.80	1.0E-06	-0.47	1.6E-02	-0.02	9.1E-01	0.05	8.3E-01	0.01	9.5E-01	0.09	6.8E-01	0.02	9.3E-01	0.34	9.2E-02	-0.15	4.6E-01
ENSCAFG0000001875	IL7R	turquoise	EC_M6	0.21	3.0E-01	0.90	4.1E-10	-0.29	1.6E-01	-0.12	5.6E-01	-0.15	4.6E-01	-0.15	4.7E-01	-0.14	4.9E-01	-0.16	4.5E-01	0.15	4.8E-01	0.03	9.0E-01
ENSCAFG0000000852	GUCY1A1	turquoise	EC_M6	0.21	3.0E-01	0.95	5.3E-14	-0.28	1.6E-01	-0.10	6.4E-01	-0.16	4.4E-01	-0.10	6.2E-01	-0.10	6.3E-01	-0.14	5.0E-01	0.13	5.3E-01	0.06	7.6E-01
ENSCAFG0000001180	PHACTR4	grey	EC_M10	0.21	3.0E-01	-0.20	3.3E-01	0.04	8.3E-01	-0.17	4.0E-01	-0.18	3.8E-01	0.11	5.9E-01	-0.24	2.3E-01	-0.14	4.9E-01	-0.01	9.5E-01	0.06	7.7E-01
ENSCAFG0000000875	ENSCAFG0000000875	grey	EC_M10	0.21	3.0E-01	0.16	4.4E-01	-0.10	6.4E-01	0.29	1.6E-01	-0.17	4.0E-01	-0.22	2.7E-01	-0.19	3.5E-01	0.14	5.0E-01	0.04	8.3E-01	0.01	9.6E-01
ENSCAFG0000000372	NRCAM	turquoise	EC_M6	0.21	3.0E-01	0.77	3.4E-06	-0.54	4.4E-03	-0.21	3.0E-01	0.23	2.7E-01	-0.14	5.0E-01	-0.08	6.9E-01	0.09	6.8E-01	0.16	4.5E-01	-0.31	1.2E-01

ENSCAFG0000003185	ENSCAFG0000003185	grey	EC_M10	0.21	3.0E-01	-0.17	3.9E-01	-0.49	1.2E-02	-0.28	1.7E-01	0.51	7.8E-03	0.24	2.4E-01	-0.14	4.9E-01	0.07	7.4E-01	0.14	5.0E-01	-0.55	3.6E-03
ENSCAFG0000001228	KALRN	turquoise	EC_M6	0.21	3.0E-01	0.69	8.6E-05	-0.17	4.1E-01	0.12	5.6E-01	-0.22	2.8E-01	-0.17	4.0E-01	-0.08	7.0E-01	-0.09	6.6E-01	0.44	2.6E-02	0.15	4.7E-01
ENSCAFG0000003178	ZNF773	grey	EC_M10	0.21	3.0E-01	-0.35	8.4E-02	0.30	1.3E-01	-0.04	8.4E-01	-0.39	4.6E-02	-0.03	9.0E-01	-0.35	8.1E-02	-0.31	1.2E-01	-0.07	7.5E-01	0.26	2.0E-01
ENSCAFG0000001262	ENSCAFG0000001262	grey	EC_M10	0.21	3.0E-01	-0.30	1.4E-01	-0.11	5.8E-01	0.31	1.2E-01	0.11	5.8E-01	-0.03	8.7E-01	0.04	8.6E-01	0.12	5.5E-01	-0.35	8.0E-02	-0.26	2.0E-01
ENSCAFG0000001583	ENSCAFG0000001583	cyan	EC_M2	0.21	3.0E-01	-0.48	1.2E-02	0.08	7.1E-01	0.11	6.1E-01	-0.07	7.4E-01	0.19	3.6E-01	0.13	5.2E-01	-0.04	8.5E-01	-0.61	8.6E-04	-0.12	5.5E-01
ENSCAFG0000001724	ADCK1	turquoise	EC_M6	0.21	3.0E-01	0.39	5.1E-02	-0.07	7.4E-01	0.01	9.7E-01	-0.24	2.4E-01	0.01	9.6E-01	-0.03	8.7E-01	0.34	9.2E-02	0.22	2.9E-01	0.13	5.4E-01
ENSCAFG0000002517	ENSCAFG0000002517	grey	EC_M10	0.21	3.0E-01	0.20	3.2E-01	0.04	8.4E-01	0.34	9.1E-02	-0.29	1.5E-01	-0.15	4.6E-01	-0.13	5.3E-01	-0.17	4.2E-01	-0.04	8.6E-01	0.24	2.5E-01
ENSCAFG0000001699	RPS6KL1	turquoise	EC_M6	0.21	3.0E-01	0.75	8.7E-06	-0.18	3.9E-01	-0.19	3.5E-01	-0.19	3.5E-01	-0.20	3.2E-01	-0.19	3.5E-01	0.14	5.0E-01	-0.07	7.2E-01	0.11	6.0E-01
ENSCAFG0000001188	ENSCAFG0000001188	turquoise	EC_M6	0.21	3.0E-01	0.45	2.0E-02	0.06	7.8E-01	-0.15	4.7E-01	-0.37	6.6E-02	-0.08	6.8E-01	-0.07	7.4E-01	-0.03	8.8E-01	-0.03	8.7E-01	0.28	1.6E-01
ENSCAFG0000002642	ENSCAFG0000002642	turquoise	EC_M6	0.21	3.0E-01	0.46	1.9E-02	0.07	7.3E-01	-0.12	5.6E-01	-0.35	7.7E-02	-0.12	5.7E-01	-0.11	6.0E-01	-0.05	8.0E-01	-0.19	3.6E-01	0.29	1.5E-01
ENSCAFG0000001833	LIG3	turquoise	EC_M6	0.21	3.0E-01	0.40	4.4E-02	-0.09	6.6E-01	0.17	4.1E-01	-0.20	3.2E-01	0.11	6.1E-01	-0.16	4.4E-01	-0.19	3.6E-01	0.04	8.5E-01	0.06	7.8E-01
ENSCAFG0000000875	FRAS1	grey	EC_M10	0.21	3.0E-01	-0.35	7.9E-02	-0.34	8.7E-02	-0.07	7.5E-01	0.31	1.2E-01	0.18	3.9E-01	-0.15	4.5E-01	0.06	7.8E-01	0.08	6.8E-01	-0.49	1.1E-02
ENSCAFG0000001106	KCTD18	grey	EC_M10	0.21	3.0E-01	-0.13	5.2E-01	0.52	5.9E-03	0.09	6.5E-01	-0.72	2.8E-05	-0.07	7.3E-01	-0.14	4.9E-01	-0.10	6.3E-01	-0.30	1.4E-01	0.62	8.1E-04
ENSCAFG0000002010	ENG	turquoise	EC_M6	0.21	3.0E-01	0.92	1.6E-11	-0.29	1.6E-01	-0.14	5.0E-01	-0.12	5.7E-01	-0.21	3.1E-01	-0.02	9.4E-01	0.03	8.9E-01	0.20	3.3E-01	0.04	8.6E-01
ENSCAFG0000000209	TMEM165	grey	EC_M10	0.21	3.0E-01	-0.24	2.3E-01	-0.61	9.9E-04	-0.13	5.4E-01	0.66	2.8E-04	0.45	2.2E-02	0.24	2.4E-01	0.06	7.7E-01	-0.15	4.6E-01	-0.76	5.8E-06
ENSCAFG0000003054	PDZRN3	grey	EC_M10	0.21	3.0E-01	-0.33	9.5E-02	-0.43	2.9E-02	0.19	3.5E-01	0.41	3.7E-02	0.20	3.3E-01	0.18	3.9E-01	-0.13	5.2E-01	0.19	3.6E-01	-0.55	3.4E-03
ENSCAFG0000000130	LRGUK	grey	EC_M10	0.21	3.0E-01	-0.36	7.4E-02	-0.45	2.0E-02	-0.20	3.2E-01	0.49	1.1E-02	0.05	7.9E-01	-0.08	7.1E-01	0.09	6.5E-01	-0.20	3.2E-01	-0.62	6.7E-04
ENSCAFG0000002525	PEAK1	grey	EC_M10	0.21	3.0E-01	0.28	1.7E-01	-0.12	5.7E-01	-0.10	6.4E-01	-0.12	5.8E-01	-0.40	4.0E-02	-0.27	1.8E-01	-0.03	9.0E-01	-0.11	5.9E-01	0.01	9.5E-01
ENSCAFG0000001271	POGZ	cyan	EC_M2	0.21	3.0E-01	0.41	3.6E-02	0.41	3.9E-02	0.02	9.1E-01	-0.83	1.6E-07	-0.14	4.9E-01	-0.04	8.4E-01	-0.08	6.9E-01	-0.13	5.2E-01	0.69	8.9E-05
ENSCAFG0000000878	PLLP	turquoise	EC_M6	0.21	3.0E-01	0.87	9.7E-09	-0.35	7.9E-02	-0.11	6.0E-01	-0.08	6.8E-01	0.23	2.5E-01	-0.10	6.3E-01	-0.12	5.5E-01	0.11	5.9E-01	-0.03	8.9E-01
ENSCAFG0000001031	TENT4A	grey	EC_M10	0.21	3.0E-01	0.25	2.2E-01	-0.06	7.7E-01	-0.12	5.8E-01	-0.17	4.0E-01	0.31	1.2E-01	0.35	8.1E-02	-0.46	1.7E-02	-0.30	1.4E-01	0.02	9.4E-01
ENSCAFG0000000042	LYZ	turquoise	EC_M6	0.21	3.1E-01	0.90	4.4E-10	-0.20	3.3E-01	0.00	9.9E-01	-0.24	2.5E-01	-0.13	5.2E-01	-0.15	4.6E-01	-0.09	6.6E-01	-0.08	7.0E-01	0.11	5.9E-01
ENSCAFG0000002053	ENSCAFG0000002053	grey	EC_M10	0.21	3.1E-01	-0.47	1.7E-02	-0.14	5.0E-01	0.26	2.0E-01	0.11	5.9E-01	0.40	4.3E-02	-0.18	3.9E-01	0.24	2.4E-01	-0.03	8.9E-01	-0.28	1.7E-01
ENSCAFG0000000293	POLD1	grey	EC_M10	0.21	3.1E-01	-0.04	8.6E-01	0.40	4.4E-02	-0.09	6.7E-01	-0.54	4.8E-03	-0.04	8.5E-01	0.27	1.8E-01	-0.01	9.5E-01	0.04	8.5E-01	0.41	3.6E-02
ENSCAFG0000000073	LYRM7	grey	EC_M10	0.21	3.1E-01	0.00	9.8E-01	-0.17	4.2E-01	-0.24	2.5E-01	0.06	7.8E-01	0.25	2.2E-01	-0.21	3.1E-01	0.25	2.2E-01	0.08	7.0E-01	-0.18	3.9E-01
ENSCAFG0000000092	DCBLD1	darkgreen	EC_M4	0.21	3.1E-01	0.60	1.2E-03	-0.78	3.1E-06	-0.16	4.4E-01	0.51	7.3E-03	0.18	3.8E-01	0.12	5.4E-01	0.10	6.3E-01	-0.17	4.0E-01	-0.62	6.7E-04
ENSCAFG0000002997	LAMTOR3	grey	EC_M10	0.21	3.1E-01	-0.15	4.7E-01	-0.56	2.8E-03	-0.32	1.1E-01	0.56	2.9E-03	0.49	1.2E-02	0.07	7.2E-01	0.22	2.7E-01	-0.01	9.7E-01	-0.70	6.0E-05
ENSCAFG0000001548	WSCD1	turquoise	EC_M6	0.21	3.1E-01	0.88	4.7E-09	-0.21	3.0E-01	-0.02	9.1E-01	-0.21	3.0E-01	-0.14	4.8E-01	-0.16	4.3E-01	-0.09	6.8E-01	-0.22	2.9E-01	0.08	7.1E-01
ENSCAFG0000002035	RIPOR1	turquoise	EC_M6	0.21	3.1E-01	0.58	2.1E-03	-0.25	2.1E-01	-0.24	2.5E-01	-0.03	8.8E-01	-0.03	8.9E-01	-0.16	4.3E-01	0.04	8.3E-01	-0.13	5.1E-01	-0.10	6.2E-01
ENSCAFG0000002362	NME1	grey	EC_M10	0.21	3.1E-01	-0.50	9.7E-03	-0.26	1.9E-01	-0.19	3.6E-01	0.38	5.2E-02	0.25	2.2E-01	0.09	6.8E-01	0.10	6.3E-01	-0.29	1.6E-01	-0.52	6.6E-03
ENSCAFG0000000707	ZNF740	cyan	EC_M2	0.21	3.1E-01	0.40	4.0E-02	0.39	4.9E-02	0.39	4.7E-02	-0.76	6.7E-06	-0.13	5.3E-01	-0.11	6.0E-01	-0.16	4.4E-01	0.01	9.5E-01	0.67	1.6E-04
ENSCAFG0000001685	STK10	turquoise	EC_M6	0.21	3.1E-01	0.88	2.4E-09	-0.38	5.7E-02	-0.09	6.8E-01	-0.05	8.0E-01	-0.16	4.3E-01	0.00	9.9E-01	-0.03	8.7E-01	-0.19	3.6E-01	-0.07	7.3E-01
ENSCAFG0000003129	ZKSCAN1	turquoise	EC_M6	0.21	3.1E-01	0.58	2.1E-03	-0.18	3.8E-01	-0.06	7.6E-01	-0.24	2.3E-01	-0.12	5.7E-01	0.19	3.6E-01	0.21	3.0E-01	0.04	8.6E-01	0.14	5.0E-01
ENSCAFG0000001745	MAP2K5	grey	EC_M10	0.21	3.1E-01	-0.05	8.3E-01	-0.36	7.0E-02	0.23	2.6E-01	0.18	3.7E-01	0.06	7.6E-01	-0.06	7.7E-01	-0.04	8.3E-01	-0.33	1.0E-01	-0.38	5.3E-02
ENSCAFG0000001190	FITM1	grey	EC_M10	0.21	3.1E-01	-0.23	2.6E-01	0.18	3.7E-01	-0.12	5.7E-01	-0.25	2.1E-01	-0.06	7.6E-01	-0.11	5.9E-01	-0.06	7.8E-01	-0.19	3.5E-01	0.15	4.8E-01
ENSCAFG0000000513	NAPB	grey	EC_M10	0.21	3.1E-01	0.32	1.1E-01	0.08	7.0E-01	0.02	9.1E-01	-0.43	3.0E-02	0.08	7.1E-01	0.23	2.7E-01	-0.08	7.1E-01	0.33	1.0E-01	0.34	9.2E-02
ENSCAFG0000000244	ENSCAFG0000000244	grey	EC_M10	0.21	3.1E-01	-0.05	8.0E-01	-0.44	2.3E-02	0.14	4.8E-01	0.35	8.2E-02	0.03	8.9E-01	0.08	6.8E-01	-0.06	7.5E-01	0.15	4.6E-01	-0.44	2.4E-02
ENSCAFG0000001152	YOD1	grey	EC_M10	0.21	3.1E-01	0.10	6.4E-01	-0.16	4.2E-01	-0.24	2.3E-01	0.03	8.7E-01	0.19	3.6E-01	-0.07	7.5E-01	0.27	1.8E-01	-0.06	7.6E-01	-0.10	6.3E-01
ENSCAFG0000001646	MAGED2	darkgreen	EC_M4	0.21	3.1E-01	0.39	4.7E-02	-0.84	9.4E-08	-0.07	7.3E-01	0.67	1.6E-04	0.21	3.0E-01	0.02	9.3E-01	-0.02	9.4E-01	0.02	9.1E-01	-0.76	6.4E-06
ENSCAFG0000000480	ENSCAFG0000000480	darkgrey	EC_M8	0.21	3.1E-01	-0.12	5.5E-01	-0.32	1.1E-01	0.17	4.2E-01	0.22	2.9E-01	0.17	4.1E-01	0.07	7.3E-01	-0.18	3.7E-01	-0.12	5.6E-01	-0.27	1.9E-01
ENSCAFG0000001249	ICA1L	grey	EC_M10	0.21	3.1E-01	0.26	2.0E-01	-0.31	1.2E-01	0.30	1.3E-01	0.04	8.6E-01	-0.04	8.4E-01	-0.47	1.4E-02	-0.08	7.1E-01	0.34	9.3E-02	-0.13	5.2E-01
ENSCAFG0000002272	ENSCAFG0000002272	grey	EC_M10	0.21	3.1E-01	0.15	4.8E-01	-0.48	1.3E-02	-0.22	2.7E-01	0.35	7.7E-02	0.36	6.7E-02	-0.13	5.1E-01	-0.13	5.3E-01	-0.07	7.5E-01	-0.46	1.8E-02
ENSCAFG0000000319	FKBP15	darkgrey	EC_M8	0.21	3.1E-01	0.65	3.3E-04	0.08	7.0E-01	0.10	6.2E-01	-0.56	3.2E-03	-0.26	1.9E-01	-0.16	4.5E-01	-0.16	4.3E-01	-0.15	4.7E-01	0.43	2.8E-02
ENSCAFG0000001464	GNG3	grey	EC_M10	0.21	3.1E-01	0.10	6.3E-01	-0.06	7.7E-01	-0.14	4.9E-01	-0.04	8.5E-01	-0.16	4.5E-01	-0.13	5.3E-01	-0.13	5.3E-01	-0.06	7.8E-01	-0.04	8.6E-01
ENSCAFG0000001456	PPP1R14B	grey	EC_M10	0.21	3.1E-01	-0.15	4.7E-01	0.40	4.4E-02	-0.19	3.4E-01	-0.46	1.7E-02	0.01	9.6E-01	-0.04	8.5E-01	0.00	9.8E-01	-0.09	6.7E-01	0.35	8.2E-02
ENSCAFG0000001183	ENSCAFG0000001183	grey	EC_M10	0.21	3.1E-01	-0.56	2.7E-03	-0.27	1.9E-01	0.09	6.7E-01	0.36	6.9E-02	0.23	2.5E-01	-0.52	6.0E-03	-0.17	4.1E-01	-0.11	5.9E-01	-0.50	9.8E-03
ENSCAFG0000000797	ENSCAFG0000000797	grey	EC_M10	0.21	3.1E-01	0.15	4.5E-01	-0.34	9.3E-02	0.32	1.1E-01	0.19	3.5E-01	0.37	6.0E-02	-0.11	5.8E-01	-0.20	3.3E-01	-0.19	3.5E-01	-0.29	1.5E-01
ENSCAFG0000000492	CCDC93	cyan	EC_M2	0.21	3.1E-01	-0.04	8.4E-01	0.48	1.3E-02	0.14	5.1E-01	-0.77	4.7E-06	-0.26	2.1E-01	-0.06	7.9E-01	-0.18	3.8E-01	-0.29	1.5E-01	0.65	3.1E-04
ENSCAFG0000000874	PLCH1	grey	EC_M10	0.21	3.1E-01	-0.10	6.1E-01	0.24	2.5E-01	0.41	3.7E-02	-0.29	1.6E-01	-0.07	7.2E-01	-0.03	8.9E-01	-0.06	7.6E-01	-0.02	9.1E-01	0.18	3.7E-01
ENSCAFG0000000173	ENSCAFG0000000173	grey	EC_M10	0.21	3.1E-01	-0.23	2.6E-01	-0.15	4.7E-01	0.45	2.2E-02	0.05	8.3E-01	-0.23	2.7E-01	-0.18	3.8E-01	0.22	2.8E-01	-0.09	6.8E-01	-0.14	4.9E-01
ENSCAFG0000002333	ENSCAFG0000002333	grey	EC_M10	0.21	3.1E-01	0.35	8.1E-02	-0.29	1.5E-01	-0.10	6.3E-01	0.09	6.8E-01	-0.10	6.3E-01	-0.08	6.9E-01	-0.10	6.3E-01	-0.13	5.4E-01	-0.15	4.8E-01
ENSCAFG0000000978	HERC6	cyan	EC_M2	0.21	3.1E-01	0.42	3.2E-02	0.43	2.8E-02	0.10	6.2E-01	-0.82	2.9E-07	-0.31	1.2E-01	-0.16	4.2E-01	-0.06	7.7E-01	0.06	7.7E-01	0.74	1.3E-05
ENSCAFG0000000615	BTG1	grey	EC_M10	0.21	3.1E-01	0.22	2.8E-01	0.33	9.5E-02	-0.05	8.0E-01	-0.65	3.6E-04	-0.12	5.5E-01	0.10	6.4E-01	-0.14	5.1E-01	-0.37	6.0E-02	0.55	3.6E-03
ENSCAFG0000001252	ENSCAFG0000001252	grey	EC_M10	0.21	3.1E-01	-0.02	9.4E-01	-0.23	2.6E-01	0.10	6.1E-01	0.08	7.0E-01	0.06	7.6E-01	-0.01	9.5E-01	-0.35	7.9E-02	-0.19	3.5E-01	-0.13	5.2E-01
ENSCAFG0000000741	MAN2A1	grey	EC_M10	0.21	3.1E-01	-0.31	1.2E-01	-0.11	5.8E-01	-0.01	9.8E-01	0.07	7.2E-01	-0.01	9.5E-01	-0.04	8.3E-01	-0.02	9.4E-01	-0.57	2.6E-03	-0.20	3.3E-01
ENSCAFG0000000441	GPR4	turquoise	EC_M6	0.21	3.1E-01	0.93	1.3E-11	-0.39	5.0E-02	-0.19	3.5E-01	-0.02	9.4E-01	-0.04	8.5E-01	-0.07	7.2E-01	-0.22	2.7E-01	0.06	7.8E-01	-0.10	6.4E-01
ENSCAFG0000000910	RBM12B	cyan	EC_M2	0.21	3.1E-01	0.26	1.9E-01	0.41	3.7E-02	0.35	7.9E-02	-0.76	5.3E-06	-0.07	7.3E-01	0.00	1.0E+00	0.10	6.2E-01	-0.11	6.0E-01	0.67	1.6E-04
ENSCAFG0000002871	ENSCAFG0000002871	grey	EC_M10	0.21	3.1E-01	0.03	8.9E-01	-0.26	2.1E-01	0.09	6.7E-01	0.12	5.5E-01	0.20	3.2E-01	-0.03	8.9E-01	0.13	5.2E-01	-0.33	1.0E-01	-0.29	1.5E-01
ENSCAFG0000001351	REXO2	darkgreen	EC_M4	0.21	3.1E-01	-0.32	1.1E-01	-0.62	7.9E-04	0.01	9.5E-01	0.67	2.0E-04	0.14	5.0E-01	-0.39	4.6E-02	0.07	7.4E-01	-0.03	8.9E-01	-0.79	1.4E-06
ENSCAFG0000000664	ENSCAFG0000000664	grey	EC_M10	0.21	3.1E-01	-0.01	9.7E-01	0.13	5.4E-01	-0.09	6.5E-01	-0.24	2.4E-01	-0.11	6.0E-01	-0.14	5.0E-01	-0.03	8.8E-01	-0.16	4.4E-01	0.16	4.3E-01
ENSCAFG0000001991	IRF8	turquoise	EC_M6	0.21	3.1E-01	0.97	1.1E-15	-0.26	2.0E-01	-0.11	5.9E-01	-0.20	3.2E-01	-0.13	5.1E-01	-0.11	5.9E-01	-0.03	8.7E-01	0.10	6.4E-01	0.09	6.7E-01
ENSCAFG0000002585	ENSCAFG0000002585	grey	EC_M10	0.21	3.1E-01	-0.05	8.0E-01	0.25	2.2E-01	0.18	3.7E-01	-0.43	2.8E-02	0.03	9.0E-01	0.08	7.1E-01	0.11	6.0E-01	-0.31	1.2E-01	0.31	1.2E-01
ENSCAFG0000000828	ENSCAFG0000000828	grey	EC_M10	0.21	3.1E-01	0.19	3.6E-01	-0.04	8.5E-01	0.07	7.3E-01	-0.13	5.2E-01	-0.09	6.6E-01	-0.21	2.9E-01	-0.05	8.0E-01	0.12	5.4E-01	0.06	7.7E-01
ENSCAFG0000001427	SOS2	turquoise	EC_M6	0.21	3.1E-01	0.74	1.9E-05	-0.05	8.0E-01	0.05	7.9E-01	-0.44	2.3E-02	-0.09	6.6E-01	-0.24	2.3E-01	-0.29	1.4E-01	-0.03	8.8E-01	0.32	1.1E-01
ENSCAFG0000003001	TRIAP1	grey	EC_M10	0.21	3.1E-01	-0.02	9.4E-01	0.24	2.3E-01	-0.08	7.0E-01	-0.40	4.5E-02	-0.16	4.3E-01	0.05	8.0E-01	0.06	7.7E-01	-0.06	7.7E-01	0.37	6.4E-02
ENSCAFG0000000843	ENTPD1	turquoise	EC_M6	0.21	3.1E-01	0.87	5.0E-09	-0.30	1.4E-01	-0.04	8.3E-01	-0.10	6.3E-01	-0.18	3.9E-01	-0.16	4.3E-01	-0.11	6.1E-01	-0.13	5.2E-01	-0.03	8.8E-01
ENSCAFG0000000949	ACCS	grey	EC_M10	0.21	3.1E-01	-0.04	8.6E-01	0.03	8.9E-01	0.13	5.3E-01	-0.24	2.4E-01	-0.20	3.3E-01	-0.02	9.3E-01	0.18	3.8E-01	0.30	1.4E-01	0.13	5.2E-01
ENSCAFG0000000200	SAMD9L	grey	EC_M10	0.21	3.1E-01	0.00	9.9E-01	-0.10	6.4E-01	0.04	8.5E-01	-0.10	6.4E-01	0.23	2.5E-01	-0.22	2.9E-01	-0.18	3.8E-01	-0.48	1.4E-02	-0.05	8.0E-01
ENSCAFG0000000611	TAF7	darkgrey	EC_M8	0.21	3.1E-01	0.39	4.8E-02	0.06	7.8E-01	0.15	4.7E-01	-0.46	1.7E-02	0.00	1.0E+00	0.11	5.9E-01	0.13	5.2E-01	-0.08	6.9E-01	0.36	7.3E-02
ENSCAFG0000001615	BCL2L13	grey	EC_M10	0.20	3.2E-01	-0.27	1.8E-01	-0.47	1.5E-02	-0.19	3.5E-01	0.45	2.1E-02	0.32	1.2E-01	-0.09	6.5E-01	0.28	1.6E-01	-0.18	3.8E-01	-0.59	1.4E-03
ENSCAFG0000000975	VANGL1	grey	EC_M10	0.20	3.2E-01	0.30	1.3E-01	0.33	9.9E-02	-0.15	4.8E-01	-0.66	2.3E-04	-0.14	5.0E-01	-0.29	1.6E-01	0.14	5.0E-01	0.01	9.5E-01	0.55	3.4E-03
ENSCAFG0000001103	ENSCAFG0000001103	grey	EC_M10	0.20	3.2E-01	-0.09	6.6E-01	-0.18	3.7E-01	-0.06	7.6E-01	0.02	9.1E-01	-0.09	6.7E-01	-0.11	5.8E-01	-0.10	6.2E-01	-0.08	6.8E-01	-0.15	4.6E-01
ENSCAFG0000000063	VARS	grey	EC_M10	0.20	3.2E-01	-0.34	8.9E-02	0.31	1.2E-01	0.08	7.1E-01	-0.28	1.7E-01	-0.03	8.9E-01	-0.12	5.6E-01	-0.19	3.6E-01	-0.33	9.6E-02	0.16	4.2E-01
ENSCAFG0000000354	ENSCAFG0000000354	grey	EC_M10	0.20	3.2E-01	-0.09	6.5E-01	-0.27	1.8E-01	0.18	3.9E-01	0.08	6.9E-01	0.29	1.6E-01	-0.11	6.1E-01	-0.07	7.3E-01	-0.12	5.5E-01	-0.19	3.6E-01
ENSCAFG0000001238	CCDC173	grey	EC_M10	0.20	3.2E-01	-0.27	1.8E-01	-0.48	1.3E-02	0.05	8.0E-01	0.46	1.9E-02	0.29	1.5E-01	-0.03	8.9E-01	-0.36	7.5E-02	-0.11	6.0E-01	-0.59	1.6E-03
ENSCAFG0000001374	TAB3	grey	EC_M10	0.20	3.2E-01	-0.10	6.4E-01	-0.66	2.6E-04	-0.17	4.1E-01	0.53	5.8E-03	0.18	3.9E-01	-0.03	8.9E-01	0.17	4.0E-01	0.17	4.0E-01	-0.67	1.7E-04
ENSCAFG0000000065	CTHRC1	turquoise	EC_M6	0.20	3.2E-01	0.85	5.5E-08	-0.29	1.5E-01	-0.14	4.8E-01	-0.11	5.8E-01	-0.19	3.4E-01	-0.19	3.5E-01	-0.23	2.7E-01	-0.03	8.7E-01	-0.02	9.4E-01
ENSCAFG0000002757	ENSCAFG0000002757	grey	EC_M10	0.20	3.2E-01	-0.02	9.2E-01	-0.15	4.6E-01	0.00	9.9E-01	0.02	9.3E-01	0.17	4.2E-01	-0.33	1.0E-01	-0.38	5.5E-02	-0.35	8.2E-02	-0.12	5.7E-01
ENSCAFG0000000991	ENSCAFG0000000991	grey	EC_M10	0.20	3.2E-01	0.48	1.2E-02	-0.61	1.1E-03	-0.35	8.3E-02	0.43	2.8E-02	0.26	2.1E-01	0.04	8.6E-01	-0.01	9.5E-01	-0.10	6.2E-01	-0.55	3.4E-03
ENSCAFG0000000398	SPATA5	grey	EC_M10	0.20	3.2E-01	-0.36	7.4E-02	-0.42	3.2E-02	-0.09	6.5E-01	0.45	2.1E-02	0.13	5.3E-01	-0.52	6.3E-03	0.18	3.8E-01	-0.21	3.0E-01	-0.55	4.0E-03
ENSCAFG0000000472	FRMD4A	grey	EC_M10	0.20	3.2E-01	0.52	5.9E-03	-0.80	7.6E-07	-0.09	6.6E-01	0.61	9.5E-04	0.06	7.5E-01	-0.12	5.7E-01	-0.15	4.7E-01	-0.02	9.1E-01	-0.70	6.5E-05
ENSCAFG0000003209	ENSCAFG0000003209	turquoise	EC_M6	0.20	3.2E-01	0.35	7.8E-02	-0.18	3.8E-01	-0.15	4.7E-01	-0.11	6.1E-01	0.51	8.3E-03	-0.12	5.5E-01	0.37	6.3E-02	-0.11	5.8E-01	0.01	9.7E-01
ENSCAFG0000001053	PUS3	grey	EC_M10	0.20	3.2E-01	-0.19	3.4E-01	-0.04	8.6E-01	-0.03	8.7E-01	-0.11	6.0E-01	0.11	5.9E-01	0.06	7.7E-01	-0.53	5.6E-03	0.06	7.8E-01	0.00	1.0E+00
ENSCAFG0000001564	TRMT5	grey	EC_M10	0.20	3.2E-01	-0.21	3.0E-01	0.39	4.9E-02	0.13	5.3E-01	-0.56	2.8E-03	0.19	3.6E-01	0.11	5.9E-01	-0.39	4.8E-02	-0.26	2.1E-01	0.45	2.0E-02
ENSCAFG0000000274	MYO6	grey	EC_M10	0.20	3.2E-01	0.31	1.2E-01	-0.28	1.7E-01	-0.02	9.4E-01	0.00	9.9E-01	-0.48	1.3E-02	-0.26	2.1E-01	0.11	5.9E-01	0.23	2.6E-01	-0.07	7.4E-01
ENSCAFG0000000404	ARHGAP12	cyan	EC_M2	0.20	3.2E-01	0.31	1.3E-01	0.35	7.7E-02	0.22	2.8E-01	-0.77	4.9E-06	-0.18	3.8E-01	-0.23	2.7E-01	0.03	8.8E-01	0.04	8.6E-01	0.66	2.4E-04
ENSCAFG0000000388	SSBP1	grey	EC_M10	0.20	3.2E-01	-0.03	9.0E-01	-0.10	6.4E-01	-0.11	5.8E-01	-0.02	9.2E-01	-0.08	7.0E-01	-0.03	8.9E-01	0.16	4.5E-01	0.31	1.3E-01	-0.08	6.9E-01
ENSCAFG0000001587	TSPAN14	turquoise	EC_M6	0.20	3.2E-01	0.92	3.1E-11	-0.29	1.6E-01	-0.08	6.9E-01	-0.12	5.5E-01	-0.20	3.3E-01	-0.07	7.4E-01	-0.04	8.3E-01	0.16	4.5E-01	0.04	8.6E-01
ENSCAFG0000000109	SMAD5	cyan	EC_M2	0.20	3.2E-01	0.02	9.2E-01	0.52	6.3E-03	0.18	3.7E-01	-0.83	1.3E-07	0.06	7.7E-01	-0.19	3.5E-01	-0.25	2.2E-01	-0.24	2.3E-01	0.68	1.5E-04
ENSCAFG0000001801	RNF138	grey	EC_M10	0.20	3.2E-01	0.21	3.1E-01	0.34	9.1E-02	0.24	2.4E-01	-0.62	7.8E-04	-0.34	8.9E-02	-0.25	2.1E-01	-0.01	9.4E-01	-0.22	2.9E-01	0.49	1.0E-02
ENSCAFG0000000697	C24H20orf96	grey	EC_M10	0.20	3.2E-01	0.07	7.4E-01	0.05	7.9E-01	-0.47	1.5E-02	-0.33	1.0E-01	0.12	5.7E-01	0.20	3.2E-01	0.11	6.0E-01	0.00	9.8E-01	0.16	4.4E-01
ENSCAFG0000000591	FERMT1	turquoise	EC_M6	0.20	3.2E-01	0.92	2.9E-11	-0.21	3.1E-01	0.08	7.1E-01	-0.24	2.4E-01	-0.15	4.7E-01	-0.14	5.1E-01	-0.16	4.4E-01	0.02	9.4E-01	0.15	4.5E-01
ENSCAFG0000000468	ENSCAFG0000000468	grey	EC_M10	0.20	3.2E-01	-0.14	4.9E-01	0.10	6.1E-01	-0.04	8.3E-01	-0.12	5.6E-01	-0.07	7.2E-01	0.00	9.9E-01	0.00	1.0E+00	-0.12	5.5E-01	0.06	7.8E-01
ENSCAFG0000000788	FFAR4	grey	EC_M10	0.20	3.2E-01	-0.14	4.9E-01	0.10	6.1E-01	-0.04	8.3E-01	-0.12	5.6E-01	-0.07	7.2E-01	0.00	9.9E-01	0.00	1.0E+00	-0.12	5.5E-01	0.06	7.8E-01
ENSCAFG0000003103	ENSCAFG0000003103	grey	EC_M10	0.20	3.2E-01	-0.14	4.9E-01	0.10	6.1E-01	-0.04	8.3E-01	-0.12	5.6E-01	-0.07	7.2E-01	0.00	9.9E-01	0.00	1.0E+00	-0.12	5.5E-01	0.06	7.8E-01
ENSCAFG0000000536	ENSCAFG0000000536	violet	EC_M7	0.20	3.2E-01	-0.04	8.4E-01	-0.23	2.7E-01	-0.08	6.8E-01	0.13	5.4E-01	0.83	1.6E-07	-0.06	7.6E-01	-0.11	6.0E-01	-0.15	4.6E-01	-0.27	1.8E-01
ENSCAFG0000000731	FGFBP3	violet	EC_M7	0.20	3.2E-01	-0.04	8.4E-01	-0.23	2.7E-01	-0.08	6.8E-01	0.13	5.4E-01	0.83	1.6E-07	-0.06	7.6E-01	-0.11	6.0E-01	-0.15	4.6E-01	-0.27	1.8E-01
ENSCAFG0000001662	ARHGAP23	turquoise	EC_M6	0.20	3.2E-01	0.80	8.4E-07	-0.48	1.3E-02	-0.27	1.8E-01	0.14	4.8E-01	-0.12	5.7E-01	-0.03	9.0E-01	-0.15	4.7E-01	0.40	4.5E-02	-0.22	2.9E-01
ENSCAFG0000003123	CAAP1	grey	EC_M10	0.20	3.2E-01	0.45	2.1E-02	0.19	3.6E-01	0.09	6.8E-01	-0.53	5.0E-03	-0.30	1.4E-01	-0.22	2.8E-01	0.11	6.0E-01	-0.34	8.7E-02	0.46	1.9E-02
ENSCAFG0000001120	SGMS2	grey	EC_M10	0.20	3.2E-01	0.56	2.9E-03	-0.66	2.8E-04	-0.23	2.6E-01	0.39	5.1E-02	-0.07	7.3E-01	-0.10	6.4E-01	0.12	5.5E-01	0.28	1.7E-01	-0.48	1.4E-02
ENSCAFG0000002180	ENSCAFG0000002180	grey	EC_M10	0.20	3.2E-01	-0.04	8.5E-01	0.31	1.2E-01	0.42	3.3E-02	-0.54	4.6E-03	0.01	9.7E-01	0.33	1.0E-01	-0.25	2.1E-01	-0.13	5.4E-01	0.43	2.9E-02
ENSCAFG0000003213	ZSCAN31	grey	EC_M10	0.20	3.2E-01	0.23	2.6E-01	-0.34	8.8E-02	0.04	8.4E-01	0.06	7.5E-01	0.35	8.0E-02	0.05	8.0E-01	-0.20	3.2E-01	0.17	3.9E-01	-0.20	3.4E-01
ENSCAFG0000000160	USP49	grey	EC_M10	0.20	3.2E-01	0.19	3.6E-01	0.24	2.3E-01	0.35	8.4E-02	-0.51	7.9E-03	-0.13	5.3E-01	-0.23	2.5E-01	0.14	4.9E-01	-0.04	8.5E-01	0.40	4.2E-02
ENSCAFG0000001135	DENND1B	grey	EC_M10	0.20	3.2E-01	0.18	3.7E-01	-0.39	5.2E-02	-0.14	4.9E-01	0.17	4.0E-01	-0.14	4.8E-01	0.26	2.0E-01	0.15	4.6E-01	0.19	3.5E-01	-0.26	2.0E-01
ENSCAFG0000002873	GEMIN8	grey	EC_M10	0.20	3.2E-01	-0.14	4.9E-01	0.33	9.5E-02	-0.20	3.3E-01	-0.51	8.3E-03	0.05	7.9E-01	-0.33	9.5E-02	-0.16	4.3E-01	0.02	9.3E-01	0.35	8.1E-02
ENSCAFG0000000214	CLOCK	darkgreen	EC_M4	0.20	3.2E-01	0.12	5.5E-01	-0.88	3.8E-09	-0.23	2.7E-01	0.80	1.2E-06	0.24	2.4E-01	0.04	8.5E-01	0.00	9.8E-01	-0.01	9.5E-01	-0.90	2.4E-10
ENSCAFG0000001654	PACRGL	grey	EC_M10	0.20	3.2E-01	0.11	6.0E-01	-0.22	2.8E-01	0.18	3.7E-01	-0.01	9.6E-01	-0.24	2.5E-01	-0.04	8.6E-01	0.14	4.8E-01	-0.39	4.7E-02	-0.14	5.0E-01
ENSCAFG0000000631	ENSCAFG0000000631	grey	EC_M10	0.20	3.2E-01	0.03	9.0E-01	-0.20	3.4E-01	-0.06	7.7E-01	0.07	7.4E-01	-0.08	6.9E-01	-0.15	4.5E-01	-0.10	6.2E-01	-0.14	4.9E-01	-0.19	3.5E-01
ENSCAFG0000000597	COG6	darkgreen	EC_M4	0.20	3.2E-01	0.12	5.6E-01	-0.74	1.5E-05	-0.03	8.9E-01	0.66	2.8E-04	0.06	7.6E-01	-0.10	6.3E-01	-0.16	4.4E-01	0.05	7.9E-01	-0.72	3.8E-05
ENSCAFG0000001142	HELZ	grey	EC_M10	0.20	3.2E-01	0.21	3.0E-01	-0.28	1.6E-01	0.13	5.2E-01	0.07	7.2E-01	0.19	3.6E-01	-0.17	4.0E-01	0.06	7.9E-01	-0.18	3.8E-01	-0.16	4.3E-01
ENSCAFG0000000617	CDC25B	grey	EC_M10	0.20	3.2E-01	-0.22	2.8E-01	0.64	3.9E-04	0.05	8.1E-01	-0.80	7.2E-07	-0.17	4.2E-01	-0.17	4.0E-01	-0.08	6.9E-01	-0.22	2.8E-01	0.69	9.9E-05
ENSCAFG0000000845	PCGF1	grey	EC_M10	0.20	3.2E-01	-0.62	7.9E-04	0.01	9.5E-01	0.26	1.9E-01	0.04	8.4E-01	0.03	8.9E-01	-0.22	2.9E-01	0.02	9.2E-01	-0.28	1.7E-01	-0.20	3.3E-01
ENSCAFG0000001779	ENSCAFG0000001779	grey	EC_M10	0.20	3.2E-01	-0.14	4.9E-01	0.15	4.7E-01	-0.18	3.9E-01	-0.22	2.8E-01	-0.05	8.2E-01	0.06	7.8E-01	-0.01	9.7E-01	-0.20	3.3E-01	0.13	5.3E-01
ENSCAFG0000000775	MAT2A	grey	EC_M10	0.20	3.2E-01	0.51	8.3E-03	-0.14	4.8E-01	0.15	4.6E-01	-0.17	4.2E-01	-0.10	6.3E-01	0.05	8.0E-01	-0.09	6.5E-01	0.20	3.3E-01	0.11	5.8E-01
ENSCAFG0000000540	LRP1B	grey	EC_M10	0.20	3.2E-01	-0.27	1.8E-01	-0.47	1.6E-02	-0.21	3.1E-01	0.47	1.4E-02	0.31	1.2E-01	0.04	8.4E-01	-0.02	9.4E-01	0.00	9.9E-01	-0.62	7.5E-04
ENSCAFG0000000669	SLCO2A1	turquoise	EC_M6	0.20	3.2E-01	0.49	1.2E-02	-0.10	6.3E-01	-0.18	3.8E-01	-0.19	3.6E-01	-0.20	3.3E-01	-0.16	4.2E-01	-0.13	5.2E-01	0.50	8.9E-03	0.11	6.0E-01

ENSCAFG0000001114	ENSCAFG0000001114	grey	EC_M10	0.20	3.2E-01	0.28	1.6E-01	0.07	7.3E-01	0.44	2.3E-02	-0.36	7.3E-02	-0.25	2.2E-01	0.12	5.6E-01	-0.20	3.3E-01	-0.01	9.7E-01	0.27	1.7E-01
ENSCAFG0000000667	SYNE4	turquoise	EC_M6	0.20	3.2E-01	0.57	2.2E-03	0.14	5.0E-01	0.14	4.9E-01	-0.50	8.5E-03	0.01	9.8E-01	-0.11	6.0E-01	-0.07	7.5E-01	0.12	5.6E-01	0.41	3.8E-02
ENSCAFG0000002965	NUPL2	turquoise	EC_M6	0.20	3.2E-01	0.53	4.9E-03	-0.10	6.2E-01	0.31	1.3E-01	-0.26	2.1E-01	-0.07	7.5E-01	-0.18	3.8E-01	0.00	9.9E-01	-0.09	6.6E-01	0.15	4.6E-01
ENSCAFG0000000329	THAP5	darkgreen	EC_M4	0.20	3.2E-01	0.35	8.1E-02	-0.82	2.3E-07	0.03	9.0E-01	0.64	4.7E-04	0.28	1.7E-01	0.05	8.2E-01	-0.14	4.8E-01	-0.18	3.7E-01	-0.76	7.7E-06
ENSCAFG0000000260	PPP6R1	turquoise	EC_M6	0.20	3.2E-01	0.51	8.2E-03	-0.22	2.8E-01	-0.07	7.3E-01	-0.02	9.1E-01	-0.18	3.7E-01	0.06	7.7E-01	-0.21	3.0E-01	-0.14	5.0E-01	-0.08	7.1E-01
ENSCAFG0000000048	TCF19	grey	EC_M10	0.20	3.3E-01	-0.35	8.2E-02	0.36	7.1E-02	0.23	2.7E-01	-0.36	7.0E-02	0.29	1.5E-01	-0.13	5.4E-01	-0.10	6.3E-01	-0.11	5.8E-01	0.27	1.7E-01
ENSCAFG0000000053	MYCT1	turquoise	EC_M6	0.20	3.3E-01	0.84	6.3E-08	-0.30	1.3E-01	-0.13	5.2E-01	-0.11	5.9E-01	-0.09	6.5E-01	-0.19	3.6E-01	0.05	8.0E-01	0.10	6.3E-01	-0.02	9.3E-01
ENSCAFG0000000673	ENSCAFG0000000673	grey	EC_M10	0.20	3.3E-01	-0.44	2.5E-02	-0.19	3.6E-01	0.13	5.4E-01	0.24	2.4E-01	0.05	8.3E-01	0.05	8.1E-01	-0.12	5.5E-01	-0.05	8.2E-01	-0.33	9.5E-02
ENSCAFG0000000758	LYVE1	turquoise	EC_M6	0.20	3.3E-01	0.91	8.4E-11	-0.53	5.0E-03	-0.20	3.3E-01	0.15	4.7E-01	0.01	9.6E-01	-0.02	9.3E-01	-0.08	6.9E-01	0.13	5.4E-01	-0.24	2.4E-01
ENSCAFG0000000424	ENSCAFG0000000424	grey	EC_M10	0.20	3.3E-01	-0.61	8.3E-04	0.08	7.0E-01	0.29	1.5E-01	-0.03	9.0E-01	-0.02	9.1E-01	-0.14	5.1E-01	0.01	9.6E-01	0.18	3.9E-01	-0.07	7.3E-01
ENSCAFG0000001741	AAGAB	grey	EC_M10	0.20	3.3E-01	0.29	1.6E-01	-0.65	2.9E-04	-0.02	9.1E-01	0.54	4.6E-03	0.15	4.6E-01	-0.15	4.6E-01	0.09	6.6E-01	0.06	7.8E-01	-0.59	1.7E-03
ENSCAFG0000001574	GRIPAP1	grey	EC_M10	0.20	3.3E-01	0.44	2.3E-02	-0.38	5.8E-02	0.20	3.2E-01	0.05	7.9E-01	0.16	4.3E-01	-0.13	5.3E-01	-0.13	5.2E-01	-0.40	4.2E-02	-0.16	4.3E-01
ENSCAFG0000000889	SERINC5	turquoise	EC_M6	0.20	3.3E-01	0.82	2.5E-07	-0.21	3.1E-01	-0.09	6.8E-01	-0.21	3.1E-01	-0.25	2.2E-01	0.14	4.9E-01	-0.01	9.7E-01	-0.13	5.1E-01	0.12	5.5E-01
ENSCAFG0000002306	RHOA	grey	EC_M10	0.20	3.3E-01	-0.06	7.6E-01	-0.60	1.3E-03	-0.29	1.5E-01	0.63	5.9E-04	0.26	2.0E-01	-0.29	1.5E-01	0.09	6.4E-01	-0.05	8.2E-01	-0.76	7.8E-06
ENSCAFG0000000483	ENSCAFG0000000483	grey	EC_M10	0.20	3.3E-01	0.30	1.4E-01	-0.22	2.9E-01	0.00	9.9E-01	-0.01	9.5E-01	-0.07	7.3E-01	-0.06	7.9E-01	-0.07	7.2E-01	0.50	9.1E-03	-0.08	7.1E-01
ENSCAFG0000002769	ENSCAFG0000002769	grey	EC_M10	0.20	3.3E-01	0.30	1.4E-01	-0.22	2.9E-01	0.00	9.9E-01	-0.01	9.5E-01	-0.07	7.3E-01	-0.06	7.9E-01	-0.07	7.2E-01	0.50	9.1E-03	-0.08	7.1E-01
ENSCAFG0000002809	ENSCAFG0000002809	grey	EC_M10	0.20	3.3E-01	-0.14	4.8E-01	0.05	8.0E-01	0.51	8.1E-03	-0.16	4.2E-01	0.36	7.1E-02	-0.12	5.5E-01	-0.20	3.3E-01	-0.23	2.6E-01	0.03	9.0E-01
ENSCAFG0000001900	TTC4	grey	EC_M10	0.20	3.3E-01	-0.55	3.3E-03	-0.03	9.0E-01	0.10	6.2E-01	0.11	6.1E-01	0.21	3.0E-01	-0.25	2.2E-01	0.00	9.9E-01	0.08	7.2E-01	-0.25	2.3E-01
ENSCAFG0000000991	ERG	turquoise	EC_M6	0.20	3.3E-01	0.71	4.9E-05	-0.75	8.5E-06	-0.19	3.5E-01	0.47	1.5E-02	0.12	5.7E-01	-0.04	8.6E-01	-0.16	4.4E-01	-0.02	9.2E-01	-0.59	1.7E-03
ENSCAFG0000000842	SELENOT	darkgreen	EC_M4	0.20	3.3E-01	-0.02	9.3E-01	-0.68	1.3E-04	-0.25	2.2E-01	0.71	5.3E-05	0.20	3.4E-01	-0.21	3.1E-01	0.17	3.9E-01	-0.04	8.6E-01	-0.76	7.0E-06
ENSCAFG0000003005	FCER2	turquoise	EC_M6	0.20	3.3E-01	0.82	3.4E-07	-0.43	2.7E-02	-0.11	6.0E-01	0.06	7.8E-01	-0.13	5.3E-01	-0.08	7.0E-01	-0.08	6.8E-01	-0.11	5.9E-01	-0.16	4.3E-01
ENSCAFG0000001747	ENSCAFG0000001747	grey	EC_M10	0.20	3.3E-01	-0.07	7.3E-01	-0.35	8.1E-02	-0.19	3.4E-01	0.27	1.8E-01	0.03	9.0E-01	-0.15	4.6E-01	0.48	1.2E-02	-0.04	8.4E-01	-0.41	3.7E-02
ENSCAFG0000000178	RTCB	grey	EC_M10	0.20	3.3E-01	0.20	3.3E-01	-0.64	4.0E-04	-0.29	1.5E-01	0.59	1.4E-03	0.33	1.0E-01	-0.03	8.7E-01	-0.11	5.8E-01	0.15	4.5E-01	-0.66	2.4E-04
ENSCAFG0000000091	SLC39A7	darkgreen	EC_M4	0.20	3.3E-01	0.12	5.7E-01	-0.70	7.4E-05	-0.05	8.0E-01	0.60	1.3E-03	0.34	9.2E-02	0.06	7.7E-01	0.19	3.6E-01	0.11	6.0E-01	-0.74	1.5E-05
ENSCAFG0000001066	ENSCAFG0000001066	grey	EC_M10	0.20	3.3E-01	-0.01	9.5E-01	-0.19	3.4E-01	0.22	2.8E-01	0.03	8.7E-01	-0.21	3.1E-01	-0.19	3.6E-01	-0.19	3.5E-01	-0.19	3.4E-01	-0.18	3.8E-01
ENSCAFG0000003273	SEC61A2	grey	EC_M10	0.20	3.3E-01	-0.30	1.4E-01	-0.05	7.9E-01	0.09	6.6E-01	-0.01	9.7E-01	0.09	6.5E-01	0.08	7.1E-01	-0.04	8.5E-01	0.20	3.2E-01	-0.06	7.7E-01
ENSCAFG0000003088	ENSCAFG0000003088	grey	EC_M10	0.20	3.3E-01	-0.09	6.5E-01	-0.23	2.7E-01	-0.05	8.0E-01	0.09	6.5E-01	0.56	3.0E-03	-0.13	5.3E-01	-0.12	5.7E-01	-0.15	4.7E-01	-0.23	2.6E-01
ENSCAFG0000001567	CHERP	grey	EC_M10	0.20	3.3E-01	0.15	4.6E-01	-0.11	5.8E-01	-0.16	4.4E-01	0.00	1.0E+00	0.01	9.7E-01	0.14	5.1E-01	-0.15	4.7E-01	-0.06	7.8E-01	-0.14	5.1E-01
ENSCAFG0000001101	PLA1A	turquoise	EC_M6	0.20	3.3E-01	0.62	7.5E-04	0.06	7.6E-01	0.15	4.7E-01	-0.47	1.5E-02	0.05	8.2E-01	-0.02	9.1E-01	0.13	5.2E-01	-0.27	1.9E-01	0.35	7.6E-02
ENSCAFG0000001562	SIX4	darkgreen	EC_M4	0.20	3.3E-01	-0.32	1.1E-01	-0.39	5.1E-02	-0.10	6.2E-01	0.42	3.4E-02	0.24	2.3E-01	-0.01	9.7E-01	-0.31	1.3E-01	-0.40	4.5E-02	-0.57	2.2E-03
ENSCAFG0000001028	ABCG1	turquoise	EC_M6	0.20	3.3E-01	0.91	1.9E-10	-0.29	1.5E-01	-0.12	5.7E-01	-0.11	6.0E-01	-0.13	5.2E-01	0.00	9.8E-01	-0.13	5.2E-01	-0.08	6.9E-01	0.00	1.0E+00
ENSCAFG0000000469	ENSCAFG0000000469	grey	EC_M10	0.20	3.3E-01	-0.26	2.0E-01	-0.16	4.3E-01	-0.14	5.0E-01	0.11	6.1E-01	-0.03	8.9E-01	0.28	1.6E-01	-0.01	9.5E-01	-0.26	2.1E-01	-0.22	2.7E-01
ENSCAFG0000000254	ENSCAFG0000000254	paleturquoise	EC_M11	0.20	3.3E-01	-0.11	6.0E-01	0.03	8.8E-01	0.62	8.1E-04	-0.16	4.4E-01	-0.08	7.0E-01	-0.09	6.6E-01	-0.11	5.8E-01	-0.03	8.9E-01	0.06	7.8E-01
ENSCAFG0000000212	MRPS9	grey	EC_M10	0.20	3.3E-01	-0.66	2.5E-04	0.30	1.4E-01	0.25	2.2E-01	-0.24	2.4E-01	0.19	3.4E-01	-0.20	3.4E-01	-0.20	3.3E-01	-0.26	1.9E-01	0.13	5.3E-01
ENSCAFG0000001406	PALD1	turquoise	EC_M6	0.20	3.3E-01	0.87	9.9E-09	-0.22	2.8E-01	-0.07	7.4E-01	-0.18	3.8E-01	-0.19	3.5E-01	-0.16	4.3E-01	-0.14	5.0E-01	-0.11	5.9E-01	0.05	8.1E-01
ENSCAFG0000000588	TMX4	turquoise	EC_M6	0.20	3.3E-01	0.42	3.4E-02	-0.35	7.6E-02	0.19	3.6E-01	0.03	8.9E-01	-0.12	5.5E-01	0.13	5.2E-01	0.11	6.0E-01	0.38	5.6E-02	-0.07	7.2E-01
ENSCAFG0000000143	UBQLN1	darkgreen	EC_M4	0.20	3.3E-01	0.33	9.9E-02	-0.80	7.2E-07	-0.31	1.2E-01	0.71	4.4E-05	0.22	2.8E-01	-0.06	7.7E-01	-0.08	7.0E-01	-0.08	7.0E-01	-0.79	1.3E-06
ENSCAFG0000001054	COL17A1	grey	EC_M10	0.20	3.3E-01	0.11	6.1E-01	0.08	6.9E-01	0.17	4.2E-01	-0.27	1.9E-01	0.06	7.7E-01	-0.07	7.2E-01	-0.45	2.1E-02	-0.29	1.6E-01	0.17	4.0E-01
ENSCAFG0000002612	ENSCAFG0000002612	turquoise	EC_M6	0.20	3.3E-01	0.41	3.7E-02	-0.30	1.4E-01	-0.23	2.5E-01	-0.02	9.3E-01	0.20	3.3E-01	-0.20	3.3E-01	-0.26	2.0E-01	0.04	8.3E-01	-0.09	6.5E-01
ENSCAFG0000001574	KDELR2	darkgreen	EC_M4	0.20	3.3E-01	0.24	2.3E-01	-0.80	9.7E-07	-0.14	4.9E-01	0.69	9.8E-05	0.44	2.4E-02	-0.01	9.6E-01	-0.20	3.2E-01	-0.22	2.8E-01	-0.83	2.0E-07
ENSCAFG0000001788	ELAC2	grey	EC_M10	0.20	3.3E-01	-0.52	6.5E-03	0.44	2.5E-02	-0.13	5.2E-01	-0.41	3.9E-02	-0.09	6.8E-01	-0.05	8.2E-01	0.27	1.9E-01	-0.05	8.1E-01	0.31	1.2E-01
ENSCAFG0000000297	PAPOLG	darkgrey	EC_M8	0.20	3.3E-01	0.34	8.7E-02	0.02	9.4E-01	0.05	8.1E-01	-0.45	2.1E-02	-0.02	9.1E-01	-0.01	9.5E-01	-0.20	3.3E-01	0.01	9.7E-01	0.30	1.4E-01
ENSCAFG0000002344	UBB	grey	EC_M10	0.20	3.3E-01	0.26	2.1E-01	-0.48	1.3E-02	-0.16	4.4E-01	0.25	2.2E-01	0.07	7.3E-01	0.27	1.8E-01	-0.09	6.7E-01	-0.15	4.6E-01	-0.34	8.5E-02
ENSCAFG0000002045	ENSCAFG0000002045	grey	EC_M10	0.20	3.3E-01	0.05	8.2E-01	-0.11	6.0E-01	-0.12	5.6E-01	-0.05	7.9E-01	-0.09	6.6E-01	-0.15	4.5E-01	-0.10	6.2E-01	-0.07	7.3E-01	-0.04	8.6E-01
ENSCAFG0000000843	ENSCAFG0000000843	grey	EC_M10	0.20	3.3E-01	-0.17	4.2E-01	-0.34	8.7E-02	0.03	8.9E-01	0.31	1.2E-01	0.10	6.2E-01	-0.23	2.6E-01	0.33	1.0E-01	-0.14	5.1E-01	-0.42	3.1E-02
ENSCAFG0000001073	TERT	grey	EC_M10	0.20	3.3E-01	-0.22	2.8E-01	-0.01	9.7E-01	0.37	6.4E-02	-0.10	6.2E-01	0.22	2.7E-01	-0.18	3.7E-01	0.09	6.8E-01	-0.12	5.7E-01	-0.03	8.9E-01
ENSCAFG0000001999	OLFM3	grey	EC_M10	0.20	3.3E-01	-0.14	5.0E-01	-0.40	4.2E-02	0.28	1.6E-01	0.34	8.5E-02	-0.19	3.6E-01	-0.23	2.6E-01	-0.17	4.2E-01	-0.15	4.8E-01	-0.46	1.8E-02
ENSCAFG0000000236	TMEM131	darkgreen	EC_M4	0.20	3.3E-01	0.35	7.5E-02	-0.71	4.7E-05	-0.07	7.4E-01	0.54	4.4E-03	0.22	2.9E-01	-0.19	3.5E-01	-0.18	3.7E-01	-0.18	3.7E-01	-0.63	5.3E-04
ENSCAFG0000000320	UTP11	turquoise	EC_M6	0.20	3.3E-01	0.46	1.7E-02	-0.32	1.1E-01	0.13	5.3E-01	-0.06	7.8E-01	-0.28	1.6E-01	0.37	6.0E-02	0.17	4.0E-01	0.13	5.1E-01	-0.07	7.4E-01
ENSCAFG0000000841	NDUFS3	grey	EC_M10	0.20	3.3E-01	-0.36	7.5E-02	0.46	1.8E-02	0.00	9.9E-01	-0.47	1.7E-02	-0.15	4.6E-01	-0.30	1.4E-01	-0.20	3.2E-01	-0.27	1.9E-01	0.40	4.3E-02
ENSCAFG0000001988	AK8	grey	EC_M10	0.20	3.3E-01	-0.13	5.4E-01	-0.02	9.3E-01	-0.21	3.1E-01	-0.07	7.3E-01	0.11	5.9E-01	-0.22	2.9E-01	0.06	7.8E-01	0.32	1.2E-01	-0.04	8.6E-01
ENSCAFG0000001054	PPFIA1	grey	EC_M10	0.20	3.3E-01	-0.15	4.5E-01	-0.09	6.7E-01	0.31	1.3E-01	-0.09	6.7E-01	-0.01	9.5E-01	-0.15	4.8E-01	-0.07	7.3E-01	0.03	8.7E-01	0.01	9.5E-01
ENSCAFG0000000748	ENSCAFG0000000748	turquoise	EC_M6	0.20	3.3E-01	0.62	8.2E-04	-0.21	3.1E-01	-0.06	7.8E-01	-0.17	4.0E-01	-0.11	6.1E-01	-0.13	5.2E-01	-0.11	6.0E-01	-0.14	4.9E-01	0.06	7.6E-01
ENSCAFG0000003126	ENSCAFG0000003126	turquoise	EC_M6	0.20	3.3E-01	0.62	8.2E-04	-0.21	3.1E-01	-0.06	7.8E-01	-0.17	4.0E-01	-0.11	6.1E-01	-0.13	5.2E-01	-0.11	6.0E-01	-0.14	4.9E-01	0.06	7.6E-01
ENSCAFG0000001721	AHSA1	grey	EC_M10	0.20	3.3E-01	-0.53	4.9E-03	-0.36	7.3E-02	0.02	9.3E-01	0.49	1.0E-02	0.49	1.1E-02	-0.02	9.1E-01	-0.01	9.8E-01	-0.22	2.8E-01	-0.59	1.5E-03
ENSCAFG0000000008	PIGN	grey	EC_M10	0.20	3.3E-01	-0.12	5.6E-01	0.05	8.2E-01	-0.05	8.1E-01	-0.18	3.7E-01	-0.15	4.6E-01	0.05	8.0E-01	-0.14	5.1E-01	-0.27	1.7E-01	0.09	6.5E-01
ENSCAFG0000000239	SHB	turquoise	EC_M6	0.20	3.3E-01	0.70	6.4E-05	-0.03	8.7E-01	-0.02	9.3E-01	-0.36	7.4E-02	-0.23	2.6E-01	-0.24	2.3E-01	0.06	7.9E-01	-0.02	9.0E-01	0.25	2.2E-01
ENSCAFG0000002556	TINF2	grey	EC_M10	0.20	3.3E-01	0.35	8.1E-02	0.48	1.3E-02	0.19	3.6E-01	-0.85	5.2E-08	-0.13	5.2E-01	0.00	9.8E-01	-0.32	1.1E-01	-0.09	6.7E-01	0.74	1.5E-05
ENSCAFG0000003046	ZNF879	cyan	EC_M2	0.20	3.3E-01	0.28	1.6E-01	0.30	1.4E-01	0.22	2.8E-01	-0.67	1.7E-04	-0.28	1.6E-01	-0.17	4.1E-01	-0.20	3.2E-01	-0.22	2.9E-01	0.57	2.5E-03
ENSCAFG0000000695	RGS20	grey	EC_M10	0.20	3.3E-01	0.24	2.3E-01	-0.64	4.2E-04	-0.22	2.8E-01	0.50	9.6E-03	0.08	6.8E-01	-0.07	7.4E-01	0.02	9.2E-01	0.04	8.5E-01	-0.61	8.3E-04
ENSCAFG0000000200	PIGO	cyan	EC_M2	0.20	3.3E-01	-0.21	3.0E-01	0.68	1.4E-04	0.27	1.9E-01	-0.87	1.2E-08	-0.04	8.3E-01	-0.17	4.0E-01	-0.04	8.5E-01	-0.01	9.6E-01	0.76	6.5E-06
ENSCAFG0000000496	XRCC2	grey	EC_M10	0.20	3.3E-01	-0.29	1.5E-01	0.08	6.9E-01	-0.04	8.6E-01	-0.07	7.4E-01	-0.05	8.3E-01	-0.16	4.3E-01	0.30	1.4E-01	0.19	3.5E-01	-0.01	9.4E-01
ENSCAFG0000003038	POLE3	grey	EC_M10	0.20	3.3E-01	0.04	8.5E-01	0.02	9.1E-01	-0.38	5.4E-02	-0.17	4.2E-01	0.26	2.0E-01	0.03	8.8E-01	0.15	4.5E-01	0.08	7.1E-01	0.06	7.9E-01
ENSCAFG0000001987	PIEZO1	turquoise	EC_M6	0.20	3.4E-01	0.66	2.2E-04	-0.04	8.4E-01	-0.13	5.2E-01	-0.31	1.3E-01	-0.29	1.6E-01	-0.33	9.5E-02	0.22	2.8E-01	0.32	1.1E-01	0.21	3.1E-01
ENSCAFG0000001299	ACE	turquoise	EC_M6	0.20	3.4E-01	0.73	2.5E-05	0.09	6.7E-01	0.03	8.9E-01	-0.49	1.1E-02	-0.22	2.8E-01	-0.27	1.8E-01	-0.10	6.1E-01	-0.06	7.8E-01	0.36	6.9E-02
ENSCAFG0000001382	SPECC1L	grey	EC_M10	0.20	3.4E-01	-0.29	1.5E-01	-0.44	2.4E-02	-0.05	8.0E-01	0.44	2.6E-02	0.13	5.3E-01	-0.11	5.8E-01	-0.05	8.0E-01	0.52	6.3E-03	-0.52	6.9E-03
ENSCAFG0000000609	NDUFAF7	grey	EC_M10	0.20	3.4E-01	-0.39	5.1E-02	-0.53	5.5E-03	-0.20	3.3E-01	0.63	5.5E-04	0.23	2.6E-01	0.00	1.0E+00	0.17	4.0E-01	0.12	5.7E-01	-0.71	4.3E-05
ENSCAFG0000002020	GOLGA1	grey	EC_M10	0.20	3.4E-01	0.06	7.6E-01	0.09	6.5E-01	-0.02	9.1E-01	-0.39	5.0E-02	-0.10	6.4E-01	-0.30	1.3E-01	-0.25	2.3E-01	0.05	8.2E-01	0.30	1.3E-01
ENSCAFG0000001601	EPHA2	turquoise	EC_M6	0.20	3.4E-01	0.62	7.5E-04	-0.58	1.7E-03	-0.24	2.3E-01	0.35	7.9E-02	-0.08	6.8E-01	-0.13	5.3E-01	0.01	9.5E-01	0.29	1.6E-01	-0.44	2.5E-02
ENSCAFG0000002869	ZNF793	grey	EC_M10	0.20	3.4E-01	0.29	1.5E-01	-0.56	2.8E-03	-0.33	1.0E-01	0.38	5.8E-02	0.04	8.7E-01	0.23	2.6E-01	0.08	7.2E-01	0.01	9.5E-01	-0.51	7.7E-03
ENSCAFG0000000004	ENSCAFG0000000004	grey	EC_M10	0.20	3.4E-01	-0.25	2.1E-01	0.08	6.9E-01	-0.12	5.7E-01	-0.20	3.3E-01	-0.13	5.4E-01	0.22	2.8E-01	-0.13	5.3E-01	-0.19	3.6E-01	0.10	6.3E-01
ENSCAFG0000000997	ARMH3	grey	EC_M10	0.20	3.4E-01	-0.28	1.7E-01	-0.48	1.3E-02	0.20	3.4E-01	0.48	1.3E-02	0.50	9.6E-03	-0.21	3.1E-01	-0.09	6.6E-01	0.08	6.8E-01	-0.59	1.4E-03
ENSCAFG0000000292	MOB1B	grey	EC_M10	0.20	3.4E-01	0.13	5.2E-01	0.02	9.1E-01	-0.10	6.2E-01	-0.19	3.5E-01	0.20	3.3E-01	-0.24	2.4E-01	-0.32	1.1E-01	0.00	1.0E+00	0.12	5.7E-01
ENSCAFG0000002961	MSS51	grey	EC_M10	0.20	3.4E-01	-0.16	4.5E-01	0.34	9.3E-02	0.49	1.1E-02	-0.48	1.2E-02	-0.16	4.4E-01	-0.29	1.5E-01	-0.33	9.6E-02	-0.37	6.1E-02	0.39	5.1E-02
ENSCAFG0000001733	LAT	grey	EC_M10	0.20	3.4E-01	-0.05	7.9E-01	-0.16	4.4E-01	-0.08	6.9E-01	0.01	9.8E-01	-0.09	6.8E-01	-0.10	6.2E-01	-0.12	5.6E-01	-0.10	6.2E-01	-0.15	4.8E-01
ENSCAFG0000000420	PSMC2	darkgreen	EC_M4	0.20	3.4E-01	-0.13	5.3E-01	-0.81	4.9E-07	-0.19	3.4E-01	0.85	3.1E-08	0.26	2.0E-01	-0.08	6.8E-01	0.09	6.7E-01	-0.09	6.5E-01	-0.94	6.4E-13
ENSCAFG0000002421	ENSCAFG0000002421	grey	EC_M10	0.20	3.4E-01	0.16	4.3E-01	-0.22	2.7E-01	-0.14	5.0E-01	0.04	8.5E-01	-0.16	4.3E-01	-0.13	5.3E-01	-0.19	3.5E-01	-0.16	4.3E-01	-0.19	3.6E-01
ENSCAFG0000000887	ENSCAFG0000000887	cyan	EC_M2	0.20	3.4E-01	-0.46	1.9E-02	0.25	2.1E-01	-0.01	9.5E-01	-0.29	1.6E-01	0.15	4.7E-01	0.23	2.5E-01	0.05	7.9E-01	-0.48	1.3E-02	0.11	6.1E-01
ENSCAFG0000001989	BANP	grey	EC_M10	0.20	3.4E-01	0.41	3.6E-02	-0.15	4.5E-01	0.16	4.4E-01	-0.25	2.2E-01	-0.05	8.3E-01	-0.01	9.7E-01	0.16	4.5E-01	-0.23	2.6E-01	0.09	6.6E-01
ENSCAFG0000000921	GMDS	grey	EC_M10	0.20	3.4E-01	0.32	1.1E-01	0.17	3.9E-01	-0.07	7.5E-01	-0.44	2.3E-02	-0.19	3.5E-01	-0.32	1.2E-01	0.09	6.8E-01	0.52	6.0E-03	0.39	5.2E-02
ENSCAFG0000001677	MYL5	grey	EC_M10	0.20	3.4E-01	0.29	1.6E-01	-0.33	9.8E-02	0.00	1.0E+00	0.10	6.1E-01	0.13	5.2E-01	0.20	3.2E-01	-0.25	2.3E-01	0.04	8.3E-01	-0.23	2.7E-01
ENSCAFG0000001071	TXLNA	grey	EC_M10	0.20	3.4E-01	0.35	7.7E-02	0.13	5.4E-01	0.11	5.9E-01	-0.38	5.5E-02	-0.02	9.4E-01	-0.19	3.5E-01	-0.26	2.0E-01	-0.30	1.3E-01	0.29	1.6E-01
ENSCAFG0000001340	BUD13	grey	EC_M10	0.20	3.4E-01	0.11	5.8E-01	0.31	1.3E-01	0.12	5.6E-01	-0.59	1.5E-03	-0.17	4.1E-01	0.04	8.6E-01	0.04	8.5E-01	0.39	4.8E-02	0.48	1.2E-02
ENSCAFG0000002363	PRSS22	turquoise	EC_M6	0.20	3.4E-01	0.92	2.6E-11	-0.30	1.3E-01	-0.14	5.0E-01	-0.12	5.6E-01	0.02	9.1E-01	-0.09	6.7E-01	-0.16	4.3E-01	0.15	4.6E-01	0.01	9.6E-01
ENSCAFG0000002574	ENSCAFG0000002574	grey	EC_M10	0.20	3.4E-01	-0.03	8.9E-01	0.22	2.9E-01	0.30	1.3E-01	-0.44	2.3E-02	0.22	2.8E-01	-0.37	6.6E-02	0.05	8.1E-01	-0.05	8.1E-01	0.31	1.3E-01
ENSCAFG0000001835	FBXO38	grey	EC_M10	0.20	3.4E-01	0.59	1.4E-03	-0.50	8.6E-03	0.08	7.1E-01	0.19	3.4E-01	0.22	2.8E-01	-0.02	9.3E-01	-0.22	2.9E-01	0.19	3.5E-01	-0.27	1.8E-01
ENSCAFG0000001042	TDP2	darkgreen	EC_M4	0.20	3.4E-01	0.20	3.3E-01	-0.73	2.0E-05	-0.02	9.2E-01	0.55	3.7E-03	0.07	7.4E-01	-0.04	8.4E-01	0.10	6.1E-01	0.03	8.8E-01	-0.65	3.4E-04
ENSCAFG0000001762	LRRC49	darkgreen	EC_M4	0.20	3.4E-01	-0.73	2.2E-05	-0.04	8.4E-01	0.10	6.3E-01	0.12	5.6E-01	0.24	2.5E-01	0.10	6.3E-01	-0.14	4.9E-01	-0.09	6.6E-01	-0.26	2.0E-01
ENSCAFG0000002306	FYB1	turquoise	EC_M6	0.20	3.4E-01	0.50	9.3E-03	-0.23	2.5E-01	0.10	6.3E-01	-0.06	7.6E-01	-0.22	2.9E-01	-0.18	3.8E-01	0.06	7.7E-01	0.18	3.8E-01	-0.03	9.0E-01
ENSCAFG0000003070	UBE2U	grey	EC_M10	0.19	3.4E-01	0.06	7.6E-01	-0.31	1.3E-01	-0.10	6.1E-01	0.24	2.5E-01	-0.08	6.9E-01	-0.10	6.3E-01	-0.07	7.5E-01	0.00	9.9E-01	-0.32	1.1E-01
ENSCAFG0000001663	ENSCAFG0000001663	grey	EC_M10	0.19	3.4E-01	-0.12	5.7E-01	0.16	4.5E-01	0.37	6.1E-02	-0.27	1.8E-01	-0.07	7.2E-01	-0.04	8.3E-01	-0.07	7.3E-01	0.47	1.4E-02	0.19	3.6E-01
ENSCAFG0000000513	LMBR1	turquoise	EC_M6	0.19	3.4E-01	0.55	3.8E-03	-0.14	4.9E-01	-0.07	7.3E-01	-0.19	3.6E-01	-0.09	6.7E-01	-0.20	3.4E-01	-0.02	9.3E-01	-0.28	1.6E-01	0.13	5.2E-01
ENSCAFG0000001801	COL4A6	turquoise	EC_M6	0.19	3.4E-01	0.82	2.7E-07	-0.12	5.5E-01	-0.09	6.6E-01	-0.31	1.2E-01	-0.08	6.9E-01	-0.08	6.9E-01	-0.09	6.6E-01	-0.15	4.7E-01	0.24	2.5E-01
ENSCAFG0000002921	ENSCAFG0000002921	grey	EC_M10	0.19	3.4E-01	0.09	6.5E-01	0.25	2.2E-01	0.32	1.2E-01	-0.47	1.6E-02	0.03	8.7E-01	0.03	8.9E-01	0.03	9.0E-01	-0.11	6.0E-01	0.33	9.6E-02
ENSCAFG0000002520	ZCCHC3	cyan	EC_M2	0.19	3.4E-01	0.20	3.3E-01	0.49	1.1E-02	0.36	6.8E-02	-0.79	2.0E-06	-0.24	2.4E-01	0.03	8.7E-01	-0.32	1.1E-01	-0.19	3.6E-01	0.68	1.2E-04
ENSCAFG0000000452	MAP3K2	grey	EC_M10	0.19	3.4E-01	-0.23	2.5E-01	-0.53	5.7E-03	-0.33	1.0E-01	0.56	2.9E-03	0.30	1.4E-01	0.15	4.5E-01	-0.07	7.4E-01	-0.04	8.5E-01	-0.66	2.3E-04
ENSCAFG0000000868	ENSCAFG0000000868	grey	EC_M10	0.19	3.4E-01	-0.48	1.3E-02	-0.26	2.0E-01	-0.24	2.5E-01	0.38	5.6E-02	0.15	4.7E-01	-0.32	1.1E-01	0.02	9.3E-01	-0.24	2.3E-01	-0.47	1.7E-02
ENSCAFG0000001746	ENSCAFG0000001746	grey	EC_M10	0.19	3.4E-01	0.27	1.8E-01	-0.32	1.1E-01	-0.13	5.2E-01	0.13	5.2E-01	-0.15	4.6E-01	-0.11	5.9E-01	-0.17	4.0E-01	-0.07	7.3E-01	-0.22	2.8E-01
ENSCAFG0000001825	SHMT1	grey	EC_M10	0.19	3.4E-01	0.54	4.2E-03	0.25	2.3E-01	-0.05	8.1E-01	-0.58	1.9E-03	-0.12	5.4E-01	0.08	7.1E-01	-0.12	5.6E-01	0.15	4.6E-01	0.54	4.0E-03
ENSCAFG0000000862	E2F5	grey	EC_M10	0.19	3.4E-01	-0.38	5.6E-02	-0.45	2.2E-02	-0.25	2.2E-01	0.49	1.1E-02	0.16	4.3E-01	0.15	4.8E-01	0.06	7.7E-01	0.07	7.4E-01	-0.63	6.2E-04
ENSCAFG0000003182	JMJD7	grey	EC_M10	0.19	3.4E-01	-0.16	4.4E-01	0.57	2.3E-03	-0.05	8.1E-01	-0.76	6.7E-06	-0.03	9.0E-01	-0.07	7.3E-01	-0.12	5.6E-01	-0.09	6.5E-01	0.67	1.8E-04
ENSCAFG0000002474	CD55	grey	EC_M10	0.19	3.4E-01	-0.30	1.3E-01	0.17	4.0E-01	0.09	6.7E-01	-0.28	1.6E-01	-0.22	2.8E-01	-0.25	2.1E-01	0.42	3.5E-02	0.17	4.2E-01	0.19	3.6E-01
ENSCAFG0000000538	SERPINH1	darkgreen	EC_M4	0.19	3.4E-01	-0.29	1.5E-01	-0.50	8.7E-03	0.02	9.1E-01	0.55	3.3E-03	0.36	6.9E-02	0.10	6.2E-01	0.04	8.3E-01	-0.03	9.0E-01	-0.71	4.1E-05
ENSCAFG0000000363	RPS11	cyan	EC_M2	0.19	3.4E-01	-0.29	1.6E-01	0.47	1.5E-02	0.18	3.8E-01	-0.64	4.6E-04	0.07	7.3E-01	0.21	3.1E-01	0.00	1.0E+00	-0.44	2.3E-02	0.48	1.3E-02
ENSCAFG0000000373	TUT4	turquoise	EC_M6	0.19	3.4E-01	0.70	6.9E-05	-0.13	5.2E-01	-0.09	6.5E-01	-0.32	1.1E-01	-0.13	5.3E-01	-0.03	8.9E-01	0.02	9.1E-01	-0.11	6.0E-01	0.20	3.2E-01
ENSCAFG0000001826	SLFN14	grey	EC_M10	0.19	3.4E-01	0.21	3.1E-01	0.01	9.5E-01	0.24	2.4E-01	-0.27	1.8E-01	-0.14	5.0E-01	0.20	3.3E-01	-0.18	3.8E-01	0.41	3.9E-02	0.21	3.1E-01
ENSCAFG0000001911	MRM3	cyan	EC_M2	0.19	3.4E-01	-0.01	9.7E-01	0.53	5.0E-03	0.23	2.5E-01	-0.76	5.8E-06	0.00	9.9E-01	0.04	8.4E-01	-0.02	9.2E-01	-0.25	2.3E-01	0.66	2.3E-04
ENSCAFG0000001670	PCNX1	grey	EC_M10	0.19	3.4E-01	0.39	4.9E-02	-0.53	5.1E-03	-0.09	6.7E-01	0.25	2.2E-01	0.18	3.7E-01	-0.15	4.5E-01	-0.17	4.1E-01	0.17	4.2E-01	-0.35	8.3E-02
ENSCAFG0000003073	MEIG1	grey	EC_M10	0.19	3.4E-01	0.02	9.2E-01	-0.24	2.3E-01	0.24	2.3E-01	0.18	3.8E-01	-0.15	4.8E-01	-0.15	4.8E-01	-0.14	4.8E-01	-0.04	8.6E-01	-0.25	2.2E-01
ENSCAFG0000002490	PLA2G4E	turquoise	EC_M6	0.19	3.4E-01	0.77	3.6E-06	-0.14	4.9E-01	0.01	9.5E-01	-0.27	1.8E-01	-0.20	3.4E-01	-0.17	4.0E-01	-0.15	4.7E-01	0.13	5.1E-01	0.16	4.2E-01
ENSCAFG0000000875	DDN	darkgreen	EC_M4	0.19	3.4E-01	-0.12	5.6E-01	-0.17	4.2E-01	-0.13	5.4E-01	0.22	2.8E-01	-0.09	6.6E-01	0.00	9.9E-01	0.01	9.7E-01	0.10	6.3E-01	-0.25	2.2E-01
ENSCAFG0000000876	SFMBT1	turquoise	EC_M6	0.19	3.4E-01	0.73	2.4E-05	-0.19	3.4E-01	-0.06	7.7E-01	-0.26	1.9E-01	-0.01	9.5E-01	0.05	8.2E-01	0.01	9.4E-01	0.42	3.5E-02	0.16	4.4E-01
ENSCAFG0000000316	PELI1	turquoise	EC_M6	0.19	3.5E-01	0.96	6.5E-15	-0.40	4.3E-02	-0.11	6.0E-01	-0.04	8.4E-01	-0.15	4.5E-01	-0.02	9.4E-01	-0.06	7.8E-01	0.12	5.7E-01	-0.06	7.8E-01
ENSCAFG0000001334	LRIG2	cyan	EC_M2	0.19	3.5E-01	-0.13	5.4E-01	0.44	2.5E-02	0.22	2.9E-01	-0.72	3.0E-05	-0.16	4.4E-01	-0.09	6.7E-01	-0.01	9.7E-01	-0.35	8.0E-02	0.56	2.7E-03
ENSCAFG0000000973	TSPAN2	turquoise	EC_M6	0.19	3.5E-01	0.84	5.8E-08	-0.27	1.8E-01	-0.08	6.9E-01	-0.16	4.3E-01	-0.13	5.2E-01	-0.13	5.3E-01	-0.17	4.0E-01	0.25	2.2E-01	0.10	6.4E-01
ENSCAFG0000002364	KRT19	paleturquoise	EC_M11	0.19	3.5E-01	-0.14	4.9E-01	0.06	7.8E-01	0.91	1.3E-10	-0.18	3.9E-01	0.18	3.9E-01	-0.08	7.1E-01	-0.11	5.9E-01	-0.15	4.6E-01	0.09	6.7E-01

ENSCAFG0000000587	RASGRP3	turquoise	EC_M6	0.19	3.5E-01	0.87	6.0E-09	-0.20	3.4E-01	-0.03	8.8E-01	-0.22	2.9E-01	-0.08	7.0E-01	-0.13	5.3E-01	-0.13	5.4E-01	-0.07	7.3E-01	0.09	6.6E-01
ENSCAFG0000000735	ADAMTS6	darkgreen	EC_M4	0.19	3.5E-01	-0.23	2.7E-01	-0.72	2.8E-05	0.00	1.0E+00	0.73	2.3E-05	0.39	4.8E-02	-0.01	9.6E-01	-0.02	9.1E-01	-0.06	7.7E-01	-0.88	4.7E-09
ENSCAFG0000001976	DENND2D	turquoise	EC_M6	0.19	3.5E-01	0.84	5.9E-08	-0.21	2.9E-01	-0.11	6.0E-01	-0.19	3.6E-01	-0.07	7.2E-01	-0.04	8.5E-01	-0.10	6.3E-01	-0.09	6.6E-01	0.07	7.3E-01
ENSCAFG0000001772	PIK3C3	grey	EC_M10	0.19	3.5E-01	-0.32	1.2E-01	-0.08	7.0E-01	0.05	8.0E-01	0.00	9.8E-01	0.05	7.9E-01	-0.12	5.6E-01	0.15	4.6E-01	-0.27	1.8E-01	-0.07	7.4E-01
ENSCAFG0000000260	ENSCAFG0000000260	grey	EC_M10	0.19	3.5E-01	0.19	3.6E-01	-0.04	8.5E-01	0.34	9.2E-02	-0.15	4.7E-01	0.14	5.1E-01	-0.10	6.3E-01	0.18	3.8E-01	-0.20	3.4E-01	0.04	8.5E-01
ENSCAFG0000002986	ENSCAFG0000002986	grey	EC_M10	0.19	3.5E-01	-0.16	4.3E-01	0.14	4.9E-01	0.12	5.4E-01	-0.23	2.6E-01	0.05	8.1E-01	-0.20	3.3E-01	0.23	2.7E-01	0.38	5.8E-02	0.19	3.4E-01
ENSCAFG0000001718	GSTZ1	grey	EC_M10	0.19	3.5E-01	-0.12	5.5E-01	0.33	9.9E-02	0.21	2.9E-01	-0.53	5.3E-03	0.20	3.4E-01	-0.22	2.8E-01	-0.28	1.7E-01	-0.03	9.0E-01	0.39	5.0E-02
ENSCAFG0000001913	RPH3AL	turquoise	EC_M6	0.19	3.5E-01	0.89	1.1E-09	-0.35	7.9E-02	-0.15	4.5E-01	-0.06	7.7E-01	-0.10	6.3E-01	-0.09	6.5E-01	-0.02	9.1E-01	0.36	6.7E-02	-0.02	9.3E-01
ENSCAFG0000000436	FOXA3	grey	EC_M10	0.19	3.5E-01	-0.09	6.5E-01	-0.09	6.6E-01	-0.14	5.0E-01	0.09	6.7E-01	0.37	6.3E-02	0.36	7.5E-02	-0.06	7.5E-01	0.00	9.8E-01	-0.19	3.6E-01
ENSCAFG0000000205	CHIC2	grey	EC_M10	0.19	3.5E-01	0.42	3.5E-02	-0.15	4.6E-01	0.09	6.7E-01	-0.17	3.9E-01	-0.05	8.2E-01	-0.26	2.0E-01	0.13	5.2E-01	-0.01	9.7E-01	0.06	7.8E-01
ENSCAFG0000000642	MAP4K3	grey	EC_M10	0.19	3.5E-01	0.53	5.0E-03	-0.65	3.3E-04	0.03	8.9E-01	0.35	7.8E-02	0.15	4.6E-01	-0.09	6.5E-01	0.04	8.6E-01	0.26	2.1E-01	-0.48	1.3E-02
ENSCAFG0000000291	ENSCAFG0000000291	grey	EC_M10	0.19	3.5E-01	-0.17	3.9E-01	-0.46	1.7E-02	-0.21	3.1E-01	0.53	5.4E-03	0.33	1.0E-01	-0.08	6.9E-01	0.06	7.8E-01	-0.01	9.6E-01	-0.68	1.5E-04
ENSCAFG0000003000	ENSCAFG0000003000	turquoise	EC_M6	0.19	3.5E-01	0.44	2.3E-02	-0.42	3.3E-02	-0.02	9.4E-01	0.17	4.2E-01	0.12	5.6E-01	0.19	3.6E-01	-0.07	7.5E-01	-0.18	3.8E-01	-0.29	1.4E-01
ENSCAFG0000002994	ZFP14	darkgrey	EC_M8	0.19	3.5E-01	0.38	5.4E-02	0.25	2.2E-01	0.07	7.2E-01	-0.64	4.4E-04	-0.26	1.9E-01	-0.09	6.6E-01	0.09	6.6E-01	0.38	5.8E-02	0.56	3.2E-03
ENSCAFG0000000998	NCOA5	grey	EC_M10	0.19	3.5E-01	0.06	7.6E-01	0.50	9.0E-03	0.00	9.8E-01	-0.75	8.7E-06	-0.10	6.2E-01	-0.40	4.2E-02	0.03	8.9E-01	0.15	4.6E-01	0.63	5.6E-04
ENSCAFG0000000683	NR1H4	grey	EC_M10	0.19	3.5E-01	0.09	6.5E-01	-0.36	7.1E-02	0.13	5.3E-01	0.19	3.6E-01	-0.10	6.3E-01	-0.20	3.4E-01	0.26	2.0E-01	0.37	6.1E-02	-0.33	1.0E-01
ENSCAFG0000000602	ENSCAFG0000000602	grey	EC_M10	0.19	3.5E-01	-0.15	4.6E-01	0.42	3.4E-02	0.28	1.7E-01	-0.63	5.1E-04	-0.01	9.8E-01	0.02	9.0E-01	0.03	8.8E-01	0.09	6.7E-01	0.53	5.8E-03
ENSCAFG0000001672	STARD8	turquoise	EC_M6	0.19	3.5E-01	0.69	1.1E-04	-0.38	5.7E-02	-0.39	4.7E-02	0.10	6.4E-01	-0.20	3.4E-01	0.17	4.0E-01	-0.11	5.8E-01	0.07	7.2E-01	-0.17	4.1E-01
ENSCAFG0000000497	ENSCAFG0000000497	turquoise	EC_M6	0.19	3.5E-01	0.62	8.0E-04	-0.36	7.5E-02	-0.14	4.8E-01	0.03	8.8E-01	-0.11	6.0E-01	-0.13	5.4E-01	-0.08	7.0E-01	-0.11	5.9E-01	-0.11	5.8E-01
ENSCAFG0000000686	ZFAND4	darkgreen	EC_M4	0.19	3.5E-01	0.10	6.3E-01	-0.69	8.5E-05	-0.09	6.5E-01	0.66	2.8E-04	0.37	6.1E-02	-0.26	2.0E-01	-0.16	4.5E-01	-0.20	3.3E-01	-0.78	2.3E-06
ENSCAFG0000002908	ARF4	darkgreen	EC_M4	0.19	3.5E-01	0.31	1.2E-01	-0.90	6.8E-10	-0.10	6.3E-01	0.75	1.3E-05	0.25	2.1E-01	-0.04	8.6E-01	0.00	9.9E-01	-0.01	9.4E-01	-0.85	3.6E-08
ENSCAFG0000000350	NOBOX	turquoise	EC_M6	0.19	3.5E-01	0.68	1.4E-04	-0.10	6.4E-01	-0.15	4.6E-01	-0.26	1.9E-01	-0.09	6.5E-01	-0.11	6.0E-01	0.30	1.4E-01	-0.02	9.3E-01	0.18	3.7E-01
ENSCAFG0000001857	ENSCAFG0000001857	grey	EC_M10	0.19	3.5E-01	-0.14	5.0E-01	-0.04	8.4E-01	0.01	9.4E-01	-0.09	6.7E-01	-0.21	3.0E-01	0.04	8.3E-01	0.06	7.7E-01	-0.42	3.5E-02	-0.05	8.1E-01
ENSCAFG0000000655	IKBIP	darkgreen	EC_M4	0.19	3.5E-01	0.09	6.5E-01	-0.86	1.2E-08	-0.14	5.0E-01	0.83	1.9E-07	0.30	1.4E-01	-0.02	9.1E-01	0.13	5.2E-01	0.03	8.9E-01	-0.89	1.1E-09
ENSCAFG0000001376	ENSCAFG0000001376	grey	EC_M10	0.19	3.5E-01	-0.05	8.1E-01	0.16	4.2E-01	0.24	2.4E-01	-0.40	4.4E-02	0.09	6.5E-01	0.08	6.8E-01	0.07	7.2E-01	0.06	7.8E-01	0.30	1.3E-01
ENSCAFG0000002848	ENSCAFG0000002848	grey	EC_M10	0.19	3.5E-01	0.44	2.5E-02	-0.10	6.4E-01	0.27	1.8E-01	-0.23	2.7E-01	0.05	7.9E-01	-0.03	8.8E-01	0.03	8.9E-01	0.48	1.3E-02	0.16	4.5E-01
ENSCAFG0000002990	HIKESHI	grey	EC_M10	0.19	3.5E-01	-0.14	5.0E-01	-0.57	2.2E-03	0.03	8.9E-01	0.55	3.3E-03	0.56	2.8E-03	-0.15	4.6E-01	0.14	5.0E-01	-0.12	5.6E-01	-0.68	1.3E-04
ENSCAFG0000000979	ENSCAFG0000000979	turquoise	EC_M6	0.19	3.5E-01	0.62	6.9E-04	-0.24	2.3E-01	0.16	4.3E-01	-0.05	7.9E-01	-0.26	2.0E-01	-0.11	5.8E-01	-0.09	6.7E-01	0.23	2.7E-01	0.04	8.4E-01
ENSCAFG0000000411	TRPC3	violet	EC_M7	0.19	3.5E-01	-0.10	6.4E-01	-0.14	5.0E-01	0.33	1.0E-01	0.01	9.5E-01	0.73	1.9E-05	-0.08	7.0E-01	-0.09	6.5E-01	-0.16	4.2E-01	-0.16	4.3E-01
ENSCAFG0000000366	TBC1D9	turquoise	EC_M6	0.19	3.5E-01	0.64	4.1E-04	-0.49	1.1E-02	-0.13	5.4E-01	0.19	3.6E-01	-0.02	9.4E-01	-0.06	7.6E-01	-0.16	4.3E-01	0.50	9.0E-03	-0.28	1.7E-01
ENSCAFG0000003254	PROKR2	darkgreen	EC_M4	0.19	3.5E-01	-0.13	5.4E-01	-0.63	5.9E-04	-0.22	2.8E-01	0.64	4.6E-04	0.40	4.5E-02	0.20	3.4E-01	-0.19	3.5E-01	-0.11	6.0E-01	-0.73	2.3E-05
ENSCAFG0000000363	ENSCAFG0000000363	grey	EC_M10	0.19	3.5E-01	0.22	2.7E-01	-0.05	7.9E-01	0.21	3.1E-01	-0.27	1.9E-01	0.01	9.7E-01	-0.39	5.2E-02	-0.38	5.5E-02	-0.18	3.9E-01	0.16	4.4E-01
ENSCAFG0000002937	UBE2S	grey	EC_M10	0.19	3.5E-01	-0.03	9.0E-01	-0.51	7.2E-03	-0.24	2.3E-01	0.56	2.7E-03	0.24	2.4E-01	0.12	5.6E-01	-0.03	8.8E-01	0.08	6.9E-01	-0.63	5.1E-04
ENSCAFG0000001225	ENSCAFG0000001225	turquoise	EC_M6	0.19	3.5E-01	0.30	1.4E-01	-0.10	6.3E-01	-0.14	5.0E-01	-0.10	6.2E-01	0.22	2.9E-01	-0.18	3.8E-01	-0.19	3.6E-01	-0.28	1.6E-01	-0.06	7.6E-01
ENSCAFG0000000061	ZDHHC14	turquoise	EC_M6	0.19	3.5E-01	0.85	2.9E-08	-0.32	1.1E-01	0.06	7.7E-01	-0.04	8.6E-01	-0.23	2.6E-01	0.02	9.3E-01	-0.20	3.2E-01	0.15	4.6E-01	-0.02	9.1E-01
ENSCAFG0000002859	ENSCAFG0000002859	turquoise	EC_M6	0.19	3.5E-01	0.65	3.2E-04	-0.32	1.1E-01	-0.10	6.3E-01	0.02	9.0E-01	0.16	4.3E-01	-0.16	4.2E-01	0.13	5.1E-01	-0.16	4.2E-01	-0.17	4.1E-01
ENSCAFG0000000685	ENSCAFG0000000685	grey	EC_M10	0.19	3.5E-01	0.25	2.2E-01	-0.15	4.6E-01	-0.21	2.9E-01	-0.08	7.0E-01	-0.26	2.1E-01	0.02	9.4E-01	-0.22	2.7E-01	-0.34	9.2E-02	-0.03	8.9E-01
ENSCAFG0000001642	TNFRSF1B	turquoise	EC_M6	0.19	3.5E-01	0.89	8.0E-10	-0.23	2.7E-01	-0.06	7.9E-01	-0.18	3.7E-01	-0.14	4.9E-01	0.02	9.3E-01	-0.18	3.7E-01	-0.03	8.9E-01	0.09	6.5E-01
ENSCAFG0000000018	STX7	darkgreen	EC_M4	0.19	3.5E-01	0.39	4.9E-02	-0.79	1.2E-06	0.06	7.8E-01	0.59	1.5E-03	0.24	2.4E-01	-0.33	9.6E-02	-0.07	7.3E-01	0.09	6.5E-01	-0.70	6.0E-05
ENSCAFG0000001654	ARHGEF9	turquoise	EC_M6	0.19	3.5E-01	0.64	4.3E-04	-0.09	6.6E-01	-0.17	4.0E-01	-0.20	3.3E-01	-0.12	5.5E-01	-0.07	7.3E-01	-0.07	7.3E-01	-0.06	7.7E-01	0.13	5.3E-01
ENSCAFG0000000834	ENSCAFG0000000834	darkgreen	EC_M4	0.19	3.5E-01	-0.05	8.0E-01	-0.66	2.3E-04	-0.10	6.3E-01	0.63	6.0E-04	0.25	2.2E-01	-0.19	3.5E-01	0.10	6.3E-01	-0.27	1.8E-01	-0.72	2.9E-05
ENSCAFG0000000073	CDC42SE2	turquoise	EC_M6	0.19	3.5E-01	0.66	2.3E-04	-0.54	4.2E-03	0.01	9.5E-01	0.30	1.4E-01	-0.13	5.2E-01	-0.25	2.2E-01	0.07	7.4E-01	0.06	7.7E-01	-0.39	5.2E-02
ENSCAFG0000003049	ENSCAFG0000003049	grey	EC_M10	0.19	3.5E-01	-0.16	4.3E-01	-0.50	9.7E-03	-0.15	4.5E-01	0.44	2.3E-02	0.46	1.8E-02	0.08	6.9E-01	-0.01	9.7E-01	-0.29	1.5E-01	-0.61	1.0E-03
ENSCAFG0000002121	ENSCAFG0000002121	grey	EC_M10	0.19	3.5E-01	0.32	1.1E-01	-0.19	3.6E-01	0.14	4.8E-01	-0.13	5.2E-01	0.32	1.1E-01	-0.20	3.2E-01	0.01	9.6E-01	0.25	2.2E-01	0.04	8.6E-01
ENSCAFG0000000487	NUB1	darkgreen	EC_M4	0.19	3.5E-01	-0.17	4.1E-01	-0.73	2.0E-05	0.08	6.9E-01	0.75	1.2E-05	0.31	1.3E-01	-0.13	5.4E-01	-0.22	2.8E-01	0.01	9.7E-01	-0.84	1.0E-07
ENSCAFG0000000255	INVS	grey	EC_M10	0.19	3.6E-01	-0.74	1.8E-05	0.10	6.2E-01	0.29	1.6E-01	-0.02	9.2E-01	0.04	8.4E-01	-0.30	1.3E-01	0.02	9.2E-01	-0.11	6.0E-01	-0.08	6.9E-01
ENSCAFG0000000795	ROBO2	grey	EC_M10	0.19	3.6E-01	0.26	2.0E-01	-0.59	1.4E-03	-0.14	5.1E-01	0.48	1.2E-02	0.15	4.8E-01	-0.18	3.7E-01	0.15	4.7E-01	-0.11	6.1E-01	-0.59	1.5E-03
ENSCAFG0000001091	ARHGAP31	darkgreen	EC_M4	0.19	3.6E-01	0.20	3.3E-01	-0.78	2.4E-06	0.05	8.1E-01	0.69	8.3E-05	0.20	3.4E-01	-0.11	5.9E-01	-0.11	5.9E-01	-0.04	8.6E-01	-0.83	1.8E-07
ENSCAFG0000000207	ENSCAFG0000000207	turquoise	EC_M6	0.19	3.6E-01	0.65	3.6E-04	0.00	9.9E-01	-0.07	7.2E-01	-0.37	6.2E-02	-0.03	8.9E-01	-0.22	2.8E-01	0.00	9.8E-01	0.07	7.2E-01	0.29	1.5E-01
ENSCAFG0000001347	PDK3	turquoise	EC_M6	0.19	3.6E-01	0.57	2.5E-03	-0.20	3.3E-01	-0.11	6.0E-01	-0.10	6.4E-01	-0.11	5.8E-01	-0.08	6.9E-01	-0.12	5.7E-01	0.58	1.9E-03	0.06	7.6E-01
ENSCAFG0000000246	NANS	darkgreen	EC_M4	0.19	3.6E-01	0.14	5.0E-01	-0.77	3.8E-06	-0.26	2.0E-01	0.75	1.3E-05	0.27	1.9E-01	-0.06	7.8E-01	-0.07	7.3E-01	-0.26	2.1E-01	-0.87	7.8E-09
ENSCAFG0000002885	ENSCAFG0000002885	grey	EC_M10	0.19	3.6E-01	0.13	5.2E-01	-0.50	9.1E-03	-0.05	8.1E-01	0.37	6.3E-02	0.18	3.8E-01	0.22	2.7E-01	0.08	7.1E-01	0.47	1.6E-02	-0.46	1.8E-02
ENSCAFG0000002986	ENSCAFG0000002986	grey	EC_M10	0.19	3.6E-01	0.24	2.4E-01	-0.32	1.1E-01	-0.14	5.0E-01	0.15	4.6E-01	0.56	2.8E-03	-0.19	3.6E-01	-0.14	4.9E-01	-0.22	2.7E-01	-0.24	2.4E-01
ENSCAFG0000000632	KCTD16	darkgreen	EC_M4	0.19	3.6E-01	0.22	2.8E-01	-0.70	6.1E-05	-0.27	1.8E-01	0.58	1.9E-03	0.16	4.3E-01	-0.20	3.3E-01	0.06	7.6E-01	0.01	9.5E-01	-0.71	5.8E-05
ENSCAFG0000002467	FBXO47	grey	EC_M10	0.19	3.6E-01	-0.08	6.9E-01	-0.31	1.3E-01	-0.03	8.8E-01	0.20	3.2E-01	0.49	1.2E-02	-0.03	8.9E-01	-0.14	5.1E-01	0.27	1.8E-01	-0.34	9.3E-02
ENSCAFG0000001721	ENSCAFG0000001721	grey	EC_M10	0.19	3.6E-01	-0.49	1.2E-02	-0.10	6.1E-01	0.04	8.4E-01	0.16	4.3E-01	0.10	6.4E-01	-0.10	6.2E-01	-0.10	6.2E-01	-0.17	4.0E-01	-0.33	1.0E-01
ENSCAFG0000000927	SERPINB1	grey	EC_M10	0.19	3.6E-01	-0.41	3.5E-02	-0.14	4.9E-01	-0.13	5.4E-01	0.19	3.5E-01	-0.12	5.5E-01	-0.27	1.8E-01	0.35	7.8E-02	0.21	3.1E-01	-0.29	1.6E-01
ENSCAFG0000001101	HEPACAM	grey	EC_M10	0.19	3.6E-01	0.26	1.9E-01	-0.56	2.8E-03	-0.06	7.7E-01	0.37	6.4E-02	0.34	8.5E-02	-0.15	4.6E-01	-0.21	3.1E-01	0.36	7.0E-02	-0.50	9.9E-03
ENSCAFG0000001546	USHBP1	turquoise	EC_M6	0.19	3.6E-01	0.93	5.4E-12	-0.22	2.9E-01	-0.10	6.1E-01	-0.21	3.0E-01	-0.14	5.0E-01	-0.13	5.3E-01	-0.13	5.1E-01	-0.07	7.4E-01	0.09	6.5E-01
ENSCAFG0000002932	WNT5A	grey	EC_M10	0.19	3.6E-01	-0.01	9.5E-01	-0.46	1.7E-02	-0.16	4.4E-01	0.42	3.1E-02	0.44	2.6E-02	0.43	3.0E-02	-0.10	6.3E-01	-0.09	6.8E-01	-0.53	5.3E-03
ENSCAFG0000003141	STYX	grey	EC_M10	0.19	3.6E-01	-0.45	2.2E-02	0.20	3.3E-01	0.13	5.3E-01	-0.22	2.7E-01	0.17	4.1E-01	-0.16	4.4E-01	-0.08	7.0E-01	-0.41	3.5E-02	0.12	5.7E-01
ENSCAFG0000002606	ENSCAFG0000002606	grey	EC_M10	0.19	3.6E-01	-0.03	9.0E-01	-0.23	2.6E-01	-0.11	5.9E-01	0.17	4.2E-01	0.65	3.3E-04	0.00	1.0E+00	-0.11	6.0E-01	-0.04	8.5E-01	-0.31	1.3E-01
ENSCAFG0000003195	ENSCAFG0000003195	grey	EC_M10	0.19	3.6E-01	0.09	6.6E-01	0.40	4.4E-02	0.12	5.7E-01	-0.70	6.5E-05	0.07	7.5E-01	0.16	4.3E-01	-0.19	3.5E-01	-0.22	2.9E-01	0.59	1.5E-03
ENSCAFG0000000832	TM4SF18	turquoise	EC_M6	0.19	3.6E-01	0.86	1.4E-08	-0.21	3.1E-01	-0.04	8.3E-01	-0.19	3.4E-01	-0.14	4.9E-01	-0.15	4.5E-01	-0.18	3.9E-01	-0.08	7.0E-01	0.06	7.6E-01
ENSCAFG0000001516	SELE	turquoise	EC_M6	0.19	3.6E-01	0.86	2.1E-08	-0.28	1.6E-01	-0.07	7.5E-01	-0.10	6.3E-01	-0.06	7.6E-01	-0.17	4.2E-01	-0.12	5.4E-01	0.14	4.8E-01	-0.01	9.7E-01
ENSCAFG0000000617	RAB11FIP1	darkgreen	EC_M4	0.19	3.6E-01	0.40	4.3E-02	-0.74	1.3E-05	-0.20	3.3E-01	0.57	2.4E-03	-0.05	7.9E-01	-0.22	2.8E-01	0.08	6.9E-01	-0.12	5.6E-01	-0.69	9.7E-05
ENSCAFG0000002406	GSTA4	grey	EC_M10	0.19	3.6E-01	-0.65	3.4E-04	0.34	9.1E-02	0.29	1.6E-01	-0.32	1.1E-01	0.08	6.8E-01	-0.26	2.0E-01	0.06	7.8E-01	0.16	4.4E-01	0.22	2.9E-01
ENSCAFG0000001634	ENSCAFG0000001634	grey	EC_M10	0.19	3.6E-01	0.21	3.0E-01	-0.38	5.5E-02	-0.09	6.7E-01	0.28	1.6E-01	-0.12	5.6E-01	-0.11	6.1E-01	-0.05	8.1E-01	-0.06	7.7E-01	-0.37	6.5E-02
ENSCAFG0000000368	CREM	turquoise	EC_M6	0.19	3.6E-01	0.86	1.5E-08	-0.45	2.2E-02	-0.17	4.1E-01	0.02	9.2E-01	-0.15	4.7E-01	-0.02	9.3E-01	-0.11	6.1E-01	0.19	3.5E-01	-0.12	5.5E-01
ENSCAFG0000000053	ENSCAFG0000000053	grey	EC_M10	0.19	3.6E-01	-0.19	3.4E-01	-0.12	5.5E-01	-0.09	6.7E-01	0.07	7.5E-01	-0.09	6.6E-01	-0.01	9.5E-01	-0.08	6.9E-01	-0.14	5.0E-01	-0.16	4.3E-01
ENSCAFG0000000210	JAK2	cyan	EC_M2	0.19	3.6E-01	0.02	9.1E-01	0.32	1.1E-01	0.21	2.9E-01	-0.60	1.3E-03	-0.19	3.6E-01	-0.04	8.4E-01	-0.17	4.1E-01	-0.41	3.7E-02	0.52	7.1E-03
ENSCAFG0000000987	ITGB6	grey	EC_M10	0.19	3.6E-01	-0.13	5.2E-01	-0.06	7.8E-01	-0.23	2.5E-01	-0.06	7.6E-01	-0.12	5.7E-01	-0.10	6.3E-01	-0.15	4.7E-01	0.31	1.3E-01	-0.02	9.2E-01
ENSCAFG0000001540	PARP11	grey	EC_M10	0.19	3.6E-01	-0.03	8.9E-01	-0.64	4.1E-04	-0.22	2.8E-01	0.57	2.2E-03	0.44	2.3E-02	-0.11	6.0E-01	0.03	8.9E-01	-0.13	5.1E-01	-0.70	7.9E-05
ENSCAFG0000002758	ENSCAFG0000002758	grey	EC_M10	0.19	3.6E-01	0.18	3.9E-01	0.07	7.2E-01	0.27	1.8E-01	-0.38	5.5E-02	-0.13	5.2E-01	0.02	9.0E-01	-0.23	2.6E-01	-0.20	3.2E-01	0.22	2.8E-01
ENSCAFG0000001380	TMEM47	turquoise	EC_M6	0.19	3.6E-01	0.77	4.8E-06	-0.51	7.2E-03	0.03	8.8E-01	0.14	5.0E-01	-0.02	9.3E-01	0.12	5.6E-01	-0.01	9.5E-01	-0.23	2.5E-01	-0.24	2.4E-01
ENSCAFG0000001178	GPER1	grey	EC_M10	0.19	3.6E-01	-0.24	2.5E-01	-0.08	6.8E-01	-0.09	6.7E-01	0.02	9.3E-01	-0.17	4.1E-01	-0.19	3.6E-01	-0.26	2.0E-01	0.29	1.5E-01	-0.14	5.1E-01
ENSCAFG0000000404	LAMA4	turquoise	EC_M6	0.19	3.6E-01	0.61	8.8E-04	-0.35	8.2E-02	-0.04	8.5E-01	0.04	8.4E-01	0.14	5.1E-01	0.24	2.5E-01	-0.42	3.3E-02	-0.27	1.9E-01	-0.13	5.3E-01
ENSCAFG0000000928	F2RL1	cyan	EC_M2	0.19	3.6E-01	-0.31	1.2E-01	0.49	1.2E-02	0.08	7.0E-01	-0.59	1.4E-03	-0.19	3.6E-01	-0.24	2.4E-01	0.10	6.4E-01	-0.10	6.1E-01	0.49	1.1E-02
ENSCAFG0000001928	ENSCAFG0000001928	grey	EC_M10	0.19	3.6E-01	0.06	7.6E-01	-0.05	8.2E-01	-0.14	5.0E-01	-0.14	5.1E-01	-0.15	4.5E-01	0.31	1.2E-01	-0.01	9.6E-01	-0.02	9.3E-01	0.06	7.6E-01
ENSCAFG0000003054	PJA1	grey	EC_M10	0.19	3.6E-01	-0.03	9.0E-01	-0.43	2.7E-02	-0.27	1.8E-01	0.35	7.8E-02	0.36	7.3E-02	0.03	8.8E-01	-0.24	2.3E-01	-0.34	9.1E-02	-0.51	8.0E-03
ENSCAFG0000000452	FGD5	turquoise	EC_M6	0.19	3.6E-01	0.91	1.5E-10	-0.29	1.6E-01	-0.12	5.7E-01	-0.12	5.6E-01	-0.11	6.0E-01	-0.19	3.5E-01	-0.11	6.1E-01	0.03	8.9E-01	0.00	9.8E-01
ENSCAFG0000000023	KIF5A	turquoise	EC_M6	0.19	3.6E-01	0.50	8.7E-03	-0.30	1.4E-01	0.11	5.8E-01	0.03	8.8E-01	0.12	5.8E-01	-0.11	5.9E-01	0.01	9.7E-01	0.13	5.3E-01	-0.12	5.6E-01
ENSCAFG0000000530	PSD3	grey	EC_M10	0.19	3.7E-01	-0.55	3.4E-03	0.12	5.7E-01	0.38	5.2E-02	-0.14	5.0E-01	0.16	4.3E-01	-0.34	8.6E-02	0.10	6.3E-01	0.05	8.0E-01	-0.01	9.7E-01
ENSCAFG0000001861	JAK1	turquoise	EC_M6	0.19	3.7E-01	0.76	5.7E-06	0.05	8.0E-01	0.07	7.5E-01	-0.48	1.3E-02	-0.31	1.3E-01	-0.29	1.5E-01	-0.16	4.4E-01	0.08	6.8E-01	0.43	3.0E-02
ENSCAFG0000001569	PRKCH	turquoise	EC_M6	0.19	3.7E-01	0.90	6.2E-10	-0.26	2.0E-01	-0.14	5.0E-01	-0.15	4.8E-01	-0.06	7.7E-01	-0.07	7.5E-01	-0.08	6.8E-01	-0.07	7.4E-01	0.02	9.3E-01
ENSCAFG0000000536	RIN2	grey	EC_M10	0.18	3.7E-01	0.33	1.0E-01	0.09	6.8E-01	0.06	7.6E-01	-0.48	1.3E-02	0.05	8.0E-01	-0.10	6.2E-01	0.06	7.7E-01	0.44	2.3E-02	0.33	9.7E-02
ENSCAFG0000001874	ENSCAFG0000001874	grey	EC_M10	0.18	3.7E-01	-0.14	4.8E-01	-0.24	2.4E-01	-0.16	4.4E-01	0.11	6.0E-01	0.41	3.5E-02	0.26	1.9E-01	-0.14	4.8E-01	-0.19	3.5E-01	-0.27	1.9E-01
ENSCAFG0000000424	SLC36A4	darkgreen	EC_M4	0.18	3.7E-01	0.25	2.1E-01	-0.86	2.3E-08	-0.11	5.9E-01	0.72	3.0E-05	0.14	4.9E-01	0.20	3.3E-01	0.02	9.3E-01	-0.14	4.9E-01	-0.81	4.3E-07
ENSCAFG0000001523	SLC19A2	grey	EC_M10	0.18	3.7E-01	-0.72	3.0E-05	0.13	5.3E-01	0.01	9.7E-01	0.02	9.4E-01	-0.06	7.9E-01	0.10	6.2E-01	0.05	8.0E-01	-0.37	6.5E-02	-0.11	6.0E-01
ENSCAFG0000000232	MGAT4A	turquoise	EC_M6	0.18	3.7E-01	0.78	3.1E-06	-0.60	1.3E-03	-0.28	1.7E-01	0.30	1.4E-01	-0.04	8.6E-01	0.12	5.5E-01	0.06	7.8E-01	-0.04	8.5E-01	-0.38	5.5E-02
ENSCAFG0000000405	ENSCAFG0000000405	grey	EC_M10	0.18	3.7E-01	-0.07	7.4E-01	0.18	3.7E-01	0.19	3.5E-01	-0.35	8.2E-02	-0.47	1.5E-02	0.05	8.0E-01	-0.41	4.0E-02	-0.49	1.0E-02	0.26	2.0E-01
ENSCAFG0000000716	SMIM15	grey	EC_M10	0.18	3.7E-01	0.00	9.9E-01	-0.50	1.0E-02	-0.16	4.3E-01	0.32	1.2E-01	0.42	3.1E-02	0.03	8.7E-01	-0.02	9.2E-01	-0.20	3.3E-01	-0.45	2.0E-02
ENSCAFG0000000798	MCTP1	turquoise	EC_M6	0.18	3.7E-01	0.79	1.2E-06	-0.10	6.4E-01	-0.09	6.6E-01	-0.33	1.0E-01	-0.08	6.9E-01	-0.08	6.9E-01	-0.09	6.5E-01	-0.15	4.6E-01	0.26	2.0E-01
ENSCAFG0000000920	NR4A2	turquoise	EC_M6	0.18	3.7E-01	0.32	1.2E-01	-0.21	3.1E-01	0.10	6.3E-01	-0.10	6.2E-01	-0.01	9.8E-01	-0.02	9.0E-01	0.06	7.6E-01	-0.01	9.7E-01	-0.07	7.5E-01
ENSCAFG0000000591	LRRC45	cyan	EC_M2	0.18	3.7E-01	0.01	9.4E-01	0.55	3.5E-03	0.30	1.4E-01	-0.83	1.6E-07	-0.12	5.5E-01	-0.06	7.9E-01	-0.10	6.3E-01	0.19	3.7E-01	0.75	1.1E-05
ENSCAFG0000001650	KLF8	grey	EC_M10	0.18	3.7E-01	-0.16	4.3E-01	0.08	7.0E-01	0.60	1.3E-03	-0.23	2.6E-01	-0.04	8.4E-01	-0.12	5.7E-01	-0.12	5.6E-01	-0.15	4.5E-01	0.11	6.1E-01
ENSCAFG0000002634	ENSCAFG0000002634	grey	EC_M10	0.18	3.7E-01	-0.16	4.3E-01	0.08	7.0E-01	0.60	1.3E-03	-0.23	2.6E-01	-0.04	8.4E-01	-0.12	5.7E-01	-0.12	5.6E-01	-0.15	4.5E-01	0.11	6.1E-01
ENSCAFG0000000877	EIF2AK4	grey	EC_M10	0.18	3.7E-01	-0.62	7.6E-04	0.27	1.8E-01	0.32	1.1E-01	-0.31	1.2E-01	-0.22	2.8E-01	0.05	7.9E-01	-0.07	7.5E-01	-0.23	2.7E-01	0.19	3.6E-01
ENSCAFG0000002935	KCNK5	turquoise	EC_M6	0.18	3.7E-01	0.85	3.4E-08	-0.09	6.5E-01	-0.07	7.4E-01	-0.32	1.1E-01	-0.10	6.4E-01	-0.21	2.9E-01	-0.23	2.6E-01	-0.23	2.6E-01	0.22	2.8E-01
ENSCAFG0000000387	TULP2	grey	EC_M10	0.18	3.7E-01	0.09	6.7E-01	0.03	8.8E-01	-0.21	3.0E-01	-0.19	3.5E-01	0.15	4.6E-01	-0.29	1.6E-01	-0.21	3.0E-01	-0.08	7.1E-01	0.06	7.6E-01
ENSCAFG0000000031	ATP23	cyan	EC_M2	0.18	3.7E-01	-0.25	2.3E-01	0.40	4.3E-02	0.19	3.5E-01	-0.55	3.9E-03	0.20	3.2E-01	-0.05	7.9E-01	-0.32	1.1E-01	-0.02	9.3E-01	0.46	1.9E-02
ENSCAFG0000001608	BRD4	turquoise	EC_M6	0.18	3.7E-01	0.50	9.5E-03	-0.41	3.9E-02	-0.13	5.4E-01	0.15	4.8E-01	0.03	8.8E-01	0.13	5.1E-01	-0.03	8.7E-01	-0.46	1.9E-02	-0.30	1.3E-01
ENSCAFG0000001080	ENSCAFG0000001080	turquoise	EC_M6	0.18	3.7E-01	0.35	8.3E-02	-0.07	7.4E-01	-0.25	2.2E-01	-0.15	4.6E-01	-0.19	3.4E-01	-0.15	4.6E-01	0.20	3.3E-01	-0.01	9.5E-01	0.09	6.7E-01
ENSCAFG0000000800	CHRM5	turquoise	EC_M6	0.18	3.7E-01	0.76	5.9E-06	-0.14	4.8E-01	-0.08	7.1E-01	-0.25	2.1E-01	-0.04	8.3E-01	-0.06	7.5E-01	-0.07	7.2E-01	-0.13	5.4E-01	0.15	4.6E-01
ENSCAFG0000001737	CCDC42	turquoise	EC_M6	0.18	3.7E-01	0.76	5.9E-06	-0.14	4.8E-01	-0.08	7.1E-01	-0.25	2.1E-01	-0.04	8.3E-01	-0.06	7.5E-01	-0.07	7.2E-01	-0.13	5.4E-01	0.15	4.6E-01
ENSCAFG0000002541	ALKAL1	turquoise	EC_M6	0.18	3.7E-01	0.76	5.9E-06	-0.14	4.8E-01	-0.08	7.1E-01	-0.25	2.1E-01	-0.04	8.3E-01	-0.06	7.5E-01	-0.07	7.2E-01	-0.13	5.4E-01	0.15	4.6E-01
ENSCAFG0000000068	ENSCAFG0000000068	grey	EC_M10	0.18	3.7E-01	-0.68	1.2E-04	0.11	5.9E-01	0.18	3.7E-01	-0.08	6.8E-01	0.23	2.5E-01	-0.16	4.3E-01	-0.05	8.0E-01	-0.14	5.0E-01	-0.06	7.5E-01
ENSCAFG0000001416	EPHB4	turquoise	EC_M6	0.18	3.7E-01	0.88	4.7E-09	-0.08	6.8E-01	-0.09	6.5E-01	-0.35	8.3E-02	-0.11	6.1E-01	-0.05	8.0E-01	-0.17	4.2E-01	-0.01	9.7E-01	0.24	2.4E-01
ENSCAFG0000000940	ENSCAFG0000000940	turquoise	EC_M6	0.18	3.7E-01	0.76	6.1E-06	-0.14	4.8E-01	-0.08	7.1E-01	-0.25	2.1E-01	-0.04	8.3E-01	-0.06	7.5E-01	-0.07	7.2E-01	-0.13	5.4E-01	0.15	4.6E-01
ENSCAFG0000000713	TOX	grey	EC_M10	0.18	3.7E-01	0.20	3.2E-01	0.30	1.4E-01	0.13	5.4E-01	-0.53	5.5E-03	-0.14	4.8E-01	0.07	7.5E-01	-0.27	1.8E-01	-0.53	5.6E-03	0.41	3.8E-02
ENSCAFG0000000230	LRRC1	darkgreen	EC_M4	0.18	3.7E-01	0.52	6.5E-03	-0.76	5.4E-06	-0.25	2.2E-01	0.56	3.1E-03	0.32	1.1E-01	-0.15	4.5E-01	0.17	4.1E-01	0.03	8.8E-01	-0.63	5.1E-04
ENSCAFG0000000180	RIC8B	turquoise	EC_M6	0.18	3.7E-01	0.49	1.1E-02	-0.08	7.0E-01	0.08	7.0E-01	-0.30	1.3E-01	0.17	4.2E-01	0.04	8.4E-01	-0.07	7.2E-01	0.07	7.2E-01	0.20	3.4E-01
ENSCAFG0000002382	PMAIP1	turquoise	EC_M6	0.18	3.7E-01	0.86	1.9E-08	-0.39	4.7E-02	0.12	5.5E-01	0.04	8.6E-01	0.11	6.0E-01	-0.14	4.9E-01	-0.21	3.0E-01	0.04	8.5E-01	-0.13	5.3E-01
ENSCAFG0000000591	TMTC2	grey	EC_M10	0.18	3.7E-01	0.20	3.3E-01	-0.63	5.9E-04	-0.30	1.4E-01	0.53	5.3E-03	-0.05	8.1E-01	0.29	1.5E-01	0.29	1.5E-01	0.34	8.8E-02	-0.60	1.2E-03
ENSCAFG0000001843	ENSCAFG0000001843	turquoise	EC_M6	0.18	3.7E-01	0.40	4.1E-02	-0.06	7.8E-01	-0.19	3.5E-01	-0.19	3.4E-01	-0.15	4.8E-01	0.41	4.0E-02	0.07	7.5E-01	-0.37	6.4E-02	0.08	6.8E-01
ENSCAFG0000003162	ZNF529	darkgrey	EC_M8	0.18	3.7E-01	0.56	2.8E-03	0.08	6.9E-01	0.29	1.5E-01	-0.50	8.6E-03	-0.22	2.8E-01	-0.17	4.1E-01	0.29	1.6E-01	-0.07	7.4E-01	0.43	3.0E-02

ENSCAFG0000001075	CCDC191	grey	EC_M10	0.18	3.7E-01	-0.20	3.2E-01	-0.21	3.1E-01	-0.05	8.0E-01	0.10	6.3E-01	-0.12	5.5E-01	-0.22	2.8E-01	0.29	1.6E-01	0.26	2.0E-01	-0.19	3.4E-01
ENSCAFG0000000493	ENSCAFG0000000493	grey	EC_M10	0.18	3.7E-01	-0.26	2.1E-01	0.16	4.2E-01	0.33	1.0E-01	-0.26	2.0E-01	-0.27	1.9E-01	-0.11	5.9E-01	-0.18	3.8E-01	-0.08	6.9E-01	0.20	3.3E-01
ENSCAFG0000002340	ENSCAFG0000002340	grey	EC_M10	0.18	3.7E-01	-0.29	1.5E-01	0.02	9.3E-01	0.16	4.4E-01	-0.03	9.0E-01	-0.25	2.2E-01	-0.27	1.9E-01	-0.26	2.1E-01	-0.24	2.3E-01	-0.02	9.3E-01
ENSCAFG0000002112	ENSCAFG0000002112	grey	EC_M10	0.18	3.7E-01	-0.10	6.3E-01	-0.20	3.3E-01	-0.10	6.2E-01	0.12	5.7E-01	-0.24	2.4E-01	0.16	4.4E-01	-0.14	4.8E-01	0.03	8.9E-01	-0.21	2.9E-01
ENSCAFG0000001165	INPP5D	turquoise	EC_M6	0.18	3.7E-01	0.91	1.0E-10	-0.24	2.4E-01	-0.13	5.4E-01	-0.18	3.8E-01	-0.07	7.2E-01	-0.10	6.4E-01	-0.17	4.2E-01	0.07	7.2E-01	0.07	7.2E-01
ENSCAFG0000001040	CPNE8	grey	EC_M10	0.18	3.7E-01	0.38	5.4E-02	-0.62	7.4E-04	-0.10	6.3E-01	0.46	1.9E-02	-0.08	7.1E-01	-0.40	4.1E-02	0.14	5.1E-01	-0.27	1.8E-01	-0.57	2.3E-03
ENSCAFG000000239	EXOSC3	grey	EC_M10	0.18	3.7E-01	-0.12	5.5E-01	0.24	2.4E-01	-0.22	2.7E-01	-0.30	1.4E-01	-0.03	8.8E-01	0.16	4.4E-01	-0.41	3.6E-02	-0.07	7.3E-01	0.24	2.3E-01
ENSCAFG0000001014	SLC45A3	turquoise	EC_M6	0.18	3.7E-01	0.42	3.2E-02	-0.44	2.5E-02	-0.15	4.7E-01	0.23	2.6E-01	-0.11	5.8E-01	0.05	8.1E-01	0.18	3.7E-01	0.25	2.1E-01	-0.34	8.8E-02
ENSCAFG0000001410	SGPL1	darkgreen	EC_M4	0.18	3.7E-01	-0.42	3.2E-02	-0.49	1.1E-02	0.01	9.6E-01	0.56	3.0E-03	0.30	1.4E-01	-0.10	6.1E-01	0.07	7.3E-01	-0.03	8.8E-01	-0.70	6.0E-05
ENSCAFG0000002950	ZNF432	cyan	EC_M2	0.18	3.7E-01	-0.31	1.3E-01	0.49	1.1E-02	0.43	2.7E-02	-0.64	3.8E-04	0.10	6.3E-01	-0.22	2.8E-01	-0.03	8.7E-01	-0.11	5.8E-01	0.53	5.7E-03
ENSCAFG0000000433	ENSCAFG0000000433	paleturquoise	EC_M11	0.18	3.7E-01	-0.13	5.4E-01	-0.15	4.7E-01	0.77	3.7E-06	0.02	9.3E-01	-0.10	6.1E-01	-0.09	6.7E-01	-0.10	6.2E-01	-0.16	4.4E-01	-0.14	4.9E-01
ENSCAFG0000002015	ENSCAFG0000002015	paleturquoise	EC_M11	0.18	3.7E-01	-0.13	5.4E-01	-0.15	4.7E-01	0.77	3.7E-06	0.02	9.3E-01	-0.10	6.1E-01	-0.09	6.7E-01	-0.10	6.2E-01	-0.16	4.4E-01	-0.14	4.9E-01
ENSCAFG0000002596	ENSCAFG0000002596	paleturquoise	EC_M11	0.18	3.7E-01	-0.13	5.4E-01	-0.15	4.7E-01	0.77	3.7E-06	0.02	9.3E-01	-0.10	6.1E-01	-0.09	6.7E-01	-0.10	6.2E-01	-0.16	4.4E-01	-0.14	4.9E-01
ENSCAFG0000002995	ENSCAFG0000002995	paleturquoise	EC_M11	0.18	3.7E-01	-0.13	5.4E-01	-0.15	4.7E-01	0.77	3.7E-06	0.02	9.3E-01	-0.10	6.1E-01	-0.09	6.7E-01	-0.10	6.2E-01	-0.16	4.4E-01	-0.14	4.9E-01
ENSCAFG0000000857	ENSCAFG0000000857	turquoise	EC_M6	0.18	3.7E-01	0.67	1.7E-04	-0.13	5.2E-01	-0.12	5.7E-01	-0.25	2.2E-01	-0.10	6.3E-01	-0.09	6.7E-01	0.01	9.7E-01	-0.08	6.9E-01	0.15	4.7E-01
ENSCAFG0000001834	ENSCAFG0000001834	turquoise	EC_M6	0.18	3.7E-01	0.67	1.7E-04	-0.13	5.2E-01	-0.12	5.7E-01	-0.25	2.2E-01	-0.10	6.3E-01	-0.09	6.7E-01	0.01	9.7E-01	-0.08	6.9E-01	0.15	4.7E-01
ENSCAFG0000000054	ENSCAFG0000000054	turquoise	EC_M6	0.18	3.7E-01	0.44	2.3E-02	-0.01	9.8E-01	0.07	7.3E-01	-0.31	1.2E-01	-0.44	2.5E-02	-0.09	6.7E-01	-0.05	8.1E-01	0.05	7.9E-01	0.25	2.1E-01
ENSCAFG0000001213	ENSCAFG0000001213	grey	EC_M10	0.18	3.7E-01	-0.21	3.0E-01	-0.38	5.7E-02	-0.11	6.1E-01	0.33	9.9E-02	0.27	1.8E-01	0.39	4.8E-02	0.27	1.8E-01	-0.13	5.2E-01	-0.46	1.9E-02
ENSCAFG0000000892	CCNT1	grey	EC_M10	0.18	3.7E-01	0.27	1.8E-01	-0.44	2.5E-02	0.02	9.2E-01	0.13	5.1E-01	0.07	7.4E-01	0.30	1.4E-01	-0.01	9.8E-01	-0.09	6.8E-01	-0.26	2.0E-01
ENSCAFG0000000997	SLC2A13	grey	EC_M10	0.18	3.7E-01	0.04	8.5E-01	-0.64	4.9E-04	-0.27	1.9E-01	0.60	1.3E-03	0.37	6.2E-02	0.05	8.1E-01	0.01	9.8E-01	-0.35	8.1E-02	-0.71	4.4E-05
ENSCAFG0000001129	RABL3	grey	EC_M10	0.18	3.7E-01	-0.14	4.9E-01	-0.18	3.8E-01	-0.07	7.3E-01	0.13	5.4E-01	0.09	6.6E-01	-0.18	3.7E-01	0.18	3.7E-01	0.38	5.4E-02	-0.18	3.9E-01
ENSCAFG0000002465	ENSCAFG0000002465	grey	EC_M10	0.18	3.7E-01	-0.18	3.9E-01	0.07	7.3E-01	0.51	8.2E-03	-0.21	3.1E-01	-0.09	6.5E-01	-0.12	5.7E-01	-0.10	6.2E-01	-0.12	5.7E-01	0.12	5.5E-01
ENSCAFG0000001987	MVD	grey	EC_M10	0.18	3.7E-01	-0.13	5.1E-01	0.45	2.1E-02	0.09	6.5E-01	-0.59	1.4E-03	-0.23	2.7E-01	-0.08	7.1E-01	-0.12	5.5E-01	-0.43	3.0E-02	0.46	1.7E-02
ENSCAFG0000003062	MYCBP	grey	EC_M10	0.18	3.7E-01	-0.32	1.1E-01	-0.33	9.5E-02	-0.05	7.9E-01	0.43	2.9E-02	0.07	7.3E-01	-0.35	7.9E-02	0.23	2.5E-01	-0.03	9.0E-01	-0.52	6.9E-03
ENSCAFG0000000430	NAALAD2	grey	EC_M10	0.18	3.7E-01	0.40	4.4E-02	0.23	2.6E-01	0.12	5.5E-01	-0.52	6.6E-03	-0.32	1.2E-01	-0.24	2.4E-01	-0.12	5.6E-01	-0.40	4.2E-02	0.47	1.4E-02
ENSCAFG0000000744	EFNA5	grey	EC_M10	0.18	3.7E-01	-0.18	3.8E-01	-0.09	6.7E-01	0.17	4.1E-01	-0.02	9.3E-01	0.12	5.4E-01	-0.13	5.3E-01	0.26	1.9E-01	0.41	3.7E-02	-0.12	5.6E-01
ENSCAFG0000000785	CAST	cyan	EC_M2	0.18	3.7E-01	-0.12	5.7E-01	0.42	3.2E-02	0.25	2.1E-01	-0.65	3.7E-04	-0.48	1.2E-02	-0.17	4.1E-01	0.20	3.4E-01	0.03	8.9E-01	0.57	2.4E-03
ENSCAFG0000000758	NCOA6	grey	EC_M10	0.18	3.7E-01	0.28	1.7E-01	0.37	6.6E-02	-0.06	7.8E-01	-0.74	1.7E-05	-0.13	5.3E-01	-0.08	6.8E-01	-0.07	7.3E-01	0.32	1.1E-01	0.61	1.0E-03
ENSCAFG0000000490	ENSCAFG0000000490	turquoise	EC_M6	0.18	3.8E-01	0.87	6.2E-09	-0.18	3.7E-01	-0.07	7.4E-01	-0.22	2.8E-01	-0.08	6.9E-01	-0.16	4.3E-01	-0.11	5.8E-01	0.15	4.6E-01	0.11	5.9E-01
ENSCAFG0000001899	DHCR24	grey	EC_M10	0.18	3.8E-01	0.07	7.4E-01	0.43	3.0E-02	0.00	9.9E-01	-0.61	9.7E-04	-0.32	1.1E-01	-0.12	5.5E-01	-0.11	5.9E-01	-0.31	1.2E-01	0.52	6.6E-03
ENSCAFG0000001373	ITGB3	darkgreen	EC_M4	0.18	3.8E-01	0.46	1.8E-02	-0.80	9.2E-07	-0.06	7.6E-01	0.61	9.3E-04	0.27	1.9E-01	0.06	7.9E-01	-0.21	2.9E-01	-0.14	5.0E-01	-0.71	5.1E-05
ENSCAFG0000000455	ENSCAFG0000000455	grey	EC_M10	0.18	3.8E-01	-0.05	8.0E-01	-0.09	6.6E-01	-0.10	6.4E-01	-0.11	5.8E-01	-0.12	5.7E-01	-0.09	6.7E-01	-0.16	4.3E-01	0.38	5.7E-02	0.04	8.3E-01
ENSCAFG0000003024	HS3ST2	grey	EC_M10	0.18	3.8E-01	0.21	3.1E-01	-0.64	3.8E-04	-0.17	4.1E-01	0.54	4.4E-03	0.03	8.8E-01	-0.12	5.7E-01	0.06	7.7E-01	-0.07	7.4E-01	-0.62	6.5E-04
ENSCAFG0000000450	C9H17orf80	grey	EC_M10	0.18	3.8E-01	0.02	9.1E-01	0.11	5.9E-01	0.01	9.7E-01	-0.28	1.7E-01	-0.43	2.8E-02	-0.12	5.7E-01	0.00	1.0E+00	0.20	3.3E-01	0.19	3.6E-01
ENSCAFG0000001903	ENSCAFG0000001903	grey	EC_M10	0.18	3.8E-01	-0.09	6.6E-01	-0.16	4.3E-01	-0.22	2.8E-01	0.03	8.8E-01	0.23	2.6E-01	-0.18	3.7E-01	-0.21	3.0E-01	0.12	5.7E-01	-0.09	6.5E-01
ENSCAFG0000000097	TCF20	turquoise	EC_M6	0.18	3.8E-01	0.52	6.6E-03	-0.11	6.0E-01	-0.04	8.6E-01	-0.29	1.5E-01	0.03	9.0E-01	-0.14	4.9E-01	-0.12	5.7E-01	-0.11	5.8E-01	0.14	5.1E-01
ENSCAFG0000000026	TNFAIP3	turquoise	EC_M6	0.18	3.8E-01	0.73	2.3E-05	0.06	7.6E-01	-0.04	8.4E-01	-0.48	1.2E-02	-0.15	4.7E-01	0.16	4.4E-01	-0.18	3.8E-01	-0.19	3.6E-01	0.36	7.1E-02
ENSCAFG0000001265	PDIK1L	turquoise	EC_M6	0.18	3.8E-01	0.43	2.6E-02	-0.21	3.1E-01	0.08	7.1E-01	-0.10	6.4E-01	-0.19	3.5E-01	0.16	4.5E-01	0.04	8.6E-01	-0.07	7.5E-01	0.03	8.7E-01
ENSCAFG0000001580	MYO5A	darkgreen	EC_M4	0.18	3.8E-01	-0.21	3.0E-01	-0.70	6.9E-05	-0.13	5.1E-01	0.75	1.2E-05	0.20	3.4E-01	0.04	8.6E-01	-0.09	6.5E-01	-0.26	2.0E-01	-0.83	2.0E-07
ENSCAFG0000001199	FAM204A	darkgreen	EC_M4	0.18	3.8E-01	-0.08	6.8E-01	-0.75	1.1E-05	-0.21	3.0E-01	0.73	2.2E-05	0.36	7.5E-02	0.18	3.7E-01	0.11	5.8E-01	-0.20	3.2E-01	-0.85	4.5E-08
ENSCAFG0000001727	PGK1	darkgreen	EC_M4	0.18	3.8E-01	0.35	8.3E-02	-0.87	7.4E-09	-0.29	1.5E-01	0.75	8.8E-06	0.14	4.8E-01	0.13	5.4E-01	0.20	3.3E-01	0.02	9.1E-01	-0.83	1.2E-07
ENSCAFG0000000440	CAB39L	turquoise	EC_M6	0.18	3.8E-01	0.65	3.2E-04	-0.25	2.1E-01	-0.16	4.4E-01	-0.11	6.1E-01	0.00	1.0E+00	-0.17	4.0E-01	0.05	8.2E-01	-0.03	8.7E-01	0.00	1.0E+00
ENSCAFG0000001072	GTF3C3	grey	EC_M10	0.18	3.8E-01	0.26	1.9E-01	0.10	6.2E-01	0.31	1.3E-01	-0.45	2.1E-02	0.30	1.3E-01	0.05	8.3E-01	0.04	8.4E-01	0.05	8.1E-01	0.33	1.0E-01
ENSCAFG0000001700	ASPHD1	grey	EC_M10	0.18	3.8E-01	-0.16	4.3E-01	-0.66	2.8E-04	-0.11	5.8E-01	0.61	8.3E-04	0.32	1.1E-01	0.14	4.8E-01	0.02	9.1E-01	0.27	1.9E-01	-0.76	6.6E-06
ENSCAFG0000000689	IL6ST	grey	EC_M10	0.18	3.8E-01	0.52	6.1E-03	0.09	6.7E-01	-0.05	8.1E-01	-0.48	1.3E-02	-0.14	5.0E-01	-0.35	8.2E-02	0.12	5.5E-01	0.13	5.2E-01	0.39	4.6E-02
ENSCAFG0000000107	TRIB1	grey	EC_M10	0.18	3.8E-01	0.25	2.3E-01	0.20	3.3E-01	0.10	6.4E-01	-0.56	2.7E-03	-0.08	7.0E-01	0.02	9.2E-01	0.01	9.5E-01	-0.25	2.2E-01	0.44	2.5E-02
ENSCAFG0000002894	LYSMD4	turquoise	EC_M6	0.18	3.8E-01	0.50	9.1E-03	-0.25	2.2E-01	0.11	5.9E-01	-0.02	9.4E-01	-0.01	9.6E-01	-0.21	3.0E-01	-0.24	2.4E-01	-0.32	1.2E-01	-0.09	6.8E-01
ENSCAFG0000000103	HDDC2	turquoise	EC_M6	0.18	3.8E-01	0.48	1.4E-02	-0.08	6.9E-01	-0.35	8.0E-02	-0.18	3.7E-01	-0.35	7.9E-02	-0.26	2.0E-01	0.14	5.1E-01	0.17	4.2E-01	0.10	6.3E-01
ENSCAFG0000000733	KIAA0355	turquoise	EC_M6	0.18	3.8E-01	0.96	2.0E-14	-0.43	2.9E-02	-0.15	4.7E-01	-0.01	9.7E-01	-0.10	6.4E-01	-0.07	7.3E-01	-0.09	6.7E-01	-0.05	7.9E-01	-0.10	6.4E-01
ENSCAFG0000000577	NKIRAS1	violet	EC_M7	0.18	3.8E-01	-0.08	6.9E-01	-0.33	1.0E-01	-0.16	4.2E-01	0.27	1.9E-01	0.63	5.2E-04	0.04	8.4E-01	-0.34	9.4E-02	-0.05	8.3E-01	-0.34	8.7E-02
ENSCAFG0000001714	ENSCAFG0000001714	darkgreen	EC_M4	0.18	3.8E-01	-0.27	1.9E-01	-0.68	1.5E-04	-0.05	8.2E-01	0.76	6.2E-06	0.30	1.4E-01	-0.05	8.1E-01	-0.02	9.3E-01	-0.25	2.2E-01	-0.89	1.2E-09
ENSCAFG0000001445	AFAP1	grey	EC_M10	0.18	3.8E-01	-0.30	1.3E-01	-0.68	1.4E-04	-0.07	7.2E-01	0.76	6.2E-06	0.19	3.5E-01	-0.17	4.2E-01	0.26	2.1E-01	0.07	7.3E-01	-0.86	1.3E-08
ENSCAFG0000000696	TRIB3	grey	EC_M10	0.18	3.8E-01	0.12	5.5E-01	-0.48	1.3E-02	-0.01	9.6E-01	0.40	4.1E-02	0.35	7.6E-02	0.01	9.5E-01	-0.04	8.6E-01	0.09	6.6E-01	-0.56	2.8E-03
ENSCAFG0000000199	CDK6	grey	EC_M10	0.18	3.8E-01	-0.16	4.5E-01	-0.41	3.6E-02	-0.15	4.7E-01	0.45	2.2E-02	0.06	7.8E-01	-0.48	1.3E-02	-0.08	6.9E-01	0.19	3.6E-01	-0.54	4.8E-03
ENSCAFG0000000279	RAD23B	grey	EC_M10	0.18	3.8E-01	-0.17	4.2E-01	-0.49	1.1E-02	-0.34	9.0E-02	0.58	2.0E-03	0.19	3.6E-01	-0.14	5.1E-01	-0.17	4.0E-01	-0.15	4.8E-01	-0.66	2.8E-04
ENSCAFG0000002889	IMPACT	grey	EC_M10	0.18	3.8E-01	-0.67	1.8E-04	0.25	2.2E-01	0.35	7.6E-02	-0.24	2.3E-01	0.10	6.4E-01	-0.37	6.3E-02	0.08	7.1E-01	-0.18	3.8E-01	0.11	5.9E-01
ENSCAFG0000000134	RPL3	cyan	EC_M2	0.18	3.8E-01	-0.39	4.8E-02	0.54	4.7E-03	0.25	2.1E-01	-0.62	7.0E-04	0.04	8.5E-01	0.08	6.9E-01	-0.06	7.8E-01	-0.47	1.5E-02	0.47	1.5E-02
ENSCAFG0000002128	ENSCAFG0000002128	grey	EC_M10	0.18	3.8E-01	-0.11	5.8E-01	-0.10	6.2E-01	0.03	8.7E-01	0.06	7.9E-01	-0.09	6.5E-01	-0.04	8.6E-01	-0.02	9.4E-01	0.02	9.1E-01	-0.17	4.1E-01
ENSCAFG0000000534	EHD3	grey	EC_M10	0.18	3.8E-01	0.09	6.6E-01	-0.69	9.4E-05	-0.09	6.8E-01	0.63	5.9E-04	0.41	3.7E-02	-0.04	8.4E-01	-0.23	2.5E-01	0.20	3.2E-01	-0.71	4.5E-05
ENSCAFG0000003078	ENSCAFG0000003078	grey	EC_M10	0.18	3.8E-01	-0.42	3.2E-02	-0.07	7.5E-01	0.10	6.1E-01	0.10	6.2E-01	0.42	3.5E-02	-0.40	4.1E-02	-0.04	8.3E-01	0.11	5.8E-01	-0.21	3.0E-01
ENSCAFG0000000961	IFIT1	turquoise	EC_M6	0.18	3.8E-01	0.75	9.7E-06	0.02	9.2E-01	0.10	6.2E-01	-0.46	1.7E-02	-0.16	4.3E-01	-0.16	4.4E-01	0.09	6.6E-01	0.39	4.8E-02	0.39	4.8E-02
ENSCAFG0000003203	APEX1	grey	EC_M10	0.18	3.8E-01	-0.28	1.6E-01	0.09	6.8E-01	0.01	9.7E-01	-0.04	8.5E-01	0.09	6.6E-01	-0.59	1.6E-03	-0.10	6.3E-01	-0.12	5.5E-01	-0.08	6.8E-01
ENSCAFG0000001690	SLC5A2	grey	EC_M10	0.18	3.8E-01	0.03	9.0E-01	-0.36	7.4E-02	-0.22	2.9E-01	0.23	2.6E-01	0.44	2.5E-02	0.41	3.7E-02	-0.24	2.3E-01	-0.23	2.6E-01	-0.36	7.0E-02
ENSCAFG0000001858	PDE4B	turquoise	EC_M6	0.18	3.8E-01	0.75	9.0E-06	-0.01	9.7E-01	0.04	8.4E-01	-0.44	2.6E-02	-0.14	4.8E-01	0.13	5.2E-01	0.15	4.8E-01	0.05	7.9E-01	0.31	1.3E-01
ENSCAFG0000000348	PNKP	grey	EC_M10	0.18	3.8E-01	0.52	6.6E-03	0.22	2.7E-01	0.13	5.4E-01	-0.57	2.4E-03	-0.07	7.3E-01	-0.03	8.9E-01	-0.01	9.6E-01	-0.01	9.8E-01	0.47	1.5E-02
ENSCAFG0000001593	PYGO1	grey	EC_M10	0.18	3.8E-01	-0.61	8.4E-04	-0.22	2.9E-01	-0.10	6.2E-01	0.32	1.1E-01	0.10	6.3E-01	0.02	9.1E-01	-0.13	5.2E-01	-0.23	2.6E-01	-0.42	3.2E-02
ENSCAFG0000001740	RGL3	grey	EC_M10	0.18	3.8E-01	0.14	5.1E-01	-0.12	5.7E-01	-0.19	3.6E-01	-0.03	8.9E-01	-0.14	5.0E-01	-0.07	7.3E-01	-0.19	3.6E-01	0.26	2.0E-01	0.02	9.3E-01
ENSCAFG0000003079	TSTD3	grey	EC_M10	0.18	3.8E-01	-0.19	3.6E-01	-0.22	2.7E-01	-0.26	1.9E-01	0.22	2.7E-01	-0.04	8.4E-01	-0.30	1.3E-01	0.33	1.0E-01	-0.21	3.0E-01	-0.33	1.0E-01
ENSCAFG0000001759	ADPRM	grey	EC_M10	0.18	3.8E-01	-0.57	2.2E-03	-0.26	2.0E-01	-0.23	2.6E-01	0.41	3.7E-02	0.16	4.4E-01	-0.09	6.5E-01	-0.09	6.5E-01	-0.06	7.8E-01	-0.52	5.9E-03
ENSCAFG0000001990	RAPGEF1	turquoise	EC_M6	0.18	3.8E-01	0.85	4.3E-08	-0.44	2.5E-02	-0.25	2.3E-01	0.10	6.2E-01	-0.02	9.4E-01	0.08	6.9E-01	-0.23	2.5E-01	-0.10	6.2E-01	-0.17	4.0E-01
ENSCAFG0000000921	DCBLD2	grey	EC_M10	0.18	3.8E-01	-0.47	1.5E-02	-0.25	2.2E-01	0.07	7.4E-01	0.31	1.3E-01	0.13	5.2E-01	-0.36	7.3E-02	-0.04	8.3E-01	0.21	2.9E-01	-0.43	2.8E-02
ENSCAFG0000002391	SLC8A3	turquoise	EC_M6	0.18	3.8E-01	0.70	7.8E-05	-0.62	7.3E-04	-0.16	4.2E-01	0.31	1.3E-01	0.01	9.5E-01	-0.18	3.7E-01	-0.11	5.9E-01	0.33	1.0E-01	-0.41	3.5E-02
ENSCAFG0000001268	RBBP7	darkgreen	EC_M4	0.18	3.8E-01	-0.27	1.9E-01	-0.62	6.6E-04	0.09	6.7E-01	0.70	7.2E-05	0.17	4.0E-01	-0.10	6.4E-01	-0.11	6.0E-01	-0.27	1.8E-01	-0.82	3.0E-07
ENSCAFG0000001233	CABP7	turquoise	EC_M6	0.18	3.8E-01	0.82	2.4E-07	-0.10	6.4E-01	-0.10	6.4E-01	-0.32	1.1E-01	-0.08	6.9E-01	-0.08	6.9E-01	-0.11	6.1E-01	-0.16	4.3E-01	0.24	2.4E-01
ENSCAFG0000000726	ENSCAFG0000000726	grey	EC_M10	0.18	3.9E-01	-0.14	5.0E-01	-0.20	3.3E-01	0.06	7.6E-01	0.10	6.4E-01	0.29	1.5E-01	0.38	5.8E-02	-0.22	2.9E-01	-0.19	3.6E-01	-0.22	2.9E-01
ENSCAFG0000003076	ZNF835	grey	EC_M10	0.18	3.9E-01	0.12	5.6E-01	0.06	7.7E-01	-0.10	6.3E-01	-0.23	2.6E-01	-0.09	6.6E-01	-0.14	4.8E-01	-0.05	8.2E-01	-0.16	4.5E-01	0.13	5.4E-01
ENSCAFG0000000270	ENSCAFG0000000270	grey	EC_M10	0.18	3.9E-01	-0.18	3.8E-01	-0.34	8.7E-02	-0.20	3.2E-01	0.37	6.3E-02	-0.01	9.7E-01	0.08	6.9E-01	0.39	4.7E-02	-0.01	9.5E-01	-0.46	1.9E-02
ENSCAFG0000001321	EHHADH	cyan	EC_M2	0.18	3.9E-01	-0.32	1.1E-01	0.62	6.7E-04	0.13	5.3E-01	-0.75	1.1E-05	-0.05	8.1E-01	-0.07	7.2E-01	-0.05	8.1E-01	-0.17	4.1E-01	0.66	2.3E-04
ENSCAFG0000000793	DIABLO	grey	EC_M10	0.18	3.9E-01	-0.10	6.3E-01	-0.69	1.0E-04	-0.09	6.6E-01	0.72	3.7E-05	0.44	2.4E-02	-0.02	9.0E-01	0.28	1.7E-01	0.18	3.8E-01	-0.79	1.9E-06
ENSCAFG0000002313	ENSCAFG0000002313	grey	EC_M10	0.18	3.9E-01	-0.10	6.4E-01	-0.02	9.4E-01	-0.09	6.6E-01	-0.08	7.0E-01	-0.09	6.5E-01	-0.06	7.8E-01	-0.13	5.4E-01	0.44	2.5E-02	0.02	9.4E-01
ENSCAFG0000001194	TARS2	grey	EC_M10	0.18	3.9E-01	-0.13	5.2E-01	0.01	9.5E-01	-0.15	4.6E-01	-0.02	9.2E-01	-0.12	5.5E-01	-0.53	5.4E-03	-0.03	8.9E-01	0.15	4.7E-01	-0.03	8.7E-01
ENSCAFG0000000474	SEPHS1	cyan	EC_M2	0.18	3.9E-01	0.29	1.5E-01	0.50	9.1E-03	0.25	2.1E-01	-0.86	1.8E-08	-0.27	1.8E-01	-0.03	9.0E-01	-0.10	6.2E-01	0.03	9.0E-01	0.77	5.0E-06
ENSCAFG0000000322	EEPD1	grey	EC_M10	0.18	3.9E-01	0.22	2.7E-01	-0.49	1.2E-02	-0.22	2.8E-01	0.35	8.1E-02	-0.01	9.6E-01	0.24	2.3E-01	-0.18	3.7E-01	0.31	1.2E-01	-0.38	5.3E-02
ENSCAFG0000000581	RAB5A	grey	EC_M10	0.18	3.9E-01	0.30	1.4E-01	-0.58	1.8E-03	0.27	1.9E-01	0.35	8.1E-02	0.25	2.3E-01	0.10	6.4E-01	-0.08	7.0E-01	-0.29	1.5E-01	-0.49	1.1E-02
ENSCAFG0000000071	ENSCAFG0000000071	grey	EC_M10	0.18	3.9E-01	-0.01	9.5E-01	-0.49	1.0E-02	-0.07	7.5E-01	0.45	2.2E-02	0.35	7.8E-02	-0.29	1.6E-01	0.13	5.3E-01	-0.04	8.4E-01	-0.51	7.7E-03
ENSCAFG0000000973	DNTTIP1	grey	EC_M10	0.18	3.9E-01	0.10	6.3E-01	-0.01	9.5E-01	0.13	5.3E-01	-0.18	3.8E-01	0.45	2.1E-02	0.14	5.0E-01	-0.48	1.2E-02	0.11	6.0E-01	0.10	6.3E-01
ENSCAFG0000000779	ENSCAFG0000000779	grey	EC_M10	0.18	3.9E-01	0.01	9.7E-01	-0.12	5.6E-01	-0.33	9.8E-02	-0.09	6.5E-01	0.30	1.4E-01	-0.05	8.0E-01	0.15	4.6E-01	-0.13	5.4E-01	-0.04	8.7E-01
ENSCAFG0000001473	ZNF674	grey	EC_M10	0.18	3.9E-01	-0.16	4.2E-01	0.31	1.3E-01	0.33	9.6E-02	-0.53	5.7E-03	0.00	9.9E-01	-0.13	5.3E-01	-0.28	1.6E-01	0.05	8.1E-01	0.42	3.1E-02
ENSCAFG0000002662	ENSCAFG0000002662	turquoise	EC_M6	0.18	3.9E-01	0.45	2.1E-02	-0.31	1.2E-01	-0.13	5.4E-01	0.04	8.5E-01	0.57	2.4E-03	0.43	2.8E-02	-0.08	7.0E-01	-0.16	4.3E-01	-0.12	5.4E-01
ENSCAFG0000002380	HERC3	turquoise	EC_M6	0.18	3.9E-01	0.68	1.2E-04	-0.66	2.2E-04	-0.12	5.4E-01	0.37	6.3E-02	0.04	8.4E-01	0.14	4.9E-01	-0.03	9.0E-01	0.03	8.9E-01	-0.42	3.2E-02
ENSCAFG0000001768	SYT4	grey	EC_M10	0.18	3.9E-01	0.00	9.9E-01	-0.51	7.5E-03	0.07	7.4E-01	0.41	3.9E-02	0.17	4.1E-01	-0.02	9.1E-01	-0.31	1.3E-01	-0.20	3.4E-01	-0.54	4.7E-03
ENSCAFG0000001477	ENSCAFG0000001477	turquoise	EC_M6	0.18	3.9E-01	0.66	2.7E-04	-0.01	9.4E-01	0.01	9.8E-01	-0.39	4.8E-02	0.21	3.0E-01	-0.23	2.5E-01	-0.17	4.1E-01	-0.02	9.1E-01	0.28	1.6E-01
ENSCAFG0000002920	EIF5A2	grey	EC_M10	0.18	3.9E-01	0.00	9.9E-01	-0.35	8.4E-02	-0.17	4.2E-01	0.22	2.7E-01	-0.12	5.6E-01	-0.23	2.7E-01	0.36	7.4E-02	0.31	1.3E-01	-0.26	2.0E-01
ENSCAFG0000001587	UCHL1	grey	EC_M10	0.18	3.9E-01	0.19	3.5E-01	-0.58	1.8E-03	-0.04	8.6E-01	0.48	1.3E-02	-0.05	8.1E-01	-0.23	2.5E-01	0.05	8.3E-01	-0.11	5.9E-01	-0.58	1.8E-03
ENSCAFG0000001199	WDR6	turquoise	EC_M6	0.18	3.9E-01	0.43	3.0E-02	0.00	1.0E+00	-0.20	3.3E-01	-0.19	3.5E-01	-0.28	1.7E-01	-0.30	1.4E-01	0.01	9.6E-01	0.01	9.7E-01	0.12	5.6E-01
ENSCAFG0000000301	UGCG	grey	EC_M10	0.18	3.9E-01	0.25	2.1E-01	-0.31	1.2E-01	0.03	8.8E-01	0.11	6.0E-01	-0.02	9.3E-01	0.36	6.9E-02	0.42	3.4E-02	-0.06	7.7E-01	-0.26	1.9E-01
ENSCAFG0000000048	NIPAL2	grey	EC_M10	0.18	3.9E-01	-0.33	1.0E-01	-0.46	1.8E-02	-0.21	3.1E-01	0.51	7.5E-03	0.33	9.9E-02	0.38	5.9E-02	-0.04	8.5E-01	-0.05	8.2E-01	-0.61	8.7E-04
ENSCAFG0000000412	KMT2E	cyan	EC_M2	0.18	3.9E-01	0.14	5.0E-01	0.51	7.5E-03	0.17	3.9E-01	-0.87	8.1E-09	-0.19	3.4E-01	-0.04	8.6E-01	0.02	9.1E-01	0.10	6.4E-01	0.75	9.2E-06
ENSCAFG0000002543	MANSC4	grey	EC_M10	0.18	3.9E-01	-0.11	5.9E-01	-0.28	1.7E-01	0.23	2.5E-01	0.19	3.6E-01	0.22	2.8E-01	-0.41	3.9E-02	-0.20	3.4E-01	-0.14	5.1E-01	-0.32	1.1E-01
ENSCAFG0000000413	EEFSEC	grey	EC_M10	0.18	3.9E-01	0.00	9.9E-01	0.19	3.6E-01	-0.47	1.4E-02	-0.31	1.3E-01	0.08	7.1E-01	-0.26	2.0E-01	-0.09	6.6E-01	0.09	6.5E-01	0.25	2.3E-01
ENSCAFG0000000552	IKBKB	turquoise	EC_M6	0.18	3.9E-01	0.68	1.2E-04	-0.22	2.7E-01	0.12	5.7E-01	-0.17	4.0E-01	-0.11	5.8E-01	-0.01	9.6E-01	-0.40	4.2E-02	0.29	1.4E-01	0.07	7.3E-01
ENSCAFG0000001331	ENSCAFG0000001331	grey	EC_M10	0.18	3.9E-01	-0.06	7.6E-01	0.16	4.2E-01	-0.03	8.7E-01	-0.40	4.1E-02	0.00	1.0E+00	-0.07	7.4E-01	-0.05	8.0E-01	-0.19	3.5E-01	0.26	2.0E-01
ENSCAFG0000001866	TNFAIP1	darkgreen	EC_M4	0.18	3.9E-01	0.45	2.0E-02	-0.79	1.4E-06	-0.36	7.0E-02	0.66	2.8E-04	0.11	5.8E-01	-0.15	4.7E-01	0.04	8.3E-01	-0.03	8.7E-01	-0.76	5.3E-06
ENSCAFG0000001766	TUBD1	grey	EC_M10	0.18	3.9E-01	0.02	9.4E-01	-0.37	6.6E-02	-0.27	1.9E-01	0.24	2.4E-01	0.00	9.9E-01	-0.11	5.8E-01	-0.04	8.6E-01	-0.28	1.6E-01	-0.36	7.0E-02
ENSCAFG0000000755	UFSP2	turquoise	EC_M6	0.18	3.9E-01	0.56	3.1E-03	-0.14	5.1E-01	0.20	3.2E-01	-0.22	2.7E-01	-0.20	3.2E-01	-0.24	2.3E-01	-0.40	4.3E-02	-0.25	2.1E-01	0.13	5.4E-01
ENSCAFG0000001613	ENSCAFG0000001613	grey	EC_M10	0.18	3.9E-01	0.16	4.4E-01	-0.11	5.8E-01	0.21	3.1E-01	-0.15	4.6E-01	0.03	8.7E-01	0.11	5.8E-01	0.00	1.0E+00	0.21	3.1E-01	0.06	7.7E-01
ENSCAFG0000001382	ADORA2A	turquoise	EC_M6	0.18	3.9E-01	0.79	1.8E-06	-0.33	9.6E-02	-0.18	3.9E-01	-0.02	9.2E-01	-0.05	7.9E-01	0.09	6.6E-01	-0.22	2.7E-01	0.31	1.2E-01	-0.05	8.0E-01
ENSCAFG0000003242	ENSCAFG0000003242	turquoise	EC_M6	0.17	3.9E-01	0.28	1.7E-01	0.12	5.6E-01	-0.17	4.1E-01	-0.42	3.4E-02	-0.12	5.4E-01	-0.12	5.6E-01	-0.09	6.6E-01	-0.04	8.5E-01	0.36	7.0E-02
ENSCAFG0000001718	ENSCAFG0000001718	grey	EC_M10	0.17	3.9E-01	-0.06	7.7E-01	0.15	4.7E-01	-0.18	3.7E-01	-0.27	1.8E-01	0.30	1.4E-01	-0.19	3.4E-01	-0.14	4.9E-01	-0.24	2.3E-01	0.14	5.0E-01
ENSCAFG0000002994	ZNF45	cyan	EC_M2	0.17	3.9E-01	0.14	4.9E-01	0.27	1.8E-01	0.14	4.9E-01	-0.58	1.9E-03	-0.14	5.0E-01	-0.12	5.6E-01	0.12	5.5E-01	0.07	7.5E-01	0.50	9.9E-03
ENSCAFG0000000058	GRHL2	violet	EC_M7	0.17	3.9E-01	-0.09	6.7E-01	-0.23	2.6E-01	-0.11	5.9E-01	0.14	4.9E-01	0.56	2.8E-03	-0.08	6.9E-01	0.41	3.7E-02	0.38	5.6E-02	-0.24	2.5E-01
ENSCAFG0000001333	SRP54	darkgreen	EC_M4	0.17	3.9E-01	-0.03	8.7E-01	-0.82	3.2E-07	0.07	7.4E-01	0.77	4.6E-06	0.26	2.1E-01	0.02	9.3E-01	0.03	8.7E-01	-0.11	6.1E-01	-0.88	2.0E-09
ENSCAFG0000001270	MRPL11	grey	EC_M10	0.17	3.9E-01	-0.21	3.0E-01	-0.28	1.6E-01	-0.08	7.1E-01	0.27	1.9E-01	0.12	5.6E-01	0.34	9.4E-02	0.25	2.1E-01	-0.20	3.2E-01	-0.37	6.4E-02
ENSCAFG0000000927	BIN3	grey	EC_M10	0.17	3.9E-01	0.01	9.6E-01	0.03	9.0E-01	0.14	4.8E-01	-0.19	3.4E-01	0.13	5.3E-01	0.13	5.4E-01	-0.64	3.9E-04	-0.13	5.2E-01	0.15	4.6E-01
ENSCAFG0000001866	KHSRP	grey	EC_M10	0.17	3.9E-01	0.18	3.8E-01	0.47	1.6E-02	0.07	7.3E-01	-0.71	5.3E-05	-0.01	9.5E-01	-0.04	8.4E-01	-0.22	2.9E-01	-0.23	2.5E-01	0.56	2.7E-03
ENSCAFG0000002390	ENSCAFG0000002390	grey	EC_M10	0.17	4.0E-01	0.03	8.7E-01	0.02	9.1E-01	0.41	3.8E-02	-0.11	6.0E-01	-0.15	4.7E-01	-0.13	5.4E-01	-0.09	6.5E-01	-0.09	6.6E-01	0.04	8.5E-01

ENSCAFG0000001760	ENSCAFG0000001760	turquoise	EC_M6	0.17	4.0E-01	0.46	1.7E-02	-0.19	3.5E-01	-0.25	2.1E-01	-0.07	7.4E-01	0.18	3.9E-01	0.03	8.8E-01	-0.38	5.6E-02	-0.22	2.8E-01	-0.04	8.6E-01
ENSCAFG0000002340	MPP3	turquoise	EC_M6	0.17	4.0E-01	0.79	1.2E-06	-0.43	2.9E-02	-0.19	3.6E-01	0.09	6.5E-01	-0.21	3.0E-01	0.08	6.8E-01	0.07	7.3E-01	0.40	4.0E-02	-0.16	4.4E-01
ENSCAFG0000001033	COQ5	grey	EC_M10	0.17	4.0E-01	-0.33	1.0E-01	0.10	6.3E-01	-0.07	7.5E-01	-0.17	4.2E-01	0.04	8.4E-01	0.01	9.6E-01	-0.13	5.4E-01	-0.16	4.3E-01	0.09	6.8E-01
ENSCAFG0000002380	ARMCX1	darkgrey	EC_M8	0.17	4.0E-01	0.22	2.7E-01	0.30	1.3E-01	0.41	4.0E-02	-0.63	5.0E-04	0.09	6.7E-01	-0.19	3.6E-01	-0.17	4.1E-01	-0.38	5.7E-02	0.53	5.0E-03
ENSCAFG0000001767	UBFD1	grey	EC_M10	0.17	4.0E-01	-0.03	8.8E-01	-0.17	4.0E-01	0.05	8.1E-01	0.11	5.8E-01	-0.33	9.6E-02	0.14	4.8E-01	-0.03	8.8E-01	0.07	7.2E-01	-0.22	2.7E-01
ENSCAFG0000003241	FLI1	turquoise	EC_M6	0.17	4.0E-01	0.91	1.5E-10	-0.28	1.6E-01	-0.08	7.1E-01	-0.12	5.6E-01	-0.13	5.3E-01	-0.20	3.3E-01	-0.01	9.5E-01	0.15	4.7E-01	0.02	9.3E-01
ENSCAFG0000002306	ENSCAFG0000002306	turquoise	EC_M6	0.17	4.0E-01	0.58	1.8E-03	-0.14	5.1E-01	-0.06	7.6E-01	-0.22	2.8E-01	-0.07	7.2E-01	-0.12	5.5E-01	-0.11	6.0E-01	-0.15	4.5E-01	0.11	6.0E-01
ENSCAFG0000000766	ZBTB38	turquoise	EC_M6	0.17	4.0E-01	0.79	1.5E-06	0.08	6.8E-01	-0.01	9.8E-01	-0.55	3.3E-03	-0.21	2.9E-01	0.14	4.9E-01	-0.17	4.1E-01	-0.05	8.0E-01	0.46	1.7E-02
ENSCAFG0000000312	MDH1	grey	EC_M10	0.17	4.0E-01	-0.39	4.7E-02	0.03	8.8E-01	-0.21	3.1E-01	0.06	7.6E-01	0.14	4.9E-01	-0.04	8.6E-01	0.01	9.7E-01	0.08	7.1E-01	-0.07	7.3E-01
ENSCAFG0000001452	NPAT	darkgrey	EC_M8	0.17	4.0E-01	0.41	3.5E-02	0.41	3.6E-02	0.07	7.4E-01	-0.82	3.7E-07	-0.10	6.4E-01	0.08	6.8E-01	-0.09	6.5E-01	-0.04	8.7E-01	0.74	1.3E-05
ENSCAFG0000001151	TOMM20	grey	EC_M10	0.17	4.0E-01	-0.20	3.3E-01	-0.39	5.0E-02	-0.09	6.7E-01	0.47	1.4E-02	0.30	1.4E-01	0.10	6.2E-01	-0.21	3.0E-01	-0.38	5.7E-02	-0.52	6.1E-03
ENSCAFG0000001073	CAB39	grey	EC_M10	0.17	4.0E-01	-0.13	5.3E-01	-0.16	4.3E-01	-0.05	8.2E-01	0.07	7.2E-01	0.02	9.2E-01	-0.21	3.0E-01	0.03	8.8E-01	-0.16	4.4E-01	-0.09	6.7E-01
ENSCAFG0000000228	THSD7A	turquoise	EC_M6	0.17	4.0E-01	0.91	6.6E-11	-0.20	3.2E-01	-0.12	5.6E-01	-0.24	2.4E-01	-0.14	5.0E-01	-0.11	5.8E-01	-0.15	4.6E-01	0.25	2.3E-01	0.17	4.2E-01
ENSCAFG0000002875	MTHFD2L	turquoise	EC_M6	0.17	4.0E-01	0.39	5.1E-02	-0.40	4.2E-02	0.22	2.8E-01	0.14	4.8E-01	-0.16	4.4E-01	-0.06	7.5E-01	-0.26	2.0E-01	0.12	5.7E-01	-0.26	2.0E-01
ENSCAFG0000001321	ENSCAFG0000001321	darkgreen	EC_M4	0.17	4.0E-01	-0.09	6.5E-01	-0.79	2.0E-06	-0.05	8.0E-01	0.83	1.6E-07	0.25	2.2E-01	-0.09	6.6E-01	0.23	2.6E-01	0.02	9.4E-01	-0.92	2.5E-11
ENSCAFG0000000602	TRIM21	cyan	EC_M2	0.17	4.0E-01	0.13	5.2E-01	0.35	8.0E-02	0.16	4.3E-01	-0.65	3.3E-04	0.04	8.3E-01	-0.20	3.2E-01	-0.07	7.2E-01	-0.21	3.1E-01	0.54	4.2E-03
ENSCAFG0000002933	ENSCAFG0000002933	grey	EC_M10	0.17	4.0E-01	-0.11	5.9E-01	0.12	5.5E-01	0.60	1.1E-03	-0.29	1.5E-01	-0.11	6.0E-01	-0.08	7.1E-01	-0.14	4.9E-01	-0.03	9.0E-01	0.25	2.2E-01
ENSCAFG0000002855	ENSCAFG0000002855	turquoise	EC_M6	0.17	4.0E-01	0.40	4.2E-02	0.06	7.9E-01	0.01	9.5E-01	-0.37	6.6E-02	0.15	4.5E-01	0.08	7.1E-01	-0.04	8.5E-01	0.14	5.1E-01	0.30	1.4E-01
ENSCAFG0000001724	INTS14	turquoise	EC_M6	0.17	4.0E-01	0.35	7.9E-02	-0.05	7.9E-01	0.15	4.6E-01	-0.23	2.5E-01	-0.02	9.2E-01	-0.16	4.4E-01	-0.22	2.8E-01	-0.46	1.7E-02	0.14	5.0E-01
ENSCAFG0000002898	ENSCAFG0000002898	grey	EC_M10	0.17	4.0E-01	-0.15	4.7E-01	-0.10	6.3E-01	-0.10	6.1E-01	-0.05	8.1E-01	0.21	3.1E-01	-0.16	4.3E-01	0.19	3.4E-01	0.10	6.4E-01	-0.10	6.2E-01
ENSCAFG0000000209	UXS1	grey	EC_M10	0.17	4.0E-01	-0.28	1.6E-01	-0.45	2.2E-02	-0.27	1.8E-01	0.55	3.4E-03	0.17	3.9E-01	-0.29	1.6E-01	0.27	1.9E-01	0.29	1.5E-01	-0.61	9.7E-04
ENSCAFG0000001280	ENSCAFG0000001280	grey	EC_M10	0.17	4.0E-01	-0.08	6.9E-01	-0.28	1.7E-01	0.30	1.4E-01	0.19	3.4E-01	0.21	3.0E-01	-0.23	2.6E-01	-0.30	1.4E-01	-0.25	2.3E-01	-0.24	2.3E-01
ENSCAFG0000002038	CBFB	grey	EC_M10	0.17	4.0E-01	-0.26	2.0E-01	0.20	3.2E-01	0.20	3.2E-01	-0.23	2.6E-01	-0.03	8.9E-01	0.10	6.4E-01	-0.37	6.1E-02	0.01	9.6E-01	0.15	4.7E-01
ENSCAFG0000001175	CD86	turquoise	EC_M6	0.17	4.0E-01	0.90	4.2E-10	-0.24	2.5E-01	-0.11	5.9E-01	-0.17	3.9E-01	-0.04	8.3E-01	-0.11	5.9E-01	-0.15	4.6E-01	0.14	4.9E-01	0.07	7.5E-01
ENSCAFG0000000609	ASTE1	cyan	EC_M2	0.17	4.0E-01	0.02	9.3E-01	0.45	2.1E-02	0.36	6.8E-02	-0.76	8.2E-06	0.06	7.6E-01	0.11	5.8E-01	-0.17	4.0E-01	-0.05	8.0E-01	0.63	5.5E-04
ENSCAFG0000000613	PXMP2	grey	EC_M10	0.17	4.0E-01	-0.16	4.4E-01	0.36	7.2E-02	0.42	3.3E-02	-0.47	1.6E-02	-0.10	6.1E-01	0.02	9.1E-01	-0.35	7.6E-02	-0.01	9.5E-01	0.37	6.4E-02
ENSCAFG0000000495	KMT2C	cyan	EC_M2	0.17	4.0E-01	0.10	6.3E-01	0.35	7.7E-02	0.09	6.6E-01	-0.66	2.3E-04	-0.32	1.2E-01	-0.12	5.5E-01	-0.14	5.1E-01	-0.13	5.3E-01	0.57	2.6E-03
ENSCAFG0000000864	TUBA1B	grey	EC_M10	0.17	4.0E-01	-0.29	1.5E-01	-0.59	1.6E-03	-0.20	3.2E-01	0.71	5.4E-05	0.12	5.5E-01	-0.19	3.5E-01	0.26	1.9E-01	-0.25	2.3E-01	-0.82	4.0E-07
ENSCAFG0000001946	TUBB4B	grey	EC_M10	0.17	4.0E-01	-0.15	4.8E-01	-0.32	1.1E-01	-0.23	2.5E-01	0.43	2.8E-02	0.03	8.8E-01	-0.26	2.1E-01	0.14	5.0E-01	0.04	8.5E-01	-0.48	1.2E-02
ENSCAFG0000000172	LINGO2	turquoise	EC_M6	0.17	4.0E-01	0.83	1.4E-07	-0.29	1.5E-01	-0.08	7.0E-01	-0.07	7.2E-01	-0.07	7.3E-01	-0.07	7.4E-01	-0.08	6.9E-01	-0.06	7.8E-01	0.02	9.2E-01
ENSCAFG0000001309	SENP5	grey	EC_M10	0.17	4.0E-01	0.29	1.5E-01	-0.60	1.3E-03	0.04	8.3E-01	0.35	7.6E-02	0.19	3.5E-01	0.18	3.9E-01	-0.08	7.0E-01	0.11	5.8E-01	-0.42	3.3E-02
ENSCAFG0000000072	ISOC1	darkgreen	EC_M4	0.17	4.0E-01	-0.39	4.8E-02	-0.44	2.3E-02	0.02	9.4E-01	0.51	7.4E-03	0.44	2.4E-02	0.14	4.8E-01	-0.13	5.2E-01	-0.27	1.8E-01	-0.67	1.9E-04
ENSCAFG0000001146	ENSCAFG0000001146	turquoise	EC_M6	0.17	4.0E-01	0.63	5.2E-04	-0.29	1.5E-01	0.28	1.7E-01	-0.03	8.9E-01	-0.15	4.6E-01	-0.14	5.1E-01	-0.16	4.2E-01	-0.22	2.8E-01	-0.06	7.6E-01
ENSCAFG0000000767	SULF1	darkgreen	EC_M4	0.17	4.0E-01	0.21	3.0E-01	-0.78	2.8E-06	-0.04	8.4E-01	0.70	6.2E-05	0.14	5.1E-01	0.04	8.5E-01	-0.18	3.7E-01	-0.29	1.5E-01	-0.80	1.0E-06
ENSCAFG0000003162	ENSCAFG0000003162	grey	EC_M10	0.17	4.0E-01	0.35	7.8E-02	-0.24	2.4E-01	-0.27	1.8E-01	0.06	7.6E-01	0.19	3.4E-01	-0.21	3.0E-01	-0.32	1.1E-01	0.11	5.8E-01	-0.13	5.2E-01
ENSCAFG0000000375	ENSCAFG0000000375	grey	EC_M10	0.17	4.0E-01	-0.10	6.2E-01	0.19	3.6E-01	0.34	9.1E-02	-0.35	7.6E-02	-0.14	4.9E-01	-0.20	3.2E-01	-0.21	3.0E-01	-0.22	2.8E-01	0.26	2.0E-01
ENSCAFG0000000403	TAL1	turquoise	EC_M6	0.17	4.0E-01	0.77	5.0E-06	-0.24	2.4E-01	-0.25	2.1E-01	-0.08	7.1E-01	-0.10	6.2E-01	-0.02	9.3E-01	-0.23	2.6E-01	-0.16	4.4E-01	-0.02	9.1E-01
ENSCAFG0000001100	C37H2orf69	grey	EC_M10	0.17	4.0E-01	-0.25	2.2E-01	0.34	8.9E-02	0.13	5.3E-01	-0.33	1.0E-01	-0.18	3.7E-01	-0.01	9.4E-01	-0.25	2.3E-01	-0.15	4.7E-01	0.28	1.6E-01
ENSCAFG0000001588	APBB2	turquoise	EC_M6	0.17	4.0E-01	0.72	3.3E-05	0.14	4.8E-01	0.02	9.1E-01	-0.59	1.6E-03	-0.25	2.1E-01	-0.17	3.9E-01	0.02	9.1E-01	0.03	8.9E-01	0.49	1.2E-02
ENSCAFG0000000782	TRAPPC11	grey	EC_M10	0.17	4.0E-01	0.21	3.0E-01	-0.51	7.5E-03	-0.20	3.2E-01	0.44	2.4E-02	0.04	8.4E-01	-0.47	1.5E-02	-0.01	9.7E-01	-0.03	9.0E-01	-0.50	9.9E-03
ENSCAFG0000001615	CPSF7	grey	EC_M10	0.17	4.0E-01	0.43	2.7E-02	0.25	2.3E-01	-0.09	6.8E-01	-0.51	7.3E-03	-0.07	7.2E-01	0.15	4.6E-01	-0.02	9.3E-01	0.00	9.9E-01	0.45	2.1E-02
ENSCAFG0000000886	SCAF4	turquoise	EC_M6	0.17	4.0E-01	0.40	4.5E-02	0.00	9.8E-01	-0.47	1.4E-02	-0.27	1.8E-01	0.14	5.0E-01	-0.16	4.4E-01	-0.13	5.2E-01	-0.18	3.7E-01	0.17	4.0E-01
ENSCAFG0000001944	NRARP	turquoise	EC_M6	0.17	4.0E-01	0.92	5.3E-11	-0.22	2.7E-01	-0.12	5.6E-01	-0.20	3.2E-01	-0.12	5.7E-01	0.12	5.6E-01	-0.15	4.7E-01	0.18	3.9E-01	0.13	5.4E-01
ENSCAFG0000000414	SLC8A2	grey	EC_M10	0.17	4.0E-01	-0.17	4.0E-01	0.29	1.6E-01	-0.11	5.8E-01	-0.38	5.5E-02	-0.11	6.0E-01	-0.05	8.0E-01	-0.11	5.9E-01	-0.08	6.8E-01	0.36	6.7E-02
ENSCAFG0000000590	NALCN	grey	EC_M10	0.17	4.0E-01	-0.19	3.6E-01	0.15	4.7E-01	0.18	3.8E-01	-0.26	2.0E-01	-0.19	3.7E-01	-0.19	3.5E-01	0.40	4.2E-02	-0.18	3.7E-01	0.17	4.2E-01
ENSCAFG0000002882	ENSCAFG0000002882	grey	EC_M10	0.17	4.0E-01	-0.36	7.5E-02	-0.03	8.8E-01	0.13	5.3E-01	0.00	9.9E-01	0.00	9.9E-01	0.01	9.7E-01	-0.04	8.3E-01	0.05	7.9E-01	-0.09	6.7E-01
ENSCAFG0000000121	NUDT3	grey	EC_M10	0.17	4.0E-01	-0.36	7.1E-02	0.10	6.4E-01	-0.01	9.6E-01	-0.10	6.4E-01	-0.32	1.1E-01	-0.41	3.9E-02	0.21	3.1E-01	-0.06	7.7E-01	-0.03	8.9E-01
ENSCAFG0000000550	ACVR2A	grey	EC_M10	0.17	4.1E-01	-0.46	1.9E-02	-0.19	3.6E-01	0.19	3.5E-01	0.25	2.1E-01	0.22	2.8E-01	-0.03	8.8E-01	0.15	4.6E-01	-0.34	9.2E-02	-0.33	1.0E-01
ENSCAFG0000001522	VWF	turquoise	EC_M6	0.17	4.1E-01	0.78	2.7E-06	-0.13	5.1E-01	-0.03	8.9E-01	-0.24	2.4E-01	-0.13	5.3E-01	-0.22	2.8E-01	-0.12	5.7E-01	-0.26	1.9E-01	0.11	5.9E-01
ENSCAFG0000003199	SLC30A1	grey	EC_M10	0.17	4.1E-01	-0.52	6.7E-03	0.08	7.1E-01	-0.13	5.2E-01	-0.05	8.1E-01	-0.02	9.1E-01	0.10	6.1E-01	-0.17	4.1E-01	-0.12	5.6E-01	-0.01	9.8E-01
ENSCAFG0000000993	TENT4B	darkgreen	EC_M4	0.17	4.1E-01	0.37	6.3E-02	-0.59	1.5E-03	0.00	9.9E-01	0.37	6.1E-02	-0.03	8.7E-01	-0.08	6.9E-01	-0.06	7.8E-01	-0.51	7.1E-03	-0.50	9.5E-03
ENSCAFG0000001038	DNAH7	grey	EC_M10	0.17	4.1E-01	0.05	8.1E-01	-0.31	1.3E-01	-0.20	3.3E-01	0.28	1.6E-01	-0.19	3.6E-01	-0.10	6.3E-01	-0.06	7.6E-01	0.30	1.3E-01	-0.32	1.2E-01
ENSCAFG0000002864	GMFB	grey	EC_M10	0.17	4.1E-01	-0.63	5.4E-04	-0.34	9.0E-02	-0.03	8.7E-01	0.49	1.1E-02	0.12	5.6E-01	-0.27	1.8E-01	0.26	2.0E-01	0.16	4.5E-01	-0.56	2.9E-03
ENSCAFG0000000399	NAMPT	magenta	EC_M13	0.17	4.1E-01	-0.20	3.3E-01	-0.16	4.3E-01	0.22	2.7E-01	0.04	8.5E-01	0.12	5.6E-01	0.17	3.9E-01	-0.29	1.4E-01	0.54	4.4E-03	-0.11	6.1E-01
ENSCAFG0000002368	BMPR2	grey	EC_M10	0.17	4.1E-01	0.19	3.6E-01	-0.77	3.7E-06	-0.12	5.7E-01	0.68	1.3E-04	0.27	1.8E-01	0.09	6.5E-01	-0.15	4.7E-01	-0.36	7.2E-02	-0.78	2.2E-06
ENSCAFG0000000926	TBC1D23	grey	EC_M10	0.17	4.1E-01	0.56	3.2E-03	-0.70	6.7E-05	-0.03	8.9E-01	0.41	3.7E-02	0.09	6.6E-01	-0.06	7.9E-01	-0.14	4.9E-01	0.24	2.3E-01	-0.50	9.7E-03
ENSCAFG0000000585	PLCB1	grey	EC_M10	0.17	4.1E-01	-0.62	7.7E-04	0.19	3.6E-01	0.17	4.1E-01	-0.18	3.8E-01	0.03	8.9E-01	-0.08	7.0E-01	-0.02	9.4E-01	0.24	2.4E-01	0.12	5.5E-01
ENSCAFG0000003075	ENSCAFG0000003075	grey	EC_M10	0.17	4.1E-01	-0.02	9.1E-01	-0.01	9.8E-01	-0.19	3.5E-01	-0.09	6.7E-01	0.15	4.7E-01	-0.24	2.5E-01	0.06	7.5E-01	-0.30	1.3E-01	-0.06	7.8E-01
ENSCAFG0000002442	ENSCAFG0000002442	violet	EC_M7	0.17	4.1E-01	-0.12	5.5E-01	-0.17	4.2E-01	0.27	1.8E-01	0.04	8.6E-01	0.69	9.4E-05	-0.13	5.1E-01	0.21	3.1E-01	0.18	3.9E-01	-0.15	4.7E-01
ENSCAFG0000002617	ENSCAFG0000002617	grey	EC_M10	0.17	4.1E-01	0.10	6.2E-01	0.07	7.2E-01	0.04	8.3E-01	-0.32	1.1E-01	0.08	7.1E-01	0.25	2.2E-01	-0.21	2.9E-01	-0.27	1.9E-01	0.19	3.4E-01
ENSCAFG0000000436	NOX4	grey	EC_M10	0.17	4.1E-01	0.41	3.5E-02	-0.45	2.1E-02	-0.30	1.3E-01	0.25	2.2E-01	0.14	4.8E-01	0.00	9.9E-01	-0.11	6.1E-01	-0.51	7.5E-03	-0.37	6.5E-02
ENSCAFG0000002996	FAM229B	violet	EC_M7	0.17	4.1E-01	-0.07	7.4E-01	-0.18	3.9E-01	-0.08	6.9E-01	0.04	8.5E-01	0.68	1.3E-04	-0.08	7.1E-01	-0.13	5.3E-01	0.36	7.3E-02	-0.15	4.6E-01
ENSCAFG0000003098	ENSCAFG0000003098	violet	EC_M7	0.17	4.1E-01	-0.07	7.4E-01	-0.18	3.9E-01	-0.08	6.9E-01	0.04	8.5E-01	0.68	1.3E-04	-0.08	7.1E-01	-0.13	5.3E-01	0.36	7.3E-02	-0.15	4.6E-01
ENSCAFG0000000682	PCBP2	cyan	EC_M2	0.17	4.1E-01	-0.02	9.4E-01	0.55	3.3E-03	0.18	3.8E-01	-0.74	1.3E-05	-0.14	4.9E-01	0.02	9.0E-01	-0.31	1.3E-01	-0.42	3.4E-02	0.63	5.1E-04
ENSCAFG0000002274	ENSCAFG0000002274	grey	EC_M10	0.17	4.1E-01	0.39	5.0E-02	-0.26	1.9E-01	0.32	1.1E-01	-0.02	9.3E-01	0.07	7.2E-01	0.10	6.2E-01	-0.13	5.2E-01	-0.06	7.8E-01	-0.10	6.4E-01
ENSCAFG0000003227	FXYD6	turquoise	EC_M6	0.17	4.1E-01	0.88	3.0E-09	-0.31	1.2E-01	-0.20	3.2E-01	-0.08	6.8E-01	-0.20	3.2E-01	-0.10	6.2E-01	-0.11	6.1E-01	0.31	1.3E-01	0.00	1.0E+00
ENSCAFG0000001210	EMID1	turquoise	EC_M6	0.17	4.1E-01	0.51	7.9E-03	-0.31	1.3E-01	-0.11	5.9E-01	0.07	7.5E-01	0.44	2.4E-02	-0.19	3.5E-01	-0.16	4.4E-01	-0.23	2.6E-01	-0.12	5.4E-01
ENSCAFG0000002414	ENSCAFG0000002414	grey	EC_M10	0.17	4.1E-01	0.14	4.9E-01	-0.37	6.1E-02	-0.13	5.2E-01	0.22	2.7E-01	0.26	2.0E-01	0.23	2.7E-01	0.56	2.6E-03	0.00	9.9E-01	-0.32	1.1E-01
ENSCAFG0000000961	CSDE1	darkgreen	EC_M4	0.17	4.1E-01	0.08	7.1E-01	-0.84	1.0E-07	-0.18	3.9E-01	0.83	1.6E-07	0.19	3.4E-01	0.00	9.9E-01	0.01	9.7E-01	0.00	9.9E-01	-0.90	3.9E-10
ENSCAFG0000003170	ATXN10	grey	EC_M10	0.17	4.1E-01	-0.18	3.7E-01	-0.39	4.6E-02	0.04	8.5E-01	0.38	5.8E-02	0.10	6.1E-01	-0.36	7.4E-02	-0.11	5.8E-01	-0.15	4.6E-01	-0.43	2.8E-02
ENSCAFG0000003220	LYPD6B	grey	EC_M10	0.17	4.1E-01	-0.15	4.8E-01	-0.28	1.7E-01	0.01	9.7E-01	0.23	2.5E-01	-0.08	7.0E-01	-0.01	9.7E-01	-0.08	7.1E-01	-0.05	8.2E-01	-0.35	8.3E-02
ENSCAFG0000001815	CDC42BPB	turquoise	EC_M6	0.17	4.1E-01	0.65	3.6E-04	-0.44	2.4E-02	-0.31	1.2E-01	0.20	3.2E-01	0.04	8.4E-01	-0.13	5.4E-01	-0.10	6.3E-01	-0.20	3.3E-01	-0.28	1.6E-01
ENSCAFG0000000510	C1H19orf54	grey	EC_M10	0.17	4.1E-01	-0.06	7.9E-01	0.40	4.5E-02	-0.24	2.4E-01	-0.57	2.2E-03	-0.12	5.5E-01	-0.16	4.3E-01	-0.27	1.7E-01	0.21	3.0E-01	0.48	1.3E-02
ENSCAFG0000000692	MIER3	grey	EC_M10	0.17	4.1E-01	0.17	4.2E-01	-0.16	4.4E-01	-0.01	9.7E-01	0.02	9.3E-01	0.04	8.5E-01	0.18	3.7E-01	-0.28	1.7E-01	-0.05	8.0E-01	-0.07	7.4E-01
ENSCAFG0000000178	MRPL2	grey	EC_M10	0.17	4.1E-01	-0.18	3.8E-01	0.31	1.2E-01	0.08	7.0E-01	-0.45	2.0E-02	0.23	2.6E-01	0.00	9.8E-01	-0.32	1.1E-01	0.05	8.0E-01	0.36	7.1E-02
ENSCAFG0000000022	GLI1	grey	EC_M10	0.17	4.1E-01	-0.22	2.8E-01	-0.10	6.2E-01	0.13	5.3E-01	-0.02	9.3E-01	0.30	1.4E-01	-0.07	7.5E-01	-0.19	3.5E-01	-0.02	9.1E-01	-0.15	4.5E-01
ENSCAFG0000003201	RAB39A	turquoise	EC_M6	0.17	4.1E-01	0.68	1.2E-04	-0.26	2.1E-01	0.05	8.2E-01	-0.11	5.8E-01	-0.15	4.6E-01	-0.15	4.7E-01	-0.22	2.8E-01	0.04	8.6E-01	-0.01	9.5E-01
ENSCAFG0000000727	PDE7A	grey	EC_M10	0.17	4.1E-01	0.28	1.7E-01	-0.51	7.3E-03	0.11	5.8E-01	0.32	1.1E-01	0.10	6.1E-01	0.08	7.1E-01	0.03	8.9E-01	0.30	1.4E-01	-0.40	4.5E-02
ENSCAFG0000000952	CUTC	grey	EC_M10	0.17	4.1E-01	0.05	8.0E-01	-0.63	6.1E-04	0.20	3.3E-01	0.52	6.5E-03	0.35	8.3E-02	0.05	8.1E-01	0.09	6.6E-01	0.09	6.5E-01	-0.62	8.1E-04
ENSCAFG0000000587	NUP98	cyan	EC_M2	0.17	4.1E-01	-0.15	4.5E-01	0.60	1.2E-03	0.10	6.2E-01	-0.84	8.3E-08	-0.10	6.4E-01	-0.20	3.2E-01	-0.07	7.3E-01	-0.09	6.8E-01	0.74	1.5E-05
ENSCAFG0000003267	ENSCAFG0000003267	grey	EC_M10	0.17	4.1E-01	-0.09	6.8E-01	-0.41	3.6E-02	0.10	6.1E-01	0.39	4.8E-02	0.31	1.2E-01	-0.06	7.6E-01	-0.06	7.9E-01	0.13	5.1E-01	-0.43	2.8E-02
ENSCAFG0000000681	RB1CC1	grey	EC_M10	0.17	4.1E-01	0.05	8.2E-01	0.12	5.7E-01	0.23	2.6E-01	-0.42	3.3E-02	-0.06	7.6E-01	-0.20	3.3E-01	-0.07	7.4E-01	-0.28	1.7E-01	0.30	1.4E-01
ENSCAFG0000000965	ENSCAFG0000000965	grey	EC_M10	0.17	4.1E-01	0.30	1.4E-01	0.12	5.5E-01	0.35	7.9E-02	-0.37	6.2E-02	-0.12	5.7E-01	-0.16	4.5E-01	-0.14	4.8E-01	-0.14	4.9E-01	0.28	1.7E-01
ENSCAFG0000000242	MEOX2	grey	EC_M10	0.17	4.1E-01	0.28	1.7E-01	0.33	9.5E-02	-0.13	5.2E-01	-0.55	3.6E-03	-0.28	1.6E-01	0.07	7.4E-01	0.02	9.3E-01	-0.36	6.7E-02	0.51	7.3E-03
ENSCAFG0000000866	ENSCAFG0000000866	grey	EC_M10	0.17	4.1E-01	-0.49	1.1E-02	-0.45	2.1E-02	-0.18	3.7E-01	0.62	7.3E-04	0.23	2.5E-01	0.06	7.6E-01	0.14	4.9E-01	-0.15	4.8E-01	-0.73	2.0E-05
ENSCAFG0000000408	UBN2	grey	EC_M10	0.17	4.1E-01	-0.36	7.1E-02	0.00	9.9E-01	0.33	9.9E-02	-0.09	6.7E-01	-0.05	8.1E-01	-0.23	2.5E-01	-0.03	9.0E-01	-0.04	8.6E-01	-0.05	8.0E-01
ENSCAFG0000001674	HDGF	grey	EC_M10	0.17	4.1E-01	-0.03	8.9E-01	0.48	1.3E-02	-0.13	5.3E-01	-0.63	6.3E-04	-0.22	2.8E-01	0.09	6.5E-01	0.25	2.1E-01	0.05	8.1E-01	0.51	7.1E-03
ENSCAFG0000001085	SYNM	turquoise	EC_M6	0.17	4.1E-01	0.81	5.0E-07	-0.25	2.2E-01	-0.28	1.6E-01	-0.06	7.7E-01	-0.17	4.1E-01	-0.01	9.5E-01	-0.13	5.3E-01	0.01	9.6E-01	0.03	8.9E-01
ENSCAFG0000000337	DGKI	grey	EC_M10	0.17	4.1E-01	-0.04	8.4E-01	-0.59	1.6E-03	-0.08	7.0E-01	0.62	7.9E-04	0.36	6.8E-02	-0.17	4.0E-01	-0.12	5.6E-01	-0.17	3.9E-01	-0.69	1.0E-04
ENSCAFG0000001395	TSPAN15	turquoise	EC_M6	0.17	4.1E-01	0.80	9.8E-07	-0.20	3.2E-01	-0.12	5.6E-01	-0.16	4.4E-01	-0.15	4.6E-01	0.00	9.9E-01	-0.16	4.3E-01	-0.07	7.2E-01	0.04	8.4E-01
ENSCAFG0000002936	ENSCAFG0000002936	grey	EC_M10	0.17	4.1E-01	-0.10	6.3E-01	-0.02	9.2E-01	-0.02	9.1E-01	-0.11	5.9E-01	-0.08	7.1E-01	-0.43	2.8E-02	0.39	5.0E-02	0.31	1.3E-01	0.03	8.8E-01
ENSCAFG0000001745	ENSCAFG0000001745	grey	EC_M10	0.17	4.1E-01	0.39	5.0E-02	0.01	9.5E-01	-0.07	7.4E-01	-0.33	9.5E-02	-0.16	4.3E-01	-0.15	4.8E-01	-0.12	5.4E-01	-0.09	6.6E-01	0.26	1.9E-01
ENSCAFG0000001133	RCC1L	grey	EC_M10	0.17	4.1E-01	-0.40	4.4E-02	0.00	9.8E-01	-0.05	8.1E-01	0.13	5.4E-01	-0.09	6.6E-01	-0.22	2.7E-01	-0.14	5.0E-01	-0.02	9.4E-01	-0.22	2.7E-01
ENSCAFG0000000203	TDRD6	grey	EC_M10	0.17	4.1E-01	-0.05	8.3E-01	-0.11	5.9E-01	-0.03	8.7E-01	-0.02	9.1E-01	-0.12	5.8E-01	-0.07	7.3E-01	-0.13	5.2E-01	-0.07	7.2E-01	-0.08	6.9E-01
ENSCAFG0000001761	UACA	darkgreen	EC_M4	0.17	4.1E-01	0.01	9.6E-01	-0.81	4.3E-07	-0.12	5.7E-01	0.79	1.5E-06	0.21	3.0E-01	-0.05	8.2E-01	-0.05	8.2E-01	0.03	8.8E-01	-0.90	5.2E-10
ENSCAFG0000001480	DNM3	turquoise	EC_M6	0.17	4.1E-01	0.42	3.1E-02	-0.35	7.6E-02	-0.09	6.8E-01	0.05	8.0E-01	0.04	8.5E-01	-0.26	2.0E-01	0.45	2.0E-02	-0.08	6.8E-01	-0.20	3.3E-01
ENSCAFG0000000584	ADAM32	grey	EC_M10	0.17	4.1E-01	-0.11	6.0E-01	-0.51	7.6E-03	-0.06	7.6E-01	0.46	1.7E-02	0.06	7.6E-01	0.16	4.3E-01	0.07	7.3E-01	-0.35	7.6E-02	-0.59	1.6E-03
ENSCAFG0000002932	SCN2B	turquoise	EC_M6	0.17	4.1E-01	0.61	1.0E-03	-0.25	2.2E-01	-0.04	8.4E-01	-0.08	7.0E-01	-0.01	9.5E-01	-0.13	5.2E-01	-0.15	4.6E-01	0.41	3.6E-02	0.01	9.5E-01
ENSCAFG0000001904	ENSCAFG0000001904	cyan	EC_M2	0.17	4.2E-01	-0.37	6.4E-02	0.33	1.0E-01	0.01	9.7E-01	-0.40	4.3E-02	0.18	3.7E-01	0.25	2.1E-01	-0.09	6.8E-01	-0.43	2.7E-02	0.24	2.3E-01
ENSCAFG0000001483	TNIP2	grey	EC_M10	0.17	4.2E-01	0.24	2.4E-01	0.06	7.8E-01	0.05	8.1E-01	-0.35	8.1E-02	0.00	9.9E-01	-0.04	8.3E-01	-0.06	7.6E-01	0.16	4.4E-01	0.25	2.3E-01
ENSCAFG0000001285	MMP21	turquoise	EC_M6	0.17	4.2E-01	0.79	1.7E-06	-0.16	4.3E-01	-0.08	6.9E-01	-0.21	3.0E-01	-0.07	7.2E-01	-0.07	7.5E-01	-0.10	6.4E-01	-0.14	5.1E-01	0.11	5.9E-01
ENSCAFG0000001876	SPEF2	turquoise	EC_M6	0.17	4.2E-01	0.79	1.7E-06	-0.16	4.3E-01	-0.08	6.9E-01	-0.21	3.0E-01	-0.07	7.2E-01	-0.07	7.5E-01	-0.10	6.4E-01	-0.14	5.1E-01	0.11	5.9E-01
ENSCAFG0000003238	ENSCAFG0000003238	turquoise	EC_M6	0.17	4.2E-01	0.79	1.7E-06	-0.16	4.3E-01	-0.08	6.9E-01	-0.21	3.0E-01	-0.07	7.2E-01	-0.07	7.5E-01	-0.10	6.4E-01	-0.14	5.1E-01	0.11	5.9E-01
ENSCAFG0000000142	GKAP1	grey	EC_M10	0.17	4.2E-01	0.52	6.8E-03	0.09	6.8E-01	0.07	7.4E-01	-0.46	1.7E-02	-0.17	4.0E-01	-0.07	7.2E-01	0.04	8.4E-01	0.08	6.9E-01	0.40	4.3E-02
ENSCAFG0000001383	MASP1	turquoise	EC_M6	0.17	4.2E-01	0.80	8.4E-07	-0.27	1.8E-01	-0.09	6.8E-01	-0.12	5.5E-01	-0.10	6.3E-01	-0.08	6.9E-01	0.12	5.6E-01	-0.13	5.2E-01	0.00	9.9E-01
ENSCAFG0000000277	ZNF462	grey	EC_M10	0.17	4.2E-01	-0.13	5.3E-01	0.20	3.3E-01	-0.09	6.7E-01	-0.32	1.1E-01	0.05	8.3E-01	0.31	1.2E-01	-0.07	7.3E-01	-0.45	2.2E-02	0.18	3.9E-01
ENSCAFG0000001525	SPNS3	turquoise	EC_M6	0.17	4.2E-01	0.72	3.3E-05	0.03	9.0E-01	-0.26	2.0E-01	-0.38	5.9E-02	-0.24	2.3E-01	-0.01	9.5E-01	-0.27	1.8E-01	-0.15	4.7E-01	0.32	1.1E-01
ENSCAFG0000001917	NSDHL	grey	EC_M10	0.17	4.2E-01	-0.42	3.3E-02	0.13	5.4E-01	0.17	4.1E-01	-0.12	5.6E-01	-0.20	3.2E-01	-0.16	4.3E-01	-0.05	8.1E-01	-0.49	1.1E-02	-0.03	8.7E-01
ENSCAFG0000000973	PLA2G4B	cyan	EC_M2	0.17	4.2E-01	0.06	7.9E-01	0.37	6.4E-02	0.36	6.7E-02	-0.66	2.7E-04	-0.28	1.6E-01	0.01	9.5E-01	0.11	5.9E-01	-0.17	4.1E-01	0.54	4.3E-03
ENSCAFG0000000988	ZNF335	grey	EC_M10	0.17	4.2E-01	0.34	9.4E-02	0.30	1.4E-01	0.05	8.1E-01	-0.57	2.2E-03	0.05	8.1E-01	-0.07	7.3E-01	0.00	9.8E-01	0.23	2.7E-01	0.49	1.1E-02
ENSCAFG0000000750	ENSCAFG0000000750	grey	EC_M10	0.17	4.2E-01	0.10	6.2E-01	-0.02	9.2E-01	0.16	4.3E-01	-0.10	6.3E-01	0.14	4.9E-01	0.37	6.6E-02	-0.14	4.9E-01	-0.05	7.9E-01	0.00	9.9E-01
ENSCAFG0000001132	ENSCAFG0000001132	grey	EC_M10	0.17	4.2E-01	-0.08	6.8E-01	-0.34	8.7E-02	0.08	7.1E-01	0.25	2.2E-01	-0.09	6.6E-01	-0.06	7.6E-01	-0.05	8.0E-01	-0.12	5.4E-01	-0.38	5.7E-02
ENSCAFG0000001164	SOX2	grey	EC_M10	0.17	4.2E-01	-0.08	6.8E-01	-0.34	8.7E-02	0.08	7.1E-01	0.25	2.2E-01	-0.09	6.6E-01	-0.06	7.6E-01	-0.05	8.0E-01	-0.12	5.4E-01	-0.38	5.7E-02
ENSCAFG0000002464	ENSCAFG0000002464	grey	EC_M10	0.17	4.2E-01	-0.20	3.3E-01	-0.18	3.8E-01	-0.22	2.9E-01	0.15	4.6E-01	0.18	3.8E-01	-0.30	1.4E-01	0.11	5.8E-01	-0.27	1.9E-01	-0.30	1.3E-01
ENSCAFG0000001654	UIMC1	cyan	EC_M2	0.17	4.2E-01	0.28	1.7E-01	0.46	1.7E-02	0.16	4.2E-01	-0.79	1.7E-06	-0.24	2.3E-01	-0.05	8.2E-01	0.00	9.9E-01	-0.15	4.7E-01	0.70	5.8E-05
ENSCAFG0000000733	CCDC73	violet	EC_M7	0.17	4.2E-01	-0.20	3.2E-01	0.13	5.2E-01	-0.07	7.5E-01	-0.14	4.9E-01	0.53	5.7E-03	-0.10	6.4E-01	-0.03	8.9E-01	-0.12	5.5E-01	0.05	7.9E-01

ENSCAFG0000000092	VGLL2	grey	EC_M10	0.17	4.2E-01	0.06	7.8E-01	-0.17	4.0E-01	-0.20	3.2E-01	0.08	6.8E-01	0.06	7.8E-01	-0.10	6.3E-01	-0.21	2.9E-01	-0.30	1.4E-01	-0.20	3.2E-01
ENSCAFG0000001329	KAT5	cyan	EC_M2	0.17	4.2E-01	-0.05	8.1E-01	0.52	7.1E-03	0.41	3.7E-02	-0.75	9.5E-06	-0.10	6.4E-01	-0.11	5.8E-01	-0.21	2.9E-01	-0.34	8.9E-02	0.64	4.0E-04
ENSCAFG0000001586	SCGB1A1	grey	EC_M10	0.17	4.2E-01	-0.12	5.5E-01	0.04	8.3E-01	-0.03	8.8E-01	-0.10	6.3E-01	-0.10	6.4E-01	-0.09	6.7E-01	-0.06	7.7E-01	-0.14	4.8E-01	0.02	9.2E-01
ENSCAFG0000003152	CROCC2	grey	EC_M10	0.17	4.2E-01	-0.12	5.5E-01	0.04	8.3E-01	-0.03	8.8E-01	-0.10	6.3E-01	-0.10	6.4E-01	-0.09	6.7E-01	-0.06	7.7E-01	-0.14	4.8E-01	0.02	9.2E-01
ENSCAFG0000001324	GPR19	grey	EC_M10	0.17	4.2E-01	0.35	8.2E-02	0.23	2.7E-01	0.24	2.4E-01	-0.50	8.6E-03	-0.13	5.1E-01	0.06	7.6E-01	-0.46	1.8E-02	0.08	7.0E-01	0.45	2.0E-02
ENSCAFG0000000745	RMND5A	cyan	EC_M2	0.17	4.2E-01	0.20	3.2E-01	0.55	3.7E-03	0.18	3.7E-01	-0.85	3.2E-08	-0.35	7.9E-02	-0.23	2.5E-01	-0.02	9.2E-01	-0.10	6.3E-01	0.81	6.5E-07
ENSCAFG0000001798	ASXL3	grey	EC_M10	0.17	4.2E-01	-0.10	6.3E-01	-0.45	2.0E-02	-0.02	9.4E-01	0.45	2.2E-02	-0.11	5.8E-01	-0.06	7.8E-01	-0.03	8.8E-01	0.01	9.5E-01	-0.53	5.3E-03
ENSCAFG0000000235	ENSCAFG0000000235	turquoise	EC_M6	0.17	4.2E-01	0.48	1.2E-02	-0.07	7.4E-01	0.23	2.6E-01	-0.21	3.1E-01	-0.32	1.1E-01	-0.42	3.3E-02	0.17	4.0E-01	-0.11	6.1E-01	0.14	4.8E-01
ENSCAFG0000001233	H2AFX	grey	EC_M10	0.17	4.2E-01	-0.43	2.7E-02	0.25	2.2E-01	0.11	6.0E-01	-0.23	2.7E-01	-0.02	9.2E-01	-0.11	5.9E-01	0.05	8.1E-01	0.01	9.8E-01	0.12	5.7E-01
ENSCAFG0000000892	NEK4	cyan	EC_M2	0.17	4.2E-01	-0.36	7.5E-02	0.28	1.6E-01	0.15	4.8E-01	-0.41	3.7E-02	-0.12	5.5E-01	-0.04	8.4E-01	-0.07	7.4E-01	0.08	6.9E-01	0.33	1.0E-01
ENSCAFG0000000966	PIK3C2B	turquoise	EC_M6	0.17	4.2E-01	0.81	4.6E-07	-0.53	5.4E-03	-0.21	3.1E-01	0.19	3.4E-01	-0.01	9.6E-01	-0.02	9.0E-01	-0.07	7.2E-01	0.24	2.4E-01	-0.29	1.5E-01
ENSCAFG0000002500	DPP4	grey	EC_M10	0.17	4.2E-01	-0.67	1.8E-04	0.18	3.8E-01	0.24	2.3E-01	-0.13	5.3E-01	0.08	7.0E-01	0.12	5.6E-01	-0.05	8.1E-01	-0.41	3.9E-02	-0.03	8.8E-01
ENSCAFG0000000430	KCNRG	turquoise	EC_M6	0.17	4.2E-01	0.51	7.3E-03	0.07	7.5E-01	0.40	4.5E-02	-0.42	3.3E-02	-0.12	5.4E-01	-0.15	4.8E-01	-0.06	7.7E-01	-0.16	4.4E-01	0.34	8.7E-02
ENSCAFG0000000840	ENSCAFG0000000840	grey	EC_M10	0.16	4.2E-01	0.12	5.6E-01	-0.42	3.4E-02	-0.08	7.0E-01	0.33	9.9E-02	-0.14	4.9E-01	-0.15	4.7E-01	-0.05	8.0E-01	-0.05	7.9E-01	-0.40	4.6E-02
ENSCAFG0000000856	GUCY1B1	turquoise	EC_M6	0.16	4.2E-01	0.66	2.4E-04	-0.23	2.7E-01	-0.23	2.5E-01	-0.08	7.0E-01	-0.12	5.7E-01	-0.05	7.9E-01	-0.02	9.3E-01	0.62	6.7E-04	0.03	8.9E-01
ENSCAFG0000001378	CCRL2	turquoise	EC_M6	0.16	4.2E-01	0.87	5.1E-09	-0.30	1.4E-01	-0.12	5.6E-01	-0.08	6.9E-01	-0.12	5.6E-01	0.16	4.4E-01	-0.10	6.2E-01	0.35	8.1E-02	0.03	8.9E-01
ENSCAFG0000002248	ENSCAFG0000002248	grey	EC_M10	0.16	4.2E-01	0.06	7.8E-01	0.07	7.2E-01	0.12	5.5E-01	-0.28	1.7E-01	-0.29	1.5E-01	0.21	3.1E-01	-0.03	8.8E-01	0.36	7.4E-02	0.20	3.2E-01
ENSCAFG0000001767	EARS2	grey	EC_M10	0.16	4.2E-01	-0.53	5.5E-03	-0.12	5.6E-01	-0.22	2.7E-01	0.22	2.8E-01	0.15	4.8E-01	-0.06	7.7E-01	0.25	2.1E-01	-0.20	3.3E-01	-0.35	8.0E-02
ENSCAFG0000001592	CCDC22	grey	EC_M10	0.16	4.2E-01	0.14	5.1E-01	0.41	3.5E-02	0.19	3.5E-01	-0.66	2.3E-04	-0.10	6.3E-01	-0.02	9.3E-01	-0.20	3.3E-01	-0.14	5.0E-01	0.56	3.0E-03
ENSCAFG0000001118	DCLRE1A	grey	EC_M10	0.16	4.2E-01	-0.35	8.0E-02	0.23	2.7E-01	0.37	6.1E-02	-0.34	9.1E-02	0.22	2.9E-01	0.00	9.9E-01	-0.16	4.3E-01	-0.06	7.6E-01	0.24	2.3E-01
ENSCAFG0000003266	LRRTM2	darkgreen	EC_M4	0.16	4.2E-01	-0.40	4.2E-02	-0.57	2.3E-03	0.02	9.3E-01	0.67	1.8E-04	0.28	1.7E-01	-0.13	5.3E-01	-0.06	7.7E-01	-0.12	5.7E-01	-0.80	9.0E-07
ENSCAFG0000001762	CARM1	grey	EC_M10	0.16	4.2E-01	-0.21	3.0E-01	0.54	4.4E-03	0.10	6.3E-01	-0.64	4.6E-04	-0.15	4.6E-01	-0.40	4.3E-02	-0.14	4.9E-01	-0.11	6.1E-01	0.52	6.5E-03
ENSCAFG0000000705	NPHP1	grey	EC_M10	0.16	4.2E-01	-0.19	3.5E-01	0.32	1.1E-01	0.19	3.5E-01	-0.51	8.2E-03	-0.17	4.0E-01	0.04	8.6E-01	0.00	9.8E-01	0.41	3.6E-02	0.45	2.2E-02
ENSCAFG0000002990	ENSCAFG0000002990	grey	EC_M10	0.16	4.2E-01	0.15	4.7E-01	0.11	5.9E-01	-0.36	7.0E-02	-0.25	2.2E-01	-0.02	9.1E-01	-0.34	9.0E-02	-0.32	1.1E-01	-0.20	3.3E-01	0.15	4.6E-01
ENSCAFG0000002910	ENSCAFG0000002910	grey	EC_M10	0.16	4.2E-01	0.28	1.6E-01	-0.03	8.8E-01	-0.16	4.3E-01	-0.20	3.3E-01	-0.09	6.7E-01	-0.11	6.0E-01	-0.10	6.3E-01	-0.12	5.6E-01	0.10	6.4E-01
ENSCAFG0000001126	SSH1	grey	EC_M10	0.16	4.2E-01	0.53	5.8E-03	0.21	3.0E-01	0.00	9.8E-01	-0.61	9.6E-04	-0.03	8.8E-01	0.06	7.8E-01	-0.05	8.2E-01	0.26	2.0E-01	0.54	4.7E-03
ENSCAFG0000003091	ENSCAFG0000003091	grey	EC_M10	0.16	4.2E-01	0.01	9.5E-01	0.03	8.9E-01	-0.07	7.3E-01	-0.09	6.5E-01	0.02	9.1E-01	0.02	9.1E-01	-0.03	9.0E-01	0.09	6.5E-01	0.01	9.6E-01
ENSCAFG0000002851	FAM206A	grey	EC_M10	0.16	4.2E-01	-0.23	2.5E-01	0.18	3.8E-01	0.30	1.3E-01	-0.28	1.6E-01	0.01	9.6E-01	-0.39	4.9E-02	-0.01	9.6E-01	-0.33	9.6E-02	0.15	4.7E-01
ENSCAFG0000003193	NRBF2	grey	EC_M10	0.16	4.2E-01	0.23	2.7E-01	-0.24	2.3E-01	0.11	6.1E-01	-0.02	9.4E-01	0.12	5.7E-01	0.15	4.6E-01	-0.24	2.4E-01	-0.62	7.8E-04	-0.13	5.2E-01
ENSCAFG0000001708	ENSCAFG0000001708	turquoise	EC_M6	0.16	4.2E-01	0.54	4.7E-03	0.19	3.6E-01	0.12	5.5E-01	-0.53	5.1E-03	0.05	8.0E-01	0.07	7.4E-01	0.11	5.9E-01	-0.24	2.4E-01	0.44	2.6E-02
ENSCAFG0000000005	ENSCAFG0000000005	grey	EC_M10	0.16	4.2E-01	-0.05	8.0E-01	0.23	2.6E-01	-0.06	7.6E-01	-0.44	2.6E-02	0.18	3.8E-01	-0.08	7.0E-01	-0.10	6.3E-01	-0.06	7.7E-01	0.32	1.1E-01
ENSCAFG0000001516	ENSCAFG0000001516	grey	EC_M10	0.16	4.2E-01	0.28	1.7E-01	-0.22	2.9E-01	0.22	2.8E-01	0.10	6.2E-01	-0.19	3.5E-01	-0.18	3.9E-01	-0.24	2.4E-01	0.06	7.8E-01	-0.16	4.2E-01
ENSCAFG0000000946	HIBCH	grey	EC_M10	0.16	4.2E-01	-0.64	4.1E-04	0.37	6.0E-02	0.26	1.9E-01	-0.34	8.7E-02	-0.03	8.8E-01	-0.23	2.6E-01	0.03	8.8E-01	-0.17	4.2E-01	0.26	2.0E-01
ENSCAFG0000000477	NAA16	cyan	EC_M2	0.16	4.3E-01	-0.08	7.1E-01	0.52	5.9E-03	0.48	1.3E-02	-0.75	1.1E-05	-0.12	5.4E-01	-0.15	4.8E-01	-0.14	4.9E-01	0.05	8.2E-01	0.67	1.7E-04
ENSCAFG0000000920	CHRM4	grey	EC_M10	0.16	4.3E-01	0.28	1.7E-01	0.13	5.2E-01	-0.11	6.0E-01	-0.42	3.4E-02	-0.16	4.5E-01	-0.21	3.1E-01	-0.14	4.8E-01	0.04	8.3E-01	0.34	8.8E-02
ENSCAFG0000001365	HMCN1	turquoise	EC_M6	0.16	4.3E-01	0.66	2.6E-04	-0.14	5.1E-01	-0.09	6.7E-01	-0.25	2.2E-01	-0.15	4.5E-01	-0.15	4.7E-01	0.25	2.1E-01	0.47	1.7E-02	0.21	3.1E-01
ENSCAFG0000003058	ENSCAFG0000003058	grey	EC_M10	0.16	4.3E-01	0.03	8.7E-01	-0.44	2.6E-02	-0.04	8.3E-01	0.38	5.5E-02	0.51	7.8E-03	-0.10	6.3E-01	-0.13	5.4E-01	-0.17	4.2E-01	-0.46	1.7E-02
ENSCAFG0000001297	MLXIPL	turquoise	EC_M6	0.16	4.3E-01	0.83	1.7E-07	-0.16	4.3E-01	0.00	9.9E-01	-0.26	2.0E-01	-0.19	3.6E-01	0.11	6.0E-01	0.02	9.4E-01	0.14	5.0E-01	0.16	4.2E-01
ENSCAFG0000002211	ENSCAFG0000002211	violet	EC_M7	0.16	4.3E-01	-0.14	5.0E-01	-0.03	8.8E-01	-0.09	6.6E-01	-0.06	7.9E-01	0.78	2.7E-06	-0.07	7.2E-01	-0.11	6.0E-01	-0.18	3.9E-01	-0.03	8.9E-01
ENSCAFG0000000239	ENSCAFG0000000239	grey	EC_M10	0.16	4.3E-01	0.31	1.2E-01	0.12	5.6E-01	0.15	4.6E-01	-0.42	3.3E-02	-0.25	2.2E-01	-0.25	2.2E-01	-0.14	5.0E-01	-0.33	9.5E-02	0.34	9.1E-02
ENSCAFG0000001209	FAM45A	grey	EC_M10	0.16	4.3E-01	0.27	1.8E-01	-0.61	9.6E-04	-0.20	3.4E-01	0.43	2.9E-02	0.25	2.1E-01	-0.16	4.4E-01	0.18	3.8E-01	-0.26	2.0E-01	-0.53	5.6E-03
ENSCAFG0000000518	SPOPL	darkgreen	EC_M4	0.16	4.3E-01	0.16	4.5E-01	-0.82	2.6E-07	-0.01	9.5E-01	0.71	4.6E-05	0.20	3.2E-01	-0.04	8.4E-01	0.03	8.9E-01	0.18	3.8E-01	-0.77	3.8E-06
ENSCAFG0000000515	THBD	grey	EC_M10	0.16	4.3E-01	0.23	2.6E-01	-0.18	3.9E-01	0.16	4.5E-01	0.04	8.7E-01	-0.08	7.1E-01	-0.37	6.1E-02	-0.22	2.7E-01	-0.22	2.8E-01	-0.09	6.7E-01
ENSCAFG0000002942	ENSCAFG0000002942	cyan	EC_M2	0.16	4.3E-01	-0.15	4.7E-01	0.31	1.3E-01	-0.12	5.7E-01	-0.48	1.4E-02	-0.13	5.3E-01	0.06	7.7E-01	0.17	4.0E-01	-0.11	6.1E-01	0.34	9.2E-02
ENSCAFG0000000060	ADM2	grey	EC_M10	0.16	4.3E-01	-0.13	5.2E-01	0.00	9.8E-01	-0.08	6.9E-01	-0.12	5.7E-01	0.00	9.8E-01	-0.10	6.3E-01	-0.08	7.1E-01	-0.11	5.8E-01	-0.01	9.6E-01
ENSCAFG0000001598	ENSCAFG0000001598	grey	EC_M10	0.16	4.3E-01	-0.13	5.2E-01	0.00	9.8E-01	-0.08	6.9E-01	-0.12	5.7E-01	0.00	9.8E-01	-0.10	6.3E-01	-0.08	7.1E-01	-0.11	5.8E-01	-0.01	9.6E-01
ENSCAFG0000003069	IL22RA1	grey	EC_M10	0.16	4.3E-01	-0.13	5.2E-01	0.00	9.8E-01	-0.08	6.9E-01	-0.12	5.7E-01	0.00	9.8E-01	-0.10	6.3E-01	-0.08	7.1E-01	-0.11	5.8E-01	-0.01	9.6E-01
ENSCAFG0000001893	CFAP53	grey	EC_M10	0.16	4.3E-01	0.19	3.4E-01	-0.16	4.3E-01	0.05	7.9E-01	-0.10	6.3E-01	-0.13	5.3E-01	-0.03	8.8E-01	0.41	3.8E-02	0.05	8.0E-01	-0.04	8.4E-01
ENSCAFG0000003223	ENSCAFG0000003223	grey	EC_M10	0.16	4.3E-01	0.30	1.3E-01	0.04	8.3E-01	0.17	4.0E-01	-0.24	2.4E-01	-0.23	2.5E-01	0.00	9.9E-01	-0.21	2.9E-01	-0.42	3.2E-02	0.13	5.3E-01
ENSCAFG0000001465	GNB3	grey	EC_M10	0.16	4.3E-01	-0.32	1.2E-01	-0.28	1.6E-01	0.15	4.7E-01	0.26	2.0E-01	0.21	3.1E-01	0.15	4.6E-01	0.02	9.2E-01	-0.31	1.2E-01	-0.43	2.7E-02
ENSCAFG0000000757	PIK3CB	grey	EC_M10	0.16	4.3E-01	-0.01	9.5E-01	-0.30	1.4E-01	-0.08	7.1E-01	0.19	3.4E-01	0.11	6.1E-01	0.32	1.1E-01	-0.25	2.2E-01	-0.57	2.5E-03	-0.27	1.9E-01
ENSCAFG0000000510	CXCR4	turquoise	EC_M6	0.16	4.3E-01	0.87	9.5E-09	-0.20	3.2E-01	-0.11	6.0E-01	-0.18	3.7E-01	-0.10	6.3E-01	-0.14	5.0E-01	-0.14	4.9E-01	-0.04	8.5E-01	0.07	7.5E-01
ENSCAFG0000001413	KIAA1143	grey	EC_M10	0.16	4.3E-01	0.12	5.7E-01	0.06	7.8E-01	0.24	2.3E-01	-0.38	5.3E-02	-0.21	3.0E-01	0.19	3.6E-01	0.13	5.4E-01	0.29	1.5E-01	0.30	1.4E-01
ENSCAFG0000001450	ZBTB37	grey	EC_M10	0.16	4.3E-01	0.28	1.6E-01	-0.19	3.6E-01	0.43	2.7E-02	-0.01	9.5E-01	0.07	7.2E-01	-0.05	7.9E-01	0.20	3.4E-01	0.08	7.2E-01	-0.06	7.8E-01
ENSCAFG0000002616	ENSCAFG0000002616	grey	EC_M10	0.16	4.3E-01	-0.17	4.1E-01	0.20	3.3E-01	-0.09	6.6E-01	-0.30	1.3E-01	-0.06	7.7E-01	-0.14	4.8E-01	-0.13	5.4E-01	-0.23	2.6E-01	0.19	3.4E-01
ENSCAFG0000000390	ENSCAFG0000000390	grey	EC_M10	0.16	4.3E-01	-0.54	4.8E-03	-0.16	4.3E-01	-0.03	8.9E-01	0.32	1.1E-01	0.13	5.3E-01	-0.32	1.1E-01	0.00	9.9E-01	-0.10	6.2E-01	-0.40	4.0E-02
ENSCAFG0000001339	LNPK	grey	EC_M10	0.16	4.3E-01	0.02	9.2E-01	-0.61	8.7E-04	-0.02	9.1E-01	0.64	4.8E-04	0.20	3.2E-01	-0.14	5.1E-01	0.05	8.2E-01	-0.06	7.8E-01	-0.65	3.7E-04
ENSCAFG0000001675	RRNAD1	grey	EC_M10	0.16	4.3E-01	0.13	5.2E-01	-0.22	2.8E-01	-0.22	2.9E-01	0.13	5.4E-01	0.20	3.2E-01	0.10	6.2E-01	0.15	4.5E-01	-0.28	1.7E-01	-0.22	2.7E-01
ENSCAFG0000001302	CEP164	grey	EC_M10	0.16	4.3E-01	-0.51	7.4E-03	-0.27	1.9E-01	0.08	7.0E-01	0.35	7.9E-02	0.13	5.2E-01	-0.16	4.3E-01	-0.11	6.1E-01	-0.21	3.0E-01	-0.43	2.9E-02
ENSCAFG0000002019	HSPA5	darkgreen	EC_M4	0.16	4.3E-01	-0.04	8.6E-01	-0.82	2.3E-07	-0.05	8.0E-01	0.82	2.2E-07	0.29	1.5E-01	0.07	7.3E-01	0.06	7.7E-01	-0.16	4.5E-01	-0.92	2.3E-11
ENSCAFG0000000618	CCNA1	grey	EC_M10	0.16	4.3E-01	0.31	1.3E-01	-0.16	4.5E-01	-0.02	9.3E-01	-0.04	8.5E-01	-0.07	7.4E-01	-0.06	7.9E-01	-0.10	6.4E-01	0.42	3.0E-02	-0.05	8.0E-01
ENSCAFG0000002520	ENSCAFG0000002520	grey	EC_M10	0.16	4.3E-01	-0.14	4.8E-01	0.24	2.5E-01	0.14	5.0E-01	-0.37	6.6E-02	-0.10	6.3E-01	-0.21	3.0E-01	0.03	8.9E-01	-0.44	2.6E-02	0.25	2.1E-01
ENSCAFG0000000410	FAM120B	grey	EC_M10	0.16	4.3E-01	0.42	3.4E-02	-0.55	3.8E-03	-0.03	8.8E-01	0.33	1.0E-01	0.29	1.5E-01	-0.08	6.8E-01	-0.08	7.2E-01	-0.03	9.0E-01	-0.39	5.1E-02
ENSCAFG0000000339	GRHL1	grey	EC_M10	0.16	4.3E-01	-0.32	1.1E-01	0.16	4.2E-01	0.32	1.1E-01	-0.29	1.5E-01	0.10	6.3E-01	0.17	4.0E-01	-0.41	3.7E-02	-0.09	6.5E-01	0.20	3.2E-01
ENSCAFG0000001818	ANKRD29	grey	EC_M10	0.16	4.3E-01	-0.16	4.3E-01	0.31	1.2E-01	-0.01	9.7E-01	-0.43	3.0E-02	-0.12	5.7E-01	-0.37	6.6E-02	0.11	6.1E-01	0.37	5.9E-02	0.36	7.4E-02
ENSCAFG0000001351	NBEAL2	turquoise	EC_M6	0.16	4.3E-01	0.78	2.4E-06	0.16	4.2E-01	0.00	1.0E+00	-0.61	8.4E-04	-0.24	2.5E-01	-0.07	7.5E-01	-0.10	6.2E-01	-0.02	9.4E-01	0.53	5.1E-03
ENSCAFG0000001001	SIAH1	grey	EC_M10	0.16	4.3E-01	0.06	7.7E-01	0.45	2.1E-02	0.30	1.4E-01	-0.66	2.4E-04	0.14	5.0E-01	-0.33	9.5E-02	-0.18	3.7E-01	0.07	7.4E-01	0.56	2.8E-03
ENSCAFG0000002887	ENSCAFG0000002887	grey	EC_M10	0.16	4.3E-01	0.16	4.5E-01	-0.19	3.5E-01	-0.08	6.8E-01	0.05	8.2E-01	0.29	1.5E-01	-0.22	2.9E-01	-0.12	5.4E-01	-0.30	1.4E-01	-0.18	3.7E-01
ENSCAFG0000002510	GPR37L1	grey	EC_M10	0.16	4.3E-01	-0.14	5.0E-01	-0.28	1.7E-01	0.45	2.2E-02	0.21	3.0E-01	0.45	2.1E-02	-0.15	4.7E-01	-0.13	5.4E-01	-0.14	4.9E-01	-0.29	1.5E-01
ENSCAFG0000001572	LIPA	turquoise	EC_M6	0.16	4.3E-01	0.75	9.5E-06	-0.33	9.5E-02	0.00	9.9E-01	0.03	8.9E-01	-0.25	2.3E-01	-0.33	9.9E-02	0.23	2.6E-01	-0.04	8.3E-01	-0.08	7.1E-01
ENSCAFG0000000607	CHFR	darkgreen	EC_M4	0.16	4.3E-01	0.29	1.5E-01	-0.80	7.6E-07	-0.34	8.6E-02	0.72	3.6E-05	0.20	3.2E-01	-0.12	5.7E-01	0.01	9.5E-01	-0.10	6.3E-01	-0.83	1.8E-07
ENSCAFG0000001307	SH3KBP1	grey	EC_M10	0.16	4.3E-01	-0.14	5.1E-01	-0.71	4.3E-05	-0.02	9.2E-01	0.71	4.7E-05	0.22	2.8E-01	-0.10	6.2E-01	-0.10	6.2E-01	0.17	4.2E-01	-0.80	1.2E-06
ENSCAFG0000001065	FAM167B	turquoise	EC_M6	0.16	4.3E-01	0.86	2.2E-08	-0.17	4.0E-01	-0.09	6.5E-01	-0.22	2.7E-01	-0.10	6.3E-01	-0.09	6.5E-01	-0.13	5.3E-01	-0.19	3.5E-01	0.10	6.3E-01
ENSCAFG0000000519	PFKFB3	grey	EC_M10	0.16	4.3E-01	0.38	5.8E-02	-0.74	1.4E-05	-0.29	1.6E-01	0.60	1.3E-03	0.14	4.9E-01	0.30	1.4E-01	0.11	5.9E-01	0.37	6.2E-02	-0.67	1.6E-04
ENSCAFG0000003021	FAM228B	grey	EC_M10	0.16	4.3E-01	-0.12	5.5E-01	-0.16	4.4E-01	-0.37	6.2E-02	0.05	8.2E-01	0.33	9.9E-02	-0.15	4.5E-01	0.02	9.1E-01	-0.12	5.5E-01	-0.15	4.8E-01
ENSCAFG0000000020	STAC3	grey	EC_M10	0.16	4.3E-01	0.02	9.3E-01	0.11	5.9E-01	-0.04	8.4E-01	-0.28	1.6E-01	-0.16	4.4E-01	0.04	8.3E-01	-0.15	4.6E-01	-0.21	2.9E-01	0.18	3.9E-01
ENSCAFG0000003060	ENSCAFG0000003060	grey	EC_M10	0.16	4.3E-01	0.13	5.3E-01	-0.22	2.8E-01	-0.03	8.9E-01	0.04	8.4E-01	0.11	5.9E-01	0.13	5.4E-01	0.19	3.5E-01	-0.05	8.2E-01	-0.14	5.0E-01
ENSCAFG0000001741	ENSCAFG0000001741	turquoise	EC_M6	0.16	4.3E-01	0.59	1.7E-03	-0.40	4.5E-02	-0.07	7.4E-01	0.11	6.1E-01	-0.18	3.7E-01	-0.16	4.3E-01	-0.19	3.6E-01	-0.21	3.0E-01	-0.22	2.8E-01
ENSCAFG0000002353	ENSCAFG0000002353	grey	EC_M10	0.16	4.3E-01	0.08	7.1E-01	0.26	2.0E-01	0.00	1.0E+00	-0.42	3.1E-02	-0.35	8.3E-02	0.04	8.5E-01	0.00	1.0E+00	-0.19	3.6E-01	0.35	7.7E-02
ENSCAFG0000001510	OTUD3	grey	EC_M10	0.16	4.3E-01	0.24	2.3E-01	-0.72	3.7E-05	-0.24	2.4E-01	0.64	4.2E-04	0.11	6.0E-01	-0.14	4.9E-01	-0.01	9.5E-01	0.23	2.6E-01	-0.69	9.5E-05
ENSCAFG0000000532	UVRAG	grey	EC_M10	0.16	4.3E-01	0.04	8.3E-01	0.08	7.1E-01	0.53	5.3E-03	-0.34	9.4E-02	0.05	8.0E-01	-0.25	2.1E-01	0.15	4.7E-01	-0.17	4.0E-01	0.21	3.1E-01
ENSCAFG0000000869	ACOT12	turquoise	EC_M6	0.16	4.3E-01	0.46	1.8E-02	-0.32	1.2E-01	-0.15	4.5E-01	0.10	6.1E-01	-0.16	4.4E-01	-0.16	4.3E-01	-0.02	9.2E-01	-0.09	6.6E-01	-0.16	4.3E-01
ENSCAFG0000000333	GPR85	grey	EC_M10	0.16	4.3E-01	0.34	9.4E-02	-0.07	7.3E-01	-0.09	6.5E-01	-0.19	3.6E-01	-0.14	4.9E-01	-0.14	5.1E-01	-0.17	4.1E-01	-0.22	2.7E-01	0.08	7.0E-01
ENSCAFG0000000599	GPCPD1	cyan	EC_M2	0.16	4.3E-01	0.05	8.1E-01	0.35	7.7E-02	0.31	1.3E-01	-0.61	1.0E-03	0.07	7.2E-01	-0.18	3.8E-01	-0.08	6.8E-01	-0.24	2.5E-01	0.51	7.7E-03
ENSCAFG0000000814	FBXW7	grey	EC_M10	0.16	4.4E-01	0.27	1.8E-01	-0.73	2.4E-05	-0.08	7.1E-01	0.58	1.7E-03	0.16	4.4E-01	0.05	8.0E-01	0.06	7.6E-01	0.03	8.9E-01	-0.65	3.5E-04
ENSCAFG0000001901	TNFAIP8L1	turquoise	EC_M6	0.16	4.4E-01	0.84	9.3E-08	-0.16	4.4E-01	-0.07	7.2E-01	-0.24	2.4E-01	-0.03	9.0E-01	-0.01	9.8E-01	-0.11	6.1E-01	0.07	7.2E-01	0.13	5.3E-01
ENSCAFG0000001458	USP5	grey	EC_M10	0.16	4.4E-01	-0.11	6.0E-01	-0.43	2.9E-02	-0.37	6.2E-02	0.53	5.8E-03	0.08	7.1E-01	-0.28	1.6E-01	0.15	4.6E-01	0.07	7.3E-01	-0.58	2.1E-03
ENSCAFG0000000835	PKIA	grey	EC_M10	0.16	4.4E-01	-0.15	4.7E-01	-0.07	7.2E-01	0.10	6.4E-01	-0.01	9.8E-01	-0.08	6.8E-01	-0.29	1.6E-01	0.16	4.3E-01	0.65	3.0E-04	-0.07	7.3E-01
ENSCAFG0000000199	DCUN1D4	grey	EC_M10	0.16	4.4E-01	-0.27	1.9E-01	-0.59	1.5E-03	-0.22	2.9E-01	0.66	2.8E-04	0.35	8.3E-02	-0.12	5.5E-01	0.23	2.5E-01	0.13	5.3E-01	-0.75	1.3E-05
ENSCAFG0000000643	SHQ1	grey	EC_M10	0.16	4.4E-01	-0.37	6.2E-02	-0.51	7.4E-03	-0.21	3.0E-01	0.66	2.7E-04	0.25	2.2E-01	-0.29	1.6E-01	-0.05	8.0E-01	0.02	9.1E-01	-0.75	1.2E-05
ENSCAFG0000000217	SYK	turquoise	EC_M6	0.16	4.4E-01	0.85	3.3E-08	-0.23	2.7E-01	-0.14	4.8E-01	-0.14	5.1E-01	-0.15	4.8E-01	-0.12	5.7E-01	-0.14	5.1E-01	-0.14	5.0E-01	0.07	7.4E-01
ENSCAFG0000003049	ENSCAFG0000003049	grey	EC_M10	0.16	4.4E-01	-0.09	6.6E-01	-0.22	2.8E-01	-0.17	4.1E-01	0.24	2.5E-01	0.21	2.9E-01	0.14	4.9E-01	-0.04	8.5E-01	-0.12	5.6E-01	-0.29	1.5E-01
ENSCAFG0000002443	ARGLU1	turquoise	EC_M6	0.16	4.4E-01	0.72	3.0E-05	-0.03	9.0E-01	0.10	6.1E-01	-0.42	3.2E-02	-0.21	3.0E-01	0.00	1.0E+00	-0.09	6.7E-01	0.31	1.2E-01	0.34	8.6E-02
ENSCAFG0000000734	BPIFB3	turquoise	EC_M6	0.16	4.4E-01	0.67	1.8E-04	-0.35	8.2E-02	-0.14	4.9E-01	0.06	7.6E-01	-0.13	5.1E-01	-0.11	5.8E-01	-0.09	6.7E-01	-0.13	5.3E-01	-0.12	5.5E-01
ENSCAFG0000000970	ENSCAFG0000000970	grey	EC_M10	0.16	4.4E-01	-0.16	4.5E-01	0.08	7.1E-01	-0.13	5.3E-01	-0.14	4.8E-01	-0.14	5.1E-01	-0.06	7.5E-01	0.47	1.5E-02	0.06	7.6E-01	0.12	5.6E-01
ENSCAFG0000000600	FASN	grey	EC_M10	0.16	4.4E-01	0.30	1.4E-01	0.15	4.6E-01	-0.10	6.2E-01	-0.34	8.6E-02	-0.40	4.1E-02	-0.11	6.0E-01	0.02	9.2E-01	-0.10	6.1E-01	0.25	2.1E-01
ENSCAFG0000003018	PASD1	turquoise	EC_M6	0.16	4.4E-01	0.82	2.6E-07	-0.17	4.2E-01	-0.08	6.8E-01	-0.23	2.5E-01	-0.08	6.8E-01	-0.10	6.4E-01	-0.10	6.3E-01	-0.15	4.8E-01	0.17	4.1E-01
ENSCAFG0000001069	LSMEM2	grey	EC_M10	0.16	4.4E-01	0.46	1.8E-02	0.19	3.6E-01	-0.09	6.5E-01	-0.48	1.3E-02	-0.12	5.5E-01	-0.18	3.8E-01	-0.08	7.0E-01	-0.23	2.7E-01	0.40	4.1E-02
ENSCAFG0000001157	MID1	grey	EC_M10	0.16	4.4E-01	0.04	8.5E-01	-0.43	2.6E-02	0.00	9.9E-01	0.35	8.1E-02	0.08	7.0E-01	-0.20	3.2E-01	0.13	5.2E-01	-0.21	3.1E-01	-0.45	2.0E-02
ENSCAFG0000000251	ABCG8	violet	EC_M7	0.16	4.4E-01	-0.11	6.1E-01	-0.34	9.0E-02	-0.06	7.6E-01	0.30	1.3E-01	0.97	7.9E-16	-0.07	7.3E-01	-0.09	6.5E-01	-0.12	5.5E-01	-0.39	4.9E-02
ENSCAFG0000001080	SEMA3F	turquoise	EC_M6	0.16	4.4E-01	0.77	3.5E-06	-0.12	5.6E-01	-0.02	9.1E-01	-0.20	3.3E-01	-0.23	2.5E-01	0.02	9.1E-01	-0.05	8.1E-01	0.24	2.3E-01	0.17	4.1E-01
ENSCAFG0000001600	ENSCAFG0000001600	grey	EC_M10	0.16	4.4E-01	-0.19	3.5E-01	-0.26	2.0E-01	-0.02	9.4E-01	0.28	1.7E-01	-0.13	5.3E-01	-0.39	5.2E-02	-0.01	9.5E-01	-0.11	5.8E-01	-0.38	5.5E-02
ENSCAFG0000000692	ANO4	grey	EC_M10	0.16	4.4E-01	0.33	1.0E-01	-0.31	1.3E-01	-0.50	9.6E-03	0.19	3.6E-01	-0.08	6.8E-01	-0.02	9.3E-01	-0.02	9.1E-01	0.13	5.4E-01	-0.20	3.3E-01
ENSCAFG0000002320	ENSCAFG0000002320	grey	EC_M10	0.16	4.4E-01	0.25	2.3E-01	0.05	8.1E-01	-0.09	6.5E-01	-0.31	1.2E-01	-0.10	6.3E-01	-0.11	6.0E-01	-0.10	6.4E-01	0.30	1.3E-01	0.21	3.1E-01
ENSCAFG0000000633	VEZT	darkgreen	EC_M4	0.16	4.4E-01	0.09	6.6E-01	-0.81	6.9E-07	-0.07	7.4E-01	0.78	2.9E-06	0.15	4.8E-01	-0.10	6.1E-01	0.10	6.1E-01	-0.06	7.8E-01	-0.86	2.2E-08
ENSCAFG0000000438	RAB38	turquoise	EC_M6	0.16	4.4E-01	0.84	7.8E-08	-0.12	5.6E-01	-0.19	3.4E-01	-0.23	2.5E-01	-0.20	3.2E-01	0.03	8.9E-01	-0.17	4.0E-01	-0.07	7.2E-01	0.14	5.1E-01
ENSCAFG0000000420	SLC1A5	grey	EC_M10	0.16	4.4E-01	-0.12	5.4E-01	-0.21	3.0E-01	0.08	7.1E-01	0.26	2.1E-01	0.26	2.0E-01	-0.22	2.7E-01	-0.12	5.7E-01	-0.17	4.2E-01	-0.39	4.9E-02
ENSCAFG0000001155	HCLS1	turquoise	EC_M6	0.16	4.4E-01	0.93	7.9E-12	-0.22	2.8E-01	-0.04	8.5E-01	-0.19	3.5E-01	-0.15	4.6E-01	-0.09	6.5E-01	-0.18	3.8E-01	0.03	9.0E-01	0.09	6.5E-01
ENSCAFG0000000219	RNF149	turquoise	EC_M6	0.16	4.4E-01	0.57	2.6E-03	-0.54	4.0E-03	-0.18	3.9E-01	0.31	1.2E-01	0.08	7.0E-01	0.21	3.0E-01	-0.17	3.9E-01	-0.31	1.2E-01	-0.36	7.2E-02
ENSCAFG0000001182	CERS6	grey	EC_M10	0.16	4.4E-01	-0.26	1.9E-01	-0.43	3.0E-02	-0.50	1.0E-02	0.51	7.2E-03	0.22	2.8E-01	-0.15	4.7E-01	0.08	6.9E-01	-0.28	1.6E-01	-0.62	6.5E-04
ENSCAFG0000002473	RP9	grey	EC_M10	0.16	4.4E-01	-0.01	9.5E-01	-0.08	6.8E-01	-0.39	4.8E-02	0.02	9.4E-01	0.09	6.8E-01	0.20	3.3E-01	-0.08	7.0E-01	-0.19	3.5E-01	-0.11	6.1E-01
ENSCAFG0000001983	PSRC1	grey	EC_M10	0.16	4.4E-01	0.39	4.6E-02	0.37	6.2E-02	-0.24	2.3E-01	-0.66	2.6E-04	-0.11	6.0E-01	0.04	8.5E-01	0.10	6.3E-01	0.06	7.6E-01	0.56	3.2E-03
ENSCAFG0000002021	PKD1L3	grey	EC_M10	0.16	4.4E-01	-0.36	7.0E-02	0.17	3.9E-01	0.24	2.3E-01	-0.23	2.6E-01	-0.05	8.0E-01	-0.20	3.3E-01	0.01	9.7E-01	-0.19	3.7E-01	0.08	7.0E-01
ENSCAFG0000001671	ENSCAFG0000001671	grey	EC_M10	0.16	4.4E-01	-0.25	2.2E-01	-0.45	2.0E-02	-0.21	2.9E-01	0.54	4.7E-03	0.23	2.5E-01	-0.14	4.9E-01	0.17	4.2E-01	0.02	9.4E-01	-0.57	2.6E-03
ENSCAFG0000000986	HERC2	grey	EC_M10	0.16	4.4E-01	-0.26	2.0E-01	-0.04	8.5E-01	-0.07	7.5E-01	-0.02	9.3E-01	-0.07	7.3E-01	-0.15	4.6E-01	-0.11	6.0E-01	-0.31	1.2E-01	-0.08	7.1E-01
ENSCAFG0000000680	NCOA4	cyan	EC_M2	0.16	4.4E-01	-0.57	2.3E-03	0.48	1.4E-02	0.09	6.7E-01	-0.48	1.3E-02	-0.04	8.4E-01	0.06	7.9E-01	0.11	5.9E-01	-0.35	7.8E-02	0.39	4.9E-02

ENSCAFG0000001804	NCOR1	grey	EC_M10	0.16	4.4E-01	0.07	7.3E-01	-0.04	8.6E-01	0.20	3.3E-01	-0.20	3.2E-01	0.08	7.0E-01	0.05	8.1E-01	0.18	3.8E-01	0.11	5.8E-01	0.04	8.3E-01
ENSCAFG0000003070	ZNF322	darkgreen	EC_M4	0.16	4.4E-01	0.23	2.6E-01	-0.68	1.4E-04	-0.18	3.9E-01	0.50	1.0E-02	0.22	2.8E-01	-0.04	8.5E-01	-0.05	8.0E-01	0.02	9.2E-01	-0.56	2.7E-03
ENSCAFG0000001154	CCDC39	grey	EC_M10	0.16	4.4E-01	-0.34	8.5E-02	0.16	4.5E-01	0.38	5.5E-02	-0.21	3.1E-01	0.13	5.2E-01	-0.26	1.9E-01	-0.09	6.8E-01	-0.01	9.7E-01	0.15	4.5E-01
ENSCAFG0000000202	CYP39A1	grey	EC_M10	0.16	4.4E-01	-0.25	2.2E-01	0.38	5.4E-02	0.42	3.1E-02	-0.53	5.0E-03	-0.26	1.9E-01	-0.07	7.3E-01	-0.08	7.0E-01	-0.37	6.5E-02	0.42	3.3E-02
ENSCAFG0000001965	MED16	turquoise	EC_M6	0.16	4.5E-01	0.40	4.0E-02	-0.05	8.2E-01	0.01	9.5E-01	-0.17	4.1E-01	-0.26	2.0E-01	0.18	3.9E-01	-0.16	4.2E-01	0.26	2.1E-01	0.08	6.8E-01
ENSCAFG0000001440	CCDC88B	grey	EC_M10	0.16	4.5E-01	-0.12	5.4E-01	0.23	2.5E-01	0.55	3.8E-03	-0.32	1.1E-01	-0.08	6.9E-01	-0.07	7.4E-01	-0.02	9.2E-01	-0.14	4.9E-01	0.23	2.5E-01
ENSCAFG0000000794	PROCR	turquoise	EC_M6	0.16	4.5E-01	0.80	7.5E-07	-0.03	8.7E-01	0.04	8.4E-01	-0.37	6.0E-02	-0.26	1.9E-01	-0.02	9.1E-01	-0.11	5.9E-01	0.37	6.3E-02	0.32	1.2E-01
ENSCAFG0000003120	RGS10	grey	EC_M10	0.16	4.5E-01	-0.28	1.7E-01	-0.18	3.7E-01	0.34	8.6E-02	0.08	6.8E-01	0.09	6.7E-01	-0.01	9.5E-01	-0.03	9.0E-01	0.00	9.9E-01	-0.25	2.3E-01
ENSCAFG0000000471	PLK3	grey	EC_M10	0.16	4.5E-01	0.12	5.5E-01	-0.68	1.2E-04	0.06	7.6E-01	0.59	1.6E-03	0.31	1.3E-01	0.08	7.1E-01	-0.02	9.1E-01	-0.10	6.4E-01	-0.73	2.7E-05
ENSCAFG0000001146	SLC9A8	turquoise	EC_M6	0.16	4.5E-01	0.66	2.5E-04	0.13	5.1E-01	0.19	3.4E-01	-0.54	4.2E-03	-0.08	6.9E-01	-0.08	7.1E-01	-0.17	4.1E-01	0.30	1.3E-01	0.49	1.2E-02
ENSCAFG0000000603	ENSCAFG0000000603	grey	EC_M10	0.16	4.5E-01	0.26	1.9E-01	0.09	6.8E-01	0.16	4.3E-01	-0.31	1.2E-01	-0.21	3.0E-01	-0.22	2.9E-01	-0.24	2.4E-01	-0.22	2.9E-01	0.22	2.8E-01
ENSCAFG0000001342	KCNK7	turquoise	EC_M6	0.16	4.5E-01	0.67	1.9E-04	0.03	8.7E-01	-0.13	5.4E-01	-0.41	3.8E-02	-0.13	5.2E-01	-0.18	3.8E-01	-0.18	3.7E-01	0.06	7.8E-01	0.30	1.3E-01
ENSCAFG0000000111	L3MBTL3	turquoise	EC_M6	0.16	4.5E-01	0.67	2.1E-04	0.11	6.0E-01	0.10	6.1E-01	-0.56	2.9E-03	-0.04	8.5E-01	-0.06	7.6E-01	0.02	9.2E-01	0.14	4.9E-01	0.45	2.2E-02
ENSCAFG0000000601	ENSCAFG0000000601	grey	EC_M10	0.16	4.5E-01	-0.18	3.9E-01	0.19	3.6E-01	-0.06	7.9E-01	-0.20	3.3E-01	-0.15	4.6E-01	-0.09	6.6E-01	-0.11	5.8E-01	-0.10	6.4E-01	0.13	5.2E-01
ENSCAFG0000001919	C6H16orf71	grey	EC_M10	0.16	4.5E-01	-0.08	6.9E-01	-0.06	7.7E-01	0.07	7.3E-01	0.02	9.0E-01	0.34	8.9E-02	0.05	8.0E-01	-0.25	2.2E-01	-0.33	1.0E-01	-0.12	5.6E-01
ENSCAFG0000003144	EREG	grey	EC_M10	0.16	4.5E-01	-0.13	5.3E-01	-0.68	1.4E-04	0.06	7.8E-01	0.70	8.1E-05	0.25	2.1E-01	-0.19	3.5E-01	-0.09	6.5E-01	0.05	8.0E-01	-0.79	1.6E-06
ENSCAFG0000000391	RASIP1	turquoise	EC_M6	0.16	4.5E-01	0.89	1.8E-09	-0.20	3.4E-01	-0.10	6.1E-01	-0.20	3.3E-01	-0.10	6.4E-01	-0.14	4.9E-01	-0.14	4.9E-01	0.07	7.2E-01	0.09	6.6E-01
ENSCAFG0000001496	ZSCAN25	cyan	EC_M2	0.16	4.5E-01	0.28	1.7E-01	0.39	4.8E-02	0.09	6.6E-01	-0.73	2.7E-05	-0.21	3.1E-01	0.07	7.3E-01	0.18	3.9E-01	-0.04	8.5E-01	0.64	4.4E-04
ENSCAFG0000001263	IQGAP1	darkgreen	EC_M4	0.16	4.5E-01	0.14	4.9E-01	-0.76	5.9E-06	-0.23	2.6E-01	0.66	2.3E-04	0.13	5.3E-01	-0.01	9.6E-01	0.05	8.3E-01	0.04	8.4E-01	-0.73	2.4E-05
ENSCAFG0000000515	ENSCAFG0000000515	grey	EC_M10	0.16	4.5E-01	-0.13	5.3E-01	-0.19	3.4E-01	-0.10	6.4E-01	0.20	3.2E-01	-0.22	2.7E-01	-0.17	4.0E-01	0.30	1.3E-01	-0.17	4.2E-01	-0.27	1.8E-01
ENSCAFG0000000194	TXNRD1	grey	EC_M10	0.16	4.5E-01	0.06	7.6E-01	-0.65	3.3E-04	-0.17	3.9E-01	0.68	1.3E-04	0.16	4.3E-01	-0.01	9.6E-01	-0.05	8.3E-01	-0.14	5.0E-01	-0.73	2.5E-05
ENSCAFG0000002464	ERAP2	turquoise	EC_M6	0.16	4.5E-01	0.71	4.9E-05	-0.61	9.7E-04	-0.20	3.2E-01	0.32	1.1E-01	0.13	5.2E-01	0.02	9.4E-01	-0.08	6.9E-01	0.03	8.9E-01	-0.42	3.4E-02
ENSCAFG0000000951	HSD17B13	grey	EC_M10	0.16	4.5E-01	-0.21	3.1E-01	0.26	2.0E-01	0.17	4.0E-01	-0.37	6.0E-02	0.11	5.9E-01	0.04	8.6E-01	0.08	7.1E-01	-0.25	2.1E-01	0.31	1.3E-01
ENSCAFG0000000292	CCDC85A	turquoise	EC_M6	0.16	4.5E-01	0.54	4.5E-03	-0.10	6.2E-01	-0.06	7.6E-01	-0.22	2.8E-01	-0.06	7.7E-01	-0.11	5.9E-01	-0.10	6.2E-01	-0.15	4.6E-01	0.12	5.7E-01
ENSCAFG0000001796	ZNF287	grey	EC_M10	0.16	4.5E-01	-0.29	1.5E-01	0.18	3.8E-01	0.22	2.8E-01	-0.31	1.2E-01	-0.11	5.8E-01	-0.23	2.5E-01	-0.09	6.6E-01	-0.18	3.8E-01	0.21	3.0E-01
ENSCAFG0000000747	IL20RB	darkgreen	EC_M4	0.16	4.5E-01	0.07	7.5E-01	-0.57	2.5E-03	-0.07	7.3E-01	0.50	8.8E-03	0.20	3.2E-01	-0.14	4.9E-01	-0.06	7.5E-01	-0.39	4.7E-02	-0.62	6.8E-04
ENSCAFG0000002870	ENSCAFG0000002870	grey	EC_M10	0.16	4.5E-01	-0.18	3.8E-01	-0.17	4.1E-01	-0.09	6.5E-01	0.11	5.8E-01	0.31	1.2E-01	-0.14	5.0E-01	-0.11	5.9E-01	-0.14	5.0E-01	-0.25	2.2E-01
ENSCAFG0000000604	CCDC57	grey	EC_M10	0.16	4.5E-01	-0.25	2.2E-01	0.28	1.6E-01	0.16	4.5E-01	-0.45	2.1E-02	-0.11	6.1E-01	-0.02	9.1E-01	-0.15	4.6E-01	-0.26	1.9E-01	0.29	1.5E-01
ENSCAFG0000001638	RBPJ	grey	EC_M10	0.16	4.5E-01	-0.54	4.2E-03	0.31	1.2E-01	0.37	6.4E-02	-0.29	1.5E-01	0.02	9.1E-01	-0.01	9.5E-01	-0.09	6.8E-01	-0.42	3.2E-02	0.23	2.6E-01
ENSCAFG0000001416	UBE2E3	grey	EC_M10	0.16	4.5E-01	0.19	3.6E-01	0.30	1.4E-01	-0.01	9.7E-01	-0.49	1.0E-02	-0.23	2.6E-01	0.21	3.0E-01	0.39	5.0E-02	0.10	6.3E-01	0.47	1.4E-02
ENSCAFG0000001796	ENSCAFG0000001796	grey	EC_M10	0.15	4.5E-01	0.19	3.4E-01	-0.08	7.1E-01	0.39	4.7E-02	-0.09	6.7E-01	-0.18	3.9E-01	-0.16	4.5E-01	-0.15	4.7E-01	-0.26	2.0E-01	0.04	8.4E-01
ENSCAFG0000000574	HBEGF	turquoise	EC_M6	0.15	4.5E-01	0.66	2.4E-04	-0.69	9.4E-05	-0.10	6.2E-01	0.46	1.9E-02	-0.02	9.1E-01	-0.10	6.1E-01	-0.21	3.0E-01	0.16	4.4E-01	-0.49	1.1E-02
ENSCAFG0000000543	SLCO2B1	turquoise	EC_M6	0.15	4.5E-01	0.82	3.3E-07	-0.28	1.6E-01	-0.12	5.7E-01	-0.08	6.9E-01	-0.10	6.2E-01	-0.09	6.5E-01	-0.07	7.3E-01	-0.11	6.0E-01	0.03	8.8E-01
ENSCAFG0000000037	ENSCAFG0000000037	grey	EC_M10	0.15	4.5E-01	-0.15	4.7E-01	-0.01	9.7E-01	-0.13	5.4E-01	-0.10	6.4E-01	-0.07	7.3E-01	-0.10	6.4E-01	-0.06	7.9E-01	-0.07	7.4E-01	0.02	9.2E-01
ENSCAFG0000002897	ENSCAFG0000002897	turquoise	EC_M6	0.15	4.5E-01	0.33	1.0E-01	-0.23	2.6E-01	-0.12	5.7E-01	0.06	7.7E-01	-0.16	4.2E-01	-0.24	2.4E-01	-0.12	5.7E-01	-0.13	5.4E-01	-0.12	5.5E-01
ENSCAFG0000003087	ZNF35	grey	EC_M10	0.15	4.5E-01	0.05	8.2E-01	0.24	2.4E-01	0.17	4.2E-01	-0.46	1.7E-02	-0.02	9.3E-01	0.11	5.8E-01	-0.31	1.2E-01	-0.01	9.7E-01	0.38	5.7E-02
ENSCAFG0000001599	DLG4	grey	EC_M10	0.15	4.5E-01	0.26	2.0E-01	-0.58	2.0E-03	-0.08	7.2E-01	0.37	6.3E-02	0.31	1.2E-01	0.07	7.5E-01	0.02	9.2E-01	0.22	2.7E-01	-0.50	8.7E-03
ENSCAFG0000001997	RNPC3	grey	EC_M10	0.15	4.5E-01	0.11	5.8E-01	-0.34	9.3E-02	-0.06	7.7E-01	0.17	4.1E-01	0.21	3.1E-01	0.20	3.2E-01	0.19	3.6E-01	-0.06	7.6E-01	-0.30	1.4E-01
ENSCAFG0000001258	ROMO1	grey	EC_M10	0.15	4.5E-01	-0.15	4.7E-01	-0.17	4.0E-01	0.28	1.7E-01	0.13	5.3E-01	0.12	5.5E-01	0.18	3.7E-01	0.18	3.9E-01	-0.20	3.3E-01	-0.21	2.9E-01
ENSCAFG0000001950	CIRBP	grey	EC_M10	0.15	4.5E-01	0.28	1.6E-01	0.33	9.7E-02	0.14	5.1E-01	-0.62	6.6E-04	0.11	6.1E-01	0.01	9.4E-01	0.04	8.6E-01	0.36	6.7E-02	0.57	2.2E-03
ENSCAFG0000001304	ENSCAFG0000001304	grey	EC_M10	0.15	4.5E-01	0.28	1.7E-01	0.39	5.0E-02	-0.04	8.4E-01	-0.64	4.4E-04	-0.25	2.2E-01	0.22	2.9E-01	0.11	5.9E-01	0.18	3.7E-01	0.57	2.2E-03
ENSCAFG0000000871	KMT2D	turquoise	EC_M6	0.15	4.5E-01	0.45	2.1E-02	0.05	8.1E-01	-0.05	8.2E-01	-0.35	7.9E-02	-0.29	1.5E-01	0.08	6.9E-01	-0.25	2.2E-01	-0.17	4.1E-01	0.29	1.6E-01
ENSCAFG0000001523	PROM1	turquoise	EC_M6	0.15	4.5E-01	0.76	6.9E-06	-0.45	2.0E-02	-0.15	4.8E-01	0.15	4.7E-01	-0.14	5.1E-01	-0.17	4.1E-01	-0.13	5.2E-01	-0.16	4.4E-01	-0.21	3.0E-01
ENSCAFG0000002417	NHSL2	turquoise	EC_M6	0.15	4.5E-01	0.51	8.1E-03	-0.29	1.5E-01	-0.21	3.0E-01	0.04	8.3E-01	-0.09	6.5E-01	0.46	1.7E-02	0.02	9.2E-01	-0.19	3.6E-01	-0.13	5.3E-01
ENSCAFG0000000394	REV3L	grey	EC_M10	0.15	4.5E-01	0.36	6.8E-02	0.06	7.7E-01	-0.07	7.4E-01	-0.43	2.8E-02	-0.01	9.7E-01	0.43	2.9E-02	0.02	9.1E-01	0.03	8.9E-01	0.32	1.2E-01
ENSCAFG0000000037	HELB	cyan	EC_M2	0.15	4.5E-01	0.28	1.7E-01	0.39	5.0E-02	0.07	7.5E-01	-0.71	5.3E-05	-0.09	6.6E-01	-0.04	8.5E-01	-0.11	6.0E-01	-0.17	4.2E-01	0.61	1.0E-03
ENSCAFG0000002002	LRRC8A	grey	EC_M10	0.15	4.5E-01	0.36	7.0E-02	-0.71	5.7E-05	-0.22	2.8E-01	0.59	1.4E-03	0.20	3.2E-01	-0.04	8.4E-01	-0.02	9.1E-01	0.30	1.3E-01	-0.67	1.6E-04
ENSCAFG0000001703	TAOK2	turquoise	EC_M6	0.15	4.5E-01	0.86	2.3E-08	-0.30	1.4E-01	-0.20	3.3E-01	-0.05	8.0E-01	-0.11	5.8E-01	-0.24	2.3E-01	0.05	7.9E-01	0.10	6.4E-01	0.00	9.9E-01
ENSCAFG0000000897	EVA1C	grey	EC_M10	0.15	4.5E-01	0.36	6.7E-02	-0.26	2.0E-01	-0.13	5.2E-01	0.08	6.9E-01	0.14	4.9E-01	-0.08	7.0E-01	0.04	8.6E-01	0.28	1.7E-01	-0.19	3.6E-01
ENSCAFG0000000864	TKT	grey	EC_M10	0.15	4.5E-01	0.31	1.2E-01	0.06	7.7E-01	-0.07	7.4E-01	-0.24	2.3E-01	-0.15	4.7E-01	0.22	2.9E-01	-0.24	2.4E-01	-0.44	2.6E-02	0.15	4.6E-01
ENSCAFG0000000820	ENSCAFG0000000820	grey	EC_M10	0.15	4.5E-01	-0.21	3.0E-01	-0.01	9.7E-01	0.16	4.3E-01	-0.05	8.0E-01	-0.11	5.9E-01	-0.18	3.8E-01	0.20	3.3E-01	-0.07	7.4E-01	-0.06	7.9E-01
ENSCAFG0000001791	CCDC69	turquoise	EC_M6	0.15	4.5E-01	0.84	9.6E-08	-0.33	1.0E-01	-0.03	9.0E-01	-0.04	8.4E-01	-0.14	5.0E-01	-0.15	4.6E-01	-0.14	4.8E-01	0.01	9.7E-01	-0.04	8.3E-01
ENSCAFG0000003162	ZNF596	grey	EC_M10	0.15	4.5E-01	0.11	5.8E-01	-0.33	9.8E-02	0.05	8.0E-01	0.20	3.2E-01	0.16	4.2E-01	-0.08	7.1E-01	0.16	4.4E-01	-0.26	2.0E-01	-0.28	1.7E-01
ENSCAFG0000002997	TRIM62	turquoise	EC_M6	0.15	4.5E-01	0.82	3.9E-07	-0.18	3.7E-01	0.01	9.4E-01	-0.17	4.1E-01	-0.11	5.9E-01	-0.30	1.4E-01	0.02	9.3E-01	0.02	9.1E-01	0.14	5.1E-01
ENSCAFG0000001075	ZDHHC23	grey	EC_M10	0.15	4.5E-01	0.10	6.1E-01	0.05	8.0E-01	-0.22	2.8E-01	-0.18	3.9E-01	-0.23	2.5E-01	-0.16	4.4E-01	0.27	1.9E-01	0.15	4.5E-01	0.11	5.9E-01
ENSCAFG0000001000	ROPN1L	grey	EC_M10	0.15	4.5E-01	-0.19	3.6E-01	-0.21	3.1E-01	0.08	7.0E-01	0.18	3.7E-01	0.09	6.5E-01	0.25	2.1E-01	-0.22	2.7E-01	-0.35	7.6E-02	-0.32	1.1E-01
ENSCAFG0000001222	NUDC	grey	EC_M10	0.15	4.5E-01	-0.24	2.4E-01	-0.60	1.1E-03	-0.32	1.1E-01	0.75	8.8E-06	0.32	1.1E-01	-0.03	8.8E-01	0.10	6.4E-01	-0.05	8.2E-01	-0.83	2.0E-07
ENSCAFG0000001297	ENSCAFG0000001297	grey	EC_M10	0.15	4.5E-01	-0.20	3.4E-01	0.01	9.5E-01	-0.15	4.6E-01	-0.13	5.1E-01	-0.12	5.6E-01	-0.09	6.7E-01	-0.13	5.2E-01	-0.06	7.6E-01	0.08	7.0E-01
ENSCAFG0000003276	ENSCAFG0000003276	cyan	EC_M2	0.15	4.5E-01	-0.22	2.9E-01	0.26	1.9E-01	0.17	4.1E-01	-0.41	4.0E-02	-0.32	1.2E-01	0.15	4.6E-01	0.04	8.6E-01	-0.38	5.3E-02	0.27	1.9E-01
ENSCAFG0000001266	DCAF17	cyan	EC_M2	0.15	4.5E-01	-0.02	9.1E-01	0.59	1.5E-03	0.03	8.9E-01	-0.81	5.9E-07	-0.37	6.5E-02	-0.20	3.2E-01	-0.24	2.4E-01	-0.14	4.9E-01	0.71	4.9E-05
ENSCAFG0000001070	ENSCAFG0000001070	cyan	EC_M2	0.15	4.5E-01	-0.17	4.2E-01	0.58	1.7E-03	0.03	8.8E-01	-0.77	4.6E-06	-0.01	9.8E-01	-0.05	8.2E-01	0.06	7.7E-01	-0.03	9.0E-01	0.70	6.9E-05
ENSCAFG0000002398	ZNF24	turquoise	EC_M6	0.15	4.6E-01	0.57	2.3E-03	-0.14	4.8E-01	-0.18	3.8E-01	-0.19	3.5E-01	-0.02	9.2E-01	0.21	3.1E-01	-0.31	1.2E-01	-0.20	3.3E-01	0.14	5.0E-01
ENSCAFG0000001257	ARCN1	grey	EC_M10	0.15	4.6E-01	-0.31	1.2E-01	-0.63	5.2E-04	-0.07	7.2E-01	0.71	4.5E-05	0.38	5.2E-02	0.00	1.0E+00	-0.13	5.2E-01	-0.40	4.4E-02	-0.84	8.5E-08
ENSCAFG0000000646	UBXN8	grey	EC_M10	0.15	4.6E-01	-0.12	5.5E-01	-0.33	9.6E-02	0.30	1.3E-01	0.24	2.4E-01	0.29	1.5E-01	-0.13	5.1E-01	-0.01	9.6E-01	0.10	6.4E-01	-0.32	1.1E-01
ENSCAFG0000000034	RUFY1	cyan	EC_M2	0.15	4.6E-01	-0.01	9.5E-01	0.56	3.1E-03	0.37	6.6E-02	-0.78	2.2E-06	-0.26	2.0E-01	-0.19	3.5E-01	-0.19	3.5E-01	0.00	9.8E-01	0.75	1.3E-05
ENSCAFG0000001470	CDC42	darkgreen	EC_M4	0.15	4.6E-01	0.07	7.3E-01	-0.69	1.1E-04	-0.19	3.5E-01	0.69	8.7E-05	0.09	6.5E-01	-0.40	4.2E-02	0.09	6.5E-01	0.07	7.3E-01	-0.76	5.7E-06
ENSCAFG0000001869	ANKRD12	turquoise	EC_M6	0.15	4.6E-01	0.46	1.9E-02	-0.19	3.6E-01	-0.04	8.4E-01	-0.16	4.4E-01	0.00	1.0E+00	0.23	2.6E-01	-0.03	9.0E-01	-0.27	1.8E-01	0.10	6.4E-01
ENSCAFG0000002450	PRRG2	turquoise	EC_M6	0.15	4.6E-01	0.39	4.8E-02	-0.16	4.3E-01	0.00	9.8E-01	-0.10	6.2E-01	-0.04	8.3E-01	0.13	5.4E-01	0.12	5.6E-01	-0.21	3.0E-01	0.00	9.8E-01
ENSCAFG0000000869	ADGRG3	turquoise	EC_M6	0.15	4.6E-01	0.85	3.3E-08	-0.15	4.6E-01	-0.08	7.1E-01	-0.27	1.9E-01	-0.08	7.0E-01	-0.09	6.6E-01	-0.13	5.4E-01	0.08	7.1E-01	0.18	3.9E-01
ENSCAFG0000001934	ISG15	turquoise	EC_M6	0.15	4.6E-01	0.56	2.6E-03	0.00	9.9E-01	-0.20	3.2E-01	-0.31	1.3E-01	0.05	8.2E-01	0.20	3.2E-01	-0.15	4.6E-01	0.09	6.5E-01	0.23	2.6E-01
ENSCAFG0000001853	ENSCAFG0000001853	grey	EC_M10	0.15	4.6E-01	-0.20	3.3E-01	-0.11	5.8E-01	-0.11	6.0E-01	0.12	5.7E-01	0.30	1.4E-01	0.48	1.4E-02	-0.04	8.3E-01	-0.13	5.1E-01	-0.21	3.1E-01
ENSCAFG0000001840	ENSCAFG0000001840	grey	EC_M10	0.15	4.6E-01	-0.03	9.0E-01	-0.50	8.8E-03	-0.44	2.6E-02	0.55	3.5E-03	0.20	3.4E-01	-0.17	4.1E-01	0.17	4.2E-01	-0.09	6.6E-01	-0.66	2.5E-04
ENSCAFG0000001537	GTPBP3	grey	EC_M10	0.15	4.6E-01	0.31	1.3E-01	0.03	8.8E-01	-0.06	7.8E-01	-0.26	2.0E-01	-0.41	3.8E-02	0.06	7.6E-01	-0.17	4.0E-01	0.37	6.1E-02	0.18	3.8E-01
ENSCAFG0000001933	TRPV1	cyan	EC_M2	0.15	4.6E-01	-0.39	4.7E-02	0.37	6.6E-02	0.09	6.6E-01	-0.42	3.1E-02	-0.25	2.1E-01	0.10	6.3E-01	-0.20	3.3E-01	-0.39	4.6E-02	0.32	1.1E-01
ENSCAFG0000000414	GATA2	turquoise	EC_M6	0.15	4.6E-01	0.86	1.5E-08	-0.06	7.7E-01	-0.07	7.4E-01	-0.32	1.1E-01	-0.31	1.3E-01	-0.08	7.2E-01	0.09	6.7E-01	0.08	7.0E-01	0.27	1.8E-01
ENSCAFG0000001216	CASP10	turquoise	EC_M6	0.15	4.6E-01	0.57	2.6E-03	-0.17	4.2E-01	-0.04	8.4E-01	-0.16	4.4E-01	-0.10	6.1E-01	-0.21	3.0E-01	0.28	1.7E-01	0.33	1.0E-01	0.05	8.1E-01
ENSCAFG0000001306	DOCK1	turquoise	EC_M6	0.15	4.6E-01	0.79	1.7E-06	-0.20	3.3E-01	-0.04	8.5E-01	-0.21	3.0E-01	-0.05	7.9E-01	-0.12	5.7E-01	-0.02	9.1E-01	-0.26	2.0E-01	0.13	5.3E-01
ENSCAFG0000001255	ENSCAFG0000001255	grey	EC_M10	0.15	4.6E-01	-0.21	3.1E-01	-0.02	9.2E-01	-0.08	7.0E-01	-0.08	7.1E-01	0.24	2.4E-01	-0.14	4.8E-01	-0.11	5.9E-01	-0.15	4.6E-01	-0.03	8.8E-01
ENSCAFG0000001821	AMOT	turquoise	EC_M6	0.15	4.6E-01	0.88	2.2E-09	-0.12	5.5E-01	-0.01	9.6E-01	-0.28	1.6E-01	-0.14	4.9E-01	-0.17	4.1E-01	-0.06	7.6E-01	-0.15	4.8E-01	0.18	3.8E-01
ENSCAFG0000003274	GSG1L	turquoise	EC_M6	0.15	4.6E-01	0.57	2.3E-03	-0.09	6.7E-01	-0.11	6.0E-01	-0.22	2.8E-01	-0.08	7.1E-01	-0.03	9.0E-01	-0.03	9.0E-01	0.52	7.0E-03	0.16	4.3E-01
ENSCAFG0000003140	ENSCAFG0000003140	darkgreen	EC_M4	0.15	4.6E-01	0.07	7.5E-01	-0.77	5.1E-06	-0.26	2.0E-01	0.75	1.0E-05	0.24	2.5E-01	0.05	8.0E-01	0.18	3.9E-01	-0.04	8.6E-01	-0.80	9.9E-07
ENSCAFG0000001410	OSBPL1A	grey	EC_M10	0.15	4.6E-01	0.07	7.4E-01	-0.31	1.2E-01	0.33	9.7E-02	0.18	3.7E-01	-0.18	3.9E-01	-0.58	1.9E-03	-0.05	7.9E-01	0.05	7.9E-01	-0.25	2.2E-01
ENSCAFG0000002860	GMFG	turquoise	EC_M6	0.15	4.6E-01	0.84	9.4E-08	-0.41	3.9E-02	-0.09	6.6E-01	0.08	7.0E-01	0.00	1.0E+00	0.00	1.0E+00	-0.34	9.0E-02	-0.19	3.5E-01	-0.15	4.7E-01
ENSCAFG0000000210	ENSCAFG0000000210	grey	EC_M10	0.15	4.6E-01	0.23	2.7E-01	0.11	6.1E-01	0.25	2.1E-01	-0.32	1.1E-01	-0.32	1.2E-01	-0.41	3.9E-02	-0.44	2.4E-02	0.04	8.4E-01	0.27	1.9E-01
ENSCAFG0000001731	SEL1L	darkgreen	EC_M4	0.15	4.6E-01	0.06	7.6E-01	-0.78	3.1E-06	0.05	8.2E-01	0.74	1.5E-05	0.14	4.9E-01	-0.09	6.6E-01	0.06	7.7E-01	-0.25	2.3E-01	-0.85	3.7E-08
ENSCAFG0000003017	ZNF229	turquoise	EC_M6	0.15	4.6E-01	0.55	3.3E-03	-0.34	8.9E-02	0.02	9.2E-01	0.03	8.9E-01	0.11	6.1E-01	0.00	9.9E-01	0.32	1.1E-01	0.40	4.1E-02	-0.11	5.8E-01
ENSCAFG0000001197	PIR	grey	EC_M10	0.15	4.6E-01	-0.06	7.7E-01	0.63	5.4E-04	0.15	4.7E-01	-0.83	1.1E-07	-0.18	3.7E-01	0.01	9.7E-01	-0.22	2.7E-01	-0.31	1.2E-01	0.75	8.4E-06
ENSCAFG0000000931	ENSCAFG0000000931	grey	EC_M10	0.15	4.6E-01	0.23	2.6E-01	0.27	1.9E-01	0.35	7.6E-02	-0.49	1.1E-02	0.02	9.2E-01	-0.11	6.1E-01	-0.15	4.8E-01	-0.11	6.0E-01	0.41	3.9E-02
ENSCAFG0000001705	ARHGEF15	turquoise	EC_M6	0.15	4.6E-01	0.86	1.7E-08	-0.15	4.6E-01	-0.14	4.9E-01	-0.23	2.5E-01	-0.15	4.5E-01	-0.16	4.3E-01	-0.08	7.0E-01	-0.10	6.1E-01	0.12	5.7E-01
ENSCAFG0000001323	SCRN3	grey	EC_M10	0.15	4.6E-01	-0.61	9.0E-04	-0.06	7.6E-01	0.17	3.9E-01	0.10	6.4E-01	0.15	4.8E-01	-0.03	8.7E-01	-0.09	6.6E-01	-0.01	9.5E-01	-0.18	3.9E-01
ENSCAFG0000001391	SLC2A3	turquoise	EC_M6	0.15	4.6E-01	0.74	1.3E-05	-0.61	9.8E-04	-0.06	7.8E-01	0.30	1.4E-01	0.02	9.2E-01	0.24	2.3E-01	-0.09	6.8E-01	0.03	8.7E-01	-0.39	5.0E-02
ENSCAFG0000000655	NEURL3	turquoise	EC_M6	0.15	4.6E-01	0.91	8.6E-11	-0.15	4.7E-01	-0.15	4.6E-01	-0.28	1.7E-01	-0.15	4.6E-01	0.07	7.3E-01	-0.17	4.2E-01	0.13	5.4E-01	0.21	3.1E-01
ENSCAFG0000001699	EIF2B2	turquoise	EC_M6	0.15	4.6E-01	0.95	4.5E-14	-0.20	3.3E-01	-0.16	4.4E-01	-0.20	3.3E-01	-0.20	3.3E-01	-0.07	7.2E-01	-0.02	9.1E-01	0.01	9.6E-01	0.13	5.4E-01
ENSCAFG0000002558	ENSCAFG0000002558	grey	EC_M10	0.15	4.6E-01	0.21	3.0E-01	-0.17	4.1E-01	-0.23	2.6E-01	0.02	9.4E-01	0.39	5.2E-02	0.22	2.9E-01	-0.20	3.4E-01	-0.07	7.2E-01	-0.11	6.0E-01
ENSCAFG0000000986	ENSCAFG0000000986	grey	EC_M10	0.15	4.6E-01	-0.19	3.4E-01	-0.03	9.0E-01	0.23	2.7E-01	0.01	9.5E-01	-0.18	3.7E-01	-0.10	6.1E-01	0.34	9.4E-02	0.10	6.3E-01	-0.03	8.9E-01
ENSCAFG0000000472	SMG9	cyan	EC_M2	0.15	4.6E-01	-0.33	9.9E-02	0.68	1.3E-04	0.20	3.2E-01	-0.79	1.9E-06	-0.02	9.3E-01	-0.15	4.5E-01	-0.14	5.1E-01	-0.21	3.1E-01	0.67	1.7E-04
ENSCAFG0000001990	SLC7A5	grey	EC_M10	0.15	4.6E-01	-0.16	4.2E-01	-0.65	3.0E-04	-0.08	7.0E-01	0.74	1.8E-05	0.29	1.5E-01	-0.14	4.8E-01	-0.07	7.4E-01	0.01	9.7E-01	-0.86	1.9E-08
ENSCAFG0000001151	LRIT3	grey	EC_M10	0.15	4.6E-01	-0.09	6.7E-01	-0.05	8.2E-01	0.14	4.9E-01	-0.05	8.0E-01	0.35	7.5E-02	-0.20	3.3E-01	-0.19	3.5E-01	-0.01	9.5E-01	-0.04	8.3E-01
ENSCAFG0000001169	JHY	turquoise	EC_M6	0.15	4.6E-01	0.42	3.2E-02	-0.37	6.4E-02	0.17	4.0E-01	0.11	6.0E-01	0.05	8.3E-01	0.07	7.3E-01	0.25	2.1E-01	0.12	5.5E-01	-0.20	3.3E-01
ENSCAFG0000002350	SETDB1	cyan	EC_M2	0.15	4.6E-01	0.30	1.3E-01	0.51	7.8E-03	0.19	3.4E-01	-0.85	3.4E-08	-0.28	1.6E-01	-0.05	8.1E-01	-0.02	9.2E-01	-0.19	3.5E-01	0.76	7.4E-06
ENSCAFG0000003241	PRKRIP1	grey	EC_M10	0.15	4.6E-01	0.19	3.4E-01	0.00	9.8E-01	-0.37	5.9E-02	-0.23	2.7E-01	0.05	8.0E-01	-0.06	7.7E-01	0.25	2.2E-01	-0.06	7.8E-01	0.17	4.2E-01
ENSCAFG0000001441	PPP1R2C	grey	EC_M10	0.15	4.6E-01	0.18	3.7E-01	-0.20	3.2E-01	-0.06	7.9E-01	0.07	7.2E-01	-0.11	6.0E-01	0.54	4.8E-03	-0.01	9.5E-01	-0.08	6.9E-01	-0.14	5.1E-01
ENSCAFG0000000545	OVOL2	grey	EC_M10	0.15	4.6E-01	-0.15	4.6E-01	0.04	8.5E-01	-0.10	6.4E-01	-0.03	8.7E-01	-0.09	6.5E-01	-0.03	8.7E-01	-0.10	6.4E-01	0.05	8.2E-01	0.05	8.1E-01
ENSCAFG0000001777	ENSCAFG0000001777	grey	EC_M10	0.15	4.6E-01	-0.37	6.6E-02	-0.07	7.3E-01	0.21	3.1E-01	0.06	7.8E-01	-0.21	2.9E-01	-0.20	3.3E-01	0.24	2.4E-01	-0.18	3.7E-01	-0.14	5.1E-01
ENSCAFG0000000407	TCTE3	turquoise	EC_M6	0.15	4.6E-01	0.40	4.4E-02	-0.02	9.4E-01	-0.11	6.0E-01	-0.23	2.6E-01	-0.07	7.3E-01	-0.03	8.8E-01	-0.11	5.8E-01	-0.09	6.5E-01	0.17	3.9E-01
ENSCAFG0000000481	ELF1	darkgrey	EC_M8	0.15	4.6E-01	0.25	2.1E-01	0.15	4.8E-01	0.17	4.1E-01	-0.51	7.7E-03	0.14	4.9E-01	-0.09	6.8E-01	-0.23	2.7E-01	0.05	8.0E-01	0.40	4.2E-02
ENSCAFG0000001903	HDGFL2	grey	EC_M10	0.15	4.7E-01	-0.06	7.5E-01	0.36	7.3E-02	0.06	7.6E-01	-0.54	4.4E-03	0.13	5.2E-01	0.02	9.0E-01	-0.01	9.7E-01	0.11	5.8E-01	0.46	1.9E-02
ENSCAFG0000002018	GAPVD1	grey	EC_M10	0.15	4.7E-01	0.26	2.1E-01	-0.73	2.6E-05	-0.05	8.2E-01	0.65	3.0E-04	0.16	4.3E-01	0.13	5.4E-01	-0.16	4.5E-01	-0.38	5.7E-02	-0.71	4.2E-05
ENSCAFG0000002007	ADAT1	grey	EC_M10	0.15	4.7E-01	0.27	1.8E-01	-0.26	2.1E-01	-0.18	3.8E-01	0.11	5.8E-01	0.26	2.0E-01	-0.14	4.9E-01	0.11	5.9E-01	0.24	2.4E-01	-0.22	2.8E-01
ENSCAFG0000001809	ENSCAFG0000001809	cyan	EC_M2	0.15	4.7E-01	-0.16	4.3E-01	0.41	3.8E-02	0.29	1.5E-01	-0.55	3.4E-03	0.07	7.2E-01	0.30	1.4E-01	-0.19	3.4E-01	-0.51	8.2E-03	0.44	2.6E-02
ENSCAFG0000000237	ZNF135	cyan	EC_M2	0.15	4.7E-01	0.35	8.1E-02	0.38	5.6E-02	0.16	4.4E-01	-0.75	9.2E-06	-0.22	2.7E-01	-0.10	6.3E-01	-0.07	7.4E-01	-0.01	9.4E-01	0.65	3.4E-04
ENSCAFG0000001325	SIK3	grey	EC_M10	0.15	4.7E-01	-0.50	9.6E-03	0.23	2.6E-01	0.18	3.8E-01	-0.21	2.9E-01	-0.12	5.6E-01	-0.48	1.4E-02	-0.06	7.6E-01	-0.18	3.8E-01	0.09	6.4E-01
ENSCAFG0000001948	WRAP73	grey	EC_M10	0.15	4.7E-01	0.40	4.2E-02	0.38	5.6E-02	0.19	3.5E-01	-0.70	6.4E-05	-0.02	9.2E-01	0.03	8.7E-01	-0.14	4.9E-01	0.14	4.8E-01	0.60	1.1E-03
ENSCAFG0000001192	HSCB	grey	EC_M10	0.15	4.7E-01	-0.51	7.8E-03	0.40	4.2E-02	0.13	5.2E-01	-0.41	4.0E-02	0.11	6.0E-01	-0.06	7.6E-01	0.00	9.9E-01	-0.12	5.6E-01	0.26	2.0E-01
ENSCAFG0000003059	ENSCAFG0000003059	grey	EC_M10	0.15	4.7E-01	-0.06	7.6E-01	-0.07	7.3E-01	-0.12	5.5E-01	0.05	7.9E-01	0.21	3.1E-01	-0.10	6.2E-01	-0.13	5.3E-01	-0.16	4.3E-01	-0.16	4.2E-01
ENSCAFG0000000590	ENSCAFG0000000590	grey	EC_M10	0.15	4.7E-01	0.04	8.5E-01	0.05	8.1E-01	-0.08	6.9E-01	-0.10	6.3E-01	0.01	9.6E-01	0.02	9.2E-01	-0.05	8.0E-01	0.08	7.1E-01	0.01	9.8E-01

ENSCAFG0000000741	WEE1	grey	EC_M10	0.15	4.7E-01	-0.30	1.4E-01	-0.10	6.4E-01	-0.04	8.3E-01	0.09	6.6E-01	0.02	9.2E-01	0.25	2.1E-01	0.00	9.9E-01	0.16	4.4E-01	-0.13	5.3E-01
ENSCAFG0000000893	NLRC5	turquoise	EC_M6	0.15	4.7E-01	0.90	4.2E-10	-0.15	4.6E-01	-0.01	9.6E-01	-0.28	1.7E-01	-0.15	4.7E-01	-0.17	4.1E-01	0.01	9.5E-01	0.21	3.0E-01	0.19	3.5E-01
ENSCAFG0000002614	ENSCAFG0000002614	grey	EC_M10	0.15	4.7E-01	0.32	1.1E-01	0.03	8.9E-01	0.08	7.1E-01	-0.24	2.4E-01	0.12	5.7E-01	-0.24	2.4E-01	0.09	6.7E-01	0.09	6.7E-01	0.22	2.8E-01
ENSCAFG0000001339	KLHL15	grey	EC_M10	0.15	4.7E-01	-0.12	5.6E-01	0.26	1.9E-01	0.10	6.4E-01	-0.44	2.3E-02	0.11	5.8E-01	-0.02	9.2E-01	-0.55	3.3E-03	-0.05	8.0E-01	0.37	6.3E-02
ENSCAFG0000001484	FAM193A	cyan	EC_M2	0.15	4.7E-01	0.26	2.0E-01	0.36	6.7E-02	0.19	3.6E-01	-0.68	1.5E-04	-0.20	3.2E-01	-0.36	7.1E-02	-0.10	6.1E-01	-0.27	1.9E-01	0.57	2.4E-03
ENSCAFG0000000004	ENSCAFG0000000004	grey	EC_M10	0.15	4.7E-01	-0.09	6.7E-01	-0.09	6.6E-01	0.00	9.9E-01	0.06	7.6E-01	-0.09	6.7E-01	-0.02	9.4E-01	-0.05	7.9E-01	-0.18	3.8E-01	-0.17	3.9E-01
ENSCAFG0000001033	MTMR10	turquoise	EC_M6	0.15	4.7E-01	0.79	1.6E-06	0.02	9.1E-01	-0.04	8.6E-01	-0.46	1.8E-02	-0.35	8.3E-02	-0.16	4.4E-01	0.18	3.9E-01	0.03	8.8E-01	0.39	4.6E-02
ENSCAFG0000000023	RMND5B	turquoise	EC_M6	0.15	4.7E-01	0.65	3.3E-04	-0.12	5.4E-01	-0.13	5.4E-01	-0.14	5.0E-01	0.14	5.0E-01	-0.05	8.1E-01	-0.41	3.5E-02	-0.02	9.1E-01	0.10	6.3E-01
ENSCAFG0000002887	ENSCAFG0000002887	cyan	EC_M2	0.15	4.7E-01	-0.04	8.4E-01	0.56	3.2E-03	0.30	1.4E-01	-0.81	4.1E-07	0.08	7.0E-01	-0.12	5.6E-01	-0.20	3.3E-01	-0.18	3.8E-01	0.70	6.2E-05
ENSCAFG0000000546	SUPT5H	grey	EC_M10	0.15	4.7E-01	-0.03	8.7E-01	-0.52	6.3E-03	-0.16	4.3E-01	0.53	5.5E-03	0.15	4.8E-01	-0.06	7.8E-01	0.11	6.1E-01	-0.11	6.0E-01	-0.60	1.1E-03
ENSCAFG0000000462	TOMM40	grey	EC_M10	0.15	4.7E-01	-0.21	2.9E-01	-0.14	4.9E-01	-0.12	5.7E-01	0.26	1.9E-01	0.16	4.4E-01	-0.10	6.3E-01	-0.02	9.3E-01	0.00	1.0E+00	-0.34	8.8E-02
ENSCAFG0000002917	ENSCAFG0000002917	grey	EC_M10	0.15	4.7E-01	-0.10	6.2E-01	-0.41	3.5E-02	0.05	8.0E-01	0.27	1.8E-01	0.26	1.9E-01	0.25	2.1E-01	0.18	3.8E-01	0.03	9.0E-01	-0.41	3.9E-02
ENSCAFG0000001862	CACHD1	grey	EC_M10	0.15	4.7E-01	0.31	1.2E-01	-0.42	3.3E-02	0.03	9.0E-01	0.36	7.2E-02	-0.04	8.3E-01	0.00	9.9E-01	-0.18	3.8E-01	-0.23	2.5E-01	-0.41	4.0E-02
ENSCAFG0000000623	PCDH12	turquoise	EC_M6	0.15	4.7E-01	0.82	2.4E-07	-0.16	4.2E-01	-0.04	8.3E-01	-0.20	3.3E-01	-0.13	5.2E-01	-0.14	4.8E-01	-0.10	6.4E-01	-0.20	3.4E-01	0.08	6.9E-01
ENSCAFG0000001072	GIT2	turquoise	EC_M6	0.15	4.7E-01	0.72	3.0E-05	-0.55	3.5E-03	-0.16	4.3E-01	0.26	2.0E-01	-0.22	2.7E-01	-0.24	2.3E-01	0.22	2.8E-01	0.03	8.8E-01	-0.30	1.4E-01
ENSCAFG0000002490	ZSCAN12	grey	EC_M10	0.15	4.7E-01	0.04	8.5E-01	-0.19	3.6E-01	-0.10	6.3E-01	0.10	6.2E-01	-0.09	6.7E-01	-0.04	8.5E-01	-0.12	5.6E-01	-0.19	3.5E-01	-0.16	4.2E-01
ENSCAFG0000001566	TMEM30B	turquoise	EC_M6	0.15	4.7E-01	0.78	3.3E-06	-0.27	1.8E-01	-0.12	5.7E-01	-0.06	7.7E-01	-0.08	6.9E-01	-0.05	8.0E-01	-0.08	7.0E-01	0.28	1.6E-01	0.02	9.3E-01
ENSCAFG0000002802	ENSCAFG0000002802	grey	EC_M10	0.15	4.7E-01	-0.28	1.6E-01	0.37	5.9E-02	-0.23	2.6E-01	-0.45	2.3E-02	-0.14	4.9E-01	-0.22	2.9E-01	0.35	8.1E-02	-0.14	5.1E-01	0.33	1.0E-01
ENSCAFG0000002156	ENSCAFG0000002156	grey	EC_M10	0.15	4.7E-01	0.00	1.0E+00	-0.12	5.7E-01	0.40	4.0E-02	-0.06	7.7E-01	0.11	5.9E-01	-0.08	7.1E-01	-0.06	7.6E-01	-0.28	1.6E-01	-0.04	8.5E-01
ENSCAFG0000001422	ARHGAP20	cyan	EC_M2	0.15	4.7E-01	-0.61	1.0E-03	0.47	1.5E-02	0.18	3.9E-01	-0.43	2.9E-02	-0.12	5.6E-01	-0.07	7.5E-01	0.12	5.7E-01	-0.33	1.0E-01	0.34	8.8E-02
ENSCAFG0000001219	ENSCAFG0000001219	grey	EC_M10	0.15	4.7E-01	-0.10	6.2E-01	0.19	3.6E-01	-0.17	4.0E-01	-0.36	7.2E-02	0.36	7.5E-02	-0.11	5.8E-01	-0.17	4.1E-01	0.17	4.0E-01	0.28	1.7E-01
ENSCAFG0000000269	LENG1	grey	EC_M10	0.15	4.7E-01	0.26	2.0E-01	0.03	8.8E-01	0.23	2.7E-01	-0.38	5.3E-02	0.01	9.8E-01	-0.04	8.3E-01	-0.22	2.8E-01	-0.02	9.1E-01	0.28	1.6E-01
ENSCAFG0000000089	PNPLA5	grey	EC_M10	0.15	4.7E-01	-0.14	5.0E-01	0.15	4.5E-01	-0.09	6.5E-01	-0.18	3.9E-01	-0.09	6.6E-01	-0.04	8.6E-01	0.07	7.3E-01	-0.08	7.0E-01	0.11	5.9E-01
ENSCAFG0000001084	ENSCAFG0000001084	grey	EC_M10	0.15	4.7E-01	-0.14	5.0E-01	0.15	4.5E-01	-0.09	6.5E-01	-0.18	3.9E-01	-0.09	6.6E-01	-0.04	8.6E-01	0.07	7.3E-01	-0.08	7.0E-01	0.11	5.9E-01
ENSCAFG0000000963	NFKBIZ	cyan	EC_M2	0.15	4.7E-01	0.05	8.1E-01	0.58	2.1E-03	0.31	1.3E-01	-0.88	2.5E-09	-0.19	3.5E-01	-0.15	4.7E-01	-0.14	4.9E-01	0.11	5.9E-01	0.79	1.3E-06
ENSCAFG0000001458	SERPINI1	grey	EC_M10	0.15	4.7E-01	-0.01	9.8E-01	-0.57	2.2E-03	-0.21	3.0E-01	0.57	2.4E-03	0.05	7.9E-01	-0.06	7.7E-01	-0.06	7.6E-01	0.34	8.9E-02	-0.59	1.4E-03
ENSCAFG0000001040	RNF19B	turquoise	EC_M6	0.15	4.7E-01	0.82	2.3E-07	-0.35	8.3E-02	0.00	9.9E-01	-0.04	8.5E-01	-0.16	4.4E-01	0.00	1.0E+00	0.00	1.0E+00	-0.11	5.8E-01	-0.05	8.1E-01
ENSCAFG0000000843	PAG1	grey	EC_M10	0.15	4.7E-01	-0.10	6.4E-01	-0.41	3.7E-02	-0.12	5.5E-01	0.46	1.8E-02	0.07	7.2E-01	-0.18	3.7E-01	-0.21	3.1E-01	-0.01	9.7E-01	-0.53	5.6E-03
ENSCAFG0000000626	ARHGAP26	turquoise	EC_M6	0.15	4.7E-01	0.72	3.3E-05	0.05	8.1E-01	-0.16	4.5E-01	-0.42	3.5E-02	-0.28	1.7E-01	0.24	2.3E-01	-0.18	3.8E-01	0.07	7.3E-01	0.39	5.1E-02
ENSCAFG0000000409	DNAJC27	grey	EC_M10	0.15	4.7E-01	-0.32	1.1E-01	0.13	5.2E-01	0.32	1.1E-01	-0.18	3.9E-01	0.16	4.5E-01	-0.05	8.0E-01	-0.39	5.1E-02	-0.16	4.2E-01	0.13	5.2E-01
ENSCAFG0000000079	ENSCAFG0000000079	grey	EC_M10	0.15	4.7E-01	0.27	1.8E-01	0.04	8.3E-01	-0.15	4.6E-01	-0.24	2.4E-01	-0.16	4.5E-01	-0.02	9.3E-01	0.09	6.6E-01	0.12	5.6E-01	0.18	3.8E-01
ENSCAFG0000003232	S100A2	grey	EC_M10	0.15	4.7E-01	-0.13	5.3E-01	-0.05	7.9E-01	-0.09	6.5E-01	0.07	7.4E-01	0.19	3.6E-01	-0.43	3.0E-02	-0.16	4.5E-01	-0.16	4.4E-01	-0.11	5.8E-01
ENSCAFG0000000494	DLEC1	grey	EC_M10	0.15	4.7E-01	-0.09	6.7E-01	0.35	7.6E-02	0.20	3.3E-01	-0.50	1.0E-02	-0.20	3.3E-01	-0.13	5.3E-01	-0.14	4.8E-01	0.13	5.3E-01	0.45	2.3E-02
ENSCAFG0000003084	ENSCAFG0000003084	violet	EC_M7	0.15	4.7E-01	-0.17	4.0E-01	-0.11	6.0E-01	-0.11	6.0E-01	0.11	5.9E-01	0.61	8.8E-04	0.50	9.5E-03	-0.04	8.6E-01	-0.16	4.2E-01	-0.17	4.1E-01
ENSCAFG0000001726	MYH10	darkgreen	EC_M4	0.15	4.7E-01	0.62	8.0E-04	-0.73	2.2E-05	0.03	8.7E-01	0.50	8.9E-03	0.15	4.6E-01	-0.10	6.2E-01	-0.13	5.2E-01	-0.13	5.1E-01	-0.59	1.4E-03
ENSCAFG0000001793	CYP1A1	turquoise	EC_M6	0.15	4.7E-01	0.54	4.5E-03	-0.25	2.2E-01	0.22	2.8E-01	0.01	9.5E-01	-0.27	1.7E-01	0.07	7.5E-01	-0.27	1.9E-01	-0.30	1.4E-01	-0.10	6.1E-01
ENSCAFG0000001454	SH3TC1	turquoise	EC_M6	0.15	4.8E-01	0.79	1.6E-06	-0.23	2.7E-01	0.03	9.0E-01	-0.13	5.3E-01	-0.24	2.3E-01	-0.26	1.9E-01	0.08	7.0E-01	0.11	6.0E-01	0.03	8.8E-01
ENSCAFG0000000144	MICALL1	turquoise	EC_M6	0.15	4.8E-01	0.77	5.3E-06	-0.64	3.8E-04	-0.26	2.1E-01	0.38	5.3E-02	-0.04	8.4E-01	0.03	8.8E-01	0.04	8.6E-01	0.08	7.1E-01	-0.45	2.2E-02
ENSCAFG0000000637	NRG1	grey	EC_M10	0.15	4.8E-01	-0.15	4.6E-01	-0.03	8.8E-01	0.21	3.1E-01	-0.08	7.1E-01	-0.26	2.0E-01	-0.03	8.7E-01	-0.01	9.6E-01	0.50	9.2E-03	0.02	9.4E-01
ENSCAFG0000001065	HYAL2	turquoise	EC_M6	0.15	4.8E-01	0.87	1.1E-08	-0.29	1.4E-01	-0.26	2.0E-01	-0.03	8.9E-01	-0.13	5.3E-01	-0.14	5.1E-01	-0.06	7.6E-01	0.05	8.0E-01	-0.06	7.6E-01
ENSCAFG0000000903	CMYA5	grey	EC_M10	0.15	4.8E-01	0.27	1.9E-01	0.24	2.4E-01	-0.19	3.5E-01	-0.48	1.4E-02	-0.15	4.7E-01	-0.12	5.6E-01	-0.10	6.2E-01	-0.18	3.9E-01	0.44	2.4E-02
ENSCAFG0000001911	ENSCAFG0000001911	grey	EC_M10	0.15	4.8E-01	-0.05	8.0E-01	-0.18	3.7E-01	-0.20	3.2E-01	0.15	4.6E-01	-0.19	3.5E-01	-0.15	4.5E-01	-0.18	3.9E-01	0.20	3.2E-01	-0.23	2.5E-01
ENSCAFG0000002127	ENSCAFG0000002127	grey	EC_M10	0.15	4.8E-01	-0.02	9.1E-01	0.42	3.2E-02	0.24	2.3E-01	-0.59	1.4E-03	-0.21	3.0E-01	0.11	6.0E-01	-0.19	3.4E-01	-0.24	2.4E-01	0.55	3.5E-03
ENSCAFG0000001590	ENSCAFG0000001590	grey	EC_M10	0.15	4.8E-01	-0.07	7.3E-01	0.30	1.4E-01	0.19	3.6E-01	-0.45	2.1E-02	0.17	4.0E-01	0.06	7.8E-01	-0.05	8.0E-01	-0.17	4.1E-01	0.38	5.9E-02
ENSCAFG0000000880	ENSCAFG0000000880	grey	EC_M10	0.15	4.8E-01	0.13	5.4E-01	-0.15	4.6E-01	0.22	2.9E-01	0.00	9.9E-01	0.17	4.1E-01	-0.38	5.7E-02	-0.43	2.8E-02	-0.10	6.1E-01	-0.09	6.8E-01
ENSCAFG0000000726	ENSCAFG0000000726	grey	EC_M10	0.15	4.8E-01	0.19	3.6E-01	-0.72	3.7E-05	-0.13	5.2E-01	0.68	1.2E-04	0.20	3.3E-01	-0.01	9.4E-01	0.08	6.8E-01	0.06	7.5E-01	-0.75	1.1E-05
ENSCAFG0000000329	RSAD2	turquoise	EC_M6	0.15	4.8E-01	0.79	1.3E-06	-0.06	7.8E-01	0.09	6.6E-01	-0.36	7.1E-02	-0.26	2.1E-01	-0.09	6.7E-01	-0.06	7.7E-01	0.18	3.9E-01	0.29	1.5E-01
ENSCAFG0000000029	METTL1	grey	EC_M10	0.15	4.8E-01	-0.30	1.4E-01	-0.43	3.0E-02	-0.15	4.5E-01	0.58	2.1E-03	0.49	1.2E-02	-0.10	6.2E-01	-0.19	3.5E-01	-0.14	4.9E-01	-0.64	3.8E-04
ENSCAFG0000003197	PRKG1	darkgreen	EC_M4	0.15	4.8E-01	-0.43	2.9E-02	-0.44	2.4E-02	0.02	9.2E-01	0.54	4.0E-03	0.25	2.1E-01	0.11	6.1E-01	0.11	6.0E-01	0.13	5.2E-01	-0.67	1.9E-04
ENSCAFG0000000757	RNF141	darkgrey	EC_M8	0.14	4.8E-01	0.58	1.8E-03	0.13	5.2E-01	0.19	3.5E-01	-0.53	5.6E-03	-0.32	1.1E-01	0.02	9.1E-01	-0.05	8.2E-01	0.18	3.7E-01	0.50	8.9E-03
ENSCAFG0000001237	SLCO1C1	grey	EC_M10	0.14	4.8E-01	-0.05	8.3E-01	-0.34	9.0E-02	0.24	2.4E-01	0.29	1.6E-01	0.33	1.0E-01	0.05	7.9E-01	-0.02	9.2E-01	0.43	2.9E-02	-0.37	6.6E-02
ENSCAFG0000002261	ENSCAFG0000002261	grey	EC_M10	0.14	4.8E-01	-0.16	4.3E-01	-0.02	9.2E-01	0.56	3.1E-03	-0.06	7.9E-01	0.60	1.2E-03	-0.12	5.6E-01	-0.16	4.5E-01	-0.20	3.3E-01	-0.02	9.1E-01
ENSCAFG0000003111	ENSCAFG0000003111	grey	EC_M10	0.14	4.8E-01	0.03	8.8E-01	-0.10	6.3E-01	-0.17	3.9E-01	0.03	9.0E-01	0.42	3.3E-02	-0.16	4.3E-01	-0.09	6.8E-01	-0.13	5.2E-01	-0.11	6.0E-01
ENSCAFG0000001855	ZBTB14	grey	EC_M10	0.14	4.8E-01	0.43	3.0E-02	0.25	2.3E-01	-0.07	7.4E-01	-0.59	1.5E-03	-0.04	8.5E-01	0.12	5.6E-01	-0.08	7.0E-01	0.21	2.9E-01	0.53	5.1E-03
ENSCAFG0000001678	DFFA	grey	EC_M10	0.14	4.8E-01	0.05	8.3E-01	0.16	4.3E-01	-0.44	2.5E-02	-0.25	2.2E-01	0.09	6.8E-01	-0.32	1.1E-01	0.08	7.1E-01	-0.05	7.9E-01	0.20	3.2E-01
ENSCAFG0000001375	CLEC14A	turquoise	EC_M6	0.14	4.8E-01	0.92	6.0E-11	-0.17	4.2E-01	-0.06	7.7E-01	-0.22	2.7E-01	-0.11	5.8E-01	-0.06	7.6E-01	-0.16	4.4E-01	0.17	4.1E-01	0.14	4.8E-01
ENSCAFG0000001176	STK39	grey	EC_M10	0.14	4.8E-01	-0.40	4.5E-02	-0.51	7.5E-03	-0.13	5.2E-01	0.64	3.8E-04	0.04	8.4E-01	-0.18	3.9E-01	0.11	5.9E-01	0.02	9.1E-01	-0.70	6.1E-05
ENSCAFG0000002372	ENSCAFG0000002372	grey	EC_M10	0.14	4.8E-01	0.03	8.8E-01	-0.69	1.0E-04	-0.12	5.6E-01	0.66	2.3E-04	0.33	9.6E-02	0.07	7.4E-01	0.29	1.5E-01	0.00	1.0E+00	-0.72	2.9E-05
ENSCAFG0000000137	JOSD1	cyan	EC_M2	0.14	4.8E-01	0.06	7.6E-01	0.39	4.7E-02	0.37	6.4E-02	-0.63	5.7E-04	-0.05	8.0E-01	-0.18	3.7E-01	-0.13	5.3E-01	-0.18	3.8E-01	0.56	2.8E-03
ENSCAFG0000002178	ENSCAFG0000002178	grey	EC_M10	0.14	4.8E-01	0.02	9.2E-01	0.01	9.5E-01	-0.08	7.1E-01	-0.04	8.4E-01	-0.01	9.6E-01	0.02	9.1E-01	-0.05	8.1E-01	0.09	6.6E-01	-0.04	8.4E-01
ENSCAFG0000003039	GPR88	grey	EC_M10	0.14	4.8E-01	0.02	9.2E-01	0.01	9.5E-01	-0.08	7.1E-01	-0.04	8.4E-01	-0.01	9.6E-01	0.02	9.1E-01	-0.05	8.1E-01	0.09	6.6E-01	-0.04	8.4E-01
ENSCAFG0000001263	ENSCAFG0000001263	darkgreen	EC_M4	0.14	4.8E-01	0.23	2.6E-01	-0.71	5.1E-05	-0.10	6.2E-01	0.66	2.3E-04	0.15	4.7E-01	-0.03	9.0E-01	-0.24	2.5E-01	-0.03	8.8E-01	-0.72	4.0E-05
ENSCAFG0000003044	KCNA6	turquoise	EC_M6	0.14	4.8E-01	0.82	2.6E-07	-0.32	1.1E-01	-0.16	4.5E-01	0.01	9.7E-01	-0.16	4.4E-01	-0.14	5.0E-01	-0.14	5.0E-01	0.07	7.5E-01	-0.07	7.4E-01
ENSCAFG0000000949	NEMP2	turquoise	EC_M6	0.14	4.8E-01	0.62	7.2E-04	-0.29	1.6E-01	0.03	8.7E-01	-0.04	8.6E-01	-0.18	3.7E-01	-0.02	9.0E-01	0.15	4.5E-01	0.16	4.3E-01	-0.02	9.2E-01
ENSCAFG0000000670	HSD17B12	grey	EC_M10	0.14	4.8E-01	-0.35	8.4E-02	-0.48	1.4E-02	-0.19	3.4E-01	0.58	1.9E-03	0.12	5.6E-01	-0.02	9.4E-01	0.03	8.8E-01	-0.53	5.0E-03	-0.68	1.4E-04
ENSCAFG0000003202	NAA20	grey	EC_M10	0.14	4.8E-01	-0.56	3.2E-03	0.29	1.5E-01	0.18	3.7E-01	-0.28	1.7E-01	0.25	2.2E-01	0.03	8.8E-01	-0.04	8.6E-01	0.19	3.6E-01	0.22	2.9E-01
ENSCAFG0000001213	CFLAR	turquoise	EC_M6	0.14	4.8E-01	0.65	3.4E-04	-0.49	1.1E-02	-0.11	6.0E-01	0.19	3.6E-01	-0.17	4.0E-01	0.23	2.5E-01	0.21	3.1E-01	-0.12	5.5E-01	-0.29	1.5E-01
ENSCAFG0000001611	ZNF280D	cyan	EC_M2	0.14	4.9E-01	0.31	1.2E-01	0.35	8.3E-02	0.29	1.6E-01	-0.72	3.0E-05	-0.18	3.9E-01	-0.07	7.4E-01	-0.06	7.6E-01	-0.11	6.0E-01	0.63	5.4E-04
ENSCAFG0000000192	C10H12orf45	grey	EC_M10	0.14	4.9E-01	-0.41	3.9E-02	-0.48	1.3E-02	0.08	7.0E-01	0.65	3.0E-04	0.32	1.1E-01	-0.23	2.6E-01	-0.06	7.9E-01	-0.06	7.5E-01	-0.73	2.3E-05
ENSCAFG0000003202	DUSP7	turquoise	EC_M6	0.14	4.9E-01	0.53	5.4E-03	0.05	8.2E-01	-0.29	1.5E-01	-0.32	1.1E-01	-0.23	2.7E-01	-0.06	7.6E-01	0.18	3.8E-01	0.40	4.4E-02	0.25	2.2E-01
ENSCAFG0000000847	MOGS	grey	EC_M10	0.14	4.9E-01	0.18	3.8E-01	-0.36	6.9E-02	-0.18	3.7E-01	0.24	2.4E-01	0.05	8.3E-01	0.25	2.3E-01	0.12	5.6E-01	-0.10	6.1E-01	-0.38	5.6E-02
ENSCAFG0000000066	EZR	grey	EC_M10	0.14	4.9E-01	-0.14	4.9E-01	-0.62	7.1E-04	-0.22	2.9E-01	0.72	3.6E-05	0.13	5.2E-01	-0.22	2.8E-01	0.00	9.9E-01	-0.35	8.3E-02	-0.79	1.9E-06
ENSCAFG0000001723	SLIRP	grey	EC_M10	0.14	4.9E-01	-0.07	7.3E-01	-0.35	8.3E-02	-0.30	1.4E-01	0.41	3.7E-02	0.36	7.0E-02	-0.08	6.9E-01	0.17	4.2E-01	0.07	7.5E-01	-0.48	1.3E-02
ENSCAFG0000001614	SLC25A18	grey	EC_M10	0.14	4.9E-01	0.10	6.1E-01	-0.22	2.8E-01	-0.09	6.5E-01	0.14	4.9E-01	0.27	1.9E-01	-0.17	4.2E-01	0.49	1.2E-02	-0.06	7.7E-01	-0.24	2.4E-01
ENSCAFG0000002357	ENSCAFG0000002357	grey	EC_M10	0.14	4.9E-01	-0.15	4.7E-01	0.21	3.0E-01	0.47	1.4E-02	-0.31	1.2E-01	-0.13	5.4E-01	-0.12	5.6E-01	0.21	3.0E-01	-0.01	9.8E-01	0.23	2.5E-01
ENSCAFG0000003276	ENSCAFG0000003276	grey	EC_M10	0.14	4.9E-01	-0.15	4.7E-01	-0.07	7.2E-01	-0.16	4.4E-01	0.09	6.6E-01	-0.14	5.0E-01	-0.03	8.9E-01	-0.10	6.2E-01	0.01	9.6E-01	-0.11	5.9E-01
ENSCAFG0000000904	IQCD	grey	EC_M10	0.14	4.9E-01	0.14	4.8E-01	-0.56	2.8E-03	-0.26	2.0E-01	0.49	1.1E-02	-0.28	1.7E-01	-0.28	1.7E-01	-0.06	7.8E-01	0.10	6.2E-01	-0.56	3.1E-03
ENSCAFG0000001075	CPT1A	darkgreen	EC_M4	0.14	4.9E-01	0.18	3.8E-01	-0.77	3.4E-06	-0.18	3.8E-01	0.76	5.5E-06	0.15	4.7E-01	-0.28	1.7E-01	0.15	4.8E-01	0.17	4.2E-01	-0.82	2.5E-07
ENSCAFG0000003230	ZNF32	grey	EC_M10	0.14	4.9E-01	0.12	5.5E-01	0.29	1.5E-01	0.03	9.0E-01	-0.53	5.0E-03	-0.08	6.9E-01	0.10	6.3E-01	-0.20	3.2E-01	0.16	4.4E-01	0.45	2.1E-02
ENSCAFG0000000362	IL15	turquoise	EC_M6	0.14	4.9E-01	0.95	2.3E-13	-0.28	1.6E-01	-0.11	6.1E-01	-0.10	6.3E-01	-0.12	5.7E-01	0.05	8.0E-01	-0.13	5.3E-01	0.00	9.9E-01	0.04	8.4E-01
ENSCAFG0000000494	ENSCAFG0000000494	paleturquoise	EC_M11	0.14	4.9E-01	0.41	3.6E-02	-0.09	6.5E-01	0.56	2.8E-03	-0.20	3.4E-01	-0.10	6.2E-01	-0.07	7.3E-01	-0.14	5.1E-01	-0.18	3.7E-01	0.12	5.5E-01
ENSCAFG0000003256	RBP1	turquoise	EC_M6	0.14	4.9E-01	0.76	5.4E-06	-0.38	5.4E-02	-0.14	5.0E-01	0.05	8.0E-01	0.08	7.0E-01	-0.03	9.0E-01	-0.15	4.6E-01	0.05	8.2E-01	-0.14	5.0E-01
ENSCAFG0000000631	ENSCAFG0000000631	turquoise	EC_M6	0.14	4.9E-01	0.60	1.2E-03	-0.44	2.3E-02	0.14	5.1E-01	0.18	3.7E-01	-0.35	7.7E-02	-0.05	8.1E-01	0.10	6.1E-01	-0.01	9.8E-01	-0.22	2.9E-01
ENSCAFG0000000285	OSBPL3	grey	EC_M10	0.14	4.9E-01	-0.02	9.4E-01	-0.64	4.3E-04	-0.05	7.9E-01	0.62	7.9E-04	0.44	2.4E-02	0.18	3.8E-01	-0.12	5.7E-01	-0.24	2.5E-01	-0.72	3.5E-05
ENSCAFG0000002733	ENSCAFG0000002733	turquoise	EC_M6	0.14	4.9E-01	0.44	2.5E-02	-0.19	3.7E-01	-0.15	4.7E-01	-0.07	7.5E-01	-0.11	5.8E-01	-0.09	6.6E-01	-0.10	6.3E-01	-0.03	9.0E-01	0.04	8.6E-01
ENSCAFG0000000269	ENSCAFG0000000269	violet	EC_M7	0.14	4.9E-01	-0.15	4.7E-01	-0.27	1.9E-01	-0.07	7.4E-01	0.22	2.9E-01	0.73	2.4E-05	-0.04	8.6E-01	-0.11	6.0E-01	-0.16	4.2E-01	-0.32	1.1E-01
ENSCAFG0000003190	ENSCAFG0000003190	grey	EC_M10	0.14	4.9E-01	0.22	2.7E-01	0.22	2.8E-01	0.42	3.5E-02	-0.51	8.1E-03	-0.28	1.7E-01	0.05	8.2E-01	-0.01	9.8E-01	0.06	7.9E-01	0.44	2.5E-02
ENSCAFG0000000551	WDR37	grey	EC_M10	0.14	4.9E-01	0.22	2.8E-01	-0.65	2.9E-04	-0.33	1.0E-01	0.65	3.5E-04	0.01	9.5E-01	0.08	7.2E-01	-0.10	6.2E-01	-0.08	6.8E-01	-0.68	1.5E-04
ENSCAFG0000001475	CHST7	turquoise	EC_M6	0.14	4.9E-01	0.63	5.0E-04	0.21	3.1E-01	-0.07	7.4E-01	-0.54	4.8E-03	-0.24	2.4E-01	-0.08	6.8E-01	-0.26	2.0E-01	-0.12	5.7E-01	0.48	1.4E-02
ENSCAFG0000001728	ENSCAFG0000001728	grey	EC_M10	0.14	4.9E-01	0.27	1.8E-01	-0.04	8.4E-01	0.34	8.9E-02	-0.16	4.4E-01	0.26	2.0E-01	-0.22	2.9E-01	-0.29	1.5E-01	0.05	8.0E-01	0.10	6.2E-01
ENSCAFG0000000087	ENSCAFG0000000087	grey	EC_M10	0.14	4.9E-01	0.27	1.8E-01	-0.35	8.4E-02	-0.30	1.3E-01	0.26	2.1E-01	-0.22	2.7E-01	0.21	2.9E-01	-0.15	4.6E-01	-0.14	5.1E-01	-0.30	1.4E-01
ENSCAFG0000002033	RANBP10	grey	EC_M10	0.14	4.9E-01	0.34	8.6E-02	0.21	3.1E-01	-0.14	4.8E-01	-0.50	8.6E-03	-0.26	2.1E-01	0.11	6.0E-01	-0.19	3.5E-01	0.20	3.3E-01	0.40	4.1E-02
ENSCAFG0000002883	ENSCAFG0000002883	grey	EC_M10	0.14	4.9E-01	0.08	6.9E-01	-0.03	8.9E-01	0.37	6.2E-02	-0.15	4.5E-01	0.19	3.4E-01	-0.07	7.5E-01	0.24	2.4E-01	0.16	4.2E-01	0.07	7.3E-01
ENSCAFG0000001065	FBXO36	grey	EC_M10	0.14	4.9E-01	-0.10	6.2E-01	-0.04	8.6E-01	-0.04	8.4E-01	0.00	9.9E-01	-0.08	6.9E-01	-0.08	7.0E-01	-0.07	7.4E-01	-0.06	7.7E-01	-0.10	6.3E-01
ENSCAFG0000002377	ZBTB8A	grey	EC_M10	0.14	4.9E-01	-0.77	4.0E-06	0.28	1.6E-01	0.13	5.2E-01	-0.20	3.3E-01	-0.07	7.2E-01	-0.04	8.5E-01	0.05	7.9E-01	-0.10	6.4E-01	0.11	5.8E-01
ENSCAFG0000000853	FAM186B	turquoise	EC_M6	0.14	4.9E-01	0.51	7.8E-03	-0.37	5.9E-02	-0.09	6.8E-01	0.13	5.4E-01	-0.23	2.6E-01	-0.25	2.2E-01	-0.01	9.7E-01	0.19	3.5E-01	-0.16	4.5E-01
ENSCAFG0000003119	SERTAD4	grey	EC_M10	0.14	4.9E-01	0.43	2.8E-02	0.11	5.8E-01	-0.05	8.1E-01	-0.37	6.4E-02	-0.05	8.2E-01	-0.09	6.7E-01	-0.40	4.0E-02	-0.32	1.1E-01	0.34	9.3E-02
ENSCAFG0000000635	DCUN1D2	grey	EC_M10	0.14	4.9E-01	-0.21	3.1E-01	-0.27	1.9E-01	-0.25	2.2E-01	0.25	2.2E-01	0.19	3.6E-01	-0.44	2.4E-02	0.17	4.1E-01	0.37	6.5E-02	-0.34	9.2E-02
ENSCAFG0000001262	ENSCAFG0000001262	grey	EC_M10	0.14	4.9E-01	0.16	4.3E-01	-0.36	7.3E-02	-0.28	1.7E-01	0.21	3.0E-01	0.15	4.6E-01	0.19	3.4E-01	0.51	8.4E-03	0.02	9.2E-01	-0.28	1.7E-01
ENSCAFG0000000238	COX5B	grey	EC_M10	0.14	4.9E-01	-0.44	2.4E-02	0.04	8.6E-01	0.15	4.7E-01	0.09	6.6E-01	0.11	6.1E-01	0.15	4.5E-01	-0.01	9.8E-01	-0.42	3.1E-02	-0.13	5.4E-01
ENSCAFG0000001897	EFCAB5	grey	EC_M10	0.14	4.9E-01	-0.08	7.1E-01	0.08	6.9E-01	-0.25	2.2E-01	-0.24	2.4E-01	-0.28	1.6E-01	-0.30	1.4E-01	0.13	5.3E-01	-0.29	1.5E-01	0.14	5.0E-01
ENSCAFG0000000838	PFN2	darkgreen	EC_M4	0.14	4.9E-01	-0.05	8.1E-01	-0.73	2.0E-05	-0.18	3.7E-01	0.81	6.0E-07	0.24	2.3E-01	-0.21	3.0E-01	0.08	6.9E-01	0.04	8.6E-01	-0.88	3.7E-09
ENSCAFG0000000418	ENSCAFG0000000418	grey	EC_M10	0.14	4.9E-01	-0.19	3.4E-01	-0.15	4.6E-01	0.37	6.5E-02	0.06	7.6E-01	0.32	1.1E-01	-0.13	5.4E-01	0.27	1.9E-01	-0.16	4.4E-01	-0.19	3.5E-01
ENSCAFG0000001118	MXRA5	grey	EC_M10	0.14	4.9E-01	-0.16	4.4E-01	0.04	8.4E-01	0.02	9.1E-01	-0.11	5.9E-01	0.14	4.9E-01	-0.15	4.6E-01	0.05	8.2E-01	-0.09	6.8E-01	-0.02	9.2E-01
ENSCAFG0000002375	GMPPB	grey	EC_M10	0.14	4.9E-01	-0.19	3.5E-01	-0.15	4.8E-01	-0.01	9.5E-01	0.14	4.8E-01	0.31	1.3E-01	0.08	7.0E-01	-0.22	2.9E-01	-0.35	7.6E-02	-0.30	1.4E-01
ENSCAFG0000000720	GGH	cyan	EC_M2	0.14	4.9E-01	-0.59	1.4E-03	0.47	1.6E-02	0.23	2.6E-01	-0.40	4.0E-02	-0.19	3.4E-01	0.09	6.5E-01	0.16	4.3E-01	-0.01	9.6E-01	0.37	6.6E-02
ENSCAFG0000001319	NRP2	grey	EC_M10	0.14	4.9E-01	0.25	2.2E-01	-0.55	3.9E-03	-0.16	4.5E-01	0.46	1.8E-02	0.19	3.6E-01	0.23	2.7E-01	-0.06	7.7E-01	-0.62	7.3E-04	-0.54	4.1E-03
ENSCAFG0000003189	EIF1AD	turquoise	EC_M6	0.14	4.9E-01	0.43	2.7E-02	-0.13	5.4E-01	-0.32	1.1E-01	-0.12	5.6E-01	-0.19	3.5E-01	-0.31	1.3E-01	0.15	4.6E-01	0.00	9.9E-01	0.00	1.0E+00
ENSCAFG0000001791	SEMA7A	grey	EC_M10	0.14	4.9E-01	0.17	4.2E-01	-0.67	1.8E-04	-0.23	2.6E-01	0.64	4.0E-04	0.25	2.2E-01	-0.27	1.9E-01	-0.15	4.8E-01	0.04	8.3E-01	-0.71	4.6E-05
ENSCAFG0000001718	SHE	turquoise	EC_M6	0.14	4.9E-01	0.89	1.2E-09	-0.22	2.9E-01	-0.14	4.8E-01	-0.17	4.2E-01	-0.09	6.6E-01	-0.13	5.4E-01	-0.15	4.6E-01	0.11	6.1E-01	0.07	7.3E-01
ENSCAFG0000003145	ENSCAFG0000003145	grey	EC_M10	0.14	4.9E-01	-0.29	1.5E-01	-0.06	7.9E-01	-0.01	9.8E-01	0.04	8.3E-01	0.45	2.1E-02	-0.03	8.9E-01	0.11	5.8E-01	-0.01	9.6E-01	-0.18	3.8E-01
ENSCAFG0000000662	EFCAB1	grey	EC_M10	0.14	4.9E-01	-0.30	1.3E-01	-0.02	9.1E-01	0.05	8.3E-01	0.00	9.8E-01	-0.23	2.5E-01	-0.13	5.2E-01	0.09	6.4E-01	0.40	4.4E-02	-0.03	8.7E-01
ENSCAFG0000000811	ENSCAFG0000000811	darkgreen	EC_M4	0.14	4.9E-01	0.13	5.2E-01	-0.79	1.3E-06	-0.35	8.2E-02	0.74	1.8E-05	0.14	4.9E-01	0.14	4.9E-01	0.24	2.3E-01	0.10	6.3E-01	-0.81	6.8E-07
ENSCAFG0000001805	DSG2	turquoise	EC_M6	0.14	4.9E-01	0.75	1.2E-05	-0.54	4.2E-03	-0.20	3.2E-01	0.28	1.7E-01	0.02	9.1E-01	-0.02	9.1E-01	0.20	3.2E-01	0.15	4.5E-01	-0.31	1.2E-01
ENSCAFG0000001694	SPOP	darkgreen	EC_M4	0.14	4.9E-01	-0.19	3.4E-01	-0.72	3.1E-05	-0.18	3.8E-01	0.81	6.4E-07	0.46	1.8E-02	0.07	7.3E-01	0.06	7.8E-01	-0.06	7.8E-01	-0.91	1.3E-10
ENSCAFG0000001696	MAZ	grey	EC_M10	0.14	4.9E-01	-0.06	7.8E-01	0.63	5.8E-04	0.09	6.5E-01	-0.82	3.6E-07	-0.02	9.1E-01	0.23	2.5E-01	-0.04	8.6E-01	0.03	8.9E-01	0.72	3.0E-05

ENSCAFG0000000726	HECTD2	darkgreen	EC_M4	0.14	4.9E-01	0.17	4.1E-01	-0.77	3.7E-06	-0.06	7.8E-01	0.72	4.0E-05	0.26	2.1E-01	0.06	7.7E-01	0.06	7.6E-01	0.20	3.3E-01	-0.82	2.9E-07
ENSCAFG0000000573	DOCK9	cyan	EC_M2	0.14	5.0E-01	0.29	1.5E-01	0.39	4.6E-02	0.07	7.3E-01	-0.71	4.2E-05	-0.20	3.2E-01	-0.01	9.5E-01	-0.16	4.3E-01	-0.47	1.5E-02	0.62	6.6E-04
ENSCAFG0000000935	TOX2	turquoise	EC_M6	0.14	5.0E-01	0.73	2.0E-05	-0.08	7.1E-01	-0.06	7.6E-01	-0.28	1.7E-01	-0.19	3.6E-01	-0.15	4.8E-01	0.18	3.8E-01	0.05	8.2E-01	0.18	3.8E-01
ENSCAFG0000001475	ENSCAFG0000001475	grey	EC_M1	0.14	5.0E-01	0.14	4.9E-01	0.28	1.6E-01	0.14	5.0E-01	-0.48	1.4E-02	-0.27	1.8E-01	-0.26	1.9E-01	0.09	6.6E-01	0.04	8.5E-01	0.42	3.2E-02
ENSCAFG0000001113	SCN2A	grey	EC_M1	0.14	5.0E-01	0.25	2.2E-01	-0.25	2.1E-01	-0.03	8.9E-01	0.12	5.6E-01	-0.03	9.0E-01	-0.05	8.1E-01	-0.23	2.6E-01	-0.34	8.7E-02	-0.22	2.7E-01
ENSCAFG0000000591	ANKRD28	grey	EC_M1	0.14	5.0E-01	-0.20	3.4E-01	0.61	9.0E-04	0.18	3.7E-01	-0.78	3.2E-06	-0.28	1.6E-01	0.10	6.2E-01	-0.01	9.6E-01	-0.22	2.7E-01	0.71	4.4E-05
ENSCAFG0000001560	ENSCAFG0000001560	grey	EC_M1	0.14	5.0E-01	-0.12	5.7E-01	-0.03	9.0E-01	-0.06	7.8E-01	-0.10	6.3E-01	-0.05	8.1E-01	-0.15	4.7E-01	-0.11	5.9E-01	-0.14	5.0E-01	-0.02	9.1E-01
ENSCAFG0000001193	PGBD1	grey	EC_M1	0.14	5.0E-01	-0.02	9.1E-01	-0.14	5.0E-01	0.03	8.7E-01	-0.03	8.8E-01	0.12	5.5E-01	-0.26	2.0E-01	-0.06	7.8E-01	0.33	9.7E-02	-0.05	8.0E-01
ENSCAFG0000000706	PPP2R3A	grey	EC_M1	0.14	5.0E-01	0.33	1.0E-01	-0.04	8.4E-01	-0.20	3.3E-01	-0.14	5.0E-01	-0.02	9.1E-01	-0.02	9.2E-01	-0.32	1.2E-01	-0.30	1.3E-01	0.09	6.5E-01
ENSCAFG0000001689	BBOF1	grey	EC_M1	0.14	5.0E-01	-0.60	1.2E-03	0.18	3.8E-01	-0.03	8.7E-01	-0.12	5.7E-01	-0.13	5.1E-01	-0.10	6.1E-01	-0.10	6.3E-01	0.02	9.3E-01	0.05	8.1E-01
ENSCAFG0000001594	ADIPOR2	turquoise	EC_M6	0.14	5.0E-01	0.66	2.7E-04	-0.42	3.1E-02	-0.19	3.6E-01	0.16	4.3E-01	-0.07	7.4E-01	0.02	9.3E-01	-0.24	2.4E-01	-0.44	2.6E-02	-0.21	3.0E-01
ENSCAFG0000000816	TIGD4	turquoise	EC_M6	0.14	5.0E-01	0.62	7.6E-04	-0.08	7.1E-01	0.32	1.2E-01	-0.29	1.6E-01	-0.31	1.2E-01	0.01	9.7E-01	0.00	9.9E-01	-0.07	7.3E-01	0.24	2.3E-01
ENSCAFG0000000138	BRPF3	turquoise	EC_M6	0.14	5.0E-01	0.83	1.3E-07	-0.35	7.6E-02	0.02	9.2E-01	-0.05	8.2E-01	0.01	9.7E-01	0.11	6.0E-01	-0.21	3.1E-01	0.13	5.2E-01	-0.01	9.7E-01
ENSCAFG0000002942	ENSCAFG0000002942	darkgreen	EC_M4	0.14	5.0E-01	-0.04	8.3E-01	-0.79	1.7E-06	-0.15	4.8E-01	0.85	5.1E-08	0.27	1.8E-01	-0.15	4.7E-01	0.12	5.4E-01	-0.16	4.2E-01	-0.94	7.5E-13
ENSCAFG0000003249	HIC2	grey	EC_M1	0.14	5.0E-01	0.04	8.3E-01	0.29	1.5E-01	0.09	6.5E-01	-0.56	3.1E-03	0.14	4.8E-01	0.38	5.6E-02	0.13	5.3E-01	-0.02	9.3E-01	0.44	2.4E-02
ENSCAFG0000000254	CAMKMT	grey	EC_M1	0.14	5.0E-01	-0.08	6.9E-01	-0.07	7.2E-01	0.00	9.9E-01	-0.07	7.5E-01	0.20	3.4E-01	-0.29	1.5E-01	-0.14	5.0E-01	-0.02	9.2E-01	-0.02	9.2E-01
ENSCAFG0000001756	CCDC88C	turquoise	EC_M6	0.14	5.0E-01	0.90	5.0E-10	-0.30	1.4E-01	-0.09	6.6E-01	-0.11	6.1E-01	-0.07	7.3E-01	-0.08	7.0E-01	-0.06	7.7E-01	0.23	2.7E-01	0.03	8.8E-01
ENSCAFG0000003240	ZSCAN1	grey	EC_M1	0.14	5.0E-01	0.25	2.1E-01	-0.04	8.5E-01	0.36	6.8E-02	-0.20	3.3E-01	-0.20	3.2E-01	-0.19	3.5E-01	-0.22	2.7E-01	0.19	3.4E-01	0.09	6.8E-01
ENSCAFG0000002615	ENSCAFG0000002615	grey	EC_M1	0.14	5.0E-01	-0.15	4.6E-01	0.10	6.2E-01	-0.10	6.1E-01	-0.17	4.1E-01	-0.10	6.1E-01	-0.09	6.6E-01	0.33	1.0E-01	0.27	1.9E-01	0.09	6.7E-01
ENSCAFG0000000630	TTI2	grey	EC_M1	0.14	5.0E-01	-0.15	4.5E-01	-0.59	1.4E-03	-0.05	8.0E-01	0.66	2.3E-04	0.42	3.3E-02	0.28	1.6E-01	0.21	3.1E-01	-0.07	7.4E-01	-0.76	5.8E-06
ENSCAFG0000001139	CLDN34	turquoise	EC_M6	0.14	5.0E-01	0.62	7.6E-04	-0.44	2.5E-02	-0.06	7.6E-01	0.21	3.0E-01	-0.16	4.4E-01	-0.41	3.9E-02	0.06	7.8E-01	-0.13	5.3E-01	-0.27	1.8E-01
ENSCAFG0000001352	CRYGS	grey	EC_M1	0.14	5.0E-01	-0.37	6.7E-02	0.16	4.4E-01	0.11	5.8E-01	-0.24	2.4E-01	0.11	5.9E-01	0.04	8.5E-01	-0.03	8.9E-01	-0.01	9.6E-01	0.14	4.9E-01
ENSCAFG0000002969	CAMLG	grey	EC_M1	0.14	5.0E-01	-0.31	1.3E-01	-0.12	5.6E-01	0.03	9.0E-01	0.04	8.5E-01	-0.03	8.7E-01	0.23	2.6E-01	-0.22	2.7E-01	-0.28	1.6E-01	-0.14	5.1E-01
ENSCAFG0000001038	ST3GAL4	grey	EC_M1	0.14	5.0E-01	-0.71	5.6E-05	0.03	8.7E-01	-0.16	4.2E-01	0.15	4.7E-01	0.35	8.3E-02	-0.01	9.8E-01	-0.12	5.6E-01	-0.19	3.4E-01	-0.24	2.4E-01
ENSCAFG0000000091	MCAT	grey	EC_M1	0.14	5.0E-01	-0.63	6.4E-04	0.10	6.4E-01	-0.09	6.5E-01	0.10	6.3E-01	0.09	6.6E-01	-0.34	9.3E-02	0.07	7.3E-01	-0.09	6.6E-01	-0.17	4.1E-01
ENSCAFG0000001026	ENSCAFG0000001026	grey	EC_M1	0.14	5.0E-01	0.24	2.3E-01	-0.12	5.7E-01	-0.29	1.5E-01	-0.02	9.4E-01	0.23	2.5E-01	-0.17	4.0E-01	0.21	3.0E-01	-0.13	5.2E-01	-0.05	8.2E-01
ENSCAFG0000002436	ENSCAFG0000002436	grey	EC_M1	0.14	5.0E-01	0.07	7.4E-01	-0.13	5.3E-01	0.02	9.3E-01	-0.01	9.7E-01	0.28	1.7E-01	0.17	4.1E-01	-0.23	2.7E-01	-0.36	7.4E-02	-0.07	7.3E-01
ENSCAFG0000001978	SLC16A4	grey	EC_M1	0.14	5.0E-01	-0.04	8.3E-01	-0.17	4.1E-01	-0.21	3.1E-01	0.22	2.9E-01	0.24	2.5E-01	-0.13	5.4E-01	-0.15	4.5E-01	-0.06	7.8E-01	-0.23	2.5E-01
ENSCAFG0000000040	PLEKHG1	turquoise	EC_M6	0.14	5.0E-01	0.90	5.9E-10	-0.44	2.6E-02	-0.15	4.6E-01	0.08	6.9E-01	-0.05	7.9E-01	-0.06	7.6E-01	-0.13	5.4E-01	-0.06	7.8E-01	-0.16	4.2E-01
ENSCAFG0000003186	ENSCAFG0000003186	turquoise	EC_M6	0.14	5.0E-01	0.92	2.2E-11	-0.22	2.8E-01	-0.07	7.2E-01	-0.18	3.7E-01	-0.11	5.9E-01	-0.09	6.6E-01	-0.13	5.4E-01	0.05	7.9E-01	0.09	6.6E-01
ENSCAFG0000000758	SNX25	grey	EC_M1	0.14	5.0E-01	-0.05	8.1E-01	-0.47	1.4E-02	0.11	6.0E-01	0.39	5.2E-02	0.01	9.7E-01	0.33	9.9E-02	0.31	1.2E-01	0.35	8.1E-02	-0.46	1.7E-02
ENSCAFG0000000185	ABCC10	grey	EC_M1	0.14	5.0E-01	0.31	1.2E-01	-0.41	3.7E-02	-0.03	8.8E-01	0.38	5.5E-02	-0.06	7.6E-01	-0.17	4.0E-01	0.04	8.5E-01	-0.15	4.5E-01	-0.44	2.4E-02
ENSCAFG0000002098	ENSCAFG0000002098	grey	EC_M1	0.14	5.0E-01	0.17	4.0E-01	-0.31	1.2E-01	-0.31	1.3E-01	0.14	5.0E-01	-0.04	8.7E-01	0.28	1.7E-01	0.22	2.8E-01	-0.30	1.3E-01	-0.23	2.6E-01
ENSCAFG0000001089	ENSCAFG0000001089	darkgreen	EC_M4	0.14	5.0E-01	0.01	9.6E-01	-0.78	2.4E-06	-0.17	4.0E-01	0.84	9.3E-08	0.27	1.8E-01	-0.14	5.1E-01	0.08	7.0E-01	-0.04	8.6E-01	-0.90	3.9E-10
ENSCAFG0000000140	PODXL	turquoise	EC_M6	0.14	5.0E-01	0.85	5.1E-08	-0.17	4.1E-01	-0.08	7.0E-01	-0.20	3.2E-01	-0.05	8.1E-01	-0.17	4.0E-01	-0.15	4.8E-01	0.13	5.1E-01	0.11	6.1E-01
ENSCAFG0000001377	ENSCAFG0000001377	turquoise	EC_M6	0.14	5.0E-01	0.78	2.3E-06	0.01	9.7E-01	-0.03	8.7E-01	-0.39	4.9E-02	-0.25	2.1E-01	-0.18	3.7E-01	-0.29	1.5E-01	-0.02	9.2E-01	0.34	9.0E-02
ENSCAFG0000001406	ENSCAFG0000001406	grey	EC_M1	0.14	5.0E-01	0.33	1.0E-01	-0.03	8.8E-01	0.04	8.6E-01	-0.19	3.4E-01	-0.36	7.5E-02	-0.56	2.9E-03	0.05	8.2E-01	-0.23	2.6E-01	0.15	4.6E-01
ENSCAFG0000000423	KIAA1217	grey	EC_M1	0.14	5.0E-01	0.25	2.3E-01	-0.64	4.4E-04	-0.22	2.8E-01	0.47	1.5E-02	0.18	3.7E-01	0.01	9.5E-01	0.11	5.9E-01	0.55	3.3E-03	-0.54	4.5E-03
ENSCAFG0000002029	MCOLN3	grey	EC_M1	0.14	5.0E-01	-0.05	7.9E-01	-0.27	1.9E-01	-0.20	3.3E-01	0.24	2.4E-01	0.31	1.2E-01	-0.07	7.5E-01	-0.12	5.5E-01	0.26	1.9E-01	-0.31	1.2E-01
ENSCAFG0000000977	THYN1	grey	EC_M1	0.14	5.0E-01	-0.53	5.0E-03	-0.27	1.9E-01	-0.17	4.0E-01	0.46	1.7E-02	0.22	2.7E-01	0.23	2.5E-01	0.08	6.9E-01	0.04	8.4E-01	-0.54	4.0E-03
ENSCAFG0000000317	AFTPH	grey	EC_M1	0.14	5.0E-01	0.46	1.9E-02	-0.11	5.9E-01	0.12	5.5E-01	-0.23	2.6E-01	-0.05	7.9E-01	0.10	6.4E-01	-0.31	1.3E-01	-0.41	3.7E-02	0.14	5.0E-01
ENSCAFG0000000105	MTSS1	turquoise	EC_M6	0.14	5.1E-01	0.70	7.9E-05	-0.25	2.2E-01	-0.11	5.8E-01	-0.04	8.4E-01	0.06	7.7E-01	0.30	1.3E-01	-0.24	2.4E-01	-0.28	1.6E-01	-0.03	8.7E-01
ENSCAFG0000002011	SH2D3C	turquoise	EC_M6	0.14	5.1E-01	0.87	1.1E-08	-0.08	7.1E-01	-0.08	7.0E-01	-0.31	1.2E-01	-0.19	3.6E-01	-0.14	4.9E-01	-0.11	6.1E-01	-0.08	7.0E-01	0.22	2.9E-01
ENSCAFG0000000736	KLKB1	turquoise	EC_M6	0.14	5.1E-01	0.72	3.4E-05	0.01	9.5E-01	-0.18	3.7E-01	-0.36	7.3E-02	-0.15	4.6E-01	-0.19	3.4E-01	0.12	5.6E-01	-0.16	4.4E-01	0.32	1.1E-01
ENSCAFG0000001274	PSMC5	grey	EC_M1	0.14	5.1E-01	-0.47	1.6E-02	-0.04	8.5E-01	-0.01	9.5E-01	0.16	4.4E-01	0.40	4.1E-02	-0.02	9.2E-01	-0.03	8.7E-01	-0.22	2.8E-01	-0.25	2.3E-01
ENSCAFG0000001393	FAU	cyan	EC_M2	0.14	5.1E-01	-0.21	3.1E-01	0.54	4.5E-03	0.21	2.9E-01	-0.69	9.5E-05	-0.10	6.4E-01	0.29	1.5E-01	0.02	9.2E-01	-0.43	2.9E-02	0.58	1.8E-03
ENSCAFG0000002271	MT-ND2	cyan	EC_M2	0.14	5.1E-01	-0.31	1.2E-01	0.18	3.7E-01	0.22	2.7E-01	-0.25	2.2E-01	0.04	8.5E-01	-0.63	5.2E-04	0.11	5.9E-01	0.03	8.8E-01	0.16	4.3E-01
ENSCAFG0000000504	ENSCAFG0000000504	grey	EC_M1	0.14	5.1E-01	-0.09	6.6E-01	0.21	3.0E-01	-0.15	4.6E-01	-0.29	1.5E-01	-0.09	6.7E-01	-0.07	7.4E-01	-0.11	6.1E-01	0.06	7.8E-01	0.26	2.0E-01
ENSCAFG0000000693	AMHR2	grey	EC_M1	0.14	5.1E-01	0.12	5.7E-01	0.01	9.6E-01	-0.17	4.1E-01	-0.17	4.1E-01	0.04	8.4E-01	0.01	9.6E-01	-0.26	2.0E-01	-0.36	7.5E-02	0.06	7.7E-01
ENSCAFG0000001642	ENSCAFG0000001642	grey	EC_M1	0.14	5.1E-01	0.12	5.7E-01	-0.10	6.3E-01	0.08	6.9E-01	-0.05	8.0E-01	0.18	3.9E-01	0.31	1.3E-01	-0.23	2.7E-01	-0.20	3.4E-01	0.05	8.2E-01
ENSCAFG0000002105	ENSCAFG0000002105	grey	EC_M1	0.14	5.1E-01	-0.05	8.1E-01	0.00	9.9E-01	0.43	3.0E-02	-0.17	4.1E-01	-0.13	5.3E-01	-0.08	7.1E-01	-0.18	3.8E-01	0.27	1.9E-01	0.11	5.9E-01
ENSCAFG0000000075	FNIP1	grey	EC_M1	0.14	5.1E-01	0.18	3.8E-01	0.12	5.6E-01	0.27	1.8E-01	-0.43	2.9E-02	-0.12	5.5E-01	0.22	2.7E-01	-0.29	1.6E-01	-0.17	4.1E-01	0.34	9.1E-02
ENSCAFG0000001298	ADAM12	grey	EC_M1	0.14	5.1E-01	-0.39	5.0E-02	-0.54	4.9E-03	-0.07	7.4E-01	0.67	1.7E-04	0.25	2.1E-01	0.08	7.1E-01	-0.03	8.9E-01	-0.32	1.1E-01	-0.79	1.8E-06
ENSCAFG0000000330	SLC13A4	grey	EC_M1	0.14	5.1E-01	-0.09	6.6E-01	-0.05	7.9E-01	-0.13	5.3E-01	0.03	9.0E-01	0.37	6.2E-02	-0.09	6.5E-01	-0.09	6.5E-01	-0.01	9.6E-01	-0.09	6.7E-01
ENSCAFG0000001346	HOXD3	grey	EC_M1	0.14	5.1E-01	0.58	1.8E-03	0.19	3.5E-01	-0.22	2.9E-01	-0.50	9.4E-03	-0.16	4.2E-01	-0.18	3.7E-01	-0.23	2.6E-01	-0.05	8.0E-01	0.49	1.2E-02
ENSCAFG0000000580	PTPRQ	grey	EC_M1	0.14	5.1E-01	0.14	4.8E-01	-0.02	9.1E-01	-0.20	3.2E-01	-0.07	7.5E-01	-0.13	5.4E-01	0.18	3.7E-01	0.40	4.1E-02	-0.04	8.6E-01	0.01	9.6E-01
ENSCAFG0000001141	MCUB	grey	EC_M1	0.14	5.1E-01	-0.51	7.8E-03	0.30	1.3E-01	0.02	9.1E-01	-0.23	2.5E-01	-0.31	1.2E-01	-0.18	3.7E-01	-0.09	6.7E-01	-0.39	4.7E-02	0.17	4.1E-01
ENSCAFG0000000903	PLCB2	turquoise	EC_M6	0.14	5.1E-01	0.88	2.2E-09	-0.18	3.7E-01	-0.09	6.6E-01	-0.22	2.9E-01	-0.09	6.7E-01	0.11	5.9E-01	-0.09	6.5E-01	0.21	3.1E-01	0.16	4.3E-01
ENSCAFG0000002918	ENSCAFG0000002918	turquoise	EC_M6	0.14	5.1E-01	0.58	2.0E-03	-0.06	7.6E-01	-0.07	7.2E-01	-0.25	2.3E-01	-0.05	8.1E-01	-0.10	6.3E-01	-0.11	5.9E-01	-0.16	4.3E-01	0.14	5.0E-01
ENSCAFG0000002272	MT-CO2	cyan	EC_M2	0.14	5.1E-01	-0.38	5.7E-02	0.46	1.9E-02	0.13	5.4E-01	-0.54	4.6E-03	0.03	8.8E-01	-0.42	3.2E-02	0.12	5.6E-01	-0.17	4.2E-01	0.44	2.3E-02
ENSCAFG0000002571	ENSCAFG0000002571	grey	EC_M1	0.14	5.1E-01	0.15	4.7E-01	0.24	2.5E-01	0.45	2.2E-02	-0.45	2.0E-02	-0.03	8.9E-01	-0.07	7.3E-01	-0.33	1.0E-01	0.00	9.8E-01	0.39	4.8E-02
ENSCAFG0000000941	TSPAN18	grey	EC_M1	0.14	5.1E-01	0.16	4.4E-01	-0.62	7.2E-04	-0.21	3.1E-01	0.59	1.7E-03	0.25	2.1E-01	0.06	7.8E-01	-0.08	6.9E-01	-0.41	3.7E-02	-0.71	4.2E-05
ENSCAFG0000002636	ENSCAFG0000002636	grey	EC_M1	0.14	5.1E-01	0.16	4.4E-01	0.01	9.8E-01	0.31	1.3E-01	-0.13	5.1E-01	-0.12	5.7E-01	-0.41	4.0E-02	-0.21	3.0E-01	0.08	6.9E-01	0.09	6.6E-01
ENSCAFG0000001497	ENSCAFG0000001497	grey	EC_M1	0.14	5.1E-01	-0.15	4.8E-01	-0.09	6.7E-01	-0.15	4.6E-01	0.02	9.4E-01	0.48	1.3E-02	0.36	7.3E-02	-0.12	5.7E-01	-0.17	4.1E-01	-0.10	6.3E-01
ENSCAFG0000000956	DNMBP	turquoise	EC_M6	0.14	5.1E-01	0.65	3.6E-04	-0.29	1.5E-01	0.04	8.4E-01	-0.06	7.8E-01	-0.10	6.1E-01	-0.09	6.5E-01	0.16	4.5E-01	-0.27	1.9E-01	-0.03	9.0E-01
ENSCAFG0000000263	PIGF	turquoise	EC_M6	0.14	5.1E-01	0.47	1.7E-02	-0.32	1.2E-01	0.05	8.1E-01	0.13	5.2E-01	-0.06	7.8E-01	0.03	9.0E-01	0.00	1.0E+00	-0.01	9.7E-01	-0.19	3.5E-01
ENSCAFG0000001783	ZNHIT3	grey	EC_M1	0.14	5.1E-01	0.21	3.1E-01	-0.03	8.7E-01	0.28	1.6E-01	-0.17	4.2E-01	0.20	3.3E-01	-0.31	1.3E-01	0.07	7.5E-01	0.11	6.1E-01	0.05	8.1E-01
ENSCAFG0000000359	ZMYM6	grey	EC_M1	0.14	5.1E-01	-0.20	3.2E-01	0.04	8.3E-01	0.03	8.7E-01	-0.17	4.1E-01	0.08	6.9E-01	-0.44	2.5E-02	-0.11	5.8E-01	-0.12	5.5E-01	0.02	9.1E-01
ENSCAFG0000000778	SMAGP	turquoise	EC_M6	0.14	5.1E-01	0.49	1.1E-02	0.12	5.7E-01	0.14	4.9E-01	-0.38	5.2E-02	-0.14	4.9E-01	0.01	9.7E-01	-0.52	6.3E-03	-0.36	7.5E-02	0.32	1.1E-01
ENSCAFG0000001768	COG7	grey	EC_M1	0.13	5.1E-01	-0.27	1.9E-01	0.21	3.0E-01	0.12	5.7E-01	-0.16	4.4E-01	0.17	4.2E-01	-0.23	2.5E-01	-0.20	3.2E-01	-0.10	6.4E-01	0.09	6.6E-01
ENSCAFG0000002470	OOEP	grey	EC_M1	0.13	5.1E-01	-0.27	1.9E-01	0.13	5.3E-01	0.27	1.7E-01	-0.19	3.6E-01	0.44	2.4E-02	-0.21	3.0E-01	0.09	6.7E-01	-0.21	3.1E-01	0.08	7.0E-01
ENSCAFG0000000622	PCDH1	turquoise	EC_M6	0.13	5.1E-01	0.87	7.7E-09	-0.33	1.0E-01	-0.08	7.0E-01	-0.02	9.3E-01	-0.01	9.6E-01	-0.18	3.8E-01	-0.16	4.2E-01	-0.01	9.5E-01	-0.08	7.0E-01
ENSCAFG0000000477	TRANK1	turquoise	EC_M6	0.13	5.1E-01	0.76	6.3E-06	0.04	8.5E-01	0.13	5.2E-01	-0.43	2.8E-02	-0.23	2.6E-01	-0.09	6.6E-01	-0.08	6.9E-01	-0.21	2.9E-01	0.34	9.2E-02
ENSCAFG0000001766	EPG5	grey	EC_M1	0.13	5.1E-01	-0.04	8.6E-01	0.11	5.9E-01	-0.02	9.3E-01	-0.23	2.5E-01	0.07	7.3E-01	-0.15	4.7E-01	-0.16	4.3E-01	-0.25	2.2E-01	0.20	3.3E-01
ENSCAFG0000000768	MFAP3L	turquoise	EC_M6	0.13	5.1E-01	0.51	8.1E-03	-0.10	6.1E-01	-0.13	5.3E-01	-0.13	5.3E-01	-0.23	2.5E-01	0.07	7.3E-01	-0.09	6.5E-01	0.53	5.0E-03	0.13	5.2E-01
ENSCAFG0000000828	ENC1	turquoise	EC_M6	0.13	5.1E-01	0.64	4.5E-04	-0.07	7.2E-01	0.08	7.1E-01	-0.31	1.2E-01	-0.22	2.9E-01	0.30	1.3E-01	-0.05	7.9E-01	-0.13	5.1E-01	0.23	2.6E-01
ENSCAFG0000000930	R3HCC1L	grey	EC_M1	0.13	5.1E-01	0.55	4.0E-03	-0.55	3.5E-03	-0.05	8.1E-01	0.32	1.1E-01	0.17	4.1E-01	-0.11	5.8E-01	0.03	8.8E-01	0.01	9.5E-01	-0.35	8.1E-02
ENSCAFG0000000183	DNPH1	cyan	EC_M2	0.13	5.1E-01	-0.25	2.2E-01	0.49	1.1E-02	-0.09	6.6E-01	-0.49	1.2E-02	0.00	9.9E-01	0.09	6.7E-01	-0.11	6.0E-01	-0.12	5.6E-01	0.39	4.9E-02
ENSCAFG0000001818	MMP28	turquoise	EC_M6	0.13	5.1E-01	0.70	5.9E-05	-0.50	9.8E-03	-0.09	6.5E-01	0.22	2.7E-01	-0.16	4.4E-01	-0.17	4.1E-01	0.14	5.1E-01	0.45	2.1E-02	-0.29	1.6E-01
ENSCAFG0000000791	ARSK	grey	EC_M1	0.13	5.1E-01	0.24	2.5E-01	0.25	2.2E-01	0.05	8.2E-01	-0.51	8.0E-03	-0.17	4.0E-01	-0.17	4.1E-01	0.30	1.4E-01	0.40	4.1E-02	0.46	1.8E-02
ENSCAFG0000001108	DKK2	grey	EC_M1	0.13	5.2E-01	0.16	4.5E-01	-0.28	1.6E-01	-0.14	5.0E-01	0.26	2.0E-01	-0.20	3.4E-01	0.07	7.5E-01	-0.22	2.9E-01	0.11	6.0E-01	-0.24	2.4E-01
ENSCAFG0000001971	POLR3K	grey	EC_M1	0.13	5.2E-01	0.06	7.6E-01	-0.43	2.7E-02	-0.43	2.7E-02	0.43	2.7E-02	0.49	1.1E-02	0.25	2.1E-01	0.00	9.8E-01	0.15	4.7E-01	-0.49	1.2E-02
ENSCAFG0000000459	ENSCAFG0000000459	grey	EC_M1	0.13	5.2E-01	-0.07	7.4E-01	0.09	6.6E-01	-0.03	8.7E-01	-0.10	6.4E-01	-0.04	8.5E-01	0.01	9.5E-01	0.01	9.5E-01	0.15	4.8E-01	0.04	8.3E-01
ENSCAFG0000002962	ACER2	grey	EC_M1	0.13	5.2E-01	0.33	1.0E-01	-0.57	2.4E-03	-0.18	3.7E-01	0.42	3.4E-02	0.25	2.2E-01	-0.10	6.1E-01	-0.01	9.4E-01	0.14	4.9E-01	-0.49	1.1E-02
ENSCAFG0000000790	LRBA	grey	EC_M1	0.13	5.2E-01	0.16	4.5E-01	0.34	8.5E-02	0.10	6.3E-01	-0.54	4.5E-03	-0.17	4.1E-01	-0.03	8.7E-01	-0.22	2.8E-01	-0.08	7.0E-01	0.49	1.1E-02
ENSCAFG0000001324	LAMC2	turquoise	EC_M6	0.13	5.2E-01	0.64	4.1E-04	0.13	5.1E-01	-0.07	7.3E-01	-0.50	9.0E-03	-0.23	2.5E-01	0.18	3.7E-01	0.01	9.8E-01	-0.31	1.2E-01	0.45	2.2E-02
ENSCAFG0000000787	ENSCAFG0000000787	grey	EC_M1	0.13	5.2E-01	-0.27	1.9E-01	0.02	9.2E-01	-0.12	5.7E-01	-0.03	8.9E-01	-0.12	5.7E-01	-0.16	4.2E-01	-0.09	6.7E-01	-0.22	2.8E-01	-0.04	8.4E-01
ENSCAFG0000001221	NOP9	grey	EC_M1	0.13	5.2E-01	-0.07	7.2E-01	0.44	2.5E-02	0.20	3.2E-01	-0.53	5.6E-03	0.02	9.1E-01	0.32	1.1E-01	0.00	9.8E-01	0.08	7.1E-01	0.51	8.2E-03
ENSCAFG0000000270	TMEM30A	darkgreen	EC_M4	0.13	5.2E-01	0.20	3.3E-01	-0.80	8.4E-07	-0.10	6.4E-01	0.74	1.5E-05	0.16	4.4E-01	0.13	5.2E-01	-0.11	6.0E-01	-0.04	8.3E-01	-0.77	3.5E-06
ENSCAFG0000001318	ENSCAFG0000001318	grey	EC_M1	0.13	5.2E-01	-0.07	7.4E-01	-0.24	2.5E-01	0.24	2.3E-01	0.27	1.7E-01	0.40	4.4E-02	-0.16	4.3E-01	-0.21	3.0E-01	-0.14	4.8E-01	-0.32	1.1E-01
ENSCAFG0000002367	ENSCAFG0000002367	grey	EC_M1	0.13	5.2E-01	0.13	5.3E-01	0.30	1.4E-01	0.06	7.8E-01	-0.53	5.3E-03	-0.10	6.2E-01	-0.25	2.2E-01	0.12	5.6E-01	-0.25	2.2E-01	0.49	1.0E-02
ENSCAFG0000002064	ENSCAFG0000002064	grey	EC_M1	0.13	5.2E-01	-0.15	4.8E-01	0.04	8.6E-01	-0.16	4.3E-01	-0.13	5.3E-01	0.45	2.0E-02	-0.11	5.9E-01	-0.11	5.8E-01	-0.21	3.1E-01	-0.01	9.5E-01
ENSCAFG0000003078	ENSCAFG0000003078	grey	EC_M1	0.13	5.2E-01	-0.12	5.5E-01	0.09	6.8E-01	-0.10	6.1E-01	-0.11	6.1E-01	0.33	1.0E-01	-0.10	6.4E-01	-0.04	8.4E-01	-0.13	5.4E-01	-0.02	9.2E-01
ENSCAFG0000002988	SRP14	darkgreen	EC_M4	0.13	5.2E-01	-0.17	4.2E-01	-0.68	1.5E-04	-0.26	2.0E-01	0.77	5.1E-06	0.32	1.1E-01	-0.17	4.2E-01	0.27	1.7E-01	-0.03	8.7E-01	-0.85	5.3E-08
ENSCAFG0000002300	ENSCAFG0000002300	turquoise	EC_M6	0.13	5.2E-01	0.90	5.8E-10	-0.13	5.3E-01	-0.07	7.5E-01	-0.26	2.0E-01	-0.18	3.7E-01	-0.12	5.7E-01	-0.17	4.0E-01	0.12	5.7E-01	0.18	3.8E-01
ENSCAFG0000001604	PLEKHG3	grey	EC_M1	0.13	5.2E-01	0.13	5.2E-01	0.09	6.7E-01	-0.01	9.7E-01	-0.24	2.5E-01	-0.20	3.2E-01	0.04	8.3E-01	-0.30	1.4E-01	0.30	1.4E-01	0.23	2.6E-01
ENSCAFG0000003059	ENSCAFG0000003059	grey	EC_M1	0.13	5.2E-01	-0.19	3.5E-01	-0.06	7.5E-01	0.39	4.9E-02	0.01	9.8E-01	-0.15	4.5E-01	0.27	1.8E-01	0.32	1.1E-01	-0.03	8.9E-01	-0.10	6.4E-01
ENSCAFG0000000874	CIAPIN1	grey	EC_M1	0.13	5.2E-01	-0.40	4.5E-02	-0.30	1.4E-01	0.15	4.5E-01	0.43	2.7E-02	0.21	3.0E-01	0.33	1.1E-01	-0.22	2.7E-01	-0.17	4.2E-01	-0.47	1.6E-02
ENSCAFG0000000299	ZNF483	grey	EC_M1	0.13	5.2E-01	-0.10	6.4E-01	0.24	2.5E-01	0.26	2.0E-01	-0.38	5.4E-02	-0.23	2.6E-01	0.06	7.9E-01	-0.05	8.1E-01	0.18	3.7E-01	0.32	1.1E-01
ENSCAFG0000000740	CEBPG	grey	EC_M1	0.13	5.2E-01	0.07	7.2E-01	-0.69	8.5E-05	0.09	6.5E-01	0.68	1.5E-04	0.43	2.8E-02	-0.17	4.1E-01	-0.03	8.7E-01	0.00	9.8E-01	-0.77	3.7E-06
ENSCAFG0000001147	PITPNC1	grey	EC_M1	0.13	5.2E-01	0.05	8.0E-01	-0.38	5.4E-02	-0.09	6.6E-01	0.33	9.8E-02	0.23	2.6E-01	-0.36	7.4E-02	-0.07	7.5E-01	0.38	5.8E-02	-0.34	8.6E-02
ENSCAFG0000000723	EEF1AKMT1	grey	EC_M1	0.13	5.2E-01	-0.37	6.5E-02	0.25	2.1E-01	0.29	1.4E-01	-0.23	2.7E-01	0.07	7.3E-01	-0.42	3.4E-02	-0.08	6.9E-01	-0.30	1.4E-01	0.16	4.5E-01
ENSCAFG0000002957	KANK3	turquoise	EC_M6	0.13	5.2E-01	0.85	4.4E-08	-0.01	9.6E-01	-0.07	7.3E-01	-0.38	5.4E-02	-0.20	3.2E-01	-0.02	9.3E-01	-0.09	6.6E-01	-0.19	3.6E-01	0.28	1.6E-01
ENSCAFG0000001404	CDCP1	darkgreen	EC_M4	0.13	5.2E-01	-0.68	1.4E-04	-0.29	1.4E-01	0.07	7.4E-01	0.46	1.9E-02	0.34	8.5E-02	-0.03	8.8E-01	-0.06	7.6E-01	-0.29	1.5E-01	-0.59	1.6E-03
ENSCAFG0000000064	ATP6V1C1	darkgreen	EC_M4	0.13	5.2E-01	-0.01	9.5E-01	-0.73	2.0E-05	0.02	9.2E-01	0.71	4.8E-05	0.37	6.7E-02	0.14	4.8E-01	-0.07	7.2E-01	-0.30	1.4E-01	-0.79	1.5E-06
ENSCAFG0000002876	KLRG1	grey	EC_M1	0.13	5.2E-01	-0.16	4.5E-01	-0.16	4.4E-01	0.36	7.4E-02	0.07	7.4E-01	0.35	8.2E-02	-0.15	4.6E-01	-0.12	5.6E-01	0.25	2.1E-01	-0.12	5.6E-01
ENSCAFG0000001479	ENSCAFG0000001479	grey	EC_M1	0.13	5.2E-01	0.18	3.7E-01	-0.24	2.4E-01	0.09	6.5E-01	0.07	7.4E-01	0.12	5.7E-01	0.24	2.4E-01	-0.12	5.5E-01	-0.05	8.2E-01	-0.16	4.3E-01
ENSCAFG0000001793	ENSCAFG0000001793	grey	EC_M1	0.13	5.2E-01	0.41	3.8E-02	0.19	3.5E-01	0.04	8.5E-01	-0.51	7.2E-03	-0.56	2.9E-03	0.10	6.2E-01	0.04	8.3E-01	0.08	7.0E-01	0.45	2.2E-02
ENSCAFG0000000791	ENSCAFG0000000791	grey	EC_M1	0.13	5.2E-01	-0.05	8.1E-01	-0.58	2.1E-03	0.26	2.0E-01	0.57	2.3E-03	0.20	3.2E-01	-0.18	3.7E-01	-0.03	8.9E-01	-0.15	4.7E-01	-0.65	3.7E-04
ENSCAFG0000000114	PKD2L2	grey	EC_M1	0.13	5.2E-01	-0.02	9.3E-01	0.09	6.5E-01	-0.11	5.9E-01	-0.16	4.5E-01	-0.01	9.5E-01	-0.01	9.6E-01	0.04	8.3E-01	0.09	6.8E-01	0.07	7.3E-01
ENSCAFG0000002868	PLEKHA3	darkgreen	EC_M4	0.13	5.2E-01	0.42	3.2E-02	-0.85	5.3E-08	-0.04	8.4E-01	0.72	3.0E-05	0.15	4.7E-01	0.06	7.7E-01	0.01	9.4E-01	0.15	4.7E-01	-0.76	8.0E-06
ENSCAFG0000001246	UCN2	grey	EC_M1	0.13	5.2E-01	0.13	5.4E-01	-0.26	1.9E-01	0.02	9.4E-01	0.16	4.2E-01	-0.09	6.7E-01	-0.10	6.2E-01	-0.11	6.0E-01	0.38	5.9E-02	-0.23	2.6E-01
ENSCAFG0000002967	ENSCAFG0000002967	grey	EC_M1	0.13	5.2E-01	0.20	3.3E-01	-0.29	1.5E-01	-0.08	7.0E-01	0.20	3.2E-01	-0.14	5.0E-01	-0.45	2.2E-02	0.10	6.1E-01	0.05	7.9E-01	-0.23	2.7E-01
ENSCAFG0000001697	ENSCAFG0000001697	grey	EC_M1	0.13	5.2E-01	-0.04	8.4E-01	-0.09	6.7E-01	0.34	8.9E-02	-0.01	9.7E-01	0.20	3.3E-01	0.21	3.0E-01	0.05	8.1E-01	-0.25	2.2E-01	-0.11	5.8E-01
ENSCAFG0000003237	EVA1A	turquoise	EC_M6	0.13	5.2E-01	0.51	7.5E-03	-0.49	1.1E-02	-0.18	3.8E-01	0.30	1.3E-01	0.00	9.9E-01	0.08	6.8E-01	0.02	9.2E-01	0.54	4.8E-03	-0.32	1.1E-01
ENSCAFG0000002975	ENSCAFG0000002975	turquoise	EC_M6	0.13	5.2E-01	0.30	1.4E-01	0.02	9.1E-01	0.43	3.0E-02	-0.30	1.4E-01	-0.16	4.4E-01	0.14	5.1E-01	-0.16	4.3E-01	-0.22	2.7E-01	0.24	2.3E-01

ENSCAFG0000002579	ENSCAFG0000002579	grey	EC_M10	0.13	5.2E-01	0.19	3.4E-01	0.22	2.8E-01	-0.37	6.3E-02	-0.43	2.7E-02	0.18	3.9E-01	0.04	8.4E-01	-0.38	5.7E-02	-0.02	9.2E-01	0.39	5.2E-02
ENSCAFG0000001884	SPIRE1	turquoise	EC_M6	0.13	5.2E-01	0.83	1.2E-07	-0.53	5.7E-03	-0.16	4.4E-01	0.20	3.3E-01	-0.05	8.1E-01	0.07	7.5E-01	-0.10	6.4E-01	-0.06	7.5E-01	-0.25	2.1E-01
ENSCAFG0000000648	ENSCAFG0000000648	grey	EC_M10	0.13	5.2E-01	-0.11	5.8E-01	-0.04	8.6E-01	-0.03	8.7E-01	-0.07	7.5E-01	-0.11	5.9E-01	-0.12	5.6E-01	-0.07	7.4E-01	-0.09	6.5E-01	-0.03	8.7E-01
ENSCAFG0000003089	ZNF502	grey	EC_M10	0.13	5.2E-01	0.27	1.9E-01	0.21	3.1E-01	0.36	6.7E-02	-0.51	7.8E-03	-0.33	1.0E-01	-0.38	5.9E-02	-0.06	7.5E-01	0.19	3.6E-01	0.44	2.6E-02
ENSCAFG0000001608	MMRN2	turquoise	EC_M6	0.13	5.2E-01	0.87	5.9E-09	-0.19	3.5E-01	-0.05	8.2E-01	-0.18	3.7E-01	-0.13	5.1E-01	-0.18	3.9E-01	-0.14	4.9E-01	0.12	5.6E-01	0.08	6.8E-01
ENSCAFG0000000811	ARRDC3	cyan	EC_M2	0.13	5.2E-01	0.03	9.0E-01	0.41	4.0E-02	0.22	2.8E-01	-0.64	3.7E-04	0.02	9.3E-01	-0.33	1.0E-01	0.11	6.1E-01	-0.06	7.9E-01	0.57	2.4E-03
ENSCAFG0000001808	AKAP10	grey	EC_M10	0.13	5.2E-01	0.20	3.3E-01	-0.26	2.0E-01	0.24	2.4E-01	-0.02	9.2E-01	0.06	7.7E-01	-0.03	8.8E-01	0.11	6.0E-01	-0.07	7.3E-01	-0.08	7.0E-01
ENSCAFG0000002634	ENSCAFG0000002634	grey	EC_M10	0.13	5.2E-01	0.04	8.4E-01	-0.48	1.4E-02	-0.31	1.2E-01	0.37	6.5E-02	0.05	8.0E-01	-0.08	7.1E-01	0.14	4.8E-01	0.20	3.2E-01	-0.48	1.3E-02
ENSCAFG0000001059	HDAC1	turquoise	EC_M6	0.13	5.2E-01	0.50	8.5E-03	-0.08	6.8E-01	0.16	4.5E-01	-0.19	3.4E-01	-0.08	6.9E-01	0.01	9.5E-01	0.25	2.2E-01	0.45	2.3E-02	0.13	5.1E-01
ENSCAFG0000000430	TRIM13	cyan	EC_M2	0.13	5.2E-01	0.35	8.3E-02	0.50	9.8E-03	0.37	6.2E-02	-0.81	6.6E-07	-0.30	1.4E-01	-0.27	1.8E-01	-0.25	2.2E-01	0.06	7.9E-01	0.73	2.1E-05
ENSCAFG0000002580	ENSCAFG0000002580	grey	EC_M10	0.13	5.2E-01	0.08	6.9E-01	0.05	8.0E-01	0.16	4.3E-01	-0.20	3.2E-01	-0.36	7.1E-02	0.22	2.8E-01	-0.06	7.6E-01	0.16	4.4E-01	0.14	4.8E-01
ENSCAFG0000003262	ENSCAFG0000003262	grey	EC_M10	0.13	5.3E-01	-0.40	4.4E-02	-0.03	8.7E-01	0.07	7.5E-01	0.10	6.2E-01	0.16	4.5E-01	-0.03	8.7E-01	-0.37	5.9E-02	-0.45	2.0E-02	-0.17	4.1E-01
ENSCAFG0000002760	ENSCAFG0000002760	grey	EC_M10	0.13	5.3E-01	0.09	6.6E-01	-0.06	7.6E-01	-0.26	2.0E-01	-0.04	8.5E-01	0.16	4.5E-01	-0.22	2.7E-01	0.20	3.3E-01	0.12	5.7E-01	0.00	9.8E-01
ENSCAFG0000001704	SLC25A35	turquoise	EC_M6	0.13	5.3E-01	0.46	1.9E-02	0.11	6.1E-01	-0.34	8.7E-02	-0.38	5.9E-02	-0.12	5.5E-01	-0.33	1.0E-01	-0.28	1.7E-01	0.18	3.9E-01	0.30	1.3E-01
ENSCAFG0000001679	PGD	grey	EC_M10	0.13	5.3E-01	-0.12	5.7E-01	0.47	1.5E-02	0.11	6.0E-01	-0.58	1.7E-03	-0.19	3.4E-01	-0.29	1.6E-01	0.04	8.4E-01	-0.65	3.0E-04	0.49	1.1E-02
ENSCAFG0000000375	ZFYVE9	grey	EC_M10	0.13	5.3E-01	0.04	8.5E-01	-0.42	3.1E-02	-0.11	6.1E-01	0.38	5.6E-02	-0.23	2.6E-01	-0.18	3.9E-01	0.24	2.4E-01	0.04	8.6E-01	-0.37	6.4E-02
ENSCAFG0000000514	GZF1	turquoise	EC_M6	0.13	5.3E-01	0.44	2.3E-02	0.22	2.9E-01	0.02	9.2E-01	-0.51	8.2E-03	0.05	8.1E-01	-0.03	9.0E-01	-0.14	4.9E-01	0.21	3.0E-01	0.45	2.2E-02
ENSCAFG0000002022	NEK6	grey	EC_M10	0.13	5.3E-01	0.45	2.0E-02	0.25	2.3E-01	0.10	6.2E-01	-0.61	8.9E-04	-0.12	5.7E-01	0.10	6.2E-01	-0.04	8.3E-01	-0.07	7.2E-01	0.49	1.0E-02
ENSCAFG0000001332	ENSCAFG0000001332	grey	EC_M10	0.13	5.3E-01	-0.03	8.9E-01	-0.21	3.0E-01	0.05	8.1E-01	0.12	5.6E-01	-0.05	8.1E-01	-0.06	7.8E-01	-0.06	7.8E-01	-0.14	5.1E-01	-0.24	2.3E-01
ENSCAFG0000001495	PRKAG3	magenta	EC_M13	0.13	5.3E-01	-0.05	8.2E-01	-0.01	9.7E-01	-0.13	5.2E-01	-0.10	6.1E-01	-0.10	6.4E-01	-0.03	9.0E-01	-0.16	4.3E-01	0.67	2.0E-04	0.09	6.5E-01
ENSCAFG0000000189	WASHC4	grey	EC_M10	0.13	5.3E-01	-0.25	2.2E-01	-0.28	1.7E-01	0.11	5.9E-01	0.17	4.0E-01	0.08	6.8E-01	-0.01	9.7E-01	0.15	4.6E-01	-0.12	5.4E-01	-0.30	1.4E-01
ENSCAFG0000000265	ABCB5	grey	EC_M10	0.13	5.3E-01	-0.04	8.4E-01	0.01	9.7E-01	-0.09	6.8E-01	-0.02	9.1E-01	-0.01	9.5E-01	0.05	8.0E-01	-0.04	8.6E-01	0.14	4.9E-01	-0.03	8.8E-01
ENSCAFG0000001515	TNIK	darkgreen	EC_M4	0.13	5.3E-01	-0.03	8.9E-01	-0.76	8.1E-06	-0.10	6.4E-01	0.77	3.5E-06	0.30	1.4E-01	0.12	5.7E-01	0.05	8.0E-01	0.05	8.1E-01	-0.87	9.2E-09
ENSCAFG0000000831	ELP3	grey	EC_M10	0.13	5.3E-01	-0.38	5.9E-02	0.16	4.3E-01	0.17	4.2E-01	-0.12	5.5E-01	0.00	9.8E-01	-0.16	4.5E-01	0.22	2.8E-01	-0.21	3.1E-01	0.05	8.1E-01
ENSCAFG0000001468	CFAP70	grey	EC_M10	0.13	5.3E-01	-0.16	4.3E-01	0.32	1.1E-01	0.46	1.9E-02	-0.43	2.9E-02	0.09	6.5E-01	-0.20	3.2E-01	-0.04	8.4E-01	-0.30	1.3E-01	0.35	8.0E-02
ENSCAFG0000001825	GREB1L	darkmagenta	EC_M12	0.13	5.3E-01	-0.12	5.5E-01	-0.07	7.3E-01	-0.16	4.4E-01	0.02	9.1E-01	0.30	1.4E-01	-0.08	7.1E-01	0.53	5.5E-03	0.10	6.2E-01	-0.11	6.0E-01
ENSCAFG0000001726	ATP7A	grey	EC_M10	0.13	5.3E-01	-0.30	1.4E-01	-0.29	1.5E-01	0.17	4.0E-01	0.26	2.0E-01	0.04	8.4E-01	-0.20	3.4E-01	-0.02	9.0E-01	-0.31	1.2E-01	-0.33	1.0E-01
ENSCAFG0000000245	ENSCAFG0000000245	grey	EC_M10	0.13	5.3E-01	-0.16	4.2E-01	0.02	9.2E-01	-0.15	4.7E-01	-0.10	6.3E-01	0.31	1.3E-01	-0.16	4.3E-01	-0.17	4.2E-01	-0.17	4.2E-01	0.00	1.0E+00
ENSCAFG0000002270	ENSCAFG0000002270	grey	EC_M10	0.13	5.3E-01	-0.09	6.8E-01	0.41	3.8E-02	0.00	9.9E-01	-0.54	4.2E-03	-0.04	8.6E-01	-0.12	5.6E-01	0.19	3.5E-01	0.15	4.6E-01	0.46	1.7E-02
ENSCAFG0000003167	ENSCAFG0000003167	grey	EC_M10	0.13	5.3E-01	-0.73	2.5E-05	0.31	1.3E-01	0.07	7.2E-01	-0.18	3.7E-01	0.16	4.3E-01	-0.02	9.3E-01	-0.01	9.7E-01	-0.24	2.4E-01	0.10	6.4E-01
ENSCAFG0000002360	ENSCAFG0000002360	turquoise	EC_M6	0.13	5.3E-01	0.87	5.5E-09	-0.21	2.9E-01	-0.13	5.3E-01	-0.16	4.5E-01	-0.08	6.9E-01	-0.16	4.4E-01	-0.12	5.4E-01	0.10	6.2E-01	0.06	7.6E-01
ENSCAFG0000001702	DAPK2	turquoise	EC_M6	0.13	5.3E-01	0.47	1.4E-02	-0.39	5.1E-02	-0.05	8.2E-01	0.13	5.3E-01	-0.14	5.1E-01	-0.10	6.2E-01	-0.06	7.8E-01	0.18	3.9E-01	-0.22	2.9E-01
ENSCAFG0000003142	RAP2A	grey	EC_M10	0.13	5.3E-01	-0.06	7.7E-01	-0.65	2.9E-04	-0.09	6.5E-01	0.66	2.7E-04	0.29	1.5E-01	-0.30	1.4E-01	0.13	5.4E-01	0.17	4.1E-01	-0.75	9.5E-06
ENSCAFG0000000960	PPM1K	grey	EC_M10	0.13	5.3E-01	0.60	1.2E-03	0.34	9.1E-02	0.15	4.6E-01	-0.70	6.9E-05	-0.31	1.2E-01	-0.20	3.3E-01	-0.08	6.8E-01	-0.01	9.6E-01	0.67	1.8E-04
ENSCAFG0000001047	FAM118B	grey	EC_M10	0.13	5.3E-01	-0.27	1.9E-01	-0.58	2.0E-03	-0.23	2.7E-01	0.67	1.7E-04	0.47	1.5E-02	-0.11	6.0E-01	0.22	2.8E-01	0.04	8.3E-01	-0.75	1.2E-05
ENSCAFG0000002988	RPS20	cyan	EC_M2	0.13	5.3E-01	-0.48	1.3E-02	0.37	6.0E-02	0.19	3.4E-01	-0.36	7.0E-02	0.06	7.6E-01	0.05	8.1E-01	-0.09	6.7E-01	-0.45	2.2E-02	0.21	3.0E-01
ENSCAFG0000000714	ELOVL7	turquoise	EC_M6	0.13	5.3E-01	0.73	2.5E-05	-0.39	4.8E-02	-0.22	2.9E-01	0.04	8.6E-01	-0.26	2.0E-01	0.14	4.8E-01	0.14	4.9E-01	0.03	8.7E-01	-0.11	5.9E-01
ENSCAFG0000000776	ZDHHC5	darkgreen	EC_M4	0.13	5.3E-01	0.60	1.3E-03	-0.74	1.3E-05	-0.25	2.1E-01	0.57	2.6E-03	0.11	6.0E-01	-0.10	6.3E-01	0.04	8.6E-01	-0.07	7.2E-01	-0.65	3.5E-04
ENSCAFG0000000619	ENSCAFG0000000619	grey	EC_M10	0.13	5.3E-01	-0.65	3.4E-04	0.46	1.9E-02	0.13	5.2E-01	-0.38	5.9E-02	-0.08	6.9E-01	-0.19	3.5E-01	0.09	6.7E-01	-0.01	9.8E-01	0.30	1.4E-01
ENSCAFG0000001334	ELL3	turquoise	EC_M6	0.13	5.3E-01	0.28	1.7E-01	-0.07	7.3E-01	-0.15	4.5E-01	-0.13	5.1E-01	0.11	6.1E-01	-0.52	6.2E-03	-0.01	9.6E-01	-0.07	7.2E-01	-0.01	9.5E-01
ENSCAFG0000002856	ENSCAFG0000002856	grey	EC_M10	0.13	5.3E-01	0.05	8.2E-01	-0.16	4.5E-01	-0.20	3.3E-01	0.06	7.6E-01	-0.17	4.2E-01	-0.18	3.8E-01	0.31	1.2E-01	-0.09	6.7E-01	-0.12	5.5E-01
ENSCAFG0000003036	ENSCAFG0000003036	cyan	EC_M2	0.13	5.3E-01	0.06	7.7E-01	0.54	4.8E-03	0.14	5.1E-01	-0.79	1.4E-06	-0.17	3.9E-01	0.01	9.6E-01	0.10	6.3E-01	-0.09	6.6E-01	0.73	2.6E-05
ENSCAFG0000000207	KDR	turquoise	EC_M6	0.13	5.3E-01	0.86	2.6E-08	-0.34	9.2E-02	-0.14	4.8E-01	-0.02	9.3E-01	-0.15	4.7E-01	-0.13	5.3E-01	0.04	8.5E-01	0.03	8.8E-01	-0.09	6.7E-01
ENSCAFG0000001070	IQCC	grey	EC_M10	0.13	5.3E-01	0.19	3.6E-01	0.29	1.6E-01	0.23	2.7E-01	-0.50	8.8E-03	-0.13	5.3E-01	0.05	8.2E-01	-0.11	6.1E-01	0.04	8.6E-01	0.45	2.3E-02
ENSCAFG0000000863	SOGA1	turquoise	EC_M6	0.13	5.3E-01	0.53	5.0E-03	-0.27	1.7E-01	-0.01	9.6E-01	0.00	9.9E-01	-0.11	5.9E-01	0.14	5.0E-01	-0.26	1.9E-01	0.46	1.7E-02	-0.03	8.7E-01
ENSCAFG0000002685	ENSCAFG0000002685	grey	EC_M10	0.13	5.3E-01	-0.06	7.8E-01	0.14	5.0E-01	-0.06	7.9E-01	-0.13	5.2E-01	-0.06	7.6E-01	-0.01	9.6E-01	-0.04	8.4E-01	-0.14	5.0E-01	0.07	7.4E-01
ENSCAFG0000001711	DCST2	violet	EC_M7	0.13	5.3E-01	-0.16	4.4E-01	0.01	9.6E-01	-0.14	4.8E-01	-0.07	7.2E-01	0.65	3.2E-04	-0.13	5.4E-01	-0.01	9.6E-01	-0.13	5.4E-01	-0.01	9.4E-01
ENSCAFG0000001117	DIS3L2	cyan	EC_M2	0.13	5.3E-01	0.23	2.6E-01	0.52	6.3E-03	0.06	7.5E-01	-0.80	7.9E-07	-0.12	5.4E-01	-0.10	6.3E-01	-0.22	2.9E-01	-0.13	5.3E-01	0.70	7.3E-05
ENSCAFG0000003019	DRAM2	turquoise	EC_M6	0.13	5.3E-01	0.66	2.3E-04	0.05	8.0E-01	0.27	1.8E-01	-0.41	4.0E-02	-0.11	6.0E-01	0.08	7.1E-01	0.01	9.7E-01	0.06	7.8E-01	0.35	8.3E-02
ENSCAFG0000003021	PEX11A	cyan	EC_M2	0.13	5.3E-01	-0.36	7.3E-02	0.37	6.1E-02	0.19	3.6E-01	-0.45	2.2E-02	-0.06	7.8E-01	0.00	9.8E-01	-0.05	8.2E-01	-0.19	3.5E-01	0.37	6.7E-02
ENSCAFG0000001882	ENSCAFG0000001882	grey	EC_M10	0.13	5.3E-01	-0.08	7.2E-01	-0.39	5.1E-02	0.22	2.8E-01	0.34	9.1E-02	0.04	8.4E-01	0.15	4.8E-01	0.11	5.8E-01	-0.22	2.8E-01	-0.42	3.1E-02
ENSCAFG0000002981	ZNF175	grey	EC_M10	0.13	5.3E-01	-0.48	1.2E-02	0.13	5.2E-01	0.08	7.1E-01	-0.19	3.4E-01	0.14	4.9E-01	0.00	9.9E-01	0.25	2.2E-01	0.19	3.4E-01	0.07	7.4E-01
ENSCAFG0000003088	ENSCAFG0000003088	grey	EC_M10	0.13	5.3E-01	0.15	4.6E-01	-0.22	2.8E-01	-0.09	6.8E-01	0.08	6.9E-01	-0.25	2.2E-01	0.07	7.4E-01	-0.06	7.8E-01	-0.34	8.8E-02	-0.13	5.2E-01
ENSCAFG0000003065	ENSCAFG0000003065	grey	EC_M10	0.13	5.3E-01	0.01	9.6E-01	0.15	4.6E-01	-0.09	6.6E-01	-0.26	1.9E-01	-0.08	7.0E-01	-0.04	8.4E-01	-0.15	4.7E-01	0.45	2.0E-02	0.22	2.8E-01
ENSCAFG0000001738	APOOL	grey	EC_M10	0.13	5.3E-01	-0.10	6.2E-01	-0.34	8.7E-02	0.19	3.6E-01	0.37	6.2E-02	-0.31	1.2E-01	-0.13	5.3E-01	-0.15	4.8E-01	-0.13	5.3E-01	-0.40	4.5E-02
ENSCAFG0000000988	GDAP2	grey	EC_M10	0.13	5.3E-01	-0.12	5.5E-01	-0.49	1.1E-02	-0.18	3.8E-01	0.54	4.6E-03	0.33	9.5E-02	-0.36	7.1E-02	0.15	4.5E-01	-0.05	8.2E-01	-0.59	1.6E-03
ENSCAFG0000000427	LRRC17	grey	EC_M10	0.13	5.3E-01	-0.07	7.3E-01	0.03	8.7E-01	-0.01	9.7E-01	-0.20	3.4E-01	0.02	9.2E-01	-0.10	6.4E-01	-0.04	8.4E-01	0.52	6.8E-03	0.14	5.0E-01
ENSCAFG0000001192	KPNA1	darkgreen	EC_M4	0.13	5.3E-01	0.25	2.2E-01	-0.86	1.7E-08	-0.23	2.6E-01	0.81	5.9E-07	0.19	3.6E-01	-0.01	9.7E-01	0.02	9.2E-01	0.15	4.6E-01	-0.85	3.8E-08
ENSCAFG0000000467	AKAP11	grey	EC_M10	0.13	5.3E-01	0.34	9.1E-02	-0.47	1.5E-02	0.01	9.7E-01	0.25	2.2E-01	-0.02	9.4E-01	0.00	9.8E-01	0.13	5.1E-01	0.27	1.9E-01	-0.29	1.5E-01
ENSCAFG0000002581	ENSCAFG0000002581	grey	EC_M10	0.13	5.3E-01	0.20	3.3E-01	0.15	4.6E-01	0.11	6.0E-01	-0.31	1.2E-01	-0.29	1.5E-01	-0.30	1.4E-01	0.01	9.5E-01	-0.28	1.6E-01	0.28	1.7E-01
ENSCAFG0000000123	KDM3B	cyan	EC_M2	0.13	5.3E-01	0.27	1.8E-01	0.45	2.0E-02	0.09	6.5E-01	-0.78	2.1E-06	-0.25	2.1E-01	-0.04	8.3E-01	-0.02	9.3E-01	-0.15	4.7E-01	0.73	2.3E-05
ENSCAFG0000000130	SLC35B4	grey	EC_M10	0.13	5.3E-01	-0.28	1.7E-01	-0.43	2.6E-02	-0.08	6.9E-01	0.58	1.8E-03	0.36	7.2E-02	0.04	8.3E-01	-0.04	8.5E-01	-0.06	7.7E-01	-0.64	4.6E-04
ENSCAFG0000000942	PTPN13	grey	EC_M10	0.13	5.4E-01	-0.70	6.3E-05	0.36	6.7E-02	0.16	4.3E-01	-0.31	1.2E-01	0.01	9.7E-01	-0.17	3.9E-01	0.14	4.9E-01	-0.04	8.6E-01	0.20	3.3E-01
ENSCAFG0000002344	ENSCAFG0000002344	grey	EC_M10	0.13	5.4E-01	-0.77	4.1E-06	0.37	6.5E-02	0.01	9.8E-01	-0.22	2.7E-01	-0.02	9.1E-01	-0.14	4.9E-01	0.12	5.6E-01	-0.05	8.0E-01	0.13	5.1E-01
ENSCAFG0000001201	SH3BP4	turquoise	EC_M6	0.13	5.4E-01	0.42	3.5E-02	-0.20	3.2E-01	-0.02	9.3E-01	-0.04	8.4E-01	0.15	4.8E-01	-0.27	1.8E-01	-0.51	8.2E-03	0.32	1.1E-01	-0.06	7.6E-01
ENSCAFG0000001783	TYK2	grey	EC_M10	0.13	5.4E-01	0.46	1.9E-02	0.36	7.0E-02	0.03	8.9E-01	-0.74	1.5E-05	-0.09	6.4E-01	0.16	4.4E-01	0.06	7.5E-01	0.19	3.4E-01	0.65	3.4E-04
ENSCAFG0000001150	ZNF202	grey	EC_M10	0.13	5.4E-01	-0.29	1.5E-01	0.43	3.0E-02	0.08	7.1E-01	-0.52	6.2E-03	-0.13	5.2E-01	-0.09	6.6E-01	0.47	1.5E-02	0.18	3.9E-01	0.43	2.7E-02
ENSCAFG0000000158	NFYA	turquoise	EC_M6	0.13	5.4E-01	0.46	1.8E-02	-0.36	6.7E-02	-0.40	4.0E-02	0.16	4.4E-01	-0.01	9.5E-01	0.09	6.5E-01	0.18	3.9E-01	-0.25	2.1E-01	-0.28	1.6E-01
ENSCAFG0000001121	ENSCAFG0000001121	grey	EC_M10	0.13	5.4E-01	-0.08	7.0E-01	-0.60	1.3E-03	-0.15	4.6E-01	0.66	2.1E-04	0.10	6.1E-01	-0.21	3.1E-01	0.00	9.8E-01	-0.11	6.0E-01	-0.73	2.5E-05
ENSCAFG0000001355	ENSCAFG0000001355	grey	EC_M10	0.13	5.4E-01	-0.18	3.9E-01	0.39	4.9E-02	0.46	1.9E-02	-0.49	1.1E-02	-0.22	2.7E-01	0.08	7.0E-01	-0.17	4.0E-01	-0.26	2.0E-01	0.45	2.1E-02
ENSCAFG0000002430	AGO4	cyan	EC_M2	0.13	5.4E-01	0.44	2.6E-02	0.48	1.2E-02	0.17	4.2E-01	-0.87	1.0E-08	-0.29	1.4E-01	-0.02	9.3E-01	0.00	9.9E-01	-0.03	8.8E-01	0.80	7.1E-07
ENSCAFG0000001785	LHX1	grey	EC_M10	0.13	5.4E-01	-0.07	7.2E-01	-0.40	4.3E-02	-0.02	9.1E-01	0.29	1.5E-01	0.32	1.1E-01	-0.03	8.8E-01	-0.15	4.5E-01	0.35	7.5E-02	-0.41	3.9E-02
ENSCAFG0000001839	CDCA4	grey	EC_M10	0.13	5.4E-01	0.01	9.5E-01	0.30	1.4E-01	0.00	1.0E+00	-0.38	5.4E-02	-0.34	8.9E-02	0.22	2.7E-01	0.15	4.6E-01	0.07	7.4E-01	0.32	1.1E-01
ENSCAFG0000001228	STRADB	grey	EC_M10	0.13	5.4E-01	0.01	9.5E-01	-0.12	5.6E-01	0.01	9.5E-01	0.07	7.5E-01	-0.16	4.3E-01	-0.35	8.3E-02	-0.11	5.8E-01	-0.63	4.9E-04	-0.17	4.1E-01
ENSCAFG0000000059	TEX43	turquoise	EC_M6	0.13	5.4E-01	0.63	6.0E-04	0.04	8.5E-01	-0.09	6.7E-01	-0.38	5.5E-02	-0.07	7.4E-01	-0.08	7.0E-01	-0.10	6.3E-01	-0.16	4.3E-01	0.34	8.7E-02
ENSCAFG0000000380	ENSCAFG0000000380	turquoise	EC_M6	0.13	5.4E-01	0.63	6.0E-04	0.04	8.5E-01	-0.09	6.7E-01	-0.38	5.5E-02	-0.07	7.4E-01	-0.08	7.0E-01	-0.10	6.3E-01	-0.16	4.3E-01	0.34	8.7E-02
ENSCAFG0000001180	CCDC172	grey	EC_M10	0.13	5.4E-01	0.16	4.5E-01	0.17	4.2E-01	-0.04	8.3E-01	-0.32	1.1E-01	0.19	3.5E-01	-0.09	6.6E-01	-0.07	7.2E-01	0.08	6.9E-01	0.26	1.9E-01
ENSCAFG0000001020	ENSCAFG0000001020	turquoise	EC_M6	0.13	5.4E-01	0.93	3.8E-12	-0.23	2.5E-01	-0.04	8.4E-01	-0.15	4.6E-01	-0.12	5.4E-01	-0.13	5.2E-01	-0.17	4.1E-01	0.02	9.3E-01	0.07	7.2E-01
ENSCAFG0000000205	ADGRF5	turquoise	EC_M6	0.13	5.4E-01	0.89	7.0E-10	-0.21	3.1E-01	-0.06	7.9E-01	-0.17	4.0E-01	-0.08	7.1E-01	-0.12	5.5E-01	-0.13	5.1E-01	-0.02	9.1E-01	0.08	7.0E-01
ENSCAFG0000002272	ENSCAFG0000002272	grey	EC_M10	0.13	5.4E-01	-0.02	9.3E-01	0.22	2.8E-01	0.31	1.2E-01	-0.37	6.4E-02	-0.03	8.9E-01	-0.46	1.8E-02	0.13	5.2E-01	0.02	9.1E-01	0.32	1.1E-01
ENSCAFG0000001706	EFNA1	turquoise	EC_M6	0.13	5.4E-01	0.79	1.3E-06	0.02	9.4E-01	0.08	6.9E-01	-0.42	3.3E-02	-0.06	7.6E-01	-0.10	6.4E-01	-0.15	4.5E-01	-0.01	9.8E-01	0.32	1.1E-01
ENSCAFG0000000023	N4BP3	turquoise	EC_M6	0.13	5.4E-01	0.95	3.8E-13	-0.27	1.8E-01	-0.15	4.6E-01	-0.10	6.4E-01	-0.11	6.0E-01	-0.11	5.8E-01	-0.08	7.0E-01	0.07	7.3E-01	0.03	8.8E-01
ENSCAFG0000002960	ENSCAFG0000002960	grey	EC_M10	0.13	5.4E-01	0.15	4.6E-01	-0.20	3.2E-01	-0.20	3.3E-01	0.10	6.4E-01	0.32	1.1E-01	0.35	8.0E-02	-0.10	6.1E-01	-0.22	2.7E-01	-0.14	4.9E-01
ENSCAFG0000000927	STAB1	turquoise	EC_M6	0.13	5.4E-01	0.78	2.2E-06	-0.08	7.1E-01	0.01	9.7E-01	-0.28	1.6E-01	-0.14	5.0E-01	-0.14	5.0E-01	-0.15	4.6E-01	0.18	3.8E-01	0.18	3.7E-01
ENSCAFG0000001561	POGK	grey	EC_M10	0.13	5.4E-01	-0.09	6.6E-01	-0.63	5.7E-04	-0.23	2.6E-01	0.62	7.2E-04	0.07	7.4E-01	-0.23	2.5E-01	0.20	3.2E-01	-0.02	9.1E-01	-0.74	1.8E-05
ENSCAFG0000000091	SHROOM1	grey	EC_M10	0.13	5.4E-01	0.49	1.1E-02	-0.47	1.5E-02	-0.11	5.8E-01	0.23	2.6E-01	0.24	2.3E-01	0.08	6.9E-01	-0.01	9.5E-01	0.38	5.3E-02	-0.32	1.2E-01
ENSCAFG0000000143	C10H22orf23	cyan	EC_M2	0.13	5.4E-01	-0.29	1.5E-01	0.60	1.1E-03	0.27	1.9E-01	-0.72	3.7E-05	0.19	3.6E-01	-0.08	7.2E-01	-0.17	4.1E-01	-0.08	6.8E-01	0.67	1.7E-04
ENSCAFG0000001868	NDUFV2	grey	EC_M10	0.13	5.4E-01	-0.14	4.9E-01	0.37	6.2E-02	0.20	3.4E-01	-0.55	3.9E-03	-0.11	5.9E-01	-0.13	5.1E-01	0.23	2.7E-01	-0.25	2.1E-01	0.47	1.5E-02
ENSCAFG0000001633	ENSCAFG0000001633	grey	EC_M10	0.13	5.4E-01	-0.02	9.3E-01	0.14	5.1E-01	0.09	6.6E-01	-0.23	2.6E-01	0.23	2.7E-01	0.27	1.9E-01	-0.13	5.2E-01	0.01	9.7E-01	0.21	3.1E-01
ENSCAFG0000000472	KCNN4	cyan	EC_M2	0.13	5.4E-01	-0.05	8.0E-01	0.61	1.0E-03	0.41	3.8E-02	-0.83	1.7E-07	-0.26	1.9E-01	-0.22	2.8E-01	-0.04	8.5E-01	-0.15	4.6E-01	0.74	1.4E-05
ENSCAFG0000002137	ENSCAFG0000002137	grey	EC_M10	0.13	5.4E-01	-0.15	4.6E-01	0.01	9.4E-01	-0.19	3.6E-01	-0.09	6.7E-01	0.30	1.4E-01	-0.14	4.8E-01	0.20	3.2E-01	0.12	5.6E-01	-0.03	9.0E-01
ENSCAFG0000000798	PLCE1	grey	EC_M10	0.13	5.4E-01	0.04	8.5E-01	-0.41	3.8E-02	-0.02	9.2E-01	0.38	5.4E-02	-0.10	6.4E-01	-0.10	6.3E-01	0.35	7.9E-02	0.27	1.8E-01	-0.44	2.6E-02
ENSCAFG0000000666	ENSCAFG0000000666	grey	EC_M10	0.13	5.4E-01	-0.10	6.1E-01	-0.37	5.9E-02	-0.11	5.8E-01	0.30	1.4E-01	0.33	1.0E-01	-0.15	4.8E-01	-0.15	4.5E-01	0.15	4.6E-01	-0.40	4.1E-02
ENSCAFG0000000150	CARD10	grey	EC_M10	0.13	5.4E-01	0.59	1.4E-03	0.30	1.4E-01	0.13	5.3E-01	-0.64	4.7E-04	-0.33	9.8E-02	-0.13	5.2E-01	-0.20	3.3E-01	0.14	5.1E-01	0.62	8.2E-04
ENSCAFG0000003026	BAMBI	grey	EC_M10	0.13	5.4E-01	0.24	2.4E-01	-0.64	4.5E-04	-0.02	9.2E-01	0.52	6.9E-03	0.46	1.9E-02	-0.09	6.8E-01	-0.16	4.4E-01	0.19	3.4E-01	-0.62	8.1E-04
ENSCAFG0000000774	TFDP2	grey	EC_M10	0.13	5.4E-01	-0.06	7.7E-01	-0.57	2.4E-03	0.04	8.4E-01	0.59	1.4E-03	0.22	2.8E-01	-0.16	4.3E-01	0.17	4.0E-01	-0.28	1.6E-01	-0.64	4.0E-04
ENSCAFG0000000600	ENSCAFG0000000600	grey	EC_M10	0.13	5.4E-01	-0.13	5.3E-01	-0.47	1.6E-02	-0.11	6.0E-01	0.44	2.6E-02	0.21	3.0E-01	0.34	9.1E-02	-0.07	7.3E-01	-0.40	4.0E-02	-0.52	7.0E-03
ENSCAFG0000000210	ENSCAFG0000000210	grey	EC_M10	0.13	5.4E-01	0.31	1.2E-01	-0.34	8.5E-02	-0.03	8.8E-01	0.14	5.0E-01	-0.14	5.0E-01	-0.09	6.5E-01	0.30	1.3E-01	-0.13	5.2E-01	-0.25	2.1E-01
ENSCAFG0000000211	MUT	grey	EC_M10	0.13	5.4E-01	-0.12	5.8E-01	0.18	3.8E-01	0.06	7.6E-01	-0.33	9.6E-02	-0.08	7.2E-01	-0.10	6.4E-01	0.10	6.2E-01	0.02	9.4E-01	0.32	1.1E-01
ENSCAFG0000000728	NUP37	cyan	EC_M2	0.13	5.4E-01	-0.39	4.9E-02	0.64	4.6E-04	0.13	5.4E-01	-0.72	3.5E-05	-0.17	4.0E-01	-0.06	7.6E-01	-0.23	2.6E-01	-0.20	3.4E-01	0.64	4.6E-04
ENSCAFG0000000221	EFHC1	turquoise	EC_M6	0.13	5.4E-01	0.64	4.4E-04	-0.36	7.3E-02	0.00	9.9E-01	0.05	7.9E-01	-0.39	5.0E-02	-0.06	7.7E-01	0.23	2.7E-01	-0.09	6.6E-01	-0.14	5.0E-01
ENSCAFG0000000497	DACH1	turquoise	EC_M6	0.12	5.4E-01	0.93	6.9E-12	-0.36	7.2E-02	-0.14	4.8E-01	-0.01	9.8E-01	-0.07	7.4E-01	-0.18	3.9E-01	-0.05	8.1E-01	0.03	8.7E-01	-0.08	7.1E-01
ENSCAFG0000002023	ENSCAFG0000002023	grey	EC_M10	0.12	5.4E-01	-0.16	4.3E-01	-0.30	1.4E-01	-0.15	4.6E-01	0.31	1.2E-01	-0.16	4.3E-01	-0.12	5.4E-01	0.35	7.5E-02	0.00	9.8E-01	-0.35	8.3E-02
ENSCAFG0000002349	ENSCAFG0000002349	grey	EC_M10	0.12	5.4E-01	0.22	2.8E-01	0.18	3.7E-01	0.15	4.7E-01	-0.50	8.9E-03	-0.19	3.4E-01	-0.03	8.7E-01	0.09	6.6E-01	-0.02	9.2E-01	0.40	4.5E-02
ENSCAFG0000002983	NIP7	grey	EC_M10	0.12	5.4E-01	0.15	4.7E-01	-0.23	2.6E-01	-0.55	3.3E-03	0.17	4.0E-01	-0.07	7.3E-01	0.13	5.3E-01	0.14	5.1E-01	-0.07	7.5E-01	-0.26	1.9E-01
ENSCAFG0000003253	ENSCAFG0000003253	grey	EC_M10	0.12	5.4E-01	0.05	7.9E-01	-0.11	6.0E-01	0.35	7.5E-02	0.04	8.6E-01	0.16	4.4E-01	-0.24	2.3E-01	0.18	3.7E-01	0.12	5.6E-01	-0.08	7.0E-01
ENSCAFG0000000504	CYP2S1	magenta	EC_M13	0.12	5.4E-01	0.25	2.3E-01	0.00	9.9E-01	0.07	7.4E-01	-0.17	4.1E-01	-0.23	2.5E-01	-0.18	3.9E-01	0.40	4.5E-02	0.45	2.1E-02	0.09	6.6E-01
ENSCAFG0000001173	SRRD	grey	EC_M10	0.12	5.4E-01	0.14	4.9E-01	-0.50	9.1E-03	-0.12	5.7E-01	0.50	8.7E-03	-0.10	6.4E-01	0.16	4.5E-01	0.21	2.9E-01	0.06	7.6E-01	-0.52	6.0E-03
ENSCAFG0000001709	FARSA	grey	EC_M10	0.12	5.4E-01	-0.36	6.7E-02	0.07	7.4E-01	-0.20	3.4E-01	0.08	7.0E-01	0.25	2.3E-01	-0.05	8.3E-01	0.04	8.3E-01	-0.01	9.8E-01	-0.14	4.9E-01
ENSCAFG0000000453	NR2C2	cyan	EC_M2	0.12	5.4E-01	0.03	8.9E-01	0.49	1.0E-02	0.29	1.5E-01	-0.71	4.4E-05	0.17	4.2E-01	-0.11	6.0E-01	-0.23	2.6E-01	0.07	7.3E-01	0.65	3.4E-04
ENSCAFG0000000828	HNF4G	grey	EC_M10	0.12	5.4E-01	-0.04	8.3E-01	-0.23	2.7E-01	-0.09	6.6E-01	0.13	5.2E-01	-0.06	7.8E-01	0.00	9.9E-01	-0.07	7.2E-01	-0.03	8.8E-01	-0.23	2.5E-01
ENSCAFG0000001675	ISG20L2	grey	EC_M10	0.12	5.5E-01	-0.07	7.5E-01	-0.55	3.4E-03	-0.26	2.0E-01	0.60	1.1E-03	0.28	1.7E-01	0.00	1.0E+00	0.15	4.7E-01	-0.18	3.9E-01	-0.71	4.6E-05
ENSCAFG0000003208	SMIM19	grey	EC_M10	0.12	5.5E-01	-0.63	5.1E-04	0.09	6.6E-01	0.16	4.2E-01	-0.02	9.0E-01	0.13	5.2E-01	-0.12	5.7E-01	0.01	9.6E-01	-0.06	7.6E-01	-0.04	8.3E-01
ENSCAFG0000002272	ENSCAFG0000002272	grey	EC_M10	0.12	5.5E-01	0.03	8.7E-01	-0.13	5.4E-01	0.17	4.1E-01	0.01	9.4E-01	0.03	8.7E-01	-0.25	2.1E-01	-0.20	3.3E-01	-0.26	2.0E-01	-0.09	6.7E-01
ENSCAFG0000003246	ENSCAFG0000003246	grey	EC_M10	0.12	5.5E-01	0.18	3.8E-01	0.23	2.6E-01	0.14	5.0E-01	-0.46	1.7E-02	0.01	9.4E-01	0.08	7.0E-01	-0.03	8.7E-01	-0.02	9.2E-01	0.43	3.0E-02
ENSCAFG0000001742	VEZF1	cyan	EC_M2	0.12	5.5E-01	0.33	9.5E-02	0.57	2.4E-03	0.13	5.3E-01	-0.90	3.7E-10	-0.11	6.0E-01	-0.08	7.1E-01	-0.07	7.3E-01	0.01	9.5E-01	0.84	9.0E-08

ENSCAFG0000000932	BPHL	grey	EC_M10	0.12	5.5E-01	-0.41	3.7E-02	-0.09	6.7E-01	-0.34	8.6E-02	0.12	5.6E-01	0.32	1.2E-01	0.11	5.9E-01	0.08	6.9E-01	-0.03	8.8E-01	-0.24	2.4E-01
ENSCAFG0000002014	DR1	grey	EC_M10	0.12	5.5E-01	0.49	1.2E-02	0.22	2.8E-01	-0.10	6.2E-01	-0.56	2.9E-03	-0.24	2.4E-01	-0.27	1.8E-01	-0.29	1.5E-01	-0.26	2.1E-01	0.50	8.8E-03
ENSCAFG0000000943	ENSCAFG0000000943	grey	EC_M10	0.12	5.5E-01	0.04	8.3E-01	-0.24	2.4E-01	-0.04	8.4E-01	0.18	3.7E-01	0.28	1.7E-01	-0.45	2.1E-02	0.32	1.1E-01	-0.13	5.1E-01	-0.27	1.8E-01
ENSCAFG0000000602	EFNB2	turquoise	EC_M6	0.12	5.5E-01	0.69	9.7E-05	-0.25	2.1E-01	-0.24	2.3E-01	-0.02	9.0E-01	-0.09	6.6E-01	-0.07	7.5E-01	-0.17	4.1E-01	0.51	7.7E-03	0.00	9.9E-01
ENSCAFG0000000343	CTTNBP2	cyan	EC_M2	0.12	5.5E-01	0.04	8.6E-01	0.58	1.8E-03	0.03	8.9E-01	-0.76	6.9E-06	-0.34	9.1E-02	0.03	8.7E-01	0.04	8.4E-01	0.03	8.9E-01	0.73	2.2E-05
ENSCAFG0000000227	SUSD3	turquoise	EC_M6	0.12	5.5E-01	0.65	3.5E-04	-0.02	9.1E-01	0.13	5.4E-01	-0.29	1.5E-01	-0.19	3.6E-01	0.11	5.9E-01	-0.14	5.1E-01	-0.20	3.3E-01	0.22	2.8E-01
ENSCAFG0000000731	KCTD6	cyan	EC_M2	0.12	5.5E-01	0.30	1.4E-01	0.22	2.8E-01	-0.04	8.5E-01	-0.53	5.7E-03	-0.42	3.4E-02	0.06	7.5E-01	-0.17	4.0E-01	-0.09	6.6E-01	0.49	1.1E-02
ENSCAFG0000000848	NDRG4	turquoise	EC_M6	0.12	5.5E-01	0.71	4.1E-05	-0.64	4.6E-04	-0.15	4.7E-01	0.36	7.1E-02	0.02	9.1E-01	-0.01	9.6E-01	0.08	7.1E-01	0.12	5.7E-01	-0.42	3.3E-02
ENSCAFG0000000807	FAM160A1	darkgreen	EC_M4	0.12	5.5E-01	0.42	3.1E-02	-0.68	1.3E-04	-0.27	1.8E-01	0.57	2.6E-03	0.17	4.1E-01	-0.07	7.2E-01	-0.19	3.6E-01	-0.22	2.7E-01	-0.65	3.2E-04
ENSCAFG0000001226	GPATCH3	grey	EC_M10	0.12	5.5E-01	0.20	3.3E-01	-0.07	7.5E-01	0.08	6.9E-01	-0.14	4.9E-01	0.22	2.7E-01	-0.01	9.5E-01	-0.32	1.1E-01	-0.19	3.5E-01	0.07	7.2E-01
ENSCAFG0000000058	TFB1M	grey	EC_M10	0.12	5.5E-01	-0.35	7.6E-02	0.33	9.8E-02	0.19	3.6E-01	-0.33	1.0E-01	-0.15	4.6E-01	0.05	8.0E-01	0.01	9.6E-01	0.20	3.3E-01	0.28	1.6E-01
ENSCAFG0000002438	ADGRG1	turquoise	EC_M6	0.12	5.5E-01	0.88	2.7E-09	-0.20	3.3E-01	-0.04	8.6E-01	-0.15	4.5E-01	-0.18	3.8E-01	-0.17	4.2E-01	-0.16	4.3E-01	0.17	3.9E-01	0.08	7.0E-01
ENSCAFG0000001167	EPB41	turquoise	EC_M6	0.12	5.5E-01	0.76	5.8E-06	-0.16	4.3E-01	0.39	4.7E-02	-0.22	2.9E-01	-0.13	5.3E-01	-0.08	7.1E-01	-0.08	6.9E-01	0.01	9.7E-01	0.14	4.9E-01
ENSCAFG0000000970	HIF1AN	darkgreen	EC_M4	0.12	5.5E-01	-0.23	2.6E-01	-0.72	3.5E-05	0.00	1.0E+00	0.83	1.6E-07	0.29	1.5E-01	-0.18	3.7E-01	0.03	9.0E-01	-0.08	6.9E-01	-0.88	1.9E-09
ENSCAFG0000001732	ENSCAFG0000001732	grey	EC_M10	0.12	5.5E-01	-0.09	6.5E-01	-0.45	2.2E-02	-0.12	5.6E-01	0.46	1.7E-02	0.70	6.5E-05	-0.08	6.9E-01	-0.05	8.1E-01	-0.06	7.7E-01	-0.51	7.6E-03
ENSCAFG0000001799	ENSCAFG0000001799	grey	EC_M10	0.12	5.5E-01	-0.09	6.5E-01	-0.45	2.2E-02	-0.12	5.6E-01	0.46	1.7E-02	0.70	6.5E-05	-0.08	6.9E-01	-0.05	8.1E-01	-0.06	7.7E-01	-0.51	7.6E-03
ENSCAFG0000000943	RPP40	grey	EC_M10	0.12	5.5E-01	0.13	5.4E-01	-0.13	5.2E-01	0.15	4.6E-01	0.03	9.0E-01	-0.20	3.4E-01	-0.42	3.1E-02	0.11	6.0E-01	0.33	9.8E-02	-0.03	8.7E-01
ENSCAFG0000001695	PKLR	grey	EC_M10	0.12	5.5E-01	-0.04	8.5E-01	0.01	9.5E-01	-0.13	5.4E-01	-0.06	7.7E-01	0.58	2.0E-03	-0.02	9.2E-01	-0.13	5.2E-01	-0.11	6.1E-01	-0.04	8.3E-01
ENSCAFG0000000560	EDEM1	grey	EC_M10	0.12	5.5E-01	0.32	1.2E-01	-0.70	7.0E-05	0.01	9.5E-01	0.54	4.5E-03	-0.05	8.0E-01	0.26	2.1E-01	-0.03	8.8E-01	0.09	6.6E-01	-0.62	7.5E-04
ENSCAFG0000001344	LSG1	darkgreen	EC_M4	0.12	5.5E-01	0.15	4.7E-01	-0.77	4.6E-06	-0.18	3.8E-01	0.76	7.6E-06	0.33	1.0E-01	-0.04	8.5E-01	0.07	7.3E-01	0.31	1.2E-01	-0.80	7.9E-07
ENSCAFG0000002302	ENSCAFG0000002302	turquoise	EC_M6	0.12	5.5E-01	0.72	3.4E-05	-0.16	4.4E-01	-0.16	4.3E-01	-0.16	4.2E-01	0.27	1.9E-01	0.08	6.9E-01	-0.13	5.4E-01	-0.18	3.7E-01	0.10	6.1E-01
ENSCAFG0000001948	MEIOB	cyan	EC_M2	0.12	5.5E-01	-0.30	1.3E-01	0.63	6.0E-04	0.36	6.7E-02	-0.77	5.1E-06	-0.23	2.6E-01	-0.04	8.6E-01	0.02	9.3E-01	-0.10	6.1E-01	0.69	8.8E-05
ENSCAFG0000000556	SEL1L2	turquoise	EC_M6	0.12	5.5E-01	0.49	1.1E-02	0.03	9.0E-01	-0.13	5.4E-01	-0.36	7.2E-02	-0.10	6.3E-01	-0.07	7.3E-01	-0.09	6.6E-01	0.37	5.9E-02	0.32	1.1E-01
ENSCAFG0000000185	ENSCAFG0000000185	grey	EC_M10	0.12	5.5E-01	0.23	2.6E-01	0.15	4.7E-01	0.29	1.6E-01	-0.33	9.4E-02	-0.20	3.4E-01	-0.19	3.5E-01	-0.22	2.8E-01	-0.03	9.0E-01	0.25	2.1E-01
ENSCAFG0000001441	LPCAT3	grey	EC_M10	0.12	5.5E-01	-0.11	5.8E-01	-0.56	2.6E-03	-0.16	4.2E-01	0.66	2.4E-04	0.26	1.9E-01	-0.19	3.4E-01	-0.20	3.2E-01	-0.32	1.1E-01	-0.76	8.1E-06
ENSCAFG0000001746	ENSCAFG0000001746	grey	EC_M10	0.12	5.5E-01	0.06	7.9E-01	-0.25	2.1E-01	-0.12	5.5E-01	0.21	2.9E-01	-0.16	4.5E-01	-0.13	5.2E-01	0.48	1.2E-02	-0.03	8.7E-01	-0.28	1.6E-01
ENSCAFG0000001304	MYOM3	turquoise	EC_M6	0.12	5.5E-01	0.80	1.1E-06	-0.27	1.8E-01	-0.11	5.9E-01	-0.06	7.7E-01	-0.11	6.0E-01	-0.11	6.1E-01	-0.08	6.8E-01	-0.12	5.7E-01	0.03	8.8E-01
ENSCAFG0000001878	ENSCAFG0000001878	grey	EC_M10	0.12	5.5E-01	-0.07	7.3E-01	-0.16	4.4E-01	-0.13	5.4E-01	0.08	6.9E-01	-0.08	6.9E-01	-0.07	7.4E-01	0.49	1.0E-02	0.04	8.4E-01	-0.17	4.0E-01
ENSCAFG0000001437	LSM12	grey	EC_M10	0.12	5.5E-01	-0.21	3.1E-01	-0.34	8.6E-02	-0.33	9.7E-02	0.42	3.5E-02	0.35	7.5E-02	-0.16	4.4E-01	0.28	1.6E-01	-0.07	7.3E-01	-0.53	4.9E-03
ENSCAFG0000001476	SLC9A7	turquoise	EC_M6	0.12	5.5E-01	0.58	2.1E-03	-0.41	3.5E-02	-0.24	2.5E-01	0.24	2.5E-01	-0.19	3.4E-01	0.20	3.2E-01	-0.16	4.2E-01	0.15	4.7E-01	-0.22	2.9E-01
ENSCAFG0000002914	FGF16	turquoise	EC_M6	0.12	5.5E-01	0.47	1.5E-02	-0.15	4.6E-01	-0.03	8.7E-01	-0.10	6.3E-01	-0.09	6.7E-01	-0.12	5.6E-01	-0.14	4.9E-01	-0.23	2.5E-01	-0.01	9.6E-01
ENSCAFG0000001085	MMAB	cyan	EC_M2	0.12	5.5E-01	-0.42	3.4E-02	0.32	1.1E-01	0.28	1.6E-01	-0.37	6.5E-02	-0.13	5.2E-01	0.24	2.4E-01	0.15	4.7E-01	-0.11	5.9E-01	0.24	2.3E-01
ENSCAFG0000002885	TOMM20L	grey	EC_M10	0.12	5.5E-01	-0.16	4.4E-01	0.14	4.8E-01	0.38	5.6E-02	-0.18	3.7E-01	-0.14	5.0E-01	0.40	4.5E-02	-0.10	6.1E-01	-0.01	9.6E-01	0.17	4.1E-01
ENSCAFG0000001349	OLR1	darkgreen	EC_M4	0.12	5.5E-01	0.42	3.1E-02	-0.74	1.4E-05	-0.15	4.7E-01	0.60	1.1E-03	0.20	3.2E-01	-0.09	6.7E-01	0.15	4.6E-01	-0.06	7.8E-01	-0.68	1.5E-04
ENSCAFG0000001431	ENSCAFG0000001431	grey	EC_M10	0.12	5.5E-01	-0.02	9.1E-01	-0.15	4.7E-01	-0.04	8.5E-01	0.06	7.9E-01	0.45	2.2E-02	0.15	4.7E-01	-0.12	5.5E-01	0.03	8.8E-01	-0.15	4.6E-01
ENSCAFG0000000411	KIAA1549	darkgreen	EC_M4	0.12	5.5E-01	0.05	7.9E-01	-0.76	8.0E-06	-0.17	4.2E-01	0.78	2.6E-06	0.10	6.4E-01	-0.09	6.7E-01	-0.05	8.0E-01	0.04	8.4E-01	-0.85	3.9E-08
ENSCAFG0000000446	GPBP1L1	grey	EC_M10	0.12	5.5E-01	0.35	8.2E-02	-0.23	2.7E-01	-0.08	7.1E-01	-0.05	8.1E-01	0.06	7.5E-01	0.41	4.0E-02	-0.27	1.9E-01	-0.24	2.4E-01	-0.04	8.4E-01
ENSCAFG0000000450	KLC3	grey	EC_M10	0.12	5.5E-01	-0.11	6.0E-01	-0.55	4.0E-03	-0.11	6.0E-01	0.58	1.7E-03	-0.18	3.8E-01	0.17	4.1E-01	0.24	2.3E-01	0.01	9.5E-01	-0.64	4.8E-04
ENSCAFG0000001065	ENSCAFG0000001065	grey	EC_M10	0.12	5.5E-01	-0.06	7.7E-01	-0.60	1.1E-03	-0.07	7.5E-01	0.61	9.7E-04	0.20	3.2E-01	-0.27	1.9E-01	0.41	3.8E-02	-0.01	9.7E-01	-0.68	1.2E-04
ENSCAFG0000001566	ATAD1	darkgreen	EC_M4	0.12	5.5E-01	-0.06	7.7E-01	-0.70	7.4E-05	-0.04	8.3E-01	0.71	4.2E-05	0.34	9.2E-02	0.05	8.2E-01	-0.02	9.1E-01	-0.32	1.1E-01	-0.77	4.4E-06
ENSCAFG0000001348	UCK2	grey	EC_M10	0.12	5.5E-01	0.07	7.5E-01	-0.46	1.9E-02	-0.20	3.2E-01	0.49	1.1E-02	0.22	2.7E-01	0.14	5.0E-01	-0.30	1.4E-01	-0.23	2.7E-01	-0.56	3.2E-03
ENSCAFG0000001596	BCL6B	turquoise	EC_M6	0.12	5.6E-01	0.85	5.3E-08	-0.17	4.1E-01	-0.08	6.9E-01	-0.19	3.7E-01	-0.06	7.8E-01	-0.17	4.0E-01	-0.14	4.8E-01	-0.03	9.0E-01	0.08	6.9E-01
ENSCAFG0000000247	TRIM14	grey	EC_M10	0.12	5.6E-01	0.24	2.3E-01	0.52	6.4E-03	0.12	5.4E-01	-0.77	5.2E-06	-0.17	4.0E-01	-0.06	7.6E-01	0.07	7.2E-01	0.05	8.1E-01	0.69	1.0E-04
ENSCAFG0000001271	SLCO4A1	turquoise	EC_M6	0.12	5.6E-01	0.91	1.3E-10	-0.24	2.3E-01	-0.16	4.3E-01	-0.10	6.2E-01	-0.12	5.7E-01	-0.03	9.0E-01	-0.13	5.4E-01	0.06	7.8E-01	0.05	8.1E-01
ENSCAFG0000003191	ENSCAFG0000003191	grey	EC_M10	0.12	5.6E-01	0.37	6.3E-02	0.10	6.3E-01	0.23	2.6E-01	-0.33	9.7E-02	-0.14	5.1E-01	-0.15	4.7E-01	0.42	3.2E-02	-0.02	9.1E-01	0.27	1.8E-01
ENSCAFG0000000062	PPP6R2	cyan	EC_M2	0.12	5.6E-01	-0.09	6.5E-01	0.64	4.1E-04	0.25	2.3E-01	-0.81	4.5E-07	-0.15	4.5E-01	-0.08	6.9E-01	-0.20	3.3E-01	-0.14	5.1E-01	0.72	3.9E-05
ENSCAFG0000000726	ENSCAFG0000000726	grey	EC_M10	0.12	5.6E-01	-0.05	8.0E-01	-0.38	5.7E-02	-0.13	5.4E-01	0.32	1.1E-01	0.32	1.1E-01	-0.12	5.5E-01	-0.13	5.2E-01	0.22	2.8E-01	-0.39	5.0E-02
ENSCAFG0000002948	KLHL11	grey	EC_M10	0.12	5.6E-01	0.06	7.6E-01	-0.33	1.0E-01	0.22	2.8E-01	0.25	2.2E-01	-0.09	6.7E-01	-0.26	2.0E-01	0.05	8.0E-01	-0.13	5.2E-01	-0.31	1.3E-01
ENSCAFG0000000102	RNF217	grey	EC_M10	0.12	5.6E-01	-0.06	7.6E-01	0.30	1.3E-01	0.04	8.5E-01	-0.42	3.1E-02	-0.23	2.5E-01	0.05	8.2E-01	0.03	8.9E-01	0.33	9.9E-02	0.41	3.9E-02
ENSCAFG0000002185	ENSCAFG0000002185	grey	EC_M10	0.12	5.6E-01	0.15	4.5E-01	0.11	6.1E-01	0.24	2.3E-01	-0.19	3.6E-01	-0.20	3.4E-01	0.05	8.3E-01	-0.23	2.6E-01	-0.12	5.6E-01	0.13	5.2E-01
ENSCAFG0000000913	SPIDR	grey	EC_M10	0.12	5.6E-01	0.03	8.7E-01	-0.62	7.1E-04	-0.20	3.4E-01	0.67	1.6E-04	0.22	2.8E-01	-0.13	5.4E-01	-0.09	6.7E-01	-0.30	1.3E-01	-0.74	1.8E-05
ENSCAFG0000000267	TSEN34	grey	EC_M10	0.12	5.6E-01	0.20	3.2E-01	0.55	3.7E-03	0.11	6.1E-01	-0.79	1.5E-06	0.06	7.9E-01	0.10	6.2E-01	-0.29	1.5E-01	-0.02	9.1E-01	0.71	4.5E-05
ENSCAFG0000001402	IL1RAP	turquoise	EC_M6	0.12	5.6E-01	0.57	2.2E-03	-0.45	2.1E-02	0.16	4.3E-01	0.21	3.1E-01	0.25	2.2E-01	-0.13	5.4E-01	-0.08	7.0E-01	-0.03	8.8E-01	-0.30	1.4E-01
ENSCAFG0000000281	CLHC1	grey	EC_M10	0.12	5.6E-01	-0.25	2.1E-01	-0.04	8.6E-01	-0.34	9.1E-02	0.02	9.3E-01	-0.02	9.4E-01	0.13	5.4E-01	0.28	1.7E-01	0.03	8.7E-01	-0.08	7.1E-01
ENSCAFG0000001906	LRRC42	grey	EC_M10	0.12	5.6E-01	0.24	2.4E-01	0.22	2.7E-01	0.09	6.5E-01	-0.51	8.5E-03	-0.37	6.2E-02	-0.16	4.2E-01	-0.03	8.8E-01	-0.14	4.8E-01	0.46	1.9E-02
ENSCAFG0000001448	ETV4	turquoise	EC_M6	0.12	5.6E-01	0.38	5.4E-02	-0.16	4.3E-01	0.02	9.1E-01	-0.06	7.7E-01	-0.12	5.6E-01	-0.09	6.6E-01	-0.14	5.1E-01	-0.18	3.8E-01	-0.07	7.3E-01
ENSCAFG0000000849	GPM6A	turquoise	EC_M6	0.12	5.6E-01	0.87	1.2E-08	-0.20	3.3E-01	-0.11	5.8E-01	-0.17	4.0E-01	-0.11	6.1E-01	-0.09	6.5E-01	-0.09	6.6E-01	0.03	8.9E-01	0.12	5.6E-01
ENSCAFG0000001345	MOV10	turquoise	EC_M6	0.12	5.6E-01	0.87	9.0E-09	0.00	1.0E+00	-0.07	7.4E-01	-0.43	2.7E-02	-0.16	4.4E-01	-0.03	8.8E-01	-0.07	7.3E-01	0.11	5.9E-01	0.35	7.7E-02
ENSCAFG0000002873	ABHD10	grey	EC_M10	0.12	5.6E-01	0.01	9.5E-01	0.46	1.8E-02	0.09	6.5E-01	-0.66	2.6E-04	-0.09	6.6E-01	-0.09	6.6E-01	0.27	1.8E-01	0.22	2.8E-01	0.58	1.9E-03
ENSCAFG0000000200	SUPT3H	grey	EC_M10	0.12	5.6E-01	-0.46	1.9E-02	-0.17	4.0E-01	-0.04	8.6E-01	0.28	1.7E-01	0.20	3.3E-01	0.03	8.8E-01	-0.01	9.5E-01	-0.02	9.4E-01	-0.32	1.1E-01
ENSCAFG0000002989	RPL13A	cyan	EC_M2	0.12	5.6E-01	-0.36	7.2E-02	0.49	1.2E-02	0.18	3.7E-01	-0.54	4.2E-03	-0.01	9.8E-01	0.25	2.2E-01	0.00	9.9E-01	-0.55	4.0E-03	0.41	3.5E-02
ENSCAFG0000002637	ENSCAFG0000002637	grey	EC_M10	0.12	5.6E-01	-0.05	8.1E-01	0.18	3.8E-01	0.40	4.1E-02	-0.33	1.0E-01	0.19	3.4E-01	-0.04	8.6E-01	-0.20	3.3E-01	-0.26	1.9E-01	0.27	1.9E-01
ENSCAFG0000003263	ENSCAFG0000003263	turquoise	EC_M6	0.12	5.6E-01	0.44	2.5E-02	-0.15	4.6E-01	-0.34	9.0E-02	-0.10	6.3E-01	-0.22	2.8E-01	0.19	3.5E-01	-0.06	7.8E-01	0.16	4.3E-01	0.01	9.8E-01
ENSCAFG0000000754	GIN1	grey	EC_M10	0.12	5.6E-01	-0.21	2.9E-01	-0.29	1.5E-01	0.15	4.7E-01	0.18	3.7E-01	0.39	4.8E-02	-0.08	6.9E-01	-0.04	8.6E-01	0.11	5.9E-01	-0.27	1.8E-01
ENSCAFG0000002587	ENSCAFG0000002587	turquoise	EC_M6	0.12	5.6E-01	0.42	3.1E-02	-0.35	7.7E-02	-0.17	4.0E-01	0.17	4.1E-01	0.28	1.6E-01	-0.10	6.4E-01	0.56	3.1E-03	0.35	8.2E-02	-0.21	3.0E-01
ENSCAFG0000000734	ENSCAFG0000000734	grey	EC_M10	0.12	5.6E-01	0.28	1.6E-01	-0.22	2.7E-01	-0.12	5.5E-01	0.03	8.9E-01	-0.08	6.8E-01	0.17	4.1E-01	-0.05	8.1E-01	-0.44	2.6E-02	-0.08	6.8E-01
ENSCAFG0000002959	POU3F2	grey	EC_M10	0.12	5.6E-01	0.22	2.8E-01	0.02	9.4E-01	0.40	4.2E-02	-0.26	2.0E-01	-0.17	4.1E-01	-0.18	3.9E-01	-0.16	4.3E-01	0.10	6.1E-01	0.19	3.5E-01
ENSCAFG0000001654	AMER1	grey	EC_M10	0.12	5.6E-01	-0.21	3.0E-01	0.55	4.0E-03	0.20	3.2E-01	-0.62	7.6E-04	-0.50	8.9E-03	-0.17	3.9E-01	0.02	9.1E-01	-0.02	9.4E-01	0.57	2.3E-03
ENSCAFG0000001717	UBE2Q1	darkgreen	EC_M4	0.12	5.6E-01	0.38	5.6E-02	-0.66	2.1E-04	-0.24	2.4E-01	0.57	2.2E-03	-0.01	9.5E-01	-0.27	1.9E-01	0.01	9.8E-01	-0.02	9.1E-01	-0.65	3.5E-04
ENSCAFG0000001660	MSN	darkgreen	EC_M4	0.12	5.6E-01	0.33	9.9E-02	-0.85	3.4E-08	-0.25	2.1E-01	0.79	1.5E-06	0.21	3.0E-01	-0.04	8.3E-01	0.16	4.5E-01	0.04	8.4E-01	-0.85	4.4E-08
ENSCAFG0000000041	AKAP12	turquoise	EC_M6	0.12	5.6E-01	0.45	2.1E-02	-0.12	5.5E-01	0.04	8.4E-01	-0.10	6.3E-01	-0.13	5.3E-01	-0.40	4.4E-02	-0.03	8.9E-01	-0.63	5.8E-04	0.02	9.4E-01
ENSCAFG0000003218	ENSCAFG0000003218	grey	EC_M10	0.12	5.6E-01	-0.09	6.7E-01	0.11	5.8E-01	-0.07	7.4E-01	-0.18	3.7E-01	-0.08	6.8E-01	-0.11	6.0E-01	-0.02	9.1E-01	-0.13	5.2E-01	0.09	6.5E-01
ENSCAFG0000000985	ENSCAFG0000000985	grey	EC_M10	0.12	5.6E-01	0.11	6.0E-01	0.03	8.7E-01	0.05	8.1E-01	-0.13	5.2E-01	0.06	7.7E-01	0.14	4.8E-01	0.45	2.0E-02	0.13	5.1E-01	0.06	7.7E-01
ENSCAFG0000000638	SLC4A11	turquoise	EC_M6	0.12	5.6E-01	0.63	6.0E-04	-0.04	8.5E-01	-0.12	5.4E-01	-0.25	2.2E-01	-0.10	6.2E-01	0.35	8.1E-02	-0.19	3.5E-01	0.26	2.1E-01	0.20	3.3E-01
ENSCAFG0000000022	ENSCAFG0000000022	grey	EC_M10	0.12	5.7E-01	-0.18	3.8E-01	-0.53	5.4E-03	-0.30	1.3E-01	0.63	5.6E-04	0.27	1.8E-01	-0.02	9.1E-01	-0.10	6.1E-01	0.09	6.6E-01	-0.69	8.3E-05
ENSCAFG0000000234	ETV1	grey	EC_M10	0.12	5.7E-01	-0.40	4.4E-02	0.06	7.7E-01	0.13	5.1E-01	-0.11	5.8E-01	0.20	3.2E-01	-0.13	5.2E-01	0.21	3.0E-01	-0.06	7.8E-01	-0.01	9.5E-01
ENSCAFG0000001783	PIGW	grey	EC_M10	0.12	5.7E-01	-0.05	8.0E-01	0.26	2.1E-01	0.26	2.0E-01	-0.38	5.5E-02	0.13	5.3E-01	0.13	5.2E-01	-0.47	1.6E-02	-0.04	8.4E-01	0.32	1.1E-01
ENSCAFG0000001775	ENSCAFG0000001775	grey	EC_M10	0.12	5.7E-01	0.20	3.2E-01	-0.01	9.4E-01	-0.04	8.4E-01	-0.15	4.8E-01	-0.08	7.0E-01	-0.10	6.2E-01	-0.07	7.5E-01	0.40	4.1E-02	0.10	6.1E-01
ENSCAFG0000002881	ENSCAFG0000002881	grey	EC_M10	0.12	5.7E-01	0.12	5.6E-01	-0.32	1.1E-01	0.19	3.6E-01	0.22	2.7E-01	0.00	9.8E-01	-0.15	4.6E-01	0.04	8.5E-01	0.49	1.0E-02	-0.29	1.4E-01
ENSCAFG0000000787	KCMF1	darkgreen	EC_M4	0.12	5.7E-01	0.51	8.4E-03	-0.73	2.4E-05	-0.22	2.9E-01	0.56	2.9E-03	0.06	7.8E-01	-0.05	8.3E-01	-0.13	5.3E-01	-0.13	5.3E-01	-0.64	4.0E-04
ENSCAFG0000000553	SRGAP3	grey	EC_M10	0.12	5.7E-01	0.08	7.1E-01	-0.04	8.4E-01	0.30	1.3E-01	-0.11	5.9E-01	-0.17	4.2E-01	-0.19	3.5E-01	-0.18	3.9E-01	0.23	2.7E-01	0.10	6.2E-01
ENSCAFG0000002611	ENSCAFG0000002611	grey	EC_M10	0.12	5.7E-01	0.38	5.9E-02	0.12	5.7E-01	0.19	3.4E-01	-0.46	1.9E-02	-0.44	2.3E-02	0.07	7.4E-01	0.07	7.3E-01	-0.02	9.1E-01	0.38	5.8E-02
ENSCAFG0000001858	FBXO4	cyan	EC_M2	0.12	5.7E-01	0.20	3.3E-01	0.49	1.2E-02	0.27	1.9E-01	-0.77	5.2E-06	-0.01	9.5E-01	-0.15	4.7E-01	-0.01	9.6E-01	0.22	2.8E-01	0.74	1.7E-05
ENSCAFG0000002331	ENSCAFG0000002331	cyan	EC_M2	0.12	5.7E-01	-0.01	9.6E-01	0.53	5.3E-03	0.35	8.3E-02	-0.72	3.3E-05	-0.41	3.9E-02	-0.01	9.4E-01	0.05	8.0E-01	0.00	9.9E-01	0.64	4.5E-04
ENSCAFG0000003028	ENSCAFG0000003028	grey	EC_M10	0.12	5.7E-01	0.18	3.8E-01	0.01	9.7E-01	-0.20	3.3E-01	-0.19	3.4E-01	0.27	1.9E-01	0.34	8.9E-02	-0.17	4.0E-01	-0.20	3.4E-01	0.12	5.6E-01
ENSCAFG0000000193	VEGFA	darkgreen	EC_M4	0.12	5.7E-01	0.14	5.1E-01	-0.68	1.4E-04	-0.12	5.5E-01	0.61	8.6E-04	0.27	1.9E-01	0.38	5.5E-02	0.14	4.8E-01	0.07	7.4E-01	-0.73	2.6E-05
ENSCAFG0000001756	ENSCAFG0000001756	grey	EC_M10	0.12	5.7E-01	-0.48	1.4E-02	-0.50	9.0E-03	-0.19	3.4E-01	0.68	1.5E-04	0.23	2.7E-01	0.02	9.3E-01	-0.06	7.9E-01	-0.09	6.5E-01	-0.74	1.6E-05
ENSCAFG0000001030	ENSCAFG0000001030	grey	EC_M10	0.12	5.7E-01	-0.12	5.5E-01	-0.07	7.3E-01	-0.10	6.4E-01	0.00	9.9E-01	-0.06	7.8E-01	-0.08	6.9E-01	-0.10	6.2E-01	-0.09	6.8E-01	-0.06	7.6E-01
ENSCAFG0000001888	PIPOX	turquoise	EC_M6	0.12	5.7E-01	0.71	4.1E-05	-0.43	2.7E-02	-0.28	1.6E-01	0.18	3.8E-01	-0.20	3.4E-01	0.04	8.3E-01	-0.06	7.9E-01	0.41	4.0E-02	-0.20	3.2E-01
ENSCAFG0000003038	ENSCAFG0000003038	grey	EC_M10	0.12	5.7E-01	-0.15	4.5E-01	0.34	8.9E-02	-0.24	2.3E-01	-0.48	1.3E-02	-0.20	3.3E-01	-0.26	2.1E-01	0.16	4.3E-01	-0.06	7.8E-01	0.40	4.4E-02
ENSCAFG0000000179	CRY1	grey	EC_M10	0.12	5.7E-01	0.07	7.4E-01	0.04	8.6E-01	-0.12	5.5E-01	-0.15	4.8E-01	0.05	8.2E-01	-0.12	5.5E-01	0.07	7.3E-01	-0.31	1.2E-01	0.09	6.6E-01
ENSCAFG0000001402	ENSCAFG0000001402	grey	EC_M10	0.12	5.7E-01	-0.18	3.7E-01	0.35	8.3E-02	0.28	1.6E-01	-0.41	3.7E-02	-0.13	5.2E-01	-0.12	5.5E-01	-0.11	5.9E-01	-0.13	5.2E-01	0.35	7.5E-02
ENSCAFG0000002344	MED31	grey	EC_M10	0.12	5.7E-01	-0.18	3.8E-01	0.31	1.2E-01	-0.42	3.3E-02	-0.39	5.1E-02	0.06	7.6E-01	-0.04	8.6E-01	0.19	3.6E-01	-0.07	7.3E-01	0.34	8.9E-02
ENSCAFG0000003148	ENSCAFG0000003148	cyan	EC_M2	0.12	5.7E-01	-0.35	7.9E-02	0.63	6.3E-04	0.05	8.2E-01	-0.64	4.6E-04	-0.06	7.6E-01	-0.02	9.4E-01	0.12	5.7E-01	-0.18	3.8E-01	0.57	2.5E-03
ENSCAFG0000001091	GSTCD	cyan	EC_M2	0.12	5.7E-01	-0.51	7.3E-03	0.54	4.2E-03	0.31	1.2E-01	-0.59	1.5E-03	0.01	9.6E-01	-0.31	1.2E-01	0.04	8.6E-01	0.00	9.9E-01	0.50	9.2E-03
ENSCAFG0000002020	KYAT3	grey	EC_M10	0.12	5.7E-01	0.05	8.1E-01	-0.29	1.5E-01	0.33	9.5E-02	0.26	2.0E-01	-0.14	4.9E-01	-0.03	8.7E-01	0.08	7.0E-01	-0.03	8.9E-01	-0.28	1.7E-01
ENSCAFG0000000862	ENSCAFG0000000862	grey	EC_M10	0.12	5.7E-01	-0.60	1.2E-03	0.15	4.7E-01	0.00	9.9E-01	-0.01	9.6E-01	0.32	1.1E-01	-0.45	2.2E-02	-0.03	8.9E-01	0.07	7.4E-01	-0.06	7.7E-01
ENSCAFG0000000326	COLEC11	grey	EC_M10	0.12	5.7E-01	-0.03	8.9E-01	-0.17	4.0E-01	-0.14	4.9E-01	0.15	4.6E-01	-0.02	9.0E-01	-0.11	5.9E-01	0.10	6.3E-01	0.17	4.1E-01	-0.25	2.1E-01
ENSCAFG0000001443	PHB2	cyan	EC_M2	0.12	5.7E-01	-0.33	9.5E-02	0.73	2.2E-05	0.14	4.9E-01	-0.78	2.7E-06	-0.23	2.6E-01	0.05	8.2E-01	-0.05	8.0E-01	-0.27	1.8E-01	0.71	5.3E-05
ENSCAFG0000001646	ALDH1A2	turquoise	EC_M6	0.12	5.7E-01	0.56	2.7E-03	-0.16	4.3E-01	-0.08	6.9E-01	-0.04	8.3E-01	-0.03	8.7E-01	-0.01	9.6E-01	-0.10	6.4E-01	0.39	4.6E-02	0.02	9.1E-01
ENSCAFG0000003144	VAMP3	turquoise	EC_M6	0.12	5.7E-01	0.64	4.6E-04	-0.35	7.7E-02	0.13	5.4E-01	0.06	7.9E-01	0.02	9.1E-01	-0.18	3.7E-01	0.02	9.2E-01	0.04	8.4E-01	-0.07	7.4E-01
ENSCAFG0000001062	LCK	turquoise	EC_M6	0.12	5.7E-01	0.55	3.5E-03	-0.06	7.9E-01	0.09	6.7E-01	-0.25	2.2E-01	-0.26	1.9E-01	0.34	8.7E-02	-0.03	8.7E-01	0.22	2.8E-01	0.21	3.0E-01
ENSCAFG0000000203	LNX1	turquoise	EC_M6	0.12	5.7E-01	0.63	6.0E-04	0.12	5.7E-01	-0.27	1.8E-01	-0.39	5.1E-02	-0.22	2.8E-01	0.14	5.0E-01	0.08	6.9E-01	0.05	7.9E-01	0.33	1.0E-01
ENSCAFG0000000215	ENSCAFG0000000215	turquoise	EC_M6	0.12	5.7E-01	0.85	3.0E-08	-0.31	1.2E-01	-0.11	5.8E-01	0.02	9.2E-01	-0.11	5.9E-01	-0.10	6.2E-01	-0.14	5.1E-01	-0.08	7.0E-01	-0.05	8.2E-01
ENSCAFG0000001890	ZNF449	cyan	EC_M2	0.12	5.7E-01	0.23	2.5E-01	0.52	5.9E-03	0.02	9.4E-01	-0.82	2.3E-07	-0.08	6.9E-01	-0.23	2.7E-01	0.06	7.6E-01	0.05	7.9E-01	0.77	4.7E-06
ENSCAFG0000000488	GTF3C2	cyan	EC_M2	0.12	5.7E-01	0.01	9.4E-01	0.66	2.4E-04	0.18	3.8E-01	-0.88	2.3E-09	-0.18	3.7E-01	-0.06	7.7E-01	-0.08	6.9E-01	0.19	3.5E-01	0.81	4.6E-07
ENSCAFG0000001088	UPK1B	magenta	EC_M13	0.12	5.7E-01	0.00	9.8E-01	0.08	7.0E-01	-0.11	6.0E-01	-0.18	3.7E-01	-0.07	7.5E-01	0.01	9.7E-01	-0.05	8.0E-01	0.67	1.6E-04	0.17	4.0E-01
ENSCAFG0000001555	L3HYPDH	cyan	EC_M2	0.12	5.7E-01	-0.59	1.7E-03	0.61	9.8E-04	0.11	5.8E-01	-0.52	6.0E-03	-0.06	7.8E-01	-0.08	7.0E-01	0.03	8.9E-01	-0.19	3.5E-01	0.46	1.8E-02
ENSCAFG0000001652	ARL5C	grey	EC_M10	0.12	5.7E-01	-0.03	9.0E-01	0.22	2.8E-01	-0.14	4.9E-01	-0.34	9.1E-02	-0.14	5.0E-01	0.16	4.5E-01	-0.17	4.1E-01	-0.14	5.1E-01	0.26	2.1E-01
ENSCAFG0000001200	WDR93	grey	EC_M10	0.12	5.7E-01	-0.11	5.8E-01	0.30	1.4E-01	-0.37	6.3E-02	-0.39	4.7E-02	0.14	4.9E-01	-0.34	8.6E-02	0.03	8.8E-01	0.12	5.5E-01	0.32	1.1E-01
ENSCAFG0000000990	DPCD	grey	EC_M10	0.12	5.7E-01	-0.69	8.2E-05	-0.18	3.8E-01	-0.06	7.6E-01	0.42	3.1E-02	0.18	3.9E-01	-0.30	1.3E-01	0.01	9.7E-01	-0.15	4.5E-01	-0.49	1.1E-02
ENSCAFG0000000398	JCAD	grey	EC_M10	0.12	5.7E-01	0.65	3.1E-04	-0.68	1.2E-04	-0.21	3.0E-01	0.47	1.6E-02	0.03	9.0E-01	-0.10	6.1E-01	-0.02	9.2E-01	0.24	2.3E-01	-0.54	4.5E-03
ENSCAFG0000001447	MEOX1	darkgreen	EC_M4	0.12	5.8E-01	0.48	1.4E-02	-0.72	3.2E-05	-0.19	3.5E-01	0.54	4.3E-03	0.08	7.0E-01	-0.08	7.1E-01	0.11	6.1E-01	-0.07	7.4E-01	-0.64	4.8E-04
ENSCAFG0000002240	ENSCAFG0000002240	grey	EC_M10	0.11	5.8E-01	0.22	2.9E-01	0.07	7.4E-01	0.15	4.6E-01	-0.27	1.8E-01	-0.19	3.5E-01	-0.19	3.5E-01	0.03	8.8E-01	0.19	3.5E-01	0.20	3.4E-01
ENSCAFG0000001744	ENSCAFG0000001744	grey	EC_M10	0.11	5.8E-01	-0.04	8.4E-01	-0.13	5.4E-01	0.00	1.0E+00	-0.03	8.9E-01	-0.13	5.3E-01	-0.10	6.3E-01	-0.15	4.5E-01	0.34	8.6E-02	-0.03	8.9E-01
ENSCAFG0000001882	STK26	turquoise	EC_M6	0.11	5.8E-01	0.71	4.2E-05	-0.16	4.3E-01	-0.18	3.8E-01	-0.11	6.0E-01	-0.19	3.6E-01	-0.11	5.8E-01	-0.03	8.9E-01	0.37	6.1E-02	0.12	5.5E-01

ENSCAFG0000001405	CCDC50	turquoise	EC_M6	0.11	5.8E-01	0.85	4.5E-08	-0.45	2.3E-02	-0.06	7.6E-01	0.10	6.1E-01	-0.06	7.7E-01	-0.03	8.9E-01	-0.02	9.1E-01	-0.13	5.2E-01	-0.15	4.8E-01
ENSCAFG0000003262	SRM	grey	EC_M10	0.11	5.8E-01	-0.35	8.2E-02	-0.30	1.3E-01	-0.18	3.8E-01	0.47	1.6E-02	0.32	1.1E-01	-0.14	4.8E-01	-0.01	9.5E-01	0.03	8.7E-01	-0.57	2.1E-03
ENSCAFG0000000917	EMILIN3	grey	EC_M10	0.11	5.8E-01	-0.22	2.9E-01	0.32	1.1E-01	0.32	1.2E-01	-0.39	4.9E-02	-0.17	3.9E-01	-0.09	6.6E-01	-0.10	6.2E-01	0.17	4.1E-01	0.33	9.6E-02
ENSCAFG0000000547	RNF169	grey	EC_M10	0.11	5.8E-01	0.00	9.8E-01	0.13	5.4E-01	-0.02	9.1E-01	-0.33	9.5E-02	0.11	6.1E-01	-0.11	6.1E-01	-0.26	1.9E-01	-0.49	1.1E-02	0.21	3.1E-01
ENSCAFG0000003191	TMEM26	turquoise	EC_M6	0.11	5.8E-01	0.66	2.5E-04	-0.08	7.1E-01	-0.13	5.4E-01	-0.26	2.1E-01	-0.17	4.0E-01	-0.14	5.0E-01	0.24	2.4E-01	0.38	5.5E-02	0.17	4.0E-01
ENSCAFG0000000757	ENSCAFG0000000757	grey	EC_M10	0.11	5.8E-01	-0.48	1.4E-02	-0.12	5.7E-01	0.18	3.9E-01	0.15	4.8E-01	0.11	5.8E-01	0.04	8.4E-01	-0.29	1.5E-01	-0.10	6.3E-01	-0.20	3.3E-01
ENSCAFG0000002980	ENSCAFG0000002980	grey	EC_M10	0.11	5.8E-01	0.00	1.0E+00	0.05	8.1E-01	-0.23	2.6E-01	-0.15	4.5E-01	0.03	8.8E-01	0.04	8.3E-01	0.07	7.2E-01	-0.02	9.4E-01	0.04	8.6E-01
ENSCAFG0000003160	XAF1	turquoise	EC_M6	0.11	5.8E-01	0.75	1.2E-05	-0.11	5.9E-01	-0.08	6.9E-01	-0.23	2.5E-01	-0.08	7.0E-01	-0.10	6.1E-01	-0.11	6.0E-01	-0.15	4.5E-01	0.19	3.5E-01
ENSCAFG0000000621	ARAP3	turquoise	EC_M6	0.11	5.8E-01	0.84	7.8E-08	-0.06	7.6E-01	-0.10	6.1E-01	-0.35	7.7E-02	-0.31	1.3E-01	-0.12	5.5E-01	-0.08	7.1E-01	0.19	3.5E-01	0.28	1.7E-01
ENSCAFG0000001562	RHNO1	grey	EC_M10	0.11	5.8E-01	-0.03	8.9E-01	-0.32	1.1E-01	-0.45	2.2E-02	0.41	3.8E-02	0.02	9.2E-01	0.13	5.3E-01	-0.22	2.7E-01	-0.20	3.2E-01	-0.43	2.8E-02
ENSCAFG0000000902	DDX54	grey	EC_M10	0.11	5.8E-01	-0.46	1.8E-02	-0.10	6.4E-01	-0.28	1.6E-01	0.30	1.3E-01	0.20	3.2E-01	-0.13	5.4E-01	0.13	5.2E-01	-0.01	9.7E-01	-0.37	6.4E-02
ENSCAFG0000001276	CYP20A1	grey	EC_M10	0.11	5.8E-01	-0.37	6.3E-02	-0.39	5.2E-02	0.11	5.9E-01	0.40	4.0E-02	0.17	4.1E-01	0.09	6.6E-01	-0.20	3.2E-01	-0.41	3.9E-02	-0.51	7.9E-03
ENSCAFG0000000822	ANKRA2	darkgreen	EC_M4	0.11	5.8E-01	0.23	2.6E-01	-0.81	6.8E-07	-0.05	8.2E-01	0.74	1.7E-05	0.39	4.7E-02	0.12	5.5E-01	0.06	7.8E-01	0.07	7.5E-01	-0.81	5.1E-07
ENSCAFG0000003221	ZNF71	turquoise	EC_M6	0.11	5.8E-01	0.88	3.7E-09	-0.28	1.6E-01	-0.28	1.7E-01	-0.06	7.6E-01	-0.10	6.3E-01	0.03	8.7E-01	0.04	8.5E-01	0.12	5.6E-01	0.02	9.3E-01
ENSCAFG0000002575	ENSCAFG0000002575	grey	EC_M10	0.11	5.8E-01	0.06	7.8E-01	0.08	7.2E-01	-0.08	7.1E-01	-0.11	5.8E-01	0.01	9.4E-01	0.01	9.5E-01	-0.06	7.8E-01	0.04	8.4E-01	0.03	8.9E-01
ENSCAFG0000002844	ENSCAFG0000002844	grey	EC_M10	0.11	5.8E-01	-0.04	8.5E-01	0.06	7.7E-01	0.15	4.7E-01	-0.11	5.9E-01	0.17	4.0E-01	0.11	5.8E-01	0.10	6.1E-01	0.07	7.4E-01	0.03	8.7E-01
ENSCAFG0000002016	BTBD8	grey	EC_M10	0.11	5.8E-01	0.57	2.3E-03	0.00	9.8E-01	-0.19	3.4E-01	-0.37	6.7E-02	-0.08	7.1E-01	-0.26	2.0E-01	-0.04	8.6E-01	-0.05	8.1E-01	0.29	1.6E-01
ENSCAFG0000002195	ENSCAFG0000002195	grey	EC_M10	0.11	5.8E-01	-0.37	6.2E-02	-0.05	8.0E-01	0.22	2.8E-01	0.03	8.7E-01	0.17	4.2E-01	0.15	4.6E-01	0.15	4.7E-01	0.14	4.8E-01	-0.14	4.8E-01
ENSCAFG0000001692	ALOX15B	grey	EC_M10	0.11	5.8E-01	-0.19	3.4E-01	-0.34	8.6E-02	-0.03	8.9E-01	0.36	6.7E-02	-0.04	8.6E-01	-0.26	2.0E-01	0.38	5.3E-02	-0.18	3.9E-01	-0.50	1.0E-02
ENSCAFG0000001197	POU2F3	turquoise	EC_M6	0.11	5.8E-01	0.78	3.2E-06	-0.10	6.3E-01	-0.09	6.6E-01	-0.24	2.3E-01	-0.08	7.0E-01	-0.10	6.3E-01	-0.12	5.6E-01	-0.17	4.1E-01	0.18	3.7E-01
ENSCAFG0000001752	TSPAN6	darkgreen	EC_M4	0.11	5.8E-01	0.24	2.3E-01	-0.83	1.3E-07	-0.27	1.8E-01	0.80	7.9E-07	0.35	8.2E-02	0.05	8.0E-01	-0.10	6.2E-01	-0.15	4.8E-01	-0.87	8.4E-09
ENSCAFG0000000647	ENSCAFG0000000647	cyan	EC_M2	0.11	5.8E-01	0.30	1.4E-01	0.54	4.2E-03	0.15	4.8E-01	-0.83	1.1E-07	-0.31	1.2E-01	-0.02	9.4E-01	-0.22	2.9E-01	-0.03	8.7E-01	0.80	9.3E-07
ENSCAFG0000000599	C15H12orf29	grey	EC_M10	0.11	5.8E-01	-0.27	1.8E-01	-0.33	9.5E-02	0.03	9.0E-01	0.37	6.2E-02	0.04	8.5E-01	-0.43	2.8E-02	0.26	1.9E-01	-0.20	3.3E-01	-0.46	1.9E-02
ENSCAFG0000000818	ARFIP1	grey	EC_M10	0.11	5.8E-01	0.27	1.8E-01	-0.44	2.3E-02	0.04	8.6E-01	0.28	1.6E-01	0.08	7.0E-01	-0.20	3.3E-01	-0.04	8.5E-01	-0.22	2.8E-01	-0.30	1.4E-01
ENSCAFG0000001513	SMURF1	darkgreen	EC_M4	0.11	5.8E-01	0.27	1.8E-01	-0.81	7.0E-07	-0.30	1.4E-01	0.73	2.3E-05	0.23	2.6E-01	0.06	7.6E-01	-0.02	9.3E-01	-0.22	2.9E-01	-0.82	3.3E-07
ENSCAFG0000000586	PLCL2	grey	EC_M10	0.11	5.8E-01	0.08	6.8E-01	0.00	9.9E-01	0.26	2.0E-01	-0.18	3.9E-01	-0.15	4.6E-01	-0.24	2.4E-01	0.31	1.3E-01	0.36	7.4E-02	0.09	6.7E-01
ENSCAFG0000001150	RRH	grey	EC_M10	0.11	5.8E-01	-0.09	6.6E-01	-0.35	8.3E-02	-0.01	9.6E-01	0.37	5.9E-02	0.29	1.5E-01	-0.11	5.8E-01	0.14	5.1E-01	0.11	6.0E-01	-0.40	4.2E-02
ENSCAFG0000001114	ESAM	turquoise	EC_M6	0.11	5.8E-01	0.85	5.5E-08	-0.20	3.3E-01	-0.14	4.8E-01	-0.14	5.1E-01	-0.12	5.7E-01	-0.11	6.0E-01	-0.18	3.7E-01	0.14	5.0E-01	0.06	7.7E-01
ENSCAFG0000001999	CMIP	turquoise	EC_M6	0.11	5.8E-01	0.80	7.2E-07	-0.28	1.7E-01	0.07	7.4E-01	-0.06	7.7E-01	-0.18	3.7E-01	-0.10	6.2E-01	-0.09	6.7E-01	-0.22	2.9E-01	-0.01	9.7E-01
ENSCAFG0000000332	LSMEM1	grey	EC_M10	0.11	5.8E-01	-0.23	2.5E-01	-0.52	6.6E-03	-0.14	5.0E-01	0.56	3.0E-03	0.37	6.5E-02	-0.10	6.2E-01	0.26	2.0E-01	-0.06	7.5E-01	-0.64	4.9E-04
ENSCAFG0000002014	COG4	grey	EC_M10	0.11	5.8E-01	0.17	4.1E-01	-0.03	9.0E-01	-0.21	3.1E-01	-0.10	6.2E-01	0.02	9.4E-01	-0.27	1.8E-01	-0.04	8.5E-01	0.07	7.5E-01	0.08	7.1E-01
ENSCAFG0000000466	PVR	grey	EC_M10	0.11	5.8E-01	0.49	1.1E-02	-0.75	1.3E-05	-0.21	3.1E-01	0.62	7.3E-04	0.12	5.4E-01	0.12	5.7E-01	0.03	8.7E-01	-0.06	7.7E-01	-0.69	9.0E-05
ENSCAFG0000002394	ENSCAFG0000002394	grey	EC_M10	0.11	5.8E-01	0.30	1.3E-01	0.00	9.9E-01	0.02	9.1E-01	-0.29	1.5E-01	-0.30	1.4E-01	-0.11	6.1E-01	0.17	4.1E-01	-0.17	4.0E-01	0.19	3.5E-01
ENSCAFG0000000123	ENSCAFG0000000123	grey	EC_M10	0.11	5.8E-01	-0.28	1.6E-01	-0.14	5.0E-01	-0.01	9.7E-01	0.25	2.2E-01	0.32	1.1E-01	-0.02	9.4E-01	-0.19	3.6E-01	-0.15	4.5E-01	-0.33	9.5E-02
ENSCAFG0000000479	LRRFIP2	grey	EC_M10	0.11	5.8E-01	0.34	8.8E-02	-0.56	2.9E-03	0.04	8.3E-01	0.40	4.1E-02	0.07	7.4E-01	-0.23	2.5E-01	0.07	7.5E-01	0.27	1.9E-01	-0.48	1.4E-02
ENSCAFG0000000121	ERCC6L2	cyan	EC_M2	0.11	5.8E-01	-0.01	9.5E-01	0.46	1.9E-02	0.21	3.1E-01	-0.69	9.3E-05	-0.09	6.5E-01	-0.09	6.6E-01	-0.08	6.9E-01	-0.39	5.0E-02	0.63	6.3E-04
ENSCAFG0000000135	PDGFB	turquoise	EC_M6	0.11	5.8E-01	0.89	7.0E-10	-0.20	3.4E-01	-0.04	8.6E-01	-0.17	4.1E-01	-0.17	4.1E-01	-0.12	5.6E-01	-0.22	2.9E-01	0.08	7.1E-01	0.09	6.5E-01
ENSCAFG0000001473	CLTCL1	darkgreen	EC_M4	0.11	5.8E-01	0.03	8.9E-01	-0.75	9.0E-06	-0.22	2.7E-01	0.80	1.2E-06	0.11	6.1E-01	-0.01	9.4E-01	-0.03	8.7E-01	0.04	8.6E-01	-0.84	6.6E-08
ENSCAFG0000000723	NOL4L	turquoise	EC_M6	0.11	5.8E-01	0.95	1.1E-13	-0.30	1.4E-01	-0.06	7.6E-01	-0.07	7.4E-01	-0.19	3.5E-01	0.00	9.8E-01	-0.10	6.2E-01	0.11	5.9E-01	0.02	9.2E-01
ENSCAFG0000003114	LAX1	grey	EC_M10	0.11	5.8E-01	0.06	7.8E-01	-0.23	2.6E-01	0.31	1.3E-01	0.14	4.8E-01	-0.14	5.1E-01	0.23	2.6E-01	-0.11	5.8E-01	-0.18	3.8E-01	-0.22	2.7E-01
ENSCAFG0000000794	FDFT1	grey	EC_M10	0.11	5.8E-01	-0.08	6.8E-01	0.31	1.3E-01	0.03	8.9E-01	-0.42	3.3E-02	-0.28	1.7E-01	-0.11	5.8E-01	-0.02	9.3E-01	-0.46	1.8E-02	0.33	1.0E-01
ENSCAFG0000001261	LMO3	grey	EC_M10	0.11	5.8E-01	-0.13	5.3E-01	0.26	2.0E-01	0.10	6.4E-01	-0.35	8.2E-02	-0.08	7.0E-01	-0.11	5.9E-01	0.14	4.9E-01	-0.08	7.1E-01	0.24	2.3E-01
ENSCAFG0000002900	ENSCAFG0000002900	grey	EC_M10	0.11	5.8E-01	0.10	6.4E-01	0.56	3.0E-03	0.13	5.1E-01	-0.78	2.7E-06	-0.01	9.6E-01	-0.05	8.3E-01	-0.16	4.4E-01	-0.07	7.2E-01	0.70	6.1E-05
ENSCAFG0000000432	KPNA3	grey	EC_M10	0.11	5.9E-01	-0.23	2.5E-01	-0.61	8.9E-04	-0.24	2.5E-01	0.76	7.2E-06	0.17	4.1E-01	-0.10	6.3E-01	-0.04	8.5E-01	-0.25	2.1E-01	-0.81	6.8E-07
ENSCAFG0000000003	ENSCAFG0000000003	grey	EC_M10	0.11	5.9E-01	-0.02	9.1E-01	-0.23	2.5E-01	0.05	8.2E-01	0.17	4.1E-01	-0.08	7.0E-01	-0.06	7.8E-01	-0.08	7.0E-01	-0.14	4.8E-01	-0.29	1.5E-01
ENSCAFG0000001489	ENSCAFG0000001489	grey	EC_M10	0.11	5.9E-01	-0.02	9.1E-01	-0.23	2.5E-01	0.05	8.2E-01	0.17	4.1E-01	-0.08	7.0E-01	-0.06	7.8E-01	-0.08	7.0E-01	-0.14	4.8E-01	-0.29	1.5E-01
ENSCAFG0000000526	SPRY2	grey	EC_M10	0.11	5.9E-01	-0.21	3.0E-01	0.64	4.7E-04	0.15	4.5E-01	-0.77	3.8E-06	-0.15	4.6E-01	-0.17	4.2E-01	-0.16	4.4E-01	0.08	7.1E-01	0.69	9.0E-05
ENSCAFG0000003171	ENSCAFG0000003171	grey	EC_M10	0.11	5.9E-01	-0.29	1.5E-01	0.32	1.1E-01	0.08	6.8E-01	-0.32	1.1E-01	0.07	7.3E-01	-0.47	1.4E-02	-0.04	8.6E-01	0.00	1.0E+00	0.27	1.9E-01
ENSCAFG0000000067	DPYS	grey	EC_M10	0.11	5.9E-01	0.03	8.7E-01	-0.23	2.6E-01	-0.14	5.1E-01	0.20	3.4E-01	-0.10	6.2E-01	-0.18	3.9E-01	-0.19	3.5E-01	-0.15	4.8E-01	-0.25	2.1E-01
ENSCAFG0000000019	SHMT2	grey	EC_M10	0.11	5.9E-01	0.14	4.8E-01	-0.62	8.2E-04	0.01	9.6E-01	0.61	8.8E-04	0.17	4.2E-01	-0.31	1.3E-01	0.06	7.7E-01	0.16	4.3E-01	-0.71	4.8E-05
ENSCAFG0000000360	GJB3	grey	EC_M10	0.11	5.9E-01	-0.52	6.1E-03	0.44	2.3E-02	0.14	5.0E-01	-0.39	5.1E-02	-0.08	7.1E-01	-0.23	2.6E-01	0.01	9.5E-01	0.03	8.9E-01	0.28	1.6E-01
ENSCAFG0000001284	IL10RA	turquoise	EC_M6	0.11	5.9E-01	0.76	7.1E-06	-0.22	2.7E-01	-0.23	2.5E-01	-0.10	6.4E-01	-0.21	3.1E-01	0.24	2.4E-01	0.09	6.7E-01	0.00	1.0E+00	0.04	8.6E-01
ENSCAFG0000002257	ENSCAFG0000002257	grey	EC_M10	0.11	5.9E-01	-0.31	1.3E-01	0.21	2.9E-01	0.24	2.4E-01	-0.20	3.3E-01	0.04	8.3E-01	0.20	3.4E-01	0.12	5.7E-01	0.03	9.0E-01	0.13	5.2E-01
ENSCAFG0000000330	NFU1	grey	EC_M10	0.11	5.9E-01	0.30	1.4E-01	-0.16	4.3E-01	0.19	3.4E-01	-0.03	8.8E-01	0.08	7.2E-01	-0.49	1.0E-02	-0.25	2.3E-01	0.28	1.7E-01	0.03	8.8E-01
ENSCAFG0000001485	RNF4	grey	EC_M10	0.11	5.9E-01	0.25	2.2E-01	-0.40	4.4E-02	-0.45	2.2E-02	0.33	9.4E-02	0.21	3.1E-01	0.00	9.9E-01	0.05	8.0E-01	0.24	2.5E-01	-0.35	7.9E-02
ENSCAFG0000000526	EMSY	grey	EC_M10	0.11	5.9E-01	0.34	9.4E-02	0.22	2.9E-01	-0.03	8.8E-01	-0.51	7.9E-03	0.07	7.2E-01	-0.21	3.0E-01	-0.07	7.4E-01	-0.18	3.8E-01	0.43	2.7E-02
ENSCAFG0000001239	ENSCAFG0000001239	cyan	EC_M2	0.11	5.9E-01	-0.35	7.8E-02	0.51	7.7E-03	0.12	5.5E-01	-0.55	3.9E-03	0.02	9.4E-01	0.25	2.2E-01	-0.05	8.2E-01	-0.49	1.1E-02	0.43	2.6E-02
ENSCAFG0000001929	ZNF263	grey	EC_M10	0.11	5.9E-01	0.05	8.1E-01	0.28	1.6E-01	0.44	2.5E-02	-0.49	1.2E-02	0.14	5.1E-01	0.09	6.6E-01	0.06	7.9E-01	0.05	8.2E-01	0.37	5.9E-02
ENSCAFG0000001685	HOXB6	turquoise	EC_M6	0.11	5.9E-01	0.57	2.5E-03	-0.39	4.7E-02	0.31	1.2E-01	0.17	4.2E-01	0.09	6.5E-01	-0.06	7.9E-01	-0.29	1.6E-01	-0.27	1.9E-01	-0.26	2.1E-01
ENSCAFG0000001504	MMP13	turquoise	EC_M6	0.11	5.9E-01	0.75	9.7E-06	-0.56	2.8E-03	-0.08	7.0E-01	0.31	1.2E-01	-0.17	4.1E-01	-0.13	5.2E-01	-0.13	5.1E-01	-0.14	5.1E-01	-0.35	7.8E-02
ENSCAFG0000003238	ERH	grey	EC_M10	0.11	5.9E-01	-0.28	1.6E-01	0.07	7.3E-01	-0.29	1.5E-01	0.06	7.6E-01	0.22	2.8E-01	-0.05	8.0E-01	-0.07	7.4E-01	0.14	5.1E-01	-0.11	5.9E-01
ENSCAFG0000001474	NAA40	grey	EC_M10	0.11	5.9E-01	-0.05	8.0E-01	-0.21	2.9E-01	0.05	8.3E-01	0.23	2.5E-01	0.05	8.3E-01	0.23	2.5E-01	0.14	5.1E-01	-0.06	7.6E-01	-0.30	1.4E-01
ENSCAFG0000000481	LLGL2	turquoise	EC_M6	0.11	5.9E-01	0.37	6.5E-02	0.08	6.9E-01	-0.21	3.0E-01	-0.34	9.0E-02	-0.04	8.5E-01	0.14	4.9E-01	-0.42	3.4E-02	0.13	5.2E-01	0.30	1.4E-01
ENSCAFG0000000656	ENSCAFG0000000656	grey	EC_M10	0.11	5.9E-01	0.07	7.3E-01	-0.10	6.4E-01	0.15	4.6E-01	-0.07	7.2E-01	-0.12	5.7E-01	0.11	5.9E-01	-0.15	4.5E-01	-0.14	5.1E-01	0.01	9.6E-01
ENSCAFG0000001753	TNRC6A	cyan	EC_M2	0.11	5.9E-01	-0.36	7.5E-02	0.41	3.8E-02	0.47	1.6E-02	-0.51	7.3E-03	-0.11	5.8E-01	-0.17	4.1E-01	-0.19	3.6E-01	-0.16	4.3E-01	0.40	4.6E-02
ENSCAFG0000000416	NDNF	darkmagenta	EC_M12	0.11	5.9E-01	-0.09	6.6E-01	-0.18	3.8E-01	-0.13	5.4E-01	0.12	5.7E-01	0.01	9.7E-01	-0.16	4.4E-01	0.62	7.5E-04	0.04	8.5E-01	-0.21	3.1E-01
ENSCAFG0000001058	ENSCAFG0000001058	grey	EC_M10	0.11	5.9E-01	-0.14	5.0E-01	-0.33	9.4E-02	-0.07	7.5E-01	0.28	1.6E-01	-0.18	3.7E-01	-0.15	4.6E-01	0.28	1.6E-01	0.00	9.9E-01	-0.33	9.6E-02
ENSCAFG0000003274	ENSCAFG0000003274	grey	EC_M10	0.11	5.9E-01	0.10	6.3E-01	-0.15	4.7E-01	0.26	2.0E-01	0.04	8.4E-01	0.01	9.8E-01	-0.07	7.5E-01	-0.03	8.8E-01	-0.38	5.7E-02	-0.11	6.0E-01
ENSCAFG0000000463	ENSCAFG0000000463	grey	EC_M10	0.11	5.9E-01	-0.30	1.3E-01	0.20	3.3E-01	0.16	4.3E-01	-0.28	1.7E-01	-0.22	2.9E-01	-0.23	2.6E-01	-0.25	2.2E-01	0.02	9.3E-01	0.25	2.2E-01
ENSCAFG0000000346	ATP6V1C2	grey	EC_M10	0.11	5.9E-01	-0.03	8.7E-01	0.10	6.4E-01	0.52	6.6E-03	-0.18	3.9E-01	-0.13	5.2E-01	-0.17	4.1E-01	-0.10	6.4E-01	-0.16	4.4E-01	0.16	4.4E-01
ENSCAFG0000000796	MMP24	turquoise	EC_M6	0.11	5.9E-01	0.65	3.5E-04	-0.33	1.1E-01	-0.15	4.7E-01	0.03	8.8E-01	0.04	8.6E-01	0.01	9.5E-01	-0.19	3.5E-01	0.50	8.6E-03	-0.02	9.1E-01
ENSCAFG0000001049	ENSCAFG0000001049	magenta	EC_M13	0.11	5.9E-01	0.40	4.4E-02	-0.31	1.2E-01	-0.05	7.9E-01	0.10	6.2E-01	0.14	5.1E-01	-0.12	5.5E-01	-0.09	6.8E-01	0.73	2.4E-05	-0.14	4.9E-01
ENSCAFG0000001012	FASTKD3	cyan	EC_M2	0.11	5.9E-01	-0.29	1.5E-01	0.36	7.3E-02	-0.15	4.5E-01	-0.33	9.7E-02	0.15	4.7E-01	-0.01	9.6E-01	-0.16	4.3E-01	-0.19	3.5E-01	0.23	2.6E-01
ENSCAFG0000000528	TIE1	turquoise	EC_M6	0.11	5.9E-01	0.85	5.0E-08	-0.12	5.5E-01	-0.02	9.1E-01	-0.23	2.6E-01	-0.12	5.7E-01	-0.13	5.3E-01	-0.12	5.5E-01	0.10	6.1E-01	0.14	4.8E-01
ENSCAFG0000001936	NOC2L	grey	EC_M10	0.11	5.9E-01	-0.09	6.7E-01	0.30	1.4E-01	-0.02	9.4E-01	-0.27	1.8E-01	-0.03	9.0E-01	0.11	6.0E-01	0.08	6.9E-01	0.09	6.5E-01	0.26	1.9E-01
ENSCAFG0000002566	ENSCAFG0000002566	grey	EC_M10	0.11	5.9E-01	0.10	6.2E-01	-0.37	6.3E-02	0.37	6.3E-02	0.31	1.3E-01	0.37	6.1E-02	0.15	4.6E-01	-0.35	7.8E-02	-0.04	8.6E-01	-0.34	8.6E-02
ENSCAFG0000003087	ENSCAFG0000003087	grey	EC_M10	0.11	5.9E-01	0.02	9.1E-01	0.01	9.7E-01	-0.15	4.6E-01	-0.05	8.0E-01	-0.07	7.3E-01	-0.15	4.6E-01	0.33	1.0E-01	-0.03	8.8E-01	-0.02	9.3E-01
ENSCAFG0000000908	ZDHHC16	grey	EC_M10	0.11	5.9E-01	-0.20	3.2E-01	-0.27	1.8E-01	-0.09	6.6E-01	0.41	3.6E-02	0.17	3.9E-01	-0.10	6.1E-01	-0.06	7.7E-01	0.16	4.3E-01	-0.46	1.8E-02
ENSCAFG0000002977	ENSCAFG0000002977	grey	EC_M10	0.11	5.9E-01	-0.36	6.7E-02	-0.18	3.8E-01	0.12	5.5E-01	0.28	1.6E-01	0.10	6.2E-01	-0.47	1.4E-02	-0.29	1.5E-01	-0.21	2.9E-01	-0.38	5.4E-02
ENSCAFG0000000988	JARID2	turquoise	EC_M6	0.11	5.9E-01	0.63	6.4E-04	-0.40	4.2E-02	0.09	6.7E-01	0.07	7.4E-01	0.14	4.9E-01	-0.15	4.7E-01	0.26	1.9E-01	0.04	8.5E-01	-0.14	4.9E-01
ENSCAFG0000002862	OLFML2A	cyan	EC_M2	0.11	6.0E-01	0.49	1.1E-02	0.46	1.7E-02	0.06	7.7E-01	-0.79	2.0E-06	-0.37	6.6E-02	-0.10	6.3E-01	-0.17	4.1E-01	-0.06	7.6E-01	0.75	8.6E-06
ENSCAFG0000000478	CASKIN2	turquoise	EC_M6	0.11	6.0E-01	0.92	1.7E-11	-0.06	7.8E-01	-0.16	4.5E-01	-0.33	9.6E-02	-0.26	2.1E-01	-0.04	8.4E-01	-0.05	8.0E-01	0.07	7.2E-01	0.30	1.4E-01
ENSCAFG0000000555	ENSCAFG0000000555	cyan	EC_M2	0.11	6.0E-01	-0.27	1.9E-01	0.52	6.3E-03	0.01	9.5E-01	-0.66	2.6E-04	0.16	4.2E-01	0.11	5.9E-01	0.14	4.9E-01	-0.16	4.4E-01	0.56	3.0E-03
ENSCAFG0000003070	ENSCAFG0000003070	grey	EC_M10	0.11	6.0E-01	0.24	2.4E-01	0.04	8.5E-01	-0.32	1.1E-01	-0.16	4.3E-01	-0.04	8.5E-01	0.22	2.8E-01	0.18	3.8E-01	-0.33	1.0E-01	0.11	5.9E-01
ENSCAFG0000001236	ENSCAFG0000001236	grey	EC_M10	0.11	6.0E-01	-0.12	5.4E-01	-0.44	2.5E-02	-0.01	9.8E-01	0.48	1.3E-02	0.01	9.6E-01	-0.48	1.4E-02	-0.02	9.4E-01	0.13	5.2E-01	-0.55	3.5E-03
ENSCAFG0000000302	HSDL2	grey	EC_M10	0.11	6.0E-01	-0.35	8.0E-02	-0.33	1.0E-01	-0.22	2.9E-01	0.46	1.8E-02	0.03	8.8E-01	-0.06	7.5E-01	0.18	3.7E-01	0.15	4.6E-01	-0.51	8.4E-03
ENSCAFG0000002984	TGIF2-RAB5IF	turquoise	EC_M6	0.11	6.0E-01	0.46	1.9E-02	-0.30	1.3E-01	-0.26	1.9E-01	0.16	4.2E-01	0.02	9.4E-01	0.16	4.2E-01	-0.43	2.7E-02	0.30	1.4E-01	-0.18	3.7E-01
ENSCAFG0000002620	ENSCAFG0000002620	violet	EC_M7	0.11	6.0E-01	-0.13	5.3E-01	-0.12	5.5E-01	-0.08	6.9E-01	0.10	6.4E-01	0.79	1.6E-06	-0.10	6.2E-01	-0.12	5.5E-01	-0.17	4.0E-01	-0.16	4.2E-01
ENSCAFG0000003144	ENSCAFG0000003144	violet	EC_M7	0.11	6.0E-01	-0.13	5.3E-01	-0.12	5.5E-01	-0.08	6.9E-01	0.10	6.4E-01	0.79	1.6E-06	-0.10	6.2E-01	-0.12	5.5E-01	-0.17	4.0E-01	-0.16	4.2E-01
ENSCAFG0000001106	FOXN3	grey	EC_M10	0.11	6.0E-01	0.23	2.6E-01	0.19	3.6E-01	0.34	9.1E-02	-0.46	1.8E-02	-0.19	3.5E-01	-0.03	8.9E-01	-0.10	6.3E-01	0.19	3.7E-01	0.41	3.9E-02
ENSCAFG0000000289	AKAP2	grey	EC_M10	0.11	6.0E-01	0.42	3.3E-02	-0.71	5.1E-05	-0.21	3.1E-01	0.60	1.1E-03	0.00	1.0E+00	-0.02	9.4E-01	0.17	4.1E-01	0.31	1.3E-01	-0.64	4.4E-04
ENSCAFG0000000460	NAT9	grey	EC_M10	0.11	6.0E-01	-0.13	5.1E-01	0.00	1.0E+00	0.25	2.2E-01	-0.10	6.4E-01	-0.02	9.1E-01	-0.03	8.8E-01	0.19	3.5E-01	0.41	3.5E-02	0.03	9.0E-01
ENSCAFG0000001253	PTPN14	grey	EC_M10	0.11	6.0E-01	0.34	9.3E-02	-0.73	2.5E-05	-0.06	7.8E-01	0.63	6.3E-04	0.04	8.5E-01	0.21	3.1E-01	-0.03	8.9E-01	0.22	2.9E-01	-0.68	1.5E-04
ENSCAFG0000001930	ENSCAFG0000001930	turquoise	EC_M6	0.11	6.0E-01	0.52	6.9E-03	0.10	6.4E-01	-0.12	5.4E-01	-0.40	4.4E-02	-0.09	6.7E-01	-0.09	6.5E-01	-0.03	8.9E-01	-0.13	5.2E-01	0.35	8.0E-02
ENSCAFG0000000519	SEPT9	grey	EC_M10	0.11	6.0E-01	0.21	2.9E-01	-0.40	4.1E-02	-0.34	9.3E-02	0.42	3.3E-02	-0.07	7.2E-01	-0.12	5.5E-01	-0.13	5.2E-01	-0.14	4.9E-01	-0.46	1.9E-02
ENSCAFG0000002915	NOVA1	grey	EC_M10	0.11	6.0E-01	-0.05	8.1E-01	0.13	5.3E-01	0.01	9.5E-01	-0.27	1.8E-01	0.07	7.3E-01	0.30	1.4E-01	0.35	7.9E-02	-0.15	4.8E-01	0.16	4.3E-01
ENSCAFG0000001061	ALDH1A3	grey	EC_M10	0.11	6.0E-01	0.07	7.3E-01	-0.16	4.3E-01	0.06	7.6E-01	0.09	6.7E-01	0.31	1.3E-01	-0.04	8.5E-01	-0.23	2.6E-01	-0.23	2.5E-01	-0.15	4.8E-01
ENSCAFG0000000989	DTNBP1	turquoise	EC_M6	0.11	6.0E-01	0.40	4.1E-02	-0.35	7.9E-02	-0.08	7.1E-01	0.16	4.2E-01	-0.01	9.5E-01	0.10	6.3E-01	0.02	9.1E-01	-0.59	1.5E-03	-0.25	2.2E-01
ENSCAFG0000002890	SLC35D1	grey	EC_M10	0.11	6.0E-01	-0.18	3.7E-01	-0.55	3.5E-03	0.14	5.1E-01	0.63	5.1E-04	0.13	5.3E-01	0.11	6.0E-01	-0.16	4.3E-01	-0.21	3.0E-01	-0.70	6.0E-05
ENSCAFG0000001310	SART1	grey	EC_M10	0.11	6.0E-01	0.24	2.5E-01	0.12	5.7E-01	0.03	8.7E-01	-0.35	7.7E-02	0.32	1.1E-01	0.42	3.1E-02	-0.07	7.2E-01	-0.09	6.7E-01	0.30	1.3E-01
ENSCAFG0000002365	ARPC4	grey	EC_M10	0.11	6.0E-01	0.21	3.0E-01	-0.62	6.9E-04	-0.21	3.0E-01	0.63	5.0E-04	-0.03	8.9E-01	-0.18	3.8E-01	-0.03	8.7E-01	-0.02	9.4E-01	-0.67	2.0E-04
ENSCAFG0000001139	RASSF8	grey	EC_M10	0.11	6.0E-01	-0.62	6.9E-04	-0.14	4.9E-01	0.13	5.3E-01	0.27	1.9E-01	0.31	1.2E-01	0.16	4.4E-01	0.01	9.6E-01	0.20	3.4E-01	-0.36	7.4E-02
ENSCAFG0000000977	ZSWIM1	turquoise	EC_M6	0.11	6.0E-01	0.46	1.9E-02	0.05	8.3E-01	-0.17	4.0E-01	-0.32	1.2E-01	0.04	8.6E-01	-0.18	3.7E-01	0.22	2.7E-01	0.56	2.8E-03	0.26	2.1E-01
ENSCAFG0000000449	CCDC174	turquoise	EC_M6	0.11	6.0E-01	0.53	5.7E-03	-0.18	3.9E-01	0.28	1.6E-01	-0.09	6.8E-01	-0.08	7.0E-01	0.03	8.9E-01	-0.06	7.8E-01	0.04	8.4E-01	0.08	7.0E-01
ENSCAFG0000000717	ZDHHC20	grey	EC_M10	0.11	6.0E-01	-0.32	1.1E-01	-0.04	8.4E-01	0.17	4.1E-01	0.11	5.9E-01	-0.01	9.8E-01	-0.45	2.1E-02	-0.01	9.6E-01	-0.30	1.4E-01	-0.14	4.8E-01
ENSCAFG0000001636	ATP6V1D	darkgreen	EC_M4	0.11	6.0E-01	-0.16	4.2E-01	-0.65	3.1E-04	0.00	9.9E-01	0.68	1.3E-04	0.21	3.1E-01	-0.15	4.7E-01	0.21	3.1E-01	-0.21	2.9E-01	-0.76	7.2E-06
ENSCAFG0000001861	CARD6	grey	EC_M10	0.11	6.0E-01	0.28	1.7E-01	0.25	2.2E-01	-0.12	5.6E-01	-0.43	3.0E-02	0.10	6.4E-01	-0.04	8.4E-01	0.03	8.7E-01	-0.46	1.8E-02	0.39	4.8E-02
ENSCAFG0000002935	FAAP24	grey	EC_M10	0.11	6.0E-01	-0.21	3.1E-01	-0.48	1.3E-02	-0.25	2.2E-01	0.59	1.5E-03	0.29	1.5E-01	0.04	8.4E-01	0.16	4.2E-01	0.03	8.9E-01	-0.65	3.4E-04
ENSCAFG0000001782	FAM199X	grey	EC_M10	0.11	6.0E-01	0.12	5.5E-01	0.17	4.1E-01	-0.07	7.4E-01	-0.34	8.5E-02	-0.52	6.2E-03	0.03	8.8E-01	-0.14	5.0E-01	0.06	7.9E-01	0.31	1.3E-01
ENSCAFG0000002322	ENSCAFG0000002322	turquoise	EC_M6	0.11	6.0E-01	0.41	3.6E-02	-0.09	6.8E-01	-0.12	5.6E-01	-0.10	6.3E-01	-0.13	5.4E-01	-0.06	7.7E-01	0.22	2.7E-01	0.47	1.6E-02	0.13	5.4E-01
ENSCAFG0000000737	NT5DC3	grey	EC_M10	0.11	6.0E-01	0.16	4.4E-01	-0.28	1.7E-01	-0.19	3.5E-01	0.21	2.9E-01	0.02	9.4E-01	-0.15	4.7E-01	0.02	9.0E-01	0.47	1.6E-02	-0.21	3.1E-01
ENSCAFG0000002007	ENSCAFG0000002007	paleturquoise	EC_M11	0.11	6.0E-01	0.51	7.4E-03	-0.04	8.4E-01	0.53	4.9E-03	-0.25	2.2E-01	-0.07	7.3E-01	-0.06	7.8E-01	-0.12	5.5E-01	-0.16	4.5E-01	0.17	4.2E-01
ENSCAFG0000003160	NUDT2	cyan	EC_M2	0.11	6.0E-01	-0.37	6.2E-02	0.55	3.5E-03	0.16	4.3E-01	-0.62	7.6E-04	0.07	7.2E-01	0.13	5.2E-01	0.14	4.9E-01	-0.21	3.1E-01	0.53	5.0E-03
ENSCAFG0000002492	ATP5PF	grey	EC_M10	0.11	6.0E-01	-0.47	1.4E-02	-0.09	6.5E-01	0.22	2.8E-01	0.26	1.9E-01	0.10	6.3E-01	-0.29	1.5E-01	-0.06	7.7E-01	0.13	5.4E-01	-0.30	1.4E-01
ENSCAFG0000000038	ENSCAFG0000000038	turquoise	EC_M6	0.11	6.0E-01	0.59	1.6E-03	0.09	6.6E-01	-0.09	6.6E-01	-0.40	4.1E-02	-0.04	8.4E-01	-0.07	7.2E-01	-0.10	6.3E-01	-0.17	4.0E-01	0.35	7.5E-02
ENSCAFG0000000533	GPR180	grey	EC_M10	0.11	6.0E-01	0.40	4.3E-02	-0.47	1.6E-02	-0.22	2.7E-01	0.33	1.0E-01	0.08	7.1E-01	0.20	3.2E-01	-0.16	4.3E-01	-0.12	5.7E-01	-0.38	5.7E-02

ENSCAFG0000003030	SDHAF2	grey	EC_M10	0.11	6.0E-01	-0.49	1.1E-02	0.06	7.7E-01	-0.18	3.9E-01	0.08	6.8E-01	0.22	2.9E-01	-0.24	2.4E-01	0.04	8.3E-01	0.03	8.8E-01	-0.18	3.9E-01
ENSCAFG0000000456	ENSCAFG0000000456	grey	EC_M10	0.11	6.0E-01	0.08	7.1E-01	-0.25	2.2E-01	-0.04	8.5E-01	0.13	5.4E-01	-0.06	7.8E-01	-0.31	1.2E-01	0.52	6.2E-03	-0.17	4.2E-01	-0.18	3.7E-01
ENSCAFG0000000742	WT1	grey	EC_M10	0.11	6.0E-01	0.06	7.7E-01	-0.37	6.3E-02	-0.13	5.2E-01	0.30	1.3E-01	0.45	2.1E-02	0.12	5.6E-01	-0.11	5.8E-01	0.09	6.5E-01	-0.37	6.6E-02
ENSCAFG0000001825	SMCR8	grey	EC_M10	0.11	6.0E-01	0.41	3.6E-02	0.10	6.2E-01	0.05	8.2E-01	-0.38	5.5E-02	-0.12	5.6E-01	-0.17	4.2E-01	-0.27	1.8E-01	-0.30	1.3E-01	0.31	1.2E-01
ENSCAFG0000000249	DYNC2LI1	grey	EC_M10	0.11	6.0E-01	-0.61	9.6E-04	0.05	8.0E-01	0.26	2.1E-01	0.00	1.0E+00	0.18	3.7E-01	-0.04	8.4E-01	0.10	6.3E-01	-0.10	6.1E-01	-0.08	6.9E-01
ENSCAFG0000001488	ENSCAFG0000001488	grey	EC_M10	0.11	6.0E-01	0.20	3.3E-01	-0.03	8.8E-01	-0.23	2.5E-01	-0.11	5.9E-01	0.18	3.7E-01	-0.20	3.3E-01	0.28	1.7E-01	0.04	8.4E-01	0.05	8.1E-01
ENSCAFG0000001464	ENSCAFG0000001464	grey	EC_M10	0.11	6.0E-01	-0.10	6.2E-01	0.29	1.5E-01	0.43	2.7E-02	-0.43	3.0E-02	0.20	3.3E-01	-0.08	7.0E-01	-0.10	6.2E-01	-0.30	1.3E-01	0.36	7.0E-02
ENSCAFG0000002866	ENSCAFG0000002866	grey	EC_M10	0.11	6.0E-01	-0.05	8.0E-01	-0.35	7.9E-02	-0.12	5.6E-01	0.35	7.6E-02	0.65	3.2E-04	-0.08	7.1E-01	-0.06	7.8E-01	-0.08	6.9E-01	-0.41	3.7E-02
ENSCAFG0000001664	NPEPPS	grey	EC_M10	0.11	6.0E-01	-0.41	3.5E-02	-0.55	3.7E-03	-0.07	7.4E-01	0.69	9.7E-05	0.28	1.6E-01	0.14	5.0E-01	0.06	7.7E-01	-0.26	2.0E-01	-0.78	2.2E-06
ENSCAFG0000000278	FAM221A	grey	EC_M10	0.11	6.0E-01	-0.31	1.2E-01	-0.35	8.1E-02	0.14	5.0E-01	0.33	1.0E-01	0.31	1.2E-01	-0.07	7.5E-01	0.24	2.3E-01	-0.05	8.1E-01	-0.41	3.9E-02
ENSCAFG0000001211	VAPB	darkgreen	EC_M4	0.11	6.0E-01	0.16	4.4E-01	-0.80	9.6E-07	-0.13	5.3E-01	0.76	6.0E-06	0.39	4.6E-02	-0.06	7.8E-01	0.02	9.4E-01	0.23	2.5E-01	-0.80	8.1E-07
ENSCAFG0000003173	ENSCAFG0000003173	grey	EC_M10	0.11	6.0E-01	-0.21	3.0E-01	-0.25	2.1E-01	0.44	2.4E-02	0.23	2.6E-01	-0.17	4.0E-01	-0.11	5.9E-01	-0.10	6.3E-01	-0.15	4.6E-01	-0.30	1.3E-01
ENSCAFG0000000254	LMBRD1	grey	EC_M10	0.11	6.0E-01	0.18	3.9E-01	-0.75	1.1E-05	-0.20	3.3E-01	0.73	2.3E-05	0.16	4.3E-01	-0.15	4.7E-01	-0.03	8.9E-01	-0.23	2.5E-01	-0.75	1.1E-05
ENSCAFG0000001440	ARMC6	grey	EC_M10	0.11	6.0E-01	-0.22	2.8E-01	-0.29	1.5E-01	-0.07	7.4E-01	0.44	2.5E-02	0.12	5.7E-01	-0.22	2.7E-01	0.14	5.0E-01	0.16	4.3E-01	-0.51	8.2E-03
ENSCAFG0000001536	FGF23	turquoise	EC_M6	0.11	6.0E-01	0.84	8.3E-08	-0.28	1.7E-01	-0.06	7.6E-01	-0.07	7.5E-01	-0.07	7.2E-01	-0.08	7.2E-01	-0.09	6.6E-01	-0.12	5.6E-01	0.03	8.7E-01
ENSCAFG0000001711	DCST1	grey	EC_M10	0.11	6.0E-01	0.06	7.6E-01	0.02	9.2E-01	0.31	1.2E-01	-0.16	4.3E-01	-0.29	1.5E-01	-0.25	2.2E-01	-0.02	9.4E-01	0.14	4.9E-01	0.11	5.8E-01
ENSCAFG0000001157	ADNP	cyan	EC_M2	0.11	6.0E-01	0.17	4.1E-01	0.56	3.1E-03	0.23	2.6E-01	-0.87	1.2E-08	-0.17	4.0E-01	-0.14	5.0E-01	-0.09	6.6E-01	0.24	2.4E-01	0.80	7.2E-07
ENSCAFG0000001490	TRIM4	grey	EC_M10	0.11	6.0E-01	-0.03	8.9E-01	0.18	3.9E-01	-0.03	8.8E-01	-0.18	3.7E-01	-0.22	2.7E-01	-0.54	4.4E-03	0.16	4.3E-01	0.21	3.0E-01	0.17	4.1E-01
ENSCAFG0000001208	TUBGCP4	grey	EC_M10	0.11	6.0E-01	0.11	5.9E-01	-0.45	2.1E-02	-0.17	4.0E-01	0.48	1.3E-02	0.09	6.5E-01	0.05	8.2E-01	-0.33	1.0E-01	0.06	7.8E-01	-0.51	8.0E-03
ENSCAFG0000002952	LBX2	grey	EC_M10	0.11	6.0E-01	-0.42	3.3E-02	0.08	6.9E-01	-0.04	8.5E-01	-0.02	9.2E-01	-0.19	3.5E-01	-0.09	6.8E-01	0.11	5.8E-01	-0.31	1.3E-01	-0.07	7.2E-01
ENSCAFG0000000975	TWNK	grey	EC_M10	0.11	6.0E-01	0.03	8.8E-01	-0.02	9.1E-01	-0.57	2.2E-03	-0.01	9.6E-01	0.32	1.1E-01	0.11	6.0E-01	-0.02	9.2E-01	-0.10	6.4E-01	-0.05	8.0E-01
ENSCAFG0000000684	LDLRAD3	grey	EC_M10	0.11	6.0E-01	0.07	7.3E-01	0.35	7.7E-02	0.09	6.8E-01	-0.53	5.4E-03	-0.17	4.2E-01	-0.23	2.5E-01	0.07	7.2E-01	0.46	1.8E-02	0.50	8.8E-03
ENSCAFG0000002010	CNN3	turquoise	EC_M6	0.11	6.0E-01	0.68	1.5E-04	-0.15	4.8E-01	-0.03	8.7E-01	-0.20	3.2E-01	-0.16	4.5E-01	-0.04	8.3E-01	0.35	8.2E-02	0.37	6.3E-02	0.14	4.8E-01
ENSCAFG0000001115	GPR156	grey	EC_M10	0.11	6.0E-01	-0.28	1.6E-01	0.31	1.3E-01	-0.01	9.8E-01	-0.37	5.9E-02	-0.02	9.2E-01	-0.37	6.6E-02	0.13	5.4E-01	0.14	4.8E-01	0.28	1.7E-01
ENSCAFG0000001417	ITGA4	turquoise	EC_M6	0.11	6.0E-01	0.54	4.3E-03	-0.43	2.7E-02	-0.07	7.4E-01	0.28	1.6E-01	-0.09	6.8E-01	-0.13	5.2E-01	-0.08	6.9E-01	-0.10	6.4E-01	-0.30	1.3E-01
ENSCAFG0000002584	ENSCAFG0000002584	grey	EC_M10	0.11	6.1E-01	-0.04	8.6E-01	0.01	9.4E-01	0.08	6.8E-01	-0.18	3.9E-01	-0.01	9.7E-01	0.22	2.8E-01	-0.07	7.4E-01	-0.15	4.7E-01	0.10	6.3E-01
ENSCAFG0000001121	RBM23	grey	EC_M10	0.11	6.1E-01	0.17	4.0E-01	0.51	8.4E-03	0.24	2.4E-01	-0.70	7.1E-05	-0.29	1.4E-01	-0.01	9.6E-01	-0.13	5.4E-01	-0.04	8.6E-01	0.65	3.2E-04
ENSCAFG0000000058	CPT1B	grey	EC_M10	0.11	6.1E-01	0.46	1.9E-02	0.26	2.1E-01	0.05	8.1E-01	-0.60	1.3E-03	-0.12	5.7E-01	-0.03	8.8E-01	-0.10	6.3E-01	0.20	3.2E-01	0.52	6.9E-03
ENSCAFG0000000399	KDM7A	grey	EC_M10	0.11	6.1E-01	0.23	2.5E-01	0.10	6.2E-01	0.17	4.2E-01	-0.40	4.4E-02	-0.09	6.6E-01	0.12	5.6E-01	-0.10	6.1E-01	-0.04	8.6E-01	0.37	6.6E-02
ENSCAFG0000002022	PSMB7	grey	EC_M10	0.11	6.1E-01	-0.23	2.7E-01	-0.09	6.7E-01	-0.14	4.8E-01	0.21	3.1E-01	-0.13	5.2E-01	-0.28	1.7E-01	0.19	3.5E-01	-0.26	1.9E-01	-0.25	2.1E-01
ENSCAFG0000002804	ENSCAFG0000002804	grey	EC_M10	0.11	6.1E-01	0.37	6.2E-02	0.01	9.8E-01	-0.09	6.7E-01	-0.21	3.1E-01	-0.12	5.7E-01	-0.06	7.8E-01	-0.01	9.6E-01	-0.05	8.1E-01	0.14	5.1E-01
ENSCAFG0000000186	ZNF804B	grey	EC_M10	0.11	6.1E-01	0.02	9.0E-01	-0.14	4.8E-01	0.07	7.2E-01	0.15	4.8E-01	0.31	1.3E-01	-0.17	4.0E-01	0.04	8.6E-01	-0.01	9.7E-01	-0.25	2.2E-01
ENSCAFG0000002865	ENSCAFG0000002865	grey	EC_M10	0.11	6.1E-01	0.20	3.2E-01	-0.09	6.6E-01	0.01	9.8E-01	-0.02	9.4E-01	-0.16	4.5E-01	-0.14	5.0E-01	-0.17	4.0E-01	-0.37	6.5E-02	-0.08	6.8E-01
ENSCAFG0000000151	CSF2RB	turquoise	EC_M6	0.11	6.1E-01	0.58	1.9E-03	-0.21	3.1E-01	-0.07	7.5E-01	-0.08	7.0E-01	-0.08	6.9E-01	-0.04	8.5E-01	-0.10	6.4E-01	-0.15	4.7E-01	0.02	9.2E-01
ENSCAFG0000000648	ENSCAFG0000000648	turquoise	EC_M6	0.11	6.1E-01	0.58	1.9E-03	-0.21	3.1E-01	-0.07	7.5E-01	-0.08	7.0E-01	-0.08	6.9E-01	-0.04	8.5E-01	-0.10	6.4E-01	-0.15	4.7E-01	0.02	9.2E-01
ENSCAFG0000002054	ENSCAFG0000002054	turquoise	EC_M6	0.11	6.1E-01	0.58	1.9E-03	-0.21	3.1E-01	-0.07	7.5E-01	-0.08	7.0E-01	-0.08	6.9E-01	-0.04	8.5E-01	-0.10	6.4E-01	-0.15	4.7E-01	0.02	9.2E-01
ENSCAFG0000001938	DIRAS1	grey	EC_M10	0.11	6.1E-01	0.17	4.0E-01	-0.32	1.1E-01	-0.03	8.7E-01	0.27	1.8E-01	-0.08	6.8E-01	-0.12	5.6E-01	0.40	4.1E-02	0.01	9.6E-01	-0.30	1.3E-01
ENSCAFG0000001739	TRIM25	cyan	EC_M2	0.11	6.1E-01	0.18	3.8E-01	0.48	1.3E-02	-0.06	7.9E-01	-0.75	9.4E-06	-0.33	1.1E-01	-0.11	5.9E-01	-0.13	5.3E-01	-0.14	5.0E-01	0.69	1.1E-04
ENSCAFG0000002878	ARL14EP	grey	EC_M10	0.11	6.1E-01	-0.02	9.0E-01	-0.48	1.3E-02	-0.26	2.0E-01	0.41	3.7E-02	0.23	2.6E-01	0.13	5.4E-01	0.22	2.7E-01	0.17	4.0E-01	-0.47	1.6E-02
ENSCAFG0000001576	RHOJ	turquoise	EC_M6	0.11	6.1E-01	0.73	2.2E-05	0.09	6.5E-01	-0.15	4.6E-01	-0.46	1.9E-02	-0.16	4.3E-01	-0.18	3.7E-01	0.11	6.0E-01	0.30	1.4E-01	0.40	4.2E-02
ENSCAFG0000001711	MTFMT	grey	EC_M10	0.11	6.1E-01	0.01	9.6E-01	-0.18	3.9E-01	-0.07	7.2E-01	0.10	6.4E-01	-0.06	7.8E-01	-0.36	7.1E-02	0.33	1.0E-01	-0.13	5.2E-01	-0.13	5.4E-01
ENSCAFG0000000806	ENSCAFG0000000806	grey	EC_M10	0.11	6.1E-01	0.29	1.5E-01	-0.36	7.4E-02	0.03	8.7E-01	0.23	2.6E-01	0.00	9.9E-01	-0.16	4.3E-01	-0.44	2.6E-02	-0.07	7.3E-01	-0.27	1.8E-01
ENSCAFG0000001488	ENSCAFG0000001488	grey	EC_M10	0.11	6.1E-01	0.07	7.4E-01	0.15	4.7E-01	0.49	1.1E-02	-0.23	2.5E-01	-0.08	6.9E-01	-0.25	2.2E-01	-0.39	5.2E-02	-0.21	2.9E-01	0.19	3.5E-01
ENSCAFG0000001062	SORCS3	grey	EC_M10	0.11	6.1E-01	-0.28	1.6E-01	-0.04	8.6E-01	-0.13	5.2E-01	0.07	7.5E-01	-0.17	4.0E-01	0.14	5.0E-01	0.07	7.2E-01	-0.10	6.4E-01	-0.11	5.8E-01
ENSCAFG0000000000	ENSCAFG0000000000	grey	EC_M10	0.11	6.1E-01	0.24	2.3E-01	-0.01	9.5E-01	-0.18	3.7E-01	-0.12	5.5E-01	0.08	6.8E-01	0.02	9.1E-01	-0.23	2.6E-01	0.04	8.4E-01	0.06	7.8E-01
ENSCAFG0000002843	ENSCAFG0000002843	grey	EC_M10	0.11	6.1E-01	0.23	2.7E-01	0.07	7.5E-01	-0.15	4.7E-01	-0.25	2.2E-01	0.33	1.0E-01	-0.19	3.5E-01	-0.12	5.7E-01	-0.19	3.5E-01	0.14	4.8E-01
ENSCAFG0000000738	COPS5	grey	EC_M10	0.11	6.1E-01	0.08	6.9E-01	-0.59	1.4E-03	0.22	2.9E-01	0.57	2.3E-03	0.25	2.2E-01	-0.24	2.3E-01	-0.16	4.3E-01	0.00	9.9E-01	-0.62	6.9E-04
ENSCAFG0000002514	ENSCAFG0000002514	grey	EC_M10	0.11	6.1E-01	-0.08	6.9E-01	-0.60	1.1E-03	0.13	5.3E-01	0.58	2.0E-03	0.24	2.5E-01	-0.18	3.8E-01	0.13	5.2E-01	-0.21	3.1E-01	-0.64	3.9E-04
ENSCAFG0000001225	AP2M1	darkgreen	EC_M4	0.11	6.1E-01	0.24	2.4E-01	-0.70	6.3E-05	-0.10	6.2E-01	0.69	8.2E-05	0.35	8.4E-02	-0.41	3.8E-02	-0.09	6.5E-01	-0.02	9.1E-01	-0.74	1.7E-05
ENSCAFG0000000142	SLC16A8	grey	EC_M10	0.11	6.1E-01	0.05	8.2E-01	0.05	8.2E-01	-0.10	6.2E-01	-0.08	6.9E-01	-0.14	5.1E-01	0.55	3.8E-03	-0.02	9.3E-01	-0.16	4.4E-01	0.05	8.1E-01
ENSCAFG0000000849	MEIS2	grey	EC_M10	0.11	6.1E-01	0.17	4.2E-01	-0.57	2.2E-03	0.24	2.3E-01	0.46	1.8E-02	0.11	6.1E-01	0.00	1.0E+00	0.19	3.6E-01	0.08	6.8E-01	-0.56	3.1E-03
ENSCAFG0000000294	HOXA2	grey	EC_M10	0.11	6.1E-01	0.06	7.7E-01	0.02	9.4E-01	0.41	3.8E-02	-0.12	5.7E-01	0.07	7.5E-01	-0.28	1.7E-01	-0.22	2.7E-01	-0.13	5.2E-01	0.03	9.0E-01
ENSCAFG0000000722	ENSCAFG0000000722	grey	EC_M10	0.10	6.1E-01	0.26	2.0E-01	-0.06	7.6E-01	0.25	2.2E-01	-0.09	6.6E-01	0.02	9.2E-01	-0.10	6.3E-01	-0.08	7.0E-01	0.01	9.8E-01	0.04	8.3E-01
ENSCAFG0000000831	TMEM131	grey	EC_M10	0.10	6.1E-01	0.21	3.1E-01	0.13	5.2E-01	0.11	5.8E-01	-0.32	1.1E-01	-0.17	4.2E-01	0.14	4.9E-01	-0.50	8.6E-03	-0.13	5.2E-01	0.24	2.4E-01
ENSCAFG0000000908	ENSCAFG0000000908	grey	EC_M10	0.10	6.1E-01	-0.18	3.7E-01	-0.12	5.5E-01	-0.13	5.2E-01	0.09	6.5E-01	-0.09	6.5E-01	-0.13	5.3E-01	-0.18	3.8E-01	0.15	4.5E-01	-0.11	5.8E-01
ENSCAFG0000002907	ENSCAFG0000002907	grey	EC_M10	0.10	6.1E-01	-0.02	9.1E-01	0.15	4.7E-01	-0.04	8.3E-01	-0.18	3.9E-01	-0.01	9.7E-01	-0.01	9.6E-01	-0.02	9.2E-01	-0.11	5.8E-01	0.12	5.6E-01
ENSCAFG0000003059	ENSCAFG0000003059	grey	EC_M10	0.10	6.1E-01	-0.08	7.1E-01	-0.36	7.0E-02	-0.18	3.8E-01	0.38	5.4E-02	0.06	7.6E-01	-0.21	3.0E-01	0.35	7.6E-02	-0.11	5.8E-01	-0.42	3.2E-02
ENSCAFG0000000498	GDPD4	grey	EC_M10	0.10	6.1E-01	-0.06	7.6E-01	-0.42	3.5E-02	-0.04	8.4E-01	0.45	2.0E-02	-0.12	5.7E-01	-0.06	7.7E-01	-0.04	8.3E-01	0.00	1.0E+00	-0.51	7.4E-03
ENSCAFG0000000918	RAPGEF3	turquoise	EC_M6	0.10	6.1E-01	0.65	3.2E-04	0.30	1.3E-01	-0.02	9.1E-01	-0.68	1.5E-04	-0.23	2.6E-01	-0.02	9.2E-01	-0.02	9.1E-01	0.26	2.0E-01	0.64	4.6E-04
ENSCAFG0000001267	ACADSB	grey	EC_M10	0.10	6.1E-01	-0.43	2.9E-02	0.27	1.8E-01	-0.06	7.7E-01	-0.24	2.3E-01	0.30	1.3E-01	-0.05	7.9E-01	-0.02	9.1E-01	0.31	1.2E-01	0.21	3.0E-01
ENSCAFG0000001434	RFXANK	turquoise	EC_M6	0.10	6.1E-01	0.64	4.4E-04	0.17	3.9E-01	0.02	9.4E-01	-0.54	4.5E-03	-0.32	1.1E-01	-0.11	5.8E-01	-0.04	8.3E-01	-0.05	8.0E-01	0.44	2.4E-02
ENSCAFG0000002315	ZKSCAN5	grey	EC_M10	0.10	6.1E-01	0.40	4.5E-02	-0.30	1.3E-01	0.22	2.9E-01	0.11	6.0E-01	-0.05	8.1E-01	0.20	3.4E-01	0.09	6.6E-01	0.38	5.3E-02	-0.14	4.9E-01
ENSCAFG0000003196	PPIC	grey	EC_M10	0.10	6.1E-01	0.33	1.0E-01	0.26	1.9E-01	0.39	4.8E-02	-0.52	6.4E-03	-0.11	6.0E-01	0.22	2.8E-01	-0.14	5.0E-01	0.06	7.7E-01	0.48	1.3E-02
ENSCAFG0000000212	PON2	grey	EC_M10	0.10	6.1E-01	0.54	4.8E-03	-0.67	1.7E-04	-0.06	7.7E-01	0.47	1.4E-02	0.07	7.4E-01	0.00	9.8E-01	0.25	2.3E-01	0.50	8.8E-03	-0.53	5.8E-03
ENSCAFG0000001204	ADAL	grey	EC_M10	0.10	6.1E-01	0.01	9.6E-01	0.11	5.8E-01	-0.24	2.4E-01	-0.35	8.3E-02	-0.07	7.4E-01	-0.06	7.9E-01	0.05	8.0E-01	-0.03	8.8E-01	0.26	2.1E-01
ENSCAFG0000001114	UNG	grey	EC_M10	0.10	6.1E-01	0.29	1.4E-01	0.25	2.2E-01	-0.18	3.7E-01	-0.38	5.2E-02	-0.01	9.5E-01	-0.21	3.0E-01	-0.49	1.0E-02	0.00	9.8E-01	0.34	9.0E-02
ENSCAFG0000000853	ENSCAFG0000000853	grey	EC_M10	0.10	6.1E-01	0.29	1.6E-01	-0.41	3.5E-02	0.19	3.5E-01	0.32	1.1E-01	0.06	7.8E-01	-0.24	2.5E-01	0.06	7.8E-01	-0.12	5.4E-01	-0.37	6.6E-02
ENSCAFG0000000441	ENSCAFG0000000441	grey	EC_M10	0.10	6.1E-01	0.19	3.5E-01	-0.09	6.7E-01	0.34	8.5E-02	-0.07	7.2E-01	-0.15	4.6E-01	-0.17	4.0E-01	-0.16	4.3E-01	0.15	4.7E-01	0.02	9.4E-01
ENSCAFG0000000138	ENSCAFG0000000138	grey	EC_M10	0.10	6.1E-01	-0.04	8.3E-01	-0.35	8.3E-02	0.15	4.8E-01	0.26	2.1E-01	0.12	5.7E-01	-0.05	8.1E-01	-0.21	3.1E-01	-0.05	7.9E-01	-0.28	1.7E-01
ENSCAFG0000000809	ENSCAFG0000000809	grey	EC_M10	0.10	6.2E-01	0.29	1.6E-01	-0.33	9.5E-02	-0.14	4.9E-01	0.22	2.8E-01	-0.16	4.4E-01	-0.10	6.2E-01	0.35	8.3E-02	0.04	8.4E-01	-0.27	1.9E-01
ENSCAFG0000001999	GAN	grey	EC_M10	0.10	6.2E-01	-0.11	6.0E-01	-0.55	3.9E-03	-0.23	2.6E-01	0.58	1.9E-03	0.28	1.6E-01	0.21	3.0E-01	-0.33	9.5E-02	-0.24	2.4E-01	-0.63	5.1E-04
ENSCAFG0000001110	NRAP	turquoise	EC_M6	0.10	6.2E-01	0.81	5.6E-07	-0.45	2.3E-02	-0.10	6.2E-01	0.19	3.6E-01	-0.12	5.5E-01	-0.11	5.9E-01	-0.11	5.9E-01	-0.12	5.4E-01	-0.23	2.6E-01
ENSCAFG0000001531	UBR4	grey	EC_M10	0.10	6.2E-01	0.13	5.1E-01	-0.60	1.2E-03	-0.15	4.5E-01	0.58	1.8E-03	0.02	9.2E-01	-0.02	9.2E-01	-0.17	4.1E-01	-0.23	2.5E-01	-0.62	7.3E-04
ENSCAFG0000001286	MORC2	grey	EC_M10	0.10	6.2E-01	0.50	9.7E-03	-0.70	6.4E-05	-0.12	5.5E-01	0.46	1.7E-02	0.10	6.3E-01	0.02	9.4E-01	0.24	2.3E-01	0.09	6.6E-01	-0.55	3.8E-03
ENSCAFG0000000865	SHROOM3	magenta	EC_M13	0.10	6.2E-01	0.27	1.8E-01	-0.18	3.9E-01	-0.14	4.8E-01	0.00	9.9E-01	0.00	9.9E-01	0.00	1.0E+00	-0.06	7.9E-01	0.62	7.6E-04	-0.04	8.4E-01
ENSCAFG0000000676	DLC1	grey	EC_M10	0.10	6.2E-01	-0.44	2.5E-02	0.51	7.5E-03	-0.07	7.3E-01	-0.45	2.2E-02	-0.24	2.5E-01	-0.17	4.0E-01	-0.12	5.7E-01	-0.23	2.7E-01	0.43	3.0E-02
ENSCAFG0000000452	TTYH2	magenta	EC_M13	0.10	6.2E-01	-0.15	4.5E-01	-0.01	9.7E-01	0.02	9.1E-01	0.00	9.9E-01	-0.05	8.1E-01	0.04	8.5E-01	-0.13	5.2E-01	0.66	2.7E-04	-0.02	9.2E-01
ENSCAFG0000000511	SEC13	darkgreen	EC_M4	0.10	6.2E-01	0.35	8.1E-02	-0.76	5.8E-06	-0.10	6.4E-01	0.69	8.8E-05	0.27	1.8E-01	0.24	2.4E-01	-0.27	1.9E-01	-0.20	3.4E-01	-0.76	7.6E-06
ENSCAFG0000000185	STEAP4	violet	EC_M7	0.10	6.2E-01	-0.16	4.4E-01	0.02	9.1E-01	-0.11	5.8E-01	-0.06	7.7E-01	0.66	2.6E-04	-0.12	5.5E-01	-0.06	7.6E-01	-0.15	4.7E-01	0.01	9.8E-01
ENSCAFG0000001472	OTUB1	grey	EC_M10	0.10	6.2E-01	0.26	1.9E-01	-0.23	2.7E-01	0.00	9.9E-01	0.14	4.8E-01	0.10	6.4E-01	0.01	9.6E-01	-0.26	2.0E-01	0.35	8.3E-02	-0.18	3.7E-01
ENSCAFG0000000604	SLC23A2	grey	EC_M10	0.10	6.2E-01	-0.27	1.8E-01	-0.42	3.3E-02	-0.20	3.3E-01	0.55	3.8E-03	0.13	5.2E-01	-0.29	1.5E-01	-0.18	3.8E-01	0.01	9.5E-01	-0.56	2.8E-03
ENSCAFG0000001003	NUAK2	turquoise	EC_M6	0.10	6.2E-01	0.71	4.2E-05	-0.49	1.1E-02	-0.23	2.6E-01	0.29	1.5E-01	0.03	8.7E-01	-0.12	5.5E-01	-0.31	1.2E-01	0.14	5.0E-01	-0.32	1.1E-01
ENSCAFG0000000140	ENSCAFG0000000140	grey	EC_M10	0.10	6.2E-01	0.01	9.6E-01	-0.21	3.0E-01	-0.08	7.1E-01	0.16	4.4E-01	0.63	5.5E-04	0.02	9.0E-01	-0.10	6.2E-01	0.47	1.5E-02	-0.22	2.7E-01
ENSCAFG0000000493	GRIK5	magenta	EC_M13	0.10	6.2E-01	-0.12	5.6E-01	0.00	1.0E+00	-0.21	2.9E-01	-0.01	9.6E-01	0.06	7.9E-01	0.30	1.4E-01	-0.20	3.3E-01	0.47	1.6E-02	0.00	9.9E-01
ENSCAFG0000000872	MROH8	grey	EC_M10	0.10	6.2E-01	0.03	9.0E-01	0.02	9.3E-01	0.25	2.1E-01	-0.08	7.1E-01	-0.24	2.4E-01	-0.44	2.3E-02	0.24	2.3E-01	-0.09	6.6E-01	0.03	8.9E-01
ENSCAFG0000000524	ENSCAFG0000000524	grey	EC_M10	0.10	6.2E-01	-0.09	6.5E-01	-0.06	7.8E-01	0.21	3.0E-01	-0.03	8.9E-01	-0.18	3.8E-01	-0.20	3.2E-01	0.15	4.7E-01	-0.21	3.1E-01	-0.08	6.8E-01
ENSCAFG0000000531	ENSCAFG0000000531	grey	EC_M10	0.10	6.2E-01	-0.22	2.7E-01	0.21	3.0E-01	0.58	2.1E-03	-0.22	2.8E-01	-0.21	3.0E-01	-0.17	4.1E-01	-0.11	5.9E-01	-0.15	4.6E-01	0.16	4.2E-01
ENSCAFG0000001182	MN1	turquoise	EC_M6	0.10	6.2E-01	0.87	7.1E-09	-0.39	4.9E-02	-0.07	7.3E-01	0.08	7.1E-01	0.05	8.0E-01	-0.12	5.4E-01	-0.15	4.7E-01	-0.06	7.8E-01	-0.11	5.9E-01
ENSCAFG0000001358	ENSCAFG0000001358	cyan	EC_M2	0.10	6.2E-01	-0.16	4.2E-01	0.46	1.7E-02	0.11	6.0E-01	-0.56	2.7E-03	0.08	6.9E-01	0.13	5.4E-01	-0.11	5.9E-01	-0.36	7.2E-02	0.46	1.8E-02
ENSCAFG0000000928	CCAR2	grey	EC_M10	0.10	6.2E-01	0.43	2.7E-02	0.37	6.3E-02	-0.07	7.2E-01	-0.58	1.7E-03	-0.12	5.6E-01	-0.04	8.4E-01	-0.18	3.9E-01	-0.03	8.9E-01	0.56	2.7E-03
ENSCAFG0000000600	PROSER1	turquoise	EC_M6	0.10	6.2E-01	0.51	7.5E-03	0.12	5.7E-01	-0.19	3.5E-01	-0.38	5.7E-02	-0.18	3.7E-01	-0.38	5.2E-02	-0.11	6.0E-01	0.16	4.4E-01	0.30	1.3E-01
ENSCAFG0000000435	NOVA2	turquoise	EC_M6	0.10	6.2E-01	0.87	6.5E-09	-0.31	1.3E-01	-0.15	4.6E-01	0.00	9.9E-01	-0.22	2.9E-01	-0.20	3.2E-01	-0.05	8.2E-01	-0.02	9.2E-01	-0.06	7.8E-01
ENSCAFG0000003257	ZBTB6	grey	EC_M10	0.10	6.2E-01	0.12	5.7E-01	-0.02	9.4E-01	0.24	2.4E-01	-0.23	2.5E-01	-0.04	8.4E-01	0.13	5.2E-01	0.01	9.7E-01	0.17	4.1E-01	0.17	4.0E-01
ENSCAFG0000000062	PRRC1	darkgreen	EC_M4	0.10	6.2E-01	0.29	1.5E-01	-0.79	1.7E-06	-0.12	5.4E-01	0.72	4.0E-05	0.37	6.4E-02	0.03	9.0E-01	-0.06	7.6E-01	-0.09	6.5E-01	-0.81	6.2E-07
ENSCAFG0000000055	ACR	grey	EC_M10	0.10	6.2E-01	-0.16	4.3E-01	-0.57	2.4E-03	-0.11	6.1E-01	0.61	9.8E-04	0.38	5.9E-02	-0.14	5.0E-01	0.29	1.5E-01	-0.05	8.0E-01	-0.67	2.0E-04
ENSCAFG0000000378	KTI12	turquoise	EC_M6	0.10	6.2E-01	0.48	1.2E-02	0.09	6.6E-01	-0.08	6.8E-01	-0.34	9.4E-02	0.10	6.2E-01	-0.22	2.7E-01	-0.19	3.6E-01	-0.31	1.2E-01	0.24	2.3E-01
ENSCAFG0000000007	RELCH	grey	EC_M10	0.10	6.2E-01	-0.38	5.8E-02	-0.52	7.1E-03	-0.03	9.0E-01	0.65	3.4E-04	0.16	4.2E-01	-0.39	4.9E-02	0.08	6.9E-01	0.07	7.2E-01	-0.70	6.9E-05
ENSCAFG0000000233	ENSCAFG0000000233	grey	EC_M10	0.10	6.2E-01	-0.04	8.6E-01	-0.46	1.9E-02	-0.02	9.1E-01	0.43	2.9E-02	0.40	4.0E-02	-0.08	6.9E-01	-0.11	6.0E-01	0.23	2.6E-01	-0.51	7.9E-03
ENSCAFG0000001679	DCAF4	grey	EC_M10	0.10	6.2E-01	0.31	1.2E-01	0.35	8.2E-02	0.06	7.6E-01	-0.58	2.1E-03	0.04	8.3E-01	0.00	1.0E+00	-0.09	6.7E-01	0.32	1.1E-01	0.51	7.1E-03
ENSCAFG0000003275	CLEC2B	grey	EC_M10	0.10	6.2E-01	-0.04	8.5E-01	-0.40	4.5E-02	-0.04	8.3E-01	0.46	1.7E-02	-0.18	3.8E-01	-0.16	4.4E-01	-0.06	7.9E-01	-0.07	7.5E-01	-0.48	1.2E-02
ENSCAFG0000002432	ENSCAFG0000002432	grey	EC_M10	0.10	6.2E-01	-0.24	2.3E-01	0.03	8.9E-01	-0.13	5.4E-01	0.00	1.0E+00	0.43	3.0E-02	-0.14	5.1E-01	-0.17	4.0E-01	-0.17	3.9E-01	-0.05	8.1E-01
ENSCAFG0000001509	ENSCAFG0000001509	grey	EC_M10	0.10	6.2E-01	-0.24	2.3E-01	-0.28	1.7E-01	0.02	9.3E-01	0.40	4.3E-02	0.21	3.1E-01	0.15	4.6E-01	0.21	3.0E-01	-0.38	5.2E-02	-0.44	2.3E-02
ENSCAFG0000001683	SMG5	grey	EC_M10	0.10	6.2E-01	-0.11	5.9E-01	-0.64	4.8E-04	-0.24	2.3E-01	0.77	5.1E-06	0.45	2.0E-02	-0.10	6.4E-01	-0.08	6.8E-01	0.09	6.8E-01	-0.78	2.7E-06
ENSCAFG0000002474	TAS2R3	paleturquoise	EC_M11	0.10	6.2E-01	-0.17	4.1E-01	0.25	2.2E-01	0.59	1.4E-03	-0.29	1.5E-01	-0.13	5.1E-01	-0.12	5.5E-01	-0.08	7.1E-01	-0.18	3.7E-01	0.23	2.5E-01
ENSCAFG0000001661	RNF111	darkgreen	EC_M4	0.10	6.3E-01	0.47	1.4E-02	-0.72	3.3E-05	-0.12	5.5E-01	0.58	2.0E-03	0.30	1.4E-01	-0.08	6.9E-01	-0.16	4.2E-01	-0.08	7.0E-01	-0.63	5.4E-04
ENSCAFG0000001821	CSF1R	grey	EC_M10	0.10	6.3E-01	-0.28	1.7E-01	-0.23	2.5E-01	-0.31	1.2E-01	0.24	2.3E-01	0.35	8.2E-02	-0.02	9.3E-01	-0.13	5.3E-01	-0.34	8.8E-02	-0.36	7.2E-02
ENSCAFG0000001057	CHSY1	darkgreen	EC_M4	0.10	6.3E-01	0.10	6.2E-01	-0.83	1.9E-07	-0.21	3.0E-01	0.83	1.5E-07	0.41	3.6E-02	0.06	7.8E-01	-0.09	6.8E-01	0.09	6.7E-01	-0.91	1.9E-10
ENSCAFG0000002118	ENSCAFG0000002118	grey	EC_M10	0.10	6.3E-01	0.04	8.4E-01	0.25	2.1E-01	0.23	2.6E-01	-0.41	3.8E-02	-0.25	2.2E-01	0.01	9.8E-01	-0.22	2.7E-01	-0.36	7.5E-02	0.30	1.4E-01
ENSCAFG0000001740	CCDC151	grey	EC_M10	0.10	6.3E-01	-0.26	2.0E-01	0.02	9.2E-01	0.03	8.7E-01	-0.01	9.6E-01	0.32	1.2E-01	0.29	1.5E-01	-0.09	6.6E-01	0.13	5.4E-01	-0.06	7.9E-01
ENSCAFG0000000616	EEA1	cyan	EC_M2	0.10	6.3E-01	-0.31	1.3E-01	0.50	9.9E-03	0.19	3.5E-01	-0.62	7.8E-04	-0.13	5.2E-01	-0.16	4.4E-01	-0.09	6.8E-01	-0.30	1.3E-01	0.57	2.2E-03
ENSCAFG0000000850	ENSCAFG0000000850	cyan	EC_M2	0.10	6.3E-01	0.20	3.2E-01	0.43	2.8E-02	0.27	1.8E-01	-0.70	8.0E-05	-0.07	7.2E-01	0.20	3.2E-01	-0.13	5.3E-01	0.03	8.7E-01	0.63	5.4E-04
ENSCAFG0000002934	ENSCAFG0000002934	turquoise	EC_M6	0.10	6.3E-01	0.86	1.5E-08	-0.15	4.6E-01	-0.08	6.9E-01	-0.19	3.5E-01	-0.12	5.5E-01	-0.12	5.7E-01	-0.14	4.9E-01	0.17	4.0E-01	0.12	5.5E-01
ENSCAFG0000000491	ENSCAFG0000000491	grey	EC_M10	0.10	6.3E-01	0.04	8.6E-01	0.00	9.9E-01	0.40	4.4E-02	-0.11	6.0E-01	-0.12	5.7E-01	-0.11	6.0E-01	0.48	1.3E-02	-0.05	8.1E-01	0.05	8.0E-01
ENSCAFG0000002984	ELMOD2	grey	EC_M10	0.10	6.3E-01	-0.14	4.9E-01	0.03	8.8E-01	0.23	2.7E-01	-0.19	3.5E-01	-0.17	3.9E-01	-0.22	2.9E-01	0.38	5.9E-02	0.18	3.7E-01	0.09	6.4E-01
ENSCAFG0000002120	ENSCAFG0000002120	darkmagenta	EC_M12	0.10	6.3E-01	-0.14	5.0E-01	-0.06	7.8E-01	-0.11	6.1E-01	0.04	8.5E-01	-0.10	6.1E-01	-0.07	7.4E-01	0.70	6.7E-05	0.15	4.6E-01	-0.09	6.6E-01
ENSCAFG0000000004	DSEL	grey	EC_M10	0.10	6.3E-01	-0.47	1.6E-02	-0.27	1.8E-01	-0.14	4.8E-01	0.41	3.5E-02	0.26	2.0E-01	0.08	7.0E-01	0.36	6.7E-02	0.30	1.3E-01	-0.50	1.0E-02

ENSCAFG0000003268	ENSCAFG0000003268	turquoise	EC_M6	0.10	6.3E-01	0.48	1.2E-02	-0.12	5.4E-01	-0.26	1.9E-01	-0.07	7.3E-01	-0.16	4.3E-01	-0.14	5.0E-01	0.42	3.3E-02	0.27	1.8E-01	0.00	9.9E-01
ENSCAFG0000000028	CDK4	cyan	EC_M2	0.10	6.3E-01	-0.34	8.8E-02	0.54	4.4E-03	0.06	7.7E-01	-0.48	1.3E-02	-0.03	9.0E-01	-0.02	9.3E-01	-0.27	1.8E-01	-0.22	2.9E-01	0.41	3.6E-02
ENSCAFG0000001569	STAMBPL1	grey	EC_M10	0.10	6.3E-01	0.18	3.8E-01	0.01	9.6E-01	-0.32	1.2E-01	-0.15	4.6E-01	0.15	4.7E-01	0.33	1.0E-01	0.03	9.0E-01	-0.36	6.7E-02	0.09	6.7E-01
ENSCAFG0000001311	UAP1	grey	EC_M10	0.10	6.3E-01	-0.60	1.1E-03	0.19	3.4E-01	0.01	9.8E-01	-0.13	5.4E-01	0.06	7.9E-01	-0.14	5.1E-01	0.20	3.3E-01	-0.33	9.5E-02	0.02	9.0E-01
ENSCAFG0000000629	RNF122	grey	EC_M10	0.10	6.3E-01	-0.02	9.1E-01	-0.67	1.6E-04	-0.09	6.5E-01	0.70	7.7E-05	0.14	4.8E-01	-0.18	3.7E-01	0.05	8.0E-01	0.09	6.6E-01	-0.78	3.1E-06
ENSCAFG0000000254	PREPL	grey	EC_M10	0.10	6.3E-01	-0.56	2.8E-03	-0.32	1.1E-01	0.02	9.2E-01	0.49	1.2E-02	0.04	8.6E-01	-0.30	1.3E-01	0.09	6.6E-01	0.22	2.9E-01	-0.54	4.5E-03
ENSCAFG0000000146	ENSCAFG0000000146	grey	EC_M10	0.10	6.3E-01	0.16	4.4E-01	-0.53	5.6E-03	-0.19	3.6E-01	0.46	1.9E-02	0.25	2.2E-01	-0.20	3.2E-01	0.06	7.7E-01	-0.13	5.2E-01	-0.51	7.2E-03
ENSCAFG0000001682	CYB5D1	grey	EC_M10	0.10	6.3E-01	-0.06	7.7E-01	-0.24	2.4E-01	0.01	9.5E-01	0.26	2.1E-01	-0.13	5.3E-01	0.05	8.2E-01	-0.23	2.5E-01	0.26	1.9E-01	-0.28	1.7E-01
ENSCAFG0000000871	NUCB2	grey	EC_M10	0.10	6.3E-01	-0.16	4.4E-01	-0.57	2.2E-03	0.16	4.3E-01	0.58	2.0E-03	0.27	1.9E-01	0.22	2.8E-01	0.10	6.3E-01	-0.03	8.7E-01	-0.65	3.2E-04
ENSCAFG0000001171	UBASH3B	grey	EC_M10	0.10	6.3E-01	0.11	5.9E-01	-0.09	6.7E-01	-0.38	5.3E-02	0.02	9.2E-01	-0.34	8.8E-02	0.15	4.6E-01	0.34	8.6E-02	0.39	4.8E-02	-0.01	9.7E-01
ENSCAFG0000000781	CLP1	grey	EC_M10	0.10	6.3E-01	0.22	2.8E-01	-0.46	1.8E-02	-0.60	1.2E-03	0.42	3.4E-02	0.19	3.5E-01	0.03	8.7E-01	0.03	8.7E-01	0.03	9.0E-01	-0.46	1.9E-02
ENSCAFG0000001092	TMEM39A	grey	EC_M10	0.10	6.3E-01	-0.10	6.2E-01	-0.04	8.5E-01	0.22	2.7E-01	-0.09	6.7E-01	0.31	1.2E-01	-0.01	9.7E-01	-0.15	4.8E-01	0.19	3.6E-01	-0.07	7.3E-01
ENSCAFG0000002489	NMT2	grey	EC_M10	0.10	6.3E-01	-0.32	1.1E-01	0.21	3.0E-01	-0.12	5.5E-01	-0.22	2.7E-01	-0.17	4.1E-01	-0.25	2.1E-01	0.24	2.4E-01	-0.44	2.4E-02	0.14	4.9E-01
ENSCAFG0000000569	ANK1	grey	EC_M10	0.10	6.3E-01	0.11	5.9E-01	-0.45	2.2E-02	0.16	4.5E-01	0.36	7.3E-02	0.20	3.3E-01	-0.37	6.1E-02	0.13	5.4E-01	-0.01	9.8E-01	-0.49	1.1E-02
ENSCAFG0000001380	OSBPL6	turquoise	EC_M6	0.10	6.3E-01	0.71	4.9E-05	-0.61	9.0E-04	-0.04	8.4E-01	0.36	7.1E-02	0.17	3.9E-01	0.07	7.3E-01	-0.03	8.8E-01	0.17	4.2E-01	-0.40	4.4E-02
ENSCAFG0000000856	WDR54	grey	EC_M10	0.10	6.3E-01	0.09	6.4E-01	-0.50	8.9E-03	-0.47	1.5E-02	0.48	1.4E-02	-0.02	9.4E-01	0.39	4.9E-02	-0.18	3.9E-01	0.05	7.9E-01	-0.51	7.6E-03
ENSCAFG0000003074	BTBD3	grey	EC_M10	0.10	6.3E-01	0.10	6.3E-01	-0.41	3.8E-02	-0.10	6.1E-01	0.31	1.2E-01	0.05	8.0E-01	-0.14	4.9E-01	-0.31	1.2E-01	-0.01	9.5E-01	-0.33	9.6E-02
ENSCAFG0000000034	ENSCAFG0000000034	grey	EC_M10	0.10	6.3E-01	-0.16	4.4E-01	0.11	5.9E-01	0.04	8.4E-01	-0.13	5.3E-01	0.00	1.0E+00	0.01	9.8E-01	-0.23	2.5E-01	-0.47	1.5E-02	0.07	7.3E-01
ENSCAFG0000003203	ENSCAFG0000003203	grey	EC_M10	0.10	6.3E-01	0.38	5.3E-02	0.17	4.1E-01	0.02	9.4E-01	-0.49	1.1E-02	-0.03	8.8E-01	0.05	7.9E-01	0.07	7.2E-01	0.03	9.0E-01	0.46	1.7E-02
ENSCAFG0000002624	ENSCAFG0000002624	grey	EC_M10	0.10	6.3E-01	0.09	6.6E-01	-0.04	8.5E-01	0.00	9.9E-01	-0.01	9.8E-01	0.39	5.1E-02	0.00	1.0E+00	0.13	5.2E-01	-0.01	9.6E-01	-0.08	6.9E-01
ENSCAFG0000001668	FAF2	grey	EC_M10	0.10	6.3E-01	-0.31	1.2E-01	-0.53	5.0E-03	-0.11	6.1E-01	0.60	1.1E-03	0.41	3.8E-02	0.01	9.5E-01	-0.08	7.0E-01	-0.20	3.2E-01	-0.70	7.6E-05
ENSCAFG0000000192	SLC41A2	grey	EC_M10	0.10	6.3E-01	0.11	5.8E-01	-0.73	2.3E-05	-0.21	2.9E-01	0.73	2.3E-05	0.41	3.9E-02	0.16	4.5E-01	-0.06	7.8E-01	-0.12	5.5E-01	-0.78	2.2E-06
ENSCAFG0000000991	PLA2G4F	grey	EC_M10	0.10	6.3E-01	0.02	9.4E-01	-0.39	4.7E-02	-0.10	6.2E-01	0.36	7.0E-02	-0.19	3.5E-01	0.35	7.9E-02	-0.05	8.0E-01	-0.03	8.8E-01	-0.37	6.4E-02
ENSCAFG0000002069	ENSCAFG0000002069	grey	EC_M10	0.10	6.3E-01	-0.20	3.2E-01	0.19	3.5E-01	0.32	1.2E-01	-0.23	2.6E-01	-0.11	6.0E-01	-0.13	5.4E-01	0.49	1.1E-02	0.05	8.1E-01	0.15	4.6E-01
ENSCAFG0000000206	KIT	turquoise	EC_M6	0.10	6.3E-01	0.83	1.3E-07	-0.20	3.4E-01	-0.06	7.6E-01	-0.13	5.2E-01	-0.14	4.9E-01	-0.16	4.5E-01	-0.16	4.3E-01	-0.11	5.9E-01	0.04	8.4E-01
ENSCAFG0000002355	OAS1	turquoise	EC_M6	0.10	6.3E-01	0.38	5.4E-02	-0.20	3.3E-01	0.14	4.8E-01	0.01	9.5E-01	0.08	7.1E-01	-0.08	6.9E-01	-0.13	5.1E-01	-0.09	6.8E-01	-0.10	6.1E-01
ENSCAFG0000001043	ACOT13	grey	EC_M10	0.10	6.3E-01	-0.55	3.6E-03	0.50	8.7E-03	0.15	4.7E-01	-0.41	3.8E-02	-0.15	4.7E-01	-0.22	2.8E-01	0.02	9.2E-01	-0.28	1.6E-01	0.37	6.6E-02
ENSCAFG0000000137	MAPK14	grey	EC_M10	0.10	6.3E-01	0.50	8.5E-03	0.05	8.1E-01	-0.20	3.2E-01	-0.39	5.0E-02	0.03	9.0E-01	0.30	1.3E-01	0.25	2.2E-01	0.37	6.6E-02	0.33	9.6E-02
ENSCAFG0000001446	EFHC2	grey	EC_M10	0.10	6.3E-01	-0.16	4.5E-01	-0.07	7.5E-01	0.12	5.7E-01	0.03	8.9E-01	0.31	1.2E-01	-0.15	4.6E-01	-0.12	5.7E-01	0.19	3.7E-01	-0.11	5.8E-01
ENSCAFG0000001204	ARIH2	grey	EC_M10	0.10	6.3E-01	0.15	4.7E-01	-0.61	9.6E-04	0.04	8.5E-01	0.55	3.3E-03	0.46	1.8E-02	0.13	5.4E-01	-0.27	1.8E-01	0.13	5.2E-01	-0.61	8.8E-04
ENSCAFG0000002444	ENSCAFG0000002444	turquoise	EC_M6	0.10	6.4E-01	0.40	4.2E-02	0.14	5.0E-01	0.08	7.0E-01	-0.37	6.4E-02	-0.26	1.9E-01	0.20	3.3E-01	0.09	6.7E-01	0.11	6.0E-01	0.32	1.1E-01
ENSCAFG0000002481	RACK1	cyan	EC_M2	0.10	6.4E-01	-0.45	2.0E-02	0.58	2.0E-03	0.12	5.7E-01	-0.56	2.8E-03	-0.04	8.6E-01	0.02	9.0E-01	0.02	9.2E-01	-0.40	4.1E-02	0.44	2.3E-02
ENSCAFG0000001773	ENSCAFG0000001773	grey	EC_M10	0.10	6.4E-01	-0.10	6.1E-01	0.23	2.6E-01	-0.08	6.9E-01	-0.25	2.2E-01	-0.01	9.6E-01	0.00	9.9E-01	-0.05	7.9E-01	0.04	8.4E-01	0.24	2.4E-01
ENSCAFG0000000937	ENSCAFG0000000937	grey	EC_M10	0.10	6.4E-01	0.00	9.9E-01	-0.08	7.0E-01	-0.33	1.0E-01	0.03	8.7E-01	0.08	7.1E-01	-0.26	2.0E-01	0.42	3.1E-02	-0.16	4.4E-01	-0.11	6.1E-01
ENSCAFG0000003056	DUSP5	turquoise	EC_M6	0.10	6.4E-01	0.51	7.5E-03	-0.45	2.1E-02	-0.15	4.7E-01	0.27	1.8E-01	-0.23	2.6E-01	0.02	9.1E-01	0.14	5.0E-01	-0.08	7.1E-01	-0.28	1.6E-01
ENSCAFG0000001292	LIMD2	turquoise	EC_M6	0.10	6.4E-01	0.55	4.0E-03	-0.48	1.3E-02	-0.18	3.8E-01	0.25	2.2E-01	0.14	5.0E-01	0.11	5.9E-01	-0.18	3.8E-01	0.08	7.0E-01	-0.35	8.2E-02
ENSCAFG0000000245	RAB23	darkgreen	EC_M4	0.10	6.4E-01	-0.57	2.5E-03	-0.33	1.1E-01	-0.16	4.2E-01	0.52	5.9E-03	0.30	1.4E-01	-0.09	6.5E-01	0.02	9.4E-01	-0.17	4.1E-01	-0.58	1.8E-03
ENSCAFG0000003137	ENSCAFG0000003137	grey	EC_M10	0.10	6.4E-01	0.15	4.6E-01	-0.15	4.7E-01	0.23	2.7E-01	0.02	9.3E-01	0.16	4.2E-01	-0.23	2.5E-01	-0.23	2.6E-01	-0.34	9.1E-02	-0.10	6.3E-01
ENSCAFG0000000584	ENSCAFG0000000584	grey	EC_M10	0.10	6.4E-01	-0.04	8.4E-01	0.49	1.2E-02	0.38	5.8E-02	-0.65	3.2E-04	-0.17	3.9E-01	0.10	6.3E-01	-0.08	7.2E-01	0.03	9.0E-01	0.61	1.0E-03
ENSCAFG0000003011	RFXAP	grey	EC_M10	0.10	6.4E-01	-0.42	3.4E-02	-0.16	4.3E-01	0.17	4.2E-01	0.19	3.6E-01	0.23	2.6E-01	-0.11	6.0E-01	-0.03	8.7E-01	-0.06	7.6E-01	-0.28	1.7E-01
ENSCAFG0000000331	ENSCAFG0000000331	darkgreen	EC_M4	0.10	6.4E-01	0.34	9.1E-02	-0.73	2.1E-05	-0.15	4.7E-01	0.67	1.7E-04	0.16	4.2E-01	-0.17	4.2E-01	-0.08	7.2E-01	-0.19	3.4E-01	-0.69	8.2E-05
ENSCAFG0000000681	TRAF6	grey	EC_M10	0.10	6.4E-01	0.45	2.2E-02	-0.58	2.1E-03	-0.22	2.8E-01	0.35	7.7E-02	0.18	3.7E-01	0.01	9.5E-01	-0.25	2.2E-01	0.03	8.7E-01	-0.42	3.4E-02
ENSCAFG0000002897	IL2RG	turquoise	EC_M6	0.10	6.4E-01	0.85	5.1E-08	-0.39	4.6E-02	-0.14	4.9E-01	0.08	6.8E-01	-0.18	3.7E-01	0.03	8.8E-01	-0.17	3.9E-01	-0.09	6.4E-01	-0.15	4.7E-01
ENSCAFG0000000163	GUCA1B	grey	EC_M10	0.10	6.4E-01	0.29	1.5E-01	0.16	4.2E-01	0.38	5.8E-02	-0.43	3.0E-02	-0.15	4.8E-01	0.31	1.2E-01	-0.15	4.7E-01	-0.13	5.4E-01	0.43	2.8E-02
ENSCAFG0000002014	GARNL3	grey	EC_M10	0.10	6.4E-01	-0.41	3.9E-02	-0.23	2.6E-01	-0.29	1.5E-01	0.39	4.8E-02	-0.03	8.8E-01	-0.02	9.0E-01	0.33	9.6E-02	0.30	1.3E-01	-0.45	2.1E-02
ENSCAFG0000001403	LARS2	grey	EC_M10	0.10	6.4E-01	-0.17	4.1E-01	0.30	1.3E-01	0.06	7.6E-01	-0.32	1.1E-01	-0.17	4.0E-01	-0.29	1.5E-01	-0.37	5.9E-02	-0.25	2.2E-01	0.29	1.5E-01
ENSCAFG0000001451	DDX49	grey	EC_M10	0.10	6.4E-01	0.01	9.7E-01	-0.40	4.3E-02	0.01	9.5E-01	0.50	1.0E-02	0.19	3.5E-01	-0.25	2.1E-01	-0.05	8.1E-01	-0.12	5.7E-01	-0.55	3.5E-03
ENSCAFG0000001750	ENSCAFG0000001750	turquoise	EC_M6	0.10	6.4E-01	0.49	1.2E-02	-0.31	1.2E-01	0.34	9.1E-02	0.11	5.9E-01	-0.13	5.2E-01	-0.11	6.1E-01	-0.07	7.4E-01	-0.07	7.3E-01	-0.12	5.7E-01
ENSCAFG0000000609	ENSCAFG0000000609	grey	EC_M10	0.10	6.4E-01	-0.21	3.1E-01	-0.21	3.0E-01	-0.16	4.5E-01	0.25	2.1E-01	-0.17	4.0E-01	-0.14	5.0E-01	0.26	2.1E-01	0.01	9.6E-01	-0.24	2.4E-01
ENSCAFG0000000961	SOX13	turquoise	EC_M6	0.10	6.4E-01	0.90	4.5E-10	-0.32	1.1E-01	-0.11	5.8E-01	-0.01	9.7E-01	-0.22	2.8E-01	0.14	5.0E-01	0.00	9.9E-01	0.25	2.1E-01	-0.03	8.7E-01
ENSCAFG0000000170	NMRK1	grey	EC_M10	0.10	6.4E-01	0.28	1.6E-01	0.19	3.5E-01	0.07	7.3E-01	-0.40	4.3E-02	-0.58	2.0E-03	-0.25	2.2E-01	0.19	3.5E-01	0.32	1.1E-01	0.41	4.0E-02
ENSCAFG0000001130	GTF2E1	turquoise	EC_M6	0.10	6.4E-01	0.40	4.2E-02	-0.06	7.8E-01	0.11	6.0E-01	-0.18	3.8E-01	0.04	8.4E-01	0.10	6.2E-01	-0.17	4.0E-01	0.42	3.3E-02	0.17	4.1E-01
ENSCAFG0000001613	ENSCAFG0000001613	cyan	EC_M2	0.10	6.4E-01	0.22	2.8E-01	0.41	3.8E-02	0.13	5.2E-01	-0.66	2.7E-04	-0.22	2.8E-01	0.25	2.1E-01	-0.40	4.3E-02	-0.15	4.5E-01	0.61	8.7E-04
ENSCAFG0000000843	ATP2A2	darkgreen	EC_M4	0.10	6.4E-01	-0.25	2.2E-01	-0.67	1.8E-04	-0.13	5.1E-01	0.81	6.4E-07	0.34	9.0E-02	0.15	4.7E-01	-0.06	7.9E-01	-0.14	5.1E-01	-0.87	5.1E-09
ENSCAFG0000002585	ENSCAFG0000002585	turquoise	EC_M6	0.10	6.4E-01	0.41	3.8E-02	0.06	7.8E-01	-0.10	6.4E-01	-0.26	1.9E-01	-0.09	6.6E-01	-0.07	7.4E-01	-0.08	7.1E-01	-0.10	6.4E-01	0.21	3.0E-01
ENSCAFG0000000584	TBC1D5	cyan	EC_M2	0.10	6.4E-01	-0.60	1.2E-03	0.64	4.4E-04	0.23	2.6E-01	-0.64	4.3E-04	-0.16	4.3E-01	-0.10	6.2E-01	0.01	9.7E-01	-0.03	8.7E-01	0.56	2.8E-03
ENSCAFG0000001897	ENSCAFG0000001897	paleturquoise	EC_M11	0.10	6.4E-01	-0.09	6.5E-01	0.07	7.4E-01	0.72	3.7E-05	-0.17	3.9E-01	-0.08	6.8E-01	-0.12	5.6E-01	-0.12	5.6E-01	-0.13	5.3E-01	0.11	5.9E-01
ENSCAFG0000002733	ENSCAFG0000002733	paleturquoise	EC_M11	0.10	6.4E-01	-0.09	6.5E-01	0.07	7.4E-01	0.72	3.7E-05	-0.17	3.9E-01	-0.08	6.8E-01	-0.12	5.6E-01	-0.12	5.6E-01	-0.13	5.3E-01	0.11	5.9E-01
ENSCAFG0000000000	ENPP1	grey	EC_M10	0.10	6.4E-01	-0.13	5.2E-01	-0.52	6.7E-03	0.24	2.3E-01	0.55	3.7E-03	0.06	7.6E-01	-0.18	3.8E-01	0.08	7.1E-01	-0.20	3.2E-01	-0.64	3.9E-04
ENSCAFG0000001326	EBF3	grey	EC_M10	0.10	6.4E-01	0.36	7.0E-02	0.25	2.3E-01	0.01	9.5E-01	-0.49	1.1E-02	-0.19	3.6E-01	-0.02	9.1E-01	-0.23	2.6E-01	-0.33	1.0E-01	0.48	1.3E-02
ENSCAFG0000002397	HACD1	darkgreen	EC_M4	0.10	6.4E-01	0.09	6.7E-01	-0.82	2.9E-07	-0.19	3.4E-01	0.82	2.5E-07	0.27	1.8E-01	-0.05	8.1E-01	0.11	5.8E-01	0.01	9.5E-01	-0.90	5.7E-10
ENSCAFG0000003244	ENSCAFG0000003244	grey	EC_M10	0.09	6.4E-01	0.22	2.7E-01	-0.21	3.1E-01	-0.22	2.7E-01	0.07	7.4E-01	0.29	1.6E-01	-0.17	4.1E-01	0.11	5.9E-01	0.09	6.7E-01	-0.14	4.9E-01
ENSCAFG0000003052	CHRAC1	grey	EC_M10	0.09	6.4E-01	-0.39	4.7E-02	-0.26	2.0E-01	-0.13	5.3E-01	0.37	6.3E-02	0.19	3.6E-01	0.25	2.1E-01	0.39	4.8E-02	-0.05	8.3E-01	-0.46	1.7E-02
ENSCAFG0000001397	BCL2A1	grey	EC_M10	0.09	6.4E-01	-0.13	5.3E-01	0.53	5.9E-03	0.11	5.8E-01	-0.62	6.6E-04	0.08	7.0E-01	-0.10	6.3E-01	-0.15	4.5E-01	-0.47	1.7E-02	0.54	4.5E-03
ENSCAFG0000000517	DNAJB6	darkgreen	EC_M4	0.09	6.5E-01	0.31	1.3E-01	-0.79	1.8E-06	-0.26	1.9E-01	0.72	3.1E-05	0.27	1.8E-01	-0.12	5.5E-01	0.15	4.5E-01	0.03	9.0E-01	-0.77	4.3E-06
ENSCAFG0000001264	PSTK	grey	EC_M10	0.09	6.5E-01	0.00	9.9E-01	-0.14	4.9E-01	0.15	4.6E-01	0.05	8.2E-01	0.00	1.0E+00	-0.07	7.3E-01	-0.04	8.5E-01	-0.23	2.6E-01	-0.07	7.5E-01
ENSCAFG0000001192	RHCG	grey	EC_M10	0.09	6.5E-01	-0.05	8.0E-01	-0.10	6.3E-01	0.17	4.1E-01	0.12	5.5E-01	-0.20	3.3E-01	-0.16	4.2E-01	-0.19	3.6E-01	-0.16	4.4E-01	-0.14	4.8E-01
ENSCAFG0000000082	P4HA2	darkgreen	EC_M4	0.09	6.5E-01	0.04	8.5E-01	-0.77	4.4E-06	-0.11	5.9E-01	0.82	3.7E-07	0.20	3.2E-01	0.13	5.2E-01	0.16	4.3E-01	0.00	9.8E-01	-0.90	4.2E-10
ENSCAFG0000001830	USP14	darkgreen	EC_M4	0.09	6.5E-01	-0.12	5.6E-01	-0.76	5.8E-06	-0.30	1.3E-01	0.89	8.1E-10	0.28	1.7E-01	0.09	6.6E-01	0.11	6.0E-01	-0.08	7.1E-01	-0.93	2.7E-12
ENSCAFG0000000880	TPRKB	grey	EC_M10	0.09	6.5E-01	-0.27	1.8E-01	-0.33	9.9E-02	0.06	7.6E-01	0.37	6.0E-02	0.16	4.3E-01	-0.13	5.1E-01	0.03	8.7E-01	0.03	8.8E-01	-0.42	3.4E-02
ENSCAFG0000001108	SLC16A6	grey	EC_M10	0.09	6.5E-01	-0.23	2.6E-01	-0.38	5.7E-02	-0.06	7.8E-01	0.52	6.7E-03	-0.24	2.4E-01	-0.21	3.0E-01	0.04	8.4E-01	0.01	9.6E-01	-0.51	8.0E-03
ENSCAFG0000000202	ENPP4	grey	EC_M10	0.09	6.5E-01	0.41	3.5E-02	0.24	2.4E-01	0.15	4.5E-01	-0.44	2.3E-02	-0.35	7.8E-02	-0.29	1.5E-01	-0.14	4.9E-01	0.06	7.5E-01	0.45	2.0E-02
ENSCAFG0000001221	KDF1	grey	EC_M10	0.09	6.5E-01	0.15	4.7E-01	0.12	5.7E-01	-0.08	6.8E-01	-0.27	1.8E-01	-0.07	7.4E-01	-0.08	6.9E-01	-0.01	9.4E-01	0.27	1.8E-01	0.21	3.1E-01
ENSCAFG0000000895	ENSCAFG0000000895	grey	EC_M10	0.09	6.5E-01	-0.04	8.5E-01	-0.21	3.1E-01	0.42	3.1E-02	0.17	4.0E-01	-0.15	4.6E-01	-0.17	3.9E-01	-0.10	6.4E-01	-0.13	5.2E-01	-0.24	2.4E-01
ENSCAFG0000000213	IL18RAP	violet	EC_M7	0.09	6.5E-01	-0.09	6.6E-01	-0.29	1.6E-01	-0.11	6.0E-01	0.29	1.5E-01	0.90	5.1E-10	-0.05	8.0E-01	-0.12	5.6E-01	-0.11	6.1E-01	-0.36	7.2E-02
ENSCAFG0000001607	ENSCAFG0000001607	darkgreen	EC_M4	0.09	6.5E-01	0.24	2.4E-01	-0.80	1.2E-06	-0.17	4.1E-01	0.74	1.7E-05	0.32	1.1E-01	0.16	4.5E-01	-0.11	5.9E-01	0.01	9.5E-01	-0.82	2.9E-07
ENSCAFG0000000582	ENSCAFG0000000582	turquoise	EC_M6	0.09	6.5E-01	0.62	6.5E-04	0.04	8.4E-01	-0.11	6.0E-01	-0.38	5.6E-02	0.22	2.7E-01	-0.09	6.5E-01	-0.14	5.0E-01	0.21	3.0E-01	0.35	8.0E-02
ENSCAFG0000002301	SLC35E3	grey	EC_M10	0.09	6.5E-01	-0.52	6.0E-03	0.54	4.4E-03	0.31	1.2E-01	-0.49	1.1E-02	0.06	7.8E-01	-0.04	8.3E-01	0.01	9.7E-01	-0.12	5.4E-01	0.47	1.6E-02
ENSCAFG0000001519	PLAU	grey	EC_M10	0.09	6.5E-01	0.27	1.8E-01	0.06	7.8E-01	-0.18	3.8E-01	-0.25	2.2E-01	-0.05	8.1E-01	0.11	5.9E-01	-0.18	3.8E-01	-0.01	9.7E-01	0.18	3.9E-01
ENSCAFG0000000982	PDZRN4	grey	EC_M10	0.09	6.5E-01	-0.22	2.9E-01	0.00	9.8E-01	-0.22	2.7E-01	-0.04	8.4E-01	-0.21	3.1E-01	-0.01	9.7E-01	0.33	1.0E-01	0.41	4.0E-02	-0.01	9.4E-01
ENSCAFG0000001185	ENSCAFG0000001185	grey	EC_M10	0.09	6.5E-01	-0.02	9.3E-01	-0.58	1.9E-03	-0.11	5.8E-01	0.65	3.4E-04	0.16	4.3E-01	0.12	5.5E-01	-0.07	7.4E-01	0.30	1.4E-01	-0.64	4.1E-04
ENSCAFG0000000717	ZNF772	grey	EC_M10	0.09	6.5E-01	0.21	3.1E-01	0.26	2.0E-01	0.01	9.7E-01	-0.49	1.0E-02	-0.25	2.3E-01	0.00	9.9E-01	-0.06	7.9E-01	-0.05	8.2E-01	0.50	9.2E-03
ENSCAFG0000001174	TCEANC	grey	EC_M10	0.09	6.5E-01	-0.12	5.5E-01	0.07	7.5E-01	0.07	7.4E-01	-0.15	4.6E-01	0.02	9.4E-01	0.07	7.4E-01	-0.60	1.2E-03	-0.03	8.7E-01	0.06	7.6E-01
ENSCAFG0000002008	ENSCAFG0000002008	violet	EC_M7	0.09	6.5E-01	0.00	9.8E-01	-0.20	3.3E-01	-0.04	8.4E-01	0.12	5.8E-01	0.78	2.4E-06	-0.03	9.0E-01	-0.10	6.2E-01	0.48	1.3E-02	-0.17	4.2E-01
ENSCAFG0000001283	RHCE	grey	EC_M10	0.09	6.5E-01	-0.14	5.0E-01	-0.16	4.2E-01	-0.21	3.0E-01	0.13	5.1E-01	-0.12	5.5E-01	-0.13	5.2E-01	0.39	5.1E-02	0.02	9.2E-01	-0.22	2.9E-01
ENSCAFG0000001049	ENSCAFG0000001049	grey	EC_M10	0.09	6.5E-01	-0.18	3.9E-01	-0.16	4.3E-01	0.18	3.9E-01	0.18	3.8E-01	0.26	2.1E-01	-0.29	1.6E-01	-0.58	1.9E-03	-0.10	6.2E-01	-0.24	2.3E-01
ENSCAFG0000001118	PRKX	turquoise	EC_M6	0.09	6.5E-01	0.74	1.6E-05	-0.05	8.2E-01	-0.14	4.8E-01	-0.28	1.7E-01	-0.29	1.5E-01	-0.26	1.9E-01	-0.01	9.8E-01	0.36	7.0E-02	0.26	2.0E-01
ENSCAFG0000000511	RNF32	cyan	EC_M2	0.09	6.5E-01	-0.63	5.9E-04	0.43	3.0E-02	0.31	1.3E-01	-0.36	6.8E-02	0.16	4.3E-01	-0.08	6.8E-01	-0.18	3.7E-01	-0.24	2.4E-01	0.31	1.2E-01
ENSCAFG0000002906	ENSCAFG0000002906	grey	EC_M10	0.09	6.5E-01	0.23	2.5E-01	-0.05	8.2E-01	-0.21	3.1E-01	-0.07	7.4E-01	0.15	4.6E-01	0.13	5.4E-01	0.02	9.3E-01	-0.32	1.1E-01	0.01	9.4E-01
ENSCAFG0000000252	COL15A1	darkgreen	EC_M4	0.09	6.5E-01	0.14	4.9E-01	-0.59	1.5E-03	-0.03	8.8E-01	0.54	4.4E-03	0.11	5.9E-01	0.14	5.0E-01	0.19	3.4E-01	0.16	4.5E-01	-0.65	3.2E-04
ENSCAFG0000000691	MAP3K1	turquoise	EC_M6	0.09	6.5E-01	0.57	2.3E-03	-0.05	8.1E-01	-0.06	7.8E-01	-0.27	1.9E-01	-0.04	8.4E-01	-0.43	2.8E-02	0.13	5.2E-01	0.29	1.5E-01	0.21	3.0E-01
ENSCAFG0000000941	CHAC1	darkgreen	EC_M4	0.09	6.5E-01	0.28	1.7E-01	-0.73	1.9E-05	-0.07	7.5E-01	0.68	1.5E-04	0.29	1.5E-01	-0.12	5.7E-01	0.02	9.1E-01	-0.17	4.1E-01	-0.78	2.7E-06
ENSCAFG0000001152	ISG20	turquoise	EC_M6	0.09	6.5E-01	0.76	6.5E-06	-0.25	2.2E-01	-0.06	7.7E-01	-0.06	7.6E-01	-0.14	5.0E-01	-0.11	5.8E-01	-0.10	6.3E-01	0.12	5.5E-01	-0.03	8.7E-01
ENSCAFG0000001658	C20H19orf57	grey	EC_M10	0.09	6.5E-01	0.15	4.6E-01	-0.05	7.9E-01	-0.15	4.7E-01	-0.12	5.7E-01	-0.17	4.0E-01	0.42	3.3E-02	0.15	4.7E-01	0.40	4.4E-02	0.10	6.4E-01
ENSCAFG0000000815	TMEM70	grey	EC_M10	0.09	6.5E-01	-0.39	4.7E-02	-0.47	1.6E-02	-0.22	2.7E-01	0.65	3.5E-04	0.22	2.7E-01	-0.04	8.4E-01	0.09	6.6E-01	-0.08	7.1E-01	-0.70	7.8E-05
ENSCAFG0000003149	BMP2	turquoise	EC_M6	0.09	6.5E-01	0.75	1.0E-05	-0.69	1.1E-04	-0.15	4.7E-01	0.44	2.3E-02	0.11	5.8E-01	-0.01	9.6E-01	-0.05	8.1E-01	0.02	9.0E-01	-0.51	8.2E-03
ENSCAFG0000000299	SMAP2	grey	EC_M10	0.09	6.5E-01	0.14	5.0E-01	-0.67	2.0E-04	-0.13	5.2E-01	0.68	1.3E-04	0.25	2.3E-01	0.01	9.7E-01	0.18	3.9E-01	0.26	2.0E-01	-0.68	1.4E-04
ENSCAFG0000000704	ENSCAFG0000000704	grey	EC_M10	0.09	6.5E-01	0.11	6.1E-01	0.01	9.5E-01	0.24	2.3E-01	-0.18	3.9E-01	-0.08	6.8E-01	-0.26	2.0E-01	-0.06	7.8E-01	0.15	4.5E-01	0.10	6.2E-01
ENSCAFG0000001740	CHM	grey	EC_M10	0.09	6.5E-01	0.28	1.6E-01	0.15	4.7E-01	0.06	7.9E-01	-0.43	2.9E-02	-0.16	4.3E-01	-0.30	1.3E-01	0.30	1.3E-01	-0.25	2.1E-01	0.34	8.5E-02
ENSCAFG0000000848	ENSCAFG0000000848	grey	EC_M10	0.09	6.5E-01	-0.59	1.6E-03	-0.44	2.4E-02	-0.12	5.7E-01	0.62	7.2E-04	0.22	2.8E-01	-0.05	8.0E-01	0.14	5.0E-01	0.14	5.0E-01	-0.68	1.5E-04
ENSCAFG0000003017	NDUFA8	grey	EC_M10	0.09	6.5E-01	0.35	7.5E-02	-0.37	6.2E-02	-0.28	1.7E-01	0.36	7.0E-02	0.02	9.3E-01	-0.11	6.1E-01	-0.16	4.3E-01	-0.13	5.4E-01	-0.33	9.9E-02
ENSCAFG0000002950	ENSCAFG0000002950	grey	EC_M10	0.09	6.5E-01	-0.11	6.0E-01	-0.31	1.3E-01	0.03	9.0E-01	0.27	1.9E-01	-0.11	6.0E-01	-0.08	6.8E-01	0.49	1.1E-02	-0.02	9.2E-01	-0.36	7.5E-02
ENSCAFG0000001468	ENSCAFG0000001468	grey	EC_M10	0.09	6.5E-01	0.15	4.6E-01	-0.09	6.6E-01	-0.16	4.3E-01	-0.01	9.5E-01	-0.19	3.4E-01	0.46	1.8E-02	0.12	5.6E-01	-0.24	2.3E-01	-0.08	6.9E-01
ENSCAFG0000001734	TOM1L1	magenta	EC_M13	0.09	6.5E-01	-0.11	5.8E-01	0.03	8.8E-01	-0.20	3.3E-01	-0.07	7.5E-01	-0.11	6.0E-01	-0.07	7.5E-01	-0.19	3.5E-01	0.49	1.0E-02	0.06	7.6E-01
ENSCAFG0000002030	SPATA1	grey	EC_M10	0.09	6.5E-01	-0.04	8.4E-01	-0.57	2.3E-03	0.07	7.2E-01	0.54	4.6E-03	-0.23	2.5E-01	-0.01	9.4E-01	0.05	8.0E-01	0.03	8.7E-01	-0.59	1.6E-03
ENSCAFG0000000961	ETNK2	grey	EC_M10	0.09	6.5E-01	0.36	6.7E-02	-0.52	6.2E-03	0.06	7.7E-01	0.39	4.7E-02	-0.02	9.1E-01	0.24	2.3E-01	-0.04	8.3E-01	0.40	4.1E-02	-0.45	2.2E-02
ENSCAFG0000001234	SEC24D	grey	EC_M10	0.09	6.5E-01	-0.13	5.1E-01	-0.73	2.2E-05	-0.14	4.9E-01	0.84	7.1E-08	0.30	1.3E-01	-0.06	7.6E-01	0.07	7.3E-01	0.18	3.9E-01	-0.92	4.3E-11
ENSCAFG0000000067	LRP12	grey	EC_M10	0.09	6.5E-01	-0.49	1.1E-02	-0.59	1.6E-03	-0.02	9.2E-01	0.76	6.4E-06	0.37	6.0E-02	0.05	7.9E-01	0.03	9.0E-01	-0.05	8.1E-01	-0.83	1.3E-07
ENSCAFG0000001391	LPP	darkgreen	EC_M4	0.09	6.6E-01	-0.24	2.3E-01	-0.71	4.9E-05	-0.12	5.4E-01	0.84	9.3E-08	0.22	2.9E-01	0.04	8.6E-01	-0.02	9.2E-01	0.15	4.8E-01	-0.87	6.6E-09
ENSCAFG0000000069	TRABD	grey	EC_M10	0.09	6.6E-01	0.25	2.1E-01	-0.04	8.4E-01	-0.27	1.8E-01	-0.05	7.9E-01	0.02	9.1E-01	-0.02	9.2E-01	0.11	5.9E-01	0.10	6.2E-01	-0.01	9.5E-01
ENSCAFG0000000113	WISP1	grey	EC_M10	0.09	6.6E-01	-0.13	5.3E-01	-0.49	1.0E-02	-0.24	2.3E-01	0.53	5.2E-03	0.03	9.0E-01	0.27	1.7E-01	-0.18	3.7E-01	-0.05	8.0E-01	-0.55	3.7E-03
ENSCAFG0000000145	MEST	grey	EC_M10	0.09	6.6E-01	-0.37	6.6E-02	-0.56	3.2E-03	-0.22	2.7E-01	0.73	2.8E-05	0.21	3.0E-01	0.04	8.5E-01	0.00	9.8E-01	-0.23	2.7E-01	-0.77	4.0E-06
ENSCAFG0000000975	ELOVL2	grey	EC_M10	0.09	6.6E-01	-0.02	9.2E-01	-0.52	6.6E-03	-0.20	3.4E-01	0.61	1.0E-03	0.29	1.4E-01	-0.11	6.0E-01	-0.11	5.9E-01	0.03	8.9E-01	-0.62	7.0E-04
ENSCAFG0000001882	ERCC4	grey	EC_M10	0.09	6.6E-01	0.07	7.4E-01	-0.37	5.9E-02	-0.11	6.0E-01	0.33	1.0E-01	-0.20	3.4E-01	-0.08	7.0E-01	0.14	4.9E-01	0.00	9.9E-01	-0.37	6.5E-02
ENSCAFG0000002957	ZNF70	grey	EC_M10	0.09	6.6E-01	0.14	4.8E-01	0.17	4.0E-01	0.31	1.3E-01	-0.37	6.3E-02	-0.25	2.3E-01	-0.20	3.2E-01	-0.46	1.7E-02	0.14	5.0E-01	0.29	1.5E-01
ENSCAFG0000001238	KLHL30	grey	EC_M10	0.09	6.6E-01	-0.03	8.8E-01	-0.23	2.5E-01	-0.18	3.7E-01	0.21	3.0E-01	0.33	1.1E-01	-0.08	7.1E-01	-0.20	3.2E-01	0.34	8.7E-02	-0.21	3.0E-01

ENSCAFG0000001597	CLCN5	grey	EC_M10	0.09	6.6E-01	0.35	7.9E-02	-0.32	1.1E-01	0.10	6.4E-01	0.09	6.7E-01	-0.02	9.4E-01	0.20	3.3E-01	-0.24	2.4E-01	0.17	4.2E-01	-0.15	4.5E-01
ENSCAFG0000001837	ENSCAFG0000001837	grey	EC_M10	0.09	6.6E-01	-0.16	4.2E-01	0.02	9.1E-01	-0.05	8.1E-01	-0.01	9.5E-01	-0.04	8.3E-01	0.03	8.9E-01	0.09	6.5E-01	0.17	4.1E-01	0.00	1.0E+00
ENSCAFG0000001445	ENSCAFG0000001445	grey	EC_M10	0.09	6.6E-01	-0.20	3.3E-01	0.26	1.9E-01	0.59	1.5E-03	-0.35	8.1E-02	-0.06	7.7E-01	-0.15	4.6E-01	-0.11	6.0E-01	-0.17	4.0E-01	0.29	1.5E-01
ENSCAFG0000001318	SP9	grey	EC_M10	0.09	6.6E-01	-0.21	3.0E-01	0.43	2.8E-02	-0.12	5.6E-01	-0.40	4.0E-02	-0.06	7.8E-01	-0.05	7.9E-01	-0.05	8.1E-01	-0.09	6.6E-01	0.41	3.6E-02
ENSCAFG0000000283	CFAP36	darkgreen	EC_M4	0.09	6.6E-01	-0.85	3.7E-08	0.09	6.8E-01	0.15	4.7E-01	0.15	4.7E-01	0.03	8.7E-01	-0.20	3.3E-01	0.01	9.5E-01	-0.20	3.2E-01	-0.19	3.6E-01
ENSCAFG0000000029	ADGRG6	grey	EC_M10	0.09	6.6E-01	0.35	8.0E-02	-0.51	7.7E-03	-0.09	6.5E-01	0.40	4.2E-02	0.02	9.2E-01	-0.27	1.8E-01	-0.22	2.8E-01	-0.43	2.8E-02	-0.47	1.7E-02
ENSCAFG0000003117	CADM4	grey	EC_M10	0.09	6.6E-01	0.53	5.4E-03	-0.67	1.8E-04	-0.19	3.5E-01	0.53	5.7E-03	-0.01	9.6E-01	0.42	3.3E-02	0.01	9.7E-01	-0.07	7.5E-01	-0.57	2.4E-03
ENSCAFG0000000255	RIMKLA	turquoise	EC_M6	0.09	6.6E-01	0.58	1.9E-03	0.14	5.0E-01	-0.20	3.2E-01	-0.40	4.3E-02	-0.15	4.7E-01	0.01	9.7E-01	-0.18	3.9E-01	0.24	2.4E-01	0.42	3.3E-02
ENSCAFG0000001073	KCNJ2	turquoise	EC_M6	0.09	6.6E-01	0.70	7.3E-05	-0.01	9.7E-01	0.04	8.3E-01	-0.30	1.3E-01	-0.17	4.1E-01	-0.06	7.6E-01	0.00	9.9E-01	-0.25	2.1E-01	0.25	2.2E-01
ENSCAFG0000000024	MAP7	grey	EC_M10	0.09	6.6E-01	-0.11	5.8E-01	-0.16	4.4E-01	0.24	2.5E-01	0.20	3.4E-01	0.15	4.7E-01	0.07	7.4E-01	-0.24	2.4E-01	-0.15	4.6E-01	-0.23	2.5E-01
ENSCAFG0000001824	PRPSAP2	grey	EC_M10	0.09	6.6E-01	0.24	2.4E-01	0.01	9.8E-01	0.09	6.6E-01	-0.25	2.3E-01	0.03	8.9E-01	-0.23	2.7E-01	-0.13	5.2E-01	-0.08	6.9E-01	0.16	4.2E-01
ENSCAFG0000000217	GADD45G	turquoise	EC_M6	0.09	6.6E-01	0.52	6.9E-03	-0.03	8.9E-01	0.21	3.0E-01	-0.23	2.5E-01	-0.13	5.3E-01	-0.04	8.5E-01	-0.06	7.6E-01	-0.06	7.8E-01	0.12	5.5E-01
ENSCAFG0000003143	MRPL18	grey	EC_M10	0.09	6.6E-01	-0.10	6.4E-01	-0.57	2.4E-03	-0.21	3.0E-01	0.59	1.5E-03	0.28	1.7E-01	-0.03	8.9E-01	0.09	6.7E-01	-0.03	8.7E-01	-0.72	4.0E-05
ENSCAFG0000000107	H2AFY	grey	EC_M10	0.09	6.6E-01	0.37	6.6E-02	-0.30	1.4E-01	-0.17	4.0E-01	0.24	2.3E-01	0.26	2.0E-01	0.05	8.0E-01	-0.21	3.0E-01	-0.33	1.0E-01	-0.29	1.5E-01
ENSCAFG0000001115	GALNT17	grey	EC_M10	0.09	6.6E-01	-0.39	4.8E-02	0.12	5.7E-01	0.15	4.6E-01	0.06	7.9E-01	-0.09	6.7E-01	0.12	5.5E-01	-0.13	5.4E-01	-0.10	6.1E-01	-0.06	7.7E-01
ENSCAFG0000002399	ENSCAFG0000002399	grey	EC_M10	0.09	6.6E-01	-0.06	7.6E-01	-0.52	6.9E-03	-0.34	8.8E-02	0.58	1.8E-03	0.05	8.0E-01	-0.03	8.9E-01	-0.03	8.7E-01	-0.24	2.3E-01	-0.60	1.1E-03
ENSCAFG0000001006	RRP9	grey	EC_M10	0.09	6.6E-01	-0.01	9.5E-01	0.12	5.5E-01	-0.07	7.2E-01	-0.12	5.7E-01	0.05	8.0E-01	-0.15	4.5E-01	-0.07	7.2E-01	0.01	9.5E-01	0.11	6.0E-01
ENSCAFG0000001487	BCS1L	grey	EC_M10	0.09	6.6E-01	-0.18	3.8E-01	-0.22	2.7E-01	0.02	9.2E-01	0.32	1.1E-01	0.23	2.5E-01	0.10	6.2E-01	-0.28	1.7E-01	0.10	6.3E-01	-0.34	8.8E-02
ENSCAFG0000002468	AGO3	darkgreen	EC_M4	0.09	6.6E-01	-0.25	2.1E-01	-0.70	6.2E-05	-0.05	8.2E-01	0.79	1.4E-06	0.26	1.9E-01	-0.04	8.5E-01	-0.04	8.6E-01	-0.17	4.0E-01	-0.85	4.7E-08
ENSCAFG0000001888	ENSCAFG0000001888	turquoise	EC_M6	0.09	6.6E-01	0.37	6.3E-02	-0.37	6.6E-02	-0.10	6.2E-01	0.23	2.7E-01	0.29	1.5E-01	-0.09	6.5E-01	0.33	9.7E-02	0.31	1.3E-01	-0.26	2.0E-01
ENSCAFG0000001839	ENSCAFG0000001839	grey	EC_M10	0.09	6.6E-01	0.29	1.6E-01	-0.10	6.2E-01	-0.05	8.2E-01	-0.04	8.6E-01	-0.08	7.1E-01	0.34	8.6E-02	-0.07	7.3E-01	0.27	1.9E-01	-0.02	9.2E-01
ENSCAFG0000003190	ENSCAFG0000003190	grey	EC_M10	0.09	6.6E-01	-0.21	3.0E-01	0.05	8.2E-01	-0.38	5.4E-02	0.07	7.5E-01	-0.16	4.4E-01	0.04	8.4E-01	0.16	4.2E-01	-0.22	2.8E-01	-0.12	5.7E-01
ENSCAFG0000001464	ZBTB40	grey	EC_M10	0.09	6.6E-01	0.19	3.5E-01	0.21	2.9E-01	0.26	2.1E-01	-0.49	1.1E-02	-0.01	9.7E-01	0.20	3.3E-01	-0.09	6.6E-01	-0.31	1.2E-01	0.40	4.3E-02
ENSCAFG0000001354	YWHAG	grey	EC_M10	0.09	6.6E-01	0.04	8.6E-01	-0.55	3.6E-03	-0.33	1.0E-01	0.66	2.5E-04	0.21	2.9E-01	-0.07	7.2E-01	0.07	7.2E-01	-0.13	5.2E-01	-0.67	1.8E-04
ENSCAFG0000000326	ARHGAP25	turquoise	EC_M6	0.09	6.6E-01	0.92	5.0E-11	-0.21	3.1E-01	-0.12	5.7E-01	-0.15	4.8E-01	-0.14	4.9E-01	-0.13	5.1E-01	0.02	9.2E-01	0.00	9.9E-01	0.08	7.0E-01
ENSCAFG0000000540	RPS3	cyan	EC_M2	0.09	6.6E-01	-0.37	6.2E-02	0.32	1.1E-01	0.08	7.1E-01	-0.27	1.8E-01	0.12	5.6E-01	0.37	6.3E-02	-0.08	6.8E-01	-0.53	5.6E-03	0.17	4.1E-01
ENSCAFG0000001876	DUS3L	cyan	EC_M2	0.09	6.6E-01	-0.31	1.2E-01	0.59	1.4E-03	0.14	5.1E-01	-0.57	2.2E-03	-0.03	9.0E-01	-0.06	7.7E-01	-0.14	4.8E-01	-0.08	6.9E-01	0.54	4.7E-03
ENSCAFG0000000538	MAP6	grey	EC_M10	0.09	6.6E-01	-0.06	7.8E-01	-0.17	4.1E-01	-0.21	3.0E-01	0.18	3.7E-01	-0.17	4.0E-01	0.16	4.3E-01	-0.11	5.8E-01	-0.07	7.3E-01	-0.18	3.7E-01
ENSCAFG0000001642	RGS14	turquoise	EC_M6	0.09	6.6E-01	0.88	3.1E-09	-0.14	4.9E-01	-0.08	7.1E-01	-0.24	2.4E-01	-0.06	7.7E-01	-0.04	8.3E-01	-0.11	5.8E-01	0.17	4.2E-01	0.17	4.0E-01
ENSCAFG0000002921	NCR3LG1	grey	EC_M10	0.09	6.6E-01	-0.14	5.1E-01	-0.61	8.6E-04	-0.13	5.2E-01	0.69	9.1E-05	-0.08	7.1E-01	-0.09	6.5E-01	0.35	8.0E-02	-0.12	5.5E-01	-0.72	3.0E-05
ENSCAFG0000003241	PLEKHA2	grey	EC_M10	0.09	6.6E-01	0.11	6.0E-01	-0.44	2.5E-02	0.07	7.4E-01	0.39	5.1E-02	0.08	7.0E-01	0.41	3.7E-02	-0.10	6.3E-01	0.32	1.1E-01	-0.37	5.9E-02
ENSCAFG0000001652	NTRK1	grey	EC_M10	0.09	6.6E-01	-0.07	7.3E-01	-0.14	4.8E-01	-0.13	5.4E-01	0.12	5.6E-01	-0.06	7.7E-01	-0.03	8.9E-01	-0.14	5.1E-01	0.07	7.4E-01	-0.14	5.0E-01
ENSCAFG0000000311	BACH2	turquoise	EC_M6	0.09	6.6E-01	0.66	2.2E-04	-0.37	6.5E-02	-0.20	3.2E-01	0.16	4.2E-01	-0.31	1.2E-01	0.03	8.8E-01	0.06	7.6E-01	0.00	9.9E-01	-0.22	2.9E-01
ENSCAFG0000000503	KLF5	grey	EC_M10	0.09	6.6E-01	0.04	8.5E-01	-0.54	4.8E-03	0.09	6.6E-01	0.55	3.7E-03	-0.06	7.7E-01	-0.11	6.0E-01	0.28	1.7E-01	0.00	9.9E-01	-0.62	8.3E-04
ENSCAFG0000001577	PLD5	grey	EC_M10	0.09	6.6E-01	-0.09	6.5E-01	-0.26	1.9E-01	0.00	9.8E-01	0.23	2.6E-01	0.12	5.6E-01	-0.12	5.4E-01	-0.12	5.6E-01	-0.11	6.0E-01	-0.31	1.2E-01
ENSCAFG0000001197	PARP14	cyan	EC_M2	0.09	6.6E-01	0.35	8.3E-02	0.37	5.9E-02	0.26	2.0E-01	-0.68	1.2E-04	-0.12	5.5E-01	-0.21	3.1E-01	-0.07	7.2E-01	0.00	1.0E+00	0.61	9.7E-04
ENSCAFG0000001899	DPP9	grey	EC_M10	0.09	6.6E-01	-0.13	5.3E-01	-0.35	7.7E-02	-0.22	2.8E-01	0.49	1.2E-02	0.17	4.1E-01	0.13	5.3E-01	0.09	6.7E-01	0.14	5.0E-01	-0.53	5.1E-03
ENSCAFG0000000961	IFIT2	turquoise	EC_M6	0.09	6.7E-01	0.44	2.5E-02	-0.03	9.0E-01	0.22	2.8E-01	-0.24	2.4E-01	-0.13	5.3E-01	-0.16	4.5E-01	0.18	3.7E-01	0.43	2.9E-02	0.21	3.1E-01
ENSCAFG0000003055	ENSCAFG0000003055	grey	EC_M10	0.09	6.7E-01	0.08	6.8E-01	-0.15	4.6E-01	0.05	8.2E-01	0.05	8.0E-01	-0.36	7.4E-02	0.15	4.6E-01	0.17	4.1E-01	0.15	4.7E-01	-0.02	9.2E-01
ENSCAFG0000001024	ENSCAFG0000001024	paleturquoise	EC_M11	0.09	6.7E-01	-0.09	6.6E-01	0.12	5.5E-01	0.97	5.5E-16	-0.21	3.1E-01	-0.06	7.9E-01	-0.06	7.7E-01	-0.09	6.6E-01	-0.10	6.4E-01	0.17	4.1E-01
ENSCAFG0000001612	CLDN7	paleturquoise	EC_M11	0.09	6.7E-01	-0.09	6.6E-01	0.12	5.5E-01	0.97	5.5E-16	-0.21	3.1E-01	-0.06	7.9E-01	-0.06	7.7E-01	-0.09	6.6E-01	-0.10	6.4E-01	0.17	4.1E-01
ENSCAFG0000000546	RBMS3	cyan	EC_M2	0.09	6.7E-01	-0.10	6.2E-01	0.53	5.5E-03	0.42	3.2E-02	-0.70	7.7E-05	-0.18	3.8E-01	-0.14	4.9E-01	0.06	7.5E-01	-0.05	8.0E-01	0.66	2.8E-04
ENSCAFG0000001940	MLST8	grey	EC_M10	0.09	6.7E-01	0.04	8.3E-01	0.45	2.0E-02	0.20	3.2E-01	-0.51	8.2E-03	-0.13	5.2E-01	-0.17	4.0E-01	-0.21	3.1E-01	0.07	7.5E-01	0.49	1.1E-02
ENSCAFG0000001117	NKAIN1	grey	EC_M10	0.09	6.7E-01	-0.11	5.9E-01	-0.27	1.9E-01	-0.08	6.8E-01	0.39	4.7E-02	0.07	7.5E-01	-0.21	3.0E-01	0.08	7.1E-01	-0.09	6.5E-01	-0.48	1.3E-02
ENSCAFG0000001525	ATP1B1	magenta	EC_M13	0.09	6.7E-01	-0.13	5.1E-01	-0.11	5.8E-01	-0.02	9.0E-01	0.10	6.3E-01	-0.14	4.9E-01	0.00	1.0E+00	0.13	5.4E-01	0.75	1.1E-05	-0.10	6.3E-01
ENSCAFG0000001238	TRAPPC4	grey	EC_M10	0.09	6.7E-01	0.05	8.2E-01	-0.38	5.5E-02	-0.06	7.7E-01	0.37	6.5E-02	0.13	5.1E-01	-0.29	1.5E-01	-0.29	1.5E-01	0.09	6.6E-01	-0.41	3.7E-02
ENSCAFG0000000110	EFR3A	grey	EC_M10	0.09	6.7E-01	0.09	6.5E-01	0.11	6.0E-01	-0.13	5.3E-01	-0.17	4.2E-01	0.23	2.5E-01	0.38	5.9E-02	-0.24	2.3E-01	-0.37	6.3E-02	0.13	5.1E-01
ENSCAFG0000000179	TMEM33	grey	EC_M10	0.09	6.7E-01	-0.16	4.3E-01	-0.55	3.7E-03	-0.15	4.7E-01	0.67	1.9E-04	0.25	2.2E-01	-0.18	3.9E-01	-0.23	2.7E-01	-0.16	4.2E-01	-0.75	1.2E-05
ENSCAFG0000000802	RPL7	cyan	EC_M2	0.09	6.7E-01	-0.49	1.1E-02	0.50	9.8E-03	0.12	5.4E-01	-0.46	1.7E-02	-0.03	8.9E-01	0.22	2.9E-01	0.06	7.7E-01	-0.43	3.0E-02	0.37	6.5E-02
ENSCAFG0000001515	ENSCAFG0000001515	cyan	EC_M2	0.09	6.7E-01	-0.51	7.4E-03	0.37	6.5E-02	-0.24	2.4E-01	-0.24	2.3E-01	0.06	7.7E-01	0.10	6.2E-01	0.18	3.8E-01	-0.03	8.7E-01	0.19	3.4E-01
ENSCAFG0000002274	MT-ND6	grey	EC_M10	0.09	6.7E-01	-0.17	4.0E-01	0.02	9.2E-01	-0.27	1.8E-01	0.02	9.3E-01	0.14	5.1E-01	0.09	6.8E-01	0.27	1.8E-01	0.20	3.3E-01	-0.02	9.4E-01
ENSCAFG0000000020	TCF21	grey	EC_M10	0.09	6.7E-01	-0.19	3.6E-01	-0.20	3.2E-01	0.15	4.7E-01	0.21	3.0E-01	0.16	4.4E-01	-0.20	3.2E-01	0.45	2.1E-02	-0.05	8.1E-01	-0.31	1.2E-01
ENSCAFG0000001915	ENSCAFG0000001915	grey	EC_M10	0.09	6.7E-01	-0.43	3.0E-02	0.12	5.5E-01	-0.31	1.3E-01	-0.10	6.3E-01	0.13	5.3E-01	0.24	2.3E-01	0.08	6.9E-01	0.14	5.0E-01	0.06	7.8E-01
ENSCAFG0000000199	OCIAD1	grey	EC_M10	0.09	6.7E-01	-0.17	4.0E-01	-0.26	1.9E-01	0.14	5.0E-01	0.21	3.1E-01	0.31	1.2E-01	-0.20	3.4E-01	0.17	4.1E-01	-0.14	4.8E-01	-0.24	2.5E-01
ENSCAFG0000000161	MED20	grey	EC_M10	0.09	6.7E-01	0.05	8.0E-01	-0.18	3.7E-01	-0.24	2.4E-01	0.19	3.5E-01	0.43	2.8E-02	-0.03	8.8E-01	0.00	9.8E-01	0.04	8.4E-01	-0.19	3.6E-01
ENSCAFG0000000738	ENSCAFG0000000738	grey	EC_M10	0.09	6.7E-01	-0.12	5.5E-01	0.28	1.6E-01	0.61	8.8E-04	-0.39	5.2E-02	-0.11	6.0E-01	-0.11	5.8E-01	-0.15	4.7E-01	-0.09	6.5E-01	0.36	6.8E-02
ENSCAFG0000000188	GABRA1	grey	EC_M10	0.09	6.7E-01	-0.26	2.0E-01	-0.36	7.3E-02	-0.17	4.2E-01	0.51	8.2E-03	0.12	5.4E-01	0.24	2.5E-01	0.28	1.6E-01	-0.07	7.4E-01	-0.51	8.1E-03
ENSCAFG0000001327	MBTPS2	grey	EC_M10	0.09	6.7E-01	-0.27	1.8E-01	-0.43	2.7E-02	0.03	8.8E-01	0.57	2.2E-03	0.15	4.6E-01	-0.41	3.7E-02	-0.16	4.5E-01	-0.28	1.7E-01	-0.63	5.7E-04
ENSCAFG0000003236	ZDHHC22	turquoise	EC_M6	0.09	6.7E-01	0.67	1.7E-04	-0.14	5.0E-01	0.16	4.3E-01	-0.18	3.8E-01	0.02	9.3E-01	-0.09	6.5E-01	-0.04	8.3E-01	-0.11	5.8E-01	0.13	5.1E-01
ENSCAFG0000001834	CCL2	turquoise	EC_M6	0.09	6.7E-01	0.70	7.3E-05	-0.23	2.5E-01	-0.21	3.0E-01	-0.01	9.7E-01	-0.13	5.4E-01	0.42	3.4E-02	-0.10	6.3E-01	0.08	7.1E-01	-0.02	9.4E-01
ENSCAFG0000001288	ARHGDIB	turquoise	EC_M6	0.09	6.7E-01	0.58	2.1E-03	-0.54	4.0E-03	-0.23	2.7E-01	0.35	8.0E-02	-0.13	5.2E-01	0.11	5.9E-01	0.34	9.3E-02	0.49	1.2E-02	-0.39	5.0E-02
ENSCAFG0000000847	ENSCAFG0000000847	grey	EC_M10	0.09	6.7E-01	0.14	4.9E-01	-0.23	2.5E-01	0.13	5.3E-01	0.14	4.9E-01	0.15	4.7E-01	-0.16	4.3E-01	0.22	2.9E-01	0.10	6.1E-01	-0.16	4.2E-01
ENSCAFG0000001252	MUC13	grey	EC_M10	0.09	6.7E-01	-0.15	4.5E-01	0.02	9.2E-01	0.30	1.4E-01	-0.02	9.1E-01	-0.13	5.1E-01	-0.13	5.3E-01	0.41	3.9E-02	-0.02	9.1E-01	-0.06	7.6E-01
ENSCAFG0000001238	RBM4	turquoise	EC_M6	0.09	6.7E-01	0.38	5.3E-02	0.26	2.0E-01	0.15	4.6E-01	-0.44	2.4E-02	-0.03	8.7E-01	-0.31	1.3E-01	0.04	8.6E-01	-0.20	3.3E-01	0.40	4.6E-02
ENSCAFG0000001071	ENSCAFG0000001071	turquoise	EC_M6	0.09	6.7E-01	0.57	2.4E-03	-0.04	8.3E-01	-0.23	2.7E-01	-0.14	5.0E-01	-0.06	7.8E-01	0.35	8.1E-02	-0.12	5.7E-01	0.30	1.3E-01	0.16	4.3E-01
ENSCAFG0000000612	ALG5	grey	EC_M10	0.09	6.7E-01	-0.44	2.4E-02	-0.55	3.4E-03	-0.07	7.5E-01	0.76	6.8E-06	0.41	3.7E-02	-0.04	8.4E-01	0.00	1.0E+00	-0.20	3.4E-01	-0.80	8.1E-07
ENSCAFG0000001368	USP28	turquoise	EC_M6	0.09	6.7E-01	0.51	7.6E-03	-0.26	2.0E-01	-0.02	9.4E-01	0.03	8.9E-01	-0.09	6.8E-01	0.17	4.1E-01	-0.41	4.0E-02	-0.54	4.8E-03	-0.09	6.6E-01
ENSCAFG0000000397	PIK3CG	turquoise	EC_M6	0.09	6.7E-01	0.84	8.3E-08	-0.14	4.9E-01	-0.06	7.7E-01	-0.22	2.7E-01	-0.07	7.4E-01	-0.04	8.3E-01	-0.11	6.0E-01	0.34	9.4E-02	0.18	3.7E-01
ENSCAFG0000001848	EMB	grey	EC_M10	0.09	6.7E-01	-0.24	2.3E-01	-0.33	1.1E-01	-0.21	3.1E-01	0.45	2.0E-02	0.09	6.6E-01	-0.10	6.4E-01	0.36	6.8E-02	0.18	3.7E-01	-0.49	1.2E-02
ENSCAFG0000001612	SYT7	turquoise	EC_M6	0.09	6.7E-01	0.93	1.3E-11	-0.25	2.3E-01	-0.08	7.0E-01	-0.08	7.0E-01	-0.08	6.9E-01	-0.04	8.6E-01	-0.09	6.8E-01	-0.04	8.6E-01	0.05	8.1E-01
ENSCAFG0000001757	HDHD2	grey	EC_M10	0.09	6.7E-01	-0.33	9.8E-02	-0.54	4.3E-03	-0.39	5.0E-02	0.69	8.7E-05	0.30	1.4E-01	0.12	5.6E-01	0.09	6.8E-01	-0.08	7.1E-01	-0.75	1.2E-05
ENSCAFG0000001524	BLZF1	darkgreen	EC_M4	0.09	6.7E-01	-0.07	7.2E-01	-0.76	8.0E-06	-0.28	1.7E-01	0.84	8.4E-08	0.33	1.0E-01	-0.11	5.9E-01	0.03	9.0E-01	-0.03	8.8E-01	-0.89	6.9E-10
ENSCAFG0000003070	UBE2N	grey	EC_M10	0.09	6.7E-01	-0.30	1.4E-01	-0.05	8.1E-01	-0.08	6.9E-01	0.15	4.5E-01	0.02	9.1E-01	-0.04	8.5E-01	-0.42	3.2E-02	-0.06	7.6E-01	-0.19	3.5E-01
ENSCAFG0000003042	ENSCAFG0000003042	grey	EC_M10	0.09	6.7E-01	-0.06	7.5E-01	-0.15	4.6E-01	0.18	3.7E-01	0.10	6.4E-01	0.35	7.9E-02	-0.35	8.4E-02	-0.12	5.6E-01	-0.03	8.8E-01	-0.22	2.8E-01
ENSCAFG0000002923	SYCE1L	grey	EC_M10	0.09	6.7E-01	0.27	1.9E-01	0.25	2.2E-01	-0.19	3.5E-01	-0.43	3.0E-02	-0.11	5.9E-01	-0.27	1.9E-01	-0.22	2.9E-01	0.04	8.3E-01	0.44	2.4E-02
ENSCAFG0000001752	SRPX2	grey	EC_M10	0.09	6.7E-01	0.04	8.5E-01	-0.66	2.6E-04	-0.27	1.9E-01	0.70	7.9E-05	0.32	1.1E-01	0.15	4.7E-01	-0.20	3.2E-01	-0.40	4.2E-02	-0.76	6.4E-06
ENSCAFG0000000341	ENSCAFG0000000341	grey	EC_M10	0.09	6.7E-01	-0.17	4.1E-01	0.34	9.3E-02	-0.06	7.6E-01	-0.32	1.1E-01	-0.06	7.8E-01	-0.03	9.0E-01	-0.05	8.3E-01	-0.17	4.1E-01	0.32	1.1E-01
ENSCAFG0000000650	ENSCAFG0000000650	grey	EC_M10	0.09	6.7E-01	-0.57	2.4E-03	-0.18	3.7E-01	0.08	6.9E-01	0.37	6.3E-02	0.02	9.2E-01	-0.07	7.2E-01	-0.06	7.7E-01	-0.26	2.0E-01	-0.41	3.5E-02
ENSCAFG0000001562	SGMS1	darkgreen	EC_M4	0.09	6.7E-01	0.08	7.0E-01	-0.70	6.2E-05	-0.29	1.6E-01	0.75	1.2E-05	0.21	3.1E-01	0.07	7.3E-01	-0.14	4.9E-01	-0.27	1.8E-01	-0.81	6.1E-07
ENSCAFG0000000968	GCNT2	grey	EC_M10	0.09	6.7E-01	0.37	6.3E-02	-0.45	2.0E-02	-0.03	8.9E-01	0.35	8.2E-02	0.12	5.6E-01	-0.01	9.7E-01	-0.23	2.6E-01	0.01	9.4E-01	-0.36	6.9E-02
ENSCAFG0000001462	IFI35	cyan	EC_M2	0.09	6.7E-01	0.30	1.4E-01	0.53	5.2E-03	0.27	1.9E-01	-0.81	6.0E-07	-0.27	1.8E-01	-0.03	8.9E-01	0.00	1.0E+00	0.06	7.7E-01	0.77	5.1E-06
ENSCAFG0000002421	ENSCAFG0000002421	grey	EC_M10	0.09	6.8E-01	-0.59	1.4E-03	0.39	4.7E-02	0.11	6.0E-01	-0.22	2.8E-01	0.20	3.2E-01	-0.01	9.5E-01	0.06	7.7E-01	-0.17	4.1E-01	0.15	4.5E-01
ENSCAFG0000000145	CEP41	grey	EC_M10	0.09	6.8E-01	-0.47	1.5E-02	-0.50	9.6E-03	-0.04	8.6E-01	0.73	2.6E-05	0.40	4.0E-02	-0.18	3.7E-01	-0.07	7.3E-01	-0.02	9.4E-01	-0.76	7.9E-06
ENSCAFG0000000264	KCNQ5	grey	EC_M10	0.09	6.8E-01	-0.19	3.6E-01	0.15	4.6E-01	-0.34	9.2E-02	-0.19	3.5E-01	-0.22	2.9E-01	-0.17	3.9E-01	0.36	7.1E-02	0.25	2.2E-01	0.16	4.4E-01
ENSCAFG0000000260	ENSCAFG0000000260	turquoise	EC_M6	0.09	6.8E-01	0.65	3.7E-04	-0.40	4.3E-02	-0.12	5.6E-01	0.18	3.8E-01	-0.12	5.7E-01	-0.09	6.7E-01	-0.04	8.6E-01	-0.04	8.4E-01	-0.18	3.8E-01
ENSCAFG0000001341	STYK1	turquoise	EC_M6	0.09	6.8E-01	0.65	3.7E-04	-0.40	4.3E-02	-0.12	5.6E-01	0.18	3.8E-01	-0.12	5.7E-01	-0.09	6.7E-01	-0.04	8.6E-01	-0.04	8.4E-01	-0.18	3.8E-01
ENSCAFG0000001516	ENSCAFG0000001516	grey	EC_M10	0.09	6.8E-01	-0.29	1.6E-01	0.44	2.4E-02	0.22	2.7E-01	-0.50	9.0E-03	-0.22	2.8E-01	-0.20	3.2E-01	-0.26	1.9E-01	0.18	3.7E-01	0.47	1.5E-02
ENSCAFG0000000309	PM20D2	grey	EC_M10	0.09	6.8E-01	-0.02	9.4E-01	-0.55	3.6E-03	-0.02	9.2E-01	0.57	2.4E-03	0.18	3.8E-01	0.18	3.8E-01	0.09	6.5E-01	0.11	5.8E-01	-0.66	2.3E-04
ENSCAFG0000002330	TWF2	grey	EC_M10	0.09	6.8E-01	0.33	9.5E-02	0.44	2.4E-02	0.06	7.6E-01	-0.64	4.3E-04	-0.17	4.2E-01	0.20	3.3E-01	-0.16	4.3E-01	-0.25	2.2E-01	0.59	1.6E-03
ENSCAFG0000003066	TTC33	cyan	EC_M2	0.09	6.8E-01	0.02	9.1E-01	0.58	1.8E-03	0.28	1.6E-01	-0.79	1.7E-06	-0.12	5.5E-01	-0.08	7.1E-01	0.13	5.4E-01	-0.18	3.7E-01	0.75	9.2E-06
ENSCAFG0000002493	MTUS2	grey	EC_M10	0.09	6.8E-01	-0.08	6.8E-01	-0.61	9.2E-04	-0.13	5.4E-01	0.69	1.1E-04	0.33	9.8E-02	0.29	1.4E-01	-0.10	6.4E-01	-0.07	7.2E-01	-0.72	4.0E-05
ENSCAFG0000001335	MGST3	cyan	EC_M2	0.09	6.8E-01	-0.31	1.3E-01	0.71	4.9E-05	0.15	4.5E-01	-0.77	3.6E-06	-0.14	5.1E-01	-0.28	1.6E-01	-0.17	4.0E-01	0.04	8.6E-01	0.73	1.9E-05
ENSCAFG0000000601	FREM2	turquoise	EC_M6	0.09	6.8E-01	0.79	1.5E-06	-0.46	1.8E-02	-0.35	7.9E-02	0.20	3.4E-01	-0.02	9.4E-01	-0.11	6.0E-01	0.18	3.8E-01	0.31	1.3E-01	-0.21	3.1E-01
ENSCAFG0000001877	ENOX2	grey	EC_M10	0.09	6.8E-01	-0.17	4.2E-01	0.34	9.2E-02	0.29	1.5E-01	-0.46	1.9E-02	-0.06	7.8E-01	-0.20	3.4E-01	0.00	9.9E-01	-0.27	1.9E-01	0.37	6.5E-02
ENSCAFG0000002941	ENSCAFG0000002941	turquoise	EC_M6	0.09	6.8E-01	0.32	1.1E-01	-0.28	1.7E-01	0.19	3.6E-01	0.05	8.0E-01	-0.07	7.3E-01	-0.06	7.6E-01	-0.17	4.0E-01	0.01	9.6E-01	-0.15	4.6E-01
ENSCAFG0000001166	SRSF4	grey	EC_M10	0.09	6.8E-01	0.59	1.5E-03	0.23	2.6E-01	-0.01	9.6E-01	-0.56	3.2E-03	0.02	9.4E-01	0.28	1.6E-01	0.00	9.9E-01	0.12	5.5E-01	0.56	3.0E-03
ENSCAFG0000002630	ENSCAFG0000002630	grey	EC_M10	0.09	6.8E-01	-0.04	8.3E-01	0.60	1.2E-03	0.16	4.3E-01	-0.72	3.6E-05	-0.28	1.7E-01	0.20	3.2E-01	-0.28	1.6E-01	-0.08	6.8E-01	0.70	6.4E-05
ENSCAFG0000001877	RBMX2	cyan	EC_M2	0.09	6.8E-01	0.10	6.2E-01	0.30	1.4E-01	0.30	1.4E-01	-0.55	3.3E-03	0.00	9.9E-01	0.06	7.7E-01	0.16	4.4E-01	0.03	9.0E-01	0.43	2.9E-02
ENSCAFG0000001385	PRKRA	grey	EC_M10	0.09	6.8E-01	0.47	1.4E-02	-0.60	1.3E-03	0.04	8.5E-01	0.47	1.6E-02	-0.07	7.5E-01	0.13	5.2E-01	-0.11	6.0E-01	-0.11	5.8E-01	-0.46	1.8E-02
ENSCAFG0000002578	ENSCAFG0000002578	grey	EC_M10	0.09	6.8E-01	0.33	1.0E-01	0.02	9.2E-01	-0.11	5.8E-01	-0.24	2.3E-01	-0.18	3.8E-01	-0.14	5.0E-01	0.22	2.9E-01	-0.01	9.7E-01	0.18	3.9E-01
ENSCAFG0000000057	ENSCAFG0000000057	grey	EC_M10	0.09	6.8E-01	-0.20	3.4E-01	-0.07	7.2E-01	-0.32	1.1E-01	0.16	4.3E-01	0.36	6.7E-02	-0.23	2.5E-01	0.12	5.5E-01	-0.14	4.9E-01	-0.19	3.4E-01
ENSCAFG0000001958	BAIAP3	grey	EC_M10	0.08	6.8E-01	0.33	1.0E-01	0.05	8.1E-01	-0.19	3.4E-01	-0.24	2.3E-01	-0.21	3.0E-01	0.15	4.7E-01	-0.21	3.1E-01	0.21	2.9E-01	0.21	3.1E-01
ENSCAFG0000002172	ENSCAFG0000002172	grey	EC_M10	0.08	6.8E-01	0.09	6.8E-01	0.03	8.8E-01	-0.31	1.3E-01	-0.05	8.1E-01	-0.30	1.4E-01	-0.20	3.2E-01	0.04	8.4E-01	0.18	3.7E-01	0.08	7.1E-01
ENSCAFG0000002039	MSH4	grey	EC_M10	0.08	6.8E-01	-0.22	2.7E-01	-0.13	5.2E-01	0.48	1.4E-02	0.13	5.4E-01	0.43	2.7E-02	-0.16	4.3E-01	-0.12	5.6E-01	-0.25	2.3E-01	-0.21	3.0E-01
ENSCAFG0000000650	UPF3A	grey	EC_M10	0.08	6.8E-01	-0.20	3.3E-01	0.04	8.6E-01	0.03	8.9E-01	-0.08	7.0E-01	-0.04	8.6E-01	-0.12	5.5E-01	0.37	6.1E-02	0.16	4.2E-01	0.05	8.1E-01
ENSCAFG0000001724	UBAP2L	grey	EC_M10	0.08	6.8E-01	-0.20	3.2E-01	0.35	7.6E-02	-0.17	4.1E-01	-0.29	1.5E-01	-0.16	4.3E-01	-0.24	2.3E-01	0.15	4.7E-01	0.12	5.7E-01	0.24	2.3E-01
ENSCAFG0000001181	ZNF217	cyan	EC_M2	0.08	6.8E-01	0.29	1.5E-01	0.49	1.1E-02	0.15	4.8E-01	-0.81	6.0E-07	-0.31	1.3E-01	-0.05	8.2E-01	0.07	7.3E-01	0.19	3.6E-01	0.77	4.9E-06
ENSCAFG0000003073	ERICH4	grey	EC_M10	0.08	6.8E-01	-0.19	3.4E-01	0.26	2.1E-01	-0.14	5.0E-01	-0.32	1.1E-01	-0.08	7.0E-01	-0.17	4.1E-01	0.30	1.4E-01	-0.06	7.7E-01	0.22	2.8E-01
ENSCAFG0000001024	METTL15	cyan	EC_M2	0.08	6.8E-01	0.09	6.7E-01	0.46	1.9E-02	0.13	5.4E-01	-0.66	2.4E-04	0.11	6.0E-01	0.10	6.4E-01	0.11	6.1E-01	0.15	4.7E-01	0.61	8.6E-04
ENSCAFG0000003108	ZFP2	grey	EC_M10	0.08	6.8E-01	0.20	3.3E-01	0.33	1.0E-01	0.17	4.1E-01	-0.53	4.9E-03	-0.20	3.2E-01	-0.28	1.7E-01	0.20	3.3E-01	0.06	7.6E-01	0.51	7.6E-03
ENSCAFG0000001695	KAT7	cyan	EC_M2	0.08	6.8E-01	0.35	7.9E-02	0.51	8.0E-03	0.31	1.2E-01	-0.81	4.0E-07	-0.23	2.6E-01	-0.04	8.4E-01	-0.11	5.9E-01	0.11	5.8E-01	0.78	3.0E-06
ENSCAFG0000000095	SNTB1	grey	EC_M10	0.08	6.8E-01	-0.16	4.3E-01	-0.27	1.8E-01	0.16	4.2E-01	0.27	1.8E-01	0.40	4.1E-02	-0.14	4.9E-01	0.39	4.9E-02	-0.06	7.8E-01	-0.34	8.5E-02
ENSCAFG0000000230	VWDE	magenta	EC_M13	0.08	6.8E-01	0.04	8.3E-01	0.09	6.7E-01	0.15	4.8E-01	-0.19	3.5E-01	-0.03	8.9E-01	-0.03	8.9E-01	-0.12	5.7E-01	0.60	1.1E-03	0.18	3.7E-01
ENSCAFG0000000979	ATP1A1	grey	EC_M10	0.08	6.8E-01	-0.07	7.4E-01	-0.38	5.7E-02	-0.16	4.4E-01	0.47	1.7E-02	0.05	7.9E-01	0.00	9.9E-01	-0.07	7.2E-01	-0.52	6.8E-03	-0.49	1.1E-02
ENSCAFG0000002627	ENSCAFG0000002627	grey	EC_M10	0.08	6.8E-01	-0.16	4.5E-01	-0.28	1.6E-01	-0.08	6.9E-01	0.29	1.6E-01	-0.14	4.9E-01	-0.11	6.1E-01	0.17	4.2E-01	-0.01	9.7E-01	-0.34	8.8E-02
ENSCAFG0000003072	PSMB9	turquoise	EC_M6	0.08	6.8E-01	0.57	2.5E-03	0.22	2.7E-01	0.06	7.8E-01	-0.49	1.2E-02	-0.12	5.6E-01	-0.05	8.1E-01	-0.03	8.9E-01	-0.09	6.5E-01	0.44	2.5E-02

ENSCAFG0000000579	ENSCAFG0000000579	grey	EC_M10	0.08	6.8E-01	-0.22	2.8E-01	0.42	3.4E-02	-0.11	6.0E-01	-0.39	4.7E-02	-0.07	7.3E-01	-0.11	5.8E-01	-0.11	5.8E-01	-0.18	3.8E-01	0.34	9.0E-02
ENSCAFG0000000003	CD226	grey	EC_M10	0.08	6.8E-01	0.11	5.9E-01	0.17	4.1E-01	0.52	6.7E-03	-0.29	1.5E-01	-0.19	3.6E-01	-0.16	4.5E-01	-0.15	4.7E-01	-0.17	4.1E-01	0.31	1.2E-01
ENSCAFG0000000918	LOXL2	darkgreen	EC_M4	0.08	6.9E-01	0.43	2.7E-02	-0.75	8.3E-06	-0.15	4.6E-01	0.64	4.4E-04	0.18	3.9E-01	0.17	4.1E-01	-0.02	9.4E-01	0.03	8.7E-01	-0.72	3.2E-05
ENSCAFG0000001092	BPNT1	grey	EC_M10	0.08	6.9E-01	-0.38	5.3E-02	-0.53	5.7E-03	-0.22	2.7E-01	0.72	3.9E-05	0.20	3.3E-01	-0.06	7.6E-01	-0.11	5.9E-01	-0.31	1.2E-01	-0.74	1.6E-05
ENSCAFG0000001095	RIN3	turquoise	EC_M6	0.08	6.9E-01	0.81	5.6E-07	0.14	5.0E-01	-0.06	7.7E-01	-0.49	1.2E-02	-0.24	2.3E-01	0.06	7.7E-01	-0.27	1.9E-01	-0.05	8.1E-01	0.45	2.1E-02
ENSCAFG0000002329	AURKC	grey	EC_M10	0.08	6.9E-01	-0.09	6.5E-01	0.06	7.8E-01	-0.13	5.1E-01	-0.13	5.3E-01	-0.06	7.8E-01	-0.09	6.5E-01	-0.02	9.1E-01	-0.08	7.1E-01	0.06	7.8E-01
ENSCAFG0000001696	AREL1	grey	EC_M10	0.08	6.9E-01	-0.29	1.4E-01	-0.29	1.5E-01	-0.13	5.2E-01	0.43	2.8E-02	0.02	9.3E-01	-0.20	3.3E-01	-0.13	5.3E-01	-0.19	3.4E-01	-0.50	8.9E-03
ENSCAFG0000001870	ACSBG2	grey	EC_M10	0.08	6.9E-01	0.03	8.9E-01	0.14	4.9E-01	-0.17	3.9E-01	-0.26	2.1E-01	-0.17	4.1E-01	-0.20	3.3E-01	0.27	1.9E-01	0.28	1.6E-01	0.19	3.4E-01
ENSCAFG0000001086	ERGIC2	darkgreen	EC_M4	0.08	6.9E-01	-0.52	7.0E-03	-0.49	1.1E-02	0.10	6.2E-01	0.68	1.2E-04	0.34	9.3E-02	0.04	8.3E-01	0.03	8.8E-01	0.05	8.3E-01	-0.72	3.3E-05
ENSCAFG0000001961	CIAO3	grey	EC_M10	0.08	6.9E-01	0.19	3.4E-01	0.45	2.0E-02	-0.07	7.3E-01	-0.65	3.0E-04	-0.23	2.6E-01	0.13	5.2E-01	-0.21	3.1E-01	-0.17	3.9E-01	0.63	5.2E-04
ENSCAFG0000003212	ENSCAFG0000003212	turquoise	EC_M6	0.08	6.9E-01	0.00	9.9E-01	-0.17	4.0E-01	-0.08	7.0E-01	0.17	4.0E-01	0.49	1.1E-02	-0.18	3.7E-01	-0.13	5.3E-01	-0.19	3.5E-01	-0.19	3.4E-01
ENSCAFG0000002913	GDNF	darkgreen	EC_M4	0.08	6.9E-01	-0.70	8.0E-05	-0.27	1.8E-01	0.09	6.7E-01	0.49	1.1E-02	0.34	9.2E-02	-0.13	5.4E-01	-0.05	7.9E-01	-0.29	1.5E-01	-0.58	1.8E-03
ENSCAFG0000001698	ITGA3	turquoise	EC_M6	0.08	6.9E-01	0.55	3.5E-03	-0.03	9.0E-01	-0.19	3.5E-01	-0.19	3.5E-01	-0.34	8.7E-02	0.14	4.9E-01	-0.14	5.0E-01	0.38	5.9E-02	0.23	2.5E-01
ENSCAFG0000002031	ENSCAFG0000002031	grey	EC_M10	0.08	6.9E-01	0.11	6.1E-01	0.23	2.5E-01	0.07	7.3E-01	-0.41	3.7E-02	-0.26	2.0E-01	0.08	7.0E-01	-0.03	9.0E-01	0.24	2.4E-01	0.36	6.9E-02
ENSCAFG0000000043	RAB3IP	darkgreen	EC_M4	0.08	6.9E-01	0.16	4.5E-01	-0.69	1.1E-04	-0.13	5.3E-01	0.67	2.0E-04	-0.03	8.8E-01	-0.14	5.1E-01	0.01	9.5E-01	-0.06	7.8E-01	-0.71	4.1E-05
ENSCAFG0000001161	RAD9A	grey	EC_M10	0.08	6.9E-01	0.10	6.4E-01	0.26	2.0E-01	-0.12	5.7E-01	-0.26	1.9E-01	-0.20	3.2E-01	-0.25	2.1E-01	-0.12	5.6E-01	-0.01	9.5E-01	0.23	2.5E-01
ENSCAFG0000000979	EDN1	turquoise	EC_M6	0.08	6.9E-01	0.81	7.0E-07	-0.62	6.9E-04	-0.22	2.7E-01	0.38	5.9E-02	-0.01	9.5E-01	-0.07	7.4E-01	0.06	7.7E-01	0.12	5.6E-01	-0.42	3.4E-02
ENSCAFG0000003158	LGALSL	turquoise	EC_M6	0.08	6.9E-01	0.80	1.2E-06	-0.26	2.0E-01	-0.20	3.2E-01	-0.04	8.4E-01	-0.19	3.6E-01	0.18	3.9E-01	-0.03	8.8E-01	0.46	1.9E-02	0.04	8.3E-01
ENSCAFG0000002129	ENSCAFG0000002129	cyan	EC_M2	0.08	6.9E-01	0.08	7.0E-01	0.51	8.1E-03	0.25	2.3E-01	-0.71	4.8E-05	-0.15	4.6E-01	-0.19	3.4E-01	-0.21	3.0E-01	0.02	9.1E-01	0.71	5.2E-05
ENSCAFG0000000931	CCDC148	grey	EC_M10	0.08	6.9E-01	-0.11	5.9E-01	-0.14	4.9E-01	0.04	8.3E-01	0.18	3.8E-01	0.03	8.9E-01	-0.18	3.7E-01	0.00	1.0E+00	0.13	5.3E-01	-0.22	2.7E-01
ENSCAFG0000000339	TES	darkgreen	EC_M4	0.08	6.9E-01	0.41	3.9E-02	-0.80	1.1E-06	-0.21	3.0E-01	0.72	2.8E-05	0.19	3.5E-01	0.05	7.9E-01	-0.02	9.1E-01	-0.19	3.5E-01	-0.78	3.0E-06
ENSCAFG0000000150	NRF1	grey	EC_M10	0.08	6.9E-01	0.54	4.0E-03	0.34	9.4E-02	0.26	2.0E-01	-0.67	2.0E-04	-0.27	1.9E-01	-0.10	6.1E-01	-0.07	7.3E-01	-0.19	3.6E-01	0.62	7.9E-04
ENSCAFG0000000946	GOT1	grey	EC_M10	0.08	6.9E-01	-0.41	3.7E-02	0.36	7.5E-02	-0.11	5.8E-01	-0.25	2.1E-01	0.11	5.8E-01	-0.20	3.2E-01	-0.13	5.2E-01	-0.13	5.3E-01	0.27	1.9E-01
ENSCAFG0000001431	SUGP1	grey	EC_M10	0.08	6.9E-01	0.20	3.2E-01	-0.18	3.7E-01	-0.01	9.8E-01	0.08	6.9E-01	-0.06	7.8E-01	-0.40	4.1E-02	0.03	8.8E-01	0.25	2.2E-01	-0.13	5.4E-01
ENSCAFG0000000055	RGS17	grey	EC_M10	0.08	6.9E-01	0.21	2.9E-01	0.27	1.8E-01	0.04	8.5E-01	-0.45	2.1E-02	-0.16	4.2E-01	-0.25	2.2E-01	0.12	5.6E-01	-0.22	2.8E-01	0.40	4.2E-02
ENSCAFG0000003271	EMCN	turquoise	EC_M6	0.08	6.9E-01	0.86	1.6E-08	-0.37	6.3E-02	-0.11	5.8E-01	0.07	7.3E-01	-0.11	5.8E-01	0.01	9.8E-01	-0.05	8.1E-01	0.07	7.5E-01	-0.13	5.3E-01
ENSCAFG0000000408	JRKL	grey	EC_M10	0.08	6.9E-01	0.02	9.2E-01	-0.30	1.3E-01	-0.20	3.4E-01	0.26	1.9E-01	-0.08	7.0E-01	-0.13	5.2E-01	0.09	6.8E-01	-0.18	3.9E-01	-0.29	1.5E-01
ENSCAFG0000002891	SNX10	darkgreen	EC_M4	0.08	6.9E-01	0.10	6.2E-01	-0.79	2.0E-06	-0.10	6.2E-01	0.79	1.5E-06	0.20	3.2E-01	0.05	8.0E-01	0.08	6.9E-01	-0.08	7.1E-01	-0.83	1.5E-07
ENSCAFG0000001518	UXT	grey	EC_M10	0.08	6.9E-01	-0.28	1.7E-01	-0.03	8.8E-01	-0.22	2.9E-01	0.11	5.9E-01	0.12	5.8E-01	-0.05	8.2E-01	-0.52	6.6E-03	-0.24	2.3E-01	-0.20	3.4E-01
ENSCAFG0000001228	ENSCAFG0000001228	grey	EC_M10	0.08	6.9E-01	-0.02	9.4E-01	-0.13	5.4E-01	-0.08	7.2E-01	0.04	8.3E-01	0.19	3.5E-01	-0.18	3.9E-01	-0.23	2.7E-01	0.13	5.3E-01	-0.09	6.4E-01
ENSCAFG0000000541	RBBP9	grey	EC_M10	0.08	6.9E-01	0.07	7.3E-01	0.27	1.8E-01	0.15	4.6E-01	-0.34	8.9E-02	-0.13	5.3E-01	-0.41	3.9E-02	0.16	4.2E-01	0.42	3.2E-02	0.32	1.1E-01
ENSCAFG0000000370	MGST2	grey	EC_M10	0.08	6.9E-01	-0.18	3.9E-01	-0.24	2.5E-01	-0.09	6.7E-01	0.38	5.8E-02	0.40	4.1E-02	-0.06	7.5E-01	-0.05	8.3E-01	-0.12	5.6E-01	-0.36	7.2E-02
ENSCAFG0000000530	ENSCAFG0000000530	grey	EC_M10	0.08	6.9E-01	-0.06	7.7E-01	0.19	3.6E-01	-0.09	6.5E-01	-0.31	1.3E-01	-0.06	7.9E-01	-0.17	4.0E-01	0.17	4.1E-01	0.35	7.6E-02	0.31	1.3E-01
ENSCAFG0000001436	ENSCAFG0000001436	magenta	EC_M13	0.08	6.9E-01	0.02	9.4E-01	0.06	7.8E-01	-0.08	7.0E-01	-0.12	5.5E-01	0.00	9.8E-01	-0.02	9.4E-01	-0.11	6.0E-01	0.53	5.4E-03	0.06	7.7E-01
ENSCAFG0000001690	IGF2BP1	grey	EC_M10	0.08	6.9E-01	-0.29	1.5E-01	0.07	7.3E-01	-0.29	1.5E-01	0.01	9.6E-01	0.07	7.4E-01	0.00	9.9E-01	-0.30	1.3E-01	-0.37	6.0E-02	-0.08	7.0E-01
ENSCAFG0000000672	MAGI1	turquoise	EC_M6	0.08	6.9E-01	0.74	1.5E-05	-0.58	1.8E-03	-0.21	3.1E-01	0.34	8.9E-02	-0.09	6.6E-01	0.00	9.9E-01	-0.18	3.7E-01	0.14	4.9E-01	-0.36	6.9E-02
ENSCAFG0000001855	WDR78	grey	EC_M10	0.08	6.9E-01	0.03	8.7E-01	-0.03	8.9E-01	0.10	6.4E-01	-0.08	6.9E-01	-0.26	2.0E-01	-0.25	2.1E-01	0.24	2.4E-01	0.33	9.6E-02	0.02	9.1E-01
ENSCAFG0000001827	PPARGC1B	cyan	EC_M2	0.08	6.9E-01	0.01	9.7E-01	0.67	1.7E-04	0.09	6.7E-01	-0.79	1.4E-06	-0.39	5.2E-02	-0.17	4.0E-01	-0.12	5.7E-01	0.00	1.0E+00	0.81	4.9E-07
ENSCAFG0000001639	LFNG	turquoise	EC_M6	0.08	6.9E-01	0.57	2.5E-03	-0.59	1.5E-03	-0.29	1.5E-01	0.46	1.8E-02	0.07	7.5E-01	-0.02	9.2E-01	-0.18	3.7E-01	0.19	3.6E-01	-0.48	1.4E-02
ENSCAFG0000000224	ENSCAFG0000000224	grey	EC_M10	0.08	6.9E-01	0.23	2.7E-01	-0.32	1.1E-01	0.35	8.0E-02	0.18	3.7E-01	0.04	8.5E-01	0.02	9.3E-01	0.01	9.6E-01	-0.13	5.1E-01	-0.20	3.2E-01
ENSCAFG0000000604	PIK3R4	grey	EC_M10	0.08	6.9E-01	-0.19	3.6E-01	-0.31	1.3E-01	-0.14	5.0E-01	0.38	5.2E-02	0.10	6.4E-01	-0.47	1.5E-02	0.02	9.3E-01	-0.12	5.5E-01	-0.40	4.4E-02
ENSCAFG0000001393	NECAP1	turquoise	EC_M6	0.08	6.9E-01	0.39	5.2E-02	-0.07	7.5E-01	0.20	3.2E-01	-0.15	4.7E-01	0.00	9.9E-01	-0.01	9.6E-01	-0.01	9.6E-01	-0.21	3.0E-01	0.13	5.4E-01
ENSCAFG0000000779	MARVELD2	turquoise	EC_M6	0.08	6.9E-01	0.58	1.8E-03	-0.33	1.0E-01	-0.21	3.1E-01	0.16	4.2E-01	-0.28	1.7E-01	0.01	9.5E-01	-0.02	9.4E-01	-0.01	9.5E-01	-0.17	4.1E-01
ENSCAFG0000000604	GPATCH11	grey	EC_M10	0.08	7.0E-01	-0.40	4.5E-02	-0.46	1.9E-02	-0.14	4.9E-01	0.57	2.1E-03	0.27	1.8E-01	-0.18	3.8E-01	0.42	3.3E-02	-0.12	5.5E-01	-0.61	8.3E-04
ENSCAFG0000002871	ENSCAFG0000002871	turquoise	EC_M6	0.08	7.0E-01	0.66	2.2E-04	0.00	9.9E-01	-0.02	9.4E-01	-0.29	1.5E-01	-0.11	6.0E-01	-0.09	6.6E-01	-0.03	8.9E-01	-0.12	5.6E-01	0.25	2.3E-01
ENSCAFG0000003267	ENSCAFG0000003267	turquoise	EC_M6	0.08	7.0E-01	0.66	2.2E-04	0.00	9.9E-01	-0.02	9.4E-01	-0.29	1.5E-01	-0.11	6.0E-01	-0.09	6.6E-01	-0.03	8.9E-01	-0.12	5.6E-01	0.25	2.3E-01
ENSCAFG0000001296	HELZ2	turquoise	EC_M6	0.08	7.0E-01	0.84	9.3E-08	0.10	6.4E-01	-0.03	8.8E-01	-0.50	8.9E-03	-0.12	5.4E-01	0.11	5.9E-01	0.02	9.4E-01	0.17	4.0E-01	0.45	2.0E-02
ENSCAFG0000002983	ENSCAFG0000002983	grey	EC_M10	0.08	7.0E-01	0.09	6.8E-01	-0.11	5.9E-01	0.02	9.2E-01	-0.02	9.2E-01	-0.37	6.5E-02	-0.31	1.2E-01	0.00	9.8E-01	-0.24	2.4E-01	0.00	9.9E-01
ENSCAFG0000001467	LAG3	grey	EC_M10	0.08	7.0E-01	-0.12	5.5E-01	-0.06	7.5E-01	-0.11	5.9E-01	0.02	9.2E-01	-0.13	5.2E-01	0.10	6.3E-01	-0.14	4.8E-01	0.19	3.5E-01	-0.07	7.5E-01
ENSCAFG0000000571	SPAST	grey	EC_M10	0.08	7.0E-01	0.41	3.5E-02	0.09	6.6E-01	-0.03	8.9E-01	-0.39	4.9E-02	0.05	8.2E-01	0.11	5.9E-01	-0.35	8.2E-02	-0.18	3.8E-01	0.35	8.4E-02
ENSCAFG0000001266	F11R	turquoise	EC_M6	0.08	7.0E-01	0.88	3.6E-09	-0.14	5.0E-01	-0.06	7.7E-01	-0.21	3.0E-01	-0.23	2.7E-01	-0.05	7.9E-01	0.14	5.0E-01	0.11	5.9E-01	0.15	4.6E-01
ENSCAFG0000001414	ADAMTS7	turquoise	EC_M6	0.08	7.0E-01	0.82	2.8E-07	-0.03	9.0E-01	-0.15	4.6E-01	-0.28	1.6E-01	-0.20	3.4E-01	0.09	6.7E-01	-0.17	4.0E-01	0.00	1.0E+00	0.23	2.7E-01
ENSCAFG0000000432	APBB1IP	darkgreen	EC_M4	0.08	7.0E-01	-0.69	9.1E-05	-0.33	1.0E-01	-0.08	6.9E-01	0.56	3.0E-03	0.32	1.1E-01	0.00	9.8E-01	0.09	6.5E-01	-0.20	3.2E-01	-0.64	4.8E-04
ENSCAFG0000000837	CCNH	grey	EC_M10	0.08	7.0E-01	-0.17	4.0E-01	0.04	8.3E-01	0.15	4.6E-01	0.00	9.9E-01	0.11	6.1E-01	0.13	5.4E-01	-0.44	2.4E-02	-0.10	6.3E-01	-0.01	9.6E-01
ENSCAFG0000003064	ENSCAFG0000003064	grey	EC_M10	0.08	7.0E-01	-0.11	5.8E-01	0.26	2.0E-01	-0.12	5.5E-01	-0.28	1.6E-01	-0.04	8.5E-01	-0.03	9.0E-01	0.02	9.4E-01	0.04	8.3E-01	0.26	2.0E-01
ENSCAFG0000003219	ZNF566	grey	EC_M10	0.08	7.0E-01	0.21	3.0E-01	0.09	6.7E-01	0.14	4.9E-01	-0.25	2.1E-01	-0.43	2.7E-02	-0.09	6.6E-01	0.37	6.0E-02	0.30	1.4E-01	0.24	2.3E-01
ENSCAFG0000000318	SLC1A4	grey	EC_M10	0.08	7.0E-01	-0.10	6.4E-01	-0.41	3.6E-02	-0.01	9.6E-01	0.51	8.4E-03	0.12	5.6E-01	-0.09	6.4E-01	-0.08	7.0E-01	0.29	1.6E-01	-0.56	2.9E-03
ENSCAFG0000001509	ARPC1A	grey	EC_M10	0.08	7.0E-01	-0.12	5.4E-01	-0.46	1.7E-02	-0.01	9.5E-01	0.60	1.3E-03	0.14	4.8E-01	0.04	8.5E-01	0.02	9.1E-01	-0.58	1.8E-03	-0.62	7.2E-04
ENSCAFG0000001571	HIF1A	turquoise	EC_M6	0.08	7.0E-01	0.71	4.1E-05	-0.48	1.3E-02	-0.07	7.3E-01	0.23	2.7E-01	-0.04	8.5E-01	0.25	2.1E-01	-0.06	7.6E-01	0.10	6.2E-01	-0.24	2.3E-01
ENSCAFG0000000060	RRM2B	darkgreen	EC_M4	0.08	7.0E-01	-0.52	6.4E-03	-0.43	2.7E-02	0.18	3.8E-01	0.55	3.9E-03	0.31	1.3E-01	0.09	6.5E-01	0.05	8.3E-01	0.03	8.8E-01	-0.62	7.5E-04
ENSCAFG0000000105	TXNDC15	grey	EC_M10	0.08	7.0E-01	0.21	3.0E-01	0.09	6.7E-01	0.04	8.6E-01	-0.22	2.9E-01	-0.42	3.1E-02	-0.10	6.4E-01	0.21	3.0E-01	0.23	2.6E-01	0.25	2.2E-01
ENSCAFG0000001280	MACO1	darkgreen	EC_M4	0.08	7.0E-01	0.08	7.0E-01	-0.68	1.3E-04	-0.19	3.5E-01	0.71	5.6E-05	0.17	4.0E-01	-0.38	5.6E-02	0.21	3.1E-01	0.13	5.2E-01	-0.74	1.6E-05
ENSCAFG0000001155	PFKFB2	grey	EC_M10	0.08	7.0E-01	-0.37	6.5E-02	0.38	5.5E-02	-0.18	3.8E-01	-0.39	5.0E-02	-0.25	2.2E-01	-0.18	3.7E-01	-0.02	9.2E-01	0.33	1.0E-01	0.37	6.3E-02
ENSCAFG0000002985	ENSCAFG0000002985	grey	EC_M10	0.08	7.0E-01	0.07	7.4E-01	0.34	9.0E-02	-0.08	7.1E-01	-0.53	5.8E-03	0.04	8.5E-01	-0.06	7.9E-01	0.21	3.0E-01	0.02	9.3E-01	0.44	2.5E-02
ENSCAFG0000001717	ATXN2L	turquoise	EC_M6	0.08	7.0E-01	0.44	2.6E-02	-0.21	2.9E-01	0.08	7.0E-01	0.04	8.5E-01	0.12	5.4E-01	-0.14	4.9E-01	0.12	5.7E-01	-0.12	5.7E-01	-0.17	4.2E-01
ENSCAFG0000000519	ENSCAFG0000000519	grey	EC_M10	0.08	7.0E-01	0.28	1.6E-01	-0.31	1.3E-01	-0.14	4.9E-01	0.25	2.1E-01	0.45	2.0E-02	0.19	3.4E-01	-0.18	3.9E-01	-0.19	3.5E-01	-0.26	1.9E-01
ENSCAFG0000000806	TBC1D12	grey	EC_M10	0.08	7.0E-01	0.22	2.7E-01	0.11	6.0E-01	0.17	3.9E-01	-0.31	1.3E-01	-0.28	1.7E-01	-0.17	4.1E-01	0.04	8.3E-01	-0.28	1.6E-01	0.30	1.4E-01
ENSCAFG0000001254	COPA	darkgreen	EC_M4	0.08	7.0E-01	-0.18	3.8E-01	-0.69	9.6E-05	-0.19	3.6E-01	0.83	1.6E-07	0.35	7.9E-02	0.12	5.7E-01	-0.09	6.6E-01	-0.06	7.5E-01	-0.90	4.7E-10
ENSCAFG0000001055	ENSCAFG0000001055	grey	EC_M10	0.08	7.0E-01	-0.36	7.4E-02	0.29	1.6E-01	-0.30	1.4E-01	-0.24	2.4E-01	0.09	6.7E-01	0.16	4.4E-01	-0.26	2.0E-01	-0.25	2.2E-01	0.20	3.3E-01
ENSCAFG0000001266	ENSCAFG0000001266	turquoise	EC_M6	0.08	7.0E-01	0.59	1.5E-03	-0.04	8.5E-01	-0.14	4.8E-01	-0.24	2.3E-01	-0.19	3.6E-01	0.10	6.1E-01	-0.02	9.0E-01	0.72	3.5E-05	0.22	2.9E-01
ENSCAFG0000000057	TIAM2	grey	EC_M10	0.08	7.0E-01	0.03	8.8E-01	0.09	6.5E-01	-0.01	9.7E-01	-0.23	2.7E-01	-0.16	4.5E-01	0.03	8.8E-01	0.04	8.6E-01	0.68	1.5E-04	0.16	4.3E-01
ENSCAFG0000000485	PROSER2	turquoise	EC_M6	0.08	7.0E-01	0.72	3.3E-05	0.00	9.9E-01	-0.18	3.7E-01	-0.26	2.1E-01	-0.18	3.8E-01	-0.16	4.3E-01	-0.22	2.7E-01	-0.24	2.3E-01	0.21	3.1E-01
ENSCAFG0000000454	AKR1A1	grey	EC_M10	0.08	7.0E-01	-0.10	6.2E-01	-0.12	5.5E-01	-0.05	7.9E-01	0.24	2.4E-01	0.14	4.8E-01	-0.02	9.2E-01	-0.04	8.6E-01	0.13	5.4E-01	-0.26	2.0E-01
ENSCAFG0000002612	ENSCAFG0000002612	grey	EC_M10	0.08	7.0E-01	0.06	7.6E-01	0.16	4.3E-01	0.01	9.4E-01	-0.23	2.7E-01	0.15	4.6E-01	-0.25	2.2E-01	-0.25	2.2E-01	-0.29	1.4E-01	0.21	3.1E-01
ENSCAFG0000003125	ZNF252	grey	EC_M10	0.08	7.0E-01	0.21	3.0E-01	0.15	4.6E-01	0.30	1.4E-01	-0.43	2.7E-02	0.10	6.2E-01	-0.15	4.7E-01	0.27	1.9E-01	0.15	4.5E-01	0.36	7.1E-02
ENSCAFG0000001694	CD2BP2	cyan	EC_M2	0.08	7.0E-01	0.16	4.3E-01	0.42	3.3E-02	0.43	2.8E-02	-0.64	4.7E-04	-0.20	3.3E-01	0.04	8.5E-01	-0.03	9.0E-01	0.13	5.4E-01	0.60	1.2E-03
ENSCAFG0000000174	GNMT	grey	EC_M10	0.08	7.0E-01	-0.13	5.1E-01	0.18	3.8E-01	-0.19	3.5E-01	-0.21	3.0E-01	0.22	2.9E-01	0.06	7.6E-01	-0.30	1.3E-01	0.09	6.5E-01	0.13	5.4E-01
ENSCAFG0000000827	ENSCAFG0000000827	grey	EC_M10	0.08	7.0E-01	-0.16	4.2E-01	-0.05	8.2E-01	-0.14	5.1E-01	0.08	6.8E-01	-0.12	5.5E-01	-0.10	6.3E-01	-0.01	9.5E-01	0.04	8.6E-01	-0.10	6.4E-01
ENSCAFG0000003193	HIST1H2BJ	grey	EC_M10	0.08	7.0E-01	0.16	4.3E-01	-0.13	5.3E-01	0.12	5.7E-01	0.10	6.2E-01	0.09	6.6E-01	-0.20	3.3E-01	0.06	7.8E-01	0.19	3.6E-01	-0.04	8.4E-01
ENSCAFG0000001405	EXOSC7	cyan	EC_M2	0.08	7.0E-01	-0.28	1.7E-01	0.70	7.7E-05	0.14	5.1E-01	-0.79	1.8E-06	-0.26	2.1E-01	-0.10	6.2E-01	0.03	8.9E-01	-0.17	4.1E-01	0.75	1.2E-05
ENSCAFG0000000816	BTBD10	grey	EC_M10	0.08	7.0E-01	-0.35	7.8E-02	-0.49	1.0E-02	-0.06	7.6E-01	0.66	2.2E-04	0.32	1.1E-01	-0.37	6.1E-02	-0.10	6.2E-01	-0.11	6.0E-01	-0.71	5.3E-05
ENSCAFG0000001559	MAEL	grey	EC_M10	0.08	7.0E-01	-0.04	8.3E-01	0.10	6.1E-01	0.29	1.6E-01	-0.21	3.1E-01	-0.13	5.4E-01	-0.03	8.9E-01	-0.11	5.9E-01	-0.17	3.9E-01	0.15	4.7E-01
ENSCAFG0000001791	CXHXorf57	grey	EC_M10	0.08	7.0E-01	-0.27	1.8E-01	-0.10	6.1E-01	0.37	6.1E-02	0.10	6.2E-01	0.28	1.7E-01	-0.10	6.3E-01	-0.05	8.2E-01	-0.30	1.3E-01	-0.21	3.0E-01
ENSCAFG0000000515	MFSD11	cyan	EC_M2	0.08	7.0E-01	-0.16	4.5E-01	0.57	2.6E-03	0.23	2.6E-01	-0.71	4.2E-05	-0.14	5.0E-01	-0.23	2.5E-01	-0.27	1.8E-01	-0.34	8.9E-02	0.65	3.4E-04
ENSCAFG0000001305	PIGX	grey	EC_M10	0.08	7.0E-01	-0.18	3.8E-01	-0.60	1.1E-03	0.03	9.0E-01	0.76	5.9E-06	0.36	7.0E-02	-0.21	3.1E-01	-0.03	8.9E-01	0.08	6.9E-01	-0.80	1.1E-06
ENSCAFG0000001299	RNF185	grey	EC_M10	0.08	7.0E-01	-0.10	6.4E-01	0.12	5.7E-01	-0.29	1.5E-01	-0.25	2.2E-01	0.03	8.7E-01	-0.20	3.2E-01	-0.23	2.7E-01	0.03	8.8E-01	0.15	4.6E-01
ENSCAFG0000000594	KCNK6	turquoise	EC_M6	0.08	7.0E-01	0.49	1.2E-02	0.06	7.6E-01	-0.05	8.2E-01	-0.36	7.1E-02	0.05	8.1E-01	0.33	1.0E-01	-0.28	1.6E-01	-0.13	5.4E-01	0.27	1.9E-01
ENSCAFG0000000986	KSR2	grey	EC_M10	0.08	7.1E-01	-0.20	3.2E-01	-0.36	7.4E-02	-0.08	7.1E-01	0.38	5.6E-02	0.50	9.6E-03	-0.15	4.5E-01	0.13	5.1E-01	-0.16	4.4E-01	-0.48	1.2E-02
ENSCAFG0000000315	ENSCAFG0000000315	grey	EC_M10	0.08	7.1E-01	0.22	2.8E-01	-0.40	4.1E-02	-0.03	9.0E-01	0.37	6.3E-02	0.19	3.5E-01	-0.13	5.2E-01	-0.20	3.4E-01	0.00	1.0E+00	-0.40	4.3E-02
ENSCAFG0000000448	SLC39A12	grey	EC_M10	0.08	7.1E-01	-0.11	6.0E-01	-0.14	4.8E-01	-0.04	8.5E-01	0.06	7.6E-01	-0.07	7.4E-01	-0.11	6.0E-01	0.33	9.7E-02	0.32	1.1E-01	-0.15	4.7E-01
ENSCAFG0000000292	SKAP2	grey	EC_M10	0.08	7.1E-01	0.04	8.3E-01	-0.65	3.4E-04	-0.23	2.5E-01	0.66	2.3E-04	0.15	4.6E-01	-0.11	6.1E-01	0.19	3.6E-01	-0.28	1.6E-01	-0.74	1.4E-05
ENSCAFG0000002608	ENSCAFG0000002608	grey	EC_M10	0.08	7.1E-01	-0.16	4.4E-01	0.23	2.5E-01	-0.15	4.7E-01	-0.32	1.1E-01	0.35	7.8E-02	-0.16	4.2E-01	-0.16	4.3E-01	-0.18	3.7E-01	0.27	1.7E-01
ENSCAFG0000001397	ENSCAFG0000001397	turquoise	EC_M6	0.08	7.1E-01	0.45	2.2E-02	-0.07	7.2E-01	0.02	9.1E-01	-0.26	2.0E-01	-0.07	7.5E-01	-0.06	7.5E-01	0.17	4.1E-01	0.17	4.2E-01	0.20	3.2E-01
ENSCAFG0000000083	ENSCAFG0000000083	grey	EC_M10	0.08	7.1E-01	-0.26	2.0E-01	0.20	3.2E-01	0.10	6.1E-01	-0.26	2.0E-01	-0.23	2.5E-01	-0.22	2.9E-01	-0.16	4.3E-01	0.13	5.3E-01	0.24	2.5E-01
ENSCAFG0000000933	TUBB2A	darkgreen	EC_M4	0.08	7.1E-01	0.08	7.1E-01	-0.73	2.7E-05	-0.30	1.4E-01	0.80	9.2E-07	0.14	5.0E-01	-0.19	3.6E-01	0.16	4.4E-01	0.10	6.3E-01	-0.86	2.1E-08
ENSCAFG0000001361	IDH1	cyan	EC_M2	0.08	7.1E-01	-0.12	5.7E-01	0.58	2.1E-03	0.21	3.0E-01	-0.70	7.9E-05	-0.36	6.9E-02	-0.48	1.4E-02	0.10	6.4E-01	-0.22	2.8E-01	0.66	2.7E-04
ENSCAFG0000003074	LRRC46	darkgreen	EC_M4	0.08	7.1E-01	-0.40	4.3E-02	-0.40	4.1E-02	0.02	9.3E-01	0.56	2.6E-03	0.30	1.3E-01	-0.14	5.1E-01	0.22	2.9E-01	0.14	5.0E-01	-0.62	7.8E-04
ENSCAFG0000003198	ENSCAFG0000003198	grey	EC_M10	0.08	7.1E-01	0.05	8.0E-01	-0.23	2.7E-01	-0.32	1.2E-01	0.16	4.4E-01	0.29	1.5E-01	0.17	4.2E-01	-0.21	2.9E-01	-0.21	3.0E-01	-0.20	3.4E-01
ENSCAFG0000000241	ENSCAFG0000000241	grey	EC_M10	0.08	7.1E-01	0.06	7.7E-01	-0.17	4.1E-01	-0.01	9.6E-01	0.11	5.8E-01	0.16	4.3E-01	-0.12	5.6E-01	0.27	1.9E-01	0.00	9.9E-01	-0.18	3.9E-01
ENSCAFG0000001364	ENSCAFG0000001364	grey	EC_M10	0.08	7.1E-01	0.13	5.4E-01	0.18	3.8E-01	0.31	1.3E-01	-0.34	8.7E-02	-0.17	4.1E-01	0.48	1.4E-02	-0.12	5.6E-01	-0.09	6.5E-01	0.33	9.6E-02
ENSCAFG0000001503	ZNF41	grey	EC_M10	0.08	7.1E-01	0.21	2.9E-01	-0.15	4.6E-01	0.27	1.8E-01	0.01	9.5E-01	0.23	2.5E-01	0.03	8.8E-01	0.10	6.3E-01	-0.06	7.6E-01	-0.10	6.4E-01
ENSCAFG0000000048	FLT4	turquoise	EC_M6	0.08	7.1E-01	0.95	2.1E-13	-0.19	3.6E-01	-0.08	7.1E-01	-0.17	4.1E-01	-0.13	5.1E-01	-0.04	8.3E-01	-0.12	5.7E-01	0.02	9.3E-01	0.13	5.3E-01
ENSCAFG0000000329	ENSCAFG0000000329	grey	EC_M10	0.08	7.1E-01	0.03	8.9E-01	0.24	2.3E-01	0.19	3.6E-01	-0.31	1.3E-01	-0.45	2.1E-02	0.13	5.2E-01	-0.15	4.7E-01	-0.47	1.5E-02	0.28	1.7E-01
ENSCAFG0000000054	ENSCAFG0000000054	grey	EC_M10	0.08	7.1E-01	0.14	4.8E-01	-0.01	9.5E-01	-0.22	2.9E-01	-0.05	8.1E-01	-0.18	3.7E-01	-0.14	5.0E-01	-0.12	5.5E-01	0.33	1.0E-01	0.05	8.3E-01
ENSCAFG0000000606	ENSCAFG0000000606	grey	EC_M10	0.08	7.1E-01	-0.06	7.5E-01	0.02	9.3E-01	0.12	5.7E-01	-0.13	5.3E-01	0.24	2.3E-01	-0.13	5.1E-01	0.00	9.9E-01	-0.16	4.3E-01	0.01	9.5E-01
ENSCAFG0000001475	ERO1A	darkgreen	EC_M4	0.08	7.1E-01	0.06	7.6E-01	-0.83	2.0E-07	-0.12	5.6E-01	0.85	3.4E-08	0.25	2.2E-01	0.13	5.2E-01	0.10	6.4E-01	-0.11	5.8E-01	-0.91	7.6E-11
ENSCAFG0000000693	TCF15	turquoise	EC_M6	0.08	7.1E-01	0.57	2.6E-03	-0.45	2.2E-02	-0.29	1.5E-01	0.34	9.1E-02	0.14	4.9E-01	0.03	8.7E-01	-0.24	2.4E-01	-0.12	5.5E-01	-0.36	6.9E-02
ENSCAFG0000000617	ENSCAFG0000000617	grey	EC_M10	0.08	7.1E-01	-0.37	6.5E-02	-0.32	1.1E-01	-0.32	1.1E-01	0.53	4.9E-03	0.14	4.8E-01	0.03	8.8E-01	0.24	2.4E-01	0.09	6.7E-01	-0.56	3.2E-03
ENSCAFG0000000367	CCNY	turquoise	EC_M6	0.08	7.1E-01	0.53	5.2E-03	-0.06	7.6E-01	-0.11	6.0E-01	-0.19	3.5E-01	-0.02	9.2E-01	-0.45	2.1E-02	0.17	4.0E-01	-0.36	6.9E-02	0.15	4.6E-01
ENSCAFG0000000746	TMED2	darkgreen	EC_M4	0.08	7.1E-01	-0.15	4.7E-01	-0.77	5.0E-06	-0.10	6.1E-01	0.88	2.2E-09	0.39	4.6E-02	0.02	9.2E-01	0.06	7.8E-01	-0.06	7.9E-01	-0.94	6.5E-13
ENSCAFG0000002972	ZNF132	grey	EC_M10	0.08	7.1E-01	0.19	3.4E-01	-0.49	1.0E-02	-0.14	5.1E-01	0.47	1.6E-02	0.19	3.5E-01	0.02	9.4E-01	-0.14	5.1E-01	-0.02	9.3E-01	-0.51	8.4E-03
ENSCAFG0000000904	RRP12	grey	EC_M10	0.08	7.1E-01	0.03	8.7E-01	-0.69	8.1E-05	-0.20	3.3E-01	0.81	5.9E-07	0.33	9.5E-02	0.11	5.9E-01	-0.03	8.8E-01	0.05	8.2E-01	-0.85	5.3E-08
ENSCAFG0000001977	ENSCAFG0000001977	turquoise	EC_M6	0.08	7.1E-01	0.33	1.0E-01	-0.06	7.6E-01	0.26	2.1E-01	-0.12	5.4E-01	-0.19	3.6E-01	-0.05	8.1E-01	-0.26	2.0E-01	0.11	5.8E-01	0.06	7.7E-01
ENSCAFG0000002031	PRMT7	grey	EC_M10	0.08	7.1E-01	-0.24	2.5E-01	-0.24	2.4E-01	-0.16	4.3E-01	0.39	4.8E-02	0.45	2.1E-02	-0.09	6.7E-01	-0.35	8.0E-02	0.02	9.2E-01	-0.42	3.1E-02
ENSCAFG0000003014	LONRF1	turquoise	EC_M6	0.08	7.1E-01	0.37	6.2E-02	-0.16	4.5E-01	-0.41	3.5E-02	0.03	8.9E-01	-0.28	1.7E-01	-0.08	6.9E-01	-0.13	5.2E-01	-0.38	5.3E-02	-0.06	7.9E-01
ENSCAFG0000000562	STK24	grey	EC_M10	0.08	7.1E-01	-0.18	3.8E-01	0.26	2.0E-01	0.00	9.8E-01	-0.35	7.9E-02	-0.02	9.2E-01	-0.22	2.8E-01	-0.02	9.4E-01	-0.09	6.5E-01	0.34	8.9E-02

ENSCAFG0000001500	GORAB	darkgreen	EC_M4	0.08	7.1E-01	0.13	5.3E-01	-0.69	9.2E-05	-0.08	6.9E-01	0.63	5.5E-04	0.37	6.3E-02	0.10	6.3E-01	0.10	6.2E-01	-0.04	8.5E-01	-0.67	1.7E-04
ENSCAFG0000000476	STAC	grey	EC_M10	0.08	7.2E-01	-0.36	7.2E-02	-0.19	3.6E-01	-0.28	1.6E-01	0.27	1.8E-01	-0.09	6.8E-01	0.18	3.8E-01	0.22	2.7E-01	0.15	4.8E-01	-0.37	6.6E-02
ENSCAFG0000003144	UBE2I	grey	EC_M10	0.07	7.2E-01	-0.15	4.8E-01	0.33	9.9E-02	0.07	7.4E-01	-0.32	1.1E-01	-0.08	6.9E-01	-0.24	2.4E-01	0.40	4.6E-02	0.03	9.0E-01	0.25	2.1E-01
ENSCAFG0000000202	VLDLR	grey	EC_M10	0.07	7.2E-01	0.47	1.5E-02	-0.79	1.5E-06	-0.04	8.5E-01	0.67	1.6E-04	0.16	4.4E-01	0.05	8.1E-01	-0.07	7.3E-01	0.25	2.3E-01	-0.71	4.3E-05
ENSCAFG0000001609	SHLD2	grey	EC_M10	0.07	7.2E-01	0.32	1.1E-01	-0.41	3.6E-02	0.15	4.5E-01	0.23	2.6E-01	-0.22	2.8E-01	0.00	9.8E-01	0.24	2.3E-01	0.00	9.9E-01	-0.27	1.8E-01
ENSCAFG0000001248	MYO3B	grey	EC_M10	0.07	7.2E-01	-0.11	5.8E-01	-0.06	7.6E-01	-0.06	7.8E-01	0.09	6.7E-01	-0.03	9.0E-01	0.04	8.4E-01	-0.05	7.9E-01	0.04	8.3E-01	-0.12	5.5E-01
ENSCAFG0000001620	TBC1D1	turquoise	EC_M6	0.07	7.2E-01	0.67	1.6E-04	-0.59	1.7E-03	-0.04	8.3E-01	0.39	5.2E-02	-0.10	6.3E-01	0.14	5.1E-01	-0.07	7.4E-01	0.34	8.5E-02	-0.40	4.0E-02
ENSCAFG0000001046	CD200	turquoise	EC_M6	0.07	7.2E-01	0.71	5.5E-05	-0.27	1.8E-01	-0.13	5.2E-01	0.01	9.7E-01	-0.13	5.4E-01	-0.02	9.1E-01	-0.12	5.7E-01	0.62	7.4E-04	-0.01	9.6E-01
ENSCAFG0000002127	ENSCAFG0000002127	darkgreen	EC_M4	0.07	7.2E-01	0.31	1.2E-01	-0.33	9.7E-02	-0.12	5.4E-01	0.33	9.4E-02	-0.10	6.2E-01	-0.05	7.9E-01	-0.04	8.5E-01	0.04	8.4E-01	-0.29	1.6E-01
ENSCAFG0000001645	RAB24	grey	EC_M10	0.07	7.2E-01	0.42	3.1E-02	0.12	5.5E-01	-0.02	9.2E-01	-0.36	7.2E-02	0.03	9.0E-01	0.06	7.8E-01	0.12	5.5E-01	0.17	4.1E-01	0.35	8.4E-02
ENSCAFG0000001886	ENSCAFG0000001886	grey	EC_M10	0.07	7.2E-01	0.18	3.7E-01	-0.21	3.0E-01	-0.08	6.8E-01	0.18	3.7E-01	-0.11	5.8E-01	-0.09	6.6E-01	-0.10	6.3E-01	-0.03	8.9E-01	-0.20	3.4E-01
ENSCAFG0000001323	RPL11	cyan	EC_M2	0.07	7.2E-01	-0.43	2.9E-02	0.48	1.2E-02	0.15	4.6E-01	-0.48	1.3E-02	0.03	9.0E-01	0.16	4.4E-01	0.11	6.1E-01	-0.48	1.4E-02	0.37	6.1E-02
ENSCAFG0000002529	ENSCAFG0000002529	turquoise	EC_M6	0.07	7.2E-01	0.58	2.0E-03	-0.20	3.3E-01	0.30	1.4E-01	-0.04	8.5E-01	-0.18	3.8E-01	-0.20	3.3E-01	0.29	1.6E-01	0.18	3.7E-01	0.00	9.9E-01
ENSCAFG0000001827	ENSCAFG0000001827	grey	EC_M10	0.07	7.2E-01	-0.14	5.0E-01	-0.06	7.6E-01	0.01	9.7E-01	0.01	9.8E-01	-0.11	5.8E-01	-0.16	4.3E-01	-0.08	6.9E-01	-0.17	4.0E-01	-0.08	6.8E-01
ENSCAFG0000000250	ENSCAFG0000000250	grey	EC_M10	0.07	7.2E-01	-0.20	3.3E-01	0.12	5.7E-01	0.31	1.2E-01	-0.11	6.0E-01	-0.13	5.3E-01	-0.11	6.0E-01	-0.09	6.6E-01	0.01	9.6E-01	0.09	6.5E-01
ENSCAFG0000000780	ENSCAFG0000000780	darkgreen	EC_M4	0.07	7.2E-01	0.00	9.9E-01	-0.14	5.0E-01	-0.11	5.9E-01	0.18	3.7E-01	-0.02	9.1E-01	-0.01	9.8E-01	-0.01	9.7E-01	0.12	5.7E-01	-0.19	3.4E-01
ENSCAFG0000000707	RET	turquoise	EC_M6	0.07	7.2E-01	0.47	1.5E-02	0.12	5.6E-01	-0.05	8.1E-01	-0.32	1.2E-01	-0.10	6.2E-01	-0.17	4.1E-01	0.12	5.7E-01	0.02	9.4E-01	0.29	1.6E-01
ENSCAFG0000001602	AKAP5	grey	EC_M10	0.07	7.2E-01	0.07	7.5E-01	-0.68	1.2E-04	-0.03	8.7E-01	0.66	2.4E-04	0.14	5.0E-01	0.21	2.9E-01	-0.13	5.3E-01	0.32	1.1E-01	-0.70	7.4E-05
ENSCAFG0000000500	TIMP4	turquoise	EC_M6	0.07	7.2E-01	0.62	6.9E-04	0.13	5.3E-01	0.15	4.8E-01	-0.45	2.1E-02	0.06	7.7E-01	-0.06	7.7E-01	0.02	9.3E-01	0.25	2.3E-01	0.42	3.3E-02
ENSCAFG0000001423	ITPRID2	grey	EC_M10	0.07	7.2E-01	0.32	1.1E-01	-0.71	4.9E-05	-0.21	3.1E-01	0.60	1.2E-03	0.09	6.6E-01	0.08	7.1E-01	0.05	8.0E-01	0.35	7.8E-02	-0.64	4.1E-04
ENSCAFG0000000020	ENSCAFG0000000020	turquoise	EC_M6	0.07	7.2E-01	0.46	1.7E-02	-0.28	1.6E-01	-0.12	5.6E-01	0.07	7.2E-01	0.34	9.3E-02	-0.16	4.4E-01	-0.16	4.3E-01	0.15	4.7E-01	-0.10	6.4E-01
ENSCAFG0000001210	RCOR3	cyan	EC_M2	0.07	7.2E-01	0.08	6.9E-01	0.46	1.9E-02	0.27	1.8E-01	-0.73	1.9E-05	-0.14	5.0E-01	-0.12	5.6E-01	0.12	5.7E-01	0.15	4.5E-01	0.68	1.3E-04
ENSCAFG0000000105	TRMT11	grey	EC_M10	0.07	7.2E-01	-0.14	5.0E-01	-0.27	1.9E-01	0.12	5.7E-01	0.20	3.3E-01	0.28	1.7E-01	0.43	2.9E-02	-0.06	7.6E-01	-0.27	1.9E-01	-0.25	2.2E-01
ENSCAFG0000000717	ENSCAFG0000000717	violet	EC_M7	0.07	7.2E-01	0.00	1.0E+00	-0.08	7.0E-01	-0.10	6.2E-01	-0.01	9.7E-01	0.53	5.2E-03	-0.06	7.7E-01	-0.16	4.5E-01	0.21	3.1E-01	-0.11	6.0E-01
ENSCAFG0000000833	ENSCAFG0000000833	grey	EC_M10	0.07	7.2E-01	-0.20	3.4E-01	-0.44	2.6E-02	-0.27	1.8E-01	0.58	1.7E-03	0.01	9.7E-01	-0.15	4.7E-01	-0.03	8.7E-01	0.18	3.8E-01	-0.56	3.1E-03
ENSCAFG0000003159	ENSCAFG0000003159	grey	EC_M10	0.07	7.2E-01	-0.41	3.8E-02	0.19	3.6E-01	0.25	2.2E-01	-0.11	6.0E-01	0.19	3.6E-01	-0.09	6.5E-01	-0.35	7.9E-02	-0.29	1.5E-01	-0.01	9.8E-01
ENSCAFG0000000487	RPL32	cyan	EC_M2	0.07	7.2E-01	-0.40	4.6E-02	0.46	1.9E-02	0.06	7.8E-01	-0.46	1.8E-02	0.09	6.6E-01	0.23	2.5E-01	0.08	6.8E-01	-0.38	5.4E-02	0.35	7.9E-02
ENSCAFG0000000068	ABRA	grey	EC_M10	0.07	7.2E-01	-0.14	5.1E-01	-0.24	2.3E-01	0.04	8.5E-01	0.20	3.4E-01	-0.11	6.0E-01	-0.09	6.5E-01	-0.11	5.9E-01	-0.18	3.8E-01	-0.29	1.5E-01
ENSCAFG0000001588	SCCPDH	grey	EC_M10	0.07	7.2E-01	-0.42	3.3E-02	-0.49	1.1E-02	-0.14	4.9E-01	0.68	1.3E-04	0.29	1.5E-01	-0.13	5.3E-01	-0.06	7.9E-01	-0.14	5.1E-01	-0.68	1.2E-04
ENSCAFG0000001310	NCBP2	grey	EC_M10	0.07	7.2E-01	-0.27	1.9E-01	-0.30	1.3E-01	-0.14	4.9E-01	0.39	5.0E-02	0.32	1.1E-01	0.10	6.2E-01	0.08	6.8E-01	0.18	3.7E-01	-0.47	1.4E-02
ENSCAFG0000001180	CIART	turquoise	EC_M6	0.07	7.2E-01	0.85	2.7E-08	-0.28	1.6E-01	-0.12	5.7E-01	-0.07	7.4E-01	-0.11	5.8E-01	0.18	3.8E-01	0.18	3.7E-01	0.06	7.7E-01	0.00	9.9E-01
ENSCAFG0000001463	RUNDC1	turquoise	EC_M6	0.07	7.2E-01	0.79	1.5E-06	-0.31	1.3E-01	0.02	9.3E-01	0.01	9.8E-01	-0.07	7.4E-01	0.16	4.3E-01	-0.02	9.4E-01	-0.05	8.1E-01	-0.03	9.0E-01
ENSCAFG0000001755	CSTF2	grey	EC_M10	0.07	7.2E-01	-0.34	9.1E-02	-0.19	3.6E-01	-0.02	9.2E-01	0.41	3.7E-02	0.04	8.3E-01	0.00	9.9E-01	0.04	8.4E-01	-0.08	7.0E-01	-0.46	1.9E-02
ENSCAFG0000000123	TAF11	grey	EC_M10	0.07	7.2E-01	-0.15	4.7E-01	-0.63	5.3E-04	-0.06	7.6E-01	0.76	6.2E-06	0.30	1.3E-01	0.07	7.3E-01	-0.03	8.8E-01	-0.11	5.9E-01	-0.80	7.7E-07
ENSCAFG0000002999	GLIPR1L2	grey	EC_M10	0.07	7.2E-01	-0.09	6.7E-01	0.21	3.0E-01	-0.05	8.2E-01	-0.30	1.4E-01	-0.14	4.9E-01	-0.09	6.8E-01	-0.11	5.8E-01	0.30	1.4E-01	0.30	1.4E-01
ENSCAFG0000000039	MAPK9	grey	EC_M10	0.07	7.2E-01	-0.04	8.3E-01	-0.52	6.0E-03	-0.36	7.0E-02	0.55	4.0E-03	-0.03	8.9E-01	0.05	7.9E-01	0.11	5.8E-01	-0.18	3.7E-01	-0.55	3.5E-03
ENSCAFG0000003251	ARPP19	grey	EC_M10	0.07	7.2E-01	0.04	8.4E-01	-0.16	4.3E-01	-0.36	6.9E-02	0.18	3.8E-01	0.16	4.2E-01	-0.10	6.3E-01	-0.23	2.7E-01	-0.22	2.9E-01	-0.24	2.4E-01
ENSCAFG0000001093	COL16A1	grey	EC_M10	0.07	7.2E-01	-0.19	3.5E-01	-0.06	7.8E-01	0.14	5.0E-01	0.02	9.1E-01	0.09	6.5E-01	0.47	1.6E-02	0.02	9.1E-01	0.17	4.0E-01	-0.13	5.2E-01
ENSCAFG0000001393	CYBB	turquoise	EC_M6	0.07	7.2E-01	0.66	2.8E-04	-0.20	3.3E-01	-0.11	6.0E-01	-0.09	6.6E-01	-0.14	4.8E-01	-0.09	6.6E-01	-0.18	3.9E-01	0.18	3.7E-01	0.08	6.9E-01
ENSCAFG0000001104	EDARADD	cyan	EC_M2	0.07	7.2E-01	0.22	2.9E-01	0.58	1.9E-03	-0.15	4.5E-01	-0.80	1.0E-06	-0.05	7.9E-01	0.00	9.8E-01	-0.11	5.8E-01	-0.24	2.4E-01	0.77	3.6E-06
ENSCAFG0000000528	ENSCAFG0000000528	grey	EC_M10	0.07	7.2E-01	0.15	4.6E-01	-0.55	3.7E-03	0.14	5.0E-01	0.55	3.6E-03	0.02	9.2E-01	-0.28	1.7E-01	0.00	9.9E-01	0.01	9.8E-01	-0.59	1.4E-03
ENSCAFG0000000750	TDRD12	grey	EC_M10	0.07	7.2E-01	-0.09	6.7E-01	0.24	2.4E-01	-0.11	6.1E-01	-0.28	1.6E-01	-0.01	9.6E-01	-0.07	7.3E-01	-0.10	6.2E-01	-0.11	6.0E-01	0.18	3.7E-01
ENSCAFG0000000009	LMAN1	darkgreen	EC_M4	0.07	7.2E-01	-0.17	4.1E-01	-0.76	6.1E-06	-0.16	4.4E-01	0.88	2.3E-09	0.36	6.8E-02	0.15	4.7E-01	-0.02	9.1E-01	-0.16	4.4E-01	-0.94	6.6E-13
ENSCAFG0000002891	RWDD3	cyan	EC_M2	0.07	7.2E-01	-0.17	4.0E-01	0.62	7.1E-04	0.10	6.2E-01	-0.76	6.7E-06	-0.11	5.9E-01	-0.20	3.2E-01	-0.08	7.0E-01	0.04	8.6E-01	0.70	7.8E-05
ENSCAFG0000000716	ENSCAFG0000000716	grey	EC_M10	0.07	7.2E-01	-0.21	3.1E-01	0.19	3.4E-01	-0.25	2.1E-01	-0.18	3.9E-01	-0.25	2.1E-01	-0.02	9.2E-01	0.01	9.4E-01	-0.08	6.9E-01	0.18	3.7E-01
ENSCAFG0000001306	GAL3ST3	turquoise	EC_M6	0.07	7.2E-01	0.57	2.6E-03	0.01	9.5E-01	-0.03	8.8E-01	-0.26	2.1E-01	-0.08	6.9E-01	-0.03	8.9E-01	-0.05	7.9E-01	0.48	1.2E-02	0.24	2.4E-01
ENSCAFG0000001089	ARRDC4	grey	EC_M10	0.07	7.2E-01	0.15	4.6E-01	0.30	1.4E-01	0.16	4.5E-01	-0.43	2.8E-02	-0.27	1.9E-01	0.17	4.1E-01	0.00	1.0E+00	0.25	2.2E-01	0.44	2.5E-02
ENSCAFG0000000869	RAPSN	grey	EC_M10	0.07	7.2E-01	0.01	9.7E-01	0.21	3.0E-01	-0.10	6.2E-01	-0.34	8.9E-02	-0.44	2.5E-02	0.02	9.3E-01	0.14	5.1E-01	0.28	1.6E-01	0.30	1.4E-01
ENSCAFG0000001758	TLE3	turquoise	EC_M6	0.07	7.2E-01	0.85	4.8E-08	-0.25	2.2E-01	-0.31	1.2E-01	-0.04	8.3E-01	0.05	8.2E-01	0.23	2.6E-01	-0.17	4.2E-01	0.12	5.5E-01	0.04	8.6E-01
ENSCAFG0000001650	PALM3	grey	EC_M10	0.07	7.3E-01	-0.35	8.2E-02	0.47	1.7E-02	0.09	6.8E-01	-0.49	1.1E-02	-0.36	6.9E-02	0.12	5.5E-01	0.20	3.3E-01	0.32	1.1E-01	0.42	3.1E-02
ENSCAFG0000003172	YAP1	grey	EC_M10	0.07	7.3E-01	-0.58	1.7E-03	-0.25	2.2E-01	0.12	5.7E-01	0.38	5.4E-02	0.08	6.8E-01	-0.22	2.9E-01	0.07	7.2E-01	0.34	8.7E-02	-0.41	3.8E-02
ENSCAFG0000000120	ENSCAFG0000000120	darkmagenta	EC_M12	0.07	7.3E-01	-0.15	4.7E-01	0.05	8.0E-01	-0.09	6.8E-01	-0.01	9.8E-01	-0.09	6.5E-01	-0.04	8.3E-01	0.73	2.4E-05	0.03	8.7E-01	0.00	9.9E-01
ENSCAFG0000000796	MAB21L2	darkmagenta	EC_M12	0.07	7.3E-01	-0.15	4.7E-01	0.05	8.0E-01	-0.09	6.8E-01	-0.01	9.8E-01	-0.09	6.5E-01	-0.04	8.3E-01	0.73	2.4E-05	0.03	8.7E-01	0.00	9.9E-01
ENSCAFG0000002465	ENSCAFG0000002465	darkmagenta	EC_M12	0.07	7.3E-01	-0.12	5.6E-01	0.15	4.8E-01	-0.12	5.5E-01	-0.11	5.9E-01	-0.11	6.0E-01	-0.06	7.8E-01	0.53	5.1E-03	-0.02	9.2E-01	0.07	7.4E-01
ENSCAFG0000000681	SDCBP2	turquoise	EC_M6	0.07	7.3E-01	0.25	2.3E-01	0.02	9.4E-01	-0.19	3.6E-01	-0.15	4.8E-01	-0.18	3.8E-01	-0.08	7.1E-01	-0.12	5.6E-01	-0.08	7.0E-01	0.16	4.5E-01
ENSCAFG0000001768	RPL7A	cyan	EC_M2	0.07	7.3E-01	-0.41	4.0E-02	0.63	5.6E-04	0.16	4.3E-01	-0.65	3.0E-04	-0.09	6.6E-01	0.12	5.6E-01	0.12	5.6E-01	-0.38	5.2E-02	0.55	3.4E-03
ENSCAFG0000001376	PTGS2	grey	EC_M10	0.07	7.3E-01	0.34	8.7E-02	-0.56	3.1E-03	-0.19	3.5E-01	0.44	2.6E-02	-0.12	5.5E-01	0.21	3.1E-01	0.17	4.1E-01	0.04	8.5E-01	-0.50	9.7E-03
ENSCAFG0000000835	GOT2	grey	EC_M10	0.07	7.3E-01	-0.52	6.6E-03	0.15	4.8E-01	0.16	4.4E-01	-0.01	9.8E-01	-0.10	6.1E-01	-0.23	2.5E-01	-0.19	3.5E-01	-0.18	3.7E-01	-0.07	7.4E-01
ENSCAFG0000000690	ANKRD55	grey	EC_M10	0.07	7.3E-01	0.36	6.9E-02	-0.34	9.0E-02	0.25	2.1E-01	0.16	4.4E-01	-0.21	3.0E-01	0.32	1.1E-01	-0.15	4.6E-01	-0.12	5.6E-01	-0.18	3.8E-01
ENSCAFG0000003182	ENSCAFG0000003182	grey	EC_M10	0.07	7.3E-01	0.15	4.7E-01	-0.12	5.5E-01	-0.18	3.8E-01	0.09	6.6E-01	-0.14	5.0E-01	-0.08	7.0E-01	-0.13	5.2E-01	-0.01	9.6E-01	-0.04	8.6E-01
ENSCAFG0000001636	TBC1D19	grey	EC_M10	0.07	7.3E-01	-0.55	3.3E-03	0.08	6.9E-01	0.17	4.1E-01	0.01	9.5E-01	0.03	8.8E-01	-0.22	2.7E-01	0.09	6.5E-01	0.26	2.1E-01	-0.11	5.9E-01
ENSCAFG0000003024	DUSP4	darkgreen	EC_M4	0.07	7.3E-01	0.45	2.0E-02	-0.67	1.7E-04	-0.15	4.8E-01	0.57	2.4E-03	0.10	6.3E-01	0.01	9.8E-01	-0.04	8.6E-01	-0.12	5.5E-01	-0.61	8.9E-04
ENSCAFG0000000074	KCNV1	grey	EC_M10	0.07	7.3E-01	-0.08	6.8E-01	-0.46	1.9E-02	0.02	9.4E-01	0.48	1.4E-02	-0.12	5.6E-01	-0.08	7.0E-01	-0.03	8.9E-01	-0.07	7.3E-01	-0.53	5.5E-03
ENSCAFG0000000265	EPCAM	grey	EC_M10	0.07	7.3E-01	-0.27	1.9E-01	0.01	9.4E-01	0.20	3.3E-01	0.03	8.9E-01	0.43	2.8E-02	0.32	1.1E-01	-0.13	5.4E-01	-0.18	3.8E-01	-0.04	8.5E-01
ENSCAFG0000001459	PYGL	turquoise	EC_M6	0.07	7.3E-01	0.68	1.3E-04	0.07	7.4E-01	0.02	9.4E-01	-0.35	7.7E-02	-0.45	2.1E-02	0.07	7.2E-01	0.01	9.5E-01	0.26	1.9E-01	0.34	8.9E-02
ENSCAFG0000002597	ENSCAFG0000002597	grey	EC_M10	0.07	7.3E-01	0.18	3.7E-01	0.35	7.9E-02	-0.03	8.9E-01	-0.58	2.1E-03	-0.33	9.7E-02	-0.03	8.8E-01	0.00	1.0E+00	0.02	9.2E-01	0.52	6.2E-03
ENSCAFG0000000679	FKBP1A	grey	EC_M10	0.07	7.3E-01	-0.03	8.9E-01	-0.29	1.5E-01	-0.01	9.7E-01	0.30	1.3E-01	0.10	6.3E-01	0.35	8.1E-02	0.26	2.0E-01	0.09	6.5E-01	-0.33	1.0E-01
ENSCAFG0000000098	DAXX	grey	EC_M10	0.07	7.3E-01	-0.13	5.1E-01	-0.33	1.0E-01	-0.10	6.2E-01	0.35	8.3E-02	0.23	2.7E-01	0.25	2.2E-01	0.35	7.8E-02	0.08	6.8E-01	-0.43	2.7E-02
ENSCAFG0000000502	HNRNPUL1	grey	EC_M10	0.07	7.3E-01	0.11	6.0E-01	0.62	7.1E-04	-0.07	7.5E-01	-0.76	6.6E-06	-0.02	9.2E-01	0.05	8.1E-01	0.02	9.2E-01	0.12	5.5E-01	0.72	3.9E-05
ENSCAFG0000003120	ENSCAFG0000003120	cyan	EC_M2	0.07	7.3E-01	0.14	5.0E-01	0.39	5.0E-02	0.24	2.4E-01	-0.62	8.0E-04	-0.21	3.0E-01	0.12	5.5E-01	-0.07	7.4E-01	0.16	4.4E-01	0.62	7.8E-04
ENSCAFG0000001789	COX10	grey	EC_M10	0.07	7.3E-01	-0.51	7.6E-03	0.34	8.5E-02	-0.24	2.5E-01	-0.20	3.3E-01	0.19	3.6E-01	-0.27	1.8E-01	-0.14	5.0E-01	-0.11	6.0E-01	0.18	3.9E-01
ENSCAFG0000000743	ENSCAFG0000000743	grey	EC_M10	0.07	7.3E-01	-0.28	1.7E-01	0.03	9.0E-01	-0.45	2.2E-02	0.09	6.8E-01	-0.10	6.1E-01	-0.15	4.7E-01	0.16	4.4E-01	-0.04	8.4E-01	-0.10	6.1E-01
ENSCAFG0000000049	ENSCAFG0000000049	grey	EC_M10	0.07	7.3E-01	-0.07	7.3E-01	-0.49	1.1E-02	-0.24	2.3E-01	0.54	4.7E-03	0.15	4.8E-01	0.30	1.3E-01	0.28	1.7E-01	-0.06	7.7E-01	-0.58	1.8E-03
ENSCAFG0000000533	ENSCAFG0000000533	grey	EC_M10	0.07	7.3E-01	0.39	5.1E-02	-0.13	5.4E-01	0.10	6.3E-01	-0.06	7.8E-01	0.12	5.5E-01	0.33	9.8E-02	-0.16	4.4E-01	-0.10	6.1E-01	0.03	8.7E-01
ENSCAFG0000001028	DZIP3	grey	EC_M10	0.07	7.3E-01	0.38	5.7E-02	0.09	6.7E-01	-0.04	8.4E-01	-0.39	4.9E-02	-0.24	2.4E-01	-0.12	5.7E-01	-0.14	5.0E-01	0.37	6.5E-02	0.36	7.1E-02
ENSCAFG0000000581	EFHB	grey	EC_M10	0.07	7.3E-01	-0.21	3.1E-01	0.06	7.6E-01	-0.10	6.4E-01	0.04	8.5E-01	-0.02	9.2E-01	-0.08	6.9E-01	-0.10	6.2E-01	-0.05	8.0E-01	-0.04	8.3E-01
ENSCAFG0000002852	ENSCAFG0000002852	grey	EC_M10	0.07	7.3E-01	-0.16	4.2E-01	-0.11	6.0E-01	0.00	9.9E-01	0.15	4.8E-01	-0.13	5.3E-01	0.52	6.5E-03	-0.01	9.6E-01	-0.16	4.4E-01	-0.18	3.8E-01
ENSCAFG0000000053	SNX24	turquoise	EC_M6	0.07	7.3E-01	0.75	9.8E-06	-0.09	6.7E-01	0.14	5.0E-01	-0.23	2.5E-01	-0.11	5.8E-01	0.17	4.0E-01	0.02	9.4E-01	-0.01	9.6E-01	0.24	2.5E-01
ENSCAFG0000000852	P2RY14	darkgreen	EC_M4	0.07	7.3E-01	0.08	7.1E-01	-0.52	6.1E-03	-0.06	7.6E-01	0.54	4.3E-03	-0.14	4.9E-01	-0.09	6.7E-01	-0.03	8.9E-01	-0.04	8.6E-01	-0.57	2.3E-03
ENSCAFG0000003199	SAR1B	grey	EC_M10	0.07	7.3E-01	0.23	2.7E-01	-0.63	5.5E-04	-0.22	2.8E-01	0.57	2.5E-03	0.24	2.3E-01	0.19	3.5E-01	-0.05	8.0E-01	-0.03	8.8E-01	-0.55	3.4E-03
ENSCAFG0000000925	PTPN22	darkgreen	EC_M4	0.07	7.3E-01	-0.01	9.7E-01	-0.71	4.3E-05	-0.24	2.5E-01	0.82	3.5E-07	0.15	4.8E-01	-0.14	4.9E-01	0.12	5.7E-01	0.04	8.3E-01	-0.84	6.6E-08
ENSCAFG0000002498	FADD	grey	EC_M10	0.07	7.3E-01	0.15	4.5E-01	0.16	4.5E-01	0.04	8.4E-01	-0.30	1.4E-01	0.07	7.2E-01	0.31	1.2E-01	0.19	3.6E-01	0.16	4.5E-01	0.32	1.1E-01
ENSCAFG0000001385	MATN2	grey	EC_M10	0.07	7.3E-01	0.29	1.6E-01	-0.42	3.5E-02	0.00	1.0E+00	0.31	1.2E-01	0.19	3.6E-01	0.16	4.5E-01	-0.18	3.9E-01	0.41	3.9E-02	-0.34	9.0E-02
ENSCAFG0000000045	TSPAN8	turquoise	EC_M6	0.07	7.3E-01	0.82	3.0E-07	-0.27	1.8E-01	0.09	6.7E-01	-0.03	8.8E-01	-0.13	5.3E-01	-0.11	5.9E-01	-0.19	3.5E-01	0.34	9.0E-02	0.02	9.3E-01
ENSCAFG0000001978	KIF1B	grey	EC_M10	0.07	7.3E-01	0.19	3.4E-01	-0.46	1.8E-02	-0.26	2.1E-01	0.38	5.8E-02	-0.10	6.1E-01	0.32	1.1E-01	-0.10	6.3E-01	-0.02	9.2E-01	-0.35	7.8E-02
ENSCAFG0000001844	ISL1	grey	EC_M10	0.07	7.3E-01	-0.24	2.4E-01	0.02	9.4E-01	0.64	4.1E-04	0.03	8.8E-01	0.10	6.4E-01	-0.13	5.3E-01	-0.10	6.4E-01	-0.13	5.4E-01	-0.09	6.7E-01
ENSCAFG0000000100	WDYHV1	grey	EC_M10	0.07	7.3E-01	-0.25	2.2E-01	0.02	9.3E-01	0.17	4.1E-01	0.04	8.5E-01	-0.08	7.0E-01	0.01	9.8E-01	0.14	4.9E-01	-0.14	4.9E-01	-0.09	6.5E-01
ENSCAFG0000000909	HPSE	grey	EC_M10	0.07	7.3E-01	-0.29	1.5E-01	0.70	8.0E-05	0.03	8.8E-01	-0.70	7.0E-05	-0.16	4.4E-01	-0.17	4.2E-01	-0.14	4.9E-01	-0.13	5.3E-01	0.69	9.9E-05
ENSCAFG0000002633	ENSCAFG0000002633	grey	EC_M10	0.07	7.3E-01	-0.16	4.5E-01	-0.07	7.3E-01	-0.16	4.3E-01	0.03	8.9E-01	0.42	3.4E-02	0.28	1.7E-01	-0.03	8.7E-01	-0.14	5.0E-01	-0.13	5.3E-01
ENSCAFG0000003112	HSPA13	darkgreen	EC_M4	0.07	7.3E-01	-0.15	4.7E-01	-0.78	2.4E-06	-0.13	5.2E-01	0.88	2.9E-09	0.31	1.2E-01	0.11	5.8E-01	0.10	6.4E-01	-0.06	7.6E-01	-0.92	3.4E-11
ENSCAFG0000001091	NR2F2	turquoise	EC_M6	0.07	7.3E-01	0.60	1.3E-03	-0.44	2.6E-02	0.08	7.0E-01	0.23	2.5E-01	-0.06	7.6E-01	-0.04	8.3E-01	0.05	8.0E-01	0.02	9.4E-01	-0.31	1.3E-01
ENSCAFG0000003227	ENSCAFG0000003227	grey	EC_M10	0.07	7.3E-01	-0.12	5.5E-01	0.12	5.4E-01	0.30	1.4E-01	-0.16	4.3E-01	-0.15	4.6E-01	-0.10	6.3E-01	-0.09	6.6E-01	0.22	2.7E-01	0.10	6.3E-01
ENSCAFG0000000845	ENSCAFG0000000845	grey	EC_M10	0.07	7.3E-01	-0.10	6.2E-01	0.33	9.6E-02	-0.15	4.7E-01	-0.45	2.0E-02	-0.04	8.3E-01	-0.12	5.5E-01	-0.17	4.0E-01	0.06	7.6E-01	0.40	4.2E-02
ENSCAFG0000001423	SF1	grey	EC_M10	0.07	7.3E-01	0.22	2.8E-01	0.34	8.6E-02	-0.08	6.9E-01	-0.45	2.1E-02	0.14	4.9E-01	0.09	6.6E-01	-0.10	6.4E-01	-0.11	5.8E-01	0.41	3.8E-02
ENSCAFG0000003052	ENSCAFG0000003052	grey	EC_M10	0.07	7.3E-01	-0.16	4.2E-01	0.12	5.5E-01	0.37	6.3E-02	-0.13	5.1E-01	0.40	4.5E-02	-0.10	6.3E-01	-0.06	7.6E-01	-0.10	6.3E-01	0.06	7.7E-01
ENSCAFG0000000418	ZC3H4	grey	EC_M10	0.07	7.3E-01	0.26	2.0E-01	0.50	8.5E-03	-0.01	9.7E-01	-0.67	1.8E-04	0.05	8.0E-01	-0.20	3.2E-01	-0.20	3.3E-01	-0.10	6.3E-01	0.65	3.3E-04
ENSCAFG0000000211	PLGRKT	grey	EC_M10	0.07	7.4E-01	-0.38	5.3E-02	-0.10	6.4E-01	0.11	5.8E-01	0.08	7.1E-01	0.26	1.9E-01	-0.27	1.8E-01	0.15	4.7E-01	-0.12	5.4E-01	-0.16	4.4E-01
ENSCAFG0000001246	FAM117B	grey	EC_M10	0.07	7.4E-01	-0.05	8.0E-01	-0.56	3.2E-03	0.06	7.7E-01	0.63	6.1E-04	0.21	3.0E-01	0.08	7.1E-01	-0.12	5.5E-01	-0.46	1.7E-02	-0.65	2.9E-04
ENSCAFG0000000275	STAP1	cyan	EC_M2	0.07	7.4E-01	0.00	1.0E+00	0.57	2.4E-03	0.32	1.1E-01	-0.73	2.4E-05	-0.20	3.3E-01	-0.10	6.3E-01	0.12	5.7E-01	-0.07	7.2E-01	0.70	7.3E-05
ENSCAFG0000000264	RIMS1	darkgreen	EC_M4	0.07	7.4E-01	0.44	2.5E-02	-0.72	3.5E-05	-0.13	5.1E-01	0.61	9.4E-04	0.38	5.4E-02	-0.05	8.0E-01	-0.11	5.8E-01	-0.07	7.3E-01	-0.68	1.4E-04
ENSCAFG0000001551	WAS	turquoise	EC_M6	0.07	7.4E-01	0.53	4.9E-03	-0.02	9.2E-01	-0.20	3.3E-01	-0.19	3.4E-01	0.16	4.4E-01	0.11	5.9E-01	0.08	7.0E-01	0.19	3.4E-01	0.18	3.7E-01
ENSCAFG0000000253	ALG2	cyan	EC_M2	0.07	7.4E-01	-0.59	1.6E-03	0.38	5.9E-02	0.21	3.1E-01	-0.28	1.6E-01	-0.03	8.7E-01	-0.26	2.0E-01	0.12	5.6E-01	-0.33	9.6E-02	0.19	3.6E-01
ENSCAFG0000001397	TMEM262	paleturquoise	EC_M11	0.07	7.4E-01	-0.04	8.6E-01	0.16	4.3E-01	0.79	1.7E-06	-0.25	2.2E-01	-0.02	9.1E-01	-0.06	7.9E-01	-0.09	6.7E-01	-0.11	5.8E-01	0.20	3.3E-01
ENSCAFG0000000451	CDC42EP4	cyan	EC_M2	0.07	7.4E-01	-0.39	4.9E-02	0.50	8.8E-03	0.03	8.8E-01	-0.44	2.6E-02	0.02	9.3E-01	0.14	4.8E-01	-0.18	3.7E-01	-0.39	5.0E-02	0.35	8.0E-02
ENSCAFG0000000500	TAMM41	cyan	EC_M2	0.07	7.4E-01	-0.27	1.8E-01	0.61	8.9E-04	0.04	8.4E-01	-0.71	5.8E-05	-0.02	9.1E-01	0.07	7.2E-01	-0.04	8.6E-01	-0.28	1.7E-01	0.65	3.1E-04
ENSCAFG0000002803	ENSCAFG0000002803	cyan	EC_M2	0.07	7.4E-01	-0.46	1.7E-02	0.46	1.9E-02	0.21	2.9E-01	-0.44	2.5E-02	0.01	9.6E-01	-0.29	1.6E-01	0.10	6.2E-01	-0.33	1.0E-01	0.34	9.0E-02
ENSCAFG0000000265	RPS9	cyan	EC_M2	0.07	7.4E-01	-0.47	1.6E-02	0.55	3.7E-03	0.21	3.0E-01	-0.53	5.5E-03	0.02	9.4E-01	0.20	3.4E-01	-0.03	8.9E-01	-0.50	9.4E-03	0.43	2.7E-02
ENSCAFG0000001254	CCDC51	grey	EC_M10	0.07	7.4E-01	-0.29	1.5E-01	0.10	6.4E-01	-0.12	5.6E-01	-0.03	8.8E-01	0.37	6.6E-02	0.06	7.8E-01	0.03	8.9E-01	-0.05	7.9E-01	-0.02	9.2E-01
ENSCAFG0000000598	KDELC1	darkgreen	EC_M4	0.07	7.4E-01	-0.17	4.1E-01	-0.70	6.7E-05	-0.09	6.6E-01	0.83	1.9E-07	0.36	7.3E-02	0.27	1.9E-01	0.01	9.7E-01	-0.15	4.7E-01	-0.87	7.2E-09
ENSCAFG0000000652	RASSF7	turquoise	EC_M6	0.07	7.4E-01	0.61	9.8E-04	-0.50	9.4E-03	-0.19	3.7E-01	0.30	1.4E-01	-0.08	7.2E-01	0.26	2.0E-01	0.07	7.3E-01	0.11	5.8E-01	-0.37	6.4E-02
ENSCAFG0000001474	ZNF384	grey	EC_M10	0.07	7.4E-01	0.53	5.6E-03	0.42	3.2E-02	0.08	7.0E-01	-0.78	3.0E-06	-0.08	6.8E-01	-0.06	7.7E-01	-0.08	6.9E-01	-0.03	8.8E-01	0.71	4.8E-05
ENSCAFG0000000468	CNTNAP5	grey	EC_M10	0.07	7.4E-01	0.15	4.5E-01	-0.58	1.9E-03	-0.22	2.9E-01	0.61	8.8E-04	0.29	1.5E-01	0.07	7.2E-01	-0.14	4.9E-01	0.19	3.5E-01	-0.63	6.4E-04
ENSCAFG0000001670	RORA	grey	EC_M10	0.07	7.4E-01	0.47	1.6E-02	-0.55	3.4E-03	0.25	2.1E-01	0.34	9.0E-02	0.19	3.6E-01	-0.03	9.0E-01	-0.08	6.9E-01	0.06	7.7E-01	-0.39	4.7E-02
ENSCAFG0000000901	PPP1R16B	turquoise	EC_M6	0.07	7.4E-01	0.88	3.7E-09	-0.50	8.6E-03	-0.24	2.4E-01	0.23	2.7E-01	-0.03	9.0E-01	0.06	7.7E-01	0.09	6.8E-01	0.12	5.6E-01	-0.26	2.0E-01
ENSCAFG0000000323	ENSCAFG0000000323	grey	EC_M10	0.07	7.4E-01	-0.34	8.7E-02	0.14	4.9E-01	0.18	3.9E-01	-0.10	6.3E-01	-0.23	2.6E-01	0.08	7.0E-01	-0.23	2.5E-01	0.02	9.3E-01	0.03	9.0E-01
ENSCAFG0000000503	ACVR2B	turquoise	EC_M6	0.07	7.4E-01	0.65	3.5E-04	-0.37	6.2E-02	0.23	2.5E-01	0.18	3.9E-01	-0.03	8.7E-01	0.08	7.0E-01	0.13	5.2E-01	0.08	6.9E-01	-0.19	3.5E-01

ENSCAFG0000001782	ENSCAFG0000001782	grey	EC_M10	0.07	7.4E-01	0.26	1.9E-01	-0.29	1.5E-01	-0.18	3.8E-01	0.24	2.4E-01	-0.27	1.8E-01	0.31	1.2E-01	0.10	6.3E-01	-0.10	6.1E-01	-0.24	2.3E-01
ENSCAFG0000000655	ITPRIPL1	turquoise	EC_M6	0.07	7.4E-01	0.69	8.4E-05	-0.23	2.5E-01	-0.12	5.7E-01	-0.02	9.3E-01	-0.25	2.2E-01	0.15	4.6E-01	0.09	6.5E-01	0.51	7.2E-03	0.01	9.8E-01
ENSCAFG0000000343	IL4I1	cyan	EC_M2	0.07	7.4E-01	0.17	4.1E-01	0.55	3.6E-03	0.15	4.8E-01	-0.76	7.2E-06	-0.16	4.4E-01	0.01	9.6E-01	-0.01	9.6E-01	-0.04	8.4E-01	0.73	2.3E-05
ENSCAFG0000002082	ENSCAFG0000002082	grey	EC_M10	0.07	7.4E-01	-0.11	5.9E-01	0.22	2.9E-01	-0.09	6.7E-01	-0.15	4.7E-01	-0.05	8.1E-01	-0.04	8.4E-01	-0.09	6.7E-01	-0.08	7.1E-01	0.13	5.2E-01
ENSCAFG0000000953	ABCC2	grey	EC_M10	0.07	7.4E-01	-0.19	3.6E-01	0.32	1.2E-01	-0.09	6.7E-01	-0.27	1.8E-01	-0.08	7.1E-01	-0.08	7.1E-01	0.00	9.9E-01	-0.14	4.9E-01	0.27	1.7E-01
ENSCAFG0000001057	SLC6A3	grey	EC_M10	0.07	7.4E-01	-0.21	3.0E-01	-0.60	1.2E-03	-0.18	3.8E-01	0.70	7.8E-05	0.45	2.0E-02	0.00	1.0E+00	0.18	3.9E-01	-0.08	7.0E-01	-0.74	1.5E-05
ENSCAFG0000000264	ENSCAFG0000000264	darkgreen	EC_M4	0.07	7.4E-01	-0.67	1.6E-04	-0.29	1.5E-01	-0.16	4.4E-01	0.54	4.7E-03	0.26	2.1E-01	-0.04	8.5E-01	0.05	8.1E-01	-0.08	7.0E-01	-0.62	8.2E-04
ENSCAFG0000000417	PRDM5	grey	EC_M10	0.07	7.4E-01	-0.05	8.1E-01	-0.09	6.8E-01	-0.14	4.8E-01	0.00	9.8E-01	-0.31	1.2E-01	0.26	2.0E-01	0.16	4.4E-01	-0.17	4.1E-01	-0.05	8.0E-01
ENSCAFG0000001177	ENSCAFG0000001177	turquoise	EC_M6	0.07	7.4E-01	0.74	1.5E-05	-0.12	5.6E-01	-0.12	5.6E-01	-0.12	5.5E-01	-0.18	3.8E-01	-0.12	5.4E-01	0.14	5.1E-01	-0.11	5.9E-01	0.09	6.6E-01
ENSCAFG0000001925	CREBBP	cyan	EC_M2	0.07	7.4E-01	0.30	1.4E-01	0.56	2.8E-03	0.17	4.1E-01	-0.85	3.7E-08	-0.25	2.2E-01	-0.06	7.6E-01	-0.14	4.9E-01	-0.16	4.5E-01	0.80	8.2E-07
ENSCAFG0000000583	SATB1	grey	EC_M10	0.07	7.4E-01	0.33	9.6E-02	-0.52	6.7E-03	-0.06	7.8E-01	0.41	3.9E-02	-0.03	8.8E-01	-0.05	7.9E-01	0.08	7.1E-01	-0.28	1.6E-01	-0.47	1.7E-02
ENSCAFG0000001671	CPLX2	grey	EC_M10	0.07	7.4E-01	-0.38	5.8E-02	0.42	3.3E-02	0.18	3.8E-01	-0.42	3.4E-02	0.36	6.8E-02	0.00	1.0E+00	-0.29	1.5E-01	-0.09	6.6E-01	0.37	6.4E-02
ENSCAFG0000001317	VPS8	grey	EC_M10	0.07	7.4E-01	-0.14	4.9E-01	-0.47	1.7E-02	-0.06	7.7E-01	0.57	2.3E-03	-0.01	9.6E-01	-0.38	5.9E-02	-0.15	4.6E-01	-0.29	1.6E-01	-0.58	2.0E-03
ENSCAFG0000002883	RAB18	turquoise	EC_M6	0.07	7.4E-01	0.61	8.4E-04	-0.14	5.1E-01	-0.17	4.2E-01	-0.16	4.5E-01	0.12	5.6E-01	0.13	5.1E-01	-0.08	7.0E-01	0.01	9.5E-01	0.14	5.0E-01
ENSCAFG0000001319	TM6SF1	turquoise	EC_M6	0.07	7.4E-01	0.86	2.6E-08	-0.33	1.0E-01	-0.07	7.4E-01	0.05	7.9E-01	-0.05	8.2E-01	0.02	9.2E-01	-0.02	9.3E-01	0.02	9.2E-01	-0.05	8.0E-01
ENSCAFG0000003180	BBS10	grey	EC_M10	0.07	7.4E-01	0.06	7.7E-01	0.08	6.9E-01	0.04	8.3E-01	-0.15	4.7E-01	0.23	2.5E-01	-0.39	4.9E-02	0.00	9.9E-01	-0.03	9.0E-01	0.13	5.4E-01
ENSCAFG0000000901	ENSCAFG0000000901	turquoise	EC_M6	0.07	7.4E-01	0.43	2.8E-02	-0.08	7.0E-01	-0.05	7.9E-01	-0.18	3.9E-01	-0.14	4.8E-01	-0.11	5.9E-01	-0.16	4.4E-01	0.11	6.1E-01	0.12	5.6E-01
ENSCAFG0000001626	CGNL1	darkgreen	EC_M4	0.07	7.4E-01	0.20	3.2E-01	-0.72	3.3E-05	-0.02	9.2E-01	0.67	1.9E-04	0.11	5.9E-01	-0.20	3.3E-01	0.19	3.4E-01	-0.10	6.2E-01	-0.77	4.3E-06
ENSCAFG0000001928	ENSCAFG0000001928	grey	EC_M10	0.07	7.4E-01	-0.57	2.5E-03	-0.01	9.6E-01	-0.17	4.0E-01	0.18	3.8E-01	0.21	3.0E-01	0.15	4.8E-01	-0.17	4.1E-01	-0.09	6.6E-01	-0.21	3.0E-01
ENSCAFG0000003042	NSG2	grey	EC_M10	0.07	7.4E-01	-0.11	6.0E-01	-0.20	3.3E-01	-0.07	7.2E-01	0.14	5.0E-01	-0.09	6.6E-01	-0.05	8.1E-01	-0.13	5.4E-01	-0.12	5.7E-01	-0.23	2.7E-01
ENSCAFG0000000546	XRRA1	grey	EC_M10	0.07	7.4E-01	-0.09	6.7E-01	0.04	8.6E-01	0.12	5.5E-01	-0.05	8.0E-01	0.09	6.7E-01	0.14	5.1E-01	-0.39	4.9E-02	-0.09	6.5E-01	0.02	9.2E-01
ENSCAFG0000000823	LIMA1	grey	EC_M10	0.07	7.4E-01	-0.46	1.7E-02	0.00	9.9E-01	-0.06	7.7E-01	0.13	5.3E-01	0.05	8.0E-01	0.26	2.0E-01	-0.03	8.7E-01	-0.55	3.4E-03	-0.20	3.2E-01
ENSCAFG0000001645	LSM8	grey	EC_M10	0.07	7.4E-01	-0.02	9.3E-01	0.39	4.7E-02	0.09	6.8E-01	-0.53	5.3E-03	-0.25	2.1E-01	-0.15	4.8E-01	0.16	4.4E-01	0.11	6.0E-01	0.44	2.3E-02
ENSCAFG0000000164	TRERF1	cyan	EC_M2	0.07	7.4E-01	-0.29	1.4E-01	0.65	3.7E-04	0.13	5.4E-01	-0.73	2.6E-05	-0.27	1.9E-01	-0.18	3.7E-01	0.11	5.8E-01	-0.01	9.6E-01	0.69	1.1E-04
ENSCAFG0000000708	LSR	cyan	EC_M2	0.07	7.4E-01	-0.33	9.5E-02	0.52	6.0E-03	0.21	3.1E-01	-0.56	2.8E-03	0.10	6.3E-01	0.22	2.7E-01	-0.22	2.9E-01	-0.06	7.6E-01	0.53	5.1E-03
ENSCAFG0000002004	WDR34	grey	EC_M10	0.07	7.4E-01	-0.36	6.9E-02	0.51	7.9E-03	0.27	1.8E-01	-0.47	1.5E-02	-0.02	9.2E-01	-0.02	9.3E-01	-0.12	5.6E-01	0.17	4.0E-01	0.43	2.8E-02
ENSCAFG0000001937	CAMKK1	grey	EC_M10	0.07	7.5E-01	0.11	6.0E-01	-0.65	3.1E-04	-0.10	6.2E-01	0.68	1.2E-04	0.25	2.2E-01	-0.19	3.5E-01	0.01	9.5E-01	0.05	8.2E-01	-0.72	3.0E-05
ENSCAFG0000003194	ENSCAFG0000003194	grey	EC_M10	0.07	7.5E-01	-0.24	2.4E-01	0.48	1.4E-02	0.16	4.2E-01	-0.49	1.2E-02	-0.11	6.0E-01	-0.12	5.6E-01	-0.05	8.0E-01	-0.21	3.0E-01	0.48	1.3E-02
ENSCAFG0000002910	NOG	grey	EC_M10	0.07	7.5E-01	-0.29	1.6E-01	-0.20	3.2E-01	-0.37	6.4E-02	0.33	9.9E-02	0.08	6.9E-01	0.21	3.0E-01	0.10	6.4E-01	0.02	9.1E-01	-0.42	3.4E-02
ENSCAFG0000002502	TRIM6	turquoise	EC_M6	0.07	7.5E-01	0.73	2.7E-05	-0.41	4.0E-02	0.05	8.0E-01	0.15	4.8E-01	-0.02	9.2E-01	-0.09	6.6E-01	0.20	3.3E-01	0.18	3.8E-01	-0.16	4.4E-01
ENSCAFG0000002048	ENSCAFG0000002048	grey	EC_M10	0.07	7.5E-01	0.30	1.4E-01	0.12	5.7E-01	-0.23	2.7E-01	-0.28	1.6E-01	0.27	1.8E-01	-0.16	4.3E-01	0.17	4.2E-01	-0.18	3.8E-01	0.26	2.0E-01
ENSCAFG0000000245	ZNF268	grey	EC_M10	0.07	7.5E-01	0.29	1.5E-01	0.06	7.9E-01	-0.03	8.9E-01	-0.32	1.1E-01	-0.25	2.1E-01	0.12	5.7E-01	0.12	5.6E-01	0.27	1.7E-01	0.31	1.3E-01
ENSCAFG0000002815	ENSCAFG0000002815	grey	EC_M10	0.07	7.5E-01	-0.08	6.8E-01	0.19	3.4E-01	-0.26	1.9E-01	-0.26	2.1E-01	-0.21	3.0E-01	0.34	8.9E-02	-0.23	2.6E-01	-0.18	3.9E-01	0.24	2.3E-01
ENSCAFG0000001111	ENSCAFG0000001111	grey	EC_M10	0.07	7.5E-01	0.07	7.4E-01	-0.50	1.0E-02	-0.05	8.1E-01	0.59	1.6E-03	0.04	8.3E-01	-0.06	7.7E-01	0.14	4.8E-01	0.03	8.9E-01	-0.55	3.5E-03
ENSCAFG0000003253	RGS18	magenta	EC_M13	0.07	7.5E-01	0.05	8.1E-01	0.00	9.9E-01	-0.04	8.5E-01	-0.03	8.7E-01	0.00	1.0E+00	0.06	7.7E-01	-0.04	8.4E-01	0.74	1.5E-05	0.04	8.6E-01
ENSCAFG0000001320	ENSCAFG0000001320	grey	EC_M10	0.07	7.5E-01	0.04	8.3E-01	-0.19	3.5E-01	-0.02	9.2E-01	0.11	5.8E-01	0.09	6.6E-01	-0.05	8.1E-01	-0.43	2.9E-02	0.02	9.3E-01	-0.17	4.1E-01
ENSCAFG0000002001	GCSH	grey	EC_M10	0.07	7.5E-01	-0.50	9.0E-03	-0.39	4.7E-02	-0.21	3.1E-01	0.62	7.5E-04	0.23	2.6E-01	-0.20	3.3E-01	0.18	3.7E-01	-0.13	5.3E-01	-0.68	1.3E-04
ENSCAFG0000001312	ZNF512B	grey	EC_M10	0.07	7.5E-01	0.13	5.3E-01	0.28	1.6E-01	0.18	3.9E-01	-0.49	1.2E-02	0.02	9.4E-01	0.09	6.5E-01	-0.15	4.6E-01	0.59	1.5E-03	0.46	1.8E-02
ENSCAFG0000000917	UBTD1	turquoise	EC_M6	0.07	7.5E-01	0.69	9.5E-05	-0.52	5.9E-03	-0.04	8.3E-01	0.32	1.1E-01	0.09	6.7E-01	-0.12	5.7E-01	0.11	6.0E-01	0.27	1.7E-01	-0.37	6.4E-02
ENSCAFG0000001205	ZSCAN29	turquoise	EC_M6	0.07	7.5E-01	0.45	2.1E-02	-0.22	2.8E-01	0.12	5.7E-01	-0.07	7.4E-01	-0.01	9.7E-01	0.09	6.8E-01	-0.08	7.0E-01	0.33	1.0E-01	0.02	9.2E-01
ENSCAFG0000000418	HEPHL1	grey	EC_M10	0.07	7.5E-01	-0.10	6.2E-01	-0.30	1.4E-01	-0.06	7.6E-01	0.33	1.0E-01	0.53	5.8E-03	-0.09	6.7E-01	-0.14	5.0E-01	-0.14	5.1E-01	-0.39	4.7E-02
ENSCAFG0000001592	ENSCAFG0000001592	grey	EC_M10	0.07	7.5E-01	0.21	3.0E-01	-0.04	8.4E-01	-0.21	3.1E-01	-0.12	5.5E-01	-0.13	5.4E-01	0.26	2.0E-01	0.19	3.6E-01	-0.05	7.9E-01	0.06	7.7E-01
ENSCAFG0000000382	MATN3	grey	EC_M10	0.07	7.5E-01	-0.23	2.7E-01	-0.54	4.8E-03	-0.02	9.1E-01	0.65	3.7E-04	0.44	2.6E-02	-0.08	7.1E-01	-0.06	7.8E-01	0.06	7.8E-01	-0.67	2.0E-04
ENSCAFG0000003033	ENSCAFG0000003033	grey	EC_M10	0.07	7.5E-01	0.09	6.5E-01	-0.13	5.2E-01	0.03	8.8E-01	0.01	9.7E-01	-0.22	2.7E-01	-0.31	1.3E-01	0.27	1.8E-01	0.27	1.9E-01	-0.10	6.4E-01
ENSCAFG0000000743	ENSCAFG0000000743	grey	EC_M10	0.07	7.5E-01	0.31	1.3E-01	-0.34	9.4E-02	-0.04	8.5E-01	0.25	2.2E-01	0.24	2.4E-01	-0.24	2.3E-01	-0.63	5.8E-04	-0.01	9.8E-01	-0.29	1.5E-01
ENSCAFG0000000284	EPB41L4E	cyan	EC_M2	0.07	7.5E-01	-0.44	2.4E-02	0.41	4.0E-02	0.04	8.6E-01	-0.40	4.3E-02	-0.06	7.9E-01	-0.21	3.1E-01	-0.17	4.2E-01	-0.10	6.3E-01	0.35	8.4E-02
ENSCAFG0000000360	DLGAP3	turquoise	EC_M6	0.07	7.5E-01	0.61	9.2E-04	-0.13	5.2E-01	-0.11	5.9E-01	-0.14	4.9E-01	-0.09	6.6E-01	-0.04	8.3E-01	-0.01	9.6E-01	-0.08	7.1E-01	0.07	7.2E-01
ENSCAFG0000002022	TXNL4B	grey	EC_M10	0.07	7.5E-01	-0.15	4.6E-01	0.28	1.7E-01	0.23	2.7E-01	-0.35	8.3E-02	0.19	3.4E-01	-0.19	3.5E-01	-0.18	3.9E-01	-0.34	9.3E-02	0.32	1.1E-01
ENSCAFG0000003158	CLYBL	cyan	EC_M2	0.07	7.5E-01	-0.17	4.1E-01	0.62	8.1E-04	0.26	1.9E-01	-0.71	4.2E-05	-0.12	5.7E-01	-0.42	3.2E-02	0.09	6.5E-01	-0.01	9.5E-01	0.70	7.8E-05
ENSCAFG0000001584	PLD2	turquoise	EC_M6	0.07	7.5E-01	0.66	2.3E-04	0.06	7.8E-01	-0.10	6.2E-01	-0.39	5.0E-02	-0.25	2.1E-01	-0.05	8.2E-01	0.04	8.5E-01	0.50	1.0E-02	0.35	8.2E-02
ENSCAFG0000000815	ENSCAFG0000000815	grey	EC_M10	0.07	7.5E-01	-0.18	3.9E-01	-0.22	2.8E-01	0.21	3.0E-01	0.17	4.0E-01	0.20	3.3E-01	-0.14	5.0E-01	0.08	6.9E-01	0.21	3.1E-01	-0.26	2.0E-01
ENSCAFG0000003056	ENSCAFG0000003056	grey	EC_M10	0.07	7.5E-01	0.38	5.6E-02	0.03	8.8E-01	0.24	2.4E-01	-0.34	9.2E-02	0.09	6.6E-01	-0.11	5.8E-01	-0.01	9.7E-01	0.09	6.7E-01	0.30	1.3E-01
ENSCAFG0000003209	ENSCAFG0000003209	turquoise	EC_M6	0.07	7.5E-01	0.85	3.5E-08	-0.07	7.2E-01	-0.04	8.6E-01	-0.27	1.8E-01	-0.14	5.1E-01	-0.08	7.1E-01	0.06	7.6E-01	0.08	7.0E-01	0.26	1.9E-01
ENSCAFG0000001976	REXO4	grey	EC_M10	0.07	7.5E-01	-0.52	7.1E-03	-0.14	4.9E-01	0.15	4.7E-01	0.37	6.2E-02	0.15	4.8E-01	-0.29	1.5E-01	-0.16	4.3E-01	-0.27	1.8E-01	-0.38	5.3E-02
ENSCAFG0000000442	NSUN6	grey	EC_M10	0.06	7.5E-01	-0.32	1.1E-01	-0.41	3.8E-02	-0.01	9.7E-01	0.54	4.5E-03	0.11	6.0E-01	0.05	8.1E-01	0.30	1.3E-01	0.14	4.9E-01	-0.58	2.0E-03
ENSCAFG0000002910	ENSCAFG0000002910	grey	EC_M10	0.06	7.5E-01	0.24	2.3E-01	-0.42	3.5E-02	0.27	1.8E-01	0.33	9.5E-02	0.29	1.5E-01	-0.06	7.8E-01	-0.27	1.9E-01	-0.04	8.3E-01	-0.37	6.0E-02
ENSCAFG0000002972	TLCD1	grey	EC_M10	0.06	7.5E-01	-0.36	7.4E-02	-0.06	7.6E-01	0.14	4.9E-01	0.27	1.9E-01	0.09	6.5E-01	-0.15	4.6E-01	-0.11	6.1E-01	0.10	6.2E-01	-0.30	1.4E-01
ENSCAFG0000000949	KLHL8	grey	EC_M10	0.06	7.5E-01	0.16	4.3E-01	0.04	8.3E-01	0.23	2.7E-01	-0.25	2.3E-01	-0.22	2.7E-01	-0.09	6.5E-01	-0.04	8.6E-01	0.21	3.0E-01	0.22	2.8E-01
ENSCAFG0000002925	CDK13	turquoise	EC_M6	0.06	7.5E-01	0.46	1.8E-02	-0.41	3.8E-02	-0.23	2.7E-01	0.25	2.2E-01	0.29	1.5E-01	-0.17	4.0E-01	0.22	2.7E-01	-0.17	4.0E-01	-0.29	1.5E-01
ENSCAFG0000000180	DDX58	turquoise	EC_M6	0.06	7.5E-01	0.47	1.5E-02	0.04	8.4E-01	0.21	3.0E-01	-0.32	1.1E-01	0.00	9.9E-01	-0.07	7.3E-01	0.02	9.1E-01	0.48	1.3E-02	0.31	1.3E-01
ENSCAFG0000001306	ENSCAFG0000001306	grey	EC_M10	0.06	7.5E-01	0.52	6.2E-03	-0.72	3.2E-05	-0.02	9.1E-01	0.59	1.5E-03	0.06	7.8E-01	0.25	2.2E-01	0.15	4.8E-01	0.22	2.9E-01	-0.59	1.4E-03
ENSCAFG0000001998	NTMT1	grey	EC_M10	0.06	7.5E-01	-0.42	3.3E-02	-0.29	1.5E-01	-0.11	5.9E-01	0.47	1.4E-02	0.03	8.7E-01	0.21	3.1E-01	0.10	6.4E-01	0.03	9.0E-01	-0.52	6.1E-03
ENSCAFG0000001199	THEMIS2	grey	EC_M10	0.06	7.6E-01	-0.17	4.1E-01	0.29	1.5E-01	-0.13	5.4E-01	-0.36	6.8E-02	-0.11	5.8E-01	-0.08	7.1E-01	-0.19	3.5E-01	0.22	2.9E-01	0.33	1.0E-01
ENSCAFG0000000222	ENSCAFG0000000222	grey	EC_M10	0.06	7.6E-01	-0.24	2.5E-01	0.06	7.8E-01	-0.03	8.8E-01	-0.03	9.0E-01	-0.12	5.7E-01	-0.09	6.7E-01	-0.06	7.7E-01	-0.21	2.9E-01	-0.02	9.3E-01
ENSCAFG0000002837	ENSCAFG0000002837	turquoise	EC_M6	0.06	7.6E-01	0.55	3.3E-03	-0.10	6.4E-01	-0.11	6.0E-01	-0.14	4.8E-01	-0.07	7.2E-01	0.00	9.9E-01	-0.04	8.5E-01	0.47	1.5E-02	0.11	6.1E-01
ENSCAFG0000001859	ENSCAFG0000001859	grey	EC_M10	0.06	7.6E-01	-0.04	8.5E-01	0.24	2.3E-01	0.25	2.2E-01	-0.35	8.1E-02	-0.20	3.2E-01	0.08	6.9E-01	-0.11	5.8E-01	-0.15	4.7E-01	0.30	1.3E-01
ENSCAFG0000002950	GLO1	darkgreen	EC_M4	0.06	7.6E-01	-0.04	8.4E-01	-0.78	3.1E-06	-0.09	6.8E-01	0.86	2.6E-08	0.16	4.4E-01	-0.13	5.3E-01	0.18	3.9E-01	-0.03	9.0E-01	-0.89	1.1E-09
ENSCAFG0000000573	GOLGA7	grey	EC_M10	0.06	7.6E-01	0.37	6.6E-02	-0.19	3.6E-01	0.22	2.9E-01	-0.06	7.6E-01	0.23	2.6E-01	-0.13	5.4E-01	-0.14	5.0E-01	-0.44	2.6E-02	0.00	9.9E-01
ENSCAFG0000003049	ENSCAFG0000003049	grey	EC_M10	0.06	7.6E-01	0.14	4.9E-01	-0.13	5.2E-01	0.29	1.5E-01	0.08	7.0E-01	0.31	1.2E-01	0.29	1.5E-01	-0.14	4.8E-01	-0.25	2.2E-01	-0.10	6.3E-01
ENSCAFG0000001284	PPEF1	grey	EC_M10	0.06	7.6E-01	-0.11	6.1E-01	0.12	5.6E-01	0.14	4.9E-01	-0.26	1.9E-01	0.18	3.8E-01	-0.14	5.0E-01	-0.09	6.6E-01	0.07	7.3E-01	0.21	3.1E-01
ENSCAFG0000000572	CLPB	darkgreen	EC_M4	0.06	7.6E-01	-0.72	3.0E-05	0.17	4.2E-01	-0.11	5.9E-01	0.05	8.1E-01	0.24	2.5E-01	-0.08	6.9E-01	0.13	5.3E-01	0.04	8.4E-01	-0.09	6.7E-01
ENSCAFG0000002495	CFL1	darkgreen	EC_M4	0.06	7.6E-01	0.11	6.0E-01	-0.74	1.9E-05	-0.26	1.9E-01	0.83	1.9E-07	0.15	4.6E-01	-0.10	6.4E-01	0.15	4.5E-01	0.03	8.9E-01	-0.86	2.1E-08
ENSCAFG0000000107	WASHC5	grey	EC_M10	0.06	7.6E-01	-0.27	1.8E-01	-0.29	1.5E-01	0.20	3.2E-01	0.33	9.7E-02	0.25	2.1E-01	-0.49	1.1E-02	-0.23	2.5E-01	-0.38	5.9E-02	-0.40	4.5E-02
ENSCAFG0000001237	ENSCAFG0000001237	grey	EC_M10	0.06	7.6E-01	-0.06	7.8E-01	0.14	4.9E-01	-0.02	9.2E-01	-0.14	5.0E-01	0.02	9.2E-01	0.04	8.4E-01	-0.07	7.5E-01	-0.24	2.3E-01	0.08	7.0E-01
ENSCAFG0000001295	FANK1	grey	EC_M10	0.06	7.6E-01	-0.19	3.6E-01	-0.55	3.3E-03	-0.13	5.3E-01	0.67	1.9E-04	0.26	2.1E-01	-0.13	5.4E-01	0.04	8.5E-01	-0.26	1.9E-01	-0.73	2.2E-05
ENSCAFG0000002427	C31H21orf62	grey	EC_M10	0.06	7.6E-01	-0.05	8.2E-01	-0.32	1.1E-01	0.06	7.7E-01	0.28	1.6E-01	-0.08	6.9E-01	-0.03	9.0E-01	-0.06	7.7E-01	-0.12	5.7E-01	-0.38	5.4E-02
ENSCAFG0000002970	LPAR6	turquoise	EC_M6	0.06	7.6E-01	0.89	1.3E-09	-0.23	2.6E-01	-0.14	5.0E-01	-0.09	6.6E-01	-0.13	5.3E-01	-0.06	7.9E-01	-0.02	9.1E-01	0.00	9.8E-01	0.07	7.5E-01
ENSCAFG0000000953	SSR1	grey	EC_M10	0.06	7.6E-01	-0.34	8.9E-02	-0.62	7.2E-04	-0.11	6.0E-01	0.84	7.6E-08	0.28	1.6E-01	0.16	4.4E-01	0.09	6.6E-01	-0.20	3.2E-01	-0.88	3.2E-09
ENSCAFG0000001194	DTX3L	cyan	EC_M2	0.06	7.6E-01	0.35	7.9E-02	0.39	5.2E-02	0.24	2.4E-01	-0.69	1.0E-04	-0.11	6.1E-01	0.07	7.5E-01	0.08	7.0E-01	0.24	2.3E-01	0.64	4.0E-04
ENSCAFG0000002870	ENSCAFG0000002870	grey	EC_M10	0.06	7.6E-01	-0.08	6.9E-01	0.12	5.6E-01	0.00	9.9E-01	-0.13	5.3E-01	-0.08	6.9E-01	-0.08	6.9E-01	-0.03	8.7E-01	0.01	9.4E-01	0.08	6.9E-01
ENSCAFG0000002694	ENSCAFG0000002694	grey	EC_M10	0.06	7.6E-01	-0.11	5.8E-01	0.16	4.3E-01	-0.06	7.7E-01	-0.23	2.6E-01	-0.10	6.1E-01	-0.12	5.5E-01	-0.11	5.9E-01	-0.16	4.5E-01	0.16	4.3E-01
ENSCAFG0000002916	VMA21	grey	EC_M10	0.06	7.6E-01	0.01	9.8E-01	-0.36	7.1E-02	-0.18	3.9E-01	0.41	3.7E-02	0.02	9.1E-01	0.32	1.1E-01	0.36	7.5E-02	-0.11	5.8E-01	-0.44	2.4E-02
ENSCAFG0000000981	KCNJ15	turquoise	EC_M6	0.06	7.6E-01	0.88	2.0E-09	-0.24	2.3E-01	-0.14	4.9E-01	-0.04	8.6E-01	-0.12	5.6E-01	0.09	6.8E-01	-0.15	4.7E-01	-0.12	5.6E-01	0.02	9.3E-01
ENSCAFG0000000925	GALNT5	grey	EC_M10	0.06	7.6E-01	0.31	1.3E-01	-0.27	1.8E-01	-0.06	7.7E-01	0.15	4.5E-01	-0.06	7.8E-01	-0.10	6.2E-01	0.33	9.5E-02	0.59	1.6E-03	-0.16	4.3E-01
ENSCAFG0000000552	USP36	grey	EC_M10	0.06	7.6E-01	-0.25	2.2E-01	0.35	8.4E-02	0.22	2.8E-01	-0.33	1.0E-01	0.26	2.0E-01	-0.11	5.9E-01	-0.09	6.8E-01	0.17	4.2E-01	0.27	1.8E-01
ENSCAFG0000001514	ANKZF1	cyan	EC_M2	0.06	7.6E-01	0.30	1.4E-01	0.36	7.0E-02	0.27	1.9E-01	-0.65	3.6E-04	-0.08	7.1E-01	0.05	7.9E-01	0.12	5.6E-01	-0.21	3.0E-01	0.59	1.6E-03
ENSCAFG0000002036	SLC9A5	grey	EC_M10	0.06	7.6E-01	-0.33	1.0E-01	0.28	1.7E-01	-0.12	5.8E-01	-0.27	1.8E-01	-0.14	5.0E-01	0.29	1.5E-01	0.32	1.2E-01	0.43	2.7E-02	0.25	2.2E-01
ENSCAFG0000001369	ALS2CL	turquoise	EC_M6	0.06	7.6E-01	0.91	6.3E-11	-0.23	2.7E-01	-0.21	3.0E-01	-0.10	6.4E-01	-0.11	5.8E-01	0.10	6.3E-01	-0.15	4.7E-01	0.18	3.7E-01	0.09	6.8E-01
ENSCAFG0000000437	SOX9	grey	EC_M10	0.06	7.6E-01	-0.26	1.9E-01	-0.23	2.7E-01	0.06	7.5E-01	0.26	2.0E-01	0.08	6.9E-01	-0.11	6.0E-01	0.34	8.7E-02	0.56	3.0E-03	-0.32	1.1E-01
ENSCAFG0000000079	PDE10A	grey	EC_M10	0.06	7.6E-01	-0.36	7.0E-02	-0.48	1.2E-02	-0.14	5.0E-01	0.65	3.4E-04	0.18	3.7E-01	0.25	2.2E-01	0.07	7.2E-01	-0.41	4.0E-02	-0.71	4.1E-05
ENSCAFG0000001240	RBM14	grey	EC_M10	0.06	7.6E-01	0.23	2.6E-01	0.28	1.6E-01	-0.18	3.8E-01	-0.38	5.2E-02	-0.18	3.8E-01	-0.09	6.6E-01	-0.07	7.4E-01	-0.07	7.3E-01	0.32	1.1E-01
ENSCAFG0000000251	ITGB8	grey	EC_M10	0.06	7.6E-01	-0.08	7.0E-01	-0.54	4.5E-03	-0.18	3.8E-01	0.60	1.1E-03	0.09	6.6E-01	0.03	8.8E-01	0.17	4.1E-01	0.52	7.0E-03	-0.61	9.0E-04
ENSCAFG0000000388	DDO	magenta	EC_M13	0.06	7.6E-01	0.04	8.3E-01	0.05	8.2E-01	-0.06	7.8E-01	-0.12	5.6E-01	-0.03	8.7E-01	0.04	8.6E-01	-0.09	6.7E-01	0.82	3.7E-07	0.14	4.9E-01
ENSCAFG0000000164	GCNT1	grey	EC_M10	0.06	7.6E-01	-0.02	9.3E-01	0.02	9.2E-01	-0.17	4.0E-01	-0.05	8.0E-01	-0.18	3.7E-01	0.08	7.0E-01	0.29	1.6E-01	-0.08	7.1E-01	-0.04	8.3E-01
ENSCAFG0000001211	MYLK	grey	EC_M10	0.06	7.6E-01	-0.14	5.0E-01	-0.54	4.4E-03	-0.16	4.3E-01	0.66	2.7E-04	0.28	1.7E-01	0.09	6.7E-01	0.15	4.7E-01	0.05	8.0E-01	-0.69	8.7E-05
ENSCAFG0000001689	FBXW11	turquoise	EC_M6	0.06	7.7E-01	0.84	1.0E-07	-0.52	6.6E-03	-0.09	6.5E-01	0.23	2.7E-01	0.15	4.8E-01	0.11	5.9E-01	-0.15	4.7E-01	0.14	5.0E-01	-0.25	2.2E-01
ENSCAFG0000001686	HOXB9	turquoise	EC_M6	0.06	7.7E-01	0.81	5.8E-07	-0.25	2.2E-01	0.08	7.1E-01	-0.04	8.6E-01	-0.12	5.5E-01	-0.10	6.4E-01	-0.12	5.6E-01	0.05	8.1E-01	0.03	8.8E-01
ENSCAFG0000001830	AFAP1L1	turquoise	EC_M6	0.06	7.7E-01	0.85	2.7E-08	-0.16	4.3E-01	-0.12	5.6E-01	-0.15	4.7E-01	-0.11	5.9E-01	-0.12	5.7E-01	-0.16	4.3E-01	-0.08	7.1E-01	0.09	6.7E-01
ENSCAFG0000000042	ARMT1	grey	EC_M10	0.06	7.7E-01	-0.02	9.1E-01	-0.67	2.0E-04	-0.08	6.9E-01	0.74	1.8E-05	0.24	2.3E-01	-0.19	3.6E-01	-0.21	3.1E-01	0.01	9.7E-01	-0.78	2.7E-06
ENSCAFG0000001965	SEC16A	grey	EC_M10	0.06	7.7E-01	0.48	1.4E-02	-0.56	3.0E-03	-0.11	5.9E-01	0.40	4.2E-02	0.07	7.5E-01	-0.10	6.1E-01	-0.22	2.9E-01	-0.24	2.3E-01	-0.48	1.3E-02
ENSCAFG0000000547	CHRDL2	grey	EC_M10	0.06	7.7E-01	-0.09	6.5E-01	0.02	9.0E-01	-0.12	5.6E-01	-0.09	6.5E-01	-0.11	6.0E-01	-0.07	7.3E-01	0.53	5.0E-03	0.14	4.8E-01	0.10	6.2E-01
ENSCAFG0000002373	GDPGP1	grey	EC_M10	0.06	7.7E-01	-0.04	8.6E-01	0.36	7.0E-02	0.34	8.6E-02	-0.38	5.2E-02	0.07	7.3E-01	0.07	7.4E-01	0.12	5.5E-01	0.06	7.8E-01	0.36	7.3E-02
ENSCAFG0000002935	LNP1	grey	EC_M10	0.06	7.7E-01	-0.09	6.6E-01	-0.35	8.4E-02	-0.18	3.9E-01	0.43	3.0E-02	0.42	3.4E-02	0.08	7.1E-01	-0.21	3.1E-01	0.15	4.7E-01	-0.44	2.5E-02
ENSCAFG0000001872	ENSCAFG0000001872	grey	EC_M10	0.06	7.7E-01	0.32	1.1E-01	-0.27	1.7E-01	0.18	3.7E-01	0.18	3.9E-01	-0.18	3.9E-01	0.10	6.2E-01	-0.21	3.0E-01	0.18	3.8E-01	-0.17	4.0E-01
ENSCAFG0000000909	SDSL	grey	EC_M10	0.06	7.7E-01	0.27	1.9E-01	-0.36	6.9E-02	-0.30	1.3E-01	0.30	1.3E-01	0.10	6.1E-01	0.19	3.6E-01	0.24	2.3E-01	0.50	9.8E-03	-0.26	1.9E-01
ENSCAFG0000001084	SLC30A10	grey	EC_M10	0.06	7.7E-01	-0.11	5.8E-01	-0.17	3.9E-01	-0.08	6.9E-01	0.15	4.6E-01	0.58	1.8E-03	0.41	3.8E-02	-0.08	7.0E-01	-0.14	4.9E-01	-0.20	3.2E-01
ENSCAFG0000002591	ENSCAFG0000002591	grey	EC_M10	0.06	7.7E-01	-0.11	5.8E-01	-0.17	3.9E-01	-0.08	6.9E-01	0.15	4.6E-01	0.58	1.8E-03	0.41	3.8E-02	-0.08	7.0E-01	-0.14	4.9E-01	-0.20	3.2E-01
ENSCAFG0000001294	TACO1	grey	EC_M10	0.06	7.7E-01	0.23	2.6E-01	-0.41	3.6E-02	0.09	6.4E-01	0.34	9.2E-02	0.00	9.9E-01	0.36	6.9E-02	-0.08	7.1E-01	-0.09	6.7E-01	-0.35	8.2E-02
ENSCAFG0000001813	SYNRG	grey	EC_M10	0.06	7.7E-01	0.10	6.1E-01	0.39	5.0E-02	0.17	3.9E-01	-0.55	3.4E-03	-0.11	5.9E-01	-0.15	4.8E-01	-0.24	2.4E-01	-0.40	4.4E-02	0.53	5.8E-03
ENSCAFG0000000222	PTPDC1	grey	EC_M10	0.06	7.7E-01	0.33	9.9E-02	-0.36	7.5E-02	0.23	2.6E-01	0.24	2.3E-01	0.17	4.0E-01	0.09	6.6E-01	-0.07	7.3E-01	0.42	3.2E-02	-0.24	2.3E-01
ENSCAFG0000001393	FAM20B	grey	EC_M10	0.06	7.7E-01	0.20	3.2E-01	-0.56	2.7E-03	-0.19	3.5E-01	0.56	2.9E-03	-0.03	8.7E-01	0.05	8.1E-01	0.13	5.2E-01	0.47	1.5E-02	-0.51	8.0E-03
ENSCAFG0000001586	NT5C3B	grey	EC_M10	0.06	7.7E-01	0.23	2.5E-01	-0.18	3.9E-01	-0.27	1.8E-01	0.16	4.5E-01	-0.24	2.3E-01	-0.24	2.4E-01	0.23	2.6E-01	0.08	7.1E-01	-0.09	6.8E-01
ENSCAFG0000001539	ENSCAFG0000001539	grey	EC_M10	0.06	7.7E-01	-0.13	5.4E-01	-0.50	9.5E-03	-0.12	5.8E-01	0.56	3.0E-03	0.35	8.1E-02	-0.11	5.9E-01	0.12	5.6E-01	-0.03	8.8E-01	-0.58	1.8E-03
ENSCAFG0000000527	TMC8	turquoise	EC_M6	0.06	7.7E-01	0.82	3.4E-07	-0.28	1.7E-01	-0.05	8.0E-01	-0.01	9.5E-01	-0.06	7.7E-01	-0.08	7.0E-01	-0.08	6.9E-01	-0.10	6.4E-01	0.02	9.4E-01
ENSCAFG0000001104	MAATS1	grey	EC_M10	0.06	7.7E-01	-0.12	5.7E-01	0.30	1.3E-01	0.27	1.8E-01	-0.33	1.0E-01	0.04	8.6E-01	0.15	4.5E-01	-0.01	9.6E-01	0.14	4.9E-01	0.36	7.5E-02
ENSCAFG0000001022	DOCK3	grey	EC_M10	0.06	7.7E-01	0.13	5.4E-01	-0.58	1.8E-03	-0.29	1.6E-01	0.52	6.0E-03	-0.08	7.0E-01	-0.21	3.0E-01	0.13	5.2E-01	0.31	1.2E-01	-0.57	2.4E-03

ENSCAFG0000002532	SOX17	turquoise	EC_M6	0.06	7.7E-01	0.85	4.8E-08	-0.22	2.8E-01	-0.23	2.6E-01	-0.06	7.7E-01	-0.12	5.7E-01	-0.09	6.8E-01	-0.12	5.7E-01	0.17	4.2E-01	0.03	8.8E-01
ENSCAFG0000002217	ENSCAFG0000002217	grey	EC_M10	0.06	7.7E-01	0.18	3.7E-01	0.00	1.0E+00	0.57	2.3E-03	-0.13	5.3E-01	0.12	5.7E-01	-0.16	4.2E-01	-0.20	3.3E-01	-0.35	7.9E-02	0.07	7.5E-01
ENSCAFG0000003033	XKR5	grey	EC_M10	0.06	7.7E-01	-0.09	6.7E-01	-0.04	8.6E-01	-0.07	7.4E-01	-0.04	8.6E-01	-0.04	8.3E-01	-0.11	6.0E-01	-0.11	5.8E-01	-0.14	5.1E-01	-0.07	7.5E-01
ENSCAFG0000000027	ARFGEF3	grey	EC_M10	0.06	7.7E-01	0.42	3.1E-02	-0.58	1.8E-03	-0.24	2.4E-01	0.50	9.7E-03	-0.14	4.9E-01	0.23	2.7E-01	0.11	5.9E-01	0.03	8.7E-01	-0.55	3.3E-03
ENSCAFG0000000673	PXDNL	grey	EC_M10	0.06	7.7E-01	0.08	7.1E-01	-0.07	7.2E-01	-0.05	8.1E-01	0.03	9.0E-01	0.02	9.2E-01	-0.03	8.7E-01	-0.23	2.6E-01	0.34	9.1E-02	0.00	9.9E-01
ENSCAFG0000000821	FHDC1	turquoise	EC_M6	0.06	7.7E-01	0.45	2.2E-02	-0.19	3.5E-01	-0.41	4.0E-02	0.05	7.9E-01	0.09	6.6E-01	0.02	9.2E-01	-0.39	4.9E-02	-0.13	5.3E-01	-0.06	7.6E-01
ENSCAFG0000000016	DMXL1	turquoise	EC_M6	0.06	7.7E-01	0.32	1.1E-01	-0.18	3.8E-01	0.20	3.2E-01	0.06	7.7E-01	-0.15	4.8E-01	-0.24	2.5E-01	-0.24	2.4E-01	-0.24	2.4E-01	-0.03	9.0E-01
ENSCAFG0000002342	ENSCAFG0000002342	darkgreen	EC_M4	0.06	7.7E-01	-0.57	2.3E-03	-0.35	8.2E-02	0.11	5.8E-01	0.53	5.1E-03	0.28	1.7E-01	0.18	3.8E-01	0.08	6.9E-01	-0.05	8.0E-01	-0.62	7.6E-04
ENSCAFG0000000157	GNAQ	darkgreen	EC_M4	0.06	7.7E-01	0.21	3.0E-01	-0.76	8.2E-06	-0.24	2.4E-01	0.78	3.0E-06	0.12	5.6E-01	0.00	1.0E+00	0.01	9.5E-01	0.29	1.5E-01	-0.77	4.1E-06
ENSCAFG0000001537	CPEB2	turquoise	EC_M6	0.06	7.7E-01	0.55	3.9E-03	-0.28	1.6E-01	-0.12	5.6E-01	0.07	7.4E-01	-0.13	5.4E-01	-0.01	9.8E-01	-0.29	1.6E-01	-0.27	1.8E-01	-0.04	8.4E-01
ENSCAFG0000000249	ANKS6	grey	EC_M10	0.06	7.7E-01	-0.03	8.8E-01	-0.16	4.2E-01	-0.28	1.6E-01	0.14	4.8E-01	0.09	6.8E-01	0.12	5.6E-01	-0.01	9.6E-01	-0.11	5.9E-01	-0.20	3.2E-01
ENSCAFG0000000520	CTNNB1	darkgreen	EC_M4	0.06	7.7E-01	0.54	4.7E-03	-0.81	6.7E-07	-0.21	3.0E-01	0.70	7.5E-05	0.20	3.4E-01	-0.05	7.9E-01	0.00	1.0E+00	0.09	6.5E-01	-0.72	2.9E-05
ENSCAFG0000000241	ENSCAFG0000000241	grey	EC_M10	0.06	7.7E-01	0.13	5.2E-01	-0.18	3.8E-01	0.32	1.1E-01	0.11	5.9E-01	-0.23	2.7E-01	0.33	9.5E-02	-0.12	5.6E-01	-0.22	2.8E-01	-0.16	4.2E-01
ENSCAFG0000001261	KCNK2	grey	EC_M10	0.06	7.8E-01	0.10	6.4E-01	-0.17	4.1E-01	0.00	9.9E-01	0.16	4.3E-01	-0.16	4.3E-01	-0.13	5.4E-01	-0.11	6.1E-01	-0.13	5.2E-01	-0.22	2.7E-01
ENSCAFG0000001704	BATF	turquoise	EC_M6	0.06	7.8E-01	0.65	3.1E-04	-0.30	1.4E-01	-0.16	4.2E-01	0.05	8.2E-01	-0.15	4.8E-01	-0.12	5.5E-01	-0.17	4.1E-01	0.18	3.8E-01	-0.07	7.5E-01
ENSCAFG0000002444	ENSCAFG0000002444	grey	EC_M10	0.06	7.8E-01	-0.07	7.3E-01	-0.08	6.8E-01	-0.08	7.0E-01	0.02	9.4E-01	-0.12	5.5E-01	-0.05	8.0E-01	0.33	1.0E-01	-0.23	2.6E-01	-0.07	7.5E-01
ENSCAFG0000000313	UGP2	cyan	EC_M2	0.06	7.8E-01	-0.54	4.8E-03	0.58	1.8E-03	0.22	2.9E-01	-0.58	1.8E-03	-0.13	5.2E-01	-0.01	9.6E-01	0.01	9.7E-01	-0.23	2.5E-01	0.54	4.6E-03
ENSCAFG0000000311	WDPCP	grey	EC_M10	0.06	7.8E-01	0.06	7.8E-01	0.01	9.8E-01	0.26	2.0E-01	0.00	9.8E-01	-0.25	2.1E-01	0.04	8.3E-01	-0.47	1.6E-02	-0.18	3.9E-01	-0.02	9.3E-01
ENSCAFG0000000256	COL19A1	grey	EC_M10	0.06	7.8E-01	-0.14	5.1E-01	-0.27	1.8E-01	0.05	8.1E-01	0.26	2.1E-01	-0.08	6.8E-01	-0.03	8.8E-01	-0.08	7.0E-01	-0.16	4.4E-01	-0.34	9.2E-02
ENSCAFG0000001002	ENSCAFG0000001002	grey	EC_M10	0.06	7.8E-01	-0.10	6.2E-01	0.05	8.3E-01	-0.24	2.3E-01	-0.01	9.6E-01	0.08	7.0E-01	-0.11	5.8E-01	-0.33	1.0E-01	0.08	6.8E-01	-0.01	9.7E-01
ENSCAFG0000000035	LTC4S	grey	EC_M10	0.06	7.8E-01	-0.16	4.3E-01	0.26	2.0E-01	-0.09	6.6E-01	-0.32	1.1E-01	-0.01	9.7E-01	-0.09	6.4E-01	-0.10	6.3E-01	-0.16	4.3E-01	0.27	1.8E-01
ENSCAFG0000000955	ENSCAFG0000000955	grey	EC_M10	0.06	7.8E-01	0.11	6.0E-01	-0.12	5.7E-01	-0.14	4.8E-01	0.05	8.2E-01	-0.14	5.0E-01	-0.13	5.3E-01	-0.08	6.9E-01	0.31	1.2E-01	-0.04	8.6E-01
ENSCAFG0000000405	CABP5	grey	EC_M10	0.06	7.8E-01	-0.27	1.8E-01	0.02	9.1E-01	-0.12	5.7E-01	-0.02	9.3E-01	0.23	2.5E-01	0.09	6.6E-01	0.38	5.4E-02	-0.16	4.3E-01	-0.07	7.5E-01
ENSCAFG0000001067	EIF3I	grey	EC_M10	0.06	7.8E-01	-0.49	1.1E-02	-0.12	5.5E-01	-0.16	4.3E-01	0.37	6.1E-02	0.15	4.8E-01	-0.16	4.5E-01	0.05	8.0E-01	-0.13	5.4E-01	-0.43	3.0E-02
ENSCAFG0000001926	TRAP1	grey	EC_M10	0.06	7.8E-01	-0.51	8.4E-03	0.28	1.6E-01	-0.04	8.5E-01	-0.07	7.4E-01	-0.14	5.1E-01	0.01	9.6E-01	-0.04	8.3E-01	-0.09	6.6E-01	0.08	6.9E-01
ENSCAFG0000001973	TMEM201	grey	EC_M10	0.06	7.8E-01	0.34	9.1E-02	0.19	3.6E-01	-0.08	6.8E-01	-0.34	9.4E-02	-0.25	2.1E-01	0.16	4.3E-01	-0.13	5.4E-01	0.24	2.3E-01	0.34	8.5E-02
ENSCAFG0000000689	TUB	grey	EC_M10	0.06	7.8E-01	-0.10	6.3E-01	-0.38	5.6E-02	-0.10	6.3E-01	0.38	5.5E-02	-0.12	5.7E-01	-0.11	6.1E-01	0.39	4.6E-02	0.14	4.9E-01	-0.46	1.8E-02
ENSCAFG0000001416	IDH3A	grey	EC_M10	0.06	7.8E-01	-0.37	6.3E-02	0.38	5.6E-02	-0.09	6.5E-01	-0.24	2.4E-01	-0.01	9.7E-01	-0.19	3.6E-01	-0.13	5.2E-01	-0.02	9.1E-01	0.28	1.7E-01
ENSCAFG0000001843	CRIP2	grey	EC_M10	0.06	7.8E-01	0.46	1.8E-02	0.42	3.4E-02	-0.08	7.1E-01	-0.66	2.5E-04	-0.24	2.4E-01	-0.27	1.9E-01	-0.08	7.0E-01	0.02	9.4E-01	0.66	2.5E-04
ENSCAFG0000001202	PPP1R8	grey	EC_M10	0.06	7.8E-01	-0.13	5.3E-01	-0.58	1.9E-03	-0.16	4.4E-01	0.71	5.6E-05	0.27	1.8E-01	-0.08	6.9E-01	0.17	4.0E-01	-0.14	5.0E-01	-0.71	4.4E-05
ENSCAFG0000001045	S100PBP	grey	EC_M10	0.06	7.8E-01	0.34	9.4E-02	-0.40	4.2E-02	0.19	3.5E-01	0.20	3.3E-01	-0.01	9.7E-01	0.28	1.7E-01	-0.12	5.4E-01	-0.10	6.4E-01	-0.25	2.2E-01
ENSCAFG0000001388	RTP4	turquoise	EC_M6	0.06	7.8E-01	0.63	5.3E-04	-0.04	8.6E-01	-0.14	4.8E-01	-0.21	3.0E-01	-0.13	5.3E-01	-0.14	4.9E-01	-0.12	5.7E-01	0.08	6.9E-01	0.16	4.3E-01
ENSCAFG0000000793	TRPC1	grey	EC_M10	0.06	7.8E-01	-0.12	5.7E-01	-0.58	1.8E-03	-0.16	4.5E-01	0.61	9.9E-04	0.31	1.2E-01	0.21	3.1E-01	0.24	2.3E-01	0.31	1.3E-01	-0.67	2.0E-04
ENSCAFG0000000113	ST13	grey	EC_M10	0.06	7.8E-01	0.24	2.5E-01	-0.41	3.6E-02	-0.29	1.5E-01	0.31	1.3E-01	0.30	1.4E-01	0.29	1.5E-01	-0.06	7.5E-01	-0.34	9.1E-02	-0.39	4.8E-02
ENSCAFG0000000301	ENSCAFG0000000301	darkgreen	EC_M4	0.06	7.8E-01	-0.17	4.2E-01	-0.73	2.7E-05	-0.22	2.7E-01	0.86	2.2E-08	0.14	4.9E-01	0.07	7.3E-01	0.17	4.0E-01	-0.03	8.8E-01	-0.89	9.5E-10
ENSCAFG0000001276	ABRAXAS2	darkgreen	EC_M4	0.06	7.8E-01	-0.09	6.7E-01	-0.70	6.2E-05	-0.17	4.2E-01	0.78	2.9E-06	0.43	2.9E-02	-0.17	4.1E-01	-0.05	8.2E-01	-0.13	5.2E-01	-0.82	3.1E-07
ENSCAFG0000000595	DPF1	grey	EC_M10	0.06	7.8E-01	-0.67	1.8E-04	0.12	5.6E-01	-0.15	4.6E-01	0.08	7.2E-01	-0.09	6.7E-01	0.27	1.8E-01	0.24	2.4E-01	-0.16	4.4E-01	-0.10	6.2E-01
ENSCAFG0000000439	ENSCAFG0000000439	grey	EC_M10	0.06	7.8E-01	0.26	2.0E-01	-0.31	1.2E-01	-0.18	3.8E-01	0.25	2.1E-01	0.19	3.6E-01	0.08	6.8E-01	0.24	2.3E-01	-0.16	4.3E-01	-0.32	1.2E-01
ENSCAFG0000001290	THEM5	grey	EC_M10	0.06	7.8E-01	0.11	6.1E-01	0.24	2.4E-01	0.24	2.4E-01	-0.40	4.5E-02	0.26	2.1E-01	-0.23	2.6E-01	0.32	1.1E-01	-0.20	3.4E-01	0.36	7.5E-02
ENSCAFG0000000088	DEPTOR	cyan	EC_M2	0.06	7.8E-01	-0.27	1.8E-01	0.37	6.6E-02	0.28	1.7E-01	-0.38	5.2E-02	0.12	5.5E-01	0.08	6.9E-01	-0.25	2.2E-01	-0.40	4.6E-02	0.32	1.1E-01
ENSCAFG0000000600	ENSCAFG0000000600	grey	EC_M10	0.06	7.8E-01	0.08	6.8E-01	-0.18	3.8E-01	0.23	2.6E-01	0.17	4.2E-01	-0.27	1.9E-01	-0.24	2.3E-01	-0.20	3.2E-01	-0.22	2.7E-01	-0.18	3.7E-01
ENSCAFG0000001916	MYO1C	darkgreen	EC_M4	0.06	7.8E-01	0.25	2.2E-01	-0.78	3.2E-06	-0.32	1.1E-01	0.81	4.9E-07	0.05	8.1E-01	0.02	9.1E-01	0.13	5.4E-01	0.19	3.5E-01	-0.82	3.2E-07
ENSCAFG0000000397	ENSCAFG0000000397	turquoise	EC_M6	0.06	7.8E-01	0.62	7.0E-04	-0.32	1.1E-01	-0.12	5.5E-01	0.13	5.2E-01	-0.11	5.9E-01	-0.08	6.9E-01	-0.06	7.6E-01	-0.07	7.4E-01	-0.15	4.8E-01
ENSCAFG0000001441	ASCC1	grey	EC_M10	0.06	7.8E-01	-0.37	6.7E-02	0.22	2.9E-01	0.14	5.0E-01	-0.21	3.0E-01	0.24	2.3E-01	-0.19	3.5E-01	-0.42	3.5E-02	-0.40	4.5E-02	0.16	4.4E-01
ENSCAFG0000001781	ANKS4B	grey	EC_M10	0.06	7.8E-01	0.20	3.4E-01	0.26	2.0E-01	-0.04	8.5E-01	-0.44	2.3E-02	-0.45	2.1E-02	0.31	1.2E-01	0.06	7.6E-01	-0.36	6.9E-02	0.42	3.1E-02
ENSCAFG0000001503	CPSF4	grey	EC_M10	0.06	7.8E-01	0.12	5.7E-01	0.37	6.4E-02	0.03	8.9E-01	-0.47	1.5E-02	0.13	5.2E-01	0.05	8.0E-01	-0.42	3.3E-02	-0.01	9.8E-01	0.40	4.3E-02
ENSCAFG0000003144	FAM237B	grey	EC_M10	0.06	7.8E-01	-0.32	1.1E-01	-0.25	2.2E-01	-0.11	5.9E-01	0.41	4.0E-02	0.08	6.9E-01	-0.19	3.5E-01	-0.10	6.1E-01	-0.47	1.5E-02	-0.41	3.8E-02
ENSCAFG0000002003	SPOUT1	grey	EC_M10	0.06	7.8E-01	-0.40	4.0E-02	0.29	1.6E-01	-0.24	2.4E-01	-0.09	6.6E-01	-0.10	6.3E-01	-0.08	6.8E-01	-0.08	7.0E-01	0.00	9.9E-01	0.11	6.1E-01
ENSCAFG0000001414	MED14	grey	EC_M10	0.06	7.8E-01	-0.21	3.0E-01	0.12	5.4E-01	-0.27	1.8E-01	-0.10	6.2E-01	0.03	8.7E-01	-0.19	3.6E-01	0.37	6.1E-02	-0.29	1.5E-01	0.06	7.6E-01
ENSCAFG0000002329	HTATSF1	grey	EC_M10	0.06	7.9E-01	0.38	5.5E-02	-0.41	3.5E-02	-0.13	5.2E-01	0.26	2.1E-01	0.08	6.9E-01	0.24	2.3E-01	0.35	7.9E-02	0.36	7.0E-02	-0.22	2.8E-01
ENSCAFG0000002968	ENSCAFG0000002968	violet	EC_M7	0.06	7.9E-01	-0.09	6.5E-01	-0.04	8.3E-01	-0.09	6.7E-01	0.02	9.3E-01	0.68	1.3E-04	-0.03	8.7E-01	-0.11	5.9E-01	-0.16	4.3E-01	-0.08	7.1E-01
ENSCAFG0000000924	ENSCAFG0000000924	grey	EC_M10	0.06	7.9E-01	-0.13	5.2E-01	-0.06	7.6E-01	-0.16	4.4E-01	0.13	5.1E-01	0.50	8.7E-03	-0.10	6.4E-01	-0.13	5.4E-01	-0.09	6.7E-01	-0.14	4.8E-01
ENSCAFG0000002015	ENSCAFG0000002015	grey	EC_M10	0.06	7.9E-01	-0.24	2.3E-01	-0.13	5.2E-01	-0.12	5.5E-01	0.12	5.7E-01	0.24	2.3E-01	0.10	6.3E-01	0.31	1.2E-01	-0.01	9.6E-01	-0.18	3.7E-01
ENSCAFG0000002906	ENSCAFG0000002906	grey	EC_M10	0.06	7.9E-01	0.28	1.7E-01	-0.48	1.2E-02	0.19	3.5E-01	0.40	4.3E-02	0.14	4.9E-01	-0.26	2.0E-01	0.25	2.2E-01	0.24	2.4E-01	-0.40	4.1E-02
ENSCAFG0000000471	FAM107B	grey	EC_M10	0.06	7.9E-01	-0.10	6.4E-01	-0.45	2.2E-02	-0.29	1.5E-01	0.50	8.7E-03	0.13	5.1E-01	0.18	3.8E-01	0.36	6.7E-02	0.33	9.8E-02	-0.58	1.9E-03
ENSCAFG0000000066	ENSCAFG0000000066	grey	EC_M10	0.06	7.9E-01	-0.05	8.2E-01	-0.03	8.7E-01	-0.22	2.8E-01	0.06	7.6E-01	-0.19	3.5E-01	-0.16	4.3E-01	-0.21	3.1E-01	-0.15	4.6E-01	-0.08	7.0E-01
ENSCAFG0000002030	CTBS	grey	EC_M10	0.06	7.9E-01	-0.11	5.8E-01	-0.44	2.4E-02	-0.12	5.6E-01	0.51	7.8E-03	0.57	2.2E-03	-0.07	7.3E-01	-0.06	7.9E-01	-0.07	7.5E-01	-0.52	6.0E-03
ENSCAFG0000001779	ENSCAFG0000001779	grey	EC_M10	0.06	7.9E-01	0.29	1.6E-01	-0.61	8.6E-04	-0.03	8.8E-01	0.48	1.2E-02	0.13	5.4E-01	0.14	4.9E-01	0.24	2.3E-01	-0.01	9.7E-01	-0.56	3.0E-03
ENSCAFG0000003176	ENSCAFG0000003176	grey	EC_M10	0.06	7.9E-01	-0.36	6.7E-02	-0.25	2.2E-01	0.15	4.7E-01	0.30	1.3E-01	0.30	1.3E-01	-0.13	5.3E-01	0.27	1.8E-01	0.23	2.6E-01	-0.36	7.4E-02
ENSCAFG0000000551	POLB	grey	EC_M10	0.06	7.9E-01	0.01	9.6E-01	-0.57	2.5E-03	-0.31	1.3E-01	0.62	7.1E-04	0.19	3.6E-01	-0.18	3.9E-01	0.01	9.7E-01	-0.18	3.7E-01	-0.67	1.8E-04
ENSCAFG0000001295	ENSCAFG0000001295	grey	EC_M10	0.06	7.9E-01	-0.12	5.7E-01	-0.40	4.3E-02	-0.14	4.8E-01	0.46	1.9E-02	0.57	2.3E-03	-0.10	6.2E-01	0.50	9.0E-03	0.04	8.6E-01	-0.47	1.5E-02
ENSCAFG0000001925	MRPL20	grey	EC_M10	0.06	7.9E-01	-0.40	4.1E-02	0.26	1.9E-01	-0.10	6.2E-01	-0.11	6.0E-01	-0.08	6.9E-01	-0.01	9.8E-01	0.11	6.1E-01	0.15	4.6E-01	0.07	7.4E-01
ENSCAFG0000000863	XRCC4	grey	EC_M10	0.06	7.9E-01	-0.43	2.8E-02	-0.49	1.2E-02	-0.05	8.2E-01	0.65	3.0E-04	0.06	7.8E-01	0.10	6.4E-01	0.34	8.9E-02	0.08	7.0E-01	-0.68	1.3E-04
ENSCAFG0000000677	ADAMTS9	turquoise	EC_M6	0.06	7.9E-01	0.83	2.1E-07	-0.18	3.7E-01	-0.04	8.6E-01	-0.10	6.1E-01	-0.10	6.3E-01	-0.17	4.2E-01	-0.10	6.1E-01	0.07	7.2E-01	0.05	7.9E-01
ENSCAFG0000000724	GNPTAB	grey	EC_M10	0.06	7.9E-01	-0.48	1.4E-02	0.20	3.2E-01	0.12	5.7E-01	-0.17	4.2E-01	-0.24	2.5E-01	0.05	8.2E-01	0.00	9.9E-01	-0.37	6.5E-02	0.12	5.7E-01
ENSCAFG0000002851	ENSCAFG0000002851	grey	EC_M10	0.06	7.9E-01	0.34	8.5E-02	-0.09	6.8E-01	0.19	3.6E-01	-0.12	5.5E-01	0.02	9.4E-01	-0.21	3.0E-01	0.21	3.1E-01	0.34	9.3E-02	0.14	4.9E-01
ENSCAFG0000002939	BASP1	grey	EC_M10	0.06	7.9E-01	0.33	9.8E-02	-0.52	6.7E-03	0.08	6.9E-01	0.47	1.6E-02	0.10	6.1E-01	0.03	8.9E-01	-0.14	4.8E-01	-0.32	1.1E-01	-0.52	6.3E-03
ENSCAFG0000001873	VAPA	grey	EC_M10	0.06	7.9E-01	0.12	5.6E-01	-0.69	1.1E-04	-0.26	2.0E-01	0.74	1.3E-05	0.19	3.6E-01	0.20	3.3E-01	-0.09	6.7E-01	-0.41	3.9E-02	-0.78	2.5E-06
ENSCAFG0000000763	CLSTN2	grey	EC_M10	0.06	7.9E-01	0.15	4.6E-01	0.00	9.9E-01	-0.01	9.4E-01	-0.09	6.8E-01	-0.10	6.4E-01	-0.06	7.8E-01	-0.10	6.3E-01	0.52	6.3E-03	0.02	9.1E-01
ENSCAFG0000000132	ATF4	turquoise	EC_M6	0.06	7.9E-01	0.50	9.5E-03	-0.23	2.5E-01	0.25	2.1E-01	-0.01	9.7E-01	0.20	3.2E-01	0.05	7.9E-01	-0.16	4.4E-01	0.04	8.5E-01	-0.08	6.8E-01
ENSCAFG0000001153	ZKSCAN8	grey	EC_M10	0.06	7.9E-01	-0.05	8.0E-01	0.13	5.2E-01	0.53	5.8E-03	-0.30	1.3E-01	-0.14	4.8E-01	0.03	8.8E-01	0.20	3.3E-01	0.16	4.4E-01	0.26	2.0E-01
ENSCAFG0000001026	MANF	grey	EC_M10	0.05	7.9E-01	-0.19	3.6E-01	-0.66	2.7E-04	-0.16	4.3E-01	0.83	1.4E-07	0.35	7.7E-02	-0.02	9.3E-01	0.13	5.4E-01	0.06	7.8E-01	-0.88	3.4E-09
ENSCAFG0000001060	ITPRIP	turquoise	EC_M6	0.05	7.9E-01	0.65	3.6E-04	-0.54	4.8E-03	-0.24	2.4E-01	0.41	3.9E-02	-0.04	8.6E-01	0.21	3.0E-01	-0.16	4.4E-01	0.18	3.7E-01	-0.40	4.2E-02
ENSCAFG0000001229	FES	turquoise	EC_M6	0.05	7.9E-01	0.77	4.7E-06	-0.12	5.6E-01	0.18	3.8E-01	-0.26	2.0E-01	-0.20	3.2E-01	0.04	8.6E-01	-0.08	7.0E-01	0.22	2.8E-01	0.20	3.4E-01
ENSCAFG0000000750	AHCY	grey	EC_M10	0.05	7.9E-01	-0.62	7.5E-04	-0.21	3.1E-01	-0.16	4.2E-01	0.47	1.5E-02	0.00	9.9E-01	-0.19	3.5E-01	0.23	2.5E-01	-0.05	8.2E-01	-0.51	7.1E-03
ENSCAFG0000000461	RAB30	cyan	EC_M2	0.05	7.9E-01	-0.43	3.0E-02	0.62	7.1E-04	0.18	3.7E-01	-0.63	5.2E-04	-0.17	4.1E-01	-0.16	4.3E-01	-0.02	9.4E-01	0.05	8.0E-01	0.60	1.1E-03
ENSCAFG0000000326	RGS3	turquoise	EC_M6	0.05	7.9E-01	0.68	1.3E-04	-0.47	1.6E-02	-0.29	1.5E-01	0.26	2.0E-01	-0.09	6.6E-01	0.13	5.1E-01	0.14	5.0E-01	0.39	4.7E-02	-0.30	1.4E-01
ENSCAFG0000000869	RHEBL1	grey	EC_M10	0.05	7.9E-01	0.14	4.9E-01	-0.07	7.5E-01	0.20	3.3E-01	0.00	9.9E-01	0.24	2.5E-01	-0.01	9.5E-01	0.17	4.2E-01	-0.24	2.3E-01	-0.03	9.0E-01
ENSCAFG0000000263	SOCS5	darkgreen	EC_M4	0.05	7.9E-01	-0.36	7.4E-02	-0.51	7.1E-03	0.05	8.0E-01	0.66	2.6E-04	0.14	4.9E-01	0.26	2.0E-01	0.06	7.6E-01	0.08	7.0E-01	-0.70	6.9E-05
ENSCAFG0000001889	MOSPD1	grey	EC_M10	0.05	7.9E-01	-0.01	9.7E-01	-0.23	2.6E-01	0.17	3.9E-01	0.12	5.5E-01	0.13	5.2E-01	-0.21	3.1E-01	0.04	8.3E-01	-0.10	6.1E-01	-0.18	3.7E-01
ENSCAFG0000000907	HOMER1	darkgreen	EC_M4	0.05	7.9E-01	0.52	6.2E-03	-0.72	3.0E-05	-0.03	8.7E-01	0.60	1.2E-03	0.04	8.6E-01	-0.10	6.4E-01	0.06	7.7E-01	-0.03	8.7E-01	-0.62	7.4E-04
ENSCAFG0000001689	ENSCAFG0000001689	grey	EC_M10	0.05	7.9E-01	0.38	5.6E-02	0.10	6.3E-01	-0.18	3.9E-01	-0.22	2.8E-01	-0.18	3.8E-01	0.25	2.2E-01	-0.11	6.0E-01	-0.20	3.4E-01	0.25	2.1E-01
ENSCAFG0000001730	RABEP2	turquoise	EC_M6	0.05	7.9E-01	0.66	2.6E-04	-0.14	5.0E-01	-0.25	2.1E-01	-0.12	5.7E-01	-0.03	8.8E-01	-0.12	5.5E-01	0.04	8.5E-01	0.19	3.5E-01	0.09	6.8E-01
ENSCAFG0000001246	SMYD2	grey	EC_M10	0.05	7.9E-01	-0.56	2.9E-03	0.03	8.9E-01	0.11	6.0E-01	0.12	5.5E-01	0.01	9.5E-01	-0.05	8.0E-01	-0.02	9.2E-01	0.42	3.2E-02	-0.18	3.9E-01
ENSCAFG0000001174	PECAM1	turquoise	EC_M6	0.05	7.9E-01	0.81	4.3E-07	-0.11	6.1E-01	-0.05	8.2E-01	-0.20	3.4E-01	-0.11	5.8E-01	-0.18	3.8E-01	-0.08	7.0E-01	0.10	6.3E-01	0.14	5.1E-01
ENSCAFG0000001553	MYO9B	darkgreen	EC_M4	0.05	7.9E-01	-0.16	4.3E-01	-0.75	1.0E-05	-0.11	6.0E-01	0.88	3.1E-09	0.32	1.1E-01	0.03	8.9E-01	0.00	1.0E+00	-0.04	8.6E-01	-0.93	4.1E-12
ENSCAFG0000002585	ENSCAFG0000002585	grey	EC_M10	0.05	7.9E-01	-0.12	5.6E-01	0.10	6.3E-01	-0.05	8.2E-01	-0.07	7.4E-01	-0.04	8.4E-01	0.01	9.7E-01	-0.05	8.0E-01	-0.16	4.3E-01	0.03	8.8E-01
ENSCAFG0000000795	MAP1B	darkgreen	EC_M4	0.05	7.9E-01	0.18	3.8E-01	-0.78	2.9E-06	-0.32	1.1E-01	0.82	2.9E-07	0.07	7.4E-01	-0.14	5.0E-01	0.15	4.7E-01	0.22	2.9E-01	-0.81	4.5E-07
ENSCAFG0000000370	ATG5	grey	EC_M10	0.05	7.9E-01	-0.08	7.0E-01	-0.06	7.7E-01	0.13	5.4E-01	0.08	7.1E-01	0.27	1.8E-01	0.11	5.8E-01	-0.24	2.4E-01	-0.11	6.1E-01	-0.09	6.6E-01
ENSCAFG0000001796	ENSCAFG0000001796	turquoise	EC_M6	0.05	7.9E-01	0.77	4.0E-06	-0.14	5.0E-01	-0.04	8.5E-01	-0.20	3.3E-01	-0.06	7.8E-01	-0.03	8.9E-01	-0.09	6.6E-01	0.53	5.7E-03	0.19	3.5E-01
ENSCAFG0000002804	ENSCAFG0000002804	turquoise	EC_M6	0.05	7.9E-01	0.77	4.0E-06	-0.14	5.0E-01	-0.04	8.5E-01	-0.20	3.3E-01	-0.06	7.8E-01	-0.03	8.9E-01	-0.09	6.6E-01	0.53	5.7E-03	0.19	3.5E-01
ENSCAFG0000002871	ENSCAFG0000002871	turquoise	EC_M6	0.05	7.9E-01	0.77	4.0E-06	-0.14	5.0E-01	-0.04	8.5E-01	-0.20	3.3E-01	-0.06	7.8E-01	-0.03	8.9E-01	-0.09	6.6E-01	0.53	5.7E-03	0.19	3.5E-01
ENSCAFG0000003229	ENSCAFG0000003229	turquoise	EC_M6	0.05	7.9E-01	0.77	4.0E-06	-0.14	5.0E-01	-0.04	8.5E-01	-0.20	3.3E-01	-0.06	7.8E-01	-0.03	8.9E-01	-0.09	6.6E-01	0.53	5.7E-03	0.19	3.5E-01
ENSCAFG0000000777	NCOA2	cyan	EC_M2	0.05	7.9E-01	-0.06	7.7E-01	0.57	2.3E-03	0.06	7.6E-01	-0.74	1.8E-05	-0.02	9.1E-01	-0.15	4.7E-01	-0.04	8.4E-01	-0.40	4.1E-02	0.70	7.8E-05
ENSCAFG0000003213	ENSCAFG0000003213	turquoise	EC_M6	0.05	7.9E-01	0.57	2.6E-03	-0.26	2.0E-01	-0.06	7.7E-01	0.12	5.5E-01	-0.03	8.7E-01	-0.05	8.3E-01	-0.08	7.1E-01	0.03	8.7E-01	-0.10	6.4E-01
ENSCAFG0000000627	P2RX2	turquoise	EC_M6	0.05	7.9E-01	0.54	4.2E-03	-0.37	6.6E-02	-0.09	6.7E-01	0.15	4.6E-01	-0.03	8.8E-01	0.33	1.0E-01	0.02	9.1E-01	0.10	6.3E-01	-0.17	4.0E-01
ENSCAFG0000000184	BAG1	darkgreen	EC_M4	0.05	8.0E-01	0.17	4.1E-01	-0.79	1.8E-06	-0.18	3.8E-01	0.80	8.9E-07	0.11	6.1E-01	0.03	8.8E-01	0.05	8.2E-01	-0.13	5.3E-01	-0.84	7.6E-08
ENSCAFG0000000721	FAM3D	turquoise	EC_M6	0.05	8.0E-01	0.80	9.5E-07	-0.16	4.3E-01	-0.12	5.7E-01	-0.12	5.7E-01	-0.10	6.3E-01	-0.07	7.2E-01	-0.12	5.5E-01	-0.10	6.2E-01	0.10	6.4E-01
ENSCAFG0000001507	FAM184B	grey	EC_M10	0.05	8.0E-01	-0.21	3.1E-01	0.16	4.3E-01	-0.04	8.5E-01	-0.15	4.6E-01	-0.15	4.6E-01	0.12	5.5E-01	-0.01	9.5E-01	0.39	4.8E-02	0.16	4.5E-01
ENSCAFG0000001405	ACHE	grey	EC_M10	0.05	8.0E-01	0.32	1.2E-01	0.07	7.3E-01	-0.17	4.0E-01	-0.18	3.8E-01	0.03	8.7E-01	-0.07	7.4E-01	-0.09	6.7E-01	-0.42	3.2E-02	0.17	3.9E-01
ENSCAFG0000001426	ATXN7L3	grey	EC_M10	0.05	8.0E-01	0.47	1.5E-02	0.21	3.1E-01	0.01	9.7E-01	-0.43	2.8E-02	0.19	3.6E-01	0.23	2.6E-01	0.04	8.3E-01	0.07	7.3E-01	0.39	5.2E-02
ENSCAFG0000000842	ZNF704	turquoise	EC_M6	0.05	8.0E-01	0.56	3.1E-03	-0.39	4.8E-02	-0.04	8.6E-01	0.19	3.6E-01	-0.11	6.0E-01	0.09	6.5E-01	-0.02	9.3E-01	0.40	4.1E-02	-0.23	2.5E-01
ENSCAFG0000001467	ELMOD1	turquoise	EC_M6	0.05	8.0E-01	0.40	4.3E-02	0.03	8.8E-01	0.47	1.5E-02	-0.26	2.0E-01	-0.13	5.3E-01	0.38	5.4E-02	-0.03	9.0E-01	-0.12	5.6E-01	0.24	2.4E-01
ENSCAFG0000002984	LASP1	grey	EC_M10	0.05	8.0E-01	0.33	1.0E-01	-0.52	6.6E-03	-0.38	5.3E-02	0.52	6.5E-03	0.03	8.7E-01	-0.07	7.4E-01	0.26	2.1E-01	-0.04	8.5E-01	-0.57	2.3E-03
ENSCAFG0000002662	ENSCAFG0000002662	grey	EC_M10	0.05	8.0E-01	0.06	7.7E-01	0.10	6.1E-01	-0.04	8.5E-01	-0.15	4.5E-01	-0.12	5.6E-01	-0.12	5.5E-01	-0.07	7.4E-01	-0.18	3.7E-01	0.09	6.5E-01
ENSCAFG0000002211	ENSCAFG0000002211	grey	EC_M10	0.05	8.0E-01	-0.33	1.0E-01	0.22	2.8E-01	0.04	8.3E-01	-0.17	4.2E-01	-0.18	3.8E-01	0.16	4.3E-01	0.26	2.0E-01	-0.07	7.3E-01	0.15	4.7E-01
ENSCAFG0000001250	TAF4	grey	EC_M10	0.05	8.0E-01	0.49	1.1E-02	0.33	9.9E-02	0.14	4.9E-01	-0.64	4.6E-04	-0.08	7.0E-01	-0.14	5.1E-01	-0.48	1.4E-02	-0.02	9.3E-01	0.61	8.6E-04
ENSCAFG0000003153	ENSCAFG0000003153	grey	EC_M10	0.05	8.0E-01	0.01	9.4E-01	0.09	6.8E-01	-0.12	5.5E-01	-0.14	4.9E-01	-0.11	5.8E-01	-0.15	4.6E-01	0.56	2.6E-03	-0.03	8.8E-01	0.12	5.6E-01
ENSCAFG0000002215	ENSCAFG0000002215	grey	EC_M10	0.05	8.0E-01	0.03	8.9E-01	0.05	7.9E-01	-0.12	5.7E-01	-0.13	5.4E-01	0.20	3.2E-01	-0.16	4.5E-01	-0.19	3.4E-01	0.28	1.7E-01	0.09	6.7E-01
ENSCAFG0000002029	WDR63	grey	EC_M10	0.05	8.0E-01	-0.11	6.0E-01	-0.19	3.5E-01	-0.12	5.8E-01	0.23	2.5E-01	-0.08	7.0E-01	-0.10	6.4E-01	0.45	2.1E-02	0.12	5.8E-01	-0.26	2.1E-01
ENSCAFG0000003265	C22orf46	grey	EC_M10	0.05	8.0E-01	-0.11	6.0E-01	0.34	8.7E-02	-0.07	7.3E-01	-0.31	1.2E-01	-0.05	7.9E-01	-0.03	8.9E-01	-0.07	7.3E-01	-0.18	3.7E-01	0.30	1.3E-01
ENSCAFG0000001166	MECR	grey	EC_M10	0.05	8.0E-01	-0.46	1.7E-02	0.24	2.4E-01	0.19	3.5E-01	-0.07	7.4E-01	-0.13	5.2E-01	-0.29	1.5E-01	-0.23	2.5E-01	-0.26	2.1E-01	0.05	8.3E-01
ENSCAFG0000001371	ENSCAFG0000001371	grey	EC_M10	0.05	8.0E-01	-0.05	8.0E-01	-0.14	5.0E-01	-0.11	5.9E-01	0.11	6.0E-01	0.53	5.7E-03	-0.03	8.8E-01	-0.01	9.7E-01	-0.07	7.2E-01	-0.20	3.4E-01
ENSCAFG0000000724	ENSCAFG0000000724	darkgreen	EC_M4	0.05	8.0E-01	-0.05	8.2E-01	-0.28	1.7E-01	-0.12	5.7E-01	0.38	5.5E-02	-0.07	7.5E-01	-0.01	9.6E-01	0.01	9.5E-01	0.17	4.1E-01	-0.35	7.9E-02
ENSCAFG0000001046	TM4SF20	darkgreen	EC_M4	0.05	8.0E-01	-0.05	8.2E-01	-0.28	1.7E-01	-0.12	5.7E-01	0.38	5.5E-02	-0.07	7.5E-01	-0.01	9.6E-01	0.01	9.5E-01	0.17	4.1E-01	-0.35	7.9E-02
ENSCAFG0000000256	ENSCAFG0000000256	grey	EC_M10	0.05	8.0E-01	-0.12	5.6E-01	-0.19	3.5E-01	-0.12	5.5E-01	0.21	3.1E-01	-0.05	8.0E-01	-0.11	6.0E-01	-0.05	8.2E-01	-0.05	8.0E-01	-0.24	2.3E-01

ENSCAFG0000003211	TAL2	grey	EC_M1C	0.05	8.0E-01	0.10	6.3E-01	-0.27	1.7E-01	-0.19	3.6E-01	0.20	3.4E-01	-0.15	4.8E-01	0.17	4.0E-01	-0.05	7.9E-01	-0.04	8.5E-01	-0.21	3.1E-01
ENSCAFG0000002551	SIX2	grey	EC_M1C	0.05	8.0E-01	0.02	9.3E-01	0.16	4.3E-01	-0.05	8.1E-01	-0.25	2.1E-01	0.20	3.2E-01	-0.21	3.1E-01	-0.32	1.1E-01	0.32	1.1E-01	0.20	3.3E-01
ENSCAFG0000001643	NUDT1	grey	EC_M1C	0.05	8.0E-01	0.08	7.1E-01	0.25	2.2E-01	0.01	9.7E-01	-0.23	2.6E-01	0.16	4.4E-01	-0.04	8.3E-01	0.13	5.4E-01	0.11	5.8E-01	0.23	2.5E-01
ENSCAFG0000002310	OAS2	grey	EC_M1C	0.05	8.0E-01	0.44	2.3E-02	0.39	5.1E-02	0.06	7.7E-01	-0.64	4.9E-04	-0.18	3.7E-01	0.14	5.1E-01	-0.14	5.1E-01	0.09	6.5E-01	0.62	8.0E-04
ENSCAFG0000000700	ENSCAFG0000000700	grey	EC_M1C	0.05	8.0E-01	-0.22	2.9E-01	-0.43	2.7E-02	-0.06	7.9E-01	0.55	3.6E-03	-0.13	5.1E-01	-0.08	7.1E-01	-0.09	6.6E-01	-0.13	5.4E-01	-0.56	3.2E-03
ENSCAFG0000000342	NUP62	grey	EC_M1C	0.05	8.0E-01	0.10	6.4E-01	0.54	4.3E-03	-0.20	3.3E-01	-0.62	8.1E-04	-0.15	4.5E-01	-0.02	9.4E-01	0.13	5.4E-01	0.01	9.6E-01	0.58	1.7E-03
ENSCAFG0000000388	APOB	grey	EC_M1C	0.05	8.0E-01	-0.06	7.9E-01	0.03	8.9E-01	-0.05	8.0E-01	-0.11	5.9E-01	-0.09	6.7E-01	-0.11	5.9E-01	-0.14	4.9E-01	-0.11	6.0E-01	0.05	7.9E-01
ENSCAFG0000002037	EXOC3L1	turquoise	EC_M6	0.05	8.0E-01	0.67	1.8E-04	-0.02	9.0E-01	-0.12	5.7E-01	-0.26	2.1E-01	-0.21	3.1E-01	-0.16	4.5E-01	-0.03	8.7E-01	0.59	1.4E-03	0.27	1.8E-01
ENSCAFG0000001973	SLC25A33	turquoise	EC_M6	0.05	8.0E-01	0.37	6.5E-02	-0.16	4.4E-01	-0.01	9.8E-01	0.00	9.8E-01	-0.03	8.9E-01	0.02	9.2E-01	-0.36	7.0E-02	0.45	2.2E-02	0.02	9.1E-01
ENSCAFG0000001238	BNIPL	grey	EC_M1C	0.05	8.0E-01	0.15	4.8E-01	-0.15	4.6E-01	-0.18	3.7E-01	0.06	7.6E-01	-0.19	3.6E-01	0.31	1.2E-01	-0.23	2.6E-01	0.21	3.0E-01	-0.11	6.0E-01
ENSCAFG0000000331	ANXA4	cyan	EC_M2	0.05	8.0E-01	-0.39	4.8E-02	0.57	2.5E-03	0.17	4.1E-01	-0.60	1.3E-03	-0.22	2.9E-01	0.00	9.9E-01	-0.05	8.1E-01	-0.18	3.9E-01	0.60	1.2E-03
ENSCAFG0000003031	HRASLS	grey	EC_M1C	0.05	8.0E-01	-0.18	3.7E-01	-0.09	6.5E-01	-0.14	4.9E-01	0.20	3.3E-01	0.43	2.7E-02	-0.11	5.8E-01	0.28	1.7E-01	-0.10	6.1E-01	-0.20	3.2E-01
ENSCAFG0000000129	AKR1B1	grey	EC_M1C	0.05	8.0E-01	-0.44	2.4E-02	-0.17	4.0E-01	-0.14	5.0E-01	0.35	8.3E-02	0.04	8.5E-01	0.14	4.9E-01	0.28	1.6E-01	0.33	9.8E-02	-0.36	6.7E-02
ENSCAFG0000001156	KCNK1	grey	EC_M1C	0.05	8.0E-01	-0.31	1.2E-01	-0.34	8.5E-02	-0.30	1.3E-01	0.53	5.3E-03	0.11	5.9E-01	0.12	5.5E-01	0.33	9.8E-02	0.23	2.6E-01	-0.52	6.8E-03
ENSCAFG0000000758	FOXL2	grey	EC_M1C	0.05	8.0E-01	0.04	8.5E-01	-0.45	2.0E-02	0.02	9.4E-01	0.50	8.6E-03	0.37	6.5E-02	-0.12	5.6E-01	-0.12	5.6E-01	-0.15	4.8E-01	-0.53	5.0E-03
ENSCAFG0000001694	ASH1L	grey	EC_M1C	0.05	8.0E-01	0.00	9.9E-01	-0.25	2.1E-01	-0.09	6.7E-01	0.22	2.9E-01	-0.04	8.5E-01	-0.02	9.4E-01	-0.25	2.2E-01	-0.25	2.1E-01	-0.24	2.4E-01
ENSCAFG0000001630	MYH9	darkgreen	EC_M4	0.05	8.0E-01	0.01	9.5E-01	-0.78	2.1E-06	-0.19	3.4E-01	0.88	5.0E-09	0.18	3.7E-01	0.13	5.4E-01	0.10	6.2E-01	0.11	5.9E-01	-0.91	1.1E-10
ENSCAFG0000001538	ADCY10	grey	EC_M1C	0.05	8.0E-01	-0.23	2.6E-01	0.10	6.2E-01	0.29	1.5E-01	-0.13	5.4E-01	0.35	7.6E-02	0.38	5.7E-02	-0.15	4.8E-01	-0.21	3.0E-01	0.11	5.8E-01
ENSCAFG0000001859	VAV1	grey	EC_M1C	0.05	8.0E-01	0.01	9.5E-01	0.36	7.3E-02	-0.06	7.7E-01	-0.45	2.2E-02	0.35	8.3E-02	0.05	8.1E-01	0.18	3.7E-01	-0.05	8.1E-01	0.42	3.3E-02
ENSCAFG0000000444	IPP	grey	EC_M1C	0.05	8.0E-01	-0.24	2.4E-01	0.23	2.6E-01	0.06	7.6E-01	-0.38	5.9E-02	-0.05	8.2E-01	-0.09	6.5E-01	0.04	8.3E-01	-0.27	1.9E-01	0.29	1.5E-01
ENSCAFG0000002432	TAF12	grey	EC_M1C	0.05	8.1E-01	-0.05	8.3E-01	-0.18	3.8E-01	0.22	2.8E-01	0.18	3.9E-01	-0.12	5.7E-01	-0.20	3.3E-01	0.20	3.3E-01	-0.30	1.4E-01	-0.22	2.7E-01
ENSCAFG0000000531	PGS1	turquoise	EC_M6	0.05	8.1E-01	0.61	8.6E-04	-0.58	1.8E-03	-0.14	5.1E-01	0.46	1.7E-02	0.04	8.5E-01	0.13	5.2E-01	0.08	7.0E-01	0.09	6.7E-01	-0.49	1.1E-02
ENSCAFG0000001842	MTA1	grey	EC_M1C	0.05	8.1E-01	0.28	1.7E-01	0.48	1.2E-02	-0.06	7.8E-01	-0.65	3.0E-04	-0.16	4.4E-01	-0.05	8.0E-01	0.09	6.5E-01	-0.09	6.7E-01	0.60	1.3E-03
ENSCAFG0000002041	CDH5	turquoise	EC_M6	0.05	8.1E-01	0.80	8.8E-07	-0.11	5.9E-01	-0.05	8.1E-01	-0.18	3.9E-01	-0.07	7.3E-01	-0.17	4.2E-01	-0.15	4.7E-01	0.12	5.7E-01	0.12	5.7E-01
ENSCAFG0000001432	ENSCAFG0000001432	paleturquoise	EC_M11	0.05	8.1E-01	-0.03	8.8E-01	0.15	4.6E-01	0.80	8.2E-07	-0.21	3.0E-01	-0.05	8.0E-01	-0.06	7.8E-01	-0.11	5.9E-01	-0.12	5.6E-01	0.16	4.3E-01
ENSCAFG0000002279	ENSCAFG0000002279	paleturquoise	EC_M11	0.05	8.1E-01	-0.03	8.8E-01	0.15	4.6E-01	0.80	8.2E-07	-0.21	3.0E-01	-0.05	8.0E-01	-0.06	7.8E-01	-0.11	5.9E-01	-0.12	5.6E-01	0.16	4.3E-01
ENSCAFG0000001193	TRPM8	grey	EC_M1C	0.05	8.1E-01	-0.20	3.4E-01	-0.29	1.6E-01	-0.17	4.1E-01	0.36	6.8E-02	0.34	9.1E-02	-0.15	4.6E-01	0.29	1.6E-01	0.27	1.9E-01	-0.39	5.2E-02
ENSCAFG0000001252	PSMA7	grey	EC_M1C	0.05	8.1E-01	-0.10	6.3E-01	-0.18	3.9E-01	-0.17	4.1E-01	0.30	1.3E-01	0.10	6.4E-01	0.07	7.4E-01	-0.26	2.0E-01	0.15	4.6E-01	-0.36	7.2E-02
ENSCAFG0000000469	HID1	turquoise	EC_M6	0.05	8.1E-01	0.90	2.4E-10	-0.28	1.7E-01	-0.19	3.5E-01	-0.01	9.7E-01	-0.18	3.9E-01	0.00	9.9E-01	0.01	9.7E-01	-0.01	9.5E-01	0.00	1.0E+0C
ENSCAFG0000001629	FAM193B	grey	EC_M1C	0.05	8.1E-01	0.03	8.8E-01	0.33	9.6E-02	-0.05	8.2E-01	-0.54	4.5E-03	-0.09	6.7E-01	0.09	6.6E-01	0.33	9.4E-02	-0.12	5.5E-01	0.43	3.0E-02
ENSCAFG0000002029	HAS3	grey	EC_M1C	0.05	8.1E-01	0.05	8.0E-01	0.37	6.0E-02	0.09	6.6E-01	-0.47	1.5E-02	-0.04	8.5E-01	0.10	6.4E-01	-0.07	7.4E-01	-0.32	1.1E-01	0.41	3.8E-02
ENSCAFG0000001750	DOCK6	turquoise	EC_M6	0.05	8.1E-01	0.84	7.1E-08	0.06	7.5E-01	-0.04	8.3E-01	-0.41	3.9E-02	-0.28	1.7E-01	-0.11	5.8E-01	0.00	1.0E+0C	-0.11	5.8E-01	0.38	5.7E-02
ENSCAFG0000001812	TMC7	turquoise	EC_M6	0.05	8.1E-01	0.84	1.1E-07	-0.51	7.4E-03	-0.08	7.1E-01	0.25	2.1E-01	-0.16	4.3E-01	0.03	8.9E-01	-0.08	7.1E-01	-0.10	6.4E-01	-0.27	1.9E-01
ENSCAFG0000001615	ENSCAFG0000001615	grey	EC_M1C	0.05	8.1E-01	0.09	6.8E-01	-0.45	2.1E-02	0.21	3.0E-01	0.36	6.9E-02	0.24	2.4E-01	0.32	1.1E-01	-0.17	4.2E-01	-0.31	1.2E-01	-0.39	4.8E-02
ENSCAFG0000000230	GNE	grey	EC_M1C	0.05	8.1E-01	-0.15	4.5E-01	-0.54	4.1E-03	0.08	6.9E-01	0.61	9.8E-04	0.13	5.2E-01	-0.11	6.1E-01	-0.13	5.2E-01	-0.04	8.3E-01	-0.66	2.5E-04
ENSCAFG0000001036	ENSCAFG0000001036	grey	EC_M1C	0.05	8.1E-01	-0.13	5.1E-01	0.00	9.9E-01	0.29	1.5E-01	0.04	8.5E-01	0.40	4.0E-02	-0.12	5.7E-01	0.15	4.7E-01	0.09	6.5E-01	-0.10	6.4E-01
ENSCAFG0000001977	ENSCAFG0000001977	grey	EC_M1C	0.05	8.1E-01	-0.14	4.8E-01	-0.21	3.1E-01	0.38	5.6E-02	0.20	3.3E-01	-0.18	3.8E-01	0.17	4.2E-01	-0.17	4.1E-01	0.02	9.2E-01	-0.22	2.8E-01
ENSCAFG0000000064	ENSCAFG0000000064	cyan	EC_M2	0.05	8.1E-01	0.08	7.0E-01	0.55	3.6E-03	0.25	2.2E-01	-0.77	4.7E-06	-0.17	4.1E-01	0.07	7.5E-01	-0.08	7.1E-01	0.08	7.1E-01	0.73	2.2E-05
ENSCAFG0000001398	CCDC103	grey	EC_M1C	0.05	8.1E-01	-0.32	1.1E-01	0.21	3.0E-01	0.28	1.6E-01	-0.19	3.4E-01	-0.32	1.1E-01	-0.10	6.1E-01	0.10	6.4E-01	0.18	3.8E-01	0.17	4.1E-01
ENSCAFG0000001219	CCDC14	grey	EC_M1C	0.05	8.1E-01	-0.36	7.0E-02	0.12	5.5E-01	0.24	2.3E-01	-0.16	4.4E-01	-0.07	7.4E-01	-0.25	2.1E-01	0.05	8.0E-01	0.31	1.3E-01	0.09	6.7E-01
ENSCAFG0000001370	PTH2R	grey	EC_M1C	0.05	8.1E-01	-0.07	7.5E-01	-0.21	3.1E-01	-0.07	7.5E-01	0.21	3.1E-01	-0.02	9.3E-01	-0.05	8.0E-01	-0.08	7.0E-01	-0.04	8.6E-01	-0.23	2.6E-01
ENSCAFG0000000215	TLN1	grey	EC_M1C	0.05	8.1E-01	-0.08	6.9E-01	-0.67	1.7E-04	-0.09	6.6E-01	0.81	5.8E-07	0.13	5.3E-01	-0.05	8.3E-01	0.20	3.3E-01	0.10	6.1E-01	-0.84	7.2E-08
ENSCAFG0000000431	POLR2D	grey	EC_M1C	0.05	8.1E-01	-0.51	8.1E-03	-0.26	2.0E-01	-0.19	3.5E-01	0.52	5.9E-03	0.37	6.1E-02	0.03	8.8E-01	0.00	1.0E+0C	-0.12	5.6E-01	-0.55	3.9E-03
ENSCAFG0000000749	RALY	grey	EC_M1C	0.05	8.1E-01	0.23	2.7E-01	0.08	7.0E-01	0.01	9.4E-01	-0.27	1.7E-01	-0.02	9.2E-01	0.28	1.6E-01	-0.21	3.1E-01	-0.16	4.2E-01	0.22	2.9E-01
ENSCAFG0000000885	TIAM1	cyan	EC_M2	0.05	8.1E-01	-0.53	5.9E-03	0.41	4.0E-02	0.05	8.2E-01	-0.32	1.2E-01	0.03	8.9E-01	0.05	8.0E-01	-0.24	2.3E-01	-0.52	6.3E-03	0.25	2.1E-01
ENSCAFG0000002428	ZFP28	grey	EC_M1C	0.05	8.1E-01	0.26	2.0E-01	0.15	4.6E-01	0.19	3.5E-01	-0.41	3.7E-02	0.29	1.5E-01	-0.07	7.2E-01	-0.14	4.9E-01	0.21	3.0E-01	0.39	5.1E-02
ENSCAFG0000001337	ENSCAFG0000001337	grey	EC_M1C	0.05	8.1E-01	-0.15	4.5E-01	-0.40	4.1E-02	-0.09	6.6E-01	0.49	1.0E-02	0.30	1.3E-01	0.23	2.6E-01	-0.11	5.8E-01	-0.01	9.7E-01	-0.48	1.3E-02
ENSCAFG0000001035	ICE1	cyan	EC_M2	0.05	8.1E-01	-0.35	8.4E-02	0.59	1.6E-03	0.19	3.5E-01	-0.63	5.0E-04	-0.11	6.1E-01	-0.19	3.7E-01	-0.13	5.4E-01	-0.30	1.3E-01	0.57	2.3E-03
ENSCAFG0000002506	ENSCAFG0000002506	turquoise	EC_M6	0.05	8.1E-01	0.94	1.3E-12	-0.17	4.1E-01	-0.06	7.9E-01	-0.16	4.3E-01	-0.27	1.8E-01	-0.03	8.8E-01	-0.04	8.6E-01	0.13	5.4E-01	0.18	3.9E-01
ENSCAFG0000001293	KCNQ2	grey	EC_M1C	0.05	8.1E-01	-0.06	7.8E-01	0.14	5.0E-01	-0.14	5.1E-01	-0.18	3.8E-01	0.08	6.9E-01	0.20	3.3E-01	0.29	1.6E-01	-0.05	7.9E-01	0.15	4.8E-01
ENSCAFG0000001380	KIF1BP	grey	EC_M1C	0.05	8.1E-01	-0.14	4.9E-01	-0.41	3.7E-02	0.18	3.8E-01	0.48	1.2E-02	0.20	3.2E-01	-0.30	1.4E-01	-0.17	4.2E-01	-0.05	8.0E-01	-0.47	1.6E-02
ENSCAFG0000000043	MYRFL	grey	EC_M1C	0.05	8.1E-01	-0.01	9.6E-01	-0.63	5.9E-04	-0.15	4.5E-01	0.69	9.4E-05	0.22	2.7E-01	0.04	8.3E-01	0.25	2.2E-01	0.03	9.0E-01	-0.73	2.1E-05
ENSCAFG0000001651	FGFR4	magenta	EC_M13	0.05	8.1E-01	0.08	7.0E-01	0.07	7.3E-01	-0.05	8.3E-01	-0.11	5.9E-01	0.03	9.0E-01	0.04	8.4E-01	-0.05	8.1E-01	0.56	3.0E-03	0.09	6.5E-01
ENSCAFG0000002591	ENSCAFG0000002591	grey	EC_M1C	0.05	8.1E-01	0.19	3.5E-01	0.29	1.6E-01	0.02	9.1E-01	-0.52	6.2E-03	0.11	5.8E-01	0.12	5.7E-01	0.15	4.7E-01	0.14	4.9E-01	0.51	7.5E-03
ENSCAFG0000000253	SLC3A1	turquoise	EC_M6	0.05	8.1E-01	0.38	5.9E-02	-0.04	8.6E-01	0.14	5.1E-01	-0.07	7.3E-01	-0.19	3.7E-01	-0.20	3.2E-01	-0.19	3.5E-01	0.16	4.4E-01	0.08	6.8E-01
ENSCAFG0000000821	ADGRV1	grey	EC_M1C	0.05	8.1E-01	0.05	7.9E-01	-0.19	3.4E-01	0.04	8.4E-01	0.18	3.7E-01	0.08	7.1E-01	-0.14	5.1E-01	0.08	6.8E-01	-0.12	5.6E-01	-0.20	3.2E-01
ENSCAFG0000001134	HLX	turquoise	EC_M6	0.05	8.1E-01	0.62	6.7E-04	-0.24	2.4E-01	0.33	9.9E-02	-0.01	9.8E-01	-0.06	7.6E-01	-0.22	2.7E-01	0.00	9.8E-01	0.11	6.0E-01	-0.01	9.7E-01
ENSCAFG0000000096	RGL2	turquoise	EC_M6	0.05	8.1E-01	0.75	9.1E-06	0.28	1.6E-01	0.05	7.9E-01	-0.63	5.2E-04	-0.31	1.3E-01	0.10	6.3E-01	-0.08	6.9E-01	0.21	3.0E-01	0.62	7.6E-04
ENSCAFG0000000624	RNF14	grey	EC_M1C	0.05	8.1E-01	0.52	6.6E-03	-0.48	1.3E-02	-0.07	7.5E-01	0.32	1.1E-01	0.03	8.8E-01	0.10	6.4E-01	-0.17	4.0E-01	0.20	3.4E-01	-0.29	1.5E-01
ENSCAFG0000001933	C5H1orf159	grey	EC_M1C	0.05	8.2E-01	0.00	1.0E+0C	0.22	2.8E-01	0.00	9.9E-01	-0.23	2.7E-01	-0.24	2.4E-01	-0.36	7.4E-02	0.15	4.5E-01	0.32	1.1E-01	0.22	2.8E-01
ENSCAFG0000001218	ENSCAFG0000001218	grey	EC_M1C	0.05	8.2E-01	-0.18	3.7E-01	0.13	5.1E-01	-0.38	5.4E-02	-0.17	4.2E-01	-0.12	5.6E-01	0.09	6.8E-01	-0.02	9.1E-01	-0.19	3.5E-01	0.10	6.3E-01
ENSCAFG0000000768	AADAT	turquoise	EC_M6	0.05	8.2E-01	0.78	2.4E-06	-0.15	4.7E-01	0.00	9.9E-01	-0.12	5.7E-01	-0.25	2.1E-01	-0.12	5.4E-01	-0.22	2.8E-01	0.06	7.8E-01	0.10	6.3E-01
ENSCAFG0000003117	CNIH1	darkgreen	EC_M4	0.05	8.2E-01	0.19	3.4E-01	-0.66	2.4E-04	-0.06	7.7E-01	0.68	1.5E-04	0.42	3.4E-02	0.07	7.5E-01	-0.09	6.6E-01	-0.28	1.6E-01	-0.75	1.1E-05
ENSCAFG0000001012	PSPH	darkgreen	EC_M4	0.05	8.2E-01	-0.58	2.0E-03	-0.26	2.0E-01	-0.05	7.9E-01	0.48	1.3E-02	0.21	3.0E-01	-0.18	3.7E-01	0.06	7.7E-01	-0.05	7.9E-01	-0.57	2.4E-03
ENSCAFG0000002678	ENSCAFG0000002678	grey	EC_M1C	0.05	8.2E-01	0.18	3.8E-01	-0.03	8.9E-01	-0.17	4.0E-01	-0.06	7.8E-01	0.17	4.1E-01	-0.11	5.8E-01	-0.14	5.0E-01	0.19	3.6E-01	-0.03	9.0E-01
ENSCAFG0000000790	ELL2	darkgreen	EC_M4	0.05	8.2E-01	0.27	1.8E-01	-0.71	5.3E-05	-0.23	2.6E-01	0.63	5.3E-04	0.14	5.0E-01	-0.04	8.3E-01	0.07	7.2E-01	0.13	5.3E-01	-0.66	2.5E-04
ENSCAFG0000001315	ARHGAP5	grey	EC_M1C	0.05	8.2E-01	-0.22	2.8E-01	0.06	7.6E-01	0.13	5.4E-01	-0.12	5.5E-01	0.17	4.0E-01	-0.34	9.3E-02	0.07	7.2E-01	-0.23	2.7E-01	0.08	6.9E-01
ENSCAFG0000003085	C15H1orf185	grey	EC_M1C	0.05	8.2E-01	0.17	4.1E-01	-0.05	8.0E-01	0.27	1.8E-01	-0.01	9.7E-01	0.03	8.7E-01	0.25	2.3E-01	0.15	4.6E-01	0.29	1.5E-01	0.05	8.2E-01
ENSCAFG0000001744	SLC27A3	cyan	EC_M2	0.05	8.2E-01	0.45	2.1E-02	0.48	1.3E-02	0.09	6.8E-01	-0.76	7.3E-06	-0.25	2.2E-01	0.18	3.7E-01	-0.17	4.1E-01	-0.22	2.8E-01	0.74	1.4E-05
ENSCAFG0000001047	GCA	grey	EC_M1C	0.05	8.2E-01	-0.29	1.5E-01	0.47	1.4E-02	0.07	7.2E-01	-0.45	2.1E-02	0.12	5.6E-01	0.07	7.5E-01	-0.38	5.5E-02	-0.07	7.2E-01	0.42	3.5E-02
ENSCAFG0000000409	ADCY3	grey	EC_M1C	0.05	8.2E-01	0.02	9.3E-01	0.07	7.4E-01	0.09	6.6E-01	-0.09	6.7E-01	-0.09	6.6E-01	0.31	1.3E-01	-0.15	4.8E-01	0.09	6.7E-01	0.02	9.2E-01
ENSCAFG0000001907	NXN	darkgreen	EC_M4	0.05	8.2E-01	0.27	1.7E-01	-0.70	6.4E-05	-0.22	2.7E-01	0.71	5.1E-05	0.17	3.9E-01	-0.16	4.3E-01	-0.07	7.3E-01	-0.18	3.9E-01	-0.77	4.2E-06
ENSCAFG0000000689	PDHX	grey	EC_M1C	0.05	8.2E-01	-0.37	6.5E-02	-0.15	4.6E-01	-0.12	5.7E-01	0.30	1.3E-01	0.11	5.8E-01	-0.07	7.3E-01	0.21	3.0E-01	-0.18	3.9E-01	-0.32	1.1E-01
ENSCAFG0000001651	ENSCAFG0000001651	turquoise	EC_M6	0.05	8.2E-01	0.18	3.7E-01	0.06	7.9E-01	-0.22	2.8E-01	-0.20	3.3E-01	-0.16	4.3E-01	0.31	1.2E-01	-0.03	8.7E-01	-0.03	8.8E-01	0.17	4.1E-01
ENSCAFG0000000101	CCDC134	grey	EC_M1C	0.05	8.2E-01	-0.34	9.0E-02	-0.05	8.2E-01	0.33	9.9E-02	0.15	4.6E-01	0.04	8.5E-01	0.39	5.0E-02	-0.01	9.7E-01	-0.05	8.2E-01	-0.15	4.7E-01
ENSCAFG0000003070	TUBGCP2	grey	EC_M1C	0.05	8.2E-01	0.09	6.5E-01	-0.15	4.6E-01	-0.06	7.8E-01	0.24	2.4E-01	0.05	8.2E-01	-0.23	2.6E-01	-0.20	3.2E-01	-0.14	4.9E-01	-0.23	2.5E-01
ENSCAFG0000001409	ENSCAFG0000001409	turquoise	EC_M6	0.05	8.2E-01	0.38	5.3E-02	-0.02	9.1E-01	0.31	1.2E-01	-0.16	4.3E-01	-0.19	3.6E-01	0.17	4.2E-01	-0.18	3.8E-01	-0.23	2.7E-01	0.10	6.2E-01
ENSCAFG0000001498	FMO4	grey	EC_M1C	0.05	8.2E-01	-0.10	6.4E-01	-0.38	5.8E-02	0.02	9.0E-01	0.39	5.0E-02	0.01	9.5E-01	-0.31	1.2E-01	0.29	1.5E-01	0.25	2.3E-01	-0.43	3.0E-02
ENSCAFG0000002878	RNFT1	cyan	EC_M2	0.05	8.2E-01	-0.22	2.7E-01	0.72	3.8E-05	0.23	2.6E-01	-0.84	7.4E-08	-0.20	3.3E-01	0.02	9.2E-01	-0.05	8.2E-01	0.04	8.6E-01	0.82	3.9E-07
ENSCAFG0000001501	SLBP	grey	EC_M1C	0.05	8.2E-01	0.07	7.5E-01	0.31	1.2E-01	-0.15	4.5E-01	-0.36	6.7E-02	0.20	3.2E-01	0.29	1.4E-01	-0.08	7.0E-01	0.20	3.3E-01	0.38	5.3E-02
ENSCAFG0000001872	RFX2	cyan	EC_M2	0.05	8.2E-01	0.16	4.5E-01	0.57	2.2E-03	0.06	7.6E-01	-0.77	3.9E-06	-0.31	1.2E-01	0.17	4.1E-01	-0.22	2.9E-01	-0.19	3.4E-01	0.74	1.6E-05
ENSCAFG0000001425	VCPKMT	grey	EC_M1C	0.05	8.2E-01	-0.49	1.1E-02	0.31	1.3E-01	0.06	7.7E-01	-0.23	2.7E-01	0.23	2.5E-01	0.07	7.2E-01	-0.19	3.6E-01	0.18	3.7E-01	0.20	3.4E-01
ENSCAFG0000001024	ARL3	darkgreen	EC_M4	0.05	8.2E-01	-0.52	7.0E-03	-0.44	2.4E-02	0.09	6.7E-01	0.59	1.4E-03	0.34	8.7E-02	0.02	9.1E-01	0.11	5.9E-01	-0.32	1.1E-01	-0.68	1.3E-04
ENSCAFG0000001047	SRPRA	darkgreen	EC_M4	0.05	8.2E-01	0.03	8.7E-01	-0.80	1.1E-06	-0.17	4.0E-01	0.88	3.9E-09	0.36	6.7E-02	-0.02	9.3E-01	0.04	8.5E-01	0.18	3.7E-01	-0.91	1.5E-10
ENSCAFG0000003091	TMEM126A	grey	EC_M1C	0.05	8.2E-01	-0.40	4.4E-02	0.19	3.4E-01	0.34	9.1E-02	-0.10	6.1E-01	0.14	5.0E-01	-0.26	1.9E-01	-0.51	8.4E-03	0.00	9.9E-01	0.07	7.2E-01
ENSCAFG0000001668	OPHN1	turquoise	EC_M6	0.05	8.2E-01	0.57	2.3E-03	0.02	9.1E-01	0.16	4.4E-01	-0.30	1.3E-01	-0.22	2.9E-01	-0.19	3.6E-01	-0.05	8.2E-01	-0.05	7.9E-01	0.28	1.6E-01
ENSCAFG0000000499	ENSCAFG0000000499	grey	EC_M1C	0.05	8.2E-01	-0.15	4.6E-01	-0.36	6.8E-02	-0.09	6.8E-01	0.36	6.9E-02	0.32	1.1E-01	0.44	2.4E-02	-0.11	6.1E-01	-0.17	4.2E-01	-0.45	2.3E-02
ENSCAFG0000002444	OASL2	grey	EC_M1C	0.05	8.2E-01	-0.06	7.8E-01	0.35	8.0E-02	0.20	3.2E-01	-0.54	4.2E-03	0.13	5.3E-01	-0.04	8.5E-01	-0.08	6.8E-01	-0.18	3.9E-01	0.46	1.9E-02
ENSCAFG0000000960	RPL24	cyan	EC_M2	0.05	8.2E-01	-0.18	3.8E-01	0.56	2.9E-03	0.24	2.3E-01	-0.65	3.4E-04	0.18	3.9E-01	0.20	3.2E-01	-0.08	6.8E-01	-0.07	7.3E-01	0.57	2.3E-03
ENSCAFG0000001984	CDH15	grey	EC_M1C	0.05	8.2E-01	-0.29	1.5E-01	0.52	7.1E-03	0.26	2.0E-01	-0.49	1.2E-02	-0.15	4.8E-01	0.23	2.6E-01	-0.13	5.3E-01	-0.13	5.2E-01	0.54	4.1E-03
ENSCAFG0000003128	ZNF620	grey	EC_M1C	0.05	8.2E-01	-0.14	5.1E-01	0.09	6.6E-01	-0.31	1.2E-01	-0.12	5.7E-01	0.26	2.1E-01	0.08	6.8E-01	0.21	3.1E-01	-0.14	4.8E-01	0.03	8.7E-01
ENSCAFG0000000326	RPS7	cyan	EC_M2	0.05	8.2E-01	-0.32	1.1E-01	0.53	5.2E-03	0.13	5.1E-01	-0.54	4.8E-03	0.08	7.1E-01	0.32	1.1E-01	-0.07	7.5E-01	-0.38	5.6E-02	0.45	2.0E-02
ENSCAFG0000001923	ENSCAFG0000001923	grey	EC_M1C	0.05	8.2E-01	0.18	3.9E-01	-0.58	1.8E-03	-0.06	7.6E-01	0.54	4.6E-03	0.32	1.1E-01	0.13	5.3E-01	0.25	2.2E-01	0.20	3.3E-01	-0.55	3.9E-03
ENSCAFG0000001322	ENSCAFG0000001322	magenta	EC_M13	0.05	8.2E-01	0.12	5.6E-01	-0.02	9.2E-01	0.05	8.1E-01	-0.06	7.9E-01	-0.17	4.1E-01	-0.06	7.5E-01	0.05	8.1E-01	0.56	2.7E-03	0.10	6.2E-01
ENSCAFG0000001858	OXCT1	cyan	EC_M2	0.05	8.2E-01	0.14	4.8E-01	0.65	3.0E-04	0.19	3.4E-01	-0.80	8.6E-07	-0.39	5.0E-02	-0.20	3.3E-01	0.02	9.2E-01	0.04	8.6E-01	0.82	2.2E-07
ENSCAFG0000002171	ENSCAFG0000002171	cyan	EC_M2	0.05	8.2E-01	-0.54	4.3E-03	0.52	6.7E-03	0.12	5.7E-01	-0.43	2.9E-02	-0.17	4.1E-01	0.15	4.7E-01	-0.10	6.4E-01	-0.01	9.6E-01	0.40	4.4E-02
ENSCAFG0000000650	SIGIRR	grey	EC_M1C	0.05	8.2E-01	0.46	1.8E-02	0.44	2.6E-02	0.06	7.6E-01	-0.69	9.2E-05	-0.22	2.9E-01	-0.06	7.7E-01	0.00	9.9E-01	-0.04	8.6E-01	0.65	3.7E-04
ENSCAFG0000001525	VCL	darkgreen	EC_M4	0.05	8.2E-01	-0.23	2.5E-01	-0.69	1.1E-04	-0.27	1.8E-01	0.86	1.9E-08	0.24	2.4E-01	0.05	8.1E-01	0.11	6.0E-01	0.17	4.2E-01	-0.88	2.3E-09
ENSCAFG0000002511	ENSCAFG0000002511	grey	EC_M1C	0.05	8.2E-01	-0.21	3.0E-01	-0.24	2.4E-01	-0.04	8.5E-01	0.40	4.3E-02	0.44	2.5E-02	-0.20	3.3E-01	-0.13	5.4E-01	-0.19	3.5E-01	-0.44	2.4E-02
ENSCAFG0000001311	RGSL1	turquoise	EC_M6	0.05	8.2E-01	0.54	4.8E-03	0.11	5.9E-01	0.21	3.1E-01	-0.37	6.5E-02	-0.19	3.5E-01	-0.21	3.0E-01	-0.19	3.6E-01	-0.04	8.3E-01	0.34	9.3E-02
ENSCAFG0000000678	NSFL1C	grey	EC_M1C	0.05	8.3E-01	0.36	7.4E-02	-0.57	2.3E-03	-0.31	1.2E-01	0.55	3.5E-03	0.10	6.1E-01	0.21	3.1E-01	0.09	6.7E-01	0.21	2.9E-01	-0.56	2.8E-03
ENSCAFG0000001957	EGFL7	turquoise	EC_M6	0.05	8.3E-01	0.92	2.2E-11	-0.34	9.3E-02	-0.12	5.6E-01	0.05	8.0E-01	-0.06	7.9E-01	-0.02	9.1E-01	-0.09	6.7E-01	0.08	7.1E-01	-0.09	6.8E-01
ENSCAFG0000000899	OTULIN	grey	EC_M1C	0.05	8.3E-01	-0.05	7.9E-01	-0.18	3.7E-01	-0.07	7.5E-01	0.18	3.8E-01	0.19	3.5E-01	0.09	6.8E-01	-0.13	5.3E-01	-0.33	1.0E-01	-0.21	3.1E-01
ENSCAFG0000000517	SEC14L1	turquoise	EC_M6	0.05	8.3E-01	0.88	2.0E-09	-0.28	1.6E-01	-0.17	4.0E-01	0.00	9.9E-01	-0.13	5.4E-01	-0.08	6.8E-01	-0.11	6.0E-01	0.29	1.5E-01	0.02	9.1E-01
ENSCAFG0000001594	PRTG	darkgreen	EC_M4	0.05	8.3E-01	-0.45	2.1E-02	-0.41	3.9E-02	-0.22	2.9E-01	0.55	3.4E-03	0.33	9.6E-02	0.10	6.2E-01	-0.08	6.8E-01	0.00	9.8E-01	-0.62	6.7E-04
ENSCAFG0000001332	ENSCAFG0000001332	grey	EC_M1C	0.05	8.3E-01	0.22	2.7E-01	0.27	1.9E-01	0.26	2.0E-01	-0.50	9.9E-03	-0.12	5.7E-01	-0.11	6.1E-01	-0.24	2.3E-01	-0.03	8.9E-01	0.43	2.8E-02
ENSCAFG0000001783	CCNK	grey	EC_M1C	0.05	8.3E-01	0.05	8.2E-01	0.22	2.8E-01	-0.11	5.8E-01	-0.21	3.0E-01	0.22	2.7E-01	-0.12	5.6E-01	-0.15	4.5E-01	-0.36	6.8E-02	0.22	2.7E-01
ENSCAFG0000001738	SMAD3	grey	EC_M1C	0.04	8.3E-01	0.54	4.0E-03	0.29	1.4E-01	0.20	3.2E-01	-0.57	2.5E-03	-0.24	2.4E-01	0.01	9.7E-01	0.02	9.1E-01	-0.03	8.9E-01	0.54	4.6E-03
ENSCAFG0000000734	VCPIP1	grey	EC_M1C	0.04	8.3E-01	0.09	6.8E-01	-0.45	2.2E-02	-0.06	7.6E-01	0.38	5.4E-02	-0.06	7.9E-01	0.09	6.7E-01	0.10	6.1E-01	-0.48	1.3E-02	-0.42	3.2E-02
ENSCAFG0000001759	SMG8	grey	EC_M1C	0.04	8.3E-01	-0.02	9.1E-01	-0.10	6.3E-01	0.17	4.0E-01	0.09	6.6E-01	0.37	6.5E-02	-0.20	3.4E-01	-0.40	4.4E-02	-0.47	1.5E-02	-0.12	5.7E-01
ENSCAFG0000001523	CCDC181	grey	EC_M1C	0.04	8.3E-01	-0.61	9.5E-04	0.08	6.8E-01	0.35	8.2E-02	0.08	7.0E-01	-0.05	8.2E-01	-0.23	2.7E-01	-0.12	5.5E-01	0.04	8.3E-01	-0.13	5.3E-01
ENSCAFG0000003016	ENSCAFG0000003016	grey	EC_M1C	0.04	8.3E-01	-0.19	3.6E-01	0.20	3.3E-01	-0.29	1.5E-01	-0.18	3.8E-01	0.02	9.1E-01	0.13	5.2E-01	0.04	8.3E-01	0.12	5.5E-01	0.16	4.4E-01
ENSCAFG0000001548	ENSCAFG0000001548	grey	EC_M1C	0.04	8.3E-01	-0.15	4.8E-01	0.01	9.5E-01	-0.15	4.8E-01	0.04	8.6E-01	-0.04	8.5E-01	-0.07	7.3E-01	-0.11	5.8E-01	0.02	9.1E-01	-0.06	7.6E-01
ENSCAFG0000001038	PCGF6	grey	EC_M1C	0.04	8.3E-01	-0.07	7.4E-01	-0.01	9.6E-01	-0.12	5.6E-01	-0.03	8.7E-01	0.29	1.5E-01	0.14	5.0E-01	-0.36	6.8E-02	0.02	9.1E-01	-0.01	9.7E-01
ENSCAFG0000000358	PHF19	grey	EC_M1C	0.04	8.3E-01	0.12	5.7E-01	0.13	5.2E-01	-0.15	4.5E-01	-0.18	3.8E-01	0.00	9.9E-01	0.34	9.2E-02	0.14	4.8E-01	0.35	8.2E-02	0.17	4.0E-01
ENSCAFG0000001707	NDEL1	grey	EC_M1C	0.04	8.3E-01	0.18	3.9E-01	-0.56	3.0E-03	-0.08	6.9E-01	0.51	7.2E-03	0.22	2.7E-01	-0.03	8.8E-01	-0.14	5.1E-01	-0.14	5.0E-01	-0.50	9.0E-03

ENSCAFG0000000174	CHRNA5	violet	EC_M7	0.04	8.3E-01	-0.16	4.2E-01	0.12	5.7E-01	-0.06	7.7E-01	-0.11	5.9E-01	0.64	4.2E-04	-0.13	5.3E-01	-0.04	8.5E-01	-0.16	4.4E-01	0.07	7.5E-01
ENSCAFG0000001803	IQCK	grey	EC_M10	0.04	8.3E-01	-0.23	2.5E-01	0.31	1.3E-01	-0.04	8.5E-01	-0.31	1.2E-01	0.06	7.6E-01	-0.41	3.5E-02	0.15	4.6E-01	-0.40	4.1E-02	0.23	2.5E-01
ENSCAFG0000000119	ENSCAFG0000000119	darkgreen	EC_M4	0.04	8.3E-01	-0.11	5.8E-01	-0.15	4.5E-01	-0.10	6.4E-01	0.28	1.6E-01	-0.11	5.9E-01	-0.04	8.6E-01	0.02	9.3E-01	-0.04	8.6E-01	-0.24	2.3E-01
ENSCAFG0000001864	ENSCAFG0000001864	darkgreen	EC_M4	0.04	8.3E-01	-0.11	5.8E-01	-0.15	4.5E-01	-0.10	6.4E-01	0.28	1.6E-01	-0.11	5.9E-01	-0.04	8.6E-01	0.02	9.3E-01	-0.04	8.6E-01	-0.24	2.3E-01
ENSCAFG0000000507	FBXL3	grey	EC_M10	0.04	8.3E-01	0.13	5.2E-01	0.40	4.2E-02	0.25	2.1E-01	-0.57	2.3E-03	-0.01	9.5E-01	-0.06	7.8E-01	-0.26	1.9E-01	-0.05	8.3E-01	0.58	1.8E-03
ENSCAFG0000002483	ENSCAFG0000002483	grey	EC_M10	0.04	8.3E-01	0.08	7.0E-01	0.42	3.5E-02	0.53	5.8E-03	-0.54	4.3E-03	-0.26	2.1E-01	-0.06	7.7E-01	0.04	8.3E-01	0.31	1.2E-01	0.56	3.2E-03
ENSCAFG0000000353	GLI3	darkgreen	EC_M4	0.04	8.3E-01	-0.72	3.1E-05	0.27	1.9E-01	0.17	4.1E-01	-0.13	5.4E-01	0.19	3.6E-01	-0.11	5.8E-01	0.06	7.7E-01	-0.31	1.2E-01	0.03	8.7E-01
ENSCAFG0000001503	UVSSA	grey	EC_M10	0.04	8.3E-01	-0.54	4.7E-03	0.32	1.1E-01	-0.01	9.7E-01	-0.26	1.9E-01	-0.09	6.7E-01	-0.20	3.4E-01	0.13	5.1E-01	-0.16	4.4E-01	0.22	2.9E-01
ENSCAFG0000003218	RNF152	turquoise	EC_M6	0.04	8.3E-01	0.41	4.0E-02	0.02	9.4E-01	-0.06	7.7E-01	-0.15	4.6E-01	-0.03	8.8E-01	-0.01	9.5E-01	-0.09	6.7E-01	-0.36	7.5E-02	0.08	7.0E-01
ENSCAFG0000002401	ENSCAFG0000002401	grey	EC_M10	0.04	8.3E-01	0.26	2.0E-01	0.21	3.1E-01	0.18	3.8E-01	-0.46	1.8E-02	-0.19	3.5E-01	-0.13	5.3E-01	0.01	9.5E-01	-0.02	9.1E-01	0.40	4.1E-02
ENSCAFG0000002340	ZNF75D	grey	EC_M10	0.04	8.3E-01	0.13	5.4E-01	0.41	3.6E-02	0.09	6.8E-01	-0.59	1.6E-03	-0.26	2.0E-01	0.18	3.9E-01	-0.13	5.1E-01	0.02	9.3E-01	0.55	3.3E-03
ENSCAFG0000001785	MAP2K4	grey	EC_M10	0.04	8.3E-01	-0.07	7.2E-01	-0.37	6.3E-02	-0.04	8.4E-01	0.47	1.4E-02	0.16	4.2E-01	0.21	2.9E-01	-0.24	2.4E-01	-0.36	7.0E-02	-0.46	1.7E-02
ENSCAFG0000001335	KIF9	grey	EC_M10	0.04	8.3E-01	0.10	6.3E-01	0.26	2.1E-01	0.02	9.2E-01	-0.36	6.9E-02	-0.19	3.6E-01	-0.03	8.9E-01	0.28	1.7E-01	0.26	2.1E-01	0.42	3.0E-02
ENSCAFG0000001071	ENSCAFG0000001071	grey	EC_M10	0.04	8.3E-01	0.22	2.7E-01	-0.18	3.9E-01	0.20	3.4E-01	0.02	9.2E-01	0.02	9.3E-01	-0.07	7.3E-01	0.03	8.9E-01	-0.35	8.0E-02	-0.03	9.0E-01
ENSCAFG0000003149	GLIS3	turquoise	EC_M6	0.04	8.3E-01	0.53	4.9E-03	0.02	9.3E-01	-0.07	7.3E-01	-0.27	1.8E-01	-0.08	6.8E-01	-0.01	9.7E-01	0.01	9.7E-01	0.36	6.8E-02	0.26	2.0E-01
ENSCAFG0000001746	PIAS1	turquoise	EC_M6	0.04	8.3E-01	0.54	4.6E-03	0.09	6.6E-01	-0.05	8.2E-01	-0.40	4.5E-02	-0.09	6.5E-01	-0.01	9.6E-01	0.02	9.0E-01	0.61	9.8E-04	0.40	4.4E-02
ENSCAFG0000000672	OGDHL	grey	EC_M10	0.04	8.3E-01	-0.55	3.6E-03	0.27	1.8E-01	-0.25	2.1E-01	-0.08	7.1E-01	0.01	9.5E-01	-0.22	2.8E-01	0.04	8.4E-01	0.14	5.0E-01	0.08	6.9E-01
ENSCAFG0000000789	HPGD	grey	EC_M10	0.04	8.3E-01	-0.35	8.0E-02	0.54	4.6E-03	0.26	2.0E-01	-0.55	3.8E-03	-0.25	2.1E-01	-0.15	4.5E-01	0.17	4.1E-01	0.25	2.3E-01	0.56	2.9E-03
ENSCAFG0000001835	ASIC2	grey	EC_M10	0.04	8.3E-01	-0.01	9.8E-01	-0.53	5.8E-03	-0.11	5.9E-01	0.53	4.9E-03	0.32	1.2E-01	0.30	1.3E-01	-0.18	3.7E-01	-0.33	1.0E-01	-0.64	4.1E-04
ENSCAFG0000000171	LRRC19	turquoise	EC_M6	0.04	8.3E-01	0.62	7.2E-04	-0.15	4.6E-01	-0.09	6.5E-01	-0.12	5.7E-01	-0.12	5.4E-01	0.31	1.2E-01	-0.11	6.0E-01	-0.18	3.9E-01	0.08	6.9E-01
ENSCAFG0000000473	BEND7	grey	EC_M10	0.04	8.3E-01	0.11	5.8E-01	-0.20	3.2E-01	-0.19	3.5E-01	0.18	3.7E-01	-0.08	7.1E-01	0.17	4.0E-01	0.08	7.0E-01	-0.43	2.9E-02	-0.20	3.2E-01
ENSCAFG0000000937	TANC1	turquoise	EC_M6	0.04	8.3E-01	0.72	4.0E-05	0.11	6.1E-01	0.16	4.4E-01	-0.42	3.2E-02	-0.21	3.1E-01	-0.21	3.0E-01	-0.02	9.2E-01	0.26	2.0E-01	0.44	2.4E-02
ENSCAFG0000001231	TMEM237	grey	EC_M10	0.04	8.3E-01	-0.24	2.4E-01	0.35	7.8E-02	0.07	7.3E-01	-0.38	5.5E-02	0.03	8.9E-01	0.19	3.5E-01	0.05	8.1E-01	0.11	6.1E-01	0.34	9.2E-02
ENSCAFG0000001189	FAM162A	darkgreen	EC_M4	0.04	8.3E-01	-0.10	6.3E-01	-0.69	8.5E-05	-0.12	5.5E-01	0.83	1.6E-07	0.42	3.1E-02	0.13	5.3E-01	0.03	8.9E-01	0.20	3.3E-01	-0.85	3.4E-08
ENSCAFG0000002908	ENSCAFG0000002908	grey	EC_M10	0.04	8.4E-01	-0.31	1.2E-01	-0.17	4.0E-01	-0.04	8.3E-01	0.28	1.6E-01	0.09	6.7E-01	0.40	4.3E-02	-0.03	8.9E-01	-0.24	2.4E-01	-0.34	9.4E-02
ENSCAFG0000001125	ENSCAFG0000001125	grey	EC_M10	0.04	8.4E-01	0.18	3.8E-01	-0.19	3.7E-01	-0.18	3.8E-01	0.23	2.6E-01	-0.15	4.7E-01	0.00	9.8E-01	-0.15	4.7E-01	0.11	6.1E-01	-0.22	2.9E-01
ENSCAFG0000001669	ANGPTL7	darkmagenta	EC_M12	0.04	8.4E-01	0.43	2.9E-02	-0.11	5.9E-01	-0.11	6.0E-01	-0.08	7.1E-01	-0.07	7.2E-01	-0.08	6.8E-01	0.74	1.5E-05	0.05	8.2E-01	0.05	8.2E-01
ENSCAFG0000001781	ZP2	violet	EC_M7	0.04	8.4E-01	-0.11	5.9E-01	-0.19	3.6E-01	-0.10	6.4E-01	0.20	3.2E-01	0.75	1.1E-05	0.57	2.2E-03	-0.04	8.4E-01	-0.11	5.9E-01	-0.23	2.6E-01
ENSCAFG0000001444	TRMT112	grey	EC_M10	0.04	8.4E-01	-0.39	4.8E-02	-0.04	8.6E-01	-0.05	8.2E-01	0.25	2.3E-01	0.16	4.4E-01	0.18	3.8E-01	0.33	1.0E-01	-0.09	6.8E-01	-0.30	1.4E-01
ENSCAFG0000002915	CALM3	grey	EC_M10	0.04	8.4E-01	-0.33	9.6E-02	0.50	9.6E-03	0.05	7.9E-01	-0.41	3.5E-02	-0.23	2.5E-01	0.04	8.3E-01	0.19	3.4E-01	0.30	1.3E-01	0.41	3.9E-02
ENSCAFG0000000247	ZNF667	cyan	EC_M2	0.04	8.4E-01	0.24	2.3E-01	0.28	1.7E-01	0.18	3.7E-01	-0.55	3.9E-03	-0.15	4.7E-01	0.31	1.3E-01	0.05	7.9E-01	-0.14	4.8E-01	0.50	9.4E-03
ENSCAFG0000002851	HOXB3	grey	EC_M10	0.04	8.4E-01	0.19	3.6E-01	-0.14	4.8E-01	0.15	4.6E-01	0.09	6.7E-01	0.08	7.0E-01	0.00	1.0E+00	-0.05	8.0E-01	0.02	9.2E-01	-0.15	4.5E-01
ENSCAFG0000001224	SPX	grey	EC_M10	0.04	8.4E-01	-0.17	4.1E-01	0.04	8.4E-01	-0.12	5.4E-01	0.04	8.3E-01	0.41	3.8E-02	-0.12	5.4E-01	-0.16	4.4E-01	-0.20	3.3E-01	-0.06	7.8E-01
ENSCAFG0000000323	INPP5B	grey	EC_M10	0.04	8.4E-01	-0.64	4.5E-04	0.19	3.6E-01	0.34	9.3E-02	-0.07	7.2E-01	0.26	2.0E-01	-0.21	3.0E-01	0.03	8.7E-01	0.07	7.3E-01	-0.02	9.4E-01
ENSCAFG0000000087	BRD2	grey	EC_M10	0.04	8.4E-01	0.48	1.2E-02	-0.57	2.2E-03	0.01	9.7E-01	0.39	4.7E-02	-0.01	9.5E-01	0.19	3.5E-01	0.19	3.5E-01	0.13	5.3E-01	-0.45	2.2E-02
ENSCAFG0000001051	PKNOX1	grey	EC_M10	0.04	8.4E-01	-0.24	2.5E-01	0.06	7.8E-01	0.14	4.9E-01	-0.01	9.4E-01	-0.19	3.5E-01	0.03	8.8E-01	0.16	4.4E-01	0.36	7.3E-02	-0.02	9.3E-01
ENSCAFG0000001763	RNF165	grey	EC_M10	0.04	8.4E-01	-0.04	8.6E-01	-0.03	8.8E-01	0.00	9.9E-01	-0.03	8.9E-01	-0.06	7.7E-01	-0.11	6.0E-01	-0.08	7.1E-01	-0.08	6.9E-01	-0.02	9.2E-01
ENSCAFG0000000385	WASF1	grey	EC_M10	0.04	8.4E-01	-0.03	8.7E-01	-0.38	5.9E-02	-0.05	7.9E-01	0.41	3.8E-02	0.05	8.0E-01	-0.09	6.6E-01	0.14	4.9E-01	0.34	8.7E-02	-0.46	1.7E-02
ENSCAFG0000000810	ENSCAFG0000000810	turquoise	EC_M6	0.04	8.4E-01	0.86	2.4E-08	-0.19	3.4E-01	0.03	8.8E-01	-0.09	6.7E-01	-0.16	4.4E-01	0.01	9.5E-01	-0.22	2.8E-01	0.15	4.6E-01	0.07	7.4E-01
ENSCAFG0000000441	ENSCAFG0000000441	grey	EC_M10	0.04	8.4E-01	0.03	8.8E-01	0.36	7.3E-02	-0.58	1.8E-03	-0.38	5.8E-02	-0.16	4.5E-01	-0.09	6.7E-01	0.18	3.9E-01	-0.03	8.7E-01	0.38	5.5E-02
ENSCAFG0000002965	ENSCAFG0000002965	grey	EC_M10	0.04	8.4E-01	0.36	7.4E-02	0.05	7.9E-01	-0.18	3.7E-01	-0.22	2.8E-01	-0.20	3.3E-01	0.24	2.4E-01	-0.18	3.8E-01	-0.19	3.5E-01	0.20	3.2E-01
ENSCAFG0000003040	LRAT	darkmagenta	EC_M12	0.04	8.4E-01	0.01	9.8E-01	-0.08	6.8E-01	-0.11	6.0E-01	0.05	8.1E-01	-0.10	6.2E-01	-0.10	6.3E-01	0.90	6.6E-10	0.12	5.7E-01	-0.08	6.9E-01
ENSCAFG0000001634	WDR26	turquoise	EC_M6	0.04	8.4E-01	0.49	1.1E-02	-0.43	2.6E-02	0.05	8.0E-01	0.20	3.3E-01	0.07	7.4E-01	-0.14	5.0E-01	0.07	7.4E-01	0.00	1.0E+00	-0.22	2.8E-01
ENSCAFG0000000990	MYLIP	turquoise	EC_M6	0.04	8.4E-01	0.83	1.2E-07	-0.19	3.6E-01	0.06	7.6E-01	-0.09	6.7E-01	-0.16	4.5E-01	0.00	9.8E-01	0.01	9.7E-01	0.13	5.3E-01	0.10	6.1E-01
ENSCAFG0000000056	ENSCAFG0000000056	darkgreen	EC_M4	0.04	8.4E-01	0.32	1.1E-01	-0.80	9.7E-07	-0.19	3.5E-01	0.78	2.1E-06	0.14	4.9E-01	0.13	5.2E-01	0.19	3.6E-01	0.10	6.1E-01	-0.80	9.8E-07
ENSCAFG0000001137	AKAP13	grey	EC_M10	0.04	8.4E-01	-0.16	4.4E-01	0.03	8.7E-01	0.10	6.4E-01	-0.14	5.1E-01	-0.23	2.6E-01	0.10	6.4E-01	0.08	6.8E-01	0.00	9.8E-01	0.10	6.2E-01
ENSCAFG0000001454	PLCB3	grey	EC_M10	0.04	8.4E-01	0.22	2.9E-01	0.04	8.4E-01	-0.30	1.3E-01	-0.08	6.8E-01	-0.16	4.3E-01	-0.02	9.3E-01	0.35	7.9E-02	0.23	2.5E-01	0.08	7.0E-01
ENSCAFG0000000001	ZNF516	turquoise	EC_M6	0.04	8.4E-01	0.63	6.3E-04	-0.46	1.9E-02	-0.15	4.6E-01	0.32	1.2E-01	-0.19	3.6E-01	0.31	1.2E-01	0.18	3.9E-01	0.14	5.1E-01	-0.32	1.2E-01
ENSCAFG0000000953	SPARCL1	turquoise	EC_M6	0.04	8.4E-01	0.93	5.5E-12	-0.24	2.3E-01	-0.10	6.3E-01	-0.06	7.8E-01	-0.21	3.0E-01	-0.08	6.8E-01	-0.08	6.9E-01	0.17	4.0E-01	0.06	7.6E-01
ENSCAFG0000002001	LRRC39	grey	EC_M10	0.04	8.4E-01	0.03	8.7E-01	-0.10	6.2E-01	-0.03	8.8E-01	0.05	8.3E-01	0.27	1.9E-01	-0.01	9.6E-01	0.19	3.4E-01	0.22	2.8E-01	-0.01	9.5E-01
ENSCAFG0000001833	DOCK11	cyan	EC_M2	0.04	8.4E-01	-0.37	6.2E-02	0.61	8.6E-04	0.33	9.5E-02	-0.68	1.4E-04	-0.17	4.1E-01	-0.15	4.8E-01	-0.05	8.2E-01	-0.30	1.4E-01	0.62	7.0E-04
ENSCAFG0000000802	ENSCAFG0000000802	grey	EC_M10	0.04	8.4E-01	-0.12	5.7E-01	-0.60	1.3E-03	0.01	9.5E-01	0.70	7.0E-05	0.08	7.0E-01	0.00	9.9E-01	0.25	2.2E-01	-0.17	4.1E-01	-0.76	6.8E-06
ENSCAFG0000000475	CLASP1	grey	EC_M10	0.04	8.4E-01	0.42	3.3E-02	-0.53	5.3E-03	-0.09	6.6E-01	0.43	2.8E-02	-0.08	7.0E-01	-0.35	8.1E-02	0.22	2.7E-01	0.00	9.9E-01	-0.43	2.9E-02
ENSCAFG0000001049	CDON	grey	EC_M10	0.04	8.4E-01	0.20	3.3E-01	0.26	2.0E-01	-0.07	7.3E-01	-0.36	7.1E-02	-0.12	5.4E-01	-0.28	1.7E-01	-0.32	1.2E-01	-0.29	1.6E-01	0.39	4.9E-02
ENSCAFG0000002956	ENSCAFG0000002956	grey	EC_M10	0.04	8.4E-01	-0.32	1.1E-01	0.01	9.4E-01	-0.22	2.8E-01	0.06	7.6E-01	-0.24	2.4E-01	0.35	8.2E-02	0.05	8.0E-01	-0.06	7.7E-01	-0.09	6.8E-01
ENSCAFG0000002370	LEMD3	grey	EC_M10	0.04	8.4E-01	0.27	1.8E-01	-0.40	4.0E-02	0.15	4.6E-01	0.28	1.7E-01	0.41	3.6E-02	0.07	7.3E-01	-0.22	2.8E-01	-0.39	4.9E-02	-0.30	1.4E-01
ENSCAFG0000001129	ENSCAFG0000001129	grey	EC_M10	0.04	8.4E-01	-0.08	7.1E-01	0.19	3.4E-01	-0.18	3.9E-01	-0.17	4.1E-01	-0.11	5.9E-01	-0.02	9.1E-01	-0.07	7.3E-01	0.44	2.5E-02	0.20	3.2E-01
ENSCAFG0000000020	TBPL1	grey	EC_M10	0.04	8.4E-01	0.33	1.0E-01	-0.37	6.2E-02	0.21	2.9E-01	0.30	1.3E-01	0.24	2.4E-01	-0.23	2.6E-01	-0.44	2.6E-02	0.03	8.9E-01	-0.33	1.0E-01
ENSCAFG0000000857	MCPH1	cyan	EC_M2	0.04	8.5E-01	0.12	5.7E-01	0.48	1.3E-02	0.03	8.8E-01	-0.69	8.5E-05	-0.07	7.2E-01	-0.03	8.8E-01	-0.05	8.0E-01	0.23	2.6E-01	0.65	2.9E-04
ENSCAFG0000001931	BICDL2	turquoise	EC_M6	0.04	8.5E-01	0.41	3.6E-02	-0.26	2.0E-01	-0.03	8.7E-01	0.08	7.1E-01	-0.13	5.3E-01	0.59	1.4E-03	-0.04	8.5E-01	-0.14	5.0E-01	-0.10	6.1E-01
ENSCAFG0000001341	ENSCAFG0000001341	grey	EC_M10	0.04	8.5E-01	0.10	6.3E-01	-0.61	9.6E-04	0.02	9.2E-01	0.65	2.9E-04	0.33	1.0E-01	-0.11	6.1E-01	-0.26	2.0E-01	-0.09	6.5E-01	-0.69	8.9E-05
ENSCAFG0000001080	TMEM39B	grey	EC_M10	0.04	8.5E-01	0.56	2.8E-03	0.25	2.2E-01	0.10	6.1E-01	-0.49	1.1E-02	0.06	7.9E-01	0.03	8.7E-01	-0.23	2.5E-01	-0.09	6.7E-01	0.49	1.1E-02
ENSCAFG0000002952	ENSCAFG0000002952	grey	EC_M10	0.04	8.5E-01	-0.12	5.7E-01	0.17	4.0E-01	-0.21	3.0E-01	-0.16	4.3E-01	0.00	1.0E+00	-0.09	6.5E-01	0.04	8.6E-01	-0.33	9.7E-02	0.14	4.9E-01
ENSCAFG0000000840	TPD52	turquoise	EC_M6	0.04	8.5E-01	0.58	2.0E-03	-0.48	1.2E-02	-0.20	3.2E-01	0.35	8.4E-02	0.08	6.9E-01	0.03	9.0E-01	-0.17	4.0E-01	-0.13	5.2E-01	-0.34	9.4E-02
ENSCAFG0000003194	PDXP	grey	EC_M10	0.04	8.5E-01	-0.13	5.2E-01	0.26	2.0E-01	-0.21	3.1E-01	-0.20	3.4E-01	0.24	2.4E-01	-0.37	6.3E-02	-0.11	5.9E-01	0.24	2.4E-01	0.21	3.1E-01
ENSCAFG0000001046	NEURL1	violet	EC_M7	0.04	8.5E-01	-0.15	4.7E-01	-0.10	6.3E-01	-0.08	7.0E-01	0.19	3.6E-01	0.64	3.9E-04	-0.06	7.9E-01	-0.11	6.0E-01	-0.04	8.3E-01	-0.17	4.1E-01
ENSCAFG0000001320	SFI1	grey	EC_M10	0.04	8.5E-01	-0.24	2.4E-01	0.55	3.8E-03	-0.15	4.5E-01	-0.58	2.0E-03	-0.09	6.8E-01	-0.10	6.2E-01	0.04	8.4E-01	0.13	5.2E-01	0.60	1.2E-03
ENSCAFG0000003235	ENSCAFG0000003235	grey	EC_M10	0.04	8.5E-01	-0.15	4.7E-01	-0.28	1.6E-01	0.24	2.4E-01	0.33	9.9E-02	0.45	2.2E-02	-0.15	4.7E-01	-0.19	3.6E-01	-0.18	3.8E-01	-0.36	7.0E-02
ENSCAFG0000000480	WBP4	turquoise	EC_M6	0.04	8.5E-01	0.48	1.3E-02	-0.32	1.2E-01	0.15	4.6E-01	0.13	5.1E-01	-0.20	3.3E-01	0.04	8.4E-01	-0.09	6.5E-01	-0.20	3.3E-01	-0.12	5.5E-01
ENSCAFG0000001128	C8H14orf93	cyan	EC_M2	0.04	8.5E-01	0.12	5.5E-01	0.69	1.1E-04	0.13	5.2E-01	-0.90	4.9E-10	-0.18	3.9E-01	-0.15	4.7E-01	-0.12	5.4E-01	-0.05	8.2E-01	0.88	4.2E-09
ENSCAFG0000002436	ENSCAFG0000002436	grey	EC_M10	0.04	8.5E-01	0.01	9.6E-01	-0.07	7.3E-01	-0.07	7.5E-01	0.10	6.4E-01	-0.01	9.5E-01	0.07	7.3E-01	-0.03	8.8E-01	0.12	5.6E-01	-0.13	5.1E-01
ENSCAFG0000001379	IFT22	grey	EC_M10	0.04	8.5E-01	-0.57	2.5E-03	0.05	8.1E-01	-0.04	8.5E-01	0.16	4.4E-01	0.15	4.8E-01	-0.14	5.1E-01	-0.03	8.9E-01	-0.07	7.4E-01	-0.21	3.0E-01
ENSCAFG0000003053	GOLPH3L	grey	EC_M10	0.04	8.5E-01	0.08	6.8E-01	0.08	6.8E-01	0.23	2.7E-01	-0.28	1.7E-01	0.10	6.3E-01	-0.13	5.2E-01	-0.01	9.7E-01	-0.20	3.4E-01	0.21	3.1E-01
ENSCAFG0000000347	TSPAN12	turquoise	EC_M6	0.04	8.5E-01	0.71	5.4E-05	-0.04	8.5E-01	0.02	9.2E-01	-0.24	2.3E-01	-0.36	6.9E-02	0.17	4.0E-01	0.17	4.1E-01	0.16	4.3E-01	0.26	2.1E-01
ENSCAFG0000000986	BRD7	darkgreen	EC_M4	0.04	8.5E-01	0.06	7.7E-01	-0.76	6.1E-06	-0.11	6.0E-01	0.79	1.4E-06	0.27	1.8E-01	-0.11	6.0E-01	-0.11	6.1E-01	-0.10	6.4E-01	-0.82	3.9E-07
ENSCAFG0000003270	SCO1	grey	EC_M10	0.04	8.5E-01	-0.45	2.1E-02	-0.06	7.8E-01	-0.28	1.7E-01	0.25	2.1E-01	0.26	2.0E-01	-0.24	2.5E-01	0.01	9.4E-01	-0.32	1.1E-01	-0.28	1.6E-01
ENSCAFG0000001412	EHD1	grey	EC_M10	0.04	8.5E-01	0.44	2.4E-02	-0.49	1.1E-02	-0.33	1.0E-01	0.47	1.5E-02	-0.08	6.9E-01	-0.24	2.3E-01	0.00	9.9E-01	-0.25	2.2E-01	-0.48	1.4E-02
ENSCAFG0000000570	ENSCAFG0000000570	grey	EC_M10	0.04	8.5E-01	0.19	3.6E-01	0.30	1.4E-01	-0.09	6.5E-01	-0.42	3.4E-02	-0.13	5.2E-01	-0.13	5.3E-01	-0.09	6.6E-01	-0.04	8.4E-01	0.43	2.9E-02
ENSCAFG0000003015	ENSCAFG0000003015	grey	EC_M10	0.04	8.5E-01	-0.59	1.7E-03	0.36	6.7E-02	0.11	5.8E-01	-0.19	3.4E-01	-0.06	7.7E-01	0.06	7.6E-01	-0.02	9.4E-01	0.06	7.6E-01	0.16	4.3E-01
ENSCAFG0000000121	GFRA3	grey	EC_M10	0.04	8.5E-01	0.22	2.8E-01	0.16	4.4E-01	-0.22	2.8E-01	-0.22	2.7E-01	-0.16	4.2E-01	0.09	6.7E-01	-0.03	8.9E-01	-0.20	3.3E-01	0.22	2.7E-01
ENSCAFG0000001190	SHTN1	grey	EC_M10	0.04	8.5E-01	-0.14	5.0E-01	-0.60	1.2E-03	-0.09	6.5E-01	0.71	5.4E-05	0.37	6.3E-02	-0.10	6.4E-01	0.16	4.4E-01	-0.03	8.7E-01	-0.75	1.2E-05
ENSCAFG0000000533	CSGALNACT1	cyan	EC_M2	0.04	8.5E-01	0.29	1.4E-01	0.41	3.9E-02	0.12	5.5E-01	-0.59	1.5E-03	-0.35	8.3E-02	0.03	8.9E-01	-0.18	3.8E-01	-0.55	3.7E-03	0.57	2.4E-03
ENSCAFG0000002880	ANKRD66	grey	EC_M10	0.04	8.5E-01	-0.28	1.6E-01	-0.24	2.3E-01	0.05	8.0E-01	0.33	9.7E-02	0.05	8.1E-01	0.31	1.2E-01	0.05	8.2E-01	-0.26	2.0E-01	-0.40	4.3E-02
ENSCAFG0000002790	ENSCAFG0000002790	grey	EC_M10	0.04	8.5E-01	-0.07	7.4E-01	0.23	2.6E-01	-0.13	5.2E-01	-0.25	2.2E-01	-0.20	3.3E-01	-0.23	2.7E-01	-0.17	4.1E-01	0.04	8.4E-01	0.22	2.7E-01
ENSCAFG0000001069	ENSCAFG0000001069	turquoise	EC_M6	0.04	8.5E-01	0.56	3.0E-03	-0.13	5.1E-01	0.32	1.1E-01	-0.11	5.8E-01	-0.20	3.4E-01	0.19	3.5E-01	-0.08	7.1E-01	0.31	1.2E-01	0.11	5.8E-01
ENSCAFG0000001104	BCL9	turquoise	EC_M6	0.04	8.5E-01	0.49	1.2E-02	-0.21	3.0E-01	0.13	5.4E-01	-0.03	8.7E-01	-0.08	7.0E-01	0.02	9.4E-01	0.27	1.8E-01	0.24	2.5E-01	-0.03	8.7E-01
ENSCAFG0000002936	ENSCAFG0000002936	grey	EC_M10	0.04	8.5E-01	0.05	8.0E-01	0.32	1.1E-01	0.05	8.2E-01	-0.42	3.5E-02	0.01	9.5E-01	-0.05	8.1E-01	-0.13	5.2E-01	-0.05	7.9E-01	0.40	4.4E-02
ENSCAFG0000001501	ZNF394	cyan	EC_M2	0.04	8.5E-01	0.25	2.2E-01	0.33	1.0E-01	0.12	5.7E-01	-0.56	2.9E-03	-0.07	7.3E-01	0.25	2.2E-01	0.16	4.3E-01	-0.16	4.3E-01	0.54	4.8E-03
ENSCAFG0000001178	ENSCAFG0000001178	grey	EC_M10	0.04	8.5E-01	-0.18	3.8E-01	0.08	7.1E-01	0.17	4.0E-01	-0.13	5.2E-01	0.13	5.3E-01	0.00	9.9E-01	-0.27	1.8E-01	-0.38	5.8E-02	0.07	7.2E-01
ENSCAFG0000002439	ENSCAFG0000002439	grey	EC_M10	0.04	8.5E-01	-0.09	6.6E-01	0.02	9.4E-01	-0.09	6.7E-01	-0.06	7.8E-01	0.39	4.8E-02	-0.14	4.9E-01	-0.11	5.8E-01	-0.18	3.9E-01	-0.01	9.5E-01
ENSCAFG0000001872	BCORL1	grey	EC_M10	0.04	8.5E-01	0.54	4.8E-03	0.26	1.9E-01	0.11	6.0E-01	-0.58	2.0E-03	-0.22	2.9E-01	0.22	2.9E-01	-0.09	6.7E-01	0.12	5.7E-01	0.56	2.8E-03
ENSCAFG0000000478	ENSCAFG0000000478	grey	EC_M10	0.04	8.5E-01	-0.08	7.0E-01	-0.46	1.8E-02	0.00	9.9E-01	0.52	7.0E-03	-0.12	5.6E-01	-0.08	7.0E-01	-0.02	9.2E-01	-0.05	8.1E-01	-0.54	4.5E-03
ENSCAFG0000001331	ENSCAFG0000001331	grey	EC_M10	0.04	8.5E-01	-0.08	7.0E-01	-0.46	1.8E-02	0.00	9.9E-01	0.52	7.0E-03	-0.12	5.6E-01	-0.08	7.0E-01	-0.02	9.2E-01	-0.05	8.1E-01	-0.54	4.5E-03
ENSCAFG0000000181	PTK7	grey	EC_M10	0.04	8.5E-01	-0.09	6.7E-01	-0.59	1.5E-03	0.04	8.5E-01	0.66	2.5E-04	0.48	1.2E-02	0.14	4.9E-01	-0.08	6.9E-01	0.01	9.5E-01	-0.73	2.6E-05
ENSCAFG0000001890	SNN	turquoise	EC_M6	0.04	8.5E-01	0.70	7.1E-05	-0.33	1.0E-01	-0.29	1.5E-01	0.15	4.7E-01	-0.14	4.9E-01	0.18	3.8E-01	-0.19	3.6E-01	-0.21	3.0E-01	-0.15	4.7E-01
ENSCAFG0000002033	CTRL	grey	EC_M10	0.04	8.5E-01	-0.10	6.2E-01	0.44	2.6E-02	0.39	5.0E-02	-0.46	1.9E-02	-0.05	8.0E-01	0.00	9.9E-01	-0.19	3.6E-01	-0.37	6.2E-02	0.42	3.5E-02
ENSCAFG0000002975	PYURF	grey	EC_M10	0.04	8.6E-01	-0.60	1.2E-03	0.23	2.6E-01	-0.22	2.9E-01	-0.02	9.1E-01	0.20	3.4E-01	0.08	6.9E-01	-0.13	5.3E-01	-0.33	1.0E-01	-0.01	9.5E-01
ENSCAFG0000000090	EFCAB6	grey	EC_M10	0.04	8.6E-01	-0.40	4.1E-02	0.32	1.1E-01	0.03	8.7E-01	-0.26	2.0E-01	0.14	4.8E-01	-0.28	1.6E-01	-0.05	8.2E-01	0.14	4.9E-01	0.23	2.6E-01
ENSCAFG0000002303	ADGRE2	grey	EC_M10	0.04	8.6E-01	-0.25	2.2E-01	-0.06	7.7E-01	-0.20	3.3E-01	0.18	3.9E-01	-0.15	4.7E-01	0.18	3.9E-01	-0.04	8.4E-01	0.18	3.9E-01	-0.23	2.6E-01
ENSCAFG0000002764	ENSCAFG0000002764	grey	EC_M10	0.04	8.6E-01	0.17	4.2E-01	0.09	6.7E-01	0.23	2.5E-01	-0.26	2.0E-01	0.12	5.5E-01	-0.27	1.8E-01	0.05	8.1E-01	-0.15	4.5E-01	0.18	3.7E-01
ENSCAFG0000000062	SNX9	cyan	EC_M2	0.04	8.6E-01	-0.11	5.9E-01	0.54	4.2E-03	0.32	1.1E-01	-0.70	5.8E-05	-0.14	4.9E-01	-0.05	8.0E-01	-0.05	7.9E-01	-0.20	3.4E-01	0.67	1.9E-04
ENSCAFG0000000610	ENSCAFG0000000610	cyan	EC_M2	0.04	8.6E-01	-0.27	1.7E-01	0.65	3.1E-04	0.23	2.7E-01	-0.68	1.5E-04	-0.04	8.5E-01	0.14	5.0E-01	-0.07	7.4E-01	-0.44	2.3E-02	0.60	1.2E-03
ENSCAFG0000000463	BCAM	turquoise	EC_M6	0.04	8.6E-01	0.77	3.5E-06	-0.01	9.5E-01	-0.11	5.9E-01	-0.30	1.4E-01	-0.19	3.5E-01	0.15	4.5E-01	0.00	9.9E-01	0.48	1.3E-02	0.30	1.4E-01
ENSCAFG0000001347	EDEM3	darkgreen	EC_M4	0.04	8.6E-01	-0.23	2.7E-01	-0.65	3.0E-04	-0.11	5.9E-01	0.78	2.6E-06	0.15	4.8E-01	0.15	4.6E-01	0.15	4.6E-01	-0.19	3.5E-01	-0.81	4.4E-07
ENSCAFG0000001373	TMPRSS5	grey	EC_M10	0.04	8.6E-01	0.49	1.1E-02	0.19	3.5E-01	0.22	2.8E-01	-0.45	2.1E-02	-0.14	4.8E-01	-0.17	4.2E-01	-0.15	4.7E-01	-0.16	4.3E-01	0.46	1.7E-02
ENSCAFG0000000517	CAPN5	grey	EC_M10	0.04	8.6E-01	0.60	1.3E-03	0.29	1.6E-01	-0.08	6.9E-01	-0.53	4.9E-03	-0.31	1.2E-01	-0.03	8.8E-01	-0.08	7.0E-01	0.01	9.5E-01	0.58	2.0E-03
ENSCAFG0000000574	NAV3	darkgreen	EC_M4	0.04	8.6E-01	-0.60	1.1E-03	-0.36	6.9E-02	0.08	7.1E-01	0.57	2.4E-03	0.15	4.6E-01	0.04	8.4E-01	-0.02	9.2E-01	-0.10	6.3E-01	-0.62	7.9E-04
ENSCAFG0000001147	POLQ	darkgreen	EC_M4	0.04	8.6E-01	-0.01	9.6E-01	-0.77	3.6E-06	-0.17	4.0E-01	0.90	3.4E-10	0.13	5.2E-01	0.00	9.8E-01	-0.03	8.9E-01	-0.11	5.9E-01	-0.90	2.3E-10
ENSCAFG0000001366	KCND3	grey	EC_M10	0.04	8.6E-01	-0.12	5.7E-01	0.49	1.1E-02	-0.11	5.9E-01	-0.51	7.7E-03	-0.26	2.0E-01	0.10	6.3E-01	0.08	7.1E-01	0.29	1.5E-01	0.56	3.1E-03
ENSCAFG0000003258	ENSCAFG0000003258	turquoise	EC_M6	0.04	8.6E-01	0.47	1.6E-02	-0.29	1.5E-01	-0.16	4.5E-01	0.19	3.6E-01	-0.12	5.5E-01	0.46	1.9E-02	-0.01	9.6E-01	0.05	8.2E-01	-0.15	4.7E-01
ENSCAFG0000001487	LRRC25	turquoise	EC_M6	0.04	8.6E-01	0.65	3.7E-04	-0.25	2.1E-01	-0.12	5.6E-01	0.04	8.3E-01	-0.11	5.9E-01	-0.12	5.7E-01	-0.08	6.9E-01	-0.10	6.2E-01	-0.02	9.1E-01
ENSCAFG0000001484	FERMT2	grey	EC_M10	0.04	8.6E-01	-0.53	5.5E-03	-0.36	7.4E-02	0.06	7.8E-01	0.57	2.3E-03	0.36	7.2E-02	0.06	7.7E-01	-0.09	6.7E-01	-0.36	7.4E-02	-0.60	1.3E-03
ENSCAFG0000003131	P3H3	darkgreen	EC_M4	0.04	8.6E-01	-0.36	7.2E-02	-0.43	3.0E-02	0.10	6.1E-01	0.57	2.5E-03	0.26	2.0E-01	0.21	2.9E-01	-0.17	4.1E-01	-0.33	9.7E-02	-0.65	3.1E-04
ENSCAFG0000000150	CDC42EP1	turquoise	EC_M6	0.04	8.6E-01	0.65	3.1E-04	-0.18	3.8E-01	0.01	9.5E-01	-0.07	7.3E-01	-0.18	3.8E-01	0.09	6.7E-01	0.10	6.4E-01	0.53	5.9E-03	0.03	8.8E-01
ENSCAFG0000002832	ENSCAFG0000002832	paleturquoise	EC_M11	0.04	8.6E-01	-0.18	3.9E-01	0.28	1.7E-01	0.56	2.9E-03	-0.25	2.2E-01	-0.08	7.1E-01	-0.09	6.6E-01	-0.11	5.9E-01	-0.19	3.5E-01	0.26	2.0E-01
ENSCAFG0000000371	CRYBG1	cyan	EC_M2	0.04	8.6E-01	0.26	2.0E-01	0.57	2.6E-03	0.03	8.9E-01	-0.77	3.7E-06	-0.34	8.7E-02	-0.20	3.3E-01	0.02	9.2E-01	0.17	4.1E-01	0.76	6.7E-06

ENSCAFG0000001010	GANC	cyan	EC_M2	0.04	8.6E-01	0.00	9.9E-01	0.33	1.0E-01	0.47	1.4E-02	-0.49	1.0E-02	-0.18	3.9E-01	-0.20	3.4E-01	0.21	2.9E-01	0.10	6.3E-01	0.46	1.7E-02
ENSCAFG0000000348	STK17A	grey	EC_M10	0.04	8.6E-01	-0.41	4.0E-02	0.46	1.8E-02	0.07	7.2E-01	-0.38	5.6E-02	-0.09	6.5E-01	-0.46	1.7E-02	-0.04	8.3E-01	-0.20	3.2E-01	0.41	3.7E-02
ENSCAFG0000001553	NAALADL2	grey	EC_M10	0.04	8.6E-01	0.09	6.6E-01	-0.20	3.3E-01	0.15	4.5E-01	0.23	2.5E-01	0.09	6.5E-01	-0.26	1.9E-01	-0.03	9.0E-01	-0.46	1.8E-02	-0.25	2.2E-01
ENSCAFG0000001447	IGFBP5	turquoise	EC_M6	0.04	8.6E-01	0.43	2.9E-02	-0.01	9.6E-01	-0.12	5.7E-01	-0.18	3.8E-01	0.11	5.8E-01	-0.05	7.9E-01	-0.14	5.1E-01	-0.22	2.8E-01	0.09	6.5E-01
ENSCAFG0000003069	ZNF593	grey	EC_M10	0.04	8.6E-01	-0.55	3.3E-03	-0.08	7.0E-01	-0.21	2.9E-01	0.35	8.4E-02	-0.04	8.4E-01	-0.13	5.4E-01	0.28	1.7E-01	-0.04	8.4E-01	-0.36	7.1E-02
ENSCAFG0000001926	SMG6	grey	EC_M10	0.04	8.6E-01	0.03	8.7E-01	-0.69	9.5E-05	-0.14	5.0E-01	0.78	2.6E-06	0.28	1.7E-01	0.03	9.0E-01	0.00	9.8E-01	-0.23	2.5E-01	-0.82	2.9E-07
ENSCAFG0000000148	TRIOBP	grey	EC_M10	0.04	8.6E-01	-0.12	5.5E-01	0.37	6.0E-02	0.03	8.8E-01	-0.35	7.6E-02	0.03	8.8E-01	-0.28	1.7E-01	0.20	3.4E-01	-0.39	5.2E-02	0.32	1.1E-01
ENSCAFG0000000184	TRPM3	magenta	EC_M13	0.04	8.6E-01	0.29	1.5E-01	-0.12	5.5E-01	-0.16	4.5E-01	-0.03	8.8E-01	-0.16	4.3E-01	-0.09	6.5E-01	0.34	9.3E-02	0.75	1.2E-05	0.00	1.0E+00
ENSCAFG0000001166	C20H3orf62	grey	EC_M10	0.04	8.6E-01	0.47	1.6E-02	-0.46	1.7E-02	-0.22	2.7E-01	0.34	8.7E-02	0.02	9.1E-01	-0.16	4.4E-01	-0.19	3.5E-01	0.09	6.7E-01	-0.33	1.0E-01
ENSCAFG0000000485	EPB41L5	grey	EC_M10	0.04	8.6E-01	0.32	1.1E-01	-0.63	6.2E-04	-0.31	1.2E-01	0.53	5.2E-03	-0.22	2.7E-01	0.08	6.9E-01	0.11	6.0E-01	0.25	2.3E-01	-0.56	2.9E-03
ENSCAFG0000001895	EMP2	cyan	EC_M2	0.04	8.6E-01	0.19	3.5E-01	0.54	4.6E-03	0.10	6.2E-01	-0.70	7.1E-05	-0.13	5.2E-01	0.12	5.5E-01	-0.09	6.5E-01	-0.25	2.1E-01	0.70	7.9E-05
ENSCAFG0000001204	PCNT	cyan	EC_M2	0.04	8.6E-01	-0.11	6.0E-01	0.68	1.2E-04	0.12	5.5E-01	-0.75	8.8E-06	-0.34	9.1E-02	0.02	9.1E-01	-0.01	9.5E-01	-0.04	8.5E-01	0.71	4.1E-05
ENSCAFG0000001115	CASP7	darkgreen	EC_M4	0.04	8.6E-01	0.45	2.0E-02	-0.68	1.2E-04	-0.18	3.8E-01	0.65	3.0E-04	0.16	4.4E-01	0.02	9.1E-01	0.02	9.1E-01	-0.13	5.4E-01	-0.67	1.8E-04
ENSCAFG0000000738	EIF2AK3	turquoise	EC_M6	0.04	8.6E-01	0.64	4.8E-04	-0.27	1.9E-01	0.09	6.7E-01	-0.07	7.2E-01	-0.01	9.7E-01	0.04	8.3E-01	0.01	9.6E-01	0.10	6.4E-01	0.03	8.7E-01
ENSCAFG0000001774	TBX4	grey	EC_M10	0.04	8.6E-01	-0.19	3.6E-01	0.45	2.3E-02	-0.11	6.0E-01	-0.41	3.6E-02	-0.04	8.3E-01	-0.05	7.9E-01	-0.06	7.8E-01	-0.09	6.7E-01	0.44	2.3E-02
ENSCAFG0000002315	C1H18orf63	grey	EC_M10	0.04	8.6E-01	-0.25	2.2E-01	0.14	5.0E-01	0.15	4.7E-01	-0.15	4.6E-01	0.27	1.8E-01	0.18	3.7E-01	-0.12	5.7E-01	-0.30	1.4E-01	0.06	7.6E-01
ENSCAFG0000003194	ENSCAFG0000003194	grey	EC_M10	0.04	8.6E-01	-0.19	3.5E-01	0.09	6.8E-01	-0.12	5.7E-01	-0.01	9.7E-01	-0.13	5.2E-01	-0.12	5.6E-01	0.04	8.3E-01	-0.09	6.5E-01	-0.01	9.5E-01
ENSCAFG0000000147	ENSCAFG0000000147	grey	EC_M10	0.04	8.6E-01	-0.23	2.6E-01	-0.23	2.7E-01	0.35	7.5E-02	0.31	1.3E-01	0.27	1.9E-01	-0.15	4.6E-01	-0.21	3.0E-01	-0.26	2.0E-01	-0.35	8.3E-02
ENSCAFG0000002287	ENSCAFG0000002287	grey	EC_M10	0.04	8.7E-01	-0.20	3.2E-01	-0.01	9.7E-01	-0.18	3.8E-01	0.04	8.3E-01	0.22	2.9E-01	0.16	4.2E-01	0.44	2.4E-02	-0.06	7.8E-01	-0.12	5.6E-01
ENSCAFG0000001843	RAI1	turquoise	EC_M6	0.03	8.7E-01	0.71	4.1E-05	-0.35	7.6E-02	-0.09	6.4E-01	0.15	4.7E-01	-0.18	3.7E-01	-0.09	6.7E-01	-0.08	7.0E-01	0.42	3.4E-02	-0.14	5.0E-01
ENSCAFG0000000041	MDM2	grey	EC_M10	0.03	8.7E-01	-0.44	2.4E-02	0.09	6.8E-01	0.26	2.0E-01	-0.03	8.7E-01	0.18	3.8E-01	0.43	2.9E-02	-0.18	3.7E-01	-0.26	2.0E-01	0.00	9.9E-01
ENSCAFG0000000116	KHDRBS3	grey	EC_M10	0.03	8.7E-01	-0.20	3.3E-01	-0.47	1.5E-02	-0.14	4.9E-01	0.54	4.2E-03	0.23	2.7E-01	-0.33	1.0E-01	0.16	4.5E-01	0.25	2.1E-01	-0.62	7.5E-04
ENSCAFG0000002418	DPEP2	grey	EC_M10	0.03	8.7E-01	0.28	1.7E-01	0.08	7.1E-01	-0.25	2.2E-01	-0.22	2.8E-01	-0.06	7.6E-01	0.11	5.9E-01	0.00	9.9E-01	0.04	8.5E-01	0.25	2.2E-01
ENSCAFG0000003060	ENSCAFG0000003060	grey	EC_M10	0.03	8.7E-01	-0.13	5.2E-01	0.21	3.0E-01	-0.17	4.0E-01	-0.19	3.4E-01	0.19	3.5E-01	-0.19	3.5E-01	-0.16	4.4E-01	-0.27	1.9E-01	0.18	3.9E-01
ENSCAFG0000000414	RPN1	darkgreen	EC_M4	0.03	8.7E-01	-0.56	3.0E-03	-0.40	4.0E-02	0.04	8.6E-01	0.70	6.9E-05	0.22	2.8E-01	-0.02	9.3E-01	0.05	8.0E-01	-0.17	4.2E-01	-0.72	2.9E-05
ENSCAFG0000000221	TRAM2	turquoise	EC_M6	0.03	8.7E-01	0.49	1.1E-02	-0.25	2.1E-01	0.10	6.2E-01	0.12	5.5E-01	-0.23	2.6E-01	-0.02	9.1E-01	0.17	4.0E-01	-0.01	9.5E-01	-0.14	5.0E-01
ENSCAFG0000000162	OPN1SW	grey	EC_M10	0.03	8.7E-01	-0.16	4.3E-01	-0.38	5.8E-02	0.04	8.5E-01	0.49	1.1E-02	0.35	8.3E-02	-0.09	6.7E-01	-0.09	6.6E-01	-0.16	4.4E-01	-0.50	8.9E-03
ENSCAFG0000000562	P2RY6	turquoise	EC_M6	0.03	8.7E-01	0.66	2.1E-04	-0.07	7.2E-01	-0.05	8.2E-01	-0.16	4.4E-01	-0.10	6.4E-01	-0.08	7.1E-01	-0.10	6.3E-01	0.50	9.2E-03	0.13	5.3E-01
ENSCAFG0000000260	RNF20	grey	EC_M10	0.03	8.7E-01	-0.18	3.8E-01	-0.26	2.0E-01	0.05	8.0E-01	0.31	1.2E-01	0.13	5.2E-01	-0.29	1.6E-01	0.03	8.7E-01	-0.16	4.4E-01	-0.29	1.6E-01
ENSCAFG0000000603	ABHD13	turquoise	EC_M6	0.03	8.7E-01	0.69	1.0E-04	-0.42	3.5E-02	-0.14	4.9E-01	0.19	3.6E-01	0.07	7.2E-01	0.12	5.5E-01	-0.02	9.4E-01	-0.07	7.4E-01	-0.20	3.2E-01
ENSCAFG0000001580	MAT1A	grey	EC_M10	0.03	8.7E-01	-0.03	8.9E-01	0.09	6.7E-01	-0.01	9.7E-01	-0.12	5.8E-01	-0.08	7.1E-01	-0.05	8.1E-01	-0.08	7.0E-01	0.41	4.0E-02	0.11	5.8E-01
ENSCAFG0000000017	FAM210A	darkmagenta	EC_M12	0.03	8.7E-01	0.08	6.9E-01	-0.22	2.9E-01	-0.05	8.0E-01	0.23	2.5E-01	-0.30	1.4E-01	0.02	9.2E-01	0.67	1.6E-04	0.07	7.4E-01	-0.25	2.1E-01
ENSCAFG0000000002	ENSCAFG0000000002	grey	EC_M10	0.03	8.7E-01	0.19	3.5E-01	0.11	5.8E-01	0.33	9.6E-02	-0.27	1.8E-01	-0.18	3.9E-01	0.08	6.9E-01	-0.20	3.3E-01	-0.16	4.4E-01	0.31	1.3E-01
ENSCAFG0000001104	PDCD6	grey	EC_M10	0.03	8.7E-01	0.19	3.6E-01	-0.42	3.1E-02	-0.23	2.6E-01	0.46	1.7E-02	-0.17	4.0E-01	-0.04	8.4E-01	0.15	4.7E-01	0.02	9.2E-01	-0.41	3.8E-02
ENSCAFG0000000203	PLA2G7	grey	EC_M10	0.03	8.7E-01	-0.57	2.6E-03	0.06	7.8E-01	0.22	2.8E-01	0.02	9.2E-01	0.06	7.9E-01	0.12	5.7E-01	0.13	5.2E-01	0.20	3.3E-01	-0.09	6.5E-01
ENSCAFG0000001251	TMOD4	turquoise	EC_M6	0.03	8.7E-01	0.65	3.0E-04	0.08	7.0E-01	0.07	7.3E-01	-0.35	7.7E-02	-0.16	4.4E-01	-0.16	4.3E-01	-0.21	3.0E-01	0.37	6.3E-02	0.38	5.8E-02
ENSCAFG0000003014	ENSCAFG0000003014	grey	EC_M10	0.03	8.7E-01	0.04	8.3E-01	0.04	8.5E-01	0.21	3.0E-01	-0.16	4.2E-01	-0.01	9.4E-01	0.03	9.0E-01	-0.12	5.5E-01	0.11	5.9E-01	0.16	4.2E-01
ENSCAFG0000002947	ENSCAFG0000002947	grey	EC_M10	0.03	8.7E-01	-0.25	2.2E-01	-0.03	9.0E-01	-0.11	5.9E-01	0.15	4.5E-01	0.18	3.7E-01	-0.12	5.7E-01	-0.22	2.9E-01	0.13	5.3E-01	-0.16	4.3E-01
ENSCAFG0000002023	ENSCAFG0000002023	grey	EC_M10	0.03	8.7E-01	-0.19	3.5E-01	0.08	6.9E-01	0.46	1.8E-02	-0.09	6.7E-01	0.51	7.4E-03	0.35	8.2E-02	-0.08	6.8E-01	-0.17	4.0E-01	0.08	7.1E-01
ENSCAFG0000000077	AGER	grey	EC_M10	0.03	8.7E-01	0.32	1.1E-01	0.04	8.4E-01	0.15	4.6E-01	-0.18	3.9E-01	-0.32	1.1E-01	-0.31	1.2E-01	0.24	2.5E-01	0.13	5.3E-01	0.19	3.6E-01
ENSCAFG0000002501	C11H5orf24	grey	EC_M10	0.03	8.7E-01	0.32	1.1E-01	-0.59	1.5E-03	-0.27	1.9E-01	0.59	1.6E-03	0.24	2.3E-01	0.23	2.7E-01	0.02	9.2E-01	-0.18	3.7E-01	-0.62	7.1E-04
ENSCAFG0000000507	SPHK1	grey	EC_M10	0.03	8.7E-01	-0.02	9.2E-01	0.42	3.2E-02	0.05	8.3E-01	-0.51	7.2E-03	0.14	5.1E-01	0.40	4.3E-02	-0.16	4.4E-01	-0.41	3.9E-02	0.48	1.3E-02
ENSCAFG0000001230	DPAGT1	grey	EC_M10	0.03	8.7E-01	-0.02	9.1E-01	-0.02	9.3E-01	-0.17	4.1E-01	0.05	8.2E-01	0.28	1.7E-01	-0.02	9.3E-01	0.11	5.8E-01	0.04	8.6E-01	-0.11	5.9E-01
ENSCAFG0000000316	BPGM	grey	EC_M10	0.03	8.7E-01	0.31	1.2E-01	-0.30	1.4E-01	-0.14	4.9E-01	0.18	3.8E-01	0.30	1.3E-01	-0.03	9.0E-01	0.09	6.5E-01	0.05	8.2E-01	-0.17	3.9E-01
ENSCAFG0000002315	ENSCAFG0000002315	grey	EC_M10	0.03	8.7E-01	0.13	5.3E-01	-0.47	1.6E-02	-0.04	8.5E-01	0.44	2.6E-02	-0.06	7.8E-01	-0.16	4.4E-01	0.04	8.3E-01	0.54	4.1E-03	-0.45	2.2E-02
ENSCAFG0000002645	ENSCAFG0000002645	violet	EC_M7	0.03	8.7E-01	-0.05	8.1E-01	-0.13	5.4E-01	-0.09	6.8E-01	0.12	5.4E-01	0.72	3.4E-05	-0.05	8.1E-01	-0.12	5.5E-01	-0.16	4.3E-01	-0.19	3.4E-01
ENSCAFG0000001454	KLHL20	grey	EC_M10	0.03	8.7E-01	-0.06	7.5E-01	-0.15	4.7E-01	0.16	4.3E-01	0.07	7.5E-01	0.21	3.0E-01	-0.22	2.9E-01	0.08	7.0E-01	-0.40	4.0E-02	-0.11	5.8E-01
ENSCAFG0000000961	SETD4	grey	EC_M10	0.03	8.7E-01	0.46	1.8E-02	0.20	3.2E-01	0.20	3.3E-01	-0.37	6.3E-02	-0.11	5.9E-01	0.10	6.1E-01	-0.12	5.7E-01	0.20	3.3E-01	0.40	4.3E-02
ENSCAFG0000001243	METTL5	grey	EC_M10	0.03	8.7E-01	-0.35	8.3E-02	0.07	7.4E-01	0.35	7.8E-02	-0.01	9.7E-01	-0.18	3.8E-01	-0.21	3.0E-01	0.29	1.6E-01	0.28	1.7E-01	-0.03	8.9E-01
ENSCAFG0000001825	PLS3	darkgreen	EC_M4	0.03	8.7E-01	-0.08	6.8E-01	-0.74	1.6E-05	-0.23	2.5E-01	0.87	9.9E-09	0.14	4.9E-01	0.12	5.7E-01	0.14	4.9E-01	0.01	9.7E-01	-0.88	4.5E-09
ENSCAFG0000001692	SNX12	grey	EC_M10	0.03	8.7E-01	-0.22	2.8E-01	-0.09	6.8E-01	-0.43	2.7E-02	0.19	3.6E-01	-0.24	2.4E-01	0.33	1.0E-01	-0.03	8.8E-01	-0.14	5.1E-01	-0.22	2.8E-01
ENSCAFG0000000992	GMPR	grey	EC_M10	0.03	8.8E-01	0.36	7.2E-02	-0.64	4.1E-04	-0.05	8.1E-01	0.62	7.1E-04	0.40	4.3E-02	0.03	8.7E-01	-0.09	6.5E-01	0.20	3.3E-01	-0.63	5.2E-04
ENSCAFG0000000975	ENSCAFG0000000975	grey	EC_M10	0.03	8.8E-01	-0.16	4.2E-01	0.14	4.9E-01	-0.15	4.7E-01	-0.19	3.6E-01	-0.07	7.3E-01	-0.17	3.9E-01	-0.15	4.6E-01	-0.20	3.3E-01	0.12	5.6E-01
ENSCAFG0000002996	APCDD1L	magenta	EC_M13	0.03	8.8E-01	-0.04	8.3E-01	0.05	7.9E-01	-0.11	6.0E-01	-0.10	6.1E-01	-0.03	8.7E-01	-0.05	8.3E-01	0.29	1.4E-01	0.69	1.1E-04	0.08	7.1E-01
ENSCAFG0000002932	ENSCAFG0000002932	grey	EC_M10	0.03	8.8E-01	-0.06	7.7E-01	0.05	8.2E-01	-0.06	7.8E-01	-0.10	6.2E-01	-0.08	7.0E-01	-0.12	5.8E-01	0.01	9.8E-01	-0.05	7.9E-01	0.05	8.2E-01
ENSCAFG0000001605	ITPKB	turquoise	EC_M6	0.03	8.8E-01	0.88	2.5E-09	-0.33	9.5E-02	-0.10	6.4E-01	0.06	7.7E-01	-0.07	7.3E-01	0.06	7.9E-01	-0.05	8.0E-01	0.00	1.0E+00	-0.09	6.7E-01
ENSCAFG0000000801	ENSCAFG0000000801	grey	EC_M10	0.03	8.8E-01	-0.11	6.1E-01	0.14	5.1E-01	0.18	3.9E-01	-0.17	4.1E-01	0.15	4.5E-01	0.07	7.4E-01	-0.28	1.7E-01	0.22	2.9E-01	0.17	4.0E-01
ENSCAFG0000001173	CADM3	turquoise	EC_M6	0.03	8.8E-01	0.84	7.4E-08	-0.28	1.7E-01	-0.06	7.6E-01	0.04	8.6E-01	-0.19	3.6E-01	-0.04	8.6E-01	-0.10	6.4E-01	0.11	5.9E-01	-0.02	9.1E-01
ENSCAFG0000002107	ENSCAFG0000002107	grey	EC_M10	0.03	8.8E-01	-0.17	4.1E-01	0.00	9.8E-01	-0.14	4.9E-01	0.02	9.2E-01	-0.05	8.0E-01	-0.15	4.8E-01	-0.05	7.9E-01	-0.10	6.4E-01	-0.05	8.1E-01
ENSCAFG0000001010	KIF13A	grey	EC_M10	0.03	8.8E-01	-0.32	1.2E-01	0.33	9.7E-02	0.09	6.5E-01	-0.32	1.1E-01	-0.28	1.6E-01	-0.25	2.1E-01	0.24	2.3E-01	-0.05	7.9E-01	0.30	1.3E-01
ENSCAFG0000000925	ENSCAFG0000000925	grey	EC_M10	0.03	8.8E-01	-0.02	9.2E-01	0.01	9.6E-01	-0.11	6.0E-01	0.00	9.9E-01	-0.04	8.4E-01	0.03	8.9E-01	0.04	8.6E-01	0.11	6.0E-01	-0.05	8.1E-01
ENSCAFG0000001096	DPYSL4	grey	EC_M10	0.03	8.8E-01	-0.02	9.2E-01	0.01	9.6E-01	-0.11	6.0E-01	0.00	9.9E-01	-0.04	8.4E-01	0.03	8.9E-01	0.04	8.6E-01	0.11	6.0E-01	-0.05	8.1E-01
ENSCAFG0000002998	ENSCAFG0000002998	grey	EC_M10	0.03	8.8E-01	-0.17	4.0E-01	0.36	7.5E-02	-0.12	5.5E-01	-0.29	1.5E-01	-0.06	7.5E-01	-0.02	9.1E-01	-0.05	8.0E-01	-0.08	7.1E-01	0.30	1.4E-01
ENSCAFG0000000833	M1AP	grey	EC_M10	0.03	8.8E-01	-0.13	5.2E-01	-0.42	3.2E-02	-0.18	3.7E-01	0.51	7.7E-03	0.11	5.8E-01	0.00	9.9E-01	-0.23	2.7E-01	-0.23	2.5E-01	-0.55	3.3E-03
ENSCAFG0000001616	SRP9	grey	EC_M10	0.03	8.8E-01	-0.20	3.3E-01	-0.43	2.7E-02	-0.43	2.8E-02	0.61	1.0E-03	0.05	8.2E-01	0.05	8.2E-01	0.40	4.3E-02	0.12	5.5E-01	-0.63	5.8E-04
ENSCAFG0000000046	ENSCAFG0000000046	grey	EC_M10	0.03	8.8E-01	-0.28	1.6E-01	-0.09	6.5E-01	0.24	2.5E-01	0.11	6.0E-01	0.06	7.7E-01	-0.32	1.2E-01	0.20	3.2E-01	0.19	3.6E-01	-0.15	4.6E-01
ENSCAFG0000003247	ENSCAFG0000003247	grey	EC_M10	0.03	8.8E-01	-0.02	9.4E-01	-0.62	7.7E-04	-0.05	8.2E-01	0.71	4.4E-05	0.31	1.2E-01	0.11	6.0E-01	-0.11	5.9E-01	-0.17	4.2E-01	-0.73	2.7E-05
ENSCAFG0000000178	ACO1	grey	EC_M10	0.03	8.8E-01	0.25	2.3E-01	0.28	1.6E-01	0.22	2.7E-01	-0.48	1.4E-02	-0.15	4.6E-01	-0.42	3.1E-02	-0.07	7.3E-01	0.07	7.4E-01	0.46	1.9E-02
ENSCAFG0000003003	PDSS2	grey	EC_M10	0.03	8.8E-01	-0.09	6.7E-01	0.18	3.8E-01	0.35	7.6E-02	-0.27	1.8E-01	-0.31	1.2E-01	-0.46	1.8E-02	-0.27	1.8E-01	0.17	3.9E-01	0.25	2.3E-01
ENSCAFG0000001412	SEC16B	grey	EC_M10	0.03	8.8E-01	-0.28	1.6E-01	-0.37	6.6E-02	0.01	9.5E-01	0.46	1.9E-02	0.28	1.6E-01	0.00	9.9E-01	0.04	8.5E-01	-0.15	4.8E-01	-0.54	4.7E-03
ENSCAFG0000000361	AMPH	grey	EC_M10	0.03	8.8E-01	-0.20	3.4E-01	-0.13	5.2E-01	-0.26	2.0E-01	0.25	2.3E-01	0.16	4.3E-01	0.36	7.2E-02	-0.14	4.9E-01	0.02	9.3E-01	-0.22	2.9E-01
ENSCAFG0000002996	RGS4	turquoise	EC_M6	0.03	8.8E-01	0.79	1.6E-06	-0.23	2.5E-01	-0.01	9.6E-01	-0.03	8.7E-01	0.00	9.9E-01	-0.03	8.8E-01	0.03	9.0E-01	0.49	1.0E-02	0.06	7.9E-01
ENSCAFG0000001122	TTBK2	grey	EC_M10	0.03	8.8E-01	0.30	1.4E-01	0.27	1.9E-01	-0.22	2.8E-01	-0.50	1.0E-02	-0.36	6.9E-02	0.12	5.5E-01	-0.03	8.8E-01	0.15	4.8E-01	0.46	1.7E-02
ENSCAFG0000000428	ENSCAFG0000000428	grey	EC_M10	0.03	8.8E-01	-0.05	8.2E-01	0.29	1.5E-01	0.07	7.2E-01	-0.36	6.8E-02	-0.33	1.0E-01	0.03	9.0E-01	-0.13	5.1E-01	-0.31	1.3E-01	0.34	9.1E-02
ENSCAFG0000000043	PPP1R18	turquoise	EC_M6	0.03	8.8E-01	0.69	9.6E-05	-0.25	2.1E-01	-0.09	6.6E-01	0.06	7.6E-01	0.06	7.8E-01	0.30	1.4E-01	0.09	6.7E-01	-0.12	5.7E-01	-0.09	6.5E-01
ENSCAFG0000000848	ENSCAFG0000000848	grey	EC_M10	0.03	8.8E-01	-0.07	7.4E-01	-0.28	1.6E-01	-0.16	4.4E-01	0.30	1.4E-01	-0.11	5.9E-01	-0.05	8.2E-01	0.00	9.9E-01	0.02	9.4E-01	-0.33	9.8E-02
ENSCAFG0000001181	COL18A1	grey	EC_M10	0.03	8.8E-01	0.45	2.0E-02	0.36	6.8E-02	0.13	5.4E-01	-0.56	3.1E-03	-0.24	2.4E-01	0.15	4.7E-01	-0.45	2.0E-02	-0.10	6.2E-01	0.54	4.3E-03
ENSCAFG0000000999	WARS2	cyan	EC_M2	0.03	8.8E-01	-0.04	8.5E-01	0.68	1.5E-04	0.13	5.2E-01	-0.81	5.5E-07	-0.25	2.2E-01	-0.18	3.7E-01	-0.13	5.2E-01	0.01	9.7E-01	0.80	1.2E-06
ENSCAFG0000001625	ENSCAFG0000001625	grey	EC_M10	0.03	8.8E-01	-0.16	4.2E-01	-0.19	3.5E-01	-0.35	8.1E-02	0.21	3.1E-01	0.07	7.2E-01	-0.03	8.8E-01	-0.29	1.5E-01	-0.38	5.4E-02	-0.24	2.4E-01
ENSCAFG0000003212	CHST11	darkgreen	EC_M4	0.03	8.8E-01	0.38	5.6E-02	-0.80	1.1E-06	-0.08	7.0E-01	0.75	1.0E-05	0.20	3.2E-01	-0.06	7.7E-01	-0.08	6.9E-01	-0.10	6.2E-01	-0.79	1.6E-06
ENSCAFG0000001117	SLC7A7	turquoise	EC_M6	0.03	8.8E-01	0.75	1.1E-05	-0.26	2.0E-01	-0.36	7.2E-02	0.06	7.9E-01	-0.13	5.4E-01	0.08	6.9E-01	-0.01	9.4E-01	-0.03	8.8E-01	-0.04	8.4E-01
ENSCAFG0000001329	IGF2BP2	grey	EC_M10	0.03	8.8E-01	0.17	4.0E-01	-0.55	3.6E-03	-0.02	9.4E-01	0.51	7.9E-03	0.17	4.0E-01	0.04	8.4E-01	-0.02	9.4E-01	-0.33	9.8E-02	-0.57	2.2E-03
ENSCAFG0000001360	SPG11	grey	EC_M10	0.03	8.8E-01	-0.40	4.2E-02	0.02	9.4E-01	0.30	1.3E-01	0.13	5.3E-01	-0.05	8.0E-01	-0.10	6.1E-01	-0.43	2.8E-02	-0.39	4.8E-02	-0.11	6.0E-01
ENSCAFG0000000468	NOS3	turquoise	EC_M6	0.03	8.8E-01	0.88	3.3E-09	-0.15	4.7E-01	-0.04	8.5E-01	-0.16	4.4E-01	-0.15	4.7E-01	-0.07	7.4E-01	-0.11	5.8E-01	0.18	3.8E-01	0.12	5.6E-01
ENSCAFG0000001716	KCNN3	turquoise	EC_M6	0.03	8.8E-01	0.81	5.4E-07	-0.35	7.7E-02	-0.15	4.6E-01	0.10	6.3E-01	-0.14	4.9E-01	-0.14	4.8E-01	-0.17	4.1E-01	0.03	8.8E-01	-0.12	5.5E-01
ENSCAFG0000003211	RCE1	grey	EC_M10	0.03	8.8E-01	0.30	1.3E-01	0.37	6.6E-02	-0.23	2.5E-01	-0.48	1.4E-02	-0.15	4.8E-01	0.11	5.9E-01	0.17	4.0E-01	0.05	8.0E-01	0.48	1.2E-02
ENSCAFG0000000432	TSPAN1	violet	EC_M7	0.03	8.8E-01	-0.13	5.3E-01	-0.06	7.6E-01	-0.08	7.0E-01	0.08	7.1E-01	0.61	8.7E-04	-0.07	7.3E-01	-0.14	5.0E-01	-0.21	3.1E-01	-0.13	5.3E-01
ENSCAFG0000002277	ENSCAFG0000002277	grey	EC_M10	0.03	8.8E-01	-0.13	5.2E-01	0.47	1.6E-02	0.41	3.5E-02	-0.54	4.2E-03	0.02	9.2E-01	0.15	4.5E-01	0.17	4.0E-01	0.09	6.7E-01	0.52	6.8E-03
ENSCAFG0000000438	RSPH6A	grey	EC_M10	0.03	8.8E-01	0.14	5.1E-01	-0.19	3.4E-01	-0.09	6.5E-01	0.19	3.5E-01	0.14	4.9E-01	-0.03	8.7E-01	0.18	3.8E-01	-0.10	6.3E-01	-0.21	3.0E-01
ENSCAFG0000000394	SPATA6	turquoise	EC_M6	0.03	8.8E-01	0.53	5.3E-03	-0.05	8.0E-01	-0.10	6.2E-01	-0.14	4.8E-01	-0.22	2.8E-01	0.23	2.6E-01	-0.22	2.9E-01	-0.36	7.4E-02	0.15	4.7E-01
ENSCAFG0000001260	PSMD2	darkgreen	EC_M4	0.03	8.8E-01	-0.12	5.7E-01	-0.70	6.4E-05	-0.20	3.3E-01	0.89	1.5E-09	0.25	2.3E-01	0.07	7.5E-01	0.03	8.9E-01	-0.01	9.5E-01	-0.88	2.0E-09
ENSCAFG0000000673	ENSCAFG0000000673	grey	EC_M10	0.03	8.9E-01	-0.08	7.1E-01	-0.15	4.7E-01	0.05	8.2E-01	0.15	4.8E-01	-0.17	4.0E-01	0.11	5.8E-01	0.21	3.0E-01	0.18	3.8E-01	-0.14	5.0E-01
ENSCAFG0000001723	ENSCAFG0000001723	grey	EC_M10	0.03	8.9E-01	-0.10	6.2E-01	-0.07	7.3E-01	0.11	6.1E-01	0.07	7.4E-01	-0.11	5.9E-01	-0.08	6.9E-01	-0.01	9.5E-01	-0.13	5.4E-01	-0.14	5.0E-01
ENSCAFG0000001221	RAB4A	grey	EC_M10	0.03	8.9E-01	-0.22	2.7E-01	0.08	6.8E-01	0.21	2.9E-01	-0.08	6.9E-01	-0.15	4.6E-01	-0.01	9.5E-01	-0.05	8.1E-01	0.12	5.7E-01	0.10	6.2E-01
ENSCAFG0000001710	GCDH	cyan	EC_M2	0.03	8.9E-01	-0.14	4.9E-01	0.68	1.4E-04	0.25	2.2E-01	-0.75	8.5E-06	-0.47	1.7E-02	-0.16	4.4E-01	0.02	9.2E-01	-0.06	7.8E-01	0.76	6.5E-06
ENSCAFG0000001256	ENSCAFG0000001256	turquoise	EC_M6	0.03	8.9E-01	0.57	2.5E-03	-0.16	4.3E-01	-0.07	7.2E-01	-0.07	7.4E-01	-0.06	7.6E-01	-0.02	9.3E-01	-0.11	6.0E-01	-0.16	4.4E-01	0.02	9.3E-01
ENSCAFG0000000827	LYSMD3	grey	EC_M10	0.03	8.9E-01	0.34	9.0E-02	-0.33	9.6E-02	0.25	2.3E-01	0.21	2.9E-01	-0.17	4.1E-01	-0.25	2.2E-01	-0.10	6.1E-01	-0.20	3.2E-01	-0.21	2.9E-01
ENSCAFG0000000089	PNPLA3	grey	EC_M10	0.03	8.9E-01	-0.27	1.8E-01	0.20	3.3E-01	0.03	8.8E-01	-0.19	3.5E-01	-0.32	1.1E-01	0.18	3.9E-01	-0.29	1.4E-01	-0.05	8.2E-01	0.12	5.4E-01
ENSCAFG0000002713	ENSCAFG0000002713	grey	EC_M10	0.03	8.9E-01	-0.32	1.1E-01	-0.03	8.8E-01	0.36	7.2E-02	0.06	7.7E-01	0.38	5.5E-02	0.13	5.3E-01	0.10	6.4E-01	-0.17	4.2E-01	-0.09	6.8E-01
ENSCAFG0000003045	ENSCAFG0000003045	turquoise	EC_M6	0.03	8.9E-01	0.72	3.2E-05	0.07	7.4E-01	0.04	8.3E-01	-0.42	3.4E-02	-0.06	7.5E-01	0.16	4.3E-01	0.04	8.5E-01	0.12	5.6E-01	0.40	4.2E-02
ENSCAFG0000000062	ENSCAFG0000000062	cyan	EC_M2	0.03	8.9E-01	0.06	7.7E-01	0.58	1.7E-03	0.33	9.6E-02	-0.76	5.9E-06	-0.36	7.1E-02	-0.16	4.3E-01	0.07	7.2E-01	0.04	8.6E-01	0.75	9.6E-06
ENSCAFG0000000514	SRSF2	grey	EC_M10	0.03	8.9E-01	-0.14	5.0E-01	0.44	2.5E-02	-0.05	8.2E-01	-0.36	7.4E-02	-0.14	5.1E-01	-0.02	9.2E-01	-0.16	4.5E-01	-0.07	7.4E-01	0.35	8.4E-02
ENSCAFG0000001285	ZSCAN2	turquoise	EC_M6	0.03	8.9E-01	0.42	3.2E-02	-0.10	6.2E-01	0.07	7.4E-01	-0.05	8.1E-01	0.10	6.4E-01	0.20	3.4E-01	-0.11	5.8E-01	0.17	4.2E-01	0.00	9.9E-01
ENSCAFG0000000815	PLSCR4	cyan	EC_M2	0.03	8.9E-01	0.15	4.5E-01	0.43	2.7E-02	0.28	1.7E-01	-0.62	7.6E-04	-0.23	2.7E-01	-0.18	3.8E-01	0.11	5.8E-01	-0.30	1.3E-01	0.60	1.3E-03
ENSCAFG0000001998	PLCG2	turquoise	EC_M6	0.03	8.9E-01	0.79	1.4E-06	-0.13	5.3E-01	-0.07	7.3E-01	-0.19	3.5E-01	-0.29	1.5E-01	0.16	4.2E-01	-0.13	5.4E-01	0.34	8.4E-02	0.18	3.8E-01
ENSCAFG0000003154	SRP19	grey	EC_M10	0.03	8.9E-01	-0.16	4.2E-01	0.01	9.5E-01	0.31	1.3E-01	0.00	9.8E-01	0.23	2.7E-01	-0.34	8.8E-02	-0.24	2.4E-01	-0.27	1.8E-01	-0.02	9.2E-01
ENSCAFG0000000055	RNF19A	darkgreen	EC_M4	0.03	8.9E-01	0.50	9.6E-03	-0.72	3.1E-05	-0.11	5.9E-01	0.60	1.1E-03	0.11	6.1E-01	-0.16	4.4E-01	0.02	9.2E-01	0.06	7.9E-01	-0.62	8.2E-04
ENSCAFG0000001767	GGA2	cyan	EC_M2	0.03	8.9E-01	0.52	6.4E-03	0.33	1.0E-01	-0.14	5.0E-01	-0.59	1.6E-03	-0.34	8.9E-02	-0.04	8.5E-01	-0.26	2.0E-01	-0.23	2.7E-01	0.59	1.5E-03
ENSCAFG0000002140	ENSCAFG0000002140	violet	EC_M7	0.03	8.9E-01	-0.03	8.7E-01	-0.19	3.6E-01	-0.08	7.1E-01	0.16	4.4E-01	0.63	5.0E-04	-0.05	8.0E-01	0.47	1.6E-02	0.50	9.4E-03	-0.18	3.9E-01
ENSCAFG0000000272	ABCA1	grey	EC_M10	0.03	8.9E-01	0.52	6.8E-03	-0.46	1.7E-02	-0.20	3.2E-01	0.37	6.2E-02	0.00	9.9E-01	-0.14	4.9E-01	-0.32	1.2E-01	-0.20	3.3E-01	-0.38	5.8E-02
ENSCAFG0000001402	TTN	grey	EC_M10	0.03	8.9E-01	-0.01	9.5E-01	-0.05	7.9E-01	-0.19	3.6E-01	0.07	7.3E-01	-0.16	4.3E-01	0.20	3.2E-01	-0.05	7.9E-01	0.02	9.4E-01	-0.03	8.7E-01
ENSCAFG0000002036	PLEKHG4	grey	EC_M10	0.03	8.9E-01	-0.44	2.4E-02	0.47	1.5E-02	0.17	4.1E-01	-0.44	2.6E-02	-0.12	5.7E-01	0.09	6.6E-01	0.17	4.2E-01	0.33	9.6E-02	0.42	3.5E-02
ENSCAFG0000000042	ZBTB2	grey	EC_M10	0.03	8.9E-01	-0.16	4.5E-01	0.51	8.3E-03	0.10	6.1E-01	-0.57	2.2E-03	-0.17	3.9E-01	-0.47	1.7E-02	-0.25	2.2E-01	-0.25	2.1E-01	0.52	5.9E-03
ENSCAFG0000002975	MMD	turquoise	EC_M6	0.03	8.9E-01	0.74	1.5E-05	-0.63	5.0E-04	-0.13	5.4E-01	0.45	2.0E-02	-0.04	8.6E-01	0.09	6.8E-01	-0.03	8.8E-01	0.33	1.0E-01	-0.44	2.3E-02
ENSCAFG0000000274	FAM126A	darkgreen	EC_M4	0.03	8.9E-01	-0.10	6.3E-01	-0.69	9.8E-05	-0.28	1.7E-01	0.83	1.5E-07	0.09	6.6E-01	0.12	5.7E-01	0.13	5.4E-01	-0.17	4.1E-01	-0.85	5.6E-08
ENSCAFG0000001028	TSPAN5	cyan	EC_M2	0.03	8.9E-01	-0.38	5.5E-02	0.64	4.2E-04	0.15	4.7E-01	-0.64	3.8E-04	-0.27	1.8E-01	-0.33	1.0E-01	0.06	7.7E-01	-0.41	3.6E-02	0.61	8.8E-04

ENSCAFG0000001775	PPM1D	cyan	EC_M2	0.03	8.9E-01	0.26	2.0E-01	0.41	3.8E-02	0.37	6.5E-02	-0.65	3.0E-04	-0.22	2.7E-01	-0.04	8.6E-01	-0.11	5.9E-01	0.18	3.9E-01	0.64	3.9E-04
ENSCAFG0000001377	PLA2G4A	grey	EC_M10	0.03	8.9E-01	-0.02	9.3E-01	0.09	6.7E-01	-0.12	5.5E-01	-0.15	4.6E-01	-0.36	7.3E-02	0.13	5.2E-01	0.35	8.4E-02	0.12	5.7E-01	0.10	6.2E-01
ENSCAFG0000001896	SSH2	cyan	EC_M2	0.03	8.9E-01	-0.10	6.4E-01	0.67	2.0E-04	0.25	2.2E-01	-0.81	4.4E-07	-0.24	2.5E-01	-0.10	6.2E-01	-0.11	5.8E-01	-0.16	4.4E-01	0.79	1.5E-06
ENSCAFG0000001232	UQCRC1	grey	EC_M10	0.03	8.9E-01	-0.31	1.2E-01	0.24	2.4E-01	-0.11	6.0E-01	-0.07	7.3E-01	-0.10	6.1E-01	-0.22	2.7E-01	-0.24	2.4E-01	-0.01	9.5E-01	0.12	5.5E-01
ENSCAFG0000001053	CARMIL1	cyan	EC_M2	0.03	8.9E-01	0.10	6.2E-01	0.63	5.4E-04	0.06	7.6E-01	-0.81	4.8E-07	-0.25	2.2E-01	-0.13	5.2E-01	0.02	9.2E-01	0.07	7.4E-01	0.85	5.2E-08
ENSCAFG0000000932	ARHGAP24	grey	EC_M10	0.03	8.9E-01	-0.09	6.5E-01	-0.58	1.9E-03	0.04	8.4E-01	0.70	7.9E-05	0.22	2.8E-01	0.33	1.0E-01	-0.04	8.5E-01	0.06	7.5E-01	-0.73	2.5E-05
ENSCAFG0000001164	ENSCAFG0000001164	grey	EC_M10	0.03	8.9E-01	-0.09	6.6E-01	0.29	1.5E-01	-0.16	4.5E-01	-0.35	8.1E-02	-0.11	6.1E-01	-0.11	6.0E-01	-0.05	8.1E-01	-0.02	9.2E-01	0.34	8.6E-02
ENSCAFG0000000153	ZDHHC21	grey	EC_M10	0.03	8.9E-01	-0.04	8.5E-01	-0.52	6.8E-03	0.09	6.6E-01	0.61	9.1E-04	0.24	2.3E-01	0.20	3.4E-01	-0.14	4.8E-01	0.00	1.0E+00	-0.57	2.6E-03
ENSCAFG0000001593	CYP4F8	turquoise	EC_M6	0.03	8.9E-01	0.33	1.0E-01	-0.01	9.6E-01	-0.15	4.7E-01	-0.12	5.5E-01	-0.04	8.5E-01	0.13	5.3E-01	-0.05	8.0E-01	0.38	5.5E-02	0.09	6.5E-01
ENSCAFG0000000818	FCHO2	cyan	EC_M2	0.03	8.9E-01	0.49	1.2E-02	0.23	2.6E-01	0.19	3.6E-01	-0.58	1.8E-03	-0.20	3.4E-01	-0.09	6.7E-01	0.00	1.0E+00	0.07	7.4E-01	0.57	2.3E-03
ENSCAFG0000003218	SGPP1	grey	EC_M10	0.03	8.9E-01	-0.71	5.7E-05	0.35	8.0E-02	0.32	1.1E-01	-0.23	2.5E-01	0.05	8.1E-01	-0.16	4.3E-01	0.08	6.9E-01	0.13	5.4E-01	0.22	2.8E-01
ENSCAFG0000000361	CLCN1	grey	EC_M10	0.03	8.9E-01	-0.25	2.2E-01	0.09	6.5E-01	-0.21	3.0E-01	0.00	9.9E-01	0.40	4.4E-02	0.25	2.2E-01	-0.09	6.5E-01	-0.23	2.5E-01	0.00	9.9E-01
ENSCAFG0000000390	FGF21	grey	EC_M10	0.03	8.9E-01	-0.11	6.1E-01	0.12	5.5E-01	0.42	3.1E-02	-0.16	4.4E-01	-0.04	8.6E-01	-0.07	7.5E-01	-0.15	4.6E-01	-0.20	3.2E-01	0.08	7.0E-01
ENSCAFG0000001165	EAF2	turquoise	EC_M6	0.03	8.9E-01	0.51	7.8E-03	-0.36	7.4E-02	-0.12	5.7E-01	0.28	1.7E-01	-0.10	6.4E-01	-0.10	6.3E-01	-0.12	5.7E-01	-0.06	7.7E-01	-0.27	1.8E-01
ENSCAFG0000003261	ENSCAFG0000003261	cyan	EC_M2	0.03	8.9E-01	-0.14	5.0E-01	0.60	1.2E-03	0.20	3.2E-01	-0.72	3.6E-05	-0.26	1.9E-01	0.06	7.7E-01	-0.02	9.2E-01	-0.08	6.9E-01	0.71	5.2E-05
ENSCAFG0000000605	SLC16A3	grey	EC_M10	0.03	8.9E-01	0.44	2.6E-02	-0.41	3.5E-02	-0.37	6.1E-02	0.35	7.7E-02	0.03	8.9E-01	0.32	1.1E-01	0.13	5.3E-01	0.30	1.4E-01	-0.36	6.7E-02
ENSCAFG0000001359	ST7L	grey	EC_M10	0.03	8.9E-01	0.07	7.2E-01	0.28	1.7E-01	-0.04	8.3E-01	-0.40	4.1E-02	-0.41	3.9E-02	-0.32	1.2E-01	0.33	1.0E-01	0.12	5.7E-01	0.34	9.0E-02
ENSCAFG0000001723	ENSCAFG0000001723	turquoise	EC_M6	0.03	8.9E-01	0.37	6.0E-02	0.24	2.4E-01	-0.15	4.7E-01	-0.45	2.2E-02	0.07	7.4E-01	-0.04	8.6E-01	0.00	9.8E-01	0.10	6.3E-01	0.43	2.8E-02
ENSCAFG0000001854	C1GALT1C1	grey	EC_M10	0.03	8.9E-01	-0.26	2.0E-01	-0.35	7.7E-02	-0.17	4.0E-01	0.53	5.5E-03	0.06	7.7E-01	0.40	4.4E-02	0.33	1.0E-01	-0.01	9.5E-01	-0.51	7.7E-03
ENSCAFG0000001654	ZNF346	grey	EC_M10	0.03	8.9E-01	-0.19	3.6E-01	0.27	1.9E-01	0.33	1.0E-01	-0.31	1.2E-01	0.04	8.4E-01	-0.14	4.9E-01	0.07	7.3E-01	0.11	6.1E-01	0.30	1.3E-01
ENSCAFG0000001173	DCUN1D1	grey	EC_M10	0.03	9.0E-01	0.16	4.4E-01	-0.46	1.9E-02	0.03	8.8E-01	0.35	8.4E-02	0.00	9.9E-01	-0.06	7.6E-01	0.09	6.6E-01	-0.01	9.7E-01	-0.40	4.5E-02
ENSCAFG0000001460	TRIM9	turquoise	EC_M6	0.03	9.0E-01	0.37	6.3E-02	-0.36	7.0E-02	-0.08	6.9E-01	0.34	9.2E-02	0.01	9.5E-01	0.03	8.9E-01	-0.27	1.8E-01	-0.20	3.3E-01	-0.37	5.9E-02
ENSCAFG0000002013	RPL12	cyan	EC_M2	0.03	9.0E-01	-0.41	3.6E-02	0.57	2.5E-03	0.15	4.5E-01	-0.54	4.5E-03	0.00	9.9E-01	0.18	3.8E-01	-0.01	9.7E-01	-0.38	5.5E-02	0.45	2.2E-02
ENSCAFG0000001400	CLDN16	grey	EC_M10	0.03	9.0E-01	-0.15	4.7E-01	-0.50	9.2E-03	-0.09	6.5E-01	0.61	1.0E-03	0.20	3.3E-01	-0.11	5.8E-01	-0.07	7.2E-01	-0.07	7.2E-01	-0.59	1.4E-03
ENSCAFG0000001241	ENSCAFG0000001241	grey	EC_M10	0.03	9.0E-01	-0.13	5.2E-01	0.20	3.2E-01	0.23	2.6E-01	-0.25	2.2E-01	0.15	4.8E-01	-0.37	6.2E-02	-0.08	6.9E-01	0.01	9.7E-01	0.23	2.6E-01
ENSCAFG0000003259	ZNF556	grey	EC_M10	0.03	9.0E-01	-0.23	2.6E-01	0.15	4.6E-01	-0.16	4.5E-01	-0.04	8.6E-01	0.37	6.5E-02	-0.10	6.2E-01	-0.06	7.9E-01	-0.12	5.5E-01	0.05	8.3E-01
ENSCAFG0000000781	ENSCAFG0000000781	grey	EC_M10	0.03	9.0E-01	-0.26	2.0E-01	-0.02	9.1E-01	-0.20	3.2E-01	0.06	7.9E-01	0.18	3.9E-01	0.13	5.3E-01	0.13	5.3E-01	-0.09	6.5E-01	-0.13	5.4E-01
ENSCAFG0000003069	ENSCAFG0000003069	turquoise	EC_M6	0.03	9.0E-01	0.46	1.8E-02	0.23	2.6E-01	0.06	7.6E-01	-0.41	3.9E-02	-0.07	7.2E-01	-0.12	5.7E-01	-0.18	3.9E-01	0.09	6.7E-01	0.43	3.0E-02
ENSCAFG0000001968	CARD9	grey	EC_M10	0.03	9.0E-01	-0.54	4.7E-03	0.34	9.1E-02	0.35	7.7E-02	-0.23	2.7E-01	-0.09	6.7E-01	-0.31	1.2E-01	0.01	9.6E-01	-0.02	9.3E-01	0.20	3.3E-01
ENSCAFG0000001710	ANKDD1A	grey	EC_M10	0.03	9.0E-01	-0.36	7.5E-02	0.34	8.9E-02	0.09	6.6E-01	-0.31	1.2E-01	-0.23	2.6E-01	0.19	3.5E-01	-0.13	5.3E-01	-0.22	2.9E-01	0.34	9.0E-02
ENSCAFG0000001233	MPP4	darkgreen	EC_M4	0.03	9.0E-01	-0.28	1.7E-01	-0.62	8.0E-04	-0.29	1.6E-01	0.83	1.5E-07	0.34	9.2E-02	-0.07	7.2E-01	0.28	1.7E-01	-0.13	5.3E-01	-0.86	2.0E-08
ENSCAFG0000001851	ENSCAFG0000001851	turquoise	EC_M6	0.03	9.0E-01	0.38	5.7E-02	-0.32	1.1E-01	-0.19	3.6E-01	0.25	2.2E-01	-0.03	9.0E-01	-0.16	4.5E-01	0.01	9.6E-01	0.02	9.4E-01	-0.27	1.9E-01
ENSCAFG0000000182	CROT	grey	EC_M10	0.03	9.0E-01	-0.18	3.9E-01	0.39	4.9E-02	0.25	2.1E-01	-0.44	2.3E-02	0.02	9.1E-01	0.04	8.6E-01	-0.22	2.7E-01	0.27	1.8E-01	0.43	2.8E-02
ENSCAFG0000002877	MRPS6	grey	EC_M10	0.03	9.0E-01	-0.43	3.0E-02	-0.38	5.9E-02	-0.08	6.9E-01	0.58	1.7E-03	0.21	3.0E-01	-0.08	6.8E-01	-0.03	8.7E-01	0.32	1.2E-01	-0.62	7.3E-04
ENSCAFG0000003220	C15H4orf46	grey	EC_M10	0.03	9.0E-01	0.13	5.4E-01	0.27	1.7E-01	0.02	9.4E-01	-0.32	1.1E-01	-0.26	2.1E-01	-0.15	4.6E-01	0.04	8.5E-01	-0.24	2.4E-01	0.33	1.0E-01
ENSCAFG0000000003	SOCS6	grey	EC_M10	0.03	9.0E-01	0.20	3.3E-01	-0.45	2.1E-02	-0.16	4.5E-01	0.36	6.9E-02	-0.03	8.9E-01	0.06	7.8E-01	-0.07	7.5E-01	-0.04	8.4E-01	-0.37	6.6E-02
ENSCAFG0000000056	MIOX	darkgreen	EC_M4	0.03	9.0E-01	-0.10	6.4E-01	-0.01	9.6E-01	-0.09	6.7E-01	0.14	4.9E-01	-0.10	6.2E-01	-0.04	8.4E-01	0.02	9.1E-01	0.02	9.2E-01	-0.12	5.6E-01
ENSCAFG0000000413	AMOTL1	grey	EC_M10	0.03	9.0E-01	0.06	7.7E-01	0.31	1.2E-01	0.40	4.2E-02	-0.41	3.8E-02	-0.38	5.6E-02	0.10	6.2E-01	0.05	8.1E-01	0.33	1.1E-01	0.40	4.0E-02
ENSCAFG0000001809	ENSCAFG0000001809	grey	EC_M10	0.03	9.0E-01	0.41	3.7E-02	-0.36	7.0E-02	0.19	3.4E-01	0.24	2.4E-01	0.08	6.8E-01	-0.18	3.8E-01	-0.17	4.1E-01	-0.25	2.3E-01	-0.26	2.0E-01
ENSCAFG0000000250	SLC2A1	grey	EC_M10	0.03	9.0E-01	0.53	5.1E-03	-0.69	9.1E-05	-0.33	1.0E-01	0.64	4.0E-04	0.07	7.4E-01	0.19	3.4E-01	0.16	4.3E-01	0.14	5.0E-01	-0.62	6.6E-04
ENSCAFG0000001322	CNKSR2	turquoise	EC_M6	0.03	9.0E-01	0.63	5.5E-04	-0.02	9.4E-01	0.23	2.6E-01	-0.24	2.3E-01	-0.22	2.8E-01	-0.22	2.9E-01	-0.27	1.9E-01	-0.01	9.6E-01	0.24	2.5E-01
ENSCAFG0000000482	ENSCAFG0000000482	grey	EC_M10	0.03	9.0E-01	0.06	7.8E-01	-0.57	2.5E-03	0.06	7.6E-01	0.61	1.0E-03	0.50	1.0E-02	0.02	9.2E-01	-0.05	8.1E-01	-0.09	6.5E-01	-0.67	1.6E-04
ENSCAFG0000000633	FUT10	grey	EC_M10	0.03	9.0E-01	-0.13	5.4E-01	0.46	1.8E-02	-0.27	1.8E-01	-0.48	1.3E-02	-0.08	7.0E-01	0.07	7.5E-01	-0.07	7.3E-01	-0.03	8.9E-01	0.51	8.2E-03
ENSCAFG0000003041	BCL2L11	grey	EC_M10	0.03	9.0E-01	0.09	6.7E-01	-0.21	2.9E-01	-0.09	6.7E-01	0.09	6.6E-01	0.32	1.1E-01	0.22	2.7E-01	-0.31	1.2E-01	0.40	4.4E-02	-0.14	4.9E-01
ENSCAFG0000001172	ENSCAFG0000001172	grey	EC_M10	0.03	9.0E-01	-0.22	2.9E-01	0.29	1.6E-01	0.52	6.6E-03	-0.26	2.0E-01	-0.13	5.3E-01	-0.07	7.4E-01	-0.02	9.3E-01	-0.17	4.0E-01	0.23	2.6E-01
ENSCAFG0000001897	ENSCAFG0000001897	turquoise	EC_M6	0.03	9.0E-01	0.45	2.0E-02	-0.36	7.2E-02	0.01	9.5E-01	0.19	3.6E-01	-0.20	3.2E-01	0.11	6.0E-01	0.00	9.9E-01	-0.10	6.2E-01	-0.22	2.7E-01
ENSCAFG0000000026	IFNGR1	turquoise	EC_M6	0.03	9.0E-01	0.63	5.8E-04	0.02	9.1E-01	-0.21	2.9E-01	-0.21	2.9E-01	-0.20	3.4E-01	0.22	2.9E-01	0.11	6.1E-01	-0.19	3.7E-01	0.23	2.7E-01
ENSCAFG0000001880	TTC23L	grey	EC_M10	0.03	9.0E-01	0.14	4.8E-01	-0.57	2.5E-03	-0.23	2.6E-01	0.57	2.3E-03	0.13	5.4E-01	0.10	6.2E-01	0.08	6.9E-01	0.09	6.5E-01	-0.64	4.7E-04
ENSCAFG0000000928	CREB3L1	grey	EC_M10	0.03	9.0E-01	-0.26	1.9E-01	-0.13	5.3E-01	0.04	8.5E-01	0.25	2.1E-01	0.27	1.8E-01	-0.15	4.6E-01	-0.01	9.6E-01	0.03	8.7E-01	-0.33	1.0E-01
ENSCAFG0000000362	LPIN1	cyan	EC_M2	0.03	9.0E-01	0.29	1.5E-01	0.44	2.4E-02	0.20	3.3E-01	-0.69	1.1E-04	-0.49	1.1E-02	0.11	5.8E-01	-0.04	8.4E-01	-0.17	4.1E-01	0.67	1.6E-04
ENSCAFG0000002408	ENSCAFG0000002408	grey	EC_M10	0.03	9.0E-01	-0.14	5.1E-01	-0.03	8.8E-01	-0.19	3.4E-01	0.13	5.3E-01	0.42	3.4E-02	-0.13	5.2E-01	-0.11	5.8E-01	-0.18	3.9E-01	-0.15	4.8E-01
ENSCAFG0000001108	ENSCAFG0000001108	grey	EC_M10	0.03	9.0E-01	0.35	7.7E-02	0.06	7.6E-01	-0.12	5.4E-01	-0.23	2.5E-01	-0.17	4.2E-01	-0.11	6.0E-01	-0.14	5.1E-01	-0.12	5.7E-01	0.24	2.3E-01
ENSCAFG0000002419	PROB1	grey	EC_M10	0.03	9.0E-01	0.18	3.9E-01	-0.10	6.3E-01	-0.14	4.9E-01	0.08	7.0E-01	-0.18	3.8E-01	-0.14	5.0E-01	0.43	2.7E-02	-0.04	8.4E-01	-0.09	6.5E-01
ENSCAFG0000001755	TEX14	darkgreen	EC_M4	0.02	9.0E-01	-0.08	7.0E-01	-0.70	6.0E-05	-0.09	6.6E-01	0.77	4.1E-06	0.23	2.5E-01	0.03	8.8E-01	0.21	3.1E-01	-0.22	2.7E-01	-0.80	1.0E-06
ENSCAFG0000002760	ENSCAFG0000002760	grey	EC_M10	0.02	9.0E-01	0.21	3.0E-01	-0.15	4.8E-01	-0.07	7.4E-01	0.10	6.4E-01	-0.10	6.4E-01	0.47	1.7E-02	-0.05	8.0E-01	-0.10	6.2E-01	-0.14	4.8E-01
ENSCAFG0000003043	ENSCAFG0000003043	grey	EC_M10	0.02	9.0E-01	-0.16	4.4E-01	0.04	8.6E-01	0.38	5.2E-02	-0.01	9.6E-01	0.46	1.7E-02	-0.14	5.1E-01	-0.15	4.7E-01	-0.20	3.3E-01	0.01	9.6E-01
ENSCAFG0000001503	ENSCAFG0000001503	grey	EC_M10	0.02	9.0E-01	-0.10	6.2E-01	0.17	4.1E-01	-0.11	5.8E-01	-0.13	5.2E-01	-0.04	8.4E-01	-0.05	8.0E-01	0.01	9.8E-01	0.05	8.0E-01	0.13	5.4E-01
ENSCAFG0000001718	NAP1L2	turquoise	EC_M6	0.02	9.0E-01	0.43	2.7E-02	-0.08	7.1E-01	0.01	9.8E-01	-0.15	4.6E-01	-0.08	7.1E-01	-0.05	8.2E-01	-0.12	5.6E-01	0.49	1.1E-02	0.14	4.8E-01
ENSCAFG0000002085	ENSCAFG0000002085	turquoise	EC_M6	0.02	9.0E-01	0.42	3.3E-02	0.12	5.7E-01	-0.33	1.0E-01	-0.25	2.1E-01	-0.02	9.4E-01	0.07	7.2E-01	0.39	4.9E-02	0.20	3.2E-01	0.26	2.0E-01
ENSCAFG0000000340	CAV1	grey	EC_M10	0.02	9.0E-01	0.29	1.5E-01	0.14	4.9E-01	-0.20	3.3E-01	-0.19	3.6E-01	-0.16	4.3E-01	-0.08	6.8E-01	0.15	4.8E-01	-0.19	3.5E-01	0.18	3.8E-01
ENSCAFG0000000491	GALK1	grey	EC_M10	0.02	9.0E-01	0.31	1.2E-01	0.50	9.3E-03	-0.12	5.7E-01	-0.67	2.0E-04	-0.30	1.4E-01	-0.09	6.6E-01	-0.14	5.1E-01	-0.16	4.5E-01	0.66	2.2E-04
ENSCAFG0000001626	ENSCAFG0000001626	grey	EC_M10	0.02	9.1E-01	-0.36	7.4E-02	-0.40	4.5E-02	-0.23	2.7E-01	0.63	5.6E-04	0.19	3.4E-01	-0.28	1.6E-01	0.22	2.7E-01	-0.04	8.4E-01	-0.66	2.2E-04
ENSCAFG0000001207	KCNH1	grey	EC_M10	0.02	9.1E-01	-0.25	2.2E-01	-0.10	6.3E-01	-0.21	2.9E-01	0.27	1.8E-01	0.10	6.1E-01	0.26	2.1E-01	0.02	9.1E-01	-0.17	4.0E-01	-0.25	2.1E-01
ENSCAFG0000002700	ENSCAFG0000002700	grey	EC_M10	0.02	9.1E-01	-0.28	1.7E-01	0.25	2.2E-01	0.12	5.6E-01	-0.24	2.5E-01	-0.25	2.2E-01	-0.18	3.7E-01	0.11	5.9E-01	0.12	5.7E-01	0.22	2.8E-01
ENSCAFG0000001740	AKAP1	magenta	EC_M13	0.02	9.1E-01	-0.12	5.7E-01	0.12	5.7E-01	0.03	9.0E-01	-0.11	5.9E-01	-0.30	1.4E-01	0.16	4.3E-01	0.33	9.9E-02	0.69	1.1E-04	0.12	5.5E-01
ENSCAFG0000001272	ENSCAFG0000001272	grey	EC_M10	0.02	9.1E-01	-0.05	8.1E-01	0.05	8.2E-01	-0.02	9.4E-01	-0.07	7.5E-01	-0.06	7.8E-01	-0.10	6.2E-01	-0.07	7.2E-01	-0.18	3.9E-01	-0.01	9.8E-01
ENSCAFG0000000366	ENSCAFG0000000366	grey	EC_M10	0.02	9.1E-01	0.03	8.7E-01	-0.10	6.4E-01	-0.06	7.7E-01	0.01	9.8E-01	-0.05	8.0E-01	-0.04	8.3E-01	-0.12	5.5E-01	0.33	9.9E-02	-0.06	7.7E-01
ENSCAFG0000001066	AMN1	turquoise	EC_M6	0.02	9.1E-01	0.54	4.8E-03	-0.37	6.2E-02	0.02	9.3E-01	0.22	2.8E-01	-0.17	4.0E-01	-0.24	2.3E-01	0.23	2.5E-01	0.08	7.0E-01	-0.20	3.2E-01
ENSCAFG0000000944	SLC10A6	cyan	EC_M2	0.02	9.1E-01	0.01	9.6E-01	0.61	8.4E-04	0.01	9.4E-01	-0.78	3.2E-06	-0.10	6.3E-01	0.07	7.4E-01	-0.15	4.8E-01	-0.04	8.4E-01	0.75	8.9E-06
ENSCAFG0000001582	TUT1	grey	EC_M10	0.02	9.1E-01	0.28	1.6E-01	0.31	1.2E-01	0.18	3.7E-01	-0.52	6.8E-03	0.02	9.1E-01	0.38	5.7E-02	-0.13	5.3E-01	0.02	9.1E-01	0.47	1.5E-02
ENSCAFG0000000713	TNFRSF19	turquoise	EC_M6	0.02	9.1E-01	0.56	3.1E-03	-0.19	3.6E-01	0.13	5.3E-01	-0.04	8.6E-01	0.00	9.9E-01	-0.13	5.2E-01	0.03	8.9E-01	0.63	5.4E-04	0.02	9.0E-01
ENSCAFG0000002188	ENSCAFG0000002188	grey	EC_M10	0.02	9.1E-01	0.18	3.8E-01	0.06	7.8E-01	-0.04	8.6E-01	-0.16	4.3E-01	-0.06	7.8E-01	-0.04	8.6E-01	-0.11	6.1E-01	0.29	1.5E-01	0.15	4.7E-01
ENSCAFG0000001354	SWT1	darkgreen	EC_M4	0.02	9.1E-01	0.25	2.1E-01	-0.69	1.0E-04	-0.08	7.0E-01	0.68	1.4E-04	0.16	4.5E-01	-0.26	2.0E-01	0.06	7.6E-01	0.09	6.8E-01	-0.66	2.7E-04
ENSCAFG0000003007	ENSCAFG0000003007	grey	EC_M10	0.02	9.1E-01	-0.16	4.3E-01	0.27	1.8E-01	-0.20	3.4E-01	-0.23	2.5E-01	-0.18	3.7E-01	0.16	4.3E-01	-0.14	4.9E-01	-0.30	1.4E-01	0.20	3.3E-01
ENSCAFG0000002737	ENSCAFG0000002737	grey	EC_M10	0.02	9.1E-01	-0.06	7.6E-01	0.09	6.6E-01	-0.11	6.1E-01	-0.12	5.5E-01	-0.01	9.7E-01	0.34	8.6E-02	-0.07	7.2E-01	0.32	1.1E-01	0.09	6.6E-01
ENSCAFG0000003074	ENSCAFG0000003074	grey	EC_M10	0.02	9.1E-01	-0.30	1.4E-01	-0.25	2.1E-01	-0.23	2.7E-01	0.39	4.9E-02	0.15	4.7E-01	0.17	4.2E-01	0.15	4.6E-01	-0.21	3.1E-01	-0.39	4.9E-02
ENSCAFG0000000460	ENSCAFG0000000460	grey	EC_M10	0.02	9.1E-01	0.25	2.1E-01	-0.60	1.1E-03	-0.06	7.6E-01	0.58	2.1E-03	0.11	5.8E-01	-0.15	4.7E-01	0.09	6.6E-01	0.04	8.6E-01	-0.57	2.4E-03
ENSCAFG0000000370	RAB33B	grey	EC_M10	0.02	9.1E-01	0.01	9.5E-01	0.07	7.5E-01	-0.05	8.1E-01	-0.16	4.3E-01	-0.03	8.9E-01	0.00	9.9E-01	0.00	9.9E-01	-0.13	5.1E-01	0.06	7.8E-01
ENSCAFG0000002971	ENSCAFG0000002971	grey	EC_M10	0.02	9.1E-01	0.10	6.2E-01	0.28	1.6E-01	-0.23	2.6E-01	-0.38	5.5E-02	-0.22	2.9E-01	0.18	3.9E-01	0.27	1.8E-01	-0.10	6.2E-01	0.36	6.8E-02
ENSCAFG0000001594	CCSER2	darkgreen	EC_M4	0.02	9.1E-01	0.03	9.0E-01	-0.79	1.8E-06	-0.05	7.9E-01	0.84	6.4E-08	0.17	4.1E-01	0.02	9.0E-01	0.05	7.9E-01	0.15	4.7E-01	-0.85	3.9E-08
ENSCAFG0000001459	UFD1	grey	EC_M10	0.02	9.1E-01	0.00	9.9E-01	-0.62	8.1E-04	-0.23	2.6E-01	0.74	1.6E-05	0.23	2.6E-01	-0.22	2.7E-01	0.08	7.1E-01	-0.01	9.6E-01	-0.77	3.4E-06
ENSCAFG0000001539	YPEL1	turquoise	EC_M6	0.02	9.1E-01	0.84	9.9E-08	-0.08	7.0E-01	-0.26	2.1E-01	-0.18	3.7E-01	0.00	9.8E-01	-0.06	7.6E-01	0.04	8.3E-01	0.03	8.8E-01	0.18	3.8E-01
ENSCAFG0000000753	ENSCAFG0000000753	grey	EC_M10	0.02	9.1E-01	-0.12	5.7E-01	-0.17	4.0E-01	0.01	9.5E-01	0.21	3.1E-01	-0.10	6.3E-01	-0.10	6.4E-01	-0.08	7.1E-01	-0.11	6.0E-01	-0.25	2.1E-01
ENSCAFG0000000448	PICALM	darkgreen	EC_M4	0.02	9.1E-01	-0.03	8.9E-01	-0.80	9.1E-07	-0.22	2.7E-01	0.91	9.5E-11	0.30	1.3E-01	0.03	9.0E-01	0.02	9.1E-01	0.03	8.8E-01	-0.92	2.4E-11
ENSCAFG0000000547	ENSCAFG0000000547	grey	EC_M10	0.02	9.1E-01	-0.07	7.3E-01	-0.20	3.3E-01	-0.10	6.1E-01	0.18	3.7E-01	-0.10	6.2E-01	-0.11	5.9E-01	-0.12	5.6E-01	0.29	1.6E-01	-0.18	3.7E-01
ENSCAFG0000000886	ENSCAFG0000000886	grey	EC_M10	0.02	9.1E-01	0.29	1.5E-01	0.10	6.2E-01	0.25	2.2E-01	-0.25	2.1E-01	-0.16	4.3E-01	-0.11	6.1E-01	-0.22	2.9E-01	-0.20	3.3E-01	0.24	2.3E-01
ENSCAFG0000002115	ENSCAFG0000002115	grey	EC_M10	0.02	9.1E-01	-0.18	3.8E-01	-0.28	1.6E-01	-0.10	6.4E-01	0.41	3.8E-02	-0.12	5.5E-01	-0.13	5.4E-01	-0.06	7.6E-01	-0.11	5.8E-01	-0.39	4.8E-02
ENSCAFG0000000595	DUS1L	grey	EC_M10	0.02	9.1E-01	0.38	5.5E-02	0.11	5.8E-01	0.15	4.7E-01	-0.22	2.9E-01	0.04	8.4E-01	0.03	9.0E-01	-0.30	1.4E-01	0.33	9.4E-02	0.25	2.1E-01
ENSCAFG0000000708	SOAT2	grey	EC_M10	0.02	9.1E-01	0.15	4.7E-01	-0.23	2.6E-01	0.07	7.3E-01	0.24	2.3E-01	0.14	5.0E-01	-0.23	2.5E-01	-0.35	8.2E-02	-0.63	6.2E-04	-0.26	2.0E-01
ENSCAFG0000001083	MVK	grey	EC_M10	0.02	9.1E-01	-0.10	6.4E-01	0.63	6.3E-04	-0.01	9.7E-01	-0.73	2.1E-05	-0.33	9.7E-02	-0.05	8.0E-01	-0.05	8.0E-01	-0.19	3.4E-01	0.69	9.1E-05
ENSCAFG0000002399	ATXN1L	turquoise	EC_M6	0.02	9.1E-01	0.70	7.1E-05	0.01	9.8E-01	-0.06	7.8E-01	-0.25	2.2E-01	-0.15	4.7E-01	0.00	1.0E+00	-0.15	4.7E-01	0.15	4.6E-01	0.30	1.4E-01
ENSCAFG0000000658	MRPL17	grey	EC_M10	0.02	9.1E-01	-0.08	7.0E-01	-0.30	1.3E-01	-0.18	3.8E-01	0.45	2.1E-02	0.27	1.8E-01	0.22	2.9E-01	-0.16	4.4E-01	-0.38	5.7E-02	-0.48	1.3E-02
ENSCAFG0000001423	L2HGDH	grey	EC_M10	0.02	9.1E-01	-0.05	8.0E-01	0.30	1.3E-01	0.17	4.0E-01	-0.38	5.7E-02	0.10	6.2E-01	0.10	6.1E-01	-0.16	4.4E-01	-0.16	4.3E-01	0.38	5.8E-02
ENSCAFG0000000077	ENSCAFG0000000077	grey	EC_M10	0.02	9.1E-01	-0.05	8.2E-01	-0.14	5.0E-01	0.41	3.9E-02	0.10	6.4E-01	-0.12	5.6E-01	-0.14	5.1E-01	-0.16	4.2E-01	0.30	1.4E-01	-0.14	4.8E-01
ENSCAFG0000000178	PRDM4	turquoise	EC_M6	0.02	9.1E-01	0.43	2.7E-02	0.16	4.4E-01	0.19	3.5E-01	-0.35	7.6E-02	-0.26	2.0E-01	-0.28	1.6E-01	0.31	1.3E-01	0.01	9.6E-01	0.34	8.9E-02
ENSCAFG0000001202	ENSCAFG0000001202	grey	EC_M10	0.02	9.1E-01	0.10	6.2E-01	0.30	1.4E-01	-0.22	2.8E-01	-0.32	1.1E-01	0.06	7.8E-01	-0.31	1.2E-01	0.12	5.5E-01	0.20	3.3E-01	0.30	1.4E-01
ENSCAFG0000000847	CCDC142	grey	EC_M10	0.02	9.1E-01	-0.06	7.7E-01	-0.24	2.4E-01	0.26	2.1E-01	0.21	3.1E-01	0.02	9.4E-01	0.07	7.5E-01	-0.12	5.7E-01	0.10	6.2E-01	-0.25	2.2E-01
ENSCAFG0000000332	KIDINS220	darkgreen	EC_M4	0.02	9.1E-01	-0.24	2.3E-01	-0.66	2.2E-04	-0.19	3.6E-01	0.80	1.1E-06	0.28	1.7E-01	0.00	9.9E-01	0.15	4.7E-01	-0.08	7.0E-01	-0.82	2.7E-07
ENSCAFG0000003087	ENSCAFG0000003087	grey	EC_M10	0.02	9.1E-01	-0.15	4.6E-01	-0.06	7.7E-01	0.32	1.1E-01	0.13	5.2E-01	0.03	8.9E-01	-0.11	5.9E-01	-0.09	6.5E-01	-0.02	9.3E-01	-0.20	3.4E-01
ENSCAFG0000000135	GOLM1	darkgreen	EC_M4	0.02	9.1E-01	0.04	8.5E-01	-0.78	2.3E-06	-0.03	8.9E-01	0.86	1.6E-08	0.29	1.5E-01	-0.04	8.4E-01	-0.01	9.5E-01	-0.13	5.4E-01	-0.90	4.0E-10
ENSCAFG0000001392	SEMA6D	turquoise	EC_M6	0.02	9.1E-01	0.49	1.1E-02	0.00	1.0E+00	-0.21	2.9E-01	-0.17	4.2E-01	-0.36	7.5E-02	0.21	3.1E-01	0.25	2.3E-01	0.53	5.3E-03	0.21	2.9E-01
ENSCAFG0000000246	TRMO	cyan	EC_M2	0.02	9.1E-01	-0.24	2.5E-01	0.58	2.1E-03	0.24	2.3E-01	-0.68	1.5E-04	-0.03	8.7E-01	-0.28	1.6E-01	-0.04	8.6E-01	-0.20	3.2E-01	0.64	4.8E-04
ENSCAFG0000000946	MTERF3	grey	EC_M10	0.02	9.1E-01	0.07	7.5E-01	0.37	6.2E-02	0.26	2.1E-01	-0.45	2.0E-02	0.16	4.4E-01	0.05	8.2E-01	-0.01	9.6E-01	0.37	6.3E-02	0.43	2.9E-02
ENSCAFG0000001864	LGALS9	turquoise	EC_M6	0.02	9.1E-01	0.79	1.9E-06	-0.12	5.6E-01	0.04	8.6E-01	-0.19	3.6E-01	-0.28	1.6E-01	0.07	7.3E-01	0.01	9.5E-01	0.10	6.1E-01	0.15	4.6E-01
ENSCAFG0000000851	PRPF40B	grey	EC_M10	0.02	9.2E-01	0.36	7.3E-02	0.32	1.1E-01	0.19	3.6E-01	-0.58	1.8E-03	0.22	2.7E-01	0.01	9.6E-01	0.16	4.5E-01	0.09	6.6E-01	0.56	3.0E-03
ENSCAFG0000003157	INKA1	turquoise	EC_M6	0.02	9.2E-01	0.76	6.9E-06	-0.03	8.9E-01	-0.24	2.3E-01	-0.19	3.4E-01	-0.12	5.5E-01	-0.03	8.8E-01	-0.26	2.0E-01	-0.01	9.5E-01	0.20	3.2E-01
ENSCAFG0000002914	PPP1R3C	darkolivegreen	EC_M9	0.02	9.2E-01	0.31	1.3E-01	-0.31	1.2E-01	0.13	5.2E-01	0.22	2.8E-01	-0.02	9.4E-01	0.67	2.0E-04	0.06	7.9E-01	0.32	1.1E-01	-0.23	2.5E-01
ENSCAFG0000002272	MT-ATP6	cyan	EC_M2	0.02	9.2E-01	-0.30	1.4E-01	0.56	3.0E-03	0.31	1.3E-01	-0.59	1.4E-03	-0.03	9.0E-01	-0.47	1.5E-02	-0.06	7.8E-01	-0.14	5.0E-01	0.57	2.5E-03
ENSCAFG0000000839	RRAS2	grey	EC_M10	0.02	9.2E-01	-0.39	4.9E-02	-0.13	5.4E-01	-0.10	6.4E-01	0.34	9.4E-02	0.15	4.6E-01	0.04	8.3E-01	0.07	7.5E-01	0.16	4.5E-01	-0.34	8.8E-02
ENSCAFG0000000730	MAPRE1	grey	EC_M10	0.02	9.2E-01	0.07	7.3E-01	-0.32	1.1E-01	-0.03	8.8E-01	0.38	5.5E-02	0.29	1.5E-01	-0.02	9.4E-01	-0.02	9.3E-01	0.32	1.1E-01	-0.39	4.6E-02
ENSCAFG0000000509	SLC25A38	grey	EC_M10	0.02	9.2E-01	-0.57	2.4E-03	0.28	1.7E-01	0.07	7.4E-01	-0.05	8.1E-01	0.00	9.9E-01	-0.24	2.3E-01	0.27	1.9E-01	0.09	6.5E-01	0.06	7.8E-01
ENSCAFG0000001163	FAM241A	turquoise	EC_M6	0.02	9.2E-01	0.74	1.5E-05	-0.19	3.5E-01	-0.12	5.7E-01	0.00	9.8E-01	-0.16	4.5E-01	-0.14	5.0E-01	-0.18	3.8E-01	0.04	8.6E-01	-0.03	8.8E-01
ENSCAFG0000001733	SBK1	turquoise	EC_M6	0.02	9.2E-01	0.36	7.2E-02	0.12	5.7E-01	0.03	8.8E-01	-0.24	2.4E-01	0.02	9.3E-01	0.09	6.5E-01	-0.27	1.8E-01	0.06	7.7E-01	0.21	3.0E-01
ENSCAFG0000000507	MYO7A	turquoise	EC_M6	0.02	9.2E-01	0.67	1.8E-04	-0.26	2.0E-01	-0.09	6.5E-01	0.06	7.8E-01	-0.13	5.4E-01	-0.06	7.7E-01	-0.15	4.8E-01	0.33	1.0E-01	-0.05	7.9E-01
ENSCAFG0000001887	CYP2J2	grey	EC_M10	0.02	9.2E-01	-0.09	6.6E-01	-0.41	3.7E-02	-0.15	4.7E-01	0.53	5.2E-03	0.29	1.6E-01	0.03	8.9E-01	0.31	1.3E-01	-0.06	7.9E-01	-0.53	5.6E-03
ENSCAFG0000000231	TSGA10	grey	EC_M10	0.02	9.2E-01	-0.05	8.0E-01	0.27	1.8E-01	0.03	8.8E-01	-0.31	1.2E-01	-0.02	9.2E-01	-0.32	1.1E-01	-0.07	7.4E-01	0.25	2.1E-01	0.29	1.6E-01

ENSCAFG0000003207	PDCD7	grey	EC_M10	0.02	9.2E-01	-0.12	5.5E-01	0.30	1.4E-01	-0.08	7.1E-01	-0.28	1.7E-01	-0.16	4.4E-01	0.10	6.3E-01	0.10	6.4E-01	-0.26	2.1E-01	0.24	2.3E-01
ENSCAFG0000001705	PPIB	grey	EC_M10	0.02	9.2E-01	-0.49	1.2E-02	-0.41	3.6E-02	0.03	8.7E-01	0.66	2.6E-04	0.23	2.6E-01	-0.01	9.7E-01	0.26	2.0E-01	0.08	6.9E-01	-0.70	7.0E-05
ENSCAFG0000001335	BDH1	grey	EC_M10	0.02	9.2E-01	0.07	7.5E-01	0.45	2.1E-02	0.22	2.9E-01	-0.56	2.9E-03	-0.09	6.6E-01	-0.18	3.9E-01	0.01	9.8E-01	0.49	1.0E-02	0.58	2.0E-03
ENSCAFG0000002861	ENSCAFG0000002861	turquoise	EC_M6	0.02	9.2E-01	0.74	1.6E-05	-0.27	1.9E-01	0.08	6.9E-01	0.03	8.9E-01	-0.30	1.4E-01	-0.08	6.8E-01	-0.09	6.7E-01	0.14	5.0E-01	-0.03	9.0E-01
ENSCAFG0000001765	SENP8	grey	EC_M10	0.02	9.2E-01	-0.34	8.9E-02	0.07	7.5E-01	-0.27	1.9E-01	0.01	9.6E-01	-0.13	5.2E-01	-0.11	5.9E-01	0.47	1.4E-02	0.18	3.8E-01	-0.09	6.6E-01
ENSCAFG0000000525	RALGAPA2	turquoise	EC_M6	0.02	9.2E-01	0.89	1.4E-09	-0.16	4.3E-01	-0.11	5.9E-01	-0.16	4.4E-01	-0.21	3.0E-01	0.03	8.8E-01	-0.22	2.9E-01	-0.02	9.1E-01	0.17	4.0E-01
ENSCAFG0000001714	PARP16	cyan	EC_M2	0.02	9.2E-01	0.11	6.0E-01	0.65	3.2E-04	0.23	2.6E-01	-0.83	1.5E-07	-0.39	5.1E-02	-0.06	7.7E-01	0.08	6.9E-01	-0.26	2.1E-01	0.79	1.4E-06
ENSCAFG0000000217	EXOC1	darkgreen	EC_M4	0.02	9.2E-01	-0.04	8.3E-01	-0.73	2.5E-05	-0.12	5.7E-01	0.84	8.7E-08	0.21	2.9E-01	-0.25	2.2E-01	-0.01	9.5E-01	-0.03	9.0E-01	-0.82	2.6E-07
ENSCAFG0000000769	VTI1B	grey	EC_M10	0.02	9.2E-01	0.19	3.5E-01	-0.06	7.8E-01	-0.08	6.9E-01	0.01	9.6E-01	-0.01	9.6E-01	0.08	7.0E-01	-0.47	1.5E-02	-0.14	4.9E-01	-0.06	7.7E-01
ENSCAFG0000003083	AOC2	grey	EC_M10	0.02	9.2E-01	0.53	5.3E-03	-0.48	1.3E-02	-0.03	8.9E-01	0.33	9.8E-02	0.18	3.8E-01	0.12	5.7E-01	0.10	6.2E-01	0.29	1.5E-01	-0.32	1.2E-01
ENSCAFG0000000150	MFNG	turquoise	EC_M6	0.02	9.2E-01	0.88	2.7E-09	-0.08	7.1E-01	-0.16	4.3E-01	-0.26	2.1E-01	-0.17	4.0E-01	-0.15	4.7E-01	-0.19	3.6E-01	0.13	5.2E-01	0.24	2.3E-01
ENSCAFG0000000564	ENSCAFG0000000564	grey	EC_M10	0.02	9.2E-01	-0.07	7.4E-01	0.33	1.0E-01	-0.15	4.5E-01	-0.35	7.8E-02	-0.09	6.8E-01	0.29	1.5E-01	-0.04	8.6E-01	0.42	3.3E-02	0.38	5.6E-02
ENSCAFG0000000223	BARX1	paleturquoise	EC_M11	0.02	9.2E-01	-0.14	5.0E-01	0.05	8.2E-01	0.94	4.8E-13	-0.07	7.4E-01	0.12	5.7E-01	-0.09	6.5E-01	-0.15	4.6E-01	-0.04	8.5E-01	0.04	8.5E-01
ENSCAFG0000001253	ABHD11	grey	EC_M10	0.02	9.2E-01	-0.17	3.9E-01	0.18	3.9E-01	-0.28	1.7E-01	-0.11	6.1E-01	0.07	7.3E-01	-0.07	7.4E-01	-0.06	7.7E-01	0.26	2.1E-01	0.09	6.7E-01
ENSCAFG0000000219	RFX8	grey	EC_M10	0.02	9.2E-01	-0.49	1.2E-02	0.35	7.8E-02	-0.08	7.0E-01	-0.25	2.2E-01	0.16	4.3E-01	-0.16	4.5E-01	0.01	9.6E-01	-0.27	1.8E-01	0.28	1.7E-01
ENSCAFG0000002771	ENSCAFG0000002771	grey	EC_M10	0.02	9.2E-01	-0.14	5.0E-01	0.21	3.1E-01	-0.08	7.0E-01	-0.17	4.0E-01	-0.11	5.9E-01	-0.09	6.5E-01	0.34	9.3E-02	0.26	2.0E-01	0.19	3.5E-01
ENSCAFG0000001305	RPS17	cyan	EC_M2	0.02	9.2E-01	-0.51	7.3E-03	0.41	3.6E-02	0.09	6.8E-01	-0.30	1.4E-01	0.11	6.1E-01	0.25	2.1E-01	0.09	6.6E-01	-0.33	1.0E-01	0.22	2.9E-01
ENSCAFG0000002032	ESRP2	grey	EC_M10	0.02	9.2E-01	0.14	4.9E-01	0.09	6.8E-01	0.30	1.3E-01	-0.15	4.6E-01	0.08	6.8E-01	0.08	7.0E-01	0.22	2.9E-01	0.26	2.0E-01	0.15	4.6E-01
ENSCAFG0000002321	ENSCAFG0000002321	grey	EC_M10	0.02	9.2E-01	-0.14	4.9E-01	0.32	1.2E-01	0.31	1.2E-01	-0.38	5.5E-02	-0.12	5.6E-01	-0.20	3.4E-01	0.17	3.9E-01	-0.13	5.3E-01	0.33	9.6E-02
ENSCAFG0000002993	CHIC1	grey	EC_M10	0.02	9.2E-01	-0.16	4.4E-01	0.28	1.7E-01	0.23	2.6E-01	-0.28	1.6E-01	-0.09	6.7E-01	0.20	3.4E-01	-0.56	2.9E-03	-0.31	1.2E-01	0.28	1.6E-01
ENSCAFG0000000882	ANKRD34B	grey	EC_M10	0.02	9.2E-01	-0.07	7.4E-01	0.21	3.1E-01	0.10	6.3E-01	-0.32	1.2E-01	0.07	7.5E-01	0.41	3.6E-02	-0.28	1.7E-01	-0.19	3.6E-01	0.28	1.7E-01
ENSCAFG0000000479	TSEN54	grey	EC_M10	0.02	9.2E-01	0.07	7.4E-01	0.59	1.6E-03	-0.07	7.5E-01	-0.69	9.4E-05	-0.26	1.9E-01	-0.06	7.6E-01	0.09	6.5E-01	-0.22	2.8E-01	0.65	3.4E-04
ENSCAFG0000001045	RIPOR2	paleturquoise	EC_M11	0.02	9.2E-01	-0.13	5.2E-01	0.33	1.0E-01	0.74	1.5E-05	-0.37	6.0E-02	-0.05	8.2E-01	-0.07	7.4E-01	-0.11	5.9E-01	-0.15	4.7E-01	0.38	5.5E-02
ENSCAFG0000000933	SV2C	grey	EC_M10	0.02	9.2E-01	-0.14	5.1E-01	-0.62	7.3E-04	-0.04	8.6E-01	0.74	1.7E-05	0.47	1.6E-02	-0.03	9.0E-01	0.00	1.0E+00	-0.15	4.6E-01	-0.77	3.6E-06
ENSCAFG0000000559	PLEKHB1	cyan	EC_M2	0.02	9.2E-01	-0.28	1.7E-01	0.52	6.6E-03	0.32	1.1E-01	-0.53	5.2E-03	-0.11	5.9E-01	-0.19	3.6E-01	-0.22	2.7E-01	-0.22	2.7E-01	0.55	3.6E-03
ENSCAFG0000003062	LAYN	turquoise	EC_M6	0.02	9.2E-01	0.58	1.8E-03	-0.21	3.0E-01	-0.12	5.5E-01	0.08	6.9E-01	-0.20	3.2E-01	0.01	9.7E-01	-0.11	5.9E-01	0.06	7.7E-01	-0.06	7.6E-01
ENSCAFG0000002918	EIF1B	grey	EC_M10	0.02	9.2E-01	0.14	4.8E-01	0.09	6.6E-01	-0.19	3.5E-01	-0.24	2.4E-01	0.14	4.9E-01	0.32	1.1E-01	-0.29	1.5E-01	-0.30	1.4E-01	0.17	4.2E-01
ENSCAFG0000003060	MED18	grey	EC_M10	0.02	9.2E-01	-0.52	6.1E-03	-0.17	4.2E-01	-0.08	6.8E-01	0.33	1.0E-01	0.33	1.0E-01	0.14	5.0E-01	0.14	5.0E-01	-0.34	9.1E-02	-0.35	7.7E-02
ENSCAFG0000000550	UGGT2	grey	EC_M10	0.02	9.2E-01	-0.48	1.4E-02	-0.07	7.3E-01	0.14	5.0E-01	0.17	4.0E-01	-0.06	7.9E-01	0.06	7.9E-01	0.48	1.3E-02	0.31	1.2E-01	-0.24	2.4E-01
ENSCAFG0000001870	ZDHHC9	darkgreen	EC_M4	0.02	9.2E-01	0.59	1.4E-03	-0.65	3.4E-04	-0.22	2.9E-01	0.54	4.0E-03	0.05	8.0E-01	-0.07	7.5E-01	0.03	8.7E-01	-0.24	2.5E-01	-0.56	2.9E-03
ENSCAFG0000001298	PLA2G3	grey	EC_M10	0.02	9.3E-01	-0.48	1.3E-02	-0.05	8.2E-01	0.05	8.0E-01	0.23	2.5E-01	-0.04	8.4E-01	0.30	1.3E-01	0.01	9.7E-01	-0.40	4.3E-02	-0.30	1.4E-01
ENSCAFG0000002626	ENSCAFG0000002626	paleturquoise	EC_M11	0.02	9.3E-01	-0.13	5.4E-01	0.11	5.8E-01	0.60	1.2E-03	-0.12	5.7E-01	0.10	6.2E-01	0.14	4.8E-01	0.07	7.3E-01	-0.20	3.3E-01	0.10	6.4E-01
ENSCAFG0000002872	FAM181B	grey	EC_M10	0.02	9.3E-01	-0.13	5.4E-01	0.13	5.3E-01	-0.11	5.8E-01	-0.09	6.5E-01	-0.05	8.1E-01	-0.10	6.4E-01	-0.11	6.0E-01	-0.06	7.7E-01	0.04	8.4E-01
ENSCAFG0000001354	DNAJB11	darkgreen	EC_M4	0.02	9.3E-01	-0.17	4.2E-01	-0.73	2.2E-05	-0.13	5.1E-01	0.92	2.8E-11	0.31	1.3E-01	-0.02	9.4E-01	0.15	4.8E-01	0.04	8.6E-01	-0.92	4.6E-11
ENSCAFG0000000842	IFT81	grey	EC_M10	0.02	9.3E-01	-0.68	1.4E-04	0.10	6.4E-01	0.31	1.2E-01	0.03	8.9E-01	0.03	8.8E-01	-0.14	4.9E-01	0.13	5.4E-01	0.05	8.2E-01	-0.07	7.4E-01
ENSCAFG0000000948	FARS2	cyan	EC_M2	0.02	9.3E-01	-0.43	2.9E-02	0.67	1.8E-04	0.15	4.6E-01	-0.65	3.2E-04	-0.08	7.1E-01	-0.08	6.9E-01	-0.18	3.7E-01	-0.18	3.9E-01	0.63	5.7E-04
ENSCAFG0000001767	PARP6	cyan	EC_M2	0.02	9.3E-01	0.24	2.5E-01	0.60	1.1E-03	0.23	2.5E-01	-0.85	4.8E-08	-0.16	4.2E-01	0.04	8.6E-01	-0.15	4.6E-01	-0.12	5.7E-01	0.82	2.6E-07
ENSCAFG0000000353	AASS	grey	EC_M10	0.02	9.3E-01	-0.23	2.7E-01	0.58	2.1E-03	0.58	2.0E-03	-0.62	6.9E-04	-0.15	4.5E-01	-0.17	4.0E-01	-0.11	6.1E-01	-0.01	9.7E-01	0.63	5.5E-04
ENSCAFG0000001933	TRPV3	grey	EC_M10	0.02	9.3E-01	-0.15	4.6E-01	0.59	1.5E-03	0.14	4.9E-01	-0.64	4.5E-04	-0.34	9.4E-02	-0.05	8.0E-01	-0.11	5.8E-01	-0.41	3.5E-02	0.63	5.7E-04
ENSCAFG0000000116	ENSCAFG0000000116	grey	EC_M10	0.02	9.3E-01	-0.22	2.7E-01	0.25	2.3E-01	0.14	5.1E-01	-0.26	2.1E-01	-0.15	4.5E-01	0.11	6.0E-01	0.11	6.0E-01	-0.03	8.7E-01	0.25	2.2E-01
ENSCAFG0000003179	RPS6KA4	grey	EC_M10	0.02	9.3E-01	0.13	5.2E-01	0.23	2.5E-01	0.01	9.6E-01	-0.24	2.4E-01	0.17	4.0E-01	-0.23	2.6E-01	-0.07	7.5E-01	0.09	6.7E-01	0.23	2.5E-01
ENSCAFG0000001875	RAB33A	grey	EC_M10	0.02	9.3E-01	-0.06	7.7E-01	0.21	3.1E-01	-0.11	5.8E-01	-0.19	3.6E-01	-0.02	9.4E-01	-0.05	8.0E-01	-0.01	9.7E-01	0.02	9.3E-01	0.16	4.2E-01
ENSCAFG0000003136	ENSCAFG0000003136	grey	EC_M10	0.02	9.3E-01	-0.12	5.7E-01	-0.12	5.5E-01	0.04	8.3E-01	0.14	5.1E-01	-0.08	7.1E-01	-0.10	6.3E-01	-0.09	6.5E-01	-0.16	4.2E-01	-0.18	3.8E-01
ENSCAFG0000001369	ST6GAL1	turquoise	EC_M6	0.02	9.3E-01	0.86	2.5E-08	-0.07	7.5E-01	-0.06	7.6E-01	-0.23	2.6E-01	-0.18	3.8E-01	-0.13	5.4E-01	-0.11	5.8E-01	0.09	6.8E-01	0.23	2.6E-01
ENSCAFG0000003249	ENSCAFG0000003249	grey	EC_M10	0.02	9.3E-01	-0.21	3.1E-01	0.13	5.1E-01	-0.14	4.9E-01	-0.15	4.6E-01	0.31	1.2E-01	-0.19	3.5E-01	0.37	6.2E-02	-0.18	3.9E-01	0.06	7.6E-01
ENSCAFG0000001221	TRAK2	turquoise	EC_M6	0.02	9.3E-01	0.44	2.5E-02	-0.26	1.9E-01	-0.11	5.9E-01	0.13	5.1E-01	-0.08	7.1E-01	-0.34	8.9E-02	-0.09	6.5E-01	0.25	2.3E-01	-0.08	7.1E-01
ENSCAFG0000001916	C5H1orf123	grey	EC_M10	0.02	9.3E-01	-0.51	7.5E-03	0.42	3.5E-02	0.22	2.8E-01	-0.27	1.9E-01	0.10	6.4E-01	-0.12	5.7E-01	-0.40	4.5E-02	-0.14	5.0E-01	0.28	1.6E-01
ENSCAFG0000000453	ZNF862	cyan	EC_M2	0.02	9.3E-01	0.21	2.9E-01	0.38	5.4E-02	0.10	6.4E-01	-0.64	4.0E-04	-0.20	3.4E-01	0.20	3.3E-01	-0.17	4.1E-01	-0.18	3.8E-01	0.59	1.4E-03
ENSCAFG0000001739	CREB3L4	grey	EC_M10	0.02	9.3E-01	0.50	9.9E-03	0.20	3.3E-01	0.06	7.8E-01	-0.37	6.1E-02	-0.19	3.6E-01	0.04	8.3E-01	-0.19	3.6E-01	-0.04	8.5E-01	0.40	4.2E-02
ENSCAFG0000001614	MSL1	grey	EC_M10	0.02	9.3E-01	0.46	1.7E-02	0.38	5.3E-02	0.12	5.7E-01	-0.69	9.8E-05	-0.41	4.0E-02	-0.03	8.8E-01	0.17	4.1E-01	-0.01	9.7E-01	0.65	3.7E-04
ENSCAFG0000002012	ENSCAFG0000002012	grey	EC_M10	0.02	9.3E-01	0.00	9.9E-01	0.01	9.5E-01	-0.02	9.4E-01	-0.05	8.0E-01	-0.06	7.8E-01	-0.10	6.2E-01	-0.10	6.4E-01	-0.11	6.1E-01	0.00	9.9E-01
ENSCAFG0000001043	IFIH1	cyan	EC_M2	0.02	9.3E-01	-0.01	9.4E-01	0.50	1.0E-02	0.28	1.7E-01	-0.63	5.0E-04	-0.26	2.0E-01	0.03	8.9E-01	-0.14	4.8E-01	-0.32	1.1E-01	0.59	1.7E-03
ENSCAFG0000000220	S1PR3	grey	EC_M10	0.02	9.3E-01	0.06	7.8E-01	0.05	8.2E-01	-0.06	7.8E-01	-0.05	8.1E-01	0.03	9.0E-01	0.04	8.6E-01	-0.04	8.5E-01	0.03	9.0E-01	0.00	9.8E-01
ENSCAFG0000000576	PLCB4	turquoise	EC_M6	0.02	9.3E-01	0.67	1.8E-04	-0.46	1.8E-02	-0.18	3.8E-01	0.34	9.0E-02	-0.24	2.3E-01	0.05	8.1E-01	0.09	6.5E-01	0.14	4.9E-01	-0.30	1.4E-01
ENSCAFG0000000820	TMEM120B	turquoise	EC_M6	0.02	9.3E-01	0.44	2.3E-02	-0.05	8.1E-01	-0.01	9.6E-01	-0.15	4.5E-01	-0.31	1.2E-01	0.08	7.1E-01	0.09	6.5E-01	0.20	3.2E-01	0.10	6.1E-01
ENSCAFG0000002660	ENSCAFG0000002660	grey	EC_M10	0.02	9.3E-01	-0.20	3.4E-01	0.04	8.3E-01	0.12	5.4E-01	-0.01	9.5E-01	0.03	8.8E-01	0.13	5.3E-01	0.23	2.6E-01	-0.14	4.9E-01	-0.06	7.5E-01
ENSCAFG0000000318	RAB1A	grey	EC_M10	0.02	9.3E-01	-0.12	5.6E-01	-0.43	2.9E-02	-0.25	2.2E-01	0.54	4.4E-03	0.29	1.5E-01	-0.02	9.4E-01	-0.17	4.0E-01	-0.37	6.1E-02	-0.55	3.6E-03
ENSCAFG0000001388	RIMKLB	grey	EC_M10	0.02	9.3E-01	0.02	9.3E-01	-0.63	5.9E-04	-0.02	9.1E-01	0.69	1.0E-04	0.23	2.7E-01	-0.12	5.4E-01	0.19	3.6E-01	0.18	3.7E-01	-0.71	5.8E-05
ENSCAFG0000000281	ENSCAFG0000000281	grey	EC_M10	0.02	9.3E-01	-0.11	6.0E-01	-0.23	2.6E-01	-0.12	5.5E-01	0.28	1.6E-01	-0.06	7.6E-01	-0.10	6.2E-01	-0.03	8.7E-01	-0.03	8.8E-01	-0.29	1.5E-01
ENSCAFG0000000840	VEGFC	turquoise	EC_M6	0.02	9.3E-01	0.50	9.2E-03	-0.04	8.6E-01	0.06	7.9E-01	-0.11	5.8E-01	-0.03	9.0E-01	0.04	8.3E-01	0.19	3.5E-01	-0.04	8.3E-01	0.13	5.3E-01
ENSCAFG0000001200	LAT2	grey	EC_M10	0.02	9.3E-01	0.22	2.8E-01	-0.01	9.7E-01	-0.18	3.7E-01	-0.12	5.6E-01	-0.15	4.8E-01	-0.18	3.8E-01	0.46	1.9E-02	0.01	9.8E-01	0.13	5.2E-01
ENSCAFG0000003175	PITX3	grey	EC_M10	0.02	9.3E-01	-0.20	3.3E-01	-0.03	8.9E-01	0.38	5.4E-02	0.04	8.3E-01	-0.07	7.5E-01	-0.07	7.2E-01	-0.15	4.7E-01	-0.14	5.1E-01	-0.04	8.6E-01
ENSCAFG0000000895	RIPK2	grey	EC_M10	0.02	9.3E-01	0.41	4.0E-02	-0.55	3.3E-03	-0.11	5.9E-01	0.47	1.5E-02	0.06	7.7E-01	-0.21	3.0E-01	0.13	5.2E-01	0.48	1.3E-02	-0.48	1.4E-02
ENSCAFG0000000149	MPDZ	grey	EC_M10	0.02	9.3E-01	0.20	3.2E-01	-0.22	2.7E-01	0.08	6.9E-01	0.06	7.8E-01	-0.11	6.1E-01	-0.12	5.5E-01	0.11	6.0E-01	0.42	3.0E-02	-0.07	7.3E-01
ENSCAFG0000000728	CRYL1	grey	EC_M10	0.02	9.4E-01	-0.41	3.7E-02	-0.21	2.9E-01	-0.07	7.3E-01	0.43	2.8E-02	0.15	4.6E-01	-0.22	2.9E-01	-0.01	9.5E-01	0.08	6.8E-01	-0.44	2.4E-02
ENSCAFG0000003227	ENSCAFG0000003227	grey	EC_M10	0.02	9.4E-01	-0.21	3.1E-01	0.07	7.4E-01	-0.15	4.5E-01	0.08	7.1E-01	0.06	7.7E-01	0.19	3.5E-01	0.12	5.6E-01	-0.21	3.0E-01	-0.07	7.2E-01
ENSCAFG0000001286	RAPH1	grey	EC_M10	0.02	9.4E-01	0.56	3.2E-03	0.07	7.3E-01	-0.25	2.1E-01	-0.26	2.0E-01	-0.26	2.0E-01	-0.16	4.4E-01	0.13	5.3E-01	0.00	1.0E+00	0.29	1.4E-01
ENSCAFG0000001172	CD34	turquoise	EC_M6	0.02	9.4E-01	0.78	2.3E-06	0.05	8.1E-01	-0.14	5.0E-01	-0.33	1.0E-01	-0.16	4.3E-01	-0.18	3.7E-01	-0.15	4.8E-01	-0.02	9.3E-01	0.31	1.2E-01
ENSCAFG0000001589	NSUN7	grey	EC_M10	0.02	9.4E-01	0.06	7.8E-01	-0.43	2.7E-02	-0.14	5.0E-01	0.48	1.3E-02	0.42	3.4E-02	0.10	6.2E-01	-0.15	4.6E-01	-0.12	5.6E-01	-0.48	1.2E-02
ENSCAFG0000003140	ENSCAFG0000003140	turquoise	EC_M6	0.02	9.4E-01	0.41	3.8E-02	0.05	8.1E-01	-0.12	5.5E-01	-0.24	2.4E-01	-0.10	6.1E-01	-0.09	6.7E-01	-0.18	3.9E-01	-0.11	5.8E-01	0.23	2.5E-01
ENSCAFG0000000008	RAB5B	grey	EC_M10	0.02	9.4E-01	0.22	2.7E-01	-0.64	4.4E-04	-0.43	3.0E-02	0.63	6.0E-04	-0.12	5.5E-01	0.11	5.9E-01	0.36	6.9E-02	0.11	5.9E-01	-0.64	4.2E-04
ENSCAFG0000001866	ENSCAFG0000001866	grey	EC_M10	0.02	9.4E-01	-0.06	7.8E-01	0.03	8.7E-01	-0.27	1.8E-01	0.00	9.9E-01	0.18	3.7E-01	0.00	1.0E+00	0.20	3.2E-01	0.21	3.0E-01	-0.03	8.8E-01
ENSCAFG0000001940	SPPL2B	grey	EC_M10	0.02	9.4E-01	-0.62	8.3E-04	0.00	9.9E-01	-0.10	6.2E-01	0.30	1.4E-01	0.17	4.1E-01	-0.15	4.6E-01	0.02	9.4E-01	-0.20	3.3E-01	-0.30	1.3E-01
ENSCAFG0000001223	MLPH	grey	EC_M10	0.02	9.4E-01	-0.04	8.3E-01	-0.10	6.2E-01	-0.12	5.7E-01	0.12	5.6E-01	-0.04	8.3E-01	-0.08	7.0E-01	-0.07	7.3E-01	-0.07	7.5E-01	-0.13	5.3E-01
ENSCAFG0000002996	ENSCAFG0000002996	grey	EC_M10	0.02	9.4E-01	-0.22	2.8E-01	-0.37	6.4E-02	-0.08	7.0E-01	0.46	1.7E-02	-0.19	3.7E-01	0.13	5.1E-01	0.22	2.8E-01	-0.12	5.6E-01	-0.49	1.2E-02
ENSCAFG0000002956	C6H1orf52	grey	EC_M10	0.02	9.4E-01	0.32	1.1E-01	-0.56	2.8E-03	-0.01	9.7E-01	0.51	7.4E-03	0.20	3.2E-01	0.18	3.8E-01	-0.02	9.1E-01	-0.22	2.7E-01	-0.50	9.3E-03
ENSCAFG0000000050	VPS13B	grey	EC_M10	0.02	9.4E-01	-0.17	4.0E-01	0.05	8.1E-01	0.03	8.7E-01	-0.01	9.7E-01	-0.05	8.2E-01	-0.21	3.1E-01	-0.27	1.8E-01	-0.20	3.3E-01	0.03	8.8E-01
ENSCAFG0000003267	ENSCAFG0000003267	turquoise	EC_M6	0.02	9.4E-01	0.40	4.5E-02	-0.10	6.1E-01	-0.09	6.7E-01	-0.08	7.1E-01	-0.11	5.8E-01	-0.14	4.9E-01	0.05	8.1E-01	0.36	6.8E-02	0.08	7.0E-01
ENSCAFG0000001348	CADM1	grey	EC_M10	0.02	9.4E-01	0.12	5.7E-01	0.06	7.7E-01	-0.29	1.5E-01	-0.17	4.1E-01	-0.27	1.9E-01	0.12	5.6E-01	0.29	1.5E-01	0.10	6.1E-01	0.10	6.4E-01
ENSCAFG0000000013	RBMS2	turquoise	EC_M6	0.02	9.4E-01	0.58	1.9E-03	-0.15	4.6E-01	0.03	8.8E-01	-0.09	6.7E-01	0.21	3.1E-01	0.17	4.0E-01	0.01	9.8E-01	-0.21	3.1E-01	0.04	8.6E-01
ENSCAFG0000002332	ENSCAFG0000002332	grey	EC_M10	0.02	9.4E-01	-0.18	3.7E-01	-0.25	2.3E-01	-0.16	4.2E-01	0.37	6.0E-02	0.43	2.7E-02	0.36	7.4E-02	0.45	2.2E-02	-0.03	9.0E-01	-0.35	8.4E-02
ENSCAFG0000000064	ENSCAFG0000000064	cyan	EC_M2	0.02	9.4E-01	-0.08	6.9E-01	0.54	4.2E-03	0.31	1.2E-01	-0.72	3.4E-05	-0.03	8.9E-01	-0.06	7.7E-01	-0.18	3.9E-01	-0.03	8.9E-01	0.70	7.5E-05
ENSCAFG0000003241	TMEM225B	cyan	EC_M2	0.02	9.4E-01	0.34	8.7E-02	0.61	8.7E-04	0.18	3.9E-01	-0.84	1.1E-07	-0.27	1.9E-01	-0.16	4.4E-01	-0.10	6.2E-01	0.10	6.1E-01	0.84	9.9E-08
ENSCAFG0000000305	INIP	grey	EC_M10	0.02	9.4E-01	-0.05	8.0E-01	-0.42	3.2E-02	0.14	4.8E-01	0.51	7.5E-03	0.27	1.9E-01	-0.18	3.7E-01	0.01	9.7E-01	0.27	1.8E-01	-0.51	7.5E-03
ENSCAFG0000001332	LUZP1	grey	EC_M10	0.02	9.4E-01	0.41	3.7E-02	-0.58	1.7E-03	-0.24	2.4E-01	0.51	7.7E-03	0.14	4.8E-01	0.35	7.8E-02	-0.12	5.5E-01	-0.23	2.7E-01	-0.51	8.2E-03
ENSCAFG0000001652	INTS1	grey	EC_M10	0.02	9.4E-01	-0.02	9.3E-01	-0.33	1.0E-01	-0.27	1.8E-01	0.51	7.5E-03	0.12	5.5E-01	-0.15	4.8E-01	-0.05	8.2E-01	0.08	6.8E-01	-0.48	1.4E-02
ENSCAFG0000001915	GLYR1	grey	EC_M10	0.02	9.4E-01	0.14	5.1E-01	0.36	6.8E-02	0.04	8.6E-01	-0.46	1.8E-02	-0.11	5.9E-01	-0.55	3.9E-03	0.06	7.8E-01	-0.19	3.6E-01	0.44	2.4E-02
ENSCAFG0000000223	ENSCAFG0000000223	grey	EC_M10	0.02	9.4E-01	-0.01	9.6E-01	0.08	7.0E-01	0.46	1.7E-02	-0.17	4.0E-01	-0.16	4.3E-01	-0.11	6.0E-01	-0.25	2.1E-01	-0.16	4.3E-01	0.17	4.2E-01
ENSCAFG0000001750	SLC5A11	grey	EC_M10	0.02	9.4E-01	-0.23	2.7E-01	0.15	4.7E-01	0.14	5.0E-01	-0.03	8.8E-01	-0.21	3.1E-01	-0.24	2.4E-01	-0.15	4.6E-01	-0.19	3.5E-01	0.06	7.7E-01
ENSCAFG0000000846	TTC31	grey	EC_M10	0.02	9.4E-01	0.31	1.3E-01	-0.34	9.3E-02	0.40	4.0E-02	0.19	3.6E-01	0.36	7.1E-02	0.04	8.6E-01	-0.08	7.1E-01	-0.02	9.3E-01	-0.24	2.3E-01
ENSCAFG0000000869	DHH	turquoise	EC_M6	0.02	9.4E-01	0.66	2.7E-04	-0.51	8.2E-03	-0.10	6.3E-01	0.32	1.1E-01	0.11	5.8E-01	0.38	5.2E-02	-0.15	4.7E-01	0.23	2.6E-01	-0.32	1.1E-01
ENSCAFG0000003215	ENSCAFG0000003215	grey	EC_M10	0.01	9.4E-01	0.16	4.2E-01	0.02	9.1E-01	-0.14	4.8E-01	-0.07	7.3E-01	-0.07	7.3E-01	-0.14	5.0E-01	0.29	1.4E-01	0.20	3.2E-01	0.07	7.3E-01
ENSCAFG0000000491	H3F3B	cyan	EC_M2	0.01	9.4E-01	-0.04	8.5E-01	0.72	3.0E-05	0.07	7.5E-01	-0.85	3.1E-08	-0.18	3.9E-01	0.06	7.9E-01	-0.13	5.2E-01	-0.17	4.0E-01	0.86	2.6E-08
ENSCAFG0000002038	ST6GALNAC3	turquoise	EC_M6	0.01	9.4E-01	0.53	5.2E-03	-0.19	3.6E-01	-0.02	9.1E-01	-0.03	9.0E-01	-0.14	5.0E-01	0.38	5.9E-02	-0.06	7.8E-01	-0.18	3.8E-01	-0.02	9.1E-01
ENSCAFG0000001645	CCDC86	grey	EC_M10	0.01	9.4E-01	0.02	9.0E-01	0.38	5.6E-02	0.26	1.9E-01	-0.36	6.8E-02	0.11	5.9E-01	0.17	4.2E-01	-0.04	8.5E-01	-0.29	1.5E-01	0.38	5.7E-02
ENSCAFG0000000533	BLVRB	grey	EC_M10	0.01	9.4E-01	0.21	3.0E-01	0.10	6.1E-01	-0.01	9.7E-01	-0.19	3.6E-01	-0.24	2.4E-01	0.38	5.6E-02	0.03	8.9E-01	-0.49	1.1E-02	0.14	4.8E-01
ENSCAFG0000000518	SLAIN1	grey	EC_M10	0.01	9.4E-01	-0.36	7.0E-02	0.57	2.6E-03	0.12	5.5E-01	-0.46	1.9E-02	-0.29	1.5E-01	-0.01	9.6E-01	-0.17	4.2E-01	-0.08	7.0E-01	0.51	7.8E-03
ENSCAFG0000002033	PSMB10	grey	EC_M10	0.01	9.4E-01	0.17	4.0E-01	0.13	5.2E-01	0.20	3.4E-01	-0.17	4.2E-01	-0.07	7.2E-01	-0.28	1.6E-01	0.04	8.6E-01	-0.18	3.8E-01	0.12	5.7E-01
ENSCAFG0000001489	GALK2	cyan	EC_M2	0.01	9.4E-01	-0.40	4.3E-02	0.66	2.5E-04	0.23	2.5E-01	-0.64	4.5E-04	-0.23	2.5E-01	-0.27	1.9E-01	0.03	8.9E-01	-0.35	7.9E-02	0.62	7.7E-04
ENSCAFG0000000537	HGSNAT	grey	EC_M10	0.01	9.4E-01	-0.02	9.4E-01	-0.58	1.9E-03	-0.24	2.3E-01	0.75	8.8E-06	0.12	5.4E-01	-0.19	3.6E-01	0.11	5.9E-01	-0.24	2.4E-01	-0.72	2.8E-05
ENSCAFG0000000509	ITIH5	turquoise	EC_M6	0.01	9.4E-01	0.72	3.7E-05	-0.14	4.8E-01	0.01	9.7E-01	-0.11	6.1E-01	-0.11	5.9E-01	-0.13	5.4E-01	0.20	3.4E-01	0.33	1.0E-01	0.07	7.3E-01
ENSCAFG0000000926	ACVR1C	darkolivegreen	EC_M9	0.01	9.4E-01	-0.04	8.5E-01	-0.02	9.1E-01	-0.09	6.5E-01	0.09	6.6E-01	-0.03	8.8E-01	0.49	1.1E-02	0.00	9.9E-01	0.15	4.7E-01	-0.08	6.8E-01
ENSCAFG0000000575	SLC4A9	magenta	EC_M13	0.01	9.4E-01	0.07	7.3E-01	0.02	9.1E-01	-0.02	9.0E-01	-0.04	8.5E-01	0.00	9.9E-01	0.06	7.7E-01	-0.05	8.2E-01	0.83	1.6E-07	0.07	7.4E-01
ENSCAFG0000001245	TREH	magenta	EC_M13	0.01	9.4E-01	0.07	7.3E-01	0.02	9.1E-01	-0.02	9.0E-01	-0.04	8.5E-01	0.00	9.9E-01	0.06	7.7E-01	-0.05	8.2E-01	0.83	1.6E-07	0.07	7.4E-01
ENSCAFG0000001292	NR1I3	magenta	EC_M13	0.01	9.4E-01	0.07	7.3E-01	0.02	9.1E-01	-0.02	9.0E-01	-0.04	8.5E-01	0.00	9.9E-01	0.06	7.7E-01	-0.05	8.2E-01	0.83	1.6E-07	0.07	7.4E-01
ENSCAFG0000003029	GRB2	grey	EC_M10	0.01	9.4E-01	0.64	4.7E-04	-0.59	1.5E-03	-0.36	7.5E-02	0.45	2.1E-02	0.18	3.7E-01	0.17	4.1E-01	0.09	6.7E-01	0.29	1.5E-01	-0.43	3.0E-02
ENSCAFG0000001736	NUP210L	grey	EC_M10	0.01	9.4E-01	0.54	4.7E-03	-0.57	2.2E-03	-0.24	2.5E-01	0.42	3.1E-02	-0.32	1.1E-01	0.01	9.7E-01	0.06	7.8E-01	0.24	2.3E-01	-0.41	3.8E-02
ENSCAFG0000002639	ENSCAFG0000002639	grey	EC_M10	0.01	9.4E-01	0.27	1.9E-01	0.15	4.5E-01	-0.15	4.5E-01	-0.30	1.4E-01	-0.20	3.2E-01	0.30	1.4E-01	-0.15	4.7E-01	-0.04	8.6E-01	0.24	2.3E-01
ENSCAFG0000000191	GTPBP2	grey	EC_M10	0.01	9.5E-01	0.56	2.7E-03	-0.57	2.4E-03	-0.02	9.3E-01	0.40	4.3E-02	0.35	7.6E-02	0.13	5.1E-01	-0.31	1.2E-01	0.08	7.1E-01	-0.44	2.6E-02
ENSCAFG0000001552	WDR1	darkgreen	EC_M4	0.01	9.5E-01	0.14	5.1E-01	-0.75	1.1E-05	-0.34	9.3E-02	0.83	1.5E-07	0.13	5.3E-01	0.08	6.9E-01	0.20	3.4E-01	0.26	2.1E-01	-0.83	1.5E-07
ENSCAFG0000001567	PQBP1	cyan	EC_M2	0.01	9.5E-01	-0.16	4.4E-01	0.69	1.1E-04	0.15	4.6E-01	-0.79	1.6E-06	-0.02	9.0E-01	0.17	3.9E-01	-0.01	9.8E-01	-0.14	5.1E-01	0.74	1.9E-05
ENSCAFG0000001914	LRP8	grey	EC_M10	0.01	9.5E-01	-0.05	8.2E-01	0.28	1.6E-01	-0.14	4.9E-01	-0.25	2.1E-01	-0.30	1.3E-01	0.18	3.8E-01	-0.16	4.3E-01	-0.63	5.6E-04	0.27	1.7E-01
ENSCAFG0000002272	ENSCAFG0000002272	grey	EC_M10	0.01	9.5E-01	-0.19	3.4E-01	-0.19	3.5E-01	0.40	4.5E-02	0.26	2.0E-01	0.36	7.4E-02	0.27	1.8E-01	-0.14	4.9E-01	-0.16	4.5E-01	-0.28	1.7E-01
ENSCAFG0000003049	FHAD1	turquoise	EC_M6	0.01	9.5E-01	0.34	8.7E-02	-0.09	6.6E-01	-0.14	5.1E-01	-0.02	9.2E-01	-0.09	6.7E-01	-0.04	8.5E-01	-0.16	4.4E-01	0.58	1.8E-03	0.06	7.7E-01
ENSCAFG0000001124	SLCO3A1	grey	EC_M10	0.01	9.5E-01	0.54	4.5E-03	-0.73	2.7E-05	-0.17	4.0E-01	0.67	1.7E-04	0.09	6.5E-01	0.23	2.6E-01	0.02	9.4E-01	-0.05	8.0E-01	-0.66	2.3E-04
ENSCAFG0000000180	TMEM263	grey	EC_M10	0.01	9.5E-01	0.27	1.8E-01	-0.52	6.4E-03	0.03	8.9E-01	0.44	2.4E-02	0.18	3.8E-01	0.32	1.1E-01	-0.01	9.8E-01	-0.23	2.5E-01	-0.52	6.7E-03

ENSCAFG0000001935	HES4	grey	EC_M10	0.01	9.5E-01	0.03	8.7E-01	0.23	2.5E-01	-0.18	3.7E-01	-0.27	1.9E-01	0.13	5.2E-01	-0.13	5.3E-01	-0.20	3.3E-01	0.03	8.8E-01	0.25	2.1E-01
ENSCAFG0000003217	ENSCAFG0000003217	grey	EC_M10	0.01	9.5E-01	0.11	6.0E-01	0.02	9.1E-01	-0.22	2.9E-01	-0.07	7.3E-01	0.02	9.2E-01	0.08	7.0E-01	0.17	4.1E-01	0.32	1.1E-01	0.04	8.3E-01
ENSCAFG0000000987	ENSCAFG0000000987	cyan	EC_M2	0.01	9.5E-01	-0.48	1.3E-02	0.52	7.1E-03	0.32	1.1E-01	-0.43	2.9E-02	0.08	6.8E-01	0.05	8.3E-01	-0.22	2.8E-01	-0.53	5.8E-03	0.37	6.6E-02
ENSCAFG0000001628	ENSCAFG0000001628	turquoise	EC_M6	0.01	9.5E-01	0.70	6.0E-05	0.16	4.3E-01	0.19	3.6E-01	-0.50	9.1E-03	-0.08	7.0E-01	-0.04	8.3E-01	-0.04	8.4E-01	0.28	1.6E-01	0.49	1.1E-02
ENSCAFG0000003259	SNRPG	grey	EC_M10	0.01	9.5E-01	0.16	4.2E-01	0.23	2.5E-01	-0.01	9.4E-01	-0.31	1.2E-01	-0.31	1.2E-01	-0.25	2.2E-01	0.32	1.1E-01	0.36	7.1E-02	0.30	1.3E-01
ENSCAFG0000002901	ENSCAFG0000002901	grey	EC_M10	0.01	9.5E-01	-0.29	1.5E-01	0.30	1.4E-01	-0.23	2.6E-01	-0.27	1.8E-01	0.38	5.4E-02	0.02	9.2E-01	-0.19	3.6E-01	-0.30	1.4E-01	0.25	2.2E-01
ENSCAFG0000001639	VPS13D	grey	EC_M10	0.01	9.5E-01	0.50	9.5E-03	-0.14	4.8E-01	0.14	5.0E-01	-0.05	8.0E-01	0.11	6.0E-01	0.05	7.9E-01	-0.51	7.7E-03	0.16	4.4E-01	0.09	6.6E-01
ENSCAFG0000001523	ANKFY1	grey	EC_M10	0.01	9.5E-01	0.12	5.8E-01	0.47	1.5E-02	-0.21	3.1E-01	-0.56	2.7E-03	-0.12	5.6E-01	-0.14	4.9E-01	-0.08	7.1E-01	-0.25	2.1E-01	0.56	3.0E-03
ENSCAFG0000003038	ENSCAFG0000003038	grey	EC_M10	0.01	9.5E-01	-0.14	4.9E-01	0.15	4.8E-01	-0.19	3.4E-01	-0.09	6.7E-01	-0.18	3.7E-01	0.16	4.4E-01	-0.16	4.4E-01	-0.22	2.8E-01	0.11	6.0E-01
ENSCAFG0000000330	MEAF6	grey	EC_M10	0.01	9.5E-01	-0.19	3.6E-01	0.23	2.5E-01	0.29	1.5E-01	-0.25	2.2E-01	0.28	1.6E-01	0.08	7.0E-01	-0.30	1.4E-01	-0.06	7.8E-01	0.22	2.7E-01
ENSCAFG0000000159	FOXP4	grey	EC_M10	0.01	9.5E-01	0.25	2.1E-01	-0.61	9.4E-04	-0.30	1.3E-01	0.63	5.6E-04	0.27	1.9E-01	0.04	8.3E-01	-0.07	7.5E-01	0.38	5.3E-02	-0.66	2.2E-04
ENSCAFG0000000411	ENSCAFG0000000411	grey	EC_M10	0.01	9.5E-01	0.17	4.1E-01	-0.13	5.4E-01	0.26	2.1E-01	0.07	7.3E-01	0.32	1.1E-01	0.11	5.9E-01	-0.43	3.0E-02	-0.05	8.2E-01	-0.08	7.0E-01
ENSCAFG0000001224	ZNF831	grey	EC_M10	0.01	9.5E-01	0.02	9.1E-01	-0.11	6.0E-01	-0.14	4.9E-01	0.17	3.9E-01	0.25	2.2E-01	0.46	1.8E-02	-0.13	5.4E-01	0.03	8.9E-01	-0.17	4.2E-01
ENSCAFG0000002016	ZBTB34	turquoise	EC_M6	0.01	9.5E-01	0.55	3.6E-03	-0.03	8.8E-01	-0.30	1.3E-01	-0.22	2.9E-01	0.10	6.2E-01	0.25	2.2E-01	-0.17	4.0E-01	0.05	8.0E-01	0.24	2.4E-01
ENSCAFG0000001575	GRID2IP	grey	EC_M10	0.01	9.5E-01	-0.25	2.2E-01	0.03	8.9E-01	0.16	4.5E-01	0.03	8.9E-01	-0.15	4.8E-01	0.19	3.6E-01	0.19	3.5E-01	0.07	7.3E-01	0.00	9.8E-01
ENSCAFG0000001481	COPS6	grey	EC_M10	0.01	9.5E-01	-0.41	3.6E-02	0.06	7.6E-01	0.32	1.1E-01	0.15	4.6E-01	-0.09	6.7E-01	0.00	1.0E+00	-0.02	9.4E-01	-0.13	5.4E-01	-0.18	3.8E-01
ENSCAFG0000001247	PDE5A	grey	EC_M10	0.01	9.5E-01	-0.07	7.4E-01	-0.64	4.7E-04	-0.16	4.4E-01	0.77	4.2E-06	0.16	4.5E-01	-0.05	8.2E-01	0.30	1.4E-01	0.08	7.0E-01	-0.79	1.3E-06
ENSCAFG0000000715	GRAMD1A	turquoise	EC_M6	0.01	9.5E-01	0.84	9.1E-08	-0.09	6.5E-01	-0.13	5.4E-01	-0.17	4.1E-01	-0.24	2.3E-01	0.14	4.9E-01	-0.15	4.5E-01	0.02	9.2E-01	0.19	3.6E-01
ENSCAFG0000000556	FLRT3	grey	EC_M10	0.01	9.5E-01	-0.34	9.2E-02	-0.04	8.3E-01	0.08	6.8E-01	0.16	4.5E-01	0.09	6.8E-01	-0.49	1.2E-02	0.21	3.0E-01	0.15	4.8E-01	-0.21	2.9E-01
ENSCAFG0000003195	ENSCAFG0000003195	grey	EC_M10	0.01	9.5E-01	-0.28	1.6E-01	-0.24	2.5E-01	-0.24	2.4E-01	0.36	7.2E-02	0.24	2.4E-01	-0.20	3.3E-01	0.36	7.0E-02	-0.02	9.3E-01	-0.33	1.0E-01
ENSCAFG0000000383	NTF4	grey	EC_M10	0.01	9.5E-01	0.08	6.8E-01	0.08	7.1E-01	-0.10	6.2E-01	-0.05	8.1E-01	-0.13	5.3E-01	-0.09	6.5E-01	-0.10	6.3E-01	0.31	1.2E-01	0.10	6.4E-01
ENSCAFG0000001229	NIPSNAP1	grey	EC_M10	0.01	9.5E-01	-0.30	1.3E-01	-0.12	5.7E-01	0.21	3.1E-01	0.19	3.6E-01	-0.08	7.1E-01	-0.35	7.6E-02	0.27	1.7E-01	0.02	9.3E-01	-0.23	2.5E-01
ENSCAFG0000002195	ENSCAFG0000002195	grey	EC_M10	0.01	9.5E-01	-0.13	5.2E-01	0.21	3.0E-01	-0.13	5.4E-01	-0.25	2.1E-01	-0.10	6.3E-01	-0.08	7.0E-01	0.44	2.5E-02	0.07	7.4E-01	0.29	1.4E-01
ENSCAFG0000000201	CLIC5	grey	EC_M10	0.01	9.5E-01	-0.38	5.9E-02	0.47	1.6E-02	0.02	9.3E-01	-0.36	7.0E-02	-0.04	8.5E-01	0.18	3.7E-01	0.06	7.8E-01	-0.13	5.2E-01	0.40	4.0E-02
ENSCAFG0000003106	TMEM267	grey	EC_M10	0.01	9.5E-01	-0.25	2.2E-01	-0.55	3.7E-03	0.00	9.8E-01	0.69	1.0E-04	0.32	1.1E-01	-0.29	1.5E-01	0.05	8.0E-01	-0.04	8.4E-01	-0.73	2.1E-05
ENSCAFG0000000562	ENSCAFG0000000562	grey	EC_M10	0.01	9.5E-01	0.38	5.9E-02	0.43	2.8E-02	0.09	6.5E-01	-0.61	9.6E-04	-0.39	4.9E-02	-0.19	3.5E-01	0.04	8.4E-01	0.00	9.8E-01	0.58	2.1E-03
ENSCAFG0000000945	POLR3D	darkgreen	EC_M4	0.01	9.5E-01	-0.08	7.0E-01	-0.66	2.4E-04	-0.14	5.0E-01	0.76	7.7E-06	0.22	2.8E-01	0.11	6.1E-01	0.22	2.9E-01	-0.14	4.9E-01	-0.81	6.1E-07
ENSCAFG0000000417	ENSCAFG0000000417	grey	EC_M10	0.01	9.5E-01	0.13	5.3E-01	0.24	2.3E-01	-0.10	6.3E-01	-0.31	1.2E-01	-0.12	5.6E-01	-0.09	6.6E-01	0.07	7.5E-01	-0.21	3.0E-01	0.31	1.3E-01
ENSCAFG0000001255	UBXN11	grey	EC_M10	0.01	9.5E-01	0.06	7.9E-01	0.24	2.4E-01	0.15	4.5E-01	-0.27	1.8E-01	0.01	9.6E-01	0.11	6.0E-01	0.02	9.3E-01	0.27	1.8E-01	0.30	1.3E-01
ENSCAFG0000002927	SOX18	turquoise	EC_M6	0.01	9.5E-01	0.85	5.0E-08	-0.10	6.1E-01	-0.12	5.6E-01	-0.16	4.2E-01	-0.13	5.4E-01	-0.03	9.0E-01	-0.17	4.1E-01	0.06	7.6E-01	0.13	5.2E-01
ENSCAFG0000001641	GNAI1	grey	EC_M10	0.01	9.5E-01	0.58	2.1E-03	-0.56	3.2E-03	-0.06	7.5E-01	0.40	4.5E-02	0.01	9.6E-01	0.03	8.8E-01	0.19	3.4E-01	-0.17	4.2E-01	-0.46	1.9E-02
ENSCAFG0000000857	CUX2	grey	EC_M10	0.01	9.5E-01	-0.01	9.6E-01	-0.31	1.3E-01	-0.23	2.5E-01	0.40	4.1E-02	-0.10	6.3E-01	-0.13	5.4E-01	-0.23	2.5E-01	0.04	8.6E-01	-0.38	5.6E-02
ENSCAFG0000003231	ENSCAFG0000003231	turquoise	EC_M6	0.01	9.6E-01	0.71	5.7E-05	-0.08	7.0E-01	-0.10	6.3E-01	-0.18	3.8E-01	-0.10	6.4E-01	0.33	1.0E-01	-0.08	7.1E-01	-0.14	4.9E-01	0.20	3.3E-01
ENSCAFG0000002901	ENSCAFG0000002901	turquoise	EC_M6	0.01	9.6E-01	0.37	6.1E-02	-0.11	5.9E-01	-0.33	1.0E-01	-0.01	9.7E-01	-0.26	1.9E-01	0.11	5.9E-01	0.07	7.3E-01	0.57	2.6E-03	0.07	7.2E-01
ENSCAFG0000001477	RCOR2	grey	EC_M10	0.01	9.6E-01	-0.37	6.4E-02	0.49	1.2E-02	0.00	1.0E+00	-0.44	2.6E-02	-0.15	4.6E-01	0.11	5.8E-01	-0.27	1.9E-01	-0.18	3.8E-01	0.42	3.1E-02
ENSCAFG0000000955	RTF1	grey	EC_M10	0.01	9.6E-01	0.13	5.3E-01	0.46	1.7E-02	0.09	6.5E-01	-0.57	2.3E-03	-0.09	6.5E-01	-0.35	7.8E-02	-0.10	6.1E-01	0.00	1.0E+00	0.61	8.6E-04
ENSCAFG0000001782	ENSCAFG0000001782	grey	EC_M10	0.01	9.6E-01	0.03	8.7E-01	0.25	2.1E-01	-0.06	7.7E-01	-0.30	1.3E-01	-0.07	7.4E-01	-0.02	9.2E-01	-0.10	6.1E-01	0.46	1.7E-02	0.31	1.2E-01
ENSCAFG0000000425	COPG1	darkgreen	EC_M4	0.01	9.6E-01	0.01	9.6E-01	-0.77	4.5E-06	-0.13	5.2E-01	0.91	6.9E-11	0.26	2.0E-01	-0.03	8.8E-01	0.04	8.4E-01	-0.09	6.7E-01	-0.93	7.0E-12
ENSCAFG0000001374	ODR4	grey	EC_M10	0.01	9.6E-01	0.14	4.9E-01	-0.35	7.5E-02	0.13	5.4E-01	0.31	1.3E-01	0.20	3.3E-01	-0.01	9.7E-01	-0.17	4.2E-01	-0.20	3.2E-01	-0.30	1.3E-01
ENSCAFG0000001365	EIF4A2	turquoise	EC_M6	0.01	9.6E-01	0.26	2.0E-01	-0.13	5.2E-01	0.06	7.9E-01	-0.06	7.8E-01	-0.23	2.6E-01	0.03	9.0E-01	0.49	1.0E-02	0.33	1.0E-01	0.07	7.4E-01
ENSCAFG0000001476	MRPS36	grey	EC_M10	0.01	9.6E-01	-0.04	8.4E-01	0.07	7.3E-01	-0.30	1.4E-01	-0.04	8.4E-01	0.27	1.7E-01	0.07	7.2E-01	-0.25	2.2E-01	-0.04	8.6E-01	0.03	8.9E-01
ENSCAFG0000000803	CEP250	cyan	EC_M2	0.01	9.6E-01	0.43	2.7E-02	0.37	6.5E-02	0.28	1.6E-01	-0.67	2.0E-04	-0.04	8.3E-01	0.04	8.4E-01	0.08	7.1E-01	0.16	4.5E-01	0.67	1.8E-04
ENSCAFG0000003270	ST8SIA6	turquoise	EC_M6	0.01	9.6E-01	0.92	1.9E-11	-0.36	6.9E-02	-0.15	4.7E-01	0.09	6.7E-01	-0.17	4.0E-01	0.02	9.4E-01	-0.02	9.2E-01	0.14	4.9E-01	-0.07	7.4E-01
ENSCAFG0000003006	ENSCAFG0000003006	grey	EC_M10	0.01	9.6E-01	-0.14	5.0E-01	-0.20	3.2E-01	-0.13	5.3E-01	0.23	2.6E-01	-0.13	5.2E-01	-0.16	4.3E-01	0.30	1.3E-01	-0.01	9.5E-01	-0.23	2.5E-01
ENSCAFG0000001719	ENSCAFG0000001719	grey	EC_M10	0.01	9.6E-01	0.34	9.1E-02	-0.05	8.1E-01	-0.02	9.1E-01	-0.11	5.9E-01	-0.03	8.7E-01	-0.05	8.1E-01	0.19	3.5E-01	0.10	6.1E-01	0.12	5.6E-01
ENSCAFG0000000454	CYP27C1	grey	EC_M10	0.01	9.6E-01	-0.37	6.4E-02	-0.48	1.2E-02	-0.06	7.7E-01	0.70	6.1E-05	0.26	2.0E-01	0.23	2.5E-01	-0.04	8.3E-01	-0.14	5.0E-01	-0.74	1.8E-05
ENSCAFG0000003096	RTN4RL2	magenta	EC_M13	0.01	9.6E-01	0.09	6.5E-01	0.07	7.4E-01	-0.03	8.7E-01	-0.09	6.5E-01	0.02	9.2E-01	0.05	8.0E-01	-0.05	8.0E-01	0.74	1.5E-05	0.11	6.0E-01
ENSCAFG0000003190	MKRN2OS	turquoise	EC_M6	0.01	9.6E-01	0.76	8.0E-06	-0.10	6.4E-01	-0.13	5.3E-01	-0.14	4.9E-01	-0.14	4.8E-01	0.20	3.2E-01	-0.15	4.8E-01	-0.22	2.9E-01	0.11	5.9E-01
ENSCAFG0000003238	DCAKD	grey	EC_M10	0.01	9.6E-01	-0.67	1.8E-04	0.39	4.9E-02	-0.16	4.2E-01	-0.19	3.6E-01	0.04	8.3E-01	-0.05	8.1E-01	-0.03	8.9E-01	-0.15	4.7E-01	0.17	3.9E-01
ENSCAFG0000001500	SOCS4	grey	EC_M10	0.01	9.6E-01	0.29	1.6E-01	0.09	6.6E-01	-0.04	8.6E-01	-0.32	1.1E-01	-0.06	7.7E-01	0.06	7.7E-01	0.17	4.1E-01	0.31	1.2E-01	0.34	8.7E-02
ENSCAFG0000000183	TTBK1	grey	EC_M10	0.01	9.6E-01	-0.05	8.0E-01	-0.58	2.0E-03	-0.17	4.1E-01	0.71	5.0E-05	0.25	2.1E-01	0.18	3.8E-01	-0.20	3.3E-01	-0.04	8.5E-01	-0.72	3.1E-05
ENSCAFG0000000068	OXR1	turquoise	EC_M6	0.01	9.6E-01	0.43	2.7E-02	-0.17	4.0E-01	0.27	1.9E-01	-0.01	9.5E-01	-0.07	7.5E-01	0.03	8.9E-01	-0.18	3.8E-01	-0.07	7.5E-01	0.01	9.4E-01
ENSCAFG0000001682	PAPLN	turquoise	EC_M6	0.01	9.6E-01	0.86	1.6E-08	-0.12	5.7E-01	-0.14	5.1E-01	-0.15	4.7E-01	-0.21	3.1E-01	0.19	3.5E-01	-0.11	6.0E-01	-0.14	4.8E-01	0.15	4.6E-01
ENSCAFG0000000977	ZSWIM3	grey	EC_M10	0.01	9.6E-01	0.03	8.8E-01	0.10	6.3E-01	0.25	2.1E-01	-0.14	5.1E-01	0.14	4.9E-01	-0.40	4.2E-02	-0.11	6.0E-01	0.33	1.0E-01	0.12	5.6E-01
ENSCAFG0000001184	ENSCAFG0000001184	turquoise	EC_M6	0.01	9.6E-01	0.50	8.9E-03	-0.35	7.6E-02	-0.12	5.7E-01	0.17	4.0E-01	0.19	3.6E-01	0.24	2.4E-01	-0.28	1.7E-01	-0.19	3.5E-01	-0.19	3.5E-01
ENSCAFG0000000991	ADCY7	grey	EC_M10	0.01	9.6E-01	0.43	2.7E-02	-0.70	7.9E-05	-0.23	2.6E-01	0.65	3.4E-04	0.07	7.4E-01	0.21	3.1E-01	0.10	6.4E-01	0.28	1.7E-01	-0.68	1.3E-04
ENSCAFG0000000711	EIF4B	cyan	EC_M2	0.01	9.6E-01	-0.22	2.7E-01	0.56	2.8E-03	0.30	1.4E-01	-0.64	3.8E-04	-0.10	6.4E-01	0.28	1.7E-01	-0.10	6.2E-01	-0.46	1.7E-02	0.58	1.7E-03
ENSCAFG0000000218	SEMA4D	turquoise	EC_M6	0.01	9.6E-01	0.73	2.1E-05	-0.15	4.7E-01	0.20	3.4E-01	-0.08	6.8E-01	-0.17	4.1E-01	0.03	8.9E-01	-0.21	3.1E-01	0.05	8.1E-01	0.12	5.7E-01
ENSCAFG0000000897	THBS4	turquoise	EC_M6	0.01	9.6E-01	0.54	4.3E-03	-0.40	4.2E-02	-0.12	5.5E-01	0.28	1.7E-01	-0.12	5.7E-01	-0.07	7.4E-01	-0.03	8.8E-01	-0.02	9.1E-01	-0.25	2.2E-01
ENSCAFG0000003167	SNAPC2	grey	EC_M10	0.01	9.6E-01	-0.23	2.6E-01	0.18	3.7E-01	-0.37	6.1E-02	-0.06	7.9E-01	0.16	4.3E-01	0.25	2.1E-01	-0.10	6.2E-01	0.09	6.6E-01	0.03	8.7E-01
ENSCAFG0000001750	SPESP1	grey	EC_M10	0.01	9.6E-01	-0.57	2.1E-03	0.20	3.3E-01	0.05	8.2E-01	-0.08	6.9E-01	0.09	6.5E-01	-0.03	8.7E-01	0.26	1.9E-01	0.00	9.9E-01	0.07	7.2E-01
ENSCAFG0000000028	CYP27B1	grey	EC_M10	0.01	9.6E-01	-0.14	5.0E-01	0.30	1.4E-01	0.12	5.7E-01	-0.31	1.2E-01	-0.24	2.3E-01	0.23	2.7E-01	-0.16	4.4E-01	-0.16	4.2E-01	0.33	1.0E-01
ENSCAFG0000000101	KLHL38	grey	EC_M10	0.01	9.6E-01	-0.12	5.5E-01	0.02	9.4E-01	-0.19	3.6E-01	-0.01	9.7E-01	-0.14	4.9E-01	-0.07	7.2E-01	-0.13	5.4E-01	0.07	7.3E-01	0.05	8.1E-01
ENSCAFG0000001732	FLRT2	grey	EC_M10	0.01	9.6E-01	0.18	3.8E-01	-0.22	2.7E-01	0.26	2.0E-01	0.13	5.4E-01	-0.13	5.2E-01	-0.22	2.9E-01	0.19	3.6E-01	0.10	6.4E-01	-0.18	3.8E-01
ENSCAFG0000000526	ENSCAFG0000000526	grey	EC_M10	0.01	9.6E-01	0.12	5.7E-01	-0.32	1.1E-01	0.36	7.4E-02	0.34	9.2E-02	0.37	6.0E-02	0.18	3.7E-01	0.11	6.0E-01	-0.23	2.5E-01	-0.33	9.9E-02
ENSCAFG0000000649	B4GALNT4	grey	EC_M10	0.01	9.7E-01	-0.06	7.6E-01	-0.04	8.5E-01	0.28	1.7E-01	0.11	6.0E-01	-0.24	2.4E-01	0.05	8.2E-01	-0.25	2.2E-01	0.50	9.0E-03	-0.10	6.3E-01
ENSCAFG0000001289	ENSCAFG0000001289	grey	EC_M10	0.01	9.7E-01	-0.29	1.6E-01	-0.15	4.8E-01	-0.07	7.4E-01	0.38	5.8E-02	-0.05	8.0E-01	-0.16	4.3E-01	0.31	1.2E-01	0.07	7.2E-01	-0.36	7.5E-02
ENSCAFG0000002863	TRIM7	cyan	EC_M2	0.01	9.7E-01	-0.07	7.3E-01	0.72	3.6E-05	-0.12	5.6E-01	-0.75	1.0E-05	-0.22	2.9E-01	-0.03	9.0E-01	-0.05	7.9E-01	0.10	6.2E-01	0.77	4.4E-06
ENSCAFG0000000881	ENSCAFG0000000881	grey	EC_M10	0.01	9.7E-01	0.28	1.7E-01	-0.65	3.4E-04	-0.21	3.0E-01	0.68	1.3E-04	0.11	6.1E-01	0.16	4.4E-01	-0.23	2.6E-01	0.23	2.6E-01	-0.67	2.0E-04
ENSCAFG0000000299	NT5E	grey	EC_M10	0.01	9.7E-01	-0.06	7.9E-01	0.31	1.2E-01	0.17	4.1E-01	-0.33	1.0E-01	-0.03	8.9E-01	-0.07	7.5E-01	0.55	3.5E-03	0.08	7.0E-01	0.30	1.3E-01
ENSCAFG0000003006	ENSCAFG0000003006	grey	EC_M10	0.01	9.7E-01	0.20	3.2E-01	0.11	6.0E-01	0.48	1.4E-02	-0.22	2.8E-01	-0.24	2.4E-01	0.06	7.5E-01	0.26	1.9E-01	-0.26	2.0E-01	0.20	3.3E-01
ENSCAFG0000000108	MYC	cyan	EC_M2	0.01	9.7E-01	-0.43	2.8E-02	0.65	3.4E-04	-0.17	4.1E-01	-0.56	2.8E-03	-0.07	7.3E-01	0.00	9.9E-01	0.11	6.1E-01	-0.30	1.3E-01	0.54	4.3E-03
ENSCAFG0000003057	ZNF570	turquoise	EC_M6	0.01	9.7E-01	0.50	9.0E-03	0.22	2.8E-01	0.10	6.3E-01	-0.45	2.2E-02	-0.27	1.8E-01	-0.32	1.1E-01	-0.06	7.7E-01	-0.20	3.3E-01	0.46	1.8E-02
ENSCAFG0000000350	ARHGEF5	cyan	EC_M2	0.01	9.7E-01	-0.14	4.9E-01	0.65	3.0E-04	0.02	9.1E-01	-0.69	9.6E-05	-0.22	2.9E-01	-0.12	5.6E-01	-0.19	3.6E-01	-0.08	7.1E-01	0.74	1.3E-05
ENSCAFG0000003102	HRAS	grey	EC_M10	0.01	9.7E-01	-0.34	9.4E-02	0.16	4.2E-01	-0.08	6.8E-01	0.05	8.1E-01	-0.05	8.2E-01	-0.27	1.9E-01	-0.08	7.1E-01	0.12	5.7E-01	-0.04	8.5E-01
ENSCAFG0000003090	RALA	turquoise	EC_M6	0.01	9.7E-01	0.43	2.7E-02	-0.30	1.4E-01	-0.42	3.2E-02	0.21	3.1E-01	0.01	9.7E-01	0.30	1.4E-01	0.07	7.2E-01	0.09	6.5E-01	-0.16	4.3E-01
ENSCAFG0000002013	BCAR3	darkgreen	EC_M4	0.01	9.7E-01	0.12	5.5E-01	-0.80	9.2E-07	-0.15	4.7E-01	0.86	1.6E-08	0.17	4.2E-01	0.10	6.3E-01	0.04	8.5E-01	0.13	5.3E-01	-0.88	2.6E-09
ENSCAFG0000000001	CTDP1	grey	EC_M10	0.01	9.7E-01	0.44	2.3E-02	0.30	1.3E-01	0.02	9.4E-01	-0.47	1.5E-02	0.03	8.7E-01	0.11	6.0E-01	0.04	8.5E-01	0.14	4.9E-01	0.50	8.8E-03
ENSCAFG0000001406	ZDHHC3	grey	EC_M10	0.01	9.7E-01	-0.33	9.9E-02	0.28	1.7E-01	0.12	5.7E-01	-0.13	5.4E-01	-0.15	4.5E-01	-0.47	1.5E-02	0.17	3.9E-01	0.04	8.4E-01	0.14	4.8E-01
ENSCAFG0000000708	UBXN2B	grey	EC_M10	0.01	9.7E-01	-0.04	8.6E-01	-0.62	6.4E-04	-0.06	7.8E-01	0.74	1.7E-05	0.23	2.6E-01	-0.26	2.0E-01	0.35	7.8E-02	-0.06	7.5E-01	-0.75	1.1E-05
ENSCAFG0000000642	GXYLT2	grey	EC_M10	0.01	9.7E-01	-0.45	2.1E-02	-0.25	2.1E-01	0.30	1.4E-01	0.40	4.3E-02	0.12	5.7E-01	0.02	9.4E-01	-0.03	9.0E-01	-0.27	1.8E-01	-0.47	1.5E-02
ENSCAFG0000003102	DAZAP2	turquoise	EC_M6	0.01	9.7E-01	0.75	8.8E-06	0.14	4.8E-01	0.06	7.7E-01	-0.46	1.7E-02	-0.13	5.3E-01	-0.16	4.2E-01	-0.07	7.2E-01	0.16	4.3E-01	0.47	1.5E-02
ENSCAFG0000000211	INSL6	grey	EC_M10	0.01	9.7E-01	-0.10	6.1E-01	-0.21	3.0E-01	-0.04	8.3E-01	0.32	1.1E-01	-0.10	6.2E-01	-0.03	8.8E-01	-0.10	6.4E-01	0.05	7.9E-01	-0.32	1.1E-01
ENSCAFG0000000243	OXER1	grey	EC_M10	0.01	9.7E-01	-0.10	6.1E-01	-0.21	3.0E-01	-0.04	8.3E-01	0.32	1.1E-01	-0.10	6.2E-01	-0.03	8.8E-01	-0.10	6.4E-01	0.05	7.9E-01	-0.32	1.1E-01
ENSCAFG0000002397	ENSCAFG0000002397	grey	EC_M10	0.01	9.7E-01	0.09	6.5E-01	0.29	1.5E-01	0.23	2.5E-01	-0.38	5.6E-02	-0.13	5.3E-01	-0.17	4.1E-01	-0.14	5.0E-01	0.06	7.8E-01	0.37	6.0E-02
ENSCAFG0000000690	VPS37A	darkgreen	EC_M4	0.01	9.7E-01	-0.01	9.6E-01	-0.77	3.8E-06	-0.23	2.5E-01	0.89	1.1E-09	0.33	9.9E-02	-0.09	6.5E-01	0.08	6.8E-01	0.06	7.6E-01	-0.91	1.5E-10
ENSCAFG0000000113	ITPR3	grey	EC_M10	0.01	9.7E-01	-0.03	8.8E-01	0.56	3.2E-03	0.07	7.2E-01	-0.64	4.5E-04	-0.29	1.5E-01	-0.18	3.8E-01	-0.20	3.3E-01	-0.34	8.7E-02	0.67	1.7E-04
ENSCAFG0000002524	ENSCAFG0000002524	grey	EC_M10	0.01	9.7E-01	-0.13	5.2E-01	-0.31	1.2E-01	-0.12	5.5E-01	0.36	7.2E-02	0.20	3.3E-01	-0.12	5.5E-01	0.29	1.6E-01	0.24	2.3E-01	-0.42	3.3E-02
ENSCAFG0000001165	ENSCAFG0000001165	darkgreen	EC_M4	0.01	9.7E-01	-0.83	1.2E-07	0.00	1.0E+00	0.08	6.9E-01	0.29	1.4E-01	0.29	1.5E-01	0.06	7.7E-01	0.06	7.7E-01	-0.24	2.3E-01	-0.34	9.3E-02
ENSCAFG0000002000	CDC14A	grey	EC_M10	0.01	9.7E-01	0.16	4.5E-01	0.22	2.8E-01	-0.04	8.6E-01	-0.28	1.7E-01	-0.11	6.0E-01	-0.11	6.0E-01	-0.05	7.9E-01	-0.02	9.3E-01	0.33	1.0E-01
ENSCAFG0000001769	GLA	grey	EC_M10	0.01	9.7E-01	-0.19	3.5E-01	-0.31	1.2E-01	0.05	8.1E-01	0.50	8.9E-03	0.14	5.0E-01	-0.30	1.3E-01	0.02	9.3E-01	-0.10	6.2E-01	-0.47	1.5E-02
ENSCAFG0000000328	RSPO1	grey	EC_M10	0.01	9.7E-01	-0.13	5.4E-01	0.37	6.5E-02	-0.13	5.3E-01	-0.33	9.9E-02	-0.05	8.3E-01	-0.07	7.2E-01	-0.01	9.6E-01	-0.09	6.6E-01	0.32	1.1E-01
ENSCAFG0000001741	STX8	grey	EC_M10	0.01	9.7E-01	-0.22	2.8E-01	-0.24	2.4E-01	0.11	5.9E-01	0.32	1.1E-01	0.30	1.4E-01	0.20	3.2E-01	0.05	8.2E-01	0.34	8.9E-02	-0.31	1.3E-01
ENSCAFG0000002554	ENSCAFG0000002554	grey	EC_M10	0.01	9.7E-01	-0.21	3.0E-01	0.48	1.3E-02	-0.10	6.3E-01	-0.42	3.2E-02	-0.07	7.4E-01	-0.08	6.9E-01	-0.04	8.5E-01	-0.18	3.7E-01	0.46	1.7E-02
ENSCAFG0000002880	ENSCAFG0000002880	turquoise	EC_M6	0.01	9.7E-01	0.63	6.2E-04	0.00	1.0E+00	-0.09	6.5E-01	-0.27	1.8E-01	-0.09	6.8E-01	-0.07	7.2E-01	0.35	7.7E-02	0.35	7.8E-02	0.29	1.5E-01
ENSCAFG0000000691	ENSCAFG0000000691	grey	EC_M10	0.01	9.7E-01	-0.30	1.3E-01	-0.34	9.4E-02	-0.36	7.3E-02	0.51	7.8E-03	0.30	1.4E-01	0.01	9.5E-01	0.00	1.0E+00	0.23	2.6E-01	-0.55	4.0E-03
ENSCAFG0000000482	ITGA9	turquoise	EC_M6	0.01	9.7E-01	0.93	4.8E-12	-0.18	3.7E-01	-0.07	7.4E-01	-0.13	5.4E-01	-0.18	3.7E-01	0.10	6.2E-01	-0.01	9.4E-01	0.19	3.5E-01	0.13	5.1E-01
ENSCAFG0000002920	ENSCAFG0000002920	grey	EC_M10	0.01	9.7E-01	-0.14	5.1E-01	0.22	2.9E-01	-0.01	9.6E-01	-0.22	2.7E-01	-0.05	8.1E-01	-0.11	6.0E-01	-0.02	9.0E-01	-0.12	5.7E-01	0.17	4.1E-01
ENSCAFG0000000436	ABI1	grey	EC_M10	0.01	9.7E-01	0.21	3.1E-01	0.48	1.3E-02	0.00	1.0E+00	-0.67	1.9E-04	-0.18	3.8E-01	-0.16	4.4E-01	0.16	4.3E-01	-0.12	5.7E-01	0.64	4.5E-04
ENSCAFG0000001174	PLEKHO1	turquoise	EC_M6	0.01	9.7E-01	0.71	4.1E-05	-0.38	5.4E-02	-0.10	6.2E-01	0.15	4.6E-01	0.00	9.9E-01	0.19	3.6E-01	-0.18	3.8E-01	0.29	1.5E-01	-0.15	4.6E-01
ENSCAFG0000002027	COL24A1	grey	EC_M10	0.01	9.7E-01	-0.04	8.4E-01	-0.28	1.6E-01	-0.07	7.3E-01	0.33	9.9E-02	-0.18	3.9E-01	-0.14	5.0E-01	-0.06	7.7E-01	-0.11	6.1E-01	-0.36	7.0E-02
ENSCAFG0000001841	RHBDL3	cyan	EC_M2	0.01	9.7E-01	0.12	5.7E-01	0.66	2.5E-04	0.15	4.8E-01	-0.81	7.0E-07	-0.28	1.6E-01	0.04	8.3E-01	-0.27	1.8E-01	-0.23	2.6E-01	0.80	9.9E-07
ENSCAFG0000000664	HOXC13	grey	EC_M10	0.01	9.7E-01	-0.16	4.4E-01	0.40	4.0E-02	-0.13	5.3E-01	-0.45	2.0E-02	-0.09	6.8E-01	-0.10	6.1E-01	-0.11	5.9E-01	-0.06	7.8E-01	0.51	8.4E-03
ENSCAFG0000001100	TINAGL1	turquoise	EC_M6	0.01	9.7E-01	0.75	9.7E-06	-0.50	1.0E-02	-0.20	3.4E-01	0.34	9.2E-02	-0.19	3.5E-01	-0.01	9.7E-01	0.05	8.2E-01	0.29	1.6E-01	-0.30	1.3E-01
ENSCAFG0000000294	ME1	grey	EC_M10	0.01	9.8E-01	0.01	9.6E-01	0.43	3.0E-02	0.18	3.8E-01	-0.57	2.4E-03	-0.28	1.7E-01	-0.24	2.4E-01	-0.14	4.8E-01	0.33	1.0E-01	0.59	1.5E-03
ENSCAFG0000002013	DDX19A	grey	EC_M10	0.01	9.8E-01	0.33	1.0E-01	-0.26	2.0E-01	0.11	6.0E-01	0.24	2.3E-01	-0.17	4.0E-01	0.44	2.3E-02	-0.08	7.0E-01	-0.33	9.5E-02	-0.21	3.1E-01
ENSCAFG0000003089	CA10	grey	EC_M10	0.01	9.8E-01	-0.03	8.8E-01	0.18	3.8E-01	-0.06	7.8E-01	-0.18	3.9E-01	-0.07	7.4E-01	0.00	9.9E-01	0.03	9.0E-01	0.50	9.0E-03	0.19	3.5E-01
ENSCAFG0000000299	ECPAS	grey	EC_M10	0.01	9.8E-01	0.30	1.3E-01	-0.16	4.4E-01	-0.15	4.6E-01	0.08	7.2E-01	-0.15	4.6E-01	0.08	7.1E-01	-0.01	9.5E-01	-0.17	3.9E-01	-0.04	8.4E-01
ENSCAFG0000002422	ENSCAFG0000002422	grey	EC_M10	0.01	9.8E-01	0.01	9.5E-01	0.14	4.9E-01	-0.02	9.2E-01	-0.14	4.9E-01	-0.07	7.4E-01	-0.03	9.0E-01	-0.09	6.6E-01	0.44	2.5E-02	0.14	4.8E-01
ENSCAFG0000000360	GJA4	turquoise	EC_M6	0.01	9.8E-01	0.82	3.5E-07	-0.08	7.2E-01	-0.10	6.2E-01	-0.22	2.7E-01	-0.09	6.7E-01	-0.08	7.1E-01	-0.11	5.8E-01	0.28	1.7E-01	0.25	2.2E-01
ENSCAFG0000003006	PLAC8B	grey	EC_M10	0.01	9.8E-01	0.05	7.9E-01	-0.27	1.8E-01	-0.14	5.1E-01	0.32	1.1E-01	-0.12	5.7E-01	-0.08	6.9E-01	0.35	8.2E-02	0.56	2.7E-03	-0.32	1.1E-01
ENSCAFG0000000959	TOMM34	grey	EC_M10	0.01	9.8E-01	-0.25	2.2E-01	0.53	5.7E-03	0.10	6.1E-01	-0.55	3.6E-03	-0.17	4.1E-01	0.09	6.5E-01	0.00	1.0E+00	0.16	4.3E-01	0.55	3.3E-03
ENSCAFG0000000605	ATP2C1	darkgreen	EC_M4	0.01	9.8E-01	0.01	9.4E-01	-0.73	2.6E-05	-0.15	4.6E-01	0.84	1.1E-07	0.10	6.2E-01	0.02	9.4E-01	0.20	3.2E-01	-0.13	5.2E-01	-0.82	2.9E-07
ENSCAFG0000000692	SETD9	grey	EC_M10	0.01	9.8E-01	-0.18	3.8E-01	0.25	2.2E-01	0.24	2.3E-01	-0.24	2.3E-01	-0.20	3.2E-01	0.04	8.5E-01	0.11	5.8E-01	0.25	2.2E-01	0.31	1.3E-01
ENSCAFG0000001244	TRAF3IP1	grey	EC_M10	0.01	9.8E-01	-0.08	7.0E-01	0.35	8.2E-02	-0.09	6.6E-01	-0.46	1.9E-02	0.05	8.0E-01	-0.01	9.6E-01	0.01	9.6E-01	-0.19	3.4E-01	0.45	2.2E-02
ENSCAFG0000000364	ALDH16A1	cyan	EC_M2	0.01	9.8E-01	0.17	4.0E-01	0.64	4.1E-04	0.09	6.8E-01	-0.83	1.4E-07	-0.28	1.7E-01	0.09	6.5E-01	-0.05	8.0E-01	0.03	8.8E-01	0.83	1.2E-07

ENSCAFG0000001892	SLC9A6	grey	EC_M10	0.01	9.8E-01	-0.04	8.3E-01	-0.52	6.4E-03	-0.15	4.6E-01	0.58	2.0E-03	0.14	5.0E-01	0.11	5.9E-01	-0.32	1.1E-01	-0.28	1.7E-01	-0.56	2.7E-03
ENSCAFG0000000547	RRBP1	grey	EC_M10	0.01	9.8E-01	-0.15	4.7E-01	-0.63	5.9E-04	-0.12	5.6E-01	0.77	5.3E-06	0.39	5.0E-02	0.10	6.2E-01	0.12	5.5E-01	-0.10	6.3E-01	-0.83	1.2E-07
ENSCAFG0000000620	FCHSD1	turquoise	EC_M6	0.01	9.8E-01	0.61	8.3E-04	-0.16	4.2E-01	0.18	3.7E-01	-0.08	6.9E-01	0.12	5.5E-01	0.06	7.8E-01	-0.11	5.8E-01	0.11	5.8E-01	0.08	7.0E-01
ENSCAFG0000001769	ENSCAFG0000001769	cyan	EC_M2	0.01	9.8E-01	0.20	3.3E-01	0.47	1.6E-02	0.18	3.7E-01	-0.66	2.1E-04	-0.20	3.3E-01	-0.05	8.2E-01	-0.10	6.1E-01	-0.34	9.2E-02	0.67	1.8E-04
ENSCAFG0000000322	PXDN	grey	EC_M10	0.01	9.8E-01	0.49	1.1E-02	-0.56	2.7E-03	-0.19	3.5E-01	0.49	1.1E-02	0.13	5.2E-01	0.18	3.7E-01	-0.10	6.3E-01	-0.40	4.1E-02	-0.49	1.1E-02
ENSCAFG0000001929	PUSL1	grey	EC_M10	0.01	9.8E-01	-0.15	4.6E-01	-0.33	1.0E-01	-0.09	6.6E-01	0.51	7.5E-03	0.02	9.2E-01	0.03	8.9E-01	0.28	1.6E-01	-0.08	7.1E-01	-0.50	8.6E-03
ENSCAFG0000000870	ZCCHC9	grey	EC_M10	0.01	9.8E-01	-0.13	5.2E-01	-0.36	7.0E-02	0.25	2.1E-01	0.42	3.3E-02	0.15	4.6E-01	0.33	1.0E-01	0.29	1.4E-01	0.30	1.4E-01	-0.40	4.5E-02
ENSCAFG0000002454	ENSCAFG0000002454	paleturquoise	EC_M11	0.01	9.8E-01	-0.14	4.9E-01	0.07	7.2E-01	0.72	2.9E-05	-0.08	6.8E-01	-0.06	7.7E-01	-0.03	8.8E-01	-0.11	6.0E-01	-0.14	5.1E-01	0.07	7.3E-01
ENSCAFG0000003004	ENSCAFG0000003004	paleturquoise	EC_M11	0.01	9.8E-01	-0.14	4.9E-01	0.07	7.2E-01	0.72	2.9E-05	-0.08	6.8E-01	-0.06	7.7E-01	-0.03	8.8E-01	-0.11	6.0E-01	-0.14	5.1E-01	0.07	7.3E-01
ENSCAFG0000001786	MYOCD	darkgreen	EC_M4	0.01	9.8E-01	-0.59	1.4E-03	-0.24	2.5E-01	-0.19	3.5E-01	0.51	8.0E-03	0.25	2.2E-01	0.18	3.8E-01	0.17	4.1E-01	0.03	9.0E-01	-0.53	5.7E-03
ENSCAFG0000000382	PGRMC2	darkgreen	EC_M4	0.00	9.8E-01	-0.02	9.2E-01	-0.72	2.9E-05	-0.11	5.9E-01	0.85	5.5E-08	0.23	2.7E-01	-0.02	9.4E-01	-0.05	8.1E-01	-0.32	1.1E-01	-0.87	1.1E-08
ENSCAFG0000001120	PUM1	cyan	EC_M2	0.00	9.8E-01	0.27	1.9E-01	0.49	1.1E-02	0.04	8.6E-01	-0.72	4.0E-05	-0.12	5.6E-01	0.04	8.4E-01	0.00	9.9E-01	-0.25	2.2E-01	0.69	9.4E-05
ENSCAFG0000001651	ENSCAFG0000001651	grey	EC_M10	0.00	9.8E-01	0.00	1.0E+00	0.46	1.8E-02	0.36	6.9E-02	-0.55	3.7E-03	-0.29	1.5E-01	-0.20	3.4E-01	0.10	6.4E-01	0.24	2.3E-01	0.54	4.8E-03
ENSCAFG0000001682	BCKDK	grey	EC_M10	0.00	9.8E-01	-0.01	9.5E-01	0.00	9.9E-01	-0.16	4.4E-01	0.13	5.3E-01	0.05	8.0E-01	-0.14	4.8E-01	-0.18	3.9E-01	0.18	3.7E-01	-0.13	5.4E-01
ENSCAFG0000003061	GATAD1	cyan	EC_M2	0.00	9.8E-01	-0.06	7.8E-01	0.63	5.2E-04	0.18	3.9E-01	-0.80	9.0E-07	-0.07	7.2E-01	-0.11	5.9E-01	0.05	8.2E-01	-0.06	7.7E-01	0.78	2.7E-06
ENSCAFG0000001701	MLH3	cyan	EC_M2	0.00	9.8E-01	-0.18	3.9E-01	0.64	4.0E-04	0.17	4.0E-01	-0.74	1.7E-05	-0.21	2.9E-01	0.07	7.3E-01	0.02	9.4E-01	0.06	7.6E-01	0.75	1.2E-05
ENSCAFG0000001933	ASPA	cyan	EC_M2	0.00	9.8E-01	-0.61	8.8E-04	0.53	5.6E-03	0.18	3.9E-01	-0.40	4.2E-02	-0.17	4.0E-01	-0.03	8.7E-01	0.02	9.2E-01	-0.47	1.6E-02	0.37	6.3E-02
ENSCAFG0000003035	ENSCAFG0000003035	cyan	EC_M2	0.00	9.8E-01	-0.60	1.2E-03	0.34	9.4E-02	0.03	8.9E-01	-0.22	2.7E-01	0.23	2.6E-01	0.10	6.4E-01	0.28	1.7E-01	0.01	9.5E-01	0.16	4.3E-01
ENSCAFG0000001316	PRPF6	grey	EC_M10	0.00	9.8E-01	0.34	8.8E-02	0.24	2.3E-01	0.40	4.0E-02	-0.46	1.8E-02	-0.35	8.0E-02	0.25	2.1E-01	-0.18	3.8E-01	0.20	3.4E-01	0.51	8.2E-03
ENSCAFG0000002855	TENT5B	cyan	EC_M2	0.00	9.8E-01	-0.46	1.8E-02	0.69	8.3E-05	0.05	8.2E-01	-0.61	8.9E-04	-0.19	3.5E-01	0.01	9.6E-01	0.12	5.7E-01	0.01	9.6E-01	0.60	1.3E-03
ENSCAFG0000000141	KIF27	grey	EC_M10	0.00	9.8E-01	-0.28	1.6E-01	0.41	3.8E-02	-0.06	7.5E-01	-0.40	4.2E-02	0.15	4.8E-01	0.04	8.5E-01	0.02	9.2E-01	-0.13	5.3E-01	0.41	3.9E-02
ENSCAFG0000000992	XPO7	grey	EC_M10	0.00	9.8E-01	-0.28	1.7E-01	-0.07	7.3E-01	-0.27	1.8E-01	0.25	2.1E-01	0.12	5.7E-01	-0.31	1.2E-01	-0.21	3.0E-01	0.11	6.1E-01	-0.20	3.2E-01
ENSCAFG0000002897	ENSCAFG0000002897	grey	EC_M10	0.00	9.8E-01	-0.02	9.3E-01	-0.33	1.0E-01	-0.02	9.2E-01	0.39	5.0E-02	-0.10	6.1E-01	-0.07	7.3E-01	-0.05	7.9E-01	-0.08	6.9E-01	-0.42	3.2E-02
ENSCAFG0000002959	DNASE1L2	grey	EC_M10	0.00	9.8E-01	-0.18	3.7E-01	-0.05	8.2E-01	-0.10	6.1E-01	0.15	4.8E-01	0.18	3.9E-01	0.35	7.7E-02	-0.15	4.6E-01	0.22	2.8E-01	-0.16	4.4E-01
ENSCAFG0000000834	SPON1	grey	EC_M10	0.00	9.8E-01	0.11	5.9E-01	0.01	9.6E-01	0.21	3.1E-01	-0.06	7.7E-01	-0.11	5.9E-01	-0.08	6.9E-01	0.16	4.4E-01	-0.09	6.6E-01	0.00	9.9E-01
ENSCAFG0000002532	CLEC4D	grey	EC_M10	0.00	9.8E-01	-0.01	9.6E-01	-0.17	4.2E-01	0.02	9.4E-01	0.15	4.5E-01	0.34	9.4E-02	-0.33	9.7E-02	-0.02	9.1E-01	0.23	2.6E-01	-0.12	5.6E-01
ENSCAFG0000000412	KPTN	grey	EC_M10	0.00	9.8E-01	0.02	9.0E-01	0.05	8.1E-01	-0.34	8.9E-02	-0.08	7.0E-01	0.14	5.0E-01	0.10	6.4E-01	0.51	8.3E-03	0.34	9.1E-02	0.07	7.5E-01
ENSCAFG0000000653	PHRF1	turquoise	EC_M6	0.00	9.8E-01	0.55	3.3E-03	-0.06	7.7E-01	-0.14	4.8E-01	-0.16	4.3E-01	-0.08	7.0E-01	0.34	8.5E-02	0.23	2.5E-01	0.04	8.3E-01	0.15	4.6E-01
ENSCAFG0000002040	TNNI3K	grey	EC_M10	0.00	9.8E-01	-0.09	6.5E-01	0.08	6.8E-01	-0.10	6.2E-01	-0.13	5.2E-01	-0.12	5.5E-01	0.41	3.7E-02	-0.09	6.5E-01	0.01	9.8E-01	0.17	4.1E-01
ENSCAFG0000000042	CPM	darkolivegreen	EC_M9	0.00	9.9E-01	-0.17	4.1E-01	0.11	5.8E-01	0.06	7.6E-01	-0.06	7.7E-01	-0.05	8.2E-01	0.73	2.1E-05	0.10	6.4E-01	0.18	3.7E-01	0.07	7.2E-01
ENSCAFG0000000015	MYO1A	grey	EC_M10	0.00	9.9E-01	0.15	4.6E-01	-0.10	6.2E-01	-0.19	3.6E-01	-0.01	9.7E-01	-0.07	7.4E-01	0.12	5.5E-01	-0.01	9.5E-01	0.14	4.9E-01	-0.03	8.9E-01
ENSCAFG0000003089	PERP	turquoise	EC_M6	0.00	9.9E-01	0.33	9.8E-02	-0.06	7.5E-01	0.03	8.7E-01	-0.02	9.0E-01	-0.28	1.7E-01	-0.18	3.7E-01	0.01	9.5E-01	-0.36	7.1E-02	0.03	8.9E-01
ENSCAFG0000001265	SNX4	magenta	EC_M13	0.00	9.9E-01	0.00	9.9E-01	-0.04	8.4E-01	0.19	3.6E-01	-0.05	8.2E-01	0.05	8.1E-01	-0.07	7.5E-01	-0.11	5.8E-01	0.67	1.9E-04	0.06	7.7E-01
ENSCAFG0000000724	IL1A	turquoise	EC_M6	0.00	9.9E-01	0.40	4.4E-02	-0.21	3.1E-01	-0.17	3.9E-01	0.09	6.5E-01	-0.14	5.0E-01	-0.10	6.1E-01	-0.08	7.1E-01	-0.03	8.9E-01	-0.05	8.0E-01
ENSCAFG0000000118	JMJD4	grey	EC_M10	0.00	9.9E-01	-0.64	4.4E-04	0.32	1.1E-01	0.10	6.2E-01	-0.10	6.4E-01	-0.08	6.9E-01	-0.15	4.6E-01	0.09	6.7E-01	-0.15	4.6E-01	0.06	7.8E-01
ENSCAFG0000003178	ENSCAFG0000003178	grey	EC_M10	0.00	9.9E-01	-0.17	4.1E-01	0.18	3.9E-01	0.07	7.3E-01	-0.11	5.9E-01	0.11	5.9E-01	0.08	6.8E-01	-0.29	1.5E-01	0.18	3.8E-01	0.17	4.1E-01
ENSCAFG0000002956	ENSCAFG0000002956	grey	EC_M10	0.00	9.9E-01	0.03	8.8E-01	0.18	3.8E-01	-0.02	9.3E-01	-0.18	3.8E-01	-0.02	9.3E-01	0.02	9.2E-01	-0.05	8.2E-01	0.47	1.4E-02	0.19	3.4E-01
ENSCAFG0000001370	ADGRA1	turquoise	EC_M6	0.00	9.9E-01	0.68	1.4E-04	-0.12	5.6E-01	-0.18	3.8E-01	-0.04	8.6E-01	-0.18	3.8E-01	0.06	7.8E-01	-0.16	4.5E-01	-0.17	4.0E-01	0.07	7.3E-01
ENSCAFG0000001418	SLC25A39	cyan	EC_M2	0.00	9.9E-01	0.01	9.6E-01	0.76	6.9E-06	0.20	3.2E-01	-0.84	6.2E-08	-0.25	2.2E-01	-0.19	3.5E-01	-0.09	6.5E-01	-0.13	5.2E-01	0.83	1.6E-07
ENSCAFG0000003272	ENSCAFG0000003272	grey	EC_M10	0.00	9.9E-01	0.33	1.0E-01	-0.43	2.7E-02	-0.11	5.8E-01	0.36	6.9E-02	-0.10	6.3E-01	-0.08	7.0E-01	-0.09	6.6E-01	0.31	1.3E-01	-0.31	1.2E-01
ENSCAFG0000002006	SLC27A4	grey	EC_M10	0.00	9.9E-01	0.22	2.8E-01	0.02	9.2E-01	-0.33	1.0E-01	0.05	8.2E-01	-0.19	3.4E-01	-0.16	4.4E-01	0.01	9.4E-01	-0.09	6.5E-01	-0.03	8.8E-01
ENSCAFG0000001640	PFN3	grey	EC_M10	0.00	9.9E-01	-0.16	4.2E-01	0.15	4.5E-01	0.55	3.7E-03	-0.09	6.7E-01	-0.13	5.3E-01	-0.10	6.2E-01	0.31	1.2E-01	-0.08	6.8E-01	0.12	5.7E-01
ENSCAFG0000001060	RGS1	grey	EC_M10	0.00	9.9E-01	0.01	9.7E-01	-0.25	2.2E-01	-0.24	2.3E-01	0.24	2.4E-01	-0.14	4.9E-01	-0.09	6.6E-01	0.24	2.4E-01	0.50	9.8E-03	-0.28	1.7E-01
ENSCAFG0000000853	IGSF10	paleturquoise	EC_M11	0.00	9.9E-01	-0.11	6.0E-01	0.26	2.0E-01	0.90	6.3E-10	-0.28	1.6E-01	-0.08	6.9E-01	-0.08	7.0E-01	-0.06	7.8E-01	-0.11	6.0E-01	0.27	1.8E-01
ENSCAFG0000000570	DPY30	grey	EC_M10	0.00	9.9E-01	-0.18	3.8E-01	-0.01	9.6E-01	-0.17	4.0E-01	0.08	7.1E-01	0.23	2.7E-01	0.08	7.0E-01	-0.02	9.3E-01	-0.03	9.0E-01	-0.12	5.6E-01
ENSCAFG0000001312	RNASEL	cyan	EC_M2	0.00	9.9E-01	0.32	1.1E-01	0.36	6.8E-02	0.24	2.4E-01	-0.61	9.0E-04	-0.32	1.1E-01	-0.12	5.6E-01	0.18	3.7E-01	0.04	8.4E-01	0.61	8.8E-04
ENSCAFG0000001979	SLC6A17	grey	EC_M10	0.00	9.9E-01	-0.09	6.5E-01	-0.10	6.3E-01	-0.30	1.4E-01	0.24	2.3E-01	0.10	6.2E-01	0.12	5.5E-01	-0.02	9.2E-01	-0.15	4.7E-01	-0.24	2.3E-01
ENSCAFG0000000017	ENSCAFG0000000017	grey	EC_M10	0.00	9.9E-01	0.18	3.8E-01	-0.09	6.7E-01	0.08	7.1E-01	-0.03	8.7E-01	0.11	6.1E-01	-0.10	6.3E-01	-0.24	2.4E-01	0.18	3.7E-01	-0.02	9.4E-01
ENSCAFG0000000172	PRPH2	grey	EC_M10	0.00	9.9E-01	-0.19	3.4E-01	0.08	6.9E-01	0.19	3.5E-01	0.03	8.9E-01	0.17	4.0E-01	0.20	3.4E-01	0.07	7.4E-01	0.13	5.4E-01	0.01	9.6E-01
ENSCAFG0000001233	TMEM206	grey	EC_M10	0.00	9.9E-01	0.57	2.5E-03	-0.50	8.7E-03	-0.15	4.8E-01	0.42	3.2E-02	0.00	9.9E-01	0.06	7.7E-01	-0.05	7.9E-01	0.13	5.2E-01	-0.38	5.6E-02
ENSCAFG0000001491	FAM227B	grey	EC_M10	0.00	9.9E-01	0.07	7.2E-01	-0.45	2.3E-02	-0.13	5.4E-01	0.49	1.1E-02	0.08	7.1E-01	0.34	8.7E-02	0.30	1.4E-01	0.00	9.9E-01	-0.47	1.6E-02
ENSCAFG0000000307	EPGN	grey	EC_M10	0.00	9.9E-01	0.26	2.0E-01	0.11	6.0E-01	0.26	2.0E-01	-0.30	1.4E-01	-0.11	5.8E-01	-0.17	4.1E-01	-0.21	3.1E-01	-0.01	9.5E-01	0.25	2.2E-01
ENSCAFG0000003253	ENSCAFG0000003253	grey	EC_M10	0.00	9.9E-01	0.24	2.4E-01	0.02	9.1E-01	-0.17	4.2E-01	-0.14	4.8E-01	-0.11	6.1E-01	-0.09	6.7E-01	-0.16	4.3E-01	0.40	4.3E-02	0.12	5.5E-01
ENSCAFG0000000638	NTN4	turquoise	EC_M6	0.00	9.9E-01	0.60	1.1E-03	0.02	9.2E-01	-0.12	5.7E-01	-0.21	3.0E-01	-0.18	3.7E-01	0.06	7.8E-01	0.00	9.8E-01	-0.19	3.4E-01	0.24	2.3E-01
ENSCAFG0000001464	SLC11A1	grey	EC_M10	0.00	9.9E-01	0.46	1.8E-02	0.26	2.0E-01	-0.28	1.6E-01	-0.47	1.6E-02	-0.23	2.6E-01	0.12	5.6E-01	0.18	3.8E-01	0.35	8.0E-02	0.51	8.2E-03
ENSCAFG0000001280	RIN1	grey	EC_M10	0.00	9.9E-01	-0.26	2.1E-01	0.49	1.1E-02	-0.02	9.4E-01	-0.45	2.1E-02	-0.32	1.1E-01	-0.14	5.0E-01	0.42	3.4E-02	0.32	1.1E-01	0.45	2.2E-02
ENSCAFG0000000444	FOSB	cyan	EC_M2	0.00	9.9E-01	-0.15	4.7E-01	0.35	8.3E-02	0.24	2.4E-01	-0.44	2.4E-02	-0.02	9.2E-01	-0.05	8.2E-01	-0.12	5.4E-01	-0.07	7.2E-01	0.40	4.1E-02
ENSCAFG0000003197	ENSCAFG0000003197	grey	EC_M10	0.00	9.9E-01	-0.07	7.4E-01	-0.13	5.4E-01	0.11	5.8E-01	0.15	4.5E-01	0.18	3.7E-01	-0.16	4.4E-01	-0.04	8.3E-01	0.25	2.1E-01	-0.17	4.0E-01
ENSCAFG0000003164	ENSCAFG0000003164	grey	EC_M10	0.00	9.9E-01	-0.15	4.7E-01	0.31	1.3E-01	0.73	2.0E-05	-0.33	1.0E-01	-0.07	7.5E-01	-0.11	5.8E-01	-0.07	7.2E-01	-0.12	5.4E-01	0.34	9.0E-02
ENSCAFG0000001289	ENSCAFG0000001289	cyan	EC_M2	0.00	9.9E-01	0.27	1.8E-01	0.35	7.7E-02	0.25	2.1E-01	-0.58	1.9E-03	-0.17	3.9E-01	0.11	6.1E-01	-0.07	7.5E-01	-0.19	3.6E-01	0.60	1.3E-03
ENSCAFG0000002599	ENSCAFG0000002599	grey	EC_M10	0.00	9.9E-01	0.03	8.9E-01	0.15	4.7E-01	0.00	9.8E-01	-0.15	4.7E-01	-0.02	9.4E-01	-0.11	6.0E-01	-0.38	5.6E-02	-0.01	9.6E-01	0.21	3.1E-01
ENSCAFG0000000921	TBX3	grey	EC_M10	0.00	9.9E-01	-0.58	2.0E-03	0.18	3.7E-01	0.27	1.8E-01	0.02	9.2E-01	0.11	6.0E-01	0.10	6.3E-01	-0.03	8.8E-01	-0.30	1.3E-01	-0.06	7.6E-01
ENSCAFG0000002889	ENSCAFG0000002889	grey	EC_M10	0.00	9.9E-01	-0.18	3.7E-01	0.56	2.7E-03	0.18	3.8E-01	-0.60	1.1E-03	-0.12	5.8E-01	-0.13	5.3E-01	-0.18	3.9E-01	0.00	1.0E+00	0.59	1.5E-03
ENSCAFG0000003099	CCDC3	magenta	EC_M13	0.00	9.9E-01	0.03	9.0E-01	-0.21	3.1E-01	-0.22	2.7E-01	0.24	2.5E-01	-0.12	5.5E-01	0.07	7.2E-01	0.36	7.4E-02	0.67	2.0E-04	-0.18	3.8E-01
ENSCAFG0000000295	BCL11A	grey	EC_M10	0.00	9.9E-01	0.05	8.2E-01	-0.48	1.4E-02	-0.22	2.9E-01	0.58	1.8E-03	0.41	4.0E-02	-0.19	3.4E-01	-0.06	7.9E-01	0.08	7.0E-01	-0.59	1.6E-03
ENSCAFG0000000605	MYO16	darkolivegreen	EC_M9	0.00	9.9E-01	0.12	5.7E-01	-0.27	1.9E-01	0.03	8.8E-01	0.19	3.4E-01	0.10	6.2E-01	0.56	2.9E-03	0.23	2.5E-01	0.22	2.8E-01	-0.22	2.8E-01
ENSCAFG0000000295	ENSCAFG0000000295	darkgreen	EC_M4	0.00	9.9E-01	-0.61	9.4E-04	0.21	3.0E-01	0.05	8.1E-01	-0.01	9.5E-01	0.08	6.9E-01	0.20	3.3E-01	0.01	9.6E-01	-0.12	5.6E-01	-0.02	9.1E-01
ENSCAFG0000002945	ENSCAFG0000002945	darkgreen	EC_M4	0.00	9.9E-01	-0.59	1.6E-03	-0.22	2.8E-01	0.02	9.4E-01	0.47	1.6E-02	0.41	3.5E-02	0.27	1.8E-01	-0.12	5.4E-01	0.02	9.1E-01	-0.50	8.8E-03
ENSCAFG0000000127	ANKS1A	turquoise	EC_M6	0.00	9.9E-01	0.77	5.2E-06	-0.40	4.1E-02	-0.22	2.8E-01	0.21	2.9E-01	-0.06	7.7E-01	0.06	7.6E-01	0.07	7.2E-01	-0.21	3.1E-01	-0.22	2.8E-01
ENSCAFG0000002682	ENSCAFG0000002682	grey	EC_M10	0.00	9.9E-01	0.08	6.8E-01	0.21	3.1E-01	-0.14	5.0E-01	-0.22	2.8E-01	-0.03	8.9E-01	-0.17	4.1E-01	-0.16	4.4E-01	0.00	1.0E+00	0.18	3.9E-01
ENSCAFG0000000515	CD93	turquoise	EC_M6	0.00	9.9E-01	0.82	2.4E-07	-0.19	3.6E-01	-0.10	6.3E-01	-0.06	7.8E-01	-0.15	4.6E-01	-0.11	5.8E-01	-0.15	4.7E-01	0.19	3.5E-01	0.03	8.8E-01
ENSCAFG0000001440	ENSCAFG0000001440	grey	EC_M10	0.00	1.0E+00	-0.11	5.9E-01	0.24	2.4E-01	-0.12	5.7E-01	-0.23	2.6E-01	-0.11	6.0E-01	-0.11	5.8E-01	-0.16	4.3E-01	-0.11	6.0E-01	0.25	2.2E-01
ENSCAFG0000002954	ZNF554	grey	EC_M10	0.00	1.0E+00	0.11	6.0E-01	0.16	4.2E-01	0.30	1.3E-01	-0.24	2.4E-01	-0.18	3.9E-01	-0.18	3.7E-01	-0.11	5.8E-01	-0.09	6.8E-01	0.23	2.7E-01
ENSCAFG0000001722	ZDHHC15	turquoise	EC_M6	0.00	1.0E+00	0.60	1.1E-03	0.19	3.6E-01	-0.26	2.1E-01	-0.41	3.8E-02	-0.20	3.2E-01	-0.13	5.2E-01	-0.25	2.2E-01	0.22	2.8E-01	0.39	4.7E-02
ENSCAFG0000003121	MANSC1	turquoise	EC_M6	0.00	1.0E+00	0.65	3.7E-04	0.15	4.5E-01	-0.07	7.2E-01	-0.36	7.1E-02	-0.39	5.2E-02	-0.30	1.3E-01	0.18	3.7E-01	0.11	5.8E-01	0.40	4.1E-02
ENSCAFG0000001198	HSPBAP1	cyan	EC_M2	0.00	1.0E+00	-0.33	9.8E-02	0.46	1.7E-02	0.22	2.8E-01	-0.52	6.6E-03	-0.15	4.8E-01	-0.12	5.7E-01	-0.19	3.5E-01	0.14	4.8E-01	0.47	1.5E-02
ENSCAFG0000003237	LRRC72	grey	EC_M10	0.00	1.0E+00	-0.16	4.3E-01	-0.19	3.5E-01	-0.08	7.0E-01	0.25	2.2E-01	0.49	1.2E-02	-0.16	4.3E-01	-0.09	6.5E-01	-0.13	5.1E-01	-0.28	1.7E-01
ENSCAFG0000002553	NT5C	cyan	EC_M2	0.00	1.0E+00	-0.38	5.5E-02	0.70	5.9E-05	0.04	8.5E-01	-0.66	2.5E-04	0.02	9.1E-01	0.17	4.2E-01	0.01	9.6E-01	-0.09	6.7E-01	0.64	3.9E-04
ENSCAFG0000000750	RGS9BP	grey	EC_M10	0.00	1.0E+00	-0.15	4.5E-01	0.18	3.9E-01	0.18	3.8E-01	-0.14	4.9E-01	0.27	1.8E-01	0.34	9.0E-02	-0.18	3.8E-01	-0.05	8.1E-01	0.17	4.0E-01
ENSCAFG0000002983	ENSCAFG0000002983	grey	EC_M10	0.00	1.0E+00	-0.15	4.7E-01	-0.12	5.6E-01	-0.17	4.0E-01	0.23	2.6E-01	-0.07	7.5E-01	-0.09	6.6E-01	-0.09	6.5E-01	0.03	8.7E-01	-0.22	2.9E-01
ENSCAFG0000003174	C10H12orf66	cyan	EC_M2	0.00	1.0E+00	-0.72	3.8E-05	0.50	1.0E-02	0.05	8.3E-01	-0.31	1.3E-01	0.07	7.5E-01	-0.11	6.1E-01	0.22	2.9E-01	-0.34	9.1E-02	0.28	1.7E-01
ENSCAFG0000001193	TTC13	grey	EC_M10	0.00	1.0E+00	-0.41	3.7E-02	-0.47	1.6E-02	-0.14	5.0E-01	0.67	1.7E-04	0.22	2.8E-01	-0.01	9.7E-01	0.09	6.6E-01	-0.08	6.8E-01	-0.64	4.0E-04
ENSCAFG0000000052	GLIPR1	grey	EC_M10	0.00	1.0E+00	-0.61	8.8E-04	0.01	9.7E-01	-0.16	4.4E-01	0.30	1.3E-01	0.04	8.3E-01	0.15	4.8E-01	0.16	4.3E-01	0.05	8.2E-01	-0.27	1.8E-01
ENSCAFG0000000457	GEMIN7	grey	EC_M10	0.00	1.0E+00	-0.39	4.7E-02	0.03	8.8E-01	0.01	9.6E-01	0.18	3.8E-01	0.16	4.3E-01	0.07	7.3E-01	-0.19	3.5E-01	-0.41	3.6E-02	-0.18	3.9E-01
ENSCAFG0000000987	MAN1A2	darkgreen	EC_M4	0.00	1.0E+00	-0.17	3.9E-01	-0.65	3.3E-04	-0.08	6.9E-01	0.81	4.2E-07	0.25	2.2E-01	0.06	7.8E-01	-0.03	8.8E-01	-0.27	1.8E-01	-0.81	6.3E-07
ENSCAFG0000000344	GPR63	grey	EC_M10	0.00	1.0E+00	0.20	3.3E-01	-0.10	6.4E-01	0.34	8.7E-02	-0.02	9.1E-01	0.07	7.5E-01	-0.10	6.3E-01	0.47	1.4E-02	0.32	1.2E-01	0.01	9.7E-01
ENSCAFG0000000666	ENSCAFG0000000666	turquoise	EC_M6	0.00	1.0E+00	0.43	2.6E-02	-0.12	5.5E-01	0.12	5.6E-01	-0.05	8.2E-01	-0.19	3.6E-01	-0.22	2.9E-01	-0.25	2.2E-01	0.01	9.7E-01	0.08	7.1E-01
ENSCAFG0000000427	MYO3A	grey	EC_M10	0.00	1.0E+00	-0.36	6.9E-02	-0.42	3.3E-02	-0.01	9.7E-01	0.62	6.9E-04	0.28	1.6E-01	-0.09	6.8E-01	-0.08	6.8E-01	-0.23	2.5E-01	-0.63	5.0E-04
ENSCAFG0000001373	GATM	grey	EC_M10	0.00	1.0E+00	0.15	4.7E-01	-0.30	1.4E-01	-0.08	6.8E-01	0.31	1.2E-01	-0.11	6.1E-01	-0.04	8.5E-01	-0.09	6.7E-01	-0.10	6.2E-01	-0.35	7.7E-02
ENSCAFG0000001489	TUBG1	grey	EC_M10	0.00	1.0E+00	0.12	5.7E-01	-0.43	2.8E-02	-0.23	2.6E-01	0.53	5.5E-03	0.10	6.1E-01	-0.05	8.2E-01	-0.16	4.2E-01	0.21	3.0E-01	-0.55	3.6E-03
ENSCAFG0000001227	SEC23IP	grey	EC_M10	0.00	1.0E+00	-0.17	4.0E-01	-0.58	1.8E-03	0.03	8.9E-01	0.68	1.3E-04	0.38	5.4E-02	-0.10	6.1E-01	-0.02	9.0E-01	-0.15	4.6E-01	-0.72	3.6E-05
ENSCAFG0000000590	FEZ2	turquoise	EC_M6	0.00	1.0E+00	0.54	4.3E-03	-0.40	4.0E-02	-0.10	6.2E-01	0.33	1.0E-01	0.06	7.6E-01	-0.06	7.8E-01	-0.09	6.7E-01	-0.10	6.4E-01	-0.26	2.0E-01
ENSCAFG0000001139	NCF1	turquoise	EC_M6	0.00	1.0E+00	0.65	3.0E-04	-0.13	5.4E-01	-0.09	6.5E-01	-0.11	6.0E-01	-0.10	6.3E-01	0.60	1.1E-03	-0.03	9.0E-01	-0.09	6.5E-01	0.13	5.3E-01
ENSCAFG0000000138	MAPK13	turquoise	EC_M6	0.00	1.0E+00	0.86	1.6E-08	-0.15	4.6E-01	-0.09	6.6E-01	-0.11	5.9E-01	-0.30	1.4E-01	-0.06	7.6E-01	0.10	6.3E-01	0.11	6.0E-01	0.12	5.7E-01
ENSCAFG0000001719	SAMD15	grey	EC_M10	0.00	1.0E+00	0.34	8.5E-02	-0.46	1.9E-02	-0.11	5.8E-01	0.40	4.5E-02	0.07	7.5E-01	-0.18	3.7E-01	-0.26	1.9E-01	0.22	2.8E-01	-0.39	5.1E-02
ENSCAFG0000002317	RNF223	grey	EC_M10	0.00	1.0E+00	-0.05	8.0E-01	0.04	8.5E-01	-0.10	6.3E-01	-0.08	6.9E-01	-0.06	7.6E-01	-0.07	7.5E-01	-0.06	7.7E-01	0.40	4.4E-02	0.08	7.0E-01
ENSCAFG0000001967	ERRFI1	grey	EC_M10	0.00	1.0E+00	-0.13	5.3E-01	-0.63	5.4E-04	-0.26	2.0E-01	0.80	9.1E-07	0.25	2.1E-01	0.07	7.4E-01	0.11	5.8E-01	0.10	6.3E-01	-0.82	2.4E-07
ENSCAFG0000000548	BFSP1	cyan	EC_M2	0.00	1.0E+00	0.20	3.3E-01	0.41	3.7E-02	0.19	3.4E-01	-0.60	1.2E-03	-0.13	5.2E-01	-0.02	9.3E-01	-0.27	1.8E-01	0.20	3.4E-01	0.63	6.4E-04
ENSCAFG0000001496	GCH1	grey	EC_M10	0.00	1.0E+00	0.43	2.9E-02	0.06	7.7E-01	0.12	5.6E-01	-0.31	1.3E-01	-0.03	8.9E-01	0.10	6.4E-01	-0.16	4.4E-01	-0.45	2.0E-02	0.30	1.4E-01
ENSCAFG0000001232	PRSS12	grey	EC_M10	0.00	1.0E+00	-0.19	3.6E-01	-0.15	4.7E-01	0.19	3.6E-01	0.25	2.1E-01	0.09	6.6E-01	0.06	7.6E-01	-0.03	8.8E-01	-0.01	9.6E-01	-0.20	3.3E-01
ENSCAFG0000000631	YIPF5	grey	EC_M10	0.00	1.0E+00	0.05	8.2E-01	-0.66	2.4E-04	-0.10	6.2E-01	0.77	5.0E-06	0.31	1.3E-01	0.11	6.0E-01	0.16	4.3E-01	-0.07	7.3E-01	-0.81	6.6E-07
ENSCAFG0000000031	ZC2HC1B	grey	EC_M10	0.00	1.0E+00	-0.04	8.5E-01	-0.24	2.3E-01	-0.16	4.4E-01	0.27	1.8E-01	0.16	4.4E-01	0.23	2.6E-01	-0.21	3.0E-01	0.13	5.4E-01	-0.27	1.7E-01
ENSCAFG0000000133	ISCA1	grey	EC_M10	0.00	1.0E+00	0.02	9.2E-01	0.12	5.7E-01	-0.35	7.6E-02	-0.08	7.0E-01	0.14	4.8E-01	-0.16	4.4E-01	0.14	5.1E-01	-0.10	6.4E-01	0.07	7.5E-01
ENSCAFG0000001045	SHANK2	turquoise	EC_M6	0.00	1.0E+00	0.43	2.8E-02	-0.19	3.5E-01	-0.13	5.3E-01	0.08	7.0E-01	-0.11	6.0E-01	-0.09	6.6E-01	-0.05	8.0E-01	-0.07	7.4E-01	-0.03	8.9E-01
ENSCAFG0000002941	ENSCAFG0000002941	grey	EC_M10	0.00	1.0E+00	-0.15	4.8E-01	0.07	7.4E-01	-0.03	8.7E-01	-0.05	8.2E-01	0.48	1.2E-02	-0.16	4.3E-01	-0.12	5.6E-01	-0.20	3.3E-01	0.02	9.4E-01
ENSCAFG0000000076	AGPAT4	grey	EC_M10	0.00	1.0E+00	0.54	4.4E-03	-0.50	9.2E-03	-0.17	4.1E-01	0.39	5.1E-02	-0.03	8.8E-01	-0.26	2.0E-01	0.30	1.4E-01	0.27	1.9E-01	-0.38	5.9E-02
ENSCAFG0000000828	GPD1	turquoise	EC_M6	0.00	1.0E+00	0.55	3.4E-03	-0.19	3.6E-01	0.30	1.3E-01	0.03	8.7E-01	-0.17	4.1E-01	0.18	3.9E-01	-0.07	7.5E-01	0.16	4.5E-01	0.03	8.7E-01
ENSCAFG0000001095	PEF1	grey	EC_M10	0.00	1.0E+00	0.18	3.7E-01	0.21	3.1E-01	-0.03	9.0E-01	-0.25	2.1E-01	-0.34	8.9E-02	-0.01	9.5E-01	-0.44	2.4E-02	-0.19	3.4E-01	0.25	2.2E-01
ENSCAFG0000000894	SFXN5	grey	EC_M10	0.00	1.0E+00	0.15	4.8E-01	-0.21	2.9E-01	-0.40	4.1E-02	0.27	1.8E-01	0.11	6.0E-01	-0.10	6.2E-01	0.10	6.3E-01	0.12	5.7E-01	-0.20	3.3E-01
ENSCAFG0000000575	FAAP100	cyan	EC_M2	0.00	1.0E+00	-0.10	6.2E-01	0.68	1.2E-04	0.17	4.0E-01	-0.76	7.0E-06	-0.02	9.3E-01	0.07	7.5E-01	0.09	6.6E-01	-0.07	7.2E-01	0.74	1.9E-05
ENSCAFG0000001697	LACTB	grey	EC_M10	0.00	1.0E+00	-0.21	3.0E-01	0.54	4.0E-03	0.09	6.5E-01	-0.54	4.3E-03	-0.18	3.8E-01	-0.23	2.5E-01	-0.17	4.0E-01	-0.56	2.9E-03	0.54	4.4E-03
ENSCAFG0000001959	TNFRSF25	turquoise	EC_M6	0.00	1.0E+00	0.74	1.5E-05	-0.34	8.7E-02	-0.06	7.5E-01	0.12	5.5E-01	-0.26	1.9E-01	0.05	7.9E-01	0.23	2.5E-01	0.39	4.9E-02	-0.11	5.8E-01
ENSCAFG0000000842	DQX1	grey	EC_M10	0.00	1.0E+00	0.05	8.0E-01	-0.25	2.2E-01	-0.05	8.1E-01	0.29	1.6E-01	0.35	8.4E-02	0.34	8.5E-02	0.17	4.1E-01	0.11	6.1E-01	-0.27	1.8E-01
ENSCAFG0000000340	DDC	grey	EC_M10	0.00	1.0E+00	-0.05	8.2E-01	0.11	5.9E-01	-0.09	6.8E-01	-0.13	5.4E-01	-0.05	8.1E-01	-0.05	8.2E-01	0.10	6.3E-01	0.01	9.4E-01	0.10	6.3E-01
ENSCAFG0000002953	LRRC7	grey	EC_M10	0.00	1.0E+00	-0.05	8.2E-01	0.11	5.9E-01	-0.09	6.8E-01	-0.13	5.4E-01	-0.05	8.1E-01	-0.05	8.2E-01	0.10	6.3E-01	0.01	9.4E-01	0.10	6.3E-01
ENSCAFG0000001815	UBE2Q2	grey	EC_M10	0.00	1.0E+00	0.08	7.1E-01	-0.64	4.0E-04	-0.28	1.7E-01	0.69	1.0E-04	0.12	5.6E-01	0.06	7.6E-01	0.33	1.0E-01	0.01	9.5E-01	-0.68	1.3E-04

ENSCAFG0000002346	ITPR1	grey	EC_M10	0.00	1.0E+00	0.38	5.4E-02	-0.56	2.8E-03	-0.29	1.5E-01	0.52	6.9E-03	-0.06	7.8E-01	0.06	7.7E-01	-0.05	8.3E-01	0.12	5.7E-01	-0.49	1.1E-02
ENSCAFG0000001525	ANO2	grey	EC_M10	0.00	1.0E+00	0.16	4.4E-01	0.09	6.8E-01	0.01	9.4E-01	-0.11	5.9E-01	0.33	9.6E-02	-0.02	9.3E-01	-0.22	2.7E-01	-0.28	1.6E-01	0.05	8.1E-01
ENSCAFG0000001212	TRAF5	grey	EC_M10	0.00	9.9E-01	-0.58	1.9E-03	-0.18	3.9E-01	0.03	8.9E-01	0.36	7.4E-02	0.01	9.6E-01	-0.02	9.1E-01	0.48	1.4E-02	0.11	5.9E-01	-0.38	5.8E-02
ENSCAFG0000001785	SLC39A6	darkgreen	EC_M4	0.00	9.9E-01	-0.80	9.0E-07	0.11	6.0E-01	0.00	1.0E+00	0.22	2.9E-01	0.06	7.8E-01	-0.17	3.9E-01	0.01	9.7E-01	-0.07	7.3E-01	-0.20	3.3E-01
ENSCAFG0000000674	PCMTD1	turquoise	EC_M6	0.00	9.9E-01	0.54	4.4E-03	-0.28	1.7E-01	0.17	4.0E-01	0.01	9.7E-01	-0.08	7.1E-01	-0.04	8.3E-01	-0.10	6.3E-01	0.28	1.6E-01	0.01	9.7E-01
ENSCAFG0000003142	PIH1D2	cyan	EC_M2	0.00	9.9E-01	-0.23	2.6E-01	0.49	1.2E-02	0.22	2.7E-01	-0.53	5.7E-03	-0.02	9.1E-01	-0.23	2.6E-01	0.18	3.8E-01	0.04	8.6E-01	0.54	4.2E-03
ENSCAFG0000000257	EDN2	grey	EC_M10	0.00	9.9E-01	-0.16	4.2E-01	0.14	4.9E-01	-0.09	6.5E-01	-0.08	7.1E-01	-0.04	8.4E-01	-0.02	9.1E-01	-0.05	8.2E-01	-0.01	9.8E-01	0.09	6.7E-01
ENSCAFG0000000010	ESYT1	grey	EC_M10	0.00	9.9E-01	-0.44	2.5E-02	0.34	8.6E-02	0.15	4.7E-01	-0.23	2.5E-01	-0.24	2.4E-01	0.03	8.8E-01	0.07	7.2E-01	0.13	5.4E-01	0.21	3.0E-01
ENSCAFG0000000591	NOTUM	turquoise	EC_M6	0.00	9.9E-01	0.49	1.1E-02	0.07	7.5E-01	-0.08	6.9E-01	-0.26	2.0E-01	-0.15	4.7E-01	-0.08	6.8E-01	0.05	8.0E-01	0.40	4.5E-02	0.24	2.4E-01
ENSCAFG0000000326	APLF	grey	EC_M10	0.00	9.9E-01	-0.46	1.9E-02	0.32	1.1E-01	0.16	4.3E-01	-0.27	1.8E-01	-0.05	8.2E-01	-0.25	2.2E-01	0.09	6.6E-01	0.41	3.6E-02	0.28	1.6E-01
ENSCAFG0000001532	TNFAIP8L3	turquoise	EC_M6	0.00	9.9E-01	0.74	1.7E-05	-0.18	3.7E-01	-0.10	6.2E-01	-0.06	7.7E-01	-0.22	2.9E-01	-0.10	6.3E-01	0.10	6.3E-01	-0.03	8.7E-01	0.06	7.7E-01
ENSCAFG0000003096	RDH14	cyan	EC_M2	0.00	9.9E-01	-0.68	1.3E-04	0.41	3.8E-02	0.32	1.1E-01	-0.26	2.1E-01	-0.01	9.7E-01	-0.13	5.4E-01	0.06	7.6E-01	-0.21	3.0E-01	0.25	2.2E-01
ENSCAFG0000001125	DNAAF5	darkgreen	EC_M4	0.00	9.9E-01	-0.44	2.5E-02	-0.30	1.3E-01	-0.13	5.2E-01	0.58	1.7E-03	0.02	9.2E-01	-0.01	9.6E-01	-0.07	7.3E-01	-0.16	4.4E-01	-0.58	1.8E-03
ENSCAFG0000000095	GJA1	grey	EC_M10	0.00	9.9E-01	-0.64	4.0E-04	0.02	9.1E-01	0.04	8.4E-01	0.18	3.7E-01	0.21	3.0E-01	-0.18	3.8E-01	0.34	8.7E-02	-0.07	7.3E-01	-0.20	3.2E-01
ENSCAFG0000000623	DCLK1	darkgreen	EC_M4	0.00	9.9E-01	-0.49	1.2E-02	-0.40	4.0E-02	-0.08	6.8E-01	0.64	4.0E-04	0.22	2.7E-01	-0.08	6.8E-01	0.23	2.5E-01	-0.03	8.7E-01	-0.69	1.1E-04
ENSCAFG0000000663	ENSCAFG0000000663	cyan	EC_M2	0.00	9.9E-01	-0.10	6.4E-01	0.76	6.7E-06	0.13	5.2E-01	-0.83	1.5E-07	-0.13	5.3E-01	0.02	9.1E-01	-0.14	4.9E-01	0.17	4.2E-01	0.87	1.2E-08
ENSCAFG0000000931	IFT52	grey	EC_M10	0.00	9.9E-01	-0.30	1.3E-01	0.21	2.9E-01	-0.18	3.7E-01	-0.10	6.2E-01	0.06	7.6E-01	-0.24	2.4E-01	-0.09	6.8E-01	0.49	1.2E-02	0.10	6.3E-01
ENSCAFG0000000938	CHST1	turquoise	EC_M6	0.00	9.9E-01	0.88	4.0E-09	-0.21	2.9E-01	-0.15	4.5E-01	-0.06	7.6E-01	-0.17	4.1E-01	0.01	9.5E-01	-0.02	9.1E-01	0.24	2.4E-01	0.05	8.1E-01
ENSCAFG0000001092	PLCXD1	grey	EC_M10	0.00	9.9E-01	0.23	2.5E-01	-0.41	3.6E-02	0.08	7.0E-01	0.40	4.2E-02	0.29	1.4E-01	-0.04	8.6E-01	-0.04	8.4E-01	0.02	9.3E-01	-0.37	6.6E-02
ENSCAFG0000001442	C5H11orf65	darkgreen	EC_M4	0.00	9.9E-01	-0.15	4.8E-01	-0.04	8.5E-01	-0.14	5.1E-01	0.14	4.9E-01	-0.05	8.0E-01	-0.10	6.1E-01	-0.08	6.9E-01	-0.01	9.7E-01	-0.12	5.7E-01
ENSCAFG0000001681	KDM6B	grey	EC_M10	0.00	9.9E-01	0.60	1.3E-03	-0.52	6.9E-03	-0.14	5.0E-01	0.33	1.0E-01	0.22	2.7E-01	0.26	2.0E-01	0.09	6.7E-01	0.32	1.1E-01	-0.36	7.2E-02
ENSCAFG0000000399	FYN	grey	EC_M10	0.00	9.9E-01	0.15	4.6E-01	-0.56	2.7E-03	-0.06	7.7E-01	0.63	6.2E-04	0.21	2.9E-01	-0.10	6.3E-01	-0.27	1.7E-01	-0.10	6.3E-01	-0.63	6.3E-04
ENSCAFG0000000016	SMAD4	grey	EC_M10	0.00	9.9E-01	0.46	1.9E-02	0.34	8.5E-02	-0.09	6.8E-01	-0.58	2.1E-03	0.08	7.0E-01	-0.21	3.0E-01	-0.27	1.9E-01	0.01	9.7E-01	0.56	2.9E-03
ENSCAFG0000000412	BBS7	cyan	EC_M2	0.00	9.9E-01	0.18	3.8E-01	0.52	7.0E-03	0.07	7.3E-01	-0.69	8.5E-05	-0.30	1.4E-01	0.09	6.6E-01	-0.21	3.1E-01	-0.16	4.4E-01	0.70	6.3E-05
ENSCAFG0000000182	ABHD17B	grey	EC_M10	0.00	9.9E-01	0.45	2.1E-02	0.16	4.3E-01	0.17	4.0E-01	-0.38	5.8E-02	-0.06	7.7E-01	-0.09	6.5E-01	-0.09	6.6E-01	-0.16	4.3E-01	0.41	3.9E-02
ENSCAFG0000000679	ENSCAFG0000000679	grey	EC_M10	0.00	9.9E-01	0.30	1.3E-01	0.22	2.8E-01	0.02	9.1E-01	-0.35	7.8E-02	-0.13	5.3E-01	0.07	7.3E-01	0.27	1.9E-01	0.33	1.0E-01	0.33	1.0E-01
ENSCAFG0000003038	RAB32	turquoise	EC_M6	0.00	9.9E-01	0.62	7.7E-04	0.06	7.6E-01	-0.10	6.2E-01	-0.26	1.9E-01	-0.24	2.3E-01	0.01	9.7E-01	-0.05	7.9E-01	-0.25	2.1E-01	0.29	1.5E-01
ENSCAFG0000002005	CNTNAP4	magenta	EC_M13	0.00	9.9E-01	0.05	8.0E-01	-0.26	2.0E-01	-0.07	7.4E-01	0.25	2.2E-01	0.10	6.3E-01	-0.06	7.7E-01	0.54	4.8E-03	0.60	1.1E-03	-0.27	1.9E-01
ENSCAFG0000000834	TM4SF1	turquoise	EC_M6	0.00	9.9E-01	0.57	2.5E-03	0.06	7.8E-01	-0.17	4.2E-01	-0.22	2.8E-01	-0.53	5.8E-03	0.11	5.9E-01	-0.05	7.9E-01	-0.04	8.5E-01	0.26	2.0E-01
ENSCAFG0000000499	SLC35F5	grey	EC_M10	0.00	9.9E-01	0.08	7.1E-01	-0.56	3.1E-03	-0.13	5.3E-01	0.63	5.1E-04	0.20	3.3E-01	0.07	7.3E-01	-0.18	3.8E-01	-0.06	7.6E-01	-0.57	2.2E-03
ENSCAFG0000000451	SPERT	grey	EC_M10	0.00	9.9E-01	0.15	4.8E-01	-0.49	1.1E-02	-0.15	4.5E-01	0.49	1.1E-02	0.53	5.1E-03	0.11	5.9E-01	-0.10	6.1E-01	0.13	5.3E-01	-0.46	1.8E-02
ENSCAFG0000000367	FZD8	turquoise	EC_M6	0.00	9.9E-01	0.83	1.1E-07	-0.10	6.1E-01	-0.23	2.6E-01	-0.17	4.2E-01	-0.06	7.9E-01	0.04	8.3E-01	-0.28	1.7E-01	0.07	7.2E-01	0.17	4.2E-01
ENSCAFG0000000936	SPINT1	grey	EC_M10	0.00	9.9E-01	0.01	9.5E-01	-0.33	9.7E-02	-0.07	7.2E-01	0.33	9.7E-02	0.05	8.1E-01	0.36	7.1E-02	-0.11	5.9E-01	0.53	5.1E-03	-0.30	1.3E-01
ENSCAFG0000001807	RCN2	grey	EC_M10	0.00	9.9E-01	-0.07	7.4E-01	-0.55	3.7E-03	0.04	8.6E-01	0.65	3.0E-04	0.14	4.9E-01	0.10	6.4E-01	-0.04	8.6E-01	0.09	6.8E-01	-0.59	1.4E-03
ENSCAFG0000001395	DYNLT3	grey	EC_M10	0.00	9.9E-01	0.03	9.0E-01	-0.55	3.4E-03	-0.11	5.8E-01	0.58	1.9E-03	0.05	8.2E-01	0.00	1.0E+00	0.06	7.8E-01	-0.13	5.4E-01	-0.54	4.3E-03
ENSCAFG0000001948	SSNA1	grey	EC_M10	0.00	9.9E-01	-0.20	3.3E-01	0.30	1.3E-01	-0.09	6.5E-01	-0.21	3.0E-01	-0.05	8.2E-01	-0.26	2.1E-01	-0.11	5.8E-01	-0.46	1.7E-02	0.17	4.1E-01
ENSCAFG0000001638	ADGRE5	grey	EC_M10	0.00	9.9E-01	-0.35	7.6E-02	-0.06	7.8E-01	-0.08	7.0E-01	0.23	2.6E-01	-0.29	1.5E-01	0.43	2.7E-02	0.12	5.7E-01	0.10	6.1E-01	-0.23	2.6E-01
ENSCAFG0000003228	HOXA3	grey	EC_M10	0.00	9.9E-01	-0.08	7.0E-01	-0.08	6.9E-01	0.04	8.3E-01	0.15	4.7E-01	0.35	7.5E-02	-0.10	6.1E-01	-0.38	5.7E-02	-0.43	2.9E-02	-0.18	3.7E-01
ENSCAFG0000000554	ENGASE	cyan	EC_M2	0.00	9.9E-01	-0.04	8.5E-01	0.62	7.2E-04	0.18	3.7E-01	-0.69	1.1E-04	-0.06	7.6E-01	-0.05	8.0E-01	0.15	4.8E-01	-0.04	8.6E-01	0.74	1.7E-05
ENSCAFG0000001037	TAGLN3	grey	EC_M10	0.00	9.8E-01	0.03	8.8E-01	-0.49	1.0E-02	0.18	3.9E-01	0.59	1.7E-03	-0.17	4.2E-01	-0.21	3.1E-01	0.24	2.3E-01	-0.05	8.2E-01	-0.60	1.2E-03
ENSCAFG0000001353	TBCCD1	grey	EC_M10	0.00	9.8E-01	0.02	9.4E-01	0.33	1.0E-01	0.24	2.4E-01	-0.45	2.1E-02	-0.01	9.8E-01	-0.32	1.1E-01	0.02	9.1E-01	0.41	3.9E-02	0.48	1.3E-02
ENSCAFG0000000274	KLHL7	grey	EC_M10	0.00	9.8E-01	0.35	8.0E-02	-0.58	1.9E-03	-0.04	8.3E-01	0.52	6.5E-03	0.25	2.3E-01	0.05	8.3E-01	0.15	4.6E-01	0.02	9.3E-01	-0.51	7.6E-03
ENSCAFG0000002300	ENSCAFG0000002300	grey	EC_M10	0.00	9.8E-01	0.22	2.9E-01	0.02	9.4E-01	-0.03	8.7E-01	-0.05	8.1E-01	0.00	1.0E+00	0.33	1.0E-01	0.16	4.3E-01	0.02	9.2E-01	0.07	7.5E-01
ENSCAFG0000000407	ENSCAFG0000000407	grey	EC_M10	0.00	9.8E-01	0.07	7.5E-01	-0.18	3.9E-01	-0.23	2.5E-01	0.16	4.2E-01	0.16	4.2E-01	0.10	6.1E-01	0.21	3.1E-01	-0.13	5.3E-01	-0.14	5.0E-01
ENSCAFG0000001694	SLC35B1	darkgreen	EC_M4	0.00	9.8E-01	-0.03	8.8E-01	-0.67	1.8E-04	-0.13	5.3E-01	0.83	1.4E-07	0.17	3.9E-01	-0.25	2.1E-01	-0.03	9.0E-01	0.05	7.9E-01	-0.81	5.2E-07
ENSCAFG0000000868	ETFDH	grey	EC_M10	0.00	9.8E-01	-0.31	1.2E-01	-0.22	2.7E-01	-0.05	8.2E-01	0.34	9.4E-02	0.07	7.2E-01	-0.31	1.3E-01	0.32	1.2E-01	0.31	1.2E-01	-0.33	9.8E-02
ENSCAFG0000000384	ENSCAFG0000000384	grey	EC_M10	0.00	9.8E-01	0.17	4.0E-01	-0.39	5.0E-02	-0.20	3.3E-01	0.41	3.6E-02	0.22	2.7E-01	0.18	3.7E-01	-0.21	3.0E-01	0.63	5.9E-04	-0.34	9.0E-02
ENSCAFG0000001858	ENSCAFG0000001858	grey	EC_M10	0.00	9.8E-01	-0.08	7.1E-01	0.10	6.1E-01	-0.09	6.8E-01	-0.08	6.8E-01	-0.06	7.6E-01	-0.07	7.3E-01	-0.07	7.4E-01	-0.01	9.7E-01	0.06	7.5E-01
ENSCAFG0000000277	RTN4	darkgreen	EC_M4	0.00	9.8E-01	-0.15	4.7E-01	-0.70	5.9E-05	-0.16	4.2E-01	0.89	1.5E-09	0.13	5.1E-01	-0.05	8.0E-01	0.18	3.7E-01	0.20	3.2E-01	-0.88	2.9E-09
ENSCAFG0000000379	RAB3B	turquoise	EC_M6	0.00	9.8E-01	0.49	1.1E-02	-0.27	1.8E-01	-0.18	3.8E-01	0.17	4.1E-01	-0.36	7.0E-02	0.31	1.2E-01	0.02	9.1E-01	-0.09	6.7E-01	-0.12	5.6E-01
ENSCAFG0000002464	KIAA1671	grey	EC_M10	0.00	9.8E-01	0.60	1.1E-03	0.20	3.3E-01	-0.03	8.9E-01	-0.41	3.6E-02	-0.38	5.7E-02	-0.25	2.2E-01	-0.06	7.5E-01	0.05	8.3E-01	0.48	1.4E-02
ENSCAFG0000001881	ENSCAFG0000001881	grey	EC_M10	0.00	9.8E-01	-0.16	4.2E-01	0.11	5.8E-01	-0.21	3.1E-01	-0.13	5.3E-01	-0.16	4.3E-01	-0.16	4.3E-01	-0.13	5.4E-01	0.18	3.7E-01	0.12	5.5E-01
ENSCAFG0000001674	FAM155B	grey	EC_M10	0.00	9.8E-01	-0.12	5.6E-01	-0.23	2.6E-01	-0.14	5.1E-01	0.37	6.7E-02	0.03	9.0E-01	-0.01	9.5E-01	-0.05	8.0E-01	-0.31	1.2E-01	-0.39	5.0E-02
ENSCAFG0000001686	LMNA	grey	EC_M10	0.00	9.8E-01	-0.33	1.0E-01	-0.16	4.4E-01	-0.16	4.3E-01	0.39	4.9E-02	-0.08	6.9E-01	0.03	8.7E-01	0.30	1.4E-01	0.05	8.3E-01	-0.38	5.8E-02
ENSCAFG0000001811	CDH2	grey	EC_M10	0.00	9.8E-01	-0.67	1.8E-04	-0.10	6.2E-01	0.06	7.6E-01	0.35	7.8E-02	0.03	8.7E-01	-0.25	2.2E-01	0.24	2.4E-01	0.33	9.7E-02	-0.37	5.9E-02
ENSCAFG0000001680	SCRN2	turquoise	EC_M6	-0.01	9.8E-01	0.68	1.3E-04	-0.07	7.3E-01	-0.31	1.3E-01	-0.18	3.8E-01	-0.26	1.9E-01	-0.03	8.9E-01	0.06	7.6E-01	0.20	3.4E-01	0.15	4.8E-01
ENSCAFG0000001628	CARD11	grey	EC_M10	-0.01	9.8E-01	0.00	1.0E+00	0.15	4.5E-01	-0.08	6.8E-01	-0.18	3.7E-01	-0.02	9.4E-01	-0.04	8.4E-01	0.07	7.5E-01	-0.02	9.2E-01	0.14	4.9E-01
ENSCAFG0000003204	COX7A2L	grey	EC_M10	-0.01	9.8E-01	-0.44	2.5E-02	-0.27	1.8E-01	-0.15	4.6E-01	0.50	1.0E-02	0.06	7.8E-01	-0.03	8.9E-01	-0.04	8.5E-01	-0.21	3.0E-01	-0.53	5.7E-03
ENSCAFG0000000568	NPTX1	cyan	EC_M2	-0.01	9.8E-01	-0.48	1.3E-02	0.46	1.7E-02	0.25	2.2E-01	-0.39	5.1E-02	-0.05	8.2E-01	-0.28	1.6E-01	-0.08	6.8E-01	0.02	9.3E-01	0.35	7.8E-02
ENSCAFG0000000258	TMEM190	grey	EC_M10	-0.01	9.8E-01	-0.22	2.8E-01	0.12	5.6E-01	0.34	9.0E-02	-0.11	6.0E-01	0.08	7.1E-01	0.15	4.6E-01	0.23	2.6E-01	0.34	8.8E-02	0.09	6.6E-01
ENSCAFG0000001200	NIF3L1	grey	EC_M10	-0.01	9.8E-01	-0.32	1.1E-01	0.16	4.4E-01	0.04	8.3E-01	-0.17	4.1E-01	0.06	7.6E-01	-0.20	3.4E-01	0.02	9.0E-01	0.14	4.9E-01	0.08	7.1E-01
ENSCAFG0000001277	YTHDF1	turquoise	EC_M6	-0.01	9.8E-01	0.74	1.6E-05	-0.20	3.2E-01	-0.11	5.8E-01	-0.04	8.5E-01	0.04	8.4E-01	-0.12	5.7E-01	0.03	8.8E-01	0.48	1.3E-02	0.04	8.3E-01
ENSCAFG0000002872	FPGT	cyan	EC_M2	-0.01	9.8E-01	-0.52	6.5E-03	0.54	4.1E-03	0.29	1.4E-01	-0.51	8.0E-03	-0.04	8.3E-01	-0.22	2.9E-01	-0.05	8.1E-01	-0.19	3.6E-01	0.47	1.4E-02
ENSCAFG0000001542	MAPK1	grey	EC_M10	-0.01	9.8E-01	0.07	7.5E-01	-0.56	2.7E-03	-0.15	4.6E-01	0.70	7.5E-05	0.05	8.1E-01	-0.30	1.4E-01	-0.01	9.5E-01	-0.09	6.7E-01	-0.68	1.5E-04
ENSCAFG0000001226	C2CD2L	grey	EC_M10	-0.01	9.8E-01	0.21	3.1E-01	-0.51	7.1E-03	-0.10	6.3E-01	0.53	5.1E-03	0.18	3.7E-01	0.16	4.3E-01	-0.02	9.1E-01	-0.18	3.8E-01	-0.55	3.7E-03
ENSCAFG0000001912	SEC14L5	grey	EC_M10	-0.01	9.8E-01	-0.04	8.6E-01	-0.15	4.5E-01	-0.20	3.3E-01	0.30	1.4E-01	-0.18	3.8E-01	0.12	5.6E-01	0.26	1.9E-01	0.08	6.9E-01	-0.21	2.9E-01
ENSCAFG0000000293	HOXA1	grey	EC_M10	-0.01	9.8E-01	0.13	5.2E-01	-0.08	6.9E-01	0.17	4.2E-01	0.05	8.0E-01	0.09	6.5E-01	0.00	9.9E-01	-0.24	2.5E-01	-0.53	5.8E-03	-0.07	7.2E-01
ENSCAFG0000002452	BORCS8	grey	EC_M10	-0.01	9.8E-01	-0.23	2.5E-01	0.03	8.7E-01	-0.22	2.8E-01	0.09	6.6E-01	-0.39	4.7E-02	-0.43	2.7E-02	0.13	5.2E-01	0.15	4.6E-01	-0.08	7.0E-01
ENSCAFG0000000163	ENSCAFG0000000163	grey	EC_M10	-0.01	9.8E-01	0.14	4.8E-01	-0.35	8.3E-02	-0.12	5.5E-01	0.43	2.9E-02	-0.14	4.9E-01	-0.10	6.2E-01	-0.07	7.3E-01	0.01	9.5E-01	-0.38	5.7E-02
ENSCAFG0000001376	ENSCAFG0000001376	grey	EC_M10	-0.01	9.8E-01	0.10	6.3E-01	-0.41	3.8E-02	0.18	3.7E-01	0.39	4.6E-02	0.22	2.8E-01	-0.01	9.4E-01	-0.10	6.2E-01	0.03	8.7E-01	-0.43	2.9E-02
ENSCAFG0000003053	ENSCAFG0000003053	grey	EC_M10	-0.01	9.8E-01	-0.14	5.1E-01	0.03	8.7E-01	-0.16	4.3E-01	-0.01	9.6E-01	0.44	2.4E-02	0.24	2.4E-01	0.27	1.8E-01	-0.15	4.7E-01	-0.04	8.5E-01
ENSCAFG0000001082	ZBTB20	darkgreen	EC_M4	-0.01	9.8E-01	-0.14	5.0E-01	-0.21	3.1E-01	0.00	9.9E-01	0.30	1.4E-01	0.03	8.8E-01	-0.08	7.0E-01	-0.22	2.9E-01	-0.03	8.7E-01	-0.29	1.6E-01
ENSCAFG0000001320	PTPRE	turquoise	EC_M6	-0.01	9.8E-01	0.61	9.1E-04	-0.33	9.8E-02	-0.17	4.1E-01	0.21	3.1E-01	-0.26	1.9E-01	0.04	8.5E-01	-0.20	3.3E-01	0.33	1.0E-01	-0.13	5.3E-01
ENSCAFG0000001494	UBA1	grey	EC_M10	-0.01	9.8E-01	0.23	2.5E-01	-0.42	3.3E-02	-0.31	1.2E-01	0.50	8.9E-03	0.23	2.5E-01	-0.13	5.1E-01	-0.08	7.1E-01	-0.25	2.2E-01	-0.52	6.6E-03
ENSCAFG0000001718	IL6R	cyan	EC_M2	-0.01	9.8E-01	0.11	5.8E-01	0.57	2.3E-03	-0.01	9.4E-01	-0.71	5.2E-05	-0.17	4.0E-01	0.06	7.6E-01	0.14	5.0E-01	0.10	6.2E-01	0.75	9.7E-06
ENSCAFG0000001100	ADPRH	turquoise	EC_M6	-0.01	9.8E-01	0.46	1.8E-02	0.01	9.5E-01	0.03	8.7E-01	-0.22	2.8E-01	-0.35	8.3E-02	-0.03	9.0E-01	-0.07	7.3E-01	-0.20	3.4E-01	0.24	2.3E-01
ENSCAFG0000001022	RPLP0	cyan	EC_M2	-0.01	9.8E-01	-0.57	2.4E-03	0.52	6.5E-03	0.09	6.6E-01	-0.37	6.5E-02	0.16	4.4E-01	0.23	2.6E-01	-0.14	5.0E-01	-0.52	6.2E-03	0.33	1.0E-01
ENSCAFG0000002628	ENSCAFG0000002628	grey	EC_M10	-0.01	9.7E-01	-0.10	6.3E-01	0.12	5.6E-01	-0.10	6.3E-01	-0.16	4.2E-01	-0.11	5.9E-01	-0.08	7.0E-01	-0.15	4.7E-01	0.37	5.9E-02	0.18	3.7E-01
ENSCAFG0000001473	LRRC31	turquoise	EC_M6	-0.01	9.7E-01	0.51	7.4E-03	-0.15	4.7E-01	-0.13	5.1E-01	-0.05	8.2E-01	-0.14	4.9E-01	0.15	4.7E-01	0.63	5.5E-04	0.41	3.6E-02	0.05	8.2E-01
ENSCAFG0000001743	USP43	grey	EC_M10	-0.01	9.7E-01	-0.16	4.4E-01	-0.26	2.0E-01	-0.14	4.9E-01	0.37	6.0E-02	0.19	3.6E-01	0.12	5.5E-01	0.31	1.3E-01	-0.10	6.4E-01	-0.39	4.9E-02
ENSCAFG0000000371	ZYG11B	grey	EC_M10	-0.01	9.7E-01	0.02	9.1E-01	0.40	4.3E-02	0.27	1.9E-01	-0.52	7.0E-03	-0.14	4.8E-01	-0.01	9.5E-01	-0.12	5.5E-01	-0.33	9.5E-02	0.52	6.7E-03
ENSCAFG0000000837	G3BP2	grey	EC_M10	-0.01	9.7E-01	0.18	3.8E-01	-0.51	8.1E-03	-0.21	2.9E-01	0.53	5.0E-03	-0.07	7.3E-01	0.10	6.4E-01	0.03	8.8E-01	-0.39	4.8E-02	-0.49	1.0E-02
ENSCAFG0000000389	HSPA4L	grey	EC_M10	-0.01	9.7E-01	-0.45	2.0E-02	-0.32	1.1E-01	0.07	7.3E-01	0.52	6.0E-03	0.15	4.6E-01	0.08	6.8E-01	0.02	9.4E-01	-0.46	1.9E-02	-0.55	3.9E-03
ENSCAFG0000003009	ENSCAFG0000003009	grey	EC_M10	-0.01	9.7E-01	-0.05	8.0E-01	0.11	5.8E-01	-0.05	8.1E-01	-0.06	7.9E-01	-0.01	9.5E-01	-0.09	6.6E-01	-0.09	6.5E-01	-0.03	8.7E-01	0.02	9.1E-01
ENSCAFG0000001343	FAM43A	turquoise	EC_M6	-0.01	9.7E-01	0.90	5.8E-10	-0.12	5.6E-01	-0.13	5.3E-01	-0.16	4.4E-01	-0.11	5.9E-01	-0.11	5.9E-01	-0.08	6.9E-01	0.08	7.1E-01	0.18	3.8E-01
ENSCAFG0000000058	GRAMD2B	turquoise	EC_M6	-0.01	9.7E-01	0.60	1.1E-03	0.20	3.2E-01	0.18	3.7E-01	-0.49	1.2E-02	-0.33	1.0E-01	-0.05	8.2E-01	-0.12	5.6E-01	-0.05	8.2E-01	0.51	8.1E-03
ENSCAFG0000002960	ZNF425	grey	EC_M10	-0.01	9.7E-01	0.31	1.2E-01	-0.62	8.2E-04	-0.18	3.7E-01	0.56	2.7E-03	0.05	7.9E-01	0.14	5.1E-01	0.06	7.8E-01	0.12	5.5E-01	-0.56	2.9E-03
ENSCAFG0000000048	CAPS2	grey	EC_M10	-0.01	9.7E-01	0.14	4.9E-01	-0.14	5.1E-01	-0.18	3.7E-01	0.06	7.6E-01	0.08	6.9E-01	-0.24	2.3E-01	-0.02	9.3E-01	0.35	7.6E-02	-0.02	9.2E-01
ENSCAFG0000002911	ENSCAFG0000002911	grey	EC_M10	-0.01	9.7E-01	0.06	7.7E-01	0.37	6.2E-02	0.22	2.8E-01	-0.45	2.0E-02	-0.09	6.6E-01	0.10	6.3E-01	-0.29	1.5E-01	-0.09	6.5E-01	0.45	2.2E-02
ENSCAFG0000000321	FHL3	grey	EC_M10	-0.01	9.7E-01	-0.15	4.5E-01	0.38	5.4E-02	0.00	9.9E-01	-0.40	4.6E-02	-0.05	8.1E-01	0.43	2.9E-02	-0.04	8.5E-01	0.13	5.4E-01	0.35	8.2E-02
ENSCAFG0000002870	ENSCAFG0000002870	grey	EC_M10	-0.01	9.7E-01	0.06	7.6E-01	0.11	6.1E-01	-0.02	9.1E-01	-0.13	5.3E-01	0.04	8.5E-01	-0.01	9.7E-01	-0.02	9.1E-01	-0.05	7.9E-01	0.10	6.2E-01
ENSCAFG0000002957	TRDV3	grey	EC_M10	-0.01	9.7E-01	0.06	7.6E-01	0.11	6.1E-01	-0.02	9.1E-01	-0.13	5.3E-01	0.04	8.5E-01	-0.01	9.7E-01	-0.02	9.1E-01	-0.05	7.9E-01	0.10	6.2E-01
ENSCAFG0000002878	GADL1	grey	EC_M10	-0.01	9.7E-01	-0.19	3.4E-01	0.30	1.3E-01	-0.13	5.2E-01	-0.19	3.6E-01	-0.09	6.7E-01	0.44	2.4E-02	0.02	9.4E-01	-0.03	8.8E-01	0.24	2.3E-01
ENSCAFG0000000149	BTBD9	grey	EC_M10	-0.01	9.7E-01	0.07	7.2E-01	-0.43	3.0E-02	-0.30	1.4E-01	0.51	8.3E-03	0.01	9.5E-01	-0.24	2.3E-01	0.09	6.7E-01	-0.09	6.6E-01	-0.49	1.1E-02
ENSCAFG0000002926	ENSCAFG0000002926	turquoise	EC_M6	-0.01	9.7E-01	0.51	7.3E-03	-0.17	4.1E-01	-0.08	7.1E-01	0.01	9.4E-01	-0.11	5.8E-01	0.18	3.7E-01	-0.08	6.9E-01	-0.12	5.6E-01	0.00	9.9E-01
ENSCAFG0000001738	PIK3R5	turquoise	EC_M6	-0.01	9.7E-01	0.69	9.6E-05	-0.33	9.7E-02	-0.15	4.5E-01	0.13	5.1E-01	-0.17	4.1E-01	0.28	1.6E-01	0.15	4.7E-01	0.44	2.5E-02	-0.07	7.2E-01
ENSCAFG0000001024	STPG2	grey	EC_M10	-0.01	9.7E-01	-0.25	2.1E-01	-0.09	6.6E-01	0.06	7.6E-01	0.19	3.5E-01	0.13	5.4E-01	-0.21	3.0E-01	0.10	6.3E-01	-0.17	4.2E-01	-0.21	3.1E-01
ENSCAFG0000002642	ENSCAFG0000002642	grey	EC_M10	-0.01	9.7E-01	0.23	2.7E-01	0.45	2.1E-02	0.08	7.1E-01	-0.61	1.0E-03	-0.19	3.5E-01	0.11	6.0E-01	0.11	5.8E-01	0.10	6.2E-01	0.62	7.3E-04
ENSCAFG0000000911	ENSCAFG0000000911	cyan	EC_M2	-0.01	9.7E-01	-0.05	8.0E-01	0.56	3.1E-03	0.23	2.6E-01	-0.69	8.2E-05	-0.28	1.7E-01	-0.07	7.2E-01	0.04	8.6E-01	-0.09	6.6E-01	0.71	4.7E-05
ENSCAFG0000000255	PPCS	grey	EC_M10	-0.01	9.7E-01	0.09	6.6E-01	-0.10	6.4E-01	-0.15	4.6E-01	0.03	8.9E-01	0.42	3.3E-02	0.03	8.7E-01	-0.21	3.0E-01	-0.28	1.7E-01	-0.02	9.4E-01
ENSCAFG0000001794	ANXA6	darkgreen	EC_M4	-0.01	9.7E-01	-0.49	1.0E-02	-0.39	5.0E-02	0.03	8.9E-01	0.65	3.0E-04	0.15	4.7E-01	-0.08	6.9E-01	0.22	2.8E-01	-0.35	8.1E-02	-0.69	8.3E-05
ENSCAFG0000001979	STRIP1	grey	EC_M10	-0.01	9.7E-01	0.29	1.6E-01	-0.40	4.5E-02	-0.11	5.8E-01	0.39	4.8E-02	0.23	2.6E-01	-0.22	2.8E-01	0.00	9.9E-01	0.04	8.6E-01	-0.33	9.9E-02
ENSCAFG0000000270	FILIP1	turquoise	EC_M6	-0.01	9.7E-01	0.68	1.5E-04	-0.59	1.3E-03	-0.28	1.6E-01	0.48	1.4E-02	-0.11	5.8E-01	0.04	8.4E-01	0.25	2.2E-01	0.22	2.7E-01	-0.44	2.5E-02
ENSCAFG0000002850	ENSCAFG0000002850	grey	EC_M10	-0.01	9.7E-01	0.34	9.0E-02	0.28	1.7E-01	0.23	2.6E-01	-0.49	1.1E-02	-0.13	5.3E-01	0.11	6.0E-01	-0.19	3.6E-01	0.30	1.4E-01	0.53	5.3E-03
ENSCAFG0000000977	NEDD9	turquoise	EC_M6	-0.01	9.7E-01	0.81	5.7E-07	-0.51	7.6E-03	-0.16	4.5E-01	0.33	9.9E-02	0.06	7.8E-01	0.07	7.3E-01	-0.04	8.3E-01	0.09	6.5E-01	-0.32	1.1E-01
ENSCAFG0000000934	SLC22A23	grey	EC_M10	-0.01	9.7E-01	0.51	7.6E-03	0.30	1.3E-01	-0.09	6.5E-01	-0.48	1.2E-02	-0.18	3.8E-01	-0.15	4.7E-01	-0.11	6.0E-01	-0.37	6.4E-02	0.52	5.9E-03
ENSCAFG0000001704	JDP2	grey	EC_M10	-0.01	9.7E-01	-0.10	6.2E-01	-0.01	9.7E-01	0.04	8.4E-01	0.06	7.6E-01	0.18	3.7E-01	0.14	4.8E-01	0.28	1.6E-01	-0.10	6.2E-01	-0.14	5.1E-01
ENSCAFG0000001725	MAGT1	grey	EC_M10	-0.01	9.7E-01	-0.40	4.6E-02	-0.27	1.9E-01	0.18	3.9E-01	0.44	2.4E-02	0.10	6.1E-01	0.14	4.8E-01	-0.02	9.1E-01	-0.21	2.9E-01	-0.42	3.2E-02
ENSCAFG0000001568	PIM2	grey	EC_M10	-0.01	9.7E-01	0.11	6.0E-01	-0.04	8.5E-01	0.16	4.3E-01	0.02	9.1E-01	0.16	4.3E-01	0.02	9.3E-01	-0.07	7.5E-01	0.38	5.3E-02	0.02	9.4E-01
ENSCAFG0000001876	DOCK7	grey	EC_M10	-0.01	9.7E-01	-0.65	3.1E-04	-0.24	2.4E-01	0.06	7.8E-01	0.48	1.4E-02	0.18	3.9E-01	-0.10	6.3E-01	0.15	4.6E-01	0.26	2.0E-01	-0.49	1.0E-02
ENSCAFG0000000942	ENSCAFG0000000942	grey	EC_M10	-0.01	9.7E-01	0.27	1.9E-01	-0.24	2.3E-01	-0.10	6.3E-01	0.14	4.8E-01	0.02	9.3E-01	0.34	9.1E-02	0.12	5.6E-01	0.15	4.7E-01	-0.16	4.3E-01
ENSCAFG0000000859	ENSCAFG0000000859	grey	EC_M10	-0.01	9.6E-01	0.12	5.6E-01	-0.26	1.9E-01	-0.16	4.4E-01	0.27	1.8E-01	-0.14	5.1E-01	-0.16	4.5E-01	-0.16	4.3E-01	-0.14	4.9E-01	-0.33	9.8E-02
ENSCAFG0000001782	CA4	turquoise	EC_M6	-0.01	9.6E-01	0.52	6.0E-03	0.04	8.6E-01	0.10	6.2E-01	-0.25	2.1E-01	-0.17	4.0E-01	-0.13	5.4E-01	0.51	8.1E-03	0.43	2.8E-02	0.26	2.0E-01
ENSCAFG0000000763	POP4	grey	EC_M10	-0.01	9.6E-01	-0.10	6.3E-01	0.20	3.3E-01	-0.30	1.3E-01	-0.18	3.7E-01	0.28	1.7E-01	0.25	2.1E-01	-0.30	1.4E-01	0.20	3.4E-01	0.17	4.2E-01
ENSCAFG0000001484	MYOZ1	grey	EC_M10	-0.01	9.6E-01	0.06	7.6E-01	0.10	6.3E-01	0.47	1.7E-02	-0.15	4.5E-01	-0.24	2.3E-01	-0.21	3.1E-01	0.05	8.1E-01	0.10	6.3E-01	0.16	4.2E-01
ENSCAFG0000003220	ENSCAFG0000003220	grey	EC_M10	-0.01	9.6E-01	0.19	3.5E-01	0.37	6.0E-02	-0.36	6.7E-02	-0.41	3.9E-02	-0.22	2.8E-01	-0.30	1.4E-01	0.04	8.6E-01	0.01	9.6E-01	0.41	3.8E-02

ENSCAFG0000001186	CMAS	darkgreen	EC_M4	-0.01	9.6E-01	-0.09	6.7E-01	-0.73	2.1E-05	-0.17	4.0E-01	0.87	1.0E-08	0.07	7.3E-01	-0.04	8.5E-01	0.25	2.3E-01	0.05	8.2E-01	-0.86	1.9E-08
ENSCAFG0000003165	ZSCAN22	grey	EC_M10	-0.01	9.6E-01	-0.17	4.0E-01	0.60	1.3E-03	0.09	6.6E-01	-0.64	4.7E-04	-0.28	1.6E-01	-0.24	2.4E-01	0.00	9.9E-01	-0.06	7.6E-01	0.68	1.4E-04
ENSCAFG0000000442	ENSCAFG0000000442	turquoise	EC_M6	-0.01	9.6E-01	0.40	4.4E-02	-0.08	6.9E-01	0.42	3.4E-02	-0.09	6.6E-01	-0.16	4.4E-01	-0.08	6.9E-01	-0.22	2.8E-01	0.16	4.2E-01	0.10	6.2E-01
ENSCAFG0000002006	KARS	cyan	EC_M2	-0.01	9.6E-01	-0.22	2.8E-01	0.71	5.3E-05	0.31	1.3E-01	-0.77	3.4E-06	-0.18	3.9E-01	-0.06	7.6E-01	-0.02	9.1E-01	-0.16	4.5E-01	0.74	1.5E-05
ENSCAFG0000000361	GAB1	grey	EC_M10	-0.01	9.6E-01	0.43	3.0E-02	0.36	6.7E-02	0.12	5.5E-01	-0.60	1.1E-03	-0.14	5.1E-01	0.06	7.8E-01	-0.27	1.8E-01	-0.01	9.6E-01	0.62	8.2E-04
ENSCAFG0000001372	OVGP1	cyan	EC_M2	-0.01	9.6E-01	0.26	1.9E-01	0.54	4.8E-03	0.18	3.7E-01	-0.76	7.2E-06	-0.17	4.0E-01	0.01	9.7E-01	0.04	8.3E-01	-0.08	6.9E-01	0.74	1.7E-05
ENSCAFG0000000156	MOCS1	grey	EC_M10	-0.01	9.6E-01	0.37	6.1E-02	-0.05	8.2E-01	0.25	2.2E-01	-0.07	7.3E-01	0.03	8.7E-01	-0.11	5.9E-01	0.14	5.0E-01	0.30	1.4E-01	0.08	7.1E-01
ENSCAFG0000000409	EHD2	grey	EC_M10	-0.01	9.6E-01	0.38	5.3E-02	0.39	5.0E-02	-0.11	5.8E-01	-0.51	7.5E-03	-0.29	1.5E-01	-0.03	8.7E-01	0.24	2.3E-01	0.19	3.5E-01	0.51	7.6E-03
ENSCAFG0000001264	ENSCAFG0000001264	turquoise	EC_M6	-0.01	9.6E-01	0.56	3.2E-03	0.06	7.7E-01	-0.09	6.7E-01	-0.27	1.8E-01	-0.15	4.7E-01	0.07	7.2E-01	0.01	9.7E-01	0.73	2.0E-05	0.29	1.5E-01
ENSCAFG0000003264	ENSCAFG0000003264	grey	EC_M10	-0.01	9.6E-01	0.14	4.9E-01	0.40	4.2E-02	0.01	9.5E-01	-0.58	2.0E-03	-0.04	8.5E-01	-0.26	1.9E-01	0.00	9.9E-01	0.30	1.4E-01	0.57	2.4E-03
ENSCAFG0000001626	DDB1	grey	EC_M10	-0.01	9.6E-01	0.25	2.2E-01	-0.34	9.0E-02	-0.39	4.8E-02	0.41	4.0E-02	-0.05	8.0E-01	0.01	9.8E-01	0.02	9.4E-01	0.34	8.7E-02	-0.30	1.3E-01
ENSCAFG0000000007	ENSCAFG0000000007	grey	EC_M10	-0.01	9.6E-01	0.30	1.4E-01	0.54	4.2E-03	0.03	8.8E-01	-0.76	7.3E-06	-0.27	1.8E-01	0.08	7.0E-01	0.01	9.6E-01	0.02	9.2E-01	0.72	2.8E-05
ENSCAFG0000002858	MS4A13	grey	EC_M10	-0.01	9.6E-01	-0.18	3.7E-01	0.11	5.9E-01	0.25	2.1E-01	-0.04	8.5E-01	0.04	8.6E-01	-0.05	8.1E-01	0.00	9.8E-01	-0.35	8.4E-02	0.00	9.8E-01
ENSCAFG0000002957	ENSCAFG0000002957	grey	EC_M10	-0.01	9.6E-01	0.07	7.3E-01	-0.32	1.1E-01	0.18	3.8E-01	0.27	1.8E-01	0.12	5.7E-01	-0.05	8.1E-01	-0.14	5.1E-01	0.13	5.3E-01	-0.29	1.6E-01
ENSCAFG0000001220	INPP5F	turquoise	EC_M6	-0.01	9.6E-01	0.67	1.6E-04	-0.36	6.9E-02	-0.29	1.5E-01	0.19	3.5E-01	-0.06	7.6E-01	0.04	8.6E-01	0.02	9.2E-01	0.39	5.1E-02	-0.11	5.9E-01
ENSCAFG0000001085	ENSCAFG0000001085	grey	EC_M10	-0.01	9.6E-01	-0.36	7.0E-02	0.25	2.2E-01	0.05	8.2E-01	-0.10	6.2E-01	0.24	2.4E-01	-0.31	1.2E-01	-0.24	2.3E-01	-0.08	7.1E-01	0.06	7.6E-01
ENSCAFG0000001126	B3GALNT2	grey	EC_M10	-0.01	9.6E-01	-0.43	2.7E-02	0.43	2.7E-02	-0.09	6.8E-01	-0.24	2.4E-01	0.03	9.0E-01	0.02	9.2E-01	0.23	2.5E-01	0.08	6.9E-01	0.29	1.6E-01
ENSCAFG0000003158	ENSCAFG0000003158	grey	EC_M10	-0.01	9.6E-01	0.17	4.1E-01	-0.68	1.4E-04	-0.22	2.7E-01	0.71	5.5E-05	0.09	6.7E-01	0.12	5.5E-01	0.37	6.5E-02	0.20	3.2E-01	-0.67	2.0E-04
ENSCAFG0000001008	CDK18	turquoise	EC_M6	-0.01	9.6E-01	0.41	3.5E-02	-0.03	8.8E-01	-0.13	5.2E-01	-0.07	7.5E-01	-0.08	6.9E-01	-0.01	9.6E-01	-0.07	7.4E-01	0.60	1.1E-03	0.11	5.9E-01
ENSCAFG0000001185	SLAMF9	grey	EC_M10	-0.01	9.6E-01	0.63	5.4E-04	0.30	1.3E-01	0.17	4.1E-01	-0.55	4.0E-03	-0.08	6.9E-01	-0.18	3.9E-01	-0.17	4.0E-01	0.05	8.0E-01	0.57	2.1E-03
ENSCAFG0000001863	DENND1C	grey	EC_M10	-0.01	9.6E-01	-0.16	4.4E-01	0.42	3.3E-02	0.43	2.8E-02	-0.44	2.3E-02	-0.11	6.0E-01	-0.07	7.3E-01	-0.12	5.7E-01	-0.10	6.2E-01	0.43	2.7E-02
ENSCAFG0000000733	NR4A1	turquoise	EC_M6	-0.01	9.6E-01	0.42	3.3E-02	-0.22	2.7E-01	-0.03	8.9E-01	0.05	8.3E-01	0.04	8.6E-01	0.13	5.4E-01	0.02	9.4E-01	-0.04	8.3E-01	-0.11	6.0E-01
ENSCAFG0000001324	CDKN1B	cyan	EC_M2	-0.01	9.6E-01	0.30	1.3E-01	0.60	1.3E-03	0.20	3.2E-01	-0.84	7.1E-08	-0.17	4.0E-01	0.04	8.4E-01	-0.14	4.8E-01	0.15	4.5E-01	0.84	1.0E-07
ENSCAFG0000003095	ENSCAFG0000003095	grey	EC_M10	-0.01	9.6E-01	0.08	7.1E-01	-0.03	8.8E-01	0.16	4.4E-01	0.02	9.1E-01	-0.08	7.0E-01	-0.35	7.7E-02	0.14	5.0E-01	0.01	9.6E-01	0.00	1.0E+00
ENSCAFG0000000239	DCAF10	grey	EC_M10	-0.01	9.6E-01	0.30	1.4E-01	-0.36	7.2E-02	0.01	9.7E-01	0.34	8.5E-02	0.21	3.1E-01	-0.17	4.2E-01	-0.46	1.7E-02	-0.41	3.5E-02	-0.32	1.1E-01
ENSCAFG0000001962	WDR24	grey	EC_M10	-0.01	9.6E-01	0.22	2.9E-01	0.24	2.3E-01	0.04	8.4E-01	-0.29	1.5E-01	-0.03	8.8E-01	-0.01	9.4E-01	-0.19	3.5E-01	0.09	6.7E-01	0.31	1.2E-01
ENSCAFG0000001435	VSIR	cyan	EC_M2	-0.01	9.6E-01	0.30	1.4E-01	0.56	3.1E-03	0.22	2.8E-01	-0.73	2.6E-05	-0.30	1.3E-01	-0.01	9.7E-01	-0.15	4.8E-01	-0.20	3.4E-01	0.76	7.1E-06
ENSCAFG0000002611	ENSCAFG0000002611	grey	EC_M10	-0.01	9.6E-01	-0.21	2.9E-01	0.20	3.3E-01	0.31	1.2E-01	-0.22	2.8E-01	-0.17	4.2E-01	0.20	3.2E-01	0.24	2.4E-01	0.06	7.5E-01	0.15	4.5E-01
ENSCAFG0000000986	BBX	grey	EC_M10	-0.01	9.6E-01	-0.04	8.3E-01	-0.52	7.0E-03	0.07	7.2E-01	0.49	1.1E-02	0.27	1.8E-01	0.05	8.0E-01	0.05	8.0E-01	0.12	5.6E-01	-0.54	4.0E-03
ENSCAFG0000000824	FAR1	darkgreen	EC_M4	-0.01	9.6E-01	-0.63	5.8E-04	-0.35	7.8E-02	-0.04	8.6E-01	0.63	5.6E-04	0.19	3.6E-01	0.06	7.7E-01	-0.07	7.4E-01	-0.13	5.4E-01	-0.65	2.9E-04
ENSCAFG0000002460	ENSCAFG0000002460	grey	EC_M10	-0.01	9.6E-01	-0.05	8.2E-01	0.13	5.4E-01	-0.11	5.8E-01	-0.15	4.5E-01	-0.10	6.2E-01	0.40	4.1E-02	-0.09	6.6E-01	0.02	9.4E-01	0.19	3.4E-01
ENSCAFG0000001328	PBX1	grey	EC_M10	-0.01	9.6E-01	-0.40	4.1E-02	-0.10	6.3E-01	0.19	3.5E-01	0.19	3.6E-01	0.39	4.8E-02	-0.01	9.4E-01	-0.26	2.0E-01	-0.51	8.0E-03	-0.26	2.0E-01
ENSCAFG0000001717	CHRNB2	turquoise	EC_M6	-0.01	9.6E-01	0.56	2.7E-03	-0.14	4.8E-01	-0.31	1.2E-01	0.09	6.5E-01	-0.10	6.2E-01	-0.16	4.4E-01	-0.09	6.6E-01	0.09	6.7E-01	-0.06	7.8E-01
ENSCAFG0000002000	DOLPP1	grey	EC_M10	-0.01	9.6E-01	-0.34	8.4E-02	-0.17	4.0E-01	0.02	9.3E-01	0.38	5.5E-02	0.25	2.2E-01	-0.43	3.0E-02	0.24	2.3E-01	-0.01	9.5E-01	-0.38	5.9E-02
ENSCAFG0000003169	ENSCAFG0000003169	grey	EC_M10	-0.01	9.5E-01	-0.32	1.1E-01	0.04	8.5E-01	-0.04	8.6E-01	0.09	6.6E-01	0.13	5.2E-01	-0.18	3.8E-01	-0.09	6.6E-01	0.19	3.5E-01	-0.01	9.7E-01
ENSCAFG0000001059	NPRL2	grey	EC_M10	-0.01	9.5E-01	0.39	5.1E-02	0.21	3.0E-01	0.24	2.3E-01	-0.40	4.6E-02	-0.14	5.1E-01	-0.13	5.1E-01	-0.11	6.1E-01	0.18	3.8E-01	0.40	4.4E-02
ENSCAFG0000001336	APOD	turquoise	EC_M6	-0.01	9.5E-01	0.82	3.0E-07	-0.05	8.2E-01	-0.07	7.2E-01	-0.21	3.1E-01	-0.06	7.6E-01	-0.10	6.1E-01	-0.12	5.7E-01	-0.14	5.0E-01	0.20	3.4E-01
ENSCAFG0000000421	MSRB2	turquoise	EC_M6	-0.01	9.5E-01	0.55	3.6E-03	-0.04	8.4E-01	0.04	8.6E-01	-0.15	4.8E-01	-0.47	1.4E-02	-0.19	3.4E-01	0.22	2.8E-01	0.14	4.8E-01	0.16	4.5E-01
ENSCAFG0000002219	ENSCAFG0000002219	grey	EC_M10	-0.01	9.5E-01	-0.13	5.2E-01	0.10	6.3E-01	-0.06	7.9E-01	-0.04	8.5E-01	-0.11	5.9E-01	0.49	1.1E-02	-0.02	9.0E-01	-0.13	5.3E-01	0.06	7.8E-01
ENSCAFG0000001015	NFKB2	turquoise	EC_M6	-0.01	9.5E-01	0.74	1.6E-05	0.01	9.5E-01	-0.24	2.3E-01	-0.21	3.1E-01	-0.20	3.4E-01	0.28	1.7E-01	-0.11	6.0E-01	0.02	9.3E-01	0.23	2.6E-01
ENSCAFG0000000677	ENSCAFG0000000677	grey	EC_M10	-0.01	9.5E-01	0.21	3.0E-01	-0.35	7.6E-02	-0.14	5.0E-01	0.30	1.3E-01	0.28	1.7E-01	0.18	3.7E-01	-0.36	6.8E-02	-0.09	6.5E-01	-0.28	1.7E-01
ENSCAFG0000000952	RREB1	grey	EC_M10	-0.01	9.5E-01	0.54	4.7E-03	0.29	1.6E-01	0.17	4.2E-01	-0.56	3.2E-03	-0.08	6.9E-01	-0.05	8.1E-01	-0.11	5.8E-01	-0.31	1.2E-01	0.53	5.3E-03
ENSCAFG0000000493	PLCD1	grey	EC_M10	-0.01	9.5E-01	-0.04	8.5E-01	0.60	1.3E-03	0.29	1.6E-01	-0.71	5.7E-05	-0.07	7.2E-01	0.16	4.3E-01	-0.21	3.1E-01	-0.06	7.8E-01	0.68	1.4E-04
ENSCAFG0000002500	NKAPL	darkolivegreen	EC_M9	-0.01	9.5E-01	-0.10	6.2E-01	-0.09	6.5E-01	-0.10	6.1E-01	0.11	6.0E-01	-0.08	7.0E-01	0.70	6.8E-05	-0.01	9.5E-01	-0.03	8.9E-01	-0.09	6.5E-01
ENSCAFG0000001390	TTC12	grey	EC_M10	-0.01	9.5E-01	-0.34	8.7E-02	0.46	1.8E-02	0.27	1.9E-01	-0.39	5.0E-02	0.03	8.8E-01	-0.26	1.9E-01	0.05	7.9E-01	0.13	5.3E-01	0.37	6.1E-02
ENSCAFG0000002913	HAPLN1	grey	EC_M10	-0.01	9.5E-01	-0.30	1.4E-01	0.27	1.9E-01	-0.09	6.5E-01	-0.22	2.8E-01	-0.25	2.3E-01	0.32	1.1E-01	-0.05	8.2E-01	-0.20	3.2E-01	0.26	2.0E-01
ENSCAFG0000001570	CYTH3	grey	EC_M10	-0.01	9.5E-01	-0.75	1.2E-05	0.45	2.0E-02	0.08	6.9E-01	-0.25	2.1E-01	-0.05	8.2E-01	0.12	5.5E-01	0.16	4.4E-01	-0.10	6.3E-01	0.23	2.5E-01
ENSCAFG0000001369	PAX9	grey	EC_M10	-0.01	9.5E-01	-0.18	3.8E-01	-0.02	9.3E-01	0.12	5.5E-01	0.16	4.2E-01	0.47	1.5E-02	-0.08	7.1E-01	-0.19	3.6E-01	0.23	2.6E-01	-0.13	5.4E-01
ENSCAFG0000002229	ENSCAFG0000002229	grey	EC_M10	-0.01	9.5E-01	-0.28	1.7E-01	0.19	3.5E-01	0.13	5.4E-01	-0.19	3.6E-01	0.03	8.8E-01	0.33	9.8E-02	0.19	3.4E-01	0.05	8.0E-01	0.20	3.2E-01
ENSCAFG0000001828	WDR44	darkgreen	EC_M4	-0.01	9.5E-01	0.15	4.7E-01	-0.67	1.8E-04	-0.03	8.9E-01	0.67	1.6E-04	0.04	8.6E-01	0.08	6.9E-01	0.15	4.6E-01	-0.16	4.4E-01	-0.67	1.9E-04
ENSCAFG0000003273	ENSCAFG0000003273	grey	EC_M10	-0.01	9.5E-01	-0.05	8.2E-01	-0.22	2.8E-01	-0.09	6.5E-01	0.25	2.2E-01	0.52	6.7E-03	-0.02	9.2E-01	0.39	4.7E-02	-0.01	9.7E-01	-0.29	1.5E-01
ENSCAFG0000003071	ENSCAFG0000003071	grey	EC_M10	-0.01	9.5E-01	-0.14	5.0E-01	-0.01	9.7E-01	0.10	6.4E-01	0.10	6.4E-01	-0.25	2.3E-01	0.16	4.2E-01	-0.20	3.2E-01	0.23	2.5E-01	-0.06	7.8E-01
ENSCAFG0000000577	CCDC137	grey	EC_M10	-0.01	9.5E-01	0.00	9.8E-01	-0.43	2.8E-02	0.22	2.8E-01	0.52	7.0E-03	0.12	5.7E-01	0.11	5.8E-01	0.22	2.7E-01	-0.10	6.4E-01	-0.52	6.8E-03
ENSCAFG0000000065	FZD6	turquoise	EC_M6	-0.01	9.5E-01	0.64	4.4E-04	-0.03	8.8E-01	-0.14	5.0E-01	-0.13	5.3E-01	-0.28	1.7E-01	-0.26	1.9E-01	0.03	9.0E-01	0.33	1.0E-01	0.17	4.0E-01
ENSCAFG0000000473	ENSCAFG0000000473	darkgreen	EC_M4	-0.01	9.5E-01	-0.13	5.2E-01	-0.55	3.8E-03	0.09	6.6E-01	0.63	5.0E-04	0.31	1.2E-01	0.31	1.2E-01	0.03	9.0E-01	-0.16	4.3E-01	-0.63	5.4E-04
ENSCAFG0000001915	CRK	grey	EC_M10	-0.01	9.5E-01	0.43	2.7E-02	-0.58	1.9E-03	-0.46	1.7E-02	0.58	1.9E-03	0.10	6.4E-01	-0.02	9.3E-01	0.13	5.4E-01	0.00	9.9E-01	-0.54	4.3E-03
ENSCAFG0000000694	CAT	grey	EC_M10	-0.01	9.5E-01	0.34	9.4E-02	0.24	2.3E-01	-0.02	9.2E-01	-0.40	4.5E-02	-0.16	4.4E-01	-0.22	2.9E-01	0.16	4.5E-01	0.50	1.0E-02	0.45	2.1E-02
ENSCAFG0000002284	ENSCAFG0000002284	magenta	EC_M13	-0.01	9.5E-01	0.06	7.6E-01	0.13	5.3E-01	-0.06	7.6E-01	-0.20	3.2E-01	-0.07	7.5E-01	-0.02	9.4E-01	-0.13	5.1E-01	0.52	6.0E-03	0.24	2.4E-01
ENSCAFG0000003135	BST2	turquoise	EC_M6	-0.01	9.5E-01	0.62	6.6E-04	0.11	6.0E-01	0.04	8.4E-01	-0.37	6.2E-02	-0.07	7.3E-01	0.21	3.0E-01	-0.06	7.6E-01	0.26	2.0E-01	0.38	5.7E-02
ENSCAFG0000002452	ENSCAFG0000002452	grey	EC_M10	-0.01	9.5E-01	-0.23	2.5E-01	-0.15	4.6E-01	-0.13	5.3E-01	0.28	1.7E-01	0.33	9.5E-02	-0.13	5.1E-01	-0.18	3.9E-01	-0.21	2.9E-01	-0.30	1.4E-01
ENSCAFG0000001048	PDE9A	turquoise	EC_M6	-0.01	9.4E-01	0.66	2.3E-04	-0.10	6.2E-01	-0.03	8.7E-01	-0.14	4.9E-01	-0.03	8.8E-01	0.19	3.6E-01	-0.25	2.1E-01	0.54	4.0E-03	0.19	3.6E-01
ENSCAFG0000000001	ATP9B	grey	EC_M10	-0.01	9.4E-01	0.26	2.0E-01	-0.31	1.2E-01	0.28	1.7E-01	0.27	1.9E-01	0.00	9.9E-01	-0.02	9.3E-01	-0.26	2.0E-01	0.04	8.5E-01	-0.27	1.8E-01
ENSCAFG0000002315	ENSCAFG0000002315	grey	EC_M10	-0.01	9.4E-01	0.43	2.7E-02	0.35	8.0E-02	0.01	9.7E-01	-0.56	2.9E-03	-0.09	6.7E-01	0.14	5.1E-01	-0.20	3.4E-01	0.07	7.4E-01	0.60	1.3E-03
ENSCAFG0000003242	ZNF619	grey	EC_M10	-0.01	9.4E-01	-0.14	5.1E-01	0.25	2.2E-01	-0.03	9.0E-01	-0.20	3.2E-01	-0.08	7.0E-01	-0.13	5.2E-01	0.25	2.1E-01	-0.05	8.1E-01	0.21	2.9E-01
ENSCAFG0000001675	IGBP1	grey	EC_M10	-0.01	9.4E-01	-0.52	6.3E-03	0.30	1.3E-01	0.38	5.7E-02	-0.23	2.5E-01	0.07	7.2E-01	-0.19	3.4E-01	-0.13	5.3E-01	-0.31	1.2E-01	0.24	2.4E-01
ENSCAFG0000000799	ENSCAFG0000000799	grey	EC_M10	-0.01	9.4E-01	0.40	4.3E-02	-0.07	7.5E-01	0.50	9.4E-03	-0.10	6.3E-01	-0.05	8.0E-01	-0.11	6.0E-01	-0.16	4.3E-01	0.26	1.9E-01	0.12	5.4E-01
ENSCAFG0000000142	BAIAP2L2	turquoise	EC_M6	-0.01	9.4E-01	0.53	5.0E-03	-0.06	7.6E-01	0.30	1.4E-01	-0.11	6.0E-01	-0.15	4.8E-01	0.15	4.6E-01	-0.18	3.7E-01	0.04	8.3E-01	0.14	5.0E-01
ENSCAFG0000000940	DLL4	turquoise	EC_M6	-0.01	9.4E-01	0.84	1.1E-07	-0.05	8.0E-01	-0.02	9.1E-01	-0.23	2.6E-01	-0.13	5.3E-01	-0.13	5.3E-01	-0.21	3.1E-01	0.09	6.5E-01	0.21	3.1E-01
ENSCAFG0000001764	SAP30L	grey	EC_M10	-0.01	9.4E-01	0.22	2.8E-01	0.01	9.6E-01	0.26	2.0E-01	-0.12	5.5E-01	-0.19	3.6E-01	-0.17	4.0E-01	-0.14	4.8E-01	0.09	6.6E-01	0.19	3.6E-01
ENSCAFG0000000308	ORC3	grey	EC_M10	-0.01	9.4E-01	-0.58	2.0E-03	0.49	1.1E-02	0.26	2.1E-01	-0.32	1.1E-01	-0.15	4.8E-01	-0.19	3.6E-01	-0.05	8.1E-01	0.13	5.3E-01	0.37	6.0E-02
ENSCAFG0000001880	KANK4	grey	EC_M10	-0.01	9.4E-01	-0.26	2.0E-01	-0.61	9.5E-04	0.06	7.6E-01	0.79	1.8E-06	0.26	2.1E-01	0.03	8.7E-01	0.06	7.8E-01	-0.17	4.2E-01	-0.83	1.8E-07
ENSCAFG0000000074	PNLDC1	grey	EC_M10	-0.01	9.4E-01	-0.17	4.1E-01	0.04	8.4E-01	-0.24	2.3E-01	0.05	8.0E-01	0.44	2.4E-02	0.10	6.1E-01	-0.23	2.6E-01	0.09	6.7E-01	-0.03	9.0E-01
ENSCAFG0000000304	KIAA1958	grey	EC_M10	-0.01	9.4E-01	0.41	3.6E-02	0.08	7.0E-01	0.11	6.0E-01	-0.22	2.9E-01	0.09	6.8E-01	0.00	9.9E-01	0.20	3.4E-01	0.37	6.1E-02	0.27	1.8E-01
ENSCAFG0000000968	SEC31B	grey	EC_M10	-0.01	9.4E-01	-0.35	7.7E-02	0.33	9.5E-02	-0.03	8.8E-01	-0.25	2.1E-01	-0.10	6.4E-01	0.10	6.1E-01	-0.06	7.7E-01	-0.50	9.4E-03	0.29	1.6E-01
ENSCAFG0000002846	TDRP	turquoise	EC_M6	-0.02	9.4E-01	0.74	1.4E-05	-0.29	1.5E-01	-0.06	7.8E-01	0.07	7.2E-01	-0.20	3.4E-01	0.15	4.6E-01	0.03	8.9E-01	0.30	1.4E-01	-0.02	9.1E-01
ENSCAFG0000000444	ZNF282	grey	EC_M10	-0.02	9.4E-01	0.11	6.0E-01	0.30	1.4E-01	0.07	7.2E-01	-0.39	5.0E-02	-0.16	4.5E-01	0.26	2.0E-01	-0.04	8.3E-01	0.22	2.7E-01	0.43	2.7E-02
ENSCAFG0000001865	IFT20	grey	EC_M10	-0.02	9.4E-01	-0.31	1.3E-01	-0.57	2.2E-03	-0.20	3.2E-01	0.79	1.9E-06	0.17	4.1E-01	-0.05	7.9E-01	0.26	2.1E-01	0.05	8.2E-01	-0.81	5.2E-07
ENSCAFG0000001370	ENSCAFG0000001370	grey	EC_M10	-0.02	9.4E-01	-0.47	1.5E-02	0.14	5.0E-01	-0.21	3.0E-01	0.14	4.9E-01	0.13	5.4E-01	-0.12	5.7E-01	0.05	8.2E-01	0.09	6.5E-01	-0.13	5.2E-01
ENSCAFG0000001918	TREX2	grey	EC_M10	-0.02	9.4E-01	-0.05	7.9E-01	-0.32	1.1E-01	-0.13	5.4E-01	0.51	8.4E-03	0.13	5.4E-01	0.06	7.8E-01	-0.02	9.2E-01	0.38	5.4E-02	-0.46	1.8E-02
ENSCAFG0000000532	CFAP57	grey	EC_M10	-0.02	9.4E-01	-0.07	7.2E-01	0.16	4.2E-01	-0.05	8.2E-01	-0.18	3.9E-01	-0.10	6.4E-01	0.00	9.9E-01	-0.10	6.2E-01	0.46	1.8E-02	0.21	3.0E-01
ENSCAFG0000001045	TARSL2	turquoise	EC_M6	-0.02	9.4E-01	0.85	2.7E-08	-0.19	3.4E-01	-0.22	2.8E-01	-0.10	6.4E-01	-0.30	1.4E-01	-0.10	6.2E-01	0.02	9.3E-01	0.23	2.6E-01	0.11	5.8E-01
ENSCAFG0000000304	SCRN1	grey	EC_M10	-0.02	9.4E-01	-0.46	1.9E-02	-0.28	1.7E-01	0.07	7.5E-01	0.46	1.9E-02	0.07	7.2E-01	0.14	4.8E-01	0.20	3.4E-01	0.16	4.4E-01	-0.48	1.4E-02
ENSCAFG0000003140	TMEM176A	grey	EC_M10	-0.02	9.4E-01	-0.21	3.0E-01	-0.11	6.0E-01	0.07	7.2E-01	0.16	4.2E-01	0.24	2.3E-01	-0.02	9.3E-01	0.24	2.3E-01	0.28	1.7E-01	-0.23	2.6E-01
ENSCAFG0000001818	ENSCAFG0000001818	grey	EC_M10	-0.02	9.4E-01	0.14	4.9E-01	0.04	8.4E-01	0.33	1.0E-01	-0.10	6.2E-01	0.15	4.6E-01	0.28	1.7E-01	-0.35	7.9E-02	0.10	6.4E-01	0.14	5.0E-01
ENSCAFG0000000368	UCP1	grey	EC_M10	-0.02	9.4E-01	-0.08	7.1E-01	0.14	4.8E-01	0.01	9.6E-01	-0.05	8.1E-01	0.07	7.3E-01	0.12	5.7E-01	0.03	8.8E-01	0.50	8.8E-03	0.06	7.7E-01
ENSCAFG0000001312	MAGI3	grey	EC_M10	-0.02	9.4E-01	0.51	7.4E-03	0.25	2.2E-01	-0.02	9.4E-01	-0.47	1.5E-02	-0.26	1.9E-01	-0.14	5.1E-01	0.25	2.1E-01	0.06	7.7E-01	0.51	7.4E-03
ENSCAFG0000001618	ENAH	darkgreen	EC_M4	-0.02	9.4E-01	-0.02	9.3E-01	-0.77	5.2E-06	-0.24	2.3E-01	0.92	2.7E-11	0.14	5.1E-01	0.13	5.1E-01	0.09	6.7E-01	0.12	5.7E-01	-0.90	6.4E-10
ENSCAFG0000001991	FOXC2	grey	EC_M10	-0.02	9.4E-01	0.38	5.4E-02	-0.38	5.9E-02	-0.10	6.1E-01	0.33	1.0E-01	-0.08	7.0E-01	-0.12	5.5E-01	0.06	7.7E-01	0.63	5.1E-04	-0.30	1.4E-01
ENSCAFG0000001488	GDF15	grey	EC_M10	-0.02	9.4E-01	0.10	6.1E-01	-0.12	5.7E-01	-0.12	5.7E-01	0.16	4.4E-01	-0.21	3.1E-01	0.16	4.5E-01	-0.13	5.4E-01	0.57	2.3E-03	-0.13	5.3E-01
ENSCAFG0000000047	C12H6orf15	grey	EC_M10	-0.02	9.4E-01	0.16	4.3E-01	-0.22	2.7E-01	-0.04	8.4E-01	0.24	2.3E-01	-0.07	7.4E-01	-0.08	7.1E-01	-0.02	9.3E-01	-0.05	8.2E-01	-0.25	2.2E-01
ENSCAFG0000002361	URM1	grey	EC_M10	-0.02	9.4E-01	0.01	9.7E-01	0.12	5.7E-01	-0.05	7.9E-01	-0.05	8.2E-01	-0.02	9.2E-01	-0.36	7.1E-02	-0.58	1.9E-03	0.10	6.3E-01	0.12	5.7E-01
ENSCAFG0000001019	RNF144B	turquoise	EC_M6	-0.02	9.4E-01	0.63	5.8E-04	-0.39	4.6E-02	-0.21	3.0E-01	0.29	1.5E-01	0.19	3.5E-01	0.05	8.2E-01	-0.06	7.6E-01	0.13	5.1E-01	-0.24	2.4E-01
ENSCAFG0000001882	ENSCAFG0000001882	grey	EC_M10	-0.02	9.4E-01	-0.19	3.6E-01	-0.64	4.5E-04	-0.06	7.5E-01	0.82	3.2E-07	0.25	2.1E-01	0.06	7.7E-01	0.04	8.3E-01	-0.31	1.3E-01	-0.85	2.8E-08
ENSCAFG0000000323	ENSCAFG0000000323	grey	EC_M10	-0.02	9.4E-01	0.04	8.5E-01	0.17	4.1E-01	-0.02	9.1E-01	-0.14	5.0E-01	-0.04	8.3E-01	0.02	9.2E-01	-0.06	7.5E-01	0.48	1.3E-02	0.16	4.4E-01
ENSCAFG0000000796	MLXIP	grey	EC_M10	-0.02	9.4E-01	0.44	2.4E-02	0.36	7.2E-02	0.22	2.7E-01	-0.60	1.3E-03	-0.15	4.6E-01	0.06	7.7E-01	0.01	9.7E-01	-0.07	7.2E-01	0.57	2.4E-03
ENSCAFG0000001332	LRP6	cyan	EC_M2	-0.02	9.4E-01	-0.28	1.7E-01	0.63	5.2E-04	0.27	1.9E-01	-0.67	1.7E-04	-0.01	9.5E-01	0.06	7.8E-01	-0.13	5.2E-01	0.00	9.9E-01	0.68	1.5E-04
ENSCAFG0000002097	ENSCAFG0000002097	paleturquoise	EC_M11	-0.02	9.4E-01	-0.01	9.5E-01	0.15	4.6E-01	0.87	1.1E-08	-0.21	3.0E-01	-0.04	8.3E-01	-0.03	8.8E-01	-0.10	6.1E-01	0.38	5.4E-02	0.23	2.6E-01
ENSCAFG0000002635	ENSCAFG0000002635	grey	EC_M10	-0.02	9.4E-01	-0.18	3.9E-01	0.30	1.3E-01	-0.17	4.1E-01	-0.28	1.7E-01	-0.12	5.7E-01	-0.12	5.8E-01	0.63	5.5E-04	0.02	9.4E-01	0.31	1.2E-01
ENSCAFG0000000422	C21H11orf54	darkgreen	EC_M4	-0.02	9.4E-01	-0.65	3.4E-04	-0.22	2.8E-01	0.09	6.7E-01	0.44	2.3E-02	0.26	2.0E-01	-0.04	8.5E-01	0.19	3.5E-01	-0.05	7.9E-01	-0.46	1.7E-02
ENSCAFG0000001517	CD9	grey	EC_M10	-0.02	9.4E-01	0.42	3.2E-02	0.45	2.2E-02	0.11	5.8E-01	-0.66	2.6E-04	-0.42	3.4E-02	0.01	9.8E-01	0.12	5.6E-01	-0.07	7.4E-01	0.68	1.4E-04
ENSCAFG0000002397	ZSWIM5	turquoise	EC_M6	-0.02	9.4E-01	0.51	7.8E-03	-0.31	1.3E-01	-0.18	3.8E-01	0.22	2.9E-01	0.30	1.4E-01	-0.13	5.3E-01	-0.15	4.6E-01	-0.11	6.0E-01	-0.17	4.1E-01
ENSCAFG0000000810	PLOD2	darkgreen	EC_M4	-0.02	9.4E-01	-0.23	2.6E-01	-0.68	1.3E-04	-0.03	8.7E-01	0.87	7.7E-09	0.25	2.2E-01	0.11	6.1E-01	0.10	6.3E-01	-0.02	9.2E-01	-0.90	5.1E-10
ENSCAFG0000001285	TDRKH	cyan	EC_M2	-0.02	9.4E-01	0.04	8.3E-01	0.67	1.9E-04	0.19	3.4E-01	-0.75	1.2E-05	-0.19	3.5E-01	0.19	3.7E-01	-0.25	2.2E-01	-0.21	3.0E-01	0.78	2.4E-06
ENSCAFG0000001108	ARNTL2	darkgreen	EC_M4	-0.02	9.4E-01	-0.60	1.3E-03	0.33	1.0E-01	0.17	4.1E-01	-0.20	3.4E-01	-0.06	7.8E-01	-0.08	6.9E-01	0.21	3.0E-01	0.44	2.3E-02	0.17	3.9E-01
ENSCAFG0000001201	NECTIN1	grey	EC_M10	-0.02	9.4E-01	-0.39	4.8E-02	0.02	9.3E-01	-0.26	2.0E-01	0.22	2.8E-01	0.05	8.2E-01	0.15	4.5E-01	0.05	8.2E-01	0.38	5.3E-02	-0.18	3.9E-01
ENSCAFG0000001015	BACE2	grey	EC_M10	-0.02	9.4E-01	0.43	2.8E-02	0.21	3.0E-01	0.16	4.2E-01	-0.40	4.3E-02	-0.19	3.6E-01	-0.38	5.4E-02	0.25	2.2E-01	0.19	3.5E-01	0.39	4.7E-02
ENSCAFG0000001443	SUGP2	grey	EC_M10	-0.02	9.3E-01	0.36	6.8E-02	0.10	6.2E-01	0.25	2.1E-01	-0.28	1.7E-01	-0.32	1.1E-01	0.20	3.3E-01	0.07	7.3E-01	0.53	5.1E-03	0.32	1.1E-01
ENSCAFG0000001882	PATJ	turquoise	EC_M6	-0.02	9.3E-01	0.80	1.0E-06	-0.18	3.9E-01	0.03	9.0E-01	-0.06	7.9E-01	-0.15	4.6E-01	0.07	7.5E-01	-0.03	9.0E-01	0.29	1.4E-01	0.11	6.1E-01
ENSCAFG0000003005	ENSCAFG0000003005	grey	EC_M10	-0.02	9.3E-01	-0.16	4.3E-01	-0.11	6.1E-01	-0.09	6.7E-01	0.16	4.2E-01	-0.11	5.9E-01	-0.12	5.7E-01	-0.07	7.3E-01	-0.08	6.8E-01	-0.20	3.3E-01
ENSCAFG0000002064	ENSCAFG0000002064	grey	EC_M10	-0.02	9.3E-01	0.02	9.4E-01	-0.02	9.1E-01	-0.16	4.3E-01	-0.05	8.2E-01	-0.20	3.4E-01	0.23	2.6E-01	-0.17	4.1E-01	-0.34	9.2E-02	-0.03	8.7E-01
ENSCAFG0000003092	ENSCAFG0000003092	grey	EC_M10	-0.02	9.3E-01	0.27	1.8E-01	-0.35	7.6E-02	0.24	2.3E-01	0.31	1.2E-01	-0.19	3.6E-01	0.20	3.3E-01	-0.14	4.9E-01	0.18	3.9E-01	-0.28	1.7E-01
ENSCAFG0000002586	ENSCAFG0000002586	grey	EC_M10	-0.02	9.3E-01	0.16	4.4E-01	0.07	7.2E-01	-0.23	2.6E-01	-0.15	4.5E-01	-0.18	3.8E-01	0.17	4.1E-01	-0.25	2.2E-01	0.20	3.4E-01	0.19	3.6E-01
ENSCAFG0000000282	SPACA6	grey	EC_M10	-0.02	9.3E-01	0.33	9.9E-02	-0.45	2.3E-02	-0.08	6.9E-01	0.36	7.1E-02	0.15	4.6E-01	0.14	4.9E-01	0.02	9.4E-01	0.29	1.6E-01	-0.35	8.0E-02
ENSCAFG0000000042	RMND1	grey	EC_M10	-0.02	9.3E-01	-0.16	4.2E-01	0.10	6.2E-01	0.37	6.2E-02	-0.08	7.1E-01	0.13	5.4E-01	-0.22	2.7E-01	0.10	6.3E-01	0.16	4.3E-01	0.10	6.4E-01
ENSCAFG0000000111	TMEM200A	grey	EC_M10	-0.02	9.3E-01	-0.24	2.4E-01	-0.61	9.3E-04	-0.13	5.4E-01	0.80	9.8E-07	0.22	2.9E-01	-0.08	7.0E-01	0.23	2.5E-01	0.24	2.5E-01	-0.79	1.3E-06
ENSCAFG0000001560	ILDR2	grey	EC_M10	-0.02	9.3E-01	-0.01	9.4E-01	0.47	1.5E-02	0.27	1.9E-01	-0.57	2.3E-03	-0.27	1.8E-01	-0.18	3.9E-01	-0.05	8.1E-01	-0.22	2.8E-01	0.60	1.3E-03

ENSCAFG0000001205	CAMK2D	grey	EC_M10	-0.02	9.3E-01	-0.67	1.9E-04	-0.12	5.6E-01	0.13	5.1E-01	0.33	1.0E-01	0.15	4.7E-01	0.10	6.2E-01	0.14	5.0E-01	0.31	1.3E-01	-0.36	7.0E-02
ENSCAFG0000000689	ENSCAFG0000000689	grey	EC_M10	-0.02	9.3E-01	-0.16	4.2E-01	0.16	4.4E-01	0.35	7.5E-02	-0.12	5.6E-01	-0.15	4.7E-01	0.38	5.5E-02	-0.11	5.9E-01	0.06	7.6E-01	0.13	5.2E-01
ENSCAFG0000000358	TRAF1	turquoise	EC_M6	-0.02	9.3E-01	0.69	1.1E-04	0.15	4.5E-01	-0.12	5.5E-01	-0.38	5.5E-02	-0.36	7.3E-02	0.11	5.9E-01	0.05	8.0E-01	0.13	5.3E-01	0.41	4.0E-02
ENSCAFG0000003069	ZBED2	grey	EC_M10	-0.02	9.3E-01	-0.18	3.8E-01	-0.55	3.4E-03	-0.19	3.5E-01	0.71	5.5E-05	0.48	1.3E-02	0.02	9.2E-01	0.06	7.9E-01	-0.06	7.5E-01	-0.74	1.3E-05
ENSCAFG0000002342	KCNE4	grey	EC_M10	-0.02	9.3E-01	-0.30	1.3E-01	-0.03	8.8E-01	0.00	9.9E-01	0.13	5.3E-01	0.09	6.7E-01	-0.01	9.5E-01	0.28	1.6E-01	-0.25	2.2E-01	-0.20	3.3E-01
ENSCAFG0000000887	HUNK	darkgreen	EC_M4	-0.02	9.3E-01	-0.08	6.9E-01	-0.31	1.2E-01	0.49	1.2E-02	0.38	5.6E-02	-0.13	5.3E-01	-0.07	7.3E-01	-0.07	7.4E-01	-0.08	7.1E-01	-0.38	5.4E-02
ENSCAFG0000001614	ENSCAFG0000001614	grey	EC_M10	-0.02	9.3E-01	-0.30	1.3E-01	-0.16	4.4E-01	0.06	7.9E-01	0.37	6.3E-02	-0.02	9.1E-01	0.00	1.0E+00	0.05	8.2E-01	-0.22	2.9E-01	-0.34	9.2E-02
ENSCAFG0000002899	ENSCAFG0000002899	grey	EC_M10	-0.02	9.3E-01	-0.14	4.9E-01	-0.13	5.1E-01	0.36	7.3E-02	0.21	3.0E-01	0.00	1.0E+00	-0.31	1.2E-01	0.19	3.4E-01	-0.17	4.0E-01	-0.26	2.1E-01
ENSCAFG0000000001	ZNF236	cyan	EC_M2	-0.02	9.3E-01	0.24	2.3E-01	0.42	3.1E-02	0.39	5.1E-02	-0.60	1.2E-03	-0.24	2.4E-01	-0.19	3.5E-01	-0.36	7.0E-02	-0.20	3.2E-01	0.59	1.4E-03
ENSCAFG0000001623	MED24	cyan	EC_M2	-0.02	9.3E-01	0.30	1.4E-01	0.49	1.2E-02	-0.03	9.0E-01	-0.64	4.1E-04	-0.28	1.7E-01	-0.23	2.6E-01	-0.09	6.5E-01	-0.31	1.2E-01	0.66	2.4E-04
ENSCAFG0000001203	ZNF710	grey	EC_M10	-0.02	9.3E-01	0.44	2.5E-02	-0.46	1.7E-02	-0.51	7.6E-03	0.41	3.5E-02	0.37	6.5E-02	0.22	2.8E-01	-0.13	5.2E-01	0.06	7.8E-01	-0.40	4.2E-02
ENSCAFG0000000834	NUGGC	grey	EC_M10	-0.02	9.3E-01	-0.10	6.4E-01	-0.02	9.3E-01	-0.12	5.6E-01	0.02	9.2E-01	-0.07	7.2E-01	-0.03	8.7E-01	0.38	5.7E-02	0.00	9.9E-01	-0.04	8.6E-01
ENSCAFG0000001327	ENSCAFG0000001327	grey	EC_M10	-0.02	9.3E-01	0.14	4.8E-01	-0.16	4.4E-01	-0.09	6.6E-01	0.21	3.0E-01	-0.11	5.9E-01	-0.08	7.0E-01	-0.10	6.1E-01	-0.01	9.7E-01	-0.17	4.0E-01
ENSCAFG0000001597	SLC16A13	turquoise	EC_M6	-0.02	9.3E-01	0.80	7.2E-07	-0.39	5.2E-02	-0.15	4.6E-01	0.18	3.7E-01	-0.11	5.8E-01	0.15	4.7E-01	0.13	5.4E-01	0.20	3.4E-01	-0.16	4.2E-01
ENSCAFG0000002139	ENSCAFG0000002139	grey	EC_M10	-0.02	9.3E-01	0.24	2.3E-01	0.33	9.9E-02	-0.15	4.5E-01	-0.44	2.6E-02	-0.20	3.2E-01	-0.19	3.6E-01	0.43	2.9E-02	-0.14	4.8E-01	0.45	2.1E-02
ENSCAFG0000000875	CCL17	grey	EC_M10	-0.02	9.3E-01	-0.15	4.6E-01	-0.55	3.8E-03	-0.13	5.3E-01	0.76	5.4E-06	0.15	4.6E-01	0.26	2.0E-01	0.08	7.0E-01	-0.22	2.8E-01	-0.74	1.8E-05
ENSCAFG0000001710	GDPD3	turquoise	EC_M6	-0.02	9.3E-01	0.60	1.1E-03	-0.05	8.1E-01	0.10	6.4E-01	-0.13	5.1E-01	0.04	8.6E-01	0.10	6.2E-01	0.11	5.9E-01	-0.01	9.7E-01	0.17	4.2E-01
ENSCAFG0000002797	ENSCAFG0000002797	grey	EC_M10	-0.02	9.3E-01	-0.14	4.8E-01	-0.03	8.7E-01	0.18	3.7E-01	0.07	7.3E-01	-0.19	3.6E-01	0.23	2.5E-01	0.22	2.8E-01	-0.14	4.9E-01	-0.10	6.1E-01
ENSCAFG0000001184	SESN2	grey	EC_M10	-0.02	9.3E-01	-0.18	3.7E-01	-0.50	9.8E-03	0.06	7.5E-01	0.65	3.6E-04	0.28	1.6E-01	-0.05	8.2E-01	0.05	8.1E-01	-0.08	7.1E-01	-0.71	5.7E-05
ENSCAFG0000001234	ABCF3	grey	EC_M10	-0.02	9.3E-01	0.19	3.5E-01	-0.07	7.3E-01	-0.07	7.4E-01	0.05	8.2E-01	0.29	1.4E-01	0.04	8.4E-01	-0.06	7.8E-01	-0.17	4.1E-01	0.01	9.7E-01
ENSCAFG0000001000	DUSP8	turquoise	EC_M6	-0.02	9.3E-01	0.77	4.4E-06	-0.02	9.1E-01	-0.06	7.7E-01	-0.18	3.8E-01	-0.06	7.8E-01	0.12	5.6E-01	0.08	7.0E-01	0.32	1.1E-01	0.22	2.7E-01
ENSCAFG0000000785	HAND2	grey	EC_M10	-0.02	9.3E-01	-0.25	2.1E-01	0.09	6.7E-01	0.34	9.3E-02	-0.02	9.2E-01	0.33	1.0E-01	-0.19	3.6E-01	-0.13	5.4E-01	-0.10	6.2E-01	0.03	9.0E-01
ENSCAFG0000000312	MACF1	grey	EC_M10	-0.02	9.3E-01	0.25	2.2E-01	-0.42	3.2E-02	-0.23	2.6E-01	0.39	5.0E-02	-0.12	5.5E-01	0.05	8.0E-01	-0.07	7.3E-01	-0.35	8.0E-02	-0.37	6.3E-02
ENSCAFG0000001514	SYN1	grey	EC_M10	-0.02	9.3E-01	-0.15	4.8E-01	-0.44	2.5E-02	-0.29	1.6E-01	0.59	1.4E-03	-0.01	9.8E-01	-0.17	4.0E-01	0.16	4.4E-01	-0.10	6.3E-01	-0.61	9.1E-04
ENSCAFG0000000709	CYP7A1	grey	EC_M10	-0.02	9.3E-01	0.30	1.4E-01	-0.23	2.6E-01	-0.18	3.8E-01	0.13	5.2E-01	-0.21	3.0E-01	0.20	3.3E-01	0.08	7.0E-01	0.34	8.5E-02	-0.11	5.9E-01
ENSCAFG0000000614	DIAPH1	grey	EC_M10	-0.02	9.3E-01	0.16	4.4E-01	0.28	1.7E-01	-0.24	2.3E-01	-0.34	9.0E-02	0.02	9.2E-01	0.11	5.8E-01	-0.41	4.0E-02	-0.21	3.1E-01	0.39	5.1E-02
ENSCAFG0000000203	UNC13B	darkgreen	EC_M4	-0.02	9.3E-01	0.03	8.7E-01	-0.75	1.1E-05	-0.07	7.4E-01	0.85	2.8E-08	0.20	3.2E-01	0.21	3.0E-01	0.07	7.3E-01	-0.19	3.5E-01	-0.86	1.9E-08
ENSCAFG0000000431	CST7	turquoise	EC_M6	-0.02	9.2E-01	0.63	5.2E-04	0.02	9.4E-01	-0.13	5.2E-01	-0.25	2.3E-01	-0.10	6.3E-01	0.15	4.7E-01	-0.13	5.2E-01	0.08	7.1E-01	0.23	2.5E-01
ENSCAFG0000001434	KIAA0232	grey	EC_M10	-0.02	9.2E-01	0.26	1.9E-01	-0.51	7.3E-03	-0.17	4.0E-01	0.51	8.4E-03	0.13	5.4E-01	0.08	7.1E-01	0.20	3.3E-01	0.35	8.3E-02	-0.45	2.1E-02
ENSCAFG0000001441	DUSP19	grey	EC_M10	-0.02	9.2E-01	-0.05	8.0E-01	-0.54	4.6E-03	-0.24	2.3E-01	0.67	2.1E-04	0.30	1.4E-01	0.12	5.7E-01	0.06	7.5E-01	-0.04	8.4E-01	-0.67	1.9E-04
ENSCAFG0000002005	PLPPR5	grey	EC_M10	-0.02	9.2E-01	-0.24	2.4E-01	-0.43	3.0E-02	0.02	9.3E-01	0.59	1.5E-03	0.20	3.2E-01	-0.03	8.8E-01	-0.07	7.5E-01	-0.11	6.0E-01	-0.59	1.5E-03
ENSCAFG0000000840	TCTN3	grey	EC_M10	-0.02	9.2E-01	0.12	5.7E-01	0.21	2.9E-01	-0.07	7.5E-01	-0.29	1.5E-01	0.09	6.6E-01	-0.01	9.8E-01	0.21	3.1E-01	0.10	6.2E-01	0.33	1.1E-01
ENSCAFG0000000719	ENSCAFG0000000719	grey	EC_M10	-0.02	9.2E-01	-0.01	9.4E-01	-0.15	4.5E-01	0.55	3.3E-03	0.20	3.4E-01	0.18	3.7E-01	-0.22	2.8E-01	0.07	7.5E-01	-0.12	5.5E-01	-0.18	3.7E-01
ENSCAFG0000000177	WASL	cyan	EC_M2	-0.02	9.2E-01	-0.02	9.3E-01	0.38	5.3E-02	0.24	2.4E-01	-0.55	3.4E-03	-0.16	4.4E-01	0.03	9.0E-01	0.12	5.5E-01	0.18	3.9E-01	0.55	3.8E-03
ENSCAFG0000000701	PFDN5	grey	EC_M10	-0.02	9.2E-01	-0.30	1.3E-01	0.24	2.4E-01	0.40	4.4E-02	-0.25	2.3E-01	0.15	4.8E-01	0.18	3.9E-01	-0.15	4.5E-01	-0.38	5.8E-02	0.24	2.3E-01
ENSCAFG0000000781	NR3C2	grey	EC_M10	-0.02	9.2E-01	0.15	4.7E-01	-0.36	7.4E-02	0.13	5.4E-01	0.30	1.4E-01	-0.02	9.4E-01	0.02	9.3E-01	0.23	2.6E-01	-0.07	7.5E-01	-0.31	1.2E-01
ENSCAFG0000001011	GAS2	turquoise	EC_M6	-0.02	9.2E-01	0.48	1.3E-02	0.06	7.6E-01	-0.11	5.9E-01	-0.24	2.3E-01	-0.08	7.0E-01	-0.09	6.7E-01	-0.06	7.7E-01	-0.13	5.4E-01	0.25	2.3E-01
ENSCAFG0000000790	WWC2	darkgreen	EC_M4	-0.02	9.2E-01	0.06	7.6E-01	-0.67	1.9E-04	-0.08	6.9E-01	0.74	1.3E-05	0.11	6.1E-01	0.02	9.4E-01	-0.08	6.8E-01	-0.19	3.6E-01	-0.75	1.2E-05
ENSCAFG0000001390	ENSCAFG0000001390	grey	EC_M10	-0.02	9.2E-01	-0.26	2.0E-01	0.27	1.8E-01	0.30	1.3E-01	-0.18	3.8E-01	0.08	6.9E-01	-0.26	2.0E-01	-0.44	2.6E-02	-0.16	4.2E-01	0.17	4.2E-01
ENSCAFG0000001203	SEMA5B	grey	EC_M10	-0.02	9.2E-01	0.09	6.8E-01	-0.16	4.5E-01	-0.12	5.7E-01	0.18	3.8E-01	-0.15	4.6E-01	0.52	6.4E-03	0.35	8.1E-02	0.23	2.5E-01	-0.16	4.2E-01
ENSCAFG0000000729	SREK1IP1	grey	EC_M10	-0.02	9.2E-01	0.31	1.2E-01	-0.35	7.9E-02	-0.24	2.3E-01	0.29	1.4E-01	-0.10	6.4E-01	0.18	3.7E-01	-0.03	9.0E-01	-0.39	4.9E-02	-0.27	1.8E-01
ENSCAFG0000000573	SFRP1	turquoise	EC_M6	-0.02	9.2E-01	0.60	1.1E-03	-0.29	1.6E-01	-0.14	4.9E-01	0.13	5.2E-01	0.04	8.4E-01	0.40	4.1E-02	-0.06	7.8E-01	0.21	2.9E-01	-0.08	7.0E-01
ENSCAFG0000000708	MTMR6	darkgreen	EC_M4	-0.02	9.2E-01	0.19	3.6E-01	-0.79	1.6E-06	-0.27	1.8E-01	0.86	2.3E-08	0.25	2.2E-01	0.06	7.9E-01	-0.04	8.5E-01	0.01	9.7E-01	-0.82	2.6E-07
ENSCAFG0000000653	DCHS1	grey	EC_M10	-0.02	9.2E-01	-0.05	8.1E-01	-0.35	8.3E-02	-0.37	5.9E-02	0.45	2.1E-02	0.35	8.2E-02	0.16	4.2E-01	-0.21	3.0E-01	-0.56	2.9E-03	-0.43	2.7E-02
ENSCAFG0000001653	ACTN1	darkgreen	EC_M4	-0.02	9.2E-01	-0.11	5.9E-01	-0.72	3.3E-05	-0.23	2.7E-01	0.91	7.4E-11	0.19	3.6E-01	0.07	7.5E-01	0.14	5.0E-01	0.20	3.3E-01	-0.91	2.1E-10
ENSCAFG0000000790	GLRX	grey	EC_M10	-0.02	9.2E-01	0.52	6.7E-03	0.33	9.8E-02	-0.01	9.6E-01	-0.53	5.1E-03	-0.40	4.6E-02	0.00	9.9E-01	-0.03	8.9E-01	0.17	4.2E-01	0.58	1.9E-03
ENSCAFG0000001833	ADRB2	turquoise	EC_M6	-0.02	9.2E-01	0.57	2.2E-03	0.11	6.1E-01	-0.19	3.5E-01	-0.23	2.7E-01	-0.18	3.8E-01	0.36	7.3E-02	0.15	4.8E-01	-0.14	4.8E-01	0.28	1.6E-01
ENSCAFG0000000605	ENSCAFG0000000605	grey	EC_M10	-0.02	9.2E-01	-0.16	4.3E-01	-0.13	5.3E-01	0.22	2.9E-01	0.16	4.5E-01	0.12	5.7E-01	-0.08	7.0E-01	0.02	9.3E-01	0.09	6.6E-01	-0.08	6.9E-01
ENSCAFG0000001678	ENSCAFG0000001678	grey	EC_M10	-0.02	9.2E-01	-0.28	1.6E-01	0.04	8.5E-01	0.32	1.2E-01	0.01	9.7E-01	0.25	2.3E-01	-0.22	2.8E-01	-0.29	1.5E-01	-0.08	7.1E-01	-0.05	8.2E-01
ENSCAFG0000001390	SERPINE1	darkgreen	EC_M4	-0.02	9.2E-01	0.35	8.3E-02	-0.77	3.6E-06	-0.25	2.1E-01	0.81	5.4E-07	0.13	5.2E-01	0.20	3.4E-01	0.08	7.1E-01	0.05	7.9E-01	-0.79	1.4E-06
ENSCAFG0000001916	GABRA3	grey	EC_M10	-0.02	9.2E-01	-0.07	7.3E-01	0.15	4.6E-01	0.43	2.8E-02	-0.15	4.8E-01	0.29	1.5E-01	0.01	9.7E-01	-0.19	3.5E-01	-0.31	1.3E-01	0.07	7.4E-01
ENSCAFG0000001832	KIF26A	turquoise	EC_M6	-0.02	9.2E-01	0.83	1.6E-07	-0.17	4.0E-01	-0.11	6.0E-01	-0.06	7.9E-01	-0.16	4.4E-01	-0.13	5.4E-01	-0.03	8.7E-01	0.26	1.9E-01	0.05	8.1E-01
ENSCAFG0000003037	ZNF514	cyan	EC_M2	-0.02	9.2E-01	0.04	8.5E-01	0.45	2.3E-02	0.19	3.4E-01	-0.62	7.4E-04	-0.27	1.8E-01	0.01	9.6E-01	-0.11	5.9E-01	0.07	7.4E-01	0.63	5.6E-04
ENSCAFG0000003116	ENSCAFG0000003116	grey	EC_M10	-0.02	9.2E-01	-0.03	8.8E-01	0.24	2.4E-01	-0.04	8.5E-01	-0.27	1.8E-01	-0.01	9.5E-01	-0.09	6.7E-01	-0.09	6.5E-01	-0.14	4.8E-01	0.26	2.0E-01
ENSCAFG0000001686	RAB25	grey	EC_M10	-0.02	9.2E-01	-0.03	8.7E-01	-0.20	3.2E-01	0.01	9.5E-01	0.23	2.7E-01	-0.07	7.5E-01	0.04	8.5E-01	-0.10	6.2E-01	0.39	4.7E-02	-0.25	2.1E-01
ENSCAFG0000000194	TMEM63B	grey	EC_M10	-0.02	9.2E-01	0.19	3.4E-01	-0.54	4.5E-03	-0.24	2.5E-01	0.60	1.1E-03	0.07	7.3E-01	0.08	7.1E-01	0.06	7.6E-01	0.28	1.6E-01	-0.59	1.7E-03
ENSCAFG0000000334	MBOAT2	grey	EC_M10	-0.02	9.2E-01	-0.52	6.9E-03	-0.42	3.0E-02	0.10	6.1E-01	0.65	3.0E-04	0.22	2.8E-01	-0.13	5.1E-01	0.02	9.4E-01	-0.17	4.0E-01	-0.65	3.1E-04
ENSCAFG0000001809	SYNPO	grey	EC_M10	-0.02	9.2E-01	0.37	6.0E-02	-0.51	7.3E-03	-0.22	2.8E-01	0.52	6.0E-03	-0.01	9.4E-01	-0.08	6.8E-01	0.02	9.1E-01	0.36	7.1E-02	-0.50	9.6E-03
ENSCAFG0000000175	GPR37	grey	EC_M10	-0.02	9.2E-01	-0.23	2.6E-01	0.05	7.9E-01	-0.13	5.3E-01	-0.01	9.7E-01	-0.10	6.4E-01	0.02	9.1E-01	-0.06	7.7E-01	-0.14	5.0E-01	-0.02	9.4E-01
ENSCAFG0000000985	ENSCAFG0000000985	grey	EC_M10	-0.02	9.2E-01	-0.09	6.6E-01	-0.59	1.6E-03	-0.15	4.6E-01	0.73	2.5E-05	0.12	5.4E-01	0.08	6.8E-01	-0.09	6.5E-01	-0.20	3.4E-01	-0.72	3.2E-05
ENSCAFG0000000571	GPAT4	turquoise	EC_M6	-0.02	9.2E-01	0.54	4.3E-03	0.04	8.5E-01	-0.03	8.9E-01	-0.27	1.9E-01	-0.07	7.4E-01	-0.16	4.2E-01	0.05	8.3E-01	0.58	1.9E-03	0.27	1.8E-01
ENSCAFG0000001911	MAMLD1	turquoise	EC_M6	-0.02	9.2E-01	0.85	3.5E-08	-0.42	3.2E-02	-0.15	4.6E-01	0.21	3.1E-01	-0.11	5.9E-01	0.08	7.0E-01	-0.09	6.6E-01	0.27	1.7E-01	-0.18	3.7E-01
ENSCAFG0000002667	ENSCAFG0000002667	grey	EC_M10	-0.02	9.2E-01	0.22	2.9E-01	0.24	2.4E-01	0.39	5.1E-02	-0.40	4.2E-02	0.25	2.3E-01	-0.11	6.0E-01	-0.24	2.4E-01	0.13	5.2E-01	0.39	5.2E-02
ENSCAFG0000001982	GNAT2	grey	EC_M10	-0.02	9.2E-01	-0.07	7.5E-01	0.23	2.5E-01	-0.09	6.7E-01	-0.25	2.2E-01	-0.05	8.1E-01	-0.09	6.8E-01	-0.14	5.1E-01	-0.06	7.8E-01	0.29	1.6E-01
ENSCAFG0000002428	TRABD2B	grey	EC_M10	-0.02	9.2E-01	-0.11	5.9E-01	0.08	6.9E-01	-0.01	9.7E-01	-0.10	6.2E-01	-0.39	4.8E-02	-0.18	3.7E-01	0.60	1.3E-03	0.34	9.3E-02	0.10	6.1E-01
ENSCAFG0000001845	ENSCAFG0000001845	grey	EC_M10	-0.02	9.2E-01	-0.27	1.8E-01	0.08	7.0E-01	0.11	5.9E-01	-0.03	8.8E-01	-0.16	4.2E-01	-0.25	2.1E-01	0.16	4.3E-01	-0.13	5.3E-01	0.04	8.4E-01
ENSCAFG0000000507	CLN5	grey	EC_M10	-0.02	9.2E-01	-0.19	3.6E-01	-0.10	6.3E-01	0.05	8.3E-01	0.26	1.9E-01	0.04	8.3E-01	-0.18	3.7E-01	0.26	1.9E-01	0.18	3.7E-01	-0.22	2.8E-01
ENSCAFG0000002002	MFSD14A	cyan	EC_M2	-0.02	9.2E-01	0.35	8.1E-02	0.48	1.4E-02	0.08	7.0E-01	-0.64	3.9E-04	-0.23	2.6E-01	0.23	2.6E-01	-0.16	4.4E-01	-0.34	9.1E-02	0.68	1.4E-04
ENSCAFG0000000310	EHBP1	cyan	EC_M2	-0.02	9.2E-01	-0.60	1.2E-03	0.58	2.0E-03	0.22	2.7E-01	-0.45	2.2E-02	0.11	6.0E-01	-0.09	6.7E-01	-0.24	2.5E-01	-0.46	1.8E-02	0.45	2.1E-02
ENSCAFG0000001135	VWA5A	turquoise	EC_M6	-0.02	9.2E-01	0.84	7.3E-08	-0.22	2.7E-01	-0.05	8.0E-01	0.02	9.1E-01	-0.24	2.5E-01	-0.09	6.8E-01	0.04	8.6E-01	-0.02	9.2E-01	0.01	9.8E-01
ENSCAFG0000000637	FAM160A2	grey	EC_M10	-0.02	9.1E-01	0.38	5.6E-02	0.33	1.0E-01	0.25	2.2E-01	-0.48	1.3E-02	-0.12	5.6E-01	0.13	5.4E-01	-0.06	7.9E-01	-0.26	2.0E-01	0.48	1.3E-02
ENSCAFG0000001292	SMTN	grey	EC_M10	-0.02	9.1E-01	0.48	1.4E-02	-0.74	1.5E-05	-0.26	2.1E-01	0.70	7.1E-05	0.15	4.7E-01	0.01	9.8E-01	0.12	5.6E-01	0.34	9.0E-02	-0.69	1.0E-04
ENSCAFG0000000870	FNIP2	cyan	EC_M2	-0.02	9.1E-01	-0.03	8.7E-01	0.64	4.0E-04	0.11	6.1E-01	-0.77	3.6E-06	-0.26	1.9E-01	0.08	7.2E-01	-0.03	8.7E-01	0.05	8.2E-01	0.80	8.6E-07
ENSCAFG0000001284	PASK	grey	EC_M10	-0.02	9.1E-01	-0.04	8.3E-01	0.65	3.5E-04	-0.23	2.6E-01	-0.64	4.2E-04	-0.21	2.9E-01	0.09	6.7E-01	-0.01	9.5E-01	-0.12	5.6E-01	0.67	1.6E-04
ENSCAFG0000000078	PBX2	grey	EC_M10	-0.02	9.1E-01	0.26	2.0E-01	0.46	1.7E-02	-0.04	8.4E-01	-0.68	1.4E-04	-0.15	4.6E-01	0.33	9.9E-02	-0.13	5.1E-01	0.29	1.5E-01	0.67	1.9E-04
ENSCAFG0000001459	ABHD12B	cyan	EC_M2	-0.02	9.1E-01	0.06	7.8E-01	0.70	6.5E-05	0.15	4.6E-01	-0.83	1.7E-07	-0.30	1.4E-01	0.04	8.4E-01	0.07	7.2E-01	-0.13	5.4E-01	0.86	1.7E-08
ENSCAFG0000001976	MED22	grey	EC_M10	-0.02	9.1E-01	0.16	4.2E-01	0.06	7.6E-01	0.16	4.5E-01	-0.02	9.2E-01	0.12	5.7E-01	-0.14	5.0E-01	-0.09	6.6E-01	0.28	1.7E-01	0.03	8.7E-01
ENSCAFG0000000825	KDM2B	cyan	EC_M2	-0.02	9.1E-01	0.25	2.2E-01	0.64	3.8E-04	0.00	9.9E-01	-0.81	5.5E-07	-0.22	2.8E-01	0.16	4.4E-01	-0.11	6.0E-01	0.02	9.3E-01	0.83	1.6E-07
ENSCAFG0000001849	CRLF3	darkgreen	EC_M4	-0.02	9.1E-01	-0.57	2.6E-03	-0.34	9.3E-02	-0.09	6.6E-01	0.63	5.6E-04	0.11	5.9E-01	-0.08	7.1E-01	0.06	7.7E-01	-0.08	7.1E-01	-0.62	6.9E-04
ENSCAFG0000000389	MPP7	grey	EC_M10	-0.02	9.1E-01	0.45	2.3E-02	-0.64	4.3E-04	0.05	8.0E-01	0.57	2.4E-03	0.00	1.0E+00	0.12	5.7E-01	-0.10	6.4E-01	-0.01	9.7E-01	-0.55	3.8E-03
ENSCAFG0000000187	STEAP2	grey	EC_M10	-0.02	9.1E-01	-0.29	1.5E-01	0.48	1.4E-02	0.19	3.5E-01	-0.44	2.5E-02	0.03	8.7E-01	-0.11	6.1E-01	-0.07	7.4E-01	-0.74	1.6E-05	0.42	3.4E-02
ENSCAFG0000001824	ABHD3	grey	EC_M10	-0.02	9.1E-01	0.08	7.0E-01	-0.50	9.1E-03	-0.08	6.8E-01	0.54	4.8E-03	-0.02	9.3E-01	-0.15	4.7E-01	0.27	1.8E-01	-0.08	6.9E-01	-0.49	1.0E-02
ENSCAFG0000001289	KLC2	grey	EC_M10	-0.02	9.1E-01	-0.01	9.5E-01	-0.57	2.6E-03	-0.08	6.9E-01	0.70	7.6E-05	0.39	4.9E-02	-0.12	5.7E-01	0.17	3.9E-01	0.21	3.0E-01	-0.67	1.7E-04
ENSCAFG0000000983	GFOD1	grey	EC_M10	-0.02	9.1E-01	0.27	1.8E-01	-0.59	1.5E-03	-0.06	7.6E-01	0.63	5.1E-04	-0.05	8.1E-01	0.12	5.5E-01	-0.07	7.3E-01	-0.09	6.5E-01	-0.58	1.9E-03
ENSCAFG0000002593	ENSCAFG0000002593	grey	EC_M10	-0.02	9.1E-01	-0.25	2.2E-01	-0.01	9.6E-01	-0.11	5.8E-01	0.20	3.3E-01	0.40	4.2E-02	0.25	2.2E-01	-0.11	5.9E-01	-0.14	4.9E-01	-0.15	4.7E-01
ENSCAFG0000000222	RGP1	turquoise	EC_M6	-0.02	9.1E-01	0.60	1.1E-03	-0.12	5.6E-01	-0.37	6.4E-02	-0.07	7.4E-01	-0.25	2.2E-01	-0.03	9.0E-01	-0.28	1.6E-01	0.15	4.6E-01	0.12	5.7E-01
ENSCAFG0000002453	ENSCAFG0000002453	darkgreen	EC_M4	-0.02	9.1E-01	0.25	2.2E-01	-0.65	2.9E-04	0.00	9.9E-01	0.65	3.2E-04	0.18	3.8E-01	0.22	2.8E-01	0.01	9.7E-01	0.02	9.3E-01	-0.64	4.1E-04
ENSCAFG0000001020	NDUFS4	grey	EC_M10	-0.02	9.1E-01	-0.36	7.3E-02	-0.01	9.6E-01	0.30	1.4E-01	0.13	5.2E-01	-0.02	9.1E-01	0.15	4.5E-01	0.19	3.5E-01	-0.11	6.0E-01	-0.11	5.9E-01
ENSCAFG0000001714	ANGEL1	grey	EC_M10	-0.02	9.1E-01	0.16	4.5E-01	-0.22	2.7E-01	-0.30	1.4E-01	0.28	1.7E-01	-0.12	5.5E-01	-0.05	8.1E-01	0.11	5.8E-01	-0.04	8.6E-01	-0.28	1.7E-01
ENSCAFG0000003224	UBE2V1	grey	EC_M10	-0.02	9.1E-01	0.12	5.5E-01	-0.55	3.7E-03	-0.38	5.6E-02	0.64	3.8E-04	0.27	1.9E-01	-0.09	6.8E-01	0.28	1.7E-01	0.41	3.6E-02	-0.63	5.9E-04
ENSCAFG0000000730	IGF1	turquoise	EC_M6	-0.02	9.1E-01	0.60	1.1E-03	0.03	8.7E-01	-0.19	3.5E-01	-0.19	3.4E-01	-0.19	3.5E-01	-0.02	9.3E-01	0.28	1.6E-01	-0.16	4.2E-01	0.21	3.0E-01
ENSCAFG0000003064	C11H5orf63	grey	EC_M10	-0.02	9.1E-01	-0.11	5.8E-01	0.30	1.4E-01	0.09	6.6E-01	-0.32	1.1E-01	0.13	5.2E-01	-0.09	6.7E-01	-0.54	4.6E-03	0.09	6.7E-01	0.34	8.6E-02
ENSCAFG0000000072	ADAMTS19	grey	EC_M10	-0.02	9.1E-01	-0.10	6.3E-01	-0.49	1.1E-02	-0.10	6.2E-01	0.61	9.5E-04	0.40	4.3E-02	0.21	3.0E-01	-0.04	8.5E-01	-0.07	7.4E-01	-0.60	1.2E-03
ENSCAFG0000002843	ENSCAFG0000002843	grey	EC_M10	-0.02	9.1E-01	-0.18	3.8E-01	0.24	2.4E-01	0.28	1.6E-01	-0.21	3.1E-01	0.31	1.2E-01	-0.15	4.5E-01	-0.14	5.0E-01	-0.11	5.9E-01	0.22	2.8E-01
ENSCAFG0000001677	ENSCAFG0000001677	grey	EC_M10	-0.02	9.1E-01	-0.39	4.7E-02	-0.27	1.8E-01	-0.19	3.6E-01	0.55	3.4E-03	0.20	3.2E-01	0.09	6.6E-01	-0.18	3.8E-01	0.00	9.9E-01	-0.54	4.2E-03
ENSCAFG0000000252	TGFBR1	darkgreen	EC_M4	-0.02	9.1E-01	0.11	6.0E-01	-0.77	5.0E-06	-0.06	7.6E-01	0.82	3.3E-07	0.17	4.1E-01	0.01	9.6E-01	0.07	7.3E-01	0.16	4.4E-01	-0.80	7.3E-07
ENSCAFG0000001546	TSPAN9	turquoise	EC_M6	-0.02	9.1E-01	0.77	4.1E-06	-0.51	8.1E-03	-0.10	6.4E-01	0.35	8.4E-02	0.07	7.4E-01	-0.07	7.5E-01	0.13	5.4E-01	0.00	9.9E-01	-0.33	9.5E-02
ENSCAFG0000000426	ENKUR	grey	EC_M10	-0.02	9.1E-01	0.40	4.4E-02	-0.21	3.0E-01	-0.19	3.6E-01	0.13	5.3E-01	-0.17	4.1E-01	-0.10	6.4E-01	-0.13	5.1E-01	0.04	8.5E-01	-0.04	8.6E-01
ENSCAFG0000000237	ENSCAFG0000000237	grey	EC_M10	-0.02	9.1E-01	0.11	5.9E-01	-0.05	8.0E-01	0.25	2.1E-01	-0.01	9.5E-01	-0.35	8.4E-02	-0.04	8.4E-01	0.26	2.0E-01	0.02	9.4E-01	0.04	8.5E-01
ENSCAFG0000001675	SETD1A	grey	EC_M10	-0.02	9.1E-01	0.36	7.2E-02	0.46	1.7E-02	0.07	7.5E-01	-0.63	5.4E-04	-0.21	3.1E-01	0.13	5.4E-01	-0.13	5.2E-01	0.00	9.9E-01	0.65	3.0E-04
ENSCAFG0000002032	NFATC3	grey	EC_M10	-0.02	9.1E-01	0.14	5.0E-01	0.30	1.3E-01	0.32	1.1E-01	-0.45	2.0E-02	0.04	8.5E-01	-0.08	7.1E-01	-0.34	9.3E-02	-0.04	8.3E-01	0.43	2.9E-02
ENSCAFG0000001863	ENSCAFG0000001863	darkmagenta	EC_M12	-0.02	9.1E-01	-0.10	6.2E-01	0.13	5.3E-01	-0.05	8.2E-01	-0.05	8.2E-01	-0.08	7.0E-01	-0.04	8.4E-01	0.66	2.3E-04	0.18	3.8E-01	0.06	7.7E-01
ENSCAFG0000002691	ENSCAFG0000002691	darkmagenta	EC_M12	-0.02	9.1E-01	-0.10	6.2E-01	0.13	5.3E-01	-0.05	8.2E-01	-0.05	8.2E-01	-0.08	7.0E-01	-0.04	8.4E-01	0.66	2.3E-04	0.18	3.8E-01	0.06	7.7E-01
ENSCAFG0000001960	GNG13	grey	EC_M10	-0.02	9.1E-01	-0.16	4.4E-01	0.36	6.7E-02	0.52	6.0E-03	-0.33	9.8E-02	-0.10	6.4E-01	0.24	2.3E-01	-0.09	6.7E-01	-0.12	5.5E-01	0.32	1.1E-01
ENSCAFG0000001876	SUPT6H	darkgreen	EC_M4	-0.02	9.0E-01	-0.01	9.8E-01	-0.69	9.3E-05	-0.26	2.0E-01	0.84	7.2E-08	0.21	3.1E-01	0.05	8.1E-01	0.02	9.3E-01	-0.20	3.4E-01	-0.84	9.5E-08
ENSCAFG0000000648	ILK	darkgreen	EC_M4	-0.02	9.0E-01	-0.22	2.8E-01	-0.63	5.3E-04	-0.21	3.1E-01	0.84	9.0E-08	0.23	2.6E-01	0.08	7.0E-01	0.06	7.9E-01	0.25	2.3E-01	-0.84	7.9E-08
ENSCAFG0000000923	CHD6	grey	EC_M10	-0.02	9.0E-01	0.25	2.3E-01	-0.60	1.3E-03	-0.06	7.8E-01	0.57	2.6E-03	0.16	4.5E-01	0.16	4.4E-01	0.02	9.2E-01	0.20	3.3E-01	-0.53	5.2E-03
ENSCAFG0000001249	TACC2	cyan	EC_M2	-0.02	9.0E-01	0.31	1.2E-01	0.56	3.0E-03	0.03	8.7E-01	-0.74	1.4E-05	-0.29	1.5E-01	-0.02	9.3E-01	0.00	1.0E+00	-0.29	1.5E-01	0.77	4.7E-06
ENSCAFG0000002060	ENSCAFG0000002060	grey	EC_M10	-0.02	9.0E-01	0.16	4.5E-01	0.22	2.9E-01	-0.17	4.0E-01	-0.32	1.1E-01	-0.18	3.8E-01	0.25	2.2E-01	0.22	2.7E-01	0.18	3.7E-01	0.39	5.1E-02
ENSCAFG0000001130	GGPS1	grey	EC_M10	-0.02	9.0E-01	0.41	3.6E-02	0.03	9.0E-01	0.44	2.5E-02	-0.19	3.6E-01	0.00	1.0E+00	-0.28	1.6E-01	-0.22	2.9E-01	-0.09	6.7E-01	0.24	2.5E-01
ENSCAFG0000000958	ENSCAFG0000000958	grey	EC_M10	-0.02	9.0E-01	-0.13	5.3E-01	0.22	2.9E-01	-0.14	5.1E-01	-0.13	5.3E-01	-0.08	7.0E-01	-0.07	7.4E-01	0.01	9.7E-01	-0.11	6.0E-01	0.17	4.0E-01
ENSCAFG0000000235	ENSCAFG0000000235	grey	EC_M10	-0.02	9.0E-01	-0.08	6.9E-01	0.08	7.1E-01	0.04	8.5E-01	-0.08	7.1E-01	-0.09	6.7E-01	-0.15	4.6E-01	-0.10	6.3E-01	-0.19	3.4E-01	0.03	9.0E-01
ENSCAFG0000000278	MEI4	grey	EC_M10	-0.03	9.0E-01	0.01	9.5E-01	-0.20	3.3E-01	-0.15	4.5E-01	0.28	1.6E-01	0.26	2.0E-01	0.04	8.3E-01	0.42	3.2E-02	-0.32	1.1E-01	-0.25	2.3E-01
ENSCAFG0000001973	PIK3CD	grey	EC_M10	-0.03	9.0E-01	-0.66	2.7E-04	0.27	1.8E-01	0.17	4.2E-01	-0.10	6.4E-01	0.05	8.2E-01	0.19	3.6E-01	-0.25	2.2E-01	-0.15	4.5E-01	0.13	5.3E-01
ENSCAFG0000000750	SBF2	cyan	EC_M2	-0.03	9.0E-01	0.07	7.2E-01	0.58	1.7E-03	0.16	4.5E-01	-0.74	1.3E-05	-0.31	1.2E-01	0.11	5.9E-01	0.06	7.7E-01	-0.19	3.5E-01	0.77	4.5E-06
ENSCAFG0000001031	ENSCAFG0000001031	grey	EC_M10	-0.03	9.0E-01	-0.48	1.3E-02	0.26	1.9E-01	-0.29	1.5E-01	-0.07	7.4E-01	-0.27	1.8E-01	-0.10	6.3E-01	0.37	6.6E-02	-0.02	9.1E-01	0.09	6.5E-01

ENSCAFG0000000270	COL12A1	grey	EC_M10	-0.03	9.0E-01	0.07	7.4E-01	-0.61	9.6E-04	-0.17	4.0E-01	0.71	5.0E-05	0.20	3.2E-01	0.32	1.1E-01	-0.16	4.3E-01	-0.23	2.6E-01	-0.69	1.1E-04
ENSCAFG0000001415	ATP13A1	grey	EC_M10	-0.03	9.0E-01	-0.10	6.3E-01	-0.36	7.1E-02	-0.24	2.3E-01	0.61	8.8E-04	0.18	3.7E-01	-0.10	6.3E-01	0.08	7.1E-01	-0.09	6.7E-01	-0.58	2.0E-03
ENSCAFG0000000432	APMAP	turquoise	EC_M6	-0.03	9.0E-01	0.49	1.1E-02	-0.13	5.2E-01	-0.09	6.5E-01	0.07	7.4E-01	-0.29	1.5E-01	0.28	1.7E-01	0.17	4.1E-01	0.05	8.2E-01	0.03	8.9E-01
ENSCAFG0000000429	SAP130	turquoise	EC_M6	-0.03	9.0E-01	0.29	1.5E-01	0.12	5.5E-01	-0.14	5.1E-01	-0.25	2.2E-01	-0.31	1.3E-01	-0.29	1.5E-01	0.32	1.1E-01	-0.27	1.8E-01	0.18	3.9E-01
ENSCAFG0000000729	TCP11L1	grey	EC_M10	-0.03	9.0E-01	0.10	6.1E-01	-0.42	3.2E-02	-0.07	7.3E-01	0.44	2.6E-02	-0.14	4.9E-01	0.05	8.1E-01	-0.05	8.0E-01	-0.32	1.1E-01	-0.45	2.2E-02
ENSCAFG0000002468	MUM1L1	grey	EC_M10	-0.03	9.0E-01	0.01	9.6E-01	-0.47	1.4E-02	0.02	9.1E-01	0.57	2.2E-03	0.40	4.1E-02	0.00	9.9E-01	0.24	2.3E-01	-0.09	6.7E-01	-0.60	1.1E-03
ENSCAFG0000000045	VARS2	grey	EC_M10	-0.03	9.0E-01	-0.11	6.1E-01	0.15	4.6E-01	-0.16	4.4E-01	-0.08	7.1E-01	-0.05	8.1E-01	-0.15	4.6E-01	0.42	3.3E-02	0.44	2.4E-02	0.09	6.7E-01
ENSCAFG0000001639	PLEK2	magenta	EC_M13	-0.03	9.0E-01	-0.12	5.7E-01	0.19	3.6E-01	0.18	3.8E-01	-0.16	4.4E-01	0.03	8.7E-01	0.01	9.5E-01	0.06	7.6E-01	0.69	8.2E-05	0.20	3.4E-01
ENSCAFG0000000765	ST3GAL5	turquoise	EC_M6	-0.03	9.0E-01	0.68	1.3E-04	-0.36	7.4E-02	-0.18	3.7E-01	0.22	2.8E-01	-0.19	3.4E-01	-0.11	5.8E-01	0.12	5.8E-01	0.03	9.0E-01	-0.18	3.9E-01
ENSCAFG0000000934	SLC40A1	grey	EC_M10	-0.03	9.0E-01	-0.18	3.7E-01	0.57	2.3E-03	0.17	4.2E-01	-0.60	1.3E-03	-0.26	2.0E-01	-0.01	9.7E-01	0.26	2.0E-01	0.54	4.1E-03	0.65	3.5E-04
ENSCAFG0000001175	TRAPPC2	grey	EC_M10	-0.03	9.0E-01	-0.12	5.5E-01	-0.50	8.7E-03	-0.07	7.3E-01	0.65	3.3E-04	0.28	1.6E-01	0.22	2.9E-01	-0.07	7.2E-01	0.08	6.9E-01	-0.68	1.2E-04
ENSCAFG0000000585	P4HB	grey	EC_M10	-0.03	9.0E-01	-0.33	1.0E-01	-0.56	3.1E-03	-0.01	9.5E-01	0.80	1.0E-06	0.22	2.7E-01	0.06	7.6E-01	0.27	1.8E-01	0.16	4.4E-01	-0.81	5.3E-07
ENSCAFG0000001190	ENSCAFG0000001190	grey	EC_M10	-0.03	9.0E-01	0.21	3.1E-01	0.08	7.2E-01	-0.07	7.2E-01	-0.18	3.8E-01	0.19	3.5E-01	0.06	7.6E-01	-0.18	3.7E-01	-0.07	7.4E-01	0.19	3.7E-01
ENSCAFG0000001979	ALX3	paleturquoise	EC_M11	-0.03	9.0E-01	-0.11	6.0E-01	0.29	1.5E-01	0.82	2.6E-07	-0.29	1.5E-01	-0.09	6.8E-01	-0.08	7.0E-01	-0.04	8.3E-01	-0.11	6.0E-01	0.29	1.5E-01
ENSCAFG0000001889	TXNDC11	grey	EC_M10	-0.03	9.0E-01	0.45	2.1E-02	-0.57	2.4E-03	0.09	6.7E-01	0.52	6.5E-03	0.07	7.3E-01	0.11	5.8E-01	0.00	1.0E+00	-0.15	4.8E-01	-0.52	6.9E-03
ENSCAFG0000001640	CALU	darkgreen	EC_M4	-0.03	9.0E-01	-0.18	3.8E-01	-0.70	6.6E-05	-0.10	6.4E-01	0.89	1.1E-09	0.30	1.4E-01	0.30	1.4E-01	0.14	5.0E-01	-0.05	8.1E-01	-0.89	8.3E-10
ENSCAFG0000001034	FAN1	grey	EC_M10	-0.03	9.0E-01	-0.08	6.8E-01	0.34	9.0E-02	0.27	1.8E-01	-0.40	4.3E-02	-0.33	1.0E-01	-0.23	2.6E-01	0.01	9.6E-01	0.17	4.1E-01	0.45	2.0E-02
ENSCAFG0000001286	ENSCAFG0000001286	grey	EC_M10	-0.03	9.0E-01	-0.53	5.4E-03	0.07	7.5E-01	-0.06	7.5E-01	0.18	3.8E-01	-0.06	7.6E-01	-0.24	2.4E-01	0.17	4.0E-01	0.03	8.7E-01	-0.15	4.5E-01
ENSCAFG0000000723	ATP6V0A2	grey	EC_M10	-0.03	9.0E-01	-0.25	2.2E-01	-0.43	3.0E-02	-0.21	3.0E-01	0.64	4.1E-04	0.16	4.2E-01	0.06	7.7E-01	-0.03	8.8E-01	0.05	8.2E-01	-0.61	9.4E-04
ENSCAFG0000002864	STAMBP	grey	EC_M10	-0.03	9.0E-01	-0.27	1.8E-01	-0.03	9.0E-01	0.13	5.2E-01	0.06	7.9E-01	-0.04	8.4E-01	0.18	3.9E-01	0.07	7.2E-01	-0.38	5.5E-02	-0.08	7.1E-01
ENSCAFG0000002994	UBE2G1	turquoise	EC_M6	-0.03	9.0E-01	0.71	5.1E-05	-0.36	6.8E-02	-0.31	1.2E-01	0.18	3.8E-01	0.15	4.7E-01	-0.16	4.5E-01	0.07	7.4E-01	0.21	2.9E-01	-0.13	5.2E-01
ENSCAFG0000001115	CFAP46	grey	EC_M10	-0.03	9.0E-01	0.14	4.9E-01	-0.66	2.3E-04	-0.28	1.7E-01	0.73	1.9E-05	0.15	4.6E-01	0.04	8.6E-01	0.30	1.3E-01	0.33	1.0E-01	-0.71	4.8E-05
ENSCAFG0000001544	DMXL2	grey	EC_M10	-0.03	9.0E-01	-0.24	2.3E-01	-0.01	9.5E-01	-0.13	5.1E-01	0.08	6.8E-01	-0.17	4.0E-01	0.16	4.3E-01	-0.20	3.3E-01	-0.22	2.8E-01	-0.04	8.3E-01
ENSCAFG0000000621	ERLIN2	grey	EC_M10	-0.03	9.0E-01	-0.31	1.2E-01	0.04	8.6E-01	0.01	9.6E-01	0.02	9.2E-01	0.04	8.3E-01	0.24	2.4E-01	-0.06	7.6E-01	-0.07	7.4E-01	0.01	9.7E-01
ENSCAFG0000002508	FADS1	grey	EC_M10	-0.03	9.0E-01	-0.33	9.8E-02	-0.27	1.9E-01	-0.03	9.0E-01	0.44	2.6E-02	0.08	6.9E-01	-0.12	5.5E-01	-0.07	7.3E-01	-0.38	5.6E-02	-0.49	1.2E-02
ENSCAFG0000000505	RNF157	grey	EC_M10	-0.03	9.0E-01	-0.03	9.0E-01	0.60	1.1E-03	-0.03	8.9E-01	-0.66	2.2E-04	-0.14	4.8E-01	0.36	7.4E-02	-0.27	1.8E-01	-0.12	5.7E-01	0.71	4.7E-05
ENSCAFG0000000789	EYA1	grey	EC_M10	-0.03	9.0E-01	-0.05	8.1E-01	0.12	5.7E-01	-0.11	6.0E-01	-0.02	9.2E-01	-0.08	7.1E-01	0.19	3.5E-01	-0.06	7.6E-01	-0.17	4.1E-01	0.03	8.9E-01
ENSCAFG0000000052	SNCAIP	turquoise	EC_M6	-0.03	9.0E-01	0.83	1.3E-07	-0.33	9.8E-02	-0.22	2.8E-01	0.13	5.1E-01	-0.01	9.5E-01	0.01	9.8E-01	-0.02	9.2E-01	0.15	4.7E-01	-0.12	5.6E-01
ENSCAFG0000000382	ZXDC	grey	EC_M10	-0.03	9.0E-01	-0.14	5.0E-01	0.40	4.3E-02	-0.27	1.8E-01	-0.34	9.3E-02	0.03	8.9E-01	-0.08	7.1E-01	-0.21	3.0E-01	0.01	9.4E-01	0.37	6.2E-02
ENSCAFG0000002946	OSGIN2	turquoise	EC_M6	-0.03	9.0E-01	0.79	1.8E-06	-0.18	3.8E-01	-0.14	4.8E-01	-0.05	8.1E-01	-0.19	3.6E-01	0.10	6.2E-01	-0.10	6.1E-01	0.55	4.0E-03	0.10	6.1E-01
ENSCAFG0000002860	ENSCAFG0000002860	grey	EC_M10	-0.03	9.0E-01	0.03	9.0E-01	-0.02	9.4E-01	0.19	3.5E-01	0.02	9.2E-01	-0.11	5.8E-01	0.21	3.0E-01	0.20	3.3E-01	0.15	4.6E-01	-0.04	8.6E-01
ENSCAFG0000001714	VASH1	turquoise	EC_M6	-0.03	9.0E-01	0.87	8.9E-09	-0.37	6.2E-02	-0.04	8.6E-01	0.16	4.2E-01	-0.17	4.0E-01	-0.01	9.6E-01	0.08	6.9E-01	0.14	4.8E-01	-0.15	4.7E-01
ENSCAFG0000001506	KIFAP3	grey	EC_M10	-0.03	9.0E-01	-0.40	4.2E-02	-0.56	3.2E-03	-0.12	5.6E-01	0.77	4.0E-06	0.30	1.4E-01	-0.04	8.4E-01	0.14	4.8E-01	-0.11	6.1E-01	-0.79	1.6E-06
ENSCAFG0000003164	FBXL7	grey	EC_M10	-0.03	9.0E-01	-0.25	2.2E-01	0.26	1.9E-01	-0.09	6.8E-01	-0.13	5.1E-01	0.08	7.0E-01	0.06	7.5E-01	-0.09	6.6E-01	-0.58	2.1E-03	0.13	5.3E-01
ENSCAFG0000000218	ENSCAFG0000000218	grey	EC_M10	-0.03	9.0E-01	-0.03	8.7E-01	0.06	7.6E-01	-0.18	3.9E-01	-0.07	7.2E-01	-0.13	5.4E-01	-0.06	7.6E-01	-0.12	5.7E-01	0.37	6.7E-02	0.10	6.4E-01
ENSCAFG0000000135	ENSCAFG0000000135	grey	EC_M10	-0.03	9.0E-01	0.01	9.7E-01	-0.07	7.4E-01	-0.52	6.4E-03	0.03	8.7E-01	-0.24	2.3E-01	-0.14	4.9E-01	0.29	1.5E-01	0.12	5.7E-01	-0.01	9.5E-01
ENSCAFG0000001345	ENSCAFG0000001345	grey	EC_M10	-0.03	9.0E-01	0.46	1.7E-02	0.30	1.4E-01	-0.21	3.1E-01	-0.48	1.3E-02	-0.17	4.1E-01	-0.17	4.1E-01	-0.25	2.1E-01	0.01	9.5E-01	0.54	4.2E-03
ENSCAFG0000001862	DAB2	grey	EC_M10	-0.03	9.0E-01	0.25	2.1E-01	-0.45	2.3E-02	-0.23	2.5E-01	0.46	1.8E-02	0.07	7.2E-01	-0.16	4.3E-01	0.07	7.2E-01	-0.03	8.9E-01	-0.45	2.1E-02
ENSCAFG0000003086	HPCAL4	magenta	EC_M13	-0.03	9.0E-01	0.20	3.3E-01	-0.20	3.2E-01	-0.01	9.6E-01	0.16	4.2E-01	0.00	9.9E-01	0.05	7.9E-01	0.15	4.6E-01	0.77	3.3E-06	-0.12	5.6E-01
ENSCAFG0000003011	ZNF599	grey	EC_M10	-0.03	9.0E-01	0.29	1.5E-01	0.33	9.7E-02	0.13	5.3E-01	-0.53	4.9E-03	-0.09	6.5E-01	-0.19	3.5E-01	0.21	3.0E-01	-0.07	7.2E-01	0.55	3.5E-03
ENSCAFG0000001828	FLII	darkgreen	EC_M4	-0.03	9.0E-01	0.37	6.7E-02	-0.57	2.6E-03	-0.24	2.3E-01	0.61	8.7E-04	-0.09	6.7E-01	0.03	8.8E-01	0.07	7.5E-01	0.03	8.8E-01	-0.58	1.9E-03
ENSCAFG0000000016	ELAC1	grey	EC_M10	-0.03	9.0E-01	-0.28	1.6E-01	0.66	2.4E-04	-0.06	7.8E-01	-0.60	1.1E-03	-0.08	6.9E-01	0.00	1.0E+00	-0.16	4.2E-01	-0.20	3.2E-01	0.62	7.0E-04
ENSCAFG0000001748	CYFIP2	grey	EC_M10	-0.03	8.9E-01	-0.30	1.4E-01	-0.31	1.2E-01	-0.24	2.3E-01	0.58	2.0E-03	0.15	4.8E-01	0.21	3.0E-01	-0.16	4.3E-01	0.18	3.7E-01	-0.52	6.4E-03
ENSCAFG0000000905	THAP9	grey	EC_M10	-0.03	8.9E-01	0.12	5.5E-01	-0.44	2.6E-02	0.11	5.9E-01	0.51	7.1E-03	0.36	7.1E-02	0.03	8.8E-01	0.00	9.8E-01	-0.30	1.3E-01	-0.49	1.1E-02
ENSCAFG0000001418	ACSBG1	grey	EC_M10	-0.03	8.9E-01	0.26	2.0E-01	0.34	9.4E-02	-0.12	5.7E-01	-0.46	1.8E-02	-0.05	8.3E-01	-0.13	5.4E-01	-0.06	7.6E-01	-0.17	4.2E-01	0.48	1.2E-02
ENSCAFG0000002955	CSKMT	grey	EC_M10	-0.03	8.9E-01	0.02	9.1E-01	0.37	5.9E-02	0.27	1.8E-01	-0.36	7.0E-02	-0.18	3.7E-01	-0.32	1.1E-01	-0.09	6.8E-01	-0.01	9.6E-01	0.41	3.9E-02
ENSCAFG0000003247	ENSCAFG0000003247	turquoise	EC_M6	-0.03	8.9E-01	0.57	2.2E-03	-0.37	6.6E-02	-0.08	7.1E-01	0.27	1.9E-01	0.02	9.2E-01	0.02	9.2E-01	-0.36	6.8E-02	-0.05	7.9E-01	-0.26	2.0E-01
ENSCAFG0000001199	RAB11FIP2	grey	EC_M10	-0.03	8.9E-01	-0.76	7.1E-06	0.44	2.4E-02	0.15	4.7E-01	-0.29	1.5E-01	0.16	4.3E-01	0.06	7.8E-01	-0.03	8.7E-01	-0.06	7.7E-01	0.29	1.6E-01
ENSCAFG0000001593	AHCTF1	grey	EC_M10	-0.03	8.9E-01	-0.10	6.1E-01	0.57	2.5E-03	0.00	1.0E+00	-0.61	9.7E-04	-0.16	4.5E-01	0.09	6.5E-01	-0.11	5.9E-01	-0.04	8.5E-01	0.63	5.0E-04
ENSCAFG0000000242	TMOD1	turquoise	EC_M6	-0.03	8.9E-01	0.69	8.2E-05	-0.25	2.3E-01	-0.13	5.4E-01	0.05	8.3E-01	-0.12	5.6E-01	0.15	4.6E-01	0.16	4.5E-01	0.68	1.4E-04	0.01	9.6E-01
ENSCAFG0000002978	C21H11orf97	grey	EC_M10	-0.03	8.9E-01	-0.13	5.3E-01	0.12	5.4E-01	-0.01	9.7E-01	-0.04	8.4E-01	-0.06	7.7E-01	0.01	9.7E-01	-0.02	9.2E-01	0.01	9.6E-01	0.04	8.4E-01
ENSCAFG0000000662	HOXC6	grey	EC_M10	-0.03	8.9E-01	-0.33	9.8E-02	0.08	6.9E-01	0.03	8.8E-01	0.03	9.0E-01	-0.01	9.8E-01	0.18	3.8E-01	-0.35	8.3E-02	-0.15	4.7E-01	-0.02	9.3E-01
ENSCAFG0000001960	SOX8	grey	EC_M10	-0.03	8.9E-01	-0.24	2.4E-01	0.12	5.7E-01	0.42	3.1E-02	0.00	9.8E-01	-0.15	4.6E-01	0.19	3.5E-01	-0.09	6.5E-01	-0.19	3.6E-01	0.01	9.4E-01
ENSCAFG0000003080	ENSCAFG0000003080	grey	EC_M10	-0.03	8.9E-01	-0.24	2.4E-01	0.46	1.9E-02	0.31	1.3E-01	-0.49	1.1E-02	-0.13	5.3E-01	-0.40	4.3E-02	-0.09	6.8E-01	-0.04	8.4E-01	0.49	1.1E-02
ENSCAFG0000000139	DDX17	cyan	EC_M2	-0.03	8.9E-01	0.46	1.7E-02	0.37	5.9E-02	0.17	4.1E-01	-0.68	1.3E-04	-0.30	1.4E-01	0.01	9.6E-01	-0.11	6.0E-01	0.11	6.0E-01	0.69	1.1E-04
ENSCAFG0000001293	MAP3K3	turquoise	EC_M6	-0.03	8.9E-01	0.81	6.7E-07	-0.08	7.0E-01	-0.04	8.3E-01	-0.16	4.4E-01	-0.32	1.1E-01	-0.16	4.5E-01	-0.10	6.2E-01	0.09	6.7E-01	0.21	3.1E-01
ENSCAFG0000001502	ENSCAFG0000001502	grey	EC_M10	-0.03	8.9E-01	0.03	9.0E-01	-0.17	4.1E-01	0.20	3.2E-01	0.18	3.9E-01	0.32	1.1E-01	-0.12	5.7E-01	-0.56	3.1E-03	0.09	6.5E-01	-0.15	4.6E-01
ENSCAFG0000001036	ALDH5A1	cyan	EC_M2	-0.03	8.9E-01	-0.05	8.0E-01	0.64	4.0E-04	-0.08	7.0E-01	-0.69	1.1E-04	-0.02	9.4E-01	0.02	9.4E-01	-0.17	4.1E-01	0.13	5.2E-01	0.74	1.9E-05
ENSCAFG0000001889	JUN	grey	EC_M10	-0.03	8.9E-01	0.24	2.4E-01	0.28	1.7E-01	0.14	4.9E-01	-0.44	2.4E-02	-0.22	2.7E-01	-0.12	5.6E-01	0.21	3.0E-01	0.30	1.3E-01	0.46	1.8E-02
ENSCAFG0000001895	CDH6	grey	EC_M10	-0.03	8.9E-01	-0.36	7.5E-02	-0.17	4.1E-01	-0.05	8.0E-01	0.32	1.1E-01	-0.09	6.6E-01	-0.24	2.3E-01	0.21	3.0E-01	0.49	1.2E-02	-0.30	1.4E-01
ENSCAFG0000003143	ENSCAFG0000003143	grey	EC_M10	-0.03	8.9E-01	0.06	7.9E-01	0.31	1.2E-01	0.16	4.3E-01	-0.40	4.1E-02	-0.11	5.9E-01	-0.15	4.6E-01	0.08	7.0E-01	0.12	5.6E-01	0.44	2.3E-02
ENSCAFG0000001300	C28H10orf90	grey	EC_M10	-0.03	8.9E-01	-0.16	4.4E-01	-0.57	2.4E-03	-0.21	3.1E-01	0.73	2.7E-05	0.16	4.3E-01	-0.10	6.3E-01	0.08	7.0E-01	-0.04	8.3E-01	-0.75	1.3E-05
ENSCAFG0000000649	PKP3	grey	EC_M10	-0.03	8.9E-01	-0.16	4.3E-01	0.33	1.0E-01	-0.02	9.4E-01	-0.30	1.4E-01	-0.23	2.6E-01	-0.20	3.3E-01	0.20	3.2E-01	0.45	2.2E-02	0.30	1.4E-01
ENSCAFG0000000395	PRKAR2B	grey	EC_M10	-0.03	8.9E-01	-0.48	1.3E-02	0.13	5.2E-01	-0.01	9.8E-01	0.00	9.9E-01	-0.07	7.5E-01	0.47	1.5E-02	0.19	3.6E-01	0.41	4.0E-02	0.00	9.9E-01
ENSCAFG0000001448	ZNF445	grey	EC_M10	-0.03	8.9E-01	0.44	2.6E-02	0.39	4.8E-02	0.06	7.8E-01	-0.60	1.3E-03	-0.14	4.9E-01	0.17	4.0E-01	0.15	4.6E-01	-0.07	7.4E-01	0.65	2.9E-04
ENSCAFG0000001345	HOXD8	grey	EC_M10	-0.03	8.9E-01	0.37	6.2E-02	0.08	6.9E-01	-0.23	2.5E-01	-0.18	3.9E-01	-0.32	1.1E-01	0.17	4.0E-01	-0.29	1.4E-01	-0.22	2.8E-01	0.25	2.2E-01
ENSCAFG0000000760	MRVI1	darkgreen	EC_M4	-0.03	8.9E-01	-0.26	2.0E-01	-0.59	1.5E-03	-0.17	4.1E-01	0.80	7.6E-07	0.31	1.3E-01	0.17	3.9E-01	0.07	7.2E-01	0.18	3.8E-01	-0.76	5.4E-06
ENSCAFG0000002958	EIF1	darkgreen	EC_M4	-0.03	8.9E-01	-0.06	7.7E-01	-0.65	3.2E-04	-0.09	6.7E-01	0.80	1.2E-06	0.30	1.4E-01	0.07	7.5E-01	0.01	9.5E-01	-0.33	9.5E-02	-0.82	3.2E-07
ENSCAFG0000000643	RIC8A	turquoise	EC_M6	-0.03	8.9E-01	0.47	1.5E-02	0.10	6.4E-01	0.02	9.3E-01	-0.16	4.4E-01	-0.15	4.6E-01	-0.14	4.8E-01	-0.32	1.1E-01	0.11	5.9E-01	0.20	3.4E-01
ENSCAFG0000002922	ENSCAFG0000002922	grey	EC_M10	-0.03	8.9E-01	-0.12	5.7E-01	0.43	2.8E-02	0.37	6.4E-02	-0.49	1.1E-02	-0.15	4.7E-01	-0.15	4.7E-01	-0.14	5.0E-01	0.15	4.8E-01	0.48	1.2E-02
ENSCAFG0000001384	NCAM1	grey	EC_M10	-0.03	8.9E-01	-0.33	1.0E-01	-0.41	3.9E-02	-0.16	4.3E-01	0.63	5.0E-04	0.19	3.4E-01	0.21	3.1E-01	0.09	6.7E-01	-0.46	1.9E-02	-0.63	5.4E-04
ENSCAFG0000000833	ENSCAFG0000000833	grey	EC_M10	-0.03	8.9E-01	0.05	7.9E-01	0.06	7.8E-01	-0.02	9.3E-01	0.02	9.3E-01	-0.07	7.4E-01	-0.13	5.2E-01	-0.10	6.3E-01	-0.09	6.5E-01	0.03	8.7E-01
ENSCAFG0000001713	TENM2	grey	EC_M10	-0.03	8.9E-01	0.03	9.0E-01	-0.69	9.8E-05	-0.03	8.7E-01	0.79	1.9E-06	0.21	3.0E-01	0.04	8.4E-01	-0.03	8.8E-01	-0.18	3.8E-01	-0.79	2.0E-06
ENSCAFG0000000996	ADAMTS15	magenta	EC_M13	-0.03	8.9E-01	-0.20	3.2E-01	0.22	2.9E-01	0.05	8.1E-01	-0.15	4.7E-01	-0.17	4.2E-01	-0.04	8.3E-01	0.45	2.1E-02	0.55	4.0E-03	0.19	3.6E-01
ENSCAFG0000000141	CSNK1E	turquoise	EC_M6	-0.03	8.9E-01	0.73	2.7E-05	0.11	5.9E-01	0.03	8.8E-01	-0.34	8.5E-02	-0.18	3.7E-01	0.17	4.2E-01	0.14	5.1E-01	0.07	7.3E-01	0.36	7.1E-02
ENSCAFG0000001495	ENSCAFG0000001495	grey	EC_M10	-0.03	8.9E-01	-0.12	5.5E-01	0.28	1.6E-01	0.00	1.0E+00	-0.26	1.9E-01	-0.01	9.7E-01	-0.13	5.3E-01	-0.58	1.7E-03	-0.22	2.8E-01	0.24	2.5E-01
ENSCAFG0000002537	AHRR	cyan	EC_M2	-0.03	8.9E-01	0.06	7.8E-01	0.67	1.8E-04	0.16	4.4E-01	-0.75	1.1E-05	-0.19	3.5E-01	-0.05	8.0E-01	-0.12	5.6E-01	-0.06	7.6E-01	0.80	7.9E-07
ENSCAFG0000000435	CCDC61	grey	EC_M10	-0.03	8.9E-01	0.33	1.0E-01	0.50	9.6E-03	-0.01	9.7E-01	-0.67	2.1E-04	-0.24	2.4E-01	-0.06	7.5E-01	0.12	5.7E-01	0.13	5.2E-01	0.66	2.4E-04
ENSCAFG0000001770	ARMCX3	darkgreen	EC_M4	-0.03	8.9E-01	-0.08	6.8E-01	-0.73	2.3E-05	-0.04	8.4E-01	0.84	6.4E-08	0.25	2.2E-01	-0.01	9.8E-01	0.12	5.6E-01	-0.06	7.6E-01	-0.86	1.8E-08
ENSCAFG0000000612	SEMA3D	grey	EC_M10	-0.03	8.9E-01	-0.04	8.5E-01	-0.06	7.6E-01	0.25	2.3E-01	0.13	5.2E-01	0.19	3.6E-01	-0.37	6.6E-02	-0.14	4.8E-01	-0.63	5.4E-04	-0.14	5.0E-01
ENSCAFG0000000954	SLC5A3	grey	EC_M10	-0.03	8.9E-01	0.37	5.9E-02	0.04	8.6E-01	-0.16	4.3E-01	-0.20	3.2E-01	-0.12	5.5E-01	0.50	9.3E-03	-0.05	8.2E-01	-0.05	8.2E-01	0.22	2.8E-01
ENSCAFG0000001092	PKNOX2	grey	EC_M10	-0.03	8.8E-01	0.05	8.2E-01	-0.16	4.4E-01	0.03	9.0E-01	0.13	5.2E-01	0.13	5.3E-01	-0.06	7.8E-01	0.14	4.9E-01	0.73	2.1E-05	-0.11	5.8E-01
ENSCAFG0000000223	ENSCAFG0000000223	grey	EC_M10	-0.03	8.8E-01	-0.31	1.2E-01	-0.10	6.3E-01	0.02	9.2E-01	0.19	3.6E-01	0.11	5.8E-01	0.16	4.2E-01	0.33	1.0E-01	0.57	2.5E-03	-0.20	3.2E-01
ENSCAFG0000000753	ANKRD27	grey	EC_M10	-0.03	8.8E-01	0.23	2.6E-01	0.34	8.5E-02	-0.06	7.8E-01	-0.43	2.9E-02	-0.11	5.8E-01	0.06	7.8E-01	-0.57	2.6E-03	-0.21	3.0E-01	0.47	1.7E-02
ENSCAFG0000003110	ENSCAFG0000003110	paleturquoise	EC_M11	-0.03	8.8E-01	-0.04	8.5E-01	0.08	7.0E-01	0.77	3.6E-06	-0.09	6.6E-01	-0.05	8.0E-01	-0.01	9.5E-01	-0.09	6.5E-01	-0.09	6.8E-01	0.08	7.1E-01
ENSCAFG0000000186	ENSCAFG0000000186	violet	EC_M7	-0.03	8.8E-01	-0.10	6.3E-01	-0.31	1.2E-01	-0.03	8.9E-01	0.43	2.7E-02	0.81	7.0E-07	-0.07	7.3E-01	-0.09	6.6E-01	-0.09	6.6E-01	-0.40	4.1E-02
ENSCAFG0000000015	TAC3	grey	EC_M10	-0.03	8.8E-01	-0.16	4.3E-01	0.06	7.8E-01	0.49	1.1E-02	-0.03	8.7E-01	-0.07	7.5E-01	-0.17	4.1E-01	-0.10	6.4E-01	-0.13	5.3E-01	0.03	9.0E-01
ENSCAFG0000001236	ENSCAFG0000001236	cyan	EC_M2	-0.03	8.8E-01	0.08	6.9E-01	0.71	4.7E-05	0.10	6.2E-01	-0.79	1.4E-06	-0.24	2.3E-01	0.14	5.1E-01	-0.13	5.3E-01	-0.24	2.4E-01	0.82	3.1E-07
ENSCAFG0000001319	NSUN5	grey	EC_M10	-0.03	8.8E-01	-0.11	6.1E-01	-0.01	9.4E-01	0.15	4.6E-01	0.09	6.5E-01	0.31	1.2E-01	0.21	3.1E-01	-0.05	8.0E-01	0.12	5.5E-01	-0.13	5.3E-01
ENSCAFG0000001169	SEZ6L	grey	EC_M10	-0.03	8.8E-01	-0.28	1.7E-01	0.47	1.6E-02	-0.13	5.3E-01	-0.43	2.9E-02	-0.06	7.6E-01	0.03	8.8E-01	-0.17	4.0E-01	0.03	8.8E-01	0.51	7.8E-03
ENSCAFG0000001004	ZNF697	darkgreen	EC_M4	-0.03	8.8E-01	0.28	1.6E-01	-0.68	1.2E-04	-0.07	7.5E-01	0.70	6.5E-05	0.09	6.6E-01	0.14	5.1E-01	-0.07	7.2E-01	-0.08	7.0E-01	-0.72	3.4E-05
ENSCAFG0000001079	SLC12A7	turquoise	EC_M6	-0.03	8.8E-01	0.89	1.8E-09	-0.35	8.4E-02	-0.12	5.5E-01	0.14	4.8E-01	-0.06	7.7E-01	-0.07	7.5E-01	-0.04	8.3E-01	0.14	5.0E-01	-0.11	5.9E-01
ENSCAFG0000001653	ADGRA3	grey	EC_M10	-0.03	8.8E-01	-0.12	5.7E-01	-0.56	2.8E-03	0.00	9.9E-01	0.76	5.3E-06	0.12	5.7E-01	0.05	8.0E-01	0.10	6.3E-01	0.03	8.9E-01	-0.73	2.6E-05
ENSCAFG0000001354	MYPN	grey	EC_M10	-0.03	8.8E-01	-0.23	2.5E-01	-0.07	7.3E-01	-0.17	4.2E-01	0.21	2.9E-01	0.13	5.3E-01	-0.21	3.0E-01	-0.10	6.2E-01	-0.19	3.5E-01	-0.21	3.0E-01
ENSCAFG0000000102	CDKN2AIPNL	grey	EC_M10	-0.03	8.8E-01	0.15	4.8E-01	0.21	3.1E-01	0.08	7.0E-01	-0.18	3.8E-01	-0.01	9.5E-01	-0.10	6.4E-01	0.28	1.6E-01	-0.21	3.0E-01	0.21	3.1E-01
ENSCAFG0000001327	ENSCAFG0000001327	grey	EC_M10	-0.03	8.8E-01	0.11	6.1E-01	-0.37	6.0E-02	0.07	7.2E-01	0.42	3.2E-02	0.38	5.9E-02	0.27	1.8E-01	-0.06	7.8E-01	0.15	4.7E-01	-0.38	5.6E-02
ENSCAFG0000001129	PSMB5	grey	EC_M10	-0.03	8.8E-01	0.01	9.8E-01	-0.44	2.4E-02	-0.21	3.1E-01	0.62	6.4E-04	0.18	3.9E-01	-0.03	9.0E-01	-0.01	9.6E-01	0.09	6.6E-01	-0.61	1.0E-03
ENSCAFG0000002814	ENSCAFG0000002814	grey	EC_M10	-0.03	8.8E-01	0.10	6.3E-01	0.14	5.0E-01	-0.04	8.6E-01	-0.14	4.8E-01	0.03	8.9E-01	-0.01	9.5E-01	-0.05	7.9E-01	-0.08	6.8E-01	0.11	6.1E-01
ENSCAFG0000002928	ENSCAFG0000002928	grey	EC_M10	-0.03	8.8E-01	0.39	4.8E-02	-0.37	5.9E-02	-0.09	6.7E-01	0.33	9.8E-02	0.25	2.1E-01	-0.12	5.7E-01	0.30	1.4E-01	0.06	7.6E-01	-0.31	1.3E-01
ENSCAFG0000000652	LRRC56	grey	EC_M10	-0.03	8.8E-01	-0.19	3.6E-01	0.46	1.8E-02	-0.20	3.3E-01	-0.44	2.6E-02	0.17	4.1E-01	0.13	5.2E-01	0.18	3.8E-01	0.40	4.2E-02	0.45	2.0E-02
ENSCAFG0000003041	STX3	turquoise	EC_M6	-0.03	8.8E-01	0.72	3.2E-05	0.02	9.3E-01	0.03	8.7E-01	-0.32	1.1E-01	-0.21	3.1E-01	0.23	2.5E-01	0.08	7.1E-01	0.18	3.8E-01	0.32	1.1E-01
ENSCAFG0000000247	CORO2A	grey	EC_M10	-0.03	8.8E-01	-0.09	6.5E-01	0.53	5.9E-03	0.21	3.1E-01	-0.54	4.6E-03	-0.51	7.9E-03	0.19	3.5E-01	0.33	9.8E-02	-0.05	8.2E-01	0.59	1.6E-03
ENSCAFG0000000033	AVPR1A	grey	EC_M10	-0.03	8.8E-01	-0.26	2.1E-01	0.23	2.6E-01	-0.15	4.6E-01	-0.08	6.8E-01	-0.10	6.1E-01	0.27	1.8E-01	-0.26	2.0E-01	-0.44	2.6E-02	0.09	6.7E-01
ENSCAFG0000001378	ENSCAFG0000001378	grey	EC_M10	-0.03	8.8E-01	-0.22	2.8E-01	0.21	3.0E-01	0.24	2.3E-01	-0.14	5.1E-01	0.08	6.9E-01	0.05	8.1E-01	0.14	5.0E-01	-0.33	1.0E-01	0.12	5.6E-01
ENSCAFG0000001846	ADAP2	grey	EC_M10	-0.03	8.8E-01	0.08	6.9E-01	-0.54	4.3E-03	-0.16	4.2E-01	0.63	5.3E-04	-0.15	4.7E-01	0.16	4.3E-01	0.42	3.1E-02	-0.11	5.9E-01	-0.63	6.2E-04
ENSCAFG0000000510	MYCBP2	grey	EC_M10	-0.03	8.8E-01	-0.41	3.7E-02	0.53	4.9E-03	0.23	2.6E-01	-0.44	2.6E-02	-0.26	2.1E-01	-0.19	3.4E-01	-0.18	3.8E-01	-0.07	7.3E-01	0.49	1.0E-02
ENSCAFG0000003225	ENSCAFG0000003225	turquoise	EC_M6	-0.03	8.8E-01	0.43	3.0E-02	-0.11	6.1E-01	-0.02	9.3E-01	0.02	9.2E-01	-0.08	6.9E-01	0.04	8.6E-01	0.25	2.2E-01	0.33	1.0E-01	0.03	8.8E-01
ENSCAFG0000000400	KCNJ14	grey	EC_M10	-0.03	8.8E-01	-0.14	5.1E-01	-0.01	9.5E-01	-0.19	3.6E-01	0.12	5.6E-01	0.29	1.5E-01	-0.14	4.9E-01	-0.08	6.8E-01	-0.19	3.6E-01	-0.12	5.5E-01
ENSCAFG0000001749	ENSCAFG0000001749	grey	EC_M10	-0.03	8.8E-01	0.09	6.7E-01	-0.11	6.0E-01	-0.07	7.4E-01	0.24	2.4E-01	-0.08	6.9E-01	-0.09	6.8E-01	-0.10	6.2E-01	-0.09	6.7E-01	-0.18	3.9E-01
ENSCAFG0000001269	ANK3	cyan	EC_M2	-0.03	8.8E-01	0.01	9.7E-01	0.55	3.4E-03	0.39	4.9E-02	-0.65	3.4E-04	-0.29	1.5E-01	-0.05	8.1E-01	-0.39	5.1E-02	-0.12	5.7E-01	0.69	1.1E-04
ENSCAFG0000001572	STK32B	cyan	EC_M2	-0.03	8.8E-01	-0.39	4.8E-02	0.49	1.0E-02	0.09	6.6E-01	-0.42	3.5E-02	0.12	5.6E-01	-0.11	5.9E-01	0.06	7.8E-01	-0.30	1.4E-01	0.37	6.6E-02
ENSCAFG0000001828	UNC45B	grey	EC_M10	-0.03	8.8E-01	-0.40	4.5E-02	-0.47	1.5E-02	-0.11	5.8E-01	0.69	9.6E-05	0.18	3.8E-01	0.34	8.7E-02	0.02	9.4E-01	0.10	6.4E-01	-0.67	2.0E-04
ENSCAFG0000003258	ENSCAFG0000003258	grey	EC_M10	-0.03	8.8E-01	0.17	4.1E-01	-0.47	1.6E-02	-0.23	2.7E-01	0.45	2.0E-02	0.35	8.2E-02	0.02	9.1E-01	0.22	2.8E-01	0.12	5.6E-01	-0.49	1.1E-02
ENSCAFG0000001767	QTRT1	cyan	EC_M2	-0.03	8.8E-01	-0.36	6.7E-02	0.56	2.8E-03	0.19	3.6E-01	-0.44	2.3E-02	-0.26	2.0E-01	0.04	8.4E-01	0.11	6.0E-01	-0.08	6.9E-01	0.46	1.9E-02
ENSCAFG0000001325	NMNAT2	grey	EC_M10	-0.03	8.8E-01	-0.03	8.9E-01	-0.53	5.6E-03	-0.12	5.6E-01	0.64	4.4E-04	0.26	2.1E-01	0.07	7.5E-01	0.33	1.0E-01	0.55	3.5E-03	-0.63	5.1E-04
ENSCAFG0000003179	RAP2B	grey	EC_M10	-0.03	8.8E-01	0.50	9.2E-03	0.37	6.6E-02	-0.03	8.8E-01	-0.58	2.0E-03	-0.31	1.2E-01	-0.14	4.8E-01	0.02	9.4E-01	0.26	1.9E-01	0.61	1.0E-03

ENSCAFG0000001976	SURF2	cyan	EC_M2	-0.03	8.8E-01	-0.40	4.1E-02	0.51	8.1E-03	0.06	7.8E-01	-0.33	9.9E-02	0.13	5.3E-01	-0.18	3.9E-01	0.08	6.9E-01	-0.19	3.6E-01	0.32	1.1E-01
ENSCAFG0000000008	ENSCAFG0000000008	grey	EC_M10	-0.03	8.8E-01	-0.03	8.8E-01	-0.22	2.8E-01	0.30	1.4E-01	0.31	1.3E-01	-0.46	1.9E-02	-0.03	9.0E-01	0.15	4.7E-01	-0.02	9.3E-01	-0.26	2.0E-01
ENSCAFG0000001331	LMX1A	grey	EC_M10	-0.03	8.8E-01	-0.19	3.4E-01	0.58	1.9E-03	-0.10	6.3E-01	-0.55	3.5E-03	-0.08	7.0E-01	-0.10	6.3E-01	-0.08	6.9E-01	-0.17	4.2E-01	0.61	9.4E-04
ENSCAFG0000003134	RHOU	magenta	EC_M13	-0.03	8.8E-01	0.10	6.2E-01	0.21	3.1E-01	0.03	9.0E-01	-0.26	2.0E-01	-0.12	5.5E-01	0.02	9.3E-01	0.28	1.6E-01	0.69	8.4E-05	0.34	9.4E-02
ENSCAFG0000000087	THBS2	grey	EC_M10	-0.03	8.8E-01	-0.41	4.0E-02	-0.50	8.9E-03	0.01	9.7E-01	0.75	1.0E-05	0.27	1.8E-01	0.03	8.8E-01	-0.02	9.2E-01	-0.24	2.3E-01	-0.78	2.2E-06
ENSCAFG0000000397	PGR	grey	EC_M10	-0.03	8.8E-01	-0.54	4.8E-03	-0.04	8.3E-01	0.22	2.9E-01	0.32	1.1E-01	0.10	6.1E-01	0.02	9.4E-01	-0.14	5.0E-01	-0.46	1.9E-02	-0.33	1.0E-01
ENSCAFG0000003178	ENSCAFG0000003178	turquoise	EC_M6	-0.03	8.8E-01	0.31	1.3E-01	-0.06	7.6E-01	-0.05	8.0E-01	-0.06	7.9E-01	-0.12	5.6E-01	0.62	7.5E-04	-0.05	8.0E-01	-0.17	4.2E-01	0.08	7.1E-01
ENSCAFG0000000584	ADAM9	grey	EC_M10	-0.03	8.8E-01	-0.06	7.7E-01	-0.50	8.9E-03	0.21	3.1E-01	0.51	7.4E-03	0.14	4.8E-01	0.08	7.0E-01	0.10	6.2E-01	-0.13	5.2E-01	-0.54	4.7E-03
ENSCAFG0000000624	CHL1	paleturquoise	EC_M11	-0.03	8.7E-01	-0.08	7.1E-01	0.33	1.0E-01	0.77	3.9E-06	-0.37	6.0E-02	-0.09	6.7E-01	-0.02	9.3E-01	0.08	7.1E-01	0.08	7.1E-01	0.36	7.0E-02
ENSCAFG0000001613	KDM5C	turquoise	EC_M6	-0.03	8.7E-01	0.69	1.0E-04	-0.32	1.1E-01	0.14	4.8E-01	0.13	5.3E-01	-0.07	7.3E-01	0.07	7.2E-01	0.01	9.6E-01	-0.04	8.4E-01	-0.12	5.7E-01
ENSCAFG0000000069	ENSCAFG0000000069	grey	EC_M10	-0.03	8.7E-01	-0.01	9.8E-01	0.06	7.6E-01	-0.14	5.1E-01	-0.01	9.7E-01	-0.16	4.4E-01	0.42	3.4E-02	-0.13	5.4E-01	-0.04	8.4E-01	0.05	7.9E-01
ENSCAFG0000001808	TMEM164	cyan	EC_M2	-0.03	8.7E-01	0.29	1.5E-01	0.65	2.9E-04	0.04	8.6E-01	-0.86	2.7E-08	-0.29	1.6E-01	-0.01	9.7E-01	-0.13	5.2E-01	-0.09	6.5E-01	0.88	3.0E-09
ENSCAFG0000002945	ENSCAFG0000002945	grey	EC_M10	-0.03	8.7E-01	-0.32	1.1E-01	0.28	1.7E-01	-0.07	7.2E-01	-0.09	6.8E-01	-0.14	5.0E-01	0.10	6.3E-01	-0.10	6.4E-01	-0.35	7.5E-02	0.09	6.7E-01
ENSCAFG0000001702	NEK9	turquoise	EC_M6	-0.03	8.7E-01	0.49	1.1E-02	-0.06	7.6E-01	-0.29	1.5E-01	-0.08	7.0E-01	-0.17	4.1E-01	0.00	9.8E-01	0.00	1.0E+00	0.25	2.1E-01	0.14	4.9E-01
ENSCAFG0000000573	SLC30A6	grey	EC_M10	-0.03	8.7E-01	-0.25	2.2E-01	-0.55	3.3E-03	-0.17	4.0E-01	0.74	1.6E-05	0.48	1.2E-02	0.03	9.0E-01	-0.06	7.7E-01	0.30	1.3E-01	-0.71	4.6E-05
ENSCAFG0000001039	PHLDB2	darkgreen	EC_M4	-0.03	8.7E-01	0.00	9.9E-01	-0.70	7.4E-05	-0.04	8.5E-01	0.80	9.6E-07	0.22	2.8E-01	0.21	2.9E-01	-0.04	8.4E-01	0.19	3.5E-01	-0.80	8.8E-07
ENSCAFG0000002270	ENSCAFG0000002270	grey	EC_M10	-0.03	8.7E-01	-0.17	4.0E-01	0.00	1.0E+00	0.01	9.5E-01	0.04	8.6E-01	-0.11	5.8E-01	0.40	4.5E-02	-0.03	9.0E-01	-0.16	4.3E-01	-0.08	7.2E-01
ENSCAFG0000003219	ENSCAFG0000003219	grey	EC_M10	-0.03	8.7E-01	-0.17	4.2E-01	0.47	1.5E-02	0.30	1.3E-01	-0.54	4.4E-03	-0.15	4.8E-01	0.35	7.8E-02	0.07	7.5E-01	-0.01	9.7E-01	0.54	4.1E-03
ENSCAFG0000001844	LPIN2	darkgreen	EC_M4	-0.03	8.7E-01	-0.67	1.7E-04	0.09	6.5E-01	0.16	4.3E-01	0.08	7.1E-01	0.22	2.9E-01	0.11	5.8E-01	0.16	4.4E-01	0.11	5.8E-01	-0.10	6.1E-01
ENSCAFG0000001754	S100A16	grey	EC_M10	-0.03	8.7E-01	0.18	3.7E-01	0.10	6.1E-01	-0.11	5.9E-01	-0.07	7.3E-01	-0.21	3.1E-01	0.16	4.3E-01	0.19	3.6E-01	-0.52	6.5E-03	0.10	6.3E-01
ENSCAFG0000001770	OTOA	cyan	EC_M2	-0.03	8.7E-01	-0.30	1.3E-01	0.38	5.5E-02	0.10	6.4E-01	-0.37	6.4E-02	-0.25	2.2E-01	-0.26	2.0E-01	0.15	4.5E-01	0.18	3.7E-01	0.37	6.0E-02
ENSCAFG0000001422	GIGYF1	cyan	EC_M2	-0.03	8.7E-01	0.11	6.0E-01	0.59	1.4E-03	0.34	9.3E-02	-0.79	2.0E-06	-0.31	1.2E-01	0.05	8.0E-01	-0.13	5.4E-01	0.02	9.4E-01	0.82	3.4E-07
ENSCAFG0000002013	FAM129B	grey	EC_M10	-0.03	8.7E-01	-0.24	2.4E-01	-0.28	1.6E-01	-0.08	7.1E-01	0.51	7.6E-03	-0.01	9.7E-01	-0.23	2.6E-01	0.27	1.9E-01	0.10	6.1E-01	-0.50	9.3E-03
ENSCAFG0000003022	RBM11	darkgreen	EC_M4	-0.03	8.7E-01	-0.08	6.9E-01	-0.62	6.6E-04	-0.26	2.0E-01	0.80	1.1E-06	0.23	2.6E-01	0.08	7.0E-01	0.20	3.4E-01	-0.14	5.0E-01	-0.81	4.1E-07
ENSCAFG0000002271	ENSCAFG0000002271	grey	EC_M10	-0.03	8.7E-01	0.07	7.2E-01	-0.11	5.9E-01	0.05	7.9E-01	0.22	2.8E-01	0.09	6.6E-01	-0.36	6.9E-02	0.01	9.7E-01	0.16	4.5E-01	-0.22	2.8E-01
ENSCAFG0000000941	NDUFAF6	grey	EC_M10	-0.03	8.7E-01	-0.27	1.8E-01	-0.55	3.6E-03	-0.28	1.6E-01	0.78	2.1E-06	0.30	1.3E-01	0.25	2.1E-01	-0.09	6.8E-01	0.05	8.1E-01	-0.74	1.6E-05
ENSCAFG0000001191	CLIP2	cyan	EC_M2	-0.03	8.7E-01	-0.22	2.9E-01	0.74	1.5E-05	0.04	8.3E-01	-0.74	1.6E-05	-0.35	7.5E-02	0.05	8.1E-01	0.07	7.4E-01	-0.04	8.6E-01	0.75	1.2E-05
ENSCAFG0000001555	PMS2	grey	EC_M10	-0.03	8.7E-01	0.14	5.0E-01	-0.43	3.0E-02	-0.10	6.4E-01	0.53	5.2E-03	0.03	8.9E-01	-0.25	2.2E-01	0.42	3.4E-02	0.39	4.6E-02	-0.46	1.7E-02
ENSCAFG0000001867	ENSCAFG0000001867	grey	EC_M10	-0.03	8.7E-01	0.10	6.4E-01	0.37	6.6E-02	0.31	1.2E-01	-0.42	3.4E-02	0.15	4.5E-01	0.14	5.0E-01	-0.26	2.0E-01	0.07	7.3E-01	0.45	2.2E-02
ENSCAFG0000003260	ENSCAFG0000003260	grey	EC_M10	-0.03	8.7E-01	-0.03	8.8E-01	0.17	4.1E-01	-0.17	4.2E-01	-0.12	5.5E-01	-0.15	4.7E-01	-0.20	3.3E-01	0.23	2.6E-01	0.13	5.4E-01	0.11	5.8E-01
ENSCAFG0000002973	CACNG7	violet	EC_M7	-0.03	8.7E-01	-0.06	7.7E-01	-0.32	1.1E-01	-0.11	5.8E-01	0.43	2.9E-02	0.83	1.4E-07	0.19	3.4E-01	-0.10	6.3E-01	-0.13	5.3E-01	-0.43	2.7E-02
ENSCAFG0000000236	ENSCAFG0000000236	grey	EC_M10	-0.03	8.7E-01	0.08	7.1E-01	0.47	1.7E-02	0.03	8.9E-01	-0.50	8.7E-03	-0.26	1.9E-01	0.01	9.4E-01	-0.15	4.7E-01	0.15	4.7E-01	0.55	3.6E-03
ENSCAFG0000000103	ENSCAFG0000000103	magenta	EC_M13	-0.03	8.7E-01	0.09	6.6E-01	0.04	8.4E-01	-0.01	9.6E-01	-0.05	8.3E-01	0.00	9.8E-01	0.06	7.8E-01	-0.05	8.1E-01	0.89	1.2E-09	0.10	6.4E-01
ENSCAFG0000000919	ENSCAFG0000000919	grey	EC_M10	-0.04	8.6E-01	-0.09	6.6E-01	-0.26	2.1E-01	0.03	8.7E-01	0.29	1.5E-01	-0.08	7.0E-01	0.00	9.9E-01	-0.09	6.7E-01	-0.14	4.8E-01	-0.35	8.2E-02
ENSCAFG0000001570	STX18	darkgreen	EC_M4	-0.04	8.6E-01	-0.43	2.7E-02	-0.37	6.2E-02	-0.05	8.0E-01	0.62	7.7E-04	0.18	3.8E-01	-0.05	8.0E-01	0.12	5.7E-01	-0.39	4.8E-02	-0.64	4.9E-04
ENSCAFG0000000380	JADE1	grey	EC_M10	-0.04	8.6E-01	0.05	8.0E-01	-0.64	4.0E-04	-0.22	2.7E-01	0.78	3.1E-06	0.19	3.6E-01	-0.09	6.7E-01	-0.14	5.0E-01	-0.24	2.4E-01	-0.75	9.4E-06
ENSCAFG0000000696	ENSCAFG0000000696	grey	EC_M10	-0.04	8.6E-01	-0.11	6.0E-01	-0.33	9.8E-02	-0.13	5.2E-01	0.45	2.1E-02	0.59	1.7E-03	0.46	1.9E-02	-0.02	9.2E-01	-0.06	7.8E-01	-0.41	3.8E-02
ENSCAFG0000003147	ENSCAFG0000003147	grey	EC_M10	-0.04	8.6E-01	-0.11	6.0E-01	-0.33	9.8E-02	-0.13	5.2E-01	0.45	2.1E-02	0.59	1.7E-03	0.46	1.9E-02	-0.02	9.2E-01	-0.06	7.8E-01	-0.41	3.8E-02
ENSCAFG0000002929	ENSCAFG0000002929	magenta	EC_M13	-0.04	8.6E-01	0.18	3.8E-01	0.23	2.7E-01	0.02	9.4E-01	-0.31	1.2E-01	-0.25	2.1E-01	0.01	9.5E-01	0.06	7.6E-01	0.85	3.6E-08	0.38	5.3E-02
ENSCAFG0000001087	IGSF11	grey	EC_M10	-0.04	8.6E-01	-0.14	5.1E-01	-0.31	1.3E-01	-0.12	5.7E-01	0.48	1.2E-02	0.06	7.7E-01	-0.08	6.9E-01	-0.11	5.8E-01	0.05	8.0E-01	-0.47	1.6E-02
ENSCAFG0000001207	MCAM	turquoise	EC_M6	-0.04	8.6E-01	0.71	5.7E-05	-0.38	5.7E-02	-0.19	3.6E-01	0.27	1.8E-01	-0.16	4.4E-01	0.20	3.2E-01	-0.15	4.8E-01	0.00	9.8E-01	-0.25	2.3E-01
ENSCAFG0000003250	ENSCAFG0000003250	grey	EC_M10	-0.04	8.6E-01	-0.21	3.1E-01	0.13	5.2E-01	-0.19	3.6E-01	0.06	7.8E-01	0.15	4.8E-01	0.00	9.9E-01	-0.13	5.2E-01	-0.13	5.2E-01	-0.04	8.4E-01
ENSCAFG0000001755	ENSCAFG0000001755	grey	EC_M10	-0.04	8.6E-01	0.05	8.1E-01	-0.05	8.2E-01	-0.03	9.0E-01	0.06	7.6E-01	0.22	2.9E-01	0.40	4.3E-02	0.11	5.9E-01	-0.13	5.3E-01	-0.03	8.8E-01
ENSCAFG0000000011	ENSCAFG0000000011	grey	EC_M10	-0.04	8.6E-01	0.10	6.2E-01	0.13	5.4E-01	-0.41	3.7E-02	-0.14	4.9E-01	-0.13	5.2E-01	0.11	5.8E-01	0.04	8.3E-01	0.04	8.5E-01	0.17	4.1E-01
ENSCAFG0000001545	HS3ST1	turquoise	EC_M6	-0.04	8.6E-01	0.38	5.6E-02	-0.20	3.2E-01	-0.19	3.5E-01	0.18	3.7E-01	0.39	4.7E-02	-0.08	7.1E-01	-0.18	3.9E-01	-0.11	6.0E-01	-0.19	3.6E-01
ENSCAFG0000001673	SIPA1L1	grey	EC_M10	-0.04	8.6E-01	-0.21	3.1E-01	-0.46	1.9E-02	0.02	9.1E-01	0.62	6.8E-04	0.04	8.6E-01	-0.13	5.2E-01	0.25	2.2E-01	0.20	3.3E-01	-0.64	4.6E-04
ENSCAFG0000000451	STAM	grey	EC_M10	-0.04	8.6E-01	0.30	1.3E-01	-0.06	7.6E-01	0.08	6.8E-01	-0.11	6.1E-01	-0.02	9.4E-01	0.14	5.1E-01	-0.20	3.3E-01	-0.24	2.3E-01	0.09	6.7E-01
ENSCAFG0000002584	ENSCAFG0000002584	grey	EC_M10	-0.04	8.6E-01	-0.15	4.5E-01	0.12	5.4E-01	0.05	8.1E-01	-0.08	6.9E-01	-0.11	5.8E-01	-0.13	5.4E-01	-0.01	9.5E-01	-0.15	4.6E-01	0.06	7.6E-01
ENSCAFG0000001164	ZFHX2	turquoise	EC_M6	-0.04	8.6E-01	0.56	3.1E-03	-0.26	2.0E-01	-0.29	1.5E-01	0.08	6.9E-01	0.13	5.2E-01	0.31	1.3E-01	-0.09	6.7E-01	-0.17	4.1E-01	-0.08	7.1E-01
ENSCAFG0000002003	TBC1D13	turquoise	EC_M6	-0.04	8.6E-01	0.41	3.8E-02	-0.22	2.8E-01	-0.12	5.7E-01	0.13	5.1E-01	-0.11	5.9E-01	0.23	2.6E-01	-0.02	9.3E-01	0.20	3.2E-01	-0.06	7.8E-01
ENSCAFG0000000602	CDS2	turquoise	EC_M6	-0.04	8.6E-01	0.57	2.3E-03	0.02	9.1E-01	-0.03	8.8E-01	-0.19	3.6E-01	0.14	5.0E-01	0.01	9.8E-01	-0.43	2.7E-02	0.31	1.3E-01	0.28	1.7E-01
ENSCAFG0000001388	ENSCAFG0000001388	grey	EC_M10	-0.04	8.6E-01	-0.66	2.2E-04	0.26	2.1E-01	0.24	2.4E-01	-0.12	5.7E-01	0.06	7.6E-01	0.02	9.4E-01	-0.10	6.3E-01	-0.14	4.9E-01	0.10	6.4E-01
ENSCAFG0000001340	YBX3	cyan	EC_M2	-0.04	8.6E-01	-0.33	1.0E-01	0.56	2.7E-03	-0.01	9.8E-01	-0.45	2.2E-02	-0.08	6.9E-01	0.12	5.6E-01	-0.10	6.2E-01	-0.54	4.6E-03	0.44	2.3E-02
ENSCAFG0000001767	ENSCAFG0000001767	paleturquoise	EC_M11	-0.04	8.6E-01	-0.12	5.6E-01	0.24	2.4E-01	0.76	5.7E-06	-0.22	2.7E-01	-0.05	8.1E-01	-0.10	6.4E-01	-0.12	5.5E-01	-0.14	4.9E-01	0.25	2.2E-01
ENSCAFG0000003140	ENSCAFG0000003140	grey	EC_M10	-0.04	8.6E-01	-0.06	7.9E-01	-0.09	6.6E-01	-0.14	5.0E-01	0.17	4.1E-01	-0.08	6.8E-01	-0.05	8.3E-01	-0.13	5.3E-01	-0.01	9.6E-01	-0.15	4.5E-01
ENSCAFG0000002008	PTGES2	cyan	EC_M2	-0.04	8.6E-01	-0.15	4.5E-01	0.76	6.9E-06	0.08	7.0E-01	-0.76	7.7E-06	-0.10	6.4E-01	0.22	2.8E-01	-0.34	9.1E-02	-0.08	7.0E-01	0.78	3.0E-06
ENSCAFG0000000085	IRF1	grey	EC_M10	-0.04	8.6E-01	0.15	4.7E-01	0.29	1.6E-01	0.31	1.2E-01	-0.35	8.0E-02	-0.06	7.5E-01	-0.21	3.1E-01	-0.25	2.2E-01	-0.21	2.9E-01	0.35	8.2E-02
ENSCAFG0000001075	CRCP	grey	EC_M10	-0.04	8.6E-01	-0.69	9.9E-05	-0.01	9.6E-01	0.03	8.7E-01	0.27	1.9E-01	0.31	1.2E-01	0.07	7.4E-01	0.14	5.1E-01	-0.15	4.5E-01	-0.28	1.6E-01
ENSCAFG0000003032	ENSCAFG0000003032	grey	EC_M10	-0.04	8.6E-01	-0.13	5.3E-01	0.47	1.6E-02	0.34	9.3E-02	-0.47	1.4E-02	0.03	8.9E-01	-0.08	7.0E-01	-0.38	5.3E-02	-0.05	8.1E-01	0.49	1.1E-02
ENSCAFG0000002247	ENSCAFG0000002247	grey	EC_M10	-0.04	8.6E-01	-0.09	6.8E-01	0.34	9.2E-02	0.03	8.7E-01	-0.36	7.3E-02	-0.01	9.5E-01	0.39	4.9E-02	-0.05	8.3E-01	0.30	1.4E-01	0.36	7.1E-02
ENSCAFG0000001816	ALDH3A2	grey	EC_M10	-0.04	8.6E-01	0.41	3.8E-02	0.42	3.2E-02	-0.09	6.5E-01	-0.57	2.3E-03	-0.33	9.5E-02	-0.15	4.7E-01	0.07	7.4E-01	0.20	3.2E-01	0.66	2.6E-04
ENSCAFG0000003005	ENSCAFG0000003005	grey	EC_M10	-0.04	8.6E-01	0.28	1.7E-01	0.22	2.7E-01	0.53	4.9E-03	-0.34	8.6E-02	-0.11	6.1E-01	0.27	1.9E-01	-0.13	5.3E-01	0.23	2.6E-01	0.40	4.1E-02
ENSCAFG0000003187	ENSCAFG0000003187	grey	EC_M10	-0.04	8.6E-01	-0.09	6.6E-01	-0.14	4.9E-01	0.03	8.9E-01	0.26	1.9E-01	-0.02	9.4E-01	0.14	4.8E-01	-0.29	1.5E-01	0.20	3.2E-01	-0.20	3.3E-01
ENSCAFG0000001578	B4GALNT3	grey	EC_M10	-0.04	8.6E-01	0.05	8.2E-01	-0.28	1.6E-01	0.27	1.8E-01	0.27	1.8E-01	-0.20	3.3E-01	0.09	6.7E-01	-0.17	4.1E-01	-0.19	3.4E-01	-0.24	2.4E-01
ENSCAFG0000003243	PPTC7	grey	EC_M10	-0.04	8.6E-01	-0.55	3.5E-03	0.11	5.8E-01	0.09	6.8E-01	0.11	6.0E-01	0.03	8.7E-01	0.12	5.7E-01	-0.26	2.0E-01	-0.30	1.4E-01	-0.06	7.9E-01
ENSCAFG0000001941	NAA10	grey	EC_M10	-0.04	8.5E-01	-0.44	2.6E-02	-0.42	3.1E-02	-0.07	7.4E-01	0.72	3.2E-05	0.16	4.4E-01	0.17	4.1E-01	0.14	4.9E-01	0.03	9.0E-01	-0.72	3.0E-05
ENSCAFG0000002162	ENSCAFG0000002162	grey	EC_M10	-0.04	8.5E-01	-0.16	4.4E-01	0.35	7.5E-02	-0.15	4.6E-01	-0.36	7.2E-02	-0.10	6.1E-01	-0.14	5.0E-01	-0.06	7.6E-01	-0.04	8.4E-01	0.41	3.8E-02
ENSCAFG0000000959	NELL2	grey	EC_M10	-0.04	8.5E-01	-0.13	5.1E-01	0.19	3.5E-01	-0.11	5.8E-01	-0.11	6.0E-01	-0.10	6.2E-01	0.49	1.1E-02	0.10	6.4E-01	-0.06	7.8E-01	0.14	5.0E-01
ENSCAFG0000000598	HEATR5B	grey	EC_M10	-0.04	8.5E-01	-0.11	5.9E-01	-0.15	4.6E-01	-0.07	7.3E-01	0.24	2.5E-01	-0.14	5.1E-01	-0.43	2.9E-02	0.00	1.0E+00	-0.34	9.0E-02	-0.20	3.3E-01
ENSCAFG0000001563	ENSCAFG0000001563	grey	EC_M10	-0.04	8.5E-01	-0.30	1.4E-01	0.12	5.6E-01	0.10	6.3E-01	0.11	5.8E-01	-0.07	7.4E-01	-0.02	9.1E-01	-0.17	4.0E-01	-0.01	9.8E-01	-0.09	6.5E-01
ENSCAFG0000003003	ENSCAFG0000003003	magenta	EC_M13	-0.04	8.5E-01	0.11	6.0E-01	0.12	5.7E-01	0.00	9.9E-01	-0.16	4.3E-01	-0.02	9.1E-01	-0.07	7.2E-01	-0.05	8.2E-01	0.88	3.5E-09	0.23	2.5E-01
ENSCAFG0000001102	COX17	grey	EC_M10	-0.04	8.5E-01	0.30	1.4E-01	-0.24	2.4E-01	-0.12	5.5E-01	0.13	5.2E-01	0.32	1.1E-01	0.06	7.6E-01	0.04	8.5E-01	0.50	9.1E-03	-0.13	5.4E-01
ENSCAFG0000000851	CSNK2A2	grey	EC_M10	-0.04	8.5E-01	0.36	6.8E-02	-0.40	4.3E-02	-0.46	1.7E-02	0.35	8.1E-02	0.15	4.8E-01	0.26	2.0E-01	0.13	5.4E-01	-0.06	7.5E-01	-0.34	8.6E-02
ENSCAFG0000002905	RAB6B	grey	EC_M10	-0.04	8.5E-01	0.45	2.1E-02	-0.27	1.8E-01	-0.27	1.8E-01	0.18	3.7E-01	-0.13	5.4E-01	-0.01	9.7E-01	0.21	3.0E-01	0.65	3.1E-04	-0.11	6.0E-01
ENSCAFG0000000145	ENSCAFG0000000145	grey	EC_M10	-0.04	8.5E-01	0.12	5.7E-01	0.02	9.1E-01	-0.13	5.3E-01	0.02	9.1E-01	-0.12	5.7E-01	-0.13	5.3E-01	-0.09	6.5E-01	-0.09	6.7E-01	-0.01	9.6E-01
ENSCAFG0000000974	ENSCAFG0000000974	grey	EC_M10	-0.04	8.5E-01	0.23	2.5E-01	0.09	6.7E-01	-0.17	4.1E-01	-0.19	3.5E-01	-0.08	7.0E-01	-0.16	4.4E-01	0.52	6.6E-03	-0.08	6.9E-01	0.17	3.9E-01
ENSCAFG0000000071	TMEM74	grey	EC_M10	-0.04	8.5E-01	0.21	2.9E-01	-0.48	1.2E-02	-0.06	7.7E-01	0.56	3.0E-03	0.19	3.6E-01	0.14	5.0E-01	-0.05	8.1E-01	0.23	2.6E-01	-0.54	4.2E-03
ENSCAFG0000000726	IL37	magenta	EC_M13	-0.04	8.5E-01	0.03	8.7E-01	-0.06	7.9E-01	-0.10	6.2E-01	0.10	6.3E-01	-0.08	6.9E-01	-0.05	8.2E-01	-0.04	8.5E-01	0.59	1.6E-03	-0.07	7.3E-01
ENSCAFG0000000675	TRMT9B	turquoise	EC_M6	-0.04	8.5E-01	0.78	2.5E-06	-0.16	4.4E-01	0.02	9.3E-01	-0.09	6.5E-01	-0.24	2.3E-01	-0.15	4.6E-01	0.12	5.6E-01	0.13	5.2E-01	0.09	6.6E-01
ENSCAFG0000001003	RYR2	grey	EC_M10	-0.04	8.5E-01	-0.13	5.1E-01	-0.35	7.6E-02	-0.01	9.6E-01	0.45	2.0E-02	-0.11	6.0E-01	-0.04	8.4E-01	-0.06	7.8E-01	-0.10	6.2E-01	-0.45	2.1E-02
ENSCAFG0000000187	TCP11L2	cyan	EC_M2	-0.04	8.5E-01	0.17	3.9E-01	0.53	5.8E-03	0.27	1.8E-01	-0.72	3.5E-05	-0.26	2.0E-01	-0.26	2.0E-01	-0.04	8.5E-01	0.00	9.9E-01	0.75	8.4E-06
ENSCAFG0000001681	PNPO	cyan	EC_M2	-0.04	8.5E-01	0.10	6.1E-01	0.72	3.5E-05	0.18	3.8E-01	-0.87	1.0E-08	-0.17	4.0E-01	-0.03	8.8E-01	0.06	7.8E-01	0.08	7.1E-01	0.90	2.4E-10
ENSCAFG0000001052	FIGN	grey	EC_M10	-0.04	8.5E-01	-0.45	2.2E-02	0.64	4.6E-04	0.30	1.3E-01	-0.53	5.1E-03	-0.41	3.8E-02	-0.24	2.5E-01	0.21	3.1E-01	-0.12	5.6E-01	0.56	3.2E-03
ENSCAFG0000002028	SYDE2	grey	EC_M10	-0.04	8.5E-01	-0.23	2.6E-01	-0.33	9.8E-02	0.23	2.5E-01	0.54	4.2E-03	-0.06	7.8E-01	-0.10	6.4E-01	-0.01	9.7E-01	0.30	1.4E-01	-0.49	1.0E-02
ENSCAFG0000001321	HSD17B7	grey	EC_M10	-0.04	8.5E-01	0.04	8.3E-01	0.51	8.2E-03	0.13	5.4E-01	-0.60	1.2E-03	-0.48	1.2E-02	-0.19	3.6E-01	0.02	9.1E-01	-0.19	3.5E-01	0.60	1.2E-03
ENSCAFG0000001960	PLEKHG5	turquoise	EC_M6	-0.04	8.5E-01	0.70	6.3E-05	-0.44	2.3E-02	-0.18	3.8E-01	0.26	1.9E-01	-0.16	4.5E-01	0.12	5.7E-01	0.12	5.7E-01	0.42	3.1E-02	-0.24	2.4E-01
ENSCAFG0000001296	DCAF7	turquoise	EC_M6	-0.04	8.5E-01	0.57	2.2E-03	0.07	7.2E-01	-0.33	1.0E-01	-0.21	3.1E-01	-0.13	5.4E-01	0.14	5.0E-01	-0.08	7.1E-01	0.19	3.5E-01	0.25	2.1E-01
ENSCAFG0000001116	PLEKHS1	magenta	EC_M13	-0.04	8.5E-01	0.01	9.6E-01	0.18	3.8E-01	-0.06	7.8E-01	-0.16	4.4E-01	-0.01	9.5E-01	0.01	9.8E-01	-0.04	8.3E-01	0.72	3.2E-05	0.21	2.9E-01
ENSCAFG0000001964	VAMP7	darkgreen	EC_M4	-0.04	8.5E-01	0.20	3.3E-01	-0.70	7.0E-05	-0.10	6.3E-01	0.76	6.0E-06	0.18	3.7E-01	-0.18	3.8E-01	0.09	6.5E-01	0.05	8.0E-01	-0.71	5.1E-05
ENSCAFG0000001452	ENO2	grey	EC_M10	-0.04	8.5E-01	0.23	2.7E-01	-0.57	2.6E-03	-0.13	5.4E-01	0.61	9.8E-04	0.14	4.8E-01	0.35	8.3E-02	-0.12	5.6E-01	-0.18	3.7E-01	-0.63	5.2E-04
ENSCAFG0000001288	RSRP1	grey	EC_M10	-0.04	8.5E-01	0.12	5.7E-01	0.37	6.3E-02	0.19	3.5E-01	-0.45	2.2E-02	-0.77	3.5E-06	0.14	4.9E-01	0.17	4.2E-01	0.25	2.2E-01	0.48	1.4E-02
ENSCAFG0000000044	SYNE1	grey	EC_M10	-0.04	8.5E-01	0.07	7.3E-01	0.21	3.0E-01	-0.08	7.0E-01	-0.26	2.1E-01	-0.13	5.3E-01	0.01	9.4E-01	-0.22	2.7E-01	-0.49	1.1E-02	0.28	1.6E-01
ENSCAFG0000000588	DND1	turquoise	EC_M6	-0.04	8.5E-01	0.47	1.5E-02	-0.25	2.2E-01	-0.18	3.7E-01	0.24	2.4E-01	-0.13	5.4E-01	-0.06	7.7E-01	-0.10	6.2E-01	0.06	7.8E-01	-0.17	4.0E-01
ENSCAFG0000002975	ENSCAFG0000002975	grey	EC_M10	-0.04	8.5E-01	0.05	8.1E-01	0.12	5.6E-01	0.18	3.9E-01	-0.13	5.3E-01	-0.31	1.3E-01	0.12	5.7E-01	-0.26	2.0E-01	0.06	7.6E-01	0.11	6.0E-01
ENSCAFG0000000905	JMY	grey	EC_M10	-0.04	8.5E-01	0.53	5.0E-03	0.23	2.7E-01	0.10	6.1E-01	-0.48	1.3E-02	-0.12	5.6E-01	-0.08	6.9E-01	-0.21	2.9E-01	-0.11	5.9E-01	0.53	5.5E-03
ENSCAFG0000000446	XPC	grey	EC_M10	-0.04	8.5E-01	-0.20	3.3E-01	0.37	6.3E-02	0.04	8.6E-01	-0.37	6.1E-02	-0.35	7.8E-02	0.12	5.7E-01	0.06	7.6E-01	-0.22	2.9E-01	0.37	6.3E-02
ENSCAFG0000003050	ENSCAFG0000003050	grey	EC_M10	-0.04	8.5E-01	-0.10	6.3E-01	0.05	8.1E-01	-0.05	8.2E-01	-0.05	7.9E-01	-0.15	4.6E-01	-0.19	3.5E-01	-0.19	3.6E-01	0.18	3.8E-01	0.03	8.8E-01
ENSCAFG0000002955	BMF	grey	EC_M10	-0.04	8.5E-01	0.53	5.7E-03	0.26	2.1E-01	0.19	3.6E-01	-0.53	5.3E-03	-0.03	8.9E-01	0.04	8.3E-01	-0.30	1.3E-01	-0.03	8.9E-01	0.52	5.9E-03
ENSCAFG0000000155	BOP1	grey	EC_M10	-0.04	8.5E-01	-0.58	1.9E-03	0.37	6.4E-02	0.00	9.8E-01	-0.09	6.6E-01	0.06	7.8E-01	-0.06	7.8E-01	-0.12	5.7E-01	0.02	9.3E-01	0.10	6.1E-01
ENSCAFG0000002441	ENSCAFG0000002441	grey	EC_M10	-0.04	8.5E-01	-0.34	8.7E-02	0.00	9.9E-01	-0.11	6.0E-01	0.16	4.5E-01	0.14	5.0E-01	0.13	5.2E-01	0.50	8.8E-03	-0.05	8.1E-01	-0.17	4.1E-01
ENSCAFG0000001541	RCSD1	grey	EC_M10	-0.04	8.5E-01	-0.17	4.2E-01	0.13	5.4E-01	0.08	7.1E-01	-0.03	8.8E-01	-0.21	3.0E-01	0.15	4.7E-01	0.10	6.2E-01	0.23	2.6E-01	0.10	6.4E-01
ENSCAFG0000000442	CDADC1	grey	EC_M10	-0.04	8.4E-01	0.11	6.0E-01	0.28	1.7E-01	0.14	5.1E-01	-0.36	7.1E-02	-0.33	9.9E-02	0.05	7.9E-01	0.03	9.0E-01	-0.11	5.9E-01	0.43	2.8E-02
ENSCAFG0000000168	C8orf33	grey	EC_M10	-0.04	8.4E-01	0.01	9.5E-01	0.55	3.9E-03	-0.13	5.3E-01	-0.55	3.4E-03	-0.22	2.9E-01	0.21	3.0E-01	-0.21	3.0E-01	-0.09	6.6E-01	0.57	2.2E-03
ENSCAFG0000000151	DNAH8	grey	EC_M10	-0.04	8.4E-01	-0.03	9.0E-01	-0.61	9.1E-04	-0.05	8.2E-01	0.73	2.0E-05	0.30	1.4E-01	-0.12	5.5E-01	0.05	8.1E-01	0.03	8.9E-01	-0.71	4.8E-05
ENSCAFG0000001665	PHKG2	grey	EC_M10	-0.04	8.4E-01	0.26	2.1E-01	0.54	4.0E-03	-0.03	8.8E-01	-0.65	3.5E-04	-0.26	1.9E-01	0.14	4.9E-01	0.03	9.0E-01	0.27	1.8E-01	0.72	3.0E-05
ENSCAFG0000002866	FAM171B	grey	EC_M10	-0.04	8.4E-01	0.19	3.6E-01	-0.39	4.8E-02	0.01	9.4E-01	0.39	5.0E-02	0.25	2.2E-01	0.13	5.2E-01	-0.07	7.3E-01	0.15	4.6E-01	-0.37	6.5E-02
ENSCAFG0000001254	PLEKHA1	turquoise	EC_M6	-0.04	8.4E-01	0.59	1.6E-03	-0.45	2.1E-02	-0.13	5.3E-01	0.26	2.0E-01	0.14	5.0E-01	0.08	7.1E-01	0.07	7.3E-01	-0.02	9.1E-01	-0.26	2.0E-01
ENSCAFG0000000462	NECTIN2	turquoise	EC_M6	-0.04	8.4E-01	0.87	5.3E-09	-0.34	8.5E-02	-0.09	6.7E-01	0.12	5.7E-01	-0.19	3.5E-01	0.07	7.2E-01	0.15	4.5E-01	0.12	5.6E-01	-0.11	6.0E-01
ENSCAFG0000003258	BCL7A	grey	EC_M10	-0.04	8.4E-01	0.37	6.2E-02	-0.05	8.2E-01	-0.20	3.4E-01	-0.13	5.2E-01	0.03	8.8E-01	0.39	5.1E-02	-0.17	4.0E-01	0.29	1.4E-01	0.18	3.8E-01
ENSCAFG0000003063	FCN1	darkgreen	EC_M4	-0.04	8.4E-01	-0.08	7.1E-01	-0.16	4.4E-01	0.55	3.9E-03	0.25	2.3E-01	-0.10	6.4E-01	-0.08	7.2E-01	-0.04	8.6E-01	-0.01	9.5E-01	-0.19	3.6E-01
ENSCAFG0000000698	C27H12orf10	cyan	EC_M2	-0.04	8.4E-01	-0.23	2.6E-01	0.56	3.1E-03	0.15	4.7E-01	-0.46	1.7E-02	-0.13	5.3E-01	0.15	4.5E-01	0.02	9.3E-01	-0.19	3.5E-01	0.43	2.8E-02
ENSCAFG0000001040	RHBDD1	grey	EC_M10	-0.04	8.4E-01	-0.24	2.3E-01	-0.41	3.8E-02	-0.21	3.1E-01	0.60	1.3E-03	0.26	2.0E-01	0.00	9.8E-01	-0.19	3.5E-01	-0.35	7.9E-02	-0.63	6.0E-04
ENSCAFG0000000757	ENSCAFG0000000757	grey	EC_M10	-0.04	8.4E-01	0.00	9.9E-01	0.02	9.1E-01	-0.09	6.5E-01	0.02	9.0E-01	-0.16	4.4E-01	-0.11	5.9E-01	0.31	1.2E-01	0.22	2.8E-01	-0.02	9.3E-01
ENSCAFG0000000039	GNL1	grey	EC_M10	-0.04	8.4E-01	0.01	9.6E-01	0.61	1.0E-03	0.28	1.6E-01	-0.63	5.8E-04	-0.07	7.3E-01	0.14	5.0E-01	-0.20	3.2E-01	0.17	4.1E-01	0.67	1.6E-04
ENSCAFG0000000050	DLA-64	grey	EC_M10	-0.04	8.4E-01	0.27	1.9E-01	-0.62	7.6E-04	-0.21	3.1E-01	0.66	2.8E-04	0.13	5.2E-01	0.36	7.0E-02	0.24	2.3E-01	0.09	6.6E-01	-0.65	3.5E-04
ENSCAFG0000001388	LANCL3	turquoise	EC_M6	-0.04	8.4E-01	0.39	5.1E-02	0.02	9.0E-01	0.39	5.2E-02	-0.09	6.7E-01	-0.05	8.2E-01	0.10	6.4E-01	-0.18	3.7E-01	0.09	6.6E-01	0.12	5.6E-01

ENSCAFG0000000798	ZNF366	grey	EC_M10	-0.04	8.4E-01	0.48	1.4E-02	0.47	1.6E-02	0.10	6.3E-01	-0.67	1.6E-04	-0.36	7.4E-02	-0.26	2.0E-01	0.13	5.4E-01	0.02	9.3E-01	0.68	1.4E-04
ENSCAFG0000001214	GBX2	grey	EC_M10	-0.04	8.4E-01	-0.11	6.1E-01	-0.39	5.0E-02	0.17	4.0E-01	0.55	4.0E-03	0.37	6.6E-02	-0.09	6.6E-01	-0.20	3.3E-01	-0.09	6.6E-01	-0.55	3.6E-03
ENSCAFG0000001430	SOX10	grey	EC_M10	-0.04	8.4E-01	-0.09	6.6E-01	-0.10	6.2E-01	-0.10	6.3E-01	0.16	4.5E-01	-0.20	3.2E-01	-0.02	9.3E-01	0.41	3.5E-02	0.28	1.6E-01	-0.18	3.9E-01
ENSCAFG0000000208	ENSCAFG0000000208	grey	EC_M10	-0.04	8.4E-01	-0.23	2.7E-01	0.02	9.1E-01	0.06	7.8E-01	0.15	4.8E-01	0.22	2.8E-01	-0.11	6.0E-01	-0.11	6.0E-01	0.04	8.4E-01	-0.13	5.4E-01
ENSCAFG0000000429	GAD2	darkgreen	EC_M4	-0.04	8.4E-01	-0.26	2.1E-01	-0.58	1.9E-03	-0.30	1.4E-01	0.82	4.0E-07	0.26	1.9E-01	0.11	6.0E-01	0.21	3.1E-01	0.01	9.6E-01	-0.80	8.0E-07
ENSCAFG0000001012	ENSCAFG0000001012	grey	EC_M10	-0.04	8.4E-01	-0.24	2.4E-01	0.06	7.9E-01	0.31	1.3E-01	0.06	7.6E-01	-0.17	4.1E-01	-0.13	5.4E-01	-0.18	3.7E-01	-0.18	3.9E-01	-0.04	8.5E-01
ENSCAFG0000000566	NEK10	grey	EC_M10	-0.04	8.4E-01	-0.49	1.2E-02	-0.17	4.1E-01	0.15	4.7E-01	0.34	9.3E-02	0.12	5.8E-01	-0.13	5.4E-01	-0.11	5.8E-01	0.21	3.0E-01	-0.35	8.1E-02
ENSCAFG0000000776	HIP1R	turquoise	EC_M6	-0.04	8.4E-01	0.76	6.4E-06	-0.17	4.0E-01	-0.08	7.0E-01	-0.06	7.6E-01	-0.20	3.2E-01	0.17	4.1E-01	-0.06	7.7E-01	0.17	4.2E-01	0.10	6.2E-01
ENSCAFG0000001457	CNPY4	grey	EC_M10	-0.04	8.4E-01	0.02	9.2E-01	-0.29	1.5E-01	0.13	5.1E-01	0.37	6.6E-02	0.07	7.4E-01	0.35	8.0E-02	0.32	1.1E-01	0.19	3.5E-01	-0.31	1.3E-01
ENSCAFG0000000307	AREG	grey	EC_M10	-0.04	8.4E-01	0.24	2.3E-01	0.12	5.6E-01	0.28	1.6E-01	-0.23	2.6E-01	-0.10	6.2E-01	-0.11	6.1E-01	-0.15	4.5E-01	-0.09	6.6E-01	0.28	1.6E-01
ENSCAFG0000002638	ENSCAFG0000002638	grey	EC_M10	-0.04	8.4E-01	-0.11	5.9E-01	-0.24	2.3E-01	-0.11	6.1E-01	0.32	1.1E-01	0.38	5.6E-02	0.42	3.2E-02	-0.05	7.9E-01	-0.10	6.4E-01	-0.30	1.4E-01
ENSCAFG0000001986	APRT	cyan	EC_M2	-0.04	8.4E-01	-0.36	6.8E-02	0.54	4.8E-03	-0.05	8.1E-01	-0.34	9.2E-02	-0.15	4.8E-01	-0.04	8.6E-01	0.07	7.3E-01	-0.14	5.0E-01	0.36	6.9E-02
ENSCAFG0000002369	ENSCAFG0000002369	grey	EC_M10	-0.04	8.4E-01	0.23	2.6E-01	-0.32	1.1E-01	-0.15	4.7E-01	0.25	2.1E-01	0.29	1.5E-01	0.25	2.1E-01	-0.23	2.6E-01	0.09	6.7E-01	-0.26	1.9E-01
ENSCAFG0000002853	ENSCAFG0000002853	turquoise	EC_M6	-0.04	8.4E-01	0.62	7.6E-04	-0.47	1.5E-02	-0.28	1.6E-01	0.41	3.7E-02	0.00	9.9E-01	0.06	7.8E-01	-0.30	1.3E-01	0.12	5.6E-01	-0.37	6.1E-02
ENSCAFG0000000500	CDK3	grey	EC_M10	-0.04	8.4E-01	-0.48	1.2E-02	0.29	1.4E-01	-0.30	1.3E-01	-0.07	7.2E-01	0.11	6.0E-01	-0.01	9.8E-01	-0.26	1.9E-01	-0.28	1.6E-01	0.12	5.7E-01
ENSCAFG0000000440	EML2	darkgreen	EC_M4	-0.04	8.4E-01	0.27	1.8E-01	-0.69	1.0E-04	-0.26	2.0E-01	0.74	1.4E-05	-0.03	9.0E-01	0.03	8.8E-01	0.06	7.6E-01	0.12	5.6E-01	-0.70	7.4E-05
ENSCAFG0000001165	ATP9A	turquoise	EC_M6	-0.04	8.4E-01	0.73	2.0E-05	-0.03	9.0E-01	-0.04	8.6E-01	-0.20	3.3E-01	-0.18	3.8E-01	-0.21	2.9E-01	-0.16	4.3E-01	0.40	4.1E-02	0.27	1.8E-01
ENSCAFG0000000792	MSC	grey	EC_M10	-0.04	8.4E-01	-0.30	1.3E-01	-0.40	4.4E-02	-0.10	6.4E-01	0.63	5.7E-04	0.28	1.6E-01	0.19	3.4E-01	-0.25	2.1E-01	-0.44	2.5E-02	-0.65	2.9E-04
ENSCAFG0000001891	OMA1	darkgreen	EC_M4	-0.04	8.4E-01	-0.66	2.6E-04	0.25	2.1E-01	0.28	1.7E-01	-0.05	7.9E-01	0.14	4.8E-01	-0.08	7.1E-01	-0.04	8.6E-01	0.11	5.8E-01	0.07	7.5E-01
ENSCAFG0000000423	HS6ST1	turquoise	EC_M6	-0.04	8.4E-01	0.67	1.7E-04	-0.22	2.8E-01	-0.18	3.9E-01	0.08	7.0E-01	0.03	8.7E-01	-0.10	6.3E-01	-0.05	8.0E-01	0.51	7.6E-03	-0.01	9.6E-01
ENSCAFG0000000994	CCSER1	grey	EC_M10	-0.04	8.3E-01	0.10	6.3E-01	-0.35	7.8E-02	-0.02	9.3E-01	0.36	7.0E-02	-0.10	6.4E-01	-0.12	5.5E-01	0.47	1.4E-02	-0.05	8.3E-01	-0.37	6.3E-02
ENSCAFG0000001436	MREG	grey	EC_M10	-0.04	8.3E-01	-0.35	7.7E-02	0.31	1.3E-01	0.11	5.8E-01	-0.15	4.5E-01	-0.30	1.4E-01	0.16	4.5E-01	0.34	8.6E-02	0.21	3.0E-01	0.19	3.6E-01
ENSCAFG0000002036	LRRC36	grey	EC_M10	-0.04	8.3E-01	0.06	7.8E-01	0.18	3.8E-01	0.45	2.3E-02	-0.24	2.3E-01	-0.39	5.1E-02	-0.19	3.5E-01	0.18	3.7E-01	0.26	2.0E-01	0.30	1.4E-01
ENSCAFG0000000914	LPIN3	cyan	EC_M2	-0.04	8.3E-01	-0.10	6.3E-01	0.81	4.4E-07	0.24	2.3E-01	-0.89	7.5E-10	-0.33	1.0E-01	0.06	7.9E-01	0.02	9.1E-01	0.02	9.3E-01	0.91	6.7E-11
ENSCAFG0000001355	PLEKHM3	grey	EC_M10	-0.04	8.3E-01	0.05	8.1E-01	-0.57	2.4E-03	-0.09	6.8E-01	0.69	8.8E-05	0.19	3.5E-01	-0.12	5.4E-01	-0.13	5.4E-01	0.16	4.5E-01	-0.65	3.1E-04
ENSCAFG0000001965	CAMTA1	grey	EC_M10	-0.04	8.3E-01	0.11	5.9E-01	-0.63	6.0E-04	-0.23	2.5E-01	0.72	3.9E-05	0.41	3.6E-02	0.35	8.4E-02	0.01	9.7E-01	-0.04	8.6E-01	-0.72	3.9E-05
ENSCAFG0000000680	FZD10	grey	EC_M10	-0.04	8.3E-01	-0.25	2.2E-01	0.45	2.2E-02	0.02	9.2E-01	-0.37	5.9E-02	-0.03	8.9E-01	-0.27	1.8E-01	-0.29	1.5E-01	-0.25	2.1E-01	0.46	1.9E-02
ENSCAFG0000001992	AIF1L	grey	EC_M10	-0.04	8.3E-01	0.32	1.2E-01	-0.15	4.7E-01	-0.14	4.8E-01	0.10	6.4E-01	-0.06	7.9E-01	0.18	3.9E-01	-0.35	8.0E-02	0.06	7.7E-01	0.00	9.9E-01
ENSCAFG0000000060	NCAPH2	grey	EC_M10	-0.04	8.3E-01	0.17	4.2E-01	0.62	7.5E-04	0.17	4.1E-01	-0.75	1.1E-05	-0.13	5.3E-01	0.25	2.1E-01	-0.16	4.3E-01	-0.35	7.6E-02	0.75	9.2E-06
ENSCAFG0000003028	ENSCAFG0000003028	grey	EC_M10	-0.04	8.3E-01	0.08	7.0E-01	0.34	9.1E-02	0.15	4.7E-01	-0.39	4.7E-02	0.16	4.4E-01	-0.14	5.0E-01	-0.21	3.0E-01	-0.40	4.5E-02	0.42	3.4E-02
ENSCAFG0000001751	NRDE2	grey	EC_M10	-0.04	8.3E-01	0.34	8.9E-02	-0.13	5.2E-01	-0.18	3.7E-01	0.07	7.3E-01	-0.09	6.5E-01	0.24	2.4E-01	-0.31	1.3E-01	0.09	6.5E-01	0.00	9.9E-01
ENSCAFG0000000033	PPM1H	grey	EC_M10	-0.04	8.3E-01	0.47	1.6E-02	-0.60	1.2E-03	-0.28	1.7E-01	0.62	6.8E-04	-0.04	8.6E-01	0.03	8.9E-01	0.01	9.7E-01	-0.11	5.9E-01	-0.55	3.7E-03
ENSCAFG0000001392	ARNT2	turquoise	EC_M6	-0.04	8.3E-01	0.60	1.1E-03	-0.43	2.8E-02	-0.10	6.2E-01	0.35	8.1E-02	-0.02	9.2E-01	0.20	3.4E-01	-0.09	6.7E-01	0.08	7.0E-01	-0.30	1.3E-01
ENSCAFG0000002580	ENSCAFG0000002580	grey	EC_M10	-0.04	8.3E-01	-0.20	3.2E-01	0.16	4.4E-01	-0.19	3.5E-01	0.01	9.5E-01	-0.11	5.9E-01	0.26	1.9E-01	-0.04	8.5E-01	-0.04	8.4E-01	0.03	8.9E-01
ENSCAFG0000001041	UNC119B	grey	EC_M10	-0.04	8.3E-01	0.02	9.1E-01	-0.48	1.4E-02	-0.41	3.6E-02	0.62	7.2E-04	0.13	5.4E-01	-0.21	3.1E-01	0.06	7.6E-01	0.21	3.1E-01	-0.54	4.3E-03
ENSCAFG0000001681	PSEN1	turquoise	EC_M6	-0.04	8.3E-01	0.41	4.0E-02	0.01	9.7E-01	-0.06	7.8E-01	-0.17	4.1E-01	-0.03	9.0E-01	-0.24	2.5E-01	-0.21	3.1E-01	-0.49	1.2E-02	0.19	3.4E-01
ENSCAFG0000002865	ENSCAFG0000002865	turquoise	EC_M6	-0.04	8.3E-01	0.47	1.6E-02	0.03	8.7E-01	0.27	1.7E-01	-0.20	3.4E-01	-0.19	3.6E-01	0.33	1.0E-01	0.00	9.8E-01	-0.09	6.7E-01	0.23	2.6E-01
ENSCAFG0000000347	ENSCAFG0000000347	darkgreen	EC_M4	-0.04	8.3E-01	-0.21	3.1E-01	-0.63	5.2E-04	-0.10	6.3E-01	0.88	3.6E-09	0.22	2.9E-01	0.29	1.5E-01	0.20	3.2E-01	0.05	8.3E-01	-0.85	3.5E-08
ENSCAFG0000000926	GOLGA7B	grey	EC_M10	-0.04	8.3E-01	0.08	7.0E-01	0.09	6.7E-01	-0.03	8.8E-01	-0.06	7.7E-01	-0.01	9.7E-01	-0.01	9.6E-01	-0.06	7.7E-01	-0.07	7.4E-01	0.04	8.6E-01
ENSCAFG0000001439	PYY	grey	EC_M10	-0.04	8.3E-01	0.08	7.0E-01	0.09	6.7E-01	-0.03	8.8E-01	-0.06	7.7E-01	-0.01	9.7E-01	-0.01	9.6E-01	-0.06	7.7E-01	-0.07	7.4E-01	0.04	8.6E-01
ENSCAFG0000002369	ANGPTL5	grey	EC_M10	-0.04	8.3E-01	0.08	7.0E-01	0.09	6.7E-01	-0.03	8.8E-01	-0.06	7.7E-01	-0.01	9.7E-01	-0.01	9.6E-01	-0.06	7.7E-01	-0.07	7.4E-01	0.04	8.6E-01
ENSCAFG0000002983	ENSCAFG0000002983	grey	EC_M10	-0.04	8.3E-01	0.08	7.0E-01	0.09	6.7E-01	-0.03	8.8E-01	-0.06	7.7E-01	-0.01	9.7E-01	-0.01	9.6E-01	-0.06	7.7E-01	-0.07	7.4E-01	0.04	8.6E-01
ENSCAFG0000003027	UBE2QL1	grey	EC_M10	-0.04	8.3E-01	-0.20	3.3E-01	-0.02	9.2E-01	-0.26	2.1E-01	0.11	5.8E-01	0.20	3.4E-01	0.17	4.1E-01	-0.20	3.2E-01	0.27	1.8E-01	-0.02	9.4E-01
ENSCAFG0000003022	ZNF471	grey	EC_M10	-0.04	8.3E-01	-0.01	9.5E-01	0.28	1.6E-01	-0.21	3.1E-01	-0.36	7.3E-02	-0.27	1.8E-01	0.12	5.8E-01	0.31	1.2E-01	0.15	4.7E-01	0.37	6.5E-02
ENSCAFG0000000325	YRDC	grey	EC_M10	-0.04	8.3E-01	0.13	5.3E-01	-0.54	4.6E-03	-0.33	9.7E-02	0.67	2.1E-04	0.33	9.6E-02	0.07	7.4E-01	0.02	9.2E-01	0.16	4.4E-01	-0.64	4.2E-04
ENSCAFG0000000087	SHISAL1	grey	EC_M10	-0.04	8.3E-01	0.53	5.4E-03	-0.38	5.4E-02	-0.11	5.9E-01	0.25	2.1E-01	0.09	6.7E-01	0.11	6.0E-01	-0.13	5.3E-01	0.56	3.0E-03	-0.22	2.7E-01
ENSCAFG0000000222	NPAS2	grey	EC_M10	-0.04	8.3E-01	0.19	3.5E-01	0.09	6.8E-01	0.02	9.4E-01	-0.08	7.0E-01	0.17	4.1E-01	0.01	9.6E-01	-0.27	1.9E-01	-0.29	1.6E-01	0.13	5.3E-01
ENSCAFG0000000384	MGAM	grey	EC_M10	-0.04	8.3E-01	0.27	1.8E-01	0.05	8.1E-01	-0.17	4.1E-01	-0.08	6.9E-01	-0.14	5.0E-01	-0.05	8.0E-01	-0.16	4.4E-01	0.08	7.0E-01	0.15	4.6E-01
ENSCAFG0000000756	ANAPC10	grey	EC_M10	-0.04	8.3E-01	-0.05	8.1E-01	-0.64	4.7E-04	-0.07	7.5E-01	0.83	2.1E-07	0.19	3.4E-01	-0.16	4.4E-01	0.08	7.0E-01	0.11	5.9E-01	-0.80	1.0E-06
ENSCAFG0000003177	TRAPPC6A	grey	EC_M10	-0.04	8.3E-01	0.08	6.8E-01	0.62	6.8E-04	-0.01	9.5E-01	-0.68	1.4E-04	-0.04	8.5E-01	0.02	9.3E-01	-0.21	3.1E-01	0.09	6.6E-01	0.69	8.3E-05
ENSCAFG0000001253	ECE2	grey	EC_M10	-0.04	8.3E-01	-0.43	3.0E-02	-0.11	5.8E-01	-0.04	8.5E-01	0.44	2.4E-02	0.13	5.2E-01	-0.19	3.4E-01	0.10	6.2E-01	-0.10	6.3E-01	-0.42	3.1E-02
ENSCAFG0000000007	ZCCHC2	turquoise	EC_M6	-0.05	8.3E-01	0.58	2.1E-03	-0.16	4.4E-01	-0.12	5.7E-01	0.00	9.9E-01	-0.25	2.2E-01	-0.04	8.6E-01	-0.30	1.4E-01	0.09	6.8E-01	0.09	6.6E-01
ENSCAFG0000000367	C15H16orf87	grey	EC_M10	-0.05	8.3E-01	0.10	6.4E-01	-0.08	6.8E-01	0.44	2.3E-02	0.03	8.7E-01	0.07	7.2E-01	0.06	7.8E-01	0.01	9.5E-01	-0.01	9.6E-01	-0.04	8.3E-01
ENSCAFG0000001081	ABCA5	grey	EC_M10	-0.05	8.3E-01	0.36	6.7E-02	0.09	6.5E-01	0.41	3.5E-02	-0.19	3.4E-01	-0.05	8.2E-01	0.11	6.1E-01	-0.16	4.5E-01	0.08	7.0E-01	0.26	1.9E-01
ENSCAFG0000001341	NFKBIA	cyan	EC_M2	-0.05	8.3E-01	0.30	1.3E-01	0.62	7.6E-04	0.14	5.1E-01	-0.84	5.8E-08	-0.20	3.2E-01	0.06	7.8E-01	-0.05	8.2E-01	0.09	6.5E-01	0.86	1.4E-08
ENSCAFG0000001492	NELFA	cyan	EC_M2	-0.05	8.3E-01	0.19	3.5E-01	0.60	1.1E-03	0.16	4.5E-01	-0.74	1.4E-05	-0.09	6.6E-01	-0.06	7.9E-01	-0.06	7.8E-01	-0.12	5.8E-01	0.74	1.4E-05
ENSCAFG0000001946	RNF151	cyan	EC_M2	-0.05	8.2E-01	-0.69	1.1E-04	0.64	3.9E-04	0.07	7.2E-01	-0.44	2.3E-02	0.06	7.7E-01	0.09	6.6E-01	-0.14	4.9E-01	-0.16	4.3E-01	0.47	1.7E-02
ENSCAFG0000001941	TRAF7	cyan	EC_M2	-0.05	8.2E-01	0.14	4.8E-01	0.68	1.2E-04	-0.05	8.0E-01	-0.78	2.1E-06	-0.23	2.5E-01	-0.10	6.3E-01	0.18	3.7E-01	0.19	3.6E-01	0.81	4.4E-07
ENSCAFG0000001177	C6H7orf50	grey	EC_M10	-0.05	8.2E-01	-0.24	2.4E-01	-0.27	1.9E-01	-0.07	7.4E-01	0.47	1.7E-02	0.26	1.9E-01	-0.06	7.8E-01	0.43	2.9E-02	0.28	1.6E-01	-0.43	3.0E-02
ENSCAFG0000003275	KIAA0408	grey	EC_M10	-0.05	8.2E-01	-0.08	6.8E-01	0.26	1.9E-01	0.53	5.1E-03	-0.27	1.8E-01	-0.13	5.4E-01	-0.14	5.1E-01	-0.15	4.6E-01	0.35	8.1E-02	0.31	1.2E-01
ENSCAFG0000000899	OTULINL	grey	EC_M10	-0.05	8.2E-01	0.20	3.3E-01	-0.29	1.5E-01	-0.14	5.0E-01	0.33	9.7E-02	0.11	5.9E-01	0.03	8.7E-01	0.13	5.4E-01	-0.10	6.1E-01	-0.29	1.5E-01
ENSCAFG0000002558	FRY	darkgreen	EC_M4	-0.05	8.2E-01	0.25	2.1E-01	-0.56	3.1E-03	-0.12	5.7E-01	0.62	7.0E-04	-0.15	4.7E-01	-0.04	8.5E-01	0.20	3.4E-01	0.32	1.1E-01	-0.56	2.6E-03
ENSCAFG0000001995	VAV3	cyan	EC_M2	-0.05	8.2E-01	-0.07	7.4E-01	0.70	7.8E-05	0.32	1.2E-01	-0.78	2.6E-06	-0.35	8.4E-02	0.09	6.7E-01	-0.06	7.7E-01	-0.34	8.8E-02	0.79	1.5E-06
ENSCAFG0000000495	TRIM47	cyan	EC_M2	-0.05	8.2E-01	0.12	5.6E-01	0.76	6.3E-06	-0.04	8.5E-01	-0.87	7.3E-09	-0.30	1.4E-01	0.05	7.9E-01	-0.10	6.4E-01	0.04	8.5E-01	0.92	3.1E-11
ENSCAFG0000002458	ENSCAFG0000002458	grey	EC_M10	-0.05	8.2E-01	-0.06	7.6E-01	0.23	2.5E-01	-0.09	6.6E-01	-0.24	2.4E-01	0.01	9.7E-01	-0.05	8.2E-01	-0.10	6.1E-01	-0.16	4.4E-01	0.21	3.1E-01
ENSCAFG0000001104	FAM207A	grey	EC_M10	-0.05	8.2E-01	0.21	3.1E-01	0.27	1.9E-01	0.09	6.7E-01	-0.23	2.5E-01	0.08	6.8E-01	0.05	8.0E-01	-0.13	5.1E-01	0.25	2.3E-01	0.33	9.9E-02
ENSCAFG0000001930	CLUH	grey	EC_M10	-0.05	8.2E-01	-0.45	2.2E-02	0.66	2.6E-04	0.05	8.1E-01	-0.50	9.0E-03	-0.17	4.2E-01	-0.17	3.9E-01	0.04	8.4E-01	0.27	1.7E-01	0.56	2.9E-03
ENSCAFG0000000319	WDR91	grey	EC_M10	-0.05	8.2E-01	0.45	2.0E-02	0.49	1.2E-02	0.18	3.7E-01	-0.74	1.5E-05	-0.34	9.1E-02	0.04	8.6E-01	-0.06	7.7E-01	0.35	8.1E-02	0.78	3.0E-06
ENSCAFG0000001796	PIN1	darkgreen	EC_M4	-0.05	8.2E-01	-0.08	6.9E-01	-0.61	9.6E-04	-0.11	5.8E-01	0.84	8.4E-08	0.05	8.1E-01	-0.04	8.4E-01	0.30	1.4E-01	0.23	2.6E-01	-0.78	2.4E-06
ENSCAFG0000001856	GRIA3	grey	EC_M10	-0.05	8.2E-01	0.24	2.4E-01	-0.02	9.1E-01	-0.05	8.0E-01	0.04	8.5E-01	0.18	3.8E-01	-0.14	5.0E-01	-0.29	1.5E-01	-0.27	1.8E-01	-0.02	9.3E-01
ENSCAFG0000003208	ENSCAFG0000003208	darkgreen	EC_M4	-0.05	8.2E-01	-0.73	2.0E-05	0.13	5.2E-01	0.04	8.5E-01	0.18	3.7E-01	0.03	8.9E-01	-0.28	1.7E-01	0.33	9.8E-02	0.02	9.1E-01	-0.18	3.9E-01
ENSCAFG0000002672	ENSCAFG0000002672	turquoise	EC_M6	-0.05	8.2E-01	0.47	1.6E-02	0.04	8.5E-01	-0.10	6.3E-01	-0.14	5.0E-01	-0.09	6.6E-01	0.27	1.8E-01	-0.10	6.2E-01	0.24	2.4E-01	0.13	5.2E-01
ENSCAFG0000003037	COMMD7	turquoise	EC_M6	-0.05	8.2E-01	0.57	2.3E-03	0.12	5.5E-01	-0.11	5.9E-01	-0.30	1.3E-01	-0.25	2.2E-01	0.18	3.8E-01	-0.08	7.1E-01	0.35	8.4E-02	0.37	6.5E-02
ENSCAFG0000001762	PRKCB	grey	EC_M10	-0.05	8.2E-01	-0.21	3.0E-01	0.24	2.3E-01	-0.08	7.0E-01	-0.03	8.7E-01	-0.05	7.9E-01	-0.07	7.3E-01	-0.05	7.9E-01	-0.08	7.0E-01	0.13	5.3E-01
ENSCAFG0000002959	ZNF527	grey	EC_M10	-0.05	8.2E-01	0.34	9.0E-02	0.15	4.6E-01	0.08	7.0E-01	-0.31	1.2E-01	-0.54	4.1E-03	0.14	4.9E-01	0.09	6.5E-01	0.24	2.3E-01	0.38	5.7E-02
ENSCAFG0000001124	ALDH3B2	grey	EC_M10	-0.05	8.2E-01	-0.09	6.7E-01	0.44	2.6E-02	-0.08	7.0E-01	-0.36	7.0E-02	-0.05	8.0E-01	-0.06	7.7E-01	-0.05	8.0E-01	-0.04	8.6E-01	0.38	5.7E-02
ENSCAFG0000000402	ZEB1	grey	EC_M10	-0.05	8.2E-01	0.42	3.2E-02	0.03	8.9E-01	-0.20	3.2E-01	-0.11	5.8E-01	-0.18	3.9E-01	-0.03	8.9E-01	-0.07	7.4E-01	-0.29	1.6E-01	0.21	3.0E-01
ENSCAFG0000000618	ADGRA2	grey	EC_M10	-0.05	8.2E-01	0.63	5.8E-04	0.34	9.3E-02	-0.21	3.0E-01	-0.52	6.3E-03	-0.26	2.0E-01	-0.03	8.7E-01	0.05	8.0E-01	0.06	7.5E-01	0.56	2.8E-03
ENSCAFG0000001176	CA14	turquoise	EC_M6	-0.05	8.2E-01	0.62	6.6E-04	-0.20	3.3E-01	-0.18	3.7E-01	0.11	5.8E-01	-0.16	4.4E-01	0.42	3.1E-02	0.12	5.7E-01	0.18	3.7E-01	-0.07	7.4E-01
ENSCAFG0000000907	CCDC9B	turquoise	EC_M6	-0.05	8.2E-01	0.50	9.7E-03	-0.11	6.0E-01	-0.01	9.7E-01	-0.06	7.6E-01	-0.13	5.2E-01	-0.01	9.7E-01	0.08	7.0E-01	0.60	1.1E-03	0.15	4.6E-01
ENSCAFG0000001934	TAX1BP3	darkgreen	EC_M4	-0.05	8.2E-01	-0.39	5.1E-02	-0.30	1.4E-01	-0.12	5.6E-01	0.54	4.0E-03	0.05	8.0E-01	0.17	4.1E-01	0.12	5.5E-01	0.17	4.2E-01	-0.54	4.8E-03
ENSCAFG0000001891	INTS6L	darkgreen	EC_M4	-0.05	8.2E-01	-0.40	4.6E-02	-0.45	2.0E-02	0.09	6.6E-01	0.69	1.0E-04	0.13	5.2E-01	-0.05	8.0E-01	0.05	8.0E-01	-0.35	7.9E-02	-0.71	5.1E-05
ENSCAFG0000000152	NFIB	cyan	EC_M2	-0.05	8.2E-01	0.24	2.3E-01	0.62	7.4E-04	-0.04	8.5E-01	-0.77	4.5E-06	-0.28	1.6E-01	-0.19	3.6E-01	0.13	5.2E-01	0.20	3.3E-01	0.80	7.4E-07
ENSCAFG0000001447	HOMER3	turquoise	EC_M6	-0.05	8.2E-01	0.90	5.9E-10	-0.07	7.3E-01	-0.14	5.1E-01	-0.20	3.3E-01	-0.20	3.2E-01	0.07	7.4E-01	-0.09	6.7E-01	0.10	6.2E-01	0.21	3.1E-01
ENSCAFG0000000836	ENSCAFG0000000836	grey	EC_M10	-0.05	8.2E-01	-0.05	7.9E-01	-0.11	5.8E-01	0.01	9.6E-01	0.15	4.7E-01	-0.07	7.4E-01	0.53	5.4E-03	-0.02	9.2E-01	-0.12	5.6E-01	-0.16	4.2E-01
ENSCAFG0000000332	COL27A1	grey	EC_M10	-0.05	8.2E-01	-0.28	1.6E-01	-0.05	8.2E-01	0.08	6.9E-01	0.15	4.5E-01	0.07	7.2E-01	0.44	2.6E-02	0.09	6.6E-01	0.28	1.7E-01	-0.18	3.8E-01
ENSCAFG0000002943	TMEM167A	darkgreen	EC_M4	-0.05	8.2E-01	-0.54	4.4E-03	-0.21	2.9E-01	-0.14	4.9E-01	0.49	1.1E-02	0.33	1.0E-01	-0.34	8.8E-02	0.16	4.4E-01	0.19	3.6E-01	-0.50	1.0E-02
ENSCAFG0000003196	NKX2-5	grey	EC_M10	-0.05	8.2E-01	0.01	9.5E-01	-0.32	1.1E-01	-0.10	6.1E-01	0.42	3.4E-02	0.68	1.5E-04	0.18	3.7E-01	-0.15	4.6E-01	0.32	1.1E-01	-0.39	4.8E-02
ENSCAFG0000001580	SDCCAG8	grey	EC_M10	-0.05	8.2E-01	-0.37	6.4E-02	0.28	1.6E-01	0.03	9.0E-01	-0.28	1.7E-01	-0.16	4.4E-01	0.02	9.3E-01	0.43	2.8E-02	0.19	3.4E-01	0.27	1.9E-01
ENSCAFG0000002551	SERPINB6	grey	EC_M10	-0.05	8.2E-01	-0.41	4.0E-02	0.61	8.4E-04	0.21	2.9E-01	-0.48	1.3E-02	-0.15	4.5E-01	-0.11	5.8E-01	0.07	7.2E-01	-0.09	6.8E-01	0.50	9.7E-03
ENSCAFG0000000801	ENSCAFG0000000801	grey	EC_M10	-0.05	8.2E-01	-0.17	4.0E-01	0.09	6.6E-01	0.32	1.1E-01	0.02	9.2E-01	0.35	8.2E-02	0.43	3.0E-02	-0.08	7.1E-01	-0.10	6.1E-01	0.02	9.3E-01
ENSCAFG0000000909	COQ2	grey	EC_M10	-0.05	8.2E-01	-0.16	4.4E-01	-0.34	9.1E-02	-0.18	3.9E-01	0.54	4.8E-03	0.06	7.7E-01	-0.22	2.7E-01	-0.06	7.7E-01	0.40	4.5E-02	-0.47	1.5E-02
ENSCAFG0000000672	KIRREL2	darkolivegreen	EC_M9	-0.05	8.2E-01	-0.07	7.4E-01	0.04	8.5E-01	-0.09	6.6E-01	0.06	7.6E-01	-0.04	8.4E-01	0.78	2.2E-06	0.03	8.9E-01	0.12	5.7E-01	-0.01	9.8E-01
ENSCAFG0000000694	ENSCAFG0000000694	darkolivegreen	EC_M9	-0.05	8.2E-01	-0.07	7.4E-01	0.04	8.5E-01	-0.09	6.6E-01	0.06	7.6E-01	-0.04	8.4E-01	0.78	2.2E-06	0.03	8.9E-01	0.12	5.7E-01	-0.01	9.8E-01
ENSCAFG0000002863	ENSCAFG0000002863	darkolivegreen	EC_M9	-0.05	8.2E-01	-0.07	7.4E-01	0.04	8.5E-01	-0.09	6.6E-01	0.06	7.6E-01	-0.04	8.4E-01	0.78	2.2E-06	0.03	8.9E-01	0.12	5.7E-01	-0.01	9.8E-01
ENSCAFG0000001283	STX6	grey	EC_M10	-0.05	8.2E-01	0.22	2.9E-01	-0.59	1.4E-03	-0.25	2.1E-01	0.66	2.4E-04	0.17	4.1E-01	0.34	8.5E-02	0.26	2.1E-01	0.13	5.1E-01	-0.61	1.0E-03
ENSCAFG0000001722	MAGEE2	grey	EC_M10	-0.05	8.2E-01	-0.59	1.6E-03	-0.11	5.9E-01	-0.06	7.8E-01	0.40	4.3E-02	0.32	1.2E-01	-0.01	9.6E-01	-0.08	6.9E-01	0.04	8.4E-01	-0.37	6.5E-02
ENSCAFG0000002762	ENSCAFG0000002762	grey	EC_M10	-0.05	8.2E-01	0.39	5.0E-02	-0.12	5.5E-01	0.51	7.1E-03	0.03	8.9E-01	-0.09	6.7E-01	-0.12	5.7E-01	0.38	5.4E-02	-0.03	8.9E-01	0.03	8.9E-01
ENSCAFG0000001631	CHRNB1	turquoise	EC_M6	-0.05	8.2E-01	0.86	1.4E-08	-0.16	4.5E-01	-0.16	4.4E-01	-0.07	7.2E-01	-0.14	4.8E-01	0.08	6.9E-01	-0.10	6.4E-01	0.01	9.7E-01	0.11	6.0E-01
ENSCAFG0000002975	C11H5orf15	grey	EC_M10	-0.05	8.2E-01	-0.01	9.5E-01	-0.67	1.9E-04	-0.16	4.3E-01	0.77	3.6E-06	0.19	3.6E-01	0.10	6.3E-01	-0.04	8.4E-01	0.04	8.5E-01	-0.74	1.9E-05
ENSCAFG0000000952	ENSCAFG0000000952	cyan	EC_M2	-0.05	8.2E-01	-0.14	5.0E-01	0.70	6.1E-05	0.08	7.1E-01	-0.69	8.2E-05	-0.04	8.6E-01	0.24	2.4E-01	-0.18	3.8E-01	-0.37	6.2E-02	0.68	1.4E-04
ENSCAFG0000000409	TTC26	cyan	EC_M2	-0.05	8.2E-01	-0.39	4.6E-02	0.59	1.4E-03	0.27	1.8E-01	-0.59	1.5E-03	-0.11	6.0E-01	-0.18	3.8E-01	-0.15	4.7E-01	0.07	7.4E-01	0.60	1.1E-03
ENSCAFG0000002882	ENSCAFG0000002882	grey	EC_M10	-0.05	8.2E-01	-0.03	9.0E-01	-0.17	4.0E-01	0.08	7.1E-01	0.26	2.0E-01	-0.24	2.4E-01	0.01	9.5E-01	0.24	2.3E-01	-0.09	6.7E-01	-0.24	2.4E-01
ENSCAFG0000001211	ACE2	grey	EC_M10	-0.05	8.2E-01	-0.23	2.7E-01	0.16	4.5E-01	0.13	5.1E-01	-0.06	7.6E-01	-0.21	3.0E-01	-0.16	4.4E-01	0.32	1.1E-01	0.20	3.3E-01	0.10	6.4E-01
ENSCAFG0000002449	ENSCAFG0000002449	violet	EC_M7	-0.05	8.2E-01	-0.05	8.0E-01	-0.24	2.4E-01	-0.04	8.4E-01	0.37	6.7E-02	0.73	2.5E-05	-0.07	7.4E-01	-0.11	5.9E-01	-0.11	5.8E-01	-0.35	8.0E-02
ENSCAFG0000000786	LACTB2	grey	EC_M10	-0.05	8.2E-01	-0.20	3.2E-01	-0.04	8.6E-01	0.03	9.0E-01	0.06	7.7E-01	0.05	8.2E-01	0.10	6.4E-01	0.03	8.9E-01	-0.21	3.0E-01	-0.04	8.5E-01
ENSCAFG0000003234	PDGFD	cyan	EC_M2	-0.05	8.2E-01	0.29	1.5E-01	0.50	8.6E-03	0.08	7.0E-01	-0.64	4.3E-04	-0.33	1.0E-01	0.26	2.1E-01	-0.26	2.0E-01	-0.25	2.2E-01	0.72	3.8E-05
ENSCAFG0000000769	GALNT18	turquoise	EC_M6	-0.05	8.2E-01	0.83	1.6E-07	-0.19	3.6E-01	-0.12	5.6E-01	-0.05	8.2E-01	-0.12	5.7E-01	-0.11	6.0E-01	-0.16	4.3E-01	0.36	6.8E-02	0.06	7.5E-01
ENSCAFG0000001706	NACC1	grey	EC_M10	-0.05	8.2E-01	0.16	4.4E-01	-0.52	5.9E-03	-0.29	1.5E-01	0.67	1.6E-04	0.07	7.4E-01	0.10	6.4E-01	0.00	1.0E+00	0.28	1.7E-01	-0.63	6.1E-04
ENSCAFG0000000637	HGF	cyan	EC_M2	-0.05	8.2E-01	-0.54	4.4E-03	0.76	7.9E-06	0.26	1.9E-01	-0.67	2.1E-04	-0.01	9.6E-01	-0.02	9.3E-01	0.09	6.7E-01	0.03	8.8E-01	0.67	1.7E-04
ENSCAFG0000000415	DNAJC1	darkgreen	EC_M4	-0.05	8.2E-01	0.00	9.9E-01	-0.68	1.1E-04	-0.13	5.3E-01	0.84	6.4E-08	0.18	3.8E-01	0.01	9.7E-01	0.05	8.2E-01	0.02	9.2E-01	-0.76	6.1E-06
ENSCAFG0000000396	FAT4	grey	EC_M10	-0.05	8.2E-01	-0.48	1.3E-02	-0.20	3.3E-01	0.00	9.9E-01	0.44	2.3E-02	0.00	9.8E-01	0.30	1.3E-01	-0.06	7.5E-01	-0.40	4.4E-02	-0.45	2.2E-02
ENSCAFG0000001096	LGALS8	grey	EC_M10	-0.05	8.1E-01	-0.12	5.5E-01	0.14	5.0E-01	0.20	3.2E-01	-0.22	2.9E-01	0.03	8.9E-01	0.36	6.9E-02	-0.12	5.6E-01	0.07	7.5E-01	0.19	3.4E-01
ENSCAFG0000001559	PCNX4	cyan	EC_M2	-0.05	8.1E-01	-0.51	7.5E-03	0.59	1.5E-03	0.08	7.0E-01	-0.50	8.9E-03	-0.08	6.9E-01	-0.25	2.2E-01	-0.07	7.3E-01	-0.02	9.2E-01	0.51	8.3E-03
ENSCAFG0000001061	BICD1	grey	EC_M10	-0.05	8.1E-01	-0.07	7.4E-01	-0.45	2.3E-02	-0.16	4.4E-01	0.57	2.4E-03	-0.01	9.6E-01	0.09	6.5E-01	0.14	4.9E-01	0.52	6.1E-03	-0.48	1.3E-02
ENSCAFG0000001779	USP32	grey	EC_M10	-0.05	8.1E-01	0.12	5.5E-01	-0.25	2.1E-01	0.03	9.0E-01	0.18	3.7E-01	0.15	4.7E-01	0.09	6.7E-01	0.01	9.8E-01	-0.04	8.3E-01	-0.18	3.9E-01

ENSCAFG0000001917	ENSCAFG0000001917	grey	EC_M10	-0.05	8.1E-01	-0.48	1.3E-02	-0.05	8.0E-01	-0.05	8.0E-01	0.20	3.3E-01	0.15	4.8E-01	0.20	3.2E-01	-0.09	6.8E-01	-0.19	3.6E-01	-0.21	3.0E-01
ENSCAFG0000001534	CC2D2A	cyan	EC_M2	-0.05	8.1E-01	-0.27	1.7E-01	0.58	1.8E-03	0.31	1.2E-01	-0.65	3.2E-04	-0.25	2.2E-01	0.03	8.7E-01	0.11	6.1E-01	0.03	8.9E-01	0.63	5.6E-04
ENSCAFG0000000037	UST	grey	EC_M10	-0.05	8.1E-01	-0.50	8.8E-03	-0.07	7.2E-01	0.27	1.8E-01	0.26	1.9E-01	-0.03	8.7E-01	-0.18	3.8E-01	0.39	4.7E-02	0.32	1.2E-01	-0.27	1.9E-01
ENSCAFG0000002874	ENSCAFG0000002874	grey	EC_M10	-0.05	8.1E-01	0.02	9.4E-01	0.24	2.4E-01	-0.24	2.4E-01	-0.23	2.6E-01	-0.18	3.8E-01	0.24	2.4E-01	0.19	3.5E-01	-0.10	6.2E-01	0.30	1.4E-01
ENSCAFG0000000720	C20H3orf67	cyan	EC_M2	-0.05	8.1E-01	-0.48	1.4E-02	0.60	1.3E-03	0.11	6.1E-01	-0.55	3.9E-03	-0.25	2.3E-01	-0.26	1.9E-01	0.06	7.9E-01	0.10	6.4E-01	0.57	2.6E-03
ENSCAFG0000000950	CDK2AP1	grey	EC_M10	-0.05	8.1E-01	-0.33	9.8E-02	0.00	9.9E-01	-0.11	5.9E-01	0.13	5.3E-01	0.37	6.1E-02	0.12	5.6E-01	0.07	7.4E-01	0.28	1.6E-01	-0.13	5.3E-01
ENSCAFG0000001336	ENSCAFG0000001336	grey	EC_M10	-0.05	8.1E-01	-0.39	4.7E-02	0.42	3.2E-02	-0.33	1.0E-01	-0.20	3.2E-01	0.13	5.4E-01	0.18	3.8E-01	-0.16	4.5E-01	-0.23	2.5E-01	0.26	2.0E-01
ENSCAFG0000000603	COL6A6	paleturquoise	EC_M11	-0.05	8.1E-01	0.01	9.5E-01	0.15	4.6E-01	0.78	2.4E-06	-0.20	3.3E-01	-0.04	8.6E-01	-0.02	9.2E-01	-0.10	6.2E-01	0.51	7.4E-03	0.24	2.4E-01
ENSCAFG0000001722	TNPO2	grey	EC_M10	-0.05	8.1E-01	-0.10	6.2E-01	-0.41	3.5E-02	-0.21	3.1E-01	0.60	1.2E-03	0.27	1.8E-01	0.06	7.8E-01	0.10	6.3E-01	0.43	3.0E-02	-0.55	3.5E-03
ENSCAFG0000001729	RNF145	grey	EC_M10	-0.05	8.1E-01	0.38	5.3E-02	-0.52	6.9E-03	-0.27	1.7E-01	0.49	1.1E-02	0.06	7.7E-01	0.12	5.6E-01	0.22	2.8E-01	0.61	1.0E-03	-0.43	2.7E-02
ENSCAFG0000000038	TRIM39	turquoise	EC_M6	-0.05	8.1E-01	0.61	1.0E-03	-0.25	2.2E-01	-0.27	1.8E-01	0.12	5.7E-01	-0.18	3.9E-01	0.04	8.6E-01	0.01	9.5E-01	0.07	7.4E-01	-0.05	8.2E-01
ENSCAFG0000001435	CASK	grey	EC_M10	-0.05	8.1E-01	-0.36	6.9E-02	0.20	3.3E-01	-0.04	8.4E-01	-0.06	7.8E-01	-0.15	4.6E-01	-0.36	7.3E-02	0.37	6.0E-02	-0.13	5.4E-01	0.06	7.9E-01
ENSCAFG0000000756	ANKRD37	grey	EC_M10	-0.05	8.1E-01	0.47	1.5E-02	-0.51	7.9E-03	-0.16	4.3E-01	0.44	2.4E-02	-0.03	8.9E-01	0.45	2.0E-02	0.15	4.7E-01	0.24	2.5E-01	-0.42	3.5E-02
ENSCAFG0000000025	SLC26A10	grey	EC_M10	-0.05	8.1E-01	-0.09	6.8E-01	0.13	5.3E-01	-0.19	3.5E-01	-0.08	7.0E-01	-0.09	6.7E-01	0.50	9.1E-03	-0.02	9.4E-01	-0.02	9.1E-01	0.07	7.3E-01
ENSCAFG0000000929	HIPK1	turquoise	EC_M6	-0.05	8.1E-01	0.67	1.7E-04	-0.25	2.3E-01	-0.19	3.5E-01	0.04	8.5E-01	0.03	9.0E-01	0.00	9.8E-01	-0.09	6.6E-01	0.17	4.1E-01	0.03	8.7E-01
ENSCAFG0000003251	C15H1orf109	grey	EC_M10	-0.05	8.1E-01	-0.42	3.1E-02	-0.07	7.5E-01	-0.02	9.3E-01	0.39	5.2E-02	-0.08	6.8E-01	0.09	6.7E-01	-0.12	5.5E-01	-0.14	5.0E-01	-0.35	8.3E-02
ENSCAFG0000000857	SLA2	grey	EC_M10	-0.05	8.1E-01	0.36	7.4E-02	0.21	2.9E-01	-0.12	5.6E-01	-0.35	8.2E-02	-0.06	7.7E-01	-0.12	5.7E-01	0.33	9.5E-02	-0.12	5.7E-01	0.38	5.3E-02
ENSCAFG0000001435	CCDC96	grey	EC_M10	-0.05	8.1E-01	-0.02	9.2E-01	0.35	7.6E-02	0.38	5.2E-02	-0.35	7.9E-02	-0.16	4.2E-01	0.09	6.7E-01	-0.16	4.3E-01	-0.01	9.8E-01	0.36	7.4E-02
ENSCAFG0000001784	MRM1	grey	EC_M10	-0.05	8.1E-01	-0.07	7.2E-01	-0.23	2.6E-01	-0.32	1.1E-01	0.42	3.4E-02	0.21	3.1E-01	-0.02	9.4E-01	0.26	2.0E-01	0.02	9.4E-01	-0.37	5.9E-02
ENSCAFG0000002445	ENSCAFG0000002445	grey	EC_M10	-0.05	8.1E-01	-0.23	2.7E-01	0.29	1.5E-01	0.21	3.1E-01	-0.26	2.0E-01	-0.16	4.4E-01	-0.28	1.7E-01	-0.03	8.7E-01	-0.47	1.6E-02	0.23	2.6E-01
ENSCAFG0000003025	FAM131C	turquoise	EC_M6	-0.05	8.1E-01	0.56	2.7E-03	0.12	5.6E-01	-0.02	9.1E-01	-0.26	2.0E-01	-0.41	4.0E-02	-0.14	5.0E-01	-0.21	3.1E-01	-0.27	1.9E-01	0.29	1.5E-01
ENSCAFG0000000067	ZFPM2	grey	EC_M10	-0.05	8.1E-01	-0.48	1.2E-02	0.16	4.4E-01	0.36	7.4E-02	-0.08	6.9E-01	0.09	6.6E-01	-0.21	3.0E-01	0.09	6.6E-01	0.04	8.5E-01	0.03	8.8E-01
ENSCAFG0000000938	OSGEPL1	grey	EC_M10	-0.05	8.1E-01	0.07	7.3E-01	0.20	3.2E-01	0.39	4.8E-02	-0.34	8.8E-02	0.07	7.4E-01	0.20	3.3E-01	-0.06	7.8E-01	0.06	7.6E-01	0.39	5.1E-02
ENSCAFG0000001448	SAV1	cyan	EC_M2	-0.05	8.1E-01	-0.25	2.2E-01	0.74	1.5E-05	0.23	2.7E-01	-0.75	1.0E-05	-0.20	3.3E-01	-0.16	4.2E-01	0.01	9.5E-01	-0.30	1.3E-01	0.77	3.6E-06
ENSCAFG0000000039	PCMT1	grey	EC_M10	-0.05	8.1E-01	-0.19	3.6E-01	-0.44	2.3E-02	-0.17	4.0E-01	0.65	3.4E-04	0.03	8.9E-01	0.33	9.8E-02	-0.13	5.2E-01	-0.17	4.2E-01	-0.57	2.6E-03
ENSCAFG0000002762	ENSCAFG0000002762	grey	EC_M10	-0.05	8.1E-01	-0.10	6.3E-01	0.23	2.7E-01	-0.04	8.4E-01	-0.14	5.0E-01	-0.05	8.1E-01	-0.01	9.7E-01	-0.09	6.6E-01	0.20	3.4E-01	0.18	3.8E-01
ENSCAFG0000000221	CDK20	grey	EC_M10	-0.05	8.1E-01	-0.05	8.2E-01	0.34	8.5E-02	-0.08	6.8E-01	-0.24	2.4E-01	0.06	7.6E-01	-0.03	9.0E-01	-0.20	3.4E-01	-0.01	9.7E-01	0.27	1.9E-01
ENSCAFG0000001038	KLHL12	grey	EC_M10	-0.05	8.1E-01	-0.02	9.2E-01	-0.19	3.4E-01	-0.11	5.9E-01	0.27	1.9E-01	0.09	6.5E-01	0.26	2.0E-01	0.13	5.2E-01	0.34	8.7E-02	-0.24	2.4E-01
ENSCAFG0000001976	STKLD1	grey	EC_M10	-0.05	8.1E-01	-0.19	3.4E-01	0.12	5.5E-01	-0.16	4.5E-01	-0.03	8.9E-01	0.27	1.8E-01	0.17	4.2E-01	-0.07	7.3E-01	0.06	7.7E-01	0.02	9.3E-01
ENSCAFG0000001873	LMBRD2	grey	EC_M10	-0.05	8.1E-01	-0.28	1.6E-01	-0.56	3.2E-03	-0.13	5.3E-01	0.76	7.5E-06	0.12	5.7E-01	0.06	7.7E-01	0.17	4.0E-01	-0.04	8.5E-01	-0.72	3.3E-05
ENSCAFG0000001506	C18H11orf95	grey	EC_M10	-0.05	8.1E-01	0.32	1.1E-01	0.57	2.2E-03	0.24	2.4E-01	-0.78	3.1E-06	-0.15	4.6E-01	0.03	9.0E-01	0.03	8.8E-01	0.22	2.7E-01	0.83	1.7E-07
ENSCAFG0000002861	ZNF174	grey	EC_M10	-0.05	8.1E-01	-0.29	1.5E-01	-0.16	4.2E-01	-0.22	2.7E-01	0.27	1.8E-01	0.18	3.8E-01	0.05	8.0E-01	-0.04	8.5E-01	0.06	7.9E-01	-0.28	1.7E-01
ENSCAFG0000001055	ENSCAFG0000001055	grey	EC_M10	-0.05	8.1E-01	-0.14	4.9E-01	-0.02	9.3E-01	-0.18	3.7E-01	0.10	6.4E-01	0.38	5.4E-02	-0.16	4.3E-01	-0.17	4.0E-01	0.13	5.3E-01	-0.07	7.2E-01
ENSCAFG0000001693	NGFR	darkgreen	EC_M4	-0.05	8.1E-01	-0.05	7.9E-01	-0.44	2.5E-02	-0.15	4.8E-01	0.59	1.4E-03	0.04	8.5E-01	-0.10	6.1E-01	0.01	9.5E-01	0.10	6.2E-01	-0.53	5.8E-03
ENSCAFG0000000769	ZNF827	grey	EC_M10	-0.05	8.1E-01	-0.16	4.4E-01	-0.30	1.3E-01	-0.29	1.5E-01	0.45	2.0E-02	0.41	3.8E-02	0.34	9.0E-02	-0.24	2.4E-01	0.11	5.9E-01	-0.42	3.4E-02
ENSCAFG0000000231	OGN	cyan	EC_M2	-0.05	8.1E-01	-0.48	1.2E-02	0.74	1.8E-05	0.32	1.1E-01	-0.66	2.1E-04	-0.22	2.8E-01	-0.17	4.0E-01	-0.06	7.8E-01	-0.16	4.3E-01	0.66	2.1E-04
ENSCAFG0000000563	ENSCAFG0000000563	grey	EC_M10	-0.05	8.1E-01	0.04	8.6E-01	0.14	4.8E-01	0.23	2.6E-01	-0.25	2.2E-01	0.16	4.3E-01	-0.11	6.0E-01	-0.12	5.6E-01	0.20	3.3E-01	0.22	2.9E-01
ENSCAFG0000000196	CAPN11	grey	EC_M10	-0.05	8.1E-01	-0.19	3.4E-01	0.20	3.2E-01	0.29	1.4E-01	-0.17	4.0E-01	-0.12	5.4E-01	0.45	2.1E-02	-0.04	8.5E-01	-0.11	5.9E-01	0.20	3.2E-01
ENSCAFG0000002848	RPS19	cyan	EC_M2	-0.05	8.1E-01	-0.31	1.3E-01	0.49	1.1E-02	0.22	2.8E-01	-0.44	2.5E-02	0.08	7.1E-01	0.47	1.5E-02	-0.03	8.7E-01	-0.36	7.3E-02	0.42	3.4E-02
ENSCAFG0000000083	TNFRSF11B	grey	EC_M10	-0.05	8.1E-01	0.29	1.5E-01	-0.12	5.5E-01	-0.14	5.0E-01	0.15	4.7E-01	-0.14	5.1E-01	0.10	6.3E-01	-0.13	5.3E-01	-0.02	9.3E-01	-0.09	6.8E-01
ENSCAFG0000002941	ENSCAFG0000002941	darkolivegreen	EC_M9	-0.05	8.1E-01	-0.14	5.0E-01	0.15	4.5E-01	-0.07	7.4E-01	-0.02	9.1E-01	-0.09	6.7E-01	0.71	4.1E-05	0.03	8.8E-01	-0.09	6.5E-01	0.09	6.6E-01
ENSCAFG0000000434	ENSCAFG0000000434	grey	EC_M10	-0.05	8.1E-01	0.33	1.0E-01	-0.45	2.1E-02	-0.22	2.9E-01	0.47	1.6E-02	0.19	3.5E-01	0.19	3.6E-01	-0.10	6.4E-01	-0.11	5.8E-01	-0.44	2.3E-02
ENSCAFG0000002853	ENSCAFG0000002853	grey	EC_M10	-0.05	8.1E-01	-0.13	5.2E-01	0.34	9.4E-02	-0.06	7.9E-01	-0.33	9.9E-02	-0.13	5.4E-01	-0.09	6.6E-01	0.25	2.2E-01	0.03	9.0E-01	0.38	5.4E-02
ENSCAFG0000001694	ALOXE3	grey	EC_M10	-0.05	8.1E-01	0.01	9.6E-01	0.20	3.3E-01	0.26	2.0E-01	-0.20	3.3E-01	-0.29	1.5E-01	0.37	6.1E-02	-0.22	2.8E-01	0.02	9.2E-01	0.26	2.1E-01
ENSCAFG0000000549	ZEB2	darkgreen	EC_M4	-0.05	8.1E-01	-0.75	1.0E-05	0.07	7.3E-01	0.13	5.3E-01	0.18	3.8E-01	0.21	3.1E-01	0.06	7.6E-01	0.16	4.3E-01	0.25	2.2E-01	-0.17	4.0E-01
ENSCAFG0000003155	ENSCAFG0000003155	grey	EC_M10	-0.05	8.1E-01	0.18	3.9E-01	-0.23	2.6E-01	-0.40	4.5E-02	0.27	1.9E-01	0.17	4.1E-01	-0.04	8.4E-01	0.30	1.4E-01	-0.15	4.5E-01	-0.21	3.1E-01
ENSCAFG0000001427	DUT	grey	EC_M10	-0.05	8.0E-01	-0.17	4.0E-01	0.36	6.8E-02	0.05	7.9E-01	-0.25	2.1E-01	0.05	8.0E-01	-0.37	6.0E-02	-0.01	9.5E-01	0.26	2.1E-01	0.30	1.3E-01
ENSCAFG0000000694	SLC5A8	grey	EC_M10	-0.05	8.0E-01	-0.17	4.1E-01	-0.04	8.4E-01	0.36	7.1E-02	0.16	4.4E-01	-0.15	4.7E-01	-0.09	6.5E-01	0.32	1.1E-01	0.07	7.2E-01	-0.13	5.3E-01
ENSCAFG0000001545	PITPNM3	magenta	EC_M13	-0.05	8.0E-01	0.25	2.1E-01	-0.05	8.1E-01	-0.05	8.1E-01	-0.01	9.5E-01	-0.05	8.2E-01	0.02	9.4E-01	-0.08	7.0E-01	0.90	2.4E-10	0.09	6.6E-01
ENSCAFG0000001360	PBLD	grey	EC_M10	-0.05	8.0E-01	0.39	5.2E-02	0.29	1.5E-01	0.08	7.1E-01	-0.45	2.2E-02	-0.29	1.5E-01	0.07	7.2E-01	-0.36	7.0E-02	0.14	5.0E-01	0.52	6.7E-03
ENSCAFG0000001531	ENSCAFG0000001531	grey	EC_M10	-0.05	8.0E-01	-0.03	8.7E-01	0.26	2.0E-01	-0.09	6.7E-01	-0.30	1.3E-01	-0.08	7.1E-01	-0.06	7.6E-01	-0.11	6.1E-01	0.50	9.2E-03	0.38	5.3E-02
ENSCAFG0000001646	ZFYVE26	grey	EC_M10	-0.05	8.0E-01	0.47	1.6E-02	-0.10	6.4E-01	0.11	5.8E-01	-0.07	7.2E-01	-0.08	7.1E-01	0.20	3.4E-01	-0.24	2.4E-01	-0.25	2.1E-01	0.12	5.6E-01
ENSCAFG0000002569	ENSCAFG0000002569	grey	EC_M10	-0.05	8.0E-01	-0.05	7.9E-01	0.00	9.9E-01	-0.02	9.1E-01	0.02	9.2E-01	-0.06	7.7E-01	-0.06	7.6E-01	-0.12	5.7E-01	-0.15	4.8E-01	-0.05	8.1E-01
ENSCAFG0000001369	C17H1orf162	grey	EC_M10	-0.05	8.0E-01	-0.04	8.3E-01	-0.19	3.6E-01	0.06	7.7E-01	0.19	3.5E-01	-0.07	7.4E-01	0.00	9.8E-01	-0.10	6.4E-01	0.37	6.3E-02	-0.20	3.3E-01
ENSCAFG0000001266	RFX5	grey	EC_M10	-0.05	8.0E-01	0.38	5.7E-02	0.37	5.9E-02	0.12	5.5E-01	-0.57	2.3E-03	-0.40	4.5E-02	0.12	5.7E-01	0.28	1.6E-01	0.05	8.1E-01	0.60	1.1E-03
ENSCAFG0000003186	HIST1H2AC	grey	EC_M10	-0.05	8.0E-01	0.09	6.8E-01	-0.55	3.4E-03	-0.09	6.7E-01	0.64	4.7E-04	0.02	9.2E-01	-0.27	1.8E-01	0.07	7.3E-01	0.37	6.1E-02	-0.55	3.3E-03
ENSCAFG0000003124	ENSCAFG0000003124	grey	EC_M10	-0.05	8.0E-01	-0.13	5.3E-01	0.13	5.3E-01	0.08	7.0E-01	-0.02	9.1E-01	-0.28	1.6E-01	-0.45	2.1E-02	0.12	5.6E-01	-0.03	8.9E-01	0.06	7.8E-01
ENSCAFG0000001428	ASB16	grey	EC_M10	-0.05	8.0E-01	-0.03	8.7E-01	0.14	4.9E-01	-0.07	7.2E-01	-0.08	6.8E-01	-0.01	9.5E-01	0.04	8.3E-01	-0.06	7.7E-01	0.02	9.2E-01	0.10	6.3E-01
ENSCAFG0000001729	TENT5D	paleturquoise	EC_M11	-0.05	8.0E-01	-0.13	5.4E-01	0.21	3.0E-01	0.70	6.1E-05	-0.19	3.5E-01	-0.04	8.3E-01	-0.07	7.3E-01	-0.14	5.0E-01	-0.18	3.8E-01	0.20	3.2E-01
ENSCAFG0000000558	AP3M2	grey	EC_M10	-0.05	8.0E-01	-0.03	9.0E-01	-0.53	5.1E-03	-0.24	2.4E-01	0.65	3.5E-04	0.00	9.9E-01	0.07	7.5E-01	-0.03	8.8E-01	0.39	4.9E-02	-0.59	1.5E-03
ENSCAFG0000001670	TBX21	grey	EC_M10	-0.05	8.0E-01	0.32	1.1E-01	0.14	5.0E-01	0.40	4.4E-02	-0.23	2.6E-01	-0.14	4.8E-01	-0.08	7.0E-01	-0.17	4.0E-01	0.20	3.2E-01	0.29	1.6E-01
ENSCAFG0000003013	ELOVL4	grey	EC_M10	-0.05	8.0E-01	-0.33	9.7E-02	-0.56	2.8E-03	-0.16	4.3E-01	0.82	2.5E-07	0.21	3.0E-01	0.15	4.6E-01	0.03	9.0E-01	-0.13	5.2E-01	-0.81	6.2E-07
ENSCAFG0000002335	ENSCAFG0000002335	grey	EC_M10	-0.05	8.0E-01	0.25	2.2E-01	0.42	3.1E-02	0.33	9.8E-02	-0.51	7.2E-03	-0.39	4.9E-02	-0.10	6.2E-01	-0.10	6.4E-01	-0.05	7.9E-01	0.59	1.5E-03
ENSCAFG0000001840	JAG2	turquoise	EC_M6	-0.05	8.0E-01	0.79	1.9E-06	-0.15	4.6E-01	-0.16	4.3E-01	-0.04	8.6E-01	-0.24	2.3E-01	0.21	3.0E-01	0.03	8.9E-01	0.43	2.8E-02	0.10	6.3E-01
ENSCAFG0000000179	MTERF2	grey	EC_M10	-0.05	8.0E-01	0.04	8.3E-01	0.17	4.2E-01	0.24	2.3E-01	-0.21	3.1E-01	-0.04	8.5E-01	-0.03	8.9E-01	-0.11	5.9E-01	-0.03	9.0E-01	0.24	2.4E-01
ENSCAFG0000001023	ARHGAP32	grey	EC_M10	-0.05	8.0E-01	-0.11	5.8E-01	0.28	1.6E-01	-0.20	3.4E-01	-0.25	2.3E-01	-0.04	8.6E-01	0.26	2.0E-01	-0.06	7.7E-01	-0.31	1.3E-01	0.22	2.7E-01
ENSCAFG0000000727	FAT1	darkgreen	EC_M4	-0.05	8.0E-01	-0.65	2.9E-04	-0.15	4.6E-01	-0.04	8.5E-01	0.46	1.7E-02	0.07	7.5E-01	0.03	8.9E-01	-0.12	5.6E-01	0.17	4.1E-01	-0.46	1.8E-02
ENSCAFG0000002384	C16H4orf47	turquoise	EC_M6	-0.05	8.0E-01	0.51	7.4E-03	0.09	6.5E-01	0.38	5.4E-02	-0.27	1.9E-01	-0.29	1.5E-01	-0.30	1.3E-01	0.03	8.8E-01	0.11	5.8E-01	0.25	2.2E-01
ENSCAFG0000001091	ENSCAFG0000001091	grey	EC_M10	-0.05	8.0E-01	0.02	9.4E-01	0.32	1.1E-01	0.06	7.8E-01	-0.32	1.1E-01	-0.25	2.3E-01	-0.36	6.9E-02	-0.15	4.6E-01	-0.35	7.9E-02	0.40	4.6E-02
ENSCAFG0000001474	ENSCAFG0000001474	grey	EC_M10	-0.05	8.0E-01	-0.12	5.6E-01	0.09	6.5E-01	0.44	2.5E-02	-0.10	6.3E-01	-0.17	4.0E-01	0.45	2.3E-02	-0.13	5.3E-01	0.17	4.0E-01	0.12	5.7E-01
ENSCAFG0000001991	FOXF1	grey	EC_M10	-0.05	8.0E-01	-0.45	2.0E-02	-0.14	5.1E-01	0.31	1.3E-01	0.34	8.7E-02	0.30	1.4E-01	0.01	9.6E-01	-0.24	2.4E-01	-0.31	1.2E-01	-0.38	5.7E-02
ENSCAFG0000000403	PDZK1IP1	turquoise	EC_M6	-0.05	8.0E-01	0.56	3.0E-03	0.02	9.1E-01	-0.20	3.3E-01	-0.08	7.0E-01	-0.21	3.1E-01	0.19	3.5E-01	-0.16	4.4E-01	-0.18	3.7E-01	0.14	4.8E-01
ENSCAFG0000001534	ADK	grey	EC_M10	-0.05	8.0E-01	-0.65	3.0E-04	0.12	5.6E-01	-0.16	4.5E-01	0.18	3.7E-01	0.04	8.5E-01	-0.01	9.5E-01	-0.16	4.4E-01	-0.13	5.3E-01	-0.14	4.9E-01
ENSCAFG0000003119	EFNB3	grey	EC_M10	-0.05	8.0E-01	-0.07	7.2E-01	-0.11	5.8E-01	0.11	5.9E-01	0.16	4.3E-01	0.27	1.8E-01	-0.26	2.0E-01	0.09	6.7E-01	0.16	4.4E-01	-0.18	3.7E-01
ENSCAFG0000000860	DNAJC22	grey	EC_M10	-0.05	8.0E-01	-0.26	1.9E-01	-0.42	3.1E-02	-0.23	2.7E-01	0.64	4.1E-04	0.11	5.8E-01	-0.13	5.4E-01	0.39	5.1E-02	-0.01	9.7E-01	-0.62	6.6E-04
ENSCAFG0000001432	B3GALNT1	grey	EC_M10	-0.05	8.0E-01	-0.57	2.2E-03	0.08	7.0E-01	0.01	9.6E-01	0.20	3.2E-01	0.02	9.4E-01	-0.07	7.3E-01	0.22	2.7E-01	-0.19	3.6E-01	-0.16	4.4E-01
ENSCAFG0000001689	CACNA1A	grey	EC_M10	-0.05	8.0E-01	-0.38	5.6E-02	0.00	1.0E+00	-0.03	8.7E-01	0.16	4.3E-01	0.30	1.4E-01	0.11	5.9E-01	-0.23	2.5E-01	-0.27	1.7E-01	-0.16	4.2E-01
ENSCAFG0000002915	ENSCAFG0000002915	grey	EC_M10	-0.05	8.0E-01	-0.04	8.6E-01	0.18	3.7E-01	0.52	7.0E-03	-0.22	2.8E-01	-0.24	2.4E-01	0.29	1.6E-01	0.00	9.9E-01	0.16	4.5E-01	0.24	2.3E-01
ENSCAFG0000003221	HEY2	magenta	EC_M13	-0.05	8.0E-01	0.20	3.4E-01	-0.05	8.1E-01	0.00	9.9E-01	-0.01	9.7E-01	-0.04	8.4E-01	-0.01	9.4E-01	0.22	2.8E-01	0.84	5.8E-08	0.05	8.0E-01
ENSCAFG0000003258	ENSCAFG0000003258	turquoise	EC_M6	-0.05	8.0E-01	0.43	2.8E-02	0.01	9.4E-01	-0.11	5.9E-01	-0.17	4.1E-01	-0.11	6.0E-01	-0.09	6.5E-01	-0.18	3.9E-01	0.22	2.9E-01	0.24	2.4E-01
ENSCAFG0000001709	TBX6	grey	EC_M10	-0.05	8.0E-01	-0.01	9.5E-01	0.02	9.3E-01	-0.28	1.6E-01	-0.03	8.9E-01	0.33	1.0E-01	0.14	4.8E-01	-0.04	8.3E-01	0.36	7.0E-02	0.07	7.2E-01
ENSCAFG0000000272	CHAC2	grey	EC_M10	-0.05	8.0E-01	0.01	9.4E-01	0.15	4.7E-01	0.18	3.8E-01	-0.06	7.7E-01	0.06	7.6E-01	0.45	2.0E-02	-0.12	5.5E-01	-0.19	3.6E-01	0.10	6.2E-01
ENSCAFG0000001333	FAM177A1	darkgreen	EC_M4	-0.05	8.0E-01	0.13	5.3E-01	-0.67	1.7E-04	-0.09	6.8E-01	0.81	4.1E-07	0.14	4.9E-01	0.11	6.1E-01	0.17	4.0E-01	0.12	5.5E-01	-0.76	6.4E-06
ENSCAFG0000000409	MGLL	turquoise	EC_M6	-0.05	7.9E-01	0.66	2.3E-04	-0.13	5.2E-01	-0.19	3.6E-01	0.00	1.0E+00	-0.16	4.3E-01	-0.16	4.4E-01	-0.15	4.7E-01	-0.13	5.3E-01	0.08	7.1E-01
ENSCAFG0000000724	CYP7B1	grey	EC_M10	-0.05	7.9E-01	-0.40	4.2E-02	-0.15	4.7E-01	0.16	4.4E-01	0.33	1.0E-01	0.03	9.0E-01	0.06	7.8E-01	0.23	2.6E-01	-0.07	7.4E-01	-0.35	7.9E-02
ENSCAFG0000002587	ENSCAFG0000002587	grey	EC_M10	-0.05	7.9E-01	0.25	2.2E-01	0.21	2.9E-01	-0.13	5.3E-01	-0.18	3.7E-01	-0.14	5.0E-01	-0.06	7.7E-01	-0.09	6.6E-01	0.31	1.3E-01	0.27	1.7E-01
ENSCAFG0000000207	CA9	grey	EC_M10	-0.05	7.9E-01	-0.11	6.0E-01	0.14	5.0E-01	-0.06	7.8E-01	-0.06	7.8E-01	-0.14	4.9E-01	0.54	4.3E-03	-0.11	5.9E-01	0.27	1.8E-01	0.06	7.7E-01
ENSCAFG0000001820	B9D1	cyan	EC_M2	-0.05	7.9E-01	-0.39	4.7E-02	0.67	1.7E-04	0.29	1.5E-01	-0.59	1.5E-03	-0.22	2.8E-01	-0.23	2.7E-01	0.14	4.9E-01	0.00	9.9E-01	0.58	1.8E-03
ENSCAFG0000000655	ENSCAFG0000000655	magenta	EC_M13	-0.05	7.9E-01	0.06	7.6E-01	0.17	4.0E-01	-0.07	7.4E-01	-0.24	2.3E-01	-0.05	8.2E-01	-0.02	9.4E-01	-0.06	7.6E-01	0.67	1.6E-04	0.29	1.5E-01
ENSCAFG0000000138	CBY1	darkgreen	EC_M4	-0.05	7.9E-01	-0.58	1.8E-03	-0.13	5.1E-01	0.09	6.7E-01	0.38	5.6E-02	0.21	3.0E-01	0.08	7.1E-01	-0.02	9.2E-01	0.12	5.7E-01	-0.36	6.8E-02
ENSCAFG0000000137	SCRIB	grey	EC_M10	-0.05	7.9E-01	0.30	1.4E-01	0.59	1.4E-03	-0.04	8.6E-01	-0.73	2.4E-05	-0.10	6.4E-01	0.13	5.4E-01	-0.17	4.0E-01	0.09	6.5E-01	0.75	1.2E-05
ENSCAFG0000000804	ENSCAFG0000000804	grey	EC_M10	-0.05	7.9E-01	-0.06	7.9E-01	-0.10	6.1E-01	-0.04	8.5E-01	0.10	6.4E-01	0.27	1.8E-01	-0.32	1.1E-01	0.09	6.4E-01	0.05	7.9E-01	-0.07	7.5E-01
ENSCAFG0000000651	DCTN6	darkgreen	EC_M4	-0.05	7.9E-01	0.00	1.0E+00	-0.73	2.2E-05	-0.14	5.0E-01	0.92	4.1E-11	0.30	1.4E-01	0.03	8.9E-01	-0.06	7.6E-01	-0.04	8.4E-01	-0.89	1.5E-09
ENSCAFG0000000797	ENSCAFG0000000797	turquoise	EC_M6	-0.05	7.9E-01	0.64	4.8E-04	-0.09	6.6E-01	-0.07	7.3E-01	-0.07	7.4E-01	-0.06	7.7E-01	-0.12	5.6E-01	-0.08	7.1E-01	-0.11	5.8E-01	0.13	5.1E-01
ENSCAFG0000000620	ENSCAFG0000000620	grey	EC_M10	-0.05	7.9E-01	-0.19	3.6E-01	-0.01	9.8E-01	-0.19	3.5E-01	0.05	8.1E-01	0.06	7.6E-01	-0.02	9.4E-01	0.08	6.9E-01	0.11	5.8E-01	-0.08	7.0E-01
ENSCAFG0000001839	CXHXorf56	grey	EC_M10	-0.05	7.9E-01	0.18	3.9E-01	-0.11	5.9E-01	0.36	7.5E-02	0.13	5.3E-01	-0.16	4.4E-01	-0.02	9.3E-01	-0.56	3.0E-03	-0.05	8.1E-01	-0.04	8.3E-01
ENSCAFG0000001780	CRYM	grey	EC_M10	-0.05	7.9E-01	-0.07	7.2E-01	0.21	3.1E-01	-0.04	8.3E-01	-0.19	3.4E-01	-0.09	6.6E-01	0.03	8.9E-01	-0.10	6.2E-01	0.00	1.0E+00	0.20	3.3E-01
ENSCAFG0000001643	PRPF19	grey	EC_M10	-0.05	7.9E-01	-0.33	1.0E-01	0.21	3.1E-01	0.04	8.3E-01	0.06	7.9E-01	-0.01	9.6E-01	-0.27	1.7E-01	-0.16	4.2E-01	-0.10	6.2E-01	0.03	8.9E-01
ENSCAFG0000001677	CASZ1	turquoise	EC_M6	-0.05	7.9E-01	0.44	2.4E-02	0.02	9.2E-01	-0.12	5.5E-01	-0.12	5.5E-01	0.28	1.7E-01	-0.10	6.2E-01	-0.16	4.4E-01	-0.13	5.4E-01	0.17	3.9E-01
ENSCAFG0000001699	PGF	turquoise	EC_M6	-0.05	7.9E-01	0.80	9.8E-07	-0.12	5.5E-01	-0.21	3.1E-01	-0.09	6.5E-01	-0.29	1.5E-01	0.19	3.6E-01	0.10	6.1E-01	0.32	1.2E-01	0.12	5.7E-01
ENSCAFG0000000506	LMO7	darkgreen	EC_M4	-0.05	7.9E-01	-0.53	5.5E-03	-0.26	2.0E-01	-0.01	9.5E-01	0.55	3.4E-03	-0.05	8.2E-01	-0.10	6.4E-01	0.23	2.6E-01	0.29	1.5E-01	-0.54	4.6E-03
ENSCAFG0000001795	COL5A3	cyan	EC_M2	-0.05	7.9E-01	-0.41	3.6E-02	0.72	3.9E-05	0.00	9.8E-01	-0.62	7.4E-04	-0.32	1.2E-01	0.04	8.5E-01	0.22	2.7E-01	0.05	8.1E-01	0.63	6.2E-04
ENSCAFG0000000951	PIDD1	cyan	EC_M2	-0.05	7.9E-01	0.28	1.7E-01	0.66	2.4E-04	0.07	7.3E-01	-0.84	8.7E-08	-0.14	4.9E-01	-0.02	9.1E-01	-0.14	4.8E-01	0.06	7.6E-01	0.85	3.3E-08
ENSCAFG0000000493	ENSCAFG0000000493	grey	EC_M10	-0.06	7.9E-01	-0.10	6.2E-01	-0.09	6.7E-01	0.35	7.7E-02	0.18	3.9E-01	0.10	6.4E-01	0.07	7.3E-01	-0.23	2.6E-01	0.08	7.0E-01	-0.16	4.4E-01
ENSCAFG0000000679	GTF3A	darkgreen	EC_M4	-0.06	7.9E-01	-0.87	1.1E-08	0.36	7.5E-02	0.16	4.3E-01	-0.04	8.6E-01	0.13	5.1E-01	-0.15	4.7E-01	0.00	1.0E+00	-0.03	8.7E-01	0.04	8.3E-01
ENSCAFG0000001230	TNS3	magenta	EC_M13	-0.06	7.9E-01	-0.09	6.5E-01	-0.18	3.8E-01	-0.14	4.9E-01	0.29	1.5E-01	-0.09	6.7E-01	-0.01	9.5E-01	0.46	1.9E-02	0.75	8.7E-06	-0.21	3.1E-01
ENSCAFG0000001898	C6H16orf72	grey	EC_M10	-0.06	7.9E-01	-0.38	5.5E-02	-0.28	1.7E-01	-0.08	6.9E-01	0.57	2.3E-03	0.11	5.8E-01	-0.16	4.4E-01	0.01	9.5E-01	-0.12	5.6E-01	-0.55	3.9E-03
ENSCAFG0000000424	KIF3C	turquoise	EC_M6	-0.06	7.9E-01	0.84	1.1E-07	-0.40	4.2E-02	-0.17	4.2E-01	0.19	3.6E-01	-0.22	2.8E-01	0.21	3.1E-01	0.07	7.5E-01	0.04	8.6E-01	-0.16	4.2E-01
ENSCAFG0000002589	ENSCAFG0000002589	grey	EC_M10	-0.06	7.9E-01	-0.04	8.3E-01	0.04	8.3E-01	-0.04	8.3E-01	-0.06	7.7E-01	-0.05	8.2E-01	-0.08	7.0E-01	-0.16	4.5E-01	0.27	1.9E-01	0.03	8.8E-01
ENSCAFG0000000275	SLC44A1	cyan	EC_M2	-0.06	7.9E-01	0.00	9.8E-01	0.78	2.2E-06	0.15	4.7E-01	-0.88	3.9E-09	-0.31	1.3E-01	0.06	7.5E-01	-0.07	7.4E-01	-0.04	8.5E-01	0.91	8.7E-11
ENSCAFG0000002473	RUFY4	grey	EC_M10	-0.06	7.9E-01	-0.21	3.0E-01	0.33	9.5E-02	0.28	1.7E-01	-0.24	2.4E-01	-0.17	4.2E-01	-0.12	5.7E-01	-0.11	5.8E-01	-0.04	8.3E-01	0.33	1.0E-01
ENSCAFG0000000894	SLIT1	magenta	EC_M13	-0.06	7.9E-01	-0.01	9.5E-01	0.22	2.8E-01	-0.07	7.4E-01	-0.19	3.6E-01	-0.02	9.3E-01	-0.01	9.5E-01	-0.04	8.5E-01	0.65	3.6E-04	0.25	2.1E-01
ENSCAFG0000001884	ADAMTS12	grey	EC_M10	-0.06	7.9E-01	0.12	5.5E-01	0.27	1.8E-01	0.10	6.2E-01	-0.25	2.1E-01	-0.28	1.6E-01	0.13	5.3E-01	-0.15	4.6E-01	-0.57	2.3E-03	0.31	1.2E-01
ENSCAFG0000000546	PFKP	darkgreen	EC_M4	-0.06	7.9E-01	0.33	1.0E-01	-0.56	3.1E-03	-0.24	2.3E-01	0.59	1.6E-03	0.07	7.4E-01	0.21	3.1E-01	0.04	8.3E-01	0.02	9.1E-01	-0.58	1.8E-03
ENSCAFG0000000371	FAM49A	turquoise	EC_M6	-0.06	7.9E-01	0.55	3.6E-03	0.21	3.1E-01	-0.09	6.8E-01	-0.34	8.8E-02	-0.17	4.1E-01	0.27	1.8E-01	-0.18	3.7E-01	-0.21	3.1E-01	0.40	4.3E-02
ENSCAFG0000002110	ENSCAFG0000002110	grey	EC_M10	-0.06	7.9E-01	-0.02	9.4E-01	-0.37	6.6E-02	-0.13	5.2E-01	0.42	3.3E-02	0.28	1.6E-01	0.12	5.6E-01	-0.21	2.9E-01	-0.24	2.5E-01	-0.38	5.5E-02

ENSCAFG0000000008	PMEL	grey	EC_M10	-0.06	7.9E-01	-0.33	1.0E-01	0.50	9.3E-03	0.13	5.2E-01	-0.42	3.4E-02	-0.27	1.9E-01	0.00	1.0E+00	0.30	1.4E-01	-0.09	6.6E-01	0.46	1.8E-02
ENSCAFG0000001519	PELI2	grey	EC_M10	-0.06	7.9E-01	0.06	7.7E-01	-0.23	2.6E-01	-0.10	6.2E-01	0.36	7.4E-02	-0.23	2.7E-01	0.01	9.5E-01	0.14	4.9E-01	0.06	7.7E-01	-0.27	1.9E-01
ENSCAFG0000000495	ATP1A3	grey	EC_M10	-0.06	7.9E-01	-0.25	2.1E-01	-0.14	4.8E-01	-0.25	2.2E-01	0.35	8.2E-02	0.08	7.1E-01	0.46	1.9E-02	-0.11	6.1E-01	-0.01	9.7E-01	-0.25	2.2E-01
ENSCAFG0000000788	RWDD4	grey	EC_M10	-0.06	7.9E-01	-0.38	5.9E-02	0.24	2.4E-01	-0.09	6.7E-01	0.02	9.3E-01	0.06	7.9E-01	-0.12	5.5E-01	0.03	9.0E-01	-0.06	7.6E-01	0.03	8.9E-01
ENSCAFG0000001323	MAP3K13	grey	EC_M10	-0.06	7.9E-01	-0.07	7.5E-01	-0.37	5.9E-02	0.04	8.6E-01	0.42	3.2E-02	0.03	8.8E-01	0.01	9.7E-01	0.04	8.3E-01	0.01	9.5E-01	-0.44	2.5E-02
ENSCAFG0000003065	ENSCAFG0000003065	grey	EC_M10	-0.06	7.9E-01	-0.25	2.1E-01	0.03	8.7E-01	0.05	8.2E-01	0.09	6.7E-01	-0.02	9.0E-01	-0.06	7.9E-01	-0.10	6.2E-01	0.44	2.4E-02	-0.01	9.7E-01
ENSCAFG0000002562	ENSCAFG0000002562	grey	EC_M10	-0.06	7.9E-01	-0.17	3.9E-01	0.50	9.0E-03	-0.04	8.4E-01	-0.43	3.0E-02	-0.10	6.4E-01	-0.07	7.3E-01	0.03	8.8E-01	-0.14	4.9E-01	0.46	1.9E-02
ENSCAFG0000002014	FUK	cyan	EC_M2	-0.06	7.9E-01	0.25	2.3E-01	0.63	5.1E-04	0.09	6.7E-01	-0.82	3.3E-07	-0.21	2.9E-01	0.00	1.0E+00	0.12	5.6E-01	0.26	1.9E-01	0.84	1.1E-07
ENSCAFG0000001805	LINGO1	grey	EC_M10	-0.06	7.9E-01	0.15	4.7E-01	0.11	5.8E-01	-0.22	2.8E-01	-0.03	9.0E-01	-0.13	5.1E-01	-0.12	5.7E-01	0.16	4.3E-01	0.23	2.6E-01	0.10	6.4E-01
ENSCAFG0000003238	ZFPM1	grey	EC_M10	-0.06	7.8E-01	0.32	1.2E-01	0.19	3.5E-01	0.28	1.7E-01	-0.24	2.3E-01	-0.47	1.5E-02	0.02	9.0E-01	-0.24	2.5E-01	0.39	5.2E-02	0.29	1.5E-01
ENSCAFG0000000588	PGAP2	grey	EC_M10	-0.06	7.8E-01	0.12	5.6E-01	0.31	1.2E-01	-0.11	5.8E-01	-0.26	2.0E-01	0.04	8.6E-01	-0.02	9.3E-01	0.18	3.7E-01	0.02	9.4E-01	0.32	1.2E-01
ENSCAFG0000001751	CALM1	darkgreen	EC_M4	-0.06	7.8E-01	0.23	2.7E-01	-0.62	6.6E-04	-0.39	5.0E-02	0.75	1.1E-05	0.12	5.6E-01	0.16	4.2E-01	0.14	5.0E-01	0.13	5.3E-01	-0.69	9.6E-05
ENSCAFG0000001509	LGALS12	grey	EC_M10	-0.06	7.8E-01	-0.05	8.2E-01	0.44	2.3E-02	-0.06	7.7E-01	-0.42	3.3E-02	-0.11	6.1E-01	-0.07	7.5E-01	-0.09	6.7E-01	0.33	9.7E-02	0.46	1.7E-02
ENSCAFG0000000355	INHBA	darkgreen	EC_M4	-0.06	7.8E-01	-0.01	9.7E-01	-0.69	8.2E-05	-0.17	4.2E-01	0.85	4.5E-08	0.22	2.7E-01	0.19	3.6E-01	0.11	6.0E-01	-0.09	6.8E-01	-0.85	3.3E-08
ENSCAFG0000000594	THSD1	turquoise	EC_M6	-0.06	7.8E-01	0.84	8.7E-08	-0.02	9.3E-01	-0.07	7.3E-01	-0.21	3.1E-01	-0.24	2.5E-01	0.01	9.5E-01	0.00	1.0E+00	-0.15	4.8E-01	0.24	2.4E-01
ENSCAFG0000002981	COMMD6	grey	EC_M10	-0.06	7.8E-01	-0.34	9.1E-02	-0.05	8.0E-01	0.01	9.4E-01	0.19	3.6E-01	-0.13	5.2E-01	-0.46	1.9E-02	0.26	2.0E-01	0.23	2.6E-01	-0.16	4.3E-01
ENSCAFG0000001251	ENSCAFG0000001251	grey	EC_M10	-0.06	7.8E-01	0.32	1.2E-01	-0.05	8.2E-01	-0.12	5.6E-01	-0.01	9.6E-01	0.30	1.4E-01	-0.09	6.6E-01	-0.21	3.0E-01	0.13	5.1E-01	-0.02	9.3E-01
ENSCAFG0000000588	DPH3	grey	EC_M10	-0.06	7.8E-01	0.03	9.0E-01	-0.24	2.4E-01	-0.11	5.8E-01	0.39	5.0E-02	0.45	2.1E-02	0.21	3.1E-01	0.40	4.4E-02	0.20	3.3E-01	-0.33	1.0E-01
ENSCAFG0000001180	SC5D	grey	EC_M10	-0.06	7.8E-01	-0.17	4.1E-01	0.08	6.9E-01	-0.01	9.4E-01	-0.01	9.7E-01	-0.19	3.7E-01	-0.04	8.4E-01	0.11	5.8E-01	-0.63	5.6E-04	0.01	9.7E-01
ENSCAFG0000003064	ENSCAFG0000003064	grey	EC_M10	-0.06	7.8E-01	-0.10	6.1E-01	0.07	7.3E-01	-0.09	6.7E-01	0.02	9.1E-01	-0.16	4.3E-01	0.46	1.7E-02	-0.05	8.2E-01	-0.19	3.5E-01	-0.03	8.9E-01
ENSCAFG0000000248	GABBR2	grey	EC_M10	-0.06	7.8E-01	0.07	7.3E-01	0.31	1.2E-01	-0.12	5.6E-01	-0.26	2.1E-01	-0.04	8.3E-01	-0.04	8.6E-01	-0.29	1.5E-01	-0.20	3.3E-01	0.35	7.8E-02
ENSCAFG0000000032	ENSCAFG0000000032	grey	EC_M10	-0.06	7.8E-01	-0.24	2.4E-01	-0.04	8.6E-01	-0.13	5.3E-01	0.23	2.6E-01	-0.16	4.3E-01	-0.15	4.7E-01	-0.06	7.7E-01	-0.11	5.8E-01	-0.20	3.2E-01
ENSCAFG0000002523	ENSCAFG0000002523	violet	EC_M7	-0.06	7.8E-01	-0.14	5.1E-01	-0.12	5.7E-01	-0.07	7.3E-01	0.19	3.5E-01	0.51	7.4E-03	-0.03	8.8E-01	-0.14	5.1E-01	-0.18	3.7E-01	-0.21	3.1E-01
ENSCAFG0000001959	G6PD	grey	EC_M10	-0.06	7.8E-01	-0.05	8.1E-01	0.71	5.2E-05	-0.08	6.8E-01	-0.66	2.2E-04	-0.17	4.0E-01	0.05	8.2E-01	-0.27	1.9E-01	-0.11	6.0E-01	0.73	2.1E-05
ENSCAFG0000000270	TFPT	grey	EC_M10	-0.06	7.8E-01	0.07	7.3E-01	-0.26	2.0E-01	0.12	5.5E-01	0.29	1.4E-01	-0.04	8.5E-01	0.17	4.1E-01	0.24	2.5E-01	0.07	7.2E-01	-0.25	2.1E-01
ENSCAFG0000002844	ENSCAFG0000002844	grey	EC_M10	-0.06	7.8E-01	-0.31	1.2E-01	0.23	2.6E-01	0.12	5.5E-01	-0.13	5.3E-01	0.28	1.7E-01	0.03	8.8E-01	0.07	7.2E-01	0.20	3.4E-01	0.18	3.8E-01
ENSCAFG0000000172	C11H9orf72	grey	EC_M10	-0.06	7.8E-01	0.38	5.8E-02	0.05	8.1E-01	0.16	4.5E-01	-0.20	3.2E-01	-0.27	1.8E-01	0.05	8.1E-01	-0.22	2.8E-01	-0.42	3.0E-02	0.24	2.3E-01
ENSCAFG0000001363	ENSCAFG0000001363	turquoise	EC_M6	-0.06	7.8E-01	0.64	4.2E-04	-0.37	6.1E-02	-0.14	5.0E-01	0.22	2.8E-01	0.06	7.7E-01	0.11	5.8E-01	-0.04	8.6E-01	0.33	9.7E-02	-0.14	5.0E-01
ENSCAFG0000002042	CDH11	grey	EC_M10	-0.06	7.8E-01	-0.18	3.8E-01	-0.41	3.5E-02	-0.11	6.1E-01	0.63	6.1E-04	0.17	4.1E-01	0.20	3.3E-01	-0.22	2.7E-01	-0.03	8.7E-01	-0.60	1.3E-03
ENSCAFG0000003044	ENSCAFG0000003044	grey	EC_M10	-0.06	7.8E-01	0.33	9.9E-02	-0.41	3.5E-02	-0.13	5.4E-01	0.43	2.8E-02	0.08	6.9E-01	0.02	9.2E-01	0.20	3.4E-01	-0.25	2.3E-01	-0.43	2.7E-02
ENSCAFG0000000510	ITPKC	grey	EC_M10	-0.06	7.8E-01	0.64	4.3E-04	0.34	8.7E-02	0.07	7.5E-01	-0.58	1.8E-03	-0.06	7.6E-01	0.08	6.8E-01	-0.15	4.5E-01	0.00	1.0E+00	0.64	4.5E-04
ENSCAFG0000000158	ENSCAFG0000000158	grey	EC_M10	-0.06	7.8E-01	-0.06	7.6E-01	-0.03	8.7E-01	-0.14	4.9E-01	0.10	6.2E-01	-0.02	9.2E-01	-0.06	7.5E-01	-0.17	4.2E-01	0.20	3.2E-01	-0.02	9.3E-01
ENSCAFG0000000421	OTUD1	grey	EC_M10	-0.06	7.8E-01	0.25	2.2E-01	0.38	5.7E-02	0.17	4.0E-01	-0.57	2.2E-03	-0.12	5.7E-01	-0.13	5.3E-01	0.25	2.1E-01	0.30	1.3E-01	0.57	2.5E-03
ENSCAFG0000001984	COL5A1	darkgreen	EC_M4	-0.06	7.8E-01	-0.52	6.0E-03	-0.42	3.1E-02	-0.01	9.7E-01	0.73	2.0E-05	0.29	1.5E-01	0.11	6.1E-01	0.04	8.3E-01	-0.06	7.6E-01	-0.76	8.1E-06
ENSCAFG0000001084	BTN2A2	grey	EC_M10	-0.06	7.8E-01	-0.06	7.8E-01	-0.48	1.2E-02	-0.15	4.6E-01	0.67	1.8E-04	0.43	3.0E-02	0.07	7.5E-01	-0.14	5.1E-01	0.12	5.7E-01	-0.62	7.1E-04
ENSCAFG0000000170	RBFOX2	grey	EC_M10	-0.06	7.8E-01	-0.25	2.2E-01	-0.34	8.6E-02	0.04	8.6E-01	0.57	2.1E-03	0.01	9.6E-01	-0.13	5.4E-01	0.23	2.5E-01	-0.02	9.3E-01	-0.60	1.3E-03
ENSCAFG0000000516	ENSCAFG0000000516	grey	EC_M10	-0.06	7.8E-01	-0.09	6.7E-01	-0.34	8.9E-02	0.10	6.4E-01	0.45	2.1E-02	-0.05	8.0E-01	-0.07	7.4E-01	-0.06	7.6E-01	-0.09	6.5E-01	-0.44	2.5E-02
ENSCAFG0000003027	ENSCAFG0000003027	grey	EC_M10	-0.06	7.8E-01	0.34	8.6E-02	0.01	9.8E-01	0.58	1.9E-03	-0.14	5.0E-01	-0.13	5.4E-01	-0.11	5.8E-01	-0.17	4.1E-01	0.06	7.5E-01	0.17	4.0E-01
ENSCAFG0000001205	IDH2	darkgreen	EC_M4	-0.06	7.8E-01	0.13	5.3E-01	-0.64	4.1E-04	-0.22	2.7E-01	0.76	7.3E-06	0.10	6.4E-01	0.44	2.4E-02	0.04	8.5E-01	0.13	5.3E-01	-0.73	2.3E-05
ENSCAFG0000003164	GNG2	turquoise	EC_M6	-0.06	7.8E-01	0.52	7.0E-03	-0.17	4.0E-01	-0.20	3.2E-01	0.11	5.9E-01	-0.27	1.8E-01	0.14	5.0E-01	-0.06	7.6E-01	-0.22	2.9E-01	-0.05	8.1E-01
ENSCAFG0000000982	IGSF9B	grey	EC_M10	-0.06	7.8E-01	-0.66	2.5E-04	0.35	7.6E-02	0.01	9.6E-01	-0.08	7.0E-01	-0.02	9.2E-01	0.28	1.6E-01	-0.04	8.6E-01	-0.20	3.3E-01	0.13	5.3E-01
ENSCAFG0000002341	TTC32	grey	EC_M10	-0.06	7.8E-01	-0.10	6.4E-01	0.19	3.4E-01	0.11	6.1E-01	-0.19	3.6E-01	-0.23	2.6E-01	0.04	8.6E-01	0.12	5.6E-01	-0.37	6.2E-02	0.25	2.1E-01
ENSCAFG0000001361	PRAME	grey	EC_M10	-0.06	7.8E-01	-0.07	7.4E-01	0.12	5.5E-01	0.25	2.3E-01	-0.05	8.0E-01	0.19	3.5E-01	-0.02	9.3E-01	-0.06	7.6E-01	-0.15	4.8E-01	0.03	9.0E-01
ENSCAFG0000002913	KAZALD1	cyan	EC_M2	-0.06	7.8E-01	-0.49	1.1E-02	0.73	1.9E-05	0.10	6.1E-01	-0.58	1.8E-03	-0.26	2.1E-01	0.13	5.2E-01	0.04	8.3E-01	-0.09	6.7E-01	0.63	5.7E-04
ENSCAFG0000001957	ACOT7	darkgreen	EC_M4	-0.06	7.8E-01	-0.12	5.5E-01	-0.68	1.5E-04	-0.25	2.3E-01	0.91	7.8E-11	0.14	5.0E-01	0.07	7.2E-01	0.16	4.5E-01	0.18	3.7E-01	-0.85	2.9E-08
ENSCAFG0000002748	ENSCAFG0000002748	grey	EC_M10	-0.06	7.8E-01	-0.16	4.5E-01	0.36	7.0E-02	-0.12	5.5E-01	-0.34	8.7E-02	-0.09	6.8E-01	-0.13	5.2E-01	-0.11	5.9E-01	-0.06	7.7E-01	0.42	3.4E-02
ENSCAFG0000002003	VAT1L	magenta	EC_M13	-0.06	7.8E-01	0.02	9.4E-01	0.08	6.9E-01	-0.10	6.1E-01	-0.05	8.0E-01	-0.07	7.4E-01	0.00	9.8E-01	0.20	3.4E-01	0.81	7.0E-07	0.10	6.1E-01
ENSCAFG0000000229	IPPK	grey	EC_M10	-0.06	7.8E-01	-0.18	3.8E-01	0.28	1.6E-01	0.19	3.4E-01	-0.15	4.5E-01	0.23	2.5E-01	0.03	8.7E-01	-0.12	5.6E-01	0.07	7.4E-01	0.22	2.8E-01
ENSCAFG0000002772	ENSCAFG0000002772	darkgreen	EC_M4	-0.06	7.8E-01	-0.09	6.8E-01	-0.10	6.2E-01	-0.12	5.8E-01	0.27	1.9E-01	-0.06	7.7E-01	-0.05	8.0E-01	-0.04	8.6E-01	0.08	6.9E-01	-0.19	3.6E-01
ENSCAFG0000002416	PRR22	grey	EC_M10	-0.06	7.8E-01	0.00	9.9E-01	0.24	2.3E-01	-0.35	8.1E-02	-0.19	3.5E-01	0.23	2.5E-01	0.16	4.5E-01	0.00	1.0E+00	-0.05	8.0E-01	0.19	3.6E-01
ENSCAFG0000002880	RNF150	grey	EC_M10	-0.06	7.8E-01	-0.13	5.2E-01	-0.35	7.6E-02	-0.13	5.3E-01	0.54	4.7E-03	0.14	5.0E-01	0.13	5.3E-01	0.19	3.5E-01	0.62	7.1E-04	-0.48	1.3E-02
ENSCAFG0000000647	ENSCAFG0000000647	grey	EC_M10	-0.06	7.8E-01	-0.34	9.0E-02	0.41	3.9E-02	0.08	7.0E-01	-0.33	1.0E-01	0.18	3.7E-01	0.16	4.3E-01	0.13	5.2E-01	0.08	7.1E-01	0.31	1.2E-01
ENSCAFG0000000775	DENND6A	grey	EC_M10	-0.06	7.8E-01	0.52	6.8E-03	0.28	1.7E-01	0.29	1.4E-01	-0.49	1.1E-02	-0.32	1.2E-01	0.09	6.6E-01	-0.01	9.5E-01	0.26	2.0E-01	0.58	1.7E-03
ENSCAFG0000000395	SF3B6	grey	EC_M10	-0.06	7.8E-01	0.05	7.9E-01	0.03	9.0E-01	-0.04	8.4E-01	0.05	8.0E-01	0.16	4.4E-01	0.16	4.2E-01	0.12	5.7E-01	-0.02	9.1E-01	-0.07	7.4E-01
ENSCAFG0000001703	MRPL27	grey	EC_M10	-0.06	7.7E-01	-0.38	5.7E-02	0.37	6.0E-02	-0.07	7.5E-01	-0.15	4.5E-01	0.08	6.9E-01	0.13	5.2E-01	0.00	9.9E-01	-0.03	8.9E-01	0.17	4.0E-01
ENSCAFG0000001859	C6	darkgreen	EC_M4	-0.06	7.7E-01	0.09	6.8E-01	-0.33	1.0E-01	-0.03	9.0E-01	0.40	4.5E-02	-0.02	9.3E-01	-0.13	5.3E-01	-0.06	7.7E-01	-0.02	9.3E-01	-0.37	6.4E-02
ENSCAFG0000001214	ARNT	cyan	EC_M2	-0.06	7.7E-01	0.28	1.6E-01	0.50	9.2E-03	0.13	5.3E-01	-0.71	4.4E-05	-0.17	3.9E-01	0.21	2.9E-01	0.05	8.0E-01	0.10	6.1E-01	0.74	1.8E-05
ENSCAFG0000000925	SFRP5	grey	EC_M10	-0.06	7.7E-01	-0.20	3.3E-01	-0.13	5.2E-01	0.17	4.1E-01	0.31	1.2E-01	0.23	2.7E-01	-0.16	4.4E-01	-0.26	2.0E-01	0.19	3.5E-01	-0.20	3.3E-01
ENSCAFG0000001771	SYNE3	cyan	EC_M2	-0.06	7.7E-01	-0.10	6.1E-01	0.82	2.5E-07	0.15	4.5E-01	-0.87	9.8E-09	-0.29	1.5E-01	0.01	9.8E-01	-0.11	5.9E-01	-0.19	3.6E-01	0.92	6.0E-11
ENSCAFG0000000289	CABS1	magenta	EC_M13	-0.06	7.7E-01	0.10	6.3E-01	-0.05	7.9E-01	-0.09	6.6E-01	0.04	8.5E-01	-0.10	6.4E-01	-0.06	7.6E-01	0.72	3.7E-05	0.52	6.8E-03	-0.02	9.1E-01
ENSCAFG0000000412	ENDOD1	grey	EC_M10	-0.06	7.7E-01	-0.36	6.9E-02	-0.07	7.5E-01	-0.16	4.4E-01	0.35	8.0E-02	-0.10	6.1E-01	0.02	9.2E-01	0.03	8.9E-01	0.20	3.2E-01	-0.23	2.6E-01
ENSCAFG0000002014	TMED5	grey	EC_M10	-0.06	7.7E-01	-0.15	4.6E-01	-0.02	9.4E-01	0.11	6.0E-01	0.11	5.9E-01	0.02	9.3E-01	-0.28	1.6E-01	-0.22	2.8E-01	-0.15	4.7E-01	-0.04	8.5E-01
ENSCAFG0000001490	LSM4	grey	EC_M10	-0.06	7.7E-01	-0.23	2.5E-01	0.58	1.7E-03	0.16	4.2E-01	-0.49	1.1E-02	-0.05	8.2E-01	-0.12	5.7E-01	0.07	7.2E-01	0.07	7.4E-01	0.47	1.4E-02
ENSCAFG0000000175	TRPM6	grey	EC_M10	-0.06	7.7E-01	-0.23	2.5E-01	0.17	4.2E-01	-0.24	2.3E-01	-0.11	6.0E-01	0.12	5.6E-01	-0.18	3.8E-01	0.16	4.3E-01	0.21	3.1E-01	0.15	4.7E-01
ENSCAFG0000001308	DNAJC5	grey	EC_M10	-0.06	7.7E-01	0.15	4.5E-01	-0.17	4.2E-01	-0.10	6.2E-01	0.27	1.7E-01	0.18	3.9E-01	-0.10	6.1E-01	0.09	6.7E-01	0.52	6.5E-03	-0.23	2.7E-01
ENSCAFG0000000069	NELFE	grey	EC_M10	-0.06	7.7E-01	0.32	1.1E-01	-0.14	5.1E-01	0.40	4.4E-02	0.15	4.7E-01	-0.10	6.1E-01	0.14	5.1E-01	-0.11	6.0E-01	-0.15	4.7E-01	-0.10	6.2E-01
ENSCAFG0000002680	ENSCAFG0000002680	grey	EC_M10	-0.06	7.7E-01	0.16	4.5E-01	-0.25	2.2E-01	-0.03	8.9E-01	0.29	1.5E-01	0.20	3.3E-01	-0.14	4.8E-01	0.27	1.9E-01	-0.17	4.0E-01	-0.31	1.2E-01
ENSCAFG0000002990	ENSCAFG0000002990	paleturquoise	EC_M11	-0.06	7.7E-01	0.01	9.6E-01	0.10	6.2E-01	0.59	1.4E-03	-0.10	6.1E-01	-0.02	9.1E-01	0.00	9.9E-01	-0.09	6.6E-01	-0.09	6.4E-01	0.09	6.7E-01
ENSCAFG0000001867	PGM1	grey	EC_M10	-0.06	7.7E-01	-0.67	2.0E-04	0.34	9.4E-02	-0.16	4.4E-01	-0.09	6.7E-01	0.11	6.0E-01	0.07	7.3E-01	-0.08	7.0E-01	-0.41	3.7E-02	0.08	7.1E-01
ENSCAFG0000001580	WNK1	grey	EC_M10	-0.06	7.7E-01	0.27	1.9E-01	-0.59	1.5E-03	-0.31	1.3E-01	0.62	7.4E-04	0.02	9.3E-01	-0.13	5.2E-01	-0.01	9.6E-01	0.06	7.7E-01	-0.56	2.7E-03
ENSCAFG0000002595	ENSCAFG0000002595	grey	EC_M10	-0.06	7.7E-01	-0.05	8.0E-01	0.29	1.5E-01	-0.01	9.6E-01	-0.21	3.1E-01	-0.06	7.6E-01	-0.01	9.8E-01	-0.05	8.0E-01	0.57	2.4E-03	0.28	1.7E-01
ENSCAFG0000000997	CAP2	grey	EC_M10	-0.06	7.7E-01	0.17	4.2E-01	-0.67	1.7E-04	-0.15	4.6E-01	0.76	7.8E-06	0.18	3.7E-01	0.11	6.1E-01	0.08	6.8E-01	0.46	1.8E-02	-0.70	7.1E-05
ENSCAFG0000000066	RSPH3	grey	EC_M10	-0.06	7.7E-01	-0.64	4.0E-04	0.47	1.6E-02	0.12	5.6E-01	-0.23	2.6E-01	0.13	5.2E-01	-0.26	1.9E-01	0.12	5.6E-01	0.05	7.9E-01	0.26	2.0E-01
ENSCAFG0000001159	ARHGAP6	magenta	EC_M13	-0.06	7.7E-01	0.37	6.5E-02	0.30	1.4E-01	-0.13	5.1E-01	-0.42	3.5E-02	-0.30	1.4E-01	0.09	6.7E-01	0.30	1.3E-01	0.59	1.6E-03	0.50	8.7E-03
ENSCAFG0000000576	RPL15	cyan	EC_M2	-0.06	7.7E-01	-0.24	2.4E-01	0.75	1.2E-05	0.19	3.4E-01	-0.68	1.5E-04	-0.03	8.7E-01	0.08	7.1E-01	-0.22	2.8E-01	-0.34	8.5E-02	0.68	1.4E-04
ENSCAFG0000001759	PIAS2	grey	EC_M10	-0.06	7.7E-01	-0.18	3.9E-01	0.62	7.9E-04	0.27	1.9E-01	-0.59	1.4E-03	-0.19	3.5E-01	-0.30	1.4E-01	0.11	6.1E-01	0.07	7.4E-01	0.66	2.6E-04
ENSCAFG0000000522	ESYT2	darkgreen	EC_M4	-0.06	7.7E-01	-0.10	6.3E-01	-0.67	1.6E-04	-0.18	3.8E-01	0.85	5.4E-08	0.18	3.9E-01	0.23	2.6E-01	0.11	5.8E-01	0.05	8.0E-01	-0.79	1.3E-06
ENSCAFG0000002926	TEN1	grey	EC_M10	-0.06	7.7E-01	-0.46	1.9E-02	0.28	1.6E-01	-0.19	3.6E-01	-0.07	7.2E-01	-0.06	7.7E-01	-0.19	3.4E-01	-0.04	8.4E-01	0.00	9.9E-01	0.11	5.9E-01
ENSCAFG0000001323	LAMC1	cyan	EC_M2	-0.06	7.7E-01	0.19	3.6E-01	0.60	1.3E-03	0.18	3.8E-01	-0.75	1.2E-05	-0.20	3.3E-01	0.07	7.5E-01	0.07	7.3E-01	-0.23	2.7E-01	0.78	3.2E-06
ENSCAFG0000001122	PRSS56	magenta	EC_M13	-0.06	7.7E-01	0.02	9.1E-01	-0.14	4.9E-01	-0.12	5.7E-01	0.20	3.2E-01	-0.06	7.7E-01	0.31	1.2E-01	-0.05	8.2E-01	0.65	3.5E-04	-0.14	5.1E-01
ENSCAFG0000000048	STK3	grey	EC_M10	-0.06	7.7E-01	-0.09	6.5E-01	0.14	4.9E-01	-0.17	4.1E-01	-0.09	6.5E-01	0.01	9.5E-01	0.14	5.0E-01	-0.05	8.0E-01	-0.22	2.8E-01	0.17	4.0E-01
ENSCAFG0000001284	THPO	grey	EC_M10	-0.06	7.7E-01	-0.25	2.1E-01	0.61	9.0E-04	0.44	2.3E-02	-0.58	1.8E-03	-0.14	4.9E-01	0.08	6.9E-01	-0.10	6.2E-01	-0.17	4.0E-01	0.66	2.5E-04
ENSCAFG0000001258	CEP85	cyan	EC_M2	-0.06	7.7E-01	-0.15	4.8E-01	0.70	7.1E-05	0.29	1.6E-01	-0.74	1.4E-05	-0.19	3.5E-01	-0.22	2.9E-01	-0.24	2.4E-01	-0.20	3.4E-01	0.79	1.6E-06
ENSCAFG0000000179	LIMCH1	turquoise	EC_M6	-0.06	7.7E-01	0.61	9.6E-04	0.19	3.5E-01	-0.04	8.4E-01	-0.36	6.7E-02	-0.22	2.9E-01	-0.14	4.8E-01	0.13	5.2E-01	-0.01	9.6E-01	0.37	5.9E-02
ENSCAFG0000001532	NDUFA9	grey	EC_M10	-0.06	7.7E-01	-0.59	1.6E-03	0.25	2.2E-01	-0.08	6.9E-01	0.06	7.7E-01	0.19	3.5E-01	-0.13	5.3E-01	-0.10	6.3E-01	0.04	8.3E-01	0.02	9.0E-01
ENSCAFG0000003099	N6AMT1	grey	EC_M10	-0.06	7.7E-01	-0.38	5.5E-02	0.46	1.7E-02	0.06	7.7E-01	-0.34	9.2E-02	-0.16	4.4E-01	-0.18	3.9E-01	-0.07	7.2E-01	-0.28	1.6E-01	0.39	4.9E-02
ENSCAFG0000001222	COL6A3	darkgreen	EC_M4	-0.06	7.7E-01	-0.81	5.3E-07	0.29	1.5E-01	0.06	7.7E-01	-0.01	9.6E-01	0.07	7.3E-01	0.33	1.0E-01	0.19	3.5E-01	0.14	5.0E-01	0.05	8.3E-01
ENSCAFG0000001312	INSYN2	turquoise	EC_M6	-0.06	7.7E-01	0.50	9.1E-03	0.08	7.1E-01	-0.12	5.5E-01	-0.23	2.5E-01	-0.10	6.2E-01	0.47	1.7E-02	-0.02	9.1E-01	-0.12	5.5E-01	0.29	1.5E-01
ENSCAFG0000000734	F11	grey	EC_M10	-0.06	7.7E-01	0.26	1.9E-01	0.29	1.6E-01	-0.16	4.4E-01	-0.37	5.9E-02	-0.10	6.4E-01	-0.15	4.6E-01	0.15	4.7E-01	-0.14	5.0E-01	0.43	2.7E-02
ENSCAFG0000000553	MBD5	grey	EC_M10	-0.06	7.7E-01	0.34	8.7E-02	-0.19	3.6E-01	0.05	8.1E-01	0.04	8.3E-01	0.10	6.1E-01	-0.05	8.0E-01	0.03	9.0E-01	0.22	2.8E-01	-0.02	9.4E-01
ENSCAFG0000001638	PHF8	grey	EC_M10	-0.06	7.7E-01	0.05	8.2E-01	0.42	3.2E-02	0.01	9.7E-01	-0.53	5.4E-03	-0.20	3.4E-01	-0.13	5.2E-01	0.01	9.8E-01	0.02	9.3E-01	0.52	5.9E-03
ENSCAFG0000001909	AFF2	grey	EC_M10	-0.06	7.7E-01	-0.47	1.4E-02	-0.11	5.8E-01	0.21	3.1E-01	0.35	7.9E-02	0.09	6.6E-01	-0.14	4.9E-01	-0.06	7.9E-01	-0.05	8.1E-01	-0.28	1.7E-01
ENSCAFG0000001844	RHOT1	grey	EC_M10	-0.06	7.7E-01	0.41	3.7E-02	-0.53	4.9E-03	-0.24	2.3E-01	0.50	9.0E-03	-0.05	8.1E-01	0.15	4.7E-01	0.18	3.7E-01	-0.13	5.1E-01	-0.44	2.6E-02
ENSCAFG0000001609	ENSCAFG0000001609	grey	EC_M10	-0.06	7.7E-01	-0.13	5.2E-01	0.39	5.1E-02	-0.18	3.8E-01	-0.29	1.4E-01	-0.03	8.8E-01	-0.10	6.3E-01	-0.14	4.8E-01	0.22	2.9E-01	0.34	9.4E-02
ENSCAFG0000000223	LONRF2	grey	EC_M10	-0.06	7.6E-01	-0.16	4.4E-01	-0.27	1.9E-01	-0.47	1.4E-02	0.43	3.0E-02	-0.11	6.0E-01	0.02	9.3E-01	0.23	2.5E-01	0.10	6.2E-01	-0.37	6.6E-02
ENSCAFG0000002954	ATF5	grey	EC_M10	-0.06	7.6E-01	-0.36	6.8E-02	-0.31	1.3E-01	0.13	5.2E-01	0.52	6.4E-03	0.24	2.4E-01	-0.17	4.2E-01	-0.05	8.2E-01	-0.01	9.5E-01	-0.53	5.6E-03
ENSCAFG0000000250	ZNF691	grey	EC_M10	-0.06	7.6E-01	0.05	8.0E-01	0.50	9.0E-03	0.32	1.2E-01	-0.51	7.8E-03	-0.15	4.6E-01	0.22	2.9E-01	0.01	9.6E-01	-0.12	5.5E-01	0.55	3.4E-03
ENSCAFG0000000748	ENSCAFG0000000748	grey	EC_M10	-0.06	7.6E-01	0.19	3.6E-01	0.35	8.2E-02	-0.16	4.3E-01	-0.41	3.6E-02	-0.11	5.8E-01	-0.16	4.3E-01	0.23	2.7E-01	0.31	1.3E-01	0.41	3.6E-02
ENSCAFG0000000393	ADCK2	cyan	EC_M2	-0.06	7.6E-01	-0.20	3.4E-01	0.75	9.2E-06	0.11	6.0E-01	-0.71	4.2E-05	-0.40	4.4E-02	-0.09	6.6E-01	0.03	8.9E-01	0.26	2.0E-01	0.77	4.8E-06
ENSCAFG0000000237	GRHPR	grey	EC_M10	-0.06	7.6E-01	-0.04	8.4E-01	0.21	3.0E-01	0.23	2.6E-01	-0.14	5.1E-01	-0.07	7.5E-01	0.01	9.6E-01	-0.24	2.4E-01	-0.22	2.7E-01	0.12	5.6E-01
ENSCAFG0000003252	COTL1	darkgreen	EC_M4	-0.06	7.6E-01	0.10	6.2E-01	-0.72	3.9E-05	-0.13	5.2E-01	0.85	4.7E-08	0.24	2.3E-01	-0.11	6.0E-01	0.14	4.9E-01	0.16	4.3E-01	-0.85	4.7E-08
ENSCAFG0000001717	ENSCAFG0000001717	grey	EC_M10	-0.06	7.6E-01	-0.08	7.1E-01	-0.09	6.7E-01	-0.03	9.0E-01	0.13	5.2E-01	-0.09	6.7E-01	-0.08	7.1E-01	0.35	7.6E-02	-0.07	7.4E-01	-0.16	4.2E-01
ENSCAFG0000002443	COLCA2	grey	EC_M10	-0.06	7.6E-01	0.04	8.5E-01	0.02	9.2E-01	0.01	9.4E-01	-0.05	8.0E-01	-0.05	8.2E-01	-0.07	7.3E-01	-0.09	6.5E-01	0.42	3.4E-02	0.07	7.3E-01
ENSCAFG0000001838	ESM1	grey	EC_M10	-0.06	7.6E-01	0.18	3.7E-01	-0.42	3.2E-02	-0.24	2.4E-01	0.45	2.2E-02	-0.02	9.1E-01	0.11	6.0E-01	0.29	1.6E-01	0.65	3.1E-04	-0.38	5.9E-02
ENSCAFG0000001745	INTS3	grey	EC_M10	-0.06	7.6E-01	0.50	9.4E-03	0.33	1.0E-01	-0.04	8.6E-01	-0.52	6.6E-03	-0.10	6.2E-01	0.20	3.4E-01	0.00	9.8E-01	0.01	9.4E-01	0.54	4.3E-03
ENSCAFG0000001014	ELK4	cyan	EC_M2	-0.06	7.6E-01	0.33	9.9E-02	0.51	8.4E-03	0.17	4.0E-01	-0.70	7.8E-05	-0.41	3.8E-02	0.15	4.6E-01	-0.01	9.5E-01	0.05	8.1E-01	0.73	2.2E-05
ENSCAFG0000000266	ENSCAFG0000000266	grey	EC_M10	-0.06	7.6E-01	-0.29	1.5E-01	0.36	7.2E-02	0.14	4.9E-01	-0.33	9.6E-02	-0.26	1.9E-01	0.19	3.6E-01	0.11	6.0E-01	0.11	6.0E-01	0.34	9.4E-02
ENSCAFG0000003245	ATP5IF1	grey	EC_M10	-0.06	7.6E-01	-0.25	2.2E-01	-0.37	6.5E-02	-0.17	4.1E-01	0.67	2.0E-04	0.18	3.9E-01	0.13	5.3E-01	0.13	5.3E-01	-0.04	8.6E-01	-0.61	8.5E-04
ENSCAFG0000001678	DNAH2	paleturquoise	EC_M11	-0.06	7.6E-01	-0.13	5.4E-01	0.08	7.0E-01	0.55	3.9E-03	-0.04	8.5E-01	-0.09	6.6E-01	0.35	8.1E-02	-0.10	6.1E-01	-0.19	3.4E-01	0.05	8.2E-01
ENSCAFG0000003216	BATF3	grey	EC_M10	-0.06	7.6E-01	0.30	1.4E-01	-0.29	1.5E-01	-0.10	6.3E-01	0.27	1.8E-01	0.36	6.7E-02	-0.11	5.8E-01	-0.22	2.7E-01	-0.19	3.6E-01	-0.26	2.0E-01
ENSCAFG0000000210	FHL2	grey	EC_M10	-0.06	7.6E-01	-0.51	7.3E-03	-0.37	6.4E-02	0.04	8.6E-01	0.65	2.9E-04	0.19	3.6E-01	0.11	6.1E-01	0.15	4.6E-01	0.22	2.8E-01	-0.65	3.7E-04
ENSCAFG0000001967	ENTR1	grey	EC_M10	-0.06	7.6E-01	0.56	3.1E-03	-0.50	8.8E-03	-0.50	9.4E-03	0.47	1.5E-02	-0.02	9.3E-01	0.20	3.4E-01	-0.04	8.5E-01	0.22	2.7E-01	-0.43	3.0E-02
ENSCAFG0000000963	CBR3	grey	EC_M10	-0.06	7.6E-01	0.27	1.9E-01	0.53	5.6E-03	-0.11	5.8E-01	-0.56	2.8E-03	-0.48	1.3E-02	-0.06	7.6E-01	-0.03	8.9E-01	-0.06	7.7E-01	0.67	1.9E-04
ENSCAFG0000001533	NPTX2	grey	EC_M10	-0.06	7.6E-01	0.43	2.7E-02	-0.55	3.8E-03	-0.27	1.8E-01	0.56	2.8E-03	0.03	8.8E-01	0.06	7.7E-01	-0.12	5.4E-01	0.42	3.3E-02	-0.47	1.5E-02
ENSCAFG0000000739	ABHD6	turquoise	EC_M6	-0.06	7.6E-01	0.60	1.1E-03	0.14	5.0E-01	0.19	3.4E-01	-0.34	9.1E-02	-0.06	7.9E-01	-0.16	4.4E-01	-0.31	1.2E-01	0.02	9.2E-01	0.37	6.5E-02
ENSCAFG0000000045	ENSCAFG0000000045	grey	EC_M10	-0.06	7.6E-01	0.12	5.7E-01	-0.08	6.8E-01	-0.03	9.0E-01	0.12	5.8E-01	-0.11	6.0E-01	-0.13	5.3E-01	-0.07	7.3E-01	-0.12	5.6E-01	-0.14	5.0E-01

ENSCAFG0000000092	GOPC	grey	EC_M10	-0.06	7.6E-01	0.25	2.2E-01	-0.24	2.4E-01	-0.08	6.8E-01	0.20	3.3E-01	0.15	4.7E-01	-0.03	9.0E-01	0.07	7.2E-01	-0.46	1.9E-02	-0.19	3.4E-01
ENSCAFG0000002566	ENSCAFG0000002566	grey	EC_M10	-0.06	7.6E-01	0.02	9.3E-01	-0.13	5.4E-01	-0.19	3.6E-01	0.27	1.9E-01	-0.15	4.6E-01	-0.11	5.8E-01	0.27	1.8E-01	0.03	8.8E-01	-0.22	2.8E-01
ENSCAFG0000001469	ENSCAFG0000001469	grey	EC_M10	-0.06	7.6E-01	-0.16	4.3E-01	0.03	9.0E-01	-0.05	8.1E-01	0.06	7.8E-01	-0.11	6.0E-01	-0.12	5.6E-01	-0.11	6.0E-01	-0.23	2.7E-01	-0.08	6.8E-01
ENSCAFG0000003113	HSBP1L1	grey	EC_M10	-0.06	7.6E-01	-0.10	6.1E-01	0.01	9.6E-01	-0.17	4.0E-01	0.10	6.2E-01	-0.05	7.9E-01	-0.05	8.0E-01	0.45	2.0E-02	0.12	5.4E-01	-0.09	6.5E-01
ENSCAFG0000000820	AGTR1	grey	EC_M10	-0.06	7.6E-01	-0.19	3.6E-01	0.54	4.8E-03	-0.11	5.9E-01	-0.45	2.0E-02	-0.07	7.4E-01	0.11	5.8E-01	-0.06	7.5E-01	-0.22	2.9E-01	0.51	7.2E-03
ENSCAFG0000000564	ENSCAFG0000000564	grey	EC_M10	-0.06	7.6E-01	0.20	3.2E-01	0.04	8.6E-01	0.39	4.7E-02	-0.09	6.7E-01	-0.12	5.5E-01	-0.21	3.0E-01	0.19	3.5E-01	-0.22	2.8E-01	0.08	7.0E-01
ENSCAFG0000003267	ENSCAFG0000003267	grey	EC_M10	-0.06	7.6E-01	-0.07	7.3E-01	0.00	9.9E-01	-0.02	9.1E-01	0.04	8.4E-01	-0.06	7.8E-01	-0.05	8.1E-01	-0.11	5.8E-01	-0.14	4.8E-01	-0.06	7.7E-01
ENSCAFG0000002830	ENSCAFG0000002830	turquoise	EC_M6	-0.06	7.6E-01	0.43	3.0E-02	0.10	6.2E-01	0.24	2.5E-01	-0.20	3.4E-01	-0.17	3.9E-01	0.22	2.8E-01	-0.14	5.0E-01	-0.22	2.9E-01	0.24	2.3E-01
ENSCAFG0000000870	CKMT2	grey	EC_M10	-0.06	7.6E-01	-0.07	7.3E-01	-0.08	6.8E-01	0.00	9.9E-01	0.12	5.7E-01	-0.09	6.5E-01	0.54	4.8E-03	-0.05	8.2E-01	-0.14	5.1E-01	-0.12	5.4E-01
ENSCAFG0000000787	DCLK2	turquoise	EC_M6	-0.06	7.6E-01	0.68	1.4E-04	-0.15	4.5E-01	-0.13	5.3E-01	0.01	9.7E-01	-0.23	2.7E-01	0.08	7.0E-01	0.15	4.6E-01	0.53	5.1E-03	0.08	7.0E-01
ENSCAFG0000000105	POLR3H	grey	EC_M10	-0.06	7.5E-01	-0.29	1.5E-01	-0.07	7.5E-01	-0.22	2.8E-01	0.35	7.8E-02	0.04	8.5E-01	0.00	1.0E+00	-0.01	9.6E-01	0.01	9.5E-01	-0.33	9.9E-02
ENSCAFG0000000068	TUBGCP6	grey	EC_M10	-0.06	7.5E-01	0.56	2.7E-03	0.35	7.6E-02	0.01	9.5E-01	-0.52	6.3E-03	-0.40	4.5E-02	0.26	2.1E-01	-0.13	5.1E-01	0.03	8.8E-01	0.57	2.2E-03
ENSCAFG0000001933	CLDN6	grey	EC_M10	-0.06	7.5E-01	-0.16	4.3E-01	-0.52	6.4E-03	-0.05	8.2E-01	0.71	5.5E-05	0.37	6.4E-02	0.18	3.9E-01	-0.08	6.9E-01	-0.10	6.4E-01	-0.71	4.2E-05
ENSCAFG0000001923	ATP2B3	grey	EC_M10	-0.06	7.5E-01	-0.11	6.1E-01	0.11	5.9E-01	-0.03	8.7E-01	0.00	1.0E+00	-0.07	7.2E-01	-0.05	8.1E-01	-0.07	7.3E-01	0.11	6.0E-01	0.09	6.5E-01
ENSCAFG0000000578	ANKHD1	cyan	EC_M2	-0.06	7.5E-01	0.03	8.7E-01	0.66	2.8E-04	0.17	4.2E-01	-0.78	3.1E-06	-0.23	2.5E-01	-0.17	4.2E-01	-0.05	8.1E-01	-0.33	9.9E-02	0.78	2.6E-06
ENSCAFG0000000560	RELT	darkgreen	EC_M4	-0.06	7.5E-01	-0.07	7.3E-01	-0.62	6.8E-04	-0.28	1.6E-01	0.86	2.4E-08	0.14	5.0E-01	0.02	9.4E-01	0.07	7.5E-01	0.13	5.4E-01	-0.80	9.9E-07
ENSCAFG0000000509	AANAT	grey	EC_M10	-0.06	7.5E-01	0.21	3.0E-01	0.38	5.7E-02	0.03	8.7E-01	-0.48	1.4E-02	0.28	1.6E-01	0.05	7.9E-01	-0.26	2.0E-01	0.08	7.0E-01	0.55	3.8E-03
ENSCAFG0000002065	ENSCAFG0000002065	grey	EC_M10	-0.06	7.5E-01	0.24	2.3E-01	0.33	9.8E-02	0.20	3.4E-01	-0.33	1.0E-01	0.11	5.8E-01	0.19	3.6E-01	-0.26	2.0E-01	-0.07	7.4E-01	0.37	6.4E-02
ENSCAFG0000001950	C5H1orf174	grey	EC_M10	-0.06	7.5E-01	0.09	6.7E-01	0.31	1.3E-01	-0.23	2.6E-01	-0.30	1.3E-01	-0.23	2.6E-01	0.13	5.2E-01	0.04	8.4E-01	0.02	9.3E-01	0.32	1.1E-01
ENSCAFG0000000663	HOXC10	grey	EC_M10	-0.06	7.5E-01	-0.17	4.0E-01	0.18	3.7E-01	-0.26	2.1E-01	-0.03	8.9E-01	-0.19	3.6E-01	0.23	2.7E-01	-0.18	3.7E-01	-0.18	3.7E-01	0.11	6.0E-01
ENSCAFG0000000867	PTK2B	grey	EC_M10	-0.06	7.5E-01	0.31	1.2E-01	0.13	5.2E-01	0.08	6.8E-01	-0.20	3.2E-01	-0.41	3.8E-02	0.08	6.8E-01	0.13	5.3E-01	0.26	2.0E-01	0.32	1.1E-01
ENSCAFG0000003011	POMK	grey	EC_M10	-0.06	7.5E-01	0.28	1.6E-01	0.13	5.3E-01	0.17	4.1E-01	-0.27	1.8E-01	0.00	9.9E-01	-0.05	8.1E-01	-0.05	8.0E-01	0.16	4.5E-01	0.31	1.3E-01
ENSCAFG0000000484	PAFAH1B3	cyan	EC_M2	-0.07	7.5E-01	-0.31	1.2E-01	0.74	1.6E-05	0.08	7.0E-01	-0.63	5.2E-04	-0.20	3.2E-01	0.13	5.3E-01	0.09	6.7E-01	-0.29	1.5E-01	0.66	2.8E-04
ENSCAFG0000000395	ENSCAFG0000000395	grey	EC_M10	-0.07	7.5E-01	-0.06	7.8E-01	-0.08	7.0E-01	0.08	6.9E-01	0.13	5.3E-01	-0.10	6.2E-01	-0.04	8.5E-01	-0.03	8.9E-01	-0.12	5.7E-01	-0.17	4.0E-01
ENSCAFG0000001682	UBTD2	cyan	EC_M2	-0.07	7.5E-01	-0.65	3.3E-04	0.37	6.2E-02	0.11	6.0E-01	-0.16	4.4E-01	0.00	9.8E-01	-0.11	6.1E-01	0.10	6.1E-01	-0.33	9.6E-02	0.21	2.9E-01
ENSCAFG0000001827	ENSCAFG0000001827	grey	EC_M10	-0.07	7.5E-01	0.18	3.7E-01	0.20	3.2E-01	0.12	5.5E-01	-0.19	3.4E-01	0.04	8.3E-01	-0.18	3.7E-01	-0.27	1.8E-01	0.43	2.7E-02	0.27	1.7E-01
ENSCAFG0000000124	PTCH1	grey	EC_M10	-0.07	7.5E-01	0.53	5.6E-03	0.28	1.7E-01	0.13	5.2E-01	-0.47	1.7E-02	-0.22	2.8E-01	0.04	8.5E-01	-0.19	3.6E-01	-0.07	7.4E-01	0.54	4.8E-03
ENSCAFG0000001795	MORC4	darkgreen	EC_M4	-0.07	7.5E-01	-0.77	4.0E-06	0.09	6.5E-01	0.11	5.8E-01	0.19	3.5E-01	0.20	3.4E-01	0.29	1.5E-01	-0.10	6.4E-01	-0.29	1.5E-01	-0.20	3.3E-01
ENSCAFG0000001927	CLUAP1	cyan	EC_M2	-0.07	7.5E-01	-0.36	7.4E-02	0.51	8.3E-03	0.24	2.4E-01	-0.51	8.1E-03	-0.28	1.7E-01	-0.15	4.5E-01	0.00	9.9E-01	-0.02	9.1E-01	0.53	5.3E-03
ENSCAFG0000003082	GCSAM	grey	EC_M10	-0.07	7.5E-01	0.17	4.1E-01	-0.44	2.4E-02	-0.03	8.7E-01	0.53	4.9E-03	-0.09	6.5E-01	-0.05	8.0E-01	-0.09	6.7E-01	0.34	8.6E-02	-0.49	1.1E-02
ENSCAFG0000000842	ENSCAFG0000000842	grey	EC_M10	-0.07	7.5E-01	-0.14	4.8E-01	0.16	4.3E-01	-0.08	6.9E-01	-0.13	5.4E-01	-0.08	7.1E-01	-0.07	7.5E-01	-0.15	4.7E-01	-0.08	7.0E-01	0.19	3.6E-01
ENSCAFG0000000668	SLC25A26	grey	EC_M10	-0.07	7.5E-01	-0.13	5.3E-01	0.51	8.5E-03	0.49	1.2E-02	-0.55	3.5E-03	-0.03	8.9E-01	0.00	9.9E-01	-0.20	3.3E-01	-0.01	9.7E-01	0.55	3.3E-03
ENSCAFG0000002727	ENSCAFG0000002727	grey	EC_M10	-0.07	7.5E-01	-0.03	8.9E-01	0.34	8.4E-02	-0.05	8.2E-01	-0.34	9.4E-02	0.02	9.1E-01	-0.03	8.8E-01	-0.06	7.6E-01	-0.12	5.6E-01	0.36	6.8E-02
ENSCAFG0000002987	ENSCAFG0000002987	grey	EC_M10	-0.07	7.5E-01	-0.03	8.9E-01	0.34	8.4E-02	-0.05	8.2E-01	-0.34	9.4E-02	0.02	9.1E-01	-0.03	8.8E-01	-0.06	7.6E-01	-0.12	5.6E-01	0.36	6.8E-02
ENSCAFG0000000192	MRPS18A	grey	EC_M10	-0.07	7.5E-01	-0.55	3.8E-03	0.09	6.6E-01	-0.14	5.1E-01	0.20	3.2E-01	0.36	6.9E-02	-0.06	7.9E-01	0.07	7.5E-01	-0.09	6.6E-01	-0.16	4.3E-01
ENSCAFG0000002381	ENSCAFG0000002381	grey	EC_M10	-0.07	7.5E-01	-0.21	3.0E-01	-0.20	3.4E-01	0.42	3.2E-02	0.32	1.1E-01	0.29	1.6E-01	-0.16	4.4E-01	0.27	1.8E-01	0.18	3.8E-01	-0.32	1.1E-01
ENSCAFG0000000897	TMEM150C	turquoise	EC_M6	-0.07	7.5E-01	0.77	3.7E-06	-0.06	7.8E-01	-0.09	6.6E-01	-0.15	4.8E-01	-0.29	1.5E-01	0.37	6.1E-02	0.22	2.7E-01	-0.04	8.7E-01	0.18	3.7E-01
ENSCAFG0000003025	ZNF333	cyan	EC_M2	-0.07	7.5E-01	-0.09	6.8E-01	0.67	1.7E-04	0.34	9.3E-02	-0.79	1.4E-06	-0.25	2.1E-01	0.04	8.3E-01	0.00	9.9E-01	0.02	9.2E-01	0.81	6.0E-07
ENSCAFG0000003150	UBAP1L	grey	EC_M10	-0.07	7.5E-01	0.07	7.2E-01	-0.19	3.5E-01	-0.23	2.6E-01	0.23	2.6E-01	-0.23	2.6E-01	0.13	5.3E-01	-0.26	2.1E-01	0.09	6.5E-01	-0.18	3.8E-01
ENSCAFG0000001930	ENSCAFG0000001930	grey	EC_M10	-0.07	7.5E-01	-0.16	4.4E-01	0.09	6.6E-01	-0.07	7.2E-01	0.07	7.3E-01	-0.13	5.1E-01	-0.10	6.4E-01	-0.10	6.2E-01	-0.15	4.6E-01	-0.04	8.5E-01
ENSCAFG0000001919	NUDT16L1	grey	EC_M10	-0.07	7.5E-01	0.18	3.7E-01	0.09	6.7E-01	-0.28	1.7E-01	-0.01	9.5E-01	0.00	9.8E-01	-0.14	5.1E-01	-0.01	9.5E-01	0.28	1.7E-01	0.09	6.6E-01
ENSCAFG0000000076	EGFL8	grey	EC_M10	-0.07	7.5E-01	0.07	7.2E-01	0.47	1.4E-02	0.09	6.4E-01	-0.58	2.0E-03	-0.13	5.2E-01	0.13	5.4E-01	0.16	4.3E-01	0.00	9.9E-01	0.61	8.7E-04
ENSCAFG0000002488	SOX4	grey	EC_M10	-0.07	7.5E-01	0.47	1.6E-02	-0.51	7.5E-03	0.00	9.9E-01	0.47	1.7E-02	0.13	5.2E-01	-0.06	7.6E-01	-0.12	5.6E-01	0.03	8.8E-01	-0.44	2.6E-02
ENSCAFG0000001249	PFKFB4	grey	EC_M10	-0.07	7.5E-01	-0.39	5.1E-02	0.43	2.8E-02	0.12	5.8E-01	-0.35	7.8E-02	-0.29	1.5E-01	0.27	1.8E-01	0.00	9.8E-01	0.08	6.8E-01	0.36	7.2E-02
ENSCAFG0000003094	ENSCAFG0000003094	grey	EC_M10	-0.07	7.5E-01	0.15	4.8E-01	0.06	7.6E-01	0.20	3.4E-01	-0.10	6.2E-01	-0.05	8.2E-01	0.34	9.4E-02	-0.08	7.1E-01	-0.02	9.1E-01	0.15	4.6E-01
ENSCAFG0000002883	ELOF1	grey	EC_M10	-0.07	7.5E-01	-0.07	7.2E-01	-0.23	2.5E-01	0.11	5.9E-01	0.47	1.6E-02	0.38	5.7E-02	-0.14	4.8E-01	-0.09	6.5E-01	0.10	6.1E-01	-0.41	3.9E-02
ENSCAFG0000000618	RELL2	grey	EC_M10	-0.07	7.5E-01	0.38	5.6E-02	0.15	4.5E-01	0.13	5.1E-01	-0.34	8.9E-02	-0.03	8.9E-01	-0.15	4.7E-01	-0.14	4.8E-01	-0.08	7.1E-01	0.38	5.2E-02
ENSCAFG0000000511	UBXN4	darkgreen	EC_M4	-0.07	7.5E-01	-0.01	9.7E-01	-0.66	2.4E-04	-0.09	6.5E-01	0.78	3.0E-06	0.21	3.1E-01	0.04	8.4E-01	0.09	6.7E-01	-0.02	9.2E-01	-0.73	2.6E-05
ENSCAFG0000001395	FAH	turquoise	EC_M6	-0.07	7.5E-01	0.40	4.5E-02	0.12	5.5E-01	-0.22	2.8E-01	-0.24	2.3E-01	-0.27	1.9E-01	-0.03	9.0E-01	0.22	2.8E-01	0.07	7.3E-01	0.25	2.1E-01
ENSCAFG0000000385	LDAH	darkgreen	EC_M4	-0.07	7.5E-01	-0.84	7.2E-08	0.02	9.1E-01	-0.10	6.1E-01	0.32	1.1E-01	0.09	6.7E-01	-0.07	7.4E-01	0.19	3.6E-01	0.04	8.5E-01	-0.29	1.6E-01
ENSCAFG0000001118	GALNT3	darkmagenta	EC_M12	-0.07	7.5E-01	0.00	9.9E-01	-0.48	1.4E-02	-0.06	7.5E-01	0.60	1.1E-03	-0.14	4.9E-01	-0.12	5.7E-01	0.61	9.0E-04	0.06	7.7E-01	-0.56	2.7E-03
ENSCAFG0000000387	ENSCAFG0000000387	turquoise	EC_M6	-0.07	7.5E-01	0.55	3.6E-03	-0.16	4.3E-01	-0.07	7.4E-01	0.05	8.1E-01	-0.06	7.8E-01	-0.09	6.6E-01	-0.01	9.5E-01	-0.05	7.9E-01	0.00	9.8E-01
ENSCAFG0000001468	MSANTD1	grey	EC_M10	-0.07	7.5E-01	0.32	1.1E-01	-0.02	9.3E-01	-0.17	4.0E-01	-0.01	9.4E-01	-0.15	4.8E-01	-0.12	5.6E-01	-0.13	5.2E-01	-0.11	5.9E-01	0.06	7.7E-01
ENSCAFG0000000356	CPT1C	turquoise	EC_M6	-0.07	7.5E-01	0.53	5.0E-03	0.06	7.6E-01	0.23	2.6E-01	-0.21	3.1E-01	-0.27	1.8E-01	0.00	1.0E+00	0.08	7.2E-01	-0.15	4.7E-01	0.22	2.8E-01
ENSCAFG0000000683	COMMD9	grey	EC_M10	-0.07	7.5E-01	0.28	1.7E-01	0.04	8.5E-01	-0.19	3.5E-01	0.02	9.3E-01	0.00	9.8E-01	-0.10	6.1E-01	-0.42	3.2E-02	-0.08	6.9E-01	0.06	7.8E-01
ENSCAFG0000001580	ENO3	grey	EC_M10	-0.07	7.4E-01	-0.05	8.1E-01	-0.16	4.4E-01	-0.34	9.0E-02	0.25	2.1E-01	0.14	4.9E-01	0.15	4.7E-01	0.13	5.3E-01	0.23	2.6E-01	-0.25	2.2E-01
ENSCAFG0000000560	TASP1	grey	EC_M10	-0.07	7.4E-01	-0.39	5.2E-02	0.27	1.8E-01	0.09	6.8E-01	-0.09	6.6E-01	0.11	6.0E-01	-0.15	4.8E-01	-0.17	3.9E-01	0.09	6.7E-01	0.14	5.0E-01
ENSCAFG0000000616	RMDN2	grey	EC_M10	-0.07	7.4E-01	-0.44	2.3E-02	-0.09	6.5E-01	0.17	4.1E-01	0.31	1.2E-01	0.21	3.1E-01	-0.15	4.8E-01	-0.12	5.7E-01	0.25	2.2E-01	-0.27	1.9E-01
ENSCAFG0000001687	FAM161B	grey	EC_M10	-0.07	7.4E-01	0.11	5.8E-01	-0.27	1.8E-01	-0.58	2.1E-03	0.29	1.5E-01	0.22	2.9E-01	0.27	1.8E-01	0.07	7.4E-01	-0.12	5.6E-01	-0.33	1.0E-01
ENSCAFG0000002009	PIP5KL1	grey	EC_M10	-0.07	7.4E-01	0.21	3.1E-01	-0.28	1.7E-01	0.11	5.8E-01	0.25	2.2E-01	-0.01	9.6E-01	0.09	6.6E-01	0.04	8.6E-01	0.05	8.0E-01	-0.23	2.6E-01
ENSCAFG0000002273	MT-ND3	cyan	EC_M2	-0.07	7.4E-01	-0.14	4.8E-01	0.64	4.0E-04	0.21	3.1E-01	-0.67	2.0E-04	-0.08	7.0E-01	-0.44	2.4E-02	-0.11	6.1E-01	-0.11	6.1E-01	0.69	8.6E-05
ENSCAFG0000003273	ENSCAFG0000003273	grey	EC_M10	-0.07	7.4E-01	-0.10	6.3E-01	-0.05	7.9E-01	-0.15	4.7E-01	0.17	4.1E-01	-0.12	5.7E-01	0.47	1.6E-02	0.05	8.1E-01	0.08	6.9E-01	-0.12	5.7E-01
ENSCAFG0000000809	ARNTL	grey	EC_M10	-0.07	7.4E-01	-0.39	4.8E-02	0.46	1.8E-02	0.07	7.5E-01	-0.32	1.1E-01	-0.17	4.0E-01	-0.05	8.1E-01	0.27	1.8E-01	0.25	2.1E-01	0.38	5.5E-02
ENSCAFG0000000513	JMJD6	grey	EC_M10	-0.07	7.4E-01	-0.18	3.7E-01	-0.53	5.0E-03	-0.13	5.2E-01	0.78	2.7E-06	0.08	7.0E-01	0.25	2.3E-01	0.03	8.9E-01	-0.30	1.4E-01	-0.75	1.2E-05
ENSCAFG0000000787	TRABD2A	grey	EC_M10	-0.07	7.4E-01	0.38	5.4E-02	-0.25	2.1E-01	-0.19	3.5E-01	0.24	2.4E-01	0.17	4.2E-01	0.15	4.8E-01	-0.15	4.7E-01	0.07	7.3E-01	-0.22	2.8E-01
ENSCAFG0000002462	ENSCAFG0000002462	turquoise	EC_M6	-0.07	7.4E-01	0.46	1.8E-02	-0.21	3.1E-01	-0.16	4.3E-01	0.12	5.6E-01	-0.14	4.9E-01	0.41	3.9E-02	0.04	8.4E-01	-0.03	8.8E-01	-0.06	7.8E-01
ENSCAFG0000001067	BANK1	grey	EC_M10	-0.07	7.4E-01	-0.04	8.6E-01	0.40	4.3E-02	0.06	7.5E-01	-0.39	4.6E-02	-0.17	4.0E-01	-0.24	2.4E-01	0.32	1.1E-01	0.23	2.5E-01	0.40	4.4E-02
ENSCAFG0000001616	FAM114A1	grey	EC_M10	-0.07	7.4E-01	0.41	3.7E-02	-0.51	8.1E-03	-0.04	8.4E-01	0.43	2.7E-02	0.18	3.9E-01	0.17	4.1E-01	-0.17	4.0E-01	-0.13	5.3E-01	-0.43	2.7E-02
ENSCAFG0000001350	FAM177B	grey	EC_M10	-0.07	7.4E-01	0.31	1.3E-01	-0.21	3.0E-01	0.34	8.6E-02	0.14	5.0E-01	-0.16	4.3E-01	-0.11	5.9E-01	-0.20	3.4E-01	0.11	5.8E-01	-0.10	6.3E-01
ENSCAFG0000000587	ARHGDIA	grey	EC_M10	-0.07	7.4E-01	0.26	1.9E-01	-0.44	2.4E-02	-0.32	1.1E-01	0.59	1.5E-03	0.01	9.5E-01	-0.03	8.8E-01	0.09	6.6E-01	0.09	6.7E-01	-0.54	4.2E-03
ENSCAFG0000000739	RPP14	grey	EC_M10	-0.07	7.4E-01	-0.06	7.6E-01	-0.04	8.4E-01	-0.26	1.9E-01	0.22	2.7E-01	-0.03	8.7E-01	0.07	7.4E-01	-0.21	3.1E-01	0.08	6.8E-01	-0.21	3.1E-01
ENSCAFG0000000136	ENSCAFG0000000136	grey	EC_M10	-0.07	7.4E-01	0.01	9.6E-01	0.30	1.3E-01	0.21	3.1E-01	-0.30	1.4E-01	0.02	9.2E-01	-0.12	5.7E-01	-0.05	8.0E-01	-0.03	8.9E-01	0.33	9.8E-02
ENSCAFG0000000776	ENSCAFG0000000776	grey	EC_M10	-0.07	7.4E-01	0.11	5.8E-01	0.10	6.2E-01	-0.25	2.2E-01	-0.07	7.3E-01	-0.23	2.6E-01	-0.22	2.8E-01	0.15	4.8E-01	0.28	1.7E-01	0.15	4.5E-01
ENSCAFG0000000872	ENSCAFG0000000872	darkmagenta	EC_M12	-0.07	7.4E-01	-0.08	6.9E-01	0.06	7.9E-01	-0.11	5.8E-01	-0.01	9.7E-01	-0.08	7.1E-01	-0.07	7.2E-01	0.80	7.1E-07	0.13	5.3E-01	0.02	9.1E-01
ENSCAFG0000000279	IRAK1BP1	cyan	EC_M2	-0.07	7.4E-01	-0.53	5.9E-03	0.60	1.1E-03	0.38	5.9E-02	-0.46	1.9E-02	0.02	9.2E-01	-0.08	7.0E-01	-0.35	8.0E-02	-0.17	4.0E-01	0.50	9.5E-03
ENSCAFG0000000383	SDC1	grey	EC_M10	-0.07	7.4E-01	-0.17	4.1E-01	-0.42	3.2E-02	-0.35	8.0E-02	0.62	7.7E-04	0.08	7.0E-01	0.32	1.1E-01	-0.05	8.1E-01	0.42	3.1E-02	-0.57	2.2E-03
ENSCAFG0000001871	PPP4R1	grey	EC_M10	-0.07	7.4E-01	0.26	2.0E-01	-0.57	2.2E-03	-0.23	2.6E-01	0.62	6.7E-04	0.25	2.2E-01	-0.12	5.4E-01	-0.22	2.9E-01	-0.35	7.6E-02	-0.61	8.8E-04
ENSCAFG0000002273	COX3	cyan	EC_M2	-0.07	7.4E-01	-0.34	8.7E-02	0.65	3.1E-04	0.24	2.4E-01	-0.59	1.5E-03	-0.03	8.9E-01	-0.40	4.4E-02	-0.07	7.3E-01	-0.16	4.3E-01	0.61	9.0E-04
ENSCAFG0000000997	ETS2	turquoise	EC_M6	-0.07	7.4E-01	0.61	8.5E-04	-0.32	1.1E-01	-0.11	5.9E-01	0.17	3.9E-01	-0.06	7.6E-01	0.27	1.8E-01	-0.02	9.2E-01	0.05	8.1E-01	-0.11	5.9E-01
ENSCAFG0000000761	PITPNM2	turquoise	EC_M6	-0.07	7.4E-01	0.83	1.2E-07	-0.01	9.5E-01	0.01	9.8E-01	-0.21	3.0E-01	-0.34	8.6E-02	0.13	5.3E-01	-0.20	3.2E-01	0.08	7.1E-01	0.28	1.7E-01
ENSCAFG0000001705	ACSF2	grey	EC_M10	-0.07	7.4E-01	-0.08	6.9E-01	0.50	9.0E-03	0.09	6.6E-01	-0.54	4.5E-03	-0.30	1.4E-01	0.01	9.7E-01	0.18	3.7E-01	0.56	2.7E-03	0.58	1.8E-03
ENSCAFG0000001994	TLDC1	grey	EC_M10	-0.07	7.4E-01	-0.03	8.8E-01	-0.53	5.9E-03	0.02	9.0E-01	0.64	4.7E-04	0.19	3.4E-01	-0.08	6.9E-01	-0.22	2.9E-01	0.10	6.3E-01	-0.65	3.2E-04
ENSCAFG0000000433	ENSCAFG0000000433	magenta	EC_M13	-0.07	7.4E-01	0.04	8.4E-01	-0.18	3.9E-01	-0.08	7.1E-01	0.28	1.6E-01	-0.05	8.1E-01	0.02	9.3E-01	-0.02	9.3E-01	0.70	7.6E-05	-0.19	3.5E-01
ENSCAFG0000000583	ZIC5	magenta	EC_M13	-0.07	7.4E-01	0.04	8.4E-01	-0.18	3.9E-01	-0.08	7.1E-01	0.28	1.6E-01	-0.05	8.1E-01	0.02	9.3E-01	-0.02	9.3E-01	0.70	7.6E-05	-0.19	3.5E-01
ENSCAFG0000002766	ENSCAFG0000002766	grey	EC_M10	-0.07	7.4E-01	0.07	7.4E-01	-0.22	2.8E-01	-0.06	7.9E-01	0.34	9.1E-02	-0.08	7.1E-01	-0.11	6.0E-01	-0.10	6.3E-01	-0.09	6.6E-01	-0.30	1.4E-01
ENSCAFG0000001876	CATSPERD	grey	EC_M10	-0.07	7.4E-01	0.14	4.8E-01	0.18	3.9E-01	-0.14	5.0E-01	-0.15	4.8E-01	-0.14	5.1E-01	-0.17	4.0E-01	-0.12	5.6E-01	-0.22	2.8E-01	0.20	3.4E-01
ENSCAFG0000001813	ENSCAFG0000001813	cyan	EC_M2	-0.07	7.4E-01	0.24	2.4E-01	0.61	9.2E-04	0.02	9.4E-01	-0.69	8.5E-05	-0.43	3.0E-02	0.03	8.7E-01	-0.04	8.5E-01	0.07	7.5E-01	0.77	5.0E-06
ENSCAFG0000002948	METTL21A	grey	EC_M10	-0.07	7.4E-01	0.08	7.0E-01	0.32	1.2E-01	-0.11	6.1E-01	-0.40	4.4E-02	0.22	2.9E-01	-0.08	7.0E-01	0.12	5.6E-01	0.43	2.9E-02	0.44	2.6E-02
ENSCAFG0000000243	TSPAN13	turquoise	EC_M6	-0.07	7.3E-01	0.84	7.4E-08	-0.22	2.8E-01	-0.14	5.1E-01	0.02	9.1E-01	-0.16	4.5E-01	0.18	3.7E-01	0.16	4.5E-01	0.11	6.1E-01	0.02	9.4E-01
ENSCAFG0000001985	C6H1orf194	grey	EC_M10	-0.07	7.3E-01	-0.32	1.1E-01	0.33	9.6E-02	0.09	6.6E-01	-0.21	2.9E-01	-0.19	3.6E-01	-0.15	4.5E-01	0.01	9.6E-01	0.02	9.2E-01	0.25	2.2E-01
ENSCAFG0000002603	ENSCAFG0000002603	grey	EC_M10	-0.07	7.3E-01	0.22	2.8E-01	0.17	4.0E-01	0.17	4.2E-01	-0.20	3.2E-01	-0.29	1.6E-01	0.16	4.2E-01	0.08	7.0E-01	-0.37	6.4E-02	0.20	3.3E-01
ENSCAFG0000000606	ENSCAFG0000000606	grey	EC_M10	-0.07	7.3E-01	-0.13	5.3E-01	0.20	3.4E-01	-0.24	2.3E-01	-0.08	6.9E-01	0.17	4.2E-01	0.05	7.9E-01	-0.20	3.3E-01	-0.20	3.3E-01	0.17	4.1E-01
ENSCAFG0000001381	FMNL1	cyan	EC_M2	-0.07	7.3E-01	-0.34	8.9E-02	0.78	2.9E-06	0.06	7.6E-01	-0.70	5.8E-05	-0.13	5.1E-01	0.01	9.7E-01	-0.14	5.1E-01	-0.22	2.7E-01	0.78	3.0E-06
ENSCAFG0000001499	MROH9	grey	EC_M10	-0.07	7.3E-01	-0.16	4.4E-01	0.21	3.0E-01	-0.08	7.1E-01	-0.17	4.2E-01	-0.10	6.3E-01	0.42	3.4E-02	-0.03	8.9E-01	-0.10	6.3E-01	0.20	3.2E-01
ENSCAFG0000001119	ST8SIA2	grey	EC_M10	-0.07	7.3E-01	-0.24	2.4E-01	-0.02	9.3E-01	-0.26	2.1E-01	0.15	4.5E-01	0.29	1.5E-01	0.29	1.5E-01	-0.02	9.4E-01	-0.18	3.9E-01	-0.14	5.0E-01
ENSCAFG0000000858	VCAN	grey	EC_M10	-0.07	7.3E-01	-0.71	5.2E-05	-0.17	4.1E-01	0.00	9.9E-01	0.50	1.0E-02	0.21	3.1E-01	0.01	9.5E-01	0.21	3.0E-01	0.22	2.7E-01	-0.50	9.8E-03
ENSCAFG0000001519	ENSCAFG0000001519	grey	EC_M10	-0.07	7.3E-01	-0.05	8.2E-01	0.08	7.0E-01	0.10	6.2E-01	-0.04	8.5E-01	-0.11	6.0E-01	0.05	8.1E-01	-0.38	5.2E-02	-0.28	1.7E-01	0.08	7.1E-01
ENSCAFG0000000313	PDE1C	grey	EC_M10	-0.07	7.3E-01	-0.13	5.1E-01	-0.08	6.9E-01	0.20	3.4E-01	0.17	4.0E-01	0.53	5.1E-03	0.02	9.1E-01	0.05	7.9E-01	0.24	2.3E-01	-0.17	4.0E-01
ENSCAFG0000003161	EFCAB8	grey	EC_M10	-0.07	7.3E-01	0.21	3.0E-01	-0.38	5.5E-02	0.01	9.6E-01	0.44	2.3E-02	-0.02	9.4E-01	-0.17	4.2E-01	0.47	1.4E-02	0.06	7.6E-01	-0.38	5.4E-02
ENSCAFG0000000086	PHF21B	grey	EC_M10	-0.07	7.3E-01	-0.28	1.7E-01	-0.13	5.4E-01	0.00	9.9E-01	0.34	9.3E-02	0.49	1.2E-02	0.06	7.8E-01	0.25	2.2E-01	0.22	2.7E-01	-0.29	1.5E-01
ENSCAFG0000000468	FAM171A1	grey	EC_M10	-0.07	7.3E-01	-0.17	4.2E-01	-0.25	2.3E-01	0.00	9.9E-01	0.44	2.4E-02	-0.03	8.8E-01	-0.45	2.0E-02	0.15	4.6E-01	-0.05	8.1E-01	-0.45	2.2E-02
ENSCAFG0000000108	FAM84B	darkgreen	EC_M4	-0.07	7.3E-01	0.07	7.2E-01	-0.16	4.4E-01	-0.08	7.1E-01	0.25	2.2E-01	-0.13	5.4E-01	-0.08	7.0E-01	-0.02	9.1E-01	-0.05	8.0E-01	-0.20	3.2E-01
ENSCAFG0000000094	FAM184A	grey	EC_M10	-0.07	7.3E-01	-0.05	8.1E-01	-0.51	8.4E-03	0.04	8.5E-01	0.68	1.5E-04	0.14	4.8E-01	-0.05	8.2E-01	0.36	6.9E-02	0.14	4.9E-01	-0.61	9.0E-04
ENSCAFG0000000850	CCDC113	grey	EC_M10	-0.07	7.3E-01	-0.54	4.1E-03	-0.41	3.6E-02	-0.08	6.9E-01	0.71	5.3E-05	0.22	2.8E-01	0.15	4.5E-01	0.05	8.2E-01	-0.25	2.2E-01	-0.71	5.6E-05
ENSCAFG0000000435	ACAD9	grey	EC_M10	-0.07	7.3E-01	-0.58	1.8E-03	-0.07	7.3E-01	0.01	9.8E-01	0.43	2.8E-02	-0.05	7.9E-01	-0.10	6.4E-01	0.10	6.2E-01	0.18	3.7E-01	-0.37	6.6E-02
ENSCAFG0000001116	LRRC58	darkgreen	EC_M4	-0.07	7.3E-01	-0.75	8.8E-06	0.32	1.2E-01	0.13	5.2E-01	-0.03	8.7E-01	0.13	5.4E-01	0.00	9.9E-01	-0.13	5.4E-01	0.31	1.2E-01	0.08	6.8E-01
ENSCAFG0000001469	GRK4	grey	EC_M10	-0.07	7.3E-01	-0.21	3.0E-01	-0.19	3.6E-01	-0.23	2.6E-01	0.34	8.8E-02	0.16	4.4E-01	-0.23	2.5E-01	0.29	1.5E-01	0.00	9.9E-01	-0.34	8.6E-02
ENSCAFG0000001134	ITGA10	grey	EC_M10	-0.07	7.3E-01	-0.23	2.6E-01	-0.03	9.0E-01	0.13	5.2E-01	0.14	5.1E-01	-0.04	8.6E-01	0.26	1.9E-01	-0.05	7.9E-01	-0.14	5.0E-01	-0.16	4.2E-01
ENSCAFG0000003103	YPEL5	darkgreen	EC_M4	-0.07	7.3E-01	0.32	1.1E-01	-0.67	1.7E-04	-0.12	5.7E-01	0.66	2.5E-04	0.25	2.2E-01	-0.07	7.2E-01	-0.05	8.0E-01	-0.09	6.7E-01	-0.63	5.1E-04
ENSCAFG0000001580	AKT3	darkgreen	EC_M4	-0.07	7.3E-01	0.35	8.2E-02	-0.69	8.8E-05	-0.26	2.0E-01	0.70	6.9E-05	0.20	3.2E-01	0.21	3.1E-01	0.04	8.3E-01	0.15	4.6E-01	-0.66	2.2E-04
ENSCAFG0000000577	ENSCAFG0000000577	grey	EC_M10	-0.07	7.3E-01	-0.03	9.0E-01	-0.35	8.4E-02	0.16	4.3E-01	0.37	6.1E-02	0.01	9.6E-01	-0.15	4.5E-01	0.27	1.7E-01	0.34	8.8E-02	-0.36	7.1E-02
ENSCAFG0000002004	PKN3	cyan	EC_M2	-0.07	7.3E-01	0.33	1.0E-01	0.64	4.0E-04	-0.02	9.2E-01	-0.79	1.7E-06	-0.33	1.1E-01	0.07	7.3E-01	-0.16	4.5E-01	-0.08	7.0E-01	0.84	1.1E-07
ENSCAFG0000000154	TTC39B	grey	EC_M10	-0.07	7.3E-01	-0.30	1.4E-01	-0.49	1.0E-02	0.11	6.0E-01	0.75	1.2E-05	0.34	9.3E-02	0.20	3.2E-01	0.00	9.8E-01	-0.21	3.1E-01	-0.74	1.8E-05
ENSCAFG0000002896	SLC25A21	turquoise	EC_M6	-0.07	7.3E-01	0.66	2.2E-04	-0.16	4.3E-01	-0.08	6.8E-01	-0.01	9.7E-01	-0.11	6.0E-01	0.19	3.5E-01	-0.11	5.9E-01	0.05	8.0E-01	0.10	6.4E-01
ENSCAFG0000001524	PLD1	darkgreen	EC_M4	-0.07	7.3E-01	-0.73	2.1E-05	0.05	8.0E-01	0.08	6.8E-01	0.23	2.6E-01	0.19	3.4E-01	0.07	7.2E-01	-0.17	4.0E-01	-0.42	3.5E-02	-0.24	2.5E-01
ENSCAFG0000000881	PNMA2	grey	EC_M10	-0.07	7.3E-01	-0.33	9.9E-02	-0.27	1.8E-01	0.19	3.5E-01	0.47	1.5E-02	0.12	5.6E-01	0.21	3.0E-01	0.00	9.8E-01	-0.06	7.7E-01	-0.46	1.9E-02

ENSCAFG0000002784	ENSCAFG0000002784	grey	EC_M10	-0.07	7.3E-01	-0.13	5.4E-01	-0.10	6.2E-01	-0.06	7.5E-01	0.19	3.4E-01	-0.11	6.0E-01	-0.08	6.8E-01	-0.06	7.7E-01	-0.16	4.2E-01	-0.21	3.0E-01
ENSCAFG0000002977	ETFRF1	grey	EC_M10	-0.07	7.3E-01	-0.33	1.0E-01	0.26	2.1E-01	0.08	6.8E-01	-0.17	4.1E-01	-0.02	9.4E-01	0.22	2.7E-01	0.10	6.4E-01	0.09	6.6E-01	0.26	2.0E-01
ENSCAFG0000001852	DHRS7B	grey	EC_M10	-0.07	7.3E-01	-0.64	4.0E-04	-0.06	7.6E-01	-0.14	4.8E-01	0.40	4.0E-02	0.09	6.5E-01	0.04	8.3E-01	0.23	2.5E-01	0.03	8.8E-01	-0.36	7.0E-02
ENSCAFG0000000316	BMPER	grey	EC_M10	-0.07	7.3E-01	-0.10	6.2E-01	-0.02	9.1E-01	0.13	5.3E-01	0.14	4.9E-01	-0.19	3.4E-01	0.05	8.1E-01	0.37	6.1E-02	0.20	3.2E-01	-0.09	6.8E-01
ENSCAFG0000003197	C1QL4	grey	EC_M10	-0.07	7.3E-01	-0.15	4.7E-01	0.14	4.9E-01	0.01	9.8E-01	0.08	6.9E-01	-0.05	8.0E-01	-0.02	9.1E-01	0.00	1.0E+00	0.01	9.4E-01	0.00	9.9E-01
ENSCAFG0000001336	KDM1A	grey	EC_M10	-0.07	7.3E-01	-0.35	7.6E-02	-0.33	1.0E-01	-0.15	4.5E-01	0.62	7.2E-04	0.06	7.6E-01	0.11	6.0E-01	-0.05	8.0E-01	-0.46	1.9E-02	-0.59	1.6E-03
ENSCAFG0000000740	TMEM232	turquoise	EC_M6	-0.07	7.3E-01	0.44	2.6E-02	-0.07	7.3E-01	-0.19	3.5E-01	-0.04	8.5E-01	-0.23	2.6E-01	-0.27	1.9E-01	0.20	3.3E-01	0.38	5.7E-02	0.10	6.3E-01
ENSCAFG0000001911	ENSCAFG0000001911	cyan	EC_M2	-0.07	7.3E-01	-0.14	5.0E-01	0.42	3.4E-02	0.58	1.8E-03	-0.48	1.3E-02	-0.07	7.3E-01	-0.17	4.1E-01	0.00	9.9E-01	0.04	8.5E-01	0.51	7.1E-03
ENSCAFG0000000717	MICU2	grey	EC_M10	-0.07	7.3E-01	-0.21	3.0E-01	0.22	2.8E-01	-0.10	6.3E-01	-0.20	3.2E-01	0.00	1.0E+00	-0.09	6.6E-01	0.21	3.0E-01	0.01	9.6E-01	0.23	2.6E-01
ENSCAFG0000002488	FOLH1	grey	EC_M10	-0.07	7.3E-01	-0.22	2.8E-01	0.22	2.7E-01	-0.08	7.0E-01	-0.11	5.9E-01	0.13	5.1E-01	0.12	5.7E-01	-0.32	1.1E-01	-0.43	2.7E-02	0.15	4.8E-01
ENSCAFG0000001822	ENSCAFG0000001822	grey	EC_M10	-0.07	7.3E-01	0.01	9.4E-01	-0.55	3.9E-03	-0.16	4.3E-01	0.70	6.8E-05	0.03	8.8E-01	0.12	5.6E-01	-0.18	3.8E-01	0.05	8.0E-01	-0.62	7.0E-04
ENSCAFG0000000488	CRYGN	grey	EC_M10	-0.07	7.3E-01	-0.13	5.2E-01	0.30	1.4E-01	-0.13	5.3E-01	-0.27	1.9E-01	-0.09	6.7E-01	-0.12	5.5E-01	0.27	1.8E-01	0.23	2.6E-01	0.24	2.4E-01
ENSCAFG0000000823	DLA-79	grey	EC_M10	-0.07	7.3E-01	-0.13	5.3E-01	-0.13	5.2E-01	-0.13	5.3E-01	0.31	1.3E-01	-0.23	2.5E-01	0.01	9.6E-01	0.41	3.9E-02	0.23	2.6E-01	-0.24	2.5E-01
ENSCAFG0000000140	PUF60	grey	EC_M10	-0.07	7.3E-01	0.03	9.0E-01	0.20	3.3E-01	-0.25	2.1E-01	-0.05	8.0E-01	0.17	4.1E-01	-0.08	7.0E-01	-0.03	8.7E-01	0.08	7.1E-01	0.12	5.7E-01
ENSCAFG0000003070	ENSCAFG0000003070	grey	EC_M10	-0.07	7.3E-01	-0.05	8.2E-01	0.37	6.6E-02	-0.05	8.1E-01	-0.35	8.0E-02	0.02	9.4E-01	-0.03	8.7E-01	-0.07	7.5E-01	-0.12	5.5E-01	0.39	5.0E-02
ENSCAFG0000000866	TMEM144	turquoise	EC_M6	-0.07	7.3E-01	0.64	4.0E-04	-0.28	1.6E-01	-0.27	1.8E-01	0.13	5.2E-01	0.18	3.9E-01	0.16	4.4E-01	0.00	1.0E+00	0.23	2.5E-01	-0.06	7.8E-01
ENSCAFG0000000355	TSKS	paleturquoise	EC_M11	-0.07	7.3E-01	-0.13	5.3E-01	0.04	8.6E-01	0.64	4.7E-04	0.04	8.3E-01	-0.16	4.4E-01	-0.21	3.0E-01	-0.15	4.7E-01	-0.25	2.2E-01	-0.06	7.7E-01
ENSCAFG0000001172	ENSCAFG0000001172	grey	EC_M10	-0.07	7.2E-01	-0.14	5.0E-01	0.16	4.3E-01	-0.03	8.7E-01	-0.13	5.3E-01	-0.06	7.8E-01	-0.07	7.2E-01	-0.12	5.7E-01	-0.17	4.1E-01	0.15	4.7E-01
ENSCAFG0000001192	CASS4	grey	EC_M10	-0.07	7.2E-01	-0.22	2.7E-01	-0.27	1.9E-01	-0.13	5.4E-01	0.42	3.4E-02	0.29	1.5E-01	-0.09	6.8E-01	0.28	1.7E-01	0.27	1.8E-01	-0.37	6.3E-02
ENSCAFG0000001042	DHCR7	grey	EC_M10	-0.07	7.2E-01	-0.29	1.5E-01	0.34	9.2E-02	-0.06	7.8E-01	-0.21	3.1E-01	-0.19	3.5E-01	0.03	8.9E-01	0.03	8.7E-01	-0.54	4.3E-03	0.18	3.7E-01
ENSCAFG0000001957	HES3	grey	EC_M10	-0.07	7.2E-01	-0.17	4.0E-01	-0.21	3.0E-01	-0.05	8.3E-01	0.33	9.4E-02	-0.12	5.5E-01	-0.10	6.4E-01	-0.05	8.1E-01	-0.12	5.5E-01	-0.31	1.3E-01
ENSCAFG0000003078	LAD1	darkgreen	EC_M4	-0.07	7.2E-01	-0.13	5.4E-01	-0.40	4.5E-02	-0.12	5.6E-01	0.58	1.8E-03	0.16	4.3E-01	-0.14	5.1E-01	0.09	6.5E-01	0.11	5.9E-01	-0.51	7.6E-03
ENSCAFG0000001125	HGD	grey	EC_M10	-0.07	7.2E-01	0.16	4.3E-01	0.48	1.4E-02	0.11	5.8E-01	-0.58	1.9E-03	-0.27	1.8E-01	-0.25	2.1E-01	0.26	2.1E-01	0.16	4.3E-01	0.62	7.4E-04
ENSCAFG0000001473	GUCY1A2	grey	EC_M10	-0.07	7.2E-01	0.14	4.9E-01	-0.04	8.5E-01	-0.08	6.8E-01	0.11	5.9E-01	-0.09	6.6E-01	0.22	2.8E-01	0.29	1.5E-01	0.23	2.6E-01	-0.06	7.6E-01
ENSCAFG0000000906	PGAM1	darkgreen	EC_M4	-0.07	7.2E-01	0.24	2.4E-01	-0.68	1.1E-04	-0.35	7.6E-02	0.82	3.1E-07	0.17	4.1E-01	0.25	2.3E-01	0.14	4.9E-01	0.13	5.3E-01	-0.77	4.9E-06
ENSCAFG0000001980	EPS8L3	grey	EC_M10	-0.07	7.2E-01	-0.20	3.2E-01	-0.29	1.5E-01	0.47	1.6E-02	0.42	3.1E-02	0.41	4.0E-02	-0.19	3.6E-01	-0.02	9.3E-01	-0.06	7.6E-01	-0.43	2.8E-02
ENSCAFG0000000021	ENSCAFG0000000021	grey	EC_M10	-0.07	7.2E-01	-0.18	3.9E-01	0.16	4.4E-01	-0.18	3.9E-01	0.00	1.0E+00	-0.16	4.4E-01	0.37	6.1E-02	0.35	8.2E-02	-0.01	9.6E-01	0.05	8.2E-01
ENSCAFG0000002005	SNX7	grey	EC_M10	-0.07	7.2E-01	-0.20	3.2E-01	-0.33	1.0E-01	0.08	7.1E-01	0.47	1.7E-02	0.27	1.8E-01	0.15	4.7E-01	0.01	9.7E-01	-0.49	1.1E-02	-0.47	1.5E-02
ENSCAFG0000000831	ASIC1	grey	EC_M10	-0.07	7.2E-01	-0.05	8.2E-01	-0.08	7.0E-01	-0.30	1.4E-01	0.20	3.3E-01	-0.27	1.8E-01	0.22	2.7E-01	0.40	4.3E-02	0.32	1.1E-01	-0.14	4.9E-01
ENSCAFG0000001673	FBXL19	grey	EC_M10	-0.07	7.2E-01	0.24	2.4E-01	0.37	6.4E-02	0.15	4.6E-01	-0.48	1.4E-02	0.00	1.0E+00	0.01	9.6E-01	-0.28	1.6E-01	0.14	4.9E-01	0.48	1.2E-02
ENSCAFG0000001843	SREBF1	cyan	EC_M2	-0.07	7.2E-01	-0.31	1.3E-01	0.58	1.7E-03	0.13	5.3E-01	-0.51	7.3E-03	0.11	6.1E-01	0.11	6.0E-01	-0.23	2.5E-01	-0.11	5.8E-01	0.49	1.2E-02
ENSCAFG0000000548	DNAJC3	grey	EC_M10	-0.07	7.2E-01	-0.26	2.0E-01	-0.57	2.2E-03	0.13	5.3E-01	0.79	1.5E-06	0.24	2.4E-01	-0.02	9.2E-01	0.07	7.2E-01	-0.11	5.8E-01	-0.74	1.9E-05
ENSCAFG0000000208	ENSCAFG0000000208	grey	EC_M10	-0.07	7.2E-01	-0.26	2.0E-01	0.12	5.7E-01	-0.07	7.4E-01	-0.07	7.5E-01	-0.12	5.7E-01	0.22	2.7E-01	-0.13	5.4E-01	-0.26	2.0E-01	0.06	7.7E-01
ENSCAFG0000000375	ENSCAFG0000000375	darkgreen	EC_M4	-0.07	7.2E-01	-0.04	8.4E-01	-0.26	2.0E-01	-0.07	7.2E-01	0.37	6.3E-02	-0.10	6.3E-01	-0.10	6.2E-01	-0.03	9.0E-01	0.00	1.0E+00	-0.31	1.2E-01
ENSCAFG0000001070	ADAMTS17	grey	EC_M10	-0.07	7.2E-01	-0.01	9.7E-01	-0.61	9.2E-04	-0.16	4.4E-01	0.78	2.5E-06	0.46	1.7E-02	0.07	7.5E-01	-0.06	7.7E-01	-0.14	4.9E-01	-0.76	6.3E-06
ENSCAFG0000001280	SLC25A12	grey	EC_M10	-0.07	7.2E-01	-0.45	2.3E-02	0.44	2.5E-02	0.00	1.0E+00	-0.26	2.1E-01	-0.22	2.7E-01	-0.41	3.7E-02	-0.14	4.9E-01	0.13	5.4E-01	0.34	8.9E-02
ENSCAFG0000001720	ABCC3	grey	EC_M10	-0.07	7.2E-01	-0.17	4.0E-01	0.21	3.0E-01	0.26	1.9E-01	-0.09	6.5E-01	-0.17	3.9E-01	0.08	6.9E-01	-0.15	4.6E-01	0.38	5.8E-02	0.21	3.0E-01
ENSCAFG0000001403	TRIP6	grey	EC_M10	-0.07	7.2E-01	-0.04	8.5E-01	0.44	2.3E-02	0.03	8.8E-01	-0.43	2.9E-02	-0.14	5.0E-01	0.20	3.3E-01	0.04	8.5E-01	0.34	8.7E-02	0.43	2.7E-02
ENSCAFG0000001264	ENSCAFG0000001264	grey	EC_M10	-0.07	7.2E-01	-0.50	1.0E-02	-0.09	6.6E-01	-0.11	5.9E-01	0.35	8.3E-02	0.13	5.4E-01	-0.19	3.6E-01	-0.12	5.5E-01	0.07	7.4E-01	-0.33	1.0E-01
ENSCAFG0000002964	ENSCAFG0000002964	paleturquoise	EC_M11	-0.07	7.2E-01	-0.13	5.3E-01	0.23	2.6E-01	0.65	3.1E-04	-0.18	3.7E-01	-0.11	6.1E-01	-0.10	6.3E-01	0.53	5.3E-03	0.00	9.9E-01	0.21	3.0E-01
ENSCAFG0000001982	CDK10	cyan	EC_M2	-0.07	7.2E-01	0.07	7.4E-01	0.60	1.1E-03	0.30	1.3E-01	-0.71	5.2E-05	-0.13	5.4E-01	-0.15	4.7E-01	0.00	9.8E-01	-0.05	8.0E-01	0.73	2.5E-05
ENSCAFG0000000877	ABCC8	darkgreen	EC_M4	-0.07	7.2E-01	-0.11	5.8E-01	0.02	9.1E-01	0.48	1.4E-02	0.07	7.3E-01	-0.09	6.6E-01	-0.08	6.8E-01	-0.06	7.6E-01	-0.06	7.6E-01	0.01	9.6E-01
ENSCAFG0000001982	SPATA33	grey	EC_M10	-0.07	7.2E-01	-0.18	3.8E-01	0.20	3.4E-01	0.31	1.2E-01	-0.05	8.2E-01	0.47	1.6E-02	0.23	2.5E-01	-0.14	4.9E-01	-0.20	3.2E-01	0.09	6.6E-01
ENSCAFG0000001726	MEGF11	grey	EC_M10	-0.07	7.2E-01	0.03	8.7E-01	-0.17	4.0E-01	-0.07	7.5E-01	0.27	1.8E-01	-0.16	4.4E-01	-0.12	5.7E-01	0.36	6.9E-02	0.35	7.6E-02	-0.24	2.4E-01
ENSCAFG0000000587	RFTN1	grey	EC_M10	-0.07	7.2E-01	0.13	5.2E-01	-0.55	3.6E-03	-0.07	7.3E-01	0.63	5.8E-04	0.06	7.8E-01	0.11	5.9E-01	0.18	3.7E-01	0.64	4.8E-04	-0.57	2.2E-03
ENSCAFG0000001947	PRDM16	magenta	EC_M13	-0.07	7.2E-01	-0.02	9.3E-01	-0.13	5.3E-01	-0.09	6.5E-01	0.24	2.5E-01	-0.04	8.5E-01	0.09	6.6E-01	-0.08	6.9E-01	0.70	6.0E-05	-0.13	5.1E-01
ENSCAFG0000000047	ENSCAFG0000000047	grey	EC_M10	-0.07	7.2E-01	-0.15	4.6E-01	-0.31	1.3E-01	-0.12	5.5E-01	0.46	1.9E-02	0.19	3.5E-01	-0.09	6.8E-01	-0.20	3.3E-01	0.17	4.1E-01	-0.38	5.4E-02
ENSCAFG0000000130	ENSCAFG0000000130	paleturquoise	EC_M11	-0.07	7.2E-01	-0.14	5.1E-01	0.21	3.0E-01	0.45	2.0E-02	-0.17	4.0E-01	-0.04	8.4E-01	-0.10	6.1E-01	-0.15	4.6E-01	-0.18	3.9E-01	0.18	3.9E-01
ENSCAFG0000002226	ENSCAFG0000002226	cyan	EC_M2	-0.07	7.2E-01	0.04	8.6E-01	0.62	8.2E-04	-0.03	8.8E-01	-0.67	1.6E-04	-0.47	1.5E-02	-0.01	9.5E-01	-0.07	7.5E-01	0.17	4.0E-01	0.73	2.5E-05
ENSCAFG0000002811	ENSCAFG0000002811	grey	EC_M10	-0.07	7.2E-01	-0.07	7.3E-01	0.16	4.4E-01	-0.07	7.3E-01	-0.12	5.6E-01	-0.11	5.9E-01	0.31	1.2E-01	0.00	9.9E-01	0.27	1.8E-01	0.15	4.5E-01
ENSCAFG0000000670	UHRF1BP1L	grey	EC_M10	-0.07	7.2E-01	-0.58	1.8E-03	-0.39	4.8E-02	0.03	9.0E-01	0.69	1.1E-04	0.27	1.7E-01	0.26	1.9E-01	0.00	1.0E+00	-0.12	5.7E-01	-0.66	2.8E-04
ENSCAFG0000000144	FRMD3	grey	EC_M10	-0.07	7.2E-01	0.07	7.3E-01	0.21	2.9E-01	0.18	3.7E-01	-0.18	3.8E-01	-0.35	7.6E-02	-0.33	9.7E-02	0.21	3.0E-01	0.13	5.3E-01	0.27	1.9E-01
ENSCAFG0000003118	ENSCAFG0000003118	grey	EC_M10	-0.07	7.2E-01	0.14	5.1E-01	0.22	2.7E-01	-0.13	5.1E-01	-0.31	1.3E-01	-0.16	4.2E-01	0.18	3.7E-01	-0.14	4.9E-01	-0.26	2.0E-01	0.34	8.8E-02
ENSCAFG0000001254	CARF	grey	EC_M10	-0.08	7.1E-01	0.27	1.8E-01	0.18	3.7E-01	0.13	5.4E-01	-0.35	7.9E-02	-0.41	3.8E-02	0.00	9.9E-01	0.06	7.6E-01	0.22	2.7E-01	0.42	3.1E-02
ENSCAFG0000000957	DSP	grey	EC_M10	-0.08	7.1E-01	-0.21	2.9E-01	-0.05	8.2E-01	-0.16	4.2E-01	0.18	3.7E-01	-0.25	2.3E-01	0.05	8.1E-01	0.06	7.6E-01	-0.03	8.8E-01	-0.18	3.8E-01
ENSCAFG0000001181	SAG	grey	EC_M10	-0.08	7.1E-01	-0.02	9.2E-01	-0.03	8.7E-01	-0.05	8.0E-01	0.07	7.2E-01	-0.15	4.5E-01	0.38	5.4E-02	0.32	1.1E-01	-0.09	6.6E-01	-0.06	7.7E-01
ENSCAFG0000002020	GBP6	grey	EC_M10	-0.08	7.1E-01	0.36	7.0E-02	0.11	6.0E-01	0.26	1.9E-01	-0.19	3.6E-01	-0.10	6.2E-01	-0.12	5.7E-01	-0.09	6.6E-01	0.11	5.8E-01	0.24	2.3E-01
ENSCAFG0000001370	KANSL1	turquoise	EC_M6	-0.08	7.1E-01	0.60	1.1E-03	0.01	9.7E-01	0.05	8.2E-01	-0.25	2.2E-01	0.07	7.5E-01	0.11	6.1E-01	-0.03	8.8E-01	-0.10	6.3E-01	0.26	2.1E-01
ENSCAFG0000002761	ENSCAFG0000002761	grey	EC_M10	-0.08	7.1E-01	0.00	9.9E-01	0.20	3.3E-01	-0.01	9.8E-01	-0.12	5.7E-01	-0.06	7.6E-01	0.05	8.3E-01	-0.03	8.8E-01	0.59	1.4E-03	0.16	4.2E-01
ENSCAFG0000000791	SPATA9	grey	EC_M10	-0.08	7.1E-01	0.40	4.3E-02	-0.08	7.1E-01	-0.14	5.0E-01	-0.01	9.5E-01	-0.12	5.6E-01	-0.03	8.9E-01	-0.15	4.6E-01	0.00	1.0E+00	0.07	7.5E-01
ENSCAFG0000001732	LCTL	magenta	EC_M13	-0.08	7.1E-01	0.10	6.2E-01	0.05	7.9E-01	0.00	1.0E+00	-0.05	8.1E-01	0.01	9.8E-01	0.06	7.9E-01	-0.05	8.1E-01	0.92	2.8E-11	0.12	5.7E-01
ENSCAFG0000001980	DBNDD1	magenta	EC_M13	-0.08	7.1E-01	0.10	6.2E-01	0.05	7.9E-01	0.00	1.0E+00	-0.05	8.1E-01	0.01	9.8E-01	0.06	7.9E-01	-0.05	8.1E-01	0.92	2.8E-11	0.12	5.7E-01
ENSCAFG0000000525	IL17RE	grey	EC_M10	-0.08	7.1E-01	-0.16	4.5E-01	0.09	6.7E-01	-0.05	8.2E-01	-0.03	9.0E-01	0.08	7.0E-01	-0.10	6.3E-01	0.08	6.9E-01	-0.20	3.3E-01	0.07	7.3E-01
ENSCAFG0000003174	ENSCAFG0000003174	grey	EC_M10	-0.08	7.1E-01	-0.04	8.5E-01	0.08	6.9E-01	-0.07	7.3E-01	0.02	9.1E-01	-0.02	9.4E-01	0.01	9.6E-01	-0.08	7.1E-01	0.04	8.6E-01	0.01	9.8E-01
ENSCAFG0000003236	ENSCAFG0000003236	grey	EC_M10	-0.08	7.1E-01	-0.04	8.5E-01	0.08	6.9E-01	-0.07	7.3E-01	0.02	9.1E-01	-0.02	9.4E-01	0.01	9.6E-01	-0.08	7.1E-01	0.04	8.6E-01	0.01	9.8E-01
ENSCAFG0000001044	ACADS	cyan	EC_M2	-0.08	7.1E-01	-0.09	6.5E-01	0.75	8.3E-06	0.11	5.9E-01	-0.76	5.8E-06	-0.26	1.9E-01	-0.11	5.8E-01	0.04	8.4E-01	0.20	3.4E-01	0.83	1.1E-07
ENSCAFG0000000259	KMT5C	grey	EC_M10	-0.08	7.1E-01	0.20	3.3E-01	0.18	3.7E-01	0.03	8.9E-01	-0.25	2.3E-01	0.05	8.0E-01	0.29	1.6E-01	0.03	8.7E-01	0.16	4.5E-01	0.27	1.9E-01
ENSCAFG0000000112	L3MBTL2	grey	EC_M10	-0.08	7.1E-01	-0.24	2.4E-01	0.05	8.2E-01	-0.16	4.2E-01	0.22	2.8E-01	0.07	7.4E-01	-0.39	5.0E-02	0.08	7.0E-01	0.25	2.3E-01	-0.13	5.1E-01
ENSCAFG0000003057	ENSCAFG0000003057	grey	EC_M10	-0.08	7.1E-01	-0.14	4.9E-01	0.09	6.7E-01	0.28	1.7E-01	-0.02	9.1E-01	-0.17	4.0E-01	0.16	4.5E-01	-0.19	3.6E-01	-0.25	2.2E-01	0.02	9.1E-01
ENSCAFG0000001010	ENSCAFG0000001010	grey	EC_M10	-0.08	7.1E-01	-0.11	6.1E-01	-0.29	1.5E-01	-0.11	5.9E-01	0.45	2.2E-02	0.35	7.7E-02	-0.11	5.9E-01	-0.05	7.9E-01	-0.01	9.7E-01	-0.41	3.7E-02
ENSCAFG0000001450	FUNDC1	grey	EC_M10	-0.08	7.1E-01	0.05	8.1E-01	-0.45	2.0E-02	-0.01	9.6E-01	0.52	6.5E-03	0.14	5.0E-01	0.11	5.9E-01	0.27	1.8E-01	-0.29	1.5E-01	-0.53	4.9E-03
ENSCAFG0000000625	NBEA	grey	EC_M10	-0.08	7.1E-01	0.42	3.2E-02	-0.43	2.7E-02	-0.25	2.1E-01	0.45	2.1E-02	-0.11	5.9E-01	-0.07	7.5E-01	0.12	5.7E-01	0.07	7.5E-01	-0.34	9.3E-02
ENSCAFG0000001904	SLITRK2	turquoise	EC_M6	-0.08	7.1E-01	0.66	2.2E-04	-0.25	2.1E-01	-0.02	9.1E-01	0.14	5.0E-01	-0.03	8.7E-01	-0.07	7.2E-01	-0.08	7.0E-01	-0.07	7.3E-01	-0.06	7.8E-01
ENSCAFG0000000172	BICRAL	grey	EC_M10	-0.08	7.1E-01	-0.58	2.0E-03	0.69	1.0E-04	0.19	3.5E-01	-0.54	4.0E-03	-0.06	7.6E-01	-0.08	7.0E-01	-0.06	7.7E-01	-0.03	8.9E-01	0.58	1.9E-03
ENSCAFG0000000609	DDHD2	cyan	EC_M2	-0.08	7.1E-01	-0.40	4.5E-02	0.46	1.8E-02	0.08	7.0E-01	-0.39	5.1E-02	-0.17	4.0E-01	-0.09	6.6E-01	0.13	5.4E-01	-0.19	3.5E-01	0.41	3.7E-02
ENSCAFG0000000228	CCIN	grey	EC_M10	-0.08	7.1E-01	0.02	9.2E-01	0.26	2.0E-01	0.01	9.7E-01	-0.30	1.4E-01	-0.37	6.3E-02	0.27	1.8E-01	0.36	7.4E-02	0.11	6.0E-01	0.35	7.6E-02
ENSCAFG0000001284	OAZ3	turquoise	EC_M6	-0.08	7.1E-01	0.52	6.3E-03	-0.04	8.6E-01	-0.09	6.6E-01	-0.09	6.5E-01	-0.10	6.4E-01	0.37	6.3E-02	-0.09	6.5E-01	0.16	4.2E-01	0.12	5.5E-01
ENSCAFG0000002514	ZNF777	grey	EC_M10	-0.08	7.1E-01	-0.35	7.7E-02	0.24	2.5E-01	0.25	2.3E-01	-0.08	7.1E-01	0.48	1.4E-02	-0.11	6.1E-01	-0.28	1.7E-01	-0.31	1.2E-01	0.07	7.2E-01
ENSCAFG0000000757	DPY19L3	grey	EC_M10	-0.08	7.1E-01	-0.21	3.0E-01	0.00	9.8E-01	0.04	8.3E-01	0.09	6.6E-01	-0.27	1.9E-01	0.05	8.0E-01	0.28	1.7E-01	0.50	9.9E-03	-0.06	7.7E-01
ENSCAFG0000001325	GPR155	grey	EC_M10	-0.08	7.1E-01	-0.22	2.8E-01	0.02	9.1E-01	-0.14	4.9E-01	0.11	5.9E-01	-0.13	5.3E-01	-0.01	9.6E-01	-0.04	8.6E-01	-0.19	3.4E-01	0.00	9.9E-01
ENSCAFG0000000581	FMNL2	cyan	EC_M2	-0.08	7.1E-01	-0.60	1.1E-03	0.53	5.6E-03	0.02	9.3E-01	-0.33	9.8E-02	0.04	8.4E-01	0.17	4.2E-01	-0.13	5.3E-01	-0.22	2.9E-01	0.39	4.9E-02
ENSCAFG0000001832	LRRC8E	grey	EC_M10	-0.08	7.1E-01	-0.27	1.8E-01	-0.04	8.3E-01	-0.16	4.3E-01	0.14	4.9E-01	0.38	5.3E-02	0.39	4.6E-02	0.05	8.2E-01	0.03	8.7E-01	-0.16	4.3E-01
ENSCAFG0000000965	DOP1B	turquoise	EC_M6	-0.08	7.1E-01	0.68	1.3E-04	0.16	4.3E-01	-0.19	3.4E-01	-0.31	1.2E-01	-0.37	6.1E-02	0.03	8.7E-01	-0.01	9.7E-01	-0.04	8.4E-01	0.41	3.8E-02
ENSCAFG0000000767	GSS	grey	EC_M10	-0.08	7.1E-01	-0.14	4.9E-01	0.34	8.7E-02	0.22	2.8E-01	-0.33	9.9E-02	-0.12	5.7E-01	-0.21	3.0E-01	0.04	8.5E-01	0.56	2.8E-03	0.36	6.9E-02
ENSCAFG0000000101	SYNGAP1	grey	EC_M10	-0.08	7.1E-01	-0.30	1.4E-01	0.43	3.0E-02	0.26	2.1E-01	-0.42	3.3E-02	-0.34	9.3E-02	0.36	7.4E-02	0.06	7.8E-01	0.24	2.3E-01	0.45	2.2E-02
ENSCAFG0000000238	FBXO10	grey	EC_M10	-0.08	7.1E-01	-0.27	1.9E-01	0.43	2.7E-02	-0.04	8.3E-01	-0.26	2.0E-01	-0.25	2.2E-01	-0.01	9.6E-01	-0.19	3.4E-01	-0.32	1.1E-01	0.29	1.5E-01
ENSCAFG0000003033	SERPINB9	turquoise	EC_M6	-0.08	7.1E-01	0.78	2.4E-06	0.12	5.6E-01	-0.01	9.5E-01	-0.37	6.5E-02	-0.20	3.2E-01	0.21	3.1E-01	0.03	8.7E-01	0.14	4.9E-01	0.44	2.6E-02
ENSCAFG0000001038	FAP	darkgreen	EC_M4	-0.08	7.1E-01	-0.44	2.5E-02	-0.21	3.0E-01	0.06	7.9E-01	0.46	1.8E-02	0.26	1.9E-01	0.31	1.3E-01	-0.09	6.6E-01	-0.49	1.1E-02	-0.49	1.2E-02
ENSCAFG0000000755	ENSCAFG0000000755	grey	EC_M10	-0.08	7.1E-01	-0.04	8.3E-01	0.05	8.3E-01	-0.02	9.1E-01	0.00	9.9E-01	0.00	9.9E-01	0.01	9.6E-01	-0.06	7.6E-01	-0.11	6.0E-01	0.00	9.9E-01
ENSCAFG0000000730	DEPDC7	grey	EC_M10	-0.08	7.1E-01	-0.20	3.2E-01	-0.40	4.1E-02	0.17	4.1E-01	0.54	4.5E-03	-0.14	4.9E-01	-0.11	6.0E-01	0.20	3.3E-01	0.16	4.5E-01	-0.52	7.1E-03
ENSCAFG0000000687	TARBP2	cyan	EC_M2	-0.08	7.1E-01	-0.32	1.1E-01	0.77	5.2E-06	0.04	8.5E-01	-0.68	1.2E-04	-0.05	8.0E-01	-0.05	7.9E-01	-0.15	4.7E-01	-0.10	6.3E-01	0.69	8.8E-05
ENSCAFG0000002026	ENSCAFG0000002026	grey	EC_M10	-0.08	7.1E-01	-0.06	7.7E-01	0.33	1.0E-01	0.05	8.2E-01	-0.31	1.2E-01	0.09	6.5E-01	-0.09	6.6E-01	-0.06	7.9E-01	-0.05	8.2E-01	0.28	1.7E-01
ENSCAFG0000001343	MDH1B	grey	EC_M10	-0.08	7.1E-01	-0.33	9.8E-02	0.25	2.1E-01	0.11	5.8E-01	-0.20	3.2E-01	-0.26	2.1E-01	-0.10	6.4E-01	0.28	1.7E-01	0.35	8.2E-02	0.23	2.6E-01
ENSCAFG0000000519	POU4F1	grey	EC_M10	-0.08	7.1E-01	0.14	4.8E-01	-0.22	2.7E-01	-0.02	9.3E-01	0.31	1.3E-01	-0.07	7.4E-01	0.45	2.2E-02	-0.03	8.8E-01	-0.06	7.8E-01	-0.23	2.6E-01
ENSCAFG0000001467	AP4M1	grey	EC_M10	-0.08	7.1E-01	-0.02	9.4E-01	0.40	4.4E-02	0.37	6.5E-02	-0.41	3.9E-02	-0.10	6.3E-01	-0.10	6.1E-01	-0.05	8.1E-01	0.13	5.3E-01	0.46	1.9E-02
ENSCAFG0000000031	LRIG3	grey	EC_M10	-0.08	7.1E-01	0.03	8.8E-01	0.63	5.8E-04	0.20	3.3E-01	-0.69	8.2E-05	-0.05	8.0E-01	0.04	8.7E-01	-0.15	4.8E-01	-0.03	8.8E-01	0.78	2.4E-06
ENSCAFG0000000696	AAAS	grey	EC_M10	-0.08	7.1E-01	0.11	6.1E-01	0.41	3.5E-02	-0.06	7.9E-01	-0.35	7.9E-02	-0.03	8.9E-01	0.18	3.8E-01	-0.12	5.5E-01	0.24	2.4E-01	0.40	4.0E-02
ENSCAFG0000000704	DNAH10	turquoise	EC_M6	-0.08	7.1E-01	0.55	3.3E-03	-0.03	8.7E-01	0.20	3.2E-01	-0.14	4.9E-01	-0.43	3.0E-02	0.08	7.1E-01	-0.10	6.1E-01	0.44	2.3E-02	0.24	2.5E-01
ENSCAFG0000001358	CCDC12	grey	EC_M10	-0.08	7.1E-01	0.43	3.0E-02	0.25	2.3E-01	-0.01	9.7E-01	-0.39	5.0E-02	-0.47	1.6E-02	0.07	7.5E-01	0.14	5.0E-01	-0.01	9.5E-01	0.42	3.3E-02
ENSCAFG0000000973	NGF	cyan	EC_M2	-0.08	7.1E-01	-0.64	3.8E-04	0.57	2.4E-03	0.30	1.4E-01	-0.36	7.2E-02	-0.13	5.2E-01	0.01	9.7E-01	-0.05	8.3E-01	-0.34	8.9E-02	0.35	8.0E-02
ENSCAFG0000000179	ALDH1A1	darkmagenta	EC_M12	-0.08	7.1E-01	-0.08	6.9E-01	0.04	8.5E-01	-0.11	6.0E-01	0.02	9.2E-01	-0.08	7.1E-01	-0.07	7.3E-01	0.89	1.4E-09	0.14	4.8E-01	0.00	9.8E-01
ENSCAFG0000001504	ENSCAFG0000001504	grey	EC_M10	-0.08	7.1E-01	-0.12	5.6E-01	-0.01	9.6E-01	0.00	9.8E-01	0.17	4.0E-01	-0.06	7.9E-01	-0.09	6.6E-01	-0.07	7.3E-01	-0.11	6.0E-01	-0.10	6.2E-01
ENSCAFG0000001993	ZDHHC7	grey	EC_M10	-0.08	7.1E-01	0.11	5.8E-01	-0.22	2.8E-01	-0.29	1.6E-01	0.38	5.7E-02	0.09	6.6E-01	-0.12	5.5E-01	-0.03	8.7E-01	-0.38	5.3E-02	-0.30	1.4E-01
ENSCAFG0000001158	NGEF	grey	EC_M10	-0.08	7.1E-01	-0.22	2.9E-01	0.13	5.3E-01	-0.08	7.0E-01	0.03	8.9E-01	-0.10	6.2E-01	0.07	7.2E-01	0.22	2.8E-01	0.61	8.5E-04	0.06	7.7E-01
ENSCAFG0000001341	ECHS1	grey	EC_M10	-0.08	7.1E-01	-0.44	2.3E-02	0.54	4.6E-03	0.11	5.8E-01	-0.45	2.0E-02	0.00	9.8E-01	-0.14	5.1E-01	0.18	3.8E-01	0.10	6.3E-01	0.45	2.0E-02
ENSCAFG0000001634	PCDH7	darkgreen	EC_M4	-0.08	7.1E-01	-0.53	5.2E-03	-0.29	1.5E-01	-0.07	7.4E-01	0.63	5.9E-04	0.22	2.7E-01	0.11	6.1E-01	0.20	3.2E-01	-0.09	6.6E-01	-0.59	1.4E-03
ENSCAFG0000000989	IFT57	darkgreen	EC_M4	-0.08	7.1E-01	-0.64	4.6E-04	-0.12	5.6E-01	0.06	7.8E-01	0.46	1.7E-02	0.10	6.3E-01	-0.02	9.3E-01	0.00	9.9E-01	0.28	1.7E-01	-0.39	4.8E-02
ENSCAFG0000002865	C9H17orf97	grey	EC_M10	-0.08	7.0E-01	0.00	9.8E-01	-0.28	1.6E-01	0.01	9.8E-01	0.33	9.7E-02	-0.04	8.3E-01	-0.03	8.8E-01	-0.12	5.6E-01	0.30	1.4E-01	-0.32	1.1E-01
ENSCAFG0000003220	PRSS53	grey	EC_M10	-0.08	7.0E-01	0.04	8.4E-01	-0.37	6.5E-02	0.02	9.1E-01	0.49	1.0E-02	0.25	2.2E-01	-0.03	9.0E-01	-0.11	5.8E-01	-0.13	5.3E-01	-0.46	1.7E-02
ENSCAFG0000002074	ENSCAFG0000002074	grey	EC_M10	-0.08	7.0E-01	-0.17	4.1E-01	0.17	4.0E-01	-0.12	5.6E-01	-0.12	5.7E-01	-0.07	7.3E-01	0.41	4.0E-02	-0.09	6.7E-01	-0.13	5.4E-01	0.17	3.9E-01
ENSCAFG0000003217	C1orf189	grey	EC_M10	-0.08	7.0E-01	0.11	6.0E-01	0.15	4.8E-01	-0.22	2.9E-01	-0.09	6.6E-01	-0.17	3.9E-01	-0.13	5.3E-01	-0.14	5.0E-01	-0.16	4.3E-01	0.15	4.6E-01
ENSCAFG0000000012	ENSCAFG0000000012	grey	EC_M10	-0.08	7.0E-01	-0.62	7.1E-04	0.35	8.1E-02	0.07	7.3E-01	-0.16	4.3E-01	-0.15	4.8E-01	-0.32	1.1E-01	0.21	3.0E-01	0.21	3.0E-01	0.22	2.8E-01
ENSCAFG0000001066	XPNPEP1	grey	EC_M10	-0.08	7.0E-01	0.17	4.1E-01	-0.32	1.1E-01	-0.14	4.9E-01	0.41	3.6E-02	0.33	9.5E-02	-0.20	3.2E-01	-0.25	2.2E-01	-0.28	1.6E-01	-0.34	8.7E-02
ENSCAFG0000001013	MFSD4A	turquoise	EC_M6	-0.08	7.0E-01	0.74	1.3E-05	-0.24	2.4E-01	-0.18	3.8E-01	0.09	6.7E-01	-0.04	8.4E-01	-0.15	4.7E-01	-0.24	2.3E-01	0.36	7.0E-02	-0.03	8.7E-01
ENSCAFG0000000138	NTRK2	grey	EC_M10	-0.08	7.0E-01	-0.18	3.7E-01	-0.46	1.9E-02	-0.20	3.2E-01	0.73	2.7E-05	-0.03	8.7E-01	0.04	8.3E-01	0.26	2.0E-01	0.07	7.3E-01	-0.62	7.5E-04
ENSCAFG0000002920	ZNF354C	grey	EC_M10	-0.08	7.0E-01	0.18	3.9E-01	-0.18	3.9E-01	-0.15	4.6E-01	0.16	4.3E-01	0.09	6.5E-01	0.20	3.2E-01	-0.17	4.1E-01	0.29	1.5E-01	-0.12	5.6E-01

ENSCAFG0000000589	SIRT7	grey	EC_M10	-0.08	7.0E-01	0.54	4.4E-03	0.42	3.1E-02	0.08	6.8E-01	-0.65	3.5E-04	-0.27	1.8E-01	0.12	5.5E-01	0.21	3.1E-01	0.20	3.2E-01	0.70	6.0E-05
ENSCAFG0000000333	ABCA13	darkgreen	EC_M4	-0.08	7.0E-01	-0.12	5.4E-01	-0.07	7.4E-01	-0.10	6.2E-01	0.27	1.9E-01	-0.10	6.3E-01	-0.07	7.2E-01	-0.02	9.2E-01	-0.06	7.7E-01	-0.17	4.1E-01
ENSCAFG0000003179	ZNF436	grey	EC_M10	-0.08	7.0E-01	0.39	4.9E-02	0.29	1.6E-01	0.17	4.0E-01	-0.50	9.2E-03	-0.05	8.1E-01	0.00	9.9E-01	0.05	8.2E-01	0.12	5.6E-01	0.52	6.9E-03
ENSCAFG0000001625	ENSCAFG0000001625	grey	EC_M10	-0.08	7.0E-01	-0.15	4.7E-01	-0.15	4.6E-01	0.30	1.4E-01	0.26	2.0E-01	-0.08	7.1E-01	-0.17	4.0E-01	-0.12	5.5E-01	-0.10	6.1E-01	-0.22	2.9E-01
ENSCAFG0000001097	ENSCAFG0000001097	grey	EC_M10	-0.08	7.0E-01	-0.15	4.8E-01	-0.21	3.1E-01	0.40	4.3E-02	0.33	1.0E-01	-0.11	5.9E-01	-0.14	5.1E-01	-0.14	4.8E-01	-0.02	9.1E-01	-0.23	2.6E-01
ENSCAFG0000000501	SLC22A13	grey	EC_M10	-0.08	7.0E-01	-0.29	1.5E-01	-0.38	5.3E-02	-0.21	3.1E-01	0.62	8.0E-04	0.27	1.9E-01	0.13	5.4E-01	0.19	3.4E-01	-0.08	7.1E-01	-0.57	2.5E-03
ENSCAFG0000002966	ENSCAFG0000002966	darkgreen	EC_M4	-0.08	7.0E-01	-0.05	8.0E-01	-0.10	6.2E-01	-0.12	5.4E-01	0.21	3.1E-01	-0.09	6.7E-01	-0.07	7.5E-01	0.10	6.4E-01	0.05	8.0E-01	-0.16	4.4E-01
ENSCAFG0000001709	SYCE2	grey	EC_M10	-0.08	7.0E-01	-0.09	6.5E-01	0.36	7.2E-02	-0.04	8.4E-01	-0.32	1.1E-01	-0.01	9.8E-01	-0.03	8.7E-01	-0.06	7.7E-01	-0.11	6.0E-01	0.39	5.1E-02
ENSCAFG0000002049	ENSCAFG0000002049	grey	EC_M10	-0.08	7.0E-01	-0.09	6.5E-01	0.36	7.2E-02	-0.04	8.4E-01	-0.32	1.1E-01	-0.01	9.8E-01	-0.03	8.7E-01	-0.06	7.7E-01	-0.11	6.0E-01	0.39	5.1E-02
ENSCAFG0000000241	AGMO	turquoise	EC_M6	-0.08	7.0E-01	0.51	8.4E-03	-0.17	4.2E-01	-0.21	3.0E-01	0.09	6.5E-01	-0.23	2.6E-01	0.25	2.2E-01	0.41	3.6E-02	-0.09	6.5E-01	-0.01	9.5E-01
ENSCAFG0000001116	ENSCAFG0000001116	grey	EC_M10	-0.08	7.0E-01	-0.23	2.6E-01	0.27	1.8E-01	-0.15	4.7E-01	-0.12	5.5E-01	-0.13	5.3E-01	0.29	1.5E-01	0.39	5.0E-02	-0.03	9.0E-01	0.20	3.4E-01
ENSCAFG0000000082	PSMB8	grey	EC_M10	-0.08	7.0E-01	0.49	1.1E-02	0.38	5.3E-02	0.26	1.9E-01	-0.53	5.8E-03	-0.37	6.3E-02	-0.07	7.5E-01	-0.08	7.1E-01	0.06	7.9E-01	0.59	1.5E-03
ENSCAFG0000003261	ENSCAFG0000003261	turquoise	EC_M6	-0.08	7.0E-01	0.39	5.1E-02	-0.18	3.7E-01	-0.17	4.2E-01	0.21	3.0E-01	-0.19	3.4E-01	-0.12	5.6E-01	-0.16	4.4E-01	-0.14	5.1E-01	-0.16	4.3E-01
ENSCAFG0000001220	ACKR3	grey	EC_M10	-0.08	7.0E-01	0.28	1.7E-01	0.58	1.8E-03	-0.03	9.0E-01	-0.66	2.5E-04	-0.29	1.5E-01	-0.16	4.4E-01	-0.25	2.2E-01	-0.24	2.4E-01	0.74	1.3E-05
ENSCAFG0000000269	TMC4	grey	EC_M10	-0.08	7.0E-01	0.21	2.9E-01	-0.06	7.7E-01	-0.04	8.4E-01	0.09	6.5E-01	-0.07	7.3E-01	-0.50	9.1E-03	-0.05	8.2E-01	0.11	5.9E-01	-0.06	7.8E-01
ENSCAFG0000000927	TSG101	darkgreen	EC_M4	-0.08	7.0E-01	0.21	3.0E-01	-0.73	2.1E-05	-0.30	1.4E-01	0.84	6.9E-08	0.20	3.2E-01	-0.10	6.1E-01	0.24	2.4E-01	0.11	5.9E-01	-0.78	2.4E-06
ENSCAFG0000000929	FBXW8	grey	EC_M10	-0.08	7.0E-01	-0.50	8.8E-03	-0.05	8.0E-01	-0.20	3.4E-01	0.35	8.2E-02	-0.02	9.1E-01	-0.14	4.8E-01	0.08	7.0E-01	-0.07	7.4E-01	-0.34	9.3E-02
ENSCAFG0000001614	TMEM82	grey	EC_M10	-0.08	7.0E-01	0.06	7.9E-01	-0.07	7.2E-01	0.41	3.9E-02	0.15	4.6E-01	-0.12	5.5E-01	0.21	3.1E-01	0.31	1.3E-01	-0.05	8.1E-01	-0.09	6.7E-01
ENSCAFG0000003160	ENSCAFG0000003160	grey	EC_M10	-0.08	7.0E-01	0.48	1.3E-02	0.22	2.8E-01	-0.15	4.6E-01	-0.38	5.7E-02	-0.17	4.2E-01	0.17	4.0E-01	-0.17	3.9E-01	-0.16	4.5E-01	0.44	2.5E-02
ENSCAFG0000000079	NOTCH4	turquoise	EC_M6	-0.08	7.0E-01	0.82	2.6E-07	-0.11	6.0E-01	-0.12	5.6E-01	-0.09	6.8E-01	-0.12	5.7E-01	-0.11	5.9E-01	-0.16	4.4E-01	0.09	6.8E-01	0.10	6.4E-01
ENSCAFG0000002339	C5H11orf87	grey	EC_M10	-0.08	7.0E-01	-0.08	6.9E-01	-0.18	3.7E-01	-0.13	5.3E-01	0.39	5.1E-02	0.38	5.9E-02	-0.07	7.4E-01	0.28	1.6E-01	-0.08	6.9E-01	-0.35	7.8E-02
ENSCAFG0000000059	MARCH3	grey	EC_M10	-0.08	7.0E-01	-0.08	6.9E-01	-0.49	1.1E-02	-0.03	8.8E-01	0.68	1.2E-04	0.22	2.8E-01	-0.15	4.8E-01	-0.17	4.1E-01	-0.23	2.7E-01	-0.70	7.8E-05
ENSCAFG0000000246	HEMGN	grey	EC_M10	-0.08	7.0E-01	-0.13	5.3E-01	0.25	2.3E-01	0.44	2.4E-02	-0.22	2.9E-01	-0.12	5.5E-01	-0.11	5.8E-01	-0.13	5.2E-01	0.23	2.5E-01	0.33	1.0E-01
ENSCAFG0000002598	ENSCAFG0000002598	grey	EC_M10	-0.08	7.0E-01	0.47	1.6E-02	-0.04	8.5E-01	-0.11	6.0E-01	-0.04	8.3E-01	-0.07	7.2E-01	0.59	1.6E-03	-0.07	7.5E-01	0.38	5.3E-02	0.09	6.7E-01
ENSCAFG0000000535	KLF6	grey	EC_M10	-0.08	7.0E-01	0.39	5.0E-02	0.43	2.9E-02	-0.15	4.6E-01	-0.55	3.4E-03	-0.39	5.1E-02	0.11	5.9E-01	0.16	4.3E-01	0.19	3.6E-01	0.62	7.6E-04
ENSCAFG0000000500	NCKAP5	darkgreen	EC_M4	-0.08	6.9E-01	-0.46	1.7E-02	-0.27	1.8E-01	0.04	8.5E-01	0.55	3.9E-03	0.27	1.7E-01	-0.06	7.7E-01	-0.03	8.9E-01	-0.42	3.5E-02	-0.57	2.2E-03
ENSCAFG0000001678	DPF3	grey	EC_M10	-0.08	6.9E-01	-0.21	2.9E-01	0.33	9.9E-02	0.27	1.8E-01	-0.22	2.9E-01	-0.12	5.7E-01	-0.14	4.9E-01	0.44	2.3E-02	-0.08	6.9E-01	0.30	1.4E-01
ENSCAFG0000001610	WDR19	cyan	EC_M2	-0.08	6.9E-01	-0.56	2.9E-03	0.64	3.9E-04	0.34	9.4E-02	-0.56	2.9E-03	-0.01	9.7E-01	-0.14	5.1E-01	-0.02	9.1E-01	-0.01	9.8E-01	0.56	3.2E-03
ENSCAFG0000001982	ATXN7L2	cyan	EC_M2	-0.08	6.9E-01	0.19	3.5E-01	0.54	4.4E-03	0.19	3.6E-01	-0.67	1.8E-04	-0.20	3.3E-01	0.05	8.0E-01	0.18	3.9E-01	0.08	6.9E-01	0.67	1.6E-04
ENSCAFG0000001520	TMEM260	grey	EC_M10	-0.08	6.9E-01	0.18	3.8E-01	-0.36	6.9E-02	-0.08	6.9E-01	0.38	5.7E-02	-0.19	3.6E-01	0.00	9.8E-01	0.51	7.5E-03	0.12	5.7E-01	-0.31	1.3E-01
ENSCAFG0000001079	ANKRD44	grey	EC_M10	-0.08	6.9E-01	-0.04	8.5E-01	-0.27	1.8E-01	-0.03	9.0E-01	0.36	7.1E-02	0.16	4.4E-01	0.03	9.0E-01	0.44	2.6E-02	0.57	2.5E-03	-0.30	1.3E-01
ENSCAFG0000001594	ZNF12	cyan	EC_M2	-0.08	6.9E-01	0.25	2.2E-01	0.45	2.1E-02	0.27	1.8E-01	-0.66	2.6E-04	-0.08	7.1E-01	0.01	9.7E-01	0.02	9.4E-01	0.12	5.5E-01	0.69	8.2E-05
ENSCAFG0000000487	TMEM177	grey	EC_M10	-0.08	6.9E-01	-0.10	6.4E-01	0.53	5.2E-03	-0.36	7.1E-02	-0.45	2.0E-02	-0.09	6.7E-01	-0.07	7.2E-01	-0.05	8.2E-01	0.01	9.5E-01	0.48	1.3E-02
ENSCAFG0000000329	LRRN3	grey	EC_M10	-0.08	6.9E-01	-0.32	1.2E-01	0.46	1.7E-02	-0.04	8.6E-01	-0.30	1.4E-01	-0.16	4.3E-01	-0.02	9.4E-01	0.01	9.7E-01	-0.20	3.4E-01	0.39	5.1E-02
ENSCAFG0000000918	CPOX	cyan	EC_M2	-0.08	6.9E-01	-0.32	1.1E-01	0.57	2.3E-03	0.16	4.3E-01	-0.56	3.2E-03	-0.07	7.5E-01	0.06	7.6E-01	-0.11	6.1E-01	-0.01	9.7E-01	0.63	6.3E-04
ENSCAFG0000000042	ENSCAFG0000000042	grey	EC_M10	-0.08	6.9E-01	-0.12	5.6E-01	0.32	1.1E-01	-0.12	5.7E-01	-0.28	1.7E-01	-0.06	7.7E-01	-0.11	6.0E-01	0.07	7.3E-01	-0.04	8.4E-01	0.31	1.3E-01
ENSCAFG0000000587	OXNAD1	grey	EC_M10	-0.08	6.9E-01	-0.25	2.1E-01	-0.16	4.4E-01	0.16	4.4E-01	0.24	2.3E-01	-0.13	5.4E-01	-0.11	5.9E-01	0.08	7.0E-01	0.05	8.0E-01	-0.21	3.1E-01
ENSCAFG0000001469	ENSCAFG0000001469	darkmagenta	EC_M12	-0.08	6.9E-01	-0.08	6.9E-01	0.03	8.9E-01	-0.11	6.1E-01	0.03	8.7E-01	-0.07	7.2E-01	-0.07	7.3E-01	0.92	2.3E-11	0.15	4.7E-01	-0.01	9.8E-01
ENSCAFG0000002432	FXYD5	grey	EC_M10	-0.08	6.9E-01	0.27	1.9E-01	-0.24	2.3E-01	-0.28	1.6E-01	0.29	1.5E-01	-0.11	5.9E-01	0.32	1.1E-01	0.07	7.5E-01	-0.09	6.5E-01	-0.25	2.1E-01
ENSCAFG0000001580	PRICKLE3	cyan	EC_M2	-0.08	6.9E-01	0.19	3.5E-01	0.63	5.1E-04	0.06	7.8E-01	-0.74	1.3E-05	0.00	9.9E-01	0.23	2.5E-01	-0.08	7.1E-01	-0.03	8.7E-01	0.78	2.1E-06
ENSCAFG0000001804	ENSCAFG0000001804	grey	EC_M10	-0.08	6.9E-01	-0.17	4.0E-01	0.12	5.5E-01	-0.40	4.4E-02	0.06	7.7E-01	0.32	1.2E-01	-0.04	8.5E-01	0.22	2.8E-01	-0.15	4.8E-01	0.04	8.6E-01
ENSCAFG0000001175	PLXNA2	turquoise	EC_M6	-0.08	6.9E-01	0.53	5.4E-03	0.05	8.2E-01	-0.37	6.3E-02	-0.14	5.0E-01	-0.33	9.5E-02	-0.06	7.8E-01	0.10	6.2E-01	0.51	7.8E-03	0.25	2.3E-01
ENSCAFG0000000995	KCNIP2	grey	EC_M10	-0.08	6.9E-01	-0.12	5.7E-01	-0.42	3.0E-02	-0.12	5.6E-01	0.55	3.8E-03	-0.18	3.9E-01	0.00	9.9E-01	0.05	8.1E-01	-0.04	8.4E-01	-0.52	6.0E-03
ENSCAFG0000001568	ZBTB49	grey	EC_M10	-0.08	6.9E-01	0.37	6.7E-02	0.34	9.4E-02	0.18	3.8E-01	-0.46	1.7E-02	0.04	8.4E-01	0.21	3.1E-01	0.06	7.7E-01	0.03	9.0E-01	0.49	1.2E-02
ENSCAFG0000000829	RNF34	grey	EC_M10	-0.08	6.9E-01	-0.31	1.3E-01	0.29	1.6E-01	0.21	3.1E-01	-0.21	3.1E-01	0.11	6.1E-01	0.10	6.3E-01	0.05	7.9E-01	0.02	9.4E-01	0.25	2.2E-01
ENSCAFG0000003042	AIFM3	grey	EC_M10	-0.08	6.9E-01	0.20	3.2E-01	-0.23	2.7E-01	0.05	8.0E-01	0.27	1.9E-01	0.04	8.5E-01	0.07	7.4E-01	0.20	3.2E-01	0.25	2.2E-01	-0.20	3.3E-01
ENSCAFG0000001326	SENP2	turquoise	EC_M6	-0.08	6.9E-01	0.39	4.9E-02	-0.11	6.1E-01	0.16	4.3E-01	-0.08	7.0E-01	0.02	9.1E-01	0.20	3.3E-01	-0.13	5.2E-01	-0.12	5.6E-01	0.11	5.8E-01
ENSCAFG0000001838	ENSCAFG0000001838	turquoise	EC_M6	-0.08	6.9E-01	0.44	2.4E-02	-0.20	3.4E-01	0.11	5.8E-01	0.11	5.9E-01	-0.25	2.1E-01	0.05	8.0E-01	-0.43	2.7E-02	0.19	3.6E-01	-0.06	7.8E-01
ENSCAFG0000001388	ENSCAFG0000001388	grey	EC_M10	-0.08	6.9E-01	-0.45	2.1E-02	0.31	1.2E-01	0.40	4.5E-02	-0.23	2.7E-01	0.04	8.5E-01	-0.13	5.2E-01	0.02	9.1E-01	-0.09	6.7E-01	0.26	2.0E-01
ENSCAFG0000000004	ENSCAFG0000000004	grey	EC_M10	-0.08	6.9E-01	0.19	3.5E-01	0.05	7.9E-01	-0.55	3.3E-03	0.00	1.0E+00	-0.03	8.8E-01	-0.03	8.7E-01	0.50	9.0E-03	-0.02	9.2E-01	0.05	8.0E-01
ENSCAFG0000001156	PARD6B	grey	EC_M10	-0.08	6.9E-01	-0.10	6.2E-01	-0.44	2.5E-02	0.12	5.4E-01	0.55	3.6E-03	0.17	4.1E-01	0.30	1.4E-01	0.14	4.9E-01	0.33	1.0E-01	-0.50	8.7E-03
ENSCAFG0000002017	MAPKAP1	grey	EC_M10	-0.08	6.9E-01	-0.17	4.1E-01	-0.13	5.4E-01	0.05	8.2E-01	0.24	2.4E-01	0.14	5.0E-01	-0.43	2.8E-02	0.06	7.6E-01	0.23	2.5E-01	-0.16	4.4E-01
ENSCAFG0000001746	MKS1	grey	EC_M10	-0.08	6.9E-01	-0.25	2.1E-01	0.46	1.8E-02	0.09	6.5E-01	-0.39	4.9E-02	-0.38	5.8E-02	-0.17	4.1E-01	0.06	7.7E-01	0.33	9.6E-02	0.47	1.6E-02
ENSCAFG0000000665	ALKBH3	grey	EC_M10	-0.08	6.9E-01	-0.16	4.3E-01	0.33	9.4E-02	0.50	9.2E-03	-0.32	1.1E-01	0.01	9.7E-01	0.18	3.8E-01	-0.39	4.9E-02	-0.18	3.9E-01	0.36	6.8E-02
ENSCAFG0000000021	TRIM36	darkgreen	EC_M4	-0.08	6.9E-01	-0.07	7.3E-01	-0.63	5.7E-04	-0.24	2.3E-01	0.83	1.3E-07	0.04	8.3E-01	-0.01	9.6E-01	0.05	8.0E-01	0.05	8.1E-01	-0.77	4.0E-06
ENSCAFG0000001816	CCL14	grey	EC_M10	-0.08	6.9E-01	-0.38	5.6E-02	-0.29	1.6E-01	0.12	5.6E-01	0.51	8.3E-03	0.08	7.0E-01	-0.22	2.9E-01	0.17	4.1E-01	-0.40	4.3E-02	-0.50	1.0E-02
ENSCAFG0000002961	AMACR	darkgreen	EC_M4	-0.08	6.9E-01	-0.52	6.7E-03	-0.27	1.8E-01	-0.19	3.5E-01	0.57	2.2E-03	0.25	2.1E-01	0.40	4.4E-02	0.13	5.3E-01	-0.05	8.0E-01	-0.53	4.9E-03
ENSCAFG0000001296	INPP5J	paleturquoise	EC_M11	-0.08	6.9E-01	0.04	8.4E-01	0.15	4.7E-01	0.67	2.0E-04	-0.18	3.8E-01	-0.03	8.9E-01	-0.01	9.7E-01	-0.10	6.3E-01	0.64	4.6E-04	0.24	2.4E-01
ENSCAFG0000001688	ITGAM	paleturquoise	EC_M11	-0.08	6.9E-01	0.04	8.4E-01	0.15	4.7E-01	0.67	2.0E-04	-0.18	3.8E-01	-0.03	8.9E-01	-0.01	9.7E-01	-0.10	6.3E-01	0.64	4.6E-04	0.24	2.4E-01
ENSCAFG0000002036	HSD11B2	grey	EC_M10	-0.08	6.9E-01	-0.39	5.0E-02	0.49	1.2E-02	0.01	9.5E-01	-0.37	6.0E-02	-0.30	1.3E-01	-0.10	6.2E-01	0.27	1.8E-01	0.08	6.8E-01	0.42	3.3E-02
ENSCAFG0000003064	ENSCAFG0000003064	grey	EC_M10	-0.08	6.9E-01	0.22	2.8E-01	-0.11	5.8E-01	-0.15	4.6E-01	0.07	7.4E-01	0.00	9.9E-01	0.10	6.3E-01	0.15	4.5E-01	-0.11	6.1E-01	-0.06	7.7E-01
ENSCAFG0000002375	GNAS	grey	EC_M10	-0.08	6.9E-01	0.52	6.1E-03	-0.47	1.4E-02	-0.24	2.3E-01	0.43	3.0E-02	0.01	9.4E-01	0.20	3.3E-01	-0.10	6.3E-01	0.41	3.5E-02	-0.38	5.8E-02
ENSCAFG0000001565	TTC9C	grey	EC_M10	-0.08	6.9E-01	0.18	3.8E-01	-0.08	6.8E-01	-0.19	3.6E-01	0.16	4.4E-01	-0.32	1.1E-01	-0.35	8.3E-02	0.03	8.9E-01	0.01	9.5E-01	-0.09	6.7E-01
ENSCAFG0000001116	UBA7	cyan	EC_M2	-0.08	6.9E-01	0.23	2.6E-01	0.71	4.4E-05	0.23	2.5E-01	-0.86	2.1E-08	-0.39	4.9E-02	-0.05	8.0E-01	-0.07	7.2E-01	-0.05	7.9E-01	0.90	4.2E-10
ENSCAFG0000001375	SSTR1	turquoise	EC_M6	-0.08	6.9E-01	0.75	1.3E-05	-0.14	5.1E-01	-0.09	6.5E-01	-0.06	7.6E-01	-0.09	6.5E-01	0.21	3.0E-01	-0.08	6.8E-01	0.12	5.6E-01	0.11	5.9E-01
ENSCAFG0000003065	CHSY3	grey	EC_M10	-0.08	6.9E-01	-0.11	5.9E-01	-0.51	7.4E-03	-0.25	2.2E-01	0.74	1.9E-05	0.12	5.6E-01	0.14	5.1E-01	0.13	5.2E-01	0.47	1.5E-02	-0.64	4.1E-04
ENSCAFG0000000606	COL4A1	grey	EC_M10	-0.08	6.9E-01	0.33	1.0E-01	-0.54	4.0E-03	-0.23	2.6E-01	0.57	2.2E-03	0.08	7.1E-01	0.49	1.0E-02	0.22	2.8E-01	-0.16	4.2E-01	-0.55	3.9E-03
ENSCAFG0000000858	CXCL10	turquoise	EC_M6	-0.08	6.9E-01	0.54	4.1E-03	-0.15	4.5E-01	0.09	6.6E-01	0.04	8.6E-01	-0.17	3.9E-01	0.21	2.9E-01	-0.14	4.9E-01	-0.17	4.2E-01	0.03	8.9E-01
ENSCAFG0000000462	ENSCAFG0000000462	grey	EC_M10	-0.08	6.9E-01	-0.18	3.7E-01	0.27	1.7E-01	0.56	2.9E-03	-0.21	3.0E-01	-0.16	4.5E-01	-0.15	4.7E-01	-0.16	4.4E-01	-0.11	5.9E-01	0.27	1.9E-01
ENSCAFG0000002994	IDNK	grey	EC_M10	-0.08	6.9E-01	0.25	2.3E-01	0.37	6.6E-02	0.11	6.0E-01	-0.44	2.5E-02	-0.12	5.5E-01	-0.14	5.0E-01	-0.13	5.3E-01	-0.01	9.4E-01	0.52	6.6E-03
ENSCAFG0000000653	LMOD3	turquoise	EC_M6	-0.08	6.9E-01	0.64	4.6E-04	-0.05	7.9E-01	-0.12	5.5E-01	-0.08	7.1E-01	-0.10	6.2E-01	-0.10	6.3E-01	-0.14	4.9E-01	-0.10	6.3E-01	0.15	4.6E-01
ENSCAFG0000001755	S100A8	grey	EC_M10	-0.08	6.9E-01	-0.19	3.6E-01	0.27	1.8E-01	-0.19	3.6E-01	-0.23	2.6E-01	0.22	2.9E-01	0.51	7.8E-03	-0.14	4.9E-01	0.02	9.3E-01	0.22	2.7E-01
ENSCAFG0000001101	SPATS2L	grey	EC_M10	-0.08	6.9E-01	-0.04	8.5E-01	-0.61	8.8E-04	-0.16	4.3E-01	0.81	5.1E-07	0.25	2.2E-01	0.25	2.2E-01	-0.09	6.8E-01	-0.20	3.3E-01	-0.78	2.1E-06
ENSCAFG0000001998	TOR1A	grey	EC_M10	-0.08	6.9E-01	0.13	5.3E-01	-0.31	1.2E-01	-0.13	5.4E-01	0.48	1.4E-02	0.19	3.7E-01	-0.12	5.5E-01	-0.22	2.7E-01	0.26	1.9E-01	-0.38	5.2E-02
ENSCAFG0000000572	RARB	darkgreen	EC_M4	-0.08	6.9E-01	0.06	7.6E-01	-0.11	5.9E-01	-0.03	9.0E-01	0.22	2.7E-01	0.00	1.0E+00	-0.07	7.4E-01	-0.14	4.8E-01	0.12	5.6E-01	-0.14	4.9E-01
ENSCAFG0000001638	DEGS1	grey	EC_M10	-0.08	6.9E-01	-0.59	1.5E-03	-0.06	7.8E-01	-0.21	3.1E-01	0.42	3.2E-02	0.22	2.8E-01	-0.20	3.2E-01	0.20	3.2E-01	-0.18	3.7E-01	-0.38	5.4E-02
ENSCAFG0000001752	PAQR5	grey	EC_M10	-0.08	6.9E-01	-0.25	2.1E-01	0.47	1.5E-02	0.21	3.1E-01	-0.40	4.4E-02	-0.17	3.9E-01	-0.15	4.7E-01	-0.24	2.4E-01	0.00	1.0E+00	0.46	1.9E-02
ENSCAFG0000000041	ATAT1	grey	EC_M10	-0.08	6.9E-01	0.24	2.4E-01	0.27	1.8E-01	0.10	6.1E-01	-0.44	2.6E-02	-0.34	8.9E-02	0.15	4.7E-01	-0.20	3.2E-01	0.23	2.7E-01	0.44	2.3E-02
ENSCAFG0000000156	BNC2	grey	EC_M10	-0.08	6.9E-01	-0.45	2.0E-02	0.29	1.5E-01	0.14	4.9E-01	-0.14	5.1E-01	0.14	4.9E-01	-0.24	2.4E-01	0.14	5.0E-01	-0.20	3.3E-01	0.10	6.3E-01
ENSCAFG0000001349	FAM129A	grey	EC_M10	-0.08	6.9E-01	-0.09	6.7E-01	-0.12	5.5E-01	-0.13	5.3E-01	0.30	1.4E-01	-0.09	6.8E-01	0.10	6.4E-01	0.08	6.9E-01	0.26	2.0E-01	-0.18	3.8E-01
ENSCAFG0000001201	EIF4H	cyan	EC_M2	-0.08	6.8E-01	-0.23	2.6E-01	0.51	8.1E-03	-0.06	7.9E-01	-0.35	7.6E-02	-0.28	1.6E-01	0.11	5.8E-01	-0.14	4.9E-01	-0.37	6.1E-02	0.40	4.5E-02
ENSCAFG0000001153	EGF	grey	EC_M10	-0.08	6.8E-01	-0.15	4.8E-01	0.43	2.7E-02	-0.16	4.4E-01	-0.42	3.2E-02	-0.09	6.5E-01	-0.10	6.4E-01	-0.05	8.2E-01	-0.06	7.5E-01	0.48	1.4E-02
ENSCAFG0000001926	ENSCAFG0000001926	grey	EC_M10	-0.08	6.8E-01	0.12	5.7E-01	0.16	4.5E-01	0.10	6.2E-01	-0.10	6.4E-01	-0.20	3.3E-01	0.13	5.3E-01	0.04	8.3E-01	0.00	1.0E+00	0.14	5.1E-01
ENSCAFG0000001827	BAG5	grey	EC_M10	-0.08	6.8E-01	-0.27	1.8E-01	-0.38	5.7E-02	-0.04	8.4E-01	0.63	6.2E-04	0.21	3.0E-01	-0.01	9.5E-01	-0.11	5.8E-01	-0.47	1.5E-02	-0.61	8.9E-04
ENSCAFG0000001586	PRXL2A	grey	EC_M10	-0.08	6.8E-01	0.17	4.1E-01	0.49	1.1E-02	0.06	7.6E-01	-0.55	3.8E-03	0.04	8.3E-01	0.02	9.3E-01	-0.13	5.3E-01	-0.29	1.4E-01	0.59	1.4E-03
ENSCAFG0000001093	NPNT	grey	EC_M10	-0.08	6.8E-01	-0.31	1.2E-01	0.25	2.1E-01	0.15	4.5E-01	-0.10	6.2E-01	-0.19	3.5E-01	-0.18	3.8E-01	0.38	5.9E-02	0.26	2.0E-01	0.17	4.1E-01
ENSCAFG0000002605	ENSCAFG0000002605	turquoise	EC_M6	-0.08	6.8E-01	0.30	1.4E-01	-0.05	7.9E-01	0.39	5.2E-02	-0.02	9.1E-01	-0.13	5.1E-01	0.42	3.1E-02	-0.13	5.2E-01	-0.21	3.0E-01	0.02	9.1E-01
ENSCAFG0000002546	TRIM65	cyan	EC_M2	-0.08	6.8E-01	0.06	7.6E-01	0.80	8.5E-07	0.11	5.8E-01	-0.89	9.8E-10	-0.24	2.3E-01	0.04	8.4E-01	-0.05	8.2E-01	0.11	5.9E-01	0.96	2.4E-14
ENSCAFG0000000725	PCGF5	grey	EC_M10	-0.08	6.8E-01	-0.15	4.7E-01	-0.52	7.0E-03	0.00	9.9E-01	0.68	1.2E-04	0.09	6.7E-01	-0.09	6.7E-01	0.23	2.5E-01	0.20	3.4E-01	-0.65	3.1E-04
ENSCAFG0000000972	ALCAM	grey	EC_M10	-0.08	6.8E-01	-0.48	1.4E-02	-0.18	3.9E-01	0.05	8.2E-01	0.39	4.9E-02	0.14	5.1E-01	0.17	4.2E-01	0.11	6.1E-01	0.48	1.2E-02	-0.37	6.5E-02
ENSCAFG0000001768	SCNN1B	turquoise	EC_M6	-0.08	6.8E-01	0.69	9.2E-05	-0.20	3.3E-01	-0.10	6.1E-01	0.06	7.9E-01	-0.08	7.0E-01	-0.11	6.1E-01	-0.08	7.0E-01	0.18	3.7E-01	0.03	8.8E-01
ENSCAFG0000000229	MITD1	grey	EC_M10	-0.08	6.8E-01	0.08	6.9E-01	0.35	8.0E-02	0.10	6.2E-01	-0.44	2.4E-02	-0.25	2.1E-01	0.08	6.9E-01	0.07	7.4E-01	-0.34	9.3E-02	0.43	2.9E-02
ENSCAFG0000001762	DDX24	darkgreen	EC_M4	-0.08	6.8E-01	-0.19	3.4E-01	-0.61	8.5E-04	-0.25	2.3E-01	0.88	3.8E-09	0.37	6.0E-02	0.14	5.0E-01	0.03	8.8E-01	0.01	9.8E-01	-0.81	4.7E-07
ENSCAFG0000001300	ADH5	grey	EC_M10	-0.08	6.8E-01	-0.08	7.1E-01	0.22	2.7E-01	0.12	5.4E-01	-0.16	4.3E-01	0.14	5.1E-01	0.24	2.4E-01	0.00	1.0E+00	0.40	4.5E-02	0.25	2.2E-01
ENSCAFG0000001283	ADAMTS4	grey	EC_M10	-0.08	6.8E-01	0.45	2.0E-02	-0.47	1.6E-02	-0.12	5.7E-01	0.46	1.9E-02	0.22	2.8E-01	0.12	5.7E-01	-0.11	5.9E-01	0.26	2.0E-01	-0.46	1.9E-02
ENSCAFG0000001525	DPT	grey	EC_M10	-0.08	6.8E-01	-0.45	2.1E-02	0.66	2.2E-04	0.14	4.9E-01	-0.48	1.4E-02	-0.23	2.5E-01	0.10	6.2E-01	0.03	8.8E-01	-0.23	2.5E-01	0.56	2.8E-03
ENSCAFG0000002020	GBP1	grey	EC_M10	-0.08	6.8E-01	0.21	3.0E-01	-0.62	6.7E-04	0.00	9.8E-01	0.72	4.0E-05	0.21	3.1E-01	0.15	4.6E-01	0.04	8.5E-01	-0.02	9.2E-01	-0.67	1.8E-04
ENSCAFG0000000913	SAA1	grey	EC_M10	-0.08	6.8E-01	-0.31	1.2E-01	0.62	7.9E-04	-0.04	8.4E-01	-0.48	1.3E-02	-0.16	4.3E-01	0.38	5.7E-02	0.03	8.9E-01	-0.21	3.1E-01	0.55	3.4E-03
ENSCAFG0000001947	ENSCAFG0000001947	grey	EC_M10	-0.08	6.8E-01	-0.56	3.1E-03	0.66	2.4E-04	-0.01	9.5E-01	-0.43	2.9E-02	-0.07	7.3E-01	-0.24	2.4E-01	-0.07	7.4E-01	0.02	9.1E-01	0.50	9.7E-03
ENSCAFG0000000671	RYK	darkgreen	EC_M4	-0.08	6.8E-01	-0.76	8.3E-06	0.38	5.3E-02	0.13	5.3E-01	-0.10	6.3E-01	-0.09	6.6E-01	-0.02	9.2E-01	0.06	7.7E-01	-0.25	2.2E-01	0.11	6.0E-01
ENSCAFG0000001526	TBX19	grey	EC_M10	-0.08	6.8E-01	-0.12	5.4E-01	0.00	9.9E-01	-0.13	5.4E-01	0.17	4.0E-01	-0.13	5.4E-01	0.16	4.2E-01	-0.22	2.7E-01	0.08	6.9E-01	-0.12	5.6E-01
ENSCAFG0000000074	ENSCAFG0000000074	grey	EC_M10	-0.08	6.8E-01	0.08	7.0E-01	0.28	1.7E-01	0.28	1.6E-01	-0.29	1.5E-01	0.26	2.0E-01	-0.17	4.2E-01	-0.10	6.1E-01	-0.11	5.8E-01	0.30	1.3E-01
ENSCAFG0000001704	STX10	grey	EC_M10	-0.08	6.8E-01	-0.05	7.9E-01	0.50	8.7E-03	0.44	2.6E-02	-0.52	6.8E-03	-0.06	7.6E-01	0.32	1.1E-01	-0.19	3.4E-01	-0.28	1.6E-01	0.52	6.7E-03
ENSCAFG0000001079	TNNT2	grey	EC_M10	-0.08	6.8E-01	0.26	1.9E-01	-0.45	2.1E-02	-0.13	5.3E-01	0.51	7.8E-03	-0.18	3.8E-01	-0.15	4.5E-01	0.38	5.3E-02	-0.05	8.1E-01	-0.46	1.7E-02
ENSCAFG0000000139	ENSCAFG0000000139	grey	EC_M10	-0.08	6.8E-01	-0.10	6.2E-01	0.30	1.4E-01	0.21	3.0E-01	-0.23	2.7E-01	-0.27	1.8E-01	-0.38	5.2E-02	0.03	8.7E-01	-0.15	4.5E-01	0.29	1.4E-01
ENSCAFG0000000382	ITGB1	darkgreen	EC_M4	-0.09	6.8E-01	-0.03	9.0E-01	-0.73	2.3E-05	-0.14	4.9E-01	0.92	6.0E-11	0.24	2.3E-01	0.27	1.8E-01	0.10	6.2E-01	-0.06	7.7E-01	-0.87	9.0E-09
ENSCAFG0000003178	RMDN1	darkgreen	EC_M4	-0.09	6.8E-01	-0.20	3.4E-01	-0.18	3.7E-01	0.08	6.8E-01	0.38	5.2E-02	-0.02	9.1E-01	-0.16	4.2E-01	0.09	6.4E-01	0.08	6.9E-01	-0.27	1.8E-01
ENSCAFG0000000104	NDUFB9	grey	EC_M10	-0.09	6.8E-01	-0.60	1.2E-03	0.49	1.1E-02	0.28	1.7E-01	-0.24	2.3E-01	0.09	6.6E-01	0.06	7.9E-01	-0.11	5.9E-01	-0.10	6.2E-01	0.32	1.2E-01
ENSCAFG0000001486	RBM10	grey	EC_M10	-0.09	6.8E-01	-0.06	7.7E-01	0.35	7.9E-02	0.06	7.7E-01	-0.31	1.3E-01	-0.04	8.5E-01	-0.01	9.7E-01	-0.06	7.7E-01	-0.18	3.8E-01	0.28	1.7E-01
ENSCAFG0000001706	KRBA2	turquoise	EC_M6	-0.09	6.8E-01	0.42	3.3E-02	-0.04	8.5E-01	0.28	1.7E-01	-0.10	6.4E-01	-0.55	3.8E-03	-0.03	8.7E-01	-0.18	3.7E-01	0.16	4.4E-01	0.17	4.0E-01
ENSCAFG0000002394	NIPSNAP3B	grey	EC_M10	-0.09	6.8E-01	0.16	4.4E-01	0.05	8.1E-01	0.27	1.7E-01	-0.10	6.3E-01	-0.20	3.2E-01	-0.28	1.7E-01	0.00	1.0E+00	-0.12	5.7E-01	0.18	3.7E-01
ENSCAFG0000001031	DYNLL1	darkgreen	EC_M4	-0.09	6.8E-01	-0.03	8.9E-01	-0.62	7.0E-04	-0.31	1.2E-01	0.83	1.9E-07	0.05	8.2E-01	0.11	5.8E-01	0.11	6.0E-01	-0.03	8.8E-01	-0.82	3.2E-07
ENSCAFG0000000192	MAD2L1BP	grey	EC_M10	-0.09	6.8E-01	0.06	7.6E-01	0.50	8.6E-03	0.22	2.8E-01	-0.55	3.6E-03	-0.17	4.1E-01	-0.07	7.4E-01	-0.42	3.4E-02	-0.06	7.9E-01	0.62	7.6E-04
ENSCAFG0000002347	ENSCAFG0000002347	grey	EC_M10	-0.09	6.8E-01	0.19	3.6E-01	-0.27	1.9E-01	-0.27	1.8E-01	0.36	7.4E-02	-0.26	2.0E-01	0.09	6.7E-01	0.07	7.2E-01	0.10	6.2E-01	-0.33	9.8E-02
ENSCAFG0000000310	ANKRD6	magenta	EC_M13	-0.09	6.8E-01	-0.05	8.2E-01	-0.01	9.4E-01	-0.01	9.5E-01	0.08	7.1E-01	0.06	7.7E-01	-0.08	7.0E-01	-0.05	8.3E-01	0.79	1.4E-06	0.03	8.9E-01
ENSCAFG0000000198	TMEM151B	grey	EC_M10	-0.09	6.8E-01	0.50	8.7E-03	0.41	4.0E-02	0.06	7.8E-01	-0.60	1.2E-03	-0.33	1.0E-01	-0.02	9.1E-01	-0.17	4.1E-01	0.48	1.4E-02	0.66	2.4E-04

ENSCAFG0000000327	ELMO1	turquoise	EC_M6	-0.09	6.8E-01	0.63	5.8E-04	0.14	4.8E-01	0.05	8.1E-01	-0.34	8.9E-02	-0.15	4.7E-01	-0.19	3.5E-01	-0.06	7.8E-01	-0.25	2.2E-01	0.36	6.9E-02
ENSCAFG0000000608	SMOX	grey	EC_M10	-0.09	6.8E-01	0.06	7.7E-01	0.33	1.0E-01	0.22	2.9E-01	-0.38	5.3E-02	-0.13	5.4E-01	-0.08	6.9E-01	0.20	3.4E-01	0.30	1.3E-01	0.42	3.2E-02
ENSCAFG0000002897	ENSCAFG0000002897	violet	EC_M7	-0.09	6.8E-01	-0.14	5.0E-01	-0.28	1.6E-01	-0.02	9.1E-01	0.43	2.9E-02	0.61	8.9E-04	-0.10	6.4E-01	-0.13	5.2E-01	-0.14	5.0E-01	-0.39	5.1E-02
ENSCAFG0000001735	ADAM19	grey	EC_M10	-0.09	6.8E-01	-0.33	9.7E-02	-0.44	2.3E-02	-0.18	3.7E-01	0.74	1.8E-05	0.16	4.3E-01	-0.13	5.4E-01	0.24	2.4E-01	-0.04	8.5E-01	-0.69	1.1E-04
ENSCAFG0000000899	USH1C	turquoise	EC_M6	-0.09	6.8E-01	0.62	7.1E-04	-0.16	4.2E-01	-0.13	5.4E-01	0.01	9.6E-01	-0.10	6.1E-01	0.21	3.1E-01	-0.13	5.3E-01	0.18	3.7E-01	0.00	9.8E-01
ENSCAFG0000000403	EMP3	cyan	EC_M2	-0.09	6.8E-01	-0.44	2.5E-02	0.83	2.1E-07	0.12	5.6E-01	-0.71	5.2E-05	-0.22	2.7E-01	-0.03	8.8E-01	0.14	5.0E-01	-0.01	9.7E-01	0.74	1.5E-05
ENSCAFG0000003237	ZNF485	grey	EC_M10	-0.09	6.8E-01	-0.23	2.5E-01	-0.36	6.8E-02	0.00	1.0E+00	0.51	8.3E-03	0.31	1.3E-01	0.06	7.6E-01	0.04	8.3E-01	-0.23	2.7E-01	-0.45	2.0E-02
ENSCAFG0000001863	KSR1	grey	EC_M10	-0.09	6.8E-01	0.64	4.7E-04	0.32	1.1E-01	0.04	8.5E-01	-0.57	2.4E-03	-0.32	1.1E-01	0.09	6.7E-01	-0.10	6.1E-01	-0.05	8.2E-01	0.60	1.3E-03
ENSCAFG0000001387	TRAPPC6B	grey	EC_M10	-0.09	6.8E-01	0.06	7.6E-01	-0.45	2.0E-02	-0.09	6.7E-01	0.58	2.0E-03	-0.03	8.8E-01	0.06	7.7E-01	0.17	4.0E-01	-0.04	8.4E-01	-0.49	1.0E-02
ENSCAFG0000000209	RCL1	cyan	EC_M2	-0.09	6.8E-01	-0.55	3.3E-03	0.67	1.8E-04	0.25	2.2E-01	-0.53	5.5E-03	-0.10	6.4E-01	0.02	9.4E-01	-0.31	1.2E-01	-0.28	1.7E-01	0.56	2.9E-03
ENSCAFG0000000915	ENSCAFG0000000915	grey	EC_M10	-0.09	6.7E-01	0.17	4.2E-01	-0.12	5.5E-01	0.16	4.4E-01	0.13	5.2E-01	-0.16	4.2E-01	0.09	6.5E-01	0.20	3.2E-01	-0.06	7.8E-01	-0.11	5.9E-01
ENSCAFG0000002034	IFI44L	grey	EC_M10	-0.09	6.7E-01	0.41	3.7E-02	0.25	2.2E-01	0.33	1.0E-01	-0.39	4.8E-02	-0.17	4.1E-01	-0.15	4.7E-01	0.14	5.1E-01	-0.07	7.3E-01	0.42	3.1E-02
ENSCAFG0000000502	TAF3	turquoise	EC_M6	-0.09	6.7E-01	0.59	1.7E-03	0.19	3.5E-01	-0.08	6.9E-01	-0.39	4.7E-02	-0.16	4.3E-01	0.29	1.5E-01	0.13	5.2E-01	0.12	5.5E-01	0.47	1.6E-02
ENSCAFG0000002627	ENSCAFG0000002627	darkgreen	EC_M4	-0.09	6.7E-01	-0.01	9.7E-01	-0.08	7.1E-01	-0.12	5.6E-01	0.18	3.9E-01	-0.06	7.7E-01	-0.06	7.6E-01	0.06	7.6E-01	0.02	9.2E-01	-0.13	5.1E-01
ENSCAFG0000001614	CASC3	turquoise	EC_M6	-0.09	6.7E-01	0.55	4.0E-03	0.03	9.0E-01	-0.24	2.3E-01	-0.10	6.4E-01	0.04	8.3E-01	-0.08	6.9E-01	0.26	2.0E-01	0.16	4.3E-01	0.18	3.8E-01
ENSCAFG0000000093	COL14A1	cyan	EC_M2	-0.09	6.7E-01	-0.22	2.7E-01	0.76	6.0E-06	0.07	7.3E-01	-0.72	2.9E-05	-0.18	3.7E-01	-0.16	4.3E-01	0.11	6.1E-01	0.17	4.0E-01	0.80	9.0E-07
ENSCAFG0000001702	ZC2HC1C	grey	EC_M10	-0.09	6.7E-01	0.09	6.6E-01	0.42	3.3E-02	0.14	4.9E-01	-0.51	7.3E-03	-0.32	1.1E-01	0.09	6.6E-01	0.20	3.4E-01	0.22	2.8E-01	0.55	3.7E-03
ENSCAFG0000000743	SMYD1	grey	EC_M10	-0.09	6.7E-01	0.00	9.9E-01	-0.19	3.4E-01	0.00	9.9E-01	0.28	1.6E-01	-0.10	6.2E-01	0.31	1.2E-01	-0.12	5.5E-01	-0.22	2.7E-01	-0.28	1.6E-01
ENSCAFG0000002317	FGA	grey	EC_M10	-0.09	6.7E-01	-0.06	7.7E-01	0.19	3.4E-01	0.29	1.6E-01	-0.11	5.8E-01	-0.18	3.7E-01	-0.18	3.7E-01	-0.24	2.5E-01	-0.08	7.0E-01	0.11	5.8E-01
ENSCAFG0000000500	MGAT5	grey	EC_M10	-0.09	6.7E-01	-0.17	3.9E-01	-0.07	7.3E-01	-0.20	3.2E-01	0.27	1.8E-01	0.02	9.0E-01	0.33	9.5E-02	-0.10	6.3E-01	-0.15	4.7E-01	-0.18	3.8E-01
ENSCAFG0000001190	PCBP3	grey	EC_M10	-0.09	6.7E-01	-0.18	3.7E-01	-0.21	2.9E-01	0.11	5.8E-01	0.35	8.1E-02	0.22	2.9E-01	-0.16	4.2E-01	0.08	7.0E-01	0.26	2.0E-01	-0.39	5.2E-02
ENSCAFG0000000572	ENSCAFG0000000572	grey	EC_M10	-0.09	6.7E-01	-0.20	3.3E-01	0.39	4.7E-02	-0.14	4.9E-01	-0.34	9.4E-02	-0.05	8.0E-01	-0.17	4.0E-01	-0.03	8.9E-01	-0.14	4.9E-01	0.36	7.0E-02
ENSCAFG0000000329	DNALI1	paleturquoise	EC_M11	-0.09	6.7E-01	0.01	9.5E-01	0.13	5.4E-01	0.79	1.4E-06	-0.14	5.0E-01	-0.04	8.3E-01	0.00	9.8E-01	-0.11	5.9E-01	0.35	8.2E-02	0.17	4.0E-01
ENSCAFG0000000160	ENSCAFG0000000160	grey	EC_M10	-0.09	6.7E-01	0.02	9.1E-01	-0.27	1.8E-01	0.24	2.4E-01	0.39	4.8E-02	-0.19	3.6E-01	-0.17	4.0E-01	-0.17	4.1E-01	-0.09	6.6E-01	-0.32	1.1E-01
ENSCAFG0000000081	TRPS1	magenta	EC_M13	-0.09	6.7E-01	-0.21	3.1E-01	0.30	1.4E-01	0.15	4.7E-01	-0.27	1.8E-01	-0.06	7.6E-01	0.09	6.7E-01	0.50	9.0E-03	0.58	1.9E-03	0.31	1.2E-01
ENSCAFG0000001505	ENSCAFG0000001505	turquoise	EC_M6	-0.09	6.7E-01	0.33	1.0E-01	-0.21	3.0E-01	-0.10	6.4E-01	0.20	3.2E-01	-0.09	6.7E-01	0.12	5.6E-01	0.21	3.0E-01	0.09	6.5E-01	-0.18	3.7E-01
ENSCAFG0000002978	CDHR4	grey	EC_M10	-0.09	6.7E-01	-0.15	4.6E-01	0.32	1.1E-01	-0.18	3.8E-01	-0.24	2.5E-01	-0.15	4.7E-01	0.40	4.3E-02	-0.05	8.0E-01	0.41	3.7E-02	0.33	1.0E-01
ENSCAFG0000001239	CDC42SE1	cyan	EC_M2	-0.09	6.7E-01	0.31	1.3E-01	0.46	1.8E-02	-0.14	5.0E-01	-0.56	2.8E-03	-0.33	1.0E-01	0.35	8.4E-02	-0.03	8.9E-01	-0.17	4.2E-01	0.62	7.6E-04
ENSCAFG0000001763	PPP4R4	darkgreen	EC_M4	-0.09	6.7E-01	-0.66	2.5E-04	-0.02	9.2E-01	0.21	2.9E-01	0.29	1.5E-01	0.13	5.3E-01	0.16	4.3E-01	-0.03	8.7E-01	-0.14	4.8E-01	-0.30	1.3E-01
ENSCAFG0000001665	AR	turquoise	EC_M6	-0.09	6.7E-01	0.62	7.5E-04	-0.08	6.9E-01	0.15	4.6E-01	-0.03	9.0E-01	-0.15	4.7E-01	-0.16	4.2E-01	0.21	3.1E-01	0.20	3.3E-01	0.10	6.2E-01
ENSCAFG0000002617	ENSCAFG0000002617	grey	EC_M10	-0.09	6.7E-01	0.18	3.7E-01	0.27	1.9E-01	-0.19	3.4E-01	-0.34	9.4E-02	0.21	3.0E-01	-0.17	4.1E-01	0.10	6.3E-01	0.44	2.4E-02	0.40	4.4E-02
ENSCAFG0000001200	ENSCAFG0000001200	grey	EC_M10	-0.09	6.7E-01	-0.09	6.8E-01	-0.32	1.1E-01	-0.22	2.7E-01	0.55	3.5E-03	0.25	2.2E-01	-0.39	4.8E-02	-0.12	5.7E-01	0.05	7.9E-01	-0.51	8.0E-03
ENSCAFG0000000909	PAIP2B	grey	EC_M10	-0.09	6.7E-01	-0.08	7.0E-01	0.10	6.3E-01	0.08	7.1E-01	-0.08	7.1E-01	0.00	9.8E-01	0.26	2.0E-01	0.35	7.6E-02	0.06	7.7E-01	0.12	5.7E-01
ENSCAFG0000002956	ENSCAFG0000002956	grey	EC_M10	-0.09	6.7E-01	0.41	3.7E-02	0.07	7.3E-01	-0.13	5.2E-01	-0.19	3.5E-01	-0.11	6.0E-01	0.41	3.8E-02	0.47	1.5E-02	-0.04	8.4E-01	0.26	2.0E-01
ENSCAFG0000000465	ITGA8	turquoise	EC_M6	-0.09	6.7E-01	0.55	3.8E-03	-0.29	1.5E-01	-0.03	8.8E-01	0.23	2.6E-01	-0.10	6.3E-01	0.34	9.1E-02	0.36	6.8E-02	0.07	7.5E-01	-0.21	3.1E-01
ENSCAFG0000001432	EPHB2	grey	EC_M10	-0.09	6.7E-01	-0.22	2.7E-01	0.17	4.0E-01	-0.15	4.7E-01	-0.02	9.3E-01	-0.08	7.1E-01	-0.12	5.5E-01	0.05	7.9E-01	0.02	9.2E-01	0.05	7.9E-01
ENSCAFG0000001445	MEPCE	grey	EC_M10	-0.09	6.7E-01	0.19	3.5E-01	0.47	1.6E-02	-0.15	4.8E-01	-0.50	1.0E-02	-0.26	2.0E-01	0.13	5.3E-01	0.01	9.5E-01	0.04	8.3E-01	0.52	6.4E-03
ENSCAFG0000003121	ENSCAFG0000003121	grey	EC_M10	-0.09	6.7E-01	-0.10	6.3E-01	0.10	6.4E-01	-0.15	4.6E-01	0.06	7.6E-01	-0.19	3.5E-01	0.16	4.4E-01	-0.19	3.5E-01	-0.13	5.1E-01	0.02	9.3E-01
ENSCAFG0000001644	TSR2	grey	EC_M10	-0.09	6.7E-01	-0.24	2.5E-01	-0.46	1.7E-02	-0.10	6.4E-01	0.69	8.8E-05	0.25	2.2E-01	0.38	5.9E-02	0.04	8.6E-01	-0.16	4.2E-01	-0.64	4.6E-04
ENSCAFG0000003007	ENSCAFG0000003007	grey	EC_M10	-0.09	6.7E-01	0.00	9.9E-01	0.19	3.5E-01	-0.04	8.4E-01	-0.16	4.5E-01	-0.03	8.9E-01	-0.12	5.7E-01	-0.13	5.3E-01	-0.16	4.3E-01	0.15	4.6E-01
ENSCAFG0000001423	FDX1	grey	EC_M10	-0.09	6.7E-01	-0.31	1.2E-01	0.18	3.7E-01	0.27	1.9E-01	-0.02	9.4E-01	-0.18	3.9E-01	0.32	1.1E-01	-0.21	3.0E-01	-0.08	6.9E-01	0.10	6.4E-01
ENSCAFG0000003072	ENSCAFG0000003072	grey	EC_M10	-0.09	6.7E-01	0.30	1.4E-01	0.13	5.3E-01	-0.21	2.9E-01	-0.19	3.5E-01	0.40	4.4E-02	0.22	2.9E-01	-0.22	2.8E-01	0.10	6.4E-01	0.19	3.4E-01
ENSCAFG0000000850	CYP2R1	grey	EC_M10	-0.09	6.7E-01	0.10	6.2E-01	-0.25	2.2E-01	0.24	2.4E-01	0.36	6.9E-02	0.09	6.8E-01	-0.06	7.7E-01	-0.28	1.7E-01	-0.07	7.4E-01	-0.27	1.8E-01
ENSCAFG0000001353	ENSCAFG0000001353	turquoise	EC_M6	-0.09	6.7E-01	0.45	2.2E-02	-0.21	2.9E-01	0.08	6.9E-01	0.16	4.3E-01	0.20	3.2E-01	-0.38	5.2E-02	-0.08	7.0E-01	-0.10	6.2E-01	-0.10	6.4E-01
ENSCAFG0000001384	UPB1	grey	EC_M10	-0.09	6.7E-01	0.24	2.5E-01	0.05	8.2E-01	0.17	4.1E-01	-0.14	4.8E-01	-0.26	2.1E-01	0.28	1.7E-01	0.35	8.4E-02	0.43	2.8E-02	0.16	4.4E-01
ENSCAFG0000000902	ASB8	grey	EC_M10	-0.09	6.7E-01	-0.30	1.3E-01	0.39	4.9E-02	-0.06	7.6E-01	-0.26	2.0E-01	0.37	6.1E-02	-0.39	5.2E-02	0.15	4.5E-01	0.04	8.6E-01	0.31	1.2E-01
ENSCAFG0000001238	ANGEL2	grey	EC_M10	-0.09	6.7E-01	-0.03	8.7E-01	0.44	2.3E-02	0.32	1.1E-01	-0.47	1.5E-02	0.01	9.7E-01	-0.16	4.5E-01	-0.04	8.5E-01	0.12	5.6E-01	0.47	1.4E-02
ENSCAFG0000002993	ENSCAFG0000002993	grey	EC_M10	-0.09	6.7E-01	0.07	7.3E-01	0.28	1.7E-01	-0.14	5.1E-01	-0.21	3.0E-01	-0.08	7.0E-01	-0.11	5.8E-01	-0.16	4.4E-01	0.29	1.4E-01	0.30	1.4E-01
ENSCAFG0000000620	PLXNC1	grey	EC_M10	-0.09	6.7E-01	-0.09	6.7E-01	-0.07	7.4E-01	-0.30	1.4E-01	0.24	2.4E-01	-0.21	2.9E-01	0.35	7.8E-02	0.07	7.4E-01	0.01	9.7E-01	-0.15	4.7E-01
ENSCAFG0000000143	KDM4C	turquoise	EC_M6	-0.09	6.7E-01	0.41	3.9E-02	-0.08	7.0E-01	-0.21	3.0E-01	-0.03	8.8E-01	0.12	5.7E-01	-0.01	9.7E-01	0.07	7.3E-01	-0.26	2.0E-01	0.07	7.5E-01
ENSCAFG0000001642	SNX8	darkgreen	EC_M4	-0.09	6.6E-01	-0.47	1.6E-02	0.04	8.6E-01	0.21	3.1E-01	0.16	4.4E-01	0.12	5.7E-01	-0.09	6.5E-01	-0.24	2.4E-01	-0.26	2.1E-01	-0.19	3.4E-01
ENSCAFG0000002359	ACOT4	grey	EC_M10	-0.09	6.6E-01	-0.16	4.4E-01	0.07	7.3E-01	-0.17	4.0E-01	0.09	6.8E-01	-0.07	7.2E-01	-0.11	5.9E-01	-0.06	7.8E-01	-0.01	9.8E-01	-0.02	9.1E-01
ENSCAFG0000003116	ZNF454	grey	EC_M10	-0.09	6.6E-01	-0.14	4.8E-01	0.47	1.4E-02	0.11	6.0E-01	-0.40	4.2E-02	0.09	6.6E-01	-0.17	4.0E-01	-0.13	5.2E-01	-0.01	9.6E-01	0.46	1.7E-02
ENSCAFG0000003186	MZF1	cyan	EC_M2	-0.09	6.6E-01	0.00	9.8E-01	0.66	2.4E-04	0.16	4.4E-01	-0.74	1.7E-05	-0.23	2.7E-01	0.14	4.9E-01	0.24	2.4E-01	0.16	4.5E-01	0.76	7.4E-06
ENSCAFG0000002024	WWP2	grey	EC_M10	-0.09	6.6E-01	0.28	1.6E-01	-0.10	6.4E-01	-0.10	6.1E-01	0.14	4.9E-01	-0.17	3.9E-01	-0.35	8.2E-02	-0.28	1.6E-01	0.06	7.6E-01	-0.06	7.6E-01
ENSCAFG0000001620	WDFY1	grey	EC_M10	-0.09	6.6E-01	0.53	5.6E-03	0.26	2.1E-01	-0.06	7.5E-01	-0.43	2.8E-02	-0.29	1.5E-01	0.15	4.6E-01	-0.29	1.6E-01	-0.04	8.6E-01	0.47	1.5E-02
ENSCAFG0000001745	GAS7	cyan	EC_M2	-0.09	6.6E-01	-0.47	1.5E-02	0.71	4.3E-05	0.13	5.2E-01	-0.57	2.2E-03	-0.25	2.1E-01	0.11	6.0E-01	-0.01	9.6E-01	-0.35	8.2E-02	0.66	2.8E-04
ENSCAFG0000000139	DMC1	grey	EC_M10	-0.09	6.6E-01	-0.09	6.6E-01	-0.37	5.9E-02	-0.09	6.7E-01	0.56	2.7E-03	-0.18	3.8E-01	0.12	5.5E-01	-0.16	4.3E-01	-0.05	8.2E-01	-0.48	1.2E-02
ENSCAFG0000001018	LGR4	cyan	EC_M2	-0.09	6.6E-01	-0.59	1.5E-03	0.71	5.2E-05	0.03	8.8E-01	-0.55	3.9E-03	-0.18	3.9E-01	0.13	5.4E-01	0.00	9.8E-01	-0.09	6.5E-01	0.59	1.6E-03
ENSCAFG0000000461	APOC1	grey	EC_M10	-0.09	6.6E-01	0.32	1.1E-01	-0.29	1.4E-01	-0.13	5.1E-01	0.37	6.0E-02	-0.12	5.5E-01	0.10	6.2E-01	-0.01	9.5E-01	-0.16	4.2E-01	-0.31	1.2E-01
ENSCAFG0000000395	ENSCAFG0000000395	darkmagenta	EC_M12	-0.09	6.6E-01	-0.07	7.5E-01	-0.03	9.0E-01	-0.07	7.3E-01	0.10	6.2E-01	-0.06	7.8E-01	-0.05	7.9E-01	0.97	3.0E-16	0.16	4.4E-01	-0.06	7.8E-01
ENSCAFG0000001169	TLR7	darkmagenta	EC_M12	-0.09	6.6E-01	-0.07	7.5E-01	-0.03	9.0E-01	-0.07	7.3E-01	0.10	6.2E-01	-0.06	7.8E-01	-0.05	7.9E-01	0.97	3.0E-16	0.16	4.4E-01	-0.06	7.8E-01
ENSCAFG0000002279	ENSCAFG0000002279	darkmagenta	EC_M12	-0.09	6.6E-01	-0.07	7.5E-01	-0.03	9.0E-01	-0.07	7.3E-01	0.10	6.2E-01	-0.06	7.8E-01	-0.05	7.9E-01	0.97	3.0E-16	0.16	4.4E-01	-0.06	7.8E-01
ENSCAFG0000002799	ENSCAFG0000002799	darkmagenta	EC_M12	-0.09	6.6E-01	-0.07	7.5E-01	-0.03	9.0E-01	-0.07	7.3E-01	0.10	6.2E-01	-0.06	7.8E-01	-0.05	7.9E-01	0.97	3.0E-16	0.16	4.4E-01	-0.06	7.8E-01
ENSCAFG0000000577	CACNB4	grey	EC_M10	-0.09	6.6E-01	-0.32	1.1E-01	0.05	8.1E-01	0.09	6.5E-01	0.18	3.8E-01	0.10	6.4E-01	0.06	7.9E-01	0.28	1.7E-01	0.24	2.4E-01	-0.11	5.9E-01
ENSCAFG0000000199	FANCG	grey	EC_M10	-0.09	6.6E-01	0.28	1.6E-01	0.51	7.6E-03	-0.29	1.5E-01	-0.57	2.2E-03	-0.09	6.4E-01	0.28	1.7E-01	0.14	4.9E-01	0.12	5.5E-01	0.66	2.7E-04
ENSCAFG0000002882	MINPP1	grey	EC_M10	-0.09	6.6E-01	-0.34	8.6E-02	-0.27	1.9E-01	-0.06	7.8E-01	0.54	4.5E-03	0.05	8.2E-01	-0.16	4.4E-01	0.18	3.9E-01	0.40	4.2E-02	-0.47	1.5E-02
ENSCAFG0000001227	ENSCAFG0000001227	grey	EC_M10	-0.09	6.6E-01	-0.58	2.1E-03	0.42	3.2E-02	-0.02	9.2E-01	-0.20	3.2E-01	-0.08	6.9E-01	-0.35	8.4E-02	0.16	4.4E-01	0.03	9.0E-01	0.28	1.7E-01
ENSCAFG0000001743	RAB3D	turquoise	EC_M6	-0.09	6.6E-01	0.83	1.3E-07	0.06	7.7E-01	-0.06	7.7E-01	-0.27	1.8E-01	-0.24	2.4E-01	0.08	7.2E-01	-0.02	9.1E-01	0.04	8.6E-01	0.35	8.0E-02
ENSCAFG0000001991	FOXL1	magenta	EC_M13	-0.09	6.6E-01	0.00	9.8E-01	-0.07	7.2E-01	-0.10	6.1E-01	0.21	3.1E-01	-0.25	2.1E-01	-0.05	8.2E-01	-0.04	8.6E-01	0.62	7.0E-04	-0.09	6.7E-01
ENSCAFG0000001379	STARD5	grey	EC_M10	-0.09	6.6E-01	0.04	8.3E-01	0.32	1.1E-01	0.17	4.0E-01	-0.28	1.7E-01	-0.19	3.5E-01	-0.33	1.0E-01	0.19	3.5E-01	0.05	8.1E-01	0.31	1.2E-01
ENSCAFG0000000058	ALDH7A1	cyan	EC_M2	-0.09	6.6E-01	-0.30	1.3E-01	0.68	1.4E-04	0.35	8.4E-02	-0.61	9.7E-04	-0.26	2.1E-01	-0.34	9.0E-02	0.09	6.4E-01	0.28	1.6E-01	0.68	1.3E-04
ENSCAFG0000000372	MGARP	darkgreen	EC_M4	-0.09	6.6E-01	-0.80	1.0E-06	0.40	4.4E-02	0.02	9.4E-01	-0.10	6.4E-01	0.14	4.9E-01	0.02	9.1E-01	0.18	3.8E-01	0.14	4.9E-01	0.14	4.9E-01
ENSCAFG0000001321	GPRC5D	grey	EC_M10	-0.09	6.6E-01	-0.28	1.7E-01	0.22	2.8E-01	0.10	6.2E-01	-0.08	7.0E-01	0.11	5.8E-01	0.36	7.4E-02	0.05	8.2E-01	0.10	6.2E-01	0.19	3.5E-01
ENSCAFG0000001956	LCN15	grey	EC_M10	-0.09	6.6E-01	-0.19	3.5E-01	-0.04	8.6E-01	-0.08	7.1E-01	0.20	3.2E-01	-0.11	5.9E-01	-0.06	7.7E-01	-0.11	5.9E-01	-0.04	8.6E-01	-0.16	4.5E-01
ENSCAFG0000001459	ITGAV	darkgreen	EC_M4	-0.09	6.6E-01	-0.30	1.4E-01	-0.58	1.9E-03	0.08	7.1E-01	0.82	3.2E-07	0.14	5.1E-01	0.16	4.3E-01	0.05	7.9E-01	0.21	3.2E-01	-0.79	1.6E-06
ENSCAFG0000001579	TPM4	darkgreen	EC_M4	-0.09	6.6E-01	-0.22	2.9E-01	-0.60	1.2E-03	-0.21	3.0E-01	0.85	4.0E-08	0.21	3.0E-01	0.24	2.4E-01	0.18	3.7E-01	0.28	1.6E-01	-0.80	9.1E-07
ENSCAFG0000001400	H2AFY2	grey	EC_M10	-0.09	6.6E-01	-0.18	3.7E-01	-0.12	5.7E-01	0.16	4.3E-01	0.23	2.5E-01	0.21	2.9E-01	0.35	7.9E-02	-0.29	1.6E-01	-0.13	5.2E-01	-0.23	2.7E-01
ENSCAFG0000000295	HOXA5	grey	EC_M10	-0.09	6.6E-01	0.18	3.8E-01	0.21	3.0E-01	-0.06	7.5E-01	-0.20	3.2E-01	-0.16	4.4E-01	0.10	6.1E-01	-0.51	8.2E-03	-0.52	6.7E-03	0.27	1.9E-01
ENSCAFG0000001853	MAP2K3	grey	EC_M10	-0.09	6.6E-01	0.10	6.4E-01	-0.15	4.8E-01	-0.20	3.3E-01	0.30	1.4E-01	-0.16	4.2E-01	-0.07	7.3E-01	0.34	9.0E-02	0.16	4.4E-01	-0.21	2.9E-01
ENSCAFG0000000646	GTSF1	grey	EC_M10	-0.09	6.6E-01	-0.10	6.1E-01	-0.03	9.0E-01	-0.01	9.5E-01	0.11	6.1E-01	-0.03	9.0E-01	0.02	9.2E-01	-0.06	7.7E-01	-0.09	6.6E-01	-0.08	6.9E-01
ENSCAFG0000000426	FBXL13	grey	EC_M10	-0.09	6.6E-01	-0.24	2.3E-01	0.30	1.4E-01	0.20	3.2E-01	-0.20	3.4E-01	-0.10	6.2E-01	-0.14	4.9E-01	0.42	3.3E-02	0.15	4.6E-01	0.24	2.3E-01
ENSCAFG0000001214	IP6K2	grey	EC_M10	-0.09	6.6E-01	-0.32	1.2E-01	0.41	3.7E-02	0.46	1.7E-02	-0.38	5.6E-02	-0.09	6.6E-01	-0.21	3.1E-01	0.06	7.9E-01	0.02	9.0E-01	0.40	4.2E-02
ENSCAFG0000000730	ENSCAFG0000000730	grey	EC_M10	-0.09	6.6E-01	-0.31	1.2E-01	0.75	1.2E-05	0.12	5.4E-01	-0.66	2.4E-04	-0.11	6.1E-01	-0.20	3.2E-01	-0.17	4.0E-01	-0.35	7.7E-02	0.70	6.0E-05
ENSCAFG0000002860	TNNC2	grey	EC_M10	-0.09	6.6E-01	0.09	6.7E-01	0.31	1.3E-01	0.04	8.3E-01	-0.31	1.2E-01	-0.34	9.4E-02	-0.29	1.5E-01	0.02	9.1E-01	0.11	6.0E-01	0.39	4.8E-02
ENSCAFG0000002843	BCKDHB	grey	EC_M10	-0.09	6.6E-01	-0.52	6.8E-03	0.57	2.2E-03	0.32	1.1E-01	-0.38	5.4E-02	-0.06	7.7E-01	-0.25	2.2E-01	-0.26	2.0E-01	0.11	6.1E-01	0.48	1.3E-02
ENSCAFG0000000496	MYD88	cyan	EC_M2	-0.09	6.6E-01	0.24	2.4E-01	0.66	2.4E-04	-0.05	7.9E-01	-0.72	2.9E-05	-0.30	1.3E-01	-0.03	9.0E-01	0.07	7.2E-01	-0.01	9.6E-01	0.80	1.1E-06
ENSCAFG0000000575	IDO1	grey	EC_M10	-0.09	6.6E-01	-0.22	2.9E-01	-0.26	2.1E-01	0.18	3.8E-01	0.45	2.0E-02	-0.08	7.1E-01	-0.28	1.6E-01	-0.14	5.1E-01	-0.13	5.3E-01	-0.39	4.7E-02
ENSCAFG0000000928	ACVR1	darkgreen	EC_M4	-0.09	6.6E-01	-0.02	9.2E-01	-0.71	5.3E-05	-0.14	4.9E-01	0.88	3.8E-09	0.31	1.2E-01	0.13	5.4E-01	-0.07	7.2E-01	-0.06	7.5E-01	-0.86	1.6E-08
ENSCAFG0000001562	FMN2	darkgreen	EC_M4	-0.09	6.6E-01	-0.56	2.7E-03	-0.11	6.0E-01	0.01	9.5E-01	0.40	4.2E-02	0.08	7.0E-01	0.20	3.2E-01	-0.01	9.8E-01	-0.66	2.7E-04	-0.36	6.9E-02
ENSCAFG0000001748	ENSCAFG0000001748	grey	EC_M10	-0.09	6.6E-01	-0.01	9.7E-01	0.48	1.4E-02	0.22	2.9E-01	-0.54	4.1E-03	0.04	8.4E-01	0.08	7.0E-01	-0.16	4.3E-01	0.02	9.3E-01	0.54	4.8E-03
ENSCAFG0000000167	RPL8	cyan	EC_M2	-0.09	6.6E-01	-0.48	1.4E-02	0.64	4.0E-04	0.08	6.9E-01	-0.47	1.6E-02	0.06	7.6E-01	0.22	2.8E-01	0.00	9.8E-01	-0.38	5.5E-02	0.45	2.0E-02
ENSCAFG0000000442	CYSLTR2	grey	EC_M10	-0.09	6.6E-01	-0.20	3.3E-01	-0.25	2.2E-01	0.20	3.2E-01	0.36	6.7E-02	0.33	9.6E-02	-0.24	2.5E-01	0.02	9.4E-01	-0.02	9.3E-01	-0.36	6.8E-02
ENSCAFG0000002627	ENSCAFG0000002627	grey	EC_M10	-0.09	6.6E-01	0.15	4.5E-01	0.18	3.9E-01	0.18	3.8E-01	-0.21	3.0E-01	-0.27	1.8E-01	0.11	5.8E-01	-0.19	3.5E-01	-0.06	7.9E-01	0.23	2.5E-01
ENSCAFG0000000040	DYRK2	darkgreen	EC_M4	-0.09	6.6E-01	-0.63	5.1E-04	-0.04	8.5E-01	0.08	7.0E-01	0.29	1.5E-01	0.22	2.8E-01	0.18	3.8E-01	0.05	8.0E-01	-0.25	2.1E-01	-0.33	1.0E-01
ENSCAFG0000001665	MPDU1	grey	EC_M10	-0.09	6.6E-01	-0.15	4.6E-01	0.33	9.8E-02	-0.04	8.6E-01	-0.13	5.4E-01	0.20	3.2E-01	-0.07	7.2E-01	-0.18	3.7E-01	-0.05	8.3E-01	0.18	3.7E-01
ENSCAFG0000000905	COL2A1	grey	EC_M10	-0.09	6.6E-01	-0.28	1.7E-01	-0.08	7.1E-01	0.11	6.0E-01	0.26	2.0E-01	0.29	1.4E-01	0.21	3.0E-01	0.08	7.1E-01	-0.22	2.8E-01	-0.23	2.6E-01
ENSCAFG0000001185	RPRD2	cyan	EC_M2	-0.09	6.6E-01	0.01	9.7E-01	0.71	5.3E-05	0.26	2.0E-01	-0.80	7.3E-07	-0.16	4.3E-01	-0.02	9.0E-01	-0.16	4.4E-01	-0.13	5.2E-01	0.85	3.7E-08
ENSCAFG0000001663	PRR14	grey	EC_M10	-0.09	6.6E-01	0.30	1.4E-01	0.48	1.4E-02	0.02	9.4E-01	-0.56	2.8E-03	-0.13	5.4E-01	0.00	9.9E-01	0.17	4.1E-01	0.36	7.3E-02	0.59	1.4E-03
ENSCAFG0000001555	DKK1	grey	EC_M10	-0.09	6.6E-01	-0.14	5.1E-01	-0.51	7.3E-03	-0.21	2.9E-01	0.76	7.8E-06	0.16	4.4E-01	0.12	5.7E-01	-0.16	4.5E-01	-0.31	1.2E-01	-0.72	3.3E-05
ENSCAFG0000001832	ABLIM3	turquoise	EC_M6	-0.09	6.6E-01	0.64	4.8E-04	-0.39	4.8E-02	-0.22	2.7E-01	0.30	1.3E-01	-0.10	6.2E-01	0.33	9.5E-02	0.30	1.3E-01	0.01	9.6E-01	-0.28	1.7E-01
ENSCAFG0000000563	P2RY2	turquoise	EC_M6	-0.09	6.6E-01	0.55	3.4E-03	-0.06	7.6E-01	-0.28	1.6E-01	0.00	9.9E-01	-0.25	2.2E-01	-0.21	3.0E-01	-0.04	8.3E-01	0.50	1.0E-02	0.13	5.2E-01
ENSCAFG0000001159	TBC1D10C	grey	EC_M10	-0.09	6.6E-01	-0.06	7.6E-01	0.59	1.6E-03	-0.05	7.9E-01	-0.62	8.2E-04	-0.21	3.1E-01	-0.10	6.4E-01	0.09	6.6E-01	0.10	6.1E-01	0.66	2.1E-04
ENSCAFG0000000924	RSBN1	grey	EC_M10	-0.09	6.6E-01	0.16	4.3E-01	0.51	7.8E-03	-0.04	8.6E-01	-0.60	1.2E-03	-0.30	1.4E-01	0.28	1.7E-01	-0.23	2.7E-01	-0.11	5.8E-01	0.66	2.5E-04
ENSCAFG0000001557	RTN1	grey	EC_M10	-0.09	6.5E-01	-0.18	3.9E-01	-0.53	5.0E-03	0.06	7.8E-01	0.76	7.3E-06	0.11	5.8E-01	-0.03	8.9E-01	0.21	3.1E-01	0.33	9.6E-02	-0.67	1.6E-04
ENSCAFG0000003030	CCR7	grey	EC_M10	-0.09	6.5E-01	-0.13	5.2E-01	0.10	6.2E-01	-0.12	5.4E-01	-0.01	9.7E-01	-0.08	7.0E-01	-0.03	9.0E-01	-0.10	6.3E-01	0.05	7.9E-01	0.09	6.7E-01
[truncated: 1,542,937 more chars]
