# Supplementary material for: Locational memory of macrovessel vascular cells is transcriptionally imprinted
Source: Sci Rep. 2023 Aug 10;13:13028. doi: 10.1038/s41598-023-38880-6 (PMC10415317; doi:10.1038/s41598-023-38880-6)
Supplement: Supplementary file 18 — Supplementary Table 9. [file 41598_2023_38880_MOESM18_ESM.pdf]

Supplemental Table 9. Module membership values of the genes in the vascular smooth muscle cell dataset. Module membership value (MM) and the corresponding p value (p.val) for all genes in the vascular smooth muscle cell WGCNA dataset. Ensembl IDs (Gene) and their gene names (Gene_sym) are provided. Each module has a color and a numeric identifier (Module_num).

Gene	Gene_sym	Module	module_num	red_MM	red_p.val	cyan_MM	cyan_p.val	yellow_MM	yellow_p.val	darkgreen_MM	darkgreen_p.val	pink_MM	pink_p.val	turquoise_MM	turquoise_p.val	violet_MM	violet_p.val	darkgrey_MM	darkgrey_p.val	darkolivegreen_MM	darkolivegreen_p.val	grey_MM	grey_p.val
ENSCAFG0000000060	CSNK2B	red	VSMC_M1	0.88	1.03E-09	0.22	2.75E-01	0.37	5.68E-02	-0.21	2.95E-01	0.12	5.38E-01	-0.13	5.22E-01	-0.19	3.42E-01	0.09	6.64E-01	-0.41	3.25E-02	-0.30	1.32E-01
ENSCAFG0000002328	ENSCAFG0000002328	red	VSMC_M1	0.87	3.61E-09	0.25	2.01E-01	0.14	4.87E-01	-0.10	6.34E-01	0.29	1.36E-01	-0.18	3.71E-01	0.15	4.58E-01	0.00	9.98E-01	-0.51	6.50E-03	-0.11	5.95E-01
ENSCAFG0000001992	COX4I1	red	VSMC_M1	0.87	4.18E-09	0.32	1.04E-01	0.25	2.16E-01	-0.02	9.39E-01	0.26	1.96E-01	-0.14	4.73E-01	-0.30	1.29E-01	-0.12	5.46E-01	-0.45	1.91E-02	-0.23	2.45E-01
ENSCAFG0000001983	WDR5	red	VSMC_M1	0.86	1.11E-08	0.60	8.41E-04	0.52	5.33E-03	-0.13	5.11E-01	-0.13	5.31E-01	-0.12	5.57E-01	-0.05	8.11E-01	-0.10	6.12E-01	-0.79	8.72E-07	0.12	5.35E-01
ENSCAFG0000003195	RPS26	red	VSMC_M1	0.85	2.01E-08	0.37	6.10E-02	0.28	1.57E-01	0.06	7.70E-01	0.16	4.25E-01	-0.30	1.22E-01	-0.12	5.65E-01	-0.18	3.75E-01	-0.54	3.60E-03	-0.07	7.45E-01
ENSCAFG0000001397	ENSCAFG0000001397	red	VSMC_M1	0.85	2.18E-08	0.32	1.04E-01	0.17	3.97E-01	0.00	9.92E-01	0.30	1.34E-01	-0.11	5.78E-01	-0.17	3.98E-01	-0.14	4.76E-01	-0.49	1.03E-02	-0.16	4.15E-01
ENSCAFG0000000236	RPS5	red	VSMC_M1	0.85	2.65E-08	0.39	4.40E-02	0.14	4.79E-01	0.13	5.13E-01	0.31	1.17E-01	-0.28	1.54E-01	-0.30	1.25E-01	-0.26	1.93E-01	-0.53	4.76E-03	-0.09	6.46E-01
ENSCAFG0000002880	ENSCAFG0000002880	red	VSMC_M1	0.84	2.99E-08	0.47	1.44E-02	0.41	3.15E-02	-0.08	7.08E-01	0.07	7.21E-01	-0.12	5.67E-01	-0.21	2.89E-01	-0.10	6.06E-01	-0.62	5.82E-04	-0.10	6.21E-01
ENSCAFG0000000258	RPL28	red	VSMC_M1	0.84	3.18E-08	0.58	1.61E-03	0.48	1.16E-02	-0.11	5.93E-01	-0.05	8.13E-01	-0.22	2.65E-01	-0.17	4.10E-01	-0.15	4.61E-01	-0.71	3.66E-05	0.04	8.50E-01
ENSCAFG0000001608	HDHD5	red	VSMC_M1	0.82	1.36E-07	0.27	1.80E-01	0.29	1.42E-01	-0.18	3.67E-01	0.11	5.87E-01	-0.17	4.05E-01	0.25	2.03E-01	0.03	8.76E-01	-0.51	6.56E-03	-0.08	6.84E-01
ENSCAFG0000002247	ENSCAFG0000002247	red	VSMC_M1	0.82	1.75E-07	0.09	6.49E-01	0.37	5.99E-02	-0.38	5.22E-02	0.16	4.24E-01	-0.20	3.30E-01	-0.06	7.73E-01	0.23	2.51E-01	-0.27	1.73E-01	-0.46	1.50E-02
ENSCAFG0000000131	RPL10A	red	VSMC_M1	0.82	1.98E-07	0.40	3.71E-02	0.45	1.81E-02	-0.15	4.63E-01	0.05	8.03E-01	-0.37	5.94E-02	-0.08	6.95E-01	-0.08	6.93E-01	-0.52	4.94E-03	-0.17	3.93E-01
ENSCAFG0000000265	EEF1A1	red	VSMC_M1	0.82	2.00E-07	0.14	4.82E-01	0.04	8.54E-01	-0.01	9.71E-01	0.49	9.98E-03	-0.34	8.28E-02	-0.32	1.06E-01	-0.05	7.94E-01	-0.28	1.55E-01	-0.37	5.52E-02
ENSCAFG0000000185	SRI	red	VSMC_M1	0.81	2.81E-07	0.14	4.90E-01	0.19	3.40E-01	-0.05	7.98E-01	0.32	1.07E-01	-0.26	1.85E-01	-0.03	8.66E-01	-0.06	7.50E-01	-0.26	1.98E-01	-0.32	9.83E-02
ENSCAFG0000001272	RPL35A	red	VSMC_M1	0.80	4.50E-07	0.35	6.95E-02	0.26	1.84E-01	0.01	9.49E-01	0.13	5.26E-01	-0.27	1.72E-01	-0.01	9.45E-01	-0.16	4.11E-01	-0.52	5.27E-03	-0.04	8.50E-01
ENSCAFG0000002372	ENSCAFG0000002372	red	VSMC_M1	0.80	4.63E-07	0.08	6.94E-01	0.19	3.31E-01	-0.08	6.89E-01	0.31	1.16E-01	-0.24	2.37E-01	-0.05	8.00E-01	0.01	9.54E-01	-0.23	2.44E-01	-0.35	7.17E-02
ENSCAFG0000001218	PPP1R11	red	VSMC_M1	0.80	5.54E-07	0.50	7.64E-03	0.50	7.38E-03	-0.13	5.27E-01	-0.11	5.89E-01	-0.14	4.89E-01	-0.28	1.51E-01	-0.07	7.41E-01	-0.64	3.34E-04	-0.01	9.71E-01
ENSCAFG0000001689	TRAPPC1	red	VSMC_M1	0.80	6.04E-07	0.30	1.29E-01	0.28	1.61E-01	-0.01	9.77E-01	0.12	5.35E-01	-0.18	3.66E-01	-0.24	2.31E-01	-0.10	6.09E-01	-0.44	2.27E-02	-0.09	6.40E-01
ENSCAFG0000001048	YARS	red	VSMC_M1	0.79	1.05E-06	0.28	1.51E-01	0.41	3.30E-02	-0.30	1.30E-01	-0.03	8.92E-01	-0.11	5.77E-01	0.20	3.22E-01	0.17	3.84E-01	-0.56	2.28E-03	-0.06	7.56E-01
ENSCAFG0000003195	ENSCAFG0000003195	red	VSMC_M1	0.79	1.17E-06	0.28	1.56E-01	0.42	2.77E-02	-0.20	3.19E-01	0.02	9.39E-01	-0.14	4.75E-01	-0.22	2.81E-01	0.10	6.29E-01	-0.45	1.77E-02	-0.23	2.56E-01
ENSCAFG0000001904	METTL22	yellow	VSMC_M3	0.78	1.38E-06	0.45	1.79E-02	0.45	1.86E-02	-0.08	6.75E-01	-0.08	6.90E-01	-0.04	8.52E-01	-0.02	9.02E-01	-0.12	5.60E-01	-0.58	1.69E-03	0.06	7.58E-01
ENSCAFG0000000320	UTP11	red	VSMC_M1	0.78	1.48E-06	0.08	7.02E-01	0.04	8.58E-01	0.01	9.66E-01	0.43	2.42E-02	-0.32	9.95E-02	-0.37	5.55E-02	-0.06	7.67E-01	-0.20	3.21E-01	-0.33	9.33E-02
ENSCAFG0000000095	POLDIP3	red	VSMC_M1	0.78	1.60E-06	0.26	1.92E-01	0.36	6.76E-02	-0.35	7.55E-02	0.12	5.40E-01	-0.23	2.51E-01	-0.01	9.58E-01	0.18	3.66E-01	-0.43	2.60E-02	-0.30	1.33E-01
ENSCAFG0000000440	SNRPD2	red	VSMC_M1	0.78	1.78E-06	0.28	1.63E-01	0.36	6.59E-02	-0.13	5.18E-01	0.04	8.46E-01	-0.20	3.12E-01	-0.14	4.83E-01	0.02	9.09E-01	-0.47	1.40E-02	-0.17	3.95E-01
ENSCAFG0000000835	GOT2	red	VSMC_M1	0.78	1.97E-06	0.28	1.58E-01	-0.09	6.63E-01	0.17	3.98E-01	0.48	1.10E-02	-0.15	4.48E-01	-0.28	1.57E-01	-0.25	2.03E-01	-0.39	4.53E-02	-0.10	6.23E-01
ENSCAFG0000001545	ENSCAFG0000001545	yellow	VSMC_M3	0.77	2.12E-06	0.57	1.74E-03	0.46	1.48E-02	-0.05	8.03E-01	-0.07	7.16E-01	-0.07	7.13E-01	-0.12	5.44E-01	-0.20	3.12E-01	-0.67	1.43E-04	0.08	6.93E-01
ENSCAFG0000000960	RPL24	red	VSMC_M1	0.77	2.67E-06	0.39	4.19E-02	0.25	2.12E-01	0.07	7.46E-01	0.11	5.92E-01	-0.23	2.47E-01	-0.15	4.55E-01	-0.17	4.01E-01	-0.55	3.21E-03	-0.01	9.49E-01
ENSCAFG0000001223	HDDC3	yellow	VSMC_M3	0.77	3.22E-06	0.53	4.73E-03	0.54	3.73E-03	-0.08	7.03E-01	-0.12	5.55E-01	-0.32	1.04E-01	-0.09	6.59E-01	-0.17	3.91E-01	-0.64	2.96E-04	0.05	8.09E-01
ENSCAFG0000002015	RPL5	red	VSMC_M1	0.76	3.53E-06	-0.02	9.12E-01	-0.03	8.84E-01	-0.03	8.80E-01	0.53	4.69E-03	-0.38	5.03E-02	-0.22	2.79E-01	0.03	8.75E-01	-0.14	4.90E-01	-0.45	1.84E-02
ENSCAFG0000000063	KLF10	red	VSMC_M1	0.76	4.20E-06	0.38	5.36E-02	0.12	5.51E-01	0.03	8.93E-01	0.27	1.66E-01	-0.01	9.53E-01	-0.18	3.59E-01	-0.12	5.66E-01	-0.56	2.33E-03	-0.03	8.70E-01
ENSCAFG0000001825	ENSCAFG0000001825	red	VSMC_M1	0.76	4.72E-06	0.13	5.17E-01	0.07	7.18E-01	-0.14	4.82E-01	0.40	4.00E-02	-0.10	6.06E-01	-0.30	1.32E-01	0.06	7.83E-01	-0.27	1.74E-01	-0.34	8.22E-02
ENSCAFG0000001671	BCL7C	yellow	VSMC_M3	0.76	4.74E-06	0.57	2.07E-03	0.79	1.19E-06	-0.35	7.64E-02	-0.38	5.34E-02	-0.14	4.83E-01	-0.11	5.79E-01	0.08	7.06E-01	-0.70	4.80E-05	-0.03	8.88E-01
ENSCAFG0000002452	BORCS8	red	VSMC_M1	0.76	4.89E-06	0.32	9.89E-02	0.18	3.76E-01	0.10	6.10E-01	0.14	4.88E-01	-0.27	1.79E-01	0.22	2.75E-01	-0.24	2.31E-01	-0.48	1.12E-02	0.14	4.72E-01
ENSCAFG0000001911	YJU2	red	VSMC_M1	0.76	5.13E-06	0.25	2.06E-01	0.34	8.37E-02	-0.19	3.42E-01	0.04	8.45E-01	-0.16	4.33E-01	-0.04	8.38E-01	0.10	6.10E-01	-0.45	1.99E-02	-0.17	4.08E-01
ENSCAFG0000000238	ACTR1B	red	VSMC_M1	0.75	5.54E-06	0.58	1.45E-03	0.42	2.83E-02	0.05	8.12E-01	-0.01	9.50E-01	-0.19	3.30E-01	-0.30	1.28E-01	-0.33	8.88E-02	-0.63	4.72E-04	0.05	8.03E-01
ENSCAFG0000000989	ENSCAFG0000000989	red	VSMC_M1	0.75	5.59E-06	-0.03	8.88E-01	-0.05	8.17E-01	0.02	9.08E-01	0.52	5.12E-03	-0.38	5.06E-02	-0.15	4.51E-01	-0.02	9.21E-01	-0.15	4.42E-01	-0.37	5.43E-02
ENSCAFG0000003246	PSENEN	red	VSMC_M1	0.75	5.67E-06	0.41	3.39E-02	0.11	5.75E-01	0.25	2.00E-01	0.19	3.32E-01	-0.16	4.22E-01	-0.42	2.74E-02	-0.36	6.71E-02	-0.50	8.25E-03	0.15	4.59E-01
ENSCAFG0000000484	PAFAH1B3	yellow	VSMC_M3	0.75	5.85E-06	0.33	8.98E-02	0.66	1.56E-04	-0.54	3.75E-03	-0.17	3.90E-01	-0.28	1.55E-01	0.11	5.92E-01	0.31	1.15E-01	-0.51	6.61E-03	-0.30	1.28E-01
ENSCAFG0000000540	RPS3	red	VSMC_M1	0.75	6.05E-06	0.32	1.03E-01	0.47	1.41E-02	-0.10	6.34E-01	-0.05	8.21E-01	-0.14	4.81E-01	-0.11	5.81E-01	-0.05	8.21E-01	-0.46	1.46E-02	-0.17	3.86E-01
ENSCAFG0000001563	ENSCAFG0000001563	yellow	VSMC_M3	0.75	6.74E-06	0.46	1.53E-02	0.64	3.65E-04	-0.28	1.53E-01	-0.28	1.62E-01	0.13	5.16E-01	-0.07	7.15E-01	0.06	7.56E-01	-0.62	6.03E-04	-0.01	9.52E-01
ENSCAFG0000000240	ALDH1B1	red	VSMC_M1	0.75	6.99E-06	0.21	2.99E-01	0.44	2.15E-02	-0.25	2.14E-01	0.08	6.76E-01	-0.24	2.20E-01	-0.29	1.36E-01	0.10	6.22E-01	-0.34	8.61E-02	-0.37	5.97E-02
ENSCAFG0000000787	ENSCAFG0000000787	red	VSMC_M1	0.75	7.55E-06	0.05	8.15E-01	0.17	3.99E-01	-0.02	9.36E-01	0.32	1.07E-01	-0.21	3.01E-01	-0.24	2.34E-01	-0.03	8.92E-01	-0.19	3.55E-01	-0.35	7.09E-02
ENSCAFG0000000346	COQ3	red	VSMC_M1	0.75	7.98E-06	0.17	3.87E-01	-0.01	9.67E-01	0.13	5.06E-01	0.39	4.25E-02	-0.41	3.55E-02	-0.15	4.61E-01	-0.17	3.84E-01	-0.34	8.16E-02	-0.06	7.58E-01
ENSCAFG0000001495	ENSCAFG0000001495	red	VSMC_M1	0.75	8.05E-06	0.15	4.53E-01	0.19	3.38E-01	-0.05	7.87E-01	0.17	3.85E-01	-0.26	1.87E-01	-0.13	5.15E-01	-0.01	9.72E-01	-0.29	1.36E-01	-0.13	5.26E-01
ENSCAFG0000003040	ENSCAFG0000003040	red	VSMC_M1	0.75	8.17E-06	0.50	8.56E-03	0.19	3.48E-01	0.19	3.31E-01	0.18	3.61E-01	-0.33	9.41E-02	-0.27	1.69E-01	-0.32	1.08E-01	-0.61	7.77E-04	0.09	6.55E-01
ENSCAFG0000001503	UVSSA	red	VSMC_M1	0.74	8.66E-06	0.43	2.68E-02	0.06	7.65E-01	0.23	2.51E-01	0.34	8.07E-02	-0.39	4.45E-02	-0.10	6.36E-01	-0.41	3.33E-02	-0.52	5.89E-03	0.10	6.03E-01
ENSCAFG0000001967	ERRFI1	red	VSMC_M1	0.74	8.93E-06	0.30	1.33E-01	0.43	2.70E-02	-0.26	1.87E-01	-0.03	8.76E-01	-0.04	8.36E-01	0.24	2.38E-01	0.10	6.20E-01	-0.50	7.70E-03	-0.11	5.87E-01
ENSCAFG0000001209	FAM45A	red	VSMC_M1	0.74	8.98E-06	0.23	2.40E-01	-0.03	8.87E-01	0.19	3.45E-01	0.45	1.95E-02	-0.16	4.29E-01	-0.45	1.82E-02	-0.27	1.75E-01	-0.32	1.02E-01	-0.18	3.77E-01
ENSCAFG0000001222	ENSCAFG0000001222	red	VSMC_M1	0.74	9.29E-06	0.23	2.51E-01	0.18	3.57E-01	-0.19	3.30E-01	0.23	2.54E-01	-0.22	2.63E-01	-0.07	7.25E-01	0.09	6.65E-01	-0.43	2.60E-02	-0.19	3.43E-01
ENSCAFG0000000691	ENSCAFG0000000691	red	VSMC_M1	0.74	9.63E-06	0.30	1.34E-01	0.07	7.46E-01	0.23	2.48E-01	0.26	1.91E-01	-0.01	9.60E-01	-0.09	6.45E-01	-0.27	1.68E-01	-0.43	2.54E-02	0.00	9.94E-01
ENSCAFG0000002013	RPL12	red	VSMC_M1	0.74	1.04E-05	0.55	3.01E-03	0.39	4.64E-02	0.00	9.89E-01	-0.04	8.46E-01	-0.24	2.25E-01	-0.09	6.66E-01	-0.19	3.53E-01	-0.70	4.18E-05	0.09	6.45E-01
ENSCAFG0000000799	RPS3A	red	VSMC_M1	0.74	1.05E-05	0.14	4.79E-01	0.11	5.77E-01	-0.09	6.59E-01	0.36	6.43E-02	-0.39	4.18E-02	-0.29	1.41E-01	-0.01	9.56E-01	-0.25	2.08E-01	-0.33	8.83E-02
ENSCAFG0000002361	SURF1	yellow	VSMC_M3	0.74	1.06E-05	0.39	4.41E-02	0.55	2.66E-03	-0.18	3.64E-01	-0.14	4.94E-01	-0.20	3.26E-01	-0.03	8.97E-01	-0.06	7.82E-01	-0.52	5.81E-03	-0.07	7.42E-01
ENSCAFG0000000016	SMAD4	red	VSMC_M1	0.74	1.09E-05	0.41	3.47E-02	0.01	9.62E-01	0.11	5.90E-01	0.44	2.15E-02	-0.15	4.41E-01	-0.25	2.11E-01	-0.29	1.38E-01	-0.47	1.40E-02	-0.06	7.69E-01
ENSCAFG0000002354	ENSCAFG0000002354	red	VSMC_M1	0.74	1.13E-05	0.08	7.08E-01	-0.06	7.58E-01	-0.09	6.61E-01	0.52	5.65E-03	-0.23	2.52E-01	-0.22	2.70E-01	0.02	9.04E-01	-0.20	3.16E-01	-0.34	8.19E-02
ENSCAFG0000001886	MYO18A	red	VSMC_M1	0.74	1.21E-05	0.44	2.31E-02	0.44	2.25E-02	-0.21	2.87E-01	-0.04	8.39E-01	-0.08	6.89E-01	0.16	4.16E-01	-0.03	8.91E-01	-0.62	5.31E-04	0.00	9.83E-01
ENSCAFG0000001691	NPM1	red	VSMC_M1	0.74	1.22E-05	0.02	9.18E-01	0.41	3.41E-02	-0.51	6.61E-03	0.08	6.81E-01	-0.05	7.94E-01	-0.17	3.92E-01	0.44	2.02E-02	-0.24	2.30E-01	-0.57	1.73E-03
ENSCAFG0000000395	SF3B6	red	VSMC_M1	0.73	1.35E-05	0.07	7.14E-01	-0.09	6.73E-01	0.03	8.70E-01	0.39	4.27E-02	-0.03	8.64E-01	-0.07	7.20E-01	0.04	8.55E-01	-0.32	1.07E-01	-0.14	4.96E-01
ENSCAFG0000001756	ENSCAFG0000001756	red	VSMC_M1	0.73	1.36E-05	-0.11	5.75E-01	0.18	3.67E-01	-0.33	9.22E-02	0.32	1.00E-01	-0.26	1.97E-01	0.21	2.86E-01	0.23	2.46E-01	-0.06	7.57E-01	-0.53	4.48E-03
ENSCAFG0000001026	RBM15B	yellow	VSMC_M3	0.73	1.37E-05	0.42	3.12E-02	0.50	8.10E-03	-0.31	1.18E-01	-0.07	7.39E-01	-0.06	7.60E-01	0.05	8.04E-01	0.10	6.30E-01	-0.60	8.75E-04	-0.13	5.08E-01
ENSCAFG0000001205	ENSA	red	VSMC_M1	0.73	1.39E-05	0.55	3.17E-03	0.38	5.36E-02	-0.18	3.72E-01	0.06	7.58E-01	-0.39	4.28E-02	-0.08	6.88E-01	-0.07	7.27E-01	-0.66	1.86E-04	-0.05	8.05E-01
ENSCAFG0000003144	UBE2I	red	VSMC_M1	0.73	1.41E-05	0.14	4.71E-01	0.33	9.04E-02	-0.18	3.74E-01	0.02	9.31E-01	0.00	9.89E-01	-0.03	8.98E-01	0.16	4.29E-01	-0.39	4.40E-02	-0.18	3.70E-01
ENSCAFG0000000354	PSMB2	red	VSMC_M1	0.73	1.60E-05	0.08	6.89E-01	0.10	6.34E-01	-0.17	4.04E-01	0.32	1.03E-01	-0.03	8.67E-01	-0.25	2.05E-01	0.13	5.22E-01	-0.24	2.37E-01	-0.36	6.43E-02
ENSCAFG0000000839	RRAS2	red	VSMC_M1	0.73	1.61E-05	0.33	9.43E-02	0.15	4.52E-01	-0.09	6.67E-01	0.19	3.45E-01	-0.17	3.84E-01	-0.04	8.44E-01	-0.06	7.83E-01	-0.54	3.91E-03	-0.05	7.87E-01
ENSCAFG0000000802	RPL7	red	VSMC_M1	0.73	1.65E-05	0.02	9.23E-01	0.07	7.37E-01	0.05	7.88E-01	0.37	6.11E-02	-0.36	6.73E-02	-0.05	8.01E-01	-0.04	8.41E-01	-0.20	3.17E-01	-0.32	1.05E-01
ENSCAFG0000000363	RPS11	red	VSMC_M1	0.73	1.69E-05	0.43	2.38E-02	0.14	4.84E-01	0.18	3.68E-01	0.27	1.77E-01	-0.41	3.43E-02	-0.28	1.61E-01	-0.36	6.91E-02	-0.47	1.39E-02	-0.02	9.06E-01
ENSCAFG0000001424	NDUFA13	red	VSMC_M1	0.73	1.79E-05	0.12	5.51E-01	0.16	4.18E-01	0.08	6.81E-01	0.14	4.97E-01	0.01	9.57E-01	-0.02	9.22E-01	-0.08	6.94E-01	-0.34	8.66E-02	-0.07	7.38E-01
ENSCAFG0000000887	ENSCAFG0000000887	red	VSMC_M1	0.73	1.83E-05	0.41	3.45E-02	0.41	3.25E-02	-0.17	3.93E-01	-0.02	9.33E-01	-0.25	2.09E-01	-0.05	8.15E-01	-0.05	8.19E-01	-0.53	4.61E-03	-0.07	7.41E-01
ENSCAFG0000002861	C15H1orf122	cyan	VSMC_M2	0.72	1.92E-05	0.35	7.15E-02	0.24	2.33E-01	0.05	7.93E-01	0.09	6.47E-01	-0.05	8.19E-01	0.08	6.76E-01	-0.17	4.00E-01	-0.52	5.40E-03	0.04	8.54E-01
ENSCAFG0000001212	GMPR2	red	VSMC_M1	0.72	1.98E-05	0.15	4.56E-01	0.13	5.18E-01	0.07	7.27E-01	0.27	1.72E-01	-0.27	1.67E-01	0.01	9.68E-01	-0.14	4.82E-01	-0.32	1.08E-01	-0.14	4.95E-01
ENSCAFG0000001950	C5H1orf174	grey	VSMC_M10	0.72	1.99E-05	0.20	3.05E-01	0.25	2.08E-01	-0.17	3.86E-01	0.18	3.72E-01	-0.06	7.55E-01	0.19	3.48E-01	0.03	8.98E-01	-0.40	3.77E-02	-0.22	2.81E-01
ENSCAFG0000003151	TIMM22	yellow	VSMC_M3	0.72	2.12E-05	0.50	7.63E-03	0.40	3.70E-02	0.00	9.94E-01	-0.07	7.12E-01	0.00	9.90E-01	-0.24	2.35E-01	-0.22	2.71E-01	-0.59	1.24E-03	0.08	6.91E-01
ENSCAFG0000000976	ACOT8	red	VSMC_M1	0.72	2.21E-05	0.28	1.59E-01	0.37	5.58E-02	-0.14	4.87E-01	0.05	8.08E-01	-0.17	3.92E-01	-0.15	4.66E-01	-0.01	9.44E-01	-0.39	4.73E-02	-0.20	3.25E-01
ENSCAFG0000001625	NDUFB7	red	VSMC_M1	0.72	2.28E-05	0.25	2.17E-01	0.31	1.16E-01	-0.14	4.79E-01	0.15	4.44E-01	-0.26	1.91E-01	-0.13	5.32E-01	0.04	8.42E-01	-0.39	4.25E-02	-0.28	1.61E-01
ENSCAFG0000003148	ENSCAFG0000003148	red	VSMC_M1	0.72	2.38E-05	0.16	4.31E-01	0.09	6.60E-01	-0.03	8.75E-01	0.30	1.25E-01	-0.30	1.31E-01	0.16	4.32E-01	0.03	8.74E-01	-0.37	6.07E-02	-0.16	4.29E-01
ENSCAFG0000001583	ENSCAFG0000001583	red	VSMC_M1	0.72	2.51E-05	0.09	6.62E-01	-0.12	5.51E-01	0.20	3.16E-01	0.54	3.57E-03	-0.30	1.22E-01	-0.20	3.17E-01	-0.21	2.93E-01	-0.23	2.56E-01	-0.24	2.18E-01
ENSCAFG0000001658	PSMB3	red	VSMC_M1	0.71	2.93E-05	0.33	9.65E-02	0.48	1.07E-02	-0.29	1.44E-01	-0.13	5.14E-01	-0.02	9.16E-01	-0.22	2.60E-01	0.15	4.70E-01	-0.47	1.26E-02	-0.14	4.92E-01
ENSCAFG0000000852	CACNA1D	red	VSMC_M1	0.71	2.99E-05	0.08	6.89E-01	0.03	8.97E-01	0.17	3.93E-01	0.37	5.58E-02	-0.26	1.83E-01	-0.28	1.58E-01	-0.18	3.64E-01	-0.20	3.14E-01	-0.17	4.01E-01
ENSCAFG0000000270	ENSCAFG0000000270	red	VSMC_M1	0.71	3.04E-05	0.18	3.82E-01	0.15	4.69E-01	-0.14	4.92E-01	0.19	3.38E-01	-0.08	6.92E-01	0.24	2.32E-01	0.01	9.77E-01	-0.36	6.70E-02	-0.05	8.10E-01
ENSCAFG0000000550	VDAC3	red	VSMC_M1	0.71	3.10E-05	0.47	1.29E-02	0.03	8.92E-01	0.20	3.20E-01	0.29	1.46E-01	-0.17	3.85E-01	-0.17	3.90E-01	-0.36	6.57E-02	-0.57	2.12E-03	0.17	4.03E-01
ENSCAFG0000003246	ENSCAFG0000003246	red	VSMC_M1	0.71	3.28E-05	0.50	8.66E-03	0.31	1.16E-01	0.04	8.50E-01	0.16	4.30E-01	-0.28	1.52E-01	-0.40	3.83E-02	-0.28	1.59E-01	-0.54	3.83E-03	-0.09	6.54E-01
ENSCAFG0000001608	BRD4	red	VSMC_M1	0.71	3.34E-05	-0.01	9.78E-01	0.22	2.59E-01	-0.35	7.50E-02	0.12	5.58E-01	0.05	8.09E-01	0.22	2.69E-01	0.30	1.30E-01	-0.29	1.42E-01	-0.24	2.22E-01
ENSCAFG0000001405	EXOSC7	red	VSMC_M1	0.71	3.49E-05	0.13	5.03E-01	0.35	7.16E-02	-0.28	1.64E-01	0.14	4.81E-01	-0.32	1.06E-01	-0.12	5.47E-01	0.16	4.25E-01	-0.30	1.28E-01	-0.45	1.85E-02
ENSCAFG0000001288	ARHGDIB	red	VSMC_M1	0.71	3.53E-05	0.26	1.97E-01	0.10	6.29E-01	-0.01	9.48E-01	0.29	1.42E-01	-0.28	1.55E-01	-0.10	6.32E-01	-0.17	3.91E-01	-0.32	1.01E-01	-0.08	6.96E-01
ENSCAFG0000000221	RPL31	red	VSMC_M1	0.71	3.73E-05	0.25	2.01E-01	0.17	4.00E-01	0.07	7.14E-01	0.17	3.92E-01	-0.26	1.93E-01	0.03	8.64E-01	-0.19	3.42E-01	-0.38	5.26E-02	0.02	9.05E-01
ENSCAFG0000001490	TRIM4	red	VSMC_M1	0.71	3.89E-05	0.16	4.35E-01	0.34	7.93E-02	-0.21	3.00E-01	0.13	5.22E-01	-0.27	1.66E-01	0.14	5.00E-01	0.05	8.17E-01	-0.26	1.93E-01	-0.34	8.54E-02
ENSCAFG0000001287	ENSCAFG0000001287	red	VSMC_M1	0.71	3.96E-05	0.47	1.40E-02	0.16	4.19E-01	0.16	4.40E-01	0.16	4.40E-01	-0.02	9.35E-01	-0.52	5.46E-03	-0.26	1.82E-01	-0.54	3.79E-03	0.11	5.81E-01
ENSCAFG0000001243	METTL5	pink	VSMC_M5	0.70	4.05E-05	-0.14	4.73E-01	-0.03	8.81E-01	-0.24	2.36E-01	0.56	2.30E-03	-0.30	1.30E-01	-0.12	5.60E-01	0.19	3.32E-01	0.00	9.98E-01	-0.62	5.94E-04
ENSCAFG0000000302	HSDL2	pink	VSMC_M5	0.70	4.19E-05	-0.06	7.58E-01	-0.12	5.67E-01	-0.02	9.17E-01	0.53	4.35E-03	0.01	9.69E-01	-0.17	3.90E-01	0.05	8.13E-01	-0.09	6.71E-01	-0.38	5.22E-02
ENSCAFG0000000220	SPIN1	red	VSMC_M1	0.70	4.35E-05	0.44	2.30E-02	0.20	3.29E-01	-0.01	9.49E-01	0.12	5.62E-01	-0.09	6.70E-01	0.21	3.04E-01	-0.17	3.86E-01	-0.59	1.34E-03	0.18	3.60E-01
ENSCAFG0000000416	PMPCB	red	VSMC_M1	0.70	4.43E-05	0.01	9.44E-01	0.30	1.33E-01	-0.31	1.15E-01	0.13	5.31E-01	-0.06	7.63E-01	-0.20	3.24E-01	0.30	1.34E-01	-0.21	3.05E-01	-0.45	1.88E-02
ENSCAFG0000000229	CLTA	red	VSMC_M1	0.70	4.45E-05	0.26	1.91E-01	0.40	4.06E-02	-0.11	5.90E-01	0.06	7.69E-01	-0.21	2.97E-01	-0.38	4.98E-02	-0.06	7.73E-01	-0.32	9.84E-02	-0.23	2.42E-01
ENSCAFG0000002226	ENSCAFG0000002226	red	VSMC_M1	0.70	4.49E-05	0.26	1.99E-01	0.30	1.32E-01	-0.07	7.40E-01	0.07	7.41E-01	-0.11	5.93E-01	0.12	5.60E-01	-0.08	6.93E-01	-0.41	3.58E-02	-0.14	4.88E-01
ENSCAFG0000001750	ENSCAFG0000001750	red	VSMC_M1	0.70	4.84E-05	-0.23	2.42E-01	0.00	9.83E-01	-0.28	1.59E-01	0.48	1.05E-02	-0.22	2.79E-01	-0.15	4.52E-01	0.33	9.46E-02	0.04	8.50E-01	-0.63	4.10E-04
ENSCAFG0000001527	ENSCAFG0000001527	pink	VSMC_M5	0.70	4.86E-05	0.03	8.98E-01	-0.26	1.90E-01	0.20	3.10E-01	0.68	1.09E-04	-0.23	2.47E-01	-0.19	3.46E-01	-0.20	3.17E-01	-0.15	4.46E-01	-0.26	1.85E-01
ENSCAFG0000003095	ENSCAFG0000003095	red	VSMC_M1	0.70	4.90E-05	-0.10	6.34E-01	-0.03	8.84E-01	-0.01	9.78E-01	0.50	7.33E-03	-0.25	2.07E-01	-0.23	2.58E-01	0.03	8.80E-01	-0.05	8.07E-01	-0.47	1.35E-02
ENSCAFG0000001370	ENSCAFG0000001370	red	VSMC_M1	0.70	4.92E-05	0.12	5.57E-01	0.56	2.64E-03	-0.49	9.14E-03	-0.13	5.09E-01	0.13	5.34E-01	-0.06	7.52E-01	0.40	3.85E-02	-0.32	9.91E-02	-0.44	2.07E-02
ENSCAFG0000000818	ARFIP1	red	VSMC_M1	0.70	4.95E-05	0.15	4.52E-01	-0.09	6.47E-01	0.29	1.48E-01	0.38	5.06E-02	-0.05	8.15E-01	-0.10	6.07E-01	-0.28	1.59E-01	-0.32	1.07E-01	0.02	9.09E-01
ENSCAFG0000000151	TST	red	VSMC_M1	0.70	4.95E-05	0.40	3.69E-02	0.18	3.76E-01	0.13	5.15E-01	0.10	6.32E-01	0.05	8.14E-01	0.05	8.17E-01	-0.27	1.81E-01	-0.53	4.56E-03	0.20	3.27E-01
ENSCAFG0000000136	ENSCAFG0000000136	red	VSMC_M1	0.70	5.01E-05	0.38	4.76E-02	-0.02	9.26E-01	0.24	2.35E-01	0.38	5.06E-02	-0.09	6.65E-01	-0.50	8.61E-03	-0.30	1.23E-01	-0.46	1.56E-02	0.01	9.53E-01
ENSCAFG0000002945	ENSCAFG0000002945	red	VSMC_M1	0.70	5.27E-05	0.26	1.83E-01	0.27	1.78E-01	-0.14	5.00E-01	0.01	9.52E-01	0.02	9.17E-01	0.09	6.51E-01	0.03	8.66E-01	-0.46	1.47E-02	0.04	8.41E-01
ENSCAFG0000002988	RPS20	red	VSMC_M1	0.70	5.27E-05	0.30	1.31E-01	0.44	2.17E-02	-0.16	4.30E-01	-0.07	7.14E-01	-0.22	2.70E-01	0.08	6.89E-01	0.03	8.98E-01	-0.48	1.04E-02	-0.11	5.71E-01
ENSCAFG0000001038	NADSYN1	cyan	VSMC_M2	0.70	5.36E-05	0.44	2.11E-02	0.37	6.07E-02	-0.10	6.27E-01	0.04	8.37E-01	-0.24	2.27E-01	-0.21	2.88E-01	-0.09	6.67E-01	-0.56	2.54E-03	-0.07	7.23E-01
ENSCAFG0000003235	ENSCAFG0000003235	red	VSMC_M1	0.70	5.64E-05	-0.01	9.79E-01	-0.01	9.57E-01	0.02	9.37E-01	0.51	6.29E-03	-0.31	1.17E-01	-0.34	8.01E-02	-0.04	8.58E-01	-0.08	6.78E-01	-0.44	2.13E-02
ENSCAFG0000001934	ENSCAFG0000001934	red	VSMC_M1	0.70	5.68E-05	0.22	2.60E-01	0.39	4.66E-02	-0.20	3.24E-01	-0.05	7.89E-01	-0.12	5.67E-01	0.11	5.91E-01	0.13	5.28E-01	-0.42	3.10E-02	-0.03	8.64E-01
ENSCAFG0000003197	ENSCAFG0000003197	yellow	VSMC_M3	0.70	5.73E-05	0.77	2.48E-06	0.57	2.04E-03	0.00	9.88E-01	-0.20	3.18E-01	-0.19	3.49E-01	-0.17	3.96E-01	-0.30	1.29E-01	-0.85	1.86E-08	0.25	2.02E-01
ENSCAFG0000000190	UBAP1	red	VSMC_M1	0.69	6.12E-05	0.14	4.93E-01	-0.23	2.51E-01	0.25	2.15E-01	0.61	7.73E-04	-0.31	1.15E-01	-0.21	2.88E-01	-0.24	2.38E-01	-0.30	1.27E-01	-0.13	5.02E-01
ENSCAFG0000002481	RACK1	red	VSMC_M1	0.69	6.40E-05	0.20	3.06E-01	0.39	4.71E-02	-0.44	2.31E-02	0.04	8.47E-01	-0.17	3.97E-01	0.00	9.90E-01	0.27	1.70E-01	-0.38	5.25E-02	-0.35	7.09E-02
ENSCAFG0000002989	RPL13A	red	VSMC_M1	0.69	7.08E-05	0.61	8.09E-04	0.38	4.95E-02	0.17	4.02E-01	-0.07	7.19E-01	-0.25	2.10E-01	-0.23	2.53E-01	-0.35	7.15E-02	-0.69	7.51E-05	0.21	2.89E-01
ENSCAFG0000000626	PCID2	red	VSMC_M1	0.69	7.20E-05	0.24	2.38E-01	-0.02	9.36E-01	0.17	3.88E-01	0.23	2.57E-01	0.08	6.75E-01	-0.02	9.12E-01	-0.18	3.71E-01	-0.46	1.54E-02	0.18	3.79E-01
ENSCAFG0000001582	PFN1	red	VSMC_M1	0.69	7.46E-05	0.10	6.09E-01	0.34	8.31E-02	-0.26	1.91E-01	0.10	6.28E-01	-0.16	4.29E-01	-0.35	7.78E-02	0.16	4.16E-01	-0.21	2.82E-01	-0.39	4.38E-02
ENSCAFG0000003190	RNF219	pink	VSMC_M5	0.69	7.53E-05	-0.18	3.65E-01	-0.14	4.74E-01	-0.11	5.79E-01	0.46	1.57E-02	0.01	9.63E-01	0.05	8.00E-01	0.20	3.28E-01	-0.10	6.21E-01	-0.31	1.12E-01
ENSCAFG0000002324	RPS15	cyan	VSMC_M2	0.69	7.62E-05	0.65	2.32E-04	0.31	1.15E-01	0.20	3.27E-01	0.00	9.84E-01	-0.20	3.12E-01	-0.45	1.87E-02	-0.39	4.42E-02	-0.70	4.96E-05	0.20	3.27E-01

ENSCAFG0000000065	DYNLT1	red	VSMC_M1	0.69	8.08E-05	0.09	6.57E-01	0.22	2.78E-01	-0.08	7.05E-01	0.08	6.74E-01	-0.04	8.28E-01	-0.19	3.47E-01	0.04	8.42E-01	-0.28	1.53E-01	-0.12	5.46E-01
ENSCAFG0000000055	RGS17	red	VSMC_M1	0.68	8.11E-05	0.37	5.92E-02	0.23	2.56E-01	-0.04	8.26E-01	0.10	6.14E-01	0.02	9.29E-01	-0.14	4.77E-01	-0.06	7.67E-01	-0.56	2.63E-03	0.00	9.89E-01
ENSCAFG0000000960	TMEM117	red	VSMC_M1	0.68	8.77E-05	0.16	4.39E-01	0.13	5.27E-01	-0.16	4.13E-01	0.24	2.32E-01	-0.05	7.94E-01	0.25	2.16E-01	0.13	5.08E-01	-0.42	2.78E-02	-0.20	3.19E-01
ENSCAFG0000000416	MEIS3	cyan	VSMC_M2	0.68	8.92E-05	0.64	2.99E-04	0.21	3.02E-01	0.31	1.16E-01	0.03	8.86E-01	-0.33	9.08E-02	-0.30	1.29E-01	-0.47	1.45E-02	-0.73	1.57E-05	0.39	4.20E-02
ENSCAFG0000002358	ENSCAFG0000002358	red	VSMC_M1	0.68	9.68E-05	-0.07	7.33E-01	-0.08	6.76E-01	0.03	8.84E-01	0.48	1.20E-02	-0.25	2.05E-01	-0.07	7.39E-01	-0.02	9.37E-01	-0.08	6.85E-01	-0.26	1.96E-01
ENSCAFG0000000755	PDCD5	red	VSMC_M1	0.68	9.69E-05	-0.15	4.47E-01	0.25	2.12E-01	-0.40	3.85E-02	0.25	2.16E-01	-0.32	1.06E-01	0.08	6.88E-01	0.39	4.60E-02	-0.08	6.81E-01	-0.60	9.15E-04
ENSCAFG0000000331	ENSCAFG0000000331	red	VSMC_M1	0.68	9.73E-05	0.22	2.67E-01	-0.11	6.01E-01	0.07	7.31E-01	0.51	7.00E-03	-0.18	3.60E-01	-0.45	1.80E-02	-0.13	5.07E-01	-0.30	1.28E-01	-0.18	3.57E-01
ENSCAFG0000001809	ENSCAFG0000001809	red	VSMC_M1	0.68	9.85E-05	0.55	2.82E-03	0.18	3.70E-01	0.35	7.51E-02	0.10	6.06E-01	-0.21	2.93E-01	-0.34	8.27E-02	-0.48	1.23E-02	-0.62	5.22E-04	0.21	2.91E-01
ENSCAFG0000001645	CCDC86	red	VSMC_M1	0.68	9.99E-05	0.11	5.91E-01	0.45	1.88E-02	-0.24	2.20E-01	-0.08	7.09E-01	0.00	9.95E-01	-0.03	8.68E-01	0.16	4.23E-01	-0.29	1.36E-01	-0.29	1.36E-01
ENSCAFG0000002581	ENSCAFG0000002581	red	VSMC_M1	0.68	1.04E-04	0.32	1.06E-01	0.13	5.23E-01	0.18	3.59E-01	0.22	2.68E-01	-0.25	2.14E-01	-0.12	5.59E-01	-0.28	1.50E-01	-0.41	3.31E-02	-0.02	9.18E-01
ENSCAFG0000000763	CTR9	red	VSMC_M1	0.68	1.04E-04	0.38	4.82E-02	0.15	4.64E-01	0.12	5.35E-01	0.17	3.97E-01	0.05	7.93E-01	-0.01	9.59E-01	-0.21	2.86E-01	-0.57	1.80E-03	0.11	5.92E-01
ENSCAFG0000002780	ENSCAFG0000002780	red	VSMC_M1	0.68	1.05E-04	0.25	2.10E-01	-0.17	3.90E-01	0.27	1.72E-01	0.54	3.31E-03	-0.23	2.59E-01	-0.16	4.21E-01	-0.32	1.09E-01	-0.35	7.01E-02	-0.09	6.44E-01
ENSCAFG0000000326	RPS7	red	VSMC_M1	0.68	1.06E-04	0.62	5.13E-04	0.50	8.48E-03	0.02	9.13E-01	-0.22	2.60E-01	-0.10	6.27E-01	-0.19	3.41E-01	-0.20	3.19E-01	-0.76	5.13E-06	0.21	2.85E-01
ENSCAFG0000001517	ELK1	red	VSMC_M1	0.68	1.08E-04	0.44	2.09E-02	0.31	1.20E-01	-0.16	4.32E-01	0.13	5.30E-01	-0.06	7.52E-01	-0.35	7.08E-02	-0.11	5.71E-01	-0.49	9.97E-03	-0.16	4.20E-01
ENSCAFG0000001691	ENSCAFG0000001691	red	VSMC_M1	0.68	1.09E-04	0.08	7.09E-01	0.11	5.77E-01	-0.04	8.36E-01	0.37	6.11E-02	-0.37	5.59E-02	-0.18	3.76E-01	-0.03	8.82E-01	-0.22	2.72E-01	-0.39	4.50E-02
ENSCAFG0000000338	OSCP1	red	VSMC_M1	0.68	1.09E-04	0.43	2.41E-02	0.08	6.99E-01	0.25	2.14E-01	0.26	1.82E-01	-0.24	2.37E-01	-0.08	7.01E-01	-0.34	7.83E-02	-0.56	2.25E-03	0.13	5.14E-01
ENSCAFG0000001004	PARP3	red	VSMC_M1	0.68	1.10E-04	0.39	4.49E-02	0.44	2.31E-02	-0.19	3.31E-01	-0.04	8.26E-01	-0.15	4.70E-01	-0.29	1.45E-01	0.03	9.01E-01	-0.50	7.35E-03	-0.12	5.57E-01
ENSCAFG0000002008	CIZ1	red	VSMC_M1	0.68	1.12E-04	0.45	1.81E-02	0.33	9.08E-02	-0.19	3.39E-01	-0.03	8.91E-01	-0.05	8.21E-01	0.21	2.91E-01	0.03	8.88E-01	-0.66	1.83E-04	0.07	7.43E-01
ENSCAFG0000001756	SMAD2	pink	VSMC_M5	0.67	1.15E-04	-0.12	5.64E-01	-0.22	2.79E-01	0.06	7.68E-01	0.63	3.89E-04	-0.08	6.98E-01	0.08	7.05E-01	-0.07	7.46E-01	-0.03	8.66E-01	-0.33	9.22E-02
ENSCAFG0000003089	RPS23	yellow	VSMC_M3	0.67	1.17E-04	0.25	1.99E-01	0.46	1.65E-02	-0.10	6.31E-01	-0.07	7.31E-01	-0.25	2.01E-01	0.03	8.95E-01	-0.05	8.15E-01	-0.39	4.51E-02	-0.13	5.29E-01
ENSCAFG0000000803	PSMD9	red	VSMC_M1	0.67	1.22E-04	0.12	5.59E-01	0.09	6.39E-01	-0.05	8.03E-01	0.38	5.35E-02	-0.45	1.80E-02	-0.18	3.64E-01	-0.01	9.53E-01	-0.23	2.58E-01	-0.34	8.43E-02
ENSCAFG0000001967	ENSCAFG0000001967	red	VSMC_M1	0.67	1.23E-04	0.62	5.94E-04	0.24	2.25E-01	0.03	8.90E-01	0.05	7.97E-01	-0.11	5.94E-01	-0.14	4.74E-01	-0.27	1.67E-01	-0.70	5.54E-05	0.20	3.25E-01
ENSCAFG0000001200	NIF3L1	red	VSMC_M1	0.67	1.29E-04	0.38	4.99E-02	0.08	7.01E-01	0.13	5.06E-01	0.18	3.76E-01	-0.12	5.66E-01	0.17	3.85E-01	-0.23	2.49E-01	-0.61	8.17E-04	0.22	2.60E-01
ENSCAFG0000001112	LRRC27	red	VSMC_M1	0.67	1.30E-04	0.23	2.44E-01	0.20	3.18E-01	-0.02	9.06E-01	0.11	5.98E-01	-0.06	7.78E-01	0.27	1.78E-01	-0.08	7.02E-01	-0.47	1.45E-02	0.03	9.01E-01
ENSCAFG0000001442	ANAPC16	red	VSMC_M1	0.67	1.30E-04	-0.04	8.44E-01	0.19	3.42E-01	-0.10	6.04E-01	0.19	3.35E-01	0.04	8.53E-01	-0.07	7.32E-01	0.16	4.23E-01	-0.15	4.52E-01	-0.36	6.77E-02
ENSCAFG0000000929	FBXW8	red	VSMC_M1	0.67	1.36E-04	0.54	3.97E-03	0.26	1.91E-01	0.15	4.53E-01	-0.05	8.22E-01	-0.07	7.19E-01	0.04	8.35E-01	-0.26	1.82E-01	-0.69	7.27E-05	0.34	8.06E-02
ENSCAFG0000001688	COQ6	yellow	VSMC_M3	0.67	1.40E-04	0.56	2.60E-03	0.49	9.14E-03	-0.01	9.58E-01	-0.21	3.00E-01	0.01	9.73E-01	-0.06	7.82E-01	-0.18	3.62E-01	-0.70	4.72E-05	0.21	2.86E-01
ENSCAFG0000001947	ENSCAFG0000001947	red	VSMC_M1	0.67	1.40E-04	0.22	2.67E-01	0.54	3.97E-03	-0.40	4.05E-02	-0.11	5.85E-01	-0.19	3.52E-01	0.02	9.37E-01	0.22	2.64E-01	-0.40	3.86E-02	-0.28	1.53E-01
ENSCAFG0000001854	MIER1	red	VSMC_M1	0.67	1.41E-04	0.15	4.69E-01	0.09	6.68E-01	-0.21	3.01E-01	0.34	7.87E-02	-0.49	9.28E-03	-0.02	9.15E-01	0.08	6.91E-01	-0.31	1.10E-01	-0.27	1.76E-01
ENSCAFG0000001954	ENSCAFG0000001954	red	VSMC_M1	0.67	1.46E-04	0.33	9.18E-02	0.54	3.59E-03	-0.24	2.35E-01	-0.18	3.74E-01	-0.26	1.85E-01	0.13	5.27E-01	0.06	7.66E-01	-0.47	1.25E-02	-0.02	9.03E-01
ENSCAFG0000003086	COMMD4	yellow	VSMC_M3	0.67	1.48E-04	0.43	2.53E-02	0.80	4.62E-07	-0.48	1.17E-02	-0.42	2.99E-02	-0.11	5.87E-01	0.06	7.64E-01	0.23	2.45E-01	-0.59	1.33E-03	-0.14	4.94E-01
ENSCAFG0000001043	ACOT13	pink	VSMC_M5	0.67	1.48E-04	-0.17	3.90E-01	-0.12	5.65E-01	-0.15	4.58E-01	0.57	2.04E-03	-0.14	4.93E-01	0.06	7.57E-01	0.22	2.80E-01	-0.06	7.70E-01	-0.51	6.85E-03
ENSCAFG0000000881	ENSCAFG0000000881	red	VSMC_M1	0.67	1.49E-04	0.41	3.38E-02	0.26	1.89E-01	-0.02	9.15E-01	0.16	4.34E-01	-0.34	8.16E-02	-0.22	2.63E-01	-0.22	2.75E-01	-0.46	1.55E-02	-0.04	8.31E-01
ENSCAFG0000000576	RPL15	cyan	VSMC_M2	0.67	1.53E-04	0.59	1.19E-03	0.24	2.35E-01	0.15	4.69E-01	-0.02	9.37E-01	-0.02	9.11E-01	-0.27	1.74E-01	-0.27	1.66E-01	-0.71	3.75E-05	0.29	1.48E-01
ENSCAFG0000000193	DCTN3	red	VSMC_M1	0.66	1.57E-04	-0.09	6.58E-01	0.07	7.13E-01	-0.03	8.64E-01	0.29	1.42E-01	-0.28	1.59E-01	0.17	3.87E-01	0.12	5.41E-01	-0.14	4.75E-01	-0.30	1.24E-01
ENSCAFG0000001576	C6H7orf26	red	VSMC_M1	0.66	1.63E-04	0.12	5.55E-01	0.17	3.98E-01	-0.28	1.54E-01	0.21	2.86E-01	-0.23	2.54E-01	0.02	9.19E-01	0.18	3.58E-01	-0.32	1.01E-01	-0.27	1.70E-01
ENSCAFG0000000097	ZBTB22	red	VSMC_M1	0.66	1.65E-04	0.50	8.40E-03	0.15	4.64E-01	0.17	4.09E-01	0.26	1.97E-01	-0.22	2.75E-01	-0.19	3.45E-01	-0.38	5.16E-02	-0.55	3.24E-03	0.00	9.87E-01
ENSCAFG0000003169	ENSCAFG0000003169	red	VSMC_M1	0.66	1.66E-04	0.39	4.45E-02	0.38	5.06E-02	-0.02	9.34E-01	-0.11	5.79E-01	0.09	6.66E-01	-0.09	6.61E-01	-0.12	5.54E-01	-0.53	4.30E-03	0.13	5.15E-01
ENSCAFG0000001803	SNUPN	red	VSMC_M1	0.66	1.72E-04	0.48	1.05E-02	0.36	6.52E-02	0.06	7.83E-01	-0.10	6.25E-01	-0.18	3.75E-01	-0.10	6.09E-01	-0.16	4.29E-01	-0.68	9.49E-05	0.20	3.15E-01
ENSCAFG0000002868	SRXN1	red	VSMC_M1	0.66	1.72E-04	0.29	1.48E-01	0.31	1.10E-01	-0.28	1.50E-01	0.00	9.84E-01	0.07	7.13E-01	0.10	6.34E-01	0.20	3.18E-01	-0.51	6.00E-03	-0.05	8.04E-01
ENSCAFG0000002679	ENSCAFG0000002679	red	VSMC_M1	0.66	1.75E-04	0.41	3.22E-02	0.30	1.23E-01	0.03	8.76E-01	0.02	9.02E-01	-0.11	5.87E-01	-0.02	9.31E-01	-0.20	3.25E-01	-0.52	5.85E-03	0.03	8.76E-01
ENSCAFG0000001952	NDUFS7	yellow	VSMC_M3	0.66	1.80E-04	0.39	4.38E-02	0.48	1.15E-02	-0.25	2.01E-01	-0.09	6.49E-01	0.02	9.14E-01	-0.03	8.90E-01	0.06	7.84E-01	-0.52	5.34E-03	-0.11	5.82E-01
ENSCAFG0000000467	CNOT10	pink	VSMC_M5	0.66	1.80E-04	-0.12	5.62E-01	-0.09	6.37E-01	-0.24	2.30E-01	0.48	1.07E-02	-0.21	2.98E-01	0.15	4.40E-01	0.23	2.52E-01	-0.10	6.03E-01	-0.34	7.88E-02
ENSCAFG0000001851	ENSCAFG0000001851	yellow	VSMC_M3	0.66	1.86E-04	0.28	1.56E-01	0.48	1.18E-02	-0.24	2.22E-01	-0.16	4.13E-01	-0.06	7.60E-01	0.05	7.94E-01	0.10	6.11E-01	-0.46	1.53E-02	-0.13	5.29E-01
ENSCAFG0000001734	STXBP4	red	VSMC_M1	0.66	1.94E-04	0.33	9.41E-02	-0.08	6.75E-01	0.13	5.23E-01	0.34	8.27E-02	-0.12	5.62E-01	0.03	8.93E-01	-0.21	2.97E-01	-0.52	5.84E-03	0.13	5.06E-01
ENSCAFG0000001336	KDM1A	red	VSMC_M1	0.66	1.97E-04	-0.04	8.28E-01	0.16	4.24E-01	-0.39	4.55E-02	0.23	2.44E-01	-0.05	8.16E-01	0.24	2.28E-01	0.30	1.34E-01	-0.18	3.76E-01	-0.37	5.78E-02
ENSCAFG0000002918	TIMP3	red	VSMC_M1	0.66	1.99E-04	0.58	1.48E-03	0.16	4.26E-01	0.10	6.11E-01	0.14	4.78E-01	-0.08	6.92E-01	-0.04	8.25E-01	-0.33	8.77E-02	-0.70	5.56E-05	0.21	2.87E-01
ENSCAFG0000000617	HDAC3	red	VSMC_M1	0.66	2.02E-04	0.33	9.69E-02	0.28	1.60E-01	-0.18	3.67E-01	0.15	4.58E-01	-0.03	8.85E-01	-0.32	1.06E-01	0.04	8.39E-01	-0.43	2.41E-02	-0.27	1.79E-01
ENSCAFG0000000605	CSNK1D	red	VSMC_M1	0.65	2.13E-04	0.24	2.20E-01	0.28	1.59E-01	-0.18	3.77E-01	0.15	4.49E-01	-0.03	8.90E-01	-0.38	5.15E-02	0.02	9.03E-01	-0.34	8.07E-02	-0.32	1.04E-01
ENSCAFG0000002003	NUDT7	pink	VSMC_M5	0.65	2.16E-04	0.12	5.63E-01	0.01	9.56E-01	-0.05	8.00E-01	0.47	1.27E-02	-0.27	1.76E-01	-0.23	2.54E-01	-0.03	8.74E-01	-0.22	2.62E-01	-0.41	3.32E-02
ENSCAFG0000002037	E2F4	red	VSMC_M1	0.65	2.19E-04	0.48	1.07E-02	0.50	8.14E-03	-0.20	3.22E-01	-0.25	2.12E-01	0.01	9.67E-01	0.07	7.38E-01	0.09	6.44E-01	-0.71	3.36E-05	0.11	5.77E-01
ENSCAFG0000001704	STX10	yellow	VSMC_M3	0.65	2.21E-04	0.70	5.34E-05	0.67	1.40E-04	-0.15	4.65E-01	-0.31	1.17E-01	-0.21	2.87E-01	-0.10	6.03E-01	-0.17	3.91E-01	-0.76	3.42E-06	0.16	4.30E-01
ENSCAFG0000001936	TBC1D24	red	VSMC_M1	0.65	2.21E-04	0.35	7.34E-02	0.24	2.21E-01	-0.11	5.92E-01	0.07	7.42E-01	-0.19	3.39E-01	0.06	7.48E-01	-0.05	7.88E-01	-0.49	9.76E-03	0.03	8.96E-01
ENSCAFG0000001987	GTF3C5	yellow	VSMC_M3	0.65	2.28E-04	0.51	6.10E-03	0.55	3.22E-03	-0.30	1.27E-01	-0.15	4.66E-01	-0.10	6.12E-01	-0.04	8.33E-01	-0.01	9.64E-01	-0.63	4.02E-04	-0.04	8.26E-01
ENSCAFG0000000052	ENSCAFG0000000052	red	VSMC_M1	0.65	2.28E-04	0.25	2.08E-01	0.05	8.10E-01	0.08	7.02E-01	0.24	2.31E-01	-0.20	3.19E-01	0.00	9.82E-01	-0.14	4.72E-01	-0.42	3.07E-02	0.01	9.78E-01
ENSCAFG0000002010	AK1	yellow	VSMC_M3	0.65	2.29E-04	0.67	1.30E-04	0.68	9.31E-05	-0.11	6.00E-01	-0.33	9.79E-02	-0.20	3.17E-01	-0.18	3.63E-01	-0.20	3.25E-01	-0.71	2.91E-05	0.15	4.45E-01
ENSCAFG0000000244	AHR	red	VSMC_M1	0.65	2.31E-04	0.21	2.86E-01	0.12	5.68E-01	-0.18	3.66E-01	0.39	4.64E-02	-0.22	2.64E-01	-0.22	2.81E-01	-0.04	8.38E-01	-0.25	2.11E-01	-0.34	8.26E-02
ENSCAFG0000000740	CEBPG	red	VSMC_M1	0.65	2.32E-04	-0.03	8.84E-01	0.12	5.49E-01	-0.07	7.12E-01	0.30	1.32E-01	-0.17	4.08E-01	0.03	8.73E-01	0.00	9.94E-01	-0.13	5.13E-01	-0.32	1.08E-01
ENSCAFG0000000487	RPL32	red	VSMC_M1	0.65	2.32E-04	0.28	1.59E-01	0.23	2.45E-01	-0.05	7.87E-01	0.06	7.55E-01	-0.15	4.70E-01	0.03	8.96E-01	-0.03	8.80E-01	-0.43	2.47E-02	-0.08	6.83E-01
ENSCAFG0000002877	ENSCAFG0000002877	red	VSMC_M1	0.65	2.39E-04	0.32	1.05E-01	0.24	2.34E-01	0.19	3.51E-01	0.02	9.32E-01	-0.11	5.99E-01	-0.20	3.17E-01	-0.27	1.70E-01	-0.44	2.05E-02	0.14	4.74E-01
ENSCAFG0000002559	HUS1	red	VSMC_M1	0.65	2.39E-04	0.14	4.87E-01	0.44	2.01E-02	-0.48	1.12E-02	-0.12	5.64E-01	-0.04	8.50E-01	-0.04	8.31E-01	0.34	7.82E-02	-0.33	9.06E-02	-0.23	2.59E-01
ENSCAFG0000001288	TMEM50A	cyan	VSMC_M2	0.65	2.40E-04	0.60	9.02E-04	0.15	4.62E-01	0.28	1.51E-01	0.12	5.65E-01	-0.13	5.07E-01	-0.58	1.39E-03	-0.48	1.04E-02	-0.62	5.16E-04	0.23	2.40E-01
ENSCAFG0000000781	SMN	red	VSMC_M1	0.65	2.56E-04	-0.11	5.83E-01	0.37	5.46E-02	-0.60	9.80E-04	0.04	8.40E-01	-0.07	7.44E-01	0.16	4.19E-01	0.61	8.27E-04	-0.16	4.12E-01	-0.56	2.43E-03
ENSCAFG0000001490	LSM4	red	VSMC_M1	0.65	2.65E-04	0.14	4.97E-01	0.21	2.94E-01	-0.21	3.03E-01	0.14	4.99E-01	0.02	9.19E-01	-0.02	9.13E-01	0.15	4.67E-01	-0.36	6.31E-02	-0.21	3.04E-01
ENSCAFG0000001184	ENSCAFG0000001184	red	VSMC_M1	0.65	2.65E-04	0.23	2.51E-01	0.30	1.27E-01	-0.31	1.12E-01	0.09	6.55E-01	-0.03	8.68E-01	-0.17	3.89E-01	0.12	5.53E-01	-0.38	5.18E-02	-0.23	2.54E-01
ENSCAFG0000000494	DLEC1	yellow	VSMC_M3	0.65	2.74E-04	0.51	6.66E-03	0.57	1.93E-03	-0.16	4.37E-01	-0.23	2.45E-01	0.15	4.56E-01	0.03	8.75E-01	-0.09	6.72E-01	-0.62	5.00E-04	0.08	6.87E-01
ENSCAFG0000001722	ENSCAFG0000001722	red	VSMC_M1	0.65	2.76E-04	0.17	4.09E-01	-0.16	4.29E-01	0.26	1.92E-01	0.50	7.36E-03	-0.10	6.04E-01	-0.21	3.04E-01	-0.33	8.86E-02	-0.26	1.95E-01	-0.05	8.12E-01
ENSCAFG0000000681	RPL21	red	VSMC_M1	0.65	2.77E-04	0.20	3.21E-01	-0.22	2.62E-01	0.39	4.54E-02	0.58	1.63E-03	-0.31	1.13E-01	-0.49	8.69E-03	-0.39	4.30E-02	-0.26	1.98E-01	-0.11	5.95E-01
ENSCAFG0000001573	ANXA11	yellow	VSMC_M3	0.65	2.80E-04	0.74	1.23E-05	0.67	1.42E-04	-0.23	2.50E-01	-0.29	1.44E-01	-0.26	1.98E-01	-0.22	2.79E-01	-0.13	5.23E-01	-0.75	6.49E-06	0.12	5.52E-01
ENSCAFG0000001548	TOP3B	red	VSMC_M1	0.64	2.99E-04	0.39	4.65E-02	0.15	4.66E-01	0.10	6.15E-01	0.15	4.48E-01	0.01	9.76E-01	-0.36	6.72E-02	-0.26	1.82E-01	-0.41	3.47E-02	0.09	6.64E-01
ENSCAFG0000000459	SLC9A3R1	yellow	VSMC_M3	0.64	3.00E-04	0.57	2.12E-03	0.52	5.25E-03	-0.08	7.06E-01	-0.22	2.79E-01	0.00	9.98E-01	-0.36	6.79E-02	-0.19	3.34E-01	-0.64	3.07E-04	0.12	5.53E-01
ENSCAFG0000001190	ENSCAFG0000001190	red	VSMC_M1	0.64	3.04E-04	0.33	9.68E-02	0.24	2.26E-01	-0.20	3.17E-01	0.19	3.47E-01	-0.27	1.80E-01	-0.08	6.93E-01	-0.02	9.28E-01	-0.42	2.83E-02	-0.22	2.75E-01
ENSCAFG0000001358	ENSCAFG0000001358	red	VSMC_M1	0.64	3.04E-04	0.28	1.52E-01	0.44	2.28E-02	-0.28	1.53E-01	0.02	9.38E-01	-0.15	4.63E-01	-0.19	3.51E-01	0.11	5.97E-01	-0.35	7.35E-02	-0.33	9.01E-02
ENSCAFG0000000254	ENSCAFG0000000254	red	VSMC_M1	0.64	3.05E-04	0.00	9.82E-01	0.49	8.94E-03	-0.51	6.62E-03	-0.04	8.37E-01	-0.22	2.77E-01	0.06	7.58E-01	0.43	2.53E-02	-0.20	3.28E-01	-0.47	1.37E-02
ENSCAFG0000000375	CC2D1B	yellow	VSMC_M3	0.64	3.07E-04	0.56	2.63E-03	0.62	5.43E-04	-0.09	6.58E-01	-0.23	2.55E-01	-0.14	4.95E-01	-0.11	6.02E-01	-0.19	3.52E-01	-0.61	6.76E-04	0.04	8.38E-01
ENSCAFG0000001569	KAT2A	red	VSMC_M1	0.64	3.08E-04	0.31	1.12E-01	0.36	6.87E-02	-0.15	4.54E-01	-0.12	5.61E-01	-0.05	7.96E-01	0.26	1.83E-01	0.06	7.56E-01	-0.57	1.93E-03	0.13	5.12E-01
ENSCAFG0000001230	ANXA9	cyan	VSMC_M2	0.64	3.08E-04	0.68	9.71E-05	0.39	4.58E-02	0.04	8.52E-01	-0.13	5.03E-01	-0.09	6.67E-01	-0.30	1.24E-01	-0.28	1.57E-01	-0.76	4.15E-06	0.29	1.40E-01
ENSCAFG0000002341	CNNM3	cyan	VSMC_M2	0.64	3.24E-04	0.62	5.17E-04	0.18	3.76E-01	0.25	2.11E-01	0.11	5.74E-01	-0.12	5.37E-01	-0.17	3.93E-01	-0.45	1.89E-02	-0.70	4.51E-05	0.25	2.12E-01
ENSCAFG0000001132	ENSCAFG0000001132	red	VSMC_M1	0.64	3.24E-04	0.21	2.95E-01	0.33	9.19E-02	-0.21	2.86E-01	0.18	3.70E-01	-0.19	3.35E-01	-0.16	4.20E-01	0.04	8.39E-01	-0.29	1.42E-01	-0.42	2.98E-02
ENSCAFG0000001891	GOLPH3	red	VSMC_M1	0.64	3.31E-04	-0.04	8.44E-01	0.05	7.97E-01	-0.20	3.27E-01	0.40	3.91E-02	-0.27	1.68E-01	0.00	9.90E-01	0.11	5.95E-01	-0.09	6.43E-01	-0.40	3.79E-02
ENSCAFG0000001715	RPS4X	red	VSMC_M1	0.64	3.41E-04	0.27	1.66E-01	0.48	1.13E-02	-0.28	1.54E-01	-0.06	7.52E-01	-0.22	2.74E-01	0.03	8.81E-01	0.11	5.80E-01	-0.42	2.92E-02	-0.29	1.48E-01
ENSCAFG0000003096	ZNF524	grey	VSMC_M10	0.64	3.48E-04	0.51	6.78E-03	0.45	1.73E-02	0.02	9.37E-01	-0.23	2.56E-01	-0.22	2.73E-01	-0.10	6.12E-01	-0.23	2.41E-01	-0.63	4.21E-04	0.30	1.32E-01
ENSCAFG0000000234	UBE2M	red	VSMC_M1	0.64	3.51E-04	-0.04	8.30E-01	0.37	5.92E-02	-0.40	4.03E-02	0.05	8.20E-01	-0.01	9.45E-01	-0.10	6.35E-01	0.32	1.05E-01	-0.14	4.85E-01	-0.52	4.96E-03
ENSCAFG0000000894	SFXN5	yellow	VSMC_M3	0.64	3.52E-04	0.36	6.39E-02	0.59	1.13E-03	-0.37	5.77E-02	-0.23	2.49E-01	-0.07	7.45E-01	0.15	4.57E-01	0.18	3.73E-01	-0.50	7.61E-03	-0.12	5.42E-01
ENSCAFG0000000049	ENSCAFG0000000049	red	VSMC_M1	0.64	3.53E-04	0.35	7.80E-02	0.11	5.87E-01	0.05	8.14E-01	0.27	1.74E-01	-0.23	2.40E-01	-0.17	4.08E-01	-0.28	1.54E-01	-0.35	7.76E-02	-0.01	9.65E-01
ENSCAFG0000000079	ENSCAFG0000000079	red	VSMC_M1	0.64	3.54E-04	0.35	7.20E-02	0.33	9.02E-02	0.02	9.34E-01	-0.10	6.18E-01	0.00	9.93E-01	0.19	3.34E-01	-0.13	5.04E-01	-0.49	9.13E-03	0.19	3.37E-01
ENSCAFG0000003149	ENSCAFG0000003149	cyan	VSMC_M2	0.64	3.59E-04	0.60	9.79E-04	0.13	5.25E-01	0.26	1.91E-01	0.11	5.69E-01	-0.05	8.09E-01	-0.32	1.00E-01	-0.45	1.81E-02	-0.65	2.12E-04	0.26	1.90E-01
ENSCAFG0000001175	RAB9A	red	VSMC_M1	0.64	3.62E-04	0.19	3.34E-01	0.19	3.38E-01	-0.27	1.75E-01	0.19	3.46E-01	-0.20	3.12E-01	0.27	1.66E-01	0.11	6.00E-01	-0.37	5.97E-02	-0.17	3.87E-01
ENSCAFG0000001730	RPL4	red	VSMC_M1	0.64	3.62E-04	0.22	2.79E-01	0.08	6.79E-01	0.10	6.37E-01	0.28	1.64E-01	-0.23	2.58E-01	-0.23	2.41E-01	-0.10	6.11E-01	-0.34	8.49E-02	-0.16	4.36E-01
ENSCAFG0000001632	VPS37C	red	VSMC_M1	0.64	3.63E-04	0.44	2.15E-02	0.27	1.72E-01	0.10	6.18E-01	0.01	9.58E-01	-0.12	5.41E-01	-0.20	3.14E-01	-0.32	1.05E-01	-0.52	4.97E-03	0.15	4.55E-01
ENSCAFG0000001565	TTC9C	red	VSMC_M1	0.64	3.68E-04	0.38	5.32E-02	0.22	2.79E-01	0.11	5.95E-01	0.00	9.97E-01	0.14	4.82E-01	-0.02	9.32E-01	-0.22	2.64E-01	-0.57	2.01E-03	0.26	1.86E-01
ENSCAFG0000002822	ENSCAFG0000002822	red	VSMC_M1	0.64	3.70E-04	0.23	2.53E-01	0.01	9.80E-01	0.10	6.09E-01	0.32	1.09E-01	-0.21	3.03E-01	-0.21	2.83E-01	-0.21	3.01E-01	-0.32	1.04E-01	-0.11	5.84E-01
ENSCAFG0000002428	TRABD2B	grey	VSMC_M10	0.64	3.71E-04	0.74	1.02E-05	0.20	3.24E-01	0.31	1.17E-01	0.04	8.38E-01	-0.23	2.44E-01	-0.62	4.92E-04	-0.49	8.89E-03	-0.78	2.05E-06	0.37	5.96E-02
ENSCAFG0000001716	PRDX2	red	VSMC_M1	0.64	3.73E-04	0.33	9.26E-02	0.34	8.44E-02	-0.08	7.00E-01	0.10	6.11E-01	-0.22	2.74E-01	-0.25	2.05E-01	-0.10	6.20E-01	-0.36	6.17E-02	-0.21	2.85E-01
ENSCAFG0000001548	NR2F6	cyan	VSMC_M2	0.63	3.74E-04	0.65	2.25E-04	0.33	8.86E-02	0.15	4.68E-01	-0.10	6.15E-01	-0.09	6.52E-01	0.10	6.09E-01	-0.37	6.10E-02	-0.74	9.25E-06	0.36	6.49E-02
ENSCAFG0000000411	TBP	pink	VSMC_M5	0.63	3.77E-04	-0.08	6.74E-01	0.17	3.93E-01	-0.47	1.24E-02	0.23	2.42E-01	-0.01	9.44E-01	0.00	9.84E-01	0.46	1.62E-02	-0.13	5.32E-01	-0.55	3.12E-03
ENSCAFG0000001519	PELI2	red	VSMC_M1	0.63	3.77E-04	-0.06	7.62E-01	-0.04	8.46E-01	-0.14	5.00E-01	0.35	7.31E-02	-0.05	8.01E-01	0.12	5.35E-01	0.09	6.46E-01	-0.14	5.02E-01	-0.22	2.79E-01
ENSCAFG0000000167	RPL8	yellow	VSMC_M3	0.63	3.77E-04	0.63	4.87E-04	0.68	1.06E-04	-0.16	4.25E-01	-0.40	3.87E-02	-0.10	6.19E-01	-0.16	4.39E-01	-0.07	7.31E-01	-0.74	1.25E-05	0.14	4.92E-01
ENSCAFG0000001610	GABARAP	cyan	VSMC_M2	0.63	3.80E-04	0.73	1.32E-05	0.44	2.25E-02	0.21	2.93E-01	-0.22	2.67E-01	-0.10	6.15E-01	-0.29	1.42E-01	-0.47	1.41E-02	-0.75	5.45E-06	0.40	3.83E-02
ENSCAFG0000001259	NDUFA10	red	VSMC_M1	0.63	3.84E-04	-0.03	8.75E-01	0.24	2.33E-01	-0.44	2.10E-02	0.13	5.11E-01	0.00	9.85E-01	0.06	7.77E-01	0.40	3.94E-02	-0.19	3.43E-01	-0.40	3.91E-02
ENSCAFG0000001714	PARP16	red	VSMC_M1	0.63	3.89E-04	0.27	1.72E-01	0.25	2.01E-01	-0.11	6.01E-01	-0.02	9.08E-01	0.10	6.27E-01	0.17	3.90E-01	0.06	7.70E-01	-0.50	7.46E-03	0.05	8.15E-01
ENSCAFG0000000488	ENSCAFG0000000488	red	VSMC_M1	0.63	3.98E-04	0.40	4.08E-02	0.37	5.99E-02	-0.14	4.78E-01	-0.13	5.11E-01	0.02	9.26E-01	-0.17	3.99E-01	-0.04	8.57E-01	-0.53	4.76E-03	0.11	5.70E-01
ENSCAFG0000001494	UBA1	red	VSMC_M1	0.63	4.00E-04	0.31	1.18E-01	0.36	6.35E-02	-0.24	2.34E-01	-0.03	8.85E-01	0.19	3.45E-01	-0.11	5.82E-01	0.06	7.77E-01	-0.40	4.09E-02	-0.09	6.44E-01
ENSCAFG0000001287	BCCIP	red	VSMC_M1	0.63	4.02E-04	0.11	5.77E-01	0.41	3.29E-02	-0.27	1.75E-01	-0.07	7.42E-01	0.15	4.55E-01	-0.06	7.61E-01	0.23	2.49E-01	-0.34	8.42E-02	-0.28	1.53E-01
ENSCAFG0000003074	LRRC46	cyan	VSMC_M2	0.63	4.13E-04	0.51	6.72E-03	0.26	1.97E-01	0.15	4.51E-01	0.01	9.63E-01	-0.21	2.97E-01	-0.15	4.56E-01	-0.32	1.08E-01	-0.63	4.49E-04	0.20	3.22E-01
ENSCAFG0000000172	ARF5	cyan	VSMC_M2	0.63	4.16E-04	0.71	2.92E-05	0.49	8.86E-03	-0.03	8.74E-01	-0.19	3.39E-01	-0.16	4.12E-01	0.07	7.43E-01	-0.24	2.20E-01	-0.78	1.30E-06	0.28	1.63E-01
ENSCAFG0000000998	ZBTB44	red	VSMC_M1	0.63	4.26E-04	0.31	1.10E-01	0.25	2.13E-01	-0.23	2.56E-01	0.26	1.87E-01	-0.32	1.02E-01	-0.16	4.23E-01	-0.03	8.92E-01	-0.34	8.05E-02	-0.31	1.16E-01
ENSCAFG0000001666	ANAPC13	red	VSMC_M1	0.63	4.37E-04	0.16	4.21E-01	0.07	7.26E-01	0.14	4.88E-01	0.20	3.10E-01	-0.35	7.30E-02	-0.11	5.83E-01	-0.17	3.93E-01	-0.30	1.22E-01	-0.03	8.93E-01
ENSCAFG0000000177	CUL7	yellow	VSMC_M3	0.63	4.37E-04	0.69	8.08E-05	0.58	1.59E-03	-0.15	4.43E-01	-0.15	4.41E-01	-0.28	1.51E-01	-0.13	5.33E-01	-0.18	3.82E-01	-0.70	4.34E-05	0.05	7.92E-01
ENSCAFG0000000209	RCL1	red	VSMC_M1	0.63	4.42E-04	0.14	4.93E-01	0.38	4.77E-02	-0.45	1.80E-02	0.10	6.19E-01	-0.14	4.76E-01	-0.03	8.94E-01	0.33	9.25E-02	-0.28	1.59E-01	-0.48	1.05E-02
ENSCAFG0000001793	ARID3B	red	VSMC_M1	0.63	4.44E-04	0.29	1.38E-01	0.38	5.03E-02	-0.18	3.57E-01	-0.10	6.11E-01	0.17	4.00E-01	0.05	7.92E-01	0.07	7.28E-01	-0.49	8.70E-03	0.00	9.99E-01
ENSCAFG0000000239	ENSCAFG0000000239	red	VSMC_M1	0.63	4.45E-04	0.38	5.35E-02	0.18	3.72E-01	0.05	8.19E-01	0.25	2.03E-01	-0.31	1.21E-01	-0.26	1.94E-01	-0.25	2.09E-01	-0.39	4.59E-02	-0.14	4.88E-01
ENSCAFG0000000154	EIF3D	red	VSMC_M1	0.63	4.45E-04	0.26	1.99E-01	0.74	8.61E-06	-0.64	3.70E-04	-0.33	9.50E-02	-0.09	6.41E-01	0.02	9.08E-01	0.42	3.03E-02	-0.40	3.77E-02	-0.36	6.69E-02
ENSCAFG0000003046	FTH1_version2	red	VSMC_M1	0.63	4.49E-04	0.20	3.06E-01	-0.05	8.20E-01	0.16	4.28E-01	0.36	6.73E-02	-0.28	1.54E-01	-0.17	4.06E-01	-0.26	1.88E-01	-0.28	1.57E-01	-0.02	9.11E-01
ENSCAFG0000000592	ENSCAFG0000000592	red	VSMC_M1	0.63	4.51E-04	0.58	1.56E-03	0.09	6.41E-01	0.21	2.85E-01	0.17	3.90E-01	-0.20	3.28E-01	-0.13	5.30E-01	-0.36	6.83E-02	-0.65	2.61E-04	0.31	1.11E-01
ENSCAFG0000000921	GMDS	red	VSMC_M1	0.63	4.56E-04	0.52	5.03E-03	0.00	9.91E-01	0.29	1.45E-01	0.22	2.76E-01	0.01	9.50E-01	-0.08	7.08E-01	-0.42	2.80E-02	-0.64	2.96E-04	0.35	7.62E-02

ENSCAFG0000001437	ENSCAFG0000001437	red	VSMC_M1	0.63	4.56E-04	0.42	2.72E-02	0.31	1.19E-01	-0.04	8.31E-01	-0.15	4.69E-01	0.13	5.21E-01	0.09	6.61E-01	-0.09	6.62E-01	-0.63	4.73E-04	0.26	1.97E-01
ENSCAFG0000000238	COX5B	red	VSMC_M1	0.63	4.57E-04	0.28	1.51E-01	0.49	9.12E-03	-0.17	3.92E-01	-0.18	3.77E-01	-0.09	6.41E-01	0.01	9.50E-01	0.07	7.22E-01	-0.46	1.64E-02	-0.10	6.12E-01
ENSCAFG0000003173	ENSCAFG0000003173	red	VSMC_M1	0.63	4.66E-04	0.29	1.46E-01	0.04	8.32E-01	0.17	4.11E-01	0.16	4.18E-01	-0.01	9.62E-01	-0.03	9.01E-01	-0.24	2.32E-01	-0.42	3.00E-02	0.15	4.58E-01
ENSCAFG0000001732	FLRT2	red	VSMC_M1	0.63	4.73E-04	0.32	1.09E-01	-0.16	4.14E-01	0.32	1.07E-01	0.45	1.73E-02	-0.14	4.78E-01	-0.27	1.69E-01	-0.36	6.60E-02	-0.39	4.63E-02	0.05	8.03E-01
ENSCAFG0000002361	URM1	red	VSMC_M1	0.63	4.74E-04	0.10	6.34E-01	0.04	8.58E-01	0.11	5.70E-01	0.22	2.67E-01	0.03	8.98E-01	-0.05	7.91E-01	-0.10	6.06E-01	-0.27	1.72E-01	-0.05	8.05E-01
ENSCAFG0000000500	ENSCAFG0000000500	yellow	VSMC_M3	0.63	4.81E-04	0.39	4.58E-02	0.28	1.64E-01	0.00	9.94E-01	0.03	8.94E-01	-0.04	8.53E-01	-0.01	9.55E-01	-0.15	4.52E-01	-0.48	1.21E-02	0.05	7.91E-01
ENSCAFG0000000840	EIF2A	red	VSMC_M1	0.63	4.83E-04	0.13	5.14E-01	0.10	6.31E-01	-0.26	1.94E-01	0.37	6.05E-02	-0.31	1.13E-01	-0.25	2.13E-01	0.10	6.11E-01	-0.19	3.47E-01	-0.39	4.48E-02
ENSCAFG0000001820	B9D1	red	VSMC_M1	0.63	4.85E-04	0.52	5.63E-03	0.19	3.39E-01	0.19	3.49E-01	0.17	3.84E-01	-0.38	5.26E-02	-0.16	4.12E-01	-0.37	5.81E-02	-0.59	1.10E-03	0.10	6.19E-01
ENSCAFG0000000054	MTRF1L	red	VSMC_M1	0.63	4.91E-04	-0.18	3.56E-01	-0.01	9.46E-01	-0.11	5.92E-01	0.36	6.55E-02	-0.10	6.33E-01	0.13	5.23E-01	0.14	4.81E-01	-0.04	8.25E-01	-0.33	9.09E-02
ENSCAFG0000001869	CLPP	cyan	VSMC_M2	0.62	4.94E-04	0.61	7.99E-04	0.29	1.45E-01	0.23	2.50E-01	-0.09	6.62E-01	-0.07	7.30E-01	-0.34	8.01E-02	-0.40	3.65E-02	-0.71	2.80E-05	0.39	4.69E-02
ENSCAFG0000001474	FKBP8	grey	VSMC_M10	0.62	5.01E-04	0.80	4.70E-07	0.32	1.04E-01	0.34	8.67E-02	-0.01	9.63E-01	-0.30	1.27E-01	-0.39	4.21E-02	-0.63	4.44E-04	-0.75	5.40E-06	0.37	5.58E-02
ENSCAFG0000001852	USP22	red	VSMC_M1	0.62	5.07E-04	0.33	9.50E-02	0.03	8.83E-01	0.10	6.12E-01	0.37	6.00E-02	-0.15	4.63E-01	-0.34	7.88E-02	-0.23	2.43E-01	-0.34	8.63E-02	-0.12	5.54E-01
ENSCAFG0000001391	LPP	red	VSMC_M1	0.62	5.22E-04	0.44	2.15E-02	0.11	5.98E-01	0.19	3.44E-01	0.27	1.75E-01	-0.14	4.87E-01	-0.53	4.44E-03	-0.41	3.20E-02	-0.41	3.35E-02	-0.01	9.42E-01
ENSCAFG0000000602	ENSCAFG0000000602	red	VSMC_M1	0.62	5.24E-04	0.33	9.36E-02	0.37	5.54E-02	-0.22	2.68E-01	-0.08	6.75E-01	-0.14	5.01E-01	0.19	3.51E-01	0.06	7.85E-01	-0.52	5.39E-03	0.02	9.40E-01
ENSCAFG0000000104	NDUFB9	yellow	VSMC_M3	0.62	5.31E-04	0.45	1.74E-02	0.62	5.32E-04	-0.07	7.46E-01	-0.31	1.13E-01	-0.12	5.68E-01	0.03	8.99E-01	-0.08	6.98E-01	-0.57	1.74E-03	0.05	7.91E-01
ENSCAFG0000002009	ZNRF1	yellow	VSMC_M3	0.62	5.32E-04	0.55	3.26E-03	0.45	1.82E-02	0.04	8.49E-01	-0.25	2.15E-01	0.15	4.52E-01	-0.16	4.25E-01	-0.22	2.63E-01	-0.67	1.49E-04	0.27	1.67E-01
ENSCAFG0000000702	CXCL12	red	VSMC_M1	0.62	5.36E-04	0.59	1.22E-03	0.51	6.48E-03	-0.23	2.51E-01	-0.25	2.08E-01	-0.07	7.11E-01	0.01	9.68E-01	0.06	7.76E-01	-0.77	2.25E-06	0.17	3.87E-01
ENSCAFG0000001390	AP1S1	red	VSMC_M1	0.62	5.40E-04	0.52	5.25E-03	0.41	3.35E-02	0.05	8.22E-01	-0.25	2.09E-01	0.01	9.53E-01	0.04	8.57E-01	-0.17	3.89E-01	-0.66	1.71E-04	0.34	8.69E-02
ENSCAFG0000000845	PCGF1	pink	VSMC_M5	0.62	5.41E-04	-0.18	3.61E-01	-0.11	5.84E-01	-0.16	4.34E-01	0.47	1.36E-02	-0.06	7.75E-01	0.03	8.78E-01	0.21	2.88E-01	-0.04	8.43E-01	-0.43	2.70E-02
ENSCAFG0000001410	SMARCB1	yellow	VSMC_M3	0.62	5.44E-04	0.18	3.58E-01	0.69	6.12E-05	-0.69	5.79E-05	-0.33	9.30E-02	0.10	6.23E-01	0.15	4.65E-01	0.54	3.73E-03	-0.40	3.62E-02	-0.31	1.15E-01
ENSCAFG0000002338	CTXN1	yellow	VSMC_M3	0.62	5.49E-04	0.42	2.83E-02	0.43	2.43E-02	-0.06	7.48E-01	-0.06	7.72E-01	-0.24	2.36E-01	-0.21	2.89E-01	-0.21	2.91E-01	-0.44	2.33E-02	-0.01	9.54E-01
ENSCAFG0000001157	ADNP	grey	VSMC_M10	0.62	5.50E-04	0.31	1.16E-01	-0.28	1.59E-01	0.30	1.24E-01	0.58	1.44E-03	-0.34	8.72E-02	-0.20	3.18E-01	-0.38	4.81E-02	-0.39	4.50E-02	0.09	6.40E-01
ENSCAFG0000001801	RNF138	red	VSMC_M1	0.62	5.51E-04	0.34	7.87E-02	0.45	1.95E-02	-0.26	1.88E-01	-0.20	3.07E-01	0.14	4.98E-01	-0.03	8.71E-01	0.13	5.31E-01	-0.57	1.92E-03	0.03	8.66E-01
ENSCAFG0000001328	GLRX3	pink	VSMC_M5	0.62	5.54E-04	-0.10	6.19E-01	0.01	9.80E-01	-0.21	2.88E-01	0.42	2.90E-02	-0.02	9.26E-01	-0.15	4.57E-01	0.19	3.50E-01	-0.01	9.71E-01	-0.53	4.62E-03
ENSCAFG0000000533	EBNA1BP2	red	VSMC_M1	0.62	5.54E-04	-0.03	8.67E-01	0.36	6.80E-02	-0.48	1.05E-02	0.08	7.08E-01	-0.15	4.68E-01	0.15	4.58E-01	0.36	6.40E-02	-0.13	5.28E-01	-0.45	1.95E-02
ENSCAFG0000000453	NR2C2	red	VSMC_M1	0.62	5.60E-04	0.32	1.05E-01	-0.09	6.52E-01	0.18	3.66E-01	0.39	4.39E-02	-0.08	6.89E-01	-0.12	5.37E-01	-0.31	1.21E-01	-0.41	3.57E-02	0.11	5.94E-01
ENSCAFG0000001442	EMG1	red	VSMC_M1	0.62	5.61E-04	0.03	8.72E-01	0.56	2.53E-03	-0.69	6.02E-05	-0.13	5.14E-01	0.00	9.90E-01	0.13	5.20E-01	0.56	2.53E-03	-0.24	2.29E-01	-0.54	3.96E-03
ENSCAFG0000001597	CLCN5	red	VSMC_M1	0.62	5.68E-04	0.38	4.90E-02	0.35	7.28E-02	-0.19	3.45E-01	-0.02	9.02E-01	0.05	8.04E-01	-0.03	8.91E-01	0.01	9.49E-01	-0.55	2.82E-03	0.03	8.74E-01
ENSCAFG0000000846	EDIL3	red	VSMC_M1	0.62	5.77E-04	-0.02	9.29E-01	0.04	8.48E-01	-0.17	3.98E-01	0.30	1.35E-01	-0.27	1.67E-01	0.39	4.22E-02	0.14	4.72E-01	-0.18	3.60E-01	-0.19	3.33E-01
ENSCAFG0000003117	CNIH1	red	VSMC_M1	0.62	5.86E-04	-0.02	9.28E-01	0.03	8.98E-01	0.10	6.27E-01	0.30	1.28E-01	-0.17	3.87E-01	-0.37	5.56E-02	-0.09	6.64E-01	-0.09	6.38E-01	-0.22	2.78E-01
ENSCAFG0000001024	ARL3	red	VSMC_M1	0.62	5.87E-04	0.27	1.73E-01	0.03	8.73E-01	0.05	8.14E-01	0.15	4.50E-01	-0.06	7.75E-01	0.08	6.93E-01	-0.09	6.69E-01	-0.49	8.82E-03	0.14	4.95E-01
ENSCAFG0000000865	ENSCAFG0000000865	red	VSMC_M1	0.62	6.00E-04	0.29	1.45E-01	0.23	2.41E-01	0.01	9.59E-01	0.02	9.11E-01	-0.15	4.59E-01	-0.14	4.95E-01	-0.13	5.21E-01	-0.40	3.77E-02	0.05	7.90E-01
ENSCAFG0000000698	C27H12orf10	yellow	VSMC_M3	0.62	6.01E-04	0.43	2.60E-02	0.79	1.13E-06	-0.36	6.84E-02	-0.50	7.46E-03	-0.02	9.34E-01	0.03	8.93E-01	0.15	4.53E-01	-0.58	1.66E-03	0.02	9.24E-01
ENSCAFG0000003015	ENSCAFG0000003015	red	VSMC_M1	0.62	6.02E-04	0.25	2.08E-01	0.26	1.84E-01	-0.18	3.65E-01	0.12	5.51E-01	-0.20	3.29E-01	-0.05	8.08E-01	0.05	8.14E-01	-0.35	7.12E-02	-0.25	2.08E-01
ENSCAFG0000001204	RAE1	red	VSMC_M1	0.62	6.05E-04	-0.22	2.81E-01	0.04	8.46E-01	-0.30	1.26E-01	0.28	1.61E-01	0.08	6.87E-01	0.09	6.45E-01	0.38	4.79E-02	-0.05	8.23E-01	-0.42	2.94E-02
ENSCAFG0000000654	ENSCAFG0000000654	pink	VSMC_M5	0.62	6.06E-04	-0.25	2.11E-01	0.13	5.20E-01	-0.46	1.58E-02	0.34	8.58E-02	-0.24	2.30E-01	-0.01	9.57E-01	0.50	8.19E-03	0.06	7.67E-01	-0.69	6.82E-05
ENSCAFG0000000345	ENSCAFG0000000345	red	VSMC_M1	0.62	6.08E-04	0.35	7.73E-02	0.22	2.68E-01	-0.21	2.82E-01	0.12	5.59E-01	-0.13	5.17E-01	-0.20	3.27E-01	-0.01	9.67E-01	-0.38	4.92E-02	-0.03	8.66E-01
ENSCAFG0000001509	ENSCAFG0000001509	red	VSMC_M1	0.62	6.09E-04	0.12	5.44E-01	0.42	2.92E-02	-0.42	2.97E-02	0.03	8.76E-01	-0.15	4.60E-01	-0.17	3.95E-01	0.22	2.74E-01	-0.23	2.50E-01	-0.39	4.49E-02
ENSCAFG0000000043	FRS2	grey	VSMC_M10	0.62	6.10E-04	0.49	9.77E-03	-0.28	1.62E-01	0.57	1.73E-03	0.47	1.28E-02	-0.24	2.20E-01	-0.25	2.09E-01	-0.68	9.09E-05	-0.54	3.39E-03	0.41	3.49E-02
ENSCAFG0000000990	DPCD	grey	VSMC_M10	0.62	6.15E-04	0.21	3.02E-01	0.26	1.92E-01	-0.02	9.20E-01	-0.06	7.50E-01	-0.09	6.53E-01	0.11	5.76E-01	0.00	9.97E-01	-0.40	3.76E-02	0.13	5.22E-01
ENSCAFG0000000717	PCCB	yellow	VSMC_M3	0.62	6.24E-04	0.19	3.40E-01	0.55	2.72E-03	-0.33	9.30E-02	-0.22	2.76E-01	-0.02	9.25E-01	0.32	9.88E-02	0.25	2.13E-01	-0.41	3.14E-02	-0.17	3.96E-01
ENSCAFG0000000867	CCNI	red	VSMC_M1	0.62	6.27E-04	0.26	1.89E-01	-0.27	1.67E-01	0.29	1.38E-01	0.68	8.19E-05	-0.27	1.77E-01	-0.36	6.45E-02	-0.42	2.89E-02	-0.25	2.04E-01	-0.12	5.39E-01
ENSCAFG0000000829	GFM2	pink	VSMC_M5	0.62	6.28E-04	-0.07	7.24E-01	-0.22	2.75E-01	0.13	5.22E-01	0.63	4.59E-04	-0.33	9.75E-02	0.06	7.66E-01	-0.08	6.83E-01	-0.12	5.59E-01	-0.33	9.23E-02
ENSCAFG0000000160	ENSCAFG0000000160	red	VSMC_M1	0.62	6.38E-04	0.37	5.89E-02	0.12	5.60E-01	0.25	2.14E-01	0.20	3.09E-01	-0.07	7.12E-01	-0.29	1.49E-01	-0.35	7.17E-02	-0.43	2.38E-02	0.02	9.23E-01
ENSCAFG0000003147	ENSCAFG0000003147	red	VSMC_M1	0.61	6.51E-04	0.15	4.66E-01	-0.06	7.47E-01	0.05	7.88E-01	0.44	2.19E-02	-0.25	2.09E-01	-0.15	4.62E-01	-0.15	4.42E-01	-0.21	2.83E-01	-0.18	3.56E-01
ENSCAFG0000001673	PRCC	cyan	VSMC_M2	0.61	6.60E-04	0.53	4.27E-03	0.01	9.58E-01	0.33	9.67E-02	0.29	1.47E-01	-0.25	2.03E-01	-0.20	3.06E-01	-0.47	1.43E-02	-0.60	1.04E-03	0.15	4.44E-01
ENSCAFG0000000452	VIM	cyan	VSMC_M2	0.61	6.62E-04	0.60	8.62E-04	0.33	9.40E-02	0.11	5.79E-01	0.01	9.70E-01	-0.43	2.67E-02	-0.32	1.04E-01	-0.26	1.95E-01	-0.64	3.17E-04	0.12	5.44E-01
ENSCAFG0000001586	ENSCAFG0000001586	cyan	VSMC_M2	0.61	6.65E-04	0.52	5.39E-03	0.25	2.07E-01	0.19	3.50E-01	-0.06	7.70E-01	0.13	5.10E-01	-0.07	7.21E-01	-0.32	1.02E-01	-0.67	1.35E-04	0.32	1.02E-01
ENSCAFG0000001999	PTPA	red	VSMC_M1	0.61	6.65E-04	-0.04	8.62E-01	0.39	4.60E-02	-0.57	1.75E-03	0.03	8.67E-01	-0.15	4.55E-01	0.19	3.51E-01	0.50	7.65E-03	-0.16	4.29E-01	-0.53	4.40E-03
ENSCAFG0000000880	ENSCAFG0000000880	red	VSMC_M1	0.61	6.69E-04	0.01	9.56E-01	0.14	4.75E-01	-0.09	6.44E-01	0.22	2.60E-01	-0.08	6.74E-01	-0.19	3.44E-01	0.07	7.36E-01	-0.15	4.49E-01	-0.32	1.05E-01
ENSCAFG0000000895	SPR	grey	VSMC_M10	0.61	6.84E-04	0.25	2.02E-01	-0.14	4.80E-01	0.28	1.60E-01	0.29	1.47E-01	0.05	8.19E-01	0.05	8.09E-01	-0.27	1.66E-01	-0.44	2.05E-02	0.27	1.79E-01
ENSCAFG0000001623	DCP2	red	VSMC_M1	0.61	6.96E-04	0.35	7.54E-02	0.51	6.81E-03	-0.21	2.87E-01	-0.25	2.00E-01	0.01	9.58E-01	-0.01	9.54E-01	0.09	6.39E-01	-0.55	3.06E-03	0.06	7.70E-01
ENSCAFG0000000830	C31H21orf91	red	VSMC_M1	0.61	7.15E-04	0.13	5.34E-01	0.11	5.86E-01	0.00	9.81E-01	0.24	2.23E-01	-0.08	6.77E-01	-0.33	9.24E-02	-0.04	8.38E-01	-0.24	2.22E-01	-0.19	3.42E-01
ENSCAFG0000003189	RITA1	red	VSMC_M1	0.61	7.17E-04	0.44	2.13E-02	0.14	4.84E-01	0.09	6.59E-01	0.05	8.23E-01	0.01	9.70E-01	-0.10	6.22E-01	-0.24	2.20E-01	-0.55	2.74E-03	0.30	1.34E-01
ENSCAFG0000003145	ENSCAFG0000003145	cyan	VSMC_M2	0.61	7.23E-04	0.57	2.00E-03	0.21	3.01E-01	0.29	1.44E-01	0.02	9.08E-01	-0.09	6.39E-01	-0.41	3.58E-02	-0.43	2.53E-02	-0.62	5.85E-04	0.23	2.39E-01
ENSCAFG0000000831	ENSCAFG0000000831	pink	VSMC_M5	0.61	7.39E-04	-0.32	1.05E-01	-0.09	6.73E-01	-0.29	1.36E-01	0.53	4.25E-03	-0.23	2.41E-01	-0.07	7.24E-01	0.36	6.44E-02	0.14	4.98E-01	-0.66	1.92E-04
ENSCAFG0000000342	GRB10	grey	VSMC_M10	0.61	7.46E-04	0.64	3.63E-04	0.05	7.93E-01	0.54	3.50E-03	0.16	4.39E-01	-0.14	4.89E-01	-0.44	2.31E-02	-0.67	1.33E-04	-0.66	1.96E-04	0.43	2.42E-02
ENSCAFG0000001476	CCDC56	grey	VSMC_M10	0.61	7.48E-04	0.58	1.52E-03	0.55	3.25E-03	-0.05	7.96E-01	-0.36	6.86E-02	-0.07	7.43E-01	0.02	9.10E-01	-0.14	4.80E-01	-0.75	7.68E-06	0.33	9.12E-02
ENSCAFG0000001374	MAP2	red	VSMC_M1	0.61	7.50E-04	0.30	1.28E-01	-0.16	4.22E-01	0.14	4.86E-01	0.52	5.51E-03	-0.27	1.66E-01	-0.26	1.99E-01	-0.27	1.74E-01	-0.37	6.08E-02	-0.02	9.09E-01
ENSCAFG0000000738	COPS5	red	VSMC_M1	0.61	7.50E-04	-0.05	8.06E-01	-0.05	7.99E-01	0.08	6.82E-01	0.46	1.58E-02	-0.30	1.24E-01	-0.05	7.95E-01	-0.09	6.73E-01	-0.10	6.18E-01	-0.33	8.96E-02
ENSCAFG0000000610	POC1B	cyan	VSMC_M2	0.61	7.51E-04	0.58	1.61E-03	0.17	4.10E-01	0.31	1.21E-01	0.13	5.13E-01	-0.24	2.24E-01	-0.25	2.16E-01	-0.48	1.16E-02	-0.60	8.29E-04	0.30	1.24E-01
ENSCAFG0000000791	ENSCAFG0000000791	red	VSMC_M1	0.61	7.52E-04	0.45	1.98E-02	-0.03	8.65E-01	0.22	2.71E-01	0.28	1.58E-01	-0.17	4.09E-01	-0.14	4.83E-01	-0.35	7.19E-02	-0.56	2.50E-03	0.24	2.30E-01
ENSCAFG0000001808	RBM22	yellow	VSMC_M3	0.61	7.56E-04	0.47	1.32E-02	0.58	1.43E-03	-0.40	3.69E-02	-0.26	1.91E-01	-0.07	7.28E-01	0.15	4.44E-01	0.14	4.93E-01	-0.59	1.07E-03	-0.02	9.37E-01
ENSCAFG0000000893	SMYD5	red	VSMC_M1	0.61	7.57E-04	-0.12	5.35E-01	0.42	2.73E-02	-0.60	1.02E-03	-0.01	9.72E-01	0.02	9.28E-01	0.07	7.44E-01	0.54	3.31E-03	-0.10	6.13E-01	-0.63	4.15E-04
ENSCAFG0000000103	HDDC2	cyan	VSMC_M2	0.61	7.58E-04	0.60	1.03E-03	0.35	6.94E-02	0.19	3.52E-01	-0.08	6.91E-01	-0.08	7.00E-01	-0.28	1.54E-01	-0.32	1.00E-01	-0.68	9.73E-05	0.17	3.90E-01
ENSCAFG0000000486	AAMDC	grey	VSMC_M10	0.61	7.66E-04	0.26	1.92E-01	0.26	1.99E-01	0.14	4.98E-01	-0.03	8.94E-01	0.11	5.74E-01	-0.01	9.43E-01	-0.16	4.22E-01	-0.42	2.99E-02	0.06	7.61E-01
ENSCAFG0000000918	ANKRD2	yellow	VSMC_M3	0.61	7.85E-04	0.51	6.45E-03	0.68	8.66E-05	-0.43	2.63E-02	-0.33	9.20E-02	0.09	6.54E-01	-0.17	4.06E-01	0.14	4.94E-01	-0.60	1.03E-03	-0.08	6.83E-01
ENSCAFG0000002598	ENSCAFG0000002598	yellow	VSMC_M3	0.61	7.85E-04	0.56	2.65E-03	0.56	2.58E-03	-0.21	3.04E-01	-0.27	1.72E-01	-0.04	8.30E-01	-0.23	2.49E-01	-0.06	7.61E-01	-0.64	3.29E-04	0.08	7.04E-01
ENSCAFG0000001747	FEM1B	pink	VSMC_M5	0.61	7.91E-04	-0.23	2.50E-01	-0.29	1.42E-01	-0.01	9.44E-01	0.68	9.78E-05	-0.10	6.35E-01	0.01	9.42E-01	0.09	6.70E-01	0.07	7.30E-01	-0.44	2.33E-02
ENSCAFG0000001812	CHST9	red	VSMC_M1	0.61	7.95E-04	0.09	6.52E-01	0.16	4.31E-01	-0.18	3.76E-01	0.24	2.33E-01	0.04	8.41E-01	0.23	2.56E-01	0.05	7.86E-01	-0.24	2.29E-01	-0.28	1.56E-01
ENSCAFG0000000877	ENSCAFG0000000877	red	VSMC_M1	0.61	7.95E-04	0.53	4.71E-03	0.23	2.45E-01	0.26	1.94E-01	-0.06	7.55E-01	-0.12	5.56E-01	-0.19	3.54E-01	-0.37	5.73E-02	-0.64	3.21E-04	0.41	3.19E-02
ENSCAFG0000001018	CUEDC2	red	VSMC_M1	0.61	7.98E-04	0.20	3.12E-01	0.29	1.39E-01	-0.29	1.45E-01	0.22	2.69E-01	-0.49	8.77E-03	-0.20	3.10E-01	0.08	6.84E-01	-0.24	2.30E-01	-0.49	9.59E-03
ENSCAFG0000000021	SGK1	red	VSMC_M1	0.61	8.06E-04	0.39	4.25E-02	0.19	3.49E-01	0.11	5.72E-01	0.00	9.88E-01	0.16	4.28E-01	-0.41	3.43E-02	-0.18	3.74E-01	-0.53	4.92E-03	0.20	3.07E-01
ENSCAFG0000001735	ENSCAFG0000001735	yellow	VSMC_M3	0.61	8.22E-04	0.38	5.23E-02	0.59	1.11E-03	-0.17	3.95E-01	-0.20	3.24E-01	-0.13	5.34E-01	-0.14	4.74E-01	-0.04	8.37E-01	-0.44	2.01E-02	-0.11	5.79E-01
ENSCAFG0000001090	B4GALT4	cyan	VSMC_M2	0.61	8.25E-04	0.71	3.89E-05	0.46	1.54E-02	-0.11	5.97E-01	-0.16	4.31E-01	-0.21	2.91E-01	-0.18	3.77E-01	-0.19	3.34E-01	-0.75	7.35E-06	0.17	3.89E-01
ENSCAFG0000001606	RARA	red	VSMC_M1	0.61	8.27E-04	0.42	3.09E-02	0.05	8.06E-01	0.23	2.41E-01	0.18	3.56E-01	-0.04	8.49E-01	-0.19	3.31E-01	-0.36	6.79E-02	-0.45	1.76E-02	0.27	1.73E-01
ENSCAFG0000001496	DYNC2H1	red	VSMC_M1	0.60	8.30E-04	0.14	4.98E-01	-0.32	1.01E-01	0.24	2.29E-01	0.65	2.31E-04	-0.29	1.48E-01	-0.15	4.69E-01	-0.29	1.44E-01	-0.21	3.05E-01	-0.07	7.46E-01
ENSCAFG0000001012	PSPH	red	VSMC_M1	0.60	8.33E-04	0.01	9.69E-01	-0.13	5.33E-01	0.20	3.06E-01	0.51	6.29E-03	-0.41	3.17E-02	0.02	9.10E-01	-0.18	3.59E-01	-0.17	4.04E-01	-0.18	3.57E-01
ENSCAFG0000000446	SDK2	red	VSMC_M1	0.60	8.42E-04	-0.02	9.10E-01	0.05	8.02E-01	-0.06	7.75E-01	0.34	7.88E-02	-0.20	3.07E-01	-0.38	5.29E-02	0.04	8.56E-01	-0.07	7.26E-01	-0.36	6.55E-02
ENSCAFG0000000365	PIH1D1	red	VSMC_M1	0.60	8.55E-04	0.50	7.35E-03	0.50	8.07E-03	-0.09	6.39E-01	-0.21	2.87E-01	-0.28	1.58E-01	0.00	9.84E-01	-0.09	6.39E-01	-0.63	4.15E-04	0.14	4.96E-01
ENSCAFG0000001341	RGL1	cyan	VSMC_M2	0.60	8.55E-04	0.54	3.51E-03	0.28	1.51E-01	0.07	7.43E-01	0.04	8.62E-01	-0.08	6.88E-01	0.04	8.50E-01	-0.27	1.69E-01	-0.60	9.38E-04	0.11	5.73E-01
ENSCAFG0000001536	BRI3	yellow	VSMC_M3	0.60	8.60E-04	0.57	1.83E-03	0.69	5.98E-05	-0.33	9.71E-02	-0.34	7.92E-02	-0.19	3.37E-01	0.07	7.19E-01	0.00	9.98E-01	-0.63	3.85E-04	0.02	9.34E-01
ENSCAFG0000000507	R3HDM1	red	VSMC_M1	0.60	8.72E-04	0.20	3.28E-01	0.15	4.62E-01	-0.12	5.64E-01	0.10	6.10E-01	-0.03	8.70E-01	0.02	9.11E-01	0.04	8.61E-01	-0.35	7.07E-02	0.05	7.94E-01
ENSCAFG0000000134	NAA35	red	VSMC_M1	0.60	8.73E-04	0.04	8.47E-01	0.27	1.72E-01	-0.30	1.24E-01	0.07	7.19E-01	-0.05	7.92E-01	0.09	6.72E-01	0.24	2.37E-01	-0.19	3.49E-01	-0.29	1.40E-01
ENSCAFG0000001952	KCNAB2	red	VSMC_M1	0.60	8.99E-04	0.09	6.49E-01	0.26	1.99E-01	-0.26	1.98E-01	0.02	9.20E-01	-0.01	9.80E-01	0.32	1.02E-01	0.26	1.96E-01	-0.35	7.01E-02	-0.11	5.85E-01
ENSCAFG0000000459	UBP1	red	VSMC_M1	0.60	9.04E-04	-0.03	8.68E-01	0.41	3.15E-02	-0.66	1.74E-04	0.03	8.68E-01	-0.04	8.33E-01	-0.01	9.44E-01	0.60	1.02E-03	-0.17	3.94E-01	-0.58	1.45E-03
ENSCAFG0000000084	FGFR1OP	red	VSMC_M1	0.60	9.16E-04	0.22	2.75E-01	0.10	6.28E-01	-0.08	7.05E-01	0.12	5.42E-01	0.02	9.12E-01	0.23	2.57E-01	-0.02	9.25E-01	-0.44	2.15E-02	0.11	5.91E-01
ENSCAFG0000000292	PGM3	red	VSMC_M1	0.60	9.22E-04	-0.09	6.51E-01	-0.09	6.42E-01	0.01	9.80E-01	0.47	1.26E-02	-0.31	1.17E-01	-0.31	1.14E-01	0.04	8.49E-01	0.00	9.87E-01	-0.38	5.35E-02
ENSCAFG0000001135	COX19	red	VSMC_M1	0.60	9.25E-04	0.15	4.49E-01	0.03	8.98E-01	0.03	8.95E-01	0.22	2.68E-01	0.01	9.43E-01	0.20	3.15E-01	-0.07	7.24E-01	-0.38	5.24E-02	-0.01	9.57E-01
ENSCAFG0000000828	ENSCAFG0000000828	grey	VSMC_M10	0.60	9.35E-04	0.09	6.70E-01	0.20	3.13E-01	-0.33	9.10E-02	0.11	5.81E-01	-0.11	5.95E-01	-0.12	5.53E-01	0.33	9.34E-02	-0.32	1.00E-01	-0.26	1.98E-01
ENSCAFG0000000375	ZFYVE9	pink	VSMC_M5	0.60	9.38E-04	-0.12	5.51E-01	-0.34	8.07E-02	0.21	2.86E-01	0.74	1.13E-05	-0.32	1.06E-01	-0.20	3.16E-01	-0.12	5.60E-01	0.01	9.48E-01	-0.38	5.25E-02
ENSCAFG0000001979	SARDH	grey	VSMC_M10	0.60	9.45E-04	0.82	1.69E-07	0.24	2.31E-01	0.41	3.37E-02	-0.02	9.15E-01	-0.20	3.17E-01	-0.20	3.27E-01	-0.67	1.36E-04	-0.83	6.26E-08	0.56	2.52E-03
ENSCAFG0000001994	USP10	red	VSMC_M1	0.60	9.46E-04	-0.08	6.91E-01	0.23	2.40E-01	-0.35	7.56E-02	0.05	8.22E-01	0.29	1.42E-01	0.14	4.92E-01	0.37	5.70E-02	-0.16	4.35E-01	-0.27	1.66E-01
ENSCAFG0000000461	RAB30	red	VSMC_M1	0.60	9.60E-04	0.47	1.24E-02	-0.01	9.59E-01	0.17	4.05E-01	0.21	3.04E-01	-0.14	4.90E-01	-0.01	9.61E-01	-0.29	1.39E-01	-0.61	6.99E-04	0.27	1.73E-01
ENSCAFG0000001091	ENSCAFG0000001091	yellow	VSMC_M3	0.60	9.80E-04	0.38	4.96E-02	0.69	6.97E-05	-0.40	3.80E-02	-0.34	8.18E-02	-0.04	8.59E-01	0.03	8.69E-01	0.19	3.39E-01	-0.54	3.44E-03	-0.12	5.46E-01
ENSCAFG0000001392	ARNT2	yellow	VSMC_M3	0.60	9.96E-04	0.42	3.04E-02	0.65	2.55E-04	-0.34	7.87E-02	-0.27	1.76E-01	0.03	8.80E-01	-0.25	2.16E-01	0.07	7.25E-01	-0.44	2.13E-02	-0.17	4.07E-01
ENSCAFG0000001368	USP28	red	VSMC_M1	0.60	1.00E-03	0.18	3.56E-01	0.05	8.20E-01	-0.15	4.61E-01	0.37	5.78E-02	-0.25	2.08E-01	-0.03	8.81E-01	0.02	9.38E-01	-0.31	1.10E-01	-0.21	2.95E-01
ENSCAFG0000000975	ELOVL2	red	VSMC_M1	0.60	1.00E-03	0.33	9.59E-02	0.48	1.12E-02	-0.35	6.92E-02	-0.01	9.69E-01	-0.18	3.61E-01	0.09	6.73E-01	0.13	5.19E-01	-0.41	3.45E-02	-0.30	1.30E-01
ENSCAFG0000003119	TMEM189	red	VSMC_M1	0.60	1.00E-03	0.11	5.75E-01	0.25	2.11E-01	-0.32	1.02E-01	0.10	6.12E-01	-0.06	7.80E-01	-0.06	7.60E-01	0.15	4.64E-01	-0.21	3.01E-01	-0.23	2.46E-01
ENSCAFG0000000823	DLA-79	grey	VSMC_M10	0.60	1.01E-03	-0.14	4.76E-01	-0.23	2.54E-01	0.19	3.35E-01	0.52	5.53E-03	-0.11	5.75E-01	0.06	7.85E-01	-0.10	6.25E-01	-0.05	7.92E-01	-0.18	3.80E-01
ENSCAFG0000000892	MLF1	pink	VSMC_M5	0.60	1.02E-03	-0.16	4.16E-01	-0.35	7.00E-02	0.23	2.44E-01	0.75	6.81E-06	-0.24	2.33E-01	-0.05	8.04E-01	-0.16	4.11E-01	0.06	7.61E-01	-0.35	7.44E-02
ENSCAFG0000001351	REXO2	red	VSMC_M1	0.60	1.02E-03	-0.26	1.97E-01	-0.07	7.46E-01	-0.04	8.50E-01	0.41	3.38E-02	-0.27	1.74E-01	0.12	5.40E-01	0.13	5.18E-01	0.03	8.72E-01	-0.36	6.92E-02
ENSCAFG0000001207	LIMK1	yellow	VSMC_M3	0.60	1.02E-03	0.62	6.28E-04	0.80	5.14E-07	-0.33	8.77E-02	-0.48	1.04E-02	-0.14	4.91E-01	-0.15	4.44E-01	0.06	7.80E-01	-0.70	5.31E-05	0.09	6.41E-01
ENSCAFG0000000970	ENSCAFG0000000970	pink	VSMC_M5	0.60	1.03E-03	0.21	2.87E-01	-0.34	8.12E-02	0.43	2.60E-02	0.65	2.14E-04	-0.23	2.42E-01	-0.35	7.37E-02	-0.42	2.76E-02	-0.29	1.48E-01	0.01	9.72E-01
ENSCAFG0000002972	C1H9orf40	yellow	VSMC_M3	0.60	1.03E-03	0.30	1.31E-01	0.56	2.26E-03	-0.53	4.55E-03	-0.22	2.67E-01	-0.19	3.31E-01	0.16	4.11E-01	0.27	1.70E-01	-0.46	1.62E-02	-0.16	4.15E-01
ENSCAFG0000000426	ENSCAFG0000000426	cyan	VSMC_M2	0.60	1.03E-03	0.55	2.97E-03	0.37	5.76E-02	0.22	2.74E-01	-0.09	6.59E-01	-0.13	5.17E-01	-0.17	4.07E-01	-0.38	5.18E-02	-0.62	5.05E-04	0.20	3.18E-01
ENSCAFG0000001939	EHMT1	yellow	VSMC_M3	0.60	1.04E-03	0.51	6.80E-03	0.62	6.14E-04	-0.11	5.80E-01	-0.36	6.33E-02	-0.22	2.67E-01	-0.05	7.87E-01	-0.10	6.31E-01	-0.65	2.21E-04	0.18	3.60E-01
ENSCAFG0000002876	ENSCAFG0000002876	cyan	VSMC_M2	0.60	1.05E-03	0.73	1.34E-05	0.55	3.04E-03	0.08	6.86E-01	-0.29	1.45E-01	-0.12	5.51E-01	-0.19	3.49E-01	-0.33	9.54E-02	-0.77	2.39E-06	0.30	1.30E-01
ENSCAFG0000002812	ENSCAFG0000002812	red	VSMC_M1	0.60	1.05E-03	0.17	3.96E-01	0.38	5.16E-02	-0.32	1.08E-01	0.02	9.10E-01	-0.19	3.51E-01	-0.08	7.00E-01	0.17	3.87E-01	-0.27	1.66E-01	-0.33	8.79E-02
ENSCAFG0000001960	HAGHL	yellow	VSMC_M3	0.60	1.05E-03	0.62	5.16E-04	0.63	4.16E-04	-0.20	3.16E-01	-0.31	1.11E-01	-0.03	8.73E-01	-0.24	2.25E-01	-0.04	8.62E-01	-0.72	1.98E-05	0.08	6.97E-01
ENSCAFG0000001139	SSPN	red	VSMC_M1	0.60	1.06E-03	0.28	1.61E-01	-0.08	6.93E-01	0.27	1.68E-01	0.33	9.39E-02	-0.11	5.96E-01	0.04	8.27E-01	-0.38	5.18E-02	-0.36	6.66E-02	0.16	4.37E-01
ENSCAFG0000000755	ENSCAFG0000000755	red	VSMC_M1	0.60	1.06E-03	0.19	3.33E-01	-0.06	7.54E-01	0.20	3.29E-01	0.37	5.69E-02	-0.03	8.75E-01	-0.42	2.72E-02	-0.23	2.50E-01	-0.25	2.09E-01	-0.07	7.14E-01
ENSCAFG0000002003	WWOX	red	VSMC_M1	0.59	1.06E-03	0.18	3.81E-01	0.39	4.53E-02	-0.37	5.85E-02	-0.05	8.02E-01	-0.01	9.63E-01	0.05	8.21E-01	0.25	2.04E-01	-0.36	6.46E-02	-0.21	2.82E-01
ENSCAFG0000003249	SF3B5	red	VSMC_M1	0.59	1.08E-03	0.26	1.96E-01	0.33	9.80E-02	-0.16	4.14E-01	-0.05	8.20E-01	0.01	9.74E-01	-0.22	2.71E-01	0.08	7.05E-01	-0.38	4.84E-02	-0.12	5.36E-01
ENSCAFG0000000011	CS	red	VSMC_M1	0.59	1.10E-03	0.46	1.46E-02	0.06	7.80E-01	0.15	4.61E-01	0.23	2.39E-01	-0.18	3.67E-01	-0.33	8.93E-02	-0.31	1.18E-01	-0.49	9.13E-03	0.10	6.07E-01
ENSCAFG0000000588	ENSCAFG0000000588	pink	VSMC_M5	0.59	1.11E-03	-0.23	2.57E-01	0.02	9.28E-01	-0.37	5.71E-02	0.39	4.43E-02	0.13	5.13E-01	-0.02	9.27E-01	0.38	4.83E-02	0.02	9.29E-01	-0.55	2.69E-03

ENSCAFG0000000645	CDK17	red	VSMC_M1	0.59	1.12E-03	-0.03	8.95E-01	-0.01	9.48E-01	0.03	8.80E-01	0.26	1.95E-01	0.33	8.91E-02	-0.08	6.99E-01	-0.02	9.18E-01	-0.16	4.16E-01	-0.18	3.57E-01
ENSCAFG0000001768	ENSCAFG0000001768	red	VSMC_M1	0.59	1.14E-03	0.11	5.73E-01	0.20	3.20E-01	-0.08	6.96E-01	0.17	4.11E-01	-0.37	5.90E-02	0.12	5.44E-01	0.03	8.78E-01	-0.24	2.21E-01	-0.26	1.84E-01
ENSCAFG0000000592	DCXR	cyan	VSMC_M2	0.59	1.15E-03	0.75	6.04E-06	0.37	6.04E-02	0.30	1.26E-01	-0.10	6.16E-01	-0.37	5.90E-02	-0.20	3.11E-01	-0.52	5.36E-03	-0.78	1.89E-06	0.39	4.73E-02
ENSCAFG0000000815	ELOC	red	VSMC_M1	0.59	1.15E-03	-0.12	5.57E-01	-0.23	2.48E-01	0.11	5.76E-01	0.48	1.13E-02	0.05	8.22E-01	-0.09	6.73E-01	-0.03	9.00E-01	-0.07	7.46E-01	-0.18	3.83E-01
ENSCAFG0000001862	ENSCAFG0000001862	pink	VSMC_M5	0.59	1.15E-03	-0.16	4.19E-01	-0.22	2.73E-01	0.01	9.45E-01	0.64	2.95E-04	-0.40	3.78E-02	-0.01	9.53E-01	0.00	9.90E-01	0.05	8.06E-01	-0.46	1.54E-02
ENSCAFG0000000011	MYL6B	cyan	VSMC_M2	0.59	1.16E-03	0.64	3.71E-04	0.39	4.32E-02	0.22	2.65E-01	-0.06	7.56E-01	-0.31	1.16E-01	-0.20	3.15E-01	-0.42	2.80E-02	-0.66	1.80E-04	0.19	3.54E-01
ENSCAFG0000001171	CCDC71	red	VSMC_M1	0.59	1.17E-03	0.37	5.85E-02	0.33	9.38E-02	-0.09	6.62E-01	-0.09	6.70E-01	-0.19	3.49E-01	0.13	5.27E-01	-0.05	7.85E-01	-0.53	4.24E-03	0.08	7.06E-01
ENSCAFG0000002957	COQ10A	red	VSMC_M1	0.59	1.17E-03	0.08	6.92E-01	0.54	3.38E-03	-0.55	3.26E-03	-0.17	3.90E-01	0.00	9.97E-01	0.27	1.80E-01	0.43	2.55E-02	-0.31	1.21E-01	-0.36	6.34E-02
ENSCAFG0000001072	GIT2	grey	VSMC_M10	0.59	1.18E-03	0.47	1.23E-02	-0.20	3.27E-01	0.66	2.02E-04	0.44	2.19E-02	-0.38	5.31E-02	-0.37	5.58E-02	-0.74	1.04E-05	-0.50	7.80E-03	0.34	7.93E-02
ENSCAFG0000001654	PCGF2	cyan	VSMC_M2	0.59	1.18E-03	0.60	8.42E-04	0.34	8.02E-02	0.00	9.84E-01	0.03	8.75E-01	-0.20	3.14E-01	-0.17	3.91E-01	-0.33	9.27E-02	-0.58	1.64E-03	0.10	6.07E-01
ENSCAFG0000000864	SPRED1	red	VSMC_M1	0.59	1.18E-03	0.22	2.74E-01	-0.22	2.77E-01	0.41	3.21E-02	0.40	3.73E-02	-0.14	4.88E-01	0.01	9.72E-01	-0.40	3.94E-02	-0.34	7.92E-02	0.20	3.06E-01
ENSCAFG0000003245	ENSCAFG0000003245	red	VSMC_M1	0.59	1.18E-03	-0.12	5.59E-01	0.01	9.69E-01	-0.03	8.64E-01	0.41	3.33E-02	-0.17	4.09E-01	-0.27	1.69E-01	0.09	6.61E-01	-0.01	9.42E-01	-0.48	1.10E-02
ENSCAFG0000001799	MID2	yellow	VSMC_M3	0.59	1.19E-03	0.40	4.05E-02	0.67	1.45E-04	-0.50	7.69E-03	-0.28	1.58E-01	-0.08	6.82E-01	0.19	3.45E-01	0.22	2.69E-01	-0.51	6.84E-03	-0.15	4.44E-01
ENSCAFG0000001241	CRTC3	red	VSMC_M1	0.59	1.19E-03	0.42	2.80E-02	0.00	9.90E-01	0.11	5.71E-01	0.32	1.04E-01	-0.11	5.89E-01	-0.35	7.12E-02	-0.30	1.30E-01	-0.44	2.04E-02	0.07	7.46E-01
ENSCAFG0000001858	ENSCAFG0000001858	yellow	VSMC_M3	0.59	1.19E-03	0.37	5.47E-02	0.46	1.49E-02	-0.18	3.58E-01	-0.16	4.36E-01	-0.08	7.00E-01	-0.31	1.14E-01	0.03	8.89E-01	-0.50	7.78E-03	-0.03	8.73E-01
ENSCAFG0000001071	KCTD3	pink	VSMC_M5	0.59	1.20E-03	-0.36	6.33E-02	-0.09	6.47E-01	-0.28	1.61E-01	0.52	5.60E-03	-0.10	6.25E-01	0.06	7.59E-01	0.32	9.84E-02	0.19	3.43E-01	-0.60	8.97E-04
ENSCAFG0000001340	ENSCAFG0000001340	red	VSMC_M1	0.59	1.20E-03	0.66	1.98E-04	0.67	1.19E-04	-0.26	1.90E-01	-0.46	1.55E-02	-0.01	9.79E-01	0.01	9.66E-01	0.01	9.53E-01	-0.83	1.05E-07	0.26	1.86E-01
ENSCAFG0000003202	GPR162	grey	VSMC_M10	0.59	1.24E-03	0.48	1.22E-02	0.33	9.16E-02	0.18	3.63E-01	-0.12	5.41E-01	-0.09	6.47E-01	-0.01	9.56E-01	-0.25	2.14E-01	-0.63	4.86E-04	0.23	2.49E-01
ENSCAFG0000000373	PARD3	red	VSMC_M1	0.59	1.25E-03	0.33	9.56E-02	0.34	8.12E-02	-0.27	1.73E-01	0.02	9.30E-01	-0.10	6.08E-01	0.25	2.00E-01	0.11	6.01E-01	-0.49	9.16E-03	-0.11	5.79E-01
ENSCAFG0000001324	CREBL2	pink	VSMC_M5	0.59	1.26E-03	0.11	5.69E-01	-0.37	5.63E-02	0.46	1.47E-02	0.67	1.15E-04	-0.30	1.28E-01	-0.19	3.40E-01	-0.44	2.30E-02	-0.21	2.91E-01	0.00	9.96E-01
ENSCAFG0000000459	CCDC90B	grey	VSMC_M10	0.59	1.26E-03	0.40	3.71E-02	-0.05	7.89E-01	0.43	2.35E-02	0.21	2.90E-01	-0.01	9.73E-01	0.02	9.34E-01	-0.46	1.52E-02	-0.57	1.83E-03	0.38	5.10E-02
ENSCAFG0000001304	EGR2	red	VSMC_M1	0.59	1.27E-03	0.38	5.32E-02	0.47	1.40E-02	-0.37	5.93E-02	-0.14	4.97E-01	-0.08	7.00E-01	0.25	2.07E-01	0.09	6.39E-01	-0.54	3.63E-03	-0.01	9.59E-01
ENSCAFG0000000495	NRBP1	red	VSMC_M1	0.59	1.27E-03	0.21	2.82E-01	0.40	4.14E-02	-0.33	9.64E-02	-0.03	8.66E-01	-0.10	6.18E-01	-0.21	2.98E-01	0.14	4.77E-01	-0.24	2.22E-01	-0.26	1.94E-01
ENSCAFG0000001898	C6H16orf72	red	VSMC_M1	0.59	1.29E-03	0.07	7.41E-01	-0.20	3.23E-01	0.19	3.34E-01	0.43	2.52E-02	0.07	7.17E-01	0.21	2.97E-01	-0.19	3.30E-01	-0.23	2.39E-01	-0.01	9.45E-01
ENSCAFG0000000270	ENSCAFG0000000270	red	VSMC_M1	0.59	1.29E-03	0.24	2.21E-01	0.37	6.10E-02	-0.10	6.21E-01	-0.05	7.88E-01	-0.06	7.50E-01	0.11	5.76E-01	-0.02	9.12E-01	-0.40	3.93E-02	-0.08	6.94E-01
ENSCAFG0000003203	APEX1	red	VSMC_M1	0.59	1.30E-03	0.05	8.03E-01	0.48	1.19E-02	-0.64	3.67E-04	-0.13	5.32E-01	0.05	8.22E-01	0.02	9.25E-01	0.55	3.27E-03	-0.29	1.41E-01	-0.44	2.27E-02
ENSCAFG0000001100	ENSCAFG0000001100	pink	VSMC_M5	0.59	1.31E-03	-0.17	3.98E-01	0.06	7.79E-01	-0.24	2.19E-01	0.37	5.54E-02	-0.20	3.08E-01	0.07	7.39E-01	0.23	2.53E-01	0.00	9.89E-01	-0.55	2.74E-03
ENSCAFG0000000379	WDR35	red	VSMC_M1	0.59	1.33E-03	0.12	5.46E-01	0.19	3.31E-01	-0.33	9.53E-02	0.19	3.35E-01	-0.30	1.34E-01	0.26	1.86E-01	0.23	2.44E-01	-0.31	1.13E-01	-0.24	2.24E-01
ENSCAFG0000001472	OTUB1	yellow	VSMC_M3	0.59	1.34E-03	0.39	4.26E-02	0.56	2.41E-03	-0.32	1.05E-01	-0.22	2.71E-01	0.11	5.75E-01	-0.22	2.68E-01	0.09	6.47E-01	-0.47	1.24E-02	-0.15	4.62E-01
ENSCAFG0000001279	PES1	red	VSMC_M1	0.59	1.34E-03	-0.03	8.80E-01	0.54	3.76E-03	-0.46	1.55E-02	-0.15	4.51E-01	-0.06	7.76E-01	0.05	8.05E-01	0.40	3.67E-02	-0.16	4.13E-01	-0.46	1.58E-02
ENSCAFG0000000451	STAM	red	VSMC_M1	0.58	1.35E-03	0.01	9.44E-01	-0.07	7.43E-01	-0.01	9.72E-01	0.48	1.19E-02	-0.17	4.00E-01	-0.17	3.94E-01	-0.13	5.09E-01	-0.08	6.80E-01	-0.32	1.04E-01
ENSCAFG0000000942	CNNM1	red	VSMC_M1	0.58	1.37E-03	0.26	1.82E-01	0.52	5.09E-03	-0.47	1.35E-02	-0.20	3.13E-01	0.10	6.03E-01	0.04	8.38E-01	0.21	3.03E-01	-0.40	3.65E-02	-0.20	3.26E-01
ENSCAFG0000001696	ENSCAFG0000001696	red	VSMC_M1	0.58	1.37E-03	0.46	1.68E-02	0.49	9.86E-03	-0.31	1.20E-01	-0.06	7.47E-01	-0.42	2.85E-02	-0.08	6.88E-01	0.06	7.71E-01	-0.47	1.32E-02	-0.15	4.45E-01
ENSCAFG0000000787	TMSB10	red	VSMC_M1	0.58	1.40E-03	-0.10	6.04E-01	0.40	3.65E-02	-0.41	3.36E-02	0.04	8.40E-01	-0.29	1.45E-01	-0.03	8.75E-01	0.33	9.11E-02	-0.03	9.00E-01	-0.58	1.67E-03
ENSCAFG0000001743	GATAD2B	red	VSMC_M1	0.58	1.42E-03	0.40	3.64E-02	0.15	4.67E-01	0.01	9.43E-01	0.15	4.41E-01	-0.07	7.27E-01	-0.14	4.87E-01	-0.19	3.52E-01	-0.47	1.41E-02	0.08	6.74E-01
ENSCAFG0000000496	FBF1	grey	VSMC_M10	0.58	1.42E-03	0.29	1.40E-01	0.02	9.24E-01	0.25	2.06E-01	0.12	5.52E-01	-0.11	5.70E-01	0.19	3.32E-01	-0.33	9.74E-02	-0.48	1.20E-02	0.36	6.20E-02
ENSCAFG0000000407	SELENOW	cyan	VSMC_M2	0.58	1.42E-03	0.45	1.73E-02	0.19	3.35E-01	0.37	5.89E-02	0.10	6.12E-01	-0.25	2.08E-01	-0.31	1.12E-01	-0.51	6.21E-03	-0.44	2.15E-02	0.13	5.15E-01
ENSCAFG0000000041	MTHFD1L	pink	VSMC_M5	0.58	1.44E-03	-0.39	4.38E-02	0.02	9.12E-01	-0.39	4.56E-02	0.36	6.24E-02	-0.07	7.27E-01	0.25	2.01E-01	0.52	5.72E-03	0.10	6.12E-01	-0.62	5.11E-04
ENSCAFG0000001462	MECOM	red	VSMC_M1	0.58	1.45E-03	0.17	4.03E-01	-0.08	6.77E-01	0.13	5.12E-01	0.38	4.84E-02	-0.27	1.80E-01	-0.28	1.54E-01	-0.11	5.88E-01	-0.24	2.33E-01	-0.08	6.86E-01
ENSCAFG0000000608	SMOX	yellow	VSMC_M3	0.58	1.45E-03	0.65	2.30E-04	0.52	5.17E-03	0.11	5.96E-01	-0.31	1.20E-01	0.06	7.61E-01	-0.07	7.39E-01	-0.28	1.50E-01	-0.77	3.05E-06	0.37	5.92E-02
ENSCAFG0000000054	ENSCAFG0000000054	yellow	VSMC_M3	0.58	1.45E-03	0.29	1.44E-01	0.47	1.34E-02	-0.16	4.17E-01	-0.20	3.23E-01	0.01	9.46E-01	-0.07	7.45E-01	0.04	8.45E-01	-0.43	2.67E-02	-0.03	8.91E-01
ENSCAFG0000000338	STK40	cyan	VSMC_M2	0.58	1.46E-03	0.49	1.03E-02	0.35	7.08E-02	0.11	5.90E-01	-0.04	8.35E-01	-0.04	8.52E-01	0.02	9.09E-01	-0.38	4.91E-02	-0.55	3.18E-03	0.17	3.89E-01
ENSCAFG0000000860	DCTN1	yellow	VSMC_M3	0.58	1.46E-03	0.44	2.18E-02	0.50	8.35E-03	-0.12	5.54E-01	-0.17	3.84E-01	-0.08	6.91E-01	-0.09	6.59E-01	-0.16	4.14E-01	-0.45	1.99E-02	0.04	8.59E-01
ENSCAFG0000001286	UROS	red	VSMC_M1	0.58	1.47E-03	0.19	3.37E-01	-0.08	6.94E-01	0.24	2.31E-01	0.36	6.73E-02	-0.21	2.87E-01	0.02	9.05E-01	-0.24	2.32E-01	-0.32	9.83E-02	0.02	9.29E-01
ENSCAFG0000000534	EHD3	cyan	VSMC_M2	0.58	1.48E-03	0.43	2.59E-02	0.29	1.43E-01	0.15	4.56E-01	-0.09	6.71E-01	0.05	7.96E-01	-0.10	6.25E-01	-0.30	1.34E-01	-0.51	6.45E-03	0.23	2.41E-01
ENSCAFG0000002883	FAM104A	pink	VSMC_M5	0.58	1.48E-03	-0.12	5.37E-01	-0.18	3.56E-01	-0.06	7.65E-01	0.52	5.53E-03	-0.11	6.01E-01	0.09	6.56E-01	0.11	5.70E-01	-0.07	7.32E-01	-0.34	8.10E-02
ENSCAFG0000002257	ENSCAFG0000002257	pink	VSMC_M5	0.58	1.49E-03	-0.19	3.35E-01	-0.27	1.79E-01	-0.05	8.19E-01	0.66	1.81E-04	-0.26	1.97E-01	0.00	9.97E-01	0.09	6.55E-01	0.06	7.59E-01	-0.45	1.99E-02
ENSCAFG0000000563	ENSCAFG0000000563	red	VSMC_M1	0.58	1.49E-03	0.26	1.84E-01	0.18	3.62E-01	-0.21	3.01E-01	0.15	4.62E-01	-0.34	7.96E-02	-0.08	6.99E-01	0.06	7.58E-01	-0.43	2.41E-02	-0.14	4.93E-01
ENSCAFG0000000134	RPL3	yellow	VSMC_M3	0.58	1.50E-03	0.53	4.87E-03	0.74	1.06E-05	-0.39	4.15E-02	-0.40	3.89E-02	-0.19	3.31E-01	-0.19	3.53E-01	0.14	4.83E-01	-0.63	4.24E-04	-0.07	7.23E-01
ENSCAFG0000001059	NPRL2	red	VSMC_M1	0.58	1.52E-03	0.30	1.24E-01	0.06	7.53E-01	0.08	6.74E-01	0.09	6.70E-01	-0.02	9.29E-01	-0.18	3.62E-01	-0.15	4.44E-01	-0.45	1.80E-02	0.22	2.66E-01
ENSCAFG0000000700	RAB28	red	VSMC_M1	0.58	1.55E-03	0.21	2.93E-01	0.30	1.27E-01	-0.09	6.41E-01	0.10	6.15E-01	-0.10	6.13E-01	-0.15	4.50E-01	-0.11	5.96E-01	-0.24	2.33E-01	-0.31	1.19E-01
ENSCAFG0000001297	MAP4	red	VSMC_M1	0.58	1.56E-03	0.28	1.55E-01	-0.12	5.44E-01	0.19	3.39E-01	0.48	1.07E-02	-0.31	1.13E-01	-0.12	5.58E-01	-0.25	2.01E-01	-0.34	8.30E-02	-0.03	8.81E-01
ENSCAFG0000000726	ENSCAFG0000000726	red	VSMC_M1	0.58	1.56E-03	-0.06	7.62E-01	0.26	1.90E-01	-0.22	2.70E-01	0.12	5.44E-01	0.19	3.31E-01	-0.07	7.30E-01	0.24	2.22E-01	-0.14	4.87E-01	-0.46	1.70E-02
ENSCAFG0000001835	ENSCAFG0000001835	red	VSMC_M1	0.58	1.59E-03	-0.09	6.73E-01	0.42	2.81E-02	-0.38	5.03E-02	-0.06	7.79E-01	-0.01	9.52E-01	-0.05	8.23E-01	0.38	5.18E-02	-0.08	7.09E-01	-0.49	9.66E-03
ENSCAFG0000000913	GPAT3	red	VSMC_M1	0.58	1.61E-03	0.53	4.47E-03	0.26	1.89E-01	0.00	9.90E-01	-0.07	7.14E-01	0.00	9.85E-01	0.12	5.65E-01	-0.15	4.69E-01	-0.72	2.61E-05	0.26	1.84E-01
ENSCAFG0000001611	CTDNEP1	red	VSMC_M1	0.58	1.62E-03	0.06	7.53E-01	0.61	7.41E-04	-0.71	3.63E-05	-0.15	4.44E-01	-0.16	4.40E-01	0.24	2.33E-01	0.50	7.97E-03	-0.20	3.08E-01	-0.50	7.65E-03
ENSCAFG0000001322	PAFAH1B2	pink	VSMC_M5	0.58	1.64E-03	-0.04	8.49E-01	-0.26	1.92E-01	0.12	5.36E-01	0.60	9.20E-04	-0.15	4.53E-01	0.02	9.35E-01	-0.16	4.16E-01	-0.06	7.62E-01	-0.23	2.43E-01
ENSCAFG0000002883	ELOF1	red	VSMC_M1	0.58	1.64E-03	0.20	3.05E-01	0.14	4.77E-01	-0.15	4.47E-01	0.08	7.02E-01	0.19	3.41E-01	-0.02	9.14E-01	0.07	7.12E-01	-0.38	5.23E-02	-0.02	9.29E-01
ENSCAFG0000001740	AKAP1	grey	VSMC_M10	0.58	1.65E-03	0.43	2.56E-02	-0.18	3.74E-01	0.53	4.62E-03	0.46	1.62E-02	-0.30	1.35E-01	-0.36	6.68E-02	-0.63	4.87E-04	-0.43	2.60E-02	0.23	2.57E-01
ENSCAFG0000003172	CPNE1	red	VSMC_M1	0.58	1.65E-03	0.60	1.02E-03	0.41	3.37E-02	-0.19	3.42E-01	-0.18	3.58E-01	0.04	8.27E-01	0.07	7.40E-01	-0.08	7.04E-01	-0.73	1.49E-05	0.26	1.88E-01
ENSCAFG0000000818	RHOF	red	VSMC_M1	0.58	1.66E-03	0.37	5.57E-02	0.40	4.09E-02	-0.01	9.47E-01	0.01	9.77E-01	-0.29	1.43E-01	-0.09	6.51E-01	-0.18	3.74E-01	-0.44	2.22E-02	-0.13	5.28E-01
ENSCAFG0000002925	ZNF837	grey	VSMC_M10	0.58	1.67E-03	0.54	3.85E-03	0.23	2.54E-01	0.16	4.29E-01	-0.05	8.14E-01	-0.03	8.73E-01	-0.19	3.46E-01	-0.28	1.52E-01	-0.68	8.70E-05	0.30	1.23E-01
ENSCAFG0000000663	ENSCAFG0000000663	yellow	VSMC_M3	0.58	1.68E-03	0.33	8.95E-02	0.66	2.00E-04	-0.50	8.40E-03	-0.22	2.62E-01	-0.13	5.09E-01	0.03	8.63E-01	0.27	1.77E-01	-0.44	2.22E-02	-0.30	1.34E-01
ENSCAFG0000001065	TUSC2	cyan	VSMC_M2	0.58	1.69E-03	0.46	1.66E-02	0.13	5.27E-01	0.34	7.87E-02	0.18	3.67E-01	-0.34	8.54E-02	-0.47	1.33E-02	-0.46	1.58E-02	-0.46	1.66E-02	0.12	5.47E-01
ENSCAFG0000002020	RABEPK	yellow	VSMC_M3	0.58	1.70E-03	0.11	5.84E-01	0.56	2.30E-03	-0.55	3.05E-03	-0.23	2.57E-01	-0.06	7.61E-01	0.20	3.26E-01	0.45	1.85E-02	-0.35	7.48E-02	-0.33	8.96E-02
ENSCAFG0000000109	ENSCAFG0000000109	red	VSMC_M1	0.57	1.71E-03	0.23	2.58E-01	-0.05	7.91E-01	0.11	5.81E-01	0.39	4.51E-02	-0.53	4.65E-03	0.06	7.70E-01	-0.23	2.50E-01	-0.33	9.21E-02	-0.05	8.14E-01
ENSCAFG0000001752	SUPT4H1	red	VSMC_M1	0.57	1.74E-03	0.37	5.78E-02	0.15	4.44E-01	0.14	4.98E-01	0.12	5.41E-01	-0.12	5.55E-01	-0.13	5.17E-01	-0.26	1.93E-01	-0.43	2.52E-02	0.11	5.78E-01
ENSCAFG0000000515	THBD	grey	VSMC_M10	0.57	1.76E-03	0.37	5.70E-02	0.33	8.88E-02	-0.03	9.00E-01	-0.05	8.19E-01	0.04	8.45E-01	-0.33	8.99E-02	-0.18	3.77E-01	-0.35	7.25E-02	0.03	8.82E-01
ENSCAFG0000001980	GSTM3	red	VSMC_M1	0.57	1.77E-03	0.17	4.08E-01	0.36	6.79E-02	-0.28	1.56E-01	-0.01	9.57E-01	-0.12	5.44E-01	-0.13	5.16E-01	0.23	2.39E-01	-0.28	1.64E-01	-0.29	1.40E-01
ENSCAFG0000001238	RBM4	red	VSMC_M1	0.57	1.78E-03	0.29	1.42E-01	0.27	1.81E-01	0.13	5.18E-01	-0.08	6.74E-01	0.19	3.33E-01	-0.14	4.90E-01	-0.13	5.02E-01	-0.46	1.69E-02	0.16	4.27E-01
ENSCAFG0000000487	PCDH17	red	VSMC_M1	0.57	1.79E-03	0.07	7.12E-01	0.04	8.54E-01	-0.02	9.17E-01	0.26	1.97E-01	-0.15	4.66E-01	-0.32	1.05E-01	0.01	9.50E-01	-0.19	3.48E-01	-0.15	4.54E-01
ENSCAFG0000001193	IMPDH2	yellow	VSMC_M3	0.57	1.79E-03	0.44	2.17E-02	0.60	8.83E-04	-0.41	3.21E-02	-0.29	1.43E-01	-0.16	4.25E-01	0.14	4.80E-01	0.23	2.50E-01	-0.62	5.83E-04	-0.07	7.23E-01
ENSCAFG0000000501	ATG7	cyan	VSMC_M2	0.57	1.82E-03	0.56	2.23E-03	0.23	2.45E-01	0.08	6.75E-01	-0.06	7.82E-01	-0.05	7.94E-01	0.01	9.74E-01	-0.28	1.61E-01	-0.72	2.48E-05	0.36	6.56E-02
ENSCAFG0000000098	DAXX	cyan	VSMC_M2	0.57	1.82E-03	0.53	4.21E-03	0.30	1.23E-01	0.24	2.34E-01	-0.08	6.89E-01	-0.03	8.86E-01	-0.06	7.53E-01	-0.43	2.48E-02	-0.62	6.15E-04	0.33	9.68E-02
ENSCAFG0000000123	TAF11	red	VSMC_M1	0.57	1.82E-03	-0.04	8.33E-01	0.26	1.84E-01	-0.17	3.90E-01	-0.02	9.24E-01	0.02	9.23E-01	0.21	3.04E-01	0.17	3.93E-01	-0.14	4.92E-01	-0.15	4.50E-01
ENSCAFG0000001711	ENSCAFG0000001711	yellow	VSMC_M3	0.57	1.83E-03	0.57	1.78E-03	0.54	3.71E-03	-0.02	9.13E-01	-0.27	1.66E-01	0.03	8.70E-01	-0.04	8.54E-01	-0.23	2.46E-01	-0.65	2.16E-04	0.18	3.78E-01
ENSCAFG0000000234	IARS	cyan	VSMC_M2	0.57	1.87E-03	0.59	1.30E-03	0.00	9.94E-01	0.30	1.25E-01	0.34	8.49E-02	-0.37	6.11E-02	-0.24	2.29E-01	-0.55	2.71E-03	-0.55	2.95E-03	0.19	3.40E-01
ENSCAFG0000001457	ACAT1	cyan	VSMC_M2	0.57	1.88E-03	0.65	2.37E-04	0.43	2.60E-02	-0.07	7.37E-01	-0.11	5.69E-01	-0.25	2.01E-01	0.02	9.07E-01	-0.17	3.98E-01	-0.75	6.39E-06	0.15	4.60E-01
ENSCAFG0000001302	STMN3	yellow	VSMC_M3	0.57	1.91E-03	0.43	2.63E-02	0.67	1.23E-04	-0.37	5.96E-02	-0.25	2.01E-01	-0.12	5.36E-01	0.15	4.68E-01	0.06	7.62E-01	-0.50	8.05E-03	-0.14	4.88E-01
ENSCAFG0000000475	MIF4GD	cyan	VSMC_M2	0.57	1.91E-03	0.65	2.17E-04	0.11	5.88E-01	0.31	1.20E-01	-0.01	9.67E-01	-0.10	6.36E-01	-0.05	7.99E-01	-0.48	1.11E-02	-0.72	2.30E-05	0.56	2.34E-03
ENSCAFG0000001252	PSMA7	red	VSMC_M1	0.57	1.91E-03	0.05	8.06E-01	0.68	1.11E-04	-0.66	1.82E-04	-0.25	2.05E-01	-0.09	6.39E-01	-0.17	4.01E-01	0.52	5.64E-03	-0.18	3.66E-01	-0.53	4.39E-03
ENSCAFG0000000109	SMAD5	grey	VSMC_M10	0.57	1.92E-03	0.58	1.38E-03	-0.04	8.36E-01	0.32	1.06E-01	0.23	2.46E-01	-0.05	7.97E-01	-0.26	1.98E-01	-0.47	1.40E-02	-0.69	6.48E-05	0.40	3.69E-02
ENSCAFG0000001921	HMG20B	yellow	VSMC_M3	0.57	1.92E-03	0.23	2.43E-01	0.62	5.07E-04	-0.35	6.99E-02	-0.31	1.15E-01	0.02	9.15E-01	0.25	2.13E-01	0.21	2.86E-01	-0.45	1.89E-02	-0.12	5.37E-01
ENSCAFG0000001947	ENSCAFG0000001947	yellow	VSMC_M3	0.57	1.93E-03	0.69	7.84E-05	0.73	1.43E-05	-0.24	2.27E-01	-0.51	6.29E-03	-0.01	9.67E-01	-0.04	8.53E-01	-0.06	7.72E-01	-0.79	1.03E-06	0.24	2.28E-01
ENSCAFG0000000747	IL20RB	red	VSMC_M1	0.57	1.94E-03	0.44	2.33E-02	0.20	3.21E-01	-0.04	8.38E-01	0.03	8.99E-01	-0.07	7.32E-01	0.10	6.24E-01	-0.14	4.77E-01	-0.58	1.43E-03	0.17	3.88E-01
ENSCAFG0000001800	ENSCAFG0000001800	red	VSMC_M1	0.57	1.95E-03	-0.10	6.33E-01	0.32	1.03E-01	-0.55	2.95E-03	0.12	5.56E-01	-0.28	1.55E-01	0.18	3.58E-01	0.45	1.99E-02	-0.12	5.40E-01	-0.51	6.67E-03
ENSCAFG0000001342	EIF2S3	pink	VSMC_M5	0.57	1.97E-03	-0.21	2.84E-01	-0.18	3.63E-01	-0.15	4.57E-01	0.59	1.25E-03	-0.22	2.78E-01	0.05	8.02E-01	0.24	2.32E-01	0.02	9.20E-01	-0.52	5.90E-03
ENSCAFG0000001119	PDGFA	yellow	VSMC_M3	0.57	1.98E-03	0.11	5.86E-01	0.65	2.33E-04	-0.64	3.65E-04	-0.25	2.05E-01	-0.13	5.32E-01	0.12	5.63E-01	0.41	3.25E-02	-0.26	1.83E-01	-0.35	7.32E-02
ENSCAFG0000000263	CRIPT	red	VSMC_M1	0.57	1.99E-03	0.31	1.18E-01	-0.03	8.84E-01	0.43	2.61E-02	0.33	9.41E-02	-0.22	2.64E-01	-0.16	4.27E-01	-0.53	4.24E-03	-0.36	6.48E-02	0.11	5.99E-01
ENSCAFG0000001795	MORC4	pink	VSMC_M5	0.57	1.99E-03	-0.03	8.87E-01	-0.10	6.32E-01	-0.06	7.59E-01	0.44	2.21E-02	-0.25	2.02E-01	0.21	3.00E-01	0.10	6.31E-01	-0.10	6.05E-01	-0.25	2.02E-01
ENSCAFG0000001933	ASPA	cyan	VSMC_M2	0.57	2.01E-03	0.50	7.97E-03	0.15	4.59E-01	0.26	1.85E-01	0.11	5.99E-01	-0.31	1.13E-01	0.00	9.81E-01	-0.37	5.68E-02	-0.60	9.04E-04	0.23	2.41E-01
ENSCAFG0000000340	KLF11	grey	VSMC_M10	0.57	2.02E-03	0.42	2.84E-02	-0.13	5.08E-01	0.53	4.22E-03	0.30	1.29E-01	-0.17	3.84E-01	-0.08	6.84E-01	-0.53	4.89E-03	-0.54	3.93E-03	0.39	4.26E-02
ENSCAFG0000000223	NPR2	red	VSMC_M1	0.57	2.02E-03	0.30	1.24E-01	0.11	5.94E-01	0.20	3.16E-01	0.21	2.83E-01	0.03	8.66E-01	-0.48	1.16E-02	-0.26	1.97E-01	-0.36	6.24E-02	-0.02	9.13E-01
ENSCAFG0000001237	ENSCAFG0000001237	red	VSMC_M1	0.57	2.03E-03	0.12	5.66E-01	0.33	9.48E-02	-0.19	3.37E-01	0.06	7.82E-01	-0.34	8.49E-02	-0.27	1.69E-01	0.05	8.21E-01	-0.18	3.61E-01	-0.29	1.47E-01
ENSCAFG0000000982	PDZRN4	yellow	VSMC_M3	0.57	2.05E-03	0.44	2.29E-02	0.57	2.13E-03	-0.34	7.89E-02	-0.27	1.72E-01	-0.08	6.85E-01	-0.02	9.13E-01	0.12	5.57E-01	-0.54	3.61E-03	0.00	9.96E-01
ENSCAFG0000003117	DCTD	grey	VSMC_M10	0.57	2.06E-03	0.13	5.27E-01	0.08	6.81E-01	-0.14	4.81E-01	0.19	3.46E-01	0.17	4.05E-01	-0.07	7.24E-01	0.10	6.32E-01	-0.27	1.73E-01	-0.18	3.67E-01
ENSCAFG0000000777	NCOA2	grey	VSMC_M10	0.57	2.08E-03	0.35	7.51E-02	-0.32	1.08E-01	0.54	3.50E-03	0.51	7.14E-03	-0.17	4.10E-01	-0.07	7.34E-01	-0.55	2.68E-03	-0.46	1.51E-02	0.33	9.15E-02
ENSCAFG0000000982	TBC1D7	red	VSMC_M1	0.57	2.11E-03	0.24	2.33E-01	-0.11	5.77E-01	0.37	5.51E-02	0.40	4.12E-02	0.02	9.30E-01	0.06	7.76E-01	-0.42	3.12E-02	-0.35	7.73E-02	0.06	7.60E-01
ENSCAFG0000001763	ENSCAFG0000001763	grey	VSMC_M10	0.56	2.14E-03	0.22	2.65E-01	-0.27	1.81E-01	0.43	2.35E-02	0.42	3.06E-02	-0.06	7.67E-01	-0.14	4.94E-01	-0.41	3.24E-02	-0.37	5.82E-02	0.25	2.06E-01
ENSCAFG0000001408	GJC1	red	VSMC_M1	0.56	2.17E-03	-0.01	9.79E-01	0.53	4.54E-03	-0.44	2.11E-02	-0.21	3.01E-01	-0.16	4.25E-01	0.34	8.06E-02	0.39	4.47E-02	-0.21	2.82E-01	-0.31	1.11E-01
ENSCAFG0000002901	NREP	red	VSMC_M1	0.56	2.17E-03	0.05	8.09E-01	0.16	4.17E-01	-0.08	6.75E-01	0.12	5.42E-01	0.20	3.24E-01	-0.05	8.18E-01	-0.03	8.63E-01	-0.20	3.27E-01	-0.13	5.21E-01
ENSCAFG0000000236	ADGRL3	grey	VSMC_M10	0.56	2.18E-03	0.69	6.81E-05	0.17	3.98E-01	0.29	1.39E-01	-0.02	9.19E-01	0.07	7.41E-01	-0.45	1.98E-02	-0.44	2.16E-02	-0.76	3.80E-06	0.44	2.17E-02
ENSCAFG0000001589	MED11	yellow	VSMC_M3	0.56	2.20E-03	0.15	4.70E-01	0.53	4.90E-03	-0.47	1.29E-02	-0.25	2.15E-01	0.11	5.91E-01	0.26	1.89E-01	0.35	6.95E-02	-0.36	6.75E-02	-0.21	3.03E-01
ENSCAFG0000001304	ADO	yellow	VSMC_M3	0.56	2.20E-03	0.53	4.50E-03	0.50	8.27E-03	-0.06	7.77E-01	-0.19	3.50E-01	-0.19	3.54E-01	-0.09	6.52E-01	-0.16	4.17E-01	-0.56	2.46E-03	0.12	5.40E-01
ENSCAFG0000002274	ENSCAFG0000002274	pink	VSMC_M5	0.56	2.20E-03	-0.04	8.59E-01	-0.05	7.92E-01	-0.19	3.48E-01	0.47	1.37E-02	-0.30	1.35E-01	-0.09	6.72E-01	0.09	6.52E-01	-0.04	8.42E-01	-0.39	4.69E-02
ENSCAFG0000003068	GLIPR2	yellow	VSMC_M3	0.56	2.22E-03	0.58	1.54E-03	0.64	3.65E-04	-0.37	5.96E-02	-0.30	1.24E-01	-0.18	3.57E-01	0.15	4.65E-01	0.06	7.74E-01	-0.68	8.15E-05	0.03	8.64E-01
ENSCAFG0000002344	MED31	red	VSMC_M1	0.56	2.22E-03	0.04	8.60E-01	0.21	2.98E-01	0.00	9.83E-01	0.10	6.33E-01	-0.14	4.98E-01	-0.06	7.78E-01	0.01	9.44E-01	-0.21	2.89E-01	-0.18	3.59E-01
ENSCAFG0000001686	ELMSAN1	cyan	VSMC_M2	0.56	2.26E-03	0.55	3.07E-03	0.18	3.81E-01	-0.04	8.58E-01	0.17	3.87E-01	-0.21	3.00E-01	-0.20	3.13E-01	-0.20	3.20E-01	-0.60	8.80E-04	0.06	7.53E-01
ENSCAFG0000003127	UQCC2	grey	VSMC_M10	0.56	2.27E-03	0.27	1.70E-01	0.28	1.56E-01	0.09	6.45E-01	-0.10	6.04E-01	-0.14	4.89E-01	0.13	5.30E-01	-0.19	3.33E-01	-0.39	4.52E-02	0.22	2.63E-01
ENSCAFG0000002901	CACYBP	pink	VSMC_M5	0.56	2.30E-03	-0.20	3.28E-01	-0.33	8.85E-02	0.20	3.25E-01	0.66	1.67E-04	-0.28	1.51E-01	-0.14	4.73E-01	-0.06	7.55E-01	0.03	8.64E-01	-0.35	7.34E-02
ENSCAFG0000000055	ENSCAFG0000000055	cyan	VSMC_M2	0.56	2.31E-03	0.60	9.39E-04	0.32	9.88E-02	0.08	7.06E-01	-0.10	6.30E-01	-0.12	5.63E-01	0.17	4.00E-01	-0.28	1.52E-01	-0.74	1.15E-05	0.31	1.18E-01
ENSCAFG0000000279	KLF4	yellow	VSMC_M3	0.56	2.34E-03	0.53	4.27E-03	0.64	3.65E-04	-0.46	1.54E-02	-0.31	1.21E-01	-0.22	2.77E-01	0.17	3.86E-01	0.13	5.15E-01	-0.66	1.71E-04	0.03	8.63E-01
ENSCAFG0000001946	ENSCAFG0000001946	red	VSMC_M1	0.56	2.35E-03	0.03	8.89E-01	0.30	1.31E-01	-0.40	3.83E-02	0.08	6.79E-01	-0.09	6.49E-01	-0.32	1.09E-01	0.32	1.03E-01	-0.12	5.62E-01	-0.47	1.41E-02
ENSCAFG0000001949	LRRC47	grey	VSMC_M10	0.56	2.35E-03	0.08	6.86E-01	0.42	3.00E-02	-0.50	7.79E-03	-0.15	4.48E-01	0.20	3.26E-01	0.37	6.05E-02	0.42	2.98E-02	-0.31	1.18E-01	-0.18	3.78E-01
ENSCAFG0000000143	POLR2F	red	VSMC_M1	0.56	2.42E-03	0.33	9.07E-02	0.32	1.07E-01	0.21	2.85E-01	-0.15	4.53E-01	0.05	7.96E-01	-0.06	7.51E-01	-0.25	2.01E-01	-0.48	1.17E-02	0.22	2.72E-01
ENSCAFG0000001543	ENSCAFG0000001543	red	VSMC_M1	0.56	2.43E-03	0.29	1.44E-01	0.15	4.44E-01	0.02	9.33E-01	0.16	4.12E-01	-0.23	2.46E-01	-0.25	2.04E-01	-0.13	5.20E-01	-0.38	4.75E-02	-0.08	6.99E-01

ENSCAFG0000001791	PMP22	red	VSMC_M1	0.56	2.43E-03	0.47	1.25E-02	0.51	6.71E-03	-0.40	3.83E-02	-0.13	5.31E-01	-0.16	4.28E-01	-0.21	2.93E-01	0.14	4.71E-01	-0.55	2.68E-03	-0.18	3.77E-01
ENSCAFG0000001755	S100A6	yellow	VSMC_M3	0.56	2.45E-03	0.47	1.28E-02	0.70	5.14E-05	-0.37	5.83E-02	-0.31	1.20E-01	-0.27	1.78E-01	0.22	2.72E-01	0.05	7.95E-01	-0.53	4.45E-03	-0.12	5.39E-01
ENSCAFG0000001631	ENSCAFG0000001631	cyan	VSMC_M2	0.56	2.45E-03	0.40	4.00E-02	0.42	2.82E-02	-0.24	2.38E-01	-0.15	4.70E-01	0.06	7.65E-01	-0.11	5.89E-01	0.00	9.98E-01	-0.45	1.93E-02	0.01	9.63E-01
ENSCAFG0000003077	ENSCAFG0000003077	red	VSMC_M1	0.56	2.47E-03	0.35	7.60E-02	0.46	1.70E-02	-0.10	6.34E-01	-0.25	2.00E-01	-0.09	6.40E-01	0.07	7.28E-01	0.01	9.61E-01	-0.53	4.13E-03	0.16	4.24E-01
ENSCAFG0000003044	ENSCAFG0000003044	red	VSMC_M1	0.56	2.48E-03	0.31	1.12E-01	0.31	1.19E-01	-0.28	1.65E-01	0.12	5.57E-01	-0.43	2.47E-02	-0.25	2.01E-01	0.01	9.57E-01	-0.30	1.28E-01	-0.24	2.24E-01
ENSCAFG0000001246	SMYD2	red	VSMC_M1	0.56	2.48E-03	0.28	1.56E-01	0.06	7.78E-01	0.20	3.22E-01	0.17	3.83E-01	0.00	9.86E-01	0.01	9.72E-01	-0.24	2.27E-01	-0.39	4.32E-02	0.10	6.36E-01
ENSCAFG0000001216	NELFCD	cyan	VSMC_M2	0.56	2.51E-03	0.51	6.20E-03	0.25	2.06E-01	0.34	8.49E-02	-0.04	8.57E-01	-0.26	1.95E-01	-0.02	9.08E-01	-0.52	5.24E-03	-0.55	2.88E-03	0.36	6.18E-02
ENSCAFG0000001074	GNAI2	cyan	VSMC_M2	0.56	2.54E-03	0.49	9.65E-03	0.33	9.40E-02	0.01	9.72E-01	-0.03	8.99E-01	-0.13	5.33E-01	-0.19	3.31E-01	-0.23	2.47E-01	-0.45	1.74E-02	0.10	6.06E-01
ENSCAFG0000001183	ENSCAFG0000001183	yellow	VSMC_M3	0.56	2.54E-03	0.17	4.03E-01	0.48	1.09E-02	-0.17	3.91E-01	-0.11	5.75E-01	-0.15	4.66E-01	-0.17	4.09E-01	0.01	9.54E-01	-0.21	2.91E-01	-0.24	2.22E-01
ENSCAFG0000001835	AKT1	yellow	VSMC_M3	0.56	2.55E-03	0.59	1.18E-03	0.85	2.21E-08	-0.39	4.17E-02	-0.53	4.64E-03	-0.05	7.88E-01	-0.13	5.21E-01	0.07	7.46E-01	-0.62	5.24E-04	0.02	9.12E-01
ENSCAFG0000003188	TSSC4	red	VSMC_M1	0.56	2.55E-03	0.42	3.11E-02	0.38	5.26E-02	0.07	7.41E-01	-0.27	1.76E-01	0.14	4.97E-01	-0.13	5.07E-01	-0.08	6.76E-01	-0.64	3.50E-04	0.29	1.45E-01
ENSCAFG0000001692	ZNF652	red	VSMC_M1	0.56	2.56E-03	0.24	2.33E-01	0.38	5.10E-02	-0.22	2.70E-01	-0.10	6.10E-01	-0.14	4.86E-01	0.02	9.02E-01	0.12	5.63E-01	-0.39	4.63E-02	-0.10	6.26E-01
ENSCAFG0000000811	ARRDC3	cyan	VSMC_M2	0.56	2.61E-03	0.36	6.72E-02	0.16	4.24E-01	-0.15	4.48E-01	0.19	3.53E-01	-0.16	4.33E-01	0.03	8.72E-01	-0.04	8.47E-01	-0.50	8.40E-03	-0.01	9.71E-01
ENSCAFG0000000633	CACNA2D1	red	VSMC_M1	0.56	2.63E-03	0.31	1.20E-01	-0.24	2.27E-01	0.38	5.13E-02	0.47	1.23E-02	-0.08	6.93E-01	-0.24	2.28E-01	-0.44	2.12E-02	-0.39	4.73E-02	0.21	2.95E-01
ENSCAFG0000000586	EIF3K	cyan	VSMC_M2	0.55	2.66E-03	0.78	1.54E-06	0.39	4.44E-02	0.23	2.51E-01	-0.23	2.46E-01	-0.10	6.18E-01	-0.18	3.76E-01	-0.45	1.99E-02	-0.84	4.21E-08	0.45	1.77E-02
ENSCAFG0000000938	CHST1	grey	VSMC_M10	0.55	2.71E-03	0.39	4.26E-02	0.12	5.59E-01	0.29	1.43E-01	0.05	8.08E-01	0.12	5.43E-01	-0.29	1.40E-01	-0.34	8.63E-02	-0.49	9.15E-03	0.32	1.01E-01
ENSCAFG0000001858	FBXO4	red	VSMC_M1	0.55	2.71E-03	0.05	8.10E-01	-0.10	6.19E-01	-0.01	9.79E-01	0.32	1.00E-01	0.02	9.09E-01	0.30	1.26E-01	-0.04	8.53E-01	-0.24	2.37E-01	-0.04	8.51E-01
ENSCAFG0000001243	SDR39U1	red	VSMC_M1	0.55	2.71E-03	0.00	9.88E-01	-0.15	4.56E-01	0.28	1.54E-01	0.53	4.18E-03	-0.20	3.06E-01	0.00	9.93E-01	-0.28	1.63E-01	-0.12	5.40E-01	-0.22	2.71E-01
ENSCAFG0000001600	ENSCAFG0000001600	cyan	VSMC_M2	0.55	2.72E-03	0.54	3.90E-03	0.15	4.48E-01	0.28	1.52E-01	0.12	5.59E-01	-0.16	4.11E-01	-0.42	3.10E-02	-0.47	1.34E-02	-0.58	1.64E-03	0.24	2.32E-01
ENSCAFG0000002517	ENSCAFG0000002517	red	VSMC_M1	0.55	2.73E-03	0.17	3.92E-01	-0.12	5.54E-01	0.00	9.89E-01	0.45	1.76E-02	-0.16	4.18E-01	-0.30	1.29E-01	-0.02	9.20E-01	-0.27	1.81E-01	-0.19	3.34E-01
ENSCAFG0000003064	ENSCAFG0000003064	red	VSMC_M1	0.55	2.73E-03	0.15	4.46E-01	0.11	5.69E-01	0.03	8.79E-01	0.18	3.73E-01	-0.30	1.27E-01	0.07	7.11E-01	-0.07	7.20E-01	-0.24	2.20E-01	-0.01	9.55E-01
ENSCAFG0000001646	MAD1L1	yellow	VSMC_M3	0.55	2.74E-03	0.18	3.81E-01	0.71	3.11E-05	-0.53	4.44E-03	-0.33	9.29E-02	0.01	9.75E-01	0.11	5.75E-01	0.39	4.60E-02	-0.36	6.28E-02	-0.33	8.82E-02
ENSCAFG0000000354	ENSCAFG0000000354	red	VSMC_M1	0.55	2.74E-03	-0.04	8.40E-01	-0.02	9.03E-01	-0.07	7.42E-01	0.35	7.12E-02	-0.27	1.69E-01	-0.02	9.02E-01	0.08	6.80E-01	-0.15	4.54E-01	-0.29	1.37E-01
ENSCAFG0000001447	C27H12orf57	yellow	VSMC_M3	0.55	2.75E-03	0.68	1.10E-04	0.72	2.49E-05	-0.18	3.78E-01	-0.38	5.03E-02	-0.35	7.36E-02	-0.16	4.20E-01	-0.16	4.28E-01	-0.70	4.73E-05	0.09	6.60E-01
ENSCAFG0000000669	STX2	grey	VSMC_M10	0.55	2.77E-03	0.20	3.23E-01	-0.03	8.65E-01	-0.03	8.79E-01	0.28	1.58E-01	-0.11	5.80E-01	0.04	8.44E-01	-0.04	8.30E-01	-0.37	6.04E-02	0.03	8.69E-01
ENSCAFG0000000346	FBXL4	pink	VSMC_M5	0.55	2.80E-03	0.17	3.86E-01	-0.39	4.32E-02	0.57	1.72E-03	0.62	5.99E-04	-0.12	5.42E-01	-0.23	2.52E-01	-0.56	2.15E-03	-0.26	1.92E-01	0.15	4.43E-01
ENSCAFG0000001024	METTL15	darkgrey	VSMC_M8	0.55	2.81E-03	-0.36	6.13E-02	0.16	4.39E-01	-0.57	1.84E-03	0.32	1.09E-01	-0.23	2.48E-01	0.29	1.42E-01	0.56	2.35E-03	0.16	4.29E-01	-0.76	4.43E-06
ENSCAFG0000000324	ENSCAFG0000000324	red	VSMC_M1	0.55	2.83E-03	0.06	7.48E-01	0.17	4.00E-01	-0.35	7.48E-02	0.23	2.53E-01	0.12	5.55E-01	-0.11	5.85E-01	0.18	3.80E-01	-0.15	4.68E-01	-0.39	4.47E-02
ENSCAFG0000000741	MAN2A1	cyan	VSMC_M2	0.55	2.84E-03	0.81	3.00E-07	0.36	6.28E-02	0.10	6.06E-01	-0.12	5.52E-01	-0.12	5.56E-01	-0.35	7.78E-02	-0.36	6.35E-02	-0.81	2.59E-07	0.37	5.61E-02
ENSCAFG0000001631	HUWE1	pink	VSMC_M5	0.55	2.84E-03	0.07	7.39E-01	-0.35	7.45E-02	0.27	1.80E-01	0.65	2.55E-04	-0.15	4.49E-01	-0.29	1.44E-01	-0.28	1.59E-01	-0.14	4.92E-01	-0.09	6.64E-01
ENSCAFG0000001136	ACTL6A	red	VSMC_M1	0.55	2.85E-03	-0.08	6.86E-01	0.09	6.47E-01	-0.10	6.21E-01	0.34	8.41E-02	-0.28	1.63E-01	-0.03	8.89E-01	0.06	7.51E-01	-0.01	9.75E-01	-0.43	2.37E-02
ENSCAFG0000001011	ENSCAFG0000001011	pink	VSMC_M5	0.55	2.89E-03	-0.11	6.00E-01	-0.05	7.98E-01	-0.18	3.58E-01	0.51	6.65E-03	-0.31	1.18E-01	-0.16	4.32E-01	0.17	4.02E-01	0.01	9.58E-01	-0.57	2.00E-03
ENSCAFG0000000732	ADHFE1	red	VSMC_M1	0.55	2.89E-03	0.37	6.10E-02	0.09	6.55E-01	0.22	2.65E-01	0.15	4.65E-01	-0.21	2.90E-01	0.05	7.98E-01	-0.39	4.15E-02	-0.39	4.42E-02	0.21	2.96E-01
ENSCAFG0000000859	NDRG3	cyan	VSMC_M2	0.55	2.92E-03	0.70	5.73E-05	0.12	5.63E-01	0.29	1.48E-01	0.06	7.74E-01	0.00	9.99E-01	-0.32	1.05E-01	-0.51	6.61E-03	-0.70	4.19E-05	0.48	1.21E-02
ENSCAFG0000001029	CISH	yellow	VSMC_M3	0.55	2.92E-03	0.65	2.21E-04	0.60	9.98E-04	-0.04	8.61E-01	-0.31	1.13E-01	-0.13	5.08E-01	-0.44	2.13E-02	-0.20	3.12E-01	-0.71	3.01E-05	0.13	5.25E-01
ENSCAFG0000000987	ENSCAFG0000000987	grey	VSMC_M10	0.55	2.95E-03	0.37	5.99E-02	-0.17	3.98E-01	0.40	3.76E-02	0.37	5.93E-02	-0.14	4.91E-01	-0.27	1.76E-01	-0.48	1.16E-02	-0.41	3.54E-02	0.19	3.46E-01
ENSCAFG0000000158	TREML2	red	VSMC_M1	0.55	2.96E-03	-0.11	5.71E-01	0.16	4.29E-01	-0.38	5.30E-02	0.22	2.77E-01	-0.23	2.53E-01	0.26	1.89E-01	0.34	8.39E-02	-0.09	6.58E-01	-0.45	1.82E-02
ENSCAFG0000003027	ENSCAFG0000003027	red	VSMC_M1	0.55	2.96E-03	-0.07	7.28E-01	0.03	8.81E-01	-0.12	5.65E-01	0.32	1.07E-01	-0.03	8.70E-01	-0.08	6.77E-01	0.14	4.94E-01	-0.12	5.40E-01	-0.36	6.33E-02
ENSCAFG0000002257	ENSCAFG0000002257	cyan	VSMC_M2	0.55	2.96E-03	0.56	2.29E-03	0.51	6.09E-03	-0.18	3.75E-01	-0.28	1.50E-01	0.00	9.90E-01	0.00	1.00E+00	-0.07	7.39E-01	-0.66	1.55E-04	0.18	3.67E-01
ENSCAFG0000000378	KTI12	yellow	VSMC_M3	0.55	3.01E-03	0.24	2.32E-01	0.67	1.26E-04	-0.40	3.66E-02	-0.38	5.10E-02	0.06	7.48E-01	-0.11	5.90E-01	0.27	1.67E-01	-0.40	3.97E-02	-0.19	3.30E-01
ENSCAFG0000001184	SYT12	yellow	VSMC_M3	0.55	3.01E-03	0.52	5.58E-03	0.63	3.83E-04	-0.20	3.10E-01	-0.39	4.72E-02	-0.08	6.84E-01	-0.16	4.14E-01	-0.04	8.38E-01	-0.56	2.45E-03	0.14	4.73E-01
ENSCAFG0000000919	FOXF2	cyan	VSMC_M2	0.55	3.02E-03	0.63	4.30E-04	0.44	2.28E-02	-0.03	8.82E-01	-0.22	2.77E-01	-0.10	6.37E-01	-0.19	3.45E-01	-0.16	4.36E-01	-0.69	6.82E-05	0.26	1.86E-01
ENSCAFG0000001195	EMC9	red	VSMC_M1	0.55	3.05E-03	0.30	1.25E-01	0.21	2.94E-01	0.05	8.23E-01	-0.07	7.25E-01	-0.07	7.18E-01	0.09	6.41E-01	-0.09	6.51E-01	-0.50	7.33E-03	0.23	2.44E-01
ENSCAFG0000000249	GALNT12	cyan	VSMC_M2	0.55	3.06E-03	0.63	4.84E-04	0.30	1.34E-01	0.24	2.22E-01	-0.08	6.79E-01	-0.25	2.15E-01	-0.31	1.19E-01	-0.41	3.29E-02	-0.66	1.66E-04	0.38	4.92E-02
ENSCAFG0000001568	MYO5C	red	VSMC_M1	0.55	3.07E-03	0.34	8.06E-02	-0.05	8.21E-01	0.38	5.31E-02	0.31	1.14E-01	-0.26	1.96E-01	-0.12	5.39E-01	-0.40	4.12E-02	-0.44	2.01E-02	0.15	4.60E-01
ENSCAFG0000000131	CTNNA1	red	VSMC_M1	0.55	3.08E-03	0.53	4.21E-03	0.47	1.39E-02	-0.16	4.36E-01	-0.12	5.49E-01	-0.16	4.32E-01	-0.09	6.37E-01	-0.08	6.92E-01	-0.55	2.88E-03	0.00	9.87E-01
ENSCAFG0000000565	HNRNPL	red	VSMC_M1	0.55	3.09E-03	0.01	9.79E-01	0.58	1.37E-03	-0.69	5.88E-05	-0.23	2.40E-01	0.01	9.54E-01	0.01	9.52E-01	0.62	5.34E-04	-0.20	3.26E-01	-0.45	1.76E-02
ENSCAFG0000001134	GSTP1	cyan	VSMC_M2	0.55	3.09E-03	0.68	1.12E-04	0.39	4.59E-02	0.32	1.02E-01	-0.17	3.85E-01	-0.25	2.01E-01	-0.19	3.49E-01	-0.52	5.88E-03	-0.70	5.70E-05	0.37	5.43E-02
ENSCAFG0000001232	WDR11	red	VSMC_M1	0.55	3.11E-03	-0.17	3.96E-01	-0.04	8.62E-01	0.09	6.64E-01	0.36	6.78E-02	-0.08	6.87E-01	0.04	8.62E-01	0.01	9.74E-01	0.02	9.10E-01	-0.28	1.59E-01
ENSCAFG0000001480	ADD1	cyan	VSMC_M2	0.55	3.13E-03	0.78	1.43E-06	0.61	7.83E-04	-0.19	3.47E-01	-0.37	5.76E-02	0.05	8.11E-01	-0.16	4.30E-01	-0.13	5.16E-01	-0.81	2.62E-07	0.29	1.49E-01
ENSCAFG0000001399	CABIN1	red	VSMC_M1	0.55	3.14E-03	0.56	2.36E-03	0.54	3.91E-03	-0.23	2.48E-01	-0.22	2.60E-01	-0.05	7.85E-01	0.14	4.72E-01	-0.03	8.76E-01	-0.64	3.67E-04	0.12	5.42E-01
ENSCAFG0000000704	HNRNPF	darkgrey	VSMC_M8	0.55	3.20E-03	-0.02	9.12E-01	0.51	6.32E-03	-0.78	1.77E-06	-0.13	5.06E-01	0.00	9.96E-01	0.27	1.81E-01	0.69	6.47E-05	-0.22	2.64E-01	-0.52	5.85E-03
ENSCAFG0000003260	DRG1	grey	VSMC_M10	0.55	3.20E-03	-0.03	8.96E-01	-0.02	9.35E-01	0.15	4.54E-01	0.16	4.20E-01	-0.11	6.00E-01	0.04	8.30E-01	-0.05	8.11E-01	-0.18	3.64E-01	0.03	8.68E-01
ENSCAFG0000001559	CPAMD8	cyan	VSMC_M2	0.55	3.21E-03	0.83	7.89E-08	0.18	3.75E-01	0.39	4.27E-02	0.05	8.00E-01	-0.27	1.74E-01	-0.44	2.31E-02	-0.66	2.01E-04	-0.78	2.00E-06	0.47	1.30E-02
ENSCAFG0000000259	TMEM150B	cyan	VSMC_M2	0.55	3.25E-03	0.60	8.73E-04	0.24	2.24E-01	0.13	5.04E-01	-0.02	9.25E-01	-0.05	8.19E-01	-0.19	3.43E-01	-0.38	5.10E-02	-0.64	3.40E-04	0.31	1.11E-01
ENSCAFG0000000113	HNRNPA0	yellow	VSMC_M3	0.55	3.25E-03	0.64	3.73E-04	0.79	1.18E-06	-0.22	2.61E-01	-0.57	2.08E-03	0.03	9.00E-01	-0.16	4.24E-01	-0.01	9.68E-01	-0.73	1.49E-05	0.24	2.24E-01
ENSCAFG0000002994	ENSCAFG0000002994	cyan	VSMC_M2	0.55	3.25E-03	0.48	1.12E-02	0.12	5.48E-01	0.28	1.53E-01	0.06	7.79E-01	-0.25	2.14E-01	-0.23	2.44E-01	-0.38	4.81E-02	-0.55	2.82E-03	0.27	1.67E-01
ENSCAFG0000001953	PAXX	grey	VSMC_M10	0.55	3.28E-03	0.86	1.08E-08	0.41	3.42E-02	0.27	1.66E-01	-0.20	3.14E-01	-0.21	2.91E-01	-0.26	1.87E-01	-0.56	2.45E-03	-0.84	3.73E-08	0.48	1.15E-02
ENSCAFG0000003011	ENSCAFG0000003011	grey	VSMC_M10	0.54	3.30E-03	0.18	3.73E-01	-0.01	9.79E-01	0.12	5.39E-01	0.16	4.38E-01	-0.04	8.27E-01	0.08	7.02E-01	-0.12	5.61E-01	-0.43	2.58E-02	0.19	3.49E-01
ENSCAFG0000001098	CFAP410	red	VSMC_M1	0.54	3.32E-03	0.41	3.43E-02	0.31	1.18E-01	-0.12	5.44E-01	-0.15	4.64E-01	0.00	9.91E-01	0.15	4.53E-01	-0.01	9.72E-01	-0.58	1.57E-03	0.29	1.47E-01
ENSCAFG0000001862	CACHD1	red	VSMC_M1	0.54	3.34E-03	0.09	6.60E-01	0.11	5.87E-01	-0.08	7.06E-01	0.05	8.15E-01	0.18	3.76E-01	0.22	2.72E-01	0.09	6.49E-01	-0.37	6.00E-02	0.07	7.29E-01
ENSCAFG0000000344	AGO1	grey	VSMC_M10	0.54	3.40E-03	0.53	4.29E-03	-0.05	8.21E-01	0.36	6.27E-02	0.33	8.83E-02	-0.08	6.80E-01	-0.38	4.88E-02	-0.54	3.85E-03	-0.51	6.07E-03	0.19	3.42E-01
ENSCAFG0000000287	TPBG	yellow	VSMC_M3	0.54	3.42E-03	-0.02	9.09E-01	0.52	4.96E-03	-0.69	5.90E-05	-0.22	2.79E-01	-0.01	9.74E-01	0.39	4.27E-02	0.61	7.58E-04	-0.21	3.01E-01	-0.35	7.39E-02
ENSCAFG0000003108	BHLHE41	red	VSMC_M1	0.54	3.42E-03	0.47	1.24E-02	0.34	8.72E-02	-0.12	5.42E-01	-0.09	6.41E-01	-0.05	7.86E-01	0.39	4.46E-02	-0.09	6.65E-01	-0.59	1.34E-03	0.22	2.73E-01
ENSCAFG0000001114	RGS9	darkgrey	VSMC_M8	0.54	3.43E-03	-0.23	2.56E-01	0.24	2.19E-01	-0.66	1.74E-04	0.20	3.27E-01	-0.11	5.73E-01	0.24	2.22E-01	0.62	6.08E-04	0.02	9.34E-01	-0.67	1.40E-04
ENSCAFG0000000160	PLIN2	grey	VSMC_M10	0.54	3.46E-03	0.41	3.42E-02	-0.18	3.78E-01	0.63	4.47E-04	0.38	4.85E-02	-0.25	2.05E-01	-0.27	1.73E-01	-0.66	1.94E-04	-0.43	2.67E-02	0.28	1.50E-01
ENSCAFG0000001715	IRF2BPL	yellow	VSMC_M3	0.54	3.46E-03	0.75	6.74E-06	0.68	8.66E-05	-0.15	4.60E-01	-0.40	3.70E-02	-0.13	5.31E-01	-0.25	2.03E-01	-0.19	3.44E-01	-0.78	1.67E-06	0.25	2.03E-01
ENSCAFG0000000254	CCDC30	red	VSMC_M1	0.54	3.49E-03	0.07	7.46E-01	-0.31	1.19E-01	0.49	1.03E-02	0.54	3.36E-03	-0.18	3.79E-01	0.03	8.97E-01	-0.41	3.22E-02	-0.20	3.08E-01	0.03	8.84E-01
ENSCAFG0000000745	GLT8D2	red	VSMC_M1	0.54	3.51E-03	0.44	2.09E-02	0.44	2.24E-02	-0.31	1.14E-01	-0.01	9.61E-01	-0.45	1.71E-02	0.11	5.96E-01	0.01	9.59E-01	-0.44	2.12E-02	-0.14	4.81E-01
ENSCAFG0000001580	MTA2	yellow	VSMC_M3	0.54	3.54E-03	0.14	4.72E-01	0.56	2.38E-03	-0.64	3.67E-04	-0.29	1.36E-01	0.02	9.28E-01	-0.01	9.79E-01	0.54	3.74E-03	-0.36	6.69E-02	-0.26	1.84E-01
ENSCAFG0000001260	IFT46	red	VSMC_M1	0.54	3.56E-03	0.23	2.52E-01	-0.18	3.79E-01	0.11	5.78E-01	0.56	2.39E-03	-0.49	9.03E-03	-0.10	6.14E-01	-0.28	1.60E-01	-0.25	2.18E-01	-0.16	4.37E-01
ENSCAFG0000000347	BLVRA	yellow	VSMC_M3	0.54	3.58E-03	0.69	6.77E-05	0.69	6.91E-05	-0.19	3.53E-01	-0.49	9.64E-03	0.00	9.97E-01	-0.21	2.84E-01	-0.07	7.41E-01	-0.77	2.28E-06	0.30	1.34E-01
ENSCAFG0000000348	KCNF1	red	VSMC_M1	0.54	3.59E-03	0.09	6.69E-01	0.08	7.00E-01	-0.19	3.39E-01	0.20	3.25E-01	-0.12	5.39E-01	0.47	1.42E-02	0.08	6.98E-01	-0.28	1.58E-01	-0.05	7.90E-01
ENSCAFG0000000334	TIA1	pink	VSMC_M5	0.54	3.60E-03	0.17	4.11E-01	0.05	8.09E-01	0.02	9.25E-01	0.35	6.96E-02	-0.47	1.25E-02	-0.02	9.06E-01	-0.09	6.39E-01	-0.27	1.72E-01	-0.28	1.61E-01
ENSCAFG0000003167	ENSCAFG0000003167	red	VSMC_M1	0.54	3.60E-03	0.37	5.68E-02	0.29	1.46E-01	-0.19	3.53E-01	0.10	6.22E-01	-0.22	2.60E-01	-0.03	8.95E-01	0.00	9.88E-01	-0.40	3.83E-02	-0.16	4.11E-01
ENSCAFG0000001562	HDAC6	cyan	VSMC_M2	0.54	3.61E-03	0.73	1.86E-05	0.34	7.86E-02	0.23	2.42E-01	-0.14	4.97E-01	-0.35	7.47E-02	-0.23	2.46E-01	-0.49	8.77E-03	-0.73	1.34E-05	0.47	1.23E-02
ENSCAFG0000001868	RAB12	red	VSMC_M1	0.54	3.62E-03	0.33	9.55E-02	0.02	9.08E-01	0.25	2.11E-01	0.23	2.40E-01	-0.27	1.67E-01	-0.30	1.33E-01	-0.34	8.39E-02	-0.39	4.51E-02	0.09	6.42E-01
ENSCAFG0000001038	CAPN3	cyan	VSMC_M2	0.54	3.63E-03	0.45	1.98E-02	0.38	5.20E-02	0.05	8.13E-01	-0.16	4.16E-01	0.00	9.83E-01	0.05	8.21E-01	-0.25	2.09E-01	-0.54	3.88E-03	0.26	1.90E-01
ENSCAFG0000001020	NDUFS4	pink	VSMC_M5	0.54	3.63E-03	-0.20	3.15E-01	-0.39	4.14E-02	0.28	1.64E-01	0.70	5.58E-05	-0.05	8.22E-01	-0.19	3.38E-01	-0.14	4.79E-01	0.08	6.87E-01	-0.32	1.00E-01
ENSCAFG0000000909	HPSE	red	VSMC_M1	0.54	3.65E-03	0.28	1.62E-01	0.16	4.38E-01	0.04	8.27E-01	0.11	5.85E-01	-0.10	6.06E-01	0.25	2.11E-01	-0.09	6.41E-01	-0.38	4.81E-02	0.05	7.95E-01
ENSCAFG0000001472	UBA52	grey	VSMC_M10	0.54	3.66E-03	0.80	6.26E-07	0.18	3.58E-01	0.44	2.14E-02	-0.07	7.39E-01	0.01	9.43E-01	-0.36	6.46E-02	-0.66	1.91E-04	-0.80	6.44E-07	0.58	1.47E-03
ENSCAFG0000003245	ATP5IF1	red	VSMC_M1	0.54	3.69E-03	0.14	4.95E-01	0.27	1.74E-01	0.10	6.15E-01	0.01	9.51E-01	-0.06	7.70E-01	-0.06	7.76E-01	-0.18	3.81E-01	-0.22	2.81E-01	-0.09	6.64E-01
ENSCAFG0000001738	APOOL	red	VSMC_M1	0.54	3.69E-03	0.18	3.64E-01	0.17	4.10E-01	0.02	9.36E-01	0.02	9.04E-01	-0.17	3.86E-01	-0.11	5.96E-01	-0.01	9.79E-01	-0.35	7.57E-02	0.02	9.39E-01
ENSCAFG0000001461	ENSCAFG0000001461	red	VSMC_M1	0.54	3.70E-03	-0.12	5.67E-01	0.23	2.57E-01	-0.29	1.44E-01	0.00	9.94E-01	-0.10	6.22E-01	0.09	6.54E-01	0.33	9.41E-02	-0.10	6.07E-01	-0.20	3.09E-01
ENSCAFG0000000307	FAM161A	red	VSMC_M1	0.54	3.74E-03	0.07	7.33E-01	0.41	3.24E-02	-0.36	6.44E-02	-0.15	4.68E-01	0.14	5.01E-01	0.24	2.24E-01	0.31	1.19E-01	-0.30	1.27E-01	-0.16	4.27E-01
ENSCAFG0000000909	LXN	pink	VSMC_M5	0.54	3.75E-03	0.16	4.39E-01	-0.23	2.51E-01	0.23	2.49E-01	0.60	8.78E-04	-0.54	3.67E-03	-0.22	2.67E-01	-0.32	1.03E-01	-0.12	5.35E-01	-0.15	4.52E-01
ENSCAFG0000003073	HIST2H2BF	grey	VSMC_M10	0.54	3.75E-03	0.11	5.72E-01	0.17	4.05E-01	0.02	9.26E-01	-0.02	9.07E-01	0.10	6.09E-01	-0.06	7.67E-01	-0.03	8.86E-01	-0.28	1.56E-01	0.13	5.27E-01
ENSCAFG0000001521	FCHO1	yellow	VSMC_M3	0.54	3.75E-03	0.64	2.83E-04	0.62	4.93E-04	-0.21	3.05E-01	-0.36	6.41E-02	0.19	3.44E-01	-0.19	3.54E-01	-0.09	6.57E-01	-0.69	7.85E-05	0.14	4.83E-01
ENSCAFG0000000062	CLIC1	red	VSMC_M1	0.54	3.75E-03	-0.17	3.96E-01	0.49	9.54E-03	-0.71	3.90E-05	-0.04	8.34E-01	-0.19	3.41E-01	0.09	6.59E-01	0.64	2.89E-04	0.00	9.87E-01	-0.67	1.13E-04
ENSCAFG0000000361	GAB1	grey	VSMC_M10	0.54	3.79E-03	0.51	6.11E-03	-0.20	3.09E-01	0.45	1.72E-02	0.42	2.81E-02	-0.13	5.07E-01	-0.17	4.00E-01	-0.60	9.17E-04	-0.55	2.78E-03	0.36	6.52E-02
ENSCAFG0000000887	TIPARP	pink	VSMC_M5	0.54	3.84E-03	-0.02	9.30E-01	-0.36	6.40E-02	0.21	2.86E-01	0.68	8.59E-05	-0.01	9.44E-01	-0.37	5.51E-02	-0.16	4.22E-01	-0.04	8.38E-01	-0.24	2.21E-01
ENSCAFG0000000851	PPP1CC	pink	VSMC_M5	0.54	3.85E-03	-0.33	9.58E-02	0.01	9.77E-01	-0.43	2.39E-02	0.36	6.13E-02	-0.04	8.52E-01	0.12	5.59E-01	0.53	4.39E-03	0.08	6.95E-01	-0.64	3.15E-04
ENSCAFG0000000418	ENSCAFG0000000418	red	VSMC_M1	0.54	3.88E-03	0.11	5.90E-01	-0.02	9.27E-01	0.17	3.91E-01	0.21	2.94E-01	-0.16	4.12E-01	0.16	4.12E-01	-0.10	6.07E-01	-0.33	9.20E-02	0.08	7.03E-01
ENSCAFG0000003220	OSGEP	yellow	VSMC_M3	0.54	3.89E-03	0.33	9.42E-02	0.83	9.31E-08	-0.63	4.12E-04	-0.57	1.96E-03	0.08	7.03E-01	0.26	1.95E-01	0.43	2.42E-02	-0.53	4.37E-03	-0.07	7.28E-01
ENSCAFG0000001981	ZNF276	red	VSMC_M1	0.54	3.89E-03	0.36	6.60E-02	0.10	6.36E-01	0.12	5.43E-01	0.10	6.33E-01	-0.19	3.37E-01	-0.18	3.73E-01	-0.17	4.05E-01	-0.46	1.67E-02	0.14	4.71E-01
ENSCAFG0000000419	ARHGAP35	red	VSMC_M1	0.54	3.90E-03	0.22	2.60E-01	-0.19	3.40E-01	0.16	4.27E-01	0.52	5.05E-03	-0.23	2.55E-01	-0.14	4.95E-01	-0.29	1.47E-01	-0.25	2.13E-01	-0.05	7.89E-01
ENSCAFG0000003101	YWHAH	grey	VSMC_M10	0.54	3.94E-03	0.01	9.57E-01	0.06	7.75E-01	-0.02	9.10E-01	0.26	1.84E-01	0.05	8.09E-01	-0.53	4.85E-03	-0.05	7.95E-01	-0.05	8.04E-01	-0.28	1.55E-01
ENSCAFG0000000450	C9H17orf80	yellow	VSMC_M3	0.54	3.97E-03	0.16	4.24E-01	0.40	3.95E-02	-0.52	5.07E-03	-0.04	8.45E-01	-0.16	4.22E-01	0.08	6.94E-01	0.38	5.21E-02	-0.30	1.23E-01	-0.29	1.39E-01
ENSCAFG0000000130	ZNF76	red	VSMC_M1	0.54	3.98E-03	0.43	2.62E-02	0.28	1.60E-01	0.04	8.31E-01	-0.18	3.66E-01	0.24	2.32E-01	-0.04	8.48E-01	-0.15	4.60E-01	-0.59	1.18E-03	0.36	6.54E-02
ENSCAFG0000001052	CDC73	red	VSMC_M1	0.54	4.02E-03	-0.18	3.82E-01	-0.36	6.27E-02	0.26	1.90E-01	0.68	1.04E-04	-0.06	7.69E-01	0.01	9.42E-01	-0.19	3.49E-01	0.06	7.71E-01	-0.27	1.74E-01
ENSCAFG0000000252	ENSCAFG0000000252	red	VSMC_M1	0.54	4.03E-03	0.07	7.18E-01	-0.11	5.89E-01	0.04	8.60E-01	0.50	8.10E-03	-0.20	3.18E-01	-0.32	1.01E-01	-0.08	6.95E-01	-0.15	4.54E-01	-0.30	1.31E-01
ENSCAFG0000000460	BIN1	cyan	VSMC_M2	0.53	4.04E-03	0.81	2.86E-07	0.52	5.62E-03	0.10	6.31E-01	-0.33	9.18E-02	-0.15	4.42E-01	-0.39	4.51E-02	-0.34	8.27E-02	-0.86	1.12E-08	0.41	3.37E-02
ENSCAFG0000002271	ENSCAFG0000002271	grey	VSMC_M10	0.53	4.05E-03	0.29	1.49E-01	-0.29	1.38E-01	0.39	4.33E-02	0.54	3.45E-03	-0.24	2.31E-01	-0.18	3.63E-01	-0.44	2.05E-02	-0.35	7.02E-02	0.10	6.29E-01
ENSCAFG0000000488	EIF2B4	red	VSMC_M1	0.53	4.06E-03	0.23	2.51E-01	0.50	7.46E-03	-0.35	7.39E-02	-0.13	5.23E-01	-0.21	2.85E-01	-0.32	1.05E-01	0.23	2.45E-01	-0.32	1.05E-01	-0.28	1.65E-01
ENSCAFG0000002035	CTCF	grey	VSMC_M10	0.53	4.09E-03	0.01	9.58E-01	0.40	4.04E-02	-0.54	3.35E-03	-0.07	7.33E-01	-0.04	8.49E-01	0.38	4.88E-02	0.46	1.66E-02	-0.26	1.88E-01	-0.32	1.03E-01
ENSCAFG0000001304	CEP19	red	VSMC_M1	0.53	4.10E-03	0.37	5.88E-02	0.01	9.66E-01	0.22	2.71E-01	0.20	3.20E-01	-0.33	9.49E-02	0.10	6.24E-01	-0.30	1.27E-01	-0.47	1.34E-02	0.22	2.71E-01
ENSCAFG0000000066	EHMT2	cyan	VSMC_M2	0.53	4.13E-03	0.83	1.07E-07	0.44	2.03E-02	-0.01	9.76E-01	-0.17	4.09E-01	-0.22	2.63E-01	-0.17	3.98E-01	-0.32	1.03E-01	-0.84	4.65E-08	0.32	9.99E-02
ENSCAFG0000001799	ENSCAFG0000001799	yellow	VSMC_M3	0.53	4.13E-03	0.31	1.22E-01	0.55	2.77E-03	-0.24	2.27E-01	-0.20	3.26E-01	-0.08	6.98E-01	0.22	2.74E-01	0.04	8.58E-01	-0.38	4.86E-02	-0.10	6.32E-01
ENSCAFG0000003108	ZFP2	red	VSMC_M1	0.53	4.14E-03	0.30	1.29E-01	-0.08	6.97E-01	0.10	6.20E-01	0.38	5.15E-02	-0.26	1.90E-01	0.07	7.12E-01	-0.22	2.61E-01	-0.40	3.89E-02	0.03	8.84E-01
ENSCAFG0000001152	IRF2BP2	red	VSMC_M1	0.53	4.16E-03	0.19	3.40E-01	0.06	7.67E-01	-0.27	1.71E-01	0.33	8.80E-02	-0.24	2.30E-01	0.03	8.83E-01	0.09	6.66E-01	-0.29	1.49E-01	-0.28	1.62E-01
ENSCAFG0000001202	DHRS9	grey	VSMC_M10	0.53	4.16E-03	0.73	1.77E-05	0.09	6.38E-01	0.34	8.70E-02	0.03	8.68E-01	-0.13	5.12E-01	-0.21	2.99E-01	-0.49	9.81E-03	-0.81	3.51E-07	0.48	1.03E-02
ENSCAFG0000000077	RNF5	grey	VSMC_M10	0.53	4.17E-03	0.30	1.28E-01	0.23	2.48E-01	-0.13	5.23E-01	0.11	5.96E-01	-0.07	7.29E-01	0.04	8.49E-01	-0.06	7.55E-01	-0.44	2.12E-02	-0.12	5.64E-01
ENSCAFG0000000769	VTI1B	red	VSMC_M1	0.53	4.26E-03	0.18	3.75E-01	0.17	4.09E-01	0.08	6.94E-01	0.10	6.12E-01	-0.36	6.54E-02	-0.08	6.78E-01	-0.16	4.36E-01	-0.30	1.31E-01	0.03	8.99E-01
ENSCAFG0000000366	GSN	cyan	VSMC_M2	0.53	4.26E-03	0.83	9.39E-08	0.44	2.07E-02	0.12	5.57E-01	-0.16	4.30E-01	-0.27	1.73E-01	-0.10	6.15E-01	-0.44	2.28E-02	-0.82	2.05E-07	0.36	6.91E-02
ENSCAFG0000002421	TRAPPC5	yellow	VSMC_M3	0.53	4.28E-03	0.40	3.69E-02	0.76	5.06E-06	-0.35	7.43E-02	-0.45	1.91E-02	0.12	5.64E-01	-0.11	5.96E-01	0.10	6.27E-01	-0.48	1.17E-02	-0.07	7.37E-01
ENSCAFG0000002303	ADGRE2	red	VSMC_M1	0.53	4.30E-03	0.28	1.62E-01	-0.08	7.04E-01	0.29	1.47E-01	0.27	1.75E-01	-0.23	2.48E-01	-0.20	3.13E-01	-0.34	8.03E-02	-0.35	7.08E-02	0.19	3.54E-01

ENSCAFG0000001669	RNF40	red	VSMC_M1	0.53	4.33E-03	0.07	7.38E-01	0.34	8.39E-02	-0.25	2.09E-01	0.05	8.07E-01	-0.24	2.28E-01	0.21	2.88E-01	0.14	4.81E-01	-0.20	3.15E-01	-0.25	2.10E-01
ENSCAFG0000002911	FAM186A	red	VSMC_M1	0.53	4.36E-03	0.25	2.01E-01	-0.02	9.41E-01	0.31	1.12E-01	0.34	8.01E-02	-0.31	1.20E-01	0.04	8.35E-01	-0.36	6.85E-02	-0.31	1.11E-01	-0.02	9.05E-01
ENSCAFG0000001134	STAU1	grey	VSMC_M10	0.53	4.40E-03	0.58	1.51E-03	-0.03	8.92E-01	0.47	1.28E-02	0.28	1.63E-01	-0.30	1.27E-01	-0.17	3.83E-01	-0.65	2.67E-04	-0.56	2.14E-03	0.36	6.76E-02
ENSCAFG0000000532	UVRAG	red	VSMC_M1	0.53	4.41E-03	0.43	2.37E-02	0.26	1.86E-01	-0.14	4.85E-01	0.03	8.77E-01	-0.04	8.26E-01	0.14	4.73E-01	-0.09	6.55E-01	-0.53	4.23E-03	0.02	9.06E-01
ENSCAFG0000000485	INTS4	pink	VSMC_M5	0.53	4.41E-03	-0.01	9.60E-01	-0.27	1.70E-01	0.21	3.05E-01	0.52	5.41E-03	0.02	9.04E-01	0.18	3.77E-01	-0.18	3.70E-01	-0.19	3.49E-01	-0.08	7.08E-01
ENSCAFG0000001999	DPH5	red	VSMC_M1	0.53	4.41E-03	-0.15	4.41E-01	-0.31	1.15E-01	0.22	2.78E-01	0.64	3.13E-04	-0.07	7.31E-01	-0.02	9.12E-01	-0.17	4.07E-01	0.00	9.82E-01	-0.29	1.39E-01
ENSCAFG0000001599	RNF216	grey	VSMC_M10	0.53	4.42E-03	0.57	1.90E-03	-0.04	8.36E-01	0.41	3.22E-02	0.20	3.25E-01	-0.22	2.73E-01	-0.10	6.30E-01	-0.52	5.10E-03	-0.62	5.36E-04	0.42	3.13E-02
ENSCAFG0000001081	PTP4A2	red	VSMC_M1	0.53	4.43E-03	-0.03	8.79E-01	0.29	1.38E-01	-0.56	2.38E-03	0.08	6.80E-01	-0.09	6.68E-01	-0.12	5.37E-01	0.46	1.58E-02	-0.11	5.78E-01	-0.49	1.01E-02
ENSCAFG0000001962	JMJD8	yellow	VSMC_M3	0.53	4.43E-03	0.40	3.83E-02	0.71	2.86E-05	-0.46	1.52E-02	-0.39	4.52E-02	-0.31	1.19E-01	-0.10	6.04E-01	0.17	4.08E-01	-0.45	1.81E-02	-0.09	6.42E-01
ENSCAFG0000000970	IRAK4	pink	VSMC_M5	0.53	4.44E-03	-0.29	1.38E-01	-0.42	2.80E-02	0.03	9.00E-01	0.70	4.07E-05	-0.19	3.38E-01	0.10	6.19E-01	0.07	7.11E-01	0.13	5.29E-01	-0.32	9.84E-02
ENSCAFG0000003252	AHNAK	red	VSMC_M1	0.53	4.47E-03	0.01	9.64E-01	0.07	7.33E-01	-0.16	4.38E-01	0.29	1.47E-01	-0.28	1.61E-01	0.20	3.15E-01	0.07	7.21E-01	-0.14	4.97E-01	-0.24	2.19E-01
ENSCAFG0000001486	RBM10	grey	VSMC_M10	0.53	4.51E-03	0.09	6.47E-01	0.35	7.08E-02	-0.51	6.35E-03	-0.10	6.32E-01	-0.01	9.64E-01	0.17	4.07E-01	0.42	3.06E-02	-0.34	8.40E-02	-0.17	3.91E-01
ENSCAFG0000002906	ENSCAFG0000002906	cyan	VSMC_M2	0.53	4.51E-03	0.53	4.53E-03	0.16	4.19E-01	0.31	1.19E-01	0.08	6.99E-01	-0.32	1.02E-01	-0.23	2.58E-01	-0.54	3.60E-03	-0.57	2.05E-03	0.25	2.09E-01
ENSCAFG0000001824	ESCO1	pink	VSMC_M5	0.53	4.59E-03	-0.11	5.78E-01	-0.27	1.67E-01	0.12	5.37E-01	0.59	1.31E-03	-0.41	3.24E-02	-0.18	3.77E-01	-0.05	8.00E-01	-0.03	9.00E-01	-0.20	3.10E-01
ENSCAFG0000002932	ZNF484	grey	VSMC_M10	0.53	4.61E-03	0.47	1.30E-02	0.00	9.88E-01	0.46	1.51E-02	0.09	6.69E-01	-0.11	5.75E-01	-0.03	8.82E-01	-0.49	8.72E-03	-0.60	9.69E-04	0.53	4.43E-03
ENSCAFG0000000236	ZNF584	red	VSMC_M1	0.53	4.65E-03	0.18	3.69E-01	0.01	9.54E-01	0.07	7.39E-01	0.20	3.18E-01	-0.17	3.99E-01	0.11	5.76E-01	-0.12	5.52E-01	-0.37	6.02E-02	0.06	7.52E-01
ENSCAFG0000001697	ENSCAFG0000001697	pink	VSMC_M5	0.53	4.69E-03	-0.26	1.94E-01	-0.14	4.95E-01	-0.08	6.79E-01	0.50	7.78E-03	-0.06	7.48E-01	-0.06	7.55E-01	0.18	3.80E-01	0.07	7.45E-01	-0.49	9.85E-03
ENSCAFG0000000680	HSPB6	cyan	VSMC_M2	0.53	4.70E-03	0.46	1.54E-02	0.43	2.61E-02	-0.07	7.42E-01	-0.18	3.83E-01	0.12	5.40E-01	-0.18	3.77E-01	-0.19	3.33E-01	-0.49	9.96E-03	0.13	5.12E-01
ENSCAFG0000001951	NPHP4	cyan	VSMC_M2	0.53	4.71E-03	0.55	2.70E-03	0.25	2.06E-01	-0.05	8.06E-01	0.01	9.43E-01	-0.04	8.26E-01	0.02	9.12E-01	-0.16	4.17E-01	-0.59	1.10E-03	0.15	4.56E-01
ENSCAFG0000001565	ZBTB3	cyan	VSMC_M2	0.53	4.71E-03	0.49	1.03E-02	0.12	5.59E-01	0.24	2.19E-01	0.03	8.65E-01	0.01	9.56E-01	0.17	4.00E-01	-0.35	7.64E-02	-0.63	4.21E-04	0.37	5.75E-02
ENSCAFG0000002924	PPP1R14A	red	VSMC_M1	0.53	4.71E-03	-0.05	7.86E-01	-0.07	7.39E-01	0.01	9.60E-01	0.32	1.07E-01	0.08	7.03E-01	-0.05	7.91E-01	0.02	9.30E-01	-0.06	7.77E-01	-0.20	3.05E-01
ENSCAFG0000001580	ENO3	grey	VSMC_M10	0.53	4.80E-03	0.27	1.75E-01	0.38	4.74E-02	-0.39	4.54E-02	-0.12	5.48E-01	0.29	1.41E-01	-0.02	9.41E-01	0.26	1.93E-01	-0.42	2.74E-02	-0.09	6.65E-01
ENSCAFG0000001951	ENSCAFG0000001951	cyan	VSMC_M2	0.53	4.82E-03	0.86	7.43E-09	0.62	5.15E-04	-0.11	5.83E-01	-0.40	3.94E-02	-0.12	5.38E-01	-0.10	6.05E-01	-0.28	1.63E-01	-0.87	2.55E-09	0.41	3.35E-02
ENSCAFG0000000609	KITLG	pink	VSMC_M5	0.53	4.83E-03	-0.21	2.97E-01	-0.33	9.21E-02	0.03	8.95E-01	0.65	2.34E-04	-0.18	3.71E-01	0.14	4.79E-01	0.01	9.62E-01	0.05	7.93E-01	-0.29	1.41E-01
ENSCAFG0000000303	ENSCAFG0000000303	red	VSMC_M1	0.53	4.84E-03	0.23	2.42E-01	0.41	3.59E-02	-0.25	2.17E-01	-0.09	6.59E-01	-0.21	2.89E-01	-0.22	2.70E-01	0.07	7.18E-01	-0.35	7.28E-02	-0.14	4.94E-01
ENSCAFG0000001263	DPP3	yellow	VSMC_M3	0.53	4.92E-03	0.43	2.51E-02	0.79	9.98E-07	-0.49	1.01E-02	-0.58	1.55E-03	0.06	7.74E-01	0.16	4.39E-01	0.29	1.39E-01	-0.60	8.46E-04	0.02	9.22E-01
ENSCAFG0000001857	GHR	grey	VSMC_M10	0.52	4.93E-03	0.44	2.02E-02	-0.17	3.98E-01	0.50	7.46E-03	0.27	1.69E-01	-0.06	7.84E-01	-0.12	5.48E-01	-0.58	1.47E-03	-0.53	4.20E-03	0.48	1.12E-02
ENSCAFG0000000585	PLCB1	grey	VSMC_M10	0.52	4.95E-03	-0.04	8.55E-01	-0.37	6.09E-02	0.27	1.68E-01	0.58	1.60E-03	-0.16	4.34E-01	0.26	1.96E-01	-0.24	2.35E-01	-0.12	5.41E-01	0.03	8.98E-01
ENSCAFG0000001926	AURKAIP1	yellow	VSMC_M3	0.52	4.97E-03	0.25	2.18E-01	0.71	2.80E-05	-0.57	1.73E-03	-0.37	5.57E-02	-0.17	4.08E-01	0.25	2.04E-01	0.39	4.55E-02	-0.40	3.86E-02	-0.24	2.26E-01
ENSCAFG0000001179	EXOC8	cyan	VSMC_M2	0.52	4.97E-03	0.34	7.99E-02	0.15	4.60E-01	0.18	3.82E-01	0.05	7.91E-01	0.05	8.15E-01	-0.10	6.04E-01	-0.28	1.55E-01	-0.46	1.67E-02	0.16	4.19E-01
ENSCAFG0000000952	CHP1	yellow	VSMC_M3	0.52	4.97E-03	0.40	3.96E-02	0.64	3.06E-04	-0.21	2.92E-01	-0.41	3.24E-02	0.02	9.31E-01	-0.16	4.25E-01	-0.01	9.52E-01	-0.47	1.41E-02	0.09	6.73E-01
ENSCAFG0000001190	PCBP3	grey	VSMC_M10	0.52	4.98E-03	0.57	2.12E-03	0.16	4.15E-01	0.16	4.13E-01	-0.05	8.03E-01	0.01	9.50E-01	-0.16	4.39E-01	-0.30	1.31E-01	-0.72	2.72E-05	0.42	2.91E-02
ENSCAFG0000001682	COPZ2	red	VSMC_M1	0.52	4.98E-03	0.31	1.13E-01	0.19	3.35E-01	0.17	3.99E-01	0.12	5.59E-01	-0.25	2.04E-01	0.01	9.66E-01	-0.34	8.26E-02	-0.36	6.24E-02	0.01	9.70E-01
ENSCAFG0000000097	TCF20	cyan	VSMC_M2	0.52	4.99E-03	0.57	1.71E-03	0.18	3.61E-01	0.17	3.98E-01	0.04	8.32E-01	-0.20	3.21E-01	0.11	6.02E-01	-0.40	3.78E-02	-0.64	3.04E-04	0.38	5.38E-02
ENSCAFG0000003263	UBL5	cyan	VSMC_M2	0.52	5.02E-03	0.42	2.94E-02	0.44	2.05E-02	-0.02	9.21E-01	-0.19	3.32E-01	0.00	9.92E-01	-0.25	2.18E-01	-0.16	4.18E-01	-0.45	1.81E-02	0.04	8.49E-01
ENSCAFG0000001495	ENSCAFG0000001495	red	VSMC_M1	0.52	5.04E-03	0.01	9.42E-01	-0.08	6.77E-01	0.07	7.18E-01	0.44	2.28E-02	-0.23	2.39E-01	-0.18	3.79E-01	-0.08	6.95E-01	-0.13	5.33E-01	-0.25	2.04E-01
ENSCAFG0000002375	GNAS	red	VSMC_M1	0.52	5.06E-03	-0.06	7.61E-01	0.25	2.08E-01	-0.20	3.28E-01	0.19	3.43E-01	-0.12	5.40E-01	-0.24	2.30E-01	0.08	6.83E-01	0.03	8.64E-01	-0.48	1.08E-02
ENSCAFG0000001913	CD99L2	yellow	VSMC_M3	0.52	5.06E-03	0.45	1.83E-02	0.59	1.25E-03	-0.23	2.42E-01	-0.27	1.77E-01	-0.10	6.08E-01	-0.23	2.44E-01	-0.05	7.85E-01	-0.48	1.05E-02	-0.02	9.04E-01
ENSCAFG0000001098	SLC25A6	yellow	VSMC_M3	0.52	5.06E-03	0.63	4.57E-04	0.80	6.76E-07	-0.46	1.62E-02	-0.48	1.07E-02	-0.11	6.02E-01	-0.05	7.97E-01	0.13	5.13E-01	-0.71	3.20E-05	0.03	8.97E-01
ENSCAFG0000000509	RHBDF2	cyan	VSMC_M2	0.52	5.08E-03	0.67	1.17E-04	0.42	3.05E-02	0.00	9.97E-01	-0.24	2.31E-01	0.07	7.31E-01	-0.20	3.25E-01	-0.24	2.32E-01	-0.75	6.13E-06	0.33	9.10E-02
ENSCAFG0000000192	MAD2L1BP	red	VSMC_M1	0.52	5.12E-03	0.20	3.17E-01	0.19	3.42E-01	0.05	8.24E-01	-0.11	5.82E-01	0.07	7.26E-01	0.09	6.41E-01	-0.03	8.69E-01	-0.43	2.38E-02	0.27	1.67E-01
ENSCAFG0000002884	JUNB	cyan	VSMC_M2	0.52	5.17E-03	0.76	4.15E-06	0.46	1.52E-02	0.01	9.73E-01	-0.20	3.21E-01	-0.18	3.72E-01	-0.14	4.98E-01	-0.28	1.52E-01	-0.83	1.10E-07	0.30	1.31E-01
ENSCAFG0000003269	FGF10	red	VSMC_M1	0.52	5.18E-03	-0.02	9.20E-01	0.11	5.91E-01	-0.12	5.39E-01	0.11	5.97E-01	-0.03	8.94E-01	-0.15	4.43E-01	0.15	4.45E-01	-0.13	5.15E-01	-0.13	5.22E-01
ENSCAFG0000000427	ENSCAFG0000000427	yellow	VSMC_M3	0.52	5.28E-03	0.32	1.03E-01	0.64	3.27E-04	-0.16	4.25E-01	-0.29	1.41E-01	-0.10	6.09E-01	-0.11	5.77E-01	-0.04	8.52E-01	-0.38	4.86E-02	-0.13	5.26E-01
ENSCAFG0000000483	ENSCAFG0000000483	yellow	VSMC_M3	0.52	5.28E-03	0.32	1.03E-01	0.64	3.27E-04	-0.16	4.25E-01	-0.29	1.41E-01	-0.10	6.09E-01	-0.11	5.77E-01	-0.04	8.52E-01	-0.38	4.86E-02	-0.13	5.26E-01
ENSCAFG0000001863	DENND1C	yellow	VSMC_M3	0.52	5.28E-03	0.32	1.03E-01	0.64	3.27E-04	-0.16	4.25E-01	-0.29	1.41E-01	-0.10	6.09E-01	-0.11	5.77E-01	-0.04	8.52E-01	-0.38	4.86E-02	-0.13	5.26E-01
ENSCAFG0000002987	C17H2orf70	yellow	VSMC_M3	0.52	5.28E-03	0.32	1.03E-01	0.64	3.27E-04	-0.16	4.25E-01	-0.29	1.41E-01	-0.10	6.09E-01	-0.11	5.77E-01	-0.04	8.52E-01	-0.38	4.86E-02	-0.13	5.26E-01
ENSCAFG0000000154	TNPO3	darkgrey	VSMC_M8	0.52	5.32E-03	-0.29	1.42E-01	0.18	3.82E-01	-0.58	1.68E-03	0.16	4.17E-01	0.16	4.16E-01	0.31	1.20E-01	0.59	1.24E-03	0.02	9.29E-01	-0.56	2.47E-03
ENSCAFG0000001511	QDPR	cyan	VSMC_M2	0.52	5.33E-03	0.31	1.18E-01	0.17	3.99E-01	0.28	1.64E-01	0.09	6.69E-01	-0.08	6.79E-01	-0.31	1.11E-01	-0.32	1.00E-01	-0.35	7.76E-02	0.03	8.85E-01
ENSCAFG0000000073	ENSCAFG0000000073	red	VSMC_M1	0.52	5.35E-03	0.19	3.31E-01	0.31	1.12E-01	-0.23	2.40E-01	-0.01	9.70E-01	0.06	7.48E-01	-0.04	8.55E-01	0.07	7.27E-01	-0.30	1.27E-01	-0.15	4.67E-01
ENSCAFG0000000482	ATRAID	cyan	VSMC_M2	0.52	5.37E-03	0.51	7.17E-03	-0.02	9.15E-01	0.32	1.05E-01	0.19	3.42E-01	-0.08	6.81E-01	-0.13	5.33E-01	-0.43	2.48E-02	-0.58	1.43E-03	0.28	1.56E-01
ENSCAFG0000000104	NCOA7	grey	VSMC_M10	0.52	5.38E-03	0.24	2.33E-01	-0.17	3.99E-01	0.23	2.56E-01	0.26	1.89E-01	0.04	8.33E-01	0.14	4.92E-01	-0.19	3.32E-01	-0.46	1.65E-02	0.32	1.04E-01
ENSCAFG0000000798	PTCD2	red	VSMC_M1	0.52	5.45E-03	-0.03	8.71E-01	0.08	6.75E-01	-0.06	7.76E-01	0.16	4.15E-01	-0.05	8.11E-01	0.20	3.30E-01	0.04	8.46E-01	-0.10	6.13E-01	-0.11	5.99E-01
ENSCAFG0000003103	YPEL5	red	VSMC_M1	0.52	5.45E-03	0.29	1.46E-01	-0.01	9.50E-01	0.17	4.09E-01	0.26	1.96E-01	0.03	8.99E-01	-0.04	8.25E-01	-0.37	5.61E-02	-0.33	9.64E-02	0.12	5.42E-01
ENSCAFG0000003187	RNF125	red	VSMC_M1	0.52	5.46E-03	-0.21	2.85E-01	0.02	9.26E-01	-0.20	3.27E-01	0.19	3.50E-01	0.22	2.79E-01	0.13	5.03E-01	0.25	2.00E-01	-0.03	9.01E-01	-0.21	2.93E-01
ENSCAFG0000000776	IRF2	grey	VSMC_M10	0.52	5.52E-03	0.51	6.00E-03	-0.16	4.12E-01	0.47	1.34E-02	0.44	2.05E-02	-0.31	1.20E-01	-0.45	1.98E-02	-0.61	7.68E-04	-0.52	5.63E-03	0.20	3.26E-01
ENSCAFG0000002480	TCFL5	grey	VSMC_M10	0.52	5.53E-03	0.37	6.12E-02	0.37	5.66E-02	-0.14	4.90E-01	-0.19	3.45E-01	-0.10	6.14E-01	0.21	2.89E-01	0.00	9.86E-01	-0.50	8.04E-03	0.22	2.62E-01
ENSCAFG0000001573	EPS15L1	grey	VSMC_M10	0.52	5.56E-03	0.46	1.56E-02	-0.28	1.50E-01	0.60	9.20E-04	0.42	3.06E-02	-0.21	2.84E-01	-0.21	2.91E-01	-0.57	1.85E-03	-0.57	1.99E-03	0.41	3.30E-02
ENSCAFG0000001523	CCDC181	red	VSMC_M1	0.52	5.57E-03	0.12	5.36E-01	0.16	4.23E-01	0.03	8.76E-01	0.05	8.10E-01	-0.11	6.01E-01	0.37	6.03E-02	-0.05	8.02E-01	-0.34	8.63E-02	0.07	7.11E-01
ENSCAFG0000001242	GABPB2	pink	VSMC_M5	0.52	5.58E-03	-0.10	6.18E-01	-0.03	8.87E-01	-0.07	7.11E-01	0.37	6.05E-02	-0.22	2.64E-01	0.08	7.01E-01	0.11	6.01E-01	-0.09	6.64E-01	-0.29	1.39E-01
ENSCAFG0000001640	TP53BP2	cyan	VSMC_M2	0.52	5.58E-03	0.56	2.60E-03	0.42	2.83E-02	-0.22	2.74E-01	-0.14	4.92E-01	0.01	9.48E-01	-0.28	1.59E-01	-0.01	9.67E-01	-0.58	1.36E-03	0.06	7.55E-01
ENSCAFG0000000121	HMGA1	red	VSMC_M1	0.52	5.61E-03	0.10	6.23E-01	0.63	4.17E-04	-0.74	8.92E-06	-0.25	2.12E-01	0.03	8.93E-01	0.06	7.60E-01	0.54	3.85E-03	-0.23	2.52E-01	-0.44	2.08E-02
ENSCAFG0000000323	INPP5B	red	VSMC_M1	0.52	5.61E-03	0.48	1.18E-02	0.29	1.48E-01	0.02	9.34E-01	0.01	9.57E-01	-0.18	3.64E-01	0.14	4.75E-01	-0.20	3.11E-01	-0.60	9.65E-04	0.17	3.98E-01
ENSCAFG0000000146	TLE1	cyan	VSMC_M2	0.52	5.62E-03	0.72	2.27E-05	0.42	3.09E-02	-0.03	8.79E-01	-0.19	3.52E-01	-0.18	3.73E-01	-0.51	6.20E-03	-0.23	2.44E-01	-0.76	5.02E-06	0.29	1.49E-01
ENSCAFG0000001469	PTGDR	grey	VSMC_M10	0.52	5.71E-03	0.57	2.09E-03	-0.09	6.57E-01	0.50	8.24E-03	0.16	4.18E-01	-0.06	7.79E-01	0.00	9.95E-01	-0.57	1.91E-03	-0.66	1.78E-04	0.56	2.58E-03
ENSCAFG0000000705	BCL2L1	yellow	VSMC_M3	0.52	5.71E-03	0.36	6.35E-02	0.68	9.63E-05	-0.33	9.41E-02	-0.46	1.51E-02	0.04	8.36E-01	0.14	4.99E-01	0.13	5.32E-01	-0.46	1.47E-02	0.07	7.29E-01
ENSCAFG0000000973	DNTTIP1	yellow	VSMC_M3	0.52	5.72E-03	0.41	3.23E-02	0.54	3.70E-03	-0.33	9.22E-02	-0.21	2.97E-01	-0.14	4.87E-01	-0.13	5.19E-01	0.14	4.96E-01	-0.53	4.63E-03	-0.11	5.86E-01
ENSCAFG0000001483	NDUFB11	grey	VSMC_M10	0.52	5.81E-03	0.25	2.14E-01	0.36	6.32E-02	-0.04	8.33E-01	-0.24	2.27E-01	0.12	5.40E-01	0.16	4.38E-01	-0.04	8.47E-01	-0.42	2.97E-02	0.23	2.53E-01
ENSCAFG0000001822	ENSCAFG0000001822	red	VSMC_M1	0.52	5.87E-03	0.02	9.22E-01	-0.12	5.49E-01	0.28	1.58E-01	0.40	3.67E-02	-0.37	5.68E-02	-0.13	5.22E-01	-0.31	1.12E-01	-0.09	6.68E-01	-0.12	5.45E-01
ENSCAFG0000001925	CREBBP	grey	VSMC_M10	0.52	5.88E-03	0.41	3.51E-02	-0.31	1.14E-01	0.47	1.29E-02	0.51	6.53E-03	-0.17	4.01E-01	-0.19	3.38E-01	-0.58	1.61E-03	-0.45	2.00E-02	0.32	9.98E-02
ENSCAFG0000000662	EFCAB1	grey	VSMC_M10	0.52	5.89E-03	0.27	1.80E-01	0.42	3.12E-02	-0.07	7.10E-01	-0.27	1.78E-01	-0.02	9.35E-01	0.25	2.09E-01	-0.07	7.28E-01	-0.41	3.32E-02	0.22	2.70E-01
ENSCAFG0000000278	ENSCAFG0000000278	yellow	VSMC_M3	0.52	5.90E-03	0.46	1.59E-02	0.50	8.46E-03	-0.06	7.81E-01	-0.27	1.78E-01	-0.06	7.47E-01	-0.14	4.98E-01	-0.15	4.52E-01	-0.54	3.65E-03	0.19	3.43E-01
ENSCAFG0000000186	TJAP1	grey	VSMC_M10	0.52	5.92E-03	0.29	1.48E-01	0.03	8.67E-01	0.26	1.87E-01	0.11	5.75E-01	0.05	8.23E-01	-0.35	6.94E-02	-0.30	1.34E-01	-0.40	3.80E-02	0.20	3.15E-01
ENSCAFG0000000547	TPPP2	cyan	VSMC_M2	0.52	5.93E-03	0.46	1.50E-02	0.20	3.10E-01	0.32	1.01E-01	0.05	8.11E-01	-0.17	3.86E-01	-0.25	2.02E-01	-0.51	6.95E-03	-0.48	1.19E-02	0.21	3.01E-01
ENSCAFG0000001590	ENSCAFG0000001590	red	VSMC_M1	0.52	5.95E-03	0.25	2.09E-01	-0.22	2.81E-01	0.33	9.67E-02	0.40	3.84E-02	-0.11	5.80E-01	-0.37	5.84E-02	-0.36	6.61E-02	-0.31	1.16E-01	0.17	3.98E-01
ENSCAFG0000000719	ENSCAFG0000000719	yellow	VSMC_M3	0.52	5.98E-03	0.65	2.60E-04	0.59	1.17E-03	0.01	9.73E-01	-0.36	6.17E-02	-0.12	5.57E-01	-0.28	1.59E-01	-0.24	2.31E-01	-0.71	3.80E-05	0.24	2.21E-01
ENSCAFG0000000350	ARHGEF5	grey	VSMC_M10	0.51	6.08E-03	0.18	3.70E-01	-0.10	6.24E-01	0.22	2.70E-01	0.25	2.08E-01	-0.08	7.02E-01	0.36	6.59E-02	-0.21	3.02E-01	-0.41	3.50E-02	0.17	3.91E-01
ENSCAFG0000003161	IFIT3	grey	VSMC_M10	0.51	6.08E-03	0.56	2.37E-03	0.07	7.26E-01	0.27	1.69E-01	0.03	8.73E-01	0.08	6.77E-01	-0.29	1.46E-01	-0.34	8.03E-02	-0.68	9.70E-05	0.39	4.16E-02
ENSCAFG0000001900	BLMH	yellow	VSMC_M3	0.51	6.12E-03	0.02	9.09E-01	0.52	5.54E-03	-0.41	3.51E-02	-0.16	4.40E-01	-0.15	4.61E-01	0.13	5.26E-01	0.33	9.09E-02	-0.24	2.33E-01	-0.36	6.19E-02
ENSCAFG0000002468	ENSCAFG0000002468	red	VSMC_M1	0.51	6.15E-03	0.23	2.56E-01	0.27	1.78E-01	-0.25	2.01E-01	0.06	7.61E-01	-0.31	1.16E-01	-0.04	8.25E-01	0.11	5.68E-01	-0.34	8.35E-02	-0.20	3.09E-01
ENSCAFG0000002492	ATP5PF	pink	VSMC_M5	0.51	6.17E-03	-0.17	3.96E-01	-0.27	1.79E-01	0.22	2.77E-01	0.64	3.39E-04	-0.31	1.19E-01	-0.26	1.90E-01	-0.13	5.09E-01	0.10	6.35E-01	-0.39	4.51E-02
ENSCAFG0000001383	CAPN1	cyan	VSMC_M2	0.51	6.17E-03	0.84	4.26E-08	0.56	2.32E-03	0.08	6.99E-01	-0.29	1.45E-01	-0.26	1.86E-01	-0.17	3.83E-01	-0.42	2.82E-02	-0.78	1.92E-06	0.34	8.75E-02
ENSCAFG0000000725	ARMC1	grey	VSMC_M10	0.51	6.23E-03	-0.14	4.88E-01	-0.25	2.05E-01	0.24	2.22E-01	0.41	3.22E-02	-0.18	3.73E-01	0.07	7.35E-01	-0.10	6.16E-01	-0.05	8.18E-01	-0.01	9.74E-01
ENSCAFG0000000545	KAT14	yellow	VSMC_M3	0.51	6.25E-03	0.15	4.43E-01	0.43	2.44E-02	-0.23	2.44E-01	-0.11	5.87E-01	-0.04	8.26E-01	0.08	6.75E-01	0.13	5.17E-01	-0.32	1.02E-01	-0.18	3.64E-01
ENSCAFG0000000534	SERTAD1	yellow	VSMC_M3	0.51	6.26E-03	0.42	3.05E-02	0.81	2.87E-07	-0.42	2.79E-02	-0.54	4.01E-03	0.04	8.35E-01	-0.08	6.75E-01	0.26	1.95E-01	-0.55	3.27E-03	-0.08	7.06E-01
ENSCAFG0000002900	CYSTM1	grey	VSMC_M10	0.51	6.28E-03	0.45	1.82E-02	-0.02	9.05E-01	0.32	1.02E-01	0.22	2.67E-01	-0.08	7.09E-01	-0.32	1.08E-01	-0.50	8.16E-03	-0.49	1.02E-02	0.22	2.74E-01
ENSCAFG0000001457	PRDX6	red	VSMC_M1	0.51	6.28E-03	-0.10	6.17E-01	0.26	1.98E-01	-0.33	9.57E-02	0.00	9.83E-01	0.19	3.52E-01	-0.07	7.24E-01	0.37	5.71E-02	-0.07	7.30E-01	-0.39	4.64E-02
ENSCAFG0000000242	ANKMY2	pink	VSMC_M5	0.51	6.29E-03	0.00	9.85E-01	-0.20	3.13E-01	0.18	3.57E-01	0.63	4.08E-04	-0.42	2.85E-02	-0.26	1.96E-01	-0.19	3.31E-01	-0.04	8.32E-01	-0.39	4.38E-02
ENSCAFG0000002381	ENSCAFG0000002381	red	VSMC_M1	0.51	6.31E-03	-0.04	8.28E-01	0.40	3.70E-02	-0.39	4.73E-02	-0.07	7.45E-01	-0.14	4.94E-01	0.43	2.38E-02	0.31	1.17E-01	-0.11	5.84E-01	-0.31	1.12E-01
ENSCAFG0000003160	ENSCAFG0000003160	yellow	VSMC_M3	0.51	6.34E-03	0.37	5.87E-02	0.68	1.08E-04	-0.20	3.06E-01	-0.34	8.55E-02	-0.12	5.48E-01	0.09	6.42E-01	-0.01	9.45E-01	-0.43	2.46E-02	-0.08	6.90E-01
ENSCAFG0000000173	ENSCAFG0000000173	red	VSMC_M1	0.51	6.35E-03	0.03	8.67E-01	0.16	4.21E-01	-0.15	4.41E-01	0.17	3.94E-01	0.00	9.92E-01	-0.04	8.40E-01	0.14	4.95E-01	-0.17	4.01E-01	-0.25	2.02E-01
ENSCAFG0000001011	TMEFF2	red	VSMC_M1	0.51	6.39E-03	0.45	1.90E-02	0.50	7.70E-03	-0.31	1.21E-01	-0.27	1.72E-01	0.01	9.72E-01	0.39	4.28E-02	0.15	4.53E-01	-0.63	4.12E-04	0.12	5.62E-01
ENSCAFG0000001117	FAM20C	grey	VSMC_M10	0.51	6.40E-03	0.11	5.76E-01	0.44	2.09E-02	-0.44	2.14E-02	-0.11	6.01E-01	-0.19	3.35E-01	0.12	5.47E-01	0.19	3.40E-01	-0.18	3.76E-01	-0.25	2.18E-01
ENSCAFG0000002476	ENSCAFG0000002476	grey	VSMC_M10	0.51	6.43E-03	0.23	2.39E-01	0.06	7.62E-01	0.11	5.76E-01	0.19	3.54E-01	0.05	8.14E-01	0.04	8.60E-01	-0.07	7.15E-01	-0.43	2.34E-02	0.02	9.20E-01
ENSCAFG0000000571	SPAST	red	VSMC_M1	0.51	6.43E-03	0.26	1.87E-01	0.09	6.48E-01	0.25	2.14E-01	0.27	1.77E-01	-0.25	2.03E-01	-0.24	2.31E-01	-0.34	8.40E-02	-0.29	1.40E-01	-0.06	7.51E-01
ENSCAFG0000001715	SHC1	cyan	VSMC_M2	0.51	6.52E-03	0.77	2.45E-06	0.48	1.20E-02	-0.08	6.76E-01	-0.23	2.56E-01	-0.26	1.94E-01	-0.08	7.08E-01	-0.23	2.48E-01	-0.78	1.48E-06	0.31	1.10E-01
ENSCAFG0000001515	TIMP1	pink	VSMC_M5	0.51	6.53E-03	0.04	8.40E-01	-0.24	2.34E-01	0.27	1.77E-01	0.54	3.49E-03	-0.30	1.28E-01	0.07	7.40E-01	-0.29	1.37E-01	-0.08	7.08E-01	-0.13	5.30E-01
ENSCAFG0000001223	PRMT2	yellow	VSMC_M3	0.51	6.53E-03	0.65	2.42E-04	0.67	1.43E-04	-0.15	4.45E-01	-0.45	1.83E-02	-0.06	7.77E-01	-0.07	7.26E-01	-0.16	4.36E-01	-0.66	1.69E-04	0.23	2.49E-01
ENSCAFG0000001612	ZBTB17	cyan	VSMC_M2	0.51	6.54E-03	0.71	3.73E-05	0.48	1.06E-02	0.01	9.80E-01	-0.21	3.02E-01	-0.10	6.03E-01	-0.37	5.49E-02	-0.23	2.54E-01	-0.77	2.30E-06	0.19	3.48E-01
ENSCAFG0000000515	ENSCAFG0000000515	red	VSMC_M1	0.51	6.54E-03	-0.22	2.60E-01	0.11	5.72E-01	-0.22	2.68E-01	0.19	3.50E-01	-0.24	2.33E-01	0.31	1.12E-01	0.24	2.21E-01	0.04	8.54E-01	-0.34	8.63E-02
ENSCAFG0000000039	MAPK9	pink	VSMC_M5	0.51	6.54E-03	-0.15	4.42E-01	-0.47	1.24E-02	0.21	3.02E-01	0.70	5.02E-05	-0.14	4.80E-01	0.02	9.11E-01	-0.12	5.48E-01	0.02	9.30E-01	-0.14	4.92E-01
ENSCAFG0000001570	DDX11	grey	VSMC_M10	0.51	6.56E-03	0.07	7.34E-01	0.48	1.11E-02	-0.47	1.23E-02	-0.20	3.19E-01	-0.10	6.10E-01	0.43	2.42E-02	0.41	3.50E-02	-0.33	9.04E-02	-0.19	3.31E-01
ENSCAFG0000001543	PORCN	cyan	VSMC_M2	0.51	6.59E-03	0.65	2.19E-04	0.38	4.80E-02	0.04	8.62E-01	-0.21	3.01E-01	-0.02	9.23E-01	-0.14	5.00E-01	-0.25	2.10E-01	-0.72	1.90E-05	0.33	9.75E-02
ENSCAFG0000001825	MARK3	grey	VSMC_M10	0.51	6.64E-03	-0.05	8.11E-01	-0.11	5.70E-01	-0.09	6.62E-01	0.44	2.11E-02	-0.18	3.78E-01	0.25	2.17E-01	0.09	6.72E-01	-0.18	3.65E-01	-0.27	1.72E-01
ENSCAFG0000001044	MRPL36	cyan	VSMC_M2	0.51	6.67E-03	0.60	1.04E-03	0.39	4.36E-02	0.04	8.57E-01	-0.25	2.07E-01	-0.17	4.09E-01	-0.10	6.23E-01	-0.23	2.50E-01	-0.66	2.07E-04	0.38	4.80E-02
ENSCAFG0000001902	GOSR1	red	VSMC_M1	0.51	6.73E-03	0.14	4.88E-01	-0.04	8.56E-01	0.26	1.92E-01	0.41	3.42E-02	-0.47	1.41E-02	-0.36	6.43E-02	-0.28	1.64E-01	-0.18	3.77E-01	-0.21	2.91E-01
ENSCAFG0000001855	ENSCAFG0000001855	pink	VSMC_M5	0.51	6.73E-03	-0.08	6.96E-01	-0.07	7.23E-01	-0.05	7.98E-01	0.49	9.53E-03	-0.49	9.50E-03	-0.21	2.83E-01	0.02	9.20E-01	0.00	9.86E-01	-0.48	1.09E-02
ENSCAFG0000000269	CNOT3	yellow	VSMC_M3	0.51	6.75E-03	0.44	2.30E-02	0.64	3.13E-04	-0.40	3.93E-02	-0.37	5.42E-02	-0.22	2.66E-01	-0.10	6.35E-01	0.18	3.74E-01	-0.56	2.32E-03	0.01	9.63E-01
ENSCAFG0000003133	OCIAD2	cyan	VSMC_M2	0.51	6.78E-03	0.59	1.12E-03	0.43	2.62E-02	0.06	7.58E-01	-0.15	4.69E-01	-0.30	1.29E-01	-0.07	7.29E-01	-0.28	1.59E-01	-0.60	9.93E-04	0.18	3.82E-01
ENSCAFG0000000274	ENSCAFG0000000274	red	VSMC_M1	0.51	6.80E-03	0.12	5.66E-01	0.11	5.86E-01	0.02	9.07E-01	0.11	6.00E-01	-0.12	5.43E-01	0.21	2.98E-01	-0.07	7.19E-01	-0.28	1.50E-01	0.00	9.84E-01
ENSCAFG0000001922	PAM16	grey	VSMC_M10	0.51	6.87E-03	0.37	5.80E-02	0.48	1.11E-02	-0.12	5.43E-01	-0.25	2.00E-01	0.11	5.73E-01	-0.08	6.82E-01	0.01	9.63E-01	-0.54	3.87E-03	0.06	7.68E-01
ENSCAFG0000000066	RSPH3	pink	VSMC_M5	0.51	6.90E-03	0.04	8.61E-01	-0.35	7.40E-02	0.16	4.11E-01	0.63	3.98E-04	-0.15	4.41E-01	-0.10	6.37E-01	-0.11	5.82E-01	-0.17	3.97E-01	-0.21	2.82E-01
ENSCAFG0000000968	HLCS	red	VSMC_M1	0.51	6.92E-03	0.29	1.37E-01	0.15	4.66E-01	-0.06	7.55E-01	0.04	8.42E-01	-0.23	2.50E-01	0.21	2.92E-01	-0.06	7.68E-01	-0.45	1.88E-02	0.20	3.28E-01
ENSCAFG0000000892	MMP16	red	VSMC_M1	0.51	6.93E-03	0.39	4.38E-02	0.03	8.83E-01	0.17	3.88E-01	0.20	3.07E-01	-0.28	1.57E-01	0.09	6.62E-01	-0.26	1.91E-01	-0.48	1.22E-02	0.20	3.28E-01
ENSCAFG0000000376	SLC41A3	pink	VSMC_M5	0.51	6.98E-03	0.05	8.07E-01	-0.11	5.83E-01	0.20	3.22E-01	0.51	6.94E-03	-0.40	4.03E-02	-0.10	6.03E-01	-0.21	3.00E-01	-0.08	6.85E-01	-0.28	1.57E-01
ENSCAFG0000001216	SYTL1	grey	VSMC_M10	0.51	7.00E-03	0.64	3.49E-04	0.06	7.58E-01	0.52	5.75E-03	0.00	1.00E+00	-0.03	8.84E-01	-0.27	1.69E-01	-0.65	2.13E-04	-0.70	4.64E-05	0.63	3.79E-04

ENSCAFG0000001624	ENSCAFG0000001624	red	VSMC_M1	0.51	7.02E-03	0.30	1.28E-01	0.31	1.12E-01	-0.11	5.76E-01	0.10	6.19E-01	-0.34	8.17E-02	-0.09	6.61E-01	-0.12	5.45E-01	-0.30	1.33E-01	-0.19	3.47E-01
ENSCAFG0000003238	DENR	pink	VSMC_M5	0.51	7.11E-03	-0.49	9.52E-03	-0.23	2.47E-01	-0.18	3.67E-01	0.60	9.65E-04	-0.14	4.75E-01	0.00	9.90E-01	0.31	1.17E-01	0.32	1.06E-01	-0.65	2.55E-04
ENSCAFG0000000422	FAM168B	cyan	VSMC_M2	0.51	7.15E-03	0.46	1.55E-02	0.10	6.27E-01	0.05	7.86E-01	0.05	7.94E-01	0.23	2.49E-01	-0.19	3.34E-01	-0.23	2.45E-01	-0.52	5.84E-03	0.26	1.93E-01
ENSCAFG0000000894	C18H11orf49	red	VSMC_M1	0.51	7.15E-03	0.40	3.97E-02	-0.05	8.19E-01	0.27	1.76E-01	0.14	4.73E-01	0.01	9.74E-01	-0.09	6.43E-01	-0.32	1.02E-01	-0.53	4.54E-03	0.41	3.50E-02
ENSCAFG0000000744	EFNA5	cyan	VSMC_M2	0.51	7.16E-03	0.55	3.20E-03	0.46	1.68E-02	-0.18	3.70E-01	-0.21	2.91E-01	-0.06	7.69E-01	-0.33	9.71E-02	-0.04	8.30E-01	-0.66	2.07E-04	0.10	6.36E-01
ENSCAFG0000000326	APLF	red	VSMC_M1	0.51	7.16E-03	0.28	1.59E-01	0.11	5.77E-01	-0.07	7.21E-01	0.22	2.76E-01	-0.35	7.19E-02	0.06	7.60E-01	0.00	9.93E-01	-0.39	4.37E-02	-0.11	5.97E-01
ENSCAFG0000001646	SEPSECS	yellow	VSMC_M3	0.50	7.23E-03	0.21	2.94E-01	0.37	5.75E-02	-0.44	2.05E-02	-0.04	8.29E-01	-0.07	7.34E-01	-0.02	9.41E-01	0.28	1.55E-01	-0.36	6.43E-02	-0.26	1.94E-01
ENSCAFG0000003121	ENSCAFG0000003121	red	VSMC_M1	0.50	7.24E-03	0.38	5.21E-02	0.26	1.98E-01	-0.06	7.69E-01	-0.06	7.63E-01	-0.09	6.38E-01	0.16	4.15E-01	-0.16	4.32E-01	-0.45	1.93E-02	0.22	2.71E-01
ENSCAFG0000000085	KIAA0930	red	VSMC_M1	0.50	7.24E-03	0.49	1.01E-02	0.33	8.91E-02	0.06	7.54E-01	-0.19	3.32E-01	0.00	9.98E-01	-0.12	5.36E-01	-0.22	2.78E-01	-0.54	3.86E-03	0.34	8.46E-02
ENSCAFG0000002537	SLC25A28	red	VSMC_M1	0.50	7.40E-03	0.10	6.29E-01	-0.10	6.21E-01	0.25	2.03E-01	0.33	9.48E-02	-0.07	7.35E-01	-0.23	2.46E-01	-0.20	3.14E-01	-0.20	3.09E-01	-0.02	9.38E-01
ENSCAFG0000001302	FBXO45	red	VSMC_M1	0.50	7.44E-03	0.11	5.86E-01	0.05	8.04E-01	-0.03	8.75E-01	0.19	3.35E-01	0.03	8.90E-01	0.02	9.29E-01	-0.01	9.79E-01	-0.28	1.63E-01	-0.10	6.21E-01
ENSCAFG0000002031	RPF1	red	VSMC_M1	0.50	7.45E-03	-0.13	5.02E-01	0.45	1.74E-02	-0.55	2.84E-03	-0.03	8.73E-01	-0.18	3.63E-01	-0.02	9.04E-01	0.52	5.83E-03	-0.03	8.75E-01	-0.61	7.39E-04
ENSCAFG0000001743	USP43	yellow	VSMC_M3	0.50	7.46E-03	0.20	3.17E-01	0.58	1.42E-03	-0.33	9.69E-02	-0.24	2.23E-01	-0.11	5.88E-01	0.37	5.71E-02	0.17	3.95E-01	-0.34	8.19E-02	-0.16	4.36E-01
ENSCAFG0000002339	ENSCAFG0000002339	red	VSMC_M1	0.50	7.46E-03	0.15	4.61E-01	0.22	2.67E-01	-0.26	1.97E-01	0.14	4.88E-01	-0.16	4.18E-01	-0.35	7.44E-02	0.13	5.18E-01	-0.15	4.41E-01	-0.34	8.04E-02
ENSCAFG0000002421	ENSCAFG0000002421	cyan	VSMC_M2	0.50	7.47E-03	0.53	4.09E-03	0.21	2.94E-01	0.11	5.71E-01	0.08	6.88E-01	-0.28	1.50E-01	-0.11	5.99E-01	-0.23	2.50E-01	-0.58	1.69E-03	0.11	5.96E-01
ENSCAFG0000000283	ENSCAFG0000000283	yellow	VSMC_M3	0.50	7.62E-03	0.23	2.55E-01	0.51	6.50E-03	-0.46	1.65E-02	-0.21	2.90E-01	-0.09	6.61E-01	0.10	6.27E-01	0.33	9.78E-02	-0.39	4.22E-02	-0.21	2.95E-01
ENSCAFG0000000814	MSRA	grey	VSMC_M10	0.50	7.62E-03	0.28	1.63E-01	0.33	8.89E-02	0.08	6.82E-01	-0.17	3.85E-01	-0.03	8.97E-01	0.09	6.43E-01	-0.16	4.12E-01	-0.40	3.94E-02	0.20	3.15E-01
ENSCAFG0000000583	RNF121	red	VSMC_M1	0.50	7.63E-03	-0.19	3.33E-01	-0.09	6.48E-01	-0.04	8.34E-01	0.32	1.01E-01	0.13	5.34E-01	-0.25	1.99E-01	0.17	3.85E-01	0.00	9.90E-01	-0.34	8.44E-02
ENSCAFG0000001422	NEMF	pink	VSMC_M5	0.50	7.65E-03	-0.06	7.59E-01	-0.13	5.09E-01	0.09	6.66E-01	0.52	5.76E-03	-0.37	6.08E-02	-0.14	4.72E-01	-0.04	8.32E-01	-0.06	7.84E-01	-0.34	8.71E-02
ENSCAFG0000000694	SLC7A2	cyan	VSMC_M2	0.50	7.67E-03	0.54	3.45E-03	0.32	1.06E-01	-0.07	7.14E-01	-0.11	5.96E-01	-0.15	4.45E-01	-0.42	2.95E-02	-0.07	7.20E-01	-0.57	1.82E-03	0.14	4.96E-01
ENSCAFG0000000098	NAGA	cyan	VSMC_M2	0.50	7.68E-03	0.68	9.03E-05	0.34	8.58E-02	-0.08	6.86E-01	-0.08	7.03E-01	-0.12	5.65E-01	-0.23	2.47E-01	-0.20	3.18E-01	-0.70	4.87E-05	0.20	3.18E-01
ENSCAFG0000000247	CORO2A	red	VSMC_M1	0.50	7.69E-03	0.43	2.45E-02	0.52	5.15E-03	-0.28	1.65E-01	-0.22	2.64E-01	-0.10	6.21E-01	0.15	4.66E-01	0.09	6.67E-01	-0.56	2.22E-03	0.00	9.83E-01
ENSCAFG0000000067	ZFPM2	cyan	VSMC_M2	0.50	7.70E-03	0.67	1.17E-04	0.34	8.59E-02	-0.03	8.81E-01	-0.12	5.38E-01	-0.15	4.56E-01	-0.53	4.42E-03	-0.24	2.32E-01	-0.69	6.23E-05	0.26	1.90E-01
ENSCAFG0000000613	PXMP2	yellow	VSMC_M3	0.50	7.71E-03	0.53	4.42E-03	0.84	3.45E-08	-0.41	3.56E-02	-0.59	1.29E-03	-0.02	9.32E-01	0.00	9.93E-01	0.15	4.55E-01	-0.64	3.43E-04	0.09	6.49E-01
ENSCAFG0000000808	ERGIC3	cyan	VSMC_M2	0.50	7.74E-03	0.77	2.88E-06	0.06	7.51E-01	0.50	8.01E-03	0.03	8.83E-01	-0.04	8.60E-01	-0.42	2.91E-02	-0.64	3.49E-04	-0.80	7.26E-07	0.56	2.24E-03
ENSCAFG0000001613	CECR2	red	VSMC_M1	0.50	7.78E-03	0.05	8.02E-01	-0.10	6.11E-01	0.13	5.15E-01	0.42	2.88E-02	-0.06	7.74E-01	-0.42	3.09E-02	-0.17	4.04E-01	-0.08	6.95E-01	-0.21	3.04E-01
ENSCAFG0000001420	DNAAF2	grey	VSMC_M10	0.50	7.80E-03	-0.09	6.60E-01	0.13	5.12E-01	-0.49	1.01E-02	0.23	2.42E-01	-0.11	5.80E-01	0.08	6.80E-01	0.51	6.95E-03	-0.13	5.28E-01	-0.51	7.07E-03
ENSCAFG0000001230	KLHL41	red	VSMC_M1	0.50	7.81E-03	-0.07	7.28E-01	0.29	1.36E-01	-0.34	8.43E-02	0.09	6.63E-01	-0.38	5.31E-02	0.24	2.22E-01	0.29	1.37E-01	-0.14	4.92E-01	-0.42	2.94E-02
ENSCAFG0000000058	GRAMD2B	darkgrey	VSMC_M8	0.50	7.82E-03	-0.26	1.86E-01	0.32	1.04E-01	-0.70	4.35E-05	0.13	5.32E-01	0.05	8.05E-01	0.27	1.77E-01	0.63	3.84E-04	0.07	7.29E-01	-0.74	1.14E-05
ENSCAFG0000001568	ZBTB49	yellow	VSMC_M3	0.50	7.84E-03	0.13	5.12E-01	0.27	1.72E-01	-0.29	1.47E-01	0.02	9.10E-01	-0.03	8.81E-01	0.31	1.19E-01	0.21	2.91E-01	-0.36	6.48E-02	-0.17	3.87E-01
ENSCAFG0000000753	ANKRD27	grey	VSMC_M10	0.50	7.85E-03	0.29	1.44E-01	0.07	7.44E-01	0.21	2.95E-01	0.02	9.04E-01	0.03	8.96E-01	-0.06	7.53E-01	-0.25	2.07E-01	-0.44	2.11E-02	0.35	7.74E-02
ENSCAFG0000000989	IFT57	red	VSMC_M1	0.50	7.86E-03	0.00	1.00E+00	-0.33	8.77E-02	0.25	2.04E-01	0.63	4.30E-04	-0.43	2.66E-02	-0.14	4.97E-01	-0.20	3.26E-01	-0.06	7.61E-01	-0.13	5.09E-01
ENSCAFG0000001141	ENSCAFG0000001141	red	VSMC_M1	0.50	7.87E-03	0.19	3.38E-01	0.03	8.77E-01	0.00	9.95E-01	0.25	2.06E-01	-0.32	1.05E-01	0.03	8.79E-01	-0.15	4.54E-01	-0.23	2.57E-01	-0.02	9.32E-01
ENSCAFG0000001298	ENSCAFG0000001298	red	VSMC_M1	0.50	7.88E-03	0.08	6.83E-01	0.22	2.73E-01	-0.08	6.87E-01	0.09	6.41E-01	0.03	8.66E-01	-0.09	6.43E-01	0.02	9.38E-01	-0.18	3.67E-01	-0.23	2.52E-01
ENSCAFG0000001953	MIDN	yellow	VSMC_M3	0.50	7.91E-03	0.52	5.67E-03	0.59	1.22E-03	-0.37	5.71E-02	-0.27	1.75E-01	0.01	9.59E-01	-0.39	4.55E-02	0.14	4.97E-01	-0.55	2.67E-03	-0.09	6.53E-01
ENSCAFG0000000002	TSHZ1	cyan	VSMC_M2	0.50	7.99E-03	0.83	7.30E-08	0.60	9.12E-04	-0.08	6.75E-01	-0.35	7.53E-02	-0.16	4.36E-01	-0.31	1.11E-01	-0.24	2.33E-01	-0.83	7.72E-08	0.29	1.46E-01
ENSCAFG0000000166	ARHGAP39	cyan	VSMC_M2	0.50	8.00E-03	0.60	1.04E-03	0.17	3.83E-01	0.29	1.36E-01	-0.05	7.87E-01	0.01	9.43E-01	-0.03	8.73E-01	-0.43	2.45E-02	-0.72	2.31E-05	0.44	2.32E-02
ENSCAFG0000002021	RPL35	grey	VSMC_M10	0.50	8.01E-03	-0.01	9.49E-01	0.33	8.85E-02	-0.31	1.21E-01	-0.10	6.31E-01	-0.11	5.98E-01	0.11	5.70E-01	0.30	1.32E-01	-0.18	3.63E-01	-0.28	1.56E-01
ENSCAFG0000002426	ATXN7L3B	grey	VSMC_M10	0.50	8.04E-03	0.15	4.68E-01	0.06	7.50E-01	0.18	3.78E-01	0.07	7.12E-01	-0.06	7.85E-01	0.01	9.76E-01	-0.18	3.64E-01	-0.28	1.65E-01	0.21	2.99E-01
ENSCAFG0000001029	HEMK1	grey	VSMC_M10	0.50	8.04E-03	0.04	8.35E-01	0.46	1.65E-02	-0.43	2.46E-02	-0.03	8.75E-01	-0.17	3.93E-01	0.15	4.69E-01	0.29	1.40E-01	-0.19	3.53E-01	-0.48	1.18E-02
ENSCAFG0000001401	AIFM2	cyan	VSMC_M2	0.50	8.06E-03	0.73	1.39E-05	0.30	1.24E-01	0.22	2.70E-01	-0.27	1.75E-01	0.13	5.16E-01	-0.18	3.82E-01	-0.43	2.60E-02	-0.81	4.02E-07	0.62	5.63E-04
ENSCAFG0000000303	ENSCAFG0000000303	yellow	VSMC_M3	0.50	8.08E-03	0.53	4.30E-03	0.54	3.43E-03	0.02	9.23E-01	-0.31	1.14E-01	-0.04	8.54E-01	-0.28	1.51E-01	-0.23	2.43E-01	-0.55	2.88E-03	0.20	3.20E-01
ENSCAFG0000000796	MMP24	yellow	VSMC_M3	0.50	8.12E-03	0.30	1.32E-01	0.64	3.00E-04	-0.19	3.43E-01	-0.31	1.19E-01	-0.06	7.65E-01	-0.05	8.02E-01	0.00	9.96E-01	-0.37	6.10E-02	-0.13	5.18E-01
ENSCAFG0000001928	SSR4	grey	VSMC_M10	0.50	8.12E-03	0.56	2.33E-03	0.04	8.52E-01	0.42	3.13E-02	0.11	5.84E-01	-0.09	6.70E-01	-0.32	1.09E-01	-0.54	3.41E-03	-0.58	1.43E-03	0.32	1.04E-01
ENSCAFG0000001654	PACRGL	red	VSMC_M1	0.50	8.12E-03	0.09	6.38E-01	0.02	9.23E-01	0.22	2.77E-01	0.23	2.46E-01	-0.07	7.18E-01	-0.16	4.28E-01	-0.18	3.79E-01	-0.18	3.67E-01	-0.06	7.49E-01
ENSCAFG0000000602	SH3BP5	cyan	VSMC_M2	0.50	8.14E-03	0.75	5.56E-06	0.55	2.95E-03	-0.07	7.30E-01	-0.32	1.03E-01	-0.03	9.00E-01	-0.08	6.98E-01	-0.19	3.54E-01	-0.83	6.28E-08	0.29	1.36E-01
ENSCAFG0000003203	ENSCAFG0000003203	red	VSMC_M1	0.50	8.14E-03	0.14	4.83E-01	-0.17	4.10E-01	0.38	5.19E-02	0.41	3.25E-02	-0.20	3.19E-01	-0.01	9.80E-01	-0.35	7.39E-02	-0.27	1.70E-01	0.03	8.70E-01
ENSCAFG0000001533	FTSJ1	red	VSMC_M1	0.50	8.15E-03	-0.09	6.65E-01	0.07	7.44E-01	-0.26	1.93E-01	0.23	2.44E-01	-0.09	6.41E-01	-0.02	9.21E-01	0.28	1.62E-01	-0.05	8.02E-01	-0.35	7.07E-02
ENSCAFG0000000656	DEAF1	yellow	VSMC_M3	0.50	8.15E-03	0.59	1.13E-03	0.56	2.23E-03	-0.06	7.69E-01	-0.34	8.56E-02	-0.14	4.84E-01	0.00	9.94E-01	-0.25	2.17E-01	-0.60	9.44E-04	0.27	1.81E-01
ENSCAFG0000003180	ENSCAFG0000003180	cyan	VSMC_M2	0.50	8.16E-03	0.67	1.47E-04	0.52	5.32E-03	-0.02	9.39E-01	-0.33	9.49E-02	-0.05	8.23E-01	0.00	9.99E-01	-0.22	2.76E-01	-0.73	1.73E-05	0.31	1.12E-01
ENSCAFG0000000057	CHKB	cyan	VSMC_M2	0.50	8.17E-03	0.43	2.46E-02	0.28	1.50E-01	-0.18	3.69E-01	0.04	8.28E-01	-0.22	2.78E-01	-0.11	5.97E-01	0.00	9.94E-01	-0.50	7.30E-03	-0.05	7.95E-01
ENSCAFG0000000563	SIRT2	grey	VSMC_M10	0.50	8.22E-03	0.85	1.45E-08	0.41	3.19E-02	0.27	1.80E-01	-0.23	2.39E-01	-0.08	6.87E-01	-0.42	3.11E-02	-0.57	2.01E-03	-0.78	1.67E-06	0.51	6.21E-03
ENSCAFG0000001092	HIST1H1C	cyan	VSMC_M2	0.50	8.24E-03	0.52	5.38E-03	0.01	9.71E-01	0.44	2.33E-02	0.12	5.36E-01	0.01	9.50E-01	-0.18	3.58E-01	-0.57	1.93E-03	-0.57	1.85E-03	0.43	2.46E-02
ENSCAFG0000001887	HPRT1	pink	VSMC_M5	0.50	8.26E-03	-0.37	5.72E-02	-0.15	4.45E-01	-0.07	7.22E-01	0.57	1.74E-03	-0.11	5.79E-01	-0.09	6.42E-01	0.17	3.89E-01	0.28	1.58E-01	-0.64	2.87E-04
ENSCAFG0000003003	ENSCAFG0000003003	red	VSMC_M1	0.50	8.28E-03	-0.07	7.29E-01	0.26	1.91E-01	-0.06	7.50E-01	0.05	7.94E-01	-0.09	6.59E-01	-0.01	9.61E-01	0.07	7.30E-01	-0.02	9.04E-01	-0.33	8.79E-02
ENSCAFG0000001296	ENSCAFG0000001296	cyan	VSMC_M2	0.50	8.35E-03	0.66	1.62E-04	0.17	3.98E-01	0.17	4.02E-01	0.10	6.15E-01	-0.24	2.32E-01	-0.46	1.60E-02	-0.47	1.38E-02	-0.59	1.08E-03	0.23	2.40E-01
ENSCAFG0000003102	HRAS	grey	VSMC_M10	0.50	8.40E-03	0.21	2.95E-01	0.51	6.79E-03	-0.31	1.12E-01	-0.35	7.68E-02	0.19	3.47E-01	0.29	1.43E-01	0.23	2.45E-01	-0.41	3.14E-02	0.03	8.66E-01
ENSCAFG0000000910	STC1	grey	VSMC_M10	0.50	8.42E-03	0.21	2.88E-01	0.17	4.03E-01	-0.13	5.04E-01	0.11	5.76E-01	0.21	3.01E-01	-0.06	7.78E-01	0.05	7.96E-01	-0.29	1.37E-01	-0.08	6.97E-01
ENSCAFG0000001745	INTS3	red	VSMC_M1	0.50	8.45E-03	0.61	7.65E-04	0.43	2.44E-02	-0.05	8.06E-01	-0.20	3.09E-01	-0.26	1.96E-01	0.15	4.49E-01	-0.22	2.60E-01	-0.69	7.87E-05	0.31	1.18E-01
ENSCAFG0000001158	SV2A	cyan	VSMC_M2	0.50	8.51E-03	0.41	3.31E-02	0.36	6.29E-02	-0.14	4.97E-01	-0.08	6.96E-01	-0.01	9.64E-01	-0.04	8.62E-01	-0.05	7.97E-01	-0.46	1.48E-02	0.07	7.35E-01
ENSCAFG0000000765	DTX4	grey	VSMC_M10	0.50	8.54E-03	0.45	1.83E-02	-0.26	1.87E-01	0.60	1.05E-03	0.35	7.08E-02	-0.25	2.04E-01	-0.19	3.30E-01	-0.63	4.38E-04	-0.49	9.28E-03	0.47	1.44E-02
ENSCAFG0000000090	TTLL12	yellow	VSMC_M3	0.50	8.59E-03	0.40	3.82E-02	0.71	3.44E-05	-0.65	2.21E-04	-0.39	4.64E-02	0.13	5.14E-01	0.07	7.19E-01	0.37	5.64E-02	-0.51	6.49E-03	-0.16	4.39E-01
ENSCAFG0000000663	TNKS	red	VSMC_M1	0.50	8.61E-03	0.23	2.43E-01	-0.24	2.21E-01	0.32	1.08E-01	0.40	3.74E-02	-0.06	7.51E-01	-0.13	5.29E-01	-0.28	1.50E-01	-0.38	4.88E-02	0.22	2.68E-01
ENSCAFG0000000265	RPS9	yellow	VSMC_M3	0.50	8.62E-03	0.65	2.47E-04	0.83	9.22E-08	-0.28	1.53E-01	-0.61	7.51E-04	-0.08	6.78E-01	-0.02	9.14E-01	0.02	9.40E-01	-0.73	1.43E-05	0.18	3.73E-01
ENSCAFG0000001258	ROMO1	yellow	VSMC_M3	0.50	8.64E-03	0.29	1.35E-01	0.73	1.69E-05	-0.32	1.08E-01	-0.45	1.90E-02	-0.09	6.56E-01	0.12	5.39E-01	0.13	5.11E-01	-0.39	4.67E-02	-0.10	6.21E-01
ENSCAFG0000000566	ECH1	cyan	VSMC_M2	0.49	8.68E-03	0.68	9.53E-05	0.16	4.34E-01	0.37	5.74E-02	0.00	9.81E-01	-0.24	2.29E-01	-0.14	4.80E-01	-0.54	3.87E-03	-0.71	3.68E-05	0.41	3.30E-02
ENSCAFG0000000788	CEP44	grey	VSMC_M10	0.49	8.72E-03	-0.10	6.24E-01	-0.07	7.20E-01	0.00	9.98E-01	0.17	3.84E-01	0.02	9.11E-01	0.28	1.57E-01	0.14	4.80E-01	-0.18	3.62E-01	0.05	8.02E-01
ENSCAFG0000001828	CSNK1A1	pink	VSMC_M5	0.49	8.75E-03	-0.15	4.45E-01	-0.50	7.49E-03	0.29	1.35E-01	0.85	1.60E-08	-0.32	1.08E-01	-0.35	7.29E-02	-0.21	2.92E-01	0.08	7.04E-01	-0.32	1.05E-01
ENSCAFG0000001773	GSKIP	pink	VSMC_M5	0.49	8.78E-03	-0.11	5.84E-01	-0.29	1.43E-01	0.29	1.44E-01	0.60	1.04E-03	0.01	9.47E-01	-0.32	9.97E-02	-0.22	2.67E-01	0.05	7.94E-01	-0.27	1.75E-01
ENSCAFG0000000154	TTC39B	grey	VSMC_M10	0.49	8.78E-03	0.24	2.21E-01	0.06	7.81E-01	-0.12	5.44E-01	0.13	5.14E-01	-0.06	7.55E-01	0.22	2.72E-01	0.00	9.88E-01	-0.42	2.74E-02	0.10	6.24E-01
ENSCAFG0000000914	CCDC32	yellow	VSMC_M3	0.49	8.80E-03	0.47	1.42E-02	0.56	2.26E-03	-0.05	8.00E-01	-0.26	1.82E-01	-0.26	1.94E-01	-0.02	9.02E-01	-0.17	4.07E-01	-0.48	1.07E-02	0.05	8.17E-01
ENSCAFG0000002852	ENSCAFG0000002852	yellow	VSMC_M3	0.49	8.83E-03	0.20	3.29E-01	0.31	1.14E-01	0.02	9.04E-01	-0.04	8.35E-01	0.09	6.73E-01	-0.16	4.23E-01	-0.12	5.44E-01	-0.25	2.04E-01	-0.07	7.30E-01
ENSCAFG0000001680	MEF2D	red	VSMC_M1	0.49	8.84E-03	0.16	4.30E-01	0.24	2.21E-01	-0.18	3.63E-01	0.08	6.83E-01	-0.15	4.49E-01	-0.13	5.32E-01	0.01	9.45E-01	-0.19	3.33E-01	-0.15	4.59E-01
ENSCAFG0000000931	ENSCAFG0000000931	red	VSMC_M1	0.49	8.86E-03	-0.05	8.16E-01	-0.31	1.18E-01	0.34	8.02E-02	0.45	1.99E-02	0.14	4.85E-01	-0.04	8.38E-01	-0.19	3.49E-01	-0.11	6.02E-01	0.07	7.19E-01
ENSCAFG0000001611	ELP5	yellow	VSMC_M3	0.49	8.88E-03	0.46	1.53E-02	0.49	9.52E-03	-0.07	7.44E-01	-0.20	3.29E-01	-0.04	8.28E-01	-0.31	1.15E-01	-0.14	4.72E-01	-0.45	1.84E-02	0.00	9.97E-01
ENSCAFG0000001906	DYM	cyan	VSMC_M2	0.49	8.89E-03	0.55	2.90E-03	0.34	8.23E-02	-0.13	5.28E-01	-0.07	7.34E-01	-0.15	4.55E-01	-0.21	2.98E-01	-0.22	2.80E-01	-0.51	6.05E-03	0.15	4.49E-01
ENSCAFG0000001968	DNLZ	red	VSMC_M1	0.49	8.90E-03	-0.14	4.94E-01	-0.32	9.85E-02	0.15	4.68E-01	0.58	1.38E-03	-0.09	6.68E-01	0.03	8.72E-01	-0.06	7.70E-01	0.00	9.81E-01	-0.24	2.25E-01
ENSCAFG0000001028	MAPKAPK3	grey	VSMC_M10	0.49	8.93E-03	0.45	1.98E-02	0.47	1.43E-02	-0.03	8.72E-01	-0.22	2.79E-01	-0.11	5.94E-01	-0.21	3.03E-01	-0.13	5.18E-01	-0.49	9.17E-03	0.08	7.06E-01
ENSCAFG0000001076	TCHP	yellow	VSMC_M3	0.49	8.99E-03	0.35	7.74E-02	0.54	3.33E-03	-0.26	1.91E-01	-0.32	1.05E-01	-0.01	9.56E-01	0.25	2.04E-01	0.10	6.12E-01	-0.55	2.99E-03	0.06	7.79E-01
ENSCAFG0000000007	PYM1	cyan	VSMC_M2	0.49	9.00E-03	0.47	1.39E-02	0.27	1.69E-01	0.08	6.87E-01	-0.06	7.74E-01	-0.26	1.94E-01	-0.11	5.93E-01	-0.20	3.20E-01	-0.58	1.36E-03	0.14	4.76E-01
ENSCAFG0000003230	ZNF32	red	VSMC_M1	0.49	9.00E-03	0.13	5.09E-01	-0.01	9.60E-01	0.07	7.11E-01	0.33	8.76E-02	-0.45	1.98E-02	0.02	9.19E-01	-0.19	3.39E-01	-0.15	4.41E-01	-0.07	7.27E-01
ENSCAFG0000000185	GUF1	pink	VSMC_M5	0.49	9.02E-03	-0.24	2.36E-01	-0.08	7.06E-01	-0.24	2.29E-01	0.38	4.81E-02	0.12	5.58E-01	0.12	5.51E-01	0.28	1.54E-01	0.04	8.59E-01	-0.40	3.88E-02
ENSCAFG0000001701	THBS3	grey	VSMC_M10	0.49	9.03E-03	0.82	1.33E-07	0.41	3.42E-02	0.18	3.72E-01	-0.20	3.06E-01	-0.25	2.11E-01	-0.48	1.19E-02	-0.47	1.43E-02	-0.77	3.22E-06	0.43	2.64E-02
ENSCAFG0000001910	RETREG1	cyan	VSMC_M2	0.49	9.08E-03	0.56	2.31E-03	0.14	4.92E-01	0.25	2.00E-01	0.06	7.51E-01	-0.43	2.38E-02	0.17	4.01E-01	-0.40	3.63E-02	-0.64	3.51E-04	0.38	5.38E-02
ENSCAFG0000001332	ENSCAFG0000001332	grey	VSMC_M10	0.49	9.12E-03	0.28	1.65E-01	-0.05	7.97E-01	0.21	3.00E-01	0.10	6.35E-01	0.07	7.12E-01	-0.05	8.23E-01	-0.19	3.50E-01	-0.47	1.27E-02	0.39	4.52E-02
ENSCAFG0000003132	CEBPB	grey	VSMC_M10	0.49	9.12E-03	0.41	3.41E-02	0.21	3.04E-01	0.28	1.57E-01	-0.07	7.43E-01	0.08	7.09E-01	0.10	6.12E-01	-0.38	4.85E-02	-0.54	3.54E-03	0.34	8.63E-02
ENSCAFG0000003222	ENSCAFG0000003222	red	VSMC_M1	0.49	9.16E-03	0.41	3.55E-02	0.00	9.86E-01	0.40	3.63E-02	0.20	3.24E-01	-0.24	2.23E-01	0.04	8.57E-01	-0.45	1.83E-02	-0.53	4.53E-03	0.27	1.68E-01
ENSCAFG0000001071	SEMA3B	cyan	VSMC_M2	0.49	9.24E-03	0.80	5.01E-07	0.49	8.88E-03	0.07	7.15E-01	-0.23	2.44E-01	-0.32	1.05E-01	-0.04	8.44E-01	-0.38	4.94E-02	-0.77	3.26E-06	0.34	7.81E-02
ENSCAFG0000001919	NUDT16L1	grey	VSMC_M10	0.49	9.30E-03	0.19	3.38E-01	-0.09	6.70E-01	0.16	4.19E-01	0.19	3.33E-01	0.16	4.28E-01	-0.01	9.51E-01	-0.14	4.85E-01	-0.36	6.70E-02	0.18	3.79E-01
ENSCAFG0000001200	WDR93	cyan	VSMC_M2	0.49	9.32E-03	0.54	3.78E-03	0.29	1.36E-01	0.26	1.85E-01	-0.14	4.94E-01	-0.05	8.04E-01	-0.08	6.96E-01	-0.39	4.64E-02	-0.60	1.06E-03	0.38	5.39E-02
ENSCAFG0000000976	ENSCAFG0000000976	red	VSMC_M1	0.49	9.33E-03	0.27	1.66E-01	0.36	6.34E-02	-0.15	4.49E-01	-0.02	9.27E-01	-0.51	6.67E-03	-0.09	6.73E-01	0.03	8.81E-01	-0.34	7.84E-02	-0.18	3.69E-01
ENSCAFG0000001030	C20H3orf18	yellow	VSMC_M3	0.49	9.34E-03	0.34	8.68E-02	0.69	5.78E-05	-0.42	2.97E-02	-0.32	1.01E-01	-0.08	6.95E-01	0.05	8.22E-01	0.12	5.63E-01	-0.36	6.23E-02	-0.21	3.00E-01
ENSCAFG0000001895	CDH6	grey	VSMC_M10	0.49	9.35E-03	0.62	5.23E-04	-0.12	5.44E-01	0.55	3.06E-03	0.32	9.90E-02	-0.37	6.03E-02	-0.62	6.03E-04	-0.71	4.00E-05	-0.55	2.98E-03	0.38	5.10E-02
ENSCAFG0000000362	RAB14	pink	VSMC_M5	0.49	9.36E-03	-0.16	4.15E-01	-0.40	3.76E-02	0.12	5.59E-01	0.69	5.88E-05	-0.21	2.94E-01	-0.11	5.85E-01	-0.05	7.93E-01	0.06	7.82E-01	-0.24	2.29E-01
ENSCAFG0000001655	MTHFR	yellow	VSMC_M3	0.49	9.39E-03	0.42	2.99E-02	0.62	5.36E-04	-0.43	2.48E-02	-0.26	1.93E-01	-0.16	4.29E-01	0.20	3.12E-01	0.12	5.54E-01	-0.47	1.44E-02	-0.11	5.75E-01
ENSCAFG0000000011	ENSCAFG0000000011	yellow	VSMC_M3	0.49	9.47E-03	0.36	6.36E-02	0.58	1.35E-03	-0.31	1.16E-01	-0.20	3.18E-01	-0.22	2.70E-01	-0.37	5.43E-02	0.07	7.15E-01	-0.39	4.59E-02	-0.28	1.61E-01
ENSCAFG0000000942	PTPN13	cyan	VSMC_M2	0.49	9.52E-03	0.55	2.91E-03	0.13	5.15E-01	-0.02	9.15E-01	0.12	5.64E-01	-0.23	2.58E-01	-0.03	9.01E-01	-0.19	3.33E-01	-0.63	4.38E-04	0.21	2.85E-01
ENSCAFG0000000478	IFT122	red	VSMC_M1	0.49	9.54E-03	0.27	1.77E-01	0.57	1.93E-03	-0.49	1.02E-02	-0.32	1.05E-01	-0.16	4.36E-01	0.23	2.46E-01	0.29	1.46E-01	-0.44	2.30E-02	-0.05	7.99E-01
ENSCAFG0000001631	ENSCAFG0000001631	grey	VSMC_M10	0.49	9.59E-03	0.28	1.56E-01	0.38	5.25E-02	0.03	8.80E-01	-0.15	4.63E-01	0.11	5.93E-01	0.16	4.40E-01	-0.10	6.03E-01	-0.41	3.15E-02	0.04	8.58E-01
ENSCAFG0000001053	ENSCAFG0000001053	grey	VSMC_M10	0.49	9.63E-03	0.19	3.45E-01	0.54	3.66E-03	-0.40	3.67E-02	-0.35	7.00E-02	0.00	9.94E-01	-0.07	7.30E-01	0.29	1.48E-01	-0.34	8.44E-02	-0.03	8.88E-01
ENSCAFG0000001577	ENSCAFG0000001577	red	VSMC_M1	0.49	9.63E-03	-0.18	3.60E-01	0.34	8.44E-02	-0.39	4.21E-02	-0.08	7.06E-01	-0.27	1.66E-01	0.05	8.22E-01	0.45	1.79E-02	-0.04	8.41E-01	-0.38	4.89E-02
ENSCAFG0000001611	H3F3A	yellow	VSMC_M3	0.49	9.64E-03	0.44	2.13E-02	0.48	1.17E-02	-0.06	7.69E-01	-0.26	1.93E-01	-0.01	9.74E-01	-0.14	4.76E-01	-0.15	4.58E-01	-0.46	1.69E-02	0.11	5.90E-01
ENSCAFG0000001788	INO80C	red	VSMC_M1	0.49	9.69E-03	-0.02	9.39E-01	0.39	4.32E-02	-0.36	6.80E-02	-0.16	4.35E-01	0.23	2.45E-01	-0.02	9.08E-01	0.31	1.13E-01	-0.16	4.25E-01	-0.25	2.05E-01
ENSCAFG0000001639	SMIM20	cyan	VSMC_M2	0.49	9.70E-03	0.41	3.19E-02	0.11	5.93E-01	0.35	7.16E-02	0.07	7.41E-01	0.05	7.90E-01	-0.03	8.81E-01	-0.44	2.11E-02	-0.45	1.73E-02	0.28	1.60E-01
ENSCAFG0000001670	YIPF6	pink	VSMC_M5	0.49	9.72E-03	-0.03	8.94E-01	-0.39	4.30E-02	0.31	1.13E-01	0.72	2.40E-05	-0.20	3.27E-01	-0.31	1.10E-01	-0.36	6.72E-02	0.01	9.59E-01	-0.22	2.72E-01
ENSCAFG0000001649	SAMD1	red	VSMC_M1	0.49	9.73E-03	0.49	1.02E-02	0.39	4.45E-02	-0.09	6.54E-01	-0.24	2.19E-01	-0.13	5.21E-01	0.21	2.99E-01	-0.02	9.31E-01	-0.66	1.91E-04	0.32	1.09E-01
ENSCAFG0000001402	IL1RAP	red	VSMC_M1	0.49	9.74E-03	0.36	6.24E-02	0.40	3.70E-02	-0.17	4.09E-01	-0.21	2.99E-01	0.18	3.67E-01	-0.43	2.58E-02	-0.02	9.40E-01	-0.46	1.68E-02	0.05	8.16E-01
ENSCAFG0000000836	SCAND1	cyan	VSMC_M2	0.49	9.81E-03	0.49	8.99E-03	0.19	3.32E-01	0.32	1.06E-01	0.01	9.46E-01	-0.17	3.84E-01	-0.40	3.83E-02	-0.45	1.81E-02	-0.48	1.23E-02	0.20	3.05E-01
ENSCAFG0000000494	WBP2	red	VSMC_M1	0.49	9.82E-03	0.42	2.74E-02	-0.23	2.53E-01	0.41	3.38E-02	0.45	2.00E-02	-0.21	3.01E-01	-0.55	2.82E-03	-0.53	4.61E-03	-0.40	4.13E-02	0.18	3.64E-01
ENSCAFG0000000713	TNFRSF19	red	VSMC_M1	0.49	9.83E-03	-0.06	7.54E-01	0.08	7.02E-01	-0.16	4.15E-01	0.20	3.14E-01	0.11	5.81E-01	-0.48	1.17E-02	0.18	3.76E-01	-0.05	8.20E-01	-0.34	8.34E-02
ENSCAFG0000000570	BAIAP2	yellow	VSMC_M3	0.49	9.87E-03	0.64	3.42E-04	0.76	3.38E-06	-0.43	2.67E-02	-0.52	5.45E-03	-0.09	6.63E-01	0.04	8.48E-01	0.12	5.43E-01	-0.71	3.04E-05	0.16	4.37E-01
ENSCAFG0000001642	WNK3	grey	VSMC_M10	0.49	9.97E-03	0.17	3.95E-01	-0.13	5.28E-01	0.29	1.49E-01	0.28	1.59E-01	-0.03	8.98E-01	-0.15	4.54E-01	-0.28	1.54E-01	-0.34	8.05E-02	0.15	4.50E-01
ENSCAFG0000000801	ENSCAFG0000000801	cyan	VSMC_M2	0.49	1.01E-02	0.35	7.13E-02	0.17	4.09E-01	0.29	1.36E-01	0.01	9.47E-01	-0.03	8.92E-01	-0.15	4.63E-01	-0.39	4.18E-02	-0.37	5.45E-02	0.20	3.25E-01
ENSCAFG0000000049	ENSCAFG0000000049	red	VSMC_M1	0.49	1.01E-02	-0.14	4.75E-01	0.00	9.86E-01	-0.11	5.87E-01	0.17	4.06E-01	0.03	8.89E-01	-0.12	5.63E-01	0.17	3.92E-01	-0.04	8.29E-01	-0.16	4.28E-01
ENSCAFG0000000863	ARHGEF26	grey	VSMC_M10	0.49	1.01E-02	0.44	2.26E-02	-0.21	2.87E-01	0.52	5.87E-03	0.37	5.73E-02	-0.32	1.04E-01	-0.26	1.98E-01	-0.54	3.91E-03	-0.51	5.99E-03	0.35	6.96E-02
ENSCAFG0000001519	TCEA2	cyan	VSMC_M2	0.49	1.01E-02	0.78	1.81E-06	0.37	5.67E-02	0.27	1.67E-01	-0.29	1.39E-01	-0.02	9.02E-01	-0.31	1.21E-01	-0.46	1.55E-02	-0.83	1.10E-07	0.60	8.87E-04

ENSCAFG0000000179	MTERF2	red	VSMC_M1	0.49	1.01E-02	-0.14	4.95E-01	-0.36	6.20E-02	0.21	3.03E-01	0.64	3.50E-04	-0.13	5.33E-01	0.13	5.33E-01	-0.10	6.29E-01	-0.06	7.62E-01	-0.23	2.41E-01
ENSCAFG0000000574	PFDN1	red	VSMC_M1	0.49	1.01E-02	0.24	2.32E-01	0.30	1.27E-01	0.09	6.53E-01	-0.03	8.66E-01	-0.21	3.03E-01	-0.25	2.10E-01	-0.15	4.42E-01	-0.31	1.20E-01	-0.02	9.08E-01
ENSCAFG0000003248	MCFD2	cyan	VSMC_M2	0.49	1.02E-02	0.71	2.92E-05	0.19	3.43E-01	0.26	1.88E-01	0.02	9.05E-01	-0.33	9.12E-02	-0.47	1.35E-02	-0.42	2.98E-02	-0.72	2.50E-05	0.29	1.36E-01
ENSCAFG0000001385	ENSCAFG0000001385	cyan	VSMC_M2	0.49	1.02E-02	0.62	6.32E-04	0.33	9.10E-02	0.25	2.06E-01	-0.19	3.30E-01	-0.05	7.89E-01	-0.03	8.91E-01	-0.40	4.03E-02	-0.69	6.42E-05	0.43	2.50E-02
ENSCAFG0000000465	BCL3	cyan	VSMC_M2	0.49	1.02E-02	0.56	2.37E-03	0.07	7.36E-01	0.19	3.51E-01	0.14	4.80E-01	-0.16	4.31E-01	-0.05	8.08E-01	-0.38	5.08E-02	-0.60	9.04E-04	0.26	1.98E-01
ENSCAFG0000000679	STARD7	grey	VSMC_M10	0.49	1.02E-02	0.30	1.22E-01	0.16	4.22E-01	0.07	7.21E-01	-0.04	8.40E-01	0.26	1.94E-01	0.01	9.70E-01	-0.05	8.11E-01	-0.46	1.46E-02	0.19	3.37E-01
ENSCAFG0000001161	ENSCAFG0000001161	grey	VSMC_M10	0.49	1.03E-02	-0.09	6.58E-01	-0.07	7.16E-01	0.27	1.79E-01	0.28	1.64E-01	0.02	9.21E-01	-0.13	5.31E-01	-0.15	4.65E-01	-0.05	8.05E-01	-0.10	6.03E-01
ENSCAFG0000001235	TATDN3	cyan	VSMC_M2	0.49	1.03E-02	0.49	9.35E-03	0.11	5.81E-01	0.38	4.84E-02	-0.03	8.95E-01	0.05	8.00E-01	-0.01	9.66E-01	-0.46	1.62E-02	-0.60	8.30E-04	0.47	1.25E-02
ENSCAFG0000000536	DGAT2	red	VSMC_M1	0.48	1.04E-02	0.30	1.28E-01	0.16	4.18E-01	-0.13	5.08E-01	0.10	6.11E-01	-0.01	9.55E-01	0.02	9.10E-01	-0.07	7.43E-01	-0.41	3.30E-02	-0.02	9.28E-01
ENSCAFG0000000216	MAP4K4	red	VSMC_M1	0.48	1.05E-02	0.02	9.29E-01	0.34	8.75E-02	-0.53	4.10E-03	0.01	9.43E-01	-0.07	7.18E-01	-0.21	2.85E-01	0.40	3.90E-02	-0.11	5.73E-01	-0.48	1.07E-02
ENSCAFG0000000529	SLITRK5	grey	VSMC_M10	0.48	1.05E-02	0.70	5.37E-05	0.23	2.47E-01	0.29	1.40E-01	-0.01	9.52E-01	-0.23	2.50E-01	-0.34	8.04E-02	-0.47	1.27E-02	-0.67	1.23E-04	0.36	6.91E-02
ENSCAFG0000001100	C37H2orf69	pink	VSMC_M5	0.48	1.05E-02	-0.31	1.12E-01	-0.16	4.15E-01	-0.09	6.68E-01	0.50	7.51E-03	-0.18	3.74E-01	-0.11	5.84E-01	0.22	2.64E-01	0.14	4.94E-01	-0.52	5.02E-03
ENSCAFG0000002024	ZFHX3	grey	VSMC_M10	0.48	1.05E-02	0.35	7.40E-02	-0.29	1.41E-01	0.46	1.49E-02	0.54	3.59E-03	-0.30	1.23E-01	-0.05	7.92E-01	-0.54	3.72E-03	-0.40	4.11E-02	0.19	3.55E-01
ENSCAFG0000001242	ENSCAFG0000001242	red	VSMC_M1	0.48	1.06E-02	0.24	2.35E-01	0.16	4.36E-01	0.16	4.38E-01	0.10	6.05E-01	-0.15	4.57E-01	-0.25	2.16E-01	-0.24	2.27E-01	-0.32	1.01E-01	-0.05	8.01E-01
ENSCAFG0000001063	ANKRD13A	grey	VSMC_M10	0.48	1.06E-02	0.59	1.16E-03	-0.15	4.51E-01	0.61	6.79E-04	0.32	1.03E-01	-0.18	3.74E-01	-0.64	3.31E-04	-0.75	6.77E-06	-0.54	3.95E-03	0.42	2.92E-02
ENSCAFG0000000789	PARVA	grey	VSMC_M10	0.48	1.06E-02	0.71	3.59E-05	0.42	2.91E-02	0.21	2.92E-01	-0.14	4.92E-01	-0.22	2.66E-01	-0.29	1.48E-01	-0.50	7.55E-03	-0.64	3.16E-04	0.27	1.67E-01
ENSCAFG0000002605	ENSCAFG0000002605	grey	VSMC_M10	0.48	1.06E-02	-0.13	5.13E-01	-0.04	8.37E-01	-0.15	4.49E-01	0.40	4.11E-02	-0.05	8.05E-01	0.07	7.40E-01	0.17	3.99E-01	-0.04	8.60E-01	-0.48	1.20E-02
ENSCAFG0000000622	DHX57	red	VSMC_M1	0.48	1.06E-02	0.02	9.11E-01	0.11	5.84E-01	-0.20	3.19E-01	0.11	5.73E-01	-0.07	7.37E-01	0.16	4.37E-01	0.13	5.14E-01	-0.25	2.17E-01	-0.10	6.07E-01
ENSCAFG0000000821	BTF3	red	VSMC_M1	0.48	1.06E-02	-0.08	6.91E-01	-0.14	4.71E-01	-0.16	4.27E-01	0.46	1.49E-02	-0.35	7.42E-02	0.05	7.91E-01	0.16	4.28E-01	-0.03	8.95E-01	-0.34	8.43E-02
ENSCAFG0000002953	LRRC7	cyan	VSMC_M2	0.48	1.06E-02	0.68	1.02E-04	0.36	6.14E-02	-0.14	4.85E-01	-0.09	6.39E-01	-0.16	4.24E-01	-0.32	1.03E-01	-0.14	4.72E-01	-0.68	9.89E-05	0.16	4.17E-01
ENSCAFG0000001038	ST3GAL4	yellow	VSMC_M3	0.48	1.07E-02	0.31	1.22E-01	0.55	2.92E-03	-0.33	8.76E-02	-0.25	2.06E-01	-0.25	2.15E-01	-0.33	8.86E-02	0.15	4.42E-01	-0.37	5.62E-02	-0.13	5.07E-01
ENSCAFG0000001506	ENSCAFG0000001506	yellow	VSMC_M3	0.48	1.07E-02	-0.03	8.82E-01	0.61	7.37E-04	-0.65	2.29E-04	-0.30	1.25E-01	0.04	8.42E-01	0.10	6.20E-01	0.55	3.17E-03	-0.18	3.64E-01	-0.42	2.95E-02
ENSCAFG0000001342	ETV5	red	VSMC_M1	0.48	1.07E-02	0.23	2.49E-01	0.12	5.59E-01	-0.01	9.50E-01	0.07	7.41E-01	0.11	5.75E-01	-0.30	1.26E-01	-0.03	8.89E-01	-0.36	6.40E-02	0.01	9.50E-01
ENSCAFG0000001646	ETV3	red	VSMC_M1	0.48	1.08E-02	0.04	8.42E-01	0.13	5.26E-01	-0.28	1.55E-01	0.25	2.06E-01	-0.08	6.99E-01	-0.29	1.44E-01	0.16	4.13E-01	-0.07	7.35E-01	-0.43	2.64E-02
ENSCAFG0000000917	CLDND1	red	VSMC_M1	0.48	1.08E-02	0.40	3.95E-02	0.17	4.05E-01	-0.01	9.42E-01	0.07	7.12E-01	0.15	4.54E-01	-0.07	7.35E-01	-0.08	7.04E-01	-0.52	5.39E-03	0.07	7.45E-01
ENSCAFG0000002965	ENSCAFG0000002965	grey	VSMC_M10	0.48	1.09E-02	0.31	1.13E-01	0.31	1.13E-01	0.13	5.18E-01	-0.14	4.95E-01	-0.03	8.97E-01	-0.14	4.79E-01	-0.23	2.52E-01	-0.39	4.15E-02	0.12	5.49E-01
ENSCAFG0000000222	ASNS	pink	VSMC_M5	0.48	1.09E-02	-0.26	1.90E-01	-0.21	2.82E-01	-0.13	5.33E-01	0.62	5.17E-04	-0.44	2.14E-02	0.08	6.91E-01	0.15	4.49E-01	0.11	5.75E-01	-0.50	7.23E-03
ENSCAFG0000001266	DCAF17	red	VSMC_M1	0.48	1.09E-02	0.13	5.15E-01	-0.11	6.02E-01	0.21	2.82E-01	0.29	1.46E-01	0.03	8.89E-01	-0.24	2.33E-01	-0.17	3.87E-01	-0.29	1.49E-01	0.05	7.92E-01
ENSCAFG0000000928	F2RL1	grey	VSMC_M10	0.48	1.10E-02	0.53	4.50E-03	-0.01	9.72E-01	0.46	1.61E-02	0.14	4.99E-01	-0.28	1.58E-01	-0.41	3.46E-02	-0.49	9.47E-03	-0.59	1.33E-03	0.41	3.21E-02
ENSCAFG0000000919	AMBRA1	red	VSMC_M1	0.48	1.10E-02	0.42	2.75E-02	0.33	9.50E-02	0.13	5.11E-01	-0.05	8.16E-01	-0.16	4.40E-01	-0.33	8.90E-02	-0.26	1.96E-01	-0.46	1.61E-02	0.01	9.71E-01
ENSCAFG0000001022	DOCK3	pink	VSMC_M5	0.48	1.12E-02	0.04	8.40E-01	-0.53	4.64E-03	0.57	1.83E-03	0.70	4.21E-05	-0.17	4.11E-01	-0.11	5.89E-01	-0.46	1.50E-02	-0.16	4.13E-01	0.08	6.82E-01
ENSCAFG0000001881	ENSCAFG0000001881	red	VSMC_M1	0.48	1.12E-02	0.33	9.15E-02	0.20	3.30E-01	-0.06	7.57E-01	0.12	5.35E-01	-0.18	3.74E-01	-0.02	9.16E-01	-0.06	7.83E-01	-0.38	4.79E-02	-0.08	6.82E-01
ENSCAFG0000000936	TFG	red	VSMC_M1	0.48	1.12E-02	-0.03	8.66E-01	-0.13	5.33E-01	-0.24	2.35E-01	0.45	1.73E-02	-0.18	3.74E-01	0.29	1.39E-01	0.14	4.80E-01	-0.09	6.66E-01	-0.26	1.93E-01
ENSCAFG0000000848	NDRG4	cyan	VSMC_M2	0.48	1.14E-02	0.50	8.09E-03	0.06	7.84E-01	0.34	8.53E-02	0.24	2.28E-01	-0.26	1.97E-01	-0.62	5.93E-04	-0.52	5.83E-03	-0.38	5.23E-02	0.07	7.17E-01
ENSCAFG0000001699	MTX1	yellow	VSMC_M3	0.48	1.14E-02	0.19	3.30E-01	0.65	2.48E-04	-0.46	1.66E-02	-0.48	1.10E-02	0.16	4.25E-01	0.17	3.96E-01	0.38	4.82E-02	-0.39	4.30E-02	-0.03	8.74E-01
ENSCAFG0000003079	ZNF250	yellow	VSMC_M3	0.48	1.14E-02	0.37	5.71E-02	0.48	1.11E-02	-0.07	7.26E-01	-0.17	4.01E-01	-0.23	2.45E-01	-0.28	1.56E-01	-0.10	6.09E-01	-0.38	5.38E-02	0.00	9.80E-01
ENSCAFG0000001922	ENSCAFG0000001922	yellow	VSMC_M3	0.48	1.14E-02	0.21	3.01E-01	0.33	9.14E-02	-0.22	2.70E-01	-0.10	6.12E-01	0.02	9.28E-01	0.01	9.50E-01	0.11	5.72E-01	-0.35	7.60E-02	-0.15	4.64E-01
ENSCAFG0000000917	EXOC2	cyan	VSMC_M2	0.48	1.16E-02	0.58	1.41E-03	0.06	7.67E-01	0.23	2.58E-01	0.13	5.04E-01	-0.08	7.06E-01	0.02	9.39E-01	-0.35	7.12E-02	-0.64	3.02E-04	0.30	1.23E-01
ENSCAFG0000002489	NMT2	cyan	VSMC_M2	0.48	1.17E-02	0.39	4.43E-02	0.21	3.03E-01	-0.09	6.40E-01	0.03	8.90E-01	-0.21	2.94E-01	-0.25	2.01E-01	-0.07	7.35E-01	-0.43	2.39E-02	0.03	8.96E-01
ENSCAFG0000000833	CAMKK2	cyan	VSMC_M2	0.48	1.17E-02	0.63	3.84E-04	0.55	3.06E-03	-0.21	2.93E-01	-0.32	9.90E-02	0.01	9.43E-01	0.07	7.26E-01	-0.01	9.47E-01	-0.72	2.37E-05	0.20	3.21E-01
ENSCAFG0000003043	ZNF74	cyan	VSMC_M2	0.48	1.17E-02	0.59	1.29E-03	0.05	8.14E-01	0.49	9.08E-03	0.06	7.66E-01	-0.14	4.95E-01	-0.22	2.69E-01	-0.60	8.64E-04	-0.64	2.83E-04	0.50	7.24E-03
ENSCAFG0000001938	ENSCAFG0000001938	cyan	VSMC_M2	0.48	1.17E-02	0.58	1.55E-03	0.31	1.21E-01	0.03	8.79E-01	-0.18	3.67E-01	-0.19	3.52E-01	-0.01	9.48E-01	-0.21	2.92E-01	-0.67	1.45E-04	0.40	3.86E-02
ENSCAFG0000000495	KMT2C	pink	VSMC_M5	0.48	1.17E-02	0.26	1.95E-01	-0.37	5.53E-02	0.53	4.29E-03	0.60	8.59E-04	-0.38	4.82E-02	-0.19	3.31E-01	-0.56	2.53E-03	-0.29	1.46E-01	0.16	4.26E-01
ENSCAFG0000001051	SYNC	cyan	VSMC_M2	0.48	1.18E-02	0.51	6.42E-03	0.04	8.27E-01	0.31	1.12E-01	0.21	3.01E-01	-0.23	2.51E-01	-0.62	5.23E-04	-0.56	2.43E-03	-0.42	2.98E-02	0.21	2.95E-01
ENSCAFG0000000461	PLXND1	yellow	VSMC_M3	0.48	1.18E-02	0.37	5.54E-02	0.61	6.96E-04	-0.59	1.35E-03	-0.33	8.97E-02	0.14	4.97E-01	0.18	3.59E-01	0.30	1.31E-01	-0.47	1.23E-02	-0.06	7.51E-01
ENSCAFG0000001675	ISG20L2	grey	VSMC_M10	0.48	1.18E-02	0.05	8.05E-01	0.21	2.94E-01	-0.21	2.96E-01	-0.10	6.32E-01	0.21	2.82E-01	0.16	4.21E-01	0.19	3.47E-01	-0.27	1.77E-01	0.05	8.12E-01
ENSCAFG0000000202	CYP39A1	pink	VSMC_M5	0.48	1.18E-02	-0.18	3.63E-01	-0.19	3.54E-01	-0.22	2.68E-01	0.54	4.02E-03	-0.40	3.90E-02	0.03	8.92E-01	0.25	2.17E-01	0.07	7.38E-01	-0.43	2.70E-02
ENSCAFG0000000621	SIGLEC1	yellow	VSMC_M3	0.48	1.18E-02	0.42	2.77E-02	0.82	1.35E-07	-0.63	4.12E-04	-0.47	1.33E-02	-0.10	6.15E-01	0.00	1.00E+00	0.32	1.03E-01	-0.48	1.07E-02	-0.21	2.82E-01
ENSCAFG0000003067	ENSCAFG0000003067	grey	VSMC_M10	0.48	1.19E-02	0.10	6.32E-01	0.10	6.14E-01	-0.19	3.45E-01	0.33	8.90E-02	-0.17	3.93E-01	-0.11	5.74E-01	0.02	9.07E-01	-0.15	4.58E-01	-0.45	1.84E-02
ENSCAFG0000000128	TCP11	red	VSMC_M1	0.48	1.19E-02	0.19	3.35E-01	0.32	1.00E-01	-0.25	2.07E-01	-0.04	8.60E-01	0.07	7.23E-01	0.01	9.59E-01	0.08	6.92E-01	-0.29	1.47E-01	-0.06	7.52E-01
ENSCAFG0000001168	ENSCAFG0000001168	grey	VSMC_M10	0.48	1.19E-02	0.40	4.10E-02	0.14	4.96E-01	-0.13	5.34E-01	0.07	7.15E-01	-0.06	7.79E-01	-0.15	4.60E-01	-0.04	8.62E-01	-0.49	9.25E-03	0.10	6.29E-01
ENSCAFG0000000162	CYHR1	cyan	VSMC_M2	0.48	1.19E-02	0.75	7.03E-06	0.28	1.55E-01	0.36	6.17E-02	-0.19	3.35E-01	0.02	9.23E-01	-0.30	1.23E-01	-0.55	3.28E-03	-0.74	1.26E-05	0.60	1.03E-03
ENSCAFG0000001073	GRAMD1C	red	VSMC_M1	0.48	1.19E-02	0.27	1.71E-01	0.14	4.83E-01	0.16	4.17E-01	0.01	9.42E-01	-0.08	7.02E-01	-0.08	6.74E-01	-0.19	3.46E-01	-0.37	5.76E-02	0.17	3.96E-01
ENSCAFG0000000749	KMT5A	red	VSMC_M1	0.48	1.20E-02	-0.19	3.36E-01	0.13	5.18E-01	-0.38	5.31E-02	0.19	3.45E-01	-0.02	9.29E-01	0.27	1.71E-01	0.34	8.00E-02	-0.01	9.63E-01	-0.48	1.21E-02
ENSCAFG0000002572	ENSCAFG0000002572	cyan	VSMC_M2	0.48	1.20E-02	0.46	1.61E-02	0.47	1.24E-02	0.07	7.31E-01	-0.29	1.38E-01	0.11	5.93E-01	0.09	6.56E-01	-0.27	1.80E-01	-0.52	5.39E-03	0.24	2.29E-01
ENSCAFG0000000387	EPS15	grey	VSMC_M10	0.48	1.20E-02	0.44	2.07E-02	-0.26	1.85E-01	0.57	2.13E-03	0.46	1.55E-02	-0.31	1.15E-01	-0.36	6.13E-02	-0.58	1.60E-03	-0.47	1.31E-02	0.28	1.61E-01
ENSCAFG0000000171	HMOX1	cyan	VSMC_M2	0.48	1.20E-02	0.61	7.66E-04	0.45	1.76E-02	-0.14	4.76E-01	-0.24	2.35E-01	0.00	9.88E-01	0.05	8.22E-01	-0.08	7.09E-01	-0.67	1.52E-04	0.16	4.17E-01
ENSCAFG0000001768	RPL7A	yellow	VSMC_M3	0.48	1.22E-02	0.32	1.08E-01	0.64	3.61E-04	-0.41	3.33E-02	-0.35	7.17E-02	-0.28	1.55E-01	0.04	8.47E-01	0.26	1.95E-01	-0.47	1.38E-02	-0.16	4.37E-01
ENSCAFG0000001417	ASTN1	cyan	VSMC_M2	0.48	1.22E-02	0.57	2.13E-03	0.47	1.23E-02	-0.14	4.86E-01	-0.27	1.74E-01	0.02	9.23E-01	-0.35	7.01E-02	-0.13	5.20E-01	-0.63	3.79E-04	0.18	3.65E-01
ENSCAFG0000001566	ITFG2	red	VSMC_M1	0.48	1.22E-02	0.23	2.53E-01	-0.18	3.65E-01	0.33	8.96E-02	0.34	7.96E-02	-0.05	7.99E-01	-0.24	2.36E-01	-0.38	5.21E-02	-0.28	1.50E-01	0.23	2.55E-01
ENSCAFG0000001942	MOB3A	red	VSMC_M1	0.48	1.23E-02	0.41	3.41E-02	0.36	6.15E-02	-0.13	5.33E-01	-0.29	1.44E-01	0.12	5.60E-01	0.05	7.88E-01	0.01	9.61E-01	-0.59	1.21E-03	0.29	1.40E-01
ENSCAFG0000002010	SLC44A3	yellow	VSMC_M3	0.47	1.23E-02	0.50	7.24E-03	0.65	2.31E-04	-0.14	4.72E-01	-0.43	2.56E-02	0.14	4.94E-01	-0.06	7.77E-01	-0.09	6.71E-01	-0.58	1.58E-03	0.11	5.91E-01
ENSCAFG0000000575	FAAP100	yellow	VSMC_M3	0.47	1.23E-02	0.50	8.41E-03	0.84	4.45E-08	-0.45	1.94E-02	-0.52	5.73E-03	-0.08	6.91E-01	-0.17	4.07E-01	0.17	3.85E-01	-0.56	2.14E-03	-0.07	7.44E-01
ENSCAFG0000000065	TULP4	grey	VSMC_M10	0.47	1.23E-02	0.43	2.50E-02	-0.19	3.43E-01	0.39	4.59E-02	0.40	3.79E-02	-0.05	8.15E-01	-0.27	1.79E-01	-0.50	8.39E-03	-0.47	1.38E-02	0.23	2.40E-01
ENSCAFG0000001487	CHCHD1	red	VSMC_M1	0.47	1.24E-02	-0.12	5.63E-01	0.21	2.96E-01	-0.20	3.12E-01	-0.04	8.62E-01	0.11	5.80E-01	0.25	2.00E-01	0.30	1.34E-01	-0.14	4.73E-01	-0.21	2.96E-01
ENSCAFG0000001103	WIPI1	red	VSMC_M1	0.47	1.24E-02	0.45	1.83E-02	0.16	4.38E-01	0.22	2.74E-01	-0.07	7.17E-01	0.21	3.05E-01	-0.34	8.43E-02	-0.36	6.20E-02	-0.50	8.47E-03	0.34	8.57E-02
ENSCAFG0000001137	AKAP13	red	VSMC_M1	0.47	1.25E-02	-0.01	9.45E-01	-0.27	1.72E-01	0.08	6.84E-01	0.55	2.84E-03	-0.22	2.72E-01	-0.07	7.40E-01	-0.10	6.26E-01	-0.10	6.11E-01	-0.13	5.13E-01
ENSCAFG0000001622	SERPINE2	cyan	VSMC_M2	0.47	1.25E-02	0.53	4.36E-03	0.21	2.94E-01	0.09	6.61E-01	0.00	9.84E-01	-0.09	6.64E-01	-0.46	1.48E-02	-0.24	2.38E-01	-0.59	1.07E-03	0.17	3.87E-01
ENSCAFG0000000477	TRANK1	cyan	VSMC_M2	0.47	1.26E-02	0.86	9.59E-09	0.28	1.61E-01	0.32	9.92E-02	-0.13	5.03E-01	-0.06	7.54E-01	-0.47	1.43E-02	-0.57	1.98E-03	-0.84	5.24E-08	0.51	7.21E-03
ENSCAFG0000001679	NAXE	grey	VSMC_M10	0.47	1.28E-02	0.58	1.43E-03	0.79	1.09E-06	-0.35	7.25E-02	-0.59	1.32E-03	0.02	9.25E-01	0.10	6.35E-01	0.04	8.39E-01	-0.65	2.67E-04	0.23	2.52E-01
ENSCAFG0000000410	EPC1	pink	VSMC_M5	0.47	1.28E-02	-0.02	9.33E-01	-0.28	1.53E-01	0.24	2.28E-01	0.64	3.57E-04	-0.20	3.10E-01	-0.14	4.81E-01	-0.27	1.77E-01	-0.01	9.45E-01	-0.25	2.01E-01
ENSCAFG0000001813	COQ7	red	VSMC_M1	0.47	1.28E-02	0.09	6.50E-01	0.10	6.27E-01	-0.23	2.43E-01	0.12	5.39E-01	-0.09	6.45E-01	0.01	9.71E-01	0.27	1.81E-01	-0.34	8.72E-02	-0.14	4.99E-01
ENSCAFG0000000946	HIBCH	grey	VSMC_M10	0.47	1.28E-02	0.46	1.65E-02	-0.16	4.14E-01	0.46	1.70E-02	0.31	1.11E-01	-0.32	1.09E-01	-0.09	6.72E-01	-0.51	6.40E-03	-0.58	1.66E-03	0.37	5.90E-02
ENSCAFG0000001941	TRAF7	cyan	VSMC_M2	0.47	1.28E-02	0.80	4.47E-07	0.57	2.12E-03	-0.01	9.49E-01	-0.44	2.25E-02	0.15	4.62E-01	-0.14	4.88E-01	-0.26	1.90E-01	-0.86	1.26E-08	0.50	7.43E-03
ENSCAFG0000000027	CCDC28A	red	VSMC_M1	0.47	1.28E-02	0.32	9.95E-02	-0.17	4.00E-01	0.32	9.87E-02	0.24	2.23E-01	0.08	6.83E-01	0.09	6.41E-01	-0.36	6.59E-02	-0.46	1.53E-02	0.39	4.36E-02
ENSCAFG0000001681	CDK5RAP3	red	VSMC_M1	0.47	1.29E-02	0.04	8.43E-01	0.45	1.80E-02	-0.55	2.75E-03	-0.09	6.63E-01	-0.32	1.09E-01	0.25	2.06E-01	0.47	1.24E-02	-0.20	3.14E-01	-0.41	3.30E-02
ENSCAFG0000001247	ITGB5	grey	VSMC_M10	0.47	1.29E-02	0.78	2.06E-06	0.22	2.70E-01	0.44	2.11E-02	-0.07	7.12E-01	-0.22	2.62E-01	-0.51	6.16E-03	-0.69	6.89E-05	-0.71	3.48E-05	0.52	5.78E-03
ENSCAFG0000001981	GSTM4	cyan	VSMC_M2	0.47	1.29E-02	0.84	3.97E-08	0.28	1.55E-01	0.30	1.23E-01	-0.12	5.47E-01	-0.33	8.78E-02	-0.14	4.71E-01	-0.52	5.77E-03	-0.84	5.71E-08	0.54	3.89E-03
ENSCAFG0000000393	ADCK2	red	VSMC_M1	0.47	1.29E-02	0.62	5.82E-04	0.23	2.43E-01	0.10	6.19E-01	-0.06	7.80E-01	-0.09	6.69E-01	0.18	3.63E-01	-0.29	1.37E-01	-0.71	2.92E-05	0.42	3.02E-02
ENSCAFG0000001618	KLF3	yellow	VSMC_M3	0.47	1.29E-02	0.32	1.08E-01	0.40	3.85E-02	-0.53	4.71E-03	-0.06	7.61E-01	-0.13	5.20E-01	-0.02	9.19E-01	0.29	1.42E-01	-0.41	3.36E-02	-0.22	2.73E-01
ENSCAFG0000001872	RFX2	yellow	VSMC_M3	0.47	1.29E-02	0.30	1.27E-01	0.37	5.59E-02	-0.32	1.04E-01	-0.10	6.09E-01	0.03	8.95E-01	0.19	3.51E-01	0.17	4.05E-01	-0.42	2.81E-02	-0.08	6.88E-01
ENSCAFG0000000114	NDRG1	red	VSMC_M1	0.47	1.29E-02	0.36	6.76E-02	0.27	1.72E-01	-0.09	6.70E-01	0.12	5.40E-01	-0.10	6.36E-01	-0.43	2.52E-02	-0.13	5.10E-01	-0.28	1.55E-01	-0.24	2.37E-01
ENSCAFG0000001500	MAPK1IP1L	pink	VSMC_M5	0.47	1.29E-02	-0.17	4.01E-01	-0.19	3.33E-01	-0.23	2.38E-01	0.57	1.98E-03	-0.18	3.75E-01	-0.18	3.62E-01	0.21	2.96E-01	0.10	6.14E-01	-0.49	9.23E-03
ENSCAFG0000002246	ENSCAFG0000002246	grey	VSMC_M10	0.47	1.30E-02	0.00	9.82E-01	0.06	7.49E-01	-0.06	7.66E-01	0.28	1.64E-01	-0.01	9.44E-01	0.12	5.45E-01	-0.04	8.41E-01	-0.06	7.76E-01	-0.32	1.02E-01
ENSCAFG0000001922	PRPF8	pink	VSMC_M5	0.47	1.30E-02	-0.27	1.81E-01	-0.36	6.36E-02	-0.11	5.94E-01	0.65	2.49E-04	-0.04	8.50E-01	-0.01	9.73E-01	0.21	2.84E-01	0.09	6.66E-01	-0.40	3.94E-02
ENSCAFG0000003106	TMEM267	grey	VSMC_M10	0.47	1.31E-02	0.14	4.87E-01	-0.05	8.18E-01	0.26	1.88E-01	0.24	2.32E-01	0.00	9.85E-01	0.14	4.77E-01	-0.27	1.67E-01	-0.32	1.04E-01	0.16	4.25E-01
ENSCAFG0000001070	IQCC	red	VSMC_M1	0.47	1.33E-02	0.38	4.78E-02	0.31	1.17E-01	-0.09	6.44E-01	-0.22	2.72E-01	0.15	4.55E-01	0.06	7.69E-01	0.04	8.57E-01	-0.59	1.22E-03	0.26	1.90E-01
ENSCAFG0000001275	MTFR1L	red	VSMC_M1	0.47	1.33E-02	0.32	1.02E-01	-0.06	7.70E-01	0.32	1.03E-01	0.20	3.15E-01	-0.05	8.13E-01	-0.11	5.71E-01	-0.38	5.38E-02	-0.37	5.93E-02	0.15	4.56E-01
ENSCAFG0000001668	ANXA2	grey	VSMC_M10	0.47	1.34E-02	-0.29	1.37E-01	0.16	4.20E-01	-0.40	3.76E-02	0.10	6.16E-01	0.02	9.17E-01	0.13	5.13E-01	0.54	3.53E-03	0.06	7.52E-01	-0.52	5.94E-03
ENSCAFG0000003050	ENSCAFG0000003050	red	VSMC_M1	0.47	1.34E-02	-0.07	7.41E-01	0.34	8.12E-02	-0.28	1.64E-01	0.05	8.16E-01	-0.35	7.58E-02	0.09	6.55E-01	0.21	2.96E-01	0.00	9.83E-01	-0.46	1.56E-02
ENSCAFG0000000757	DPY19L3	cyan	VSMC_M2	0.47	1.34E-02	0.80	7.06E-07	0.19	3.37E-01	0.20	3.14E-01	0.02	9.14E-01	-0.28	1.57E-01	-0.40	3.96E-02	-0.48	1.17E-02	-0.76	4.62E-06	0.38	5.23E-02
ENSCAFG0000000785	CAST	cyan	VSMC_M2	0.47	1.35E-02	0.73	1.89E-05	0.22	2.66E-01	0.15	4.43E-01	0.02	9.40E-01	-0.30	1.23E-01	-0.44	2.17E-02	-0.43	2.67E-02	-0.65	2.23E-04	0.33	9.72E-02
ENSCAFG0000001458	ARL4D	red	VSMC_M1	0.47	1.35E-02	0.54	3.87E-03	0.25	2.08E-01	-0.05	7.96E-01	0.00	9.84E-01	-0.21	2.98E-01	-0.15	4.58E-01	-0.17	3.91E-01	-0.56	2.48E-03	0.10	6.24E-01
ENSCAFG0000001170	B3GALT1	grey	VSMC_M10	0.47	1.35E-02	-0.06	7.53E-01	-0.21	2.94E-01	0.03	8.71E-01	0.42	3.12E-02	0.00	9.88E-01	-0.42	3.13E-02	0.00	9.81E-01	-0.01	9.70E-01	-0.19	3.55E-01
ENSCAFG0000001348	CADM1	grey	VSMC_M10	0.47	1.36E-02	-0.02	9.30E-01	0.30	1.23E-01	-0.58	1.44E-03	0.11	5.78E-01	-0.24	2.19E-01	0.36	6.60E-02	0.40	3.71E-02	-0.08	7.07E-01	-0.49	9.82E-03
ENSCAFG0000000912	TMEM67	grey	VSMC_M10	0.47	1.36E-02	0.26	1.87E-01	-0.06	7.63E-01	0.24	2.25E-01	0.14	4.94E-01	-0.07	7.13E-01	0.25	2.04E-01	-0.27	1.76E-01	-0.44	2.18E-02	0.38	4.85E-02
ENSCAFG0000000824	FAR1	pink	VSMC_M5	0.47	1.36E-02	0.10	6.24E-01	-0.53	4.42E-03	0.65	2.68E-04	0.70	5.09E-05	-0.23	2.45E-01	-0.35	7.77E-02	-0.52	5.50E-03	-0.19	3.43E-01	0.13	5.04E-01
ENSCAFG0000000208	ENSCAFG0000000208	pink	VSMC_M5	0.47	1.36E-02	0.01	9.64E-01	0.02	9.30E-01	0.00	1.00E+00	0.42	2.97E-02	-0.43	2.39E-02	-0.15	4.41E-01	-0.03	8.80E-01	-0.05	8.06E-01	-0.45	1.89E-02
ENSCAFG0000000607	NAXD	red	VSMC_M1	0.47	1.36E-02	0.38	4.82E-02	-0.11	5.93E-01	0.52	5.83E-03	0.19	3.32E-01	-0.13	5.27E-01	-0.16	4.12E-01	-0.53	4.59E-03	-0.45	1.88E-02	0.41	3.17E-02
ENSCAFG0000001118	ARFGEF2	red	VSMC_M1	0.47	1.37E-02	0.13	5.33E-01	0.12	5.53E-01	-0.09	6.48E-01	0.06	7.69E-01	0.10	6.32E-01	0.04	8.31E-01	-0.04	8.37E-01	-0.20	3.16E-01	0.09	6.42E-01
ENSCAFG0000000472	JPT1	yellow	VSMC_M3	0.47	1.37E-02	0.34	8.55E-02	0.45	1.86E-02	-0.10	6.11E-01	-0.22	2.65E-01	-0.31	1.11E-01	0.11	5.73E-01	0.05	7.91E-01	-0.50	8.54E-03	0.01	9.50E-01
ENSCAFG0000001178	SPRTN	grey	VSMC_M10	0.47	1.37E-02	-0.01	9.66E-01	-0.10	6.28E-01	-0.07	7.14E-01	0.31	1.10E-01	0.04	8.45E-01	-0.26	1.86E-01	0.13	5.12E-01	-0.17	3.96E-01	-0.20	3.23E-01
ENSCAFG0000001698	PER1	yellow	VSMC_M3	0.47	1.38E-02	0.24	2.25E-01	0.59	1.32E-03	-0.56	2.65E-03	-0.22	2.75E-01	-0.03	8.87E-01	0.02	9.26E-01	0.34	8.28E-02	-0.31	1.15E-01	-0.29	1.46E-01
ENSCAFG0000001771	METTL9	red	VSMC_M1	0.47	1.38E-02	0.32	9.91E-02	0.09	6.72E-01	-0.09	6.71E-01	0.21	2.92E-01	-0.31	1.16E-01	0.15	4.46E-01	-0.07	7.15E-01	-0.40	3.75E-02	-0.04	8.48E-01
ENSCAFG0000000399	PARP12	cyan	VSMC_M2	0.47	1.38E-02	0.63	4.25E-04	0.18	3.66E-01	0.28	1.55E-01	0.09	6.48E-01	-0.33	9.45E-02	-0.11	5.89E-01	-0.51	6.75E-03	-0.62	6.02E-04	0.24	2.21E-01
ENSCAFG0000001788	EVL	grey	VSMC_M10	0.47	1.39E-02	0.81	3.24E-07	0.28	1.54E-01	0.43	2.44E-02	-0.12	5.51E-01	-0.09	6.71E-01	-0.32	1.05E-01	-0.67	1.36E-04	-0.75	7.48E-06	0.49	9.71E-03
ENSCAFG0000001007	LSP1	yellow	VSMC_M3	0.47	1.40E-02	0.47	1.31E-02	0.64	3.36E-04	-0.45	1.90E-02	-0.34	8.20E-02	-0.09	6.47E-01	0.21	2.85E-01	0.23	2.41E-01	-0.60	8.83E-04	-0.06	7.69E-01
ENSCAFG0000001699	EIF2B2	red	VSMC_M1	0.47	1.40E-02	0.42	2.84E-02	0.31	1.20E-01	-0.05	8.17E-01	-0.20	3.10E-01	0.12	5.56E-01	-0.12	5.39E-01	-0.06	7.58E-01	-0.51	6.09E-03	0.33	9.64E-02
ENSCAFG0000000923	GNAO1	cyan	VSMC_M2	0.47	1.41E-02	0.32	1.08E-01	0.16	4.35E-01	0.35	7.44E-02	0.04	8.38E-01	0.03	8.64E-01	-0.27	1.68E-01	-0.43	2.69E-02	-0.36	6.38E-02	0.14	4.86E-01
ENSCAFG0000001732	ENSCAFG0000001732	red	VSMC_M1	0.47	1.41E-02	0.02	9.40E-01	0.17	4.08E-01	-0.15	4.52E-01	0.13	5.15E-01	-0.27	1.77E-01	-0.07	7.36E-01	0.15	4.62E-01	-0.16	4.38E-01	-0.30	1.28E-01
ENSCAFG0000000385	ENSCAFG0000000385	yellow	VSMC_M3	0.47	1.41E-02	0.51	7.22E-03	0.61	7.20E-04	-0.15	4.49E-01	-0.33	9.64E-02	-0.06	7.74E-01	0.12	5.47E-01	-0.08	6.97E-01	-0.55	3.18E-03	0.09	6.41E-01
ENSCAFG0000000492	TMEM40	grey	VSMC_M10	0.47	1.41E-02	0.73	1.88E-05	0.19	3.39E-01	0.32	1.09E-01	-0.08	6.95E-01	-0.06	7.55E-01	-0.26	1.99E-01	-0.50	8.01E-03	-0.74	9.72E-06	0.51	6.96E-03
ENSCAFG0000000148	TRIOBP	red	VSMC_M1	0.47	1.41E-02	0.03	8.77E-01	0.64	3.33E-04	-0.71	3.10E-05	-0.26	1.88E-01	-0.07	7.43E-01	-0.07	7.19E-01	0.56	2.38E-03	-0.14	4.73E-01	-0.50	7.28E-03
ENSCAFG0000001869	TWSG1	grey	VSMC_M10	0.47	1.43E-02	0.60	8.90E-04	-0.16	4.22E-01	0.59	1.34E-03	0.23	2.56E-01	-0.18	3.71E-01	-0.27	1.75E-01	-0.68	1.05E-04	-0.60	8.70E-04	0.61	8.09E-04
ENSCAFG0000000428	ENSCAFG0000000428	red	VSMC_M1	0.47	1.44E-02	-0.02	9.18E-01	-0.01	9.78E-01	0.12	5.61E-01	0.21	3.02E-01	-0.03	8.84E-01	-0.18	3.59E-01	-0.02	9.17E-01	-0.11	5.72E-01	-0.10	6.33E-01
ENSCAFG0000001906	SMAD7	pink	VSMC_M5	0.47	1.44E-02	-0.20	3.09E-01	-0.25	2.12E-01	-0.04	8.61E-01	0.54	3.52E-03	-0.20	3.05E-01	0.13	5.22E-01	0.19	3.52E-01	0.02	9.29E-01	-0.34	8.08E-02

ENSCAFG0000001367	RAB36	grey	VSMC_M1C	0.47	1.45E-02	0.21	3.05E-01	0.43	2.34E-02	-0.08	6.92E-01	-0.23	2.48E-01	-0.05	8.19E-01	-0.06	7.55E-01	0.01	9.63E-01	-0.33	9.65E-02	-0.04	8.48E-01
ENSCAFG0000001518	GLB1L	grey	VSMC_M1C	0.47	1.45E-02	0.61	6.51E-04	-0.12	5.42E-01	0.57	1.76E-03	0.24	2.27E-01	-0.20	3.21E-01	-0.22	2.67E-01	-0.69	7.38E-05	-0.65	2.18E-04	0.49	9.55E-03
ENSCAFG0000000115	ZFAT	red	VSMC_M1	0.47	1.45E-02	0.45	1.89E-02	0.12	5.62E-01	0.16	4.21E-01	0.15	4.57E-01	-0.07	7.20E-01	-0.09	6.50E-01	-0.36	6.83E-02	-0.45	1.81E-02	0.17	3.97E-01
ENSCAFG0000000335	ENSCAFG0000000335	red	VSMC_M1	0.46	1.46E-02	0.10	6.10E-01	0.16	4.33E-01	0.03	8.97E-01	0.11	5.74E-01	-0.07	7.46E-01	0.18	3.80E-01	-0.08	6.85E-01	-0.26	1.97E-01	-0.07	7.13E-01
ENSCAFG0000002660	ENSCAFG0000002660	red	VSMC_M1	0.46	1.46E-02	0.01	9.77E-01	0.07	7.21E-01	0.00	9.83E-01	0.23	2.54E-01	-0.25	2.00E-01	-0.10	6.14E-01	-0.07	7.24E-01	-0.06	7.54E-01	-0.21	2.91E-01
ENSCAFG0000003189	EIF1AD	grey	VSMC_M1C	0.46	1.46E-02	-0.39	4.72E-02	0.01	9.42E-01	-0.45	1.79E-02	0.26	1.88E-01	0.07	7.47E-01	-0.01	9.76E-01	0.55	3.02E-03	0.17	3.95E-01	-0.55	2.86E-03
ENSCAFG0000001798	PPCDC	cyan	VSMC_M2	0.46	1.46E-02	0.58	1.38E-03	0.39	4.72E-02	0.05	8.06E-01	-0.24	2.21E-01	0.05	8.07E-01	0.02	9.17E-01	-0.26	1.90E-01	-0.68	1.09E-04	0.40	3.99E-02
ENSCAFG0000001961	RPUSD1	grey	VSMC_M1C	0.46	1.47E-02	0.38	4.95E-02	0.58	1.37E-03	-0.34	8.03E-02	-0.48	1.11E-02	0.10	6.10E-01	0.41	3.30E-02	0.19	3.47E-01	-0.58	1.54E-03	0.22	2.71E-01
ENSCAFG0000001361	DTX2	cyan	VSMC_M2	0.46	1.47E-02	0.75	6.65E-06	0.27	1.80E-01	0.32	1.01E-01	-0.13	5.21E-01	-0.19	3.50E-01	-0.11	5.79E-01	-0.56	2.54E-03	-0.76	4.14E-06	0.55	3.09E-03
ENSCAFG0000003057	OSER1	yellow	VSMC_M3	0.46	1.48E-02	0.14	4.89E-01	0.60	9.67E-04	-0.40	3.62E-02	-0.31	1.11E-01	-0.03	8.67E-01	0.19	3.50E-01	0.32	1.01E-01	-0.30	1.23E-01	-0.26	1.90E-01
ENSCAFG0000001027	CARS	yellow	VSMC_M3	0.46	1.48E-02	0.44	2.17E-02	0.64	3.56E-04	-0.17	3.87E-01	-0.38	4.77E-02	-0.08	7.08E-01	-0.03	8.94E-01	-0.03	8.69E-01	-0.56	2.34E-03	0.07	7.20E-01
ENSCAFG0000001273	IQCG	yellow	VSMC_M3	0.46	1.49E-02	0.30	1.23E-01	0.49	8.73E-03	-0.35	6.92E-02	-0.27	1.80E-01	-0.17	4.03E-01	0.30	1.32E-01	0.15	4.40E-01	-0.47	1.31E-02	0.04	8.55E-01
ENSCAFG0000001066	SLC16A14	yellow	VSMC_M3	0.46	1.49E-02	0.17	4.06E-01	0.48	1.05E-02	-0.20	3.11E-01	-0.17	3.97E-01	-0.17	4.02E-01	-0.06	7.54E-01	0.11	6.01E-01	-0.28	1.51E-01	-0.19	3.50E-01
ENSCAFG0000001034	MED1C	grey	VSMC_M1C	0.46	1.49E-02	0.46	1.47E-02	0.02	9.18E-01	0.49	9.49E-03	0.13	5.20E-01	-0.28	1.62E-01	-0.35	7.30E-02	-0.57	2.08E-03	-0.47	1.42E-02	0.34	7.98E-02
ENSCAFG0000001458	USP5	red	VSMC_M1	0.46	1.50E-02	0.09	6.42E-01	0.54	3.62E-03	-0.54	3.75E-03	-0.30	1.31E-01	0.10	6.25E-01	0.04	8.61E-01	0.49	1.03E-02	-0.26	1.85E-01	-0.29	1.48E-01
ENSCAFG0000001942	HCFC1	grey	VSMC_M1C	0.46	1.51E-02	0.04	8.55E-01	0.13	5.33E-01	-0.32	1.03E-01	0.05	8.13E-01	0.21	3.01E-01	0.00	9.98E-01	0.31	1.16E-01	-0.25	2.06E-01	-0.10	6.29E-01
ENSCAFG0000000798	SUCLG1	darkgrey	VSMC_M8	0.46	1.51E-02	-0.11	5.71E-01	0.48	1.19E-02	-0.65	2.42E-04	-0.10	6.12E-01	-0.09	6.69E-01	0.15	4.51E-01	0.60	1.04E-03	-0.02	9.29E-01	-0.56	2.40E-03
ENSCAFG0000000403	TAL1	red	VSMC_M1	0.46	1.52E-02	-0.32	1.04E-01	0.02	9.02E-01	-0.30	1.34E-01	0.31	1.20E-01	0.19	3.49E-01	0.03	8.79E-01	0.31	1.15E-01	0.15	4.51E-01	-0.52	5.34E-03
ENSCAFG0000000361	ZYX	yellow	VSMC_M3	0.46	1.52E-02	0.51	6.29E-03	0.59	1.09E-03	-0.21	2.84E-01	-0.30	1.30E-01	-0.13	5.13E-01	-0.26	1.88E-01	-0.08	6.77E-01	-0.44	2.14E-02	0.04	8.48E-01
ENSCAFG0000003157	ENSCAFG0000003157	red	VSMC_M1	0.46	1.52E-02	0.28	1.53E-01	0.45	1.87E-02	-0.19	3.34E-01	-0.25	2.12E-01	0.07	7.14E-01	0.08	6.78E-01	0.05	8.12E-01	-0.35	7.02E-02	0.05	8.06E-01
ENSCAFG0000000979	ENSCAFG0000000979	red	VSMC_M1	0.46	1.52E-02	0.08	6.85E-01	0.06	7.54E-01	-0.23	2.45E-01	0.18	3.72E-01	0.05	7.93E-01	0.11	5.96E-01	0.14	4.97E-01	-0.22	2.65E-01	-0.13	5.06E-01
ENSCAFG0000000138	UHRF2	grey	VSMC_M1C	0.46	1.53E-02	0.27	1.78E-01	-0.06	7.69E-01	0.18	3.80E-01	0.27	1.78E-01	-0.16	4.30E-01	0.04	8.49E-01	-0.22	2.73E-01	-0.41	3.57E-02	0.16	4.31E-01
ENSCAFG0000001011	NIPSNAP2	pink	VSMC_M5	0.46	1.53E-02	-0.34	8.08E-02	-0.49	8.89E-03	0.15	4.47E-01	0.85	2.44E-08	-0.17	4.02E-01	-0.15	4.54E-01	-0.03	8.93E-01	0.24	2.32E-01	-0.49	1.02E-02
ENSCAFG0000001361	IDH1	yellow	VSMC_M3	0.46	1.53E-02	0.40	3.96E-02	0.57	2.00E-03	-0.48	1.04E-02	-0.20	3.27E-01	-0.19	3.31E-01	0.13	5.34E-01	0.15	4.47E-01	-0.44	2.11E-02	-0.17	4.03E-01
ENSCAFG0000000223	ENSCAFG0000000223	cyan	VSMC_M2	0.46	1.54E-02	0.69	6.66E-05	0.38	4.76E-02	0.01	9.68E-01	-0.25	2.02E-01	-0.13	5.06E-01	-0.34	7.82E-02	-0.20	3.13E-01	-0.72	2.40E-05	0.38	4.96E-02
ENSCAFG0000000872	MROH8	grey	VSMC_M1C	0.46	1.55E-02	-0.13	5.17E-01	0.31	1.15E-01	-0.41	3.17E-02	-0.08	7.03E-01	0.19	3.47E-01	0.16	4.26E-01	0.40	3.83E-02	-0.10	6.14E-01	-0.28	1.53E-01
ENSCAFG0000000123	ENSCAFG0000000123	red	VSMC_M1	0.46	1.55E-02	-0.16	4.14E-01	0.10	6.19E-01	-0.07	7.42E-01	0.03	8.92E-01	0.17	4.06E-01	0.05	8.00E-01	0.20	3.06E-01	-0.07	7.47E-01	-0.12	5.44E-01
ENSCAFG0000002534	AVPI1	grey	VSMC_M1C	0.46	1.56E-02	0.51	6.17E-03	0.40	3.92E-02	-0.04	8.28E-01	-0.31	1.16E-01	0.11	5.96E-01	0.08	6.85E-01	-0.14	4.74E-01	-0.64	2.90E-04	0.44	2.04E-02
ENSCAFG0000001576	TFE3	grey	VSMC_M1C	0.46	1.56E-02	0.67	1.16E-04	-0.01	9.49E-01	0.44	2.01E-02	0.13	5.23E-01	-0.12	5.41E-01	-0.14	4.74E-01	-0.61	7.59E-04	-0.70	4.29E-05	0.54	3.92E-03
ENSCAFG0000000312	MDH1	pink	VSMC_M5	0.46	1.57E-02	-0.12	5.42E-01	-0.46	1.45E-02	0.25	2.00E-01	0.73	1.80E-05	-0.23	2.43E-01	-0.18	3.67E-01	-0.13	5.33E-01	0.05	7.91E-01	-0.21	3.05E-01
ENSCAFG0000001138	ENSCAFG0000001138	cyan	VSMC_M2	0.46	1.58E-02	0.80	4.26E-07	0.50	8.42E-03	0.09	6.39E-01	-0.42	3.02E-02	-0.09	6.57E-01	-0.20	3.25E-01	-0.34	8.14E-02	-0.86	7.76E-09	0.59	1.12E-03
ENSCAFG0000000746	ZNF341	red	VSMC_M1	0.46	1.58E-02	0.31	1.19E-01	-0.31	1.13E-01	0.48	1.22E-02	0.42	3.13E-02	-0.20	3.25E-01	-0.08	7.01E-01	-0.48	1.12E-02	-0.41	3.41E-02	0.39	4.40E-02
ENSCAFG0000001928	IDH3G	cyan	VSMC_M2	0.46	1.58E-02	0.64	3.26E-04	0.44	2.16E-02	-0.09	6.49E-01	-0.26	1.87E-01	-0.12	5.38E-01	-0.31	1.14E-01	-0.18	3.79E-01	-0.60	9.08E-04	0.29	1.43E-01
ENSCAFG0000002270	ENSCAFG0000002270	grey	VSMC_M1C	0.46	1.59E-02	0.35	7.37E-02	-0.27	1.75E-01	0.42	2.74E-02	0.47	1.39E-02	-0.19	3.45E-01	-0.26	1.97E-01	-0.49	1.03E-02	-0.39	4.62E-02	0.16	4.37E-01
ENSCAFG0000001685	DNAL1	red	VSMC_M1	0.46	1.59E-02	0.10	6.25E-01	0.19	3.33E-01	-0.09	6.43E-01	0.05	7.95E-01	0.10	6.26E-01	0.18	3.76E-01	-0.01	9.59E-01	-0.23	2.50E-01	-0.07	7.40E-01
ENSCAFG0000001477	RCOR2	grey	VSMC_M1C	0.46	1.59E-02	0.19	3.34E-01	0.54	3.97E-03	-0.56	2.44E-03	-0.31	1.12E-01	0.12	5.60E-01	0.24	2.30E-01	0.43	2.70E-02	-0.42	3.10E-02	-0.10	6.08E-01
ENSCAFG0000001831	RFFL	red	VSMC_M1	0.46	1.60E-02	0.42	3.04E-02	0.19	3.54E-01	0.10	6.18E-01	0.11	5.82E-01	-0.30	1.33E-01	0.11	5.92E-01	-0.27	1.70E-01	-0.45	1.92E-02	0.11	5.96E-01
ENSCAFG0000003268	ENSCAFG0000003268	red	VSMC_M1	0.46	1.61E-02	0.21	3.02E-01	0.07	7.43E-01	0.12	5.53E-01	0.10	6.18E-01	-0.22	2.69E-01	-0.18	3.78E-01	-0.20	3.08E-01	-0.26	1.85E-01	0.03	8.82E-01
ENSCAFG0000003020	CDK9	cyan	VSMC_M2	0.46	1.61E-02	0.78	1.45E-06	0.30	1.22E-01	0.32	1.07E-01	-0.24	2.22E-01	0.04	8.47E-01	-0.13	5.13E-01	-0.50	8.09E-03	-0.83	8.59E-08	0.61	7.34E-04
ENSCAFG0000000511	RNF32	red	VSMC_M1	0.46	1.61E-02	0.15	4.47E-01	-0.15	4.59E-01	0.09	6.64E-01	0.39	4.41E-02	-0.17	4.08E-01	0.01	9.50E-01	-0.14	4.85E-01	-0.27	1.80E-01	-0.04	8.47E-01
ENSCAFG0000001531	UBR4	pink	VSMC_M5	0.46	1.62E-02	0.06	7.75E-01	-0.46	1.58E-02	0.45	1.85E-02	0.68	1.09E-04	-0.19	3.32E-01	-0.30	1.27E-01	-0.44	2.03E-02	-0.08	6.96E-01	0.04	8.60E-01
ENSCAFG0000000571	ENSCAFG0000000571	red	VSMC_M1	0.46	1.63E-02	0.29	1.41E-01	-0.25	2.12E-01	0.35	7.04E-02	0.38	4.94E-02	-0.28	1.63E-01	-0.20	3.25E-01	-0.41	3.14E-02	-0.34	7.86E-02	0.26	1.90E-01
ENSCAFG0000003060	ENSCAFG0000003060	red	VSMC_M1	0.46	1.65E-02	0.44	2.26E-02	0.18	3.71E-01	0.26	1.95E-01	0.06	7.80E-01	-0.08	6.74E-01	-0.45	1.73E-02	-0.39	4.19E-02	-0.46	1.53E-02	0.08	6.87E-01
ENSCAFG0000000774	PRIMPOL	grey	VSMC_M1C	0.46	1.65E-02	0.11	6.00E-01	-0.33	9.17E-02	0.33	9.58E-02	0.47	1.38E-02	-0.06	7.55E-01	0.24	2.35E-01	-0.28	1.61E-01	-0.28	1.61E-01	0.21	2.96E-01
ENSCAFG0000000595	SETMAR	grey	VSMC_M1C	0.46	1.65E-02	0.15	4.46E-01	-0.12	5.56E-01	0.25	2.11E-01	0.22	2.66E-01	0.09	6.72E-01	-0.08	7.03E-01	-0.20	3.18E-01	-0.36	6.69E-02	0.21	2.95E-01
ENSCAFG0000001862	EVI2B	grey	VSMC_M1C	0.46	1.65E-02	0.11	6.01E-01	-0.29	1.43E-01	0.26	1.96E-01	0.40	3.84E-02	0.02	9.22E-01	-0.02	9.08E-01	-0.24	2.22E-01	-0.26	1.98E-01	0.20	3.22E-01
ENSCAFG0000003212	RBM38	grey	VSMC_M1C	0.46	1.66E-02	0.57	2.12E-03	0.64	3.69E-04	-0.16	4.28E-01	-0.54	3.63E-03	0.09	6.52E-01	0.01	9.58E-01	-0.04	8.42E-01	-0.68	9.96E-05	0.41	3.47E-02
ENSCAFG0000001381	ZNHIT1	yellow	VSMC_M3	0.46	1.66E-02	0.53	4.11E-03	0.66	1.74E-04	-0.15	4.53E-01	-0.36	6.13E-02	-0.18	3.66E-01	-0.13	5.24E-01	-0.12	5.51E-01	-0.57	1.85E-03	0.03	8.70E-01
ENSCAFG0000000359	IRF3	cyan	VSMC_M2	0.46	1.66E-02	0.85	1.64E-08	0.62	5.03E-04	-0.08	6.91E-01	-0.35	7.21E-02	-0.23	2.45E-01	-0.20	3.14E-01	-0.31	1.11E-01	-0.79	1.01E-06	0.27	1.79E-01
ENSCAFG0000002861	ZNF174	grey	VSMC_M1C	0.46	1.67E-02	0.15	4.41E-01	-0.29	1.43E-01	0.59	1.25E-03	0.46	1.51E-02	-0.43	2.59E-02	-0.18	3.72E-01	-0.55	2.77E-03	-0.23	2.57E-01	0.19	3.36E-01
ENSCAFG0000001637	ENSCAFG0000001637	pink	VSMC_M5	0.46	1.67E-02	-0.05	7.87E-01	-0.27	1.67E-01	0.04	8.48E-01	0.50	7.44E-03	-0.32	1.04E-01	-0.09	6.58E-01	0.05	8.05E-01	-0.08	6.96E-01	-0.13	5.09E-01
ENSCAFG0000002502	TRIM6	grey	VSMC_M1C	0.46	1.67E-02	0.58	1.38E-03	0.00	9.95E-01	0.48	1.18E-02	0.12	5.63E-01	-0.05	8.01E-01	-0.33	8.96E-02	-0.60	8.63E-04	-0.61	7.13E-04	0.44	2.03E-02
ENSCAFG0000003098	DUSP22	grey	VSMC_M1C	0.46	1.68E-02	0.50	8.30E-03	-0.14	5.01E-01	0.61	7.67E-04	0.27	1.77E-01	-0.07	7.25E-01	-0.18	3.69E-01	-0.75	6.98E-06	-0.50	8.62E-03	0.47	1.25E-02
ENSCAFG0000000149	MPDZ	grey	VSMC_M1C	0.46	1.68E-02	0.65	2.74E-04	-0.04	8.44E-01	0.43	2.48E-02	0.20	3.23E-01	-0.13	5.32E-01	-0.40	3.69E-02	-0.56	2.36E-03	-0.66	1.74E-04	0.41	3.32E-02
ENSCAFG0000000093	PACSIN2	cyan	VSMC_M2	0.46	1.68E-02	0.49	9.63E-03	0.28	1.51E-01	0.22	2.64E-01	-0.19	3.33E-01	0.02	9.24E-01	-0.07	7.34E-01	-0.27	1.76E-01	-0.66	1.63E-04	0.39	4.20E-02
ENSCAFG0000000385	LDAH	grey	VSMC_M1C	0.46	1.68E-02	0.08	7.03E-01	-0.40	3.70E-02	0.29	1.43E-01	0.59	1.16E-03	-0.14	4.73E-01	0.04	8.38E-01	-0.27	1.74E-01	-0.18	3.77E-01	0.07	7.40E-01
ENSCAFG0000000351	ENSCAFG0000000351	grey	VSMC_M1C	0.46	1.68E-02	0.36	6.72E-02	0.01	9.52E-01	0.29	1.37E-01	0.12	5.63E-01	0.06	7.62E-01	-0.21	2.91E-01	-0.35	7.49E-02	-0.49	1.03E-02	0.28	1.65E-01
ENSCAFG0000000752	ENSCAFG0000000752	red	VSMC_M1	0.46	1.68E-02	0.03	8.96E-01	0.29	1.44E-01	-0.25	2.16E-01	0.05	8.11E-01	-0.19	3.32E-01	0.03	8.65E-01	0.18	3.60E-01	-0.16	4.35E-01	-0.38	4.79E-02
ENSCAFG0000000239	ANKRD39	red	VSMC_M1	0.46	1.69E-02	0.36	6.48E-02	0.29	1.45E-01	0.02	9.40E-01	-0.13	5.30E-01	0.23	2.41E-01	0.01	9.63E-01	-0.11	5.87E-01	-0.48	1.19E-02	0.13	5.13E-01
ENSCAFG0000000137	DNAL4	red	VSMC_M1	0.46	1.69E-02	0.20	3.29E-01	-0.06	7.74E-01	0.07	7.45E-01	0.30	1.34E-01	-0.19	3.32E-01	0.09	6.68E-01	-0.12	5.51E-01	-0.35	7.51E-02	0.04	8.50E-01
ENSCAFG0000000457	GEMIN7	yellow	VSMC_M3	0.46	1.69E-02	0.29	1.38E-01	0.76	4.49E-06	-0.49	9.52E-03	-0.56	2.65E-03	0.18	3.66E-01	0.08	6.98E-01	0.32	1.06E-01	-0.47	1.25E-02	-0.02	9.02E-01
ENSCAFG0000000618	ENSCAFG0000000618	yellow	VSMC_M3	0.46	1.70E-02	0.11	5.95E-01	0.78	1.93E-06	-0.63	3.88E-04	-0.48	1.16E-02	0.14	4.72E-01	0.23	2.41E-01	0.47	1.27E-02	-0.26	1.98E-01	-0.31	1.11E-01
ENSCAFG0000000003	DOK6	darkgreen	VSMC_M4	0.46	1.70E-02	0.45	1.73E-02	-0.28	1.50E-01	0.75	6.36E-06	0.37	5.85E-02	-0.20	3.12E-01	-0.35	7.45E-02	-0.74	9.07E-06	-0.46	1.46E-02	0.49	1.01E-02
ENSCAFG0000001978	RBM15	red	VSMC_M1	0.46	1.71E-02	0.25	2.18E-01	0.21	2.98E-01	0.02	9.34E-01	-0.15	4.42E-01	0.28	1.58E-01	0.03	8.71E-01	-0.03	8.85E-01	-0.42	2.80E-02	0.23	2.49E-01
ENSCAFG0000000565	SLC26A11	cyan	VSMC_M2	0.45	1.71E-02	0.74	9.28E-06	0.30	1.24E-01	0.11	5.97E-01	-0.09	6.70E-01	-0.15	4.42E-01	-0.14	4.84E-01	-0.45	1.79E-02	-0.66	1.55E-04	0.38	4.99E-02
ENSCAFG0000000502	XYLB	grey	VSMC_M1C	0.45	1.71E-02	0.44	2.24E-02	0.08	7.05E-01	0.25	2.01E-01	0.12	5.45E-01	0.17	4.00E-01	-0.04	8.37E-01	-0.44	2.04E-02	-0.49	1.02E-02	0.24	2.24E-01
ENSCAFG0000002015	EVI5	red	VSMC_M1	0.45	1.71E-02	-0.01	9.72E-01	-0.37	5.60E-02	0.22	2.60E-01	0.62	4.99E-04	-0.04	8.29E-01	-0.24	2.19E-01	-0.15	4.57E-01	-0.09	6.48E-01	-0.12	5.35E-01
ENSCAFG0000000778	ARHGAP10	grey	VSMC_M1C	0.45	1.71E-02	0.58	1.62E-03	-0.17	3.93E-01	0.60	8.53E-04	0.28	1.63E-01	-0.30	1.34E-01	-0.19	3.38E-01	-0.73	1.44E-05	-0.56	2.14E-03	0.55	3.28E-03
ENSCAFG0000001571	ENSCAFG0000001571	red	VSMC_M1	0.45	1.71E-02	-0.14	4.96E-01	0.55	2.77E-03	-0.59	1.16E-03	-0.19	3.39E-01	-0.11	5.97E-01	0.24	2.25E-01	0.54	3.36E-03	-0.06	7.74E-01	-0.58	1.41E-03
ENSCAFG0000000256	COL19A1	yellow	VSMC_M3	0.45	1.72E-02	0.13	5.09E-01	0.49	9.46E-03	-0.19	3.49E-01	-0.17	4.06E-01	-0.12	5.47E-01	-0.02	9.31E-01	0.05	8.09E-01	-0.22	2.67E-01	-0.21	2.94E-01
ENSCAFG0000001820	ABCC1	cyan	VSMC_M2	0.45	1.72E-02	0.68	8.90E-05	0.60	9.28E-04	-0.25	2.02E-01	-0.34	8.56E-02	-0.05	8.09E-01	0.03	8.90E-01	-0.06	7.56E-01	-0.73	1.79E-05	0.16	4.13E-01
ENSCAFG0000002377	TSPYL4	grey	VSMC_M1C	0.45	1.73E-02	0.12	5.37E-01	0.20	3.25E-01	-0.27	1.74E-01	0.13	5.03E-01	-0.07	7.26E-01	0.05	7.93E-01	0.09	6.40E-01	-0.16	4.25E-01	-0.27	1.65E-01
ENSCAFG0000000878	WWP1	pink	VSMC_M5	0.45	1.74E-02	-0.21	2.96E-01	-0.50	8.63E-03	0.19	3.53E-01	0.82	1.66E-07	-0.19	3.40E-01	-0.15	4.56E-01	-0.11	5.98E-01	0.17	3.96E-01	-0.34	8.07E-02
ENSCAFG0000000595	MCM8	grey	VSMC_M1C	0.45	1.74E-02	-0.04	8.32E-01	0.43	2.51E-02	-0.50	8.20E-03	-0.12	5.66E-01	-0.03	8.72E-01	0.28	1.62E-01	0.48	1.20E-02	-0.21	2.95E-01	-0.40	3.78E-02
ENSCAFG0000001486	ENSCAFG0000001486	pink	VSMC_M5	0.45	1.74E-02	-0.23	2.49E-01	-0.14	4.95E-01	-0.29	1.39E-01	0.55	3.15E-03	-0.35	7.70E-02	-0.10	6.23E-01	0.25	2.09E-01	0.16	4.32E-01	-0.58	1.53E-03
ENSCAFG0000001523	PROM1	red	VSMC_M1	0.45	1.74E-02	0.04	8.25E-01	0.04	8.38E-01	-0.06	7.48E-01	0.16	4.18E-01	0.13	5.19E-01	-0.23	2.50E-01	0.07	7.19E-01	-0.16	4.12E-01	-0.09	6.45E-01
ENSCAFG0000001010	KIF13A	grey	VSMC_M1C	0.45	1.74E-02	0.64	2.91E-04	0.11	5.90E-01	0.32	1.09E-01	0.12	5.39E-01	-0.47	1.42E-02	-0.45	1.75E-02	-0.49	9.79E-03	-0.61	8.09E-04	0.32	1.03E-01
ENSCAFG0000001899	TTC22	grey	VSMC_M1C	0.45	1.74E-02	0.01	9.42E-01	0.01	9.50E-01	0.21	3.04E-01	0.22	2.66E-01	-0.17	3.92E-01	-0.20	3.07E-01	-0.18	3.81E-01	-0.11	5.90E-01	-0.08	6.77E-01
ENSCAFG0000000274	SPTBN1	grey	VSMC_M1C	0.45	1.75E-02	0.75	7.11E-06	0.18	3.75E-01	0.39	4.50E-02	0.00	9.95E-01	-0.13	5.31E-01	-0.63	4.17E-04	-0.57	1.83E-03	-0.66	2.03E-04	0.41	3.40E-02
ENSCAFG0000002751	ENSCAFG0000002751	red	VSMC_M1	0.45	1.76E-02	0.13	5.31E-01	0.18	3.80E-01	0.01	9.66E-01	0.04	8.27E-01	-0.06	7.73E-01	-0.06	7.55E-01	-0.06	7.57E-01	-0.17	3.83E-01	-0.07	7.34E-01
ENSCAFG0000001465	ENSCAFG0000001465	grey	VSMC_M1C	0.45	1.76E-02	-0.18	3.82E-01	0.31	1.19E-01	-0.51	6.01E-03	0.01	9.51E-01	-0.25	2.18E-01	0.19	3.31E-01	0.48	1.18E-02	-0.04	8.56E-01	-0.49	9.32E-03
ENSCAFG0000002550	GTDC1	cyan	VSMC_M2	0.45	1.76E-02	0.60	9.20E-04	0.21	2.82E-01	0.21	3.03E-01	0.04	8.48E-01	-0.16	4.33E-01	-0.27	1.78E-01	-0.40	4.02E-02	-0.57	1.71E-03	0.17	3.97E-01
ENSCAFG0000002038	TRADD	yellow	VSMC_M3	0.45	1.76E-02	0.25	2.13E-01	0.45	1.75E-02	-0.11	5.78E-01	-0.07	7.23E-01	-0.31	1.19E-01	-0.43	2.61E-02	-0.07	7.45E-01	-0.23	2.45E-01	-0.24	2.31E-01
ENSCAFG0000001189	WDR5B	red	VSMC_M1	0.45	1.77E-02	-0.02	9.35E-01	0.14	4.86E-01	-0.22	2.72E-01	0.21	2.94E-01	-0.33	9.27E-02	-0.17	3.95E-01	0.14	4.79E-01	-0.02	9.16E-01	-0.37	5.61E-02
ENSCAFG0000001321	HSD17B7	grey	VSMC_M1C	0.45	1.78E-02	0.08	7.07E-01	0.10	6.24E-01	-0.24	2.38E-01	0.27	1.72E-01	-0.15	4.41E-01	0.33	9.26E-02	0.11	5.81E-01	-0.19	3.49E-01	-0.34	8.16E-02
ENSCAFG0000001675	IGBP1	pink	VSMC_M5	0.45	1.78E-02	0.20	3.09E-01	-0.26	1.90E-01	0.34	8.62E-02	0.53	4.23E-03	-0.55	3.18E-03	-0.31	1.18E-01	-0.34	7.81E-02	-0.24	2.28E-01	-0.05	7.91E-01
ENSCAFG0000001167	ENSCAFG0000001167	yellow	VSMC_M3	0.45	1.78E-02	0.35	6.96E-02	0.59	1.10E-03	-0.32	1.02E-01	-0.33	9.59E-02	-0.04	8.32E-01	-0.06	7.58E-01	0.08	6.93E-01	-0.40	4.01E-02	0.00	9.81E-01
ENSCAFG0000000495	PAK1	red	VSMC_M1	0.45	1.78E-02	0.14	4.84E-01	0.60	8.58E-04	-0.76	4.80E-06	-0.22	2.81E-01	-0.19	3.30E-01	-0.01	9.63E-01	0.54	3.73E-03	-0.25	2.17E-01	-0.44	2.01E-02
ENSCAFG0000001550	DAAM1	grey	VSMC_M1C	0.45	1.78E-02	0.32	1.04E-01	-0.22	2.64E-01	0.46	1.68E-02	0.36	6.88E-02	-0.09	6.42E-01	-0.16	4.33E-01	-0.51	6.61E-03	-0.43	2.41E-02	0.34	7.89E-02
ENSCAFG0000001125	LEF1	cyan	VSMC_M2	0.45	1.79E-02	0.15	4.56E-01	0.11	5.95E-01	0.27	1.72E-01	0.09	6.47E-01	-0.07	7.11E-01	0.25	2.05E-01	-0.29	1.42E-01	-0.27	1.69E-01	0.13	5.27E-01
ENSCAFG0000001803	VPS35L	grey	VSMC_M1C	0.45	1.80E-02	0.21	3.01E-01	0.28	1.65E-01	-0.35	7.68E-02	0.03	8.93E-01	-0.36	6.87E-02	0.18	3.60E-01	0.11	5.83E-01	-0.27	1.80E-01	-0.14	4.99E-01
ENSCAFG0000000983	PLTP	grey	VSMC_M1C	0.45	1.80E-02	-0.21	2.82E-01	0.20	3.19E-01	-0.37	5.50E-02	0.13	5.12E-01	0.10	6.26E-01	0.12	5.53E-01	0.39	4.47E-02	0.04	8.38E-01	-0.56	2.37E-03
ENSCAFG0000000704	ENSCAFG0000000704	grey	VSMC_M1C	0.45	1.80E-02	0.67	1.44E-04	0.18	3.64E-01	0.39	4.44E-02	-0.09	6.42E-01	0.06	7.83E-01	-0.32	1.01E-01	-0.49	1.02E-02	-0.73	1.75E-05	0.52	5.65E-03
ENSCAFG0000001067	EIF3I	red	VSMC_M1	0.45	1.81E-02	0.03	8.93E-01	0.26	1.93E-01	-0.33	9.31E-02	0.01	9.78E-01	0.03	8.91E-01	-0.26	1.82E-01	0.27	1.69E-01	-0.11	5.77E-01	-0.33	9.08E-02
ENSCAFG0000001887	PLAC1	cyan	VSMC_M2	0.45	1.81E-02	0.61	6.43E-04	0.33	9.63E-02	-0.01	9.55E-01	-0.12	5.49E-01	0.04	8.56E-01	-0.15	4.50E-01	-0.25	2.01E-01	-0.65	2.18E-04	0.20	3.06E-01
ENSCAFG0000001723	FBXW9	yellow	VSMC_M3	0.45	1.82E-02	0.34	8.07E-02	0.60	9.32E-04	-0.27	1.78E-01	-0.27	1.75E-01	-0.14	4.81E-01	-0.07	7.22E-01	0.03	8.93E-01	-0.38	4.85E-02	-0.10	6.23E-01
ENSCAFG0000003222	DLA-12	grey	VSMC_M1C	0.45	1.83E-02	0.40	4.06E-02	-0.03	8.70E-01	0.34	8.22E-02	0.31	1.11E-01	-0.21	2.84E-01	-0.29	1.40E-01	-0.53	4.63E-03	-0.34	7.97E-02	0.12	5.56E-01
ENSCAFG0000001968	CARD9	yellow	VSMC_M3	0.45	1.83E-02	0.65	2.44E-04	0.71	3.25E-05	-0.05	8.21E-01	-0.54	3.84E-03	-0.02	9.20E-01	-0.14	4.99E-01	-0.17	3.85E-01	-0.69	5.82E-05	0.32	1.06E-01
ENSCAFG0000000019	COMMD1C	grey	VSMC_M1C	0.45	1.84E-02	0.37	5.76E-02	0.43	2.65E-02	-0.04	8.36E-01	-0.26	1.95E-01	0.02	9.29E-01	-0.01	9.80E-01	-0.09	6.57E-01	-0.49	9.33E-03	0.11	5.72E-01
ENSCAFG0000002980	DUSP26	grey	VSMC_M1C	0.45	1.84E-02	0.44	2.11E-02	-0.24	2.19E-01	0.63	4.61E-04	0.36	6.74E-02	-0.12	5.50E-01	-0.31	1.12E-01	-0.69	7.33E-05	-0.42	2.86E-02	0.43	2.64E-02
ENSCAFG0000000772	SLC4A8	pink	VSMC_M5	0.45	1.84E-02	-0.22	2.67E-01	-0.27	1.78E-01	0.14	4.77E-01	0.59	1.17E-03	-0.21	3.00E-01	-0.23	2.54E-01	-0.10	6.20E-01	0.14	4.71E-01	-0.42	2.85E-02
ENSCAFG0000001564	TAF6L	yellow	VSMC_M3	0.45	1.84E-02	0.47	1.34E-02	0.52	4.99E-03	0.00	9.99E-01	-0.25	2.09E-01	-0.08	7.01E-01	-0.20	3.07E-01	-0.19	3.43E-01	-0.47	1.36E-02	0.03	8.94E-01
ENSCAFG0000003071	BTBD19	red	VSMC_M1	0.45	1.85E-02	0.31	1.16E-01	0.01	9.45E-01	0.18	3.67E-01	0.29	1.44E-01	-0.37	5.56E-02	-0.07	7.44E-01	-0.39	4.62E-02	-0.26	1.92E-01	0.03	9.00E-01
ENSCAFG0000000692	RBCK1	cyan	VSMC_M2	0.45	1.86E-02	0.62	5.85E-04	0.56	2.23E-03	-0.15	4.63E-01	-0.20	3.09E-01	-0.28	1.62E-01	-0.14	4.79E-01	-0.19	3.41E-01	-0.56	2.18E-03	-0.01	9.75E-01
ENSCAFG0000002887	ENSCAFG0000002887	red	VSMC_M1	0.45	1.86E-02	0.20	3.25E-01	-0.10	6.25E-01	0.32	1.02E-01	0.23	2.52E-01	-0.06	7.59E-01	0.11	5.86E-01	-0.28	1.52E-01	-0.36	6.48E-02	0.22	2.69E-01
ENSCAFG0000000007	MMP19	cyan	VSMC_M2	0.45	1.86E-02	0.68	9.86E-05	0.50	8.12E-03	-0.20	3.23E-01	-0.23	2.49E-01	-0.14	4.88E-01	-0.05	8.13E-01	-0.11	5.69E-01	-0.70	4.51E-05	0.13	5.24E-01
ENSCAFG0000001390	ENSCAFG0000001390	cyan	VSMC_M2	0.45	1.86E-02	0.43	2.53E-02	0.41	3.22E-02	-0.13	5.20E-01	-0.21	2.97E-01	-0.24	2.37E-01	-0.31	1.18E-01	-0.07	7.30E-01	-0.47	1.36E-02	0.11	6.02E-01
ENSCAFG0000001628	DNAJB1	grey	VSMC_M1C	0.45	1.87E-02	0.11	5.88E-01	0.43	2.52E-02	-0.52	4.94E-03	-0.21	2.98E-01	-0.13	5.08E-01	0.20	3.16E-01	0.43	2.58E-02	-0.32	1.04E-01	-0.17	4.01E-01
ENSCAFG0000001157	SOX5	cyan	VSMC_M2	0.45	1.88E-02	0.61	7.13E-04	0.47	1.23E-02	-0.14	4.88E-01	-0.29	1.46E-01	-0.13	5.10E-01	-0.48	1.21E-02	-0.11	5.78E-01	-0.62	5.76E-04	0.24	2.19E-01
ENSCAFG0000003169	ENSCAFG0000003169	red	VSMC_M1	0.45	1.88E-02	-0.06	7.74E-01	0.27	1.81E-01	-0.28	1.64E-01	0.00	9.88E-01	-0.15	4.65E-01	-0.20	3.29E-01	0.24	2.31E-01	-0.04	8.30E-01	-0.29	1.38E-01
ENSCAFG0000001730	SNAPC5	grey	VSMC_M1C	0.45	1.89E-02	0.06	7.61E-01	-0.09	6.57E-01	0.29	1.44E-01	0.13	5.18E-01	0.13	5.10E-01	-0.05	8.06E-01	-0.20	3.09E-01	-0.23	2.42E-01	0.27	1.68E-01
ENSCAFG0000001488	RNF25	grey	VSMC_M1C	0.45	1.91E-02	0.08	6.78E-01	0.19	3.49E-01	-0.17	3.87E-01	-0.09	6.38E-01	0.21	2.83E-01	-0.16	4.16E-01	0.18	3.73E-01	-0.24	2.35E-01	0.02	9.21E-01
ENSCAFG0000000873	COQ9	cyan	VSMC_M2	0.45	1.92E-02	0.59	1.26E-03	0.06	7.80E-01	0.33	9.16E-02	0.11	5.89E-01	-0.20	3.08E-01	-0.04	8.27E-01	-0.51	6.55E-03	-0.63	4.53E-04	0.45	1.93E-02
ENSCAFG0000002848	RPS19	cyan	VSMC_M2	0.45	1.92E-02	0.67	1.22E-04	0.50	7.85E-03	0.18	3.59E-01	-0.39	4.54E-02	-0.05	7.89E-01	-0.03	8.70E-01	-0.38	4.96E-02	-0.73	1.31E-05	0.44	2.23E-02
ENSCAFG0000001236	VPS11	cyan	VSMC_M2	0.45	1.92E-02	0.65	2.13E-04	0.31	1.17E-01	0.11	5.98E-01	-0.06	7.81E-01	-0.27	1.81E-01	-0.17	3.97E-01	-0.37	5.98E-02	-0.62	5.86E-04	0.25	2.17E-01
ENSCAFG0000000868	PRKCD	cyan	VSMC_M2	0.45	1.92E-02	0.47	1.38E-02	0.35	7.11E-02	-0.08	6.85E-01	-0.09	6.57E-01	-0.21	3.03E-01	-0.53	4.70E-03	-0.09	6.70E-01	-0.44	2.03E-02	0.00	9.89E-01

ENSCAFG0000000546	DZIP1	cyan	VSMC_M2	0.45	1.92E-02	0.42	2.75E-02	0.13	5.29E-01	-0.05	8.14E-01	0.13	5.32E-01	-0.22	2.78E-01	0.11	5.69E-01	-0.11	5.82E-01	-0.52	4.93E-03	0.10	6.34E-01
ENSCAFG0000000066	DCAF13	red	VSMC_M1	0.45	1.92E-02	0.10	6.28E-01	-0.12	5.48E-01	0.11	5.81E-01	0.43	2.66E-02	-0.31	1.17E-01	-0.27	1.67E-01	-0.08	6.98E-01	-0.16	4.14E-01	-0.19	3.44E-01
ENSCAFG0000001852	TMEM11	red	VSMC_M1	0.45	1.93E-02	0.33	9.22E-02	-0.12	5.57E-01	0.20	3.26E-01	0.29	1.40E-01	-0.03	9.01E-01	-0.32	1.09E-01	-0.32	9.87E-02	-0.38	4.80E-02	0.11	5.80E-01
ENSCAFG0000001084	GAP43	grey	VSMC_M10	0.45	1.93E-02	0.17	3.91E-01	0.39	4.71E-02	-0.38	4.98E-02	-0.17	4.00E-01	0.09	6.52E-01	0.57	1.92E-03	0.21	2.87E-01	-0.38	5.11E-02	-0.03	8.83E-01
ENSCAFG0000000396	RPL18	cyan	VSMC_M2	0.45	1.94E-02	0.67	1.16E-04	0.52	5.32E-03	0.13	5.34E-01	-0.39	4.66E-02	-0.05	8.23E-01	-0.30	1.23E-01	-0.32	1.06E-01	-0.68	9.05E-05	0.33	8.79E-02
ENSCAFG0000000259	KMT5C	red	VSMC_M1	0.45	1.94E-02	0.20	3.26E-01	0.20	3.16E-01	0.08	6.89E-01	-0.02	9.23E-01	-0.12	5.35E-01	0.02	9.08E-01	-0.17	3.91E-01	-0.30	1.29E-01	0.10	6.23E-01
ENSCAFG0000000167	CDKN2A	grey	VSMC_M10	0.45	1.95E-02	0.61	7.18E-04	-0.04	8.56E-01	0.62	5.22E-04	0.21	2.85E-01	-0.24	2.24E-01	-0.44	2.15E-02	-0.78	1.25E-06	-0.52	5.44E-03	0.40	4.08E-02
ENSCAFG0000002006	KARS	grey	VSMC_M10	0.45	1.96E-02	0.69	7.57E-05	0.24	2.28E-01	0.25	2.10E-01	-0.08	6.78E-01	-0.10	6.21E-01	-0.65	2.58E-04	-0.41	3.15E-02	-0.66	1.59E-04	0.33	9.72E-02
ENSCAFG0000002897	HS3ST3B1	cyan	VSMC_M2	0.45	1.96E-02	0.47	1.28E-02	0.35	7.54E-02	-0.17	4.04E-01	-0.14	4.84E-01	0.16	4.17E-01	-0.21	2.93E-01	-0.03	8.65E-01	-0.54	3.73E-03	0.10	6.16E-01
ENSCAFG0000001176	ANK2	grey	VSMC_M10	0.45	1.96E-02	0.72	2.19E-05	-0.03	8.83E-01	0.54	3.30E-03	0.11	5.98E-01	-0.17	3.85E-01	-0.14	5.00E-01	-0.66	1.56E-04	-0.75	5.72E-06	0.61	7.45E-04
ENSCAFG0000000716	NDUFAF2	grey	VSMC_M10	0.45	1.96E-02	-0.08	7.04E-01	0.13	5.11E-01	-0.18	3.61E-01	0.05	8.11E-01	0.06	7.72E-01	0.12	5.39E-01	0.25	2.06E-01	-0.18	3.57E-01	-0.21	2.88E-01
ENSCAFG0000001705	DPM3	yellow	VSMC_M3	0.45	1.96E-02	0.35	6.99E-02	0.54	4.03E-03	-0.18	3.73E-01	-0.25	2.05E-01	-0.11	5.82E-01	-0.18	3.66E-01	-0.03	8.88E-01	-0.35	7.56E-02	-0.11	5.93E-01
ENSCAFG0000000577	CACNB4	grey	VSMC_M10	0.45	1.97E-02	0.16	4.29E-01	-0.09	6.68E-01	0.20	3.28E-01	0.37	5.62E-02	-0.24	2.24E-01	-0.12	5.53E-01	-0.26	1.93E-01	-0.23	2.54E-01	-0.05	8.06E-01
ENSCAFG0000003190	ENSCAFG0000003190	grey	VSMC_M10	0.45	1.97E-02	0.40	3.79E-02	0.49	9.39E-03	-0.10	6.36E-01	-0.43	2.67E-02	0.17	3.86E-01	0.08	6.91E-01	-0.02	9.33E-01	-0.55	2.68E-03	0.37	5.68E-02
ENSCAFG0000001979	KCNC4	grey	VSMC_M10	0.45	1.97E-02	0.72	2.51E-05	0.29	1.48E-01	0.39	4.44E-02	-0.12	5.65E-01	-0.12	5.39E-01	-0.34	7.91E-02	-0.63	4.40E-04	-0.68	1.07E-04	0.41	3.47E-02
ENSCAFG0000000050	DLA-64	grey	VSMC_M10	0.45	1.99E-02	0.41	3.41E-02	-0.21	3.03E-01	0.48	1.08E-02	0.43	2.49E-02	-0.21	2.92E-01	-0.24	2.28E-01	-0.65	2.67E-04	-0.34	8.37E-02	0.24	2.30E-01
ENSCAFG0000001018	ENSCAFG0000001018	red	VSMC_M1	0.45	1.99E-02	0.14	4.88E-01	0.16	4.30E-01	-0.05	7.91E-01	0.01	9.66E-01	0.09	6.65E-01	0.06	7.64E-01	0.01	9.64E-01	-0.31	1.12E-01	0.01	9.56E-01
ENSCAFG0000001159	HCCS	yellow	VSMC_M3	0.45	1.99E-02	0.27	1.72E-01	0.68	9.60E-05	-0.41	3.34E-02	-0.49	8.97E-03	0.12	5.50E-01	-0.01	9.61E-01	0.30	1.28E-01	-0.44	2.01E-02	-0.02	9.24E-01
ENSCAFG0000000807	STAU2	red	VSMC_M1	0.45	2.00E-02	0.10	6.04E-01	-0.22	2.63E-01	0.33	8.82E-02	0.50	8.56E-03	-0.33	9.33E-02	0.06	7.57E-01	-0.39	4.16E-02	-0.09	6.46E-01	0.03	8.82E-01
ENSCAFG0000002599	ENSCAFG0000002599	grey	VSMC_M10	0.45	2.00E-02	0.11	5.98E-01	0.38	5.26E-02	-0.27	1.72E-01	-0.13	5.26E-01	0.04	8.52E-01	0.21	3.01E-01	0.16	4.13E-01	-0.22	2.62E-01	-0.19	3.38E-01
ENSCAFG0000002581	ENSCAFG0000002581	yellow	VSMC_M3	0.45	2.00E-02	0.35	6.96E-02	0.59	1.07E-03	-0.27	1.72E-01	-0.34	8.50E-02	0.12	5.64E-01	-0.20	3.12E-01	0.07	7.23E-01	-0.39	4.40E-02	-0.04	8.28E-01
ENSCAFG0000001085	ENSCAFG0000001085	grey	VSMC_M10	0.45	2.00E-02	0.21	3.00E-01	0.06	7.71E-01	-0.02	9.28E-01	0.15	4.53E-01	0.11	6.02E-01	0.15	4.70E-01	-0.03	8.66E-01	-0.37	5.71E-02	-0.06	7.79E-01
ENSCAFG0000000216	ENSCAFG0000000216	cyan	VSMC_M2	0.45	2.00E-02	0.64	3.30E-04	0.44	2.25E-02	0.04	8.56E-01	-0.31	1.14E-01	-0.04	8.58E-01	-0.33	9.57E-02	-0.33	9.22E-02	-0.64	3.06E-04	0.40	4.04E-02
ENSCAFG0000002037	ELMO3	red	VSMC_M1	0.44	2.00E-02	0.52	5.37E-03	0.37	5.75E-02	-0.01	9.63E-01	-0.33	9.21E-02	0.26	1.90E-01	0.18	3.65E-01	-0.13	5.18E-01	-0.68	1.02E-04	0.42	2.81E-02
ENSCAFG0000001727	DIS3L	pink	VSMC_M5	0.44	2.01E-02	-0.16	4.15E-01	-0.03	8.81E-01	-0.19	3.45E-01	0.38	4.83E-02	-0.17	3.92E-01	0.21	3.05E-01	0.24	2.21E-01	-0.04	8.30E-01	-0.48	1.22E-02
ENSCAFG0000000788	SLC12A6	red	VSMC_M1	0.44	2.02E-02	0.46	1.52E-02	-0.23	2.49E-01	0.39	4.41E-02	0.38	5.34E-02	-0.06	7.73E-01	-0.23	2.47E-01	-0.56	2.52E-03	-0.49	1.01E-02	0.38	5.36E-02
ENSCAFG0000000189	COMMD8	grey	VSMC_M10	0.44	2.02E-02	-0.12	5.53E-01	0.25	2.17E-01	-0.49	9.12E-03	0.10	6.19E-01	-0.09	6.63E-01	0.01	9.80E-01	0.50	7.34E-03	-0.06	7.85E-01	-0.54	3.52E-03
ENSCAFG0000001366	ARHGEF10	cyan	VSMC_M2	0.44	2.04E-02	0.75	7.42E-06	0.45	1.90E-02	0.00	9.95E-01	-0.34	8.76E-02	0.01	9.76E-01	-0.05	8.05E-01	-0.26	1.82E-01	-0.82	1.53E-07	0.51	6.72E-03
ENSCAFG0000001806	HMG20A	red	VSMC_M1	0.44	2.04E-02	0.00	9.84E-01	-0.02	9.10E-01	0.13	5.26E-01	0.19	3.46E-01	-0.15	4.51E-01	-0.01	9.52E-01	0.00	9.98E-01	-0.15	4.62E-01	-0.04	8.42E-01
ENSCAFG0000000884	CPNE3	pink	VSMC_M5	0.44	2.05E-02	-0.04	8.60E-01	-0.48	1.20E-02	0.37	5.94E-02	0.76	3.94E-06	-0.18	3.76E-01	-0.24	2.30E-01	-0.29	1.44E-01	0.02	9.02E-01	-0.18	3.76E-01
ENSCAFG0000001214	SCN4A	yellow	VSMC_M3	0.44	2.06E-02	0.22	2.67E-01	0.62	6.01E-04	-0.21	2.96E-01	-0.32	1.01E-01	-0.08	7.00E-01	-0.02	9.19E-01	0.07	7.28E-01	-0.31	1.15E-01	-0.15	4.68E-01
ENSCAFG0000001905	UBXN6	cyan	VSMC_M2	0.44	2.06E-02	0.53	4.64E-03	0.15	4.51E-01	0.31	1.15E-01	0.16	4.12E-01	-0.41	3.31E-02	-0.47	1.45E-02	-0.57	1.91E-03	-0.39	4.15E-02	0.11	5.83E-01
ENSCAFG0000000610	DUSP6	grey	VSMC_M10	0.44	2.06E-02	-0.15	4.65E-01	-0.08	6.83E-01	-0.20	3.13E-01	0.39	4.40E-02	-0.11	5.86E-01	-0.16	4.35E-01	0.21	2.89E-01	0.07	7.47E-01	-0.43	2.37E-02
ENSCAFG0000002014	GARNL3	red	VSMC_M1	0.44	2.06E-02	0.40	3.97E-02	0.27	1.69E-01	0.08	7.04E-01	-0.24	2.20E-01	0.05	8.00E-01	0.17	3.98E-01	-0.13	5.21E-01	-0.58	1.36E-03	0.40	3.92E-02
ENSCAFG0000000748	RILPL2	grey	VSMC_M10	0.44	2.07E-02	0.05	8.07E-01	0.08	6.77E-01	-0.17	3.96E-01	0.11	5.91E-01	0.19	3.53E-01	-0.01	9.56E-01	0.17	3.93E-01	-0.21	2.82E-01	-0.15	4.55E-01
ENSCAFG0000000858	SPATS2	red	VSMC_M1	0.44	2.08E-02	0.17	3.87E-01	0.10	6.05E-01	-0.06	7.59E-01	0.27	1.71E-01	-0.19	3.39E-01	-0.50	8.36E-03	-0.02	9.32E-01	-0.17	3.99E-01	-0.32	1.01E-01
ENSCAFG0000001093	CHKA	grey	VSMC_M10	0.44	2.08E-02	0.53	4.89E-03	0.71	3.00E-05	-0.32	1.01E-01	-0.56	2.49E-03	-0.04	8.46E-01	-0.06	7.84E-01	0.06	7.81E-01	-0.61	8.10E-04	0.24	2.37E-01
ENSCAFG0000000576	ARL5A	grey	VSMC_M10	0.44	2.08E-02	-0.12	5.56E-01	-0.28	1.61E-01	0.25	2.16E-01	0.48	1.20E-02	-0.26	1.84E-01	-0.02	9.06E-01	-0.09	6.65E-01	-0.06	7.65E-01	-0.05	8.02E-01
ENSCAFG0000000323	MEIS1	cyan	VSMC_M2	0.44	2.08E-02	0.49	8.84E-03	0.19	3.37E-01	0.06	7.62E-01	-0.04	8.31E-01	-0.08	6.94E-01	-0.62	5.87E-04	-0.20	3.13E-01	-0.54	3.31E-03	0.18	3.60E-01
ENSCAFG0000000468	FAM171A1	red	VSMC_M1	0.44	2.09E-02	0.22	2.76E-01	0.21	2.98E-01	-0.08	6.98E-01	-0.01	9.59E-01	0.00	9.92E-01	-0.37	5.50E-02	0.05	8.06E-01	-0.35	7.43E-02	-0.05	8.01E-01
ENSCAFG0000001104	BCL9	cyan	VSMC_M2	0.44	2.09E-02	0.55	2.82E-03	-0.08	6.75E-01	0.36	6.92E-02	0.28	1.62E-01	0.01	9.60E-01	-0.39	4.73E-02	-0.46	1.60E-02	-0.54	3.51E-03	0.25	2.02E-01
ENSCAFG0000000688	EIF3F	cyan	VSMC_M2	0.44	2.09E-02	0.69	7.41E-05	0.38	5.15E-02	0.31	1.12E-01	-0.22	2.79E-01	-0.12	5.35E-01	-0.49	9.56E-03	-0.51	6.39E-03	-0.67	1.43E-04	0.38	4.94E-02
ENSCAFG0000001652	NTRK1	cyan	VSMC_M2	0.44	2.10E-02	0.45	1.94E-02	0.08	6.87E-01	0.42	3.13E-02	0.07	7.23E-01	-0.25	2.11E-01	-0.18	3.79E-01	-0.52	5.12E-03	-0.46	1.47E-02	0.32	1.06E-01
ENSCAFG0000001534	ADK	grey	VSMC_M10	0.44	2.11E-02	-0.01	9.57E-01	0.44	2.33E-02	-0.57	1.89E-03	-0.04	8.49E-01	-0.20	3.21E-01	0.29	1.47E-01	0.42	2.86E-02	-0.11	5.80E-01	-0.49	9.58E-03
ENSCAFG0000000618	SOCS2	red	VSMC_M1	0.44	2.11E-02	0.43	2.69E-02	0.32	1.06E-01	-0.18	3.77E-01	-0.09	6.72E-01	0.11	5.73E-01	0.33	9.08E-02	-0.01	9.44E-01	-0.55	3.17E-03	0.12	5.63E-01
ENSCAFG0000002840	ENSCAFG0000002840	red	VSMC_M1	0.44	2.12E-02	0.12	5.56E-01	0.19	3.48E-01	-0.12	5.50E-01	0.09	6.43E-01	-0.26	1.97E-01	0.10	6.21E-01	0.05	8.20E-01	-0.20	3.23E-01	-0.13	5.15E-01
ENSCAFG0000002567	ENSCAFG0000002567	pink	VSMC_M5	0.44	2.12E-02	-0.13	5.09E-01	-0.15	4.45E-01	0.00	9.83E-01	0.56	2.46E-03	-0.29	1.47E-01	-0.27	1.79E-01	-0.04	8.59E-01	0.11	5.78E-01	-0.46	1.54E-02
ENSCAFG0000002396	SLC25A1	red	VSMC_M1	0.44	2.12E-02	0.32	1.04E-01	0.37	5.97E-02	-0.38	4.96E-02	0.01	9.78E-01	-0.33	8.80E-02	0.24	2.20E-01	0.09	6.60E-01	-0.34	8.62E-02	-0.16	4.25E-01
ENSCAFG0000000781	LPCAT4	cyan	VSMC_M2	0.44	2.13E-02	0.73	1.83E-05	0.33	9.17E-02	0.12	5.65E-01	-0.16	4.26E-01	-0.36	6.61E-02	-0.07	7.26E-01	-0.33	9.43E-02	-0.76	4.72E-06	0.40	3.88E-02
ENSCAFG0000000720	GGH	darkgrey	VSMC_M8	0.44	2.13E-02	-0.16	4.22E-01	0.39	4.72E-02	-0.76	3.91E-06	-0.02	9.24E-01	-0.07	7.40E-01	0.29	1.49E-01	0.70	4.40E-05	-0.04	8.39E-01	-0.58	1.57E-03
ENSCAFG0000003044	ENSCAFG0000003044	red	VSMC_M1	0.44	2.13E-02	0.21	2.91E-01	0.11	5.97E-01	-0.06	7.72E-01	0.05	8.11E-01	0.43	2.61E-02	0.07	7.14E-01	0.01	9.66E-01	-0.36	6.22E-02	0.03	8.77E-01
ENSCAFG0000000188	GABRA1	grey	VSMC_M10	0.44	2.14E-02	0.44	2.22E-02	0.03	8.97E-01	0.44	2.13E-02	0.10	6.08E-01	-0.34	8.75E-02	-0.43	2.64E-02	-0.46	1.58E-02	-0.50	7.55E-03	0.34	8.76E-02
ENSCAFG0000000255	INVS	red	VSMC_M1	0.44	2.14E-02	0.19	3.54E-01	0.04	8.26E-01	-0.10	6.18E-01	0.37	5.70E-02	-0.51	6.38E-03	-0.36	6.13E-02	-0.06	7.71E-01	-0.19	3.30E-01	-0.36	6.54E-02
ENSCAFG0000001546	KCNMA1	grey	VSMC_M10	0.44	2.15E-02	-0.13	5.16E-01	0.06	7.79E-01	-0.25	2.07E-01	0.20	3.24E-01	0.13	5.33E-01	0.35	7.25E-02	0.30	1.31E-01	-0.13	5.17E-01	-0.30	1.28E-01
ENSCAFG0000000868	CCNG2	grey	VSMC_M10	0.44	2.17E-02	0.55	2.89E-03	-0.22	2.60E-01	0.67	1.41E-04	0.38	4.77E-02	-0.39	4.49E-02	-0.37	5.96E-02	-0.81	3.35E-07	-0.50	8.32E-03	0.44	2.13E-02
ENSCAFG0000000694	ENSCAFG0000000694	pink	VSMC_M5	0.44	2.18E-02	-0.34	8.10E-02	-0.38	4.95E-02	-0.05	7.98E-01	0.67	1.49E-04	-0.10	6.30E-01	-0.16	4.18E-01	0.23	2.57E-01	0.20	3.28E-01	-0.48	1.08E-02
ENSCAFG0000000247	TRIM14	cyan	VSMC_M2	0.44	2.18E-02	0.57	1.73E-03	0.48	1.18E-02	-0.08	6.88E-01	-0.30	1.30E-01	-0.01	9.69E-01	-0.07	7.39E-01	-0.04	8.54E-01	-0.70	5.03E-05	0.23	2.57E-01
ENSCAFG0000000843	C30H15orf41	red	VSMC_M1	0.44	2.18E-02	0.31	1.15E-01	0.07	7.30E-01	0.01	9.47E-01	0.25	2.08E-01	-0.46	1.46E-02	0.16	4.29E-01	-0.14	4.79E-01	-0.38	5.17E-02	-0.07	7.46E-01
ENSCAFG0000000111	L3MBTL3	grey	VSMC_M10	0.44	2.19E-02	0.58	1.45E-03	0.02	9.10E-01	0.37	5.70E-02	0.08	6.77E-01	0.01	9.76E-01	-0.18	3.72E-01	-0.52	5.11E-03	-0.63	4.31E-04	0.43	2.48E-02
ENSCAFG0000001464	ENSCAFG0000001464	red	VSMC_M1	0.44	2.19E-02	0.11	5.70E-01	-0.24	2.22E-01	0.32	1.05E-01	0.46	1.60E-02	-0.03	8.90E-01	-0.06	7.65E-01	-0.29	1.45E-01	-0.23	2.54E-01	-0.03	8.97E-01
ENSCAFG0000001998	NTMT1	yellow	VSMC_M3	0.44	2.21E-02	0.42	3.09E-02	0.75	6.89E-06	-0.28	1.52E-01	-0.55	3.08E-03	0.11	5.70E-01	-0.15	4.56E-01	0.11	5.94E-01	-0.51	6.16E-03	0.01	9.45E-01
ENSCAFG0000000015	HSD17B4	grey	VSMC_M10	0.44	2.21E-02	0.08	6.95E-01	-0.12	5.42E-01	-0.03	8.85E-01	0.34	8.37E-02	0.14	4.82E-01	0.32	1.08E-01	-0.03	8.70E-01	-0.27	1.75E-01	-0.04	8.36E-01
ENSCAFG0000000305	PLEKHA8	yellow	VSMC_M3	0.44	2.21E-02	0.41	3.42E-02	0.60	9.59E-04	-0.56	2.60E-03	-0.23	2.46E-01	-0.14	4.80E-01	-0.15	4.48E-01	0.22	2.81E-01	-0.42	2.72E-02	-0.21	2.92E-01
ENSCAFG0000001531	GPR161	grey	VSMC_M10	0.44	2.21E-02	0.30	1.25E-01	0.36	6.74E-02	-0.20	3.09E-01	-0.14	4.81E-01	-0.09	6.53E-01	0.11	5.94E-01	0.04	8.49E-01	-0.38	5.00E-02	0.07	7.22E-01
ENSCAFG0000000068	TUBGCP6	grey	VSMC_M10	0.44	2.21E-02	0.27	1.76E-01	0.20	3.25E-01	-0.01	9.48E-01	0.12	5.63E-01	-0.04	8.30E-01	-0.12	5.42E-01	-0.13	5.04E-01	-0.27	1.75E-01	-0.12	5.53E-01
ENSCAFG0000003142	NOP10	red	VSMC_M1	0.44	2.22E-02	-0.19	3.45E-01	0.16	4.16E-01	-0.31	1.18E-01	0.14	4.87E-01	-0.09	6.62E-01	0.09	6.70E-01	0.33	9.51E-02	0.06	7.74E-01	-0.48	1.23E-02
ENSCAFG0000000599	C15H12orf29	grey	VSMC_M10	0.44	2.23E-02	-0.06	7.75E-01	0.29	1.43E-01	-0.41	3.47E-02	0.15	4.47E-01	-0.17	3.99E-01	-0.09	6.53E-01	0.27	1.68E-01	-0.02	9.24E-01	-0.62	5.42E-04
ENSCAFG0000001817	RPS15A	red	VSMC_M1	0.44	2.23E-02	-0.20	3.28E-01	0.00	9.84E-01	-0.14	4.84E-01	0.35	7.64E-02	-0.42	2.84E-02	0.15	4.70E-01	0.14	4.77E-01	0.07	7.26E-01	-0.49	9.13E-03
ENSCAFG0000000848	VPS29	pink	VSMC_M5	0.44	2.23E-02	0.01	9.65E-01	-0.37	5.80E-02	0.39	4.57E-02	0.63	3.92E-04	-0.40	3.99E-02	-0.34	8.72E-02	-0.35	7.66E-02	0.01	9.77E-01	-0.12	5.58E-01
ENSCAFG0000000686	ANGPT4	cyan	VSMC_M2	0.44	2.24E-02	0.70	5.66E-05	0.35	7.19E-02	0.18	3.68E-01	-0.13	5.17E-01	-0.11	5.78E-01	-0.10	6.23E-01	-0.44	2.11E-02	-0.69	6.54E-05	0.35	7.60E-02
ENSCAFG0000001636	TBC1D19	grey	VSMC_M10	0.44	2.25E-02	-0.25	2.09E-01	0.30	1.26E-01	-0.43	2.55E-02	0.05	8.04E-01	0.09	6.56E-01	0.15	4.43E-01	0.38	4.86E-02	0.09	6.58E-01	-0.56	2.58E-03
ENSCAFG0000000735	BTAF1	grey	VSMC_M10	0.44	2.27E-02	-0.20	3.17E-01	0.03	8.69E-01	-0.43	2.53E-02	0.33	9.24E-02	-0.06	7.83E-01	0.03	8.81E-01	0.40	3.72E-02	0.02	9.04E-01	-0.56	2.61E-03
ENSCAFG0000001967	PARK7	grey	VSMC_M10	0.44	2.27E-02	0.17	4.00E-01	-0.33	8.85E-02	0.67	1.18E-04	0.51	6.43E-03	-0.22	2.72E-01	-0.20	3.22E-01	-0.62	6.32E-04	-0.19	3.36E-01	0.17	4.00E-01
ENSCAFG0000003238	ZFPM1	cyan	VSMC_M2	0.44	2.27E-02	0.58	1.59E-03	0.41	3.25E-02	-0.01	9.48E-01	-0.26	1.86E-01	0.01	9.52E-01	-0.61	7.24E-04	-0.13	5.05E-01	-0.63	4.18E-04	0.21	2.82E-01
ENSCAFG0000001913	PPL	yellow	VSMC_M3	0.44	2.28E-02	0.59	1.12E-03	0.60	1.03E-03	-0.33	8.88E-02	-0.34	8.69E-02	-0.03	8.82E-01	0.26	1.90E-01	0.07	7.24E-01	-0.68	9.43E-05	0.09	6.45E-01
ENSCAFG0000000209	NCK2	grey	VSMC_M10	0.44	2.28E-02	0.62	6.24E-04	0.15	4.46E-01	0.44	2.29E-02	-0.08	6.94E-01	-0.10	6.27E-01	-0.52	5.72E-03	-0.51	6.40E-03	-0.64	3.11E-04	0.49	1.01E-02
ENSCAFG0000001158	NGEF	yellow	VSMC_M3	0.44	2.28E-02	0.36	6.56E-02	0.57	1.84E-03	-0.49	9.19E-03	-0.28	1.51E-01	-0.04	8.45E-01	0.12	5.45E-01	0.25	2.10E-01	-0.44	2.07E-02	-0.12	5.65E-01
ENSCAFG0000000095	PFDN6	red	VSMC_M1	0.44	2.28E-02	-0.14	4.84E-01	0.11	5.99E-01	-0.01	9.55E-01	0.08	6.97E-01	0.00	9.90E-01	0.28	1.56E-01	0.09	6.46E-01	-0.06	7.69E-01	-0.15	4.70E-01
ENSCAFG0000000440	ME3	cyan	VSMC_M2	0.44	2.28E-02	0.52	4.96E-03	0.35	7.65E-02	0.25	2.04E-01	-0.11	5.87E-01	-0.28	1.63E-01	-0.24	2.18E-01	-0.41	3.28E-02	-0.47	1.27E-02	0.19	3.46E-01
ENSCAFG0000000730	IGF1	cyan	VSMC_M2	0.44	2.29E-02	0.68	1.10E-04	0.62	5.79E-04	-0.18	3.79E-01	-0.38	4.98E-02	-0.15	4.42E-01	-0.25	2.11E-01	-0.13	5.03E-01	-0.66	1.87E-04	0.17	3.92E-01
ENSCAFG0000001704	SNX1	grey	VSMC_M10	0.44	2.29E-02	0.14	4.87E-01	-0.24	2.30E-01	0.24	2.23E-01	0.44	2.01E-02	-0.29	1.42E-01	0.24	2.25E-01	-0.27	1.79E-01	-0.25	2.11E-01	0.11	5.93E-01
ENSCAFG0000000954	SLC5A3	cyan	VSMC_M2	0.44	2.31E-02	0.35	7.54E-02	0.26	1.84E-01	0.22	2.80E-01	-0.04	8.56E-01	0.02	9.10E-01	-0.32	1.08E-01	-0.31	1.19E-01	-0.38	4.85E-02	0.14	4.81E-01
ENSCAFG0000000392	DBP	cyan	VSMC_M2	0.44	2.31E-02	0.63	4.18E-04	0.12	5.61E-01	0.26	1.87E-01	0.13	5.16E-01	-0.44	2.15E-02	-0.06	7.51E-01	-0.54	3.73E-03	-0.55	2.77E-03	0.33	9.13E-02
ENSCAFG0000001554	TBL1XR1	grey	VSMC_M10	0.44	2.32E-02	0.01	9.79E-01	0.13	5.20E-01	-0.23	2.41E-01	0.17	3.93E-01	0.09	6.47E-01	0.35	7.36E-02	0.19	3.33E-01	-0.14	4.73E-01	-0.30	1.25E-01
ENSCAFG0000000728	ENSCAFG0000000728	red	VSMC_M1	0.44	2.33E-02	0.36	6.36E-02	0.29	1.43E-01	-0.16	4.39E-01	0.00	9.82E-01	-0.10	6.27E-01	-0.28	1.60E-01	-0.01	9.63E-01	-0.38	5.29E-02	-0.15	4.67E-01
ENSCAFG0000000278	MEI4	red	VSMC_M1	0.44	2.33E-02	0.31	1.20E-01	-0.07	7.14E-01	0.26	1.92E-01	0.15	4.70E-01	-0.08	6.74E-01	0.09	6.50E-01	-0.31	1.18E-01	-0.36	6.46E-02	0.36	6.30E-02
ENSCAFG0000001993	ABL1	cyan	VSMC_M2	0.44	2.33E-02	0.74	8.91E-06	0.44	2.30E-02	0.07	7.47E-01	-0.29	1.49E-01	0.04	8.26E-01	-0.03	8.98E-01	-0.34	8.73E-02	-0.78	1.36E-06	0.43	2.51E-02
ENSCAFG0000000982	HTATIP2	yellow	VSMC_M3	0.44	2.33E-02	0.43	2.42E-02	0.46	1.69E-02	-0.13	5.27E-01	-0.14	4.76E-01	-0.06	7.49E-01	-0.02	9.13E-01	-0.12	5.62E-01	-0.44	2.08E-02	-0.04	8.25E-01
ENSCAFG0000000695	SP1	red	VSMC_M1	0.43	2.34E-02	0.06	7.49E-01	-0.01	9.62E-01	-0.31	1.15E-01	0.31	1.20E-01	-0.24	2.25E-01	0.13	5.29E-01	0.24	2.26E-01	-0.19	3.43E-01	-0.22	2.72E-01
ENSCAFG0000000300	PTGR1	yellow	VSMC_M3	0.43	2.34E-02	0.61	6.58E-04	0.73	1.28E-05	-0.44	2.03E-02	-0.53	4.88E-03	-0.03	8.86E-01	0.16	4.36E-01	0.17	3.84E-01	-0.71	3.30E-05	0.13	5.15E-01
ENSCAFG0000003203	CCDC58	grey	VSMC_M10	0.43	2.34E-02	-0.19	3.35E-01	0.22	2.60E-01	-0.43	2.59E-02	0.19	3.33E-01	-0.20	3.22E-01	0.09	6.58E-01	0.39	4.43E-02	0.03	8.93E-01	-0.63	4.32E-04
ENSCAFG0000001880	KANK4	red	VSMC_M1	0.43	2.34E-02	0.56	2.48E-03	0.23	2.53E-01	0.10	6.22E-01	-0.17	4.01E-01	0.14	4.96E-01	-0.12	5.67E-01	-0.34	8.63E-02	-0.62	5.48E-04	0.47	1.27E-02
ENSCAFG0000000220	CREB3	darkgreen	VSMC_M4	0.43	2.35E-02	0.40	3.70E-02	-0.28	1.64E-01	0.73	1.83E-05	0.33	8.93E-02	-0.09	6.65E-01	-0.35	7.76E-02	-0.73	1.48E-05	-0.42	3.07E-02	0.44	2.07E-02
ENSCAFG0000003121	OAF	yellow	VSMC_M3	0.43	2.35E-02	0.57	1.73E-03	0.68	9.25E-05	-0.35	7.63E-02	-0.47	1.36E-02	0.04	8.62E-01	0.06	7.72E-01	0.14	4.99E-01	-0.68	9.56E-05	0.10	6.06E-01
ENSCAFG0000000334	MBOAT2	grey	VSMC_M10	0.43	2.35E-02	-0.06	7.73E-01	-0.18	3.77E-01	-0.09	6.58E-01	0.36	6.33E-02	0.03	8.94E-01	0.01	9.51E-01	0.08	6.79E-01	-0.14	4.95E-01	-0.16	4.40E-01
ENSCAFG0000000706	ITGB7	yellow	VSMC_M3	0.43	2.35E-02	0.39	4.66E-02	0.60	8.47E-04	-0.34	8.65E-02	-0.29	1.46E-01	-0.34	8.14E-02	-0.01	9.50E-01	0.10	6.13E-01	-0.43	2.48E-02	-0.10	6.32E-01
ENSCAFG0000000277	PPP2R1A	grey	VSMC_M10	0.43	2.36E-02	0.53	4.71E-03	0.24	2.25E-01	0.22	2.65E-01	-0.10	6.20E-01	0.03	8.81E-01	-0.67	1.53E-04	-0.34	8.75E-02	-0.49	8.98E-03	0.24	2.35E-01
ENSCAFG0000001431	UNC5B	darkgreen	VSMC_M4	0.43	2.37E-02	0.48	1.10E-02	-0.10	6.15E-01	0.56	2.31E-03	0.10	6.04E-01	0.07	7.15E-01	-0.05	8.16E-01	-0.58	1.64E-03	-0.57	1.94E-03	0.60	1.05E-03
ENSCAFG0000000831	ANAPC5	grey	VSMC_M10	0.43	2.37E-02	-0.14	5.02E-01	-0.20	3.12E-01	-0.03	8.99E-01	0.35	7.21E-02	0.12	5.59E-01	0.26	1.97E-01	0.17	3.91E-01	-0.09	6.49E-01	-0.09	6.57E-01
ENSCAFG0000001377	STOX1	grey	VSMC_M10	0.43	2.37E-02	0.75	6.89E-06	0.11	5.71E-01	0.40	3.65E-02	-0.02	9.17E-01	-0.05	8.17E-01	-0.43	2.34E-02	-0.52	5.19E-03	-0.76	4.92E-06	0.50	7.47E-03
ENSCAFG0000003274	GSG1L	yellow	VSMC_M3	0.43	2.38E-02	0.34	8.54E-02	0.65	2.69E-04	-0.22	2.66E-01	-0.34	8.49E-02	-0.06	7.51E-01	0.10	6.32E-01	0.02	9.38E-01	-0.40	3.91E-02	-0.07	7.17E-01
ENSCAFG0000000985	RANBP9	pink	VSMC_M5	0.43	2.38E-02	-0.16	4.21E-01	-0.38	5.26E-02	0.09	6.62E-01	0.71	3.34E-05	-0.26	1.84E-01	-0.13	5.25E-01	-0.10	6.09E-01	0.14	4.78E-01	-0.33	9.26E-02
ENSCAFG0000001913	MAP2K2	yellow	VSMC_M3	0.43	2.39E-02	0.47	1.34E-02	0.85	2.85E-08	-0.40	3.82E-02	-0.57	1.95E-03	-0.02	9.26E-01	-0.10	6.08E-01	0.09	6.45E-01	-0.47	1.31E-02	-0.03	8.99E-01
ENSCAFG0000001874	ENSCAFG0000001874	red	VSMC_M1	0.43	2.39E-02	0.22	2.64E-01	-0.03	8.88E-01	0.13	5.24E-01	0.16	4.12E-01	-0.02	9.25E-01	-0.35	7.25E-02	-0.10	6.24E-01	-0.33	9.28E-02	0.12	5.57E-01
ENSCAFG0000000818	PLSCR1	cyan	VSMC_M2	0.43	2.39E-02	0.45	1.79E-02	-0.06	7.57E-01	0.25	2.14E-01	0.23	2.51E-01	0.04	8.59E-01	-0.25	2.00E-01	-0.32	1.01E-01	-0.53	4.58E-03	0.14	4.83E-01
ENSCAFG0000002993	CHIC1	cyan	VSMC_M2	0.43	2.40E-02	0.42	3.05E-02	0.20	3.10E-01	0.31	1.14E-01	-0.08	6.79E-01	-0.08	6.90E-01	-0.08	6.96E-01	-0.43	2.57E-02	-0.49	9.05E-03	0.34	8.54E-02
ENSCAFG0000001197	PIR	yellow	VSMC_M3	0.43	2.40E-02	0.44	2.14E-02	0.53	4.14E-03	-0.29	1.48E-01	-0.30	1.33E-01	-0.17	4.09E-01	0.24	2.31E-01	0.16	4.34E-01	-0.59	1.22E-03	-0.01	9.61E-01
ENSCAFG0000001156	PARD6B	darkgreen	VSMC_M4	0.43	2.41E-02	0.51	6.54E-03	-0.06	7.61E-01	0.61	7.28E-04	0.10	6.15E-01	0.09	6.43E-01	-0.29	1.37E-01	-0.70	4.20E-05	-0.52	5.63E-03	0.54	3.43E-03
ENSCAFG0000000543	EID2	grey	VSMC_M10	0.43	2.41E-02	0.34	8.47E-02	0.54	3.48E-03	-0.22	2.73E-01	-0.35	7.63E-02	0.04	8.58E-01	0.00	9.81E-01	0.04	8.37E-01	-0.47	1.35E-02	0.02	9.25E-01
ENSCAFG0000001224	SPX	yellow	VSMC_M3	0.43	2.41E-02	0.23	2.48E-01	0.61	6.91E-04	-0.21	3.00E-01	-0.32	1.01E-01	-0.08	6.98E-01	-0.06	7.59E-01	0.05	7.98E-01	-0.28	1.54E-01	-0.15	4.67E-01
ENSCAFG0000001940	LSM7	grey	VSMC_M10	0.43	2.43E-02	0.26	1.94E-01	0.64	3.37E-04	-0.33	9.79E-02	-0.42	2.98E-02	-0.14	4.97E-01	0.11	5.89E-01	0.19	3.51E-01	-0.39	4.59E-02	-0.06	7.65E-01
ENSCAFG0000003177	ENSCAFG0000003177	grey	VSMC_M10	0.43	2.44E-02	0.00	9.83E-01	0.00	9.85E-01	-0.07	7.47E-01	0.21	2.82E-01	-0.13	5.14E-01	0.07	7.24E-01	0.06	7.71E-01	-0.19	3.30E-01	-0.12	5.61E-01
ENSCAFG0000003074	RGS19	cyan	VSMC_M2	0.43	2.44E-02	0.55	3.17E-03	0.36	6.30E-02	0.01	9.60E-01	-0.13	5.10E-01	-0.15	4.53E-01	-0.14	4.92E-01	-0.25	2.17E-01	-0.57	1.73E-03	0.15	4.65E-01
ENSCAFG0000001158	ENPEP	grey	VSMC_M10	0.43	2.44E-02	-0.10	6.11E-01	-0.21	2.94E-01	0.19	3.35E-01	0.30	1.24E-01	0.14	4.98E-01	-0.10	6.10E-01	-0.08	6.88E-01	-0.09	6.67E-01	0.02	9.37E-01
ENSCAFG0000000587	ANAPC11	cyan	VSMC_M2	0.43	2.45E-02	0.77	2.30E-06	0.67	1.49E-04	-0.05	8.10E-01	-0.52	5.95E-03	-0.08	6.73E-01	0.06	7.57E-01	-0.24	2.26E-01	-0.81	3.82E-07	0.44	2.04E-02
ENSCAFG0000001973	SLC25A33	grey	VSMC_M10	0.43	2.47E-02	0.40	3.63E-02	0.53	4.92E-03	-0.22	2.79E-01	-0.36	6.18E-02	0.08	6.74E-01	-0.19	3.34E-01	0.04	8.27E-01	-0.46	1.53E-02	0.15	4.61E-01

ENSCAFG0000003152	ENSCAFG0000003152	grey	VSMC_M10	0.43	2.47E-02	0.43	2.67E-02	-0.16	4.25E-01	0.52	5.54E-03	0.27	1.81E-01	-0.16	4.17E-01	-0.01	9.44E-01	-0.55	3.19E-03	-0.48	1.09E-02	0.35	7.62E-02
ENSCAFG0000001731	MBTD1	red	VSMC_M1	0.43	2.47E-02	-0.21	2.87E-01	-0.38	4.77E-02	0.18	3.82E-01	0.66	1.68E-04	-0.16	4.15E-01	0.13	5.15E-01	-0.08	6.77E-01	0.08	7.04E-01	-0.28	1.65E-01
ENSCAFG0000001919	TJP3	yellow	VSMC_M3	0.43	2.48E-02	0.34	8.50E-02	0.69	6.55E-05	-0.21	2.91E-01	-0.41	3.39E-02	-0.08	6.76E-01	0.16	4.21E-01	0.02	9.39E-01	-0.40	3.98E-02	-0.05	8.23E-01
ENSCAFG0000003144	EREG	grey	VSMC_M10	0.43	2.48E-02	-0.06	7.60E-01	0.13	5.14E-01	-0.30	1.30E-01	0.16	4.36E-01	-0.11	6.01E-01	-0.36	6.46E-02	0.21	2.96E-01	0.05	8.08E-01	-0.35	7.65E-02
ENSCAFG0000003093	ENSCAFG0000003093	cyan	VSMC_M2	0.43	2.48E-02	0.33	9.41E-02	0.11	5.69E-01	0.40	4.02E-02	0.03	8.88E-01	-0.02	9.36E-01	-0.02	9.16E-01	-0.45	2.00E-02	-0.40	3.70E-02	0.25	2.13E-01
ENSCAFG0000003173	TNFSF13B	grey	VSMC_M10	0.43	2.48E-02	0.60	9.26E-04	0.54	3.79E-03	-0.03	8.99E-01	-0.48	1.10E-02	-0.04	8.48E-01	0.12	5.46E-01	-0.17	4.08E-01	-0.70	4.91E-05	0.48	1.16E-02
ENSCAFG0000000708	FAM110B	cyan	VSMC_M2	0.43	2.48E-02	0.76	5.11E-06	0.03	8.96E-01	0.59	1.15E-03	0.01	9.78E-01	-0.17	3.85E-01	-0.43	2.47E-02	-0.72	2.11E-05	-0.77	2.91E-06	0.69	5.96E-05
ENSCAFG0000001547	DES	grey	VSMC_M10	0.43	2.49E-02	0.60	8.28E-04	0.00	9.90E-01	0.44	2.12E-02	0.14	4.94E-01	-0.04	8.60E-01	-0.45	1.86E-02	-0.61	7.55E-04	-0.56	2.25E-03	0.43	2.54E-02
ENSCAFG0000001479	RP2	red	VSMC_M1	0.43	2.49E-02	0.22	2.62E-01	0.27	1.76E-01	0.08	6.96E-01	-0.05	7.92E-01	-0.15	4.53E-01	0.11	5.93E-01	-0.12	5.64E-01	-0.33	9.54E-02	0.08	6.91E-01
ENSCAFG0000002896	ENSCAFG0000002896	red	VSMC_M1	0.43	2.49E-02	-0.20	3.24E-01	0.25	2.08E-01	-0.30	1.26E-01	0.03	8.91E-01	-0.04	8.29E-01	-0.01	9.77E-01	0.34	8.39E-02	0.01	9.61E-01	-0.42	3.11E-02
ENSCAFG0000000778	SMAGP	grey	VSMC_M10	0.43	2.49E-02	-0.02	9.14E-01	0.34	8.25E-02	-0.19	3.35E-01	-0.11	5.93E-01	0.10	6.10E-01	0.15	4.49E-01	0.11	5.93E-01	-0.11	5.73E-01	-0.22	2.72E-01
ENSCAFG0000001804	SNX33	cyan	VSMC_M2	0.43	2.50E-02	0.87	5.00E-09	0.30	1.32E-01	0.34	8.62E-02	-0.18	3.62E-01	-0.15	4.53E-01	-0.22	2.68E-01	-0.61	7.52E-04	-0.83	6.63E-08	0.61	7.71E-04
ENSCAFG0000000969	NDUFB8	red	VSMC_M1	0.43	2.50E-02	-0.23	2.59E-01	0.03	8.89E-01	-0.06	7.72E-01	0.26	1.83E-01	-0.08	6.75E-01	0.03	8.75E-01	0.23	2.55E-01	0.04	8.48E-01	-0.41	3.19E-02
ENSCAFG0000001688	UBE2Z	pink	VSMC_M5	0.43	2.50E-02	-0.21	2.98E-01	-0.29	1.49E-01	0.14	4.93E-01	0.69	7.77E-05	-0.33	9.51E-02	-0.20	3.29E-01	-0.08	6.86E-01	0.18	3.71E-01	-0.50	8.43E-03
ENSCAFG0000001909	MPND	cyan	VSMC_M2	0.43	2.50E-02	0.71	3.60E-05	0.49	9.28E-03	0.24	2.33E-01	-0.35	6.98E-02	-0.15	4.48E-01	-0.09	6.54E-01	-0.49	1.03E-02	-0.69	7.80E-05	0.47	1.26E-02
ENSCAFG0000001280	MPZL3	yellow	VSMC_M3	0.43	2.51E-02	0.21	2.97E-01	0.60	8.56E-04	-0.23	2.45E-01	-0.32	1.07E-01	-0.05	7.86E-01	0.19	3.48E-01	0.08	6.84E-01	-0.32	1.03E-01	-0.14	4.99E-01
ENSCAFG0000000586	NDUFA2	red	VSMC_M1	0.43	2.51E-02	0.24	2.18E-01	0.11	5.91E-01	0.23	2.46E-01	0.13	5.20E-01	-0.19	3.53E-01	-0.10	6.36E-01	-0.24	2.27E-01	-0.34	8.00E-02	-0.01	9.70E-01
ENSCAFG0000000480	WBP4	grey	VSMC_M10	0.43	2.51E-02	-0.20	3.23E-01	0.22	2.60E-01	-0.34	7.97E-02	0.17	3.93E-01	-0.36	6.77E-02	-0.07	7.21E-01	0.28	1.52E-01	0.12	5.65E-01	-0.59	1.23E-03
ENSCAFG0000000544	TIMM50	grey	VSMC_M10	0.43	2.51E-02	0.18	3.77E-01	0.49	1.01E-02	-0.29	1.45E-01	-0.39	4.37E-02	0.26	1.90E-01	0.17	4.09E-01	0.30	1.30E-01	-0.40	3.94E-02	0.05	8.01E-01
ENSCAFG0000002344	ENSCAFG0000002344	grey	VSMC_M10	0.43	2.52E-02	0.17	3.84E-01	0.34	8.20E-02	-0.36	6.47E-02	-0.02	9.19E-01	-0.22	2.60E-01	0.10	6.36E-01	0.12	5.58E-01	-0.20	3.15E-01	-0.22	2.62E-01
ENSCAFG0000001023	TRIM8	cyan	VSMC_M2	0.43	2.52E-02	0.80	5.16E-07	0.41	3.44E-02	0.22	2.66E-01	-0.27	1.69E-01	-0.09	6.43E-01	-0.53	4.43E-03	-0.48	1.13E-02	-0.74	1.08E-05	0.45	1.83E-02
ENSCAFG0000000622	PCDH1	grey	VSMC_M10	0.43	2.53E-02	0.49	9.64E-03	0.04	8.58E-01	0.42	3.11E-02	-0.02	9.08E-01	0.21	2.97E-01	-0.21	2.91E-01	-0.43	2.52E-02	-0.59	1.11E-03	0.53	4.73E-03
ENSCAFG0000000948	FARS2	red	VSMC_M1	0.43	2.54E-02	0.33	9.16E-02	-0.07	7.11E-01	0.09	6.47E-01	0.26	1.93E-01	-0.13	5.30E-01	0.09	6.59E-01	-0.21	2.84E-01	-0.37	5.89E-02	0.12	5.42E-01
ENSCAFG0000000324	ALAD	red	VSMC_M1	0.43	2.55E-02	0.43	2.34E-02	0.41	3.58E-02	-0.08	7.08E-01	-0.22	2.62E-01	-0.43	2.34E-02	0.12	5.43E-01	-0.08	7.00E-01	-0.55	2.87E-03	0.23	2.45E-01
ENSCAFG0000001920	HMOX2	red	VSMC_M1	0.43	2.55E-02	-0.03	8.86E-01	0.45	1.95E-02	-0.42	2.78E-02	-0.13	5.09E-01	0.02	9.03E-01	-0.19	3.30E-01	0.37	5.96E-02	-0.04	8.40E-01	-0.45	1.76E-02
ENSCAFG0000002106	ENSCAFG0000002106	red	VSMC_M1	0.43	2.55E-02	0.23	2.56E-01	0.30	1.23E-01	0.04	8.52E-01	-0.14	4.98E-01	0.05	8.16E-01	0.04	8.60E-01	-0.07	7.26E-01	-0.41	3.39E-02	0.08	6.96E-01
ENSCAFG0000001691	RIT1	grey	VSMC_M10	0.43	2.56E-02	0.40	3.89E-02	0.67	1.26E-04	-0.31	1.20E-01	-0.55	2.81E-03	0.02	9.32E-01	0.26	1.87E-01	0.16	4.24E-01	-0.57	1.81E-03	0.22	2.75E-01
ENSCAFG0000000800	SBSPON	cyan	VSMC_M2	0.43	2.56E-02	0.71	3.55E-05	0.31	1.20E-01	0.23	2.50E-01	-0.17	3.88E-01	-0.22	2.79E-01	-0.09	6.49E-01	-0.43	2.64E-02	-0.73	1.76E-05	0.47	1.43E-02
ENSCAFG0000001614	KLHL5	grey	VSMC_M10	0.43	2.56E-02	0.40	3.96E-02	0.14	4.94E-01	0.09	6.56E-01	0.12	5.55E-01	-0.10	6.03E-01	-0.07	7.14E-01	-0.26	1.89E-01	-0.46	1.56E-02	0.09	6.70E-01
ENSCAFG0000001182	CERS6	grey	VSMC_M10	0.43	2.58E-02	0.13	5.25E-01	0.06	7.82E-01	0.00	9.96E-01	0.06	7.48E-01	0.01	9.57E-01	0.38	5.34E-02	-0.02	9.18E-01	-0.31	1.15E-01	0.12	5.51E-01
ENSCAFG0000000055	ACR	red	VSMC_M1	0.43	2.58E-02	0.13	5.21E-01	0.48	1.05E-02	-0.30	1.31E-01	-0.30	1.30E-01	0.05	8.14E-01	-0.02	9.03E-01	0.21	2.82E-01	-0.25	2.05E-01	-0.13	5.30E-01
ENSCAFG0000001754	S100A16	yellow	VSMC_M3	0.43	2.59E-02	0.34	7.87E-02	0.80	6.27E-07	-0.68	9.99E-05	-0.54	4.00E-03	0.09	6.47E-01	0.20	3.23E-01	0.43	2.37E-02	-0.48	1.08E-02	-0.17	3.92E-01
ENSCAFG0000000251	ITGB8	cyan	VSMC_M2	0.43	2.59E-02	0.44	2.06E-02	0.23	2.38E-01	-0.16	4.29E-01	-0.05	7.92E-01	-0.04	8.47E-01	-0.37	6.08E-02	-0.04	8.42E-01	-0.47	1.28E-02	0.12	5.58E-01
ENSCAFG0000001082	ZBTB20	grey	VSMC_M10	0.43	2.59E-02	0.69	6.57E-05	0.18	3.82E-01	0.30	1.23E-01	-0.07	7.25E-01	-0.14	4.71E-01	-0.26	1.84E-01	-0.49	8.99E-03	-0.71	3.66E-05	0.54	3.30E-03
ENSCAFG0000001178	GPER1	grey	VSMC_M10	0.43	2.60E-02	-0.42	2.98E-02	0.11	5.81E-01	-0.52	5.98E-03	0.29	1.45E-01	-0.26	1.94E-01	0.30	1.27E-01	0.53	4.68E-03	0.26	1.82E-01	-0.74	1.08E-05
ENSCAFG0000000568	PHLDA1	yellow	VSMC_M3	0.43	2.61E-02	0.48	1.06E-02	0.66	1.62E-04	-0.29	1.37E-01	-0.48	1.23E-02	-0.02	9.03E-01	-0.26	1.96E-01	0.05	8.04E-01	-0.58	1.60E-03	0.12	5.64E-01
ENSCAFG0000001991	ENSCAFG0000001991	yellow	VSMC_M3	0.43	2.61E-02	0.42	2.95E-02	0.71	3.02E-05	-0.29	1.43E-01	-0.47	1.28E-02	-0.09	6.69E-01	-0.09	6.50E-01	0.06	7.53E-01	-0.50	8.53E-03	0.02	9.12E-01
ENSCAFG0000001633	KAZN	cyan	VSMC_M2	0.43	2.62E-02	0.75	8.03E-06	0.57	1.92E-03	0.12	5.58E-01	-0.42	2.84E-02	0.00	9.82E-01	-0.20	3.11E-01	-0.37	5.51E-02	-0.74	1.07E-05	0.44	2.22E-02
ENSCAFG0000001312	SAMD10	cyan	VSMC_M2	0.43	2.64E-02	0.50	7.22E-03	0.28	1.53E-01	0.21	2.97E-01	-0.08	7.04E-01	0.01	9.56E-01	-0.04	8.60E-01	-0.36	6.22E-02	-0.52	5.03E-03	0.21	2.90E-01
ENSCAFG0000003188	ZNF200	grey	VSMC_M10	0.43	2.64E-02	0.13	5.33E-01	-0.01	9.64E-01	0.26	1.90E-01	0.14	4.79E-01	0.04	8.62E-01	-0.19	3.41E-01	-0.18	3.73E-01	-0.26	1.91E-01	0.06	7.55E-01
ENSCAFG0000000950	ABI3BP	red	VSMC_M1	0.43	2.65E-02	0.43	2.63E-02	0.43	2.59E-02	-0.27	1.72E-01	-0.22	2.63E-01	-0.06	7.57E-01	0.48	1.08E-02	0.11	5.95E-01	-0.57	1.78E-03	0.11	6.02E-01
ENSCAFG0000001939	MORN1	grey	VSMC_M10	0.43	2.65E-02	0.20	3.16E-01	0.39	4.33E-02	-0.17	3.84E-01	-0.25	2.11E-01	0.19	3.37E-01	0.01	9.66E-01	0.09	6.67E-01	-0.32	1.03E-01	0.02	9.05E-01
ENSCAFG0000001936	ITGAE	red	VSMC_M1	0.43	2.65E-02	0.21	2.92E-01	0.02	9.32E-01	0.15	4.45E-01	0.27	1.80E-01	-0.24	2.25E-01	-0.07	7.35E-01	-0.24	2.34E-01	-0.29	1.42E-01	-0.09	6.38E-01
ENSCAFG0000001253	BTBD16	yellow	VSMC_M3	0.43	2.65E-02	0.22	2.73E-01	0.66	2.02E-04	-0.26	1.91E-01	-0.37	5.58E-02	-0.06	7.58E-01	0.08	6.94E-01	0.11	5.74E-01	-0.32	1.00E-01	-0.14	4.78E-01
ENSCAFG0000000124	REEP2	red	VSMC_M1	0.43	2.66E-02	0.33	9.20E-02	0.05	8.13E-01	0.25	2.15E-01	0.11	5.69E-01	-0.02	9.19E-01	-0.19	3.46E-01	-0.37	5.56E-02	-0.30	1.24E-01	0.18	3.72E-01
ENSCAFG0000003188	ENSCAFG0000003188	red	VSMC_M1	0.43	2.66E-02	0.15	4.45E-01	-0.09	6.42E-01	0.12	5.52E-01	0.29	1.49E-01	-0.14	4.74E-01	-0.13	5.02E-01	-0.21	2.92E-01	-0.19	3.32E-01	-0.03	8.88E-01
ENSCAFG0000001940	DPH7	cyan	VSMC_M2	0.43	2.69E-02	0.67	1.42E-04	0.18	3.82E-01	0.35	7.24E-02	-0.06	7.84E-01	-0.06	7.75E-01	-0.13	5.21E-01	-0.62	5.18E-04	-0.65	2.59E-04	0.53	4.57E-03
ENSCAFG0000001061	BICD1	pink	VSMC_M5	0.43	2.70E-02	-0.29	1.41E-01	-0.44	2.15E-02	0.24	2.28E-01	0.72	2.53E-05	-0.23	2.50E-01	-0.17	4.01E-01	-0.13	5.06E-01	0.22	2.63E-01	-0.31	1.16E-01
ENSCAFG0000002990	C1QTNF2	grey	VSMC_M10	0.43	2.70E-02	0.13	5.08E-01	0.54	4.02E-03	-0.51	7.09E-03	-0.28	1.53E-01	-0.18	3.70E-01	0.53	4.55E-03	0.39	4.34E-02	-0.30	1.32E-01	-0.15	4.46E-01
ENSCAFG0000000139	KCTD20	red	VSMC_M1	0.43	2.71E-02	-0.16	4.29E-01	0.19	3.46E-01	-0.22	2.66E-01	0.13	5.16E-01	0.06	7.83E-01	-0.08	6.88E-01	0.24	2.38E-01	0.04	8.57E-01	-0.47	1.36E-02
ENSCAFG0000001371	ORAI2	cyan	VSMC_M2	0.43	2.71E-02	0.64	3.36E-04	0.33	9.16E-02	-0.01	9.48E-01	-0.17	3.86E-01	-0.09	6.60E-01	-0.04	8.26E-01	-0.24	2.26E-01	-0.63	4.55E-04	0.35	7.19E-02
ENSCAFG0000000741	SNTA1	cyan	VSMC_M2	0.42	2.72E-02	0.61	7.15E-04	0.45	1.86E-02	-0.03	8.78E-01	-0.21	2.98E-01	-0.26	1.92E-01	-0.44	2.07E-02	-0.29	1.46E-01	-0.51	6.58E-03	0.20	3.17E-01
ENSCAFG0000000091	MCAT	grey	VSMC_M10	0.42	2.72E-02	0.20	3.09E-01	0.51	6.22E-03	-0.28	1.60E-01	-0.43	2.41E-02	0.20	3.14E-01	0.36	6.81E-02	0.20	3.28E-01	-0.43	2.48E-02	0.16	4.12E-01
ENSCAFG0000002143	ENSCAFG0000002143	grey	VSMC_M10	0.42	2.73E-02	-0.04	8.43E-01	0.31	1.16E-01	-0.22	2.81E-01	-0.05	8.14E-01	-0.14	4.91E-01	0.09	6.69E-01	0.17	3.92E-01	-0.10	6.32E-01	-0.24	2.24E-01
ENSCAFG0000003242	ZNF619	pink	VSMC_M5	0.42	2.73E-02	0.06	7.51E-01	-0.08	6.80E-01	0.04	8.43E-01	0.46	1.71E-02	-0.18	3.57E-01	-0.16	4.19E-01	-0.12	5.37E-01	-0.03	8.68E-01	-0.36	6.49E-02
ENSCAFG0000002558	ENSCAFG0000002558	pink	VSMC_M5	0.42	2.74E-02	-0.03	8.97E-01	-0.27	1.66E-01	0.01	9.53E-01	0.55	2.96E-03	-0.27	1.80E-01	-0.21	2.96E-01	-0.02	9.27E-01	-0.05	8.19E-01	-0.28	1.64E-01
ENSCAFG0000001905	ENSCAFG0000001905	red	VSMC_M1	0.42	2.75E-02	0.23	2.46E-01	-0.04	8.61E-01	0.08	7.06E-01	0.11	5.71E-01	-0.06	7.52E-01	0.06	7.72E-01	-0.10	6.14E-01	-0.41	3.33E-02	0.19	3.54E-01
ENSCAFG0000000449	LCP1	grey	VSMC_M10	0.42	2.75E-02	0.22	2.63E-01	-0.19	3.55E-01	0.45	1.99E-02	0.28	1.51E-01	0.13	5.19E-01	-0.30	1.33E-01	-0.50	7.70E-03	-0.26	1.83E-01	0.28	1.53E-01
ENSCAFG0000001422	ENSCAFG0000001422	grey	VSMC_M10	0.42	2.77E-02	0.32	1.06E-01	0.13	5.17E-01	0.04	8.37E-01	0.04	8.34E-01	0.02	9.35E-01	-0.36	6.13E-02	-0.14	4.86E-01	-0.36	6.41E-02	0.08	6.76E-01
ENSCAFG0000001301	CYB561	cyan	VSMC_M2	0.42	2.77E-02	0.81	3.46E-07	0.52	5.02E-03	0.08	6.97E-01	-0.44	2.23E-02	0.04	8.59E-01	-0.10	6.09E-01	-0.36	6.27E-02	-0.82	1.37E-07	0.53	4.68E-03
ENSCAFG0000000710	ENSCAFG0000000710	red	VSMC_M1	0.42	2.78E-02	0.25	2.16E-01	0.16	4.17E-01	0.05	7.90E-01	0.03	8.96E-01	-0.01	9.74E-01	-0.23	2.46E-01	-0.07	7.27E-01	-0.31	1.14E-01	0.00	9.89E-01
ENSCAFG0000000264	KCNQ5	cyan	VSMC_M2	0.42	2.78E-02	0.64	3.52E-04	0.45	2.00E-02	-0.02	9.36E-01	-0.38	4.92E-02	-0.24	2.25E-01	0.04	8.28E-01	-0.16	4.38E-01	-0.74	9.64E-06	0.49	8.73E-03
ENSCAFG0000000385	WASF1	grey	VSMC_M10	0.42	2.78E-02	0.32	1.09E-01	-0.09	6.51E-01	0.28	1.52E-01	0.17	4.04E-01	-0.14	4.73E-01	-0.16	4.28E-01	-0.33	9.18E-02	-0.40	3.82E-02	0.36	6.46E-02
ENSCAFG0000002041	CTH	darkgrey	VSMC_M8	0.42	2.79E-02	-0.51	6.21E-03	0.11	5.93E-01	-0.64	2.92E-04	0.28	1.52E-01	-0.05	8.01E-01	0.28	1.64E-01	0.71	2.88E-05	0.28	1.55E-01	-0.81	2.32E-07
ENSCAFG0000002062	ENSCAFG0000002062	yellow	VSMC_M3	0.42	2.79E-02	0.41	3.29E-02	0.61	7.71E-04	-0.25	2.06E-01	-0.34	8.66E-02	-0.14	4.90E-01	-0.26	1.90E-01	-0.04	8.55E-01	-0.38	5.29E-02	-0.02	9.10E-01
ENSCAFG0000001075	CCDC191	grey	VSMC_M10	0.42	2.79E-02	-0.08	7.09E-01	-0.14	4.92E-01	0.22	2.80E-01	0.33	9.26E-02	-0.27	1.66E-01	0.19	3.40E-01	-0.18	3.65E-01	-0.03	8.76E-01	-0.01	9.45E-01
ENSCAFG0000003155	ENSCAFG0000003155	red	VSMC_M1	0.42	2.80E-02	0.25	2.01E-01	0.11	5.84E-01	0.12	5.47E-01	0.19	3.36E-01	-0.22	2.69E-01	-0.18	3.62E-01	-0.23	2.41E-01	-0.29	1.40E-01	-0.05	8.17E-01
ENSCAFG0000000076	MAP3K4	grey	VSMC_M10	0.42	2.81E-02	0.34	7.85E-02	0.05	8.19E-01	0.29	1.48E-01	-0.01	9.78E-01	0.08	6.79E-01	0.03	8.90E-01	-0.25	2.09E-01	-0.52	4.95E-03	0.40	4.10E-02
ENSCAFG0000001592	CCDC22	yellow	VSMC_M3	0.42	2.82E-02	0.32	1.05E-01	0.66	1.56E-04	-0.51	6.98E-03	-0.48	1.07E-02	0.03	8.87E-01	0.05	8.07E-01	0.28	1.64E-01	-0.44	2.23E-02	0.01	9.69E-01
ENSCAFG0000000554	UCP3	pink	VSMC_M5	0.42	2.82E-02	-0.22	2.63E-01	-0.20	3.07E-01	-0.04	8.45E-01	0.40	3.90E-02	-0.01	9.65E-01	0.30	1.24E-01	0.12	5.38E-01	-0.01	9.45E-01	-0.16	4.15E-01
ENSCAFG0000001397	C8H14orf28	red	VSMC_M1	0.42	2.82E-02	0.11	6.01E-01	-0.07	7.18E-01	0.16	4.35E-01	0.24	2.27E-01	-0.19	3.33E-01	0.08	6.94E-01	-0.18	3.56E-01	-0.19	3.38E-01	0.08	6.78E-01
ENSCAFG0000002518	EPS8L2	grey	VSMC_M10	0.42	2.84E-02	0.62	5.22E-04	0.08	7.07E-01	0.54	3.89E-03	0.06	7.70E-01	-0.11	5.81E-01	-0.14	4.84E-01	-0.73	1.34E-05	-0.56	2.22E-03	0.51	6.00E-03
ENSCAFG0000001863	TUBB4A	red	VSMC_M1	0.42	2.84E-02	0.34	8.41E-02	-0.13	5.22E-01	0.59	1.20E-03	0.16	4.16E-01	-0.03	8.85E-01	-0.05	8.13E-01	-0.57	1.82E-03	-0.39	4.40E-02	0.47	1.35E-02
ENSCAFG0000001984	ANKRD11	grey	VSMC_M10	0.42	2.84E-02	0.12	5.63E-01	0.06	7.57E-01	-0.04	8.56E-01	0.16	4.21E-01	-0.06	7.52E-01	0.01	9.74E-01	-0.01	9.69E-01	-0.30	1.26E-01	-0.02	9.29E-01
ENSCAFG0000000763	UQCRFS1	yellow	VSMC_M3	0.42	2.84E-02	0.13	5.25E-01	0.49	8.77E-03	-0.38	4.87E-02	-0.23	2.39E-01	0.16	4.17E-01	0.17	4.04E-01	0.30	1.26E-01	-0.31	1.17E-01	-0.26	1.96E-01
ENSCAFG0000001621	MICAL3	darkgreen	VSMC_M4	0.42	2.84E-02	0.46	1.53E-02	-0.19	3.51E-01	0.68	1.09E-04	0.24	2.34E-01	0.03	8.85E-01	-0.33	9.72E-02	-0.65	2.42E-04	-0.54	3.73E-03	0.52	5.62E-03
ENSCAFG0000002373	GDPGP1	grey	VSMC_M10	0.42	2.85E-02	0.26	1.83E-01	0.19	3.49E-01	-0.13	5.17E-01	0.05	8.18E-01	0.04	8.35E-01	0.09	6.67E-01	0.06	7.59E-01	-0.38	5.06E-02	-0.11	5.74E-01
ENSCAFG0000000436	IRF2BP1	red	VSMC_M1	0.42	2.85E-02	0.43	2.63E-02	0.18	3.74E-01	0.35	7.38E-02	0.02	9.11E-01	-0.11	5.75E-01	-0.19	3.42E-01	-0.47	1.35E-02	-0.48	1.07E-02	0.22	2.72E-01
ENSCAFG0000001195	NDUFAF3	cyan	VSMC_M2	0.42	2.85E-02	0.47	1.28E-02	0.49	1.03E-02	-0.05	7.86E-01	-0.24	2.28E-01	-0.22	2.72E-01	0.08	7.01E-01	-0.18	3.65E-01	-0.52	5.78E-03	0.12	5.52E-01
ENSCAFG0000000741	PPWD1	pink	VSMC_M5	0.42	2.85E-02	-0.38	4.96E-02	-0.11	5.80E-01	-0.19	3.32E-01	0.43	2.43E-02	-0.23	2.49E-01	0.35	7.47E-02	0.34	8.75E-02	0.14	5.01E-01	-0.48	1.18E-02
ENSCAFG0000000701	PFDN5	grey	VSMC_M10	0.42	2.85E-02	0.40	3.71E-02	-0.05	8.16E-01	0.56	2.32E-03	0.22	2.60E-01	-0.37	5.94E-02	-0.21	2.90E-01	-0.66	1.62E-04	-0.36	6.51E-02	0.27	1.78E-01
ENSCAFG0000000583	ENSCAFG0000000583	yellow	VSMC_M3	0.42	2.86E-02	0.32	1.08E-01	0.60	8.64E-04	-0.21	2.92E-01	-0.34	7.88E-02	-0.05	8.10E-01	-0.05	8.10E-01	0.04	8.60E-01	-0.39	4.24E-02	-0.07	7.40E-01
ENSCAFG0000001481	JADE3	pink	VSMC_M5	0.42	2.86E-02	0.09	6.68E-01	-0.29	1.48E-01	0.42	2.76E-02	0.57	1.79E-03	-0.25	2.00E-01	-0.44	2.13E-02	-0.46	1.51E-02	-0.09	6.60E-01	-0.08	6.87E-01
ENSCAFG0000001591	JUP	cyan	VSMC_M2	0.42	2.86E-02	0.84	2.99E-08	0.14	4.93E-01	0.46	1.70E-02	0.02	9.26E-01	-0.24	2.23E-01	-0.60	1.05E-03	-0.72	2.72E-05	-0.73	1.27E-05	0.52	5.72E-03
ENSCAFG0000000676	DLC1	pink	VSMC_M5	0.42	2.86E-02	0.03	8.95E-01	-0.39	4.48E-02	0.50	7.67E-03	0.56	2.45E-03	-0.20	3.11E-01	-0.32	1.03E-01	-0.38	5.34E-02	-0.04	8.44E-01	0.00	9.96E-01
ENSCAFG0000001925	ATAD3A	grey	VSMC_M10	0.42	2.87E-02	-0.14	4.78E-01	0.44	2.06E-02	-0.45	1.71E-02	-0.22	2.67E-01	0.14	4.80E-01	0.04	8.57E-01	0.53	4.06E-03	-0.10	6.14E-01	-0.39	4.22E-02
ENSCAFG0000001689	BBOF1	grey	VSMC_M10	0.42	2.88E-02	0.41	3.19E-02	0.04	8.48E-01	0.24	2.26E-01	0.03	8.77E-01	0.04	8.58E-01	-0.06	7.59E-01	-0.26	1.97E-01	-0.56	2.37E-03	0.40	3.73E-02
ENSCAFG0000003115	PAIP2	pink	VSMC_M5	0.42	2.88E-02	0.07	7.27E-01	-0.60	1.04E-03	0.55	2.68E-03	0.81	2.38E-07	-0.36	6.61E-02	-0.36	6.30E-02	-0.52	5.10E-03	-0.08	6.84E-01	0.01	9.46E-01
ENSCAFG0000002540	ZNF684	grey	VSMC_M10	0.42	2.89E-02	0.25	2.04E-01	0.17	4.02E-01	-0.05	8.15E-01	-0.14	4.99E-01	0.18	3.59E-01	-0.05	8.19E-01	0.02	9.20E-01	-0.43	2.63E-02	0.30	1.24E-01
ENSCAFG0000002015	IL34	cyan	VSMC_M2	0.42	2.92E-02	0.85	1.55E-08	0.60	1.03E-03	-0.01	9.46E-01	-0.46	1.65E-02	-0.02	9.12E-01	-0.07	7.42E-01	-0.33	9.29E-02	-0.83	1.06E-07	0.48	1.07E-02
ENSCAFG0000000475	CLASP1	pink	VSMC_M5	0.42	2.93E-02	0.09	6.70E-01	-0.50	7.88E-03	0.40	4.02E-02	0.75	5.79E-06	-0.18	3.68E-01	-0.07	7.43E-01	-0.40	4.05E-02	-0.13	5.04E-01	-0.01	9.41E-01
ENSCAFG0000001333	ATF2	pink	VSMC_M5	0.42	2.93E-02	0.02	9.13E-01	-0.44	2.15E-02	0.22	2.67E-01	0.71	3.64E-05	-0.23	2.54E-01	-0.08	6.77E-01	-0.27	1.76E-01	-0.09	6.45E-01	-0.11	5.86E-01
ENSCAFG0000001058	CYB561D2	red	VSMC_M1	0.42	2.93E-02	0.37	5.54E-02	0.00	9.91E-01	0.19	3.44E-01	0.16	4.31E-01	-0.20	3.12E-01	-0.34	8.51E-02	-0.27	1.78E-01	-0.41	3.41E-02	0.15	4.59E-01
ENSCAFG0000000437	ACBD5	pink	VSMC_M5	0.42	2.94E-02	-0.29	1.36E-01	-0.23	2.58E-01	0.09	6.53E-01	0.62	5.14E-04	-0.40	4.03E-02	-0.06	7.50E-01	-0.01	9.46E-01	0.23	2.44E-01	-0.54	3.63E-03
ENSCAFG0000000956	RIOK1	grey	VSMC_M10	0.42	2.94E-02	-0.20	3.18E-01	0.15	4.53E-01	-0.33	9.47E-02	0.16	4.16E-01	-0.19	3.33E-01	0.11	5.81E-01	0.38	4.81E-02	-0.01	9.78E-01	-0.48	1.05E-02
ENSCAFG0000000640	ENSCAFG0000000640	grey	VSMC_M10	0.42	2.94E-02	0.43	2.56E-02	0.63	4.07E-04	-0.31	1.17E-01	-0.42	3.11E-02	0.11	5.70E-01	0.12	5.54E-01	0.13	5.10E-01	-0.53	4.32E-03	0.05	7.96E-01
ENSCAFG0000001704	ZMYM3	cyan	VSMC_M2	0.42	2.94E-02	0.62	5.00E-04	-0.09	6.47E-01	0.51	6.28E-03	0.24	2.22E-01	-0.09	6.54E-01	-0.21	2.96E-01	-0.68	9.89E-05	-0.59	1.10E-03	0.41	3.30E-02
ENSCAFG0000003238	ERH	darkgrey	VSMC_M8	0.42	2.94E-02	-0.27	1.68E-01	0.25	2.07E-01	-0.41	3.45E-02	-0.02	9.28E-01	0.14	4.85E-01	0.14	4.75E-01	0.55	3.05E-03	0.06	7.68E-01	-0.46	1.66E-02
ENSCAFG0000003037	TSPAN4	cyan	VSMC_M2	0.42	2.95E-02	0.38	5.06E-02	0.43	2.40E-02	-0.34	8.36E-02	-0.18	3.79E-01	-0.13	5.20E-01	-0.20	3.06E-01	0.08	6.84E-01	-0.36	6.34E-02	-0.07	7.21E-01
ENSCAFG0000000865	ENSCAFG0000000865	red	VSMC_M1	0.42	2.95E-02	0.09	6.42E-01	-0.13	5.30E-01	0.18	3.62E-01	0.33	9.81E-02	0.02	9.19E-01	-0.27	1.76E-01	-0.21	2.86E-01	-0.16	4.29E-01	-0.01	9.58E-01
ENSCAFG0000002002	DYNLRB2	red	VSMC_M1	0.42	2.96E-02	0.30	1.32E-01	-0.07	7.12E-01	0.18	3.71E-01	0.19	3.32E-01	-0.03	8.76E-01	0.15	4.65E-01	-0.20	3.10E-01	-0.44	2.30E-02	0.19	3.51E-01
ENSCAFG0000003261	C4orf48	cyan	VSMC_M2	0.42	2.96E-02	0.37	5.68E-02	0.28	1.61E-01	0.27	1.68E-01	-0.03	8.82E-01	-0.34	8.44E-02	-0.26	1.85E-01	-0.41	3.19E-02	-0.34	8.68E-02	0.13	5.23E-01
ENSCAFG0000002863	IGFBP6	yellow	VSMC_M3	0.42	2.96E-02	0.46	1.53E-02	0.82	1.41E-07	-0.59	1.25E-03	-0.46	1.53E-02	-0.29	1.42E-01	-0.02	9.40E-01	0.23	2.41E-01	-0.45	1.94E-02	-0.17	4.10E-01
ENSCAFG0000001200	ARL4C	red	VSMC_M1	0.42	2.97E-02	0.06	7.60E-01	0.63	4.18E-04	-0.74	1.18E-05	-0.32	1.03E-01	-0.21	2.99E-01	-0.13	5.22E-01	0.58	1.42E-03	-0.19	3.46E-01	-0.40	3.74E-02
ENSCAFG0000001186	PCK2	yellow	VSMC_M3	0.42	2.97E-02	0.32	1.07E-01	0.67	1.49E-04	-0.55	2.70E-03	-0.34	8.41E-02	-0.21	2.95E-01	0.17	3.87E-01	0.32	1.07E-01	-0.45	1.98E-02	-0.18	3.75E-01
ENSCAFG0000001464	ZBTB40	grey	VSMC_M10	0.42	2.97E-02	0.52	5.04E-03	-0.23	2.54E-01	0.63	4.31E-04	0.36	6.31E-02	-0.14	4.96E-01	-0.19	3.50E-01	-0.76	5.12E-06	-0.55	3.15E-03	0.46	1.66E-02
ENSCAFG0000001132	ENSCAFG0000001132	yellow	VSMC_M3	0.42	2.98E-02	0.16	4.24E-01	0.60	8.99E-04	-0.54	3.57E-03	-0.40	4.05E-02	0.10	6.06E-01	0.05	8.14E-01	0.44	2.22E-02	-0.34	8.39E-02	-0.15	4.59E-01
ENSCAFG0000000103	ENSCAFG0000000103	pink	VSMC_M5	0.42	2.99E-02	-0.47	1.28E-02	-0.02	9.25E-01	-0.39	4.53E-02	0.33	8.90E-02	-0.01	9.75E-01	0.05	7.94E-01	0.52	5.12E-03	0.30	1.34E-01	-0.70	5.03E-05
ENSCAFG0000000229	ENSCAFG0000000229	red	VSMC_M1	0.42	3.00E-02	0.31	1.12E-01	0.15	4.43E-01	0.11	5.82E-01	0.08	6.97E-01	0.24	2.23E-01	-0.14	4.76E-01	-0.26	1.90E-01	-0.30	1.30E-01	0.08	7.08E-01
ENSCAFG0000001050	ENSCAFG0000001050	grey	VSMC_M10	0.42	3.00E-02	0.02	9.16E-01	0.26	1.93E-01	-0.17	3.95E-01	0.00	9.92E-01	0.12	5.63E-01	0.15	4.60E-01	0.09	6.47E-01	-0.07	7.19E-01	-0.24	2.29E-01
ENSCAFG0000002032	ENSCAFG0000002032	red	VSMC_M1	0.42	3.02E-02	0.28	1.55E-01	0.19	3.44E-01	0.19	3.48E-01	-0.08	7.02E-01	-0.02	9.35E-01	0.09	6.63E-01	-0.22	2.60E-01	-0.41	3.44E-02	0.20	3.29E-01
ENSCAFG0000001922	GLIS2	cyan	VSMC_M2	0.42	3.03E-02	0.69	5.86E-05	0.31	1.14E-01	0.08	6.81E-01	-0.11	5.74E-01	-0.12	5.61E-01	0.09	6.64E-01	-0.35	6.94E-02	-0.72	2.02E-05	0.35	7.47E-02
ENSCAFG0000001520	TMEM260	grey	VSMC_M10	0.42	3.04E-02	0.10	6.19E-01	-0.31	1.15E-01	0.23	2.57E-01	0.54	4.02E-03	-0.30	1.31E-01	0.17	3.93E-01	-0.31	1.20E-01	-0.14	5.01E-01	0.06	7.65E-01
ENSCAFG0000001953	PTGDS	cyan	VSMC_M2	0.42	3.04E-02	0.90	2.75E-10	0.41	3.54E-02	0.24	2.37E-01	-0.24	2.27E-01	-0.28	1.60E-01	-0.27	1.67E-01	-0.54	3.96E-03	-0.83	8.66E-08	0.53	4.72E-03
ENSCAFG0000003190	IMP4	darkgrey	VSMC_M8	0.42	3.04E-02	-0.21	2.87E-01	0.37	6.04E-02	-0.77	2.61E-06	-0.02	9.39E-01	-0.13	5.32E-01	0.27	1.68E-01	0.66	1.59E-04	0.06	7.75E-01	-0.60	9.52E-04
ENSCAFG0000001047	GCA	red	VSMC_M1	0.42	3.05E-02	0.23	2.49E-01	0.18	3.79E-01	-0.03	8.82E-01	-0.01	9.62E-01	-0.45	1.84E-02	0.15	4.47E-01	-0.05	8.00E-01	-0.36	6.21E-02	0.16	4.31E-01

ENSCAFG0000000344	CUL1	pink	VSMC_M5	0.42	3.05E-02	-0.45	1.96E-02	-0.22	2.65E-01	-0.36	6.23E-02	0.49	9.08E-03	0.11	5.75E-01	0.19	3.52E-01	0.51	7.17E-03	0.23	2.48E-01	-0.58	1.57E-03
ENSCAFG0000000529	WNT11	grey	VSMC_M10	0.42	3.07E-02	0.22	2.61E-01	0.13	5.29E-01	0.19	3.35E-01	0.05	8.20E-01	0.16	4.23E-01	-0.25	2.13E-01	-0.26	1.83E-01	-0.29	1.46E-01	0.06	7.82E-01
ENSCAFG0000001962	MPP1	grey	VSMC_M10	0.42	3.07E-02	0.39	4.58E-02	0.67	1.26E-04	-0.41	3.22E-02	-0.52	5.44E-03	0.06	7.49E-01	0.12	5.58E-01	0.23	2.42E-01	-0.54	3.59E-03	0.15	4.52E-01
ENSCAFG0000001595	ENSCAFG0000001595	grey	VSMC_M10	0.42	3.07E-02	0.30	1.23E-01	0.32	1.08E-01	-0.11	5.95E-01	-0.07	7.46E-01	0.16	4.22E-01	-0.04	8.25E-01	-0.01	9.53E-01	-0.38	5.35E-02	-0.05	8.15E-01
ENSCAFG0000001454	FBN1	grey	VSMC_M10	0.42	3.08E-02	-0.04	8.24E-01	0.13	5.30E-01	-0.39	4.23E-02	0.22	2.76E-01	-0.09	6.48E-01	0.48	1.11E-02	0.27	1.68E-01	-0.08	6.78E-01	-0.34	8.66E-02
ENSCAFG0000002975	ENSCAFG0000002975	grey	VSMC_M10	0.42	3.09E-02	0.43	2.60E-02	0.41	3.40E-02	-0.01	9.78E-01	-0.21	2.95E-01	-0.09	6.57E-01	-0.23	2.49E-01	-0.15	4.41E-01	-0.48	1.19E-02	0.07	7.22E-01
ENSCAFG0000001791	WDR25	red	VSMC_M1	0.42	3.12E-02	0.23	2.52E-01	-0.10	6.28E-01	0.18	3.81E-01	0.41	3.37E-02	-0.19	3.32E-01	-0.19	3.39E-01	-0.31	1.21E-01	-0.23	2.55E-01	-0.13	5.05E-01
ENSCAFG0000001172	EGFL6	cyan	VSMC_M2	0.42	3.13E-02	0.66	1.95E-04	0.53	4.12E-03	-0.16	4.23E-01	-0.31	1.11E-01	-0.13	5.18E-01	-0.23	2.52E-01	-0.10	6.08E-01	-0.65	2.26E-04	0.18	3.70E-01
ENSCAFG0000001904	SSBP3	grey	VSMC_M10	0.42	3.13E-02	0.33	8.94E-02	-0.06	7.62E-01	0.24	2.24E-01	0.23	2.47E-01	-0.10	6.03E-01	-0.10	6.21E-01	-0.29	1.41E-01	-0.36	6.87E-02	0.23	2.42E-01
ENSCAFG0000003266	LRRTM2	grey	VSMC_M10	0.41	3.14E-02	-0.20	3.25E-01	0.00	9.98E-01	-0.32	1.01E-01	0.20	3.18E-01	0.04	8.30E-01	0.12	5.66E-01	0.40	3.79E-02	-0.03	9.00E-01	-0.30	1.34E-01
ENSCAFG0000000575	ENSCAFG0000000575	red	VSMC_M1	0.41	3.16E-02	-0.02	9.24E-01	-0.01	9.42E-01	0.06	7.50E-01	0.20	3.12E-01	0.11	5.87E-01	0.14	4.90E-01	-0.10	6.13E-01	-0.08	7.04E-01	-0.08	6.88E-01
ENSCAFG0000002097	ENSCAFG0000002097	pink	VSMC_M5	0.41	3.16E-02	-0.15	4.70E-01	-0.25	2.14E-01	-0.07	7.11E-01	0.59	1.12E-03	-0.22	2.76E-01	-0.14	4.97E-01	0.11	5.84E-01	0.03	8.87E-01	-0.43	2.36E-02
ENSCAFG0000000893	RAB11FIP5	grey	VSMC_M10	0.41	3.17E-02	0.05	8.05E-01	0.37	5.91E-02	-0.56	2.46E-03	0.00	1.00E+00	0.20	3.10E-01	0.15	4.47E-01	0.32	1.01E-01	-0.11	5.79E-01	-0.42	2.78E-02
ENSCAFG0000002311	NFIA	cyan	VSMC_M2	0.41	3.17E-02	0.72	2.33E-05	0.00	9.90E-01	0.45	1.72E-02	0.10	6.33E-01	-0.16	4.26E-01	-0.49	1.03E-02	-0.61	6.97E-04	-0.70	5.60E-05	0.50	8.01E-03
ENSCAFG0000001424	YJEFN3	yellow	VSMC_M3	0.41	3.17E-02	0.36	6.35E-02	0.56	2.36E-03	-0.15	4.41E-01	-0.33	9.58E-02	0.06	7.51E-01	0.00	9.92E-01	-0.02	9.03E-01	-0.43	2.47E-02	0.00	9.85E-01
ENSCAFG0000002371	SARNP	grey	VSMC_M10	0.41	3.18E-02	-0.14	4.81E-01	0.47	1.45E-02	-0.51	6.21E-03	-0.17	4.08E-01	-0.09	6.39E-01	0.16	4.21E-01	0.47	1.25E-02	-0.03	8.87E-01	-0.42	3.07E-02
ENSCAFG0000001903	ABAT	grey	VSMC_M10	0.41	3.19E-02	0.64	3.00E-04	0.59	1.19E-03	-0.08	6.75E-01	-0.52	5.26E-03	0.17	3.84E-01	-0.03	8.71E-01	-0.19	3.41E-01	-0.68	8.28E-05	0.45	1.97E-02
ENSCAFG0000003033	ENSCAFG0000003033	red	VSMC_M1	0.41	3.19E-02	0.25	2.13E-01	-0.01	9.68E-01	0.16	4.40E-01	0.25	2.00E-01	-0.07	7.35E-01	-0.50	7.37E-03	-0.21	2.94E-01	-0.28	1.62E-01	-0.07	7.34E-01
ENSCAFG0000001684	HOXB2	cyan	VSMC_M2	0.41	3.19E-02	0.58	1.50E-03	0.41	3.29E-02	-0.06	7.61E-01	-0.31	1.14E-01	0.15	4.51E-01	-0.47	1.45E-02	-0.14	4.96E-01	-0.65	2.59E-04	0.34	8.48E-02
ENSCAFG0000001563	TMEM223	cyan	VSMC_M2	0.41	3.20E-02	0.54	3.70E-03	0.49	1.03E-02	-0.08	7.09E-01	-0.41	3.52E-02	0.14	4.75E-01	0.08	6.95E-01	-0.10	6.10E-01	-0.67	1.49E-04	0.33	9.02E-02
ENSCAFG0000001783	MYO19	yellow	VSMC_M3	0.41	3.20E-02	0.12	5.61E-01	0.73	1.53E-05	-0.69	6.66E-05	-0.49	9.16E-03	0.09	6.58E-01	0.11	5.72E-01	0.58	1.48E-03	-0.30	1.28E-01	-0.24	2.35E-01
ENSCAFG0000000325	MANEAL	cyan	VSMC_M2	0.41	3.20E-02	0.79	1.14E-06	0.54	3.59E-03	-0.10	6.30E-01	-0.33	9.23E-02	-0.06	7.66E-01	-0.24	2.27E-01	-0.22	2.72E-01	-0.79	7.61E-07	0.27	1.81E-01
ENSCAFG0000000433	ENSCAFG0000000433	grey	VSMC_M10	0.41	3.20E-02	0.15	4.51E-01	0.43	2.55E-02	-0.23	2.38E-01	-0.22	2.62E-01	0.28	1.51E-01	0.10	6.32E-01	0.11	5.68E-01	-0.21	2.85E-01	-0.13	5.22E-01
ENSCAFG0000002898	ENSCAFG0000002898	grey	VSMC_M10	0.41	3.20E-02	0.77	2.48E-06	0.03	8.77E-01	0.42	3.09E-02	0.05	8.21E-01	-0.17	3.91E-01	-0.23	2.43E-01	-0.58	1.42E-03	-0.81	3.82E-07	0.61	7.99E-04
ENSCAFG0000001956	AGPAT2	grey	VSMC_M10	0.41	3.21E-02	-0.11	5.80E-01	-0.21	3.01E-01	0.19	3.52E-01	0.30	1.34E-01	0.25	2.09E-01	0.02	9.18E-01	-0.09	6.66E-01	-0.05	7.86E-01	0.02	9.09E-01
ENSCAFG0000002585	ENSCAFG0000002585	grey	VSMC_M10	0.41	3.22E-02	0.33	9.39E-02	0.51	6.57E-03	-0.27	1.75E-01	-0.29	1.41E-01	-0.12	5.64E-01	-0.04	8.40E-01	0.13	5.08E-01	-0.40	3.69E-02	-0.03	8.66E-01
ENSCAFG0000000491	H3F3B	yellow	VSMC_M3	0.41	3.22E-02	0.19	3.41E-01	0.67	1.38E-04	-0.65	2.23E-04	-0.39	4.16E-02	-0.11	5.72E-01	0.31	1.15E-01	0.49	1.01E-02	-0.38	4.97E-02	-0.18	3.60E-01
ENSCAFG0000001125	DNAAF5	pink	VSMC_M5	0.41	3.23E-02	-0.13	5.34E-01	-0.30	1.29E-01	0.34	7.94E-02	0.52	4.97E-03	-0.12	5.37E-01	-0.17	4.03E-01	-0.29	1.39E-01	0.08	6.87E-01	-0.11	5.97E-01
ENSCAFG0000001985	CBFA2T3	cyan	VSMC_M2	0.41	3.23E-02	0.59	1.22E-03	0.51	6.27E-03	-0.05	8.00E-01	-0.32	1.05E-01	0.10	6.20E-01	-0.40	3.84E-02	-0.19	3.35E-01	-0.63	4.82E-04	0.18	3.74E-01
ENSCAFG0000001650	LRRC71	yellow	VSMC_M3	0.41	3.27E-02	0.39	4.45E-02	0.63	4.02E-04	-0.30	1.34E-01	-0.45	1.93E-02	-0.08	6.86E-01	0.10	6.32E-01	0.06	7.53E-01	-0.46	1.63E-02	0.12	5.46E-01
ENSCAFG0000001366	ENSCAFG0000001366	grey	VSMC_M10	0.41	3.28E-02	0.72	2.27E-05	0.18	3.76E-01	0.45	1.76E-02	-0.10	6.30E-01	-0.04	8.32E-01	-0.07	7.20E-01	-0.63	4.61E-04	-0.72	1.99E-05	0.60	9.72E-04
ENSCAFG0000000214	IL1R1	yellow	VSMC_M3	0.41	3.28E-02	0.29	1.49E-01	0.39	4.70E-02	-0.43	2.52E-02	-0.11	5.68E-01	-0.06	7.49E-01	0.25	2.12E-01	0.31	1.17E-01	-0.43	2.46E-02	-0.16	4.23E-01
ENSCAFG0000001851	ENSCAFG0000001851	red	VSMC_M1	0.41	3.28E-02	-0.28	1.60E-01	0.18	3.63E-01	-0.48	1.13E-02	0.25	2.04E-01	-0.14	4.91E-01	-0.04	8.30E-01	0.43	2.55E-02	0.16	4.12E-01	-0.71	3.05E-05
ENSCAFG0000000123	KDM3B	grey	VSMC_M10	0.41	3.28E-02	0.48	1.07E-02	-0.26	1.83E-01	0.47	1.33E-02	0.46	1.59E-02	-0.13	5.24E-01	-0.42	2.88E-02	-0.61	7.79E-04	-0.43	2.53E-02	0.27	1.73E-01
ENSCAFG0000000526	SPRY2	grey	VSMC_M10	0.41	3.28E-02	0.24	2.32E-01	0.46	1.71E-02	-0.34	8.42E-02	-0.13	5.31E-01	-0.20	3.29E-01	0.26	1.98E-01	0.22	2.80E-01	-0.38	5.07E-02	-0.22	2.79E-01
ENSCAFG0000000102	MEI1	red	VSMC_M1	0.41	3.28E-02	0.23	2.50E-01	0.25	2.08E-01	0.02	9.21E-01	0.03	8.70E-01	0.08	7.04E-01	0.08	6.85E-01	-0.13	5.30E-01	-0.33	9.51E-02	-0.09	6.43E-01
ENSCAFG0000001335	MGST3	yellow	VSMC_M3	0.41	3.28E-02	0.15	4.41E-01	0.61	8.08E-04	-0.59	1.15E-03	-0.18	3.74E-01	-0.09	6.70E-01	0.15	4.56E-01	0.35	7.68E-02	-0.19	3.48E-01	-0.49	9.01E-03
ENSCAFG0000001971	UBAC1	yellow	VSMC_M3	0.41	3.29E-02	0.43	2.63E-02	0.56	2.34E-03	-0.34	8.64E-02	-0.24	2.29E-01	-0.21	2.94E-01	0.15	4.55E-01	0.08	7.07E-01	-0.46	1.47E-02	-0.09	6.57E-01
ENSCAFG0000001590	ARRB2	cyan	VSMC_M2	0.41	3.29E-02	0.62	5.75E-04	0.34	8.40E-02	0.03	8.80E-01	-0.19	3.30E-01	-0.23	2.45E-01	-0.36	6.55E-02	-0.24	2.38E-01	-0.62	5.13E-04	0.36	6.17E-02
ENSCAFG0000000889	SERINC5	grey	VSMC_M10	0.41	3.29E-02	-0.03	9.00E-01	0.22	2.73E-01	-0.35	6.92E-02	-0.06	7.58E-01	0.45	2.00E-02	0.26	1.89E-01	0.28	1.55E-01	-0.13	5.30E-01	-0.11	5.81E-01
ENSCAFG0000001578	WDR45	cyan	VSMC_M2	0.41	3.30E-02	0.58	1.43E-03	0.38	4.91E-02	-0.05	7.89E-01	-0.22	2.75E-01	-0.04	8.60E-01	-0.13	5.21E-01	-0.27	1.79E-01	-0.53	4.63E-03	0.28	1.64E-01
ENSCAFG0000001529	ASMTL	yellow	VSMC_M3	0.41	3.30E-02	0.32	9.85E-02	0.48	1.10E-02	-0.28	1.58E-01	-0.31	1.18E-01	-0.14	4.77E-01	0.09	6.73E-01	0.08	6.85E-01	-0.40	3.68E-02	0.09	6.38E-01
ENSCAFG0000002306	NDST1	yellow	VSMC_M3	0.41	3.31E-02	0.56	2.23E-03	0.63	4.35E-04	-0.38	4.74E-02	-0.43	2.50E-02	-0.04	8.42E-01	0.10	6.22E-01	0.01	9.51E-01	-0.61	6.91E-04	0.19	3.42E-01
ENSCAFG0000001501	ZNF394	yellow	VSMC_M3	0.41	3.31E-02	0.08	7.04E-01	0.53	4.86E-03	-0.32	1.05E-01	-0.18	3.70E-01	-0.05	8.04E-01	-0.07	7.30E-01	0.20	3.19E-01	-0.16	4.25E-01	-0.35	7.71E-02
ENSCAFG0000000534	OGG1	yellow	VSMC_M3	0.41	3.31E-02	0.10	6.28E-01	0.48	1.15E-02	-0.58	1.63E-03	-0.12	5.64E-01	-0.15	4.46E-01	0.11	5.78E-01	0.36	6.38E-02	-0.19	3.32E-01	-0.44	2.22E-02
ENSCAFG0000002996	APCDD1L	yellow	VSMC_M3	0.41	3.31E-02	0.34	8.67E-02	0.68	1.05E-04	-0.19	3.35E-01	-0.40	3.91E-02	-0.10	6.13E-01	-0.18	3.73E-01	0.01	9.64E-01	-0.39	4.25E-02	-0.07	7.34E-01
ENSCAFG0000000477	EPM2AIP1	grey	VSMC_M10	0.41	3.32E-02	0.50	8.25E-03	0.12	5.46E-01	0.15	4.68E-01	-0.02	9.26E-01	-0.08	6.87E-01	-0.28	1.51E-01	-0.19	3.46E-01	-0.62	5.73E-04	0.26	1.85E-01
ENSCAFG0000000225	ICK	red	VSMC_M1	0.41	3.32E-02	0.40	3.73E-02	-0.21	2.99E-01	0.23	2.40E-01	0.49	9.53E-03	-0.39	4.41E-02	-0.31	1.12E-01	-0.40	3.81E-02	-0.33	9.58E-02	0.08	7.05E-01
ENSCAFG0000002913	KAZALD1	red	VSMC_M1	0.41	3.33E-02	0.45	1.88E-02	0.43	2.44E-02	-0.24	2.33E-01	-0.22	2.60E-01	-0.02	9.28E-01	0.25	2.05E-01	0.08	6.92E-01	-0.59	1.16E-03	0.13	5.16E-01
ENSCAFG0000002002	CDYL2	red	VSMC_M1	0.41	3.33E-02	0.34	7.92E-02	-0.23	2.39E-01	0.53	4.46E-03	0.30	1.28E-01	0.10	6.29E-01	-0.34	8.33E-02	-0.58	1.41E-03	-0.31	1.11E-01	0.40	3.69E-02
ENSCAFG0000001401	FKBP3	pink	VSMC_M5	0.41	3.33E-02	-0.35	7.70E-02	-0.32	1.06E-01	0.03	8.81E-01	0.67	1.39E-04	-0.24	2.25E-01	-0.21	2.97E-01	0.12	5.64E-01	0.22	2.81E-01	-0.59	1.16E-03
ENSCAFG0000000935	POC5	pink	VSMC_M5	0.41	3.33E-02	-0.17	3.87E-01	-0.52	5.24E-03	0.36	6.44E-02	0.78	2.00E-06	-0.09	6.43E-01	-0.25	2.07E-01	-0.22	2.62E-01	0.09	6.70E-01	-0.25	2.05E-01
ENSCAFG0000003190	ZNF790	red	VSMC_M1	0.41	3.33E-02	0.17	4.00E-01	0.20	3.26E-01	-0.14	4.83E-01	-0.04	8.32E-01	0.15	4.66E-01	-0.10	6.03E-01	0.04	8.44E-01	-0.28	1.57E-01	0.01	9.69E-01
ENSCAFG0000001002	ENSCAFG0000001002	cyan	VSMC_M2	0.41	3.33E-02	0.54	3.85E-03	0.25	2.08E-01	0.31	1.19E-01	-0.13	5.09E-01	0.02	9.28E-01	-0.08	6.87E-01	-0.51	6.35E-03	-0.55	2.96E-03	0.41	3.41E-02
ENSCAFG0000001707	ENSCAFG0000001707	cyan	VSMC_M2	0.41	3.34E-02	0.57	1.73E-03	0.33	9.40E-02	0.16	4.31E-01	-0.22	2.74E-01	0.25	2.15E-01	-0.35	7.04E-02	-0.30	1.34E-01	-0.60	8.80E-04	0.38	5.01E-02
ENSCAFG0000003178	ENSCAFG0000003178	red	VSMC_M1	0.41	3.34E-02	0.18	3.74E-01	0.36	6.39E-02	-0.36	6.56E-02	0.00	9.87E-01	-0.49	9.41E-03	0.08	7.07E-01	0.17	4.08E-01	-0.24	2.35E-01	-0.31	1.16E-01
ENSCAFG0000000279	RAD23B	grey	VSMC_M10	0.41	3.34E-02	0.08	6.78E-01	-0.40	3.76E-02	0.49	1.03E-02	0.48	1.19E-02	-0.08	7.02E-01	0.03	8.88E-01	-0.35	7.73E-02	-0.22	2.60E-01	0.21	2.91E-01
ENSCAFG0000000177	ANXA1	pink	VSMC_M5	0.41	3.34E-02	-0.16	4.19E-01	-0.50	8.18E-03	0.25	2.11E-01	0.66	1.62E-04	0.00	9.82E-01	-0.16	4.23E-01	-0.07	7.13E-01	0.03	8.96E-01	-0.17	3.95E-01
ENSCAFG0000001379	STARD5	grey	VSMC_M10	0.41	3.35E-02	0.09	6.54E-01	0.11	5.90E-01	0.06	7.82E-01	0.05	8.09E-01	-0.19	3.34E-01	0.15	4.55E-01	-0.08	6.95E-01	-0.24	2.27E-01	0.09	6.58E-01
ENSCAFG0000002903	ENSCAFG0000002903	yellow	VSMC_M3	0.41	3.35E-02	0.12	5.62E-01	0.40	3.85E-02	-0.10	6.20E-01	-0.18	3.80E-01	-0.08	6.82E-01	0.09	6.46E-01	0.03	8.69E-01	-0.23	2.40E-01	-0.09	6.71E-01
ENSCAFG0000001581	PADI2	grey	VSMC_M10	0.41	3.37E-02	-0.08	6.85E-01	0.10	6.31E-01	-0.38	4.81E-02	0.16	4.16E-01	0.11	6.01E-01	0.19	3.51E-01	0.31	1.18E-01	-0.11	6.00E-01	-0.33	9.69E-02
ENSCAFG0000001198	ABCB11	grey	VSMC_M10	0.41	3.38E-02	0.52	5.41E-03	0.09	6.60E-01	0.27	1.71E-01	0.03	8.77E-01	-0.20	3.21E-01	-0.27	1.67E-01	-0.37	5.51E-02	-0.54	3.61E-03	0.34	8.04E-02
ENSCAFG0000002094	ENSCAFG0000002094	yellow	VSMC_M3	0.41	3.38E-02	0.45	1.72E-02	0.58	1.36E-03	-0.04	8.60E-01	-0.33	9.60E-02	-0.15	4.65E-01	-0.22	2.75E-01	-0.22	2.73E-01	-0.47	1.43E-02	0.09	6.53E-01
ENSCAFG0000001829	XAB2	grey	VSMC_M10	0.41	3.38E-02	0.48	1.12E-02	0.60	8.96E-04	-0.21	3.04E-01	-0.39	4.70E-02	0.09	6.60E-01	-0.10	6.07E-01	-0.02	9.23E-01	-0.54	3.29E-03	0.06	7.81E-01
ENSCAFG0000003237	PEG10	grey	VSMC_M10	0.41	3.39E-02	0.08	6.78E-01	0.23	2.56E-01	-0.48	1.16E-02	0.00	9.92E-01	0.00	9.88E-01	0.12	5.36E-01	0.44	2.31E-02	-0.25	2.05E-01	-0.20	3.16E-01
ENSCAFG0000000373	ELF2	grey	VSMC_M10	0.41	3.39E-02	-0.02	9.07E-01	-0.28	1.58E-01	0.15	4.63E-01	0.47	1.24E-02	-0.13	5.02E-01	0.22	2.62E-01	-0.10	6.21E-01	-0.07	7.35E-01	-0.01	9.69E-01
ENSCAFG0000000447	CACNB2	grey	VSMC_M10	0.41	3.40E-02	0.00	9.98E-01	-0.21	2.98E-01	0.23	2.43E-01	0.33	9.54E-02	-0.01	9.52E-01	-0.02	9.23E-01	-0.17	3.84E-01	-0.12	5.38E-01	0.16	4.15E-01
ENSCAFG0000000042	ZBTB2	grey	VSMC_M10	0.41	3.40E-02	0.43	2.54E-02	0.23	2.54E-01	-0.19	3.30E-01	-0.14	4.87E-01	-0.05	7.92E-01	0.10	6.05E-01	0.04	8.60E-01	-0.56	2.53E-03	0.29	1.37E-01
ENSCAFG0000000199	CDK6	grey	VSMC_M10	0.41	3.41E-02	0.40	3.86E-02	0.02	9.18E-01	0.53	4.54E-03	0.07	7.29E-01	-0.04	8.59E-01	-0.41	3.50E-02	-0.50	7.28E-03	-0.44	2.20E-02	0.33	9.73E-02
ENSCAFG0000001244	SLC16A9	cyan	VSMC_M2	0.41	3.41E-02	0.38	5.39E-02	0.11	5.83E-01	0.37	5.54E-02	0.05	7.92E-01	-0.18	3.64E-01	0.11	5.74E-01	-0.47	1.43E-02	-0.45	1.89E-02	0.29	1.39E-01
ENSCAFG0000000969	AP2A2	yellow	VSMC_M3	0.41	3.42E-02	0.55	2.80E-03	0.68	9.11E-05	-0.37	5.42E-02	-0.43	2.57E-02	-0.14	4.90E-01	-0.15	4.42E-01	0.07	7.40E-01	-0.51	6.00E-03	0.06	7.57E-01
ENSCAFG0000002922	ZNF770	pink	VSMC_M5	0.41	3.42E-02	-0.25	2.04E-01	-0.41	3.42E-02	0.07	7.10E-01	0.79	7.61E-07	-0.29	1.44E-01	-0.05	8.19E-01	-0.05	8.13E-01	0.19	3.37E-01	-0.48	1.05E-02
ENSCAFG0000003035	ENSCAFG0000003035	red	VSMC_M1	0.41	3.42E-02	0.08	6.81E-01	0.56	2.17E-03	-0.48	1.14E-02	-0.23	2.46E-01	-0.04	8.52E-01	0.05	8.08E-01	0.33	9.14E-02	-0.15	4.50E-01	-0.44	2.21E-02
ENSCAFG0000001197	PARL	pink	VSMC_M5	0.41	3.42E-02	-0.44	2.25E-02	-0.05	8.13E-01	-0.23	2.52E-01	0.43	2.38E-02	-0.12	5.60E-01	-0.10	6.25E-01	0.37	6.06E-02	0.32	1.07E-01	-0.75	7.31E-06
ENSCAFG0000001340	YBX3	grey	VSMC_M10	0.41	3.42E-02	-0.07	7.41E-01	0.49	1.03E-02	-0.71	3.86E-05	-0.12	5.60E-01	0.00	9.82E-01	0.05	8.16E-01	0.52	5.97E-03	-0.03	9.01E-01	-0.57	2.02E-03
ENSCAFG0000001416	UBE2E3	pink	VSMC_M5	0.41	3.44E-02	0.11	5.74E-01	-0.45	1.98E-02	0.50	8.07E-03	0.57	1.73E-03	-0.16	4.37E-01	-0.37	5.82E-02	-0.37	5.57E-02	-0.19	3.30E-01	0.13	5.08E-01
ENSCAFG0000000371	ZYG11B	grey	VSMC_M10	0.41	3.46E-02	0.14	4.82E-01	-0.17	3.85E-01	0.10	6.06E-01	0.44	2.23E-02	-0.11	5.69E-01	0.18	3.81E-01	-0.16	4.30E-01	-0.23	2.56E-01	-0.07	7.21E-01
ENSCAFG0000000339	GRHL1	grey	VSMC_M10	0.41	3.48E-02	0.22	2.71E-01	0.15	4.56E-01	-0.15	4.55E-01	0.03	8.95E-01	0.09	6.59E-01	0.06	7.65E-01	0.02	9.03E-01	-0.36	6.14E-02	0.02	9.10E-01
ENSCAFG0000000192	ENSCAFG0000000192	red	VSMC_M1	0.41	3.48E-02	0.24	2.18E-01	0.21	2.95E-01	-0.04	8.50E-01	-0.06	7.61E-01	-0.08	6.76E-01	-0.20	3.22E-01	-0.08	6.86E-01	-0.28	1.59E-01	0.05	8.11E-01
ENSCAFG0000003021	SDC4	cyan	VSMC_M2	0.41	3.48E-02	0.45	1.80E-02	0.53	4.62E-03	-0.21	3.04E-01	-0.36	6.52E-02	0.02	9.30E-01	-0.19	3.44E-01	0.06	7.48E-01	-0.51	7.09E-03	0.09	6.48E-01
ENSCAFG0000001212	GRK5	cyan	VSMC_M2	0.41	3.48E-02	0.59	1.22E-03	0.38	5.10E-02	-0.17	3.94E-01	-0.21	2.95E-01	-0.06	7.70E-01	0.16	4.21E-01	-0.07	7.16E-01	-0.67	1.24E-04	0.27	1.72E-01
ENSCAFG0000000558	AP3M2	grey	VSMC_M10	0.41	3.49E-02	0.47	1.26E-02	-0.09	6.38E-01	0.32	1.05E-01	0.15	4.52E-01	-0.11	5.79E-01	-0.16	4.21E-01	-0.45	1.85E-02	-0.53	4.23E-03	0.51	6.32E-03
ENSCAFG0000000777	CGGBP1	pink	VSMC_M5	0.41	3.49E-02	-0.12	5.51E-01	-0.30	1.28E-01	0.10	6.09E-01	0.62	5.44E-04	-0.07	7.22E-01	0.12	5.66E-01	-0.09	6.70E-01	0.04	8.35E-01	-0.32	1.09E-01
ENSCAFG0000000412	RUVBL1	darkgrey	VSMC_M8	0.41	3.51E-02	-0.22	2.80E-01	0.52	5.14E-03	-0.71	3.32E-05	-0.19	3.45E-01	0.02	9.10E-01	0.21	3.02E-01	0.73	1.52E-05	-0.01	9.68E-01	-0.62	5.46E-04
ENSCAFG0000001017	KDM1B	yellow	VSMC_M3	0.41	3.52E-02	0.44	2.33E-02	0.71	3.51E-05	-0.52	5.73E-03	-0.46	1.48E-02	-0.18	3.68E-01	0.10	6.11E-01	0.33	9.57E-02	-0.58	1.49E-03	-0.01	9.57E-01
ENSCAFG0000000013	TXNL1	darkgrey	VSMC_M8	0.41	3.52E-02	-0.39	4.53E-02	0.11	5.76E-01	-0.54	3.34E-03	0.30	1.25E-01	-0.27	1.81E-01	-0.02	9.07E-01	0.59	1.14E-03	0.24	2.26E-01	-0.82	1.25E-07
ENSCAFG0000002410	CHST8	cyan	VSMC_M2	0.41	3.52E-02	0.45	1.95E-02	0.20	3.12E-01	0.34	8.76E-02	-0.07	7.15E-01	0.01	9.52E-01	-0.11	5.83E-01	-0.40	3.70E-02	-0.52	5.71E-03	0.33	8.93E-02
ENSCAFG0000001600	KRT10	yellow	VSMC_M3	0.41	3.53E-02	0.14	4.87E-01	0.40	3.91E-02	-0.47	1.42E-02	-0.09	6.62E-01	0.01	9.57E-01	-0.05	7.88E-01	0.34	8.31E-02	-0.26	1.83E-01	-0.38	4.84E-02
ENSCAFG0000000931	TMEM45A	grey	VSMC_M10	0.41	3.53E-02	-0.02	9.38E-01	0.24	2.21E-01	-0.29	1.48E-01	0.10	6.20E-01	-0.37	5.63E-02	0.33	9.16E-02	0.22	2.62E-01	-0.11	5.94E-01	-0.35	6.92E-02
ENSCAFG0000002010	ALG14	red	VSMC_M1	0.41	3.53E-02	0.27	1.73E-01	0.08	7.06E-01	0.05	7.90E-01	-0.06	7.84E-01	0.08	6.91E-01	0.28	1.53E-01	-0.04	8.45E-01	-0.49	9.38E-03	0.38	5.39E-02
ENSCAFG0000003193	SLC35C1	grey	VSMC_M10	0.41	3.55E-02	0.35	7.12E-02	-0.11	5.98E-01	0.08	7.10E-01	0.24	2.32E-01	-0.16	4.36E-01	0.11	5.99E-01	-0.20	3.08E-01	-0.43	2.53E-02	0.24	2.33E-01
ENSCAFG0000000452	MAP3K2	pink	VSMC_M5	0.41	3.55E-02	-0.41	3.46E-02	-0.18	3.62E-01	-0.07	7.27E-01	0.48	1.12E-02	-0.23	2.48E-01	-0.26	1.82E-01	0.22	2.81E-01	0.29	1.45E-01	-0.53	4.70E-03
ENSCAFG0000001474	NAA40	grey	VSMC_M10	0.41	3.55E-02	0.00	9.99E-01	0.24	2.34E-01	-0.41	3.14E-02	-0.08	7.05E-01	0.07	7.44E-01	0.51	6.62E-03	0.36	6.39E-02	-0.23	2.42E-01	-0.10	6.30E-01
ENSCAFG0000000652	ZNF382	grey	VSMC_M10	0.41	3.55E-02	0.07	7.31E-01	-0.49	1.00E-02	0.48	1.21E-02	0.59	1.31E-03	-0.05	8.04E-01	-0.14	4.99E-01	-0.42	2.79E-02	-0.19	3.50E-01	0.21	2.88E-01
ENSCAFG0000001587	TSPAN14	turquoise	VSMC_M6	0.41	3.56E-02	0.25	2.15E-01	0.37	5.42E-02	-0.20	3.21E-01	-0.27	1.66E-01	0.61	7.84E-04	-0.11	5.86E-01	0.13	5.27E-01	-0.37	6.10E-02	0.08	6.78E-01
ENSCAFG0000002532	ENSCAFG0000002532	grey	VSMC_M10	0.41	3.57E-02	0.00	9.87E-01	-0.03	8.79E-01	-0.13	5.09E-01	0.29	1.43E-01	-0.11	5.72E-01	-0.34	8.38E-02	0.16	4.30E-01	-0.07	7.39E-01	-0.30	1.29E-01
ENSCAFG0000002001	ATMIN	cyan	VSMC_M2	0.41	3.57E-02	0.50	7.24E-03	-0.15	4.64E-01	0.34	8.71E-02	0.25	2.00E-01	-0.06	7.79E-01	-0.27	1.78E-01	-0.44	2.17E-02	-0.51	6.36E-03	0.40	4.04E-02
ENSCAFG0000001011	TSPAN32	pink	VSMC_M5	0.41	3.57E-02	-0.24	2.34E-01	-0.42	2.82E-02	0.12	5.62E-01	0.69	6.27E-05	-0.24	2.36E-01	0.27	1.74E-01	0.03	8.75E-01	0.04	8.39E-01	-0.32	1.06E-01
ENSCAFG0000000710	PDE4D	yellow	VSMC_M3	0.41	3.57E-02	0.27	1.66E-01	0.39	4.69E-02	-0.31	1.16E-01	-0.03	8.65E-01	-0.25	2.14E-01	-0.36	6.33E-02	0.10	6.33E-01	-0.28	1.61E-01	-0.24	2.37E-01
ENSCAFG0000000097	TAPBP	cyan	VSMC_M2	0.41	3.58E-02	0.89	4.67E-10	0.38	5.06E-02	0.16	4.17E-01	-0.22	2.59E-01	-0.19	3.36E-01	-0.44	2.09E-02	-0.47	1.34E-02	-0.83	1.02E-07	0.47	1.24E-02
ENSCAFG0000002907	ZNF614	red	VSMC_M1	0.41	3.58E-02	0.15	4.62E-01	-0.12	5.48E-01	0.18	3.56E-01	0.34	7.91E-02	0.00	9.92E-01	-0.11	5.75E-01	-0.25	2.03E-01	-0.16	4.17E-01	-0.06	7.57E-01
ENSCAFG0000002228	ENSCAFG0000002228	red	VSMC_M1	0.41	3.61E-02	0.29	1.44E-01	0.44	2.05E-02	-0.15	4.47E-01	-0.30	1.29E-01	0.12	5.41E-01	0.04	8.24E-01	0.13	5.21E-01	-0.47	1.37E-02	0.14	4.83E-01
ENSCAFG0000001525	FAM129C	yellow	VSMC_M3	0.41	3.61E-02	0.61	6.94E-04	0.64	3.41E-04	-0.06	7.79E-01	-0.40	3.63E-02	-0.15	4.44E-01	-0.25	2.17E-01	-0.18	3.76E-01	-0.62	5.25E-04	0.17	3.91E-01
ENSCAFG0000001054	RNPEP	cyan	VSMC_M2	0.40	3.63E-02	0.64	3.30E-04	0.24	2.23E-01	0.25	2.13E-01	-0.14	4.96E-01	-0.16	4.15E-01	-0.03	9.00E-01	-0.39	4.23E-02	-0.68	8.39E-05	0.50	7.71E-03
ENSCAFG0000000340	ADD2	grey	VSMC_M10	0.40	3.64E-02	0.30	1.26E-01	-0.01	9.44E-01	0.51	7.20E-03	0.10	6.10E-01	0.00	9.88E-01	-0.08	6.81E-01	-0.51	6.08E-03	-0.40	3.74E-02	0.35	7.39E-02
ENSCAFG0000003052	RPS19BP1	grey	VSMC_M10	0.40	3.64E-02	-0.34	7.87E-02	0.31	1.10E-01	-0.47	1.29E-02	0.03	9.00E-01	-0.06	7.56E-01	0.16	4.15E-01	0.47	1.29E-02	0.19	3.32E-01	-0.59	1.16E-03
ENSCAFG0000001271	EXTL1	yellow	VSMC_M3	0.40	3.64E-02	0.35	7.51E-02	0.53	4.16E-03	-0.18	3.68E-01	-0.28	1.51E-01	-0.18	3.61E-01	0.13	5.08E-01	0.02	9.21E-01	-0.43	2.40E-02	0.03	8.85E-01
ENSCAFG0000001017	PHKG1	cyan	VSMC_M2	0.40	3.65E-02	0.60	1.03E-03	0.22	2.67E-01	0.26	1.99E-01	-0.10	6.34E-01	0.02	9.40E-01	-0.25	2.09E-01	-0.41	3.15E-02	-0.57	2.08E-03	0.39	4.51E-02
ENSCAFG0000001249	SCNM1	pink	VSMC_M5	0.40	3.65E-02	-0.21	2.91E-01	-0.20	3.07E-01	0.14	4.98E-01	0.53	4.24E-03	-0.34	8.55E-02	-0.06	7.83E-01	-0.08	7.09E-01	0.14	4.74E-01	-0.39	4.44E-02
ENSCAFG0000000918	RAPGEF3	grey	VSMC_M10	0.40	3.67E-02	0.09	6.63E-01	0.36	6.14E-02	-0.32	9.96E-02	-0.19	3.54E-01	0.41	3.33E-02	0.05	8.08E-01	0.27	1.67E-01	-0.21	3.04E-01	-0.15	4.62E-01
ENSCAFG0000000549	CMC1	grey	VSMC_M10	0.40	3.67E-02	0.32	9.91E-02	0.21	2.96E-01	0.16	4.33E-01	-0.04	8.41E-01	-0.40	3.92E-02	-0.15	4.50E-01	-0.26	1.83E-01	-0.32	1.00E-01	0.12	5.42E-01
ENSCAFG0000002519	CASP8	grey	VSMC_M10	0.40	3.67E-02	-0.18	3.81E-01	0.17	3.98E-01	-0.41	3.44E-02	0.08	6.88E-01	-0.07	7.29E-01	0.68	9.79E-05	0.38	4.83E-02	-0.07	7.44E-01	-0.29	1.38E-01
ENSCAFG0000000110	PTPRK	cyan	VSMC_M2	0.40	3.69E-02	0.70	4.38E-05	0.01	9.75E-01	0.42	3.06E-02	0.11	5.85E-01	-0.21	3.03E-01	-0.68	8.12E-05	-0.59	1.33E-03	-0.64	3.38E-04	0.45	1.93E-02
ENSCAFG0000000490	CLNS1A	grey	VSMC_M10	0.40	3.69E-02	0.27	1.77E-01	0.52	5.52E-03	-0.28	1.53E-01	-0.42	3.13E-02	-0.12	5.56E-01	0.12	5.50E-01	0.17	3.99E-01	-0.44	2.05E-02	0.15	4.58E-01
ENSCAFG0000000879	DUSP11	pink	VSMC_M5	0.40	3.70E-02	-0.13	5.10E-01	-0.39	4.22E-02	0.06	7.79E-01	0.64	3.47E-04	-0.18	3.81E-01	-0.02	9.13E-01	-0.02	9.40E-01	0.03	8.72E-01	-0.25	2.08E-01
ENSCAFG0000000183	DNPH1	yellow	VSMC_M3	0.40	3.70E-02	0.29	1.49E-01	0.72	2.31E-05	-0.59	1.30E-03	-0.50	7.81E-03	0.11	5.84E-01	0.28	1.61E-01	0.41	3.24E-02	-0.49	9.64E-03	-0.11	5.79E-01

ENSCAFG0000000591	ITGBL1	cyan	VSMC_M2	0.40	3.71E-02	0.82	1.96E-07	0.22	2.81E-01	0.41	3.48E-02	-0.09	6.65E-01	-0.26	1.85E-01	-0.41	3.40E-02	-0.57	1.72E-03	-0.80	4.30E-07	0.51	6.73E-03
ENSCAFG0000001398	RALGPS2	pink	VSMC_M5	0.40	3.71E-02	-0.34	8.30E-02	-0.45	1.73E-02	0.06	7.62E-01	0.66	1.77E-04	0.07	7.26E-01	-0.03	8.68E-01	0.14	4.71E-01	0.16	4.24E-01	-0.31	1.18E-01
ENSCAFG0000000437	SOX9	grey	VSMC_M1C	0.40	3.71E-02	-0.05	8.23E-01	0.23	2.58E-01	-0.49	8.73E-03	0.10	6.03E-01	-0.09	6.43E-01	0.31	1.21E-01	0.30	1.22E-01	-0.05	8.20E-01	-0.34	7.86E-02
ENSCAFG0000002340	MPP3	grey	VSMC_M1C	0.40	3.73E-02	0.26	1.94E-01	-0.07	7.47E-01	0.22	2.77E-01	0.31	1.11E-01	-0.29	1.41E-01	-0.35	7.24E-02	-0.33	9.18E-02	-0.22	2.69E-01	0.02	9.35E-01
ENSCAFG0000001668	CTBP1	yellow	VSMC_M3	0.40	3.74E-02	0.71	3.71E-05	0.80	7.16E-07	-0.35	7.29E-02	-0.61	6.54E-04	0.00	9.95E-01	-0.13	5.26E-01	0.04	8.61E-01	-0.76	3.50E-06	0.25	2.13E-01
ENSCAFG0000001684	PAQR6	cyan	VSMC_M2	0.40	3.74E-02	0.81	3.33E-07	0.43	2.39E-02	0.27	1.72E-01	-0.40	3.97E-02	-0.02	9.19E-01	-0.22	2.80E-01	-0.51	7.19E-03	-0.78	1.43E-06	0.67	1.26E-04
ENSCAFG0000002873	ABHD10	grey	VSMC_M1C	0.40	3.75E-02	0.34	8.20E-02	-0.02	9.36E-01	0.38	5.10E-02	-0.01	9.50E-01	0.07	7.11E-01	-0.03	8.85E-01	-0.39	4.69E-02	-0.46	1.47E-02	0.55	3.18E-03
ENSCAFG0000001596	RNASEK	cyan	VSMC_M2	0.40	3.76E-02	0.54	3.75E-03	0.45	1.82E-02	-0.09	6.73E-01	-0.28	1.58E-01	0.15	4.54E-01	-0.32	1.01E-01	-0.18	3.56E-01	-0.52	5.01E-03	0.17	3.93E-01
ENSCAFG0000000881	ENSCAFG0000000881	red	VSMC_M1	0.40	3.77E-02	0.17	3.86E-01	0.03	8.68E-01	0.21	2.89E-01	0.12	5.43E-01	0.04	8.47E-01	0.15	4.64E-01	-0.23	2.58E-01	-0.28	1.58E-01	0.06	7.49E-01
ENSCAFG0000001495	RPL22L1	darkgrey	VSMC_M8	0.40	3.79E-02	-0.21	3.00E-01	0.39	4.58E-02	-0.76	3.70E-06	0.00	9.81E-01	-0.15	4.68E-01	0.03	8.97E-01	0.71	2.84E-05	0.03	8.78E-01	-0.72	2.31E-05
ENSCAFG0000001843	RAI1	grey	VSMC_M1C	0.40	3.81E-02	0.32	1.03E-01	0.10	6.21E-01	0.03	8.86E-01	0.00	9.95E-01	0.06	7.62E-01	-0.09	6.46E-01	-0.17	4.06E-01	-0.39	4.54E-02	0.28	1.52E-01
ENSCAFG0000001189	DCAF11	cyan	VSMC_M2	0.40	3.81E-02	0.66	1.77E-04	0.68	8.58E-05	-0.17	3.84E-01	-0.47	1.39E-02	-0.03	8.64E-01	0.19	3.51E-01	-0.17	4.02E-01	-0.69	7.78E-05	0.30	1.30E-01
ENSCAFG0000000189	PTAR1	grey	VSMC_M1C	0.40	3.83E-02	0.12	5.59E-01	-0.37	5.82E-02	0.48	1.13E-02	0.38	5.04E-02	-0.01	9.55E-01	0.07	7.44E-01	-0.38	5.16E-02	-0.27	1.78E-01	0.37	5.99E-02
ENSCAFG0000001466	ENSCAFG0000001466	red	VSMC_M1	0.40	3.84E-02	0.19	3.39E-01	0.12	5.41E-01	0.06	7.65E-01	0.10	6.29E-01	-0.02	9.02E-01	-0.25	2.12E-01	-0.14	4.88E-01	-0.24	2.32E-01	-0.08	7.00E-01
ENSCAFG0000000862	STBD1	cyan	VSMC_M2	0.40	3.84E-02	0.33	9.29E-02	-0.05	8.22E-01	0.52	5.35E-03	0.10	6.19E-01	-0.11	5.78E-01	-0.14	4.83E-01	-0.56	2.38E-03	-0.34	8.56E-02	0.43	2.53E-02
ENSCAFG0000003186	ENSCAFG0000003186	red	VSMC_M1	0.40	3.84E-02	-0.35	7.77E-02	-0.11	5.76E-01	-0.06	7.71E-01	0.33	9.44E-02	0.21	2.92E-01	0.11	5.96E-01	0.16	4.15E-01	0.15	4.42E-01	-0.38	4.87E-02
ENSCAFG0000002819	ENSCAFG0000002819	grey	VSMC_M1C	0.40	3.84E-02	0.55	2.96E-03	0.55	3.22E-03	0.02	9.10E-01	-0.42	2.73E-02	-0.19	3.53E-01	-0.12	5.54E-01	-0.21	2.86E-01	-0.59	1.26E-03	0.33	9.63E-02
ENSCAFG0000001706	INO80E	red	VSMC_M1	0.40	3.84E-02	0.09	6.54E-01	0.39	4.24E-02	-0.36	6.41E-02	-0.28	1.62E-01	0.36	6.17E-02	-0.01	9.79E-01	0.30	1.22E-01	-0.23	2.39E-01	-0.07	7.40E-01
ENSCAFG0000001277	CTBP2	yellow	VSMC_M3	0.40	3.84E-02	0.48	1.12E-02	0.56	2.40E-03	-0.43	2.64E-02	-0.41	3.31E-02	0.15	4.60E-01	-0.10	6.32E-01	0.18	3.68E-01	-0.57	1.90E-03	0.17	4.01E-01
ENSCAFG0000001444	PPP1R35	yellow	VSMC_M3	0.40	3.86E-02	0.56	2.34E-03	0.68	1.03E-04	-0.23	2.55E-01	-0.53	4.07E-03	0.02	9.11E-01	0.27	1.77E-01	-0.01	9.43E-01	-0.67	1.22E-04	0.29	1.40E-01
ENSCAFG0000001770	OTOA	grey	VSMC_M1C	0.40	3.86E-02	0.30	1.23E-01	0.03	8.87E-01	0.20	3.22E-01	0.15	4.65E-01	-0.27	1.79E-01	-0.14	4.95E-01	-0.23	2.38E-01	-0.36	6.76E-02	0.17	3.97E-01
ENSCAFG0000000430	TRIM13	cyan	VSMC_M2	0.40	3.86E-02	0.45	1.92E-02	-0.02	9.04E-01	0.39	4.40E-02	0.08	6.74E-01	-0.07	7.28E-01	-0.19	3.39E-01	-0.47	1.37E-02	-0.52	5.92E-03	0.41	3.24E-02
ENSCAFG0000001004	ENSCAFG0000001004	grey	VSMC_M1C	0.40	3.86E-02	0.11	5.96E-01	0.31	1.12E-01	-0.17	4.06E-01	-0.09	6.38E-01	-0.05	8.14E-01	0.26	1.87E-01	0.09	6.41E-01	-0.26	1.87E-01	-0.10	6.12E-01
ENSCAFG0000001467	ENSCAFG0000001467	grey	VSMC_M1C	0.40	3.87E-02	-0.09	6.65E-01	-0.01	9.45E-01	-0.17	3.89E-01	0.27	1.69E-01	0.22	2.64E-01	-0.51	7.18E-03	0.17	3.98E-01	0.06	7.80E-01	-0.38	5.13E-02
ENSCAFG0000001997	ENSCAFG0000001997	red	VSMC_M1	0.40	3.87E-02	0.11	5.87E-01	-0.04	8.50E-01	-0.01	9.60E-01	0.32	1.09E-01	-0.16	4.39E-01	-0.34	8.43E-02	-0.07	7.14E-01	-0.09	6.51E-01	-0.29	1.44E-01
ENSCAFG0000001469	CRLF1	violet	VSMC_M7	0.40	3.87E-02	-0.20	3.14E-01	0.05	7.98E-01	-0.25	2.13E-01	0.16	4.15E-01	-0.10	6.13E-01	0.67	1.24E-04	0.30	1.24E-01	-0.03	8.99E-01	-0.23	2.56E-01
ENSCAFG0000001444	TRMT112	yellow	VSMC_M3	0.40	3.87E-02	0.31	1.13E-01	0.74	1.04E-05	-0.52	5.83E-03	-0.56	2.50E-03	-0.03	8.74E-01	0.21	2.87E-01	0.38	4.89E-02	-0.50	7.30E-03	-0.07	7.45E-01
ENSCAFG0000000249	ANKS6	grey	VSMC_M1C	0.40	3.88E-02	0.35	7.50E-02	0.41	3.19E-02	-0.07	7.23E-01	-0.29	1.36E-01	-0.12	5.67E-01	-0.24	2.26E-01	-0.06	7.63E-01	-0.40	4.13E-02	0.18	3.67E-01
ENSCAFG0000001426	UBTF	yellow	VSMC_M3	0.40	3.88E-02	0.04	8.50E-01	0.66	1.58E-04	-0.50	8.05E-03	-0.36	6.61E-02	-0.10	6.21E-01	-0.03	8.89E-01	0.45	1.88E-02	-0.17	3.99E-01	-0.40	3.91E-02
ENSCAFG0000002920	ENSCAFG0000002920	grey	VSMC_M1C	0.40	3.89E-02	0.10	6.37E-01	-0.01	9.45E-01	0.26	1.94E-01	0.15	4.43E-01	-0.02	9.32E-01	-0.14	4.96E-01	-0.24	2.35E-01	-0.14	4.78E-01	0.09	6.63E-01
ENSCAFG0000001445	NDP	yellow	VSMC_M3	0.40	3.89E-02	0.17	3.87E-01	0.51	6.44E-03	-0.52	5.70E-03	-0.18	3.61E-01	-0.20	3.15E-01	0.17	3.94E-01	0.35	7.51E-02	-0.24	2.37E-01	-0.33	8.85E-02
ENSCAFG0000000398	MTPAP	grey	VSMC_M1C	0.40	3.89E-02	-0.16	4.27E-01	-0.39	4.46E-02	0.41	3.35E-02	0.53	4.44E-03	-0.06	7.57E-01	0.04	8.54E-01	-0.30	1.33E-01	0.03	8.71E-01	0.01	9.43E-01
ENSCAFG0000000644	PURG	grey	VSMC_M1C	0.40	3.89E-02	0.24	2.25E-01	0.00	9.93E-01	0.28	1.64E-01	0.12	5.38E-01	-0.41	3.44E-02	0.06	7.55E-01	-0.39	4.52E-02	-0.30	1.27E-01	0.32	1.00E-01
ENSCAFG0000001690	TLN2	grey	VSMC_M1C	0.40	3.89E-02	0.10	6.26E-01	0.30	1.31E-01	-0.35	7.09E-02	-0.10	6.28E-01	-0.09	6.45E-01	0.61	7.83E-04	0.30	1.29E-01	-0.34	8.27E-02	-0.08	7.00E-01
ENSCAFG0000001365	TRIM69	pink	VSMC_M5	0.40	3.89E-02	-0.15	4.57E-01	-0.42	2.96E-02	0.17	3.84E-01	0.61	7.07E-04	-0.31	1.16E-01	-0.10	6.06E-01	-0.03	8.94E-01	0.04	8.42E-01	-0.14	4.72E-01
ENSCAFG0000001204	PCNT	yellow	VSMC_M3	0.40	3.90E-02	0.51	7.15E-03	0.72	2.13E-05	-0.36	6.15E-02	-0.54	3.39E-03	0.17	3.89E-01	0.17	3.95E-01	0.15	4.41E-01	-0.65	2.38E-04	0.15	4.44E-01
ENSCAFG0000001569	NSG1	grey	VSMC_M1C	0.40	3.90E-02	0.14	4.93E-01	0.01	9.69E-01	0.18	3.71E-01	0.24	2.24E-01	-0.09	6.49E-01	-0.20	3.28E-01	-0.15	4.68E-01	-0.15	4.42E-01	-0.13	5.07E-01
ENSCAFG0000000326	COLEC11	cyan	VSMC_M2	0.40	3.90E-02	0.74	1.11E-05	0.52	5.33E-03	-0.07	7.30E-01	-0.36	6.52E-02	-0.05	7.99E-01	-0.39	4.69E-02	-0.24	2.31E-01	-0.72	2.28E-05	0.36	6.50E-02
ENSCAFG0000001755	S100A5	yellow	VSMC_M3	0.40	3.91E-02	0.53	4.23E-03	0.65	2.70E-04	-0.24	2.21E-01	-0.45	1.86E-02	-0.25	2.09E-01	-0.03	8.94E-01	0.02	9.10E-01	-0.60	9.72E-04	0.16	4.29E-01
ENSCAFG0000001469	MLF2	yellow	VSMC_M3	0.40	3.92E-02	0.47	1.26E-02	0.65	2.24E-04	-0.35	7.21E-02	-0.52	5.36E-03	0.09	6.68E-01	-0.09	6.38E-01	0.17	3.84E-01	-0.56	2.29E-03	0.19	3.32E-01
ENSCAFG0000001660	SLIT2	cyan	VSMC_M2	0.40	3.93E-02	0.70	5.04E-05	0.28	1.63E-01	0.21	2.97E-01	-0.25	2.04E-01	0.00	9.84E-01	-0.42	2.96E-02	-0.34	7.81E-02	-0.75	6.04E-06	0.53	4.81E-03
ENSCAFG0000003233	ENSCAFG0000003233	grey	VSMC_M1C	0.40	3.94E-02	0.01	9.50E-01	-0.15	4.59E-01	0.19	3.56E-01	0.38	4.91E-02	-0.30	1.28E-01	0.30	1.30E-01	-0.19	3.51E-01	-0.13	5.11E-01	-0.06	7.65E-01
ENSCAFG0000001191	CHEK2	grey	VSMC_M1C	0.40	3.94E-02	0.03	8.76E-01	0.40	4.13E-02	-0.49	1.01E-02	-0.25	2.04E-01	0.19	3.41E-01	0.53	4.44E-03	0.45	1.72E-02	-0.30	1.24E-01	-0.08	6.89E-01
ENSCAFG0000003198	ENSCAFG0000003198	yellow	VSMC_M3	0.40	3.94E-02	0.37	5.65E-02	0.61	6.92E-04	-0.13	5.24E-01	-0.37	6.11E-02	-0.04	8.25E-01	0.16	4.16E-01	-0.06	7.52E-01	-0.41	3.14E-02	0.04	8.54E-01
ENSCAFG0000002975	PYURF	grey	VSMC_M1C	0.40	3.95E-02	0.01	9.54E-01	0.40	3.88E-02	-0.65	2.75E-04	-0.06	7.66E-01	-0.12	5.61E-01	0.29	1.50E-01	0.45	1.86E-02	-0.12	5.57E-01	-0.41	3.54E-02
ENSCAFG0000001792	C20H19orf66	yellow	VSMC_M3	0.40	3.95E-02	0.51	6.21E-03	0.74	9.15E-06	-0.35	7.04E-02	-0.58	1.65E-03	0.31	1.18E-01	-0.08	6.76E-01	0.09	6.65E-01	-0.58	1.39E-03	0.16	4.36E-01
ENSCAFG0000000128	LY6E	grey	VSMC_M1C	0.40	3.96E-02	0.40	3.82E-02	0.07	7.13E-01	0.41	3.45E-02	0.00	9.83E-01	-0.02	9.32E-01	-0.24	2.25E-01	-0.45	1.79E-02	-0.50	8.45E-03	0.39	4.23E-02
ENSCAFG0000000428	FAM185A	red	VSMC_M1	0.40	3.96E-02	0.08	6.94E-01	0.10	6.29E-01	-0.30	1.35E-01	0.23	2.46E-01	-0.10	6.31E-01	0.10	6.15E-01	0.15	4.48E-01	-0.15	4.67E-01	-0.32	1.08E-01
ENSCAFG0000001838	PLD4	grey	VSMC_M1C	0.40	3.97E-02	0.04	8.30E-01	0.30	1.24E-01	-0.31	1.12E-01	-0.08	6.95E-01	-0.27	1.74E-01	0.00	9.97E-01	0.29	1.40E-01	-0.18	3.77E-01	-0.17	3.99E-01
ENSCAFG0000000835	PHF20	grey	VSMC_M1C	0.40	3.97E-02	0.32	1.03E-01	-0.30	1.34E-01	0.53	4.89E-03	0.50	8.07E-03	-0.33	9.12E-02	-0.08	6.90E-01	-0.51	6.99E-03	-0.38	5.16E-02	0.16	4.24E-01
ENSCAFG0000000040	DYRK2	red	VSMC_M1	0.40	3.98E-02	-0.04	8.55E-01	0.45	1.89E-02	-0.60	1.05E-03	-0.08	6.95E-01	-0.22	2.66E-01	-0.17	4.01E-01	0.42	2.88E-02	-0.01	9.70E-01	-0.52	5.93E-03
ENSCAFG0000002333	JUND	yellow	VSMC_M3	0.40	3.98E-02	0.51	6.29E-03	0.69	6.23E-05	-0.28	1.59E-01	-0.42	3.05E-02	-0.19	3.48E-01	-0.03	8.98E-01	-0.05	7.91E-01	-0.49	9.78E-03	0.05	8.11E-01
ENSCAFG0000000888	TLL1	red	VSMC_M1	0.40	3.98E-02	0.04	8.40E-01	0.14	4.93E-01	-0.27	1.67E-01	0.06	7.82E-01	0.16	4.19E-01	-0.33	8.98E-02	0.23	2.54E-01	-0.14	4.95E-01	-0.23	2.41E-01
ENSCAFG000000016	MEX3C	pink	VSMC_M5	0.40	3.99E-02	-0.01	9.76E-01	-0.48	1.18E-02	0.32	1.05E-01	0.64	3.70E-04	-0.04	8.36E-01	-0.32	1.08E-01	-0.28	1.64E-01	-0.07	7.30E-01	-0.03	8.99E-01
ENSCAFG0000001979	UBE4B	red	VSMC_M1	0.40	3.99E-02	0.18	3.70E-01	0.05	8.10E-01	-0.11	5.93E-01	0.24	2.29E-01	-0.14	5.00E-01	0.14	4.83E-01	-0.08	6.90E-01	-0.23	2.55E-01	-0.02	9.38E-01
ENSCAFG0000001455	DHX8	grey	VSMC_M1C	0.40	4.00E-02	-0.31	1.22E-01	0.16	4.35E-01	-0.42	2.88E-02	0.18	3.79E-01	0.15	4.63E-01	-0.14	4.75E-01	0.47	1.42E-02	0.16	4.11E-01	-0.63	4.38E-04
ENSCAFG0000001766	PTRH2	darkgrey	VSMC_M8	0.40	4.00E-02	-0.29	1.43E-01	0.11	5.99E-01	-0.60	9.52E-04	0.17	3.91E-01	0.08	6.86E-01	-0.08	6.82E-01	0.62	5.26E-04	0.10	6.16E-01	-0.59	1.09E-03
ENSCAFG0000001075	QTRT2	grey	VSMC_M1C	0.40	4.01E-02	-0.08	7.01E-01	0.18	3.63E-01	-0.38	4.96E-02	-0.04	8.46E-01	0.18	3.64E-01	0.19	3.35E-01	0.42	3.08E-02	-0.14	4.79E-01	-0.19	3.40E-01
ENSCAFG0000000245	XPA	red	VSMC_M1	0.40	4.01E-02	0.41	3.19E-02	0.33	8.81E-02	0.05	8.00E-01	-0.26	1.91E-01	-0.02	9.37E-01	0.19	3.43E-01	-0.22	2.62E-01	-0.50	8.54E-03	0.39	4.24E-02
ENSCAFG0000001478	CEP152	grey	VSMC_M1C	0.40	4.03E-02	-0.19	3.40E-01	0.22	2.61E-01	-0.43	2.45E-02	-0.07	7.18E-01	0.20	3.24E-01	0.45	1.81E-02	0.53	4.91E-03	-0.10	6.05E-01	-0.23	2.48E-01
ENSCAFG0000000573	NRG2	red	VSMC_M1	0.40	4.03E-02	-0.12	5.63E-01	-0.17	3.97E-01	0.05	7.90E-01	0.37	5.49E-02	0.05	8.19E-01	0.23	2.52E-01	0.00	9.90E-01	-0.07	7.29E-01	-0.18	3.68E-01
ENSCAFG0000002556	A2M	grey	VSMC_M1C	0.40	4.04E-02	0.27	1.71E-01	-0.17	3.85E-01	0.45	1.90E-02	0.24	2.35E-01	0.10	6.27E-01	-0.57	1.88E-03	-0.42	2.72E-02	-0.32	1.03E-01	0.26	1.95E-01
ENSCAFG0000001653	POLR2A	yellow	VSMC_M3	0.40	4.04E-02	0.38	5.00E-02	0.44	2.31E-02	-0.34	8.39E-02	-0.18	3.69E-01	-0.01	9.55E-01	-0.20	3.22E-01	0.10	6.11E-01	-0.39	4.62E-02	-0.06	7.64E-01
ENSCAFG0000001435	TADA2B	grey	VSMC_M1C	0.40	4.05E-02	0.54	4.03E-03	0.65	2.60E-04	-0.15	4.63E-01	-0.56	2.29E-03	0.12	5.55E-01	-0.22	2.75E-01	0.01	9.71E-01	-0.65	2.40E-04	0.27	1.67E-01
ENSCAFG0000001559	NXF1	grey	VSMC_M1C	0.40	4.05E-02	0.34	8.27E-02	0.24	2.22E-01	-0.13	5.21E-01	-0.02	9.36E-01	-0.22	2.74E-01	0.04	8.49E-01	-0.06	7.64E-01	-0.39	4.49E-02	0.04	8.42E-01
ENSCAFG0000001917	CPT2	cyan	VSMC_M2	0.40	4.06E-02	0.77	2.27E-06	0.29	1.47E-01	0.16	4.27E-01	-0.06	7.67E-01	-0.35	7.03E-02	-0.24	2.32E-01	-0.46	1.49E-02	-0.71	3.50E-05	0.35	7.61E-02
ENSCAFG0000000644	LTA4H	red	VSMC_M1	0.40	4.06E-02	0.40	3.71E-02	0.30	1.33E-01	-0.07	7.27E-01	-0.15	4.54E-01	-0.25	2.12E-01	0.26	1.97E-01	-0.11	5.68E-01	-0.47	1.44E-02	0.25	2.16E-01
ENSCAFG0000002009	LDHD	cyan	VSMC_M2	0.40	4.07E-02	0.76	4.75E-06	0.27	1.71E-01	0.17	3.95E-01	-0.18	3.62E-01	0.02	9.40E-01	-0.04	8.40E-01	-0.38	4.98E-02	-0.81	3.94E-07	0.54	3.84E-03
ENSCAFG0000001086	GPAM	grey	VSMC_M1C	0.40	4.08E-02	0.26	1.97E-01	-0.13	5.07E-01	0.33	9.52E-02	0.20	3.17E-01	-0.01	9.58E-01	0.26	1.90E-01	-0.32	1.02E-01	-0.43	2.37E-02	0.35	7.46E-02
ENSCAFG0000000822	ANKRA2	grey	VSMC_M1C	0.40	4.08E-02	-0.11	5.76E-01	-0.35	7.15E-02	0.27	1.72E-01	0.55	3.24E-03	0.07	7.39E-01	-0.38	4.97E-02	-0.22	2.77E-01	0.05	8.16E-01	-0.14	5.00E-01
ENSCAFG0000000667	SYNE4	grey	VSMC_M1C	0.40	4.14E-02	0.26	1.92E-01	-0.07	7.35E-01	0.16	4.33E-01	0.25	2.03E-01	-0.01	9.53E-01	-0.07	7.31E-01	-0.20	3.07E-01	-0.39	4.30E-02	0.06	7.49E-01
ENSCAFG0000000632	KCTD16	red	VSMC_M1	0.40	4.14E-02	0.30	1.28E-01	0.16	4.23E-01	0.15	4.63E-01	0.08	7.00E-01	-0.08	6.85E-01	-0.20	3.12E-01	-0.19	3.41E-01	-0.40	4.09E-02	0.05	7.97E-01
ENSCAFG0000000136	ENSCAFG0000000136	cyan	VSMC_M2	0.40	4.14E-02	0.82	1.23E-07	0.13	5.06E-01	0.38	5.30E-02	-0.01	9.75E-01	-0.14	4.98E-01	-0.37	5.88E-02	-0.59	1.24E-03	-0.75	6.42E-06	0.53	4.31E-03
ENSCAFG0000000545	ENSCAFG0000000545	grey	VSMC_M1C	0.40	4.14E-02	0.40	3.81E-02	0.07	7.24E-01	0.18	3.57E-01	-0.02	9.35E-01	0.15	4.47E-01	0.07	7.17E-01	-0.28	1.52E-01	-0.54	3.96E-03	0.38	4.95E-02
ENSCAFG0000000878	GMPS	grey	VSMC_M1C	0.39	4.14E-02	-0.04	8.26E-01	0.38	5.10E-02	-0.64	3.70E-04	-0.04	8.24E-01	-0.01	9.70E-01	0.24	2.28E-01	0.52	4.96E-03	-0.10	6.31E-01	-0.49	1.02E-02
ENSCAFG0000000710	CAPRIN1	pink	VSMC_M5	0.39	4.15E-02	0.05	8.01E-01	-0.28	1.50E-01	0.12	5.68E-01	0.62	5.23E-04	-0.31	1.10E-01	-0.36	6.67E-02	-0.09	6.67E-01	-0.08	6.89E-01	-0.32	1.01E-01
ENSCAFG0000000360	SMIM12	yellow	VSMC_M3	0.39	4.17E-02	0.07	7.19E-01	0.40	3.97E-02	-0.48	1.18E-02	-0.16	4.26E-01	0.07	7.16E-01	0.21	3.05E-01	0.35	7.24E-02	-0.22	2.62E-01	-0.29	1.44E-01
ENSCAFG0000000150	UBE2H	pink	VSMC_M5	0.39	4.17E-02	0.14	4.97E-01	-0.51	6.29E-03	0.69	6.48E-05	0.66	1.60E-04	-0.23	2.52E-01	-0.27	1.75E-01	-0.65	2.16E-04	-0.13	5.23E-01	0.23	2.54E-01
ENSCAFG0000001440	MPP2	grey	VSMC_M1C	0.39	4.19E-02	0.13	5.14E-01	0.15	4.53E-01	0.03	8.70E-01	0.12	5.38E-01	-0.16	4.24E-01	-0.04	8.61E-01	-0.10	6.18E-01	-0.21	2.95E-01	-0.18	3.68E-01
ENSCAFG0000001993	FAM102B	cyan	VSMC_M2	0.39	4.19E-02	0.58	1.69E-03	0.41	3.54E-02	-0.11	5.80E-01	-0.27	1.81E-01	-0.01	9.72E-01	0.03	8.66E-01	-0.12	5.61E-01	-0.63	4.17E-04	0.30	1.24E-01
ENSCAFG0000001995	NCS1	cyan	VSMC_M2	0.39	4.21E-02	0.37	6.08E-02	0.06	7.60E-01	0.41	3.16E-02	0.04	8.35E-01	-0.19	3.52E-01	-0.27	1.74E-01	-0.46	1.57E-02	-0.38	5.21E-02	0.35	7.49E-02
ENSCAFG0000002973	ENSCAFG0000002973	yellow	VSMC_M3	0.39	4.22E-02	0.44	2.32E-02	0.55	3.17E-03	-0.31	1.21E-01	-0.31	1.16E-01	-0.14	4.76E-01	-0.09	6.61E-01	0.06	7.64E-01	-0.51	6.99E-03	-0.06	7.83E-01
ENSCAFG0000003047	ENSCAFG0000003047	cyan	VSMC_M2	0.39	4.22E-02	0.61	7.94E-04	0.40	3.85E-02	0.00	9.93E-01	-0.22	2.72E-01	-0.25	2.06E-01	-0.30	1.27E-01	-0.24	2.30E-01	-0.61	8.20E-04	0.29	1.42E-01
ENSCAFG0000000439	TMEM135	darkgreen	VSMC_M4	0.39	4.24E-02	0.08	6.76E-01	-0.45	1.73E-02	0.60	9.21E-04	0.54	3.41E-03	-0.06	7.68E-01	-0.01	9.57E-01	-0.54	3.77E-03	-0.14	5.01E-01	0.29	1.44E-01
ENSCAFG0000001213	ENSCAFG0000001213	grey	VSMC_M1C	0.39	4.24E-02	0.01	9.79E-01	0.38	5.24E-02	-0.27	1.74E-01	-0.13	5.25E-01	-0.14	5.00E-01	0.19	3.46E-01	0.17	3.91E-01	-0.08	6.95E-01	-0.24	2.28E-01
ENSCAFG0000000123	GNG5	red	VSMC_M1	0.39	4.24E-02	0.02	9.30E-01	0.04	8.49E-01	0.05	7.90E-01	0.23	2.50E-01	-0.09	6.44E-01	-0.10	6.21E-01	-0.05	7.87E-01	-0.14	4.82E-01	-0.27	1.76E-01
ENSCAFG0000000457	ZNF296	yellow	VSMC_M3	0.39	4.25E-02	0.01	9.70E-01	0.35	7.30E-02	-0.48	1.09E-02	-0.12	5.53E-01	0.18	3.59E-01	0.36	6.78E-02	0.38	5.16E-02	-0.19	3.43E-01	-0.28	1.55E-01
ENSCAFG0000000945	BTG2	pink	VSMC_M5	0.39	4.27E-02	0.32	1.03E-01	-0.27	1.68E-01	0.39	4.21E-02	0.43	2.37E-02	-0.16	4.23E-01	-0.18	3.80E-01	-0.53	4.58E-03	-0.34	8.67E-02	0.23	2.41E-01
ENSCAFG0000001587	ERC1	red	VSMC_M1	0.39	4.27E-02	0.00	9.91E-01	-0.18	3.77E-01	0.06	7.49E-01	0.46	1.65E-02	-0.14	4.73E-01	-0.27	1.68E-01	-0.04	8.40E-01	-0.06	7.81E-01	-0.25	2.04E-01
ENSCAFG0000000725	UBA2	darkgrey	VSMC_M8	0.39	4.28E-02	-0.52	5.58E-03	0.07	7.47E-01	-0.62	5.39E-04	0.26	1.93E-01	0.03	8.95E-01	0.09	6.39E-01	0.75	5.61E-06	0.29	1.43E-01	-0.78	1.48E-06
ENSCAFG0000001927	NAA60	grey	VSMC_M1C	0.39	4.28E-02	0.54	4.00E-03	-0.03	8.78E-01	0.35	7.72E-02	0.15	4.64E-01	0.03	8.74E-01	-0.28	1.61E-01	-0.50	7.26E-03	-0.59	1.19E-03	0.35	6.93E-02
ENSCAFG0000001550	LYSMD2	cyan	VSMC_M2	0.39	4.30E-02	0.51	7.20E-03	0.33	9.76E-02	0.15	4.65E-01	-0.20	3.27E-01	0.21	3.04E-01	0.07	7.39E-01	-0.34	8.66E-02	-0.55	2.76E-03	0.34	7.99E-02
ENSCAFG0000003223	ENSCAFG0000003223	grey	VSMC_M1C	0.39	4.31E-02	0.28	1.62E-01	0.18	3.82E-01	0.16	4.36E-01	-0.11	5.86E-01	-0.07	7.45E-01	-0.02	9.36E-01	-0.20	3.27E-01	-0.37	5.46E-02	0.32	9.95E-02
ENSCAFG0000001321	MUS81	cyan	VSMC_M2	0.39	4.32E-02	0.55	3.03E-03	0.35	7.66E-02	0.01	9.46E-01	-0.26	1.91E-01	-0.04	8.53E-01	0.08	7.10E-01	-0.17	3.87E-01	-0.67	1.17E-04	0.43	2.35E-02
ENSCAFG0000001704	JDP2	grey	VSMC_M1C	0.39	4.32E-02	0.24	2.22E-01	0.10	6.31E-01	0.17	3.85E-01	0.11	6.00E-01	-0.38	5.38E-02	0.37	5.57E-02	-0.24	2.20E-01	-0.37	5.94E-02	0.14	4.74E-01
ENSCAFG0000001851	LAMP2	grey	VSMC_M1C	0.39	4.33E-02	0.59	1.35E-03	-0.28	1.57E-01	0.62	6.29E-04	0.44	2.31E-02	-0.26	1.98E-01	-0.38	5.29E-02	-0.77	2.54E-06	-0.52	5.36E-03	0.42	2.94E-02
ENSCAFG0000001833	INF2	yellow	VSMC_M3	0.39	4.34E-02	0.67	1.52E-04	0.83	7.96E-08	-0.30	1.23E-01	-0.60	9.75E-04	-0.15	4.53E-01	-0.01	9.45E-01	-0.04	8.57E-01	-0.66	1.70E-04	0.19	3.47E-01
ENSCAFG0000001245	CCDC6	grey	VSMC_M1C	0.39	4.35E-02	0.03	8.87E-01	-0.13	5.24E-01	0.06	7.73E-01	0.27	1.75E-01	-0.04	8.46E-01	0.19	3.43E-01	-0.04	8.37E-01	-0.19	3.32E-01	0.01	9.53E-01
ENSCAFG0000001158	FXR1	pink	VSMC_M5	0.39	4.35E-02	-0.05	8.23E-01	-0.35	7.36E-02	0.37	6.11E-02	0.67	1.23E-04	-0.45	1.75E-02	-0.20	3.16E-01	-0.35	7.31E-02	0.07	7.23E-01	-0.23	2.48E-01
ENSCAFG0000000684	LDLRAD3	yellow	VSMC_M3	0.39	4.36E-02	0.30	1.34E-01	0.48	1.05E-02	-0.48	1.12E-02	-0.18	3.79E-01	-0.08	6.85E-01	0.02	9.09E-01	0.31	1.20E-01	-0.37	5.42E-02	-0.24	2.20E-01
ENSCAFG0000003010	EVA1B	yellow	VSMC_M3	0.39	4.37E-02	0.43	2.67E-02	0.61	7.65E-04	-0.33	9.55E-02	-0.36	6.70E-02	-0.17	3.97E-01	0.18	3.69E-01	0.05	8.00E-01	-0.43	2.49E-02	0.04	8.39E-01
ENSCAFG0000000444	TMEM43	yellow	VSMC_M3	0.39	4.38E-02	0.45	1.78E-02	0.70	4.14E-05	-0.57	1.79E-03	-0.50	8.15E-03	-0.05	7.98E-01	0.09	6.53E-01	0.32	1.09E-01	-0.57	2.10E-03	0.01	9.47E-01
ENSCAFG0000000834	SPON1	cyan	VSMC_M2	0.39	4.38E-02	0.57	2.03E-03	0.20	3.08E-01	0.02	9.18E-01	0.00	9.88E-01	-0.23	2.58E-01	-0.20	3.24E-01	-0.33	9.71E-02	-0.55	2.82E-03	0.23	2.46E-01
ENSCAFG0000000419	AP2S1	grey	VSMC_M1C	0.39	4.39E-02	0.34	8.34E-02	0.29	1.43E-01	-0.06	7.55E-01	-0.20	3.07E-01	0.24	2.18E-01	-0.19	3.32E-01	-0.05	8.07E-01	-0.39	4.44E-02	0.16	4.38E-01
ENSCAFG0000001610	WDR19	grey	VSMC_M1C	0.39	4.39E-02	0.46	1.50E-02	-0.12	5.52E-01	0.46	1.50E-02	0.30	1.28E-01	-0.26	1.90E-01	0.01	9.57E-01	-0.57	1.75E-03	-0.51	6.66E-03	0.31	1.18E-01
ENSCAFG0000001219	LDHB	red	VSMC_M1	0.39	4.39E-02	0.07	7.26E-01	0.27	1.77E-01	-0.20	3.17E-01	0.01	9.48E-01	-0.26	1.96E-01	-0.13	5.12E-01	0.17	3.91E-01	-0.12	5.55E-01	-0.28	1.54E-01
ENSCAFG0000000198	AARS2	yellow	VSMC_M3	0.39	4.39E-02	0.09	6.47E-01	0.65	2.56E-04	-0.65	2.75E-04	-0.40	4.02E-02	0.06	7.84E-01	0.30	1.27E-01	0.55	3.18E-03	-0.30	1.29E-01	-0.26	1.87E-01
ENSCAFG0000001108	CD99	grey	VSMC_M1C	0.39	4.40E-02	0.49	8.86E-03	-0.23	2.43E-01	0.69	7.45E-05	0.36	6.87E-02	-0.27	1.70E-01	-0.44	2.01E-02	-0.74	9.09E-06	-0.44	2.04E-02	0.38	4.82E-02
ENSCAFG0000001817	INSR	grey	VSMC_M1C	0.39	4.41E-02	0.29	1.42E-01	0.06	7.77E-01	0.26	1.97E-01	0.15	4.57E-01	-0.07	7.46E-01	-0.39	4.69E-02	-0.35	7.08E-02	-0.30	1.29E-01	0.11	5.75E-01
ENSCAFG0000002525	FOCAD	grey	VSMC_M1C	0.39	4.41E-02	-0.03	8.71E-01	-0.14	4.80E-01	-0.08	6.82E-01	0.32	1.02E-01	-0.04	8.34E-01	0.21	2.86E-01	0.02	9.05E-01	-0.07	7.21E-01	-0.05	8.01E-01
ENSCAFG0000001256	ENSCAFG0000001256	cyan	VSMC_M2	0.39	4.41E-02	0.48	1.08E-02	0.31	1.12E-01	0.18	3.79E-01	-0.24	2.25E-01	0.08	6.88E-01	-0.07	7.41E-01	-0.28	1.55E-01	-0.55	3.15E-03	0.37	5.98E-02
ENSCAFG0000000848	WBP1	grey	VSMC_M1C	0.39	4.42E-02	0.67	1.23E-04	-0.06	7.60E-01	0.55	3.26E-03	0.21	2.87E-01	-0.13	5.34E-01	-0.38	5.15E-02	-0.74	1.11E-05	-0.57	2.13E-03	0.42	2.87E-02
ENSCAFG0000001765	PSTPIP2	pink	VSMC_M5	0.39	4.42E-02	0.12	5.47E-01	-0.31	1.21E-01	0.26	1.91E-01	0.58	1.49E-03	-0.37	5.72E-02	-0.16	4.40E-01	-0.30	1.23E-01	-0.09	6.62E-01	-0.14	4.93E-01
ENSCAFG0000000953	NRAS	darkgrey	VSMC_M8	0.39	4.45E-02	-0.36	6.19E-02	0.25	2.01E-01	-0.71	2.94E-05	0.06	7.58E-01	-0.03	8.81E-01	0.17	4.06E-01	0.72	2.46E-05	0.19	3.33E-01	-0.66	1.65E-04
ENSCAFG0000000682	PCBP2	grey	VSMC_M1C	0.39	4.45E-02	-0.02	9.13E-01	0.29	1.38E-01	-0.58	1.54E-03	0.02	9.34E-01	0.06	7.51E-01	-0.01	9.72E-01	0.51	6.05E-03	-0.08	7.01E-01	-0.49	9.35E-03
ENSCAFG0000002961	FBXO6	yellow	VSMC_M3	0.39	4.47E-02	0.81	3.95E-07	0.70	5.72E-05	-0.11	5.92E-01	-0.58	1.45E-03	-0.03	8.95E-01	-0.15	4.66E-01	-0.23	2.51E-01	-0.78	1.48E-06	0.47	1.36E-02

ENSCAFG0000000503	KLF5	grey	VSMC_M10	0.39	4.47E-02	0.23	2.46E-01	0.00	9.88E-01	0.09	6.59E-01	0.02	9.09E-01	0.06	7.84E-01	0.16	4.15E-01	-0.12	5.53E-01	-0.43	2.56E-02	0.35	7.22E-02
ENSCAFG0000000095	CYB5R3	cyan	VSMC_M2	0.39	4.47E-02	0.65	2.71E-04	0.10	6.16E-01	0.34	7.80E-02	0.13	5.26E-01	-0.33	9.76E-02	-0.31	1.20E-01	-0.62	5.57E-04	-0.51	6.81E-03	0.32	1.07E-01
ENSCAFG0000000510	MYCBP2	grey	VSMC_M10	0.39	4.48E-02	0.55	3.21E-03	-0.19	3.55E-01	0.46	1.69E-02	0.31	1.19E-01	-0.23	2.53E-01	-0.15	4.66E-01	-0.59	1.17E-03	-0.58	1.48E-03	0.41	3.36E-02
ENSCAFG0000002213	ENSCAFG0000002213	red	VSMC_M1	0.39	4.48E-02	0.15	4.45E-01	0.17	4.04E-01	-0.17	3.96E-01	0.00	9.83E-01	-0.03	8.83E-01	0.03	8.74E-01	0.09	6.64E-01	-0.23	2.55E-01	-0.06	7.57E-01
ENSCAFG0000000088	DEPTOR	cyan	VSMC_M2	0.39	4.49E-02	0.69	5.95E-05	0.50	8.34E-03	-0.06	7.84E-01	-0.34	7.86E-02	-0.06	7.66E-01	-0.10	6.12E-01	-0.19	3.40E-01	-0.77	3.28E-06	0.29	1.43E-01
ENSCAFG0000000867	SSBP2	grey	VSMC_M10	0.39	4.49E-02	0.29	1.48E-01	-0.50	8.13E-03	0.66	1.73E-04	0.63	4.56E-04	-0.24	2.35E-01	-0.22	2.64E-01	-0.66	1.63E-04	-0.31	1.14E-01	0.31	1.13E-01
ENSCAFG0000001798	DYNC1H1	pink	VSMC_M5	0.39	4.50E-02	0.00	9.94E-01	-0.43	2.60E-02	0.47	1.30E-02	0.61	7.67E-04	-0.18	3.58E-01	-0.26	1.87E-01	-0.42	2.87E-02	-0.03	8.91E-01	0.02	9.29E-01
ENSCAFG0000000235	ZBTB45	cyan	VSMC_M2	0.39	4.50E-02	0.53	4.60E-03	0.33	8.86E-02	0.09	6.45E-01	-0.30	1.26E-01	0.16	4.34E-01	0.08	6.92E-01	-0.20	3.15E-01	-0.66	1.81E-04	0.45	1.79E-02
ENSCAFG0000001016	FIBIN	cyan	VSMC_M2	0.39	4.51E-02	0.46	1.52E-02	0.34	7.97E-02	0.06	7.70E-01	-0.18	3.64E-01	0.06	7.51E-01	-0.20	3.07E-01	-0.32	1.07E-01	-0.47	1.24E-02	0.24	2.37E-01
ENSCAFG0000001588	ENSCAFG0000001588	grey	VSMC_M10	0.39	4.52E-02	0.03	9.01E-01	0.50	8.53E-03	-0.62	4.93E-04	-0.17	4.00E-01	-0.10	6.20E-01	0.13	5.15E-01	0.50	8.53E-03	-0.19	3.56E-01	-0.40	3.64E-02
ENSCAFG0000000422	ENSCAFG0000000422	grey	VSMC_M10	0.39	4.53E-02	0.72	2.10E-05	0.12	5.42E-01	0.47	1.32E-02	-0.09	6.57E-01	-0.01	9.66E-01	-0.41	3.57E-02	-0.59	1.25E-03	-0.74	8.94E-06	0.58	1.57E-03
ENSCAFG0000001985	TRAPPC2L	pink	VSMC_M5	0.39	4.53E-02	-0.02	9.14E-01	-0.29	1.35E-01	0.08	6.76E-01	0.48	1.08E-02	-0.11	5.92E-01	-0.14	4.85E-01	-0.08	6.80E-01	-0.06	7.61E-01	-0.12	5.64E-01
ENSCAFG0000001785	SPARC	red	VSMC_M1	0.39	4.53E-02	-0.30	1.31E-01	-0.05	7.88E-01	-0.12	5.64E-01	0.31	1.12E-01	-0.06	7.48E-01	-0.14	4.82E-01	0.14	4.93E-01	0.18	3.65E-01	-0.40	3.66E-02
ENSCAFG0000001419	PBX4	yellow	VSMC_M3	0.39	4.54E-02	0.20	3.29E-01	0.31	1.13E-01	0.05	7.95E-01	-0.12	5.55E-01	0.07	7.29E-01	0.02	9.23E-01	-0.11	5.80E-01	-0.28	1.60E-01	-0.02	9.04E-01
ENSCAFG0000000133	TSTA3	yellow	VSMC_M3	0.39	4.54E-02	0.60	9.71E-04	0.63	4.14E-04	-0.02	9.10E-01	-0.38	4.82E-02	-0.24	2.28E-01	-0.12	5.62E-01	-0.28	1.65E-01	-0.53	4.35E-03	0.18	3.62E-01
ENSCAFG0000002466	OXLD1	cyan	VSMC_M2	0.39	4.56E-02	0.60	9.44E-04	0.39	4.66E-02	0.12	5.48E-01	-0.20	3.20E-01	0.02	9.16E-01	-0.21	3.03E-01	-0.36	6.85E-02	-0.55	3.23E-03	0.21	2.88E-01
ENSCAFG0000000657	ENSCAFG0000000657	grey	VSMC_M10	0.39	4.56E-02	0.35	7.14E-02	0.28	1.62E-01	-0.07	7.44E-01	0.02	9.26E-01	-0.19	3.33E-01	-0.36	6.24E-02	-0.15	4.52E-01	-0.32	1.02E-01	-0.06	7.77E-01
ENSCAFG0000001273	PFDN2	yellow	VSMC_M3	0.39	4.56E-02	0.10	6.35E-01	0.57	1.87E-03	-0.38	5.12E-02	-0.30	1.31E-01	0.05	7.86E-01	-0.08	6.98E-01	0.29	1.43E-01	-0.23	2.39E-01	-0.30	1.24E-01
ENSCAFG0000001696	PDK2	cyan	VSMC_M2	0.39	4.56E-02	0.66	2.04E-04	0.37	6.10E-02	-0.10	6.11E-01	-0.12	5.49E-01	-0.11	5.72E-01	-0.21	2.98E-01	-0.22	2.61E-01	-0.58	1.61E-03	0.14	5.00E-01
ENSCAFG0000002822	ENSCAFG0000002822	cyan	VSMC_M2	0.39	4.56E-02	0.38	4.79E-02	0.26	1.89E-01	0.06	7.59E-01	-0.09	6.47E-01	-0.15	4.51E-01	-0.25	2.18E-01	-0.18	3.80E-01	-0.45	1.79E-02	0.14	4.78E-01
ENSCAFG0000002984	TGIF2-RAB5IF	grey	VSMC_M10	0.39	4.57E-02	-0.25	2.08E-01	0.04	8.54E-01	-0.30	1.25E-01	0.20	3.07E-01	0.12	5.53E-01	-0.07	7.15E-01	0.39	4.37E-02	0.09	6.71E-01	-0.49	8.91E-03
ENSCAFG0000000678	MTIF3	red	VSMC_M1	0.39	4.57E-02	0.31	1.20E-01	0.16	4.39E-01	-0.09	6.40E-01	0.05	8.19E-01	-0.14	4.73E-01	0.07	7.26E-01	0.02	9.21E-01	-0.47	1.39E-02	0.00	9.84E-01
ENSCAFG0000000130	DEF6	yellow	VSMC_M3	0.39	4.58E-02	0.36	6.77E-02	0.65	2.11E-04	-0.65	2.41E-04	-0.34	7.90E-02	-0.23	2.42E-01	0.09	6.54E-01	0.37	5.55E-02	-0.41	3.58E-02	-0.18	3.57E-01
ENSCAFG0000001049	ENSCAFG0000001049	darkgrey	VSMC_M8	0.39	4.59E-02	-0.44	2.21E-02	0.05	7.90E-01	-0.59	1.26E-03	0.36	6.54E-02	-0.19	3.43E-01	0.03	8.81E-01	0.61	7.89E-04	0.27	1.68E-01	-0.86	1.11E-08
ENSCAFG0000000806	ENSCAFG0000000806	grey	VSMC_M10	0.39	4.59E-02	0.42	2.88E-02	-0.04	8.48E-01	0.36	6.31E-02	0.25	2.10E-01	-0.51	6.40E-03	-0.42	3.11E-02	-0.44	2.12E-02	-0.41	3.37E-02	0.17	3.86E-01
ENSCAFG0000003017	NDUFA8	grey	VSMC_M10	0.39	4.59E-02	-0.28	1.64E-01	0.15	4.57E-01	-0.32	1.03E-01	0.20	3.13E-01	-0.01	9.54E-01	-0.03	8.94E-01	0.31	1.18E-01	0.20	3.26E-01	-0.65	2.38E-04
ENSCAFG0000001057	TMEM115	cyan	VSMC_M2	0.39	4.60E-02	0.50	7.81E-03	0.38	4.84E-02	0.02	9.18E-01	-0.22	2.71E-01	-0.06	7.56E-01	-0.05	8.22E-01	-0.21	2.92E-01	-0.55	2.80E-03	0.19	3.36E-01
ENSCAFG0000000347	AKT1S1	cyan	VSMC_M2	0.39	4.60E-02	0.77	2.79E-06	0.42	2.75E-02	0.08	6.86E-01	-0.34	8.61E-02	0.10	6.36E-01	-0.42	2.90E-02	-0.38	4.84E-02	-0.72	2.16E-05	0.46	1.51E-02
ENSCAFG0000001250	ENSCAFG0000001250	red	VSMC_M1	0.39	4.62E-02	0.34	8.65E-02	0.20	3.07E-01	0.19	3.52E-01	-0.12	5.64E-01	0.08	7.06E-01	-0.08	7.08E-01	-0.24	2.26E-01	-0.44	2.08E-02	0.30	1.33E-01
ENSCAFG0000000560	PAK4	cyan	VSMC_M2	0.39	4.62E-02	0.47	1.44E-02	0.11	5.85E-01	0.42	2.95E-02	-0.09	6.47E-01	-0.06	7.70E-01	-0.44	2.17E-02	-0.44	2.07E-02	-0.52	5.14E-03	0.46	1.70E-02
ENSCAFG0000000802	SLC9A9	grey	VSMC_M10	0.39	4.63E-02	0.43	2.42E-02	-0.06	7.50E-01	0.31	1.16E-01	0.07	7.41E-01	0.06	7.68E-01	-0.04	8.25E-01	-0.31	1.16E-01	-0.59	1.10E-03	0.44	2.06E-02
ENSCAFG0000000958	CHID1	grey	VSMC_M10	0.39	4.63E-02	0.65	2.54E-04	0.24	2.19E-01	0.41	3.27E-02	-0.22	2.72E-01	0.01	9.78E-01	-0.24	2.28E-01	-0.52	5.19E-03	-0.71	3.39E-05	0.52	5.41E-03
ENSCAFG0000000558	ENSCAFG0000000558	grey	VSMC_M10	0.39	4.63E-02	-0.13	5.15E-01	0.03	8.88E-01	-0.34	8.03E-02	0.23	2.44E-01	-0.27	1.75E-01	0.03	8.88E-01	0.36	6.64E-02	-0.06	7.84E-01	-0.38	5.39E-02
ENSCAFG0000001935	ELOB	cyan	VSMC_M2	0.39	4.64E-02	0.63	4.43E-04	0.43	2.47E-02	0.26	1.97E-01	-0.29	1.47E-01	-0.09	6.53E-01	-0.21	2.88E-01	-0.48	1.17E-02	-0.58	1.49E-03	0.39	4.71E-02
ENSCAFG0000002302	BIRC2	pink	VSMC_M5	0.39	4.64E-02	-0.35	6.97E-02	-0.43	2.63E-02	0.15	4.49E-01	0.71	3.24E-05	0.00	9.97E-01	0.00	9.97E-01	-0.02	9.13E-01	0.30	1.33E-01	-0.38	5.12E-02
ENSCAFG0000000517	CAPN5	cyan	VSMC_M2	0.39	4.65E-02	0.50	8.46E-03	0.32	1.00E-01	-0.04	8.46E-01	-0.14	4.82E-01	0.30	1.31E-01	0.03	8.64E-01	-0.19	3.37E-01	-0.48	1.19E-02	0.19	3.36E-01
ENSCAFG0000000747	CHMP4B	cyan	VSMC_M2	0.39	4.65E-02	0.66	2.01E-04	0.37	5.43E-02	0.09	6.49E-01	-0.19	3.55E-01	-0.27	1.72E-01	-0.51	6.53E-03	-0.34	8.19E-02	-0.54	3.47E-03	0.27	1.78E-01
ENSCAFG0000000607	RAB20	yellow	VSMC_M3	0.39	4.66E-02	0.36	6.56E-02	0.57	1.81E-03	-0.28	1.63E-01	-0.47	1.31E-02	0.27	1.78E-01	0.13	5.06E-01	0.15	4.63E-01	-0.49	9.81E-03	0.13	5.05E-01
ENSCAFG0000001703	ENSCAFG0000001703	yellow	VSMC_M3	0.39	4.67E-02	0.41	3.39E-02	0.42	2.73E-02	-0.18	3.73E-01	-0.19	3.37E-01	0.06	7.56E-01	0.11	5.96E-01	-0.07	7.19E-01	-0.41	3.25E-02	0.07	7.46E-01
ENSCAFG0000000836	P2RX4	red	VSMC_M1	0.39	4.69E-02	0.28	1.51E-01	0.14	4.95E-01	0.09	6.55E-01	-0.04	8.37E-01	-0.06	7.67E-01	-0.44	2.05E-02	-0.17	4.09E-01	-0.30	1.26E-01	0.16	4.12E-01
ENSCAFG0000001391	PTS	red	VSMC_M1	0.39	4.69E-02	0.14	4.88E-01	-0.18	3.63E-01	0.45	1.84E-02	0.29	1.36E-01	-0.08	7.00E-01	0.07	7.20E-01	-0.46	1.48E-02	-0.18	3.69E-01	0.24	2.31E-01
ENSCAFG0000001433	BLOC1S4	yellow	VSMC_M3	0.39	4.70E-02	0.22	2.73E-01	0.68	8.80E-05	-0.38	5.20E-02	-0.36	6.42E-02	-0.10	6.04E-01	-0.02	9.36E-01	0.18	3.77E-01	-0.23	2.42E-01	-0.27	1.68E-01
ENSCAFG0000000350	FUZ	grey	VSMC_M10	0.39	4.73E-02	0.24	2.30E-01	0.09	6.71E-01	0.27	1.71E-01	0.17	3.89E-01	-0.28	1.65E-01	-0.16	4.29E-01	-0.35	7.59E-02	-0.25	2.17E-01	0.02	9.27E-01
ENSCAFG0000000314	ENSCAFG0000000314	red	VSMC_M1	0.39	4.73E-02	0.10	6.12E-01	-0.28	1.54E-01	0.31	1.17E-01	0.48	1.18E-02	-0.31	1.18E-01	0.03	9.01E-01	-0.32	1.05E-01	-0.16	4.17E-01	0.03	8.90E-01
ENSCAFG0000002495	RNF130	grey	VSMC_M10	0.38	4.74E-02	0.74	9.48E-06	0.11	5.71E-01	0.34	8.55E-02	-0.06	7.47E-01	-0.08	6.96E-01	-0.01	9.77E-01	-0.54	3.34E-03	-0.73	1.35E-05	0.62	6.05E-04
ENSCAFG0000001794	CSK	yellow	VSMC_M3	0.38	4.74E-02	0.27	1.68E-01	0.74	1.14E-05	-0.55	2.98E-03	-0.51	6.72E-03	0.09	6.57E-01	0.15	4.46E-01	0.41	3.55E-02	-0.44	2.04E-02	-0.16	4.37E-01
ENSCAFG0000001614	ENSCAFG0000001614	grey	VSMC_M10	0.38	4.74E-02	-0.14	4.73E-01	0.01	9.60E-01	-0.03	8.85E-01	0.07	7.38E-01	0.24	2.33E-01	0.43	2.70E-02	0.11	5.94E-01	-0.13	5.28E-01	0.01	9.68E-01
ENSCAFG0000000405	SRPK2	pink	VSMC_M5	0.38	4.75E-02	-0.17	3.91E-01	-0.46	1.53E-02	0.21	3.03E-01	0.70	5.19E-05	-0.15	4.54E-01	0.15	4.54E-01	-0.07	7.25E-01	-0.01	9.55E-01	-0.22	2.79E-01
ENSCAFG0000003271	DNAJC19	grey	VSMC_M10	0.38	4.78E-02	0.03	8.81E-01	0.26	1.89E-01	-0.20	3.11E-01	0.08	6.84E-01	-0.19	3.47E-01	0.08	6.82E-01	0.12	5.35E-01	-0.09	6.65E-01	-0.42	2.80E-02
ENSCAFG0000000941	CDYL	grey	VSMC_M10	0.38	4.78E-02	-0.53	4.78E-03	-0.08	6.87E-01	-0.39	4.71E-02	0.35	7.47E-02	0.18	3.68E-01	0.09	6.53E-01	0.50	8.24E-03	0.33	9.15E-02	-0.62	4.95E-04
ENSCAFG0000000035	C10H12orf56	grey	VSMC_M10	0.38	4.79E-02	0.15	4.41E-01	0.10	6.19E-01	-0.05	8.15E-01	0.21	3.03E-01	-0.22	2.61E-01	-0.19	3.54E-01	-0.09	6.73E-01	-0.15	4.53E-01	-0.23	2.42E-01
ENSCAFG0000001711	SPG21	red	VSMC_M1	0.38	4.80E-02	0.42	2.76E-02	0.03	8.77E-01	0.19	3.37E-01	0.13	5.20E-01	-0.13	5.18E-01	0.18	3.67E-01	-0.35	7.39E-02	-0.50	8.34E-03	0.27	1.78E-01
ENSCAFG0000003216	ZNF792	grey	VSMC_M10	0.38	4.80E-02	0.55	2.93E-03	0.50	7.44E-03	-0.07	7.13E-01	-0.51	6.74E-03	0.11	5.91E-01	0.07	7.13E-01	-0.09	6.51E-01	-0.66	1.55E-04	0.53	4.74E-03
ENSCAFG0000000337	CPSF3	pink	VSMC_M5	0.38	4.80E-02	-0.26	1.84E-01	-0.44	2.26E-02	0.06	7.66E-01	0.73	1.84E-05	-0.15	4.46E-01	-0.12	5.65E-01	0.08	6.84E-01	0.12	5.35E-01	-0.39	4.37E-02
ENSCAFG0000000705	PLAG1	grey	VSMC_M10	0.38	4.81E-02	0.12	5.39E-01	0.38	5.26E-02	-0.22	2.80E-01	-0.05	7.89E-01	-0.38	4.79E-02	0.10	6.13E-01	0.13	5.18E-01	-0.18	3.64E-01	-0.29	1.40E-01
ENSCAFG0000000461	HPDL	grey	VSMC_M10	0.38	4.81E-02	0.30	1.23E-01	0.68	1.10E-04	-0.37	5.51E-02	-0.56	2.33E-03	0.05	8.17E-01	-0.06	7.78E-01	0.27	1.80E-01	-0.47	1.43E-02	0.09	6.65E-01
ENSCAFG0000001674	HDGF	darkgrey	VSMC_M8	0.38	4.82E-02	0.01	9.74E-01	0.71	2.90E-05	-0.85	1.51E-08	-0.35	7.71E-02	-0.09	6.51E-01	0.22	2.76E-01	0.66	2.04E-04	-0.12	5.51E-01	-0.56	2.30E-03
ENSCAFG0000000231	RNF38	pink	VSMC_M5	0.38	4.82E-02	0.12	5.45E-01	-0.52	5.89E-03	0.57	2.11E-03	0.67	1.19E-04	-0.12	5.57E-01	-0.26	1.90E-01	-0.51	6.12E-03	-0.09	6.43E-01	0.12	5.49E-01
ENSCAFG0000001988	GTF3C4	grey	VSMC_M10	0.38	4.84E-02	0.45	1.80E-02	-0.15	4.45E-01	0.50	8.36E-03	0.30	1.23E-01	-0.39	4.24E-02	-0.30	1.31E-01	-0.57	1.92E-03	-0.44	2.13E-02	0.37	5.86E-02
ENSCAFG0000000355	CADPS2	cyan	VSMC_M2	0.38	4.85E-02	0.67	1.43E-04	0.13	5.16E-01	0.13	5.25E-01	0.05	8.09E-01	-0.07	7.40E-01	-0.20	3.22E-01	-0.41	3.48E-02	-0.61	6.99E-04	0.33	8.82E-02
ENSCAFG0000001730	STON2	cyan	VSMC_M2	0.38	4.85E-02	0.71	3.30E-05	0.28	1.53E-01	0.06	7.63E-01	-0.12	5.37E-01	-0.04	8.36E-01	0.20	3.13E-01	-0.32	9.96E-02	-0.74	9.94E-06	0.40	3.94E-02
ENSCAFG0000001058	BOC	cyan	VSMC_M2	0.38	4.86E-02	0.69	7.68E-05	0.18	3.57E-01	0.08	7.05E-01	0.01	9.60E-01	-0.14	4.85E-01	-0.17	3.89E-01	-0.31	1.17E-01	-0.65	2.12E-04	0.29	1.49E-01
ENSCAFG0000002894	ELN	yellow	VSMC_M3	0.38	4.86E-02	0.17	3.95E-01	0.68	9.51E-05	-0.74	1.12E-05	-0.30	1.22E-01	-0.01	9.70E-01	0.19	3.54E-01	0.54	3.37E-03	-0.27	1.69E-01	-0.44	2.08E-02
ENSCAFG0000001406	EFTUD2	darkgrey	VSMC_M8	0.38	4.87E-02	-0.07	7.32E-01	0.31	1.13E-01	-0.64	3.35E-04	-0.03	8.76E-01	-0.14	4.96E-01	0.23	2.39E-01	0.58	1.67E-03	-0.09	6.53E-01	-0.40	3.71E-02
ENSCAFG0000002413	ENSCAFG0000002413	yellow	VSMC_M3	0.38	4.87E-02	0.17	3.83E-01	0.49	9.50E-03	-0.21	2.97E-01	-0.22	2.77E-01	-0.06	7.76E-01	0.06	7.67E-01	0.00	9.81E-01	-0.24	2.29E-01	-0.12	5.35E-01
ENSCAFG0000003124	ENSCAFG0000003124	grey	VSMC_M10	0.38	4.87E-02	-0.28	1.55E-01	-0.33	8.78E-02	0.16	4.16E-01	0.52	5.44E-03	-0.17	3.89E-01	0.01	9.57E-01	0.06	7.70E-01	0.09	6.53E-01	-0.28	1.64E-01
ENSCAFG0000001349	OLR1	grey	VSMC_M10	0.38	4.87E-02	0.24	2.31E-01	-0.06	7.79E-01	0.32	1.09E-01	0.07	7.14E-01	0.33	9.67E-02	-0.39	4.27E-02	-0.29	1.41E-01	-0.37	6.00E-02	0.31	1.20E-01
ENSCAFG0000001844	CRIP1	red	VSMC_M1	0.38	4.87E-02	0.39	4.20E-02	0.16	4.26E-01	0.22	2.69E-01	0.04	8.34E-01	-0.28	1.56E-01	0.22	2.63E-01	-0.37	6.05E-02	-0.41	3.21E-02	0.12	5.41E-01
ENSCAFG0000000883	TRAFD1	grey	VSMC_M10	0.38	4.88E-02	0.55	2.74E-03	-0.04	8.28E-01	0.40	3.70E-02	0.14	4.94E-01	-0.10	6.27E-01	-0.39	4.33E-02	-0.54	3.68E-03	-0.52	5.02E-03	0.44	2.11E-02
ENSCAFG0000001165	SSH3	cyan	VSMC_M2	0.38	4.89E-02	0.89	3.30E-10	0.19	3.46E-01	0.48	1.19E-02	-0.08	6.80E-01	-0.15	4.64E-01	-0.57	1.72E-03	-0.74	8.66E-06	-0.78	1.36E-06	0.59	1.17E-03
ENSCAFG0000000026	RPA3	grey	VSMC_M10	0.38	4.90E-02	0.32	9.84E-02	0.57	1.75E-03	-0.24	2.37E-01	-0.43	2.62E-02	0.03	8.73E-01	0.22	2.81E-01	0.09	6.72E-01	-0.43	2.54E-02	0.11	5.96E-01
ENSCAFG0000001440	ENSCAFG0000001440	red	VSMC_M1	0.38	4.90E-02	0.20	3.20E-01	0.30	1.31E-01	-0.08	7.07E-01	-0.16	4.13E-01	0.04	8.29E-01	-0.16	4.31E-01	0.07	7.41E-01	-0.32	1.01E-01	0.09	6.57E-01
ENSCAFG0000001786	EML1	yellow	VSMC_M3	0.38	4.92E-02	0.34	8.60E-02	0.46	1.46E-02	-0.38	4.81E-02	-0.15	4.68E-01	-0.24	2.18E-01	-0.13	5.13E-01	0.14	4.85E-01	-0.35	7.01E-02	-0.22	2.71E-01
ENSCAFG0000000490	CAND2	grey	VSMC_M10	0.38	4.92E-02	0.27	1.76E-01	-0.07	7.11E-01	0.48	1.16E-02	0.09	6.49E-01	0.14	4.96E-01	-0.13	5.32E-01	-0.50	7.24E-03	-0.36	6.46E-02	0.42	3.13E-02
ENSCAFG0000001512	CFAP300	red	VSMC_M1	0.38	4.92E-02	0.02	9.25E-01	-0.20	3.16E-01	0.29	1.37E-01	0.45	1.96E-02	-0.07	7.34E-01	-0.21	2.98E-01	-0.19	3.32E-01	-0.10	6.22E-01	-0.20	3.15E-01
ENSCAFG0000000878	PLLP	grey	VSMC_M10	0.38	4.93E-02	0.34	8.67E-02	0.30	1.29E-01	0.17	4.03E-01	-0.18	3.67E-01	0.27	1.73E-01	-0.13	5.29E-01	-0.28	1.60E-01	-0.41	3.54E-02	0.18	3.62E-01
ENSCAFG0000001649	MAFK	grey	VSMC_M10	0.38	4.93E-02	0.25	2.03E-01	0.10	6.04E-01	0.35	7.23E-02	-0.02	9.14E-01	0.12	5.49E-01	-0.04	8.50E-01	-0.43	2.70E-02	-0.32	1.02E-01	0.32	1.05E-01
ENSCAFG0000000827	SORBS1	grey	VSMC_M10	0.38	4.93E-02	0.45	1.72E-02	0.01	9.79E-01	0.22	2.62E-01	0.17	4.02E-01	-0.04	8.29E-01	-0.59	1.32E-03	-0.40	3.96E-02	-0.45	1.94E-02	0.20	3.21E-01
ENSCAFG0000001800	ATG4A	darkgreen	VSMC_M4	0.38	4.94E-02	0.31	1.21E-01	-0.14	4.72E-01	0.57	1.93E-03	0.13	5.32E-01	0.16	4.12E-01	-0.03	8.94E-01	-0.52	5.20E-03	-0.41	3.15E-02	0.53	4.16E-03
ENSCAFG0000000798	ENSCAFG0000000798	red	VSMC_M1	0.38	4.94E-02	0.06	7.83E-01	0.17	4.08E-01	-0.21	3.01E-01	0.19	3.52E-01	-0.11	5.94E-01	-0.27	1.74E-01	0.12	5.57E-01	-0.12	5.66E-01	-0.35	6.96E-02
ENSCAFG0000001849	CD320	grey	VSMC_M10	0.38	4.95E-02	0.46	1.48E-02	0.54	3.70E-03	-0.16	4.15E-01	-0.36	6.89E-02	-0.05	7.89E-01	-0.19	3.38E-01	-0.04	8.46E-01	-0.47	1.34E-02	0.10	6.04E-01
ENSCAFG0000002384	MYPOP	red	VSMC_M1	0.38	4.95E-02	0.19	3.37E-01	0.29	1.48E-01	-0.06	7.84E-01	-0.15	4.69E-01	0.08	7.08E-01	0.17	3.98E-01	-0.10	6.26E-01	-0.26	1.87E-01	0.09	6.48E-01
ENSCAFG0000001990	KLHDC4	red	VSMC_M1	0.38	4.97E-02	0.07	7.39E-01	0.42	2.82E-02	-0.24	2.28E-01	-0.30	1.25E-01	0.15	4.69E-01	0.27	1.79E-01	0.23	2.48E-01	-0.20	3.06E-01	-0.01	9.74E-01
ENSCAFG0000001050	TM2D3	cyan	VSMC_M2	0.38	4.98E-02	0.70	4.34E-05	0.12	5.35E-01	0.46	1.64E-02	-0.15	4.66E-01	0.00	9.82E-01	-0.10	6.28E-01	-0.64	3.40E-04	-0.70	5.52E-05	0.74	1.01E-05
ENSCAFG0000001173	DCUN1D1	pink	VSMC_M5	0.38	4.99E-02	-0.16	4.16E-01	-0.27	1.67E-01	0.17	3.89E-01	0.60	9.71E-04	-0.36	6.45E-02	-0.19	3.54E-01	-0.11	5.68E-01	0.10	6.30E-01	-0.33	8.97E-02
ENSCAFG0000001814	DUSP14	grey	VSMC_M10	0.38	5.00E-02	0.04	8.27E-01	0.12	5.38E-01	-0.03	8.63E-01	0.06	7.65E-01	-0.07	7.29E-01	-0.14	4.79E-01	-0.02	9.32E-01	-0.09	6.51E-01	-0.13	5.15E-01
ENSCAFG0000001877	PROCA1	grey	VSMC_M10	0.38	5.01E-02	0.39	4.73E-02	0.13	5.31E-01	0.16	4.18E-01	-0.17	3.89E-01	0.07	7.32E-01	0.12	5.66E-01	-0.16	4.26E-01	-0.54	3.36E-03	0.51	6.21E-03
ENSCAFG0000000572	PSD2	red	VSMC_M1	0.38	5.02E-02	0.38	4.78E-02	0.26	1.95E-01	0.04	8.26E-01	-0.04	8.30E-01	-0.33	9.10E-02	0.16	4.34E-01	-0.22	2.61E-01	-0.41	3.27E-02	0.13	5.32E-01
ENSCAFG0000001059	GSTO1	yellow	VSMC_M3	0.38	5.03E-02	0.06	7.58E-01	0.71	3.35E-05	-0.67	1.18E-04	-0.47	1.45E-02	0.09	6.52E-01	-0.05	8.20E-01	0.57	1.73E-03	-0.20	3.10E-01	-0.37	6.04E-02
ENSCAFG0000000841	NCAM2	grey	VSMC_M10	0.38	5.03E-02	0.23	2.57E-01	0.12	5.46E-01	0.17	3.96E-01	0.05	8.19E-01	-0.17	4.05E-01	-0.04	8.47E-01	-0.18	3.81E-01	-0.32	1.00E-01	0.08	7.00E-01
ENSCAFG0000001605	ITPKB	red	VSMC_M1	0.38	5.05E-02	0.48	1.11E-02	0.00	9.89E-01	0.29	1.49E-01	0.08	6.94E-01	-0.07	7.41E-01	-0.33	9.64E-02	-0.40	3.74E-02	-0.49	9.40E-03	0.40	3.95E-02
ENSCAFG0000000390	AMD1	darkgrey	VSMC_M8	0.38	5.05E-02	-0.22	2.76E-01	0.30	1.26E-01	-0.65	2.75E-04	0.04	8.52E-01	0.03	8.93E-01	-0.03	8.63E-01	0.59	1.13E-03	0.10	6.19E-01	-0.65	2.19E-04
ENSCAFG0000000730	TNKS2	pink	VSMC_M5	0.38	5.06E-02	-0.44	2.07E-02	-0.50	7.40E-03	0.20	3.07E-01	0.76	4.68E-06	-0.08	6.93E-01	-0.16	4.38E-01	0.02	9.22E-01	0.32	1.07E-01	-0.41	3.15E-02
ENSCAFG0000001345	MOV10	cyan	VSMC_M2	0.38	5.06E-02	0.81	3.21E-07	0.58	1.60E-03	-0.01	9.67E-01	-0.40	3.80E-02	-0.01	9.52E-01	-0.26	1.97E-01	-0.28	1.51E-01	-0.79	1.10E-06	0.36	6.33E-02
ENSCAFG0000001171	ZBED9	pink	VSMC_M5	0.38	5.07E-02	-0.46	1.58E-02	-0.48	1.14E-02	0.10	6.12E-01	0.72	2.35E-05	-0.02	9.21E-01	0.02	9.10E-01	0.11	5.69E-01	0.29	1.44E-01	-0.41	3.15E-02
ENSCAFG0000001964	TMLHE	grey	VSMC_M10	0.38	5.07E-02	0.33	8.94E-02	0.06	7.81E-01	0.28	1.60E-01	0.10	6.23E-01	-0.34	7.90E-02	-0.20	3.11E-01	-0.40	4.07E-02	-0.37	5.74E-02	0.24	2.32E-01
ENSCAFG0000001802	GPRC5B	cyan	VSMC_M2	0.38	5.07E-02	0.88	1.54E-09	0.29	1.38E-01	0.36	6.82E-02	-0.25	2.00E-01	-0.08	6.95E-01	-0.44	2.07E-02	-0.59	1.17E-03	-0.86	6.14E-09	0.67	1.18E-04
ENSCAFG0000001468	ENSCAFG0000001468	yellow	VSMC_M3	0.38	5.08E-02	0.25	2.09E-01	0.87	3.33E-09	-0.58	1.47E-03	-0.56	2.57E-03	-0.10	6.09E-01	0.15	4.60E-01	0.38	5.31E-02	-0.35	7.72E-02	-0.27	1.78E-01
ENSCAFG0000000731	QSER1	grey	VSMC_M10	0.38	5.09E-02	-0.13	5.31E-01	-0.39	4.53E-02	0.14	4.79E-01	0.57	1.72E-03	0.00	1.00E+00	-0.05	8.13E-01	-0.12	5.45E-01	0.01	9.42E-01	-0.08	7.06E-01
ENSCAFG0000000681	SDCBP2	grey	VSMC_M10	0.38	5.09E-02	0.20	3.09E-01	0.07	7.43E-01	-0.18	3.77E-01	0.14	4.93E-01	-0.15	4.55E-01	-0.26	1.92E-01	0.08	6.80E-01	-0.25	2.14E-01	-0.04	8.26E-01
ENSCAFG0000000100	CUTA	cyan	VSMC_M2	0.38	5.09E-02	0.73	1.82E-05	0.49	9.54E-03	0.05	8.11E-01	-0.28	1.53E-01	-0.15	4.63E-01	-0.15	4.41E-01	-0.39	4.32E-02	-0.67	1.53E-04	0.29	1.49E-01
ENSCAFG0000000877	ENSCAFG0000000877	red	VSMC_M1	0.38	5.09E-02	0.18	3.66E-01	0.27	1.66E-01	-0.02	9.21E-01	0.09	6.38E-01	-0.50	7.54E-03	-0.06	7.70E-01	-0.14	5.01E-01	-0.16	4.12E-01	-0.25	2.06E-01
ENSCAFG0000000567	RNF213	cyan	VSMC_M2	0.38	5.10E-02	0.87	4.44E-09	0.32	9.94E-02	0.25	2.16E-01	-0.21	2.82E-01	0.04	8.30E-01	-0.20	3.26E-01	-0.56	2.33E-03	-0.84	5.54E-08	0.58	1.58E-03
ENSCAFG0000001062	SPICE1	grey	VSMC_M10	0.38	5.11E-02	-0.09	6.50E-01	-0.01	9.43E-01	-0.23	2.48E-01	0.28	1.64E-01	-0.06	7.57E-01	0.32	1.04E-01	0.20	3.15E-01	-0.10	6.27E-01	-0.24	2.33E-01
ENSCAFG0000001120	PUM1	pink	VSMC_M5	0.38	5.11E-02	0.27	1.68E-01	-0.46	1.61E-02	0.57	1.71E-03	0.55	2.84E-03	-0.07	7.16E-01	-0.35	7.34E-02	-0.53	4.36E-03	-0.28	1.65E-01	0.33	9.48E-02
ENSCAFG0000000201	ENSCAFG0000000201	cyan	VSMC_M2	0.38	5.11E-02	0.56	2.23E-03	0.26	1.86E-01	0.31	1.11E-01	-0.15	4.46E-01	-0.17	3.87E-01	-0.31	1.14E-01	-0.45	1.97E-02	-0.58	1.55E-03	0.37	6.01E-02
ENSCAFG0000001212	LRP2	darkgreen	VSMC_M4	0.38	5.12E-02	0.21	2.97E-01	-0.38	5.02E-02	0.66	1.67E-04	0.44	2.06E-02	-0.07	7.27E-01	-0.36	6.46E-02	-0.55	3.13E-03	-0.28	1.59E-01	0.31	1.21E-01
ENSCAFG0000000405	ENSCAFG0000000405	grey	VSMC_M10	0.38	5.12E-02	0.01	9.53E-01	0.05	7.88E-01	-0.15	4.51E-01	0.18	3.80E-01	0.24	2.35E-01	-0.03	9.01E-01	0.09	6.73E-01	-0.07	7.46E-01	-0.23	2.44E-01
ENSCAFG0000000760	URI1	pink	VSMC_M5	0.38	5.13E-02	0.13	5.31E-01	-0.11	5.69E-01	0.01	9.70E-01	0.48	1.06E-02	-0.48	1.22E-02	-0.26	1.89E-01	-0.11	5.83E-01	-0.09	6.48E-01	-0.32	1.05E-01
ENSCAFG0000001981	ENSCAFG0000001981	cyan	VSMC_M2	0.38	5.13E-02	0.73	1.73E-05	0.33	9.35E-02	0.14	4.74E-01	-0.27	1.81E-01	0.17	3.99E-01	-0.44	2.04E-02	-0.35	7.78E-02	-0.75	8.10E-06	0.51	6.32E-03
ENSCAFG0000000157	PSAT1	grey	VSMC_M10	0.38	5.13E-02	-0.10	6.16E-01	0.24	2.27E-01	-0.35	7.64E-02	-0.14	4.81E-01	0.13	5.18E-01	0.38	4.76E-02	0.42	3.04E-02	-0.21	2.94E-01	-0.09	6.69E-01
ENSCAFG0000003241	PRKRIP1	grey	VSMC_M10	0.38	5.14E-02	0.29	1.39E-01	0.19	3.49E-01	-0.04	8.55E-01	-0.14	4.75E-01	0.12	5.51E-01	0.22	2.69E-01	-0.02	9.30E-01	-0.47	1.30E-02	0.30	1.35E-01
ENSCAFG0000003181	TOMM7	red	VSMC_M1	0.38	5.15E-02	-0.24	2.37E-01	-0.07	7.36E-01	0.08	7.08E-01	0.34	8.25E-02	-0.18	3.67E-01	0.15	4.58E-01	0.00	9.94E-01	0.11	5.93E-01	-0.33	9.73E-02
ENSCAFG0000000093	MCM9	grey	VSMC_M10	0.38	5.15E-02	0.32	1.02E-01	-0.07	7.20E-01	0.45	1.77E-02	0.13	5.25E-01	0.11	5.90E-01	0.06	7.81E-01	-0.53	4.37E-03	-0.39	4.41E-02	0.44	2.07E-02
ENSCAFG0000000863	CCDC158	darkgreen	VSMC_M4	0.38	5.16E-02	0.14	4.95E-01	-0.36	6.54E-02	0.55	2.72E-03	0.50	7.64E-03	-0.16	4.21E-01	-0.05	7.98E-01	-0.50	7.71E-03	-0.20	3.29E-01	0.17	3.86E-01
ENSCAFG0000000218	SECISBP2	yellow	VSMC_M3	0.38	5.16E-02	-0.04	8.26E-01	0.61	7.68E-04	-0.70	5.18E-05	-0.36	6.91E-02	0.17	4.01E-01	0.30	1.29E-01	0.61	7.12E-04	-0.19	3.46E-01	-0.38	5.33E-02

ENSCAFG0000000408	NOP53	cyan	VSMC_M2	0.38	5.16E-02	0.82	1.33E-07	0.55	3.18E-03	0.20	3.23E-01	-0.47	1.37E-02	-0.12	5.35E-01	-0.25	2.16E-01	-0.43	2.39E-02	-0.82	1.23E-07	0.58	1.37E-03
ENSCAFG0000001680	SCRN2	cyan	VSMC_M2	0.38	5.17E-02	0.65	2.39E-04	0.26	1.88E-01	0.39	4.52E-02	-0.24	2.32E-01	0.03	8.87E-01	-0.01	9.60E-01	-0.50	7.36E-03	-0.71	2.93E-05	0.63	4.21E-04
ENSCAFG0000000637	ENSCAFG0000000637	cyan	VSMC_M2	0.38	5.17E-02	0.56	2.26E-03	0.23	2.41E-01	0.19	3.40E-01	-0.05	7.99E-01	-0.29	1.41E-01	-0.29	1.48E-01	-0.29	1.48E-01	-0.55	3.18E-03	0.26	1.95E-01
ENSCAFG0000000533	CRNKL1	grey	VSMC_M10	0.38	5.18E-02	0.25	2.06E-01	-0.13	5.13E-01	0.23	2.54E-01	0.27	1.68E-01	-0.21	2.94E-01	0.06	7.66E-01	-0.31	1.21E-01	-0.31	1.14E-01	0.25	2.03E-01
ENSCAFG0000001135	DISP1	cyan	VSMC_M2	0.38	5.18E-02	0.48	1.14E-02	0.01	9.48E-01	0.45	1.71E-02	0.03	8.67E-01	0.13	5.24E-01	0.12	5.65E-01	-0.56	2.23E-03	-0.54	3.60E-03	0.51	6.53E-03
ENSCAFG0000000641	WRN	grey	VSMC_M10	0.38	5.19E-02	-0.22	2.62E-01	-0.05	7.99E-01	-0.36	6.30E-02	0.28	1.50E-01	-0.20	3.13E-01	0.39	4.24E-02	0.43	2.65E-02	0.01	9.65E-01	-0.31	1.10E-01
ENSCAFG0000000689	TUB	grey	VSMC_M10	0.38	5.19E-02	0.06	7.80E-01	-0.10	6.06E-01	0.25	2.15E-01	0.26	1.97E-01	-0.09	6.38E-01	-0.09	6.56E-01	-0.16	4.13E-01	-0.17	3.90E-01	0.07	7.46E-01
ENSCAFG0000000783	TFCP2	grey	VSMC_M10	0.38	5.20E-02	0.21	2.86E-01	0.03	8.88E-01	-0.11	5.87E-01	0.25	2.02E-01	-0.24	2.35E-01	0.00	9.98E-01	-0.01	9.76E-01	-0.28	1.63E-01	-0.13	5.18E-01
ENSCAFG0000001456	TRMT44	red	VSMC_M1	0.38	5.21E-02	0.14	4.84E-01	0.25	2.01E-01	-0.13	5.04E-01	-0.06	7.49E-01	-0.14	4.93E-01	-0.05	8.20E-01	0.04	8.40E-01	-0.23	2.39E-01	0.03	8.85E-01
ENSCAFG0000002388	BCAS4	cyan	VSMC_M2	0.38	5.22E-02	0.83	8.17E-08	0.39	4.45E-02	0.18	3.67E-01	-0.21	2.96E-01	-0.33	9.72E-02	-0.24	2.33E-01	-0.50	7.66E-03	-0.75	5.39E-06	0.44	2.10E-02
ENSCAFG0000000048	DLA88	grey	VSMC_M10	0.38	5.24E-02	0.34	8.48E-02	-0.15	4.69E-01	0.56	2.38E-03	0.29	1.49E-01	0.01	9.67E-01	-0.26	1.91E-01	-0.59	1.33E-03	-0.35	7.54E-02	0.24	2.20E-01
ENSCAFG0000003091	TMEM126A	grey	VSMC_M10	0.38	5.24E-02	0.33	9.44E-02	0.32	1.01E-01	-0.20	3.12E-01	-0.12	5.51E-01	0.11	5.71E-01	0.00	9.84E-01	0.03	8.70E-01	-0.44	2.11E-02	0.00	9.97E-01
ENSCAFG0000001032	SRD5A1	grey	VSMC_M10	0.38	5.25E-02	0.37	5.71E-02	-0.20	3.20E-01	0.41	3.55E-02	0.30	1.24E-01	-0.05	7.88E-01	-0.17	3.95E-01	-0.52	5.98E-03	-0.34	8.32E-02	0.33	8.88E-02
ENSCAFG0000000151	MPST	yellow	VSMC_M3	0.38	5.25E-02	0.69	6.15E-05	0.79	8.22E-07	-0.32	1.09E-01	-0.62	6.06E-04	-0.13	5.14E-01	-0.13	5.05E-01	0.00	9.93E-01	-0.71	3.25E-05	0.23	2.55E-01
ENSCAFG0000001740	ENSCAFG0000001740	yellow	VSMC_M3	0.38	5.25E-02	0.42	2.82E-02	0.60	1.06E-03	-0.16	4.16E-01	-0.39	4.71E-02	-0.08	6.98E-01	-0.19	3.52E-01	-0.04	8.38E-01	-0.45	1.90E-02	0.03	8.98E-01
ENSCAFG0000001912	FAM57A	grey	VSMC_M10	0.38	5.26E-02	0.44	2.04E-02	-0.22	2.77E-01	0.46	1.69E-02	0.22	2.75E-01	0.12	5.67E-01	-0.38	4.98E-02	-0.49	1.03E-02	-0.50	8.47E-03	0.45	1.76E-02
ENSCAFG0000000310	MDN1	grey	VSMC_M10	0.38	5.26E-02	-0.10	6.19E-01	-0.17	3.85E-01	0.13	5.19E-01	0.37	5.64E-02	-0.11	5.93E-01	-0.08	6.77E-01	-0.09	6.72E-01	0.01	9.68E-01	-0.12	5.51E-01
ENSCAFG0000000578	HGS	cyan	VSMC_M2	0.38	5.26E-02	0.61	6.90E-04	0.43	2.44E-02	-0.04	8.55E-01	-0.25	2.00E-01	-0.15	4.51E-01	-0.30	1.34E-01	-0.22	2.71E-01	-0.57	1.90E-03	0.24	2.22E-01
ENSCAFG0000003188	ENSCAFG0000003188	yellow	VSMC_M3	0.38	5.27E-02	0.45	1.92E-02	0.57	1.79E-03	-0.06	7.67E-01	-0.34	8.38E-02	-0.09	6.53E-01	-0.02	9.16E-01	-0.13	5.02E-01	-0.47	1.28E-02	0.10	6.37E-01
ENSCAFG0000003088	BMT2	pink	VSMC_M5	0.38	5.28E-02	-0.10	6.09E-01	-0.64	2.89E-04	0.55	3.04E-03	0.84	4.80E-08	-0.20	3.12E-01	-0.19	3.44E-01	-0.46	1.59E-02	0.08	7.04E-01	-0.01	9.76E-01
ENSCAFG0000000319	FKBP15	grey	VSMC_M10	0.38	5.28E-02	0.28	1.54E-01	-0.20	3.21E-01	0.33	9.53E-02	0.32	1.03E-01	-0.17	4.05E-01	0.05	7.94E-01	-0.39	4.59E-02	-0.33	9.49E-02	0.28	1.51E-01
ENSCAFG0000002340	ENSCAFG0000002340	grey	VSMC_M10	0.38	5.29E-02	-0.06	7.62E-01	0.26	1.96E-01	-0.26	1.94E-01	0.09	6.67E-01	-0.14	4.79E-01	0.27	1.75E-01	0.17	3.91E-01	-0.06	7.60E-01	-0.40	3.82E-02
ENSCAFG0000001310	ATF7IP	pink	VSMC_M5	0.38	5.30E-02	0.08	7.09E-01	-0.50	8.42E-03	0.45	1.98E-02	0.75	7.19E-06	-0.29	1.37E-01	-0.36	6.20E-02	-0.44	2.07E-02	-0.07	7.33E-01	-0.03	8.82E-01
ENSCAFG0000000768	RNF181	grey	VSMC_M10	0.38	5.30E-02	0.33	8.91E-02	0.00	9.99E-01	0.55	3.08E-03	0.09	6.50E-01	-0.06	7.52E-01	-0.03	8.88E-01	-0.61	6.67E-04	-0.34	8.26E-02	0.35	6.94E-02
ENSCAFG0000000410	FAM120B	yellow	VSMC_M3	0.38	5.31E-02	0.25	2.14E-01	0.44	2.07E-02	-0.20	3.20E-01	-0.21	3.01E-01	-0.02	9.34E-01	0.12	5.63E-01	0.03	8.89E-01	-0.33	9.06E-02	-0.04	8.35E-01
ENSCAFG0000000356	YAE1	red	VSMC_M1	0.38	5.32E-02	0.12	5.61E-01	-0.28	1.56E-01	0.41	3.43E-02	0.52	5.10E-03	-0.33	9.47E-02	-0.39	4.72E-02	-0.38	4.94E-02	-0.09	6.41E-01	-0.07	7.26E-01
ENSCAFG0000000180	DDX58	darkgreen	VSMC_M4	0.38	5.34E-02	0.66	1.80E-04	-0.19	3.53E-01	0.64	3.03E-04	0.17	4.02E-01	-0.02	9.19E-01	-0.41	3.40E-02	-0.74	1.17E-05	-0.65	2.64E-04	0.70	4.38E-05
ENSCAFG0000001061	HSF2BP	grey	VSMC_M10	0.38	5.35E-02	0.39	4.66E-02	0.49	9.39E-03	-0.11	5.70E-01	-0.42	2.75E-02	0.08	7.00E-01	0.16	4.20E-01	0.04	8.57E-01	-0.53	4.63E-03	0.29	1.42E-01
ENSCAFG0000000662	WDR62	darkgrey	VSMC_M8	0.38	5.35E-02	-0.19	3.34E-01	0.59	1.13E-03	-0.85	2.27E-08	-0.32	1.02E-01	0.09	6.63E-01	0.45	2.00E-02	0.82	1.96E-07	-0.06	7.55E-01	-0.51	6.99E-03
ENSCAFG0000001507	GAPDH	pink	VSMC_M5	0.38	5.37E-02	-0.28	1.62E-01	-0.25	2.07E-01	0.07	7.21E-01	0.57	1.96E-03	-0.25	2.09E-01	-0.15	4.66E-01	0.02	9.10E-01	0.25	2.14E-01	-0.46	1.52E-02
ENSCAFG0000003139	MPZL1	pink	VSMC_M5	0.38	5.38E-02	0.12	5.43E-01	-0.23	2.48E-01	0.24	2.33E-01	0.51	6.08E-03	-0.34	8.21E-02	-0.08	7.00E-01	-0.31	1.12E-01	-0.12	5.58E-01	-0.13	5.26E-01
ENSCAFG0000001305	DUSP12	grey	VSMC_M10	0.38	5.39E-02	0.06	7.75E-01	-0.09	6.62E-01	0.17	3.90E-01	0.28	1.50E-01	-0.09	6.41E-01	-0.27	1.78E-01	-0.19	3.33E-01	-0.07	7.43E-01	-0.07	7.16E-01
ENSCAFG0000001053	CARMIL1	grey	VSMC_M10	0.37	5.40E-02	0.72	2.46E-05	0.10	6.34E-01	0.24	2.22E-01	-0.01	9.65E-01	-0.01	9.47E-01	-0.27	1.81E-01	-0.41	3.38E-02	-0.73	1.35E-05	0.46	1.55E-02
ENSCAFG0000002006	DPYD	cyan	VSMC_M2	0.37	5.40E-02	0.79	7.70E-07	0.21	2.85E-01	0.30	1.24E-01	-0.07	7.38E-01	-0.27	1.76E-01	-0.13	5.28E-01	-0.53	4.17E-03	-0.76	4.72E-06	0.49	9.17E-03
ENSCAFG0000000528	SLITRK6	cyan	VSMC_M2	0.37	5.41E-02	0.60	8.52E-04	0.53	4.62E-03	-0.14	4.88E-01	-0.28	1.50E-01	-0.11	5.81E-01	-0.19	3.41E-01	-0.11	5.75E-01	-0.57	1.93E-03	0.14	4.81E-01
ENSCAFG0000000121	NUDT3	yellow	VSMC_M3	0.37	5.41E-02	0.17	3.98E-01	0.74	8.52E-06	-0.54	3.30E-03	-0.49	1.03E-02	-0.10	6.03E-01	0.10	6.20E-01	0.36	6.85E-02	-0.29	1.37E-01	-0.20	3.08E-01
ENSCAFG0000001796	ZNF287	red	VSMC_M1	0.37	5.41E-02	-0.10	6.22E-01	-0.31	1.12E-01	0.26	1.91E-01	0.45	1.88E-02	-0.05	7.96E-01	-0.06	7.65E-01	-0.20	3.07E-01	-0.02	9.16E-01	0.00	9.85E-01
ENSCAFG0000000275	MALSU1	red	VSMC_M1	0.37	5.41E-02	0.29	1.44E-01	0.17	4.04E-01	0.10	6.06E-01	0.14	4.91E-01	-0.34	8.17E-02	-0.43	2.38E-02	-0.22	2.67E-01	-0.26	1.95E-01	-0.13	5.16E-01
ENSCAFG0000000836	ENSCAFG0000000836	grey	VSMC_M10	0.37	5.42E-02	0.05	7.86E-01	-0.11	5.75E-01	0.03	8.80E-01	0.36	6.80E-02	-0.22	2.78E-01	0.03	8.86E-01	-0.02	9.19E-01	-0.15	4.53E-01	-0.22	2.72E-01
ENSCAFG0000001966	INPP5E	cyan	VSMC_M2	0.37	5.44E-02	0.64	2.86E-04	0.36	6.46E-02	0.07	7.43E-01	-0.35	7.07E-02	0.13	5.17E-01	0.01	9.69E-01	-0.23	2.47E-01	-0.73	1.59E-05	0.56	2.62E-03
ENSCAFG0000001715	RNASEH2A	grey	VSMC_M10	0.37	5.44E-02	0.09	6.41E-01	0.65	2.38E-04	-0.61	6.72E-04	-0.34	8.33E-02	-0.03	9.01E-01	0.31	1.19E-01	0.48	1.09E-02	-0.26	1.83E-01	-0.39	4.69E-02
ENSCAFG0000001343	ZNF511	yellow	VSMC_M3	0.37	5.44E-02	0.16	4.38E-01	0.73	1.87E-05	-0.57	1.79E-03	-0.48	1.04E-02	0.09	6.56E-01	0.00	9.83E-01	0.42	3.02E-02	-0.25	2.08E-01	-0.29	1.38E-01
ENSCAFG0000001999	GAN	pink	VSMC_M5	0.37	5.45E-02	-0.44	2.20E-02	-0.14	4.96E-01	-0.24	2.37E-01	0.48	1.19E-02	0.01	9.67E-01	-0.04	8.42E-01	0.33	9.58E-02	0.29	1.43E-01	-0.66	2.00E-04
ENSCAFG0000001361	FRMD8	cyan	VSMC_M2	0.37	5.46E-02	0.44	2.18E-02	0.34	7.91E-02	-0.14	4.76E-01	-0.07	7.23E-01	-0.15	4.49E-01	0.02	9.24E-01	-0.19	3.44E-01	-0.43	2.69E-02	0.06	7.71E-01
ENSCAFG0000001696	AREL1	grey	VSMC_M10	0.37	5.47E-02	0.20	3.17E-01	-0.26	1.89E-01	0.40	3.88E-02	0.29	1.45E-01	0.03	8.63E-01	-0.15	4.62E-01	-0.35	7.61E-02	-0.35	7.53E-02	0.28	1.56E-01
ENSCAFG0000001264	ENSCAFG0000001264	grey	VSMC_M10	0.37	5.48E-02	0.08	6.93E-01	0.23	2.48E-01	-0.06	7.55E-01	0.00	9.95E-01	0.06	7.51E-01	-0.02	9.22E-01	-0.08	6.76E-01	-0.14	5.00E-01	-0.12	5.68E-01
ENSCAFG0000002869	ENSCAFG0000002869	red	VSMC_M1	0.37	5.48E-02	0.12	5.57E-01	-0.13	5.31E-01	0.24	2.28E-01	0.41	3.20E-02	-0.33	9.44E-02	-0.48	1.06E-02	-0.27	1.81E-01	-0.07	7.29E-01	-0.18	3.59E-01
ENSCAFG0000001485	RNF4	grey	VSMC_M10	0.37	5.48E-02	-0.29	1.46E-01	0.04	8.30E-01	-0.38	4.82E-02	0.22	2.65E-01	0.16	4.33E-01	0.02	9.06E-01	0.46	1.48E-02	0.13	5.09E-01	-0.50	7.70E-03
ENSCAFG0000001016	PRDM10	red	VSMC_M1	0.37	5.48E-02	0.19	3.50E-01	0.15	4.46E-01	-0.23	2.43E-01	0.22	2.68E-01	-0.30	1.34E-01	-0.45	1.87E-02	0.06	7.85E-01	-0.17	4.09E-01	-0.37	6.07E-02
ENSCAFG0000001165	ENSCAFG0000001165	darkgrey	VSMC_M8	0.37	5.49E-02	-0.06	7.60E-01	0.62	6.31E-04	-0.87	3.68E-09	-0.34	8.49E-02	0.05	8.15E-01	0.35	7.21E-02	0.74	1.06E-05	-0.15	4.58E-01	-0.47	1.26E-02
ENSCAFG0000000490	KDM4A	grey	VSMC_M10	0.37	5.51E-02	0.34	8.48E-02	-0.21	3.02E-01	0.47	1.32E-02	0.34	7.86E-02	-0.22	2.60E-01	-0.01	9.75E-01	-0.54	3.50E-03	-0.43	2.52E-02	0.33	8.99E-02
ENSCAFG0000000569	CHMP6	darkgrey	VSMC_M8	0.37	5.51E-02	-0.21	2.98E-01	0.27	1.74E-01	-0.58	1.59E-03	0.06	7.66E-01	-0.25	2.14E-01	0.16	4.17E-01	0.56	2.56E-03	0.09	6.68E-01	-0.58	1.39E-03
ENSCAFG0000001090	INTS12	grey	VSMC_M10	0.37	5.55E-02	-0.01	9.61E-01	-0.08	6.96E-01	-0.10	6.08E-01	0.20	3.17E-01	0.07	7.38E-01	0.16	4.39E-01	0.15	4.62E-01	-0.19	3.52E-01	-0.05	8.02E-01
ENSCAFG0000002683	ENSCAFG0000002683	yellow	VSMC_M3	0.37	5.55E-02	0.43	2.48E-02	0.65	2.25E-04	-0.38	5.29E-02	-0.42	2.94E-02	0.08	6.76E-01	0.08	6.91E-01	0.15	4.64E-01	-0.53	4.16E-03	-0.07	7.30E-01
ENSCAFG0000000160	CPSF1	grey	VSMC_M10	0.37	5.55E-02	0.73	1.39E-05	0.74	8.58E-06	-0.19	3.55E-01	-0.69	6.31E-05	0.17	4.01E-01	-0.08	6.85E-01	-0.08	6.73E-01	-0.81	2.72E-07	0.47	1.32E-02
ENSCAFG0000001283	ENSCAFG0000001283	grey	VSMC_M10	0.37	5.56E-02	0.47	1.40E-02	-0.07	7.41E-01	0.45	1.92E-02	0.29	1.36E-01	-0.47	1.44E-02	-0.34	8.63E-02	-0.64	3.09E-04	-0.38	5.39E-02	0.23	2.41E-01
ENSCAFG0000000679	C18H11orf74	red	VSMC_M1	0.37	5.56E-02	0.31	1.20E-01	0.09	6.68E-01	0.15	4.62E-01	-0.03	8.97E-01	0.00	9.91E-01	0.15	4.41E-01	-0.13	5.21E-01	-0.48	1.07E-02	0.32	1.09E-01
ENSCAFG0000000679	ENSCAFG0000000679	darkgrey	VSMC_M8	0.37	5.58E-02	-0.06	7.64E-01	0.67	1.49E-04	-0.85	2.16E-08	-0.39	4.50E-02	0.13	5.15E-01	0.32	1.04E-01	0.72	1.97E-05	-0.15	4.56E-01	-0.49	1.02E-02
ENSCAFG0000001381	FMNL1	yellow	VSMC_M3	0.37	5.60E-02	0.15	4.64E-01	0.32	1.06E-01	-0.41	3.42E-02	-0.13	5.32E-01	-0.04	8.52E-01	0.37	5.88E-02	0.30	1.32E-01	-0.33	9.22E-02	-0.11	5.71E-01
ENSCAFG0000001218	DVL3	grey	VSMC_M10	0.37	5.61E-02	0.78	1.32E-06	0.12	5.62E-01	0.33	9.39E-02	0.00	9.83E-01	-0.07	7.37E-01	-0.38	5.32E-02	-0.64	3.19E-04	-0.69	5.95E-05	0.53	4.11E-03
ENSCAFG0000001661	ENSCAFG0000001661	cyan	VSMC_M2	0.37	5.62E-02	0.45	1.95E-02	0.09	6.42E-01	0.35	7.47E-02	-0.01	9.45E-01	0.19	3.54E-01	-0.11	5.92E-01	-0.53	4.72E-03	-0.42	3.13E-02	0.40	3.62E-02
ENSCAFG0000001650	PALM3	yellow	VSMC_M3	0.37	5.63E-02	0.19	3.50E-01	0.71	3.68E-05	-0.70	5.03E-05	-0.37	5.50E-02	-0.14	4.72E-01	0.20	3.10E-01	0.50	7.94E-03	-0.28	1.52E-01	-0.33	8.99E-02
ENSCAFG0000002877	ENSCAFG0000002877	yellow	VSMC_M3	0.37	5.63E-02	0.16	4.23E-01	0.42	2.85E-02	-0.51	6.10E-03	-0.16	4.33E-01	-0.13	5.16E-01	0.12	5.49E-01	0.35	7.31E-02	-0.24	2.30E-01	-0.25	2.01E-01
ENSCAFG0000001863	ENSCAFG0000001863	red	VSMC_M1	0.37	5.63E-02	0.31	1.13E-01	0.30	1.26E-01	-0.24	2.28E-01	-0.08	6.78E-01	-0.13	5.11E-01	0.04	8.26E-01	0.09	6.48E-01	-0.39	4.35E-02	-0.01	9.66E-01
ENSCAFG0000000621	ERLIN2	red	VSMC_M1	0.37	5.65E-02	0.23	2.56E-01	0.01	9.66E-01	0.20	3.12E-01	0.28	1.55E-01	-0.38	4.88E-02	-0.02	9.16E-01	-0.28	1.56E-01	-0.25	2.12E-01	-0.06	7.82E-01
ENSCAFG0000000497	CELF2	grey	VSMC_M10	0.37	5.65E-02	0.73	1.84E-05	0.10	6.32E-01	0.48	1.08E-02	-0.02	9.30E-01	-0.34	8.40E-02	-0.05	7.86E-01	-0.62	5.54E-04	-0.74	9.29E-06	0.59	1.35E-03
ENSCAFG0000001315	FIBP	cyan	VSMC_M2	0.37	5.65E-02	0.61	7.09E-04	0.29	1.40E-01	0.04	8.33E-01	-0.11	5.68E-01	0.03	8.81E-01	-0.05	7.90E-01	-0.31	1.12E-01	-0.64	3.45E-04	0.27	1.76E-01
ENSCAFG0000000611	EXOSC8	darkgrey	VSMC_M8	0.37	5.65E-02	-0.47	1.27E-02	-0.01	9.68E-01	-0.50	7.28E-03	0.36	6.52E-02	-0.11	5.95E-01	0.00	9.98E-01	0.60	9.36E-04	0.29	1.37E-01	-0.78	1.56E-06
ENSCAFG0000000313	CCM2	yellow	VSMC_M3	0.37	5.74E-02	0.36	6.53E-02	0.42	2.98E-02	-0.21	2.82E-01	-0.26	1.88E-01	-0.12	5.47E-01	-0.15	4.42E-01	0.14	4.95E-01	-0.44	2.06E-02	-0.01	9.77E-01
ENSCAFG0000001739	TRIM25	yellow	VSMC_M3	0.37	5.75E-02	0.60	8.74E-04	0.58	1.61E-03	-0.34	8.05E-02	-0.43	2.50E-02	0.05	7.91E-01	0.30	1.28E-01	0.05	7.91E-01	-0.72	2.27E-05	0.27	1.75E-01
ENSCAFG0000000989	POLL	grey	VSMC_M10	0.37	5.75E-02	0.54	3.55E-03	0.40	4.11E-02	-0.01	9.49E-01	-0.30	1.30E-01	-0.15	4.70E-01	0.12	5.50E-01	-0.15	4.66E-01	-0.61	7.82E-04	0.43	2.67E-02
ENSCAFG0000000430	SPRYD7	pink	VSMC_M5	0.37	5.76E-02	-0.35	6.95E-02	-0.48	1.17E-02	0.26	1.83E-01	0.78	1.89E-06	-0.23	2.48E-01	-0.28	1.52E-01	-0.10	6.27E-01	0.28	1.59E-01	-0.47	1.31E-02
ENSCAFG0000002854	ENSCAFG0000002854	yellow	VSMC_M3	0.37	5.78E-02	0.24	2.21E-01	0.45	1.72E-02	-0.01	9.57E-01	-0.24	2.36E-01	-0.11	5.99E-01	-0.18	3.60E-01	-0.08	6.96E-01	-0.30	1.27E-01	-0.01	9.47E-01
ENSCAFG0000002347	ENSCAFG0000002347	turquoise	VSMC_M6	0.37	5.78E-02	-0.02	9.20E-01	-0.06	7.69E-01	0.00	9.83E-01	0.13	5.12E-01	0.18	3.57E-01	0.07	7.39E-01	0.03	8.88E-01	-0.18	3.82E-01	0.02	9.27E-01
ENSCAFG0000000130	LRGUK	pink	VSMC_M5	0.37	5.79E-02	-0.10	6.12E-01	-0.15	4.40E-01	-0.10	6.24E-01	0.39	4.41E-02	-0.18	3.67E-01	0.13	5.32E-01	0.08	7.05E-01	-0.04	8.29E-01	-0.29	1.37E-01
ENSCAFG0000000374	EPHB6	darkgreen	VSMC_M4	0.37	5.79E-02	0.61	6.97E-04	0.03	8.90E-01	0.57	1.81E-03	0.00	9.95E-01	-0.10	6.24E-01	-0.29	1.44E-01	-0.67	1.24E-04	-0.63	4.84E-04	0.63	4.67E-04
ENSCAFG0000001171	GMEB1	grey	VSMC_M10	0.37	5.79E-02	-0.04	8.28E-01	0.13	5.09E-01	-0.48	1.09E-02	0.22	2.81E-01	-0.16	4.24E-01	0.19	3.41E-01	0.37	5.73E-02	-0.06	7.71E-01	-0.48	1.17E-02
ENSCAFG0000000741	WEE1	darkgrey	VSMC_M8	0.37	5.80E-02	-0.29	1.41E-01	0.29	1.43E-01	-0.62	5.86E-04	0.01	9.56E-01	0.01	9.74E-01	0.47	1.34E-02	0.62	5.19E-04	0.06	7.48E-01	-0.57	1.74E-03
ENSCAFG0000000799	ENSCAFG0000000799	grey	VSMC_M10	0.37	5.81E-02	0.07	7.31E-01	0.00	9.86E-01	0.14	4.91E-01	0.17	3.97E-01	-0.16	4.11E-01	-0.16	4.26E-01	-0.10	6.22E-01	-0.16	4.25E-01	0.00	9.85E-01
ENSCAFG0000002899	ENSCAFG0000002899	grey	VSMC_M10	0.37	5.81E-02	0.34	8.00E-02	-0.07	7.45E-01	0.35	7.37E-02	0.08	6.81E-01	0.17	3.93E-01	-0.27	1.80E-01	-0.36	6.77E-02	-0.47	1.28E-02	0.37	5.91E-02
ENSCAFG0000000927	EGR3	yellow	VSMC_M3	0.37	5.83E-02	0.27	1.68E-01	0.38	5.11E-02	-0.46	1.58E-02	-0.11	5.75E-01	0.05	7.93E-01	0.24	2.35E-01	0.28	1.53E-01	-0.36	6.91E-02	-0.17	4.00E-01
ENSCAFG0000003035	ENSCAFG0000003035	cyan	VSMC_M2	0.37	5.83E-02	0.46	1.61E-02	0.23	2.55E-01	0.29	1.47E-01	-0.17	3.97E-01	-0.10	6.20E-01	-0.07	7.17E-01	-0.37	5.98E-02	-0.54	3.41E-03	0.48	1.15E-02
ENSCAFG0000001681	KDM6B	grey	VSMC_M10	0.37	5.84E-02	0.46	1.70E-02	0.09	6.56E-01	0.26	1.89E-01	-0.08	7.07E-01	0.14	4.78E-01	-0.29	1.41E-01	-0.38	5.21E-02	-0.54	3.54E-03	0.50	8.31E-03
ENSCAFG0000001222	COL6A3	grey	VSMC_M10	0.37	5.85E-02	0.16	4.20E-01	0.33	9.66E-02	-0.44	2.09E-02	-0.06	7.48E-01	-0.12	5.40E-01	0.61	7.69E-04	0.28	1.55E-01	-0.33	9.01E-02	-0.12	5.63E-01
ENSCAFG0000001837	SLC25A43	grey	VSMC_M10	0.37	5.85E-02	0.29	1.38E-01	-0.17	3.99E-01	0.16	4.32E-01	0.37	5.61E-02	-0.03	8.70E-01	0.06	7.75E-01	-0.23	2.46E-01	-0.38	5.37E-02	0.07	7.20E-01
ENSCAFG0000001710	PLEKHO2	yellow	VSMC_M3	0.37	5.85E-02	0.54	3.43E-03	0.61	7.68E-04	-0.32	1.03E-01	-0.42	3.11E-02	0.09	6.40E-01	-0.29	1.40E-01	0.08	7.03E-01	-0.55	2.84E-03	0.05	8.21E-01
ENSCAFG0000001899	SLC6A4	yellow	VSMC_M3	0.37	5.86E-02	0.44	2.31E-02	0.59	1.10E-03	-0.18	3.71E-01	-0.31	1.11E-01	-0.04	8.35E-01	-0.06	7.82E-01	-0.05	7.92E-01	-0.43	2.67E-02	0.01	9.48E-01
ENSCAFG0000000551	POLB	darkgreen	VSMC_M4	0.37	5.86E-02	0.02	9.33E-01	-0.40	3.77E-02	0.52	5.16E-03	0.56	2.58E-03	-0.07	7.22E-01	-0.30	1.31E-01	-0.46	1.58E-02	-0.02	9.37E-01	0.03	8.97E-01
ENSCAFG0000001686	ENSCAFG0000001686	grey	VSMC_M10	0.37	5.87E-02	0.55	3.28E-03	0.54	3.56E-03	-0.18	3.61E-01	-0.41	3.56E-02	-0.13	5.23E-01	0.11	5.76E-01	-0.01	9.79E-01	-0.65	2.43E-04	0.29	1.48E-01
ENSCAFG0000000348	PNKP	yellow	VSMC_M3	0.37	5.87E-02	0.38	4.99E-02	0.73	1.28E-05	-0.46	1.57E-02	-0.48	1.13E-02	0.09	6.55E-01	-0.01	9.44E-01	0.19	3.33E-01	-0.44	2.20E-02	-0.06	7.50E-01
ENSCAFG0000000274	HEBP2	grey	VSMC_M10	0.37	5.90E-02	0.08	6.89E-01	0.19	3.52E-01	-0.10	6.10E-01	-0.11	5.78E-01	-0.07	7.20E-01	0.16	4.37E-01	0.13	5.11E-01	-0.27	1.66E-01	0.07	7.33E-01
ENSCAFG0000001018	DEK	darkgrey	VSMC_M8	0.37	5.91E-02	-0.42	2.81E-02	0.20	3.23E-01	-0.64	2.84E-04	0.14	4.77E-01	-0.06	7.76E-01	0.34	7.84E-02	0.73	1.27E-05	0.22	2.80E-01	-0.69	7.25E-05
ENSCAFG0000002903	C20H19orf53	grey	VSMC_M10	0.37	5.91E-02	0.06	7.73E-01	0.26	1.86E-01	-0.09	6.56E-01	-0.07	7.46E-01	0.15	4.52E-01	0.03	9.00E-01	0.04	8.30E-01	-0.17	3.97E-01	-0.18	3.68E-01
ENSCAFG0000002321	ENSCAFG0000002321	cyan	VSMC_M2	0.37	5.93E-02	0.46	1.50E-02	0.33	8.86E-02	-0.02	9.20E-01	-0.12	5.39E-01	-0.14	4.88E-01	-0.29	1.43E-01	-0.18	3.59E-01	-0.44	2.32E-02	0.15	4.41E-01
ENSCAFG0000000733	REEP5	cyan	VSMC_M2	0.37	5.94E-02	0.58	1.43E-03	0.35	7.01E-02	0.35	7.42E-02	-0.29	1.41E-01	-0.02	9.28E-01	-0.39	4.37E-02	-0.51	6.27E-03	-0.55	3.25E-03	0.44	2.09E-02
ENSCAFG0000001667	LCORL	grey	VSMC_M10	0.37	5.96E-02	-0.01	9.60E-01	0.31	1.21E-01	-0.33	9.59E-02	0.10	6.14E-01	-0.36	6.18E-02	0.28	1.50E-01	0.19	3.53E-01	0.00	9.96E-01	-0.42	2.93E-02
ENSCAFG0000001684	HOXB4	cyan	VSMC_M2	0.37	5.96E-02	0.55	2.86E-03	0.31	1.17E-01	-0.01	9.42E-01	-0.17	4.05E-01	0.06	7.75E-01	-0.35	7.35E-02	-0.18	3.64E-01	-0.57	2.02E-03	0.24	2.37E-01
ENSCAFG0000000789	CDKN2AIP	grey	VSMC_M10	0.37	5.97E-02	0.01	9.71E-01	0.12	5.48E-01	-0.01	9.44E-01	-0.09	6.69E-01	0.33	8.78E-02	0.14	4.73E-01	0.08	6.77E-01	-0.19	3.32E-01	0.10	6.12E-01
ENSCAFG0000002326	GAS1	cyan	VSMC_M2	0.37	5.97E-02	0.65	2.10E-04	0.69	5.90E-05	-0.31	1.19E-01	-0.48	1.21E-02	-0.11	5.75E-01	0.06	7.54E-01	0.00	9.85E-01	-0.67	1.30E-04	0.17	3.85E-01
ENSCAFG0000001637	DHRS3	grey	VSMC_M10	0.37	5.97E-02	0.62	5.07E-04	0.58	1.47E-03	-0.05	8.01E-01	-0.48	1.06E-02	0.04	8.29E-01	-0.15	4.41E-01	-0.21	2.82E-01	-0.62	5.39E-04	0.39	4.71E-02
ENSCAFG0000002525	PEAK1	grey	VSMC_M10	0.37	5.98E-02	0.23	2.54E-01	-0.35	6.94E-02	0.49	9.29E-03	0.40	3.78E-02	0.06	7.56E-01	0.04	8.33E-01	-0.45	1.83E-02	-0.29	1.42E-01	0.33	9.05E-02
ENSCAFG0000001966	R3HDM4	cyan	VSMC_M2	0.37	6.00E-02	0.52	5.81E-03	0.36	6.83E-02	0.08	6.80E-01	-0.23	2.55E-01	0.03	8.74E-01	0.13	5.32E-01	-0.19	3.44E-01	-0.60	9.24E-04	0.32	1.09E-01
ENSCAFG0000000188	APPL2	cyan	VSMC_M2	0.37	6.02E-02	0.43	2.46E-02	-0.10	6.26E-01	0.29	1.48E-01	0.28	1.51E-01	-0.28	1.57E-01	0.01	9.74E-01	-0.46	1.65E-02	-0.47	1.45E-02	0.29	1.37E-01
ENSCAFG0000001658	ENSCAFG0000001658	grey	VSMC_M10	0.37	6.03E-02	0.25	2.13E-01	0.25	2.18E-01	0.07	7.24E-01	-0.11	5.77E-01	-0.27	1.81E-01	-0.09	6.42E-01	-0.12	5.53E-01	-0.30	1.33E-01	0.15	4.66E-01
ENSCAFG0000000095	RPS18	grey	VSMC_M10	0.37	6.03E-02	0.05	8.02E-01	0.12	5.54E-01	-0.06	7.77E-01	-0.01	9.59E-01	-0.06	7.52E-01	0.09	6.50E-01	0.13	5.23E-01	-0.26	1.90E-01	-0.08	6.92E-01
ENSCAFG0000003153	ENSCAFG0000003153	cyan	VSMC_M2	0.37	6.04E-02	0.80	5.62E-07	0.65	2.44E-04	-0.10	6.09E-01	-0.45	1.96E-02	-0.20	3.30E-01	-0.11	5.79E-01	-0.30	1.32E-01	-0.71	3.02E-05	0.32	1.03E-01
ENSCAFG0000001934	TAX1BP3	grey	VSMC_M10	0.37	6.04E-02	0.41	3.45E-02	0.50	7.57E-03	-0.14	4.84E-01	-0.29	1.38E-01	-0.20	3.11E-01	0.03	8.72E-01	-0.03	8.69E-01	-0.41	3.33E-02	0.03	8.69E-01
ENSCAFG0000000532	GALNT14	yellow	VSMC_M3	0.37	6.04E-02	0.09	6.48E-01	0.54	3.45E-03	-0.25	2.00E-01	-0.29	1.47E-01	-0.14	5.01E-01	-0.04	8.33E-01	0.15	4.51E-01	-0.18	3.79E-01	-0.21	3.03E-01
ENSCAFG0000001615	NR1D1	red	VSMC_M1	0.37	6.05E-02	0.08	7.07E-01	-0.08	6.87E-01	-0.12	5.54E-01	0.32	1.04E-01	-0.18	3.78E-01	0.09	6.56E-01	0.08	7.01E-01	-0.15	4.58E-01	-0.10	6.35E-01
ENSCAFG0000001462	VAT1	cyan	VSMC_M2	0.37	6.05E-02	0.82	1.53E-07	0.70	4.55E-05	-0.18	3.73E-01	-0.50	7.93E-03	-0.10	6.08E-01	-0.12	5.53E-01	-0.23	2.57E-01	-0.75	6.05E-06	0.32	1.01E-01
ENSCAFG0000003088	ZBTB47	cyan	VSMC_M2	0.37	6.06E-02	0.79	8.89E-07	0.16	4.38E-01	0.51	6.26E-03	-0.03	8.85E-01	-0.18	3.69E-01	-0.53	4.85E-03	-0.77	2.81E-06	-0.64	2.83E-04	0.54	3.62E-03
ENSCAFG0000000766	USP39	grey	VSMC_M10	0.37	6.07E-02	-0.26	1.94E-01	-0.19	3.38E-01	-0.11	5.76E-01	0.35	7.16E-02	-0.05	8.01E-01	0.06	7.66E-01	0.29	1.46E-01	0.10	6.07E-01	-0.24	2.24E-01
ENSCAFG0000000234	INPP4A	grey	VSMC_M10	0.37	6.08E-02	0.35	7.23E-02	-0.08	7.02E-01	0.53	4.61E-03	0.26	1.99E-01	-0.15	4.45E-01	-0.22	2.70E-01	-0.62	5.29E-04	-0.30	1.34E-01	0.27	1.76E-01
ENSCAFG0000000693	AMHR2	grey	VSMC_M10	0.37	6.08E-02	0.23	2.56E-01	0.45	1.97E-02	-0.26	1.95E-01	-0.15	4.52E-01	-0.16	4.26E-01	0.00	9.82E-01	0.04	8.39E-01	-0.26	1.83E-01	-0.21	2.96E-01
ENSCAFG0000000473	BEND7	red	VSMC_M1	0.37	6.08E-02	0.24	2.24E-01	0.18	3.61E-01	-0.10	6.06E-01	0.12	5.58E-01	-0.38	4.93E-02	-0.22	2.67E-01	-0.03	8.73E-01	-0.28	1.61E-01	-0.23	2.51E-01
ENSCAFG0000001514	TAPBPL	grey	VSMC_M10	0.37	6.08E-02	0.83	1.00E-07	0.23	2.39E-01	0.34	8.51E-02	-0.19	3.45E-01	0.02	9.03E-01	-0.18	3.63E-01	-0.54	3.74E-03	-0.83	7.09E-08	0.63	4.28E-04

ENSCAFG0000000895	RIPK2	grey	VSMC_M10	0.37	6.09E-02	0.56	2.37E-03	0.06	7.48E-01	0.32	1.06E-01	0.08	6.76E-01	-0.17	4.03E-01	-0.38	5.27E-02	-0.49	9.62E-03	-0.57	1.87E-03	0.29	1.43E-01
ENSCAFG0000000560	RELT	grey	VSMC_M10	0.37	6.11E-02	-0.43	2.43E-02	0.06	7.79E-01	-0.38	5.36E-02	0.11	5.80E-01	0.20	3.22E-01	0.17	3.84E-01	0.51	6.02E-03	0.22	2.60E-01	-0.42	2.90E-02
ENSCAFG0000002465	ENSCAFG0000002465	yellow	VSMC_M3	0.36	6.12E-02	0.29	1.36E-01	0.56	2.36E-03	-0.20	3.07E-01	-0.31	1.15E-01	-0.02	9.21E-01	-0.01	9.79E-01	0.02	9.37E-01	-0.34	7.85E-02	-0.06	7.77E-01
ENSCAFG0000000941	NDUFAF6	grey	VSMC_M10	0.36	6.12E-02	-0.01	9.73E-01	-0.26	1.88E-01	0.38	5.28E-02	0.38	5.18E-02	-0.13	5.11E-01	0.02	9.12E-01	-0.34	7.88E-02	-0.05	8.05E-01	0.11	6.00E-01
ENSCAFG0000001365	DPF2	cyan	VSMC_M2	0.36	6.13E-02	0.60	8.34E-04	0.06	7.52E-01	0.24	2.37E-01	-0.03	8.80E-01	-0.21	2.83E-01	-0.20	3.22E-01	-0.37	6.07E-02	-0.64	3.21E-04	0.50	8.04E-03
ENSCAFG0000001373	MIPOL1	grey	VSMC_M10	0.36	6.13E-02	0.01	9.48E-01	-0.40	4.12E-02	0.13	5.13E-01	0.56	2.49E-03	-0.25	2.11E-01	0.14	4.92E-01	-0.14	4.85E-01	-0.12	5.54E-01	0.03	8.92E-01
ENSCAFG0000001635	DBN1	yellow	VSMC_M3	0.36	6.13E-02	0.40	3.74E-02	0.86	1.09E-08	-0.67	1.36E-04	-0.56	2.21E-03	-0.17	3.87E-01	-0.06	7.51E-01	0.39	4.67E-02	-0.46	1.58E-02	-0.21	2.96E-01
ENSCAFG0000000768	VAMP5	grey	VSMC_M10	0.36	6.14E-02	0.40	3.68E-02	0.61	6.97E-04	-0.21	2.96E-01	-0.36	6.78E-02	-0.06	7.65E-01	0.19	3.39E-01	0.00	9.88E-01	-0.44	2.03E-02	0.02	9.37E-01
ENSCAFG0000002991	CLIC3	cyan	VSMC_M2	0.36	6.14E-02	0.83	9.00E-08	0.37	5.81E-02	0.12	5.61E-01	-0.26	1.89E-01	-0.17	4.10E-01	-0.32	1.06E-01	-0.37	5.82E-02	-0.79	1.11E-06	0.48	1.09E-02
ENSCAFG0000000548	POLD3	darkgrey	VSMC_M8	0.36	6.15E-02	-0.34	8.50E-02	0.22	2.71E-01	-0.52	5.92E-03	0.01	9.45E-01	-0.10	6.32E-01	0.42	3.03E-02	0.61	7.33E-04	0.04	8.47E-01	-0.49	1.01E-02
ENSCAFG0000000841	EPB41L1	grey	VSMC_M10	0.36	6.15E-02	0.13	5.30E-01	-0.17	4.06E-01	0.35	7.40E-02	0.36	6.77E-02	-0.25	2.06E-01	-0.21	2.93E-01	-0.27	1.81E-01	-0.22	2.74E-01	0.00	9.85E-01
ENSCAFG0000000076	EGFL8	red	VSMC_M1	0.36	6.15E-02	0.34	8.23E-02	0.09	6.62E-01	0.07	7.26E-01	-0.02	9.17E-01	-0.10	6.14E-01	-0.04	8.25E-01	-0.16	4.32E-01	-0.43	2.66E-02	0.34	8.59E-02
ENSCAFG0000000412	BBS7	grey	VSMC_M10	0.36	6.16E-02	-0.20	3.14E-01	0.00	9.94E-01	-0.08	6.88E-01	0.21	2.96E-01	-0.10	6.13E-01	0.29	1.49E-01	0.18	3.60E-01	-0.02	9.18E-01	-0.22	2.75E-01
ENSCAFG0000000214	IL1RL1	red	VSMC_M1	0.36	6.16E-02	0.30	1.26E-01	-0.17	4.02E-01	0.34	8.07E-02	0.23	2.40E-01	-0.10	6.19E-01	-0.31	1.15E-01	-0.44	2.23E-02	-0.35	7.00E-02	0.31	1.20E-01
ENSCAFG0000001319	NSUN5	grey	VSMC_M10	0.36	6.18E-02	0.38	4.99E-02	0.69	7.16E-05	-0.34	8.64E-02	-0.62	6.00E-04	0.21	2.99E-01	0.18	3.83E-01	0.15	4.57E-01	-0.54	3.98E-03	0.27	1.76E-01
ENSCAFG0000001892	KDM4B	cyan	VSMC_M2	0.36	6.19E-02	0.50	7.57E-03	0.26	1.89E-01	0.21	2.93E-01	0.03	8.99E-01	-0.20	3.07E-01	-0.28	1.60E-01	-0.49	9.81E-03	-0.35	7.58E-02	0.12	5.65E-01
ENSCAFG0000001150	RRH	grey	VSMC_M10	0.36	6.20E-02	-0.03	8.82E-01	-0.06	7.50E-01	0.14	4.72E-01	0.15	4.69E-01	0.13	5.03E-01	0.09	6.42E-01	-0.12	5.55E-01	-0.10	6.21E-01	0.11	5.86E-01
ENSCAFG0000002990	HIKESHI	grey	VSMC_M10	0.36	6.20E-02	0.14	4.98E-01	0.13	5.14E-01	0.17	4.04E-01	0.13	5.05E-01	-0.32	9.89E-02	-0.03	8.77E-01	-0.19	3.50E-01	-0.19	3.37E-01	-0.11	6.01E-01
ENSCAFG0000001411	MORF4L1	pink	VSMC_M5	0.36	6.22E-02	-0.49	9.98E-03	-0.24	2.19E-01	-0.16	4.31E-01	0.56	2.47E-03	-0.13	5.23E-01	-0.04	8.62E-01	0.34	7.95E-02	0.37	5.88E-02	-0.63	4.78E-04
ENSCAFG0000000859	ENSCAFG0000000859	red	VSMC_M1	0.36	6.24E-02	0.12	5.53E-01	-0.12	5.59E-01	0.18	3.76E-01	0.15	4.62E-01	-0.05	7.95E-01	0.23	2.49E-01	-0.15	4.54E-01	-0.23	2.46E-01	0.23	2.59E-01
ENSCAFG0000000141	CSNK1E	grey	VSMC_M10	0.36	6.25E-02	0.20	3.07E-01	-0.03	8.81E-01	0.25	2.16E-01	0.21	2.95E-01	0.26	1.97E-01	-0.47	1.26E-02	-0.27	1.68E-01	-0.18	3.62E-01	-0.03	8.96E-01
ENSCAFG0000000575	IDO1	grey	VSMC_M10	0.36	6.25E-02	0.35	7.04E-02	-0.10	6.32E-01	0.38	5.04E-02	0.16	4.38E-01	-0.11	5.94E-01	-0.64	3.61E-04	-0.39	4.22E-02	-0.36	6.64E-02	0.28	1.59E-01
ENSCAFG0000001410	PCBD1	cyan	VSMC_M2	0.36	6.26E-02	0.83	9.49E-08	0.18	3.57E-01	0.54	3.49E-03	-0.09	6.71E-01	-0.24	2.26E-01	-0.40	3.71E-02	-0.75	6.48E-06	-0.72	2.02E-05	0.59	1.15E-03
ENSCAFG0000000582	IL18BP	red	VSMC_M1	0.36	6.27E-02	0.24	2.34E-01	0.29	1.38E-01	-0.26	1.83E-01	0.01	9.73E-01	-0.18	3.63E-01	-0.24	2.19E-01	0.12	5.50E-01	-0.30	1.32E-01	-0.25	2.03E-01
ENSCAFG0000001657	SEPT1	grey	VSMC_M10	0.36	6.27E-02	0.24	2.36E-01	-0.30	1.24E-01	0.28	1.51E-01	0.54	3.70E-03	-0.16	4.37E-01	-0.18	3.65E-01	-0.31	1.11E-01	-0.23	2.44E-01	0.01	9.72E-01
ENSCAFG0000000241	TDRD7	grey	VSMC_M10	0.36	6.29E-02	0.65	2.29E-04	-0.15	4.67E-01	0.65	2.12E-04	0.17	3.86E-01	-0.16	4.12E-01	-0.08	6.84E-01	-0.78	1.32E-06	-0.66	2.07E-04	0.68	8.13E-05
ENSCAFG0000001047	FAM118B	red	VSMC_M1	0.36	6.29E-02	0.25	2.07E-01	0.08	6.78E-01	0.04	8.34E-01	0.00	9.86E-01	0.04	8.45E-01	0.44	2.04E-02	-0.12	5.60E-01	-0.41	3.38E-02	0.23	2.38E-01
ENSCAFG0000001176	STK39	pink	VSMC_M5	0.36	6.29E-02	-0.23	2.53E-01	-0.39	4.65E-02	0.20	3.22E-01	0.57	1.95E-03	-0.16	4.20E-01	0.03	8.77E-01	-0.10	6.28E-01	0.15	4.58E-01	-0.18	3.67E-01
ENSCAFG0000001252	ENSCAFG0000001252	grey	VSMC_M10	0.36	6.29E-02	-0.02	9.34E-01	0.03	8.90E-01	0.11	5.95E-01	0.20	3.06E-01	-0.02	9.33E-01	-0.11	5.81E-01	-0.09	6.63E-01	-0.04	8.40E-01	-0.19	3.52E-01
ENSCAFG0000000002	CYB5A	cyan	VSMC_M2	0.36	6.30E-02	0.65	2.53E-04	0.26	1.84E-01	0.13	5.14E-01	-0.21	2.88E-01	0.03	8.84E-01	-0.60	1.04E-03	-0.29	1.42E-01	-0.68	9.87E-05	0.39	4.18E-02
ENSCAFG0000000784	TIMM10	red	VSMC_M1	0.36	6.30E-02	-0.07	7.31E-01	0.44	2.29E-02	-0.44	2.02E-02	-0.18	3.76E-01	0.29	1.45E-01	-0.17	3.85E-01	0.41	3.61E-02	-0.05	8.12E-01	-0.46	1.51E-02
ENSCAFG0000000751	NLN	grey	VSMC_M10	0.36	6.30E-02	0.38	5.20E-02	-0.22	2.64E-01	0.57	1.99E-03	0.27	1.80E-01	-0.15	4.51E-01	0.00	9.99E-01	-0.59	1.07E-03	-0.45	1.88E-02	0.50	7.24E-03
ENSCAFG0000001720	CCNG1	darkgreen	VSMC_M4	0.36	6.32E-02	0.42	2.73E-02	-0.36	6.76E-02	0.75	6.23E-06	0.39	4.73E-02	-0.08	7.04E-01	-0.45	1.99E-02	-0.75	7.73E-06	-0.42	3.05E-02	0.50	8.42E-03
ENSCAFG0000001116	LRRC58	grey	VSMC_M10	0.36	6.32E-02	0.31	1.12E-01	0.29	1.39E-01	-0.11	5.89E-01	-0.16	4.26E-01	0.14	4.77E-01	0.01	9.59E-01	-0.04	8.25E-01	-0.37	5.42E-02	0.14	4.83E-01
ENSCAFG0000001406	ZDHHC3	yellow	VSMC_M3	0.36	6.35E-02	0.36	6.74E-02	0.63	4.31E-04	-0.37	5.59E-02	-0.44	2.31E-02	0.02	9.37E-01	-0.15	4.66E-01	0.14	4.75E-01	-0.39	4.14E-02	-0.05	8.11E-01
ENSCAFG0000000002	TIMM21	grey	VSMC_M10	0.36	6.36E-02	-0.29	1.44E-01	0.20	3.23E-01	-0.37	5.57E-02	-0.04	8.39E-01	0.02	9.09E-01	0.24	2.32E-01	0.49	1.03E-02	0.00	9.90E-01	-0.30	1.30E-01
ENSCAFG0000002317	RBM8A	yellow	VSMC_M3	0.36	6.36E-02	0.06	7.83E-01	0.59	1.11E-03	-0.43	2.54E-02	-0.48	1.07E-02	0.00	9.88E-01	0.15	4.54E-01	0.41	3.38E-02	-0.25	2.03E-01	-0.06	7.76E-01
ENSCAFG0000001821	PDGFRB	cyan	VSMC_M2	0.36	6.37E-02	0.71	3.33E-05	0.56	2.51E-03	-0.23	2.43E-01	-0.33	8.92E-02	-0.19	3.49E-01	0.07	7.34E-01	-0.12	5.41E-01	-0.70	4.50E-05	0.24	2.37E-01
ENSCAFG0000002915	CALM3	cyan	VSMC_M2	0.36	6.38E-02	0.46	1.64E-02	0.29	1.37E-01	0.17	4.04E-01	-0.19	3.32E-01	-0.01	9.62E-01	-0.32	1.00E-01	-0.17	4.05E-01	-0.54	3.45E-03	0.19	3.44E-01
ENSCAFG0000001086	SCN3A	red	VSMC_M1	0.36	6.39E-02	0.12	5.59E-01	0.11	5.70E-01	-0.09	6.53E-01	-0.04	8.26E-01	0.19	3.38E-01	-0.41	3.20E-02	0.05	8.08E-01	-0.21	2.99E-01	0.07	7.44E-01
ENSCAFG0000001283	DUSP18	grey	VSMC_M10	0.36	6.39E-02	0.41	3.15E-02	0.13	5.03E-01	0.00	9.86E-01	-0.10	6.07E-01	0.13	5.06E-01	-0.02	9.17E-01	-0.15	4.63E-01	-0.49	1.03E-02	0.37	5.85E-02
ENSCAFG0000001690	KHDC4	grey	VSMC_M10	0.36	6.40E-02	0.40	3.92E-02	0.20	3.23E-01	0.10	6.16E-01	0.05	8.05E-01	-0.33	9.15E-02	-0.12	5.65E-01	-0.34	8.62E-02	-0.41	3.38E-02	0.10	6.22E-01
ENSCAFG0000001566	ZMIZ1	red	VSMC_M1	0.36	6.40E-02	0.40	3.80E-02	-0.07	7.31E-01	0.27	1.75E-01	0.15	4.51E-01	0.14	4.93E-01	-0.29	1.42E-01	-0.38	4.86E-02	-0.41	3.36E-02	0.33	9.17E-02
ENSCAFG0000001057	U2AF1	darkgrey	VSMC_M8	0.36	6.40E-02	-0.30	1.26E-01	0.50	8.25E-03	-0.79	1.04E-06	-0.15	4.54E-01	0.12	5.35E-01	0.15	4.61E-01	0.77	2.73E-06	0.13	5.17E-01	-0.76	3.79E-06
ENSCAFG0000001354	AIDA	grey	VSMC_M10	0.36	6.41E-02	-0.25	2.18E-01	0.03	8.75E-01	-0.43	2.57E-02	0.27	1.68E-01	-0.12	5.48E-01	0.41	3.56E-02	0.43	2.51E-02	0.08	6.85E-01	-0.48	1.08E-02
ENSCAFG0000002899	ENSCAFG0000002899	yellow	VSMC_M3	0.36	6.42E-02	0.38	5.18E-02	0.68	9.80E-05	-0.32	1.07E-01	-0.45	1.79E-02	-0.07	7.11E-01	0.39	4.45E-02	0.10	6.28E-01	-0.46	1.64E-02	0.01	9.41E-01
ENSCAFG0000000952	ENSCAFG0000000952	yellow	VSMC_M3	0.36	6.43E-02	0.52	5.54E-03	0.75	6.58E-06	-0.25	2.15E-01	-0.51	7.10E-03	-0.17	3.89E-01	-0.12	5.51E-01	-0.03	8.66E-01	-0.50	7.49E-03	0.01	9.64E-01
ENSCAFG0000001915	EEF2	cyan	VSMC_M2	0.36	6.45E-02	0.87	3.92E-09	0.52	5.52E-03	0.18	3.60E-01	-0.43	2.70E-02	-0.05	8.03E-01	-0.35	7.20E-02	-0.46	1.65E-02	-0.84	5.94E-08	0.53	4.28E-03
ENSCAFG0000001562	RAB5C	grey	VSMC_M10	0.36	6.45E-02	0.64	3.10E-04	-0.07	7.33E-01	0.54	3.34E-03	0.07	7.26E-01	0.02	9.27E-01	-0.44	2.24E-02	-0.64	3.64E-04	-0.61	7.39E-04	0.59	1.23E-03
ENSCAFG0000001652	SPIN4	cyan	VSMC_M2	0.36	6.45E-02	0.57	1.77E-03	0.50	7.59E-03	-0.14	4.89E-01	-0.31	1.16E-01	-0.27	1.67E-01	-0.02	9.08E-01	-0.09	6.50E-01	-0.57	1.77E-03	0.17	3.87E-01
ENSCAFG0000001240	RPS6KA1	grey	VSMC_M10	0.36	6.47E-02	-0.26	1.84E-01	0.24	2.25E-01	-0.30	1.27E-01	-0.05	8.13E-01	0.29	1.41E-01	0.03	8.86E-01	0.40	4.12E-02	0.10	6.28E-01	-0.36	6.84E-02
ENSCAFG0000001362	TAF1A	grey	VSMC_M10	0.36	6.47E-02	0.19	3.37E-01	0.46	1.61E-02	-0.17	3.91E-01	-0.31	1.21E-01	-0.08	6.81E-01	0.02	9.12E-01	0.08	6.80E-01	-0.29	1.37E-01	0.05	8.07E-01
ENSCAFG0000001568	ENSCAFG0000001568	darkgrey	VSMC_M8	0.36	6.48E-02	-0.07	7.16E-01	0.61	6.85E-04	-0.64	3.22E-04	-0.25	2.09E-01	-0.15	4.43E-01	0.21	3.04E-01	0.57	1.73E-03	-0.05	8.16E-01	-0.51	6.51E-03
ENSCAFG0000002021	NR6A1	grey	VSMC_M10	0.36	6.48E-02	0.19	3.51E-01	-0.01	9.50E-01	0.05	8.12E-01	0.08	6.79E-01	-0.07	7.47E-01	0.21	2.89E-01	-0.02	9.17E-01	-0.30	1.25E-01	0.21	2.96E-01
ENSCAFG0000001893	GIT1	yellow	VSMC_M3	0.36	6.48E-02	0.54	3.61E-03	0.62	6.17E-04	-0.27	1.79E-01	-0.38	5.10E-02	0.05	7.93E-01	-0.10	6.32E-01	0.01	9.75E-01	-0.59	1.28E-03	0.05	8.12E-01
ENSCAFG0000001040	CPNE8	pink	VSMC_M5	0.36	6.50E-02	-0.08	7.08E-01	-0.60	1.02E-03	0.57	1.85E-03	0.77	2.79E-06	-0.28	1.50E-01	-0.08	7.04E-01	-0.49	9.07E-03	0.04	8.49E-01	0.01	9.63E-01
ENSCAFG0000000952	RREB1	grey	VSMC_M10	0.36	6.50E-02	0.55	2.96E-03	-0.12	5.51E-01	0.58	1.51E-03	0.13	5.12E-01	0.13	5.29E-01	-0.37	5.42E-02	-0.64	2.86E-04	-0.59	1.27E-03	0.59	1.12E-03
ENSCAFG0000003056	DUSP5	yellow	VSMC_M3	0.36	6.51E-02	0.53	4.18E-03	0.58	1.46E-03	-0.24	2.30E-01	-0.43	2.54E-02	0.07	7.28E-01	-0.40	3.84E-02	0.04	8.29E-01	-0.54	3.39E-03	0.11	5.72E-01
ENSCAFG0000000446	ENSCAFG0000000446	pink	VSMC_M5	0.36	6.51E-02	-0.28	1.55E-01	-0.21	2.85E-01	0.05	8.15E-01	0.53	4.50E-03	0.01	9.60E-01	0.06	7.78E-01	0.02	9.23E-01	0.22	2.70E-01	-0.40	3.76E-02
ENSCAFG0000000143	KDM4C	grey	VSMC_M10	0.36	6.51E-02	0.54	3.89E-03	-0.11	5.98E-01	0.47	1.27E-02	0.25	2.06E-01	-0.41	3.32E-02	-0.02	9.12E-01	-0.65	2.33E-04	-0.51	6.21E-03	0.41	3.14E-02
ENSCAFG0000000658	ENSCAFG0000000658	darkgrey	VSMC_M8	0.36	6.55E-02	-0.07	7.29E-01	0.71	3.35E-05	-0.83	8.12E-08	-0.39	4.53E-02	0.04	8.39E-01	0.06	7.47E-01	0.75	7.81E-06	-0.08	6.97E-01	-0.59	1.23E-03
ENSCAFG0000001493	PSMC3IP	darkgrey	VSMC_M8	0.36	6.56E-02	-0.09	6.43E-01	0.47	1.37E-02	-0.63	4.08E-04	-0.28	1.62E-01	0.11	5.71E-01	0.45	1.81E-02	0.64	3.55E-04	-0.18	3.69E-01	-0.31	1.15E-01
ENSCAFG0000000350	MRPL32	darkgrey	VSMC_M8	0.36	6.56E-02	-0.30	1.24E-01	0.38	5.23E-02	-0.62	5.78E-04	-0.09	6.42E-01	0.06	7.77E-01	0.28	1.62E-01	0.66	1.55E-04	0.09	6.57E-01	-0.59	1.15E-03
ENSCAFG0000001190	ASB9	yellow	VSMC_M3	0.36	6.56E-02	0.43	2.48E-02	0.69	7.21E-05	-0.44	2.28E-02	-0.47	1.24E-02	0.10	6.19E-01	0.10	6.18E-01	0.20	3.22E-01	-0.53	4.17E-03	-0.03	8.68E-01
ENSCAFG0000000656	ARHGAP22	yellow	VSMC_M3	0.36	6.57E-02	0.43	2.60E-02	0.83	7.95E-08	-0.61	6.93E-04	-0.58	1.64E-03	-0.17	4.01E-01	0.20	3.25E-01	0.30	1.33E-01	-0.52	5.29E-03	-0.05	7.93E-01
ENSCAFG0000000519	SEPT9	yellow	VSMC_M3	0.36	6.59E-02	0.25	2.08E-01	0.64	3.60E-04	-0.48	1.08E-02	-0.39	4.72E-02	-0.11	5.88E-01	-0.25	2.00E-01	0.27	1.81E-01	-0.28	1.63E-01	-0.20	3.25E-01
ENSCAFG0000002325	ERBB4	yellow	VSMC_M3	0.36	6.60E-02	0.55	3.28E-03	0.53	4.41E-03	0.00	9.94E-01	-0.39	4.31E-02	0.05	8.20E-01	-0.36	6.30E-02	-0.19	3.36E-01	-0.58	1.65E-03	0.27	1.71E-01
ENSCAFG0000000596	TRMT6	grey	VSMC_M10	0.36	6.62E-02	-0.11	5.76E-01	0.09	6.72E-01	-0.09	6.63E-01	0.21	2.87E-01	0.01	9.68E-01	-0.21	3.03E-01	0.15	4.58E-01	-0.01	9.61E-01	-0.45	1.93E-02
ENSCAFG0000003188	PCDHGB1	pink	VSMC_M5	0.36	6.62E-02	0.01	9.75E-01	-0.18	3.67E-01	0.05	7.87E-01	0.56	2.54E-03	-0.40	4.01E-02	-0.18	3.74E-01	-0.07	7.43E-01	0.00	9.92E-01	-0.42	2.86E-02
ENSCAFG0000000841	KBTBD4	cyan	VSMC_M2	0.36	6.62E-02	0.44	2.32E-02	0.28	1.55E-01	-0.08	6.95E-01	-0.05	7.93E-01	-0.22	2.78E-01	0.07	7.20E-01	-0.10	6.23E-01	-0.46	1.48E-02	0.04	8.31E-01
ENSCAFG0000000546	XRRA1	grey	VSMC_M10	0.36	6.64E-02	0.16	4.18E-01	-0.43	2.37E-02	0.51	6.69E-03	0.59	1.34E-03	-0.18	3.69E-01	0.04	8.31E-01	-0.53	4.63E-03	-0.22	2.64E-01	0.20	3.17E-01
ENSCAFG0000001267	RPS21	yellow	VSMC_M3	0.36	6.65E-02	0.59	1.25E-03	0.72	2.26E-05	-0.09	6.40E-01	-0.54	3.76E-03	-0.16	4.28E-01	-0.03	8.83E-01	-0.19	3.46E-01	-0.57	1.91E-03	0.20	3.13E-01
ENSCAFG0000002164	ENSCAFG0000002164	cyan	VSMC_M2	0.36	6.65E-02	0.47	1.32E-02	0.52	5.81E-03	-0.15	4.62E-01	-0.45	1.74E-02	-0.02	9.18E-01	0.09	6.57E-01	-0.01	9.62E-01	-0.57	1.99E-03	0.31	1.22E-01
ENSCAFG0000001276	CLCN2	cyan	VSMC_M2	0.36	6.65E-02	0.61	7.84E-04	0.01	9.45E-01	0.30	1.23E-01	0.11	5.69E-01	-0.06	7.81E-01	-0.18	3.72E-01	-0.43	2.56E-02	-0.62	5.60E-04	0.34	7.92E-02
ENSCAFG0000000343	NOL10	darkgrey	VSMC_M8	0.36	6.66E-02	-0.45	1.72E-02	0.15	4.61E-01	-0.60	9.10E-04	0.19	3.41E-01	-0.11	5.87E-01	0.05	8.04E-01	0.62	5.30E-04	0.31	1.21E-01	-0.72	2.38E-05
ENSCAFG0000001594	PRTG	red	VSMC_M1	0.36	6.68E-02	0.01	9.65E-01	0.16	4.34E-01	0.03	8.82E-01	-0.04	8.34E-01	-0.08	6.81E-01	-0.06	7.53E-01	0.03	8.94E-01	-0.14	4.93E-01	0.06	7.71E-01
ENSCAFG0000001968	AXIN1	cyan	VSMC_M2	0.36	6.68E-02	0.72	2.19E-05	0.61	8.02E-04	-0.04	8.34E-01	-0.49	9.44E-03	0.00	9.84E-01	-0.21	2.88E-01	-0.22	2.59E-01	-0.74	1.03E-05	0.37	5.65E-02
ENSCAFG0000000173	GCC1	grey	VSMC_M10	0.36	6.69E-02	0.38	5.14E-02	0.00	9.87E-01	0.41	3.31E-02	-0.02	9.20E-01	0.20	3.07E-01	0.01	9.57E-01	-0.46	1.48E-02	-0.48	1.20E-02	0.52	5.94E-03
ENSCAFG0000000038	ARG1	red	VSMC_M1	0.36	6.70E-02	0.19	3.34E-01	-0.14	4.88E-01	0.13	5.05E-01	0.28	1.56E-01	-0.22	2.59E-01	-0.13	5.08E-01	-0.21	2.99E-01	-0.19	3.42E-01	0.13	5.30E-01
ENSCAFG0000000297	CEP162	grey	VSMC_M10	0.36	6.71E-02	0.44	2.30E-02	0.01	9.55E-01	0.26	1.84E-01	0.05	8.01E-01	-0.08	6.75E-01	0.04	8.27E-01	-0.34	8.58E-02	-0.53	4.66E-03	0.41	3.37E-02
ENSCAFG0000001494	CYP27A1	grey	VSMC_M10	0.36	6.73E-02	0.18	3.57E-01	0.18	3.70E-01	-0.18	3.62E-01	0.05	8.00E-01	-0.10	6.32E-01	0.17	3.98E-01	0.01	9.77E-01	-0.21	2.85E-01	-0.13	5.34E-01
ENSCAFG0000000721	ASXL1	grey	VSMC_M10	0.36	6.74E-02	0.30	1.31E-01	0.41	3.45E-02	-0.21	2.87E-01	-0.15	4.61E-01	-0.04	8.39E-01	-0.30	1.33E-01	0.06	7.61E-01	-0.32	1.08E-01	-0.10	6.05E-01
ENSCAFG0000000255	SIX2	grey	VSMC_M10	0.36	6.76E-02	0.40	4.11E-02	0.08	6.90E-01	0.10	6.33E-01	0.02	9.26E-01	-0.13	5.27E-01	0.31	1.12E-01	-0.17	4.00E-01	-0.57	2.04E-03	0.30	1.34E-01
ENSCAFG0000002316	INCA1	cyan	VSMC_M2	0.36	6.78E-02	0.48	1.20E-02	0.34	8.67E-02	0.17	3.86E-01	-0.22	2.80E-01	0.05	8.12E-01	-0.01	9.77E-01	-0.32	1.04E-01	-0.54	3.36E-03	0.34	8.07E-02
ENSCAFG0000001437	ENSCAFG0000001437	grey	VSMC_M10	0.36	6.78E-02	0.06	7.75E-01	-0.20	3.09E-01	0.09	6.66E-01	0.44	2.31E-02	-0.21	2.90E-01	-0.27	1.68E-01	-0.12	5.52E-01	-0.10	6.07E-01	-0.14	4.99E-01
ENSCAFG0000001964	RAB39B	red	VSMC_M1	0.36	6.78E-02	0.45	1.87E-02	0.25	2.10E-01	-0.04	8.56E-01	-0.16	4.20E-01	-0.01	9.70E-01	0.24	2.31E-01	-0.14	4.88E-01	-0.58	1.42E-03	0.36	6.73E-02
ENSCAFG0000002273	ND4L	pink	VSMC_M5	0.36	6.78E-02	-0.34	8.59E-02	-0.52	5.88E-03	0.17	3.83E-01	0.75	8.15E-06	-0.20	3.25E-01	0.11	5.83E-01	-0.04	8.61E-01	0.21	2.90E-01	-0.33	8.85E-02
ENSCAFG0000001366	KCND3	cyan	VSMC_M2	0.36	6.79E-02	0.50	8.50E-03	0.37	5.72E-02	-0.09	6.51E-01	-0.22	2.80E-01	-0.18	3.68E-01	-0.22	2.81E-01	-0.10	6.12E-01	-0.51	6.61E-03	0.19	3.36E-01
ENSCAFG0000000655	MAPK8	pink	VSMC_M5	0.36	6.81E-02	-0.13	5.05E-01	-0.32	9.96E-02	0.09	6.46E-01	0.47	1.39E-02	0.09	6.38E-01	-0.35	7.62E-02	0.05	7.87E-01	0.02	9.38E-01	-0.16	4.32E-01
ENSCAFG0000002981	COMMD6	grey	VSMC_M10	0.36	6.82E-02	0.30	1.29E-01	0.08	7.05E-01	0.11	5.93E-01	0.05	8.22E-01	0.14	4.78E-01	-0.18	3.79E-01	-0.18	3.79E-01	-0.37	5.88E-02	0.13	5.16E-01
ENSCAFG0000002966	HNRNPLL	pink	VSMC_M5	0.36	6.83E-02	-0.47	1.25E-02	-0.24	2.30E-01	-0.09	6.68E-01	0.53	4.16E-03	-0.30	1.28E-01	0.03	8.74E-01	0.20	3.22E-01	0.34	8.27E-02	-0.52	5.39E-03
ENSCAFG0000002406	GSTA4	cyan	VSMC_M2	0.36	6.83E-02	0.56	2.39E-03	0.28	1.53E-01	0.28	1.65E-01	-0.14	4.96E-01	0.14	4.82E-01	-0.42	2.73E-02	-0.38	4.86E-02	-0.54	3.45E-03	0.28	1.59E-01
ENSCAFG0000000838	SLC38A7	cyan	VSMC_M2	0.36	6.83E-02	0.57	1.96E-03	0.08	6.79E-01	0.19	3.30E-01	0.11	5.75E-01	-0.20	3.05E-01	-0.01	9.51E-01	-0.48	1.06E-02	-0.50	7.70E-03	0.31	1.16E-01
ENSCAFG0000001414	ZNF385B	yellow	VSMC_M3	0.36	6.83E-02	0.49	1.02E-02	0.45	1.84E-02	-0.17	4.09E-01	-0.24	2.33E-01	-0.23	2.38E-01	-0.69	7.93E-05	-0.04	8.42E-01	-0.43	2.47E-02	0.01	9.60E-01
ENSCAFG0000001645	RAB24	yellow	VSMC_M3	0.36	6.84E-02	0.56	2.65E-03	0.63	3.97E-04	-0.17	3.91E-01	-0.42	2.99E-02	-0.08	6.83E-01	-0.15	4.57E-01	-0.09	6.39E-01	-0.55	2.68E-03	0.10	6.03E-01
ENSCAFG0000001121	ENSCAFG0000001121	grey	VSMC_M10	0.36	6.85E-02	-0.16	4.18E-01	0.43	2.68E-02	-0.54	3.31E-03	-0.16	4.17E-01	0.02	9.06E-01	-0.08	6.78E-01	0.53	4.29E-03	0.05	8.00E-01	-0.48	1.09E-02
ENSCAFG0000000650	NEDD1	pink	VSMC_M5	0.36	6.85E-02	-0.49	9.69E-03	-0.41	3.48E-02	-0.14	4.77E-01	0.70	5.31E-05	-0.14	4.79E-01	0.10	6.19E-01	0.31	1.13E-01	0.30	1.33E-01	-0.52	5.54E-03
ENSCAFG0000000798	ZNF366	grey	VSMC_M10	0.36	6.86E-02	0.08	6.78E-01	0.12	5.68E-01	-0.21	2.82E-01	0.12	5.57E-01	0.11	6.00E-01	0.36	6.45E-02	0.19	3.46E-01	-0.24	2.28E-01	-0.23	2.43E-01
ENSCAFG0000001811	CHRDL1	grey	VSMC_M10	0.36	6.87E-02	0.56	2.48E-03	-0.26	1.82E-01	0.61	7.20E-04	0.43	2.48E-02	-0.41	3.48E-02	-0.48	1.12E-02	-0.77	2.10E-06	-0.44	2.20E-02	0.37	5.50E-02
ENSCAFG0000002897	ENSCAFG0000002897	yellow	VSMC_M3	0.36	6.88E-02	0.30	1.26E-01	0.51	6.03E-03	-0.12	5.44E-01	-0.31	1.20E-01	-0.13	5.26E-01	-0.11	5.99E-01	-0.11	5.69E-01	-0.35	7.50E-02	0.00	9.85E-01
ENSCAFG0000000338	IAH1	cyan	VSMC_M2	0.36	6.89E-02	0.47	1.36E-02	0.39	4.62E-02	-0.10	6.13E-01	-0.37	5.51E-02	0.12	5.54E-01	0.02	9.36E-01	-0.06	7.61E-01	-0.57	1.77E-03	0.42	3.06E-02
ENSCAFG0000001318	PDCD1	grey	VSMC_M10	0.36	6.89E-02	0.29	1.37E-01	0.55	2.96E-03	-0.24	2.34E-01	-0.33	9.66E-02	-0.23	2.49E-01	-0.03	8.64E-01	0.07	7.11E-01	-0.37	5.63E-02	0.03	8.88E-01
ENSCAFG0000000330	RNF144A	cyan	VSMC_M2	0.36	6.90E-02	0.80	6.65E-07	0.42	3.12E-02	0.18	3.69E-01	-0.32	1.08E-01	-0.11	5.98E-01	-0.32	1.00E-01	-0.45	1.79E-02	-0.74	8.64E-06	0.53	4.05E-03
ENSCAFG0000001669	ICE2	grey	VSMC_M10	0.36	6.90E-02	-0.19	3.34E-01	-0.19	3.50E-01	-0.10	6.29E-01	0.35	6.93E-02	-0.05	7.97E-01	0.38	4.92E-02	0.18	3.82E-01	-0.06	7.64E-01	-0.17	4.03E-01
ENSCAFG0000002005	FRRS1	grey	VSMC_M10	0.36	6.90E-02	0.27	1.66E-01	-0.07	7.47E-01	0.50	7.79E-03	0.14	4.87E-01	-0.24	2.36E-01	-0.09	6.53E-01	-0.47	1.24E-02	-0.34	8.52E-02	0.32	1.05E-01
ENSCAFG0000001181	RHOD	red	VSMC_M1	0.36	6.91E-02	0.05	7.85E-01	0.06	7.76E-01	0.13	5.04E-01	0.19	3.30E-01	-0.05	8.07E-01	-0.45	1.80E-02	-0.13	5.29E-01	-0.08	7.00E-01	-0.28	1.61E-01
ENSCAFG0000002883	ENSCAFG0000002883	red	VSMC_M1	0.36	6.91E-02	0.28	1.52E-01	0.23	2.50E-01	-0.05	7.90E-01	-0.23	2.50E-01	0.11	5.96E-01	0.18	3.82E-01	-0.03	8.77E-01	-0.45	1.92E-02	0.34	8.25E-02
ENSCAFG0000003268	ENSCAFG0000003268	grey	VSMC_M10	0.36	6.91E-02	0.20	3.20E-01	0.23	2.57E-01	-0.31	1.11E-01	-0.07	7.19E-01	0.26	1.98E-01	0.07	7.38E-01	0.21	2.91E-01	-0.31	1.19E-01	-0.09	6.62E-01
ENSCAFG0000001832	ABLIM3	darkgreen	VSMC_M4	0.36	6.92E-02	0.48	1.04E-02	-0.29	1.47E-01	0.77	2.23E-06	0.29	1.39E-01	0.06	7.69E-01	-0.31	1.10E-01	-0.80	4.48E-07	-0.50	7.22E-03	0.57	2.06E-03
ENSCAFG0000000691	MAP3K12	cyan	VSMC_M2	0.35	6.93E-02	0.87	4.66E-09	0.42	3.08E-02	0.23	2.52E-01	-0.31	1.15E-01	-0.26	1.92E-01	-0.29	1.42E-01	-0.56	2.31E-03	-0.81	2.86E-07	0.55	3.00E-03
ENSCAFG0000000603	TMEM230	grey	VSMC_M10	0.35	6.96E-02	0.30	1.30E-01	0.23	2.47E-01	-0.13	5.15E-01	-0.10	6.34E-01	0.09	6.40E-01	-0.06	7.55E-01	-0.02	9.06E-01	-0.39	4.58E-02	0.03	8.79E-01
ENSCAFG0000002957	ENSCAFG0000002957	grey	VSMC_M10	0.35	6.96E-02	-0.17	3.89E-01	0.02	9.38E-01	-0.24	2.30E-01	0.30	1.34E-01	-0.33	9.26E-02	0.10	6.12E-01	0.19	3.34E-01	0.04	8.38E-01	-0.40	3.92E-02
ENSCAFG0000000986	HERC2	grey	VSMC_M10	0.35	6.97E-02	0.26	1.84E-01	-0.18	3.71E-01	0.47	1.36E-02	0.30	1.29E-01	-0.15	4.53E-01	-0.13	5.20E-01	-0.54	3.91E-03	-0.27	1.77E-01	0.28	1.59E-01
ENSCAFG0000000319	RRAGC	cyan	VSMC_M2	0.35	6.99E-02	0.45	1.77E-02	0.23	2.47E-01	-0.10	6.18E-01	0.06	7.51E-01	-0.28	1.52E-01	-0.08	6.81E-01	-0.24	2.23E-01	-0.40	4.06E-02	0.01	9.69E-01
ENSCAFG0000001805	DSG2	grey	VSMC_M10	0.35	7.00E-02	0.19	3.47E-01	-0.45	1.84E-02	0.58	1.38E-03	0.52	5.55E-03	0.08	6.84E-01	-0.60	8.98E-04	-0.50	7.28E-03	-0.18	3.77E-01	0.23	2.41E-01
ENSCAFG0000000617	RAB11FIP1	cyan	VSMC_M2	0.35	7.01E-02	0.54	3.52E-03	0.37	5.46E-02	-0.12	5.52E-01	-0.23	2.55E-01	-0.10	6.23E-01	-0.61	7.39E-04	-0.08	6.96E-01	-0.54	3.97E-03	0.14	4.88E-01

ENSCAFG0000001836	ENSCAFG0000001836	cyan	VSMC_M2	0.35	7.02E-02	0.41	3.61E-02	-0.08	7.02E-01	0.52	4.93E-03	0.08	7.09E-01	0.04	8.62E-01	-0.40	3.74E-02	-0.52	4.96E-03	-0.39	4.32E-02	0.50	7.78E-03
ENSCAFG0000000057	ENSCAFG0000000057	grey	VSMC_M10	0.35	7.03E-02	0.15	4.66E-01	0.01	9.52E-01	-0.21	2.97E-01	0.19	3.41E-01	0.00	9.95E-01	-0.08	7.07E-01	0.12	5.48E-01	-0.22	2.60E-01	-0.16	4.11E-01
ENSCAFG0000001679	CREBRF	grey	VSMC_M10	0.35	7.03E-02	0.37	5.45E-02	-0.38	4.90E-02	0.70	4.35E-05	0.50	7.67E-03	-0.27	1.76E-01	-0.13	5.30E-01	-0.78	1.48E-06	-0.36	6.84E-02	0.39	4.37E-02
ENSCAFG0000000542	DYRK1B	grey	VSMC_M10	0.35	7.04E-02	0.78	1.32E-06	0.23	2.50E-01	0.51	6.19E-03	-0.15	4.42E-01	-0.23	2.38E-01	-0.28	1.61E-01	-0.76	5.11E-06	-0.71	3.09E-05	0.67	1.16E-04
ENSCAFG0000001585	PSMB6	red	VSMC_M1	0.35	7.04E-02	-0.16	4.38E-01	0.45	1.72E-02	-0.43	2.34E-02	-0.28	1.56E-01	0.11	5.68E-01	0.16	4.23E-01	0.46	1.61E-02	-0.01	9.59E-01	-0.30	1.24E-01
ENSCAFG0000002987	ZNF180	red	VSMC_M1	0.35	7.05E-02	0.20	3.13E-01	0.36	6.28E-02	-0.20	3.27E-01	-0.01	9.58E-01	-0.30	1.33E-01	0.04	8.51E-01	0.04	8.41E-01	-0.23	2.49E-01	-0.26	1.85E-01
ENSCAFG0000000116	IBA57	cyan	VSMC_M2	0.35	7.06E-02	0.56	2.61E-03	0.29	1.47E-01	0.16	4.11E-01	-0.20	3.07E-01	-0.01	9.72E-01	0.11	5.85E-01	-0.31	1.11E-01	-0.65	2.52E-04	0.42	3.00E-02
ENSCAFG0000000593	RFNG	cyan	VSMC_M2	0.35	7.07E-02	0.54	3.36E-03	0.52	5.79E-03	0.09	6.57E-01	-0.36	6.75E-02	-0.05	7.95E-01	-0.11	6.01E-01	-0.31	1.19E-01	-0.53	4.90E-03	0.27	1.67E-01
ENSCAFG0000000353	GLI3	cyan	VSMC_M2	0.35	7.07E-02	0.74	1.12E-05	0.43	2.46E-02	-0.08	6.83E-01	-0.28	1.59E-01	-0.10	6.21E-01	-0.47	1.32E-02	-0.16	4.32E-01	-0.73	1.66E-05	0.27	1.66E-01
ENSCAFG0000000991	NPM3	yellow	VSMC_M3	0.35	7.10E-02	0.19	3.46E-01	0.77	2.89E-06	-0.68	1.05E-04	-0.62	5.39E-04	0.17	3.86E-01	0.31	1.18E-01	0.51	6.68E-03	-0.38	5.28E-02	-0.10	6.13E-01
ENSCAFG0000003099	CCDC3	grey	VSMC_M10	0.35	7.10E-02	0.74	9.08E-06	0.17	3.99E-01	0.45	1.73E-02	-0.11	5.93E-01	-0.01	9.74E-01	-0.42	2.78E-02	-0.61	8.11E-04	-0.70	4.68E-05	0.56	2.17E-03
ENSCAFG0000001965	WDR90	cyan	VSMC_M2	0.35	7.10E-02	0.64	3.71E-04	0.65	2.73E-04	-0.17	3.85E-01	-0.41	3.59E-02	-0.29	1.50E-01	0.08	6.79E-01	-0.14	5.02E-01	-0.63	4.38E-04	0.19	3.35E-01
ENSCAFG0000000815	PLSCR4	grey	VSMC_M10	0.35	7.11E-02	0.24	2.23E-01	0.35	7.00E-02	-0.16	4.34E-01	-0.15	4.48E-01	-0.06	7.67E-01	-0.01	9.71E-01	0.05	7.88E-01	-0.37	5.98E-02	-0.04	8.57E-01
ENSCAFG0000000041	MRPS18B	darkgrey	VSMC_M8	0.35	7.12E-02	-0.08	6.82E-01	0.43	2.44E-02	-0.69	6.97E-05	-0.06	7.53E-01	-0.06	7.66E-01	-0.03	8.67E-01	0.57	1.77E-03	-0.01	9.69E-01	-0.67	1.48E-04
ENSCAFG0000000418	ENSCAFG0000000418	grey	VSMC_M10	0.35	7.14E-02	0.33	9.59E-02	-0.18	3.60E-01	0.49	9.17E-03	0.33	8.80E-02	-0.31	1.18E-01	0.13	5.05E-01	-0.51	6.59E-03	-0.39	4.24E-02	0.29	1.44E-01
ENSCAFG0000001790	EIF3G	yellow	VSMC_M3	0.35	7.16E-02	0.38	4.83E-02	0.70	4.73E-05	-0.35	7.78E-02	-0.55	2.82E-03	-0.06	7.52E-01	-0.11	5.91E-01	0.24	2.24E-01	-0.49	9.70E-03	0.03	8.81E-01
ENSCAFG0000000576	TSPAN10	yellow	VSMC_M3	0.35	7.17E-02	0.30	1.26E-01	0.54	3.81E-03	-0.13	5.22E-01	-0.29	1.41E-01	0.12	5.46E-01	-0.03	8.68E-01	-0.04	8.62E-01	-0.32	9.81E-02	-0.04	8.36E-01
ENSCAFG0000000179	TMEM3	pink	VSMC_M5	0.35	7.17E-02	-0.20	3.19E-01	-0.42	3.05E-02	0.07	7.11E-01	0.64	2.98E-04	-0.16	4.23E-01	-0.32	1.04E-01	0.06	7.78E-01	0.11	5.77E-01	-0.34	8.62E-02
ENSCAFG0000001976	SURF2	grey	VSMC_M10	0.35	7.18E-02	0.21	2.90E-01	0.41	3.60E-02	-0.19	3.37E-01	-0.37	5.98E-02	0.11	5.94E-01	0.39	4.64E-02	0.11	5.73E-01	-0.43	2.64E-02	0.20	3.19E-01
ENSCAFG0000000497	DACH1	grey	VSMC_M10	0.35	7.20E-02	-0.13	5.10E-01	0.09	6.68E-01	-0.08	6.98E-01	0.14	4.72E-01	-0.14	4.80E-01	0.42	3.06E-02	0.07	7.32E-01	0.01	9.78E-01	-0.18	3.71E-01
ENSCAFG0000000795	ROBO2	grey	VSMC_M10	0.35	7.21E-02	-0.08	7.02E-01	0.01	9.77E-01	-0.35	7.19E-02	0.23	2.52E-01	-0.01	9.60E-01	-0.12	5.65E-01	0.33	9.12E-02	-0.08	7.09E-01	-0.32	9.89E-02
ENSCAFG0000000416	MRE11	pink	VSMC_M5	0.35	7.22E-02	-0.29	1.44E-01	-0.41	3.22E-02	0.12	5.63E-01	0.68	1.04E-04	-0.27	1.66E-01	0.23	2.55E-01	0.03	8.92E-01	0.11	5.97E-01	-0.32	1.00E-01
ENSCAFG0000000413	EFR3B	red	VSMC_M1	0.35	7.22E-02	0.37	5.87E-02	0.34	8.22E-02	0.08	6.82E-01	-0.34	8.74E-02	0.35	7.20E-02	0.03	8.85E-01	-0.11	6.00E-01	-0.53	4.72E-03	0.36	6.75E-02
ENSCAFG0000001325	SIK3	grey	VSMC_M10	0.35	7.23E-02	0.36	6.21E-02	-0.19	3.44E-01	0.30	1.24E-01	0.29	1.42E-01	-0.15	4.54E-01	0.26	1.95E-01	-0.38	5.39E-02	-0.47	1.40E-02	0.35	7.13E-02
ENSCAFG0000001543	DACT1	darkgreen	VSMC_M4	0.35	7.23E-02	0.51	6.17E-03	-0.22	2.70E-01	0.73	1.34E-05	0.30	1.29E-01	-0.19	3.53E-01	-0.62	5.62E-04	-0.78	2.06E-06	-0.45	1.74E-02	0.47	1.41E-02
ENSCAFG0000000689	PDHX	grey	VSMC_M10	0.35	7.25E-02	-0.10	6.25E-01	-0.34	8.19E-02	0.42	3.12E-02	0.41	3.51E-02	0.19	3.39E-01	0.11	5.75E-01	-0.30	1.31E-01	-0.08	6.78E-01	0.13	5.29E-01
ENSCAFG0000000940	DLL4	yellow	VSMC_M3	0.35	7.25E-02	0.35	7.62E-02	0.52	5.06E-03	-0.14	4.76E-01	-0.36	6.14E-02	0.40	4.13E-02	-0.14	4.80E-01	-0.01	9.62E-01	-0.40	4.00E-02	0.02	9.22E-01
ENSCAFG0000000375	ENSCAFG0000000375	pink	VSMC_M5	0.35	7.26E-02	-0.18	3.63E-01	-0.28	1.60E-01	0.06	7.54E-01	0.47	1.44E-02	0.13	5.18E-01	-0.11	5.96E-01	0.07	7.32E-01	0.05	8.18E-01	-0.28	1.60E-01
ENSCAFG0000001696	MAZ	darkgrey	VSMC_M8	0.35	7.26E-02	0.00	9.98E-01	0.53	4.27E-03	-0.76	4.71E-06	-0.20	3.20E-01	-0.11	6.00E-01	0.30	1.22E-01	0.59	1.12E-03	-0.15	4.63E-01	-0.48	1.12E-02
ENSCAFG0000000679	GTF3A	cyan	VSMC_M2	0.35	7.27E-02	0.76	4.53E-06	0.06	7.55E-01	0.49	8.83E-03	0.08	6.80E-01	-0.42	2.87E-02	-0.41	3.42E-02	-0.74	8.33E-06	-0.64	3.53E-04	0.50	8.42E-03
ENSCAFG0000000143	PI16	grey	VSMC_M10	0.35	7.29E-02	0.22	2.65E-01	0.29	1.36E-01	-0.32	1.05E-01	-0.06	7.53E-01	-0.07	7.24E-01	0.28	1.51E-01	0.18	3.66E-01	-0.31	1.18E-01	-0.07	7.18E-01
ENSCAFG0000000932	DNAJC17	yellow	VSMC_M3	0.35	7.29E-02	0.11	5.84E-01	0.58	1.41E-03	-0.48	1.04E-02	-0.45	1.85E-02	0.09	6.70E-01	0.28	1.51E-01	0.43	2.63E-02	-0.32	1.05E-01	-0.11	5.91E-01
ENSCAFG0000000786	LACTB2	cyan	VSMC_M2	0.35	7.30E-02	0.57	2.12E-03	0.08	6.78E-01	0.10	6.15E-01	0.12	5.65E-01	-0.24	2.18E-01	-0.20	3.08E-01	-0.40	3.91E-02	-0.47	1.31E-02	0.26	1.94E-01
ENSCAFG0000001329	RNASEH2C	yellow	VSMC_M3	0.35	7.31E-02	0.12	5.39E-01	0.67	1.29E-04	-0.50	7.49E-03	-0.55	2.70E-03	0.18	3.57E-01	0.33	9.53E-02	0.44	2.03E-02	-0.32	1.03E-01	-0.02	9.04E-01
ENSCAFG0000001793	CYP1A1	red	VSMC_M1	0.35	7.31E-02	0.43	2.54E-02	0.22	2.62E-01	-0.10	6.24E-01	0.04	8.48E-01	-0.18	3.78E-01	-0.22	2.80E-01	-0.08	7.08E-01	-0.44	2.32E-02	-0.04	8.34E-01
ENSCAFG0000000062	ENSCAFG0000000062	cyan	VSMC_M2	0.35	7.32E-02	0.39	4.20E-02	0.37	6.07E-02	-0.13	5.25E-01	-0.31	1.21E-01	0.14	4.85E-01	0.27	1.78E-01	0.02	9.38E-01	-0.57	1.83E-03	0.27	1.81E-01
ENSCAFG0000000746	ENSCAFG0000000746	red	VSMC_M1	0.35	7.36E-02	0.14	4.82E-01	0.04	8.24E-01	0.02	9.34E-01	0.04	8.31E-01	0.14	4.99E-01	0.19	3.41E-01	0.01	9.70E-01	-0.34	8.68E-02	0.13	5.34E-01
ENSCAFG0000001662	ENSCAFG0000001662	grey	VSMC_M10	0.35	7.40E-02	0.20	3.07E-01	0.32	1.09E-01	0.02	9.03E-01	-0.22	2.77E-01	0.07	7.31E-01	-0.10	6.03E-01	-0.07	7.13E-01	-0.30	1.27E-01	0.05	8.01E-01
ENSCAFG0000000632	ENSCAFG0000000632	grey	VSMC_M10	0.35	7.42E-02	0.17	3.92E-01	0.39	4.19E-02	-0.27	1.71E-01	-0.19	3.52E-01	-0.20	3.27E-01	0.00	9.97E-01	0.22	2.81E-01	-0.32	1.06E-01	-0.07	7.36E-01
ENSCAFG0000001400	SNX15	grey	VSMC_M10	0.35	7.43E-02	0.10	6.05E-01	0.41	3.37E-02	-0.50	7.37E-03	-0.30	1.28E-01	0.01	9.43E-01	0.16	4.31E-01	0.38	4.90E-02	-0.26	1.89E-01	-0.01	9.64E-01
ENSCAFG0000000800	EIF6	red	VSMC_M1	0.35	7.44E-02	-0.04	8.29E-01	0.46	1.64E-02	-0.55	2.80E-03	-0.31	1.16E-01	0.20	3.18E-01	0.08	6.74E-01	0.53	4.20E-03	-0.12	5.41E-01	-0.26	1.95E-01
ENSCAFG0000001808	TMEM16	cyan	VSMC_M2	0.35	7.44E-02	0.48	1.23E-02	0.37	5.76E-02	-0.16	4.33E-01	-0.15	4.43E-01	0.34	7.93E-02	-0.14	4.78E-01	-0.08	7.07E-01	-0.43	2.42E-02	0.05	8.20E-01
ENSCAFG0000000561	ARL8B	pink	VSMC_M5	0.35	7.45E-02	-0.36	6.25E-02	-0.41	3.39E-02	0.00	9.81E-01	0.68	8.26E-05	-0.04	8.44E-01	0.13	5.14E-01	0.10	6.11E-01	0.26	1.94E-01	-0.47	1.33E-02
ENSCAFG0000001355	ENSCAFG0000001355	grey	VSMC_M10	0.35	7.45E-02	0.67	1.34E-04	0.15	4.59E-01	0.26	1.96E-01	-0.05	7.92E-01	0.00	9.85E-01	-0.33	9.67E-02	-0.47	1.28E-02	-0.71	3.53E-05	0.43	2.47E-02
ENSCAFG0000000697	C24H20orf9	yellow	VSMC_M3	0.35	7.46E-02	0.22	2.77E-01	0.52	5.57E-03	-0.17	3.90E-01	-0.35	7.75E-02	-0.05	8.06E-01	0.04	8.54E-01	0.10	6.21E-01	-0.35	7.78E-02	0.01	9.79E-01
ENSCAFG0000002508	PAXBP1	grey	VSMC_M10	0.35	7.47E-02	-0.21	2.99E-01	0.35	7.48E-02	-0.44	2.28E-02	-0.08	6.80E-01	-0.14	4.84E-01	-0.11	5.79E-01	0.44	2.01E-02	0.04	8.62E-01	-0.46	1.53E-02
ENSCAFG0000001434	RFXANK	grey	VSMC_M10	0.35	7.48E-02	0.06	7.81E-01	-0.12	5.44E-01	-0.10	6.16E-01	0.30	1.26E-01	-0.04	8.36E-01	-0.06	7.76E-01	0.12	5.68E-01	-0.17	3.88E-01	-0.13	5.20E-01
ENSCAFG0000000132	TEAD3	cyan	VSMC_M2	0.35	7.49E-02	0.46	1.54E-02	0.31	1.20E-01	0.05	7.89E-01	-0.04	8.47E-01	-0.30	1.24E-01	-0.50	7.58E-03	-0.31	1.12E-01	-0.35	6.99E-02	0.02	9.24E-01
ENSCAFG0000001580	MAT1A	grey	VSMC_M10	0.35	7.50E-02	0.11	5.73E-01	0.17	3.89E-01	0.04	8.45E-01	-0.01	9.44E-01	0.36	6.48E-02	-0.07	7.32E-01	-0.13	5.18E-01	-0.22	2.60E-01	-0.06	7.50E-01
ENSCAFG0000001726	C7H1orf43	cyan	VSMC_M2	0.35	7.51E-02	0.51	6.86E-03	0.21	2.93E-01	0.22	2.70E-01	0.03	8.76E-01	-0.26	1.82E-01	-0.08	6.81E-01	-0.38	4.87E-02	-0.50	7.56E-03	0.16	4.16E-01
ENSCAFG0000000906	COPS4	pink	VSMC_M5	0.35	7.52E-02	-0.57	2.11E-03	-0.44	2.30E-02	0.02	9.16E-01	0.69	6.53E-05	-0.16	4.14E-01	0.11	5.83E-01	0.21	2.99E-01	0.39	4.15E-02	-0.48	1.11E-02
ENSCAFG0000000105	POLR3H	red	VSMC_M1	0.35	7.53E-02	0.32	1.01E-01	0.26	1.82E-01	-0.01	9.51E-01	-0.20	3.06E-01	0.27	1.70E-01	-0.08	6.86E-01	-0.05	8.10E-01	-0.48	1.17E-02	0.20	3.19E-01
ENSCAFG0000001473	COX8A	grey	VSMC_M10	0.35	7.53E-02	0.20	3.15E-01	0.34	7.93E-02	0.02	9.30E-01	-0.23	2.44E-01	0.04	8.32E-01	0.05	8.11E-01	-0.03	8.75E-01	-0.31	1.18E-01	0.05	7.92E-01
ENSCAFG0000000880	CCDC65	grey	VSMC_M10	0.35	7.54E-02	0.21	3.01E-01	0.12	5.53E-01	0.12	5.66E-01	-0.04	8.35E-01	0.09	6.51E-01	0.24	2.33E-01	-0.14	4.86E-01	-0.33	8.77E-02	0.27	1.77E-01
ENSCAFG0000000977	ZSWIM3	red	VSMC_M1	0.35	7.54E-02	0.33	9.71E-02	-0.06	7.77E-01	0.43	2.38E-02	0.10	6.15E-01	-0.05	8.18E-01	-0.20	3.07E-01	-0.46	1.62E-02	-0.39	4.18E-02	0.39	4.34E-02
ENSCAFG0000001156	PPP1CA	yellow	VSMC_M3	0.35	7.55E-02	0.62	5.59E-04	0.93	4.96E-12	-0.49	9.52E-03	-0.76	4.29E-06	0.03	8.99E-01	0.12	5.63E-01	0.16	4.23E-01	-0.67	1.13E-04	0.17	3.86E-01
ENSCAFG0000000205	PDGFRA	yellow	VSMC_M3	0.35	7.55E-02	0.42	3.05E-02	0.44	2.06E-02	-0.29	1.49E-01	-0.18	3.56E-01	-0.23	2.42E-01	-0.18	3.78E-01	0.14	4.75E-01	-0.43	2.64E-02	-0.10	6.20E-01
ENSCAFG0000000912	BBS2	cyan	VSMC_M2	0.35	7.56E-02	0.52	5.72E-03	-0.01	9.74E-01	0.18	3.62E-01	0.18	3.71E-01	-0.34	8.23E-02	-0.09	6.60E-01	-0.31	1.12E-01	-0.49	8.72E-03	0.24	2.32E-01
ENSCAFG0000003218	RNF152	grey	VSMC_M10	0.35	7.60E-02	0.11	5.80E-01	0.62	5.57E-04	-0.63	4.84E-04	-0.23	2.59E-01	-0.18	3.70E-01	0.16	4.31E-01	0.38	4.81E-02	-0.17	3.92E-01	-0.48	1.20E-02
ENSCAFG0000001722	SPTLC2	pink	VSMC_M5	0.35	7.60E-02	-0.42	2.84E-02	-0.16	4.24E-01	-0.16	4.19E-01	0.50	7.70E-03	-0.17	3.86E-01	-0.07	7.17E-01	0.23	2.57E-01	0.39	4.50E-02	-0.64	3.32E-04
ENSCAFG0000001283	RHCE	yellow	VSMC_M3	0.35	7.60E-02	0.19	3.43E-01	0.59	1.24E-03	-0.22	2.81E-01	-0.35	7.22E-02	-0.05	8.02E-01	-0.03	8.73E-01	0.09	6.68E-01	-0.26	1.90E-01	-0.12	5.49E-01
ENSCAFG0000001220	ZRSR2	grey	VSMC_M10	0.35	7.61E-02	-0.06	7.61E-01	0.11	5.77E-01	-0.03	8.97E-01	0.24	2.25E-01	-0.28	1.59E-01	-0.05	7.90E-01	-0.05	8.12E-01	0.03	8.96E-01	-0.41	3.59E-02
ENSCAFG0000001461	HIRA	grey	VSMC_M10	0.35	7.62E-02	0.08	7.04E-01	0.26	1.96E-01	-0.44	2.26E-02	-0.12	5.50E-01	0.24	2.22E-01	0.18	3.80E-01	0.42	2.81E-02	-0.27	1.74E-01	-0.12	5.60E-01
ENSCAFG0000001317	OLA1	grey	VSMC_M10	0.35	7.62E-02	0.03	8.83E-01	-0.24	2.19E-01	0.18	3.69E-01	0.38	4.92E-02	-0.19	3.42E-01	0.21	2.95E-01	-0.08	6.87E-01	-0.17	4.08E-01	0.02	9.11E-01
ENSCAFG0000003073	ENSCAFG0000003073	cyan	VSMC_M2	0.35	7.63E-02	0.37	5.62E-02	0.39	4.40E-02	-0.19	3.55E-01	-0.09	6.47E-01	-0.50	8.39E-03	-0.29	1.48E-01	-0.08	6.75E-01	-0.29	1.41E-01	-0.08	6.86E-01
ENSCAFG0000001661	MRI1	cyan	VSMC_M2	0.35	7.64E-02	0.58	1.56E-03	0.46	1.68E-02	-0.05	8.12E-01	-0.36	6.36E-02	0.16	4.31E-01	-0.23	2.45E-01	-0.15	4.52E-01	-0.67	1.18E-04	0.28	1.64E-01
ENSCAFG0000001934	CTNS	yellow	VSMC_M3	0.35	7.64E-02	0.58	1.60E-03	0.58	1.54E-03	-0.35	6.93E-02	-0.36	6.56E-02	-0.08	6.94E-01	0.15	4.47E-01	0.01	9.73E-01	-0.60	1.00E-03	0.13	5.31E-01
ENSCAFG0000000427	LRRC17	cyan	VSMC_M2	0.35	7.64E-02	0.74	9.68E-06	0.36	6.79E-02	0.06	7.52E-01	-0.23	2.53E-01	-0.24	2.19E-01	-0.32	1.07E-01	-0.31	1.16E-01	-0.71	3.41E-05	0.40	4.00E-02
ENSCAFG0000001187	DOK5	grey	VSMC_M10	0.35	7.65E-02	0.12	5.62E-01	-0.01	9.52E-01	0.19	3.34E-01	0.15	4.68E-01	0.13	5.33E-01	-0.01	9.63E-01	-0.18	3.63E-01	-0.26	1.85E-01	0.08	7.00E-01
ENSCAFG0000001377	PLA2G4A	grey	VSMC_M10	0.35	7.66E-02	0.26	1.84E-01	-0.18	3.65E-01	0.30	1.27E-01	0.36	6.33E-02	-0.07	7.25E-01	0.16	4.11E-01	-0.33	9.12E-02	-0.29	1.48E-01	0.14	4.95E-01
ENSCAFG0000000266	FBXO11	pink	VSMC_M5	0.35	7.66E-02	-0.24	2.25E-01	-0.25	2.15E-01	-0.13	5.12E-01	0.61	8.26E-04	-0.29	1.48E-01	-0.34	8.36E-02	0.18	3.81E-01	0.19	3.37E-01	-0.57	1.93E-03
ENSCAFG0000001335	KIF9	grey	VSMC_M10	0.35	7.66E-02	0.10	6.35E-01	-0.11	6.01E-01	0.42	2.88E-02	0.19	3.31E-01	-0.13	5.26E-01	0.12	5.38E-01	-0.38	4.89E-02	-0.18	3.64E-01	0.24	2.33E-01
ENSCAFG0000000715	ENSCAFG0000000715	pink	VSMC_M5	0.35	7.67E-02	-0.23	2.43E-01	0.00	9.98E-01	-0.35	7.60E-02	0.39	4.65E-02	-0.25	2.16E-01	0.17	3.93E-01	0.35	7.60E-02	0.15	4.53E-01	-0.65	2.48E-04
ENSCAFG0000000750	AHCY	grey	VSMC_M10	0.35	7.70E-02	-0.56	2.53E-03	-0.05	8.20E-01	-0.30	1.34E-01	0.32	1.04E-01	-0.10	6.23E-01	0.38	5.31E-02	0.47	1.26E-02	0.36	6.39E-02	-0.65	2.59E-04
ENSCAFG0000001298	ENSCAFG0000001298	grey	VSMC_M10	0.35	7.70E-02	0.03	8.94E-01	0.26	1.91E-01	-0.22	2.62E-01	-0.20	3.12E-01	0.19	3.53E-01	0.17	3.86E-01	0.26	1.86E-01	-0.24	2.29E-01	0.02	9.27E-01
ENSCAFG0000001999	CRAT	cyan	VSMC_M2	0.35	7.71E-02	0.81	2.45E-07	0.43	2.44E-02	0.19	3.43E-01	-0.26	1.87E-01	-0.24	2.32E-01	-0.10	6.23E-01	-0.51	6.92E-03	-0.72	2.66E-05	0.42	2.73E-02
ENSCAFG0000000008	RAB5B	cyan	VSMC_M2	0.35	7.71E-02	0.28	1.50E-01	0.15	4.61E-01	0.29	1.37E-01	0.01	9.66E-01	-0.13	5.10E-01	-0.17	4.04E-01	-0.43	2.48E-02	-0.26	1.84E-01	0.19	3.50E-01
ENSCAFG0000001742	VEZF1	grey	VSMC_M10	0.35	7.73E-02	0.27	1.72E-01	-0.14	4.86E-01	0.00	9.81E-01	0.33	9.12E-02	-0.16	4.12E-01	-0.07	7.45E-01	-0.07	7.22E-01	-0.35	6.95E-02	0.00	9.92E-01
ENSCAFG0000000332	PCBP1	grey	VSMC_M10	0.35	7.73E-02	0.04	8.45E-01	0.38	4.96E-02	-0.55	2.97E-03	-0.20	3.28E-01	0.07	7.12E-01	0.17	3.97E-01	0.52	5.08E-03	-0.23	2.48E-01	-0.23	2.57E-01
ENSCAFG0000000104	XRCC6	grey	VSMC_M10	0.35	7.74E-02	0.09	6.67E-01	0.20	3.12E-01	-0.16	4.18E-01	0.03	8.75E-01	-0.17	3.95E-01	0.04	8.59E-01	0.13	5.31E-01	-0.13	5.11E-01	-0.25	2.03E-01
ENSCAFG0000000550	RPS16	cyan	VSMC_M2	0.35	7.75E-02	0.74	1.01E-05	0.42	3.12E-02	0.30	1.25E-01	-0.38	5.31E-02	-0.05	7.91E-01	-0.36	6.29E-02	-0.48	1.16E-02	-0.71	2.87E-05	0.51	6.29E-03
ENSCAFG0000002947	GADD45A	grey	VSMC_M10	0.35	7.76E-02	0.09	6.53E-01	-0.23	2.44E-01	0.34	8.02E-02	0.40	4.00E-02	-0.16	4.32E-01	0.00	9.94E-01	-0.35	7.15E-02	-0.21	2.94E-01	0.11	5.78E-01
ENSCAFG0000001560	RPS24	red	VSMC_M1	0.35	7.77E-02	-0.09	6.68E-01	0.21	3.03E-01	-0.13	5.17E-01	0.02	9.07E-01	-0.21	2.86E-01	0.33	8.96E-02	0.13	5.31E-01	-0.04	8.45E-01	-0.26	1.92E-01
ENSCAFG0000003043	ENSCAFG0000003043	grey	VSMC_M10	0.35	7.79E-02	0.26	1.85E-01	0.00	9.89E-01	0.34	7.83E-02	0.13	5.11E-01	0.16	4.40E-01	0.02	9.13E-01	-0.37	5.47E-02	-0.28	1.54E-01	0.24	2.38E-01
ENSCAFG0000001400	RPGR	cyan	VSMC_M2	0.35	7.79E-02	0.61	6.72E-04	0.45	1.75E-02	-0.14	4.95E-01	-0.24	2.30E-01	-0.22	2.75E-01	-0.61	8.17E-04	-0.14	4.83E-01	-0.56	2.14E-03	0.13	5.08E-01
ENSCAFG0000001497	ENSCAFG0000001497	cyan	VSMC_M2	0.34	7.81E-02	0.86	8.59E-09	0.14	4.98E-01	0.44	2.30E-02	-0.03	8.66E-01	-0.10	6.07E-01	-0.46	1.58E-02	-0.67	1.33E-04	-0.77	2.95E-06	0.57	2.06E-03
ENSCAFG0000001700	PPP1R9B	cyan	VSMC_M2	0.34	7.83E-02	0.72	2.31E-05	0.66	1.81E-04	-0.30	1.24E-01	-0.44	2.12E-02	-0.16	4.18E-01	-0.03	8.72E-01	-0.12	5.54E-01	-0.66	1.66E-04	0.21	2.99E-01
ENSCAFG0000000532	TADA3	grey	VSMC_M10	0.34	7.85E-02	0.63	4.33E-04	0.27	1.79E-01	0.28	1.59E-01	-0.07	7.28E-01	-0.52	5.39E-03	-0.12	5.40E-01	-0.52	5.26E-03	-0.57	1.75E-03	0.37	6.09E-02
ENSCAFG0000001178	ZFP64	grey	VSMC_M10	0.34	7.87E-02	0.51	7.18E-03	0.06	7.61E-01	0.27	1.70E-01	0.04	8.37E-01	0.03	8.87E-01	-0.06	7.52E-01	-0.37	5.82E-02	-0.60	9.93E-04	0.35	6.99E-02
ENSCAFG0000001615	BCL2L13	grey	VSMC_M10	0.34	7.87E-02	-0.01	9.77E-01	0.24	2.26E-01	-0.25	2.09E-01	0.05	8.09E-01	0.02	9.26E-01	0.23	2.39E-01	0.17	3.91E-01	-0.11	5.79E-01	-0.32	9.88E-02
ENSCAFG0000000395	ENSCAFG0000000395	grey	VSMC_M10	0.34	7.89E-02	0.22	2.77E-01	-0.24	2.35E-01	0.52	5.23E-03	0.34	8.61E-02	-0.07	7.18E-01	0.10	6.04E-01	-0.55	2.97E-03	-0.25	2.01E-01	0.32	1.07E-01
ENSCAFG0000000454	AKR1A1	grey	VSMC_M10	0.34	7.90E-02	-0.15	4.52E-01	0.23	2.49E-01	-0.48	1.12E-02	0.13	5.29E-01	-0.13	5.09E-01	-0.07	7.10E-01	0.43	2.71E-02	0.11	5.71E-01	-0.64	3.59E-04
ENSCAFG0000001908	SH3GL1	cyan	VSMC_M2	0.34	7.91E-02	0.60	1.00E-03	0.54	3.56E-03	-0.24	2.21E-01	-0.44	2.25E-02	-0.02	9.05E-01	-0.27	1.79E-01	0.02	9.32E-01	-0.63	4.57E-04	0.29	1.37E-01
ENSCAFG0000002347	RPS27A	grey	VSMC_M10	0.34	7.92E-02	-0.04	8.33E-01	0.15	4.64E-01	0.02	9.19E-01	0.00	9.87E-01	-0.03	8.94E-01	0.11	5.75E-01	0.06	7.72E-01	-0.13	5.12E-01	-0.18	3.77E-01
ENSCAFG0000000693	AACS	yellow	VSMC_M3	0.34	7.93E-02	-0.04	8.34E-01	0.66	1.96E-04	-0.81	2.44E-07	-0.30	1.31E-01	0.04	8.37E-01	0.34	8.45E-02	0.60	9.25E-04	-0.04	8.58E-01	-0.55	3.07E-03
ENSCAFG0000000081	DLA-DOB	grey	VSMC_M10	0.34	7.94E-02	0.35	6.96E-02	0.62	5.31E-04	-0.37	5.48E-02	-0.43	2.54E-02	-0.06	7.74E-01	0.23	2.51E-01	0.20	3.24E-01	-0.47	1.32E-02	0.02	9.19E-01
ENSCAFG0000002033	ADGRL2	grey	VSMC_M10	0.34	7.95E-02	0.32	1.05E-01	0.24	2.22E-01	0.01	9.47E-01	-0.05	8.01E-01	-0.23	2.42E-01	-0.61	7.04E-04	-0.11	5.76E-01	-0.27	1.66E-01	0.01	9.62E-01
ENSCAFG0000000980	PHACTR1	cyan	VSMC_M2	0.34	7.95E-02	0.88	9.70E-10	0.33	9.22E-02	0.26	1.83E-01	-0.23	2.47E-01	-0.23	2.51E-01	-0.17	3.86E-01	-0.51	6.54E-03	-0.85	1.40E-08	0.58	1.51E-03
ENSCAFG0000002033	PSKH1	grey	VSMC_M10	0.34	7.95E-02	0.40	3.63E-02	-0.01	9.61E-01	0.57	2.08E-03	0.09	6.73E-01	-0.17	4.08E-01	-0.12	5.54E-01	-0.66	1.83E-04	-0.40	3.72E-02	0.43	2.36E-02
ENSCAFG0000000433	ENSCAFG0000000433	cyan	VSMC_M2	0.34	7.97E-02	0.41	3.17E-02	-0.05	7.94E-01	0.25	2.00E-01	0.27	1.66E-01	-0.26	1.91E-01	-0.04	8.41E-01	-0.38	4.90E-02	-0.44	2.14E-02	0.14	4.74E-01
ENSCAFG0000001303	SF3B2	darkgrey	VSMC_M8	0.34	7.98E-02	0.06	7.63E-01	0.81	3.68E-07	-0.80	6.38E-07	-0.48	1.09E-02	-0.12	5.57E-01	0.18	3.72E-01	0.66	2.05E-04	-0.20	3.16E-01	-0.47	1.41E-02
ENSCAFG0000000877	FAM192A	grey	VSMC_M10	0.34	8.02E-02	0.14	4.78E-01	0.01	9.76E-01	0.19	3.50E-01	0.06	7.58E-01	-0.20	3.09E-01	0.17	3.93E-01	-0.18	3.56E-01	-0.24	2.29E-01	0.25	2.13E-01
ENSCAFG0000001173	SRRD	yellow	VSMC_M3	0.34	8.03E-02	0.12	5.37E-01	0.48	1.07E-02	-0.29	1.35E-01	-0.17	3.83E-01	-0.12	5.64E-01	-0.06	7.74E-01	0.15	4.45E-01	-0.22	2.73E-01	-0.30	1.26E-01
ENSCAFG0000001997	OSGIN1	cyan	VSMC_M2	0.34	8.03E-02	0.70	4.61E-05	0.36	6.78E-02	0.02	9.18E-01	-0.34	8.18E-02	0.06	7.81E-01	-0.25	2.11E-01	-0.17	3.92E-01	-0.76	3.85E-06	0.47	1.33E-02
ENSCAFG0000000131	FANCE	yellow	VSMC_M3	0.34	8.04E-02	0.51	7.11E-03	0.74	9.97E-06	-0.40	4.14E-02	-0.60	1.06E-03	-0.10	6.28E-01	0.11	5.95E-01	0.16	4.21E-01	-0.57	1.89E-03	0.15	4.68E-01
ENSCAFG0000000000	TXNL4A	darkgrey	VSMC_M8	0.34	8.04E-02	-0.18	3.77E-01	0.49	8.94E-03	-0.63	3.98E-04	-0.21	2.87E-01	0.08	6.92E-01	0.14	5.00E-01	0.60	1.00E-03	-0.01	9.59E-01	-0.53	4.43E-03
ENSCAFG0000001022	SUFU	grey	VSMC_M10	0.34	8.05E-02	0.35	7.51E-02	-0.23	2.44E-01	0.54	3.75E-03	0.37	5.68E-02	-0.30	1.24E-01	-0.23	2.57E-01	-0.62	5.13E-04	-0.34	8.71E-02	0.28	1.52E-01
ENSCAFG0000000978	NEURL2	grey	VSMC_M10	0.34	8.07E-02	0.18	3.74E-01	0.24	2.22E-01	-0.25	2.00E-01	0.00	9.95E-01	0.11	5.86E-01	-0.09	6.70E-01	0.11	5.99E-01	-0.25	2.17E-01	-0.18	3.82E-01
ENSCAFG0000001612	SDE2	yellow	VSMC_M3	0.34	8.07E-02	0.02	9.09E-01	0.68	9.30E-05	-0.49	8.67E-03	-0.41	3.39E-02	0.09	6.65E-01	0.02	9.24E-01	0.38	4.79E-02	-0.10	6.03E-01	-0.34	8.47E-02
ENSCAFG0000002881	ENSCAFG0000002881	grey	VSMC_M10	0.34	8.09E-02	0.58	1.60E-03	0.17	4.00E-01	0.30	1.24E-01	-0.13	5.12E-01	0.00	9.97E-01	-0.68	1.09E-04	-0.41	3.55E-02	-0.55	2.75E-03	0.41	3.17E-02
ENSCAFG0000000829	ENSCAFG0000000829	yellow	VSMC_M3	0.34	8.09E-02	0.43	2.42E-02	0.44	2.00E-02	-0.21	2.90E-01	-0.20	3.13E-01	-0.14	4.88E-01	0.00	9.92E-01	0.03	8.95E-01	-0.46	1.49E-02	-0.01	9.63E-01
ENSCAFG0000002961	TM2D2	grey	VSMC_M10	0.34	8.11E-02	0.45	1.88E-02	0.06	7.54E-01	0.47	1.42E-02	0.01	9.69E-01	-0.27	1.81E-01	-0.50	7.61E-03	-0.57	1.76E-03	-0.40	3.77E-02	0.39	4.69E-02
ENSCAFG0000002379	E2F6	grey	VSMC_M10	0.34	8.11E-02	-0.15	4.58E-01	0.03	8.78E-01	-0.37	6.05E-02	0.13	5.28E-01	0.15	4.53E-01	0.23	2.53E-01	0.37	5.89E-02	-0.01	9.55E-01	-0.20	3.09E-01
ENSCAFG0000000506	WDR48	grey	VSMC_M10	0.34	8.12E-02	0.09	6.47E-01	-0.19	3.33E-01	0.40	3.70E-02	0.24	2.23E-01	-0.02	9.06E-01	0.17	3.87E-01	-0.39	4.21E-02	-0.23	2.56E-01	0.30	1.31E-01
ENSCAFG0000002915	NOVA1	grey	VSMC_M10	0.34	8.13E-02	0.22	2.79E-01	0.22	2.60E-01	-0.15	4.50E-01	-0.10	6.11E-01	-0.06	7.50E-01	-0.10	6.03E-01	0.06	7.66E-01	-0.32	9.98E-02	0.11	5.92E-01
ENSCAFG0000001575	CNP	yellow	VSMC_M3	0.34	8.13E-02	0.73	1.44E-05	0.75	7.03E-06	-0.15	4.63E-01	-0.60	1.06E-03	-0.04	8.56E-01	-0.05	8.17E-01	-0.17	4.10E-01	-0.76	4.97E-06	0.29	1.46E-01

ENSCAFG0000000952	CUTC	grey	VSMC_M10	0.34	8.14E-02	0.09	6.70E-01	0.33	9.27E-02	-0.06	7.81E-01	-0.24	2.21E-01	0.21	3.01E-01	0.21	2.98E-01	0.07	7.47E-01	-0.26	1.89E-01	0.12	5.64E-01
ENSCAFG0000000023	DCTN2	yellow	VSMC_M3	0.34	8.14E-02	0.31	1.13E-01	0.58	1.44E-03	-0.46	1.53E-02	-0.20	3.11E-01	-0.28	1.63E-01	-0.10	6.08E-01	0.20	3.23E-01	-0.29	1.41E-01	-0.36	6.83E-02
ENSCAFG0000003025	ENSCAFG0000003025	grey	VSMC_M10	0.34	8.15E-02	-0.05	8.02E-01	-0.31	1.11E-01	0.30	1.29E-01	0.43	2.51E-02	-0.25	2.08E-01	0.08	6.90E-01	-0.18	3.79E-01	-0.12	5.45E-01	0.06	7.73E-01
ENSCAFG0000001230	PIGV	grey	VSMC_M10	0.34	8.15E-02	0.29	1.42E-01	0.42	2.89E-02	-0.34	7.96E-02	-0.23	2.49E-01	-0.17	3.86E-01	0.28	1.62E-01	0.10	6.20E-01	-0.36	6.25E-02	0.03	8.98E-01
ENSCAFG0000000536	PLD3	cyan	VSMC_M2	0.34	8.18E-02	0.88	1.21E-09	0.13	5.32E-01	0.53	4.92E-03	-0.04	8.31E-01	-0.24	2.38E-01	-0.35	7.52E-02	-0.79	8.66E-07	-0.77	2.11E-06	0.68	1.09E-04
ENSCAFG0000001630	ARAP2	grey	VSMC_M10	0.34	8.19E-02	0.49	9.32E-03	-0.14	5.01E-01	0.41	3.55E-02	0.23	2.44E-01	0.02	9.31E-01	-0.64	3.33E-04	-0.46	1.67E-02	-0.51	6.95E-03	0.29	1.47E-01
ENSCAFG0000001586	NT5C3B	grey	VSMC_M10	0.34	8.20E-02	0.03	8.85E-01	-0.05	8.13E-01	0.17	4.04E-01	0.30	1.34E-01	-0.30	1.23E-01	-0.20	3.10E-01	-0.21	2.96E-01	0.04	8.42E-01	-0.19	3.50E-01
ENSCAFG0000001988	CYBA	grey	VSMC_M10	0.34	8.22E-02	0.64	3.19E-04	0.18	3.73E-01	0.43	2.37E-02	-0.21	3.05E-01	-0.06	7.85E-01	-0.29	1.49E-01	-0.50	7.86E-03	-0.68	9.87E-05	0.59	1.07E-03
ENSCAFG0000000286	PPP4R3B	grey	VSMC_M10	0.34	8.22E-02	0.10	6.23E-01	-0.05	8.07E-01	0.10	6.16E-01	0.15	4.50E-01	0.11	5.93E-01	0.06	7.55E-01	-0.08	6.89E-01	-0.26	1.92E-01	0.16	4.18E-01
ENSCAFG0000001499	FMO3	grey	VSMC_M10	0.34	8.23E-02	0.71	2.98E-05	0.20	3.08E-01	0.35	7.70E-02	-0.16	4.35E-01	-0.03	8.84E-01	-0.26	1.93E-01	-0.54	3.37E-03	-0.75	7.54E-06	0.56	2.62E-03
ENSCAFG0000001933	CLDN6	yellow	VSMC_M3	0.34	8.24E-02	0.09	6.39E-01	0.55	3.08E-03	-0.50	8.30E-03	-0.40	3.76E-02	0.35	7.47E-02	0.03	8.87E-01	0.39	4.72E-02	-0.20	3.05E-01	-0.08	6.96E-01
ENSCAFG0000001174	MCCC1	grey	VSMC_M10	0.34	8.25E-02	0.00	9.89E-01	0.48	1.08E-02	-0.41	3.25E-02	-0.21	2.84E-01	0.04	8.46E-01	0.23	2.39E-01	0.38	4.95E-02	-0.16	4.18E-01	-0.36	6.88E-02
ENSCAFG0000001285	TDRKH	grey	VSMC_M10	0.34	8.26E-02	0.37	5.91E-02	0.48	1.08E-02	-0.15	4.60E-01	-0.46	1.53E-02	-0.01	9.56E-01	0.25	2.07E-01	0.09	6.48E-01	-0.56	2.18E-03	0.32	1.06E-01
ENSCAFG0000001080	PDCD4	grey	VSMC_M10	0.34	8.27E-02	0.81	2.35E-07	0.15	4.41E-01	0.43	2.48E-02	-0.11	5.94E-01	-0.14	4.88E-01	-0.01	9.73E-01	-0.67	1.54E-04	-0.77	2.50E-06	0.65	2.24E-04
ENSCAFG0000001233	NENF	cyan	VSMC_M2	0.34	8.29E-02	0.57	1.96E-03	0.52	5.82E-03	0.09	6.73E-01	-0.41	3.16E-02	0.13	5.22E-01	-0.07	7.26E-01	-0.36	6.37E-02	-0.54	3.59E-03	0.35	7.35E-02
ENSCAFG0000001620	TCF12	cyan	VSMC_M2	0.34	8.29E-02	0.40	4.01E-02	0.07	7.10E-01	-0.09	6.40E-01	0.19	3.46E-01	-0.26	1.91E-01	-0.09	6.52E-01	-0.09	6.45E-01	-0.43	2.65E-02	-0.03	8.92E-01
ENSCAFG0000001766	SLC14A1	darkgreen	VSMC_M4	0.34	8.30E-02	0.58	1.41E-03	-0.25	2.15E-01	0.77	3.17E-06	0.28	1.51E-01	-0.18	3.82E-01	-0.52	5.62E-03	-0.78	1.54E-06	-0.55	3.02E-03	0.57	1.99E-03
ENSCAFG0000000712	SCN1B	cyan	VSMC_M2	0.34	8.31E-02	0.72	1.96E-05	0.45	1.99E-02	0.08	6.83E-01	-0.41	3.15E-02	0.11	5.87E-01	-0.02	9.40E-01	-0.35	7.33E-02	-0.69	7.05E-05	0.57	1.74E-03
ENSCAFG0000001979	STRIP1	grey	VSMC_M10	0.34	8.31E-02	0.32	9.92E-02	-0.19	3.33E-01	0.46	1.62E-02	0.38	5.25E-02	-0.09	6.54E-01	-0.53	4.14E-03	-0.50	7.64E-03	-0.26	1.97E-01	0.12	5.61E-01
ENSCAFG0000002307	ENSCAFG0000002307	grey	VSMC_M10	0.34	8.32E-02	0.21	2.92E-01	0.32	1.08E-01	-0.27	1.76E-01	-0.08	6.80E-01	-0.23	2.45E-01	0.10	6.09E-01	0.06	7.76E-01	-0.22	2.64E-01	-0.07	7.11E-01
ENSCAFG0000001118	DCLRE1A	pink	VSMC_M5	0.34	8.32E-02	-0.07	7.46E-01	-0.43	2.46E-02	0.34	8.44E-02	0.56	2.31E-03	-0.04	8.53E-01	0.13	5.16E-01	-0.26	1.93E-01	-0.09	6.57E-01	0.02	9.11E-01
ENSCAFG0000000056	ZNF608	yellow	VSMC_M3	0.34	8.35E-02	0.31	1.12E-01	0.41	3.20E-02	-0.48	1.18E-02	-0.16	4.36E-01	-0.02	9.06E-01	0.09	6.54E-01	0.31	1.20E-01	-0.35	6.96E-02	-0.16	4.18E-01
ENSCAFG0000000470	HSPA14	grey	VSMC_M10	0.34	8.36E-02	-0.39	4.22E-02	-0.06	7.76E-01	-0.16	4.32E-01	0.41	3.52E-02	-0.14	4.91E-01	-0.12	5.51E-01	0.27	1.78E-01	0.30	1.27E-01	-0.67	1.24E-04
ENSCAFG0000001197	POU2F3	grey	VSMC_M10	0.34	8.36E-02	0.16	4.23E-01	0.45	1.81E-02	-0.18	3.63E-01	-0.28	1.52E-01	0.45	1.96E-02	-0.17	4.06E-01	0.12	5.57E-01	-0.25	2.15E-01	-0.11	5.90E-01
ENSCAFG0000001738	RPS27	cyan	VSMC_M2	0.34	8.37E-02	0.51	6.17E-03	0.30	1.32E-01	0.21	3.01E-01	-0.22	2.73E-01	-0.13	5.26E-01	-0.03	8.92E-01	-0.36	6.24E-02	-0.51	6.19E-03	0.33	8.85E-02
ENSCAFG0000001115	STARD9	cyan	VSMC_M2	0.34	8.37E-02	0.40	4.02E-02	-0.20	3.25E-01	0.30	1.24E-01	0.38	4.82E-02	-0.15	4.66E-01	-0.27	1.72E-01	-0.46	1.60E-02	-0.31	1.12E-01	0.19	3.38E-01
ENSCAFG0000000031	ATP23	grey	VSMC_M10	0.34	8.39E-02	-0.03	8.99E-01	-0.22	2.62E-01	0.01	9.43E-01	0.44	2.28E-02	0.00	9.93E-01	0.12	5.53E-01	0.00	9.82E-01	-0.08	6.87E-01	-0.18	3.64E-01
ENSCAFG0000000942	ENSCAFG0000000942	grey	VSMC_M10	0.34	8.40E-02	0.09	6.52E-01	-0.36	6.45E-02	0.24	2.26E-01	0.54	3.54E-03	-0.20	3.21E-01	0.14	4.78E-01	-0.26	1.83E-01	-0.14	4.87E-01	0.07	7.18E-01
ENSCAFG0000002445	ENSCAFG0000002445	grey	VSMC_M10	0.34	8.41E-02	-0.05	7.92E-01	0.02	9.14E-01	0.23	2.44E-01	0.17	4.10E-01	-0.13	5.16E-01	-0.07	7.41E-01	-0.13	5.08E-01	-0.03	9.00E-01	-0.09	6.47E-01
ENSCAFG0000000335	TMEM268	grey	VSMC_M10	0.34	8.42E-02	0.61	7.20E-04	0.27	1.67E-01	0.18	3.60E-01	-0.29	1.41E-01	0.22	2.80E-01	0.06	7.50E-01	-0.35	7.05E-02	-0.72	2.41E-05	0.62	5.49E-04
ENSCAFG0000000178	KLC4	yellow	VSMC_M3	0.34	8.46E-02	0.22	2.76E-01	0.37	6.11E-02	-0.02	9.32E-01	-0.10	6.03E-01	-0.09	6.70E-01	-0.13	5.31E-01	-0.21	3.04E-01	-0.19	3.31E-01	-0.08	7.03E-01
ENSCAFG0000000017	ENSCAFG0000000017	grey	VSMC_M10	0.34	8.46E-02	0.25	2.11E-01	0.26	1.88E-01	0.09	6.39E-01	-0.20	3.12E-01	-0.05	7.98E-01	-0.06	7.79E-01	-0.15	4.46E-01	-0.35	7.07E-02	0.27	1.69E-01
ENSCAFG0000000282	NFYC	grey	VSMC_M10	0.34	8.47E-02	-0.05	7.98E-01	-0.30	1.26E-01	0.17	3.88E-01	0.33	9.81E-02	0.15	4.67E-01	0.10	6.08E-01	-0.06	7.74E-01	-0.14	4.86E-01	0.17	3.94E-01
ENSCAFG0000000609	DDHD2	grey	VSMC_M10	0.34	8.48E-02	0.28	1.62E-01	-0.30	1.27E-01	0.45	1.83E-02	0.36	6.79E-02	0.08	6.98E-01	0.20	3.27E-01	-0.48	1.14E-02	-0.34	8.10E-02	0.40	3.62E-02
ENSCAFG0000001628	PLSCR3	cyan	VSMC_M2	0.34	8.49E-02	0.58	1.37E-03	0.31	1.17E-01	0.00	9.89E-01	-0.23	2.52E-01	0.09	6.69E-01	0.04	8.27E-01	-0.24	2.24E-01	-0.64	3.13E-04	0.36	6.39E-02
ENSCAFG0000001409	BCOR	grey	VSMC_M10	0.34	8.50E-02	-0.04	8.38E-01	0.21	2.85E-01	-0.43	2.35E-02	0.02	9.31E-01	0.19	3.41E-01	0.08	6.93E-01	0.38	4.99E-02	-0.07	7.32E-01	-0.27	1.72E-01
ENSCAFG0000001430	ATIC	yellow	VSMC_M3	0.34	8.52E-02	0.36	6.90E-02	0.47	1.37E-02	-0.24	2.24E-01	-0.19	3.34E-01	-0.19	3.38E-01	0.17	3.92E-01	0.05	8.18E-01	-0.39	4.57E-02	-0.09	6.50E-01
ENSCAFG0000000304	SCRN1	grey	VSMC_M10	0.34	8.54E-02	0.44	2.07E-02	-0.13	5.23E-01	0.51	6.52E-03	0.28	1.58E-01	-0.21	2.90E-01	-0.17	3.89E-01	-0.64	3.21E-04	-0.39	4.22E-02	0.34	8.33E-02
ENSCAFG0000000585	MCRIP1	red	VSMC_M1	0.34	8.55E-02	0.45	1.80E-02	0.50	8.12E-03	0.00	9.88E-01	-0.40	3.66E-02	-0.07	7.34E-01	0.11	5.76E-01	-0.14	5.00E-01	-0.54	3.46E-03	0.29	1.43E-01
ENSCAFG0000000311	WDPCP	yellow	VSMC_M3	0.34	8.55E-02	0.10	6.30E-01	0.52	5.83E-03	-0.32	1.03E-01	-0.25	2.05E-01	-0.15	4.53E-01	0.08	7.05E-01	0.27	1.72E-01	-0.21	2.99E-01	-0.23	2.56E-01
ENSCAFG0000000495	ACAA1	cyan	VSMC_M2	0.34	8.58E-02	0.57	1.72E-03	0.30	1.35E-01	0.19	3.44E-01	-0.08	6.97E-01	-0.31	1.18E-01	0.06	7.68E-01	-0.36	6.64E-02	-0.57	1.92E-03	0.22	2.76E-01
ENSCAFG0000002905	ENSCAFG0000002905	grey	VSMC_M10	0.34	8.58E-02	0.02	9.15E-01	0.14	4.94E-01	-0.17	3.95E-01	0.17	3.87E-01	-0.12	5.35E-01	0.08	7.10E-01	0.09	6.54E-01	-0.07	7.42E-01	-0.41	3.22E-02
ENSCAFG0000001981	VPS9D1	grey	VSMC_M10	0.34	8.59E-02	0.45	1.95E-02	0.62	5.29E-04	-0.20	3.17E-01	-0.53	4.85E-03	-0.16	4.12E-01	0.04	8.27E-01	-0.01	9.61E-01	-0.49	8.90E-03	0.31	1.13E-01
ENSCAFG0000002012	GCLM	darkgrey	VSMC_M8	0.34	8.60E-02	-0.25	2.09E-01	0.43	2.49E-02	-0.63	4.88E-04	-0.25	2.09E-01	0.20	3.16E-01	0.44	2.02E-02	0.67	1.13E-04	0.01	9.62E-01	-0.39	4.67E-02
ENSCAFG0000001557	RTN1	grey	VSMC_M10	0.34	8.60E-02	-0.30	1.29E-01	-0.32	1.03E-01	0.27	1.78E-01	0.45	1.76E-02	0.13	5.04E-01	-0.14	4.81E-01	-0.04	8.38E-01	0.13	5.30E-01	-0.23	2.58E-01
ENSCAFG0000000035	MAML1	grey	VSMC_M10	0.34	8.61E-02	0.29	1.39E-01	0.20	3.26E-01	-0.25	2.15E-01	-0.05	8.16E-01	-0.15	4.67E-01	0.17	3.89E-01	0.05	8.14E-01	-0.41	3.25E-02	0.08	6.77E-01
ENSCAFG0000001941	PNPLA7	cyan	VSMC_M2	0.34	8.61E-02	0.74	9.50E-06	0.28	1.64E-01	0.29	1.39E-01	-0.05	8.23E-01	-0.34	8.39E-02	-0.42	2.78E-02	-0.60	9.92E-04	-0.59	1.16E-03	0.30	1.25E-01
ENSCAFG0000000125	EGR1	red	VSMC_M1	0.34	8.67E-02	0.36	6.47E-02	0.17	3.96E-01	-0.03	8.97E-01	-0.01	9.66E-01	-0.13	5.09E-01	0.34	8.48E-02	-0.10	6.13E-01	-0.51	7.17E-03	0.19	3.35E-01
ENSCAFG0000000428	LURAP1	cyan	VSMC_M2	0.34	8.67E-02	0.72	2.17E-05	0.39	4.41E-02	0.10	6.31E-01	-0.32	1.02E-01	0.04	8.39E-01	-0.01	9.45E-01	-0.34	8.47E-02	-0.73	1.56E-05	0.53	4.58E-03
ENSCAFG0000000022	GLI1	grey	VSMC_M10	0.34	8.68E-02	-0.05	8.08E-01	0.45	1.73E-02	-0.63	4.09E-04	-0.13	5.19E-01	-0.16	4.22E-01	0.41	3.27E-02	0.45	1.86E-02	-0.06	7.67E-01	-0.38	5.37E-02
ENSCAFG0000001870	ZDHHC9	cyan	VSMC_M2	0.34	8.69E-02	0.59	1.23E-03	0.18	3.73E-01	0.29	1.45E-01	-0.14	4.78E-01	-0.06	7.62E-01	-0.16	4.36E-01	-0.42	2.82E-02	-0.65	2.79E-04	0.52	5.33E-03
ENSCAFG0000000192	MRPS18A	yellow	VSMC_M3	0.34	8.70E-02	0.33	9.02E-02	0.73	1.41E-05	-0.41	3.53E-02	-0.56	2.28E-03	0.02	9.32E-01	0.00	9.82E-01	0.23	2.50E-01	-0.40	3.72E-02	0.00	9.86E-01
ENSCAFG0000000458	TSC22D1	red	VSMC_M1	0.34	8.70E-02	0.19	3.36E-01	0.08	6.80E-01	-0.16	4.32E-01	0.15	4.63E-01	-0.17	4.00E-01	0.48	1.18E-02	-0.01	9.59E-01	-0.26	1.97E-01	0.02	9.29E-01
ENSCAFG0000000513	LTBP4	cyan	VSMC_M2	0.34	8.72E-02	0.84	4.45E-08	0.54	3.60E-03	-0.08	6.97E-01	-0.37	5.46E-02	-0.12	5.66E-01	-0.24	2.20E-01	-0.25	2.01E-01	-0.79	1.13E-06	0.37	5.54E-02
ENSCAFG0000001002	ENSCAFG0000001002	grey	VSMC_M10	0.34	8.72E-02	0.28	1.51E-01	0.45	1.95E-02	-0.03	8.79E-01	-0.36	6.47E-02	-0.20	3.08E-01	0.11	5.79E-01	-0.10	6.29E-01	-0.34	8.69E-02	0.24	2.36E-01
ENSCAFG0000002022	PSMB7	grey	VSMC_M10	0.34	8.74E-02	-0.56	2.23E-03	-0.08	6.85E-01	-0.26	1.83E-01	0.28	1.57E-01	0.00	9.89E-01	0.26	1.92E-01	0.48	1.12E-02	0.34	8.39E-02	-0.56	2.17E-03
ENSCAFG0000003210	FAM180B	cyan	VSMC_M2	0.34	8.74E-02	0.64	3.38E-04	0.57	1.98E-03	-0.24	2.27E-01	-0.43	2.69E-02	-0.16	4.14E-01	0.10	6.05E-01	-0.06	7.82E-01	-0.68	9.10E-05	0.25	2.05E-01
ENSCAFG0000000479	RNF220	yellow	VSMC_M3	0.34	8.76E-02	0.46	1.55E-02	0.63	4.03E-04	-0.21	2.87E-01	-0.51	6.23E-03	-0.14	4.86E-01	-0.12	5.62E-01	0.01	9.66E-01	-0.51	6.75E-03	0.21	2.87E-01
ENSCAFG0000000061	DDAH2	cyan	VSMC_M2	0.33	8.77E-02	0.82	1.95E-07	0.40	3.77E-02	0.28	1.57E-01	-0.32	1.05E-01	-0.19	3.33E-01	-0.05	8.05E-01	-0.50	8.10E-03	-0.79	8.46E-07	0.60	1.02E-03
ENSCAFG0000000996	PSMD14	darkgrey	VSMC_M8	0.33	8.77E-02	-0.58	1.61E-03	0.04	8.28E-01	-0.43	2.46E-02	0.29	1.44E-01	-0.18	3.76E-01	0.11	5.71E-01	0.61	6.92E-04	0.39	4.24E-02	-0.78	1.25E-06
ENSCAFG0000000584	TBC1D5	cyan	VSMC_M2	0.33	8.80E-02	0.40	3.92E-02	0.06	7.62E-01	-0.03	8.69E-01	0.23	2.55E-01	-0.42	2.83E-02	0.04	8.30E-01	-0.23	2.49E-01	-0.39	4.52E-02	0.03	8.74E-01
ENSCAFG0000000145	TBC1D22B	grey	VSMC_M10	0.33	8.80E-02	0.38	5.33E-02	-0.15	4.50E-01	0.38	5.32E-02	0.14	4.72E-01	0.13	5.04E-01	-0.01	9.75E-01	-0.40	3.96E-02	-0.49	8.85E-03	0.47	1.31E-02
ENSCAFG0000000399	FGF2	red	VSMC_M1	0.33	8.81E-02	0.35	7.79E-02	0.16	4.33E-01	0.16	4.12E-01	-0.11	6.01E-01	-0.07	7.42E-01	0.22	2.75E-01	-0.22	2.70E-01	-0.46	1.49E-02	0.34	8.62E-02
ENSCAFG0000000996	ADAMTS15	grey	VSMC_M10	0.33	8.81E-02	0.72	2.28E-05	0.28	1.63E-01	0.42	2.90E-02	-0.22	2.73E-01	0.19	3.39E-01	-0.41	3.52E-02	-0.55	2.70E-03	-0.68	1.12E-04	0.52	5.06E-03
ENSCAFG0000002886	XIRP1	darkgreen	VSMC_M4	0.33	8.82E-02	0.48	1.06E-02	-0.20	3.16E-01	0.58	1.61E-03	0.20	3.22E-01	-0.02	9.34E-01	-0.32	1.06E-01	-0.58	1.35E-03	-0.52	4.97E-03	0.57	1.74E-03
ENSCAFG0000001044	FOXRED1	grey	VSMC_M10	0.33	8.84E-02	0.08	6.99E-01	0.28	1.62E-01	-0.48	1.17E-02	-0.12	5.68E-01	0.12	5.35E-01	-0.05	7.86E-01	0.40	3.90E-02	-0.24	2.18E-01	-0.18	3.58E-01
ENSCAFG0000000637	HGF	darkgrey	VSMC_M8	0.33	8.84E-02	-0.02	9.23E-01	0.51	6.72E-03	-0.70	5.33E-05	-0.23	2.53E-01	0.04	8.43E-01	0.46	1.48E-02	0.59	1.21E-03	-0.15	4.49E-01	-0.40	3.85E-02
ENSCAFG0000000711	EIF4B	yellow	VSMC_M3	0.33	8.84E-02	0.62	5.06E-04	0.71	3.51E-05	-0.31	1.14E-01	-0.48	1.09E-02	-0.25	2.00E-01	-0.07	7.32E-01	-0.02	9.37E-01	-0.61	6.93E-04	0.11	5.95E-01
ENSCAFG0000002315	IER2	red	VSMC_M1	0.33	8.87E-02	0.50	7.84E-03	0.44	2.00E-02	-0.06	7.71E-01	-0.30	1.26E-01	-0.08	6.98E-01	0.22	2.81E-01	-0.18	3.70E-01	-0.58	1.63E-03	0.31	1.14E-01
ENSCAFG0000000114	ST3GAL1	cyan	VSMC_M2	0.33	8.88E-02	0.48	1.09E-02	0.34	7.92E-02	0.10	6.37E-01	-0.12	5.40E-01	-0.01	9.48E-01	-0.48	1.11E-02	-0.25	2.13E-01	-0.41	3.61E-02	0.03	8.80E-01
ENSCAFG0000000568	NPTX1	cyan	VSMC_M2	0.33	8.88E-02	0.69	6.06E-05	0.56	2.34E-03	-0.20	3.14E-01	-0.40	3.93E-02	0.00	9.98E-01	-0.23	2.58E-01	-0.11	5.72E-01	-0.70	4.93E-05	0.29	1.40E-01
ENSCAFG0000000514	FNDC4	grey	VSMC_M10	0.33	8.89E-02	-0.02	9.34E-01	0.23	2.55E-01	-0.24	2.26E-01	0.02	9.18E-01	-0.02	9.07E-01	-0.58	1.54E-03	0.22	2.81E-01	0.02	9.28E-01	-0.35	7.22E-02
ENSCAFG0000000589	RYR1	grey	VSMC_M10	0.33	8.91E-02	0.44	2.23E-02	0.52	5.89E-03	-0.13	5.34E-01	-0.38	5.07E-02	0.13	5.16E-01	-0.20	3.28E-01	-0.14	4.77E-01	-0.39	4.54E-02	0.16	4.17E-01
ENSCAFG0000001880	TRAF4	cyan	VSMC_M2	0.33	8.91E-02	0.80	4.84E-07	0.58	1.45E-03	0.07	7.13E-01	-0.55	2.74E-03	0.05	8.19E-01	-0.36	6.17E-02	-0.34	8.04E-02	-0.79	1.16E-06	0.58	1.64E-03
ENSCAFG0000001974	CTNNBIP1	red	VSMC_M1	0.33	8.92E-02	0.28	1.56E-01	-0.01	9.58E-01	0.27	1.73E-01	0.27	1.77E-01	-0.29	1.48E-01	-0.17	4.03E-01	-0.31	1.17E-01	-0.28	1.55E-01	0.00	9.84E-01
ENSCAFG0000000630	MORN2	yellow	VSMC_M3	0.33	8.92E-02	-0.09	6.47E-01	0.38	5.06E-02	-0.21	3.02E-01	-0.15	4.62E-01	-0.06	7.85E-01	0.04	8.35E-01	0.21	2.83E-01	-0.01	9.58E-01	-0.32	1.02E-01
ENSCAFG0000001198	HSPBAP1	pink	VSMC_M5	0.33	8.93E-02	0.13	5.24E-01	-0.43	2.52E-02	0.56	2.57E-03	0.60	9.28E-04	-0.31	1.19E-01	-0.42	3.09E-02	-0.54	3.88E-03	-0.09	6.38E-01	0.09	6.68E-01
ENSCAFG0000000639	ACKR4	cyan	VSMC_M2	0.33	8.95E-02	0.79	8.87E-07	0.12	5.42E-01	0.40	4.06E-02	-0.05	7.93E-01	-0.18	3.56E-01	-0.58	1.65E-03	-0.61	7.00E-04	-0.72	2.41E-05	0.53	4.21E-03
ENSCAFG0000003230	ENSCAFG0000003230	grey	VSMC_M10	0.33	8.95E-02	-0.26	1.82E-01	-0.13	5.06E-01	-0.21	2.98E-01	0.35	7.59E-02	0.03	8.99E-01	0.25	2.12E-01	0.29	1.39E-01	0.07	7.40E-01	-0.37	6.05E-02
ENSCAFG0000000491	GALK1	yellow	VSMC_M3	0.33	8.95E-02	0.45	1.95E-02	0.65	2.20E-04	-0.39	4.38E-02	-0.47	1.42E-02	0.06	7.84E-01	0.18	3.78E-01	0.14	4.83E-01	-0.53	4.51E-03	0.01	9.41E-01
ENSCAFG0000000253	ALG2	grey	VSMC_M10	0.33	8.96E-02	0.29	1.41E-01	-0.15	4.43E-01	0.49	1.01E-02	0.10	6.16E-01	0.01	9.73E-01	0.04	8.26E-01	-0.46	1.47E-02	-0.41	3.35E-02	0.56	2.33E-03
ENSCAFG0000003009	ENSCAFG0000003009	red	VSMC_M1	0.33	8.97E-02	0.06	7.61E-01	0.52	5.60E-03	-0.40	4.00E-02	-0.29	1.47E-01	-0.05	8.12E-01	0.00	1.00E+00	0.32	1.01E-01	-0.16	4.36E-01	-0.33	9.44E-02
ENSCAFG0000000780	RETSAT	grey	VSMC_M10	0.33	8.97E-02	-0.10	6.14E-01	0.31	1.15E-01	-0.56	2.24E-03	0.08	7.09E-01	-0.41	3.28E-02	0.39	4.50E-02	0.42	2.89E-02	-0.01	9.75E-01	-0.53	4.39E-03
ENSCAFG0000000532	AKR1E2	red	VSMC_M1	0.33	8.98E-02	0.23	2.52E-01	0.12	5.65E-01	0.04	8.25E-01	-0.07	7.39E-01	-0.07	7.37E-01	0.34	8.47E-02	-0.13	5.11E-01	-0.29	1.38E-01	0.27	1.69E-01
ENSCAFG0000002978	SAAL1	darkgrey	VSMC_M8	0.33	8.99E-02	-0.33	9.59E-02	0.11	5.72E-01	-0.54	3.50E-03	0.25	1.99E-01	-0.28	1.55E-01	0.01	9.73E-01	0.54	3.41E-03	0.25	2.03E-01	-0.67	1.34E-04
ENSCAFG0000002010	ENSCAFG0000002010	grey	VSMC_M10	0.33	9.01E-02	0.12	5.67E-01	0.12	5.55E-01	-0.04	8.43E-01	-0.12	5.38E-01	0.00	9.99E-01	0.22	2.72E-01	0.07	7.37E-01	-0.29	1.49E-01	0.26	1.91E-01
ENSCAFG0000000453	AGBL5	cyan	VSMC_M2	0.33	9.03E-02	0.51	6.83E-03	0.17	4.03E-01	0.39	4.65E-02	-0.16	4.19E-01	-0.01	9.75E-01	-0.10	6.07E-01	-0.51	6.53E-03	-0.51	6.29E-03	0.55	3.27E-03
ENSCAFG0000001341	MTX2	pink	VSMC_M5	0.33	9.03E-02	-0.64	3.33E-04	-0.41	3.38E-02	-0.19	3.33E-01	0.71	4.02E-05	-0.10	6.20E-01	0.19	3.39E-01	0.37	5.96E-02	0.49	9.14E-03	-0.67	1.45E-04
ENSCAFG0000001659	NANOS3	yellow	VSMC_M3	0.33	9.03E-02	0.36	6.52E-02	0.44	2.14E-02	0.04	8.51E-01	-0.27	1.77E-01	-0.14	4.97E-01	-0.05	7.88E-01	-0.23	2.51E-01	-0.39	4.51E-02	0.14	4.86E-01
ENSCAFG0000000511	MYRIP	yellow	VSMC_M3	0.33	9.04E-02	0.65	2.76E-04	0.64	3.49E-04	-0.01	9.58E-01	-0.48	1.18E-02	-0.09	6.47E-01	-0.20	3.25E-01	-0.22	2.78E-01	-0.65	2.40E-04	0.27	1.68E-01
ENSCAFG0000000407	ENSCAFG0000000407	red	VSMC_M1	0.33	9.04E-02	-0.17	3.94E-01	0.12	5.59E-01	-0.22	2.76E-01	0.01	9.42E-01	0.11	5.90E-01	-0.06	7.61E-01	0.23	2.58E-01	0.03	8.70E-01	-0.16	4.32E-01
ENSCAFG0000001107	ADARB1	grey	VSMC_M10	0.33	9.05E-02	0.17	3.91E-01	-0.03	8.69E-01	0.21	2.97E-01	0.12	5.57E-01	0.15	4.69E-01	-0.04	8.27E-01	-0.31	1.15E-01	-0.16	4.32E-01	0.21	2.82E-01
ENSCAFG0000001546	ZNF518B	pink	VSMC_M5	0.33	9.05E-02	-0.30	1.33E-01	-0.45	1.88E-02	0.09	6.72E-01	0.72	2.24E-05	-0.16	4.30E-01	-0.36	6.17E-02	0.04	8.29E-01	0.24	2.29E-01	-0.46	1.53E-02
ENSCAFG0000001446	KCNK4	grey	VSMC_M10	0.33	9.05E-02	0.57	1.87E-03	0.10	6.18E-01	0.40	4.12E-02	-0.02	9.41E-01	-0.21	3.00E-01	-0.27	1.78E-01	-0.53	4.32E-03	-0.55	2.79E-03	0.43	2.45E-02
ENSCAFG0000003167	POLR2M	grey	VSMC_M10	0.33	9.06E-02	0.20	3.08E-01	-0.35	7.50E-02	0.51	6.15E-03	0.48	1.19E-02	-0.20	3.06E-01	-0.05	8.03E-01	-0.47	1.24E-02	-0.31	1.15E-01	0.18	3.66E-01
ENSCAFG0000001969	PALM	grey	VSMC_M10	0.33	9.06E-02	0.62	5.01E-04	0.09	6.54E-01	0.49	8.87E-03	-0.02	9.33E-01	-0.03	8.98E-01	-0.13	5.08E-01	-0.60	9.89E-04	-0.66	2.08E-04	0.47	1.40E-02
ENSCAFG0000000332	C10H2orf42	cyan	VSMC_M2	0.33	9.06E-02	0.51	6.82E-03	-0.08	7.05E-01	0.34	7.89E-02	0.22	2.80E-01	-0.24	2.29E-01	0.04	8.52E-01	-0.59	1.13E-03	-0.47	1.25E-02	0.42	3.12E-02
ENSCAFG0000000446	XPC	grey	VSMC_M10	0.33	9.06E-02	-0.06	7.56E-01	-0.11	6.02E-01	0.26	1.86E-01	0.26	1.90E-01	-0.04	8.48E-01	0.07	7.21E-01	-0.10	6.06E-01	-0.10	6.37E-01	-0.11	6.01E-01
ENSCAFG0000001970	FGF22	grey	VSMC_M10	0.33	9.07E-02	0.21	3.02E-01	0.11	5.77E-01	-0.10	6.35E-01	0.13	5.34E-01	-0.16	4.24E-01	0.15	4.65E-01	-0.04	8.53E-01	-0.30	1.30E-01	-0.10	6.29E-01
ENSCAFG0000000793	CTSB	cyan	VSMC_M2	0.33	9.07E-02	0.86	5.90E-09	0.09	6.72E-01	0.58	1.53E-03	0.00	1.00E+00	-0.24	2.33E-01	-0.50	8.21E-03	-0.81	2.37E-07	-0.73	1.79E-05	0.63	4.74E-04
ENSCAFG0000002029	MCOLN3	grey	VSMC_M10	0.33	9.08E-02	0.09	6.60E-01	0.22	2.74E-01	-0.18	3.82E-01	0.04	8.51E-01	-0.08	7.02E-01	0.07	7.47E-01	0.02	9.28E-01	-0.15	4.69E-01	-0.18	3.57E-01
ENSCAFG0000002034	TSNAXIP1	grey	VSMC_M10	0.33	9.09E-02	0.32	1.03E-01	0.31	1.17E-01	-0.02	9.05E-01	-0.26	1.89E-01	0.14	4.81E-01	0.10	6.32E-01	-0.06	7.79E-01	-0.46	1.52E-02	0.31	1.17E-01
ENSCAFG0000000714	ELOVL7	grey	VSMC_M10	0.33	9.09E-02	0.36	6.84E-02	-0.33	9.05E-02	0.52	4.98E-03	0.49	9.56E-03	-0.16	4.32E-01	-0.18	3.80E-01	-0.68	1.05E-04	-0.25	2.08E-01	0.27	1.72E-01
ENSCAFG0000001570	FH	darkgrey	VSMC_M8	0.33	9.10E-02	-0.43	2.53E-02	0.24	2.34E-01	-0.53	4.75E-03	0.05	7.95E-01	-0.08	6.96E-01	0.32	1.08E-01	0.65	2.16E-04	0.20	3.23E-01	-0.70	5.68E-05
ENSCAFG0000000403	CCDC114	cyan	VSMC_M2	0.33	9.14E-02	0.66	1.88E-04	0.54	3.86E-03	-0.10	6.31E-01	-0.41	3.14E-02	0.10	6.09E-01	0.17	3.91E-01	-0.13	5.17E-01	-0.71	2.85E-05	0.34	8.45E-02
ENSCAFG0000000385	RHOB	cyan	VSMC_M2	0.33	9.16E-02	0.52	5.89E-03	0.29	1.39E-01	0.16	4.30E-01	-0.19	3.42E-01	0.14	4.79E-01	-0.20	3.21E-01	-0.37	5.86E-02	-0.54	3.64E-03	0.35	7.54E-02
ENSCAFG0000001732	ENSCAFG0000001732	yellow	VSMC_M3	0.33	9.17E-02	0.26	1.89E-01	0.67	1.43E-04	-0.32	1.06E-01	-0.44	2.03E-02	-0.23	2.49E-01	-0.04	8.25E-01	0.16	4.40E-01	-0.32	1.07E-01	-0.11	5.72E-01
ENSCAFG0000001107	ENSCAFG0000001107	cyan	VSMC_M2	0.33	9.19E-02	0.78	2.00E-06	0.53	4.08E-03	0.13	5.18E-01	-0.49	9.72E-03	0.10	6.29E-01	-0.18	3.63E-01	-0.36	6.53E-02	-0.80	4.66E-07	0.58	1.38E-03
ENSCAFG0000001638	RBPJ	grey	VSMC_M10	0.33	9.20E-02	-0.11	6.02E-01	0.11	5.69E-01	-0.35	7.10E-02	0.06	7.65E-01	0.10	6.24E-01	0.34	8.70E-02	0.28	1.60E-01	-0.07	7.45E-01	-0.18	3.76E-01
ENSCAFG0000002480	ENSCAFG0000002480	grey	VSMC_M10	0.33	9.24E-02	-0.01	9.70E-01	-0.23	2.52E-01	0.03	8.72E-01	0.43	2.45E-02	-0.14	4.94E-01	-0.05	7.94E-01	-0.07	7.26E-01	0.00	9.95E-01	-0.16	4.38E-01
ENSCAFG0000001688	ATP5MC1	darkgrey	VSMC_M8	0.33	9.26E-02	-0.27	1.78E-01	0.25	2.15E-01	-0.59	1.23E-03	-0.06	7.68E-01	0.15	4.42E-01	0.42	2.86E-02	0.66	1.87E-04	0.02	9.12E-01	-0.49	9.40E-03
ENSCAFG0000000649	TAF10	grey	VSMC_M10	0.33	9.27E-02	0.52	5.41E-03	0.09	6.63E-01	0.34	8.71E-02	-0.03	8.92E-01	-0.20	3.28E-01	-0.17	4.04E-01	-0.52	5.39E-03	-0.48	1.17E-02	0.44	2.14E-02
ENSCAFG0000000257	NAT14	cyan	VSMC_M2	0.33	9.28E-02	0.60	8.85E-04	0.30	1.32E-01	0.23	2.55E-01	-0.34	8.68E-02	0.01	9.78E-01	0.03	8.98E-01	-0.37	6.06E-02	-0.67	1.17E-04	0.61	6.52E-04
ENSCAFG0000000556	UCP2	yellow	VSMC_M3	0.33	9.29E-02	0.60	9.73E-04	0.71	3.82E-05	-0.50	8.41E-03	-0.48	1.15E-02	-0.05	7.87E-01	0.04	8.46E-01	0.19	3.33E-01	-0.63	4.49E-04	0.05	8.20E-01
ENSCAFG0000002928	GLI4	cyan	VSMC_M2	0.33	9.29E-02	0.76	4.20E-06	0.61	7.04E-04	-0.04	8.48E-01	-0.53	4.15E-03	-0.04	8.29E-01	0.05	7.97E-01	-0.27	1.77E-01	-0.79	1.05E-06	0.49	8.88E-03
ENSCAFG0000002588	ENSCAFG0000002588	grey	VSMC_M10	0.33	9.30E-02	0.13	5.22E-01	-0.03	8.74E-01	0.11	5.79E-01	0.09	6.68E-01	0.05	7.90E-01	0.10	6.35E-01	-0.08	6.97E-01	-0.26	1.92E-01	0.20	3.17E-01

ENSCAFG0000001408	ENSCAFG0000001408	turquoise	VSMC_M6	0.33	9.31E-02	0.01	9.49E-01	0.18	3.74E-01	-0.24	2.21E-01	0.06	7.66E-01	0.35	7.41E-02	-0.09	6.66E-01	0.19	3.33E-01	-0.12	5.67E-01	-0.31	1.22E-01
ENSCAFG0000001393	FAU	red	VSMC_M1	0.33	9.34E-02	0.23	2.57E-01	0.37	5.46E-02	-0.10	6.07E-01	-0.11	5.86E-01	-0.27	1.81E-01	-0.22	2.67E-01	-0.04	8.31E-01	-0.19	3.31E-01	-0.18	3.58E-01
ENSCAFG0000000949	DENND2C	grey	VSMC_M10	0.33	9.35E-02	-0.06	7.54E-01	-0.35	7.33E-02	0.46	1.56E-02	0.36	6.57E-02	0.05	8.11E-01	0.19	3.42E-01	-0.27	1.77E-01	-0.15	4.41E-01	0.20	3.12E-01
ENSCAFG0000000615	BTG1	grey	VSMC_M10	0.33	9.35E-02	-0.13	5.30E-01	0.28	1.63E-01	-0.58	1.62E-03	0.17	3.98E-01	-0.27	1.76E-01	0.09	6.52E-01	0.39	4.52E-02	0.07	7.39E-01	-0.68	1.11E-04
ENSCAFG0000001979	AHCYL1	darkgreen	VSMC_M4	0.33	9.35E-02	0.49	9.84E-03	-0.32	1.05E-01	0.79	8.46E-07	0.33	9.25E-02	-0.12	5.57E-01	-0.29	1.48E-01	-0.76	3.86E-06	-0.47	1.29E-02	0.54	3.36E-03
ENSCAFG0000001609	RADIL	grey	VSMC_M10	0.33	9.38E-02	-0.07	7.46E-01	0.48	1.19E-02	-0.56	2.62E-03	-0.27	1.73E-01	0.04	8.60E-01	0.67	1.20E-04	0.46	1.48E-02	-0.17	3.88E-01	-0.20	3.12E-01
ENSCAFG0000001494	RAB3A	grey	VSMC_M10	0.33	9.38E-02	0.35	7.16E-02	-0.30	1.26E-01	0.55	3.26E-03	0.28	1.61E-01	-0.02	9.27E-01	-0.11	5.95E-01	-0.52	5.78E-03	-0.45	1.72E-02	0.47	1.36E-02
ENSCAFG0000001706	NACC1	grey	VSMC_M10	0.33	9.39E-02	0.32	1.04E-01	0.55	2.84E-03	-0.25	2.06E-01	-0.41	3.56E-02	0.22	2.74E-01	-0.39	4.29E-02	0.11	5.73E-01	-0.35	7.43E-02	-0.03	8.89E-01
ENSCAFG0000001170	MNDA	darkgrey	VSMC_M8	0.33	9.39E-02	-0.10	6.31E-01	0.42	3.08E-02	-0.69	6.71E-05	-0.22	2.70E-01	0.07	7.34E-01	0.21	2.97E-01	0.62	5.39E-04	-0.11	5.68E-01	-0.37	5.74E-02
ENSCAFG0000000275	SLC44A1	cyan	VSMC_M2	0.33	9.40E-02	0.52	5.68E-03	0.08	7.03E-01	0.06	7.64E-01	0.11	5.82E-01	-0.23	2.59E-01	0.19	3.47E-01	-0.28	1.60E-01	-0.51	6.65E-03	0.24	2.22E-01
ENSCAFG0000001712	SGF29	yellow	VSMC_M3	0.33	9.40E-02	0.30	1.27E-01	0.38	5.38E-02	-0.36	6.70E-02	-0.19	3.35E-01	0.04	8.42E-01	0.02	9.17E-01	0.20	3.25E-01	-0.38	4.90E-02	-0.04	8.40E-01
ENSCAFG0000003226	DHRS1	grey	VSMC_M10	0.33	9.41E-02	0.32	1.00E-01	-0.28	1.55E-01	0.37	5.85E-02	0.39	4.41E-02	-0.09	6.60E-01	-0.15	4.64E-01	-0.40	3.74E-02	-0.34	7.91E-02	0.25	2.09E-01
ENSCAFG0000000757	ENSCAFG0000000757	yellow	VSMC_M3	0.33	9.42E-02	0.35	7.71E-02	0.42	2.88E-02	-0.32	1.00E-01	-0.20	3.11E-01	-0.10	6.37E-01	0.10	6.29E-01	0.13	5.06E-01	-0.37	5.89E-02	-0.05	8.19E-01
ENSCAFG0000001761	OTUB2	grey	VSMC_M10	0.33	9.43E-02	0.26	1.99E-01	-0.05	8.12E-01	0.24	2.29E-01	0.11	5.89E-01	-0.05	7.90E-01	-0.03	8.90E-01	-0.29	1.48E-01	-0.35	7.29E-02	0.33	9.09E-02
ENSCAFG0000001712	FLAD1	yellow	VSMC_M3	0.33	9.44E-02	0.47	1.33E-02	0.62	5.92E-04	-0.34	8.00E-02	-0.52	5.40E-03	0.06	7.51E-01	0.34	8.67E-02	0.10	6.03E-01	-0.56	2.25E-03	0.25	2.08E-01
ENSCAFG0000001660	SMOC1	cyan	VSMC_M2	0.33	9.44E-02	0.57	1.73E-03	0.37	5.94E-02	-0.10	6.27E-01	-0.23	2.49E-01	0.36	6.24E-02	-0.12	5.45E-01	-0.15	4.70E-01	-0.57	1.90E-03	0.24	2.22E-01
ENSCAFG0000001617	THRA	grey	VSMC_M10	0.33	9.45E-02	0.27	1.81E-01	0.08	6.74E-01	0.06	7.84E-01	0.14	4.82E-01	-0.11	5.73E-01	-0.11	5.91E-01	-0.22	2.72E-01	-0.26	1.90E-01	-0.01	9.52E-01
ENSCAFG0000001447	GPR137	grey	VSMC_M10	0.33	9.49E-02	0.68	8.32E-05	0.15	4.57E-01	0.29	1.38E-01	-0.15	4.44E-01	0.04	8.57E-01	-0.12	5.55E-01	-0.50	7.27E-03	-0.72	1.92E-05	0.66	1.90E-04
ENSCAFG0000001805	ANKRD9	yellow	VSMC_M3	0.33	9.50E-02	0.42	3.04E-02	0.52	5.38E-03	-0.03	8.69E-01	-0.45	1.88E-02	-0.03	8.70E-01	-0.02	9.04E-01	-0.15	4.63E-01	-0.44	2.14E-02	0.27	1.74E-01
ENSCAFG0000001085	SYNM	grey	VSMC_M10	0.33	9.50E-02	0.09	6.39E-01	0.32	9.81E-02	-0.25	2.00E-01	-0.11	5.72E-01	0.30	1.32E-01	0.12	5.39E-01	0.10	6.32E-01	-0.16	4.38E-01	-0.12	5.66E-01
ENSCAFG0000001563	MNAT1	darkgrey	VSMC_M8	0.33	9.52E-02	-0.38	4.97E-02	0.10	6.19E-01	-0.58	1.69E-03	0.14	4.96E-01	0.05	8.23E-01	0.46	1.50E-02	0.61	6.47E-04	0.18	3.72E-01	-0.53	4.13E-03
ENSCAFG0000001137	LIX1L	yellow	VSMC_M3	0.33	9.53E-02	0.20	3.06E-01	0.63	4.05E-04	-0.46	1.46E-02	-0.28	1.53E-01	-0.17	4.03E-01	0.13	5.31E-01	0.17	3.90E-01	-0.19	3.45E-01	-0.31	1.18E-01
ENSCAFG0000001461	ARPC2	pink	VSMC_M5	0.33	9.54E-02	-0.40	3.62E-02	-0.17	3.93E-01	-0.25	2.08E-01	0.52	5.92E-03	-0.34	8.18E-02	-0.30	1.31E-01	0.34	7.80E-02	0.36	6.28E-02	-0.68	1.03E-04
ENSCAFG0000002684	ENSCAFG0000002684	grey	VSMC_M10	0.33	9.55E-02	0.14	4.88E-01	0.44	2.28E-02	-0.24	2.32E-01	-0.26	1.90E-01	-0.17	3.95E-01	0.13	5.31E-01	0.16	4.15E-01	-0.25	2.15E-01	-0.04	8.49E-01
ENSCAFG0000000403	PUS7	darkgrey	VSMC_M8	0.33	9.59E-02	-0.52	5.59E-03	0.12	5.38E-01	-0.71	3.25E-05	0.19	3.32E-01	0.04	8.50E-01	0.39	4.39E-02	0.81	2.78E-07	0.28	1.51E-01	-0.79	1.20E-06
ENSCAFG0000003073	ENSCAFG0000003073	grey	VSMC_M10	0.33	9.60E-02	0.34	8.73E-02	0.17	3.94E-01	0.06	7.61E-01	-0.01	9.41E-01	0.00	9.97E-01	-0.13	5.34E-01	-0.16	4.16E-01	-0.37	6.04E-02	0.14	4.84E-01
ENSCAFG0000001840	DRC3	grey	VSMC_M10	0.33	9.62E-02	0.22	2.79E-01	0.37	5.87E-02	-0.01	9.67E-01	-0.33	9.14E-02	0.10	6.21E-01	0.33	9.20E-02	0.01	9.69E-01	-0.41	3.57E-02	0.26	1.92E-01
ENSCAFG0000002945	SNAI3	grey	VSMC_M10	0.33	9.63E-02	0.17	4.08E-01	0.45	1.86E-02	-0.22	2.66E-01	-0.28	1.62E-01	-0.11	5.68E-01	0.43	2.36E-02	0.09	6.53E-01	-0.30	1.34E-01	0.00	9.95E-01
ENSCAFG0000001237	VASH2	grey	VSMC_M10	0.33	9.65E-02	-0.21	3.04E-01	0.10	6.30E-01	-0.30	1.31E-01	0.14	4.96E-01	0.07	7.20E-01	-0.37	5.40E-02	0.33	9.57E-02	0.09	6.69E-01	-0.43	2.39E-02
ENSCAFG0000000625	NBEA	pink	VSMC_M5	0.33	9.67E-02	0.02	9.10E-01	-0.56	2.14E-03	0.50	8.43E-03	0.75	6.59E-06	-0.21	3.03E-01	0.09	6.53E-01	-0.47	1.45E-02	-0.08	6.80E-01	0.06	7.57E-01
ENSCAFG0000001762	DHX40	pink	VSMC_M5	0.33	9.68E-02	0.02	9.04E-01	-0.13	5.32E-01	0.18	3.74E-01	0.51	7.01E-03	-0.43	2.56E-02	-0.22	2.79E-01	-0.26	1.90E-01	0.05	7.96E-01	-0.34	8.51E-02
ENSCAFG0000002005	GLE1	darkgrey	VSMC_M8	0.33	9.69E-02	-0.38	4.92E-02	0.28	1.62E-01	-0.70	5.52E-05	0.07	7.47E-01	-0.20	3.28E-01	0.41	3.39E-02	0.73	1.52E-05	0.17	3.89E-01	-0.73	1.85E-05
ENSCAFG0000001613	ILVBL	yellow	VSMC_M3	0.33	9.69E-02	0.74	9.26E-06	0.85	2.38E-08	-0.30	1.25E-01	-0.66	1.96E-04	-0.05	8.18E-01	-0.02	9.16E-01	-0.05	7.86E-01	-0.74	1.01E-05	0.24	2.23E-01
ENSCAFG0000003166	CNRIP1	grey	VSMC_M10	0.33	9.70E-02	0.10	6.26E-01	0.50	7.41E-03	-0.62	5.83E-04	-0.23	2.54E-01	-0.01	9.77E-01	0.61	7.13E-04	0.41	3.57E-02	-0.23	2.39E-01	-0.27	1.67E-01
ENSCAFG0000003047	TGIF2	grey	VSMC_M10	0.33	9.70E-02	0.33	8.87E-02	0.32	9.96E-02	0.03	8.64E-01	-0.22	2.65E-01	0.13	5.33E-01	-0.11	5.72E-01	-0.08	7.05E-01	-0.42	2.87E-02	0.08	6.78E-01
ENSCAFG0000001081	LRRC28	cyan	VSMC_M2	0.33	9.70E-02	0.59	1.29E-03	0.00	9.94E-01	0.40	3.84E-02	0.16	4.17E-01	-0.27	1.66E-01	-0.17	4.09E-01	-0.58	1.51E-03	-0.53	4.27E-03	0.31	1.11E-01
ENSCAFG0000003122	MARVELD1	yellow	VSMC_M3	0.33	9.72E-02	0.44	2.03E-02	0.70	5.36E-05	-0.25	2.06E-01	-0.51	6.11E-03	-0.19	3.50E-01	0.08	6.92E-01	0.00	9.85E-01	-0.46	1.65E-02	0.16	4.12E-01
ENSCAFG0000000612	SEMA3D	grey	VSMC_M10	0.33	9.72E-02	0.12	5.62E-01	-0.15	4.47E-01	0.21	2.95E-01	0.21	2.85E-01	0.14	4.75E-01	0.22	2.61E-01	-0.13	5.15E-01	-0.27	1.81E-01	0.18	3.70E-01
ENSCAFG0000000886	TGM2	cyan	VSMC_M2	0.33	9.72E-02	0.84	5.07E-08	0.34	7.91E-02	0.27	1.67E-01	-0.22	2.63E-01	-0.07	7.11E-01	-0.64	3.36E-04	-0.58	1.65E-03	-0.71	3.52E-05	0.46	1.53E-02
ENSCAFG0000000567	ARAP1	cyan	VSMC_M2	0.33	9.73E-02	0.81	2.55E-07	0.66	1.87E-04	-0.14	4.92E-01	-0.46	1.60E-02	-0.12	5.56E-01	-0.19	3.54E-01	-0.22	2.79E-01	-0.76	5.10E-06	0.31	1.11E-01
ENSCAFG0000000446	GPBP1L1	grey	VSMC_M10	0.33	9.73E-02	0.25	2.11E-01	0.02	9.37E-01	0.39	4.43E-02	0.15	4.55E-01	-0.09	6.65E-01	-0.22	2.70E-01	-0.50	7.77E-03	-0.21	3.01E-01	0.14	4.73E-01
ENSCAFG0000000618	ADGRA2	cyan	VSMC_M2	0.33	9.73E-02	0.78	1.28E-06	0.55	3.07E-03	-0.15	4.62E-01	-0.39	4.59E-02	-0.05	8.08E-01	-0.13	5.33E-01	-0.23	2.54E-01	-0.71	3.62E-05	0.35	7.49E-02
ENSCAFG0000000993	ATXN1	grey	VSMC_M10	0.33	9.75E-02	0.63	3.97E-04	-0.04	8.47E-01	0.38	4.85E-02	0.11	5.80E-01	-0.01	9.51E-01	-0.40	4.09E-02	-0.55	2.90E-03	-0.60	9.14E-04	0.50	8.28E-03
ENSCAFG0000000173	GRM8	grey	VSMC_M10	0.33	9.76E-02	0.10	6.14E-01	-0.13	5.06E-01	0.12	5.59E-01	0.30	1.23E-01	-0.14	4.74E-01	-0.23	2.42E-01	-0.06	7.66E-01	-0.20	3.24E-01	0.03	8.69E-01
ENSCAFG0000001942	MKNK2	yellow	VSMC_M3	0.33	9.77E-02	0.26	1.96E-01	0.81	2.46E-07	-0.63	4.04E-04	-0.56	2.43E-03	0.07	7.43E-01	0.21	3.00E-01	0.41	3.24E-02	-0.38	5.11E-02	-0.17	4.10E-01
ENSCAFG0000001818	MMP28	darkgreen	VSMC_M4	0.33	9.77E-02	0.44	2.17E-02	-0.05	7.91E-01	0.56	2.60E-03	0.04	8.30E-01	0.14	4.75E-01	-0.02	9.26E-01	-0.62	5.19E-04	-0.49	8.73E-03	0.55	2.84E-03
ENSCAFG0000000653	ENSCAFG0000000653	cyan	VSMC_M2	0.33	9.78E-02	0.64	2.86E-04	0.16	4.15E-01	0.25	2.01E-01	-0.10	6.23E-01	-0.01	9.60E-01	-0.01	9.46E-01	-0.41	3.59E-02	-0.66	1.88E-04	0.48	1.11E-02
ENSCAFG0000001407	CTSH	grey	VSMC_M10	0.33	9.79E-02	0.59	1.07E-03	-0.10	6.21E-01	0.63	4.50E-04	0.19	3.54E-01	-0.23	2.54E-01	-0.43	2.51E-02	-0.79	1.13E-06	-0.52	5.29E-03	0.51	6.78E-03
ENSCAFG0000001531	SLC27A1	grey	VSMC_M10	0.33	9.80E-02	0.83	1.19E-07	0.26	1.99E-01	0.29	1.45E-01	-0.14	4.97E-01	-0.10	6.12E-01	-0.36	6.12E-02	-0.64	3.61E-04	-0.72	2.66E-05	0.54	3.50E-03
ENSCAFG0000000766	EIF4G2	pink	VSMC_M5	0.33	9.80E-02	-0.60	9.24E-04	-0.17	3.86E-01	-0.43	2.41E-02	0.53	4.56E-03	-0.12	5.60E-01	0.04	8.58E-01	0.57	2.12E-03	0.45	1.76E-02	-0.84	3.59E-08
ENSCAFG0000000162	KIFC2	cyan	VSMC_M2	0.33	9.80E-02	0.80	5.30E-07	0.60	9.83E-04	0.07	7.30E-01	-0.53	4.41E-03	0.01	9.58E-01	-0.06	7.76E-01	-0.36	6.20E-02	-0.77	2.43E-06	0.58	1.57E-03
ENSCAFG0000000343	UFL1	pink	VSMC_M5	0.33	9.81E-02	-0.29	1.46E-01	-0.46	1.56E-02	0.16	4.29E-01	0.66	1.79E-04	-0.20	3.23E-01	-0.06	7.52E-01	0.04	8.38E-01	0.14	4.97E-01	-0.23	2.46E-01
ENSCAFG0000001077	RABGEF1	pink	VSMC_M5	0.32	9.82E-02	-0.20	3.28E-01	-0.57	2.11E-03	0.51	6.69E-03	0.71	2.81E-05	-0.18	3.61E-01	-0.03	8.88E-01	-0.38	4.85E-02	0.15	4.41E-01	-0.05	8.01E-01
ENSCAFG0000000074	TBC1D22A	cyan	VSMC_M2	0.32	9.83E-02	0.72	2.32E-05	0.36	6.91E-02	0.15	4.55E-01	-0.21	2.90E-01	-0.22	2.79E-01	-0.17	3.96E-01	-0.43	2.42E-02	-0.68	1.05E-04	0.36	6.21E-02
ENSCAFG0000000156	MOCS1	grey	VSMC_M10	0.32	9.83E-02	0.40	3.92E-02	0.22	2.81E-01	-0.06	7.68E-01	-0.05	7.96E-01	-0.05	7.91E-01	-0.04	8.57E-01	-0.14	4.82E-01	-0.41	3.33E-02	0.16	4.28E-01
ENSCAFG0000000541	OSBPL10	cyan	VSMC_M2	0.32	9.83E-02	0.71	3.63E-05	0.11	5.94E-01	0.37	5.89E-02	-0.12	5.55E-01	-0.11	5.89E-01	-0.09	6.41E-01	-0.57	2.13E-03	-0.75	6.24E-06	0.69	7.62E-05
ENSCAFG0000002271	ENSCAFG0000002271	grey	VSMC_M10	0.32	9.84E-02	-0.25	2.03E-01	0.24	2.37E-01	-0.54	3.86E-03	0.04	8.51E-01	-0.11	5.73E-01	0.21	2.91E-01	0.48	1.06E-02	0.15	4.49E-01	-0.50	8.07E-03
ENSCAFG0000001697	MED12	cyan	VSMC_M2	0.32	9.89E-02	0.47	1.45E-02	-0.07	7.38E-01	0.18	3.69E-01	0.15	4.68E-01	0.14	4.81E-01	-0.38	4.89E-02	-0.30	1.28E-01	-0.50	8.58E-03	0.33	9.68E-02
ENSCAFG0000000312	MACF1	pink	VSMC_M5	0.32	9.91E-02	0.29	1.36E-01	-0.49	9.29E-03	0.67	1.37E-04	0.56	2.26E-03	-0.22	2.68E-01	-0.41	3.52E-02	-0.68	9.86E-05	-0.28	1.52E-01	0.35	6.95E-02
ENSCAFG0000000061	UBR5	pink	VSMC_M5	0.32	9.91E-02	-0.23	2.56E-01	-0.64	3.65E-04	0.48	1.21E-02	0.77	2.91E-06	0.02	9.15E-01	-0.11	5.82E-01	-0.29	1.35E-01	0.13	5.06E-01	-0.06	7.78E-01
ENSCAFG0000000318	RAB1A	pink	VSMC_M5	0.32	9.92E-02	-0.45	1.76E-02	-0.45	1.82E-02	0.13	5.29E-01	0.68	9.76E-05	-0.06	7.69E-01	-0.10	6.15E-01	0.10	6.11E-01	0.36	6.39E-02	-0.50	7.24E-03
ENSCAFG0000001382	VPS26A	grey	VSMC_M10	0.32	9.92E-02	0.57	1.99E-03	-0.19	3.44E-01	0.62	5.38E-04	0.32	1.07E-01	-0.13	5.27E-01	-0.44	2.28E-02	-0.75	5.89E-06	-0.45	1.83E-02	0.40	3.62E-02
ENSCAFG0000000187	GNPDA2	grey	VSMC_M10	0.32	9.93E-02	-0.26	1.94E-01	-0.12	5.48E-01	-0.29	1.47E-01	0.43	2.67E-02	-0.17	3.90E-01	0.10	6.11E-01	0.33	9.59E-02	0.14	5.02E-01	-0.57	1.75E-03
ENSCAFG0000000590	PYCR1	yellow	VSMC_M3	0.32	9.94E-02	0.34	7.81E-02	0.81	2.32E-07	-0.56	2.29E-03	-0.55	3.00E-03	-0.05	8.11E-01	-0.06	7.60E-01	0.33	8.92E-02	-0.40	3.80E-02	-0.19	3.48E-01
ENSCAFG0000000774	ENSCAFG0000000774	grey	VSMC_M10	0.32	9.96E-02	-0.12	5.51E-01	-0.03	8.75E-01	-0.05	8.03E-01	0.29	1.42E-01	-0.14	4.77E-01	-0.08	6.97E-01	0.05	7.91E-01	0.05	8.04E-01	-0.30	1.30E-01
ENSCAFG0000002520	ENSCAFG0000002520	yellow	VSMC_M3	0.32	9.99E-02	0.54	3.63E-03	0.63	3.76E-04	-0.01	9.62E-01	-0.54	3.41E-03	0.11	5.79E-01	0.03	8.66E-01	-0.20	3.12E-01	-0.57	2.00E-03	0.35	7.24E-02
ENSCAFG0000000715	ZC3H8	grey	VSMC_M10	0.32	1.00E-01	0.06	7.84E-01	0.14	4.80E-01	-0.23	2.50E-01	-0.03	8.75E-01	0.00	9.85E-01	0.17	4.11E-01	0.15	4.62E-01	-0.23	2.58E-01	-0.01	9.50E-01
ENSCAFG0000001177	EGLN1	pink	VSMC_M5	0.32	1.00E-01	0.15	4.49E-01	-0.53	4.14E-03	0.64	3.44E-04	0.73	1.73E-05	-0.33	9.31E-02	-0.23	2.53E-01	-0.66	2.09E-04	-0.07	7.18E-01	0.15	4.59E-01
ENSCAFG0000000316	BBS9	cyan	VSMC_M2	0.32	1.00E-01	0.57	1.87E-03	0.04	8.31E-01	0.34	7.96E-02	-0.06	7.60E-01	0.05	7.90E-01	0.11	5.86E-01	-0.48	1.14E-02	-0.68	1.05E-04	0.63	4.77E-04
ENSCAFG0000002935	KCNK5	grey	VSMC_M10	0.32	1.00E-01	0.29	1.49E-01	0.52	5.10E-03	-0.32	9.86E-02	-0.44	2.08E-02	0.41	3.21E-02	0.24	2.31E-01	0.15	4.68E-01	-0.40	3.67E-02	0.10	6.30E-01
ENSCAFG0000001615	TMEM156	pink	VSMC_M5	0.32	1.00E-01	-0.25	2.03E-01	-0.29	1.44E-01	-0.08	7.06E-01	0.46	1.51E-02	0.06	7.48E-01	0.03	8.85E-01	0.16	4.26E-01	0.10	6.19E-01	-0.30	1.26E-01
ENSCAFG0000001515	CAPZB	cyan	VSMC_M2	0.32	1.01E-01	0.22	2.70E-01	0.19	3.39E-01	0.17	3.89E-01	0.03	8.82E-01	-0.05	8.02E-01	-0.15	4.57E-01	-0.27	1.76E-01	-0.17	3.87E-01	-0.07	7.40E-01
ENSCAFG0000001462	ENSCAFG0000001462	grey	VSMC_M10	0.32	1.01E-01	0.44	2.13E-02	0.54	3.77E-03	-0.15	4.56E-01	-0.40	4.10E-02	-0.10	6.14E-01	-0.12	5.63E-01	-0.06	7.53E-01	-0.46	1.49E-02	0.15	4.44E-01
ENSCAFG0000002938	ENSCAFG0000002938	grey	VSMC_M10	0.32	1.01E-01	-0.07	7.39E-01	0.13	5.07E-01	-0.20	3.13E-01	0.07	7.18E-01	-0.01	9.74E-01	-0.09	6.39E-01	0.15	4.41E-01	0.02	9.30E-01	-0.30	1.31E-01
ENSCAFG0000001229	NIPSNAP1	grey	VSMC_M10	0.32	1.01E-01	0.30	1.28E-01	0.48	1.14E-02	-0.12	5.44E-01	-0.42	2.77E-02	-0.05	8.09E-01	0.07	7.46E-01	0.02	9.32E-01	-0.39	4.67E-02	0.28	1.64E-01
ENSCAFG0000001401	BATF2	grey	VSMC_M10	0.32	1.01E-01	0.54	3.60E-03	-0.04	8.43E-01	0.41	3.39E-02	0.12	5.67E-01	0.02	9.29E-01	-0.27	1.70E-01	-0.48	1.21E-02	-0.54	3.50E-03	0.41	3.24E-02
ENSCAFG0000000593	PSMD8	pink	VSMC_M5	0.32	1.01E-01	-0.44	2.30E-02	-0.28	1.61E-01	-0.06	7.59E-01	0.49	1.01E-02	-0.04	8.30E-01	-0.10	6.27E-01	0.23	2.44E-01	0.32	1.05E-01	-0.50	7.67E-03
ENSCAFG0000001166	DHRS4	cyan	VSMC_M2	0.32	1.01E-01	0.58	1.65E-03	0.27	1.80E-01	0.27	1.78E-01	-0.06	7.58E-01	-0.25	2.02E-01	-0.21	2.95E-01	-0.45	1.76E-02	-0.56	2.15E-03	0.24	2.31E-01
ENSCAFG0000000187	POLR1C	darkgrey	VSMC_M8	0.32	1.01E-01	-0.15	4.57E-01	0.44	2.07E-02	-0.60	8.96E-04	-0.23	2.41E-01	0.19	3.41E-01	0.32	1.03E-01	0.60	8.64E-04	-0.06	7.49E-01	-0.42	3.00E-02
ENSCAFG0000001264	ENSCAFG0000001264	cyan	VSMC_M2	0.32	1.02E-01	0.74	1.07E-05	0.17	3.97E-01	0.39	4.73E-02	-0.19	3.42E-01	0.16	4.37E-01	-0.43	2.55E-02	-0.58	1.50E-03	-0.72	2.13E-05	0.67	1.28E-04
ENSCAFG0000001687	FAM161B	yellow	VSMC_M3	0.32	1.02E-01	0.20	3.28E-01	0.43	2.35E-02	-0.26	1.91E-01	-0.26	1.94E-01	0.03	8.81E-01	0.25	2.09E-01	0.20	3.24E-01	-0.38	5.17E-02	-0.03	8.66E-01
ENSCAFG0000001193	TRPM8	grey	VSMC_M10	0.32	1.02E-01	0.19	3.31E-01	0.32	1.02E-01	-0.15	4.59E-01	-0.12	5.47E-01	0.02	9.27E-01	0.12	5.53E-01	-0.01	9.73E-01	-0.27	1.70E-01	-0.05	8.06E-01
ENSCAFG0000000022	YTHDC2	grey	VSMC_M10	0.32	1.02E-01	-0.31	1.17E-01	-0.15	4.45E-01	-0.33	8.96E-02	0.38	5.39E-02	0.02	9.35E-01	0.12	5.56E-01	0.39	4.19E-02	0.07	7.12E-01	-0.40	3.83E-02
ENSCAFG0000000094	MRPL13	grey	VSMC_M10	0.32	1.02E-01	-0.39	4.47E-02	0.04	8.28E-01	-0.26	1.86E-01	0.13	5.31E-01	0.11	5.98E-01	0.08	6.83E-01	0.45	1.85E-02	0.20	3.25E-01	-0.43	2.36E-02
ENSCAFG0000001481	COL3A1	grey	VSMC_M10	0.32	1.02E-01	0.31	1.20E-01	-0.02	9.21E-01	0.34	7.85E-02	0.15	4.53E-01	-0.15	4.55E-01	-0.65	2.52E-04	-0.43	2.38E-02	-0.28	1.59E-01	0.18	3.56E-01
ENSCAFG0000001801	CENPV	cyan	VSMC_M2	0.32	1.02E-01	0.65	2.52E-04	0.47	1.33E-02	-0.04	8.58E-01	-0.32	1.08E-01	0.08	7.07E-01	0.05	8.08E-01	-0.19	3.45E-01	-0.68	9.08E-05	0.27	1.78E-01
ENSCAFG0000003208	SMIM19	pink	VSMC_M5	0.32	1.02E-01	-0.07	7.41E-01	-0.41	3.52E-02	0.25	2.15E-01	0.71	3.69E-05	-0.46	1.70E-02	-0.17	4.10E-01	-0.28	1.59E-01	0.12	5.47E-01	-0.23	2.42E-01
ENSCAFG0000000222	PDCL3	grey	VSMC_M10	0.32	1.02E-01	-0.49	8.78E-03	0.11	5.94E-01	-0.46	1.67E-02	0.26	1.96E-01	-0.25	2.00E-01	0.11	5.98E-01	0.55	2.97E-03	0.35	7.29E-02	-0.81	3.13E-07
ENSCAFG0000001354	YWHAG	grey	VSMC_M10	0.32	1.02E-01	0.00	9.85E-01	-0.36	6.54E-02	0.30	1.22E-01	0.35	7.69E-02	0.22	2.75E-01	-0.10	6.23E-01	-0.15	4.65E-01	-0.14	4.88E-01	0.18	3.63E-01
ENSCAFG0000003076	STARD4	grey	VSMC_M10	0.32	1.02E-01	-0.02	9.21E-01	0.39	4.18E-02	-0.50	8.55E-03	-0.22	2.70E-01	0.20	3.16E-01	0.36	6.59E-02	0.40	4.01E-02	-0.14	4.77E-01	-0.20	3.20E-01
ENSCAFG0000001648	PRKACA	cyan	VSMC_M2	0.32	1.02E-01	0.59	1.28E-03	0.42	2.94E-02	0.01	9.61E-01	-0.33	8.87E-02	0.06	7.49E-01	0.00	9.83E-01	-0.18	3.69E-01	-0.58	1.64E-03	0.31	1.13E-01
ENSCAFG0000000471	ENSCAFG0000000471	grey	VSMC_M10	0.32	1.03E-01	0.24	2.26E-01	-0.26	1.83E-01	0.34	8.31E-02	0.40	3.99E-02	-0.22	2.73E-01	-0.10	6.25E-01	-0.32	9.98E-02	-0.30	1.33E-01	0.13	5.15E-01
ENSCAFG0000001744	SLC27A3	yellow	VSMC_M3	0.32	1.03E-01	0.32	9.83E-02	0.69	8.08E-05	-0.46	1.67E-02	-0.55	2.92E-03	0.15	4.58E-01	0.27	1.79E-01	0.32	1.01E-01	-0.49	1.01E-02	0.08	6.79E-01
ENSCAFG0000000868	ENSCAFG0000000868	grey	VSMC_M10	0.32	1.03E-01	0.02	9.40E-01	0.17	4.02E-01	-0.11	6.00E-01	0.15	4.64E-01	-0.02	9.22E-01	-0.05	8.24E-01	0.08	7.05E-01	-0.09	6.60E-01	-0.34	8.32E-02
ENSCAFG0000001829	XRCC3	red	VSMC_M1	0.32	1.03E-01	0.25	2.07E-01	0.09	6.72E-01	0.14	4.98E-01	-0.01	9.58E-01	0.00	9.92E-01	0.01	9.59E-01	-0.13	5.05E-01	-0.42	3.05E-02	0.18	3.75E-01
ENSCAFG0000001302	WDR53	grey	VSMC_M10	0.32	1.03E-01	-0.14	4.90E-01	0.07	7.44E-01	0.00	9.81E-01	0.06	7.60E-01	-0.26	1.88E-01	0.00	9.89E-01	0.16	4.18E-01	-0.02	9.23E-01	-0.13	5.25E-01
ENSCAFG0000001939	ZMYND19	grey	VSMC_M10	0.32	1.03E-01	-0.08	6.91E-01	0.46	1.53E-02	-0.39	4.68E-02	-0.26	1.84E-01	-0.08	6.81E-01	0.10	6.25E-01	0.34	8.29E-02	-0.06	7.65E-01	-0.25	2.06E-01
ENSCAFG0000003164	GNG2	grey	VSMC_M10	0.32	1.03E-01	-0.25	2.04E-01	0.20	3.12E-01	-0.55	3.27E-03	0.09	6.51E-01	0.12	5.38E-01	0.35	7.33E-02	0.49	9.12E-03	0.11	5.78E-01	-0.51	6.16E-03
ENSCAFG0000003247	ISCA2	grey	VSMC_M10	0.32	1.04E-01	0.43	2.44E-02	0.05	8.20E-01	0.38	5.11E-02	-0.06	7.56E-01	-0.15	4.51E-01	-0.21	2.99E-01	-0.44	2.06E-02	-0.48	1.04E-02	0.58	1.62E-03
ENSCAFG0000000221	CDK20	grey	VSMC_M10	0.32	1.04E-01	0.27	1.81E-01	0.31	1.13E-01	-0.09	6.54E-01	-0.26	1.94E-01	0.17	3.95E-01	0.38	4.92E-02	0.07	7.12E-01	-0.48	1.12E-02	0.20	3.16E-01
ENSCAFG0000001593	PYGO1	pink	VSMC_M5	0.32	1.04E-01	-0.54	4.02E-03	-0.40	3.86E-02	-0.15	4.56E-01	0.69	7.62E-05	-0.17	4.11E-01	0.23	2.58E-01	0.31	1.13E-01	0.37	5.90E-02	-0.63	3.76E-04
ENSCAFG0000001732	UTP18	grey	VSMC_M10	0.32	1.04E-01	-0.04	8.41E-01	0.59	1.34E-03	-0.63	4.13E-04	-0.35	7.66E-02	0.21	2.84E-01	0.12	5.61E-01	0.56	2.31E-03	-0.13	5.34E-01	-0.41	3.47E-02
ENSCAFG0000003132	ENSCAFG0000003132	pink	VSMC_M5	0.32	1.04E-01	-0.29	1.39E-01	-0.07	7.39E-01	-0.32	1.04E-01	0.48	1.06E-02	-0.46	1.46E-02	-0.07	7.45E-01	0.31	1.16E-01	0.27	1.80E-01	-0.71	3.49E-05
ENSCAFG0000003248	LY96	red	VSMC_M1	0.32	1.04E-01	0.40	4.08E-02	0.13	5.09E-01	0.23	2.43E-01	-0.03	8.93E-01	-0.13	5.20E-01	-0.16	4.15E-01	-0.39	4.18E-02	-0.35	7.01E-02	0.23	2.54E-01
ENSCAFG0000000854	MCRS1	yellow	VSMC_M3	0.32	1.04E-01	0.17	4.09E-01	0.59	1.25E-03	-0.59	1.16E-03	-0.43	2.35E-02	0.14	4.86E-01	0.15	4.61E-01	0.51	6.51E-03	-0.29	1.43E-01	-0.11	5.78E-01
ENSCAFG0000001539	ENSCAFG0000001539	pink	VSMC_M5	0.32	1.04E-01	-0.27	1.67E-01	-0.20	3.17E-01	-0.01	9.55E-01	0.43	2.51E-02	0.05	8.15E-01	-0.21	3.02E-01	0.11	5.84E-01	0.20	3.29E-01	-0.44	2.20E-02
ENSCAFG0000000775	GALNT6	darkgrey	VSMC_M8	0.32	1.04E-01	-0.47	1.33E-02	0.19	3.43E-01	-0.62	5.37E-04	0.12	5.40E-01	-0.01	9.47E-01	0.45	1.80E-02	0.66	1.64E-04	0.29	1.40E-01	-0.71	3.31E-05
ENSCAFG0000001524	TRRAP	red	VSMC_M1	0.32	1.04E-01	0.17	4.06E-01	-0.31	1.15E-01	0.27	1.68E-01	0.35	7.72E-02	0.08	6.98E-01	-0.15	4.42E-01	-0.25	2.04E-01	-0.26	1.83E-01	0.28	1.64E-01
ENSCAFG0000001787	ENSCAFG0000001787	cyan	VSMC_M2	0.32	1.04E-01	0.69	7.38E-05	0.29	1.46E-01	0.23	2.57E-01	-0.28	1.50E-01	0.03	8.78E-01	-0.06	7.56E-01	-0.45	1.79E-02	-0.70	4.52E-05	0.58	1.39E-03
ENSCAFG0000000504	ENSCAFG0000000504	yellow	VSMC_M3	0.32	1.04E-01	0.29	1.41E-01	0.50	7.32E-03	-0.06	7.70E-01	-0.24	2.19E-01	-0.08	6.86E-01	-0.12	5.46E-01	-0.18	3.78E-01	-0.24	2.24E-01	-0.01	9.44E-01
ENSCAFG0000000538	FNTA	pink	VSMC_M5	0.32	1.05E-01	-0.58	1.58E-03	-0.26	1.97E-01	-0.26	1.99E-01	0.65	2.54E-04	-0.23	2.58E-01	0.02	9.02E-01	0.39	4.53E-02	0.48	1.14E-02	-0.85	2.16E-08
ENSCAFG0000000684	TRIM44	grey	VSMC_M10	0.32	1.05E-01	0.32	1.02E-01	-0.01	9.42E-01	0.28	1.63E-01	0.09	6.38E-01	-0.12	5.66E-01	0.01	9.49E-01	-0.32	1.09E-01	-0.42	3.00E-02	0.29	1.40E-01
ENSCAFG0000000588	RHOG	cyan	VSMC_M2	0.32	1.06E-01	0.34	8.68E-02	0.30	1.33E-01	-0.13	5.24E-01	-0.19	3.52E-01	0.29	1.36E-01	0.05	8.19E-01	-0.03	8.85E-01	-0.37	5.91E-02	0.08	6.87E-01
ENSCAFG0000001087	TET2	pink	VSMC_M5	0.32	1.06E-01	-0.37	5.86E-02	-0.31	1.16E-01	-0.06	7.62E-01	0.56	2.20E-03	0.09	6.46E-01	0.31	1.14E-01	0.18	3.70E-01	0.19	3.37E-01	-0.42	2.98E-02
ENSCAFG0000001113	INPP5A	grey	VSMC_M10	0.32	1.06E-01	0.18	3.70E-01	-0.32	1.02E-01	0.51	7.15E-03	0.53	4.18E-03	-0.28	1.63E-01	-0.34	8.69E-02	-0.59	1.21E-03	-0.09	6.53E-01	0.06	7.52E-01
ENSCAFG0000001634	WDR26	pink	VSMC_M5	0.32	1.06E-01	0.11	5.73E-01	-0.61	7.16E-04	0.66	1.67E-04	0.78	1.99E-06	-0.22	2.69E-01	-0.46	1.56E-02	-0.62	4.97E-04	-0.05	7.98E-01	0.07	7.14E-01

ENSCAFG0000002815	ENSCAFG0000002815	grey	VSMC_M10	0.32	1.06E-01	0.26	1.95E-01	0.48	1.16E-02	-0.13	5.33E-01	-0.38	5.30E-02	0.02	9.13E-01	-0.02	9.27E-01	0.00	9.94E-01	-0.31	1.15E-01	0.11	5.94E-01
ENSCAFG0000000070	CRELD2	yellow	VSMC_M3	0.32	1.06E-01	0.04	8.54E-01	0.54	3.76E-03	-0.36	6.33E-02	-0.45	1.98E-02	0.16	4.31E-01	0.22	2.73E-01	0.41	3.26E-02	-0.25	2.14E-01	-0.08	6.90E-01
ENSCAFG0000000583	SATB1	darkgreen	VSMC_M4	0.32	1.06E-01	0.25	2.03E-01	-0.38	5.30E-02	0.61	8.21E-04	0.42	2.85E-02	-0.27	1.74E-01	-0.34	8.20E-02	-0.55	3.28E-03	-0.29	1.44E-01	0.32	1.03E-01
ENSCAFG0000000647	PKDCC	cyan	VSMC_M2	0.32	1.06E-01	0.84	5.86E-08	0.41	3.23E-02	0.16	4.36E-01	-0.30	1.34E-01	-0.17	3.95E-01	-0.54	3.32E-03	-0.42	2.87E-02	-0.79	1.01E-06	0.44	2.20E-02
ENSCAFG0000000991	ENSCAFG0000000991	cyan	VSMC_M2	0.32	1.06E-01	0.77	3.00E-06	0.57	1.79E-03	-0.10	6.15E-01	-0.42	3.09E-02	-0.19	3.48E-01	0.07	7.16E-01	-0.29	1.49E-01	-0.74	9.98E-06	0.40	3.94E-02
ENSCAFG0000002271	ENSCAFG0000002271	grey	VSMC_M10	0.32	1.06E-01	0.25	2.13E-01	0.52	5.37E-03	-0.25	2.18E-01	-0.42	2.80E-02	0.15	4.45E-01	-0.02	9.29E-01	0.17	3.95E-01	-0.36	6.37E-02	0.05	7.86E-01
ENSCAFG0000002095	ENSCAFG0000002095	yellow	VSMC_M3	0.32	1.06E-01	0.14	4.93E-01	0.70	5.38E-05	-0.59	1.22E-03	-0.46	1.52E-02	0.01	9.45E-01	0.10	6.04E-01	0.42	2.75E-02	-0.25	2.13E-01	-0.31	1.19E-01
ENSCAFG0000001069	ENSCAFG0000001069	grey	VSMC_M10	0.32	1.06E-01	0.25	2.14E-01	0.27	1.67E-01	-0.14	4.98E-01	0.02	9.04E-01	-0.15	4.46E-01	-0.01	9.71E-01	-0.05	7.95E-01	-0.26	1.97E-01	-0.17	4.03E-01
ENSCAFG0000001942	TNFRSF14	cyan	VSMC_M2	0.32	1.07E-01	0.77	2.46E-06	0.44	2.14E-02	0.06	7.58E-01	-0.39	4.43E-02	-0.07	7.13E-01	-0.32	1.03E-01	-0.29	1.39E-01	-0.78	1.28E-06	0.51	6.35E-03
ENSCAFG0000001795	DTNA	grey	VSMC_M10	0.32	1.07E-01	-0.27	1.80E-01	-0.33	8.80E-02	0.24	2.38E-01	0.47	1.28E-02	0.00	9.98E-01	0.03	8.64E-01	-0.16	4.22E-01	0.16	4.11E-01	-0.11	5.70E-01
ENSCAFG0000000308	PABPC4	darkgrey	VSMC_M8	0.32	1.07E-01	-0.07	7.10E-01	0.48	1.11E-02	-0.69	5.88E-05	-0.32	9.85E-02	0.28	1.56E-01	0.09	6.50E-01	0.69	7.96E-05	-0.13	5.05E-01	-0.33	9.26E-02
ENSCAFG0000000843	AAR2	grey	VSMC_M10	0.32	1.07E-01	-0.23	2.39E-01	-0.05	8.00E-01	-0.27	1.71E-01	0.15	4.52E-01	0.26	1.97E-01	0.24	2.26E-01	0.42	3.08E-02	0.00	9.93E-01	-0.25	2.01E-01
ENSCAFG0000000130	GSDMD	cyan	VSMC_M2	0.32	1.07E-01	0.81	3.44E-07	0.58	1.62E-03	0.08	7.02E-01	-0.52	5.03E-03	-0.03	8.82E-01	-0.05	7.87E-01	-0.30	1.27E-01	-0.83	6.54E-08	0.53	4.51E-03
ENSCAFG0000001277	B4GAT1	cyan	VSMC_M2	0.32	1.07E-01	0.59	1.28E-03	0.21	2.95E-01	0.21	3.02E-01	-0.18	3.63E-01	-0.13	5.10E-01	0.10	6.06E-01	-0.43	2.49E-02	-0.60	8.52E-04	0.54	3.32E-03
ENSCAFG0000001319	MAGEF1	cyan	VSMC_M2	0.32	1.07E-01	0.54	3.61E-03	0.10	6.08E-01	0.27	1.81E-01	-0.06	7.71E-01	-0.11	5.76E-01	0.12	5.46E-01	-0.40	4.04E-02	-0.65	2.81E-04	0.49	8.67E-03
ENSCAFG0000000609	ASTE1	grey	VSMC_M10	0.32	1.08E-01	0.10	6.30E-01	0.24	2.24E-01	-0.20	3.13E-01	-0.13	5.30E-01	0.03	8.97E-01	0.37	5.65E-02	0.15	4.48E-01	-0.23	2.56E-01	0.07	7.19E-01
ENSCAFG0000000553	ZFP36	cyan	VSMC_M2	0.32	1.08E-01	0.82	1.29E-07	0.32	1.06E-01	0.16	4.29E-01	-0.22	2.76E-01	-0.09	6.39E-01	-0.28	1.57E-01	-0.44	2.14E-02	-0.79	7.67E-07	0.51	6.63E-03
ENSCAFG0000000428	WDFY2	yellow	VSMC_M3	0.32	1.08E-01	0.72	2.64E-05	0.73	1.59E-05	-0.08	6.88E-01	-0.55	2.90E-03	0.05	8.13E-01	-0.18	3.62E-01	-0.25	2.12E-01	-0.64	3.15E-04	0.27	1.80E-01
ENSCAFG0000001800	GAREM1	grey	VSMC_M10	0.32	1.08E-01	0.14	4.97E-01	-0.22	2.79E-01	0.29	1.37E-01	0.20	3.06E-01	0.00	9.99E-01	0.36	6.24E-02	-0.25	2.05E-01	-0.31	1.14E-01	0.38	5.10E-02
ENSCAFG0000002033	PSMB10	yellow	VSMC_M3	0.32	1.08E-01	0.55	3.17E-03	0.78	1.30E-06	-0.43	2.44E-02	-0.64	3.56E-04	0.15	4.59E-01	0.02	9.17E-01	0.16	4.28E-01	-0.62	5.16E-04	0.11	5.99E-01
ENSCAFG0000001454	HTRA3	grey	VSMC_M10	0.32	1.08E-01	-0.18	3.72E-01	0.41	3.16E-02	-0.67	1.38E-04	-0.11	5.95E-01	-0.06	7.77E-01	0.70	4.92E-05	0.56	2.59E-03	0.02	9.30E-01	-0.47	1.39E-02
ENSCAFG0000001136	ENSCAFG0000001136	grey	VSMC_M10	0.32	1.08E-01	0.37	5.66E-02	-0.26	1.97E-01	0.63	4.02E-04	0.37	5.93E-02	-0.34	8.43E-02	0.05	8.22E-01	-0.73	1.80E-05	-0.34	8.37E-02	0.41	3.49E-02
ENSCAFG0000003006	PLAC8B	grey	VSMC_M10	0.32	1.08E-01	0.73	1.33E-05	0.27	1.80E-01	0.33	8.89E-02	-0.18	3.73E-01	-0.26	1.85E-01	-0.50	8.52E-03	-0.53	4.36E-03	-0.62	5.32E-04	0.48	1.10E-02
ENSCAFG0000003157	INKA1	cyan	VSMC_M2	0.32	1.09E-01	0.43	2.58E-02	0.32	9.84E-02	0.15	4.43E-01	-0.12	5.64E-01	-0.05	7.88E-01	-0.06	7.61E-01	-0.39	4.15E-02	-0.33	9.06E-02	0.10	6.16E-01
ENSCAFG0000000881	PNMA2	grey	VSMC_M10	0.32	1.09E-01	-0.04	8.57E-01	-0.20	3.26E-01	0.19	3.36E-01	0.28	1.51E-01	-0.28	1.65E-01	0.00	9.85E-01	-0.19	3.44E-01	-0.02	9.37E-01	0.11	5.93E-01
ENSCAFG0000000965	ENSCAFG0000000965	pink	VSMC_M5	0.32	1.09E-01	-0.07	7.24E-01	-0.10	6.09E-01	0.04	8.40E-01	0.34	8.20E-02	-0.35	7.67E-02	-0.12	5.44E-01	0.00	9.93E-01	0.06	7.69E-01	-0.28	1.61E-01
ENSCAFG0000000367	ENSCAFG0000000367	red	VSMC_M1	0.32	1.09E-01	0.10	6.25E-01	0.11	5.75E-01	-0.16	4.32E-01	-0.09	6.51E-01	0.07	7.14E-01	0.38	4.82E-02	0.14	4.98E-01	-0.35	7.77E-02	0.15	4.49E-01
ENSCAFG0000001320	SIDT2	grey	VSMC_M10	0.32	1.09E-01	0.61	7.91E-04	0.00	9.93E-01	0.43	2.63E-02	0.10	6.22E-01	-0.06	7.60E-01	-0.64	3.09E-04	-0.66	1.91E-04	-0.47	1.38E-02	0.39	4.34E-02
ENSCAFG0000000765	SMAD1	pink	VSMC_M5	0.32	1.09E-01	0.04	8.43E-01	-0.59	1.11E-03	0.58	1.45E-03	0.68	9.97E-05	0.00	9.81E-01	-0.03	8.96E-01	-0.41	3.53E-02	-0.15	4.57E-01	0.14	4.86E-01
ENSCAFG0000002374	ENSCAFG0000002374	grey	VSMC_M10	0.32	1.09E-01	0.19	3.39E-01	0.57	1.92E-03	-0.42	3.00E-02	-0.21	2.89E-01	-0.20	3.26E-01	0.19	3.39E-01	0.21	2.84E-01	-0.22	2.61E-01	-0.37	5.46E-02
ENSCAFG0000000459	PTER	grey	VSMC_M10	0.31	1.10E-01	0.00	1.00E+00	0.22	2.60E-01	-0.24	2.30E-01	-0.23	2.57E-01	0.42	2.99E-02	0.35	7.01E-02	0.25	2.12E-01	-0.20	3.08E-01	0.08	6.95E-01
ENSCAFG0000000554	C1QTNF1	grey	VSMC_M10	0.31	1.10E-01	0.59	1.12E-03	-0.04	8.45E-01	0.45	1.95E-02	0.14	4.92E-01	-0.18	3.70E-01	-0.09	6.72E-01	-0.59	1.31E-03	-0.58	1.46E-03	0.50	8.20E-03
ENSCAFG0000000100	TNFRSF13C	grey	VSMC_M10	0.31	1.10E-01	0.16	4.17E-01	0.44	2.11E-02	-0.40	3.82E-02	-0.20	3.13E-01	-0.02	9.05E-01	0.44	2.05E-02	0.22	2.59E-01	-0.26	1.92E-01	-0.19	3.48E-01
ENSCAFG0000000985	PPM1M	yellow	VSMC_M3	0.31	1.10E-01	0.67	1.43E-04	0.75	5.68E-06	-0.21	3.04E-01	-0.54	3.59E-03	-0.27	1.70E-01	-0.11	5.94E-01	-0.17	4.02E-01	-0.58	1.36E-03	0.20	3.06E-01
ENSCAFG0000000413	AMOTL1	darkgrey	VSMC_M8	0.31	1.10E-01	-0.13	5.05E-01	0.27	1.77E-01	-0.64	2.97E-04	0.03	8.74E-01	-0.05	7.91E-01	0.28	1.64E-01	0.57	2.11E-03	0.02	9.30E-01	-0.49	9.39E-03
ENSCAFG0000001866	ENSCAFG0000001866	yellow	VSMC_M3	0.31	1.11E-01	0.29	1.39E-01	0.52	5.55E-03	-0.09	6.70E-01	-0.33	9.23E-02	-0.13	5.20E-01	0.07	7.37E-01	-0.05	8.05E-01	-0.33	9.65E-02	0.08	7.00E-01
ENSCAFG0000002450	ENSCAFG0000002450	grey	VSMC_M10	0.31	1.11E-01	0.24	2.21E-01	0.22	2.64E-01	-0.08	6.93E-01	-0.09	6.41E-01	0.20	3.30E-01	-0.08	6.99E-01	-0.02	9.27E-01	-0.27	1.75E-01	0.01	9.54E-01
ENSCAFG0000002932	WNT5A	grey	VSMC_M10	0.31	1.11E-01	-0.11	5.81E-01	0.24	2.28E-01	-0.47	1.37E-02	-0.07	7.19E-01	0.10	6.20E-01	0.30	1.32E-01	0.48	1.16E-02	-0.07	7.28E-01	-0.22	2.79E-01
ENSCAFG0000002673	ENSCAFG0000002673	yellow	VSMC_M3	0.31	1.11E-01	0.46	1.69E-02	0.66	2.05E-04	-0.18	3.61E-01	-0.44	2.12E-02	-0.10	6.19E-01	-0.17	3.90E-01	-0.07	7.20E-01	-0.48	1.21E-02	0.08	6.94E-01
ENSCAFG0000000482	ENSCAFG0000000482	pink	VSMC_M5	0.31	1.11E-01	-0.37	6.01E-02	-0.12	5.39E-01	0.00	9.82E-01	0.40	4.04E-02	-0.17	3.93E-01	-0.14	5.00E-01	0.10	6.06E-01	0.30	1.33E-01	-0.45	1.73E-02
ENSCAFG0000000967	LRRN2	grey	VSMC_M10	0.31	1.11E-01	0.40	4.04E-02	0.03	8.66E-01	0.35	7.36E-02	-0.03	8.95E-01	0.11	5.71E-01	0.16	4.20E-01	-0.47	1.42E-02	-0.48	1.10E-02	0.51	6.67E-03
ENSCAFG0000001882	DHRS13	cyan	VSMC_M2	0.31	1.12E-01	0.76	4.37E-06	0.63	4.23E-04	-0.01	9.43E-01	-0.48	1.10E-02	-0.15	4.47E-01	-0.09	6.45E-01	-0.29	1.37E-01	-0.69	7.84E-05	0.34	8.01E-02
ENSCAFG0000002042	TK2	cyan	VSMC_M2	0.31	1.12E-01	0.73	1.50E-05	0.40	3.83E-02	0.22	2.66E-01	-0.37	5.79E-02	-0.06	7.79E-01	0.12	5.38E-01	-0.42	2.72E-02	-0.75	8.04E-06	0.60	9.93E-04
ENSCAFG0000001687	ZNF410	grey	VSMC_M10	0.31	1.12E-01	-0.06	7.79E-01	0.19	3.36E-01	-0.20	3.06E-01	-0.04	8.42E-01	0.17	4.09E-01	0.20	3.18E-01	0.23	2.57E-01	-0.10	6.27E-01	-0.15	4.51E-01
ENSCAFG0000000546	RBMS3	grey	VSMC_M10	0.31	1.12E-01	0.66	1.82E-04	0.00	9.98E-01	0.34	7.83E-02	0.11	5.72E-01	-0.31	1.13E-01	-0.38	5.16E-02	-0.54	3.47E-03	-0.60	1.06E-03	0.39	4.29E-02
ENSCAFG0000001328	PBX1	yellow	VSMC_M3	0.31	1.12E-01	0.21	2.90E-01	0.52	5.84E-03	-0.55	2.93E-03	-0.21	3.03E-01	-0.13	5.20E-01	-0.46	1.48E-02	0.29	1.41E-01	-0.18	3.82E-01	-0.34	8.00E-02
ENSCAFG0000000239	SHB	grey	VSMC_M10	0.31	1.12E-01	0.14	4.92E-01	-0.42	2.82E-02	0.48	1.19E-02	0.53	4.26E-03	0.02	9.33E-01	-0.35	7.15E-02	-0.38	4.74E-02	-0.20	3.07E-01	0.05	8.06E-01
ENSCAFG0000000395	FKBP1B	red	VSMC_M1	0.31	1.12E-01	0.21	2.91E-01	-0.16	4.15E-01	0.33	9.46E-02	0.33	9.42E-02	-0.19	3.37E-01	-0.28	1.52E-01	-0.39	4.35E-02	-0.19	3.30E-01	0.08	6.93E-01
ENSCAFG0000000039	ENSCAFG0000000039	cyan	VSMC_M2	0.31	1.12E-01	0.47	1.40E-02	0.11	5.85E-01	0.38	5.27E-02	-0.05	8.08E-01	0.02	9.04E-01	0.10	6.02E-01	-0.49	9.67E-03	-0.50	8.29E-03	0.46	1.70E-02
ENSCAFG0000000074	ENSCAFG0000000074	grey	VSMC_M10	0.31	1.12E-01	-0.03	8.85E-01	0.24	2.24E-01	-0.28	1.50E-01	0.00	9.91E-01	-0.13	5.20E-01	0.13	5.17E-01	0.15	4.48E-01	0.01	9.53E-01	-0.27	1.74E-01
ENSCAFG0000000659	TBCB	yellow	VSMC_M3	0.31	1.13E-01	0.13	5.15E-01	0.57	1.86E-03	-0.56	2.49E-03	-0.30	1.23E-01	0.01	9.75E-01	0.10	6.20E-01	0.38	5.06E-02	-0.23	2.57E-01	-0.35	7.76E-02
ENSCAFG0000000102	JADE2	yellow	VSMC_M3	0.31	1.13E-01	0.61	7.87E-04	0.67	1.43E-04	-0.32	1.00E-01	-0.45	1.97E-02	-0.08	6.81E-01	-0.13	5.10E-01	0.02	9.05E-01	-0.61	7.73E-04	0.10	6.12E-01
ENSCAFG0000001519	SCNN1A	red	VSMC_M1	0.31	1.13E-01	0.24	2.20E-01	-0.29	1.40E-01	0.37	5.66E-02	0.36	6.13E-02	-0.16	4.35E-01	0.12	5.61E-01	-0.43	2.56E-02	-0.23	2.50E-01	0.28	1.50E-01
ENSCAFG0000000958	CHUK	darkgrey	VSMC_M8	0.31	1.13E-01	-0.27	1.80E-01	0.18	3.68E-01	-0.55	3.01E-03	-0.02	9.34E-01	0.17	3.87E-01	0.38	4.75E-02	0.60	9.30E-04	0.04	8.28E-01	-0.37	5.72E-02
ENSCAFG0000001798	ASXL3	grey	VSMC_M10	0.31	1.14E-01	-0.01	9.59E-01	0.00	9.83E-01	-0.13	5.19E-01	0.20	3.25E-01	-0.12	5.61E-01	-0.17	3.99E-01	0.09	6.71E-01	0.01	9.52E-01	-0.17	3.90E-01
ENSCAFG0000001051	DDX25	grey	VSMC_M10	0.31	1.14E-01	0.24	2.22E-01	0.01	9.76E-01	0.07	7.33E-01	0.28	1.62E-01	-0.19	3.37E-01	0.02	9.11E-01	-0.24	2.19E-01	-0.23	2.57E-01	-0.09	6.49E-01
ENSCAFG0000002021	ZNF821	darkgreen	VSMC_M4	0.31	1.14E-01	0.52	4.99E-03	0.02	9.34E-01	0.63	4.88E-04	-0.04	8.52E-01	0.05	8.04E-01	-0.27	1.76E-01	-0.70	4.21E-05	-0.50	7.42E-03	0.58	1.40E-03
ENSCAFG0000002450	ENSCAFG0000002450	grey	VSMC_M10	0.31	1.14E-01	-0.34	7.90E-02	-0.06	7.77E-01	-0.36	6.55E-02	0.26	1.88E-01	0.04	8.31E-01	0.36	6.82E-02	0.47	1.45E-02	0.09	6.46E-01	-0.45	1.77E-02
ENSCAFG0000001723	ENSCAFG0000001723	grey	VSMC_M10	0.31	1.14E-01	0.09	6.58E-01	0.41	3.27E-02	-0.27	1.75E-01	-0.26	1.82E-01	-0.14	4.95E-01	0.01	9.59E-01	0.23	2.56E-01	-0.25	2.08E-01	-0.11	5.73E-01
ENSCAFG0000000049	OSR2	grey	VSMC_M10	0.31	1.14E-01	0.29	1.47E-01	0.15	4.62E-01	-0.11	5.71E-01	0.08	6.86E-01	-0.07	7.46E-01	0.36	6.24E-02	-0.03	8.76E-01	-0.38	5.23E-02	0.01	9.66E-01
ENSCAFG0000000160	SLC39A4	grey	VSMC_M10	0.31	1.14E-01	0.00	9.82E-01	-0.10	6.10E-01	-0.07	7.44E-01	0.33	9.73E-02	-0.25	2.09E-01	-0.01	9.41E-01	0.01	9.43E-01	-0.05	7.95E-01	-0.22	2.69E-01
ENSCAFG0000000457	ANKRD42	grey	VSMC_M10	0.31	1.14E-01	0.11	5.73E-01	-0.22	2.71E-01	0.09	6.68E-01	0.32	1.07E-01	-0.12	5.37E-01	0.13	5.14E-01	-0.17	4.05E-01	-0.20	3.23E-01	0.14	4.71E-01
ENSCAFG0000002217	ENSCAFG0000002217	grey	VSMC_M10	0.31	1.14E-01	0.07	7.46E-01	0.39	4.22E-02	-0.23	2.48E-01	-0.28	1.57E-01	0.08	6.89E-01	0.27	1.78E-01	0.23	2.56E-01	-0.23	2.41E-01	-0.05	7.92E-01
ENSCAFG0000002918	SCAMP4	cyan	VSMC_M2	0.31	1.15E-01	0.66	1.91E-04	0.52	5.07E-03	-0.08	7.10E-01	-0.52	5.70E-03	0.07	7.19E-01	-0.02	9.26E-01	-0.11	5.89E-01	-0.77	3.04E-06	0.52	5.36E-03
ENSCAFG0000000041	MDM1	grey	VSMC_M10	0.31	1.15E-01	-0.04	8.50E-01	0.35	7.71E-02	-0.55	3.26E-03	-0.14	4.88E-01	0.21	2.87E-01	0.38	5.07E-02	0.43	2.34E-02	-0.14	4.84E-01	-0.25	2.07E-01
ENSCAFG0000000927	TSG101	pink	VSMC_M5	0.31	1.15E-01	-0.46	1.61E-02	-0.36	6.75E-02	0.06	7.81E-01	0.60	9.47E-04	-0.10	6.32E-01	-0.20	3.14E-01	0.08	6.82E-01	0.38	4.80E-02	-0.51	6.15E-03
ENSCAFG0000002892	NDUFAF8	grey	VSMC_M10	0.31	1.15E-01	0.41	3.44E-02	0.18	3.74E-01	0.21	2.97E-01	-0.20	3.24E-01	0.07	7.27E-01	0.15	4.65E-01	-0.28	1.51E-01	-0.48	1.12E-02	0.48	1.10E-02
ENSCAFG0000000063	HSPA1L	red	VSMC_M1	0.31	1.15E-01	0.16	4.22E-01	0.15	4.61E-01	-0.14	4.77E-01	-0.09	6.46E-01	-0.09	6.54E-01	0.15	4.62E-01	0.05	7.93E-01	-0.28	1.64E-01	0.16	4.39E-01
ENSCAFG0000000509	SLC25A38	grey	VSMC_M10	0.31	1.16E-01	-0.14	5.00E-01	0.21	3.03E-01	-0.51	6.30E-03	-0.11	6.00E-01	0.40	3.91E-02	0.30	1.34E-01	0.54	3.90E-03	-0.09	6.45E-01	-0.21	3.02E-01
ENSCAFG0000001194	RTF2	grey	VSMC_M10	0.31	1.16E-01	-0.14	4.92E-01	0.27	1.73E-01	-0.25	2.07E-01	-0.01	9.75E-01	-0.05	7.94E-01	-0.15	4.56E-01	0.29	1.43E-01	0.08	6.87E-01	-0.44	2.02E-02
ENSCAFG0000000503	KIN	grey	VSMC_M10	0.31	1.16E-01	-0.02	9.25E-01	-0.20	3.20E-01	0.25	2.09E-01	0.19	3.51E-01	0.00	9.94E-01	-0.09	6.70E-01	-0.14	4.98E-01	-0.12	5.50E-01	0.22	2.76E-01
ENSCAFG0000003069	ENSCAFG0000003069	grey	VSMC_M10	0.31	1.16E-01	0.10	6.27E-01	0.04	8.56E-01	-0.17	3.86E-01	0.09	6.71E-01	0.21	3.01E-01	-0.09	6.72E-01	0.18	3.71E-01	-0.22	2.78E-01	-0.10	6.23E-01
ENSCAFG0000000493	ENSCAFG0000000493	grey	VSMC_M10	0.31	1.16E-01	0.24	2.24E-01	0.18	3.77E-01	0.03	8.87E-01	-0.17	4.04E-01	0.24	2.21E-01	-0.10	6.33E-01	-0.08	6.90E-01	-0.34	8.20E-02	0.29	1.44E-01
ENSCAFG0000000114	AKAP7	grey	VSMC_M10	0.31	1.16E-01	0.22	2.72E-01	-0.25	2.02E-01	0.47	1.40E-02	0.20	3.26E-01	0.01	9.65E-01	-0.07	7.22E-01	-0.38	4.92E-02	-0.36	6.58E-02	0.51	6.64E-03
ENSCAFG0000000886	CPE	cyan	VSMC_M2	0.31	1.16E-01	0.71	2.85E-05	-0.09	6.67E-01	0.64	3.67E-04	0.13	5.24E-01	-0.29	1.49E-01	-0.59	1.24E-03	-0.75	5.93E-06	-0.61	7.37E-04	0.58	1.59E-03
ENSCAFG0000001039	AK2	yellow	VSMC_M3	0.31	1.16E-01	0.09	6.55E-01	0.64	2.87E-04	-0.45	1.75E-02	-0.30	1.23E-01	-0.11	5.92E-01	-0.09	6.53E-01	0.30	1.24E-01	-0.15	4.60E-01	-0.43	2.60E-02
ENSCAFG0000002391	LOXL1	yellow	VSMC_M3	0.31	1.17E-01	0.16	4.29E-01	0.51	6.32E-03	-0.66	1.68E-04	-0.22	2.80E-01	-0.19	3.39E-01	0.04	8.29E-01	0.40	4.00E-02	-0.18	3.81E-01	-0.31	1.13E-01
ENSCAFG0000001777	GPRASP2	grey	VSMC_M10	0.31	1.17E-01	0.32	9.86E-02	-0.31	1.15E-01	0.62	5.81E-04	0.40	3.91E-02	-0.34	7.94E-02	-0.22	2.67E-01	-0.64	2.82E-04	-0.31	1.11E-01	0.34	8.18E-02
ENSCAFG0000000098	SMDT1	grey	VSMC_M10	0.31	1.17E-01	0.13	5.29E-01	0.05	8.11E-01	0.23	2.44E-01	0.10	6.15E-01	-0.31	1.12E-01	-0.29	1.36E-01	-0.24	2.25E-01	-0.10	6.10E-01	0.02	9.36E-01
ENSCAFG0000001423	ARVCF	grey	VSMC_M10	0.31	1.17E-01	0.65	2.26E-04	0.05	7.98E-01	0.56	2.50E-03	0.01	9.72E-01	-0.07	7.11E-01	-0.07	7.31E-01	-0.75	5.74E-06	-0.56	2.23E-03	0.59	1.09E-03
ENSCAFG0000000541	FBL	darkgrey	VSMC_M8	0.31	1.18E-01	-0.03	8.76E-01	0.77	2.80E-06	-0.83	7.34E-08	-0.48	1.18E-02	0.10	6.25E-01	0.17	4.03E-01	0.72	2.33E-05	-0.13	5.34E-01	-0.53	4.88E-03
ENSCAFG0000000441	ARL5B	grey	VSMC_M10	0.31	1.18E-01	-0.07	7.38E-01	0.03	8.63E-01	-0.16	4.27E-01	0.11	5.71E-01	0.06	7.47E-01	0.03	8.83E-01	0.12	5.50E-01	-0.09	6.52E-01	-0.07	7.37E-01
ENSCAFG0000002642	ENSCAFG0000002642	yellow	VSMC_M3	0.31	1.18E-01	0.27	1.73E-01	0.48	1.11E-02	-0.37	6.01E-02	-0.26	1.89E-01	0.10	6.36E-01	-0.01	9.57E-01	0.23	2.51E-01	-0.30	1.31E-01	-0.17	4.08E-01
ENSCAFG0000000758	NCOA6	grey	VSMC_M10	0.31	1.18E-01	0.34	8.39E-02	-0.06	7.51E-01	0.20	3.22E-01	0.21	2.83E-01	-0.33	8.86E-02	-0.14	4.75E-01	-0.34	8.31E-02	-0.34	8.07E-02	0.25	2.09E-01
ENSCAFG0000000679	RASL11A	grey	VSMC_M10	0.31	1.18E-01	0.59	1.19E-03	0.23	2.57E-01	0.25	2.15E-01	-0.13	5.28E-01	-0.10	6.31E-01	-0.74	1.10E-05	-0.43	2.61E-02	-0.50	8.51E-03	0.32	1.04E-01
ENSCAFG0000000789	CLIP1	grey	VSMC_M10	0.31	1.18E-01	0.29	1.42E-01	0.32	1.04E-01	-0.29	1.47E-01	-0.12	5.60E-01	-0.18	3.74E-01	0.13	5.32E-01	0.07	7.43E-01	-0.34	8.17E-02	0.04	8.49E-01
ENSCAFG0000000841	C33H3orf38	grey	VSMC_M10	0.31	1.18E-01	-0.08	6.98E-01	-0.04	8.39E-01	-0.19	3.43E-01	0.13	5.15E-01	-0.01	9.43E-01	0.47	1.26E-02	0.24	2.35E-01	-0.14	4.75E-01	-0.05	8.00E-01
ENSCAFG0000002180	ENSCAFG0000002180	grey	VSMC_M10	0.31	1.18E-01	0.19	3.43E-01	0.42	2.85E-02	-0.14	4.82E-01	-0.32	9.95E-02	-0.25	2.17E-01	0.04	8.55E-01	0.13	5.14E-01	-0.29	1.46E-01	0.04	8.33E-01
ENSCAFG0000000968	PAK1IP1	darkgrey	VSMC_M8	0.31	1.18E-01	-0.53	4.31E-03	-0.04	8.44E-01	-0.54	3.34E-03	0.29	1.43E-01	0.06	7.52E-01	0.32	1.09E-01	0.66	1.81E-04	0.28	1.50E-01	-0.67	1.43E-04
ENSCAFG0000002474	CD55	cyan	VSMC_M2	0.31	1.18E-01	0.76	4.47E-06	-0.07	7.24E-01	0.66	1.81E-04	0.05	7.95E-01	-0.09	6.66E-01	-0.63	3.90E-04	-0.78	1.74E-06	-0.69	7.62E-05	0.68	8.91E-05
ENSCAFG0000000689	ENSCAFG0000000689	grey	VSMC_M10	0.31	1.18E-01	0.16	4.21E-01	0.09	6.69E-01	-0.08	7.09E-01	0.03	8.78E-01	0.00	9.97E-01	0.26	1.91E-01	-0.03	8.91E-01	-0.27	1.66E-01	0.07	7.31E-01
ENSCAFG0000001049	ENSCAFG0000001049	cyan	VSMC_M2	0.31	1.18E-01	0.56	2.41E-03	0.22	2.77E-01	0.21	2.85E-01	-0.13	5.26E-01	-0.01	9.43E-01	-0.48	1.05E-02	-0.33	9.47E-02	-0.51	6.69E-03	0.32	1.02E-01
ENSCAFG0000000321	SEPT7	pink	VSMC_M5	0.31	1.18E-01	-0.05	8.07E-01	-0.34	8.14E-02	0.28	1.53E-01	0.66	1.72E-04	-0.59	1.16E-03	-0.34	8.70E-02	-0.24	2.37E-01	0.10	6.06E-01	-0.32	1.01E-01
ENSCAFG0000000177	ENSCAFG0000000177	grey	VSMC_M10	0.31	1.18E-01	-0.10	6.34E-01	-0.13	5.20E-01	-0.04	8.59E-01	0.31	1.15E-01	-0.26	1.92E-01	0.08	6.84E-01	0.14	4.94E-01	0.00	9.93E-01	-0.21	3.02E-01
ENSCAFG0000000697	TCEA1	darkgrey	VSMC_M8	0.31	1.19E-01	-0.24	2.21E-01	0.37	5.77E-02	-0.69	6.94E-05	-0.02	9.24E-01	-0.18	3.72E-01	0.18	3.80E-01	0.61	7.02E-04	0.10	6.25E-01	-0.63	4.02E-04
ENSCAFG0000003003	PDSS2	grey	VSMC_M10	0.31	1.19E-01	0.42	2.89E-02	0.50	7.25E-03	-0.17	3.95E-01	-0.31	1.10E-01	0.04	8.24E-01	0.16	4.31E-01	-0.07	7.11E-01	-0.41	3.17E-02	0.13	5.08E-01
ENSCAFG0000001528	ENSCAFG0000001528	pink	VSMC_M5	0.31	1.19E-01	-0.38	5.30E-02	-0.29	1.46E-01	-0.19	3.55E-01	0.52	5.82E-03	-0.10	6.25E-01	0.07	7.32E-01	0.29	1.38E-01	0.24	2.25E-01	-0.51	7.12E-03
ENSCAFG0000001949	MRPS34	grey	VSMC_M10	0.31	1.19E-01	0.50	8.54E-03	0.64	3.35E-04	-0.14	4.94E-01	-0.61	7.11E-04	0.20	3.24E-01	0.15	4.43E-01	-0.05	8.14E-01	-0.62	6.32E-04	0.35	7.23E-02
ENSCAFG0000001968	MRPL28	yellow	VSMC_M3	0.31	1.19E-01	0.29	1.43E-01	0.85	2.18E-08	-0.46	1.68E-02	-0.63	4.43E-04	0.06	7.65E-01	0.10	6.22E-01	0.28	1.58E-01	-0.38	5.14E-02	-0.13	5.06E-01
ENSCAFG0000000287	UBE3D	grey	VSMC_M10	0.31	1.19E-01	0.38	4.96E-02	-0.10	6.05E-01	0.35	7.31E-02	0.05	8.08E-01	0.04	8.57E-01	-0.01	9.57E-01	-0.36	6.87E-02	-0.50	8.11E-03	0.56	2.16E-03
ENSCAFG0000000997	RBBP5	grey	VSMC_M10	0.31	1.19E-01	0.05	7.96E-01	0.25	2.15E-01	-0.12	5.54E-01	-0.10	6.25E-01	0.01	9.56E-01	0.34	8.38E-02	0.08	6.93E-01	-0.16	4.24E-01	0.03	8.85E-01
ENSCAFG0000000056	MAPK8IP2	grey	VSMC_M10	0.31	1.20E-01	0.28	1.51E-01	0.47	1.25E-02	-0.12	5.65E-01	-0.35	7.77E-02	0.14	5.00E-01	0.07	7.18E-01	-0.02	9.04E-01	-0.39	4.72E-02	0.16	4.11E-01
ENSCAFG0000002552	ENSCAFG0000002552	yellow	VSMC_M3	0.31	1.20E-01	0.17	3.93E-01	0.58	1.48E-03	-0.66	1.75E-04	-0.33	9.72E-02	0.06	7.53E-01	0.36	6.14E-02	0.42	2.92E-02	-0.29	1.47E-01	-0.27	1.78E-01
ENSCAFG0000000965	TFAP2A	grey	VSMC_M10	0.31	1.20E-01	0.01	9.73E-01	0.08	7.06E-01	-0.15	4.49E-01	0.08	6.83E-01	-0.16	4.30E-01	0.08	6.83E-01	0.11	5.72E-01	-0.04	8.54E-01	-0.15	4.48E-01
ENSCAFG0000000524	WDR60	grey	VSMC_M10	0.31	1.21E-01	0.54	3.52E-03	0.04	8.50E-01	0.43	2.65E-02	0.04	8.61E-01	-0.03	8.76E-01	-0.23	2.49E-01	-0.50	8.65E-03	-0.53	4.56E-03	0.47	1.33E-02
ENSCAFG0000000759	GGT7	cyan	VSMC_M2	0.31	1.21E-01	0.53	4.81E-03	-0.01	9.79E-01	0.30	1.24E-01	0.11	5.73E-01	-0.16	4.15E-01	-0.25	2.02E-01	-0.51	6.08E-03	-0.44	2.03E-02	0.37	5.46E-02
ENSCAFG0000000744	PXMP4	grey	VSMC_M10	0.31	1.21E-01	0.34	8.43E-02	0.14	4.88E-01	-0.12	5.39E-01	-0.11	5.83E-01	0.04	8.62E-01	0.29	1.43E-01	0.00	9.95E-01	-0.45	1.72E-02	0.29	1.46E-01
ENSCAFG0000001280	SLC25A12	darkgreen	VSMC_M4	0.31	1.21E-01	-0.14	4.78E-01	-0.54	3.66E-03	0.56	2.55E-03	0.60	8.35E-04	-0.11	5.89E-01	0.20	3.20E-01	-0.36	6.73E-02	0.02	9.31E-01	0.13	5.07E-01
ENSCAFG0000000158	NFYA	grey	VSMC_M10	0.31	1.21E-01	0.08	6.85E-01	-0.18	3.69E-01	0.48	1.10E-02	0.30	1.33E-01	-0.17	3.99E-01	-0.16	4.32E-01	-0.44	2.05E-02	-0.12	5.39E-01	0.18	3.80E-01
ENSCAFG0000001457	VEGFB	cyan	VSMC_M2	0.31	1.21E-01	0.79	1.00E-06	0.64	3.56E-04	-0.07	7.21E-01	-0.47	1.23E-02	-0.14	4.81E-01	-0.21	2.86E-01	-0.33	9.00E-02	-0.67	1.22E-04	0.35	6.94E-02
ENSCAFG0000001541	COMTD1	yellow	VSMC_M3	0.31	1.21E-01	0.46	1.63E-02	0.70	4.27E-05	-0.14	4.78E-01	-0.55	2.76E-03	0.11	5.95E-01	0.15	4.49E-01	-0.11	5.87E-01	-0.45	1.72E-02	0.18	3.57E-01
ENSCAFG0000001149	SGSM1	cyan	VSMC_M2	0.31	1.21E-01	0.54	3.96E-03	0.37	5.96E-02	0.06	7.72E-01	-0.30	1.35E-01	0.22	2.75E-01	-0.13	5.09E-01	-0.25	2.00E-01	-0.57	2.08E-03	0.38	4.86E-02
ENSCAFG0000000240	ENSCAFG0000000240	grey	VSMC_M10	0.31	1.21E-01	0.14	4.84E-01	0.41	3.32E-02	-0.25	2.11E-01	-0.19	3.43E-01	-0.02	9.14E-01	0.13	5.32E-01	0.11	5.87E-01	-0.21	3.02E-01	-0.12	5.63E-01
ENSCAFG0000003133	ENSCAFG0000003133	yellow	VSMC_M3	0.31	1.21E-01	0.21	2.82E-01	0.66	1.58E-04	-0.34	8.08E-02	-0.47	1.36E-02	-0.10	6.19E-01	0.34	8.57E-02	0.10	6.16E-01	-0.29	1.45E-01	-0.05	8.16E-01
ENSCAFG0000001477	WNK4	cyan	VSMC_M2	0.31	1.21E-01	0.69	6.90E-05	0.61	7.10E-04	-0.13	5.10E-01	-0.58	1.52E-03	0.11	5.99E-01	0.03	8.95E-01	-0.14	4.91E-01	-0.70	5.64E-05	0.46	1.59E-02
ENSCAFG0000001281	ABI2	pink	VSMC_M5	0.31	1.21E-01	-0.13	5.13E-01	-0.54	3.64E-03	0.24	2.37E-01	0.73	1.86E-05	-0.10	6.33E-01	-0.34	8.55E-02	-0.15	4.65E-01	0.09	6.56E-01	-0.24	2.26E-01

ENSCAFG0000003174	FBXO42	grey	VSMC_M10	0.31	1.22E-01	0.25	2.01E-01	0.04	8.54E-01	0.25	2.07E-01	0.01	9.73E-01	0.03	8.99E-01	0.14	5.01E-01	-0.35	7.00E-02	-0.30	1.28E-01	0.33	9.24E-02
ENSCAFG0000001823	IL13RA2	red	VSMC_M1	0.31	1.22E-01	0.21	2.99E-01	-0.06	7.69E-01	0.09	6.73E-01	0.18	3.77E-01	-0.20	3.24E-01	-0.25	1.99E-01	-0.08	6.89E-01	-0.25	2.14E-01	0.13	5.10E-01
ENSCAFG0000000347	VOPP1	red	VSMC_M1	0.31	1.22E-01	0.37	6.03E-02	0.01	9.49E-01	0.19	3.42E-01	0.10	6.25E-01	-0.14	4.83E-01	-0.03	8.78E-01	-0.35	6.95E-02	-0.32	1.05E-01	0.24	2.38E-01
ENSCAFG0000003207	SAP18	grey	VSMC_M10	0.31	1.22E-01	-0.02	9.07E-01	0.26	1.83E-01	-0.10	6.18E-01	-0.20	3.17E-01	0.05	7.98E-01	0.32	1.07E-01	0.18	3.71E-01	-0.13	5.10E-01	-0.03	8.82E-01
ENSCAFG0000001841	PACS2	yellow	VSMC_M3	0.30	1.22E-01	0.40	4.06E-02	0.42	2.75E-02	-0.18	3.72E-01	-0.25	2.06E-01	0.13	5.11E-01	0.28	1.50E-01	-0.06	7.83E-01	-0.38	5.11E-02	0.14	4.97E-01
ENSCAFG0000000486	RECQL5	red	VSMC_M1	0.30	1.22E-01	0.27	1.69E-01	0.00	9.93E-01	0.28	1.55E-01	0.20	3.20E-01	-0.08	6.74E-01	-0.08	6.94E-01	-0.40	3.78E-02	-0.28	1.53E-01	0.05	8.15E-01
ENSCAFG0000003020	KCTD1	grey	VSMC_M10	0.30	1.22E-01	0.42	2.84E-02	-0.21	2.95E-01	0.59	1.13E-03	0.40	3.72E-02	-0.30	1.22E-01	-0.41	3.57E-02	-0.70	4.38E-05	-0.31	1.17E-01	0.22	2.71E-01
ENSCAFG0000001453	COPE	yellow	VSMC_M3	0.30	1.22E-01	0.30	1.29E-01	0.70	5.24E-05	-0.33	8.85E-02	-0.52	5.87E-03	-0.02	9.31E-01	-0.23	2.44E-01	0.16	4.34E-01	-0.35	7.41E-02	-0.07	7.44E-01
ENSCAFG0000001601	ACADVL	grey	VSMC_M10	0.30	1.22E-01	0.09	6.47E-01	0.37	5.78E-02	-0.06	7.83E-01	-0.20	3.17E-01	-0.15	4.51E-01	-0.21	2.83E-01	0.11	5.92E-01	-0.17	3.95E-01	-0.14	4.72E-01
ENSCAFG0000000984	FAM13A	cyan	VSMC_M2	0.30	1.22E-01	0.37	5.83E-02	0.09	6.60E-01	0.32	9.89E-02	-0.16	4.25E-01	0.15	4.58E-01	-0.28	1.62E-01	-0.33	9.10E-02	-0.44	2.25E-02	0.48	1.04E-02
ENSCAFG0000000420	FKRP	cyan	VSMC_M2	0.30	1.22E-01	0.61	6.77E-04	0.23	2.46E-01	0.38	4.92E-02	-0.24	2.33E-01	-0.13	5.23E-01	-0.18	3.60E-01	-0.54	3.36E-03	-0.59	1.24E-03	0.61	6.79E-04
ENSCAFG0000000611	PRKD3	pink	VSMC_M5	0.30	1.22E-01	-0.43	2.37E-02	-0.14	4.91E-01	-0.47	1.44E-02	0.43	2.47E-02	0.08	6.75E-01	-0.01	9.49E-01	0.55	2.79E-03	0.25	2.08E-01	-0.68	8.13E-05
ENSCAFG0000000141	SRSF3	darkgrey	VSMC_M8	0.30	1.22E-01	-0.45	1.98E-02	0.35	6.93E-02	-0.78	1.80E-06	-0.07	7.43E-01	0.15	4.62E-01	0.25	2.05E-01	0.86	1.14E-08	0.22	2.65E-01	-0.77	2.12E-06
ENSCAFG0000000184	B4GALT1	yellow	VSMC_M3	0.30	1.22E-01	0.18	3.67E-01	0.50	7.34E-03	-0.66	1.78E-04	-0.18	3.56E-01	-0.25	2.12E-01	-0.03	8.65E-01	0.45	1.80E-02	-0.20	3.12E-01	-0.37	5.53E-02
ENSCAFG0000001551	USE1	cyan	VSMC_M2	0.30	1.23E-01	0.57	2.11E-03	0.23	2.41E-01	0.12	5.55E-01	-0.25	2.00E-01	0.13	5.30E-01	0.11	5.75E-01	-0.28	1.51E-01	-0.63	3.91E-04	0.49	8.82E-03
ENSCAFG0000000438	ENSCAFG0000000438	cyan	VSMC_M2	0.30	1.23E-01	0.59	1.19E-03	0.24	2.29E-01	0.21	2.90E-01	-0.18	3.56E-01	-0.03	8.88E-01	-0.08	6.91E-01	-0.36	6.39E-02	-0.65	2.49E-04	0.41	3.23E-02
ENSCAFG0000000545	NEU3	grey	VSMC_M10	0.30	1.23E-01	0.36	6.54E-02	-0.07	7.26E-01	0.47	1.26E-02	-0.01	9.80E-01	0.21	2.98E-01	-0.07	7.25E-01	-0.53	4.43E-03	-0.43	2.71E-02	0.61	6.47E-04
ENSCAFG0000000203	ENSCAFG0000000203	grey	VSMC_M10	0.30	1.23E-01	-0.03	8.91E-01	-0.32	1.08E-01	0.17	4.09E-01	0.50	8.07E-03	-0.19	3.40E-01	0.02	9.40E-01	-0.13	5.10E-01	-0.06	7.49E-01	-0.09	6.67E-01
ENSCAFG0000001788	DCUN1D3	grey	VSMC_M10	0.30	1.23E-01	0.34	8.08E-02	-0.17	4.00E-01	0.36	6.28E-02	0.22	2.60E-01	0.14	4.73E-01	-0.01	9.60E-01	-0.38	4.84E-02	-0.45	1.71E-02	0.28	1.51E-01
ENSCAFG0000002962	ACER2	grey	VSMC_M10	0.30	1.23E-01	-0.08	6.83E-01	0.17	3.90E-01	-0.48	1.20E-02	0.10	6.07E-01	0.09	6.58E-01	0.12	5.41E-01	0.32	1.07E-01	-0.05	7.98E-01	-0.38	4.87E-02
ENSCAFG0000001163	IQCB1	darkgrey	VSMC_M8	0.30	1.23E-01	-0.19	3.49E-01	0.14	4.75E-01	-0.47	1.25E-02	0.08	6.91E-01	-0.02	9.28E-01	0.09	6.71E-01	0.54	3.52E-03	0.00	9.89E-01	-0.48	1.17E-02
ENSCAFG0000000263	SOCS5	yellow	VSMC_M3	0.30	1.23E-01	0.33	9.22E-02	0.45	1.85E-02	-0.32	9.97E-02	-0.11	5.71E-01	-0.31	1.13E-01	-0.37	5.39E-02	0.10	6.27E-01	-0.27	1.68E-01	-0.30	1.31E-01
ENSCAFG0000001927	DVL1	cyan	VSMC_M2	0.30	1.23E-01	0.54	3.83E-03	0.33	9.46E-02	0.05	8.21E-01	-0.14	4.71E-01	-0.12	5.36E-01	-0.04	8.56E-01	-0.32	1.03E-01	-0.53	4.14E-03	0.24	2.29E-01
ENSCAFG0000001416	GRN	cyan	VSMC_M2	0.30	1.24E-01	0.91	2.41E-11	0.28	1.59E-01	0.40	3.79E-02	-0.18	3.75E-01	-0.17	3.98E-01	-0.38	5.03E-02	-0.73	1.39E-05	-0.78	1.91E-06	0.61	7.16E-04
ENSCAFG0000002864	ENSCAFG0000002864	yellow	VSMC_M3	0.30	1.24E-01	0.37	6.10E-02	0.59	1.07E-03	-0.15	4.50E-01	-0.40	3.71E-02	-0.17	3.97E-01	-0.19	3.31E-01	-0.08	6.92E-01	-0.37	5.46E-02	0.07	7.42E-01
ENSCAFG0000003150	ENSCAFG0000003150	pink	VSMC_M5	0.30	1.24E-01	-0.04	8.33E-01	-0.36	6.79E-02	0.18	3.79E-01	0.58	1.39E-03	-0.25	2.15E-01	0.09	6.40E-01	-0.20	3.15E-01	-0.03	8.93E-01	-0.12	5.41E-01
ENSCAFG0000003075	FLYWCH2	red	VSMC_M1	0.30	1.24E-01	0.37	5.62E-02	-0.06	7.56E-01	0.31	1.17E-01	0.14	4.80E-01	0.02	9.33E-01	-0.17	4.01E-01	-0.36	6.67E-02	-0.38	5.12E-02	0.30	1.31E-01
ENSCAFG0000000322	ENSCAFG0000000322	darkgrey	VSMC_M8	0.30	1.24E-01	-0.50	7.59E-03	0.01	9.80E-01	-0.55	2.76E-03	0.23	2.57E-01	0.05	7.88E-01	0.28	1.63E-01	0.70	5.54E-05	0.26	1.91E-01	-0.68	1.09E-04
ENSCAFG0000000894	GLT8D1	grey	VSMC_M10	0.30	1.24E-01	-0.06	7.48E-01	-0.11	5.89E-01	-0.28	1.61E-01	0.34	7.86E-02	-0.13	5.26E-01	-0.16	4.34E-01	0.28	1.63E-01	-0.01	9.63E-01	-0.36	6.70E-02
ENSCAFG0000002408	BLOC1S3	grey	VSMC_M10	0.30	1.24E-01	0.39	4.73E-02	0.01	9.48E-01	0.37	5.65E-02	-0.05	8.10E-01	0.10	6.18E-01	-0.06	7.66E-01	-0.42	2.94E-02	-0.45	1.85E-02	0.56	2.49E-03
ENSCAFG0000000682	LIN37	grey	VSMC_M10	0.30	1.24E-01	0.36	6.55E-02	0.19	3.35E-01	0.04	8.45E-01	0.00	9.99E-01	-0.08	6.93E-01	-0.36	6.64E-02	-0.21	2.99E-01	-0.30	1.28E-01	0.04	8.39E-01
ENSCAFG0000000362	LPIN1	grey	VSMC_M10	0.30	1.25E-01	-0.19	3.41E-01	-0.05	7.92E-01	-0.24	2.25E-01	0.28	1.58E-01	0.08	7.05E-01	0.64	3.49E-04	0.24	2.19E-01	0.01	9.45E-01	-0.31	1.15E-01
ENSCAFG0000000933	CES1	grey	VSMC_M10	0.30	1.25E-01	0.09	6.46E-01	-0.30	1.22E-01	0.38	5.34E-02	0.41	3.38E-02	-0.14	5.00E-01	0.14	4.74E-01	-0.32	1.04E-01	-0.14	4.81E-01	0.16	4.37E-01
ENSCAFG0000002416	PRR22	grey	VSMC_M10	0.30	1.25E-01	0.02	9.14E-01	0.27	1.77E-01	-0.28	1.54E-01	-0.04	8.44E-01	0.10	6.06E-01	-0.01	9.77E-01	0.20	3.06E-01	-0.07	7.35E-01	-0.22	2.75E-01
ENSCAFG0000000798	PLCE1	grey	VSMC_M10	0.30	1.25E-01	0.59	1.34E-03	0.02	9.24E-01	0.36	6.60E-02	0.07	7.21E-01	-0.14	4.96E-01	0.02	9.12E-01	-0.49	1.01E-02	-0.60	8.59E-04	0.44	2.13E-02
ENSCAFG0000001708	PPP4C	yellow	VSMC_M3	0.30	1.25E-01	0.48	1.05E-02	0.78	1.66E-06	-0.43	2.54E-02	-0.68	9.04E-05	0.23	2.44E-01	-0.12	5.60E-01	0.24	2.37E-01	-0.57	1.80E-03	0.13	5.05E-01
ENSCAFG0000001526	TBX19	grey	VSMC_M10	0.30	1.25E-01	0.31	1.18E-01	0.45	1.97E-02	-0.01	9.52E-01	-0.25	2.07E-01	-0.11	5.99E-01	-0.03	8.69E-01	-0.15	4.42E-01	-0.28	1.55E-01	0.05	7.88E-01
ENSCAFG0000003080	IKZF4	grey	VSMC_M10	0.30	1.25E-01	0.49	9.86E-03	-0.11	5.91E-01	0.49	1.01E-02	0.29	1.40E-01	-0.33	9.05E-02	-0.29	1.41E-01	-0.67	1.46E-04	-0.38	5.36E-02	0.30	1.34E-01
ENSCAFG0000001753	SPC24	darkgrey	VSMC_M8	0.30	1.25E-01	-0.46	1.52E-02	0.37	5.81E-02	-0.80	4.76E-07	-0.06	7.59E-01	-0.05	8.00E-01	0.30	1.28E-01	0.88	1.35E-09	0.23	2.51E-01	-0.77	3.34E-06
ENSCAFG0000000696	TRIB3	grey	VSMC_M10	0.30	1.25E-01	0.36	6.35E-02	0.31	1.13E-01	-0.20	3.11E-01	-0.16	4.13E-01	-0.05	7.87E-01	0.24	2.21E-01	-0.02	9.19E-01	-0.48	1.14E-02	0.17	3.89E-01
ENSCAFG0000001148	PABPN1	yellow	VSMC_M3	0.30	1.26E-01	0.47	1.31E-02	0.58	1.37E-03	-0.33	9.74E-02	-0.47	1.24E-02	0.18	3.69E-01	0.13	5.12E-01	0.17	4.02E-01	-0.58	1.67E-03	0.17	4.11E-01
ENSCAFG0000000221	ENSCAFG0000000221	cyan	VSMC_M2	0.30	1.26E-01	0.55	2.69E-03	0.18	3.57E-01	0.35	7.27E-02	-0.09	6.50E-01	-0.06	7.57E-01	0.04	8.46E-01	-0.55	2.73E-03	-0.55	2.75E-03	0.44	2.12E-02
ENSCAFG0000000382	MATN3	grey	VSMC_M10	0.30	1.26E-01	-0.28	1.63E-01	-0.37	5.96E-02	0.17	3.87E-01	0.43	2.47E-02	0.02	9.21E-01	0.22	2.61E-01	0.10	6.23E-01	0.02	9.07E-01	-0.10	6.23E-01
ENSCAFG0000002432	ENSCAFG0000002432	grey	VSMC_M10	0.30	1.26E-01	0.28	1.58E-01	0.25	2.08E-01	0.18	3.78E-01	-0.20	3.19E-01	-0.05	8.21E-01	0.11	5.80E-01	-0.21	3.04E-01	-0.31	1.16E-01	0.25	2.00E-01
ENSCAFG0000001316	PRPF6	yellow	VSMC_M3	0.30	1.26E-01	0.30	1.22E-01	0.80	4.35E-07	-0.42	2.92E-02	-0.60	9.42E-04	0.00	9.80E-01	-0.04	8.52E-01	0.23	2.53E-01	-0.33	9.15E-02	-0.08	7.03E-01
ENSCAFG0000000006	ENSCAFG0000000006	cyan	VSMC_M2	0.30	1.26E-01	0.45	1.82E-02	0.22	2.60E-01	0.15	4.57E-01	-0.18	3.70E-01	-0.09	6.61E-01	0.24	2.29E-01	-0.29	1.39E-01	-0.52	5.35E-03	0.40	3.75E-02
ENSCAFG0000003227	CINP	grey	VSMC_M10	0.30	1.26E-01	0.21	2.84E-01	0.39	4.56E-02	-0.05	8.03E-01	-0.29	1.38E-01	0.02	9.13E-01	0.10	6.14E-01	-0.09	6.69E-01	-0.29	1.40E-01	0.13	5.29E-01
ENSCAFG0000000970	ENSCAFG0000000970	grey	VSMC_M10	0.30	1.26E-01	-0.07	7.46E-01	0.19	3.55E-01	-0.28	1.51E-01	0.03	8.85E-01	-0.19	3.36E-01	-0.29	1.36E-01	0.29	1.48E-01	0.01	9.64E-01	-0.29	1.37E-01
ENSCAFG0000002005	ODF2	darkgrey	VSMC_M8	0.30	1.26E-01	-0.25	2.02E-01	0.43	2.38E-02	-0.75	7.68E-06	-0.16	4.23E-01	-0.16	4.39E-01	0.49	1.02E-02	0.77	2.78E-06	0.03	8.73E-01	-0.54	3.68E-03
ENSCAFG0000000047	PSORS1C2	pink	VSMC_M5	0.30	1.26E-01	-0.16	4.38E-01	-0.23	2.56E-01	-0.12	5.40E-01	0.45	1.92E-02	-0.23	2.42E-01	0.14	4.81E-01	0.16	4.34E-01	-0.02	9.16E-01	-0.28	1.53E-01
ENSCAFG0000000746	RHPN2	cyan	VSMC_M2	0.30	1.27E-01	0.77	2.48E-06	0.37	5.53E-02	0.00	9.87E-01	-0.25	2.10E-01	-0.25	2.04E-01	-0.18	3.56E-01	-0.29	1.42E-01	-0.71	2.98E-05	0.41	3.44E-02
ENSCAFG0000000878	RSPRY1	pink	VSMC_M5	0.30	1.27E-01	-0.47	1.27E-02	-0.36	6.27E-02	-0.07	7.45E-01	0.51	6.21E-03	-0.09	6.71E-01	0.04	8.26E-01	0.23	2.56E-01	0.31	1.22E-01	-0.42	3.07E-02
ENSCAFG0000001527	PGLS	cyan	VSMC_M2	0.30	1.27E-01	0.52	5.69E-03	0.27	1.80E-01	0.11	5.76E-01	-0.07	7.24E-01	-0.34	8.21E-02	-0.43	2.65E-02	-0.40	3.78E-02	-0.39	4.31E-02	0.16	4.34E-01
ENSCAFG0000000791	DNAH6	grey	VSMC_M10	0.30	1.27E-01	0.13	5.07E-01	0.10	6.30E-01	-0.12	5.52E-01	0.10	6.09E-01	-0.05	7.95E-01	-0.07	7.32E-01	0.07	7.29E-01	-0.17	3.85E-01	-0.07	7.27E-01
ENSCAFG0000002875	MTHFD2L	grey	VSMC_M10	0.30	1.27E-01	0.12	5.42E-01	-0.11	5.90E-01	0.23	2.54E-01	0.20	3.18E-01	-0.10	6.24E-01	-0.25	2.12E-01	-0.29	1.47E-01	-0.14	5.01E-01	0.13	5.15E-01
ENSCAFG0000000465	GLB1	grey	VSMC_M10	0.30	1.27E-01	0.31	1.16E-01	-0.31	1.10E-01	0.57	2.05E-03	0.40	3.80E-02	-0.31	1.14E-01	0.02	9.11E-01	-0.59	1.08E-03	-0.31	1.19E-01	0.37	5.50E-02
ENSCAFG0000001187	HSPA12A	grey	VSMC_M10	0.30	1.27E-01	0.14	4.81E-01	-0.35	7.11E-02	0.49	9.24E-03	0.34	8.59E-02	0.18	3.68E-01	0.14	4.71E-01	-0.44	2.29E-02	-0.23	2.38E-01	0.39	4.49E-02
ENSCAFG0000001654	CLCN6	cyan	VSMC_M2	0.30	1.28E-01	0.71	3.47E-05	0.50	7.53E-03	-0.13	5.29E-01	-0.36	6.51E-02	-0.12	5.61E-01	-0.06	7.57E-01	-0.24	2.33E-01	-0.65	2.20E-04	0.35	7.79E-02
ENSCAFG0000000644	TEX15	grey	VSMC_M10	0.30	1.28E-01	0.07	7.31E-01	-0.04	8.25E-01	0.25	2.09E-01	0.07	7.40E-01	0.03	8.81E-01	0.28	1.53E-01	-0.22	2.64E-01	-0.21	2.97E-01	0.28	1.50E-01
ENSCAFG0000001513	LDB2	cyan	VSMC_M2	0.30	1.28E-01	0.80	4.84E-07	0.29	1.44E-01	0.30	1.23E-01	-0.17	3.93E-01	-0.03	8.99E-01	-0.38	5.05E-02	-0.54	3.30E-03	-0.71	3.29E-05	0.45	1.79E-02
ENSCAFG0000001126	SSH1	grey	VSMC_M10	0.30	1.28E-01	0.34	7.93E-02	0.13	5.20E-01	0.18	3.66E-01	-0.11	5.84E-01	0.12	5.60E-01	-0.12	5.57E-01	-0.20	3.09E-01	-0.44	2.29E-02	0.32	1.07E-01
ENSCAFG0000000058	ENSCAFG0000000058	pink	VSMC_M5	0.30	1.28E-01	-0.57	1.80E-03	-0.13	5.15E-01	-0.32	1.09E-01	0.52	5.72E-03	-0.24	2.31E-01	0.00	9.83E-01	0.41	3.20E-02	0.50	7.80E-03	-0.82	1.57E-07
ENSCAFG0000001974	CLSTN1	cyan	VSMC_M2	0.30	1.28E-01	0.71	2.91E-05	0.00	9.98E-01	0.72	1.91E-05	-0.01	9.63E-01	-0.07	7.31E-01	-0.36	6.31E-02	-0.82	1.22E-07	-0.64	3.21E-04	0.72	2.58E-05
ENSCAFG0000003014	ENSCAFG0000003014	cyan	VSMC_M2	0.30	1.29E-01	0.66	1.88E-04	0.34	8.35E-02	0.16	4.19E-01	-0.35	7.36E-02	0.08	6.75E-01	-0.22	2.73E-01	-0.35	7.17E-02	-0.69	6.56E-05	0.47	1.24E-02
ENSCAFG0000001325	NMNAT2	cyan	VSMC_M2	0.30	1.29E-01	0.61	7.44E-04	0.31	1.19E-01	0.32	1.08E-01	-0.29	1.37E-01	0.07	7.28E-01	-0.23	2.50E-01	-0.50	8.60E-03	-0.55	2.69E-03	0.54	3.86E-03
ENSCAFG0000001293	KCNQ2	red	VSMC_M1	0.30	1.29E-01	0.28	1.58E-01	0.23	2.39E-01	0.14	4.85E-01	-0.13	5.31E-01	0.05	8.15E-01	-0.08	6.85E-01	-0.17	4.08E-01	-0.38	4.78E-02	0.20	3.08E-01
ENSCAFG0000000139	ENSCAFG0000000139	grey	VSMC_M10	0.30	1.29E-01	0.00	9.80E-01	-0.08	7.02E-01	0.25	2.12E-01	0.14	5.00E-01	-0.05	8.10E-01	0.12	5.62E-01	-0.11	5.70E-01	-0.17	3.85E-01	0.05	7.94E-01
ENSCAFG0000001504	ATG14	darkgreen	VSMC_M4	0.30	1.29E-01	0.59	1.06E-03	0.10	6.29E-01	0.55	2.89E-03	-0.10	6.18E-01	-0.08	6.89E-01	-0.19	3.53E-01	-0.64	2.93E-04	-0.64	3.53E-04	0.64	3.65E-04
ENSCAFG0000001215	ARSJ	grey	VSMC_M10	0.30	1.29E-01	-0.26	1.93E-01	0.14	4.81E-01	-0.49	8.98E-03	0.17	4.05E-01	-0.22	2.71E-01	0.09	6.53E-01	0.38	4.81E-02	0.23	2.42E-01	-0.52	5.62E-03
ENSCAFG0000001970	RHBDF1	cyan	VSMC_M2	0.30	1.30E-01	0.68	1.05E-04	0.42	2.75E-02	0.11	5.69E-01	-0.38	5.26E-02	0.03	8.99E-01	-0.14	4.97E-01	-0.33	9.41E-02	-0.66	1.58E-04	0.49	1.01E-02
ENSCAFG0000002473	TRIM24	grey	VSMC_M10	0.30	1.30E-01	-0.40	3.75E-02	-0.13	5.04E-01	-0.31	1.16E-01	0.39	4.54E-02	0.28	1.58E-01	0.08	7.07E-01	0.44	2.30E-02	0.25	2.00E-01	-0.59	1.15E-03
ENSCAFG0000000138	CBY1	grey	VSMC_M10	0.30	1.30E-01	0.25	2.05E-01	0.36	6.46E-02	0.11	5.72E-01	-0.25	2.01E-01	-0.09	6.39E-01	0.15	4.40E-01	-0.20	3.22E-01	-0.35	7.23E-02	0.19	3.45E-01
ENSCAFG0000000008	PMEL	cyan	VSMC_M2	0.30	1.30E-01	0.49	9.05E-03	0.28	1.62E-01	0.28	1.51E-01	-0.22	2.79E-01	-0.01	9.51E-01	-0.16	4.21E-01	-0.46	1.47E-02	-0.48	1.18E-02	0.40	3.64E-02
ENSCAFG0000001140	TXNIP	darkgreen	VSMC_M4	0.30	1.30E-01	0.44	2.23E-02	-0.32	9.86E-02	0.70	5.28E-05	0.35	7.32E-02	-0.10	6.22E-01	-0.11	6.00E-01	-0.72	1.89E-05	-0.46	1.63E-02	0.54	3.54E-03
ENSCAFG0000000765	SPSB4	grey	VSMC_M10	0.30	1.30E-01	-0.01	9.63E-01	0.47	1.30E-02	-0.72	1.95E-05	-0.27	1.78E-01	0.13	5.28E-01	0.48	1.16E-02	0.57	1.89E-03	-0.17	4.05E-01	-0.27	1.74E-01
ENSCAFG0000001770	ARMCX6	grey	VSMC_M10	0.30	1.31E-01	-0.10	6.09E-01	0.20	3.10E-01	-0.33	9.62E-02	0.03	8.73E-01	-0.23	2.42E-01	0.42	2.71E-02	0.27	1.73E-01	-0.05	8.09E-01	-0.32	1.02E-01
ENSCAFG0000001579	EML3	yellow	VSMC_M3	0.30	1.31E-01	0.47	1.38E-02	0.80	4.19E-07	-0.54	3.84E-03	-0.58	1.53E-03	0.00	9.81E-01	-0.05	8.13E-01	0.28	1.63E-01	-0.49	8.86E-03	-0.03	8.95E-01
ENSCAFG0000001122	IP6K1	cyan	VSMC_M2	0.30	1.31E-01	0.74	1.07E-05	0.36	6.65E-02	0.31	1.21E-01	-0.40	4.04E-02	0.06	7.71E-01	-0.13	5.20E-01	-0.50	8.12E-03	-0.73	1.60E-05	0.73	1.36E-05
ENSCAFG0000002309	CCDC160	cyan	VSMC_M2	0.30	1.31E-01	0.65	2.24E-04	-0.12	5.60E-01	0.66	1.98E-04	0.08	7.08E-01	-0.09	6.55E-01	-0.45	1.76E-02	-0.72	2.12E-05	-0.64	3.11E-04	0.69	7.83E-05
ENSCAFG0000002908	HRCT1	cyan	VSMC_M2	0.30	1.31E-01	0.51	6.27E-03	0.32	1.04E-01	-0.02	9.32E-01	-0.22	2.62E-01	0.04	8.51E-01	-0.08	7.05E-01	-0.19	3.55E-01	-0.52	5.52E-03	0.25	2.03E-01
ENSCAFG0000000869	RAPSN	yellow	VSMC_M3	0.30	1.31E-01	0.25	2.08E-01	0.56	2.48E-03	-0.45	1.92E-02	-0.36	6.23E-02	-0.05	8.12E-01	0.13	5.07E-01	0.32	9.97E-02	-0.31	1.16E-01	-0.08	6.83E-01
ENSCAFG0000001414	ADAMTS7	cyan	VSMC_M2	0.30	1.32E-01	0.82	1.46E-07	0.40	3.67E-02	0.09	6.63E-01	-0.29	1.46E-01	-0.17	4.06E-01	-0.32	1.03E-01	-0.43	2.53E-02	-0.73	1.59E-05	0.48	1.20E-02
ENSCAFG0000000510	ITPKC	grey	VSMC_M10	0.30	1.32E-01	0.33	8.82E-02	0.37	5.62E-02	-0.05	8.00E-01	-0.29	1.35E-01	0.29	1.42E-01	-0.04	8.29E-01	-0.06	7.84E-01	-0.45	1.99E-02	0.25	2.05E-01
ENSCAFG0000000694	CAT	grey	VSMC_M10	0.30	1.32E-01	0.61	7.62E-04	0.03	8.63E-01	0.54	3.92E-03	-0.01	9.70E-01	0.03	8.80E-01	-0.04	8.59E-01	-0.65	2.33E-04	-0.56	2.30E-03	0.56	2.17E-03
ENSCAFG0000000724	ENSCAFG0000000724	grey	VSMC_M10	0.30	1.32E-01	0.48	1.05E-02	0.53	4.37E-03	-0.24	2.29E-01	-0.38	4.99E-02	0.16	4.39E-01	-0.06	7.81E-01	0.06	7.60E-01	-0.52	5.98E-03	0.15	4.54E-01
ENSCAFG0000003089	PERP	grey	VSMC_M10	0.30	1.32E-01	0.15	4.40E-01	-0.38	4.98E-02	0.50	8.49E-03	0.38	4.95E-02	-0.12	5.39E-01	-0.26	1.86E-01	-0.38	4.78E-02	-0.22	2.70E-01	0.26	1.90E-01
ENSCAFG0000002013	FAM129B	darkgrey	VSMC_M8	0.30	1.32E-01	-0.10	6.35E-01	0.67	1.19E-04	-0.83	1.13E-07	-0.38	5.16E-02	0.04	8.42E-01	0.19	3.52E-01	0.71	3.51E-05	-0.02	9.40E-01	-0.56	2.35E-03
ENSCAFG0000001042	DCPS	yellow	VSMC_M3	0.30	1.32E-01	0.21	2.83E-01	0.45	2.00E-02	-0.51	6.64E-03	-0.29	1.44E-01	0.11	5.82E-01	0.13	5.29E-01	0.39	4.26E-02	-0.36	6.36E-02	-0.07	7.29E-01
ENSCAFG0000000378	FOXO3	grey	VSMC_M10	0.30	1.32E-01	0.44	2.30E-02	-0.22	2.73E-01	0.51	6.61E-03	0.41	3.38E-02	-0.24	2.31E-01	-0.63	4.21E-04	-0.62	5.99E-04	-0.31	1.13E-01	0.19	3.52E-01
ENSCAFG0000000165	IMPDH1	grey	VSMC_M10	0.30	1.32E-01	-0.34	7.89E-02	-0.10	6.15E-01	-0.03	8.92E-01	0.27	1.79E-01	0.04	8.40E-01	-0.04	8.52E-01	0.11	6.01E-01	0.30	1.27E-01	-0.31	1.12E-01
ENSCAFG0000001076	IPO8	pink	VSMC_M5	0.30	1.32E-01	-0.53	4.07E-03	-0.22	2.80E-01	-0.28	1.50E-01	0.51	6.45E-03	-0.25	2.12E-01	0.33	9.78E-02	0.41	3.57E-02	0.41	3.40E-02	-0.58	1.35E-03
ENSCAFG0000003263	ENSCAFG0000003263	pink	VSMC_M5	0.30	1.32E-01	0.04	8.59E-01	-0.70	5.59E-05	0.65	2.26E-04	0.81	2.82E-07	-0.33	8.96E-02	-0.27	1.69E-01	-0.54	3.69E-03	-0.06	7.85E-01	0.11	5.75E-01
ENSCAFG0000001125	ENSCAFG0000001125	grey	VSMC_M10	0.30	1.33E-01	0.08	7.06E-01	0.30	1.25E-01	-0.28	1.52E-01	-0.14	4.73E-01	0.10	6.06E-01	-0.15	4.44E-01	0.20	3.27E-01	-0.19	3.39E-01	-0.08	7.01E-01
ENSCAFG0000001943	BTBD2	grey	VSMC_M10	0.30	1.33E-01	0.84	3.34E-08	0.43	2.48E-02	0.24	2.34E-01	-0.36	6.89E-02	-0.06	7.51E-01	-0.32	1.06E-01	-0.53	4.65E-03	-0.75	7.76E-06	0.59	1.14E-03
ENSCAFG0000003262	ENSCAFG0000003262	yellow	VSMC_M3	0.30	1.33E-01	0.59	1.23E-03	0.40	3.79E-02	0.15	4.41E-01	-0.38	5.01E-02	-0.02	9.35E-01	-0.17	3.91E-01	-0.33	9.05E-02	-0.59	1.20E-03	0.52	5.11E-03
ENSCAFG0000001633	MIEN1	grey	VSMC_M10	0.30	1.33E-01	0.30	1.35E-01	-0.14	5.01E-01	0.30	1.30E-01	0.30	1.34E-01	-0.35	7.54E-02	-0.17	4.00E-01	-0.36	6.30E-02	-0.32	1.09E-01	0.11	5.80E-01
ENSCAFG0000000707	IMPAD1	pink	VSMC_M5	0.30	1.33E-01	-0.47	1.40E-02	-0.27	1.73E-01	-0.02	9.27E-01	0.59	1.32E-03	-0.24	2.18E-01	-0.10	6.26E-01	0.19	3.31E-01	0.39	4.68E-02	-0.64	3.10E-04
ENSCAFG0000000993	ENSCAFG0000000993	grey	VSMC_M10	0.30	1.33E-01	-0.18	3.63E-01	-0.36	6.63E-02	0.03	8.70E-01	0.56	2.58E-03	-0.33	9.71E-02	0.10	6.10E-01	0.09	6.62E-01	0.04	8.28E-01	-0.24	2.19E-01
ENSCAFG0000001687	UBQLN4	cyan	VSMC_M2	0.30	1.33E-01	0.43	2.61E-02	0.21	3.02E-01	-0.03	8.91E-01	-0.17	4.02E-01	0.03	9.01E-01	-0.02	9.24E-01	-0.05	8.19E-01	-0.53	4.60E-03	0.32	1.01E-01
ENSCAFG0000002916	ENSCAFG0000002916	darkgrey	VSMC_M8	0.30	1.33E-01	-0.11	5.73E-01	0.29	1.45E-01	-0.59	1.18E-03	-0.11	5.92E-01	0.16	4.36E-01	0.21	3.04E-01	0.59	1.17E-03	-0.05	8.19E-01	-0.40	4.01E-02
ENSCAFG0000003185	ENSCAFG0000003185	grey	VSMC_M10	0.30	1.34E-01	-0.03	8.95E-01	-0.15	4.64E-01	-0.10	6.09E-01	0.40	4.01E-02	-0.18	3.69E-01	0.08	7.03E-01	0.13	5.06E-01	-0.08	6.96E-01	-0.27	1.78E-01
ENSCAFG0000001479	ENSCAFG0000001479	grey	VSMC_M10	0.30	1.34E-01	-0.17	4.00E-01	0.38	5.21E-02	-0.49	9.30E-03	-0.19	3.49E-01	0.09	6.49E-01	0.12	5.35E-01	0.46	1.64E-02	0.06	7.51E-01	-0.39	4.38E-02
ENSCAFG0000000918	KCNJ3	grey	VSMC_M10	0.30	1.34E-01	0.35	7.34E-02	0.06	7.63E-01	0.28	1.58E-01	-0.01	9.54E-01	0.36	6.68E-02	-0.26	1.95E-01	-0.31	1.11E-01	-0.36	6.70E-02	0.30	1.24E-01
ENSCAFG0000000726	ACOX2	grey	VSMC_M10	0.30	1.34E-01	0.07	7.15E-01	0.06	7.83E-01	-0.28	1.55E-01	0.24	2.26E-01	-0.15	4.49E-01	-0.13	5.06E-01	0.21	3.02E-01	-0.10	6.20E-01	-0.38	4.81E-02
ENSCAFG0000000723	PDCD2L	yellow	VSMC_M3	0.30	1.34E-01	0.17	3.94E-01	0.70	4.79E-05	-0.47	1.33E-02	-0.52	5.68E-03	-0.09	6.43E-01	0.08	6.93E-01	0.29	1.38E-01	-0.27	1.68E-01	-0.09	6.62E-01
ENSCAFG0000000521	ULK4	darkgrey	VSMC_M8	0.30	1.34E-01	-0.42	3.02E-02	0.18	3.78E-01	-0.68	1.07E-04	0.14	4.72E-01	-0.08	6.91E-01	0.39	4.54E-02	0.70	5.44E-05	0.22	2.61E-01	-0.71	3.95E-05
ENSCAFG0000001175	RCC1	darkgrey	VSMC_M8	0.30	1.34E-01	-0.35	6.96E-02	0.33	8.87E-02	-0.78	1.84E-06	-0.08	6.93E-01	0.12	5.66E-01	0.34	8.38E-02	0.83	9.86E-08	0.11	5.71E-01	-0.65	2.11E-04
ENSCAFG0000003220	ENSCAFG0000003220	red	VSMC_M1	0.30	1.34E-01	0.31	1.21E-01	0.12	5.42E-01	0.15	4.54E-01	-0.15	4.42E-01	0.13	5.13E-01	0.17	4.09E-01	-0.12	5.40E-01	-0.45	1.82E-02	0.41	3.59E-02
ENSCAFG0000001460	NUDT13	cyan	VSMC_M2	0.30	1.34E-01	0.60	1.05E-03	0.17	4.07E-01	0.43	2.69E-02	-0.19	3.47E-01	0.11	5.93E-01	-0.22	2.69E-01	-0.57	1.80E-03	-0.62	6.22E-04	0.62	5.42E-04
ENSCAFG0000001230	TNS3	cyan	VSMC_M2	0.30	1.35E-01	0.78	1.30E-06	-0.07	7.36E-01	0.65	2.20E-04	0.08	7.07E-01	-0.14	4.83E-01	-0.59	1.12E-03	-0.80	4.76E-07	-0.70	4.94E-05	0.67	1.41E-04
ENSCAFG0000000742	CBFA2T2	grey	VSMC_M10	0.30	1.35E-01	0.51	6.08E-03	0.12	5.52E-01	0.23	2.42E-01	-0.02	9.25E-01	-0.10	6.29E-01	-0.16	4.20E-01	-0.44	2.27E-02	-0.54	3.68E-03	0.34	8.09E-02
ENSCAFG0000001043	CACNA2D2	cyan	VSMC_M2	0.30	1.35E-01	0.61	7.21E-04	0.43	2.39E-02	-0.06	7.66E-01	-0.42	3.03E-02	0.01	9.69E-01	0.28	1.56E-01	-0.13	5.09E-01	-0.67	1.21E-04	0.48	1.11E-02
ENSCAFG0000001151	DET1	grey	VSMC_M10	0.30	1.35E-01	0.03	8.94E-01	-0.25	2.01E-01	0.00	9.84E-01	0.31	1.14E-01	0.13	5.20E-01	0.16	4.18E-01	0.02	9.31E-01	-0.16	4.12E-01	0.11	5.98E-01
ENSCAFG0000000036	SQSTM1	yellow	VSMC_M3	0.30	1.35E-01	0.59	1.31E-03	0.89	4.23E-10	-0.56	2.29E-03	-0.66	1.92E-04	-0.01	9.57E-01	-0.01	9.72E-01	0.20	3.15E-01	-0.56	2.25E-03	0.00	9.83E-01
ENSCAFG0000000984	PCIF1	yellow	VSMC_M3	0.29	1.35E-01	0.12	5.45E-01	0.52	5.35E-03	-0.62	6.34E-04	-0.28	1.60E-01	-0.17	3.98E-01	0.08	7.06E-01	0.47	1.41E-02	-0.16	4.24E-01	-0.28	1.59E-01
ENSCAFG0000000682	SNPH	grey	VSMC_M10	0.29	1.35E-01	0.19	3.55E-01	-0.21	3.00E-01	0.34	7.93E-02	0.28	1.54E-01	-0.01	9.51E-01	0.16	4.34E-01	-0.39	4.65E-02	-0.27	1.70E-01	0.27	1.71E-01

ENSCAFG0000000488	CRYGN	grey	VSMC_M10	0.29	1.35E-01	0.49	8.96E-03	-0.04	8.49E-01	0.59	1.27E-03	0.09	6.67E-01	-0.19	3.39E-01	-0.33	9.34E-02	-0.62	6.07E-04	-0.46	1.60E-02	0.45	2.00E-02
ENSCAFG0000000522	PLB1	cyan	VSMC_M2	0.29	1.35E-01	0.77	2.91E-06	0.53	4.40E-03	-0.02	9.40E-01	-0.49	9.08E-03	0.02	9.11E-01	-0.27	1.69E-01	-0.26	1.86E-01	-0.80	5.40E-07	0.49	9.06E-03
ENSCAFG0000002950	ENSCAFG0000002950	grey	VSMC_M10	0.29	1.35E-01	0.13	5.06E-01	0.26	1.89E-01	-0.20	3.28E-01	0.02	9.10E-01	-0.16	4.16E-01	-0.02	9.19E-01	0.03	8.81E-01	-0.19	3.46E-01	-0.24	2.30E-01
ENSCAFG0000001750	ANP32A	darkgrey	VSMC_M8	0.29	1.36E-01	-0.12	5.43E-01	0.59	1.11E-03	-0.77	3.02E-06	-0.30	1.26E-01	-0.08	7.07E-01	0.31	1.21E-01	0.70	4.25E-05	-0.04	8.44E-01	-0.52	5.48E-03
ENSCAFG0000001965	MED16	yellow	VSMC_M3	0.29	1.36E-01	0.44	2.05E-02	0.73	1.67E-05	-0.50	7.26E-03	-0.57	2.06E-03	0.15	4.69E-01	-0.03	8.63E-01	0.22	2.68E-01	-0.46	1.56E-02	0.05	8.19E-01
ENSCAFG0000002017	TGFBR3	yellow	VSMC_M3	0.29	1.36E-01	0.49	9.77E-03	0.49	9.97E-03	-0.29	1.45E-01	-0.31	1.19E-01	-0.20	3.20E-01	0.32	9.87E-02	0.10	6.05E-01	-0.55	2.90E-03	0.10	6.19E-01
ENSCAFG0000001773	ENSCAFG0000001773	pink	VSMC_M5	0.29	1.36E-01	-0.22	2.69E-01	-0.16	4.19E-01	-0.23	2.44E-01	0.47	1.33E-02	-0.20	3.20E-01	0.02	9.36E-01	0.24	2.27E-01	0.16	4.32E-01	-0.50	7.92E-03
ENSCAFG0000000714	TM9SF4	grey	VSMC_M10	0.29	1.36E-01	0.32	1.08E-01	-0.32	1.09E-01	0.45	1.99E-02	0.34	7.94E-02	-0.06	7.84E-01	-0.16	4.14E-01	-0.51	6.60E-03	-0.29	1.43E-01	0.35	7.73E-02
ENSCAFG0000001173	CADM3	violet	VSMC_M7	0.29	1.37E-01	0.03	8.84E-01	0.32	1.03E-01	-0.28	1.50E-01	-0.18	3.70E-01	0.16	4.27E-01	0.60	8.80E-04	0.21	2.82E-01	-0.22	2.63E-01	-0.06	7.49E-01
ENSCAFG0000003259	SNRPG	red	VSMC_M1	0.29	1.37E-01	0.09	6.47E-01	0.30	1.34E-01	-0.08	6.83E-01	-0.05	8.03E-01	-0.32	1.05E-01	0.06	7.82E-01	-0.05	8.20E-01	-0.10	6.03E-01	-0.14	4.74E-01
ENSCAFG0000000851	GABPA	pink	VSMC_M5	0.29	1.37E-01	-0.07	7.44E-01	-0.31	1.13E-01	-0.04	8.51E-01	0.58	1.47E-03	-0.24	2.28E-01	0.12	5.44E-01	0.05	8.02E-01	-0.03	8.90E-01	-0.28	1.53E-01
ENSCAFG0000001392	PLCD3	cyan	VSMC_M2	0.29	1.37E-01	0.60	9.21E-04	0.33	9.43E-02	0.09	6.64E-01	-0.24	2.30E-01	0.16	4.11E-01	-0.08	6.78E-01	-0.36	6.56E-02	-0.51	6.40E-03	0.40	4.09E-02
ENSCAFG0000002925	ENSCAFG0000002925	grey	VSMC_M10	0.29	1.37E-01	-0.11	5.82E-01	-0.13	5.14E-01	-0.23	2.56E-01	0.27	1.67E-01	0.00	9.81E-01	0.25	2.03E-01	0.25	2.16E-01	-0.07	7.41E-01	-0.18	3.74E-01
ENSCAFG0000000768	MFAP3L	grey	VSMC_M10	0.29	1.37E-01	-0.12	5.53E-01	-0.19	3.37E-01	0.24	2.34E-01	0.25	2.14E-01	0.35	7.68E-02	0.12	5.38E-01	-0.08	6.77E-01	0.00	9.88E-01	0.04	8.24E-01
ENSCAFG0000000925	PTPN22	darkgreen	VSMC_M4	0.29	1.37E-01	0.09	6.64E-01	-0.55	2.91E-03	0.60	9.48E-04	0.48	1.11E-02	0.15	4.57E-01	-0.15	4.61E-01	-0.49	9.26E-03	-0.20	3.23E-01	0.40	3.72E-02
ENSCAFG0000000827	ENSCAFG0000000827	grey	VSMC_M10	0.29	1.37E-01	0.43	2.64E-02	0.61	6.61E-04	-0.26	1.87E-01	-0.54	3.40E-03	0.05	8.00E-01	-0.22	2.67E-01	0.13	5.30E-01	-0.53	4.26E-03	0.20	3.12E-01
ENSCAFG0000000444	ENSCAFG0000000444	grey	VSMC_M10	0.29	1.38E-01	0.32	1.01E-01	0.33	9.49E-02	0.11	5.73E-01	-0.19	3.34E-01	0.12	5.58E-01	-0.40	3.98E-02	-0.19	3.44E-01	-0.30	1.22E-01	0.13	5.03E-01
ENSCAFG0000001031	ELMO2	grey	VSMC_M10	0.29	1.38E-01	-0.37	5.86E-02	-0.13	5.16E-01	-0.22	2.63E-01	0.49	8.90E-03	-0.36	6.55E-02	-0.05	7.94E-01	0.32	1.05E-01	0.28	1.53E-01	-0.69	6.89E-05
ENSCAFG0000001136	ENSCAFG0000001136	cyan	VSMC_M2	0.29	1.38E-01	0.41	3.20E-02	0.10	6.27E-01	-0.10	6.26E-01	0.17	4.04E-01	-0.29	1.35E-01	-0.10	6.13E-01	-0.14	4.77E-01	-0.35	7.21E-02	0.01	9.73E-01
ENSCAFG0000003059	ENSCAFG0000003059	grey	VSMC_M10	0.29	1.38E-01	0.35	7.29E-02	0.01	9.45E-01	-0.03	8.88E-01	0.25	2.13E-01	-0.20	3.09E-01	-0.28	1.63E-01	-0.13	5.26E-01	-0.30	1.22E-01	-0.10	6.07E-01
ENSCAFG0000001514	SYN1	grey	VSMC_M10	0.29	1.38E-01	-0.04	8.31E-01	-0.23	2.45E-01	-0.03	8.71E-01	0.40	4.07E-02	0.08	7.09E-01	0.28	1.62E-01	0.06	7.70E-01	-0.07	7.21E-01	-0.19	3.34E-01
ENSCAFG0000000170	SND1	yellow	VSMC_M3	0.29	1.38E-01	0.27	1.71E-01	0.63	3.97E-04	-0.46	1.56E-02	-0.51	6.52E-03	0.09	6.53E-01	0.03	8.94E-01	0.32	1.03E-01	-0.40	3.77E-02	-0.01	9.77E-01
ENSCAFG0000001796	ENSCAFG0000001796	grey	VSMC_M10	0.29	1.38E-01	0.07	7.33E-01	0.48	1.11E-02	-0.41	3.33E-02	-0.29	1.35E-01	0.11	5.94E-01	-0.05	8.19E-01	0.28	1.53E-01	-0.16	4.36E-01	-0.26	1.95E-01
ENSCAFG0000000173	CNPY3	cyan	VSMC_M2	0.29	1.38E-01	0.59	1.26E-03	0.38	5.31E-02	0.09	6.68E-01	-0.23	2.44E-01	-0.23	2.58E-01	-0.15	4.55E-01	-0.34	8.59E-02	-0.54	3.64E-03	0.25	2.14E-01
ENSCAFG0000000171	TEK	grey	VSMC_M10	0.29	1.38E-01	0.63	4.04E-04	0.07	7.19E-01	0.36	6.86E-02	-0.11	5.99E-01	0.30	1.35E-01	-0.50	7.75E-03	-0.47	1.23E-02	-0.65	2.11E-04	0.56	2.53E-03
ENSCAFG0000001073	NFKB1	cyan	VSMC_M2	0.29	1.38E-01	0.58	1.51E-03	0.13	5.23E-01	0.15	4.59E-01	-0.11	5.90E-01	0.27	1.74E-01	-0.21	2.90E-01	-0.28	1.56E-01	-0.66	2.08E-04	0.43	2.36E-02
ENSCAFG0000000780	ENSCAFG0000000780	grey	VSMC_M10	0.29	1.38E-01	0.19	3.45E-01	-0.02	9.08E-01	0.24	2.30E-01	0.09	6.49E-01	-0.19	3.38E-01	0.03	8.64E-01	-0.22	2.65E-01	-0.28	1.60E-01	0.29	1.46E-01
ENSCAFG0000000917	RAD54B	grey	VSMC_M10	0.29	1.39E-01	-0.18	3.71E-01	0.10	6.05E-01	-0.21	2.91E-01	-0.07	7.12E-01	0.14	4.98E-01	0.51	6.45E-03	0.34	8.63E-02	-0.10	6.17E-01	0.00	9.92E-01
ENSCAFG0000002369	ENSCAFG0000002369	grey	VSMC_M10	0.29	1.39E-01	-0.09	6.49E-01	0.33	9.44E-02	-0.24	2.28E-01	-0.11	5.78E-01	-0.13	5.34E-01	0.21	2.88E-01	0.19	3.41E-01	-0.02	9.31E-01	-0.25	2.03E-01
ENSCAFG0000000849	MEIS2	darkgreen	VSMC_M4	0.29	1.39E-01	0.46	1.50E-02	-0.24	2.38E-01	0.71	3.78E-05	0.24	2.37E-01	-0.04	8.55E-01	-0.54	3.32E-03	-0.74	1.24E-05	-0.44	2.01E-02	0.51	6.47E-03
ENSCAFG0000003253	RGS18	yellow	VSMC_M3	0.29	1.39E-01	0.29	1.49E-01	0.56	2.22E-03	-0.16	4.13E-01	-0.33	9.07E-02	-0.06	7.71E-01	-0.08	6.96E-01	0.01	9.72E-01	-0.33	9.39E-02	-0.02	9.03E-01
ENSCAFG0000001281	PPOX	yellow	VSMC_M3	0.29	1.39E-01	0.19	3.44E-01	0.64	3.10E-04	-0.37	6.00E-02	-0.39	4.30E-02	0.01	9.74E-01	0.02	9.34E-01	0.13	5.06E-01	-0.16	4.36E-01	-0.16	4.14E-01
ENSCAFG0000001953	STK11	yellow	VSMC_M3	0.29	1.39E-01	0.55	3.19E-03	0.71	3.38E-05	-0.14	4.77E-01	-0.68	9.11E-05	0.25	2.13E-01	0.06	7.62E-01	-0.01	9.75E-01	-0.65	2.57E-04	0.38	5.16E-02
ENSCAFG0000001648	ARHGEF11	grey	VSMC_M10	0.29	1.39E-01	0.68	8.39E-05	0.03	8.79E-01	0.43	2.71E-02	0.04	8.42E-01	-0.22	2.78E-01	-0.26	1.84E-01	-0.58	1.49E-03	-0.63	4.12E-04	0.50	8.32E-03
ENSCAFG0000001822	ENSCAFG0000001822	red	VSMC_M1	0.29	1.40E-01	0.32	1.07E-01	-0.08	6.79E-01	0.10	6.04E-01	0.22	2.78E-01	0.01	9.73E-01	-0.24	2.21E-01	-0.23	2.56E-01	-0.33	8.86E-02	0.10	6.06E-01
ENSCAFG0000001467	PLEKHH3	cyan	VSMC_M2	0.29	1.40E-01	0.44	2.25E-02	0.42	2.95E-02	-0.23	2.47E-01	-0.09	6.60E-01	-0.33	9.69E-02	-0.02	9.08E-01	-0.05	8.23E-01	-0.38	5.36E-02	-0.17	3.94E-01
ENSCAFG0000001606	WIPI2	grey	VSMC_M10	0.29	1.40E-01	-0.04	8.27E-01	0.03	8.64E-01	0.04	8.62E-01	0.27	1.78E-01	-0.18	3.68E-01	-0.20	3.07E-01	-0.12	5.40E-01	0.11	5.78E-01	-0.39	4.52E-02
ENSCAFG0000001959	G6PD	darkgrey	VSMC_M8	0.29	1.40E-01	0.06	7.53E-01	0.69	7.70E-05	-0.81	3.83E-07	-0.47	1.39E-02	0.07	7.29E-01	0.33	9.28E-02	0.64	3.20E-04	-0.21	3.04E-01	-0.33	9.69E-02
ENSCAFG0000000470	ASIC3	grey	VSMC_M10	0.29	1.40E-01	-0.07	7.44E-01	-0.06	7.62E-01	-0.21	2.93E-01	0.17	3.97E-01	0.17	3.87E-01	0.14	4.91E-01	0.26	1.82E-01	-0.13	5.11E-01	-0.15	4.64E-01
ENSCAFG0000001143	SCN9A	pink	VSMC_M5	0.29	1.41E-01	0.24	2.38E-01	-0.35	7.20E-02	0.31	1.18E-01	0.59	1.35E-03	-0.39	4.23E-02	-0.35	7.60E-02	-0.39	4.71E-02	-0.18	3.64E-01	0.04	8.50E-01
ENSCAFG0000001438	TMEM101	cyan	VSMC_M2	0.29	1.41E-01	0.72	2.06E-05	0.56	2.20E-03	-0.04	8.36E-01	-0.55	3.02E-03	0.03	8.99E-01	-0.15	4.59E-01	-0.19	3.32E-01	-0.74	1.02E-05	0.48	1.17E-02
ENSCAFG0000001783	CCNK	pink	VSMC_M5	0.29	1.41E-01	-0.38	5.31E-02	-0.19	3.48E-01	-0.22	2.62E-01	0.45	1.87E-02	-0.06	7.48E-01	-0.08	6.81E-01	0.37	5.80E-02	0.25	2.00E-01	-0.61	6.65E-04
ENSCAFG0000001725	ENSCAFG0000001725	cyan	VSMC_M2	0.29	1.41E-01	0.91	6.49E-11	0.26	1.99E-01	0.39	4.36E-02	-0.18	3.72E-01	-0.16	4.29E-01	-0.45	1.77E-02	-0.71	3.63E-05	-0.76	4.52E-06	0.63	4.44E-04
ENSCAFG0000001549	DLG5	cyan	VSMC_M2	0.29	1.41E-01	0.57	1.82E-03	0.62	5.40E-04	-0.19	3.38E-01	-0.49	9.61E-03	0.15	4.57E-01	-0.05	7.86E-01	-0.10	6.06E-01	-0.53	4.26E-03	0.30	1.27E-01
ENSCAFG0000000992	XPO7	grey	VSMC_M10	0.29	1.41E-01	-0.09	6.48E-01	-0.08	6.92E-01	-0.33	9.17E-02	0.35	7.55E-02	-0.01	9.60E-01	0.29	1.38E-01	0.25	2.02E-01	-0.01	9.55E-01	-0.37	5.96E-02
ENSCAFG0000000480	ENSCAFG0000000480	red	VSMC_M1	0.29	1.41E-01	0.18	3.68E-01	0.29	1.45E-01	-0.12	5.40E-01	-0.03	8.93E-01	-0.10	6.09E-01	-0.11	5.75E-01	0.03	8.80E-01	-0.19	3.49E-01	-0.23	2.56E-01
ENSCAFG0000000650	RBPMS	grey	VSMC_M10	0.29	1.41E-01	0.52	5.89E-03	-0.17	4.04E-01	0.54	3.92E-03	0.20	3.20E-01	0.07	7.12E-01	-0.68	1.10E-04	-0.69	8.05E-05	-0.42	3.12E-02	0.43	2.34E-02
ENSCAFG0000002194	ENSCAFG0000002194	cyan	VSMC_M2	0.29	1.41E-01	0.55	2.90E-03	0.46	1.60E-02	0.02	9.26E-01	-0.33	9.34E-02	-0.17	4.08E-01	-0.03	8.67E-01	-0.30	1.25E-01	-0.50	7.27E-03	0.28	1.61E-01
ENSCAFG0000000569	RPTOR	grey	VSMC_M10	0.29	1.42E-01	0.41	3.56E-02	0.56	2.54E-03	-0.21	2.94E-01	-0.49	8.98E-03	0.33	9.11E-02	-0.17	3.99E-01	-0.03	8.73E-01	-0.43	2.49E-02	0.20	3.11E-01
ENSCAFG0000003063	ENSCAFG0000003063	grey	VSMC_M10	0.29	1.42E-01	0.35	7.67E-02	0.35	7.23E-02	0.02	9.15E-01	-0.25	2.01E-01	-0.13	5.07E-01	-0.04	8.33E-01	-0.21	3.04E-01	-0.35	7.02E-02	0.16	4.19E-01
ENSCAFG0000001257	ZNF148	pink	VSMC_M5	0.29	1.42E-01	-0.30	1.25E-01	-0.33	9.38E-02	0.16	4.20E-01	0.59	1.15E-03	-0.20	3.30E-01	-0.36	6.75E-02	-0.01	9.77E-01	0.27	1.73E-01	-0.45	1.93E-02
ENSCAFG0000001894	PLIN3	grey	VSMC_M10	0.29	1.42E-01	0.58	1.35E-03	0.00	9.88E-01	0.45	1.82E-02	0.04	8.50E-01	0.27	1.73E-01	-0.20	3.12E-01	-0.51	6.31E-03	-0.59	1.12E-03	0.51	6.97E-03
ENSCAFG0000001092	PKNOX2	cyan	VSMC_M2	0.29	1.42E-01	0.87	4.68E-09	0.14	4.92E-01	0.52	5.82E-03	-0.10	6.21E-01	-0.20	3.10E-01	-0.58	1.38E-03	-0.73	1.52E-05	-0.74	9.60E-06	0.65	2.77E-04
ENSCAFG0000001500	CDK16	darkgrey	VSMC_M8	0.29	1.42E-01	-0.26	1.97E-01	0.10	6.35E-01	-0.60	9.13E-04	0.10	6.37E-01	0.17	4.04E-01	0.24	2.30E-01	0.61	6.80E-04	0.06	7.62E-01	-0.41	3.25E-02
ENSCAFG0000002494	KBTBD7	grey	VSMC_M10	0.29	1.42E-01	0.08	6.87E-01	0.29	1.48E-01	-0.11	5.71E-01	-0.24	2.36E-01	0.20	3.08E-01	0.12	5.52E-01	0.14	5.00E-01	-0.23	2.42E-01	0.13	5.31E-01
ENSCAFG0000001625	MCC	cyan	VSMC_M2	0.29	1.42E-01	0.56	2.33E-03	0.40	3.84E-02	-0.08	6.94E-01	-0.28	1.58E-01	0.10	6.09E-01	-0.32	1.02E-01	-0.21	2.91E-01	-0.52	5.45E-03	0.26	1.85E-01
ENSCAFG0000000966	MATN4	grey	VSMC_M10	0.29	1.43E-01	0.74	1.06E-05	0.01	9.53E-01	0.45	1.77E-02	0.01	9.51E-01	-0.05	8.23E-01	-0.36	6.25E-02	-0.59	1.22E-03	-0.72	2.39E-05	0.62	5.24E-04
ENSCAFG0000001858	STAG2	pink	VSMC_M5	0.29	1.43E-01	-0.42	3.09E-02	-0.27	1.74E-01	-0.18	3.80E-01	0.60	8.98E-04	-0.17	4.05E-01	0.26	1.84E-01	0.22	2.77E-01	0.32	1.03E-01	-0.52	5.06E-03
ENSCAFG0000000642	APBB1	cyan	VSMC_M2	0.29	1.43E-01	0.69	5.85E-05	0.44	2.32E-02	0.06	7.64E-01	-0.25	2.09E-01	-0.21	2.84E-01	-0.13	5.21E-01	-0.31	1.17E-01	-0.61	7.78E-04	0.26	1.83E-01
ENSCAFG0000003253	RCHY1	red	VSMC_M1	0.29	1.43E-01	-0.06	7.61E-01	-0.19	3.34E-01	0.11	5.96E-01	0.34	8.50E-02	-0.24	2.20E-01	-0.13	5.12E-01	-0.06	7.83E-01	-0.03	8.86E-01	-0.12	5.59E-01
ENSCAFG0000000456	NUFIP1	darkgrey	VSMC_M8	0.29	1.43E-01	-0.54	3.46E-03	-0.06	7.73E-01	-0.43	2.42E-02	0.21	2.92E-01	-0.06	7.53E-01	0.34	8.51E-02	0.57	1.78E-03	0.29	1.38E-01	-0.49	8.92E-03
ENSCAFG0000001050	NDUFV3	grey	VSMC_M10	0.29	1.43E-01	-0.02	9.19E-01	0.35	7.35E-02	-0.47	1.27E-02	-0.17	3.95E-01	0.07	7.30E-01	0.04	8.33E-01	0.42	3.06E-02	-0.08	7.00E-01	-0.29	1.37E-01
ENSCAFG0000001811	CDH2	darkgreen	VSMC_M4	0.29	1.44E-01	0.39	4.37E-02	-0.35	7.11E-02	0.65	2.78E-04	0.29	1.40E-01	-0.16	4.36E-01	-0.49	1.01E-02	-0.58	1.43E-03	-0.44	2.26E-02	0.52	5.60E-03
ENSCAFG0000001202	ENSCAFG0000001202	cyan	VSMC_M2	0.29	1.44E-01	0.93	3.59E-12	0.25	2.10E-01	0.34	7.81E-02	-0.18	3.72E-01	-0.21	2.92E-01	-0.37	5.47E-02	-0.64	2.86E-04	-0.82	1.69E-07	0.63	4.88E-04
ENSCAFG0000001049	CDON	grey	VSMC_M10	0.29	1.44E-01	0.22	2.71E-01	-0.20	3.27E-01	0.27	1.71E-01	0.30	1.32E-01	-0.18	3.64E-01	0.33	9.51E-02	-0.40	4.07E-02	-0.25	2.02E-01	0.23	2.50E-01
ENSCAFG0000001200	IRF9	grey	VSMC_M10	0.29	1.44E-01	0.82	1.78E-07	0.38	5.21E-02	0.41	3.47E-02	-0.37	5.68E-02	0.04	8.45E-01	-0.19	3.33E-01	-0.64	3.10E-04	-0.76	4.79E-06	0.67	1.23E-04
ENSCAFG0000002486	ENSCAFG0000002486	grey	VSMC_M10	0.29	1.44E-01	-0.14	4.91E-01	-0.12	5.51E-01	0.20	3.07E-01	0.24	2.18E-01	-0.06	7.61E-01	0.39	4.29E-02	-0.11	5.97E-01	-0.03	8.84E-01	-0.01	9.45E-01
ENSCAFG0000001051	PKNOX1	grey	VSMC_M10	0.29	1.44E-01	-0.14	4.71E-01	-0.04	8.57E-01	-0.32	1.03E-01	0.16	4.17E-01	0.01	9.42E-01	0.41	3.38E-02	0.35	7.23E-02	-0.02	9.29E-01	-0.18	3.66E-01
ENSCAFG0000001654	MYLPF	grey	VSMC_M10	0.29	1.45E-01	0.19	3.44E-01	0.03	8.68E-01	-0.06	7.81E-01	0.14	4.86E-01	-0.07	7.32E-01	-0.09	6.49E-01	-0.02	9.38E-01	-0.21	2.88E-01	0.01	9.70E-01
ENSCAFG0000002870	ENSCAFG0000002870	grey	VSMC_M10	0.29	1.45E-01	0.19	3.44E-01	0.03	8.68E-01	-0.06	7.81E-01	0.14	4.86E-01	-0.07	7.32E-01	-0.09	6.49E-01	-0.02	9.38E-01	-0.21	2.88E-01	0.01	9.70E-01
ENSCAFG0000001603	HSPA2	red	VSMC_M1	0.29	1.45E-01	0.29	1.49E-01	-0.23	2.49E-01	0.53	4.70E-03	0.24	2.19E-01	-0.22	2.69E-01	-0.12	5.53E-01	-0.56	2.63E-03	-0.28	1.50E-01	0.46	1.57E-02
ENSCAFG0000000776	EDNRA	red	VSMC_M1	0.29	1.45E-01	0.18	3.72E-01	0.08	6.79E-01	-0.13	5.14E-01	0.07	7.33E-01	0.02	9.26E-01	0.35	7.49E-02	-0.01	9.42E-01	-0.23	2.49E-01	0.07	7.19E-01
ENSCAFG0000001000	ROPN1L	grey	VSMC_M10	0.29	1.45E-01	0.49	8.99E-03	0.64	3.38E-04	-0.27	1.72E-01	-0.52	5.85E-03	0.16	4.32E-01	0.09	6.45E-01	0.08	6.86E-01	-0.51	6.21E-03	0.23	2.39E-01
ENSCAFG0000001165	TIFA	grey	VSMC_M10	0.29	1.45E-01	0.26	1.98E-01	-0.17	4.04E-01	0.24	2.37E-01	0.15	4.65E-01	0.16	4.29E-01	0.02	9.37E-01	-0.23	2.46E-01	-0.36	6.57E-02	0.30	1.34E-01
ENSCAFG0000001438	PSAP	cyan	VSMC_M2	0.29	1.45E-01	0.88	1.51E-09	0.24	2.36E-01	0.44	2.34E-02	-0.14	4.84E-01	-0.18	3.75E-01	-0.57	1.89E-03	-0.74	1.06E-05	-0.71	3.02E-05	0.57	2.01E-03
ENSCAFG0000002999	DDAH1	pink	VSMC_M5	0.29	1.46E-01	-0.03	8.64E-01	-0.47	1.27E-02	0.51	6.94E-03	0.50	8.10E-03	-0.16	4.12E-01	-0.37	5.94E-02	-0.40	3.76E-02	-0.05	7.92E-01	0.16	4.39E-01
ENSCAFG0000001859	NF1	pink	VSMC_M5	0.29	1.46E-01	0.17	4.02E-01	-0.57	1.78E-03	0.56	2.37E-03	0.71	3.02E-05	-0.25	2.17E-01	-0.19	3.44E-01	-0.56	2.54E-03	-0.18	3.71E-01	0.17	3.87E-01
ENSCAFG0000002875	PLPP3	cyan	VSMC_M2	0.29	1.46E-01	0.53	4.24E-03	0.50	7.98E-03	-0.19	3.50E-01	-0.36	6.90E-02	0.15	4.61E-01	0.01	9.59E-01	0.05	8.19E-01	-0.58	1.55E-03	0.13	5.32E-01
ENSCAFG0000000285	ETFB	grey	VSMC_M10	0.29	1.46E-01	0.25	2.04E-01	0.34	8.58E-02	0.12	5.58E-01	-0.19	3.38E-01	-0.02	9.21E-01	-0.01	9.49E-01	-0.16	4.33E-01	-0.26	1.90E-01	0.04	8.37E-01
ENSCAFG0000000552	TIMP2	cyan	VSMC_M2	0.29	1.46E-01	0.76	4.37E-06	0.21	2.82E-01	0.30	1.25E-01	-0.04	8.60E-01	-0.31	1.22E-01	-0.38	4.74E-02	-0.59	1.08E-03	-0.61	7.09E-04	0.37	5.41E-02
ENSCAFG0000000489	C19H2orf76	grey	VSMC_M10	0.29	1.46E-01	0.04	8.47E-01	0.07	7.13E-01	-0.17	3.90E-01	-0.07	7.20E-01	0.08	6.86E-01	0.40	3.86E-02	0.18	3.58E-01	-0.24	2.30E-01	0.15	4.59E-01
ENSCAFG0000000354	AP2A1	cyan	VSMC_M2	0.29	1.46E-01	0.89	8.04E-10	0.60	8.66E-04	-0.05	7.92E-01	-0.53	4.86E-03	-0.06	7.80E-01	-0.26	1.87E-01	-0.27	1.76E-01	-0.83	8.94E-08	0.51	6.74E-03
ENSCAFG0000001589	ENSCAFG0000001589	cyan	VSMC_M2	0.29	1.47E-01	0.51	6.35E-03	0.19	3.35E-01	0.18	3.77E-01	-0.06	7.81E-01	-0.25	2.00E-01	-0.14	4.88E-01	-0.35	7.46E-02	-0.47	1.34E-02	0.31	1.11E-01
ENSCAFG0000000870	CCDC102A	yellow	VSMC_M3	0.29	1.47E-01	0.58	1.36E-03	0.76	3.38E-06	-0.25	2.05E-01	-0.59	1.26E-03	-0.12	5.52E-01	-0.08	6.76E-01	0.01	9.71E-01	-0.58	1.57E-03	0.13	5.23E-01
ENSCAFG0000000488	GTF3C2	yellow	VSMC_M3	0.29	1.47E-01	0.18	3.58E-01	0.42	2.75E-02	-0.55	3.13E-03	-0.25	2.09E-01	-0.03	8.97E-01	0.09	6.39E-01	0.43	2.62E-02	-0.26	1.96E-01	-0.14	4.77E-01
ENSCAFG0000001301	RNF168	grey	VSMC_M10	0.29	1.47E-01	-0.25	2.16E-01	0.32	9.88E-02	-0.45	1.82E-02	-0.03	8.92E-01	-0.23	2.57E-01	0.13	5.03E-01	0.45	1.75E-02	0.08	6.82E-01	-0.50	8.10E-03
ENSCAFG0000000756	ANAPC10	pink	VSMC_M5	0.29	1.47E-01	-0.42	3.12E-02	-0.51	6.60E-03	0.28	1.55E-01	0.71	3.76E-05	-0.35	6.95E-02	-0.16	4.23E-01	-0.12	5.49E-01	0.35	7.51E-02	-0.31	1.14E-01
ENSCAFG0000000911	ENSCAFG0000000911	cyan	VSMC_M2	0.29	1.47E-01	0.49	1.02E-02	0.23	2.47E-01	0.00	9.91E-01	-0.06	7.63E-01	-0.08	7.02E-01	0.08	7.01E-01	-0.30	1.31E-01	-0.48	1.10E-02	0.22	2.70E-01
ENSCAFG0000001385	FYCO1	cyan	VSMC_M2	0.29	1.47E-01	0.81	2.49E-07	0.16	4.29E-01	0.42	3.12E-02	-0.04	8.33E-01	-0.23	2.39E-01	-0.30	1.27E-01	-0.75	5.97E-06	-0.66	1.67E-04	0.57	1.81E-03
ENSCAFG0000003073	MEIG1	yellow	VSMC_M3	0.29	1.47E-01	0.32	9.94E-02	0.56	2.15E-03	-0.21	2.88E-01	-0.36	6.31E-02	-0.04	8.42E-01	-0.09	6.56E-01	0.05	7.93E-01	-0.33	9.34E-02	-0.02	9.23E-01
ENSCAFG0000001161	MSL3	pink	VSMC_M5	0.29	1.48E-01	-0.36	6.41E-02	-0.48	1.09E-02	0.07	7.28E-01	0.68	9.45E-05	-0.22	2.74E-01	0.19	3.40E-01	0.02	9.10E-01	0.26	1.87E-01	-0.31	1.21E-01
ENSCAFG0000000024	PHYKPL	cyan	VSMC_M2	0.29	1.48E-01	0.63	3.91E-04	0.36	6.73E-02	0.17	3.94E-01	-0.40	4.09E-02	0.07	7.14E-01	0.06	7.78E-01	-0.33	9.30E-02	-0.66	1.78E-04	0.60	9.17E-04
ENSCAFG0000001753	ILF2	darkgrey	VSMC_M8	0.29	1.48E-01	-0.34	8.35E-02	0.35	6.99E-02	-0.81	3.62E-07	-0.10	6.24E-01	0.04	8.45E-01	0.28	1.52E-01	0.88	1.74E-09	0.11	5.75E-01	-0.66	1.56E-04
ENSCAFG0000001458	DNAJC4	grey	VSMC_M10	0.29	1.48E-01	-0.06	7.58E-01	-0.05	7.94E-01	0.09	6.54E-01	0.22	2.61E-01	-0.12	5.41E-01	-0.07	7.40E-01	0.02	9.32E-01	-0.06	7.65E-01	-0.20	3.17E-01
ENSCAFG0000001005	CTSD	cyan	VSMC_M2	0.29	1.48E-01	0.90	1.01E-10	0.62	5.33E-04	0.01	9.72E-01	-0.51	7.02E-03	-0.13	5.14E-01	-0.17	4.01E-01	-0.40	4.06E-02	-0.80	4.84E-07	0.50	8.51E-03
ENSCAFG0000002956	DIO3	grey	VSMC_M10	0.29	1.48E-01	-0.31	1.14E-01	-0.15	4.61E-01	-0.13	5.12E-01	0.36	6.42E-02	0.09	6.58E-01	-0.27	1.81E-01	0.15	4.69E-01	0.29	1.44E-01	-0.43	2.35E-02
ENSCAFG0000001895	ANKRD13B	yellow	VSMC_M3	0.29	1.48E-01	0.44	2.29E-02	0.79	9.90E-07	-0.35	6.96E-02	-0.61	6.84E-04	-0.01	9.77E-01	-0.08	6.83E-01	0.10	6.24E-01	-0.43	2.35E-02	0.05	7.90E-01
ENSCAFG0000001671	CPLX2	cyan	VSMC_M2	0.29	1.49E-01	0.62	6.08E-04	0.56	2.18E-03	-0.15	4.68E-01	-0.52	4.97E-03	0.24	2.34E-01	0.07	7.14E-01	-0.12	5.59E-01	-0.70	5.54E-05	0.45	1.93E-02
ENSCAFG0000003104	UBE2D2	pink	VSMC_M5	0.29	1.49E-01	-0.57	1.91E-03	-0.48	1.15E-02	0.02	9.23E-01	0.79	9.46E-07	-0.16	4.34E-01	-0.03	8.94E-01	0.15	4.56E-01	0.50	7.37E-03	-0.68	9.49E-05
ENSCAFG0000001563	SLC2A9	grey	VSMC_M10	0.29	1.49E-01	0.66	1.62E-04	0.05	8.11E-01	0.52	5.25E-03	-0.02	9.05E-01	0.03	8.81E-01	-0.39	4.24E-02	-0.62	6.33E-04	-0.65	2.20E-04	0.56	2.25E-03
ENSCAFG0000001861	JAK1	grey	VSMC_M10	0.29	1.49E-01	0.58	1.46E-03	-0.05	8.16E-01	0.32	1.01E-01	0.17	3.90E-01	-0.11	5.68E-01	-0.38	5.11E-02	-0.50	7.42E-03	-0.51	6.19E-03	0.30	1.27E-01
ENSCAFG0000000008	SUOX	grey	VSMC_M10	0.29	1.49E-01	0.62	5.45E-04	-0.16	4.30E-01	0.50	8.38E-03	0.24	2.19E-01	-0.14	4.99E-01	-0.14	4.85E-01	-0.64	3.17E-04	-0.57	1.81E-03	0.45	1.74E-02
ENSCAFG0000000848	PDE3B	yellow	VSMC_M3	0.29	1.49E-01	0.37	6.04E-02	0.53	4.78E-03	-0.51	7.07E-03	-0.26	1.95E-01	-0.13	5.33E-01	-0.22	2.80E-01	0.26	1.92E-01	-0.37	5.79E-02	-0.17	4.00E-01
ENSCAFG0000001683	SNX11	pink	VSMC_M5	0.29	1.49E-01	-0.41	3.54E-02	-0.42	2.96E-02	0.16	4.15E-01	0.60	1.04E-03	0.08	7.07E-01	-0.04	8.57E-01	0.01	9.80E-01	0.30	1.27E-01	-0.42	2.99E-02
ENSCAFG0000001347	FAM89B	cyan	VSMC_M2	0.29	1.49E-01	0.56	2.37E-03	0.28	1.62E-01	0.31	1.21E-01	-0.24	2.37E-01	0.09	6.61E-01	-0.23	2.59E-01	-0.53	4.86E-03	-0.45	2.00E-02	0.45	1.80E-02
ENSCAFG0000000409	TTC26	grey	VSMC_M10	0.29	1.50E-01	0.28	1.59E-01	-0.10	6.21E-01	0.16	4.33E-01	0.10	6.10E-01	-0.08	6.92E-01	0.37	5.80E-02	-0.21	2.88E-01	-0.42	2.80E-02	0.42	2.71E-02
ENSCAFG0000000496	ENSCAFG0000000496	grey	VSMC_M10	0.28	1.50E-01	0.42	2.91E-02	0.61	7.43E-04	-0.27	1.81E-01	-0.46	1.60E-02	0.09	6.59E-01	0.13	5.15E-01	0.02	9.14E-01	-0.48	1.17E-02	0.05	8.01E-01
ENSCAFG0000001423	ENSCAFG0000001423	yellow	VSMC_M3	0.28	1.50E-01	0.48	1.12E-02	0.50	8.03E-03	0.01	9.80E-01	-0.38	4.94E-02	0.03	8.95E-01	0.03	8.63E-01	-0.21	2.91E-01	-0.51	7.17E-03	0.25	2.09E-01
ENSCAFG0000000914	CHST14	cyan	VSMC_M2	0.28	1.50E-01	0.54	3.34E-03	-0.04	8.48E-01	0.31	1.16E-01	0.08	6.78E-01	0.00	9.92E-01	-0.23	2.40E-01	-0.41	3.55E-02	-0.57	2.09E-03	0.46	1.64E-02
ENSCAFG0000000073	TCP1	darkgrey	VSMC_M8	0.28	1.50E-01	-0.61	6.68E-04	-0.06	7.84E-01	-0.50	8.64E-03	0.29	1.41E-01	0.08	6.83E-01	0.02	9.09E-01	0.67	1.16E-04	0.44	2.15E-02	-0.73	1.66E-05
ENSCAFG0000000169	PCSK5	pink	VSMC_M5	0.28	1.50E-01	-0.20	3.07E-01	-0.48	1.09E-02	0.30	1.22E-01	0.69	6.57E-05	-0.05	7.97E-01	-0.21	3.04E-01	-0.23	2.58E-01	0.17	4.07E-01	-0.22	2.80E-01
ENSCAFG0000003000	COA7	grey	VSMC_M10	0.28	1.51E-01	0.11	5.92E-01	0.29	1.47E-01	-0.25	2.17E-01	-0.28	1.53E-01	0.07	7.25E-01	0.17	4.08E-01	0.27	1.78E-01	-0.31	1.21E-01	0.11	5.75E-01
ENSCAFG0000001301	THAP4	yellow	VSMC_M3	0.28	1.51E-01	0.36	6.54E-02	0.71	3.28E-05	-0.29	1.48E-01	-0.50	8.19E-03	-0.10	6.04E-01	-0.03	8.66E-01	0.05	7.98E-01	-0.37	5.60E-02	0.01	9.76E-01
ENSCAFG0000002353	SRRT	darkgrey	VSMC_M8	0.28	1.51E-01	-0.27	1.76E-01	0.45	1.78E-02	-0.79	1.16E-06	-0.15	4.43E-01	0.03	9.01E-01	0.20	3.06E-01	0.80	4.87E-07	0.04	8.39E-01	-0.67	1.13E-04
ENSCAFG0000002496	ENSCAFG0000002496	grey	VSMC_M10	0.28	1.51E-01	-0.09	6.57E-01	-0.15	4.65E-01	0.07	7.27E-01	0.42	2.86E-02	-0.38	4.81E-02	0.21	2.94E-01	-0.12	5.50E-01	0.04	8.47E-01	-0.28	1.58E-01
ENSCAFG0000000163	MLLT3	darkgreen	VSMC_M4	0.28	1.51E-01	0.62	4.98E-04	-0.06	7.68E-01	0.65	2.21E-04	0.05	8.04E-01	-0.11	5.92E-01	-0.07	7.32E-01	-0.75	6.11E-06	-0.59	1.18E-03	0.71	3.67E-05

ENSCAFG0000002908	GATA4	cyan	VSMC_M2	0.28	1.51E-01	0.72	2.12E-05	-0.06	7.75E-01	0.65	2.23E-04	0.04	8.42E-01	-0.06	7.76E-01	-0.50	8.47E-03	-0.74	9.38E-06	-0.69	7.54E-05	0.67	1.49E-04
ENSCAFG0000001436	TMEM161A	cyan	VSMC_M2	0.28	1.52E-01	0.76	3.67E-06	0.54	3.67E-03	0.01	9.73E-01	-0.56	2.19E-03	0.15	4.58E-01	-0.21	2.90E-01	-0.24	2.19E-01	-0.76	3.46E-06	0.57	2.00E-03
ENSCAFG0000001505	MAEA	cyan	VSMC_M2	0.28	1.52E-01	0.75	7.04E-06	0.30	1.35E-01	0.27	1.68E-01	-0.32	1.05E-01	0.13	5.31E-01	-0.08	6.98E-01	-0.51	7.07E-03	-0.72	2.04E-05	0.65	2.28E-04
ENSCAFG0000001496	HSD17B1	yellow	VSMC_M3	0.28	1.52E-01	0.58	1.37E-03	0.64	2.81E-04	-0.27	1.73E-01	-0.53	4.82E-03	0.24	2.24E-01	0.00	9.86E-01	-0.01	9.65E-01	-0.64	2.82E-04	0.23	2.45E-01
ENSCAFG0000001276	ENSCAFG0000001276	grey	VSMC_M10	0.28	1.52E-01	0.82	1.46E-07	0.17	3.99E-01	0.52	5.90E-03	-0.16	4.26E-01	-0.02	9.24E-01	-0.39	4.46E-02	-0.73	1.81E-05	-0.77	3.00E-06	0.67	1.16E-04
ENSCAFG0000001593	FOXP3	grey	VSMC_M10	0.28	1.52E-01	0.13	5.16E-01	-0.14	4.76E-01	0.31	1.19E-01	0.28	1.60E-01	0.18	3.72E-01	-0.21	2.85E-01	-0.23	2.39E-01	-0.16	4.12E-01	-0.01	9.61E-01
ENSCAFG0000001554	C1QBP	yellow	VSMC_M3	0.28	1.53E-01	0.07	7.45E-01	0.73	1.35E-05	-0.67	1.14E-04	-0.60	9.51E-04	0.16	4.18E-01	0.31	1.20E-01	0.61	6.69E-04	-0.30	1.30E-01	-0.23	2.48E-01
ENSCAFG0000000566	STARD10	cyan	VSMC_M2	0.28	1.53E-01	0.52	5.62E-03	0.39	4.51E-02	0.05	8.04E-01	-0.19	3.32E-01	-0.17	3.95E-01	-0.46	1.66E-02	-0.31	1.17E-01	-0.40	3.99E-02	0.09	6.69E-01
ENSCAFG0000000109	PHF5A	darkgrey	VSMC_M8	0.28	1.53E-01	-0.24	2.20E-01	0.38	4.84E-02	-0.61	6.50E-04	-0.17	3.96E-01	0.07	7.16E-01	-0.01	9.49E-01	0.68	1.06E-04	0.09	6.40E-01	-0.56	2.24E-03
ENSCAFG0000002274	MT-CYB	grey	VSMC_M10	0.28	1.54E-01	-0.08	7.03E-01	-0.42	2.78E-02	0.12	5.59E-01	0.57	1.82E-03	-0.13	5.06E-01	0.18	3.61E-01	-0.11	5.88E-01	-0.02	9.13E-01	-0.07	7.22E-01
ENSCAFG0000000311	TMEM17	grey	VSMC_M10	0.28	1.54E-01	0.27	1.72E-01	-0.01	9.71E-01	0.29	1.44E-01	-0.03	8.72E-01	0.21	3.01E-01	0.14	5.00E-01	-0.27	1.71E-01	-0.43	2.64E-02	0.39	4.46E-02
ENSCAFG0000000950	CDK2AP1	grey	VSMC_M10	0.28	1.54E-01	0.09	6.72E-01	0.16	4.21E-01	0.01	9.72E-01	-0.13	5.30E-01	0.16	4.26E-01	0.08	6.94E-01	-0.07	7.45E-01	-0.16	4.17E-01	0.15	4.47E-01
ENSCAFG0000001685	HOXB5	grey	VSMC_M10	0.28	1.54E-01	0.13	5.21E-01	0.26	1.94E-01	-0.38	5.33E-02	-0.14	4.82E-01	0.20	3.23E-01	-0.32	1.08E-01	0.29	1.49E-01	-0.23	2.43E-01	-0.11	6.01E-01
ENSCAFG0000000151	KCTD17	cyan	VSMC_M2	0.28	1.54E-01	0.75	5.67E-06	0.47	1.37E-02	0.11	5.90E-01	-0.40	3.83E-02	-0.07	7.39E-01	-0.14	5.00E-01	-0.32	1.03E-01	-0.78	1.73E-06	0.51	6.71E-03
ENSCAFG0000001043	TIRAP	cyan	VSMC_M2	0.28	1.55E-01	0.48	1.10E-02	0.13	5.28E-01	0.51	7.18E-03	-0.08	6.91E-01	-0.28	1.64E-01	-0.17	4.07E-01	-0.65	2.52E-04	-0.43	2.42E-02	0.51	6.32E-03
ENSCAFG0000001903	ENSCAFG0000001903	grey	VSMC_M10	0.28	1.55E-01	0.08	7.06E-01	0.39	4.19E-02	-0.33	9.66E-02	-0.21	3.03E-01	0.39	4.35E-02	0.12	5.65E-01	0.25	2.10E-01	-0.17	4.09E-01	-0.26	1.83E-01
ENSCAFG0000002624	ENSCAFG0000002624	grey	VSMC_M10	0.28	1.55E-01	0.41	3.36E-02	0.69	8.04E-05	-0.28	1.53E-01	-0.56	2.52E-03	0.12	5.59E-01	0.23	2.45E-01	0.12	5.53E-01	-0.52	5.67E-03	0.16	4.18E-01
ENSCAFG0000000301	ENSCAFG0000000301	grey	VSMC_M10	0.28	1.55E-01	0.29	1.40E-01	0.42	3.01E-02	-0.10	6.33E-01	-0.30	1.34E-01	0.32	1.08E-01	0.10	6.20E-01	0.02	9.04E-01	-0.38	5.32E-02	0.09	6.53E-01
ENSCAFG0000001647	NSD1	grey	VSMC_M10	0.28	1.55E-01	-0.31	1.10E-01	0.00	9.86E-01	-0.38	5.00E-02	0.24	2.26E-01	0.05	8.16E-01	0.29	1.47E-01	0.40	3.90E-02	0.12	5.67E-01	-0.43	2.42E-02
ENSCAFG0000001700	NFIX	yellow	VSMC_M3	0.28	1.55E-01	0.61	6.46E-04	0.47	1.41E-02	-0.25	2.09E-01	-0.25	2.08E-01	-0.36	6.65E-02	0.08	6.93E-01	-0.06	7.61E-01	-0.57	1.87E-03	0.15	4.55E-01
ENSCAFG0000002040	CRYZ	grey	VSMC_M10	0.28	1.55E-01	0.36	6.26E-02	-0.14	4.89E-01	0.31	1.16E-01	0.33	9.66E-02	-0.10	6.25E-01	-0.08	7.04E-01	-0.41	3.22E-02	-0.36	6.25E-02	0.10	6.16E-01
ENSCAFG0000000088	SAMM50	yellow	VSMC_M3	0.28	1.56E-01	0.25	2.01E-01	0.82	1.23E-07	-0.70	5.57E-05	-0.64	3.13E-04	0.16	4.21E-01	0.28	1.62E-01	0.47	1.31E-02	-0.38	5.20E-02	-0.16	4.20E-01
ENSCAFG0000000762	PLEKHF1	yellow	VSMC_M3	0.28	1.56E-01	0.46	1.50E-02	0.68	8.19E-05	-0.43	2.50E-02	-0.47	1.23E-02	-0.15	4.47E-01	0.30	1.23E-01	0.18	3.64E-01	-0.53	4.65E-03	0.02	9.37E-01
ENSCAFG0000001483	SH3BP2	yellow	VSMC_M3	0.28	1.56E-01	0.34	8.52E-02	0.59	1.11E-03	-0.45	1.89E-02	-0.48	1.10E-02	0.23	2.44E-01	0.37	5.58E-02	0.28	1.63E-01	-0.52	5.27E-03	0.09	6.41E-01
ENSCAFG0000000387	NUCB1	cyan	VSMC_M2	0.28	1.56E-01	0.75	7.59E-06	-0.05	8.06E-01	0.73	1.61E-05	0.09	6.59E-01	-0.19	3.49E-01	-0.49	8.90E-03	-0.91	2.40E-11	-0.61	7.05E-04	0.65	2.52E-04
ENSCAFG0000000621	SEMA3A	grey	VSMC_M10	0.28	1.56E-01	0.08	6.80E-01	0.44	2.09E-02	-0.34	8.18E-02	-0.39	4.61E-02	0.44	2.33E-02	0.50	8.38E-03	0.28	1.59E-01	-0.27	1.71E-01	0.03	8.75E-01
ENSCAFG0000001915	GLYR1	grey	VSMC_M10	0.28	1.57E-01	0.16	4.26E-01	0.05	8.00E-01	0.04	8.54E-01	0.09	6.59E-01	-0.04	8.60E-01	-0.04	8.35E-01	0.00	9.91E-01	-0.25	2.16E-01	-0.08	6.77E-01
ENSCAFG0000000642	PUS1	yellow	VSMC_M3	0.28	1.57E-01	0.31	1.14E-01	0.63	4.24E-04	-0.54	3.36E-03	-0.56	2.37E-03	0.22	2.68E-01	0.19	3.48E-01	0.32	1.01E-01	-0.45	1.99E-02	0.12	5.36E-01
ENSCAFG0000000788	RWDD4	grey	VSMC_M10	0.28	1.57E-01	0.09	6.67E-01	-0.05	7.99E-01	0.32	9.94E-02	0.10	6.12E-01	-0.11	5.77E-01	-0.23	2.59E-01	-0.24	2.33E-01	-0.12	5.57E-01	0.15	4.41E-01
ENSCAFG0000000087	RTL6	cyan	VSMC_M2	0.28	1.57E-01	0.52	5.72E-03	0.63	4.53E-04	-0.19	3.54E-01	-0.51	6.79E-03	0.15	4.70E-01	-0.19	3.54E-01	-0.04	8.56E-01	-0.57	1.74E-03	0.19	3.38E-01
ENSCAFG0000001000	MOB2	cyan	VSMC_M2	0.28	1.57E-01	0.46	1.62E-02	0.27	1.76E-01	0.04	8.56E-01	-0.04	8.62E-01	-0.15	4.68E-01	-0.06	7.78E-01	-0.28	1.59E-01	-0.35	7.33E-02	0.05	8.00E-01
ENSCAFG0000000461	APOE	cyan	VSMC_M2	0.28	1.57E-01	0.76	4.45E-06	0.56	2.47E-03	0.03	8.70E-01	-0.47	1.45E-02	0.05	8.07E-01	-0.40	4.07E-02	-0.32	1.08E-01	-0.72	2.41E-05	0.41	3.26E-02
ENSCAFG0000001719	SAMD15	cyan	VSMC_M2	0.28	1.58E-01	0.45	1.79E-02	0.21	3.04E-01	0.35	7.42E-02	-0.19	3.40E-01	-0.01	9.73E-01	0.05	8.20E-01	-0.44	2.10E-02	-0.48	1.18E-02	0.47	1.32E-02
ENSCAFG0000000414	RPN1	darkgrey	VSMC_M8	0.28	1.58E-01	-0.16	4.35E-01	0.36	6.88E-02	-0.60	1.01E-03	-0.17	3.92E-01	0.03	8.95E-01	-0.15	4.50E-01	0.63	4.31E-04	-0.03	8.94E-01	-0.45	1.93E-02
ENSCAFG0000001578	RAB8A	grey	VSMC_M10	0.28	1.58E-01	-0.42	2.86E-02	-0.15	4.60E-01	-0.30	1.28E-01	0.44	2.24E-02	-0.23	2.47E-01	0.34	8.11E-02	0.40	3.72E-02	0.28	1.58E-01	-0.64	3.07E-04
ENSCAFG0000003170	ATXN10	grey	VSMC_M10	0.28	1.58E-01	0.36	6.38E-02	-0.15	4.51E-01	0.55	2.88E-03	0.13	5.29E-01	-0.01	9.55E-01	-0.04	8.51E-01	-0.52	5.82E-03	-0.41	3.21E-02	0.46	1.47E-02
ENSCAFG0000000905	JMY	darkgreen	VSMC_M4	0.28	1.59E-01	0.34	8.07E-02	-0.37	5.40E-02	0.77	2.11E-06	0.43	2.53E-02	-0.14	4.89E-01	-0.25	2.15E-01	-0.79	1.08E-06	-0.29	1.44E-01	0.46	1.56E-02
ENSCAFG0000000282	LCA5	pink	VSMC_M5	0.28	1.59E-01	-0.23	2.47E-01	-0.27	1.77E-01	0.02	9.15E-01	0.52	5.65E-03	-0.07	7.42E-01	0.03	8.73E-01	-0.01	9.48E-01	0.16	4.12E-01	-0.34	7.81E-02
ENSCAFG0000000349	PTOV1	cyan	VSMC_M2	0.28	1.59E-01	0.82	1.61E-07	0.35	7.59E-02	0.20	3.19E-01	-0.22	2.74E-01	-0.20	3.11E-01	-0.30	1.26E-01	-0.50	8.46E-03	-0.70	5.61E-05	0.42	3.08E-02
ENSCAFG0000001416	MDGA2	grey	VSMC_M10	0.28	1.59E-01	-0.12	5.67E-01	-0.37	5.83E-02	0.31	1.10E-01	0.41	3.45E-02	-0.23	2.53E-01	-0.25	2.14E-01	-0.11	5.94E-01	-0.01	9.77E-01	0.03	8.95E-01
ENSCAFG0000000575	SLC4A9	cyan	VSMC_M2	0.28	1.59E-01	0.52	5.03E-03	0.28	1.64E-01	0.14	4.84E-01	-0.22	2.61E-01	0.23	2.57E-01	-0.29	1.48E-01	-0.25	2.15E-01	-0.55	2.70E-03	0.33	9.40E-02
ENSCAFG0000000806	TBC1D12	grey	VSMC_M10	0.28	1.59E-01	0.09	6.69E-01	0.10	6.10E-01	0.11	5.92E-01	0.11	5.86E-01	0.00	1.00E+00	-0.28	1.62E-01	-0.14	4.96E-01	-0.02	9.24E-01	-0.15	4.58E-01
ENSCAFG0000003057	ENSCAFG0000003057	grey	VSMC_M10	0.28	1.60E-01	-0.16	4.31E-01	0.18	3.78E-01	-0.23	2.52E-01	-0.03	8.87E-01	0.19	3.38E-01	-0.10	6.20E-01	0.27	1.66E-01	0.03	8.98E-01	-0.24	2.19E-01
ENSCAFG0000000391	KLHL29	grey	VSMC_M10	0.28	1.60E-01	0.05	7.90E-01	-0.05	8.14E-01	0.19	3.36E-01	0.17	4.04E-01	0.01	9.70E-01	0.04	8.31E-01	-0.24	2.19E-01	-0.05	7.96E-01	0.05	8.06E-01
ENSCAFG0000000988	GDAP2	grey	VSMC_M10	0.28	1.60E-01	0.03	8.99E-01	-0.14	5.01E-01	0.11	5.87E-01	0.25	2.08E-01	-0.05	8.00E-01	0.02	9.07E-01	-0.04	8.48E-01	-0.17	3.84E-01	0.05	8.15E-01
ENSCAFG0000001418	ENSCAFG0000001418	grey	VSMC_M10	0.28	1.60E-01	0.39	4.56E-02	0.20	3.20E-01	0.02	9.36E-01	-0.08	7.05E-01	-0.15	4.66E-01	0.06	7.50E-01	-0.19	3.38E-01	-0.37	5.44E-02	0.16	4.12E-01
ENSCAFG0000000052	ENSCAFG0000000052	red	VSMC_M1	0.28	1.60E-01	-0.02	9.24E-01	0.16	4.16E-01	-0.04	8.24E-01	0.12	5.66E-01	-0.25	2.17E-01	0.16	4.35E-01	0.06	7.67E-01	-0.04	8.38E-01	-0.28	1.53E-01
ENSCAFG0000001923	MMP23	grey	VSMC_M10	0.28	1.60E-01	0.69	6.93E-05	0.13	5.13E-01	0.45	1.97E-02	-0.17	4.00E-01	-0.01	9.54E-01	-0.21	2.96E-01	-0.56	2.47E-03	-0.73	1.88E-05	0.65	2.81E-04
ENSCAFG0000000154	ENSCAFG0000000154	cyan	VSMC_M2	0.28	1.60E-01	0.66	1.69E-04	0.31	1.21E-01	0.21	2.91E-01	-0.21	2.98E-01	-0.22	2.68E-01	-0.10	6.13E-01	-0.50	8.42E-03	-0.59	1.10E-03	0.49	9.03E-03
ENSCAFG0000000160	ENSCAFG0000000160	grey	VSMC_M10	0.28	1.60E-01	0.24	2.21E-01	0.61	8.08E-04	-0.35	7.27E-02	-0.51	6.37E-03	0.16	4.31E-01	0.28	1.59E-01	0.20	3.05E-01	-0.37	6.08E-02	0.15	4.43E-01
ENSCAFG0000002359	SLC8A1	darkgreen	VSMC_M4	0.28	1.60E-01	0.23	2.59E-01	-0.36	6.87E-02	0.63	3.80E-04	0.37	5.83E-02	0.04	8.47E-01	-0.53	4.04E-03	-0.56	2.21E-03	-0.24	2.34E-01	0.35	7.02E-02
ENSCAFG0000003107	ZFP36L2	grey	VSMC_M10	0.28	1.61E-01	0.28	1.62E-01	0.25	2.13E-01	-0.21	2.92E-01	-0.06	7.52E-01	0.01	9.46E-01	0.40	3.65E-02	0.09	6.60E-01	-0.35	7.51E-02	0.00	9.86E-01
ENSCAFG0000000472	KCNN4	darkgrey	VSMC_M8	0.28	1.61E-01	-0.15	4.66E-01	0.54	3.53E-03	-0.73	1.33E-05	-0.24	2.22E-01	-0.14	4.75E-01	0.56	2.51E-03	0.65	2.32E-04	-0.03	8.87E-01	-0.54	3.78E-03
ENSCAFG0000000587	ARHGDIA	darkgrey	VSMC_M8	0.28	1.61E-01	-0.14	4.90E-01	0.57	1.71E-03	-0.65	2.46E-04	-0.35	7.09E-02	-0.06	7.74E-01	0.14	4.86E-01	0.60	9.63E-04	0.03	8.90E-01	-0.45	1.97E-02
ENSCAFG0000000230	OMD	grey	VSMC_M10	0.28	1.61E-01	0.32	1.01E-01	-0.17	4.10E-01	0.34	8.27E-02	0.29	1.40E-01	-0.34	8.20E-02	0.20	3.09E-01	-0.36	6.23E-02	-0.33	9.24E-02	0.23	2.58E-01
ENSCAFG0000001775	UQCRC2	grey	VSMC_M10	0.28	1.62E-01	-0.35	7.02E-02	-0.23	2.41E-01	-0.17	3.87E-01	0.34	7.93E-02	0.18	3.77E-01	0.18	3.57E-01	0.34	8.63E-02	0.14	4.79E-01	-0.33	8.90E-02
ENSCAFG0000001866	POLDIP2	darkgrey	VSMC_M8	0.28	1.62E-01	-0.06	7.55E-01	0.52	5.81E-03	-0.72	2.19E-05	-0.33	9.57E-02	0.06	7.76E-01	0.41	3.31E-02	0.65	2.35E-04	-0.13	5.11E-01	-0.39	4.67E-02
ENSCAFG0000002882	ENSCAFG0000002882	grey	VSMC_M10	0.28	1.62E-01	0.04	8.25E-01	-0.10	6.19E-01	-0.14	4.98E-01	0.31	1.18E-01	-0.05	7.94E-01	0.04	8.50E-01	0.06	7.60E-01	-0.09	6.44E-01	-0.19	3.47E-01
ENSCAFG0000002356	DMD	grey	VSMC_M10	0.28	1.62E-01	0.01	9.48E-01	-0.14	4.95E-01	-0.01	9.64E-01	0.32	1.03E-01	-0.17	3.94E-01	-0.50	7.25E-03	-0.06	7.48E-01	0.04	8.60E-01	-0.20	3.19E-01
ENSCAFG0000000704	DNAH10	grey	VSMC_M10	0.28	1.62E-01	-0.22	2.80E-01	0.01	9.74E-01	-0.02	9.27E-01	0.14	4.71E-01	0.07	7.19E-01	0.19	3.42E-01	0.14	5.00E-01	0.06	7.66E-01	-0.19	3.46E-01
ENSCAFG0000000783	UBE2L6	grey	VSMC_M10	0.28	1.62E-01	0.07	7.17E-01	0.02	9.18E-01	0.00	9.89E-01	0.20	3.06E-01	-0.13	5.12E-01	0.12	5.57E-01	0.01	9.74E-01	-0.18	3.62E-01	-0.25	2.18E-01
ENSCAFG0000000117	C14H1orf35	cyan	VSMC_M2	0.28	1.63E-01	0.46	1.53E-02	0.38	5.33E-02	0.18	3.70E-01	-0.27	1.75E-01	-0.26	1.96E-01	-0.02	9.09E-01	-0.31	1.20E-01	-0.52	5.19E-03	0.28	1.51E-01
ENSCAFG0000000588	PCYT2	yellow	VSMC_M3	0.28	1.63E-01	0.30	1.34E-01	0.70	4.56E-05	-0.61	6.62E-04	-0.46	1.50E-02	-0.09	6.59E-01	0.35	6.97E-02	0.31	1.18E-01	-0.35	7.68E-02	-0.12	5.40E-01
ENSCAFG0000001885	PTPRS	cyan	VSMC_M2	0.28	1.63E-01	0.94	2.91E-13	0.38	5.19E-02	0.25	2.15E-01	-0.37	5.72E-02	-0.06	7.58E-01	-0.27	1.73E-01	-0.51	6.14E-03	-0.90	1.41E-10	0.71	3.06E-05
ENSCAFG0000002911	ENSCAFG0000002911	grey	VSMC_M10	0.28	1.63E-01	0.13	5.14E-01	-0.08	6.95E-01	-0.03	8.79E-01	0.16	4.16E-01	0.23	2.56E-01	-0.07	7.21E-01	0.01	9.74E-01	-0.19	3.45E-01	0.01	9.64E-01
ENSCAFG0000000372	QRSL1	grey	VSMC_M10	0.28	1.63E-01	0.02	9.12E-01	0.38	5.25E-02	-0.17	3.89E-01	-0.19	3.42E-01	0.06	7.82E-01	0.05	7.99E-01	0.12	5.60E-01	-0.11	5.68E-01	-0.13	5.20E-01
ENSCAFG0000001740	CHM	pink	VSMC_M5	0.28	1.63E-01	0.13	5.12E-01	-0.54	3.63E-03	0.46	1.45E-02	0.73	1.61E-05	-0.27	1.81E-01	-0.26	1.84E-01	-0.53	4.45E-03	-0.08	7.09E-01	0.05	8.04E-01
ENSCAFG0000001239	ENSCAFG0000001239	grey	VSMC_M10	0.28	1.64E-01	0.30	1.23E-01	0.35	7.34E-02	0.01	9.71E-01	-0.32	1.09E-01	-0.23	2.41E-01	0.27	1.70E-01	-0.07	7.13E-01	-0.42	3.12E-02	0.23	2.40E-01
ENSCAFG0000001051	OASL	grey	VSMC_M10	0.28	1.64E-01	0.32	1.09E-01	-0.27	1.66E-01	0.26	1.85E-01	0.30	1.30E-01	0.10	6.29E-01	-0.15	4.66E-01	-0.30	1.32E-01	-0.37	5.82E-02	0.32	1.03E-01
ENSCAFG0000003005	ZNF234	grey	VSMC_M10	0.28	1.64E-01	0.26	1.94E-01	-0.18	3.77E-01	0.35	7.43E-02	0.30	1.30E-01	-0.05	8.14E-01	-0.24	2.29E-01	-0.42	2.82E-02	-0.24	2.27E-01	0.12	5.64E-01
ENSCAFG0000001489	ENSCAFG0000001489	grey	VSMC_M10	0.28	1.64E-01	-0.12	5.67E-01	0.00	9.87E-01	-0.13	5.17E-01	0.17	4.03E-01	-0.09	6.69E-01	-0.11	5.98E-01	0.15	4.48E-01	0.09	6.73E-01	-0.24	2.26E-01
ENSCAFG0000003258	ENSCAFG0000003258	grey	VSMC_M10	0.28	1.64E-01	0.07	7.42E-01	0.37	5.77E-02	-0.29	1.45E-01	-0.20	3.09E-01	-0.18	3.72E-01	0.19	3.30E-01	0.24	2.30E-01	-0.16	4.17E-01	-0.08	6.84E-01
ENSCAFG0000001650	DHX15	darkgrey	VSMC_M8	0.28	1.64E-01	-0.50	7.68E-03	0.23	2.48E-01	-0.70	5.14E-05	-0.02	9.13E-01	0.11	5.89E-01	0.22	2.63E-01	0.80	5.10E-07	0.28	1.51E-01	-0.62	5.66E-04
ENSCAFG0000000687	MARCH8	grey	VSMC_M10	0.28	1.64E-01	0.54	3.66E-03	0.05	8.22E-01	0.52	5.20E-03	0.09	6.67E-01	-0.25	2.12E-01	-0.17	3.87E-01	-0.71	3.12E-05	-0.45	1.81E-02	0.41	3.28E-02
ENSCAFG0000001342	ACAP2	pink	VSMC_M5	0.28	1.64E-01	-0.32	1.07E-01	-0.16	4.40E-01	-0.17	3.83E-01	0.49	9.91E-03	-0.20	3.23E-01	0.05	7.88E-01	0.24	2.33E-01	0.24	2.38E-01	-0.53	4.57E-03
ENSCAFG0000001777	NEO1	cyan	VSMC_M2	0.28	1.64E-01	0.77	3.28E-06	0.12	5.60E-01	0.45	1.98E-02	-0.10	6.31E-01	-0.06	7.55E-01	-0.73	1.72E-05	-0.59	1.20E-03	-0.70	5.34E-05	0.54	3.49E-03
ENSCAFG0000001821	CSF1R	grey	VSMC_M10	0.28	1.65E-01	0.25	2.17E-01	0.54	3.69E-03	-0.20	3.08E-01	-0.41	3.51E-02	0.11	6.00E-01	0.35	7.61E-02	0.03	8.75E-01	-0.37	5.88E-02	0.12	5.67E-01
ENSCAFG0000001842	MTA1	cyan	VSMC_M2	0.28	1.65E-01	0.66	1.81E-04	0.64	2.98E-04	-0.20	3.23E-01	-0.51	6.14E-03	-0.11	5.95E-01	-0.23	2.53E-01	-0.05	8.18E-01	-0.69	5.95E-05	0.26	1.95E-01
ENSCAFG0000000948	ENSCAFG0000000948	red	VSMC_M1	0.28	1.65E-01	0.13	5.30E-01	0.00	9.97E-01	0.13	5.07E-01	-0.06	7.76E-01	0.22	2.61E-01	0.20	3.19E-01	-0.11	5.88E-01	-0.24	2.37E-01	0.28	1.51E-01
ENSCAFG0000000550	ENSCAFG0000000550	darkgrey	VSMC_M8	0.28	1.65E-01	-0.13	5.23E-01	0.34	8.68E-02	-0.45	1.93E-02	-0.23	2.49E-01	0.18	3.66E-01	0.24	2.33E-01	0.56	2.26E-03	-0.12	5.65E-01	-0.25	2.03E-01
ENSCAFG0000001234	ZMAT5	grey	VSMC_M10	0.28	1.65E-01	0.24	2.30E-01	0.53	4.44E-03	-0.14	4.86E-01	-0.46	1.67E-02	-0.18	3.65E-01	0.02	9.24E-01	0.11	5.72E-01	-0.34	8.18E-02	0.13	5.26E-01
ENSCAFG0000000308	GARS	darkgrey	VSMC_M8	0.28	1.65E-01	-0.32	1.08E-01	0.50	8.31E-03	-0.82	1.79E-07	-0.19	3.40E-01	0.01	9.53E-01	0.24	2.37E-01	0.75	5.63E-06	0.16	4.38E-01	-0.66	2.09E-04
ENSCAFG0000001441	DUSP19	grey	VSMC_M10	0.28	1.65E-01	-0.04	8.32E-01	-0.35	7.33E-02	0.14	4.84E-01	0.39	4.73E-02	-0.01	9.53E-01	0.08	6.85E-01	-0.02	9.10E-01	-0.10	6.26E-01	0.09	6.47E-01
ENSCAFG0000001838	ENSCAFG0000001838	yellow	VSMC_M3	0.28	1.65E-01	0.45	1.88E-02	0.58	1.62E-03	-0.27	1.76E-01	-0.47	1.44E-02	-0.02	9.05E-01	0.39	4.17E-02	0.06	7.54E-01	-0.57	1.93E-03	0.21	2.84E-01
ENSCAFG0000001981	AMPD2	yellow	VSMC_M3	0.27	1.65E-01	0.55	2.74E-03	0.63	4.64E-04	-0.32	9.91E-02	-0.43	2.36E-02	-0.12	5.36E-01	-0.45	1.79E-02	0.05	8.12E-01	-0.49	9.16E-03	0.04	8.45E-01
ENSCAFG0000000418	SAE1	darkgrey	VSMC_M8	0.27	1.66E-01	-0.03	8.95E-01	0.66	1.61E-04	-0.73	1.45E-05	-0.48	1.06E-02	0.02	9.19E-01	0.26	1.89E-01	0.69	6.19E-05	-0.15	4.40E-01	-0.36	6.39E-02
ENSCAFG0000001615	CASP14	yellow	VSMC_M3	0.27	1.66E-01	0.45	1.81E-02	0.49	9.19E-03	-0.20	3.29E-01	-0.36	6.43E-02	-0.09	6.58E-01	0.26	1.90E-01	0.04	8.44E-01	-0.54	3.47E-03	0.14	4.82E-01
ENSCAFG0000002349	ENSCAFG0000002349	grey	VSMC_M10	0.27	1.66E-01	-0.12	5.38E-01	-0.43	2.53E-02	0.20	3.09E-01	0.58	1.56E-03	-0.31	1.13E-01	0.14	4.81E-01	-0.14	4.88E-01	0.00	9.96E-01	-0.08	6.97E-01
ENSCAFG0000001421	KLHDC2	pink	VSMC_M5	0.27	1.66E-01	-0.28	1.64E-01	-0.42	3.10E-02	0.05	8.02E-01	0.71	3.21E-05	-0.15	4.46E-01	-0.37	5.92E-02	0.05	8.00E-01	0.28	1.59E-01	-0.51	6.21E-03
ENSCAFG0000001320	ENSCAFG0000001320	cyan	VSMC_M2	0.27	1.66E-01	0.66	1.80E-04	0.28	1.59E-01	0.34	8.08E-02	-0.26	1.88E-01	-0.15	4.67E-01	-0.11	5.80E-01	-0.54	3.61E-03	-0.65	2.80E-04	0.52	5.37E-03
ENSCAFG0000001217	TIAL1	pink	VSMC_M5	0.27	1.67E-01	-0.55	2.76E-03	-0.24	2.25E-01	-0.33	8.86E-02	0.56	2.57E-03	-0.15	4.59E-01	0.17	3.86E-01	0.49	9.43E-03	0.36	6.33E-02	-0.74	8.98E-06
ENSCAFG0000000756	CEP70	grey	VSMC_M10	0.27	1.67E-01	-0.32	1.09E-01	-0.27	1.81E-01	0.02	9.29E-01	0.50	8.30E-03	-0.38	5.35E-02	0.24	2.23E-01	0.13	5.23E-01	0.18	3.74E-01	-0.36	6.31E-02
ENSCAFG0000001287	WDR73	grey	VSMC_M10	0.27	1.67E-01	0.21	2.95E-01	-0.19	3.52E-01	0.18	3.72E-01	0.14	4.84E-01	0.09	6.41E-01	0.07	7.35E-01	-0.18	3.72E-01	-0.32	1.09E-01	0.37	6.05E-02
ENSCAFG0000001411	CABYR	darkgrey	VSMC_M8	0.27	1.67E-01	-0.05	8.17E-01	0.29	1.37E-01	-0.44	2.31E-02	-0.16	4.33E-01	-0.14	4.84E-01	0.15	4.44E-01	0.51	6.30E-03	-0.19	3.32E-01	-0.24	2.35E-01
ENSCAFG0000002928	SAT1	cyan	VSMC_M2	0.27	1.67E-01	0.71	2.98E-05	-0.07	7.11E-01	0.61	8.13E-04	0.13	5.13E-01	-0.20	3.24E-01	-0.58	1.67E-03	-0.76	4.38E-06	-0.61	8.10E-04	0.56	2.41E-03
ENSCAFG0000001386	LRRC75B	grey	VSMC_M10	0.27	1.67E-01	0.37	5.45E-02	0.35	7.69E-02	0.09	6.67E-01	-0.17	4.08E-01	-0.28	1.64E-01	-0.27	1.80E-01	-0.17	3.90E-01	-0.39	4.48E-02	0.08	6.86E-01
ENSCAFG0000001661	ZNF668	grey	VSMC_M10	0.27	1.67E-01	0.34	8.02E-02	0.13	5.02E-01	0.01	9.66E-01	-0.13	5.27E-01	0.02	9.34E-01	0.10	6.02E-01	-0.09	6.51E-01	-0.47	1.35E-02	0.38	5.37E-02
ENSCAFG0000000719	ASPH	grey	VSMC_M10	0.27	1.67E-01	0.21	3.05E-01	-0.24	2.37E-01	0.33	8.80E-02	0.36	6.22E-02	-0.25	2.01E-01	-0.43	2.63E-02	-0.26	1.92E-01	-0.26	1.98E-01	0.04	8.25E-01
ENSCAFG0000000058	ALDH7A1	grey	VSMC_M10	0.27	1.67E-01	0.38	4.84E-02	0.20	3.22E-01	0.26	1.87E-01	-0.07	7.39E-01	-0.09	6.71E-01	-0.13	5.09E-01	-0.32	1.05E-01	-0.40	3.99E-02	0.19	3.51E-01
ENSCAFG0000001016	TPMT	grey	VSMC_M10	0.27	1.68E-01	0.16	4.32E-01	0.22	2.80E-01	-0.29	1.46E-01	-0.13	5.24E-01	-0.02	9.20E-01	0.08	6.74E-01	0.31	1.20E-01	-0.32	1.07E-01	-0.01	9.41E-01
ENSCAFG0000001159	RLBP1	cyan	VSMC_M2	0.27	1.68E-01	0.49	9.13E-03	0.38	4.81E-02	0.19	3.33E-01	-0.22	2.71E-01	-0.06	7.55E-01	-0.14	4.73E-01	-0.38	5.27E-02	-0.42	2.74E-02	0.26	1.89E-01
ENSCAFG0000001202	ENSCAFG0000001202	yellow	VSMC_M3	0.27	1.68E-01	0.16	4.32E-01	0.74	8.55E-06	-0.66	1.66E-04	-0.57	1.93E-03	-0.06	7.79E-01	0.35	7.58E-02	0.50	7.84E-03	-0.34	8.75E-02	-0.16	4.18E-01
ENSCAFG0000000473	ENSCAFG0000000473	cyan	VSMC_M2	0.27	1.68E-01	0.43	2.65E-02	0.35	7.56E-02	0.12	5.41E-01	-0.30	1.29E-01	0.12	5.55E-01	-0.06	7.57E-01	-0.30	1.33E-01	-0.47	1.45E-02	0.32	1.08E-01
ENSCAFG0000001026	WBP1L	darkgreen	VSMC_M4	0.27	1.68E-01	0.49	8.70E-03	-0.12	5.47E-01	0.70	4.97E-05	0.11	5.74E-01	-0.14	4.80E-01	-0.27	1.79E-01	-0.74	1.14E-05	-0.51	7.05E-03	0.58	1.61E-03
ENSCAFG0000001470	PTGER2	grey	VSMC_M10	0.27	1.68E-01	-0.22	2.60E-01	0.29	1.43E-01	-0.38	4.77E-02	-0.15	4.55E-01	0.30	1.23E-01	0.38	4.95E-02	0.44	2.05E-02	0.06	7.51E-01	-0.22	2.71E-01
ENSCAFG0000001431	SUGP1	grey	VSMC_M10	0.27	1.68E-01	0.28	1.55E-01	0.31	1.14E-01	-0.10	6.19E-01	-0.08	7.06E-01	-0.11	5.80E-01	-0.53	4.08E-03	-0.05	8.21E-01	-0.25	2.12E-01	-0.19	3.32E-01
ENSCAFG0000001514	ANKZF1	grey	VSMC_M10	0.27	1.68E-01	0.45	1.81E-02	0.20	3.07E-01	0.27	1.72E-01	-0.10	6.37E-01	-0.17	3.85E-01	-0.01	9.44E-01	-0.43	2.43E-02	-0.48	1.08E-02	0.30	1.26E-01
ENSCAFG0000000368	DKKL1	grey	VSMC_M10	0.27	1.68E-01	0.16	4.28E-01	-0.08	6.81E-01	0.50	8.18E-03	0.07	7.26E-01	0.06	7.58E-01	-0.01	9.49E-01	-0.40	4.00E-02	-0.23	2.46E-01	0.34	8.36E-02
ENSCAFG0000001270	GATA5	cyan	VSMC_M2	0.27	1.69E-01	0.76	3.75E-06	0.19	3.41E-01	0.43	2.59E-02	-0.16	4.18E-01	-0.16	4.23E-01	-0.68	9.72E-05	-0.57	2.06E-03	-0.71	3.21E-05	0.54	3.70E-03
ENSCAFG0000000957	ERLIN1	grey	VSMC_M10	0.27	1.69E-01	-0.18	3.71E-01	0.17	3.91E-01	-0.49	9.78E-03	-0.01	9.66E-01	0.00	9.82E-01	0.30	1.30E-01	0.49	9.35E-03	0.00	9.89E-01	-0.33	9.50E-02
ENSCAFG0000000733	ATG101	darkgrey	VSMC_M8	0.27	1.69E-01	-0.07	7.28E-01	0.69	5.81E-05	-0.71	3.07E-05	-0.46	1.46E-02	0.09	6.54E-01	0.25	2.09E-01	0.64	3.48E-04	-0.06	7.78E-01	-0.45	1.73E-02
ENSCAFG0000002576	ENSCAFG0000002576	grey	VSMC_M10	0.27	1.69E-01	0.06	7.81E-01	0.13	5.32E-01	-0.30	1.33E-01	-0.01	9.58E-01	-0.16	4.27E-01	0.07	7.38E-01	0.25	2.15E-01	-0.20	3.06E-01	-0.05	8.14E-01
ENSCAFG0000001936	NTN3	grey	VSMC_M10	0.27	1.69E-01	0.15	4.53E-01	-0.08	6.77E-01	0.27	1.72E-01	0.10	6.31E-01	0.09	6.61E-01	-0.03	8.74E-01	-0.25	2.10E-01	-0.19	3.33E-01	0.25	2.06E-01
ENSCAFG0000000866	SEPT11	grey	VSMC_M10	0.27	1.69E-01	-0.03	8.97E-01	0.11	5.69E-01	-0.25	2.03E-01	0.03	8.98E-01	0.01	9.69E-01	0.48	1.10E-02	0.30	1.32E-01	-0.17	3.96E-01	-0.13	5.28E-01
ENSCAFG0000000131	EEF1D	yellow	VSMC_M3	0.27	1.69E-01	0.71	3.72E-05	0.77	2.66E-06	-0.10	6.28E-01	-0.69	7.32E-05	-0.11	5.81E-01	-0.05	7.97E-01	-0.17	4.03E-01	-0.71	2.97E-05	0.37	5.60E-02
ENSCAFG0000001343	XXYLT1	grey	VSMC_M10	0.27	1.69E-01	0.37	6.04E-02	-0.22	2.72E-01	0.54	3.82E-03	0.31	1.10E-01	-0.30	1.27E-01	-0.28	1.63E-01	-0.58	1.39E-03	-0.32	1.08E-01	0.34	8.01E-02

ENSCAFG0000002872	ENSCAFG0000002872	red	VSMC_M1	0.27	1.69E-01	0.26	1.98E-01	-0.03	8.79E-01	0.14	4.75E-01	0.15	4.40E-01	0.14	4.94E-01	-0.12	5.62E-01	-0.19	3.38E-01	-0.30	1.31E-01	0.04	8.31E-01
ENSCAFG0000000295	BCL11A	turquoise	VSMC_M6	0.27	1.69E-01	0.10	6.17E-01	0.04	8.37E-01	0.08	7.00E-01	-0.03	8.81E-01	0.24	2.34E-01	-0.06	7.78E-01	-0.10	6.06E-01	-0.19	3.52E-01	0.16	4.16E-01
ENSCAFG0000001827	ENSCAFG0000001827	grey	VSMC_M10	0.27	1.70E-01	0.03	8.98E-01	0.14	4.76E-01	-0.15	4.41E-01	-0.09	6.65E-01	-0.04	8.24E-01	0.04	8.53E-01	0.12	5.35E-01	-0.11	5.69E-01	0.08	6.75E-01
ENSCAFG0000001170	CEP95	pink	VSMC_M5	0.27	1.70E-01	-0.13	5.20E-01	-0.19	3.40E-01	0.03	8.89E-01	0.45	1.86E-02	-0.38	4.90E-02	-0.03	8.74E-01	-0.01	9.75E-01	0.08	6.94E-01	-0.31	1.15E-01
ENSCAFG0000001996	TAF1C	grey	VSMC_M10	0.27	1.70E-01	0.29	1.38E-01	0.40	3.61E-02	-0.33	8.82E-02	-0.33	8.88E-02	0.21	2.85E-01	-0.07	7.27E-01	0.16	4.34E-01	-0.31	1.19E-01	0.06	7.81E-01
ENSCAFG0000001205	PDIA5	grey	VSMC_M10	0.27	1.70E-01	0.11	5.93E-01	0.57	2.06E-03	-0.70	4.70E-05	-0.35	6.94E-02	-0.03	8.63E-01	0.02	9.32E-01	0.55	3.13E-03	-0.24	2.37E-01	-0.29	1.43E-01
ENSCAFG0000000366	SLC17A7	cyan	VSMC_M2	0.27	1.70E-01	0.29	1.40E-01	0.12	5.59E-01	0.37	6.11E-02	-0.07	7.10E-01	0.06	7.79E-01	-0.01	9.66E-01	-0.42	3.03E-02	-0.35	7.64E-02	0.33	9.48E-02
ENSCAFG0000000417	PRDM5	pink	VSMC_M5	0.27	1.70E-01	0.15	4.51E-01	-0.05	7.89E-01	0.00	9.81E-01	0.37	5.43E-02	-0.29	1.43E-01	-0.35	7.00E-02	-0.08	6.84E-01	-0.14	4.78E-01	-0.30	1.26E-01
ENSCAFG0000001778	KEAP1	grey	VSMC_M10	0.27	1.70E-01	0.40	3.86E-02	0.53	4.06E-03	-0.05	7.85E-01	-0.52	5.63E-03	0.19	3.45E-01	-0.11	5.89E-01	-0.03	8.67E-01	-0.44	2.01E-02	0.29	1.48E-01
ENSCAFG0000002306	APOL5	cyan	VSMC_M2	0.27	1.70E-01	0.67	1.19E-04	0.23	2.46E-01	0.08	6.87E-01	-0.14	4.73E-01	-0.02	9.08E-01	0.14	4.81E-01	-0.33	9.46E-02	-0.70	5.53E-05	0.45	1.93E-02
ENSCAFG0000001307	PIK3IP1	grey	VSMC_M10	0.27	1.70E-01	0.78	1.48E-06	0.09	6.61E-01	0.47	1.34E-02	-0.03	8.75E-01	-0.18	3.57E-01	-0.32	1.08E-01	-0.71	3.76E-05	-0.67	1.30E-04	0.60	1.05E-03
ENSCAFG0000001747	ZKSCAN2	darkgreen	VSMC_M4	0.27	1.70E-01	0.17	4.04E-01	-0.50	7.75E-03	0.54	3.94E-03	0.47	1.31E-02	0.00	9.86E-01	-0.04	8.32E-01	-0.44	2.01E-02	-0.30	1.30E-01	0.42	3.06E-02
ENSCAFG0000003114	ENSCAFG0000003114	pink	VSMC_M5	0.27	1.70E-01	-0.47	1.41E-02	-0.47	1.26E-02	0.11	5.82E-01	0.68	8.97E-05	0.02	9.35E-01	-0.11	5.69E-01	0.05	8.16E-01	0.38	5.38E-02	-0.48	1.20E-02
ENSCAFG0000001424	ENSCAFG0000001424	grey	VSMC_M10	0.27	1.71E-01	-0.48	1.16E-02	-0.21	2.94E-01	-0.06	7.60E-01	0.36	6.27E-02	-0.07	7.39E-01	0.09	6.46E-01	0.24	2.25E-01	0.31	1.11E-01	-0.35	7.70E-02
ENSCAFG0000001720	ENSCAFG0000001720	yellow	VSMC_M3	0.27	1.71E-01	0.69	6.31E-05	0.66	1.77E-04	-0.22	2.66E-01	-0.60	1.05E-03	0.03	8.76E-01	-0.18	3.69E-01	-0.10	6.16E-01	-0.65	2.53E-04	0.36	6.42E-02
ENSCAFG0000000007	ENSCAFG0000000007	yellow	VSMC_M3	0.27	1.71E-01	0.25	2.04E-01	0.63	4.34E-04	-0.66	2.06E-04	-0.49	1.01E-02	0.03	8.92E-01	0.24	2.26E-01	0.48	1.19E-02	-0.37	5.50E-02	-0.07	7.33E-01
ENSCAFG0000001354	SWT1	grey	VSMC_M10	0.27	1.71E-01	-0.25	2.17E-01	0.03	8.62E-01	-0.27	1.67E-01	0.18	3.70E-01	-0.03	8.75E-01	0.21	2.86E-01	0.33	9.21E-02	0.07	7.29E-01	-0.34	8.20E-02
ENSCAFG0000001240	UMPS	darkgrey	VSMC_M8	0.27	1.71E-01	-0.41	3.15E-02	-0.05	7.94E-01	-0.50	7.42E-03	0.30	1.35E-01	0.02	9.16E-01	0.31	1.13E-01	0.60	9.03E-04	0.18	3.82E-01	-0.62	5.03E-04
ENSCAFG0000000338	SH3D21	grey	VSMC_M10	0.27	1.72E-01	0.61	6.47E-04	0.48	1.19E-02	-0.12	5.53E-01	-0.47	1.31E-02	0.04	8.41E-01	0.08	6.99E-01	-0.16	4.33E-01	-0.65	2.35E-04	0.49	8.91E-03
ENSCAFG0000001078	RBM20	darkgreen	VSMC_M4	0.27	1.72E-01	0.46	1.66E-02	-0.14	4.76E-01	0.68	9.63E-05	0.09	6.58E-01	0.11	5.88E-01	-0.05	7.95E-01	-0.71	2.80E-05	-0.47	1.43E-02	0.62	5.79E-04
ENSCAFG0000003076	FAM210B	grey	VSMC_M10	0.27	1.72E-01	0.58	1.57E-03	-0.30	1.29E-01	0.71	2.85E-05	0.34	8.54E-02	-0.14	4.85E-01	-0.35	7.49E-02	-0.83	7.83E-08	-0.49	1.03E-02	0.57	2.11E-03
ENSCAFG0000002523	ENTPD5	grey	VSMC_M10	0.27	1.72E-01	0.21	2.89E-01	-0.10	6.22E-01	0.15	4.50E-01	0.12	5.63E-01	0.04	8.38E-01	-0.04	8.29E-01	-0.20	3.28E-01	-0.30	1.24E-01	0.29	1.37E-01
ENSCAFG0000000255	SRBD1	grey	VSMC_M10	0.27	1.72E-01	0.04	8.25E-01	-0.17	4.02E-01	0.00	9.87E-01	0.26	1.99E-01	0.00	9.94E-01	0.39	4.44E-02	0.08	6.93E-01	-0.26	1.85E-01	0.04	8.50E-01
ENSCAFG0000001953	FBXW5	cyan	VSMC_M2	0.27	1.72E-01	0.93	4.09E-12	0.25	2.00E-01	0.38	4.80E-02	-0.26	1.89E-01	-0.04	8.42E-01	-0.38	5.12E-02	-0.68	1.08E-04	-0.84	5.82E-08	0.73	1.48E-05
ENSCAFG0000000458	RBSN	grey	VSMC_M10	0.27	1.72E-01	0.37	5.98E-02	-0.10	6.14E-01	0.35	7.31E-02	0.15	4.61E-01	0.13	5.17E-01	-0.64	2.83E-04	-0.41	3.45E-02	-0.35	7.39E-02	0.18	3.59E-01
ENSCAFG0000000517	HYI	grey	VSMC_M10	0.27	1.72E-01	0.42	3.13E-02	0.35	7.48E-02	0.02	9.12E-01	-0.17	3.92E-01	-0.11	5.97E-01	0.21	2.82E-01	-0.21	2.98E-01	-0.49	9.86E-03	0.16	4.11E-01
ENSCAFG0000000514	SHKBP1	cyan	VSMC_M2	0.27	1.72E-01	0.54	3.55E-03	0.33	9.33E-02	-0.05	7.89E-01	-0.27	1.68E-01	-0.05	8.19E-01	-0.05	8.06E-01	-0.14	4.98E-01	-0.55	2.77E-03	0.32	1.05E-01
ENSCAFG0000000670	FLT1	grey	VSMC_M10	0.27	1.72E-01	-0.19	3.31E-01	0.17	4.05E-01	-0.43	2.38E-02	-0.04	8.34E-01	0.29	1.44E-01	0.05	8.00E-01	0.36	6.25E-02	0.10	6.17E-01	-0.25	2.06E-01
ENSCAFG0000000481	ELF1	darkgreen	VSMC_M4	0.27	1.72E-01	0.33	9.77E-02	-0.55	3.11E-03	0.78	1.59E-06	0.61	6.45E-04	-0.33	8.82E-02	-0.43	2.37E-02	-0.79	8.23E-07	-0.27	1.77E-01	0.39	4.28E-02
ENSCAFG0000000824	ENSCAFG0000000824	grey	VSMC_M10	0.27	1.73E-01	0.25	2.08E-01	0.56	2.56E-03	-0.35	7.30E-02	-0.54	4.03E-03	0.10	6.35E-01	0.32	1.07E-01	0.30	1.25E-01	-0.44	2.05E-02	0.19	3.31E-01
ENSCAFG0000000752	CBR4	grey	VSMC_M10	0.27	1.73E-01	-0.06	7.62E-01	-0.09	6.65E-01	0.00	9.84E-01	0.29	1.50E-01	0.05	8.01E-01	0.11	6.02E-01	0.01	9.79E-01	-0.03	8.86E-01	-0.17	3.97E-01
ENSCAFG0000002040	NAE1	grey	VSMC_M10	0.27	1.73E-01	-0.19	3.34E-01	-0.35	7.76E-02	-0.02	9.21E-01	0.64	3.67E-04	-0.19	3.34E-01	0.01	9.46E-01	0.09	6.48E-01	0.11	5.95E-01	-0.46	1.53E-02
ENSCAFG0000001809	ENSCAFG0000001809	grey	VSMC_M10	0.27	1.74E-01	0.19	3.45E-01	-0.17	3.97E-01	0.32	1.02E-01	0.40	3.71E-02	-0.27	1.77E-01	-0.14	4.73E-01	-0.41	3.44E-02	-0.14	4.85E-01	-0.06	7.81E-01
ENSCAFG0000000454	ENSCAFG0000000454	grey	VSMC_M10	0.27	1.74E-01	0.30	1.34E-01	0.08	7.08E-01	0.10	6.20E-01	-0.07	7.31E-01	0.10	6.05E-01	0.26	1.96E-01	-0.14	4.71E-01	-0.43	2.37E-02	0.33	9.26E-02
ENSCAFG0000000223	MIOS	darkgrey	VSMC_M8	0.27	1.74E-01	-0.33	9.11E-02	0.37	5.65E-02	-0.76	3.98E-06	-0.09	6.60E-01	0.07	7.15E-01	0.11	5.83E-01	0.75	7.69E-06	0.17	3.95E-01	-0.69	7.05E-05
ENSCAFG0000000571	CXXC5	yellow	VSMC_M3	0.27	1.74E-01	0.63	4.10E-04	0.66	1.65E-04	-0.10	6.08E-01	-0.61	6.95E-04	0.00	9.97E-01	-0.21	2.98E-01	-0.09	6.63E-01	-0.65	2.15E-04	0.38	5.13E-02
ENSCAFG0000003050	ENSCAFG0000003050	grey	VSMC_M10	0.27	1.74E-01	0.06	7.55E-01	0.47	1.43E-02	-0.54	3.51E-03	-0.24	2.35E-01	-0.12	5.58E-01	0.13	5.23E-01	0.37	5.67E-02	-0.12	5.66E-01	-0.30	1.32E-01
ENSCAFG0000001424	CEACAM1	cyan	VSMC_M2	0.27	1.74E-01	0.69	7.52E-05	-0.09	6.49E-01	0.66	1.69E-04	0.12	5.58E-01	-0.22	2.60E-01	-0.35	7.28E-02	-0.84	5.24E-08	-0.59	1.14E-03	0.65	2.54E-04
ENSCAFG0000001479	SHC4	grey	VSMC_M10	0.27	1.74E-01	0.41	3.55E-02	0.28	1.62E-01	0.13	5.32E-01	-0.32	1.06E-01	0.19	3.37E-01	0.21	2.87E-01	-0.26	1.93E-01	-0.50	7.67E-03	0.54	3.34E-03
ENSCAFG0000000342	ENSCAFG0000000342	grey	VSMC_M10	0.27	1.75E-01	0.09	6.68E-01	0.43	2.46E-02	-0.37	6.11E-02	-0.21	3.02E-01	-0.19	3.31E-01	0.23	2.44E-01	0.23	2.43E-01	-0.16	4.13E-01	-0.21	3.04E-01
ENSCAFG0000001716	ADAR	cyan	VSMC_M2	0.27	1.75E-01	0.90	1.48E-10	0.44	2.17E-02	0.23	2.51E-01	-0.41	3.34E-02	0.05	8.07E-01	-0.36	6.24E-02	-0.53	4.62E-03	-0.85	1.71E-08	0.66	1.88E-04
ENSCAFG0000000974	MRPL43	yellow	VSMC_M3	0.27	1.75E-01	0.68	9.71E-05	0.75	5.41E-06	-0.17	4.03E-01	-0.73	1.67E-05	0.05	8.12E-01	-0.09	6.62E-01	-0.07	7.16E-01	-0.74	1.14E-05	0.42	2.91E-02
ENSCAFG0000002328	SMIM14	pink	VSMC_M5	0.27	1.76E-01	-0.06	7.60E-01	-0.50	7.71E-03	0.36	6.32E-02	0.70	5.37E-05	-0.28	1.65E-01	-0.25	2.07E-01	-0.37	5.89E-02	0.10	6.20E-01	-0.15	4.49E-01
ENSCAFG0000000067	DPYS	grey	VSMC_M10	0.27	1.76E-01	-0.14	4.82E-01	0.31	1.21E-01	-0.30	1.28E-01	-0.12	5.55E-01	-0.12	5.59E-01	0.25	2.05E-01	0.24	2.29E-01	0.07	7.17E-01	-0.24	2.22E-01
ENSCAFG0000000107	H2AFY	yellow	VSMC_M3	0.27	1.76E-01	0.31	1.20E-01	0.66	2.00E-04	-0.50	7.45E-03	-0.49	1.02E-02	-0.03	8.78E-01	-0.11	6.01E-01	0.34	7.84E-02	-0.36	6.37E-02	-0.12	5.50E-01
ENSCAFG0000000436	MAGI2	pink	VSMC_M5	0.27	1.76E-01	0.18	3.77E-01	-0.58	1.42E-03	0.63	4.65E-04	0.67	1.53E-04	-0.09	6.51E-01	-0.39	4.17E-02	-0.63	4.09E-04	-0.12	5.35E-01	0.19	3.47E-01
ENSCAFG0000000972	CYFIP1	grey	VSMC_M10	0.27	1.76E-01	0.02	9.35E-01	0.06	7.67E-01	0.09	6.50E-01	0.04	8.47E-01	0.28	1.65E-01	0.30	1.27E-01	-0.12	5.45E-01	-0.08	6.74E-01	0.05	8.13E-01
ENSCAFG0000000721	ENSCAFG0000000721	grey	VSMC_M10	0.27	1.76E-01	-0.26	1.93E-01	-0.07	7.45E-01	-0.35	7.78E-02	0.31	1.22E-01	0.10	6.13E-01	0.10	6.15E-01	0.40	3.85E-02	0.12	5.35E-01	-0.47	1.28E-02
ENSCAFG0000000498	CD79A	yellow	VSMC_M3	0.27	1.76E-01	0.15	4.61E-01	0.61	8.13E-04	-0.31	1.21E-01	-0.49	9.74E-03	0.17	3.86E-01	0.18	3.65E-01	0.21	2.89E-01	-0.26	1.92E-01	-0.08	6.86E-01
ENSCAFG0000001997	HSBP1	cyan	VSMC_M2	0.27	1.77E-01	0.67	1.47E-04	0.38	5.11E-02	0.17	3.83E-01	-0.44	2.03E-02	0.13	5.34E-01	-0.02	9.15E-01	-0.38	5.35E-02	-0.72	1.99E-05	0.64	3.66E-04
ENSCAFG0000001020	BDNF	grey	VSMC_M10	0.27	1.77E-01	0.23	2.49E-01	-0.18	3.56E-01	0.40	4.12E-02	0.22	2.60E-01	0.08	7.09E-01	-0.73	1.41E-05	-0.42	3.12E-02	-0.20	3.13E-01	0.22	2.72E-01
ENSCAFG0000000721	DIMT1	grey	VSMC_M10	0.27	1.77E-01	0.33	9.06E-02	0.68	9.95E-05	-0.34	8.56E-02	-0.66	1.98E-04	0.28	1.54E-01	0.23	2.42E-01	0.25	2.14E-01	-0.50	7.72E-03	0.21	2.84E-01
ENSCAFG0000000864	FAM98B	darkgrey	VSMC_M8	0.27	1.77E-01	-0.45	1.84E-02	0.24	2.31E-01	-0.56	2.61E-03	0.08	6.84E-01	-0.18	3.76E-01	0.33	9.58E-02	0.61	7.88E-04	0.29	1.43E-01	-0.72	1.93E-05
ENSCAFG0000001609	ENSCAFG0000001609	yellow	VSMC_M3	0.27	1.77E-01	0.03	9.00E-01	0.63	4.16E-04	-0.81	2.81E-07	-0.32	1.02E-01	0.02	9.04E-01	0.08	7.07E-01	0.59	1.31E-03	-0.06	7.56E-01	-0.48	1.15E-02
ENSCAFG0000002546	DHRS2	grey	VSMC_M10	0.27	1.77E-01	0.69	6.96E-05	0.14	4.77E-01	0.48	1.10E-02	-0.18	3.77E-01	-0.14	4.86E-01	-0.49	8.98E-03	-0.62	6.02E-04	-0.64	2.92E-04	0.66	1.67E-04
ENSCAFG0000002844	ENSCAFG0000002844	cyan	VSMC_M2	0.27	1.77E-01	0.57	1.73E-03	0.49	8.71E-03	0.00	9.88E-01	-0.44	2.02E-02	0.05	8.13E-01	-0.02	9.04E-01	-0.22	2.69E-01	-0.56	2.22E-03	0.35	7.35E-02
ENSCAFG0000000587	RFTN1	cyan	VSMC_M2	0.27	1.77E-01	0.59	1.33E-03	0.02	9.19E-01	0.54	3.90E-03	0.02	9.08E-01	-0.06	7.69E-01	-0.58	1.62E-03	-0.59	1.24E-03	-0.51	6.59E-03	0.43	2.44E-02
ENSCAFG0000001075	MAP2K6	grey	VSMC_M10	0.27	1.77E-01	0.40	3.73E-02	0.51	7.04E-03	-0.22	2.66E-01	-0.45	1.91E-02	0.42	3.10E-02	0.27	1.80E-01	0.09	6.55E-01	-0.48	1.18E-02	0.21	3.05E-01
ENSCAFG0000002031	SLC7A6OS	grey	VSMC_M10	0.27	1.77E-01	0.23	2.51E-01	0.45	1.77E-02	-0.24	2.32E-01	-0.40	3.89E-02	-0.02	9.10E-01	0.22	2.65E-01	0.17	3.89E-01	-0.41	3.41E-02	0.19	3.42E-01
ENSCAFG0000000988	JARID2	grey	VSMC_M10	0.27	1.78E-01	0.17	4.02E-01	-0.07	7.43E-01	-0.04	8.46E-01	0.31	1.16E-01	0.12	5.67E-01	-0.16	4.19E-01	-0.09	6.54E-01	-0.18	3.58E-01	-0.14	4.79E-01
ENSCAFG0000000781	NR3C2	pink	VSMC_M5	0.27	1.78E-01	0.05	8.18E-01	-0.43	2.68E-02	0.38	4.90E-02	0.65	2.09E-04	-0.31	1.12E-01	-0.33	9.78E-02	-0.39	4.49E-02	0.01	9.47E-01	-0.12	5.48E-01
ENSCAFG0000000075	TNXB	grey	VSMC_M10	0.27	1.79E-01	0.41	3.20E-02	0.08	6.85E-01	0.08	6.81E-01	0.04	8.29E-01	-0.01	9.72E-01	0.38	5.06E-02	-0.25	2.14E-01	-0.47	1.24E-02	0.22	2.81E-01
ENSCAFG0000000650	ENSCAFG0000000650	darkgrey	VSMC_M8	0.27	1.79E-01	-0.11	5.88E-01	0.34	8.53E-02	-0.61	7.22E-04	-0.11	5.78E-01	0.03	9.01E-01	0.02	9.39E-01	0.58	1.39E-03	0.00	9.87E-01	-0.40	3.90E-02
ENSCAFG0000000897	EVA1C	turquoise	VSMC_M6	0.27	1.79E-01	0.24	2.27E-01	0.37	5.66E-02	-0.12	5.55E-01	-0.31	1.10E-01	0.55	3.22E-03	0.28	1.51E-01	0.09	6.69E-01	-0.37	5.87E-02	0.16	4.23E-01
ENSCAFG0000000017	ENSCAFG0000000017	cyan	VSMC_M2	0.27	1.79E-01	0.54	3.49E-03	0.42	2.91E-02	-0.06	7.74E-01	-0.31	1.16E-01	-0.09	6.39E-01	-0.22	2.64E-01	-0.22	2.61E-01	-0.50	7.68E-03	0.23	2.46E-01
ENSCAFG0000001501	LGALS3	yellow	VSMC_M3	0.27	1.79E-01	0.07	7.21E-01	0.58	1.57E-03	-0.79	1.04E-06	-0.25	2.05E-01	-0.14	4.80E-01	0.27	1.69E-01	0.55	3.06E-03	-0.14	4.84E-01	-0.45	1.78E-02
ENSCAFG0000000647	ENSCAFG0000000647	yellow	VSMC_M3	0.27	1.79E-01	0.18	3.80E-01	0.51	6.13E-03	-0.69	6.60E-05	-0.31	1.12E-01	0.00	9.80E-01	0.06	7.72E-01	0.55	2.90E-03	-0.25	2.04E-01	-0.26	1.90E-01
ENSCAFG0000001379	IFT22	grey	VSMC_M10	0.27	1.80E-01	-0.25	2.12E-01	0.27	1.68E-01	-0.45	1.90E-02	-0.07	7.27E-01	-0.15	4.48E-01	0.34	8.26E-02	0.40	3.92E-02	0.13	5.26E-01	-0.36	6.22E-02
ENSCAFG0000001717	ATXN2L	grey	VSMC_M10	0.27	1.80E-01	0.32	1.01E-01	0.25	2.13E-01	-0.20	3.23E-01	-0.16	4.14E-01	0.00	9.89E-01	0.21	2.94E-01	0.07	7.30E-01	-0.49	9.01E-03	0.21	2.84E-01
ENSCAFG0000000719	KIF2A	darkgrey	VSMC_M8	0.27	1.80E-01	-0.46	1.62E-02	0.14	4.91E-01	-0.74	1.16E-05	0.13	5.26E-01	0.04	8.26E-01	0.42	3.01E-02	0.75	6.02E-06	0.25	2.00E-01	-0.67	1.18E-04
ENSCAFG0000001307	FUCA1	cyan	VSMC_M2	0.27	1.80E-01	0.75	6.35E-06	0.06	7.78E-01	0.59	1.20E-03	-0.04	8.57E-01	-0.18	3.63E-01	-0.30	1.32E-01	-0.76	4.04E-06	-0.65	2.66E-04	0.63	4.59E-04
ENSCAFG0000001614	ENSCAFG0000001614	yellow	VSMC_M3	0.27	1.80E-01	0.48	1.04E-02	0.71	3.65E-05	-0.37	5.53E-02	-0.54	3.35E-03	0.05	7.88E-01	-0.06	7.78E-01	0.14	4.75E-01	-0.50	8.41E-03	0.04	8.35E-01
ENSCAFG0000003091	NEU2	grey	VSMC_M10	0.27	1.80E-01	0.31	1.10E-01	0.57	2.11E-03	-0.23	2.53E-01	-0.38	4.99E-02	-0.14	4.97E-01	-0.27	1.72E-01	0.11	5.86E-01	-0.35	7.49E-02	-0.02	9.17E-01
ENSCAFG0000001634	PCDH7	grey	VSMC_M10	0.27	1.80E-01	0.40	4.11E-02	0.20	3.29E-01	0.00	9.80E-01	-0.13	5.31E-01	-0.11	5.94E-01	-0.36	6.72E-02	-0.17	4.04E-01	-0.33	9.01E-02	0.26	1.86E-01
ENSCAFG0000000649	B4GALNT4	yellow	VSMC_M3	0.27	1.81E-01	-0.03	8.89E-01	0.50	7.55E-03	-0.49	9.87E-03	-0.43	2.60E-02	0.13	5.09E-01	0.19	3.43E-01	0.47	1.24E-02	-0.14	4.98E-01	-0.14	4.77E-01
ENSCAFG0000003138	C1QTNF7	grey	VSMC_M10	0.27	1.81E-01	0.55	3.24E-03	0.11	5.82E-01	0.39	4.34E-02	-0.05	8.05E-01	-0.03	8.88E-01	-0.35	7.28E-02	-0.51	6.05E-03	-0.53	4.50E-03	0.36	6.33E-02
ENSCAFG0000000474	SEPHS1	yellow	VSMC_M3	0.27	1.81E-01	0.08	6.92E-01	0.34	8.74E-02	-0.55	2.89E-03	-0.14	4.81E-01	-0.03	8.76E-01	-0.08	6.87E-01	0.43	2.45E-02	-0.14	4.95E-01	-0.23	2.42E-01
ENSCAFG0000000697	SOX12	grey	VSMC_M10	0.27	1.81E-01	0.12	5.47E-01	0.51	6.16E-03	-0.27	1.66E-01	-0.28	1.51E-01	-0.17	3.86E-01	0.11	5.91E-01	0.17	3.94E-01	-0.13	5.25E-01	-0.18	3.66E-01
ENSCAFG0000001606	MYRF	cyan	VSMC_M2	0.27	1.81E-01	0.73	1.36E-05	0.11	5.89E-01	0.48	1.21E-02	-0.13	5.03E-01	0.00	9.87E-01	-0.70	5.34E-05	-0.61	7.85E-04	-0.68	9.27E-05	0.61	7.75E-04
ENSCAFG0000001824	AP2B1	grey	VSMC_M10	0.27	1.82E-01	0.55	2.72E-03	-0.26	1.98E-01	0.67	1.14E-04	0.35	7.57E-02	-0.34	8.65E-02	-0.46	1.58E-02	-0.81	2.95E-07	-0.40	4.02E-02	0.47	1.27E-02
ENSCAFG0000001664	ENSCAFG0000001664	grey	VSMC_M10	0.27	1.82E-01	0.55	3.02E-03	0.01	9.44E-01	0.24	2.34E-01	0.09	6.72E-01	-0.04	8.41E-01	-0.41	3.16E-02	-0.42	3.09E-02	-0.49	9.39E-03	0.31	1.11E-01
ENSCAFG0000002308	NABP2	yellow	VSMC_M3	0.26	1.82E-01	0.52	5.33E-03	0.73	1.73E-05	-0.37	5.88E-02	-0.49	9.09E-03	-0.28	1.64E-01	-0.15	4.68E-01	0.04	8.30E-01	-0.48	1.18E-02	0.03	8.83E-01
ENSCAFG0000000329	LRRN3	grey	VSMC_M10	0.26	1.82E-01	-0.17	3.94E-01	-0.18	3.68E-01	0.08	6.98E-01	0.27	1.72E-01	-0.18	3.71E-01	0.16	4.12E-01	0.07	7.13E-01	0.07	7.33E-01	-0.10	6.32E-01
ENSCAFG0000001650	UBQLN2	cyan	VSMC_M2	0.26	1.82E-01	0.42	2.73E-02	-0.11	6.02E-01	0.12	5.50E-01	0.19	3.40E-01	-0.18	3.80E-01	-0.30	1.25E-01	-0.29	1.42E-01	-0.41	3.36E-02	0.27	1.73E-01
ENSCAFG0000001666	GTF2A2	pink	VSMC_M5	0.26	1.82E-01	-0.42	3.07E-02	-0.34	8.54E-02	0.10	6.21E-01	0.53	4.06E-03	-0.27	1.78E-01	-0.14	4.73E-01	0.05	8.23E-01	0.34	8.24E-02	-0.36	6.88E-02
ENSCAFG0000001786	ISLR	grey	VSMC_M10	0.26	1.82E-01	0.07	7.28E-01	0.45	1.85E-02	-0.54	3.73E-03	-0.20	3.23E-01	-0.25	2.06E-01	0.45	1.94E-02	0.40	3.78E-02	-0.19	3.47E-01	-0.32	1.04E-01
ENSCAFG0000001855	ADAMTS10	grey	VSMC_M10	0.26	1.83E-01	-0.15	4.67E-01	0.03	8.70E-01	-0.26	1.83E-01	0.19	3.38E-01	-0.14	4.74E-01	0.30	1.27E-01	0.15	4.53E-01	0.10	6.12E-01	-0.29	1.38E-01
ENSCAFG0000001572	LIPA	grey	VSMC_M10	0.26	1.83E-01	0.26	1.85E-01	-0.25	2.08E-01	0.37	5.92E-02	0.43	2.46E-02	-0.18	3.74E-01	-0.15	4.47E-01	-0.45	1.91E-02	-0.17	3.93E-01	0.07	7.23E-01
ENSCAFG0000000874	EPHA3	cyan	VSMC_M2	0.26	1.83E-01	0.75	5.59E-06	0.12	5.50E-01	0.45	1.84E-02	-0.03	8.77E-01	-0.27	1.78E-01	-0.78	1.48E-06	-0.63	4.01E-04	-0.60	8.33E-04	0.46	1.66E-02
ENSCAFG0000001854	MYO1F	grey	VSMC_M10	0.26	1.83E-01	-0.16	4.16E-01	0.13	5.24E-01	-0.08	6.91E-01	0.03	8.96E-01	0.10	6.37E-01	0.05	8.10E-01	0.15	4.55E-01	0.01	9.54E-01	-0.25	2.17E-01
ENSCAFG0000000487	PTPN4	pink	VSMC_M5	0.26	1.83E-01	-0.18	3.58E-01	-0.42	2.92E-02	0.34	8.18E-02	0.44	2.15E-02	0.15	4.59E-01	-0.12	5.48E-01	-0.13	5.14E-01	0.03	8.81E-01	0.08	6.81E-01
ENSCAFG0000000810	ENSCAFG0000000810	grey	VSMC_M10	0.26	1.83E-01	-0.19	3.30E-01	-0.16	4.30E-01	-0.23	2.45E-01	0.39	4.58E-02	0.13	5.31E-01	-0.03	8.75E-01	0.25	2.10E-01	0.13	5.34E-01	-0.44	2.17E-02
ENSCAFG0000001568	OTUD5	grey	VSMC_M10	0.26	1.83E-01	0.47	1.32E-02	-0.21	2.94E-01	0.58	1.62E-03	0.31	1.21E-01	-0.22	2.76E-01	-0.50	8.60E-03	-0.70	4.50E-05	-0.31	1.10E-01	0.36	6.88E-02
ENSCAFG0000000007	DGKA	pink	VSMC_M5	0.26	1.83E-01	0.14	5.01E-01	-0.50	7.78E-03	0.54	3.85E-03	0.57	1.80E-03	-0.13	5.06E-01	-0.10	6.03E-01	-0.54	3.38E-03	-0.10	6.14E-01	0.26	1.91E-01
ENSCAFG0000000164	GCNT1	cyan	VSMC_M2	0.26	1.84E-01	0.60	1.02E-03	0.30	1.25E-01	0.21	2.88E-01	-0.16	4.19E-01	-0.18	3.73E-01	-0.22	2.60E-01	-0.37	5.83E-02	-0.51	6.34E-03	0.30	1.27E-01
ENSCAFG0000000686	THOC7	grey	VSMC_M10	0.26	1.84E-01	-0.03	8.74E-01	-0.32	1.05E-01	0.12	5.50E-01	0.46	1.69E-02	-0.23	2.39E-01	0.19	3.50E-01	-0.06	7.68E-01	-0.03	8.91E-01	-0.06	7.79E-01
ENSCAFG0000001871	ENSCAFG0000001871	grey	VSMC_M10	0.26	1.84E-01	0.34	7.91E-02	0.48	1.20E-02	-0.26	1.82E-01	-0.30	1.34E-01	-0.26	1.83E-01	-0.13	5.30E-01	0.08	7.08E-01	-0.38	5.20E-02	-0.02	9.19E-01
ENSCAFG0000002031	ENSCAFG0000002031	grey	VSMC_M10	0.26	1.84E-01	0.33	9.09E-02	0.26	1.84E-01	0.03	9.00E-01	-0.24	2.36E-01	0.17	4.09E-01	-0.05	8.12E-01	-0.02	9.09E-01	-0.45	1.96E-02	0.30	1.32E-01
ENSCAFG0000001593	CYP4F8	yellow	VSMC_M3	0.26	1.84E-01	0.48	1.18E-02	0.59	1.10E-03	-0.41	3.49E-02	-0.44	2.24E-02	-0.09	6.54E-01	0.18	3.82E-01	0.12	5.46E-01	-0.51	6.60E-03	0.14	4.78E-01
ENSCAFG0000000301	TRIL	cyan	VSMC_M2	0.26	1.84E-01	0.53	4.22E-03	0.45	1.89E-02	-0.26	1.85E-01	-0.44	2.24E-02	0.13	5.27E-01	0.10	6.03E-01	0.08	6.88E-01	-0.62	6.13E-04	0.35	7.26E-02
ENSCAFG0000002571	ENSCAFG0000002571	yellow	VSMC_M3	0.26	1.84E-01	0.42	2.77E-02	0.68	9.78E-05	-0.19	3.44E-01	-0.53	4.48E-03	0.09	6.47E-01	-0.20	3.25E-01	0.02	9.08E-01	-0.43	2.38E-02	0.11	5.68E-01
ENSCAFG0000001180	ICAM2	grey	VSMC_M10	0.26	1.84E-01	0.30	1.26E-01	0.02	9.10E-01	0.23	2.38E-01	-0.06	7.72E-01	0.53	4.05E-03	-0.29	1.42E-01	-0.25	2.06E-01	-0.39	4.44E-02	0.35	7.68E-02
ENSCAFG0000001133	MARC2	grey	VSMC_M10	0.26	1.85E-01	0.29	1.45E-01	0.10	6.22E-01	0.18	3.68E-01	-0.08	7.05E-01	0.05	8.10E-01	0.07	7.26E-01	-0.15	4.63E-01	-0.31	1.11E-01	0.30	1.25E-01
ENSCAFG0000001785	ENSCAFG0000001785	grey	VSMC_M10	0.26	1.85E-01	-0.12	5.57E-01	-0.06	7.75E-01	-0.30	1.25E-01	0.34	8.22E-02	-0.33	9.02E-02	-0.08	7.04E-01	0.25	2.06E-01	0.06	7.53E-01	-0.48	1.13E-02
ENSCAFG0000000516	ENSCAFG0000000516	grey	VSMC_M10	0.26	1.85E-01	-0.06	7.80E-01	0.16	4.14E-01	-0.14	4.89E-01	-0.06	7.85E-01	-0.05	8.07E-01	0.01	9.57E-01	0.19	3.41E-01	-0.08	6.95E-01	-0.14	4.89E-01
ENSCAFG0000001201	EIF4H	darkgrey	VSMC_M8	0.26	1.86E-01	-0.36	6.39E-02	0.19	3.39E-01	-0.65	2.33E-04	0.10	6.14E-01	-0.16	4.14E-01	0.12	5.42E-01	0.70	5.28E-05	0.25	2.13E-01	-0.66	2.00E-04
ENSCAFG0000001116	USP30	grey	VSMC_M10	0.26	1.86E-01	0.32	1.06E-01	0.23	2.55E-01	-0.06	7.74E-01	-0.13	5.11E-01	0.19	3.52E-01	-0.03	9.00E-01	-0.18	3.70E-01	-0.35	7.49E-02	0.20	3.19E-01
ENSCAFG0000000713	MIPEP	grey	VSMC_M10	0.26	1.86E-01	-0.21	2.86E-01	-0.04	8.31E-01	-0.30	1.28E-01	0.39	4.15E-02	-0.20	3.29E-01	0.11	6.02E-01	0.24	2.22E-01	0.13	5.26E-01	-0.59	1.35E-03
ENSCAFG0000001207	SLC25A20	grey	VSMC_M10	0.26	1.86E-01	0.28	1.55E-01	-0.25	2.01E-01	0.48	1.11E-02	0.14	4.75E-01	0.06	7.62E-01	0.15	4.49E-01	-0.37	5.96E-02	-0.43	2.41E-02	0.55	3.03E-03
ENSCAFG0000001929	MNT	yellow	VSMC_M3	0.26	1.86E-01	0.53	4.79E-03	0.63	4.53E-04	-0.42	2.76E-02	-0.37	5.48E-02	-0.14	4.93E-01	-0.06	7.71E-01	0.07	7.28E-01	-0.47	1.33E-02	-0.01	9.65E-01
ENSCAFG0000001995	HMCN2	grey	VSMC_M10	0.26	1.86E-01	0.20	3.11E-01	-0.28	1.51E-01	0.40	3.71E-02	0.27	1.78E-01	0.19	3.42E-01	-0.42	2.83E-02	-0.35	7.60E-02	-0.20	3.26E-01	0.27	1.67E-01
ENSCAFG0000002037	FHOD1	grey	VSMC_M10	0.26	1.86E-01	0.17	3.99E-01	0.35	7.24E-02	0.01	9.73E-01	-0.23	2.43E-01	0.23	2.56E-01	0.19	3.45E-01	-0.01	9.78E-01	-0.28	1.61E-01	-0.02	9.35E-01
ENSCAFG0000003256	NKIRAS2	grey	VSMC_M10	0.26	1.86E-01	-0.45	1.74E-02	-0.17	4.05E-01	-0.19	3.37E-01	0.40	3.72E-02	0.04	8.39E-01	0.03	8.88E-01	0.22	2.78E-01	0.38	5.10E-02	-0.53	4.17E-03
ENSCAFG0000001193	ARHGEF12	cyan	VSMC_M2	0.26	1.86E-01	0.47	1.24E-02	0.00	9.93E-01	0.19	3.52E-01	0.18	3.75E-01	-0.19	3.51E-01	-0.32	1.02E-01	-0.38	4.98E-02	-0.33	8.94E-02	0.21	2.85E-01
ENSCAFG0000001709	ZNF609	grey	VSMC_M10	0.26	1.86E-01	0.50	8.33E-03	0.01	9.47E-01	0.39	4.70E-02	-0.02	9.34E-01	0.06	7.85E-01	-0.25	2.07E-01	-0.49	9.28E-03	-0.51	6.12E-03	0.50	8.03E-03
ENSCAFG0000001670	IDUA	grey	VSMC_M10	0.26	1.86E-01	0.78	1.41E-06	0.18	3.76E-01	0.40	3.80E-02	-0.10	6.12E-01	-0.15	4.64E-01	-0.32	1.08E-01	-0.71	3.41E-05	-0.66	1.96E-04	0.57	2.05E-03

ENSCAFG0000000246	NANS	darkgrey	VSMC_M8	0.26	1.86E-01	-0.37	5.87E-02	0.37	5.88E-02	-0.66	1.69E-04	-0.10	6.24E-01	0.13	5.28E-01	0.25	2.13E-01	0.72	1.97E-05	0.16	4.16E-01	-0.70	5.00E-05
ENSCAFG0000001333	SRP54	pink	VSMC_M5	0.26	1.86E-01	-0.41	3.46E-02	-0.30	1.23E-01	0.05	7.88E-01	0.51	6.23E-03	-0.25	2.01E-01	-0.29	1.49E-01	0.14	4.92E-01	0.29	1.45E-01	-0.44	2.27E-02
ENSCAFG0000001731	SEL1L	grey	VSMC_M10	0.26	1.87E-01	0.06	7.75E-01	-0.22	2.68E-01	0.10	6.21E-01	0.39	4.22E-02	-0.21	3.02E-01	-0.19	3.54E-01	-0.21	2.84E-01	-0.08	6.93E-01	-0.05	8.13E-01
ENSCAFG0000000658	PCED1A	cyan	VSMC_M2	0.26	1.87E-01	0.70	4.84E-05	0.06	7.79E-01	0.42	2.91E-02	-0.12	5.56E-01	0.07	7.13E-01	-0.07	7.18E-01	-0.57	1.91E-03	-0.72	2.61E-05	0.71	3.34E-05
ENSCAFG0000001968	SLC45A1	grey	VSMC_M10	0.26	1.87E-01	0.29	1.37E-01	0.17	4.09E-01	0.11	5.88E-01	-0.02	9.28E-01	0.02	9.03E-01	-0.20	3.10E-01	-0.20	3.08E-01	-0.30	1.33E-01	0.04	8.35E-01
ENSCAFG0000000589	MAFG	grey	VSMC_M10	0.26	1.87E-01	0.04	8.43E-01	0.33	9.26E-02	-0.33	9.28E-02	-0.02	9.03E-01	-0.06	7.78E-01	0.12	5.66E-01	0.23	2.46E-01	-0.13	5.05E-01	-0.40	3.92E-02
ENSCAFG0000001316	SP3	grey	VSMC_M10	0.26	1.87E-01	-0.21	3.02E-01	-0.04	8.46E-01	-0.25	2.06E-01	0.29	1.39E-01	-0.26	1.97E-01	0.20	3.15E-01	0.31	1.17E-01	0.13	5.29E-01	-0.33	8.84E-02
ENSCAFG0000000138	ENSCAFG0000000138	grey	VSMC_M10	0.26	1.87E-01	0.40	3.98E-02	0.19	3.47E-01	0.25	2.17E-01	-0.29	1.35E-01	0.34	8.30E-02	0.16	4.24E-01	-0.30	1.34E-01	-0.47	1.42E-02	0.56	2.22E-03
ENSCAFG0000001487	MXD4	cyan	VSMC_M2	0.26	1.87E-01	0.88	1.64E-09	0.29	1.44E-01	0.41	3.36E-02	-0.22	2.63E-01	-0.11	6.02E-01	-0.32	1.05E-01	-0.68	8.85E-05	-0.77	2.58E-06	0.61	7.32E-04
ENSCAFG0000001000	PSMG1	pink	VSMC_M5	0.26	1.88E-01	-0.03	8.72E-01	-0.47	1.26E-02	0.49	9.96E-03	0.65	2.72E-04	-0.23	2.43E-01	-0.40	3.94E-02	-0.40	3.70E-02	0.07	7.23E-01	-0.15	4.56E-01
ENSCAFG0000000847	MRPL39	darkgrey	VSMC_M8	0.26	1.88E-01	-0.54	3.57E-03	0.01	9.68E-01	-0.53	4.30E-03	0.27	1.72E-01	-0.06	7.71E-01	0.22	2.66E-01	0.68	9.26E-05	0.32	1.03E-01	-0.77	2.82E-06
ENSCAFG0000001022	ENSCAFG0000001022	grey	VSMC_M10	0.26	1.88E-01	0.00	9.86E-01	0.46	1.56E-02	-0.70	5.35E-05	-0.31	1.18E-01	-0.05	8.08E-01	0.25	2.09E-01	0.57	1.82E-03	-0.18	3.60E-01	-0.22	2.69E-01
ENSCAFG0000003252	PTPRN2	red	VSMC_M1	0.26	1.88E-01	0.08	6.92E-01	-0.09	6.39E-01	0.23	2.58E-01	0.26	1.95E-01	0.04	8.33E-01	-0.16	4.11E-01	-0.25	2.14E-01	-0.10	6.22E-01	-0.07	7.21E-01
ENSCAFG0000000538	PARP2	darkgrey	VSMC_M8	0.26	1.88E-01	-0.21	3.02E-01	0.33	8.97E-02	-0.66	1.87E-04	-0.21	2.94E-01	0.11	5.94E-01	0.49	9.26E-03	0.70	4.85E-05	-0.07	7.36E-01	-0.31	1.18E-01
ENSCAFG0000000960	ZC3H11A	pink	VSMC_M5	0.26	1.88E-01	-0.04	8.40E-01	-0.66	1.63E-04	0.60	9.33E-04	0.79	9.64E-07	-0.24	2.23E-01	-0.38	5.25E-02	-0.48	1.22E-02	0.06	7.77E-01	0.03	8.66E-01
ENSCAFG0000000871	RAPGEF2	grey	VSMC_M10	0.26	1.88E-01	-0.19	3.41E-01	-0.32	1.00E-01	0.05	8.12E-01	0.43	2.52E-02	0.23	2.41E-01	-0.21	2.89E-01	0.12	5.65E-01	0.07	7.20E-01	-0.20	3.23E-01
ENSCAFG0000001774	TBX4	grey	VSMC_M10	0.26	1.88E-01	-0.04	8.36E-01	0.07	7.36E-01	-0.21	3.01E-01	0.08	6.90E-01	-0.13	5.14E-01	0.37	5.42E-02	0.23	2.45E-01	-0.11	5.98E-01	-0.12	5.38E-01
ENSCAFG0000001276	ACBD6	darkgrey	VSMC_M8	0.26	1.88E-01	-0.11	5.87E-01	0.33	9.68E-02	-0.64	3.32E-04	-0.09	6.50E-01	-0.13	5.31E-01	0.41	3.36E-02	0.58	1.49E-03	-0.08	7.10E-01	-0.37	5.53E-02
ENSCAFG0000002444	ENSCAFG0000002444	grey	VSMC_M10	0.26	1.89E-01	0.39	4.40E-02	0.47	1.44E-02	-0.08	6.77E-01	-0.35	7.64E-02	-0.09	6.39E-01	0.11	5.88E-01	-0.17	4.01E-01	-0.40	3.82E-02	0.20	3.13E-01
ENSCAFG0000000078	PBX2	yellow	VSMC_M3	0.26	1.89E-01	0.24	2.20E-01	0.52	5.00E-03	-0.47	1.38E-02	-0.28	1.57E-01	-0.11	5.86E-01	0.07	7.33E-01	0.27	1.71E-01	-0.28	1.51E-01	-0.23	2.41E-01
ENSCAFG0000000436	ABI1	grey	VSMC_M10	0.26	1.89E-01	0.12	5.57E-01	0.03	8.76E-01	0.07	7.27E-01	0.20	3.21E-01	-0.33	9.12E-02	-0.10	6.12E-01	-0.18	3.67E-01	-0.01	9.79E-01	-0.11	5.96E-01
ENSCAFG0000002029	DAB2IP	grey	VSMC_M10	0.26	1.89E-01	0.67	1.22E-04	0.04	8.26E-01	0.67	1.19E-04	-0.04	8.30E-01	-0.25	2.18E-01	-0.35	7.69E-02	-0.80	6.48E-07	-0.57	1.89E-03	0.66	1.83E-04
ENSCAFG0000001461	RND2	cyan	VSMC_M2	0.26	1.89E-01	0.50	7.90E-03	0.38	5.05E-02	0.02	9.27E-01	-0.25	2.09E-01	0.20	3.30E-01	-0.03	8.97E-01	-0.22	2.80E-01	-0.46	1.62E-02	0.26	1.84E-01
ENSCAFG0000001679	PGD	yellow	VSMC_M3	0.26	1.89E-01	0.12	5.35E-01	0.64	3.32E-04	-0.72	1.95E-05	-0.44	2.15E-02	-0.02	9.34E-01	0.59	1.33E-03	0.50	7.62E-03	-0.25	2.11E-01	-0.21	2.98E-01
ENSCAFG0000001199	TFAP2C	grey	VSMC_M10	0.26	1.89E-01	-0.03	8.64E-01	0.13	5.27E-01	-0.41	3.44E-02	0.10	6.05E-01	-0.06	7.72E-01	0.50	8.62E-03	0.34	8.49E-02	-0.12	5.47E-01	-0.28	1.61E-01
ENSCAFG0000000456	PRDX1	darkgrey	VSMC_M8	0.26	1.90E-01	-0.27	1.66E-01	0.57	1.89E-03	-0.80	6.51E-07	-0.31	1.19E-01	0.08	6.90E-01	0.30	1.25E-01	0.79	1.15E-06	0.12	5.46E-01	-0.66	1.89E-04
ENSCAFG0000000175	MEA1	cyan	VSMC_M2	0.26	1.90E-01	0.68	1.05E-04	0.34	8.29E-02	0.27	1.66E-01	-0.28	1.56E-01	-0.18	3.78E-01	0.05	8.18E-01	-0.47	1.38E-02	-0.64	3.05E-04	0.50	8.27E-03
ENSCAFG0000001791	CXHXorf57	yellow	VSMC_M3	0.26	1.90E-01	0.17	3.84E-01	0.55	3.16E-03	-0.30	1.34E-01	-0.42	2.93E-02	0.12	5.40E-01	-0.06	7.72E-01	0.25	2.12E-01	-0.26	1.85E-01	-0.05	7.86E-01
ENSCAFG0000001591	RAB3IL1	yellow	VSMC_M3	0.26	1.90E-01	0.70	4.13E-05	0.75	6.54E-06	-0.39	4.33E-02	-0.57	1.99E-03	-0.16	4.23E-01	-0.02	9.05E-01	0.04	8.60E-01	-0.69	8.01E-05	0.19	3.43E-01
ENSCAFG0000000472	PDCD6IP	pink	VSMC_M5	0.26	1.91E-01	-0.20	3.30E-01	-0.57	1.81E-03	0.26	1.83E-01	0.80	4.17E-07	-0.50	7.22E-03	-0.06	7.57E-01	-0.20	3.29E-01	0.19	3.40E-01	-0.23	2.50E-01
ENSCAFG0000000006	CD63	yellow	VSMC_M3	0.26	1.91E-01	0.62	5.00E-04	0.78	1.56E-06	-0.23	2.52E-01	-0.64	3.37E-04	-0.01	9.72E-01	-0.28	1.62E-01	-0.08	6.99E-01	-0.55	2.91E-03	0.18	3.67E-01
ENSCAFG0000000958	TXNDC5	grey	VSMC_M10	0.26	1.91E-01	0.65	2.72E-04	0.12	5.57E-01	0.51	6.84E-03	-0.06	7.78E-01	-0.09	6.38E-01	-0.55	2.85E-03	-0.62	5.98E-04	-0.56	2.23E-03	0.46	1.68E-02
ENSCAFG0000000390	FAF1	grey	VSMC_M10	0.26	1.91E-01	-0.27	1.79E-01	0.14	4.94E-01	-0.44	2.08E-02	0.02	9.19E-01	-0.07	7.35E-01	0.44	2.18E-02	0.52	5.43E-03	0.13	5.17E-01	-0.33	9.60E-02
ENSCAFG0000000641	TMEM108	cyan	VSMC_M2	0.26	1.91E-01	0.75	6.26E-06	0.04	8.58E-01	0.49	9.77E-03	-0.05	8.20E-01	-0.22	2.67E-01	-0.43	2.65E-02	-0.65	2.38E-04	-0.73	1.42E-05	0.67	1.40E-04
ENSCAFG0000001071	ENSCAFG0000001071	grey	VSMC_M10	0.26	1.92E-01	0.28	1.64E-01	-0.25	2.13E-01	0.52	5.01E-03	0.26	1.90E-01	0.06	7.63E-01	-0.08	6.83E-01	-0.58	1.51E-03	-0.27	1.67E-01	0.35	7.51E-02
ENSCAFG0000000672	SYT9	grey	VSMC_M10	0.26	1.92E-01	0.09	6.43E-01	0.38	4.94E-02	-0.45	1.77E-02	-0.10	6.26E-01	-0.22	2.77E-01	0.54	3.38E-03	0.22	2.75E-01	-0.16	4.40E-01	-0.24	2.37E-01
ENSCAFG0000001095	SDHA	yellow	VSMC_M3	0.26	1.92E-01	0.49	9.70E-03	0.57	2.09E-03	-0.32	9.89E-02	-0.37	5.81E-02	0.11	5.79E-01	0.05	7.91E-01	0.12	5.57E-01	-0.51	6.19E-03	-0.02	9.14E-01
ENSCAFG0000001250	TAF4	grey	VSMC_M10	0.26	1.92E-01	0.04	8.48E-01	0.19	3.37E-01	-0.18	3.80E-01	-0.14	4.83E-01	0.11	5.98E-01	0.18	3.69E-01	0.17	3.99E-01	-0.18	3.58E-01	0.11	5.76E-01
ENSCAFG0000000314	AVL9	grey	VSMC_M10	0.26	1.92E-01	-0.13	5.19E-01	-0.38	5.09E-02	0.14	5.00E-01	0.49	8.83E-03	0.19	3.42E-01	-0.05	8.16E-01	-0.04	8.54E-01	0.02	9.36E-01	-0.08	7.02E-01
ENSCAFG0000001710	ENSCAFG0000001710	grey	VSMC_M10	0.26	1.92E-01	0.69	7.26E-05	0.25	2.12E-01	0.30	1.22E-01	-0.26	1.99E-01	0.06	7.57E-01	-0.34	8.42E-02	-0.44	2.06E-02	-0.64	3.24E-04	0.55	2.82E-03
ENSCAFG0000001940	PGP	yellow	VSMC_M3	0.26	1.93E-01	0.35	7.17E-02	0.61	7.36E-04	-0.40	3.93E-02	-0.45	1.78E-02	0.01	9.72E-01	-0.02	9.35E-01	0.17	4.03E-01	-0.37	5.70E-02	0.02	9.22E-01
ENSCAFG0000001963	VBP1	grey	VSMC_M10	0.26	1.93E-01	-0.28	1.53E-01	0.34	8.36E-02	-0.31	1.22E-01	-0.09	6.40E-01	-0.01	9.55E-01	0.04	8.26E-01	0.37	5.45E-02	0.17	4.10E-01	-0.46	1.54E-02
ENSCAFG0000003216	ENSCAFG0000003216	grey	VSMC_M10	0.26	1.93E-01	0.25	2.07E-01	0.22	2.61E-01	-0.08	6.86E-01	-0.17	4.03E-01	0.06	7.61E-01	0.02	9.23E-01	-0.03	8.88E-01	-0.27	1.70E-01	0.14	4.86E-01
ENSCAFG0000000736	EIF3M	darkgrey	VSMC_M8	0.26	1.93E-01	-0.29	1.37E-01	0.32	1.05E-01	-0.74	1.24E-05	-0.04	8.55E-01	-0.04	8.38E-01	0.08	6.85E-01	0.72	2.15E-05	0.16	4.24E-01	-0.70	4.20E-05
ENSCAFG0000001607	DAGLA	cyan	VSMC_M2	0.26	1.93E-01	0.84	5.06E-08	0.24	2.36E-01	0.41	3.48E-02	-0.20	3.08E-01	0.07	7.14E-01	-0.36	6.17E-02	-0.64	3.44E-04	-0.75	6.63E-06	0.60	1.01E-03
ENSCAFG0000000298	MYBPC2	grey	VSMC_M10	0.26	1.93E-01	0.81	2.50E-07	0.29	1.36E-01	0.30	1.31E-01	-0.31	1.17E-01	0.04	8.52E-01	-0.19	3.31E-01	-0.51	6.68E-03	-0.79	8.14E-07	0.65	2.16E-04
ENSCAFG0000000556	SAMD4B	cyan	VSMC_M2	0.26	1.94E-01	0.68	1.10E-04	0.41	3.31E-02	0.08	6.87E-01	-0.27	1.76E-01	-0.14	4.89E-01	-0.56	2.44E-03	-0.37	5.82E-02	-0.55	2.91E-03	0.31	1.20E-01
ENSCAFG0000000227	CARD19	cyan	VSMC_M2	0.26	1.94E-01	0.77	3.07E-06	0.37	5.75E-02	0.03	8.70E-01	-0.33	9.50E-02	-0.12	5.41E-01	-0.06	7.54E-01	-0.31	1.16E-01	-0.74	1.26E-05	0.52	5.08E-03
ENSCAFG0000000246	THADA	darkgrey	VSMC_M8	0.26	1.94E-01	-0.31	1.12E-01	0.20	3.12E-01	-0.47	1.35E-02	0.07	7.46E-01	-0.15	4.46E-01	0.26	1.99E-01	0.53	4.26E-03	0.18	3.77E-01	-0.54	3.79E-03
ENSCAFG0000001312	MAGI3	grey	VSMC_M10	0.26	1.94E-01	0.07	7.26E-01	-0.41	3.33E-02	0.38	5.36E-02	0.41	3.42E-02	-0.04	8.55E-01	0.30	1.29E-01	-0.26	1.91E-01	-0.22	2.80E-01	0.32	1.03E-01
ENSCAFG0000001454	KLHL20	grey	VSMC_M10	0.26	1.94E-01	0.29	1.36E-01	-0.35	7.15E-02	0.65	2.25E-04	0.41	3.19E-02	-0.24	2.19E-01	-0.02	9.29E-01	-0.69	5.81E-05	-0.32	1.08E-01	0.41	3.52E-02
ENSCAFG0000001805	TECPR2	grey	VSMC_M10	0.26	1.94E-01	0.44	2.33E-02	-0.17	3.97E-01	0.60	1.04E-03	0.20	3.16E-01	-0.02	9.25E-01	-0.04	8.38E-01	-0.65	2.75E-04	-0.45	1.86E-02	0.44	2.09E-02
ENSCAFG0000000491	ENSCAFG0000000491	grey	VSMC_M10	0.26	1.94E-01	0.27	1.80E-01	-0.13	5.28E-01	0.16	4.39E-01	0.22	2.70E-01	0.08	6.89E-01	-0.08	7.07E-01	-0.17	3.92E-01	-0.34	8.76E-02	0.09	6.39E-01
ENSCAFG0000000440	NINL	grey	VSMC_M10	0.26	1.94E-01	0.39	4.30E-02	0.43	2.58E-02	-0.02	9.27E-01	-0.36	6.40E-02	0.28	1.53E-01	-0.17	4.08E-01	-0.13	5.14E-01	-0.36	6.56E-02	0.20	3.15E-01
ENSCAFG0000000184	TRPM3	cyan	VSMC_M2	0.26	1.94E-01	0.65	2.74E-04	-0.07	7.25E-01	0.69	7.19E-05	0.04	8.33E-01	-0.28	1.64E-01	-0.41	3.34E-02	-0.75	6.26E-06	-0.61	8.22E-04	0.68	1.12E-04
ENSCAFG0000002007	ENSCAFG0000002007	grey	VSMC_M10	0.26	1.94E-01	-0.03	9.00E-01	-0.02	9.24E-01	0.15	4.51E-01	0.32	1.02E-01	-0.46	1.58E-02	-0.21	2.98E-01	-0.17	4.04E-01	0.06	7.85E-01	-0.29	1.49E-01
ENSCAFG0000003275	CDIPT	grey	VSMC_M10	0.26	1.94E-01	0.54	3.81E-03	0.39	4.26E-02	0.05	7.97E-01	-0.46	1.58E-02	0.32	1.01E-01	-0.18	3.56E-01	-0.19	3.35E-01	-0.55	3.22E-03	0.47	1.37E-02
ENSCAFG0000000588	DND1	yellow	VSMC_M3	0.26	1.95E-01	0.24	2.19E-01	0.63	4.68E-04	-0.26	1.91E-01	-0.47	1.43E-02	-0.11	5.74E-01	-0.04	8.39E-01	0.14	4.88E-01	-0.34	8.48E-02	-0.04	8.43E-01
ENSCAFG0000001096	LGALS8	cyan	VSMC_M2	0.26	1.95E-01	0.51	6.50E-03	0.02	9.27E-01	0.11	5.93E-01	0.18	3.78E-01	-0.31	1.11E-01	-0.28	1.51E-01	-0.35	7.30E-02	-0.37	5.76E-02	0.19	3.44E-01
ENSCAFG0000002983	SMIM8	grey	VSMC_M10	0.26	1.95E-01	0.05	7.98E-01	-0.10	6.30E-01	0.08	6.84E-01	0.10	6.19E-01	-0.10	6.16E-01	0.40	3.86E-02	-0.05	7.86E-01	-0.23	2.43E-01	0.21	2.85E-01
ENSCAFG0000000645	ARFIP2	grey	VSMC_M10	0.26	1.95E-01	0.05	8.07E-01	0.30	1.27E-01	-0.23	2.42E-01	-0.15	4.41E-01	-0.02	9.26E-01	-0.06	7.72E-01	0.18	3.79E-01	-0.07	7.26E-01	-0.15	4.43E-01
ENSCAFG0000000041	C12H6orf136	grey	VSMC_M10	0.26	1.96E-01	0.04	8.50E-01	-0.08	6.85E-01	-0.06	7.78E-01	0.14	4.92E-01	0.23	2.47E-01	-0.07	7.44E-01	0.06	7.55E-01	-0.09	6.62E-01	-0.03	8.73E-01
ENSCAFG0000002585	ENSCAFG0000002585	yellow	VSMC_M3	0.26	1.96E-01	0.28	1.50E-01	0.58	1.49E-03	-0.36	6.45E-02	-0.45	1.99E-02	-0.11	6.02E-01	0.22	2.63E-01	0.24	2.19E-01	-0.37	5.73E-02	0.01	9.48E-01
ENSCAFG0000000611	LSM1	grey	VSMC_M10	0.26	1.96E-01	0.29	1.38E-01	0.03	8.94E-01	0.19	3.42E-01	0.05	7.95E-01	0.00	9.82E-01	-0.18	3.71E-01	-0.27	1.78E-01	-0.30	1.24E-01	0.08	6.85E-01
ENSCAFG0000003242	RARRES1	grey	VSMC_M10	0.26	1.96E-01	0.59	1.12E-03	-0.03	8.93E-01	0.54	3.39E-03	0.12	5.64E-01	-0.39	4.15E-02	-0.07	7.12E-01	-0.67	1.14E-04	-0.48	1.05E-02	0.49	9.11E-03
ENSCAFG0000000494	PCDH9	darkgreen	VSMC_M4	0.26	1.97E-01	-0.21	2.83E-01	-0.49	9.69E-03	0.43	2.43E-02	0.58	1.43E-03	0.00	9.86E-01	-0.07	7.33E-01	-0.25	2.16E-01	0.10	6.11E-01	-0.05	7.94E-01
ENSCAFG0000000869	PPID	darkgrey	VSMC_M8	0.26	1.97E-01	-0.49	9.51E-03	0.18	3.75E-01	-0.60	8.68E-04	0.12	5.51E-01	-0.25	2.18E-01	0.42	2.82E-02	0.65	2.64E-04	0.30	1.29E-01	-0.73	1.55E-05
ENSCAFG0000001323	SH3GL3	grey	VSMC_M10	0.26	1.97E-01	-0.07	7.34E-01	0.09	6.66E-01	-0.12	5.40E-01	-0.10	6.30E-01	0.29	1.47E-01	0.40	4.07E-02	0.16	4.27E-01	-0.16	4.28E-01	0.14	4.87E-01
ENSCAFG0000001404	LRRC20	grey	VSMC_M10	0.26	1.97E-01	0.07	7.19E-01	-0.04	8.44E-01	0.04	8.48E-01	-0.01	9.48E-01	0.15	4.41E-01	0.12	5.35E-01	0.08	6.79E-01	-0.23	2.42E-01	0.20	3.29E-01
ENSCAFG0000001871	ALDOC	grey	VSMC_M10	0.26	1.97E-01	0.22	2.68E-01	-0.34	8.41E-02	0.53	4.63E-03	0.54	3.37E-03	-0.41	3.40E-02	-0.27	1.72E-01	-0.58	1.53E-03	-0.10	6.07E-01	0.06	7.55E-01
ENSCAFG0000000282	SH3BGRL2	grey	VSMC_M10	0.26	1.97E-01	0.29	1.42E-01	-0.13	5.27E-01	0.38	5.07E-02	0.06	7.59E-01	0.41	3.39E-02	0.10	6.13E-01	-0.32	1.03E-01	-0.42	2.98E-02	0.43	2.54E-02
ENSCAFG0000000946	PRELP	grey	VSMC_M10	0.26	1.98E-01	0.44	2.12E-02	-0.35	7.67E-02	0.64	3.07E-04	0.38	4.89E-02	-0.23	2.44E-01	0.05	8.08E-01	-0.74	9.48E-06	-0.40	3.92E-02	0.51	6.65E-03
ENSCAFG0000000981	NOD2	grey	VSMC_M10	0.26	1.98E-01	0.73	1.30E-05	0.22	2.66E-01	0.36	6.74E-02	-0.28	1.51E-01	0.16	4.18E-01	-0.27	1.76E-01	-0.53	4.86E-03	-0.76	3.80E-06	0.70	4.26E-05
ENSCAFG0000001180	CCDC172	grey	VSMC_M10	0.26	1.98E-01	-0.07	7.24E-01	-0.04	8.32E-01	-0.01	9.48E-01	0.24	2.29E-01	-0.08	7.09E-01	-0.27	1.69E-01	0.08	7.01E-01	-0.01	9.42E-01	-0.27	1.81E-01
ENSCAFG0000002522	ACVRL1	grey	VSMC_M10	0.26	1.98E-01	0.15	4.66E-01	0.28	1.50E-01	-0.22	2.71E-01	-0.03	8.75E-01	-0.47	1.43E-02	0.12	5.53E-01	0.07	7.12E-01	-0.13	5.25E-01	-0.22	2.69E-01
ENSCAFG0000001866	TNFAIP1	grey	VSMC_M10	0.26	1.98E-01	0.04	8.32E-01	-0.07	7.32E-01	-0.14	4.71E-01	0.22	2.71E-01	0.17	4.02E-01	-0.26	1.89E-01	0.07	7.31E-01	-0.04	8.32E-01	-0.19	3.34E-01
ENSCAFG0000002538	ENSCAFG0000002538	grey	VSMC_M10	0.26	1.98E-01	-0.06	7.53E-01	-0.03	8.64E-01	-0.05	8.21E-01	0.29	1.44E-01	-0.38	5.06E-02	0.11	5.86E-01	-0.02	9.05E-01	0.06	7.58E-01	-0.26	1.83E-01
ENSCAFG0000002007	CFDP1	grey	VSMC_M10	0.26	1.99E-01	-0.20	3.14E-01	0.40	3.63E-02	-0.55	3.16E-03	-0.19	3.42E-01	-0.12	5.52E-01	0.38	5.13E-02	0.53	4.13E-03	0.01	9.56E-01	-0.43	2.64E-02
ENSCAFG0000000637	EP400	grey	VSMC_M10	0.26	1.99E-01	0.18	3.80E-01	-0.27	1.70E-01	0.38	5.24E-02	0.32	1.07E-01	0.22	2.61E-01	-0.22	2.76E-01	-0.39	4.64E-02	-0.19	3.35E-01	0.23	2.39E-01
ENSCAFG0000001944	SLC9A3R2	cyan	VSMC_M2	0.26	1.99E-01	0.71	2.85E-05	0.49	9.07E-03	0.24	2.19E-01	-0.49	1.03E-02	0.13	5.28E-01	-0.12	5.57E-01	-0.41	3.49E-02	-0.69	6.09E-05	0.58	1.65E-03
ENSCAFG0000003227	ENSCAFG0000003227	grey	VSMC_M10	0.26	1.99E-01	0.03	8.94E-01	0.22	2.66E-01	-0.10	6.11E-01	-0.06	7.81E-01	0.04	8.43E-01	-0.17	3.90E-01	0.02	9.11E-01	-0.02	9.21E-01	-0.18	3.56E-01
ENSCAFG0000003005	ENSCAFG0000003005	grey	VSMC_M10	0.26	1.99E-01	0.24	2.27E-01	0.32	1.03E-01	-0.24	2.35E-01	-0.11	5.80E-01	-0.18	3.82E-01	-0.25	2.05E-01	0.03	8.97E-01	-0.22	2.75E-01	-0.13	5.10E-01
ENSCAFG0000002492	DGAT1	cyan	VSMC_M2	0.25	1.99E-01	0.32	1.02E-01	0.33	9.30E-02	-0.13	5.27E-01	-0.36	6.65E-02	0.29	1.49E-01	0.15	4.40E-01	0.00	9.98E-01	-0.44	2.33E-02	0.31	1.14E-01
ENSCAFG0000001950	GAMT	grey	VSMC_M10	0.25	2.00E-01	0.57	1.72E-03	-0.23	2.43E-01	0.67	1.22E-04	0.32	1.06E-01	-0.31	1.14E-01	-0.34	8.19E-02	-0.81	2.75E-07	-0.44	2.13E-02	0.47	1.24E-02
ENSCAFG0000001455	ACOX3	pink	VSMC_M5	0.25	2.00E-01	0.04	8.24E-01	-0.41	3.22E-02	0.48	1.15E-02	0.58	1.62E-03	-0.29	1.47E-01	0.01	9.73E-01	-0.42	3.02E-02	-0.03	8.88E-01	0.05	8.13E-01
ENSCAFG0000000980	ENSCAFG0000000980	grey	VSMC_M10	0.25	2.00E-01	0.37	5.92E-02	0.19	3.49E-01	0.18	3.77E-01	-0.24	2.30E-01	0.15	4.46E-01	0.14	4.95E-01	-0.19	3.55E-01	-0.48	1.08E-02	0.42	3.08E-02
ENSCAFG0000000996	CHRM3	grey	VSMC_M10	0.25	2.00E-01	0.28	1.53E-01	0.11	5.70E-01	-0.12	5.35E-01	-0.01	9.50E-01	0.15	4.61E-01	-0.22	2.66E-01	-0.02	9.04E-01	-0.27	1.66E-01	0.08	6.90E-01
ENSCAFG0000001300	GMEB2	yellow	VSMC_M3	0.25	2.01E-01	0.48	1.18E-02	0.84	5.16E-08	-0.42	2.97E-02	-0.77	3.03E-06	0.29	1.38E-01	-0.01	9.64E-01	0.20	3.14E-01	-0.57	1.97E-03	0.17	3.90E-01
ENSCAFG0000000280	ACTL7A	grey	VSMC_M10	0.25	2.01E-01	0.38	4.91E-02	0.37	5.44E-02	0.06	7.50E-01	-0.36	6.39E-02	-0.17	3.90E-01	0.06	7.61E-01	-0.20	3.15E-01	-0.42	2.84E-02	0.45	1.96E-02
ENSCAFG0000000925	SFRP5	cyan	VSMC_M2	0.25	2.01E-01	0.82	1.61E-07	0.56	2.19E-03	0.05	8.12E-01	-0.48	1.12E-02	-0.13	5.31E-01	-0.28	1.53E-01	-0.36	6.67E-02	-0.77	2.60E-06	0.48	1.21E-02
ENSCAFG0000001701	NLGN3	grey	VSMC_M10	0.25	2.01E-01	0.18	3.68E-01	0.27	1.76E-01	0.02	9.30E-01	-0.16	4.16E-01	-0.02	9.20E-01	0.19	3.55E-01	-0.14	4.94E-01	-0.19	3.55E-01	0.14	4.96E-01
ENSCAFG0000001920	MGRN1	grey	VSMC_M10	0.25	2.01E-01	0.79	7.53E-07	0.28	1.62E-01	0.45	1.77E-02	-0.36	6.33E-02	0.11	5.99E-01	-0.20	3.19E-01	-0.65	2.23E-04	-0.78	1.55E-06	0.80	4.52E-07
ENSCAFG0000003220	C15H4orf46	cyan	VSMC_M2	0.25	2.01E-01	0.38	5.19E-02	0.35	7.60E-02	-0.12	5.48E-01	-0.32	1.02E-01	0.05	7.92E-01	0.16	4.37E-01	0.01	9.75E-01	-0.47	1.26E-02	0.23	2.40E-01
ENSCAFG0000001218	ENSCAFG0000001218	grey	VSMC_M10	0.25	2.01E-01	0.04	8.38E-01	-0.06	7.69E-01	0.06	7.68E-01	0.14	4.78E-01	-0.22	2.62E-01	0.18	3.69E-01	-0.05	8.04E-01	-0.17	4.00E-01	0.09	6.54E-01
ENSCAFG0000001263	PI4KB	darkgreen	VSMC_M4	0.25	2.01E-01	0.58	1.47E-03	-0.23	2.41E-01	0.71	2.89E-05	0.23	2.46E-01	-0.06	7.75E-01	-0.42	2.87E-02	-0.78	1.66E-06	-0.51	6.24E-03	0.58	1.35E-03
ENSCAFG0000002013	AARS	grey	VSMC_M10	0.25	2.01E-01	-0.18	3.70E-01	0.27	1.68E-01	-0.29	1.38E-01	-0.15	4.55E-01	0.16	4.20E-01	0.11	5.98E-01	0.33	9.31E-02	-0.04	8.35E-01	-0.21	2.84E-01
ENSCAFG0000000144	PIM1	grey	VSMC_M10	0.25	2.02E-01	0.11	5.85E-01	0.26	1.96E-01	-0.29	1.42E-01	-0.07	7.30E-01	-0.08	6.75E-01	-0.13	5.33E-01	0.20	3.14E-01	-0.22	2.63E-01	-0.14	4.91E-01
ENSCAFG0000000020	TBPL1	grey	VSMC_M10	0.25	2.02E-01	0.28	1.64E-01	0.02	9.06E-01	0.34	8.37E-02	-0.09	6.53E-01	0.14	4.75E-01	0.27	1.78E-01	-0.38	4.95E-02	-0.34	8.04E-02	0.51	6.70E-03
ENSCAFG0000003070	TUBGCP2	yellow	VSMC_M3	0.25	2.02E-01	0.39	4.37E-02	0.71	3.56E-05	-0.50	8.61E-03	-0.61	7.52E-04	0.18	3.62E-01	0.09	6.44E-01	0.32	1.07E-01	-0.49	9.98E-03	0.06	7.85E-01
ENSCAFG0000001563	TMEM179B	red	VSMC_M1	0.25	2.02E-01	0.40	3.77E-02	-0.06	7.65E-01	0.41	3.47E-02	-0.02	9.16E-01	0.10	6.22E-01	-0.07	7.36E-01	-0.46	1.68E-02	-0.45	1.90E-02	0.52	5.96E-03
ENSCAFG0000001028	TSPAN5	yellow	VSMC_M3	0.25	2.02E-01	0.21	3.02E-01	0.48	1.18E-02	-0.53	4.04E-03	-0.26	1.82E-01	-0.21	3.01E-01	0.45	1.74E-02	0.40	3.88E-02	-0.30	1.34E-01	-0.19	3.37E-01
ENSCAFG0000000773	LSM6	yellow	VSMC_M3	0.25	2.03E-01	0.08	7.02E-01	0.42	2.85E-02	-0.14	4.73E-01	-0.25	2.04E-01	-0.04	8.27E-01	0.10	6.17E-01	0.08	6.89E-01	-0.17	3.92E-01	-0.17	3.84E-01
ENSCAFG0000000191	PIP5K1B	darkgreen	VSMC_M4	0.25	2.03E-01	0.53	4.52E-03	-0.24	2.20E-01	0.77	2.56E-06	0.18	3.74E-01	-0.08	6.83E-01	-0.52	5.28E-03	-0.76	4.58E-06	-0.48	1.21E-02	0.61	6.61E-04
ENSCAFG0000001899	CDH10	grey	VSMC_M10	0.25	2.03E-01	-0.05	8.12E-01	0.07	7.13E-01	-0.10	6.28E-01	-0.04	8.50E-01	-0.03	8.67E-01	-0.06	7.78E-01	0.14	4.89E-01	-0.09	6.48E-01	-0.04	8.43E-01
ENSCAFG0000001086	BRD9	yellow	VSMC_M3	0.25	2.03E-01	0.04	8.34E-01	0.72	2.38E-05	-0.58	1.66E-03	-0.57	1.97E-03	0.14	4.81E-01	0.28	1.58E-01	0.51	6.65E-03	-0.17	3.85E-01	-0.19	3.42E-01
ENSCAFG0000000949	ACCS	yellow	VSMC_M3	0.25	2.03E-01	0.51	6.88E-03	0.56	2.22E-03	-0.30	1.29E-01	-0.40	4.00E-02	0.00	9.81E-01	0.05	8.11E-01	0.02	9.33E-01	-0.47	1.27E-02	0.15	4.57E-01
ENSCAFG0000000292	CCDC85A	grey	VSMC_M10	0.25	2.04E-01	0.43	2.49E-02	0.43	2.60E-02	0.13	5.12E-01	-0.38	4.92E-02	-0.01	9.75E-01	-0.05	7.89E-01	-0.21	2.96E-01	-0.50	7.35E-03	0.34	8.75E-02
ENSCAFG0000000917	UBTD1	cyan	VSMC_M2	0.25	2.04E-01	0.62	6.32E-04	0.33	9.24E-02	0.22	2.65E-01	-0.24	2.19E-01	-0.03	8.77E-01	-0.44	2.31E-02	-0.48	1.17E-02	-0.52	5.76E-03	0.38	4.86E-02
ENSCAFG0000001561	KIF1C	grey	VSMC_M10	0.25	2.04E-01	0.75	5.80E-06	0.33	8.88E-02	0.36	6.63E-02	-0.22	2.78E-01	-0.15	4.65E-01	-0.44	2.24E-02	-0.62	6.18E-04	-0.60	1.01E-03	0.43	2.46E-02
ENSCAFG0000000122	PACSIN1	yellow	VSMC_M3	0.25	2.04E-01	0.55	3.06E-03	0.71	2.78E-05	-0.37	5.41E-02	-0.51	6.41E-03	-0.12	5.42E-01	-0.13	5.28E-01	0.07	7.30E-01	-0.49	9.31E-03	0.05	8.07E-01
ENSCAFG0000000178	RTCB	yellow	VSMC_M3	0.25	2.04E-01	0.05	8.21E-01	0.59	1.34E-03	-0.58	1.44E-03	-0.53	4.38E-03	0.22	2.64E-01	0.16	4.15E-01	0.54	3.77E-03	-0.20	3.06E-01	-0.08	6.95E-01
ENSCAFG0000001332	ENSCAFG0000001332	grey	VSMC_M10	0.25	2.04E-01	0.25	2.10E-01	0.53	4.45E-03	-0.22	2.61E-01	-0.41	3.52E-02	0.37	5.87E-02	-0.13	5.24E-01	0.09	6.57E-01	-0.27	1.66E-01	0.01	9.61E-01
ENSCAFG0000000692	KCNIP3	grey	VSMC_M10	0.25	2.04E-01	-0.18	3.73E-01	-0.12	5.54E-01	-0.19	3.34E-01	0.38	5.30E-02	-0.18	3.76E-01	0.14	4.96E-01	0.13	5.09E-01	0.12	5.50E-01	-0.47	1.26E-02
ENSCAFG0000001120	NDUFB4	grey	VSMC_M10	0.25	2.04E-01	-0.01	9.60E-01	0.34	7.90E-02	-0.20	3.30E-01	-0.33	9.05E-02	0.06	7.50E-01	0.08	7.07E-01	0.24	2.36E-01	-0.14	4.93E-01	0.04	8.47E-01
ENSCAFG0000002011	GLG1	darkgreen	VSMC_M4	0.25	2.04E-01	0.33	9.10E-02	-0.20	3.09E-01	0.51	6.41E-03	0.29	1.40E-01	-0.23	2.40E-01	-0.85	2.36E-08	-0.56	2.49E-03	-0.21	2.99E-01	0.21	2.85E-01
ENSCAFG0000001185	KLHL24	grey	VSMC_M10	0.25	2.04E-01	0.36	6.85E-02	-0.33	8.96E-02	0.56	2.53E-03	0.48	1.13E-02	-0.19	3.49E-01	-0.20	3.29E-01	-0.72	2.66E-05	-0.26	1.95E-01	0.27	1.80E-01

ENSCAFG0000000092	TTLL1	darkgrey	VSMC_M8	0.25	2.05E-01	-0.35	7.32E-02	0.31	1.10E-01	-0.66	2.09E-04	0.01	9.44E-01	-0.08	6.85E-01	0.14	4.81E-01	0.63	4.70E-04	0.25	2.16E-01	-0.78	1.57E-06
ENSCAFG0000003053	ENSCAFG0000003053	grey	VSMC_M1C	0.25	2.05E-01	0.34	7.96E-02	0.00	9.88E-01	0.49	9.72E-03	0.02	9.25E-01	0.06	7.69E-01	0.05	8.22E-01	-0.49	1.03E-02	-0.37	5.96E-02	0.41	3.29E-02
ENSCAFG0000000545	ENSCAFG0000000545	red	VSMC_M1	0.25	2.05E-01	0.23	2.58E-01	0.36	6.62E-02	-0.10	6.15E-01	-0.10	6.02E-01	-0.36	6.12E-02	-0.12	5.45E-01	-0.04	8.51E-01	-0.21	2.87E-01	-0.16	4.19E-01
ENSCAFG0000003252	ENSCAFG0000003252	grey	VSMC_M1C	0.25	2.05E-01	0.11	5.99E-01	-0.28	1.53E-01	0.36	6.40E-02	0.21	2.89E-01	0.19	3.42E-01	0.02	9.22E-01	-0.29	1.42E-01	-0.19	3.56E-01	0.36	6.58E-02
ENSCAFG0000000159	ADCK5	grey	VSMC_M1C	0.25	2.05E-01	0.29	1.39E-01	0.55	2.68E-03	-0.18	3.66E-01	-0.52	5.08E-03	0.17	3.83E-01	-0.09	6.44E-01	0.11	5.71E-01	-0.36	6.92E-02	0.14	4.99E-01
ENSCAFG0000001488	BMP4	cyan	VSMC_M2	0.25	2.05E-01	0.68	9.43E-05	-0.18	3.58E-01	0.78	1.52E-06	0.17	3.99E-01	-0.11	5.93E-01	-0.61	6.97E-04	-0.87	3.22E-09	-0.57	1.97E-03	0.65	2.67E-04
ENSCAFG0000002014	DR1	red	VSMC_M1	0.25	2.05E-01	-0.01	9.47E-01	-0.04	8.30E-01	0.15	4.54E-01	0.16	4.35E-01	0.13	5.12E-01	-0.07	7.19E-01	-0.10	6.09E-01	-0.05	7.88E-01	-0.12	5.48E-01
ENSCAFG0000001372	MYL4	red	VSMC_M1	0.25	2.05E-01	0.26	1.94E-01	0.12	5.60E-01	0.23	2.48E-01	-0.04	8.58E-01	-0.15	4.63E-01	-0.14	4.76E-01	-0.23	2.58E-01	-0.30	1.29E-01	0.20	3.12E-01
ENSCAFG0000002998	RNF139	pink	VSMC_M5	0.25	2.05E-01	-0.12	5.39E-01	-0.50	7.62E-03	0.29	1.40E-01	0.68	9.15E-05	-0.28	1.57E-01	-0.03	8.97E-01	-0.19	3.35E-01	0.06	7.71E-01	-0.15	4.55E-01
ENSCAFG0000001934	AGRN	cyan	VSMC_M2	0.25	2.05E-01	0.60	9.19E-04	-0.24	2.19E-01	0.69	6.79E-05	0.25	2.10E-01	-0.11	5.78E-01	-0.40	3.75E-02	-0.75	5.52E-06	-0.55	2.80E-03	0.58	1.58E-03
ENSCAFG0000001191	CSTF1	grey	VSMC_M1C	0.25	2.06E-01	0.18	3.61E-01	0.37	6.07E-02	-0.06	7.59E-01	-0.30	1.30E-01	0.15	4.57E-01	0.06	7.81E-01	0.02	9.09E-01	-0.32	9.88E-02	0.14	4.72E-01
ENSCAFG0000002758	ENSCAFG0000002758	grey	VSMC_M1C	0.25	2.06E-01	0.12	5.44E-01	0.26	1.98E-01	-0.19	3.34E-01	-0.26	1.91E-01	0.27	1.67E-01	0.07	7.34E-01	0.18	3.57E-01	-0.31	1.12E-01	0.12	5.58E-01
ENSCAFG0000002370	GRHPR	yellow	VSMC_M3	0.25	2.06E-01	0.64	2.88E-04	0.71	3.51E-05	-0.31	1.20E-01	-0.63	4.16E-04	0.01	9.64E-01	0.02	9.04E-01	-0.01	9.78E-01	-0.64	3.37E-04	0.29	1.36E-01
ENSCAFG0000000876	CX3CL1	yellow	VSMC_M3	0.25	2.06E-01	0.38	5.37E-02	0.65	2.77E-04	-0.58	1.67E-03	-0.41	3.32E-02	-0.01	9.51E-01	-0.27	1.66E-01	0.30	1.34E-01	-0.33	9.54E-02	-0.20	3.13E-01
ENSCAFG0000003255	BOLA1	yellow	VSMC_M3	0.25	2.06E-01	0.26	1.86E-01	0.67	1.52E-04	-0.44	2.23E-02	-0.45	1.75E-02	-0.08	7.08E-01	-0.13	5.05E-01	0.28	1.58E-01	-0.27	1.69E-01	-0.19	3.34E-01
ENSCAFG0000000108	FAM84B	grey	VSMC_M1C	0.25	2.06E-01	0.38	5.04E-02	0.45	1.89E-02	-0.04	8.25E-01	-0.37	5.84E-02	0.23	2.48E-01	0.13	5.23E-01	-0.09	6.64E-01	-0.48	1.13E-02	0.25	2.03E-01
ENSCAFG0000001627	HSD17B1C	grey	VSMC_M1C	0.25	2.06E-01	0.35	7.64E-02	0.17	4.03E-01	-0.18	3.60E-01	-0.02	9.14E-01	-0.05	8.09E-01	-0.07	7.35E-01	0.07	7.40E-01	-0.38	5.16E-02	-0.03	8.76E-01
ENSCAFG0000001349	PCYT1B	violet	VSMC_M7	0.25	2.06E-01	-0.09	6.68E-01	0.09	6.57E-01	-0.10	6.14E-01	0.00	9.98E-01	-0.07	7.37E-01	0.68	9.05E-05	0.11	5.83E-01	-0.11	5.70E-01	-0.03	8.87E-01
ENSCAFG0000001990	ZCCHC14	cyan	VSMC_M2	0.25	2.06E-01	0.61	7.35E-04	0.20	3.13E-01	0.03	8.89E-01	-0.06	7.51E-01	-0.16	4.32E-01	-0.19	3.31E-01	-0.22	2.73E-01	-0.60	1.02E-03	0.25	2.02E-01
ENSCAFG0000000078	TTC38	cyan	VSMC_M2	0.25	2.06E-01	0.83	1.16E-07	0.60	8.40E-04	-0.02	9.41E-01	-0.55	2.93E-03	0.02	9.33E-01	-0.30	1.27E-01	-0.30	1.34E-01	-0.76	3.59E-06	0.51	6.26E-03
ENSCAFG0000001068	SLC39A8	turquoise	VSMC_M6	0.25	2.06E-01	0.15	4.67E-01	0.47	1.23E-02	-0.39	4.39E-02	-0.41	3.38E-02	0.57	1.98E-03	-0.01	9.61E-01	0.25	2.00E-01	-0.21	2.93E-01	-0.06	7.61E-01
ENSCAFG0000001469	GRK4	grey	VSMC_M1C	0.25	2.06E-01	0.15	4.68E-01	-0.03	8.65E-01	-0.01	9.52E-01	0.21	3.01E-01	-0.22	2.65E-01	-0.42	2.77E-02	-0.05	8.02E-01	-0.12	5.49E-01	-0.04	8.29E-01
ENSCAFG0000000416	NDNF	grey	VSMC_M1C	0.25	2.06E-01	0.09	6.65E-01	0.02	9.12E-01	-0.11	5.69E-01	0.12	5.66E-01	0.18	3.77E-01	-0.13	5.31E-01	0.11	5.71E-01	-0.16	4.39E-01	-0.05	8.15E-01
ENSCAFG0000003016	COMMD2	pink	VSMC_M5	0.25	2.06E-01	-0.55	3.07E-03	-0.27	1.67E-01	-0.23	2.47E-01	0.59	1.07E-03	-0.19	3.48E-01	0.06	7.62E-01	0.40	3.66E-02	0.41	3.60E-02	-0.77	2.96E-06
ENSCAFG0000000753	ENSCAFG0000000753	grey	VSMC_M1C	0.25	2.07E-01	0.23	2.56E-01	0.27	1.78E-01	-0.09	6.41E-01	-0.05	7.95E-01	0.17	3.86E-01	0.05	7.98E-01	-0.03	8.73E-01	-0.24	2.35E-01	-0.10	6.30E-01
ENSCAFG0000003023	ENSCAFG0000003023	grey	VSMC_M1C	0.25	2.07E-01	0.32	1.07E-01	0.58	1.60E-03	-0.31	1.20E-01	-0.40	4.14E-02	-0.11	6.00E-01	0.14	4.74E-01	0.11	6.02E-01	-0.34	8.52E-02	0.07	7.37E-01
ENSCAFG0000000439	ENSCAFG0000000439	pink	VSMC_M5	0.25	2.07E-01	-0.43	2.43E-02	-0.44	2.19E-02	-0.05	8.16E-01	0.66	2.03E-04	-0.05	7.88E-01	0.05	8.04E-01	0.24	2.32E-01	0.34	8.42E-02	-0.50	7.78E-03
ENSCAFG0000000416	BMI1	grey	VSMC_M1C	0.25	2.07E-01	0.08	7.00E-01	-0.20	3.10E-01	0.43	2.34E-02	0.25	2.05E-01	-0.13	5.28E-01	0.00	9.94E-01	-0.36	6.16E-02	-0.15	4.65E-01	0.21	2.97E-01
ENSCAFG0000000755	ESYT3	darkgreen	VSMC_M4	0.25	2.07E-01	-0.07	7.26E-01	-0.42	2.76E-02	0.42	2.95E-02	0.48	1.09E-02	-0.17	4.08E-01	-0.04	8.27E-01	-0.26	1.88E-01	0.02	9.38E-01	0.11	5.78E-01
ENSCAFG0000000570	TOP2B	pink	VSMC_M5	0.25	2.07E-01	-0.26	1.92E-01	-0.27	1.71E-01	-0.14	4.71E-01	0.51	6.73E-03	-0.21	3.04E-01	0.27	1.78E-01	0.20	3.20E-01	0.12	5.59E-01	-0.36	6.88E-02
ENSCAFG0000000883	RPRD1B	grey	VSMC_M1C	0.25	2.07E-01	0.24	2.28E-01	-0.08	7.10E-01	0.44	2.20E-02	0.05	8.18E-01	-0.03	8.78E-01	-0.03	8.91E-01	-0.41	3.40E-02	-0.31	1.13E-01	0.43	2.38E-02
ENSCAFG0000000893	ENSCAFG0000000893	pink	VSMC_M5	0.25	2.07E-01	-0.36	6.16E-02	-0.08	6.77E-01	-0.13	5.21E-01	0.34	8.03E-02	-0.12	5.37E-01	-0.14	4.94E-01	0.27	1.78E-01	0.28	1.54E-01	-0.52	5.03E-03
ENSCAFG0000000578	ENSCAFG0000000578	grey	VSMC_M1C	0.25	2.07E-01	0.15	4.64E-01	-0.06	7.65E-01	0.13	5.32E-01	0.16	4.34E-01	0.00	9.90E-01	-0.02	9.36E-01	-0.19	3.48E-01	-0.24	2.30E-01	0.02	9.26E-01
ENSCAFG0000000246	SNX13	darkgreen	VSMC_M4	0.25	2.08E-01	0.09	6.63E-01	-0.49	9.17E-03	0.63	4.24E-04	0.49	1.01E-02	-0.05	7.97E-01	-0.20	3.13E-01	-0.54	3.72E-03	-0.17	3.94E-01	0.33	8.82E-02
ENSCAFG0000001967	ENTR1	cyan	VSMC_M2	0.25	2.08E-01	0.58	1.62E-03	0.50	7.75E-03	-0.12	5.45E-01	-0.54	3.90E-03	-0.05	8.03E-01	0.11	5.74E-01	-0.08	7.07E-01	-0.68	9.78E-05	0.49	9.67E-03
ENSCAFG0000001763	TMED1	yellow	VSMC_M3	0.25	2.08E-01	0.05	7.96E-01	0.57	1.74E-03	-0.33	9.00E-02	-0.36	6.86E-02	-0.21	2.85E-01	-0.10	6.08E-01	0.27	1.80E-01	-0.09	6.69E-01	-0.29	1.37E-01
ENSCAFG0000001266	GPC1	turquoise	VSMC_M6	0.25	2.08E-01	0.33	8.83E-02	0.24	2.18E-01	0.10	6.17E-01	-0.32	1.02E-01	0.52	5.38E-03	0.31	1.17E-01	-0.15	4.67E-01	-0.50	8.62E-03	0.44	2.20E-02
ENSCAFG0000000275	GPNMB	cyan	VSMC_M2	0.25	2.08E-01	0.77	3.16E-06	0.27	1.67E-01	0.13	5.05E-01	-0.10	6.13E-01	-0.25	1.99E-01	-0.27	1.81E-01	-0.49	8.89E-03	-0.61	7.30E-04	0.34	8.69E-02
ENSCAFG0000001114	SNRNP40	darkgrey	VSMC_M8	0.25	2.08E-01	-0.20	3.06E-01	0.56	2.62E-03	-0.80	4.62E-07	-0.30	1.29E-01	0.04	8.35E-01	0.20	3.09E-01	0.78	1.40E-06	0.04	8.31E-01	-0.61	7.65E-04
ENSCAFG0000002761	ENSCAFG0000002761	grey	VSMC_M1C	0.25	2.08E-01	0.37	5.66E-02	0.58	1.41E-03	-0.21	2.94E-01	-0.40	3.68E-02	-0.05	7.95E-01	0.11	6.02E-01	0.00	9.83E-01	-0.38	5.22E-02	0.07	7.27E-01
ENSCAFG0000001720	VIPAS39	grey	VSMC_M1C	0.25	2.08E-01	-0.44	2.32E-02	-0.44	2.17E-02	0.02	9.18E-01	0.65	2.45E-04	0.02	9.13E-01	0.05	8.09E-01	0.06	7.60E-01	0.36	6.81E-02	-0.47	1.33E-02
ENSCAFG0000000601	EAF1	pink	VSMC_M5	0.25	2.09E-01	-0.31	1.15E-01	-0.22	2.64E-01	-0.03	8.95E-01	0.51	6.97E-03	-0.27	1.80E-01	-0.24	2.32E-01	0.07	7.20E-01	0.34	8.56E-02	-0.53	4.55E-03
ENSCAFG0000001047	NOTCH2	pink	VSMC_M5	0.25	2.09E-01	-0.02	9.27E-01	-0.45	1.80E-02	0.31	1.16E-01	0.58	1.37E-03	-0.08	6.84E-01	-0.16	4.36E-01	-0.33	9.80E-02	0.05	8.21E-01	0.02	9.02E-01
ENSCAFG0000001117	NR5A2	grey	VSMC_M1C	0.25	2.09E-01	0.11	5.73E-01	0.46	1.60E-02	-0.18	3.67E-01	-0.36	6.14E-02	0.53	4.08E-03	0.12	5.40E-01	0.11	5.72E-01	-0.20	3.12E-01	-0.06	7.72E-01
ENSCAFG0000001434	TBC1D14	grey	VSMC_M1C	0.25	2.09E-01	0.04	8.34E-01	-0.02	9.25E-01	0.01	9.52E-01	0.20	3.23E-01	-0.14	4.97E-01	0.26	1.97E-01	-0.01	9.46E-01	-0.10	6.27E-01	-0.18	3.77E-01
ENSCAFG0000000727	FAT1	pink	VSMC_M5	0.25	2.09E-01	-0.10	6.07E-01	-0.65	2.66E-04	0.59	1.24E-03	0.75	6.51E-06	-0.22	2.75E-01	-0.31	1.11E-01	-0.48	1.16E-02	0.11	5.70E-01	0.05	8.22E-01
ENSCAFG0000001990	UCK1	grey	VSMC_M1C	0.25	2.09E-01	0.11	5.70E-01	0.29	1.41E-01	-0.14	4.84E-01	-0.23	2.50E-01	0.13	5.16E-01	0.28	1.51E-01	0.03	9.01E-01	-0.19	3.42E-01	0.02	9.08E-01
ENSCAFG0000000002	ZADH2	cyan	VSMC_M2	0.25	2.09E-01	0.52	4.95E-03	0.34	8.74E-02	0.09	6.67E-01	-0.37	5.56E-02	-0.02	9.07E-01	0.05	7.95E-01	-0.18	3.76E-01	-0.56	2.44E-03	0.52	5.95E-03
ENSCAFG0000001221	RAB4A	grey	VSMC_M1C	0.25	2.09E-01	0.11	5.90E-01	0.05	7.98E-01	0.12	5.39E-01	0.02	9.25E-01	-0.07	7.38E-01	0.17	4.09E-01	-0.09	6.42E-01	-0.22	2.68E-01	0.20	3.05E-01
ENSCAFG0000000210	ENSCAFG0000000210	grey	VSMC_M1C	0.25	2.10E-01	0.02	9.10E-01	0.37	6.01E-02	-0.34	7.94E-02	-0.19	3.31E-01	-0.13	5.04E-01	0.32	1.06E-01	0.31	1.20E-01	-0.18	3.56E-01	-0.11	5.75E-01
ENSCAFG0000000217	ENSCAFG0000000217	darkgrey	VSMC_M8	0.25	2.10E-01	-0.25	2.12E-01	0.44	2.14E-02	-0.58	1.46E-03	-0.20	3.16E-01	-0.02	9.05E-01	0.27	1.76E-01	0.61	6.76E-04	0.05	8.03E-01	-0.53	4.22E-03
ENSCAFG0000000907	RUNX1T1	grey	VSMC_M1C	0.25	2.10E-01	-0.39	4.43E-02	-0.29	1.43E-01	-0.21	3.00E-01	0.50	7.40E-03	-0.03	8.80E-01	0.41	3.17E-02	0.20	3.25E-01	0.27	1.74E-01	-0.38	4.99E-02
ENSCAFG0000001490	POLN	grey	VSMC_M1C	0.25	2.11E-01	0.39	4.62E-02	0.19	3.45E-01	0.19	3.46E-01	-0.20	3.30E-01	0.02	9.33E-01	-0.10	6.02E-01	-0.33	9.26E-02	-0.37	5.79E-02	0.38	5.05E-02
ENSCAFG0000001677	CASZ1	grey	VSMC_M1C	0.25	2.11E-01	0.61	7.40E-04	-0.06	7.52E-01	0.42	2.92E-02	0.05	8.22E-01	0.03	8.81E-01	-0.42	2.74E-02	-0.53	4.24E-03	-0.63	4.73E-04	0.55	3.15E-03
ENSCAFG0000002374	PTPRR	yellow	VSMC_M3	0.25	2.11E-01	0.17	3.95E-01	0.41	3.26E-02	-0.44	2.11E-02	-0.28	1.56E-01	-0.04	8.35E-01	0.34	8.65E-02	0.28	1.62E-01	-0.29	1.43E-01	-0.03	9.00E-01
ENSCAFG0000000186	KLF9	cyan	VSMC_M2	0.25	2.11E-01	0.73	1.37E-05	0.63	4.25E-04	-0.08	7.01E-01	-0.65	2.81E-04	0.02	9.17E-01	-0.10	6.04E-01	-0.20	3.20E-01	-0.73	1.42E-05	0.55	2.99E-03
ENSCAFG0000000765	C29H8orf34	grey	VSMC_M1C	0.25	2.11E-01	-0.13	5.24E-01	-0.03	8.94E-01	-0.18	3.62E-01	0.12	5.58E-01	0.23	2.41E-01	-0.02	9.25E-01	0.25	2.03E-01	-0.02	9.14E-01	-0.16	4.25E-01
ENSCAFG0000003206	RAB11B	cyan	VSMC_M2	0.25	2.12E-01	0.86	5.89E-09	0.18	3.73E-01	0.56	2.33E-03	-0.25	2.12E-01	-0.01	9.61E-01	-0.38	5.01E-02	-0.74	1.06E-05	-0.79	9.87E-07	0.80	5.56E-07
ENSCAFG0000002901	CD164	pink	VSMC_M5	0.25	2.12E-01	0.20	3.13E-01	-0.34	7.95E-02	0.25	2.13E-01	0.57	1.99E-03	-0.43	2.49E-02	-0.30	1.26E-01	-0.34	8.48E-02	-0.10	6.26E-01	-0.03	8.81E-01
ENSCAFG0000000439	MAST2	darkgrey	VSMC_M8	0.25	2.12E-01	-0.10	6.18E-01	0.48	1.07E-02	-0.75	8.03E-06	-0.28	1.64E-01	0.13	5.34E-01	0.05	7.90E-01	0.68	8.76E-05	-0.06	7.80E-01	-0.49	9.69E-03
ENSCAFG0000000102	CDKN2AIPNL	red	VSMC_M1	0.25	2.12E-01	0.02	9.38E-01	0.06	7.58E-01	-0.10	6.10E-01	0.03	9.01E-01	0.15	4.65E-01	0.24	2.18E-01	0.10	6.03E-01	-0.14	4.83E-01	-0.03	8.98E-01
ENSCAFG0000001283	SLC35E4	grey	VSMC_M1C	0.25	2.12E-01	0.00	9.88E-01	0.07	7.47E-01	-0.37	5.63E-02	0.08	7.01E-01	0.09	6.48E-01	0.16	4.39E-01	0.32	1.08E-01	-0.13	5.33E-01	-0.24	2.34E-01
ENSCAFG0000002483	KIF21B	grey	VSMC_M1C	0.25	2.12E-01	0.24	2.35E-01	0.59	1.10E-03	-0.57	2.04E-03	-0.43	2.67E-02	0.18	3.77E-01	0.32	1.04E-01	0.34	8.31E-02	-0.35	7.64E-02	-0.09	6.71E-01
ENSCAFG0000000968	SYS1	cyan	VSMC_M2	0.25	2.12E-01	0.57	1.83E-03	0.08	6.91E-01	0.34	8.70E-02	-0.13	5.17E-01	0.18	3.76E-01	-0.16	4.16E-01	-0.43	2.43E-02	-0.58	1.70E-03	0.56	2.55E-03
ENSCAFG0000001963	RHBDL1	grey	VSMC_M1C	0.25	2.12E-01	0.55	2.74E-03	0.07	7.17E-01	0.44	2.00E-02	0.01	9.71E-01	-0.12	5.67E-01	-0.19	3.49E-01	-0.58	1.57E-03	-0.51	6.05E-03	0.36	6.52E-02
ENSCAFG0000001923	ENSCAFG0000001923	yellow	VSMC_M3	0.25	2.12E-01	-0.10	6.28E-01	0.66	2.05E-04	-0.71	3.70E-05	-0.51	6.11E-03	0.23	2.57E-01	0.34	7.86E-02	0.65	2.11E-04	-0.10	6.13E-01	-0.29	1.35E-01
ENSCAFG0000000599	IL5RA	cyan	VSMC_M2	0.25	2.12E-01	0.38	5.21E-02	0.13	5.29E-01	-0.07	7.39E-01	-0.02	9.31E-01	-0.08	7.07E-01	-0.14	4.79E-01	-0.07	7.47E-01	-0.37	5.64E-02	0.14	4.71E-01
ENSCAFG0000000790	ROBO1	grey	VSMC_M1C	0.25	2.13E-01	0.31	1.15E-01	-0.38	5.18E-02	0.51	6.39E-03	0.34	7.91E-02	0.04	8.44E-01	-0.16	4.14E-01	-0.55	3.27E-03	-0.35	7.39E-02	0.48	1.10E-02
ENSCAFG0000002949	TP53RK	grey	VSMC_M1C	0.25	2.13E-01	0.05	7.86E-01	0.45	1.97E-02	-0.30	1.23E-01	-0.16	4.14E-01	-0.09	6.61E-01	-0.06	7.75E-01	0.21	3.03E-01	-0.07	7.32E-01	-0.40	3.78E-02
ENSCAFG0000000077	AGPAT1	grey	VSMC_M1C	0.25	2.13E-01	0.51	6.46E-03	-0.11	5.80E-01	0.61	7.73E-04	0.19	3.49E-01	-0.20	3.16E-01	-0.29	1.40E-01	-0.74	1.24E-05	-0.44	2.33E-02	0.43	2.39E-02
ENSCAFG0000000862	P2RY1	grey	VSMC_M1C	0.25	2.13E-01	-0.13	5.20E-01	0.26	1.96E-01	-0.21	2.85E-01	-0.13	5.24E-01	0.00	9.89E-01	-0.19	3.41E-01	0.26	1.92E-01	0.01	9.62E-01	-0.21	2.87E-01
ENSCAFG0000001068	DENND5B	pink	VSMC_M5	0.25	2.13E-01	-0.54	3.44E-03	-0.29	1.37E-01	-0.31	1.12E-01	0.49	8.76E-03	0.13	5.17E-01	0.21	3.05E-01	0.41	3.33E-02	0.37	5.94E-02	-0.57	1.85E-03
ENSCAFG0000003098	TSNAX-DISC1	pink	VSMC_M5	0.25	2.13E-01	-0.27	1.79E-01	-0.40	4.05E-02	0.23	2.51E-01	0.54	3.64E-03	-0.09	6.71E-01	0.01	9.54E-01	-0.08	6.80E-01	0.12	5.47E-01	-0.11	5.75E-01
ENSCAFG0000001627	FYTTD1	grey	VSMC_M1C	0.25	2.14E-01	-0.10	6.29E-01	0.12	5.44E-01	-0.18	3.81E-01	0.21	2.89E-01	-0.31	1.16E-01	0.03	8.75E-01	0.09	6.62E-01	0.08	6.99E-01	-0.47	1.24E-02
ENSCAFG0000001937	CAMKK1	grey	VSMC_M1C	0.25	2.14E-01	0.08	6.82E-01	-0.13	5.06E-01	0.46	1.64E-02	0.05	7.87E-01	0.21	2.83E-01	-0.11	5.95E-01	-0.34	8.00E-02	-0.18	3.57E-01	0.41	3.59E-02
ENSCAFG0000000011	RNF41	darkgreen	VSMC_M4	0.25	2.14E-01	0.40	4.13E-02	-0.32	1.04E-01	0.78	1.77E-06	0.27	1.71E-01	-0.15	4.52E-01	-0.22	2.73E-01	-0.80	7.00E-07	-0.38	5.38E-02	0.61	6.47E-04
ENSCAFG0000001559	DHRS7	darkgreen	VSMC_M4	0.25	2.14E-01	0.20	3.15E-01	-0.34	8.65E-02	0.75	8.22E-06	0.38	5.20E-02	-0.08	6.92E-01	-0.27	1.71E-01	-0.62	5.14E-04	-0.20	3.19E-01	0.29	1.47E-01
ENSCAFG0000001132	ENSCAFG0000001132	grey	VSMC_M1C	0.25	2.14E-01	0.21	2.86E-01	-0.13	5.20E-01	0.23	2.54E-01	0.13	5.30E-01	0.06	7.75E-01	0.09	6.71E-01	-0.23	2.52E-01	-0.28	1.56E-01	0.24	2.33E-01
ENSCAFG0000001408	SLC2A11	cyan	VSMC_M2	0.25	2.15E-01	0.63	4.91E-04	0.24	2.27E-01	0.10	6.24E-01	-0.20	3.08E-01	-0.24	2.32E-01	0.06	7.83E-01	-0.33	8.80E-02	-0.60	8.37E-04	0.50	8.27E-03
ENSCAFG0000001819	RASL10B	cyan	VSMC_M2	0.25	2.15E-01	0.55	3.20E-03	0.56	2.28E-03	-0.20	3.19E-01	-0.53	4.64E-03	0.19	3.43E-01	0.06	7.76E-01	0.00	9.83E-01	-0.57	1.75E-03	0.30	1.30E-01
ENSCAFG0000002523	GRASP	cyan	VSMC_M2	0.25	2.15E-01	0.51	7.03E-03	0.53	4.09E-03	-0.18	3.58E-01	-0.50	7.58E-03	0.04	8.25E-01	-0.17	3.92E-01	0.05	7.98E-01	-0.60	9.01E-04	0.27	1.81E-01
ENSCAFG0000000817	MRPL19	grey	VSMC_M1C	0.25	2.15E-01	-0.41	3.32E-02	-0.11	5.88E-01	-0.14	4.85E-01	0.37	5.57E-02	-0.24	2.21E-01	-0.03	8.62E-01	0.22	2.72E-01	0.37	5.93E-02	-0.57	1.74E-03
ENSCAFG0000000451	CDC42EP4	darkgrey	VSMC_M8	0.25	2.15E-01	-0.10	6.16E-01	0.56	2.45E-03	-0.71	3.29E-05	-0.37	5.77E-02	-0.03	8.86E-01	0.57	1.96E-03	0.60	1.00E-03	-0.04	8.54E-01	-0.30	1.35E-01
ENSCAFG0000001327	DUSP16	grey	VSMC_M1C	0.25	2.15E-01	0.36	6.88E-02	-0.05	8.12E-01	0.14	4.81E-01	0.13	5.05E-01	-0.02	9.20E-01	-0.56	2.32E-03	-0.15	4.42E-01	-0.35	7.60E-02	0.11	5.91E-01
ENSCAFG0000002039	CIAO2B	grey	VSMC_M1C	0.25	2.15E-01	0.23	2.51E-01	0.19	3.31E-01	-0.01	9.72E-01	-0.18	3.63E-01	0.19	3.49E-01	-0.11	5.93E-01	-0.04	8.39E-01	-0.30	1.28E-01	0.16	4.33E-01
ENSCAFG0000000476	STAC	grey	VSMC_M1C	0.25	2.15E-01	-0.49	9.14E-03	-0.04	8.49E-01	-0.36	6.46E-02	0.25	2.05E-01	-0.13	5.08E-01	0.51	6.11E-03	0.52	5.17E-03	0.27	1.69E-01	-0.55	3.16E-03
ENSCAFG0000003055	ENSCAFG0000003055	grey	VSMC_M1C	0.25	2.16E-01	0.35	6.96E-02	0.18	3.65E-01	0.08	6.94E-01	-0.04	8.53E-01	0.17	3.85E-01	-0.15	4.62E-01	-0.30	1.33E-01	-0.35	7.37E-02	0.16	4.35E-01
ENSCAFG0000000210	ENSCAFG0000000210	grey	VSMC_M1C	0.25	2.16E-01	0.08	6.76E-01	-0.05	8.02E-01	0.27	1.71E-01	0.03	8.90E-01	0.12	5.51E-01	-0.09	6.55E-01	-0.11	5.90E-01	-0.24	2.22E-01	0.18	3.77E-01
ENSCAFG0000003054	ENSCAFG0000003054	red	VSMC_M1	0.25	2.16E-01	0.46	1.66E-02	-0.14	4.94E-01	0.56	2.65E-03	0.09	6.64E-01	0.05	8.19E-01	-0.39	4.54E-02	-0.62	5.99E-04	-0.42	2.92E-02	0.54	3.78E-03
ENSCAFG0000001774	TBX2	grey	VSMC_M1C	0.25	2.16E-01	0.25	2.07E-01	-0.09	6.46E-01	0.39	4.15E-02	0.22	2.63E-01	-0.06	7.52E-01	-0.73	1.63E-05	-0.42	2.87E-02	-0.14	5.01E-01	0.11	5.71E-01
ENSCAFG0000001825	GREB1L	turquoise	VSMC_M6	0.25	2.16E-01	0.23	2.55E-01	0.47	1.40E-02	-0.18	3.80E-01	-0.35	7.62E-02	0.59	1.30E-03	-0.02	9.35E-01	0.08	7.00E-01	-0.28	1.65E-01	-0.01	9.64E-01
ENSCAFG0000002043	WLS	darkgreen	VSMC_M4	0.25	2.16E-01	0.15	4.42E-01	-0.44	2.22E-02	0.68	8.81E-05	0.53	4.12E-03	-0.15	4.50E-01	-0.59	1.14E-03	-0.67	1.35E-04	-0.05	8.05E-01	0.17	4.07E-01
ENSCAFG0000002992	TSC22D3	grey	VSMC_M1C	0.25	2.17E-01	0.23	2.45E-01	0.13	5.11E-01	-0.04	8.29E-01	0.05	8.03E-01	-0.24	2.18E-01	0.06	7.71E-01	-0.12	5.48E-01	-0.31	1.20E-01	0.03	8.91E-01
ENSCAFG0000001655	ENSCAFG0000001655	grey	VSMC_M1C	0.25	2.17E-01	-0.03	8.79E-01	0.09	6.64E-01	0.10	6.25E-01	-0.02	9.34E-01	-0.02	9.25E-01	0.12	5.40E-01	-0.02	9.26E-01	-0.05	8.09E-01	0.05	8.01E-01
ENSCAFG0000002444	ENSCAFG0000002444	grey	VSMC_M1C	0.25	2.17E-01	0.17	3.97E-01	0.22	2.61E-01	0.16	4.26E-01	-0.19	3.33E-01	0.14	4.84E-01	0.06	7.52E-01	-0.14	4.74E-01	-0.23	2.56E-01	0.22	2.65E-01
ENSCAFG0000001181	MRPS21	grey	VSMC_M1C	0.25	2.17E-01	-0.04	8.47E-01	-0.01	9.77E-01	0.03	8.87E-01	0.10	6.05E-01	-0.04	8.57E-01	0.21	3.00E-01	0.04	8.55E-01	-0.12	5.46E-01	-0.14	4.78E-01
ENSCAFG0000000175	PPP2R5D	darkgrey	VSMC_M8	0.25	2.17E-01	-0.39	4.59E-02	0.21	2.94E-01	-0.68	8.09E-05	0.13	5.13E-01	-0.08	6.93E-01	0.34	8.17E-02	0.70	4.79E-05	0.25	2.09E-01	-0.77	2.20E-06
ENSCAFG0000000150	NRF1	grey	VSMC_M1C	0.25	2.17E-01	0.02	9.18E-01	-0.11	5.99E-01	0.08	6.91E-01	0.27	1.80E-01	-0.06	7.77E-01	-0.19	3.50E-01	-0.09	6.43E-01	-0.03	8.68E-01	-0.09	6.56E-01
ENSCAFG0000003275	CLEC2B	grey	VSMC_M1C	0.25	2.17E-01	-0.23	2.41E-01	-0.04	8.62E-01	-0.16	4.22E-01	0.26	1.89E-01	0.04	8.30E-01	0.04	8.56E-01	0.18	3.65E-01	0.15	4.67E-01	-0.45	1.84E-02
ENSCAFG0000002510	GPR37L1	grey	VSMC_M1C	0.25	2.17E-01	0.39	4.19E-02	0.47	1.38E-02	-0.16	4.25E-01	-0.25	2.14E-01	-0.18	3.76E-01	-0.30	1.35E-01	-0.10	6.11E-01	-0.29	1.45E-01	-0.04	8.26E-01
ENSCAFG0000000039	PCMT1	pink	VSMC_M5	0.25	2.18E-01	0.00	9.89E-01	-0.64	3.50E-04	0.63	3.94E-04	0.67	1.17E-04	-0.19	3.46E-01	-0.24	2.29E-01	-0.44	2.08E-02	-0.02	9.13E-01	0.16	4.33E-01
ENSCAFG0000001153	ZKSCAN8	grey	VSMC_M1C	0.25	2.18E-01	0.44	2.31E-02	-0.22	2.79E-01	0.43	2.50E-02	0.39	4.65E-02	-0.01	9.73E-01	-0.15	4.68E-01	-0.57	1.82E-03	-0.37	5.98E-02	0.22	2.61E-01
ENSCAFG0000001040	TJP1	cyan	VSMC_M2	0.25	2.18E-01	0.51	6.15E-03	-0.15	4.64E-01	0.27	1.72E-01	0.28	1.57E-01	-0.27	1.66E-01	-0.23	2.56E-01	-0.44	2.09E-02	-0.46	1.51E-02	0.31	1.17E-01
ENSCAFG0000000582	ACSS3	grey	VSMC_M1C	0.25	2.18E-01	0.72	2.13E-05	0.00	9.95E-01	0.46	1.48E-02	0.03	8.63E-01	-0.31	1.21E-01	-0.13	5.16E-01	-0.63	4.36E-04	-0.63	4.49E-04	0.62	5.25E-04
ENSCAFG0000000048	STK3	grey	VSMC_M1C	0.25	2.18E-01	-0.16	4.29E-01	-0.34	8.61E-02	0.16	4.34E-01	0.44	2.22E-02	-0.03	8.97E-01	0.55	3.14E-03	-0.08	7.06E-01	0.03	8.94E-01	-0.03	8.73E-01
ENSCAFG0000001843	SREBF1	grey	VSMC_M1C	0.25	2.18E-01	0.18	3.68E-01	0.51	6.24E-03	-0.64	3.05E-04	-0.27	1.66E-01	0.00	9.90E-01	0.28	1.63E-01	0.37	5.96E-02	-0.26	1.84E-01	-0.19	3.50E-01
ENSCAFG0000001888	ZFR	pink	VSMC_M5	0.24	2.18E-01	-0.28	1.62E-01	-0.60	8.61E-04	0.36	6.23E-02	0.74	1.09E-05	-0.22	2.70E-01	0.20	3.07E-01	-0.18	3.64E-01	0.16	4.34E-01	-0.10	6.18E-01
ENSCAFG0000001919	ANKS3	grey	VSMC_M1C	0.24	2.18E-01	0.28	1.51E-01	0.63	4.49E-04	-0.23	2.48E-01	-0.62	5.65E-04	0.15	4.56E-01	0.25	2.12E-01	0.14	4.90E-01	-0.45	1.91E-02	0.21	3.03E-01
ENSCAFG0000001513	CEP126	grey	VSMC_M1C	0.24	2.18E-01	0.06	7.79E-01	0.34	8.43E-02	-0.14	4.85E-01	-0.18	3.73E-01	-0.41	3.32E-02	0.28	1.65E-01	0.07	7.39E-01	-0.14	4.93E-01	-0.03	8.77E-01
ENSCAFG0000001790	ENSCAFG0000001790	yellow	VSMC_M3	0.24	2.18E-01	0.31	1.21E-01	0.44	2.17E-02	-0.28	1.50E-01	-0.22	2.76E-01	-0.03	8.67E-01	-0.09	6.48E-01	0.07	7.26E-01	-0.27	1.72E-01	-0.15	4.50E-01
ENSCAFG0000001892	DAB1	grey	VSMC_M1C	0.24	2.18E-01	-0.14	4.75E-01	-0.17	4.10E-01	-0.12	5.35E-01	0.47	1.31E-02	-0.14	4.80E-01	-0.07	7.36E-01	0.11	5.72E-01	0.10	6.22E-01	-0.52	5.03E-03
ENSCAFG0000002022	ENSCAFG0000002022	grey	VSMC_M1C	0.24	2.18E-01	-0.14	4.75E-01	-0.17	4.10E-01	-0.12	5.35E-01	0.47	1.31E-02	-0.14	4.80E-01	-0.07	7.36E-01	0.11	5.72E-01	0.10	6.22E-01	-0.52	5.03E-03
ENSCAFG0000002051	ENSCAFG0000002051	grey	VSMC_M1C	0.24	2.18E-01	-0.14	4.75E-01	-0.17	4.10E-01	-0.12	5.35E-01	0.47	1.31E-02	-0.14	4.80E-01	-0.07	7.36E-01	0.11	5.72E-01	0.10	6.22E-01	-0.52	5.03E-03
ENSCAFG0000000030	CTDSP2	yellow	VSMC_M3	0.24	2.19E-01	0.64	3.02E-04	0.54	3.36E-03	-0.20	3.19E-01	-0.35	7.25E-02	-0.21	2.83E-01	-0.02	9.32E-01	-0.11	5.83E-01	-0.58	1.41E-03	0.14	4.72E-01
ENSCAFG0000001992	AIF1L	cyan	VSMC_M2	0.24	2.19E-01	0.29	1.45E-01	0.20	3.25E-01	0.21	2.99E-01	-0.12	5.54E-01	0.11	5.87E-01	-0.08	6.84E-01	-0.40	4.02E-02	-0.25	2.02E-01	0.23	2.38E-01
ENSCAFG0000001925	FNDC10	cyan	VSMC_M2	0.24	2.19E-01	0.53	4.50E-03	0.25	2.10E-01	0.11	5.80E-01	-0.29	1.41E-01	0.07	7.33E-01	0.16	4.33E-01	-0.25	2.05E-01	-0.56	2.63E-03	0.54	3.29E-03

ENSCAFG0000001703	ENSCAFG0000001703	grey	VSMC_M10	0.24	2.19E-01	0.00	9.82E-01	-0.15	4.56E-01	0.05	8.14E-01	0.22	2.66E-01	-0.09	6.38E-01	0.08	6.82E-01	-0.06	7.73E-01	-0.07	7.32E-01	0.00	9.90E-01
ENSCAFG0000001886	ENSCAFG0000001886	grey	VSMC_M10	0.24	2.19E-01	-0.27	1.69E-01	-0.10	6.19E-01	-0.19	3.42E-01	0.23	2.54E-01	-0.13	5.28E-01	0.04	8.47E-01	0.28	1.56E-01	0.14	4.84E-01	-0.26	1.89E-01
ENSCAFG0000000839	PLRG1	red	VSMC_M1	0.24	2.19E-01	0.04	8.24E-01	0.02	9.35E-01	0.05	7.92E-01	0.04	8.61E-01	-0.10	6.15E-01	0.28	1.59E-01	-0.05	7.92E-01	-0.19	3.30E-01	0.08	6.99E-01
ENSCAFG0000000593	STRN	darkgrey	VSMC_M8	0.24	2.19E-01	-0.32	1.09E-01	0.29	1.38E-01	-0.77	2.29E-06	0.00	9.89E-01	-0.06	7.59E-01	0.33	9.21E-02	0.73	1.56E-05	0.18	3.78E-01	-0.62	6.12E-04
ENSCAFG0000001980	TCF25	cyan	VSMC_M2	0.24	2.19E-01	0.67	1.24E-04	0.61	6.90E-04	-0.14	4.71E-01	-0.45	1.88E-02	-0.15	4.55E-01	-0.42	3.11E-02	-0.12	5.51E-01	-0.58	1.54E-03	0.12	5.40E-01
ENSCAFG0000003219	MAP1A	grey	VSMC_M10	0.24	2.19E-01	-0.24	2.31E-01	0.03	8.85E-01	-0.31	1.11E-01	0.21	2.86E-01	-0.26	1.83E-01	0.47	1.38E-02	0.34	8.38E-02	0.15	4.52E-01	-0.41	3.24E-02
ENSCAFG0000002398	TMEM121	grey	VSMC_M10	0.24	2.20E-01	0.32	1.01E-01	0.42	2.86E-02	-0.23	2.58E-01	-0.40	3.67E-02	0.20	3.12E-01	0.38	5.29E-02	0.07	7.33E-01	-0.45	1.97E-02	0.24	2.25E-01
ENSCAFG0000001180	CFAP45	grey	VSMC_M10	0.24	2.20E-01	0.20	3.25E-01	0.40	3.69E-02	-0.09	6.58E-01	-0.36	6.51E-02	-0.12	5.35E-01	-0.07	7.30E-01	0.00	9.95E-01	-0.24	2.29E-01	0.18	3.80E-01
ENSCAFG0000001456	PPP1R14B	darkgrey	VSMC_M8	0.24	2.20E-01	-0.34	8.21E-02	0.42	3.01E-02	-0.80	5.37E-07	-0.12	5.38E-01	-0.09	6.68E-01	0.15	4.59E-01	0.79	1.00E-06	0.19	3.31E-01	-0.76	4.30E-06
ENSCAFG0000001918	HAUS7	darkgrey	VSMC_M8	0.24	2.20E-01	-0.22	2.60E-01	0.53	4.43E-03	-0.58	1.60E-03	-0.31	1.18E-01	0.14	4.90E-01	0.19	3.33E-01	0.62	5.66E-04	0.07	7.46E-01	-0.47	1.42E-02
ENSCAFG0000003083	PTGES	grey	VSMC_M10	0.24	2.21E-01	0.53	4.15E-03	0.02	9.39E-01	0.33	9.48E-02	-0.02	9.40E-01	0.10	6.21E-01	0.18	3.71E-01	-0.43	2.52E-02	-0.59	1.20E-03	0.49	9.50E-03
ENSCAFG0000001554	RPAIN	pink	VSMC_M5	0.24	2.21E-01	0.30	1.22E-01	-0.38	4.96E-02	0.49	8.87E-03	0.56	2.33E-03	-0.45	1.71E-02	-0.21	2.83E-01	-0.57	1.88E-03	-0.22	2.73E-01	0.13	5.23E-01
ENSCAFG0000001434	KIAA0232	grey	VSMC_M10	0.24	2.21E-01	0.14	4.79E-01	-0.47	1.25E-02	0.52	5.48E-03	0.56	2.15E-03	-0.38	4.91E-02	-0.11	5.74E-01	-0.48	1.08E-02	-0.15	4.43E-01	0.20	3.12E-01
ENSCAFG0000001264	PSTK	yellow	VSMC_M3	0.24	2.21E-01	0.39	4.42E-02	0.37	5.43E-02	-0.28	1.51E-01	-0.14	4.85E-01	0.02	9.32E-01	-0.22	2.73E-01	0.05	8.01E-01	-0.35	7.56E-02	-0.11	5.76E-01
ENSCAFG0000002965	NUPL2	grey	VSMC_M10	0.24	2.21E-01	0.32	9.82E-02	0.11	5.72E-01	0.33	8.86E-02	-0.12	5.36E-01	0.01	9.58E-01	-0.07	7.19E-01	-0.42	3.09E-02	-0.36	6.59E-02	0.43	2.54E-02
ENSCAFG0000001634	ENSCAFG0000001634	grey	VSMC_M10	0.24	2.21E-01	-0.07	7.45E-01	-0.26	1.86E-01	0.07	7.27E-01	0.39	4.35E-02	-0.21	2.89E-01	0.00	9.90E-01	-0.05	7.91E-01	0.02	9.17E-01	-0.10	6.24E-01
ENSCAFG0000001396	BCO2	grey	VSMC_M10	0.24	2.22E-01	0.15	4.48E-01	0.35	7.05E-02	-0.22	2.71E-01	-0.22	2.62E-01	-0.05	7.91E-01	0.33	9.63E-02	0.08	6.74E-01	-0.25	2.15E-01	0.03	8.70E-01
ENSCAFG0000001780	NPTN	darkgreen	VSMC_M4	0.24	2.22E-01	-0.07	7.36E-01	-0.64	3.02E-04	0.76	3.80E-06	0.72	2.13E-05	-0.21	2.83E-01	-0.36	6.66E-02	-0.62	5.64E-04	0.09	6.63E-01	0.11	5.99E-01
ENSCAFG0000001416	CDC42BPG	cyan	VSMC_M2	0.24	2.22E-01	0.40	3.82E-02	0.25	2.16E-01	0.28	1.61E-01	-0.20	3.25E-01	0.10	6.09E-01	-0.03	8.73E-01	-0.40	3.88E-02	-0.38	5.22E-02	0.37	5.75E-02
ENSCAFG0000001166	SIPA1L2	cyan	VSMC_M2	0.24	2.22E-01	0.77	2.29E-06	0.05	7.93E-01	0.64	3.61E-04	-0.10	6.07E-01	-0.03	9.01E-01	-0.44	2.03E-02	-0.75	7.09E-06	-0.72	2.06E-05	0.73	1.36E-05
ENSCAFG0000000409	MGLL	yellow	VSMC_M3	0.24	2.22E-01	0.52	5.36E-03	0.58	1.54E-03	-0.15	4.41E-01	-0.50	7.27E-03	0.24	2.22E-01	-0.22	2.74E-01	-0.13	5.34E-01	-0.49	9.55E-03	0.23	2.52E-01
ENSCAFG0000003263	ZNF283	grey	VSMC_M10	0.24	2.22E-01	0.13	5.32E-01	0.21	2.84E-01	-0.21	2.96E-01	-0.13	5.25E-01	0.01	9.45E-01	-0.07	7.46E-01	0.11	5.80E-01	-0.22	2.74E-01	0.00	9.84E-01
ENSCAFG0000002880	PDZD11	grey	VSMC_M10	0.24	2.22E-01	0.04	8.43E-01	0.42	2.79E-02	-0.48	1.06E-02	-0.26	1.95E-01	0.00	9.83E-01	0.05	7.87E-01	0.39	4.39E-02	-0.13	5.34E-01	-0.28	1.60E-01
ENSCAFG0000002466	ENSCAFG0000002466	red	VSMC_M1	0.24	2.23E-01	-0.03	8.70E-01	0.00	9.90E-01	0.02	9.30E-01	0.06	7.72E-01	0.28	1.64E-01	0.04	8.36E-01	-0.01	9.42E-01	-0.08	7.05E-01	-0.01	9.72E-01
ENSCAFG0000000885	SOD1	yellow	VSMC_M3	0.24	2.23E-01	0.30	1.32E-01	0.50	7.67E-03	-0.23	2.51E-01	-0.24	2.35E-01	-0.31	1.14E-01	0.02	9.11E-01	0.03	8.89E-01	-0.23	2.40E-01	-0.13	5.09E-01
ENSCAFG0000000349	HECW1	grey	VSMC_M10	0.24	2.23E-01	0.08	6.77E-01	0.19	3.44E-01	-0.30	1.28E-01	-0.09	6.65E-01	0.09	6.40E-01	0.42	3.10E-02	0.26	1.85E-01	-0.22	2.65E-01	0.00	9.91E-01
ENSCAFG0000000519	POU4F1	grey	VSMC_M10	0.24	2.23E-01	-0.14	4.85E-01	-0.07	7.36E-01	-0.11	5.81E-01	0.21	2.84E-01	-0.12	5.55E-01	-0.17	3.92E-01	0.12	5.48E-01	0.15	4.61E-01	-0.23	2.56E-01
ENSCAFG0000001038	DNAH7	darkgreen	VSMC_M4	0.24	2.23E-01	0.12	5.60E-01	-0.45	1.73E-02	0.75	7.97E-06	0.52	5.92E-03	-0.27	1.67E-01	-0.45	1.90E-02	-0.62	6.07E-04	-0.09	6.39E-01	0.21	3.04E-01
ENSCAFG0000003059	NFKBIL1	cyan	VSMC_M2	0.24	2.24E-01	0.53	4.25E-03	0.31	1.21E-01	0.01	9.49E-01	-0.26	1.94E-01	0.02	9.21E-01	0.14	4.85E-01	-0.19	3.32E-01	-0.60	8.41E-04	0.39	4.59E-02
ENSCAFG0000001111	GYG2	darkgreen	VSMC_M4	0.24	2.24E-01	0.07	7.31E-01	-0.32	1.03E-01	0.58	1.61E-03	0.33	9.77E-02	-0.01	9.51E-01	0.11	6.00E-01	-0.46	1.70E-02	-0.16	4.18E-01	0.28	1.62E-01
ENSCAFG0000001650	IL27RA	cyan	VSMC_M2	0.24	2.24E-01	0.72	2.66E-05	0.48	1.13E-02	0.00	9.91E-01	-0.54	3.96E-03	0.20	3.19E-01	-0.11	5.85E-01	-0.21	2.89E-01	-0.72	2.06E-05	0.58	1.48E-03
ENSCAFG0000001711	IFT43	darkgreen	VSMC_M4	0.24	2.24E-01	0.41	3.40E-02	-0.12	5.65E-01	0.69	6.68E-05	0.11	5.79E-01	-0.20	3.23E-01	-0.35	7.44E-02	-0.70	5.44E-05	-0.37	5.39E-02	0.51	7.13E-03
ENSCAFG0000001991	FOXF1	grey	VSMC_M10	0.24	2.24E-01	0.46	1.58E-02	0.32	9.90E-02	-0.18	3.61E-01	-0.23	2.46E-01	0.11	5.83E-01	-0.35	7.75E-02	-0.03	8.78E-01	-0.51	6.49E-03	0.16	4.12E-01
ENSCAFG0000000317	DPY19L1	pink	VSMC_M5	0.24	2.25E-01	-0.44	2.15E-02	-0.26	1.86E-01	-0.15	4.58E-01	0.48	1.12E-02	-0.16	4.34E-01	-0.01	9.70E-01	0.29	1.41E-01	0.36	6.36E-02	-0.55	2.74E-03
ENSCAFG0000001683	TMEM79	yellow	VSMC_M3	0.24	2.25E-01	0.19	3.42E-01	0.41	3.18E-02	-0.02	9.02E-01	-0.28	1.53E-01	-0.07	7.40E-01	0.00	9.82E-01	-0.03	8.98E-01	-0.25	2.02E-01	0.02	9.30E-01
ENSCAFG0000000439	FZD4	cyan	VSMC_M2	0.24	2.25E-01	0.70	4.08E-05	-0.07	7.38E-01	0.62	5.12E-04	0.07	7.28E-01	-0.16	4.39E-01	-0.53	4.47E-03	-0.75	7.44E-06	-0.60	9.60E-04	0.63	4.85E-04
ENSCAFG0000000170	CARNMT1	grey	VSMC_M10	0.24	2.25E-01	-0.06	7.69E-01	0.09	6.57E-01	-0.21	2.85E-01	0.08	6.98E-01	-0.16	4.36E-01	-0.01	9.68E-01	0.21	2.85E-01	-0.06	7.54E-01	-0.23	2.47E-01
ENSCAFG0000002391	SLC8A3	cyan	VSMC_M2	0.24	2.25E-01	0.56	2.58E-03	0.28	1.63E-01	0.25	2.06E-01	-0.26	1.84E-01	0.11	5.98E-01	-0.55	2.99E-03	-0.40	4.14E-02	-0.49	9.00E-03	0.43	2.55E-02
ENSCAFG0000000330	ZC3H12A	yellow	VSMC_M3	0.24	2.25E-01	0.58	1.50E-03	0.58	1.45E-03	-0.22	2.69E-01	-0.42	2.73E-02	-0.06	7.50E-01	-0.10	6.19E-01	-0.07	7.30E-01	-0.57	1.99E-03	0.21	3.02E-01
ENSCAFG0000000540	GPD1L	grey	VSMC_M10	0.24	2.25E-01	0.40	3.92E-02	-0.02	9.18E-01	0.24	2.26E-01	0.06	7.59E-01	-0.16	4.23E-01	0.29	1.37E-01	-0.33	8.89E-02	-0.45	1.77E-02	0.39	4.26E-02
ENSCAFG0000002478	ARL16	grey	VSMC_M10	0.24	2.26E-01	0.50	7.60E-03	-0.03	8.82E-01	0.30	1.30E-01	0.05	8.17E-01	0.00	9.92E-01	-0.30	1.26E-01	-0.49	1.02E-02	-0.43	2.48E-02	0.40	4.04E-02
ENSCAFG0000000801	SH3D19	grey	VSMC_M10	0.24	2.26E-01	0.55	3.11E-03	-0.05	7.89E-01	0.42	3.04E-02	0.18	3.65E-01	-0.52	5.76E-03	-0.19	3.54E-01	-0.57	2.02E-03	-0.44	2.14E-02	0.36	6.67E-02
ENSCAFG0000003034	ENSCAFG0000003034	grey	VSMC_M10	0.24	2.26E-01	0.08	6.80E-01	0.22	2.80E-01	-0.01	9.53E-01	-0.07	7.41E-01	-0.20	3.10E-01	0.08	7.00E-01	0.01	9.53E-01	-0.14	4.88E-01	-0.04	8.59E-01
ENSCAFG0000001863	ROR1	cyan	VSMC_M2	0.24	2.27E-01	0.78	1.37E-06	0.14	4.75E-01	0.38	5.18E-02	-0.10	6.10E-01	-0.27	1.73E-01	-0.43	2.59E-02	-0.55	2.81E-03	-0.72	1.96E-05	0.55	2.82E-03
ENSCAFG0000000195	SLAIN2	grey	VSMC_M10	0.24	2.27E-01	-0.09	6.38E-01	-0.28	1.60E-01	0.04	8.39E-01	0.33	9.76E-02	-0.01	9.43E-01	-0.06	7.71E-01	-0.02	9.21E-01	0.05	8.21E-01	0.04	8.35E-01
ENSCAFG0000000395	PRKAR2B	grey	VSMC_M10	0.24	2.27E-01	-0.26	1.92E-01	0.08	6.96E-01	-0.48	1.13E-02	0.21	2.89E-01	-0.24	2.27E-01	0.42	3.02E-02	0.41	3.37E-02	0.19	3.39E-01	-0.48	1.08E-02
ENSCAFG0000001605	SLC29A4	grey	VSMC_M10	0.24	2.27E-01	0.62	5.95E-04	0.20	3.06E-01	0.41	3.47E-02	-0.18	3.77E-01	-0.22	2.61E-01	-0.25	2.18E-01	-0.53	4.26E-03	-0.60	9.36E-04	0.57	2.04E-03
ENSCAFG0000001789	YY1	grey	VSMC_M10	0.24	2.27E-01	0.60	8.82E-04	0.00	9.94E-01	0.58	1.70E-03	0.08	6.97E-01	-0.16	4.25E-01	-0.43	2.65E-02	-0.73	1.67E-05	-0.50	8.55E-03	0.51	7.18E-03
ENSCAFG0000001522	CYB5D2	grey	VSMC_M10	0.24	2.27E-01	0.51	7.16E-03	0.56	2.55E-03	-0.09	6.39E-01	-0.62	6.31E-04	0.18	3.67E-01	-0.02	9.24E-01	-0.08	6.91E-01	-0.56	2.18E-03	0.50	8.28E-03
ENSCAFG0000001499	ENSCAFG0000001499	yellow	VSMC_M3	0.24	2.27E-01	0.51	6.16E-03	0.61	8.10E-04	-0.16	4.12E-01	-0.56	2.40E-03	-0.04	8.50E-01	0.39	4.19E-02	-0.07	7.34E-01	-0.58	1.50E-03	0.35	7.13E-02
ENSCAFG0000000661	SUCLG2	grey	VSMC_M10	0.24	2.27E-01	0.40	3.62E-02	-0.10	6.28E-01	0.35	7.40E-02	0.13	5.31E-01	-0.11	5.75E-01	-0.45	1.98E-02	-0.37	5.65E-02	-0.40	3.77E-02	0.35	7.09E-02
ENSCAFG0000003044	MGAT3	turquoise	VSMC_M6	0.24	2.27E-01	0.09	6.57E-01	0.43	2.43E-02	-0.25	2.17E-01	-0.30	1.27E-01	0.50	7.44E-03	-0.10	6.14E-01	0.16	4.26E-01	-0.11	5.73E-01	-0.12	5.39E-01
ENSCAFG0000003147	ENSCAFG0000003147	yellow	VSMC_M3	0.24	2.28E-01	0.22	2.62E-01	0.40	4.10E-02	-0.30	1.29E-01	-0.22	2.68E-01	0.04	8.30E-01	0.13	5.03E-01	0.18	3.62E-01	-0.28	1.54E-01	-0.11	5.90E-01
ENSCAFG0000002005	CNTNAP4	grey	VSMC_M10	0.24	2.28E-01	0.67	1.35E-04	0.17	4.09E-01	0.27	1.75E-01	-0.14	4.78E-01	0.11	5.79E-01	-0.42	2.83E-02	-0.43	2.69E-02	-0.61	6.55E-04	0.53	4.68E-03
ENSCAFG0000000560	XDH	pink	VSMC_M5	0.24	2.28E-01	-0.01	9.60E-01	-0.59	1.09E-03	0.60	9.38E-04	0.55	3.01E-03	0.05	7.89E-01	-0.11	6.01E-01	-0.39	4.70E-02	-0.11	5.95E-01	0.21	2.87E-01
ENSCAFG0000001922	FZR1	grey	VSMC_M10	0.24	2.28E-01	-0.01	9.72E-01	0.19	3.48E-01	-0.12	5.46E-01	-0.12	5.45E-01	0.18	3.62E-01	-0.03	8.70E-01	0.10	6.04E-01	-0.09	6.73E-01	-0.04	8.44E-01
ENSCAFG0000001595	USP27X	yellow	VSMC_M3	0.24	2.28E-01	0.47	1.30E-02	0.71	3.96E-05	-0.33	9.26E-02	-0.57	1.78E-03	0.15	4.41E-01	0.04	8.30E-01	0.09	6.61E-01	-0.50	7.89E-03	0.10	6.10E-01
ENSCAFG0000000085	IRF1	cyan	VSMC_M2	0.24	2.28E-01	0.66	1.77E-04	0.56	2.45E-03	-0.25	2.14E-01	-0.43	2.35E-02	0.04	8.28E-01	-0.08	6.80E-01	-0.01	9.42E-01	-0.69	7.05E-05	0.23	2.39E-01
ENSCAFG0000000932	RIPK1	grey	VSMC_M10	0.24	2.28E-01	0.38	4.93E-02	0.37	5.50E-02	-0.07	7.14E-01	-0.28	1.65E-01	-0.08	7.08E-01	0.17	4.01E-01	-0.07	7.18E-01	-0.42	3.09E-02	0.23	2.50E-01
ENSCAFG0000001950	DFFB	grey	VSMC_M10	0.24	2.28E-01	0.13	5.11E-01	0.34	8.22E-02	-0.41	3.29E-02	-0.06	7.80E-01	-0.21	3.03E-01	-0.01	9.75E-01	0.30	1.31E-01	-0.21	2.86E-01	-0.31	1.17E-01
ENSCAFG0000000806	ENSCAFG0000000806	grey	VSMC_M10	0.24	2.29E-01	-0.43	2.56E-02	-0.23	2.49E-01	-0.16	4.40E-01	0.37	5.69E-02	-0.04	8.43E-01	0.14	4.95E-01	0.33	9.12E-02	0.23	2.51E-01	-0.43	2.71E-02
ENSCAFG0000002993	ENSCAFG0000002993	grey	VSMC_M10	0.24	2.29E-01	0.28	1.64E-01	-0.24	2.29E-01	0.73	1.62E-05	0.28	1.59E-01	-0.15	4.58E-01	-0.25	2.02E-01	-0.70	4.60E-05	-0.24	2.32E-01	0.38	5.14E-02
ENSCAFG0000001502	ENSCAFG0000001502	grey	VSMC_M10	0.24	2.29E-01	0.34	8.06E-02	0.12	5.56E-01	0.03	8.65E-01	0.08	6.92E-01	-0.14	4.85E-01	-0.04	8.26E-01	-0.15	4.65E-01	-0.32	1.08E-01	-0.01	9.63E-01
ENSCAFG0000001904	CDH18	darkgreen	VSMC_M4	0.24	2.29E-01	0.33	8.86E-02	-0.30	1.30E-01	0.68	1.05E-04	0.25	2.02E-01	-0.01	9.76E-01	-0.04	8.42E-01	-0.67	1.31E-04	-0.37	5.74E-02	0.57	1.79E-03
ENSCAFG0000000529	ENSCAFG0000000529	grey	VSMC_M10	0.24	2.29E-01	-0.07	7.13E-01	0.30	1.28E-01	-0.27	1.70E-01	-0.28	1.51E-01	-0.06	7.53E-01	0.44	2.27E-02	0.35	7.39E-02	-0.17	4.05E-01	0.02	9.19E-01
ENSCAFG0000001604	TEX9	pink	VSMC_M5	0.24	2.29E-01	-0.20	3.17E-01	-0.55	2.94E-03	0.22	2.76E-01	0.71	2.94E-05	-0.31	1.12E-01	0.03	8.96E-01	-0.11	5.95E-01	0.14	4.72E-01	-0.23	2.40E-01
ENSCAFG0000001991	STXBP3	pink	VSMC_M5	0.24	2.29E-01	-0.16	4.24E-01	-0.48	1.22E-02	0.30	1.24E-01	0.66	1.69E-04	-0.36	6.50E-02	0.01	9.60E-01	-0.22	2.75E-01	0.12	5.46E-01	-0.16	4.24E-01
ENSCAFG0000001552	ZWINT	darkgrey	VSMC_M8	0.24	2.29E-01	-0.48	1.10E-02	0.25	2.15E-01	-0.66	1.72E-04	0.02	9.30E-01	-0.11	5.94E-01	0.20	3.12E-01	0.80	5.76E-07	0.29	1.50E-01	-0.73	1.77E-05
ENSCAFG0000001767	UBFD1	grey	VSMC_M10	0.24	2.30E-01	-0.14	4.88E-01	0.17	3.94E-01	-0.22	2.77E-01	0.01	9.41E-01	-0.25	2.13E-01	-0.01	9.65E-01	0.18	3.72E-01	0.07	7.24E-01	-0.28	1.54E-01
ENSCAFG0000000463	FDXR	yellow	VSMC_M3	0.24	2.30E-01	0.30	1.26E-01	0.55	3.07E-03	-0.41	3.26E-02	-0.46	1.70E-02	0.18	3.75E-01	0.26	1.98E-01	0.29	1.46E-01	-0.42	3.03E-02	0.10	6.15E-01
ENSCAFG0000000707	ENSCAFG0000000707	grey	VSMC_M10	0.24	2.30E-01	0.20	3.18E-01	0.37	5.64E-02	-0.31	1.20E-01	-0.35	7.58E-02	0.09	6.64E-01	0.06	7.54E-01	0.21	2.97E-01	-0.30	1.30E-01	0.13	5.13E-01
ENSCAFG0000001987	CTU2	yellow	VSMC_M3	0.24	2.30E-01	0.49	1.00E-02	0.72	2.71E-05	-0.23	2.53E-01	-0.56	2.36E-03	-0.19	3.38E-01	-0.13	5.15E-01	0.06	7.83E-01	-0.51	6.34E-03	0.10	6.29E-01
ENSCAFG0000000388	DDO	grey	VSMC_M10	0.24	2.31E-01	0.46	1.47E-02	0.42	2.85E-02	0.00	9.98E-01	-0.33	9.14E-02	-0.28	1.57E-01	-0.33	9.23E-02	-0.16	4.19E-01	-0.48	1.16E-02	0.26	1.89E-01
ENSCAFG0000002549	C9H17orf58	grey	VSMC_M10	0.24	2.31E-01	0.13	5.15E-01	-0.09	6.50E-01	0.34	7.80E-02	-0.01	9.78E-01	0.46	1.51E-02	0.00	9.82E-01	-0.24	2.18E-01	-0.22	2.78E-01	0.43	2.40E-02
ENSCAFG0000000027	ARFGEF3	pink	VSMC_M5	0.24	2.31E-01	0.00	9.97E-01	-0.45	1.89E-02	0.56	2.45E-03	0.55	2.70E-03	-0.19	3.45E-01	-0.10	6.17E-01	-0.48	1.15E-02	0.00	9.87E-01	0.14	4.84E-01
ENSCAFG0000001122	AUTS2	grey	VSMC_M10	0.24	2.31E-01	0.62	4.97E-04	0.05	7.87E-01	0.29	1.37E-01	0.09	6.38E-01	-0.31	1.11E-01	-0.14	4.85E-01	-0.55	2.76E-03	-0.54	3.68E-03	0.35	7.76E-02
ENSCAFG0000000674	PRAG1	turquoise	VSMC_M6	0.24	2.31E-01	0.05	8.13E-01	0.05	8.01E-01	-0.14	4.96E-01	-0.04	8.35E-01	0.48	1.10E-02	0.40	3.89E-02	0.08	6.94E-01	-0.16	4.18E-01	0.08	7.10E-01
ENSCAFG0000003137	UBXN2A	pink	VSMC_M5	0.24	2.32E-01	-0.44	2.12E-02	-0.30	1.25E-01	-0.22	2.65E-01	0.55	3.22E-03	0.05	8.13E-01	-0.01	9.66E-01	0.39	4.59E-02	0.30	1.34E-01	-0.63	4.43E-04
ENSCAFG0000000237	ZNF135	grey	VSMC_M10	0.24	2.32E-01	0.33	8.77E-02	-0.19	3.42E-01	0.34	8.51E-02	0.12	5.48E-01	0.14	4.82E-01	-0.17	3.91E-01	-0.35	7.09E-02	-0.39	4.45E-02	0.45	1.73E-02
ENSCAFG0000000092	ROS1	grey	VSMC_M10	0.24	2.32E-01	0.17	4.05E-01	0.40	3.93E-02	-0.18	3.81E-01	-0.28	1.62E-01	0.14	4.75E-01	-0.25	2.08E-01	0.14	4.98E-01	-0.24	2.34E-01	-0.06	7.64E-01
ENSCAFG0000001155	BPTF	pink	VSMC_M5	0.24	2.32E-01	-0.04	8.24E-01	-0.61	8.05E-04	0.46	1.53E-02	0.65	2.34E-04	-0.11	5.76E-01	0.04	8.46E-01	-0.38	5.12E-02	-0.03	8.68E-01	0.16	4.23E-01
ENSCAFG0000001354	HSPB1	darkgreen	VSMC_M4	0.24	2.32E-01	0.06	7.77E-01	-0.51	6.09E-03	0.70	5.64E-05	0.56	2.24E-03	-0.12	5.48E-01	-0.31	1.22E-01	-0.65	2.26E-04	-0.01	9.69E-01	0.21	2.99E-01
ENSCAFG0000000504	IFT172	grey	VSMC_M10	0.24	2.32E-01	0.40	3.78E-02	0.26	1.97E-01	-0.07	7.28E-01	-0.05	8.17E-01	-0.20	3.26E-01	0.13	5.30E-01	-0.14	4.93E-01	-0.45	1.79E-02	0.06	7.78E-01
ENSCAFG0000001042	IRX2	grey	VSMC_M10	0.24	2.32E-01	-0.11	5.96E-01	0.10	6.11E-01	-0.11	5.93E-01	-0.05	7.88E-01	0.22	2.80E-01	0.11	5.93E-01	0.10	6.10E-01	-0.01	9.45E-01	-0.02	9.33E-01
ENSCAFG0000002399	ATXN1L	grey	VSMC_M10	0.24	2.32E-01	0.10	6.20E-01	-0.11	5.68E-01	0.30	1.23E-01	0.17	3.87E-01	0.09	6.44E-01	-0.47	1.41E-02	-0.34	8.76E-02	-0.10	6.36E-01	0.13	5.29E-01
ENSCAFG0000001435	CASK	grey	VSMC_M10	0.24	2.33E-01	0.05	7.98E-01	-0.10	6.26E-01	0.11	5.94E-01	0.23	2.47E-01	-0.28	1.51E-01	0.57	2.11E-03	-0.07	7.22E-01	-0.16	4.25E-01	0.06	7.53E-01
ENSCAFG0000001232	MINDY1	cyan	VSMC_M2	0.24	2.33E-01	0.89	4.28E-10	0.30	1.26E-01	0.38	5.18E-02	-0.30	1.22E-01	-0.06	7.77E-01	-0.26	1.88E-01	-0.61	6.66E-04	-0.81	2.32E-07	0.70	4.67E-05
ENSCAFG0000000673	TTC17	grey	VSMC_M10	0.24	2.33E-01	0.11	5.92E-01	-0.62	5.04E-04	0.69	6.59E-05	0.65	2.76E-04	-0.22	2.69E-01	-0.12	5.60E-01	-0.63	4.39E-04	-0.15	4.41E-01	0.34	8.08E-02
ENSCAFG0000001249	LYSMD1	grey	VSMC_M10	0.24	2.33E-01	0.17	3.99E-01	0.20	3.08E-01	0.16	4.13E-01	-0.12	5.58E-01	0.02	9.22E-01	-0.41	3.31E-02	-0.22	2.59E-01	-0.15	4.54E-01	0.12	5.62E-01
ENSCAFG0000002421	ENSCAFG0000002421	yellow	VSMC_M3	0.24	2.33E-01	0.30	1.27E-01	0.72	2.07E-05	-0.52	5.04E-03	-0.53	4.12E-03	0.00	9.97E-01	0.39	4.58E-02	0.33	8.82E-02	-0.39	4.49E-02	-0.11	5.90E-01
ENSCAFG0000000431	PPP5C	darkgrey	VSMC_M8	0.24	2.33E-01	-0.48	1.20E-02	0.02	9.26E-01	-0.62	6.30E-04	0.23	2.41E-01	0.03	8.66E-01	0.27	1.68E-01	0.71	3.36E-05	0.28	1.62E-01	-0.71	3.60E-05
ENSCAFG0000000683	SLC15A4	cyan	VSMC_M2	0.24	2.33E-01	0.66	1.92E-04	0.37	5.44E-02	0.03	8.75E-01	-0.16	4.13E-01	-0.29	1.39E-01	-0.13	5.12E-01	-0.35	6.95E-02	-0.57	1.83E-03	0.22	2.63E-01
ENSCAFG0000003101	FAM180A	cyan	VSMC_M2	0.24	2.34E-01	0.76	3.40E-06	0.30	1.25E-01	0.14	4.92E-01	-0.25	2.04E-01	0.09	6.62E-01	-0.13	5.18E-01	-0.42	2.88E-02	-0.72	2.12E-05	0.50	8.54E-03
ENSCAFG0000000587	MAP4K1	grey	VSMC_M10	0.24	2.34E-01	-0.06	7.81E-01	0.17	3.84E-01	-0.10	6.10E-01	-0.05	7.94E-01	-0.10	6.35E-01	-0.04	8.62E-01	0.15	4.59E-01	-0.04	8.50E-01	-0.13	5.24E-01
ENSCAFG0000002024	RABGAP1	grey	VSMC_M10	0.24	2.34E-01	0.17	4.07E-01	-0.34	7.94E-02	0.36	6.92E-02	0.50	8.44E-03	-0.27	1.70E-01	0.06	7.51E-01	-0.46	1.61E-02	-0.20	3.17E-01	0.13	5.08E-01
ENSCAFG0000001576	GANAB	cyan	VSMC_M2	0.24	2.34E-01	0.70	4.54E-05	0.27	1.77E-01	0.26	1.95E-01	-0.31	1.10E-01	0.10	6.13E-01	-0.47	1.38E-02	-0.45	1.85E-02	-0.70	5.06E-05	0.60	1.05E-03
ENSCAFG0000001361	HNRNPH3	darkgrey	VSMC_M8	0.24	2.34E-01	-0.42	3.01E-02	0.34	8.48E-02	-0.77	2.12E-06	-0.04	8.27E-01	-0.02	9.10E-01	0.29	1.36E-01	0.81	4.11E-07	0.23	2.49E-01	-0.74	8.61E-06
ENSCAFG0000003162	ZNF529	grey	VSMC_M10	0.24	2.34E-01	0.26	1.91E-01	-0.27	1.78E-01	0.35	7.17E-02	0.32	9.83E-02	-0.09	6.45E-01	0.11	5.86E-01	-0.36	6.52E-02	-0.33	9.23E-02	0.29	1.46E-01
ENSCAFG0000001196	RFC2	darkgrey	VSMC_M8	0.24	2.34E-01	-0.11	5.70E-01	0.68	1.03E-04	-0.84	3.11E-08	-0.45	1.91E-02	0.05	8.04E-01	0.36	6.20E-02	0.78	1.53E-06	-0.05	8.13E-01	-0.48	1.07E-02
ENSCAFG0000000459	TMCC1	darkgreen	VSMC_M4	0.24	2.34E-01	0.34	8.47E-02	-0.55	3.17E-03	0.81	4.11E-07	0.53	4.24E-03	-0.13	5.30E-01	-0.25	2.04E-01	-0.78	1.25E-06	-0.31	1.11E-01	0.47	1.36E-02
ENSCAFG0000003196	NKX2-5	grey	VSMC_M10	0.24	2.35E-01	0.04	8.54E-01	-0.11	5.70E-01	0.27	1.76E-01	0.01	9.75E-01	0.53	4.67E-03	-0.16	4.29E-01	-0.19	3.44E-01	-0.14	4.74E-01	0.29	1.39E-01
ENSCAFG0000001912	SIRT6	yellow	VSMC_M3	0.24	2.35E-01	0.62	6.09E-04	0.61	6.93E-04	-0.02	9.32E-01	-0.58	1.35E-03	-0.09	6.68E-01	0.08	6.98E-01	-0.18	3.73E-01	-0.67	1.38E-04	0.42	2.92E-02
ENSCAFG0000001483	COL5A2	grey	VSMC_M10	0.24	2.35E-01	0.08	6.89E-01	0.00	9.87E-01	0.15	4.52E-01	0.17	3.99E-01	-0.25	2.09E-01	-0.29	1.43E-01	-0.29	1.45E-01	-0.05	8.06E-01	-0.01	9.73E-01
ENSCAFG0000000012	ONECUT2	grey	VSMC_M10	0.24	2.36E-01	0.36	6.91E-02	0.10	6.11E-01	0.03	8.99E-01	-0.02	9.26E-01	-0.10	6.26E-01	0.16	4.37E-01	-0.13	5.07E-01	-0.36	6.18E-02	0.21	2.92E-01
ENSCAFG0000000059	APOM	grey	VSMC_M10	0.24	2.36E-01	0.47	1.45E-02	0.68	8.10E-05	-0.18	3.64E-01	-0.59	1.18E-03	0.10	6.08E-01	0.06	7.55E-01	-0.05	8.10E-01	-0.46	1.54E-02	0.21	2.84E-01
ENSCAFG0000000539	CMTM6	cyan	VSMC_M2	0.24	2.36E-01	0.62	6.35E-04	0.45	2.00E-02	0.11	6.00E-01	-0.45	1.77E-02	-0.06	7.63E-01	-0.21	2.86E-01	-0.29	1.48E-01	-0.65	2.22E-04	0.46	1.49E-02
ENSCAFG0000000889	KIAA1755	cyan	VSMC_M2	0.24	2.36E-01	0.44	2.27E-02	0.21	2.85E-01	-0.14	4.87E-01	-0.07	7.19E-01	-0.06	7.67E-01	0.09	6.68E-01	-0.03	8.66E-01	-0.41	3.33E-02	0.17	3.83E-01
ENSCAFG0000002998	ENSCAFG0000002998	grey	VSMC_M10	0.24	2.36E-01	-0.03	8.90E-01	-0.11	5.84E-01	-0.13	5.17E-01	0.41	3.30E-02	-0.15	4.64E-01	-0.09	6.39E-01	0.08	6.78E-01	0.01	9.57E-01	-0.44	2.04E-02
ENSCAFG0000002004	MON1B	cyan	VSMC_M2	0.24	2.36E-01	0.50	7.74E-03	0.19	3.44E-01	0.22	2.72E-01	-0.23	2.53E-01	0.06	7.79E-01	-0.29	1.44E-01	-0.36	6.29E-02	-0.48	1.21E-02	0.52	5.65E-03
ENSCAFG0000001891	OMA1	red	VSMC_M1	0.24	2.37E-01	-0.06	7.52E-01	-0.12	5.61E-01	0.13	5.25E-01	0.14	4.79E-01	0.08	6.97E-01	0.00	1.00E+00	-0.10	6.13E-01	-0.04	8.39E-01	0.05	7.86E-01
ENSCAFG0000000982	IGSF9B	grey	VSMC_M10	0.24	2.37E-01	0.20	3.26E-01	0.27	1.67E-01	-0.24	2.28E-01	-0.12	5.58E-01	-0.05	8.10E-01	0.39	4.51E-02	0.08	6.83E-01	-0.31	1.15E-01	-0.02	9.18E-01
ENSCAFG0000003137	ENSCAFG0000003137	grey	VSMC_M10	0.24	2.37E-01	0.62	5.21E-04	0.01	9.64E-01	0.54	3.81E-03	0.05	7.95E-01	-0.29	1.42E-01	-0.35	7.26E-02	-0.68	1.06E-04	-0.55	2.67E-03	0.47	1.26E-02
ENSCAFG0000000529	ENSCAFG0000000529	pink	VSMC_M5	0.24	2.37E-01	-0.01	9.46E-01	-0.01	9.69E-01	-0.15	4.61E-01	0.32	1.06E-01	-0.27	1.79E-01	-0.26	1.87E-01	0.11	5.93E-01	0.05	8.01E-01	-0.47	1.25E-02
ENSCAFG0000001994	TLDC1	grey	VSMC_M10	0.24	2.37E-01	-0.11	5.72E-01	-0.11	5.86E-01	0.23	2.51E-01	0.30	1.27E-01	-0.05	7.89E-01	0.00	9.99E-01	-0.15	4.46E-01	0.09	6.39E-01	-0.21	2.82E-01
ENSCAFG0000000647	DNHD1	darkgreen	VSMC_M4	0.24	2.37E-01	0.23	2.51E-01	-0.24	2.36E-01	0.43	2.37E-02	0.19	3.44E-01	0.15	4.45E-01	0.14	4.97E-01	-0.42	2.79E-02	-0.32	1.03E-01	0.44	2.32E-02
ENSCAFG0000001042	DHCR7	yellow	VSMC_M3	0.24	2.37E-01	0.27	1.76E-01	0.53	4.33E-03	-0.52	5.50E-03	-0.24	2.34E-01	-0.26	1.95E-01	0.18	3.71E-01	0.19	3.55E-01	-0.24	2.26E-01	-0.21	2.90E-01

ENSCAFG0000000037	PPIL4	pink	VSMC_M5	0.24	2.38E-01	-0.30	1.30E-01	-0.58	1.40E-03	0.15	4.47E-01	0.69	6.80E-05	-0.16	4.34E-01	0.05	7.87E-01	0.00	9.89E-01	0.21	2.93E-01	-0.14	4.85E-01
ENSCAFG0000000221	TRAM2	grey	VSMC_M1C	0.24	2.38E-01	0.59	1.10E-03	-0.17	4.10E-01	0.61	8.27E-04	0.16	4.15E-01	-0.13	5.07E-01	-0.58	1.40E-03	-0.69	6.59E-05	-0.56	2.33E-03	0.53	4.11E-03
ENSCAFG0000000830	SMARCD1	grey	VSMC_M1C	0.24	2.38E-01	0.43	2.47E-02	0.72	2.77E-05	-0.23	2.40E-01	-0.69	6.69E-05	0.18	3.63E-01	0.02	9.32E-01	0.04	8.38E-01	-0.50	8.00E-03	0.30	1.31E-01
ENSCAFG0000000737	PEPD	yellow	VSMC_M3	0.24	2.38E-01	0.70	4.97E-05	0.74	1.10E-05	-0.32	9.84E-02	-0.61	8.13E-04	-0.06	7.57E-01	-0.18	3.59E-01	-0.05	8.14E-01	-0.62	5.74E-04	0.24	2.21E-01
ENSCAFG0000000253	EPN1	yellow	VSMC_M3	0.24	2.38E-01	0.48	1.09E-02	0.76	4.62E-06	-0.38	5.30E-02	-0.71	2.78E-05	0.06	7.57E-01	-0.03	8.71E-01	0.10	6.21E-01	-0.53	4.05E-03	0.22	2.76E-01
ENSCAFG0000002339	C5H11orf87	grey	VSMC_M1C	0.23	2.38E-01	-0.25	2.05E-01	-0.30	1.28E-01	0.14	4.72E-01	0.37	5.75E-02	0.15	4.49E-01	-0.28	1.63E-01	0.02	9.05E-01	0.10	6.19E-01	-0.15	4.53E-01
ENSCAFG0000001675	CRABP2	yellow	VSMC_M3	0.23	2.38E-01	0.24	2.22E-01	0.61	7.14E-04	-0.66	2.07E-04	-0.34	8.05E-02	-0.12	5.57E-01	0.34	8.34E-02	0.43	2.50E-02	-0.32	1.09E-01	-0.25	2.15E-01
ENSCAFG0000002843	ENSCAFG0000002843	grey	VSMC_M1C	0.23	2.39E-01	0.29	1.37E-01	0.55	2.74E-03	-0.26	1.87E-01	-0.45	2.00E-02	-0.06	7.48E-01	0.27	1.68E-01	0.12	5.43E-01	-0.38	5.16E-02	0.08	6.95E-01
ENSCAFG0000000890	ARFGAP2	grey	VSMC_M1C	0.23	2.39E-01	0.16	4.22E-01	0.45	1.91E-02	-0.37	5.79E-02	-0.34	7.96E-02	-0.03	8.78E-01	0.14	4.88E-01	0.36	6.73E-02	-0.32	1.02E-01	-0.09	6.71E-01
ENSCAFG0000001641	PLEKHH1	grey	VSMC_M1C	0.23	2.39E-01	0.45	1.97E-02	0.05	8.01E-01	0.48	1.19E-02	-0.09	6.41E-01	-0.01	9.42E-01	-0.17	4.04E-01	-0.51	6.85E-03	-0.50	8.31E-03	0.55	3.19E-03
ENSCAFG0000003149	ENSCAFG0000003149	grey	VSMC_M1C	0.23	2.39E-01	0.41	3.23E-02	0.36	6.73E-02	-0.06	7.83E-01	-0.31	1.15E-01	0.00	9.85E-01	0.17	3.84E-01	-0.14	4.79E-01	-0.45	1.81E-02	0.37	5.73E-02
ENSCAFG0000000300	USP34	pink	VSMC_M5	0.23	2.39E-01	-0.10	6.14E-01	-0.39	4.54E-02	0.07	7.26E-01	0.62	5.38E-04	-0.14	4.72E-01	0.04	8.57E-01	-0.09	6.65E-01	0.06	7.54E-01	-0.23	2.47E-01
ENSCAFG0000001963	F8	pink	VSMC_M5	0.23	2.39E-01	-0.20	3.23E-01	-0.35	7.11E-02	0.13	5.14E-01	0.50	7.74E-03	-0.18	3.59E-01	-0.21	2.83E-01	-0.09	6.63E-01	0.14	4.89E-01	-0.22	2.62E-01
ENSCAFG0000001669	ENSCAFG0000001669	grey	VSMC_M1C	0.23	2.40E-01	0.31	1.19E-01	0.10	6.09E-01	0.16	4.32E-01	-0.12	5.38E-01	0.18	3.71E-01	-0.30	1.27E-01	-0.17	4.05E-01	-0.36	6.84E-02	0.28	1.60E-01
ENSCAFG0000003069	IL22RA1	cyan	VSMC_M2	0.23	2.40E-01	0.71	3.70E-05	0.42	2.80E-02	0.06	7.84E-01	-0.41	3.29E-02	-0.14	5.00E-01	-0.25	2.04E-01	-0.29	1.47E-01	-0.74	1.12E-05	0.51	6.30E-03
ENSCAFG0000002627	ENSCAFG0000002627	grey	VSMC_M1C	0.23	2.40E-01	-0.22	2.77E-01	0.02	9.09E-01	-0.27	1.79E-01	0.11	5.93E-01	-0.07	7.33E-01	0.31	1.16E-01	0.25	2.13E-01	0.11	5.86E-01	-0.23	2.40E-01
ENSCAFG0000000847	GPN3	grey	VSMC_M1C	0.23	2.40E-01	-0.42	2.76E-02	0.23	2.43E-01	-0.40	3.79E-02	0.04	8.61E-01	0.10	6.33E-01	0.11	5.87E-01	0.51	6.78E-03	0.30	1.29E-01	-0.69	6.18E-05
ENSCAFG0000000072	ADAMTS19	grey	VSMC_M1C	0.23	2.40E-01	0.02	9.40E-01	-0.26	1.87E-01	0.46	1.58E-02	0.25	2.11E-01	0.15	4.55E-01	-0.24	2.19E-01	-0.33	8.87E-02	-0.12	5.58E-01	0.23	2.56E-01
ENSCAFG0000000142	GKAP1	grey	VSMC_M1C	0.23	2.40E-01	-0.18	3.77E-01	-0.10	6.20E-01	-0.08	6.82E-01	0.31	1.13E-01	-0.42	3.12E-02	0.25	2.08E-01	0.14	4.99E-01	0.08	7.05E-01	-0.26	1.91E-01
ENSCAFG0000000161	RPS6	yellow	VSMC_M3	0.23	2.40E-01	0.47	1.27E-02	0.67	1.38E-04	-0.16	4.16E-01	-0.58	1.50E-03	0.06	7.70E-01	0.09	6.67E-01	-0.02	9.25E-01	-0.53	4.07E-03	0.15	4.40E-01
ENSCAFG0000001224	ADCY1	cyan	VSMC_M2	0.23	2.40E-01	0.59	1.23E-03	0.40	3.76E-02	0.12	5.66E-01	-0.36	6.44E-02	-0.09	6.42E-01	-0.03	8.66E-01	-0.34	7.85E-02	-0.56	2.31E-03	0.51	6.77E-03
ENSCAFG0000001244	DDX6	grey	VSMC_M1C	0.23	2.40E-01	-0.43	2.66E-02	-0.10	6.32E-01	-0.22	2.75E-01	0.42	3.06E-02	-0.10	6.03E-01	0.02	9.14E-01	0.33	8.93E-02	0.33	9.06E-02	-0.66	1.55E-04
ENSCAFG0000000114	SLC25A17	pink	VSMC_M5	0.23	2.41E-01	-0.21	3.01E-01	-0.45	1.99E-02	0.45	1.89E-02	0.60	9.35E-04	0.07	7.27E-01	-0.21	2.98E-01	-0.32	1.08E-01	0.23	2.44E-01	-0.19	3.49E-01
ENSCAFG0000000514	GZF1	grey	VSMC_M1C	0.23	2.41E-01	-0.10	6.11E-01	0.15	4.68E-01	-0.34	8.36E-02	0.17	3.86E-01	-0.19	3.30E-01	-0.07	7.33E-01	0.24	2.23E-01	0.08	6.95E-01	-0.46	1.64E-02
ENSCAFG0000002346	ACOT6	cyan	VSMC_M2	0.23	2.41E-01	0.65	2.53E-04	0.46	1.66E-02	0.06	7.53E-01	-0.46	1.54E-02	0.07	7.34E-01	-0.03	8.90E-01	-0.28	1.64E-01	-0.69	5.99E-05	0.52	5.90E-03
ENSCAFG0000001878	RAB34	yellow	VSMC_M3	0.23	2.41E-01	0.12	5.41E-01	0.48	1.22E-02	-0.29	1.39E-01	-0.46	1.67E-02	-0.09	6.44E-01	0.18	3.60E-01	0.29	1.37E-01	-0.29	1.42E-01	0.09	6.43E-01
ENSCAFG0000001084	ENSCAFG0000001084	pink	VSMC_M5	0.23	2.41E-01	-0.19	3.46E-01	-0.32	1.02E-01	0.18	3.80E-01	0.49	9.49E-03	-0.21	3.02E-01	-0.19	3.51E-01	-0.02	9.15E-01	0.11	5.87E-01	-0.28	1.52E-01
ENSCAFG0000000261	DNAAF3	cyan	VSMC_M2	0.23	2.41E-01	0.50	7.37E-03	0.45	1.86E-02	-0.03	8.68E-01	-0.32	1.02E-01	-0.01	9.55E-01	0.07	7.21E-01	-0.20	3.11E-01	-0.46	1.59E-02	0.22	2.66E-01
ENSCAFG0000000411	KIAA1549	grey	VSMC_M1C	0.23	2.41E-01	0.41	3.44E-02	0.32	1.07E-01	-0.05	7.89E-01	-0.24	2.26E-01	0.15	4.58E-01	0.19	3.55E-01	-0.12	5.50E-01	-0.49	1.00E-02	0.27	1.79E-01
ENSCAFG0000002383	ACAD11	darkgreen	VSMC_M4	0.23	2.41E-01	0.37	5.85E-02	-0.29	1.48E-01	0.69	6.88E-05	0.35	7.06E-02	-0.20	3.13E-01	-0.51	6.00E-03	-0.70	4.50E-05	-0.29	1.44E-01	0.36	6.29E-02
ENSCAFG0000000762	ENSCAFG0000000762	darkgrey	VSMC_M8	0.23	2.41E-01	-0.05	8.20E-01	0.44	2.03E-02	-0.68	8.48E-05	-0.20	3.23E-01	-0.10	6.17E-01	0.39	4.63E-02	0.61	7.37E-04	-0.13	5.34E-01	-0.42	2.74E-02
ENSCAFG0000002520	CA2	grey	VSMC_M1C	0.23	2.42E-01	0.24	2.28E-01	0.47	1.32E-02	-0.27	1.70E-01	-0.31	1.15E-01	-0.14	4.82E-01	-0.17	3.93E-01	0.17	3.90E-01	-0.28	1.51E-01	-0.05	7.96E-01
ENSCAFG0000000658	MRPL17	grey	VSMC_M1C	0.23	2.42E-01	-0.37	5.71E-02	0.19	3.43E-01	-0.44	2.28E-02	0.09	6.45E-01	0.05	8.01E-01	0.10	6.22E-01	0.48	1.22E-02	0.31	1.22E-01	-0.65	2.42E-04
ENSCAFG0000001095	RIN3	yellow	VSMC_M3	0.23	2.42E-01	0.52	5.36E-03	0.47	1.42E-02	-0.18	3.67E-01	-0.30	1.22E-01	-0.01	9.50E-01	-0.50	8.31E-03	-0.12	5.37E-01	-0.39	4.45E-02	0.07	7.39E-01
ENSCAFG0000000902	ASB8	grey	VSMC_M1C	0.23	2.42E-01	0.02	9.08E-01	0.12	5.39E-01	-0.06	7.63E-01	0.13	5.04E-01	-0.19	3.30E-01	-0.16	4.20E-01	0.01	9.58E-01	-0.03	8.69E-01	-0.30	1.24E-01
ENSCAFG0000001317	HOMER2	grey	VSMC_M1C	0.23	2.43E-01	0.24	2.21E-01	-0.08	6.90E-01	0.37	6.00E-02	0.03	8.65E-01	0.04	8.40E-01	-0.02	9.02E-01	-0.32	1.07E-01	-0.36	6.42E-02	0.39	4.69E-02
ENSCAFG0000001327	E2F2	grey	VSMC_M1C	0.23	2.43E-01	0.13	5.34E-01	0.41	3.19E-02	-0.45	1.93E-02	-0.35	7.44E-02	0.16	4.16E-01	-0.06	7.71E-01	0.42	2.89E-02	-0.24	2.26E-01	-0.01	9.49E-01
ENSCAFG0000003003	ENSCAFG0000003003	grey	VSMC_M1C	0.23	2.43E-01	-0.26	1.88E-01	0.04	8.53E-01	-0.23	2.58E-01	0.06	7.55E-01	-0.11	5.84E-01	0.29	1.36E-01	0.32	1.03E-01	0.06	7.55E-01	-0.20	3.13E-01
ENSCAFG0000001625	ENSCAFG0000001625	grey	VSMC_M1C	0.23	2.43E-01	0.21	2.89E-01	0.04	8.38E-01	0.04	8.49E-01	0.04	8.50E-01	0.25	2.05E-01	-0.06	7.81E-01	-0.04	8.27E-01	-0.34	8.50E-02	0.10	6.34E-01
ENSCAFG0000002864	STAMBP	grey	VSMC_M1C	0.23	2.43E-01	0.21	2.88E-01	-0.19	3.37E-01	0.46	1.54E-02	0.19	3.31E-01	0.01	9.64E-01	0.11	5.94E-01	-0.45	1.74E-02	-0.27	1.68E-01	0.42	2.96E-02
ENSCAFG0000001731	ACP5	cyan	VSMC_M2	0.23	2.43E-01	0.84	3.05E-08	0.59	1.26E-03	0.11	6.01E-01	-0.55	3.05E-03	0.02	9.35E-01	-0.26	1.93E-01	-0.39	4.16E-02	-0.79	8.75E-07	0.55	2.89E-03
ENSCAFG0000000393	ENSCAFG0000000393	pink	VSMC_M5	0.23	2.43E-01	-0.38	5.33E-02	-0.68	8.27E-05	0.36	6.73E-02	0.81	2.48E-07	-0.26	1.95E-01	-0.15	4.46E-01	-0.20	3.14E-01	0.34	8.41E-02	-0.19	3.50E-01
ENSCAFG0000003009	ENSCAFG0000003009	grey	VSMC_M1C	0.23	2.43E-01	0.04	8.62E-01	0.19	3.38E-01	-0.17	4.00E-01	0.06	7.69E-01	-0.23	2.47E-01	0.00	9.82E-01	0.11	5.76E-01	-0.04	8.26E-01	-0.31	1.19E-01
ENSCAFG0000001636	ATP6V1D	grey	VSMC_M1C	0.23	2.43E-01	0.06	7.68E-01	-0.12	5.53E-01	0.09	6.70E-01	0.38	4.94E-02	-0.30	1.25E-01	-0.10	6.14E-01	-0.24	2.29E-01	0.03	8.94E-01	-0.24	2.29E-01
ENSCAFG0000002025	NOB1	darkgrey	VSMC_M8	0.23	2.43E-01	-0.44	2.07E-02	0.14	4.79E-01	-0.66	2.08E-04	0.03	9.01E-01	0.20	3.16E-01	0.31	1.16E-01	0.78	1.40E-06	0.21	2.91E-01	-0.62	5.74E-04
ENSCAFG0000001972	CAMSAP1	grey	VSMC_M1C	0.23	2.43E-01	0.19	3.52E-01	0.13	5.23E-01	-0.02	9.16E-01	0.16	4.32E-01	-0.38	5.01E-02	-0.03	8.99E-01	-0.16	4.14E-01	-0.16	4.28E-01	-0.17	3.91E-01
ENSCAFG0000000886	PACSIN3	yellow	VSMC_M3	0.23	2.44E-01	0.51	6.58E-03	0.79	8.06E-07	-0.43	2.41E-02	-0.58	1.36E-03	-0.08	6.84E-01	0.13	5.17E-01	0.14	4.78E-01	-0.50	7.42E-03	0.05	7.88E-01
ENSCAFG0000001255	SF3A1	yellow	VSMC_M3	0.23	2.44E-01	0.43	2.60E-02	0.70	5.04E-05	-0.56	2.48E-03	-0.58	1.42E-03	0.02	9.09E-01	0.15	4.58E-01	0.32	1.08E-01	-0.51	7.09E-03	0.10	6.24E-01
ENSCAFG0000001331	ENSCAFG0000001331	grey	VSMC_M1C	0.23	2.44E-01	-0.07	7.43E-01	-0.16	4.29E-01	0.21	2.99E-01	0.42	3.12E-02	-0.10	6.07E-01	-0.12	5.40E-01	-0.21	2.91E-01	0.11	5.78E-01	-0.20	3.07E-01
ENSCAFG0000001980	GAS8	yellow	VSMC_M3	0.23	2.44E-01	0.16	4.39E-01	0.53	4.43E-03	-0.43	2.57E-02	-0.47	1.24E-02	0.27	1.69E-01	0.06	7.72E-01	0.43	2.51E-02	-0.30	1.27E-01	-0.07	7.46E-01
ENSCAFG0000000669	LRFN3	grey	VSMC_M1C	0.23	2.44E-01	-0.03	8.98E-01	-0.20	3.26E-01	0.17	3.93E-01	0.41	3.48E-02	-0.23	2.46E-01	0.07	7.10E-01	-0.21	2.97E-01	0.08	6.74E-01	-0.12	5.67E-01
ENSCAFG0000002537	EXO5	darkgreen	VSMC_M4	0.23	2.44E-01	0.48	1.21E-02	-0.12	5.51E-01	0.66	2.01E-04	0.02	9.25E-01	0.12	5.39E-01	-0.16	4.27E-01	-0.61	7.57E-04	-0.53	4.74E-03	0.64	2.88E-04
ENSCAFG0000001980	ENSCAFG0000001980	grey	VSMC_M1C	0.23	2.44E-01	0.79	9.50E-07	0.25	2.07E-01	0.25	2.05E-01	-0.18	3.71E-01	-0.15	4.44E-01	-0.39	4.46E-02	-0.46	1.46E-02	-0.71	3.07E-05	0.50	7.46E-03
ENSCAFG0000000947	EXT2	cyan	VSMC_M2	0.23	2.44E-01	0.66	1.59E-04	-0.07	7.25E-01	0.57	1.86E-03	0.16	4.29E-01	-0.37	5.93E-02	-0.51	6.48E-03	-0.72	2.02E-05	-0.52	5.12E-03	0.45	1.82E-02
ENSCAFG0000000643	RIC8A	yellow	VSMC_M3	0.23	2.45E-01	0.16	4.16E-01	0.51	6.66E-03	-0.22	2.68E-01	-0.26	1.88E-01	-0.14	4.93E-01	-0.10	6.17E-01	0.08	7.09E-01	-0.17	3.94E-01	-0.22	2.63E-01
ENSCAFG0000000235	ENSCAFG0000000235	grey	VSMC_M1C	0.23	2.45E-01	0.28	1.51E-01	0.40	3.96E-02	-0.21	2.91E-01	-0.23	2.52E-01	0.09	6.55E-01	-0.08	6.75E-01	0.10	6.26E-01	-0.32	1.01E-01	-0.05	8.08E-01
ENSCAFG0000002585	ENSCAFG0000002585	grey	VSMC_M1C	0.23	2.45E-01	-0.10	6.02E-01	-0.30	1.25E-01	0.24	2.35E-01	0.38	5.00E-02	-0.11	5.88E-01	-0.14	4.80E-01	-0.16	4.30E-01	0.10	6.30E-01	-0.04	8.25E-01
ENSCAFG0000000829	NUP160	darkgrey	VSMC_M8	0.23	2.45E-01	-0.53	4.24E-03	-0.07	7.44E-01	-0.48	1.11E-02	0.26	1.87E-01	-0.04	8.47E-01	0.36	6.43E-02	0.66	1.93E-04	0.29	1.47E-01	-0.57	1.94E-03
ENSCAFG0000000720	LATS2	darkgreen	VSMC_M4	0.23	2.45E-01	0.37	5.76E-02	-0.43	2.66E-02	0.84	4.27E-08	0.50	7.88E-03	-0.26	1.86E-01	-0.50	8.54E-03	-0.85	2.25E-08	-0.28	1.60E-01	0.40	4.11E-02
ENSCAFG0000002342	ENSCAFG0000002342	grey	VSMC_M1C	0.23	2.46E-01	0.16	4.32E-01	0.05	7.91E-01	-0.10	6.31E-01	0.11	5.90E-01	0.00	9.90E-01	0.01	9.41E-01	0.01	9.47E-01	-0.17	4.02E-01	0.00	9.80E-01
ENSCAFG0000000918	HARBI1	grey	VSMC_M1C	0.23	2.46E-01	0.08	6.78E-01	-0.30	1.23E-01	0.27	1.74E-01	0.32	1.07E-01	0.10	6.13E-01	0.17	3.86E-01	-0.28	1.65E-01	-0.19	3.41E-01	0.22	2.69E-01
ENSCAFG0000000502	ENSCAFG0000000502	grey	VSMC_M1C	0.23	2.46E-01	-0.22	2.72E-01	0.22	2.67E-01	-0.56	2.41E-03	-0.05	8.02E-01	0.17	3.90E-01	0.40	3.75E-02	0.53	4.84E-03	0.04	8.40E-01	-0.43	2.40E-02
ENSCAFG0000001799	ACACA	grey	VSMC_M1C	0.23	2.46E-01	0.06	7.55E-01	0.23	2.56E-01	-0.41	3.48E-02	0.04	8.53E-01	-0.05	7.87E-01	0.17	4.10E-01	0.18	3.74E-01	-0.12	5.62E-01	-0.24	2.19E-01
ENSCAFG0000000385	HS1BP3	cyan	VSMC_M2	0.23	2.46E-01	0.82	1.30E-07	0.66	2.02E-04	-0.13	5.15E-01	-0.55	3.16E-03	0.00	9.91E-01	-0.12	5.53E-01	-0.26	1.88E-01	-0.73	1.48E-05	0.43	2.45E-02
ENSCAFG0000002490	PLA2G4E	yellow	VSMC_M3	0.23	2.46E-01	0.53	4.68E-03	0.62	5.13E-04	-0.24	2.18E-01	-0.55	2.98E-03	0.07	7.11E-01	-0.22	2.78E-01	0.00	9.89E-01	-0.52	5.70E-03	0.17	3.88E-01
ENSCAFG0000001651	RFX1	cyan	VSMC_M2	0.23	2.46E-01	0.43	2.39E-02	0.16	4.30E-01	0.14	4.84E-01	-0.13	5.16E-01	-0.08	6.88E-01	0.17	3.95E-01	-0.27	1.66E-01	-0.52	5.47E-03	0.44	2.21E-02
ENSCAFG0000000769	VAMP8	darkgrey	VSMC_M8	0.23	2.46E-01	-0.38	4.89E-02	0.19	3.55E-01	-0.59	1.27E-03	0.03	8.77E-01	0.08	6.92E-01	0.15	4.49E-01	0.63	4.73E-04	0.24	2.36E-01	-0.61	6.68E-04
ENSCAFG0000000527	ENSCAFG0000000527	grey	VSMC_M1C	0.23	2.47E-01	-0.02	9.13E-01	-0.03	8.85E-01	0.10	6.25E-01	0.14	4.82E-01	0.11	5.91E-01	0.11	5.86E-01	-0.10	6.16E-01	-0.04	8.61E-01	-0.05	8.20E-01
ENSCAFG0000001313	FOSL1	darkgrey	VSMC_M8	0.23	2.47E-01	-0.22	2.62E-01	0.52	5.49E-03	-0.74	1.21E-05	-0.35	7.28E-02	0.21	2.86E-01	0.03	8.68E-01	0.79	1.23E-06	0.05	8.06E-01	-0.51	6.49E-03
ENSCAFG0000001851	MRPS30	grey	VSMC_M1C	0.23	2.47E-01	-0.15	4.54E-01	0.23	2.52E-01	-0.54	3.87E-03	-0.17	3.99E-01	0.04	8.38E-01	0.40	3.91E-02	0.55	2.68E-03	-0.07	7.46E-01	-0.15	4.46E-01
ENSCAFG0000001056	NEPRO	grey	VSMC_M1C	0.23	2.47E-01	-0.06	7.84E-01	0.04	8.44E-01	-0.05	8.08E-01	0.11	5.68E-01	-0.01	9.79E-01	0.15	4.58E-01	0.11	5.88E-01	-0.10	6.29E-01	-0.14	4.83E-01
ENSCAFG0000001041	TMPRSS7	darkgreen	VSMC_M4	0.23	2.47E-01	0.15	4.66E-01	-0.45	1.87E-02	0.56	2.53E-03	0.47	1.25E-02	-0.22	2.80E-01	-0.34	8.11E-02	-0.45	1.75E-02	-0.14	4.90E-01	0.27	1.79E-01
ENSCAFG0000001532	AKAP3	grey	VSMC_M1C	0.23	2.47E-01	0.03	8.99E-01	0.05	7.93E-01	-0.17	4.02E-01	0.16	4.36E-01	-0.02	9.05E-01	0.09	6.56E-01	0.11	5.72E-01	-0.12	5.59E-01	-0.25	2.10E-01
ENSCAFG0000002001	GCSH	grey	VSMC_M1C	0.23	2.47E-01	0.22	2.81E-01	0.02	9.13E-01	0.43	2.44E-02	-0.06	7.56E-01	0.06	7.62E-01	-0.04	8.56E-01	-0.40	4.02E-02	-0.28	1.52E-01	0.37	5.76E-02
ENSCAFG0000000767	ABCB9	grey	VSMC_M1C	0.23	2.47E-01	0.29	1.48E-01	0.47	1.45E-02	-0.31	1.16E-01	-0.41	3.45E-02	0.03	8.64E-01	0.09	6.41E-01	0.15	4.58E-01	-0.34	8.04E-02	0.16	4.24E-01
ENSCAFG0000002053	ENSCAFG0000002053	yellow	VSMC_M3	0.23	2.47E-01	0.21	2.88E-01	0.37	5.40E-02	-0.44	2.17E-02	-0.22	2.78E-01	0.04	8.46E-01	0.03	8.69E-01	0.34	8.57E-02	-0.28	1.56E-01	-0.14	4.87E-01
ENSCAFG0000000456	CUBN	grey	VSMC_M1C	0.23	2.47E-01	0.26	1.86E-01	-0.29	1.47E-01	0.32	1.03E-01	0.21	2.91E-01	0.01	9.76E-01	0.07	7.47E-01	-0.29	1.38E-01	-0.38	5.16E-02	0.46	1.64E-02
ENSCAFG0000000200	PIGO	yellow	VSMC_M3	0.23	2.48E-01	0.13	5.06E-01	0.70	4.42E-05	-0.72	2.10E-05	-0.50	7.85E-03	0.12	5.54E-01	0.37	5.72E-02	0.52	5.20E-03	-0.28	1.58E-01	-0.25	2.08E-01
ENSCAFG0000002386	ZNF205	cyan	VSMC_M2	0.23	2.48E-01	0.75	7.72E-06	0.63	4.42E-04	-0.10	6.22E-01	-0.59	1.24E-03	0.12	5.62E-01	0.05	7.98E-01	-0.18	3.73E-01	-0.75	5.48E-06	0.50	7.91E-03
ENSCAFG0000001590	SZRD1	grey	VSMC_M1C	0.23	2.48E-01	0.10	6.03E-01	0.42	3.07E-02	-0.32	1.05E-01	-0.11	5.96E-01	-0.11	5.99E-01	0.00	1.00E+00	0.17	4.02E-01	-0.13	5.15E-01	-0.40	3.87E-02
ENSCAFG0000000480	ERI3	red	VSMC_M1	0.23	2.48E-01	0.31	1.16E-01	0.02	9.11E-01	0.19	3.31E-01	-0.05	8.20E-01	0.08	7.01E-01	0.19	3.38E-01	-0.30	1.30E-01	-0.31	1.18E-01	0.37	5.42E-02
ENSCAFG0000001184	TTC28	pink	VSMC_M5	0.23	2.48E-01	0.33	9.37E-02	-0.40	3.65E-02	0.45	1.83E-02	0.55	3.03E-03	-0.25	2.09E-01	-0.25	2.05E-01	-0.50	7.39E-03	-0.25	2.02E-01	0.21	2.84E-01
ENSCAFG0000002934	PLN	darkgreen	VSMC_M4	0.23	2.48E-01	0.20	3.25E-01	-0.46	1.52E-02	0.67	1.48E-04	0.48	1.12E-02	-0.14	4.88E-01	-0.20	3.06E-01	-0.57	1.75E-03	-0.19	3.32E-01	0.34	7.87E-02
ENSCAFG0000000892	NEK4	darkgreen	VSMC_M4	0.23	2.48E-01	0.12	5.66E-01	-0.37	5.77E-02	0.43	2.68E-02	0.34	8.52E-02	0.01	9.44E-01	0.15	4.50E-01	-0.35	7.33E-02	-0.26	1.89E-01	0.33	9.27E-02
ENSCAFG0000001671	TP53	cyan	VSMC_M2	0.23	2.48E-01	0.73	1.75E-05	0.35	7.24E-02	0.01	9.64E-01	-0.36	6.18E-02	0.19	3.41E-01	-0.07	7.45E-01	-0.22	2.71E-01	-0.77	2.50E-06	0.49	9.64E-03
ENSCAFG0000002478	ANKRD40	grey	VSMC_M1C	0.23	2.49E-01	-0.09	6.59E-01	-0.25	2.12E-01	0.20	3.13E-01	0.38	5.16E-02	-0.13	5.07E-01	-0.18	3.76E-01	-0.21	2.90E-01	0.12	5.60E-01	-0.11	5.74E-01
ENSCAFG0000001552	WDR74	grey	VSMC_M1C	0.23	2.49E-01	-0.09	6.50E-01	0.41	3.38E-02	-0.31	1.10E-01	-0.26	1.95E-01	-0.03	8.76E-01	0.21	2.88E-01	0.31	1.17E-01	-0.02	9.29E-01	-0.25	2.11E-01
ENSCAFG0000000092	HSD17B8	grey	VSMC_M1C	0.23	2.49E-01	0.21	2.92E-01	0.34	8.24E-02	-0.26	1.96E-01	-0.04	8.49E-01	-0.25	2.10E-01	0.25	2.00E-01	0.02	9.33E-01	-0.17	3.99E-01	-0.25	2.08E-01
ENSCAFG0000000883	ANXA3	darkgreen	VSMC_M4	0.23	2.49E-01	0.04	8.27E-01	-0.53	4.10E-03	0.70	5.71E-05	0.64	3.65E-04	-0.24	2.20E-01	-0.10	6.26E-01	-0.55	2.99E-03	-0.04	8.36E-01	0.10	6.08E-01
ENSCAFG0000001579	GPKOW	yellow	VSMC_M3	0.23	2.49E-01	0.15	4.67E-01	0.49	1.01E-02	-0.21	2.96E-01	-0.22	2.59E-01	-0.04	8.48E-01	0.24	2.32E-01	0.05	8.01E-01	-0.19	3.38E-01	-0.22	2.79E-01
ENSCAFG0000001065	FBXO36	grey	VSMC_M1C	0.23	2.49E-01	-0.17	3.93E-01	0.03	8.82E-01	-0.22	2.74E-01	0.09	6.53E-01	-0.14	4.71E-01	0.45	1.80E-02	0.25	2.05E-01	0.06	7.52E-01	-0.19	3.50E-01
ENSCAFG0000000231	ARL4A	grey	VSMC_M1C	0.23	2.49E-01	-0.20	3.20E-01	-0.02	9.27E-01	0.04	8.24E-01	-0.03	8.88E-01	0.29	1.43E-01	0.20	3.19E-01	0.12	5.59E-01	0.02	9.29E-01	0.05	7.89E-01
ENSCAFG0000000938	NISCH	cyan	VSMC_M2	0.23	2.49E-01	0.91	5.62E-11	0.33	8.98E-02	0.31	1.19E-01	-0.31	1.18E-01	-0.08	6.97E-01	-0.51	7.00E-03	-0.57	1.90E-03	-0.80	4.24E-07	0.62	5.91E-04
ENSCAFG0000000726	ENSCAFG0000000726	grey	VSMC_M1C	0.23	2.49E-01	0.20	3.11E-01	0.04	8.62E-01	-0.01	9.67E-01	0.07	7.43E-01	-0.13	5.04E-01	-0.21	2.94E-01	-0.10	6.26E-01	-0.15	4.49E-01	0.06	7.61E-01
ENSCAFG0000003244	ENSCAFG0000003244	grey	VSMC_M1C	0.23	2.50E-01	0.22	2.64E-01	0.26	1.84E-01	-0.05	8.08E-01	-0.14	4.87E-01	-0.12	5.68E-01	-0.01	9.65E-01	0.01	9.48E-01	-0.31	1.19E-01	0.12	5.55E-01
ENSCAFG0000001185	TBCEL	grey	VSMC_M1C	0.23	2.50E-01	0.39	4.67E-02	-0.01	9.47E-01	0.12	5.44E-01	0.12	5.61E-01	0.04	8.32E-01	-0.07	7.41E-01	-0.34	8.21E-02	-0.38	5.16E-02	0.24	2.19E-01
ENSCAFG0000000855	ZNF319	grey	VSMC_M1C	0.23	2.50E-01	0.39	4.20E-02	0.05	8.06E-01	0.09	6.54E-01	-0.12	5.65E-01	0.07	7.32E-01	0.05	8.22E-01	-0.25	2.17E-01	-0.43	2.46E-02	0.47	1.26E-02
ENSCAFG0000001338	PPM1J	grey	VSMC_M1C	0.23	2.50E-01	-0.08	6.87E-01	-0.22	2.73E-01	-0.04	8.25E-01	0.31	1.14E-01	-0.13	5.05E-01	0.11	5.75E-01	0.06	7.64E-01	0.00	9.86E-01	-0.12	5.59E-01
ENSCAFG0000001985	C6H1orf194	grey	VSMC_M1C	0.23	2.50E-01	0.15	4.61E-01	0.01	9.43E-01	-0.09	6.48E-01	0.13	5.18E-01	-0.13	5.22E-01	0.14	4.98E-01	0.00	9.83E-01	-0.20	3.08E-01	-0.07	7.23E-01
ENSCAFG0000001403	ENSCAFG0000001403	grey	VSMC_M1C	0.23	2.50E-01	0.40	3.82E-02	-0.11	5.89E-01	0.33	9.50E-02	0.27	1.69E-01	-0.20	3.17E-01	-0.15	4.56E-01	-0.40	3.89E-02	-0.34	8.70E-02	0.14	4.94E-01
ENSCAFG0000000779	ENSCAFG0000000779	grey	VSMC_M1C	0.23	2.50E-01	0.05	8.19E-01	-0.20	3.15E-01	0.26	1.96E-01	0.22	2.79E-01	-0.02	9.05E-01	-0.03	8.85E-01	-0.20	3.17E-01	-0.11	5.90E-01	0.18	3.71E-01
ENSCAFG0000000766	CLCN3	darkgreen	VSMC_M4	0.23	2.50E-01	0.31	1.19E-01	-0.47	1.38E-02	0.75	6.01E-06	0.46	1.57E-02	-0.08	7.10E-01	-0.50	8.25E-03	-0.68	9.28E-05	-0.31	1.12E-01	0.41	3.61E-02
ENSCAFG0000001057	DAPP1	darkgreen	VSMC_M4	0.23	2.50E-01	0.42	3.12E-02	-0.28	1.62E-01	0.81	2.60E-07	0.22	2.60E-01	-0.22	2.64E-01	-0.26	1.94E-01	-0.78	1.39E-06	-0.40	3.75E-02	0.57	1.74E-03
ENSCAFG0000000653	COPZ1	grey	VSMC_M1C	0.23	2.50E-01	-0.14	4.96E-01	0.34	8.30E-02	-0.59	1.12E-03	0.02	9.17E-01	-0.51	6.04E-03	0.03	8.87E-01	0.49	9.69E-03	0.07	7.19E-01	-0.64	3.12E-04
ENSCAFG0000001078	TNNI1	grey	VSMC_M1C	0.23	2.50E-01	0.39	4.19E-02	-0.13	5.13E-01	0.40	3.97E-02	0.14	4.80E-01	0.00	9.87E-01	0.01	9.60E-01	-0.52	5.51E-03	-0.43	2.52E-02	0.43	2.35E-02
ENSCAFG0000001347	FRMD5	grey	VSMC_M1C	0.23	2.51E-01	0.70	4.90E-05	0.02	9.20E-01	0.50	7.74E-03	-0.06	7.53E-01	-0.20	3.06E-01	-0.22	2.78E-01	-0.61	6.69E-04	-0.66	1.73E-04	0.66	2.04E-04
ENSCAFG0000000849	GINS3	grey	VSMC_M1C	0.23	2.51E-01	0.00	9.87E-01	0.23	2.45E-01	-0.51	7.07E-03	0.01	9.64E-01	-0.10	6.07E-01	0.38	5.29E-02	0.43	2.51E-02	-0.14	4.71E-01	-0.37	5.72E-02
ENSCAFG0000001421	MAP4K2	grey	VSMC_M1C	0.23	2.51E-01	0.19	3.51E-01	0.03	8.96E-01	-0.06	7.71E-01	0.04	8.55E-01	0.09	6.43E-01	0.01	9.80E-01	-0.03	8.90E-01	-0.19	3.41E-01	0.13	5.13E-01
ENSCAFG0000000562	ENSCAFG0000000562	grey	VSMC_M1C	0.23	2.51E-01	0.03	8.80E-01	0.38	4.87E-02	-0.35	6.95E-02	-0.15	4.52E-01	0.42	3.06E-02	-0.09	6.73E-01	0.25	2.00E-01	-0.07	7.33E-01	-0.40	3.62E-02
ENSCAFG0000001181	ZNF217	grey	VSMC_M1C	0.23	2.51E-01	0.32	1.04E-01	-0.15	4.51E-01	0.14	4.87E-01	0.29	1.43E-01	-0.17	3.93E-01	-0.04	8.54E-01	-0.23	2.47E-01	-0.33	9.81E-02	0.11	5.71E-01
ENSCAFG0000000557	ENSCAFG0000000557	darkgreen	VSMC_M4	0.23	2.51E-01	0.45	1.77E-02	-0.20	3.21E-01	0.59	1.11E-03	0.22	2.64E-01	-0.09	6.61E-01	-0.80	4.61E-07	-0.63	3.88E-04	-0.34	8.54E-02	0.37	5.97E-02
ENSCAFG0000003015	NDUFS6	grey	VSMC_M1C	0.23	2.51E-01	0.13	5.34E-01	0.05	7.98E-01	0.35	7.60E-02	-0.05	8.18E-01	-0.14	4.71E-01	-0.10	6.14E-01	-0.29	1.36E-01	-0.17	4.10E-01	0.25	2.02E-01
ENSCAFG0000001649	ZFP36L1	cyan	VSMC_M2	0.23	2.51E-01	0.85	1.94E-08	0.10	6.35E-01	0.42	2.74E-02	-0.12	5.53E-01	-0.10	6.33E-01	-0.23	2.43E-01	-0.64	3.09E-04	-0.78	1.83E-06	0.73	1.58E-05
ENSCAFG0000001618	PLEKHM2	cyan	VSMC_M2	0.23	2.51E-01	0.86	1.28E-08	0.62	4.92E-04	-0.13	5.21E-01	-0.51	7.09E-03	-0.11	5.94E-01	-0.28	1.62E-01	-0.26	1.90E-01	-0.75	7.70E-06	0.39	4.50E-02
ENSCAFG0000000479	TSEN54	red	VSMC_M1	0.23	2.52E-01	0.02	9.15E-01	-0.22	2.66E-01	0.33	9.34E-02	0.38	5.07E-02	-0.02	9.33E-01	-0.03	8.89E-01	-0.34	7.89E-02	-0.06	7.68E-01	-0.07	7.13E-01
ENSCAFG0000002306	FYB1	grey	VSMC_M1C	0.23	2.52E-01	0.31	1.17E-01	0.27	1.80E-01	-0.03	8.84E-01	-0.23	2.44E-01	0.05	8.08E-01	0.02	9.03E-01	-0.02	9.38E-01	-0.38	5.04E-02	0.21	2.97E-01

ENSCAFG0000000667	ENSCAFG0000000667	grey	VSMC_M10	0.23	2.52E-01	0.05	8.11E-01	-0.07	7.38E-01	-0.05	7.89E-01	0.24	2.23E-01	-0.13	5.08E-01	0.01	9.54E-01	0.06	7.85E-01	-0.12	5.66E-01	-0.15	4.40E-01
ENSCAFG0000001066	ENSCAFG0000001066	grey	VSMC_M10	0.23	2.52E-01	0.09	6.50E-01	0.42	3.07E-02	-0.24	2.23E-01	-0.23	2.39E-01	0.17	3.98E-01	-0.15	4.52E-01	0.19	3.41E-01	-0.12	5.62E-01	-0.24	2.32E-01
ENSCAFG0000002027	ZNHIT6	grey	VSMC_M10	0.23	2.52E-01	-0.16	4.16E-01	0.13	5.27E-01	0.02	9.33E-01	0.05	8.02E-01	-0.17	3.88E-01	-0.06	7.81E-01	-0.01	9.54E-01	0.15	4.54E-01	-0.24	2.19E-01
ENSCAFG0000003018	ENSCAFG0000003018	grey	VSMC_M10	0.23	2.52E-01	0.33	9.04E-02	0.19	3.49E-01	0.19	3.35E-01	-0.21	2.93E-01	0.02	9.07E-01	0.00	9.95E-01	-0.24	2.32E-01	-0.38	4.90E-02	0.47	1.37E-02
ENSCAFG0000000510	C1H19orf54	grey	VSMC_M10	0.23	2.52E-01	0.24	2.24E-01	0.15	4.56E-01	-0.05	7.89E-01	-0.07	7.28E-01	-0.10	6.17E-01	0.19	3.50E-01	-0.17	4.04E-01	-0.22	2.80E-01	0.21	2.93E-01
ENSCAFG0000000209	UXS1	grey	VSMC_M10	0.23	2.52E-01	-0.05	8.09E-01	-0.09	6.72E-01	0.00	9.92E-01	0.14	5.02E-01	-0.19	3.52E-01	-0.10	6.16E-01	0.18	3.74E-01	-0.09	6.55E-01	-0.09	6.63E-01
ENSCAFG0000000016	NAB2	yellow	VSMC_M3	0.23	2.53E-01	0.29	1.35E-01	0.80	4.20E-07	-0.70	4.62E-05	-0.64	2.93E-04	0.01	9.61E-01	-0.08	6.85E-01	0.45	1.90E-02	-0.33	8.91E-02	-0.13	5.03E-01
ENSCAFG0000001889	MOSPD1	cyan	VSMC_M2	0.23	2.53E-01	0.60	1.04E-03	0.25	2.08E-01	0.24	2.25E-01	-0.12	5.35E-01	-0.04	8.37E-01	-0.21	2.85E-01	-0.46	1.58E-02	-0.52	5.31E-03	0.32	1.08E-01
ENSCAFG0000001512	TMCO4	cyan	VSMC_M2	0.23	2.53E-01	0.82	1.73E-07	0.28	1.60E-01	0.26	1.85E-01	-0.21	2.83E-01	-0.10	6.12E-01	-0.17	4.09E-01	-0.53	4.46E-03	-0.72	2.18E-05	0.50	7.62E-03
ENSCAFG0000001073	CAB39	pink	VSMC_M5	0.23	2.54E-01	-0.51	6.02E-03	-0.53	4.48E-03	0.06	7.75E-01	0.77	3.26E-06	-0.01	9.55E-01	0.00	9.82E-01	0.10	6.22E-01	0.46	1.51E-02	-0.54	3.98E-03
ENSCAFG0000001403	TRIP6	grey	VSMC_M10	0.23	2.54E-01	0.37	5.66E-02	0.38	4.84E-02	-0.04	8.53E-01	-0.27	1.68E-01	0.05	7.86E-01	-0.15	4.64E-01	-0.09	6.56E-01	-0.42	2.84E-02	0.12	5.39E-01
ENSCAFG0000000700	ENSCAFG0000000700	grey	VSMC_M10	0.23	2.54E-01	0.08	6.75E-01	0.36	6.63E-02	-0.26	1.83E-01	-0.16	4.16E-01	-0.06	7.65E-01	0.25	2.01E-01	0.11	5.72E-01	-0.14	4.72E-01	-0.08	6.86E-01
ENSCAFG0000002036	ZDHHC1	grey	VSMC_M10	0.23	2.54E-01	0.60	9.02E-04	-0.20	3.21E-01	0.67	1.51E-04	0.21	2.94E-01	-0.24	2.32E-01	-0.46	1.56E-02	-0.79	1.07E-06	-0.46	1.51E-02	0.55	2.72E-03
ENSCAFG0000003193	ENSCAFG0000003193	grey	VSMC_M10	0.23	2.54E-01	-0.01	9.60E-01	0.17	3.92E-01	-0.23	2.56E-01	-0.10	6.03E-01	0.01	9.59E-01	-0.02	9.13E-01	0.27	1.71E-01	-0.11	5.72E-01	-0.12	5.67E-01
ENSCAFG0000002464	ENSCAFG0000002464	yellow	VSMC_M3	0.23	2.54E-01	0.37	5.62E-02	0.46	1.64E-02	-0.23	2.38E-01	-0.34	7.97E-02	-0.18	3.56E-01	-0.01	9.68E-01	0.09	6.38E-01	-0.40	3.75E-02	0.13	5.23E-01
ENSCAFG0000001300	C28H10orf90	darkolivegreen	VSMC_M9	0.23	2.54E-01	-0.43	2.52E-02	-0.06	7.58E-01	-0.26	1.92E-01	0.27	1.66E-01	0.05	8.23E-01	0.33	9.19E-02	0.26	1.91E-01	0.27	1.70E-01	-0.43	2.41E-02
ENSCAFG0000001953	C8G	yellow	VSMC_M3	0.23	2.55E-01	0.58	1.66E-03	0.58	1.56E-03	-0.03	8.64E-01	-0.51	6.61E-03	-0.17	3.90E-01	-0.24	2.26E-01	-0.18	3.76E-01	-0.55	2.89E-03	0.29	1.39E-01
ENSCAFG0000002039	CDH16	grey	VSMC_M10	0.23	2.55E-01	0.13	5.13E-01	0.07	7.35E-01	-0.12	5.56E-01	0.07	7.25E-01	0.02	9.04E-01	0.04	8.28E-01	0.07	7.45E-01	-0.16	4.11E-01	-0.01	9.80E-01
ENSCAFG0000000186	ZNF804B	grey	VSMC_M10	0.23	2.55E-01	-0.18	3.58E-01	0.35	7.59E-02	-0.65	2.46E-04	-0.22	2.67E-01	0.19	3.31E-01	0.51	6.40E-03	0.59	1.22E-03	-0.02	9.16E-01	-0.29	1.49E-01
ENSCAFG0000002899	TTC1	pink	VSMC_M5	0.23	2.55E-01	-0.26	1.91E-01	-0.45	1.81E-02	0.21	2.85E-01	0.75	6.62E-06	-0.56	2.49E-03	-0.28	1.61E-01	-0.11	5.86E-01	0.29	1.49E-01	-0.46	1.69E-02
ENSCAFG0000000022	TMED9	darkgrey	VSMC_M8	0.23	2.55E-01	-0.15	4.56E-01	0.59	1.19E-03	-0.66	1.92E-04	-0.38	5.01E-02	0.06	7.60E-01	-0.02	9.41E-01	0.64	3.10E-04	0.04	8.56E-01	-0.47	1.41E-02
ENSCAFG0000001823	ENSCAFG0000001823	grey	VSMC_M10	0.23	2.56E-01	0.73	1.56E-05	0.08	6.77E-01	0.44	2.31E-02	-0.03	8.81E-01	-0.19	3.43E-01	-0.69	7.52E-05	-0.64	3.15E-04	-0.61	6.79E-04	0.48	1.15E-02
ENSCAFG0000001149	MRPL46	grey	VSMC_M10	0.23	2.56E-01	-0.18	3.80E-01	0.12	5.55E-01	-0.21	2.93E-01	0.11	5.93E-01	0.20	3.13E-01	0.07	7.18E-01	0.26	1.84E-01	0.06	7.61E-01	-0.44	2.29E-02
ENSCAFG0000000129	HSPA9	darkgrey	VSMC_M8	0.23	2.56E-01	-0.42	2.79E-02	0.33	8.85E-02	-0.80	5.71E-07	-0.08	7.00E-01	0.07	7.18E-01	0.29	1.42E-01	0.80	4.43E-07	0.22	2.66E-01	-0.68	9.68E-05
ENSCAFG0000002498	TMEM38B	pink	VSMC_M5	0.23	2.56E-01	0.17	3.85E-01	-0.36	6.54E-02	0.47	1.45E-02	0.40	3.87E-02	-0.08	7.05E-01	-0.20	3.17E-01	-0.53	4.72E-03	-0.19	3.55E-01	0.28	1.56E-01
ENSCAFG0000002240	ENSCAFG0000002240	grey	VSMC_M10	0.23	2.56E-01	0.08	7.06E-01	0.13	5.29E-01	-0.26	1.90E-01	0.00	9.93E-01	0.15	4.50E-01	-0.21	2.87E-01	0.22	2.62E-01	-0.09	6.68E-01	-0.09	6.65E-01
ENSCAFG0000001734	THG1L	grey	VSMC_M10	0.23	2.56E-01	-0.20	3.20E-01	-0.05	8.14E-01	-0.09	6.57E-01	0.19	3.54E-01	-0.11	5.84E-01	0.35	6.94E-02	0.10	6.26E-01	0.11	5.77E-01	-0.13	5.18E-01
ENSCAFG0000002967	ENSCAFG0000002967	grey	VSMC_M10	0.23	2.56E-01	0.20	3.21E-01	0.42	2.96E-02	-0.21	2.93E-01	-0.36	6.23E-02	0.15	4.66E-01	0.11	6.00E-01	0.19	3.53E-01	-0.29	1.46E-01	0.09	6.47E-01
ENSCAFG0000001091	ARHGAP31	red	VSMC_M1	0.23	2.56E-01	-0.11	5.87E-01	-0.32	1.09E-01	0.18	3.67E-01	0.43	2.56E-02	0.08	6.75E-01	-0.50	7.67E-03	-0.20	3.20E-01	0.12	5.38E-01	-0.12	5.66E-01
ENSCAFG0000000609	PLPP5	grey	VSMC_M10	0.23	2.57E-01	0.27	1.68E-01	0.28	1.57E-01	-0.07	7.22E-01	-0.13	5.22E-01	-0.12	5.64E-01	0.11	5.96E-01	-0.06	7.80E-01	-0.33	9.07E-02	-0.03	8.93E-01
ENSCAFG0000001981	SPATA2L	grey	VSMC_M10	0.23	2.57E-01	0.38	5.20E-02	0.14	4.89E-01	0.00	9.86E-01	-0.20	3.08E-01	0.02	9.21E-01	0.02	9.03E-01	-0.10	6.03E-01	-0.49	9.60E-03	0.47	1.23E-02
ENSCAFG0000001725	DENND4A	grey	VSMC_M10	0.23	2.57E-01	-0.34	8.46E-02	-0.31	1.16E-01	-0.05	7.95E-01	0.36	6.39E-02	0.21	3.03E-01	-0.07	7.40E-01	0.21	2.94E-01	0.17	4.10E-01	-0.21	2.85E-01
ENSCAFG0000001323	LAMC1	grey	VSMC_M10	0.23	2.57E-01	0.59	1.21E-03	-0.09	6.38E-01	0.44	2.25E-02	0.11	5.93E-01	0.16	4.12E-01	-0.46	1.62E-02	-0.64	3.71E-04	-0.46	1.60E-02	0.51	6.09E-03
ENSCAFG0000000006	RDH5	grey	VSMC_M10	0.23	2.57E-01	0.21	2.86E-01	0.02	9.34E-01	0.14	4.99E-01	0.15	4.51E-01	0.07	7.28E-01	-0.03	8.72E-01	-0.20	3.29E-01	-0.26	1.89E-01	-0.05	8.12E-01
ENSCAFG0000000663	SNAI2	darkgrey	VSMC_M8	0.23	2.57E-01	-0.34	8.09E-02	0.18	3.71E-01	-0.60	9.58E-04	0.05	8.10E-01	0.10	6.17E-01	-0.12	5.51E-01	0.62	5.20E-04	0.22	2.64E-01	-0.61	7.91E-04
ENSCAFG0000001423	FDX1	grey	VSMC_M10	0.23	2.57E-01	0.29	1.49E-01	0.51	6.45E-03	-0.13	5.27E-01	-0.39	4.58E-02	-0.10	6.21E-01	-0.06	7.71E-01	-0.05	8.18E-01	-0.31	1.21E-01	0.08	6.79E-01
ENSCAFG0000000137	JOSD1	grey	VSMC_M10	0.23	2.57E-01	0.31	1.10E-01	0.45	1.94E-02	-0.19	3.30E-01	-0.20	3.18E-01	-0.01	9.56E-01	-0.04	8.56E-01	0.01	9.77E-01	-0.29	1.44E-01	-0.15	4.57E-01
ENSCAFG0000002033	CTRL	yellow	VSMC_M3	0.23	2.58E-01	0.18	3.80E-01	0.53	4.22E-03	-0.44	2.17E-02	-0.51	6.50E-03	0.23	2.48E-01	0.15	4.46E-01	0.35	6.95E-02	-0.32	9.92E-02	0.11	5.77E-01
ENSCAFG0000000388	TXNRD3	grey	VSMC_M10	0.23	2.58E-01	-0.10	6.04E-01	0.35	7.02E-02	-0.38	5.14E-02	-0.19	3.42E-01	0.05	8.18E-01	0.45	1.76E-02	0.40	3.73E-02	-0.06	7.55E-01	-0.28	1.53E-01
ENSCAFG0000003140	ENSCAFG0000003140	grey	VSMC_M10	0.23	2.58E-01	0.04	8.43E-01	-0.10	6.25E-01	0.11	5.97E-01	0.18	3.64E-01	-0.13	5.20E-01	-0.20	3.08E-01	-0.10	6.12E-01	-0.01	9.59E-01	-0.02	9.25E-01
ENSCAFG0000000039	GNL1	yellow	VSMC_M3	0.23	2.58E-01	0.60	8.64E-04	0.83	8.36E-08	-0.32	1.03E-01	-0.79	9.47E-07	0.08	6.93E-01	0.00	9.84E-01	0.07	7.24E-01	-0.61	7.55E-04	0.30	1.30E-01
ENSCAFG0000001173	GRK2	grey	VSMC_M10	0.23	2.58E-01	-0.08	6.82E-01	-0.25	2.09E-01	0.07	7.32E-01	0.44	2.19E-02	-0.29	1.48E-01	-0.03	8.78E-01	-0.10	6.17E-01	0.07	7.26E-01	-0.15	4.52E-01
ENSCAFG0000001294	DLX2	pink	VSMC_M5	0.23	2.58E-01	-0.24	2.22E-01	-0.77	2.80E-06	0.54	3.69E-03	0.78	1.92E-06	-0.02	9.31E-01	-0.05	8.03E-01	-0.32	1.04E-01	0.16	4.35E-01	0.08	6.83E-01
ENSCAFG0000000791	TRPC4AP	grey	VSMC_M10	0.23	2.58E-01	0.30	1.30E-01	0.02	9.19E-01	0.10	6.28E-01	0.03	8.79E-01	0.12	5.59E-01	-0.41	3.54E-02	-0.14	4.99E-01	-0.28	1.52E-01	0.14	5.01E-01
ENSCAFG0000000851	CFAP20	grey	VSMC_M10	0.23	2.58E-01	-0.10	6.08E-01	-0.25	2.10E-01	0.06	7.51E-01	0.19	3.44E-01	0.08	6.95E-01	0.32	1.01E-01	0.09	6.64E-01	-0.09	6.68E-01	0.16	4.35E-01
ENSCAFG0000001537	GTPBP3	yellow	VSMC_M3	0.23	2.59E-01	0.40	3.98E-02	0.55	3.01E-03	-0.15	4.42E-01	-0.47	1.44E-02	0.14	4.98E-01	0.29	1.46E-01	-0.04	8.59E-01	-0.50	7.83E-03	0.26	1.95E-01
ENSCAFG0000002167	ENSCAFG0000002167	grey	VSMC_M10	0.23	2.59E-01	-0.29	1.46E-01	0.02	9.30E-01	-0.40	3.71E-02	0.19	3.39E-01	0.01	9.68E-01	0.03	8.83E-01	0.44	2.22E-02	0.16	4.19E-01	-0.52	5.83E-03
ENSCAFG0000001327	ENSCAFG0000001327	red	VSMC_M1	0.23	2.59E-01	-0.13	5.12E-01	-0.13	5.34E-01	-0.03	8.77E-01	0.27	1.72E-01	0.13	5.26E-01	-0.15	4.70E-01	0.06	7.85E-01	0.05	8.09E-01	-0.29	1.43E-01
ENSCAFG0000000016	ME2	pink	VSMC_M5	0.23	2.59E-01	-0.17	3.96E-01	-0.32	1.03E-01	0.04	8.26E-01	0.57	2.11E-03	-0.27	1.79E-01	-0.17	4.05E-01	0.09	6.52E-01	0.15	4.70E-01	-0.39	4.21E-02
ENSCAFG0000000626	ARHGAP26	grey	VSMC_M10	0.23	2.59E-01	0.31	1.11E-01	-0.01	9.43E-01	0.31	1.19E-01	0.03	8.87E-01	0.11	5.69E-01	0.23	2.45E-01	-0.40	3.74E-02	-0.36	6.30E-02	0.36	6.47E-02
ENSCAFG0000002588	ENSCAFG0000002588	yellow	VSMC_M3	0.22	2.59E-01	0.15	4.44E-01	0.52	5.46E-03	-0.36	6.89E-02	-0.36	6.33E-02	0.05	8.18E-01	0.10	6.19E-01	0.25	2.17E-01	-0.24	2.25E-01	-0.19	3.38E-01
ENSCAFG0000001618	ENSCAFG0000001618	cyan	VSMC_M2	0.22	2.59E-01	0.50	8.62E-03	0.30	1.26E-01	0.01	9.58E-01	-0.20	3.14E-01	0.19	3.44E-01	0.07	7.26E-01	-0.17	3.84E-01	-0.55	2.70E-03	0.26	1.82E-01
ENSCAFG0000000925	GALNT5	grey	VSMC_M10	0.22	2.59E-01	-0.09	6.71E-01	0.00	9.86E-01	0.04	8.38E-01	-0.07	7.45E-01	0.17	3.89E-01	-0.03	8.69E-01	0.12	5.57E-01	-0.11	5.92E-01	0.15	4.60E-01
ENSCAFG0000000755	MAP1LC3A	darkgreen	VSMC_M4	0.22	2.59E-01	0.53	4.19E-03	-0.03	8.81E-01	0.55	2.75E-03	0.06	7.57E-01	-0.07	7.24E-01	-0.57	1.72E-03	-0.70	4.83E-05	-0.37	5.55E-02	0.45	1.73E-02
ENSCAFG0000000604	CCDC57	yellow	VSMC_M3	0.22	2.60E-01	0.49	9.62E-03	0.70	5.72E-05	-0.20	3.20E-01	-0.59	1.21E-03	-0.07	7.27E-01	0.12	5.60E-01	-0.03	8.80E-01	-0.50	8.22E-03	0.26	1.95E-01
ENSCAFG0000000394	MFSD2B	grey	VSMC_M10	0.22	2.60E-01	0.24	2.34E-01	0.04	8.30E-01	0.02	9.41E-01	0.01	9.48E-01	-0.11	5.78E-01	-0.20	3.16E-01	-0.07	7.13E-01	-0.30	1.31E-01	0.14	4.74E-01
ENSCAFG0000002049	ENSCAFG0000002049	cyan	VSMC_M2	0.22	2.60E-01	0.60	8.57E-04	0.59	1.22E-03	-0.10	6.11E-01	-0.58	1.70E-03	0.20	3.13E-01	-0.09	6.48E-01	-0.18	3.80E-01	-0.60	8.80E-04	0.42	2.81E-02
ENSCAFG0000000017	LRP1	grey	VSMC_M10	0.22	2.60E-01	0.44	2.02E-02	-0.02	9.08E-01	0.40	3.89E-02	0.10	6.09E-01	-0.10	6.36E-01	-0.36	6.57E-02	-0.57	1.87E-03	-0.36	6.44E-02	0.35	7.51E-02
ENSCAFG0000000872	BACH1	grey	VSMC_M10	0.22	2.60E-01	0.29	1.49E-01	0.40	3.98E-02	-0.34	8.15E-02	-0.17	3.96E-01	-0.13	5.29E-01	-0.36	6.58E-02	0.20	3.26E-01	-0.27	1.77E-01	-0.24	2.19E-01
ENSCAFG0000000208	CD2AP	pink	VSMC_M5	0.22	2.60E-01	-0.59	1.33E-03	-0.47	1.29E-02	-0.18	3.60E-01	0.67	1.18E-04	-0.02	9.30E-01	-0.07	7.44E-01	0.38	4.81E-02	0.44	2.05E-02	-0.61	7.97E-04
ENSCAFG0000001745	ENSCAFG0000001745	yellow	VSMC_M3	0.22	2.60E-01	0.27	1.74E-01	0.53	4.29E-03	-0.15	4.68E-01	-0.39	4.53E-02	0.29	1.37E-01	0.01	9.79E-01	-0.03	8.96E-01	-0.28	1.64E-01	0.03	8.82E-01
ENSCAFG0000001827	PPARGC1B	grey	VSMC_M10	0.22	2.60E-01	0.33	9.01E-02	0.44	2.33E-02	-0.21	2.95E-01	-0.45	1.90E-02	0.60	9.94E-04	0.18	3.64E-01	0.14	4.97E-01	-0.48	1.19E-02	0.26	1.84E-01
ENSCAFG0000001204	IPO4	darkgrey	VSMC_M8	0.22	2.61E-01	0.00	9.94E-01	0.68	9.76E-05	-0.80	6.51E-07	-0.54	3.69E-03	0.23	2.47E-01	0.21	2.97E-01	0.69	6.92E-05	-0.14	4.92E-01	-0.33	9.06E-02
ENSCAFG0000000263	RHOQ	pink	VSMC_M5	0.22	2.61E-01	-0.34	8.65E-02	-0.09	6.66E-01	-0.29	1.43E-01	0.47	1.35E-02	-0.40	4.13E-02	-0.01	9.64E-01	0.18	3.71E-01	0.39	4.72E-02	-0.69	7.18E-05
ENSCAFG0000000550	PGM2L1	grey	VSMC_M10	0.22	2.61E-01	0.23	2.51E-01	-0.16	4.28E-01	0.32	1.05E-01	0.30	1.33E-01	-0.38	4.96E-02	0.14	4.73E-01	-0.43	2.48E-02	-0.19	3.39E-01	0.18	3.73E-01
ENSCAFG0000002010	CNN3	cyan	VSMC_M2	0.22	2.61E-01	0.78	1.93E-06	0.32	1.00E-01	0.15	4.63E-01	-0.27	1.77E-01	-0.14	4.81E-01	-0.11	5.73E-01	-0.37	5.47E-02	-0.70	5.54E-05	0.48	1.20E-02
ENSCAFG0000001009	ENSCAFG0000001009	grey	VSMC_M10	0.22	2.61E-01	-0.28	1.55E-01	0.04	8.45E-01	-0.05	8.03E-01	0.07	7.14E-01	0.12	5.60E-01	-0.10	6.19E-01	0.25	2.16E-01	0.13	5.10E-01	-0.32	1.08E-01
ENSCAFG0000000701	TGS1	grey	VSMC_M10	0.22	2.61E-01	-0.35	7.54E-02	0.08	7.05E-01	-0.20	3.13E-01	0.09	6.39E-01	0.03	8.84E-01	0.15	4.46E-01	0.34	8.17E-02	0.22	2.70E-01	-0.39	4.73E-02
ENSCAFG0000000185	ABCC10	grey	VSMC_M10	0.22	2.61E-01	0.26	1.91E-01	0.38	4.83E-02	-0.16	4.24E-01	-0.35	7.24E-02	0.22	2.80E-01	-0.32	1.03E-01	0.06	7.65E-01	-0.29	1.49E-01	0.14	4.83E-01
ENSCAFG0000000131	TIGD5	yellow	VSMC_M3	0.22	2.61E-01	0.43	2.54E-02	0.62	5.73E-04	-0.16	4.39E-01	-0.49	9.59E-03	0.08	6.84E-01	-0.14	4.76E-01	-0.15	4.56E-01	-0.37	5.67E-02	0.15	4.47E-01
ENSCAFG0000001746	NAP1L3	pink	VSMC_M5	0.22	2.61E-01	-0.36	6.87E-02	-0.22	2.65E-01	-0.16	4.17E-01	0.40	3.81E-02	-0.17	4.02E-01	-0.10	6.27E-01	0.24	2.37E-01	0.30	1.22E-01	-0.40	3.90E-02
ENSCAFG0000000397	SPRY1	grey	VSMC_M10	0.22	2.61E-01	0.17	4.05E-01	0.36	6.61E-02	-0.17	4.02E-01	-0.19	3.45E-01	-0.03	8.69E-01	0.54	3.82E-03	0.12	5.37E-01	-0.26	1.82E-01	-0.07	7.44E-01
ENSCAFG0000001630	VWCE	cyan	VSMC_M2	0.22	2.61E-01	0.79	1.16E-06	0.59	1.23E-03	0.06	7.64E-01	-0.58	1.58E-03	0.02	9.17E-01	-0.16	4.17E-01	-0.29	1.40E-01	-0.74	1.09E-05	0.52	4.98E-03
ENSCAFG0000000615	EIF4EBP1	yellow	VSMC_M3	0.22	2.61E-01	0.21	3.01E-01	0.34	8.26E-02	-0.36	6.52E-02	-0.15	4.57E-01	-0.29	1.42E-01	0.04	8.38E-01	0.21	3.00E-01	-0.29	1.36E-01	-0.12	5.35E-01
ENSCAFG0000002447	ENSCAFG0000002447	grey	VSMC_M10	0.22	2.62E-01	0.18	3.58E-01	-0.14	4.79E-01	0.06	7.76E-01	0.19	3.43E-01	0.16	4.18E-01	0.09	6.57E-01	-0.07	7.47E-01	-0.30	1.25E-01	0.13	5.14E-01
ENSCAFG0000001520	STK16	grey	VSMC_M10	0.22	2.62E-01	0.29	1.38E-01	0.37	5.40E-02	0.02	9.17E-01	-0.31	1.16E-01	-0.08	6.80E-01	-0.09	6.37E-01	-0.13	5.10E-01	-0.32	9.88E-02	0.19	3.35E-01
ENSCAFG0000001292	LIMD2	grey	VSMC_M10	0.22	2.62E-01	0.38	4.88E-02	0.53	4.32E-03	-0.11	5.77E-01	-0.50	8.21E-03	0.40	3.89E-02	-0.01	9.75E-01	0.02	9.16E-01	-0.45	1.88E-02	0.20	3.13E-01
ENSCAFG0000001534	STAT5A	cyan	VSMC_M2	0.22	2.62E-01	0.62	5.89E-04	0.42	2.79E-02	-0.08	7.06E-01	-0.40	3.81E-02	-0.21	2.86E-01	0.16	4.18E-01	-0.15	4.67E-01	-0.68	9.10E-05	0.52	5.80E-03
ENSCAFG0000001083	SHOC2	pink	VSMC_M5	0.22	2.62E-01	-0.44	2.23E-02	-0.60	9.57E-04	0.17	4.03E-01	0.88	1.09E-09	-0.23	2.56E-01	-0.08	6.80E-01	-0.04	8.28E-01	0.43	2.69E-02	-0.51	6.94E-03
ENSCAFG0000001474	ANXA7	grey	VSMC_M10	0.22	2.62E-01	-0.30	1.33E-01	0.02	9.04E-01	-0.47	1.24E-02	0.15	4.56E-01	-0.11	5.91E-01	0.37	5.86E-02	0.44	2.07E-02	0.17	3.97E-01	-0.38	5.08E-02
ENSCAFG0000000029	METTL1	darkgrey	VSMC_M8	0.22	2.62E-01	-0.28	1.58E-01	0.42	2.99E-02	-0.75	8.02E-06	-0.18	3.65E-01	0.09	6.64E-01	0.33	8.78E-02	0.75	8.25E-06	0.11	6.00E-01	-0.64	2.95E-04
ENSCAFG0000000601	FREM2	grey	VSMC_M10	0.22	2.62E-01	0.21	2.90E-01	-0.12	5.50E-01	0.51	6.17E-03	0.20	3.08E-01	0.10	6.35E-01	-0.39	4.67E-02	-0.47	1.29E-02	-0.20	3.18E-01	0.13	5.10E-01
ENSCAFG0000000750	SBF2	grey	VSMC_M10	0.22	2.63E-01	0.35	6.95E-02	-0.46	1.60E-02	0.67	1.14E-04	0.51	5.98E-03	-0.25	2.14E-01	-0.08	6.97E-01	-0.68	1.01E-04	-0.34	8.10E-02	0.43	2.62E-02
ENSCAFG0000001689	TGFB1I1	grey	VSMC_M10	0.22	2.63E-01	0.09	6.53E-01	0.22	2.60E-01	-0.26	1.93E-01	0.04	8.42E-01	-0.23	2.47E-01	-0.20	3.22E-01	0.07	7.25E-01	0.01	9.53E-01	-0.28	1.60E-01
ENSCAFG0000001650	KLF8	grey	VSMC_M10	0.22	2.63E-01	-0.12	5.55E-01	-0.30	1.23E-01	0.13	5.21E-01	0.53	4.66E-03	-0.17	4.03E-01	0.12	5.63E-01	-0.10	6.20E-01	0.09	6.47E-01	-0.24	2.32E-01
ENSCAFG0000001005	ENSCAFG0000001005	pink	VSMC_M5	0.22	2.63E-01	-0.33	8.91E-02	-0.22	2.65E-01	-0.26	1.98E-01	0.42	3.02E-02	-0.01	9.50E-01	0.05	8.22E-01	0.33	9.68E-02	0.22	2.77E-01	-0.47	1.39E-02
ENSCAFG0000000615	PANK2	grey	VSMC_M10	0.22	2.63E-01	0.14	4.94E-01	-0.14	4.96E-01	0.28	1.53E-01	0.09	6.47E-01	0.14	4.83E-01	-0.01	9.76E-01	-0.23	2.51E-01	-0.24	2.24E-01	0.28	1.58E-01
ENSCAFG0000002462	ZCCHC7	red	VSMC_M1	0.22	2.64E-01	-0.04	8.42E-01	-0.26	1.91E-01	0.30	1.26E-01	0.26	1.98E-01	-0.01	9.47E-01	0.14	4.85E-01	-0.22	2.74E-01	-0.12	5.52E-01	0.19	3.49E-01
ENSCAFG0000001173	TRNAU1AP	grey	VSMC_M10	0.22	2.64E-01	-0.19	3.42E-01	-0.62	5.19E-04	0.30	1.23E-01	0.71	2.88E-05	-0.16	4.39E-01	-0.30	1.28E-01	-0.13	5.05E-01	0.13	5.29E-01	-0.13	5.17E-01
ENSCAFG0000001949	HAGH	cyan	VSMC_M2	0.22	2.64E-01	0.68	8.95E-05	0.18	3.67E-01	0.54	3.52E-03	-0.25	2.08E-01	0.06	7.71E-01	-0.21	2.89E-01	-0.69	6.71E-05	-0.63	4.71E-04	0.71	2.78E-05
ENSCAFG0000001407	CCDC141	cyan	VSMC_M2	0.22	2.64E-01	0.69	7.94E-05	-0.13	5.25E-01	0.72	2.69E-05	0.10	6.16E-01	-0.16	4.19E-01	-0.43	2.63E-02	-0.83	9.13E-08	-0.60	9.92E-04	0.68	9.41E-05
ENSCAFG0000002330	TWF2	yellow	VSMC_M3	0.22	2.64E-01	0.32	1.02E-01	0.72	2.19E-05	-0.62	6.32E-04	-0.62	5.33E-04	0.18	3.58E-01	0.32	1.08E-01	0.39	4.28E-02	-0.40	3.78E-02	0.07	7.11E-01
ENSCAFG0000002092	ENSCAFG0000002092	grey	VSMC_M10	0.22	2.65E-01	0.07	7.36E-01	0.23	2.46E-01	0.02	9.04E-01	-0.15	4.49E-01	0.24	2.21E-01	-0.22	2.70E-01	-0.04	8.30E-01	-0.07	7.27E-01	-0.04	8.28E-01
ENSCAFG0000000321	SPRED2	yellow	VSMC_M3	0.22	2.65E-01	0.35	7.06E-02	0.47	1.29E-02	-0.47	1.37E-02	-0.39	4.17E-02	0.15	4.62E-01	0.12	5.51E-01	0.26	1.94E-01	-0.46	1.50E-02	0.07	7.22E-01
ENSCAFG0000001816	HRH4	grey	VSMC_M10	0.22	2.65E-01	0.05	8.24E-01	0.05	8.10E-01	-0.06	7.84E-01	0.03	8.65E-01	-0.19	3.33E-01	-0.17	3.85E-01	0.04	8.41E-01	-0.11	5.69E-01	-0.03	8.63E-01
ENSCAFG0000003212	ENSCAFG0000003212	grey	VSMC_M10	0.22	2.65E-01	0.11	5.80E-01	-0.43	2.35E-02	0.53	4.05E-03	0.51	6.03E-03	-0.38	5.16E-02	-0.49	9.77E-03	-0.51	7.21E-03	-0.08	7.05E-01	0.09	6.50E-01
ENSCAFG0000000139	DDX17	pink	VSMC_M5	0.22	2.65E-01	0.18	3.70E-01	-0.48	1.19E-02	0.43	2.59E-02	0.61	7.13E-04	-0.26	1.94E-01	-0.13	5.09E-01	-0.46	1.64E-02	-0.16	4.29E-01	0.16	4.19E-01
ENSCAFG0000000147	ENSCAFG0000000147	grey	VSMC_M10	0.22	2.66E-01	-0.17	4.07E-01	-0.01	9.79E-01	-0.20	3.24E-01	0.14	4.71E-01	-0.17	4.10E-01	0.25	2.07E-01	0.26	1.83E-01	0.03	8.70E-01	-0.20	3.20E-01
ENSCAFG0000002597	ENSCAFG0000002597	grey	VSMC_M10	0.22	2.66E-01	-0.19	3.44E-01	-0.28	1.56E-01	-0.04	8.60E-01	0.48	1.12E-02	-0.21	2.88E-01	0.20	3.28E-01	0.08	6.88E-01	0.12	5.41E-01	-0.30	1.31E-01
ENSCAFG0000001513	NBL1	cyan	VSMC_M2	0.22	2.66E-01	0.82	1.34E-07	0.40	3.91E-02	0.31	1.15E-01	-0.35	7.08E-02	-0.25	2.03E-01	-0.48	1.19E-02	-0.57	2.03E-03	-0.67	1.32E-04	0.57	2.00E-03
ENSCAFG0000000407	ENSCAFG0000000407	grey	VSMC_M10	0.22	2.66E-01	-0.25	2.10E-01	-0.04	8.31E-01	-0.32	1.09E-01	0.24	2.29E-01	-0.14	4.79E-01	0.08	6.94E-01	0.37	5.45E-02	0.12	5.60E-01	-0.43	2.56E-02
ENSCAFG0000002544	KCNH2	grey	VSMC_M10	0.22	2.66E-01	0.30	1.24E-01	-0.23	2.57E-01	0.47	1.43E-02	0.24	2.27E-01	0.06	7.74E-01	-0.12	5.52E-01	-0.39	4.67E-02	-0.33	9.34E-02	0.37	5.58E-02
ENSCAFG0000002887	ENSCAFG0000002887	grey	VSMC_M10	0.22	2.66E-01	-0.04	8.32E-01	-0.03	8.94E-01	-0.16	4.20E-01	0.15	4.44E-01	-0.09	6.69E-01	0.02	9.24E-01	0.19	3.55E-01	-0.07	7.39E-01	-0.10	6.18E-01
ENSCAFG0000000251	TWISTNB	darkgrey	VSMC_M8	0.22	2.66E-01	-0.48	1.22E-02	0.10	6.14E-01	-0.55	2.75E-03	0.16	4.14E-01	-0.04	8.40E-01	0.05	8.02E-01	0.67	1.44E-04	0.34	8.55E-02	-0.78	1.47E-06
ENSCAFG0000000104	TRMT12	yellow	VSMC_M3	0.22	2.67E-01	0.23	2.50E-01	0.58	1.60E-03	-0.18	3.76E-01	-0.38	4.89E-02	0.13	5.19E-01	-0.14	4.96E-01	-0.02	9.20E-01	-0.21	2.92E-01	-0.11	5.93E-01
ENSCAFG0000001423	ITPRID2	grey	VSMC_M10	0.22	2.67E-01	0.53	4.34E-03	-0.12	5.43E-01	0.53	4.38E-03	0.10	6.08E-01	-0.05	8.00E-01	-0.30	1.30E-01	-0.68	8.23E-05	-0.42	2.77E-02	0.57	1.85E-03
ENSCAFG0000001205	CAMK2D	darkgreen	VSMC_M4	0.22	2.67E-01	0.36	6.81E-02	-0.53	4.48E-03	0.85	1.45E-08	0.50	7.42E-03	-0.13	5.06E-01	-0.54	4.02E-03	-0.80	4.90E-07	-0.29	1.38E-01	0.49	9.01E-03
ENSCAFG0000000307	AREG	grey	VSMC_M10	0.22	2.67E-01	0.09	6.43E-01	0.01	9.52E-01	-0.14	4.81E-01	0.21	2.92E-01	-0.07	7.34E-01	-0.11	5.74E-01	0.04	8.49E-01	-0.07	7.10E-01	-0.18	3.76E-01
ENSCAFG0000000184	CRIP3	grey	VSMC_M10	0.22	2.67E-01	0.14	5.00E-01	0.36	6.55E-02	-0.11	5.96E-01	-0.22	2.69E-01	0.16	4.40E-01	-0.20	3.20E-01	0.06	7.74E-01	-0.18	3.64E-01	-0.05	8.21E-01
ENSCAFG0000001367	MBIP	grey	VSMC_M10	0.22	2.68E-01	-0.29	1.41E-01	-0.10	6.15E-01	-0.16	4.36E-01	0.35	7.77E-02	-0.09	6.47E-01	0.09	6.48E-01	0.14	4.96E-01	0.31	1.14E-01	-0.48	1.04E-02
ENSCAFG0000000482	ITGA9	darkgreen	VSMC_M4	0.22	2.68E-01	0.40	3.63E-02	-0.18	3.58E-01	0.57	2.02E-03	0.26	1.90E-01	0.18	3.79E-01	-0.42	3.01E-02	-0.68	1.11E-04	-0.32	1.08E-01	0.28	1.56E-01
ENSCAFG0000000988	CD47	darkgreen	VSMC_M4	0.22	2.68E-01	0.44	2.20E-02	-0.37	5.88E-02	0.77	2.51E-06	0.39	4.30E-02	-0.28	1.57E-01	-0.20	3.25E-01	-0.76	4.14E-06	-0.39	4.27E-02	0.46	1.70E-02
ENSCAFG0000001580	SDCCAG8	grey	VSMC_M10	0.22	2.69E-01	0.30	1.30E-01	0.18	3.60E-01	0.04	8.52E-01	-0.14	5.00E-01	-0.21	3.04E-01	0.42	2.86E-02	-0.10	6.04E-01	-0.43	2.66E-02	0.33	9.41E-02
ENSCAFG0000000776	HIP1R	grey	VSMC_M10	0.22	2.69E-01	0.58	1.54E-03	0.10	6.05E-01	0.52	5.56E-03	-0.04	8.36E-01	-0.14	4.79E-01	-0.62	6.38E-04	-0.70	4.48E-05	-0.43	2.69E-02	0.45	1.78E-02
ENSCAFG0000003084	ENSCAFG0000003084	grey	VSMC_M10	0.22	2.69E-01	0.14	4.88E-01	0.46	1.67E-02	-0.25	2.00E-01	-0.30	1.24E-01	-0.16	4.32E-01	0.01	9.78E-01	0.09	6.69E-01	-0.16	4.15E-01	-0.07	7.35E-01
ENSCAFG0000002021	PKD1L3	grey	VSMC_M10	0.22	2.69E-01	0.28	1.60E-01	0.44	2.29E-02	-0.22	2.73E-01	-0.36	6.38E-02	0.03	8.88E-01	0.23	2.57E-01	0.03	9.01E-01	-0.33	9.60E-02	0.14	5.00E-01

ENSCAFG0000000109	TGFBI	cyan	VSMC_M2	0.22	2.69E-01	0.60	9.65E-04	0.50	7.51E-03	-0.14	4.97E-01	-0.39	4.60E-02	-0.03	8.74E-01	-0.62	5.84E-04	-0.16	4.27E-01	-0.53	4.68E-03	0.19	3.35E-01
ENSCAFG0000001608	PARP1	grey	VSMC_M10	0.22	2.69E-01	0.82	1.78E-07	0.30	1.24E-01	0.33	9.42E-02	-0.34	8.29E-02	-0.08	7.04E-01	-0.33	9.34E-02	-0.50	7.94E-03	-0.78	1.65E-06	0.70	5.19E-05
ENSCAFG0000002307	AFF3	darkgreen	VSMC_M4	0.22	2.69E-01	0.39	4.17E-02	-0.42	2.94E-02	0.73	1.64E-05	0.34	8.65E-02	-0.13	5.20E-01	-0.17	4.01E-01	-0.66	1.73E-04	-0.43	2.71E-02	0.61	7.38E-04
ENSCAFG0000000191	GTPBP2	grey	VSMC_M10	0.22	2.69E-01	-0.23	2.59E-01	-0.22	2.77E-01	0.02	9.24E-01	0.40	3.79E-02	0.09	6.42E-01	-0.02	9.19E-01	-0.06	7.68E-01	0.16	4.12E-01	-0.26	1.93E-01
ENSCAFG0000000979	GXYLT1	pink	VSMC_M5	0.22	2.69E-01	-0.56	2.35E-03	-0.21	2.88E-01	-0.31	1.14E-01	0.46	1.51E-02	-0.04	8.35E-01	-0.05	8.13E-01	0.49	8.82E-03	0.45	1.94E-02	-0.74	1.12E-05
ENSCAFG0000000377	NRP1	cyan	VSMC_M2	0.22	2.70E-01	0.72	2.08E-05	-0.05	8.13E-01	0.58	1.47E-03	0.00	9.92E-01	-0.14	4.82E-01	-0.39	4.21E-02	-0.67	1.32E-04	-0.68	9.64E-05	0.68	9.51E-05
ENSCAFG0000001539	ENSCAFG0000001539	grey	VSMC_M10	0.22	2.70E-01	0.24	2.20E-01	0.20	3.11E-01	-0.18	3.65E-01	-0.11	5.84E-01	-0.03	8.65E-01	0.37	6.06E-02	0.07	7.14E-01	-0.29	1.41E-01	0.09	6.53E-01
ENSCAFG0000000621	ARAP3	yellow	VSMC_M3	0.22	2.70E-01	0.39	4.27E-02	0.72	2.17E-05	-0.59	1.20E-03	-0.56	2.48E-03	0.21	2.87E-01	-0.21	2.86E-01	0.32	1.09E-01	-0.39	4.73E-02	-0.05	7.86E-01
ENSCAFG0000003169	CRADD	grey	VSMC_M10	0.22	2.70E-01	0.14	4.74E-01	-0.34	7.81E-02	0.33	8.82E-02	0.45	1.90E-02	-0.22	2.61E-01	0.23	2.51E-01	-0.34	8.05E-02	-0.22	2.75E-01	0.16	4.21E-01
ENSCAFG0000001185	ENSCAFG0000001185	pink	VSMC_M5	0.22	2.70E-01	-0.01	9.60E-01	-0.26	1.85E-01	0.34	8.32E-02	0.46	1.69E-02	-0.50	8.27E-03	-0.17	3.83E-01	-0.32	9.95E-02	0.04	8.31E-01	-0.08	7.06E-01
ENSCAFG0000001048	STN1	grey	VSMC_M10	0.22	2.71E-01	0.51	6.30E-03	-0.09	6.51E-01	0.58	1.42E-03	-0.03	8.92E-01	0.06	7.66E-01	-0.22	2.63E-01	-0.54	3.44E-03	-0.55	2.69E-03	0.67	1.35E-04
ENSCAFG0000000264	TTC7A	yellow	VSMC_M3	0.22	2.71E-01	0.32	1.02E-01	0.79	9.01E-07	-0.66	1.84E-04	-0.59	1.20E-03	0.03	8.86E-01	0.20	3.07E-01	0.33	8.78E-02	-0.31	1.14E-01	-0.13	5.34E-01
ENSCAFG0000003037	C12H6orf141	grey	VSMC_M10	0.22	2.71E-01	0.06	7.65E-01	0.28	1.53E-01	-0.22	2.67E-01	-0.05	8.03E-01	-0.10	6.08E-01	-0.02	9.38E-01	0.09	6.58E-01	-0.06	7.65E-01	-0.36	6.39E-02
ENSCAFG0000000631	ENSCAFG0000000631	grey	VSMC_M10	0.22	2.71E-01	0.31	1.13E-01	0.32	1.05E-01	-0.03	8.78E-01	-0.21	2.91E-01	0.28	1.63E-01	-0.15	4.53E-01	-0.10	6.30E-01	-0.29	1.46E-01	0.14	4.86E-01
ENSCAFG0000001275	FAM53B	cyan	VSMC_M2	0.22	2.71E-01	0.87	2.83E-09	0.55	3.13E-03	0.11	5.98E-01	-0.49	9.23E-03	-0.07	7.27E-01	-0.06	7.81E-01	-0.42	2.94E-02	-0.82	1.63E-07	0.56	2.36E-03
ENSCAFG0000002003	SPOUT1	grey	VSMC_M10	0.22	2.71E-01	0.01	9.45E-01	0.38	5.18E-02	-0.37	5.53E-02	-0.40	3.72E-02	0.18	3.66E-01	0.39	4.41E-02	0.36	6.58E-02	-0.23	2.39E-01	0.12	5.48E-01
ENSCAFG0000000180	RIC8B	grey	VSMC_M10	0.22	2.71E-01	0.04	8.34E-01	-0.26	1.89E-01	0.49	9.98E-03	0.20	3.22E-01	0.13	5.08E-01	-0.16	4.36E-01	-0.41	3.22E-02	-0.11	5.73E-01	0.35	7.63E-02
ENSCAFG0000001712	SULT1A1	yellow	VSMC_M3	0.22	2.72E-01	0.47	1.23E-02	0.62	5.66E-04	-0.40	4.04E-02	-0.45	1.88E-02	-0.03	8.72E-01	0.09	6.56E-01	0.10	6.34E-01	-0.40	3.98E-02	0.02	9.20E-01
ENSCAFG0000002844	ENSCAFG0000002844	grey	VSMC_M10	0.22	2.72E-01	0.18	3.67E-01	0.04	8.27E-01	0.12	5.37E-01	0.09	6.55E-01	0.00	9.92E-01	0.02	9.04E-01	-0.15	4.56E-01	-0.24	2.30E-01	-0.01	9.58E-01
ENSCAFG0000003029	GRB2	grey	VSMC_M10	0.22	2.72E-01	0.29	1.35E-01	0.40	3.91E-02	-0.10	6.36E-01	-0.45	1.90E-02	0.21	2.94E-01	0.18	3.73E-01	0.06	7.80E-01	-0.37	5.50E-02	0.27	1.73E-01
ENSCAFG0000000121	RPS10	grey	VSMC_M10	0.22	2.72E-01	0.46	1.69E-02	0.38	4.99E-02	0.18	3.64E-01	-0.38	4.89E-02	0.02	9.17E-01	0.05	8.08E-01	-0.29	1.42E-01	-0.49	9.74E-03	0.36	6.13E-02
ENSCAFG0000001695	KAT7	grey	VSMC_M10	0.22	2.72E-01	-0.21	2.95E-01	0.17	3.92E-01	-0.30	1.28E-01	-0.02	9.16E-01	-0.17	3.90E-01	0.37	5.40E-02	0.33	8.90E-02	0.03	8.80E-01	-0.22	2.73E-01
ENSCAFG0000000542	POLR3F	darkgrey	VSMC_M8	0.22	2.72E-01	-0.48	1.17E-02	0.18	3.66E-01	-0.67	1.14E-04	0.07	7.30E-01	0.02	9.18E-01	0.13	5.05E-01	0.75	8.12E-06	0.32	1.09E-01	-0.79	1.22E-06
ENSCAFG0000002272	MT-CO2	pink	VSMC_M5	0.22	2.72E-01	-0.22	2.75E-01	-0.66	1.63E-04	0.39	4.40E-02	0.82	1.40E-07	-0.31	1.19E-01	-0.12	5.61E-01	-0.29	1.40E-01	0.20	3.23E-01	-0.18	3.59E-01
ENSCAFG0000000471	FAM107B	grey	VSMC_M10	0.22	2.73E-01	0.32	9.83E-02	-0.12	5.68E-01	0.35	7.32E-02	0.16	4.25E-01	-0.18	3.79E-01	0.12	5.45E-01	-0.43	2.51E-02	-0.37	6.03E-02	0.34	8.24E-02
ENSCAFG0000003161	EFCAB8	grey	VSMC_M10	0.22	2.73E-01	-0.22	2.63E-01	-0.38	5.10E-02	0.04	8.34E-01	0.53	4.08E-03	-0.29	1.39E-01	-0.14	4.76E-01	0.09	6.43E-01	0.12	5.57E-01	-0.23	2.47E-01
ENSCAFG0000001090	FAR2	grey	VSMC_M10	0.22	2.73E-01	-0.21	3.04E-01	0.09	6.72E-01	-0.37	5.55E-02	-0.06	7.57E-01	0.31	1.18E-01	0.02	9.38E-01	0.46	1.51E-02	-0.03	8.71E-01	-0.17	3.99E-01
ENSCAFG0000000583	ENSCAFG0000000583	grey	VSMC_M10	0.22	2.73E-01	0.08	6.87E-01	-0.05	7.89E-01	0.28	1.59E-01	-0.03	8.90E-01	0.12	5.36E-01	0.00	9.80E-01	-0.18	3.62E-01	-0.16	4.26E-01	0.35	6.96E-02
ENSCAFG0000000879	NPY1R	grey	VSMC_M10	0.22	2.73E-01	0.29	1.42E-01	-0.01	9.75E-01	0.37	5.57E-02	-0.02	9.37E-01	0.17	3.87E-01	-0.47	1.32E-02	-0.39	4.72E-02	-0.30	1.30E-01	0.36	6.14E-02
ENSCAFG0000000452	MARK4	cyan	VSMC_M2	0.22	2.73E-01	0.56	2.53E-03	0.04	8.46E-01	0.24	2.37E-01	0.07	7.32E-01	0.03	8.66E-01	-0.43	2.71E-02	-0.45	1.73E-02	-0.45	1.71E-02	0.29	1.49E-01
ENSCAFG0000000816	TMEM154	pink	VSMC_M5	0.22	2.73E-01	0.04	8.43E-01	-0.67	1.40E-04	0.71	3.06E-05	0.67	1.16E-04	-0.10	6.29E-01	-0.06	7.82E-01	-0.58	1.47E-03	-0.06	7.74E-01	0.29	1.48E-01
ENSCAFG0000001469	ENSCAFG0000001469	cyan	VSMC_M2	0.22	2.73E-01	0.26	1.92E-01	0.11	5.89E-01	-0.12	5.52E-01	-0.01	9.47E-01	-0.07	7.22E-01	0.11	5.80E-01	0.03	8.82E-01	-0.31	1.17E-01	0.10	6.34E-01
ENSCAFG0000001640	TMEM132A	grey	VSMC_M10	0.22	2.74E-01	0.73	1.60E-05	0.20	3.27E-01	0.38	4.82E-02	-0.11	5.89E-01	-0.34	7.86E-02	-0.33	9.32E-02	-0.66	1.58E-04	-0.55	2.77E-03	0.52	5.48E-03
ENSCAFG0000001956	LCN8	darkgreen	VSMC_M4	0.22	2.74E-01	-0.12	5.54E-01	-0.57	1.88E-03	0.57	1.94E-03	0.61	6.59E-04	-0.12	5.63E-01	-0.17	4.10E-01	-0.39	4.24E-02	0.05	7.98E-01	0.11	6.02E-01
ENSCAFG0000002504	ENSCAFG0000002504	pink	VSMC_M5	0.22	2.74E-01	-0.05	8.02E-01	-0.69	7.44E-05	0.61	8.14E-04	0.85	1.38E-08	-0.36	6.62E-02	-0.37	5.70E-02	-0.50	8.57E-03	0.10	6.24E-01	-0.05	7.90E-01
ENSCAFG0000000717	CHCHD5	grey	VSMC_M10	0.22	2.74E-01	0.13	5.25E-01	0.17	3.99E-01	0.01	9.69E-01	0.03	8.85E-01	-0.07	7.18E-01	-0.10	6.14E-01	-0.03	8.90E-01	-0.15	4.64E-01	-0.20	3.06E-01
ENSCAFG0000002069	ENSCAFG0000002069	grey	VSMC_M10	0.22	2.74E-01	0.20	3.06E-01	0.16	4.38E-01	0.25	2.13E-01	-0.06	7.81E-01	-0.20	3.06E-01	0.00	9.82E-01	-0.29	1.40E-01	-0.24	2.23E-01	0.18	3.74E-01
ENSCAFG0000001928	INTS11	grey	VSMC_M10	0.22	2.75E-01	0.29	1.44E-01	0.30	1.33E-01	-0.30	1.24E-01	-0.12	5.52E-01	-0.21	2.83E-01	-0.02	9.37E-01	0.07	7.12E-01	-0.24	2.21E-01	-0.04	8.38E-01
ENSCAFG0000000672	ENSCAFG0000000672	grey	VSMC_M10	0.22	2.75E-01	0.41	3.30E-02	0.36	6.75E-02	-0.19	3.52E-01	-0.30	1.26E-01	0.08	7.02E-01	-0.12	5.59E-01	-0.02	9.17E-01	-0.42	2.72E-02	0.26	1.91E-01
ENSCAFG0000002886	ZNF22	grey	VSMC_M10	0.22	2.75E-01	-0.11	6.01E-01	-0.32	1.01E-01	0.13	5.33E-01	0.34	8.30E-02	-0.18	3.64E-01	0.17	3.98E-01	-0.03	8.83E-01	-0.02	9.09E-01	0.05	7.89E-01
ENSCAFG0000000803	DIP2B	pink	VSMC_M5	0.22	2.75E-01	0.20	3.22E-01	-0.44	2.32E-02	0.50	7.52E-03	0.55	3.26E-03	-0.14	4.72E-01	-0.44	2.21E-02	-0.57	2.04E-03	-0.07	7.25E-01	0.19	3.41E-01
ENSCAFG0000000069	ANGPT1	darkgreen	VSMC_M4	0.22	2.76E-01	0.51	7.05E-03	-0.34	7.88E-02	0.80	6.87E-07	0.27	1.68E-01	-0.21	3.03E-01	-0.27	1.80E-01	-0.75	5.37E-06	-0.51	7.01E-03	0.64	3.68E-04
ENSCAFG0000002998	C18H11orf91	grey	VSMC_M10	0.22	2.76E-01	0.12	5.50E-01	-0.05	8.10E-01	0.04	8.43E-01	0.09	6.41E-01	-0.15	4.67E-01	0.05	8.22E-01	-0.04	8.50E-01	-0.18	3.70E-01	0.11	5.88E-01
ENSCAFG0000000027	NHSL1	grey	VSMC_M10	0.22	2.76E-01	-0.03	8.91E-01	0.11	5.78E-01	-0.31	1.14E-01	0.10	6.22E-01	0.08	6.78E-01	0.11	6.01E-01	0.11	5.92E-01	0.03	8.63E-01	-0.24	2.18E-01
ENSCAFG0000000756	SREK1	grey	VSMC_M10	0.22	2.76E-01	0.00	9.99E-01	-0.21	2.92E-01	0.01	9.53E-01	0.39	4.70E-02	-0.24	2.21E-01	0.30	1.23E-01	-0.01	9.60E-01	-0.12	5.40E-01	-0.06	7.60E-01
ENSCAFG0000000112	L3MBTL2	grey	VSMC_M10	0.22	2.77E-01	0.20	3.29E-01	0.45	1.84E-02	-0.14	4.71E-01	-0.42	2.86E-02	-0.02	9.24E-01	0.28	1.54E-01	0.18	3.70E-01	-0.37	5.58E-02	0.18	3.68E-01
ENSCAFG0000001142	DYRK3	grey	VSMC_M10	0.22	2.77E-01	-0.26	1.88E-01	-0.04	8.57E-01	-0.25	2.07E-01	0.23	2.44E-01	-0.07	7.47E-01	0.24	2.30E-01	0.29	1.42E-01	0.18	3.62E-01	-0.40	4.00E-02
ENSCAFG0000001966	PER3	grey	VSMC_M10	0.22	2.77E-01	0.64	3.12E-04	0.10	6.05E-01	0.33	9.37E-02	0.01	9.42E-01	-0.47	1.34E-02	-0.26	1.97E-01	-0.49	9.80E-03	-0.57	1.93E-03	0.41	3.43E-02
ENSCAFG0000000249	PTP4A1	pink	VSMC_M5	0.22	2.77E-01	-0.41	3.15E-02	-0.60	1.06E-03	0.17	4.01E-01	0.81	2.28E-07	-0.14	4.92E-01	-0.14	4.79E-01	-0.01	9.74E-01	0.36	6.54E-02	-0.46	1.51E-02
ENSCAFG0000000431	POLR2D	pink	VSMC_M5	0.22	2.77E-01	-0.31	1.14E-01	-0.15	4.70E-01	0.00	9.83E-01	0.36	6.14E-02	-0.03	8.76E-01	-0.31	1.17E-01	0.05	8.06E-01	0.31	1.17E-01	-0.40	3.75E-02
ENSCAFG0000003099	N6AMT1	grey	VSMC_M10	0.22	2.77E-01	-0.17	3.88E-01	0.13	5.05E-01	-0.15	4.56E-01	0.12	5.62E-01	-0.22	2.77E-01	0.00	9.98E-01	0.06	7.62E-01	0.17	4.07E-01	-0.39	4.60E-02
ENSCAFG0000001849	ENSCAFG0000001849	grey	VSMC_M10	0.22	2.78E-01	0.53	4.76E-03	0.50	7.27E-03	0.08	6.88E-01	-0.58	1.57E-03	0.12	5.49E-01	0.21	3.00E-01	-0.26	1.85E-01	-0.58	1.51E-03	0.57	1.90E-03
ENSCAFG0000001924	ABCD1	cyan	VSMC_M2	0.22	2.78E-01	0.67	1.48E-04	0.58	1.37E-03	-0.18	3.82E-01	-0.58	1.66E-03	0.33	8.78E-02	-0.18	3.61E-01	-0.08	6.84E-01	-0.65	2.38E-04	0.42	3.10E-02
ENSCAFG0000000954	PNPLA2	cyan	VSMC_M2	0.22	2.78E-01	0.55	2.92E-03	0.27	1.79E-01	0.13	5.07E-01	-0.06	7.58E-01	-0.25	2.11E-01	-0.46	1.59E-02	-0.41	3.37E-02	-0.36	6.81E-02	0.14	4.85E-01
ENSCAFG0000000887	MIS18A	darkgrey	VSMC_M8	0.22	2.78E-01	-0.29	1.36E-01	0.40	3.67E-02	-0.69	5.76E-05	-0.29	1.36E-01	0.15	4.47E-01	0.52	5.93E-03	0.77	2.79E-06	0.03	8.89E-01	-0.36	6.76E-02
ENSCAFG0000003024	DUSP4	grey	VSMC_M10	0.22	2.78E-01	-0.43	2.45E-02	0.16	4.22E-01	-0.42	3.01E-02	-0.01	9.71E-01	0.19	3.34E-01	0.60	8.58E-04	0.47	1.37E-02	0.22	2.66E-01	-0.43	2.47E-02
ENSCAFG0000000445	MED4	pink	VSMC_M5	0.22	2.78E-01	-0.12	5.43E-01	-0.01	9.64E-01	-0.14	4.86E-01	0.25	2.16E-01	-0.15	4.48E-01	-0.24	2.31E-01	0.20	3.26E-01	0.14	4.81E-01	-0.44	2.06E-02
ENSCAFG0000000453	ZNF862	grey	VSMC_M10	0.22	2.78E-01	0.18	3.77E-01	0.33	8.87E-02	-0.35	7.34E-02	-0.10	6.28E-01	-0.18	3.71E-01	0.43	2.53E-02	0.10	6.36E-01	-0.22	2.64E-01	-0.14	4.97E-01
ENSCAFG0000003124	ENSCAFG0000003124	grey	VSMC_M10	0.22	2.78E-01	0.10	6.32E-01	0.29	1.43E-01	-0.26	1.98E-01	-0.28	1.58E-01	0.24	2.28E-01	0.11	5.75E-01	0.19	3.53E-01	-0.20	3.06E-01	0.04	8.59E-01
ENSCAFG0000000440	ENSCAFG0000000440	grey	VSMC_M10	0.22	2.79E-01	-0.05	8.19E-01	-0.03	8.64E-01	-0.15	4.61E-01	0.18	3.73E-01	-0.06	7.65E-01	-0.03	8.90E-01	0.17	3.98E-01	-0.05	7.93E-01	-0.10	6.10E-01
ENSCAFG0000000475	ETHE1	cyan	VSMC_M2	0.22	2.79E-01	0.87	3.24E-09	0.62	6.03E-04	0.08	6.97E-01	-0.56	2.21E-03	-0.01	9.54E-01	-0.42	2.93E-02	-0.39	4.22E-02	-0.76	3.57E-06	0.50	7.88E-03
ENSCAFG0000003166	ENSCAFG0000003166	red	VSMC_M1	0.22	2.79E-01	0.06	7.73E-01	-0.04	8.45E-01	0.08	6.98E-01	0.04	8.42E-01	0.11	5.72E-01	0.31	1.17E-01	-0.14	4.98E-01	-0.15	4.49E-01	0.20	3.24E-01
ENSCAFG0000000439	SIX5	yellow	VSMC_M3	0.22	2.80E-01	0.61	7.16E-04	0.48	1.20E-02	0.16	4.40E-01	-0.34	8.60E-02	-0.09	6.61E-01	-0.37	5.91E-02	-0.43	2.47E-02	-0.50	8.31E-03	0.31	1.18E-01
ENSCAFG0000000749	RALY	yellow	VSMC_M3	0.22	2.80E-01	0.10	6.21E-01	0.78	1.90E-06	-0.84	3.48E-08	-0.50	8.34E-03	-0.12	5.46E-01	0.26	1.83E-01	0.61	6.93E-04	-0.16	4.26E-01	-0.42	3.07E-02
ENSCAFG0000000875	PRKAG1	grey	VSMC_M10	0.22	2.80E-01	0.18	3.82E-01	-0.16	4.37E-01	0.15	4.56E-01	0.28	1.51E-01	-0.10	6.33E-01	0.09	6.59E-01	-0.23	2.47E-01	-0.15	4.62E-01	0.03	8.66E-01
ENSCAFG0000001569	ZCCHC24	cyan	VSMC_M2	0.22	2.80E-01	0.80	5.35E-07	0.29	1.42E-01	0.19	3.52E-01	-0.17	3.89E-01	-0.28	1.61E-01	-0.29	1.41E-01	-0.53	4.74E-03	-0.64	2.90E-04	0.45	1.81E-02
ENSCAFG0000001759	PIAS2	grey	VSMC_M10	0.22	2.80E-01	0.06	7.85E-01	-0.46	1.47E-02	0.29	1.41E-01	0.43	2.38E-02	0.09	6.60E-01	-0.05	8.15E-01	-0.22	2.60E-01	-0.14	4.89E-01	0.21	2.86E-01
ENSCAFG0000000742	SWAP70	yellow	VSMC_M3	0.22	2.80E-01	0.24	2.22E-01	0.39	4.51E-02	-0.39	4.29E-02	-0.13	5.31E-01	-0.21	2.95E-01	0.16	4.35E-01	0.16	4.19E-01	-0.24	2.34E-01	-0.18	3.76E-01
ENSCAFG0000001809	AMMECR1	grey	VSMC_M10	0.22	2.81E-01	0.02	9.16E-01	0.10	6.22E-01	-0.15	4.65E-01	-0.05	7.92E-01	0.10	6.33E-01	0.41	3.15E-02	0.13	5.09E-01	-0.16	4.15E-01	0.03	8.89E-01
ENSCAFG0000002037	ZZZ3	pink	VSMC_M5	0.22	2.81E-01	-0.04	8.28E-01	-0.35	7.02E-02	0.33	9.49E-02	0.52	5.71E-03	-0.20	3.25E-01	-0.39	4.34E-02	-0.26	1.94E-01	-0.02	9.13E-01	-0.12	5.39E-01
ENSCAFG0000001828	FLII	cyan	VSMC_M2	0.22	2.81E-01	0.61	6.46E-04	0.33	9.03E-02	0.24	2.37E-01	-0.29	1.43E-01	-0.09	6.41E-01	-0.39	4.52E-02	-0.51	7.01E-03	-0.47	1.34E-02	0.44	2.06E-02
ENSCAFG0000000591	LRRC45	yellow	VSMC_M3	0.22	2.81E-01	0.41	3.27E-02	0.72	1.95E-05	-0.48	1.05E-02	-0.55	2.82E-03	-0.06	7.85E-01	0.29	1.49E-01	0.25	2.09E-01	-0.48	1.09E-02	0.00	9.97E-01
ENSCAFG0000003066	TTC33	cyan	VSMC_M2	0.22	2.81E-01	0.48	1.13E-02	0.28	1.51E-01	0.00	9.93E-01	-0.07	7.24E-01	-0.43	2.65E-02	0.11	5.95E-01	-0.23	2.58E-01	-0.43	2.55E-02	0.06	7.81E-01
ENSCAFG0000001066	ANO1	pink	VSMC_M5	0.22	2.81E-01	-0.53	4.08E-03	-0.33	9.02E-02	-0.16	4.28E-01	0.49	9.63E-03	0.15	4.48E-01	0.21	2.89E-01	0.36	6.87E-02	0.39	4.39E-02	-0.52	5.31E-03
ENSCAFG0000002996	ENSCAFG0000002996	red	VSMC_M1	0.22	2.81E-01	0.15	4.48E-01	0.12	5.52E-01	0.10	6.26E-01	0.03	8.66E-01	-0.23	2.41E-01	-0.19	3.43E-01	-0.21	2.84E-01	-0.10	6.06E-01	-0.04	8.25E-01
ENSCAFG0000002846	ENSCAFG0000002846	grey	VSMC_M10	0.22	2.81E-01	0.50	8.52E-03	0.16	4.20E-01	0.43	2.65E-02	-0.24	2.19E-01	0.00	9.96E-01	-0.19	3.39E-01	-0.49	9.42E-03	-0.55	2.79E-03	0.57	1.81E-03
ENSCAFG0000002906	ENSCAFG0000002906	yellow	VSMC_M3	0.21	2.82E-01	0.34	7.97E-02	0.82	1.79E-07	-0.40	4.12E-02	-0.68	9.53E-05	0.00	9.96E-01	0.20	3.24E-01	0.19	3.44E-01	-0.41	3.18E-02	0.04	8.35E-01
ENSCAFG0000000272	ABCA1	darkgreen	VSMC_M4	0.21	2.82E-01	0.51	6.50E-03	-0.32	1.05E-01	0.82	2.26E-07	0.23	2.55E-01	0.14	4.96E-01	-0.56	2.31E-03	-0.79	8.33E-07	-0.46	1.56E-02	0.63	4.27E-04
ENSCAFG0000001394	C1QL1	grey	VSMC_M10	0.21	2.83E-01	0.01	9.48E-01	0.21	3.04E-01	-0.35	6.93E-02	-0.07	7.22E-01	0.08	6.95E-01	0.32	1.01E-01	0.18	3.71E-01	-0.09	6.67E-01	-0.14	4.91E-01
ENSCAFG0000002592	ENSCAFG0000002592	yellow	VSMC_M3	0.21	2.83E-01	0.27	1.69E-01	0.64	3.72E-04	-0.36	6.44E-02	-0.51	6.59E-03	0.04	8.26E-01	0.24	2.32E-01	0.21	3.02E-01	-0.39	4.26E-02	0.00	9.86E-01
ENSCAFG0000001819	ENSCAFG0000001819	darkgreen	VSMC_M4	0.21	2.83E-01	0.11	5.80E-01	-0.46	1.65E-02	0.63	3.79E-04	0.48	1.23E-02	-0.14	5.01E-01	0.05	8.11E-01	-0.53	4.63E-03	-0.13	5.27E-01	0.29	1.37E-01
ENSCAFG0000001010	GANC	grey	VSMC_M10	0.21	2.83E-01	0.25	2.01E-01	-0.24	2.30E-01	0.30	1.33E-01	0.24	2.26E-01	-0.03	8.63E-01	0.05	8.08E-01	-0.35	7.24E-02	-0.32	9.98E-02	0.35	7.14E-02
ENSCAFG0000000788	ING2	grey	VSMC_M10	0.21	2.83E-01	-0.11	5.75E-01	0.03	8.82E-01	-0.21	2.90E-01	0.06	7.59E-01	-0.10	6.04E-01	0.09	6.54E-01	0.29	1.43E-01	-0.09	6.43E-01	-0.17	4.08E-01
ENSCAFG0000001603	TNRC18	yellow	VSMC_M3	0.21	2.83E-01	0.69	6.15E-05	0.54	3.72E-03	0.08	6.86E-01	-0.53	4.45E-03	0.07	7.38E-01	-0.26	1.92E-01	-0.28	1.56E-01	-0.66	1.58E-04	0.54	3.69E-03
ENSCAFG0000000985	ENSCAFG0000000985	grey	VSMC_M10	0.21	2.83E-01	-0.15	4.56E-01	-0.09	6.41E-01	-0.23	2.47E-01	0.24	2.21E-01	0.03	8.82E-01	0.01	9.76E-01	0.19	3.54E-01	0.17	3.84E-01	-0.26	1.82E-01
ENSCAFG0000002368	PCDHGA1	darkgreen	VSMC_M4	0.21	2.84E-01	0.53	4.46E-03	0.02	9.32E-01	0.54	3.35E-03	0.00	9.98E-01	-0.09	6.43E-01	-0.16	4.34E-01	-0.72	2.30E-05	-0.46	1.57E-02	0.56	2.63E-03
ENSCAFG0000002887	CAVIN1	yellow	VSMC_M3	0.21	2.84E-01	0.35	7.21E-02	0.75	5.41E-06	-0.31	1.21E-01	-0.63	4.86E-04	-0.04	8.33E-01	-0.09	6.40E-01	0.10	6.34E-01	-0.33	8.86E-02	0.04	8.60E-01
ENSCAFG0000001616	EPHX1	grey	VSMC_M10	0.21	2.84E-01	0.77	2.48E-06	0.15	4.63E-01	0.56	2.53E-03	-0.20	3.15E-01	-0.07	7.10E-01	-0.15	4.61E-01	-0.73	1.47E-05	-0.69	5.96E-05	0.74	8.49E-06
ENSCAFG0000001037	TAGLN3	grey	VSMC_M10	0.21	2.84E-01	-0.20	3.06E-01	-0.17	3.96E-01	0.13	5.26E-01	0.28	1.60E-01	0.02	9.28E-01	-0.55	2.81E-03	0.02	9.15E-01	0.15	4.66E-01	-0.26	1.94E-01
ENSCAFG0000003132	C18H11orf68	cyan	VSMC_M2	0.21	2.84E-01	0.57	1.95E-03	0.41	3.20E-02	-0.14	4.84E-01	-0.40	3.84E-02	0.16	4.26E-01	-0.21	2.87E-01	-0.09	6.43E-01	-0.52	5.19E-03	0.31	1.19E-01
ENSCAFG0000001151	ENSCAFG0000001151	pink	VSMC_M5	0.21	2.84E-01	0.04	8.35E-01	-0.40	3.92E-02	0.38	5.23E-02	0.51	6.34E-03	-0.19	3.54E-01	0.15	4.70E-01	-0.37	5.87E-02	-0.08	6.91E-01	0.03	8.95E-01
ENSCAFG0000001644	PRELID1	darkgrey	VSMC_M8	0.21	2.84E-01	-0.26	1.85E-01	0.52	5.79E-03	-0.73	1.56E-05	-0.30	1.32E-01	-0.03	8.63E-01	-0.02	9.24E-01	0.73	1.85E-05	0.13	5.14E-01	-0.58	1.69E-03
ENSCAFG0000000278	FAM221A	grey	VSMC_M10	0.21	2.84E-01	0.01	9.49E-01	-0.37	5.90E-02	0.50	7.61E-03	0.46	1.69E-02	-0.26	1.86E-01	-0.30	1.26E-01	-0.42	2.93E-02	0.01	9.54E-01	0.11	5.88E-01
ENSCAFG0000002022	DENND1A	grey	VSMC_M10	0.21	2.85E-01	0.10	6.22E-01	-0.19	3.31E-01	0.01	9.64E-01	0.23	2.46E-01	-0.09	6.44E-01	0.39	4.16E-02	-0.03	8.73E-01	-0.24	2.32E-01	0.16	4.22E-01
ENSCAFG0000001739	ENSCAFG0000001739	grey	VSMC_M10	0.21	2.85E-01	0.06	7.55E-01	0.02	9.07E-01	0.07	7.45E-01	0.12	5.54E-01	-0.20	3.06E-01	0.20	3.16E-01	-0.15	4.51E-01	-0.11	5.91E-01	-0.06	7.57E-01
ENSCAFG0000003231	ENSCAFG0000003231	turquoise	VSMC_M6	0.21	2.85E-01	0.26	1.99E-01	0.32	9.89E-02	0.02	9.19E-01	-0.25	2.07E-01	0.49	9.45E-03	-0.18	3.63E-01	-0.11	5.78E-01	-0.26	1.95E-01	0.08	6.93E-01
ENSCAFG0000001246	OSM	grey	VSMC_M10	0.21	2.85E-01	0.05	8.15E-01	0.08	7.08E-01	-0.06	7.67E-01	0.05	8.02E-01	-0.10	6.34E-01	-0.14	4.84E-01	0.07	7.22E-01	-0.10	6.33E-01	-0.10	6.27E-01
ENSCAFG0000002778	ENSCAFG0000002778	grey	VSMC_M10	0.21	2.85E-01	0.05	8.15E-01	0.08	7.08E-01	-0.06	7.67E-01	0.05	8.02E-01	-0.10	6.34E-01	-0.14	4.84E-01	0.07	7.22E-01	-0.10	6.33E-01	-0.10	6.27E-01
ENSCAFG0000003022	RBM11	grey	VSMC_M10	0.21	2.86E-01	-0.32	1.03E-01	-0.38	5.12E-02	0.25	2.13E-01	0.41	3.51E-02	0.23	2.50E-01	0.05	7.94E-01	-0.08	7.04E-01	0.16	4.28E-01	-0.08	7.01E-01
ENSCAFG0000000158	ENSCAFG0000000158	grey	VSMC_M10	0.21	2.86E-01	0.23	2.57E-01	0.01	9.76E-01	0.04	8.37E-01	0.02	9.18E-01	-0.09	6.55E-01	0.10	6.09E-01	-0.02	9.16E-01	-0.30	1.23E-01	0.22	2.69E-01
ENSCAFG0000001202	PPP1R8	red	VSMC_M1	0.21	2.86E-01	-0.35	7.00E-02	-0.20	3.07E-01	-0.04	8.40E-01	0.26	1.97E-01	0.18	3.69E-01	-0.15	4.46E-01	0.23	2.52E-01	0.22	2.65E-01	-0.21	2.98E-01
ENSCAFG0000000930	ESRP1	pink	VSMC_M5	0.21	2.87E-01	-0.30	1.26E-01	-0.52	5.60E-03	0.34	8.51E-02	0.52	5.21E-03	0.04	8.62E-01	0.14	4.77E-01	-0.16	4.23E-01	0.15	4.57E-01	0.05	8.15E-01
ENSCAFG0000001613	GPS2	grey	VSMC_M10	0.21	2.87E-01	0.41	3.51E-02	0.50	8.13E-03	-0.35	6.95E-02	-0.39	4.24E-02	-0.06	7.57E-01	0.27	1.72E-01	0.15	4.51E-01	-0.45	1.77E-02	0.11	5.73E-01
ENSCAFG0000002497	LRRN4	cyan	VSMC_M2	0.21	2.87E-01	0.82	1.24E-07	0.22	2.67E-01	0.36	6.17E-02	-0.25	2.11E-01	-0.10	6.17E-01	-0.47	1.27E-02	-0.58	1.56E-03	-0.77	2.45E-06	0.63	3.79E-04
ENSCAFG0000001771	EEF2K	yellow	VSMC_M3	0.21	2.87E-01	0.37	5.58E-02	0.48	1.23E-02	-0.42	2.97E-02	-0.30	1.31E-01	-0.12	5.60E-01	0.32	1.02E-01	0.12	5.49E-01	-0.39	4.50E-02	0.06	7.48E-01
ENSCAFG0000000740	TLR3	cyan	VSMC_M2	0.21	2.87E-01	0.68	9.26E-05	-0.12	5.60E-01	0.61	6.93E-04	0.14	4.89E-01	-0.17	4.10E-01	-0.68	8.81E-05	-0.74	8.40E-06	-0.58	1.54E-03	0.53	4.61E-03
ENSCAFG0000003059	ENSCAFG0000003059	grey	VSMC_M10	0.21	2.88E-01	-0.10	6.24E-01	0.09	6.61E-01	-0.07	7.29E-01	0.04	8.50E-01	-0.23	2.47E-01	-0.03	8.81E-01	0.06	7.76E-01	0.03	8.80E-01	-0.15	4.45E-01
ENSCAFG0000001081	NCOA3	darkgreen	VSMC_M4	0.21	2.88E-01	-0.06	7.77E-01	-0.61	7.03E-04	0.60	1.04E-03	0.62	5.61E-04	-0.14	4.83E-01	-0.11	5.69E-01	-0.48	1.21E-02	0.04	8.53E-01	0.26	1.88E-01
ENSCAFG0000002498	FADD	grey	VSMC_M10	0.21	2.88E-01	0.02	9.04E-01	0.49	9.55E-03	-0.48	1.13E-02	-0.14	5.00E-01	-0.26	1.98E-01	-0.08	7.04E-01	0.33	8.77E-02	-0.02	9.14E-01	-0.55	2.83E-03
ENSCAFG0000001755	S100A4	yellow	VSMC_M3	0.21	2.88E-01	0.24	2.27E-01	0.69	6.07E-05	-0.59	1.20E-03	-0.41	3.50E-02	-0.19	3.36E-01	0.43	2.50E-02	0.29	1.36E-01	-0.23	2.49E-01	-0.23	2.46E-01
ENSCAFG0000000706	NUP58	grey	VSMC_M10	0.21	2.89E-01	-0.66	2.06E-04	-0.23	2.43E-01	-0.30	1.25E-01	0.38	5.31E-02	0.07	7.11E-01	0.08	6.90E-01	0.54	3.59E-03	0.45	1.87E-02	-0.55	3.19E-03
ENSCAFG0000001743	SRSF1	darkgrey	VSMC_M8	0.21	2.89E-01	-0.31	1.22E-01	0.37	5.70E-02	-0.72	2.63E-05	-0.14	4.85E-01	0.14	4.86E-01	0.39	4.69E-02	0.77	2.61E-06	0.08	7.04E-01	-0.61	6.61E-04
ENSCAFG0000001250	TP53BP1	darkgreen	VSMC_M4	0.21	2.89E-01	0.23	2.41E-01	-0.30	1.24E-01	0.66	2.04E-04	0.40	3.90E-02	-0.28	1.54E-01	-0.19	3.45E-01	-0.59	1.09E-03	-0.23	2.54E-01	0.23	2.43E-01
ENSCAFG0000001692	FOXO4	grey	VSMC_M10	0.21	2.89E-01	0.34	8.47E-02	-0.23	2.54E-01	0.36	6.35E-02	0.25	2.16E-01	-0.15	4.49E-01	0.13	5.02E-01	-0.41	3.35E-02	-0.39	4.56E-02	0.38	5.00E-02
ENSCAFG0000003041	RGS16	yellow	VSMC_M3	0.21	2.89E-01	0.29	1.40E-01	0.46	1.59E-02	0.04	8.27E-01	-0.39	4.46E-02	0.27	1.66E-01	-0.01	9.62E-01	-0.13	5.23E-01	-0.32	1.00E-01	0.15	4.45E-01
ENSCAFG0000001240	COL7A1	grey	VSMC_M10	0.21	2.90E-01	-0.17	3.97E-01	0.45	1.79E-02	-0.59	1.14E-03	-0.29	1.41E-01	0.09	6.45E-01	0.58	1.57E-03	0.47	1.24E-02	0.01	9.47E-01	-0.29	1.44E-01

ENSCAFG0000000333	EPHA7	grey	VSMC_M10	0.21	2.90E-01	0.22	2.77E-01	0.11	5.75E-01	-0.08	7.03E-01	0.02	9.37E-01	-0.11	5.80E-01	-0.21	2.94E-01	0.03	8.68E-01	-0.25	2.03E-01	0.05	8.02E-01
ENSCAFG0000001125	INTS13	grey	VSMC_M10	0.21	2.90E-01	-0.30	1.27E-01	-0.30	1.34E-01	-0.06	7.61E-01	0.41	3.18E-02	0.11	5.93E-01	0.48	1.05E-02	0.19	3.35E-01	0.07	7.44E-01	-0.21	2.97E-01
ENSCAFG0000001518	ENSCAFG0000001518	yellow	VSMC_M3	0.21	2.91E-01	-0.01	9.42E-01	0.72	2.47E-05	-0.64	3.71E-04	-0.65	2.34E-04	0.28	1.51E-01	0.34	8.63E-02	0.62	6.06E-04	-0.19	3.55E-01	-0.17	4.05E-01
ENSCAFG0000001128	STS	cyan	VSMC_M2	0.21	2.91E-01	0.59	1.26E-03	0.42	3.09E-02	0.00	9.85E-01	-0.44	2.26E-02	0.19	3.40E-01	0.05	7.99E-01	-0.23	2.44E-01	-0.64	2.92E-04	0.46	1.62E-02
ENSCAFG0000000979	HIVEP1	grey	VSMC_M10	0.21	2.92E-01	0.56	2.37E-03	-0.19	3.36E-01	0.51	6.06E-03	0.17	4.11E-01	-0.01	9.59E-01	0.00	9.90E-01	-0.62	5.64E-04	-0.57	2.13E-03	0.58	1.66E-03
ENSCAFG0000000308	ORC3	grey	VSMC_M10	0.21	2.92E-01	0.12	5.44E-01	0.03	8.70E-01	0.22	2.70E-01	-0.03	8.68E-01	0.09	6.67E-01	-0.19	3.39E-01	-0.22	2.67E-01	-0.14	4.90E-01	0.28	1.54E-01
ENSCAFG0000000388	TTC39A	grey	VSMC_M10	0.21	2.92E-01	0.73	1.48E-05	0.18	3.82E-01	0.37	5.89E-02	-0.11	5.85E-01	-0.15	4.41E-01	-0.22	2.78E-01	-0.65	2.41E-04	-0.58	1.54E-03	0.52	5.83E-03
ENSCAFG0000000766	ENSCAFG0000000766	grey	VSMC_M10	0.21	2.92E-01	-0.02	9.10E-01	0.25	2.14E-01	-0.08	7.07E-01	-0.07	7.16E-01	-0.17	3.84E-01	-0.02	9.22E-01	0.13	5.06E-01	-0.04	8.35E-01	-0.22	2.74E-01
ENSCAFG0000000340	CAPZA2	pink	VSMC_M5	0.21	2.92E-01	-0.60	1.06E-03	-0.30	1.34E-01	-0.29	1.38E-01	0.63	3.99E-04	-0.18	3.61E-01	0.05	8.03E-01	0.38	5.02E-02	0.56	2.26E-03	-0.79	9.38E-07
ENSCAFG0000000941	TP53INP1	grey	VSMC_M10	0.21	2.92E-01	0.72	2.35E-05	0.15	4.43E-01	0.51	6.79E-03	-0.23	2.54E-01	-0.01	9.59E-01	-0.19	3.49E-01	-0.67	1.31E-04	-0.66	1.89E-04	0.74	9.21E-06
ENSCAFG0000001323	RPL11	grey	VSMC_M10	0.21	2.92E-01	0.36	6.38E-02	0.55	3.23E-03	-0.16	4.34E-01	-0.45	1.75E-02	-0.10	6.15E-01	0.11	5.78E-01	0.02	9.23E-01	-0.41	3.47E-02	0.11	5.79E-01
ENSCAFG0000000822	ENSCAFG0000000822	grey	VSMC_M10	0.21	2.92E-01	-0.01	9.66E-01	-0.27	1.81E-01	0.34	7.85E-02	0.42	2.96E-02	-0.14	4.77E-01	-0.15	4.44E-01	-0.32	9.84E-02	0.09	6.69E-01	-0.08	7.07E-01
ENSCAFG0000002360	ZNF622	grey	VSMC_M10	0.21	2.93E-01	-0.19	3.34E-01	0.23	2.56E-01	-0.14	4.73E-01	-0.06	7.75E-01	-0.18	3.60E-01	-0.19	3.41E-01	0.19	3.34E-01	0.15	4.46E-01	-0.30	1.35E-01
ENSCAFG0000001933	C5H1orf159	yellow	VSMC_M3	0.21	2.93E-01	0.03	8.83E-01	0.59	1.28E-03	-0.52	5.58E-03	-0.50	7.71E-03	0.04	8.60E-01	0.30	1.32E-01	0.47	1.33E-02	-0.20	3.08E-01	-0.05	7.89E-01
ENSCAFG0000001817	LAMA3	grey	VSMC_M10	0.21	2.93E-01	0.67	1.50E-04	-0.12	5.65E-01	0.61	7.79E-04	0.02	9.12E-01	-0.08	6.90E-01	-0.28	1.57E-01	-0.65	2.35E-04	-0.68	1.12E-04	0.72	2.00E-05
ENSCAFG0000001330	INO80D	red	VSMC_M1	0.21	2.93E-01	0.19	3.36E-01	-0.02	9.03E-01	0.17	4.09E-01	0.04	8.61E-01	0.03	8.64E-01	-0.01	9.50E-01	-0.24	2.29E-01	-0.27	1.72E-01	0.23	2.52E-01
ENSCAFG0000003144	ENSCAFG0000003144	grey	VSMC_M10	0.21	2.93E-01	-0.07	7.38E-01	-0.23	2.45E-01	0.06	7.85E-01	0.47	1.39E-02	-0.16	4.39E-01	-0.10	6.10E-01	-0.04	8.24E-01	0.04	8.46E-01	-0.36	6.73E-02
ENSCAFG0000001251	LSM14B	yellow	VSMC_M3	0.21	2.94E-01	0.48	1.12E-02	0.57	1.72E-03	-0.17	4.05E-01	-0.49	1.01E-02	-0.14	4.82E-01	0.02	9.07E-01	-0.04	8.54E-01	-0.49	9.29E-03	0.26	1.91E-01
ENSCAFG0000001683	NUMB	darkgreen	VSMC_M4	0.21	2.94E-01	0.42	3.08E-02	-0.23	2.43E-01	0.75	6.05E-06	0.19	3.42E-01	-0.07	7.18E-01	-0.17	3.98E-01	-0.76	5.24E-06	-0.34	7.81E-02	0.55	2.98E-03
ENSCAFG0000001774	BCAS3	grey	VSMC_M10	0.21	2.94E-01	0.28	1.61E-01	-0.31	1.18E-01	0.56	2.59E-03	0.47	1.34E-02	-0.20	3.17E-01	-0.42	2.91E-02	-0.67	1.48E-04	-0.12	5.58E-01	0.11	5.77E-01
ENSCAFG0000000121	PTK2	grey	VSMC_M10	0.21	2.94E-01	0.53	4.04E-03	0.27	1.79E-01	0.25	2.06E-01	-0.30	1.31E-01	0.25	2.05E-01	-0.49	9.78E-03	-0.32	9.96E-02	-0.58	1.61E-03	0.42	3.00E-02
ENSCAFG0000000473	ZNF428	grey	VSMC_M10	0.21	2.94E-01	0.50	7.36E-03	0.25	2.09E-01	0.25	2.02E-01	-0.13	5.21E-01	-0.12	5.56E-01	-0.23	2.40E-01	-0.35	7.30E-02	-0.48	1.05E-02	0.25	2.11E-01
ENSCAFG0000001329	KAT5	yellow	VSMC_M3	0.21	2.94E-01	0.22	2.70E-01	0.70	5.23E-05	-0.62	6.39E-04	-0.46	1.58E-02	0.00	9.96E-01	0.18	3.60E-01	0.37	5.74E-02	-0.23	2.48E-01	-0.25	2.15E-01
ENSCAFG0000001736	NUP210L	grey	VSMC_M10	0.21	2.95E-01	0.34	8.51E-02	0.22	2.72E-01	0.04	8.35E-01	-0.09	6.71E-01	0.06	7.69E-01	-0.02	9.23E-01	-0.17	4.03E-01	-0.30	1.23E-01	0.04	8.37E-01
ENSCAFG0000000005	ENSCAFG0000000005	cyan	VSMC_M2	0.21	2.95E-01	0.44	2.07E-02	0.31	1.11E-01	-0.13	5.18E-01	-0.14	4.90E-01	-0.43	2.51E-02	-0.10	6.10E-01	-0.08	6.77E-01	-0.39	4.69E-02	0.02	9.20E-01
ENSCAFG0000001065	HYAL2	turquoise	VSMC_M6	0.21	2.95E-01	0.43	2.66E-02	0.15	4.60E-01	0.15	4.54E-01	-0.27	1.71E-01	0.67	1.14E-04	-0.09	6.72E-01	-0.21	2.96E-01	-0.49	9.61E-03	0.51	6.93E-03
ENSCAFG0000003087	ENSCAFG0000003087	grey	VSMC_M10	0.21	2.95E-01	-0.19	3.40E-01	-0.39	4.32E-02	0.18	3.76E-01	0.59	1.14E-03	-0.15	4.69E-01	-0.08	6.86E-01	-0.08	7.00E-01	0.16	4.24E-01	-0.33	8.86E-02
ENSCAFG0000000593	CATSPERG	cyan	VSMC_M2	0.21	2.96E-01	0.52	5.20E-03	0.41	3.60E-02	0.07	7.13E-01	-0.38	5.26E-02	-0.14	4.95E-01	0.14	4.81E-01	-0.20	3.15E-01	-0.55	3.21E-03	0.35	7.61E-02
ENSCAFG0000000330	NFU1	pink	VSMC_M5	0.21	2.97E-01	-0.39	4.15E-02	-0.56	2.22E-03	0.39	4.57E-02	0.76	4.26E-06	-0.33	8.94E-02	-0.10	6.21E-01	-0.18	3.81E-01	0.36	6.67E-02	-0.33	9.25E-02
ENSCAFG0000001794	PPP2R5C	grey	VSMC_M10	0.21	2.97E-01	0.66	1.77E-04	0.11	5.78E-01	0.29	1.38E-01	-0.17	4.04E-01	0.00	9.93E-01	0.12	5.51E-01	-0.40	3.80E-02	-0.70	5.61E-05	0.65	2.34E-04
ENSCAFG0000001702	SLIT3	darkgreen	VSMC_M4	0.21	2.97E-01	0.36	6.20E-02	-0.18	3.59E-01	0.55	2.70E-03	0.15	4.58E-01	0.01	9.71E-01	0.31	1.13E-01	-0.61	6.66E-04	-0.41	3.31E-02	0.54	3.86E-03
ENSCAFG0000001410	OSBPL1A	grey	VSMC_M10	0.21	2.97E-01	0.55	2.94E-03	-0.22	2.59E-01	0.54	3.42E-03	0.29	1.45E-01	-0.30	1.30E-01	-0.67	1.33E-04	-0.61	6.54E-04	-0.47	1.29E-02	0.40	4.08E-02
ENSCAFG0000002512	ENSCAFG0000002512	grey	VSMC_M10	0.21	2.97E-01	-0.16	4.35E-01	-0.12	5.51E-01	-0.17	3.95E-01	0.39	4.24E-02	-0.06	7.52E-01	0.03	8.63E-01	0.17	4.04E-01	0.10	6.03E-01	-0.49	9.47E-03
ENSCAFG0000001660	MLLT6	cyan	VSMC_M2	0.21	2.97E-01	0.57	1.81E-03	0.53	4.79E-03	-0.16	4.16E-01	-0.45	1.90E-02	0.17	3.98E-01	-0.08	6.99E-01	-0.04	8.30E-01	-0.57	2.02E-03	0.20	3.11E-01
ENSCAFG0000003197	PRKG1	darkgreen	VSMC_M4	0.21	2.98E-01	0.15	4.52E-01	-0.49	8.71E-03	0.65	2.51E-04	0.56	2.64E-03	-0.05	8.08E-01	-0.54	3.80E-03	-0.65	2.78E-04	-0.08	6.94E-01	0.22	2.81E-01
ENSCAFG0000002992	ENSCAFG0000002992	grey	VSMC_M10	0.21	2.98E-01	-0.29	1.36E-01	-0.21	3.01E-01	-0.16	4.17E-01	0.29	1.44E-01	0.11	5.79E-01	0.06	7.49E-01	0.28	1.63E-01	0.15	4.66E-01	-0.26	1.84E-01
ENSCAFG0000003220	TYW3	grey	VSMC_M10	0.21	2.98E-01	-0.32	1.02E-01	0.35	7.39E-02	-0.51	7.03E-03	-0.06	7.63E-01	-0.12	5.60E-01	0.03	8.83E-01	0.52	4.96E-03	0.23	2.47E-01	-0.65	2.49E-04
ENSCAFG0000000848	JAM2	grey	VSMC_M10	0.21	2.98E-01	0.02	9.09E-01	0.12	5.56E-01	-0.35	7.11E-02	0.11	5.93E-01	-0.14	5.02E-01	0.27	1.81E-01	0.28	1.58E-01	-0.09	6.56E-01	-0.32	1.07E-01
ENSCAFG0000000970	HIF1AN	grey	VSMC_M10	0.21	2.98E-01	-0.28	1.64E-01	-0.18	3.76E-01	0.06	7.59E-01	0.45	1.72E-02	-0.15	4.43E-01	-0.12	5.53E-01	-0.01	9.49E-01	0.24	2.19E-01	-0.53	4.34E-03
ENSCAFG0000000846	ENSCAFG0000000846	grey	VSMC_M10	0.21	2.98E-01	0.10	6.26E-01	-0.16	4.38E-01	0.03	8.87E-01	0.33	9.10E-02	0.13	5.05E-01	-0.20	3.05E-01	-0.04	8.31E-01	-0.11	5.71E-01	-0.08	6.78E-01
ENSCAFG0000001779	PDE4A	grey	VSMC_M10	0.21	2.99E-01	0.13	5.31E-01	0.18	3.56E-01	0.17	3.84E-01	-0.01	9.68E-01	-0.12	5.36E-01	-0.44	2.03E-02	-0.19	3.38E-01	-0.05	8.07E-01	-0.10	6.22E-01
ENSCAFG0000001984	RPL13	cyan	VSMC_M2	0.21	2.99E-01	0.76	5.26E-06	0.39	4.19E-02	0.25	2.00E-01	-0.47	1.27E-02	0.07	7.19E-01	-0.30	1.26E-01	-0.43	2.57E-02	-0.76	3.57E-06	0.65	2.73E-04
ENSCAFG0000000032	USP15	grey	VSMC_M10	0.21	2.99E-01	-0.33	9.43E-02	-0.45	1.79E-02	0.07	7.24E-01	0.50	8.06E-03	0.04	8.61E-01	0.49	1.02E-02	0.13	5.17E-01	0.11	5.79E-01	-0.11	5.96E-01
ENSCAFG0000002911	ZNF354B	grey	VSMC_M10	0.21	2.99E-01	-0.12	5.44E-01	-0.45	1.78E-02	0.24	2.22E-01	0.53	4.04E-03	-0.21	2.99E-01	0.33	9.00E-02	-0.14	4.76E-01	0.02	9.25E-01	0.03	8.67E-01
ENSCAFG0000000660	POLR2I	grey	VSMC_M10	0.21	2.99E-01	0.33	8.76E-02	0.52	5.92E-03	-0.23	2.48E-01	-0.47	1.30E-02	0.08	6.85E-01	0.24	2.32E-01	0.09	6.67E-01	-0.45	1.97E-02	0.18	3.75E-01
ENSCAFG0000000289	ENSCAFG0000000289	grey	VSMC_M10	0.21	2.99E-01	0.32	1.03E-01	-0.26	1.96E-01	0.42	2.85E-02	0.28	1.54E-01	-0.14	4.88E-01	-0.26	1.97E-01	-0.38	5.04E-02	-0.34	8.01E-02	0.37	5.74E-02
ENSCAFG0000000723	NOL4L	grey	VSMC_M10	0.21	2.99E-01	0.29	1.39E-01	-0.43	2.46E-02	0.63	3.79E-04	0.37	6.01E-02	0.31	1.17E-01	-0.32	1.04E-01	-0.60	9.80E-04	-0.29	1.35E-01	0.47	1.26E-02
ENSCAFG0000001602	AKAP5	cyan	VSMC_M2	0.21	2.99E-01	0.72	1.91E-05	0.28	1.51E-01	0.31	1.13E-01	-0.36	6.35E-02	0.07	7.24E-01	-0.14	4.86E-01	-0.52	5.55E-03	-0.65	2.18E-04	0.75	5.79E-06
ENSCAFG0000000489	SNX17	yellow	VSMC_M3	0.21	3.00E-01	0.55	3.13E-03	0.59	1.18E-03	0.05	8.21E-01	-0.50	7.60E-03	-0.08	6.87E-01	-0.33	8.81E-02	-0.26	1.88E-01	-0.44	2.18E-02	0.27	1.72E-01
ENSCAFG0000001872	SKP2	darkgreen	VSMC_M4	0.21	3.00E-01	0.41	3.57E-02	-0.45	1.94E-02	0.70	4.29E-05	0.37	5.59E-02	-0.13	5.25E-01	-0.23	2.53E-01	-0.61	7.14E-04	-0.43	2.63E-02	0.53	4.60E-03
ENSCAFG0000001134	ITPR2	darkolivegreen	VSMC_M9	0.21	3.00E-01	-0.51	6.00E-03	-0.42	2.72E-02	-0.02	9.40E-01	0.58	1.54E-03	-0.02	9.23E-01	0.33	9.07E-02	0.15	4.65E-01	0.37	6.08E-02	-0.37	5.84E-02
ENSCAFG0000000053	SNX2	pink	VSMC_M5	0.21	3.00E-01	-0.33	9.18E-02	-0.32	9.97E-02	-0.05	8.12E-01	0.63	4.32E-04	-0.43	2.38E-02	-0.23	2.52E-01	0.05	8.12E-01	0.38	5.05E-02	-0.52	5.27E-03
ENSCAFG0000001940	ARHGAP4	grey	VSMC_M10	0.21	3.00E-01	0.30	1.31E-01	0.32	1.07E-01	-0.03	8.79E-01	-0.23	2.40E-01	-0.16	4.31E-01	-0.12	5.43E-01	-0.07	7.17E-01	-0.29	1.43E-01	0.15	4.46E-01
ENSCAFG0000001755	ENSCAFG0000001755	grey	VSMC_M10	0.21	3.00E-01	-0.04	8.33E-01	0.30	1.22E-01	-0.39	4.47E-02	0.01	9.49E-01	-0.15	4.48E-01	0.12	5.46E-01	0.20	3.25E-01	0.04	8.62E-01	-0.44	2.04E-02
ENSCAFG0000000761	SLCO6A1	grey	VSMC_M10	0.21	3.00E-01	-0.10	6.14E-01	-0.08	7.07E-01	-0.18	3.58E-01	0.34	7.99E-02	-0.15	4.49E-01	0.05	7.94E-01	0.16	4.17E-01	0.06	7.52E-01	-0.46	1.53E-02
ENSCAFG0000001797	MPI	grey	VSMC_M10	0.21	3.00E-01	-0.06	7.77E-01	0.02	9.32E-01	0.13	5.34E-01	0.22	2.61E-01	0.03	8.81E-01	-0.13	5.26E-01	-0.15	4.47E-01	0.09	6.68E-01	-0.30	1.30E-01
ENSCAFG0000000474	TMUB1	yellow	VSMC_M3	0.21	3.00E-01	0.69	7.22E-05	0.61	6.69E-04	0.03	8.74E-01	-0.63	4.11E-04	0.17	4.01E-01	-0.14	5.00E-01	-0.24	2.26E-01	-0.68	8.27E-05	0.55	2.85E-03
ENSCAFG0000001234	HMBS	darkgrey	VSMC_M8	0.21	3.01E-01	-0.20	3.18E-01	0.25	2.08E-01	-0.42	2.87E-02	-0.08	6.92E-01	-0.07	7.30E-01	0.24	2.37E-01	0.52	5.87E-03	0.01	9.73E-01	-0.41	3.56E-02
ENSCAFG0000000417	C5AR1	grey	VSMC_M10	0.21	3.01E-01	-0.35	6.95E-02	-0.46	1.51E-02	0.21	2.88E-01	0.55	3.14E-03	-0.14	4.96E-01	0.19	3.46E-01	-0.04	8.53E-01	0.20	3.08E-01	-0.13	5.24E-01
ENSCAFG0000000603	ZNF605	pink	VSMC_M5	0.21	3.01E-01	0.08	6.75E-01	-0.43	2.57E-02	0.51	6.35E-03	0.48	1.22E-02	-0.08	6.98E-01	-0.14	4.75E-01	-0.46	1.53E-02	-0.13	5.04E-01	0.17	4.10E-01
ENSCAFG0000001570	STX18	grey	VSMC_M10	0.21	3.01E-01	-0.01	9.45E-01	-0.07	7.29E-01	0.11	5.74E-01	0.08	6.74E-01	-0.22	2.80E-01	0.52	4.96E-03	-0.09	6.52E-01	-0.17	3.87E-01	0.20	3.23E-01
ENSCAFG0000002614	ENSCAFG0000002614	grey	VSMC_M10	0.21	3.01E-01	-0.06	7.61E-01	0.13	5.16E-01	-0.06	7.69E-01	-0.08	7.04E-01	0.10	6.22E-01	0.11	5.71E-01	0.12	5.64E-01	-0.11	5.88E-01	-0.01	9.46E-01
ENSCAFG0000001740	SCPEP1	grey	VSMC_M10	0.21	3.01E-01	0.75	7.06E-06	0.07	7.45E-01	0.43	2.59E-02	-0.03	8.90E-01	-0.15	4.67E-01	-0.15	4.59E-01	-0.74	1.14E-05	-0.63	4.52E-04	0.59	1.13E-03
ENSCAFG0000003158	LGALSL	grey	VSMC_M10	0.21	3.01E-01	-0.26	1.85E-01	-0.23	2.41E-01	-0.15	4.52E-01	0.41	3.51E-02	0.08	6.95E-01	0.38	4.90E-02	0.15	4.62E-01	0.20	3.13E-01	-0.25	2.02E-01
ENSCAFG0000001884	MBNL3	grey	VSMC_M10	0.21	3.01E-01	0.28	1.55E-01	0.06	7.63E-01	0.02	9.29E-01	-0.06	7.74E-01	0.05	8.04E-01	-0.41	3.44E-02	-0.07	7.18E-01	-0.35	7.18E-02	0.21	2.99E-01
ENSCAFG0000000093	SLC35F1	grey	VSMC_M10	0.21	3.02E-01	0.27	1.77E-01	0.55	3.20E-03	-0.30	1.28E-01	-0.46	1.48E-02	0.12	5.40E-01	0.14	4.91E-01	0.08	6.99E-01	-0.31	1.18E-01	0.04	8.32E-01
ENSCAFG0000000295	SNAP91	darkgreen	VSMC_M4	0.21	3.02E-01	0.49	9.12E-03	-0.23	2.46E-01	0.71	3.28E-05	0.10	6.02E-01	0.26	1.82E-01	-0.43	2.48E-02	-0.69	6.31E-05	-0.46	1.51E-02	0.67	1.48E-04
ENSCAFG0000001045	TARSL2	darkgreen	VSMC_M4	0.21	3.02E-01	0.47	1.26E-02	-0.31	1.14E-01	0.81	3.44E-07	0.29	1.38E-01	-0.10	6.35E-01	-0.15	4.51E-01	-0.82	1.39E-07	-0.43	2.54E-02	0.57	1.86E-03
ENSCAFG0000000045	RAB21	grey	VSMC_M10	0.21	3.02E-01	0.00	9.85E-01	0.14	4.88E-01	0.16	4.38E-01	0.00	9.90E-01	-0.09	6.41E-01	0.17	4.02E-01	-0.16	4.33E-01	-0.06	7.61E-01	-0.02	9.18E-01
ENSCAFG0000001041	UNC119B	red	VSMC_M1	0.21	3.02E-01	0.22	2.66E-01	0.00	9.97E-01	0.20	3.07E-01	0.03	8.76E-01	0.25	2.16E-01	0.00	9.89E-01	-0.27	1.70E-01	-0.27	1.73E-01	0.21	2.98E-01
ENSCAFG0000001199	FAM204A	grey	VSMC_M10	0.21	3.02E-01	-0.44	2.21E-02	0.10	6.07E-01	-0.26	1.91E-01	0.12	5.37E-01	-0.15	4.49E-01	0.30	1.31E-01	0.32	1.09E-01	0.36	6.71E-02	-0.46	1.61E-02
ENSCAFG0000000692	SETD9	grey	VSMC_M10	0.21	3.02E-01	-0.18	3.82E-01	0.32	1.01E-01	-0.39	4.64E-02	-0.18	3.60E-01	0.01	9.76E-01	0.18	3.64E-01	0.43	2.45E-02	0.03	8.73E-01	-0.25	2.15E-01
ENSCAFG0000000407	NCOA1	pink	VSMC_M5	0.21	3.02E-01	0.10	6.06E-01	-0.48	1.05E-02	0.51	6.87E-03	0.52	5.32E-03	-0.16	4.29E-01	0.02	9.16E-01	-0.50	8.48E-03	-0.10	6.16E-01	0.32	1.07E-01
ENSCAFG0000001380	KIF1BP	grey	VSMC_M10	0.21	3.03E-01	-0.49	9.40E-03	-0.46	1.53E-02	0.01	9.63E-01	0.58	1.64E-03	-0.19	3.35E-01	0.28	1.60E-01	0.23	2.45E-01	0.29	1.40E-01	-0.33	8.94E-02
ENSCAFG0000001445	MAOB	darkgreen	VSMC_M4	0.21	3.03E-01	0.31	1.17E-01	-0.47	1.38E-02	0.86	1.05E-08	0.43	2.56E-02	-0.07	7.33E-01	-0.30	1.32E-01	-0.76	4.95E-06	-0.29	1.44E-01	0.47	1.43E-02
ENSCAFG0000000393	HBP1	cyan	VSMC_M2	0.21	3.03E-01	0.54	3.97E-03	-0.08	7.08E-01	0.45	1.91E-02	0.20	3.26E-01	-0.14	4.98E-01	-0.22	2.78E-01	-0.70	4.44E-05	-0.39	4.56E-02	0.37	5.84E-02
ENSCAFG0000000554	ENGASE	red	VSMC_M1	0.21	3.03E-01	0.25	2.18E-01	0.23	2.45E-01	-0.05	8.03E-01	-0.23	2.52E-01	0.21	2.93E-01	0.23	2.57E-01	0.01	9.67E-01	-0.39	4.65E-02	0.23	2.47E-01
ENSCAFG0000001178	ZFAND2A	pink	VSMC_M5	0.21	3.03E-01	-0.53	4.12E-03	-0.34	8.22E-02	-0.12	5.43E-01	0.48	1.06E-02	0.01	9.54E-01	0.11	5.88E-01	0.38	5.23E-02	0.37	5.73E-02	-0.51	6.42E-03
ENSCAFG0000002517	ENSCAFG0000002517	grey	VSMC_M10	0.21	3.03E-01	0.26	1.93E-01	0.40	3.99E-02	-0.30	1.24E-01	-0.20	3.12E-01	0.16	4.33E-01	0.10	6.03E-01	0.14	4.87E-01	-0.34	8.38E-02	-0.13	5.16E-01
ENSCAFG0000000203	PUM3	darkgrey	VSMC_M8	0.21	3.04E-01	-0.66	1.70E-04	-0.03	8.78E-01	-0.54	3.91E-03	0.29	1.36E-01	-0.04	8.41E-01	0.05	7.99E-01	0.71	3.53E-05	0.49	9.40E-03	-0.82	2.06E-07
ENSCAFG0000003017	RNF8	grey	VSMC_M10	0.21	3.04E-01	-0.48	1.09E-02	-0.05	8.10E-01	-0.33	9.01E-02	0.26	1.89E-01	0.03	9.01E-01	0.27	1.73E-01	0.48	1.09E-02	0.32	1.05E-01	-0.64	2.88E-04
ENSCAFG0000000356	CPT1C	yellow	VSMC_M3	0.21	3.04E-01	0.52	5.08E-03	0.43	2.37E-02	-0.29	1.39E-01	-0.26	1.88E-01	-0.07	7.24E-01	-0.17	4.04E-01	0.05	8.05E-01	-0.46	1.47E-02	0.06	7.70E-01
ENSCAFG0000000676	API5	darkgrey	VSMC_M8	0.21	3.04E-01	-0.29	1.47E-01	0.25	2.00E-01	-0.65	2.62E-04	-0.04	8.53E-01	0.16	4.23E-01	0.28	1.50E-01	0.68	1.04E-04	0.15	4.66E-01	-0.56	2.23E-03
ENSCAFG0000000627	FGD6	grey	VSMC_M10	0.21	3.04E-01	0.28	1.56E-01	-0.15	4.66E-01	0.33	9.76E-02	0.05	8.15E-01	-0.04	8.38E-01	-0.58	1.61E-03	-0.31	1.18E-01	-0.32	1.03E-01	0.40	3.82E-02
ENSCAFG0000000360	GJB3	darkgrey	VSMC_M8	0.21	3.04E-01	-0.24	2.34E-01	0.49	9.52E-03	-0.89	3.13E-10	-0.23	2.56E-01	0.04	8.44E-01	0.26	1.99E-01	0.83	7.77E-08	0.09	6.63E-01	-0.64	3.37E-04
ENSCAFG0000003030	SDHAF2	grey	VSMC_M10	0.21	3.04E-01	-0.23	2.47E-01	0.16	4.28E-01	-0.40	3.84E-02	-0.04	8.31E-01	-0.03	8.83E-01	-0.05	8.02E-01	0.48	1.14E-02	0.07	7.12E-01	-0.37	5.66E-02
ENSCAFG0000001110	NDUFV1	yellow	VSMC_M3	0.21	3.04E-01	0.33	9.43E-02	0.87	4.63E-09	-0.55	2.72E-03	-0.70	5.62E-05	0.05	8.21E-01	0.19	3.34E-01	0.35	7.74E-02	-0.39	4.72E-02	-0.08	7.01E-01
ENSCAFG0000001488	ENSCAFG0000001488	yellow	VSMC_M3	0.21	3.04E-01	0.33	9.46E-02	0.69	7.93E-05	-0.33	9.74E-02	-0.55	3.21E-03	-0.29	1.38E-01	-0.19	3.54E-01	0.18	3.82E-01	-0.35	7.20E-02	-0.01	9.71E-01
ENSCAFG0000000166	RBM28	grey	VSMC_M10	0.21	3.05E-01	-0.29	1.41E-01	-0.15	4.50E-01	-0.05	7.87E-01	0.30	1.35E-01	-0.06	7.50E-01	0.29	1.45E-01	0.21	2.95E-01	0.09	6.43E-01	-0.28	1.63E-01
ENSCAFG0000001669	NOP16	yellow	VSMC_M3	0.21	3.05E-01	0.10	6.23E-01	0.56	2.24E-03	-0.27	1.66E-01	-0.37	5.45E-02	0.04	8.32E-01	0.02	9.27E-01	0.22	2.69E-01	-0.16	4.19E-01	-0.21	2.86E-01
ENSCAFG0000000808	GATB	yellow	VSMC_M3	0.20	3.05E-01	0.08	6.78E-01	0.60	9.12E-04	-0.44	2.06E-02	-0.31	1.19E-01	-0.10	6.28E-01	0.09	6.50E-01	0.26	1.91E-01	-0.12	5.43E-01	-0.36	6.80E-02
ENSCAFG0000000930	ENSCAFG0000000930	grey	VSMC_M10	0.20	3.05E-01	0.41	3.40E-02	-0.11	5.80E-01	0.63	3.94E-04	0.04	8.28E-01	-0.18	3.70E-01	-0.23	2.53E-01	-0.60	9.07E-04	-0.41	3.29E-02	0.62	6.31E-04
ENSCAFG0000000792	ATF1	darkgrey	VSMC_M8	0.20	3.05E-01	-0.13	5.18E-01	0.30	1.31E-01	-0.64	2.88E-04	-0.06	7.77E-01	-0.11	5.72E-01	0.34	8.10E-02	0.55	3.26E-03	-0.02	9.16E-01	-0.39	4.37E-02
ENSCAFG0000000514	MNX1	grey	VSMC_M10	0.20	3.06E-01	-0.18	3.64E-01	-0.10	6.06E-01	-0.06	7.48E-01	0.23	2.39E-01	-0.10	6.03E-01	-0.12	5.35E-01	0.08	7.04E-01	0.20	3.19E-01	-0.24	2.32E-01
ENSCAFG0000003153	ENSCAFG0000003153	grey	VSMC_M10	0.20	3.06E-01	-0.18	3.64E-01	-0.10	6.06E-01	-0.06	7.48E-01	0.23	2.39E-01	-0.10	6.03E-01	-0.12	5.35E-01	0.08	7.04E-01	0.20	3.19E-01	-0.24	2.32E-01
ENSCAFG0000003273	ENSCAFG0000003273	grey	VSMC_M10	0.20	3.06E-01	-0.18	3.64E-01	-0.10	6.06E-01	-0.06	7.48E-01	0.23	2.39E-01	-0.10	6.03E-01	-0.12	5.35E-01	0.08	7.04E-01	0.20	3.19E-01	-0.24	2.32E-01
ENSCAFG0000000021	ENSCAFG0000000021	grey	VSMC_M10	0.20	3.06E-01	-0.28	1.60E-01	0.11	5.79E-01	-0.27	1.71E-01	0.02	9.26E-01	0.03	8.80E-01	0.14	4.79E-01	0.27	1.78E-01	0.20	3.24E-01	-0.38	4.98E-02
ENSCAFG0000001732	BRWD3	grey	VSMC_M10	0.20	3.06E-01	-0.26	1.92E-01	-0.43	2.70E-02	0.16	4.26E-01	0.55	2.70E-03	-0.03	8.79E-01	-0.04	8.29E-01	-0.01	9.76E-01	0.14	4.99E-01	-0.17	3.84E-01
ENSCAFG0000001858	ENSCAFG0000001858	grey	VSMC_M10	0.20	3.07E-01	0.13	5.21E-01	-0.29	1.40E-01	0.31	1.14E-01	0.38	4.97E-02	-0.17	4.05E-01	-0.23	2.48E-01	-0.36	6.45E-02	-0.09	6.46E-01	0.05	8.12E-01
ENSCAFG0000000745	NCK1	darkgreen	VSMC_M4	0.20	3.07E-01	0.41	3.33E-02	-0.27	1.66E-01	0.69	7.18E-05	0.23	2.39E-01	-0.28	1.50E-01	-0.12	5.54E-01	-0.69	8.06E-05	-0.36	6.91E-02	0.56	2.31E-03
ENSCAFG0000001535	ENSCAFG0000001535	grey	VSMC_M10	0.20	3.07E-01	-0.21	3.02E-01	-0.06	7.72E-01	-0.13	5.14E-01	0.17	4.05E-01	-0.07	7.18E-01	0.30	1.23E-01	0.18	3.62E-01	0.06	7.63E-01	-0.17	3.96E-01
ENSCAFG0000001701	HERC1	pink	VSMC_M5	0.20	3.07E-01	-0.22	2.72E-01	-0.53	4.70E-03	0.29	1.41E-01	0.70	4.72E-05	-0.15	4.41E-01	-0.15	4.57E-01	-0.18	3.64E-01	0.19	3.35E-01	-0.25	2.15E-01
ENSCAFG0000002950	GLO1	grey	VSMC_M10	0.20	3.07E-01	-0.08	6.87E-01	-0.30	1.24E-01	0.39	4.68E-02	0.38	4.98E-02	0.08	6.86E-01	-0.22	2.69E-01	-0.33	9.54E-02	0.10	6.27E-01	-0.01	9.41E-01
ENSCAFG0000000129	RHPN1	red	VSMC_M1	0.20	3.07E-01	0.41	3.30E-02	0.09	6.60E-01	0.27	1.76E-01	-0.17	3.99E-01	0.02	9.27E-01	0.12	5.64E-01	-0.34	8.18E-02	-0.42	2.93E-02	0.54	3.37E-03
ENSCAFG0000000942	MMP2	cyan	VSMC_M2	0.20	3.07E-01	0.74	1.06E-05	0.06	7.64E-01	0.57	2.11E-03	-0.02	9.14E-01	-0.31	1.13E-01	-0.65	2.61E-04	-0.73	1.67E-05	-0.60	1.02E-03	0.55	3.26E-03
ENSCAFG0000000430	AMMECR1L	grey	VSMC_M10	0.20	3.07E-01	-0.31	1.18E-01	-0.41	3.27E-02	0.16	4.25E-01	0.57	1.92E-03	-0.18	3.73E-01	-0.31	1.15E-01	-0.10	6.04E-01	0.30	1.34E-01	-0.27	1.70E-01
ENSCAFG0000000632	LAMP1	cyan	VSMC_M2	0.20	3.08E-01	0.78	1.95E-06	-0.02	9.12E-01	0.53	4.71E-03	0.04	8.28E-01	-0.20	3.14E-01	-0.40	3.85E-02	-0.80	7.15E-07	-0.61	7.93E-04	0.63	4.86E-04
ENSCAFG0000001836	TMEM98	cyan	VSMC_M2	0.20	3.08E-01	0.69	6.92E-05	0.38	5.19E-02	0.33	9.17E-02	-0.48	1.08E-02	0.16	4.31E-01	-0.23	2.45E-01	-0.47	1.25E-02	-0.69	7.73E-05	0.71	3.21E-05
ENSCAFG0000002926	TEN1	grey	VSMC_M10	0.20	3.08E-01	0.26	1.85E-01	0.14	4.98E-01	-0.07	7.30E-01	-0.08	6.99E-01	-0.20	3.07E-01	0.13	5.21E-01	0.00	9.98E-01	-0.37	5.59E-02	0.10	6.21E-01
ENSCAFG0000002383	PTGFRN	cyan	VSMC_M2	0.20	3.08E-01	0.84	3.45E-08	0.30	1.30E-01	0.25	2.17E-01	-0.26	1.96E-01	-0.05	7.88E-01	-0.27	1.81E-01	-0.55	3.11E-03	-0.76	4.43E-06	0.56	2.27E-03
ENSCAFG0000001008	MYO1B	darkgreen	VSMC_M4	0.20	3.08E-01	0.32	9.87E-02	-0.38	5.04E-02	0.71	3.05E-05	0.34	8.74E-02	-0.31	1.18E-01	0.00	9.86E-01	-0.66	1.58E-04	-0.32	1.09E-01	0.54	3.59E-03
ENSCAFG0000001192	ASB11	grey	VSMC_M10	0.20	3.08E-01	0.48	1.22E-02	-0.09	6.38E-01	0.54	3.47E-03	0.05	8.23E-01	-0.02	9.30E-01	-0.42	2.88E-02	-0.62	6.22E-04	-0.40	3.68E-02	0.53	4.06E-03
ENSCAFG0000000370	RAB33B	pink	VSMC_M5	0.20	3.08E-01	-0.23	2.45E-01	-0.56	2.60E-03	0.22	2.74E-01	0.73	1.73E-05	-0.08	6.77E-01	-0.22	2.79E-01	-0.10	6.25E-01	0.22	2.73E-01	-0.32	1.02E-01
ENSCAFG0000001861	PRKAA1	pink	VSMC_M5	0.20	3.08E-01	-0.18	3.82E-01	-0.65	2.16E-04	0.54	3.69E-03	0.65	2.44E-04	-0.03	8.80E-01	-0.02	9.31E-01	-0.34	8.39E-02	0.11	5.85E-01	0.15	4.62E-01
ENSCAFG0000000144	FRMD3	turquoise	VSMC_M6	0.20	3.08E-01	-0.06	7.50E-01	-0.11	5.90E-01	0.16	4.37E-01	0.09	6.41E-01	0.52	5.88E-03	0.03	8.72E-01	-0.05	7.91E-01	-0.08	6.96E-01	0.05	8.04E-01
ENSCAFG0000000534	ENSCAFG0000000534	grey	VSMC_M10	0.20	3.08E-01	0.39	4.16E-02	0.31	1.19E-01	-0.17	3.87E-01	-0.28	1.55E-01	0.23	2.52E-01	0.22	2.79E-01	-0.01	9.79E-01	-0.48	1.13E-02	0.33	9.67E-02

ENSCAFG0000001713	NUPR1	cyan	VSMC_M2	0.20	3.09E-01	0.83	8.25E-08	0.46	1.54E-02	0.17	4.10E-01	-0.41	3.31E-02	-0.23	2.50E-01	-0.20	3.20E-01	-0.45	1.77E-02	-0.75	6.47E-06	0.53	4.53E-03
ENSCAFG0000001082	ENSCAFG0000001082	cyan	VSMC_M2	0.20	3.09E-01	0.62	6.21E-04	0.61	6.68E-04	-0.21	2.96E-01	-0.59	1.13E-03	0.21	3.02E-01	-0.16	4.34E-01	-0.06	7.63E-01	-0.60	8.39E-04	0.38	5.25E-02
ENSCAFG0000000932	PDLIM2	cyan	VSMC_M2	0.20	3.09E-01	0.69	7.75E-05	-0.08	6.79E-01	0.72	1.97E-05	0.10	6.35E-01	-0.25	2.01E-01	-0.55	3.17E-03	-0.81	3.29E-07	-0.56	2.46E-03	0.59	1.13E-03
ENSCAFG0000001675	RRNAD1	yellow	VSMC_M3	0.20	3.09E-01	-0.06	7.68E-01	0.56	2.35E-03	-0.46	1.69E-02	-0.39	4.20E-02	0.16	4.15E-01	0.01	9.76E-01	0.44	2.01E-02	-0.03	8.94E-01	-0.27	1.76E-01
ENSCAFG0000001012	CD81	cyan	VSMC_M2	0.20	3.10E-01	0.76	4.97E-06	0.06	7.75E-01	0.59	1.30E-03	-0.02	9.03E-01	-0.19	3.44E-01	-0.48	1.13E-02	-0.79	7.79E-07	-0.60	9.32E-04	0.60	9.99E-04
ENSCAFG0000001856	MYH11	grey	VSMC_M10	0.20	3.11E-01	0.19	3.46E-01	-0.37	5.99E-02	0.47	1.24E-02	0.43	2.39E-02	-0.04	8.56E-01	-0.47	1.31E-02	-0.42	2.89E-02	-0.14	4.95E-01	0.17	3.97E-01
ENSCAFG0000000295	ENSCAFG0000000295	grey	VSMC_M10	0.20	3.11E-01	0.29	1.45E-01	-0.08	6.99E-01	0.16	4.29E-01	0.00	9.81E-01	0.06	7.68E-01	-0.07	7.15E-01	-0.24	2.31E-01	-0.34	8.08E-02	0.42	2.91E-02
ENSCAFG0000002006	CERCAM	cyan	VSMC_M2	0.20	3.11E-01	0.65	2.50E-04	0.37	5.63E-02	0.08	6.96E-01	-0.27	1.70E-01	-0.30	1.29E-01	0.16	4.38E-01	-0.42	2.88E-02	-0.56	2.32E-03	0.44	2.21E-02
ENSCAFG0000001698	GBA	cyan	VSMC_M2	0.20	3.11E-01	0.79	1.16E-06	0.10	6.05E-01	0.43	2.47E-02	-0.09	6.63E-01	-0.21	2.86E-01	-0.44	2.10E-02	-0.73	1.52E-05	-0.61	6.47E-04	0.62	5.53E-04
ENSCAFG0000001657	ENSCAFG0000001657	darkgrey	VSMC_M8	0.20	3.11E-01	0.05	8.06E-01	0.55	2.73E-03	-0.73	1.77E-05	-0.40	4.07E-02	0.00	9.90E-01	0.30	1.25E-01	0.58	1.49E-03	-0.16	4.14E-01	-0.27	1.75E-01
ENSCAFG0000000812	TENM3	grey	VSMC_M10	0.20	3.11E-01	-0.36	6.19E-02	-0.06	7.72E-01	-0.27	1.79E-01	0.11	5.92E-01	0.38	5.01E-02	0.00	9.85E-01	0.39	4.65E-02	0.15	4.64E-01	-0.23	2.39E-01
ENSCAFG0000002302	ARRDC5	pink	VSMC_M5	0.20	3.12E-01	-0.24	2.30E-01	-0.27	1.79E-01	0.20	3.26E-01	0.41	3.43E-02	0.13	5.29E-01	-0.33	8.88E-02	-0.06	7.68E-01	0.19	3.39E-01	-0.31	1.16E-01
ENSCAFG0000001501	ENSCAFG0000001501	grey	VSMC_M10	0.20	3.12E-01	0.22	2.78E-01	0.41	3.47E-02	-0.27	1.79E-01	-0.33	8.90E-02	-0.17	3.97E-01	-0.04	8.45E-01	0.23	2.39E-01	-0.29	1.39E-01	0.08	6.94E-01
ENSCAFG0000002026	ENSCAFG0000002026	grey	VSMC_M10	0.20	3.12E-01	0.04	8.38E-01	0.15	4.56E-01	-0.24	2.29E-01	-0.10	6.31E-01	0.02	9.23E-01	0.20	3.09E-01	0.19	3.55E-01	-0.21	3.02E-01	-0.03	8.88E-01
ENSCAFG0000000856	GUCY1B1	grey	VSMC_M10	0.20	3.12E-01	0.03	8.70E-01	0.26	1.82E-01	-0.18	3.72E-01	-0.21	2.93E-01	0.15	4.61E-01	0.64	2.81E-04	0.15	4.51E-01	-0.19	3.37E-01	0.09	6.52E-01
ENSCAFG0000002539	VXN	grey	VSMC_M10	0.20	3.12E-01	0.10	6.21E-01	-0.09	6.62E-01	0.38	4.77E-02	0.20	3.12E-01	-0.05	7.86E-01	-0.17	3.90E-01	-0.38	5.16E-02	-0.07	7.25E-01	0.11	5.80E-01
ENSCAFG0000000565	METTL3	yellow	VSMC_M3	0.20	3.12E-01	0.27	1.71E-01	0.60	9.68E-04	-0.44	2.04E-02	-0.47	1.41E-02	-0.02	9.04E-01	-0.02	9.37E-01	0.27	1.78E-01	-0.36	6.38E-02	-0.01	9.52E-01
ENSCAFG0000001951	DPP7	cyan	VSMC_M2	0.20	3.12E-01	0.79	7.54E-07	0.38	5.34E-02	0.10	6.24E-01	-0.30	1.22E-01	-0.16	4.15E-01	-0.09	6.49E-01	-0.49	1.03E-02	-0.65	2.72E-04	0.50	7.77E-03
ENSCAFG0000001186	CMAS	grey	VSMC_M10	0.20	3.12E-01	-0.28	1.51E-01	-0.40	4.12E-02	0.35	7.51E-02	0.57	1.82E-03	-0.34	8.57E-02	0.01	9.53E-01	-0.25	2.03E-01	0.26	1.98E-01	-0.26	1.97E-01
ENSCAFG0000001067	HECW2	grey	VSMC_M10	0.20	3.12E-01	0.02	9.14E-01	-0.03	8.77E-01	-0.23	2.39E-01	0.16	4.28E-01	0.16	4.32E-01	0.15	4.53E-01	0.16	4.39E-01	-0.03	8.96E-01	-0.10	6.29E-01
ENSCAFG0000001174	TFIP11	pink	VSMC_M5	0.20	3.12E-01	-0.41	3.59E-02	-0.25	2.07E-01	-0.09	6.63E-01	0.38	4.94E-02	-0.06	7.64E-01	0.28	1.59E-01	0.17	4.06E-01	0.31	1.15E-01	-0.38	4.91E-02
ENSCAFG0000001281	CDKL5	grey	VSMC_M10	0.20	3.13E-01	0.22	2.81E-01	-0.10	6.14E-01	0.28	1.60E-01	0.16	4.14E-01	0.05	7.90E-01	-0.14	4.76E-01	-0.36	6.26E-02	-0.15	4.61E-01	0.18	3.61E-01
ENSCAFG0000000775	CASP3	grey	VSMC_M10	0.20	3.13E-01	0.07	7.29E-01	0.23	2.44E-01	-0.17	4.02E-01	-0.06	7.85E-01	0.13	5.14E-01	-0.21	2.85E-01	0.07	7.23E-01	-0.08	6.97E-01	-0.19	3.32E-01
ENSCAFG0000001033	COQ5	grey	VSMC_M10	0.20	3.13E-01	-0.27	1.71E-01	-0.17	3.98E-01	-0.04	8.46E-01	0.20	3.17E-01	0.06	7.70E-01	0.31	1.11E-01	0.23	2.51E-01	0.06	7.71E-01	-0.14	4.91E-01
ENSCAFG0000001543	ENSCAFG0000001543	grey	VSMC_M10	0.20	3.13E-01	-0.12	5.34E-01	0.08	6.79E-01	-0.40	3.98E-02	0.06	7.58E-01	-0.15	4.60E-01	0.31	1.10E-01	0.42	3.07E-02	-0.04	8.42E-01	-0.34	8.03E-02
ENSCAFG0000000261	TNNI3	grey	VSMC_M10	0.20	3.13E-01	0.21	2.95E-01	0.43	2.63E-02	-0.25	2.16E-01	-0.36	6.38E-02	-0.21	3.01E-01	-0.12	5.45E-01	0.19	3.47E-01	-0.26	1.97E-01	0.05	8.14E-01
ENSCAFG0000000611	TAF7	grey	VSMC_M10	0.20	3.13E-01	0.10	6.05E-01	0.40	3.90E-02	-0.37	5.74E-02	-0.21	2.95E-01	-0.07	7.44E-01	-0.06	7.68E-01	0.21	3.00E-01	-0.11	5.84E-01	-0.23	2.54E-01
ENSCAFG0000002910	ENSCAFG0000002910	grey	VSMC_M10	0.20	3.14E-01	0.18	3.74E-01	-0.36	6.49E-02	0.31	1.15E-01	0.40	3.90E-02	-0.14	4.87E-01	0.07	7.12E-01	-0.33	8.80E-02	-0.23	2.42E-01	0.24	2.23E-01
ENSCAFG0000003065	ENSCAFG0000003065	yellow	VSMC_M3	0.20	3.14E-01	0.41	3.58E-02	0.58	1.70E-03	-0.09	6.61E-01	-0.49	9.30E-03	0.14	4.86E-01	-0.28	1.57E-01	-0.07	7.34E-01	-0.40	4.10E-02	0.17	4.00E-01
ENSCAFG0000001789	ZNF396	grey	VSMC_M10	0.20	3.14E-01	-0.16	4.28E-01	-0.12	5.53E-01	0.00	9.87E-01	0.19	3.51E-01	-0.25	2.09E-01	0.27	1.71E-01	0.06	7.78E-01	0.02	9.26E-01	-0.05	8.02E-01
ENSCAFG0000000251	ENSCAFG0000000251	yellow	VSMC_M3	0.20	3.14E-01	0.36	6.79E-02	0.70	4.18E-05	-0.35	7.34E-02	-0.60	9.38E-04	0.20	3.21E-01	-0.20	3.17E-01	0.15	4.41E-01	-0.38	5.25E-02	0.10	6.34E-01
ENSCAFG0000001180	TSHZ2	yellow	VSMC_M3	0.20	3.14E-01	0.37	5.66E-02	0.72	2.67E-05	-0.51	7.08E-03	-0.67	1.49E-04	0.42	2.96E-02	0.05	7.97E-01	0.34	8.52E-02	-0.46	1.66E-02	0.10	6.17E-01
ENSCAFG0000002343	SOWAHC	cyan	VSMC_M2	0.20	3.15E-01	0.61	7.08E-04	0.14	4.84E-01	0.47	1.23E-02	-0.17	4.01E-01	-0.01	9.61E-01	-0.22	2.72E-01	-0.57	1.72E-03	-0.57	1.93E-03	0.60	1.05E-03
ENSCAFG0000001174	LAMB2	cyan	VSMC_M2	0.20	3.15E-01	0.85	2.64E-08	0.10	6.28E-01	0.54	3.52E-03	-0.09	6.69E-01	-0.22	2.81E-01	-0.38	5.33E-02	-0.80	6.87E-07	-0.69	7.96E-05	0.69	6.62E-05
ENSCAFG0000001626	ENSCAFG0000001626	pink	VSMC_M5	0.20	3.15E-01	-0.45	1.71E-02	-0.26	1.88E-01	-0.17	4.08E-01	0.48	1.23E-02	0.07	7.12E-01	-0.07	7.40E-01	0.33	9.51E-02	0.38	5.34E-02	-0.59	1.12E-03
ENSCAFG0000000073	EBAG9	grey	VSMC_M10	0.20	3.15E-01	-0.05	7.89E-01	-0.02	9.39E-01	0.13	5.21E-01	0.10	6.05E-01	-0.18	3.67E-01	0.21	2.88E-01	-0.04	8.60E-01	-0.05	8.14E-01	0.00	9.87E-01
ENSCAFG0000002941	CARD8	cyan	VSMC_M2	0.20	3.15E-01	0.52	4.95E-03	0.20	3.21E-01	-0.07	7.10E-01	-0.10	6.32E-01	-0.08	6.83E-01	-0.17	3.88E-01	-0.10	6.05E-01	-0.47	1.38E-02	0.24	2.37E-01
ENSCAFG0000000916	ARSB	pink	VSMC_M5	0.20	3.15E-01	-0.05	8.22E-01	-0.68	9.48E-05	0.64	3.36E-04	0.76	4.67E-06	-0.13	5.30E-01	-0.11	5.76E-01	-0.52	5.13E-03	0.05	8.00E-01	0.08	6.91E-01
ENSCAFG0000000256	FOXJ3	grey	VSMC_M10	0.20	3.15E-01	-0.39	4.45E-02	-0.65	2.13E-04	0.21	3.01E-01	0.74	9.81E-06	0.02	9.36E-01	0.10	6.33E-01	-0.06	7.75E-01	0.28	1.52E-01	-0.18	3.79E-01
ENSCAFG0000001012	SNRPN	pink	VSMC_M5	0.20	3.15E-01	-0.35	7.26E-02	-0.21	2.99E-01	0.03	8.64E-01	0.44	2.16E-02	-0.11	5.71E-01	-0.44	2.27E-02	0.07	7.26E-01	0.36	6.66E-02	-0.52	5.70E-03
ENSCAFG0000000429	RNASEH2B	darkgrey	VSMC_M8	0.20	3.16E-01	-0.21	2.93E-01	0.48	1.06E-02	-0.88	2.35E-09	-0.24	2.32E-01	0.03	8.79E-01	0.38	5.11E-02	0.81	3.11E-07	0.08	6.74E-01	-0.58	1.59E-03
ENSCAFG0000000043	RAB3IP	grey	VSMC_M10	0.20	3.16E-01	-0.07	7.12E-01	-0.27	1.70E-01	0.29	1.44E-01	0.34	8.57E-02	-0.24	2.32E-01	0.24	2.29E-01	-0.23	2.52E-01	0.00	9.90E-01	0.10	6.36E-01
ENSCAFG0000000870	ADGRG5	grey	VSMC_M10	0.20	3.16E-01	0.14	4.89E-01	0.06	7.75E-01	-0.11	5.98E-01	0.09	6.47E-01	0.03	8.93E-01	0.05	8.01E-01	0.02	9.10E-01	-0.14	4.71E-01	0.00	9.85E-01
ENSCAFG0000002853	ENSCAFG0000002853	grey	VSMC_M10	0.20	3.16E-01	0.14	4.89E-01	0.06	7.75E-01	-0.11	5.98E-01	0.09	6.47E-01	0.03	8.93E-01	0.05	8.01E-01	0.02	9.10E-01	-0.14	4.71E-01	0.00	9.85E-01
ENSCAFG0000001216	CASP10	cyan	VSMC_M2	0.20	3.16E-01	0.62	5.34E-04	-0.10	6.26E-01	0.62	5.13E-04	0.06	7.56E-01	-0.12	5.45E-01	-0.34	8.16E-02	-0.62	6.31E-04	-0.62	5.48E-04	0.57	1.81E-03
ENSCAFG0000000520	NXPH2	darkgreen	VSMC_M4	0.20	3.16E-01	0.54	3.89E-03	-0.35	6.95E-02	0.83	7.75E-08	0.33	9.04E-02	-0.17	4.00E-01	-0.33	8.93E-02	-0.83	7.05E-08	-0.48	1.10E-02	0.61	7.30E-04
ENSCAFG0000000705	NPHP1	grey	VSMC_M10	0.20	3.16E-01	0.06	7.81E-01	-0.09	6.54E-01	0.00	9.95E-01	0.31	1.16E-01	-0.43	2.61E-02	0.13	5.10E-01	-0.10	6.07E-01	-0.03	8.92E-01	-0.12	5.51E-01
ENSCAFG0000000860	SOX6	grey	VSMC_M10	0.20	3.16E-01	-0.19	3.44E-01	-0.25	2.13E-01	0.17	4.09E-01	0.27	1.65E-01	0.22	2.67E-01	-0.02	9.40E-01	-0.12	5.55E-01	0.11	5.99E-01	-0.04	8.50E-01
ENSCAFG0000001460	FERMT3	grey	VSMC_M10	0.20	3.16E-01	0.00	9.89E-01	0.01	9.50E-01	-0.25	2.13E-01	0.20	3.06E-01	-0.07	7.33E-01	-0.03	8.88E-01	0.21	3.04E-01	-0.06	7.73E-01	-0.34	8.05E-02
ENSCAFG0000001066	ENSCAFG0000001066	grey	VSMC_M10	0.20	3.17E-01	0.25	2.05E-01	-0.20	3.07E-01	0.31	1.15E-01	0.18	3.72E-01	0.31	1.12E-01	-0.21	2.82E-01	-0.28	1.56E-01	-0.28	1.53E-01	0.27	1.70E-01
ENSCAFG0000001020	GCN1	darkgrey	VSMC_M8	0.20	3.17E-01	-0.23	2.57E-01	0.27	1.80E-01	-0.54	3.50E-03	-0.10	6.15E-01	0.01	9.50E-01	0.08	6.77E-01	0.53	4.76E-03	0.13	5.15E-01	-0.38	4.74E-02
ENSCAFG0000001677	ENSCAFG0000001677	grey	VSMC_M10	0.20	3.17E-01	0.28	1.57E-01	0.29	1.43E-01	-0.01	9.77E-01	-0.31	1.21E-01	0.25	2.00E-01	0.09	6.65E-01	-0.10	6.16E-01	-0.33	9.47E-02	0.29	1.37E-01
ENSCAFG0000000406	ENSCAFG0000000406	grey	VSMC_M10	0.20	3.17E-01	0.03	8.66E-01	-0.31	1.16E-01	0.20	3.24E-01	0.53	4.28E-03	-0.18	3.71E-01	-0.18	3.61E-01	-0.23	2.59E-01	0.00	9.94E-01	-0.20	3.11E-01
ENSCAFG0000001934	ISG15	cyan	VSMC_M2	0.20	3.17E-01	0.36	6.37E-02	0.13	5.14E-01	0.44	2.22E-02	-0.19	3.42E-01	0.09	6.58E-01	0.08	7.03E-01	-0.45	1.76E-02	-0.44	2.01E-02	0.46	1.52E-02
ENSCAFG0000001773	POLR3E	grey	VSMC_M10	0.20	3.17E-01	0.04	8.57E-01	0.11	5.77E-01	-0.06	7.83E-01	-0.15	4.42E-01	0.25	2.00E-01	0.16	4.23E-01	0.12	5.37E-01	-0.24	2.27E-01	0.22	2.80E-01
ENSCAFG0000000032	ENSCAFG0000000032	grey	VSMC_M10	0.20	3.17E-01	-0.06	7.77E-01	0.30	1.25E-01	-0.39	4.46E-02	-0.18	3.82E-01	-0.31	1.20E-01	0.19	3.37E-01	0.33	9.75E-02	-0.05	8.03E-01	-0.16	4.33E-01
ENSCAFG0000001892	DEXI	cyan	VSMC_M2	0.20	3.18E-01	0.59	1.17E-03	0.15	4.57E-01	0.46	1.65E-02	-0.28	1.58E-01	0.08	6.84E-01	-0.20	3.18E-01	-0.55	2.90E-03	-0.56	2.62E-03	0.72	2.07E-05
ENSCAFG0000000872	TET3	pink	VSMC_M5	0.20	3.18E-01	-0.14	4.98E-01	-0.57	2.02E-03	0.36	6.61E-02	0.75	5.34E-06	-0.03	8.91E-01	-0.04	8.31E-01	-0.34	8.58E-02	0.14	4.71E-01	-0.17	3.99E-01
ENSCAFG0000001525	NME7	grey	VSMC_M10	0.20	3.18E-01	-0.01	9.52E-01	-0.08	6.89E-01	-0.02	9.33E-01	0.05	7.96E-01	0.15	4.41E-01	0.40	4.12E-02	0.12	5.66E-01	-0.20	3.18E-01	0.17	4.04E-01
ENSCAFG0000002515	TTC9	grey	VSMC_M10	0.20	3.18E-01	0.13	5.14E-01	0.57	1.92E-03	-0.40	3.79E-02	-0.46	1.58E-02	0.19	3.53E-01	0.24	2.26E-01	0.21	2.84E-01	-0.18	3.76E-01	-0.05	8.09E-01
ENSCAFG0000001735	SPATA7	pink	VSMC_M5	0.20	3.18E-01	-0.08	6.85E-01	-0.41	3.38E-02	0.14	4.85E-01	0.69	7.09E-05	-0.31	1.17E-01	-0.23	2.49E-01	-0.16	4.33E-01	0.13	5.12E-01	-0.33	9.03E-02
ENSCAFG0000003135	ENSCAFG0000003135	yellow	VSMC_M3	0.20	3.18E-01	0.40	3.99E-02	0.39	4.68E-02	0.19	3.32E-01	-0.30	1.26E-01	-0.07	7.12E-01	-0.03	8.81E-01	-0.34	8.62E-02	-0.35	7.30E-02	0.28	1.52E-01
ENSCAFG0000003075	ENSCAFG0000003075	cyan	VSMC_M2	0.20	3.18E-01	0.63	4.68E-04	0.62	5.81E-04	-0.22	2.77E-01	-0.60	9.01E-04	0.00	9.87E-01	0.14	4.79E-01	-0.06	7.62E-01	-0.62	4.98E-04	0.42	2.95E-02
ENSCAFG0000000100	CENPM	darkgrey	VSMC_M8	0.20	3.18E-01	-0.14	5.02E-01	0.68	9.51E-05	-0.90	1.47E-10	-0.50	7.92E-03	0.11	5.91E-01	0.43	2.68E-02	0.81	2.50E-07	-0.04	8.40E-01	-0.43	2.62E-02
ENSCAFG0000002947	ENSCAFG0000002947	grey	VSMC_M10	0.20	3.18E-01	0.31	1.22E-01	0.40	3.73E-02	-0.13	5.26E-01	-0.36	6.68E-02	-0.03	8.78E-01	-0.24	2.24E-01	-0.03	8.65E-01	-0.30	1.35E-01	0.18	3.67E-01
ENSCAFG0000001598	ENSCAFG0000001598	grey	VSMC_M10	0.20	3.18E-01	-0.19	3.41E-01	-0.04	8.38E-01	-0.12	5.60E-01	0.16	4.29E-01	-0.16	4.32E-01	-0.19	3.54E-01	0.17	4.01E-01	0.20	3.24E-01	-0.26	1.90E-01
ENSCAFG0000000572	RARB	grey	VSMC_M10	0.20	3.18E-01	0.29	1.38E-01	0.36	6.38E-02	-0.02	9.07E-01	-0.37	5.41E-02	0.23	2.58E-01	0.00	9.87E-01	-0.07	7.38E-01	-0.32	1.03E-01	0.23	2.47E-01
ENSCAFG0000002905	TRIB2	yellow	VSMC_M3	0.20	3.18E-01	0.52	5.95E-03	0.35	7.09E-02	-0.05	8.03E-01	-0.27	1.72E-01	-0.14	4.91E-01	0.33	9.69E-02	-0.09	6.46E-01	-0.59	1.28E-03	0.27	1.67E-01
ENSCAFG0000001733	SMAD6	darkgreen	VSMC_M4	0.20	3.19E-01	0.41	3.45E-02	-0.42	2.90E-02	0.88	1.05E-09	0.37	6.10E-02	-0.05	8.22E-01	-0.41	3.41E-02	-0.82	1.32E-07	-0.37	5.74E-02	0.56	2.32E-03
ENSCAFG0000003148	ENSCAFG0000003148	grey	VSMC_M10	0.20	3.19E-01	0.48	1.12E-02	0.69	7.00E-05	-0.30	1.26E-01	-0.50	7.70E-03	-0.03	8.87E-01	0.21	2.89E-01	0.09	6.57E-01	-0.50	8.00E-03	0.05	7.89E-01
ENSCAFG0000000492	CCDC93	grey	VSMC_M10	0.20	3.19E-01	0.03	8.72E-01	0.08	7.00E-01	-0.31	1.17E-01	0.19	3.47E-01	-0.27	1.78E-01	0.34	8.08E-02	0.15	4.42E-01	-0.05	7.89E-01	-0.27	1.67E-01
ENSCAFG0000002326	PRIM2	darkgrey	VSMC_M8	0.20	3.19E-01	-0.52	5.56E-03	0.26	1.91E-01	-0.65	2.13E-04	-0.10	6.09E-01	0.15	4.64E-01	0.65	2.24E-04	0.76	3.43E-06	0.27	1.80E-01	-0.54	3.35E-03
ENSCAFG0000000142	BAIAP2L2	grey	VSMC_M10	0.20	3.19E-01	0.03	8.78E-01	0.03	8.80E-01	-0.10	6.31E-01	0.10	6.37E-01	0.16	4.34E-01	-0.26	1.85E-01	0.13	5.29E-01	-0.08	6.97E-01	-0.19	3.37E-01
ENSCAFG0000001403	PPA1	cyan	VSMC_M2	0.20	3.19E-01	0.54	3.35E-03	-0.08	7.10E-01	0.55	3.08E-03	0.06	7.53E-01	-0.02	9.02E-01	-0.54	3.86E-03	-0.58	1.62E-03	-0.50	7.65E-03	0.45	1.95E-02
ENSCAFG0000002982	VPS26B	cyan	VSMC_M2	0.20	3.19E-01	0.60	1.06E-03	0.31	1.20E-01	0.25	2.05E-01	-0.17	3.99E-01	-0.21	2.96E-01	-0.17	3.86E-01	-0.46	1.49E-02	-0.50	7.76E-03	0.28	1.61E-01
ENSCAFG0000001567	PQBP1	cyan	VSMC_M2	0.20	3.19E-01	0.79	7.61E-07	0.55	3.19E-03	-0.04	8.51E-01	-0.45	1.90E-02	-0.17	4.08E-01	-0.31	1.16E-01	-0.29	1.48E-01	-0.69	7.86E-05	0.35	7.08E-02
ENSCAFG0000000690	LMO1	cyan	VSMC_M2	0.20	3.19E-01	0.65	2.35E-04	0.28	1.64E-01	0.35	6.93E-02	-0.28	1.63E-01	-0.24	2.35E-01	-0.20	3.27E-01	-0.53	4.47E-03	-0.58	1.38E-03	0.56	2.44E-03
ENSCAFG0000001857	XIAP	pink	VSMC_M5	0.20	3.20E-01	-0.47	1.43E-02	-0.29	1.37E-01	-0.10	6.19E-01	0.55	2.82E-03	-0.29	1.42E-01	0.02	9.34E-01	0.23	2.56E-01	0.38	5.09E-02	-0.54	3.45E-03
ENSCAFG0000001211	RHBDD3	cyan	VSMC_M2	0.20	3.20E-01	0.92	9.62E-12	0.49	9.19E-03	0.26	1.89E-01	-0.48	1.06E-02	-0.06	7.49E-01	-0.29	1.42E-01	-0.59	1.08E-03	-0.79	1.10E-06	0.67	1.16E-04
ENSCAFG0000000414	ENSCAFG0000000414	grey	VSMC_M10	0.20	3.20E-01	0.03	8.74E-01	-0.13	5.14E-01	0.06	7.54E-01	0.23	2.42E-01	-0.22	2.77E-01	0.12	5.55E-01	0.02	9.34E-01	-0.11	5.95E-01	-0.07	7.20E-01
ENSCAFG0000001711	CORO1A	yellow	VSMC_M3	0.20	3.20E-01	0.41	3.58E-02	0.66	1.98E-04	-0.53	4.69E-03	-0.58	1.57E-03	0.20	3.08E-01	0.19	3.37E-01	0.32	1.05E-01	-0.52	5.61E-03	0.11	5.91E-01
ENSCAFG0000000927	DCLRE1B	darkgrey	VSMC_M8	0.20	3.20E-01	-0.49	9.98E-03	-0.10	6.22E-01	-0.48	1.18E-02	0.29	1.45E-01	0.01	9.43E-01	0.28	1.53E-01	0.65	2.76E-04	0.28	1.55E-01	-0.59	1.15E-03
ENSCAFG0000003258	METTL7A	grey	VSMC_M10	0.20	3.20E-01	0.74	1.07E-05	0.24	2.38E-01	0.27	1.74E-01	-0.25	2.17E-01	-0.03	8.76E-01	-0.16	4.31E-01	-0.49	9.16E-03	-0.73	1.59E-05	0.58	1.49E-03
ENSCAFG0000001490	SH3BP1	yellow	VSMC_M3	0.20	3.20E-01	0.11	5.88E-01	0.63	4.49E-04	-0.59	1.30E-03	-0.45	1.96E-02	-0.10	6.10E-01	0.19	3.41E-01	0.47	1.32E-02	-0.17	3.93E-01	-0.24	2.30E-01
ENSCAFG0000000871	ENSCAFG0000000871	grey	VSMC_M10	0.20	3.20E-01	-0.48	1.17E-02	-0.25	2.10E-01	-0.04	8.55E-01	0.32	1.07E-01	0.14	4.96E-01	0.36	6.71E-02	0.21	3.03E-01	0.23	2.42E-01	-0.24	2.19E-01
ENSCAFG0000001162	CLCF1	yellow	VSMC_M3	0.20	3.20E-01	0.14	4.73E-01	0.42	2.74E-02	-0.51	6.01E-03	-0.18	3.60E-01	-0.07	7.11E-01	-0.15	4.48E-01	0.30	1.23E-01	-0.10	6.18E-01	-0.31	1.21E-01
ENSCAFG0000002110	ENSCAFG0000002110	grey	VSMC_M10	0.20	3.20E-01	-0.10	6.25E-01	-0.12	5.52E-01	-0.13	5.23E-01	0.31	1.22E-01	0.24	2.33E-01	0.16	4.33E-01	0.13	5.05E-01	0.02	9.39E-01	-0.32	1.04E-01
ENSCAFG0000003264	ENSCAFG0000003264	pink	VSMC_M5	0.20	3.21E-01	-0.29	1.45E-01	-0.46	1.57E-02	0.37	5.50E-02	0.65	2.42E-04	-0.24	2.32E-01	-0.31	1.14E-01	-0.24	2.31E-01	0.27	1.72E-01	-0.30	1.35E-01
ENSCAFG0000001906	LRRC42	grey	VSMC_M10	0.20	3.21E-01	0.01	9.69E-01	0.33	9.31E-02	-0.42	2.74E-02	-0.11	5.82E-01	-0.06	7.74E-01	0.39	4.61E-02	0.38	5.25E-02	-0.12	5.66E-01	-0.30	1.27E-01
ENSCAFG0000000432	APMAP	grey	VSMC_M10	0.20	3.21E-01	0.23	2.45E-01	0.41	3.56E-02	-0.15	4.63E-01	-0.26	1.90E-01	-0.02	9.02E-01	-0.42	2.88E-02	0.05	8.19E-01	-0.22	2.65E-01	-0.07	7.26E-01
ENSCAFG0000003178	RMDN1	pink	VSMC_M5	0.20	3.21E-01	-0.19	3.50E-01	-0.56	2.34E-03	0.47	1.26E-02	0.65	2.17E-04	0.09	6.42E-01	-0.39	4.37E-02	-0.32	1.05E-01	0.20	3.26E-01	-0.10	6.12E-01
ENSCAFG0000000569	DNAJC18	grey	VSMC_M10	0.20	3.21E-01	-0.01	9.41E-01	-0.59	1.11E-03	0.62	5.25E-04	0.54	3.85E-03	0.05	8.20E-01	0.06	7.54E-01	-0.51	6.69E-03	-0.05	8.20E-01	0.33	9.55E-02
ENSCAFG0000000581	SRA1	grey	VSMC_M10	0.20	3.21E-01	0.42	2.88E-02	0.62	5.52E-04	-0.28	1.60E-01	-0.49	1.01E-02	-0.13	5.22E-01	0.01	9.64E-01	0.11	5.78E-01	-0.47	1.37E-02	0.00	9.83E-01
ENSCAFG0000002021	ENSCAFG0000002021	grey	VSMC_M10	0.20	3.21E-01	0.41	3.40E-02	0.30	1.28E-01	0.06	7.66E-01	-0.25	2.07E-01	-0.04	8.50E-01	-0.29	1.45E-01	-0.14	4.85E-01	-0.37	5.45E-02	0.21	2.92E-01
ENSCAFG0000000549	ZEB2	pink	VSMC_M5	0.20	3.22E-01	-0.03	8.63E-01	-0.64	3.12E-04	0.70	5.47E-05	0.67	1.47E-04	-0.20	3.13E-01	-0.09	6.61E-01	-0.54	3.92E-03	0.03	8.64E-01	0.19	3.45E-01
ENSCAFG0000000044	SYNE1	pink	VSMC_M5	0.20	3.22E-01	-0.15	4.40E-01	-0.52	5.15E-03	0.42	2.81E-02	0.56	2.39E-03	0.05	8.20E-01	-0.06	7.65E-01	-0.32	1.07E-01	0.06	7.67E-01	0.07	7.32E-01
ENSCAFG0000000220	TBC1D8	grey	VSMC_M10	0.20	3.22E-01	0.58	1.40E-03	-0.05	7.91E-01	0.47	1.41E-02	-0.03	8.99E-01	-0.08	6.94E-01	0.05	8.23E-01	-0.54	3.99E-03	-0.62	6.31E-04	0.68	8.97E-05
ENSCAFG0000001289	PARD3B	cyan	VSMC_M2	0.20	3.22E-01	0.41	3.14E-02	0.17	4.08E-01	-0.10	6.37E-01	-0.07	7.35E-01	-0.16	4.15E-01	0.30	1.28E-01	-0.11	5.93E-01	-0.45	1.72E-02	0.22	2.69E-01
ENSCAFG0000000264	ENSCAFG0000000264	grey	VSMC_M10	0.20	3.22E-01	0.16	4.32E-01	-0.23	2.39E-01	0.36	6.46E-02	0.28	1.53E-01	-0.12	5.61E-01	-0.03	8.98E-01	-0.41	3.39E-02	-0.14	5.01E-01	0.24	2.29E-01
ENSCAFG0000000198	SLC29A1	darkgrey	VSMC_M8	0.20	3.22E-01	-0.20	3.20E-01	0.59	1.31E-03	-0.92	2.39E-11	-0.38	4.90E-02	0.16	4.28E-01	0.29	1.45E-01	0.87	4.48E-09	0.03	8.87E-01	-0.55	3.06E-03
ENSCAFG0000000004	ENSCAFG0000000004	grey	VSMC_M10	0.20	3.23E-01	0.10	6.11E-01	-0.08	7.05E-01	0.07	7.25E-01	0.11	6.01E-01	0.31	1.11E-01	0.07	7.16E-01	-0.04	8.28E-01	-0.18	3.61E-01	0.13	5.32E-01
ENSCAFG0000001220	INPP5F	darkgreen	VSMC_M4	0.20	3.23E-01	0.32	1.05E-01	-0.46	1.46E-02	0.75	7.21E-06	0.44	2.19E-02	-0.01	9.54E-01	-0.41	3.14E-02	-0.71	3.60E-05	-0.27	1.68E-01	0.43	2.60E-02
ENSCAFG0000003204	COX7A2L	grey	VSMC_M10	0.20	3.23E-01	-0.21	2.92E-01	0.06	7.79E-01	-0.18	3.68E-01	0.07	7.14E-01	-0.09	6.50E-01	0.10	6.32E-01	0.19	3.31E-01	0.16	4.29E-01	-0.27	1.77E-01
ENSCAFG0000001402	ENSCAFG0000001402	cyan	VSMC_M2	0.20	3.23E-01	0.57	2.13E-03	0.56	2.56E-03	-0.10	6.02E-01	-0.55	2.91E-03	0.11	5.92E-01	0.02	9.40E-01	-0.12	5.43E-01	-0.56	2.56E-03	0.38	4.77E-02
ENSCAFG0000002978	ENSCAFG0000002978	grey	VSMC_M10	0.20	3.23E-01	-0.09	6.50E-01	-0.28	1.62E-01	0.01	9.67E-01	0.31	1.15E-01	0.16	4.16E-01	-0.03	8.64E-01	0.10	6.37E-01	-0.04	8.30E-01	-0.08	7.01E-01
ENSCAFG0000001034	NECTIN3	grey	VSMC_M10	0.20	3.24E-01	-0.34	7.92E-02	0.02	9.10E-01	-0.54	3.39E-03	0.14	4.96E-01	0.09	6.41E-01	0.21	2.98E-01	0.51	6.63E-03	0.18	3.81E-01	-0.44	2.31E-02
ENSCAFG0000002964	ENSCAFG0000002964	grey	VSMC_M10	0.20	3.24E-01	0.18	3.61E-01	0.53	4.24E-03	-0.39	4.65E-02	-0.50	8.30E-03	0.24	2.26E-01	0.31	1.16E-01	0.30	1.32E-01	-0.37	6.00E-02	0.05	8.13E-01
ENSCAFG0000000716	CHD7	darkgreen	VSMC_M4	0.20	3.24E-01	0.54	3.58E-03	-0.13	5.12E-01	0.58	1.56E-03	0.05	7.86E-01	0.16	4.40E-01	-0.61	7.62E-04	-0.63	4.55E-04	-0.49	9.23E-03	0.58	1.65E-03
ENSCAFG0000000066	FNDC1	yellow	VSMC_M3	0.20	3.24E-01	0.63	4.01E-04	0.65	2.53E-04	-0.39	4.31E-02	-0.45	1.92E-02	-0.15	4.66E-01	-0.11	5.98E-01	0.04	8.41E-01	-0.58	1.50E-03	0.09	6.45E-01
ENSCAFG0000001741	AAGAB	grey	VSMC_M10	0.20	3.24E-01	0.05	8.11E-01	0.02	9.20E-01	0.18	3.56E-01	-0.03	8.73E-01	0.13	5.14E-01	0.23	2.55E-01	-0.18	3.58E-01	-0.11	5.86E-01	0.23	2.48E-01
ENSCAFG0000000981	SFXN3	darkgrey	VSMC_M8	0.20	3.24E-01	-0.26	1.85E-01	0.44	2.14E-02	-0.84	4.98E-08	-0.17	3.84E-01	-0.16	4.24E-01	0.40	3.78E-02	0.77	2.57E-06	0.14	4.79E-01	-0.60	8.60E-04
ENSCAFG0000001163	ENSCAFG0000001163	grey	VSMC_M10	0.20	3.24E-01	0.59	1.16E-03	0.10	6.13E-01	0.51	6.10E-03	-0.10	6.28E-01	-0.11	5.80E-01	0.00	9.83E-01	-0.67	1.54E-04	-0.55	3.01E-03	0.54	3.30E-03
ENSCAFG0000001896	RBMX	darkgrey	VSMC_M8	0.20	3.25E-01	-0.40	3.93E-02	0.09	6.44E-01	-0.58	1.35E-03	0.13	5.04E-01	-0.14	4.93E-01	0.17	3.98E-01	0.68	8.35E-05	0.25	2.09E-01	-0.63	4.91E-04
ENSCAFG0000001330	SAXO2	grey	VSMC_M10	0.20	3.25E-01	0.12	5.53E-01	-0.13	5.34E-01	0.33	9.05E-02	0.21	3.01E-01	0.00	9.96E-01	0.11	5.72E-01	-0.38	4.80E-02	-0.06	7.64E-01	0.11	5.82E-01
ENSCAFG0000000796	MRPS27	grey	VSMC_M10	0.20	3.25E-01	0.20	3.07E-01	0.11	5.71E-01	0.19	3.41E-01	-0.14	4.78E-01	-0.11	5.83E-01	-0.11	5.79E-01	-0.12	5.52E-01	-0.33	9.14E-02	0.32	1.07E-01
ENSCAFG0000000715	ENSCAFG0000000715	cyan	VSMC_M2	0.20	3.25E-01	0.56	2.32E-03	-0.09	6.55E-01	0.64	3.41E-04	0.03	8.73E-01	-0.06	7.77E-01	-0.69	7.43E-05	-0.67	1.51E-04	-0.50	7.64E-03	0.53	4.58E-03

ENSCAFG0000003142	PIH1D2	grey	VSMC_M1C	0.20	3.25E-01	0.13	5.31E-01	0.46	1.63E-02	-0.20	3.26E-01	-0.39	4.31E-02	0.12	5.52E-01	0.44	2.29E-02	0.08	7.06E-01	-0.21	2.83E-01	0.13	5.24E-01
ENSCAFG0000001593	PPP1R3F	grey	VSMC_M1C	0.20	3.25E-01	-0.10	6.34E-01	-0.26	1.90E-01	0.17	4.09E-01	0.27	1.76E-01	0.22	2.68E-01	0.06	7.83E-01	-0.08	6.85E-01	-0.03	8.94E-01	0.08	6.88E-01
ENSCAFG0000003084	PXDC1	grey	VSMC_M1C	0.20	3.25E-01	0.01	9.69E-01	-0.26	1.82E-01	0.25	2.01E-01	0.35	7.48E-02	0.10	6.19E-01	-0.57	1.99E-03	-0.24	2.26E-01	0.07	7.40E-01	-0.04	8.57E-01
ENSCAFG0000001965	ARID3A	grey	VSMC_M1C	0.20	3.26E-01	0.24	2.24E-01	0.51	6.42E-03	-0.48	1.08E-02	-0.36	6.60E-02	0.24	2.30E-01	-0.28	1.54E-01	0.36	6.22E-02	-0.27	1.66E-01	-0.19	3.50E-01
ENSCAFG0000001323	PTPN23	yellow	VSMC_M3	0.20	3.26E-01	0.69	6.14E-05	0.72	2.13E-05	-0.22	2.68E-01	-0.67	1.52E-04	-0.07	7.41E-01	-0.21	2.88E-01	-0.12	5.60E-01	-0.63	4.87E-04	0.35	7.04E-02
ENSCAFG0000003155	MBD6	cyan	VSMC_M2	0.20	3.26E-01	0.67	1.47E-04	0.29	1.41E-01	0.06	7.58E-01	-0.31	1.12E-01	0.11	5.69E-01	-0.21	3.01E-01	-0.32	1.03E-01	-0.65	2.68E-04	0.55	3.20E-03
ENSCAFG0000001912	MTM1	grey	VSMC_M1C	0.20	3.26E-01	0.02	9.08E-01	-0.37	5.77E-02	0.37	6.09E-02	0.35	7.15E-02	0.08	6.79E-01	0.11	5.97E-01	-0.27	1.79E-01	-0.13	5.26E-01	0.23	2.46E-01
ENSCAFG0000002195	ENSCAFG0000002195	grey	VSMC_M1C	0.20	3.26E-01	0.27	1.70E-01	0.47	1.38E-02	-0.17	3.87E-01	-0.39	4.65E-02	0.35	7.17E-02	-0.07	7.40E-01	-0.01	9.60E-01	-0.27	1.66E-01	0.05	7.86E-01
ENSCAFG0000001938	SGTA	grey	VSMC_M1C	0.20	3.26E-01	0.34	7.99E-02	0.43	2.48E-02	0.02	9.09E-01	-0.42	3.12E-02	0.09	6.52E-01	-0.06	7.57E-01	-0.08	6.82E-01	-0.43	2.53E-02	0.20	3.06E-01
ENSCAFG0000001672	WRAP53	grey	VSMC_M1C	0.20	3.26E-01	0.41	3.28E-02	0.50	7.43E-03	-0.23	2.41E-01	-0.59	1.14E-03	0.24	2.21E-01	0.16	4.19E-01	0.09	6.43E-01	-0.53	4.19E-03	0.43	2.53E-02
ENSCAFG0000001937	THOP1	darkgrey	VSMC_M8	0.20	3.26E-01	-0.10	6.09E-01	0.67	1.53E-04	-0.79	7.30E-07	-0.57	1.81E-03	0.22	2.69E-01	0.44	2.06E-02	0.73	1.33E-05	-0.12	5.51E-01	-0.27	1.81E-01
ENSCAFG0000003173	ENSCAFG0000003173	grey	VSMC_M1C	0.20	3.27E-01	-0.36	6.37E-02	-0.14	4.82E-01	-0.16	4.28E-01	0.26	1.98E-01	-0.08	6.98E-01	-0.02	9.29E-01	0.27	1.66E-01	0.26	1.97E-01	-0.31	1.13E-01
ENSCAFG0000001421	TANGO2	grey	VSMC_M1C	0.20	3.27E-01	0.19	3.35E-01	-0.16	4.38E-01	0.58	1.58E-03	0.23	2.59E-01	0.03	8.93E-01	0.06	7.51E-01	-0.57	2.02E-03	-0.19	3.55E-01	0.22	2.68E-01
ENSCAFG0000001924	SERPINF2	grey	VSMC_M1C	0.20	3.27E-01	0.39	4.58E-02	0.18	3.67E-01	0.08	6.97E-01	-0.22	2.70E-01	0.06	7.75E-01	0.14	4.91E-01	-0.19	3.51E-01	-0.49	9.30E-03	0.39	4.45E-02
ENSCAFG0000001728	TIPIN	grey	VSMC_M1C	0.20	3.27E-01	-0.46	1.55E-02	-0.07	7.29E-01	-0.27	1.67E-01	0.20	3.12E-01	0.04	8.47E-01	0.11	5.98E-01	0.46	1.49E-02	0.26	1.98E-01	-0.45	1.93E-02
ENSCAFG0000001538	CCND2	darkgreen	VSMC_M4	0.20	3.27E-01	0.51	6.48E-03	-0.17	3.84E-01	0.72	2.32E-05	0.06	7.77E-01	0.15	4.60E-01	-0.12	5.61E-01	-0.72	2.75E-05	-0.55	3.25E-03	0.69	6.34E-05
ENSCAFG0000000164	TRERF1	cyan	VSMC_M2	0.20	3.27E-01	0.67	1.19E-04	0.26	1.93E-01	0.15	4.49E-01	-0.17	3.92E-01	-0.22	2.62E-01	-0.06	7.76E-01	-0.34	8.60E-02	-0.64	3.07E-04	0.40	3.81E-02
ENSCAFG0000001553	NAALADL2	pink	VSMC_M5	0.20	3.28E-01	-0.02	9.29E-01	-0.54	3.89E-03	0.55	2.71E-03	0.62	6.27E-04	-0.16	4.16E-01	0.05	7.89E-01	-0.42	3.09E-02	-0.03	8.83E-01	0.08	7.01E-01
ENSCAFG0000001097	RGMA	cyan	VSMC_M2	0.20	3.28E-01	0.56	2.61E-03	0.44	2.04E-02	-0.18	3.69E-01	-0.26	1.83E-01	-0.11	5.77E-01	0.04	8.27E-01	-0.07	7.12E-01	-0.52	5.65E-03	0.10	6.09E-01
ENSCAFG0000003176	C12H6orf132	grey	VSMC_M1C	0.20	3.28E-01	-0.10	6.26E-01	-0.09	6.40E-01	-0.07	7.23E-01	0.30	1.30E-01	-0.10	6.22E-01	0.32	1.03E-01	0.07	7.34E-01	0.03	8.91E-01	-0.31	1.17E-01
ENSCAFG0000002287	ENSCAFG0000002287	grey	VSMC_M1C	0.20	3.28E-01	-0.04	8.34E-01	0.33	9.58E-02	-0.40	4.00E-02	-0.22	2.61E-01	0.28	1.63E-01	0.25	2.01E-01	0.26	1.83E-01	-0.02	9.24E-01	-0.12	5.61E-01
ENSCAFG0000000526	ENSCAFG0000000526	cyan	VSMC_M2	0.20	3.28E-01	0.53	4.84E-03	0.31	1.21E-01	0.18	3.65E-01	-0.39	4.27E-02	0.19	3.51E-01	0.14	4.99E-01	-0.33	8.77E-02	-0.54	3.50E-03	0.63	3.80E-04
ENSCAFG0000001997	ENSCAFG0000001997	grey	VSMC_M1C	0.20	3.28E-01	0.04	8.58E-01	0.08	7.08E-01	0.03	8.81E-01	0.02	9.26E-01	-0.11	5.96E-01	-0.05	8.15E-01	-0.01	9.70E-01	-0.12	5.57E-01	-0.07	7.20E-01
ENSCAFG0000001156	USP19	grey	VSMC_M1C	0.20	3.29E-01	0.77	3.34E-06	-0.04	8.32E-01	0.62	6.21E-04	-0.03	8.70E-01	-0.10	6.26E-01	-0.24	2.33E-01	-0.79	1.08E-06	-0.69	7.51E-05	0.79	9.73E-07
ENSCAFG0000001575	RCC2	yellow	VSMC_M3	0.20	3.29E-01	0.20	3.05E-01	0.79	9.38E-07	-0.78	1.37E-06	-0.64	3.14E-04	0.26	1.88E-01	0.16	4.27E-01	0.56	2.21E-03	-0.32	1.07E-01	-0.19	3.51E-01
ENSCAFG0000002985	ENSCAFG0000002985	grey	VSMC_M1C	0.20	3.29E-01	0.01	9.49E-01	0.48	1.12E-02	-0.49	8.91E-03	-0.24	2.33E-01	0.06	7.74E-01	-0.08	7.03E-01	0.38	4.81E-02	-0.03	8.74E-01	-0.39	4.64E-02
ENSCAFG0000000203	PLA2G7	cyan	VSMC_M2	0.20	3.29E-01	0.85	2.69E-08	0.49	1.00E-02	0.01	9.75E-01	-0.44	2.20E-02	-0.15	4.54E-01	-0.49	8.86E-03	-0.30	1.23E-01	-0.76	4.20E-06	0.47	1.39E-02
ENSCAFG0000001626	CGNL1	cyan	VSMC_M2	0.20	3.30E-01	0.62	6.21E-04	-0.14	4.99E-01	0.73	1.34E-05	0.12	5.37E-01	-0.27	1.70E-01	-0.72	2.25E-05	-0.80	4.58E-07	-0.49	8.77E-03	0.58	1.70E-03
ENSCAFG0000001605	MNS1	grey	VSMC_M1C	0.19	3.30E-01	-0.25	2.18E-01	0.21	3.03E-01	-0.39	4.52E-02	0.03	8.97E-01	-0.09	6.44E-01	0.40	4.13E-02	0.38	5.30E-02	0.09	6.39E-01	-0.45	1.83E-02
ENSCAFG0000001191	CLIP2	yellow	VSMC_M3	0.19	3.30E-01	0.32	1.08E-01	0.66	1.58E-04	-0.64	3.52E-04	-0.44	2.11E-02	-0.31	1.13E-01	0.07	7.16E-01	0.36	6.73E-02	-0.29	1.39E-01	-0.16	4.39E-01
ENSCAFG0000000018	MOXD1	cyan	VSMC_M2	0.19	3.30E-01	0.74	1.17E-05	0.01	9.76E-01	0.57	1.97E-03	0.05	8.22E-01	-0.27	1.66E-01	-0.52	4.94E-03	-0.73	1.35E-05	-0.61	6.93E-04	0.54	3.44E-03
ENSCAFG0000001308	DNAJC5	grey	VSMC_M1C	0.19	3.30E-01	0.15	4.61E-01	0.15	4.46E-01	-0.05	8.18E-01	-0.17	3.86E-01	0.16	4.31E-01	-0.36	6.77E-02	0.05	8.19E-01	-0.16	4.24E-01	0.10	6.11E-01
ENSCAFG0000001821	EIF5	darkgrey	VSMC_M8	0.19	3.30E-01	-0.62	6.06E-04	0.00	9.85E-01	-0.60	8.71E-04	0.18	3.69E-01	0.16	4.12E-01	0.35	6.95E-02	0.76	3.38E-06	0.38	5.12E-02	-0.70	5.05E-05
ENSCAFG0000001285	RAB1B	grey	VSMC_M1C	0.19	3.30E-01	0.07	7.19E-01	0.19	3.33E-01	-0.14	4.96E-01	0.03	8.78E-01	0.02	9.29E-01	-0.20	3.30E-01	0.08	6.83E-01	-0.03	8.78E-01	-0.30	1.23E-01
ENSCAFG0000002964	PIGK	darkgreen	VSMC_M4	0.19	3.30E-01	0.04	8.50E-01	-0.37	5.65E-02	0.54	3.83E-03	0.42	2.76E-02	-0.18	3.57E-01	-0.71	3.36E-05	-0.41	3.59E-02	0.00	9.82E-01	0.06	7.77E-01
ENSCAFG0000001845	MED9	grey	VSMC_M1C	0.19	3.30E-01	0.20	3.16E-01	-0.24	2.23E-01	0.18	3.63E-01	0.24	2.23E-01	0.02	9.16E-01	-0.07	7.29E-01	-0.19	3.45E-01	-0.27	1.72E-01	0.20	3.29E-01
ENSCAFG0000000046	ENSCAFG0000000046	grey	VSMC_M1C	0.19	3.30E-01	-0.04	8.28E-01	0.13	5.18E-01	-0.05	8.10E-01	-0.05	8.05E-01	-0.14	4.97E-01	-0.10	6.08E-01	0.08	7.05E-01	-0.03	8.78E-01	-0.05	7.94E-01
ENSCAFG0000001110	SNAP23	grey	VSMC_M1C	0.19	3.30E-01	-0.16	4.23E-01	0.13	5.03E-01	-0.40	3.91E-02	0.06	7.68E-01	0.00	9.90E-01	0.19	3.51E-01	0.37	5.55E-02	0.02	9.40E-01	-0.40	3.95E-02
ENSCAFG0000003065	ENSCAFG0000003065	grey	VSMC_M1C	0.19	3.30E-01	0.08	6.89E-01	-0.35	7.31E-02	0.28	1.53E-01	0.41	3.49E-02	0.06	7.50E-01	-0.06	7.55E-01	-0.26	1.95E-01	-0.10	6.07E-01	0.00	9.82E-01
ENSCAFG0000001969	NPRL3	cyan	VSMC_M2	0.19	3.31E-01	0.70	5.27E-05	0.22	2.78E-01	0.12	5.43E-01	-0.22	2.80E-01	-0.03	8.96E-01	0.02	9.15E-01	-0.40	3.66E-02	-0.65	2.72E-04	0.52	4.97E-03
ENSCAFG0000000533	CAMK1	cyan	VSMC_M2	0.19	3.31E-01	0.74	1.02E-05	0.33	9.49E-02	0.20	3.14E-01	-0.33	9.45E-02	-0.23	2.40E-01	-0.31	1.16E-01	-0.40	3.63E-02	-0.66	1.97E-04	0.57	1.83E-03
ENSCAFG0000000131	DAPK1	darkgreen	VSMC_M4	0.19	3.31E-01	0.47	1.36E-02	-0.30	1.31E-01	0.70	4.97E-05	0.20	3.25E-01	0.14	4.84E-01	-0.09	6.66E-01	-0.74	1.12E-05	-0.49	8.90E-03	0.66	1.63E-04
ENSCAFG0000001918	ZNF275	grey	VSMC_M1C	0.19	3.31E-01	0.24	2.35E-01	0.16	4.22E-01	0.19	3.34E-01	-0.10	6.17E-01	0.20	3.19E-01	-0.29	1.36E-01	-0.20	3.25E-01	-0.27	1.69E-01	0.13	5.31E-01
ENSCAFG0000000689	IL6ST	pink	VSMC_M5	0.19	3.31E-01	0.14	4.83E-01	-0.67	1.15E-04	0.72	2.12E-05	0.71	3.55E-05	-0.09	6.61E-01	-0.16	4.32E-01	-0.64	2.93E-04	-0.14	4.99E-01	0.28	1.57E-01
ENSCAFG0000000378	PCDH10	grey	VSMC_M1C	0.19	3.31E-01	-0.19	3.43E-01	-0.34	8.04E-02	0.28	1.53E-01	0.39	4.49E-02	-0.16	4.32E-01	-0.09	6.54E-01	-0.12	5.51E-01	0.14	4.84E-01	-0.06	7.81E-01
ENSCAFG0000002834	ENSCAFG0000002834	grey	VSMC_M1C	0.19	3.32E-01	-0.09	6.38E-01	-0.20	3.13E-01	0.18	3.80E-01	0.13	5.21E-01	0.23	2.51E-01	0.19	3.51E-01	-0.07	7.34E-01	-0.02	9.20E-01	0.23	2.55E-01
ENSCAFG0000000483	ENSCAFG0000000483	pink	VSMC_M5	0.19	3.32E-01	-0.26	1.82E-01	-0.32	1.03E-01	0.07	7.12E-01	0.40	3.86E-02	-0.04	8.30E-01	-0.01	9.61E-01	0.06	7.54E-01	0.19	3.48E-01	-0.23	2.45E-01
ENSCAFG0000001751	PCDH19	grey	VSMC_M1C	0.19	3.32E-01	-0.18	3.74E-01	-0.02	9.40E-01	-0.39	4.49E-02	0.19	3.35E-01	0.08	6.92E-01	0.47	1.25E-02	0.34	8.34E-02	0.01	9.51E-01	-0.34	8.24E-02
ENSCAFG0000000756	LRP2BP	grey	VSMC_M1C	0.19	3.32E-01	-0.23	2.50E-01	-0.16	4.14E-01	-0.06	7.59E-01	0.28	1.62E-01	-0.05	7.91E-01	-0.07	7.11E-01	0.14	4.85E-01	0.10	6.16E-01	-0.20	3.29E-01
ENSCAFG0000000396	ENSCAFG0000000396	grey	VSMC_M1C	0.19	3.32E-01	-0.16	4.35E-01	0.15	4.54E-01	-0.14	4.94E-01	-0.10	6.27E-01	-0.07	7.41E-01	0.07	7.27E-01	0.22	2.77E-01	0.04	8.44E-01	-0.08	6.94E-01
ENSCAFG0000001166	MYO18B	red	VSMC_M1	0.19	3.32E-01	-0.19	3.31E-01	-0.23	2.52E-01	0.15	4.46E-01	0.23	2.44E-01	0.23	2.45E-01	-0.34	7.83E-02	-0.03	8.99E-01	0.15	4.64E-01	-0.06	7.74E-01
ENSCAFG0000001804	NCOR1	grey	VSMC_M1C	0.19	3.32E-01	0.43	2.35E-02	0.06	7.80E-01	0.16	4.23E-01	0.04	8.57E-01	-0.04	8.43E-01	0.05	7.95E-01	-0.34	8.51E-02	-0.42	2.85E-02	0.35	7.37E-02
ENSCAFG0000001809	SYNPO	darkgreen	VSMC_M4	0.19	3.32E-01	0.41	3.47E-02	-0.08	6.82E-01	0.44	2.07E-02	0.17	4.07E-01	-0.25	2.16E-01	-0.75	6.95E-06	-0.54	3.76E-03	-0.25	2.18E-01	0.25	2.05E-01
ENSCAFG0000001859	C6	cyan	VSMC_M2	0.19	3.32E-01	0.74	1.02E-05	0.30	1.30E-01	0.20	3.09E-01	-0.35	7.10E-02	0.07	7.14E-01	-0.47	1.39E-02	-0.42	3.05E-02	-0.70	4.07E-05	0.59	1.35E-03
ENSCAFG0000003024	CA11	cyan	VSMC_M2	0.19	3.32E-01	0.52	5.58E-03	0.20	3.21E-01	0.21	2.90E-01	-0.23	2.41E-01	0.04	8.28E-01	-0.08	7.09E-01	-0.37	6.11E-02	-0.52	5.85E-03	0.48	1.07E-02
ENSCAFG0000002926	THY1	grey	VSMC_M1C	0.19	3.33E-01	0.40	3.76E-02	0.42	2.87E-02	-0.28	1.52E-01	-0.26	1.89E-01	-0.10	6.08E-01	0.53	4.13E-03	0.02	9.12E-01	-0.42	2.94E-02	0.10	6.25E-01
ENSCAFG0000000135	AGTPBP1	grey	VSMC_M1C	0.19	3.33E-01	0.37	5.98E-02	-0.15	4.57E-01	0.39	4.19E-02	0.00	9.86E-01	0.22	2.81E-01	-0.26	1.83E-01	-0.38	4.83E-02	-0.45	1.89E-02	0.60	8.35E-04
ENSCAFG0000001062	RRP1B	yellow	VSMC_M3	0.19	3.33E-01	0.01	9.53E-01	0.69	5.77E-05	-0.66	1.85E-04	-0.51	6.18E-03	0.03	8.87E-01	0.28	1.56E-01	0.61	7.17E-04	-0.20	3.10E-01	-0.32	1.09E-01
ENSCAFG0000001447	BCHE	grey	VSMC_M1C	0.19	3.33E-01	-0.29	1.47E-01	0.00	9.87E-01	-0.02	9.15E-01	-0.02	9.17E-01	0.22	2.76E-01	0.26	1.88E-01	0.16	4.24E-01	0.14	4.81E-01	-0.07	7.19E-01
ENSCAFG0000001253	ABHD11	grey	VSMC_M1C	0.19	3.33E-01	0.35	7.08E-02	0.43	2.46E-02	0.04	8.31E-01	-0.46	1.52E-02	0.18	3.78E-01	0.20	3.30E-01	-0.13	5.14E-01	-0.40	3.99E-02	0.40	3.93E-02
ENSCAFG0000001287	CD28	grey	VSMC_M1C	0.19	3.33E-01	-0.08	6.83E-01	-0.09	6.58E-01	-0.17	3.87E-01	0.34	8.42E-02	-0.12	5.38E-01	-0.02	9.17E-01	0.15	4.46E-01	0.04	8.55E-01	-0.43	2.44E-02
ENSCAFG0000001859	LAMA1	grey	VSMC_M1C	0.19	3.33E-01	0.15	4.56E-01	0.37	5.43E-02	-0.03	8.68E-01	-0.32	9.81E-02	0.30	1.33E-01	0.32	9.98E-02	0.00	9.93E-01	-0.23	2.39E-01	0.07	7.13E-01
ENSCAFG0000000512	SFMBT2	grey	VSMC_M1C	0.19	3.33E-01	0.59	1.26E-03	0.18	3.58E-01	0.23	2.58E-01	-0.16	4.34E-01	0.00	9.95E-01	-0.45	1.83E-02	-0.44	2.28E-02	-0.53	4.22E-03	0.42	3.00E-02
ENSCAFG0000000309	COMMD1	pink	VSMC_M5	0.19	3.34E-01	-0.47	1.36E-02	-0.34	8.03E-02	-0.32	1.05E-01	0.53	4.46E-03	-0.10	6.04E-01	0.37	5.78E-02	0.46	1.64E-02	0.29	1.40E-01	-0.51	6.99E-03
ENSCAFG0000001167	TMEM200B	darkgrey	VSMC_M8	0.19	3.34E-01	-0.18	3.70E-01	0.25	2.06E-01	-0.60	9.92E-04	-0.09	6.40E-01	0.05	8.06E-01	0.00	9.93E-01	0.62	5.99E-04	0.03	8.76E-01	-0.39	4.49E-02
ENSCAFG0000000310	EHBP1	grey	VSMC_M1C	0.19	3.34E-01	0.06	7.72E-01	0.18	3.76E-01	-0.40	3.81E-02	-0.09	6.58E-01	0.00	9.84E-01	0.14	4.95E-01	0.37	5.95E-02	-0.26	1.88E-01	-0.10	6.24E-01
ENSCAFG0000003065	CHSY3	grey	VSMC_M1C	0.19	3.35E-01	-0.19	3.52E-01	-0.38	5.11E-02	0.33	9.76E-02	0.41	3.57E-02	0.12	5.68E-01	-0.32	1.04E-01	-0.19	3.31E-01	0.13	5.02E-01	0.01	9.56E-01
ENSCAFG0000001014	ELK4	grey	VSMC_M1C	0.19	3.35E-01	0.49	9.39E-03	0.41	3.17E-02	-0.20	3.07E-01	-0.42	3.13E-02	0.21	2.89E-01	0.04	8.58E-01	-0.05	8.13E-01	-0.50	7.71E-03	0.36	6.45E-02
ENSCAFG0000002315	ENSCAFG0000002315	cyan	VSMC_M2	0.19	3.35E-01	0.68	9.46E-05	0.01	9.51E-01	0.49	1.01E-02	0.01	9.48E-01	-0.23	2.52E-01	-0.81	3.29E-07	-0.64	3.03E-04	-0.55	3.23E-03	0.49	9.60E-03
ENSCAFG0000000215	ENSCAFG0000000215	grey	VSMC_M1C	0.19	3.36E-01	-0.24	2.26E-01	-0.11	5.89E-01	-0.23	2.59E-01	0.36	6.34E-02	-0.13	5.17E-01	0.29	1.39E-01	0.26	1.92E-01	0.13	5.19E-01	-0.52	5.20E-03
ENSCAFG0000001435	NMD3	darkgrey	VSMC_M8	0.19	3.36E-01	-0.53	4.20E-03	0.01	9.49E-01	-0.54	3.60E-03	0.21	3.04E-01	0.05	7.93E-01	0.13	5.19E-01	0.70	4.65E-05	0.34	8.21E-02	-0.70	4.30E-05
ENSCAFG0000001305	ATG4B	cyan	VSMC_M2	0.19	3.36E-01	0.59	1.30E-03	0.42	3.00E-02	0.26	1.85E-01	-0.49	9.60E-03	-0.06	7.82E-01	-0.27	1.73E-01	-0.39	4.54E-02	-0.58	1.46E-03	0.59	1.22E-03
ENSCAFG0000001831	RAD51D	grey	VSMC_M1C	0.19	3.36E-01	0.35	7.40E-02	0.25	2.03E-01	0.33	9.59E-02	-0.23	2.52E-01	-0.08	7.02E-01	0.01	9.64E-01	-0.40	3.87E-02	-0.34	7.94E-02	0.38	5.27E-02
ENSCAFG0000002589	ENSCAFG0000002589	yellow	VSMC_M3	0.19	3.36E-01	0.31	1.17E-01	0.56	2.57E-03	-0.11	5.89E-01	-0.49	9.84E-03	0.02	9.24E-01	0.17	3.97E-01	-0.05	7.96E-01	-0.35	7.25E-02	0.18	3.63E-01
ENSCAFG0000001070	CCDC28B	yellow	VSMC_M3	0.19	3.36E-01	0.34	8.64E-02	0.44	2.05E-02	0.13	5.29E-01	-0.39	4.32E-02	0.04	8.55E-01	-0.07	7.19E-01	-0.26	1.93E-01	-0.31	1.13E-01	0.29	1.37E-01
ENSCAFG0000003185	CBX8	grey	VSMC_M1C	0.19	3.36E-01	0.15	4.65E-01	-0.36	6.61E-02	0.33	8.80E-02	0.26	1.91E-01	0.06	7.74E-01	0.18	3.74E-01	-0.26	1.90E-01	-0.27	1.71E-01	0.42	2.90E-02
ENSCAFG0000001561	TULP3	grey	VSMC_M1C	0.19	3.36E-01	0.32	1.04E-01	-0.30	1.26E-01	0.38	4.78E-02	0.27	1.74E-01	-0.18	3.75E-01	0.17	4.04E-01	-0.41	3.34E-02	-0.38	5.09E-02	0.50	8.07E-03
ENSCAFG0000000927	CRTAC1	grey	VSMC_M1C	0.19	3.37E-01	-0.19	3.45E-01	0.31	1.18E-01	-0.47	1.43E-02	-0.20	3.08E-01	0.40	3.79E-02	0.47	1.25E-02	0.39	4.21E-02	0.01	9.52E-01	-0.19	3.34E-01
ENSCAFG0000000531	ENSCAFG0000000531	turquoise	VSMC_M6	0.19	3.37E-01	0.00	1.00E+00	-0.03	8.86E-01	0.04	8.39E-01	0.05	8.07E-01	0.14	4.91E-01	-0.22	2.77E-01	0.01	9.53E-01	-0.14	4.86E-01	0.03	9.01E-01
ENSCAFG0000003158	ENSCAFG0000003158	cyan	VSMC_M2	0.19	3.37E-01	0.54	3.38E-03	0.07	7.23E-01	0.19	3.50E-01	0.01	9.73E-01	0.00	9.84E-01	-0.29	1.43E-01	-0.44	2.12E-02	-0.40	4.12E-02	0.33	8.80E-02
ENSCAFG0000001921	ENSCAFG0000001921	darkgrey	VSMC_M8	0.19	3.37E-01	-0.03	8.81E-01	0.62	5.39E-04	-0.76	3.90E-06	-0.44	2.06E-02	0.20	3.25E-01	0.21	3.02E-01	0.71	3.78E-05	-0.11	5.75E-01	-0.41	3.51E-02
ENSCAFG0000002320	ENSCAFG0000002320	yellow	VSMC_M3	0.19	3.37E-01	0.56	2.32E-03	0.81	3.28E-07	-0.48	1.15E-02	-0.75	7.69E-06	0.02	9.28E-01	0.25	2.10E-01	0.15	4.59E-01	-0.60	1.05E-03	0.26	1.92E-01
ENSCAFG0000002468	AGO3	pink	VSMC_M5	0.19	3.38E-01	-0.33	9.58E-02	-0.41	3.37E-02	-0.02	9.12E-01	0.66	1.80E-04	-0.15	4.42E-01	-0.01	9.45E-01	0.08	6.83E-01	0.36	6.73E-02	-0.47	1.33E-02
ENSCAFG0000001023	ARHGAP32	grey	VSMC_M1C	0.19	3.38E-01	0.08	6.82E-01	-0.05	8.18E-01	-0.03	8.88E-01	0.08	6.98E-01	-0.10	6.32E-01	0.43	2.57E-02	-0.02	9.05E-01	-0.20	3.05E-01	0.19	3.39E-01
ENSCAFG0000000953	SSR1	pink	VSMC_M5	0.19	3.38E-01	-0.46	1.49E-02	-0.30	1.28E-01	-0.07	7.26E-01	0.47	1.24E-02	-0.08	6.77E-01	-0.13	5.28E-01	0.33	8.93E-02	0.31	1.15E-01	-0.54	3.42E-03
ENSCAFG0000000436	ACSS1	cyan	VSMC_M2	0.19	3.38E-01	0.86	1.27E-08	0.54	3.60E-03	0.05	8.06E-01	-0.48	1.07E-02	-0.09	6.57E-01	-0.17	4.07E-01	-0.37	5.49E-02	-0.80	4.71E-07	0.50	8.41E-03
ENSCAFG0000000297	ENSCAFG0000000297	darkgreen	VSMC_M4	0.19	3.38E-01	0.17	3.84E-01	-0.39	4.37E-02	0.75	5.80E-06	0.41	3.24E-02	-0.13	5.24E-01	-0.22	2.69E-01	-0.65	2.45E-04	-0.16	4.17E-01	0.30	1.35E-01
ENSCAFG0000001139	CLDN34	grey	VSMC_M1C	0.19	3.38E-01	0.25	2.10E-01	-0.17	3.88E-01	0.58	1.38E-03	0.05	8.09E-01	0.17	3.89E-01	-0.27	1.78E-01	-0.49	9.62E-03	-0.29	1.45E-01	0.50	7.30E-03
ENSCAFG0000002954	UMAD1	grey	VSMC_M1C	0.19	3.38E-01	0.35	7.20E-02	-0.12	5.63E-01	0.44	2.17E-02	-0.04	8.42E-01	0.01	9.44E-01	-0.02	9.29E-01	-0.46	1.59E-02	-0.40	3.77E-02	0.69	7.36E-05
ENSCAFG0000001954	TELO2	yellow	VSMC_M3	0.19	3.39E-01	0.49	9.25E-03	0.83	7.27E-08	-0.37	5.83E-02	-0.77	2.71E-06	0.15	4.70E-01	0.08	7.02E-01	0.15	4.52E-01	-0.57	1.93E-03	0.23	2.50E-01
ENSCAFG0000001504	DDOST	grey	VSMC_M1C	0.19	3.39E-01	0.36	6.63E-02	0.03	8.94E-01	0.32	1.06E-01	-0.01	9.54E-01	0.00	9.97E-01	-0.51	6.16E-03	-0.34	8.53E-02	-0.34	8.39E-02	0.24	2.35E-01
ENSCAFG0000003138	ZNF555	grey	VSMC_M1C	0.19	3.39E-01	0.06	7.71E-01	-0.15	4.64E-01	0.19	3.51E-01	0.16	4.19E-01	-0.14	4.98E-01	-0.10	6.09E-01	-0.10	6.14E-01	-0.14	4.72E-01	0.18	3.70E-01
ENSCAFG0000001589	NSUN7	grey	VSMC_M1C	0.19	3.39E-01	-0.41	3.16E-02	-0.44	2.16E-02	0.11	5.69E-01	0.57	1.76E-03	-0.22	2.81E-01	0.15	4.60E-01	0.05	8.14E-01	0.30	1.24E-01	-0.30	1.28E-01
ENSCAFG0000000499	GPR39	darkgreen	VSMC_M4	0.19	3.39E-01	0.45	1.95E-02	-0.22	2.73E-01	0.78	1.41E-06	0.17	3.91E-01	-0.17	3.97E-01	-0.33	9.20E-02	-0.75	8.04E-06	-0.41	3.36E-02	0.55	2.72E-03
ENSCAFG0000001762	CNOT8	grey	VSMC_M1C	0.19	3.39E-01	-0.27	1.67E-01	-0.15	4.54E-01	-0.36	6.57E-02	0.38	5.38E-02	0.07	7.39E-01	-0.08	6.86E-01	0.37	6.07E-02	0.24	2.37E-01	-0.51	6.70E-03
ENSCAFG0000001872	ENSCAFG0000001872	yellow	VSMC_M3	0.19	3.39E-01	0.21	2.92E-01	0.68	1.04E-04	-0.35	7.00E-02	-0.53	4.58E-03	0.14	4.87E-01	0.19	3.52E-01	0.15	4.59E-01	-0.26	1.92E-01	-0.06	7.71E-01
ENSCAFG0000000602	EFNB2	cyan	VSMC_M2	0.19	3.39E-01	0.60	1.03E-03	-0.14	4.82E-01	0.76	4.18E-06	0.04	8.35E-01	0.07	7.34E-01	-0.47	1.30E-02	-0.78	1.49E-06	-0.55	3.12E-03	0.69	6.76E-05
ENSCAFG0000003222	ENSCAFG0000003222	grey	VSMC_M1C	0.19	3.39E-01	-0.49	9.04E-03	-0.06	7.64E-01	-0.35	7.78E-02	0.21	2.87E-01	-0.09	6.48E-01	0.24	2.33E-01	0.51	6.86E-03	0.32	1.06E-01	-0.51	6.54E-03
ENSCAFG0000001350	LTBP3	cyan	VSMC_M2	0.19	3.39E-01	0.84	4.72E-08	0.28	1.56E-01	0.44	2.27E-02	-0.23	2.38E-01	-0.15	4.62E-01	-0.48	1.13E-02	-0.73	1.81E-05	-0.64	3.60E-04	0.61	7.43E-04
ENSCAFG0000001967	RAB11FIP3	cyan	VSMC_M2	0.19	3.39E-01	0.72	2.21E-05	0.55	2.89E-03	0.09	6.50E-01	-0.51	6.58E-03	0.04	8.58E-01	0.04	8.28E-01	-0.33	8.96E-02	-0.73	1.38E-05	0.50	7.52E-03
ENSCAFG0000001363	B2M	cyan	VSMC_M2	0.19	3.39E-01	0.84	4.13E-08	0.07	7.14E-01	0.62	5.83E-04	-0.14	4.93E-01	-0.03	8.73E-01	-0.48	1.05E-02	-0.80	5.47E-07	-0.74	9.40E-06	0.75	8.16E-06
ENSCAFG0000003240	ZSCAN1	yellow	VSMC_M3	0.19	3.39E-01	0.46	1.52E-02	0.59	1.31E-03	-0.07	7.22E-01	-0.54	3.67E-03	0.13	5.19E-01	-0.21	3.01E-01	-0.12	5.41E-01	-0.47	1.24E-02	0.25	2.10E-01
ENSCAFG0000003111	COA1	grey	VSMC_M1C	0.19	3.39E-01	0.18	3.58E-01	-0.16	4.14E-01	0.35	7.28E-02	0.36	6.34E-02	-0.40	3.75E-02	-0.20	3.28E-01	-0.43	2.51E-02	-0.08	6.78E-01	-0.03	8.79E-01
ENSCAFG0000000681	RB1CC1	pink	VSMC_M5	0.19	3.40E-01	0.33	9.66E-02	-0.40	3.93E-02	0.55	2.68E-03	0.55	3.17E-03	-0.45	1.98E-02	-0.37	5.93E-02	-0.67	1.52E-04	-0.18	3.56E-01	0.26	1.86E-01
ENSCAFG0000000585	TMCO6	grey	VSMC_M1C	0.19	3.40E-01	0.17	3.86E-01	0.12	5.41E-01	-0.20	3.28E-01	0.07	7.13E-01	-0.19	3.31E-01	0.07	7.25E-01	-0.02	9.30E-01	-0.13	5.18E-01	-0.08	6.87E-01
ENSCAFG0000002014	ENSCAFG0000002014	grey	VSMC_M1C	0.19	3.41E-01	0.16	4.28E-01	-0.09	6.46E-01	0.22	2.68E-01	0.22	2.81E-01	-0.40	3.98E-02	0.02	9.08E-01	-0.26	1.97E-01	-0.20	3.12E-01	0.09	6.47E-01
ENSCAFG0000001859	ARHGAP28	darkgreen	VSMC_M4	0.19	3.41E-01	0.56	2.24E-03	-0.32	1.00E-01	0.81	3.40E-07	0.28	1.56E-01	-0.13	5.04E-01	-0.31	1.22E-01	-0.81	2.72E-07	-0.50	7.61E-03	0.62	5.26E-04
ENSCAFG0000000578	ANKHD1	cyan	VSMC_M2	0.19	3.41E-01	0.66	1.80E-04	0.05	7.92E-01	0.30	1.33E-01	-0.03	8.88E-01	-0.16	4.38E-01	-0.17	4.09E-01	-0.52	5.68E-03	-0.60	8.38E-04	0.57	1.98E-03
ENSCAFG0000003154	PRELID2	darkgreen	VSMC_M4	0.19	3.41E-01	0.26	1.85E-01	-0.43	2.63E-02	0.65	2.10E-04	0.38	5.30E-02	0.09	6.39E-01	-0.32	1.09E-01	-0.64	3.38E-04	-0.25	2.16E-01	0.44	2.24E-02
ENSCAFG0000002493	MTUS2	grey	VSMC_M1C	0.19	3.41E-01	-0.17	4.02E-01	0.41	3.60E-02	-0.62	6.10E-04	-0.25	2.10E-01	0.28	1.63E-01	-0.06	7.68E-01	0.51	7.10E-03	0.12	5.60E-01	-0.38	4.96E-02
ENSCAFG0000002490	ZSCAN12	grey	VSMC_M1C	0.19	3.41E-01	0.00	9.95E-01	0.30	1.27E-01	-0.07	7.35E-01	-0.17	4.06E-01	0.19	3.53E-01	-0.06	7.68E-01	0.07	7.43E-01	-0.06	7.63E-01	-0.06	7.77E-01
ENSCAFG0000000177	WASL	pink	VSMC_M5	0.19	3.41E-01	-0.18	3.58E-01	-0.77	3.00E-06	0.60	9.13E-04	0.82	1.90E-07	-0.25	2.04E-01	-0.16	4.40E-01	-0.39	4.59E-02	0.13	5.12E-01	0.07	7.13E-01
ENSCAFG0000001497	EIF4G3	darkgreen	VSMC_M4	0.19	3.41E-01	0.55	3.24E-03	-0.42	3.07E-02	0.80	5.43E-07	0.40	3.89E-02	-0.13	5.05E-01	-0.46	1.50E-02	-0.86	8.59E-09	-0.45	1.86E-02	0.58	1.37E-03
ENSCAFG0000000957	DSP	grey	VSMC_M1C	0.19	3.41E-01	0.32	1.07E-01	-0.15	4.51E-01	0.35	7.27E-02	0.13	5.08E-01	-0.18	3.70E-01	0.03	8.74E-01	-0.41	3.26E-02	-0.31	1.20E-01	0.37	5.69E-02
ENSCAFG0000001736	KANK2	cyan	VSMC_M2	0.19	3.42E-01	0.87	4.28E-09	0.24	2.35E-01	0.44	2.06E-02	-0.20	3.19E-01	-0.21	2.84E-01	-0.34	8.54E-02	-0.73	1.38E-05	-0.69	7.85E-05	0.65	2.23E-04
ENSCAFG0000000267	TSEN34	grey	VSMC_M1C	0.19	3.42E-01	0.11	5.99E-01	0.65	2.61E-04	-0.67	1.41E-04	-0.43	2.38E-02	0.08	6.90E-01	0.11	5.69E-01	0.57	1.89E-03	-0.19	3.41E-01	-0.38	4.97E-02

ENSCAFG00000026039	ENSCAFG00000026039	grey	VSMC_M10	0.19	3.43E-01	-0.04	8.40E-01	-0.38	5.07E-02	0.41	3.25E-02	0.40	3.76E-02	-0.17	3.96E-01	0.17	3.96E-01	-0.31	1.18E-01	0.02	9.29E-01	0.13	5.19E-01
ENSCAFG00000031107	ENSCAFG00000031107	grey	VSMC_M10	0.19	3.43E-01	0.07	7.38E-01	0.09	6.46E-01	-0.16	4.16E-01	0.03	9.01E-01	-0.05	7.96E-01	0.22	2.67E-01	0.11	5.76E-01	-0.09	6.59E-01	-0.03	8.91E-01
ENSCAFG00000002106	FEM1C	grey	VSMC_M10	0.19	3.43E-01	0.05	7.96E-01	0.06	7.58E-01	-0.16	4.18E-01	0.05	7.86E-01	0.06	7.81E-01	-0.04	8.35E-01	0.07	7.29E-01	-0.08	6.82E-01	-0.08	6.93E-01
ENSCAFG00000004830	ENSCAFG00000004830	grey	VSMC_M10	0.19	3.43E-01	0.17	3.93E-01	0.48	1.08E-02	-0.15	4.70E-01	-0.33	9.21E-02	-0.11	5.93E-01	0.10	6.31E-01	-0.06	7.57E-01	-0.15	4.58E-01	0.00	9.98E-01
ENSCAFG00000026214	ENSCAFG00000026214	grey	VSMC_M10	0.19	3.43E-01	0.25	2.11E-01	0.42	2.73E-02	-0.17	3.92E-01	-0.43	2.53E-02	0.37	5.71E-02	0.14	4.84E-01	0.17	4.05E-01	-0.41	3.36E-02	0.22	2.69E-01
ENSCAFG00000003471	ENSCAFG00000003471	grey	VSMC_M10	0.19	3.43E-01	-0.12	5.43E-01	-0.22	2.80E-01	0.06	7.51E-01	0.29	1.37E-01	-0.02	9.40E-01	0.15	4.49E-01	0.03	8.88E-01	0.03	8.88E-01	-0.01	9.47E-01
ENSCAFG00000014441	TSC22D4	cyan	VSMC_M2	0.19	3.44E-01	0.66	1.86E-04	0.24	2.27E-01	0.36	6.34E-02	-0.24	2.22E-01	0.04	8.40E-01	-0.27	1.68E-01	-0.55	3.04E-03	-0.58	1.56E-03	0.56	2.39E-03
ENSCAFG00000014569	PDCD10	grey	VSMC_M10	0.19	3.44E-01	-0.41	3.14E-02	-0.08	6.76E-01	-0.08	6.98E-01	0.26	1.90E-01	0.09	6.65E-01	-0.15	4.47E-01	0.19	3.52E-01	0.38	4.79E-02	-0.44	2.24E-02
ENSCAFG00000007264	CSTF3	grey	VSMC_M10	0.19	3.44E-01	-0.11	5.71E-01	0.00	9.88E-01	-0.19	3.30E-01	0.15	4.42E-01	-0.09	6.69E-01	0.32	1.07E-01	0.17	3.84E-01	-0.06	7.75E-01	-0.19	3.49E-01
ENSCAFG00000013638	ENSCAFG00000013638	grey	VSMC_M10	0.19	3.44E-01	0.26	1.90E-01	0.26	1.85E-01	-0.13	5.32E-01	-0.09	6.71E-01	0.02	9.20E-01	-0.09	6.52E-01	0.03	8.84E-01	-0.29	1.42E-01	-0.09	6.54E-01
ENSCAFG00000018031	KNOP1	grey	VSMC_M10	0.19	3.44E-01	-0.13	5.25E-01	0.29	1.37E-01	-0.49	9.26E-03	-0.15	4.52E-01	-0.01	9.65E-01	0.15	4.62E-01	0.43	2.58E-02	0.01	9.66E-01	-0.35	7.46E-02
ENSCAFG00000006834	PSMF1	grey	VSMC_M10	0.19	3.44E-01	0.35	7.67E-02	0.06	7.84E-01	0.04	8.60E-01	0.03	8.81E-01	0.07	7.30E-01	0.01	9.62E-01	-0.18	3.71E-01	-0.36	6.87E-02	0.13	5.34E-01
ENSCAFG00000014230	WDR61	grey	VSMC_M10	0.19	3.44E-01	-0.30	1.24E-01	-0.03	8.99E-01	-0.18	3.75E-01	0.07	7.45E-01	0.16	4.33E-01	0.01	9.65E-01	0.29	1.46E-01	0.17	4.07E-01	-0.25	2.04E-01
ENSCAFG00000000450	TSPAN8	grey	VSMC_M10	0.19	3.45E-01	0.29	1.42E-01	0.19	3.49E-01	-0.13	5.22E-01	-0.10	6.28E-01	-0.08	7.04E-01	0.01	9.56E-01	0.04	8.40E-01	-0.30	1.35E-01	0.13	5.14E-01
ENSCAFG00000005481	ARHGEF40	yellow	VSMC_M3	0.19	3.45E-01	0.55	2.89E-03	0.68	8.62E-05	-0.15	4.58E-01	-0.62	5.47E-04	0.17	4.02E-01	-0.11	5.72E-01	-0.16	4.36E-01	-0.50	8.25E-03	0.30	1.28E-01
ENSCAFG00000013030	PDHA1	darkgrey	VSMC_M8	0.19	3.45E-01	-0.39	4.51E-02	0.31	1.21E-01	-0.80	6.50E-07	-0.09	6.58E-01	0.13	5.34E-01	0.48	1.10E-02	0.84	3.98E-08	0.20	3.12E-01	-0.63	3.79E-04
ENSCAFG00000004731	DGKH	pink	VSMC_M5	0.19	3.45E-01	-0.28	1.55E-01	-0.40	3.78E-02	0.11	5.91E-01	0.50	7.96E-03	0.18	3.76E-01	0.15	4.50E-01	0.00	9.99E-01	0.19	3.48E-01	-0.19	3.45E-01
ENSCAFG0000001850	MPRIP	grey	VSMC_M10	0.19	3.45E-01	-0.06	7.55E-01	0.05	8.22E-01	0.04	8.24E-01	0.23	2.41E-01	-0.09	6.68E-01	-0.23	2.53E-01	-0.18	3.64E-01	0.19	3.30E-01	-0.30	1.34E-01
ENSCAFG00000002070	FAM32A	grey	VSMC_M10	0.19	3.45E-01	0.12	5.37E-01	0.16	4.39E-01	-0.20	3.28E-01	0.01	9.45E-01	0.19	3.38E-01	-0.07	7.18E-01	0.10	6.29E-01	-0.17	3.95E-01	-0.20	3.24E-01
ENSCAFG00000018140	TADA2A	darkgrey	VSMC_M8	0.19	3.45E-01	-0.33	9.38E-02	0.25	2.01E-01	-0.68	9.11E-05	0.00	9.97E-01	0.01	9.50E-01	0.47	1.42E-02	0.66	1.60E-04	0.16	4.18E-01	-0.55	2.68E-03
ENSCAFG00000032621	ENSCAFG00000032625	darkgrey	VSMC_M8	0.19	3.46E-01	-0.11	5.84E-01	0.49	1.00E-02	-0.69	6.11E-05	-0.25	2.13E-01	-0.05	7.91E-01	-0.19	3.46E-01	0.58	1.44E-03	0.07	7.32E-01	-0.52	5.84E-03
ENSCAFG00000009702	FAM160B2	grey	VSMC_M10	0.19	3.46E-01	0.44	2.04E-02	-0.04	8.56E-01	0.38	5.15E-02	-0.12	5.63E-01	0.13	5.09E-01	-0.06	7.59E-01	-0.41	3.40E-02	-0.51	6.20E-03	0.68	9.96E-05
ENSCAFG00000008087	PINX1	yellow	VSMC_M3	0.19	3.46E-01	0.09	6.64E-01	0.54	3.53E-03	-0.53	4.71E-03	-0.55	3.27E-03	0.26	1.85E-01	0.39	4.23E-02	0.43	2.47E-02	-0.27	1.74E-01	0.06	7.62E-01
ENSCAFG00000030050	CCSAP	grey	VSMC_M10	0.19	3.46E-01	-0.39	4.60E-02	-0.02	9.09E-01	-0.23	2.49E-01	0.13	5.23E-01	-0.03	8.82E-01	0.43	2.36E-02	0.48	1.11E-02	0.13	5.34E-01	-0.32	1.09E-01
ENSCAFG00000014817	ISYNA1	yellow	VSMC_M3	0.19	3.46E-01	-0.21	2.93E-01	0.38	5.09E-02	-0.64	3.57E-04	-0.17	3.90E-01	0.19	3.52E-01	0.55	2.98E-03	0.55	3.19E-03	0.10	6.14E-01	-0.40	3.80E-02
ENSCAFG00000006511	PTDSS2	yellow	VSMC_M3	0.19	3.46E-01	0.51	6.20E-03	0.65	2.49E-04	-0.20	3.09E-01	-0.57	2.07E-03	0.18	3.72E-01	-0.10	6.18E-01	-0.02	9.35E-01	-0.54	3.38E-03	0.16	4.13E-01
ENSCAFG0000000775	DENND6A	grey	VSMC_M10	0.19	3.46E-01	0.16	4.14E-01	-0.25	2.04E-01	0.48	1.20E-02	0.22	2.77E-01	0.12	5.64E-01	-0.24	2.34E-01	-0.47	1.42E-02	-0.10	6.33E-01	0.33	9.27E-02
ENSCAFG00000018021	GP2	darkgreen	VSMC_M4	0.19	3.46E-01	0.20	3.18E-01	-0.43	2.58E-02	0.73	1.83E-05	0.41	3.42E-02	-0.13	5.07E-01	-0.57	1.79E-03	-0.68	9.33E-05	-0.10	6.23E-01	0.33	9.07E-02
ENSCAFG00000004630	BCAM	grey	VSMC_M10	0.19	3.46E-01	0.61	6.45E-04	0.17	4.07E-01	0.50	7.47E-03	-0.10	6.13E-01	-0.05	8.02E-01	-0.42	2.93E-02	-0.68	8.93E-05	-0.46	1.46E-02	0.42	2.77E-02
ENSCAFG00000023490	ZBTB43	red	VSMC_M1	0.19	3.47E-01	0.03	8.74E-01	0.14	5.01E-01	-0.04	8.46E-01	-0.13	5.08E-01	0.31	1.10E-01	0.08	7.10E-01	0.02	9.20E-01	-0.10	6.19E-01	0.06	7.48E-01
ENSCAFG00000029401	ENSCAFG00000029401	yellow	VSMC_M3	0.19	3.47E-01	0.44	2.19E-02	0.92	2.02E-11	-0.50	8.29E-03	-0.86	1.29E-08	0.10	6.31E-01	0.14	4.84E-01	0.27	1.74E-01	-0.51	6.39E-03	0.16	4.19E-01
ENSCAFG00000007608	SH3RF1	darkgreen	VSMC_M4	0.19	3.47E-01	0.20	3.20E-01	-0.33	9.35E-02	0.62	5.20E-04	0.28	1.65E-01	0.18	3.70E-01	-0.41	3.37E-02	-0.51	6.60E-03	-0.24	2.32E-01	0.37	5.85E-02
ENSCAFG00000009770	ZSWIM1	grey	VSMC_M10	0.19	3.47E-01	0.20	3.05E-01	0.36	6.39E-02	-0.21	2.96E-01	-0.42	3.10E-02	0.15	4.49E-01	0.20	3.26E-01	0.16	4.11E-01	-0.32	1.06E-01	0.30	1.25E-01
ENSCAFG00000021715	ENSCAFG00000021715	grey	VSMC_M10	0.19	3.47E-01	-0.15	4.68E-01	-0.13	5.32E-01	-0.16	4.24E-01	0.41	3.56E-02	-0.06	7.65E-01	0.04	8.39E-01	0.13	5.12E-01	0.12	5.63E-01	-0.48	1.21E-02
ENSCAFG00000020497	ENSCAFG00000020497	grey	VSMC_M10	0.19	3.47E-01	-0.15	4.68E-01	-0.13	5.32E-01	-0.16	4.24E-01	0.41	3.57E-02	-0.06	7.66E-01	0.04	8.39E-01	0.13	5.12E-01	0.12	5.63E-01	-0.48	1.21E-02
ENSCAFG00000029970	TMEM250	cyan	VSMC_M2	0.19	3.48E-01	0.65	2.27E-04	0.33	9.38E-02	0.05	7.93E-01	-0.38	4.79E-02	0.03	8.68E-01	0.06	7.53E-01	-0.19	3.32E-01	-0.73	1.33E-05	0.57	1.85E-03
ENSCAFG0000000339	ENSCAFG0000000339	grey	VSMC_M10	0.19	3.48E-01	-0.20	3.27E-01	0.12	5.44E-01	-0.27	1.78E-01	0.10	6.15E-01	-0.24	2.36E-01	0.08	7.09E-01	0.24	2.27E-01	0.18	3.75E-01	-0.48	1.19E-02
ENSCAFG00000017018	COL1A1	darkgrey	VSMC_M8	0.19	3.48E-01	-0.28	1.50E-01	0.42	3.13E-02	-0.67	1.40E-04	-0.22	2.64E-01	0.14	4.75E-01	0.30	1.34E-01	0.57	1.72E-03	0.15	4.66E-01	-0.45	2.00E-02
ENSCAFG00000005111	CYGB	grey	VSMC_M10	0.19	3.48E-01	0.34	7.96E-02	0.24	2.23E-01	-0.16	4.34E-01	-0.18	3.56E-01	-0.08	7.03E-01	0.22	2.73E-01	0.07	7.40E-01	-0.37	5.77E-02	0.19	3.54E-01
ENSCAFG00000000521	SNCAIP	grey	VSMC_M10	0.19	3.49E-01	-0.24	2.25E-01	0.32	1.05E-01	-0.47	1.23E-02	-0.07	7.47E-01	-0.25	2.02E-01	0.38	5.11E-02	0.47	1.37E-02	0.18	3.78E-01	-0.47	1.35E-02
ENSCAFG00000006651	ADGRD1	cyan	VSMC_M2	0.19	3.49E-01	0.77	2.72E-06	-0.02	9.11E-01	0.65	2.58E-04	0.02	9.24E-01	-0.24	2.25E-01	-0.23	2.43E-01	-0.83	1.03E-07	-0.65	2.16E-04	0.68	9.95E-05
ENSCAFG0000002954	ATF5	grey	VSMC_M10	0.19	3.49E-01	0.54	3.36E-03	0.09	6.65E-01	0.29	1.46E-01	-0.15	4.48E-01	-0.10	6.28E-01	0.15	4.48E-01	-0.42	2.99E-02	-0.63	3.86E-04	0.59	1.12E-03
ENSCAFG00000007021	NAT10	yellow	VSMC_M3	0.19	3.49E-01	0.05	7.97E-01	0.65	2.68E-04	-0.51	6.13E-03	-0.52	5.05E-03	0.22	2.78E-01	0.29	1.44E-01	0.48	1.15E-02	-0.22	2.72E-01	-0.22	2.65E-01
ENSCAFG00000012740	DIDO1	grey	VSMC_M10	0.19	3.49E-01	0.18	3.67E-01	0.20	3.24E-01	-0.31	1.10E-01	-0.06	7.80E-01	0.14	4.97E-01	0.20	3.22E-01	0.18	3.80E-01	-0.31	1.15E-01	-0.04	8.32E-01
ENSCAFG00000029141	BEND3	grey	VSMC_M10	0.19	3.49E-01	0.33	9.33E-02	0.28	1.56E-01	-0.23	2.47E-01	-0.24	2.36E-01	0.40	4.13E-02	-0.22	2.66E-01	0.13	5.17E-01	-0.40	4.09E-02	0.10	6.35E-01
ENSCAFG00000009551	CRACR2B	cyan	VSMC_M2	0.19	3.50E-01	0.55	2.85E-03	0.40	4.03E-02	0.27	1.68E-01	-0.40	3.72E-02	0.14	4.79E-01	0.01	9.58E-01	-0.48	1.21E-02	-0.50	7.25E-03	0.48	1.05E-02
ENSCAFG00000011057	TRAIP	darkgrey	VSMC_M8	0.19	3.50E-01	-0.47	1.28E-02	0.19	3.54E-01	-0.61	6.53E-04	-0.07	7.33E-01	0.06	7.74E-01	0.48	1.21E-02	0.77	2.89E-06	0.22	2.73E-01	-0.47	1.24E-02
ENSCAFG00000018620	GPR108	grey	VSMC_M10	0.19	3.50E-01	0.77	2.23E-06	0.12	5.42E-01	0.54	3.43E-03	-0.13	5.04E-01	-0.19	3.48E-01	-0.35	6.96E-02	-0.76	3.37E-06	-0.65	2.62E-04	0.67	1.20E-04
ENSCAFG00000006663	KBTBD8	grey	VSMC_M10	0.19	3.50E-01	0.26	1.93E-01	-0.14	4.74E-01	0.18	3.58E-01	0.08	6.80E-01	0.12	5.44E-01	0.29	1.44E-01	-0.21	2.99E-01	-0.41	3.42E-02	0.41	3.28E-02
ENSCAFG00000009289	SRSF6	grey	VSMC_M10	0.19	3.50E-01	0.18	3.69E-01	0.37	5.85E-02	-0.38	4.79E-02	-0.33	9.56E-02	0.29	1.37E-01	0.24	2.23E-01	0.36	6.74E-02	-0.35	7.14E-02	0.07	7.45E-01
ENSCAFG00000029410	ENSCAFG00000029410	pink	VSMC_M5	0.19	3.50E-01	-0.38	5.37E-02	-0.48	1.09E-02	0.12	5.35E-01	0.61	6.54E-04	-0.37	5.70E-02	-0.16	4.29E-01	0.02	9.38E-01	0.32	9.94E-02	-0.27	1.79E-01
ENSCAFG00000004757	PHLDB3	cyan	VSMC_M2	0.19	3.50E-01	0.48	1.05E-02	0.23	2.56E-01	0.22	2.62E-01	-0.28	1.59E-01	-0.05	7.99E-01	-0.03	9.00E-01	-0.27	1.65E-01	-0.50	8.62E-03	0.48	1.05E-02
ENSCAFG00000019257	HIC1	yellow	VSMC_M3	0.19	3.51E-01	0.03	9.00E-01	0.62	6.22E-04	-0.58	1.65E-03	-0.52	5.32E-03	0.29	1.39E-01	0.10	6.19E-01	0.50	7.42E-03	-0.18	3.82E-01	-0.20	3.25E-01
ENSCAFG00000013920	ABL2	grey	VSMC_M10	0.19	3.51E-01	-0.06	7.82E-01	0.35	7.40E-02	-0.45	1.72E-02	-0.15	4.59E-01	0.00	9.86E-01	-0.07	7.14E-01	0.39	4.32E-02	-0.08	6.89E-01	-0.38	5.03E-02
ENSCAFG00000032594	PEX12	grey	VSMC_M10	0.19	3.51E-01	-0.05	7.99E-01	-0.02	9.32E-01	0.26	1.88E-01	0.14	4.81E-01	-0.24	2.28E-01	0.03	8.72E-01	-0.24	2.28E-01	0.00	9.90E-01	-0.02	9.34E-01
ENSCAFG00000006927	PRR13	grey	VSMC_M10	0.19	3.51E-01	0.38	5.37E-02	-0.07	7.46E-01	0.45	1.73E-02	0.11	5.85E-01	-0.02	9.29E-01	-0.52	5.77E-03	-0.56	2.60E-03	-0.24	2.23E-01	0.27	1.74E-01
ENSCAFG0000000227	PHF14	grey	VSMC_M10	0.19	3.52E-01	0.39	4.66E-02	-0.03	9.01E-01	0.30	1.25E-01	0.14	4.84E-01	-0.38	5.35E-02	0.01	9.48E-01	-0.38	5.21E-02	-0.42	2.83E-02	0.26	1.95E-01
ENSCAFG00000005401	SEC23B	darkgrey	VSMC_M8	0.19	3.52E-01	-0.43	2.48E-02	-0.03	8.96E-01	-0.49	9.90E-03	0.24	2.21E-01	-0.17	3.96E-01	0.11	5.91E-01	0.55	2.76E-03	0.33	9.14E-02	-0.58	1.48E-03
ENSCAFG00000019204	CDIP1	grey	VSMC_M10	0.19	3.52E-01	0.55	2.66E-03	0.16	4.39E-01	0.23	2.41E-01	-0.15	4.48E-01	0.24	2.37E-01	-0.22	2.63E-01	-0.41	3.60E-02	-0.54	3.80E-03	0.41	3.19E-02
ENSCAFG00000010617	ALDH1A3	grey	VSMC_M10	0.19	3.52E-01	0.26	1.87E-01	0.43	2.62E-02	-0.27	1.81E-01	-0.43	2.51E-02	0.49	8.88E-03	-0.02	9.18E-01	0.09	6.64E-01	-0.30	1.24E-01	0.17	4.07E-01
ENSCAFG00000015047	IFFO1	grey	VSMC_M10	0.19	3.53E-01	0.36	6.65E-02	0.30	1.31E-01	-0.12	5.56E-01	-0.22	2.71E-01	-0.10	6.15E-01	0.16	4.34E-01	-0.08	6.80E-01	-0.42	3.02E-02	0.19	3.49E-01
ENSCAFG00000000417	NUP107	darkgrey	VSMC_M8	0.19	3.53E-01	-0.56	2.59E-03	0.20	3.20E-01	-0.63	3.74E-04	-0.04	8.28E-01	0.07	7.31E-01	0.40	3.78E-02	0.80	7.05E-07	0.30	1.27E-01	-0.61	6.93E-04
ENSCAFG00000015880	ENSCAFG00000015880	grey	VSMC_M10	0.19	3.53E-01	0.23	2.47E-01	-0.10	6.25E-01	0.25	2.04E-01	0.09	6.44E-01	-0.19	3.44E-01	0.00	9.99E-01	-0.33	9.36E-02	-0.26	1.96E-01	0.31	1.20E-01
ENSCAFG00000019897	SETX	pink	VSMC_M5	0.19	3.53E-01	0.04	8.57E-01	-0.49	9.35E-03	0.60	9.13E-04	0.57	2.12E-03	-0.15	4.41E-01	-0.09	6.68E-01	-0.54	3.54E-03	-0.04	8.53E-01	0.18	3.56E-01
ENSCAFG00000023527	ENSCAFG00000023527	grey	VSMC_M10	0.19	3.53E-01	-0.21	2.89E-01	0.12	5.37E-01	-0.08	6.96E-01	0.08	6.79E-01	-0.11	5.76E-01	0.09	6.69E-01	0.08	6.94E-01	0.17	3.84E-01	-0.40	3.88E-02
ENSCAFG00000005859	P4HB	yellow	VSMC_M3	0.19	3.54E-01	0.48	1.17E-02	0.77	2.08E-06	-0.44	2.24E-02	-0.62	6.36E-04	-0.19	3.49E-01	-0.26	1.93E-01	0.18	3.65E-01	-0.46	1.64E-02	0.00	9.81E-01
ENSCAFG00000012083	TUBGCP4	grey	VSMC_M10	0.19	3.54E-01	0.15	4.59E-01	0.32	1.06E-01	-0.14	4.71E-01	-0.31	1.10E-01	0.05	7.86E-01	0.28	1.59E-01	0.13	5.13E-01	-0.24	2.20E-01	0.12	5.41E-01
ENSCAFG00000016560	CC2D1A	grey	VSMC_M10	0.19	3.54E-01	0.46	1.47E-02	0.38	4.76E-02	-0.20	3.10E-01	-0.29	1.48E-01	-0.14	4.79E-01	0.03	8.71E-01	-0.05	8.09E-01	-0.48	1.20E-02	0.23	2.51E-01
ENSCAFG00000029095	ENSCAFG00000029095	grey	VSMC_M10	0.19	3.54E-01	0.34	8.37E-02	-0.12	5.62E-01	0.33	9.25E-02	0.11	6.01E-01	-0.07	7.31E-01	-0.09	6.53E-01	-0.38	5.19E-02	-0.40	3.82E-02	0.37	5.63E-02
ENSCAFG00000024511	ENSCAFG00000024511	grey	VSMC_M10	0.19	3.54E-01	0.06	7.73E-01	0.08	6.82E-01	-0.20	3.06E-01	-0.01	9.48E-01	0.19	3.44E-01	0.12	5.38E-01	0.16	4.23E-01	-0.14	4.82E-01	-0.01	9.66E-01
ENSCAFG00000032614	ENSCAFG00000032614	grey	VSMC_M10	0.19	3.55E-01	0.11	5.76E-01	0.14	4.85E-01	0.06	7.76E-01	-0.12	5.55E-01	0.01	9.60E-01	-0.17	4.01E-01	-0.04	8.50E-01	-0.15	4.58E-01	0.11	5.86E-01
ENSCAFG00000009550	RTF1	grey	VSMC_M10	0.19	3.55E-01	0.06	7.54E-01	0.09	6.49E-01	0.01	9.56E-01	-0.02	9.02E-01	-0.17	4.00E-01	0.04	8.62E-01	0.05	7.99E-01	-0.13	5.30E-01	0.02	9.11E-01
ENSCAFG00000004211	MSRB2	grey	VSMC_M10	0.19	3.55E-01	0.37	6.03E-02	0.03	8.65E-01	0.40	4.00E-02	-0.12	5.36E-01	0.04	8.52E-01	-0.20	3.10E-01	-0.38	5.18E-02	-0.44	2.09E-02	0.50	7.46E-03
ENSCAFG00000015841	PLD2	cyan	VSMC_M2	0.19	3.55E-01	0.53	4.33E-03	0.38	5.14E-02	-0.16	4.13E-01	-0.28	1.52E-01	-0.15	4.54E-01	-0.15	4.47E-01	-0.07	7.19E-01	-0.50	7.72E-03	0.27	1.77E-01
ENSCAFG00000030901	TRIM59	darkgrey	VSMC_M8	0.19	3.55E-01	-0.43	2.65E-02	0.00	9.87E-01	-0.54	3.38E-03	0.30	1.23E-01	-0.24	2.36E-01	0.26	1.94E-01	0.59	1.16E-03	0.27	1.81E-01	-0.70	4.38E-05
ENSCAFG00000004620	TOMM40	darkgrey	VSMC_M8	0.19	3.55E-01	-0.25	2.07E-01	0.37	5.93E-02	-0.57	1.77E-03	-0.31	1.16E-01	0.41	3.39E-02	0.26	1.93E-01	0.69	6.33E-05	0.04	8.51E-01	-0.37	5.83E-02
ENSCAFG00000016154	TMEM63A	grey	VSMC_M10	0.19	3.55E-01	0.39	4.28E-02	-0.13	5.05E-01	0.54	3.78E-03	0.14	4.71E-01	-0.42	3.10E-02	0.03	8.66E-01	-0.60	1.04E-03	-0.33	8.95E-02	0.49	1.02E-02
ENSCAFG00000008047	ENSCAFG00000008047	cyan	VSMC_M2	0.19	3.55E-01	0.48	1.16E-02	0.39	4.44E-02	-0.07	7.14E-01	-0.34	8.05E-02	-0.06	7.50E-01	-0.19	3.47E-01	-0.08	6.86E-01	-0.46	1.55E-02	0.25	2.05E-01
ENSCAFG00000012801	RIN1	grey	VSMC_M10	0.19	3.55E-01	0.27	1.79E-01	0.40	3.92E-02	-0.34	8.06E-02	-0.37	5.43E-02	0.13	5.09E-01	0.16	4.35E-01	0.22	2.81E-01	-0.34	8.12E-02	0.13	5.07E-01
ENSCAFG0000003049	ENSCAFG0000003049	grey	VSMC_M10	0.18	3.56E-01	-0.12	5.43E-01	0.06	7.71E-01	-0.26	1.98E-01	0.03	8.68E-01	0.26	1.94E-01	0.46	1.48E-02	0.27	1.68E-01	0.02	9.15E-01	-0.13	5.30E-01
ENSCAFG0000001654	ZNF346	pink	VSMC_M5	0.18	3.56E-01	-0.09	6.45E-01	-0.53	4.41E-03	0.38	4.92E-02	0.69	6.95E-05	-0.33	9.39E-02	0.00	9.92E-01	-0.30	1.30E-01	0.04	8.61E-01	-0.13	5.06E-01
ENSCAFG00000009150	OGFOD1	red	VSMC_M1	0.18	3.56E-01	0.07	7.37E-01	0.02	9.26E-01	0.08	6.97E-01	-0.11	5.75E-01	0.27	1.74E-01	0.40	4.13E-02	-0.01	9.60E-01	-0.31	1.10E-01	0.29	1.40E-01
ENSCAFG00000004650	ENSCAFG00000004650	grey	VSMC_M10	0.18	3.56E-01	-0.02	9.18E-01	-0.42	2.84E-02	0.32	9.91E-02	0.45	1.95E-02	-0.08	6.90E-01	-0.24	2.31E-01	-0.19	3.30E-01	-0.05	7.91E-01	0.08	6.82E-01
ENSCAFG00000009631	CBR3	grey	VSMC_M10	0.18	3.56E-01	0.45	1.81E-02	0.24	2.30E-01	0.11	5.97E-01	-0.27	1.78E-01	0.01	9.77E-01	0.41	3.47E-02	-0.20	3.30E-01	-0.49	9.27E-03	0.46	1.52E-02
ENSCAFG00000000870	PARVG	grey	VSMC_M10	0.18	3.56E-01	0.28	1.57E-01	0.04	8.34E-01	0.00	9.91E-01	0.04	8.61E-01	-0.08	6.83E-01	-0.12	5.40E-01	-0.20	3.05E-01	-0.26	1.87E-01	0.15	4.50E-01
ENSCAFG0000000997	CAP2	grey	VSMC_M10	0.18	3.56E-01	0.48	1.18E-02	-0.31	1.17E-01	0.68	1.04E-04	0.33	9.57E-02	0.01	9.77E-01	-0.51	6.83E-03	-0.78	1.38E-06	-0.34	8.35E-02	0.48	1.05E-02
ENSCAFG00000017149	IGDCC3	grey	VSMC_M10	0.18	3.56E-01	-0.19	3.41E-01	0.12	5.61E-01	-0.27	1.66E-01	0.14	4.76E-01	-0.08	6.78E-01	-0.22	2.69E-01	0.26	1.93E-01	0.19	3.49E-01	-0.48	1.21E-02
ENSCAFG00000005993	LHFPL6	grey	VSMC_M10	0.18	3.56E-01	0.77	3.35E-06	0.01	9.73E-01	0.56	2.31E-03	0.00	9.97E-01	-0.12	5.43E-01	-0.09	6.71E-01	-0.80	6.10E-07	-0.67	1.42E-04	0.67	1.43E-04
ENSCAFG00000018760	SLC25A14	grey	VSMC_M10	0.18	3.56E-01	0.28	1.61E-01	0.06	7.84E-01	0.21	2.98E-01	-0.18	3.76E-01	0.00	9.80E-01	0.09	6.70E-01	-0.20	3.21E-01	-0.35	7.08E-02	0.54	3.47E-03
ENSCAFG00000011890	FAM162A	pink	VSMC_M5	0.18	3.57E-01	-0.16	4.17E-01	-0.49	1.03E-02	0.50	7.32E-03	0.64	3.59E-04	-0.33	9.45E-02	-0.22	2.69E-01	-0.38	5.38E-02	0.22	2.65E-01	-0.14	4.78E-01
ENSCAFG00000013051	RPS17	grey	VSMC_M10	0.18	3.57E-01	0.13	5.20E-01	0.46	1.60E-02	-0.17	3.87E-01	-0.42	2.83E-02	-0.08	7.10E-01	0.26	1.98E-01	0.12	5.67E-01	-0.23	2.49E-01	0.04	8.26E-01
ENSCAFG00000012927	ALPK3	grey	VSMC_M10	0.18	3.57E-01	0.07	7.11E-01	-0.49	9.20E-03	0.60	8.78E-04	0.50	7.88E-03	-0.27	1.67E-01	-0.31	1.14E-01	-0.48	1.10E-02	-0.05	7.98E-01	0.22	2.80E-01
ENSCAFG00000016574	PIP4K2B	yellow	VSMC_M3	0.18	3.57E-01	0.48	1.07E-02	0.45	1.76E-02	-0.23	2.53E-01	-0.34	8.43E-02	0.11	5.91E-01	0.31	1.12E-01	0.01	9.44E-01	-0.51	6.68E-03	0.16	4.32E-01
ENSCAFG00000020039	ZER1	grey	VSMC_M10	0.18	3.57E-01	0.72	2.15E-05	0.09	6.66E-01	0.45	1.78E-02	-0.09	6.52E-01	0.00	9.86E-01	-0.23	2.55E-01	-0.68	9.23E-05	-0.65	2.65E-04	0.65	2.53E-04
ENSCAFG00000015158	ENSCAFG00000015158	grey	VSMC_M10	0.18	3.57E-01	0.03	8.77E-01	-0.38	5.34E-02	0.21	2.88E-01	0.30	1.29E-01	0.01	9.75E-01	0.08	7.03E-01	-0.14	4.72E-01	-0.12	5.37E-01	0.27	1.76E-01
ENSCAFG00000013059	SLC2A4RG	cyan	VSMC_M2	0.18	3.58E-01	0.63	4.49E-04	0.37	5.73E-02	0.24	2.22E-01	-0.27	1.73E-01	-0.32	1.07E-01	-0.31	1.21E-01	-0.49	9.11E-03	-0.48	1.20E-02	0.41	3.16E-02
ENSCAFG00000000151	TAC3	grey	VSMC_M10	0.18	3.58E-01	0.01	9.52E-01	-0.23	2.52E-01	0.30	1.26E-01	0.25	2.05E-01	-0.17	4.11E-01	-0.17	3.83E-01	-0.17	4.05E-01	-0.09	6.53E-01	0.08	6.78E-01
ENSCAFG00000028839	RAB18	pink	VSMC_M5	0.18	3.58E-01	-0.09	6.41E-01	-0.67	1.41E-04	0.55	2.92E-03	0.71	3.04E-05	-0.25	2.10E-01	-0.32	1.01E-01	-0.42	2.98E-02	0.09	6.63E-01	0.08	6.84E-01
ENSCAFG00000020380	AK5	darkgreen	VSMC_M4	0.18	3.59E-01	0.24	2.21E-01	-0.20	3.16E-01	0.51	6.46E-03	0.18	3.67E-01	0.07	7.14E-01	-0.84	5.73E-08	-0.46	1.48E-02	-0.20	3.12E-01	0.26	1.97E-01
ENSCAFG00000002580	PLPPR1	grey	VSMC_M10	0.18	3.59E-01	-0.42	2.99E-02	-0.21	3.00E-01	-0.23	2.40E-01	0.35	7.57E-02	0.18	3.72E-01	0.35	7.54E-02	0.36	6.64E-02	0.23	2.57E-01	-0.44	2.05E-02
ENSCAFG0000001374	SLC30A4	grey	VSMC_M10	0.18	3.59E-01	-0.02	9.08E-01	0.26	1.88E-01	-0.23	2.53E-01	-0.15	4.48E-01	0.03	8.91E-01	0.10	6.34E-01	0.22	2.72E-01	-0.08	6.92E-01	-0.05	8.06E-01
ENSCAFG00000014239	SF1	darkgrey	VSMC_M8	0.18	3.59E-01	-0.11	5.86E-01	0.43	2.36E-02	-0.78	1.93E-06	-0.24	2.21E-01	0.05	7.99E-01	0.16	4.16E-01	0.68	9.09E-05	-0.01	9.65E-01	-0.45	1.94E-02
ENSCAFG00000001377	GTPBP1	grey	VSMC_M10	0.18	3.59E-01	0.37	5.50E-02	0.12	5.55E-01	0.19	3.43E-01	-0.03	8.92E-01	-0.28	1.60E-01	0.15	4.65E-01	-0.23	2.56E-01	-0.39	4.37E-02	0.25	2.08E-01
ENSCAFG00000006578	COX7A1	grey	VSMC_M10	0.18	3.60E-01	0.18	3.79E-01	0.58	1.64E-03	-0.35	7.23E-02	-0.36	6.73E-02	-0.03	8.96E-01	0.13	5.05E-01	0.13	5.27E-01	-0.16	4.37E-01	-0.22	2.64E-01
ENSCAFG00000028551	TENT5B	yellow	VSMC_M3	0.18	3.60E-01	0.12	5.46E-01	0.64	3.70E-04	-0.77	2.73E-06	-0.39	4.22E-02	-0.11	5.99E-01	0.00	9.80E-01	0.54	3.36E-03	-0.14	4.99E-01	-0.40	4.14E-02
ENSCAFG00000026610	ENSCAFG00000026610	grey	VSMC_M10	0.18	3.60E-01	0.05	7.98E-01	0.04	8.61E-01	-0.13	5.26E-01	0.07	7.41E-01	-0.05	7.88E-01	0.28	1.61E-01	0.11	5.90E-01	-0.14	4.76E-01	-0.02	9.38E-01
ENSCAFG00000004700	CDNF	grey	VSMC_M10	0.18	3.60E-01	0.40	4.03E-02	0.29	1.47E-01	0.09	6.49E-01	-0.24	2.30E-01	0.07	7.27E-01	0.13	5.13E-01	-0.25	2.14E-01	-0.39	4.55E-02	0.23	2.40E-01
ENSCAFG00000018523	NATD1	grey	VSMC_M10	0.18	3.60E-01	0.55	3.00E-03	0.08	6.87E-01	0.35	7.64E-02	-0.07	7.12E-01	-0.03	8.91E-01	-0.17	3.96E-01	-0.54	3.69E-03	-0.52	5.36E-03	0.48	1.05E-02
ENSCAFG00000008811	TTI1	grey	VSMC_M10	0.18	3.61E-01	0.10	6.06E-01	0.07	7.25E-01	-0.03	8.72E-01	-0.11	5.90E-01	0.19	3.52E-01	0.36	6.16E-02	0.07	7.17E-01	-0.30	1.24E-01	0.23	2.51E-01
ENSCAFG00000019001	TTC4	grey	VSMC_M10	0.18	3.61E-01	0.00	9.83E-01	0.12	5.36E-01	-0.09	6.69E-01	-0.11	5.81E-01	0.00	9.96E-01	0.25	2.10E-01	0.12	5.54E-01	-0.14	4.75E-01	0.06	7.59E-01
ENSCAFG0000001759	UBR7	pink	VSMC_M5	0.18	3.61E-01	0.00	1.00E+00	-0.42	3.11E-02	0.33	9.23E-02	0.52	5.95E-03	-0.32	1.06E-01	0.00	9.82E-01	-0.23	2.57E-01	0.00	9.93E-01	-0.03	8.90E-01
ENSCAFG00000013211	ENSCAFG00000013211	grey	VSMC_M10	0.18	3.61E-01	-0.37	5.80E-02	-0.23	2.44E-01	-0.24	2.29E-01	0.41	3.49E-02	0.06	7.77E-01	-0.08	6.94E-01	0.29	1.48E-01	0.30	1.26E-01	-0.48	1.21E-02
ENSCAFG00000012141	IP6K2	grey	VSMC_M10	0.18	3.61E-01	0.38	5.36E-02	-0.10	6.20E-01	0.13	5.23E-01	0.12	5.58E-01	0.04	8.39E-01	-0.15	4.69E-01	-0.19	3.35E-01	-0.41	3.32E-02	0.21	3.02E-01
ENSCAFG00000011771	C6H7orf50	grey	VSMC_M10	0.18	3.61E-01	-0.01	9.44E-01	0.40	4.02E-02	-0.30	1.30E-01	-0.22	2.61E-01	-0.18	3.82E-01	-0.06	7.82E-01	0.33	9.67E-02	-0.09	6.52E-01	-0.27	1.78E-01
ENSCAFG00000001311	CHCHD3	darkgrey	VSMC_M8	0.18	3.61E-01	-0.40	3.99E-02	0.43	2.49E-02	-0.76	3.76E-06	-0.11	5.92E-01	-0.06	7.78E-01	0.31	1.18E-01	0.78	1.71E-06	0.26	1.86E-01	-0.80	6.41E-07
ENSCAFG00000017144	VASH1	grey	VSMC_M10	0.18	3.61E-01	-0.37	5.90E-02	-0.11	5.86E-01	0.01	9.73E-01	0.34	7.95E-02	0.05	8.01E-01	0.04	8.28E-01	0.04	8.59E-01	0.35	7.72E-02	-0.46	1.53E-02

ENSCAFG0000000003	CD226	grey	VSMC_M10	0.18	3.61E-01	0.24	2.31E-01	0.47	1.27E-02	-0.11	5.68E-01	-0.30	1.33E-01	-0.01	9.58E-01	-0.06	7.76E-01	-0.10	6.17E-01	-0.17	3.88E-01	0.02	9.24E-01
ENSCAFG0000001698	RAB8B	grey	VSMC_M10	0.18	3.62E-01	0.25	2.05E-01	0.22	2.80E-01	-0.17	3.99E-01	-0.03	8.94E-01	-0.34	8.53E-02	0.37	5.83E-02	0.02	9.23E-01	-0.28	1.52E-01	-0.06	7.51E-01
ENSCAFG0000002470	OOEP	grey	VSMC_M10	0.18	3.62E-01	0.45	1.95E-02	0.30	1.35E-01	0.08	7.09E-01	-0.40	3.67E-02	0.31	1.11E-01	-0.10	6.29E-01	-0.13	5.14E-01	-0.52	5.90E-03	0.46	1.60E-02
ENSCAFG0000003208	GTF2H4	cyan	VSMC_M2	0.18	3.62E-01	0.52	5.14E-03	-0.24	2.31E-01	0.51	6.07E-03	0.20	3.19E-01	-0.11	6.00E-01	-0.12	5.51E-01	-0.62	4.94E-04	-0.47	1.30E-02	0.59	1.11E-03
ENSCAFG0000001958	UNKL	cyan	VSMC_M2	0.18	3.62E-01	0.43	2.51E-02	0.29	1.41E-01	0.28	1.56E-01	-0.25	2.02E-01	-0.22	2.77E-01	0.03	8.96E-01	-0.40	3.97E-02	-0.40	3.83E-02	0.41	3.19E-02
ENSCAFG0000001836	YES1	grey	VSMC_M10	0.18	3.62E-01	-0.23	2.51E-01	-0.53	4.40E-03	0.31	1.11E-01	0.49	1.01E-02	0.31	1.13E-01	0.05	7.99E-01	-0.13	5.08E-01	0.09	6.73E-01	0.07	7.17E-01
ENSCAFG0000001460	LRPAP1	grey	VSMC_M10	0.18	3.62E-01	0.72	2.76E-05	0.27	1.78E-01	0.54	3.49E-03	-0.33	9.36E-02	-0.08	6.74E-01	-0.38	4.92E-02	-0.67	1.30E-04	-0.65	2.35E-04	0.67	1.33E-04
ENSCAFG0000001585	MFAP2	grey	VSMC_M10	0.18	3.62E-01	-0.10	6.10E-01	0.40	3.94E-02	-0.59	1.09E-03	-0.21	3.00E-01	-0.03	8.74E-01	0.64	3.12E-04	0.45	1.92E-02	0.03	8.95E-01	-0.30	1.28E-01
ENSCAFG0000001322	ENSCAFG0000001322	cyan	VSMC_M2	0.18	3.63E-01	0.45	1.72E-02	0.27	1.80E-01	0.27	1.72E-01	-0.37	5.41E-02	0.21	2.90E-01	-0.22	2.61E-01	-0.37	5.84E-02	-0.52	5.24E-03	0.55	3.19E-03
ENSCAFG0000000024	MTFR2	darkgrey	VSMC_M8	0.18	3.63E-01	-0.34	8.00E-02	0.21	2.85E-01	-0.68	1.12E-04	-0.09	6.67E-01	0.10	6.11E-01	0.38	4.83E-02	0.76	3.89E-06	0.08	6.81E-01	-0.45	1.79E-02
ENSCAFG0000001475	ENSCAFG0000001475	grey	VSMC_M10	0.18	3.63E-01	-0.01	9.48E-01	0.05	7.90E-01	-0.17	3.97E-01	0.02	9.25E-01	-0.07	7.25E-01	0.11	5.72E-01	0.16	4.34E-01	-0.09	6.40E-01	-0.06	7.70E-01
ENSCAFG0000000053	ENSCAFG0000000053	grey	VSMC_M10	0.18	3.63E-01	-0.06	7.71E-01	-0.12	5.53E-01	0.04	8.38E-01	0.20	3.11E-01	-0.09	6.58E-01	-0.14	4.93E-01	-0.02	9.09E-01	0.08	6.76E-01	-0.06	7.49E-01
ENSCAFG0000000721	YTHDF3	pink	VSMC_M5	0.18	3.64E-01	-0.04	8.32E-01	-0.48	1.12E-02	0.22	2.60E-01	0.62	4.93E-04	-0.18	3.80E-01	-0.36	6.67E-02	-0.25	2.03E-01	0.10	6.31E-01	-0.09	6.40E-01
ENSCAFG0000000256	ENSCAFG0000000256	cyan	VSMC_M2	0.18	3.64E-01	0.70	4.03E-05	0.41	3.53E-02	0.20	3.27E-01	-0.52	5.85E-03	0.08	6.87E-01	-0.10	6.35E-01	-0.39	4.61E-02	-0.74	1.02E-05	0.70	5.16E-05
ENSCAFG0000001245	ENSCAFG0000001245	grey	VSMC_M10	0.18	3.64E-01	0.38	5.33E-02	0.10	6.03E-01	0.43	2.55E-02	-0.13	5.23E-01	-0.22	2.76E-01	0.08	6.97E-01	-0.51	6.66E-03	-0.38	5.22E-02	0.49	9.05E-03
ENSCAFG0000000208	ENSCAFG0000000208	grey	VSMC_M10	0.18	3.64E-01	0.40	4.12E-02	0.29	1.49E-01	0.20	3.14E-01	-0.38	4.76E-02	-0.04	8.54E-01	0.17	3.84E-01	-0.27	1.69E-01	-0.44	2.34E-02	0.51	6.47E-03
ENSCAFG0000001739	COIL	grey	VSMC_M10	0.18	3.64E-01	-0.40	3.95E-02	-0.24	2.34E-01	0.04	8.61E-01	0.28	1.53E-01	0.24	2.19E-01	-0.17	4.02E-01	0.20	3.16E-01	0.26	1.88E-01	-0.23	2.44E-01
ENSCAFG0000002952	ENSCAFG0000002952	grey	VSMC_M10	0.18	3.64E-01	-0.06	7.61E-01	0.26	1.93E-01	-0.19	3.51E-01	-0.26	1.93E-01	0.10	6.07E-01	-0.02	9.04E-01	0.23	2.42E-01	-0.10	6.37E-01	0.01	9.68E-01
ENSCAFG0000001342	KCNK7	grey	VSMC_M10	0.18	3.64E-01	0.24	2.25E-01	0.00	9.84E-01	0.01	9.44E-01	0.06	7.60E-01	-0.10	6.06E-01	-0.01	9.77E-01	-0.05	7.93E-01	-0.31	1.21E-01	0.16	4.30E-01
ENSCAFG0000001977	LRIF1	pink	VSMC_M5	0.18	3.64E-01	-0.16	4.29E-01	-0.11	5.94E-01	-0.15	4.69E-01	0.40	3.89E-02	-0.10	6.22E-01	-0.05	7.86E-01	0.10	6.09E-01	0.17	3.84E-01	-0.53	4.81E-03
ENSCAFG0000001434	KDELC2	grey	VSMC_M10	0.18	3.65E-01	0.55	2.99E-03	-0.28	1.57E-01	0.64	2.91E-04	0.23	2.59E-01	-0.08	7.07E-01	-0.13	5.26E-01	-0.71	3.65E-05	-0.53	4.48E-03	0.60	9.95E-04
ENSCAFG0000000757	TP53INP2	cyan	VSMC_M2	0.18	3.65E-01	0.49	1.01E-02	0.26	1.99E-01	0.22	2.75E-01	-0.31	1.15E-01	0.07	7.11E-01	-0.27	1.70E-01	-0.29	1.43E-01	-0.50	8.46E-03	0.45	1.75E-02
ENSCAFG0000000384	MGAM	grey	VSMC_M10	0.18	3.66E-01	0.26	1.89E-01	0.04	8.51E-01	0.04	8.44E-01	0.06	7.66E-01	-0.02	9.21E-01	-0.07	7.25E-01	-0.12	5.52E-01	-0.24	2.21E-01	0.14	4.71E-01
ENSCAFG0000000887	ANTXR2	pink	VSMC_M5	0.18	3.66E-01	-0.21	2.99E-01	-0.25	2.04E-01	0.10	6.20E-01	0.45	1.73E-02	-0.42	2.81E-02	0.11	5.91E-01	-0.11	5.92E-01	0.24	2.27E-01	-0.27	1.72E-01
ENSCAFG0000000876	WNT10B	yellow	VSMC_M3	0.18	3.66E-01	0.52	5.43E-03	0.84	3.06E-08	-0.37	5.51E-02	-0.72	2.50E-05	-0.07	7.29E-01	-0.14	4.86E-01	0.07	7.35E-01	-0.48	1.09E-02	0.09	6.58E-01
ENSCAFG0000002123	ENSCAFG0000002123	yellow	VSMC_M3	0.18	3.67E-01	0.53	4.91E-03	0.60	1.05E-03	-0.17	3.94E-01	-0.51	6.45E-03	-0.01	9.42E-01	-0.14	4.94E-01	-0.09	6.67E-01	-0.49	9.85E-03	0.15	4.58E-01
ENSCAFG0000002404	ENSCAFG0000002404	cyan	VSMC_M2	0.18	3.67E-01	0.45	1.90E-02	0.30	1.27E-01	0.21	2.87E-01	-0.34	8.25E-02	0.11	5.74E-01	0.02	9.16E-01	-0.28	1.50E-01	-0.47	1.44E-02	0.43	2.45E-02
ENSCAFG0000001247	SEMA6C	cyan	VSMC_M2	0.18	3.67E-01	0.62	5.85E-04	0.23	2.56E-01	0.27	1.67E-01	-0.09	6.72E-01	-0.22	2.64E-01	-0.21	2.99E-01	-0.50	7.85E-03	-0.51	6.95E-03	0.25	2.01E-01
ENSCAFG0000000410	ZC3HAV1	grey	VSMC_M10	0.18	3.67E-01	0.74	1.07E-05	0.21	2.91E-01	0.39	4.31E-02	-0.26	1.86E-01	0.03	8.91E-01	-0.24	2.36E-01	-0.60	9.91E-04	-0.68	8.29E-05	0.69	7.33E-05
ENSCAFG0000000525	WDR43	darkgrey	VSMC_M8	0.18	3.67E-01	-0.49	9.49E-03	0.32	1.08E-01	-0.62	5.78E-04	-0.18	3.73E-01	0.15	4.47E-01	0.31	1.18E-01	0.74	8.72E-06	0.25	2.01E-01	-0.54	3.52E-03
ENSCAFG0000001318	GSG1	cyan	VSMC_M2	0.18	3.67E-01	0.48	1.13E-02	0.28	1.60E-01	-0.15	4.41E-01	-0.22	2.62E-01	-0.10	6.26E-01	0.07	7.26E-01	-0.08	6.77E-01	-0.46	1.63E-02	0.25	2.09E-01
ENSCAFG0000000742	WT1	cyan	VSMC_M2	0.18	3.67E-01	0.52	4.97E-03	0.46	1.50E-02	-0.11	6.01E-01	-0.44	2.16E-02	0.05	7.99E-01	-0.13	5.20E-01	-0.09	6.42E-01	-0.55	2.87E-03	0.34	8.33E-02
ENSCAFG0000000310	ANKRD6	turquoise	VSMC_M6	0.18	3.67E-01	-0.02	9.09E-01	0.02	9.38E-01	-0.10	6.27E-01	0.00	9.84E-01	0.35	7.44E-02	-0.18	3.77E-01	0.03	8.94E-01	-0.07	7.36E-01	0.00	9.81E-01
ENSCAFG0000001386	AXDND1	grey	VSMC_M10	0.18	3.67E-01	0.36	6.60E-02	0.29	1.40E-01	0.00	9.89E-01	-0.29	1.38E-01	0.23	2.58E-01	0.25	2.08E-01	-0.11	5.76E-01	-0.43	2.56E-02	0.34	8.53E-02
ENSCAFG0000001902	ACOT11	grey	VSMC_M10	0.18	3.67E-01	-0.35	7.64E-02	0.01	9.43E-01	-0.22	2.77E-01	0.14	4.82E-01	0.17	3.91E-01	0.16	4.34E-01	0.30	1.25E-01	0.20	3.08E-01	-0.45	1.91E-02
ENSCAFG0000001341	MFAP1	grey	VSMC_M10	0.18	3.68E-01	-0.09	6.61E-01	0.27	1.67E-01	-0.33	9.26E-02	-0.02	9.03E-01	-0.08	6.96E-01	0.20	3.12E-01	0.25	2.08E-01	0.01	9.45E-01	-0.42	3.12E-02
ENSCAFG0000001077	MEF2A	pink	VSMC_M5	0.18	3.68E-01	0.29	1.36E-01	-0.48	1.15E-02	0.52	5.94E-03	0.48	1.18E-02	0.07	7.41E-01	-0.37	5.98E-02	-0.56	2.36E-03	-0.21	2.83E-01	0.36	6.12E-02
ENSCAFG0000003085	ENSCAFG0000003085	grey	VSMC_M10	0.18	3.68E-01	0.21	2.99E-01	0.42	2.80E-02	-0.15	4.42E-01	-0.41	3.57E-02	0.19	3.31E-01	-0.13	5.09E-01	0.07	7.16E-01	-0.25	2.15E-01	0.14	5.01E-01
ENSCAFG0000001181	SAG	grey	VSMC_M10	0.18	3.68E-01	0.28	1.58E-01	0.45	1.93E-02	-0.11	5.71E-01	-0.33	8.88E-02	-0.14	4.93E-01	0.28	1.54E-01	-0.13	5.10E-01	-0.27	1.73E-01	0.14	4.92E-01
ENSCAFG0000001377	MAP3K14	yellow	VSMC_M3	0.18	3.68E-01	0.48	1.14E-02	0.45	1.72E-02	-0.35	6.97E-02	-0.35	7.04E-02	0.21	2.90E-01	-0.10	6.08E-01	0.07	7.36E-01	-0.42	2.82E-02	0.12	5.62E-01
ENSCAFG0000001868	MLLT1	cyan	VSMC_M2	0.18	3.69E-01	0.71	3.18E-05	0.57	1.84E-03	-0.24	2.36E-01	-0.58	1.45E-03	0.13	5.14E-01	0.00	9.84E-01	-0.04	8.42E-01	-0.74	1.23E-05	0.48	1.18E-02
ENSCAFG0000001919	SLC43A2	darkgreen	VSMC_M4	0.18	3.69E-01	0.49	1.03E-02	-0.21	2.82E-01	0.66	1.76E-04	0.08	6.94E-01	0.07	7.44E-01	-0.17	4.06E-01	-0.69	6.25E-05	-0.45	1.73E-02	0.70	4.56E-05
ENSCAFG0000001644	FGD1	grey	VSMC_M10	0.18	3.69E-01	0.37	5.67E-02	0.08	7.05E-01	0.13	5.16E-01	-0.02	9.38E-01	-0.27	1.65E-01	-0.06	7.62E-01	-0.15	4.49E-01	-0.40	4.00E-02	0.27	1.77E-01
ENSCAFG0000000838	ENSCAFG0000000838	yellow	VSMC_M3	0.18	3.69E-01	0.21	3.01E-01	0.79	1.11E-06	-0.68	1.09E-04	-0.61	6.56E-04	0.06	7.74E-01	0.11	6.00E-01	0.47	1.24E-02	-0.28	1.63E-01	-0.20	3.25E-01
ENSCAFG0000001243	CTSF	darkgreen	VSMC_M4	0.18	3.69E-01	0.48	1.05E-02	-0.19	3.49E-01	0.53	4.10E-03	0.24	2.23E-01	-0.28	1.60E-01	-0.14	4.82E-01	-0.72	2.55E-05	-0.32	1.08E-01	0.43	2.68E-02
ENSCAFG0000001462	IFI35	yellow	VSMC_M3	0.18	3.69E-01	0.71	3.20E-05	0.64	3.32E-04	-0.04	8.57E-01	-0.60	8.57E-04	-0.06	7.51E-01	0.07	7.24E-01	-0.30	1.31E-01	-0.66	1.94E-04	0.48	1.19E-02
ENSCAFG0000000260	ENSCAFG0000000260	grey	VSMC_M10	0.18	3.69E-01	0.14	4.73E-01	0.05	8.19E-01	0.06	7.50E-01	-0.05	8.21E-01	0.02	9.36E-01	0.06	7.77E-01	-0.05	8.23E-01	-0.28	1.62E-01	0.24	2.23E-01
ENSCAFG0000000177	BPIFC	grey	VSMC_M10	0.18	3.70E-01	0.37	5.92E-02	0.25	1.99E-01	-0.22	2.65E-01	-0.15	4.45E-01	-0.12	5.65E-01	0.00	9.93E-01	0.04	8.47E-01	-0.35	7.23E-02	0.17	3.95E-01
ENSCAFG0000000585	GGACT	grey	VSMC_M10	0.18	3.70E-01	0.18	3.57E-01	-0.17	4.02E-01	0.28	1.58E-01	0.15	4.66E-01	0.18	3.79E-01	0.18	3.82E-01	-0.31	1.12E-01	-0.28	1.62E-01	0.25	2.07E-01
ENSCAFG0000002307	POC1A	darkgrey	VSMC_M8	0.18	3.70E-01	-0.35	7.53E-02	0.43	2.47E-02	-0.82	2.18E-07	-0.31	1.20E-01	0.24	2.34E-01	0.51	6.61E-03	0.86	8.86E-09	0.10	6.28E-01	-0.47	1.44E-02
ENSCAFG0000002915	ZNF385A	yellow	VSMC_M3	0.18	3.70E-01	0.48	1.10E-02	0.57	2.07E-03	-0.37	5.50E-02	-0.38	4.76E-02	-0.20	3.06E-01	0.17	3.86E-01	0.06	7.57E-01	-0.47	1.37E-02	0.02	9.23E-01
ENSCAFG0000000757	PIGU	grey	VSMC_M10	0.18	3.70E-01	0.16	4.20E-01	0.24	2.32E-01	-0.28	1.63E-01	-0.24	2.20E-01	0.07	7.11E-01	0.18	3.70E-01	0.18	3.61E-01	-0.27	1.80E-01	0.12	5.48E-01
ENSCAFG0000000624	CHL1	grey	VSMC_M10	0.18	3.70E-01	0.30	1.33E-01	0.30	1.25E-01	-0.18	3.75E-01	-0.31	1.15E-01	0.27	1.75E-01	-0.26	1.84E-01	0.04	8.45E-01	-0.39	4.59E-02	0.20	3.16E-01
ENSCAFG0000000328	GNL2	darkgrey	VSMC_M8	0.18	3.70E-01	-0.32	9.90E-02	0.34	8.05E-02	-0.52	5.96E-03	-0.07	7.18E-01	-0.01	9.41E-01	-0.18	3.65E-01	0.57	1.81E-03	0.21	2.83E-01	-0.68	8.15E-05
ENSCAFG0000001565	PCSK1N	darkgreen	VSMC_M4	0.18	3.71E-01	0.53	4.21E-03	-0.06	7.80E-01	0.58	1.67E-03	0.07	7.22E-01	-0.22	2.72E-01	-0.39	4.64E-02	-0.68	8.85E-05	-0.39	4.60E-02	0.45	1.95E-02
ENSCAFG0000002910	ENSCAFG0000002910	grey	VSMC_M10	0.18	3.71E-01	0.21	2.86E-01	0.08	7.00E-01	-0.05	8.00E-01	0.04	8.49E-01	-0.09	6.44E-01	-0.15	4.55E-01	0.00	9.89E-01	-0.24	2.34E-01	0.07	7.12E-01
ENSCAFG0000002306	RHOA	grey	VSMC_M10	0.18	3.72E-01	0.16	4.26E-01	0.45	1.81E-02	-0.42	2.86E-02	-0.37	5.85E-02	0.18	3.80E-01	-0.21	2.93E-01	0.26	1.89E-01	-0.15	4.55E-01	-0.09	6.43E-01
ENSCAFG0000001572	DHX58	cyan	VSMC_M2	0.18	3.72E-01	0.72	2.47E-05	-0.13	5.29E-01	0.62	5.20E-04	0.10	6.06E-01	-0.10	6.31E-01	-0.46	1.61E-02	-0.75	6.31E-06	-0.65	2.50E-04	0.62	6.20E-04
ENSCAFG0000001067	BANK1	grey	VSMC_M10	0.18	3.72E-01	0.25	2.14E-01	0.20	3.24E-01	0.04	8.39E-01	-0.25	2.01E-01	0.19	3.37E-01	0.29	1.38E-01	-0.04	8.50E-01	-0.34	8.15E-02	0.37	5.58E-02
ENSCAFG0000001983	RXRA	grey	VSMC_M10	0.18	3.72E-01	0.81	2.70E-07	0.15	4.64E-01	0.43	2.50E-02	-0.23	2.48E-01	0.00	9.99E-01	-0.21	2.94E-01	-0.61	7.88E-04	-0.75	7.18E-06	0.77	2.82E-06
ENSCAFG0000001254	VPS72	grey	VSMC_M10	0.18	3.73E-01	0.69	7.45E-05	0.33	8.93E-02	0.20	3.25E-01	-0.35	7.50E-02	-0.18	3.61E-01	0.00	9.93E-01	-0.38	4.98E-02	-0.73	1.54E-05	0.56	2.32E-03
ENSCAFG0000003099	INSYN1	grey	VSMC_M10	0.18	3.73E-01	0.53	4.53E-03	-0.10	6.04E-01	0.65	2.62E-04	0.04	8.43E-01	-0.05	8.22E-01	-0.18	3.57E-01	-0.75	5.85E-06	-0.47	1.35E-02	0.66	2.01E-04
ENSCAFG0000001286	ENSCAFG0000001286	grey	VSMC_M10	0.18	3.74E-01	-0.15	4.43E-01	0.22	2.81E-01	-0.25	2.16E-01	-0.19	3.39E-01	0.25	2.09E-01	0.27	1.75E-01	0.37	5.78E-02	-0.05	7.95E-01	-0.15	4.63E-01
ENSCAFG0000000598	HEATR5B	grey	VSMC_M10	0.18	3.74E-01	0.30	1.24E-01	-0.54	3.48E-03	0.76	4.98E-06	0.46	1.50E-02	-0.04	8.58E-01	-0.04	8.48E-01	-0.73	1.71E-05	-0.30	1.33E-01	0.57	1.93E-03
ENSCAFG0000002541	ALKAL1	turquoise	VSMC_M6	0.18	3.74E-01	0.03	8.95E-01	-0.44	2.16E-02	0.46	1.46E-02	0.41	3.29E-02	0.16	4.27E-01	-0.21	2.86E-01	-0.44	2.29E-02	-0.08	6.94E-01	0.23	2.59E-01
ENSCAFG0000002633	ENSCAFG0000002633	grey	VSMC_M10	0.18	3.74E-01	0.20	3.28E-01	0.01	9.75E-01	-0.09	6.42E-01	0.10	6.18E-01	0.04	8.49E-01	-0.06	7.76E-01	0.02	9.32E-01	-0.20	3.21E-01	0.02	9.25E-01
ENSCAFG0000000725	DRAM1	grey	VSMC_M10	0.18	3.74E-01	0.76	5.17E-06	0.25	2.14E-01	0.28	1.59E-01	-0.27	1.77E-01	-0.07	7.16E-01	-0.03	8.65E-01	-0.50	8.09E-03	-0.70	4.54E-05	0.62	5.44E-04
ENSCAFG0000000845	CELF1	grey	VSMC_M10	0.18	3.74E-01	-0.52	5.28E-03	-0.30	1.32E-01	-0.02	9.27E-01	0.40	4.07E-02	0.17	3.89E-01	0.04	8.30E-01	0.18	3.82E-01	0.43	2.55E-02	-0.36	6.54E-02
ENSCAFG0000000153	SHARPIN	yellow	VSMC_M3	0.18	3.74E-01	0.57	1.84E-03	0.70	4.83E-05	-0.20	3.05E-01	-0.61	6.71E-04	-0.01	9.47E-01	-0.19	3.45E-01	-0.08	6.94E-01	-0.51	6.36E-03	0.22	2.73E-01
ENSCAFG0000000090	KPNA5	pink	VSMC_M5	0.18	3.75E-01	-0.38	4.89E-02	-0.51	6.93E-03	0.30	1.26E-01	0.70	4.66E-05	-0.23	2.45E-01	-0.16	4.28E-01	-0.11	5.85E-01	0.36	6.82E-02	-0.36	6.49E-02
ENSCAFG0000001596	ENSCAFG0000001596	grey	VSMC_M10	0.18	3.75E-01	0.59	1.09E-03	0.12	5.55E-01	0.34	8.25E-02	-0.08	7.02E-01	0.04	8.47E-01	-0.27	1.81E-01	-0.54	3.55E-03	-0.48	1.14E-02	0.41	3.58E-02
ENSCAFG0000001664	NPEPPS	grey	VSMC_M10	0.18	3.75E-01	0.41	3.29E-02	-0.24	2.20E-01	0.56	2.46E-03	0.35	7.51E-02	-0.11	5.91E-01	-0.57	2.12E-03	-0.71	3.74E-05	-0.21	2.84E-01	0.25	2.01E-01
ENSCAFG0000001484	MYOZ1	grey	VSMC_M10	0.18	3.75E-01	-0.32	1.08E-01	-0.23	2.56E-01	-0.05	7.94E-01	0.24	2.25E-01	0.17	4.04E-01	0.39	4.25E-02	0.20	3.09E-01	0.14	4.89E-01	-0.10	6.23E-01
ENSCAFG0000000264	ENSCAFG0000000264	darkgrey	VSMC_M8	0.18	3.75E-01	-0.54	4.01E-03	0.01	9.52E-01	-0.51	6.79E-03	0.19	3.38E-01	-0.17	3.91E-01	0.40	3.97E-02	0.59	1.35E-03	0.40	3.84E-02	-0.60	8.46E-04
ENSCAFG0000003162	ENSCAFG0000003162	grey	VSMC_M10	0.18	3.75E-01	0.13	5.04E-01	0.34	8.76E-02	-0.09	6.47E-01	-0.25	2.05E-01	-0.06	7.53E-01	0.05	8.00E-01	0.06	7.52E-01	-0.26	1.86E-01	0.06	7.53E-01
ENSCAFG0000000438	ABHD12	darkgreen	VSMC_M4	0.18	3.76E-01	0.56	2.60E-03	-0.07	7.33E-01	0.65	2.29E-04	0.01	9.43E-01	-0.27	1.81E-01	-0.49	8.73E-03	-0.73	1.39E-05	-0.46	1.65E-02	0.59	1.27E-03
ENSCAFG0000001436	MREG	grey	VSMC_M10	0.18	3.76E-01	0.00	9.84E-01	0.39	4.32E-02	-0.38	5.36E-02	-0.18	3.70E-01	-0.12	5.45E-01	0.60	9.18E-04	0.25	1.99E-01	-0.11	5.99E-01	-0.25	2.13E-01
ENSCAFG0000001622	ACAP1	grey	VSMC_M10	0.18	3.76E-01	0.75	6.85E-06	0.20	3.13E-01	0.30	1.25E-01	-0.24	2.22E-01	0.07	7.35E-01	-0.05	8.03E-01	-0.54	3.94E-03	-0.71	2.86E-05	0.69	7.94E-05
ENSCAFG0000001941	LINGO3	grey	VSMC_M10	0.18	3.76E-01	0.24	2.31E-01	0.17	3.86E-01	-0.15	4.68E-01	-0.10	6.37E-01	-0.06	7.53E-01	-0.14	5.00E-01	0.06	7.70E-01	-0.27	1.76E-01	0.06	7.53E-01
ENSCAFG0000000568	ENDOV	pink	VSMC_M5	0.18	3.76E-01	-0.25	2.11E-01	-0.07	7.41E-01	-0.12	5.39E-01	0.29	1.48E-01	-0.29	1.41E-01	-0.27	1.79E-01	0.13	5.12E-01	0.29	1.45E-01	-0.45	1.79E-02
ENSCAFG0000001325	GPR155	grey	VSMC_M10	0.18	3.76E-01	-0.05	7.91E-01	-0.47	1.39E-02	0.55	3.02E-03	0.55	2.85E-03	-0.11	5.97E-01	0.13	5.10E-01	-0.48	1.19E-02	0.03	8.92E-01	0.07	7.11E-01
ENSCAFG0000001480	DNM3	grey	VSMC_M10	0.18	3.76E-01	-0.02	9.41E-01	-0.13	5.20E-01	-0.04	8.37E-01	0.14	4.71E-01	0.26	1.92E-01	0.21	2.94E-01	0.18	3.75E-01	-0.12	5.46E-01	-0.01	9.74E-01
ENSCAFG0000001697	DLX3	grey	VSMC_M10	0.18	3.77E-01	-0.01	9.42E-01	0.12	5.36E-01	0.13	5.10E-01	-0.10	6.09E-01	0.15	4.48E-01	0.39	4.35E-02	-0.07	7.43E-01	-0.09	6.64E-01	0.08	6.88E-01
ENSCAFG0000003187	ENSCAFG0000003187	grey	VSMC_M10	0.18	3.77E-01	0.21	2.85E-01	-0.07	7.42E-01	0.09	6.53E-01	0.14	4.88E-01	0.05	7.90E-01	-0.28	1.61E-01	-0.14	4.75E-01	-0.26	1.87E-01	0.08	6.87E-01
ENSCAFG0000001202	REC8	cyan	VSMC_M2	0.18	3.77E-01	0.64	3.06E-04	0.02	9.05E-01	0.36	6.69E-02	-0.08	6.79E-01	-0.16	4.21E-01	-0.01	9.42E-01	-0.52	5.63E-03	-0.58	1.61E-03	0.66	1.55E-04
ENSCAFG0000002164	ENSCAFG0000002164	grey	VSMC_M10	0.18	3.78E-01	-0.11	5.79E-01	-0.17	4.00E-01	0.10	6.37E-01	0.25	2.15E-01	-0.12	5.52E-01	-0.15	4.43E-01	-0.07	7.47E-01	0.14	4.86E-01	-0.10	6.02E-01
ENSCAFG0000001291	CLIC4	grey	VSMC_M10	0.18	3.78E-01	0.32	1.08E-01	-0.10	6.30E-01	0.31	1.13E-01	0.27	1.66E-01	-0.37	5.64E-02	0.06	7.77E-01	-0.50	8.16E-03	-0.20	3.13E-01	0.16	4.30E-01
ENSCAFG0000002439	ENSCAFG0000002439	grey	VSMC_M10	0.18	3.78E-01	0.25	2.05E-01	0.09	6.48E-01	0.31	1.17E-01	-0.03	8.69E-01	-0.27	1.81E-01	-0.27	1.69E-01	-0.31	1.10E-01	-0.23	2.47E-01	0.20	3.19E-01
ENSCAFG0000001989	BANP	grey	VSMC_M10	0.18	3.78E-01	0.16	4.37E-01	-0.14	4.85E-01	0.17	3.99E-01	0.16	4.38E-01	-0.02	9.26E-01	0.26	1.97E-01	-0.18	3.69E-01	-0.28	1.52E-01	0.29	1.39E-01
ENSCAFG0000002554	ENSCAFG0000002554	grey	VSMC_M10	0.18	3.78E-01	0.20	3.07E-01	-0.40	3.75E-02	0.59	1.31E-03	0.38	5.13E-02	0.01	9.64E-01	-0.34	8.08E-02	-0.61	8.25E-04	-0.12	5.60E-01	0.36	6.65E-02
ENSCAFG0000000326	FBXO48	grey	VSMC_M10	0.18	3.79E-01	0.32	1.00E-01	0.17	3.89E-01	0.02	9.15E-01	-0.12	5.45E-01	0.07	7.19E-01	-0.07	7.30E-01	-0.14	5.00E-01	-0.36	6.91E-02	0.25	2.17E-01
ENSCAFG0000000786	ENSCAFG0000000786	grey	VSMC_M10	0.18	3.80E-01	0.02	9.34E-01	0.10	6.14E-01	-0.11	5.74E-01	0.00	9.81E-01	-0.02	9.33E-01	-0.02	9.02E-01	0.13	5.13E-01	-0.07	7.18E-01	-0.10	6.25E-01
ENSCAFG0000001441	PPP1R2C	grey	VSMC_M10	0.18	3.80E-01	0.02	9.34E-01	0.10	6.14E-01	-0.11	5.74E-01	0.00	9.81E-01	-0.02	9.33E-01	-0.02	9.02E-01	0.13	5.13E-01	-0.07	7.18E-01	-0.10	6.25E-01
ENSCAFG0000003224	ENSCAFG0000003224	grey	VSMC_M10	0.18	3.80E-01	0.02	9.34E-01	0.10	6.14E-01	-0.11	5.74E-01	0.00	9.81E-01	-0.02	9.33E-01	-0.02	9.02E-01	0.13	5.13E-01	-0.07	7.18E-01	-0.10	6.25E-01
ENSCAFG0000000388	SSBP1	grey	VSMC_M10	0.18	3.80E-01	-0.11	5.84E-01	0.35	7.09E-02	-0.37	5.88E-02	-0.12	5.62E-01	-0.02	9.14E-01	0.20	3.15E-01	0.34	8.51E-02	0.00	9.94E-01	-0.43	2.49E-02
ENSCAFG0000002433	ZBTB7A	cyan	VSMC_M2	0.18	3.80E-01	0.65	2.75E-04	0.47	1.29E-02	0.09	6.42E-01	-0.46	1.46E-02	0.09	6.73E-01	-0.40	4.06E-02	-0.39	4.67E-02	-0.52	5.45E-03	0.44	2.24E-02
ENSCAFG0000001466	CTDSP1	yellow	VSMC_M3	0.18	3.80E-01	0.68	1.06E-04	0.48	1.06E-02	-0.19	3.35E-01	-0.36	6.73E-02	-0.20	3.09E-01	0.10	6.16E-01	-0.15	4.59E-01	-0.61	8.15E-04	0.31	1.12E-01
ENSCAFG0000000164	GPT	grey	VSMC_M10	0.18	3.80E-01	0.34	8.34E-02	0.31	1.21E-01	-0.01	9.77E-01	-0.18	3.62E-01	-0.13	5.22E-01	-0.07	7.17E-01	-0.08	6.89E-01	-0.39	4.35E-02	0.08	6.95E-01
ENSCAFG0000002045	ENSCAFG0000002045	grey	VSMC_M10	0.18	3.80E-01	0.00	9.88E-01	0.10	6.31E-01	-0.16	4.32E-01	-0.01	9.55E-01	-0.11	5.95E-01	0.33	9.78E-02	0.17	4.01E-01	-0.10	6.32E-01	-0.04	8.43E-01
ENSCAFG0000002877	ENSCAFG0000002877	grey	VSMC_M10	0.18	3.81E-01	0.47	1.34E-02	-0.14	4.77E-01	0.48	1.17E-02	0.03	8.79E-01	0.13	5.18E-01	-0.30	1.31E-01	-0.55	2.67E-03	-0.45	1.81E-02	0.60	1.04E-03
ENSCAFG0000001189	ENO4	grey	VSMC_M10	0.18	3.81E-01	-0.21	2.93E-01	-0.14	4.88E-01	-0.18	3.76E-01	0.38	5.13E-02	-0.12	5.37E-01	0.23	2.59E-01	0.20	3.19E-01	0.11	5.81E-01	-0.48	1.10E-02
ENSCAFG0000000659	ANKS1B	grey	VSMC_M10	0.18	3.81E-01	-0.15	4.49E-01	0.30	1.30E-01	-0.39	4.21E-02	-0.19	3.54E-01	0.02	9.19E-01	0.49	9.20E-03	0.37	5.53E-02	0.03	8.65E-01	-0.18	3.78E-01
ENSCAFG0000000060	ARID1B	grey	VSMC_M10	0.18	3.81E-01	0.25	2.09E-01	0.19	3.33E-01	0.06	7.54E-01	-0.06	7.67E-01	-0.22	2.63E-01	-0.01	9.66E-01	-0.11	5.70E-01	-0.24	2.23E-01	0.07	7.41E-01
ENSCAFG0000001982	GNAI3	grey	VSMC_M10	0.18	3.81E-01	-0.49	9.78E-03	0.08	6.90E-01	-0.49	9.77E-03	0.24	2.37E-01	-0.21	3.04E-01	-0.26	1.92E-01	0.56	2.14E-03	0.44	2.07E-02	-0.84	3.58E-08
ENSCAFG0000001271	POGZ	grey	VSMC_M10	0.18	3.81E-01	0.30	1.34E-01	0.12	5.42E-01	0.04	8.52E-01	-0.20	3.20E-01	0.05	8.00E-01	0.18	3.61E-01	-0.15	4.60E-01	-0.39	4.58E-02	0.49	8.88E-03
ENSCAFG0000001038	POP5	grey	VSMC_M10	0.18	3.82E-01	-0.04	8.32E-01	0.45	1.76E-02	-0.46	1.65E-02	-0.28	1.58E-01	0.09	6.68E-01	0.52	5.89E-03	0.37	5.79E-02	-0.12	5.62E-01	-0.31	1.18E-01
ENSCAFG0000000903	PFKM	grey	VSMC_M10	0.18	3.82E-01	0.53	4.54E-03	-0.05	8.04E-01	0.57	1.98E-03	0.10	6.23E-01	-0.20	3.24E-01	0.01	9.49E-01	-0.68	8.65E-05	-0.49	8.85E-03	0.48	1.23E-02
ENSCAFG0000002380	KRT25	cyan	VSMC_M2	0.18	3.82E-01	0.55	2.83E-03	0.18	3.79E-01	-0.04	8.59E-01	-0.12	5.54E-01	-0.10	6.25E-01	-0.19	3.32E-01	-0.24	2.33E-01	-0.49	1.02E-02	0.31	1.21E-01
ENSCAFG0000001918	PODN	cyan	VSMC_M2	0.18	3.82E-01	0.78	1.83E-06	0.00	9.98E-01	0.57	1.86E-03	0.05	8.10E-01	-0.33	9.01E-02	-0.27	1.69E-01	-0.81	2.53E-07	-0.61	7.39E-04	0.61	7.07E-04
ENSCAFG0000000399	NUDT6	red	VSMC_M1	0.18	3.82E-01	0.15	4.50E-01	-0.02	9.11E-01	0.18	3.60E-01	-0.07	7.32E-01	0.29	1.46E-01	0.32	1.05E-01	-0.20	3.27E-01	-0.27	1.65E-01	0.36	6.84E-02
ENSCAFG0000003082	POMGNT2	grey	VSMC_M10	0.18	3.82E-01	0.39	4.33E-02	0.09	6.62E-01	0.30	1.27E-01	-0.29	1.47E-01	0.26	1.97E-01	0.05	8.06E-01	-0.27	1.74E-01	-0.50	8.09E-03	0.66	1.63E-04
ENSCAFG0000003274	MXD1	pink	VSMC_M5	0.18	3.83E-01	-0.28	1.53E-01	-0.31	1.16E-01	-0.01	9.62E-01	0.48	1.15E-02	-0.13	5.03E-01	-0.10	6.21E-01	0.08	6.83E-01	0.27	1.77E-01	-0.42	3.02E-02
ENSCAFG0000000714	FBXO3	darkgreen	VSMC_M4	0.17	3.83E-01	0.32	1.07E-01	-0.27	1.81E-01	0.71	3.16E-05	0.19	3.39E-01	-0.15	4.49E-01	-0.26	1.91E-01	-0.64	3.67E-04	-0.27	1.66E-01	0.54	3.60E-03
ENSCAFG0000000422	C21H11orf54	grey	VSMC_M10	0.17	3.83E-01	-0.02	9.13E-01	-0.16	4.31E-01	0.01	9.53E-01	0.33	9.30E-02	-0.17	3.85E-01	0.11	5.86E-01	0.03	8.93E-01	-0.10	6.08E-01	-0.18	3.77E-01
ENSCAFG0000001911	MRM3	grey	VSMC_M10	0.17	3.84E-01	0.06	7.49E-01	0.24	2.30E-01	-0.21	2.87E-01	-0.25	2.07E-01	-0.03	8.96E-01	0.37	5.56E-02	0.26	1.96E-01	-0.27	1.75E-01	0.09	6.58E-01
ENSCAFG0000001863	PTPRM	pink	VSMC_M5	0.17	3.84E-01	-0.33	8.86E-02	-0.56	2.49E-03	0.31	1.14E-01	0.57	1.98E-03	0.16	4.29E-01	0.29	1.36E-01	-0.10	6.09E-01	0.18	3.62E-01	-0.03	8.90E-01

ENSCAFG0000001350	NFE2L2	grey	VSMC_M10	0.17	3.84E-01	0.28	1.57E-01	-0.15	4.45E-01	0.22	2.67E-01	0.25	2.18E-01	-0.13	5.07E-01	0.37	5.80E-02	-0.27	1.77E-01	-0.31	1.19E-01	0.20	3.16E-01
ENSCAFG0000000792	MSC	darkgrey	VSMC_M8	0.17	3.84E-01	-0.37	5.65E-02	0.45	1.80E-02	-0.79	9.61E-07	-0.20	3.25E-01	0.18	3.59E-01	0.16	4.12E-01	0.71	3.96E-05	0.26	1.86E-01	-0.68	9.49E-05
ENSCAFG0000000453	SLC25A30	grey	VSMC_M10	0.17	3.86E-01	0.28	1.65E-01	0.30	1.25E-01	-0.08	6.74E-01	-0.25	2.09E-01	-0.10	6.11E-01	-0.15	4.57E-01	-0.04	8.53E-01	-0.29	1.41E-01	0.07	7.37E-01
ENSCAFG0000001702	XYLT2	cyan	VSMC_M2	0.17	3.86E-01	0.41	3.38E-02	0.61	7.50E-04	-0.37	5.95E-02	-0.51	6.48E-03	0.02	9.21E-01	0.04	8.47E-01	0.19	3.53E-01	-0.50	7.50E-03	0.06	7.50E-01
ENSCAFG0000001840	ENSCAFG0000001840	grey	VSMC_M10	0.17	3.86E-01	-0.44	2.02E-02	-0.16	4.22E-01	-0.33	9.01E-02	0.38	5.03E-02	0.10	6.17E-01	-0.21	2.91E-01	0.45	1.92E-02	0.38	4.99E-02	-0.67	1.48E-04
ENSCAFG0000000616	CYP1B1	darkgreen	VSMC_M4	0.17	3.87E-01	0.19	3.53E-01	-0.49	1.03E-02	0.65	2.46E-04	0.40	4.09E-02	0.03	8.97E-01	0.15	4.45E-01	-0.61	6.47E-04	-0.22	2.62E-01	0.52	5.10E-03
ENSCAFG0000003167	SNAPC2	yellow	VSMC_M3	0.17	3.87E-01	-0.02	9.36E-01	0.61	7.71E-04	-0.60	9.61E-04	-0.46	1.56E-02	-0.02	9.16E-01	0.17	4.08E-01	0.57	1.79E-03	-0.12	5.45E-01	-0.28	1.54E-01
ENSCAFG0000001722	ENSCAFG0000001722	grey	VSMC_M10	0.17	3.87E-01	-0.24	2.33E-01	-0.24	2.34E-01	-0.28	1.58E-01	0.34	8.16E-02	-0.07	7.22E-01	0.33	9.70E-02	0.40	3.81E-02	0.08	7.07E-01	-0.27	1.70E-01
ENSCAFG0000001255	ENSCAFG0000001255	grey	VSMC_M10	0.17	3.87E-01	0.27	1.66E-01	0.47	1.35E-02	-0.16	4.35E-01	-0.41	3.59E-02	0.25	2.02E-01	0.02	9.15E-01	0.00	1.00E+00	-0.32	1.05E-01	0.12	5.45E-01
ENSCAFG0000000507	MYO7A	grey	VSMC_M10	0.17	3.88E-01	0.26	1.83E-01	0.51	6.66E-03	-0.34	8.39E-02	-0.45	1.98E-02	0.15	4.67E-01	-0.07	7.47E-01	0.26	1.85E-01	-0.35	7.07E-02	0.03	8.97E-01
ENSCAFG0000000038	ENSCAFG0000000038	grey	VSMC_M10	0.17	3.88E-01	-0.06	7.61E-01	-0.25	2.09E-01	0.17	4.11E-01	0.24	2.36E-01	0.14	4.98E-01	-0.31	1.13E-01	-0.09	6.44E-01	-0.02	9.11E-01	0.06	7.52E-01
ENSCAFG0000002408	ENSCAFG0000002408	grey	VSMC_M10	0.17	3.88E-01	-0.04	8.58E-01	-0.14	4.77E-01	-0.02	9.19E-01	0.37	5.83E-02	-0.13	5.10E-01	-0.12	5.56E-01	0.00	9.83E-01	0.02	9.09E-01	-0.36	6.85E-02
ENSCAFG0000001914	ENSCAFG0000001914	cyan	VSMC_M2	0.17	3.89E-01	0.55	3.02E-03	0.38	4.97E-02	-0.05	8.15E-01	-0.27	1.67E-01	-0.29	1.40E-01	0.08	6.90E-01	-0.15	4.42E-01	-0.54	3.92E-03	0.24	2.35E-01
ENSCAFG0000001437	TXNRD2	yellow	VSMC_M3	0.17	3.89E-01	0.19	3.48E-01	0.45	1.85E-02	-0.43	2.68E-02	-0.31	1.15E-01	0.17	4.00E-01	0.10	6.36E-01	0.25	2.02E-01	-0.16	4.23E-01	-0.12	5.42E-01
ENSCAFG0000000530	ENSCAFG0000000530	grey	VSMC_M10	0.17	3.89E-01	0.57	1.73E-03	0.19	3.46E-01	0.27	1.73E-01	-0.25	2.18E-01	0.08	6.74E-01	-0.12	5.56E-01	-0.44	2.26E-02	-0.53	4.10E-03	0.56	2.51E-03
ENSCAFG0000000924	VIRMA	pink	VSMC_M5	0.17	3.89E-01	-0.39	4.27E-02	-0.27	1.68E-01	-0.20	3.21E-01	0.56	2.56E-03	0.02	9.15E-01	-0.05	8.24E-01	0.29	1.42E-01	0.30	1.28E-01	-0.59	1.22E-03
ENSCAFG0000001104	PDCD6	grey	VSMC_M10	0.17	3.89E-01	-0.32	1.04E-01	0.09	6.38E-01	-0.21	2.97E-01	0.03	8.63E-01	-0.07	7.45E-01	0.21	2.85E-01	0.32	1.04E-01	0.17	3.83E-01	-0.41	3.16E-02
ENSCAFG0000001218	NLRX1	grey	VSMC_M10	0.17	3.89E-01	0.62	5.86E-04	0.54	3.48E-03	-0.02	9.06E-01	-0.62	5.55E-04	0.28	1.51E-01	0.19	3.35E-01	-0.17	3.88E-01	-0.67	1.30E-04	0.59	1.30E-03
ENSCAFG0000001240	FZD7	grey	VSMC_M10	0.17	3.89E-01	0.67	1.13E-04	0.05	7.90E-01	0.42	2.86E-02	-0.06	7.57E-01	0.06	7.73E-01	-0.60	9.28E-04	-0.61	6.60E-04	-0.56	2.35E-03	0.52	5.62E-03
ENSCAFG0000001077	CISD2	grey	VSMC_M10	0.17	3.89E-01	-0.25	2.17E-01	0.02	9.07E-01	-0.33	9.65E-02	-0.02	9.20E-01	0.21	2.93E-01	0.21	2.92E-01	0.43	2.50E-02	0.05	8.16E-01	-0.15	4.55E-01
ENSCAFG0000000648	ENSCAFG0000000648	grey	VSMC_M10	0.17	3.90E-01	-0.27	1.68E-01	-0.09	6.51E-01	-0.16	4.22E-01	0.19	3.46E-01	0.00	9.88E-01	-0.04	8.50E-01	0.23	2.56E-01	0.20	3.09E-01	-0.25	2.04E-01
ENSCAFG0000000190	ATP10D	cyan	VSMC_M2	0.17	3.90E-01	0.83	6.22E-08	0.10	6.03E-01	0.48	1.14E-02	-0.14	5.00E-01	-0.10	6.24E-01	-0.57	1.72E-03	-0.66	1.62E-04	-0.74	1.25E-05	0.67	1.36E-04
ENSCAFG0000001207	KCNH1	grey	VSMC_M10	0.17	3.90E-01	-0.04	8.48E-01	0.23	2.54E-01	-0.50	8.45E-03	-0.09	6.68E-01	-0.10	6.19E-01	0.32	1.09E-01	0.46	1.59E-02	-0.07	7.14E-01	-0.29	1.46E-01
ENSCAFG0000000794	SSRP1	darkgrey	VSMC_M8	0.17	3.91E-01	-0.29	1.49E-01	0.58	1.52E-03	-0.92	1.64E-11	-0.37	5.89E-02	0.09	6.66E-01	0.40	3.72E-02	0.91	7.37E-11	0.10	6.07E-01	-0.59	1.07E-03
ENSCAFG0000000251	SVBP	grey	VSMC_M10	0.17	3.91E-01	-0.12	5.39E-01	-0.13	5.29E-01	0.07	7.40E-01	0.10	6.12E-01	0.06	7.55E-01	0.06	7.54E-01	0.12	5.68E-01	-0.01	9.77E-01	0.08	6.97E-01
ENSCAFG0000000301	UGCG	pink	VSMC_M5	0.17	3.91E-01	0.02	9.32E-01	-0.53	4.33E-03	0.60	1.02E-03	0.55	2.76E-03	-0.17	4.03E-01	-0.27	1.81E-01	-0.42	3.08E-02	-0.02	9.31E-01	0.14	4.85E-01
ENSCAFG0000001434	FN1	grey	VSMC_M10	0.17	3.91E-01	-0.14	4.95E-01	-0.11	5.92E-01	-0.17	3.93E-01	0.36	6.31E-02	-0.30	1.28E-01	-0.36	6.47E-02	0.05	8.05E-01	0.24	2.35E-01	-0.39	4.25E-02
ENSCAFG0000000379	C19H4orf33	grey	VSMC_M10	0.17	3.91E-01	-0.23	2.48E-01	-0.71	3.49E-05	0.45	1.92E-02	0.70	4.04E-05	0.14	4.86E-01	0.00	9.98E-01	-0.22	2.67E-01	0.09	6.46E-01	0.06	7.74E-01
ENSCAFG0000001136	ENSCAFG0000001136	cyan	VSMC_M2	0.17	3.91E-01	0.44	2.32E-02	0.42	2.92E-02	-0.12	5.65E-01	-0.40	3.89E-02	0.30	1.32E-01	-0.03	8.95E-01	-0.04	8.39E-01	-0.47	1.36E-02	0.25	2.11E-01
ENSCAFG0000001221	KDF1	grey	VSMC_M10	0.17	3.91E-01	-0.07	7.36E-01	-0.30	1.24E-01	0.36	6.64E-02	0.33	9.18E-02	-0.15	4.52E-01	-0.19	3.33E-01	-0.19	3.42E-01	0.01	9.53E-01	0.05	8.12E-01
ENSCAFG0000001784	ENSCAFG0000001784	red	VSMC_M1	0.17	3.92E-01	0.24	2.20E-01	0.29	1.36E-01	0.05	7.93E-01	-0.08	6.98E-01	-0.23	2.48E-01	-0.06	7.82E-01	-0.22	2.80E-01	-0.16	4.21E-01	-0.10	6.37E-01
ENSCAFG0000001838	ESM1	grey	VSMC_M10	0.17	3.92E-01	-0.03	8.96E-01	0.54	4.03E-03	-0.61	7.56E-04	-0.40	3.80E-02	0.47	1.30E-02	0.09	6.41E-01	0.54	3.97E-03	-0.07	7.32E-01	-0.29	1.37E-01
ENSCAFG0000001177	APH1A	cyan	VSMC_M2	0.17	3.92E-01	0.73	1.27E-05	0.34	8.03E-02	0.27	1.71E-01	-0.43	2.57E-02	0.20	3.15E-01	-0.10	6.14E-01	-0.47	1.38E-02	-0.70	4.12E-05	0.67	1.22E-04
ENSCAFG0000001548	SCG3	red	VSMC_M1	0.17	3.92E-01	0.07	7.33E-01	-0.22	2.79E-01	0.28	1.64E-01	0.11	5.83E-01	0.34	8.60E-02	0.26	1.92E-01	-0.19	3.48E-01	-0.20	3.13E-01	0.29	1.36E-01
ENSCAFG0000000510	RPSA	grey	VSMC_M10	0.17	3.92E-01	0.21	2.97E-01	0.51	7.01E-03	-0.35	7.30E-02	-0.37	5.61E-02	-0.03	8.89E-01	0.21	2.82E-01	0.16	4.13E-01	-0.23	2.57E-01	-0.09	6.38E-01
ENSCAFG0000002444	MEX3A	grey	VSMC_M10	0.17	3.92E-01	0.49	9.10E-03	0.06	7.53E-01	0.44	2.14E-02	-0.16	4.37E-01	0.26	1.89E-01	-0.42	2.91E-02	-0.50	7.79E-03	-0.50	7.84E-03	0.57	1.83E-03
ENSCAFG0000000213	ENSCAFG0000000213	grey	VSMC_M10	0.17	3.92E-01	-0.61	8.18E-04	-0.23	2.52E-01	-0.35	7.43E-02	0.42	2.80E-02	-0.13	5.34E-01	0.14	4.84E-01	0.57	2.10E-03	0.44	2.23E-02	-0.69	7.36E-05
ENSCAFG0000001120	DAG1	grey	VSMC_M10	0.17	3.93E-01	0.53	4.07E-03	-0.26	1.91E-01	0.65	2.61E-04	0.31	1.12E-01	-0.30	1.31E-01	-0.36	6.51E-02	-0.78	1.77E-06	-0.37	5.89E-02	0.46	1.63E-02
ENSCAFG0000000757	FAIM	grey	VSMC_M10	0.17	3.93E-01	0.07	7.24E-01	0.13	5.21E-01	-0.19	3.52E-01	0.03	8.74E-01	-0.15	4.58E-01	0.13	5.11E-01	0.16	4.38E-01	-0.16	4.16E-01	-0.18	3.75E-01
ENSCAFG0000000067	PLXNB2	cyan	VSMC_M2	0.17	3.93E-01	0.83	9.85E-08	0.15	4.49E-01	0.60	8.78E-04	-0.24	2.27E-01	-0.14	4.96E-01	-0.43	2.69E-02	-0.75	6.52E-06	-0.75	6.98E-06	0.82	2.16E-07
ENSCAFG0000001194	VEGFD	cyan	VSMC_M2	0.17	3.93E-01	0.74	1.11E-05	-0.12	5.67E-01	0.69	6.04E-05	0.03	8.76E-01	-0.16	4.22E-01	-0.43	2.35E-02	-0.81	3.58E-07	-0.66	2.09E-04	0.74	1.08E-05
ENSCAFG0000000098	SERINC1	pink	VSMC_M5	0.17	3.93E-01	-0.30	1.28E-01	-0.74	8.91E-06	0.55	2.68E-03	0.87	5.65E-09	-0.22	2.68E-01	-0.27	1.81E-01	-0.39	4.35E-02	0.34	8.44E-02	-0.18	3.71E-01
ENSCAFG0000002855	SLC25A45	grey	VSMC_M10	0.17	3.93E-01	-0.01	9.79E-01	-0.15	4.59E-01	0.09	6.69E-01	0.21	2.92E-01	-0.11	5.86E-01	-0.17	3.95E-01	-0.04	8.59E-01	-0.03	8.67E-01	0.02	9.37E-01
ENSCAFG0000001800	MAN2C1	cyan	VSMC_M2	0.17	3.93E-01	0.67	1.45E-04	0.42	2.76E-02	0.15	4.60E-01	-0.47	1.43E-02	0.08	7.06E-01	-0.32	9.83E-02	-0.29	1.37E-01	-0.63	3.99E-04	0.55	2.93E-03
ENSCAFG0000001197	KIF7	cyan	VSMC_M2	0.17	3.93E-01	0.63	4.75E-04	0.38	5.23E-02	0.26	1.91E-01	-0.32	1.04E-01	0.03	8.63E-01	-0.06	7.76E-01	-0.43	2.48E-02	-0.59	1.29E-03	0.41	3.51E-02
ENSCAFG0000001162	PITX2	grey	VSMC_M10	0.17	3.93E-01	-0.05	8.17E-01	-0.23	2.47E-01	0.30	1.28E-01	0.26	1.95E-01	-0.19	3.37E-01	-0.58	1.55E-03	-0.20	3.20E-01	0.08	6.90E-01	0.02	9.36E-01
ENSCAFG0000001241	FAM13C	grey	VSMC_M10	0.17	3.94E-01	-0.02	9.11E-01	-0.15	4.56E-01	0.15	4.58E-01	0.34	8.58E-02	-0.26	1.87E-01	0.28	1.56E-01	-0.16	4.23E-01	0.02	9.17E-01	-0.07	7.40E-01
ENSCAFG0000001046	AGFG1	cyan	VSMC_M2	0.17	3.94E-01	0.42	2.76E-02	-0.11	5.80E-01	0.11	5.81E-01	0.15	4.51E-01	-0.03	8.77E-01	-0.29	1.45E-01	-0.22	2.71E-01	-0.39	4.46E-02	0.24	2.37E-01
ENSCAFG0000000120	HABP4	grey	VSMC_M10	0.17	3.94E-01	-0.05	8.03E-01	-0.03	8.67E-01	-0.04	8.32E-01	0.22	2.66E-01	-0.02	9.14E-01	0.11	6.01E-01	-0.05	8.02E-01	0.09	6.71E-01	-0.23	2.43E-01
ENSCAFG0000000170	APOL6	grey	VSMC_M10	0.17	3.94E-01	0.64	3.52E-04	0.08	6.74E-01	0.57	2.02E-03	-0.14	5.02E-01	-0.08	6.90E-01	-0.16	4.29E-01	-0.65	2.68E-04	-0.62	6.38E-04	0.66	1.57E-04
ENSCAFG0000000674	OLFML1	grey	VSMC_M10	0.17	3.94E-01	0.66	1.78E-04	0.01	9.64E-01	0.53	4.59E-03	-0.04	8.59E-01	-0.10	6.21E-01	-0.03	8.98E-01	-0.65	2.60E-04	-0.62	5.01E-04	0.59	1.30E-03
ENSCAFG0000003226	WNT2	grey	VSMC_M10	0.17	3.94E-01	-0.23	2.52E-01	0.39	4.59E-02	-0.66	1.65E-04	-0.21	2.97E-01	0.03	8.72E-01	0.48	1.21E-02	0.56	2.21E-03	0.15	4.47E-01	-0.42	2.92E-02
ENSCAFG0000000238	FRMPD1	darkgreen	VSMC_M4	0.17	3.94E-01	0.29	1.39E-01	-0.55	2.71E-03	0.86	6.61E-09	0.50	8.06E-03	-0.21	3.04E-01	-0.16	4.26E-01	-0.77	2.75E-06	-0.29	1.49E-01	0.48	1.06E-02
ENSCAFG0000000348	ING3	grey	VSMC_M10	0.17	3.94E-01	-0.26	1.94E-01	0.01	9.79E-01	-0.07	7.23E-01	0.15	4.48E-01	-0.33	9.22E-02	0.39	4.64E-02	0.10	6.02E-01	0.17	3.95E-01	-0.26	1.98E-01
ENSCAFG0000000935	PYROXD2	cyan	VSMC_M2	0.17	3.94E-01	0.69	7.89E-05	-0.22	2.78E-01	0.76	3.87E-06	0.13	5.17E-01	-0.20	3.17E-01	-0.47	1.38E-02	-0.87	4.97E-09	-0.57	1.98E-03	0.74	1.16E-05
ENSCAFG0000000021	CCDC112	grey	VSMC_M10	0.17	3.94E-01	0.31	1.18E-01	0.36	6.44E-02	-0.35	7.69E-02	-0.30	1.23E-01	0.15	4.41E-01	0.39	4.32E-02	0.20	3.08E-01	-0.45	1.81E-02	0.16	4.23E-01
ENSCAFG0000001176	OFD1	grey	VSMC_M10	0.17	3.95E-01	0.38	5.03E-02	0.13	5.13E-01	0.12	5.57E-01	-0.11	5.90E-01	0.00	9.85E-01	0.13	5.26E-01	-0.22	2.76E-01	-0.48	1.20E-02	0.32	1.02E-01
ENSCAFG0000000743	SMYD1	grey	VSMC_M10	0.17	3.95E-01	-0.18	3.58E-01	0.04	8.57E-01	-0.23	2.51E-01	0.04	8.41E-01	-0.09	6.41E-01	0.31	1.15E-01	0.30	1.27E-01	0.08	6.82E-01	-0.16	4.29E-01
ENSCAFG0000000725	PCGF5	darkgreen	VSMC_M4	0.17	3.95E-01	0.43	2.67E-02	-0.37	5.86E-02	0.80	6.38E-07	0.39	4.20E-02	-0.27	1.78E-01	-0.47	1.32E-02	-0.79	7.82E-07	-0.32	9.83E-02	0.42	2.84E-02
ENSCAFG0000000939	PPP3CC	pink	VSMC_M5	0.17	3.95E-01	-0.09	6.38E-01	-0.44	2.33E-02	0.32	1.09E-01	0.56	2.51E-03	-0.11	5.90E-01	-0.12	5.42E-01	-0.22	2.63E-01	0.06	7.73E-01	-0.07	7.20E-01
ENSCAFG0000000489	DBI	darkgrey	VSMC_M8	0.17	3.95E-01	-0.37	5.49E-02	0.20	3.17E-01	-0.65	2.26E-04	-0.08	6.84E-01	0.15	4.41E-01	0.39	4.67E-02	0.76	4.92E-06	0.14	4.76E-01	-0.48	1.07E-02
ENSCAFG0000001758	KATNAL2	pink	VSMC_M5	0.17	3.95E-01	-0.21	2.94E-01	-0.32	9.86E-02	0.04	8.55E-01	0.51	6.23E-03	-0.16	4.28E-01	0.21	2.82E-01	-0.02	9.28E-01	0.22	2.75E-01	-0.28	1.57E-01
ENSCAFG0000001082	ACTN2	darkgreen	VSMC_M4	0.17	3.96E-01	0.39	4.15E-02	-0.35	7.00E-02	0.81	4.08E-07	0.22	2.70E-01	0.05	7.91E-01	-0.35	7.25E-02	-0.74	9.72E-06	-0.41	3.39E-02	0.66	2.04E-04
ENSCAFG0000001683	KAT8	cyan	VSMC_M2	0.17	3.96E-01	0.63	4.37E-04	0.38	5.10E-02	0.00	9.98E-01	-0.44	2.19E-02	0.22	2.79E-01	0.05	8.15E-01	-0.14	4.85E-01	-0.66	1.98E-04	0.52	5.68E-03
ENSCAFG0000001205	MCL1	grey	VSMC_M10	0.17	3.96E-01	0.24	2.26E-01	0.27	1.72E-01	-0.10	6.21E-01	-0.11	5.85E-01	-0.07	7.41E-01	0.04	8.32E-01	-0.11	5.69E-01	-0.24	2.25E-01	0.04	8.37E-01
ENSCAFG0000003037	ENSCAFG0000003037	grey	VSMC_M10	0.17	3.96E-01	0.13	5.02E-01	0.18	3.78E-01	-0.04	8.38E-01	-0.02	9.07E-01	-0.23	2.47E-01	0.25	2.14E-01	-0.06	7.84E-01	-0.18	3.75E-01	-0.01	9.80E-01
ENSCAFG0000000014	STARD6	grey	VSMC_M10	0.17	3.97E-01	0.22	2.68E-01	-0.23	2.40E-01	0.44	2.19E-02	0.05	7.90E-01	0.09	6.57E-01	0.14	4.92E-01	-0.34	8.45E-02	-0.34	8.39E-02	0.59	1.11E-03
ENSCAFG0000000138	NTRK2	grey	VSMC_M10	0.17	3.97E-01	0.02	9.17E-01	-0.15	4.58E-01	0.13	5.31E-01	0.17	3.87E-01	-0.16	4.18E-01	-0.03	8.97E-01	-0.08	6.98E-01	-0.04	8.54E-01	0.11	5.96E-01
ENSCAFG0000000965	HR	darkgrey	VSMC_M8	0.17	3.97E-01	-0.40	4.01E-02	0.30	1.23E-01	-0.75	6.12E-06	0.03	8.74E-01	-0.12	5.39E-01	0.34	7.81E-02	0.70	4.71E-05	0.28	1.61E-01	-0.79	7.65E-07
ENSCAFG0000000124	PTCH1	grey	VSMC_M10	0.17	3.97E-01	0.24	2.19E-01	-0.31	1.10E-01	0.48	1.16E-02	0.35	7.01E-02	0.01	9.56E-01	-0.30	1.31E-01	-0.59	1.10E-03	-0.14	4.85E-01	0.25	2.04E-01
ENSCAFG0000001062	SORCS1	red	VSMC_M1	0.17	3.98E-01	-0.03	8.67E-01	0.00	9.95E-01	-0.08	7.00E-01	0.03	8.69E-01	0.07	7.14E-01	-0.55	3.03E-03	0.11	5.98E-01	0.01	9.60E-01	-0.10	6.33E-01
ENSCAFG0000001675	ORAI3	cyan	VSMC_M2	0.17	3.98E-01	0.86	7.44E-09	0.42	2.92E-02	0.18	3.66E-01	-0.45	1.95E-02	0.10	6.09E-01	-0.16	4.18E-01	-0.53	4.37E-03	-0.78	1.45E-06	0.69	7.60E-05
ENSCAFG0000000550	UGGT2	grey	VSMC_M10	0.17	3.98E-01	0.51	7.07E-03	-0.11	5.72E-01	0.35	7.45E-02	0.30	1.30E-01	-0.45	1.82E-02	-0.47	1.43E-02	-0.52	4.96E-03	-0.35	7.14E-02	0.18	3.79E-01
ENSCAFG0000000833	ENSCAFG0000000833	grey	VSMC_M10	0.17	3.98E-01	0.27	1.67E-01	-0.16	4.26E-01	0.29	1.36E-01	0.18	3.75E-01	-0.10	6.26E-01	-0.25	2.13E-01	-0.29	1.41E-01	-0.27	1.81E-01	0.26	1.91E-01
ENSCAFG0000001110	PDE6D	grey	VSMC_M10	0.17	3.98E-01	0.21	2.98E-01	0.10	6.24E-01	-0.12	5.47E-01	0.12	5.62E-01	-0.15	4.47E-01	-0.02	9.18E-01	-0.02	9.08E-01	-0.21	2.95E-01	-0.18	3.60E-01
ENSCAFG0000001763	TIMM8A	darkgrey	VSMC_M8	0.17	3.98E-01	-0.34	8.52E-02	0.19	3.40E-01	-0.55	3.28E-03	-0.09	6.42E-01	0.13	5.06E-01	0.31	1.19E-01	0.64	3.29E-04	0.11	5.85E-01	-0.42	2.73E-02
ENSCAFG0000001011	GAS2	grey	VSMC_M10	0.17	3.98E-01	0.22	2.61E-01	0.09	6.54E-01	-0.10	6.33E-01	0.01	9.44E-01	-0.10	6.22E-01	-0.13	5.09E-01	0.03	9.01E-01	-0.19	3.47E-01	0.06	7.83E-01
ENSCAFG0000001115	CFAP46	grey	VSMC_M10	0.17	3.98E-01	0.36	6.38E-02	-0.09	6.49E-01	0.50	7.39E-03	0.02	9.15E-01	-0.01	9.41E-01	0.03	8.70E-01	-0.54	3.68E-03	-0.39	4.33E-02	0.53	4.13E-03
ENSCAFG0000000384	RUVBL2	darkgrey	VSMC_M8	0.17	3.98E-01	-0.11	5.95E-01	0.46	1.46E-02	-0.72	2.46E-05	-0.27	1.73E-01	-0.04	8.56E-01	0.38	4.94E-02	0.68	1.05E-04	-0.06	7.64E-01	-0.42	2.83E-02
ENSCAFG0000000425	PRTFDC1	grey	VSMC_M10	0.17	3.99E-01	-0.53	4.64E-03	-0.16	4.35E-01	-0.05	8.15E-01	0.33	9.49E-02	0.05	7.97E-01	0.12	5.54E-01	0.26	1.96E-01	0.41	3.57E-02	-0.50	8.38E-03
ENSCAFG0000002274	ENSCAFG0000002274	cyan	VSMC_M2	0.17	3.99E-01	0.42	2.97E-02	0.19	3.53E-01	-0.12	5.42E-01	-0.07	7.24E-01	0.00	9.87E-01	-0.03	8.79E-01	-0.05	8.07E-01	-0.37	5.95E-02	0.18	3.70E-01
ENSCAFG0000000004	DSEL	darkgreen	VSMC_M4	0.17	3.99E-01	0.06	7.51E-01	-0.53	4.54E-03	0.58	1.67E-03	0.47	1.27E-02	-0.02	9.39E-01	-0.03	8.78E-01	-0.40	3.93E-02	-0.14	4.91E-01	0.37	5.54E-02
ENSCAFG0000002415	ARF6	darkgrey	VSMC_M8	0.17	4.00E-01	0.13	5.29E-01	0.60	1.02E-03	-0.69	5.77E-05	-0.45	1.88E-02	0.14	4.85E-01	0.25	2.05E-01	0.59	1.09E-03	-0.28	1.56E-01	-0.27	1.68E-01
ENSCAFG0000000374	PRPF38A	grey	VSMC_M10	0.17	4.00E-01	-0.08	6.77E-01	0.28	1.56E-01	-0.19	3.54E-01	-0.19	3.48E-01	-0.05	8.09E-01	0.28	1.55E-01	0.30	1.34E-01	-0.08	7.07E-01	-0.13	5.32E-01
ENSCAFG0000001876	CATSPERD	grey	VSMC_M10	0.17	4.00E-01	-0.01	9.77E-01	-0.30	1.31E-01	0.25	2.01E-01	0.29	1.38E-01	-0.16	4.35E-01	0.14	4.77E-01	-0.21	3.02E-01	-0.09	6.39E-01	0.20	3.14E-01
ENSCAFG0000002899	MTPN	pink	VSMC_M5	0.17	4.00E-01	-0.45	1.72E-02	-0.51	5.99E-03	0.10	6.30E-01	0.71	3.02E-05	-0.11	5.82E-01	-0.25	2.10E-01	0.04	8.46E-01	0.47	1.28E-02	-0.48	1.19E-02
ENSCAFG0000001762	CARM1	yellow	VSMC_M3	0.17	4.00E-01	0.25	2.14E-01	0.79	8.70E-07	-0.70	5.44E-05	-0.71	3.63E-05	0.14	4.78E-01	0.17	3.85E-01	0.51	6.31E-03	-0.33	8.90E-02	-0.05	7.97E-01
ENSCAFG0000000144	GRINA	cyan	VSMC_M2	0.17	4.00E-01	0.94	6.10E-13	0.56	2.39E-03	0.09	6.58E-01	-0.58	1.44E-03	0.01	9.56E-01	-0.26	1.98E-01	-0.43	2.55E-02	-0.84	3.93E-08	0.64	2.88E-04
ENSCAFG0000001504	FBXO34	darkgreen	VSMC_M4	0.17	4.00E-01	0.31	1.12E-01	-0.21	2.90E-01	0.71	3.94E-05	0.15	4.65E-01	-0.09	6.44E-01	-0.24	2.33E-01	-0.63	3.96E-04	-0.33	8.98E-02	0.47	1.26E-02
ENSCAFG0000002856	JCHAIN	grey	VSMC_M10	0.17	4.01E-01	0.14	4.99E-01	-0.23	2.44E-01	0.27	1.65E-01	0.40	3.65E-02	-0.23	2.39E-01	-0.29	1.44E-01	-0.44	2.08E-02	0.01	9.42E-01	0.01	9.68E-01
ENSCAFG0000000477	KIF2C	darkgrey	VSMC_M8	0.17	4.01E-01	-0.41	3.28E-02	0.29	1.41E-01	-0.68	8.70E-05	-0.16	4.39E-01	0.08	6.91E-01	0.57	2.05E-03	0.76	4.06E-06	0.15	4.69E-01	-0.46	1.65E-02
ENSCAFG0000001848	CERS4	cyan	VSMC_M2	0.17	4.01E-01	0.78	1.70E-06	0.41	3.45E-02	0.12	5.46E-01	-0.47	1.31E-02	0.08	6.98E-01	-0.23	2.44E-01	-0.29	1.40E-01	-0.74	9.25E-06	0.61	8.21E-04
ENSCAFG0000003236	ZDHHC22	pink	VSMC_M5	0.17	4.01E-01	0.19	3.33E-01	-0.40	3.79E-02	0.48	1.05E-02	0.40	3.76E-02	0.29	1.38E-01	-0.04	8.52E-01	-0.45	1.73E-02	-0.21	2.88E-01	0.24	2.34E-01
ENSCAFG0000000038	LATS1	pink	VSMC_M5	0.17	4.01E-01	-0.48	1.09E-02	-0.50	8.06E-03	-0.05	7.92E-01	0.77	3.12E-06	-0.34	8.65E-02	0.07	7.43E-01	0.18	3.66E-01	0.42	3.11E-02	-0.55	3.18E-03
ENSCAFG0000001929	ACAP3	yellow	VSMC_M3	0.17	4.01E-01	0.58	1.43E-03	0.82	1.69E-07	-0.21	2.83E-01	-0.78	1.80E-06	0.07	7.41E-01	0.02	9.23E-01	-0.03	8.89E-01	-0.59	1.13E-03	0.32	1.06E-01
ENSCAFG0000001647	PSMG3	darkgrey	VSMC_M8	0.17	4.01E-01	-0.21	2.82E-01	0.49	8.86E-03	-0.61	6.75E-04	-0.32	9.87E-02	0.14	4.81E-01	0.25	2.05E-01	0.56	2.65E-03	0.10	6.06E-01	-0.49	1.00E-02
ENSCAFG0000001839	NDC80	darkgrey	VSMC_M8	0.17	4.01E-01	-0.52	5.85E-03	0.19	3.50E-01	-0.73	1.39E-05	-0.05	8.20E-01	0.13	5.24E-01	0.46	1.71E-02	0.87	5.03E-09	0.26	1.94E-01	-0.57	2.04E-03
ENSCAFG0000000419	ENSCAFG0000000419	grey	VSMC_M10	0.17	4.01E-01	0.00	9.89E-01	0.16	4.24E-01	-0.29	1.44E-01	-0.04	8.33E-01	-0.06	7.61E-01	0.31	1.12E-01	0.29	1.36E-01	-0.15	4.43E-01	-0.06	7.83E-01
ENSCAFG0000001042	ENSCAFG0000001042	yellow	VSMC_M3	0.17	4.02E-01	0.46	1.61E-02	0.61	7.99E-04	-0.35	7.05E-02	-0.54	3.60E-03	0.08	6.85E-01	0.26	1.96E-01	0.13	5.32E-01	-0.52	5.62E-03	0.23	2.48E-01
ENSCAFG0000001401	LIMD1	yellow	VSMC_M3	0.17	4.02E-01	0.45	1.98E-02	0.71	3.63E-05	-0.56	2.21E-03	-0.55	2.75E-03	0.02	9.02E-01	0.26	1.96E-01	0.23	2.43E-01	-0.46	1.68E-02	0.04	8.38E-01
ENSCAFG0000002008	ZFP1	grey	VSMC_M10	0.17	4.02E-01	0.06	7.73E-01	0.25	2.08E-01	-0.28	1.61E-01	-0.21	2.89E-01	0.15	4.62E-01	0.08	6.75E-01	0.20	3.20E-01	-0.12	5.48E-01	0.05	8.02E-01
ENSCAFG0000000617	CDC25B	darkgrey	VSMC_M8	0.17	4.03E-01	-0.10	6.25E-01	0.64	3.27E-04	-0.93	3.18E-12	-0.42	3.01E-02	0.01	9.65E-01	0.46	1.63E-02	0.80	5.00E-07	-0.04	8.27E-01	-0.48	1.12E-02
ENSCAFG0000001037	ENSCAFG0000001037	grey	VSMC_M10	0.17	4.03E-01	0.02	9.29E-01	0.14	4.99E-01	-0.17	3.88E-01	0.00	9.96E-01	0.00	9.88E-01	0.12	5.53E-01	0.10	6.28E-01	-0.08	7.04E-01	-0.19	3.54E-01
ENSCAFG0000000004	ENSCAFG0000000004	grey	VSMC_M10	0.17	4.03E-01	0.26	1.90E-01	0.19	3.47E-01	0.15	4.68E-01	-0.13	5.22E-01	-0.06	7.51E-01	-0.02	9.35E-01	-0.23	2.55E-01	-0.22	2.69E-01	0.22	2.73E-01
ENSCAFG0000003228	HOXA3	grey	VSMC_M10	0.17	4.03E-01	0.39	4.58E-02	0.35	7.39E-02	-0.21	2.89E-01	-0.37	5.91E-02	0.16	4.14E-01	-0.27	1.74E-01	0.09	6.69E-01	-0.46	1.47E-02	0.25	2.05E-01
ENSCAFG0000003036	ENSCAFG0000003036	yellow	VSMC_M3	0.17	4.04E-01	0.51	6.19E-03	0.75	6.10E-06	-0.26	1.89E-01	-0.71	3.12E-05	0.04	8.43E-01	-0.04	8.58E-01	-0.01	9.56E-01	-0.51	6.03E-03	0.29	1.39E-01
ENSCAFG0000001481	PPP3CB	grey	VSMC_M10	0.17	4.04E-01	-0.21	2.91E-01	0.28	1.57E-01	-0.51	6.51E-03	0.10	6.34E-01	-0.36	6.50E-02	0.01	9.70E-01	0.41	3.51E-02	0.23	2.57E-01	-0.66	1.77E-04
ENSCAFG0000000978	YAF2	yellow	VSMC_M3	0.17	4.04E-01	0.30	1.33E-01	0.42	3.10E-02	0.05	8.23E-01	-0.27	1.72E-01	-0.08	7.03E-01	-0.24	2.28E-01	-0.13	5.08E-01	-0.27	1.67E-01	0.02	9.22E-01
ENSCAFG0000001327	ENSCAFG0000001327	cyan	VSMC_M2	0.17	4.04E-01	0.69	7.34E-05	0.22	2.81E-01	0.15	4.56E-01	-0.24	2.33E-01	-0.08	6.95E-01	-0.04	8.57E-01	-0.34	8.05E-02	-0.72	2.45E-05	0.57	2.13E-03
ENSCAFG0000001603	PSEN2	grey	VSMC_M10	0.17	4.04E-01	0.79	1.01E-06	0.18	3.64E-01	0.44	2.12E-02	-0.26	1.87E-01	0.10	6.07E-01	-0.21	2.89E-01	-0.59	1.34E-03	-0.75	7.51E-06	0.75	6.29E-06
ENSCAFG0000001225	ENSCAFG0000001225	grey	VSMC_M10	0.17	4.05E-01	-0.10	6.37E-01	-0.01	9.76E-01	-0.06	7.68E-01	0.13	5.32E-01	-0.14	4.75E-01	-0.16	4.13E-01	0.07	7.23E-01	0.10	6.11E-01	-0.16	4.34E-01
ENSCAFG0000002429	PJVK	grey	VSMC_M10	0.17	4.05E-01	-0.07	7.32E-01	-0.23	2.41E-01	0.12	5.38E-01	0.26	1.83E-01	-0.12	5.52E-01	0.11	5.89E-01	-0.11	5.89E-01	0.05	8.00E-01	0.03	8.79E-01
ENSCAFG0000001446	PTPN6	grey	VSMC_M10	0.17	4.05E-01	-0.06	7.76E-01	-0.33	8.85E-02	0.32	1.06E-01	0.36	6.74E-02	-0.13	5.15E-01	0.13	5.13E-01	-0.17	3.92E-01	-0.04	8.37E-01	0.11	5.91E-01
ENSCAFG0000001150	NOL11	darkgrey	VSMC_M8	0.17	4.06E-01	-0.45	1.84E-02	-0.08	7.08E-01	-0.33	8.85E-02	0.13	5.19E-01	0.07	7.26E-01	0.24	2.27E-01	0.56	2.58E-03	0.16	4.22E-01	-0.37	5.73E-02
ENSCAFG0000003036	ENSCAFG0000003036	grey	VSMC_M10	0.17	4.06E-01	0.31	1.11E-01	-0.12	5.49E-01	0.35	7.05E-02	0.20	3.15E-01	-0.29	1.49E-01	-0.11	5.84E-01	-0.37	5.47E-02	-0.33	9.09E-02	0.22	2.65E-01
ENSCAFG0000000324	WDR92	grey	VSMC_M10	0.17	4.06E-01	-0.23	2.41E-01	0.00	9.98E-01	0.05	7.89E-01	0.13	5.08E-01	-0.10	6.12E-01	-0.04	8.41E-01	0.04	8.51E-01	0.19	3.41E-01	-0.23	2.53E-01

ENSCAFG0000001662	UNC5A	grey	VSMC_M10	0.17	4.06E-01	0.41	3.17E-02	0.33	9.14E-02	-0.22	2.67E-01	-0.31	1.18E-01	0.20	3.30E-01	0.13	5.18E-01	0.04	8.34E-01	-0.48	1.13E-02	0.20	3.10E-01
ENSCAFG0000001930	ENSCAFG0000001930	grey	VSMC_M10	0.17	4.06E-01	0.13	5.34E-01	-0.03	8.87E-01	0.16	4.25E-01	0.08	6.96E-01	-0.11	5.72E-01	-0.17	3.85E-01	-0.12	5.37E-01	-0.15	4.48E-01	0.08	7.01E-01
ENSCAFG0000001238	SLC37A4	grey	VSMC_M10	0.17	4.06E-01	0.09	6.69E-01	0.14	4.88E-01	-0.27	1.81E-01	-0.02	9.15E-01	-0.11	5.90E-01	0.23	2.52E-01	0.23	2.49E-01	-0.13	5.05E-01	-0.12	5.61E-01
ENSCAFG0000000295	ENSCAFG0000000295	grey	VSMC_M10	0.17	4.06E-01	0.14	4.84E-01	0.25	2.07E-01	-0.13	5.30E-01	-0.15	4.43E-01	0.25	2.13E-01	-0.15	4.44E-01	0.05	8.02E-01	-0.17	3.86E-01	-0.05	8.07E-01
ENSCAFG0000000271	DNAH11	grey	VSMC_M10	0.17	4.07E-01	0.15	4.59E-01	0.42	2.90E-02	-0.31	1.10E-01	-0.36	6.63E-02	-0.22	2.79E-01	0.30	1.28E-01	0.25	2.18E-01	-0.23	2.52E-01	0.10	6.25E-01
ENSCAFG0000001715	HDAC8	grey	VSMC_M10	0.17	4.07E-01	0.06	7.74E-01	0.14	4.87E-01	-0.11	5.91E-01	0.04	8.25E-01	0.11	5.76E-01	0.16	4.22E-01	-0.02	9.19E-01	-0.08	6.79E-01	-0.22	2.65E-01
ENSCAFG0000000666	ALKBH6	grey	VSMC_M10	0.17	4.07E-01	0.41	3.60E-02	0.09	6.53E-01	0.21	2.91E-01	-0.01	9.62E-01	0.00	9.89E-01	-0.36	6.30E-02	-0.36	6.51E-02	-0.31	1.19E-01	0.15	4.44E-01
ENSCAFG0000001799	C30H15orf39	yellow	VSMC_M3	0.17	4.08E-01	0.24	2.20E-01	0.68	8.76E-05	-0.69	7.25E-05	-0.62	5.68E-04	0.25	2.08E-01	0.27	1.79E-01	0.51	7.07E-03	-0.40	4.07E-02	-0.02	9.06E-01
ENSCAFG0000000360	RRAS	cyan	VSMC_M2	0.17	4.08E-01	0.36	6.26E-02	0.14	4.80E-01	0.38	5.25E-02	-0.07	7.42E-01	-0.19	3.31E-01	-0.29	1.49E-01	-0.55	3.25E-03	-0.23	2.55E-01	0.26	1.82E-01
ENSCAFG0000002984	ELMOD2	pink	VSMC_M5	0.17	4.08E-01	0.10	6.02E-01	-0.34	7.92E-02	0.36	6.81E-02	0.51	6.62E-03	-0.43	2.46E-02	0.00	9.82E-01	-0.35	7.21E-02	-0.12	5.57E-01	0.02	9.13E-01
ENSCAFG0000000226	FAM120A	darkgrey	VSMC_M8	0.17	4.09E-01	-0.21	2.86E-01	0.23	2.55E-01	-0.74	9.12E-06	0.00	9.84E-01	0.04	8.36E-01	0.23	2.51E-01	0.66	1.69E-04	0.11	5.91E-01	-0.48	1.21E-02
ENSCAFG0000001741	DENND4B	cyan	VSMC_M2	0.17	4.09E-01	0.72	2.72E-05	0.52	5.60E-03	-0.06	7.65E-01	-0.49	9.36E-03	0.06	7.65E-01	-0.03	8.63E-01	-0.22	2.81E-01	-0.68	1.05E-04	0.41	3.58E-02
ENSCAFG0000002020	SCAI	grey	VSMC_M10	0.17	4.09E-01	-0.07	7.33E-01	0.28	1.54E-01	-0.39	4.41E-02	-0.12	5.64E-01	-0.06	7.64E-01	0.19	3.36E-01	0.31	1.16E-01	0.01	9.68E-01	-0.21	2.84E-01
ENSCAFG0000001925	MRPL20	yellow	VSMC_M3	0.17	4.09E-01	0.09	6.60E-01	0.76	3.70E-06	-0.58	1.56E-03	-0.69	6.07E-05	-0.04	8.51E-01	0.28	1.63E-01	0.49	8.84E-03	-0.23	2.41E-01	-0.11	5.92E-01
ENSCAFG0000003276	PCBD2	grey	VSMC_M10	0.17	4.09E-01	-0.18	3.82E-01	0.01	9.57E-01	-0.04	8.32E-01	0.20	3.06E-01	-0.04	8.43E-01	-0.22	2.81E-01	0.06	7.85E-01	0.15	4.67E-01	-0.36	6.62E-02
ENSCAFG0000001409	ENSCAFG0000001409	grey	VSMC_M10	0.17	4.09E-01	0.03	8.80E-01	0.29	1.47E-01	-0.23	2.56E-01	-0.21	2.83E-01	-0.12	5.59E-01	0.32	1.06E-01	0.11	5.89E-01	-0.06	7.48E-01	-0.02	9.35E-01
ENSCAFG0000001365	ENSCAFG0000001365	grey	VSMC_M10	0.17	4.09E-01	0.00	9.91E-01	-0.05	7.95E-01	-0.07	7.25E-01	0.16	4.23E-01	-0.06	7.51E-01	-0.12	5.67E-01	0.07	7.19E-01	-0.06	7.48E-01	-0.06	7.68E-01
ENSCAFG0000003028	ENSCAFG0000003028	grey	VSMC_M10	0.17	4.09E-01	0.00	9.91E-01	-0.05	7.95E-01	-0.07	7.25E-01	0.16	4.23E-01	-0.06	7.51E-01	-0.12	5.67E-01	0.07	7.19E-01	-0.06	7.48E-01	-0.06	7.68E-01
ENSCAFG0000001327	WIPF1	cyan	VSMC_M2	0.17	4.09E-01	0.44	2.02E-02	0.57	1.84E-03	-0.33	9.05E-02	-0.55	2.95E-03	-0.01	9.77E-01	0.47	1.31E-02	0.10	6.30E-01	-0.50	7.55E-03	0.33	9.76E-02
ENSCAFG0000000304	KIAA1958	grey	VSMC_M10	0.17	4.10E-01	0.41	3.40E-02	0.52	5.74E-03	-0.10	6.21E-01	-0.57	1.93E-03	0.34	8.06E-02	-0.04	8.62E-01	-0.01	9.79E-01	-0.50	8.26E-03	0.38	5.24E-02
ENSCAFG0000002033	ENSCAFG0000002033	grey	VSMC_M10	0.17	4.10E-01	-0.23	2.42E-01	0.01	9.64E-01	-0.41	3.54E-02	0.08	6.78E-01	0.20	3.16E-01	0.16	4.14E-01	0.50	8.52E-03	0.09	6.71E-01	-0.35	7.74E-02
ENSCAFG0000000322	EEPD1	cyan	VSMC_M2	0.17	4.10E-01	0.74	1.05E-05	0.49	9.42E-03	0.03	8.63E-01	-0.54	3.36E-03	0.09	6.55E-01	-0.32	1.07E-01	-0.31	1.15E-01	-0.69	7.51E-05	0.59	1.34E-03
ENSCAFG0000001122	ENSCAFG0000001122	grey	VSMC_M10	0.17	4.10E-01	-0.33	9.34E-02	-0.07	7.24E-01	-0.37	6.04E-02	0.35	6.97E-02	-0.36	6.21E-02	0.04	8.52E-01	0.32	1.04E-01	0.33	9.14E-02	-0.62	5.90E-04
ENSCAFG0000001453	GPC2	cyan	VSMC_M2	0.17	4.10E-01	0.49	9.07E-03	0.28	1.50E-01	0.33	9.57E-02	-0.39	4.28E-02	0.13	5.14E-01	-0.18	3.66E-01	-0.40	3.77E-02	-0.48	1.13E-02	0.59	1.16E-03
ENSCAFG0000000305	ENSCAFG0000000305	grey	VSMC_M10	0.17	4.10E-01	0.15	4.49E-01	0.25	2.05E-01	-0.06	7.51E-01	-0.15	4.53E-01	0.06	7.54E-01	-0.05	8.06E-01	-0.07	7.33E-01	-0.12	5.49E-01	0.04	8.35E-01
ENSCAFG0000000763	CLSTN2	grey	VSMC_M10	0.17	4.10E-01	0.30	1.25E-01	0.28	1.54E-01	-0.14	4.90E-01	-0.32	1.00E-01	0.26	1.87E-01	-0.26	1.94E-01	0.06	7.51E-01	-0.37	5.63E-02	0.27	1.81E-01
ENSCAFG0000000387	METTL24	darkgreen	VSMC_M4	0.17	4.10E-01	0.22	2.77E-01	-0.54	3.84E-03	0.77	2.73E-06	0.50	8.55E-03	-0.19	3.40E-01	-0.28	1.60E-01	-0.68	9.66E-05	-0.21	3.04E-01	0.42	2.86E-02
ENSCAFG0000001532	NDUFA9	grey	VSMC_M10	0.17	4.11E-01	0.11	5.78E-01	0.49	9.23E-03	-0.43	2.68E-02	-0.44	2.23E-02	0.16	4.32E-01	0.37	6.03E-02	0.34	8.32E-02	-0.24	2.35E-01	-0.07	7.33E-01
ENSCAFG0000001888	FGGY	grey	VSMC_M10	0.16	4.11E-01	0.30	1.35E-01	-0.13	5.10E-01	0.46	1.69E-02	0.17	4.02E-01	-0.24	2.32E-01	0.14	4.92E-01	-0.56	2.65E-03	-0.28	1.60E-01	0.36	6.34E-02
ENSCAFG0000001607	IGFBP4	grey	VSMC_M10	0.16	4.11E-01	0.76	5.28E-06	0.21	2.97E-01	0.41	3.61E-02	-0.20	3.21E-01	-0.16	4.37E-01	-0.06	7.77E-01	-0.62	5.75E-04	-0.69	6.61E-05	0.62	5.71E-04
ENSCAFG0000002382	PMAIP1	turquoise	VSMC_M6	0.16	4.11E-01	-0.05	8.02E-01	0.20	3.07E-01	-0.15	4.55E-01	-0.19	3.33E-01	0.40	4.00E-02	-0.06	7.83E-01	0.11	5.97E-01	-0.03	8.81E-01	0.00	1.00E+00
ENSCAFG0000002432	TAF12	grey	VSMC_M10	0.16	4.11E-01	0.26	1.87E-01	0.44	2.05E-02	-0.08	6.90E-01	-0.54	3.84E-03	-0.03	8.69E-01	0.13	5.31E-01	-0.01	9.78E-01	-0.35	7.46E-02	0.39	4.69E-02
ENSCAFG0000001496	ENSCAFG0000001496	darkgrey	VSMC_M8	0.16	4.11E-01	-0.16	4.24E-01	0.67	1.40E-04	-0.84	4.54E-08	-0.53	4.78E-03	0.17	3.98E-01	0.31	1.10E-01	0.77	3.01E-06	0.03	8.89E-01	-0.43	2.55E-02
ENSCAFG0000001714	ENSCAFG0000001714	grey	VSMC_M10	0.16	4.12E-01	-0.11	5.99E-01	0.33	9.52E-02	-0.23	2.44E-01	-0.20	3.14E-01	-0.03	8.76E-01	0.06	7.58E-01	0.21	2.86E-01	-0.02	9.17E-01	-0.23	2.53E-01
ENSCAFG0000000691	MAP3K1	darkgreen	VSMC_M4	0.16	4.12E-01	0.55	2.86E-03	-0.15	4.65E-01	0.71	2.93E-05	0.03	8.67E-01	0.04	8.36E-01	-0.21	2.95E-01	-0.71	3.83E-05	-0.56	2.27E-03	0.73	1.48E-05
ENSCAFG0000001566	TIMM17B	grey	VSMC_M10	0.16	4.12E-01	0.04	8.29E-01	0.46	1.61E-02	-0.37	5.77E-02	-0.33	9.58E-02	0.13	5.12E-01	-0.14	4.94E-01	0.30	1.30E-01	-0.08	6.80E-01	-0.29	1.46E-01
ENSCAFG0000002029	TANGO6	grey	VSMC_M10	0.16	4.12E-01	0.29	1.36E-01	-0.06	7.59E-01	0.34	8.23E-02	0.04	8.32E-01	-0.09	6.58E-01	-0.10	6.27E-01	-0.34	8.54E-02	-0.34	7.86E-02	0.37	5.97E-02
ENSCAFG0000000056	ENSCAFG0000000056	grey	VSMC_M10	0.16	4.12E-01	0.10	6.03E-01	-0.06	7.53E-01	0.12	5.63E-01	0.18	3.68E-01	-0.29	1.48E-01	-0.45	1.80E-02	-0.13	5.29E-01	-0.14	5.00E-01	-0.02	9.33E-01
ENSCAFG0000001218	ZNRD1	yellow	VSMC_M3	0.16	4.13E-01	0.42	2.84E-02	0.58	1.47E-03	-0.03	8.74E-01	-0.56	2.28E-03	0.11	5.76E-01	0.09	6.44E-01	-0.09	6.73E-01	-0.48	1.19E-02	0.28	1.58E-01
ENSCAFG0000001798	SCAMP5	grey	VSMC_M10	0.16	4.13E-01	0.65	2.49E-04	-0.03	8.97E-01	0.59	1.11E-03	-0.07	7.18E-01	0.07	7.41E-01	-0.24	2.19E-01	-0.76	3.72E-06	-0.56	2.55E-03	0.74	1.23E-05
ENSCAFG0000001268	CCDC157	grey	VSMC_M10	0.16	4.13E-01	0.52	5.24E-03	-0.13	5.29E-01	0.40	3.95E-02	0.04	8.58E-01	-0.07	7.25E-01	-0.01	9.69E-01	-0.50	7.59E-03	-0.54	3.70E-03	0.67	1.53E-04
ENSCAFG0000000830	ZNF395	grey	VSMC_M10	0.16	4.14E-01	0.07	7.21E-01	-0.12	5.62E-01	0.00	9.96E-01	0.36	6.58E-02	-0.41	3.27E-02	-0.01	9.44E-01	-0.11	5.74E-01	0.03	8.77E-01	-0.18	3.76E-01
ENSCAFG0000001270	MRPL11	darkgrey	VSMC_M8	0.16	4.14E-01	-0.10	6.24E-01	0.53	4.70E-03	-0.70	4.25E-05	-0.42	3.13E-02	0.00	9.98E-01	0.42	2.79E-02	0.61	7.07E-04	-0.05	8.21E-01	-0.30	1.30E-01
ENSCAFG0000001991	FOXC2	cyan	VSMC_M2	0.16	4.14E-01	0.52	5.56E-03	0.34	8.23E-02	0.12	5.64E-01	-0.32	9.85E-02	0.02	9.30E-01	-0.60	8.29E-04	-0.25	2.11E-01	-0.42	2.91E-02	0.34	8.14E-02
ENSCAFG0000000171	TOM1	yellow	VSMC_M3	0.16	4.14E-01	0.41	3.26E-02	0.63	4.05E-04	-0.46	1.69E-02	-0.44	2.23E-02	-0.09	6.71E-01	-0.42	2.72E-02	0.15	4.60E-01	-0.31	1.20E-01	-0.12	5.48E-01
ENSCAFG0000000688	CD44	darkgrey	VSMC_M8	0.16	4.14E-01	-0.55	3.19E-03	-0.01	9.62E-01	-0.60	9.35E-04	0.24	2.19E-01	-0.16	4.12E-01	0.46	1.62E-02	0.63	4.37E-04	0.42	2.99E-02	-0.67	1.19E-04
ENSCAFG0000001611	FOXK1	yellow	VSMC_M3	0.16	4.15E-01	0.38	5.28E-02	0.37	5.49E-02	-0.32	9.90E-02	-0.22	2.77E-01	-0.01	9.78E-01	-0.50	8.09E-03	0.07	7.25E-01	-0.29	1.46E-01	-0.04	8.30E-01
ENSCAFG0000000281	HAS1	grey	VSMC_M10	0.16	4.15E-01	0.71	2.95E-05	0.27	1.78E-01	0.24	2.29E-01	-0.34	8.47E-02	-0.17	3.88E-01	-0.38	4.75E-02	-0.39	4.64E-02	-0.66	2.03E-04	0.60	9.40E-04
ENSCAFG0000000849	DLGAP4	grey	VSMC_M10	0.16	4.15E-01	-0.01	9.62E-01	0.28	1.54E-01	-0.39	4.64E-02	-0.02	9.31E-01	-0.46	1.48E-02	0.10	6.23E-01	0.17	3.90E-01	0.07	7.33E-01	-0.32	9.92E-02
ENSCAFG0000001523	SLC19A2	darkgrey	VSMC_M8	0.16	4.15E-01	-0.31	1.16E-01	0.44	2.25E-02	-0.82	1.73E-07	-0.27	1.68E-01	0.04	8.43E-01	0.59	1.28E-03	0.82	1.61E-07	0.11	6.00E-01	-0.49	9.53E-03
ENSCAFG0000001349	ENSCAFG0000001349	pink	VSMC_M5	0.16	4.15E-01	-0.03	8.85E-01	-0.47	1.39E-02	0.53	4.18E-03	0.57	1.78E-03	-0.26	1.93E-01	0.01	9.62E-01	-0.51	6.07E-03	0.04	8.62E-01	0.06	7.77E-01
ENSCAFG0000002025	NFAT5	pink	VSMC_M5	0.16	4.15E-01	0.15	4.70E-01	-0.65	2.75E-04	0.74	1.23E-05	0.62	5.84E-04	0.06	7.63E-01	-0.24	2.35E-01	-0.69	6.49E-05	-0.11	5.68E-01	0.37	5.85E-02
ENSCAFG0000000515	BRK1	grey	VSMC_M10	0.16	4.16E-01	0.13	5.09E-01	-0.35	7.72E-02	0.50	8.65E-03	0.30	1.32E-01	-0.04	8.26E-01	-0.07	7.33E-01	-0.39	4.58E-02	-0.16	4.37E-01	0.26	1.88E-01
ENSCAFG0000002519	ATN1	cyan	VSMC_M2	0.16	4.16E-01	0.63	4.86E-04	-0.16	4.37E-01	0.66	2.09E-04	0.20	3.16E-01	-0.25	2.05E-01	-0.69	8.05E-05	-0.81	3.80E-07	-0.43	2.45E-02	0.48	1.11E-02
ENSCAFG0000001125	POLR3C	grey	VSMC_M10	0.16	4.16E-01	-0.04	8.44E-01	0.12	5.56E-01	-0.47	1.35E-02	0.03	8.99E-01	-0.13	5.12E-01	0.07	7.47E-01	0.39	4.45E-02	-0.04	8.55E-01	-0.28	1.55E-01
ENSCAFG0000000336	PTN	grey	VSMC_M10	0.16	4.16E-01	-0.26	1.87E-01	0.01	9.57E-01	-0.30	1.28E-01	0.17	3.89E-01	-0.05	7.99E-01	0.16	4.22E-01	0.18	3.63E-01	0.24	2.31E-01	-0.33	9.39E-02
ENSCAFG0000000160	TOMM6	grey	VSMC_M10	0.16	4.16E-01	0.08	7.01E-01	0.29	1.41E-01	-0.09	6.67E-01	-0.40	3.72E-02	0.38	5.02E-02	0.41	3.37E-02	0.13	5.20E-01	-0.26	1.95E-01	0.29	1.38E-01
ENSCAFG0000000597	FGFR1	cyan	VSMC_M2	0.16	4.17E-01	0.37	5.58E-02	0.21	2.87E-01	0.08	6.90E-01	-0.05	8.21E-01	-0.27	1.78E-01	-0.62	4.93E-04	-0.24	2.30E-01	-0.19	3.45E-01	-0.01	9.50E-01
ENSCAFG0000000304	TRIT1	grey	VSMC_M10	0.16	4.17E-01	-0.17	3.91E-01	0.06	7.61E-01	-0.09	6.51E-01	-0.11	5.78E-01	0.13	5.19E-01	0.32	1.01E-01	0.19	3.39E-01	-0.01	9.43E-01	0.05	8.24E-01
ENSCAFG0000001880	RAD1	pink	VSMC_M5	0.16	4.17E-01	-0.55	3.24E-03	-0.38	5.14E-02	0.09	6.56E-01	0.53	4.45E-03	0.04	8.35E-01	0.01	9.79E-01	0.13	5.03E-01	0.42	2.90E-02	-0.48	1.15E-02
ENSCAFG0000001010	ENSCAFG0000001010	grey	VSMC_M10	0.16	4.17E-01	-0.19	3.33E-01	0.11	5.77E-01	-0.18	3.61E-01	0.03	8.87E-01	-0.04	8.49E-01	-0.16	4.31E-01	0.18	3.62E-01	0.18	3.82E-01	-0.26	1.91E-01
ENSCAFG0000000794	FDFT1	yellow	VSMC_M3	0.16	4.18E-01	-0.24	2.34E-01	0.31	1.22E-01	-0.67	1.23E-04	-0.02	9.12E-01	-0.08	6.94E-01	0.55	2.70E-03	0.57	1.99E-03	0.16	4.38E-01	-0.57	2.11E-03
ENSCAFG0000003271	C7H1orf53	grey	VSMC_M10	0.16	4.18E-01	0.41	3.18E-02	0.22	2.80E-01	0.07	7.22E-01	-0.30	1.34E-01	-0.04	8.46E-01	0.19	3.39E-01	-0.15	4.48E-01	-0.47	1.27E-02	0.49	9.44E-03
ENSCAFG0000001593	DNAAF4	grey	VSMC_M10	0.16	4.18E-01	-0.32	9.84E-02	-0.04	8.33E-01	-0.30	1.32E-01	0.24	2.22E-01	-0.12	5.53E-01	0.42	3.04E-02	0.29	1.45E-01	0.25	2.18E-01	-0.43	2.60E-02
ENSCAFG0000003163	ENSCAFG0000003163	grey	VSMC_M10	0.16	4.18E-01	0.11	5.86E-01	0.15	4.58E-01	-0.20	3.23E-01	-0.11	5.80E-01	-0.08	6.78E-01	0.10	6.02E-01	0.15	4.64E-01	-0.21	2.94E-01	0.02	9.14E-01
ENSCAFG0000000211	C10H2orf49	grey	VSMC_M10	0.16	4.18E-01	0.13	5.09E-01	-0.49	9.28E-03	0.65	2.55E-04	0.34	8.01E-02	0.25	2.18E-01	-0.09	6.39E-01	-0.46	1.67E-02	-0.27	1.75E-01	0.47	1.25E-02
ENSCAFG0000002436	ENSCAFG0000002436	pink	VSMC_M5	0.16	4.18E-01	-0.07	7.13E-01	-0.46	1.68E-02	0.34	8.47E-02	0.55	3.06E-03	-0.24	2.28E-01	0.06	7.49E-01	-0.28	1.55E-01	0.10	6.10E-01	0.02	9.03E-01
ENSCAFG0000000167	COMMD5	yellow	VSMC_M3	0.16	4.18E-01	0.60	8.91E-04	0.49	9.93E-03	0.22	2.61E-01	-0.56	2.33E-03	0.06	7.68E-01	0.07	7.18E-01	-0.38	4.97E-02	-0.62	6.18E-04	0.60	8.78E-04
ENSCAFG0000001632	ENSCAFG0000001632	grey	VSMC_M10	0.16	4.19E-01	0.38	4.99E-02	0.08	6.97E-01	0.20	3.06E-01	0.02	9.35E-01	-0.33	8.83E-02	-0.46	1.65E-02	-0.39	4.19E-02	-0.26	1.90E-01	0.21	3.04E-01
ENSCAFG0000000868	ETFDH	grey	VSMC_M10	0.16	4.19E-01	0.45	2.00E-02	-0.21	2.96E-01	0.57	1.83E-03	0.16	4.27E-01	-0.08	7.03E-01	-0.21	2.82E-01	-0.65	2.29E-04	-0.40	3.96E-02	0.52	5.11E-03
ENSCAFG0000000837	RNF13	pink	VSMC_M5	0.16	4.19E-01	-0.02	9.27E-01	-0.72	2.20E-05	0.65	2.32E-04	0.75	5.81E-06	-0.17	4.07E-01	-0.02	9.06E-01	-0.54	3.33E-03	0.03	8.74E-01	0.23	2.56E-01
ENSCAFG0000001677	VPS13C	grey	VSMC_M10	0.16	4.19E-01	0.03	8.84E-01	-0.37	5.44E-02	0.18	3.81E-01	0.49	1.00E-02	-0.06	7.82E-01	0.13	5.34E-01	-0.19	3.42E-01	-0.08	7.00E-01	-0.01	9.47E-01
ENSCAFG0000002851	ENSCAFG0000002851	grey	VSMC_M10	0.16	4.19E-01	0.01	9.43E-01	0.11	5.78E-01	0.00	9.93E-01	0.08	6.99E-01	0.01	9.67E-01	0.10	6.07E-01	0.04	8.55E-01	-0.10	6.32E-01	-0.22	2.60E-01
ENSCAFG0000001505	ENSCAFG0000001505	grey	VSMC_M10	0.16	4.19E-01	0.33	9.19E-02	0.05	8.00E-01	0.33	9.05E-02	0.09	6.73E-01	-0.11	6.01E-01	-0.56	2.23E-03	-0.44	2.03E-02	-0.19	3.53E-01	0.08	6.95E-01
ENSCAFG0000000670	ENSCAFG0000000670	grey	VSMC_M10	0.16	4.20E-01	0.15	4.40E-01	0.05	7.92E-01	-0.08	6.96E-01	0.08	6.85E-01	-0.13	5.27E-01	-0.13	5.05E-01	0.04	8.34E-01	-0.18	3.79E-01	-0.12	5.46E-01
ENSCAFG0000001461	C26H22orf39	yellow	VSMC_M3	0.16	4.20E-01	0.51	5.99E-03	0.58	1.59E-03	-0.01	9.80E-01	-0.49	1.01E-02	0.00	9.83E-01	-0.13	5.33E-01	-0.22	2.60E-01	-0.48	1.17E-02	0.21	2.95E-01
ENSCAFG0000003040	ZNF624	grey	VSMC_M10	0.16	4.20E-01	0.19	3.42E-01	0.53	4.04E-03	-0.20	3.05E-01	-0.45	1.96E-02	0.03	8.80E-01	-0.06	7.64E-01	0.11	6.01E-01	-0.24	2.19E-01	-0.04	8.34E-01
ENSCAFG0000000509	AANAT	grey	VSMC_M10	0.16	4.20E-01	0.39	4.25E-02	0.37	5.74E-02	0.13	5.22E-01	-0.35	7.29E-02	-0.11	5.78E-01	0.18	3.64E-01	-0.21	2.98E-01	-0.46	1.55E-02	0.31	1.22E-01
ENSCAFG0000001284	PPEF1	darkgreen	VSMC_M4	0.16	4.20E-01	-0.01	9.71E-01	-0.45	1.85E-02	0.45	1.89E-02	0.41	3.33E-02	-0.22	2.77E-01	0.05	7.91E-01	-0.34	8.75E-02	-0.05	8.03E-01	0.30	1.33E-01
ENSCAFG0000001342	ALDH9A1	grey	VSMC_M10	0.16	4.20E-01	0.20	3.19E-01	0.11	5.79E-01	0.22	2.66E-01	-0.05	8.00E-01	0.05	7.98E-01	-0.03	8.77E-01	-0.23	2.54E-01	-0.23	2.47E-01	0.12	5.58E-01
ENSCAFG0000002847	ENSCAFG0000002847	cyan	VSMC_M2	0.16	4.21E-01	0.62	5.22E-04	0.40	4.09E-02	0.25	2.11E-01	-0.34	8.72E-02	-0.07	7.21E-01	-0.32	1.05E-01	-0.46	1.59E-02	-0.50	7.35E-03	0.35	7.15E-02
ENSCAFG0000000051	ATP6V1G2	grey	VSMC_M10	0.16	4.21E-01	-0.02	9.29E-01	-0.28	1.54E-01	0.11	5.77E-01	0.42	2.81E-02	-0.21	2.95E-01	-0.23	2.57E-01	-0.04	8.39E-01	-0.01	9.61E-01	-0.16	4.37E-01
ENSCAFG0000000710	ANAPC1	darkgrey	VSMC_M8	0.16	4.21E-01	-0.48	1.22E-02	0.23	2.42E-01	-0.72	1.99E-05	-0.08	6.77E-01	0.25	2.01E-01	0.43	2.54E-02	0.79	7.94E-07	0.26	1.96E-01	-0.56	2.43E-03
ENSCAFG0000001475	TFPI	darkgrey	VSMC_M8	0.16	4.21E-01	-0.35	7.44E-02	0.28	1.52E-01	-0.76	3.88E-06	-0.10	6.35E-01	0.32	1.06E-01	0.39	4.57E-02	0.71	3.52E-05	0.23	2.57E-01	-0.55	2.81E-03
ENSCAFG0000002369	ERCC6L	darkgrey	VSMC_M8	0.16	4.21E-01	-0.38	5.33E-02	0.43	2.50E-02	-0.85	1.49E-08	-0.27	1.66E-01	0.11	5.74E-01	0.44	2.11E-02	0.91	3.12E-11	0.15	4.51E-01	-0.56	2.28E-03
ENSCAFG0000001608	AKAP8	grey	VSMC_M10	0.16	4.21E-01	0.18	3.57E-01	0.44	2.09E-02	-0.36	6.54E-02	-0.30	1.35E-01	0.00	9.85E-01	-0.11	5.73E-01	0.22	2.64E-01	-0.18	3.79E-01	-0.13	5.23E-01
ENSCAFG0000000337	DGKI	grey	VSMC_M10	0.16	4.22E-01	-0.05	7.97E-01	0.43	2.64E-02	-0.38	5.07E-02	-0.34	8.06E-02	0.29	1.46E-01	0.36	6.80E-02	0.25	2.11E-01	-0.07	7.25E-01	-0.10	6.30E-01
ENSCAFG0000001333	ENSCAFG0000001333	turquoise	VSMC_M6	0.16	4.22E-01	0.03	8.82E-01	0.44	2.14E-02	-0.23	2.48E-01	-0.36	6.82E-02	0.45	1.92E-02	0.02	9.29E-01	0.19	3.52E-01	-0.09	6.71E-01	-0.11	5.78E-01
ENSCAFG0000001096	MRPS35	grey	VSMC_M10	0.16	4.22E-01	-0.45	1.73E-02	-0.06	7.63E-01	-0.37	5.72E-02	0.20	3.19E-01	-0.14	5.02E-01	0.37	5.54E-02	0.50	7.23E-03	0.27	1.69E-01	-0.42	2.73E-02
ENSCAFG0000001761	TIMM29	grey	VSMC_M10	0.16	4.22E-01	0.35	7.61E-02	0.41	3.14E-02	-0.08	6.94E-01	-0.42	2.75E-02	-0.13	5.28E-01	0.11	5.77E-01	-0.04	8.51E-01	-0.42	3.12E-02	0.31	1.21E-01
ENSCAFG0000000065	ENSCAFG0000000065	grey	VSMC_M10	0.16	4.22E-01	0.16	4.29E-01	0.41	3.57E-02	-0.16	4.30E-01	-0.33	9.08E-02	0.06	7.57E-01	-0.19	3.39E-01	0.08	6.93E-01	-0.17	3.93E-01	-0.06	7.76E-01
ENSCAFG0000001216	NCKIPSD	cyan	VSMC_M2	0.16	4.22E-01	0.48	1.15E-02	0.41	3.28E-02	-0.16	4.36E-01	-0.29	1.37E-01	0.04	8.43E-01	0.04	8.62E-01	-0.16	4.18E-01	-0.37	5.83E-02	0.16	4.12E-01
ENSCAFG0000001554	GMPPA	cyan	VSMC_M2	0.16	4.22E-01	0.58	1.70E-03	0.27	1.76E-01	-0.08	6.86E-01	-0.16	4.13E-01	-0.16	4.18E-01	-0.20	3.22E-01	-0.13	5.22E-01	-0.54	3.44E-03	0.20	3.12E-01
ENSCAFG0000000509	ITIH5	darkgreen	VSMC_M4	0.16	4.22E-01	0.56	2.44E-03	-0.20	3.11E-01	0.69	6.68E-05	0.17	3.89E-01	-0.21	2.98E-01	-0.19	3.56E-01	-0.73	1.46E-05	-0.52	5.26E-03	0.58	1.55E-03
ENSCAFG0000002873	ZFP3	grey	VSMC_M10	0.16	4.23E-01	0.40	3.82E-02	-0.11	5.69E-01	0.55	3.13E-03	0.08	7.02E-01	-0.03	8.79E-01	-0.17	4.03E-01	-0.60	8.39E-04	-0.40	4.04E-02	0.52	5.80E-03
ENSCAFG0000000112	EP300	darkgreen	VSMC_M4	0.16	4.23E-01	0.55	2.69E-03	-0.23	2.39E-01	0.63	4.90E-04	0.10	6.15E-01	0.06	7.49E-01	-0.17	4.06E-01	-0.69	7.37E-05	-0.54	3.36E-03	0.78	2.02E-06
ENSCAFG0000001949	NUBP2	yellow	VSMC_M3	0.16	4.23E-01	0.27	1.78E-01	0.75	8.21E-06	-0.42	2.88E-02	-0.55	2.77E-03	-0.08	7.01E-01	-0.02	9.17E-01	0.22	2.64E-01	-0.23	2.52E-01	-0.19	3.36E-01
ENSCAFG0000001425	COMT	grey	VSMC_M10	0.16	4.23E-01	0.65	2.39E-04	0.23	2.43E-01	0.40	3.99E-02	-0.22	2.70E-01	0.10	6.36E-01	-0.19	3.37E-01	-0.63	4.67E-04	-0.52	5.08E-03	0.51	7.14E-03
ENSCAFG0000002967	NAF1	pink	VSMC_M5	0.16	4.24E-01	-0.51	6.81E-03	-0.34	8.29E-02	-0.30	1.33E-01	0.57	1.80E-03	-0.02	9.04E-01	-0.06	7.72E-01	0.40	3.74E-02	0.42	2.72E-02	-0.71	3.38E-05
ENSCAFG0000001939	ARRDC1	grey	VSMC_M10	0.16	4.24E-01	0.54	3.50E-03	0.45	1.72E-02	-0.13	5.07E-01	-0.48	1.22E-02	-0.11	5.70E-01	0.15	4.70E-01	-0.04	8.59E-01	-0.54	3.36E-03	0.44	2.04E-02
ENSCAFG0000001371	ALKBH4	yellow	VSMC_M3	0.16	4.24E-01	0.28	1.62E-01	0.59	1.11E-03	-0.16	4.36E-01	-0.58	1.50E-03	0.13	5.08E-01	0.02	9.19E-01	0.03	9.01E-01	-0.30	1.22E-01	0.13	5.28E-01
ENSCAFG0000000594	HACL1	grey	VSMC_M10	0.16	4.24E-01	0.41	3.49E-02	0.42	2.94E-02	-0.19	3.51E-01	-0.31	1.16E-01	-0.02	9.28E-01	0.08	7.07E-01	0.06	7.68E-01	-0.42	3.13E-02	0.08	7.06E-01
ENSCAFG0000001670	SLC26A1	grey	VSMC_M10	0.16	4.24E-01	0.20	3.29E-01	0.43	2.35E-02	-0.05	8.19E-01	-0.42	3.08E-02	0.30	1.30E-01	0.14	5.00E-01	-0.05	8.02E-01	-0.24	2.26E-01	0.10	6.12E-01
ENSCAFG0000001284	IL10RA	grey	VSMC_M10	0.16	4.24E-01	0.22	2.80E-01	0.47	1.33E-02	-0.08	7.04E-01	-0.34	8.16E-02	0.04	8.36E-01	0.05	8.09E-01	-0.07	7.43E-01	-0.20	3.20E-01	0.06	7.83E-01
ENSCAFG0000001092	KMT5B	pink	VSMC_M5	0.16	4.25E-01	-0.34	8.06E-02	-0.71	3.32E-05	0.33	8.92E-02	0.84	4.15E-08	-0.27	1.75E-01	-0.09	6.71E-01	-0.16	4.18E-01	0.29	1.44E-01	-0.22	2.67E-01
ENSCAFG0000001943	TMEM187	grey	VSMC_M10	0.16	4.25E-01	0.36	6.43E-02	0.56	2.40E-03	-0.16	4.30E-01	-0.63	4.83E-04	0.03	8.79E-01	0.19	3.36E-01	0.04	8.42E-01	-0.46	1.46E-02	0.41	3.30E-02
ENSCAFG0000000056	SNX31	grey	VSMC_M10	0.16	4.25E-01	0.06	7.66E-01	-0.05	8.02E-01	0.26	1.92E-01	-0.04	8.46E-01	0.41	3.38E-02	-0.13	5.15E-01	-0.19	3.52E-01	-0.15	4.47E-01	0.24	2.21E-01
ENSCAFG0000002938	TIGAR	grey	VSMC_M10	0.16	4.25E-01	-0.45	1.75E-02	-0.18	3.59E-01	-0.10	6.26E-01	0.25	2.07E-01	0.12	5.35E-01	-0.10	6.14E-01	0.34	8.13E-02	0.30	1.26E-01	-0.40	3.80E-02
ENSCAFG0000002977	BORCS5	grey	VSMC_M10	0.16	4.25E-01	0.27	1.71E-01	0.04	8.31E-01	0.43	2.37E-02	-0.10	6.29E-01	0.16	4.34E-01	-0.24	2.36E-01	-0.39	4.64E-02	-0.24	2.38E-01	0.36	6.65E-02
ENSCAFG0000000292	SKAP2	grey	VSMC_M10	0.16	4.26E-01	0.05	7.98E-01	0.08	7.07E-01	-0.18	3.78E-01	0.11	5.80E-01	-0.22	2.67E-01	-0.08	7.02E-01	0.10	6.24E-01	-0.09	6.41E-01	-0.15	4.61E-01
ENSCAFG0000000590	ENSCAFG0000000590	grey	VSMC_M10	0.16	4.26E-01	0.11	5.71E-01	0.06	7.63E-01	-0.11	5.72E-01	0.07	7.24E-01	0.05	7.88E-01	0.09	6.53E-01	0.03	8.71E-01	-0.11	5.71E-01	0.00	9.91E-01
ENSCAFG0000002324	CILP2	grey	VSMC_M10	0.16	4.26E-01	0.21	3.01E-01	0.10	6.19E-01	0.14	4.99E-01	-0.03	8.83E-01	0.04	8.26E-01	0.39	4.22E-02	-0.22	2.72E-01	-0.23	2.54E-01	0.24	2.24E-01
ENSCAFG0000001564	FOXM1	darkgrey	VSMC_M8	0.16	4.26E-01	-0.47	1.37E-02	0.29	1.36E-01	-0.81	2.75E-07	-0.11	5.82E-01	0.09	6.53E-01	0.47	1.39E-02	0.91	7.40E-11	0.25	2.07E-01	-0.64	3.21E-04
ENSCAFG0000002637	ENSCAFG0000002637	grey	VSMC_M10	0.16	4.26E-01	0.01	9.57E-01	0.05	7.88E-01	-0.18	3.57E-01	0.01	9.63E-01	0.10	6.22E-01	-0.06	7.82E-01	0.27	1.79E-01	-0.15	4.61E-01	-0.10	6.24E-01
ENSCAFG0000002036	ATP6V0D1	cyan	VSMC_M2	0.16	4.26E-01	0.67	1.30E-04	0.25	2.16E-01	0.28	1.58E-01	-0.32	1.07E-01	0.11	5.90E-01	-0.26	1.87E-01	-0.49	9.49E-03	-0.57	1.72E-03	0.59	1.24E-03
ENSCAFG0000000482	GLI2	grey	VSMC_M10	0.16	4.27E-01	0.12	5.40E-01	0.27	1.78E-01	-0.37	5.71E-02	-0.17	3.98E-01	-0.04	8.38E-01	0.21	2.90E-01	0.21	2.90E-01	-0.15	4.53E-01	0.00	9.85E-01

ENSCAFG0000001152	AEN	grey	VSMC_M10	0.16	4.27E-01	-0.13	5.09E-01	0.12	5.52E-01	-0.13	5.18E-01	-0.16	4.27E-01	0.31	1.16E-01	0.43	2.70E-02	0.30	1.22E-01	-0.15	4.52E-01	0.00	9.83E-01
ENSCAFG0000001175	TPST2	darkgreen	VSMC_M4	0.16	4.27E-01	0.33	9.49E-02	-0.42	2.74E-02	0.87	3.23E-09	0.34	7.82E-02	0.01	9.42E-01	-0.38	4.95E-02	-0.82	1.38E-07	-0.27	1.69E-01	0.53	4.60E-03
ENSCAFG0000001249	TACC2	cyan	VSMC_M2	0.16	4.27E-01	0.76	4.22E-06	0.45	1.74E-02	-0.07	7.25E-01	-0.41	3.40E-02	-0.07	7.11E-01	-0.32	1.09E-01	-0.25	2.03E-01	-0.67	1.39E-04	0.43	2.37E-02
ENSCAFG0000002435	CENPP	darkgrey	VSMC_M8	0.16	4.27E-01	-0.25	2.07E-01	0.45	1.81E-02	-0.78	1.87E-06	-0.36	6.46E-02	0.26	1.90E-01	0.44	2.14E-02	0.80	5.88E-07	0.04	8.50E-01	-0.41	3.45E-02
ENSCAFG0000000069	SELENOO	grey	VSMC_M10	0.16	4.27E-01	0.30	1.31E-01	0.56	2.56E-03	-0.21	2.94E-01	-0.60	9.41E-04	0.38	5.02E-02	-0.03	8.72E-01	0.11	5.80E-01	-0.35	7.11E-02	0.23	2.48E-01
ENSCAFG0000000161	VPS28	yellow	VSMC_M3	0.16	4.28E-01	0.61	6.52E-04	0.50	7.46E-03	0.13	5.04E-01	-0.41	3.24E-02	-0.16	4.33E-01	-0.40	3.96E-02	-0.41	3.21E-02	-0.46	1.68E-02	0.32	1.04E-01
ENSCAFG0000002026	ENSCAFG0000002026	grey	VSMC_M10	0.16	4.28E-01	0.38	4.86E-02	0.04	8.45E-01	0.46	1.51E-02	-0.11	5.83E-01	0.11	5.92E-01	0.01	9.58E-01	-0.42	2.74E-02	-0.41	3.54E-02	0.54	3.84E-03
ENSCAFG0000002961	AMACR	grey	VSMC_M10	0.16	4.28E-01	0.21	2.95E-01	-0.19	3.35E-01	0.36	6.45E-02	0.21	2.83E-01	-0.16	4.19E-01	0.07	7.40E-01	-0.42	2.86E-02	-0.17	3.93E-01	0.29	1.38E-01
ENSCAFG0000001194	PARP9	grey	VSMC_M10	0.16	4.28E-01	0.74	8.31E-06	0.05	7.94E-01	0.55	3.10E-03	-0.14	4.81E-01	0.07	7.19E-01	-0.12	5.66E-01	-0.69	7.54E-05	-0.71	3.21E-05	0.76	4.09E-06
ENSCAFG0000000418	TMEM160	grey	VSMC_M10	0.16	4.29E-01	0.49	9.66E-03	0.42	2.93E-02	0.05	7.91E-01	-0.37	6.04E-02	0.00	9.99E-01	-0.12	5.55E-01	-0.25	2.03E-01	-0.44	2.12E-02	0.17	3.87E-01
ENSCAFG0000001701	SGCA	darkgreen	VSMC_M4	0.16	4.29E-01	0.54	3.59E-03	-0.23	2.56E-01	0.83	8.00E-08	0.11	5.94E-01	0.12	5.64E-01	-0.34	7.92E-02	-0.86	1.13E-08	-0.48	1.13E-02	0.73	1.87E-05
ENSCAFG0000000882	ANKRD34B	grey	VSMC_M10	0.16	4.29E-01	0.33	8.90E-02	0.59	1.11E-03	-0.26	1.93E-01	-0.54	3.57E-03	0.17	4.00E-01	0.26	1.88E-01	0.05	7.95E-01	-0.35	7.19E-02	0.16	4.20E-01
ENSCAFG0000002329	HTATSF1	grey	VSMC_M10	0.16	4.29E-01	0.09	6.68E-01	0.47	1.33E-02	-0.25	2.02E-01	-0.43	2.64E-02	-0.07	7.32E-01	0.40	3.68E-02	0.21	3.02E-01	-0.21	2.82E-01	0.05	8.05E-01
ENSCAFG0000000642	GXYLT2	grey	VSMC_M10	0.16	4.29E-01	-0.01	9.64E-01	-0.26	1.85E-01	0.17	4.04E-01	0.38	5.03E-02	-0.27	1.76E-01	-0.75	6.21E-06	-0.08	6.83E-01	0.04	8.54E-01	-0.15	4.47E-01
ENSCAFG0000002418	SERPINF1	cyan	VSMC_M2	0.16	4.29E-01	0.79	8.20E-07	0.05	8.14E-01	0.53	4.56E-03	-0.06	7.62E-01	-0.19	3.49E-01	-0.32	9.99E-02	-0.73	1.43E-05	-0.69	5.95E-05	0.65	2.43E-04
ENSCAFG0000000968	PIGT	grey	VSMC_M10	0.16	4.30E-01	0.52	5.85E-03	-0.40	3.85E-02	0.77	2.81E-06	0.33	9.57E-02	-0.16	4.38E-01	-0.40	3.94E-02	-0.83	8.96E-08	-0.41	3.35E-02	0.63	3.90E-04
ENSCAFG0000000933	C30H15orf62	cyan	VSMC_M2	0.16	4.30E-01	0.53	4.53E-03	0.25	2.03E-01	0.29	1.45E-01	-0.25	2.10E-01	-0.04	8.35E-01	-0.34	8.40E-02	-0.41	3.33E-02	-0.43	2.53E-02	0.41	3.48E-02
ENSCAFG0000002001	DBT	pink	VSMC_M5	0.16	4.30E-01	-0.34	8.09E-02	-0.42	3.05E-02	-0.14	4.79E-01	0.65	2.20E-04	-0.14	4.72E-01	0.04	8.45E-01	0.21	2.97E-01	0.28	1.59E-01	-0.54	3.40E-03
ENSCAFG0000000472	SUMO2	grey	VSMC_M10	0.16	4.30E-01	-0.02	9.06E-01	-0.21	2.94E-01	0.10	6.06E-01	0.44	2.19E-02	-0.09	6.64E-01	-0.24	2.33E-01	-0.06	7.76E-01	0.04	8.39E-01	-0.35	7.54E-02
ENSCAFG0000003137	PYGO2	cyan	VSMC_M2	0.16	4.30E-01	0.67	1.51E-04	0.18	3.62E-01	0.45	1.86E-02	-0.27	1.81E-01	0.05	8.18E-01	-0.08	6.84E-01	-0.62	5.38E-04	-0.62	5.22E-04	0.68	8.32E-05
ENSCAFG0000000470	ENSCAFG0000000470	grey	VSMC_M10	0.16	4.30E-01	-0.03	8.95E-01	0.39	4.46E-02	-0.17	3.87E-01	-0.34	8.21E-02	0.05	8.06E-01	0.40	3.94E-02	0.20	3.26E-01	-0.12	5.57E-01	-0.08	7.06E-01
ENSCAFG0000001008	FAM173B	grey	VSMC_M10	0.16	4.31E-01	-0.16	4.34E-01	0.21	2.97E-01	-0.30	1.35E-01	-0.09	6.66E-01	0.00	9.90E-01	0.40	3.75E-02	0.33	8.88E-02	-0.03	8.65E-01	-0.24	2.22E-01
ENSCAFG0000000390	ENSCAFG0000000390	grey	VSMC_M10	0.16	4.31E-01	0.11	5.84E-01	0.43	2.66E-02	-0.41	3.21E-02	-0.39	4.58E-02	-0.08	6.75E-01	0.62	5.29E-04	0.31	1.15E-01	-0.29	1.40E-01	0.04	8.39E-01
ENSCAFG0000000183	CUL9	cyan	VSMC_M2	0.16	4.31E-01	0.87	2.71E-09	0.24	2.32E-01	0.30	1.22E-01	-0.28	1.57E-01	-0.01	9.65E-01	-0.28	1.52E-01	-0.58	1.39E-03	-0.78	1.98E-06	0.72	2.18E-05
ENSCAFG0000000404	HDAC2	grey	VSMC_M10	0.16	4.31E-01	-0.49	9.87E-03	-0.06	7.71E-01	-0.39	4.51E-02	0.38	5.09E-02	-0.06	7.53E-01	-0.04	8.34E-01	0.49	1.03E-02	0.42	3.10E-02	-0.80	5.62E-07
ENSCAFG0000001045	CALHM1	grey	VSMC_M10	0.16	4.31E-01	-0.23	2.38E-01	-0.02	9.24E-01	-0.21	2.91E-01	0.06	7.80E-01	0.27	1.77E-01	0.40	4.03E-02	0.30	1.25E-01	0.06	7.68E-01	-0.15	4.55E-01
ENSCAFG0000001576	AP1M1	yellow	VSMC_M3	0.16	4.31E-01	0.13	5.22E-01	0.63	4.90E-04	-0.42	2.79E-02	-0.59	1.28E-03	-0.01	9.47E-01	0.06	7.52E-01	0.35	7.26E-02	-0.18	3.64E-01	0.01	9.41E-01
ENSCAFG0000001934	FLYWCH1	cyan	VSMC_M2	0.16	4.32E-01	0.55	3.14E-03	0.27	1.69E-01	0.32	9.99E-02	-0.35	7.46E-02	0.15	4.66E-01	-0.13	5.07E-01	-0.45	1.90E-02	-0.54	3.90E-03	0.61	7.46E-04
ENSCAFG0000001554	STX5	grey	VSMC_M10	0.16	4.32E-01	-0.11	5.69E-01	0.27	1.72E-01	-0.44	2.22E-02	-0.07	7.44E-01	-0.42	2.72E-02	0.20	3.09E-01	0.36	6.19E-02	0.10	6.34E-01	-0.35	7.65E-02
ENSCAFG0000001335	PTCHD1	cyan	VSMC_M2	0.16	4.32E-01	0.63	4.54E-04	0.30	1.28E-01	0.22	2.71E-01	-0.36	6.70E-02	0.21	2.86E-01	-0.37	5.59E-02	-0.36	6.29E-02	-0.63	4.75E-04	0.51	7.05E-03
ENSCAFG0000003212	C1QTNF6	grey	VSMC_M10	0.16	4.32E-01	-0.23	2.39E-01	0.13	5.10E-01	-0.49	9.77E-03	0.04	8.26E-01	0.19	3.36E-01	-0.06	7.53E-01	0.37	5.63E-02	0.21	2.92E-01	-0.41	3.32E-02
ENSCAFG0000000482	SLC6A9	cyan	VSMC_M2	0.16	4.32E-01	0.63	4.60E-04	0.34	7.89E-02	-0.12	5.48E-01	-0.23	2.47E-01	-0.20	3.09E-01	-0.19	3.36E-01	-0.22	2.80E-01	-0.56	2.35E-03	0.25	2.04E-01
ENSCAFG0000001325	MRC2	yellow	VSMC_M3	0.16	4.33E-01	0.54	3.55E-03	0.73	1.31E-05	-0.46	1.70E-02	-0.62	5.78E-04	-0.15	4.60E-01	0.15	4.41E-01	0.11	6.02E-01	-0.55	3.23E-03	0.20	3.20E-01
ENSCAFG0000001687	CALCOCO2	darkgreen	VSMC_M4	0.16	4.33E-01	0.31	1.12E-01	-0.36	6.18E-02	0.59	1.34E-03	0.41	3.15E-02	-0.21	3.05E-01	-0.40	3.64E-02	-0.68	8.67E-05	-0.14	4.84E-01	0.31	1.16E-01
ENSCAFG0000002430	RPL37	grey	VSMC_M10	0.16	4.33E-01	0.44	2.20E-02	0.36	6.56E-02	-0.10	6.32E-01	-0.33	8.83E-02	-0.15	4.58E-01	-0.08	6.78E-01	-0.06	7.74E-01	-0.48	1.18E-02	0.24	2.19E-01
ENSCAFG0000001559	PCNX4	darkgreen	VSMC_M4	0.16	4.34E-01	0.05	8.07E-01	-0.43	2.56E-02	0.59	1.23E-03	0.41	3.47E-02	-0.09	6.70E-01	0.08	7.07E-01	-0.44	2.07E-02	-0.15	4.55E-01	0.31	1.11E-01
ENSCAFG0000000148	ZFAND3	grey	VSMC_M10	0.16	4.34E-01	0.28	1.62E-01	-0.37	5.51E-02	0.64	3.49E-04	0.45	1.82E-02	-0.14	4.87E-01	-0.03	8.67E-01	-0.67	1.17E-04	-0.23	2.42E-01	0.25	2.08E-01
ENSCAFG0000002479	RNH1	yellow	VSMC_M3	0.16	4.34E-01	0.54	3.51E-03	0.94	6.67E-13	-0.47	1.38E-02	-0.83	6.22E-08	0.00	9.93E-01	0.00	9.95E-01	0.15	4.46E-01	-0.51	5.98E-03	0.13	5.07E-01
ENSCAFG0000001339	KLHL15	pink	VSMC_M5	0.16	4.34E-01	-0.10	6.08E-01	-0.42	2.72E-02	0.15	4.70E-01	0.52	5.52E-03	-0.03	8.72E-01	-0.16	4.20E-01	-0.10	6.08E-01	0.11	5.86E-01	-0.14	4.96E-01
ENSCAFG0000002874	ENSCAFG0000002874	grey	VSMC_M10	0.16	4.34E-01	-0.09	6.61E-01	-0.15	4.65E-01	0.09	6.66E-01	0.22	2.63E-01	-0.11	5.95E-01	-0.16	4.23E-01	-0.07	7.24E-01	0.13	5.10E-01	-0.10	6.22E-01
ENSCAFG0000000894	SLIT1	grey	VSMC_M10	0.16	4.35E-01	0.28	1.59E-01	0.16	4.31E-01	-0.12	5.40E-01	-0.13	5.26E-01	-0.11	5.84E-01	-0.11	5.80E-01	0.06	7.63E-01	-0.33	9.74E-02	0.12	5.35E-01
ENSCAFG0000001465	CDCA3	darkgrey	VSMC_M8	0.16	4.35E-01	-0.44	2.22E-02	0.42	2.95E-02	-0.90	2.04E-10	-0.19	3.48E-01	0.09	6.45E-01	0.34	8.20E-02	0.95	4.76E-14	0.23	2.43E-01	-0.73	1.62E-05
ENSCAFG0000003130	ENSCAFG0000003130	grey	VSMC_M10	0.16	4.35E-01	0.65	2.31E-04	0.17	4.07E-01	0.42	2.75E-02	-0.16	4.19E-01	-0.12	5.50E-01	-0.17	3.88E-01	-0.57	1.97E-03	-0.59	1.21E-03	0.48	1.18E-02
ENSCAFG0000002713	ENSCAFG0000002713	grey	VSMC_M10	0.16	4.35E-01	0.42	2.73E-02	0.52	5.43E-03	-0.10	6.07E-01	-0.47	1.27E-02	0.02	9.16E-01	0.08	6.75E-01	-0.06	7.66E-01	-0.45	1.84E-02	0.21	2.83E-01
ENSCAFG0000000935	ENSCAFG0000000935	grey	VSMC_M10	0.16	4.35E-01	0.08	7.04E-01	0.18	3.77E-01	-0.06	7.55E-01	-0.13	5.22E-01	-0.20	3.20E-01	-0.05	8.14E-01	0.05	8.09E-01	-0.17	3.96E-01	-0.01	9.49E-01
ENSCAFG0000000485	ENSCAFG0000000485	grey	VSMC_M10	0.16	4.36E-01	0.07	7.27E-01	-0.29	1.41E-01	0.30	1.32E-01	0.36	6.88E-02	-0.03	8.84E-01	0.02	9.20E-01	-0.24	2.19E-01	-0.11	5.91E-01	0.00	9.91E-01
ENSCAFG0000003142	GNA14	grey	VSMC_M10	0.16	4.36E-01	0.31	1.20E-01	-0.31	1.20E-01	0.49	9.36E-03	0.24	2.27E-01	-0.07	7.11E-01	-0.30	1.29E-01	-0.44	2.16E-02	-0.31	1.19E-01	0.47	1.35E-02
ENSCAFG0000001672	STARD8	grey	VSMC_M10	0.16	4.36E-01	-0.30	1.27E-01	-0.25	2.14E-01	0.05	7.94E-01	0.42	3.11E-02	0.04	8.44E-01	0.25	2.15E-01	0.10	6.10E-01	0.18	3.66E-01	-0.36	6.17E-02
ENSCAFG0000003017	ENSCAFG0000003017	grey	VSMC_M10	0.16	4.36E-01	-0.13	5.21E-01	-0.11	5.87E-01	-0.13	5.06E-01	0.33	9.67E-02	-0.10	6.15E-01	-0.11	5.84E-01	0.13	5.11E-01	0.10	6.34E-01	-0.44	2.31E-02
ENSCAFG0000001142	PTGIS	cyan	VSMC_M2	0.16	4.36E-01	0.50	8.48E-03	0.03	8.69E-01	0.35	7.59E-02	0.13	5.05E-01	-0.41	3.36E-02	-0.56	2.23E-03	-0.50	7.89E-03	-0.30	1.34E-01	0.15	4.57E-01
ENSCAFG0000000391	BCAP29	grey	VSMC_M10	0.16	4.36E-01	-0.48	1.14E-02	-0.11	5.74E-01	0.00	9.87E-01	0.24	2.20E-01	-0.10	6.34E-01	0.24	2.27E-01	0.21	3.04E-01	0.34	8.76E-02	-0.44	2.23E-02
ENSCAFG0000001718	NAP1L2	grey	VSMC_M10	0.16	4.36E-01	0.03	8.94E-01	0.06	7.72E-01	-0.17	4.04E-01	0.03	8.90E-01	-0.02	9.16E-01	0.18	3.77E-01	0.16	4.26E-01	-0.08	6.92E-01	-0.04	8.56E-01
ENSCAFG0000001758	TMEM251	pink	VSMC_M5	0.16	4.36E-01	-0.07	7.35E-01	-0.40	3.94E-02	0.16	4.13E-01	0.57	1.75E-03	-0.43	2.58E-02	-0.29	1.42E-01	-0.14	4.96E-01	0.06	7.83E-01	-0.18	3.77E-01
ENSCAFG0000001073	ZMYND8	pink	VSMC_M5	0.16	4.37E-01	0.05	8.09E-01	-0.51	6.02E-03	0.46	1.62E-02	0.51	6.81E-03	-0.05	8.06E-01	-0.15	4.70E-01	-0.41	3.14E-02	-0.04	8.59E-01	0.25	2.08E-01
ENSCAFG0000001928	SGSM2	cyan	VSMC_M2	0.16	4.37E-01	0.65	2.77E-04	0.00	9.88E-01	0.56	2.57E-03	0.01	9.74E-01	-0.19	3.48E-01	-0.60	8.83E-04	-0.71	3.10E-05	-0.47	1.38E-02	0.53	4.58E-03
ENSCAFG0000000562	BHLHE40	grey	VSMC_M10	0.16	4.37E-01	0.11	5.83E-01	0.22	2.64E-01	-0.34	8.57E-02	-0.01	9.67E-01	-0.18	3.71E-01	0.29	1.48E-01	0.14	4.90E-01	-0.16	4.39E-01	-0.14	4.98E-01
ENSCAFG0000001593	EPHA4	grey	VSMC_M10	0.16	4.37E-01	0.22	2.65E-01	-0.10	6.18E-01	0.54	3.79E-03	-0.05	8.13E-01	0.03	8.68E-01	-0.07	7.30E-01	-0.43	2.36E-02	-0.28	1.50E-01	0.55	3.03E-03
ENSCAFG0000001208	SEMA4B	cyan	VSMC_M2	0.16	4.38E-01	0.44	2.10E-02	0.09	6.50E-01	0.00	9.93E-01	-0.04	8.41E-01	-0.11	5.84E-01	-0.33	9.45E-02	-0.14	4.88E-01	-0.41	3.20E-02	0.19	3.41E-01
ENSCAFG0000001643	MIIP	yellow	VSMC_M3	0.16	4.38E-01	0.31	1.13E-01	0.75	7.43E-06	-0.45	1.97E-02	-0.69	7.89E-05	-0.01	9.58E-01	0.16	4.28E-01	0.33	9.07E-02	-0.42	3.06E-02	0.07	7.41E-01
ENSCAFG0000001542	MAPK1	grey	VSMC_M10	0.16	4.38E-01	-0.42	3.08E-02	-0.20	3.26E-01	-0.39	4.67E-02	0.44	2.23E-02	-0.14	4.98E-01	0.30	1.35E-01	0.36	6.16E-02	0.35	6.96E-02	-0.53	4.66E-03
ENSCAFG0000000687	TARBP2	yellow	VSMC_M3	0.16	4.38E-01	0.41	3.51E-02	0.90	1.85E-10	-0.63	4.29E-04	-0.74	9.09E-06	-0.11	5.83E-01	0.04	8.56E-01	0.32	1.00E-01	-0.40	4.03E-02	-0.04	8.56E-01
ENSCAFG0000002457	ENSCAFG0000002457	grey	VSMC_M10	0.16	4.39E-01	-0.13	5.25E-01	0.18	3.57E-01	-0.34	7.99E-02	-0.23	2.51E-01	0.18	3.61E-01	0.09	6.73E-01	0.39	4.26E-02	-0.05	8.05E-01	-0.07	7.31E-01
ENSCAFG0000003189	HPCAL1	darkgrey	VSMC_M8	0.16	4.39E-01	-0.51	6.07E-03	0.33	9.26E-02	-0.80	4.82E-07	-0.08	6.92E-01	0.02	9.30E-01	0.51	6.94E-03	0.78	1.34E-06	0.39	4.49E-02	-0.73	1.83E-05
ENSCAFG0000000529	TK1	darkgrey	VSMC_M8	0.16	4.39E-01	-0.43	2.50E-02	0.45	1.99E-02	-0.92	2.15E-11	-0.22	2.60E-01	0.09	6.38E-01	0.41	3.56E-02	0.95	7.99E-14	0.25	2.09E-01	-0.70	4.10E-05
ENSCAFG0000002773	ENSCAFG0000002773	grey	VSMC_M10	0.16	4.39E-01	0.03	9.01E-01	0.09	6.47E-01	-0.10	6.08E-01	0.02	9.06E-01	-0.01	9.46E-01	-0.02	9.30E-01	0.09	6.39E-01	-0.06	7.75E-01	-0.09	6.57E-01
ENSCAFG0000000972	ALCAM	grey	VSMC_M10	0.16	4.39E-01	-0.15	4.55E-01	-0.45	1.79E-02	0.28	1.54E-01	0.64	3.70E-04	-0.35	7.03E-02	-0.47	1.29E-02	-0.28	1.55E-01	0.25	2.00E-01	-0.22	2.77E-01
ENSCAFG0000001372	SHF	grey	VSMC_M10	0.16	4.39E-01	0.18	3.66E-01	-0.36	6.91E-02	0.62	5.34E-04	0.30	1.29E-01	-0.08	6.97E-01	-0.34	7.90E-02	-0.56	2.20E-03	-0.21	2.86E-01	0.40	3.86E-02
ENSCAFG0000001948	SSNA1	yellow	VSMC_M3	0.16	4.40E-01	0.09	6.42E-01	0.69	7.45E-05	-0.49	8.75E-03	-0.56	2.53E-03	0.02	9.07E-01	0.39	4.52E-02	0.36	6.18E-02	-0.16	4.14E-01	-0.13	5.06E-01
ENSCAFG0000002138	ENSCAFG0000002138	darkgreen	VSMC_M4	0.15	4.41E-01	0.00	9.94E-01	-0.41	3.54E-02	0.39	4.29E-02	0.42	2.75E-02	-0.14	5.00E-01	0.16	4.21E-01	-0.31	1.20E-01	-0.08	7.04E-01	0.14	4.93E-01
ENSCAFG0000000309	ENSCAFG0000000309	grey	VSMC_M10	0.15	4.41E-01	-0.03	9.00E-01	-0.17	3.93E-01	0.09	6.71E-01	0.33	9.78E-02	-0.11	5.96E-01	-0.11	5.76E-01	-0.02	9.04E-01	0.00	9.82E-01	-0.18	3.78E-01
ENSCAFG0000000281	MPP6	grey	VSMC_M10	0.15	4.41E-01	-0.07	7.18E-01	0.16	4.18E-01	-0.30	1.30E-01	-0.13	5.06E-01	0.25	2.15E-01	0.22	2.68E-01	0.37	5.70E-02	-0.14	4.89E-01	-0.12	5.63E-01
ENSCAFG0000001535	KAT6B	pink	VSMC_M5	0.15	4.41E-01	0.08	6.96E-01	-0.29	1.48E-01	0.37	5.71E-02	0.44	2.30E-02	-0.39	4.70E-02	0.09	6.56E-01	-0.41	3.54E-02	-0.07	7.30E-01	0.10	6.11E-01
ENSCAFG0000001491	STK36	grey	VSMC_M10	0.15	4.41E-01	0.28	1.61E-01	-0.02	9.36E-01	0.14	4.89E-01	-0.11	5.82E-01	0.29	1.37E-01	0.31	1.15E-01	-0.20	3.23E-01	-0.36	6.76E-02	0.48	1.20E-02
ENSCAFG0000002612	ENSCAFG0000002612	grey	VSMC_M10	0.15	4.41E-01	-0.29	1.36E-01	-0.49	9.22E-03	0.23	2.49E-01	0.58	1.45E-03	-0.22	2.80E-01	0.02	9.35E-01	-0.05	7.99E-01	0.18	3.56E-01	-0.21	2.87E-01
ENSCAFG0000001065	AGPAT3	darkgreen	VSMC_M4	0.15	4.41E-01	0.50	7.30E-03	-0.26	1.88E-01	0.81	3.03E-07	0.24	2.35E-01	-0.24	2.25E-01	-0.32	1.04E-01	-0.86	9.47E-09	-0.41	3.58E-02	0.59	1.26E-03
ENSCAFG0000001876	PIEZO2	cyan	VSMC_M2	0.15	4.42E-01	0.57	1.85E-03	-0.22	2.70E-01	0.70	5.14E-05	0.15	4.63E-01	-0.13	5.23E-01	-0.73	1.46E-05	-0.74	1.07E-05	-0.47	1.30E-02	0.60	1.06E-03
ENSCAFG0000000095	MTBP	darkgrey	VSMC_M8	0.15	4.42E-01	-0.38	5.20E-02	0.31	1.11E-01	-0.69	6.06E-05	-0.15	4.68E-01	0.19	3.51E-01	0.40	3.72E-02	0.74	1.01E-05	0.16	4.15E-01	-0.57	1.91E-03
ENSCAFG0000000595	DUS1L	darkgrey	VSMC_M8	0.15	4.42E-01	-0.12	5.60E-01	0.69	6.74E-05	-0.75	7.61E-06	-0.45	1.81E-02	-0.06	7.79E-01	0.15	4.56E-01	0.65	2.30E-04	0.05	7.90E-01	-0.50	7.27E-03
ENSCAFG0000002961	ENSCAFG0000002961	grey	VSMC_M10	0.15	4.42E-01	0.16	4.21E-01	0.33	9.22E-02	-0.31	1.17E-01	-0.23	2.39E-01	-0.34	8.66E-02	-0.05	8.12E-01	0.19	3.47E-01	-0.15	4.46E-01	0.03	8.94E-01
ENSCAFG0000001818	TNFAIP2	grey	VSMC_M10	0.15	4.43E-01	0.45	1.72E-02	0.43	2.48E-02	-0.21	2.84E-01	-0.46	1.70E-02	0.42	3.00E-02	-0.27	1.66E-01	0.04	8.42E-01	-0.45	1.84E-02	0.26	1.96E-01
ENSCAFG0000000655	APAF1	pink	VSMC_M5	0.15	4.43E-01	0.02	9.13E-01	-0.27	1.79E-01	0.36	6.64E-02	0.44	2.04E-02	-0.34	8.04E-02	-0.09	6.48E-01	-0.34	8.71E-02	-0.03	8.65E-01	-0.06	7.82E-01
ENSCAFG0000001077	KHDRBS1	darkgrey	VSMC_M8	0.15	4.43E-01	-0.08	7.05E-01	0.57	1.99E-03	-0.78	1.48E-06	-0.43	2.66E-02	-0.06	7.66E-01	0.29	1.44E-01	0.76	3.38E-06	-0.11	5.85E-01	-0.37	5.61E-02
ENSCAFG0000000749	EIF2S2	darkolivegreen	VSMC_M9	0.15	4.43E-01	-0.68	1.02E-04	-0.35	7.70E-02	-0.07	7.43E-01	0.50	7.97E-03	-0.11	6.00E-01	-0.06	7.67E-01	0.32	1.06E-01	0.55	3.03E-03	-0.60	1.02E-03
ENSCAFG0000003212	ENSCAFG0000003212	turquoise	VSMC_M6	0.15	4.43E-01	0.03	8.80E-01	0.06	7.48E-01	-0.12	5.67E-01	-0.04	8.42E-01	0.52	5.98E-03	0.26	1.88E-01	0.12	5.58E-01	-0.11	5.99E-01	0.01	9.57E-01
ENSCAFG0000000105	HINT3	grey	VSMC_M10	0.15	4.43E-01	0.21	2.90E-01	-0.25	2.16E-01	0.30	1.23E-01	0.23	2.43E-01	-0.16	4.40E-01	0.14	4.76E-01	-0.36	6.63E-02	-0.19	3.36E-01	0.32	1.05E-01
ENSCAFG0000001931	THOC6	darkgrey	VSMC_M8	0.15	4.44E-01	0.00	9.96E-01	0.55	2.84E-03	-0.70	4.97E-05	-0.34	8.09E-02	-0.06	7.68E-01	0.46	1.64E-02	0.60	9.44E-04	-0.11	5.81E-01	-0.36	6.48E-02
ENSCAFG0000000940	ENSCAFG0000000940	grey	VSMC_M10	0.15	4.44E-01	-0.23	2.44E-01	-0.11	5.85E-01	-0.12	5.55E-01	0.20	3.13E-01	0.03	8.89E-01	0.04	8.26E-01	0.20	3.27E-01	0.11	6.01E-01	-0.18	3.58E-01
ENSCAFG0000002584	ENSCAFG0000002584	grey	VSMC_M10	0.15	4.44E-01	-0.23	2.44E-01	-0.11	5.85E-01	-0.12	5.55E-01	0.20	3.13E-01	0.03	8.89E-01	0.04	8.26E-01	0.20	3.27E-01	0.11	6.01E-01	-0.18	3.58E-01
ENSCAFG0000000961	CSDE1	pink	VSMC_M5	0.15	4.44E-01	-0.22	2.64E-01	-0.70	4.39E-05	0.53	4.19E-03	0.80	4.41E-07	-0.18	3.56E-01	-0.34	7.92E-02	-0.37	5.77E-02	0.28	1.52E-01	-0.13	5.06E-01
ENSCAFG0000002905	UCHL3	grey	VSMC_M10	0.15	4.44E-01	-0.17	4.03E-01	-0.02	9.16E-01	-0.04	8.40E-01	0.06	7.49E-01	-0.16	4.15E-01	-0.01	9.74E-01	0.17	3.83E-01	0.04	8.59E-01	-0.13	5.22E-01
ENSCAFG0000001583	CHRNE	cyan	VSMC_M2	0.15	4.44E-01	0.63	4.43E-04	0.30	1.24E-01	0.44	2.20E-02	-0.40	3.89E-02	-0.03	8.73E-01	-0.35	7.22E-02	-0.57	2.00E-03	-0.56	2.51E-03	0.65	2.54E-04
ENSCAFG0000001128	C8H14orf93	yellow	VSMC_M3	0.15	4.44E-01	0.48	1.06E-02	0.60	9.39E-04	-0.19	3.51E-01	-0.52	5.41E-03	-0.04	8.35E-01	0.30	1.24E-01	-0.04	8.36E-01	-0.51	6.20E-03	0.29	1.42E-01
ENSCAFG0000000779	POU6F1	cyan	VSMC_M2	0.15	4.44E-01	0.62	5.98E-04	0.55	2.75E-03	-0.17	4.08E-01	-0.52	5.60E-03	-0.04	8.33E-01	-0.21	3.00E-01	-0.12	5.61E-01	-0.54	3.91E-03	0.39	4.39E-02
ENSCAFG0000001815	ZNF521	yellow	VSMC_M3	0.15	4.45E-01	0.15	4.45E-01	0.37	5.49E-02	-0.62	5.68E-04	-0.18	3.61E-01	0.19	3.35E-01	0.11	5.69E-01	0.43	2.38E-02	-0.20	3.13E-01	-0.26	1.88E-01
ENSCAFG0000001426	GATAD2A	yellow	VSMC_M3	0.15	4.45E-01	0.36	6.41E-02	0.67	1.26E-04	-0.57	1.73E-03	-0.56	2.58E-03	0.28	1.53E-01	-0.17	3.90E-01	0.35	7.68E-02	-0.38	5.36E-02	-0.06	7.52E-01
ENSCAFG0000002764	ENSCAFG0000002764	grey	VSMC_M10	0.15	4.45E-01	-0.10	6.07E-01	-0.06	7.82E-01	-0.16	4.35E-01	0.22	2.80E-01	0.02	9.08E-01	0.02	9.39E-01	0.17	4.07E-01	0.04	8.61E-01	-0.30	1.30E-01
ENSCAFG0000001331	ENSCAFG0000001331	pink	VSMC_M5	0.15	4.45E-01	-0.08	6.98E-01	-0.49	9.99E-03	0.52	5.04E-03	0.47	1.27E-02	-0.05	8.03E-01	-0.08	7.03E-01	-0.35	7.02E-02	0.01	9.73E-01	0.18	3.72E-01
ENSCAFG0000001244	NOP58	darkgrey	VSMC_M8	0.15	4.45E-01	-0.46	1.50E-02	0.28	1.54E-01	-0.75	8.27E-06	-0.11	5.94E-01	0.13	5.15E-01	0.12	5.45E-01	0.84	3.48E-08	0.24	2.19E-01	-0.68	1.11E-04
ENSCAFG0000001909	IDS	grey	VSMC_M10	0.15	4.45E-01	0.54	3.58E-03	-0.24	2.23E-01	0.54	4.03E-03	0.31	1.20E-01	-0.33	9.10E-02	-0.40	3.84E-02	-0.72	2.28E-05	-0.35	7.09E-02	0.44	2.15E-02
ENSCAFG0000000457	ENSCAFG0000000457	darkgrey	VSMC_M8	0.15	4.45E-01	-0.37	5.44E-02	0.48	1.06E-02	-0.71	3.00E-05	-0.33	9.51E-02	0.17	3.84E-01	0.38	4.74E-02	0.76	4.85E-06	0.17	4.09E-01	-0.53	4.66E-03
ENSCAFG0000001127	PUDP	grey	VSMC_M10	0.15	4.46E-01	0.22	2.62E-01	0.11	5.75E-01	-0.01	9.67E-01	-0.26	1.98E-01	0.34	8.34E-02	0.13	5.30E-01	0.00	9.97E-01	-0.35	7.76E-02	0.41	3.28E-02
ENSCAFG0000000547	ENSCAFG0000000547	grey	VSMC_M10	0.15	4.46E-01	0.27	1.80E-01	0.34	8.54E-02	-0.10	6.07E-01	-0.32	1.06E-01	0.13	5.03E-01	-0.29	1.39E-01	0.06	7.63E-01	-0.29	1.38E-01	0.17	4.08E-01
ENSCAFG0000000616	ENSCAFG0000000616	grey	VSMC_M10	0.15	4.47E-01	-0.17	4.06E-01	-0.04	8.51E-01	-0.21	3.04E-01	0.15	4.57E-01	-0.05	8.18E-01	0.45	1.78E-02	0.22	2.63E-01	0.07	7.46E-01	-0.15	4.59E-01
ENSCAFG0000000789	PLS1	grey	VSMC_M10	0.15	4.47E-01	0.39	4.41E-02	0.25	2.11E-01	-0.13	5.32E-01	-0.29	1.43E-01	0.05	8.03E-01	0.05	7.93E-01	0.01	9.65E-01	-0.46	1.68E-02	0.32	1.01E-01
ENSCAFG0000000287	ENSCAFG0000000287	grey	VSMC_M10	0.15	4.47E-01	0.14	4.76E-01	-0.19	3.36E-01	0.22	2.78E-01	0.13	5.23E-01	-0.07	7.22E-01	-0.23	2.46E-01	-0.20	3.27E-01	-0.16	4.15E-01	0.25	2.05E-01
ENSCAFG0000001152	YOD1	grey	VSMC_M10	0.15	4.47E-01	0.23	2.40E-01	0.34	8.57E-02	-0.01	9.59E-01	-0.47	1.43E-02	0.37	5.64E-02	0.09	6.40E-01	0.03	8.77E-01	-0.37	6.02E-02	0.43	2.38E-02
ENSCAFG0000003081	NUDT8	cyan	VSMC_M2	0.15	4.48E-01	0.48	1.10E-02	0.09	6.47E-01	0.27	1.75E-01	-0.10	6.19E-01	-0.08	6.97E-01	0.11	5.70E-01	-0.51	6.73E-03	-0.41	3.16E-02	0.50	7.51E-03
ENSCAFG0000000841	NDUFS3	grey	VSMC_M10	0.15	4.48E-01	0.27	1.81E-01	0.45	1.98E-02	-0.14	4.96E-01	-0.40	4.08E-02	0.06	7.66E-01	-0.12	5.54E-01	0.06	7.62E-01	-0.28	1.59E-01	0.08	7.06E-01
ENSCAFG0000000152	NFIB	cyan	VSMC_M2	0.15	4.48E-01	0.61	6.79E-04	0.18	3.81E-01	0.12	5.37E-01	-0.19	3.36E-01	-0.06	7.84E-01	-0.06	7.80E-01	-0.24	2.32E-01	-0.62	5.76E-04	0.45	1.81E-02
ENSCAFG0000001055	ENSCAFG0000001055	grey	VSMC_M10	0.15	4.48E-01	0.29	1.40E-01	0.24	2.30E-01	0.08	6.97E-01	-0.24	2.25E-01	0.28	1.55E-01	-0.23	2.44E-01	-0.20	3.15E-01	-0.24	2.33E-01	0.19	3.41E-01
ENSCAFG0000002001	RTCA	grey	VSMC_M10	0.15	4.48E-01	-0.41	3.56E-02	-0.32	1.01E-01	0.19	3.39E-01	0.56	2.46E-03	-0.23	2.40E-01	-0.03	8.95E-01	-0.08	7.03E-01	0.43	2.65E-02	-0.47	1.30E-02
ENSCAFG0000001315	D2HGDH	grey	VSMC_M10	0.15	4.48E-01	0.47	1.23E-02	0.08	6.95E-01	0.50	8.43E-03	-0.08	6.98E-01	-0.19	3.51E-01	-0.15	4.44E-01	-0.60	9.57E-04	-0.42	3.12E-02	0.49	9.75E-03
ENSCAFG0000002901	ENSCAFG0000002901	grey	VSMC_M10	0.15	4.48E-01	0.38	5.27E-02	0.25	2.11E-01	0.13	5.05E-01	-0.22	2.71E-01	0.10	6.07E-01	-0.08	6.75E-01	-0.27	1.79E-01	-0.35	7.10E-02	0.36	6.55E-02
ENSCAFG0000000508	RBM33	grey	VSMC_M10	0.15	4.48E-01	-0.18	3.81E-01	0.22	2.75E-01	-0.39	4.43E-02	-0.09	6.70E-01	0.04	8.46E-01	0.03	8.89E-01	0.36	6.84E-02	0.14	4.88E-01	-0.24	2.36E-01
ENSCAFG0000000966	NUDT18	grey	VSMC_M10	0.15	4.48E-01	0.32	1.03E-01	0.39	4.16E-02	0.15	4.67E-01	-0.43	2.60E-02	0.28	1.51E-01	-0.17	4.06E-01	-0.20	3.19E-01	-0.35	7.26E-02	0.34	8.50E-02
ENSCAFG0000000573	GOLGA7	pink	VSMC_M5	0.15	4.48E-01	-0.14	5.00E-01	-0.60	9.98E-04	0.39	4.49E-02	0.79	7.96E-07	-0.35	7.77E-02	-0.18	3.70E-01	-0.32	1.01E-01	0.17	3.86E-01	-0.24	2.37E-01

ENSCAFG0000001483	PLCD4	grey	VSMC_M10	0.15	4.48E-01	0.66	1.76E-04	0.15	4.63E-01	0.19	3.53E-01	-0.16	4.37E-01	-0.09	6.52E-01	-0.24	2.21E-01	-0.32	1.00E-01	-0.60	9.10E-04	0.48	1.10E-02
ENSCAFG0000000307	EPGN	grey	VSMC_M10	0.15	4.48E-01	-0.07	7.31E-01	0.07	7.31E-01	-0.21	2.96E-01	0.02	9.14E-01	-0.09	6.48E-01	0.29	1.46E-01	0.26	1.97E-01	-0.09	6.55E-01	-0.08	6.74E-01
ENSCAFG0000001635	ERBB2	cyan	VSMC_M2	0.15	4.48E-01	0.85	1.62E-08	0.36	6.70E-02	0.26	1.85E-01	-0.29	1.41E-01	-0.25	2.11E-01	-0.23	2.41E-01	-0.57	1.95E-03	-0.72	1.91E-05	0.55	2.73E-03
ENSCAFG0000000316	BMPER	grey	VSMC_M10	0.15	4.48E-01	0.16	4.22E-01	-0.44	2.26E-02	0.43	2.35E-02	0.40	3.83E-02	0.33	9.39E-02	-0.15	4.57E-01	-0.35	7.59E-02	-0.24	2.23E-01	0.28	1.63E-01
ENSCAFG0000000496	MYD88	grey	VSMC_M10	0.15	4.48E-01	0.35	7.78E-02	0.04	8.40E-01	-0.02	9.39E-01	0.02	9.07E-01	0.02	9.11E-01	-0.04	8.39E-01	-0.02	9.05E-01	-0.40	3.90E-02	0.12	5.62E-01
ENSCAFG0000000005	SERPINB8	grey	VSMC_M10	0.15	4.49E-01	0.16	4.33E-01	0.06	7.64E-01	0.04	8.40E-01	-0.05	7.99E-01	-0.07	7.31E-01	0.34	7.93E-02	-0.11	5.78E-01	-0.28	1.61E-01	0.19	3.37E-01
ENSCAFG0000001141	MCUB	grey	VSMC_M10	0.15	4.49E-01	0.12	5.50E-01	-0.13	5.28E-01	0.45	1.80E-02	0.17	3.94E-01	-0.02	9.34E-01	-0.28	1.61E-01	-0.32	1.09E-01	-0.15	4.41E-01	0.08	7.01E-01
ENSCAFG0000001437	LSM12	grey	VSMC_M10	0.15	4.49E-01	-0.19	3.34E-01	0.18	3.82E-01	-0.28	1.61E-01	0.01	9.61E-01	0.04	8.36E-01	0.15	4.56E-01	0.33	9.58E-02	0.07	7.43E-01	-0.47	1.31E-02
ENSCAFG0000000196	PHF24	grey	VSMC_M10	0.15	4.49E-01	0.08	6.84E-01	0.15	4.48E-01	-0.24	2.29E-01	0.06	7.71E-01	-0.20	3.14E-01	0.15	4.40E-01	0.12	5.51E-01	-0.14	4.75E-01	-0.20	3.15E-01
ENSCAFG0000001052	PCSK6	cyan	VSMC_M2	0.15	4.49E-01	0.78	1.53E-06	0.01	9.79E-01	0.60	8.71E-04	-0.10	6.33E-01	-0.05	8.02E-01	-0.41	3.43E-02	-0.75	5.78E-06	-0.72	2.29E-05	0.75	7.33E-06
ENSCAFG0000001742	MRPS23	darkgrey	VSMC_M8	0.15	4.49E-01	-0.47	1.26E-02	0.07	7.11E-01	-0.47	1.24E-02	0.16	4.23E-01	0.02	9.25E-01	-0.13	5.31E-01	0.56	2.23E-03	0.38	4.98E-02	-0.72	1.95E-05
ENSCAFG0000002803	ENSCAFG0000002803	grey	VSMC_M10	0.15	4.49E-01	0.24	2.36E-01	0.47	1.45E-02	-0.39	4.54E-02	-0.44	2.18E-02	0.31	1.15E-01	0.09	6.39E-01	0.29	1.37E-01	-0.35	6.94E-02	0.04	8.42E-01
ENSCAFG0000001951	ADAMTSL5	grey	VSMC_M10	0.15	4.50E-01	-0.03	8.96E-01	0.15	4.48E-01	-0.06	7.83E-01	-0.01	9.48E-01	-0.22	2.67E-01	0.25	2.02E-01	0.00	9.98E-01	0.05	8.23E-01	-0.06	7.66E-01
ENSCAFG0000002790	ENSCAFG0000002790	grey	VSMC_M10	0.15	4.50E-01	0.08	6.81E-01	0.09	6.68E-01	-0.09	6.45E-01	0.13	5.18E-01	-0.43	2.36E-02	-0.12	5.35E-01	-0.08	6.84E-01	0.00	9.95E-01	-0.24	2.24E-01
ENSCAFG0000001068	ENSCAFG0000001068	darkgrey	VSMC_M8	0.15	4.50E-01	-0.03	8.72E-01	0.68	9.45E-05	-0.73	1.79E-05	-0.53	4.10E-03	0.21	2.93E-01	0.08	6.89E-01	0.66	2.08E-04	-0.09	6.72E-01	-0.38	4.74E-02
ENSCAFG0000003128	ENSCAFG0000003128	yellow	VSMC_M3	0.15	4.50E-01	-0.25	2.07E-01	0.44	2.27E-02	-0.45	1.78E-02	-0.34	8.22E-02	0.08	6.96E-01	0.36	6.30E-02	0.53	4.82E-03	0.05	7.86E-01	-0.33	9.49E-02
ENSCAFG0000000535	KLF6	grey	VSMC_M10	0.15	4.50E-01	-0.08	7.04E-01	0.39	4.53E-02	-0.36	6.16E-02	-0.22	2.61E-01	0.05	7.94E-01	0.20	3.16E-01	0.24	2.28E-01	0.06	7.72E-01	-0.23	2.48E-01
ENSCAFG0000001315	ARHGAP5	pink	VSMC_M5	0.15	4.50E-01	0.02	9.31E-01	-0.74	8.57E-06	0.67	1.29E-04	0.77	2.83E-06	-0.20	3.12E-01	-0.04	8.44E-01	-0.55	2.77E-03	-0.03	8.99E-01	0.22	2.63E-01
ENSCAFG0000000678	NSFL1C	grey	VSMC_M10	0.15	4.50E-01	-0.21	2.98E-01	0.16	4.21E-01	-0.12	5.67E-01	0.01	9.59E-01	-0.23	2.57E-01	-0.15	4.67E-01	0.20	3.28E-01	0.18	3.56E-01	-0.40	4.01E-02
ENSCAFG0000000696	UTP20	darkgrey	VSMC_M8	0.15	4.50E-01	-0.40	3.70E-02	0.04	8.38E-01	-0.34	8.52E-02	-0.01	9.46E-01	0.10	6.16E-01	0.24	2.38E-01	0.55	2.97E-03	0.13	5.23E-01	-0.27	1.76E-01
ENSCAFG0000003030	ENSCAFG0000003030	turquoise	VSMC_M6	0.15	4.51E-01	-0.10	6.25E-01	0.02	9.12E-01	-0.11	5.74E-01	0.02	9.13E-01	0.23	2.52E-01	-0.08	6.77E-01	0.16	4.21E-01	-0.03	9.01E-01	-0.08	6.80E-01
ENSCAFG0000000123	UHRF1BP1	grey	VSMC_M10	0.15	4.51E-01	0.23	2.59E-01	-0.20	3.06E-01	0.34	8.03E-02	0.19	3.33E-01	0.07	7.20E-01	-0.25	2.06E-01	-0.37	5.95E-02	-0.15	4.46E-01	0.30	1.23E-01
ENSCAFG0000001143	ENSCAFG0000001143	grey	VSMC_M10	0.15	4.51E-01	-0.48	1.08E-02	-0.52	5.14E-03	0.05	8.09E-01	0.58	1.39E-03	0.08	7.03E-01	0.27	1.71E-01	0.14	4.71E-01	0.36	6.46E-02	-0.28	1.54E-01
ENSCAFG0000001649	SOD3	grey	VSMC_M10	0.15	4.51E-01	0.67	1.15E-04	0.05	7.86E-01	0.33	9.11E-02	-0.09	6.41E-01	-0.07	7.28E-01	-0.08	6.93E-01	-0.55	2.71E-03	-0.64	2.85E-04	0.58	1.57E-03
ENSCAFG0000000945	FMOD	darkgreen	VSMC_M4	0.15	4.51E-01	0.43	2.57E-02	-0.47	1.23E-02	0.83	6.22E-08	0.39	4.56E-02	-0.10	6.26E-01	-0.33	9.79E-02	-0.82	1.83E-07	-0.40	3.98E-02	0.60	8.53E-04
ENSCAFG0000001662	SYNJ2BP	grey	VSMC_M10	0.15	4.51E-01	0.39	4.16E-02	-0.35	7.64E-02	0.60	9.36E-04	0.18	3.81E-01	-0.02	9.18E-01	0.00	9.93E-01	-0.62	6.01E-04	-0.44	2.08E-02	0.71	2.88E-05
ENSCAFG0000000472	CDK5	grey	VSMC_M10	0.15	4.51E-01	0.37	5.83E-02	0.25	2.11E-01	0.08	6.89E-01	-0.14	4.74E-01	0.01	9.64E-01	-0.01	9.65E-01	-0.35	7.77E-02	-0.24	2.30E-01	0.17	3.95E-01
ENSCAFG0000001705	ACSF2	yellow	VSMC_M3	0.15	4.51E-01	0.16	4.25E-01	0.49	8.81E-03	-0.46	1.63E-02	-0.27	1.70E-01	0.06	7.81E-01	0.05	8.11E-01	0.29	1.40E-01	-0.13	5.18E-01	-0.31	1.21E-01
ENSCAFG0000001132	PRKCA	darkgreen	VSMC_M4	0.15	4.52E-01	0.43	2.44E-02	-0.29	1.43E-01	0.64	3.56E-04	0.25	2.00E-01	-0.27	1.78E-01	-0.46	1.52E-02	-0.61	6.75E-04	-0.38	5.13E-02	0.47	1.27E-02
ENSCAFG0000001274	OAT	grey	VSMC_M10	0.15	4.52E-01	-0.17	3.87E-01	0.00	9.95E-01	-0.21	2.95E-01	0.20	3.07E-01	-0.14	4.89E-01	0.15	4.43E-01	0.18	3.58E-01	0.21	2.92E-01	-0.33	9.34E-02
ENSCAFG0000001241	RPS6KC1	pink	VSMC_M5	0.15	4.52E-01	-0.58	1.57E-03	-0.53	4.35E-03	0.16	4.27E-01	0.74	1.21E-05	-0.22	2.63E-01	0.19	3.46E-01	0.05	7.88E-01	0.48	1.20E-02	-0.51	6.99E-03
ENSCAFG0000001604	ENSCAFG0000001604	grey	VSMC_M10	0.15	4.52E-01	0.07	7.12E-01	0.33	9.75E-02	-0.32	9.99E-02	-0.15	4.43E-01	-0.01	9.79E-01	0.18	3.58E-01	0.23	2.42E-01	-0.16	4.26E-01	-0.24	2.29E-01
ENSCAFG0000001111	FABP3	yellow	VSMC_M3	0.15	4.52E-01	-0.12	5.63E-01	0.49	9.44E-03	-0.81	3.31E-07	-0.19	3.36E-01	-0.15	4.52E-01	0.45	1.84E-02	0.61	7.36E-04	0.05	7.97E-01	-0.55	3.03E-03
ENSCAFG0000001755	S100A8	grey	VSMC_M10	0.15	4.52E-01	0.15	4.64E-01	0.37	5.76E-02	-0.32	9.90E-02	-0.33	9.61E-02	0.03	8.72E-01	0.31	1.20E-01	0.14	4.73E-01	-0.18	3.63E-01	0.11	5.80E-01
ENSCAFG0000001389	SOAT1	grey	VSMC_M10	0.15	4.53E-01	0.35	7.65E-02	0.30	1.35E-01	-0.13	5.16E-01	-0.09	6.46E-01	-0.20	3.19E-01	-0.20	3.07E-01	-0.16	4.34E-01	-0.22	2.67E-01	-0.07	7.36E-01
ENSCAFG0000001947	TMEM203	grey	VSMC_M10	0.15	4.53E-01	0.35	7.74E-02	0.27	1.70E-01	0.12	5.39E-01	-0.41	3.31E-02	0.24	2.26E-01	0.18	3.72E-01	-0.23	2.42E-01	-0.41	3.29E-02	0.55	2.80E-03
ENSCAFG0000001091	RBM6	yellow	VSMC_M3	0.15	4.54E-01	-0.09	6.70E-01	0.49	1.03E-02	-0.47	1.28E-02	-0.44	2.26E-02	0.06	7.69E-01	0.52	4.97E-03	0.46	1.62E-02	-0.09	6.48E-01	-0.10	6.21E-01
ENSCAFG0000002013	ST3GAL2	grey	VSMC_M10	0.15	4.54E-01	-0.15	4.53E-01	-0.37	5.84E-02	0.16	4.24E-01	0.43	2.46E-02	0.08	7.06E-01	0.17	3.89E-01	-0.16	4.35E-01	0.16	4.37E-01	-0.02	9.08E-01
ENSCAFG0000001154	COA6	grey	VSMC_M10	0.15	4.54E-01	0.11	5.93E-01	-0.24	2.30E-01	0.46	1.54E-02	0.22	2.75E-01	0.04	8.59E-01	0.20	3.24E-01	-0.42	2.93E-02	-0.10	6.33E-01	0.35	7.32E-02
ENSCAFG0000001765	SENP8	yellow	VSMC_M3	0.15	4.54E-01	0.53	4.40E-03	0.57	1.74E-03	-0.07	7.34E-01	-0.59	1.23E-03	0.11	5.94E-01	0.11	5.73E-01	-0.13	5.13E-01	-0.55	3.07E-03	0.40	3.87E-02
ENSCAFG0000002863	TRIM7	yellow	VSMC_M3	0.15	4.54E-01	0.16	4.35E-01	0.60	9.50E-04	-0.65	2.66E-04	-0.49	9.88E-03	0.17	3.86E-01	0.32	1.03E-01	0.51	6.63E-03	-0.26	1.91E-01	-0.14	4.81E-01
ENSCAFG0000003231	ZNF470	grey	VSMC_M10	0.15	4.54E-01	0.14	4.75E-01	0.18	3.57E-01	0.02	9.05E-01	-0.20	3.17E-01	-0.03	8.87E-01	0.11	5.70E-01	-0.10	6.24E-01	-0.18	3.60E-01	0.30	1.26E-01
ENSCAFG0000002993	ENSCAFG0000002993	grey	VSMC_M10	0.15	4.54E-01	0.30	1.29E-01	-0.14	4.93E-01	0.35	7.15E-02	0.11	5.77E-01	-0.09	6.41E-01	0.05	8.03E-01	-0.34	8.30E-02	-0.31	1.17E-01	0.36	6.67E-02
ENSCAFG0000001152	AP1G2	yellow	VSMC_M3	0.15	4.55E-01	0.25	2.18E-01	0.33	8.78E-02	-0.39	4.72E-02	-0.19	3.34E-01	-0.03	8.77E-01	-0.04	8.54E-01	0.27	1.74E-01	-0.25	2.02E-01	-0.14	4.79E-01
ENSCAFG0000001433	NRXN2	grey	VSMC_M10	0.15	4.55E-01	0.27	1.78E-01	0.56	2.44E-03	-0.28	1.61E-01	-0.55	3.12E-03	0.26	1.98E-01	0.26	1.91E-01	0.13	5.17E-01	-0.34	8.29E-02	0.18	3.75E-01
ENSCAFG0000000470	TCTEX1D4	grey	VSMC_M10	0.15	4.55E-01	0.40	3.67E-02	-0.01	9.43E-01	0.43	2.45E-02	0.10	6.26E-01	-0.07	7.43E-01	-0.11	5.68E-01	-0.60	8.97E-04	-0.30	1.29E-01	0.31	1.13E-01
ENSCAFG0000001665	AR	cyan	VSMC_M2	0.15	4.55E-01	0.84	5.07E-08	0.16	4.26E-01	0.41	3.17E-02	-0.26	1.96E-01	-0.07	7.29E-01	-0.29	1.38E-01	-0.59	1.21E-03	-0.78	2.05E-06	0.74	1.10E-05
ENSCAFG0000000787	KCMF1	pink	VSMC_M5	0.15	4.55E-01	-0.58	1.44E-03	-0.54	3.37E-03	-0.08	6.89E-01	0.80	5.45E-07	-0.20	3.16E-01	-0.05	8.09E-01	0.25	2.16E-01	0.55	3.17E-03	-0.68	1.12E-04
ENSCAFG0000001486	ZFYVE28	grey	VSMC_M10	0.15	4.55E-01	0.67	1.48E-04	-0.12	5.53E-01	0.60	8.64E-04	0.09	6.51E-01	-0.35	7.64E-02	-0.28	1.57E-01	-0.70	4.07E-05	-0.58	1.45E-03	0.60	9.00E-04
ENSCAFG0000001812	ARSI	yellow	VSMC_M3	0.15	4.55E-01	0.52	5.76E-03	0.69	6.08E-05	-0.49	8.73E-03	-0.60	9.28E-04	0.10	6.29E-01	-0.15	4.45E-01	0.18	3.60E-01	-0.53	4.48E-03	0.12	5.52E-01
ENSCAFG0000001706	RPL26	grey	VSMC_M10	0.15	4.56E-01	0.00	9.83E-01	0.49	9.47E-03	-0.52	5.34E-03	-0.26	1.90E-01	0.08	6.77E-01	-0.17	4.07E-01	0.39	4.72E-02	0.05	8.06E-01	-0.46	1.66E-02
ENSCAFG0000000429	CCDC146	grey	VSMC_M10	0.15	4.56E-01	0.24	2.28E-01	-0.34	7.97E-02	0.33	9.66E-02	0.29	1.42E-01	-0.24	2.28E-01	-0.02	9.33E-01	-0.31	1.15E-01	-0.25	2.13E-01	0.37	5.69E-02
ENSCAFG0000000910	VDR	grey	VSMC_M10	0.15	4.56E-01	-0.27	1.78E-01	-0.07	7.37E-01	-0.25	2.08E-01	0.17	3.86E-01	0.23	2.45E-01	0.30	1.33E-01	0.31	1.16E-01	0.13	5.16E-01	-0.24	2.31E-01
ENSCAFG0000001966	PIGQ	cyan	VSMC_M2	0.15	4.56E-01	0.47	1.35E-02	0.52	5.58E-03	-0.07	7.43E-01	-0.48	1.11E-02	0.01	9.44E-01	-0.45	1.81E-02	-0.15	4.69E-01	-0.36	6.83E-02	0.23	2.59E-01
ENSCAFG0000000140	MKLN1	pink	VSMC_M5	0.15	4.56E-01	-0.53	4.13E-03	-0.48	1.17E-02	0.06	7.66E-01	0.71	3.59E-05	-0.35	7.45E-02	0.17	3.87E-01	0.07	7.42E-01	0.47	1.39E-02	-0.53	4.79E-03
ENSCAFG0000001427	RDX	darkgreen	VSMC_M4	0.15	4.56E-01	-0.10	6.16E-01	-0.53	4.56E-03	0.66	1.73E-04	0.59	1.24E-03	-0.33	8.92E-02	-0.41	3.41E-02	-0.52	5.86E-03	0.15	4.55E-01	0.06	7.56E-01
ENSCAFG0000000538	ENSCAFG0000000538	grey	VSMC_M10	0.15	4.57E-01	0.08	7.09E-01	-0.14	4.82E-01	0.16	4.39E-01	0.12	5.38E-01	0.07	7.15E-01	-0.13	5.14E-01	-0.07	7.36E-01	-0.13	5.18E-01	0.11	5.77E-01
ENSCAFG0000001999	S1PR1	turquoise	VSMC_M6	0.15	4.57E-01	0.19	3.55E-01	0.43	2.59E-02	-0.35	7.44E-02	-0.38	5.17E-02	0.73	1.43E-05	-0.18	3.63E-01	0.26	1.87E-01	-0.23	2.57E-01	-0.04	8.33E-01
ENSCAFG0000001835	DRG2	darkgrey	VSMC_M8	0.15	4.57E-01	-0.19	3.42E-01	0.46	1.62E-02	-0.69	6.88E-05	-0.32	1.09E-01	0.04	8.45E-01	0.20	3.22E-01	0.58	1.49E-03	0.07	7.16E-01	-0.43	2.71E-02
ENSCAFG0000001736	TMEM100	grey	VSMC_M10	0.15	4.58E-01	-0.25	2.13E-01	0.25	2.16E-01	-0.44	2.16E-02	-0.08	7.02E-01	-0.08	6.75E-01	0.37	5.69E-02	0.42	2.97E-02	0.19	3.42E-01	-0.38	5.03E-02
ENSCAFG0000000342	NUP62	darkgrey	VSMC_M8	0.15	4.58E-01	0.03	8.97E-01	0.70	5.49E-05	-0.73	1.44E-05	-0.54	3.37E-03	0.10	6.16E-01	0.21	2.91E-01	0.66	1.71E-04	-0.13	5.27E-01	-0.32	9.93E-02
ENSCAFG0000001332	PHEX	cyan	VSMC_M2	0.15	4.58E-01	0.74	1.23E-05	0.35	7.69E-02	0.18	3.66E-01	-0.31	1.11E-01	-0.11	5.91E-01	-0.45	1.76E-02	-0.41	3.33E-02	-0.67	1.29E-04	0.42	2.73E-02
ENSCAFG0000001420	IL12A	grey	VSMC_M10	0.15	4.58E-01	0.37	6.02E-02	0.22	2.70E-01	0.06	7.69E-01	-0.34	8.68E-02	0.15	4.42E-01	0.10	6.14E-01	-0.14	4.99E-01	-0.47	1.24E-02	0.52	5.33E-03
ENSCAFG0000000908	NUP93	darkgrey	VSMC_M8	0.15	4.58E-01	-0.02	9.16E-01	0.65	2.47E-04	-0.75	6.92E-06	-0.55	3.03E-03	0.23	2.53E-01	0.32	1.07E-01	0.71	3.84E-05	-0.14	4.78E-01	-0.26	1.84E-01
ENSCAFG0000001505	ENSCAFG0000001505	cyan	VSMC_M2	0.15	4.58E-01	0.26	1.86E-01	0.28	1.64E-01	0.19	3.45E-01	-0.23	2.53E-01	-0.22	2.69E-01	-0.25	2.16E-01	-0.30	1.33E-01	-0.22	2.69E-01	0.21	2.98E-01
ENSCAFG0000001429	MAU2	grey	VSMC_M10	0.15	4.58E-01	0.36	6.89E-02	-0.08	6.80E-01	0.24	2.32E-01	-0.05	7.89E-01	0.13	5.33E-01	-0.05	8.10E-01	-0.25	2.05E-01	-0.41	3.53E-02	0.54	3.36E-03
ENSCAFG0000001270	PSMB4	yellow	VSMC_M3	0.15	4.58E-01	0.16	4.23E-01	0.55	2.84E-03	-0.25	2.08E-01	-0.49	9.64E-03	0.17	3.96E-01	0.06	7.69E-01	0.21	2.85E-01	-0.23	2.49E-01	-0.10	6.26E-01
ENSCAFG0000000359	ZMYM6	grey	VSMC_M10	0.15	4.59E-01	0.31	1.15E-01	-0.37	5.40E-02	0.47	1.41E-02	0.30	1.29E-01	-0.01	9.54E-01	0.01	9.79E-01	-0.52	5.38E-03	-0.33	9.06E-02	0.49	8.88E-03
ENSCAFG0000002900	ENSCAFG0000002900	cyan	VSMC_M2	0.15	4.59E-01	0.42	2.82E-02	0.37	5.71E-02	-0.02	9.40E-01	-0.36	6.92E-02	0.14	4.72E-01	-0.12	5.65E-01	-0.11	5.93E-01	-0.42	2.80E-02	0.25	2.05E-01
ENSCAFG0000000233	ENSCAFG0000000233	grey	VSMC_M10	0.15	4.59E-01	-0.38	5.30E-02	-0.20	3.24E-01	-0.23	2.42E-01	0.33	9.80E-02	-0.01	9.45E-01	0.10	6.15E-01	0.35	6.98E-02	0.25	2.14E-01	-0.35	7.48E-02
ENSCAFG0000000215	TLN1	grey	VSMC_M10	0.15	4.59E-01	0.53	4.93E-03	0.12	5.47E-01	0.36	6.30E-02	-0.06	7.62E-01	-0.12	5.42E-01	-0.69	7.97E-05	-0.57	1.77E-03	-0.32	1.01E-01	0.36	6.22E-02
ENSCAFG0000000435	CCDC61	grey	VSMC_M10	0.15	4.59E-01	0.16	4.22E-01	0.06	7.84E-01	-0.07	7.25E-01	-0.13	5.11E-01	0.13	5.27E-01	0.38	5.22E-02	0.06	7.75E-01	-0.31	1.11E-01	0.28	1.59E-01
ENSCAFG0000000845	CCNJ	grey	VSMC_M10	0.15	4.60E-01	-0.54	3.66E-03	-0.07	7.43E-01	-0.47	1.38E-02	0.22	2.77E-01	0.23	2.48E-01	-0.06	7.48E-01	0.57	2.01E-03	0.45	1.93E-02	-0.60	8.88E-04
ENSCAFG0000001118	GALNT3	darkgreen	VSMC_M4	0.15	4.60E-01	0.39	4.40E-02	-0.28	1.62E-01	0.75	7.25E-06	0.15	4.56E-01	0.18	3.73E-01	-0.33	8.94E-02	-0.72	2.45E-05	-0.37	5.49E-02	0.57	2.03E-03
ENSCAFG0000000033	ADAMTS2	cyan	VSMC_M2	0.15	4.60E-01	0.44	2.11E-02	0.19	3.50E-01	-0.02	9.15E-01	-0.09	6.60E-01	0.02	9.20E-01	0.09	6.55E-01	-0.29	1.36E-01	-0.37	5.67E-02	0.29	1.49E-01
ENSCAFG0000000679	FKBP1A	grey	VSMC_M10	0.15	4.60E-01	0.17	4.07E-01	0.24	2.29E-01	-0.03	9.01E-01	-0.08	7.02E-01	0.02	9.35E-01	-0.13	5.07E-01	-0.12	5.46E-01	-0.09	6.62E-01	-0.04	8.57E-01
ENSCAFG0000001481	CNOT9	darkgrey	VSMC_M8	0.15	4.60E-01	-0.50	8.01E-03	0.20	3.15E-01	-0.67	1.23E-04	-0.10	6.09E-01	0.22	2.76E-01	0.25	2.00E-01	0.81	2.90E-07	0.25	2.11E-01	-0.56	2.32E-03
ENSCAFG0000001667	MED6	grey	VSMC_M10	0.15	4.60E-01	-0.20	3.15E-01	-0.17	3.83E-01	0.14	5.01E-01	0.34	8.65E-02	-0.22	2.65E-01	-0.09	6.68E-01	-0.08	6.85E-01	0.17	3.94E-01	-0.31	1.11E-01
ENSCAFG0000001722	WFIKKN2	grey	VSMC_M10	0.15	4.60E-01	0.66	2.07E-04	0.27	1.77E-01	0.30	1.24E-01	-0.32	1.01E-01	0.00	9.99E-01	-0.58	1.59E-03	-0.45	1.87E-02	-0.59	1.08E-03	0.53	4.34E-03
ENSCAFG0000002981	ENSCAFG0000002981	grey	VSMC_M10	0.15	4.61E-01	0.20	3.23E-01	-0.04	8.24E-01	0.13	5.26E-01	0.04	8.40E-01	-0.13	5.04E-01	-0.22	2.70E-01	-0.17	4.05E-01	-0.17	4.02E-01	0.14	4.72E-01
ENSCAFG0000001634	ZBTB4	darkgreen	VSMC_M4	0.15	4.61E-01	0.61	6.56E-04	-0.30	1.35E-01	0.81	2.90E-07	0.25	2.12E-01	-0.15	4.68E-01	-0.45	1.95E-02	-0.92	1.42E-11	-0.46	1.61E-02	0.68	9.20E-05
ENSCAFG0000001410	SESTD1	grey	VSMC_M10	0.15	4.61E-01	-0.07	7.11E-01	-0.02	9.25E-01	-0.24	2.30E-01	0.29	1.47E-01	-0.18	3.63E-01	-0.08	7.01E-01	0.09	6.55E-01	0.19	3.35E-01	-0.38	5.31E-02
ENSCAFG0000003087	ENSCAFG0000003087	grey	VSMC_M10	0.15	4.61E-01	-0.18	3.66E-01	0.21	2.98E-01	-0.21	2.96E-01	-0.08	6.83E-01	0.04	8.51E-01	0.12	5.50E-01	0.32	1.03E-01	0.01	9.48E-01	-0.32	9.86E-02
ENSCAFG0000002436	ENSCAFG0000002436	grey	VSMC_M10	0.15	4.61E-01	-0.21	2.83E-01	-0.15	4.64E-01	-0.03	8.83E-01	0.22	2.77E-01	-0.06	7.67E-01	0.08	7.08E-01	0.09	6.67E-01	0.16	4.33E-01	-0.14	4.98E-01
ENSCAFG0000001334	TRA2B	darkgrey	VSMC_M8	0.15	4.61E-01	-0.45	1.97E-02	0.36	6.47E-02	-0.79	1.16E-06	-0.18	3.66E-01	0.06	7.49E-01	0.32	1.06E-01	0.87	3.28E-09	0.23	2.43E-01	-0.62	5.53E-04
ENSCAFG0000003043	ENSCAFG0000003043	grey	VSMC_M10	0.15	4.62E-01	0.27	1.70E-01	0.00	9.88E-01	0.15	4.43E-01	0.01	9.75E-01	-0.10	6.12E-01	-0.24	2.23E-01	-0.21	2.90E-01	-0.28	1.56E-01	0.22	2.79E-01
ENSCAFG0000000738	STAG1	darkgrey	VSMC_M8	0.15	4.62E-01	-0.72	2.14E-05	-0.09	6.60E-01	-0.56	2.50E-03	0.38	5.25E-02	-0.19	3.50E-01	0.29	1.44E-01	0.69	6.71E-05	0.58	1.69E-03	-0.84	4.89E-08
ENSCAFG0000003071	ENSCAFG0000003071	grey	VSMC_M10	0.15	4.63E-01	0.18	3.71E-01	0.53	4.23E-03	-0.26	1.91E-01	-0.39	4.58E-02	0.00	9.96E-01	0.41	3.33E-02	0.08	6.87E-01	-0.18	3.70E-01	0.00	9.80E-01
ENSCAFG0000001172	HPS4	grey	VSMC_M10	0.15	4.63E-01	0.50	8.09E-03	0.15	4.48E-01	0.21	2.97E-01	-0.15	4.46E-01	0.13	5.19E-01	0.10	6.16E-01	-0.36	6.66E-02	-0.49	9.58E-03	0.42	3.05E-02
ENSCAFG0000000168	ELAVL2	pink	VSMC_M5	0.15	4.63E-01	0.04	8.30E-01	-0.48	1.08E-02	0.50	8.22E-03	0.48	1.05E-02	0.08	6.95E-01	-0.21	2.84E-01	-0.40	3.82E-02	-0.06	7.59E-01	0.24	2.29E-01
ENSCAFG0000002006	TRUB2	darkgrey	VSMC_M8	0.15	4.63E-01	0.16	4.39E-01	0.83	1.06E-07	-0.84	3.01E-08	-0.66	1.83E-04	0.20	3.23E-01	0.18	3.69E-01	0.65	2.62E-04	-0.24	2.21E-01	-0.30	1.32E-01
ENSCAFG0000000657	WDFY4	cyan	VSMC_M2	0.15	4.63E-01	0.51	6.30E-03	0.37	5.71E-02	0.12	5.52E-01	-0.36	6.60E-02	0.17	3.84E-01	-0.07	7.42E-01	-0.31	1.15E-01	-0.52	5.52E-03	0.39	4.50E-02
ENSCAFG0000000518	XRN2	darkgrey	VSMC_M8	0.15	4.64E-01	-0.44	2.10E-02	0.08	6.97E-01	-0.62	5.03E-04	0.16	4.35E-01	0.01	9.77E-01	0.07	7.25E-01	0.71	3.52E-05	0.32	1.01E-01	-0.69	5.94E-05
ENSCAFG0000001870	XPNPEP2	cyan	VSMC_M2	0.15	4.64E-01	0.60	1.03E-03	0.46	1.57E-02	-0.20	3.23E-01	-0.45	1.91E-02	-0.14	4.81E-01	-0.08	7.04E-01	-0.03	8.82E-01	-0.62	5.91E-04	0.34	7.98E-02
ENSCAFG0000000303	PPIE	darkgrey	VSMC_M8	0.15	4.64E-01	-0.26	1.87E-01	0.36	6.28E-02	-0.64	3.59E-04	-0.21	2.95E-01	0.17	3.99E-01	0.40	4.07E-02	0.61	6.62E-04	0.11	5.73E-01	-0.40	3.89E-02
ENSCAFG0000001830	LLGL1	cyan	VSMC_M2	0.15	4.64E-01	0.71	3.41E-05	0.28	1.64E-01	0.16	4.21E-01	-0.37	5.59E-02	0.15	4.61E-01	-0.03	8.65E-01	-0.35	7.04E-02	-0.70	4.10E-05	0.68	8.47E-05
ENSCAFG0000000524	NET1	darkgrey	VSMC_M8	0.15	4.65E-01	-0.27	1.74E-01	0.49	8.78E-03	-0.81	2.76E-07	-0.21	2.85E-01	0.11	5.71E-01	0.20	3.25E-01	0.77	2.27E-06	0.17	3.94E-01	-0.71	3.35E-05
ENSCAFG0000000358	PRMT1	darkgrey	VSMC_M8	0.15	4.65E-01	-0.28	1.50E-01	0.49	9.51E-03	-0.84	4.40E-08	-0.31	1.10E-01	0.27	1.73E-01	0.15	4.44E-01	0.83	7.27E-08	0.14	5.02E-01	-0.62	5.40E-04
ENSCAFG0000000809	ARNTL	grey	VSMC_M10	0.15	4.65E-01	0.10	6.26E-01	-0.14	4.86E-01	-0.10	6.18E-01	0.28	1.55E-01	0.06	7.74E-01	-0.19	3.48E-01	0.04	8.38E-01	-0.09	6.69E-01	-0.15	4.67E-01
ENSCAFG0000000556	SEL1L2	yellow	VSMC_M3	0.15	4.65E-01	0.50	8.37E-03	0.49	1.01E-02	0.02	9.10E-01	-0.46	1.67E-02	-0.12	5.36E-01	-0.30	1.30E-01	-0.28	1.61E-01	-0.41	3.22E-02	0.27	1.67E-01
ENSCAFG0000000571	CEP131	yellow	VSMC_M3	0.15	4.65E-01	0.51	6.67E-03	0.80	5.59E-07	-0.37	5.95E-02	-0.64	3.15E-04	-0.09	6.66E-01	-0.03	8.74E-01	0.10	6.28E-01	-0.50	7.66E-03	0.08	6.77E-01
ENSCAFG0000000230	ENSCAFG0000000230	pink	VSMC_M5	0.15	4.66E-01	-0.07	7.22E-01	-0.64	3.52E-04	0.48	1.15E-02	0.77	2.74E-06	-0.28	1.64E-01	-0.12	5.44E-01	-0.41	3.50E-02	0.10	6.20E-01	-0.02	9.18E-01
ENSCAFG0000000918	ENSCAFG0000000918	grey	VSMC_M10	0.15	4.67E-01	0.17	3.94E-01	0.36	6.91E-02	-0.13	5.13E-01	-0.22	2.72E-01	-0.02	9.12E-01	0.23	2.48E-01	-0.02	9.19E-01	-0.19	3.51E-01	-0.05	8.19E-01
ENSCAFG0000000705	RARG	cyan	VSMC_M2	0.15	4.67E-01	0.53	4.83E-03	0.41	3.36E-02	-0.24	2.35E-01	-0.37	5.91E-02	-0.22	2.76E-01	0.29	1.40E-01	-0.02	9.34E-01	-0.52	5.27E-03	0.34	8.26E-02
ENSCAFG0000001147	PPP1R3E	grey	VSMC_M10	0.15	4.67E-01	0.42	2.92E-02	0.10	6.11E-01	0.10	6.22E-01	-0.21	2.86E-01	0.11	5.82E-01	0.09	6.69E-01	-0.19	3.38E-01	-0.46	1.50E-02	0.45	1.96E-02
ENSCAFG0000000997	ADAMTS8	grey	VSMC_M10	0.15	4.68E-01	0.47	1.33E-02	-0.18	3.78E-01	0.49	8.71E-03	0.14	4.96E-01	-0.17	4.07E-01	-0.15	4.52E-01	-0.54	3.87E-03	-0.40	3.64E-02	0.52	5.31E-03
ENSCAFG0000001825	SMCR8	grey	VSMC_M10	0.15	4.68E-01	0.16	4.39E-01	-0.07	7.40E-01	0.23	2.55E-01	-0.08	6.98E-01	0.11	5.81E-01	-0.15	4.67E-01	-0.20	3.06E-01	-0.21	2.86E-01	0.42	3.07E-02
ENSCAFG0000000917	EMILIN3	grey	VSMC_M10	0.15	4.68E-01	0.17	3.94E-01	0.56	2.44E-03	-0.27	1.70E-01	-0.54	3.32E-03	0.34	8.31E-02	0.08	6.98E-01	0.15	4.60E-01	-0.23	2.42E-01	0.03	9.01E-01
ENSCAFG0000003053	GOLPH3L	pink	VSMC_M5	0.15	4.68E-01	0.10	6.11E-01	-0.56	2.50E-03	0.43	2.49E-02	0.58	1.48E-03	-0.27	1.80E-01	0.05	7.96E-01	-0.41	3.54E-02	-0.12	5.47E-01	0.24	2.21E-01
ENSCAFG0000001689	ARMC5	yellow	VSMC_M3	0.15	4.69E-01	0.31	1.17E-01	0.50	7.71E-03	-0.33	8.83E-02	-0.44	2.16E-02	-0.10	6.35E-01	0.25	2.18E-01	0.15	4.40E-01	-0.31	1.17E-01	0.09	6.44E-01
ENSCAFG0000001920	UBALD1	grey	VSMC_M10	0.15	4.69E-01	0.43	2.68E-02	-0.02	9.09E-01	0.17	4.05E-01	0.09	6.68E-01	-0.01	9.45E-01	-0.18	3.72E-01	-0.35	6.97E-02	-0.34	7.96E-02	0.26	1.83E-01
ENSCAFG0000001428	ATP5S	grey	VSMC_M10	0.15	4.69E-01	0.31	1.22E-01	0.04	8.43E-01	0.42	3.08E-02	-0.15	4.50E-01	0.10	6.18E-01	-0.08	7.08E-01	-0.44	2.29E-02	-0.35	7.47E-02	0.56	2.37E-03
ENSCAFG0000002760	ENSCAFG0000002760	grey	VSMC_M10	0.15	4.69E-01	0.18	3.82E-01	0.33	9.20E-02	-0.12	5.61E-01	-0.31	1.12E-01	0.14	4.92E-01	-0.22	2.77E-01	0.09	6.57E-01	-0.23	2.49E-01	0.08	6.82E-01
ENSCAFG0000001996	DNAAF1	cyan	VSMC_M2	0.15	4.69E-01	0.41	3.39E-02	0.30	1.29E-01	0.24	2.26E-01	-0.20	3.22E-01	-0.35	7.25E-02	-0.20	3.17E-01	-0.41	3.52E-02	-0.33	9.50E-02	0.22	2.60E-01
ENSCAFG0000003059	ENSCAFG0000003059	grey	VSMC_M10	0.15	4.69E-01	0.44	2.16E-02	0.48	1.13E-02	-0.05	8.05E-01	-0.49	1.02E-02	0.30	1.28E-01	0.00	9.86E-01	-0.08	6.97E-01	-0.44	2.33E-02	0.25	2.11E-01
ENSCAFG0000001260	ZNF687	grey	VSMC_M10	0.15	4.70E-01	0.71	3.49E-05	0.36	6.54E-02	0.24	2.19E-01	-0.37	5.47E-02	0.02	9.28E-01	-0.18	3.78E-01	-0.47	1.26E-02	-0.65	2.13E-04	0.57	2.12E-03

ENSCAFG0000001174	ANP32E	darkgrey	VSMC_M8	0.15	4.70E-01	-0.61	6.52E-04	0.07	7.32E-01	-0.47	1.31E-02	0.15	4.52E-01	0.12	5.62E-01	0.21	3.00E-01	0.69	7.04E-05	0.45	1.96E-02	-0.73	1.52E-05
ENSCAFG0000000383	DLD	grey	VSMC_M10	0.15	4.70E-01	0.12	5.35E-01	0.04	8.40E-01	-0.03	8.79E-01	0.02	9.18E-01	-0.10	6.21E-01	0.32	1.05E-01	0.04	8.47E-01	-0.20	3.05E-01	0.08	6.90E-01
ENSCAFG0000001822	ZNF358	yellow	VSMC_M3	0.15	4.70E-01	0.50	8.20E-03	0.74	8.57E-06	-0.50	8.20E-03	-0.59	1.20E-03	-0.08	6.77E-01	0.11	5.99E-01	0.11	5.71E-01	-0.41	3.14E-02	0.05	8.14E-01
ENSCAFG0000000235	ENSCAFG0000000235	grey	VSMC_M10	0.15	4.70E-01	0.14	4.93E-01	0.06	7.81E-01	0.02	9.02E-01	-0.05	8.01E-01	-0.11	5.88E-01	0.06	7.81E-01	0.10	6.06E-01	-0.28	1.64E-01	0.13	5.25E-01
ENSCAFG0000001093	MON1A	grey	VSMC_M10	0.14	4.71E-01	0.41	3.55E-02	-0.12	5.59E-01	0.40	3.81E-02	0.01	9.46E-01	0.10	6.35E-01	-0.03	9.01E-01	-0.46	1.60E-02	-0.46	1.55E-02	0.60	9.72E-04
ENSCAFG0000001918	PITPNA	grey	VSMC_M10	0.14	4.71E-01	0.14	4.93E-01	-0.09	6.69E-01	0.36	6.20E-02	0.02	9.03E-01	-0.04	8.24E-01	0.02	9.35E-01	-0.32	1.01E-01	-0.17	3.84E-01	0.31	1.12E-01
ENSCAFG0000000921	R3HCC1	yellow	VSMC_M3	0.14	4.72E-01	0.17	3.96E-01	0.70	4.74E-05	-0.47	1.25E-02	-0.47	1.31E-02	0.04	8.59E-01	0.13	5.20E-01	0.32	1.05E-01	-0.16	4.12E-01	-0.27	1.81E-01
ENSCAFG0000000576	PLCB4	darkgreen	VSMC_M4	0.14	4.72E-01	-0.07	7.19E-01	-0.53	4.76E-03	0.52	5.35E-03	0.49	9.71E-03	0.11	5.82E-01	-0.05	7.92E-01	-0.38	4.87E-02	0.03	8.80E-01	0.23	2.45E-01
ENSCAFG0000000693	HAUS5	darkgrey	VSMC_M8	0.14	4.72E-01	-0.07	7.25E-01	0.51	5.98E-03	-0.63	3.84E-04	-0.43	2.44E-02	0.14	4.96E-01	0.36	6.82E-02	0.65	2.18E-04	-0.13	5.11E-01	-0.20	3.09E-01
ENSCAFG0000001333	RXRG	grey	VSMC_M10	0.14	4.72E-01	-0.17	4.01E-01	-0.04	8.35E-01	-0.22	2.74E-01	0.14	4.88E-01	-0.08	6.77E-01	0.40	3.92E-02	0.25	2.04E-01	0.07	7.37E-01	-0.15	4.63E-01
ENSCAFG0000001736	NIPAL4	grey	VSMC_M10	0.14	4.72E-01	-0.25	2.07E-01	0.24	2.18E-01	-0.35	6.96E-02	-0.17	3.83E-01	0.32	1.05E-01	0.26	1.93E-01	0.37	6.00E-02	0.19	3.38E-01	-0.23	2.49E-01
ENSCAFG0000001274	ENSCAFG0000001274	grey	VSMC_M10	0.14	4.73E-01	0.49	9.65E-03	0.12	5.35E-01	0.19	3.46E-01	-0.23	2.47E-01	0.23	2.47E-01	0.07	7.13E-01	-0.34	8.63E-02	-0.50	7.69E-03	0.57	1.76E-03
ENSCAFG0000001913	RFLNB	grey	VSMC_M10	0.14	4.74E-01	0.17	4.11E-01	0.20	3.23E-01	0.13	5.20E-01	-0.15	4.61E-01	-0.08	7.09E-01	-0.21	2.82E-01	-0.10	6.21E-01	-0.15	4.63E-01	0.17	4.01E-01
ENSCAFG0000002028	SYDE2	darkgreen	VSMC_M4	0.14	4.74E-01	-0.09	6.47E-01	-0.70	5.43E-05	0.77	2.39E-06	0.72	1.97E-05	-0.31	1.13E-01	-0.20	3.25E-01	-0.60	8.58E-04	0.10	6.09E-01	0.14	4.72E-01
ENSCAFG0000000317	AFTPH	grey	VSMC_M10	0.14	4.74E-01	-0.01	9.50E-01	-0.19	3.40E-01	0.26	1.89E-01	0.27	1.77E-01	-0.14	4.72E-01	0.17	3.86E-01	-0.28	1.65E-01	-0.04	8.55E-01	0.12	5.56E-01
ENSCAFG0000002865	ENSCAFG0000002865	grey	VSMC_M10	0.14	4.74E-01	0.20	3.27E-01	-0.28	1.62E-01	0.36	6.34E-02	0.22	2.61E-01	-0.05	8.02E-01	-0.22	2.70E-01	-0.28	1.58E-01	-0.31	1.11E-01	0.33	8.83E-02
ENSCAFG0000002334	ENSCAFG0000002334	darkgrey	VSMC_M8	0.14	4.75E-01	-0.23	2.52E-01	0.40	3.64E-02	-0.76	3.61E-06	-0.22	2.67E-01	0.18	3.60E-01	0.27	1.78E-01	0.67	1.35E-04	0.11	5.95E-01	-0.50	7.94E-03
ENSCAFG0000000554	CANT1	cyan	VSMC_M2	0.14	4.75E-01	0.52	5.95E-03	0.42	2.96E-02	0.02	9.30E-01	-0.41	3.36E-02	0.03	8.80E-01	-0.04	8.27E-01	-0.22	2.80E-01	-0.53	4.77E-03	0.36	6.58E-02
ENSCAFG0000000850	TCTN1	pink	VSMC_M5	0.14	4.75E-01	-0.10	6.24E-01	-0.62	5.36E-04	0.55	3.17E-03	0.65	2.21E-04	-0.21	2.89E-01	-0.27	1.76E-01	-0.36	6.54E-02	0.04	8.26E-01	0.02	9.25E-01
ENSCAFG0000000104	PMM1	grey	VSMC_M10	0.14	4.75E-01	0.38	4.96E-02	0.04	8.53E-01	0.30	1.29E-01	0.05	7.87E-01	-0.35	7.08E-02	-0.18	3.82E-01	-0.42	3.04E-02	-0.29	1.43E-01	0.24	2.35E-01
ENSCAFG0000000847	BLNK	darkgreen	VSMC_M4	0.14	4.75E-01	0.37	5.98E-02	-0.26	1.98E-01	0.62	5.95E-04	0.24	2.30E-01	-0.03	8.73E-01	-0.35	7.16E-02	-0.70	4.11E-05	-0.29	1.41E-01	0.46	1.55E-02
ENSCAFG0000002025	NQO1	grey	VSMC_M10	0.14	4.75E-01	-0.05	8.10E-01	0.32	1.07E-01	-0.41	3.52E-02	-0.36	6.84E-02	0.42	2.99E-02	0.43	2.53E-02	0.43	2.71E-02	-0.11	5.85E-01	0.01	9.71E-01
ENSCAFG0000000929	RNFT2	grey	VSMC_M10	0.14	4.75E-01	0.66	2.07E-04	0.11	5.97E-01	0.50	7.26E-03	-0.16	4.36E-01	-0.06	7.77E-01	0.03	8.99E-01	-0.66	1.79E-04	-0.59	1.09E-03	0.63	4.90E-04
ENSCAFG0000000581	ENSCAFG0000000581	darkgrey	VSMC_M8	0.14	4.76E-01	-0.05	8.21E-01	0.78	1.72E-06	-0.75	7.03E-06	-0.61	8.14E-04	0.06	7.52E-01	0.23	2.55E-01	0.65	2.80E-04	-0.06	7.54E-01	-0.41	3.48E-02
ENSCAFG0000003041	ENSCAFG0000003041	grey	VSMC_M10	0.14	4.76E-01	-0.25	2.10E-01	-0.06	7.48E-01	-0.29	1.35E-01	0.24	2.33E-01	-0.15	4.62E-01	0.23	2.41E-01	0.31	1.17E-01	0.19	3.53E-01	-0.39	4.33E-02
ENSCAFG0000001483	DGCR2	cyan	VSMC_M2	0.14	4.76E-01	0.82	1.38E-07	0.49	8.67E-03	0.15	4.48E-01	-0.54	3.68E-03	-0.06	7.80E-01	-0.04	8.41E-01	-0.47	1.44E-02	-0.75	7.06E-06	0.67	1.13E-04
ENSCAFG0000000482	SLC30A3	yellow	VSMC_M3	0.14	4.76E-01	0.18	3.73E-01	0.62	5.44E-04	-0.50	8.09E-03	-0.49	9.93E-03	-0.02	9.21E-01	0.13	5.06E-01	0.36	6.51E-02	-0.26	1.89E-01	-0.12	5.43E-01
ENSCAFG0000002862	RNF103	pink	VSMC_M5	0.14	4.77E-01	-0.46	1.50E-02	-0.63	4.16E-04	0.23	2.52E-01	0.78	1.25E-06	-0.11	5.84E-01	0.04	8.42E-01	-0.07	7.40E-01	0.45	1.98E-02	-0.35	7.13E-02
ENSCAFG0000002881	CFD	grey	VSMC_M10	0.14	4.77E-01	0.17	3.83E-01	-0.27	1.68E-01	0.55	2.90E-03	0.22	2.70E-01	-0.07	7.27E-01	-0.06	7.68E-01	-0.44	2.30E-02	-0.22	2.67E-01	0.36	6.71E-02
ENSCAFG0000000377	AFG1L	pink	VSMC_M5	0.14	4.78E-01	-0.29	1.36E-01	-0.43	2.63E-02	0.03	8.99E-01	0.63	4.31E-04	-0.26	1.82E-01	0.05	7.92E-01	0.07	7.37E-01	0.22	2.69E-01	-0.41	3.56E-02
ENSCAFG0000003097	GYPC	turquoise	VSMC_M6	0.14	4.78E-01	0.50	7.25E-03	0.31	1.12E-01	-0.09	6.66E-01	-0.34	8.50E-02	0.42	2.79E-02	-0.22	2.67E-01	-0.06	7.63E-01	-0.53	4.32E-03	0.31	1.18E-01
ENSCAFG0000002868	JAGN1	yellow	VSMC_M3	0.14	4.78E-01	0.09	6.65E-01	0.53	4.05E-03	-0.43	2.35E-02	-0.42	2.72E-02	0.17	3.89E-01	-0.09	6.73E-01	0.33	9.55E-02	-0.11	5.73E-01	-0.22	2.81E-01
ENSCAFG0000001625	ENSCAFG0000001625	darkgreen	VSMC_M4	0.14	4.79E-01	0.21	2.91E-01	-0.34	7.90E-02	0.51	6.48E-03	0.32	1.08E-01	-0.10	6.10E-01	-0.18	3.62E-01	-0.46	1.56E-02	-0.19	3.32E-01	0.31	1.21E-01
ENSCAFG0000001831	CD209	darkgreen	VSMC_M4	0.14	4.79E-01	0.21	2.91E-01	-0.34	7.90E-02	0.51	6.48E-03	0.32	1.08E-01	-0.10	6.10E-01	-0.18	3.62E-01	-0.46	1.56E-02	-0.19	3.32E-01	0.31	1.21E-01
ENSCAFG0000000964	PLEKHA6	grey	VSMC_M10	0.14	4.79E-01	0.34	7.87E-02	0.24	2.32E-01	-0.02	9.05E-01	-0.23	2.50E-01	-0.03	8.73E-01	-0.60	9.19E-04	-0.07	7.10E-01	-0.32	1.05E-01	0.18	3.66E-01
ENSCAFG0000000963	TYRO3	yellow	VSMC_M3	0.14	4.79E-01	0.62	6.24E-04	0.55	3.21E-03	0.05	8.14E-01	-0.59	1.33E-03	-0.04	8.49E-01	-0.22	2.73E-01	-0.19	3.51E-01	-0.59	1.07E-03	0.49	9.16E-03
ENSCAFG0000001472	KXD1	grey	VSMC_M10	0.14	4.79E-01	0.73	1.29E-05	0.23	2.58E-01	0.28	1.54E-01	-0.24	2.25E-01	-0.04	8.52E-01	-0.19	3.51E-01	-0.53	4.75E-03	-0.60	9.97E-04	0.55	2.71E-03
ENSCAFG0000000932	ARHGAP24	grey	VSMC_M10	0.14	4.79E-01	-0.19	3.34E-01	-0.37	5.55E-02	0.36	6.30E-02	0.52	5.45E-03	-0.07	7.41E-01	0.16	4.20E-01	-0.29	1.40E-01	0.18	3.77E-01	-0.16	4.38E-01
ENSCAFG0000002372	ENSCAFG0000002372	darkgreen	VSMC_M4	0.14	4.79E-01	0.24	2.38E-01	-0.39	4.24E-02	0.75	5.93E-06	0.35	7.78E-02	0.01	9.75E-01	-0.39	4.49E-02	-0.63	4.05E-04	-0.20	3.16E-01	0.35	7.37E-02
ENSCAFG0000001607	BMPR1A	grey	VSMC_M10	0.14	4.79E-01	0.48	1.05E-02	-0.30	1.35E-01	0.60	1.02E-03	0.30	1.31E-01	-0.31	1.12E-01	-0.58	1.55E-03	-0.67	1.13E-04	-0.34	8.33E-02	0.45	1.92E-02
ENSCAFG0000002322	CCL16	grey	VSMC_M10	0.14	4.80E-01	0.14	4.73E-01	-0.06	7.60E-01	0.22	2.60E-01	0.08	6.78E-01	-0.11	5.74E-01	-0.18	3.81E-01	-0.18	3.62E-01	-0.16	4.18E-01	0.13	5.13E-01
ENSCAFG0000001909	ENSCAFG0000001909	grey	VSMC_M10	0.14	4.80E-01	0.14	4.94E-01	-0.10	6.10E-01	0.38	4.80E-02	0.04	8.35E-01	0.00	9.92E-01	-0.20	3.22E-01	-0.35	7.30E-02	-0.17	4.02E-01	0.33	8.86E-02
ENSCAFG0000001044	GMNN	darkgrey	VSMC_M8	0.14	4.80E-01	-0.41	3.26E-02	0.48	1.14E-02	-0.80	6.67E-07	-0.27	1.66E-01	-0.04	8.32E-01	0.33	9.42E-02	0.87	3.36E-09	0.22	2.71E-01	-0.66	1.58E-04
ENSCAFG0000002026	ENSCAFG0000002026	grey	VSMC_M10	0.14	4.80E-01	0.41	3.44E-02	0.30	1.34E-01	-0.07	7.39E-01	-0.32	1.08E-01	0.05	7.90E-01	-0.02	9.13E-01	-0.09	6.43E-01	-0.44	2.24E-02	0.30	1.31E-01
ENSCAFG0000000394	DENND2A	cyan	VSMC_M2	0.14	4.80E-01	0.86	1.14E-08	0.13	5.30E-01	0.53	4.92E-03	-0.19	3.42E-01	-0.01	9.57E-01	-0.36	6.13E-02	-0.72	1.95E-05	-0.74	1.03E-05	0.75	6.38E-06
ENSCAFG0000003020	ENSCAFG0000003020	grey	VSMC_M10	0.14	4.81E-01	-0.34	8.24E-02	-0.11	5.88E-01	-0.21	3.03E-01	0.22	2.65E-01	-0.08	6.96E-01	0.01	9.65E-01	0.31	1.12E-01	0.21	2.98E-01	-0.30	1.31E-01
ENSCAFG0000000101	SREBF2	yellow	VSMC_M3	0.14	4.81E-01	0.29	1.44E-01	0.67	1.30E-04	-0.64	3.48E-04	-0.42	2.88E-02	-0.12	5.42E-01	0.27	1.70E-01	0.30	1.23E-01	-0.27	1.81E-01	-0.20	3.16E-01
ENSCAFG0000000921	MORN4	grey	VSMC_M10	0.14	4.81E-01	0.24	2.26E-01	0.10	6.05E-01	0.04	8.54E-01	0.02	9.24E-01	0.01	9.79E-01	0.19	3.39E-01	-0.18	3.76E-01	-0.26	1.89E-01	0.00	9.84E-01
ENSCAFG0000001176	GTF2IRD1	grey	VSMC_M10	0.14	4.81E-01	-0.22	2.81E-01	0.14	4.99E-01	-0.30	1.32E-01	-0.11	5.69E-01	0.17	3.87E-01	0.05	8.11E-01	0.28	1.61E-01	0.14	4.83E-01	-0.17	3.96E-01
ENSCAFG0000002349	C17H1orf56	grey	VSMC_M10	0.14	4.82E-01	-0.16	4.15E-01	-0.23	2.59E-01	-0.10	6.22E-01	0.19	3.31E-01	0.13	5.31E-01	0.16	4.21E-01	0.20	3.25E-01	0.03	9.00E-01	-0.02	9.32E-01
ENSCAFG0000000995	ENSCAFG0000000995	grey	VSMC_M10	0.14	4.82E-01	0.26	1.93E-01	0.34	7.95E-02	0.02	9.16E-01	-0.35	7.76E-02	-0.10	6.14E-01	-0.06	7.59E-01	-0.12	5.52E-01	-0.26	1.85E-01	0.31	1.13E-01
ENSCAFG0000000154	HGH1	yellow	VSMC_M3	0.14	4.82E-01	0.37	5.92E-02	0.85	1.81E-08	-0.41	3.57E-02	-0.80	6.30E-07	0.16	4.34E-01	0.11	5.92E-01	0.20	3.08E-01	-0.41	3.50E-02	0.12	5.40E-01
ENSCAFG0000000061	LY6G6C	grey	VSMC_M10	0.14	4.82E-01	0.07	7.19E-01	-0.18	3.63E-01	0.28	1.52E-01	0.17	3.97E-01	-0.12	5.67E-01	0.22	2.68E-01	-0.23	2.51E-01	-0.13	5.06E-01	0.18	3.75E-01
ENSCAFG0000003223	ENSCAFG0000003223	grey	VSMC_M10	0.14	4.83E-01	0.07	7.42E-01	0.39	4.22E-02	-0.40	3.71E-02	-0.40	4.07E-02	0.35	7.26E-02	0.53	4.84E-03	0.35	7.14E-02	-0.23	2.48E-01	0.05	8.06E-01
ENSCAFG0000000020	TCF21	cyan	VSMC_M2	0.14	4.83E-01	0.49	8.74E-03	-0.11	5.69E-01	0.66	1.57E-04	0.00	9.81E-01	0.08	6.95E-01	-0.60	8.98E-04	-0.64	3.44E-04	-0.46	1.47E-02	0.56	2.26E-03
ENSCAFG0000001019	CCDC34	grey	VSMC_M10	0.14	4.83E-01	-0.06	7.65E-01	0.38	5.28E-02	-0.44	2.04E-02	-0.40	3.88E-02	0.33	9.70E-02	0.63	3.78E-04	0.45	1.74E-02	-0.14	5.01E-01	0.01	9.49E-01
ENSCAFG0000001318	ENSCAFG0000001318	grey	VSMC_M10	0.14	4.83E-01	-0.09	6.65E-01	0.35	7.38E-02	-0.40	4.10E-02	-0.09	6.56E-01	-0.07	7.29E-01	0.11	6.00E-01	0.30	1.24E-01	0.07	7.36E-01	-0.47	1.30E-02
ENSCAFG0000000300	CREB5	grey	VSMC_M10	0.14	4.84E-01	-0.01	9.58E-01	-0.07	7.37E-01	-0.03	8.73E-01	0.16	4.30E-01	-0.10	6.12E-01	0.22	2.74E-01	-0.04	8.36E-01	0.02	9.21E-01	-0.02	9.40E-01
ENSCAFG0000000921	DCBLD2	darkgreen	VSMC_M4	0.14	4.84E-01	0.45	1.84E-02	-0.46	1.64E-02	0.90	2.82E-10	0.37	5.89E-02	-0.18	3.78E-01	-0.39	4.26E-02	-0.84	3.26E-08	-0.40	3.92E-02	0.61	7.53E-04
ENSCAFG0000003250	ENSCAFG0000003250	pink	VSMC_M5	0.14	4.84E-01	-0.07	7.29E-01	-0.37	5.72E-02	0.44	2.05E-02	0.40	3.75E-02	-0.07	7.28E-01	-0.01	9.49E-01	-0.37	6.12E-02	0.04	8.52E-01	0.08	6.85E-01
ENSCAFG0000001893	FHL1	grey	VSMC_M10	0.14	4.84E-01	0.19	3.53E-01	-0.23	2.52E-01	0.34	8.26E-02	0.40	3.98E-02	-0.49	9.76E-03	-0.09	6.47E-01	-0.47	1.32E-02	-0.03	8.99E-01	0.08	7.08E-01
ENSCAFG0000003197	ENSCAFG0000003197	grey	VSMC_M10	0.14	4.84E-01	0.26	1.92E-01	-0.12	5.37E-01	0.13	5.21E-01	0.11	5.90E-01	-0.18	3.62E-01	-0.24	2.37E-01	-0.14	4.73E-01	-0.26	1.85E-01	0.22	2.64E-01
ENSCAFG0000000520	EMC3	grey	VSMC_M10	0.14	4.85E-01	0.24	2.29E-01	0.21	3.01E-01	-0.09	6.53E-01	-0.27	1.72E-01	0.33	8.79E-02	-0.22	2.77E-01	0.03	8.81E-01	-0.31	1.10E-01	0.15	4.56E-01
ENSCAFG0000000830	ENSCAFG0000000830	pink	VSMC_M5	0.14	4.85E-01	-0.11	5.94E-01	-0.38	5.18E-02	0.44	2.24E-02	0.42	2.76E-02	-0.03	8.91E-01	-0.21	2.91E-01	-0.30	1.22E-01	0.07	7.31E-01	0.00	9.99E-01
ENSCAFG0000001565	SLC38A6	grey	VSMC_M10	0.14	4.85E-01	0.39	4.66E-02	0.45	1.83E-02	-0.17	4.00E-01	-0.50	8.16E-03	-0.06	7.54E-01	0.46	1.68E-02	0.03	8.71E-01	-0.50	7.94E-03	0.37	6.06E-02
ENSCAFG0000000081	TRPS1	darkgreen	VSMC_M4	0.14	4.85E-01	0.36	6.53E-02	-0.30	1.34E-01	0.71	3.29E-05	0.35	7.73E-02	-0.27	1.75E-01	-0.29	1.49E-01	-0.72	2.11E-05	-0.26	1.82E-01	0.38	5.00E-02
ENSCAFG0000000188	CLDN12	grey	VSMC_M10	0.14	4.86E-01	-0.08	6.75E-01	-0.10	6.28E-01	-0.21	2.89E-01	0.12	5.44E-01	0.12	5.44E-01	0.08	6.88E-01	0.22	2.65E-01	0.00	9.91E-01	-0.08	6.94E-01
ENSCAFG0000000885	TIAM1	grey	VSMC_M10	0.14	4.86E-01	-0.05	8.02E-01	0.46	1.65E-02	-0.56	2.29E-03	-0.37	5.42E-02	0.27	1.78E-01	0.72	2.14E-05	0.50	8.22E-03	-0.14	4.98E-01	-0.17	4.09E-01
ENSCAFG0000000745	DDX55	darkgrey	VSMC_M8	0.14	4.86E-01	-0.25	2.05E-01	0.53	4.31E-03	-0.74	9.06E-06	-0.29	1.37E-01	0.01	9.74E-01	0.15	4.41E-01	0.70	5.00E-05	0.12	5.67E-01	-0.66	1.84E-04
ENSCAFG0000000050	VPS13B	pink	VSMC_M5	0.14	4.86E-01	0.08	6.85E-01	-0.60	8.41E-04	0.64	2.91E-04	0.60	8.79E-04	-0.24	2.31E-01	-0.22	2.67E-01	-0.58	1.59E-03	-0.06	7.64E-01	0.27	1.69E-01
ENSCAFG0000001561	ENSCAFG0000001561	grey	VSMC_M10	0.14	4.86E-01	0.22	2.66E-01	-0.10	6.16E-01	0.06	7.76E-01	0.16	4.21E-01	-0.02	9.26E-01	-0.14	5.01E-01	-0.10	6.20E-01	-0.23	2.46E-01	0.07	7.17E-01
ENSCAFG0000003186	HIST1H2AC	grey	VSMC_M10	0.14	4.87E-01	0.39	4.30E-02	0.09	6.51E-01	0.40	3.99E-02	-0.24	2.36E-01	0.09	6.41E-01	-0.21	2.86E-01	-0.40	3.67E-02	-0.45	1.80E-02	0.55	2.95E-03
ENSCAFG0000000116	ENSCAFG0000000116	grey	VSMC_M10	0.14	4.87E-01	0.13	5.23E-01	0.23	2.46E-01	-0.04	8.33E-01	-0.20	3.27E-01	-0.17	4.08E-01	0.26	1.95E-01	0.06	7.63E-01	-0.25	2.15E-01	0.14	4.92E-01
ENSCAFG0000001779	ENSCAFG0000001779	grey	VSMC_M10	0.14	4.87E-01	-0.03	8.79E-01	-0.47	1.25E-02	0.29	1.49E-01	0.49	9.79E-03	-0.01	9.73E-01	0.02	9.27E-01	-0.19	3.49E-01	-0.04	8.40E-01	0.12	5.65E-01
ENSCAFG0000003160	NUDT2	grey	VSMC_M10	0.14	4.87E-01	0.03	8.72E-01	-0.29	1.42E-01	0.25	2.08E-01	0.39	4.31E-02	-0.31	1.16E-01	-0.03	8.63E-01	-0.24	2.35E-01	-0.03	8.72E-01	-0.06	7.56E-01
ENSCAFG0000002596	ENSCAFG0000002596	grey	VSMC_M10	0.14	4.87E-01	0.01	9.62E-01	-0.08	6.87E-01	0.09	6.41E-01	0.14	4.86E-01	0.00	9.88E-01	-0.05	8.01E-01	-0.04	8.25E-01	-0.07	7.44E-01	0.03	8.85E-01
ENSCAFG0000001412	EHD1	grey	VSMC_M10	0.14	4.87E-01	0.40	3.93E-02	-0.16	4.15E-01	0.56	2.39E-03	-0.01	9.61E-01	0.33	9.12E-02	-0.09	6.63E-01	-0.57	2.12E-03	-0.43	2.59E-02	0.59	1.20E-03
ENSCAFG0000000415	RAB7A	grey	VSMC_M10	0.14	4.88E-01	0.01	9.45E-01	0.11	5.85E-01	-0.18	3.79E-01	0.10	6.10E-01	-0.17	4.08E-01	-0.34	8.07E-02	0.06	7.81E-01	0.11	5.83E-01	-0.30	1.23E-01
ENSCAFG0000001877	LONP1	grey	VSMC_M10	0.14	4.88E-01	0.32	9.94E-02	0.51	7.19E-03	-0.01	9.56E-01	-0.53	4.47E-03	0.12	5.42E-01	0.16	4.22E-01	-0.12	5.53E-01	-0.35	7.56E-02	0.36	6.42E-02
ENSCAFG0000000434	SETDB2	grey	VSMC_M10	0.14	4.88E-01	-0.51	6.23E-03	-0.47	1.45E-02	-0.04	8.61E-01	0.66	1.80E-04	-0.02	9.13E-01	0.02	9.09E-01	0.15	4.58E-01	0.42	3.07E-02	-0.50	8.11E-03
ENSCAFG0000000421	OTUD1	grey	VSMC_M10	0.14	4.88E-01	-0.54	3.85E-03	-0.33	9.71E-02	-0.06	7.85E-01	0.54	4.00E-03	-0.31	1.14E-01	0.24	2.27E-01	0.14	4.78E-01	0.44	2.24E-02	-0.50	7.24E-03
ENSCAFG0000001190	SHTN1	darkgreen	VSMC_M4	0.14	4.88E-01	0.40	3.70E-02	-0.25	2.04E-01	0.57	1.82E-03	0.18	3.61E-01	-0.17	3.83E-01	-0.18	3.62E-01	-0.58	1.46E-03	-0.40	3.97E-02	0.51	6.62E-03
ENSCAFG0000001655	C9H17orf98	grey	VSMC_M10	0.14	4.89E-01	0.38	5.11E-02	-0.12	5.39E-01	0.31	1.21E-01	0.10	6.18E-01	-0.07	7.27E-01	-0.19	3.35E-01	-0.31	1.14E-01	-0.37	5.68E-02	0.41	3.57E-02
ENSCAFG0000001833	SH3TC2	darkgreen	VSMC_M4	0.14	4.89E-01	0.41	3.56E-02	-0.41	3.14E-02	0.88	1.08E-09	0.31	1.15E-01	-0.08	6.74E-01	-0.29	1.37E-01	-0.82	1.78E-07	-0.38	5.36E-02	0.62	5.88E-04
ENSCAFG0000000500	TIMP4	grey	VSMC_M10	0.14	4.89E-01	0.37	5.94E-02	0.47	1.25E-02	-0.10	6.37E-01	-0.45	1.86E-02	-0.09	6.61E-01	-0.05	8.21E-01	-0.01	9.56E-01	-0.40	3.75E-02	0.18	3.58E-01
ENSCAFG0000000946	OPTC	pink	VSMC_M5	0.14	4.89E-01	-0.19	3.46E-01	-0.57	1.76E-03	0.38	5.15E-02	0.53	4.36E-03	0.11	5.98E-01	0.24	2.31E-01	-0.24	2.20E-01	0.08	6.93E-01	0.16	4.31E-01
ENSCAFG0000000080	ENSCAFG0000000080	grey	VSMC_M10	0.14	4.89E-01	0.24	2.22E-01	0.14	4.79E-01	-0.07	7.36E-01	-0.06	7.71E-01	-0.06	7.48E-01	-0.11	5.69E-01	-0.08	7.08E-01	-0.22	2.59E-01	0.08	6.96E-01
ENSCAFG0000000074	SYBU	turquoise	VSMC_M6	0.14	4.89E-01	0.21	2.97E-01	0.33	9.23E-02	-0.16	4.31E-01	-0.38	5.34E-02	0.56	2.14E-03	0.25	2.06E-01	0.11	5.76E-01	-0.33	9.30E-02	0.24	2.35E-01
ENSCAFG0000002389	ZSCAN30	darkgreen	VSMC_M4	0.14	4.89E-01	0.11	5.70E-01	-0.38	5.21E-02	0.52	5.42E-03	0.22	2.78E-01	0.19	3.49E-01	0.30	1.34E-01	-0.41	3.18E-02	-0.24	2.28E-01	0.52	5.52E-03
ENSCAFG0000001001	N4BP1	darkgreen	VSMC_M4	0.14	4.89E-01	0.43	2.43E-02	-0.48	1.15E-02	0.80	5.36E-07	0.42	2.75E-02	-0.22	2.61E-01	-0.26	1.82E-01	-0.84	5.66E-08	-0.37	5.65E-02	0.57	2.09E-03
ENSCAFG0000001222	MCMBP	grey	VSMC_M10	0.14	4.89E-01	-0.09	6.64E-01	0.34	7.92E-02	-0.64	2.94E-04	-0.23	2.39E-01	0.15	4.42E-01	0.02	9.17E-01	0.51	6.31E-03	-0.02	9.19E-01	-0.34	8.16E-02
ENSCAFG0000001004	GFRA2	cyan	VSMC_M2	0.14	4.90E-01	0.55	2.97E-03	0.20	3.11E-01	0.20	3.30E-01	-0.20	3.07E-01	-0.21	2.90E-01	-0.26	1.98E-01	-0.34	8.56E-02	-0.51	6.12E-03	0.40	3.66E-02
ENSCAFG0000003191	ENSCAFG0000003191	grey	VSMC_M10	0.14	4.90E-01	0.15	4.46E-01	0.17	4.10E-01	-0.21	2.98E-01	-0.10	6.03E-01	0.00	9.90E-01	0.37	5.79E-02	0.12	5.40E-01	-0.21	2.91E-01	0.06	7.56E-01
ENSCAFG0000001184	QARS	yellow	VSMC_M3	0.14	4.90E-01	0.45	2.00E-02	0.53	4.46E-03	-0.07	7.38E-01	-0.38	4.77E-02	-0.22	2.63E-01	-0.28	1.64E-01	-0.18	3.66E-01	-0.29	1.43E-01	0.07	7.23E-01
ENSCAFG0000000003	ENSCAFG0000000003	darkgreen	VSMC_M4	0.14	4.90E-01	0.30	1.32E-01	-0.37	5.85E-02	0.67	1.48E-04	0.26	1.86E-01	0.19	3.51E-01	0.01	9.45E-01	-0.59	1.28E-03	-0.29	1.36E-01	0.50	8.46E-03
ENSCAFG0000001831	EVI5L	darkgreen	VSMC_M4	0.14	4.91E-01	0.45	1.95E-02	-0.19	3.31E-01	0.74	1.18E-05	0.11	6.00E-01	0.05	8.04E-01	-0.27	1.65E-01	-0.79	1.05E-06	-0.38	5.36E-02	0.57	2.06E-03
ENSCAFG0000000165	MFSD3	grey	VSMC_M10	0.14	4.91E-01	0.56	2.58E-03	0.73	1.71E-05	-0.23	2.59E-01	-0.70	4.38E-05	-0.04	8.33E-01	0.00	9.95E-01	-0.04	8.62E-01	-0.51	6.36E-03	0.34	7.87E-02
ENSCAFG0000000093	CEP85L	grey	VSMC_M10	0.14	4.91E-01	-0.10	6.21E-01	0.17	4.10E-01	-0.44	2.23E-02	-0.17	3.88E-01	0.21	3.02E-01	0.10	6.30E-01	0.47	1.29E-02	-0.04	8.52E-01	-0.08	6.84E-01
ENSCAFG0000001839	ENSCAFG0000001839	grey	VSMC_M10	0.14	4.91E-01	0.13	5.04E-01	-0.26	1.87E-01	0.36	6.64E-02	0.31	1.15E-01	-0.08	6.85E-01	-0.13	5.07E-01	-0.30	1.23E-01	-0.15	4.70E-01	0.22	2.76E-01
ENSCAFG0000003160	GRPEL2	grey	VSMC_M10	0.14	4.92E-01	-0.43	2.36E-02	-0.16	4.25E-01	-0.06	7.54E-01	0.20	3.26E-01	0.14	4.98E-01	0.06	7.68E-01	0.23	2.54E-01	0.32	1.01E-01	-0.27	1.73E-01
ENSCAFG0000000758	MAST4	grey	VSMC_M10	0.14	4.92E-01	-0.34	8.37E-02	-0.41	3.52E-02	0.33	9.61E-02	0.48	1.19E-02	-0.14	4.82E-01	-0.14	4.87E-01	-0.18	3.60E-01	0.29	1.46E-01	-0.13	5.16E-01
ENSCAFG0000003198	C9H9orf16	grey	VSMC_M10	0.14	4.92E-01	0.79	9.74E-07	0.24	2.30E-01	0.44	2.32E-02	-0.34	8.08E-02	0.01	9.80E-01	-0.22	2.79E-01	-0.67	1.43E-04	-0.68	8.62E-05	0.77	2.60E-06
ENSCAFG0000000536	RIN2	grey	VSMC_M10	0.14	4.92E-01	0.04	8.25E-01	0.46	1.53E-02	-0.45	1.84E-02	-0.35	7.09E-02	0.10	6.07E-01	0.32	1.03E-01	0.40	3.83E-02	-0.16	4.34E-01	-0.13	5.05E-01
ENSCAFG0000001746	CALML4	grey	VSMC_M10	0.14	4.92E-01	-0.05	7.94E-01	0.06	7.83E-01	-0.20	3.13E-01	0.02	9.39E-01	0.20	3.13E-01	0.46	1.68E-02	0.18	3.64E-01	-0.10	6.27E-01	-0.11	5.97E-01
ENSCAFG0000002931	FAM241B	grey	VSMC_M10	0.14	4.92E-01	0.20	3.07E-01	-0.03	8.70E-01	0.47	1.28E-02	-0.05	7.95E-01	0.15	4.55E-01	0.02	9.11E-01	-0.47	1.25E-02	-0.18	3.56E-01	0.43	2.64E-02
ENSCAFG0000001817	HEATR9	darkgreen	VSMC_M4	0.14	4.92E-01	-0.01	9.74E-01	-0.55	2.90E-03	0.68	1.12E-04	0.54	3.88E-03	-0.10	6.15E-01	-0.14	4.72E-01	-0.52	5.38E-03	0.00	9.92E-01	0.23	2.55E-01
ENSCAFG0000001838	HSPB3	darkgreen	VSMC_M4	0.14	4.92E-01	-0.01	9.74E-01	-0.55	2.90E-03	0.68	1.12E-04	0.54	3.88E-03	-0.10	6.15E-01	-0.14	4.72E-01	-0.52	5.38E-03	0.00	9.92E-01	0.23	2.55E-01
ENSCAFG0000002063	ENSCAFG0000002063	darkgreen	VSMC_M4	0.14	4.92E-01	-0.01	9.74E-01	-0.55	2.90E-03	0.68	1.12E-04	0.54	3.88E-03	-0.10	6.15E-01	-0.14	4.72E-01	-0.52	5.38E-03	0.00	9.92E-01	0.23	2.55E-01
ENSCAFG0000002934	ENSCAFG0000002934	darkgreen	VSMC_M4	0.14	4.92E-01	-0.01	9.74E-01	-0.55	2.90E-03	0.68	1.12E-04	0.54	3.88E-03	-0.10	6.15E-01	-0.14	4.72E-01	-0.52	5.38E-03	0.00	9.92E-01	0.23	2.55E-01
ENSCAFG0000002941	ENSCAFG0000002941	darkgreen	VSMC_M4	0.14	4.92E-01	-0.01	9.74E-01	-0.55	2.90E-03	0.68	1.12E-04	0.54	3.88E-03	-0.10	6.15E-01	-0.14	4.72E-01	-0.52	5.38E-03	0.00	9.92E-01	0.23	2.55E-01
ENSCAFG0000001803	IMP3	cyan	VSMC_M2	0.14	4.93E-01	0.48	1.10E-02	0.63	4.21E-04	-0.24	2.31E-01	-0.66	2.07E-04	0.18	3.62E-01	0.06	7.82E-01	0.10	6.36E-01	-0.56	2.20E-03	0.29	1.44E-01
ENSCAFG0000003080	RNLS	cyan	VSMC_M2	0.14	4.93E-01	0.77	2.54E-06	0.18	3.75E-01	0.49	1.03E-02	-0.19	3.31E-01	-0.10	6.23E-01	-0.30	1.29E-01	-0.68	1.10E-04	-0.65	2.52E-04	0.60	8.40E-04
ENSCAFG0000001026	EIF2D	grey	VSMC_M10	0.14	4.93E-01	-0.37	6.01E-02	0.09	6.67E-01	-0.44	2.27E-02	0.16	4.40E-01	-0.19	3.33E-01	0.14	4.93E-01	0.53	4.76E-03	0.26	1.98E-01	-0.66	1.66E-04
ENSCAFG0000001976	CEPT1	pink	VSMC_M5	0.14	4.93E-01	-0.17	3.86E-01	-0.31	1.11E-01	0.09	6.58E-01	0.47	1.35E-02	-0.36	6.78E-02	-0.02	9.21E-01	-0.09	6.53E-01	0.20	3.06E-01	-0.24	2.31E-01
ENSCAFG0000000331	ANXA4	grey	VSMC_M10	0.14	4.93E-01	-0.16	4.34E-01	-0.29	1.49E-01	0.31	1.19E-01	0.30	1.24E-01	-0.22	2.77E-01	0.17	4.08E-01	-0.16	4.40E-01	0.11	5.98E-01	0.04	8.59E-01
ENSCAFG0000001901	PMM2	grey	VSMC_M10	0.14	4.93E-01	-0.32	1.05E-01	0.02	9.02E-01	-0.47	1.34E-02	0.12	5.43E-01	0.07	7.26E-01	0.23	2.41E-01	0.51	6.32E-03	0.14	4.90E-01	-0.44	2.16E-02

ENSCAFG0000000569	SPATA24	grey	VSMC_M10	0.14	4.93E-01	0.17	3.99E-01	0.15	4.57E-01	0.20	3.11E-01	-0.20	3.09E-01	0.07	7.20E-01	0.11	5.90E-01	-0.22	2.70E-01	-0.16	4.14E-01	0.31	1.21E-01
ENSCAFG0000001439	XRCC5	grey	VSMC_M10	0.14	4.94E-01	-0.61	7.92E-04	-0.08	7.06E-01	-0.34	8.53E-02	0.21	3.01E-01	0.15	4.51E-01	0.46	1.52E-02	0.55	2.94E-03	0.36	6.68E-02	-0.57	1.94E-03
ENSCAFG0000000875	FRAS1	grey	VSMC_M10	0.14	4.94E-01	0.18	3.82E-01	0.37	5.44E-02	-0.35	7.32E-02	-0.22	2.79E-01	-0.10	6.26E-01	0.23	2.57E-01	0.23	2.43E-01	-0.24	2.28E-01	-0.14	4.73E-01
ENSCAFG0000000281	CLHC1	red	VSMC_M1	0.14	4.94E-01	0.23	2.56E-01	-0.15	4.63E-01	0.11	5.71E-01	0.19	3.38E-01	0.19	3.45E-01	0.19	3.42E-01	-0.25	2.11E-01	-0.23	2.46E-01	0.21	2.97E-01
ENSCAFG0000001432	PCOLCE	cyan	VSMC_M2	0.14	4.94E-01	0.82	1.26E-07	0.64	2.90E-04	-0.13	5.13E-01	-0.61	6.62E-04	-0.14	4.97E-01	-0.13	5.15E-01	-0.18	3.56E-01	-0.77	3.03E-06	0.47	1.33E-02
ENSCAFG0000001040	RNF19B	yellow	VSMC_M3	0.14	4.94E-01	-0.08	6.90E-01	0.50	8.02E-03	-0.73	1.79E-05	-0.22	2.61E-01	-0.02	9.04E-01	0.26	1.82E-01	0.57	1.89E-03	0.06	7.55E-01	-0.55	3.10E-03
ENSCAFG0000002911	FAM76A	pink	VSMC_M5	0.14	4.94E-01	0.08	7.10E-01	-0.42	3.02E-02	0.41	3.20E-02	0.55	3.00E-03	-0.30	1.22E-01	-0.31	1.17E-01	-0.41	3.43E-02	0.00	9.99E-01	0.03	8.79E-01
ENSCAFG0000001892	TP53I13	grey	VSMC_M10	0.14	4.95E-01	0.63	4.19E-04	0.60	1.05E-03	0.03	8.91E-01	-0.68	8.80E-05	0.10	6.35E-01	0.05	7.88E-01	-0.24	2.34E-01	-0.65	2.62E-04	0.61	6.96E-04
ENSCAFG0000000097	ENSCAFG0000000097	grey	VSMC_M10	0.14	4.95E-01	-0.14	4.87E-01	-0.19	3.46E-01	0.06	7.82E-01	0.22	2.65E-01	-0.12	5.60E-01	-0.22	2.75E-01	-0.06	7.84E-01	0.11	5.79E-01	-0.07	7.29E-01
ENSCAFG0000000280	ENSCAFG0000000280	grey	VSMC_M10	0.14	4.95E-01	0.35	7.67E-02	-0.11	5.77E-01	0.49	9.55E-03	-0.09	6.60E-01	0.18	3.64E-01	0.01	9.46E-01	-0.49	1.02E-02	-0.40	3.69E-02	0.73	1.32E-05
ENSCAFG0000001189	SLC19A1	yellow	VSMC_M3	0.14	4.95E-01	0.54	4.02E-03	0.68	9.43E-05	-0.40	3.74E-02	-0.66	1.75E-04	0.03	8.70E-01	-0.08	6.90E-01	0.13	5.28E-01	-0.53	4.10E-03	0.26	1.95E-01
ENSCAFG0000001111	COPS7B	grey	VSMC_M10	0.14	4.96E-01	-0.25	2.17E-01	-0.40	3.79E-02	0.25	2.00E-01	0.40	3.72E-02	-0.14	4.87E-01	0.02	9.14E-01	-0.10	6.25E-01	0.14	4.75E-01	0.02	9.10E-01
ENSCAFG0000003254	PROKR2	red	VSMC_M1	0.14	4.96E-01	-0.13	5.14E-01	0.25	1.99E-01	-0.36	6.47E-02	-0.24	2.37E-01	0.22	2.63E-01	-0.27	1.74E-01	0.42	2.77E-02	0.03	8.97E-01	-0.20	3.08E-01
ENSCAFG0000000177	LMOD2	grey	VSMC_M10	0.14	4.96E-01	0.51	6.21E-03	0.11	5.74E-01	0.45	1.96E-02	-0.20	3.16E-01	0.04	8.62E-01	-0.23	2.39E-01	-0.54	3.81E-03	-0.47	1.38E-02	0.64	2.98E-04
ENSCAFG0000000402	KIAA1109	pink	VSMC_M5	0.14	4.97E-01	-0.11	5.78E-01	-0.53	4.66E-03	0.53	4.92E-03	0.54	3.78E-03	-0.18	3.77E-01	0.09	6.47E-01	-0.42	2.84E-02	0.06	7.53E-01	0.18	3.78E-01
ENSCAFG0000000735	ENSCAFG0000000735	pink	VSMC_M5	0.14	4.97E-01	-0.36	6.70E-02	-0.35	7.23E-02	-0.07	7.41E-01	0.46	1.53E-02	0.07	7.15E-01	-0.10	6.10E-01	0.25	2.00E-01	0.29	1.39E-01	-0.43	2.48E-02
ENSCAFG0000000795	TNKS1BP1	yellow	VSMC_M3	0.14	4.97E-01	0.68	8.11E-05	0.80	4.53E-07	-0.13	5.05E-01	-0.79	1.21E-06	-0.03	8.93E-01	-0.18	3.70E-01	-0.16	4.37E-01	-0.61	7.88E-04	0.40	3.70E-02
ENSCAFG0000000698	NCOR2	darkgreen	VSMC_M4	0.14	4.97E-01	0.66	1.58E-04	0.02	9.13E-01	0.57	2.12E-03	-0.05	8.02E-01	-0.06	7.59E-01	-0.51	6.69E-03	-0.76	5.21E-06	-0.48	1.06E-02	0.61	6.62E-04
ENSCAFG0000002503	BTN1A1	grey	VSMC_M10	0.14	4.97E-01	-0.22	2.66E-01	-0.40	3.84E-02	0.23	2.59E-01	0.42	3.01E-02	-0.18	3.77E-01	-0.10	6.07E-01	-0.10	6.26E-01	0.16	4.32E-01	0.00	9.92E-01
ENSCAFG0000001345	TSEN15	pink	VSMC_M5	0.14	4.97E-01	-0.27	1.70E-01	-0.45	1.85E-02	0.13	5.11E-01	0.56	2.38E-03	-0.11	5.96E-01	0.21	2.98E-01	0.01	9.42E-01	0.15	4.54E-01	-0.19	3.38E-01
ENSCAFG0000001909	FSD1	grey	VSMC_M10	0.14	4.98E-01	-0.43	2.66E-02	-0.13	5.11E-01	-0.20	3.17E-01	0.43	2.60E-02	-0.22	2.69E-01	0.10	6.26E-01	0.28	1.62E-01	0.41	3.33E-02	-0.66	1.73E-04
ENSCAFG0000001324	LAMC2	turquoise	VSMC_M6	0.14	4.98E-01	-0.05	8.02E-01	0.09	6.53E-01	-0.28	1.61E-01	-0.04	8.57E-01	0.77	2.58E-06	0.11	5.90E-01	0.26	1.92E-01	-0.05	8.04E-01	-0.17	3.99E-01
ENSCAFG0000001783	ZNHIT3	grey	VSMC_M10	0.14	4.98E-01	-0.21	2.98E-01	0.36	6.49E-02	-0.47	1.33E-02	-0.19	3.36E-01	0.16	4.13E-01	0.00	9.83E-01	0.51	7.12E-03	0.06	7.52E-01	-0.48	1.09E-02
ENSCAFG0000000028	ENSCAFG0000000028	grey	VSMC_M10	0.14	4.98E-01	0.33	8.93E-02	0.15	4.47E-01	0.09	6.65E-01	-0.10	6.12E-01	-0.30	1.23E-01	-0.17	3.89E-01	-0.15	4.54E-01	-0.31	1.12E-01	0.19	3.48E-01
ENSCAFG0000000659	ENSCAFG0000000659	darkgrey	VSMC_M8	0.14	4.98E-01	-0.52	5.84E-03	-0.12	5.44E-01	-0.48	1.13E-02	0.33	8.88E-02	-0.04	8.30E-01	0.40	3.78E-02	0.60	9.69E-04	0.33	9.31E-02	-0.63	3.76E-04
ENSCAFG0000002421	ENSCAFG0000002421	grey	VSMC_M10	0.14	4.98E-01	-0.01	9.42E-01	0.11	5.90E-01	-0.09	6.72E-01	0.08	6.98E-01	-0.37	5.69E-02	-0.42	2.90E-02	0.10	6.35E-01	0.04	8.39E-01	-0.30	1.32E-01
ENSCAFG0000001710	ADAM15	cyan	VSMC_M2	0.14	4.98E-01	0.87	4.14E-09	0.13	5.14E-01	0.51	6.72E-03	-0.17	3.88E-01	-0.12	5.56E-01	-0.30	1.27E-01	-0.74	1.11E-05	-0.73	1.43E-05	0.73	1.57E-05
ENSCAFG0000000007	RELCH	pink	VSMC_M5	0.14	4.98E-01	-0.43	2.65E-02	-0.53	4.30E-03	0.34	8.72E-02	0.66	2.05E-04	0.01	9.73E-01	-0.05	8.14E-01	-0.11	5.70E-01	0.33	8.77E-02	-0.31	1.16E-01
ENSCAFG0000002599	ENSCAFG0000002599	yellow	VSMC_M3	0.14	4.99E-01	0.49	9.20E-03	0.66	1.86E-04	-0.23	2.47E-01	-0.70	5.65E-05	0.05	7.91E-01	-0.04	8.37E-01	0.08	6.82E-01	-0.56	2.41E-03	0.31	1.17E-01
ENSCAFG0000001879	ENSCAFG0000001879	grey	VSMC_M10	0.14	4.99E-01	0.49	9.82E-03	-0.03	8.90E-01	0.27	1.66E-01	0.06	7.62E-01	-0.20	3.21E-01	-0.19	3.52E-01	-0.35	7.39E-02	-0.47	1.29E-02	0.29	1.44E-01
ENSCAFG0000000998	ZNF423	grey	VSMC_M10	0.14	4.99E-01	0.63	3.97E-04	0.07	7.30E-01	0.37	5.60E-02	-0.16	4.39E-01	0.12	5.58E-01	-0.30	1.30E-01	-0.49	9.38E-03	-0.61	7.44E-04	0.62	5.93E-04
ENSCAFG0000000957	CPN1	grey	VSMC_M10	0.14	4.99E-01	-0.03	8.66E-01	-0.29	1.42E-01	0.24	2.19E-01	0.23	2.45E-01	-0.23	2.53E-01	0.06	7.49E-01	-0.17	3.98E-01	-0.07	7.41E-01	0.21	2.93E-01
ENSCAFG0000001688	ITGAM	grey	VSMC_M10	0.14	4.99E-01	-0.07	7.32E-01	-0.28	1.62E-01	0.31	1.15E-01	0.29	1.38E-01	-0.15	4.43E-01	-0.19	3.31E-01	-0.17	3.96E-01	0.06	7.69E-01	0.03	8.68E-01
ENSCAFG0000003261	ENSCAFG0000003261	grey	VSMC_M10	0.14	5.00E-01	0.12	5.42E-01	-0.36	6.92E-02	0.58	1.52E-03	0.46	1.69E-02	-0.26	1.96E-01	-0.01	9.76E-01	-0.51	6.31E-03	-0.10	6.20E-01	0.11	5.79E-01
ENSCAFG0000000572	ENSCAFG0000000572	grey	VSMC_M10	0.14	5.00E-01	0.04	8.61E-01	-0.13	5.26E-01	0.16	4.23E-01	0.14	4.95E-01	-0.13	5.20E-01	-0.19	3.54E-01	-0.14	4.75E-01	-0.02	9.21E-01	0.05	7.92E-01
ENSCAFG0000000790	LRBA	pink	VSMC_M5	0.14	5.00E-01	0.01	9.72E-01	-0.54	3.88E-03	0.61	8.27E-04	0.66	1.71E-04	-0.34	7.86E-02	-0.18	3.71E-01	-0.57	2.11E-03	0.07	7.34E-01	0.05	8.14E-01
ENSCAFG0000002018	GAPVD1	grey	VSMC_M10	0.14	5.00E-01	-0.50	7.22E-03	-0.29	1.39E-01	0.14	4.86E-01	0.30	1.24E-01	0.08	7.05E-01	0.12	5.61E-01	0.07	7.12E-01	0.36	6.55E-02	-0.22	2.69E-01
ENSCAFG0000000418	ENSCAFG0000000418	grey	VSMC_M10	0.14	5.00E-01	-0.12	5.68E-01	0.23	2.40E-01	-0.21	2.84E-01	-0.18	3.79E-01	0.26	1.95E-01	0.43	2.38E-02	0.23	2.45E-01	-0.02	9.05E-01	-0.10	6.32E-01
ENSCAFG0000001339	ENSCAFG0000001339	grey	VSMC_M10	0.14	5.00E-01	0.27	1.71E-01	0.15	4.61E-01	-0.02	9.02E-01	-0.23	2.54E-01	-0.06	7.82E-01	0.13	5.12E-01	-0.01	9.49E-01	-0.36	6.15E-02	0.40	3.85E-02
ENSCAFG0000001782	CA4	grey	VSMC_M10	0.14	5.00E-01	0.41	3.29E-02	0.40	3.66E-02	-0.10	6.17E-01	-0.39	4.55E-02	0.44	2.06E-02	-0.11	5.69E-01	-0.09	6.38E-01	-0.40	4.09E-02	0.24	2.33E-01
ENSCAFG0000000504	KLF12	grey	VSMC_M10	0.14	5.00E-01	-0.32	1.02E-01	-0.53	4.19E-03	0.06	7.56E-01	0.65	2.76E-04	-0.04	8.42E-01	-0.03	8.85E-01	0.03	8.86E-01	0.26	1.92E-01	-0.30	1.32E-01
ENSCAFG0000000940	ECI2	grey	VSMC_M10	0.14	5.01E-01	-0.51	6.36E-03	0.00	9.85E-01	-0.29	1.48E-01	0.24	2.34E-01	-0.10	6.28E-01	0.20	3.14E-01	0.43	2.61E-02	0.45	1.71E-02	-0.57	1.91E-03
ENSCAFG0000002022	TXNL4B	grey	VSMC_M10	0.14	5.01E-01	0.03	8.67E-01	-0.21	2.86E-01	0.23	2.42E-01	0.42	2.84E-02	-0.28	1.62E-01	-0.35	7.25E-02	-0.31	1.21E-01	0.06	7.66E-01	-0.17	4.03E-01
ENSCAFG0000000750	TDRD12	grey	VSMC_M10	0.14	5.01E-01	0.26	1.94E-01	0.13	5.15E-01	-0.15	4.55E-01	-0.04	8.25E-01	0.02	9.15E-01	0.20	3.06E-01	-0.03	8.97E-01	-0.26	1.85E-01	0.14	5.00E-01
ENSCAFG0000000610	BAG4	grey	VSMC_M10	0.14	5.01E-01	-0.03	8.97E-01	-0.13	5.03E-01	0.06	7.57E-01	0.30	1.33E-01	0.03	8.81E-01	-0.01	9.60E-01	-0.06	7.52E-01	-0.03	8.76E-01	-0.17	3.93E-01
ENSCAFG0000001076	SMC3	darkgrey	VSMC_M8	0.14	5.01E-01	-0.53	4.41E-03	0.16	4.24E-01	-0.73	1.54E-05	0.07	7.17E-01	-0.03	8.69E-01	0.42	2.80E-02	0.83	7.69E-08	0.32	1.03E-01	-0.71	3.73E-05
ENSCAFG0000001961	CHTF18	darkgrey	VSMC_M8	0.14	5.01E-01	-0.32	1.02E-01	0.49	8.89E-03	-0.82	2.04E-07	-0.36	6.78E-02	0.19	3.49E-01	0.38	5.15E-02	0.89	3.29E-10	0.10	6.28E-01	-0.52	5.07E-03
ENSCAFG0000001149	RNF114	grey	VSMC_M10	0.14	5.02E-01	0.46	1.61E-02	0.09	6.71E-01	0.33	9.79E-02	-0.30	1.29E-01	0.28	1.65E-01	0.04	8.58E-01	-0.40	3.99E-02	-0.53	4.88E-03	0.75	6.39E-06
ENSCAFG0000001797	COX5A	grey	VSMC_M10	0.14	5.02E-01	-0.05	8.22E-01	0.08	6.97E-01	-0.21	2.90E-01	-0.07	7.39E-01	-0.08	7.09E-01	0.29	1.43E-01	0.21	2.83E-01	-0.08	7.05E-01	-0.02	9.08E-01
ENSCAFG0000000571	ZDHHC17	pink	VSMC_M5	0.14	5.02E-01	-0.21	3.03E-01	-0.67	1.25E-04	0.37	5.87E-02	0.83	1.16E-07	-0.26	1.99E-01	-0.18	3.59E-01	-0.25	2.13E-01	0.20	3.13E-01	-0.18	3.66E-01
ENSCAFG0000001366	GNAZ	cyan	VSMC_M2	0.14	5.02E-01	0.49	8.83E-03	0.43	2.40E-02	-0.10	6.31E-01	-0.43	2.50E-02	0.00	9.86E-01	-0.01	9.54E-01	-0.07	7.42E-01	-0.50	7.33E-03	0.28	1.57E-01
ENSCAFG0000001691	GNGT2	turquoise	VSMC_M6	0.14	5.02E-01	0.10	6.27E-01	0.44	2.09E-02	-0.18	3.61E-01	-0.36	6.26E-02	0.63	4.33E-04	0.13	5.33E-01	0.07	7.22E-01	-0.12	5.36E-01	-0.02	9.25E-01
ENSCAFG0000001251	PEX19	grey	VSMC_M10	0.14	5.02E-01	0.45	1.73E-02	-0.07	7.29E-01	0.60	9.86E-04	-0.09	6.51E-01	0.15	4.65E-01	-0.08	7.08E-01	-0.63	4.78E-04	-0.46	1.54E-02	0.73	1.31E-05
ENSCAFG0000001793	ENSCAFG0000001793	grey	VSMC_M10	0.13	5.02E-01	0.02	9.23E-01	0.30	1.28E-01	-0.17	3.89E-01	-0.25	2.00E-01	0.08	7.02E-01	0.16	4.18E-01	0.09	6.41E-01	-0.07	7.30E-01	-0.05	7.94E-01
ENSCAFG0000003039	MRPS15	darkgrey	VSMC_M8	0.13	5.02E-01	-0.42	3.09E-02	0.26	1.94E-01	-0.54	3.81E-03	-0.07	7.14E-01	0.00	1.00E+00	0.24	2.31E-01	0.60	8.44E-04	0.27	1.77E-01	-0.61	7.86E-04
ENSCAFG0000000967	ENSCAFG0000000967	grey	VSMC_M10	0.13	5.02E-01	-0.26	1.96E-01	-0.23	2.43E-01	0.00	1.00E+00	0.34	8.59E-02	-0.15	4.68E-01	-0.10	6.18E-01	0.13	5.02E-01	0.13	5.14E-01	-0.28	1.51E-01
ENSCAFG0000000174	PEX6	grey	VSMC_M10	0.13	5.02E-01	0.46	1.60E-02	-0.07	7.24E-01	0.43	2.64E-02	0.10	6.22E-01	-0.21	3.04E-01	0.02	9.21E-01	-0.56	2.27E-03	-0.40	3.73E-02	0.40	3.67E-02
ENSCAFG0000002317	ENSCAFG0000002317	cyan	VSMC_M2	0.13	5.03E-01	0.47	1.26E-02	0.23	2.48E-01	-0.14	5.01E-01	-0.16	4.13E-01	0.02	9.40E-01	-0.03	8.72E-01	-0.04	8.35E-01	-0.42	2.88E-02	0.23	2.46E-01
ENSCAFG0000001471	REX1BD	grey	VSMC_M10	0.13	5.03E-01	0.11	5.96E-01	0.61	8.20E-04	-0.57	1.92E-03	-0.31	1.19E-01	-0.27	1.69E-01	-0.11	6.00E-01	0.39	4.66E-02	-0.10	6.33E-01	-0.46	1.57E-02
ENSCAFG0000000602	TRIM21	cyan	VSMC_M2	0.13	5.03E-01	0.61	7.06E-04	0.36	6.76E-02	0.10	6.26E-01	-0.33	9.70E-02	0.06	7.48E-01	-0.38	4.93E-02	-0.30	1.34E-01	-0.53	4.67E-03	0.37	5.76E-02
ENSCAFG0000000666	ENSCAFG0000000666	grey	VSMC_M10	0.13	5.03E-01	0.09	6.73E-01	0.35	7.32E-02	-0.26	1.82E-01	-0.36	6.63E-02	0.19	3.31E-01	0.25	2.18E-01	0.19	3.35E-01	-0.18	3.74E-01	0.11	5.69E-01
ENSCAFG0000001784	ATOX1	cyan	VSMC_M2	0.13	5.03E-01	0.60	8.56E-04	0.49	8.73E-03	0.06	7.72E-01	-0.59	1.12E-03	0.05	8.04E-01	-0.04	8.48E-01	-0.22	2.70E-01	-0.61	6.82E-04	0.48	1.19E-02
ENSCAFG0000000229	IPPK	grey	VSMC_M10	0.13	5.03E-01	-0.52	5.48E-03	-0.29	1.40E-01	-0.05	7.93E-01	0.30	1.24E-01	0.29	1.36E-01	0.04	8.58E-01	0.32	1.01E-01	0.36	6.54E-02	-0.33	9.23E-02
ENSCAFG0000002383	GPRIN1	grey	VSMC_M10	0.13	5.03E-01	0.09	6.73E-01	0.56	2.63E-03	-0.65	2.53E-04	-0.52	5.00E-03	0.27	1.76E-01	0.50	7.61E-03	0.51	6.91E-03	-0.23	2.50E-01	-0.03	8.71E-01
ENSCAFG0000002537	AHRR	grey	VSMC_M10	0.13	5.03E-01	-0.27	1.79E-01	0.31	1.14E-01	-0.46	1.46E-02	-0.12	5.60E-01	-0.10	6.32E-01	0.52	5.37E-03	0.46	1.54E-02	0.13	5.07E-01	-0.45	1.96E-02
ENSCAFG0000000119	AGO2	grey	VSMC_M10	0.13	5.04E-01	-0.04	8.42E-01	0.18	3.72E-01	-0.24	2.20E-01	-0.13	5.06E-01	0.00	9.98E-01	-0.30	1.30E-01	0.24	2.22E-01	0.05	8.00E-01	-0.12	5.61E-01
ENSCAFG0000003236	MPV17	cyan	VSMC_M2	0.13	5.04E-01	0.56	2.35E-03	0.08	6.81E-01	0.14	4.95E-01	-0.17	3.89E-01	0.07	7.17E-01	-0.19	3.35E-01	-0.34	8.45E-02	-0.51	6.41E-03	0.52	5.51E-03
ENSCAFG0000001404	PPP2R5B	grey	VSMC_M10	0.13	5.04E-01	0.46	1.50E-02	0.02	9.41E-01	0.40	3.68E-02	-0.03	8.86E-01	0.09	6.64E-01	-0.22	2.71E-01	-0.61	7.25E-04	-0.34	7.96E-02	0.53	4.52E-03
ENSCAFG0000002390	RNF212B	grey	VSMC_M10	0.13	5.04E-01	0.01	9.62E-01	-0.05	8.14E-01	-0.10	6.22E-01	0.22	2.67E-01	-0.10	6.24E-01	0.11	5.96E-01	0.08	7.04E-01	-0.01	9.54E-01	-0.25	2.16E-01
ENSCAFG0000000850	ENSCAFG0000000850	grey	VSMC_M10	0.13	5.04E-01	-0.15	4.61E-01	-0.09	6.48E-01	-0.02	9.26E-01	0.21	2.82E-01	-0.01	9.56E-01	0.04	8.35E-01	0.00	9.84E-01	0.20	3.26E-01	-0.22	2.80E-01
ENSCAFG0000000761	ST8SIA4	grey	VSMC_M10	0.13	5.05E-01	-0.55	2.94E-03	0.00	9.88E-01	-0.47	1.37E-02	0.23	2.52E-01	-0.02	9.03E-01	-0.17	3.93E-01	0.50	7.42E-03	0.48	1.04E-02	-0.73	1.86E-05
ENSCAFG0000001588	INCENP	darkgrey	VSMC_M8	0.13	5.05E-01	-0.35	7.72E-02	0.51	6.34E-03	-0.84	2.97E-08	-0.39	4.21E-02	0.21	2.85E-01	0.41	3.26E-02	0.89	4.32E-10	0.12	5.35E-01	-0.49	1.02E-02
ENSCAFG0000002397	ENSCAFG0000002397	darkgrey	VSMC_M8	0.13	5.05E-01	-0.36	6.14E-02	0.36	6.82E-02	-0.69	6.94E-05	-0.17	3.94E-01	0.06	7.84E-01	0.40	3.79E-02	0.72	2.23E-05	0.19	3.38E-01	-0.54	3.44E-03
ENSCAFG0000002543	MANSC4	grey	VSMC_M10	0.13	5.05E-01	-0.07	7.21E-01	-0.27	1.78E-01	0.10	6.37E-01	0.26	1.88E-01	-0.16	4.36E-01	0.33	9.21E-02	-0.05	8.04E-01	-0.01	9.42E-01	0.15	4.64E-01
ENSCAFG0000000037	ENSCAFG0000000037	grey	VSMC_M10	0.13	5.05E-01	-0.17	3.89E-01	-0.13	5.15E-01	-0.12	5.55E-01	0.21	2.89E-01	-0.13	5.30E-01	-0.02	9.07E-01	0.13	5.25E-01	0.16	4.29E-01	-0.19	3.45E-01
ENSCAFG0000003049	FHAD1	grey	VSMC_M10	0.13	5.05E-01	-0.08	6.94E-01	0.29	1.45E-01	-0.31	1.19E-01	-0.20	3.18E-01	0.37	5.58E-02	0.55	2.95E-03	0.25	2.06E-01	-0.03	8.94E-01	-0.09	6.38E-01
ENSCAFG0000001317	NPAS3	darkgreen	VSMC_M4	0.13	5.06E-01	0.50	7.25E-03	-0.12	5.62E-01	0.67	1.33E-04	-0.06	7.64E-01	0.02	9.06E-01	-0.21	3.00E-01	-0.71	2.90E-05	-0.48	1.15E-02	0.76	3.49E-06
ENSCAFG0000002861	ENSCAFG0000002861	grey	VSMC_M10	0.13	5.06E-01	0.50	8.65E-03	0.57	1.97E-03	-0.16	4.22E-01	-0.58	1.55E-03	0.02	9.05E-01	0.24	2.36E-01	-0.07	7.30E-01	-0.52	5.36E-03	0.36	6.38E-02
ENSCAFG0000003252	COTL1	grey	VSMC_M10	0.13	5.06E-01	-0.20	3.14E-01	-0.08	6.82E-01	-0.09	6.41E-01	0.19	3.47E-01	0.31	1.19E-01	-0.29	1.39E-01	0.06	7.51E-01	0.24	2.19E-01	-0.30	1.35E-01
ENSCAFG0000000996	CTNND2	turquoise	VSMC_M6	0.13	5.06E-01	-0.11	5.91E-01	-0.12	5.54E-01	-0.01	9.48E-01	0.23	2.44E-01	0.16	4.17E-01	-0.15	4.70E-01	0.03	8.95E-01	0.00	9.94E-01	-0.20	3.29E-01
ENSCAFG0000003054	TRIR	yellow	VSMC_M3	0.13	5.06E-01	0.44	2.11E-02	0.59	1.32E-03	-0.05	8.10E-01	-0.53	4.07E-03	-0.02	9.39E-01	-0.24	2.25E-01	-0.12	5.47E-01	-0.40	3.95E-02	0.21	2.92E-01
ENSCAFG0000003078	ENSCAFG0000003078	grey	VSMC_M10	0.13	5.06E-01	0.11	5.93E-01	0.13	5.13E-01	-0.15	4.55E-01	-0.06	7.82E-01	-0.10	6.13E-01	0.34	8.18E-02	0.12	5.42E-01	-0.20	3.13E-01	0.05	7.92E-01
ENSCAFG0000002119	ENSCAFG0000002119	grey	VSMC_M10	0.13	5.06E-01	0.29	1.45E-01	0.19	3.45E-01	-0.13	5.33E-01	-0.15	4.66E-01	-0.09	6.70E-01	0.10	6.12E-01	-0.03	8.78E-01	-0.33	9.38E-02	0.18	3.70E-01
ENSCAFG0000000389	AGK	grey	VSMC_M10	0.13	5.06E-01	0.06	7.83E-01	0.25	2.16E-01	-0.13	5.17E-01	-0.26	1.87E-01	-0.01	9.55E-01	0.11	5.98E-01	0.19	3.38E-01	-0.19	3.41E-01	0.12	5.46E-01
ENSCAFG0000000105	TRMT11	grey	VSMC_M10	0.13	5.06E-01	-0.30	1.22E-01	0.30	1.30E-01	-0.54	3.86E-03	-0.12	5.55E-01	0.09	6.64E-01	0.01	9.48E-01	0.51	6.23E-03	0.26	1.90E-01	-0.54	3.53E-03
ENSCAFG0000000230	LRRC1	pink	VSMC_M5	0.13	5.07E-01	-0.46	1.50E-02	-0.20	3.06E-01	-0.28	1.50E-01	0.42	2.75E-02	-0.19	3.50E-01	0.17	3.86E-01	0.35	7.57E-02	0.41	3.58E-02	-0.60	9.15E-04
ENSCAFG0000000388	PLEKHA4	yellow	VSMC_M3	0.13	5.07E-01	0.56	2.54E-03	0.79	1.01E-06	-0.41	3.29E-02	-0.78	1.27E-06	0.09	6.58E-01	0.14	5.00E-01	0.12	5.61E-01	-0.60	8.56E-04	0.35	7.69E-02
ENSCAFG0000001404	CLEC3B	cyan	VSMC_M2	0.13	5.07E-01	0.81	3.31E-07	0.46	1.60E-02	0.05	8.03E-01	-0.48	1.20E-02	0.00	9.97E-01	-0.29	1.38E-01	-0.29	1.40E-01	-0.74	1.19E-05	0.53	4.43E-03
ENSCAFG0000001756	TRIM37	grey	VSMC_M10	0.13	5.07E-01	-0.12	5.35E-01	0.14	4.77E-01	-0.35	7.33E-02	0.17	4.09E-01	-0.36	6.84E-02	-0.02	9.27E-01	0.31	1.22E-01	0.09	6.65E-01	-0.54	3.63E-03
ENSCAFG0000001757	HDHD2	yellow	VSMC_M3	0.13	5.07E-01	-0.26	1.86E-01	0.42	3.02E-02	-0.44	2.15E-02	-0.29	1.49E-01	0.12	5.56E-01	0.02	9.24E-01	0.51	6.28E-03	0.16	4.17E-01	-0.41	3.49E-02
ENSCAFG0000000096	ZHX2	red	VSMC_M1	0.13	5.08E-01	0.47	1.31E-02	0.25	1.99E-01	0.23	2.49E-01	-0.32	1.02E-01	0.10	6.07E-01	0.03	8.75E-01	-0.37	5.67E-02	-0.47	1.26E-02	0.49	9.49E-03
ENSCAFG0000002857	NXT2	grey	VSMC_M10	0.13	5.08E-01	-0.48	1.18E-02	-0.15	4.54E-01	-0.33	8.98E-02	0.32	1.01E-01	-0.23	2.55E-01	0.26	1.94E-01	0.50	7.72E-03	0.36	6.32E-02	-0.54	3.49E-03
ENSCAFG0000003206	ENSCAFG0000003206	grey	VSMC_M10	0.13	5.08E-01	-0.35	7.65E-02	-0.12	5.63E-01	-0.34	8.05E-02	0.18	3.65E-01	-0.09	6.67E-01	0.30	1.28E-01	0.42	2.97E-02	0.21	2.86E-01	-0.31	1.10E-01
ENSCAFG0000000048	ENSCAFG0000000048	grey	VSMC_M10	0.13	5.08E-01	0.28	1.63E-01	0.42	2.72E-02	-0.20	3.29E-01	-0.43	2.46E-02	0.31	1.15E-01	0.04	8.34E-01	0.12	5.66E-01	-0.36	6.44E-02	0.16	4.36E-01
ENSCAFG0000001857	ZNF131	grey	VSMC_M10	0.13	5.08E-01	-0.33	9.63E-02	0.22	2.63E-01	-0.40	3.70E-02	-0.05	7.94E-01	0.04	8.59E-01	-0.16	4.18E-01	0.42	2.76E-02	0.25	2.00E-01	-0.51	6.70E-03
ENSCAFG0000002602	ENSCAFG0000002602	grey	VSMC_M10	0.13	5.09E-01	-0.33	9.44E-02	-0.50	8.58E-03	0.28	1.63E-01	0.48	1.11E-02	0.20	3.20E-01	0.10	6.06E-01	-0.05	7.87E-01	0.16	4.15E-01	-0.03	8.70E-01
ENSCAFG0000000850	CCDC113	grey	VSMC_M10	0.13	5.09E-01	0.24	2.21E-01	0.03	8.99E-01	0.02	9.05E-01	0.03	8.99E-01	0.01	9.45E-01	0.09	6.39E-01	-0.25	2.04E-01	-0.21	2.93E-01	0.18	3.61E-01
ENSCAFG0000001764	HAUS1	darkgrey	VSMC_M8	0.13	5.09E-01	-0.38	4.80E-02	0.13	5.29E-01	-0.51	6.35E-03	-0.03	8.66E-01	0.09	6.61E-01	0.40	4.05E-02	0.63	4.39E-04	0.20	3.16E-01	-0.42	2.99E-02
ENSCAFG0000002359	MFSD9	grey	VSMC_M10	0.13	5.09E-01	0.37	5.77E-02	-0.21	2.99E-01	0.65	2.79E-04	0.12	5.58E-01	-0.09	6.53E-01	-0.31	1.19E-01	-0.68	8.93E-05	-0.31	1.20E-01	0.58	1.41E-03
ENSCAFG0000001433	HDAC5	cyan	VSMC_M2	0.13	5.09E-01	0.40	4.13E-02	0.30	1.31E-01	0.00	9.94E-01	-0.08	7.08E-01	-0.31	1.19E-01	-0.08	6.94E-01	-0.29	1.43E-01	-0.24	2.34E-01	0.02	9.40E-01
ENSCAFG0000002934	ENSCAFG0000002934	grey	VSMC_M10	0.13	5.10E-01	0.19	3.37E-01	-0.01	9.76E-01	0.24	2.29E-01	0.14	4.89E-01	-0.38	5.24E-02	-0.05	7.99E-01	-0.35	7.05E-02	-0.12	5.42E-01	0.12	5.54E-01
ENSCAFG0000000184	ZNF318	grey	VSMC_M10	0.13	5.10E-01	-0.04	8.37E-01	-0.27	1.78E-01	-0.03	8.82E-01	0.30	1.26E-01	-0.03	8.83E-01	0.08	7.01E-01	-0.04	8.41E-01	0.03	8.96E-01	0.04	8.37E-01
ENSCAFG0000000883	ZFYVE16	pink	VSMC_M5	0.13	5.10E-01	-0.35	7.56E-02	-0.59	1.33E-03	0.12	5.45E-01	0.78	1.36E-06	-0.21	2.97E-01	-0.03	8.63E-01	-0.08	6.87E-01	0.33	9.27E-02	-0.35	7.23E-02
ENSCAFG0000001172	ATRNL1	cyan	VSMC_M2	0.13	5.10E-01	0.69	5.80E-05	-0.13	5.09E-01	0.77	2.47E-06	0.03	8.65E-01	-0.15	4.49E-01	-0.42	2.78E-02	-0.83	9.53E-08	-0.62	5.94E-04	0.74	1.11E-05
ENSCAFG0000001244	AEBP2	grey	VSMC_M10	0.13	5.10E-01	-0.19	3.40E-01	-0.53	4.55E-03	0.33	9.28E-02	0.60	1.04E-03	-0.24	2.35E-01	0.02	9.13E-01	-0.27	1.77E-01	0.17	3.97E-01	-0.02	9.30E-01
ENSCAFG0000000515	GCKR	grey	VSMC_M10	0.13	5.10E-01	0.17	4.07E-01	-0.04	8.40E-01	0.18	3.63E-01	0.07	7.29E-01	-0.07	7.33E-01	-0.51	6.33E-03	-0.14	4.77E-01	-0.15	4.62E-01	0.09	6.61E-01
ENSCAFG0000002016	ZBTB34	grey	VSMC_M10	0.13	5.11E-01	0.10	6.26E-01	0.34	7.86E-02	-0.22	2.67E-01	-0.22	2.71E-01	0.13	5.14E-01	-0.15	4.55E-01	0.16	4.33E-01	-0.13	5.15E-01	-0.17	3.88E-01
ENSCAFG0000000605	ENSCAFG0000000605	grey	VSMC_M10	0.13	5.11E-01	0.41	3.55E-02	0.24	2.37E-01	-0.08	6.96E-01	-0.31	1.16E-01	0.13	5.20E-01	0.15	4.41E-01	-0.09	6.54E-01	-0.45	1.80E-02	0.36	6.71E-02
ENSCAFG0000001124	PRMT5	darkgrey	VSMC_M8	0.13	5.11E-01	-0.46	1.53E-02	0.18	3.67E-01	-0.64	2.93E-04	-0.01	9.58E-01	-0.03	8.82E-01	0.62	5.89E-04	0.76	5.15E-06	0.21	2.97E-01	-0.59	1.22E-03
ENSCAFG0000001777	ENSCAFG0000001777	cyan	VSMC_M2	0.13	5.11E-01	0.47	1.43E-02	0.26	1.92E-01	-0.12	5.62E-01	-0.21	2.98E-01	-0.12	5.59E-01	0.03	8.85E-01	-0.03	8.77E-01	-0.46	1.67E-02	0.26	1.99E-01
ENSCAFG0000002956	ENSCAFG0000002956	grey	VSMC_M10	0.13	5.11E-01	-0.10	6.34E-01	-0.13	5.24E-01	0.06	7.83E-01	0.20	3.06E-01	-0.06	7.59E-01	-0.09	6.44E-01	-0.05	7.99E-01	0.15	4.69E-01	-0.10	6.27E-01
ENSCAFG0000000134	FKBP5	darkgrey	VSMC_M8	0.13	5.11E-01	-0.04	8.25E-01	0.69	7.66E-05	-0.89	7.16E-10	-0.49	8.79E-03	0.04	8.59E-01	0.51	6.64E-03	0.76	3.83E-06	-0.10	6.23E-01	-0.42	3.00E-02
ENSCAFG0000001746	ENSCAFG0000001746	grey	VSMC_M10	0.13	5.11E-01	-0.22	2.75E-01	0.18	3.65E-01	-0.41	3.17E-02	0.03	8.90E-01	0.05	8.19E-01	0.23	2.47E-01	0.39	4.40E-02	0.10	6.22E-01	-0.42	2.99E-02
ENSCAFG0000000306	SLC35A1	grey	VSMC_M10	0.13	5.12E-01	-0.38	4.96E-02	-0.14	4.97E-01	-0.28	1.55E-01	0.41	3.50E-02	-0.14	4.88E-01	0.03	8.72E-01	0.26	1.93E-01	0.33	9.28E-02	-0.57	1.84E-03
ENSCAFG0000003097	ENSCAFG0000003097	grey	VSMC_M10	0.13	5.12E-01	-0.10	6.27E-01	-0.28	1.64E-01	0.00	9.93E-01	0.22	2.70E-01	0.04	8.46E-01	0.22	2.68E-01	0.07	7.17E-01	-0.09	6.51E-01	0.14	4.88E-01
ENSCAFG0000003231	ENSCAFG0000003231	darkgreen	VSMC_M4	0.13	5.12E-01	-0.10	6.03E-01	-0.49	9.72E-03	0.57	1.86E-03	0.52	5.24E-03	-0.39	4.41E-02	-0.10	6.05E-01	-0.37	5.55E-02	0.07	7.17E-01	0.07	7.14E-01

ENSCAFG0000000829	RNF34	darkgreen	VSMC_M4	0.13	5.12E-01	-0.10	6.30E-01	-0.46	1.69E-02	0.50	8.51E-03	0.54	3.39E-03	-0.14	5.02E-01	-0.14	4.89E-01	-0.45	1.95E-02	0.19	3.42E-01	-0.04	8.60E-01
ENSCAFG0000001677	HSD3B7	cyan	VSMC_M2	0.13	5.12E-01	0.56	2.53E-03	0.29	1.43E-01	0.10	6.16E-01	-0.05	4.45E-01	-0.48	1.04E-02	-0.04	8.27E-01	-0.34	8.42E-02	-0.43	2.50E-02	0.24	2.32E-01
ENSCAFG0000002878	ENSCAFG0000002878	grey	VSMC_M10	0.13	5.12E-01	0.06	7.58E-01	0.00	9.98E-01	-0.15	4.67E-01	0.14	4.94E-01	-0.12	5.64E-01	-0.15	4.64E-01	0.10	6.26E-01	-0.07	7.36E-01	-0.24	2.33E-01
ENSCAFG0000001112	ENSCAFG0000001112	grey	VSMC_M10	0.13	5.13E-01	0.16	4.14E-01	-0.24	2.20E-01	0.53	4.56E-03	0.16	4.24E-01	0.03	8.72E-01	-0.15	4.60E-01	-0.44	2.20E-02	-0.15	4.59E-01	0.40	3.92E-02
ENSCAFG0000000534	SERTAD3	grey	VSMC_M10	0.13	5.13E-01	0.10	6.18E-01	-0.10	6.07E-01	0.11	5.87E-01	0.18	3.79E-01	-0.29	1.46E-01	-0.31	1.11E-01	-0.08	6.81E-01	-0.06	7.73E-01	0.01	9.71E-01
ENSCAFG0000001911	ENSCAFG0000001911	grey	VSMC_M10	0.13	5.13E-01	-0.02	9.27E-01	-0.09	6.67E-01	0.03	8.99E-01	0.08	6.98E-01	0.05	7.88E-01	0.26	1.94E-01	0.04	8.43E-01	-0.07	7.30E-01	0.13	5.08E-01
ENSCAFG0000000069	DXO	grey	VSMC_M10	0.13	5.14E-01	0.14	4.87E-01	0.47	1.30E-02	-0.42	3.04E-02	-0.26	1.95E-01	-0.21	2.92E-01	-0.12	5.65E-01	0.26	1.94E-01	-0.09	6.60E-01	-0.28	1.62E-01
ENSCAFG0000000298	TBX18	darkgreen	VSMC_M4	0.13	5.14E-01	0.56	2.62E-03	-0.22	2.74E-01	0.69	5.75E-05	0.13	5.29E-01	-0.23	2.42E-01	-0.11	5.82E-01	-0.71	3.65E-05	-0.49	9.21E-03	0.70	5.25E-05
ENSCAFG0000001487	BCS1L	grey	VSMC_M10	0.13	5.14E-01	-0.24	2.20E-01	0.34	8.04E-02	-0.16	4.38E-01	-0.31	1.20E-01	0.01	9.69E-01	0.09	6.59E-01	0.06	7.74E-01	-0.23	2.42E-01	0.06	7.64E-01
ENSCAFG0000001599	LIAS	grey	VSMC_M10	0.13	5.14E-01	-0.02	9.14E-01	0.06	7.50E-01	0.21	2.82E-01	-0.02	9.03E-01	-0.08	6.76E-01	0.13	5.31E-01	-0.13	5.06E-01	-0.06	7.63E-01	0.05	7.94E-01
ENSCAFG0000001182	PRPF3	darkgrey	VSMC_M8	0.13	5.14E-01	-0.35	7.31E-02	0.21	2.99E-01	-0.44	2.08E-02	-0.06	7.81E-01	-0.23	2.45E-01	0.20	3.10E-01	0.53	4.79E-03	0.20	3.11E-01	-0.38	5.08E-02
ENSCAFG0000001458	SERPINI1	grey	VSMC_M10	0.13	5.15E-01	-0.37	5.42E-02	-0.39	4.59E-02	0.12	5.65E-01	0.34	8.62E-02	0.21	3.01E-01	0.22	2.64E-01	0.12	5.46E-01	0.24	2.24E-01	-0.01	9.66E-01
ENSCAFG0000001735	SOX30	grey	VSMC_M10	0.13	5.15E-01	-0.20	3.17E-01	-0.18	3.73E-01	0.03	8.95E-01	0.16	4.34E-01	0.21	2.85E-01	0.08	6.94E-01	0.08	6.84E-01	0.08	6.74E-01	0.01	9.80E-01
ENSCAFG0000001681	SP2	yellow	VSMC_M3	0.13	5.15E-01	0.17	3.88E-01	0.43	2.47E-02	-0.54	3.65E-03	-0.32	9.91E-02	0.07	7.13E-01	0.20	3.17E-01	0.37	5.65E-02	-0.19	3.36E-01	-0.09	6.52E-01
ENSCAFG0000003117	ST6GALNAC6	cyan	VSMC_M2	0.13	5.16E-01	0.48	1.18E-02	0.28	1.62E-01	-0.04	8.47E-01	-0.11	5.75E-01	-0.23	2.53E-01	-0.41	3.49E-02	-0.23	2.40E-01	-0.29	1.41E-01	0.03	8.75E-01
ENSCAFG0000002969	ZNF517	grey	VSMC_M10	0.13	5.16E-01	0.32	1.09E-01	-0.06	7.64E-01	0.41	3.40E-02	-0.05	8.16E-01	0.11	5.96E-01	-0.23	2.59E-01	-0.39	4.17E-02	-0.31	1.10E-01	0.45	1.99E-02
ENSCAFG0000001051	ENSCAFG0000001051	turquoise	VSMC_M6	0.13	5.16E-01	0.05	7.94E-01	0.00	9.85E-01	-0.05	8.04E-01	0.02	9.37E-01	0.62	5.97E-04	-0.07	7.16E-01	0.07	7.41E-01	-0.10	6.26E-01	0.02	9.14E-01
ENSCAFG0000001244	USP53	darkgreen	VSMC_M4	0.13	5.16E-01	0.36	6.81E-02	-0.51	6.21E-03	0.81	2.53E-07	0.44	2.01E-02	-0.19	3.38E-01	-0.29	1.48E-01	-0.79	1.18E-06	-0.32	1.00E-01	0.56	2.50E-03
ENSCAFG0000001468	ENSCAFG0000001468	grey	VSMC_M10	0.13	5.16E-01	-0.31	1.19E-01	-0.13	5.07E-01	-0.01	9.48E-01	0.35	6.99E-02	-0.15	4.44E-01	0.07	7.39E-01	0.05	7.86E-01	0.34	7.86E-02	-0.38	4.87E-02
ENSCAFG0000001067	EYA2	darkgreen	VSMC_M4	0.13	5.16E-01	0.44	2.05E-02	-0.25	2.06E-01	0.68	1.08E-04	0.24	2.35E-01	-0.36	6.54E-02	-0.01	9.46E-01	-0.67	1.35E-04	-0.40	4.11E-02	0.48	1.22E-02
ENSCAFG0000002468	MUM1L1	darkgreen	VSMC_M4	0.13	5.16E-01	0.23	2.51E-01	-0.28	1.50E-01	0.54	3.77E-03	0.20	3.08E-01	-0.06	7.74E-01	0.06	7.74E-01	-0.54	3.32E-03	-0.25	2.10E-01	0.51	6.34E-03
ENSCAFG0000000299	TAX1BP1	pink	VSMC_M5	0.13	5.16E-01	-0.38	5.38E-02	-0.69	7.89E-05	0.50	7.27E-03	0.82	2.07E-07	-0.20	3.20E-01	-0.28	1.53E-01	-0.32	1.02E-01	0.38	5.13E-02	-0.25	2.01E-01
ENSCAFG0000000924	ENSCAFG0000000924	grey	VSMC_M10	0.13	5.17E-01	-0.16	4.32E-01	-0.05	8.05E-01	-0.33	9.61E-02	0.20	3.09E-01	-0.25	2.04E-01	0.16	4.27E-01	0.30	1.23E-01	0.07	7.29E-01	-0.29	1.42E-01
ENSCAFG0000001545	ENSCAFG0000001545	pink	VSMC_M5	0.13	5.17E-01	-0.16	4.23E-01	-0.23	2.49E-01	0.02	9.23E-01	0.47	1.36E-02	-0.33	9.31E-02	-0.09	6.60E-01	0.01	9.52E-01	0.17	3.85E-01	-0.44	2.25E-02
ENSCAFG0000000203	LNX1	grey	VSMC_M10	0.13	5.17E-01	0.02	9.17E-01	0.19	3.31E-01	-0.38	5.05E-02	0.01	9.73E-01	-0.03	8.97E-01	0.23	2.55E-01	0.37	5.74E-02	-0.09	6.70E-01	-0.35	7.48E-02
ENSCAFG0000000953	ABCC2	grey	VSMC_M10	0.13	5.17E-01	-0.41	3.44E-02	-0.27	1.76E-01	-0.11	5.97E-01	0.37	5.97E-02	-0.08	6.90E-01	0.05	7.98E-01	0.23	2.49E-01	0.26	1.92E-01	-0.29	1.46E-01
ENSCAFG0000001869	NIPBL	pink	VSMC_M5	0.13	5.17E-01	-0.35	6.95E-02	-0.62	6.33E-04	0.22	2.79E-01	0.80	5.43E-07	-0.43	2.52E-02	-0.17	3.98E-01	-0.09	6.41E-01	0.32	1.03E-01	-0.34	8.67E-02
ENSCAFG0000000577	CCDC137	yellow	VSMC_M3	0.13	5.17E-01	0.04	8.54E-01	0.60	8.79E-04	-0.36	6.77E-02	-0.59	1.28E-03	0.22	2.62E-01	0.06	7.63E-01	0.31	1.20E-01	-0.13	5.07E-01	-0.03	8.81E-01
ENSCAFG0000001304	LIMK2	grey	VSMC_M10	0.13	5.17E-01	0.19	3.32E-01	0.15	4.58E-01	0.24	2.28E-01	-0.17	3.95E-01	0.26	1.86E-01	-0.33	9.63E-02	-0.29	1.40E-01	-0.21	2.95E-01	0.20	3.22E-01
ENSCAFG0000003168	ZNF391	grey	VSMC_M10	0.13	5.18E-01	0.03	8.97E-01	-0.08	6.82E-01	0.04	8.50E-01	0.14	4.97E-01	-0.12	5.59E-01	0.15	4.56E-01	-0.02	9.24E-01	-0.16	4.33E-01	0.01	9.65E-01
ENSCAFG0000003119	EFNB3	grey	VSMC_M10	0.13	5.18E-01	0.19	3.48E-01	-0.37	5.42E-02	0.32	1.02E-01	0.39	4.56E-02	0.01	9.61E-01	0.09	6.39E-01	-0.34	8.13E-02	-0.20	3.25E-01	0.22	2.67E-01
ENSCAFG0000000890	BPI	cyan	VSMC_M2	0.13	5.18E-01	0.44	2.24E-02	0.30	1.25E-01	-0.15	4.55E-01	-0.28	1.64E-01	0.00	9.86E-01	0.32	1.01E-01	0.02	9.32E-01	-0.46	1.64E-02	0.27	1.69E-01
ENSCAFG0000000914	ARHGAP1	cyan	VSMC_M2	0.13	5.18E-01	0.67	1.51E-04	0.32	1.07E-01	0.15	4.53E-01	-0.25	2.00E-01	-0.06	7.50E-01	-0.70	4.57E-05	-0.44	2.10E-02	-0.48	1.09E-02	0.32	1.08E-01
ENSCAFG0000000839	SCARA3	grey	VSMC_M10	0.13	5.18E-01	-0.29	1.47E-01	0.21	2.82E-01	-0.63	4.34E-04	-0.05	7.94E-01	0.12	5.58E-01	0.64	3.26E-04	0.52	5.82E-03	0.16	4.37E-01	-0.39	4.43E-02
ENSCAFG0000000025	MAP3K5	grey	VSMC_M10	0.13	5.18E-01	0.43	2.59E-02	0.12	5.43E-01	0.15	4.52E-01	-0.08	6.74E-01	0.04	8.55E-01	-0.16	4.30E-01	-0.25	2.05E-01	-0.43	2.47E-02	0.24	2.27E-01
ENSCAFG0000003194	ENSCAFG0000003194	grey	VSMC_M10	0.13	5.19E-01	0.70	4.28E-05	0.14	4.93E-01	0.20	3.19E-01	-0.18	3.63E-01	-0.14	4.84E-01	-0.11	5.75E-01	-0.42	2.89E-02	-0.63	3.83E-04	0.55	3.19E-03
ENSCAFG0000003010	ATP6V1FNB	grey	VSMC_M10	0.13	5.19E-01	0.39	4.41E-02	0.35	7.26E-02	0.12	5.44E-01	-0.43	2.56E-02	0.23	2.56E-01	-0.02	9.07E-01	-0.15	4.67E-01	-0.41	3.42E-02	0.46	1.63E-02
ENSCAFG0000000863	SOGA1	turquoise	VSMC_M6	0.13	5.19E-01	-0.20	3.29E-01	0.12	5.62E-01	-0.09	6.45E-01	-0.07	7.28E-01	0.13	5.22E-01	-0.05	7.97E-01	0.12	5.65E-01	0.08	6.75E-01	-0.10	6.27E-01
ENSCAFG0000001786	RPRD1A	grey	VSMC_M10	0.13	5.19E-01	0.00	9.83E-01	-0.27	1.77E-01	0.48	1.23E-02	0.37	5.77E-02	-0.08	6.96E-01	0.01	9.79E-01	-0.45	1.89E-02	-0.02	9.41E-01	0.09	6.61E-01
ENSCAFG0000002905	RAB6B	grey	VSMC_M10	0.13	5.19E-01	0.69	6.36E-05	-0.03	8.74E-01	0.57	1.92E-03	-0.12	5.35E-01	0.02	9.02E-01	-0.21	2.85E-01	-0.67	1.28E-04	-0.65	2.50E-04	0.81	3.26E-07
ENSCAFG0000001614	SLC25A18	turquoise	VSMC_M6	0.13	5.19E-01	-0.16	4.33E-01	-0.04	8.48E-01	-0.13	5.19E-01	0.11	5.75E-01	-0.04	8.59E-01	-0.09	6.46E-01	0.18	3.82E-01	0.06	7.81E-01	-0.15	4.59E-01
ENSCAFG0000002882	UBE2V2	pink	VSMC_M5	0.13	5.19E-01	-0.67	1.24E-04	-0.52	5.51E-03	-0.05	7.88E-01	0.77	2.61E-06	-0.19	3.54E-01	-0.11	5.85E-01	0.28	1.55E-01	0.63	4.65E-04	-0.74	8.82E-06
ENSCAFG0000001637	IQCE	grey	VSMC_M10	0.13	5.19E-01	-0.21	2.99E-01	-0.14	4.94E-01	-0.12	5.39E-01	0.25	2.13E-01	-0.17	3.95E-01	-0.21	2.88E-01	0.15	4.52E-01	0.20	3.29E-01	-0.24	2.24E-01
ENSCAFG0000000714	FBLN7	darkgreen	VSMC_M4	0.13	5.19E-01	0.36	6.28E-02	-0.40	4.09E-02	0.85	1.37E-08	0.36	6.92E-02	-0.18	3.62E-01	-0.43	2.42E-02	-0.78	1.97E-06	-0.30	1.30E-01	0.45	1.78E-02
ENSCAFG0000000086	AFDN	cyan	VSMC_M2	0.13	5.20E-01	0.66	1.91E-04	0.09	6.54E-01	0.44	2.08E-02	-0.20	3.20E-01	0.03	8.95E-01	-0.65	2.40E-04	-0.51	6.63E-03	-0.61	6.66E-04	0.62	6.19E-04
ENSCAFG0000001172	ENSCAFG0000001172	grey	VSMC_M10	0.13	5.20E-01	-0.05	8.02E-01	0.45	1.94E-02	-0.31	1.13E-01	-0.26	1.91E-01	-0.13	5.13E-01	0.09	6.52E-01	0.25	2.06E-01	0.03	9.00E-01	-0.31	1.20E-01
ENSCAFG0000001139	WSCD2	darkgreen	VSMC_M4	0.13	5.20E-01	-0.13	5.04E-01	-0.49	9.89E-03	0.46	1.53E-02	0.54	3.90E-03	-0.15	4.55E-01	0.22	2.76E-01	-0.29	1.39E-01	0.06	7.62E-01	0.00	9.80E-01
ENSCAFG0000001649	RPL19	grey	VSMC_M10	0.13	5.20E-01	0.06	7.73E-01	0.26	1.96E-01	-0.01	9.71E-01	-0.19	3.34E-01	-0.13	5.26E-01	0.11	5.99E-01	0.00	9.87E-01	-0.13	5.29E-01	-0.04	8.31E-01
ENSCAFG0000000213	IL18R1	darkgreen	VSMC_M4	0.13	5.20E-01	0.22	2.70E-01	-0.36	6.38E-02	0.61	7.57E-04	0.23	2.56E-01	0.05	8.19E-01	-0.38	4.88E-02	-0.52	5.31E-03	-0.24	2.25E-01	0.46	1.63E-02
ENSCAFG0000000260	HSPBP1	yellow	VSMC_M3	0.13	5.21E-01	0.19	3.37E-01	0.76	4.06E-06	-0.54	3.36E-03	-0.78	1.44E-06	0.27	1.68E-01	0.22	2.60E-01	0.46	1.50E-02	-0.33	9.04E-02	0.08	6.98E-01
ENSCAFG0000003130	ENSCAFG0000003130	cyan	VSMC_M2	0.13	5.21E-01	0.76	4.99E-06	0.15	4.68E-01	0.57	1.99E-03	-0.20	3.09E-01	-0.29	1.35E-01	-0.37	5.85E-02	-0.75	6.27E-06	-0.65	2.27E-04	0.70	5.45E-05
ENSCAFG0000000553	MBD5	pink	VSMC_M5	0.13	5.21E-01	0.04	8.53E-01	-0.62	6.19E-04	0.50	8.27E-03	0.66	1.83E-04	-0.11	5.92E-01	-0.22	2.81E-01	-0.44	2.23E-02	0.03	8.77E-01	0.08	7.01E-01
ENSCAFG0000000817	JPH1	darkgreen	VSMC_M4	0.13	5.21E-01	-0.05	7.89E-01	-0.58	1.55E-03	0.61	6.68E-04	0.48	1.10E-02	-0.02	9.06E-01	-0.15	4.47E-01	-0.38	4.90E-02	0.01	9.75E-01	0.29	1.44E-01
ENSCAFG0000001211	NDUFB3	grey	VSMC_M10	0.13	5.21E-01	0.03	8.74E-01	0.18	3.80E-01	0.00	9.89E-01	-0.17	3.91E-01	-0.15	4.47E-01	0.04	8.28E-01	0.04	8.39E-01	-0.11	5.96E-01	0.00	9.96E-01
ENSCAFG0000001740	CRTC2	grey	VSMC_M10	0.13	5.21E-01	0.49	9.49E-03	0.47	1.33E-02	-0.26	1.88E-01	-0.45	1.98E-02	0.10	6.26E-01	-0.08	6.81E-01	0.05	7.90E-01	-0.45	1.80E-02	0.23	2.45E-01
ENSCAFG0000001267	SELENBP1	grey	VSMC_M10	0.13	5.21E-01	0.66	1.98E-04	0.30	1.33E-01	0.25	2.03E-01	-0.29	1.36E-01	-0.30	1.29E-01	-0.16	4.35E-01	-0.43	2.56E-02	-0.56	2.62E-03	0.51	6.55E-03
ENSCAFG0000001237	FGFR2	darkgreen	VSMC_M4	0.13	5.22E-01	0.08	6.89E-01	-0.61	7.60E-04	0.80	4.23E-07	0.50	8.40E-03	0.02	9.33E-01	-0.20	3.13E-01	-0.62	5.47E-04	-0.11	6.00E-01	0.45	1.91E-02
ENSCAFG0000001262	ADRM1	yellow	VSMC_M3	0.13	5.22E-01	0.25	2.18E-01	0.80	5.85E-07	-0.58	1.37E-03	-0.72	2.13E-05	0.17	4.07E-01	-0.03	8.79E-01	0.43	2.45E-02	-0.29	1.41E-01	-0.12	5.43E-01
ENSCAFG0000001946	RNF208	grey	VSMC_M10	0.13	5.22E-01	0.19	3.37E-01	-0.16	4.28E-01	0.22	2.63E-01	0.14	4.82E-01	-0.13	5.11E-01	-0.01	9.44E-01	-0.24	2.32E-01	-0.20	3.19E-01	0.27	1.67E-01
ENSCAFG0000001878	RPL23A	grey	VSMC_M10	0.13	5.22E-01	0.09	6.42E-01	0.38	5.28E-02	-0.29	1.47E-01	-0.35	7.70E-02	-0.09	6.71E-01	0.26	1.98E-01	0.27	1.72E-01	-0.22	2.78E-01	-0.05	7.93E-01
ENSCAFG0000000745	KRCC1	grey	VSMC_M10	0.13	5.22E-01	-0.04	8.40E-01	-0.34	8.10E-02	0.15	4.60E-01	0.42	3.11E-02	-0.19	3.51E-01	0.35	7.55E-02	-0.11	5.71E-01	-0.09	6.51E-01	0.00	9.97E-01
ENSCAFG0000000276	EML6	darkgreen	VSMC_M4	0.13	5.22E-01	0.42	3.07E-02	-0.31	1.13E-01	0.65	2.48E-04	0.23	2.51E-01	-0.18	3.62E-01	-0.03	8.85E-01	-0.63	3.94E-04	-0.40	3.74E-02	0.60	1.04E-03
ENSCAFG0000001959	FAM3A	cyan	VSMC_M2	0.13	5.22E-01	0.56	2.65E-03	0.28	1.56E-01	0.38	5.10E-02	-0.39	4.17E-02	0.03	8.91E-01	-0.28	1.58E-01	-0.45	2.00E-02	-0.53	4.05E-03	0.61	7.47E-04
ENSCAFG0000000060	NCAPH2	yellow	VSMC_M3	0.13	5.23E-01	-0.09	6.50E-01	0.70	4.50E-05	-0.67	1.14E-04	-0.47	1.45E-02	-0.15	4.54E-01	0.23	2.42E-01	0.53	4.47E-03	0.07	7.42E-01	-0.48	1.17E-02
ENSCAFG0000001845	ENSCAFG0000001845	grey	VSMC_M10	0.13	5.23E-01	-0.09	6.49E-01	-0.02	9.02E-01	-0.25	2.11E-01	0.02	9.35E-01	0.09	6.63E-01	0.23	2.49E-01	0.21	2.83E-01	-0.01	9.51E-01	-0.01	9.60E-01
ENSCAFG0000000325	C11H9orf43	grey	VSMC_M10	0.13	5.23E-01	-0.20	3.08E-01	-0.07	7.23E-01	-0.04	8.34E-01	0.10	6.33E-01	-0.08	6.85E-01	0.21	2.83E-01	0.14	4.78E-01	0.06	7.55E-01	-0.05	8.11E-01
ENSCAFG0000000614	QPCT	darkgreen	VSMC_M4	0.13	5.23E-01	0.32	1.07E-01	-0.42	2.95E-02	0.79	1.06E-06	0.31	1.11E-01	0.05	8.16E-01	-0.53	4.11E-03	-0.79	9.73E-07	-0.26	1.90E-01	0.53	4.19E-03
ENSCAFG0000002054	ENSCAFG0000002054	darkgreen	VSMC_M4	0.13	5.23E-01	0.09	6.70E-01	-0.38	4.91E-02	0.51	6.12E-03	0.35	7.19E-02	-0.11	5.88E-01	-0.17	4.06E-01	-0.41	3.30E-02	-0.11	5.89E-01	0.24	2.34E-01
ENSCAFG0000000549	ENSCAFG0000000549	cyan	VSMC_M2	0.13	5.23E-01	0.67	1.37E-04	0.39	4.36E-02	0.10	6.34E-01	-0.47	1.34E-02	0.22	2.74E-01	-0.09	6.66E-01	-0.29	1.40E-01	-0.65	2.59E-04	0.54	3.29E-03
ENSCAFG0000002904	ENSCAFG0000002904	grey	VSMC_M10	0.13	5.23E-01	-0.12	5.36E-01	0.42	3.05E-02	-0.38	4.77E-02	-0.34	7.83E-02	-0.11	5.73E-01	0.54	3.32E-03	0.31	1.11E-01	0.04	8.58E-01	-0.15	4.48E-01
ENSCAFG0000000168	ENSCAFG0000000168	grey	VSMC_M10	0.13	5.23E-01	0.10	6.34E-01	0.36	6.13E-02	-0.21	2.91E-01	-0.28	1.64E-01	0.16	4.37E-01	0.10	6.12E-01	0.04	8.25E-01	-0.11	5.92E-01	-0.02	9.25E-01
ENSCAFG0000002849	HOXC5	yellow	VSMC_M3	0.13	5.23E-01	0.10	6.19E-01	0.49	9.48E-03	-0.42	3.08E-02	-0.47	1.44E-02	0.02	9.31E-01	-0.01	9.67E-01	0.34	8.49E-02	-0.17	4.06E-01	0.06	7.53E-01
ENSCAFG0000000918	NT5DC2	darkgrey	VSMC_M8	0.13	5.24E-01	-0.10	6.33E-01	0.52	5.25E-03	-0.78	1.54E-06	-0.28	1.50E-01	-0.10	6.03E-01	0.15	4.60E-01	0.66	1.77E-04	0.07	7.42E-01	-0.49	9.83E-03
ENSCAFG0000001121	HADH	cyan	VSMC_M2	0.13	5.24E-01	0.74	8.73E-06	-0.05	8.02E-01	0.66	1.96E-04	-0.03	8.85E-01	-0.18	3.61E-01	-0.58	1.48E-03	-0.81	3.03E-07	-0.62	5.32E-04	0.68	9.45E-05
ENSCAFG0000000031	LRIG3	darkolivegreen	VSMC_M9	0.13	5.24E-01	-0.44	2.27E-02	-0.27	1.68E-01	0.00	9.91E-01	0.35	6.95E-02	0.06	7.65E-01	0.59	1.17E-03	0.13	5.10E-01	0.27	1.80E-01	-0.23	2.56E-01
ENSCAFG0000002443	COLCA2	grey	VSMC_M10	0.13	5.25E-01	0.38	4.96E-02	0.13	5.29E-01	-0.06	7.70E-01	-0.05	8.15E-01	-0.06	7.83E-01	-0.18	3.56E-01	-0.32	9.84E-02	-0.32	1.04E-01	0.21	2.89E-01
ENSCAFG0000000043	GFPT2	grey	VSMC_M10	0.13	5.25E-01	0.29	1.44E-01	0.37	5.96E-02	-0.24	2.36E-01	-0.33	9.47E-02	0.17	3.87E-01	0.00	9.91E-01	0.15	4.51E-01	-0.40	4.10E-02	0.04	8.27E-01
ENSCAFG0000000433	EBPL	grey	VSMC_M10	0.13	5.25E-01	0.42	2.88E-02	0.09	6.72E-01	0.45	1.97E-02	-0.10	6.22E-01	0.10	6.22E-01	-0.51	6.14E-03	-0.49	9.00E-03	-0.35	7.59E-02	0.35	7.52E-02
ENSCAFG0000000750	ENSCAFG0000000750	yellow	VSMC_M3	0.13	5.26E-01	0.50	8.11E-03	0.65	2.57E-04	-0.08	6.99E-01	-0.66	1.58E-04	0.15	4.50E-01	-0.10	6.35E-01	-0.12	5.68E-01	-0.47	1.32E-02	0.35	7.65E-02
ENSCAFG0000001811	TCOF1	grey	VSMC_M10	0.13	5.26E-01	-0.05	7.96E-01	0.18	3.75E-01	-0.35	7.77E-02	-0.16	4.32E-01	0.09	6.60E-01	0.23	2.56E-01	0.40	4.06E-02	-0.17	3.91E-01	-0.09	6.61E-01
ENSCAFG0000002933	ENSCAFG0000002933	grey	VSMC_M10	0.13	5.26E-01	0.21	2.83E-01	0.56	2.19E-03	-0.25	2.05E-01	-0.51	6.19E-03	0.42	2.82E-02	0.11	5.79E-01	0.04	8.52E-01	-0.21	2.91E-01	0.06	7.65E-01
ENSCAFG0000001485	COPS2	pink	VSMC_M5	0.13	5.26E-01	-0.37	6.00E-02	-0.78	1.28E-06	0.52	5.47E-03	0.84	5.92E-08	-0.22	2.69E-01	0.10	6.14E-01	-0.29	1.38E-01	0.32	1.09E-01	-0.07	7.44E-01
ENSCAFG0000003199	ENSCAFG0000003199	grey	VSMC_M10	0.13	5.26E-01	0.51	6.47E-03	0.05	8.14E-01	0.38	5.17E-02	-0.03	8.88E-01	-0.10	6.06E-01	-0.70	5.59E-05	-0.51	6.22E-03	-0.35	7.03E-02	0.34	8.51E-02
ENSCAFG0000003257	ZFP69B	grey	VSMC_M10	0.13	5.26E-01	-0.05	8.22E-01	0.12	5.55E-01	-0.06	7.48E-01	0.02	9.24E-01	-0.32	9.86E-02	0.12	5.59E-01	0.03	8.66E-01	0.04	8.39E-01	-0.11	5.76E-01
ENSCAFG0000001094	CCDC91	grey	VSMC_M10	0.13	5.26E-01	-0.43	2.35E-02	-0.35	6.98E-02	0.15	4.67E-01	0.46	1.65E-02	-0.20	3.23E-01	0.16	4.29E-01	0.05	8.21E-01	0.29	1.41E-01	-0.23	2.49E-01
ENSCAFG0000001986	ENSCAFG0000001986	cyan	VSMC_M2	0.13	5.27E-01	0.57	1.71E-03	0.24	2.37E-01	0.02	9.07E-01	-0.34	8.23E-02	0.06	7.75E-01	0.18	3.71E-01	-0.18	3.77E-01	-0.61	6.54E-04	0.62	5.21E-04
ENSCAFG0000000424	ASXL2	pink	VSMC_M5	0.13	5.27E-01	-0.14	4.74E-01	-0.39	4.25E-02	0.24	2.35E-01	0.59	1.25E-03	-0.33	9.12E-02	-0.04	8.52E-01	-0.21	2.94E-01	0.16	4.20E-01	-0.23	2.52E-01
ENSCAFG0000001456	LAMTOR4	yellow	VSMC_M3	0.13	5.27E-01	0.06	7.69E-01	0.55	3.09E-03	-0.31	1.21E-01	-0.47	1.23E-02	-0.08	6.76E-01	-0.05	8.02E-01	0.19	3.45E-01	-0.04	8.33E-01	-0.11	5.83E-01
ENSCAFG0000001060	RGS1	grey	VSMC_M10	0.13	5.27E-01	0.01	9.55E-01	-0.34	7.97E-02	0.43	2.44E-02	0.26	1.86E-01	-0.02	9.05E-01	-0.38	4.81E-02	-0.33	9.52E-02	0.04	8.43E-01	0.22	2.60E-01
ENSCAFG0000000451	SPERT	grey	VSMC_M10	0.13	5.27E-01	0.01	9.50E-01	-0.07	7.35E-01	-0.09	6.72E-01	0.20	3.27E-01	0.13	5.22E-01	-0.14	4.91E-01	0.09	6.59E-01	-0.06	7.68E-01	-0.27	1.72E-01
ENSCAFG0000001710	TGFB3	darkgreen	VSMC_M4	0.13	5.28E-01	0.55	2.76E-03	-0.23	2.54E-01	0.76	3.51E-06	0.11	5.90E-01	0.04	8.33E-01	-0.08	6.93E-01	-0.77	2.19E-06	-0.53	4.69E-03	0.74	1.15E-05
ENSCAFG0000002507	ARSE	violet	VSMC_M7	0.13	5.28E-01	-0.21	2.89E-01	0.08	6.93E-01	-0.11	5.70E-01	-0.12	5.44E-01	0.17	4.09E-01	0.62	6.25E-04	0.23	2.58E-01	0.01	9.49E-01	0.00	9.94E-01
ENSCAFG0000000950	ENSCAFG0000000950	grey	VSMC_M10	0.13	5.28E-01	-0.15	4.66E-01	-0.16	4.13E-01	-0.01	9.71E-01	0.27	1.69E-01	-0.10	6.05E-01	0.26	1.98E-01	0.03	8.98E-01	0.10	6.35E-01	-0.17	3.94E-01
ENSCAFG0000002401	TLR1	darkgreen	VSMC_M4	0.13	5.29E-01	0.32	1.00E-01	-0.47	1.32E-02	0.81	3.43E-07	0.37	5.76E-02	-0.15	4.49E-01	-0.29	1.41E-01	-0.73	1.72E-05	-0.28	1.59E-01	0.53	4.08E-03
ENSCAFG0000000219	CNOT11	grey	VSMC_M10	0.13	5.29E-01	0.14	4.76E-01	-0.14	4.91E-01	0.37	5.57E-02	0.06	7.64E-01	0.13	5.22E-01	0.01	9.63E-01	-0.33	9.12E-02	-0.19	3.41E-01	0.36	6.50E-02
ENSCAFG0000001039	IRS1	grey	VSMC_M10	0.13	5.29E-01	0.10	6.18E-01	0.20	3.13E-01	-0.21	2.84E-01	0.00	9.98E-01	-0.05	7.86E-01	0.17	3.94E-01	0.03	8.82E-01	-0.05	8.20E-01	-0.12	5.61E-01
ENSCAFG0000001280	MUC2C	pink	VSMC_M5	0.13	5.29E-01	-0.24	2.32E-01	-0.45	1.74E-02	0.24	2.33E-01	0.53	4.18E-03	-0.10	6.36E-01	-0.33	9.70E-02	-0.04	8.55E-01	0.21	2.93E-01	-0.17	3.84E-01
ENSCAFG0000001923	WDR81	cyan	VSMC_M2	0.13	5.30E-01	0.82	2.07E-07	0.54	3.48E-03	-0.01	9.57E-01	-0.53	4.08E-03	0.05	7.99E-01	-0.06	7.51E-01	-0.36	6.13E-02	-0.72	2.53E-05	0.56	2.43E-03
ENSCAFG0000000208	ENSCAFG0000000208	grey	VSMC_M10	0.13	5.30E-01	0.20	3.17E-01	0.22	2.72E-01	-0.07	7.15E-01	-0.22	2.69E-01	0.23	2.58E-01	-0.21	2.99E-01	-0.06	7.53E-01	-0.23	2.51E-01	0.15	4.62E-01
ENSCAFG0000001347	EDEM3	grey	VSMC_M10	0.13	5.30E-01	-0.33	8.84E-02	-0.52	5.81E-03	0.16	4.28E-01	0.68	1.07E-04	-0.22	2.74E-01	-0.24	2.35E-01	-0.11	5.92E-01	0.40	4.13E-02	-0.31	1.18E-01
ENSCAFG0000000211	TGFBRAP1	grey	VSMC_M10	0.13	5.30E-01	0.21	2.83E-01	0.11	5.85E-01	0.11	5.94E-01	-0.04	8.55E-01	-0.13	5.07E-01	0.07	7.36E-01	-0.15	4.67E-01	-0.27	1.69E-01	0.12	5.39E-01
ENSCAFG0000000260	TMEM86E	grey	VSMC_M10	0.13	5.30E-01	0.21	3.00E-01	0.10	6.26E-01	0.09	6.69E-01	-0.14	4.73E-01	0.01	9.73E-01	-0.15	4.56E-01	-0.16	4.13E-01	-0.18	3.57E-01	0.27	1.77E-01
ENSCAFG0000001690	ALDH6A1	grey	VSMC_M10	0.13	5.30E-01	0.66	1.78E-04	0.01	9.51E-01	0.45	1.75E-02	-0.05	8.18E-01	-0.14	5.01E-01	-0.04	8.53E-01	-0.62	5.70E-04	-0.59	1.32E-03	0.61	7.31E-04
ENSCAFG0000001064	CSTB	cyan	VSMC_M2	0.13	5.30E-01	0.51	6.69E-03	0.56	2.32E-03	-0.15	4.45E-01	-0.50	7.47E-03	0.03	8.84E-01	-0.15	4.43E-01	-0.10	6.29E-01	-0.44	2.30E-02	0.20	3.27E-01
ENSCAFG0000000338	THRAP3	darkgrey	VSMC_M8	0.13	5.30E-01	-0.18	3.79E-01	0.43	2.70E-02	-0.80	5.84E-07	-0.22	2.64E-01	0.01	9.69E-01	0.19	3.30E-01	0.74	1.16E-05	0.08	6.80E-01	-0.53	4.23E-03
ENSCAFG0000000049	ENSCAFG0000000049	grey	VSMC_M10	0.13	5.30E-01	-0.12	5.38E-01	-0.18	3.63E-01	-0.02	9.32E-01	0.24	2.26E-01	-0.15	4.57E-01	-0.31	1.19E-01	0.10	6.08E-01	0.08	6.87E-01	-0.10	6.30E-01
ENSCAFG0000000087	ENSCAFG0000000087	grey	VSMC_M10	0.13	5.30E-01	0.03	8.63E-01	-0.34	8.07E-02	0.32	1.04E-01	0.33	9.29E-02	0.22	2.66E-01	-0.07	7.31E-01	-0.25	2.05E-01	-0.13	5.24E-01	0.18	3.80E-01
ENSCAFG0000001820	EPN2	cyan	VSMC_M2	0.13	5.30E-01	0.69	6.52E-05	0.15	4.61E-01	0.30	1.28E-01	-0.20	3.27E-01	0.02	9.24E-01	-0.36	6.18E-02	-0.51	6.93E-03	-0.59	1.09E-03	0.58	1.36E-03
ENSCAFG0000000626	ENSCAFG0000000626	grey	VSMC_M10	0.13	5.30E-01	-0.26	1.90E-01	0.03	8.89E-01	-0.45	1.86E-02	0.10	6.33E-01	-0.26	1.90E-01	0.05	7.96E-01	0.46	1.52E-02	0.13	5.09E-01	-0.40	3.85E-02
ENSCAFG0000003013	GBA2	yellow	VSMC_M3	0.13	5.31E-01	0.30	1.29E-01	0.69	7.64E-05	-0.39	4.51E-02	-0.55	2.98E-03	0.23	2.57E-01	0.02	9.39E-01	0.22	2.75E-01	-0.33	8.93E-02	-0.06	7.83E-01
ENSCAFG0000001786	ENSCAFG0000001786	yellow	VSMC_M3	0.13	5.31E-01	0.30	1.26E-01	0.66	2.09E-04	-0.38	5.30E-02	-0.52	5.92E-03	-0.12	5.60E-01	-0.08	7.08E-01	0.21	2.84E-01	-0.33	9.74E-02	-0.07	7.40E-01
ENSCAFG0000002421	USP4	darkgrey	VSMC_M8	0.13	5.31E-01	-0.20	3.21E-01	0.60	1.00E-03	-0.86	1.15E-08	-0.37	5.62E-02	0.11	6.01E-01	0.17	3.99E-01	0.76	5.09E-06	0.13	5.31E-01	-0.61	7.73E-04
ENSCAFG0000001230	DPAGT1	grey	VSMC_M10	0.13	5.31E-01	-0.25	2.11E-01	0.10	6.31E-01	-0.46	1.56E-02	-0.02	9.22E-01	0.11	5.79E-01	0.30	1.23E-01	0.50	8.14E-03	0.07	7.41E-01	-0.31	1.16E-01
ENSCAFG0000002009	NAIF1	grey	VSMC_M10	0.13	5.31E-01	0.25	2.17E-01	0.00	9.84E-01	0.48	1.18E-02	-0.12	5.57E-01	0.08	7.03E-01	-0.15	4.54E-01	-0.50	8.24E-03	-0.22	2.70E-01	0.53	4.53E-03
ENSCAFG0000003273	SEC61A2	grey	VSMC_M10	0.13	5.31E-01	-0.17	3.91E-01	-0.41	3.30E-02	0.25	2.15E-01	0.35	7.70E-02	0.08	7.06E-01	0.02	9.25E-01	-0.02	9.15E-01	0.08	6.94E-01	0.08	7.00E-01
ENSCAFG0000001699	USP3	pink	VSMC_M5	0.13	5.32E-01	0.12	5.37E-01	-0.56	2.64E-03	0.40	4.01E-02	0.64	2.99E-04	-0.24	2.34E-01	-0.35	7.19E-02	-0.39	4.44E-02	-0.07	7.41E-01	0.09	6.60E-01

ENSCAFG0000002875	OVCA2	grey	VSMC_M10	0.13	5.32E-01	0.29	1.47E-01	0.26	1.91E-01	-0.17	3.99E-01	-0.20	3.09E-01	0.12	5.66E-01	0.05	8.07E-01	0.04	8.38E-01	-0.31	1.16E-01	0.03	9.01E-01
ENSCAFG0000001326	KLHL34	grey	VSMC_M10	0.13	5.32E-01	0.11	5.97E-01	0.61	7.73E-04	-0.55	2.94E-03	-0.40	3.73E-02	-0.06	7.68E-01	0.02	9.11E-01	0.45	1.80E-02	-0.10	6.22E-01	-0.33	8.86E-02
ENSCAFG0000000327	ELMO1	cyan	VSMC_M2	0.13	5.32E-01	0.75	6.05E-06	0.38	4.93E-02	0.00	9.98E-01	-0.41	3.42E-02	0.06	7.78E-01	-0.19	3.52E-01	-0.24	2.32E-01	-0.72	1.97E-05	0.51	6.77E-03
ENSCAFG0000000589	SIRT7	grey	VSMC_M10	0.13	5.32E-01	0.56	2.46E-03	0.12	5.58E-01	0.30	1.25E-01	-0.10	6.34E-01	-0.20	3.05E-01	-0.08	6.91E-01	-0.46	1.59E-02	-0.52	5.14E-03	0.44	2.05E-02
ENSCAFG0000000068	ENSCAFG0000000068	cyan	VSMC_M2	0.13	5.32E-01	0.82	2.24E-07	0.47	1.24E-02	0.00	9.87E-01	-0.45	1.84E-02	-0.16	4.14E-01	-0.06	7.61E-01	-0.29	1.38E-01	-0.74	9.34E-06	0.50	8.28E-03
ENSCAFG0000001508	PLA2G16	grey	VSMC_M10	0.13	5.33E-01	0.65	2.58E-04	0.14	4.74E-01	0.51	6.06E-03	-0.09	6.56E-01	-0.15	4.51E-01	-0.19	3.53E-01	-0.70	4.27E-05	-0.51	6.68E-03	0.47	1.26E-02
ENSCAFG0000001586	PRXL2A	yellow	VSMC_M3	0.13	5.33E-01	0.49	9.09E-03	0.73	1.31E-05	-0.34	8.74E-02	-0.60	9.23E-04	-0.04	8.47E-01	0.12	5.40E-01	0.04	8.25E-01	-0.45	1.76E-02	0.09	6.44E-01
ENSCAFG0000001797	ENSCAFG0000001797	grey	VSMC_M10	0.13	5.33E-01	0.13	5.17E-01	-0.29	1.42E-01	0.50	8.28E-03	0.31	1.15E-01	-0.06	7.68E-01	-0.17	3.94E-01	-0.49	9.81E-03	-0.06	7.70E-01	0.14	4.72E-01
ENSCAFG0000000209	CASD1	darkgreen	VSMC_M4	0.13	5.33E-01	0.29	1.35E-01	-0.46	1.55E-02	0.72	2.36E-05	0.38	4.99E-02	-0.17	4.01E-01	-0.05	7.99E-01	-0.70	5.06E-05	-0.29	1.41E-01	0.54	3.59E-03
ENSCAFG0000000444	ZNF282	yellow	VSMC_M3	0.13	5.33E-01	0.46	1.55E-02	0.60	1.02E-03	-0.02	9.34E-01	-0.49	9.91E-03	-0.08	7.08E-01	-0.14	4.85E-01	-0.19	3.47E-01	-0.40	3.86E-02	0.17	3.89E-01
ENSCAFG0000002016	VAC14	yellow	VSMC_M3	0.13	5.33E-01	0.27	1.72E-01	0.62	5.41E-04	-0.56	2.46E-03	-0.35	7.12E-02	-0.23	2.46E-01	-0.08	6.74E-01	0.24	2.34E-01	-0.18	3.80E-01	-0.28	1.62E-01
ENSCAFG0000000814	FBXW7	grey	VSMC_M10	0.13	5.34E-01	0.09	6.53E-01	-0.21	2.83E-01	0.54	3.58E-03	0.06	7.55E-01	0.32	1.02E-01	-0.09	6.44E-01	-0.46	1.61E-02	-0.15	4.47E-01	0.43	2.64E-02
ENSCAFG0000000839	DPH6	pink	VSMC_M5	0.13	5.34E-01	-0.28	1.50E-01	-0.52	5.59E-03	0.51	6.32E-03	0.51	6.76E-03	-0.12	5.56E-01	-0.10	6.31E-01	-0.30	1.33E-01	0.24	2.21E-01	0.00	9.85E-01
ENSCAFG0000000042	RMND1	grey	VSMC_M10	0.12	5.35E-01	0.11	5.81E-01	-0.29	1.44E-01	0.54	3.92E-03	0.39	4.43E-02	-0.26	1.82E-01	-0.33	9.15E-02	-0.45	1.79E-02	-0.04	8.32E-01	0.09	6.72E-01
ENSCAFG0000003276	ENSCAFG0000003276	grey	VSMC_M10	0.12	5.35E-01	0.51	6.12E-03	0.35	7.67E-02	-0.03	8.83E-01	-0.38	4.88E-02	0.11	5.68E-01	0.00	9.90E-01	-0.09	6.43E-01	-0.59	1.12E-03	0.36	6.36E-02
ENSCAFG0000000203	FIP1L1	grey	VSMC_M10	0.12	5.35E-01	-0.06	7.78E-01	0.20	3.08E-01	-0.47	1.26E-02	-0.06	7.61E-01	-0.02	9.26E-01	-0.12	5.58E-01	0.50	8.10E-03	-0.01	9.69E-01	-0.34	8.58E-02
ENSCAFG0000000878	ARL2BP	grey	VSMC_M10	0.12	5.36E-01	-0.10	6.11E-01	-0.38	5.09E-02	0.36	6.41E-02	0.30	1.27E-01	0.39	4.29E-02	-0.15	4.54E-01	-0.20	3.23E-01	0.03	8.90E-01	0.10	6.14E-01
ENSCAFG0000000769	GALNT18	cyan	VSMC_M2	0.12	5.36E-01	0.69	6.54E-05	0.48	1.06E-02	-0.06	7.50E-01	-0.45	1.78E-02	-0.13	5.25E-01	-0.20	3.28E-01	-0.23	2.44E-01	-0.61	7.93E-04	0.42	2.96E-02
ENSCAFG0000001756	RAD51C	grey	VSMC_M10	0.12	5.36E-01	-0.22	2.65E-01	-0.01	9.59E-01	-0.12	5.42E-01	0.09	6.53E-01	-0.09	6.50E-01	0.02	9.02E-01	0.18	3.71E-01	0.16	4.36E-01	-0.22	2.63E-01
ENSCAFG0000000624	GNPDA1	yellow	VSMC_M3	0.12	5.36E-01	0.48	1.12E-02	0.63	4.73E-04	-0.43	2.45E-02	-0.57	2.06E-03	0.21	2.97E-01	0.28	1.60E-01	0.19	3.54E-01	-0.54	3.30E-03	0.17	4.02E-01
ENSCAFG0000000109	TEF	cyan	VSMC_M2	0.12	5.36E-01	0.74	8.29E-06	0.22	2.81E-01	0.32	1.08E-01	-0.17	3.85E-01	-0.26	1.95E-01	-0.35	6.98E-02	-0.58	1.66E-03	-0.60	1.06E-03	0.53	4.82E-03
ENSCAFG0000000709	ENSCAFG0000000709	grey	VSMC_M10	0.12	5.36E-01	0.43	2.40E-02	-0.01	9.44E-01	0.25	2.16E-01	0.05	8.16E-01	-0.07	7.11E-01	-0.18	3.65E-01	-0.33	9.28E-02	-0.36	6.29E-02	0.33	9.10E-02
ENSCAFG0000000835	SFRP2	cyan	VSMC_M2	0.12	5.37E-01	0.66	2.08E-04	-0.09	6.55E-01	0.62	5.04E-04	-0.02	9.02E-01	-0.22	2.77E-01	-0.36	6.72E-02	-0.66	1.86E-04	-0.63	4.26E-04	0.68	8.95E-05
ENSCAFG0000003193	ENSCAFG0000003193	grey	VSMC_M10	0.12	5.37E-01	-0.18	3.82E-01	0.02	9.20E-01	-0.19	3.51E-01	-0.09	6.59E-01	0.17	4.08E-01	0.57	1.71E-03	0.30	1.31E-01	-0.08	6.89E-01	0.07	7.35E-01
ENSCAFG0000001824	SNRPD1	darkgrey	VSMC_M8	0.12	5.37E-01	-0.35	7.18E-02	0.39	4.36E-02	-0.73	1.78E-05	-0.21	3.04E-01	-0.03	8.95E-01	0.39	4.35E-02	0.73	1.82E-05	0.18	3.72E-01	-0.56	2.44E-03
ENSCAFG0000001195	ENSCAFG0000001195	darkgrey	VSMC_M8	0.12	5.37E-01	-0.69	6.64E-05	-0.07	7.27E-01	-0.48	1.18E-02	0.34	8.12E-02	-0.17	4.05E-01	0.36	6.89E-02	0.65	2.41E-04	0.51	6.06E-03	-0.84	5.50E-08
ENSCAFG0000000445	ABHD5	grey	VSMC_M10	0.12	5.37E-01	-0.30	1.24E-01	-0.08	7.06E-01	-0.41	3.26E-02	0.33	9.10E-02	-0.03	8.66E-01	-0.10	6.13E-01	0.35	7.04E-02	0.31	1.19E-01	-0.56	2.41E-03
ENSCAFG0000000306	MTURN	grey	VSMC_M10	0.12	5.37E-01	0.60	9.56E-04	0.34	8.17E-02	0.23	2.58E-01	-0.40	4.10E-02	-0.08	6.99E-01	0.15	4.46E-01	-0.43	2.67E-02	-0.60	9.14E-04	0.65	2.32E-04
ENSCAFG0000001013	APLP2	grey	VSMC_M10	0.12	5.37E-01	0.48	1.22E-02	0.09	6.44E-01	0.44	2.13E-02	-0.10	6.05E-01	-0.20	3.20E-01	-0.08	6.93E-01	-0.47	1.30E-02	-0.43	2.41E-02	0.42	2.96E-02
ENSCAFG0000000272	ASB3	grey	VSMC_M10	0.12	5.37E-01	0.02	9.26E-01	0.07	7.23E-01	0.03	8.63E-01	-0.07	7.46E-01	-0.09	6.49E-01	0.19	3.51E-01	-0.05	7.93E-01	-0.04	8.59E-01	0.12	5.67E-01
ENSCAFG0000001396	ENSCAFG0000001396	grey	VSMC_M10	0.12	5.38E-01	-0.36	6.79E-02	-0.17	4.03E-01	-0.15	4.52E-01	0.27	1.72E-01	-0.05	7.87E-01	0.09	6.70E-01	0.22	2.78E-01	0.32	9.85E-02	-0.31	1.17E-01
ENSCAFG0000001776	TPGS2	grey	VSMC_M10	0.12	5.38E-01	0.07	7.22E-01	0.24	2.29E-01	-0.13	5.25E-01	-0.06	7.64E-01	-0.06	7.52E-01	-0.29	1.39E-01	0.00	9.90E-01	0.06	7.73E-01	-0.20	3.13E-01
ENSCAFG0000000496	XRCC2	grey	VSMC_M10	0.12	5.38E-01	-0.07	7.16E-01	0.03	8.68E-01	-0.32	1.05E-01	0.08	7.05E-01	-0.03	8.79E-01	0.31	1.11E-01	0.30	1.30E-01	-0.06	7.54E-01	-0.21	3.04E-01
ENSCAFG0000001448	BAD	yellow	VSMC_M3	0.12	5.38E-01	0.20	3.12E-01	0.57	1.91E-03	-0.35	7.77E-02	-0.50	8.63E-03	-0.09	6.45E-01	0.20	3.17E-01	0.29	1.38E-01	-0.28	1.52E-01	0.00	9.95E-01
ENSCAFG0000000016	DMXL1	pink	VSMC_M5	0.12	5.38E-01	-0.25	2.01E-01	-0.72	2.19E-05	0.56	2.18E-03	0.72	2.70E-05	0.04	8.50E-01	-0.01	9.59E-01	-0.35	7.65E-02	0.18	3.72E-01	0.05	7.91E-01
ENSCAFG0000002409	FAT3	darkgreen	VSMC_M4	0.12	5.38E-01	0.28	1.65E-01	-0.32	1.08E-01	0.65	2.39E-04	0.27	1.71E-01	-0.18	3.62E-01	-0.22	2.76E-01	-0.58	1.62E-03	-0.24	2.20E-01	0.42	3.01E-02
ENSCAFG0000000245	ENSCAFG0000000245	grey	VSMC_M10	0.12	5.39E-01	-0.20	3.21E-01	0.08	7.08E-01	-0.34	8.45E-02	0.11	5.74E-01	-0.18	3.60E-01	0.15	4.64E-01	0.29	1.49E-01	0.17	4.08E-01	-0.32	1.09E-01
ENSCAFG0000000668	SDHAF1	yellow	VSMC_M3	0.12	5.39E-01	0.42	3.06E-02	0.71	3.35E-05	-0.34	8.09E-02	-0.65	2.21E-04	-0.04	8.35E-01	-0.01	9.48E-01	0.08	6.89E-01	-0.41	3.47E-02	0.13	5.05E-01
ENSCAFG0000001870	ACSBG2	grey	VSMC_M10	0.12	5.39E-01	0.21	2.91E-01	0.13	5.25E-01	-0.09	6.40E-01	-0.14	4.77E-01	-0.08	6.86E-01	0.02	9.11E-01	-0.03	8.63E-01	-0.20	3.26E-01	0.16	4.29E-01
ENSCAFG0000003062	MYCBP	grey	VSMC_M10	0.12	5.39E-01	0.04	8.51E-01	-0.37	6.03E-02	0.27	1.78E-01	0.33	8.99E-02	0.28	1.63E-01	0.15	4.63E-01	-0.17	3.95E-01	-0.13	5.22E-01	0.16	4.40E-01
ENSCAFG0000003002	ENSCAFG0000003002	grey	VSMC_M10	0.12	5.39E-01	-0.04	8.44E-01	0.00	9.83E-01	-0.18	3.56E-01	0.20	3.29E-01	-0.19	3.51E-01	0.10	6.24E-01	0.18	3.59E-01	-0.02	9.17E-01	-0.32	9.91E-02
ENSCAFG0000001961	CIAO3	grey	VSMC_M10	0.12	5.40E-01	0.06	7.51E-01	0.54	3.43E-03	-0.41	3.23E-02	-0.40	4.14E-02	0.05	8.16E-01	0.21	3.05E-01	0.29	1.42E-01	-0.13	5.04E-01	-0.22	2.73E-01
ENSCAFG0000001574	ENSCAFG0000001574	cyan	VSMC_M2	0.12	5.40E-01	0.62	5.17E-04	0.37	5.47E-02	0.28	1.65E-01	-0.38	4.84E-02	-0.04	8.38E-01	0.02	9.33E-01	-0.48	1.21E-02	-0.57	1.94E-03	0.53	4.73E-03
ENSCAFG0000001069	ADD3	grey	VSMC_M10	0.12	5.40E-01	0.31	1.18E-01	-0.08	6.98E-01	0.16	4.17E-01	0.04	8.51E-01	0.02	9.40E-01	0.47	1.35E-02	-0.17	3.92E-01	-0.40	3.79E-02	0.34	8.53E-02
ENSCAFG0000000038	GRIP1	darkgreen	VSMC_M4	0.12	5.40E-01	0.51	6.38E-03	-0.31	1.11E-01	0.75	6.08E-06	0.16	4.35E-01	-0.15	4.63E-01	-0.40	3.98E-02	-0.68	9.35E-05	-0.49	9.18E-03	0.70	4.83E-05
ENSCAFG0000000652	RASSF7	grey	VSMC_M10	0.12	5.40E-01	0.20	3.12E-01	0.08	6.87E-01	0.09	6.62E-01	-0.11	5.76E-01	0.25	2.00E-01	0.12	5.59E-01	-0.12	5.65E-01	-0.25	2.11E-01	0.22	2.73E-01
ENSCAFG0000001721	SH2B1	grey	VSMC_M10	0.12	5.41E-01	0.53	4.36E-03	0.16	4.14E-01	0.38	5.14E-02	-0.19	3.48E-01	-0.14	4.74E-01	-0.18	3.75E-01	-0.48	1.11E-02	-0.48	1.16E-02	0.53	4.06E-03
ENSCAFG0000002402	ENSCAFG0000002402	yellow	VSMC_M3	0.12	5.41E-01	0.25	2.01E-01	0.71	3.63E-05	-0.54	3.57E-03	-0.68	1.12E-04	0.15	4.53E-01	0.05	8.22E-01	0.34	8.55E-02	-0.27	1.70E-01	0.04	8.28E-01
ENSCAFG0000001197	PARP14	grey	VSMC_M10	0.12	5.41E-01	0.75	8.01E-06	0.15	4.41E-01	0.46	1.53E-02	-0.31	1.17E-01	0.10	6.23E-01	-0.12	5.61E-01	-0.60	8.36E-04	-0.76	5.23E-06	0.83	1.04E-07
ENSCAFG0000000516	ENSCAFG0000000516	grey	VSMC_M10	0.12	5.41E-01	0.04	8.58E-01	0.10	6.17E-01	-0.08	7.01E-01	-0.04	8.24E-01	-0.06	7.72E-01	-0.17	3.86E-01	0.10	6.33E-01	-0.07	7.20E-01	-0.06	7.58E-01
ENSCAFG0000000032	EPM2A	grey	VSMC_M10	0.12	5.41E-01	0.20	3.07E-01	-0.26	1.82E-01	0.42	3.04E-02	0.44	2.28E-02	-0.15	4.49E-01	-0.36	6.12E-02	-0.47	1.30E-02	-0.11	5.74E-01	0.00	9.89E-01
ENSCAFG0000001471	PIANP	cyan	VSMC_M2	0.12	5.42E-01	0.45	1.77E-02	0.22	2.80E-01	0.32	1.02E-01	-0.31	1.10E-01	0.21	2.99E-01	-0.23	2.55E-01	-0.37	5.46E-02	-0.46	1.49E-02	0.52	5.94E-03
ENSCAFG0000000513	RPL14	grey	VSMC_M10	0.12	5.42E-01	-0.07	7.27E-01	0.27	1.79E-01	-0.17	3.83E-01	-0.12	5.42E-01	-0.22	2.73E-01	0.16	4.13E-01	0.17	3.86E-01	0.02	9.19E-01	-0.30	1.27E-01
ENSCAFG0000000276	IGF2BP3	grey	VSMC_M10	0.12	5.42E-01	0.21	2.91E-01	-0.08	7.03E-01	0.19	3.54E-01	0.03	8.94E-01	0.05	7.86E-01	-0.13	5.16E-01	-0.14	4.93E-01	-0.18	3.60E-01	0.30	1.24E-01
ENSCAFG0000000869	RHEBL1	grey	VSMC_M10	0.12	5.43E-01	-0.03	8.88E-01	0.18	3.68E-01	-0.42	2.83E-02	-0.05	8.20E-01	-0.04	8.26E-01	0.33	9.44E-02	0.27	1.80E-01	-0.06	7.65E-01	-0.24	2.23E-01
ENSCAFG0000001380	ENSCAFG0000001380	grey	VSMC_M10	0.12	5.43E-01	0.20	3.07E-01	-0.06	7.73E-01	0.15	4.51E-01	0.20	3.14E-01	-0.19	3.49E-01	-0.20	3.13E-01	-0.21	3.00E-01	-0.17	3.99E-01	-0.07	7.40E-01
ENSCAFG0000001031	ENSCAFG0000001031	grey	VSMC_M10	0.12	5.43E-01	0.03	8.84E-01	0.24	2.18E-01	0.06	7.84E-01	-0.15	4.49E-01	-0.18	3.63E-01	0.23	2.41E-01	-0.03	8.69E-01	-0.07	7.43E-01	-0.04	8.31E-01
ENSCAFG0000002397	HACD1	pink	VSMC_M5	0.12	5.43E-01	-0.28	1.52E-01	-0.70	5.32E-05	0.57	1.99E-03	0.72	2.00E-05	0.01	9.42E-01	-0.31	1.14E-01	-0.36	6.47E-02	0.29	1.39E-01	-0.05	7.94E-01
ENSCAFG0000001529	YDJC	grey	VSMC_M10	0.12	5.43E-01	0.41	3.24E-02	-0.11	5.92E-01	0.30	1.25E-01	0.04	8.54E-01	-0.15	4.47E-01	-0.09	6.62E-01	-0.37	6.03E-02	-0.40	3.66E-02	0.49	8.82E-03
ENSCAFG0000001305	ZGPAT	yellow	VSMC_M3	0.12	5.43E-01	0.50	7.51E-03	0.55	3.05E-03	0.08	6.83E-01	-0.50	8.20E-03	0.10	6.26E-01	-0.29	1.49E-01	-0.23	2.46E-01	-0.44	2.13E-02	0.26	1.90E-01
ENSCAFG0000001087	SULF2	grey	VSMC_M10	0.12	5.43E-01	0.66	1.88E-04	0.26	1.98E-01	0.18	3.75E-01	-0.35	7.55E-02	0.16	4.36E-01	-0.04	8.30E-01	-0.39	4.43E-02	-0.69	6.53E-05	0.59	1.19E-03
ENSCAFG0000001543	ABHD8	grey	VSMC_M10	0.12	5.43E-01	0.34	7.80E-02	0.65	2.76E-04	-0.29	1.48E-01	-0.47	1.24E-02	-0.07	7.42E-01	-0.25	2.17E-01	0.12	5.61E-01	-0.32	1.09E-01	-0.11	5.86E-01
ENSCAFG0000001576	SPAG7	grey	VSMC_M10	0.12	5.43E-01	0.05	8.17E-01	0.48	1.18E-02	-0.46	1.65E-02	-0.28	1.54E-01	-0.14	4.96E-01	0.05	8.10E-01	0.29	1.45E-01	-0.01	9.47E-01	-0.37	5.77E-02
ENSCAFG0000001708	ENSCAFG0000001708	grey	VSMC_M10	0.12	5.43E-01	0.16	4.24E-01	0.24	2.21E-01	-0.07	7.47E-01	-0.30	1.26E-01	-0.01	9.67E-01	-0.07	7.43E-01	0.04	8.57E-01	-0.24	2.23E-01	0.29	1.49E-01
ENSCAFG0000000852	GUCY1A1	grey	VSMC_M10	0.12	5.43E-01	0.14	5.00E-01	0.44	2.32E-02	-0.19	3.36E-01	-0.36	6.31E-02	0.28	1.52E-01	0.65	2.72E-04	0.08	6.86E-01	-0.20	3.12E-01	0.06	7.52E-01
ENSCAFG0000003011	DDIT3	grey	VSMC_M10	0.12	5.43E-01	0.10	6.15E-01	0.04	8.26E-01	0.17	3.95E-01	-0.07	7.25E-01	-0.03	8.91E-01	-0.01	9.69E-01	-0.27	1.73E-01	-0.15	4.59E-01	0.29	1.47E-01
ENSCAFG0000001577	HSH2D	grey	VSMC_M10	0.12	5.44E-01	0.18	3.74E-01	-0.01	9.56E-01	0.19	3.49E-01	0.04	8.33E-01	-0.09	6.41E-01	-0.19	3.47E-01	-0.17	3.96E-01	-0.18	3.81E-01	0.12	5.46E-01
ENSCAFG0000000778	MYH7B	grey	VSMC_M10	0.12	5.44E-01	0.11	5.75E-01	0.29	1.39E-01	-0.08	6.78E-01	-0.14	4.91E-01	0.00	9.95E-01	0.06	7.52E-01	0.02	9.03E-01	-0.08	6.81E-01	-0.09	6.68E-01
ENSCAFG0000001774	AP1M2	grey	VSMC_M10	0.12	5.44E-01	0.41	3.48E-02	0.02	9.34E-01	0.14	4.88E-01	-0.07	7.19E-01	0.28	1.64E-01	-0.19	3.38E-01	-0.28	1.62E-01	-0.39	4.71E-02	0.35	7.63E-02
ENSCAFG0000003214	BAALC	grey	VSMC_M10	0.12	5.45E-01	0.15	4.47E-01	-0.34	8.31E-02	0.51	6.57E-03	0.17	4.04E-01	0.27	1.70E-01	0.11	5.74E-01	-0.40	4.14E-02	-0.22	2.75E-01	0.53	4.61E-03
ENSCAFG0000001520	ENSCAFG0000001520	grey	VSMC_M10	0.12	5.45E-01	0.52	5.49E-03	0.03	8.91E-01	0.54	3.55E-03	-0.15	4.58E-01	-0.09	6.56E-01	-0.34	8.55E-02	-0.54	4.00E-03	-0.51	6.70E-03	0.60	1.01E-03
ENSCAFG0000002033	LCAT	cyan	VSMC_M2	0.12	5.45E-01	0.60	9.29E-04	-0.28	1.62E-01	0.81	3.23E-07	0.17	3.96E-01	-0.05	7.91E-01	-0.49	9.96E-03	-0.88	2.07E-09	-0.48	1.07E-02	0.68	8.34E-05
ENSCAFG0000000521	IL17RC	cyan	VSMC_M2	0.12	5.45E-01	0.66	1.78E-04	0.57	1.72E-03	-0.05	8.01E-01	-0.51	6.24E-03	0.09	6.44E-01	-0.27	1.66E-01	-0.26	1.88E-01	-0.57	1.88E-03	0.33	8.87E-02
ENSCAFG0000000541	RBBP9	grey	VSMC_M10	0.12	5.45E-01	-0.07	7.41E-01	-0.09	6.44E-01	0.20	3.12E-01	0.03	8.85E-01	-0.01	9.75E-01	0.09	6.46E-01	-0.01	9.60E-01	-0.03	8.96E-01	0.11	6.00E-01
ENSCAFG0000000091	GDF9	grey	VSMC_M10	0.12	5.46E-01	0.35	7.24E-02	0.24	2.37E-01	0.19	3.33E-01	-0.24	2.23E-01	-0.01	9.58E-01	0.18	3.67E-01	-0.27	1.74E-01	-0.38	5.13E-02	0.35	7.15E-02
ENSCAFG0000001320	HEBP1	darkgreen	VSMC_M4	0.12	5.46E-01	0.34	8.15E-02	-0.51	6.55E-03	0.84	5.34E-08	0.49	1.02E-02	-0.15	4.56E-01	-0.24	2.34E-01	-0.81	2.29E-07	-0.26	1.86E-01	0.44	2.15E-02
ENSCAFG0000001533	MVB12A	yellow	VSMC_M3	0.12	5.46E-01	0.32	1.00E-01	0.62	5.21E-04	-0.34	8.33E-02	-0.42	2.87E-02	-0.17	3.90E-01	0.01	9.74E-01	0.11	5.70E-01	-0.23	2.49E-01	-0.13	5.28E-01
ENSCAFG0000001477	ELL	grey	VSMC_M10	0.12	5.46E-01	0.34	8.02E-02	0.36	6.28E-02	-0.12	5.40E-01	-0.45	1.95E-02	-0.02	9.27E-01	0.01	9.70E-01	0.00	9.81E-01	-0.40	3.98E-02	0.37	5.77E-02
ENSCAFG0000003274	UBL3	grey	VSMC_M10	0.12	5.46E-01	-0.54	3.74E-03	-0.65	2.36E-04	0.21	2.88E-01	0.71	2.84E-05	0.07	7.22E-01	0.24	2.28E-01	0.03	8.77E-01	0.44	2.11E-02	-0.26	1.83E-01
ENSCAFG0000000228	GCLC	pink	VSMC_M5	0.12	5.46E-01	-0.19	3.31E-01	-0.37	6.04E-02	0.11	5.89E-01	0.48	1.05E-02	-0.16	4.37E-01	-0.17	4.09E-01	0.00	9.94E-01	0.18	3.65E-01	-0.25	2.00E-01
ENSCAFG0000001968	RERE	cyan	VSMC_M2	0.12	5.46E-01	0.78	1.59E-06	0.09	6.61E-01	0.44	2.02E-02	-0.09	6.63E-01	-0.19	3.54E-01	-0.57	1.87E-03	-0.69	7.05E-05	-0.59	1.35E-03	0.58	1.48E-03
ENSCAFG0000001066	XPNPEP1	grey	VSMC_M10	0.12	5.46E-01	0.43	2.70E-02	0.11	5.91E-01	0.35	7.28E-02	-0.08	6.86E-01	-0.21	2.99E-01	-0.32	1.05E-01	-0.38	5.03E-02	-0.39	4.35E-02	0.29	1.39E-01
ENSCAFG0000000962	ENSCAFG0000000962	grey	VSMC_M10	0.12	5.47E-01	0.20	3.17E-01	-0.21	3.03E-01	0.25	2.12E-01	0.22	2.65E-01	-0.18	3.68E-01	-0.26	1.88E-01	-0.18	3.78E-01	-0.24	2.21E-01	0.16	4.32E-01
ENSCAFG0000002486	PTRHD1	grey	VSMC_M10	0.12	5.47E-01	0.44	2.24E-02	0.32	1.04E-01	0.07	7.47E-01	-0.41	3.23E-02	0.17	4.02E-01	-0.11	5.81E-01	-0.19	3.54E-01	-0.42	3.01E-02	0.48	1.13E-02
ENSCAFG0000001571	UBXN1	yellow	VSMC_M3	0.12	5.47E-01	0.41	3.23E-02	0.75	6.02E-06	-0.34	8.21E-02	-0.59	1.23E-03	-0.19	3.50E-01	0.05	8.04E-01	0.09	6.43E-01	-0.38	5.22E-02	0.01	9.52E-01
ENSCAFG0000002880	MRPL34	yellow	VSMC_M3	0.12	5.47E-01	0.50	8.04E-03	0.70	5.47E-05	-0.18	3.61E-01	-0.63	4.38E-04	0.06	7.77E-01	-0.19	3.55E-01	0.00	9.82E-01	-0.49	9.45E-03	0.18	3.63E-01
ENSCAFG0000002020	KYAT3	grey	VSMC_M10	0.12	5.48E-01	-0.35	7.68E-02	0.00	9.96E-01	-0.26	1.84E-01	0.13	5.08E-01	0.05	7.99E-01	0.04	8.47E-01	0.32	1.02E-01	0.32	1.08E-01	-0.44	2.01E-02
ENSCAFG0000000123	PTP4A3	grey	VSMC_M10	0.12	5.48E-01	0.45	1.80E-02	0.52	5.46E-03	-0.19	3.39E-01	-0.57	1.83E-03	0.13	5.34E-01	0.26	1.93E-01	-0.05	8.12E-01	-0.46	1.59E-02	0.40	3.77E-02
ENSCAFG0000002032	DUS2	grey	VSMC_M10	0.12	5.48E-01	-0.04	8.59E-01	-0.01	9.49E-01	0.08	7.07E-01	0.20	3.26E-01	-0.08	6.76E-01	-0.24	2.23E-01	-0.05	8.03E-01	0.10	6.36E-01	-0.24	2.29E-01
ENSCAFG0000000946	MARCH7	grey	VSMC_M10	0.12	5.49E-01	-0.23	2.52E-01	-0.26	1.85E-01	-0.26	1.99E-01	0.40	3.88E-02	0.01	9.51E-01	-0.27	1.75E-01	0.32	1.01E-01	0.19	3.34E-01	-0.40	3.99E-02
ENSCAFG0000002443	ARGLU1	grey	VSMC_M10	0.12	5.49E-01	0.11	5.99E-01	-0.04	8.54E-01	0.04	8.47E-01	0.05	8.17E-01	0.06	7.56E-01	-0.10	6.06E-01	0.04	8.37E-01	-0.25	2.17E-01	0.07	7.37E-01
ENSCAFG0000001885	TARS	darkolivegreen	VSMC_M9	0.12	5.49E-01	-0.69	6.52E-05	-0.23	2.39E-01	-0.27	1.67E-01	0.40	3.82E-02	-0.06	7.71E-01	0.05	8.08E-01	0.52	5.53E-03	0.49	1.03E-02	-0.66	1.93E-04
ENSCAFG0000001682	TMEM8B	grey	VSMC_M10	0.12	5.49E-01	0.25	2.05E-01	0.28	1.54E-01	0.15	4.69E-01	-0.38	5.16E-02	0.20	3.10E-01	-0.23	2.57E-01	-0.15	4.55E-01	-0.26	1.86E-01	0.37	5.88E-02
ENSCAFG0000000843	PAG1	grey	VSMC_M10	0.12	5.49E-01	0.29	1.42E-01	-0.12	5.67E-01	0.42	3.06E-02	-0.04	8.48E-01	0.17	3.96E-01	-0.07	7.36E-01	-0.38	4.96E-02	-0.37	6.11E-02	0.55	2.89E-03
ENSCAFG0000001083	TRIP13	darkgrey	VSMC_M8	0.12	5.49E-01	-0.52	5.61E-03	0.21	2.88E-01	-0.80	6.79E-07	-0.06	7.79E-01	0.16	4.24E-01	0.48	1.11E-02	0.91	5.42E-11	0.29	1.40E-01	-0.66	2.05E-04
ENSCAFG0000001295	FANK1	turquoise	VSMC_M6	0.12	5.50E-01	-0.24	2.34E-01	-0.14	4.78E-01	-0.04	8.49E-01	0.11	5.91E-01	0.39	4.44E-02	0.24	2.23E-01	0.11	5.74E-01	0.08	6.91E-01	-0.02	9.17E-01
ENSCAFG0000000885	HECTD4	darkgreen	VSMC_M4	0.12	5.50E-01	0.22	2.59E-01	-0.61	7.23E-04	0.85	2.06E-08	0.58	1.41E-03	-0.18	3.79E-01	-0.35	7.78E-02	-0.81	3.08E-07	-0.14	4.73E-01	0.41	3.26E-02
ENSCAFG0000000921	TBX3	darkgrey	VSMC_M8	0.12	5.50E-01	-0.31	1.10E-01	0.10	6.35E-01	-0.52	5.93E-03	-0.03	8.66E-01	0.21	2.99E-01	0.33	9.50E-02	0.58	1.66E-03	0.14	4.93E-01	-0.31	1.11E-01
ENSCAFG0000000260	ENSCAFG0000000260	grey	VSMC_M10	0.12	5.50E-01	-0.01	9.74E-01	-0.05	8.19E-01	-0.12	5.48E-01	0.17	3.86E-01	-0.17	4.10E-01	-0.09	6.61E-01	0.04	8.53E-01	-0.04	8.26E-01	-0.24	2.27E-01
ENSCAFG0000000333	NR1H2	grey	VSMC_M10	0.12	5.50E-01	0.77	2.13E-06	0.14	4.90E-01	0.60	8.29E-04	-0.20	3.24E-01	-0.18	3.74E-01	-0.26	1.85E-01	-0.85	1.91E-08	-0.61	7.78E-04	0.75	5.37E-06
ENSCAFG0000001296	HELZ2	cyan	VSMC_M2	0.12	5.50E-01	0.70	5.45E-05	0.56	2.63E-03	-0.01	9.45E-01	-0.62	6.03E-04	0.32	1.08E-01	-0.28	1.52E-01	-0.25	2.08E-01	-0.66	1.69E-04	0.53	4.09E-03
ENSCAFG0000000471	NIFK	darkgrey	VSMC_M8	0.12	5.50E-01	-0.60	1.04E-03	0.09	6.57E-01	-0.63	4.24E-04	0.11	5.71E-01	0.12	5.48E-01	-0.04	8.44E-01	0.77	2.11E-06	0.46	1.70E-02	-0.79	8.01E-07
ENSCAFG0000000318	RHBDL2	grey	VSMC_M10	0.12	5.51E-01	0.29	1.48E-01	-0.09	6.48E-01	0.52	5.08E-03	0.06	7.76E-01	-0.29	1.43E-01	-0.29	1.41E-01	-0.53	4.20E-03	-0.21	2.84E-01	0.45	1.88E-02
ENSCAFG0000000768	MMAA	grey	VSMC_M10	0.12	5.51E-01	-0.07	7.26E-01	0.24	2.26E-01	-0.38	5.12E-02	0.02	9.21E-01	-0.15	4.69E-01	0.27	1.67E-01	0.28	1.59E-01	0.03	8.84E-01	-0.45	1.85E-02
ENSCAFG0000000532	CFAP57	grey	VSMC_M10	0.12	5.51E-01	-0.02	9.31E-01	0.15	4.45E-01	-0.19	3.42E-01	-0.09	6.66E-01	-0.03	8.87E-01	0.13	5.07E-01	0.22	2.80E-01	-0.07	7.21E-01	-0.10	6.37E-01
ENSCAFG0000001924	MIB2	grey	VSMC_M10	0.12	5.51E-01	0.41	3.45E-02	0.35	7.37E-02	-0.05	7.95E-01	-0.32	1.01E-01	-0.12	5.50E-01	0.01	9.53E-01	-0.09	6.69E-01	-0.49	9.22E-03	0.28	1.62E-01
ENSCAFG0000000432	WDR33	darkgrey	VSMC_M8	0.12	5.51E-01	-0.33	8.93E-02	0.19	3.53E-01	-0.59	1.25E-03	-0.07	7.43E-01	0.32	1.02E-01	0.07	7.29E-01	0.65	2.26E-04	0.18	3.78E-01	-0.52	5.05E-03
ENSCAFG0000001365	EIF4A2	darkgreen	VSMC_M4	0.12	5.52E-01	0.22	2.71E-01	-0.41	3.49E-02	0.77	2.94E-06	0.36	6.30E-02	-0.29	1.40E-01	-0.19	3.33E-01	-0.73	1.85E-05	-0.22	2.77E-01	0.45	1.88E-02
ENSCAFG0000001690	CNTROB	grey	VSMC_M10	0.12	5.52E-01	-0.30	1.29E-01	-0.10	6.05E-01	0.00	9.87E-01	0.22	2.61E-01	-0.04	8.61E-01	0.28	1.50E-01	0.14	4.82E-01	0.15	4.42E-01	-0.25	2.07E-01
ENSCAFG0000003015	PPM1E	darkgreen	VSMC_M4	0.12	5.53E-01	0.30	1.26E-01	-0.31	1.14E-01	0.59	1.08E-03	0.23	2.54E-01	-0.18	3.70E-01	-0.14	4.72E-01	-0.52	5.05E-03	-0.29	1.39E-01	0.47	1.27E-02
ENSCAFG0000001402	TYSND1	grey	VSMC_M10	0.12	5.53E-01	-0.07	7.31E-01	0.29	1.36E-01	-0.42	2.82E-02	-0.15	4.57E-01	-0.08	7.09E-01	-0.21	2.93E-01	0.35	7.49E-02	0.07	7.30E-01	-0.27	1.69E-01
ENSCAFG0000001368	RFC4	darkgrey	VSMC_M8	0.12	5.54E-01	-0.39	4.52E-02	0.47	1.25E-02	-0.83	1.02E-07	-0.34	8.44E-02	0.13	5.29E-01	0.55	2.94E-03	0.89	5.74E-10	0.15	4.47E-01	-0.55	3.07E-03
ENSCAFG0000001643	MRM2	grey	VSMC_M10	0.12	5.54E-01	0.35	7.06E-02	0.54	3.71E-03	-0.29	1.45E-01	-0.59	1.21E-03	0.35	7.28E-02	0.08	6.98E-01	0.12	5.51E-01	-0.44	2.07E-02	0.27	1.74E-01
ENSCAFG0000001542	ZNF503	cyan	VSMC_M2	0.12	5.54E-01	0.63	4.01E-04	0.44	2.07E-02	-0.08	7.07E-01	-0.36	6.34E-02	-0.13	5.12E-01	-0.50	8.33E-03	-0.16	4.23E-01	-0.52	4.94E-03	0.25	2.16E-01
ENSCAFG0000002995	PKIB	turquoise	VSMC_M6	0.12	5.54E-01	-0.06	7.55E-01	0.06	7.77E-01	-0.20	3.28E-01	-0.02	9.11E-01	0.56	2.38E-03	-0.06	7.48E-01	0.23	2.44E-01	0.00	9.98E-01	-0.07	7.27E-01
ENSCAFG0000000363	FLT3LG	cyan	VSMC_M2	0.12	5.54E-01	0.77	2.71E-06	0.40	3.98E-02	0.21	2.99E-01	-0.43	2.59E-02	-0.13	5.30E-01	0.09	6.58E-01	-0.46	1.47E-02	-0.69	7.07E-05	0.66	1.90E-04

ENSCAFG0000001210	RAB22A	grey	VSMC_M10	0.12	5.54E-01	-0.06	7.78E-01	0.10	6.07E-01	-0.38	5.04E-02	0.17	3.89E-01	-0.17	3.97E-01	-0.13	5.27E-01	0.21	2.85E-01	0.10	6.21E-01	-0.49	9.56E-03
ENSCAFG0000000087	BRD2	grey	VSMC_M10	0.12	5.55E-01	-0.60	1.02E-03	-0.59	1.26E-03	0.03	8.95E-01	0.73	1.64E-05	-0.15	4.71E-01	0.08	6.92E-01	0.17	3.92E-01	0.47	1.25E-02	-0.46	1.63E-02
ENSCAFG0000000665	ALKBH3	grey	VSMC_M10	0.12	5.55E-01	0.28	1.54E-01	0.69	6.60E-05	-0.47	1.36E-02	-0.54	3.95E-03	-0.04	8.35E-01	0.00	9.99E-01	0.26	1.90E-01	-0.23	2.42E-01	-0.12	5.35E-01
ENSCAFG0000000301	ENSCAFG0000000301	pink	VSMC_M5	0.12	5.55E-01	-0.38	5.04E-02	-0.35	7.58E-02	0.15	4.52E-01	0.56	2.21E-03	-0.33	9.18E-02	-0.31	1.11E-01	-0.05	7.93E-01	0.48	1.23E-02	-0.47	1.37E-02
ENSCAFG0000000678	ENSCAFG0000000678	yellow	VSMC_M3	0.12	5.55E-01	0.01	9.54E-01	0.68	1.01E-04	-0.65	2.46E-04	-0.53	4.50E-03	0.02	9.15E-01	0.32	1.00E-01	0.56	2.16E-03	-0.14	4.74E-01	-0.24	2.23E-01
ENSCAFG0000000932	WDR75	darkolivegreen	VSMC_M9	0.12	5.55E-01	-0.73	1.77E-05	-0.51	6.94E-03	-0.19	3.54E-01	0.69	6.48E-05	-0.16	4.25E-01	0.15	4.51E-01	0.45	1.78E-02	0.56	2.52E-03	-0.67	1.29E-04
ENSCAFG0000000992	GMPR	grey	VSMC_M10	0.12	5.55E-01	0.32	9.97E-02	0.15	4.49E-01	0.27	1.80E-01	-0.21	2.84E-01	0.10	6.09E-01	-0.05	7.95E-01	-0.33	9.04E-02	-0.30	1.28E-01	0.39	4.29E-02
ENSCAFG0000002004	SPTAN1	darkgreen	VSMC_M4	0.12	5.56E-01	0.62	5.29E-04	-0.24	2.28E-01	0.87	2.87E-09	0.13	5.16E-01	-0.10	6.10E-01	-0.38	4.81E-02	-0.91	3.54E-11	-0.51	6.60E-03	0.74	1.16E-05
ENSCAFG0000000479	ARMH1	cyan	VSMC_M2	0.12	5.56E-01	0.67	1.46E-04	0.42	3.03E-02	0.05	8.17E-01	-0.45	1.95E-02	-0.07	7.14E-01	0.15	4.41E-01	-0.29	1.50E-01	-0.65	2.37E-04	0.51	6.65E-03
ENSCAFG0000003017	RFK	darkgreen	VSMC_M4	0.12	5.56E-01	0.19	3.39E-01	-0.50	7.56E-03	0.76	4.30E-06	0.52	4.93E-03	-0.18	3.61E-01	-0.42	2.97E-02	-0.66	1.80E-04	-0.12	5.46E-01	0.24	2.22E-01
ENSCAFG0000001675	TARDBP	grey	VSMC_M10	0.12	5.57E-01	0.05	7.94E-01	0.19	3.43E-01	-0.12	5.50E-01	-0.29	1.41E-01	0.33	9.33E-02	0.44	2.05E-02	0.17	3.83E-01	-0.29	1.50E-01	0.27	1.73E-01
ENSCAFG0000000966	PIK3C2B	cyan	VSMC_M2	0.12	5.57E-01	0.76	5.18E-06	-0.08	7.00E-01	0.53	4.75E-03	0.00	9.99E-01	-0.06	7.59E-01	-0.41	3.35E-02	-0.67	1.30E-04	-0.67	1.13E-04	0.70	5.70E-05
ENSCAFG0000002893	ENSCAFG0000002893	cyan	VSMC_M2	0.12	5.57E-01	0.53	4.24E-03	0.36	6.14E-02	-0.09	6.43E-01	-0.39	4.30E-02	-0.16	4.26E-01	0.00	9.89E-01	-0.10	6.08E-01	-0.51	6.40E-03	0.42	2.83E-02
ENSCAFG0000002019	HSPA5	grey	VSMC_M10	0.12	5.57E-01	-0.45	2.00E-02	-0.15	4.64E-01	-0.12	5.38E-01	0.25	2.16E-01	0.00	9.85E-01	0.05	8.23E-01	0.33	9.65E-02	0.25	2.02E-01	-0.39	4.14E-02
ENSCAFG0000000058	GRHL2	darkgreen	VSMC_M4	0.12	5.57E-01	-0.05	7.90E-01	-0.48	1.15E-02	0.57	1.82E-03	0.43	2.48E-02	0.15	4.66E-01	0.08	6.92E-01	-0.41	3.53E-02	0.02	9.39E-01	0.21	2.90E-01
ENSCAFG0000001422	IKZF2	grey	VSMC_M10	0.12	5.57E-01	0.28	1.50E-01	-0.44	2.26E-02	0.52	5.38E-03	0.34	8.74E-02	-0.04	8.46E-01	0.02	9.11E-01	-0.46	1.70E-02	-0.32	1.01E-01	0.48	1.10E-02
ENSCAFG0000001211	MYLK	darkgreen	VSMC_M4	0.12	5.57E-01	0.46	1.49E-02	-0.20	3.22E-01	0.70	5.44E-05	0.13	5.31E-01	0.03	8.71E-01	-0.61	6.44E-04	-0.74	1.20E-05	-0.37	5.44E-02	0.53	4.45E-03
ENSCAFG0000002521	FAM111B	darkgrey	VSMC_M8	0.12	5.57E-01	-0.31	1.17E-01	-0.02	9.14E-01	-0.46	1.50E-02	0.22	2.69E-01	-0.18	3.59E-01	0.08	6.87E-01	0.54	4.03E-03	0.22	2.62E-01	-0.56	2.48E-03
ENSCAFG0000001560	ILDR2	grey	VSMC_M10	0.12	5.57E-01	0.55	2.78E-03	0.13	5.13E-01	0.31	1.17E-01	-0.24	2.20E-01	0.03	8.68E-01	0.13	5.34E-01	-0.42	3.13E-02	-0.57	1.75E-03	0.57	1.99E-03
ENSCAFG0000001306	GAL3ST3	grey	VSMC_M10	0.12	5.57E-01	0.30	1.26E-01	0.26	1.95E-01	-0.19	3.55E-01	-0.20	3.28E-01	-0.05	7.94E-01	0.10	6.13E-01	0.07	7.43E-01	-0.29	1.42E-01	0.13	5.26E-01
ENSCAFG0000001955	PHPT1	cyan	VSMC_M2	0.12	5.57E-01	0.61	6.80E-04	0.39	4.21E-02	0.26	1.86E-01	-0.53	4.07E-03	0.02	9.12E-01	-0.15	4.70E-01	-0.37	5.74E-02	-0.61	7.96E-04	0.63	4.26E-04
ENSCAFG0000001418	MGAT2	grey	VSMC_M10	0.12	5.57E-01	-0.51	6.44E-03	-0.03	8.91E-01	-0.45	1.83E-02	0.25	2.18E-01	-0.10	6.14E-01	-0.20	3.24E-01	0.57	1.88E-03	0.47	1.40E-02	-0.72	2.17E-05
ENSCAFG0000002271	ENSCAFG0000002271	grey	VSMC_M10	0.12	5.58E-01	0.09	6.38E-01	0.49	9.40E-03	-0.36	6.23E-02	-0.44	2.01E-02	-0.18	3.56E-01	0.25	2.00E-01	0.29	1.44E-01	-0.19	3.51E-01	0.05	8.08E-01
ENSCAFG0000000118	BRD8	darkgreen	VSMC_M4	0.12	5.58E-01	0.16	4.36E-01	-0.46	1.59E-02	0.68	9.80E-05	0.39	4.27E-02	-0.07	7.45E-01	-0.46	1.57E-02	-0.63	4.25E-04	-0.12	5.49E-01	0.37	5.59E-02
ENSCAFG0000002219	ENSCAFG0000002219	cyan	VSMC_M2	0.12	5.58E-01	0.43	2.38E-02	0.53	4.11E-03	-0.15	4.41E-01	-0.52	5.22E-03	0.23	2.43E-01	-0.09	6.56E-01	-0.04	8.52E-01	-0.46	1.47E-02	0.23	2.38E-01
ENSCAFG0000001249	ICA1L	grey	VSMC_M10	0.12	5.58E-01	-0.54	3.72E-03	-0.41	3.45E-02	0.08	6.96E-01	0.42	3.07E-02	-0.04	8.39E-01	0.21	2.84E-01	0.14	5.01E-01	0.40	4.08E-02	-0.25	2.14E-01
ENSCAFG0000000995	KCNIP2	grey	VSMC_M10	0.12	5.58E-01	0.15	4.50E-01	0.21	2.94E-01	-0.16	4.27E-01	-0.16	4.38E-01	0.03	8.95E-01	-0.03	8.66E-01	0.07	7.31E-01	-0.17	4.10E-01	-0.01	9.70E-01
ENSCAFG0000001116	VSIG2	darkgreen	VSMC_M4	0.12	5.59E-01	0.07	7.11E-01	-0.50	7.69E-03	0.65	2.58E-04	0.46	1.48E-02	-0.11	5.76E-01	-0.17	4.06E-01	-0.59	1.17E-03	-0.06	7.66E-01	0.29	1.40E-01
ENSCAFG0000001463	FRMD6	darkgrey	VSMC_M8	0.12	5.59E-01	-0.39	4.50E-02	0.10	6.18E-01	-0.67	1.52E-04	0.11	5.88E-01	-0.06	7.67E-01	0.47	1.30E-02	0.65	2.15E-04	0.23	2.42E-01	-0.53	4.11E-03
ENSCAFG0000002135	ENSCAFG0000002135	grey	VSMC_M10	0.12	5.59E-01	0.18	3.71E-01	0.30	1.31E-01	-0.24	2.28E-01	-0.34	8.34E-02	0.23	2.40E-01	0.08	6.77E-01	0.11	5.85E-01	-0.21	2.98E-01	0.22	2.74E-01
ENSCAFG0000003238	IL17B	grey	VSMC_M10	0.12	5.59E-01	0.26	1.94E-01	0.13	5.12E-01	-0.05	8.23E-01	-0.15	4.54E-01	0.26	1.91E-01	-0.33	9.27E-02	-0.04	8.43E-01	-0.30	1.28E-01	0.17	3.89E-01
ENSCAFG0000001349	RNF2	pink	VSMC_M5	0.12	5.59E-01	0.16	4.15E-01	-0.52	5.83E-03	0.58	1.60E-03	0.57	1.83E-03	-0.09	6.45E-01	-0.31	1.19E-01	-0.56	2.47E-03	-0.11	5.98E-01	0.19	3.51E-01
ENSCAFG0000000761	PITPNM2	grey	VSMC_M10	0.12	5.59E-01	0.08	6.88E-01	-0.20	3.22E-01	0.18	3.70E-01	0.26	1.86E-01	0.22	2.79E-01	-0.19	3.33E-01	-0.23	2.47E-01	0.00	9.83E-01	0.01	9.76E-01
ENSCAFG0000001725	ATRX	pink	VSMC_M5	0.12	5.60E-01	-0.08	7.03E-01	-0.71	2.96E-05	0.73	1.37E-05	0.75	7.90E-06	-0.26	1.83E-01	-0.22	2.60E-01	-0.56	2.61E-03	0.09	6.58E-01	0.13	5.04E-01
ENSCAFG0000001236	ALS2	pink	VSMC_M5	0.12	5.60E-01	0.07	7.10E-01	-0.48	1.12E-02	0.42	2.94E-02	0.51	6.57E-03	-0.07	7.24E-01	-0.15	4.54E-01	-0.39	4.29E-02	-0.07	7.38E-01	0.13	5.14E-01
ENSCAFG0000002000	EXTL2	darkgreen	VSMC_M4	0.12	5.60E-01	0.61	7.56E-04	-0.05	8.19E-01	0.52	5.57E-03	-0.09	6.66E-01	-0.02	9.20E-01	-0.01	9.47E-01	-0.61	8.01E-04	-0.58	1.37E-03	0.80	6.78E-07
ENSCAFG0000001904	ENSCAFG0000001904	cyan	VSMC_M2	0.12	5.60E-01	0.43	2.40E-02	0.22	2.74E-01	0.10	6.36E-01	-0.23	2.49E-01	-0.16	4.27E-01	0.00	9.96E-01	-0.25	1.99E-01	-0.39	4.39E-02	0.33	9.56E-02
ENSCAFG0000000071	NUDCD1	grey	VSMC_M10	0.12	5.60E-01	-0.63	4.91E-04	-0.24	2.26E-01	-0.23	2.56E-01	0.43	2.38E-02	-0.08	6.78E-01	0.25	2.01E-01	0.45	1.80E-02	0.46	1.63E-02	-0.62	5.12E-04
ENSCAFG0000001453	LRRC23	grey	VSMC_M10	0.12	5.61E-01	-0.14	4.83E-01	0.00	9.92E-01	-0.13	5.28E-01	0.07	7.41E-01	-0.07	7.31E-01	-0.17	4.07E-01	0.13	5.14E-01	0.16	4.19E-01	-0.18	3.57E-01
ENSCAFG0000002344	FBP2	grey	VSMC_M10	0.12	5.61E-01	0.15	4.63E-01	0.06	7.54E-01	-0.15	4.43E-01	-0.09	6.63E-01	0.12	5.36E-01	0.14	4.76E-01	0.12	5.63E-01	-0.27	1.66E-01	0.16	4.34E-01
ENSCAFG0000000318	CEP68	grey	VSMC_M10	0.12	5.61E-01	0.33	9.36E-02	0.36	6.38E-02	0.00	9.88E-01	-0.43	2.66E-02	0.08	6.98E-01	0.18	3.79E-01	-0.14	4.86E-01	-0.36	6.19E-02	0.40	3.95E-02
ENSCAFG0000001089	ARRDC4	darkgreen	VSMC_M4	0.12	5.61E-01	0.17	3.86E-01	-0.34	8.60E-02	0.43	2.39E-02	0.25	2.02E-01	-0.01	9.50E-01	0.22	2.59E-01	-0.38	4.74E-02	-0.26	1.90E-01	0.44	2.19E-02
ENSCAFG0000002013	LRSAM1	darkgreen	VSMC_M4	0.12	5.61E-01	0.52	5.59E-03	-0.11	5.84E-01	0.55	2.91E-03	0.04	8.34E-01	-0.18	3.64E-01	0.06	7.76E-01	-0.65	2.52E-04	-0.49	9.98E-03	0.68	8.55E-05
ENSCAFG0000000857	MCPH1	grey	VSMC_M10	0.12	5.61E-01	0.25	2.10E-01	0.48	1.04E-02	-0.53	4.44E-03	-0.43	2.70E-02	0.12	5.38E-01	0.28	1.64E-01	0.37	5.92E-02	-0.38	5.36E-02	0.04	8.46E-01
ENSCAFG0000001571	UQCC3	yellow	VSMC_M3	0.12	5.62E-01	0.00	9.82E-01	0.62	4.98E-04	-0.49	8.97E-03	-0.62	5.44E-04	0.12	5.67E-01	0.20	3.20E-01	0.44	2.15E-02	-0.13	5.32E-01	-0.05	8.01E-01
ENSCAFG0000001282	TCN2	cyan	VSMC_M2	0.12	5.62E-01	0.77	2.63E-06	-0.06	7.77E-01	0.69	6.79E-05	-0.05	8.16E-01	-0.02	9.14E-01	-0.42	2.99E-02	-0.89	6.73E-10	-0.62	5.08E-04	0.78	1.45E-06
ENSCAFG0000003144	VAMP3	grey	VSMC_M10	0.12	5.62E-01	-0.10	6.19E-01	-0.27	1.79E-01	0.14	4.79E-01	0.48	1.14E-02	-0.21	2.94E-01	-0.22	2.64E-01	-0.17	4.06E-01	0.24	2.32E-01	-0.32	9.92E-02
ENSCAFG0000001596	UBE2K	darkgrey	VSMC_M8	0.12	5.62E-01	-0.71	3.83E-05	-0.06	7.71E-01	-0.52	5.56E-03	0.28	1.64E-01	0.03	8.77E-01	0.14	4.93E-01	0.68	9.99E-05	0.59	1.15E-03	-0.80	4.85E-07
ENSCAFG0000000187	NUAK1	grey	VSMC_M10	0.12	5.62E-01	0.19	3.46E-01	-0.04	8.47E-01	0.23	2.38E-01	0.05	8.17E-01	0.04	8.24E-01	-0.83	7.03E-08	-0.23	2.50E-01	-0.14	4.86E-01	0.08	7.07E-01
ENSCAFG0000002374	GCNA	grey	VSMC_M10	0.12	5.62E-01	0.31	1.20E-01	0.09	6.63E-01	-0.10	6.07E-01	-0.02	9.32E-01	0.17	3.87E-01	0.11	5.97E-01	-0.02	9.22E-01	-0.33	8.99E-02	0.07	7.18E-01
ENSCAFG0000000282	YTHDC1	grey	VSMC_M10	0.12	5.63E-01	-0.10	6.16E-01	-0.20	3.15E-01	0.19	3.54E-01	0.32	9.89E-02	-0.20	3.16E-01	0.20	3.19E-01	-0.17	3.97E-01	0.02	9.33E-01	-0.06	7.82E-01
ENSCAFG0000000891	RASGEF1B	grey	VSMC_M10	0.12	5.63E-01	-0.01	9.54E-01	0.47	1.38E-02	-0.62	5.76E-04	-0.35	7.27E-02	0.19	3.36E-01	0.47	1.33E-02	0.46	1.63E-02	-0.09	6.39E-01	-0.21	2.94E-01
ENSCAFG0000000231	MLIP	cyan	VSMC_M2	0.12	5.63E-01	0.48	1.08E-02	0.13	5.30E-01	0.05	8.17E-01	-0.16	4.23E-01	-0.11	5.80E-01	-0.25	2.06E-01	-0.13	5.24E-01	-0.50	7.75E-03	0.36	6.26E-02
ENSCAFG0000002380	ARMCX1	yellow	VSMC_M3	0.12	5.63E-01	0.57	1.86E-03	0.66	1.92E-04	-0.10	6.30E-01	-0.68	9.19E-05	0.02	9.34E-01	-0.26	1.84E-01	-0.13	5.10E-01	-0.51	6.07E-03	0.39	4.30E-02
ENSCAFG0000000874	MAPKAPK5	grey	VSMC_M10	0.12	5.63E-01	-0.21	2.83E-01	-0.02	9.13E-01	-0.02	9.10E-01	0.22	2.72E-01	-0.08	6.94E-01	-0.06	7.77E-01	0.03	8.70E-01	0.17	3.84E-01	-0.37	5.86E-02
ENSCAFG0000000586	PLCL2	grey	VSMC_M10	0.12	5.63E-01	0.16	4.12E-01	0.23	2.44E-01	-0.22	2.68E-01	-0.15	4.70E-01	-0.04	8.30E-01	-0.37	6.00E-02	0.17	3.94E-01	-0.24	2.28E-01	-0.09	6.50E-01
ENSCAFG0000000174	GNMT	grey	VSMC_M10	0.12	5.63E-01	-0.07	7.20E-01	-0.19	3.45E-01	-0.07	7.28E-01	0.26	1.96E-01	0.23	2.47E-01	0.04	8.35E-01	0.08	6.84E-01	0.04	8.37E-01	-0.12	5.37E-01
ENSCAFG0000000432	TMEM60	red	VSMC_M1	0.12	5.64E-01	0.11	5.80E-01	-0.06	7.62E-01	0.17	4.03E-01	-0.03	8.85E-01	0.17	3.97E-01	0.32	9.93E-02	-0.19	3.53E-01	-0.20	3.24E-01	0.33	9.43E-02
ENSCAFG0000000176	PWP1	darkgrey	VSMC_M8	0.12	5.64E-01	-0.55	2.73E-03	-0.13	5.25E-01	-0.35	7.27E-02	0.20	3.29E-01	0.14	4.87E-01	0.27	1.73E-01	0.63	4.18E-04	0.31	1.14E-01	-0.50	7.35E-03
ENSCAFG0000002957	RGS12	grey	VSMC_M10	0.12	5.64E-01	0.19	3.52E-01	-0.15	4.69E-01	0.53	4.73E-03	0.04	8.62E-01	0.26	1.94E-01	0.00	9.94E-01	-0.44	2.06E-02	-0.23	2.53E-01	0.43	2.40E-02
ENSCAFG0000002700	ENSCAFG0000002700	grey	VSMC_M10	0.12	5.64E-01	0.01	9.69E-01	-0.02	9.41E-01	-0.13	5.11E-01	0.00	9.93E-01	0.13	5.12E-01	0.05	8.20E-01	0.13	5.12E-01	-0.14	4.86E-01	0.07	7.17E-01
ENSCAFG0000001468	MSANTD1	grey	VSMC_M10	0.12	5.64E-01	0.12	5.62E-01	0.10	6.15E-01	-0.12	5.41E-01	-0.07	7.37E-01	-0.07	7.36E-01	-0.15	4.68E-01	0.10	6.20E-01	-0.15	4.65E-01	0.02	9.23E-01
ENSCAFG0000002033	RANBP10	darkgrey	VSMC_M8	0.12	5.65E-01	-0.56	2.45E-03	0.07	7.46E-01	-0.60	8.69E-04	0.18	3.62E-01	-0.06	7.54E-01	0.19	3.30E-01	0.73	1.50E-05	0.41	3.58E-02	-0.83	6.58E-08
ENSCAFG0000002394	ENSCAFG0000002394	grey	VSMC_M10	0.12	5.65E-01	0.29	1.39E-01	0.04	8.38E-01	0.26	1.85E-01	-0.14	4.76E-01	0.34	7.82E-02	-0.08	6.92E-01	-0.31	1.16E-01	-0.33	9.63E-02	0.44	2.11E-02
ENSCAFG0000000722	HTR7	grey	VSMC_M10	0.12	5.65E-01	0.25	2.09E-01	0.21	2.99E-01	-0.08	6.88E-01	-0.09	6.51E-01	-0.01	9.59E-01	-0.56	2.56E-03	0.00	9.91E-01	-0.18	3.80E-01	-0.07	7.40E-01
ENSCAFG0000000191	ALDH1L2	darkgrey	VSMC_M8	0.12	5.65E-01	-0.41	3.25E-02	0.15	4.45E-01	-0.59	1.09E-03	0.01	9.67E-01	0.02	9.23E-01	0.17	3.84E-01	0.69	7.50E-05	0.20	3.08E-01	-0.58	1.60E-03
ENSCAFG0000000537	C1H19orf47	grey	VSMC_M10	0.12	5.65E-01	0.39	4.60E-02	0.40	3.94E-02	-0.06	7.73E-01	-0.45	1.78E-02	0.08	6.91E-01	0.13	5.27E-01	-0.05	8.06E-01	-0.47	1.33E-02	0.44	2.31E-02
ENSCAFG0000000610	NEK11	grey	VSMC_M10	0.12	5.65E-01	0.17	3.98E-01	-0.10	6.31E-01	0.25	2.03E-01	-0.07	7.45E-01	0.12	5.51E-01	0.02	9.23E-01	-0.20	3.17E-01	-0.29	1.41E-01	0.49	9.01E-03
ENSCAFG0000001228	STRADB	grey	VSMC_M10	0.12	5.66E-01	0.09	6.66E-01	-0.25	2.07E-01	0.28	1.59E-01	0.23	2.38E-01	0.03	8.86E-01	0.14	4.74E-01	-0.29	1.42E-01	-0.16	4.32E-01	0.15	4.50E-01
ENSCAFG0000000043	MYRFL	grey	VSMC_M10	0.12	5.66E-01	-0.34	8.11E-02	-0.33	9.56E-02	0.04	8.48E-01	0.48	1.22E-02	-0.18	3.59E-01	0.10	6.36E-01	0.04	8.37E-01	0.30	1.34E-01	-0.28	1.56E-01
ENSCAFG0000000712	LMO2	grey	VSMC_M10	0.12	5.66E-01	0.32	1.04E-01	0.15	4.52E-01	-0.07	7.35E-01	-0.19	3.39E-01	0.44	2.25E-02	-0.13	5.24E-01	0.06	7.72E-01	-0.35	7.33E-02	0.20	3.27E-01
ENSCAFG0000000248	TBC1D2	cyan	VSMC_M2	0.12	5.66E-01	0.60	8.31E-04	0.60	1.01E-03	-0.08	7.02E-01	-0.67	1.15E-04	0.08	6.92E-01	0.00	9.85E-01	-0.09	6.45E-01	-0.63	4.67E-04	0.51	6.28E-03
ENSCAFG0000003257	ENSCAFG0000003257	grey	VSMC_M10	0.12	5.66E-01	-0.10	6.29E-01	-0.06	7.57E-01	-0.10	6.06E-01	0.26	1.86E-01	-0.12	5.41E-01	0.30	1.31E-01	0.02	9.05E-01	0.11	5.79E-01	-0.29	1.38E-01
ENSCAFG0000000251	C15H1orf50	darkgreen	VSMC_M4	0.12	5.67E-01	0.27	1.81E-01	-0.47	1.38E-02	0.70	5.08E-05	0.48	1.04E-02	-0.20	3.29E-01	-0.34	8.62E-02	-0.65	2.28E-04	-0.22	2.60E-01	0.34	8.31E-02
ENSCAFG0000003213	ENSCAFG0000003213	grey	VSMC_M10	0.12	5.67E-01	0.09	6.42E-01	0.13	5.20E-01	-0.18	3.82E-01	-0.08	6.96E-01	-0.03	8.95E-01	0.10	6.34E-01	0.14	4.86E-01	-0.15	4.50E-01	0.02	9.37E-01
ENSCAFG0000000861	ENSCAFG0000000861	grey	VSMC_M10	0.12	5.67E-01	-0.31	1.11E-01	-0.43	2.64E-02	0.15	4.57E-01	0.55	2.86E-03	-0.16	4.19E-01	-0.07	7.37E-01	-0.01	9.73E-01	0.25	2.01E-01	-0.31	1.14E-01
ENSCAFG0000001807	ADORA2B	grey	VSMC_M10	0.11	5.68E-01	0.19	3.37E-01	0.24	2.23E-01	-0.11	5.97E-01	-0.27	1.73E-01	-0.09	6.43E-01	-0.03	8.95E-01	0.04	8.61E-01	-0.23	2.48E-01	0.14	4.95E-01
ENSCAFG0000001119	NID1	darkgreen	VSMC_M4	0.11	5.69E-01	0.47	1.45E-02	-0.42	3.10E-02	0.89	5.87E-10	0.27	1.80E-01	0.02	9.16E-01	-0.44	2.00E-02	-0.88	1.97E-09	-0.40	4.13E-02	0.70	4.98E-05
ENSCAFG0000001397	ZFPL1	yellow	VSMC_M3	0.11	5.69E-01	0.28	1.63E-01	0.50	8.35E-03	-0.48	1.20E-02	-0.40	3.73E-02	-0.18	3.81E-01	0.12	5.68E-01	0.28	1.62E-01	-0.27	1.77E-01	-0.03	9.00E-01
ENSCAFG0000001167	EPB41	grey	VSMC_M10	0.11	5.69E-01	0.23	2.57E-01	-0.15	4.69E-01	0.26	1.84E-01	0.19	3.53E-01	0.20	3.06E-01	-0.06	7.81E-01	-0.25	2.13E-01	-0.27	1.81E-01	0.18	3.81E-01
ENSCAFG0000002957	ZNF70	grey	VSMC_M10	0.11	5.69E-01	0.15	4.56E-01	0.17	4.00E-01	-0.19	3.40E-01	0.05	7.86E-01	-0.12	5.44E-01	0.11	5.93E-01	0.03	9.00E-01	-0.13	5.34E-01	-0.26	1.83E-01
ENSCAFG0000001840	FST	darkgreen	VSMC_M4	0.11	5.69E-01	0.46	1.52E-02	-0.41	3.34E-02	0.89	3.12E-10	0.26	1.88E-01	-0.07	7.39E-01	-0.35	7.13E-02	-0.83	8.38E-08	-0.41	3.25E-02	0.70	5.63E-05
ENSCAFG0000000913	SAA1	grey	VSMC_M10	0.11	5.70E-01	0.61	6.76E-04	0.05	8.11E-01	0.55	2.99E-03	-0.14	4.74E-01	-0.18	3.57E-01	-0.47	1.24E-02	-0.62	5.75E-04	-0.54	3.81E-03	0.61	7.61E-04
ENSCAFG0000000120	CDC14B	cyan	VSMC_M2	0.11	5.70E-01	0.58	1.64E-03	0.34	8.47E-02	0.08	6.99E-01	-0.38	4.98E-02	0.13	5.23E-01	0.11	5.92E-01	-0.28	1.54E-01	-0.55	3.16E-03	0.49	9.04E-03
ENSCAFG0000000983	CD101	grey	VSMC_M10	0.11	5.70E-01	-0.32	9.98E-02	-0.17	3.96E-01	-0.27	1.76E-01	0.39	4.63E-02	-0.12	5.58E-01	0.26	1.98E-01	0.33	9.57E-02	0.21	2.97E-01	-0.52	5.07E-03
ENSCAFG0000002761	ENSCAFG0000002761	grey	VSMC_M10	0.11	5.70E-01	-0.03	8.91E-01	-0.19	3.56E-01	0.22	2.69E-01	0.17	3.91E-01	-0.09	6.38E-01	0.15	4.54E-01	-0.15	4.69E-01	-0.05	7.88E-01	0.12	5.52E-01
ENSCAFG0000003065	ENSCAFG0000003065	grey	VSMC_M10	0.11	5.70E-01	0.12	5.51E-01	0.52	5.24E-03	-0.24	2.21E-01	-0.54	3.33E-03	0.26	1.91E-01	0.23	2.51E-01	0.17	3.86E-01	-0.23	2.55E-01	0.10	6.27E-01
ENSCAFG0000000157	GNAQ	darkgreen	VSMC_M4	0.11	5.70E-01	0.21	2.89E-01	-0.55	2.79E-03	0.77	3.32E-06	0.41	3.27E-02	0.03	8.72E-01	-0.08	6.87E-01	-0.69	7.34E-05	-0.23	2.51E-01	0.59	1.27E-03
ENSCAFG0000002499	ENSCAFG0000002499	grey	VSMC_M10	0.11	5.70E-01	0.25	2.04E-01	0.07	7.12E-01	-0.04	8.36E-01	-0.06	7.85E-01	0.29	1.47E-01	0.08	6.92E-01	0.00	9.88E-01	-0.30	1.34E-01	0.21	2.83E-01
ENSCAFG0000000888	FGF5	grey	VSMC_M10	0.11	5.71E-01	-0.38	4.91E-02	0.06	7.75E-01	-0.38	4.85E-02	-0.05	7.90E-01	0.34	8.15E-02	0.46	1.56E-02	0.43	2.55E-02	0.19	3.32E-01	-0.20	3.25E-01
ENSCAFG0000000257	SSC5D	yellow	VSMC_M3	0.11	5.71E-01	0.44	2.14E-02	0.60	8.60E-04	-0.35	7.65E-02	-0.58	1.63E-03	-0.07	7.11E-01	0.31	1.18E-01	0.12	5.50E-01	-0.47	1.30E-02	0.26	1.94E-01
ENSCAFG0000000471	KCTD2	grey	VSMC_M10	0.11	5.72E-01	-0.19	3.49E-01	-0.11	5.94E-01	0.14	4.93E-01	0.00	9.96E-01	-0.04	8.52E-01	0.02	9.12E-01	0.01	9.52E-01	0.04	8.35E-01	0.12	5.43E-01
ENSCAFG0000000247	ENSCAFG0000000247	grey	VSMC_M10	0.11	5.72E-01	-0.21	2.91E-01	0.27	1.82E-01	-0.32	1.06E-01	-0.18	3.82E-01	0.29	1.38E-01	0.36	6.36E-02	0.32	1.04E-01	0.11	5.79E-01	-0.23	2.51E-01
ENSCAFG0000001731	SPNS1	cyan	VSMC_M2	0.11	5.72E-01	0.60	1.06E-03	0.42	3.10E-02	-0.13	5.34E-01	-0.45	1.93E-02	0.28	1.64E-01	-0.09	6.70E-01	-0.16	4.23E-01	-0.55	2.68E-03	0.38	4.86E-02
ENSCAFG0000001606	DVL2	grey	VSMC_M10	0.11	5.73E-01	0.74	1.15E-05	0.11	5.97E-01	0.37	5.46E-02	-0.18	3.80E-01	-0.13	5.07E-01	-0.09	6.50E-01	-0.61	8.00E-04	-0.69	5.78E-05	0.72	2.08E-05
ENSCAFG0000000371	ENSCAFG0000000371	grey	VSMC_M10	0.11	5.73E-01	-0.18	3.74E-01	0.04	8.58E-01	-0.25	2.05E-01	-0.02	9.26E-01	0.49	9.62E-03	0.20	3.25E-01	0.33	9.78E-02	0.04	8.42E-01	-0.14	4.74E-01
ENSCAFG0000000819	SPAG4	grey	VSMC_M10	0.11	5.73E-01	0.33	9.42E-02	0.03	8.93E-01	0.48	1.14E-02	-0.05	8.10E-01	-0.17	3.92E-01	-0.28	1.53E-01	-0.54	3.30E-03	-0.25	2.10E-01	0.37	5.80E-02
ENSCAFG0000000875	DPYSL2	grey	VSMC_M10	0.11	5.73E-01	0.57	1.73E-03	-0.03	8.88E-01	0.36	6.81E-02	0.11	5.87E-01	-0.26	1.88E-01	-0.05	8.06E-01	-0.53	4.37E-03	-0.46	1.71E-02	0.38	5.28E-02
ENSCAFG0000000109	ASAP1	grey	VSMC_M10	0.11	5.73E-01	0.15	4.64E-01	-0.25	2.10E-01	0.46	1.66E-02	0.24	2.31E-01	-0.34	7.96E-02	0.25	2.11E-01	-0.37	5.65E-02	-0.13	5.09E-01	0.29	1.36E-01
ENSCAFG0000000211	MUT	red	VSMC_M1	0.11	5.73E-01	-0.05	8.11E-01	-0.18	3.71E-01	0.26	1.92E-01	0.04	8.50E-01	0.22	2.66E-01	0.11	5.92E-01	-0.16	4.37E-01	-0.06	7.83E-01	0.31	1.15E-01
ENSCAFG0000000396	FAT4	grey	VSMC_M10	0.11	5.73E-01	-0.45	1.80E-02	-0.35	7.63E-02	-0.08	6.86E-01	0.57	1.90E-03	-0.17	4.05E-01	0.09	6.41E-01	0.10	6.20E-01	0.49	9.11E-03	-0.48	1.19E-02
ENSCAFG0000003125	DDA1	grey	VSMC_M10	0.11	5.74E-01	-0.13	5.20E-01	-0.28	1.51E-01	0.48	1.14E-02	0.33	9.07E-02	-0.16	4.14E-01	-0.38	4.79E-02	-0.40	3.78E-02	0.18	3.69E-01	0.00	9.84E-01
ENSCAFG0000000071	BRD1	grey	VSMC_M10	0.11	5.74E-01	0.43	2.54E-02	0.10	6.13E-01	0.36	6.77E-02	-0.05	8.16E-01	-0.27	1.73E-01	-0.19	3.49E-01	-0.44	2.20E-02	-0.43	2.54E-02	0.34	8.00E-02
ENSCAFG0000002047	ENSCAFG0000002047	grey	VSMC_M10	0.11	5.74E-01	0.48	1.21E-02	-0.11	5.79E-01	0.39	4.61E-02	0.00	9.81E-01	0.16	4.17E-01	-0.26	1.85E-01	-0.40	4.14E-02	-0.50	8.39E-03	0.55	3.09E-03
ENSCAFG0000001113	SCN2A	grey	VSMC_M10	0.11	5.74E-01	-0.39	4.66E-02	-0.25	2.00E-01	-0.15	4.46E-01	0.28	1.54E-01	0.18	3.60E-01	-0.01	9.74E-01	0.34	8.69E-02	0.26	1.85E-01	-0.27	1.81E-01
ENSCAFG0000000121	ENSCAFG0000000121	pink	VSMC_M5	0.11	5.75E-01	-0.36	6.65E-02	-0.21	2.90E-01	0.02	9.39E-01	0.40	3.73E-02	-0.22	2.74E-01	0.05	7.95E-01	0.15	4.67E-01	0.28	1.50E-01	-0.48	1.21E-02
ENSCAFG0000002037	USP33	pink	VSMC_M5	0.11	5.75E-01	-0.29	1.39E-01	-0.39	4.48E-02	-0.06	7.63E-01	0.64	2.92E-04	-0.15	4.54E-01	-0.16	4.15E-01	0.12	5.51E-01	0.30	1.25E-01	-0.50	7.84E-03
ENSCAFG0000003006	ENSCAFG0000003006	grey	VSMC_M10	0.11	5.75E-01	-0.01	9.69E-01	-0.20	3.24E-01	0.06	7.48E-01	0.25	2.11E-01	0.07	7.28E-01	0.22	2.77E-01	-0.01	9.49E-01	-0.06	7.74E-01	-0.09	6.46E-01
ENSCAFG0000000111	ARHGAP18	cyan	VSMC_M2	0.11	5.75E-01	0.61	6.83E-04	-0.02	9.40E-01	0.65	2.33E-04	-0.02	9.04E-01	-0.11	5.82E-01	-0.42	2.94E-02	-0.72	2.61E-05	-0.52	5.77E-03	0.52	5.70E-03
ENSCAFG0000001601	EPHA2	cyan	VSMC_M2	0.11	5.75E-01	0.54	3.67E-03	0.22	2.60E-01	0.42	3.01E-02	-0.35	7.08E-02	0.26	1.95E-01	-0.32	1.06E-01	-0.46	1.58E-02	-0.54	3.70E-03	0.58	1.61E-03
ENSCAFG0000001420	KLHDC1	grey	VSMC_M10	0.11	5.75E-01	-0.14	4.71E-01	-0.11	6.00E-01	-0.03	8.83E-01	0.40	3.71E-02	-0.26	1.86E-01	-0.31	1.11E-01	-0.02	9.05E-01	0.20	3.05E-01	-0.48	1.14E-02
ENSCAFG0000001870	RALBP1	grey	VSMC_M10	0.11	5.75E-01	-0.12	5.35E-01	0.40	3.96E-02	-0.41	3.50E-02	-0.28	1.63E-01	-0.07	7.11E-01	0.20	3.22E-01	0.35	7.27E-02	0.03	8.91E-01	-0.20	3.05E-01
ENSCAFG0000000328	ZNF618	grey	VSMC_M10	0.11	5.75E-01	0.23	2.58E-01	0.04	8.34E-01	-0.05	8.13E-01	-0.12	5.49E-01	0.49	9.60E-03	-0.01	9.80E-01	0.05	8.06E-01	-0.34	7.91E-02	0.25	2.05E-01
ENSCAFG0000000090	EFCAB6	grey	VSMC_M10	0.11	5.76E-01	0.00	9.93E-01	-0.17	3.96E-01	0.34	8.29E-02	0.04	8.38E-01	0.14	4.80E-01	0.35	7.14E-02	-0.22	2.66E-01	-0.10	6.32E-01	0.33	9.12E-02
ENSCAFG0000002044	ENSCAFG0000002044	grey	VSMC_M10	0.11	5.76E-01	0.20	3.26E-01	0.16	4.17E-01	-0.09	6.71E-01	-0.10	6.14E-01	-0.07	7.33E-01	-0.20	3.15E-01	0.05	8.09E-01	-0.19	3.40E-01	0.03	8.75E-01
ENSCAFG0000002849	ENSCAFG0000002849	grey	VSMC_M10	0.11	5.76E-01	0.15	4.53E-01	0.08	6.78E-01	-0.16	4.27E-01	-0.02	9.26E-01	0.04	8.29E-01	0.02	9.20E-01	0.09	6.56E-01	-0.18	3.66E-01	0.05	7.97E-01

ENSCAFG0000001277	FTSJ3	yellow	VSMC_M3	0.11	5.76E-01	-0.30	1.35E-01	0.45	1.82E-02	-0.49	1.02E-02	-0.39	4.49E-02	0.13	5.26E-01	0.27	1.71E-01	0.55	3.23E-03	0.11	5.93E-01	-0.31	1.13E-01
ENSCAFG0000001917	NSDHL	yellow	VSMC_M3	0.11	5.76E-01	-0.11	5.76E-01	0.43	2.50E-02	-0.69	6.83E-05	-0.22	2.68E-01	-0.02	9.05E-01	0.64	2.95E-04	0.50	7.49E-03	0.03	8.71E-01	-0.39	4.34E-02
ENSCAFG0000000028	CDK4	yellow	VSMC_M3	0.11	5.76E-01	0.32	1.05E-01	0.70	5.54E-05	-0.65	2.46E-04	-0.64	3.43E-04	0.17	3.87E-01	0.16	4.12E-01	0.48	1.12E-02	-0.42	2.75E-02	-0.06	7.81E-01
ENSCAFG0000000525	RALGAPA2	darkgreen	VSMC_M4	0.11	5.76E-01	0.36	6.47E-02	-0.32	1.08E-01	0.72	1.99E-05	0.20	3.16E-01	0.34	8.64E-02	-0.12	5.46E-01	-0.74	8.60E-06	-0.33	9.08E-02	0.61	7.81E-04
ENSCAFG0000001899	DHCR24	yellow	VSMC_M3	0.11	5.76E-01	0.00	9.83E-01	0.57	1.94E-03	-0.79	1.24E-06	-0.35	7.07E-02	0.09	6.65E-01	0.49	1.02E-02	0.56	2.43E-03	-0.05	8.07E-01	-0.38	5.04E-02
ENSCAFG0000001195	ENSCAFG0000001195	grey	VSMC_M10	0.11	5.76E-01	-0.22	2.75E-01	-0.01	9.76E-01	-0.20	3.16E-01	0.05	8.05E-01	-0.21	2.94E-01	0.21	2.92E-01	0.26	1.89E-01	0.12	5.67E-01	-0.26	1.88E-01
ENSCAFG0000002052	ENSCAFG0000002052	grey	VSMC_M10	0.11	5.77E-01	0.04	8.47E-01	-0.20	3.06E-01	0.10	6.28E-01	0.15	4.57E-01	0.25	2.13E-01	-0.22	2.66E-01	-0.05	7.88E-01	-0.06	7.80E-01	0.08	6.90E-01
ENSCAFG0000002850	ENSCAFG0000002850	grey	VSMC_M10	0.11	5.77E-01	-0.29	1.47E-01	-0.14	5.01E-01	-0.13	5.17E-01	0.14	4.99E-01	0.17	4.02E-01	-0.10	6.19E-01	0.30	1.32E-01	0.21	2.97E-01	-0.24	2.23E-01
ENSCAFG0000002920	EIF5A2	pink	VSMC_M5	0.11	5.78E-01	-0.20	3.10E-01	-0.57	1.81E-03	0.37	5.40E-02	0.70	5.02E-05	-0.16	4.36E-01	-0.30	1.22E-01	-0.23	2.47E-01	0.21	2.90E-01	-0.26	1.87E-01
ENSCAFG0000002970	ENSCAFG0000002970	pink	VSMC_M5	0.11	5.78E-01	-0.26	1.99E-01	-0.44	2.03E-02	0.15	4.69E-01	0.63	4.75E-04	-0.29	1.41E-01	-0.34	8.25E-02	-0.02	9.13E-01	0.27	1.77E-01	-0.43	2.62E-02
ENSCAFG0000000866	PLEKHA7	grey	VSMC_M10	0.11	5.78E-01	0.40	4.09E-02	0.25	2.00E-01	-0.08	6.87E-01	-0.28	1.53E-01	0.05	8.13E-01	-0.18	3.74E-01	0.00	9.97E-01	-0.44	2.03E-02	0.28	1.58E-01
ENSCAFG0000001752	PAQR5	grey	VSMC_M10	0.11	5.78E-01	-0.01	9.47E-01	0.11	5.93E-01	-0.11	5.93E-01	-0.04	8.28E-01	-0.10	6.13E-01	-0.10	6.35E-01	0.17	3.88E-01	-0.06	7.48E-01	-0.08	6.84E-01
ENSCAFG0000001459	TRPT1	grey	VSMC_M10	0.11	5.78E-01	0.33	8.84E-02	0.08	7.07E-01	0.09	6.48E-01	-0.16	4.23E-01	0.02	9.08E-01	0.35	7.40E-02	-0.18	3.65E-01	-0.43	2.66E-02	0.50	7.98E-03
ENSCAFG0000000370	ATG5	grey	VSMC_M10	0.11	5.78E-01	-0.18	3.77E-01	-0.05	8.18E-01	-0.27	1.67E-01	0.12	5.39E-01	0.16	4.33E-01	0.34	8.05E-02	0.35	7.56E-02	0.06	7.84E-01	-0.19	3.32E-01
ENSCAFG0000000711	PCM1	pink	VSMC_M5	0.11	5.78E-01	-0.51	6.24E-03	-0.66	1.72E-04	0.29	1.42E-01	0.77	2.84E-06	-0.20	3.12E-01	0.24	2.20E-01	-0.02	9.35E-01	0.39	4.46E-02	-0.28	1.51E-01
ENSCAFG0000001941	RENBP	cyan	VSMC_M2	0.11	5.79E-01	0.84	5.60E-08	0.49	9.86E-03	0.19	3.54E-01	-0.46	1.59E-02	-0.19	3.54E-01	-0.07	7.17E-01	-0.52	5.07E-03	-0.69	7.73E-05	0.57	1.86E-03
ENSCAFG0000000657	MEDAG	darkgrey	VSMC_M8	0.11	5.79E-01	-0.24	2.23E-01	0.48	1.19E-02	-0.85	2.79E-08	-0.24	2.34E-01	-0.04	8.24E-01	0.08	6.98E-01	0.74	1.04E-05	0.17	3.85E-01	-0.66	1.74E-04
ENSCAFG0000000455	ENSCAFG0000000455	grey	VSMC_M10	0.11	5.80E-01	0.25	2.07E-01	0.07	7.27E-01	0.20	3.28E-01	-0.12	5.42E-01	-0.08	7.05E-01	-0.26	1.94E-01	-0.30	1.30E-01	-0.16	4.18E-01	0.33	8.82E-02
ENSCAFG0000001962	ENSCAFG0000001962	darkgrey	VSMC_M8	0.11	5.80E-01	-0.36	6.35E-02	0.44	2.16E-02	-0.71	3.89E-05	-0.36	6.61E-02	0.24	2.25E-01	0.22	2.71E-01	0.82	1.80E-07	0.15	4.53E-01	-0.46	1.65E-02
ENSCAFG0000001285	ZSCAN2	grey	VSMC_M10	0.11	5.80E-01	0.29	1.44E-01	-0.23	2.58E-01	0.50	8.47E-03	0.26	1.90E-01	-0.10	6.08E-01	-0.29	1.36E-01	-0.50	7.54E-03	-0.19	3.36E-01	0.20	3.05E-01
ENSCAFG0000001099	ACACB	pink	VSMC_M5	0.11	5.80E-01	-0.21	2.85E-01	-0.55	2.75E-03	0.40	3.69E-02	0.63	4.89E-04	-0.13	5.24E-01	0.08	6.96E-01	-0.28	1.63E-01	0.21	2.91E-01	-0.10	6.04E-01
ENSCAFG0000001863	KSR1	grey	VSMC_M10	0.11	5.80E-01	0.74	9.89E-06	0.13	5.22E-01	0.38	4.85E-02	-0.15	4.64E-01	-0.22	2.73E-01	-0.18	3.59E-01	-0.58	1.70E-03	-0.65	2.20E-04	0.54	3.58E-03
ENSCAFG0000003058	ENSCAFG0000003058	grey	VSMC_M10	0.11	5.80E-01	-0.13	5.23E-01	-0.18	3.68E-01	0.16	4.30E-01	0.14	4.94E-01	-0.06	7.48E-01	0.27	1.76E-01	0.00	9.92E-01	-0.01	9.69E-01	0.08	6.92E-01
ENSCAFG0000001298	H2AFJ	yellow	VSMC_M3	0.11	5.80E-01	0.56	2.47E-03	0.63	4.66E-04	-0.07	7.38E-01	-0.55	2.80E-03	-0.03	8.92E-01	-0.32	1.00E-01	-0.18	3.63E-01	-0.44	2.01E-02	0.23	2.57E-01
ENSCAFG0000001584	ATP13A2	grey	VSMC_M10	0.11	5.81E-01	0.64	2.99E-04	0.29	1.46E-01	0.43	2.46E-02	-0.32	1.07E-01	-0.05	8.18E-01	-0.25	2.12E-01	-0.63	3.94E-04	-0.55	3.27E-03	0.56	2.47E-03
ENSCAFG0000000646	GTSF1	darkgreen	VSMC_M4	0.11	5.81E-01	0.13	5.25E-01	-0.47	1.32E-02	0.67	1.27E-04	0.44	2.10E-02	-0.09	6.42E-01	-0.02	9.26E-01	-0.57	2.11E-03	-0.10	6.08E-01	0.34	8.47E-02
ENSCAFG0000002953	BLOC1S2	darkgrey	VSMC_M8	0.11	5.81E-01	-0.41	3.27E-02	0.00	9.88E-01	-0.41	3.37E-02	0.16	4.31E-01	0.15	4.57E-01	-0.15	4.46E-01	0.55	3.13E-03	0.26	1.84E-01	-0.58	1.35E-03
ENSCAFG0000000577	NKIRAS1	pink	VSMC_M5	0.11	5.81E-01	-0.04	8.30E-01	-0.38	5.07E-02	0.38	4.90E-02	0.50	8.33E-03	-0.18	3.63E-01	-0.22	2.80E-01	-0.32	9.97E-02	0.03	8.76E-01	-0.11	5.75E-01
ENSCAFG0000001712	SLC51B	cyan	VSMC_M2	0.11	5.81E-01	0.50	7.41E-03	0.25	2.09E-01	0.18	3.74E-01	-0.23	2.54E-01	-0.13	5.30E-01	0.02	9.18E-01	-0.41	3.21E-02	-0.41	3.54E-02	0.45	1.91E-02
ENSCAFG0000000442	CYSLTR2	grey	VSMC_M10	0.11	5.81E-01	0.05	8.13E-01	0.16	4.16E-01	-0.16	4.27E-01	-0.08	7.02E-01	-0.01	9.78E-01	-0.04	8.31E-01	0.14	5.00E-01	-0.07	7.16E-01	-0.07	7.31E-01
ENSCAFG0000000412	KMT2E	yellow	VSMC_M3	0.11	5.81E-01	0.33	9.65E-02	0.25	2.07E-01	-0.32	1.02E-01	-0.16	4.18E-01	-0.04	8.30E-01	-0.01	9.57E-01	0.15	4.47E-01	-0.30	1.30E-01	0.06	7.56E-01
ENSCAFG0000003037	COMMD7	grey	VSMC_M10	0.11	5.81E-01	-0.21	2.98E-01	-0.17	4.01E-01	0.05	7.99E-01	0.26	1.96E-01	-0.01	9.72E-01	0.01	9.44E-01	0.09	6.69E-01	0.14	4.72E-01	-0.28	1.54E-01
ENSCAFG0000001416	IDH3A	grey	VSMC_M10	0.11	5.82E-01	-0.41	3.23E-02	-0.14	4.97E-01	-0.27	1.72E-01	0.32	9.89E-02	0.21	2.93E-01	0.20	3.08E-01	0.37	6.02E-02	0.31	1.11E-01	-0.60	8.97E-04
ENSCAFG0000000210	SGCE	grey	VSMC_M10	0.11	5.82E-01	0.47	1.26E-02	-0.30	1.26E-01	0.59	1.27E-03	0.25	2.15E-01	-0.38	4.98E-02	-0.38	5.11E-02	-0.62	5.04E-04	-0.40	4.02E-02	0.53	4.41E-03
ENSCAFG0000001781	NMUR2	grey	VSMC_M10	0.11	5.82E-01	0.60	8.53E-04	-0.08	6.95E-01	0.51	6.36E-03	0.03	8.81E-01	-0.23	2.46E-01	-0.46	1.60E-02	-0.59	1.07E-03	-0.57	2.12E-03	0.54	3.30E-03
ENSCAFG0000001208	ABCC5	grey	VSMC_M10	0.11	5.82E-01	-0.36	6.56E-02	0.12	5.65E-01	-0.43	2.40E-02	0.08	6.75E-01	0.10	6.29E-01	-0.11	5.91E-01	0.43	2.50E-02	0.37	5.68E-02	-0.60	8.83E-04
ENSCAFG0000003106	ENSCAFG0000003106	grey	VSMC_M10	0.11	5.84E-01	0.14	4.80E-01	0.06	7.84E-01	-0.10	6.15E-01	-0.06	7.53E-01	0.24	2.35E-01	-0.06	7.48E-01	0.01	9.75E-01	-0.17	4.06E-01	0.09	6.40E-01
ENSCAFG0000001086	ERGIC2	grey	VSMC_M10	0.11	5.84E-01	-0.19	3.44E-01	-0.15	4.43E-01	0.17	3.92E-01	0.17	4.07E-01	0.07	7.43E-01	-0.12	5.44E-01	-0.04	8.39E-01	0.16	4.25E-01	0.01	9.55E-01
ENSCAFG0000001853	CUL4B	grey	VSMC_M10	0.11	5.84E-01	0.29	1.39E-01	0.01	9.64E-01	-0.09	6.72E-01	0.10	6.22E-01	-0.31	1.21E-01	0.08	7.04E-01	-0.09	6.61E-01	-0.23	2.53E-01	0.15	4.56E-01
ENSCAFG0000002988	SLC25A36	pink	VSMC_M5	0.11	5.84E-01	-0.21	2.94E-01	-0.66	1.92E-04	0.43	2.56E-02	0.82	1.97E-07	-0.42	2.80E-02	-0.08	6.85E-01	-0.35	7.52E-02	0.27	1.79E-01	-0.15	4.60E-01
ENSCAFG0000001335	SIPA1	yellow	VSMC_M3	0.11	5.85E-01	0.16	4.12E-01	0.74	1.21E-05	-0.68	1.11E-04	-0.70	5.59E-05	0.37	5.76E-02	0.17	4.08E-01	0.51	6.09E-03	-0.24	2.27E-01	-0.07	7.27E-01
ENSCAFG0000003181	UBE2R2	grey	VSMC_M10	0.11	5.85E-01	0.12	5.35E-01	-0.23	2.57E-01	0.34	8.25E-02	0.39	4.33E-02	-0.38	5.15E-02	-0.53	4.35E-03	-0.41	3.15E-02	-0.01	9.58E-01	-0.07	7.17E-01
ENSCAFG0000001399	ENSCAFG0000001399	grey	VSMC_M10	0.11	5.85E-01	0.07	7.24E-01	0.29	1.49E-01	-0.21	2.96E-01	-0.08	6.75E-01	-0.21	3.02E-01	0.13	5.11E-01	0.07	7.38E-01	-0.04	8.45E-01	-0.19	3.55E-01
ENSCAFG0000003225	ENSCAFG0000003225	red	VSMC_M1	0.11	5.85E-01	0.18	3.76E-01	0.12	5.66E-01	0.21	2.83E-01	-0.13	5.29E-01	-0.14	4.91E-01	0.10	6.18E-01	-0.28	1.64E-01	-0.17	3.95E-01	0.28	1.59E-01
ENSCAFG0000002876	CST3	darkgreen	VSMC_M4	0.11	5.85E-01	0.59	1.31E-03	-0.13	5.20E-01	0.79	8.51E-07	0.01	9.69E-01	-0.09	6.39E-01	-0.27	1.80E-01	-0.84	4.49E-08	-0.49	8.89E-03	0.70	4.56E-05
ENSCAFG0000001943	TSC2	cyan	VSMC_M2	0.11	5.86E-01	0.67	1.40E-04	0.45	1.86E-02	0.20	3.10E-01	-0.53	4.73E-03	0.04	8.42E-01	-0.29	1.43E-01	-0.43	2.41E-02	-0.54	3.56E-03	0.61	7.03E-04
ENSCAFG0000000626	PCLO	darkgreen	VSMC_M4	0.11	5.86E-01	0.29	1.42E-01	-0.28	1.53E-01	0.72	2.76E-05	0.11	5.95E-01	0.11	5.88E-01	-0.06	7.59E-01	-0.62	5.35E-04	-0.34	8.61E-02	0.65	2.31E-04
ENSCAFG0000002860	ENSCAFG0000002860	grey	VSMC_M10	0.11	5.86E-01	0.12	5.67E-01	-0.17	4.06E-01	0.53	4.59E-03	0.08	6.83E-01	0.05	7.92E-01	0.11	5.86E-01	-0.50	8.33E-03	-0.12	5.49E-01	0.42	2.86E-02
ENSCAFG0000001954	MAMDC4	grey	VSMC_M10	0.11	5.87E-01	0.10	6.34E-01	-0.06	7.49E-01	0.29	1.37E-01	0.02	9.10E-01	-0.26	1.87E-01	0.10	6.07E-01	-0.27	1.71E-01	-0.10	6.04E-01	0.29	1.40E-01
ENSCAFG0000002694	ENSCAFG0000002694	yellow	VSMC_M3	0.11	5.87E-01	0.47	1.41E-02	0.42	3.07E-02	0.11	5.96E-01	-0.36	6.66E-02	-0.09	6.62E-01	-0.06	7.67E-01	-0.29	1.36E-01	-0.42	3.04E-02	0.32	1.03E-01
ENSCAFG0000001790	ENSCAFG0000001790	grey	VSMC_M10	0.11	5.87E-01	0.03	8.80E-01	-0.02	9.28E-01	0.11	5.95E-01	0.02	9.21E-01	0.03	8.77E-01	-0.10	6.33E-01	-0.14	4.87E-01	-0.01	9.80E-01	0.14	4.98E-01
ENSCAFG0000000873	FSIP1	grey	VSMC_M10	0.11	5.87E-01	0.02	9.21E-01	-0.29	1.42E-01	0.30	1.28E-01	0.34	7.87E-02	-0.08	6.88E-01	0.01	9.49E-01	-0.22	2.75E-01	-0.01	9.48E-01	0.00	9.95E-01
ENSCAFG0000000137	SUN2	grey	VSMC_M10	0.11	5.87E-01	0.29	1.48E-01	0.44	2.19E-02	-0.22	2.74E-01	-0.27	1.81E-01	-0.40	4.13E-02	0.25	2.08E-01	-0.03	8.82E-01	-0.19	3.34E-01	0.01	9.79E-01
ENSCAFG0000001816	ALDH3A2	darkgrey	VSMC_M8	0.11	5.87E-01	-0.18	3.56E-01	0.51	6.62E-03	-0.69	7.40E-05	-0.41	3.53E-02	0.09	6.72E-01	0.22	2.71E-01	0.66	1.83E-04	0.10	6.35E-01	-0.33	9.17E-02
ENSCAFG0000001706	EFNA1	grey	VSMC_M10	0.11	5.87E-01	0.14	4.93E-01	-0.32	1.02E-01	0.17	4.01E-01	0.44	2.05E-02	-0.13	5.30E-01	-0.47	1.41E-02	-0.22	2.67E-01	-0.05	8.21E-01	-0.11	5.90E-01
ENSCAFG0000001721	ENSCAFG0000001721	grey	VSMC_M10	0.11	5.88E-01	-0.04	8.25E-01	0.40	3.87E-02	-0.34	7.88E-02	-0.34	8.19E-02	-0.14	4.76E-01	0.24	2.30E-01	0.28	1.65E-01	-0.08	6.95E-01	-0.06	7.67E-01
ENSCAFG0000001678	STX4	grey	VSMC_M10	0.11	5.88E-01	0.13	5.06E-01	0.64	2.88E-04	-0.57	1.95E-03	-0.52	5.56E-03	0.03	8.87E-01	0.61	7.72E-04	0.35	7.38E-02	-0.23	2.45E-01	-0.11	5.70E-01
ENSCAFG0000001321	GPRC5A	darkgreen	VSMC_M4	0.11	5.88E-01	0.13	5.26E-01	-0.51	7.03E-03	0.71	3.09E-05	0.34	8.31E-02	0.19	3.38E-01	-0.26	1.93E-01	-0.56	2.38E-03	-0.22	2.73E-01	0.48	1.05E-02
ENSCAFG0000000323	NUP205	darkgrey	VSMC_M8	0.11	5.88E-01	-0.44	2.07E-02	0.24	2.20E-01	-0.79	8.74E-07	-0.02	9.16E-01	0.10	6.28E-01	0.32	1.02E-01	0.82	1.20E-07	0.26	1.86E-01	-0.70	4.37E-05
ENSCAFG0000000242	BZW2	darkolivegreen	VSMC_M9	0.11	5.88E-01	-0.59	1.23E-03	-0.08	6.92E-01	-0.30	1.34E-01	0.31	1.21E-01	-0.08	6.97E-01	-0.30	1.28E-01	0.47	1.29E-02	0.54	3.67E-03	-0.78	1.80E-06
ENSCAFG0000001278	NHS	darkgrey	VSMC_M8	0.11	5.89E-01	-0.48	1.16E-02	0.13	5.15E-01	-0.68	9.11E-05	0.13	5.03E-01	0.00	9.84E-01	0.58	1.44E-03	0.65	2.19E-04	0.36	6.55E-02	-0.68	9.20E-05
ENSCAFG0000001586	ENSCAFG0000001586	grey	VSMC_M10	0.11	5.89E-01	0.28	1.54E-01	0.51	6.22E-03	-0.33	9.77E-02	-0.53	4.91E-03	0.28	1.60E-01	0.22	2.62E-01	0.24	2.34E-01	-0.33	9.58E-02	0.11	5.73E-01
ENSCAFG0000003208	ENSCAFG0000003208	grey	VSMC_M10	0.11	5.89E-01	0.20	3.09E-01	-0.05	8.04E-01	0.15	4.63E-01	0.22	2.71E-01	-0.26	1.84E-01	-0.11	5.92E-01	-0.25	2.11E-01	-0.14	4.88E-01	-0.05	8.01E-01
ENSCAFG0000000827	PTPRJ	grey	VSMC_M10	0.11	5.89E-01	0.03	8.88E-01	-0.48	1.09E-02	0.68	1.02E-04	0.33	9.63E-02	0.23	2.48E-01	-0.28	1.60E-01	-0.50	7.34E-03	-0.05	8.10E-01	0.39	4.35E-02
ENSCAFG0000000487	TMEM177	grey	VSMC_M10	0.11	5.89E-01	0.41	3.24E-02	0.29	1.46E-01	0.03	8.69E-01	-0.28	1.58E-01	0.02	9.35E-01	0.08	6.79E-01	-0.15	4.53E-01	-0.46	1.67E-02	0.25	2.05E-01
ENSCAFG0000000180	KIAA1324L	cyan	VSMC_M2	0.11	5.89E-01	0.52	5.68E-03	0.16	4.13E-01	0.30	1.28E-01	-0.26	1.90E-01	0.07	7.24E-01	0.07	7.24E-01	-0.38	5.14E-02	-0.53	4.41E-03	0.56	2.21E-03
ENSCAFG0000000178	ENSCAFG0000000178	cyan	VSMC_M2	0.11	5.89E-01	0.52	5.09E-03	0.26	1.84E-01	0.15	4.45E-01	-0.34	8.71E-02	0.13	5.08E-01	-0.01	9.43E-01	-0.25	2.18E-01	-0.60	1.06E-03	0.49	9.77E-03
ENSCAFG0000000675	PPFIBP2	cyan	VSMC_M2	0.11	5.90E-01	0.87	3.39E-09	0.29	1.38E-01	0.35	7.35E-02	-0.36	6.71E-02	-0.05	7.88E-01	-0.25	2.02E-01	-0.63	4.84E-04	-0.74	1.01E-05	0.76	4.92E-06
ENSCAFG0000003092	GIPC2	grey	VSMC_M10	0.11	5.90E-01	-0.39	4.33E-02	-0.29	1.47E-01	-0.12	5.39E-01	0.40	3.76E-02	-0.13	5.33E-01	0.15	4.46E-01	0.25	2.05E-01	0.26	1.91E-01	-0.36	6.65E-02
ENSCAFG0000000864	TUBA1B	darkgrey	VSMC_M8	0.11	5.90E-01	-0.80	4.70E-07	-0.13	5.11E-01	-0.51	7.07E-03	0.32	1.02E-01	0.03	8.88E-01	0.39	4.59E-02	0.75	6.78E-06	0.62	5.18E-04	-0.82	2.02E-07
ENSCAFG0000000272	CDCA7L	darkgrey	VSMC_M8	0.11	5.90E-01	-0.21	2.84E-01	0.53	4.19E-03	-0.84	3.60E-08	-0.33	9.70E-02	-0.05	8.22E-01	0.25	2.04E-01	0.84	3.24E-08	0.07	7.11E-01	-0.61	7.24E-04
ENSCAFG0000001100	TYW5	grey	VSMC_M10	0.11	5.90E-01	-0.55	3.25E-03	0.00	9.92E-01	-0.21	2.82E-01	0.05	8.19E-01	0.08	6.78E-01	0.31	1.14E-01	0.38	5.29E-02	0.36	6.13E-02	-0.33	9.48E-02
ENSCAFG0000003191	ENSCAFG0000003191	grey	VSMC_M10	0.11	5.91E-01	-0.16	4.32E-01	0.12	5.58E-01	-0.13	5.10E-01	-0.08	7.04E-01	0.05	7.96E-01	0.34	7.89E-02	0.19	3.41E-01	0.05	7.91E-01	-0.15	4.69E-01
ENSCAFG0000000077	ENSCAFG0000000077	grey	VSMC_M10	0.11	5.91E-01	-0.15	4.58E-01	0.02	9.36E-01	-0.28	1.61E-01	0.10	6.31E-01	0.13	5.27E-01	0.40	4.12E-02	0.32	9.83E-02	0.06	7.74E-01	-0.29	1.45E-01
ENSCAFG0000003202	DUSP7	darkgrey	VSMC_M8	0.11	5.91E-01	-0.04	8.33E-01	0.55	2.90E-03	-0.75	7.71E-06	-0.40	3.63E-02	0.12	5.59E-01	-0.03	8.71E-01	0.68	8.42E-05	0.00	9.84E-01	-0.44	2.24E-02
ENSCAFG0000000877	ENSCAFG0000000877	grey	VSMC_M10	0.11	5.91E-01	0.10	6.12E-01	-0.04	8.37E-01	0.28	1.63E-01	0.03	8.97E-01	0.10	6.03E-01	0.00	9.90E-01	-0.28	1.62E-01	-0.15	4.45E-01	0.22	2.68E-01
ENSCAFG0000000404	ZSWIM9	grey	VSMC_M10	0.11	5.91E-01	0.29	1.44E-01	0.21	3.02E-01	-0.02	9.28E-01	-0.09	6.42E-01	-0.05	7.87E-01	0.15	4.55E-01	-0.07	7.21E-01	-0.32	1.01E-01	0.03	8.98E-01
ENSCAFG0000000591	ENSCAFG0000000591	turquoise	VSMC_M6	0.11	5.91E-01	0.21	3.00E-01	0.03	8.75E-01	0.10	6.10E-01	-0.04	8.27E-01	0.48	1.11E-02	-0.17	4.04E-01	-0.12	5.66E-01	-0.17	3.89E-01	0.17	4.07E-01
ENSCAFG0000003098	POPDC2	grey	VSMC_M10	0.11	5.91E-01	0.15	4.48E-01	-0.28	1.50E-01	0.39	4.70E-02	0.20	3.13E-01	0.15	4.41E-01	-0.12	5.68E-01	-0.38	5.01E-02	-0.18	3.81E-01	0.31	1.16E-01
ENSCAFG0000003244	MARVELD3	grey	VSMC_M10	0.11	5.91E-01	0.22	2.63E-01	0.04	8.25E-01	0.37	5.93E-02	-0.12	5.62E-01	0.37	5.89E-02	-0.18	3.56E-01	-0.32	9.81E-02	-0.23	2.54E-01	0.28	1.51E-01
ENSCAFG0000002475	TRIM26	cyan	VSMC_M2	0.11	5.91E-01	0.51	6.67E-03	0.27	1.79E-01	0.17	3.91E-01	-0.15	4.66E-01	-0.13	5.18E-01	-0.02	9.32E-01	-0.39	4.34E-02	-0.42	2.71E-02	0.19	3.35E-01
ENSCAFG0000001615	ENSCAFG0000001615	grey	VSMC_M10	0.11	5.92E-01	-0.29	1.40E-01	0.22	2.76E-01	-0.32	1.07E-01	0.06	7.70E-01	-0.09	6.56E-01	-0.19	3.50E-01	0.25	2.09E-01	0.34	8.63E-02	-0.60	1.05E-03
ENSCAFG0000001164	SOX2	grey	VSMC_M10	0.11	5.93E-01	-0.38	5.31E-02	-0.07	7.39E-01	-0.21	2.96E-01	0.07	7.25E-01	0.34	8.49E-02	0.17	3.90E-01	0.35	7.61E-02	0.25	2.14E-01	-0.25	2.09E-01
ENSCAFG0000001399	NKAPD1	pink	VSMC_M5	0.11	5.93E-01	-0.60	8.93E-04	-0.39	4.70E-02	-0.17	3.99E-01	0.63	4.50E-04	-0.11	5.72E-01	0.04	8.33E-01	0.32	1.03E-01	0.50	7.45E-03	-0.69	6.90E-05
ENSCAFG0000000233	ENSCAFG0000000233	grey	VSMC_M10	0.11	5.93E-01	0.35	7.48E-02	0.09	6.47E-01	-0.09	6.57E-01	-0.11	5.73E-01	-0.16	4.32E-01	0.16	4.34E-01	-0.03	8.69E-01	-0.36	6.33E-02	0.28	1.55E-01
ENSCAFG0000000422	MOB3C	grey	VSMC_M10	0.11	5.93E-01	0.37	5.46E-02	-0.04	8.55E-01	0.51	6.35E-03	0.01	9.46E-01	-0.09	6.66E-01	-0.41	3.49E-02	-0.52	5.71E-03	-0.30	1.23E-01	0.32	1.06E-01
ENSCAFG0000001126	ENSCAFG0000001126	grey	VSMC_M10	0.11	5.93E-01	-0.06	7.51E-01	0.10	6.28E-01	0.05	7.89E-01	-0.06	7.75E-01	0.05	8.13E-01	0.29	1.46E-01	-0.01	9.58E-01	-0.04	8.58E-01	-0.02	9.34E-01
ENSCAFG0000001121	RBM23	darkgrey	VSMC_M8	0.11	5.93E-01	-0.18	3.57E-01	0.30	1.33E-01	-0.65	2.59E-04	-0.15	4.43E-01	0.17	3.98E-01	0.16	4.18E-01	0.69	6.17E-05	0.02	9.36E-01	-0.51	7.00E-03
ENSCAFG0000001026	RAP1GDS1	grey	VSMC_M10	0.11	5.93E-01	-0.37	5.62E-02	-0.53	4.64E-03	0.03	8.99E-01	0.60	8.81E-04	-0.18	3.71E-01	0.10	6.17E-01	0.15	4.66E-01	0.26	1.99E-01	-0.29	1.44E-01
ENSCAFG0000001061	H2AFZ	darkgrey	VSMC_M8	0.11	5.93E-01	-0.44	2.27E-02	0.51	6.58E-03	-0.88	2.20E-09	-0.36	6.38E-02	0.14	4.83E-01	0.37	5.68E-02	0.93	4.21E-12	0.24	2.30E-01	-0.63	4.31E-04
ENSCAFG0000001588	FKBP10	cyan	VSMC_M2	0.11	5.94E-01	0.69	7.23E-05	0.52	5.93E-03	0.06	7.85E-01	-0.52	5.59E-03	-0.21	2.99E-01	-0.44	2.05E-02	-0.26	1.89E-01	-0.60	1.05E-03	0.43	2.40E-02
ENSCAFG0000001360	SPG11	grey	VSMC_M10	0.11	5.94E-01	0.30	1.24E-01	0.01	9.51E-01	0.09	6.72E-01	0.04	8.44E-01	-0.11	5.94E-01	0.20	3.20E-01	-0.29	1.41E-01	-0.29	1.40E-01	0.26	1.83E-01
ENSCAFG0000003184	ENSCAFG0000003184	cyan	VSMC_M2	0.11	5.94E-01	0.59	1.19E-03	0.45	1.82E-02	-0.26	1.91E-01	-0.45	1.92E-02	0.08	7.06E-01	-0.05	7.90E-01	0.03	8.82E-01	-0.58	1.45E-03	0.29	1.38E-01
ENSCAFG0000000496	CCT8L2	grey	VSMC_M10	0.11	5.94E-01	0.06	7.52E-01	0.18	3.59E-01	-0.17	3.83E-01	-0.16	4.11E-01	0.22	2.76E-01	0.28	1.57E-01	0.18	3.59E-01	-0.14	4.72E-01	0.00	9.91E-01
ENSCAFG0000000225	TMEM8B	darkgreen	VSMC_M4	0.11	5.94E-01	0.17	3.92E-01	-0.21	3.03E-01	0.44	2.31E-02	0.16	4.24E-01	0.04	8.24E-01	0.20	3.28E-01	-0.44	2.06E-02	-0.21	2.85E-01	0.43	2.63E-02
ENSCAFG0000001126	CSE1L	darkgrey	VSMC_M8	0.11	5.94E-01	-0.73	1.48E-05	-0.21	2.92E-01	-0.50	7.84E-03	0.35	7.34E-02	0.07	7.43E-01	0.28	1.64E-01	0.74	9.37E-06	0.53	4.82E-03	-0.71	2.81E-05
ENSCAFG0000001120	REM2	grey	VSMC_M10	0.11	5.95E-01	0.15	4.69E-01	0.00	1.00E+00	-0.07	7.10E-01	0.18	3.70E-01	-0.08	7.01E-01	0.10	6.19E-01	-0.04	8.55E-01	-0.12	5.62E-01	-0.10	6.29E-01
ENSCAFG0000001242	SYNPO2	darkgreen	VSMC_M4	0.11	5.95E-01	0.44	2.13E-02	-0.44	2.16E-02	0.92	1.80E-11	0.34	7.93E-02	-0.12	5.62E-01	-0.33	9.38E-02	-0.91	5.69E-11	-0.33	9.77E-02	0.65	2.35E-04
ENSCAFG0000003062	CMTM4	grey	VSMC_M10	0.11	5.95E-01	0.12	5.66E-01	-0.27	1.68E-01	0.61	7.68E-04	0.30	1.29E-01	-0.05	7.93E-01	-0.32	1.07E-01	-0.56	2.59E-03	-0.05	8.04E-01	0.17	3.89E-01
ENSCAFG0000001196	DALRD3	grey	VSMC_M10	0.11	5.95E-01	0.00	9.95E-01	0.22	2.78E-01	-0.48	1.20E-02	-0.05	8.18E-01	-0.20	3.12E-01	0.11	5.96E-01	0.41	3.23E-02	-0.03	8.65E-01	-0.36	6.33E-02
ENSCAFG0000001376	ENSCAFG0000001376	grey	VSMC_M10	0.11	5.96E-01	0.22	2.81E-01	-0.12	5.53E-01	0.26	1.89E-01	0.17	3.88E-01	-0.05	8.19E-01	-0.14	4.97E-01	-0.27	1.77E-01	-0.23	2.53E-01	0.17	3.99E-01
ENSCAFG0000001919	MRPL54	grey	VSMC_M10	0.11	5.96E-01	0.32	1.03E-01	0.26	1.92E-01	0.18	3.77E-01	-0.23	2.47E-01	-0.07	7.18E-01	-0.05	8.02E-01	-0.26	1.94E-01	-0.28	1.57E-01	0.18	3.67E-01
ENSCAFG0000000189	MAMDC2	grey	VSMC_M10	0.11	5.96E-01	-0.50	8.03E-03	-0.57	1.89E-03	0.15	4.59E-01	0.60	9.06E-04	0.14	4.74E-01	0.29	1.41E-01	0.07	7.46E-01	0.41	3.59E-02	-0.23	2.44E-01
ENSCAFG0000001236	FLVCR1	darkgrey	VSMC_M8	0.11	5.96E-01	-0.22	2.65E-01	0.22	2.77E-01	-0.52	5.48E-03	-0.09	6.55E-01	0.42	3.05E-02	0.44	2.17E-02	0.57	2.11E-03	0.05	8.23E-01	-0.40	3.67E-02
ENSCAFG0000001172	POLG2	grey	VSMC_M10	0.11	5.97E-01	-0.34	8.45E-02	0.10	6.18E-01	-0.24	2.20E-01	0.05	7.88E-01	-0.20	3.29E-01	0.31	1.19E-01	0.31	1.19E-01	0.24	2.26E-01	-0.42	2.91E-02
ENSCAFG0000001267	SCFD1	pink	VSMC_M5	0.11	5.97E-01	-0.54	3.65E-03	-0.61	7.65E-04	0.08	6.90E-01	0.84	3.73E-08	-0.37	5.90E-02	-0.19	3.44E-01	0.11	5.92E-01	0.51	6.27E-03	-0.59	1.13E-03
ENSCAFG0000000981	CTSA	cyan	VSMC_M2	0.11	5.97E-01	0.76	3.71E-06	0.23	2.41E-01	0.28	1.59E-01	-0.23	2.55E-01	-0.16	4.15E-01	-0.47	1.36E-02	-0.60	8.41E-04	-0.54	3.66E-03	0.54	3.57E-03
ENSCAFG0000000270	PRPF31	grey	VSMC_M10	0.11	5.97E-01	-0.18	3.68E-01	0.29	1.38E-01	-0.51	6.90E-03	-0.17	4.06E-01	-0.06	7.70E-01	0.04	8.44E-01	0.46	1.52E-02	0.16	4.31E-01	-0.37	5.75E-02
ENSCAFG0000000730	GPI	grey	VSMC_M10	0.11	5.97E-01	0.30	1.22E-01	0.00	9.92E-01	0.41	3.45E-02	0.10	6.32E-01	-0.22	2.77E-01	-0.08	6.87E-01	-0.53	4.14E-03	-0.13	5.02E-01	0.22	2.74E-01
ENSCAFG0000001948	TPRG1L	grey	VSMC_M10	0.11	5.98E-01	0.54	4.02E-03	0.08	6.78E-01	0.33	8.87E-02	-0.15	4.68E-01	-0.17	4.04E-01	-0.02	9.05E-01	-0.44	2.20E-02	-0.45	1.97E-02	0.51	6.86E-03
ENSCAFG0000000208	ENSCAFG0000000208	grey	VSMC_M10	0.11	5.98E-01	-0.28	1.59E-01	-0.07	7.20E-01	-0.05	8.23E-01	0.10	6.05E-01	0.02	9.13E-01	0.24	2.22E-01	0.06	7.85E-01	0.23	2.52E-01	-0.15	4.69E-01
ENSCAFG0000002973	CACNG7	grey	VSMC_M10	0.11	5.98E-01	-0.09	6.58E-01	-0.11	5.73E-01	-0.12	5.45E-01	0.26	1.82E-01	0.30	1.22E-01	0.00	9.91E-01	0.05	8.24E-01	0.07	7.39E-01	-0.32	1.08E-01
ENSCAFG0000003120	ENSCAFG0000003120	grey	VSMC_M10	0.11	5.98E-01	0.00	9.96E-01	0.12	5.52E-01	-0.17	4.08E-01	-0.09	6.60E-01	-0.08	7.07E-01	0.25	2.14E-01	0.18	3.60E-01	-0.08	6.96E-01	-0.02	9.33E-01
ENSCAFG0000000346	ATP6V1C2	grey	VSMC_M10	0.11	5.98E-01	-0.14	4.93E-01	0.02	9.40E-01	-0.14	4.82E-01	-0.01	9.48E-01	0.20	3.22E-01	0.33	9.01E-02	0.26	1.89E-01	-0.06	7.74E-01	-0.02	9.22E-01
ENSCAFG0000002511	JPH2	grey	VSMC_M10	0.11	5.99E-01	-0.03	8.97E-01	-0.28	1.60E-01	0.43	2.55E-02	0.29	1.45E-01	0.15	4.43E-01	-0.32	1.01E-01	-0.32	1.05E-01	0.04	8.49E-01	0.10	6.03E-01
ENSCAFG0000000063	ENSCAFG0000000063	grey	VSMC_M10	0.11	5.99E-01	0.18	3.79E-01	0.15	4.48E-01	-0.04	8.40E-01	-0.07	7.19E-01	0.02	9.31E-01	-0.01	9.44E-01	-0.04	8.42E-01	-0.18	3.70E-01	-0.03	8.94E-01

ENSCAFG0000001925	DPH1	grey	VSMC_M10	0.11	6.00E-01	-0.16	4.31E-01	0.37	5.81E-02	-0.61	7.15E-04	-0.20	3.22E-01	0.19	3.32E-01	-0.09	6.66E-01	0.47	1.28E-02	0.16	4.27E-01	-0.48	1.06E-02
ENSCAFG0000000155	TXN2	grey	VSMC_M10	0.11	6.00E-01	-0.05	8.13E-01	0.37	5.87E-02	-0.22	2.79E-01	-0.27	1.74E-01	0.05	7.86E-01	0.31	1.13E-01	0.14	4.74E-01	0.00	9.89E-01	-0.20	3.29E-01
ENSCAFG0000001788	NRK	darkgreen	VSMC_M4	0.11	6.00E-01	-0.03	8.70E-01	-0.46	1.59E-02	0.54	3.36E-03	0.44	2.21E-02	-0.02	9.30E-01	-0.02	9.12E-01	-0.40	3.99E-02	0.02	9.34E-01	0.19	3.34E-01
ENSCAFG0000000415	DNMT3A	grey	VSMC_M10	0.11	6.00E-01	-0.16	4.29E-01	-0.26	1.96E-01	0.25	2.06E-01	0.42	2.94E-02	-0.06	7.68E-01	-0.11	6.01E-01	-0.20	3.20E-01	0.17	4.04E-01	-0.23	2.43E-01
ENSCAFG0000003106	LYRM1	grey	VSMC_M10	0.11	6.00E-01	0.32	1.03E-01	-0.35	7.04E-02	0.65	2.40E-04	0.31	1.18E-01	-0.25	2.18E-01	-0.04	8.31E-01	-0.69	6.94E-05	-0.28	1.56E-01	0.46	1.46E-02
ENSCAFG0000000006	ITGA7	grey	VSMC_M10	0.11	6.00E-01	0.44	2.28E-02	0.14	5.00E-01	0.20	3.15E-01	-0.24	2.35E-01	0.17	4.01E-01	0.26	1.88E-01	-0.23	2.40E-01	-0.49	1.01E-02	0.52	5.01E-03
ENSCAFG0000001986	ENSCAFG0000001986	grey	VSMC_M10	0.11	6.01E-01	0.09	6.43E-01	0.23	2.53E-01	-0.17	3.96E-01	-0.12	5.46E-01	-0.46	1.51E-02	-0.20	3.20E-01	0.08	6.89E-01	-0.11	5.72E-01	-0.10	6.25E-01
ENSCAFG0000001191	COL6A1	yellow	VSMC_M3	0.11	6.01E-01	0.40	3.87E-02	0.78	1.38E-06	-0.49	1.03E-02	-0.58	1.46E-03	-0.27	1.73E-01	0.21	2.90E-01	0.15	4.53E-01	-0.33	9.45E-02	-0.05	8.10E-01
ENSCAFG0000001837	PGRMC1	darkgreen	VSMC_M4	0.11	6.01E-01	0.29	1.44E-01	-0.34	8.72E-02	0.60	1.01E-03	0.32	1.01E-01	-0.40	3.67E-02	-0.07	7.30E-01	-0.59	1.12E-03	-0.18	3.77E-01	0.38	4.79E-02
ENSCAFG0000000153	TSPAN33	grey	VSMC_M10	0.11	6.01E-01	0.01	9.44E-01	-0.07	7.33E-01	-0.02	9.37E-01	0.10	6.22E-01	-0.08	6.83E-01	0.10	6.33E-01	0.04	8.57E-01	-0.08	6.95E-01	0.05	8.11E-01
ENSCAFG0000001937	ABCA3	grey	VSMC_M10	0.11	6.01E-01	0.48	1.20E-02	-0.08	6.95E-01	0.43	2.38E-02	-0.05	8.19E-01	-0.05	8.07E-01	0.12	5.40E-01	-0.51	7.15E-03	-0.49	9.98E-03	0.64	3.46E-04
ENSCAFG0000001331	SIRT1	grey	VSMC_M10	0.11	6.02E-01	-0.45	1.96E-02	-0.38	5.29E-02	-0.12	5.44E-01	0.37	5.71E-02	0.09	6.47E-01	0.30	1.26E-01	0.32	1.02E-01	0.26	1.94E-01	-0.21	2.90E-01
ENSCAFG0000000277	ENSCAFG0000000277	grey	VSMC_M10	0.10	6.02E-01	0.07	7.45E-01	-0.03	8.75E-01	0.19	3.54E-01	-0.05	7.99E-01	0.12	5.45E-01	0.13	5.31E-01	-0.16	4.28E-01	-0.13	5.16E-01	0.30	1.34E-01
ENSCAFG0000001341	NFKBIA	cyan	VSMC_M2	0.10	6.03E-01	0.76	5.20E-06	0.04	8.57E-01	0.57	1.76E-03	-0.19	3.31E-01	0.10	6.22E-01	-0.33	9.11E-02	-0.67	1.32E-04	-0.72	2.01E-05	0.79	1.03E-06
ENSCAFG0000000740	THNSL2	grey	VSMC_M10	0.10	6.03E-01	0.06	7.67E-01	-0.06	7.69E-01	0.22	2.72E-01	0.02	9.30E-01	-0.04	8.50E-01	0.21	2.85E-01	-0.29	1.38E-01	-0.05	8.15E-01	0.31	1.21E-01
ENSCAFG0000000640	CAVIN3	yellow	VSMC_M3	0.10	6.03E-01	0.30	1.35E-01	0.81	3.04E-07	-0.49	1.02E-02	-0.77	2.75E-06	0.27	1.71E-01	-0.03	8.86E-01	0.31	1.10E-01	-0.32	1.06E-01	-0.01	9.49E-01
ENSCAFG0000001952	NPDC1	grey	VSMC_M10	0.10	6.03E-01	0.57	1.91E-03	0.04	8.59E-01	0.42	3.06E-02	-0.10	6.30E-01	-0.06	7.73E-01	-0.32	1.03E-01	-0.58	1.66E-03	-0.52	5.37E-03	0.57	1.75E-03
ENSCAFG0000001638	STARD3	cyan	VSMC_M2	0.10	6.03E-01	0.30	1.23E-01	0.54	3.45E-03	-0.19	3.41E-01	-0.54	3.56E-03	0.14	4.89E-01	-0.06	7.66E-01	0.05	7.94E-01	-0.33	9.42E-02	0.17	4.10E-01
ENSCAFG0000001112	GSK3B	pink	VSMC_M5	0.10	6.03E-01	-0.51	6.41E-03	-0.46	1.56E-02	-0.03	8.63E-01	0.71	3.53E-05	-0.15	4.63E-01	-0.20	3.21E-01	0.17	4.11E-01	0.54	3.34E-03	-0.66	2.04E-04
ENSCAFG0000001665	FAM81A	darkgrey	VSMC_M8	0.10	6.03E-01	-0.55	2.96E-03	0.25	2.06E-01	-0.80	5.65E-07	-0.02	9.27E-01	0.01	9.45E-01	0.66	1.89E-04	0.82	1.69E-07	0.38	5.33E-02	-0.72	1.90E-05
ENSCAFG0000000066	ENSCAFG0000000066	grey	VSMC_M10	0.10	6.03E-01	0.27	1.68E-01	0.21	2.91E-01	0.12	5.50E-01	-0.23	2.43E-01	0.21	2.83E-01	0.10	6.13E-01	-0.27	1.80E-01	-0.25	2.02E-01	0.30	1.27E-01
ENSCAFG0000001369	LRWD1	yellow	VSMC_M3	0.10	6.03E-01	0.12	5.67E-01	0.68	8.50E-05	-0.39	4.22E-02	-0.68	1.07E-04	0.22	2.62E-01	0.08	6.91E-01	0.29	1.37E-01	-0.19	3.39E-01	0.01	9.79E-01
ENSCAFG0000001033	NSUN2	darkgrey	VSMC_M8	0.10	6.04E-01	-0.51	7.17E-03	0.35	7.11E-02	-0.67	1.49E-04	-0.18	3.59E-01	0.19	3.53E-01	0.20	3.23E-01	0.79	1.09E-06	0.33	9.04E-02	-0.71	3.44E-05
ENSCAFG0000000463	FBXL2	grey	VSMC_M10	0.10	6.04E-01	0.12	5.68E-01	-0.39	4.31E-02	0.44	2.13E-02	0.42	3.02E-02	0.05	8.00E-01	-0.09	6.52E-01	-0.44	2.13E-02	-0.09	6.70E-01	0.14	5.02E-01
ENSCAFG0000002984	ENSCAFG0000002984	yellow	VSMC_M3	0.10	6.04E-01	0.29	1.45E-01	0.60	8.76E-04	-0.14	4.81E-01	-0.62	4.96E-04	0.17	3.93E-01	-0.01	9.47E-01	0.04	8.33E-01	-0.36	6.63E-02	0.20	3.10E-01
ENSCAFG0000001405	CCDC50	pink	VSMC_M5	0.10	6.04E-01	-0.04	8.31E-01	-0.50	7.28E-03	0.38	5.21E-02	0.61	6.58E-04	-0.14	5.00E-01	-0.34	8.13E-02	-0.31	1.10E-01	0.11	5.80E-01	-0.17	3.92E-01
ENSCAFG0000001012	ENSCAFG0000001012	grey	VSMC_M10	0.10	6.05E-01	-0.05	7.92E-01	-0.12	5.64E-01	-0.04	8.33E-01	0.26	1.85E-01	-0.13	5.23E-01	-0.17	3.87E-01	0.05	7.86E-01	0.03	8.68E-01	-0.32	1.08E-01
ENSCAFG0000000579	ZNF385D	grey	VSMC_M10	0.10	6.05E-01	0.02	9.15E-01	0.15	4.48E-01	-0.32	1.06E-01	-0.05	7.94E-01	-0.13	5.27E-01	0.47	1.32E-02	0.20	3.11E-01	-0.06	7.65E-01	-0.06	7.49E-01
ENSCAFG0000001348	KLF7	grey	VSMC_M10	0.10	6.05E-01	-0.02	9.27E-01	0.46	1.64E-02	-0.56	2.43E-03	-0.32	1.01E-01	0.22	2.77E-01	0.27	1.81E-01	0.45	1.99E-02	-0.10	6.20E-01	-0.22	2.65E-01
ENSCAFG0000000696	ENSCAFG0000000696	grey	VSMC_M10	0.10	6.05E-01	-0.07	7.45E-01	-0.37	5.41E-02	0.34	8.49E-02	0.46	1.48E-02	-0.12	5.43E-01	-0.08	6.77E-01	-0.23	2.46E-01	0.05	8.17E-01	-0.08	6.90E-01
ENSCAFG0000003042	ENSCAFG0000003042	pink	VSMC_M5	0.10	6.06E-01	-0.55	2.91E-03	-0.20	3.20E-01	-0.27	1.80E-01	0.46	1.69E-02	0.00	9.87E-01	0.02	9.09E-01	0.33	8.89E-02	0.55	3.16E-03	-0.72	1.99E-05
ENSCAFG0000000831	TMEM131I	grey	VSMC_M10	0.10	6.06E-01	-0.09	6.61E-01	0.17	3.85E-01	-0.40	3.71E-02	-0.16	4.34E-01	0.37	5.62E-02	0.26	1.96E-01	0.35	6.97E-02	-0.08	6.91E-01	-0.16	4.31E-01
ENSCAFG0000000163	PPP1R16A	grey	VSMC_M10	0.10	6.06E-01	0.35	7.48E-02	0.38	5.20E-02	0.05	7.99E-01	-0.28	1.55E-01	-0.19	3.41E-01	-0.22	2.81E-01	-0.14	4.95E-01	-0.32	1.03E-01	0.12	5.44E-01
ENSCAFG0000002859	ENSCAFG0000002859	grey	VSMC_M10	0.10	6.06E-01	-0.05	8.10E-01	-0.07	7.45E-01	-0.13	5.22E-01	0.10	6.36E-01	0.13	5.03E-01	0.03	8.77E-01	0.06	7.52E-01	0.00	9.91E-01	-0.01	9.67E-01
ENSCAFG0000000299	SMAP2	pink	VSMC_M5	0.10	6.07E-01	-0.35	7.57E-02	-0.48	1.21E-02	0.20	3.08E-01	0.57	1.95E-03	-0.09	6.49E-01	0.25	2.17E-01	-0.07	7.15E-01	0.31	1.16E-01	-0.26	1.85E-01
ENSCAFG0000001204	ADAL	pink	VSMC_M5	0.10	6.07E-01	-0.21	2.95E-01	-0.54	3.63E-03	0.39	4.66E-02	0.55	2.66E-03	-0.08	6.85E-01	0.14	4.81E-01	-0.24	2.32E-01	0.21	3.05E-01	0.05	8.13E-01
ENSCAFG0000002996	RGS4	grey	VSMC_M10	0.10	6.07E-01	0.29	1.44E-01	-0.32	1.08E-01	0.51	6.39E-03	0.23	2.44E-01	0.27	1.81E-01	-0.24	2.28E-01	-0.45	1.82E-02	-0.26	1.92E-01	0.44	2.03E-02
ENSCAFG0000001386	PLOD3	grey	VSMC_M10	0.10	6.07E-01	0.67	1.14E-04	0.11	5.97E-01	0.57	1.84E-03	-0.16	4.32E-01	-0.02	9.30E-01	-0.62	6.30E-04	-0.73	1.29E-05	-0.53	4.29E-03	0.59	1.35E-03
ENSCAFG0000003220	ENSCAFG0000003220	grey	VSMC_M10	0.10	6.07E-01	0.00	9.99E-01	0.05	7.86E-01	-0.28	1.55E-01	0.09	6.54E-01	-0.07	7.19E-01	0.20	3.27E-01	0.16	4.39E-01	-0.03	8.82E-01	-0.25	2.15E-01
ENSCAFG0000000100	FBXO32	cyan	VSMC_M2	0.10	6.07E-01	0.46	1.55E-02	0.21	2.84E-01	-0.08	6.94E-01	-0.10	6.07E-01	-0.09	6.41E-01	-0.18	3.77E-01	-0.25	2.12E-01	-0.34	8.25E-02	0.15	4.52E-01
ENSCAFG0000001088	PPP2R2D	grey	VSMC_M10	0.10	6.07E-01	0.25	2.07E-01	0.29	1.43E-01	-0.18	3.61E-01	-0.10	6.06E-01	-0.30	1.24E-01	-0.11	5.96E-01	-0.05	7.87E-01	-0.11	5.74E-01	-0.05	8.09E-01
ENSCAFG0000000708	LSR	grey	VSMC_M10	0.10	6.08E-01	0.34	8.39E-02	0.52	5.28E-03	-0.11	6.00E-01	-0.50	7.87E-03	0.25	2.13E-01	0.18	3.73E-01	-0.07	7.14E-01	-0.34	8.61E-02	0.22	2.67E-01
ENSCAFG0000000597	ALX1	grey	VSMC_M10	0.10	6.08E-01	0.10	6.36E-01	0.33	9.78E-02	-0.21	3.04E-01	-0.26	1.99E-01	0.21	2.87E-01	0.12	5.49E-01	0.10	6.31E-01	-0.16	4.31E-01	0.01	9.80E-01
ENSCAFG0000000338	FOXP2	grey	VSMC_M10	0.10	6.08E-01	-0.07	7.13E-01	-0.24	2.24E-01	0.27	1.80E-01	0.25	2.07E-01	0.10	6.18E-01	-0.33	9.59E-02	-0.16	4.26E-01	0.08	6.95E-01	0.01	9.73E-01
ENSCAFG0000002905	ENSCAFG0000002905	darkgreen	VSMC_M4	0.10	6.08E-01	0.40	3.82E-02	-0.43	2.51E-02	0.79	1.11E-06	0.30	1.32E-01	0.01	9.69E-01	-0.36	6.73E-02	-0.75	7.91E-06	-0.36	6.39E-02	0.60	8.60E-04
ENSCAFG0000000085	NUP50	darkgrey	VSMC_M8	0.10	6.08E-01	-0.39	4.41E-02	0.09	6.65E-01	-0.66	1.56E-04	0.10	6.26E-01	-0.03	8.92E-01	0.28	1.54E-01	0.75	5.98E-06	0.23	2.43E-01	-0.64	3.02E-04
ENSCAFG0000002939	BASP1	grey	VSMC_M10	0.10	6.08E-01	0.34	8.28E-02	0.24	2.30E-01	0.21	3.00E-01	-0.26	1.97E-01	0.02	9.20E-01	-0.05	7.86E-01	-0.32	1.06E-01	-0.35	7.45E-02	0.30	1.32E-01
ENSCAFG0000001915	PIAS4	grey	VSMC_M10	0.10	6.09E-01	0.49	1.03E-02	0.32	1.09E-01	-0.08	7.08E-01	-0.43	2.38E-02	0.19	3.47E-01	-0.08	7.02E-01	-0.12	5.56E-01	-0.51	6.70E-03	0.51	7.02E-03
ENSCAFG0000001686	PNMA1	grey	VSMC_M10	0.10	6.09E-01	0.31	1.13E-01	0.09	6.66E-01	0.31	1.21E-01	-0.18	3.81E-01	0.27	1.71E-01	0.10	6.29E-01	-0.28	1.61E-01	-0.39	4.15E-02	0.43	2.41E-02
ENSCAFG0000001745	MAP2K5	grey	VSMC_M10	0.10	6.09E-01	0.38	5.08E-02	-0.14	4.85E-01	0.37	6.01E-02	0.12	5.64E-01	-0.46	1.53E-02	0.06	7.67E-01	-0.45	1.73E-02	-0.36	6.19E-02	0.45	1.87E-02
ENSCAFG0000000603	PCNA	darkgrey	VSMC_M8	0.10	6.09E-01	-0.41	3.51E-02	0.34	8.12E-02	-0.73	1.48E-05	-0.25	2.10E-01	0.18	3.63E-01	0.52	5.31E-03	0.86	8.73E-09	0.15	4.59E-01	-0.48	1.17E-02
ENSCAFG0000001153	EGF	grey	VSMC_M10	0.10	6.09E-01	0.30	1.26E-01	0.03	8.75E-01	0.12	5.52E-01	-0.03	8.69E-01	-0.07	7.44E-01	0.27	1.79E-01	-0.19	3.46E-01	-0.29	1.45E-01	0.27	1.65E-01
ENSCAFG0000001175	ATG16L1	grey	VSMC_M10	0.10	6.09E-01	-0.18	3.81E-01	0.06	7.62E-01	-0.03	8.98E-01	0.07	7.30E-01	0.10	6.24E-01	-0.28	1.58E-01	0.04	8.58E-01	0.16	4.18E-01	-0.34	8.76E-02
ENSCAFG0000002850	CD59	darkgreen	VSMC_M4	0.10	6.09E-01	0.37	5.48E-02	-0.32	1.02E-01	0.69	7.65E-05	0.37	5.50E-02	-0.25	2.15E-01	-0.37	5.68E-02	-0.69	7.58E-05	-0.22	2.76E-01	0.30	1.26E-01
ENSCAFG0000000849	RAD9B	grey	VSMC_M10	0.10	6.10E-01	-0.11	6.00E-01	-0.20	3.17E-01	-0.01	9.49E-01	0.31	1.11E-01	-0.17	3.93E-01	0.09	6.72E-01	-0.03	8.64E-01	0.04	8.36E-01	-0.11	5.83E-01
ENSCAFG0000000377	SNX3	yellow	VSMC_M3	0.10	6.10E-01	0.50	8.12E-03	0.60	1.01E-03	-0.03	8.68E-01	-0.52	5.94E-03	-0.06	7.83E-01	0.12	5.58E-01	-0.22	2.59E-01	-0.45	1.97E-02	0.23	2.40E-01
ENSCAFG0000001626	ENSCAFG0000001626	grey	VSMC_M10	0.10	6.10E-01	0.05	8.05E-01	0.12	5.59E-01	0.08	6.87E-01	0.02	9.13E-01	0.18	3.81E-01	-0.31	1.21E-01	-0.05	7.92E-01	-0.03	8.65E-01	-0.17	3.85E-01
ENSCAFG0000001842	FBN3	grey	VSMC_M10	0.10	6.10E-01	0.20	3.15E-01	0.09	6.50E-01	-0.04	8.33E-01	-0.02	9.29E-01	-0.09	6.37E-01	-0.16	4.16E-01	0.00	9.83E-01	-0.22	2.65E-01	0.10	6.19E-01
ENSCAFG0000001567	CHERP	yellow	VSMC_M3	0.10	6.11E-01	0.52	5.62E-03	0.74	8.89E-06	-0.36	6.90E-02	-0.79	1.13E-06	0.19	3.45E-01	0.06	7.75E-01	0.15	4.68E-01	-0.57	1.88E-03	0.32	1.06E-01
ENSCAFG0000001039	FAM189A1	turquoise	VSMC_M6	0.10	6.11E-01	0.24	2.36E-01	0.13	5.04E-01	0.10	6.07E-01	-0.22	2.75E-01	0.28	1.60E-01	-0.04	8.33E-01	-0.23	2.54E-01	-0.30	1.26E-01	0.33	9.39E-02
ENSCAFG0000001196	PSME2	yellow	VSMC_M3	0.10	6.11E-01	0.32	1.08E-01	0.72	2.10E-05	-0.48	1.18E-02	-0.58	1.51E-03	-0.07	7.16E-01	-0.05	8.21E-01	0.22	2.73E-01	-0.28	1.51E-01	-0.11	5.72E-01
ENSCAFG0000001588	ENSCAFG0000001588	cyan	VSMC_M2	0.10	6.11E-01	0.46	1.56E-02	0.37	5.74E-02	0.14	4.75E-01	-0.38	5.29E-02	-0.17	3.88E-01	0.23	2.58E-01	-0.25	2.08E-01	-0.44	2.27E-02	0.44	2.16E-02
ENSCAFG0000002765	ENSCAFG0000002765	darkgreen	VSMC_M4	0.10	6.11E-01	-0.06	7.51E-01	-0.52	5.58E-03	0.59	1.07E-03	0.48	1.06E-02	-0.10	6.37E-01	0.06	7.57E-01	-0.43	2.69E-02	0.02	9.28E-01	0.20	3.10E-01
ENSCAFG0000001204	ADAMTSL4	grey	VSMC_M10	0.10	6.11E-01	0.74	8.40E-06	-0.03	8.66E-01	0.54	3.39E-03	-0.02	9.15E-01	-0.16	4.21E-01	-0.19	3.37E-01	-0.78	1.68E-06	-0.61	8.25E-04	0.70	5.27E-05
ENSCAFG0000001397	BCL2A1	grey	VSMC_M10	0.10	6.12E-01	-0.03	8.85E-01	0.40	3.76E-02	-0.38	5.00E-02	-0.40	3.62E-02	0.30	1.27E-01	0.49	9.80E-03	0.37	5.59E-02	-0.18	3.66E-01	-0.01	9.55E-01
ENSCAFG0000000977	ENSCAFG0000000977	grey	VSMC_M10	0.10	6.12E-01	0.39	4.58E-02	0.25	2.12E-01	0.11	5.88E-01	-0.35	7.63E-02	0.09	6.71E-01	-0.04	8.39E-01	-0.17	4.02E-01	-0.45	1.81E-02	0.47	1.37E-02
ENSCAFG0000000364	STARD3NL	grey	VSMC_M10	0.10	6.12E-01	-0.25	2.08E-01	-0.14	4.92E-01	0.02	9.06E-01	0.21	3.03E-01	-0.04	8.42E-01	-0.03	8.68E-01	0.09	6.63E-01	0.14	4.94E-01	-0.20	3.05E-01
ENSCAFG0000000294	HOXA2	grey	VSMC_M10	0.10	6.13E-01	0.36	6.27E-02	0.20	3.12E-01	-0.19	3.36E-01	-0.20	3.09E-01	0.06	7.76E-01	-0.27	1.78E-01	0.04	8.58E-01	-0.40	3.74E-02	0.16	4.12E-01
ENSCAFG0000001621	MAX	grey	VSMC_M10	0.10	6.13E-01	0.27	1.68E-01	0.11	5.72E-01	-0.19	3.43E-01	-0.10	6.05E-01	0.05	8.01E-01	-0.02	9.20E-01	-0.01	9.48E-01	-0.24	2.22E-01	0.16	4.19E-01
ENSCAFG0000001512	NCAPD2	darkgrey	VSMC_M8	0.10	6.13E-01	-0.42	3.11E-02	0.36	6.58E-02	-0.87	3.75E-09	-0.19	3.44E-01	0.10	6.33E-01	0.45	1.99E-02	0.92	6.07E-12	0.20	3.15E-01	-0.63	4.49E-04
ENSCAFG0000000119	ENSCAFG0000000119	grey	VSMC_M10	0.10	6.13E-01	-0.04	8.54E-01	0.04	8.58E-01	-0.06	7.80E-01	-0.03	8.63E-01	0.51	6.87E-03	-0.12	5.54E-01	0.13	5.30E-01	-0.03	8.90E-01	-0.06	7.48E-01
ENSCAFG0000000310	LYRM2	grey	VSMC_M10	0.10	6.13E-01	0.24	2.33E-01	0.15	4.55E-01	0.06	7.76E-01	-0.29	1.44E-01	-0.05	8.10E-01	0.09	6.55E-01	-0.11	5.94E-01	-0.30	1.25E-01	0.46	1.46E-02
ENSCAFG0000000981	JAM3	darkgreen	VSMC_M4	0.10	6.13E-01	0.25	2.06E-01	-0.46	1.50E-02	0.83	8.63E-08	0.33	9.35E-02	-0.03	8.89E-01	0.02	9.39E-01	-0.76	4.51E-06	-0.23	2.49E-01	0.62	5.92E-04
ENSCAFG0000001001	PCBP4	yellow	VSMC_M3	0.10	6.13E-01	0.46	1.53E-02	0.58	1.65E-03	-0.34	8.45E-02	-0.56	2.44E-03	0.04	8.40E-01	-0.07	7.41E-01	0.12	5.52E-01	-0.42	3.06E-02	0.21	2.83E-01
ENSCAFG0000000646	FOXP1	grey	VSMC_M10	0.10	6.13E-01	0.00	9.85E-01	-0.01	9.49E-01	-0.15	4.43E-01	0.24	2.33E-01	-0.27	1.76E-01	-0.54	3.92E-03	0.08	6.78E-01	0.10	6.29E-01	-0.33	9.75E-02
ENSCAFG0000000458	TMEM176B	cyan	VSMC_M2	0.10	6.13E-01	0.59	1.17E-03	-0.07	7.37E-01	0.62	5.66E-04	0.01	9.62E-01	-0.12	5.49E-01	-0.45	1.89E-02	-0.63	3.80E-04	-0.54	3.85E-03	0.54	3.72E-03
ENSCAFG0000001962	THAP3	grey	VSMC_M10	0.10	6.13E-01	0.20	3.29E-01	0.47	1.34E-02	-0.32	1.08E-01	-0.42	2.89E-02	0.03	8.94E-01	0.12	5.47E-01	0.21	2.85E-01	-0.32	9.89E-02	0.03	8.67E-01
ENSCAFG0000002541	ENSCAFG0000002541	turquoise	VSMC_M6	0.10	6.14E-01	0.42	2.92E-02	0.47	1.27E-02	-0.08	7.04E-01	-0.54	3.67E-03	0.61	6.52E-04	-0.18	3.80E-01	0.01	9.52E-01	-0.46	1.51E-02	0.27	1.76E-01
ENSCAFG0000000080	FBLN1	grey	VSMC_M10	0.10	6.14E-01	0.44	2.13E-02	0.28	1.55E-01	0.02	9.04E-01	-0.30	1.33E-01	-0.05	8.04E-01	0.41	3.23E-02	-0.12	5.42E-01	-0.49	9.62E-03	0.37	6.06E-02
ENSCAFG0000001145	PSMD12	darkolivegreen	VSMC_M9	0.10	6.14E-01	-0.59	1.25E-03	-0.23	2.53E-01	-0.22	2.68E-01	0.40	3.62E-02	0.02	9.09E-01	0.02	9.30E-01	0.41	3.30E-02	0.50	7.33E-03	-0.66	1.78E-04
ENSCAFG0000002306	ENSCAFG0000002306	grey	VSMC_M10	0.10	6.14E-01	0.03	8.64E-01	0.28	1.50E-01	-0.15	4.50E-01	-0.26	1.94E-01	0.22	2.81E-01	0.14	4.79E-01	0.15	4.55E-01	-0.15	4.69E-01	-0.01	9.45E-01
ENSCAFG0000000629	NR3C1	pink	VSMC_M5	0.10	6.14E-01	0.21	2.90E-01	-0.53	4.78E-03	0.55	3.12E-03	0.56	2.17E-03	-0.17	3.88E-01	0.12	5.48E-01	-0.53	4.30E-03	-0.17	3.84E-01	0.26	1.91E-01
ENSCAFG0000000201	FAM214B	darkgreen	VSMC_M4	0.10	6.15E-01	0.35	7.61E-02	-0.24	2.28E-01	0.69	7.76E-05	0.15	4.59E-01	-0.05	8.21E-01	-0.45	1.85E-02	-0.75	6.94E-06	-0.21	2.87E-01	0.49	9.04E-03
ENSCAFG0000003211	ENSCAFG0000003211	grey	VSMC_M10	0.10	6.15E-01	-0.23	2.57E-01	0.09	6.55E-01	-0.22	2.67E-01	-0.06	7.57E-01	-0.15	4.58E-01	0.06	7.71E-01	0.32	1.07E-01	0.17	3.96E-01	-0.22	2.67E-01
ENSCAFG0000000628	ENSCAFG0000000628	grey	VSMC_M10	0.10	6.16E-01	-0.01	9.59E-01	0.11	5.68E-01	-0.03	8.90E-01	-0.12	5.66E-01	0.26	1.90E-01	0.39	4.52E-02	0.07	7.23E-01	-0.12	5.50E-01	0.14	4.76E-01
ENSCAFG0000003179	RAP2B	grey	VSMC_M10	0.10	6.16E-01	0.38	4.77E-02	0.16	4.12E-01	0.11	5.89E-01	-0.16	4.21E-01	-0.05	8.12E-01	-0.24	2.33E-01	-0.09	6.45E-01	-0.40	4.14E-02	0.19	3.39E-01
ENSCAFG0000000276	FSD1L	grey	VSMC_M10	0.10	6.16E-01	-0.18	3.70E-01	0.19	3.45E-01	-0.64	3.30E-04	-0.02	9.15E-01	0.10	6.14E-01	0.24	2.35E-01	0.54	3.97E-03	0.10	6.20E-01	-0.42	2.82E-02
ENSCAFG0000000766	ZBTB38	yellow	VSMC_M3	0.10	6.16E-01	0.47	1.28E-02	0.55	3.06E-03	-0.34	7.97E-02	-0.40	3.75E-02	0.13	5.20E-01	0.06	7.85E-01	0.01	9.47E-01	-0.39	4.59E-02	0.07	7.34E-01
ENSCAFG0000002364	KRT19	grey	VSMC_M10	0.10	6.17E-01	0.26	1.98E-01	0.07	7.19E-01	-0.04	8.50E-01	-0.01	9.56E-01	-0.10	6.14E-01	-0.17	3.90E-01	-0.13	5.03E-01	-0.16	4.14E-01	0.10	6.13E-01
ENSCAFG0000002012	TOR2A	grey	VSMC_M10	0.10	6.17E-01	0.14	4.77E-01	0.37	5.78E-02	-0.37	5.66E-02	-0.24	2.30E-01	-0.26	1.98E-01	-0.28	1.63E-01	0.22	2.76E-01	-0.13	5.13E-01	-0.16	4.11E-01
ENSCAFG0000001986	PPP1R26	darkgreen	VSMC_M4	0.10	6.17E-01	0.64	3.29E-04	-0.21	2.89E-01	0.80	6.30E-07	0.10	6.15E-01	-0.12	5.52E-01	-0.49	1.02E-02	-0.90	9.21E-11	-0.52	5.80E-03	0.75	5.53E-06
ENSCAFG0000001312	ZNF512B	cyan	VSMC_M2	0.10	6.17E-01	0.57	1.81E-03	0.39	4.24E-02	0.13	5.10E-01	-0.47	1.29E-02	0.07	7.34E-01	-0.51	6.73E-03	-0.24	2.21E-01	-0.49	9.52E-03	0.49	9.47E-03
ENSCAFG0000001542	BHLHA15	grey	VSMC_M10	0.10	6.17E-01	0.24	2.30E-01	0.44	2.17E-02	-0.16	4.35E-01	-0.47	1.28E-02	0.37	5.62E-02	-0.16	4.24E-01	0.15	4.67E-01	-0.28	1.50E-01	0.12	5.62E-01
ENSCAFG0000001433	SLC29A3	grey	VSMC_M10	0.10	6.17E-01	0.15	4.66E-01	0.11	5.74E-01	-0.23	2.49E-01	-0.18	3.64E-01	0.26	1.89E-01	-0.03	8.64E-01	0.18	3.72E-01	-0.20	3.24E-01	0.19	3.42E-01
ENSCAFG0000002318	ENSCAFG0000002318	grey	VSMC_M10	0.10	6.17E-01	0.09	6.72E-01	0.11	5.71E-01	-0.12	5.64E-01	-0.04	8.28E-01	-0.31	1.14E-01	-0.17	4.05E-01	0.10	6.37E-01	-0.09	6.65E-01	0.01	9.46E-01
ENSCAFG0000000000	ENPP1	yellow	VSMC_M3	0.10	6.18E-01	-0.29	1.40E-01	0.30	1.35E-01	-0.72	1.90E-05	-0.03	8.84E-01	-0.11	5.83E-01	0.35	7.74E-02	0.61	6.87E-04	0.22	2.73E-01	-0.60	8.67E-04
ENSCAFG0000001251	TMOD4	grey	VSMC_M10	0.10	6.18E-01	0.26	1.89E-01	0.03	8.80E-01	0.39	4.68E-02	-0.10	6.27E-01	0.01	9.45E-01	-0.26	1.83E-01	-0.33	9.41E-02	-0.29	1.36E-01	0.38	5.28E-02
ENSCAFG0000001562	SGMS1	grey	VSMC_M10	0.10	6.18E-01	-0.17	4.09E-01	-0.14	4.79E-01	-0.19	3.32E-01	0.27	1.66E-01	-0.11	5.95E-01	-0.38	5.10E-02	0.29	1.45E-01	0.13	5.31E-01	-0.34	7.82E-02
ENSCAFG0000001830	USP14	pink	VSMC_M5	0.10	6.18E-01	-0.77	3.14E-06	-0.39	4.66E-02	-0.16	4.11E-01	0.58	1.69E-03	-0.04	8.59E-01	-0.02	9.33E-01	0.42	2.87E-02	0.69	7.88E-05	-0.75	7.92E-06
ENSCAFG0000002475	ENSCAFG0000002475	pink	VSMC_M5	0.10	6.19E-01	-0.46	1.57E-02	-0.48	1.22E-02	-0.08	6.77E-01	0.60	9.72E-04	-0.01	9.75E-01	0.02	9.18E-01	0.24	2.29E-01	0.41	3.22E-02	-0.46	1.57E-02
ENSCAFG0000001766	SIGLEC15	cyan	VSMC_M2	0.10	6.19E-01	0.36	6.40E-02	0.43	2.35E-02	0.06	7.64E-01	-0.41	3.35E-02	-0.15	4.56E-01	-0.23	2.47E-01	-0.15	4.45E-01	-0.32	1.03E-01	0.25	2.01E-01
ENSCAFG0000000959	PCNP	darkolivegreen	VSMC_M9	0.10	6.19E-01	-0.61	6.93E-04	-0.33	9.27E-02	-0.12	5.60E-01	0.52	5.72E-03	-0.08	6.91E-01	0.10	6.23E-01	0.34	8.40E-02	0.51	6.41E-03	-0.56	2.61E-03
ENSCAFG0000001746	PIAS1	pink	VSMC_M5	0.10	6.19E-01	-0.30	1.22E-01	-0.61	6.57E-04	0.37	5.97E-02	0.73	1.64E-05	-0.12	5.40E-01	-0.19	3.31E-01	-0.15	4.55E-01	0.33	9.50E-02	-0.27	1.79E-01
ENSCAFG0000003134	RHOU	grey	VSMC_M10	0.10	6.20E-01	0.03	8.65E-01	0.09	6.56E-01	0.02	9.39E-01	-0.18	3.79E-01	0.31	1.17E-01	0.55	3.25E-03	-0.03	8.92E-01	-0.15	4.47E-01	0.30	1.30E-01
ENSCAFG0000001543	ANKLE1	darkgrey	VSMC_M8	0.10	6.20E-01	-0.28	1.54E-01	0.56	2.14E-03	-0.91	8.41E-11	-0.37	5.70E-02	0.08	7.04E-01	0.27	1.69E-01	0.88	1.73E-09	0.14	4.97E-01	-0.64	3.72E-04
ENSCAFG0000000578	LAMTOR1	yellow	VSMC_M3	0.10	6.20E-01	0.56	2.17E-03	0.62	5.74E-04	-0.36	6.53E-02	-0.57	1.73E-03	0.07	7.26E-01	-0.06	7.57E-01	0.05	8.02E-01	-0.51	6.70E-03	0.21	2.94E-01
ENSCAFG0000002015	SF3B3	darkgrey	VSMC_M8	0.10	6.20E-01	-0.48	1.08E-02	0.02	9.04E-01	-0.61	7.93E-04	0.10	6.24E-01	0.24	2.30E-01	0.42	2.90E-02	0.80	6.50E-07	0.25	2.14E-01	-0.58	1.48E-03
ENSCAFG0000001211	URB2	grey	VSMC_M10	0.10	6.20E-01	0.07	7.30E-01	0.11	5.74E-01	-0.31	1.13E-01	-0.15	4.43E-01	0.32	1.02E-01	0.20	3.20E-01	0.22	2.80E-01	-0.19	3.54E-01	0.08	6.94E-01
ENSCAFG0000001011	MTRR	grey	VSMC_M10	0.10	6.20E-01	-0.19	3.37E-01	-0.43	2.38E-02	0.14	4.99E-01	0.46	1.49E-02	0.01	9.70E-01	0.22	2.81E-01	-0.02	9.28E-01	0.04	8.62E-01	-0.03	8.96E-01
ENSCAFG0000000690	VPS37A	pink	VSMC_M5	0.10	6.21E-01	-0.31	1.17E-01	-0.70	5.68E-05	0.47	1.40E-02	0.71	3.92E-05	-0.10	6.28E-01	-0.43	2.50E-02	-0.26	1.92E-01	0.28	1.51E-01	-0.07	7.29E-01
ENSCAFG0000001314	ENSCAFG0000001314	yellow	VSMC_M3	0.10	6.21E-01	0.48	1.13E-02	0.53	4.65E-03	-0.17	4.04E-01	-0.59	1.25E-03	0.08	7.06E-01	0.18	3.83E-01	-0.01	9.72E-01	-0.55	3.16E-03	0.38	4.94E-02
ENSCAFG0000002903	PDLIM3	darkgreen	VSMC_M4	0.10	6.21E-01	0.45	1.82E-02	-0.39	4.38E-02	0.90	1.26E-10	0.26	1.93E-01	-0.11	5.94E-01	-0.49	9.22E-03	-0.85	2.64E-08	-0.37	5.83E-02	0.66	1.62E-04
ENSCAFG0000001266	RFX5	cyan	VSMC_M2	0.10	6.22E-01	0.61	6.78E-04	0.33	9.26E-02	0.35	7.11E-02	-0.46	1.67E-02	0.10	6.30E-01	0.07	7.35E-01	-0.49	8.92E-03	-0.60	9.91E-04	0.69	6.78E-05
ENSCAFG0000001452	STAG3	grey	VSMC_M10	0.10	6.22E-01	-0.29	1.46E-01	0.03	8.68E-01	-0.31	1.22E-01	0.04	8.54E-01	-0.11	5.93E-01	-0.08	6.89E-01	0.42	3.12E-02	0.17	4.09E-01	-0.26	1.91E-01
ENSCAFG0000001747	TTC8	pink	VSMC_M5	0.10	6.22E-01	-0.23	2.56E-01	-0.62	5.05E-04	0.24	2.28E-01	0.76	5.25E-06	-0.31	1.19E-01	-0.05	7.95E-01	-0.14	4.72E-01	0.25	2.18E-01	-0.26	1.91E-01
ENSCAFG0000001268	RNPEPL1	cyan	VSMC_M2	0.10	6.22E-01	0.47	1.44E-02	0.60	1.04E-03	-0.19	3.38E-01	-0.53	4.10E-03	0.01	9.45E-01	-0.08	7.02E-01	-0.11	5.82E-01	-0.36	6.47E-02	0.22	2.67E-01

ENSCAFG0000001203	ZNF710	darkgreen	VSMC_M4	0.10	6.23E-01	0.16	4.20E-01	-0.34	7.93E-02	0.56	2.34E-03	0.26	1.96E-01	0.12	5.46E-01	-0.07	7.43E-01	-0.44	2.32E-02	-0.18	3.75E-01	0.37	5.40E-02
ENSCAFG0000001987	MVD	yellow	VSMC_M3	0.10	6.23E-01	0.00	9.94E-01	0.65	2.22E-04	-0.76	4.14E-06	-0.48	1.16E-02	0.07	7.37E-01	0.47	1.34E-02	0.57	1.78E-03	-0.06	7.64E-01	-0.34	8.71E-02
ENSCAFG0000001383	KANSL1L	grey	VSMC_M10	0.10	6.23E-01	0.45	1.73E-02	-0.30	1.34E-01	0.45	1.94E-02	0.39	4.35E-02	-0.34	8.70E-02	-0.51	6.91E-03	-0.60	9.67E-04	-0.28	1.51E-01	0.26	1.85E-01
ENSCAFG0000002601	ENSCAFG0000002601	grey	VSMC_M10	0.10	6.23E-01	0.07	7.47E-01	0.01	9.72E-01	0.00	9.82E-01	-0.01	9.46E-01	-0.11	5.86E-01	0.14	4.81E-01	0.00	9.93E-01	-0.17	3.96E-01	0.15	4.66E-01
ENSCAFG0000001349	FAM129A	darkgreen	VSMC_M4	0.10	6.23E-01	0.62	5.97E-04	-0.29	1.46E-01	0.81	2.49E-07	0.23	2.46E-01	-0.22	2.74E-01	-0.42	2.93E-02	-0.88	1.49E-09	-0.49	9.25E-03	0.67	1.39E-04
ENSCAFG0000001272	EPS8	grey	VSMC_M10	0.10	6.24E-01	0.26	1.86E-01	0.35	7.56E-02	-0.42	3.12E-02	-0.20	3.11E-01	-0.36	6.51E-02	0.47	1.39E-02	0.18	3.77E-01	-0.27	1.78E-01	-0.02	9.12E-01
ENSCAFG0000001834	ENSCAFG0000001834	grey	VSMC_M10	0.10	6.24E-01	-0.17	4.03E-01	-0.02	9.05E-01	-0.25	2.06E-01	0.22	2.80E-01	-0.04	8.33E-01	0.25	2.08E-01	0.24	2.28E-01	0.10	6.35E-01	-0.40	3.82E-02
ENSCAFG0000002344	UBB	grey	VSMC_M10	0.10	6.24E-01	-0.15	4.49E-01	0.11	5.87E-01	-0.03	8.64E-01	0.02	9.40E-01	-0.22	2.64E-01	0.42	2.86E-02	0.13	5.24E-01	0.06	7.62E-01	-0.24	2.19E-01
ENSCAFG0000000649	PKP3	grey	VSMC_M10	0.10	6.24E-01	0.10	6.08E-01	0.14	4.75E-01	0.06	7.81E-01	-0.22	2.78E-01	0.12	5.67E-01	0.57	1.73E-03	0.00	9.80E-01	-0.25	2.02E-01	0.24	2.22E-01
ENSCAFG0000001474	ZNF384	grey	VSMC_M10	0.10	6.24E-01	0.27	1.72E-01	-0.12	5.42E-01	-0.06	7.85E-01	0.17	4.05E-01	-0.09	6.48E-01	-0.02	9.05E-01	-0.06	7.83E-01	-0.27	1.72E-01	0.15	4.41E-01
ENSCAFG0000000025	ARHGEF25	cyan	VSMC_M2	0.10	6.24E-01	0.54	3.72E-03	0.29	1.39E-01	0.34	8.51E-02	-0.29	1.35E-01	-0.27	1.66E-01	-0.19	3.45E-01	-0.47	1.33E-02	-0.41	3.30E-02	0.45	1.95E-02
ENSCAFG0000002377	ZBTB8A	grey	VSMC_M10	0.10	6.24E-01	-0.29	1.46E-01	-0.07	7.24E-01	-0.46	1.60E-02	0.28	1.56E-01	-0.31	1.13E-01	0.18	3.79E-01	0.45	1.97E-02	0.24	2.31E-01	-0.48	1.09E-02
ENSCAFG0000001021	KIF18A	darkgrey	VSMC_M8	0.10	6.25E-01	-0.60	8.82E-04	0.00	9.93E-01	-0.59	1.08E-03	0.22	2.80E-01	-0.11	5.98E-01	0.40	3.70E-02	0.75	7.81E-06	0.42	3.10E-02	-0.71	3.50E-05
ENSCAFG0000002762	ENSCAFG0000002762	grey	VSMC_M10	0.10	6.25E-01	0.21	2.89E-01	0.22	2.80E-01	-0.20	3.23E-01	-0.16	4.38E-01	0.00	9.96E-01	0.20	3.08E-01	0.13	5.17E-01	-0.25	2.14E-01	0.06	7.53E-01
ENSCAFG0000000541	RNF170	cyan	VSMC_M2	0.10	6.25E-01	0.44	2.26E-02	0.23	2.54E-01	0.39	4.30E-02	-0.22	2.59E-01	-0.04	8.54E-01	-0.24	2.20E-01	-0.53	4.58E-03	-0.35	7.44E-02	0.37	5.76E-02
ENSCAFG0000000829	CHDH	grey	VSMC_M10	0.10	6.25E-01	0.18	3.60E-01	0.29	1.41E-01	-0.27	1.66E-01	-0.28	1.60E-01	0.07	7.33E-01	0.43	2.51E-02	0.17	3.89E-01	-0.28	1.59E-01	0.14	4.95E-01
ENSCAFG0000000530	BIRC5	darkgrey	VSMC_M8	0.10	6.26E-01	-0.52	5.55E-03	0.40	3.94E-02	-0.91	8.31E-11	-0.20	3.06E-01	0.07	7.19E-01	0.43	2.64E-02	0.96	7.54E-16	0.33	9.34E-02	-0.74	9.77E-06
ENSCAFG0000000747	ENSCAFG0000000747	cyan	VSMC_M2	0.10	6.26E-01	0.58	1.57E-03	0.45	1.81E-02	0.01	9.50E-01	-0.40	3.65E-02	-0.22	2.61E-01	-0.15	4.69E-01	-0.29	1.42E-01	-0.50	7.31E-03	0.28	1.54E-01
ENSCAFG0000000405	HS3ST5	grey	VSMC_M10	0.10	6.26E-01	-0.26	1.94E-01	-0.03	8.63E-01	-0.17	3.95E-01	0.03	8.98E-01	0.13	5.23E-01	-0.12	5.46E-01	0.28	1.57E-01	0.20	3.28E-01	-0.18	3.77E-01
ENSCAFG0000001381	SRGN	grey	VSMC_M10	0.10	6.26E-01	-0.39	4.49E-02	0.11	5.99E-01	-0.50	7.25E-03	0.10	6.08E-01	0.13	5.23E-01	-0.25	1.99E-01	0.49	9.11E-03	0.37	6.09E-02	-0.63	4.84E-04
ENSCAFG0000000477	AGAP3	cyan	VSMC_M2	0.10	6.26E-01	0.60	9.52E-04	0.14	4.75E-01	0.03	8.76E-01	-0.16	4.21E-01	-0.22	2.71E-01	-0.17	4.08E-01	-0.22	2.80E-01	-0.58	1.52E-03	0.42	2.77E-02
ENSCAFG0000003096	ENSCAFG0000003096	turquoise	VSMC_M6	0.10	6.26E-01	-0.22	2.65E-01	-0.11	5.88E-01	-0.06	7.63E-01	0.12	5.61E-01	0.49	9.73E-03	-0.11	5.94E-01	0.13	5.19E-01	0.21	2.90E-01	-0.18	3.68E-01
ENSCAFG0000003128	ZNF620	grey	VSMC_M10	0.10	6.27E-01	-0.31	1.11E-01	0.25	2.07E-01	-0.45	1.96E-02	-0.09	6.70E-01	0.25	2.14E-01	0.07	7.41E-01	0.47	1.25E-02	0.29	1.35E-01	-0.53	4.35E-03
ENSCAFG0000001579	CRMP1	grey	VSMC_M10	0.10	6.27E-01	-0.19	3.38E-01	0.01	9.80E-01	-0.18	3.75E-01	0.05	7.94E-01	-0.07	7.15E-01	0.14	5.00E-01	0.20	3.16E-01	0.15	4.43E-01	-0.19	3.36E-01
ENSCAFG0000001884	PRELID3A	grey	VSMC_M10	0.10	6.27E-01	-0.09	6.41E-01	0.40	4.13E-02	-0.52	5.57E-03	-0.29	1.43E-01	-0.16	4.17E-01	0.49	8.90E-03	0.44	2.07E-02	-0.01	9.58E-01	-0.20	3.22E-01
ENSCAFG0000001225	ENSCAFG0000001225	grey	VSMC_M10	0.10	6.27E-01	0.01	9.52E-01	0.11	5.98E-01	-0.01	9.66E-01	-0.04	8.54E-01	-0.06	7.62E-01	0.06	7.75E-01	-0.02	9.18E-01	-0.04	8.26E-01	0.03	9.00E-01
ENSCAFG0000000075	GRAMD4	grey	VSMC_M10	0.10	6.27E-01	0.70	4.67E-05	0.34	8.21E-02	0.25	2.17E-01	-0.47	1.36E-02	0.09	6.63E-01	0.03	8.88E-01	-0.48	1.10E-02	-0.68	9.84E-05	0.77	2.81E-06
ENSCAFG0000000420	MKNK1	grey	VSMC_M10	0.10	6.28E-01	0.71	3.84E-05	0.22	2.65E-01	0.28	1.59E-01	-0.25	2.04E-01	-0.02	9.27E-01	-0.57	1.86E-03	-0.49	9.28E-03	-0.60	9.29E-04	0.51	6.26E-03
ENSCAFG0000001667	NCAPG	darkgrey	VSMC_M8	0.10	6.28E-01	-0.48	1.07E-02	0.38	4.82E-02	-0.91	5.01E-11	-0.20	3.24E-01	0.10	6.28E-01	0.39	4.48E-02	0.97	1.57E-16	0.29	1.44E-01	-0.70	5.17E-05
ENSCAFG0000000382	PGRMC2	grey	VSMC_M10	0.10	6.28E-01	-0.09	6.44E-01	-0.08	6.78E-01	-0.08	6.84E-01	0.31	1.11E-01	-0.27	1.69E-01	-0.69	7.06E-05	0.00	9.94E-01	0.24	2.26E-01	-0.42	3.07E-02
ENSCAFG0000000183	SMU1	grey	VSMC_M10	0.10	6.28E-01	-0.01	9.59E-01	0.23	2.51E-01	-0.45	1.76E-02	0.04	8.58E-01	-0.34	8.03E-02	-0.16	4.14E-01	0.28	1.51E-01	0.07	7.29E-01	-0.48	1.17E-02
ENSCAFG0000002925	ENSCAFG0000002925	grey	VSMC_M10	0.10	6.28E-01	0.12	5.39E-01	-0.03	8.80E-01	0.23	2.49E-01	0.00	9.89E-01	-0.09	6.52E-01	0.17	3.86E-01	-0.23	2.56E-01	-0.17	4.11E-01	0.26	1.86E-01
ENSCAFG0000000862	E2F5	grey	VSMC_M10	0.10	6.29E-01	-0.37	5.69E-02	-0.53	4.33E-03	0.32	1.08E-01	0.63	3.99E-04	-0.33	8.77E-02	-0.28	1.52E-01	-0.11	5.70E-01	0.39	4.69E-02	-0.30	1.30E-01
ENSCAFG0000000580	NUMA1	yellow	VSMC_M3	0.10	6.29E-01	0.24	2.21E-01	0.76	3.78E-06	-0.62	5.42E-04	-0.57	1.78E-03	-0.02	9.28E-01	0.00	9.96E-01	0.34	8.69E-02	-0.18	3.69E-01	-0.17	3.91E-01
ENSCAFG0000000544	TGFBR2	grey	VSMC_M10	0.10	6.29E-01	0.74	1.19E-05	0.09	6.47E-01	0.38	5.20E-02	-0.14	4.94E-01	-0.16	4.28E-01	-0.06	7.52E-01	-0.54	3.65E-03	-0.66	1.66E-04	0.61	7.86E-04
ENSCAFG0000001844	ISL1	grey	VSMC_M10	0.10	6.30E-01	0.16	4.35E-01	0.09	6.72E-01	-0.16	4.23E-01	-0.12	5.36E-01	0.36	6.31E-02	0.04	8.62E-01	0.03	8.99E-01	-0.21	2.95E-01	0.14	4.97E-01
ENSCAFG0000000735	LSM14A	pink	VSMC_M5	0.10	6.30E-01	-0.53	4.81E-03	-0.36	6.40E-02	-0.16	4.35E-01	0.54	3.36E-03	-0.15	4.68E-01	0.30	1.32E-01	0.33	9.16E-02	0.43	2.47E-02	-0.56	2.34E-03
ENSCAFG0000000586	TMTC4	grey	VSMC_M10	0.10	6.30E-01	0.50	7.85E-03	-0.02	9.38E-01	0.38	5.22E-02	0.06	7.72E-01	0.00	9.85E-01	-0.37	6.00E-02	-0.49	1.03E-02	-0.41	3.39E-02	0.30	1.25E-01
ENSCAFG0000001635	BRAT1	cyan	VSMC_M2	0.10	6.31E-01	0.68	1.07E-04	0.30	1.31E-01	0.22	2.77E-01	-0.39	4.31E-02	0.12	5.42E-01	-0.10	6.10E-01	-0.43	2.39E-02	-0.63	4.11E-04	0.64	3.56E-04
ENSCAFG0000002874	ENSCAFG0000002874	pink	VSMC_M5	0.10	6.31E-01	-0.16	4.22E-01	-0.48	1.18E-02	0.37	6.09E-02	0.47	1.25E-02	0.11	5.76E-01	0.04	8.55E-01	-0.23	2.44E-01	0.13	5.09E-01	0.09	6.62E-01
ENSCAFG0000000738	ENSCAFG0000000738	grey	VSMC_M10	0.10	6.31E-01	-0.04	8.33E-01	0.08	6.77E-01	-0.23	2.42E-01	-0.05	7.93E-01	-0.05	8.14E-01	0.06	7.78E-01	0.24	2.35E-01	0.01	9.73E-01	-0.08	7.00E-01
ENSCAFG0000001716	PMVK	yellow	VSMC_M3	0.10	6.31E-01	0.38	4.96E-02	0.66	1.76E-04	-0.16	4.14E-01	-0.63	4.87E-04	0.00	9.86E-01	0.26	1.86E-01	-0.04	8.40E-01	-0.36	6.43E-02	0.24	2.32E-01
ENSCAFG0000001795	TNIP1	grey	VSMC_M10	0.10	6.31E-01	0.07	7.14E-01	0.27	1.69E-01	-0.16	4.13E-01	-0.09	6.53E-01	0.12	5.63E-01	-0.41	3.26E-02	0.10	6.16E-01	0.01	9.70E-01	-0.32	1.07E-01
ENSCAFG0000003150	ENSCAFG0000003150	grey	VSMC_M10	0.10	6.31E-01	-0.23	2.58E-01	-0.12	5.53E-01	-0.16	4.32E-01	0.22	2.76E-01	-0.07	7.25E-01	0.07	7.31E-01	0.18	3.58E-01	0.22	2.72E-01	-0.22	2.64E-01
ENSCAFG0000000227	SUSD3	grey	VSMC_M10	0.10	6.32E-01	-0.05	8.21E-01	-0.22	2.72E-01	0.20	3.08E-01	0.23	2.46E-01	-0.06	7.78E-01	-0.13	5.05E-01	-0.13	5.16E-01	-0.01	9.44E-01	0.06	7.80E-01
ENSCAFG0000003258	ENSCAFG0000003258	grey	VSMC_M10	0.10	6.32E-01	0.05	8.11E-01	0.02	9.32E-01	-0.07	7.40E-01	0.04	8.35E-01	0.29	1.44E-01	0.11	5.87E-01	0.08	7.06E-01	-0.08	6.90E-01	-0.11	5.98E-01
ENSCAFG0000002811	ENSCAFG0000002811	grey	VSMC_M10	0.10	6.32E-01	-0.01	9.70E-01	-0.01	9.70E-01	-0.12	5.41E-01	0.07	7.26E-01	-0.06	7.80E-01	0.15	4.65E-01	0.13	5.12E-01	-0.08	6.88E-01	-0.03	8.87E-01
ENSCAFG0000000948	CPQ	darkgreen	VSMC_M4	0.10	6.32E-01	0.33	8.86E-02	-0.32	1.02E-01	0.84	3.61E-08	0.27	1.67E-01	-0.20	3.27E-01	-0.33	9.80E-02	-0.80	7.24E-07	-0.25	2.04E-01	0.46	1.45E-02
ENSCAFG0000001121	TBRG1	grey	VSMC_M10	0.10	6.32E-01	0.45	1.97E-02	0.20	3.17E-01	0.33	9.16E-02	-0.16	4.25E-01	-0.24	2.33E-01	-0.29	1.39E-01	-0.50	8.42E-03	-0.32	9.84E-02	0.29	1.44E-01
ENSCAFG0000001313	CCDC85B	yellow	VSMC_M3	0.10	6.32E-01	0.56	2.29E-03	0.59	1.33E-03	-0.02	9.19E-01	-0.48	1.16E-02	-0.24	2.32E-01	0.00	9.97E-01	-0.28	1.54E-01	-0.46	1.63E-02	0.21	3.03E-01
ENSCAFG0000000592	BTD	grey	VSMC_M10	0.10	6.32E-01	0.31	1.15E-01	-0.15	4.69E-01	0.31	1.10E-01	0.17	3.93E-01	-0.07	7.11E-01	-0.47	1.37E-02	-0.36	6.50E-02	-0.18	3.72E-01	0.13	5.32E-01
ENSCAFG0000000274	ZNF331	darkgreen	VSMC_M4	0.10	6.32E-01	0.44	2.01E-02	-0.39	4.45E-02	0.74	1.13E-05	0.34	7.80E-02	-0.22	2.66E-01	-0.23	2.49E-01	-0.75	5.46E-06	-0.39	4.16E-02	0.55	2.86E-03
ENSCAFG0000001780	CDC37	yellow	VSMC_M3	0.10	6.32E-01	0.30	1.23E-01	0.64	2.94E-04	-0.25	2.06E-01	-0.61	7.72E-04	0.01	9.68E-01	-0.23	2.59E-01	0.13	5.31E-01	-0.26	1.95E-01	0.06	7.49E-01
ENSCAFG0000001405	MID1IP1	yellow	VSMC_M3	0.10	6.33E-01	0.27	1.71E-01	0.41	3.47E-02	-0.38	4.95E-02	-0.23	2.47E-01	-0.05	8.00E-01	-0.02	9.29E-01	0.12	5.41E-01	-0.22	2.60E-01	-0.12	5.54E-01
ENSCAFG0000000034	ENSCAFG0000000034	grey	VSMC_M10	0.10	6.33E-01	0.06	7.56E-01	-0.43	2.70E-02	0.44	2.32E-02	0.41	3.26E-02	-0.27	1.69E-01	0.12	5.66E-01	-0.39	4.37E-02	-0.06	7.60E-01	0.18	3.69E-01
ENSCAFG0000003260	LURAP1L	grey	VSMC_M10	0.10	6.34E-01	-0.25	2.02E-01	-0.57	1.91E-03	0.40	3.88E-02	0.57	1.83E-03	-0.03	8.85E-01	0.45	1.73E-02	-0.27	1.69E-01	0.16	4.18E-01	0.06	7.84E-01
ENSCAFG0000003035	ENSCAFG0000003035	darkgreen	VSMC_M4	0.10	6.34E-01	0.07	7.11E-01	-0.57	1.74E-03	0.79	9.84E-07	0.51	5.99E-03	-0.10	6.06E-01	-0.18	3.74E-01	-0.64	3.54E-04	-0.05	7.94E-01	0.34	8.33E-02
ENSCAFG0000002376	ENSCAFG0000002376	yellow	VSMC_M3	0.10	6.34E-01	0.13	5.15E-01	0.84	4.05E-08	-0.70	5.55E-05	-0.70	4.58E-05	0.04	8.29E-01	0.25	2.11E-01	0.54	3.90E-03	-0.20	3.05E-01	-0.22	2.77E-01
ENSCAFG0000000400	GRWD1	darkgrey	VSMC_M8	0.10	6.34E-01	-0.18	3.79E-01	0.47	1.25E-02	-0.59	1.16E-03	-0.46	1.64E-02	0.28	1.62E-01	0.10	6.22E-01	0.62	5.04E-04	0.01	9.55E-01	-0.23	2.52E-01
ENSCAFG0000002961	MSS51	grey	VSMC_M10	0.10	6.35E-01	-0.08	6.95E-01	0.15	4.68E-01	-0.36	6.57E-02	-0.16	4.21E-01	0.12	5.49E-01	0.33	9.29E-02	0.42	2.92E-02	-0.06	7.66E-01	-0.01	9.56E-01
ENSCAFG0000000745	RMND5A	darkgreen	VSMC_M4	0.10	6.35E-01	0.17	3.96E-01	-0.59	1.20E-03	0.71	3.16E-05	0.61	6.95E-04	-0.17	3.89E-01	-0.30	1.35E-01	-0.65	2.09E-04	-0.06	7.49E-01	0.27	1.73E-01
ENSCAFG0000001916	ROGDI	grey	VSMC_M10	0.10	6.35E-01	0.38	5.04E-02	0.08	6.97E-01	0.14	5.02E-01	-0.30	1.32E-01	0.27	1.81E-01	-0.04	8.39E-01	-0.19	3.39E-01	-0.44	2.31E-02	0.63	4.36E-04
ENSCAFG0000000918	TBX5	grey	VSMC_M10	0.10	6.35E-01	0.49	1.03E-02	0.04	8.26E-01	0.23	2.58E-01	-0.10	6.11E-01	-0.16	4.38E-01	-0.16	4.30E-01	-0.27	1.66E-01	-0.49	1.01E-02	0.46	1.59E-02
ENSCAFG0000000995	TMEM81	grey	VSMC_M10	0.10	6.36E-01	-0.21	2.97E-01	-0.22	2.67E-01	0.03	8.82E-01	0.18	3.76E-01	0.18	3.72E-01	0.31	1.18E-01	0.14	4.86E-01	0.03	8.65E-01	0.04	8.38E-01
ENSCAFG0000002474	TAS2R3	grey	VSMC_M10	0.10	6.36E-01	0.33	9.22E-02	0.00	9.96E-01	0.17	3.95E-01	-0.02	9.10E-01	-0.09	6.60E-01	-0.17	3.88E-01	-0.32	1.07E-01	-0.30	1.34E-01	0.31	1.21E-01
ENSCAFG0000002426	RNASE13	grey	VSMC_M10	0.10	6.36E-01	0.44	2.16E-02	0.17	4.00E-01	0.24	2.20E-01	-0.24	2.20E-01	0.10	6.04E-01	-0.43	2.71E-02	-0.31	1.14E-01	-0.43	2.56E-02	0.46	1.67E-02
ENSCAFG0000000060	ENSCAFG0000000060	grey	VSMC_M10	0.10	6.36E-01	0.50	8.19E-03	0.15	4.57E-01	0.27	1.72E-01	-0.29	1.40E-01	0.22	2.81E-01	-0.17	3.87E-01	-0.39	4.31E-02	-0.44	2.04E-02	0.58	1.68E-03
ENSCAFG0000000843	ENTPD1	darkgreen	VSMC_M4	0.10	6.36E-01	0.12	5.53E-01	-0.49	8.68E-03	0.78	1.65E-06	0.40	3.68E-02	-0.14	4.72E-01	-0.20	3.25E-01	-0.59	1.12E-03	-0.13	5.31E-01	0.39	4.35E-02
ENSCAFG0000002388	ENSCAFG0000002388	grey	VSMC_M10	0.10	6.36E-01	0.01	9.69E-01	-0.28	1.58E-01	0.12	5.37E-01	0.16	4.15E-01	0.19	3.34E-01	0.25	2.15E-01	0.04	8.25E-01	-0.18	3.64E-01	0.24	2.38E-01
ENSCAFG0000000689	ENSCAFG0000000689	grey	VSMC_M10	0.10	6.36E-01	0.08	6.75E-01	-0.06	7.83E-01	0.12	5.36E-01	0.07	7.46E-01	-0.09	6.67E-01	-0.02	9.24E-01	-0.13	5.33E-01	-0.09	6.40E-01	0.14	4.81E-01
ENSCAFG0000003210	GON7	yellow	VSMC_M3	0.10	6.36E-01	0.36	6.55E-02	0.62	5.17E-04	-0.11	5.99E-01	-0.70	4.24E-05	0.34	8.51E-02	0.18	3.75E-01	0.00	9.90E-01	-0.43	2.56E-02	0.34	8.57E-02
ENSCAFG0000000542	DZANK1	grey	VSMC_M10	0.10	6.37E-01	0.23	2.40E-01	-0.10	6.37E-01	0.28	1.52E-01	-0.04	8.61E-01	0.03	8.96E-01	0.36	6.52E-02	-0.32	1.08E-01	-0.32	1.07E-01	0.53	4.14E-03
ENSCAFG0000001990	ENSCAFG0000001990	darkgreen	VSMC_M4	0.10	6.37E-01	0.39	4.17E-02	-0.22	2.67E-01	0.67	1.22E-04	0.24	2.23E-01	-0.14	4.82E-01	-0.40	3.64E-02	-0.75	5.93E-06	-0.24	2.26E-01	0.33	8.92E-02
ENSCAFG0000000353	AASS	cyan	VSMC_M2	0.10	6.37E-01	0.61	8.05E-04	0.20	3.20E-01	0.00	9.89E-01	-0.20	3.22E-01	-0.03	8.87E-01	-0.16	4.33E-01	-0.26	1.93E-01	-0.51	6.07E-03	0.40	3.85E-02
ENSCAFG0000001828	ENSCAFG0000001828	grey	VSMC_M10	0.10	6.37E-01	-0.60	8.63E-04	-0.03	8.91E-01	-0.27	1.74E-01	0.20	3.17E-01	-0.01	9.73E-01	0.19	3.52E-01	0.39	4.69E-02	0.54	3.41E-03	-0.59	1.13E-03
ENSCAFG0000000843	ENSCAFG0000000843	grey	VSMC_M10	0.10	6.37E-01	0.08	6.88E-01	0.21	2.89E-01	-0.03	8.63E-01	-0.38	4.89E-02	0.39	4.61E-02	0.16	4.14E-01	0.02	9.02E-01	-0.21	3.05E-01	0.36	6.33E-02
ENSCAFG0000000994	ABHD14B	cyan	VSMC_M2	0.09	6.38E-01	0.59	1.26E-03	0.32	1.01E-01	0.19	3.43E-01	-0.52	5.56E-03	0.18	3.59E-01	-0.03	8.81E-01	-0.31	1.15E-01	-0.62	5.20E-04	0.75	6.38E-06
ENSCAFG0000003027	ENSCAFG0000003027	grey	VSMC_M10	0.09	6.38E-01	-0.12	5.62E-01	-0.06	7.48E-01	-0.13	5.21E-01	0.26	1.83E-01	-0.07	7.36E-01	0.28	1.60E-01	0.05	7.88E-01	0.14	5.00E-01	-0.31	1.12E-01
ENSCAFG0000003071	HIST1H2AB	grey	VSMC_M10	0.09	6.38E-01	0.16	4.34E-01	-0.09	6.44E-01	0.04	8.39E-01	0.05	7.86E-01	0.06	7.51E-01	0.00	9.89E-01	-0.02	9.20E-01	-0.20	3.23E-01	0.13	5.26E-01
ENSCAFG0000000419	ENSCAFG0000000419	grey	VSMC_M10	0.09	6.38E-01	0.45	1.89E-02	0.43	2.70E-02	-0.08	7.00E-01	-0.40	3.77E-02	0.23	2.46E-01	0.21	2.83E-01	-0.13	5.33E-01	-0.46	1.56E-02	0.26	1.92E-01
ENSCAFG0000001717	PHKA1	grey	VSMC_M10	0.09	6.38E-01	0.41	3.15E-02	-0.35	7.00E-02	0.67	1.52E-04	0.39	4.73E-02	-0.39	4.40E-02	-0.32	1.06E-01	-0.73	1.66E-05	-0.28	1.64E-01	0.44	2.23E-02
ENSCAFG0000000861	GLRB	cyan	VSMC_M2	0.09	6.38E-01	0.56	2.30E-03	-0.23	2.57E-01	0.74	8.88E-06	0.21	2.82E-01	-0.30	1.27E-01	-0.74	8.60E-06	-0.81	2.44E-07	-0.38	5.28E-02	0.50	8.20E-03
ENSCAFG0000001261	LMO3	cyan	VSMC_M2	0.09	6.39E-01	0.44	2.23E-02	0.30	1.24E-01	-0.17	4.02E-01	-0.27	1.67E-01	-0.13	5.05E-01	-0.31	1.11E-01	0.06	7.78E-01	-0.40	3.96E-02	0.20	3.23E-01
ENSCAFG0000003022	ENSCAFG0000003022	yellow	VSMC_M3	0.09	6.39E-01	-0.13	5.05E-01	0.41	3.32E-02	-0.35	7.29E-02	-0.39	4.19E-02	0.05	8.05E-01	0.21	2.90E-01	0.37	5.74E-02	-0.02	9.11E-01	-0.15	4.62E-01
ENSCAFG0000001609	ENSCAFG0000001609	grey	VSMC_M10	0.09	6.39E-01	-0.37	5.70E-02	-0.37	5.73E-02	-0.14	4.86E-01	0.43	2.64E-02	-0.12	5.65E-01	-0.08	6.77E-01	0.33	9.02E-02	0.25	2.12E-01	-0.35	7.65E-02
ENSCAFG0000000390	IZUMO1	grey	VSMC_M10	0.09	6.39E-01	0.41	3.42E-02	0.50	7.76E-03	-0.08	6.89E-01	-0.41	3.50E-02	0.13	5.16E-01	-0.15	4.69E-01	-0.11	5.96E-01	-0.34	8.20E-02	0.08	6.84E-01
ENSCAFG0000000101	TRDN	darkgreen	VSMC_M4	0.09	6.39E-01	0.10	6.35E-01	-0.61	6.72E-04	0.86	6.10E-09	0.53	4.26E-03	-0.12	5.55E-01	-0.19	3.37E-01	-0.70	5.07E-05	-0.07	7.15E-01	0.39	4.15E-02
ENSCAFG0000000495	RAF1	grey	VSMC_M10	0.09	6.40E-01	0.16	4.33E-01	-0.43	2.46E-02	0.43	2.36E-02	0.50	7.34E-03	-0.24	2.30E-01	-0.01	9.56E-01	-0.44	2.07E-02	-0.09	6.60E-01	0.09	6.62E-01
ENSCAFG0000000600	FASN	yellow	VSMC_M3	0.09	6.40E-01	0.14	4.76E-01	0.69	6.60E-05	-0.79	8.46E-07	-0.51	7.09E-03	0.03	8.67E-01	0.15	4.59E-01	0.52	5.07E-03	-0.15	4.46E-01	-0.27	1.66E-01
ENSCAFG0000001889	TAOK1	grey	VSMC_M10	0.09	6.40E-01	-0.17	4.00E-01	-0.30	1.23E-01	0.31	1.13E-01	0.39	4.48E-02	-0.13	5.13E-01	0.04	8.49E-01	-0.28	1.52E-01	0.18	3.68E-01	-0.06	7.66E-01
ENSCAFG0000000014	RDH16	grey	VSMC_M10	0.09	6.41E-01	0.34	8.59E-02	0.02	9.29E-01	0.24	2.38E-01	-0.02	9.30E-01	-0.08	7.00E-01	-0.17	3.93E-01	-0.25	2.12E-01	-0.30	1.26E-01	0.27	1.66E-01
ENSCAFG0000001228	ENSCAFG0000001228	turquoise	VSMC_M6	0.09	6.41E-01	0.11	5.71E-01	0.34	8.40E-02	-0.08	6.94E-01	-0.32	1.05E-01	0.41	3.41E-02	0.10	6.20E-01	0.05	7.94E-01	-0.14	4.87E-01	0.07	7.14E-01
ENSCAFG0000000635	CDKL4	grey	VSMC_M10	0.09	6.41E-01	0.11	5.81E-01	-0.15	4.48E-01	0.13	5.32E-01	0.07	7.40E-01	0.20	3.23E-01	0.27	1.70E-01	-0.11	5.85E-01	-0.23	2.43E-01	0.31	1.17E-01
ENSCAFG0000000944	UQCRB	grey	VSMC_M10	0.09	6.41E-01	-0.61	7.62E-04	-0.16	4.32E-01	-0.30	1.28E-01	0.37	5.84E-02	-0.16	4.21E-01	0.16	4.25E-01	0.46	1.55E-02	0.51	7.17E-03	-0.77	2.67E-06
ENSCAFG0000003201	ENSCAFG0000003201	darkgreen	VSMC_M4	0.09	6.41E-01	0.05	7.99E-01	-0.64	2.87E-04	0.78	1.85E-06	0.60	9.56E-04	-0.07	7.44E-01	-0.19	3.48E-01	-0.64	3.61E-04	-0.01	9.47E-01	0.27	1.67E-01
ENSCAFG0000001958	TSR3	yellow	VSMC_M3	0.09	6.41E-01	0.28	1.50E-01	0.59	1.29E-03	-0.17	3.98E-01	-0.47	1.26E-02	-0.02	9.10E-01	-0.15	4.47E-01	-0.02	9.22E-01	-0.25	2.13E-01	-0.01	9.42E-01
ENSCAFG0000001221	GAS2L1	cyan	VSMC_M2	0.09	6.41E-01	0.34	8.61E-02	0.68	8.14E-05	-0.29	1.43E-01	-0.53	4.59E-03	-0.16	4.14E-01	-0.11	5.88E-01	0.03	8.83E-01	-0.22	2.70E-01	-0.04	8.50E-01
ENSCAFG0000003143	ENSCAFG0000003143	grey	VSMC_M10	0.09	6.41E-01	0.30	1.34E-01	0.25	2.08E-01	0.17	3.98E-01	-0.35	7.43E-02	0.02	9.39E-01	0.32	9.90E-02	-0.22	2.63E-01	-0.38	5.01E-02	0.45	1.86E-02
ENSCAFG0000000203	SCFD2	grey	VSMC_M10	0.09	6.41E-01	0.31	1.12E-01	0.30	1.30E-01	0.06	7.54E-01	-0.34	7.81E-02	0.12	5.57E-01	-0.03	8.97E-01	-0.10	6.17E-01	-0.37	5.79E-02	0.25	2.14E-01
ENSCAFG0000001109	AMZ2	darkgreen	VSMC_M4	0.09	6.42E-01	0.41	3.46E-02	-0.23	2.50E-01	0.55	3.23E-03	0.23	2.55E-01	-0.03	8.72E-01	-0.09	6.51E-01	-0.61	7.20E-04	-0.37	5.99E-02	0.35	7.56E-02
ENSCAFG0000002333	ENSCAFG0000002333	grey	VSMC_M10	0.09	6.42E-01	0.04	8.54E-01	0.06	7.70E-01	-0.12	5.63E-01	-0.05	8.09E-01	-0.09	6.53E-01	0.31	1.21E-01	0.06	7.59E-01	-0.12	5.49E-01	0.06	7.62E-01
ENSCAFG0000000339	TES	darkolivegreen	VSMC_M9	0.09	6.42E-01	-0.29	1.46E-01	-0.07	7.20E-01	-0.26	1.89E-01	0.25	2.11E-01	0.05	7.88E-01	0.08	7.09E-01	0.17	3.83E-01	0.34	8.56E-02	-0.37	5.69E-02
ENSCAFG0000000507	FBXL3	pink	VSMC_M5	0.09	6.43E-01	-0.27	1.70E-01	-0.19	3.50E-01	-0.18	3.64E-01	0.48	1.13E-02	-0.32	1.08E-01	-0.14	4.78E-01	0.19	3.40E-01	0.32	9.85E-02	-0.62	5.71E-04
ENSCAFG0000000549	ZNF219	grey	VSMC_M10	0.09	6.43E-01	0.70	4.69E-05	0.31	1.21E-01	0.34	8.60E-02	-0.33	9.38E-02	0.02	9.36E-01	-0.25	2.15E-01	-0.56	2.35E-03	-0.57	1.86E-03	0.53	4.11E-03
ENSCAFG0000001346	LRRC15	grey	VSMC_M10	0.09	6.43E-01	-0.03	8.65E-01	-0.16	4.37E-01	0.06	7.78E-01	0.27	1.74E-01	-0.31	1.12E-01	-0.06	7.55E-01	-0.09	6.72E-01	0.12	5.39E-01	-0.06	7.56E-01
ENSCAFG0000000172	TBCC	yellow	VSMC_M3	0.09	6.43E-01	0.31	1.16E-01	0.84	4.40E-08	-0.60	8.97E-04	-0.74	1.26E-05	0.04	8.58E-01	0.02	9.31E-01	0.34	8.33E-02	-0.29	1.35E-01	-0.07	7.20E-01
ENSCAFG0000001003	RYR2	grey	VSMC_M10	0.09	6.43E-01	0.09	6.63E-01	0.04	8.58E-01	0.09	6.61E-01	-0.06	7.62E-01	0.27	1.73E-01	0.13	5.14E-01	-0.05	8.20E-01	-0.13	5.06E-01	0.10	6.07E-01
ENSCAFG0000000755	ENSCAFG0000000755	grey	VSMC_M10	0.09	6.43E-01	0.13	5.15E-01	0.20	3.27E-01	-0.04	8.43E-01	-0.26	1.89E-01	0.04	8.59E-01	0.50	7.85E-03	0.00	9.92E-01	-0.27	1.78E-01	0.28	1.51E-01
ENSCAFG0000001700	BORCS6	grey	VSMC_M10	0.09	6.44E-01	0.23	2.51E-01	0.62	5.73E-04	-0.28	1.59E-01	-0.62	6.05E-04	-0.08	7.10E-01	0.19	3.37E-01	0.19	3.52E-01	-0.28	1.54E-01	0.14	4.73E-01
ENSCAFG0000002951	ENSCAFG0000002951	grey	VSMC_M10	0.09	6.44E-01	-0.07	7.13E-01	-0.38	5.39E-02	0.29	1.44E-01	0.46	1.47E-02	0.00	9.88E-01	-0.19	3.42E-01	-0.19	3.53E-01	0.04	8.35E-01	-0.13	5.05E-01
ENSCAFG0000000759	PAM	darkgreen	VSMC_M4	0.09	6.44E-01	0.30	1.34E-01	-0.30	1.29E-01	0.64	2.90E-04	0.21	2.98E-01	0.21	2.82E-01	0.08	6.79E-01	-0.61	6.87E-04	-0.31	1.16E-01	0.51	6.22E-03
ENSCAFG0000000440	CAB39L	cyan	VSMC_M2	0.09	6.44E-01	0.54	3.42E-03	0.43	2.62E-02	0.01	9.42E-01	-0.41	3.15E-02	0.09	6.45E-01	-0.36	6.91E-02	-0.13	5.17E-01	-0.50	7.47E-03	0.25	2.07E-01
ENSCAFG0000001246	TWIST2	yellow	VSMC_M3	0.09	6.45E-01	0.21	2.86E-01	0.55	2.90E-03	-0.52	5.32E-03	-0.53	4.92E-03	0.15	4.66E-01	0.39	4.19E-02	0.33	9.10E-02	-0.30	1.23E-01	0.03	8.81E-01
ENSCAFG0000001943	NTHL1	grey	VSMC_M10	0.09	6.46E-01	0.01	9.49E-01	0.08	6.84E-01	-0.24	2.30E-01	-0.09	6.38E-01	0.30	1.28E-01	0.47	1.34E-02	0.16	4.14E-01	-0.14	4.73E-01	0.04	8.27E-01
ENSCAFG0000002487	DEFB124	grey	VSMC_M10	0.09	6.46E-01	-0.22	2.66E-01	0.00	9.88E-01	-0.17	3.83E-01	0.05	7.99E-01	-0.07	7.27E-01	-0.04	8.57E-01	0.23	2.50E-01	0.20	3.16E-01	-0.22	2.73E-01
ENSCAFG0000000489	DEDD2	grey	VSMC_M10	0.09	6.46E-01	0.46	1.52E-02	0.06	7.70E-01	0.15	4.67E-01	0.02	9.17E-01	-0.05	8.22E-01	-0.10	6.16E-01	-0.36	6.55E-02	-0.34	8.08E-02	0.21	3.00E-01

ENSCAFG0000000292	MOB1B	grey	VSMC_M10	0.09	6.46E-01	0.14	4.87E-01	-0.03	8.87E-01	-0.02	9.27E-01	-0.09	6.67E-01	0.15	4.62E-01	0.02	9.18E-01	0.06	7.56E-01	-0.24	2.35E-01	0.27	1.65E-01
ENSCAFG0000002303	ENSCAFG0000002303	grey	VSMC_M10	0.09	6.46E-01	0.00	9.98E-01	-0.08	6.99E-01	0.02	9.38E-01	-0.01	9.57E-01	0.21	2.98E-01	-0.05	7.94E-01	0.08	6.90E-01	-0.09	6.67E-01	0.11	5.98E-01
ENSCAFG0000001092	ACSL5	grey	VSMC_M10	0.09	6.46E-01	0.47	1.31E-02	0.11	5.73E-01	0.22	2.63E-01	-0.26	1.93E-01	0.44	2.16E-02	0.18	3.76E-01	-0.23	2.49E-01	-0.58	1.47E-03	0.53	4.31E-03
ENSCAFG0000001336	ENSCAFG0000001336	yellow	VSMC_M3	0.09	6.46E-01	0.08	6.94E-01	0.67	1.27E-04	-0.59	1.26E-03	-0.67	1.54E-04	0.17	3.97E-01	0.30	1.30E-01	0.50	8.60E-03	-0.18	3.77E-01	-0.04	8.47E-01
ENSCAFG0000001550	SPEG	darkgreen	VSMC_M4	0.09	6.47E-01	0.50	7.73E-03	-0.16	4.28E-01	0.76	3.87E-06	0.08	6.88E-01	-0.11	5.83E-01	-0.56	2.45E-03	-0.80	5.73E-07	-0.38	4.85E-02	0.57	1.91E-03
ENSCAFG0000001281	DDX42	grey	VSMC_M10	0.09	6.47E-01	-0.04	8.35E-01	0.05	8.04E-01	0.17	3.93E-01	0.09	6.41E-01	-0.21	2.90E-01	-0.23	2.51E-01	-0.09	6.71E-01	0.08	6.96E-01	-0.17	3.84E-01
ENSCAFG0000000561	KIF5C	grey	VSMC_M10	0.09	6.47E-01	-0.48	1.04E-02	-0.17	4.07E-01	-0.09	6.42E-01	0.40	3.81E-02	-0.25	2.06E-01	0.18	3.75E-01	0.19	3.39E-01	0.50	7.54E-03	-0.57	1.99E-03
ENSCAFG0000003096	COX14	grey	VSMC_M10	0.09	6.47E-01	0.11	5.82E-01	-0.08	6.86E-01	-0.07	7.45E-01	0.21	3.03E-01	-0.21	2.99E-01	-0.20	3.20E-01	-0.04	8.24E-01	-0.04	8.47E-01	-0.13	5.03E-01
ENSCAFG0000000762	PREX2	grey	VSMC_M10	0.09	6.47E-01	-0.32	1.01E-01	-0.12	5.54E-01	-0.21	2.92E-01	0.16	4.35E-01	0.34	8.62E-02	0.23	2.38E-01	0.24	2.26E-01	0.18	3.57E-01	-0.22	2.80E-01
ENSCAFG0000001655	EXD2	grey	VSMC_M10	0.09	6.47E-01	0.13	5.27E-01	0.12	5.44E-01	0.07	7.15E-01	0.05	7.91E-01	-0.23	2.51E-01	0.01	9.73E-01	-0.17	3.96E-01	-0.15	4.50E-01	-0.13	5.12E-01
ENSCAFG0000000549	KCNE3	darkgreen	VSMC_M4	0.09	6.47E-01	0.57	1.87E-03	-0.29	1.47E-01	0.73	1.84E-05	0.15	4.68E-01	-0.20	3.06E-01	-0.27	1.78E-01	-0.75	5.66E-06	-0.52	5.43E-03	0.71	3.30E-05
ENSCAFG0000001207	SEC22A	grey	VSMC_M10	0.09	6.47E-01	0.03	8.94E-01	-0.54	3.30E-03	0.46	1.56E-02	0.45	1.72E-02	0.03	8.95E-01	0.16	4.37E-01	-0.33	9.57E-02	-0.12	5.48E-01	0.30	1.34E-01
ENSCAFG0000000624	MCF2L	grey	VSMC_M10	0.09	6.48E-01	0.28	1.55E-01	-0.06	7.83E-01	0.22	2.65E-01	-0.03	8.75E-01	0.02	9.17E-01	0.31	1.18E-01	-0.32	9.97E-02	-0.33	9.40E-02	0.49	1.03E-02
ENSCAFG0000001972	H6PD	cyan	VSMC_M2	0.09	6.48E-01	0.81	3.79E-07	-0.02	9.36E-01	0.56	2.45E-03	-0.08	6.90E-01	-0.15	4.63E-01	-0.35	7.03E-02	-0.74	1.03E-05	-0.67	1.13E-04	0.76	4.39E-06
ENSCAFG0000001037	A3GALT2	grey	VSMC_M10	0.09	6.48E-01	0.05	7.91E-01	0.11	5.79E-01	-0.23	2.41E-01	-0.10	6.16E-01	-0.07	7.13E-01	0.20	3.15E-01	0.21	3.04E-01	-0.13	5.15E-01	0.00	9.82E-01
ENSCAFG0000002938	ENSCAFG0000002938	yellow	VSMC_M3	0.09	6.48E-01	0.10	6.35E-01	0.57	2.05E-03	-0.53	4.14E-03	-0.34	8.21E-02	-0.31	1.12E-01	-0.15	4.50E-01	0.32	1.03E-01	-0.01	9.76E-01	-0.34	8.25E-02
ENSCAFG0000001731	ZWILCH	darkgrey	VSMC_M8	0.09	6.48E-01	-0.53	4.22E-03	0.06	7.84E-01	-0.55	2.80E-03	0.04	8.36E-01	0.12	5.55E-01	0.27	1.76E-01	0.75	8.11E-06	0.31	1.16E-01	-0.55	2.74E-03
ENSCAFG0000000742	PJA2	pink	VSMC_M5	0.09	6.48E-01	-0.60	1.05E-03	-0.46	1.58E-02	0.00	9.90E-01	0.70	4.89E-05	-0.20	3.19E-01	-0.13	5.14E-01	0.18	3.74E-01	0.62	5.99E-04	-0.65	2.38E-04
ENSCAFG0000001367	SORD	grey	VSMC_M10	0.09	6.48E-01	-0.53	4.13E-03	-0.29	1.48E-01	-0.01	9.79E-01	0.37	5.70E-02	-0.11	5.97E-01	0.55	3.01E-03	0.24	2.32E-01	0.35	7.53E-02	-0.36	6.47E-02
ENSCAFG0000000913	MMS19	cyan	VSMC_M2	0.09	6.48E-01	0.72	2.69E-05	0.14	4.92E-01	0.29	1.38E-01	-0.21	2.82E-01	0.01	9.57E-01	-0.01	9.75E-01	-0.50	7.36E-03	-0.66	1.78E-04	0.67	1.25E-04
ENSCAFG0000003033	CPTP	grey	VSMC_M10	0.09	6.49E-01	0.64	3.59E-04	0.31	1.17E-01	0.08	7.00E-01	-0.34	8.37E-02	0.04	8.56E-01	-0.06	7.62E-01	-0.35	7.05E-02	-0.54	3.99E-03	0.53	4.55E-03
ENSCAFG0000001357	HTR3B	grey	VSMC_M10	0.09	6.49E-01	-0.11	6.02E-01	-0.06	7.54E-01	-0.14	4.78E-01	0.25	2.06E-01	-0.19	3.34E-01	-0.07	7.19E-01	0.13	5.32E-01	0.10	6.27E-01	-0.35	7.34E-02
ENSCAFG0000000498	GDPD4	grey	VSMC_M10	0.09	6.49E-01	0.19	3.30E-01	0.19	3.34E-01	-0.13	5.21E-01	-0.19	3.44E-01	-0.06	7.63E-01	0.41	3.30E-02	0.04	8.47E-01	-0.23	2.56E-01	0.11	5.70E-01
ENSCAFG0000003054	PJA1	darkgrey	VSMC_M8	0.09	6.49E-01	-0.44	2.09E-02	-0.01	9.73E-01	-0.49	8.93E-03	0.21	2.84E-01	-0.15	4.61E-01	0.47	1.28E-02	0.58	1.65E-03	0.31	1.11E-01	-0.63	4.25E-04
ENSCAFG0000001819	ALG13	grey	VSMC_M10	0.09	6.49E-01	-0.48	1.15E-02	-0.16	4.14E-01	-0.34	8.38E-02	0.27	1.74E-01	0.17	3.98E-01	0.18	3.73E-01	0.47	1.30E-02	0.32	1.09E-01	-0.49	9.59E-03
ENSCAFG0000001623	FUT8	pink	VSMC_M5	0.09	6.50E-01	0.04	8.55E-01	-0.61	6.67E-04	0.64	3.15E-04	0.58	1.69E-03	-0.33	9.54E-02	-0.19	3.44E-01	-0.46	1.64E-02	-0.04	8.42E-01	0.25	2.07E-01
ENSCAFG0000000610	ANKLE2	darkgreen	VSMC_M4	0.09	6.50E-01	-0.15	4.57E-01	-0.60	1.01E-03	0.70	4.83E-05	0.57	2.12E-03	-0.04	8.27E-01	-0.12	5.44E-01	-0.55	3.25E-03	0.16	4.30E-01	0.20	3.11E-01
ENSCAFG0000000580	PTPRQ	grey	VSMC_M10	0.09	6.50E-01	-0.13	5.07E-01	-0.13	5.28E-01	0.12	5.58E-01	0.12	5.51E-01	-0.09	6.50E-01	0.63	3.85E-04	-0.02	9.04E-01	-0.01	9.55E-01	0.13	5.18E-01
ENSCAFG0000002039	RRAD	grey	VSMC_M10	0.09	6.50E-01	0.26	1.91E-01	0.30	1.29E-01	0.21	2.92E-01	-0.26	1.89E-01	0.03	8.70E-01	-0.06	7.79E-01	-0.20	3.12E-01	-0.26	1.96E-01	0.18	3.79E-01
ENSCAFG0000001336	APOD	grey	VSMC_M10	0.09	6.51E-01	-0.03	8.95E-01	-0.21	2.94E-01	0.14	4.96E-01	0.31	1.20E-01	0.21	3.03E-01	0.12	5.43E-01	-0.11	5.88E-01	0.01	9.71E-01	-0.15	4.65E-01
ENSCAFG0000003179	RPS6KA4	grey	VSMC_M10	0.09	6.51E-01	0.62	5.57E-04	0.66	1.62E-04	-0.14	4.89E-01	-0.75	5.37E-06	0.06	7.70E-01	-0.01	9.64E-01	-0.04	8.56E-01	-0.63	4.48E-04	0.54	3.90E-03
ENSCAFG0000000790	GLRX	grey	VSMC_M10	0.09	6.51E-01	0.65	2.77E-04	0.67	1.52E-04	-0.12	5.65E-01	-0.73	1.38E-05	0.07	7.37E-01	-0.09	6.67E-01	-0.11	5.69E-01	-0.59	1.11E-03	0.50	7.24E-03
ENSCAFG0000001807	ZNF317	grey	VSMC_M10	0.09	6.51E-01	-0.22	2.70E-01	0.04	8.35E-01	-0.09	6.42E-01	0.12	5.59E-01	-0.08	7.02E-01	0.07	7.40E-01	0.10	6.15E-01	0.18	3.73E-01	-0.32	9.92E-02
ENSCAFG0000002584	ENSCAFG0000002584	cyan	VSMC_M2	0.09	6.51E-01	0.38	4.87E-02	0.46	1.59E-02	-0.11	5.96E-01	-0.46	1.57E-02	0.46	1.68E-02	0.03	8.74E-01	0.00	9.90E-01	-0.39	4.24E-02	0.27	1.70E-01
ENSCAFG0000001851	ENSCAFG0000001851	grey	VSMC_M10	0.09	6.51E-01	0.09	6.69E-01	0.32	1.05E-01	-0.17	3.94E-01	-0.28	1.52E-01	-0.24	2.37E-01	0.06	7.75E-01	0.12	5.57E-01	-0.14	4.84E-01	0.09	6.65E-01
ENSCAFG0000001644	RDH11	grey	VSMC_M10	0.09	6.51E-01	-0.11	6.02E-01	0.15	4.61E-01	-0.30	1.34E-01	-0.01	9.75E-01	-0.05	8.01E-01	0.25	2.08E-01	0.23	2.59E-01	0.10	6.05E-01	-0.30	1.30E-01
ENSCAFG0000000083	ENSCAFG0000000083	grey	VSMC_M10	0.09	6.51E-01	0.53	4.36E-03	0.48	1.21E-02	-0.02	9.41E-01	-0.47	1.34E-02	-0.09	6.53E-01	0.09	6.63E-01	-0.27	1.76E-01	-0.44	2.08E-02	0.41	3.42E-02
ENSCAFG0000001480	EID1	yellow	VSMC_M3	0.09	6.51E-01	0.27	1.74E-01	0.64	3.30E-04	-0.19	3.32E-01	-0.57	1.94E-03	0.08	6.78E-01	-0.04	8.27E-01	0.05	7.86E-01	-0.29	1.49E-01	0.05	8.01E-01
ENSCAFG0000001021	MBOAT1	darkolivegreen	VSMC_M9	0.09	6.51E-01	-0.56	2.40E-03	-0.07	7.24E-01	-0.51	6.77E-03	0.25	2.06E-01	0.03	8.93E-01	0.69	6.33E-05	0.58	1.57E-03	0.40	4.14E-02	-0.59	1.19E-03
ENSCAFG0000001629	NLGN2	grey	VSMC_M10	0.09	6.52E-01	0.37	6.05E-02	-0.27	1.67E-01	0.33	9.20E-02	0.21	2.89E-01	0.07	7.26E-01	0.04	8.25E-01	-0.41	3.25E-02	-0.31	1.18E-01	0.44	2.02E-02
ENSCAFG0000000958	ENSCAFG0000000958	turquoise	VSMC_M6	0.09	6.52E-01	0.21	2.93E-01	0.13	5.14E-01	-0.10	6.03E-01	-0.13	5.20E-01	0.52	5.39E-03	0.13	5.21E-01	0.06	7.84E-01	-0.22	2.65E-01	0.13	5.33E-01
ENSCAFG0000000163	FOXH1	grey	VSMC_M10	0.09	6.52E-01	0.07	7.38E-01	-0.43	2.61E-02	0.51	6.01E-03	0.36	6.38E-02	-0.12	5.45E-01	-0.11	5.95E-01	-0.42	2.84E-02	-0.04	8.35E-01	0.27	1.72E-01
ENSCAFG0000001959	TNFRSF25	grey	VSMC_M10	0.09	6.53E-01	0.36	6.88E-02	-0.30	1.31E-01	0.58	1.43E-03	0.29	1.49E-01	-0.24	2.31E-01	-0.33	9.27E-02	-0.60	9.59E-04	-0.21	2.99E-01	0.35	7.23E-02
ENSCAFG0000001896	SSH2	grey	VSMC_M10	0.09	6.53E-01	-0.19	3.42E-01	-0.20	3.19E-01	-0.02	9.23E-01	0.24	2.37E-01	0.02	9.27E-01	-0.01	9.54E-01	0.18	3.68E-01	0.09	6.70E-01	-0.26	1.83E-01
ENSCAFG0000000383	NTF4	grey	VSMC_M10	0.09	6.53E-01	-0.05	7.97E-01	0.08	6.96E-01	-0.10	6.12E-01	-0.04	8.58E-01	-0.08	7.00E-01	-0.16	4.32E-01	0.16	4.33E-01	0.01	9.44E-01	-0.11	5.72E-01
ENSCAFG0000003267	ENSCAFG0000003267	grey	VSMC_M10	0.09	6.53E-01	-0.05	7.97E-01	0.08	6.96E-01	-0.10	6.12E-01	-0.04	8.58E-01	-0.08	7.00E-01	-0.16	4.32E-01	0.16	4.33E-01	0.01	9.44E-01	-0.11	5.72E-01
ENSCAFG0000003189	TENT5A	grey	VSMC_M10	0.09	6.53E-01	0.00	9.82E-01	0.07	7.21E-01	-0.25	2.09E-01	-0.04	8.26E-01	0.18	3.67E-01	-0.39	4.47E-02	0.31	1.19E-01	-0.11	5.73E-01	-0.15	4.40E-01
ENSCAFG0000000872	POLR2C	yellow	VSMC_M3	0.09	6.53E-01	-0.10	6.04E-01	0.49	1.01E-02	-0.38	5.15E-02	-0.43	2.41E-02	0.16	4.24E-01	0.03	8.87E-01	0.38	5.09E-02	0.03	8.80E-01	-0.23	2.49E-01
ENSCAFG0000003133	MED21	grey	VSMC_M10	0.09	6.53E-01	0.02	9.25E-01	-0.45	1.73E-02	0.41	3.25E-02	0.35	7.12E-02	0.08	6.91E-01	-0.31	1.11E-01	-0.27	1.75E-01	-0.04	8.44E-01	0.23	2.46E-01
ENSCAFG0000003232	S100A2	grey	VSMC_M10	0.09	6.53E-01	0.12	5.40E-01	0.10	6.23E-01	0.08	6.80E-01	-0.12	5.36E-01	0.23	2.49E-01	0.26	1.98E-01	-0.08	6.86E-01	-0.16	4.27E-01	0.17	3.90E-01
ENSCAFG0000001243	MYOZ2	grey	VSMC_M10	0.09	6.53E-01	0.39	4.34E-02	-0.22	2.71E-01	0.51	7.03E-03	0.12	5.38E-01	-0.20	3.07E-01	-0.09	6.52E-01	-0.46	1.48E-02	-0.38	4.95E-02	0.56	2.15E-03
ENSCAFG0000001930	CLUH	grey	VSMC_M10	0.09	6.53E-01	0.33	9.22E-02	0.53	4.42E-03	-0.16	4.31E-01	-0.63	4.27E-04	0.43	2.38E-02	-0.07	7.24E-01	0.11	5.92E-01	-0.40	4.02E-02	0.28	1.52E-01
ENSCAFG0000002908	ENSCAFG0000002908	grey	VSMC_M10	0.09	6.54E-01	0.21	2.90E-01	0.47	1.29E-02	-0.38	4.74E-02	-0.34	8.50E-02	0.05	8.12E-01	-0.04	8.34E-01	0.12	5.43E-01	-0.17	4.03E-01	-0.10	6.03E-01
ENSCAFG0000001494	PRRC2C	grey	VSMC_M10	0.09	6.54E-01	-0.25	2.10E-01	-0.68	8.28E-05	0.38	4.97E-02	0.66	1.56E-04	-0.01	9.51E-01	-0.23	2.42E-01	-0.17	4.08E-01	0.21	2.90E-01	-0.03	8.65E-01
ENSCAFG0000000577	BIRC6	pink	VSMC_M5	0.09	6.54E-01	-0.12	5.56E-01	-0.78	1.54E-06	0.69	6.67E-05	0.73	1.28E-05	-0.08	6.96E-01	-0.32	1.05E-01	-0.50	8.04E-03	0.15	4.65E-01	0.19	3.41E-01
ENSCAFG0000003081	PCDHB15	grey	VSMC_M10	0.09	6.55E-01	0.46	1.63E-02	-0.16	4.16E-01	0.59	1.22E-03	-0.03	8.79E-01	-0.08	7.00E-01	-0.11	5.87E-01	-0.64	3.61E-04	-0.42	2.97E-02	0.76	5.17E-06
ENSCAFG0000003257	ENSCAFG0000003257	grey	VSMC_M10	0.09	6.55E-01	0.16	4.14E-01	0.31	1.20E-01	-0.30	1.24E-01	-0.39	4.47E-02	0.26	1.87E-01	0.09	6.48E-01	0.19	3.32E-01	-0.24	2.24E-01	0.11	5.98E-01
ENSCAFG0000000428	ENSCAFG0000000428	grey	VSMC_M10	0.09	6.55E-01	0.00	9.97E-01	0.52	5.39E-03	-0.62	5.84E-04	-0.32	1.03E-01	0.03	8.92E-01	0.18	3.69E-01	0.48	1.23E-02	-0.01	9.46E-01	-0.39	4.36E-02
ENSCAFG0000000875	ENSCAFG0000000875	grey	VSMC_M10	0.09	6.55E-01	-0.06	7.82E-01	0.03	8.89E-01	-0.11	6.02E-01	-0.01	9.57E-01	0.03	8.78E-01	0.28	1.51E-01	0.14	4.97E-01	-0.02	9.14E-01	0.01	9.60E-01
ENSCAFG0000000386	FTL	yellow	VSMC_M3	0.09	6.55E-01	0.66	1.78E-04	0.77	2.46E-06	-0.21	2.94E-01	-0.80	6.59E-07	0.10	6.03E-01	0.07	7.11E-01	-0.05	8.02E-01	-0.64	2.92E-04	0.38	4.74E-02
ENSCAFG0000001730	CD19	grey	VSMC_M10	0.09	6.55E-01	0.12	5.66E-01	0.05	7.92E-01	-0.08	6.91E-01	-0.02	9.09E-01	-0.16	4.19E-01	-0.21	2.83E-01	-0.01	9.43E-01	-0.08	7.05E-01	0.03	8.79E-01
ENSCAFG0000001297	ENSCAFG0000001297	grey	VSMC_M10	0.09	6.55E-01	0.30	1.25E-01	-0.12	5.51E-01	0.23	2.55E-01	0.09	6.65E-01	-0.13	5.19E-01	-0.15	4.58E-01	-0.33	9.22E-02	-0.25	2.02E-01	0.32	1.02E-01
ENSCAFG0000001869	ALKBH7	yellow	VSMC_M3	0.09	6.55E-01	0.56	2.35E-03	0.77	2.41E-06	-0.29	1.41E-01	-0.81	3.46E-07	0.14	4.77E-01	-0.11	5.95E-01	0.07	7.47E-01	-0.56	2.54E-03	0.27	1.81E-01
ENSCAFG0000000025	SLC35D3	darkgreen	VSMC_M4	0.09	6.55E-01	0.15	4.49E-01	-0.29	1.45E-01	0.49	1.01E-02	0.23	2.55E-01	-0.05	8.05E-01	0.24	2.36E-01	-0.41	3.61E-02	-0.18	3.73E-01	0.37	5.77E-02
ENSCAFG0000000832	RBM39	grey	VSMC_M10	0.09	6.56E-01	-0.12	5.40E-01	-0.18	3.74E-01	-0.08	6.88E-01	0.25	2.06E-01	-0.07	7.25E-01	0.37	5.53E-02	0.14	4.96E-01	-0.04	8.57E-01	-0.07	7.40E-01
ENSCAFG0000000702	ENSCAFG0000000702	darkgreen	VSMC_M4	0.09	6.56E-01	0.29	1.49E-01	-0.41	3.23E-02	0.70	4.38E-05	0.32	1.05E-01	-0.12	5.53E-01	-0.24	2.31E-01	-0.63	4.47E-04	-0.25	2.05E-01	0.45	1.76E-02
ENSCAFG0000001056	BSDC1	grey	VSMC_M10	0.09	6.56E-01	0.33	9.27E-02	-0.37	6.09E-02	0.67	1.33E-04	0.33	9.08E-02	0.02	9.38E-01	-0.06	7.82E-01	-0.73	1.46E-05	-0.23	2.45E-01	0.44	2.19E-02
ENSCAFG0000003003	ENSCAFG0000003003	grey	VSMC_M10	0.09	6.56E-01	-0.13	5.33E-01	-0.18	3.61E-01	0.06	7.63E-01	0.15	4.59E-01	0.31	1.20E-01	0.28	1.55E-01	0.06	7.66E-01	0.00	9.95E-01	0.09	6.48E-01
ENSCAFG0000001848	ATAD5	darkgrey	VSMC_M8	0.09	6.56E-01	-0.69	6.94E-05	-0.04	8.35E-01	-0.59	1.29E-03	0.17	4.06E-01	0.16	4.24E-01	0.31	1.18E-01	0.82	1.40E-07	0.47	1.24E-02	-0.69	6.16E-05
ENSCAFG0000002127	ENSCAFG0000002127	grey	VSMC_M10	0.09	6.57E-01	0.06	7.61E-01	0.14	5.01E-01	-0.27	1.76E-01	-0.02	9.36E-01	-0.12	5.36E-01	0.03	8.96E-01	0.22	2.66E-01	-0.08	6.88E-01	-0.19	3.41E-01
ENSCAFG0000000448	SLC6A6	turquoise	VSMC_M6	0.09	6.57E-01	-0.28	1.59E-01	-0.13	5.21E-01	-0.15	4.55E-01	0.22	2.67E-01	0.52	4.98E-03	0.30	1.28E-01	0.15	4.57E-01	0.23	2.41E-01	-0.27	1.77E-01
ENSCAFG0000000170	NMRK1	grey	VSMC_M10	0.09	6.58E-01	0.25	2.03E-01	-0.25	2.09E-01	0.38	4.97E-02	0.26	1.97E-01	0.01	9.51E-01	-0.03	8.97E-01	-0.37	5.91E-02	-0.27	1.75E-01	0.25	2.14E-01
ENSCAFG0000001761	LARP6	grey	VSMC_M10	0.09	6.58E-01	-0.36	6.89E-02	-0.08	7.08E-01	-0.08	7.06E-01	0.26	1.84E-01	-0.33	9.72E-02	0.42	2.90E-02	0.10	6.16E-01	0.28	1.53E-01	-0.36	6.30E-02
ENSCAFG0000000740	CDK5RAP1	pink	VSMC_M5	0.09	6.58E-01	-0.50	8.11E-03	-0.28	1.51E-01	-0.25	2.06E-01	0.49	9.06E-03	-0.08	6.82E-01	0.05	7.96E-01	0.42	3.00E-02	0.38	4.87E-02	-0.62	5.04E-04
ENSCAFG0000000450	SSPO	grey	VSMC_M10	0.09	6.58E-01	0.24	2.27E-01	0.20	3.27E-01	-0.12	5.60E-01	-0.20	3.21E-01	-0.06	7.69E-01	0.32	9.95E-02	0.02	9.37E-01	-0.26	1.90E-01	0.14	4.83E-01
ENSCAFG0000001927	GNA15	grey	VSMC_M10	0.09	6.58E-01	0.24	2.27E-01	0.20	3.27E-01	-0.12	5.60E-01	-0.20	3.21E-01	-0.06	7.69E-01	0.32	9.95E-02	0.02	9.37E-01	-0.26	1.90E-01	0.14	4.83E-01
ENSCAFG0000001694	LTBP2	darkgrey	VSMC_M8	0.09	6.59E-01	-0.25	2.00E-01	0.50	7.54E-03	-0.81	4.07E-07	-0.25	2.12E-01	-0.11	5.95E-01	0.14	4.77E-01	0.67	1.31E-04	0.23	2.58E-01	-0.63	4.20E-04
ENSCAFG0000000260	PPP6R1	darkgrey	VSMC_M8	0.09	6.59E-01	-0.10	6.36E-01	0.64	3.35E-04	-0.75	5.76E-06	-0.49	8.86E-03	0.10	6.34E-01	0.01	9.45E-01	0.65	2.55E-04	0.02	9.28E-01	-0.41	3.41E-02
ENSCAFG0000001541	CREG1	darkgrey	VSMC_M8	0.09	6.59E-01	-0.11	5.88E-01	0.47	1.42E-02	-0.71	3.26E-05	-0.44	2.18E-02	0.25	2.12E-01	0.31	1.18E-01	0.71	3.41E-05	-0.10	6.36E-01	-0.27	1.65E-01
ENSCAFG0000000837	ENSCAFG0000000837	grey	VSMC_M10	0.09	6.59E-01	0.06	7.49E-01	-0.19	3.46E-01	0.10	6.11E-01	0.19	3.33E-01	-0.54	3.50E-03	0.00	9.89E-01	-0.11	5.92E-01	-0.07	7.37E-01	0.13	5.26E-01
ENSCAFG0000001296	SEPT2	grey	VSMC_M10	0.09	6.59E-01	0.17	4.03E-01	-0.06	7.70E-01	0.20	3.11E-01	0.21	2.86E-01	-0.38	4.92E-02	-0.51	6.79E-03	-0.23	2.49E-01	-0.02	9.04E-01	-0.14	4.95E-01
ENSCAFG0000001432	PPM1L	grey	VSMC_M10	0.09	6.59E-01	0.02	9.09E-01	0.47	1.42E-02	-0.49	9.41E-03	-0.29	1.39E-01	-0.04	8.43E-01	0.41	3.31E-02	0.31	1.11E-01	-0.08	7.00E-01	-0.26	1.87E-01
ENSCAFG0000001031	ENSCAFG0000001031	grey	VSMC_M10	0.09	6.59E-01	0.40	3.98E-02	0.26	1.87E-01	0.27	1.80E-01	-0.40	3.80E-02	0.21	3.05E-01	0.13	5.32E-01	-0.28	1.51E-01	-0.46	1.64E-02	0.59	1.08E-03
ENSCAFG0000000965	TWF1	pink	VSMC_M5	0.09	6.60E-01	-0.64	3.35E-04	-0.47	1.27E-02	-0.02	9.11E-01	0.65	2.38E-04	-0.10	6.26E-01	-0.16	4.33E-01	0.30	1.28E-01	0.53	4.06E-03	-0.61	8.20E-04
ENSCAFG0000000181	PTK7	grey	VSMC_M10	0.09	6.60E-01	-0.31	1.19E-01	0.04	8.26E-01	-0.35	7.46E-02	-0.06	7.84E-01	0.56	2.45E-03	0.27	1.74E-01	0.36	6.45E-02	0.17	3.95E-01	-0.17	4.02E-01
ENSCAFG0000002025	ENSCAFG0000002025	grey	VSMC_M10	0.09	6.60E-01	-0.20	3.13E-01	-0.18	3.73E-01	0.17	3.99E-01	0.22	2.74E-01	-0.06	7.70E-01	0.09	6.50E-01	-0.08	6.96E-01	0.17	4.09E-01	-0.03	8.72E-01
ENSCAFG0000002156	ENSCAFG0000002156	grey	VSMC_M10	0.09	6.60E-01	-0.21	2.97E-01	-0.06	7.85E-01	-0.34	8.24E-02	0.16	4.18E-01	0.28	1.52E-01	0.10	6.22E-01	0.39	4.32E-02	0.13	5.29E-01	-0.35	6.93E-02
ENSCAFG0000000206	SLC1A1	grey	VSMC_M10	0.09	6.60E-01	0.03	8.93E-01	-0.34	8.67E-02	0.54	3.64E-03	0.33	9.32E-02	-0.37	5.76E-02	-0.42	3.00E-02	-0.41	3.15E-02	0.07	7.14E-01	0.13	5.02E-01
ENSCAFG0000000921	ENSCAFG0000000921	pink	VSMC_M5	0.09	6.61E-01	-0.58	1.66E-03	-0.45	1.87E-02	0.01	9.47E-01	0.67	1.44E-04	-0.22	2.80E-01	-0.06	7.54E-01	0.15	4.62E-01	0.60	9.57E-04	-0.61	7.02E-04
ENSCAFG0000000346	ENSCAFG0000000346	grey	VSMC_M10	0.09	6.61E-01	0.02	9.04E-01	-0.02	9.12E-01	-0.23	2.55E-01	-0.05	8.02E-01	0.40	4.07E-02	0.12	5.47E-01	0.25	2.01E-01	-0.13	5.22E-01	0.06	7.59E-01
ENSCAFG0000000021	PGGT1B	grey	VSMC_M10	0.09	6.61E-01	-0.02	9.12E-01	0.27	1.70E-01	-0.18	3.79E-01	-0.35	7.74E-02	0.07	7.44E-01	0.22	2.62E-01	0.18	3.63E-01	-0.10	6.18E-01	0.20	3.15E-01
ENSCAFG0000001014	CCT6A	darkgrey	VSMC_M8	0.09	6.61E-01	-0.73	1.62E-05	-0.05	7.98E-01	-0.54	3.90E-03	0.23	2.56E-01	0.08	6.90E-01	0.22	2.66E-01	0.75	6.19E-06	0.56	2.62E-03	-0.79	1.17E-06
ENSCAFG0000001995	VAV3	grey	VSMC_M10	0.09	6.62E-01	-0.11	5.93E-01	0.16	4.33E-01	-0.48	1.14E-02	0.04	8.60E-01	0.12	5.40E-01	-0.21	2.93E-01	0.43	2.37E-02	0.09	6.45E-01	-0.52	5.29E-03
ENSCAFG0000003195	ENSCAFG0000003195	grey	VSMC_M10	0.09	6.63E-01	-0.06	7.63E-01	-0.31	1.12E-01	0.31	1.10E-01	0.28	1.61E-01	-0.03	8.93E-01	0.07	7.45E-01	-0.17	3.87E-01	0.01	9.79E-01	0.14	4.97E-01
ENSCAFG0000002851	HOXB3	grey	VSMC_M10	0.09	6.63E-01	0.40	4.11E-02	0.21	2.89E-01	-0.12	5.62E-01	-0.21	2.93E-01	0.11	6.02E-01	-0.55	3.04E-03	-0.02	9.14E-01	-0.38	5.08E-02	0.16	4.39E-01
ENSCAFG0000002284	BCKDHB	cyan	VSMC_M2	0.09	6.63E-01	0.55	3.13E-03	0.62	5.46E-04	-0.27	1.71E-01	-0.65	2.58E-04	-0.04	8.30E-01	0.09	6.59E-01	0.03	8.71E-01	-0.53	4.32E-03	0.39	4.30E-02
ENSCAFG0000001958	BAIAP3	grey	VSMC_M10	0.09	6.64E-01	0.29	1.36E-01	0.06	7.57E-01	0.37	5.97E-02	-0.14	4.94E-01	-0.15	4.68E-01	0.00	9.91E-01	-0.30	1.23E-01	-0.34	8.50E-02	0.41	3.57E-02
ENSCAFG0000001037	ENSCAFG0000001037	grey	VSMC_M10	0.09	6.64E-01	0.22	2.65E-01	-0.15	4.51E-01	0.42	2.85E-02	0.09	6.43E-01	-0.05	8.05E-01	-0.32	1.04E-01	-0.52	4.94E-03	-0.13	5.34E-01	0.38	4.94E-02
ENSCAFG0000000581	KAT2B	cyan	VSMC_M2	0.09	6.64E-01	0.68	1.11E-04	0.02	9.07E-01	0.37	5.50E-02	0.05	8.11E-01	-0.42	3.10E-02	-0.53	4.86E-03	-0.62	6.34E-04	-0.47	1.42E-02	0.40	3.69E-02
ENSCAFG0000001460	TRIM9	grey	VSMC_M10	0.09	6.64E-01	-0.10	6.29E-01	0.41	3.39E-02	-0.26	1.94E-01	-0.40	3.66E-02	0.33	9.07E-02	0.62	5.06E-04	0.28	1.54E-01	-0.06	7.54E-01	-0.05	7.91E-01
ENSCAFG0000000595	SLC25A15	grey	VSMC_M10	0.09	6.64E-01	-0.14	4.72E-01	0.29	1.44E-01	-0.46	1.67E-02	-0.21	3.05E-01	0.20	3.16E-01	-0.17	4.06E-01	0.36	6.73E-02	0.11	5.69E-01	-0.29	1.40E-01
ENSCAFG0000003076	SLC25A29	grey	VSMC_M10	0.09	6.64E-01	0.62	5.35E-04	0.06	7.74E-01	0.35	7.51E-02	-0.03	8.80E-01	-0.11	5.92E-01	-0.36	6.85E-02	-0.54	3.96E-03	-0.50	8.35E-03	0.35	7.38E-02
ENSCAFG0000003112	ENSCAFG0000003112	darkgrey	VSMC_M8	0.09	6.64E-01	-0.43	2.37E-02	0.27	1.71E-01	-0.42	2.72E-02	-0.22	2.70E-01	0.15	4.49E-01	0.22	2.70E-01	0.58	1.55E-03	0.28	1.64E-01	-0.40	3.67E-02
ENSCAFG0000001142	ACIN1	grey	VSMC_M10	0.09	6.65E-01	0.20	3.26E-01	0.03	8.72E-01	0.00	9.84E-01	0.07	7.27E-01	-0.22	2.68E-01	0.01	9.79E-01	-0.07	7.31E-01	-0.25	2.07E-01	0.02	9.03E-01
ENSCAFG0000000228	RECK	darkgreen	VSMC_M4	0.09	6.65E-01	0.47	1.39E-02	-0.41	3.21E-02	0.88	1.00E-09	0.29	1.38E-01	-0.08	6.75E-01	-0.20	3.17E-01	-0.85	1.94E-08	-0.40	3.75E-02	0.68	9.75E-05
ENSCAFG0000001516	ENSCAFG0000001516	grey	VSMC_M10	0.09	6.65E-01	0.37	5.61E-02	-0.12	5.61E-01	0.35	7.70E-02	0.01	9.71E-01	0.08	6.74E-01	-0.17	4.08E-01	-0.29	1.35E-01	-0.38	5.14E-02	0.46	1.54E-02
ENSCAFG0000001203	ARPIN	yellow	VSMC_M3	0.09	6.66E-01	0.55	3.16E-03	0.60	9.82E-04	-0.18	3.60E-01	-0.62	5.62E-04	0.00	9.95E-01	0.27	1.75E-01	-0.10	6.17E-01	-0.54	3.73E-03	0.42	2.83E-02
ENSCAFG0000001381	ENSCAFG0000001381	grey	VSMC_M10	0.09	6.66E-01	-0.06	7.56E-01	-0.05	8.08E-01	-0.01	9.42E-01	0.24	2.25E-01	-0.08	6.92E-01	0.09	6.47E-01	0.05	8.05E-01	0.04	8.46E-01	-0.33	9.12E-02
ENSCAFG0000000002	ZNF407	grey	VSMC_M10	0.09	6.66E-01	0.05	8.15E-01	0.25	2.07E-01	-0.15	4.57E-01	-0.08	6.76E-01	-0.08	7.10E-01	0.02	9.38E-01	0.05	8.01E-01	-0.11	5.98E-01	-0.17	3.99E-01
ENSCAFG0000000720	C20H3orf67	pink	VSMC_M5	0.09	6.67E-01	-0.15	4.49E-01	-0.23	2.39E-01	0.01	9.46E-01	0.46	1.63E-02	-0.22	2.69E-01	0.08	6.73E-01	0.01	9.77E-01	0.16	4.39E-01	-0.31	1.11E-01
ENSCAFG0000000691	MTMR7	grey	VSMC_M10	0.09	6.67E-01	0.01	9.66E-01	0.15	4.60E-01	-0.26	1.99E-01	-0.15	4.54E-01	0.09	6.48E-01	-0.08	6.93E-01	0.22	2.79E-01	-0.06	7.72E-01	0.01	9.48E-01
ENSCAFG0000003147	ZNF791	grey	VSMC_M10	0.09	6.67E-01	0.09	6.64E-01	-0.28	1.56E-01	0.56	2.30E-03	0.32	1.08E-01	-0.14	4.74E-01	-0.33	8.91E-02	-0.46	1.62E-02	-0.07	7.16E-01	0.15	4.68E-01
ENSCAFG0000001709	TRIP4	darkolivegreen	VSMC_M9	0.09	6.67E-01	-0.72	2.04E-05	-0.34	8.53E-02	-0.34	7.92E-02	0.57	1.76E-03	-0.29	1.47E-01	0.31	1.19E-01	0.56	2.17E-03	0.58	1.47E-03	-0.78	1.65E-06
ENSCAFG0000001567	LYAR	darkgrey	VSMC_M8	0.09	6.67E-01	-0.38	5.06E-02	0.23	2.59E-01	-0.61	8.22E-04	-0.14	4.99E-01	0.21	3.04E-01	0.50	7.58E-03	0.67	1.36E-04	0.15	4.47E-01	-0.42	2.92E-02
ENSCAFG0000001994	KLHL36	grey	VSMC_M10	0.09	6.67E-01	0.65	2.56E-04	-0.04	8.26E-01	0.56	2.66E-03	-0.13	5.32E-01	0.11	5.95E-01	-0.21	2.90E-01	-0.66	1.78E-04	-0.57	1.82E-03	0.75	7.36E-06
ENSCAFG0000000566	ENSCAFG0000000566	grey	VSMC_M10	0.09	6.67E-01	-0.35	7.59E-02	-0.08	6.97E-01	-0.16	4.11E-01	0.09	6.41E-01	0.19	3.49E-01	0.20	3.08E-01	0.33	9.27E-02	0.22	2.76E-01	-0.18	3.64E-01

ENSCAFG0000000758	PTCD3	pink	VSMC_M5	0.09	6.68E-01	-0.51	6.84E-03	-0.34	8.20E-02	-0.24	2.27E-01	0.47	1.27E-02	0.05	7.96E-01	0.12	5.35E-01	0.47	1.41E-02	0.34	8.32E-02	-0.57	1.99E-03
ENSCAFG0000003201	ENSCAFG0000003201	yellow	VSMC_M3	0.09	6.68E-01	0.18	3.77E-01	0.67	1.30E-04	-0.36	6.43E-02	-0.55	2.81E-03	-0.02	9.24E-01	0.14	4.78E-01	0.26	1.88E-01	-0.23	2.40E-01	-0.13	5.16E-01
ENSCAFG0000002960	DMAC1	grey	VSMC_M10	0.09	6.68E-01	0.00	9.80E-01	0.33	9.81E-02	-0.16	4.36E-01	-0.30	1.29E-01	0.03	8.85E-01	0.09	6.40E-01	0.14	4.76E-01	-0.05	7.96E-01	0.02	9.29E-01
ENSCAFG0000001777	ARMCX5	grey	VSMC_M10	0.09	6.68E-01	0.07	7.16E-01	-0.18	3.63E-01	0.08	6.80E-01	0.11	5.92E-01	0.11	5.93E-01	0.09	6.52E-01	-0.04	8.34E-01	-0.15	4.56E-01	0.19	3.41E-01
ENSCAFG0000001149	SNAI1	grey	VSMC_M10	0.09	6.68E-01	-0.18	3.68E-01	0.03	8.72E-01	-0.37	6.11E-02	0.07	7.15E-01	0.10	6.29E-01	0.40	3.98E-02	0.40	4.05E-02	0.02	9.26E-01	-0.27	1.79E-01
ENSCAFG0000000304	ENSCAFG0000000304	grey	VSMC_M10	0.09	6.69E-01	-0.05	7.95E-01	-0.31	1.22E-01	0.24	2.27E-01	0.25	2.11E-01	0.24	2.20E-01	0.19	3.45E-01	-0.09	6.61E-01	-0.04	8.59E-01	0.12	5.52E-01
ENSCAFG0000001198	SPP2	darkgrey	VSMC_M8	0.09	6.69E-01	-0.35	6.94E-02	0.40	4.09E-02	-0.68	9.41E-05	-0.14	4.87E-01	-0.15	4.63E-01	0.14	4.91E-01	0.63	3.81E-04	0.31	1.16E-01	-0.70	5.44E-05
ENSCAFG0000000766	PXYLP1	darkgrey	VSMC_M8	0.09	6.69E-01	-0.45	1.86E-02	0.14	4.84E-01	-0.69	7.10E-05	0.09	6.72E-01	0.08	6.96E-01	0.43	2.38E-02	0.67	1.47E-04	0.33	8.92E-02	-0.66	2.04E-04
ENSCAFG0000001839	TTBK1	grey	VSMC_M10	0.09	6.70E-01	-0.26	1.91E-01	-0.19	3.49E-01	-0.16	4.36E-01	0.33	9.65E-02	-0.19	3.30E-01	0.29	1.39E-01	0.18	3.76E-01	0.23	2.53E-01	-0.28	1.55E-01
ENSCAFG0000001986	CDT1	darkgrey	VSMC_M8	0.09	6.71E-01	-0.39	4.18E-02	0.46	1.47E-02	-0.90	1.39E-10	-0.33	9.04E-02	0.16	4.23E-01	0.41	3.21E-02	0.95	4.56E-14	0.19	3.38E-01	-0.59	1.34E-03
ENSCAFG0000001658	MAD2L2	yellow	VSMC_M3	0.09	6.71E-01	0.21	2.85E-01	0.81	2.67E-07	-0.66	1.88E-04	-0.81	2.38E-07	0.19	3.53E-01	0.29	1.48E-01	0.48	1.04E-02	-0.30	1.25E-01	0.03	8.90E-01
ENSCAFG0000001341	ECHS1	cyan	VSMC_M2	0.09	6.71E-01	0.69	5.83E-05	0.51	6.66E-03	0.14	4.73E-01	-0.55	3.09E-03	-0.06	7.75E-01	-0.25	2.05E-01	-0.43	2.62E-02	-0.57	1.86E-03	0.52	5.21E-03
ENSCAFG0000002499	TMOD3	darkgrey	VSMC_M8	0.09	6.72E-01	-0.31	1.12E-01	0.26	1.85E-01	-0.58	1.66E-03	-0.17	4.05E-01	0.30	1.29E-01	0.66	1.64E-04	0.63	4.38E-04	0.15	4.70E-01	-0.33	8.80E-02
ENSCAFG0000001002	LONP2	grey	VSMC_M10	0.09	6.73E-01	0.53	4.45E-03	-0.13	5.27E-01	0.43	2.52E-02	0.09	6.44E-01	-0.31	1.13E-01	-0.05	8.18E-01	-0.51	7.10E-03	-0.53	4.47E-03	0.49	9.96E-03
ENSCAFG0000000581	ENSCAFG0000000581	grey	VSMC_M10	0.09	6.73E-01	-0.24	2.29E-01	0.11	6.01E-01	-0.35	7.08E-02	-0.10	6.34E-01	0.24	2.21E-01	0.16	4.32E-01	0.45	1.84E-02	0.07	7.40E-01	-0.24	2.20E-01
ENSCAFG0000000906	TMEM106C	grey	VSMC_M10	0.09	6.73E-01	0.27	1.69E-01	0.03	8.90E-01	0.02	9.07E-01	0.06	7.56E-01	-0.26	1.86E-01	-0.31	1.22E-01	-0.13	5.04E-01	-0.13	5.18E-01	0.03	8.84E-01
ENSCAFG0000003058	ZNF782	darkgreen	VSMC_M4	0.09	6.73E-01	0.06	7.75E-01	-0.46	1.54E-02	0.70	5.27E-05	0.35	7.05E-02	0.00	9.88E-01	-0.17	4.05E-01	-0.57	2.08E-03	-0.05	8.16E-01	0.38	5.32E-02
ENSCAFG0000002904	ENSCAFG0000002904	darkgreen	VSMC_M4	0.09	6.73E-01	-0.02	9.08E-01	-0.46	1.59E-02	0.54	3.42E-03	0.45	1.82E-02	-0.01	9.43E-01	-0.01	9.41E-01	-0.43	2.63E-02	0.03	8.78E-01	0.20	3.23E-01
ENSCAFG0000002023	LMO4	darkgreen	VSMC_M4	0.09	6.73E-01	0.45	1.78E-02	-0.44	2.14E-02	0.85	1.51E-08	0.32	9.99E-02	-0.26	1.97E-01	-0.43	2.57E-02	-0.83	1.13E-07	-0.36	6.50E-02	0.65	2.74E-04
ENSCAFG0000000023	N4BP3	grey	VSMC_M10	0.08	6.73E-01	0.05	7.87E-01	-0.17	3.93E-01	0.10	6.35E-01	0.05	8.06E-01	0.26	1.98E-01	0.11	6.00E-01	0.01	9.67E-01	-0.20	3.25E-01	0.25	2.05E-01
ENSCAFG0000000722	FAM107A	grey	VSMC_M10	0.08	6.73E-01	0.43	2.51E-02	-0.16	4.17E-01	0.44	2.27E-02	0.09	6.56E-01	-0.15	4.67E-01	0.22	2.79E-01	-0.49	1.01E-02	-0.40	4.03E-02	0.53	4.16E-03
ENSCAFG0000001050	ENSCAFG0000001050	grey	VSMC_M10	0.08	6.73E-01	-0.12	5.43E-01	-0.19	3.32E-01	0.03	8.86E-01	0.31	1.10E-01	-0.08	6.99E-01	0.32	1.06E-01	-0.02	9.33E-01	0.08	6.99E-01	-0.19	3.46E-01
ENSCAFG0000000641	NCKAP1L	grey	VSMC_M10	0.08	6.74E-01	-0.07	7.30E-01	-0.28	1.54E-01	0.44	2.29E-02	0.20	3.26E-01	0.12	5.51E-01	0.20	3.06E-01	-0.29	1.36E-01	-0.02	9.15E-01	0.25	2.12E-01
ENSCAFG0000003245	MAFA	darkgreen	VSMC_M4	0.08	6.74E-01	-0.04	8.51E-01	-0.45	1.79E-02	0.53	4.85E-03	0.44	2.29E-02	0.01	9.41E-01	0.03	8.92E-01	-0.40	4.08E-02	0.03	8.68E-01	0.20	3.30E-01
ENSCAFG0000002038	ENSCAFG0000002038	grey	VSMC_M10	0.08	6.74E-01	0.46	1.52E-02	0.20	3.09E-01	0.18	3.70E-01	-0.18	3.79E-01	-0.16	4.22E-01	-0.05	8.15E-01	-0.37	6.08E-02	-0.43	2.70E-02	0.31	1.16E-01
ENSCAFG0000000102	TPD52L1	darkgreen	VSMC_M4	0.08	6.74E-01	0.33	9.43E-02	-0.27	1.67E-01	0.73	1.63E-05	0.13	5.12E-01	0.07	7.19E-01	-0.34	8.35E-02	-0.63	4.20E-04	-0.36	6.59E-02	0.56	2.54E-03
ENSCAFG0000003003	ENSCAFG0000003003	grey	VSMC_M10	0.08	6.75E-01	-0.16	4.31E-01	-0.01	9.75E-01	-0.27	1.71E-01	0.09	6.66E-01	-0.08	6.94E-01	0.02	9.09E-01	0.24	2.20E-01	0.04	8.29E-01	-0.29	1.47E-01
ENSCAFG0000002555	SP7	grey	VSMC_M10	0.08	6.75E-01	0.02	9.25E-01	-0.56	2.53E-03	0.46	1.49E-02	0.47	1.39E-02	-0.03	8.76E-01	-0.13	5.33E-01	-0.39	4.17E-02	-0.08	7.06E-01	0.32	1.05E-01
ENSCAFG0000000648	GTF2E2	grey	VSMC_M10	0.08	6.75E-01	-0.20	3.24E-01	0.28	1.60E-01	-0.43	2.46E-02	-0.21	3.05E-01	-0.10	6.11E-01	0.50	7.53E-03	0.48	1.06E-02	0.00	9.91E-01	-0.25	2.08E-01
ENSCAFG0000001852	ZNF414	grey	VSMC_M10	0.08	6.75E-01	0.30	1.26E-01	0.12	5.44E-01	0.02	9.24E-01	-0.22	2.66E-01	0.18	3.66E-01	0.11	6.01E-01	-0.04	8.32E-01	-0.41	3.34E-02	0.29	1.39E-01
ENSCAFG0000000528	ENSCAFG0000000528	grey	VSMC_M10	0.08	6.75E-01	0.20	3.22E-01	-0.06	7.75E-01	0.12	5.60E-01	0.00	9.87E-01	0.14	4.77E-01	-0.20	3.26E-01	-0.13	5.14E-01	-0.19	3.35E-01	0.30	1.34E-01
ENSCAFG0000002074	ENSCAFG0000002074	grey	VSMC_M10	0.08	6.75E-01	-0.07	7.24E-01	0.15	4.67E-01	-0.19	3.47E-01	-0.09	6.39E-01	-0.03	8.72E-01	0.23	2.45E-01	0.21	2.93E-01	0.02	9.33E-01	-0.11	5.98E-01
ENSCAFG0000001926	DNASE1	grey	VSMC_M10	0.08	6.76E-01	0.10	6.07E-01	-0.05	8.12E-01	0.09	6.59E-01	0.04	8.44E-01	0.01	9.66E-01	0.26	1.90E-01	-0.06	7.48E-01	-0.16	4.15E-01	0.17	4.00E-01
ENSCAFG0000001183	IGSF9	yellow	VSMC_M3	0.08	6.77E-01	0.02	9.03E-01	0.53	4.18E-03	-0.46	1.52E-02	-0.43	2.49E-02	-0.17	3.87E-01	0.25	2.17E-01	0.32	1.09E-01	-0.05	7.87E-01	-0.12	5.56E-01
ENSCAFG0000000330	SNIP1	grey	VSMC_M10	0.08	6.77E-01	-0.15	4.53E-01	-0.39	4.24E-02	0.21	2.85E-01	0.34	8.51E-02	0.17	3.98E-01	-0.17	3.96E-01	-0.08	7.06E-01	0.08	6.76E-01	0.04	8.61E-01
ENSCAFG0000001984	SARS	yellow	VSMC_M3	0.08	6.77E-01	0.15	4.41E-01	0.71	3.59E-05	-0.64	3.41E-04	-0.65	2.12E-04	0.08	7.02E-01	0.34	8.59E-02	0.47	1.25E-02	-0.28	1.52E-01	-0.04	8.24E-01
ENSCAFG0000001833	DOCK11	yellow	VSMC_M3	0.08	6.77E-01	0.47	1.24E-02	0.46	1.49E-02	-0.41	3.40E-02	-0.33	9.43E-02	-0.22	2.74E-01	0.10	6.08E-01	0.09	6.39E-01	-0.40	3.62E-02	0.09	6.52E-01
ENSCAFG0000000708	MTMR6	pink	VSMC_M5	0.08	6.77E-01	-0.40	3.74E-02	-0.70	4.39E-05	0.48	1.17E-02	0.66	1.82E-04	0.02	9.31E-01	-0.20	3.06E-01	-0.24	2.23E-01	0.38	5.06E-02	-0.03	8.72E-01
ENSCAFG0000000071	CYP21A2	grey	VSMC_M10	0.08	6.77E-01	-0.14	4.87E-01	-0.15	4.57E-01	-0.09	6.71E-01	0.06	7.79E-01	0.15	4.63E-01	0.13	5.31E-01	0.16	4.14E-01	0.02	9.30E-01	0.09	6.37E-01
ENSCAFG0000001760	UNC79	grey	VSMC_M10	0.08	6.78E-01	0.13	5.19E-01	-0.49	9.48E-03	0.62	5.46E-04	0.42	2.85E-02	-0.11	5.89E-01	-0.07	7.47E-01	-0.51	6.34E-03	-0.13	5.31E-01	0.37	5.89E-02
ENSCAFG0000001338	PCNX3	grey	VSMC_M10	0.08	6.78E-01	0.51	6.77E-03	0.19	3.34E-01	-0.01	9.49E-01	-0.29	1.37E-01	0.29	1.49E-01	0.02	9.10E-01	-0.19	3.55E-01	-0.53	4.67E-03	0.51	7.07E-03
ENSCAFG0000001293	RHOBTB1	grey	VSMC_M10	0.08	6.78E-01	0.35	7.63E-02	0.14	4.91E-01	0.10	6.11E-01	-0.20	3.05E-01	0.13	5.07E-01	0.14	4.90E-01	-0.31	1.13E-01	-0.31	1.19E-01	0.44	2.21E-02
ENSCAFG0000003245	ENSCAFG0000003245	grey	VSMC_M10	0.08	6.78E-01	0.30	1.28E-01	-0.23	2.42E-01	0.30	1.33E-01	0.27	1.77E-01	-0.16	4.38E-01	-0.03	8.62E-01	-0.39	4.38E-02	-0.27	1.66E-01	0.29	1.45E-01
ENSCAFG0000000448	SLC39A12	grey	VSMC_M10	0.08	6.78E-01	-0.23	2.41E-01	-0.13	5.23E-01	-0.14	4.90E-01	0.20	3.23E-01	-0.11	5.72E-01	0.12	5.50E-01	0.23	2.57E-01	0.13	5.05E-01	-0.17	3.92E-01
ENSCAFG0000002990	ENSCAFG0000002990	grey	VSMC_M10	0.08	6.78E-01	-0.14	4.88E-01	0.28	1.55E-01	-0.40	3.94E-02	-0.15	4.66E-01	0.07	7.17E-01	-0.13	5.13E-01	0.42	2.93E-02	0.10	6.27E-01	-0.37	6.09E-02
ENSCAFG0000000293	POLD1	darkgrey	VSMC_M8	0.08	6.78E-01	-0.16	4.38E-01	0.47	1.26E-02	-0.67	1.51E-04	-0.41	3.35E-02	0.20	3.27E-01	0.47	1.37E-02	0.73	1.29E-05	-0.07	7.37E-01	-0.29	1.41E-01
ENSCAFG0000000403	HIPK2	darkgreen	VSMC_M4	0.08	6.78E-01	0.27	1.66E-01	-0.34	7.88E-02	0.66	1.98E-04	0.27	1.72E-01	0.03	8.76E-01	-0.76	4.02E-06	-0.64	3.06E-04	-0.15	4.59E-01	0.37	5.46E-02
ENSCAFG0000001737	ANKFN1	cyan	VSMC_M2	0.08	6.78E-01	0.43	2.57E-02	0.28	1.54E-01	-0.14	4.89E-01	-0.29	1.42E-01	-0.08	6.74E-01	0.16	4.21E-01	-0.01	9.47E-01	-0.42	2.81E-02	0.24	2.25E-01
ENSCAFG0000000062	PPP6R2	cyan	VSMC_M2	0.08	6.79E-01	0.73	1.53E-05	0.64	3.19E-04	-0.09	6.52E-01	-0.62	6.33E-04	-0.05	7.87E-01	-0.10	6.16E-01	-0.20	3.13E-01	-0.65	2.19E-04	0.41	3.44E-02
ENSCAFG0000003136	ENSCAFG0000003136	grey	VSMC_M10	0.08	6.79E-01	-0.27	1.73E-01	-0.03	8.79E-01	-0.17	4.08E-01	0.02	9.08E-01	0.40	4.07E-02	0.41	3.22E-02	0.24	2.27E-01	0.18	3.62E-01	-0.18	3.82E-01
ENSCAFG0000001818	ENSCAFG0000001818	grey	VSMC_M10	0.08	6.79E-01	-0.08	6.97E-01	0.23	2.42E-01	-0.38	5.13E-02	-0.05	8.11E-01	-0.06	7.85E-01	0.07	7.40E-01	0.32	1.08E-01	0.02	9.32E-01	-0.42	2.93E-02
ENSCAFG0000002577	ENSCAFG0000002577	grey	VSMC_M10	0.08	6.79E-01	0.33	9.42E-02	0.52	5.30E-03	-0.09	6.64E-01	-0.57	2.03E-03	0.38	4.74E-02	0.22	2.72E-01	-0.01	9.58E-01	-0.38	5.30E-02	0.25	2.04E-01
ENSCAFG0000001473	ZNF674	grey	VSMC_M10	0.08	6.79E-01	0.24	2.24E-01	-0.01	9.45E-01	0.12	5.65E-01	0.14	4.95E-01	-0.06	7.77E-01	0.04	8.39E-01	-0.24	2.37E-01	-0.23	2.50E-01	0.03	8.93E-01
ENSCAFG0000000206	COL1A2	darkgrey	VSMC_M8	0.08	6.79E-01	-0.05	8.14E-01	0.67	1.16E-04	-0.78	1.32E-06	-0.51	6.96E-03	0.03	8.84E-01	0.29	1.49E-01	0.58	1.48E-03	-0.03	8.88E-01	-0.34	7.94E-02
ENSCAFG0000000970	PAX2	grey	VSMC_M10	0.08	6.79E-01	0.04	8.38E-01	-0.06	7.56E-01	0.08	6.99E-01	0.08	6.86E-01	-0.05	8.04E-01	-0.10	6.20E-01	-0.10	6.25E-01	0.00	9.97E-01	0.04	8.61E-01
ENSCAFG0000000991	FBXW4	grey	VSMC_M10	0.08	6.79E-01	0.40	3.79E-02	0.04	8.33E-01	0.13	5.16E-01	-0.01	9.71E-01	-0.26	1.94E-01	-0.05	8.07E-01	-0.36	6.31E-02	-0.27	1.80E-01	0.33	9.26E-02
ENSCAFG0000000760	SCN8A	darkgreen	VSMC_M4	0.08	6.80E-01	0.04	8.59E-01	-0.38	5.06E-02	0.63	4.76E-04	0.33	9.24E-02	-0.12	5.63E-01	-0.59	1.06E-03	-0.49	8.93E-03	0.02	9.32E-01	0.19	3.41E-01
ENSCAFG0000001862	DAB2	darkgreen	VSMC_M4	0.08	6.80E-01	0.32	1.02E-01	-0.50	8.09E-03	0.86	6.38E-09	0.37	5.58E-02	-0.21	2.99E-01	-0.35	7.19E-02	-0.76	4.27E-06	-0.29	1.48E-01	0.58	1.40E-03
ENSCAFG0000000468	EIF2B3	grey	VSMC_M10	0.08	6.80E-01	-0.57	2.04E-03	0.06	7.48E-01	-0.33	9.34E-02	0.09	6.59E-01	-0.06	7.64E-01	0.00	9.95E-01	0.51	7.06E-03	0.45	1.95E-02	-0.65	2.25E-04
ENSCAFG0000001192	HSD11B1	grey	VSMC_M10	0.08	6.80E-01	0.16	4.23E-01	0.16	4.19E-01	-0.08	7.08E-01	-0.13	5.26E-01	-0.13	5.33E-01	-0.22	2.78E-01	0.07	7.14E-01	-0.22	2.81E-01	0.05	8.12E-01
ENSCAFG0000001838	PSMD11	darkgrey	VSMC_M8	0.08	6.81E-01	-0.49	1.00E-02	0.21	3.01E-01	-0.58	1.62E-03	-0.11	5.68E-01	0.06	7.73E-01	0.14	4.91E-01	0.72	2.18E-05	0.36	6.90E-02	-0.57	1.94E-03
ENSCAFG0000001141	RPL36AL	yellow	VSMC_M3	0.08	6.81E-01	0.25	2.03E-01	0.77	2.52E-06	-0.41	3.31E-02	-0.70	4.63E-05	0.00	9.89E-01	0.18	3.68E-01	0.26	1.95E-01	-0.28	1.52E-01	-0.04	8.62E-01
ENSCAFG0000002401	ENSCAFG0000002401	grey	VSMC_M10	0.08	6.81E-01	-0.16	4.33E-01	-0.26	1.82E-01	0.26	1.90E-01	0.43	2.65E-02	-0.16	4.30E-01	-0.30	1.32E-01	-0.25	2.17E-01	0.26	1.98E-01	-0.29	1.45E-01
ENSCAFG0000001556	CSTF2T	yellow	VSMC_M3	0.08	6.81E-01	0.30	1.25E-01	0.81	3.43E-07	-0.45	1.89E-02	-0.83	1.18E-07	0.09	6.42E-01	0.25	2.12E-01	0.28	1.56E-01	-0.37	5.45E-02	0.14	4.87E-01
ENSCAFG0000001472	FLRT1	grey	VSMC_M10	0.08	6.81E-01	0.28	1.64E-01	-0.16	4.23E-01	0.31	1.13E-01	0.06	7.74E-01	0.06	7.78E-01	0.22	2.73E-01	-0.30	1.29E-01	-0.33	9.66E-02	0.43	2.53E-02
ENSCAFG0000001691	SYT11	grey	VSMC_M10	0.08	6.81E-01	0.28	1.58E-01	0.16	4.29E-01	0.15	4.68E-01	-0.01	9.51E-01	-0.39	4.70E-02	-0.06	7.76E-01	-0.30	1.28E-01	-0.12	5.44E-01	0.08	6.91E-01
ENSCAFG0000000907	PLBD2	cyan	VSMC_M2	0.08	6.82E-01	0.81	2.27E-07	0.10	6.03E-01	0.50	7.84E-03	-0.14	4.92E-01	-0.20	3.20E-01	-0.28	1.60E-01	-0.79	9.44E-07	-0.64	3.36E-04	0.70	5.64E-05
ENSCAFG0000000290	KLK4	grey	VSMC_M10	0.08	6.82E-01	0.09	6.60E-01	-0.12	5.54E-01	0.07	7.11E-01	0.10	6.15E-01	0.13	5.13E-01	0.21	2.84E-01	0.01	9.58E-01	-0.19	3.42E-01	0.09	6.52E-01
ENSCAFG0000002309	PRMT6	grey	VSMC_M10	0.08	6.82E-01	0.41	3.15E-02	0.53	4.38E-03	-0.18	3.72E-01	-0.47	1.37E-02	0.19	3.38E-01	-0.04	8.46E-01	0.02	9.22E-01	-0.42	2.77E-02	0.11	6.02E-01
ENSCAFG0000001126	AJUBA	grey	VSMC_M10	0.08	6.83E-01	0.20	3.19E-01	0.29	1.37E-01	0.12	5.65E-01	-0.22	2.64E-01	-0.16	4.37E-01	-0.18	3.68E-01	-0.13	5.07E-01	-0.23	2.39E-01	0.09	6.62E-01
ENSCAFG0000001285	RERG	cyan	VSMC_M2	0.08	6.83E-01	0.79	1.22E-06	0.10	6.34E-01	0.56	2.43E-03	-0.21	2.85E-01	-0.11	5.88E-01	-0.52	5.91E-03	-0.71	2.90E-05	-0.65	2.10E-04	0.76	4.90E-06
ENSCAFG0000002587	ENSCAFG0000002587	grey	VSMC_M10	0.08	6.83E-01	0.18	3.82E-01	0.22	2.73E-01	-0.20	3.19E-01	-0.17	3.96E-01	-0.01	9.70E-01	0.26	1.85E-01	0.13	5.06E-01	-0.24	2.38E-01	0.10	6.26E-01
ENSCAFG0000001236	SLCO1A2	grey	VSMC_M10	0.08	6.85E-01	0.52	5.61E-03	0.16	4.13E-01	0.33	9.81E-02	-0.29	1.43E-01	-0.15	4.67E-01	-0.36	6.43E-02	-0.34	7.93E-02	-0.49	1.03E-02	0.52	5.77E-03
ENSCAFG0000000680	FZD10	violet	VSMC_M7	0.08	6.86E-01	-0.08	6.83E-01	0.21	3.01E-01	-0.42	2.73E-02	-0.18	3.83E-01	0.18	3.56E-01	0.78	1.99E-06	0.44	2.13E-02	-0.09	6.72E-01	-0.15	4.65E-01
ENSCAFG0000001419	TRMT2A	yellow	VSMC_M3	0.08	6.86E-01	0.71	3.27E-05	0.67	1.47E-04	0.00	9.83E-01	-0.66	2.04E-04	-0.14	4.86E-01	-0.12	5.60E-01	-0.25	2.11E-01	-0.65	2.58E-04	0.41	3.24E-02
ENSCAFG0000002634	ENSCAFG0000002634	grey	VSMC_M10	0.08	6.87E-01	0.18	3.68E-01	0.21	2.96E-01	-0.26	1.91E-01	-0.17	4.03E-01	-0.03	8.95E-01	0.34	8.56E-02	0.12	5.64E-01	-0.15	4.64E-01	0.09	6.66E-01
ENSCAFG0000002009	FAM102A	cyan	VSMC_M2	0.08	6.87E-01	0.55	3.18E-03	0.43	2.41E-02	-0.24	2.34E-01	-0.45	1.89E-02	0.02	9.03E-01	0.16	4.20E-01	0.02	9.04E-01	-0.55	2.87E-03	0.38	5.04E-02
ENSCAFG0000002466	ENSCAFG0000002466	grey	VSMC_M10	0.08	6.87E-01	-0.14	4.81E-01	0.01	9.63E-01	-0.19	3.50E-01	0.06	7.59E-01	-0.02	9.18E-01	0.10	6.20E-01	0.19	3.52E-01	0.16	4.30E-01	-0.17	4.02E-01
ENSCAFG0000001688	ARHGEF2	yellow	VSMC_M3	0.08	6.87E-01	0.17	3.88E-01	0.71	3.09E-05	-0.64	3.08E-04	-0.56	2.66E-03	0.02	9.21E-01	0.10	6.04E-01	0.35	7.36E-02	-0.17	4.00E-01	-0.16	4.34E-01
ENSCAFG0000001698	FAM189B	darkgreen	VSMC_M4	0.08	6.87E-01	0.64	2.82E-04	0.03	8.73E-01	0.53	4.68E-03	-0.04	8.45E-01	-0.18	3.61E-01	-0.47	1.34E-02	-0.72	2.67E-05	-0.43	2.35E-02	0.53	4.59E-03
ENSCAFG0000001025	CIP2A	darkgrey	VSMC_M8	0.08	6.88E-01	-0.55	3.01E-03	0.30	1.26E-01	-0.81	2.48E-07	-0.17	4.02E-01	0.16	4.34E-01	0.44	2.08E-02	0.92	1.22E-11	0.34	8.12E-02	-0.64	3.29E-04
ENSCAFG0000001583	SDHB	grey	VSMC_M10	0.08	6.88E-01	-0.02	9.39E-01	-0.29	1.42E-01	0.14	4.96E-01	0.40	3.73E-02	-0.03	8.63E-01	-0.39	4.43E-02	-0.14	4.83E-01	0.13	5.27E-01	-0.19	3.39E-01
ENSCAFG0000001964	RHOT2	cyan	VSMC_M2	0.08	6.88E-01	0.71	3.11E-05	0.53	4.17E-03	0.05	7.88E-01	-0.60	1.04E-03	-0.01	9.75E-01	-0.44	2.08E-02	-0.30	1.32E-01	-0.58	1.54E-03	0.54	3.49E-03
ENSCAFG0000000867	RXFP1	darkgreen	VSMC_M4	0.08	6.88E-01	0.45	1.83E-02	-0.43	2.55E-02	0.91	2.66E-11	0.30	1.24E-01	-0.11	5.85E-01	-0.33	9.50E-02	-0.86	8.20E-09	-0.38	5.12E-02	0.65	2.56E-04
ENSCAFG0000001420	SIK2	grey	VSMC_M10	0.08	6.88E-01	0.15	4.48E-01	-0.24	2.33E-01	0.33	9.30E-02	0.09	6.47E-01	0.17	4.05E-01	0.21	2.92E-01	-0.31	1.18E-01	-0.18	3.78E-01	0.45	1.81E-02
ENSCAFG0000000342	VRK3	grey	VSMC_M10	0.08	6.88E-01	0.64	3.42E-04	-0.03	8.69E-01	0.55	3.27E-03	-0.11	5.73E-01	-0.04	8.49E-01	-0.07	7.26E-01	-0.65	2.34E-04	-0.63	4.30E-04	0.75	6.30E-06
ENSCAFG0000000343	COBL	cyan	VSMC_M2	0.08	6.89E-01	0.69	7.26E-05	0.10	6.37E-01	0.63	4.72E-04	-0.16	4.28E-01	-0.23	2.55E-01	-0.36	6.84E-02	-0.76	3.38E-06	-0.57	2.13E-03	0.65	2.13E-04
ENSCAFG0000001252	TLK1	pink	VSMC_M5	0.08	6.89E-01	-0.24	2.33E-01	-0.51	6.27E-03	0.23	2.54E-01	0.72	2.70E-05	-0.23	2.56E-01	-0.32	9.97E-02	-0.14	5.00E-01	0.31	1.21E-01	-0.40	4.01E-02
ENSCAFG0000001299	RNF185	grey	VSMC_M10	0.08	6.89E-01	0.07	7.32E-01	0.56	2.18E-03	-0.50	7.93E-03	-0.40	3.98E-02	0.06	7.62E-01	-0.02	9.32E-01	0.37	5.85E-02	-0.11	5.94E-01	-0.29	1.42E-01
ENSCAFG0000002784	ENSCAFG0000002784	grey	VSMC_M10	0.08	6.89E-01	-0.37	6.09E-02	-0.20	3.20E-01	-0.13	5.31E-01	0.20	3.28E-01	0.18	3.66E-01	0.36	6.90E-02	0.24	2.21E-01	0.26	1.95E-01	-0.18	3.70E-01
ENSCAFG0000002902	COL6A2	yellow	VSMC_M3	0.08	6.90E-01	0.34	7.97E-02	0.67	1.47E-04	-0.40	4.00E-02	-0.48	1.12E-02	-0.26	1.98E-01	0.38	5.26E-02	0.09	6.38E-01	-0.29	1.49E-01	-0.03	9.00E-01
ENSCAFG0000001033	TMEM183A	yellow	VSMC_M3	0.08	6.90E-01	0.00	9.92E-01	0.68	1.09E-04	-0.85	1.43E-08	-0.53	4.24E-03	-0.06	7.56E-01	0.23	2.55E-01	0.65	2.17E-04	-0.07	7.26E-01	-0.35	7.41E-02
ENSCAFG0000000943	RPP40	grey	VSMC_M10	0.08	6.90E-01	0.16	4.22E-01	0.24	2.31E-01	-0.22	2.65E-01	-0.35	7.31E-02	0.43	2.61E-02	0.34	8.18E-02	0.18	3.64E-01	-0.30	1.33E-01	0.26	1.89E-01
ENSCAFG0000000386	BAX	cyan	VSMC_M2	0.08	6.90E-01	0.47	1.28E-02	0.46	1.54E-02	-0.02	9.37E-01	-0.40	3.62E-02	-0.02	9.13E-01	0.15	4.66E-01	-0.14	4.93E-01	-0.46	1.48E-02	0.20	3.07E-01
ENSCAFG0000002039	MSH4	grey	VSMC_M10	0.08	6.90E-01	0.00	9.83E-01	0.03	8.70E-01	-0.08	6.95E-01	0.01	9.74E-01	-0.13	5.11E-01	-0.21	3.04E-01	0.08	6.99E-01	0.00	9.87E-01	-0.05	7.86E-01
ENSCAFG0000000801	ENSCAFG0000000801	grey	VSMC_M10	0.08	6.91E-01	-0.20	3.18E-01	-0.37	5.48E-02	0.23	2.41E-01	0.36	6.92E-02	-0.10	6.13E-01	0.31	1.21E-01	-0.16	4.20E-01	0.08	6.82E-01	0.08	7.09E-01
ENSCAFG0000001059	RGS2	darkgreen	VSMC_M4	0.08	6.91E-01	0.24	2.22E-01	-0.39	4.60E-02	0.80	5.09E-07	0.29	1.42E-01	-0.24	2.30E-01	-0.44	2.33E-02	-0.75	7.51E-06	-0.14	4.99E-01	0.49	9.38E-03
ENSCAFG0000001046	ENSCAFG0000001046	grey	VSMC_M10	0.08	6.92E-01	-0.20	3.10E-01	-0.11	6.02E-01	-0.06	7.54E-01	0.11	5.85E-01	-0.14	4.96E-01	-0.02	9.35E-01	0.19	3.54E-01	0.12	5.37E-01	-0.08	7.02E-01
ENSCAFG0000001148	AMT	grey	VSMC_M10	0.08	6.92E-01	0.23	2.51E-01	0.13	5.17E-01	-0.03	8.84E-01	0.02	9.39E-01	-0.19	3.37E-01	0.08	6.80E-01	-0.10	6.08E-01	-0.18	3.56E-01	-0.01	9.60E-01
ENSCAFG0000000479	NUDT5	grey	VSMC_M10	0.08	6.92E-01	-0.23	2.57E-01	0.19	3.41E-01	-0.40	3.77E-02	0.01	9.48E-01	-0.17	4.06E-01	0.18	3.69E-01	0.46	1.57E-02	0.18	3.75E-01	-0.57	1.97E-03
ENSCAFG0000003009	C1D	grey	VSMC_M10	0.08	6.92E-01	-0.17	4.03E-01	-0.24	2.21E-01	0.18	3.79E-01	0.16	4.35E-01	0.11	5.78E-01	0.09	6.41E-01	-0.01	9.79E-01	0.08	7.04E-01	0.14	4.75E-01
ENSCAFG0000000369	STOM	cyan	VSMC_M2	0.08	6.92E-01	0.69	6.36E-05	-0.26	1.89E-01	0.78	1.72E-06	0.13	5.34E-01	-0.07	7.18E-01	-0.35	7.00E-02	-0.89	4.88E-10	-0.57	1.90E-03	0.78	1.64E-06
ENSCAFG0000002394	NIPSNAP3B	grey	VSMC_M10	0.08	6.92E-01	0.55	3.04E-03	0.03	8.80E-01	0.44	2.14E-02	-0.13	5.17E-01	-0.09	6.58E-01	-0.01	9.64E-01	-0.62	5.28E-04	-0.47	1.33E-02	0.64	3.26E-04
ENSCAFG0000001326	ENSCAFG0000001326	grey	VSMC_M10	0.08	6.93E-01	0.17	3.88E-01	0.09	6.52E-01	0.13	5.05E-01	0.03	8.69E-01	-0.25	2.03E-01	0.02	9.22E-01	-0.26	1.96E-01	-0.12	5.53E-01	-0.02	9.28E-01
ENSCAFG0000003199	MSANTD4	grey	VSMC_M10	0.08	6.93E-01	0.19	3.48E-01	0.29	1.45E-01	0.17	3.88E-01	-0.20	3.12E-01	-0.16	4.27E-01	-0.18	3.70E-01	-0.28	1.55E-01	-0.11	5.75E-01	0.06	7.59E-01
ENSCAFG0000000937	SORBS3	cyan	VSMC_M2	0.08	6.93E-01	0.78	1.52E-06	0.08	6.81E-01	0.60	9.63E-04	-0.14	4.72E-01	-0.29	1.42E-01	-0.40	4.14E-02	-0.77	2.60E-06	-0.59	1.09E-03	0.70	4.32E-05
ENSCAFG0000001812	ENSCAFG0000001812	cyan	VSMC_M2	0.08	6.93E-01	0.46	1.62E-02	0.07	7.30E-01	0.12	5.41E-01	-0.09	6.47E-01	-0.15	4.66E-01	-0.34	8.17E-02	-0.32	1.04E-01	-0.36	6.34E-02	0.35	7.65E-02
ENSCAFG0000000015	MYO1A	grey	VSMC_M10	0.08	6.93E-01	0.26	1.88E-01	0.32	1.06E-01	-0.30	1.28E-01	-0.28	1.58E-01	-0.10	6.03E-01	0.10	6.32E-01	0.11	5.88E-01	-0.23	2.45E-01	0.13	5.16E-01
ENSCAFG0000003263	ENSCAFG0000003263	grey	VSMC_M10	0.08	6.93E-01	-0.08	6.82E-01	0.24	2.27E-01	-0.43	2.66E-02	-0.20	3.24E-01	0.05	8.09E-01	0.09	6.42E-01	0.40	3.71E-02	0.03	8.75E-01	-0.16	4.35E-01
ENSCAFG0000000351	TLR4	grey	VSMC_M10	0.08	6.93E-01	-0.24	2.30E-01	-0.05	7.98E-01	-0.25	2.08E-01	0.17	3.90E-01	0.51	6.23E-03	0.34	8.53E-02	0.29	1.36E-01	0.14	4.88E-01	-0.40	4.07E-02
ENSCAFG0000002373	BCL7B	grey	VSMC_M10	0.08	6.94E-01	-0.47	1.25E-02	-0.19	3.32E-01	-0.26	1.93E-01	0.31	1.13E-01	0.03	8.94E-01	-0.26	1.97E-01	0.39	4.55E-02	0.42	2.90E-02	-0.56	2.28E-03
ENSCAFG0000000738	CYP4V2	cyan	VSMC_M2	0.08	6.94E-01	0.74	1.04E-05	0.05	8.22E-01	0.54	3.32E-03	-0.09	6.58E-01	0.04	8.39E-01	-0.44	2.31E-02	-0.73	1.61E-05	-0.58	1.60E-03	0.62	6.39E-04
ENSCAFG0000000707	CADPS	grey	VSMC_M10	0.08	6.94E-01	0.40	4.12E-02	0.25	2.04E-01	-0.12	5.51E-01	-0.24	2.23E-01	-0.07	7.29E-01	0.23	2.41E-01	-0.03	8.84E-01	-0.37	5.82E-02	0.22	2.64E-01
ENSCAFG0000003064	LYPLA2	grey	VSMC_M10	0.08	6.94E-01	0.26	1.93E-01	0.40	3.63E-02	-0.29	1.39E-01	-0.30	1.31E-01	0.09	6.44E-01	-0.40	3.91E-02	0.12	5.37E-01	-0.15	4.51E-01	-0.14	4.80E-01
ENSCAFG0000003211	ENSCAFG0000003211	grey	VSMC_M10	0.08	6.94E-01	-0.06	7.65E-01	-0.50	7.67E-03	0.41	3.48E-02	0.48	1.06E-02	-0.34	8.64E-02	0.14	5.01E-01	-0.28	1.56E-01	0.01	9.77E-01	0.11	5.70E-01
ENSCAFG0000003182	ENSCAFG0000003182	darkgreen	VSMC_M4	0.08	6.94E-01	0.35	7.65E-02	-0.51	6.15E-03	0.91	7.65E-11	0.38	5.16E-02	-0.06	7.48E-01	-0.52	5.91E-03	-0.82	1.66E-07	-0.30	1.31E-01	0.59	1.29E-03

ENSCAFG0000000762	PIK3R1	grey	VSMC_M10	0.08	6.95E-01	0.20	3.25E-01	-0.17	3.84E-01	0.23	2.40E-01	0.15	4.63E-01	-0.17	3.84E-01	-0.11	5.68E-01	-0.22	2.78E-01	-0.17	4.06E-01	0.23	2.53E-01
ENSCAFG0000001959	UBL4A	grey	VSMC_M10	0.08	6.95E-01	0.37	6.00E-02	0.06	7.63E-01	0.25	2.09E-01	-0.23	2.58E-01	0.09	6.69E-01	0.08	6.79E-01	-0.30	1.28E-01	-0.45	1.76E-02	0.58	1.62E-03
ENSCAFG0000001768	GSC	grey	VSMC_M10	0.08	6.95E-01	0.24	2.19E-01	0.49	8.98E-03	-0.39	4.54E-02	-0.50	8.50E-03	0.15	4.45E-01	0.21	2.84E-01	0.23	2.53E-01	-0.32	1.03E-01	0.13	5.06E-01
ENSCAFG0000000864	ENSCAFG0000000864	grey	VSMC_M10	0.08	6.95E-01	0.02	9.08E-01	-0.12	5.36E-01	0.14	4.84E-01	0.13	5.16E-01	0.01	9.52E-01	-0.05	8.09E-01	-0.09	6.63E-01	-0.03	8.79E-01	0.10	6.12E-01
ENSCAFG0000000557	CBX4	grey	VSMC_M10	0.08	6.96E-01	0.23	2.47E-01	-0.14	4.93E-01	0.47	1.27E-02	0.19	3.33E-01	-0.27	1.65E-01	0.07	7.27E-01	-0.51	6.75E-03	-0.24	2.33E-01	0.26	1.90E-01
ENSCAFG0000002035	ENKD1	grey	VSMC_M10	0.08	6.96E-01	0.03	8.94E-01	0.11	5.76E-01	0.04	8.43E-01	-0.17	4.02E-01	0.01	9.60E-01	0.53	4.91E-03	0.01	9.61E-01	-0.16	4.22E-01	0.24	2.19E-01
ENSCAFG0000000019	EYA4	grey	VSMC_M10	0.08	6.96E-01	0.38	5.38E-02	0.06	7.65E-01	0.23	2.42E-01	-0.09	6.45E-01	-0.06	7.52E-01	-0.83	9.97E-08	-0.31	1.17E-01	-0.24	2.34E-01	0.26	1.85E-01
ENSCAFG0000000999	ANKRD33B	grey	VSMC_M10	0.08	6.96E-01	-0.15	4.67E-01	0.12	5.65E-01	-0.05	8.09E-01	-0.09	6.67E-01	0.23	2.39E-01	0.29	1.38E-01	0.05	8.04E-01	0.13	5.31E-01	0.01	9.64E-01
ENSCAFG0000001745	EML5	grey	VSMC_M10	0.08	6.96E-01	-0.09	6.66E-01	-0.37	5.51E-02	0.25	2.08E-01	0.21	2.82E-01	-0.03	8.89E-01	0.08	7.08E-01	-0.13	5.07E-01	-0.01	9.55E-01	0.33	9.68E-02
ENSCAFG0000001938	SKI	grey	VSMC_M10	0.08	6.97E-01	0.45	1.79E-02	-0.08	6.93E-01	0.35	7.43E-02	0.07	7.47E-01	0.00	9.97E-01	0.02	9.18E-01	-0.42	2.79E-02	-0.36	6.13E-02	0.45	1.86E-02
ENSCAFG0000000281	ENSCAFG0000000281	turquoise	VSMC_M6	0.08	6.97E-01	-0.23	2.44E-01	-0.08	7.06E-01	-0.11	5.98E-01	0.08	7.04E-01	0.52	5.39E-03	-0.02	9.34E-01	0.18	3.76E-01	0.21	2.89E-01	-0.18	3.73E-01
ENSCAFG0000001308	PATZ1	grey	VSMC_M10	0.08	6.97E-01	0.26	1.91E-01	0.42	2.91E-02	-0.16	4.22E-01	-0.29	1.45E-01	-0.21	2.85E-01	-0.24	2.38E-01	0.05	7.91E-01	-0.23	2.42E-01	-0.12	5.37E-01
ENSCAFG0000000499	DMAC2	grey	VSMC_M10	0.08	6.97E-01	0.05	7.87E-01	0.44	2.03E-02	-0.39	4.64E-02	-0.30	1.29E-01	-0.12	5.46E-01	-0.25	2.02E-01	0.31	1.10E-01	0.00	9.82E-01	-0.28	1.59E-01
ENSCAFG0000001234	PRUNE1	cyan	VSMC_M2	0.08	6.97E-01	0.80	5.50E-07	0.36	6.51E-02	0.36	6.48E-02	-0.41	3.29E-02	-0.06	7.54E-01	-0.33	8.83E-02	-0.60	9.02E-04	-0.64	3.24E-04	0.65	2.70E-04
ENSCAFG0000001416	EPHB4	grey	VSMC_M10	0.08	6.97E-01	0.64	3.30E-04	0.30	1.26E-01	0.34	8.10E-02	-0.43	2.35E-02	0.53	4.25E-03	-0.33	9.14E-02	-0.48	1.08E-02	-0.55	2.75E-03	0.61	7.77E-04
ENSCAFG0000000816	SETD1B	yellow	VSMC_M3	0.08	6.98E-01	0.38	5.27E-02	0.66	1.77E-04	-0.21	3.01E-01	-0.57	1.92E-03	-0.05	8.15E-01	-0.43	2.42E-02	-0.01	9.67E-01	-0.28	1.50E-01	0.08	6.78E-01
ENSCAFG0000001267	TRIM63	darkgreen	VSMC_M4	0.08	6.98E-01	0.48	1.10E-02	-0.18	3.75E-01	0.65	2.46E-04	0.12	5.64E-01	-0.19	3.35E-01	-0.44	2.33E-02	-0.76	4.06E-06	-0.37	6.01E-02	0.50	7.36E-03
ENSCAFG0000001841	SEPT6	darkgrey	VSMC_M8	0.08	6.98E-01	-0.37	5.40E-02	0.41	3.17E-02	-0.82	1.79E-07	-0.19	3.47E-01	0.02	9.13E-01	0.30	1.24E-01	0.72	2.22E-05	0.33	9.53E-02	-0.65	2.33E-04
ENSCAFG0000001278	BRMS1	yellow	VSMC_M3	0.08	6.98E-01	0.18	3.59E-01	0.70	4.88E-05	-0.49	9.60E-03	-0.64	3.42E-04	0.00	9.91E-01	0.04	8.45E-01	0.33	8.81E-02	-0.21	2.88E-01	-0.06	7.76E-01
ENSCAFG0000000355	KIAA0319L	darkgreen	VSMC_M4	0.08	6.98E-01	0.41	3.45E-02	-0.38	5.06E-02	0.82	1.63E-07	0.33	9.52E-02	-0.24	2.34E-01	-0.49	1.01E-02	-0.83	1.11E-07	-0.30	1.29E-01	0.51	6.91E-03
ENSCAFG0000000696	AAAS	darkgrey	VSMC_M8	0.08	6.99E-01	-0.04	8.25E-01	0.58	1.63E-03	-0.76	4.19E-06	-0.44	2.11E-02	0.11	5.77E-01	0.25	2.16E-01	0.71	3.01E-05	-0.08	7.02E-01	-0.36	6.82E-02
ENSCAFG0000001492	CHD4	darkgrey	VSMC_M8	0.08	6.99E-01	-0.20	3.10E-01	0.36	6.37E-02	-0.72	2.66E-05	-0.19	3.53E-01	-0.01	9.77E-01	0.31	1.17E-01	0.65	2.20E-04	0.16	4.39E-01	-0.47	1.30E-02
ENSCAFG0000000991	ENSCAFG0000000991	yellow	VSMC_M3	0.08	6.99E-01	0.46	1.71E-02	0.53	4.70E-03	-0.33	9.65E-02	-0.50	7.64E-03	0.10	6.25E-01	-0.01	9.43E-01	0.08	6.86E-01	-0.40	3.71E-02	0.11	5.70E-01
ENSCAFG0000000490	ENSCAFG0000000490	grey	VSMC_M10	0.08	7.00E-01	0.13	5.27E-01	-0.03	9.01E-01	0.18	3.65E-01	0.00	9.98E-01	-0.08	6.82E-01	-0.21	3.05E-01	-0.14	4.99E-01	-0.14	4.88E-01	0.14	4.93E-01
ENSCAFG0000000556	FLRT3	pink	VSMC_M5	0.08	7.00E-01	-0.09	6.39E-01	-0.64	3.34E-04	0.58	1.67E-03	0.61	6.62E-04	-0.27	1.78E-01	-0.09	6.41E-01	-0.44	2.11E-02	0.02	9.06E-01	0.15	4.47E-01
ENSCAFG0000003265	EXOSC5	grey	VSMC_M10	0.08	7.01E-01	0.05	8.11E-01	0.38	5.33E-02	-0.32	9.96E-02	-0.32	1.02E-01	0.24	2.29E-01	0.29	1.48E-01	0.26	1.95E-01	-0.13	5.16E-01	-0.09	6.56E-01
ENSCAFG0000000361	CLCN1	grey	VSMC_M10	0.08	7.01E-01	-0.13	5.33E-01	0.07	7.40E-01	-0.24	2.29E-01	-0.06	7.56E-01	0.33	9.34E-02	-0.01	9.43E-01	0.30	1.29E-01	0.04	8.33E-01	-0.11	5.71E-01
ENSCAFG0000003247	ENSCAFG0000003247	grey	VSMC_M10	0.08	7.01E-01	-0.06	7.84E-01	-0.45	1.84E-02	0.48	1.22E-02	0.31	1.12E-01	0.03	8.65E-01	0.06	7.60E-01	-0.35	7.48E-02	0.04	8.42E-01	0.35	7.48E-02
ENSCAFG0000000654	UBA3	pink	VSMC_M5	0.08	7.01E-01	-0.51	6.01E-03	-0.70	4.61E-05	0.29	1.41E-01	0.77	2.59E-06	-0.16	4.39E-01	0.07	7.27E-01	-0.03	8.99E-01	0.46	1.71E-02	-0.31	1.22E-01
ENSCAFG0000001151	AIP	yellow	VSMC_M3	0.08	7.01E-01	0.62	5.85E-04	0.61	6.42E-04	-0.02	9.32E-01	-0.66	1.88E-04	-0.07	7.22E-01	-0.12	5.61E-01	-0.16	4.24E-01	-0.58	1.50E-03	0.42	3.00E-02
ENSCAFG0000001046	CD200	grey	VSMC_M10	0.08	7.01E-01	0.24	2.20E-01	-0.12	5.52E-01	0.36	6.60E-02	0.21	2.89E-01	0.10	6.28E-01	-0.59	1.27E-03	-0.42	2.77E-02	-0.08	6.80E-01	0.03	8.91E-01
ENSCAFG0000001469	ENSCAFG0000001469	grey	VSMC_M10	0.08	7.01E-01	-0.24	2.33E-01	0.31	1.13E-01	-0.30	1.33E-01	-0.26	1.93E-01	-0.12	5.51E-01	0.17	3.96E-01	0.36	6.72E-02	0.12	5.56E-01	-0.25	2.11E-01
ENSCAFG0000001477	GPR160	darkgreen	VSMC_M4	0.08	7.01E-01	0.27	1.81E-01	-0.13	5.18E-01	0.38	5.11E-02	0.03	8.84E-01	0.06	7.69E-01	0.32	1.04E-01	-0.46	1.53E-02	-0.28	1.52E-01	0.54	3.35E-03
ENSCAFG0000001740	CCDC151	grey	VSMC_M10	0.08	7.01E-01	0.21	3.04E-01	0.44	2.30E-02	-0.22	2.65E-01	-0.39	4.26E-02	-0.04	8.57E-01	0.01	9.44E-01	0.20	3.27E-01	-0.23	2.46E-01	0.07	7.13E-01
ENSCAFG0000003170	ENSCAFG0000003170	cyan	VSMC_M2	0.08	7.02E-01	0.74	1.11E-05	0.32	1.08E-01	0.17	3.92E-01	-0.41	3.60E-02	0.09	6.66E-01	-0.51	6.49E-03	-0.32	1.01E-01	-0.65	2.16E-04	0.53	4.65E-03
ENSCAFG0000002008	LCN2	grey	VSMC_M10	0.08	7.02E-01	0.11	5.79E-01	-0.32	1.02E-01	0.45	1.94E-02	0.20	3.25E-01	0.07	7.20E-01	0.43	2.37E-02	-0.39	4.61E-02	-0.16	4.24E-01	0.43	2.52E-02
ENSCAFG0000002026	TERF2	grey	VSMC_M10	0.08	7.02E-01	0.35	7.37E-02	0.20	3.15E-01	0.27	1.76E-01	-0.27	1.68E-01	-0.02	9.06E-01	-0.03	8.70E-01	-0.30	1.29E-01	-0.39	4.66E-02	0.44	2.10E-02
ENSCAFG0000000536	INTS10	grey	VSMC_M10	0.08	7.02E-01	0.16	4.30E-01	0.05	7.86E-01	-0.08	6.98E-01	-0.14	4.88E-01	0.12	5.51E-01	0.51	6.91E-03	0.09	6.56E-01	-0.28	1.50E-01	0.30	1.31E-01
ENSCAFG0000001884	ENSCAFG0000001884	grey	VSMC_M10	0.08	7.02E-01	0.26	1.92E-01	0.36	6.45E-02	-0.16	4.31E-01	-0.35	6.94E-02	-0.14	5.01E-01	-0.21	2.92E-01	0.05	7.99E-01	-0.30	1.30E-01	0.15	4.54E-01
ENSCAFG0000000195	ENSCAFG0000000195	grey	VSMC_M10	0.08	7.02E-01	-0.07	7.34E-01	0.41	3.39E-02	-0.36	6.48E-02	-0.41	3.39E-02	0.25	2.06E-01	0.22	2.79E-01	0.34	7.98E-02	-0.06	7.81E-01	-0.04	8.56E-01
ENSCAFG0000002229	ENSCAFG0000002229	grey	VSMC_M10	0.08	7.02E-01	-0.09	6.58E-01	0.36	6.67E-02	-0.34	8.65E-02	-0.27	1.72E-01	-0.11	6.01E-01	0.14	4.77E-01	0.30	1.22E-01	0.02	9.34E-01	-0.30	1.34E-01
ENSCAFG0000000085	UNC93A	grey	VSMC_M10	0.08	7.02E-01	-0.05	8.07E-01	-0.37	6.04E-02	0.40	4.02E-02	0.31	1.12E-01	-0.14	4.88E-01	-0.03	8.77E-01	-0.22	2.80E-01	-0.06	7.69E-01	0.20	3.13E-01
ENSCAFG0000000898	EXOC6B	grey	VSMC_M10	0.08	7.03E-01	-0.35	7.62E-02	-0.61	7.50E-04	0.09	6.66E-01	0.65	2.33E-04	0.03	8.72E-01	-0.04	8.56E-01	0.08	6.76E-01	0.29	1.49E-01	-0.28	1.64E-01
ENSCAFG0000000187	CFAP69	grey	VSMC_M10	0.08	7.03E-01	-0.27	1.74E-01	-0.04	8.35E-01	-0.30	1.27E-01	0.06	7.74E-01	-0.18	3.77E-01	0.26	1.94E-01	0.36	6.74E-02	0.14	4.94E-01	-0.15	4.65E-01
ENSCAFG0000000911	SLC25A37	grey	VSMC_M10	0.08	7.04E-01	0.41	3.57E-02	0.44	2.09E-02	-0.33	9.28E-02	-0.48	1.06E-02	0.08	6.78E-01	-0.33	9.70E-02	0.15	4.51E-01	-0.40	3.67E-02	0.22	2.66E-01
ENSCAFG0000000950	PHF7	turquoise	VSMC_M6	0.08	7.04E-01	-0.01	9.42E-01	0.02	9.09E-01	-0.14	4.94E-01	-0.06	7.67E-01	0.41	3.56E-02	0.07	7.42E-01	0.16	4.36E-01	-0.04	8.42E-01	0.02	9.10E-01
ENSCAFG0000001629	GIPC1	cyan	VSMC_M2	0.08	7.04E-01	0.79	7.64E-07	0.33	9.74E-02	0.35	7.53E-02	-0.45	1.79E-02	-0.05	7.96E-01	0.00	9.83E-01	-0.55	2.77E-03	-0.71	3.22E-05	0.78	2.02E-06
ENSCAFG0000000256	TEX10	grey	VSMC_M10	0.08	7.04E-01	-0.33	8.80E-02	0.01	9.57E-01	-0.36	6.68E-02	0.09	6.67E-01	0.12	5.64E-01	0.30	1.29E-01	0.40	4.07E-02	0.20	3.23E-01	-0.33	8.98E-02
ENSCAFG0000001878	GNAL	grey	VSMC_M10	0.08	7.04E-01	-0.11	5.77E-01	-0.32	9.81E-02	0.32	1.03E-01	0.31	1.13E-01	-0.09	6.61E-01	0.32	1.06E-01	-0.23	2.53E-01	0.11	5.86E-01	0.12	5.59E-01
ENSCAFG0000000999	WARS2	grey	VSMC_M10	0.08	7.04E-01	0.04	8.39E-01	0.00	9.84E-01	-0.03	8.92E-01	0.00	9.99E-01	0.08	6.78E-01	0.52	5.52E-03	0.02	9.39E-01	-0.18	3.79E-01	0.13	5.09E-01
ENSCAFG0000001961	ABCA7	grey	VSMC_M10	0.08	7.04E-01	0.01	9.57E-01	-0.16	4.23E-01	0.26	1.91E-01	0.11	5.88E-01	0.13	5.20E-01	-0.03	8.89E-01	-0.16	4.16E-01	-0.06	7.70E-01	0.18	3.70E-01
ENSCAFG0000003114	LAX1	grey	VSMC_M10	0.08	7.04E-01	0.29	1.38E-01	0.08	6.94E-01	0.10	6.05E-01	-0.04	8.46E-01	-0.04	8.58E-01	-0.11	5.75E-01	-0.16	4.36E-01	-0.25	2.17E-01	0.19	3.54E-01
ENSCAFG0000002458	ENSCAFG0000002458	turquoise	VSMC_M6	0.08	7.05E-01	-0.26	1.94E-01	-0.15	4.44E-01	-0.06	7.79E-01	0.13	5.02E-01	0.58	1.57E-03	-0.06	7.51E-01	0.18	3.65E-01	0.13	5.14E-01	-0.14	4.96E-01
ENSCAFG0000001074	NAV1	darkgrey	VSMC_M8	0.08	7.05E-01	-0.42	3.13E-02	0.23	2.41E-01	-0.70	4.94E-05	0.02	9.08E-01	0.13	5.25E-01	0.01	9.57E-01	0.61	7.52E-04	0.39	4.18E-02	-0.72	2.59E-05
ENSCAFG0000000834	NUGGC	grey	VSMC_M10	0.08	7.05E-01	-0.26	1.84E-01	-0.03	8.83E-01	-0.25	2.12E-01	0.06	7.78E-01	-0.07	7.14E-01	0.45	1.81E-02	0.29	1.43E-01	0.18	3.82E-01	-0.20	3.27E-01
ENSCAFG0000000140	ENSCAFG0000000140	grey	VSMC_M10	0.08	7.05E-01	-0.40	4.09E-02	-0.54	3.75E-03	0.16	4.37E-01	0.63	4.71E-04	0.06	7.77E-01	0.18	3.69E-01	-0.06	7.52E-01	0.37	6.04E-02	-0.26	1.86E-01
ENSCAFG0000001495	IFI30	cyan	VSMC_M2	0.08	7.05E-01	0.80	6.38E-07	0.42	3.02E-02	0.19	3.36E-01	-0.48	1.08E-02	-0.06	7.56E-01	-0.29	1.48E-01	-0.44	2.29E-02	-0.70	5.67E-05	0.60	9.29E-04
ENSCAFG0000000547	CHRDL2	grey	VSMC_M10	0.08	7.05E-01	0.12	5.50E-01	0.15	4.55E-01	-0.15	4.63E-01	-0.16	4.32E-01	-0.10	6.33E-01	0.14	4.90E-01	0.15	4.61E-01	-0.20	3.09E-01	0.05	8.15E-01
ENSCAFG0000003272	PRIMA1	grey	VSMC_M10	0.08	7.06E-01	-0.04	8.48E-01	-0.26	1.94E-01	0.23	2.47E-01	0.16	4.35E-01	0.47	1.37E-02	0.19	3.44E-01	-0.18	3.82E-01	-0.07	7.40E-01	0.18	3.71E-01
ENSCAFG0000000538	AKT2	grey	VSMC_M10	0.08	7.06E-01	0.58	1.52E-03	0.41	3.59E-02	-0.02	9.06E-01	-0.55	3.17E-03	0.14	4.99E-01	-0.01	9.72E-01	-0.18	3.69E-01	-0.58	1.63E-03	0.59	1.31E-03
ENSCAFG0000001806	ACSL4	darkgrey	VSMC_M8	0.08	7.06E-01	-0.18	3.82E-01	0.31	1.17E-01	-0.66	1.69E-04	-0.09	6.57E-01	-0.13	5.30E-01	0.06	7.69E-01	0.52	5.10E-03	0.16	4.37E-01	-0.48	1.08E-02
ENSCAFG0000001101	SPATS2L	darkolivegreen	VSMC_M9	0.08	7.06E-01	-0.66	1.95E-04	-0.51	6.99E-03	0.00	9.88E-01	0.62	5.97E-04	0.08	7.05E-01	0.26	1.88E-01	0.19	3.43E-01	0.60	1.05E-03	-0.45	1.87E-02
ENSCAFG0000001430	SOX10	grey	VSMC_M10	0.08	7.07E-01	-0.07	7.28E-01	0.01	9.56E-01	-0.35	7.79E-02	0.14	4.96E-01	-0.09	6.64E-01	0.25	2.15E-01	0.25	2.04E-01	0.02	9.31E-01	-0.28	1.62E-01
ENSCAFG0000000067	SOD2	darkgreen	VSMC_M4	0.08	7.07E-01	0.35	7.57E-02	-0.48	1.09E-02	0.76	4.13E-06	0.48	1.12E-02	-0.16	4.33E-01	-0.35	7.79E-02	-0.81	4.07E-07	-0.19	3.44E-01	0.39	4.71E-02
ENSCAFG0000003221	HEY2	grey	VSMC_M10	0.08	7.07E-01	0.23	2.52E-01	0.05	7.96E-01	0.01	9.72E-01	0.01	9.74E-01	-0.06	7.54E-01	-0.13	5.12E-01	-0.15	4.62E-01	-0.16	4.39E-01	0.15	4.60E-01
ENSCAFG0000000790	ELL2	darkgreen	VSMC_M4	0.08	7.07E-01	0.22	2.74E-01	-0.44	2.15E-02	0.78	1.31E-06	0.38	5.10E-02	-0.11	5.98E-01	-0.64	3.46E-04	-0.70	5.27E-05	-0.12	5.37E-01	0.37	5.41E-02
ENSCAFG0000000479	MTRF1	grey	VSMC_M10	0.08	7.07E-01	-0.25	2.00E-01	-0.47	1.36E-02	0.07	7.21E-01	0.52	5.86E-03	-0.04	8.25E-01	0.02	9.36E-01	0.10	6.18E-01	0.16	4.23E-01	-0.21	2.99E-01
ENSCAFG0000001999	OLFM3	grey	VSMC_M10	0.08	7.07E-01	-0.14	4.88E-01	-0.22	2.69E-01	0.07	7.29E-01	0.26	1.90E-01	-0.27	1.67E-01	-0.23	2.41E-01	0.01	9.73E-01	0.12	5.50E-01	-0.11	5.89E-01
ENSCAFG0000001076	MANBA	yellow	VSMC_M3	0.08	7.08E-01	0.36	6.23E-02	0.67	1.22E-04	-0.61	6.58E-04	-0.52	5.30E-03	-0.14	4.86E-01	-0.14	4.77E-01	0.33	9.29E-02	-0.31	1.19E-01	-0.11	5.82E-01
ENSCAFG0000000397	PGR	darkgrey	VSMC_M8	0.08	7.08E-01	-0.73	1.32E-05	-0.09	6.47E-01	-0.56	2.50E-03	0.31	1.11E-01	-0.08	6.85E-01	0.30	1.26E-01	0.66	1.64E-04	0.64	3.07E-04	-0.82	1.37E-07
ENSCAFG0000000541	PNP	cyan	VSMC_M2	0.08	7.08E-01	0.66	1.87E-04	0.58	1.50E-03	-0.06	7.72E-01	-0.51	7.16E-03	0.05	8.09E-01	-0.13	5.31E-01	-0.19	3.53E-01	-0.54	3.96E-03	0.25	2.04E-01
ENSCAFG0000000391	MAMSTR	grey	VSMC_M10	0.08	7.08E-01	0.39	4.54E-02	0.06	7.49E-01	0.08	7.00E-01	-0.18	3.57E-01	0.20	3.17E-01	0.06	7.53E-01	-0.13	5.32E-01	-0.41	3.40E-02	0.49	8.90E-03
ENSCAFG0000000266	ENSCAFG0000000266	grey	VSMC_M10	0.08	7.09E-01	0.06	7.77E-01	0.01	9.41E-01	-0.15	4.60E-01	-0.10	6.18E-01	0.02	9.16E-01	0.03	8.66E-01	0.18	3.71E-01	-0.14	4.74E-01	0.17	4.02E-01
ENSCAFG0000002913	HAPLN1	violet	VSMC_M7	0.08	7.09E-01	-0.40	3.63E-02	0.08	6.84E-01	-0.42	2.96E-02	0.05	8.11E-01	-0.09	6.43E-01	0.85	1.90E-08	0.50	8.62E-03	0.22	2.63E-01	-0.44	2.29E-02
ENSCAFG0000000859	NUP54	grey	VSMC_M10	0.08	7.09E-01	-0.45	1.71E-02	-0.54	3.93E-03	0.00	1.00E+00	0.49	9.52E-03	0.07	7.47E-01	0.04	8.53E-01	0.27	1.79E-01	0.32	1.05E-01	-0.20	3.29E-01
ENSCAFG0000001109	XG	cyan	VSMC_M2	0.08	7.10E-01	0.67	1.45E-04	0.37	5.84E-02	0.20	3.19E-01	-0.35	7.48E-02	-0.17	3.99E-01	0.12	5.61E-01	-0.46	1.59E-02	-0.56	2.33E-03	0.46	1.51E-02
ENSCAFG0000001329	SKA1	pink	VSMC_M5	0.07	7.10E-01	-0.35	7.38E-02	-0.39	4.34E-02	0.07	7.12E-01	0.52	5.32E-03	-0.18	3.60E-01	-0.20	3.11E-01	0.09	6.69E-01	0.28	1.57E-01	-0.38	5.24E-02
ENSCAFG0000000696	TMEM147	cyan	VSMC_M2	0.07	7.10E-01	0.61	6.70E-04	0.51	6.23E-03	0.01	9.50E-01	-0.62	6.38E-04	0.25	2.12E-01	-0.13	5.18E-01	-0.26	1.89E-01	-0.56	2.51E-03	0.48	1.16E-02
ENSCAFG0000001146	ENSCAFG0000001146	grey	VSMC_M10	0.07	7.10E-01	0.10	6.07E-01	0.23	2.58E-01	-0.21	2.85E-01	-0.17	4.10E-01	-0.04	8.51E-01	0.32	9.82E-02	0.20	3.21E-01	-0.18	3.71E-01	0.02	9.31E-01
ENSCAFG0000003049	ZNF7	grey	VSMC_M10	0.07	7.11E-01	0.15	4.41E-01	0.40	3.75E-02	-0.47	1.32E-02	-0.38	4.83E-02	0.15	4.48E-01	0.21	2.89E-01	0.34	8.48E-02	-0.20	3.22E-01	-0.03	8.75E-01
ENSCAFG0000000285	CYCS	darkgrey	VSMC_M8	0.07	7.11E-01	-0.68	1.12E-04	0.22	2.72E-01	-0.73	1.33E-05	-0.09	6.48E-01	0.22	2.68E-01	0.43	2.60E-02	0.90	2.19E-10	0.45	1.92E-02	-0.71	2.78E-05
ENSCAFG0000001455	ANKRD45	grey	VSMC_M10	0.07	7.11E-01	-0.17	4.10E-01	-0.03	8.99E-01	-0.20	3.26E-01	-0.01	9.77E-01	0.07	7.25E-01	0.01	9.64E-01	0.29	1.37E-01	0.08	6.77E-01	-0.04	8.57E-01
ENSCAFG0000001693	NPC2	cyan	VSMC_M2	0.07	7.11E-01	0.85	1.92E-08	0.45	1.91E-02	0.14	4.82E-01	-0.48	1.21E-02	-0.09	6.61E-01	-0.25	2.14E-01	-0.53	4.57E-03	-0.67	1.16E-04	0.62	5.32E-04
ENSCAFG0000001192	YEATS2	darkgreen	VSMC_M4	0.07	7.11E-01	0.14	4.86E-01	-0.56	2.18E-03	0.60	9.02E-04	0.50	7.71E-03	-0.15	4.43E-01	-0.32	1.04E-01	-0.45	1.88E-02	-0.18	3.74E-01	0.26	1.82E-01
ENSCAFG0000001180	CIART	grey	VSMC_M10	0.07	7.12E-01	0.45	1.99E-02	0.20	3.30E-01	0.15	4.43E-01	-0.20	3.07E-01	-0.01	9.68E-01	0.15	4.47E-01	-0.27	1.76E-01	-0.48	1.21E-02	0.37	5.67E-02
ENSCAFG0000002279	ENSCAFG0000002279	grey	VSMC_M10	0.07	7.12E-01	0.02	9.30E-01	0.06	7.50E-01	-0.14	4.83E-01	-0.01	9.74E-01	0.02	9.11E-01	0.00	9.86E-01	0.12	5.46E-01	-0.02	9.22E-01	-0.03	8.74E-01
ENSCAFG0000001867	PGM1	yellow	VSMC_M3	0.07	7.12E-01	0.33	9.01E-02	0.70	5.67E-05	-0.53	4.15E-03	-0.61	7.06E-04	-0.03	8.86E-01	0.26	1.99E-01	0.22	2.77E-01	-0.30	1.33E-01	0.02	9.12E-01
ENSCAFG0000000512	NUMBL	grey	VSMC_M10	0.07	7.12E-01	0.48	1.15E-02	0.46	1.48E-02	-0.26	1.97E-01	-0.47	1.24E-02	0.14	4.91E-01	-0.29	1.41E-01	0.12	5.67E-01	-0.44	2.06E-02	0.19	3.46E-01
ENSCAFG0000001840	UBE2A	pink	VSMC_M5	0.07	7.12E-01	-0.56	2.16E-03	-0.25	2.08E-01	-0.14	4.98E-01	0.48	1.21E-02	-0.24	2.27E-01	-0.15	4.56E-01	0.24	2.33E-01	0.60	9.05E-04	-0.65	2.76E-04
ENSCAFG0000001574	DNAJC7	darkgrey	VSMC_M8	0.07	7.12E-01	-0.19	3.40E-01	0.38	5.10E-02	-0.67	1.45E-04	-0.11	5.91E-01	-0.16	4.24E-01	0.23	2.52E-01	0.60	1.00E-03	0.12	5.59E-01	-0.66	1.94E-04
ENSCAFG0000001468	HTT	grey	VSMC_M10	0.07	7.12E-01	-0.34	8.25E-02	-0.29	1.48E-01	-0.02	9.26E-01	0.25	2.09E-01	0.26	1.87E-01	-0.17	3.90E-01	0.15	4.50E-01	0.27	1.71E-01	-0.12	5.48E-01
ENSCAFG0000001579	WDR89	grey	VSMC_M10	0.07	7.12E-01	-0.25	2.01E-01	-0.21	2.91E-01	0.18	3.67E-01	0.25	2.13E-01	-0.01	9.52E-01	0.33	9.62E-02	-0.02	9.10E-01	0.10	6.34E-01	-0.10	6.23E-01
ENSCAFG0000001617	IQSEC2	grey	VSMC_M10	0.07	7.12E-01	0.49	8.72E-03	0.02	9.24E-01	0.27	1.69E-01	-0.17	3.88E-01	0.04	8.61E-01	-0.14	4.98E-01	-0.40	3.75E-02	-0.48	1.19E-02	0.65	2.21E-04
ENSCAFG0000000651	EML4	darkgreen	VSMC_M4	0.07	7.12E-01	0.15	4.46E-01	-0.49	9.02E-03	0.73	1.37E-05	0.41	3.41E-02	0.05	8.13E-01	-0.43	2.42E-02	-0.58	1.65E-03	-0.15	4.65E-01	0.32	1.04E-01
ENSCAFG0000000343	IL4I1	grey	VSMC_M10	0.07	7.13E-01	-0.14	4.88E-01	0.34	8.24E-02	-0.32	1.08E-01	-0.37	6.03E-02	0.19	3.31E-01	0.30	1.27E-01	0.31	1.14E-01	0.06	7.55E-01	-0.07	7.22E-01
ENSCAFG0000001166	HSPA8	grey	VSMC_M10	0.07	7.13E-01	-0.52	5.98E-03	-0.27	1.69E-01	-0.09	6.66E-01	0.26	1.93E-01	0.08	7.09E-01	0.09	6.50E-01	0.39	4.45E-02	0.29	1.40E-01	-0.30	1.31E-01
ENSCAFG0000001518	UXT	grey	VSMC_M10	0.07	7.13E-01	0.09	6.43E-01	-0.05	8.19E-01	0.20	3.07E-01	-0.11	5.82E-01	0.20	3.28E-01	-0.07	7.25E-01	-0.04	8.38E-01	-0.21	3.00E-01	0.29	1.43E-01
ENSCAFG0000001462	DOK7	grey	VSMC_M10	0.07	7.13E-01	0.25	2.12E-01	-0.02	9.19E-01	0.40	3.68E-02	-0.07	7.27E-01	0.16	4.11E-01	0.29	1.44E-01	-0.45	1.89E-02	-0.26	1.89E-01	0.44	2.17E-02
ENSCAFG0000003229	ENSCAFG0000003229	grey	VSMC_M10	0.07	7.13E-01	0.22	2.68E-01	0.19	3.47E-01	-0.25	2.15E-01	-0.21	2.93E-01	0.19	3.35E-01	0.18	3.79E-01	0.20	3.16E-01	-0.32	1.01E-01	0.17	3.86E-01
ENSCAFG0000001313	ENSCAFG0000001313	grey	VSMC_M10	0.07	7.14E-01	0.00	9.89E-01	0.17	3.84E-01	-0.20	3.23E-01	-0.16	4.21E-01	-0.08	6.75E-01	0.00	9.98E-01	0.23	2.45E-01	-0.08	6.83E-01	-0.07	7.25E-01
ENSCAFG0000001049	YARS2	grey	VSMC_M10	0.07	7.14E-01	0.00	9.92E-01	0.36	6.89E-02	-0.25	2.12E-01	-0.28	1.56E-01	0.09	6.64E-01	-0.31	1.11E-01	0.20	3.07E-01	0.02	9.40E-01	-0.20	3.19E-01
ENSCAFG0000002611	ENSCAFG0000002611	grey	VSMC_M10	0.07	7.14E-01	0.06	7.80E-01	0.23	2.56E-01	-0.11	6.02E-01	-0.25	2.14E-01	0.19	3.40E-01	0.05	8.08E-01	0.13	5.09E-01	-0.14	4.82E-01	0.08	6.85E-01
ENSCAFG0000001995	NTNG1	violet	VSMC_M7	0.07	7.14E-01	-0.03	8.85E-01	0.13	5.26E-01	-0.13	5.07E-01	-0.10	6.14E-01	-0.05	8.12E-01	0.61	7.44E-04	0.11	5.79E-01	-0.04	8.54E-01	-0.02	9.38E-01
ENSCAFG0000002983	ENSCAFG0000002983	violet	VSMC_M7	0.07	7.14E-01	-0.03	8.85E-01	0.13	5.26E-01	-0.13	5.07E-01	-0.10	6.14E-01	-0.05	8.12E-01	0.61	7.44E-04	0.11	5.79E-01	-0.04	8.54E-01	-0.02	9.38E-01
ENSCAFG0000000590	NALCN	grey	VSMC_M10	0.07	7.14E-01	0.05	8.19E-01	0.03	8.94E-01	-0.24	2.25E-01	0.02	9.22E-01	0.11	6.01E-01	0.04	8.28E-01	0.29	1.36E-01	-0.17	3.86E-01	-0.08	6.93E-01
ENSCAFG0000001405	TSPAN7	violet	VSMC_M7	0.07	7.14E-01	-0.24	2.32E-01	0.14	4.77E-01	-0.32	1.09E-01	-0.20	3.09E-01	0.13	5.03E-01	0.81	3.54E-07	0.38	5.17E-02	0.03	8.80E-01	0.01	9.45E-01
ENSCAFG0000001263	CNKSR1	grey	VSMC_M10	0.07	7.14E-01	0.03	8.90E-01	-0.33	8.77E-02	0.45	1.77E-02	0.44	2.30E-02	-0.11	5.97E-01	-0.23	2.46E-01	-0.45	1.95E-02	0.05	8.02E-01	-0.03	8.75E-01
ENSCAFG0000001678	ENSCAFG0000001678	grey	VSMC_M10	0.07	7.14E-01	0.02	9.33E-01	-0.17	3.99E-01	0.19	3.52E-01	0.18	3.68E-01	-0.11	5.88E-01	0.34	8.73E-02	-0.12	5.44E-01	-0.09	6.46E-01	0.03	9.01E-01
ENSCAFG0000000673	ENSCAFG0000000673	darkgreen	VSMC_M4	0.07	7.15E-01	-0.10	6.34E-01	-0.47	1.44E-02	0.51	7.03E-03	0.42	2.95E-02	-0.09	6.72E-01	0.19	3.49E-01	-0.34	8.35E-02	0.03	8.93E-01	0.18	3.82E-01
ENSCAFG0000000920	KNL1	darkgrey	VSMC_M8	0.07	7.15E-01	-0.54	3.62E-03	0.29	1.46E-01	-0.88	2.23E-09	-0.09	6.55E-01	0.09	6.56E-01	0.38	5.04E-02	0.96	1.43E-15	0.35	7.30E-02	-0.75	5.61E-06
ENSCAFG0000003237	LRRC72	grey	VSMC_M10	0.07	7.15E-01	0.41	3.43E-02	-0.10	6.25E-01	0.30	1.26E-01	0.03	8.69E-01	-0.12	5.46E-01	-0.08	6.74E-01	-0.41	3.46E-02	-0.39	4.67E-02	0.47	1.38E-02
ENSCAFG0000001685	STK10	grey	VSMC_M10	0.07	7.15E-01	0.42	2.97E-02	0.50	7.61E-03	-0.25	2.06E-01	-0.51	6.88E-03	0.20	3.26E-01	-0.23	2.40E-01	0.11	5.74E-01	-0.46	1.61E-02	0.21	2.82E-01
ENSCAFG0000000440	EML2	grey	VSMC_M10	0.07	7.16E-01	0.22	2.76E-01	-0.09	6.49E-01	0.43	2.46E-02	-0.03	8.88E-01	0.18	3.63E-01	-0.60	9.05E-04	-0.42	3.03E-02	-0.16	4.23E-01	0.34	8.36E-02
ENSCAFG0000002427	ENSCAFG0000002427	grey	VSMC_M10	0.07	7.16E-01	0.38	5.39E-02	0.15	4.61E-01	0.24	2.19E-01	-0.22	2.75E-01	0.15	4.57E-01	-0.02	9.21E-01	-0.21	2.88E-01	-0.44	2.26E-02	0.37	5.75E-02
ENSCAFG0000002272	MT-ATP8	grey	VSMC_M10	0.07	7.16E-01	-0.48	1.11E-02	-0.43	2.53E-02	-0.01	9.51E-01	0.53	4.67E-03	-0.14	4.94E-01	0.29	1.36E-01	0.20	3.10E-01	0.39	4.17E-02	-0.40	3.93E-02
ENSCAFG0000001867	WDR70	darkgrey	VSMC_M8	0.07	7.17E-01	-0.66	1.65E-04	-0.07	7.20E-01	-0.51	7.04E-03	0.25	2.02E-01	0.04	8.28E-01	0.25	2.01E-01	0.72	2.73E-05	0.49	8.86E-03	-0.77	3.34E-06

ENSCAFG0000001365	CTTNBP2NL	grey	VSMC_M1C	0.07	7.17E-01	0.26	1.89E-01	-0.27	1.76E-01	0.40	3.89E-02	0.13	5.28E-01	0.21	2.82E-01	0.14	4.73E-01	-0.45	1.77E-02	-0.28	1.61E-01	0.56	2.51E-03
ENSCAFG0000001750	ENSCAFG0000001750	grey	VSMC_M1C	0.07	7.17E-01	0.25	2.09E-01	0.20	3.06E-01	-0.18	3.70E-01	-0.22	2.70E-01	0.47	1.43E-02	-0.08	7.08E-01	0.12	5.54E-01	-0.27	1.72E-01	0.13	5.16E-01
ENSCAFG0000000016	STAT6	cyan	VSMC_M2	0.07	7.17E-01	0.62	5.37E-04	0.32	1.08E-01	0.27	1.72E-01	-0.26	1.90E-01	-0.02	9.35E-01	-0.33	9.18E-02	-0.52	5.09E-03	-0.42	3.02E-02	0.36	6.36E-02
ENSCAFG0000001746	ENSCAFG0000001746	grey	VSMC_M1C	0.07	7.17E-01	0.02	9.28E-01	0.12	5.66E-01	-0.27	1.78E-01	-0.14	4.99E-01	0.38	5.12E-02	0.10	6.06E-01	0.30	1.30E-01	-0.14	4.96E-01	0.02	9.28E-01
ENSCAFG0000002579	ENSCAFG0000002579	grey	VSMC_M1C	0.07	7.17E-01	-0.09	6.66E-01	0.16	4.21E-01	-0.23	2.49E-01	-0.11	5.85E-01	0.01	9.80E-01	0.41	3.19E-02	0.24	2.26E-01	0.04	8.48E-01	-0.10	6.08E-01
ENSCAFG0000000341	ENSCAFG0000000341	grey	VSMC_M1C	0.07	7.18E-01	0.05	8.01E-01	0.13	5.07E-01	-0.17	4.05E-01	-0.08	6.75E-01	-0.08	6.88E-01	-0.22	2.67E-01	0.16	4.21E-01	-0.02	9.02E-01	-0.06	7.84E-01
ENSCAFG0000000078	GTSE1	darkgrey	VSMC_M8	0.07	7.18E-01	-0.48	1.22E-02	0.43	2.39E-02	-0.88	2.17E-09	-0.26	1.83E-01	0.10	6.21E-01	0.39	4.53E-02	0.95	5.15E-14	0.28	1.56E-01	-0.67	1.26E-04
ENSCAFG0000002597	ENSCAFG0000002597	grey	VSMC_M1C	0.07	7.18E-01	0.00	9.84E-01	0.11	5.94E-01	-0.20	3.13E-01	-0.08	6.97E-01	-0.04	8.42E-01	0.18	3.67E-01	0.21	3.00E-01	-0.07	7.31E-01	-0.03	8.76E-01
ENSCAFG0000000659	HMGB1	darkgrey	VSMC_M8	0.07	7.18E-01	-0.60	8.34E-04	0.27	1.72E-01	-0.85	1.62E-08	-0.05	7.90E-01	0.02	9.28E-01	0.37	5.76E-02	0.96	4.26E-15	0.43	2.39E-02	-0.83	8.23E-08
ENSCAFG0000002016	BTBD8	grey	VSMC_M1C	0.07	7.18E-01	-0.35	7.65E-02	-0.50	7.51E-03	0.19	3.31E-01	0.54	3.67E-03	0.02	9.40E-01	0.34	8.10E-02	-0.04	8.39E-01	0.20	3.15E-01	-0.11	5.96E-01
ENSCAFG0000001924	SLC6A8	cyan	VSMC_M2	0.07	7.18E-01	0.83	7.18E-08	0.23	2.46E-01	0.35	7.31E-02	-0.35	7.16E-02	-0.02	9.25E-01	-0.38	4.92E-02	-0.63	4.34E-04	-0.68	9.25E-05	0.78	2.02E-06
ENSCAFG0000001908	FMR1	grey	VSMC_M1C	0.07	7.19E-01	0.14	4.81E-01	-0.28	1.58E-01	0.43	2.39E-02	0.28	1.65E-01	-0.05	8.20E-01	-0.27	1.72E-01	-0.34	8.41E-02	-0.14	4.88E-01	0.23	2.54E-01
ENSCAFG0000001315	ENSCAFG0000001315	darkgrey	VSMC_M8	0.07	7.20E-01	-0.26	1.95E-01	0.49	9.95E-03	-0.76	4.05E-06	-0.29	1.43E-01	0.28	1.63E-01	0.28	1.58E-01	0.74	8.89E-06	0.12	5.43E-01	-0.61	6.72E-04
ENSCAFG0000001455	GAL3ST4	grey	VSMC_M1C	0.07	7.20E-01	-0.07	7.15E-01	-0.08	6.79E-01	0.15	4.62E-01	0.01	9.61E-01	0.04	8.45E-01	-0.12	5.51E-01	-0.10	6.25E-01	-0.02	9.38E-01	0.19	3.49E-01
ENSCAFG0000000069	IGF2R	grey	VSMC_M1C	0.07	7.20E-01	0.77	3.32E-06	0.18	3.73E-01	0.36	6.91E-02	-0.22	2.66E-01	-0.08	7.08E-01	-0.27	1.77E-01	-0.66	1.77E-04	-0.63	4.79E-04	0.67	1.14E-04
ENSCAFG0000000925	CYTIP	grey	VSMC_M1C	0.07	7.20E-01	-0.29	1.48E-01	0.00	9.83E-01	-0.25	2.04E-01	0.03	8.68E-01	0.17	4.08E-01	0.28	1.59E-01	0.34	7.97E-02	0.14	4.82E-01	-0.20	3.28E-01
ENSCAFG0000001325	MKI67	darkgrey	VSMC_M8	0.07	7.20E-01	-0.52	5.28E-03	0.32	1.01E-01	-0.90	1.09E-10	-0.11	5.82E-01	0.06	7.84E-01	0.35	7.05E-02	0.95	2.41E-14	0.36	6.52E-02	-0.76	3.95E-06
ENSCAFG0000000635	PDS5B	darkolivegreen	VSMC_M9	0.07	7.21E-01	-0.53	4.70E-03	-0.27	1.77E-01	-0.26	1.89E-01	0.43	2.62E-02	-0.13	5.28E-01	0.00	9.84E-01	0.46	1.60E-02	0.40	3.64E-02	-0.56	2.14E-03
ENSCAFG0000002332	METTL18	grey	VSMC_M1C	0.07	7.21E-01	-0.24	2.20E-01	-0.24	2.22E-01	0.07	7.43E-01	0.37	5.94E-02	-0.19	3.34E-01	0.06	7.62E-01	0.01	9.63E-01	0.18	3.63E-01	-0.18	3.65E-01
ENSCAFG0000003021	FAM228B	grey	VSMC_M1C	0.07	7.21E-01	0.29	1.42E-01	0.24	2.21E-01	0.12	5.64E-01	-0.32	1.05E-01	0.29	1.47E-01	0.26	1.98E-01	-0.22	2.68E-01	-0.32	1.06E-01	0.39	4.39E-02
ENSCAFG0000001100	ADPRH	grey	VSMC_M1C	0.07	7.21E-01	0.29	1.49E-01	0.36	6.28E-02	-0.10	6.06E-01	-0.26	1.90E-01	-0.10	6.27E-01	0.12	5.44E-01	-0.09	6.60E-01	-0.20	3.08E-01	0.09	6.51E-01
ENSCAFG0000002131	ENSCAFG0000002131	grey	VSMC_M1C	0.07	7.21E-01	-0.04	8.29E-01	0.09	6.65E-01	-0.21	2.91E-01	-0.07	7.35E-01	-0.04	8.52E-01	0.08	6.99E-01	0.21	2.88E-01	-0.03	8.69E-01	-0.04	8.34E-01
ENSCAFG0000003068	ADPRHL2	cyan	VSMC_M2	0.07	7.21E-01	0.69	7.64E-05	0.37	6.07E-02	0.17	4.07E-01	-0.35	7.12E-02	-0.04	8.42E-01	-0.06	7.53E-01	-0.43	2.49E-02	-0.60	8.87E-04	0.48	1.21E-02
ENSCAFG0000003228	ENSCAFG0000003228	grey	VSMC_M1C	0.07	7.22E-01	0.04	8.36E-01	0.08	6.79E-01	-0.07	7.17E-01	0.01	9.47E-01	-0.29	1.40E-01	-0.21	2.82E-01	0.06	7.52E-01	-0.07	7.28E-01	-0.16	4.28E-01
ENSCAFG0000001958	PLXNA3	grey	VSMC_M1C	0.07	7.22E-01	-0.23	2.38E-01	-0.25	2.01E-01	0.28	1.57E-01	0.31	1.18E-01	-0.02	9.27E-01	-0.40	3.83E-02	-0.15	4.58E-01	0.30	1.34E-01	-0.19	3.38E-01
ENSCAFG0000001926	SLX4	grey	VSMC_M1C	0.07	7.22E-01	0.01	9.60E-01	-0.16	4.35E-01	0.22	2.78E-01	0.04	8.54E-01	-0.17	3.90E-01	0.33	9.67E-02	-0.11	5.90E-01	-0.18	3.80E-01	0.34	8.71E-02
ENSCAFG0000001730	RABEP2	yellow	VSMC_M3	0.07	7.22E-01	0.24	2.28E-01	0.60	9.50E-04	-0.16	4.32E-01	-0.58	1.39E-03	0.19	3.55E-01	-0.01	9.47E-01	0.07	7.13E-01	-0.25	2.15E-01	0.07	7.22E-01
ENSCAFG0000001703	RANGRF	yellow	VSMC_M3	0.07	7.22E-01	0.09	6.65E-01	0.66	1.76E-04	-0.49	9.93E-03	-0.65	2.10E-04	0.20	3.13E-01	0.42	2.85E-02	0.36	6.75E-02	-0.19	3.49E-01	0.05	8.01E-01
ENSCAFG0000000707	ENSCAFG0000000707	grey	VSMC_M1C	0.07	7.22E-01	0.33	9.56E-02	0.36	6.89E-02	-0.27	1.77E-01	-0.37	5.56E-02	0.23	2.49E-01	0.18	3.57E-01	0.09	6.42E-01	-0.35	7.58E-02	0.24	2.26E-01
ENSCAFG0000000364	ALDH16A1	yellow	VSMC_M3	0.07	7.23E-01	0.29	1.43E-01	0.82	2.07E-07	-0.65	2.62E-04	-0.74	8.56E-06	0.10	6.19E-01	0.40	4.11E-02	0.48	1.22E-02	-0.39	4.67E-02	-0.01	9.69E-01
ENSCAFG0000001890	ENSCAFG0000001890	grey	VSMC_M1C	0.07	7.23E-01	-0.05	8.17E-01	-0.21	2.99E-01	0.11	5.91E-01	0.24	2.24E-01	-0.05	8.00E-01	0.10	6.12E-01	-0.07	7.14E-01	0.02	9.33E-01	0.05	7.92E-01
ENSCAFG0000002942	ENSCAFG0000002942	grey	VSMC_M1C	0.07	7.23E-01	-0.12	5.54E-01	-0.52	5.74E-03	0.47	1.39E-02	0.39	4.27E-02	0.24	2.32E-01	-0.30	1.31E-01	-0.21	2.87E-01	0.05	8.19E-01	0.12	5.46E-01
ENSCAFG0000001890	SNN	cyan	VSMC_M2	0.07	7.23E-01	0.52	5.47E-03	0.47	1.28E-02	-0.05	8.06E-01	-0.59	1.23E-03	0.19	3.38E-01	0.13	5.14E-01	-0.17	3.92E-01	-0.49	9.49E-03	0.49	1.02E-02
ENSCAFG0000000239	ZSCAN18	cyan	VSMC_M2	0.07	7.23E-01	0.71	3.51E-05	-0.12	5.62E-01	0.72	2.68E-05	0.00	9.90E-01	-0.14	4.95E-01	-0.47	1.27E-02	-0.85	2.39E-08	-0.60	9.93E-04	0.75	7.21E-06
ENSCAFG0000001793	ERI2	grey	VSMC_M1C	0.07	7.23E-01	-0.21	2.84E-01	-0.04	8.25E-01	-0.20	3.26E-01	0.17	3.84E-01	-0.17	4.03E-01	0.38	4.98E-02	0.26	1.90E-01	0.10	6.27E-01	-0.31	1.21E-01
ENSCAFG0000000190	FAM189A2	grey	VSMC_M1C	0.07	7.24E-01	0.32	9.84E-02	-0.05	8.07E-01	0.51	6.91E-03	-0.13	5.32E-01	0.57	1.99E-03	0.02	9.09E-01	-0.51	6.17E-03	-0.35	7.45E-02	0.58	1.55E-03
ENSCAFG0000000976	ENSCAFG0000000976	grey	VSMC_M1C	0.07	7.24E-01	0.08	7.00E-01	0.04	8.24E-01	0.07	7.44E-01	-0.12	5.43E-01	0.07	7.25E-01	0.30	1.33E-01	-0.04	8.59E-01	-0.11	5.77E-01	0.26	1.93E-01
ENSCAFG0000003264	MRRF	grey	VSMC_M1C	0.07	7.24E-01	-0.40	3.62E-02	-0.22	2.65E-01	-0.24	2.37E-01	0.21	2.97E-01	0.37	6.02E-02	0.37	5.55E-02	0.40	3.73E-02	0.19	3.39E-01	-0.28	1.60E-01
ENSCAFG0000002357	ENSCAFG0000002357	grey	VSMC_M1C	0.07	7.24E-01	0.19	3.50E-01	0.09	6.46E-01	-0.07	7.42E-01	-0.11	5.92E-01	-0.12	5.54E-01	0.02	9.37E-01	-0.02	9.13E-01	-0.15	4.43E-01	0.12	5.47E-01
ENSCAFG0000000365	NBAS	darkgrey	VSMC_M8	0.07	7.25E-01	-0.53	4.71E-03	-0.08	6.80E-01	-0.38	4.87E-02	0.26	1.95E-01	0.11	5.89E-01	0.20	3.14E-01	0.56	2.48E-03	0.36	6.76E-02	-0.66	1.88E-04
ENSCAFG0000000023	ENSCAFG0000000023	grey	VSMC_M1C	0.07	7.25E-01	-0.11	5.87E-01	-0.17	4.05E-01	0.21	3.02E-01	0.26	1.85E-01	-0.18	3.60E-01	-0.15	4.56E-01	-0.27	1.67E-01	0.21	2.96E-01	-0.06	7.84E-01
ENSCAFG0000002886	ENSCAFG0000002886	grey	VSMC_M1C	0.07	7.25E-01	-0.05	7.90E-01	-0.08	6.77E-01	0.11	5.79E-01	0.12	5.55E-01	0.11	5.81E-01	-0.32	1.05E-01	-0.11	5.97E-01	0.13	5.10E-01	-0.09	6.66E-01
ENSCAFG0000002924	ABT1	darkgrey	VSMC_M8	0.07	7.25E-01	-0.65	2.52E-04	0.13	5.27E-01	-0.73	1.30E-05	0.06	7.53E-01	0.05	7.98E-01	0.38	4.96E-02	0.88	2.05E-09	0.45	1.80E-02	-0.80	5.92E-07
ENSCAFG0000000991	PLA2G4F	darkgreen	VSMC_M4	0.07	7.25E-01	0.02	9.07E-01	-0.40	3.71E-02	0.52	5.54E-03	0.35	7.14E-02	-0.09	6.63E-01	-0.12	5.56E-01	-0.37	5.77E-02	-0.03	8.94E-01	0.25	2.10E-01
ENSCAFG0000000261	SYT5	darkgreen	VSMC_M4	0.07	7.25E-01	0.14	5.02E-01	-0.48	1.19E-02	0.68	9.19E-05	0.43	2.56E-02	-0.04	8.47E-01	-0.09	6.59E-01	-0.57	1.84E-03	-0.10	6.37E-01	0.36	6.74E-02
ENSCAFG0000000625	SPRY4	pink	VSMC_M5	0.07	7.26E-01	-0.21	2.86E-01	-0.55	3.19E-03	0.26	1.91E-01	0.55	3.18E-03	0.18	3.58E-01	-0.17	4.07E-01	-0.12	5.47E-01	0.20	3.17E-01	-0.11	5.82E-01
ENSCAFG0000001065	FAM167B	grey	VSMC_M1C	0.07	7.26E-01	0.09	6.51E-01	0.17	3.91E-01	-0.14	4.88E-01	-0.18	3.77E-01	-0.07	7.44E-01	0.37	6.08E-02	0.09	6.38E-01	-0.16	4.23E-01	0.05	7.98E-01
ENSCAFG0000002240	ENSCAFG0000002240	grey	VSMC_M1C	0.07	7.26E-01	0.09	6.51E-01	0.17	3.91E-01	-0.14	4.88E-01	-0.18	3.77E-01	-0.07	7.44E-01	0.37	6.08E-02	0.09	6.38E-01	-0.16	4.23E-01	0.05	7.98E-01
ENSCAFG0000002981	ENSCAFG0000002981	grey	VSMC_M1C	0.07	7.26E-01	0.09	6.51E-01	0.17	3.91E-01	-0.14	4.88E-01	-0.18	3.77E-01	-0.07	7.44E-01	0.37	6.08E-02	0.09	6.38E-01	-0.16	4.23E-01	0.05	7.98E-01
ENSCAFG0000000530	LBH	darkgreen	VSMC_M4	0.07	7.26E-01	0.50	7.63E-03	-0.32	1.06E-01	0.88	1.60E-09	0.23	2.39E-01	-0.24	2.23E-01	-0.31	1.17E-01	-0.87	3.28E-09	-0.39	4.19E-02	0.62	5.72E-04
ENSCAFG0000000770	ENSCAFG0000000770	darkgrey	VSMC_M8	0.07	7.26E-01	-0.52	5.17E-03	0.38	5.28E-02	-0.89	3.14E-10	-0.19	3.34E-01	0.07	7.13E-01	0.34	8.30E-02	0.97	1.05E-16	0.34	7.83E-02	-0.74	1.13E-05
ENSCAFG0000003006	ENSCAFG0000003006	grey	VSMC_M1C	0.07	7.26E-01	0.07	7.18E-01	0.44	2.27E-02	-0.28	1.64E-01	-0.42	3.10E-02	0.31	1.20E-01	0.07	7.45E-01	0.19	3.46E-01	-0.11	5.92E-01	0.00	9.88E-01
ENSCAFG0000001871	PIGS	darkgreen	VSMC_M4	0.07	7.27E-01	0.67	1.44E-04	-0.12	5.66E-01	0.78	1.72E-06	-0.01	9.53E-01	-0.09	6.48E-01	-0.33	8.81E-02	-0.91	6.06E-11	-0.52	5.10E-03	0.76	3.50E-06
ENSCAFG0000002973	RPUSD3	grey	VSMC_M1C	0.07	7.27E-01	0.15	4.46E-01	-0.19	3.45E-01	0.31	1.16E-01	0.01	9.55E-01	0.28	1.58E-01	0.11	5.77E-01	-0.29	1.46E-01	-0.25	2.14E-01	0.54	3.36E-03
ENSCAFG0000000556	EPC2	pink	VSMC_M5	0.07	7.27E-01	-0.29	1.49E-01	-0.28	1.62E-01	-0.18	3.69E-01	0.49	9.82E-03	-0.13	5.17E-01	0.06	7.54E-01	0.27	1.75E-01	0.19	3.38E-01	-0.47	1.32E-02
ENSCAFG0000000289	CABS1	grey	VSMC_M1C	0.07	7.27E-01	0.14	4.72E-01	-0.01	9.58E-01	0.04	8.41E-01	-0.02	9.37E-01	0.17	3.87E-01	-0.18	3.82E-01	0.02	9.39E-01	-0.23	2.59E-01	0.18	3.79E-01
ENSCAFG0000000685	TMEM132E	grey	VSMC_M1C	0.07	7.27E-01	0.40	3.66E-02	0.20	3.13E-01	0.29	1.44E-01	-0.37	6.03E-02	0.17	3.96E-01	0.06	7.62E-01	-0.37	5.58E-02	-0.43	2.40E-02	0.60	8.81E-04
ENSCAFG0000000429	SAP130	pink	VSMC_M5	0.07	7.27E-01	-0.36	6.34E-02	-0.44	2.06E-02	-0.06	7.79E-01	0.59	1.14E-03	0.09	6.41E-01	-0.31	1.12E-01	0.20	3.24E-01	0.31	1.16E-01	-0.49	1.00E-02
ENSCAFG0000001630	EFHD2	yellow	VSMC_M3	0.07	7.27E-01	-0.08	6.89E-01	0.70	4.64E-05	-0.64	3.70E-04	-0.58	1.61E-03	0.06	7.49E-01	0.33	9.03E-02	0.53	4.14E-03	-0.02	9.20E-01	-0.30	1.27E-01
ENSCAFG0000000282	ENSCAFG0000000282	yellow	VSMC_M3	0.07	7.27E-01	0.25	2.10E-01	0.77	3.13E-06	-0.45	1.75E-02	-0.79	1.07E-06	0.23	2.43E-01	0.16	4.25E-01	0.31	1.17E-01	-0.29	1.42E-01	0.13	5.24E-01
ENSCAFG0000000445	KRBA1	grey	VSMC_M1C	0.07	7.28E-01	0.41	3.41E-02	-0.01	9.45E-01	0.34	8.72E-02	-0.07	7.31E-01	-0.12	5.45E-01	-0.21	2.82E-01	-0.42	3.04E-02	-0.40	3.61E-02	0.46	1.71E-02
ENSCAFG0000000434	ENSCAFG0000000434	grey	VSMC_M1C	0.07	7.28E-01	0.19	3.51E-01	0.18	3.68E-01	-0.06	7.74E-01	-0.07	7.11E-01	-0.22	2.63E-01	-0.37	6.08E-02	-0.07	7.25E-01	-0.09	6.57E-01	-0.08	7.01E-01
ENSCAFG0000001739	PRKCSH	cyan	VSMC_M2	0.07	7.29E-01	0.82	1.32E-07	0.37	5.87E-02	0.41	3.32E-02	-0.42	2.73E-02	-0.21	2.90E-01	-0.35	7.78E-02	-0.67	1.34E-04	-0.68	8.92E-05	0.69	6.38E-05
ENSCAFG0000000929	IQGAP2	grey	VSMC_M1C	0.07	7.29E-01	-0.22	2.77E-01	0.16	4.19E-01	-0.51	7.15E-03	-0.08	6.91E-01	0.41	3.35E-02	0.43	2.38E-02	0.53	4.92E-03	0.02	9.40E-01	-0.31	1.22E-01
ENSCAFG0000000780	ENSCAFG0000000780	grey	VSMC_M1C	0.07	7.29E-01	0.25	2.06E-01	0.25	2.15E-01	-0.18	3.71E-01	-0.27	1.68E-01	-0.01	9.62E-01	0.30	1.27E-01	0.08	6.79E-01	-0.29	1.36E-01	0.15	4.55E-01
ENSCAFG0000003032	ENSCAFG0000003032	grey	VSMC_M1C	0.07	7.29E-01	0.53	4.28E-03	0.45	1.82E-02	-0.12	5.65E-01	-0.47	1.26E-02	-0.15	4.58E-01	-0.25	2.09E-01	-0.04	8.56E-01	-0.49	8.87E-03	0.34	8.30E-02
ENSCAFG0000001372	OVGP1	grey	VSMC_M1C	0.07	7.30E-01	0.11	5.97E-01	0.45	1.87E-02	-0.33	9.00E-02	-0.27	1.71E-01	-0.15	4.65E-01	-0.03	8.83E-01	0.26	1.90E-01	-0.09	6.50E-01	-0.22	2.68E-01
ENSCAFG0000000583	ASPSCR1	grey	VSMC_M1C	0.07	7.30E-01	0.74	1.10E-05	0.48	1.17E-02	0.23	2.49E-01	-0.55	2.92E-03	0.01	9.70E-01	-0.20	3.25E-01	-0.46	1.62E-02	-0.63	4.91E-04	0.57	1.90E-03
ENSCAFG0000000339	MED25	grey	VSMC_M1C	0.07	7.30E-01	0.57	2.03E-03	0.02	9.26E-01	0.39	4.33E-02	-0.14	4.97E-01	-0.12	5.63E-01	-0.17	3.97E-01	-0.56	2.53E-03	-0.45	1.73E-02	0.67	1.40E-04
ENSCAFG0000000294	ME1	grey	VSMC_M1C	0.07	7.30E-01	0.28	1.55E-01	-0.27	1.76E-01	0.36	6.14E-02	0.37	5.97E-02	-0.21	3.03E-01	0.11	5.81E-01	-0.44	2.07E-02	-0.20	3.12E-01	0.15	4.44E-01
ENSCAFG0000002418	DPEP2	grey	VSMC_M1C	0.07	7.30E-01	0.22	2.74E-01	-0.17	3.85E-01	0.29	1.47E-01	0.03	8.88E-01	-0.25	2.18E-01	0.03	8.67E-01	-0.27	1.78E-01	-0.22	2.69E-01	0.44	2.02E-02
ENSCAFG0000003180	ISPD	grey	VSMC_M1C	0.07	7.30E-01	-0.27	1.68E-01	-0.25	2.03E-01	0.05	7.91E-01	0.26	1.97E-01	0.05	8.08E-01	0.25	2.01E-01	0.11	5.74E-01	0.11	5.78E-01	-0.12	5.42E-01
ENSCAFG0000000825	KDM2B	yellow	VSMC_M3	0.07	7.31E-01	0.18	3.69E-01	0.39	4.47E-02	-0.48	1.05E-02	-0.25	2.06E-01	0.17	4.02E-01	0.09	6.57E-01	0.35	7.49E-02	-0.21	2.98E-01	-0.23	2.55E-01
ENSCAFG0000000504	CYP2S1	cyan	VSMC_M2	0.07	7.31E-01	0.53	4.39E-03	0.16	4.11E-01	0.11	5.89E-01	-0.28	1.64E-01	0.09	6.42E-01	-0.12	5.52E-01	-0.19	3.38E-01	-0.54	3.35E-03	0.50	7.55E-03
ENSCAFG0000002405	GGN	turquoise	VSMC_M6	0.07	7.31E-01	-0.09	6.55E-01	0.12	5.44E-01	-0.09	6.48E-01	-0.17	3.83E-01	0.34	8.42E-02	0.10	6.07E-01	0.16	4.31E-01	-0.06	7.52E-01	0.03	9.00E-01
ENSCAFG0000000943	BAZ2B	pink	VSMC_M5	0.07	7.31E-01	-0.27	1.71E-01	-0.49	9.53E-03	0.40	3.64E-02	0.56	2.19E-03	0.01	9.51E-01	-0.16	4.13E-01	-0.21	2.97E-01	0.30	1.28E-01	-0.17	3.97E-01
ENSCAFG0000001907	CTIF	darkgreen	VSMC_M4	0.07	7.31E-01	0.38	4.86E-02	-0.21	2.93E-01	0.69	7.38E-05	0.22	2.70E-01	-0.23	2.50E-01	-0.64	3.71E-04	-0.75	7.93E-06	-0.19	3.33E-01	0.36	6.71E-02
ENSCAFG0000001497	NAGLU	cyan	VSMC_M2	0.07	7.32E-01	0.74	1.07E-05	0.43	2.42E-02	0.09	6.53E-01	-0.35	7.25E-02	-0.28	1.52E-01	-0.44	2.12E-02	-0.43	2.44E-02	-0.53	4.43E-03	0.36	6.69E-02
ENSCAFG0000001035	CYB5R1	grey	VSMC_M1C	0.07	7.32E-01	0.51	7.04E-03	0.01	9.52E-01	0.55	2.70E-03	-0.06	7.59E-01	-0.02	9.27E-01	-0.40	3.76E-02	-0.62	6.24E-04	-0.37	5.88E-02	0.45	1.86E-02
ENSCAFG0000000427	MYO3A	grey	VSMC_M1C	0.07	7.32E-01	-0.30	1.30E-01	-0.22	2.66E-01	0.04	8.38E-01	0.16	4.25E-01	0.36	6.53E-02	-0.38	4.85E-02	0.16	4.34E-01	0.21	3.03E-01	-0.14	4.82E-01
ENSCAFG0000000715	ENSCAFG0000000715	grey	VSMC_M1C	0.07	7.32E-01	-0.02	9.13E-01	0.21	2.89E-01	-0.30	1.29E-01	-0.09	6.72E-01	-0.06	7.61E-01	0.02	9.14E-01	0.28	1.61E-01	-0.05	7.88E-01	-0.32	9.86E-02
ENSCAFG0000001484	FAM193A	grey	VSMC_M1C	0.07	7.32E-01	0.29	1.44E-01	-0.09	6.70E-01	0.32	1.01E-01	-0.02	9.07E-01	-0.09	6.39E-01	0.16	4.13E-01	-0.33	9.34E-02	-0.40	4.06E-02	0.52	5.01E-03
ENSCAFG0000000680	NCOA4	grey	VSMC_M1C	0.07	7.32E-01	0.27	1.68E-01	-0.19	3.30E-01	0.37	5.73E-02	0.27	1.78E-01	-0.36	6.49E-02	-0.09	6.50E-01	-0.45	1.99E-02	-0.16	4.13E-01	0.20	3.17E-01
ENSCAFG0000001506	C18H11orf95	yellow	VSMC_M3	0.07	7.33E-01	0.61	6.48E-04	0.49	9.62E-03	0.15	4.43E-01	-0.53	4.46E-03	0.17	3.99E-01	-0.34	8.18E-02	-0.36	6.14E-02	-0.49	9.33E-03	0.48	1.09E-02
ENSCAFG0000001182	B3GNT5	grey	VSMC_M1C	0.07	7.33E-01	-0.41	3.51E-02	-0.74	1.06E-05	0.37	6.00E-02	0.67	1.50E-04	0.09	6.52E-01	-0.17	4.00E-01	-0.09	6.52E-01	0.31	1.15E-01	-0.06	7.71E-01
ENSCAFG0000001924	NFIC	grey	VSMC_M1C	0.07	7.33E-01	0.32	1.05E-01	0.58	1.66E-03	-0.24	2.35E-01	-0.58	1.70E-03	0.17	4.01E-01	-0.29	1.48E-01	0.14	4.94E-01	-0.29	1.38E-01	0.10	6.12E-01
ENSCAFG0000001777	ENSCAFG0000001777	grey	VSMC_M1C	0.07	7.33E-01	0.05	7.93E-01	-0.01	9.62E-01	0.22	2.78E-01	0.10	6.23E-01	-0.33	9.56E-02	-0.16	4.23E-01	-0.20	3.08E-01	0.02	9.39E-01	0.02	9.35E-01
ENSCAFG0000001505	MMP1	darkgreen	VSMC_M4	0.07	7.34E-01	0.32	1.04E-01	-0.44	2.30E-02	0.83	6.41E-08	0.32	1.02E-01	-0.14	5.01E-01	-0.28	1.59E-01	-0.73	1.30E-05	-0.26	1.85E-01	0.53	4.91E-03
ENSCAFG0000001656	ENSCAFG0000001656	grey	VSMC_M1C	0.07	7.34E-01	0.18	3.72E-01	0.07	7.45E-01	-0.09	6.51E-01	-0.03	8.81E-01	0.00	9.85E-01	-0.03	8.93E-01	-0.03	8.67E-01	-0.18	3.69E-01	0.11	5.97E-01
ENSCAFG0000002320	ENSCAFG0000002320	violet	VSMC_M7	0.07	7.34E-01	-0.09	6.49E-01	0.35	7.57E-02	-0.27	1.79E-01	-0.31	1.21E-01	-0.04	8.61E-01	0.44	2.33E-02	0.28	1.63E-01	-0.03	8.67E-01	-0.12	5.59E-01
ENSCAFG0000000065	FZD6	grey	VSMC_M1C	0.07	7.34E-01	-0.05	8.03E-01	-0.31	1.20E-01	0.31	1.14E-01	0.27	1.79E-01	0.13	5.32E-01	0.03	8.95E-01	-0.14	4.92E-01	-0.03	8.94E-01	0.07	7.23E-01
ENSCAFG0000001639	DCAF15	darkgrey	VSMC_M8	0.07	7.35E-01	-0.27	1.65E-01	0.43	2.53E-02	-0.68	8.12E-05	-0.24	2.27E-01	0.14	4.97E-01	0.08	7.01E-01	0.67	1.44E-04	0.19	3.34E-01	-0.59	1.27E-03
ENSCAFG0000000829	HEXB	cyan	VSMC_M2	0.07	7.35E-01	0.65	2.79E-04	-0.24	2.22E-01	0.77	3.20E-06	0.17	3.86E-01	-0.16	4.23E-01	-0.44	2.17E-02	-0.91	4.61E-11	-0.46	1.55E-02	0.66	1.61E-04
ENSCAFG0000002806	ENSCAFG0000002806	grey	VSMC_M1C	0.07	7.35E-01	0.00	9.89E-01	-0.04	8.55E-01	-0.01	9.60E-01	0.08	7.02E-01	-0.15	4.42E-01	0.31	1.13E-01	0.08	6.98E-01	-0.08	6.87E-01	-0.07	7.22E-01
ENSCAFG0000001668	FAF2	darkgreen	VSMC_M4	0.07	7.35E-01	-0.10	6.29E-01	-0.67	1.30E-04	0.61	6.50E-04	0.61	7.33E-04	-0.16	4.21E-01	-0.12	5.39E-01	-0.42	2.78E-02	0.03	8.74E-01	0.19	3.51E-01
ENSCAFG0000000022	MARS	grey	VSMC_M1C	0.07	7.36E-01	-0.39	4.57E-02	-0.12	5.57E-01	-0.12	5.61E-01	0.06	7.70E-01	0.14	4.72E-01	0.26	1.85E-01	0.35	7.10E-02	0.15	4.44E-01	-0.16	4.28E-01
ENSCAFG0000000073	ENSCAFG0000000073	grey	VSMC_M1C	0.07	7.36E-01	-0.29	1.45E-01	-0.13	5.34E-01	-0.04	8.35E-01	0.28	1.58E-01	0.10	6.15E-01	-0.19	3.49E-01	0.10	6.30E-01	0.32	1.03E-01	-0.40	3.95E-02
ENSCAFG0000000386	CDC40	pink	VSMC_M5	0.07	7.36E-01	-0.43	2.52E-02	-0.29	1.43E-01	-0.12	5.40E-01	0.53	4.84E-03	-0.22	2.69E-01	-0.27	1.67E-01	0.21	2.84E-01	0.44	2.30E-02	-0.66	2.06E-04
ENSCAFG0000000241	AGMO	pink	VSMC_M5	0.07	7.36E-01	-0.22	2.60E-01	-0.43	2.34E-02	0.23	2.48E-01	0.50	7.65E-03	-0.24	2.33E-01	0.11	5.88E-01	-0.16	4.15E-01	0.22	2.79E-01	-0.18	3.66E-01
ENSCAFG0000000986	KSR2	grey	VSMC_M1C	0.07	7.36E-01	0.13	5.24E-01	-0.11	5.90E-01	0.17	3.95E-01	0.05	8.10E-01	-0.13	5.18E-01	-0.17	3.90E-01	-0.11	5.87E-01	-0.20	3.15E-01	0.21	3.03E-01
ENSCAFG0000000905	COL2A1	turquoise	VSMC_M6	0.07	7.36E-01	-0.06	7.84E-01	0.15	4.58E-01	-0.13	5.27E-01	-0.10	6.13E-01	0.27	1.75E-01	0.12	5.47E-01	0.06	7.63E-01	0.01	9.43E-01	-0.13	5.25E-01
ENSCAFG0000001816	CCL14	grey	VSMC_M1C	0.07	7.37E-01	0.20	3.16E-01	-0.01	9.55E-01	-0.02	9.28E-01	-0.05	7.91E-01	0.13	5.17E-01	0.26	1.89E-01	-0.06	7.70E-01	-0.24	2.32E-01	0.29	1.40E-01
ENSCAFG0000002857	XK	grey	VSMC_M1C	0.07	7.37E-01	-0.26	1.97E-01	-0.63	4.52E-04	0.49	1.02E-02	0.51	6.29E-03	0.19	3.52E-01	0.11	5.94E-01	-0.30	1.31E-01	0.20	3.07E-01	0.21	2.88E-01
ENSCAFG0000001447	IGFBP5	yellow	VSMC_M3	0.07	7.37E-01	0.49	8.72E-03	0.53	4.84E-03	-0.30	1.26E-01	-0.49	9.86E-03	-0.10	6.30E-01	0.26	1.87E-01	0.12	5.42E-01	-0.54	3.89E-03	0.20	3.07E-01
ENSCAFG0000001852	RAB11FIP4	pink	VSMC_M5	0.07	7.37E-01	-0.02	9.27E-01	-0.37	5.61E-02	0.24	2.24E-01	0.41	3.58E-02	-0.12	5.45E-01	-0.01	9.52E-01	-0.17	3.99E-01	-0.03	8.67E-01	0.00	9.94E-01
ENSCAFG0000000664	ENSCAFG0000000664	grey	VSMC_M1C	0.07	7.37E-01	0.19	3.50E-01	0.19	3.47E-01	-0.20	3.24E-01	-0.14	4.74E-01	-0.02	9.11E-01	-0.06	7.77E-01	0.13	5.11E-01	-0.15	4.49E-01	0.06	7.83E-01
ENSCAFG0000000480	ENSCAFG0000000480	grey	VSMC_M1C	0.07	7.38E-01	0.08	6.95E-01	-0.03	8.89E-01	0.05	7.95E-01	-0.04	8.45E-01	0.20	3.09E-01	0.09	6.41E-01	0.03	8.85E-01	-0.11	5.94E-01	0.19	3.41E-01
ENSCAFG0000000241	ENSCAFG0000000241	grey	VSMC_M1C	0.07	7.38E-01	-0.17	3.97E-01	-0.36	6.34E-02	0.16	4.37E-01	0.36	6.67E-02	0.05	8.01E-01	-0.18	3.69E-01	-0.01	9.57E-01	0.16	4.25E-01	-0.08	6.81E-01
ENSCAFG0000000830	AQR	pink	VSMC_M5	0.07	7.39E-01	-0.62	5.10E-04	-0.45	1.90E-02	-0.21	2.86E-01	0.59	1.31E-03	0.00	9.94E-01	0.37	6.07E-02	0.40	4.05E-02	0.45	1.79E-02	-0.57	1.75E-03
ENSCAFG0000000153	TLE4	darkgreen	VSMC_M4	0.07	7.39E-01	0.51	6.10E-03	-0.20	3.17E-01	0.65	2.56E-04	0.16	4.36E-01	-0.41	3.45E-02	-0.36	6.52E-02	-0.68	8.25E-05	-0.39	4.40E-02	0.52	5.91E-03
ENSCAFG0000001555	NUP88	yellow	VSMC_M3	0.07	7.39E-01	-0.02	9.02E-01	0.43	2.39E-02	-0.40	4.12E-02	-0.46	1.66E-02	0.14	4.93E-01	0.35	7.18E-02	0.44	2.16E-02	-0.18	3.56E-01	-0.10	6.31E-01
ENSCAFG0000000835	WWTR1	cyan	VSMC_M2	0.07	7.39E-01	0.65	2.52E-04	0.04	8.35E-01	0.39	4.19E-02	-0.12	5.57E-01	0.20	3.10E-01	0.03	8.81E-01	-0.59	1.07E-03	-0.51	6.54E-03	0.61	7.08E-04
ENSCAFG0000001006	RRP9	darkgrey	VSMC_M8	0.07	7.39E-01	-0.17	3.84E-01	0.65	2.50E-04	-0.78	1.62E-06	-0.47	1.37E-02	0.07	7.16E-01	0.10	6.22E-01	0.67	1.13E-04	0.09	6.65E-01	-0.55	2.88E-03
ENSCAFG0000001415	TBC1D2B	grey	VSMC_M1C	0.07	7.39E-01	0.21	3.00E-01	0.39	4.39E-02	-0.36	6.19E-02	-0.30	1.23E-01	-0.13	5.05E-01	0.65	2.58E-04	0.16	4.14E-01	-0.26	1.86E-01	0.05	8.07E-01
ENSCAFG0000001933	TRPV3	grey	VSMC_M1C	0.07	7.39E-01	-0.34	8.12E-02	-0.09	6.40E-01	-0.25	2.02E-01	0.21	2.92E-01	0.23	2.49E-01	0.30	1.26E-01	0.37	5.98E-02	0.21	3.00E-01	-0.48	1.21E-02
ENSCAFG0000001622	PEX26	grey	VSMC_M1C	0.07	7.40E-01	0.10	6.18E-01	-0.38	5.04E-02	0.27	1.67E-01	0.43	2.62E-02	-0.13	5.12E-01	-0.08	6.76E-01	-0.34	8.39E-02	-0.10	6.11E-01	0.10	6.32E-01

ENSCAFG0000000114	ENSCAFG0000000114	cyan	VSMC_M2	0.07	7.40E-01	0.76	4.02E-06	0.38	4.89E-02	0.17	3.91E-01	-0.38	5.04E-02	-0.08	6.76E-01	-0.41	3.29E-02	-0.45	1.77E-02	-0.62	5.81E-04	0.45	1.78E-02
ENSCAFG0000000828	HNF4G	grey	VSMC_M10	0.07	7.40E-01	-0.43	2.57E-02	-0.26	1.85E-01	-0.10	6.09E-01	0.28	1.59E-01	0.36	6.53E-02	0.04	8.31E-01	0.26	1.90E-01	0.27	1.68E-01	-0.25	2.05E-01
ENSCAFG0000001089	COQ10B	grey	VSMC_M10	0.07	7.40E-01	-0.41	3.18E-02	-0.13	5.23E-01	-0.16	4.15E-01	0.11	5.86E-01	0.17	3.84E-01	0.10	6.18E-01	0.35	7.38E-02	0.20	3.19E-01	-0.28	1.56E-01
ENSCAFG0000001306	ENSCAFG0000001306	grey	VSMC_M10	0.07	7.40E-01	0.68	9.94E-05	0.14	4.97E-01	0.34	8.34E-02	-0.15	4.62E-01	-0.15	4.59E-01	-0.01	9.49E-01	-0.56	2.57E-03	-0.59	1.08E-03	0.50	8.01E-03
ENSCAFG0000000595	DPF1	grey	VSMC_M10	0.07	7.40E-01	-0.25	2.02E-01	0.28	1.50E-01	-0.37	5.54E-02	-0.16	4.12E-01	0.19	3.48E-01	0.11	5.99E-01	0.45	1.83E-02	0.11	5.83E-01	-0.39	4.73E-02
ENSCAFG0000000652	TMPO	darkgrey	VSMC_M8	0.07	7.41E-01	-0.52	5.27E-03	0.31	1.12E-01	-0.86	1.21E-08	-0.15	4.47E-01	0.12	5.61E-01	0.49	9.73E-03	0.94	2.33E-13	0.31	1.15E-01	-0.67	1.38E-04
ENSCAFG0000000807	ENSCAFG0000000807	grey	VSMC_M10	0.07	7.41E-01	0.23	2.53E-01	-0.32	1.02E-01	0.40	3.72E-02	0.21	2.91E-01	0.09	6.61E-01	-0.07	7.22E-01	-0.38	4.80E-02	-0.26	1.91E-01	0.46	1.52E-02
ENSCAFG0000002867	ZNF23	grey	VSMC_M10	0.07	7.41E-01	0.23	2.53E-01	-0.06	7.84E-01	0.27	1.70E-01	-0.06	7.66E-01	-0.09	6.66E-01	0.25	2.01E-01	-0.26	1.89E-01	-0.31	1.18E-01	0.50	7.57E-03
ENSCAFG0000001620	TBC1D1	grey	VSMC_M10	0.07	7.41E-01	0.17	3.94E-01	0.06	7.61E-01	0.06	7.76E-01	0.09	6.66E-01	0.00	9.90E-01	-0.50	7.67E-03	-0.24	2.24E-01	0.03	8.80E-01	-0.08	7.02E-01
ENSCAFG0000001326	PCMTD2	darkgreen	VSMC_M4	0.07	7.41E-01	0.23	2.40E-01	-0.46	1.47E-02	0.74	9.06E-06	0.51	6.55E-03	-0.23	2.58E-01	-0.49	9.91E-03	-0.79	1.23E-06	-0.06	7.54E-01	0.26	1.89E-01
ENSCAFG0000003096	RTN4RL2	turquoise	VSMC_M6	0.07	7.41E-01	-0.06	7.63E-01	0.11	5.84E-01	-0.16	4.31E-01	-0.09	6.41E-01	0.46	1.66E-02	0.29	1.45E-01	0.18	3.70E-01	0.00	9.88E-01	-0.06	7.70E-01
ENSCAFG0000003238	DCAKD	pink	VSMC_M5	0.07	7.41E-01	-0.49	8.96E-03	-0.28	1.58E-01	-0.35	7.34E-02	0.45	1.72E-02	-0.08	6.82E-01	0.33	9.42E-02	0.43	2.68E-02	0.42	3.06E-02	-0.56	2.24E-03
ENSCAFG0000000904	SYNJ1	grey	VSMC_M10	0.07	7.42E-01	0.38	4.86E-02	0.09	6.61E-01	0.05	7.97E-01	-0.06	7.72E-01	-0.14	4.97E-01	-0.16	4.32E-01	-0.14	4.97E-01	-0.38	4.80E-02	0.23	2.48E-01
ENSCAFG0000001376	PTGS2	darkolivegreen	VSMC_M9	0.07	7.42E-01	-0.47	1.24E-02	-0.10	6.10E-01	-0.20	3.26E-01	0.24	2.25E-01	0.04	8.33E-01	0.28	1.53E-01	0.28	1.51E-01	0.45	1.96E-02	-0.42	2.89E-02
ENSCAFG0000001917	SLC1A7	grey	VSMC_M10	0.07	7.42E-01	0.70	5.19E-05	0.22	2.68E-01	0.27	1.70E-01	-0.23	2.43E-01	-0.03	8.77E-01	-0.34	8.18E-02	-0.45	1.74E-02	-0.61	7.51E-04	0.47	1.41E-02
ENSCAFG0000000189	WASHC4	grey	VSMC_M10	0.07	7.42E-01	0.19	3.49E-01	-0.47	1.41E-02	0.54	3.56E-03	0.47	1.42E-02	-0.18	3.81E-01	-0.09	6.68E-01	-0.58	1.67E-03	-0.17	3.88E-01	0.33	9.66E-02
ENSCAFG0000000035	MGAT4B	cyan	VSMC_M2	0.07	7.42E-01	0.49	9.26E-03	0.56	2.27E-03	-0.26	1.96E-01	-0.40	3.64E-02	-0.04	8.31E-01	-0.16	4.18E-01	-0.04	8.24E-01	-0.32	1.01E-01	0.02	9.07E-01
ENSCAFG0000001968	TMEM8A	grey	VSMC_M10	0.07	7.42E-01	0.80	5.16E-07	0.33	9.35E-02	0.33	9.10E-02	-0.44	2.31E-02	-0.04	8.54E-01	-0.29	1.42E-01	-0.59	1.11E-03	-0.66	1.60E-04	0.76	4.00E-06
ENSCAFG0000000569	NAP1L1	darkgrey	VSMC_M8	0.07	7.42E-01	-0.26	1.90E-01	0.56	2.53E-03	-0.89	7.97E-10	-0.33	8.79E-02	0.02	9.14E-01	0.28	1.58E-01	0.82	1.76E-07	0.16	4.23E-01	-0.64	3.62E-04
ENSCAFG0000002383	ENSCAFG0000002383	grey	VSMC_M10	0.07	7.42E-01	0.65	2.69E-04	0.11	6.00E-01	0.37	5.42E-02	-0.30	1.24E-01	0.13	5.22E-01	-0.27	1.70E-01	-0.42	2.95E-02	-0.67	1.30E-04	0.72	2.52E-05
ENSCAFG0000001492	NELFA	grey	VSMC_M10	0.07	7.42E-01	0.12	5.51E-01	0.06	7.82E-01	-0.12	5.53E-01	0.09	6.63E-01	-0.02	9.28E-01	-0.17	3.93E-01	0.10	6.16E-01	-0.12	5.42E-01	-0.22	2.79E-01
ENSCAFG0000001635	DDX39A	darkgrey	VSMC_M8	0.07	7.42E-01	-0.30	1.25E-01	0.62	6.22E-04	-0.90	1.39E-10	-0.47	1.40E-02	0.19	3.51E-01	0.29	1.44E-01	0.91	3.46E-11	0.15	4.49E-01	-0.59	1.20E-03
ENSCAFG0000001963	NOTCH1	turquoise	VSMC_M6	0.07	7.42E-01	0.16	4.17E-01	0.33	9.44E-02	-0.14	4.75E-01	-0.31	1.19E-01	0.77	3.34E-06	-0.01	9.62E-01	0.09	6.39E-01	-0.19	3.50E-01	-0.01	9.59E-01
ENSCAFG0000003216	MYL12B	darkgreen	VSMC_M4	0.07	7.43E-01	-0.20	3.22E-01	-0.41	3.34E-02	0.55	2.91E-03	0.46	1.58E-02	-0.17	3.84E-01	-0.28	1.52E-01	-0.44	2.12E-02	0.27	1.68E-01	-0.06	7.84E-01
ENSCAFG0000000302	CHN2	darkgreen	VSMC_M4	0.07	7.43E-01	0.52	5.82E-03	-0.38	5.29E-02	0.73	1.48E-05	0.26	1.94E-01	-0.05	8.12E-01	-0.54	3.62E-03	-0.76	3.92E-06	-0.41	3.21E-02	0.60	9.98E-04
ENSCAFG0000000526	ENSCAFG0000000526	grey	VSMC_M10	0.07	7.43E-01	0.13	5.22E-01	0.02	9.30E-01	0.08	6.84E-01	-0.05	7.91E-01	0.00	9.92E-01	0.18	3.78E-01	-0.17	3.92E-01	-0.15	4.65E-01	0.17	3.89E-01
ENSCAFG0000000735	SGK3	darkgrey	VSMC_M8	0.07	7.43E-01	-0.52	5.58E-03	-0.04	8.53E-01	-0.55	3.09E-03	0.27	1.80E-01	-0.16	4.25E-01	0.11	5.80E-01	0.63	4.41E-04	0.48	1.10E-02	-0.72	2.56E-05
ENSCAFG0000001591	PELP1	yellow	VSMC_M3	0.07	7.43E-01	0.30	1.32E-01	0.57	1.71E-03	-0.37	5.86E-02	-0.65	2.50E-04	0.28	1.62E-01	-0.06	7.65E-01	0.30	1.23E-01	-0.39	4.20E-02	0.16	4.39E-01
ENSCAFG0000003113	HSBP1L1	grey	VSMC_M10	0.07	7.44E-01	-0.06	7.75E-01	-0.06	7.68E-01	-0.06	7.59E-01	0.08	7.08E-01	0.13	5.28E-01	-0.04	8.25E-01	0.08	6.88E-01	0.03	8.64E-01	0.01	9.44E-01
ENSCAFG0000000526	CIDEC	grey	VSMC_M10	0.07	7.44E-01	0.23	2.39E-01	0.05	8.09E-01	0.09	6.39E-01	-0.13	5.27E-01	0.10	6.24E-01	-0.17	4.00E-01	-0.12	5.38E-01	-0.29	1.45E-01	0.25	2.12E-01
ENSCAFG0000000641	SMPD1	cyan	VSMC_M2	0.07	7.44E-01	0.81	2.48E-07	0.08	6.78E-01	0.68	1.10E-04	-0.24	2.25E-01	0.00	9.81E-01	-0.35	7.50E-02	-0.85	2.03E-08	-0.68	8.64E-05	0.86	1.36E-08
ENSCAFG0000000478	CASKIN2	grey	VSMC_M10	0.07	7.44E-01	0.37	5.89E-02	-0.01	9.69E-01	0.55	3.22E-03	-0.04	8.44E-01	0.03	8.65E-01	-0.21	3.02E-01	-0.64	3.42E-04	-0.25	2.00E-01	0.50	8.05E-03
ENSCAFG0000000984	TIGD2	grey	VSMC_M10	0.07	7.44E-01	-0.09	6.47E-01	0.24	2.37E-01	-0.45	1.71E-02	-0.02	9.05E-01	0.04	8.25E-01	0.13	5.13E-01	0.39	4.28E-02	0.04	8.35E-01	-0.48	1.19E-02
ENSCAFG0000001905	TCEANC2	grey	VSMC_M10	0.07	7.45E-01	0.09	6.70E-01	-0.40	3.62E-02	0.21	3.01E-01	0.47	1.42E-02	-0.01	9.45E-01	0.17	3.94E-01	-0.22	2.61E-01	-0.10	6.06E-01	0.03	9.00E-01
ENSCAFG0000001034	PHC2	darkgrey	VSMC_M8	0.07	7.45E-01	-0.28	1.53E-01	0.37	5.46E-02	-0.56	2.38E-03	-0.20	3.12E-01	-0.08	6.93E-01	-0.24	2.25E-01	0.59	1.10E-03	0.26	1.88E-01	-0.60	8.94E-04
ENSCAFG0000003048	YIF1A	grey	VSMC_M10	0.07	7.45E-01	0.05	7.90E-01	0.48	1.22E-02	-0.48	1.16E-02	-0.40	3.62E-02	0.03	8.71E-01	-0.20	3.30E-01	0.41	3.21E-02	-0.10	6.16E-01	-0.23	2.51E-01
ENSCAFG0000000988	ENSCAFG0000000988	grey	VSMC_M10	0.07	7.45E-01	0.36	6.60E-02	0.05	7.98E-01	0.26	1.96E-01	-0.12	5.64E-01	0.22	2.80E-01	-0.30	1.35E-01	-0.29	1.37E-01	-0.34	8.32E-02	0.31	1.10E-01
ENSCAFG0000000595	ENSCAFG0000000595	grey	VSMC_M10	0.07	7.45E-01	-0.04	8.36E-01	0.07	7.30E-01	-0.22	2.79E-01	-0.02	9.15E-01	-0.21	2.83E-01	0.22	2.65E-01	0.24	2.18E-01	-0.03	8.99E-01	-0.13	5.26E-01
ENSCAFG0000001797	NOL4	grey	VSMC_M10	0.07	7.45E-01	0.24	2.24E-01	-0.26	1.82E-01	0.42	2.72E-02	0.22	2.63E-01	-0.05	7.87E-01	-0.10	6.07E-01	-0.49	9.62E-03	-0.19	3.37E-01	0.34	7.97E-02
ENSCAFG0000001157	HIST2H2AC	yellow	VSMC_M3	0.07	7.45E-01	0.16	4.25E-01	0.66	1.73E-04	-0.56	2.51E-03	-0.66	1.80E-04	0.30	1.34E-01	0.40	3.65E-02	0.47	1.27E-02	-0.27	1.70E-01	0.01	9.52E-01
ENSCAFG0000001271	KIF1A	cyan	VSMC_M2	0.07	7.45E-01	0.74	9.44E-06	-0.05	8.22E-01	0.70	4.57E-05	-0.10	6.35E-01	-0.10	6.12E-01	-0.34	8.03E-02	-0.88	2.13E-09	-0.61	7.93E-04	0.80	4.35E-07
ENSCAFG0000001112	ENSCAFG0000001112	grey	VSMC_M10	0.07	7.45E-01	-0.40	3.97E-02	-0.21	2.82E-01	0.00	9.91E-01	0.28	1.61E-01	-0.05	8.23E-01	-0.02	9.20E-01	0.15	4.58E-01	0.34	8.59E-02	-0.30	1.26E-01
ENSCAFG0000001839	CXHXorf56	grey	VSMC_M10	0.07	7.46E-01	0.16	4.28E-01	0.03	8.67E-01	0.28	1.50E-01	-0.19	3.47E-01	-0.01	9.60E-01	-0.16	4.33E-01	-0.26	1.94E-01	-0.19	3.39E-01	0.37	5.52E-02
ENSCAFG0000000680	PRICKLE2	cyan	VSMC_M2	0.07	7.46E-01	0.75	8.13E-06	-0.05	7.97E-01	0.71	2.92E-05	-0.09	6.60E-01	-0.17	4.06E-01	-0.31	1.11E-01	-0.82	2.06E-07	-0.63	4.88E-04	0.81	2.61E-07
ENSCAFG0000001971	POLRMT	cyan	VSMC_M2	0.07	7.46E-01	0.55	2.87E-03	0.68	1.03E-04	-0.27	1.81E-01	-0.73	1.47E-05	0.26	1.96E-01	-0.13	5.33E-01	0.03	8.68E-01	-0.56	2.19E-03	0.34	7.98E-02
ENSCAFG0000001110	GPR137B	grey	VSMC_M10	0.07	7.46E-01	0.61	6.50E-04	-0.26	1.97E-01	0.63	4.76E-04	0.10	6.04E-01	-0.09	6.45E-01	-0.28	1.60E-01	-0.77	3.27E-06	-0.51	7.09E-03	0.74	8.62E-06
ENSCAFG0000001881	ERAL1	grey	VSMC_M10	0.07	7.46E-01	0.09	6.73E-01	0.20	3.14E-01	-0.33	9.55E-02	-0.14	4.93E-01	0.15	4.45E-01	0.28	1.61E-01	0.29	1.47E-01	-0.18	3.74E-01	-0.15	4.60E-01
ENSCAFG0000001392	SUSD2	grey	VSMC_M10	0.07	7.46E-01	0.51	6.84E-03	0.10	6.13E-01	0.40	3.71E-02	-0.15	4.53E-01	-0.01	9.50E-01	-0.02	9.27E-01	-0.49	9.01E-03	-0.42	2.75E-02	0.56	2.23E-03
ENSCAFG0000001678	DPF3	grey	VSMC_M10	0.07	7.47E-01	-0.08	6.91E-01	0.06	7.80E-01	-0.09	6.44E-01	-0.07	7.35E-01	0.34	7.83E-02	-0.15	4.53E-01	0.18	3.71E-01	0.02	9.09E-01	-0.09	6.39E-01
ENSCAFG0000002362	BSCL2	grey	VSMC_M10	0.07	7.47E-01	0.73	1.73E-05	0.22	2.68E-01	0.32	1.05E-01	-0.36	6.68E-02	0.16	4.23E-01	-0.40	3.72E-02	-0.53	4.48E-03	-0.61	6.64E-04	0.69	6.67E-05
ENSCAFG0000001319	BTBD1	grey	VSMC_M10	0.07	7.47E-01	0.36	6.19E-02	-0.07	7.40E-01	0.10	6.31E-01	0.21	2.97E-01	-0.21	3.04E-01	-0.29	1.37E-01	-0.23	2.46E-01	-0.24	2.36E-01	0.02	9.11E-01
ENSCAFG0000001055	ZBTB8OS	grey	VSMC_M10	0.07	7.47E-01	0.08	6.92E-01	0.50	7.87E-03	-0.28	1.62E-01	-0.43	2.58E-02	-0.09	6.60E-01	0.07	7.36E-01	0.18	3.72E-01	-0.11	5.88E-01	-0.09	6.57E-01
ENSCAFG0000001681	ERGIC1	cyan	VSMC_M2	0.07	7.47E-01	0.40	4.08E-02	0.31	1.13E-01	-0.17	3.98E-01	-0.39	4.27E-02	0.08	7.01E-01	0.43	2.50E-02	-0.03	8.86E-01	-0.42	2.89E-02	0.39	4.28E-02
ENSCAFG0000003085	ENSCAFG0000003085	grey	VSMC_M10	0.06	7.47E-01	-0.21	2.88E-01	-0.10	6.04E-01	-0.04	8.45E-01	0.24	2.19E-01	0.01	9.48E-01	0.08	6.87E-01	0.09	6.71E-01	0.17	3.91E-01	-0.38	5.08E-02
ENSCAFG0000000793	EDEM2	cyan	VSMC_M2	0.06	7.48E-01	0.47	1.43E-02	0.62	6.29E-04	-0.17	4.10E-01	-0.67	1.49E-04	0.29	1.37E-01	0.24	2.36E-01	-0.02	9.02E-01	-0.48	1.16E-02	0.33	9.09E-02
ENSCAFG0000000975	SALL1	grey	VSMC_M10	0.06	7.48E-01	-0.17	4.07E-01	-0.21	2.83E-01	0.06	7.63E-01	0.23	2.59E-01	0.17	3.86E-01	0.32	9.90E-02	-0.05	7.87E-01	0.12	5.44E-01	0.03	8.78E-01
ENSCAFG0000001048	PKP2	grey	VSMC_M10	0.06	7.48E-01	-0.19	3.51E-01	-0.61	7.28E-04	0.62	5.56E-04	0.56	2.52E-03	0.01	9.77E-01	-0.43	2.66E-02	-0.48	1.16E-02	0.20	3.06E-01	0.11	5.89E-01
ENSCAFG0000000246	ANP32B	darkgrey	VSMC_M8	0.06	7.48E-01	-0.26	1.95E-01	0.62	5.41E-04	-0.80	4.61E-07	-0.46	1.53E-02	0.07	7.15E-01	0.41	3.42E-02	0.76	4.07E-06	0.11	5.96E-01	-0.53	4.66E-03
ENSCAFG0000001558	LRRC9	grey	VSMC_M10	0.06	7.48E-01	0.19	3.34E-01	-0.03	8.79E-01	0.36	6.59E-02	-0.11	5.87E-01	-0.03	8.92E-01	0.09	6.59E-01	-0.34	8.30E-02	-0.21	2.96E-01	0.49	9.38E-03
ENSCAFG0000001668	CLTB	yellow	VSMC_M3	0.06	7.49E-01	0.50	7.26E-03	0.67	1.35E-04	-0.21	2.92E-01	-0.66	1.92E-04	-0.08	6.82E-01	0.01	9.56E-01	-0.03	8.76E-01	-0.47	1.43E-02	0.22	2.59E-01
ENSCAFG0000000233	ENSCAFG0000000233	yellow	VSMC_M3	0.06	7.49E-01	0.53	4.10E-03	0.76	4.58E-06	-0.25	2.01E-01	-0.82	2.00E-07	0.08	6.87E-01	-0.11	5.72E-01	0.05	7.94E-01	-0.53	4.13E-03	0.35	7.24E-02
ENSCAFG0000001364	WNT2B	cyan	VSMC_M2	0.06	7.49E-01	0.48	1.10E-02	0.62	5.31E-04	-0.24	2.29E-01	-0.67	1.22E-04	0.38	5.30E-02	0.12	5.55E-01	0.07	7.23E-01	-0.50	7.93E-03	0.30	1.33E-01
ENSCAFG0000001217	INTS7	darkgrey	VSMC_M8	0.06	7.49E-01	-0.52	5.36E-03	0.12	5.35E-01	-0.72	2.38E-05	0.17	3.94E-01	-0.12	5.51E-01	0.08	7.01E-01	0.74	8.91E-06	0.45	1.84E-02	-0.89	4.40E-10
ENSCAFG0000003042	ZNHIT2	yellow	VSMC_M3	0.06	7.50E-01	0.25	2.07E-01	0.70	4.37E-05	-0.34	8.29E-02	-0.71	2.98E-05	0.05	8.03E-01	0.16	4.14E-01	0.16	4.13E-01	-0.25	2.12E-01	0.17	4.04E-01
ENSCAFG0000001721	TUFM	yellow	VSMC_M3	0.06	7.50E-01	0.26	1.83E-01	0.73	1.49E-05	-0.71	3.01E-05	-0.64	3.34E-04	-0.05	8.01E-01	0.07	7.10E-01	0.51	6.12E-03	-0.32	1.08E-01	-0.16	4.27E-01
ENSCAFG0000001336	ENSCAFG0000001336	grey	VSMC_M10	0.06	7.50E-01	-0.33	9.11E-02	-0.03	8.75E-01	-0.21	2.99E-01	0.03	8.85E-01	-0.11	5.81E-01	0.39	4.28E-02	0.25	2.17E-01	0.20	3.21E-01	-0.11	5.85E-01
ENSCAFG0000000006	KDSR	grey	VSMC_M10	0.06	7.50E-01	0.68	9.24E-05	-0.14	4.98E-01	0.69	6.52E-05	0.01	9.50E-01	-0.18	3.60E-01	-0.14	4.93E-01	-0.86	7.62E-09	-0.55	2.66E-03	0.82	2.25E-07
ENSCAFG0000003038	ENSCAFG0000003038	grey	VSMC_M10	0.06	7.50E-01	-0.28	1.62E-01	-0.11	5.81E-01	-0.11	6.01E-01	0.14	4.99E-01	-0.04	8.37E-01	-0.10	6.04E-01	0.22	2.81E-01	0.18	3.74E-01	-0.19	3.43E-01
ENSCAFG0000000001	ENSCAFG0000000001	turquoise	VSMC_M6	0.06	7.51E-01	-0.04	8.55E-01	-0.06	7.50E-01	-0.03	8.67E-01	0.06	7.75E-01	-0.04	8.28E-01	-0.15	4.64E-01	-0.04	8.54E-01	-0.02	9.33E-01	0.01	9.65E-01
ENSCAFG0000001445	AFAP1	grey	VSMC_M10	0.06	7.51E-01	-0.21	2.83E-01	-0.47	1.35E-02	0.53	4.53E-03	0.41	3.32E-02	0.08	7.06E-01	-0.41	3.33E-02	-0.34	8.33E-02	0.21	2.87E-01	0.06	7.63E-01
ENSCAFG0000002272	ENSCAFG0000002272	grey	VSMC_M10	0.06	7.51E-01	-0.12	5.51E-01	-0.23	2.45E-01	0.11	5.94E-01	0.23	2.50E-01	-0.19	3.55E-01	0.06	7.55E-01	-0.10	6.33E-01	0.14	4.95E-01	0.05	7.90E-01
ENSCAFG0000000159	FOXP4	grey	VSMC_M10	0.06	7.51E-01	0.23	2.41E-01	0.14	5.01E-01	0.07	7.47E-01	-0.13	5.21E-01	-0.03	8.62E-01	-0.79	1.22E-06	-0.12	5.65E-01	-0.14	4.72E-01	0.07	7.17E-01
ENSCAFG0000002241	ENSCAFG0000002241	turquoise	VSMC_M6	0.06	7.52E-01	0.14	4.74E-01	-0.04	8.37E-01	0.09	6.39E-01	-0.02	9.13E-01	0.33	8.90E-02	-0.19	3.34E-01	-0.11	5.84E-01	-0.10	6.17E-01	0.13	5.12E-01
ENSCAFG0000002128	ENSCAFG0000002128	grey	VSMC_M10	0.06	7.52E-01	0.19	3.35E-01	0.21	2.88E-01	-0.17	4.06E-01	-0.22	2.64E-01	0.02	9.34E-01	0.40	3.91E-02	0.08	6.86E-01	-0.22	2.65E-01	0.12	5.56E-01
ENSCAFG0000003027	ZNF140	grey	VSMC_M10	0.06	7.52E-01	0.21	2.97E-01	-0.34	7.92E-02	0.33	8.86E-02	0.18	3.66E-01	0.10	6.11E-01	0.04	8.47E-01	-0.31	1.14E-01	-0.23	2.58E-01	0.49	9.38E-03
ENSCAFG0000000227	GCM1	pink	VSMC_M5	0.06	7.52E-01	-0.09	6.49E-01	-0.54	3.55E-03	0.48	1.04E-02	0.54	3.36E-03	0.06	7.51E-01	-0.07	7.44E-01	-0.40	4.10E-02	0.07	7.18E-01	0.09	6.44E-01
ENSCAFG0000002028	TTLL11	grey	VSMC_M10	0.06	7.53E-01	0.25	2.04E-01	0.10	6.17E-01	0.08	6.83E-01	0.07	7.16E-01	-0.34	8.60E-02	-0.19	3.38E-01	-0.25	2.00E-01	-0.11	5.70E-01	-0.12	5.45E-01
ENSCAFG0000000352	TCAF1	darkgreen	VSMC_M4	0.06	7.53E-01	0.56	2.39E-03	-0.12	5.58E-01	0.65	2.50E-04	-0.04	8.52E-01	-0.02	9.27E-01	-0.20	3.13E-01	-0.64	3.39E-04	-0.55	3.22E-03	0.68	1.11E-04
ENSCAFG0000000069	NELFE	yellow	VSMC_M3	0.06	7.53E-01	-0.12	5.49E-01	0.53	4.78E-03	-0.58	1.40E-03	-0.29	1.48E-01	-0.03	8.80E-01	0.06	7.73E-01	0.52	5.75E-03	0.09	6.71E-01	-0.57	2.04E-03
ENSCAFG0000001942	IZUMO4	yellow	VSMC_M3	0.06	7.53E-01	0.44	2.07E-02	0.56	2.28E-03	-0.21	2.84E-01	-0.47	1.33E-02	-0.07	7.44E-01	-0.21	2.90E-01	0.00	9.90E-01	-0.36	6.45E-02	0.05	8.13E-01
ENSCAFG0000000445	NDUFB6	grey	VSMC_M10	0.06	7.54E-01	0.38	4.76E-02	0.32	1.01E-01	0.12	5.63E-01	-0.50	7.37E-03	0.31	1.11E-01	0.19	3.31E-01	-0.19	3.31E-01	-0.46	1.61E-02	0.55	2.82E-03
ENSCAFG0000001663	PRR14	yellow	VSMC_M3	0.06	7.54E-01	0.52	4.94E-03	0.84	3.88E-08	-0.45	1.85E-02	-0.86	1.25E-08	0.11	5.92E-01	0.07	7.31E-01	0.18	3.79E-01	-0.55	2.92E-03	0.30	1.33E-01
ENSCAFG0000001007	ENSCAFG0000001007	grey	VSMC_M10	0.06	7.54E-01	-0.05	8.01E-01	0.01	9.72E-01	-0.13	5.25E-01	0.05	8.06E-01	-0.09	6.38E-01	-0.06	7.82E-01	0.15	4.41E-01	0.01	9.47E-01	-0.10	6.28E-01
ENSCAFG0000001895	ENSCAFG0000001895	grey	VSMC_M10	0.06	7.55E-01	0.21	2.89E-01	0.19	3.40E-01	-0.20	3.11E-01	-0.17	3.92E-01	-0.08	6.85E-01	-0.16	4.25E-01	0.10	6.36E-01	-0.14	4.92E-01	0.05	8.03E-01
ENSCAFG0000002321	ABCA2	cyan	VSMC_M2	0.06	7.55E-01	0.75	7.09E-06	0.35	7.51E-02	0.13	5.18E-01	-0.32	9.81E-02	-0.11	5.99E-01	-0.17	3.86E-01	-0.48	1.18E-02	-0.55	2.82E-03	0.51	7.15E-03
ENSCAFG0000000375	ENSCAFG0000000375	grey	VSMC_M10	0.06	7.55E-01	0.22	2.68E-01	-0.10	6.37E-01	0.43	2.41E-02	-0.04	8.38E-01	0.01	9.70E-01	-0.25	2.15E-01	-0.44	2.23E-02	-0.17	3.90E-01	0.50	8.18E-03
ENSCAFG0000000852	GPR87	darkgreen	VSMC_M4	0.06	7.56E-01	0.15	4.53E-01	-0.61	6.47E-04	0.93	4.55E-12	0.50	7.79E-03	-0.12	5.50E-01	-0.21	2.92E-01	-0.76	3.56E-06	-0.11	5.75E-01	0.47	1.36E-02
ENSCAFG0000001504	ENSCAFG0000001504	grey	VSMC_M10	0.06	7.56E-01	-0.20	3.18E-01	-0.04	8.30E-01	-0.14	4.85E-01	0.07	7.43E-01	0.07	7.31E-01	0.02	9.08E-01	0.21	2.86E-01	0.10	6.24E-01	-0.13	5.13E-01
ENSCAFG0000001511	ARAF	grey	VSMC_M10	0.06	7.57E-01	0.44	2.08E-02	-0.23	2.50E-01	0.68	1.04E-04	0.01	9.63E-01	0.02	9.13E-01	-0.11	5.87E-01	-0.72	2.17E-05	-0.40	3.90E-02	0.75	6.35E-06
ENSCAFG0000000687	ENSCAFG0000000687	grey	VSMC_M10	0.06	7.57E-01	-0.02	9.19E-01	-0.34	8.75E-02	0.28	1.50E-01	0.18	3.79E-01	0.32	1.05E-01	-0.07	7.19E-01	-0.10	6.36E-01	-0.05	8.19E-01	0.32	1.05E-01
ENSCAFG0000000090	SCUBE1	darkgreen	VSMC_M4	0.06	7.57E-01	0.08	7.04E-01	-0.55	2.97E-03	0.78	1.55E-06	0.45	1.94E-02	-0.10	6.10E-01	-0.20	3.14E-01	-0.62	5.88E-04	-0.06	7.63E-01	0.37	5.53E-02
ENSCAFG0000001701	TMEM219	grey	VSMC_M10	0.06	7.57E-01	0.16	4.39E-01	0.21	3.01E-01	-0.17	3.84E-01	-0.14	4.90E-01	-0.25	2.02E-01	0.18	3.57E-01	0.08	6.82E-01	-0.16	4.13E-01	0.02	9.37E-01
ENSCAFG0000000282	SPACA6	grey	VSMC_M10	0.06	7.57E-01	0.15	4.67E-01	0.41	3.54E-02	-0.09	6.67E-01	-0.31	1.20E-01	0.00	9.97E-01	-0.04	8.42E-01	0.06	7.64E-01	-0.09	6.65E-01	-0.11	6.01E-01
ENSCAFG0000000683	NR1H4	grey	VSMC_M10	0.06	7.57E-01	0.25	2.14E-01	0.21	3.01E-01	-0.05	8.18E-01	-0.10	6.03E-01	0.28	1.51E-01	-0.43	2.55E-02	-0.03	8.89E-01	-0.22	2.70E-01	-0.06	7.82E-01
ENSCAFG0000001977	ENSCAFG0000001977	grey	VSMC_M10	0.06	7.57E-01	0.23	2.39E-01	0.33	8.79E-02	-0.01	9.65E-01	-0.41	3.47E-02	0.11	5.80E-01	-0.03	8.89E-01	-0.10	6.37E-01	-0.21	2.93E-01	0.35	7.24E-02
ENSCAFG0000001091	NR2F2	grey	VSMC_M10	0.06	7.57E-01	0.79	1.14E-06	0.09	6.53E-01	0.46	1.68E-02	-0.14	4.74E-01	-0.09	6.72E-01	-0.56	2.30E-03	-0.69	7.86E-05	-0.64	2.96E-04	0.63	4.41E-04
ENSCAFG0000001885	HS6ST2	grey	VSMC_M10	0.06	7.57E-01	0.38	5.15E-02	0.13	5.03E-01	0.04	8.52E-01	-0.20	3.10E-01	0.24	2.35E-01	0.02	9.05E-01	-0.14	4.96E-01	-0.36	6.29E-02	0.35	7.61E-02
ENSCAFG0000001138	SHROOM2	grey	VSMC_M10	0.06	7.58E-01	0.51	6.10E-03	0.14	4.79E-01	0.32	1.01E-01	-0.21	2.98E-01	0.04	8.27E-01	-0.85	1.80E-08	-0.44	2.13E-02	-0.37	5.58E-02	0.40	3.82E-02
ENSCAFG0000000481	DMAP1	yellow	VSMC_M3	0.06	7.58E-01	-0.05	8.10E-01	0.61	7.67E-04	-0.53	4.69E-03	-0.44	2.07E-02	-0.02	9.38E-01	-0.06	7.49E-01	0.37	5.76E-02	0.09	6.54E-01	-0.36	6.50E-02
ENSCAFG0000000408	ERMARD	grey	VSMC_M10	0.06	7.58E-01	0.25	2.12E-01	-0.14	4.78E-01	0.49	1.03E-02	0.21	2.90E-01	-0.13	5.18E-01	-0.11	6.00E-01	-0.58	1.61E-03	-0.17	3.84E-01	0.21	3.03E-01
ENSCAFG0000001282	KIAA1614	grey	VSMC_M10	0.06	7.58E-01	0.37	5.70E-02	0.40	4.07E-02	0.00	9.81E-01	-0.43	2.65E-02	-0.07	7.43E-01	0.07	7.13E-01	-0.09	6.45E-01	-0.42	2.89E-02	0.26	1.98E-01
ENSCAFG0000001500	HP1BP3	pink	VSMC_M5	0.06	7.58E-01	-0.49	9.15E-03	-0.60	8.46E-04	0.04	8.62E-01	0.74	9.76E-06	-0.36	6.56E-02	0.09	6.61E-01	0.19	3.36E-01	0.42	2.97E-02	-0.45	1.72E-02
ENSCAFG0000001750	DOCK6	cyan	VSMC_M2	0.06	7.58E-01	0.68	1.03E-04	0.10	6.16E-01	0.52	5.33E-03	-0.24	2.31E-01	0.26	1.97E-01	-0.08	6.76E-01	-0.66	1.58E-04	-0.58	1.52E-03	0.75	6.04E-06
ENSCAFG0000000196	SH3RF3	darkgreen	VSMC_M4	0.06	7.58E-01	0.49	8.75E-03	-0.22	2.65E-01	0.59	1.13E-03	0.03	8.64E-01	0.11	5.79E-01	-0.10	6.30E-01	-0.60	8.61E-04	-0.48	1.21E-02	0.73	1.49E-05
ENSCAFG0000000619	CPNE4	darkgreen	VSMC_M4	0.06	7.59E-01	0.36	6.41E-02	-0.35	7.51E-02	0.72	2.39E-05	0.18	3.58E-01	-0.02	9.28E-01	-0.27	1.75E-01	-0.63	3.91E-04	-0.36	6.71E-02	0.60	8.51E-04
ENSCAFG0000003061	GATAD1	grey	VSMC_M10	0.06	7.59E-01	0.10	6.19E-01	0.08	7.09E-01	-0.12	5.52E-01	-0.11	5.82E-01	0.09	6.67E-01	0.58	1.48E-03	0.07	7.27E-01	-0.23	2.49E-01	0.21	2.85E-01
ENSCAFG0000000928	CREB3L1	darkgrey	VSMC_M8	0.06	7.59E-01	-0.07	7.32E-01	0.46	1.52E-02	-0.60	8.58E-04	-0.44	2.17E-02	0.18	3.78E-01	-0.16	4.37E-01	0.59	1.33E-03	-0.07	7.18E-01	-0.23	2.49E-01
ENSCAFG0000000686	PAMR1	cyan	VSMC_M2	0.06	7.59E-01	0.62	5.05E-04	0.41	3.28E-02	-0.14	4.91E-01	-0.36	6.14E-02	-0.06	7.61E-01	-0.02	9.32E-01	-0.17	4.04E-01	-0.53	4.40E-03	0.26	1.85E-01
ENSCAFG0000001454	PLCB3	yellow	VSMC_M3	0.06	7.60E-01	0.62	5.47E-04	0.62	5.27E-04	0.00	9.99E-01	-0.72	2.64E-05	0.06	7.76E-01	0.14	4.88E-01	-0.26	1.99E-01	-0.60	8.42E-04	0.54	3.92E-03
ENSCAFG0000001745	GAS7	grey	VSMC_M10	0.06	7.60E-01	0.05	7.91E-01	0.33	8.98E-02	-0.50	8.52E-03	-0.27	1.78E-01	0.06	7.52E-01	0.69	7.19E-05	0.41	3.32E-02	-0.17	3.85E-01	-0.12	5.60E-01
ENSCAFG0000000042	ENSCAFG0000000042	grey	VSMC_M10	0.06	7.60E-01	-0.11	5.86E-01	0.10	6.13E-01	-0.20	3.28E-01	-0.06	7.69E-01	-0.01	9.60E-01	0.20	3.08E-01	0.25	2.11E-01	0.04	8.30E-01	-0.13	5.32E-01
ENSCAFG0000001398	COL13A1	grey	VSMC_M10	0.06	7.60E-01	0.15	4.57E-01	-0.18	3.81E-01	0.24	2.24E-01	0.04	8.57E-01	0.36	6.30E-02	-0.38	5.09E-02	-0.17	4.09E-01	-0.21	2.87E-01	0.31	1.18E-01
ENSCAFG0000000013	ENSCAFG0000000013	darkgrey	VSMC_M8	0.06	7.60E-01	-0.26	1.97E-01	0.54	3.39E-03	-0.70	4.74E-05	-0.46	1.48E-02	0.30	1.27E-01	0.41	3.23E-02	0.73	1.57E-05	0.09	6.40E-01	-0.42	2.77E-02
ENSCAFG0000003028	CDR2L	darkgrey	VSMC_M8	0.06	7.60E-01	-0.16	4.12E-01	0.57	1.74E-03	-0.85	2.64E-08	-0.41	3.48E-02	0.10	6.21E-01	0.12	5.42E-01	0.73	1.36E-05	0.11	5.68E-01	-0.49	9.99E-03
ENSCAFG0000000948	SEMA3G	grey	VSMC_M10	0.06	7.60E-01	0.44	2.02E-02	0.22	2.65E-01	-0.03	8.98E-01	-0.30	1.35E-01	-0.09	6.60E-01	0.02	9.21E-01	-0.18	3.56E-01	-0.43	2.54E-02	0.35	7.48E-02
ENSCAFG0000000559	ENSCAFG0000000559	grey	VSMC_M10	0.06	7.60E-01	-0.19	3.54E-01	-0.06	7.53E-01	-0.04	8.58E-01	0.10	6.36E-01	-0.15	4.44E-01	0.12	5.54E-01	0.09	6.73E-01	0.17	3.95E-01	-0.10	6.09E-01

ENSCAFG0000000784	GBE1	grey	VSMC_M10	0.06	7.61E-01	-0.44	2.15E-02	-0.10	6.07E-01	-0.33	9.31E-02	0.40	4.03E-02	-0.24	2.24E-01	0.12	5.52E-01	0.39	4.68E-02	0.44	2.05E-02	-0.72	2.75E-05
ENSCAFG0000001820	SLC6A7	grey	VSMC_M10	0.06	7.61E-01	0.36	6.81E-02	0.26	1.86E-01	-0.07	7.14E-01	-0.29	1.44E-01	-0.10	6.30E-01	0.25	2.07E-01	0.02	9.15E-01	-0.39	4.25E-02	0.29	1.43E-01
ENSCAFG0000002628	ENSCAFG0000002628	grey	VSMC_M10	0.06	7.61E-01	-0.26	1.86E-01	0.00	9.83E-01	-0.24	2.29E-01	0.00	9.88E-01	-0.04	8.39E-01	0.37	5.42E-02	0.33	8.98E-02	0.11	5.71E-01	-0.16	4.33E-01
ENSCAFG0000001931	BICDL2	grey	VSMC_M10	0.06	7.62E-01	0.36	6.19E-02	0.21	2.92E-01	-0.08	6.74E-01	-0.26	1.83E-01	-0.09	6.52E-01	0.14	4.77E-01	-0.13	5.10E-01	-0.37	6.01E-02	0.26	1.90E-01
ENSCAFG0000001026	ENSCAFG0000001026	grey	VSMC_M10	0.06	7.62E-01	0.12	5.42E-01	0.07	7.22E-01	-0.17	4.00E-01	-0.01	9.51E-01	-0.11	5.91E-01	0.10	6.35E-01	0.08	6.83E-01	-0.10	6.32E-01	0.05	8.08E-01
ENSCAFG0000000375	OSTM1	grey	VSMC_M10	0.06	7.62E-01	0.41	3.55E-02	-0.27	1.69E-01	0.55	3.16E-03	0.26	1.88E-01	-0.12	5.64E-01	-0.57	2.02E-03	-0.64	3.03E-04	-0.27	1.71E-01	0.39	4.43E-02
ENSCAFG0000003167	COL8A1	cyan	VSMC_M2	0.06	7.62E-01	0.66	1.68E-04	-0.13	5.29E-01	0.71	3.71E-05	0.08	6.96E-01	-0.22	2.78E-01	-0.66	1.66E-04	-0.84	3.89E-08	-0.45	1.83E-02	0.62	6.26E-04
ENSCAFG0000001117	DIS3L2	grey	VSMC_M10	0.06	7.63E-01	0.09	6.57E-01	0.21	3.03E-01	-0.27	1.78E-01	-0.21	3.04E-01	0.24	2.25E-01	0.08	7.09E-01	0.15	4.45E-01	-0.05	8.15E-01	0.06	7.68E-01
ENSCAFG0000002910	NOG	grey	VSMC_M10	0.06	7.63E-01	-0.15	4.58E-01	0.04	8.42E-01	-0.02	9.22E-01	-0.12	5.43E-01	0.07	7.11E-01	0.46	1.63E-02	0.14	4.96E-01	-0.02	9.29E-01	0.10	6.27E-01
ENSCAFG0000000045	VARS2	darkgrey	VSMC_M8	0.06	7.63E-01	-0.19	3.40E-01	0.58	1.70E-03	-0.66	1.85E-04	-0.48	1.07E-02	0.21	3.03E-01	0.37	5.40E-02	0.64	2.98E-04	0.07	7.34E-01	-0.35	7.23E-02
ENSCAFG0000000833	ENSCAFG0000000833	grey	VSMC_M10	0.06	7.63E-01	0.17	4.05E-01	-0.05	8.18E-01	0.38	4.96E-02	0.00	9.99E-01	-0.14	4.77E-01	-0.03	8.67E-01	-0.33	9.37E-02	-0.15	4.40E-01	0.35	7.22E-02
ENSCAFG0000000506	PAXIP1	grey	VSMC_M10	0.06	7.63E-01	-0.18	3.82E-01	-0.19	3.41E-01	-0.20	3.25E-01	0.19	3.41E-01	0.05	7.95E-01	0.01	9.74E-01	0.33	9.41E-02	0.06	7.53E-01	-0.18	3.57E-01
ENSCAFG0000000444	FOSB	darkgrey	VSMC_M8	0.06	7.64E-01	-0.33	9.40E-02	0.26	1.84E-01	-0.64	3.33E-04	-0.11	6.02E-01	0.13	5.16E-01	0.25	2.07E-01	0.55	2.81E-03	0.24	2.37E-01	-0.52	5.10E-03
ENSCAFG0000000072	ACAT2	yellow	VSMC_M3	0.06	7.64E-01	-0.21	2.90E-01	0.45	1.92E-02	-0.77	2.68E-06	-0.24	2.24E-01	0.02	9.20E-01	0.59	1.27E-03	0.64	3.32E-04	0.13	5.27E-01	-0.52	5.91E-03
ENSCAFG0000001574	WASHC1	yellow	VSMC_M3	0.06	7.64E-01	0.47	1.30E-02	0.71	3.07E-05	-0.54	3.84E-03	-0.69	6.14E-05	0.02	9.03E-01	0.21	3.01E-01	0.24	2.22E-01	-0.46	1.58E-02	0.21	3.04E-01
ENSCAFG0000001748	ENSCAFG0000001748	grey	VSMC_M10	0.06	7.64E-01	0.32	9.83E-02	-0.13	5.03E-01	0.32	9.91E-02	0.00	9.95E-01	0.19	3.41E-01	-0.20	3.28E-01	-0.34	8.29E-02	-0.29	1.47E-01	0.51	6.48E-03
ENSCAFG0000000641	LZTS3	cyan	VSMC_M2	0.06	7.64E-01	0.49	9.13E-03	0.33	9.69E-02	0.25	2.06E-01	-0.45	1.72E-02	0.03	8.90E-01	-0.23	2.57E-01	-0.43	2.71E-02	-0.39	4.70E-02	0.58	1.43E-03
ENSCAFG0000000223	CHST10	grey	VSMC_M10	0.06	7.64E-01	0.27	1.73E-01	-0.22	2.75E-01	0.46	1.52E-02	0.17	3.98E-01	-0.15	4.55E-01	-0.12	5.64E-01	-0.57	1.92E-03	-0.14	4.91E-01	0.43	2.40E-02
ENSCAFG0000000026	TNFAIP3	grey	VSMC_M10	0.06	7.65E-01	0.43	2.40E-02	-0.14	4.71E-01	0.51	7.01E-03	-0.05	8.19E-01	0.39	4.38E-02	-0.34	8.23E-02	-0.49	9.25E-03	-0.45	1.91E-02	0.61	7.12E-04
ENSCAFG0000000900	DECR1	cyan	VSMC_M2	0.06	7.65E-01	0.58	1.57E-03	0.01	9.43E-01	0.45	1.99E-02	-0.12	5.59E-01	-0.14	4.93E-01	-0.10	6.16E-01	-0.62	5.74E-04	-0.47	1.26E-02	0.67	1.38E-04
ENSCAFG0000001428	GNB2	yellow	VSMC_M3	0.06	7.65E-01	0.06	7.66E-01	0.71	3.65E-05	-0.68	9.12E-05	-0.61	8.17E-04	0.17	3.94E-01	-0.01	9.62E-01	0.54	4.02E-03	-0.06	7.73E-01	-0.28	1.63E-01
ENSCAFG0000000515	MFSD11	yellow	VSMC_M3	0.06	7.65E-01	0.28	1.64E-01	0.34	8.38E-02	-0.43	2.46E-02	-0.23	2.44E-01	-0.14	4.79E-01	0.04	8.34E-01	0.17	3.93E-01	-0.22	2.64E-01	-0.01	9.49E-01
ENSCAFG0000000062	DENND6B	grey	VSMC_M10	0.06	7.65E-01	0.50	7.70E-03	0.21	2.83E-01	0.16	4.16E-01	-0.37	5.77E-02	0.23	2.41E-01	0.13	5.07E-01	-0.31	1.13E-01	-0.52	5.52E-03	0.64	3.09E-04
ENSCAFG0000001953	MUM1	grey	VSMC_M10	0.06	7.66E-01	0.63	3.77E-04	0.16	4.29E-01	0.43	2.63E-02	-0.25	2.05E-01	-0.02	9.21E-01	0.00	9.89E-01	-0.52	5.96E-03	-0.61	6.95E-04	0.64	3.04E-04
ENSCAFG0000000235	CHMP2A	grey	VSMC_M10	0.06	7.66E-01	0.36	6.41E-02	0.28	1.59E-01	-0.02	9.16E-01	-0.19	3.37E-01	-0.30	1.24E-01	0.04	8.37E-01	-0.19	3.34E-01	-0.26	1.93E-01	0.07	7.39E-01
ENSCAFG0000000172	C11H9orf72	pink	VSMC_M5	0.06	7.66E-01	-0.24	2.24E-01	-0.60	1.04E-03	0.24	2.31E-01	0.67	1.30E-04	-0.21	2.95E-01	0.07	7.15E-01	-0.12	5.56E-01	0.20	3.27E-01	-0.14	4.75E-01
ENSCAFG0000001588	ZMYND15	grey	VSMC_M10	0.06	7.66E-01	0.12	5.48E-01	-0.33	9.17E-02	0.42	3.13E-02	0.22	2.70E-01	0.24	2.28E-01	-0.26	1.89E-01	-0.33	9.41E-02	-0.11	5.73E-01	0.33	9.06E-02
ENSCAFG0000001590	FTH1	grey	VSMC_M10	0.06	7.67E-01	-0.25	2.16E-01	0.08	7.07E-01	-0.04	8.62E-01	-0.01	9.69E-01	0.13	5.10E-01	0.10	6.19E-01	0.17	3.83E-01	0.14	5.02E-01	-0.30	1.34E-01
ENSCAFG0000001099	PRKAR1A	grey	VSMC_M10	0.06	7.67E-01	-0.17	4.02E-01	0.30	1.23E-01	-0.32	1.07E-01	-0.23	2.42E-01	0.33	9.20E-02	-0.09	6.67E-01	0.34	8.64E-02	0.10	6.29E-01	-0.31	1.14E-01
ENSCAFG0000001309	UCKL1	cyan	VSMC_M2	0.06	7.67E-01	0.84	4.20E-08	0.65	2.48E-04	-0.03	8.68E-01	-0.66	1.87E-04	0.08	6.86E-01	-0.11	5.92E-01	-0.34	8.28E-02	-0.71	3.50E-05	0.54	3.41E-03
ENSCAFG0000000730	DEPDC7	grey	VSMC_M10	0.06	7.67E-01	0.17	3.98E-01	-0.34	7.94E-02	0.57	1.71E-03	0.23	2.59E-01	-0.29	1.44E-01	-0.21	2.94E-01	-0.50	7.42E-03	-0.17	3.94E-01	0.46	1.52E-02
ENSCAFG0000003127	ENSCAFG0000003127	grey	VSMC_M10	0.06	7.67E-01	-0.10	6.12E-01	0.02	9.36E-01	-0.19	3.46E-01	0.02	9.06E-01	0.06	7.76E-01	0.29	1.37E-01	0.23	2.57E-01	-0.01	9.74E-01	-0.07	7.27E-01
ENSCAFG0000001283	CNIH2	grey	VSMC_M10	0.06	7.67E-01	-0.32	9.93E-02	-0.03	8.72E-01	-0.29	1.42E-01	0.04	8.59E-01	0.27	1.75E-01	0.27	1.72E-01	0.40	4.00E-02	0.18	3.64E-01	-0.20	3.12E-01
ENSCAFG0000001313	SMARCC1	darkgrey	VSMC_M8	0.06	7.67E-01	-0.50	7.67E-03	0.26	1.95E-01	-0.78	1.82E-06	-0.07	7.41E-01	0.12	5.47E-01	0.18	3.78E-01	0.83	7.80E-08	0.38	5.00E-02	-0.74	9.85E-06
ENSCAFG0000002917	ENSCAFG0000002917	grey	VSMC_M10	0.06	7.69E-01	-0.28	1.64E-01	0.09	6.65E-01	-0.32	1.02E-01	-0.03	8.91E-01	-0.16	4.29E-01	0.19	3.42E-01	0.46	1.68E-02	0.10	6.37E-01	-0.32	1.09E-01
ENSCAFG0000001748	LCMT1	grey	VSMC_M10	0.06	7.69E-01	-0.39	4.29E-02	-0.07	7.19E-01	-0.42	2.77E-02	0.30	1.26E-01	-0.10	6.14E-01	0.27	1.82E-01	0.43	2.46E-02	0.38	4.82E-02	-0.65	2.25E-04
ENSCAFG0000001151	LRIT3	grey	VSMC_M10	0.06	7.69E-01	0.02	9.05E-01	0.08	6.74E-01	-0.14	4.89E-01	-0.08	6.80E-01	-0.11	5.86E-01	-0.16	4.22E-01	0.14	4.93E-01	-0.03	8.88E-01	-0.04	8.47E-01
ENSCAFG0000000133	ISCA1	grey	VSMC_M10	0.06	7.69E-01	0.24	2.27E-01	-0.15	4.46E-01	0.52	5.98E-03	0.15	4.52E-01	0.19	3.51E-01	-0.36	6.24E-02	-0.49	8.99E-03	-0.20	3.10E-01	0.21	2.95E-01
ENSCAFG0000000413	CWC15	darkolivegreen	VSMC_M9	0.06	7.70E-01	-0.72	2.32E-05	-0.38	5.08E-02	-0.32	1.02E-01	0.57	1.90E-03	0.02	9.33E-01	0.12	5.55E-01	0.58	1.49E-03	0.59	1.16E-03	-0.79	1.24E-06
ENSCAFG0000000743	LRP3	yellow	VSMC_M3	0.06	7.70E-01	0.69	6.29E-05	0.57	1.93E-03	0.19	3.52E-01	-0.65	2.64E-04	0.12	5.66E-01	-0.30	1.29E-01	-0.39	4.18E-02	-0.61	7.73E-04	0.56	2.65E-03
ENSCAFG0000001704	FOS	grey	VSMC_M10	0.06	7.70E-01	0.35	7.47E-02	0.19	3.32E-01	0.01	9.56E-01	-0.13	5.03E-01	-0.04	8.57E-01	0.21	2.99E-01	-0.25	2.11E-01	-0.32	1.01E-01	0.22	2.60E-01
ENSCAFG0000000070	EIF3E	grey	VSMC_M10	0.06	7.70E-01	0.38	4.77E-02	0.03	8.91E-01	0.25	2.09E-01	-0.01	9.74E-01	-0.36	6.17E-02	-0.17	3.88E-01	-0.37	5.51E-02	-0.23	2.39E-01	0.30	1.30E-01
ENSCAFG0000003033	SERPINB9	grey	VSMC_M10	0.06	7.70E-01	0.73	1.64E-05	0.06	7.59E-01	0.51	6.28E-03	-0.19	3.50E-01	0.12	5.42E-01	-0.30	1.34E-01	-0.70	4.10E-05	-0.59	1.33E-03	0.76	5.19E-06
ENSCAFG0000003116	GID4	grey	VSMC_M10	0.06	7.70E-01	-0.25	2.09E-01	-0.33	9.55E-02	0.14	4.92E-01	0.28	1.52E-01	0.16	4.32E-01	0.37	5.79E-02	-0.06	7.78E-01	0.17	3.87E-01	0.01	9.64E-01
ENSCAFG0000001795	LMAN1L	grey	VSMC_M10	0.06	7.71E-01	0.16	4.32E-01	-0.24	2.29E-01	0.37	5.64E-02	0.17	4.09E-01	-0.01	9.48E-01	-0.12	5.49E-01	-0.32	1.09E-01	-0.15	4.63E-01	0.27	1.67E-01
ENSCAFG0000002404	ENSCAFG0000002404	grey	VSMC_M10	0.06	7.71E-01	0.01	9.73E-01	-0.25	2.16E-01	0.17	4.00E-01	0.16	4.31E-01	0.40	3.97E-02	-0.03	8.74E-01	-0.05	7.92E-01	-0.09	6.70E-01	0.20	3.20E-01
ENSCAFG0000000797	TERF1	darkgrey	VSMC_M8	0.06	7.71E-01	-0.40	3.84E-02	0.04	8.36E-01	-0.50	7.43E-03	-0.03	8.68E-01	0.16	4.22E-01	0.56	2.29E-03	0.64	3.14E-04	0.18	3.72E-01	-0.30	1.35E-01
ENSCAFG0000000229	MITD1	grey	VSMC_M10	0.06	7.71E-01	0.32	1.06E-01	-0.12	5.51E-01	0.40	3.72E-02	-0.01	9.60E-01	-0.30	1.27E-01	0.09	6.60E-01	-0.42	2.89E-02	-0.33	9.31E-02	0.59	1.29E-03
ENSCAFG0000000760	FLNB	grey	VSMC_M10	0.06	7.72E-01	-0.22	2.64E-01	0.39	4.52E-02	-0.66	1.94E-04	-0.29	1.45E-01	0.28	1.59E-01	-0.06	7.56E-01	0.54	3.85E-03	0.18	3.64E-01	-0.40	4.09E-02
ENSCAFG0000000074	PNLDC1	grey	VSMC_M10	0.06	7.72E-01	-0.24	2.21E-01	-0.05	8.16E-01	-0.27	1.71E-01	0.12	5.68E-01	-0.08	7.06E-01	0.14	4.97E-01	0.29	1.46E-01	0.23	2.50E-01	-0.19	3.38E-01
ENSCAFG0000000216	NFIL3	grey	VSMC_M10	0.06	7.72E-01	0.08	7.06E-01	-0.06	7.48E-01	0.07	7.28E-01	0.08	6.93E-01	-0.09	6.52E-01	0.13	5.33E-01	-0.11	5.88E-01	-0.18	3.66E-01	0.15	4.66E-01
ENSCAFG0000000163	GUCA1B	grey	VSMC_M10	0.06	7.73E-01	-0.21	2.90E-01	-0.17	4.10E-01	0.00	9.93E-01	0.20	3.20E-01	-0.08	7.08E-01	0.10	6.25E-01	0.10	6.04E-01	0.13	5.12E-01	-0.11	5.74E-01
ENSCAFG0000000552	P4HA3	yellow	VSMC_M3	0.06	7.74E-01	0.15	4.64E-01	0.57	1.75E-03	-0.72	2.11E-05	-0.45	1.88E-02	-0.06	7.77E-01	0.15	4.49E-01	0.52	4.96E-03	-0.18	3.65E-01	-0.21	2.98E-01
ENSCAFG0000000978	ADTRP	grey	VSMC_M10	0.06	7.74E-01	0.10	6.19E-01	0.11	5.82E-01	-0.13	5.05E-01	-0.11	5.76E-01	-0.09	6.71E-01	-0.12	5.60E-01	0.05	7.89E-01	-0.12	5.59E-01	0.06	7.72E-01
ENSCAFG0000000426	THNSL1	darkgreen	VSMC_M4	0.06	7.74E-01	0.42	2.85E-02	-0.20	3.17E-01	0.66	1.85E-04	0.05	7.91E-01	-0.18	3.63E-01	0.19	3.40E-01	-0.69	7.43E-05	-0.40	3.82E-02	0.70	5.09E-05
ENSCAFG0000001841	RHBDL3	grey	VSMC_M10	0.06	7.74E-01	0.12	5.51E-01	0.08	6.88E-01	-0.03	8.85E-01	-0.10	6.14E-01	0.01	9.64E-01	0.34	8.45E-02	0.00	9.84E-01	-0.17	4.00E-01	0.15	4.44E-01
ENSCAFG0000000619	SPEF1	grey	VSMC_M10	0.06	7.75E-01	-0.02	9.13E-01	0.16	4.29E-01	-0.06	7.56E-01	-0.16	4.31E-01	0.16	4.31E-01	-0.06	7.85E-01	0.06	7.83E-01	0.04	8.58E-01	0.04	8.52E-01
ENSCAFG0000000066	EZR	darkgrey	VSMC_M8	0.06	7.75E-01	-0.65	2.75E-04	0.29	1.45E-01	-0.79	7.55E-07	-0.09	6.61E-01	0.03	8.97E-01	0.32	1.08E-01	0.91	3.66E-11	0.49	9.24E-03	-0.82	1.64E-07
ENSCAFG0000000467	HECTD3	yellow	VSMC_M3	0.06	7.75E-01	0.16	4.24E-01	0.65	2.44E-04	-0.51	7.03E-03	-0.63	4.91E-04	0.11	5.87E-01	-0.06	7.70E-01	0.34	8.36E-02	-0.14	4.72E-01	0.01	9.59E-01
ENSCAFG0000001005	SYT8	grey	VSMC_M10	0.06	7.76E-01	0.30	1.23E-01	-0.19	3.49E-01	0.36	6.65E-02	0.16	4.33E-01	-0.06	7.80E-01	-0.11	5.69E-01	-0.45	2.00E-02	-0.24	2.31E-01	0.35	7.56E-02
ENSCAFG0000001744	IL4R	grey	VSMC_M10	0.06	7.76E-01	-0.35	7.75E-02	0.16	4.26E-01	-0.31	1.11E-01	-0.09	6.58E-01	0.16	4.15E-01	0.59	1.18E-03	0.41	3.22E-02	0.18	3.78E-01	-0.33	9.16E-02
ENSCAFG0000000736	PXK	grey	VSMC_M10	0.06	7.76E-01	0.14	4.87E-01	-0.08	6.87E-01	-0.10	6.18E-01	0.15	4.56E-01	-0.05	8.16E-01	0.33	9.17E-02	0.03	9.01E-01	-0.20	3.12E-01	0.04	8.52E-01
ENSCAFG0000001531	ENSCAFG0000001531	grey	VSMC_M10	0.06	7.76E-01	0.49	9.81E-03	0.35	7.77E-02	-0.02	9.26E-01	-0.45	1.83E-02	0.12	5.47E-01	0.07	7.25E-01	-0.17	4.01E-01	-0.45	1.72E-02	0.47	1.23E-02
ENSCAFG0000002002	LRRC8A	grey	VSMC_M10	0.06	7.76E-01	0.01	9.58E-01	-0.20	3.19E-01	0.25	2.13E-01	0.08	6.95E-01	0.35	7.44E-02	-0.12	5.49E-01	-0.24	2.33E-01	-0.06	7.76E-01	0.29	1.46E-01
ENSCAFG0000002871	ENSCAFG0000002871	grey	VSMC_M10	0.06	7.77E-01	0.22	2.78E-01	0.20	3.17E-01	-0.12	5.43E-01	-0.16	4.11E-01	-0.06	7.69E-01	0.39	4.26E-02	0.03	8.91E-01	-0.21	2.91E-01	0.12	5.62E-01
ENSCAFG0000000350	NOBOX	grey	VSMC_M10	0.06	7.77E-01	0.22	2.77E-01	0.20	3.17E-01	-0.12	5.43E-01	-0.16	4.11E-01	-0.06	7.69E-01	0.39	4.26E-02	0.03	8.91E-01	-0.21	2.91E-01	0.12	5.62E-01
ENSCAFG0000001210	SFXN4	darkgrey	VSMC_M8	0.06	7.77E-01	-0.28	1.61E-01	0.52	5.70E-03	-0.77	2.94E-06	-0.35	7.54E-02	0.23	2.48E-01	0.43	2.56E-02	0.74	9.37E-06	0.17	4.10E-01	-0.54	3.43E-03
ENSCAFG0000003110	ENSCAFG0000003110	grey	VSMC_M10	0.06	7.77E-01	-0.47	1.28E-02	-0.25	2.05E-01	-0.16	4.17E-01	0.30	1.22E-01	-0.07	7.11E-01	0.33	8.87E-02	0.31	1.17E-01	0.29	1.48E-01	-0.29	1.47E-01
ENSCAFG0000000925	ENSCAFG0000000925	grey	VSMC_M10	0.06	7.77E-01	0.29	1.36E-01	0.27	1.79E-01	0.06	7.57E-01	-0.22	2.79E-01	0.12	5.60E-01	-0.30	1.24E-01	-0.15	4.41E-01	-0.26	1.96E-01	0.10	6.28E-01
ENSCAFG0000002990	ENSCAFG0000002990	grey	VSMC_M10	0.06	7.77E-01	-0.05	8.15E-01	-0.13	5.08E-01	0.10	6.33E-01	0.09	6.38E-01	-0.07	7.40E-01	-0.12	5.38E-01	-0.01	9.69E-01	0.02	9.30E-01	0.07	7.35E-01
ENSCAFG0000000586	IK	grey	VSMC_M10	0.06	7.77E-01	-0.14	4.97E-01	0.17	4.05E-01	-0.27	1.66E-01	-0.05	8.17E-01	-0.15	4.46E-01	-0.15	4.45E-01	0.33	9.33E-02	0.11	5.88E-01	-0.38	4.86E-02
ENSCAFG0000001234	FASTKD1	grey	VSMC_M10	0.06	7.77E-01	-0.21	3.05E-01	-0.52	5.58E-03	0.15	4.65E-01	0.44	2.04E-02	0.03	8.63E-01	0.13	5.10E-01	0.01	9.59E-01	0.09	6.63E-01	0.10	6.17E-01
ENSCAFG0000000928	SLC38A1	grey	VSMC_M10	0.06	7.77E-01	-0.32	1.08E-01	-0.16	4.14E-01	-0.36	6.60E-02	0.17	3.95E-01	0.33	9.32E-02	0.44	2.12E-02	0.43	2.35E-02	0.11	6.01E-01	-0.21	2.98E-01
ENSCAFG0000000955	ENSCAFG0000000955	grey	VSMC_M10	0.06	7.77E-01	0.19	3.48E-01	0.11	5.83E-01	-0.11	5.84E-01	-0.10	6.36E-01	0.07	7.41E-01	0.02	9.06E-01	-0.06	7.70E-01	-0.17	4.00E-01	0.13	5.16E-01
ENSCAFG0000002272	MT-CO1	pink	VSMC_M5	0.06	7.78E-01	0.19	3.36E-01	-0.54	3.60E-03	0.50	8.65E-03	0.53	4.87E-03	-0.21	2.83E-01	-0.19	3.44E-01	-0.50	8.22E-03	-0.13	5.04E-01	0.26	1.88E-01
ENSCAFG0000002886	GPX8	grey	VSMC_M10	0.06	7.78E-01	-0.42	3.11E-02	-0.08	6.90E-01	-0.17	4.08E-01	0.22	2.73E-01	-0.04	8.37E-01	0.28	1.58E-01	0.32	9.96E-02	0.25	2.16E-01	-0.47	1.34E-02
ENSCAFG0000000017	ENSCAFG0000000017	grey	VSMC_M10	0.06	7.78E-01	0.24	2.20E-01	0.34	8.08E-02	-0.18	3.70E-01	-0.44	2.27E-02	0.10	6.07E-01	0.08	6.97E-01	0.10	6.06E-01	-0.33	8.95E-02	0.34	8.62E-02
ENSCAFG0000003014	ENSCAFG0000003014	grey	VSMC_M10	0.06	7.78E-01	0.09	6.44E-01	0.33	9.51E-02	-0.17	3.87E-01	-0.44	2.12E-02	0.19	3.49E-01	0.47	1.34E-02	0.10	6.06E-01	-0.24	2.25E-01	0.29	1.46E-01
ENSCAFG0000003036	ENSCAFG0000003036	grey	VSMC_M10	0.06	7.78E-01	0.16	4.17E-01	-0.18	3.71E-01	0.17	3.93E-01	0.24	2.23E-01	-0.13	5.15E-01	-0.12	5.43E-01	-0.18	3.69E-01	-0.14	4.81E-01	0.12	5.51E-01
ENSCAFG0000000068	HDAC10	yellow	VSMC_M3	0.06	7.78E-01	0.63	3.96E-04	0.73	1.42E-05	-0.16	4.33E-01	-0.74	9.75E-06	0.13	5.12E-01	-0.05	8.08E-01	-0.17	3.98E-01	-0.56	2.63E-03	0.41	3.22E-02
ENSCAFG0000000380	SCLT1	pink	VSMC_M5	0.06	7.78E-01	-0.52	5.39E-03	-0.28	1.62E-01	-0.34	8.26E-02	0.41	3.55E-02	-0.15	4.64E-01	0.35	7.11E-02	0.52	5.92E-03	0.36	6.62E-02	-0.54	4.01E-03
ENSCAFG0000000831	HPS3	grey	VSMC_M10	0.06	7.78E-01	0.53	4.26E-03	0.06	7.75E-01	0.28	1.64E-01	-0.07	7.27E-01	-0.13	5.05E-01	0.12	5.63E-01	-0.46	1.50E-02	-0.45	1.89E-02	0.52	5.45E-03
ENSCAFG0000000193	VEGFA	pink	VSMC_M5	0.06	7.78E-01	-0.32	9.88E-02	-0.43	2.46E-02	0.24	2.23E-01	0.58	1.45E-03	-0.20	3.07E-01	0.01	9.72E-01	-0.20	3.15E-01	0.34	8.51E-02	-0.24	2.19E-01
ENSCAFG0000003156	ZPR1	darkolivegreen	VSMC_M9	0.06	7.79E-01	-0.70	4.44E-05	-0.23	2.43E-01	-0.35	7.06E-02	0.38	4.80E-02	-0.10	6.24E-01	0.19	3.50E-01	0.59	1.32E-03	0.57	1.89E-03	-0.72	2.08E-05
ENSCAFG0000001839	CDCA4	darkgrey	VSMC_M8	0.06	7.79E-01	-0.37	5.99E-02	0.43	2.60E-02	-0.81	3.45E-07	-0.27	1.68E-01	0.06	7.55E-01	0.36	6.66E-02	0.84	3.10E-08	0.20	3.13E-01	-0.61	7.04E-04
ENSCAFG0000001695	KCNIP1	grey	VSMC_M10	0.06	7.79E-01	0.23	2.47E-01	0.17	4.09E-01	-0.26	1.96E-01	-0.02	9.39E-01	-0.19	3.41E-01	0.21	2.98E-01	0.08	6.83E-01	-0.23	2.41E-01	-0.08	6.97E-01
ENSCAFG0000001933	PKMYT1	darkgrey	VSMC_M8	0.06	7.79E-01	-0.45	1.96E-02	0.43	2.55E-02	-0.91	4.11E-11	-0.27	1.73E-01	0.11	5.84E-01	0.38	4.75E-02	0.96	2.70E-15	0.27	1.77E-01	-0.67	1.46E-04
ENSCAFG0000002748	ENSCAFG0000002748	grey	VSMC_M10	0.06	7.79E-01	-0.13	5.16E-01	-0.11	5.81E-01	0.06	7.61E-01	0.07	7.35E-01	0.27	1.70E-01	0.23	2.42E-01	0.00	9.82E-01	0.11	6.01E-01	-0.01	9.52E-01
ENSCAFG0000001303	DHX30	grey	VSMC_M10	0.06	7.80E-01	-0.20	3.25E-01	0.07	7.35E-01	-0.09	6.51E-01	-0.15	4.41E-01	0.45	1.79E-02	0.04	8.62E-01	0.25	2.02E-01	0.01	9.70E-01	-0.04	8.55E-01
ENSCAFG0000001368	PIKFYVE	grey	VSMC_M10	0.06	7.80E-01	-0.19	3.50E-01	-0.12	5.59E-01	0.03	8.81E-01	0.07	7.17E-01	0.03	8.64E-01	0.36	6.28E-02	0.00	9.86E-01	0.13	5.24E-01	0.12	5.56E-01
ENSCAFG0000000121	HSD17B3	grey	VSMC_M10	0.06	7.80E-01	-0.15	4.50E-01	-0.08	6.92E-01	-0.04	8.57E-01	0.14	4.86E-01	-0.06	7.78E-01	0.23	2.49E-01	0.01	9.62E-01	0.13	5.19E-01	-0.05	7.98E-01
ENSCAFG0000002414	HEXIM1	cyan	VSMC_M2	0.06	7.80E-01	0.43	2.35E-02	0.35	7.24E-02	-0.07	7.11E-01	-0.16	4.19E-01	-0.27	1.68E-01	-0.14	4.95E-01	-0.14	4.72E-01	-0.34	7.95E-02	-0.01	9.61E-01
ENSCAFG0000001656	GALNT16	grey	VSMC_M10	0.06	7.80E-01	0.15	4.48E-01	0.38	4.86E-02	-0.32	9.98E-02	-0.21	2.99E-01	-0.22	2.76E-01	0.36	6.62E-02	0.16	4.20E-01	-0.13	5.07E-01	-0.17	3.91E-01
ENSCAFG0000000898	HERPUD1	darkgreen	VSMC_M4	0.06	7.80E-01	0.10	6.30E-01	-0.44	2.17E-02	0.54	3.56E-03	0.36	6.60E-02	-0.09	6.51E-01	0.29	1.47E-01	-0.51	7.02E-03	-0.16	4.28E-01	0.42	3.02E-02
ENSCAFG0000002462	TMEM252	grey	VSMC_M10	0.06	7.80E-01	0.42	3.08E-02	0.18	3.82E-01	0.34	8.55E-02	-0.26	1.89E-01	0.14	4.71E-01	0.06	7.77E-01	-0.43	2.62E-02	-0.41	3.36E-02	0.47	1.41E-02
ENSCAFG0000002977	ETFRF1	grey	VSMC_M10	0.06	7.81E-01	0.11	5.80E-01	0.07	7.17E-01	0.19	3.37E-01	0.04	8.51E-01	-0.28	1.62E-01	-0.05	8.17E-01	-0.20	3.17E-01	-0.03	8.87E-01	-0.08	6.93E-01
ENSCAFG0000002954	ENSCAFG0000002954	grey	VSMC_M10	0.06	7.81E-01	-0.10	6.15E-01	0.06	7.69E-01	-0.21	2.92E-01	0.00	9.91E-01	-0.03	8.63E-01	0.07	7.39E-01	0.22	2.65E-01	0.03	8.66E-01	-0.13	5.18E-01
ENSCAFG0000001740	GTF3C1	darkgreen	VSMC_M4	0.06	7.81E-01	0.40	3.67E-02	-0.20	3.18E-01	0.72	2.33E-05	0.12	5.53E-01	-0.06	7.53E-01	-0.38	5.23E-02	-0.72	1.92E-05	-0.35	7.35E-02	0.52	5.33E-03
ENSCAFG0000001344	KLRA1	grey	VSMC_M10	0.06	7.81E-01	0.15	4.61E-01	0.21	2.86E-01	-0.16	4.28E-01	-0.24	2.30E-01	0.20	3.26E-01	-0.20	3.07E-01	0.17	4.11E-01	-0.18	3.64E-01	0.04	8.41E-01
ENSCAFG0000003104	PLAGL2	grey	VSMC_M10	0.06	7.81E-01	0.02	9.16E-01	-0.07	7.21E-01	0.25	2.00E-01	-0.04	8.61E-01	0.25	2.17E-01	-0.03	8.68E-01	-0.23	2.47E-01	-0.06	7.82E-01	0.23	2.42E-01
ENSCAFG0000000250	ENSCAFG0000000250	grey	VSMC_M10	0.06	7.81E-01	-0.06	7.56E-01	-0.01	9.65E-01	-0.17	3.83E-01	0.10	6.22E-01	-0.10	6.19E-01	0.07	7.22E-01	0.11	5.76E-01	0.09	6.66E-01	-0.19	3.39E-01
ENSCAFG0000001728	ENSCAFG0000001728	grey	VSMC_M10	0.06	7.82E-01	0.39	4.67E-02	-0.10	6.06E-01	0.32	1.05E-01	-0.01	9.76E-01	0.06	7.49E-01	0.08	6.78E-01	-0.39	4.52E-02	-0.36	6.23E-02	0.42	2.95E-02
ENSCAFG0000003235	UBE2D1	grey	VSMC_M10	0.06	7.82E-01	0.01	9.53E-01	-0.05	8.06E-01	-0.02	9.05E-01	0.11	5.74E-01	0.02	9.11E-01	0.23	2.46E-01	-0.10	6.21E-01	-0.02	9.12E-01	0.01	9.60E-01
ENSCAFG0000000459	ENSCAFG0000000459	grey	VSMC_M10	0.06	7.82E-01	-0.24	2.25E-01	-0.02	9.14E-01	-0.23	2.51E-01	0.06	7.78E-01	-0.13	5.21E-01	0.49	9.89E-03	0.22	2.80E-01	0.17	3.87E-01	-0.16	4.32E-01
ENSCAFG0000001256	SH3BGRL3	grey	VSMC_M10	0.06	7.82E-01	0.40	3.86E-02	0.54	3.53E-03	-0.05	8.19E-01	-0.59	1.22E-03	-0.08	6.99E-01	-0.03	8.75E-01	-0.09	6.41E-01	-0.37	5.60E-02	0.40	4.02E-02
ENSCAFG0000001902	LDOC1	yellow	VSMC_M3	0.06	7.83E-01	0.49	1.03E-02	0.41	3.56E-02	0.18	3.73E-01	-0.47	1.25E-02	0.14	4.82E-01	-0.22	2.71E-01	-0.37	5.88E-02	-0.42	2.93E-02	0.48	1.21E-02
ENSCAFG0000000663	JAKMIP2	grey	VSMC_M10	0.06	7.83E-01	-0.49	8.87E-03	-0.29	1.36E-01	-0.13	5.07E-01	0.43	2.68E-02	-0.13	5.05E-01	0.04	8.59E-01	0.26	1.89E-01	0.44	2.24E-02	-0.47	1.33E-02
ENSCAFG0000000120	KIF20A	darkgrey	VSMC_M8	0.06	7.83E-01	-0.38	4.74E-02	0.47	1.44E-02	-0.94	6.26E-13	-0.31	1.19E-01	0.11	5.97E-01	0.43	2.44E-02	0.94	1.77E-13	0.22	2.73E-01	-0.61	6.65E-04
ENSCAFG0000001038	APBA2	yellow	VSMC_M3	0.06	7.83E-01	0.35	7.50E-02	0.45	1.72E-02	-0.28	1.60E-01	-0.38	5.27E-02	0.10	6.13E-01	0.21	3.00E-01	0.06	7.57E-01	-0.33	9.18E-02	0.10	6.35E-01
ENSCAFG0000000064	ENSCAFG0000000064	cyan	VSMC_M2	0.06	7.83E-01	0.38	5.29E-02	0.43	2.48E-02	0.05	8.15E-01	-0.48	1.06E-02	0.19	3.45E-01	0.04	8.47E-01	-0.20	3.19E-01	-0.37	5.72E-02	0.38	5.32E-02
ENSCAFG0000000905	SLC26A7	darkgreen	VSMC_M4	0.06	7.83E-01	0.18	3.80E-01	-0.57	2.11E-03	0.87	2.88E-09	0.43	2.60E-02	-0.04	8.50E-01	-0.16	4.12E-01	-0.71	3.64E-05	-0.16	4.26E-01	0.50	8.29E-03
ENSCAFG0000002541	ENSCAFG0000002541	grey	VSMC_M10	0.06	7.83E-01	0.04	8.37E-01	-0.27	1.71E-01	0.27	1.80E-01	0.33	8.96E-02	-0.13	5.32E-01	-0.16	4.19E-01	-0.25	2.12E-01	0.00	9.86E-01	-0.03	8.99E-01
ENSCAFG0000000359	HACE1	grey	VSMC_M10	0.06	7.83E-01	-0.22	2.77E-01	-0.59	1.08E-03	0.29	1.47E-01	0.60	1.02E-03	-0.17	3.90E-01	-0.18	3.80E-01	-0.19	3.48E-01	0.23	2.46E-01	0.03	8.69E-01
ENSCAFG0000003078	LAD1	grey	VSMC_M10	0.06	7.83E-01	0.16	4.39E-01	0.02	9.11E-01	0.14	4.73E-01	-0.04	8.37E-01	-0.06	7.55E-01	-0.22	2.78E-01	-0.12	5.48E-01	-0.15	4.61E-01	0.13	5.24E-01
ENSCAFG0000000507	HTR5A	turquoise	VSMC_M6	0.06	7.84E-01	-0.23	2.55E-01	-0.07	7.46E-01	-0.14	4.79E-01	0.07	7.36E-01	0.45	1.76E-02	-0.01	9.59E-01	0.22	2.64E-01	0.12	5.59E-01	-0.14	4.98E-01
ENSCAFG0000000981	IGSF3	turquoise	VSMC_M6	0.06	7.84E-01	-0.35	7.05E-02	0.02	9.27E-01	-0.34	8.24E-02	-0.06	7.81E-01	0.57	1.94E-03	0.15	4.53E-01	0.47	1.45E-02	0.20	3.13E-01	-0.22	2.63E-01

ENSCAFG0000000110	LAMA2	darkgreen	VSMC_M4	0.06	7.84E-01	0.06	7.50E-01	-0.42	3.00E-02	0.52	5.35E-03	0.43	2.41E-02	-0.33	9.20E-02	0.24	2.28E-01	-0.51	7.02E-03	-0.07	7.36E-01	0.27	1.73E-01
ENSCAFG0000002427	C31H21orf62	grey	VSMC_M10	0.06	7.84E-01	0.19	3.30E-01	-0.21	2.86E-01	0.34	8.29E-02	0.10	6.26E-01	0.17	3.91E-01	-0.36	6.48E-02	-0.37	5.68E-02	-0.19	3.43E-01	0.37	5.93E-02
ENSCAFG0000001209	CIB1	grey	VSMC_M10	0.06	7.85E-01	0.03	8.67E-01	0.52	4.98E-03	-0.40	3.67E-02	-0.32	1.04E-01	-0.22	2.68E-01	-0.04	8.42E-01	0.28	1.63E-01	0.05	8.19E-01	-0.37	5.55E-02
ENSCAFG0000001165	ATP9A	darkgreen	VSMC_M4	0.06	7.85E-01	0.61	6.77E-04	-0.20	3.20E-01	0.78	1.25E-06	0.04	8.55E-01	0.11	5.93E-01	-0.35	7.26E-02	-0.86	1.22E-08	-0.51	6.51E-03	0.76	4.08E-06
ENSCAFG0000000992	ENSCAFG0000000992	grey	VSMC_M10	0.06	7.85E-01	0.15	4.51E-01	0.17	3.90E-01	-0.15	4.63E-01	-0.15	4.45E-01	-0.13	5.30E-01	-0.02	9.24E-01	0.09	6.60E-01	-0.14	4.89E-01	0.05	7.88E-01
ENSCAFG0000000397	ENSCAFG0000000397	grey	VSMC_M10	0.06	7.85E-01	-0.19	3.46E-01	-0.02	9.24E-01	-0.17	3.88E-01	0.07	7.11E-01	-0.10	6.16E-01	0.09	6.64E-01	0.27	1.78E-01	0.10	6.29E-01	-0.17	3.90E-01
ENSCAFG0000000270	FILIP1	darkgreen	VSMC_M4	0.06	7.85E-01	0.58	1.39E-03	-0.32	1.09E-01	0.82	1.64E-07	0.13	5.11E-01	-0.01	9.48E-01	-0.53	4.70E-03	-0.82	2.04E-07	-0.50	7.89E-03	0.74	1.15E-05
ENSCAFG0000003047	ENSCAFG0000003047	grey	VSMC_M10	0.06	7.85E-01	-0.10	6.35E-01	-0.25	2.03E-01	0.23	2.49E-01	0.27	1.81E-01	-0.09	6.51E-01	0.26	1.94E-01	-0.23	2.53E-01	0.13	5.05E-01	0.09	6.70E-01
ENSCAFG0000000420	VSTM5	grey	VSMC_M10	0.06	7.85E-01	0.05	8.00E-01	0.04	8.35E-01	-0.16	4.20E-01	0.06	7.56E-01	-0.14	4.87E-01	0.28	1.56E-01	0.06	7.68E-01	-0.08	7.02E-01	-0.16	4.35E-01
ENSCAFG0000001808	SPECC1	darkgreen	VSMC_M4	0.05	7.85E-01	0.52	5.94E-03	-0.38	4.91E-02	0.81	2.92E-07	0.30	1.25E-01	-0.20	3.28E-01	-0.46	1.48E-02	-0.90	2.00E-10	-0.38	4.93E-02	0.62	5.71E-04
ENSCAFG0000001463	RPL27	grey	VSMC_M10	0.05	7.86E-01	0.39	4.17E-02	0.39	4.71E-02	0.13	5.15E-01	-0.52	5.69E-03	0.00	9.93E-01	0.06	7.74E-01	-0.18	3.61E-01	-0.41	3.48E-02	0.39	4.33E-02
ENSCAFG0000000495	TRIM47	darkgrey	VSMC_M8	0.05	7.86E-01	-0.16	4.29E-01	0.42	3.02E-02	-0.77	2.95E-06	-0.27	1.71E-01	0.10	6.36E-01	0.52	5.83E-03	0.67	1.28E-04	0.09	6.60E-01	-0.43	2.50E-02
ENSCAFG0000000743	ENSCAFG0000000743	darkolivegreen	VSMC_M9	0.05	7.86E-01	-0.45	1.97E-02	-0.23	2.53E-01	-0.12	5.56E-01	0.40	3.84E-02	-0.24	2.24E-01	0.17	3.99E-01	0.14	4.94E-01	0.46	1.58E-02	-0.43	2.42E-02
ENSCAFG0000001986	APRT	yellow	VSMC_M3	0.05	7.86E-01	0.23	2.50E-01	0.71	3.82E-05	-0.36	6.21E-02	-0.79	1.16E-06	0.20	3.06E-01	0.28	1.65E-01	0.29	1.39E-01	-0.33	8.77E-02	0.21	2.91E-01
ENSCAFG0000001880	TTC23L	grey	VSMC_M10	0.05	7.86E-01	0.30	1.34E-01	0.04	8.35E-01	-0.05	8.22E-01	-0.14	4.72E-01	0.00	9.97E-01	-0.07	7.27E-01	-0.05	7.90E-01	-0.31	1.14E-01	0.33	9.01E-02
ENSCAFG0000001385	TLNRD1	grey	VSMC_M10	0.05	7.86E-01	0.21	2.96E-01	0.57	1.98E-03	-0.21	3.03E-01	-0.62	6.12E-04	0.34	8.03E-02	-0.18	3.64E-01	0.12	5.47E-01	-0.25	2.03E-01	0.13	5.08E-01
ENSCAFG0000000553	GTPBP4	darkgrey	VSMC_M8	0.05	7.86E-01	-0.27	1.66E-01	0.56	2.17E-03	-0.84	3.99E-08	-0.47	1.44E-02	0.13	5.34E-01	0.31	1.20E-01	0.87	5.84E-09	0.10	6.08E-01	-0.50	8.02E-03
ENSCAFG0000000864	TKT	yellow	VSMC_M3	0.05	7.87E-01	0.16	4.33E-01	0.53	4.73E-03	-0.51	6.51E-03	-0.31	1.15E-01	-0.19	3.38E-01	-0.36	6.82E-02	0.28	1.64E-01	-0.03	8.92E-01	-0.36	6.81E-02
ENSCAFG0000000975	VANGL1	grey	VSMC_M10	0.05	7.87E-01	0.10	6.11E-01	0.02	9.29E-01	-0.01	9.54E-01	-0.16	4.20E-01	0.24	2.21E-01	0.05	8.13E-01	0.07	7.11E-01	-0.21	2.96E-01	0.30	1.27E-01
ENSCAFG0000000772	GGCX	cyan	VSMC_M2	0.05	7.87E-01	0.58	1.62E-03	0.44	2.01E-02	-0.02	9.27E-01	-0.50	7.46E-03	-0.01	9.80E-01	0.19	3.53E-01	-0.17	3.87E-01	-0.59	1.10E-03	0.47	1.40E-02
ENSCAFG0000001295	ENSCAFG0000001295	grey	VSMC_M10	0.05	7.88E-01	0.05	8.19E-01	-0.11	5.84E-01	0.16	4.13E-01	0.04	8.40E-01	-0.02	9.32E-01	0.46	1.58E-02	-0.11	5.96E-01	-0.12	5.55E-01	0.20	3.26E-01
ENSCAFG0000001284	PASK	grey	VSMC_M10	0.05	7.88E-01	-0.02	9.10E-01	0.19	3.30E-01	-0.26	1.91E-01	-0.23	2.55E-01	-0.03	8.93E-01	0.42	2.89E-02	0.35	7.58E-02	-0.19	3.30E-01	0.05	8.22E-01
ENSCAFG0000002495	ENSCAFG0000002495	turquoise	VSMC_M6	0.05	7.88E-01	-0.19	3.45E-01	-0.04	8.47E-01	-0.16	4.25E-01	0.11	5.89E-01	0.13	5.17E-01	0.20	3.24E-01	0.18	3.56E-01	0.12	5.60E-01	-0.13	5.08E-01
ENSCAFG0000001905	ENSCAFG0000001905	darkgreen	VSMC_M4	0.05	7.88E-01	-0.01	9.61E-01	-0.43	2.65E-02	0.54	3.54E-03	0.36	6.47E-02	-0.06	7.63E-01	-0.18	3.77E-01	-0.40	3.75E-02	0.01	9.53E-01	0.21	2.85E-01
ENSCAFG0000001273	NIT1	cyan	VSMC_M2	0.05	7.88E-01	0.81	2.82E-07	0.26	1.88E-01	0.33	9.23E-02	-0.31	1.12E-01	-0.16	4.20E-01	-0.45	1.93E-02	-0.59	1.30E-03	-0.64	3.69E-04	0.60	1.02E-03
ENSCAFG0000000584	LTBP1	pink	VSMC_M5	0.05	7.88E-01	-0.32	1.05E-01	-0.35	7.21E-02	0.18	3.73E-01	0.52	5.41E-03	-0.16	4.14E-01	-0.51	6.45E-03	-0.07	7.18E-01	0.43	2.42E-02	-0.41	3.37E-02
ENSCAFG0000001190	QRICH1	grey	VSMC_M10	0.05	7.88E-01	0.16	4.33E-01	-0.34	8.14E-02	0.33	9.81E-02	0.22	2.76E-01	0.09	6.71E-01	-0.25	2.15E-01	-0.33	9.30E-02	-0.14	4.76E-01	0.36	6.33E-02
ENSCAFG0000001247	PLEKHA5	pink	VSMC_M5	0.05	7.89E-01	-0.26	1.83E-01	-0.32	9.86E-02	-0.17	3.85E-01	0.48	1.15E-02	-0.06	7.61E-01	0.27	1.81E-01	0.21	2.90E-01	0.20	3.08E-01	-0.31	1.15E-01
ENSCAFG0000000861	PDGFC	darkgreen	VSMC_M4	0.05	7.89E-01	0.56	2.53E-03	-0.25	2.14E-01	0.79	8.38E-07	0.17	3.90E-01	-0.32	1.06E-01	-0.22	2.65E-01	-0.87	5.03E-09	-0.43	2.56E-02	0.66	1.59E-04
ENSCAFG0000001117	FSTL1	pink	VSMC_M5	0.05	7.89E-01	0.04	8.42E-01	-0.53	4.52E-03	0.49	1.01E-02	0.60	1.03E-03	-0.24	2.37E-01	-0.04	8.25E-01	-0.51	6.97E-03	0.09	6.63E-01	0.11	5.90E-01
ENSCAFG0000000108	MYC	grey	VSMC_M10	0.05	7.89E-01	-0.33	9.72E-02	0.28	1.63E-01	-0.51	6.41E-03	-0.11	5.70E-01	-0.02	9.32E-01	0.39	4.53E-02	0.48	1.10E-02	0.20	3.28E-01	-0.41	3.35E-02
ENSCAFG0000001578	B3GAT3	cyan	VSMC_M2	0.05	7.90E-01	0.54	3.34E-03	0.56	2.50E-03	-0.04	8.53E-01	-0.64	3.67E-04	0.07	7.46E-01	-0.25	2.00E-01	-0.18	3.81E-01	-0.47	1.25E-02	0.42	2.76E-02
ENSCAFG0000000107	TRIB1	grey	VSMC_M10	0.05	7.90E-01	0.24	2.20E-01	0.47	1.35E-02	-0.50	8.50E-03	-0.40	3.81E-02	0.03	8.93E-01	0.21	2.99E-01	0.29	1.38E-01	-0.31	1.12E-01	0.02	9.32E-01
ENSCAFG0000001707	TRMT1	yellow	VSMC_M3	0.05	7.90E-01	0.30	1.35E-01	0.71	3.24E-05	-0.37	5.98E-02	-0.69	6.47E-05	0.07	7.11E-01	0.16	4.27E-01	0.24	2.35E-01	-0.37	5.69E-02	0.14	4.73E-01
ENSCAFG0000001642	TNFRSF8	grey	VSMC_M10	0.05	7.90E-01	0.06	7.72E-01	-0.20	3.17E-01	0.33	9.26E-02	0.19	3.31E-01	-0.09	6.60E-01	-0.24	2.21E-01	-0.30	1.33E-01	-0.01	9.76E-01	0.16	4.19E-01
ENSCAFG0000001805	ODF3L1	grey	VSMC_M10	0.05	7.90E-01	0.32	1.03E-01	0.17	3.92E-01	-0.06	7.68E-01	-0.21	2.84E-01	-0.08	7.03E-01	0.20	3.08E-01	-0.16	4.24E-01	-0.31	1.21E-01	0.24	2.24E-01
ENSCAFG0000002273	ND5	pink	VSMC_M5	0.05	7.91E-01	-0.13	5.23E-01	-0.69	7.20E-05	0.37	5.77E-02	0.74	1.24E-05	-0.26	1.84E-01	-0.03	8.85E-01	-0.32	1.08E-01	0.16	4.35E-01	0.01	9.74E-01
ENSCAFG0000000146	ENSCAFG0000000146	grey	VSMC_M10	0.05	7.91E-01	0.18	3.78E-01	-0.15	4.64E-01	0.19	3.31E-01	0.14	4.92E-01	0.19	3.32E-01	-0.07	7.31E-01	-0.13	5.26E-01	-0.22	2.60E-01	0.15	4.50E-01
ENSCAFG0000000487	WDR86	darkgreen	VSMC_M4	0.05	7.91E-01	0.50	7.54E-03	-0.36	6.51E-02	0.87	3.36E-09	0.20	3.23E-01	-0.08	7.04E-01	-0.28	1.50E-01	-0.81	2.27E-07	-0.43	2.60E-02	0.70	4.71E-05
ENSCAFG0000001223	ENSCAFG0000001223	grey	VSMC_M10	0.05	7.91E-01	0.17	4.03E-01	0.28	1.57E-01	0.03	8.81E-01	-0.38	5.25E-02	-0.10	6.29E-01	0.10	6.14E-01	-0.02	9.06E-01	-0.24	2.30E-01	0.24	2.21E-01
ENSCAFG0000002359	ENSCAFG0000002359	darkgreen	VSMC_M4	0.05	7.91E-01	0.45	1.82E-02	-0.27	1.70E-01	0.65	2.57E-04	0.27	1.81E-01	-0.12	5.46E-01	-0.35	7.47E-02	-0.75	7.50E-06	-0.28	1.52E-01	0.40	4.09E-02
ENSCAFG0000001876	DUS3L	yellow	VSMC_M3	0.05	7.91E-01	0.07	7.20E-01	0.68	8.12E-05	-0.58	1.69E-03	-0.55	2.71E-03	0.01	9.71E-01	-0.20	3.18E-01	0.44	2.21E-02	-0.10	6.32E-01	-0.31	1.19E-01
ENSCAFG0000001075	CRCP	grey	VSMC_M10	0.05	7.92E-01	-0.47	1.41E-02	-0.01	9.48E-01	-0.22	2.62E-01	0.05	8.15E-01	-0.05	8.18E-01	0.39	4.37E-02	0.39	4.45E-02	0.26	1.94E-01	-0.33	8.81E-02
ENSCAFG0000000560	SPTLC3	grey	VSMC_M10	0.05	7.92E-01	0.54	3.38E-03	-0.09	6.60E-01	0.37	5.68E-02	0.01	9.57E-01	0.00	9.93E-01	-0.01	9.74E-01	-0.45	1.92E-02	-0.49	9.27E-03	0.53	4.65E-03
ENSCAFG0000001599	DCP1B	grey	VSMC_M10	0.05	7.92E-01	0.44	2.26E-02	-0.24	2.34E-01	0.56	2.33E-03	0.06	7.56E-01	-0.14	4.86E-01	-0.08	6.82E-01	-0.59	1.09E-03	-0.41	3.44E-02	0.70	4.61E-05
ENSCAFG0000000881	RND1	grey	VSMC_M10	0.05	7.92E-01	0.48	1.15E-02	0.14	4.91E-01	0.40	4.01E-02	-0.25	2.18E-01	0.02	9.12E-01	0.19	3.44E-01	-0.48	1.05E-02	-0.46	1.69E-02	0.63	4.86E-04
ENSCAFG0000001751	NPR1	turquoise	VSMC_M6	0.05	7.92E-01	0.26	1.89E-01	0.37	5.81E-02	-0.19	3.41E-01	-0.45	1.99E-02	0.73	1.54E-05	0.01	9.60E-01	0.13	5.31E-01	-0.29	1.41E-01	0.22	2.61E-01
ENSCAFG0000002975	ENSCAFG0000002975	grey	VSMC_M10	0.05	7.93E-01	-0.41	3.48E-02	-0.22	2.73E-01	0.22	2.63E-01	0.27	1.65E-01	-0.01	9.45E-01	-0.11	6.01E-01	-0.03	8.87E-01	0.40	4.05E-02	-0.28	1.50E-01
ENSCAFG0000001883	TM2D1	darkgreen	VSMC_M4	0.05	7.93E-01	0.30	1.29E-01	-0.32	1.02E-01	0.71	3.16E-05	0.18	3.62E-01	0.03	8.68E-01	-0.40	3.96E-02	-0.66	1.80E-04	-0.21	2.94E-01	0.50	8.19E-03
ENSCAFG0000000941	HNF4A	darkgreen	VSMC_M4	0.05	7.93E-01	0.14	4.95E-01	-0.55	3.12E-03	0.82	1.41E-07	0.46	1.71E-02	-0.10	6.27E-01	-0.19	3.37E-01	-0.68	8.43E-05	-0.09	6.39E-01	0.41	3.52E-02
ENSCAFG0000000029	ENSCAFG0000000029	grey	VSMC_M10	0.05	7.93E-01	-0.28	1.60E-01	-0.06	7.58E-01	-0.27	1.69E-01	0.19	3.44E-01	-0.05	7.99E-01	0.35	7.57E-02	0.32	1.05E-01	0.18	3.58E-01	-0.39	4.63E-02
ENSCAFG0000001236	ENSCAFG0000001236	pink	VSMC_M5	0.05	7.94E-01	-0.12	5.52E-01	-0.58	1.49E-03	0.37	5.83E-02	0.54	3.72E-03	0.03	8.98E-01	-0.16	4.15E-01	-0.25	2.09E-01	0.07	7.17E-01	0.01	9.51E-01
ENSCAFG0000001806	RCOR1	grey	VSMC_M10	0.05	7.94E-01	-0.28	1.51E-01	-0.46	1.48E-02	0.25	2.08E-01	0.54	3.40E-03	-0.05	8.03E-01	-0.17	3.98E-01	-0.08	6.81E-01	0.26	1.93E-01	-0.22	2.64E-01
ENSCAFG0000001734	KCNK10	darkgreen	VSMC_M4	0.05	7.95E-01	0.20	3.29E-01	-0.41	3.46E-02	0.71	3.16E-05	0.28	1.51E-01	-0.21	3.02E-01	-0.23	2.48E-01	-0.54	3.41E-03	-0.22	2.66E-01	0.46	1.47E-02
ENSCAFG0000001194	DTX3L	grey	VSMC_M10	0.05	7.96E-01	0.66	1.69E-04	0.21	3.00E-01	0.39	4.70E-02	-0.32	1.01E-01	-0.12	5.36E-01	-0.12	5.53E-01	-0.55	2.66E-03	-0.59	1.08E-03	0.73	1.65E-05
ENSCAFG0000001885	CEP76	darkgrey	VSMC_M8	0.05	7.96E-01	-0.65	2.40E-04	-0.24	2.36E-01	-0.44	2.04E-02	0.39	4.28E-02	0.21	3.02E-01	0.23	2.46E-01	0.63	4.74E-04	0.49	9.63E-03	-0.70	5.18E-05
ENSCAFG0000003259	MRPL50	grey	VSMC_M10	0.05	7.96E-01	-0.17	3.99E-01	-0.17	3.92E-01	-0.14	4.75E-01	0.10	6.24E-01	0.26	1.91E-01	0.23	2.44E-01	0.28	1.50E-01	-0.01	9.66E-01	-0.06	7.56E-01
ENSCAFG0000002990	ENSCAFG0000002990	grey	VSMC_M10	0.05	7.97E-01	0.11	5.83E-01	0.35	7.20E-02	-0.36	6.91E-02	-0.40	3.95E-02	0.13	5.07E-01	0.24	2.22E-01	0.31	1.16E-01	-0.22	2.72E-01	-0.01	9.75E-01
ENSCAFG0000000785	HMGB2	darkgrey	VSMC_M8	0.05	7.97E-01	-0.51	6.57E-03	0.26	1.97E-01	-0.73	1.65E-05	-0.16	4.32E-01	0.10	6.04E-01	0.56	2.17E-03	0.85	2.38E-08	0.28	1.53E-01	-0.53	4.41E-03
ENSCAFG0000001749	ENSCAFG0000001749	grey	VSMC_M10	0.05	7.97E-01	0.21	3.00E-01	0.24	2.35E-01	-0.19	3.38E-01	-0.26	1.83E-01	0.03	8.78E-01	0.32	9.93E-02	0.11	5.81E-01	-0.25	2.05E-01	0.13	5.28E-01
ENSCAFG0000001345	HOXD8	grey	VSMC_M10	0.05	7.97E-01	-0.27	1.70E-01	0.25	2.00E-01	-0.37	6.03E-02	-0.16	4.11E-01	0.11	5.99E-01	0.54	3.95E-03	0.32	9.92E-02	0.20	3.06E-01	-0.27	1.81E-01
ENSCAFG0000001604	TNS4	violet	VSMC_M7	0.05	7.98E-01	0.02	9.15E-01	0.19	3.47E-01	-0.20	3.26E-01	-0.19	3.38E-01	0.02	9.36E-01	0.55	3.24E-03	0.17	4.01E-01	-0.10	6.16E-01	0.02	9.29E-01
ENSCAFG0000000697	ABTB2	grey	VSMC_M10	0.05	7.98E-01	0.69	7.18E-05	0.16	4.35E-01	0.44	2.17E-02	-0.32	1.07E-01	-0.13	5.12E-01	-0.20	3.14E-01	-0.58	1.59E-03	-0.60	9.32E-04	0.80	6.90E-07
ENSCAFG0000001062	SORCS3	grey	VSMC_M10	0.05	7.98E-01	0.34	8.59E-02	0.14	4.75E-01	-0.04	8.55E-01	-0.17	3.99E-01	-0.04	8.61E-01	-0.11	5.99E-01	-0.08	7.08E-01	-0.30	1.23E-01	0.20	3.27E-01
ENSCAFG0000000252	P3H1	cyan	VSMC_M2	0.05	7.99E-01	0.57	1.80E-03	0.60	9.07E-04	-0.15	4.67E-01	-0.73	1.51E-05	0.17	3.97E-01	-0.19	3.52E-01	-0.06	7.81E-01	-0.57	2.09E-03	0.51	6.54E-03
ENSCAFG0000000897	LRP4	cyan	VSMC_M2	0.05	7.99E-01	0.51	6.94E-03	0.51	5.98E-03	-0.27	1.76E-01	-0.49	9.47E-03	0.08	6.89E-01	-0.06	7.52E-01	0.02	9.22E-01	-0.44	2.05E-02	0.26	1.82E-01
ENSCAFG0000000414	ENSCAFG0000000414	grey	VSMC_M10	0.05	7.99E-01	0.06	7.80E-01	-0.41	3.41E-02	0.48	1.19E-02	0.36	6.46E-02	0.15	4.62E-01	-0.25	2.12E-01	-0.37	5.74E-02	0.01	9.77E-01	0.19	3.54E-01
ENSCAFG0000001310	SART1	yellow	VSMC_M3	0.05	7.99E-01	-0.04	8.26E-01	0.76	4.22E-06	-0.67	1.42E-04	-0.65	2.31E-04	-0.02	9.20E-01	0.21	3.01E-01	0.57	1.82E-03	-0.03	8.75E-01	-0.29	1.44E-01
ENSCAFG0000001271	ARHGAP30	grey	VSMC_M10	0.05	8.00E-01	-0.14	4.91E-01	-0.02	9.02E-01	-0.07	7.36E-01	0.07	7.40E-01	-0.07	7.15E-01	0.05	7.97E-01	0.03	8.87E-01	0.20	3.10E-01	-0.11	5.98E-01
ENSCAFG0000000133	SIL1	grey	VSMC_M10	0.05	8.00E-01	0.27	1.70E-01	-0.10	6.37E-01	0.54	3.59E-03	0.01	9.45E-01	-0.27	1.79E-01	-0.29	1.45E-01	-0.50	8.29E-03	-0.22	2.73E-01	0.40	4.04E-02
ENSCAFG0000001101	HEPACAM	grey	VSMC_M10	0.05	8.01E-01	0.16	4.26E-01	-0.01	9.53E-01	0.08	6.84E-01	0.01	9.76E-01	-0.05	8.12E-01	-0.15	4.66E-01	-0.11	5.82E-01	-0.09	6.60E-01	0.13	5.13E-01
ENSCAFG0000000774	RIOK2	darkolivegreen	VSMC_M9	0.05	8.01E-01	-0.66	1.88E-04	-0.24	2.23E-01	-0.33	9.59E-02	0.47	1.28E-02	-0.26	1.84E-01	-0.02	9.07E-01	0.50	8.38E-03	0.62	6.31E-04	-0.81	2.88E-07
ENSCAFG0000001611	ZNF280D	darkgreen	VSMC_M4	0.05	8.02E-01	0.06	7.56E-01	-0.60	8.57E-04	0.65	2.30E-04	0.47	1.43E-02	-0.07	7.23E-01	-0.13	5.26E-01	-0.54	3.79E-03	-0.05	7.98E-01	0.49	8.98E-03
ENSCAFG0000000460	SERP2	yellow	VSMC_M3	0.05	8.02E-01	0.21	2.86E-01	0.62	5.54E-04	-0.29	1.37E-01	-0.58	1.57E-03	0.10	6.23E-01	0.37	6.05E-02	0.14	4.91E-01	-0.25	2.17E-01	0.09	6.66E-01
ENSCAFG0000003012	ZNF572	darkgreen	VSMC_M4	0.05	8.02E-01	0.16	4.25E-01	-0.33	8.87E-02	0.62	4.95E-04	0.18	3.80E-01	0.06	7.80E-01	0.17	3.90E-01	-0.54	4.02E-03	-0.21	2.99E-01	0.58	1.49E-03
ENSCAFG0000000990	MYLIP	grey	VSMC_M10	0.05	8.03E-01	0.41	3.40E-02	0.01	9.67E-01	0.14	4.98E-01	-0.09	6.54E-01	0.34	8.17E-02	0.09	6.47E-01	-0.23	2.53E-01	-0.40	3.86E-02	0.45	1.82E-02
ENSCAFG0000000878	MSH3	darkgrey	VSMC_M8	0.05	8.03E-01	-0.61	7.36E-04	-0.05	8.09E-01	-0.62	6.14E-04	0.25	2.02E-01	-0.10	6.32E-01	0.22	2.77E-01	0.69	7.90E-05	0.49	9.72E-03	-0.76	3.43E-06
ENSCAFG0000001285	MMP21	grey	VSMC_M10	0.05	8.03E-01	-0.05	8.00E-01	0.03	8.97E-01	-0.15	4.51E-01	-0.06	7.62E-01	-0.12	5.49E-01	-0.11	5.71E-01	0.21	2.94E-01	-0.04	8.56E-01	-0.04	8.62E-01
ENSCAFG0000001645	ADGRL1	darkgreen	VSMC_M4	0.05	8.03E-01	0.48	1.04E-02	-0.33	9.11E-02	0.86	8.43E-09	0.27	1.81E-01	-0.15	4.54E-01	-0.35	7.55E-02	-0.91	7.70E-11	-0.31	1.10E-01	0.59	1.09E-03
ENSCAFG0000001398	CCDC103	grey	VSMC_M10	0.05	8.03E-01	-0.06	7.76E-01	-0.08	6.90E-01	0.05	7.97E-01	-0.03	8.76E-01	0.06	7.77E-01	0.17	3.89E-01	0.05	8.09E-01	-0.03	8.79E-01	0.17	3.88E-01
ENSCAFG0000003146	ICAM1	darkgreen	VSMC_M4	0.05	8.04E-01	0.27	1.75E-01	-0.19	3.48E-01	0.53	4.14E-03	0.09	6.65E-01	0.03	8.77E-01	-0.69	6.90E-05	-0.54	3.46E-03	-0.17	3.87E-01	0.36	6.22E-02
ENSCAFG0000000610	ENSCAFG0000000610	yellow	VSMC_M3	0.05	8.04E-01	0.62	5.95E-04	0.55	2.86E-03	-0.03	8.68E-01	-0.64	3.10E-04	0.06	7.76E-01	-0.01	9.41E-01	-0.15	4.59E-01	-0.61	7.78E-04	0.45	1.99E-02
ENSCAFG0000001857	ENSCAFG0000001857	yellow	VSMC_M3	0.05	8.04E-01	-0.23	2.56E-01	0.48	1.19E-02	-0.78	1.28E-06	-0.27	1.67E-01	-0.02	9.35E-01	0.63	4.31E-04	0.67	1.44E-04	0.14	4.78E-01	-0.50	7.65E-03
ENSCAFG0000000536	ACKR2	grey	VSMC_M10	0.05	8.05E-01	0.20	3.08E-01	-0.10	6.32E-01	0.21	2.82E-01	-0.02	9.25E-01	0.17	3.95E-01	0.21	3.03E-01	-0.14	4.88E-01	-0.32	1.02E-01	0.40	3.68E-02
ENSCAFG0000000932	BPHL	grey	VSMC_M10	0.05	8.05E-01	0.47	1.39E-02	0.19	3.56E-01	-0.01	9.57E-01	-0.20	3.09E-01	-0.10	6.26E-01	-0.04	8.28E-01	-0.20	3.07E-01	-0.39	4.55E-02	0.36	6.41E-02
ENSCAFG0000001278	PTPRO	grey	VSMC_M10	0.05	8.05E-01	0.06	7.57E-01	0.03	8.86E-01	0.03	8.81E-01	0.04	8.44E-01	-0.07	7.18E-01	-0.14	4.75E-01	-0.09	6.38E-01	0.00	9.82E-01	0.02	9.27E-01
ENSCAFG0000003032	ENSCAFG0000003032	darkgrey	VSMC_M8	0.05	8.05E-01	-0.48	1.07E-02	0.27	1.65E-01	-0.82	2.10E-07	-0.13	5.18E-01	0.03	8.67E-01	0.42	2.80E-02	0.90	1.20E-10	0.31	1.16E-01	-0.65	2.46E-04
ENSCAFG0000001482	RGN	turquoise	VSMC_M6	0.05	8.05E-01	-0.05	8.18E-01	0.09	6.53E-01	-0.18	3.79E-01	-0.14	4.92E-01	0.46	1.48E-02	0.42	2.89E-02	0.21	2.88E-01	-0.06	7.52E-01	0.01	9.53E-01
ENSCAFG0000000403	EMP3	yellow	VSMC_M3	0.05	8.05E-01	0.31	1.11E-01	0.63	4.02E-04	-0.56	2.59E-03	-0.58	1.65E-03	-0.21	3.00E-01	0.43	2.38E-02	0.32	1.03E-01	-0.32	9.94E-02	0.04	8.59E-01
ENSCAFG0000002843	ENSCAFG0000002843	grey	VSMC_M10	0.05	8.05E-01	0.01	9.61E-01	-0.28	1.61E-01	0.35	7.44E-02	0.20	3.14E-01	-0.12	5.37E-01	-0.14	4.75E-01	-0.21	3.00E-01	0.01	9.42E-01	0.20	3.17E-01
ENSCAFG0000000369	TEAD2	grey	VSMC_M10	0.05	8.06E-01	0.59	1.25E-03	0.01	9.63E-01	0.47	1.45E-02	-0.07	7.12E-01	-0.13	5.14E-01	-0.47	1.34E-02	-0.65	2.62E-04	-0.40	3.99E-02	0.51	6.03E-03
ENSCAFG0000003043	ENSCAFG0000003043	grey	VSMC_M10	0.05	8.06E-01	0.12	5.53E-01	0.44	2.01E-02	-0.37	5.87E-02	-0.25	2.08E-01	-0.24	2.30E-01	0.07	7.41E-01	0.18	3.71E-01	-0.06	7.59E-01	-0.28	1.54E-01
ENSCAFG0000000712	HCK	grey	VSMC_M10	0.05	8.06E-01	-0.41	3.26E-02	-0.18	3.76E-01	-0.27	1.78E-01	0.36	6.30E-02	-0.07	7.19E-01	0.53	4.44E-03	0.31	1.17E-01	0.32	1.06E-01	-0.49	9.72E-03
ENSCAFG0000000299	ANKRD17	grey	VSMC_M10	0.05	8.06E-01	-0.47	1.44E-02	-0.67	1.50E-04	0.24	2.25E-01	0.62	5.27E-04	0.14	4.91E-01	-0.05	8.02E-01	0.03	8.86E-01	0.39	4.56E-02	-0.16	4.12E-01
ENSCAFG0000001219	BAG3	grey	VSMC_M10	0.05	8.06E-01	0.32	1.02E-01	-0.22	2.76E-01	0.47	1.37E-02	0.30	1.28E-01	-0.33	9.37E-02	-0.25	2.14E-01	-0.55	2.99E-03	-0.17	4.10E-01	0.19	3.45E-01
ENSCAFG0000000096	FSTL4	grey	VSMC_M10	0.05	8.06E-01	-0.02	9.33E-01	0.05	7.99E-01	-0.24	2.21E-01	-0.11	5.82E-01	0.12	5.50E-01	0.02	9.25E-01	0.23	2.42E-01	-0.01	9.58E-01	0.06	7.66E-01
ENSCAFG0000000281	ELP1	grey	VSMC_M10	0.05	8.06E-01	-0.56	2.51E-03	-0.13	5.07E-01	-0.28	1.54E-01	0.30	1.34E-01	-0.15	4.50E-01	0.35	7.31E-02	0.41	3.14E-02	0.40	3.89E-02	-0.53	4.83E-03
ENSCAFG0000000937	CRY2	grey	VSMC_M10	0.05	8.07E-01	0.13	5.07E-01	0.01	9.52E-01	-0.04	8.28E-01	0.14	4.71E-01	-0.37	5.89E-02	0.08	6.90E-01	-0.03	8.63E-01	-0.07	7.25E-01	-0.09	6.44E-01
ENSCAFG0000001130	GET4	grey	VSMC_M10	0.05	8.07E-01	-0.03	8.75E-01	-0.01	9.73E-01	-0.19	3.42E-01	-0.11	5.97E-01	0.18	3.80E-01	0.11	5.82E-01	0.23	2.57E-01	-0.09	6.61E-01	0.13	5.31E-01
ENSCAFG0000001187	MOSPD2	grey	VSMC_M10	0.05	8.07E-01	0.63	4.37E-04	0.08	6.97E-01	0.28	1.52E-01	-0.01	9.42E-01	-0.26	1.86E-01	-0.13	5.11E-01	-0.48	1.11E-02	-0.48	1.07E-02	0.36	6.83E-02
ENSCAFG0000000652	FRMD4B	grey	VSMC_M10	0.05	8.07E-01	0.60	8.44E-04	0.04	8.51E-01	0.42	2.90E-02	-0.22	2.62E-01	0.21	3.04E-01	-0.49	9.34E-03	-0.48	1.14E-02	-0.56	2.63E-03	0.64	3.42E-04
ENSCAFG0000001921	MFSD12	cyan	VSMC_M2	0.05	8.07E-01	0.45	1.93E-02	0.57	1.90E-03	-0.13	5.15E-01	-0.63	3.95E-04	0.31	1.17E-01	0.15	4.52E-01	-0.06	7.71E-01	-0.47	1.28E-02	0.35	7.22E-02
ENSCAFG0000003092	ENSCAFG0000003092	grey	VSMC_M10	0.05	8.07E-01	0.02	9.28E-01	0.12	5.60E-01	-0.09	6.62E-01	-0.09	6.59E-01	-0.11	5.93E-01	-0.21	2.97E-01	0.14	4.93E-01	-0.06	7.67E-01	-0.04	8.35E-01
ENSCAFG0000001926	TRAP1	yellow	VSMC_M3	0.05	8.07E-01	0.15	4.56E-01	0.62	5.01E-04	-0.53	4.23E-03	-0.66	1.63E-04	0.17	3.89E-01	0.35	7.26E-02	0.44	2.29E-02	-0.26	1.87E-01	0.08	7.04E-01
ENSCAFG0000001435	AGFG2	turquoise	VSMC_M6	0.05	8.08E-01	0.03	8.67E-01	0.35	7.24E-02	-0.44	2.31E-02	-0.38	4.88E-02	0.65	2.32E-04	0.33	8.82E-02	0.35	7.00E-02	-0.15	4.60E-01	-0.02	9.30E-01
ENSCAFG0000001699	SPDL1	darkgrey	VSMC_M8	0.05	8.08E-01	-0.64	3.28E-04	0.01	9.60E-01	-0.68	9.44E-05	0.13	5.07E-01	0.00	9.91E-01	0.40	3.92E-02	0.88	2.07E-09	0.42	2.79E-02	-0.74	1.25E-05
ENSCAFG0000000175	TRPM6	grey	VSMC_M10	0.05	8.08E-01	0.30	1.34E-01	0.51	6.28E-03	-0.35	7.24E-02	-0.54	3.50E-03	-0.13	5.11E-01	0.19	3.52E-01	0.22	2.76E-01	-0.35	7.16E-02	0.19	3.53E-01
ENSCAFG0000001884	SNX29	cyan	VSMC_M2	0.05	8.08E-01	0.51	6.26E-03	0.47	1.26E-02	-0.03	8.95E-01	-0.40	3.97E-02	-0.01	9.43E-01	-0.17	4.01E-01	-0.31	1.15E-01	-0.40	3.95E-02	0.25	2.17E-01
ENSCAFG0000000120	ENSCAFG0000000120	grey	VSMC_M10	0.05	8.08E-01	0.06	7.70E-01	0.01	9.41E-01	-0.06	7.65E-01	0.03	8.76E-01	-0.09	6.50E-01	-0.18	3.75E-01	0.07	7.20E-01	-0.11	5.69E-01	0.03	8.73E-01
ENSCAFG0000001047	SH3PXD2A	grey	VSMC_M10	0.05	8.08E-01	0.03	8.82E-01	0.02	9.37E-01	-0.04	8.47E-01	0.11	5.76E-01	-0.10	6.33E-01	-0.67	1.33E-04	-0.07	7.36E-01	0.13	5.27E-01	-0.20	3.22E-01
ENSCAFG0000003054	ENSCAFG0000003054	turquoise	VSMC_M6	0.05	8.09E-01	-0.10	6.24E-01	-0.11	5.72E-01	-0.02	9.31E-01	0.06	7.59E-01	0.46	1.62E-02	-0.10	6.16E-01	0.00	9.85E-01	0.02	9.22E-01	0.00	9.88E-01
ENSCAFG0000001480	ESS2	grey	VSMC_M10	0.05	8.09E-01	0.30	1.23E-01	0.13	5.20E-01	0.35	7.03E-02	-0.24	2.24E-01	0.06	7.50E-01	0.06	7.72E-01	-0.37	5.80E-02	-0.36	6.12E-02	0.51	6.15E-03
ENSCAFG0000001799	ENSCAFG0000001799	grey	VSMC_M10	0.05	8.09E-01	-0.37	5.48E-02	-0.44	2.26E-02	0.11	5.92E-01	0.51	6.81E-03	-0.03	8.87E-01	-0.07	7.19E-01	0.08	6.97E-01	0.32	1.02E-01	-0.23	2.58E-01
ENSCAFG0000003084	ENSCAFG0000003084	grey	VSMC_M10	0.05	8.10E-01	0.02	9.07E-01	0.17	3.98E-01	-0.45	1.73E-02	-0.07	7.40E-01	-0.19	3.52E-01	0.16	4.17E-01	0.33	9.52E-02	-0.02	9.31E-01	-0.21	2.95E-01
ENSCAFG0000001474	ENSCAFG0000001474	grey	VSMC_M10	0.05	8.10E-01	-0.08	6.81E-01	0.12	5.52E-01	-0.21	2.86E-01	-0.07	7.32E-01	0.10	6.25E-01	0.37	6.02E-02	0.23	2.39E-01	0.00	9.90E-01	-0.09	6.51E-01
ENSCAFG0000001418	SLC25A39	yellow	VSMC_M3	0.05	8.10E-01	0.41	3.43E-02	0.79	7.64E-07	-0.66	2.01E-04	-0.74	1.22E-05	0.18	3.56E-01	-0.01	9.41E-01	0.37	6.10E-02	-0.39	4.56E-02	-0.01	9.63E-01

ENSCAFG0000001697	MVP	yellow	VSMC_M3	0.05	8.11E-01	0.56	2.36E-03	0.73	1.53E-05	-0.20	3.25E-01	-0.69	7.14E-05	-0.04	8.29E-01	-0.12	5.56E-01	-0.05	8.11E-01	-0.45	1.88E-02	0.24	2.26E-01
ENSCAFG0000001807	ZSWIM7	darkgrey	VSMC_M8	0.05	8.11E-01	-0.35	7.49E-02	0.16	4.26E-01	-0.49	9.59E-03	-0.01	9.77E-01	0.19	3.45E-01	0.23	2.58E-01	0.56	2.52E-03	0.23	2.50E-01	-0.58	1.37E-03
ENSCAFG0000001299	PPIP5K1	darkgreen	VSMC_M4	0.05	8.11E-01	-0.05	8.03E-01	-0.60	9.78E-04	0.66	1.71E-04	0.60	9.23E-04	-0.19	3.32E-01	0.03	8.64E-01	-0.56	2.47E-03	0.11	5.85E-01	0.15	4.43E-01
ENSCAFG0000003165	ZSCAN22	grey	VSMC_M10	0.05	8.11E-01	0.31	1.16E-01	-0.14	4.92E-01	0.30	1.25E-01	0.09	6.70E-01	0.05	7.88E-01	0.00	9.88E-01	-0.41	3.58E-02	-0.28	1.54E-01	0.37	5.85E-02
ENSCAFG0000002952	LBX2	grey	VSMC_M10	0.05	8.11E-01	-0.02	9.38E-01	0.38	4.95E-02	-0.35	7.20E-02	-0.36	6.84E-02	0.18	3.60E-01	0.33	9.36E-02	0.30	1.31E-01	-0.11	5.91E-01	-0.10	6.02E-01
ENSCAFG0000000026	IFNGR1	darkgreen	VSMC_M4	0.05	8.12E-01	0.18	3.65E-01	-0.61	8.18E-04	0.92	1.41E-11	0.51	6.57E-03	-0.15	4.46E-01	-0.23	2.51E-01	-0.81	3.38E-07	-0.09	6.57E-01	0.47	1.28E-02
ENSCAFG0000001366	CDC42EP2	grey	VSMC_M10	0.05	8.12E-01	0.68	1.06E-04	0.08	7.09E-01	0.38	5.01E-02	-0.24	2.26E-01	0.09	6.51E-01	-0.24	2.22E-01	-0.48	1.22E-02	-0.61	7.10E-04	0.71	3.09E-05
ENSCAFG0000000994	CCSER1	grey	VSMC_M10	0.05	8.12E-01	0.00	1.00E+00	-0.41	3.61E-02	0.38	5.06E-02	0.33	8.94E-02	-0.13	5.31E-01	0.03	8.78E-01	-0.30	1.33E-01	-0.06	7.77E-01	0.27	1.76E-01
ENSCAFG0000001192	CASS4	grey	VSMC_M10	0.05	8.12E-01	0.26	1.99E-01	-0.27	1.75E-01	0.55	2.90E-03	0.18	3.64E-01	-0.13	5.24E-01	-0.26	1.94E-01	-0.54	3.99E-03	-0.18	3.81E-01	0.42	2.73E-02
ENSCAFG0000000887	BUB1B	darkgrey	VSMC_M8	0.05	8.12E-01	-0.45	1.99E-02	0.41	3.17E-02	-0.92	1.23E-11	-0.26	1.93E-01	0.14	4.91E-01	0.43	2.54E-02	0.96	6.03E-16	0.26	1.95E-01	-0.66	1.76E-04
ENSCAFG0000001115	ENSCAFG0000001115	violet	VSMC_M7	0.05	8.12E-01	-0.05	7.91E-01	0.15	4.58E-01	-0.18	3.65E-01	-0.13	5.04E-01	0.03	8.79E-01	0.65	2.21E-04	0.17	3.97E-01	-0.02	9.29E-01	-0.02	9.04E-01
ENSCAFG0000002804	ENSCAFG0000002804	violet	VSMC_M7	0.05	8.12E-01	-0.05	7.91E-01	0.15	4.58E-01	-0.18	3.65E-01	-0.13	5.04E-01	0.03	8.79E-01	0.65	2.21E-04	0.17	3.97E-01	-0.02	9.29E-01	-0.02	9.04E-01
ENSCAFG0000001925	VWA1	grey	VSMC_M10	0.05	8.13E-01	0.33	9.59E-02	-0.08	6.93E-01	0.46	1.57E-02	-0.10	6.32E-01	0.19	3.35E-01	-0.07	7.20E-01	-0.48	1.20E-02	-0.33	9.23E-02	0.63	3.96E-04
ENSCAFG0000002612	ENSCAFG0000002612	grey	VSMC_M10	0.05	8.13E-01	0.07	7.24E-01	0.06	7.58E-01	-0.07	7.13E-01	-0.01	9.75E-01	-0.12	5.67E-01	-0.24	2.26E-01	-0.01	9.78E-01	0.03	8.67E-01	0.00	9.91E-01
ENSCAFG0000001415	FAM171A2	darkgreen	VSMC_M4	0.05	8.13E-01	0.26	1.98E-01	-0.39	4.51E-02	0.74	9.11E-06	0.31	1.15E-01	-0.19	3.38E-01	-0.28	1.55E-01	-0.77	2.23E-06	-0.17	4.06E-01	0.51	6.57E-03
ENSCAFG0000001772	BDKRB1	grey	VSMC_M10	0.05	8.13E-01	0.17	3.84E-01	0.38	5.13E-02	-0.40	3.90E-02	-0.39	4.61E-02	0.29	1.38E-01	-0.06	7.53E-01	0.26	1.85E-01	-0.26	1.97E-01	0.01	9.72E-01
ENSCAFG0000000092	GOPC	grey	VSMC_M10	0.05	8.13E-01	-0.34	7.90E-02	-0.20	3.20E-01	-0.26	1.95E-01	0.37	6.06E-02	-0.06	7.54E-01	0.24	2.36E-01	0.35	7.79E-02	0.26	1.86E-01	-0.52	5.81E-03
ENSCAFG0000001609	SHLD2	grey	VSMC_M10	0.05	8.13E-01	-0.22	2.63E-01	-0.42	2.74E-02	0.35	7.11E-02	0.56	2.29E-03	-0.38	5.07E-02	-0.09	6.72E-01	-0.30	1.25E-01	0.30	1.24E-01	-0.15	4.68E-01
ENSCAFG0000003039	AP3S2	grey	VSMC_M10	0.05	8.13E-01	-0.46	1.67E-02	-0.07	7.35E-01	-0.48	1.04E-02	0.22	2.79E-01	0.12	5.58E-01	0.36	6.40E-02	0.55	3.19E-03	0.35	7.61E-02	-0.62	5.43E-04
ENSCAFG0000000224	HINT2	grey	VSMC_M10	0.05	8.13E-01	0.11	5.95E-01	0.14	4.77E-01	0.04	8.37E-01	-0.12	5.54E-01	-0.17	4.10E-01	0.47	1.31E-02	-0.08	6.85E-01	-0.19	3.40E-01	0.09	6.56E-01
ENSCAFG0000001470	CDC42	grey	VSMC_M10	0.05	8.13E-01	-0.27	1.71E-01	0.09	6.54E-01	-0.29	1.41E-01	0.09	6.59E-01	-0.01	9.68E-01	0.07	7.28E-01	0.25	2.07E-01	0.33	8.92E-02	-0.54	3.59E-03
ENSCAFG0000001127	ZNF639	grey	VSMC_M10	0.05	8.13E-01	-0.02	9.41E-01	0.13	5.21E-01	-0.03	8.74E-01	-0.10	6.26E-01	0.05	7.96E-01	0.16	4.27E-01	0.08	6.80E-01	-0.05	8.01E-01	0.03	8.88E-01
ENSCAFG0000000944	TRIM33	darkgrey	VSMC_M8	0.05	8.13E-01	-0.24	2.19E-01	0.17	4.01E-01	-0.52	5.39E-03	0.01	9.78E-01	-0.13	5.21E-01	0.16	4.16E-01	0.59	1.12E-03	0.17	3.83E-01	-0.47	1.30E-02
ENSCAFG0000001054	PPFIA1	grey	VSMC_M10	0.05	8.15E-01	0.04	8.35E-01	0.11	5.73E-01	-0.10	6.20E-01	-0.15	4.46E-01	-0.05	7.96E-01	-0.13	5.19E-01	0.15	4.56E-01	-0.07	7.37E-01	0.08	6.78E-01
ENSCAFG0000002363	ENSCAFG0000002363	grey	VSMC_M10	0.05	8.15E-01	-0.15	4.48E-01	0.03	8.67E-01	-0.25	2.18E-01	-0.01	9.64E-01	0.12	5.50E-01	0.04	8.37E-01	0.34	7.99E-02	0.06	7.51E-01	-0.18	3.73E-01
ENSCAFG0000000594	ENSCAFG0000000594	grey	VSMC_M10	0.05	8.15E-01	0.25	2.05E-01	0.20	3.28E-01	-0.01	9.74E-01	-0.21	2.97E-01	0.07	7.24E-01	-0.21	2.92E-01	-0.09	6.50E-01	-0.26	1.89E-01	0.19	3.44E-01
ENSCAFG0000000371	RTN4IP1	grey	VSMC_M10	0.05	8.15E-01	0.11	5.95E-01	0.51	6.48E-03	-0.22	2.64E-01	-0.47	1.30E-02	0.16	4.16E-01	0.26	1.82E-01	0.17	4.01E-01	-0.17	4.07E-01	-0.02	9.35E-01
ENSCAFG0000002926	ENSCAFG0000002926	grey	VSMC_M10	0.05	8.15E-01	0.22	2.77E-01	0.15	4.57E-01	-0.11	5.84E-01	-0.26	1.94E-01	0.36	6.29E-02	0.00	9.83E-01	0.08	6.97E-01	-0.29	1.37E-01	0.26	1.84E-01
ENSCAFG0000000677	ENSCAFG0000000677	grey	VSMC_M10	0.05	8.15E-01	-0.08	6.86E-01	-0.39	4.35E-02	0.42	2.81E-02	0.32	1.07E-01	-0.15	4.44E-01	-0.14	4.90E-01	-0.35	7.33E-02	0.12	5.58E-01	0.16	4.16E-01
ENSCAFG0000001355	PLEKHM3	grey	VSMC_M10	0.05	8.15E-01	0.02	9.34E-01	-0.25	2.16E-01	0.39	4.27E-02	0.18	3.79E-01	0.23	2.55E-01	0.01	9.58E-01	-0.40	4.06E-02	-0.05	7.87E-01	0.26	1.98E-01
ENSCAFG0000002037	HSF4	grey	VSMC_M10	0.05	8.16E-01	0.22	2.65E-01	-0.09	6.49E-01	0.17	3.94E-01	0.19	3.31E-01	-0.25	2.10E-01	-0.08	6.89E-01	-0.21	2.89E-01	-0.15	4.48E-01	-0.01	9.59E-01
ENSCAFG0000000473	FASTK	grey	VSMC_M10	0.05	8.16E-01	0.22	2.64E-01	0.13	5.19E-01	0.06	7.78E-01	-0.28	1.62E-01	0.09	6.38E-01	0.35	7.16E-02	-0.17	3.91E-01	-0.26	1.88E-01	0.48	1.04E-02
ENSCAFG0000001117	ALDH3B1	grey	VSMC_M10	0.05	8.16E-01	-0.15	4.64E-01	0.10	6.13E-01	-0.21	2.89E-01	-0.11	5.83E-01	0.18	3.62E-01	0.58	1.63E-03	0.21	2.99E-01	0.01	9.57E-01	-0.09	6.70E-01
ENSCAFG0000001368	ENSCAFG0000001368	grey	VSMC_M10	0.05	8.16E-01	0.00	9.92E-01	0.12	5.62E-01	-0.01	9.79E-01	-0.12	5.52E-01	0.20	3.15E-01	0.05	8.12E-01	0.04	8.47E-01	-0.03	8.75E-01	-0.11	5.73E-01
ENSCAFG0000003198	HIST1H2BE	darkgrey	VSMC_M8	0.05	8.16E-01	-0.19	3.34E-01	0.51	6.45E-03	-0.63	4.31E-04	-0.52	4.96E-03	0.34	8.32E-02	0.23	2.38E-01	0.66	1.78E-04	0.04	8.37E-01	-0.20	3.11E-01
ENSCAFG0000001876	DOCK7	grey	VSMC_M10	0.05	8.16E-01	-0.42	3.12E-02	-0.09	6.43E-01	-0.49	8.97E-03	0.34	8.34E-02	-0.17	3.88E-01	-0.08	6.99E-01	0.51	6.63E-03	0.38	4.81E-02	-0.68	8.35E-05
ENSCAFG0000000093	COL14A1	grey	VSMC_M10	0.05	8.16E-01	0.00	9.86E-01	0.38	5.24E-02	-0.53	4.76E-03	-0.26	1.82E-01	-0.10	6.18E-01	0.67	1.35E-04	0.44	2.04E-02	-0.11	5.69E-01	-0.21	2.90E-01
ENSCAFG0000002363	PRSS22	turquoise	VSMC_M6	0.05	8.17E-01	-0.26	1.97E-01	-0.10	6.34E-01	-0.12	5.56E-01	0.07	7.29E-01	0.59	1.25E-03	0.06	7.52E-01	0.24	2.35E-01	0.13	5.13E-01	-0.13	5.14E-01
ENSCAFG0000002947	ENSCAFG0000002947	grey	VSMC_M10	0.05	8.17E-01	-0.04	8.50E-01	0.25	2.15E-01	-0.37	5.40E-02	-0.30	1.34E-01	0.16	4.20E-01	0.09	6.61E-01	0.41	3.30E-02	-0.08	6.78E-01	-0.06	7.60E-01
ENSCAFG0000000096	ENSCAFG0000000096	turquoise	VSMC_M6	0.05	8.17E-01	-0.23	2.42E-01	-0.05	7.96E-01	-0.14	4.85E-01	0.06	7.83E-01	0.55	2.93E-03	0.07	7.29E-01	0.19	3.36E-01	0.22	2.63E-01	-0.17	3.93E-01
ENSCAFG0000000930	ENSCAFG0000000930	grey	VSMC_M10	0.05	8.17E-01	0.30	1.22E-01	0.46	1.49E-02	-0.23	2.51E-01	-0.56	2.24E-03	0.31	1.17E-01	0.18	3.61E-01	0.08	6.76E-01	-0.35	7.62E-02	0.30	1.29E-01
ENSCAFG0000000860	SH2B3	violet	VSMC_M7	0.05	8.18E-01	-0.48	1.14E-02	-0.05	8.09E-01	-0.40	3.74E-02	0.11	5.80E-01	0.21	2.85E-01	0.72	2.70E-05	0.45	1.99E-02	0.31	1.11E-01	-0.34	8.48E-02
ENSCAFG0000001412	SEC16B	grey	VSMC_M10	0.05	8.18E-01	0.15	4.44E-01	0.22	2.72E-01	-0.13	5.24E-01	-0.18	3.74E-01	0.13	5.02E-01	0.00	9.90E-01	0.15	4.53E-01	-0.22	2.63E-01	0.02	9.10E-01
ENSCAFG0000002986	ENSCAFG0000002986	grey	VSMC_M10	0.05	8.18E-01	-0.40	3.73E-02	-0.18	3.78E-01	-0.17	3.84E-01	0.22	2.78E-01	-0.09	6.56E-01	0.01	9.63E-01	0.30	1.29E-01	0.29	1.44E-01	-0.27	1.71E-01
ENSCAFG0000000582	ENSCAFG0000000582	grey	VSMC_M10	0.05	8.18E-01	-0.37	5.83E-02	-0.13	5.22E-01	-0.16	4.23E-01	0.11	5.87E-01	0.45	1.73E-02	0.42	3.06E-02	0.29	1.48E-01	0.21	2.90E-01	-0.19	3.47E-01
ENSCAFG0000000283	RIMS3	grey	VSMC_M10	0.05	8.19E-01	-0.16	4.23E-01	-0.43	2.53E-02	0.39	4.18E-02	0.34	8.31E-02	0.12	5.67E-01	-0.07	7.20E-01	-0.22	2.69E-01	0.11	5.84E-01	0.17	3.92E-01
ENSCAFG0000001791	P2RY11	darkgrey	VSMC_M8	0.05	8.19E-01	-0.18	3.64E-01	0.39	4.56E-02	-0.66	1.88E-04	-0.30	1.27E-01	0.18	3.80E-01	0.34	8.59E-02	0.62	6.14E-04	0.05	8.01E-01	-0.38	5.36E-02
ENSCAFG0000001538	ADCY10	grey	VSMC_M10	0.05	8.19E-01	0.25	2.00E-01	0.03	8.87E-01	0.08	7.04E-01	-0.06	7.68E-01	-0.01	9.70E-01	-0.07	7.24E-01	-0.13	5.31E-01	-0.22	2.68E-01	0.24	2.37E-01
ENSCAFG0000003200	ENSCAFG0000003200	pink	VSMC_M5	0.05	8.19E-01	-0.35	7.13E-02	-0.67	1.27E-04	0.26	1.92E-01	0.70	4.80E-05	-0.04	8.30E-01	-0.21	2.86E-01	-0.05	7.96E-01	0.31	1.13E-01	-0.20	3.23E-01
ENSCAFG0000000353	SIM1	cyan	VSMC_M2	0.05	8.19E-01	0.51	6.72E-03	0.25	2.17E-01	-0.08	7.02E-01	-0.26	1.86E-01	-0.07	7.42E-01	-0.17	4.06E-01	-0.09	6.71E-01	-0.45	1.75E-02	0.28	1.50E-01
ENSCAFG0000000924	SLC38A4	grey	VSMC_M10	0.05	8.19E-01	0.11	5.86E-01	0.20	3.22E-01	-0.22	2.63E-01	-0.19	3.36E-01	0.28	1.57E-01	0.27	1.75E-01	0.20	3.17E-01	-0.15	4.44E-01	0.06	7.51E-01
ENSCAFG0000000087	ENSCAFG0000000087	grey	VSMC_M10	0.05	8.19E-01	-0.06	7.57E-01	-0.21	2.89E-01	0.14	4.89E-01	0.19	3.40E-01	0.12	5.35E-01	-0.11	5.95E-01	-0.06	7.49E-01	0.07	7.20E-01	-0.04	8.54E-01
ENSCAFG0000000901	ENSCAFG0000000901	darkgrey	VSMC_M8	0.05	8.20E-01	-0.51	6.54E-03	0.00	9.84E-01	-0.38	5.00E-02	0.10	6.22E-01	0.07	7.29E-01	0.37	5.42E-02	0.60	1.02E-03	0.27	1.66E-01	-0.50	7.34E-03
ENSCAFG0000001400	ANGPTL1	grey	VSMC_M10	0.05	8.20E-01	0.23	2.46E-01	0.06	7.66E-01	0.08	6.81E-01	-0.04	8.42E-01	-0.22	2.61E-01	-0.49	8.77E-03	-0.18	3.58E-01	-0.16	4.33E-01	0.11	5.96E-01
ENSCAFG0000001726	ENSCAFG0000001726	darkgrey	VSMC_M8	0.05	8.21E-01	-0.43	2.68E-02	0.43	2.38E-02	-0.89	8.33E-10	-0.27	1.73E-01	0.07	7.11E-01	0.41	3.39E-02	0.93	4.41E-12	0.26	1.92E-01	-0.66	2.01E-04
ENSCAFG0000001507	HDC	grey	VSMC_M10	0.05	8.21E-01	-0.08	6.98E-01	0.04	8.57E-01	-0.14	4.95E-01	0.01	9.56E-01	-0.08	7.09E-01	-0.21	2.96E-01	0.11	5.81E-01	0.14	4.91E-01	-0.09	6.57E-01
ENSCAFG0000001870	SLC1A3	darkolivegreen	VSMC_M9	0.05	8.21E-01	-0.77	3.25E-06	-0.31	1.15E-01	-0.37	5.76E-02	0.49	1.02E-02	-0.03	8.95E-01	0.20	3.16E-01	0.58	1.68E-03	0.64	2.91E-04	-0.77	3.11E-06
ENSCAFG0000001530	SDF2L1	grey	VSMC_M10	0.05	8.22E-01	0.25	2.15E-01	0.42	3.13E-02	0.00	9.85E-01	-0.48	1.06E-02	0.24	2.34E-01	-0.09	6.41E-01	-0.03	8.76E-01	-0.28	1.53E-01	0.21	3.04E-01
ENSCAFG0000000325	EIPR1	yellow	VSMC_M3	0.05	8.22E-01	0.56	2.51E-03	0.49	8.82E-03	-0.28	1.51E-01	-0.50	8.58E-03	-0.01	9.48E-01	-0.10	6.24E-01	0.02	9.37E-01	-0.48	1.19E-02	0.24	2.24E-01
ENSCAFG0000000394	SPATA6	grey	VSMC_M10	0.05	8.22E-01	-0.33	9.57E-02	-0.34	8.44E-02	0.14	4.75E-01	0.46	1.66E-02	-0.31	1.13E-01	0.20	3.09E-01	-0.06	7.77E-01	0.34	8.71E-02	-0.25	2.14E-01
ENSCAFG0000000977	TTC3	grey	VSMC_M10	0.05	8.23E-01	-0.22	2.62E-01	-0.17	3.91E-01	-0.30	1.30E-01	0.45	1.82E-02	-0.28	1.62E-01	-0.20	3.21E-01	0.23	2.58E-01	0.31	1.21E-01	-0.55	2.69E-03
ENSCAFG0000000029	VTA1	pink	VSMC_M5	0.05	8.23E-01	-0.60	9.41E-04	-0.70	4.51E-05	0.12	5.47E-01	0.82	1.35E-07	-0.22	2.79E-01	0.26	1.87E-01	0.09	6.59E-01	0.53	4.82E-03	-0.45	1.95E-02
ENSCAFG0000000606	TMTC3	pink	VSMC_M5	0.04	8.24E-01	-0.28	1.57E-01	-0.87	5.11E-09	0.54	3.85E-03	0.85	1.75E-08	-0.20	3.29E-01	-0.14	4.79E-01	-0.34	7.93E-02	0.27	1.81E-01	0.02	9.14E-01
ENSCAFG0000000132	ATF4	grey	VSMC_M10	0.04	8.24E-01	-0.42	2.80E-02	-0.37	5.41E-02	0.12	5.67E-01	0.34	8.03E-02	0.05	8.08E-01	0.33	9.01E-02	0.03	8.92E-01	0.25	2.12E-01	-0.08	6.88E-01
ENSCAFG0000000865	SHROOM3	darkgreen	VSMC_M4	0.04	8.25E-01	0.29	1.37E-01	-0.45	1.94E-02	0.78	1.88E-06	0.28	1.61E-01	0.00	9.97E-01	-0.11	5.95E-01	-0.65	2.33E-04	-0.30	1.30E-01	0.64	3.01E-04
ENSCAFG0000001853	SERBP1	darkgrey	VSMC_M8	0.04	8.25E-01	-0.24	2.25E-01	0.60	8.31E-04	-0.78	1.58E-06	-0.53	4.64E-03	0.23	2.54E-01	0.37	5.71E-02	0.80	5.38E-07	0.07	7.27E-01	-0.40	3.63E-02
ENSCAFG0000000603	COL6A6	grey	VSMC_M10	0.04	8.25E-01	0.31	1.18E-01	0.03	8.88E-01	0.14	4.84E-01	-0.09	6.61E-01	-0.07	7.45E-01	-0.20	3.08E-01	-0.27	1.68E-01	-0.27	1.71E-01	0.30	1.23E-01
ENSCAFG0000000201	SMARCA2	grey	VSMC_M10	0.04	8.25E-01	0.10	6.14E-01	0.10	6.23E-01	-0.08	6.84E-01	0.05	8.19E-01	-0.24	2.24E-01	-0.22	2.68E-01	-0.14	4.74E-01	0.09	6.58E-01	-0.07	7.20E-01
ENSCAFG0000000956	ATP2B4	pink	VSMC_M5	0.04	8.25E-01	-0.28	1.65E-01	-0.66	1.72E-04	0.46	1.48E-02	0.72	2.71E-05	-0.03	8.78E-01	0.21	2.93E-01	-0.39	4.33E-02	0.31	1.13E-01	-0.04	8.61E-01
ENSCAFG0000002449	ENSCAFG0000002449	grey	VSMC_M10	0.04	8.25E-01	0.20	3.19E-01	0.20	3.14E-01	-0.16	4.38E-01	-0.20	3.18E-01	0.02	9.23E-01	0.40	3.88E-02	0.05	8.23E-01	-0.21	3.05E-01	0.12	5.37E-01
ENSCAFG0000002958	GCHFR	cyan	VSMC_M2	0.04	8.25E-01	0.77	3.12E-06	0.38	4.99E-02	0.19	3.35E-01	-0.43	2.63E-02	-0.05	8.09E-01	-0.30	1.25E-01	-0.48	1.12E-02	-0.61	6.65E-04	0.56	2.19E-03
ENSCAFG0000001217	TMEM222	darkgreen	VSMC_M4	0.04	8.26E-01	0.34	8.41E-02	-0.20	3.17E-01	0.68	9.68E-05	0.10	6.22E-01	0.10	6.23E-01	-0.16	4.40E-01	-0.66	1.80E-04	-0.27	1.67E-01	0.51	6.49E-03
ENSCAFG0000002969	CAMLG	grey	VSMC_M10	0.04	8.26E-01	0.17	3.88E-01	0.02	9.15E-01	0.16	4.27E-01	-0.19	3.35E-01	0.16	4.22E-01	0.27	1.67E-01	-0.17	4.09E-01	-0.23	2.56E-01	0.47	1.42E-02
ENSCAFG0000001654	AMER1	yellow	VSMC_M3	0.04	8.26E-01	0.11	5.73E-01	0.40	3.76E-02	-0.65	2.63E-04	-0.30	1.29E-01	-0.02	9.39E-01	0.24	2.37E-01	0.46	1.53E-02	-0.16	4.25E-01	-0.20	3.07E-01
ENSCAFG0000001123	SDC3	darkgreen	VSMC_M4	0.04	8.27E-01	0.65	2.68E-04	-0.03	8.99E-01	0.58	1.49E-03	-0.05	8.17E-01	0.06	7.50E-01	-0.76	3.42E-06	-0.73	1.65E-05	-0.44	2.06E-02	0.57	2.13E-03
ENSCAFG0000001253	ECE2	grey	VSMC_M10	0.04	8.27E-01	0.16	4.24E-01	0.17	3.99E-01	0.15	4.58E-01	-0.32	1.05E-01	0.34	8.39E-02	0.19	3.52E-01	-0.15	4.64E-01	-0.26	1.90E-01	0.40	3.73E-02
ENSCAFG0000002399	ENSCAFG0000002399	darkgrey	VSMC_M8	0.04	8.27E-01	-0.39	4.61E-02	0.28	1.58E-01	-0.57	1.90E-03	-0.17	3.85E-01	-0.08	7.07E-01	0.49	9.04E-03	0.59	1.15E-03	0.26	1.86E-01	-0.47	1.34E-02
ENSCAFG0000001473	GUCY1A2	turquoise	VSMC_M6	0.04	8.27E-01	-0.15	4.41E-01	-0.03	8.69E-01	-0.15	4.44E-01	0.02	9.24E-01	0.39	4.20E-02	0.04	8.29E-01	0.15	4.53E-01	0.06	7.70E-01	-0.06	7.51E-01
ENSCAFG0000002320	WTAP	darkgrey	VSMC_M8	0.04	8.28E-01	-0.41	3.17E-02	0.24	2.37E-01	-0.70	4.80E-05	-0.05	7.89E-01	0.02	9.16E-01	0.19	3.42E-01	0.77	2.62E-06	0.29	1.45E-01	-0.64	3.16E-04
ENSCAFG0000003051	ENSCAFG0000003051	grey	VSMC_M10	0.04	8.28E-01	0.02	9.21E-01	0.03	8.73E-01	-0.16	4.27E-01	-0.05	7.98E-01	0.20	3.23E-01	-0.02	9.40E-01	0.15	4.43E-01	-0.06	7.52E-01	-0.03	8.98E-01
ENSCAFG0000002789	ENSCAFG0000002789	grey	VSMC_M10	0.04	8.29E-01	-0.16	4.35E-01	-0.02	9.27E-01	-0.13	5.34E-01	0.08	6.94E-01	0.00	9.88E-01	0.21	2.99E-01	0.10	6.30E-01	0.12	5.46E-01	-0.08	7.05E-01
ENSCAFG0000001097	ENSCAFG0000001097	turquoise	VSMC_M6	0.04	8.29E-01	-0.20	3.09E-01	-0.06	7.82E-01	-0.12	5.60E-01	0.02	9.04E-01	0.52	5.58E-03	-0.08	6.79E-01	0.21	2.88E-01	0.09	6.41E-01	-0.11	5.77E-01
ENSCAFG0000000019	RPS12	grey	VSMC_M10	0.04	8.30E-01	0.22	2.71E-01	0.52	5.04E-03	-0.28	1.51E-01	-0.61	7.26E-04	0.07	7.36E-01	0.36	6.34E-02	0.22	2.77E-01	-0.33	8.98E-02	0.20	3.26E-01
ENSCAFG0000000846	FMNL3	darkgrey	VSMC_M8	0.04	8.30E-01	-0.45	1.77E-02	0.43	2.36E-02	-0.87	2.86E-09	-0.21	3.05E-01	0.10	6.36E-01	0.35	7.47E-02	0.83	6.53E-08	0.37	5.79E-02	-0.75	7.95E-06
ENSCAFG0000001397	ENSCAFG0000001397	darkgreen	VSMC_M4	0.04	8.30E-01	0.36	6.24E-02	-0.37	5.61E-02	0.65	2.52E-04	0.34	8.48E-02	-0.12	5.51E-01	-0.16	4.11E-01	-0.67	1.47E-04	-0.30	1.25E-01	0.40	4.07E-02
ENSCAFG0000001456	ENSCAFG0000001456	grey	VSMC_M10	0.04	8.30E-01	-0.08	6.83E-01	-0.04	8.30E-01	-0.10	6.12E-01	0.00	9.87E-01	0.28	1.55E-01	0.01	9.76E-01	0.15	4.64E-01	0.08	6.81E-01	0.03	8.95E-01
ENSCAFG0000003260	RASD2	grey	VSMC_M10	0.04	8.31E-01	-0.05	8.07E-01	0.35	7.55E-02	-0.25	2.16E-01	-0.39	4.52E-02	0.31	1.12E-01	0.35	7.17E-02	0.28	1.64E-01	-0.08	6.81E-01	-0.01	9.69E-01
ENSCAFG0000000444	ITM2B	darkgreen	VSMC_M4	0.04	8.31E-01	0.44	2.27E-02	-0.45	1.93E-02	0.88	1.46E-09	0.32	1.09E-01	-0.02	9.38E-01	-0.48	1.17E-02	-0.92	1.14E-11	-0.30	1.26E-01	0.62	5.21E-04
ENSCAFG0000000366	ENSCAFG0000000366	grey	VSMC_M10	0.04	8.31E-01	0.05	8.10E-01	-0.09	6.58E-01	0.07	7.28E-01	0.08	6.93E-01	-0.04	8.61E-01	0.05	7.95E-01	-0.09	6.47E-01	-0.08	6.99E-01	0.15	4.44E-01
ENSCAFG0000000056	PABPC1	grey	VSMC_M10	0.04	8.31E-01	0.48	1.18E-02	0.13	5.14E-01	-0.07	7.46E-01	-0.20	3.19E-01	0.10	6.34E-01	-0.21	2.83E-01	-0.11	6.00E-01	-0.47	1.29E-02	0.35	7.34E-02
ENSCAFG0000000643	SHQ1	pink	VSMC_M5	0.04	8.31E-01	-0.54	3.60E-03	-0.66	1.55E-04	0.35	7.60E-02	0.68	1.12E-04	-0.23	2.48E-01	-0.23	2.39E-01	-0.03	8.93E-01	0.49	9.87E-03	-0.25	2.16E-01
ENSCAFG0000001695	KIF22	darkgrey	VSMC_M8	0.04	8.31E-01	-0.41	3.17E-02	0.46	1.54E-02	-0.89	4.23E-10	-0.31	1.19E-01	0.13	5.24E-01	0.44	2.03E-02	0.94	4.95E-13	0.23	2.51E-01	-0.64	3.64E-04
ENSCAFG0000000103	ENSCAFG0000000103	cyan	VSMC_M2	0.04	8.32E-01	0.49	9.91E-03	0.21	2.92E-01	-0.05	8.04E-01	-0.22	2.78E-01	-0.05	8.07E-01	-0.14	4.78E-01	-0.11	5.80E-01	-0.41	3.34E-02	0.27	1.66E-01
ENSCAFG0000003108	RGS8	cyan	VSMC_M2	0.04	8.32E-01	0.49	9.91E-03	0.21	2.92E-01	-0.05	8.04E-01	-0.22	2.78E-01	-0.05	8.07E-01	-0.14	4.78E-01	-0.11	5.80E-01	-0.41	3.34E-02	0.27	1.66E-01
ENSCAFG0000001377	ENSCAFG0000001377	grey	VSMC_M10	0.04	8.32E-01	0.41	3.48E-02	-0.02	9.31E-01	0.39	4.17E-02	-0.08	6.90E-01	0.23	2.56E-01	-0.49	9.32E-03	-0.42	2.89E-02	-0.31	1.12E-01	0.45	1.83E-02
ENSCAFG0000001215	NUP133	darkgrey	VSMC_M8	0.04	8.32E-01	-0.52	5.43E-03	-0.07	7.10E-01	-0.32	1.02E-01	0.11	5.79E-01	0.14	5.01E-01	0.50	7.73E-03	0.56	2.54E-03	0.27	1.69E-01	-0.39	4.19E-02
ENSCAFG0000003004	ENSCAFG0000003004	grey	VSMC_M10	0.04	8.32E-01	0.20	3.11E-01	-0.18	3.72E-01	0.14	4.89E-01	0.16	4.32E-01	0.10	6.22E-01	0.28	1.63E-01	-0.21	2.86E-01	-0.22	2.79E-01	0.28	1.64E-01
ENSCAFG0000002246	ENSCAFG0000002246	grey	VSMC_M10	0.04	8.33E-01	0.09	6.59E-01	-0.06	7.61E-01	-0.05	8.22E-01	0.07	7.32E-01	0.25	2.16E-01	0.06	7.71E-01	0.06	7.75E-01	-0.17	4.05E-01	0.02	9.03E-01
ENSCAFG0000000790	WWC2	darkgreen	VSMC_M4	0.04	8.33E-01	0.02	9.12E-01	-0.59	1.18E-03	0.80	7.25E-07	0.49	9.90E-03	-0.03	8.90E-01	-0.47	1.34E-02	-0.63	4.63E-04	0.03	8.96E-01	0.32	1.04E-01
ENSCAFG0000003217	ENSCAFG0000003217	grey	VSMC_M10	0.04	8.33E-01	0.72	2.11E-05	0.54	3.40E-03	0.15	4.58E-01	-0.67	1.41E-04	0.08	6.97E-01	-0.22	2.68E-01	-0.38	5.35E-02	-0.62	5.20E-04	0.61	7.72E-04
ENSCAFG0000001815	CDC42BPB	grey	VSMC_M10	0.04	8.34E-01	0.22	2.62E-01	0.31	1.11E-01	-0.01	9.78E-01	-0.25	1.99E-01	0.36	6.91E-02	-0.18	3.70E-01	-0.05	7.97E-01	-0.21	2.95E-01	-0.01	9.52E-01
ENSCAFG0000000851	CSNK2A2	grey	VSMC_M10	0.04	8.34E-01	0.07	7.40E-01	-0.13	5.10E-01	0.28	1.63E-01	0.18	3.58E-01	0.00	9.90E-01	0.02	9.20E-01	-0.27	1.78E-01	-0.09	6.57E-01	0.03	8.94E-01
ENSCAFG0000001803	PTPN9	pink	VSMC_M5	0.04	8.34E-01	-0.37	5.97E-02	-0.38	4.92E-02	0.06	7.81E-01	0.54	3.49E-03	-0.27	1.80E-01	-0.23	2.58E-01	0.09	6.42E-01	0.43	2.47E-02	-0.48	1.15E-02
ENSCAFG0000000176	RORB	grey	VSMC_M10	0.04	8.34E-01	0.35	7.06E-02	0.25	2.02E-01	-0.10	6.24E-01	-0.28	1.56E-01	-0.12	5.52E-01	0.16	4.30E-01	0.00	9.90E-01	-0.39	4.23E-02	0.26	1.96E-01
ENSCAFG0000001679	DCAF4	grey	VSMC_M10	0.04	8.34E-01	-0.08	6.92E-01	-0.08	6.88E-01	-0.12	5.66E-01	-0.01	9.52E-01	0.21	2.99E-01	0.39	4.25E-02	0.24	2.34E-01	-0.13	5.29E-01	0.15	4.51E-01
ENSCAFG0000001447	HOMER3	cyan	VSMC_M2	0.04	8.35E-01	0.69	5.79E-05	0.54	3.63E-03	-0.01	9.68E-01	-0.60	9.19E-04	0.20	3.27E-01	-0.38	5.07E-02	-0.26	1.82E-01	-0.59	1.22E-03	0.46	1.47E-02
ENSCAFG0000000787	ENSCAFG0000000787	grey	VSMC_M10	0.04	8.35E-01	0.17	4.06E-01	0.24	2.25E-01	-0.04	8.53E-01	-0.08	6.85E-01	0.01	9.79E-01	-0.13	5.09E-01	-0.09	6.48E-01	-0.05	7.93E-01	-0.14	4.75E-01
ENSCAFG0000003025	FAM131C	grey	VSMC_M10	0.04	8.35E-01	-0.25	2.12E-01	0.25	2.06E-01	-0.37	5.81E-02	-0.22	2.75E-01	0.34	8.00E-02	0.28	1.59E-01	0.42	2.92E-02	0.10	6.13E-01	-0.28	1.56E-01
ENSCAFG0000001115	GPR156	grey	VSMC_M10	0.04	8.36E-01	0.02	9.23E-01	0.13	5.16E-01	0.03	8.67E-01	-0.24	2.24E-01	0.16	4.12E-01	0.43	2.50E-02	-0.03	8.73E-01	-0.11	5.89E-01	0.25	2.17E-01
ENSCAFG0000001751	MTMR4	yellow	VSMC_M3	0.04	8.36E-01	0.17	3.87E-01	0.39	4.46E-02	-0.52	5.22E-03	-0.25	2.05E-01	-0.15	4.58E-01	0.24	2.29E-01	0.37	6.02E-02	-0.17	3.92E-01	-0.19	3.50E-01
ENSCAFG0000001022	C2CD2	grey	VSMC_M10	0.04	8.36E-01	0.24	2.19E-01	0.48	1.16E-02	-0.39	4.43E-02	-0.44	2.27E-02	0.08	6.77E-01	0.61	7.47E-04	0.17	3.83E-01	-0.29	1.36E-01	0.10	6.23E-01
ENSCAFG0000000638	SLC4A11	turquoise	VSMC_M6	0.04	8.36E-01	-0.01	9.80E-01	0.02	9.37E-01	-0.06	7.64E-01	-0.09	6.45E-01	0.80	5.85E-07	0.02	9.16E-01	0.12	5.66E-01	-0.06	7.68E-01	0.04	8.26E-01
ENSCAFG0000000926	SLC38A2	grey	VSMC_M10	0.04	8.36E-01	-0.21	2.90E-01	-0.06	7.55E-01	-0.39	4.56E-02	0.21	2.82E-01	0.02	9.34E-01	0.08	7.09E-01	0.29	1.48E-01	0.23	2.41E-01	-0.30	1.30E-01
ENSCAFG0000003178	ENSCAFG0000003178	turquoise	VSMC_M6	0.04	8.36E-01	0.01	9.53E-01	-0.04	8.27E-01	0.14	4.98E-01	-0.05	8.19E-01	0.31	1.20E-01	0.28	1.57E-01	-0.04	8.40E-01	-0.10	6.10E-01	0.15	4.66E-01
ENSCAFG0000001229	FES	cyan	VSMC_M2	0.04	8.36E-01	0.59	1.29E-03	0.59	1.13E-03	-0.23	2.53E-01	-0.56	2.33E-03	-0.04	8.46E-01	-0.30	1.33E-01	-0.05	8.15E-01	-0.46	1.61E-02	0.22	2.60E-01
ENSCAFG0000000422	ENSCAFG0000000422	grey	VSMC_M10	0.04	8.37E-01	-0.08	6.99E-01	0.29	1.43E-01	-0.39	4.58E-02	-0.17	4.09E-01	0.09	6.73E-01	-0.29	1.41E-01	0.32	9.97E-02	0.09	6.52E-01	-0.29	1.43E-01

ENSCAFG0000003031	HRASLS	turquoise	VSMC_M6	0.04	8.37E-01	0.05	8.10E-01	-0.01	9.61E-01	-0.02	9.36E-01	-0.04	8.42E-01	0.46	1.61E-02	0.10	6.15E-01	0.04	8.35E-01	-0.12	5.56E-01	0.11	5.91E-01
ENSCAFG0000002921	NCR3LG1	pink	VSMC_M5	0.04	8.37E-01	-0.35	7.38E-02	-0.76	4.50E-06	0.44	2.02E-02	0.88	1.12E-09	-0.41	3.27E-02	-0.07	7.35E-01	-0.30	1.29E-01	0.41	3.16E-02	-0.23	2.43E-01
ENSCAFG0000002274	C10H2orf73	grey	VSMC_M10	0.04	8.37E-01	0.18	3.59E-01	0.00	9.92E-01	0.04	8.31E-01	-0.05	7.88E-01	-0.14	4.74E-01	0.29	1.42E-01	-0.14	4.85E-01	-0.17	3.99E-01	0.28	1.57E-01
ENSCAFG0000001178	ENSCAFG0000001178	grey	VSMC_M10	0.04	8.37E-01	0.42	2.76E-02	-0.05	7.89E-01	0.26	1.82E-01	-0.17	4.06E-01	0.29	1.44E-01	0.00	9.89E-01	-0.28	1.51E-01	-0.46	1.60E-02	0.66	1.83E-04
ENSCAFG0000000263	PIGF	grey	VSMC_M10	0.04	8.37E-01	-0.44	2.22E-02	-0.41	3.49E-02	0.12	5.44E-01	0.56	2.33E-03	-0.11	6.00E-01	0.05	8.19E-01	0.06	7.71E-01	0.37	6.01E-02	-0.46	1.67E-02
ENSCAFG0000001318	DDR2	darkgreen	VSMC_M4	0.04	8.37E-01	0.49	9.73E-03	-0.35	6.98E-02	0.78	1.64E-06	0.26	1.99E-01	-0.04	8.57E-01	-0.31	1.11E-01	-0.84	5.31E-08	-0.38	5.17E-02	0.59	1.12E-03
ENSCAFG0000000382	ZXDC	grey	VSMC_M10	0.04	8.37E-01	0.11	5.89E-01	-0.04	8.55E-01	0.09	6.45E-01	0.12	5.38E-01	0.07	7.31E-01	0.07	7.37E-01	-0.11	5.98E-01	-0.12	5.46E-01	-0.02	9.36E-01
ENSCAFG0000001455	ENSCAFG0000001455	grey	VSMC_M10	0.04	8.38E-01	0.37	6.06E-02	0.07	7.11E-01	0.30	1.25E-01	-0.20	3.14E-01	0.13	5.25E-01	-0.20	3.26E-01	-0.27	1.68E-01	-0.38	4.91E-02	0.46	1.57E-02
ENSCAFG0000001685	CHD3	darkgreen	VSMC_M4	0.04	8.38E-01	0.22	2.70E-01	-0.24	2.24E-01	0.62	6.04E-04	0.25	2.12E-01	0.36	3.05E-02	-0.55	3.15E-03	-0.61	8.03E-04	-0.27	7.38E-01	0.30	1.29E-01
ENSCAFG0000001154	CCDC39	grey	VSMC_M10	0.04	8.38E-01	0.11	5.70E-01	-0.21	2.96E-01	0.22	2.77E-01	0.12	5.58E-01	-0.23	2.46E-01	0.28	1.57E-01	-0.21	3.04E-01	-0.21	2.94E-01	0.39	4.56E-02
ENSCAFG0000000174	CHRNA5	grey	VSMC_M10	0.04	8.38E-01	-0.06	7.69E-01	0.16	4.21E-01	-0.37	6.10E-02	-0.09	6.39E-01	-0.15	4.45E-01	0.36	6.41E-02	0.39	4.41E-02	-0.05	8.11E-01	-0.22	2.67E-01
ENSCAFG0000000539	DYNC1LI1	grey	VSMC_M10	0.04	8.39E-01	-0.06	7.85E-01	0.34	7.93E-02	-0.25	2.12E-01	-0.46	1.64E-02	0.28	1.55E-01	0.20	3.06E-01	0.26	1.90E-01	-0.06	7.77E-01	0.17	3.98E-01
ENSCAFG0000003074	FDPS	yellow	VSMC_M3	0.04	8.39E-01	-0.03	8.82E-01	0.60	1.05E-03	-0.79	1.14E-06	-0.39	4.35E-02	-0.01	9.57E-01	0.48	1.06E-02	0.57	1.91E-03	0.00	9.99E-01	-0.40	3.68E-02
ENSCAFG0000001679	VKORC1	grey	VSMC_M10	0.04	8.39E-01	0.27	1.65E-01	-0.04	8.52E-01	0.41	3.30E-02	-0.11	5.72E-01	-0.19	3.50E-01	-0.16	4.33E-01	-0.39	4.23E-02	-0.32	1.08E-01	0.47	1.41E-02
ENSCAFG0000001820	TMEM241	grey	VSMC_M10	0.04	8.39E-01	-0.39	4.52E-02	-0.09	6.54E-01	-0.39	4.73E-02	0.28	1.58E-01	0.07	7.44E-01	0.15	4.56E-01	0.49	9.96E-03	0.28	1.61E-01	-0.62	5.43E-04
ENSCAFG0000001909	STAP2	grey	VSMC_M10	0.04	8.39E-01	-0.01	9.54E-01	-0.01	9.68E-01	-0.18	3.74E-01	0.06	7.81E-01	-0.04	8.59E-01	0.18	3.78E-01	0.15	4.51E-01	0.01	9.64E-01	-0.01	9.79E-01
ENSCAFG0000000783	TCF7L1	darkgreen	VSMC_M4	0.04	8.39E-01	0.46	1.65E-02	-0.29	1.39E-01	0.59	1.07E-03	0.12	5.37E-01	-0.02	9.09E-01	0.14	4.83E-01	-0.62	5.88E-04	-0.43	2.54E-02	0.70	5.50E-05
ENSCAFG0000003122	ENSCAFG0000003122	grey	VSMC_M10	0.04	8.39E-01	-0.51	6.85E-03	-0.48	1.13E-02	-0.10	6.17E-01	0.56	2.40E-03	-0.30	1.33E-01	0.37	5.95E-02	0.27	1.73E-01	0.40	3.65E-02	-0.36	6.78E-02
ENSCAFG0000000885	CPNE2	grey	VSMC_M10	0.04	8.39E-01	0.53	4.64E-03	0.19	3.36E-01	0.27	1.67E-01	-0.33	9.05E-02	0.25	2.03E-01	0.10	6.23E-01	-0.41	3.38E-02	-0.49	1.02E-02	0.63	3.89E-04
ENSCAFG0000001380	OSBPL6	grey	VSMC_M10	0.04	8.40E-01	-0.08	6.99E-01	-0.31	1.19E-01	0.24	2.19E-01	0.39	4.56E-02	0.03	8.81E-01	-0.35	7.35E-02	-0.17	3.98E-01	0.10	6.08E-01	-0.13	5.19E-01
ENSCAFG0000002036	PLEKHG4	darkgrey	VSMC_M8	0.04	8.40E-01	-0.12	5.36E-01	0.44	2.20E-02	-0.70	4.60E-05	-0.32	1.08E-01	-0.03	8.63E-01	0.52	5.35E-03	0.61	7.14E-04	0.00	9.85E-01	-0.31	1.17E-01
ENSCAFG0000001496	ATP8B4	darkolivegreen	VSMC_M9	0.04	8.40E-01	-0.46	1.70E-02	-0.35	7.27E-02	0.04	8.57E-01	0.38	5.16E-02	0.07	7.34E-01	0.37	5.75E-02	0.11	5.97E-01	0.36	6.17E-02	-0.20	3.21E-01
ENSCAFG0000002274	ENSCAFG0000002274	grey	VSMC_M10	0.04	8.41E-01	-0.31	1.18E-01	-0.03	8.85E-01	-0.08	6.82E-01	0.18	3.59E-01	0.08	6.79E-01	-0.02	9.09E-01	0.19	3.38E-01	0.27	1.80E-01	-0.49	9.69E-03
ENSCAFG0000002284	ENSCAFG0000002284	pink	VSMC_M5	0.04	8.41E-01	-0.14	4.96E-01	-0.49	9.33E-03	0.47	1.41E-02	0.49	9.26E-03	-0.18	3.64E-01	0.00	9.94E-01	-0.27	1.71E-01	0.09	6.71E-01	0.03	8.82E-01
ENSCAFG0000003109	ENSCAFG0000003109	grey	VSMC_M10	0.04	8.42E-01	-0.23	2.40E-01	-0.29	1.36E-01	-0.07	7.17E-01	0.38	5.12E-02	0.06	7.81E-01	0.04	8.53E-01	0.07	7.33E-01	0.19	3.42E-01	-0.26	1.82E-01
ENSCAFG0000003007	ENSCAFG0000003007	grey	VSMC_M10	0.04	8.42E-01	0.12	5.60E-01	-0.15	4.68E-01	0.09	6.71E-01	0.13	5.02E-01	-0.12	5.66E-01	0.05	8.01E-01	-0.23	2.47E-01	-0.12	5.47E-01	0.21	2.92E-01
ENSCAFG0000002534	SPCS3	darkgrey	VSMC_M8	0.04	8.42E-01	-0.66	1.97E-04	0.10	6.36E-01	-0.72	2.39E-05	0.06	7.74E-01	0.03	8.68E-01	0.17	3.98E-01	0.89	7.31E-10	0.49	9.12E-03	-0.79	1.22E-06
ENSCAFG0000000170	MCM5	darkgrey	VSMC_M8	0.04	8.43E-01	-0.37	5.40E-02	0.50	7.74E-03	-0.93	2.73E-12	-0.36	6.78E-02	0.13	5.06E-01	0.36	6.58E-02	0.95	7.24E-14	0.22	2.62E-01	-0.61	6.96E-04
ENSCAFG0000001079	TRPV4	grey	VSMC_M10	0.04	8.43E-01	-0.38	4.85E-02	0.19	3.49E-01	-0.53	4.17E-03	-0.09	6.51E-01	0.08	6.88E-01	0.42	3.02E-02	0.49	1.20E-02	0.30	1.33E-01	-0.37	5.51E-02
ENSCAFG0000000235	TRIM28	darkgrey	VSMC_M8	0.04	8.43E-01	-0.11	5.87E-01	0.60	9.12E-04	-0.86	1.30E-08	-0.52	5.75E-03	0.19	3.55E-01	0.17	3.93E-01	0.82	2.13E-07	-0.02	9.39E-01	-0.42	2.90E-02
ENSCAFG0000000840	VEGFC	grey	VSMC_M10	0.04	8.44E-01	-0.33	9.15E-02	-0.07	7.11E-01	-0.20	3.18E-01	0.10	6.20E-01	0.24	2.33E-01	0.07	7.41E-01	0.24	2.22E-01	0.29	1.41E-01	-0.30	1.31E-01
ENSCAFG0000001212	ENSCAFG0000001212	grey	VSMC_M10	0.04	8.44E-01	-0.26	1.95E-01	-0.07	7.14E-01	-0.15	4.46E-01	0.01	9.77E-01	0.40	4.03E-02	-0.16	4.18E-01	0.28	1.61E-01	0.19	3.45E-01	-0.16	4.36E-01
ENSCAFG0000000384	OSBPL9	pink	VSMC_M5	0.04	8.44E-01	-0.08	6.77E-01	-0.71	3.27E-05	0.61	6.94E-04	0.63	4.34E-04	0.08	6.75E-01	-0.23	2.58E-01	-0.49	9.01E-03	0.12	5.49E-01	0.22	2.60E-01
ENSCAFG0000001575	ZDHHC4	grey	VSMC_M10	0.04	8.44E-01	0.09	6.39E-01	-0.28	1.56E-01	0.43	2.66E-02	0.13	5.12E-01	0.17	3.95E-01	-0.29	1.44E-01	-0.30	1.28E-01	-0.10	6.15E-01	0.37	5.98E-02
ENSCAFG0000001256	PSMD4	darkgrey	VSMC_M8	0.04	8.44E-01	-0.50	8.32E-03	0.08	7.00E-01	-0.58	1.51E-03	0.12	5.61E-01	-0.20	3.29E-01	0.31	1.14E-01	0.66	1.59E-04	0.39	4.57E-02	-0.68	8.70E-05
ENSCAFG0000001094	MCTP2	turquoise	VSMC_M6	0.04	8.44E-01	-0.25	2.01E-01	-0.12	5.50E-01	-0.10	6.31E-01	0.11	5.99E-01	0.60	9.47E-04	0.04	8.57E-01	0.20	3.29E-01	0.15	4.66E-01	-0.13	5.23E-01
ENSCAFG0000001229	IGFBP3	turquoise	VSMC_M6	0.04	8.44E-01	-0.25	2.01E-01	-0.12	5.50E-01	-0.10	6.31E-01	0.11	5.99E-01	0.60	9.47E-04	0.04	8.57E-01	0.20	3.29E-01	0.15	4.66E-01	-0.13	5.23E-01
ENSCAFG0000001629	FAM193B	grey	VSMC_M10	0.04	8.44E-01	0.31	1.14E-01	-0.01	9.70E-01	0.36	6.72E-02	-0.15	4.65E-01	0.15	4.66E-01	0.10	6.12E-01	-0.48	1.22E-02	-0.31	1.11E-01	0.57	1.99E-03
ENSCAFG0000000522	SZT2	grey	VSMC_M10	0.04	8.44E-01	0.68	1.12E-04	-0.06	7.65E-01	0.56	2.33E-03	-0.08	6.98E-01	-0.04	8.44E-01	-0.21	2.97E-01	-0.74	1.21E-05	-0.60	9.35E-04	0.76	3.98E-06
ENSCAFG0000003248	EN1	cyan	VSMC_M2	0.04	8.45E-01	0.44	2.33E-02	0.34	8.61E-02	0.04	8.56E-01	-0.34	8.70E-02	-0.19	3.46E-01	0.44	2.04E-02	-0.20	3.24E-01	-0.45	1.87E-02	0.38	5.20E-02
ENSCAFG0000000782	TRAPPC11	grey	VSMC_M10	0.04	8.45E-01	-0.46	1.67E-02	-0.55	2.81E-03	0.16	4.36E-01	0.61	7.19E-04	-0.09	6.52E-01	-0.16	4.25E-01	0.06	7.79E-01	0.43	2.63E-02	-0.29	1.42E-01
ENSCAFG0000001878	ENSCAFG0000001878	cyan	VSMC_M2	0.04	8.45E-01	0.45	1.73E-02	0.25	2.03E-01	-0.12	5.60E-01	-0.28	1.51E-01	-0.01	9.67E-01	-0.08	6.93E-01	-0.03	8.85E-01	-0.42	2.91E-02	0.26	1.89E-01
ENSCAFG0000000863	XRCC4	grey	VSMC_M10	0.04	8.45E-01	0.10	6.32E-01	0.29	1.45E-01	-0.22	2.66E-01	-0.40	3.78E-02	-0.08	6.81E-01	0.18	3.72E-01	0.19	3.40E-01	-0.17	4.05E-01	0.25	2.12E-01
ENSCAFG0000000838	FAIM2	grey	VSMC_M10	0.04	8.45E-01	-0.04	8.46E-01	0.41	3.32E-02	-0.29	1.47E-01	-0.23	2.54E-01	-0.19	3.54E-01	0.09	6.40E-01	0.15	4.58E-01	0.12	5.49E-01	-0.29	1.37E-01
ENSCAFG0000000015	ENSCAFG0000000015	grey	VSMC_M10	0.04	8.45E-01	0.10	6.31E-01	-0.40	3.67E-02	0.33	8.99E-02	0.24	2.20E-01	0.26	1.86E-01	-0.07	7.36E-01	-0.25	2.01E-01	-0.14	4.79E-01	0.33	9.17E-02
ENSCAFG0000002028	BCL10	grey	VSMC_M10	0.04	8.45E-01	-0.29	1.43E-01	-0.21	3.00E-01	0.12	5.42E-01	0.24	2.25E-01	-0.01	9.59E-01	-0.11	5.70E-01	-0.05	8.17E-01	0.25	2.08E-01	-0.14	4.95E-01
ENSCAFG0000001950	CIRBP	grey	VSMC_M10	0.04	8.46E-01	-0.13	5.05E-01	0.39	4.61E-02	-0.38	4.82E-02	-0.33	9.20E-02	0.18	3.81E-01	-0.08	6.99E-01	0.41	3.27E-02	0.10	6.18E-01	-0.34	7.83E-02
ENSCAFG0000000094	A4GALT	grey	VSMC_M10	0.04	8.46E-01	0.41	3.42E-02	0.08	6.96E-01	0.22	2.74E-01	-0.16	4.16E-01	0.12	5.63E-01	0.25	2.13E-01	-0.36	6.20E-02	-0.38	5.05E-02	0.52	5.44E-03
ENSCAFG0000000476	SLC25A19	darkgrey	VSMC_M8	0.04	8.46E-01	-0.48	1.17E-02	-0.02	9.24E-01	-0.49	9.57E-03	-0.02	9.20E-01	0.39	4.31E-02	0.39	4.47E-02	0.67	1.54E-04	0.26	1.83E-01	-0.34	8.38E-02
ENSCAFG0000000960	PPM1K	grey	VSMC_M10	0.04	8.46E-01	-0.24	2.28E-01	-0.05	8.19E-01	-0.06	7.48E-01	0.26	1.84E-01	-0.09	6.43E-01	-0.02	9.18E-01	0.07	7.31E-01	0.25	2.15E-01	-0.46	1.51E-02
ENSCAFG0000000699	CCDC92	cyan	VSMC_M2	0.04	8.47E-01	0.58	1.51E-03	0.15	4.70E-01	0.15	4.56E-01	-0.20	3.19E-01	-0.16	4.28E-01	0.07	7.25E-01	-0.35	7.78E-02	-0.47	1.31E-02	0.53	4.23E-03
ENSCAFG0000001872	BCORL1	pink	VSMC_M5	0.04	8.47E-01	0.17	3.98E-01	-0.35	7.07E-02	0.51	6.06E-03	0.30	1.35E-01	-0.01	9.79E-01	-0.03	8.63E-01	-0.51	6.55E-03	-0.20	3.30E-01	0.34	8.75E-02
ENSCAFG0000002350	SETDB1	grey	VSMC_M10	0.04	8.48E-01	-0.06	7.83E-01	-0.32	1.00E-01	0.19	3.39E-01	0.48	1.11E-02	-0.31	1.13E-01	0.30	1.26E-01	-0.21	2.94E-01	0.10	6.25E-01	-0.14	4.83E-01
ENSCAFG0000001199	ENSCAFG0000001199	grey	VSMC_M10	0.04	8.49E-01	0.34	8.74E-02	0.25	2.02E-01	-0.01	9.68E-01	-0.44	2.01E-02	0.35	6.93E-02	0.08	6.94E-01	0.00	9.94E-01	-0.44	2.17E-02	0.52	5.73E-03
ENSCAFG0000001982	CYB561D1	grey	VSMC_M10	0.04	8.49E-01	0.47	1.42E-02	0.26	1.84E-01	0.20	3.21E-01	-0.29	1.49E-01	-0.12	5.62E-01	-0.12	5.61E-01	-0.41	3.30E-02	-0.37	5.70E-02	0.43	2.63E-02
ENSCAFG0000001768	SCNN1B	grey	VSMC_M10	0.04	8.49E-01	0.18	3.76E-01	-0.30	1.25E-01	0.46	1.56E-02	0.18	3.77E-01	0.34	8.06E-02	0.05	8.12E-01	-0.37	5.75E-02	-0.19	3.39E-01	0.39	4.62E-02
ENSCAFG0000001976	MED22	grey	VSMC_M10	0.04	8.49E-01	0.09	6.69E-01	0.48	1.14E-02	-0.45	1.96E-02	-0.54	3.36E-03	0.41	3.53E-02	0.22	2.63E-01	0.42	2.84E-02	-0.23	2.54E-01	0.06	7.57E-01
ENSCAFG0000001003	NUAK2	grey	VSMC_M10	0.04	8.50E-01	-0.42	3.11E-02	-0.09	6.54E-01	-0.12	5.47E-01	0.12	5.67E-01	0.22	2.61E-01	0.16	4.19E-01	0.25	2.17E-01	0.39	4.47E-02	-0.28	1.50E-01
ENSCAFG0000000489	RHEB	pink	VSMC_M5	0.04	8.50E-01	-0.31	1.10E-01	-0.66	2.06E-04	0.50	7.38E-03	0.74	1.18E-05	-0.22	2.75E-01	-0.18	3.73E-01	-0.28	1.54E-01	0.36	6.25E-02	-0.23	2.51E-01
ENSCAFG0000001052	FIGN	grey	VSMC_M10	0.04	8.50E-01	0.34	8.20E-02	0.00	9.89E-01	0.51	6.24E-03	-0.20	3.24E-01	0.24	2.35E-01	0.06	7.57E-01	-0.50	7.56E-03	-0.32	1.07E-01	0.64	3.54E-04
ENSCAFG0000001915	CRK	grey	VSMC_M10	0.04	8.51E-01	-0.07	7.30E-01	0.17	4.06E-01	-0.25	2.03E-01	-0.09	6.45E-01	-0.30	1.35E-01	0.16	4.33E-01	0.23	2.53E-01	0.02	9.05E-01	-0.09	6.70E-01
ENSCAFG0000000399	BBS12	pink	VSMC_M5	0.04	8.51E-01	-0.35	7.42E-02	-0.60	8.69E-04	0.34	8.54E-02	0.64	3.26E-04	-0.21	2.82E-01	0.05	7.89E-01	-0.07	7.10E-01	0.25	2.05E-01	-0.22	2.63E-01
ENSCAFG0000001871	ENSCAFG0000001871	darkgreen	VSMC_M4	0.04	8.51E-01	-0.10	6.22E-01	-0.45	1.72E-02	0.51	6.77E-03	0.39	4.45E-02	-0.12	5.54E-01	-0.14	4.79E-01	-0.34	8.41E-02	0.12	5.56E-01	0.15	4.62E-01
ENSCAFG0000003092	ENSCAFG0000003092	grey	VSMC_M10	0.04	8.51E-01	0.20	3.24E-01	0.25	2.10E-01	-0.05	7.89E-01	-0.23	2.42E-01	-0.14	4.73E-01	0.41	3.54E-02	-0.04	8.36E-01	-0.26	1.99E-01	0.21	2.94E-01
ENSCAFG0000000219	CKS2	darkgrey	VSMC_M8	0.04	8.51E-01	-0.37	5.76E-02	0.38	4.81E-02	-0.73	1.74E-05	-0.35	7.00E-02	0.05	8.05E-01	0.46	1.51E-02	0.79	8.14E-07	0.18	3.61E-01	-0.35	6.98E-02
ENSCAFG0000000873	MOB1A	grey	VSMC_M10	0.04	8.51E-01	0.13	5.15E-01	-0.06	7.71E-01	0.07	7.16E-01	-0.14	4.97E-01	0.28	1.52E-01	0.39	4.32E-02	-0.05	8.04E-01	-0.30	1.31E-01	0.46	1.67E-02
ENSCAFG0000000498	TSEN2	darkgrey	VSMC_M8	0.04	8.51E-01	-0.47	1.30E-02	-0.12	5.67E-01	-0.50	8.35E-03	0.26	1.83E-01	-0.10	6.11E-01	0.37	5.55E-02	0.64	3.31E-04	0.33	9.29E-02	-0.54	3.40E-03
ENSCAFG0000000600	ENSCAFG0000000600	grey	VSMC_M10	0.04	8.52E-01	0.14	4.84E-01	0.00	9.97E-01	0.04	8.35E-01	0.04	8.51E-01	0.24	2.36E-01	-0.21	2.92E-01	-0.09	6.50E-01	-0.05	8.17E-01	0.07	7.12E-01
ENSCAFG0000000206	CCDC107	yellow	VSMC_M3	0.04	8.52E-01	0.43	2.70E-02	0.50	7.56E-03	0.09	6.43E-01	-0.52	5.61E-03	-0.01	9.65E-01	-0.07	7.25E-01	-0.23	2.42E-01	-0.36	6.65E-02	0.32	1.06E-01
ENSCAFG0000000202	ENPP4	grey	VSMC_M10	0.04	8.52E-01	0.18	3.63E-01	-0.50	7.72E-03	0.67	1.42E-04	0.28	1.53E-01	0.16	4.29E-01	-0.04	8.48E-01	-0.55	3.14E-03	-0.24	2.29E-01	0.57	1.81E-03
ENSCAFG0000003146	ENSCAFG0000003146	grey	VSMC_M10	0.04	8.53E-01	0.12	5.59E-01	-0.50	8.45E-03	0.47	1.38E-02	0.49	8.78E-03	-0.08	6.81E-01	-0.18	3.82E-01	-0.40	3.88E-02	-0.05	8.00E-01	0.10	6.30E-01
ENSCAFG0000001844	LPIN2	grey	VSMC_M10	0.04	8.53E-01	0.38	5.38E-02	0.16	4.22E-01	0.01	9.49E-01	-0.07	7.19E-01	-0.16	4.29E-01	-0.59	1.14E-03	-0.18	3.76E-01	-0.24	2.29E-01	0.01	9.47E-01
ENSCAFG0000000122	FAM53C	grey	VSMC_M10	0.04	8.53E-01	0.57	2.04E-03	-0.25	2.03E-01	0.65	2.42E-04	0.11	5.76E-01	0.06	7.63E-01	-0.26	1.82E-01	-0.77	2.26E-06	-0.46	1.51E-02	0.70	5.72E-05
ENSCAFG0000001414	MED14	grey	VSMC_M10	0.04	8.53E-01	0.26	1.93E-01	-0.62	5.96E-04	0.71	3.66E-05	0.50	8.01E-03	-0.18	3.82E-01	-0.11	5.86E-01	-0.62	5.42E-04	-0.26	1.98E-01	0.51	7.14E-03
ENSCAFG0000001768	RIT2	grey	VSMC_M10	0.04	8.53E-01	-0.05	7.95E-01	0.18	3.65E-01	-0.42	3.08E-02	-0.03	8.74E-01	-0.03	8.82E-01	0.34	8.16E-02	0.37	5.41E-02	0.03	8.64E-01	-0.27	1.77E-01
ENSCAFG0000000179	LIMCH1	darkgreen	VSMC_M4	0.04	8.53E-01	0.38	5.06E-02	-0.44	2.04E-02	0.88	9.54E-10	0.30	1.32E-01	-0.06	7.82E-01	-0.17	4.07E-01	-0.80	5.20E-07	-0.33	9.37E-02	0.64	3.41E-04
ENSCAFG0000001504	MMP13	grey	VSMC_M10	0.04	8.53E-01	0.19	3.45E-01	-0.46	1.66E-02	0.59	1.08E-03	0.37	5.80E-02	0.08	6.92E-01	-0.13	5.29E-01	-0.59	1.29E-03	-0.13	5.21E-01	0.37	5.49E-02
ENSCAFG0000001099	PPFIBP1	grey	VSMC_M10	0.04	8.53E-01	-0.05	7.88E-01	0.07	7.27E-01	-0.33	8.86E-02	-0.03	8.63E-01	0.45	1.89E-02	-0.13	5.19E-01	0.28	1.55E-01	0.07	7.33E-01	-0.18	3.70E-01
ENSCAFG0000001316	AKAP6	darkgreen	VSMC_M4	0.04	8.53E-01	0.06	7.59E-01	-0.58	1.69E-03	0.74	8.42E-06	0.41	3.38E-02	0.17	4.00E-01	0.04	8.55E-01	-0.57	1.77E-03	-0.11	5.69E-01	0.48	1.08E-02
ENSCAFG0000000592	FGF14	darkgreen	VSMC_M4	0.04	8.53E-01	0.12	5.64E-01	-0.45	1.84E-02	0.67	1.44E-04	0.35	7.33E-02	-0.09	6.38E-01	-0.14	4.76E-01	-0.53	4.42E-03	-0.10	6.20E-01	0.37	5.90E-02
ENSCAFG0000002522	ENSCAFG0000002522	grey	VSMC_M10	0.04	8.54E-01	0.23	2.57E-01	-0.11	5.96E-01	0.15	4.48E-01	-0.04	8.56E-01	0.27	1.78E-01	0.10	6.03E-01	-0.20	3.18E-01	-0.28	1.59E-01	0.45	1.98E-02
ENSCAFG0000003162	FAM200A	grey	VSMC_M10	0.04	8.54E-01	0.02	9.39E-01	-0.05	8.01E-01	0.24	2.23E-01	0.20	3.28E-01	-0.25	2.00E-01	-0.28	1.51E-01	-0.26	1.88E-01	0.09	6.46E-01	-0.12	5.43E-01
ENSCAFG0000000893	NLRC5	grey	VSMC_M10	0.04	8.54E-01	0.68	1.05E-04	0.13	5.14E-01	0.42	2.80E-02	-0.32	1.09E-01	0.30	1.28E-01	-0.05	8.07E-01	-0.58	1.69E-03	-0.66	1.66E-04	0.77	2.88E-06
ENSCAFG0000001938	DIRAS1	grey	VSMC_M10	0.04	8.54E-01	0.23	2.56E-01	-0.12	5.43E-01	0.31	1.18E-01	0.05	7.97E-01	-0.02	9.05E-01	0.17	4.05E-01	-0.28	1.56E-01	-0.24	2.19E-01	0.34	7.98E-02
ENSCAFG0000002861	ENSCAFG0000002861	grey	VSMC_M10	0.04	8.54E-01	0.27	1.70E-01	0.45	1.79E-02	-0.18	3.60E-01	-0.50	8.55E-03	-0.19	3.43E-01	0.14	4.94E-01	0.02	9.18E-01	-0.25	2.09E-01	0.25	2.09E-01
ENSCAFG0000000914	RBM19	darkgrey	VSMC_M8	0.04	8.54E-01	-0.44	2.20E-02	0.26	1.83E-01	-0.58	1.70E-03	-0.25	2.09E-01	0.19	3.47E-01	0.23	2.46E-01	0.71	3.64E-05	0.27	1.69E-01	-0.41	3.32E-02
ENSCAFG0000001358	ENSCAFG0000001358	cyan	VSMC_M2	0.04	8.54E-01	0.49	9.32E-03	0.21	2.87E-01	-0.05	8.05E-01	-0.21	2.87E-01	-0.05	8.07E-01	-0.14	4.81E-01	-0.11	5.76E-01	-0.41	3.48E-02	0.27	1.66E-01
ENSCAFG0000000389	HSD17B14	grey	VSMC_M10	0.04	8.54E-01	0.04	8.40E-01	0.07	7.39E-01	-0.10	6.36E-01	-0.21	3.02E-01	0.51	6.29E-03	0.00	9.93E-01	0.06	7.51E-01	-0.07	7.40E-01	0.23	2.50E-01
ENSCAFG0000000252	COL15A1	yellow	VSMC_M3	0.04	8.55E-01	0.21	3.00E-01	0.41	3.25E-02	-0.37	5.81E-02	-0.27	1.66E-01	-0.09	6.68E-01	-0.30	1.24E-01	0.17	4.07E-01	-0.12	5.59E-01	-0.11	5.84E-01
ENSCAFG0000001846	RNF135	grey	VSMC_M10	0.04	8.55E-01	0.52	5.96E-03	0.19	3.31E-01	0.42	3.05E-02	-0.24	2.27E-01	-0.23	2.31E-01	-0.16	4.27E-01	-0.55	2.74E-03	-0.41	3.24E-02	0.45	1.80E-02
ENSCAFG0000001921	DNAJA3	grey	VSMC_M10	0.04	8.55E-01	-0.38	5.31E-02	-0.13	5.03E-01	-0.25	2.12E-01	0.23	2.52E-01	-0.28	1.54E-01	0.19	3.48E-01	0.37	6.03E-02	0.26	1.94E-01	-0.43	2.45E-02
ENSCAFG0000002375	ADAMTSL2	darkgreen	VSMC_M4	0.04	8.55E-01	0.17	3.97E-01	-0.26	1.88E-01	0.45	1.99E-02	0.19	3.54E-01	0.06	7.53E-01	-0.01	9.43E-01	-0.42	2.91E-02	-0.16	4.20E-01	0.36	6.62E-02
ENSCAFG0000000932	LOXL4	grey	VSMC_M10	0.04	8.55E-01	0.20	3.29E-01	0.29	1.49E-01	-0.20	3.28E-01	-0.36	6.15E-02	0.36	6.34E-02	0.18	3.75E-01	-0.01	9.61E-01	-0.21	2.89E-01	0.25	2.14E-01
ENSCAFG0000000425	PRKD2	grey	VSMC_M10	0.04	8.55E-01	0.64	3.21E-04	0.03	8.69E-01	0.52	5.46E-03	-0.17	3.89E-01	0.10	6.21E-01	-0.20	3.14E-01	-0.72	2.13E-05	-0.53	4.48E-03	0.77	2.60E-06
ENSCAFG0000001767	ENSCAFG0000001767	grey	VSMC_M10	0.04	8.56E-01	0.09	6.72E-01	0.16	4.17E-01	-0.17	4.00E-01	-0.16	4.23E-01	-0.15	4.59E-01	-0.11	5.94E-01	0.22	2.80E-01	-0.19	3.33E-01	0.04	8.59E-01
ENSCAFG0000000537	SLC24A3	grey	VSMC_M10	0.04	8.56E-01	-0.31	1.13E-01	-0.54	3.66E-03	0.48	1.18E-02	0.49	9.24E-03	0.04	8.54E-01	-0.36	6.55E-02	-0.29	1.35E-01	0.32	9.83E-02	-0.04	8.59E-01
ENSCAFG0000001836	ENSCAFG0000001836	grey	VSMC_M10	0.04	8.56E-01	0.15	4.43E-01	0.15	4.51E-01	-0.12	5.65E-01	-0.14	4.95E-01	0.00	9.83E-01	0.19	3.39E-01	0.00	9.94E-01	-0.13	5.28E-01	0.13	5.31E-01
ENSCAFG0000000341	ODC1	darkgrey	VSMC_M8	0.04	8.56E-01	-0.56	2.25E-03	0.04	8.30E-01	-0.34	8.13E-02	-0.04	8.37E-01	0.38	4.95E-02	0.36	6.29E-02	0.62	5.91E-04	0.32	1.04E-01	-0.44	2.31E-02
ENSCAFG0000001921	C20H19orf71	violet	VSMC_M7	0.04	8.56E-01	-0.06	7.70E-01	0.15	4.61E-01	-0.19	3.49E-01	-0.14	4.93E-01	0.06	7.83E-01	0.63	4.03E-04	0.18	3.69E-01	-0.01	9.58E-01	-0.03	8.98E-01
ENSCAFG0000002878	GADL1	violet	VSMC_M7	0.04	8.56E-01	-0.06	7.70E-01	0.15	4.61E-01	-0.19	3.49E-01	-0.14	4.93E-01	0.06	7.83E-01	0.63	4.03E-04	0.18	3.69E-01	-0.01	9.58E-01	-0.03	8.98E-01
ENSCAFG0000002920	ENSCAFG0000002920	violet	VSMC_M7	0.04	8.56E-01	-0.06	7.70E-01	0.15	4.61E-01	-0.19	3.49E-01	-0.14	4.93E-01	0.06	7.83E-01	0.63	4.03E-04	0.18	3.69E-01	-0.01	9.58E-01	-0.03	8.98E-01
ENSCAFG0000003260	ENSCAFG0000003260	violet	VSMC_M7	0.04	8.56E-01	-0.06	7.70E-01	0.15	4.61E-01	-0.19	3.49E-01	-0.14	4.93E-01	0.06	7.83E-01	0.63	4.03E-04	0.18	3.69E-01	-0.01	9.58E-01	-0.03	8.98E-01
ENSCAFG0000000081	SFT2D1	pink	VSMC_M5	0.04	8.57E-01	-0.09	6.70E-01	-0.49	9.56E-03	0.27	1.74E-01	0.55	2.72E-03	-0.06	7.67E-01	-0.39	4.17E-02	-0.22	2.60E-01	0.14	4.72E-01	-0.15	4.51E-01
ENSCAFG0000001922	VASN	yellow	VSMC_M3	0.04	8.57E-01	0.52	5.95E-03	0.74	1.01E-05	-0.55	3.01E-03	-0.70	4.94E-05	0.03	8.95E-01	-0.09	6.38E-01	0.25	2.02E-01	-0.50	8.60E-03	0.12	5.58E-01
ENSCAFG0000002538	PML	cyan	VSMC_M2	0.04	8.57E-01	0.71	3.59E-05	0.56	2.36E-03	-0.13	5.22E-01	-0.61	6.79E-04	0.24	2.21E-01	-0.20	3.17E-01	-0.20	3.19E-01	-0.61	7.44E-04	0.46	1.61E-02
ENSCAFG0000000564	TOX4	grey	VSMC_M10	0.04	8.57E-01	-0.57	1.82E-03	-0.37	5.96E-02	-0.24	2.37E-01	0.41	3.30E-02	0.11	5.85E-01	0.00	9.98E-01	0.43	2.36E-02	0.49	1.03E-02	-0.49	9.03E-03
ENSCAFG0000001490	IQCN	grey	VSMC_M10	0.04	8.58E-01	-0.21	2.96E-01	0.11	5.80E-01	-0.28	1.62E-01	-0.08	6.86E-01	0.04	8.59E-01	0.28	1.51E-01	0.29	1.42E-01	0.19	3.34E-01	-0.18	3.59E-01
ENSCAFG0000000270	TFPT	grey	VSMC_M10	0.04	8.58E-01	0.27	1.78E-01	0.31	1.10E-01	-0.07	7.13E-01	-0.31	1.20E-01	-0.24	2.28E-01	0.04	8.27E-01	-0.02	9.41E-01	-0.25	2.16E-01	0.16	4.25E-01
ENSCAFG0000001436	PECR	grey	VSMC_M10	0.04	8.58E-01	0.29	1.37E-01	0.15	4.52E-01	0.20	3.10E-01	-0.28	1.61E-01	-0.25	2.11E-01	0.21	3.01E-01	-0.26	1.82E-01	-0.36	6.61E-02	0.53	4.63E-03
ENSCAFG0000003184	NUP35	darkolivegreen	VSMC_M9	0.04	8.58E-01	-0.70	4.82E-05	-0.48	1.04E-02	-0.21	2.90E-01	0.58	1.58E-03	-0.06	7.75E-01	0.16	4.28E-01	0.50	7.24E-03	0.52	5.35E-03	-0.58	1.54E-03
ENSCAFG0000000520	TSKU	grey	VSMC_M10	0.04	8.58E-01	0.41	3.22E-02	0.60	9.18E-04	-0.45	1.93E-02	-0.57	1.90E-03	0.25	2.12E-01	-0.22	2.77E-01	0.26	1.91E-01	-0.37	5.82E-02	0.05	8.20E-01
ENSCAFG0000001177	TEX2	grey	VSMC_M10	0.04	8.58E-01	0.03	8.84E-01	-0.22	2.76E-01	0.06	7.70E-01	0.31	1.19E-01	-0.03	8.93E-01	0.13	5.05E-01	-0.08	6.85E-01	0.05	8.22E-01	-0.08	6.78E-01
ENSCAFG0000000358	ZMYM4	pink	VSMC_M5	0.04	8.59E-01	-0.09	6.65E-01	-0.68	9.70E-05	0.67	1.40E-04	0.75	8.13E-06	-0.34	7.97E-02	-0.26	1.97E-01	-0.59	1.08E-03	0.17	3.99E-01	0.07	7.36E-01
ENSCAFG0000001987	RNF166	cyan	VSMC_M2	0.04	8.59E-01	0.41	3.37E-02	0.52	5.14E-03	-0.19	3.34E-01	-0.54	3.39E-03	0.14	4.89E-01	-0.15	4.43E-01	0.00	9.83E-01	-0.39	4.63E-02	0.19	3.47E-01
ENSCAFG0000001226	DTL	darkgrey	VSMC_M8	0.04	8.59E-01	-0.55	2.67E-03	0.23	2.42E-01	-0.82	1.87E-07	-0.10	6.02E-01	0.09	6.65E-01	0.43	2.41E-02	0.95	2.24E-14	0.34	8.27E-02	-0.69	7.55E-05
ENSCAFG0000001721	AHSA1	darkgrey	VSMC_M8	0.04	8.60E-01	-0.51	6.40E-03	0.28	1.54E-01	-0.57	1.91E-03	-0.05	7.86E-01	-0.04	8.52E-01	0.24	2.22E-01	0.61	6.42E-04	0.43	2.40E-02	-0.76	4.13E-06

ENSCAFG0000000817	PTH	darkgreen	VSMC_M4	0.04	8.60E-01	0.11	5.91E-01	-0.46	1.53E-02	0.67	1.49E-04	0.34	7.82E-02	-0.12	5.44E-01	-0.03	8.90E-01	-0.52	5.91E-03	-0.12	5.43E-01	0.38	5.22E-02
ENSCAFG0000001248	MYO3B	grey	VSMC_M10	0.04	8.60E-01	0.05	7.95E-01	0.09	6.39E-01	-0.07	7.35E-01	-0.12	5.51E-01	-0.10	6.30E-01	-0.14	4.82E-01	0.02	9.12E-01	-0.09	6.63E-01	0.03	8.74E-01
ENSCAFG0000000469	HID1	grey	VSMC_M10	0.04	8.61E-01	0.49	1.02E-02	0.09	6.40E-01	0.43	2.37E-02	-0.31	1.16E-01	0.54	3.75E-03	-0.12	5.49E-01	-0.44	2.28E-02	-0.46	1.46E-02	0.67	1.28E-04
ENSCAFG0000000607	CHFR	darkgrey	VSMC_M8	0.04	8.61E-01	-0.47	1.29E-02	0.18	3.60E-01	-0.50	8.22E-03	-0.09	6.53E-01	0.21	2.89E-01	0.06	7.72E-01	0.58	1.41E-03	0.38	5.08E-02	-0.51	7.20E-03
ENSCAFG0000000521	KIZ	grey	VSMC_M10	0.04	8.61E-01	-0.23	2.38E-01	-0.41	3.60E-02	0.02	9.35E-01	0.38	4.90E-02	0.11	5.99E-01	-0.06	7.70E-01	0.11	5.93E-01	0.17	3.90E-01	-0.12	5.42E-01
ENSCAFG0000002118	ENSCAFG0000002118	grey	VSMC_M10	0.04	8.61E-01	0.02	9.19E-01	0.02	9.31E-01	0.02	9.22E-01	-0.04	8.37E-01	-0.19	3.37E-01	-0.05	7.97E-01	0.03	8.71E-01	-0.06	7.53E-01	0.09	6.47E-01
ENSCAFG0000001083	MVK	yellow	VSMC_M3	0.04	8.61E-01	0.37	5.42E-02	0.39	4.42E-02	-0.39	4.50E-02	-0.33	9.11E-02	0.02	9.14E-01	0.29	1.37E-01	0.16	4.24E-01	-0.39	4.63E-02	0.11	5.96E-01
ENSCAFG0000000033	ENSCAFG0000000033	darkgrey	VSMC_M8	0.04	8.62E-01	-0.30	1.29E-01	0.09	6.54E-01	-0.46	1.51E-02	-0.08	6.84E-01	0.08	7.05E-01	0.22	2.71E-01	0.61	7.40E-04	0.16	4.37E-01	-0.30	1.35E-01
ENSCAFG0000000562	DIP2C	grey	VSMC_M10	0.04	8.62E-01	0.27	1.67E-01	0.48	1.16E-02	-0.38	5.28E-02	-0.49	8.81E-03	0.15	4.54E-01	-0.34	8.09E-02	0.25	2.05E-01	-0.26	1.84E-01	0.03	8.70E-01
ENSCAFG0000000797	U2SURP	darkolivegreen	VSMC_M9	0.04	8.62E-01	-0.76	3.73E-06	-0.18	3.60E-01	-0.38	4.93E-02	0.39	4.64E-02	-0.11	5.80E-01	0.20	3.07E-01	0.61	6.72E-04	0.61	7.57E-04	-0.81	2.41E-07
ENSCAFG0000001998	ENSCAFG0000001998	grey	VSMC_M10	0.03	8.63E-01	0.35	7.13E-02	0.18	3.58E-01	0.00	9.83E-01	-0.23	2.59E-01	0.23	2.53E-01	-0.02	9.07E-01	-0.08	6.85E-01	-0.33	9.06E-02	0.32	1.01E-01
ENSCAFG0000003128	ZNF134	grey	VSMC_M10	0.03	8.63E-01	-0.11	5.76E-01	-0.47	1.36E-02	0.28	1.65E-01	0.34	8.50E-02	0.18	3.80E-01	0.08	7.07E-01	-0.13	5.09E-01	0.05	8.02E-01	0.24	2.38E-01
ENSCAFG0000001552	TMOD2	grey	VSMC_M10	0.03	8.63E-01	-0.01	9.78E-01	-0.36	6.12E-02	0.35	7.76E-02	0.26	1.88E-01	0.20	3.09E-01	0.04	8.44E-01	-0.27	1.67E-01	-0.06	7.73E-01	0.30	1.35E-01
ENSCAFG0000001512	ENSCAFG0000001512	cyan	VSMC_M2	0.03	8.63E-01	0.66	2.08E-04	0.62	5.30E-04	-0.11	5.84E-01	-0.73	1.48E-05	0.16	4.27E-01	-0.17	3.90E-01	-0.14	4.87E-01	-0.59	1.10E-03	0.48	1.06E-02
ENSCAFG0000000254	ERP44	grey	VSMC_M10	0.03	8.64E-01	0.26	1.89E-01	-0.26	1.83E-01	0.35	7.56E-02	0.29	1.38E-01	-0.23	2.38E-01	-0.58	1.69E-03	-0.40	3.65E-02	-0.19	3.38E-01	0.12	5.65E-01
ENSCAFG0000001885	PSMG2	grey	VSMC_M10	0.03	8.64E-01	-0.08	7.04E-01	0.10	6.15E-01	-0.42	2.96E-02	-0.05	8.10E-01	0.05	7.95E-01	0.34	7.92E-02	0.41	3.57E-02	0.02	9.39E-01	-0.22	2.69E-01
ENSCAFG0000002370	ENSCAFG0000002370	grey	VSMC_M10	0.03	8.64E-01	0.12	5.41E-01	0.18	3.72E-01	-0.17	3.86E-01	-0.21	2.83E-01	-0.05	8.03E-01	0.59	1.29E-03	0.12	5.56E-01	-0.20	3.10E-01	0.10	6.04E-01
ENSCAFG0000001199	UTP25	darkgrey	VSMC_M8	0.03	8.64E-01	-0.26	1.99E-01	0.13	5.30E-01	-0.44	2.09E-02	-0.04	8.41E-01	0.10	6.11E-01	0.32	1.00E-01	0.54	3.70E-03	0.09	6.67E-01	-0.38	5.06E-02
ENSCAFG0000001667	MAP3K9	grey	VSMC_M10	0.03	8.64E-01	-0.05	8.01E-01	-0.08	6.88E-01	0.04	8.56E-01	0.12	5.62E-01	-0.02	9.04E-01	-0.06	7.48E-01	-0.09	6.50E-01	0.15	4.54E-01	-0.02	9.40E-01
ENSCAFG0000001070	ENSCAFG0000001070	grey	VSMC_M10	0.03	8.64E-01	0.46	1.68E-02	0.46	1.62E-02	0.00	9.96E-01	-0.57	1.87E-03	0.04	8.36E-01	-0.10	6.09E-01	-0.17	3.99E-01	-0.42	2.73E-02	0.50	8.18E-03
ENSCAFG0000000681	TRAF6	grey	VSMC_M10	0.03	8.64E-01	0.06	7.76E-01	-0.23	2.42E-01	-0.01	9.78E-01	0.33	8.91E-02	-0.15	4.67E-01	-0.51	6.29E-03	-0.10	6.06E-01	0.09	6.39E-01	-0.14	4.81E-01
ENSCAFG0000001917	TBXA2R	darkolivegreen	VSMC_M9	0.03	8.65E-01	-0.49	9.59E-03	-0.14	4.85E-01	-0.26	1.83E-01	0.26	1.83E-01	-0.03	8.85E-01	0.72	2.07E-05	0.33	8.92E-02	0.38	5.14E-02	-0.43	2.50E-02
ENSCAFG0000001400	H2AFY2	grey	VSMC_M10	0.03	8.65E-01	0.17	4.02E-01	0.29	1.46E-01	-0.27	1.78E-01	-0.37	5.57E-02	-0.05	7.86E-01	-0.04	8.40E-01	0.22	2.81E-01	-0.25	2.04E-01	0.19	3.43E-01
ENSCAFG0000000179	ALDH1A1	grey	VSMC_M10	0.03	8.65E-01	0.52	4.95E-03	-0.11	5.90E-01	0.50	8.44E-03	-0.01	9.67E-01	-0.11	5.94E-01	-0.24	2.35E-01	-0.54	3.57E-03	-0.45	1.84E-02	0.56	2.29E-03
ENSCAFG0000001707	CSNK1G1	grey	VSMC_M10	0.03	8.65E-01	-0.03	8.92E-01	0.02	9.04E-01	-0.12	5.37E-01	-0.02	9.08E-01	0.20	3.10E-01	0.17	3.84E-01	0.16	4.17E-01	-0.09	6.70E-01	0.02	9.39E-01
ENSCAFG0000000656	NCAPH	darkgrey	VSMC_M8	0.03	8.66E-01	-0.54	3.90E-03	0.32	1.01E-01	-0.87	2.80E-09	-0.17	3.91E-01	0.11	5.82E-01	0.46	1.70E-02	0.97	2.94E-17	0.35	7.49E-02	-0.70	5.09E-05
ENSCAFG0000000251	ABCG8	grey	VSMC_M10	0.03	8.66E-01	0.37	5.52E-02	0.09	6.66E-01	0.06	7.68E-01	-0.16	4.18E-01	-0.06	7.52E-01	-0.16	4.32E-01	-0.18	3.77E-01	-0.36	6.76E-02	0.30	1.26E-01
ENSCAFG0000003149	ZNF461	grey	VSMC_M10	0.03	8.67E-01	0.17	4.10E-01	-0.32	9.93E-02	0.45	1.74E-02	0.06	7.67E-01	0.32	9.82E-02	-0.04	8.25E-01	-0.32	1.02E-01	-0.25	2.07E-01	0.59	1.30E-03
ENSCAFG0000000298	KIAA1841	darkgrey	VSMC_M8	0.03	8.67E-01	-0.30	1.31E-01	-0.04	8.26E-01	-0.43	2.47E-02	0.08	6.87E-01	0.00	9.86E-01	0.40	3.89E-02	0.54	3.89E-03	0.14	4.86E-01	-0.29	1.39E-01
ENSCAFG0000001577	CCDC120	grey	VSMC_M10	0.03	8.67E-01	0.28	1.54E-01	0.01	9.55E-01	0.28	1.59E-01	-0.05	8.02E-01	0.18	3.68E-01	-0.18	3.69E-01	-0.38	4.77E-02	-0.22	2.73E-01	0.28	1.53E-01
ENSCAFG0000000618	RELL2	grey	VSMC_M10	0.03	8.67E-01	0.50	7.85E-03	0.50	7.34E-03	0.03	8.97E-01	-0.63	4.51E-04	0.05	8.20E-01	0.23	2.44E-01	-0.16	4.35E-01	-0.55	2.67E-03	0.54	3.48E-03
ENSCAFG0000000885	CACNB3	cyan	VSMC_M2	0.03	8.68E-01	0.74	1.07E-05	-0.05	7.97E-01	0.63	4.27E-04	0.00	9.82E-01	-0.23	2.46E-01	-0.54	3.44E-03	-0.80	6.49E-07	-0.53	4.46E-03	0.63	3.94E-04
ENSCAFG0000000901	ENSCAFG0000000901	grey	VSMC_M10	0.03	8.68E-01	0.19	3.37E-01	0.35	7.77E-02	0.01	9.45E-01	-0.41	3.41E-02	0.13	5.34E-01	0.19	3.39E-01	-0.12	5.38E-01	-0.18	3.75E-01	0.25	2.14E-01
ENSCAFG0000002375	CCDC59	grey	VSMC_M10	0.03	8.69E-01	-0.25	1.99E-01	0.05	8.23E-01	-0.38	5.18E-02	0.10	6.28E-01	-0.07	7.35E-01	0.26	1.86E-01	0.40	3.90E-02	0.16	4.39E-01	-0.42	2.78E-02
ENSCAFG0000000432	ADGRF3	red	VSMC_M1	0.03	8.69E-01	0.10	6.27E-01	-0.10	6.37E-01	0.10	6.30E-01	0.02	9.06E-01	0.10	6.27E-01	0.33	9.46E-02	-0.11	5.95E-01	-0.19	3.34E-01	0.23	2.48E-01
ENSCAFG0000000553	MED29	grey	VSMC_M10	0.03	8.69E-01	0.38	4.88E-02	0.10	6.13E-01	0.31	1.17E-01	-0.09	6.44E-01	0.07	7.45E-01	-0.08	7.03E-01	-0.42	2.85E-02	-0.26	1.91E-01	0.21	2.90E-01
ENSCAFG0000000825	RBM12	darkgrey	VSMC_M8	0.03	8.69E-01	-0.46	1.63E-02	-0.07	7.21E-01	-0.44	2.08E-02	0.07	7.25E-01	0.28	1.61E-01	-0.05	7.86E-01	0.61	6.68E-04	0.33	9.23E-02	-0.43	2.59E-02
ENSCAFG0000001185	LRFN4	yellow	VSMC_M3	0.03	8.69E-01	0.18	3.70E-01	0.66	1.77E-04	-0.70	5.33E-05	-0.60	8.66E-04	0.13	5.17E-01	0.22	2.76E-01	0.51	6.36E-03	-0.21	2.85E-01	-0.12	5.51E-01
ENSCAFG0000001579	NINJ2	grey	VSMC_M10	0.03	8.69E-01	0.32	1.04E-01	-0.20	3.05E-01	0.44	2.10E-02	0.05	7.92E-01	-0.07	7.28E-01	-0.27	1.82E-01	-0.47	1.45E-02	-0.21	2.82E-01	0.54	3.45E-03
ENSCAFG0000001228	ENSCAFG0000001228	pink	VSMC_M5	0.03	8.69E-01	-0.58	1.61E-03	-0.42	2.91E-02	-0.09	6.63E-01	0.63	3.93E-04	-0.38	5.10E-02	-0.05	8.17E-01	0.21	2.86E-01	0.56	2.45E-03	-0.68	9.57E-05
ENSCAFG0000000150	OPLAH	cyan	VSMC_M2	0.03	8.70E-01	0.79	9.02E-07	0.44	2.23E-02	0.10	6.16E-01	-0.44	2.10E-02	-0.28	1.56E-01	-0.26	1.89E-01	-0.37	5.64E-02	-0.64	3.19E-04	0.52	5.95E-03
ENSCAFG0000003189	ATP6V0E1	darkgreen	VSMC_M4	0.03	8.70E-01	0.51	7.08E-03	-0.28	1.58E-01	0.67	1.40E-04	0.14	4.80E-01	0.00	9.82E-01	-0.37	5.82E-02	-0.78	1.28E-06	-0.36	6.68E-02	0.65	2.11E-04
ENSCAFG0000001880	IMPA2	grey	VSMC_M10	0.03	8.70E-01	0.12	5.37E-01	0.10	6.30E-01	0.35	7.54E-02	-0.17	3.93E-01	0.16	4.20E-01	0.09	6.50E-01	-0.34	8.13E-02	-0.11	5.69E-01	0.34	8.17E-02
ENSCAFG0000001853	DLGAP1	grey	VSMC_M10	0.03	8.70E-01	0.00	1.00E+00	0.12	5.51E-01	-0.23	2.55E-01	-0.09	6.64E-01	-0.03	8.73E-01	0.23	2.46E-01	0.19	3.47E-01	-0.03	8.72E-01	0.02	9.08E-01
ENSCAFG0000001866	KHSRP	yellow	VSMC_M3	0.03	8.70E-01	0.18	3.79E-01	0.68	8.54E-05	-0.61	7.79E-04	-0.71	3.32E-05	0.23	2.48E-01	0.05	8.01E-01	0.45	1.87E-02	-0.24	2.37E-01	0.02	9.31E-01
ENSCAFG0000000480	KBTBD6	grey	VSMC_M10	0.03	8.70E-01	0.06	7.61E-01	-0.15	4.67E-01	0.02	9.15E-01	0.05	8.18E-01	0.13	5.27E-01	0.13	5.28E-01	0.07	7.40E-01	-0.13	5.13E-01	0.18	3.79E-01
ENSCAFG0000001663	ENSCAFG0000001663	grey	VSMC_M10	0.03	8.71E-01	0.30	1.26E-01	-0.01	9.60E-01	-0.03	8.96E-01	0.07	7.18E-01	-0.08	7.02E-01	-0.37	5.45E-02	-0.09	6.41E-01	-0.22	2.66E-01	0.03	8.93E-01
ENSCAFG0000000072	ISOC1	darkgrey	VSMC_M8	0.03	8.71E-01	-0.47	1.24E-02	0.33	8.91E-02	-0.74	1.18E-05	-0.11	5.69E-01	0.11	5.96E-01	0.03	8.67E-01	0.76	4.28E-06	0.40	3.68E-02	-0.76	3.61E-06
ENSCAFG0000001422	ARHGAP20	darkgreen	VSMC_M4	0.03	8.71E-01	0.46	1.60E-02	-0.25	2.10E-01	0.66	1.64E-04	0.04	8.57E-01	-0.04	8.28E-01	-0.16	4.39E-01	-0.61	7.00E-04	-0.47	1.42E-02	0.72	2.36E-05
ENSCAFG0000001261	GORASP2	grey	VSMC_M10	0.03	8.71E-01	-0.28	1.62E-01	-0.12	5.42E-01	-0.23	2.43E-01	0.21	2.94E-01	-0.09	6.44E-01	-0.38	4.83E-02	0.28	1.65E-01	0.33	9.17E-02	-0.38	4.81E-02
ENSCAFG0000001239	ERFE	cyan	VSMC_M2	0.03	8.71E-01	0.44	2.08E-02	0.26	1.95E-01	-0.13	5.27E-01	-0.29	1.46E-01	0.00	9.97E-01	-0.07	7.46E-01	-0.01	9.42E-01	-0.41	3.31E-02	0.25	2.02E-01
ENSCAFG0000003053	HSPB8	grey	VSMC_M10	0.03	8.71E-01	0.44	2.12E-02	-0.20	3.12E-01	0.58	1.61E-03	-0.01	9.75E-01	0.21	2.83E-01	-0.23	2.41E-01	-0.68	9.85E-05	-0.38	4.94E-02	0.72	2.45E-05
ENSCAFG0000002012	DNTTIP2	darkolivegreen	VSMC_M9	0.03	8.72E-01	-0.69	5.84E-05	-0.24	2.37E-01	-0.47	1.40E-02	0.43	2.54E-02	-0.13	5.20E-01	0.10	6.27E-01	0.64	3.03E-04	0.57	1.84E-03	-0.79	1.09E-06
ENSCAFG0000002050	ENSCAFG0000002050	grey	VSMC_M10	0.03	8.72E-01	-0.08	6.88E-01	-0.54	3.89E-03	0.37	5.81E-02	0.40	3.96E-02	0.25	2.04E-01	-0.06	7.57E-01	-0.18	3.68E-01	0.02	9.12E-01	0.22	2.77E-01
ENSCAFG0000000088	ENSCAFG0000000088	grey	VSMC_M10	0.03	8.72E-01	-0.02	9.31E-01	-0.09	6.40E-01	0.19	3.34E-01	0.06	7.72E-01	-0.28	1.50E-01	-0.04	8.29E-01	-0.15	4.66E-01	0.05	8.19E-01	0.13	5.04E-01
ENSCAFG0000000642	MAP4K3	pink	VSMC_M5	0.03	8.72E-01	-0.07	7.44E-01	-0.79	8.51E-07	0.82	1.48E-07	0.75	5.44E-06	-0.19	3.31E-01	-0.33	9.71E-02	-0.61	6.80E-04	0.10	6.09E-01	0.21	2.95E-01
ENSCAFG0000001705	KRTCAP2	grey	VSMC_M10	0.03	8.73E-01	0.66	1.78E-04	0.30	1.26E-01	0.35	7.22E-02	-0.49	9.11E-03	0.13	5.27E-01	-0.31	1.20E-01	-0.50	8.30E-03	-0.59	1.28E-03	0.71	3.28E-05
ENSCAFG0000003147	ENSCAFG0000003147	grey	VSMC_M10	0.03	8.73E-01	-0.05	7.85E-01	-0.08	6.80E-01	0.00	9.91E-01	0.11	5.77E-01	-0.06	7.85E-01	-0.12	5.47E-01	-0.03	8.66E-01	0.05	8.07E-01	0.00	9.92E-01
ENSCAFG0000000695	RGS20	grey	VSMC_M10	0.03	8.73E-01	0.15	4.63E-01	0.16	4.38E-01	-0.03	8.89E-01	-0.25	2.06E-01	0.27	1.72E-01	-0.06	7.52E-01	0.00	9.88E-01	-0.16	4.30E-01	0.26	1.91E-01
ENSCAFG0000001546	PIMREG	darkgrey	VSMC_M8	0.03	8.74E-01	-0.41	3.37E-02	0.50	7.78E-03	-0.93	3.03E-12	-0.33	8.78E-02	0.12	5.49E-01	0.32	1.05E-01	0.95	1.06E-13	0.25	2.08E-01	-0.67	1.40E-04
ENSCAFG0000003269	ENSCAFG0000003269	grey	VSMC_M10	0.03	8.74E-01	0.19	3.35E-01	-0.08	6.75E-01	0.23	2.54E-01	0.00	9.97E-01	0.26	1.91E-01	-0.24	2.37E-01	-0.25	2.07E-01	-0.12	5.51E-01	0.28	1.59E-01
ENSCAFG0000000604	SLC23A2	grey	VSMC_M10	0.03	8.74E-01	-0.56	2.27E-03	-0.12	5.38E-01	-0.27	1.81E-01	0.15	4.46E-01	0.34	8.08E-02	0.20	3.26E-01	0.47	1.27E-02	0.45	1.92E-02	-0.48	1.15E-02
ENSCAFG0000002949	ENSCAFG0000002949	grey	VSMC_M10	0.03	8.74E-01	0.42	2.97E-02	0.25	2.13E-01	-0.08	7.02E-01	-0.32	1.02E-01	-0.01	9.64E-01	0.15	4.65E-01	-0.14	4.75E-01	-0.40	3.94E-02	0.34	7.81E-02
ENSCAFG0000000746	IDE	grey	VSMC_M10	0.03	8.74E-01	-0.30	1.30E-01	-0.43	2.68E-02	0.01	9.51E-01	0.49	9.64E-03	-0.16	4.38E-01	0.05	8.12E-01	-0.01	9.62E-01	0.32	9.84E-02	-0.17	3.83E-01
ENSCAFG0000000616	RMDN2	grey	VSMC_M10	0.03	8.74E-01	-0.41	3.52E-02	-0.32	1.04E-01	0.09	6.51E-01	0.50	8.44E-03	-0.21	2.93E-01	-0.19	3.36E-01	0.07	7.38E-01	0.40	3.77E-02	-0.55	2.77E-03
ENSCAFG0000003256	EMD	cyan	VSMC_M2	0.03	8.75E-01	0.30	1.23E-01	0.46	1.46E-02	-0.18	3.70E-01	-0.52	5.93E-03	0.04	8.53E-01	-0.01	9.52E-01	-0.03	8.78E-01	-0.22	2.74E-01	0.24	2.33E-01
ENSCAFG0000001571	RAC1	darkgrey	VSMC_M8	0.03	8.75E-01	-0.68	9.30E-05	0.09	6.64E-01	-0.64	3.37E-04	0.13	5.03E-01	0.01	9.43E-01	0.22	2.65E-01	0.77	2.40E-06	0.59	1.22E-03	-0.88	1.79E-09
ENSCAFG0000000762	C1H19orf12	grey	VSMC_M10	0.03	8.75E-01	0.49	1.02E-02	0.32	9.94E-02	-0.05	8.00E-01	-0.35	6.94E-02	0.07	7.37E-01	0.25	2.10E-01	-0.18	3.63E-01	-0.43	2.69E-02	0.40	3.76E-02
ENSCAFG0000000288	KLK13	grey	VSMC_M10	0.03	8.75E-01	0.00	1.00E+00	0.00	9.82E-01	0.01	9.46E-01	-0.05	7.95E-01	-0.15	4.48E-01	-0.28	1.62E-01	-0.01	9.75E-01	0.03	8.74E-01	0.01	9.49E-01
ENSCAFG0000000220	KIAA1211	grey	VSMC_M10	0.03	8.75E-01	0.37	5.76E-02	0.37	5.94E-02	-0.14	5.01E-01	-0.47	1.25E-02	0.33	8.90E-02	-0.18	3.60E-01	0.00	9.97E-01	-0.42	2.75E-02	0.33	9.04E-02
ENSCAFG0000001419	MOSPD3	grey	VSMC_M10	0.03	8.75E-01	0.62	5.55E-04	0.26	1.90E-01	0.28	1.55E-01	-0.40	3.90E-02	0.23	2.57E-01	-0.10	6.22E-01	-0.44	2.02E-02	-0.55	3.06E-03	0.58	1.41E-03
ENSCAFG0000001118	SIAE	darkgreen	VSMC_M4	0.03	8.75E-01	0.35	7.31E-02	-0.47	1.32E-02	0.80	4.94E-07	0.35	7.78E-02	-0.08	6.86E-01	-0.27	1.71E-01	-0.83	9.56E-08	-0.21	2.85E-01	0.60	8.76E-04
ENSCAFG0000000094	MAN1A1	darkgrey	VSMC_M8	0.03	8.75E-01	-0.30	1.23E-01	0.34	8.41E-02	-0.73	1.79E-05	-0.12	5.51E-01	-0.17	3.95E-01	0.31	1.20E-01	0.63	3.88E-04	0.31	1.19E-01	-0.60	9.00E-04
ENSCAFG0000001646	FBXL20	darkgreen	VSMC_M4	0.03	8.75E-01	0.10	6.29E-01	-0.56	2.24E-03	0.85	2.66E-08	0.52	5.74E-03	-0.28	1.63E-01	-0.21	2.83E-01	-0.76	4.47E-06	0.01	9.43E-01	0.35	7.00E-02
ENSCAFG0000000241	DST	pink	VSMC_M5	0.03	8.76E-01	-0.31	1.11E-01	-0.63	4.82E-04	0.40	4.11E-02	0.68	1.09E-04	-0.25	2.02E-01	-0.11	5.92E-01	-0.29	1.39E-01	0.32	1.09E-01	-0.09	6.49E-01
ENSCAFG0000000223	BARX1	turquoise	VSMC_M6	0.03	8.77E-01	-0.03	8.89E-01	-0.13	5.05E-01	0.05	8.15E-01	0.07	7.22E-01	0.43	2.47E-02	-0.22	2.68E-01	-0.09	6.51E-01	0.00	9.86E-01	0.01	9.71E-01
ENSCAFG0000000832	PEX2	grey	VSMC_M10	0.03	8.77E-01	0.41	3.14E-02	0.34	7.81E-02	0.09	6.70E-01	-0.39	4.19E-02	0.07	7.16E-01	0.02	9.23E-01	-0.24	2.25E-01	-0.38	4.79E-02	0.38	4.84E-02
ENSCAFG0000001987	PIEZO1	grey	VSMC_M10	0.03	8.77E-01	0.44	2.08E-02	0.10	6.23E-01	0.37	6.10E-02	-0.24	2.24E-01	0.34	8.10E-02	-0.44	2.06E-02	-0.36	6.53E-02	-0.42	2.87E-02	0.47	1.42E-02
ENSCAFG0000001180	SC5D	grey	VSMC_M10	0.03	8.78E-01	0.13	5.11E-01	-0.19	3.30E-01	0.26	1.99E-01	0.24	2.22E-01	-0.31	1.17E-01	0.34	8.13E-02	-0.33	8.93E-02	-0.09	6.57E-01	0.18	3.75E-01
ENSCAFG0000000178	PRDM4	grey	VSMC_M10	0.03	8.78E-01	-0.24	2.19E-01	-0.17	3.97E-01	0.14	4.73E-01	0.00	9.97E-01	0.22	2.81E-01	0.04	8.25E-01	0.06	7.54E-01	0.09	6.64E-01	0.13	5.32E-01
ENSCAFG0000000514	SRSF2	darkgrey	VSMC_M8	0.03	8.78E-01	-0.21	2.98E-01	0.52	5.04E-03	-0.74	1.08E-05	-0.43	2.37E-02	0.23	2.54E-01	0.01	9.42E-01	0.75	7.40E-06	0.11	5.71E-01	-0.50	7.51E-03
ENSCAFG0000001661	RNF111	grey	VSMC_M10	0.03	8.78E-01	-0.15	4.45E-01	-0.50	8.05E-03	0.43	2.49E-02	0.33	9.20E-02	0.10	6.36E-01	-0.07	7.37E-01	-0.30	1.23E-01	0.09	6.37E-01	0.29	1.47E-01
ENSCAFG0000000161	CCND3	grey	VSMC_M10	0.03	8.78E-01	0.11	6.00E-01	-0.11	5.90E-01	0.43	2.47E-02	0.01	9.80E-01	0.05	8.08E-01	-0.29	1.37E-01	-0.33	9.45E-02	-0.08	6.76E-01	0.26	1.91E-01
ENSCAFG0000000748	ENSCAFG0000000748	grey	VSMC_M10	0.03	8.78E-01	0.09	6.71E-01	0.14	4.73E-01	-0.10	6.31E-01	-0.19	3.51E-01	0.00	9.82E-01	0.41	3.17E-02	0.09	6.56E-01	-0.15	4.45E-01	0.13	5.03E-01
ENSCAFG0000001990	SLC7A5	grey	VSMC_M10	0.03	8.78E-01	-0.20	3.06E-01	-0.05	7.96E-01	-0.08	6.92E-01	-0.02	9.11E-01	0.20	3.10E-01	0.27	1.80E-01	0.13	5.26E-01	0.06	7.79E-01	0.06	7.70E-01
ENSCAFG0000000675	POLR1D	yellow	VSMC_M3	0.03	8.79E-01	0.12	5.50E-01	0.69	7.74E-05	-0.55	2.91E-03	-0.64	3.40E-04	0.12	5.65E-01	0.22	2.62E-01	0.36	6.42E-02	-0.14	4.75E-01	-0.09	6.44E-01
ENSCAFG0000001396	VPS51	yellow	VSMC_M3	0.03	8.79E-01	0.65	2.22E-04	0.77	2.98E-06	-0.29	1.49E-01	-0.74	9.13E-06	0.15	4.53E-01	-0.03	8.86E-01	-0.06	7.56E-01	-0.55	2.81E-03	0.30	1.28E-01
ENSCAFG0000000086	SMOC2	darkgreen	VSMC_M4	0.03	8.79E-01	0.43	2.48E-02	-0.31	1.19E-01	0.76	4.16E-06	0.15	4.52E-01	0.02	9.26E-01	-0.01	9.41E-01	-0.72	2.35E-05	-0.42	2.82E-02	0.66	1.74E-04
ENSCAFG0000000722	ENSCAFG0000000722	grey	VSMC_M10	0.03	8.79E-01	-0.15	4.49E-01	-0.22	2.75E-01	0.04	8.60E-01	0.31	1.17E-01	-0.14	4.80E-01	0.13	5.29E-01	-0.06	7.82E-01	0.15	4.46E-01	-0.18	3.60E-01
ENSCAFG0000001873	KIAA0100	grey	VSMC_M10	0.03	8.80E-01	-0.45	1.92E-02	-0.42	2.78E-02	-0.11	5.84E-01	0.46	1.48E-02	0.13	5.20E-01	0.27	1.78E-01	0.26	1.85E-01	0.34	8.47E-02	-0.30	1.28E-01
ENSCAFG0000000701	ENSCAFG0000000701	grey	VSMC_M10	0.03	8.80E-01	0.15	4.66E-01	0.18	3.68E-01	-0.20	3.13E-01	-0.30	1.27E-01	0.27	1.68E-01	0.25	2.00E-01	0.13	5.28E-01	-0.21	2.84E-01	0.20	3.11E-01
ENSCAFG0000001407	ENSCAFG0000001407	grey	VSMC_M10	0.03	8.80E-01	0.15	4.55E-01	0.35	7.31E-02	-0.16	4.28E-01	-0.43	2.46E-02	0.29	1.39E-01	0.25	2.09E-01	0.14	4.80E-01	-0.27	1.75E-01	0.21	2.84E-01
ENSCAFG0000001681	PSEN1	grey	VSMC_M10	0.03	8.80E-01	0.47	1.29E-02	-0.08	6.84E-01	0.61	7.92E-04	0.08	6.74E-01	-0.30	1.25E-01	-0.24	2.36E-01	-0.75	6.56E-06	-0.31	1.13E-01	0.46	1.55E-02
ENSCAFG0000001594	ALOX15	cyan	VSMC_M2	0.03	8.81E-01	0.65	2.56E-04	0.28	1.54E-01	0.05	7.93E-01	-0.37	5.74E-02	-0.04	8.59E-01	-0.35	7.09E-02	-0.21	2.87E-01	-0.58	1.59E-03	0.46	1.68E-02
ENSCAFG0000000039	NUP43	darkgrey	VSMC_M8	0.03	8.81E-01	-0.59	1.31E-03	-0.23	2.39E-01	-0.35	7.77E-02	0.26	1.90E-01	0.08	7.04E-01	0.36	6.84E-02	0.60	8.47E-04	0.38	5.25E-02	-0.44	2.07E-02
ENSCAFG0000000977	THYN1	darkgrey	VSMC_M8	0.03	8.81E-01	-0.21	2.82E-01	0.33	9.31E-02	-0.80	6.44E-07	-0.25	2.03E-01	0.10	6.23E-01	0.33	8.83E-02	0.75	6.42E-06	0.09	6.70E-01	-0.41	3.26E-02
ENSCAFG0000001184	SESN2	darkgreen	VSMC_M4	0.03	8.81E-01	0.17	3.96E-01	-0.18	3.79E-01	0.43	2.57E-02	0.05	7.99E-01	0.01	9.74E-01	0.21	3.03E-01	-0.44	2.22E-02	-0.24	2.31E-01	0.51	6.42E-03
ENSCAFG0000001255	SS18L1	grey	VSMC_M10	0.03	8.81E-01	0.23	2.39E-01	0.10	6.08E-01	0.33	9.51E-02	-0.11	5.75E-01	0.10	6.17E-01	-0.33	8.95E-02	-0.46	1.65E-02	-0.13	5.14E-01	0.24	2.24E-01
ENSCAFG0000002432	NOL3	grey	VSMC_M10	0.03	8.81E-01	0.42	2.96E-02	0.48	1.20E-02	0.03	9.01E-01	-0.61	6.58E-04	0.06	7.64E-01	-0.01	9.78E-01	-0.15	4.58E-01	-0.37	5.50E-02	0.51	6.82E-03
ENSCAFG0000001137	CDK2AP2	yellow	VSMC_M3	0.03	8.81E-01	0.05	8.05E-01	0.55	2.73E-03	-0.29	1.46E-01	-0.51	6.77E-03	0.11	5.68E-01	0.13	5.17E-01	0.19	3.44E-01	-0.09	6.58E-01	-0.07	7.35E-01
ENSCAFG0000001568	STK11IP	grey	VSMC_M10	0.03	8.81E-01	0.30	1.26E-01	0.22	2.79E-01	0.10	6.14E-01	-0.20	3.28E-01	-0.11	6.02E-01	-0.11	5.89E-01	-0.18	3.64E-01	-0.31	1.14E-01	0.13	5.30E-01
ENSCAFG0000001093	GTPBP6	grey	VSMC_M10	0.03	8.82E-01	0.14	4.76E-01	0.47	1.27E-02	-0.39	4.33E-02	-0.47	1.41E-02	0.39	4.19E-02	0.07	7.19E-01	0.25	2.15E-01	-0.18	3.67E-01	0.02	9.09E-01
ENSCAFG0000001386	NMT1	grey	VSMC_M10	0.03	8.82E-01	-0.34	8.48E-02	0.02	9.16E-01	-0.25	2.15E-01	-0.01	9.42E-01	0.21	2.82E-01	0.15	4.52E-01	0.38	4.80E-02	0.28	1.58E-01	-0.23	2.41E-01
ENSCAFG0000002585	ENSCAFG0000002585	grey	VSMC_M10	0.03	8.82E-01	0.28	1.62E-01	0.15	4.56E-01	-0.09	6.49E-01	-0.20	3.25E-01	-0.01	9.66E-01	0.06	7.62E-01	0.03	8.66E-01	-0.32	9.88E-02	0.22	2.72E-01
ENSCAFG0000000190	KIF24	darkgrey	VSMC_M8	0.03	8.83E-01	-0.54	3.71E-03	0.08	6.83E-01	-0.66	1.94E-04	-0.02	9.05E-01	0.07	7.31E-01	0.57	1.78E-03	0.83	7.11E-08	0.30	1.31E-01	-0.51	6.58E-03
ENSCAFG0000000323	ENSCAFG0000000323	grey	VSMC_M10	0.03	8.83E-01	-0.20	3.29E-01	0.17	3.85E-01	-0.23	2.41E-01	-0.18	3.76E-01	0.11	5.92E-01	0.20	3.08E-01	0.25	2.15E-01	0.18	3.63E-01	-0.12	5.43E-01
ENSCAFG0000001656	ENSCAFG0000001656	grey	VSMC_M10	0.03	8.83E-01	0.46	1.51E-02	0.12	5.38E-01	0.33	9.05E-02	-0.15	4.46E-01	-0.15	4.62E-01	-0.27	1.66E-01	-0.37	5.63E-02	-0.41	3.40E-02	0.33	8.90E-02
ENSCAFG0000000447	EED	grey	VSMC_M10	0.03	8.83E-01	-0.11	5.97E-01	-0.24	2.23E-01	0.40	3.86E-02	0.23	2.52E-01	-0.14	4.98E-01	-0.13	5.03E-01	-0.32	1.09E-01	0.11	5.88E-01	0.08	6.79E-01
ENSCAFG0000001289	HAT1	darkgrey	VSMC_M8	0.03	8.83E-01	-0.68	9.73E-05	-0.09	6.67E-01	-0.48	1.13E-02	0.17	3.90E-01	0.06	7.74E-01	0.53	4.61E-03	0.71	3.73E-05	0.47	1.44E-02	-0.56	2.20E-03
ENSCAFG0000001453	OIT3	grey	VSMC_M10	0.03	8.83E-01	0.64	3.10E-04	0.07	7.27E-01	0.53	4.90E-03	-0.21	2.97E-01	-0.10	6.33E-01	-0.19	3.51E-01	-0.69	7.77E-05	-0.54	3.70E-03	0.68	1.01E-04
ENSCAFG0000001594	ARHGEF19	grey	VSMC_M10	0.03	8.84E-01	0.05	8.13E-01	0.20	3.09E-01	-0.17	3.98E-01	0.02	9.17E-01	-0.31	1.11E-01	0.28	1.59E-01	0.02	9.06E-01	0.04	8.29E-01	-0.27	1.70E-01
ENSCAFG0000001555	HAUS8	darkgrey	VSMC_M8	0.03	8.84E-01	-0.42	2.81E-02	0.37	5.54E-02	-0.74	1.14E-05	-0.27	1.74E-01	-0.04	8.27E-01	0.16	4.22E-01	0.79	8.50E-07	0.27	1.67E-01	-0.56	2.53E-03
ENSCAFG0000001767	GGA2	grey	VSMC_M10	0.03	8.85E-01	0.36	6.47E-02	0.07	7.29E-01	0.08	6.96E-01	0.04	8.37E-01	-0.28	1.52E-01	-0.06	7.71E-01	-0.31	1.22E-01	-0.21	2.89E-01	0.15	4.60E-01
ENSCAFG0000000149	KLHDC10	pink	VSMC_M5	0.03	8.85E-01	-0.39	4.16E-02	-0.54	3.39E-03	0.13	5.10E-01	0.56	2.56E-03	-0.07	7.31E-01	-0.09	6.58E-01	0.00	9.80E-01	0.37	6.03E-02	-0.22	2.66E-01
ENSCAFG0000001833	COLEC12	grey	VSMC_M10	0.03	8.85E-01	0.36	6.77E-02	0.40	4.01E-02	-0.09	6.52E-01	-0.52	5.45E-03	0.45	1.77E-02	0.07	7.46E-01	0.02	9.11E-01	-0.40	3.71E-02	0.32	9.97E-02
ENSCAFG0000001324	ENSCAFG0000001324	cyan	VSMC_M2	0.03	8.86E-01	0.68	1.05E-04	0.46	1.58E-02	-0.02	9.08E-01	-0.51	6.03E-03	0.01	9.45E-01	0.09	6.50E-01	-0.29	1.44E-01	-0.61	6.66E-04	0.56	2.59E-03

ENSCAFG0000000373	TUT4	grey	VSMC_M10	0.03	8.86E-01	-0.26	1.84E-01	-0.67	1.21E-04	0.25	2.02E-01	0.68	9.14E-05	0.07	7.35E-01	0.02	9.40E-01	-0.13	5.15E-01	0.26	1.82E-01	-0.08	6.80E-01
ENSCAFG0000002680	ENSCAFG0000002680	grey	VSMC_M10	0.03	8.86E-01	0.24	2.24E-01	0.05	8.18E-01	-0.03	8.86E-01	0.00	9.90E-01	-0.06	7.71E-01	-0.15	4.41E-01	-0.14	5.01E-01	-0.19	3.35E-01	0.02	9.29E-01
ENSCAFG0000001746	NSMCE1	grey	VSMC_M10	0.03	8.86E-01	-0.04	8.35E-01	-0.03	8.72E-01	0.04	8.60E-01	0.15	4.48E-01	-0.13	5.21E-01	0.29	1.37E-01	-0.01	9.64E-01	0.04	8.24E-01	-0.18	3.61E-01
ENSCAFG0000000959	PKD2	grey	VSMC_M10	0.03	8.86E-01	0.45	1.98E-02	-0.39	4.20E-02	0.69	6.37E-05	0.29	1.38E-01	-0.17	4.07E-01	-0.11	5.90E-01	-0.75	5.42E-06	-0.36	6.50E-02	0.61	7.92E-04
ENSCAFG0000001990	JPH3	grey	VSMC_M10	0.03	8.86E-01	-0.18	3.74E-01	-0.50	7.22E-03	0.38	5.19E-02	0.51	6.93E-03	-0.14	4.90E-01	0.16	4.38E-01	-0.22	2.79E-01	0.12	5.36E-01	-0.03	8.95E-01
ENSCAFG0000001318	EGLN3	grey	VSMC_M10	0.03	8.87E-01	0.10	6.07E-01	-0.38	5.37E-02	0.63	3.92E-04	0.15	4.58E-01	0.40	4.05E-02	0.06	7.83E-01	-0.53	4.61E-03	-0.14	4.93E-01	0.56	2.44E-03
ENSCAFG0000001334	ELL3	cyan	VSMC_M2	0.03	8.87E-01	0.74	8.81E-06	0.46	1.67E-02	0.15	4.49E-01	-0.60	9.08E-04	-0.03	8.88E-01	-0.04	8.27E-01	-0.38	4.90E-02	-0.68	1.07E-04	0.74	1.07E-05
ENSCAFG0000003270	ST8SIA6	grey	VSMC_M10	0.03	8.88E-01	0.34	8.52E-02	0.09	6.70E-01	0.08	6.92E-01	-0.18	3.68E-01	0.17	4.00E-01	-0.33	9.54E-02	-0.16	4.27E-01	-0.29	1.38E-01	0.33	9.76E-02
ENSCAFG0000000101	PPP2CA	darkolivegreen	VSMC_M9	0.03	8.88E-01	-0.74	9.24E-06	-0.37	5.48E-02	-0.19	3.41E-01	0.47	1.25E-02	0.16	4.14E-01	-0.05	8.06E-01	0.42	2.77E-02	0.66	2.05E-04	-0.61	7.65E-04
ENSCAFG0000002891	RWDD3	grey	VSMC_M10	0.03	8.88E-01	0.30	1.24E-01	-0.05	8.00E-01	0.24	2.26E-01	-0.05	8.09E-01	0.15	4.46E-01	0.12	5.45E-01	-0.27	1.67E-01	-0.35	7.07E-02	0.43	2.34E-02
ENSCAFG0000002369	PHETA1	grey	VSMC_M10	0.03	8.88E-01	0.27	1.68E-01	0.54	3.70E-03	-0.24	2.21E-01	-0.60	9.52E-04	0.02	9.13E-01	0.18	3.72E-01	0.07	7.45E-01	-0.28	1.53E-01	0.31	1.19E-01
ENSCAFG0000002897	ENSCAFG0000002897	grey	VSMC_M10	0.03	8.89E-01	-0.26	1.84E-01	-0.23	2.42E-01	-0.06	7.79E-01	0.23	2.54E-01	0.26	1.95E-01	0.19	3.30E-01	0.17	3.86E-01	0.13	5.32E-01	-0.18	3.57E-01
ENSCAFG0000001998	PLCG2	darkgreen	VSMC_M4	0.03	8.89E-01	0.42	3.07E-02	-0.35	7.41E-02	0.78	1.31E-06	0.21	3.02E-01	0.07	7.42E-01	-0.32	1.03E-01	-0.73	1.65E-05	-0.35	7.12E-02	0.59	1.12E-03
ENSCAFG0000002575	ENSCAFG0000002575	violet	VSMC_M7	0.03	8.89E-01	-0.04	8.30E-01	0.14	4.91E-01	-0.17	3.97E-01	-0.11	5.79E-01	0.03	8.69E-01	0.65	2.51E-04	0.13	5.18E-01	0.00	9.87E-01	-0.02	9.36E-01
ENSCAFG0000001835	IL13RA1	cyan	VSMC_M2	0.03	8.89E-01	0.69	7.18E-05	-0.04	8.30E-01	0.50	8.45E-03	0.01	9.68E-01	-0.18	3.63E-01	-0.59	1.07E-03	-0.66	1.79E-04	-0.52	5.32E-03	0.52	5.53E-03
ENSCAFG0000001588	SCCPDH	grey	VSMC_M10	0.03	8.89E-01	-0.41	3.15E-02	0.04	8.25E-01	-0.19	3.53E-01	0.05	7.97E-01	-0.09	6.51E-01	-0.05	7.98E-01	0.28	1.51E-01	0.40	3.76E-02	-0.39	4.37E-02
ENSCAFG0000001750	SPESP1	grey	VSMC_M10	0.03	8.89E-01	0.04	8.29E-01	-0.27	1.73E-01	0.22	2.66E-01	0.29	1.37E-01	-0.01	9.72E-01	-0.14	4.98E-01	-0.30	1.26E-01	0.00	9.86E-01	0.11	5.69E-01
ENSCAFG0000000485	CIC	yellow	VSMC_M3	0.03	8.90E-01	0.45	1.82E-02	0.67	1.14E-04	-0.57	1.74E-03	-0.57	1.76E-03	-0.05	8.00E-01	0.12	5.36E-01	0.24	2.18E-01	-0.39	4.16E-02	0.05	7.96E-01
ENSCAFG0000003110	ENSCAFG0000003110	grey	VSMC_M10	0.03	8.90E-01	0.02	9.22E-01	0.24	2.23E-01	-0.06	7.78E-01	-0.22	2.62E-01	-0.05	7.91E-01	0.13	5.25E-01	-0.06	7.58E-01	0.03	8.68E-01	0.07	7.29E-01
ENSCAFG0000002809	ENSCAFG0000002809	grey	VSMC_M10	0.03	8.91E-01	0.04	8.49E-01	0.03	8.77E-01	-0.09	6.53E-01	-0.03	8.71E-01	-0.09	6.49E-01	0.11	5.95E-01	0.01	9.63E-01	-0.01	9.65E-01	0.03	8.74E-01
ENSCAFG0000000851	CHMP4C	grey	VSMC_M10	0.03	8.91E-01	0.12	5.66E-01	-0.03	8.98E-01	0.06	7.70E-01	-0.04	8.49E-01	0.20	3.18E-01	-0.14	5.01E-01	-0.07	7.31E-01	-0.10	6.26E-01	0.18	3.63E-01
ENSCAFG0000002286	ENSCAFG0000002286	grey	VSMC_M10	0.03	8.91E-01	0.00	9.83E-01	0.30	1.35E-01	-0.37	5.91E-02	-0.27	1.74E-01	0.08	7.02E-01	0.23	2.53E-01	0.33	9.07E-02	-0.06	7.71E-01	-0.17	4.05E-01
ENSCAFG0000001323	PISD	grey	VSMC_M10	0.03	8.91E-01	0.74	8.52E-06	0.16	4.31E-01	0.37	5.95E-02	-0.24	2.18E-01	-0.14	4.78E-01	-0.49	8.71E-03	-0.57	1.93E-03	-0.62	5.84E-04	0.64	2.90E-04
ENSCAFG0000000869	ACOT12	grey	VSMC_M10	0.03	8.92E-01	0.07	7.45E-01	0.05	8.13E-01	0.03	8.63E-01	-0.04	8.33E-01	-0.04	8.43E-01	0.00	9.97E-01	-0.04	8.57E-01	-0.06	7.59E-01	0.07	7.32E-01
ENSCAFG0000000048	CAPS2	grey	VSMC_M10	0.03	8.92E-01	0.21	2.99E-01	-0.19	3.45E-01	0.20	3.08E-01	0.23	2.43E-01	-0.24	2.34E-01	-0.14	4.93E-01	-0.30	1.31E-01	-0.14	5.00E-01	0.05	7.99E-01
ENSCAFG0000001566	GNB5	grey	VSMC_M10	0.03	8.92E-01	0.18	3.81E-01	-0.03	8.69E-01	0.42	2.84E-02	-0.05	8.24E-01	-0.01	9.44E-01	-0.06	7.81E-01	-0.40	3.93E-02	-0.09	6.49E-01	0.34	8.08E-02
ENSCAFG0000000794	ENSCAFG0000000794	grey	VSMC_M10	0.03	8.92E-01	-0.25	2.14E-01	-0.03	8.83E-01	-0.27	1.73E-01	0.11	5.79E-01	0.01	9.67E-01	-0.02	9.34E-01	0.35	7.75E-02	0.19	3.45E-01	-0.33	9.64E-02
ENSCAFG0000001693	AHSP	grey	VSMC_M10	0.03	8.92E-01	0.24	2.19E-01	0.09	6.39E-01	-0.11	5.88E-01	-0.13	5.32E-01	0.36	6.57E-02	0.04	8.43E-01	0.04	8.49E-01	-0.25	2.15E-01	0.20	3.27E-01
ENSCAFG0000001346	C7H1orf21	grey	VSMC_M10	0.03	8.92E-01	0.30	1.28E-01	-0.21	3.02E-01	0.48	1.14E-02	0.23	2.46E-01	-0.43	2.68E-02	0.05	8.08E-01	-0.51	6.12E-03	-0.25	2.15E-01	0.31	1.16E-01
ENSCAFG0000000755	MRPL35	grey	VSMC_M10	0.03	8.93E-01	-0.34	8.73E-02	-0.11	5.93E-01	-0.16	4.20E-01	0.06	7.56E-01	0.15	4.64E-01	0.17	4.06E-01	0.29	1.40E-01	0.24	2.20E-01	-0.21	2.96E-01
ENSCAFG0000000614	TUBGCP3	darkolivegreen	VSMC_M9	0.03	8.93E-01	-0.68	9.21E-05	-0.37	5.91E-02	-0.09	6.58E-01	0.38	5.11E-02	0.21	3.00E-01	0.22	2.59E-01	0.35	7.20E-02	0.51	6.97E-03	-0.42	2.86E-02
ENSCAFG0000000087	SHISAL1	grey	VSMC_M10	0.03	8.93E-01	0.07	7.12E-01	-0.28	1.61E-01	0.18	3.65E-01	0.24	2.34E-01	0.27	1.79E-01	-0.11	5.92E-01	-0.14	4.84E-01	-0.08	6.86E-01	0.07	7.29E-01
ENSCAFG0000001177	ENSCAFG0000001177	turquoise	VSMC_M6	0.03	8.93E-01	0.08	6.90E-01	0.10	6.35E-01	-0.13	5.33E-01	-0.08	7.04E-01	0.30	1.23E-01	-0.13	5.34E-01	0.09	6.71E-01	-0.02	9.23E-01	0.04	8.56E-01
ENSCAFG0000001751	PSMC1	darkgrey	VSMC_M8	0.03	8.93E-01	-0.60	9.67E-04	-0.03	8.83E-01	-0.49	9.04E-03	0.23	2.52E-01	-0.21	3.03E-01	0.08	6.73E-01	0.64	3.25E-04	0.55	2.74E-03	-0.78	1.64E-06
ENSCAFG0000001717	CHRNB2	grey	VSMC_M10	0.03	8.93E-01	-0.01	9.64E-01	0.14	4.86E-01	-0.17	4.07E-01	-0.07	7.13E-01	0.02	9.31E-01	0.31	1.13E-01	0.12	5.55E-01	-0.06	7.54E-01	-0.11	5.95E-01
ENSCAFG0000001702	ZC2HC1C	grey	VSMC_M10	0.03	8.93E-01	-0.06	7.69E-01	0.29	1.41E-01	-0.31	1.10E-01	-0.05	8.06E-01	-0.25	2.03E-01	-0.08	6.87E-01	0.21	2.87E-01	0.07	7.16E-01	-0.44	2.09E-02
ENSCAFG0000000678	LNX2	grey	VSMC_M10	0.03	8.93E-01	-0.11	5.75E-01	-0.27	1.79E-01	0.00	9.94E-01	0.25	2.01E-01	0.00	9.94E-01	0.49	9.89E-03	0.05	8.22E-01	-0.02	9.16E-01	0.08	7.00E-01
ENSCAFG0000001935	HES4	grey	VSMC_M10	0.03	8.94E-01	-0.28	1.59E-01	-0.10	6.16E-01	-0.29	1.43E-01	0.22	2.73E-01	0.30	1.25E-01	0.47	1.34E-02	0.33	9.47E-02	0.19	3.40E-01	-0.34	8.06E-02
ENSCAFG0000001952	SAPCD2	grey	VSMC_M10	0.03	8.94E-01	-0.31	1.17E-01	-0.03	8.83E-01	-0.34	8.23E-02	0.03	8.99E-01	0.20	3.21E-01	0.43	2.65E-02	0.48	1.06E-02	0.15	4.60E-01	-0.26	1.95E-01
ENSCAFG0000000047	CCHCR1	yellow	VSMC_M3	0.03	8.94E-01	0.04	8.38E-01	0.71	3.37E-05	-0.54	3.61E-03	-0.58	1.59E-03	-0.12	5.39E-01	0.17	3.85E-01	0.44	2.33E-02	-0.10	6.32E-01	-0.23	2.39E-01
ENSCAFG0000001576	RHOJ	grey	VSMC_M10	0.03	8.94E-01	0.21	2.86E-01	0.11	5.73E-01	0.14	5.01E-01	-0.14	4.85E-01	0.40	3.71E-02	-0.46	1.46E-02	-0.11	6.00E-01	-0.21	2.91E-01	0.07	7.29E-01
ENSCAFG0000003229	ENSCAFG0000003229	grey	VSMC_M10	0.03	8.95E-01	0.09	6.64E-01	-0.03	8.92E-01	0.18	3.71E-01	-0.04	8.62E-01	-0.08	7.09E-01	0.41	3.16E-02	-0.16	4.18E-01	-0.12	5.58E-01	0.21	3.00E-01
ENSCAFG0000000838	TSC22D2	turquoise	VSMC_M6	0.03	8.95E-01	0.03	8.69E-01	0.11	5.84E-01	-0.05	7.99E-01	-0.22	2.60E-01	0.34	8.75E-02	-0.18	3.58E-01	0.04	8.41E-01	-0.13	5.15E-01	0.21	2.98E-01
ENSCAFG0000002287	ENSCAFG0000002287	grey	VSMC_M10	0.03	8.95E-01	-0.18	3.61E-01	0.07	7.14E-01	-0.36	6.38E-02	0.03	8.66E-01	-0.10	6.11E-01	0.25	2.01E-01	0.37	5.57E-02	0.18	3.70E-01	-0.28	1.58E-01
ENSCAFG0000001425	NYX	cyan	VSMC_M2	0.03	8.96E-01	0.42	3.12E-02	0.52	5.40E-03	-0.04	8.41E-01	-0.59	1.13E-03	0.10	6.24E-01	-0.06	7.51E-01	-0.04	8.38E-01	-0.44	2.13E-02	0.31	1.20E-01
ENSCAFG0000001272	ENSCAFG0000001272	grey	VSMC_M10	0.03	8.96E-01	0.05	8.07E-01	-0.06	7.80E-01	0.09	6.69E-01	0.05	8.21E-01	-0.10	6.32E-01	-0.22	2.80E-01	-0.08	6.87E-01	0.03	8.66E-01	0.04	8.24E-01
ENSCAFG0000000724	GNPTAB	cyan	VSMC_M2	0.03	8.96E-01	0.61	7.71E-04	0.34	8.06E-02	-0.09	6.68E-01	-0.35	7.66E-02	-0.16	4.21E-01	-0.46	1.48E-02	-0.13	5.32E-01	-0.48	1.11E-02	0.32	1.09E-01
ENSCAFG0000002802	ENSCAFG0000002802	grey	VSMC_M10	0.03	8.96E-01	0.34	7.91E-02	0.12	5.62E-01	0.00	9.83E-01	-0.15	4.68E-01	-0.05	8.14E-01	0.15	4.54E-01	-0.06	7.56E-01	-0.34	7.89E-02	0.28	1.50E-01
ENSCAFG0000000506	LMO7	darkgreen	VSMC_M4	0.03	8.96E-01	0.40	4.10E-02	-0.41	3.46E-02	0.84	4.67E-08	0.24	2.33E-01	-0.09	6.54E-01	-0.56	2.14E-03	-0.73	1.32E-05	-0.33	9.05E-02	0.60	9.22E-04
ENSCAFG0000001247	PDE5A	pink	VSMC_M5	0.03	8.97E-01	-0.22	2.76E-01	-0.70	4.46E-05	0.70	4.98E-05	0.67	1.38E-04	-0.07	7.39E-01	-0.32	1.04E-01	-0.46	1.61E-02	0.21	3.01E-01	0.03	8.71E-01
ENSCAFG0000000968	GCNT2	grey	VSMC_M10	0.03	8.97E-01	-0.14	4.95E-01	-0.07	7.22E-01	-0.06	7.72E-01	0.04	8.45E-01	0.12	5.55E-01	-0.23	2.39E-01	0.09	6.57E-01	0.18	3.62E-01	-0.05	8.20E-01
ENSCAFG0000001776	APPBP2	pink	VSMC_M5	0.03	8.97E-01	-0.23	2.49E-01	-0.80	6.36E-07	0.51	6.41E-03	0.79	1.01E-06	-0.19	3.35E-01	-0.10	6.37E-01	-0.32	1.02E-01	0.20	3.11E-01	0.01	9.58E-01
ENSCAFG0000003019	DRAM2	grey	VSMC_M10	0.03	8.97E-01	0.02	9.22E-01	-0.02	9.27E-01	-0.17	3.87E-01	0.06	7.73E-01	-0.04	8.41E-01	0.15	4.52E-01	0.12	5.59E-01	-0.01	9.80E-01	-0.03	8.75E-01
ENSCAFG0000000228	ENSCAFG0000000228	grey	VSMC_M10	0.03	8.98E-01	0.13	5.06E-01	0.18	3.70E-01	-0.15	4.70E-01	-0.22	2.71E-01	0.00	9.90E-01	0.40	3.71E-02	0.00	9.83E-01	-0.18	3.78E-01	0.13	5.23E-01
ENSCAFG0000001893	ENSCAFG0000001893	grey	VSMC_M10	0.03	8.98E-01	-0.24	2.36E-01	-0.33	8.90E-02	0.15	4.52E-01	0.31	1.16E-01	-0.02	9.04E-01	-0.19	3.36E-01	0.04	8.43E-01	0.16	4.29E-01	-0.11	5.95E-01
ENSCAFG0000003248	ENSCAFG0000003248	darkgreen	VSMC_M4	0.03	8.98E-01	0.39	4.35E-02	-0.50	7.97E-03	0.84	3.49E-08	0.28	1.55E-01	-0.01	9.50E-01	-0.41	3.32E-02	-0.78	1.81E-06	-0.35	7.24E-02	0.72	2.14E-05
ENSCAFG0000001752	RNF43	grey	VSMC_M10	0.03	8.98E-01	0.21	2.92E-01	-0.19	3.52E-01	0.40	3.81E-02	0.11	5.78E-01	-0.04	8.50E-01	-0.19	3.37E-01	-0.33	8.84E-02	-0.20	3.26E-01	0.33	9.42E-02
ENSCAFG0000000609	ENSCAFG0000000609	grey	VSMC_M10	0.03	8.98E-01	-0.15	4.45E-01	0.08	7.05E-01	-0.27	1.72E-01	-0.06	7.63E-01	0.04	8.43E-01	0.20	3.20E-01	0.31	1.22E-01	0.05	7.98E-01	-0.11	6.02E-01
ENSCAFG0000003039	ENSCAFG0000003039	grey	VSMC_M10	0.03	8.98E-01	0.06	7.74E-01	0.49	9.42E-03	-0.29	1.43E-01	-0.45	1.87E-02	0.04	8.57E-01	-0.11	5.84E-01	0.16	4.25E-01	0.01	9.52E-01	-0.05	8.01E-01
ENSCAFG0000002901	ENSCAFG0000002901	grey	VSMC_M10	0.03	8.98E-01	0.46	1.47E-02	0.28	1.60E-01	0.06	7.67E-01	-0.41	3.47E-02	0.07	7.11E-01	-0.02	9.36E-01	-0.14	4.96E-01	-0.44	2.04E-02	0.46	1.65E-02
ENSCAFG0000001207	ENSCAFG0000001207	grey	VSMC_M10	0.03	8.98E-01	0.39	4.25E-02	0.13	5.32E-01	0.39	4.15E-02	-0.27	1.80E-01	0.28	1.55E-01	-0.12	5.39E-01	-0.41	3.45E-02	-0.40	4.05E-02	0.45	1.94E-02
ENSCAFG0000001855	ENSCAFG0000001855	grey	VSMC_M10	0.03	8.98E-01	-0.06	7.78E-01	-0.42	2.74E-02	0.34	8.43E-02	0.45	1.82E-02	-0.03	8.93E-01	-0.31	1.19E-01	-0.31	1.12E-01	0.16	4.30E-01	-0.04	8.46E-01
ENSCAFG0000000340	PDIA4	yellow	VSMC_M3	0.03	8.99E-01	0.55	3.02E-03	0.49	9.95E-03	0.08	6.84E-01	-0.58	1.57E-03	-0.09	6.65E-01	-0.18	3.61E-01	-0.21	3.01E-01	-0.55	2.96E-03	0.46	1.56E-02
ENSCAFG0000000909	ENSCAFG0000000909	grey	VSMC_M10	0.03	8.99E-01	-0.13	5.18E-01	-0.06	7.52E-01	-0.03	8.94E-01	0.04	8.39E-01	-0.04	8.47E-01	-0.02	9.17E-01	0.08	7.04E-01	0.16	4.26E-01	-0.06	7.79E-01
ENSCAFG0000003068	ENSCAFG0000003068	cyan	VSMC_M2	0.03	8.99E-01	0.41	3.47E-02	0.55	2.78E-03	-0.25	2.15E-01	-0.49	9.62E-03	0.02	9.15E-01	-0.04	8.49E-01	-0.02	9.23E-01	-0.33	9.80E-02	0.11	5.97E-01
ENSCAFG0000000367	CCDC155	grey	VSMC_M10	0.03	8.99E-01	0.15	4.56E-01	0.11	5.80E-01	-0.06	7.58E-01	-0.14	4.77E-01	-0.04	8.30E-01	-0.08	6.91E-01	0.02	9.10E-01	-0.18	3.65E-01	0.08	6.76E-01
ENSCAFG0000001790	SLC25A47	grey	VSMC_M10	0.03	8.99E-01	0.15	4.56E-01	0.11	5.80E-01	-0.06	7.58E-01	-0.14	4.77E-01	-0.04	8.30E-01	-0.08	6.91E-01	0.02	9.10E-01	-0.18	3.65E-01	0.08	6.76E-01
ENSCAFG0000001958	FAM50A	yellow	VSMC_M3	0.03	8.99E-01	0.06	7.82E-01	0.76	3.49E-06	-0.70	5.17E-05	-0.70	4.83E-05	-0.03	8.76E-01	0.16	4.36E-01	0.56	2.43E-03	-0.11	5.99E-01	-0.19	3.30E-01
ENSCAFG0000000074	KCNV1	grey	VSMC_M10	0.03	8.99E-01	0.05	8.02E-01	0.20	3.17E-01	-0.32	1.07E-01	-0.18	3.77E-01	0.03	8.73E-01	0.29	1.36E-01	0.26	1.91E-01	-0.10	6.25E-01	0.00	9.91E-01
ENSCAFG0000000946	NKX2-3	turquoise	VSMC_M6	0.03	8.99E-01	-0.06	7.84E-01	-0.09	6.46E-01	-0.01	9.51E-01	0.03	8.89E-01	0.35	7.19E-02	-0.11	5.83E-01	-0.05	8.07E-01	0.00	9.88E-01	0.04	8.44E-01
ENSCAFG0000000790	SLC11A2	yellow	VSMC_M3	0.03	8.99E-01	0.08	7.03E-01	0.67	1.22E-04	-0.55	2.87E-03	-0.63	4.74E-04	0.23	2.53E-01	0.09	6.41E-01	0.43	2.65E-02	-0.13	5.17E-01	-0.15	4.59E-01
ENSCAFG0000002487	ENSCAFG0000002487	grey	VSMC_M10	0.03	8.99E-01	0.20	3.27E-01	0.49	9.85E-03	-0.15	4.61E-01	-0.57	1.99E-03	0.29	1.48E-01	0.01	9.60E-01	0.11	5.90E-01	-0.26	1.90E-01	0.19	3.54E-01
ENSCAFG0000000194	GALT	grey	VSMC_M10	0.03	8.99E-01	0.62	4.92E-04	0.03	8.65E-01	0.49	9.11E-03	-0.15	4.61E-01	-0.06	7.62E-01	-0.28	1.60E-01	-0.65	2.66E-04	-0.45	1.95E-02	0.68	9.28E-05
ENSCAFG0000000464	ENSCAFG0000000464	grey	VSMC_M10	0.03	9.00E-01	0.17	3.99E-01	0.11	5.95E-01	-0.12	5.53E-01	-0.12	5.44E-01	0.17	4.11E-01	-0.15	4.63E-01	0.01	9.46E-01	-0.18	3.56E-01	0.09	6.41E-01
ENSCAFG0000001685	SLC25A44	grey	VSMC_M10	0.03	9.00E-01	0.26	1.96E-01	-0.39	4.15E-02	0.46	1.47E-02	0.21	3.02E-01	0.07	7.42E-01	-0.15	4.44E-01	-0.42	3.06E-02	-0.24	2.21E-01	0.52	5.53E-03
ENSCAFG0000001864	SLC25A23	grey	VSMC_M10	0.02	9.03E-01	0.31	1.18E-01	-0.16	4.36E-01	0.48	1.09E-02	0.00	9.86E-01	-0.06	7.75E-01	-0.32	1.04E-01	-0.47	1.31E-02	-0.23	2.47E-01	0.51	6.72E-03
ENSCAFG0000001067	ETFBKMT	yellow	VSMC_M3	0.02	9.03E-01	0.37	5.40E-02	0.64	3.57E-04	-0.26	1.98E-01	-0.77	2.32E-06	0.34	7.90E-02	0.11	6.01E-01	0.15	4.57E-01	-0.44	2.16E-02	0.36	6.47E-02
ENSCAFG0000002551	SERPINB6	cyan	VSMC_M2	0.02	9.03E-01	0.62	5.23E-04	0.70	4.09E-05	-0.24	2.37E-01	-0.78	1.91E-06	0.16	4.27E-01	-0.14	4.78E-01	-0.02	9.19E-01	-0.58	1.54E-03	0.38	5.27E-02
ENSCAFG0000000263	RDH13	grey	VSMC_M10	0.02	9.03E-01	-0.29	1.42E-01	0.13	5.16E-01	-0.31	1.14E-01	-0.12	5.46E-01	0.38	5.29E-02	0.42	2.78E-02	0.45	1.87E-02	0.09	6.71E-01	-0.27	1.75E-01
ENSCAFG0000000916	AMFR	grey	VSMC_M10	0.02	9.03E-01	0.25	2.07E-01	-0.01	9.51E-01	0.40	4.09E-02	-0.08	6.95E-01	0.00	9.99E-01	-0.33	8.82E-02	-0.41	3.23E-02	-0.18	3.56E-01	0.32	1.09E-01
ENSCAFG0000000932	UEVLD	pink	VSMC_M5	0.02	9.03E-01	0.15	4.44E-01	-0.47	1.36E-02	0.41	3.33E-02	0.52	5.92E-03	-0.28	1.51E-01	-0.27	1.81E-01	-0.43	2.47E-02	-0.05	8.16E-01	0.13	5.12E-01
ENSCAFG0000002508	KLHL23	grey	VSMC_M10	0.02	9.03E-01	-0.27	1.71E-01	-0.42	3.09E-02	0.00	9.86E-01	0.41	3.24E-02	0.06	7.67E-01	0.25	2.08E-01	0.10	6.04E-01	0.21	2.88E-01	-0.14	5.01E-01
ENSCAFG0000001526	MYBBP1A	darkgrey	VSMC_M8	0.02	9.04E-01	-0.29	1.42E-01	0.54	3.59E-03	-0.78	1.75E-06	-0.49	8.71E-03	0.27	1.66E-01	0.21	2.95E-01	0.84	3.41E-08	0.11	5.74E-01	-0.45	1.81E-02
ENSCAFG0000001093	NPNT	darkgreen	VSMC_M4	0.02	9.04E-01	0.31	1.20E-01	-0.36	6.81E-02	0.64	3.59E-04	0.39	4.30E-02	-0.33	9.47E-02	-0.57	1.84E-03	-0.64	2.95E-04	-0.11	5.88E-01	0.25	2.08E-01
ENSCAFG0000001601	FSCN1	darkgrey	VSMC_M8	0.02	9.04E-01	-0.24	2.33E-01	0.55	2.70E-03	-0.87	3.01E-09	-0.44	2.08E-02	0.26	1.89E-01	0.22	2.70E-01	0.85	1.42E-08	0.14	4.85E-01	-0.51	6.69E-03
ENSCAFG0000002416	FJX1	grey	VSMC_M10	0.02	9.04E-01	0.43	2.61E-02	0.26	1.89E-01	0.02	9.31E-01	-0.37	5.92E-02	0.12	5.50E-01	0.08	6.79E-01	-0.24	2.23E-01	-0.36	6.19E-02	0.45	1.80E-02
ENSCAFG0000001572	STK32B	grey	VSMC_M10	0.02	9.04E-01	-0.19	3.37E-01	0.30	1.32E-01	-0.49	9.88E-03	-0.17	4.08E-01	-0.07	7.35E-01	0.40	3.77E-02	0.45	1.91E-02	0.09	6.55E-01	-0.38	5.21E-02
ENSCAFG0000000875	DDN	grey	VSMC_M10	0.02	9.04E-01	-0.07	7.28E-01	0.16	4.27E-01	-0.28	1.60E-01	-0.19	3.40E-01	-0.05	8.05E-01	0.41	3.26E-02	0.31	1.14E-01	-0.02	9.29E-01	-0.04	8.45E-01
ENSCAFG0000000138	ENSCAFG0000000138	cyan	VSMC_M2	0.02	9.04E-01	0.53	4.83E-03	0.46	1.52E-02	0.01	9.48E-01	-0.46	1.47E-02	0.16	4.23E-01	-0.13	5.16E-01	-0.20	3.21E-01	-0.47	1.36E-02	0.27	1.72E-01
ENSCAFG0000000155	PSIP1	darkgrey	VSMC_M8	0.02	9.04E-01	-0.42	2.74E-02	0.20	3.10E-01	-0.68	1.09E-04	-0.02	9.26E-01	-0.10	6.17E-01	0.39	4.20E-02	0.76	4.83E-06	0.28	1.63E-01	-0.63	4.41E-04
ENSCAFG0000000832	TM4SF18	turquoise	VSMC_M6	0.02	9.05E-01	-0.19	3.32E-01	0.04	8.52E-01	-0.16	4.32E-01	-0.07	7.39E-01	0.89	8.17E-10	0.02	9.37E-01	0.24	2.30E-01	0.12	5.67E-01	-0.18	3.74E-01
ENSCAFG0000001161	SF3B4	darkgrey	VSMC_M8	0.02	9.05E-01	-0.06	7.67E-01	0.45	1.86E-02	-0.71	3.62E-05	-0.36	6.57E-02	0.09	6.65E-01	0.07	7.28E-01	0.65	2.34E-04	-0.02	9.04E-01	-0.34	8.45E-02
ENSCAFG0000001487	DDHD1	grey	VSMC_M10	0.02	9.05E-01	-0.03	8.99E-01	0.18	3.74E-01	-0.35	7.64E-02	-0.11	5.75E-01	-0.33	8.93E-02	0.13	5.15E-01	0.32	1.06E-01	-0.03	8.73E-01	-0.18	3.78E-01
ENSCAFG0000000962	ENSCAFG0000000962	grey	VSMC_M10	0.02	9.05E-01	0.01	9.62E-01	0.12	5.60E-01	-0.27	1.66E-01	-0.14	4.72E-01	-0.06	7.50E-01	0.40	3.93E-02	0.18	3.56E-01	-0.09	6.68E-01	0.03	8.71E-01
ENSCAFG0000000325	YRDC	darkgrey	VSMC_M8	0.02	9.06E-01	-0.48	1.17E-02	0.17	3.91E-01	-0.48	1.14E-02	-0.12	5.64E-01	0.34	8.12E-02	0.17	3.90E-01	0.57	1.97E-03	0.36	6.59E-02	-0.50	7.48E-03
ENSCAFG0000000140	NRBP2	grey	VSMC_M10	0.02	9.06E-01	0.59	1.31E-03	-0.12	5.41E-01	0.56	2.22E-03	-0.03	8.85E-01	-0.09	6.61E-01	-0.17	4.05E-01	-0.70	5.10E-05	-0.48	1.12E-02	0.70	4.23E-05
ENSCAFG0000001044	ACADS	cyan	VSMC_M2	0.02	9.06E-01	0.42	2.80E-02	0.37	5.77E-02	0.07	7.41E-01	-0.54	3.66E-03	0.11	5.96E-01	-0.31	1.15E-01	-0.07	7.33E-01	-0.46	1.67E-02	0.43	2.38E-02
ENSCAFG0000001015	SUMF2	grey	VSMC_M10	0.02	9.07E-01	0.24	2.28E-01	0.33	9.23E-02	-0.04	8.55E-01	-0.17	3.97E-01	-0.27	1.76E-01	-0.29	1.45E-01	-0.14	4.95E-01	-0.06	7.73E-01	-0.11	5.77E-01
ENSCAFG0000001701	ENSCAFG0000001701	darkolivegreen	VSMC_M9	0.02	9.07E-01	-0.53	4.85E-03	-0.17	4.00E-01	-0.16	4.38E-01	0.30	1.32E-01	-0.09	6.64E-01	0.35	7.54E-02	0.20	3.14E-01	0.52	5.43E-03	-0.43	2.57E-02
ENSCAFG0000000041	AKAP12	cyan	VSMC_M2	0.02	9.07E-01	0.52	5.88E-03	0.51	6.53E-03	-0.09	6.67E-01	-0.57	1.73E-03	0.22	2.79E-01	-0.27	1.81E-01	-0.07	7.24E-01	-0.48	1.07E-02	0.31	1.10E-01
ENSCAFG0000001089	ENSCAFG0000001089	grey	VSMC_M10	0.02	9.07E-01	0.14	4.96E-01	-0.36	6.23E-02	0.58	1.57E-03	0.34	8.41E-02	-0.18	3.70E-01	-0.17	4.03E-01	-0.55	2.68E-03	-0.06	7.52E-01	0.26	1.86E-01
ENSCAFG0000001032	OSBPL5	cyan	VSMC_M2	0.02	9.07E-01	0.74	9.41E-06	0.46	1.69E-02	0.03	8.78E-01	-0.51	6.32E-03	0.01	9.64E-01	0.16	4.13E-01	-0.32	1.01E-01	-0.68	1.03E-04	0.59	1.34E-03
ENSCAFG0000000440	MYO7B	grey	VSMC_M10	0.02	9.07E-01	0.41	3.36E-02	0.07	7.28E-01	0.11	5.95E-01	-0.15	4.41E-01	-0.12	5.44E-01	0.04	8.32E-01	-0.16	4.23E-01	-0.42	2.74E-02	0.41	3.47E-02
ENSCAFG0000000010	ENSCAFG0000000010	grey	VSMC_M10	0.02	9.07E-01	0.32	1.09E-01	0.27	1.71E-01	-0.16	4.16E-01	-0.33	9.08E-02	0.16	4.15E-01	-0.33	8.93E-02	0.05	8.11E-01	-0.32	1.03E-01	0.19	3.40E-01
ENSCAFG0000001764	MYO9A	grey	VSMC_M10	0.02	9.08E-01	-0.44	2.29E-02	-0.39	4.73E-02	-0.02	9.06E-01	0.61	6.90E-04	-0.20	3.29E-01	0.02	9.30E-01	0.10	6.04E-01	0.42	3.09E-02	-0.54	3.69E-03
ENSCAFG0000003267	FGF13	darkgreen	VSMC_M4	0.02	9.08E-01	0.37	6.00E-02	-0.46	1.61E-02	0.90	2.41E-10	0.33	9.34E-02	-0.09	6.46E-01	-0.13	5.22E-01	-0.84	3.43E-08	-0.30	1.31E-01	0.63	4.00E-04
ENSCAFG0000000348	PQLC3	darkgreen	VSMC_M4	0.02	9.08E-01	0.07	7.19E-01	-0.49	9.98E-03	0.62	6.34E-04	0.54	3.98E-03	-0.29	1.42E-01	-0.28	1.59E-01	-0.59	1.26E-03	0.07	7.26E-01	0.11	5.97E-01
ENSCAFG0000001643	CAPN8	grey	VSMC_M10	0.02	9.08E-01	-0.22	2.66E-01	-0.37	5.43E-02	0.26	1.95E-01	0.33	9.53E-02	-0.09	6.68E-01	0.37	5.96E-02	-0.07	7.19E-01	0.10	6.35E-01	0.09	6.61E-01
ENSCAFG0000000754	ENSCAFG0000000754	grey	VSMC_M10	0.02	9.08E-01	-0.18	3.68E-01	-0.41	3.22E-02	0.22	2.75E-01	0.38	5.01E-02	-0.28	1.60E-01	0.12	5.58E-01	-0.11	5.98E-01	0.06	7.50E-01	-0.02	9.18E-01
ENSCAFG0000002946	OSGIN2	darkgreen	VSMC_M4	0.02	9.09E-01	0.50	7.61E-03	-0.15	4.49E-01	0.71	3.44E-05	-0.08	6.95E-01	0.38	4.85E-02	-0.12	5.40E-01	-0.70	4.45E-05	-0.45	1.72E-02	0.80	6.10E-07
ENSCAFG0000000104	DESI1	darkgrey	VSMC_M8	0.02	9.09E-01	-0.39	4.48E-02	0.25	2.05E-01	-0.67	1.15E-04	-0.03	8.96E-01	-0.13	5.25E-01	-0.09	6.59E-01	0.62	6.02E-04	0.37	5.49E-02	-0.70	5.25E-05
ENSCAFG0000000778	CHMP2B	grey	VSMC_M10	0.02	9.09E-01	-0.35	7.58E-02	-0.44	2.10E-02	0.20	3.13E-01	0.64	3.58E-04	-0.41	3.57E-02	0.07	7.35E-01	-0.10	6.35E-01	0.41	3.21E-02	-0.42	2.90E-02
ENSCAFG0000001067	ZNF496	cyan	VSMC_M2	0.02	9.09E-01	0.50	8.63E-03	0.44	2.07E-02	-0.24	2.22E-01	-0.45	1.74E-02	0.00	1.00E+00	-0.06	7.81E-01	0.01	9.51E-01	-0.46	1.67E-02	0.32	1.02E-01
ENSCAFG0000000102	RNF217	grey	VSMC_M10	0.02	9.09E-01	-0.19	3.55E-01	-0.21	3.05E-01	0.05	8.06E-01	0.25	2.09E-01	0.12	5.51E-01	0.10	6.18E-01	-0.01	9.50E-01	0.20	3.15E-01	-0.16	4.15E-01
ENSCAFG0000001269	CAPN10	yellow	VSMC_M3	0.02	9.10E-01	0.43	2.56E-02	0.74	9.25E-06	-0.19	3.40E-01	-0.74	1.21E-05	0.02	9.39E-01	-0.24	2.29E-01	0.01	9.55E-01	-0.38	4.82E-02	0.19	3.34E-01
ENSCAFG0000001601	ENSCAFG0000001601	yellow	VSMC_M3	0.02	9.10E-01	0.07	7.46E-01	0.48	1.20E-02	-0.33	9.03E-02	-0.49	8.75E-03	-0.04	8.26E-01	0.16	4.32E-01	0.24	2.19E-01	-0.13	5.31E-01	-0.01	9.52E-01

ENSCAFG0000000362	IL15	grey	VSMC_M1C	0.02	9.10E-01	0.25	2.00E-01	0.16	4.17E-01	-0.10	6.08E-01	-0.19	3.31E-01	0.06	7.83E-01	0.04	8.61E-01	0.02	9.34E-01	-0.24	2.25E-01	0.15	4.41E-01
ENSCAFG0000003304	ZNF292	pink	VSMC_M5	0.02	9.10E-01	-0.33	9.49E-02	-0.59	1.31E-03	0.39	4.19E-02	0.68	8.17E-05	-0.38	4.97E-02	-0.21	2.93E-01	-0.27	1.73E-01	0.41	3.44E-02	-0.18	3.78E-01
ENSCAFG0000002300	ENSCAFG0000002300	turquoise	VSMC_M6	0.02	9.10E-01	0.33	9.56E-02	0.28	1.61E-01	-0.07	7.41E-01	-0.37	5.75E-02	0.82	2.08E-07	-0.18	3.79E-01	-0.02	9.08E-01	-0.33	9.55E-02	0.25	2.04E-01
ENSCAFG0000000754	MPPED2	grey	VSMC_M1C	0.02	9.10E-01	-0.32	1.02E-01	-0.41	3.56E-02	0.22	2.75E-01	0.31	1.17E-01	0.23	2.57E-01	0.11	5.71E-01	-0.01	9.71E-01	0.23	2.55E-01	0.09	6.50E-01
ENSCAFG0000001057	SLC6A3	grey	VSMC_M1C	0.02	9.11E-01	-0.13	5.15E-01	-0.41	3.19E-02	0.42	3.08E-02	0.41	3.42E-02	-0.13	5.14E-01	-0.19	3.32E-01	-0.37	5.97E-02	0.26	1.96E-01	0.08	6.79E-01
ENSCAFG0000000283	TMEM245	darkgreen	VSMC_M4	0.02	9.11E-01	0.46	1.63E-02	-0.30	1.25E-01	0.73	1.67E-05	0.17	4.01E-01	0.00	9.99E-01	-0.28	1.53E-01	-0.81	3.18E-07	-0.32	9.83E-02	0.59	1.13E-03
ENSCAFG0000000725	ENSCAFG0000000725	grey	VSMC_M1C	0.02	9.11E-01	0.22	2.71E-01	-0.06	7.50E-01	0.03	8.74E-01	0.02	9.35E-01	-0.19	3.33E-01	0.11	5.76E-01	-0.09	6.43E-01	-0.28	1.57E-01	0.22	2.81E-01
ENSCAFG0000000291	UTP3	grey	VSMC_M1C	0.02	9.11E-01	-0.19	3.38E-01	0.22	2.71E-01	-0.13	5.32E-01	-0.20	3.06E-01	0.01	9.61E-01	0.11	5.78E-01	0.21	3.04E-01	0.08	6.94E-01	-0.11	5.93E-01
ENSCAFG0000000567	SALL2	darkgreen	VSMC_M4	0.02	9.11E-01	0.62	6.11E-04	-0.28	1.62E-01	0.82	1.77E-07	0.13	5.19E-01	-0.13	5.10E-01	-0.33	9.11E-02	-0.89	7.45E-10	-0.52	5.96E-03	0.75	7.55E-06
ENSCAFG0000001412	ENSCAFG0000001412	grey	VSMC_M1C	0.02	9.11E-01	0.00	9.91E-01	-0.10	6.05E-01	0.20	3.19E-01	0.13	5.32E-01	0.01	9.73E-01	0.16	4.31E-01	-0.10	6.35E-01	-0.02	9.05E-01	-0.01	9.46E-01
ENSCAFG0000003104	MLNR	grey	VSMC_M1C	0.02	9.11E-01	0.21	2.94E-01	0.52	5.57E-03	-0.34	8.10E-02	-0.62	5.94E-04	0.23	2.54E-01	0.34	8.05E-02	0.23	2.54E-01	-0.26	1.91E-01	0.23	2.55E-01
ENSCAFG0000003088	ENSCAFG0000003088	darkgreen	VSMC_M4	0.02	9.12E-01	-0.18	3.60E-01	-0.39	4.56E-02	0.52	5.23E-03	0.35	7.30E-02	-0.05	7.89E-01	-0.40	3.77E-02	-0.33	9.66E-02	0.23	2.57E-01	-0.02	9.34E-01
ENSCAFG0000000961	IFIT1	darkgreen	VSMC_M4	0.02	9.12E-01	0.47	1.26E-02	-0.21	2.92E-01	0.61	7.45E-04	0.04	8.33E-01	0.10	6.21E-01	-0.24	2.30E-01	-0.63	4.89E-04	-0.42	2.83E-02	0.64	3.36E-04
ENSCAFG0000000928	CCAR2	yellow	VSMC_M3	0.02	9.12E-01	0.29	1.37E-01	0.64	3.56E-04	-0.48	1.21E-02	-0.66	1.75E-04	0.09	6.46E-01	0.02	9.06E-01	0.36	6.19E-02	-0.33	9.31E-02	0.06	7.73E-01
ENSCAFG0000001728	ADRA1B	darkgreen	VSMC_M4	0.02	9.12E-01	0.45	1.98E-02	-0.37	5.76E-02	0.87	3.49E-09	0.18	3.83E-01	-0.06	7.79E-01	-0.44	2.08E-02	-0.79	8.81E-07	-0.37	5.88E-02	0.70	4.77E-05
ENSCAFG0000001551	ENSCAFG0000001551	grey	VSMC_M1C	0.02	9.12E-01	-0.04	8.45E-01	0.00	9.96E-01	-0.09	6.41E-01	-0.05	8.00E-01	0.31	1.20E-01	-0.03	8.84E-01	0.16	4.23E-01	-0.02	9.38E-01	0.01	9.44E-01
ENSCAFG0000001392	SEMA6D	grey	VSMC_M1C	0.02	9.12E-01	0.05	8.06E-01	-0.56	2.21E-03	0.58	1.42E-03	0.43	2.61E-02	0.10	6.31E-01	-0.14	4.75E-01	-0.40	3.63E-02	-0.04	8.32E-01	0.30	1.27E-01
ENSCAFG0000000553	LGALS3BP	cyan	VSMC_M2	0.02	9.12E-01	0.84	3.18E-08	0.24	2.19E-01	0.52	5.01E-03	-0.34	8.07E-02	-0.15	4.66E-01	-0.43	2.37E-02	-0.76	5.28E-06	-0.66	1.89E-04	0.74	1.01E-05
ENSCAFG0000001026	SLC35C2	yellow	VSMC_M3	0.02	9.13E-01	-0.04	8.42E-01	0.66	1.64E-04	-0.69	7.14E-05	-0.62	5.84E-04	0.27	1.74E-01	0.14	4.78E-01	0.55	2.68E-03	-0.02	9.30E-01	-0.22	2.76E-01
ENSCAFG0000001755	GPR68	grey	VSMC_M1C	0.02	9.13E-01	-0.21	2.97E-01	0.16	4.23E-01	-0.46	1.49E-02	-0.18	3.70E-01	0.57	1.78E-03	0.31	1.15E-01	0.43	2.39E-02	0.07	7.47E-01	-0.19	3.47E-01
ENSCAFG0000000842	SELENOT	darkgrey	VSMC_M8	0.02	9.13E-01	-0.59	1.21E-03	-0.17	4.07E-01	-0.46	1.65E-02	0.36	6.67E-02	-0.15	4.50E-01	0.17	3.87E-01	0.58	1.46E-03	0.55	2.98E-03	-0.68	9.84E-05
ENSCAFG0000000426	ENKUR	darkgreen	VSMC_M4	0.02	9.13E-01	-0.07	7.32E-01	-0.40	3.82E-02	0.47	1.41E-02	0.32	1.09E-01	-0.06	7.78E-01	0.04	8.53E-01	-0.31	1.13E-01	0.03	8.89E-01	0.19	3.42E-01
ENSCAFG0000000774	CDK7	grey	VSMC_M1C	0.02	9.13E-01	-0.19	3.53E-01	0.28	1.63E-01	-0.32	1.02E-01	-0.30	1.32E-01	0.15	4.66E-01	0.13	5.19E-01	0.34	7.88E-02	0.13	5.12E-01	-0.12	5.40E-01
ENSCAFG0000003079	TSTD3	pink	VSMC_M5	0.02	9.13E-01	-0.35	7.39E-02	-0.49	9.40E-03	0.17	3.84E-01	0.58	1.61E-03	-0.40	3.96E-02	0.13	5.14E-01	0.01	9.67E-01	0.32	1.03E-01	-0.25	2.03E-01
ENSCAFG0000002879	ENSCAFG0000002879	grey	VSMC_M1C	0.02	9.13E-01	0.20	3.22E-01	0.21	2.88E-01	-0.23	2.49E-01	-0.22	2.80E-01	0.05	8.21E-01	0.02	9.33E-01	0.17	3.86E-01	-0.19	3.34E-01	0.10	6.06E-01
ENSCAFG0000001375	MEX3B	yellow	VSMC_M3	0.02	9.13E-01	0.18	3.80E-01	0.58	1.69E-03	-0.37	5.71E-02	-0.53	4.23E-03	0.25	2.06E-01	0.01	9.71E-01	0.17	3.97E-01	-0.16	4.21E-01	-0.03	8.84E-01
ENSCAFG0000000597	SIPA1L3	darkgrey	VSMC_M8	0.02	9.14E-01	-0.29	1.49E-01	0.48	1.12E-02	-0.82	1.79E-07	-0.35	7.67E-02	0.20	3.13E-01	0.21	2.82E-01	0.74	9.02E-06	0.19	3.53E-01	-0.54	3.29E-03
ENSCAFG0000002955	BMF	cyan	VSMC_M2	0.02	9.14E-01	0.45	1.82E-02	0.21	2.90E-01	-0.12	5.35E-01	-0.23	2.55E-01	0.01	9.55E-01	-0.22	2.66E-01	-0.15	4.68E-01	-0.33	8.96E-02	0.30	1.28E-01
ENSCAFG0000000360	DLGAP3	grey	VSMC_M1C	0.02	9.14E-01	0.34	8.36E-02	0.25	2.07E-01	-0.15	4.55E-01	-0.30	1.25E-01	0.01	9.80E-01	-0.07	7.19E-01	0.03	8.64E-01	-0.33	9.04E-02	0.20	3.29E-01
ENSCAFG0000002361	ANKRD54	grey	VSMC_M1C	0.02	9.14E-01	-0.22	2.70E-01	0.10	6.07E-01	-0.51	7.20E-03	0.03	8.68E-01	-0.05	8.08E-01	0.30	1.27E-01	0.46	1.52E-02	0.14	4.99E-01	-0.47	1.40E-02
ENSCAFG0000001001	SIAH1	grey	VSMC_M1C	0.02	9.14E-01	0.17	3.87E-01	-0.39	4.25E-02	0.44	2.23E-02	0.38	5.14E-02	-0.11	5.83E-01	0.02	9.25E-01	-0.41	3.48E-02	-0.15	4.55E-01	0.19	3.43E-01
ENSCAFG0000001310	GLUL	grey	VSMC_M1C	0.02	9.14E-01	0.21	2.84E-01	0.08	6.79E-01	0.11	5.70E-01	-0.24	2.37E-01	0.23	2.50E-01	0.04	8.59E-01	-0.07	7.44E-01	-0.24	2.27E-01	0.39	4.65E-02
ENSCAFG0000000859	ENSCAFG0000000859	darkgreen	VSMC_M4	0.02	9.14E-01	0.36	6.46E-02	-0.13	5.11E-01	0.50	7.52E-03	0.06	7.76E-01	-0.16	4.20E-01	-0.55	2.68E-03	-0.54	3.94E-03	-0.25	2.16E-01	0.40	3.96E-02
ENSCAFG0000000422	STRN4	grey	VSMC_M1C	0.02	9.15E-01	0.21	2.87E-01	0.37	5.75E-02	-0.26	1.96E-01	-0.36	6.57E-02	0.17	4.05E-01	-0.37	5.74E-02	0.16	4.16E-01	-0.12	5.55E-01	-0.04	8.59E-01
ENSCAFG0000000175	GPR37	grey	VSMC_M1C	0.02	9.15E-01	0.35	7.73E-02	0.19	3.51E-01	-0.07	7.30E-01	-0.20	3.28E-01	-0.06	7.83E-01	-0.12	5.41E-01	-0.04	8.57E-01	-0.32	1.08E-01	0.19	3.45E-01
ENSCAFG0000000952	NUDT9	darkolivegreen	VSMC_M9	0.02	9.15E-01	-0.55	2.92E-03	-0.10	6.15E-01	-0.36	6.36E-02	0.28	1.65E-01	-0.02	9.07E-01	0.09	6.49E-01	0.52	5.84E-03	0.50	8.60E-03	-0.69	6.27E-05
ENSCAFG0000001794	MAPRE2	darkgreen	VSMC_M4	0.02	9.15E-01	0.28	1.52E-01	-0.45	1.79E-02	0.73	1.88E-05	0.40	3.74E-02	-0.17	4.00E-01	-0.27	1.72E-01	-0.72	2.24E-05	-0.16	4.22E-01	0.45	1.87E-02
ENSCAFG0000001858	OXCT1	grey	VSMC_M1C	0.02	9.16E-01	0.66	1.94E-04	0.26	1.97E-01	0.35	7.38E-02	-0.40	3.96E-02	0.13	5.10E-01	0.01	9.71E-01	-0.49	9.18E-03	-0.58	1.68E-03	0.72	2.51E-05
ENSCAFG0000000570	DPY30	grey	VSMC_M1C	0.02	9.16E-01	-0.08	7.03E-01	0.13	5.31E-01	-0.04	8.51E-01	-0.15	4.41E-01	-0.18	3.71E-01	0.12	5.54E-01	0.05	8.20E-01	-0.02	9.13E-01	0.04	8.48E-01
ENSCAFG0000000927	BIN3	grey	VSMC_M1C	0.02	9.16E-01	0.42	2.83E-02	0.44	2.22E-02	0.02	9.08E-01	-0.54	3.55E-03	0.03	8.79E-01	-0.20	3.25E-01	-0.17	4.04E-01	-0.33	8.83E-02	0.41	3.25E-02
ENSCAFG0000001010	TEX264	grey	VSMC_M1C	0.02	9.16E-01	0.54	4.01E-03	-0.10	6.28E-01	0.56	2.16E-03	0.08	6.89E-01	-0.30	1.26E-01	-0.32	9.82E-02	-0.68	9.49E-05	-0.37	6.10E-02	0.47	1.35E-02
ENSCAFG0000001854	NNT	darkgrey	VSMC_M8	0.02	9.17E-01	-0.16	4.15E-01	0.45	1.99E-02	-0.76	4.49E-06	-0.26	1.92E-01	0.03	8.98E-01	0.18	3.60E-01	0.64	3.25E-04	0.17	4.09E-01	-0.50	8.30E-03
ENSCAFG0000000473	TENM4	darkgreen	VSMC_M4	0.02	9.18E-01	0.12	5.41E-01	-0.41	3.39E-02	0.60	1.01E-03	0.34	8.34E-02	-0.03	8.70E-01	-0.20	3.05E-01	-0.53	4.89E-03	-0.10	6.14E-01	0.32	1.01E-01
ENSCAFG0000000160	ENSCAFG0000000160	grey	VSMC_M1C	0.02	9.18E-01	0.23	2.46E-01	0.04	8.37E-01	0.09	6.60E-01	-0.16	4.26E-01	0.19	3.38E-01	-0.09	6.44E-01	-0.08	6.81E-01	-0.25	2.00E-01	0.29	1.47E-01
ENSCAFG0000003101	ENSCAFG0000003101	grey	VSMC_M1C	0.02	9.18E-01	0.32	9.96E-02	0.26	1.85E-01	-0.15	4.56E-01	-0.37	6.06E-02	0.04	8.52E-01	0.24	2.31E-01	0.03	8.88E-01	-0.39	4.36E-02	0.35	7.22E-02
ENSCAFG0000001281	ARFGAP1	darkgrey	VSMC_M8	0.02	9.18E-01	-0.12	5.36E-01	0.63	4.89E-04	-0.76	3.64E-06	-0.45	1.83E-02	0.01	9.59E-01	0.06	7.56E-01	0.70	5.24E-05	0.09	6.50E-01	-0.55	2.78E-03
ENSCAFG0000003008	ENSCAFG0000003008	darkgrey	VSMC_M8	0.02	9.18E-01	-0.53	4.60E-03	0.32	1.00E-01	-0.90	2.93E-10	-0.16	4.13E-01	0.18	3.71E-01	0.42	2.92E-02	0.97	6.98E-17	0.35	7.21E-02	-0.73	1.52E-05
ENSCAFG0000002989	FGF9	darkgreen	VSMC_M4	0.02	9.19E-01	0.14	4.88E-01	-0.44	2.00E-02	0.68	1.08E-04	0.30	1.25E-01	0.14	4.82E-01	-0.33	9.66E-02	-0.59	1.35E-03	-0.12	5.67E-01	0.44	2.22E-02
ENSCAFG0000000418	ZC3H4	grey	VSMC_M1C	0.02	9.19E-01	0.09	6.57E-01	0.26	1.82E-01	-0.25	2.15E-01	-0.11	5.77E-01	0.10	6.28E-01	-0.03	8.72E-01	0.13	5.19E-01	-0.07	7.42E-01	-0.17	4.03E-01
ENSCAFG0000000780	OCLN	darkgreen	VSMC_M4	0.02	9.19E-01	-0.08	6.96E-01	-0.39	4.38E-02	0.46	1.47E-02	0.35	7.28E-02	-0.03	8.82E-01	0.24	2.28E-01	-0.36	6.62E-02	0.09	6.67E-01	0.21	3.00E-01
ENSCAFG0000000866	ENSCAFG0000000866	darkgrey	VSMC_M8	0.02	9.19E-01	-0.59	1.16E-03	-0.09	6.39E-01	-0.58	1.47E-03	0.22	2.70E-01	0.07	7.44E-01	0.16	4.34E-01	0.71	2.87E-05	0.47	1.45E-02	-0.67	1.25E-04
ENSCAFG0000001635	ENSCAFG0000001635	grey	VSMC_M1C	0.02	9.19E-01	0.03	9.01E-01	-0.08	7.10E-01	0.02	9.36E-01	0.13	5.07E-01	-0.05	8.08E-01	-0.12	5.35E-01	-0.09	6.69E-01	0.02	9.02E-01	0.04	8.27E-01
ENSCAFG0000000802	ENSCAFG0000000802	grey	VSMC_M1C	0.02	9.20E-01	-0.34	8.53E-02	-0.36	6.44E-02	0.29	1.41E-01	0.36	6.37E-02	-0.03	8.96E-01	-0.15	4.56E-01	-0.13	5.24E-01	0.32	1.08E-01	-0.13	5.08E-01
ENSCAFG0000001201	P4HTM	darkgreen	VSMC_M4	0.02	9.20E-01	0.61	7.42E-04	-0.12	5.61E-01	0.69	8.00E-05	0.04	8.36E-01	-0.12	5.60E-01	-0.50	8.28E-03	-0.77	2.76E-06	-0.43	2.63E-02	0.57	1.92E-03
ENSCAFG0000000462	MAPRE3	grey	VSMC_M1C	0.02	9.21E-01	0.18	3.79E-01	-0.29	1.37E-01	0.60	1.05E-03	0.06	7.52E-01	0.07	7.34E-01	-0.25	2.10E-01	-0.53	4.64E-03	-0.14	4.82E-01	0.55	2.84E-03
ENSCAFG0000000000	PARD6G	turquoise	VSMC_M6	0.02	9.21E-01	0.09	6.44E-01	0.39	4.29E-02	-0.31	1.16E-01	-0.52	5.23E-03	0.62	5.23E-04	-0.07	7.35E-01	0.25	2.00E-01	-0.16	4.28E-01	0.14	4.81E-01
ENSCAFG0000002857	RTN4RL1	grey	VSMC_M1C	0.02	9.21E-01	-0.09	6.71E-01	0.12	5.42E-01	-0.22	2.60E-01	-0.17	3.94E-01	0.25	2.00E-01	0.54	3.36E-03	0.25	2.05E-01	-0.02	9.39E-01	-0.02	9.29E-01
ENSCAFG0000000244	BEND6	grey	VSMC_M1C	0.02	9.21E-01	-0.23	2.45E-01	0.04	8.35E-01	-0.58	1.64E-03	0.04	8.34E-01	0.11	5.69E-01	0.45	1.85E-02	0.53	4.60E-03	0.11	5.70E-01	-0.32	1.06E-01
ENSCAFG0000000036	TBC1D30	grey	VSMC_M1C	0.02	9.21E-01	0.17	3.90E-01	0.43	2.42E-02	-0.11	5.88E-01	-0.49	8.82E-03	0.40	3.63E-02	0.33	9.07E-02	0.05	8.23E-01	-0.24	2.25E-01	0.19	3.42E-01
ENSCAFG0000000449	ERCC3	grey	VSMC_M1C	0.02	9.21E-01	-0.34	8.76E-02	-0.27	1.71E-01	0.08	7.05E-01	0.27	1.66E-01	0.06	7.79E-01	-0.08	6.81E-01	0.09	6.72E-01	0.39	4.72E-02	-0.19	3.50E-01
ENSCAFG0000001059	HDAC1	grey	VSMC_M1C	0.02	9.21E-01	-0.10	6.23E-01	-0.19	3.45E-01	0.07	7.42E-01	0.23	2.40E-01	0.03	8.96E-01	-0.13	5.28E-01	0.02	9.18E-01	0.14	4.74E-01	-0.19	3.50E-01
ENSCAFG0000003103	EMP1	violet	VSMC_M7	0.02	9.21E-01	-0.33	9.80E-02	-0.08	6.88E-01	-0.26	1.86E-01	0.15	4.54E-01	-0.09	6.50E-01	0.87	3.33E-09	0.26	1.82E-01	0.19	3.35E-01	-0.23	2.55E-01
ENSCAFG0000001489	CDKN3	darkgrey	VSMC_M8	0.02	9.22E-01	-0.45	1.81E-02	-0.01	9.56E-01	-0.60	1.05E-03	0.10	6.03E-01	0.08	6.90E-01	0.37	5.84E-02	0.70	5.05E-05	0.30	1.33E-01	-0.52	5.24E-03
ENSCAFG0000001717	ENSCAFG0000001717	grey	VSMC_M1C	0.02	9.22E-01	-0.19	3.39E-01	-0.04	8.53E-01	-0.14	4.74E-01	0.06	7.70E-01	0.32	1.07E-01	-0.04	8.47E-01	0.20	3.11E-01	0.15	4.53E-01	-0.13	5.13E-01
ENSCAFG0000001076	TESMIN	grey	VSMC_M1C	0.02	9.23E-01	-0.02	9.41E-01	-0.11	5.93E-01	0.25	2.16E-01	0.01	9.65E-01	-0.12	5.39E-01	0.12	5.52E-01	-0.09	6.69E-01	-0.05	8.22E-01	0.17	3.83E-01
ENSCAFG0000001938	PRKCZ	grey	VSMC_M1C	0.02	9.23E-01	-0.38	4.79E-02	0.11	6.01E-01	-0.32	1.00E-01	-0.01	9.61E-01	0.12	5.39E-01	0.03	8.65E-01	0.38	4.98E-02	0.36	6.30E-02	-0.43	2.56E-02
ENSCAFG0000001915	ATCAY	darkgreen	VSMC_M4	0.02	9.24E-01	-0.10	6.18E-01	-0.40	3.62E-02	0.44	2.07E-02	0.29	1.42E-01	0.55	2.71E-03	-0.02	9.05E-01	-0.26	1.85E-01	0.06	7.58E-01	0.17	3.85E-01
ENSCAFG0000002812	ENSCAFG0000002812	grey	VSMC_M1C	0.02	9.24E-01	0.05	7.89E-01	-0.07	7.36E-01	-0.13	5.13E-01	0.10	6.14E-01	0.13	5.19E-01	0.00	9.90E-01	0.10	6.07E-01	-0.10	6.18E-01	-0.11	5.83E-01
ENSCAFG0000000985	GPRIN3	darkgreen	VSMC_M4	0.02	9.24E-01	0.34	8.62E-02	-0.42	2.95E-02	0.89	3.44E-10	0.27	1.75E-01	-0.05	8.02E-01	-0.26	1.85E-01	-0.85	2.16E-08	-0.26	1.90E-01	0.63	4.63E-04
ENSCAFG0000003117	ENSCAFG0000003117	grey	VSMC_M1C	0.02	9.24E-01	0.19	3.30E-01	-0.26	1.97E-01	0.36	6.27E-02	0.11	5.84E-01	0.15	4.56E-01	0.08	6.93E-01	-0.33	8.88E-02	-0.24	2.31E-01	0.48	1.17E-02
ENSCAFG0000000667	SPINK5	violet	VSMC_M7	0.02	9.24E-01	-0.12	5.35E-01	0.09	6.50E-01	-0.15	4.63E-01	-0.16	4.17E-01	0.30	1.33E-01	0.72	2.00E-05	0.18	3.66E-01	-0.06	7.80E-01	0.10	6.07E-01
ENSCAFG0000002907	ENSCAFG0000002907	grey	VSMC_M1C	0.02	9.24E-01	0.50	7.85E-03	0.10	6.33E-01	0.13	5.04E-01	-0.14	4.87E-01	-0.06	7.85E-01	-0.16	4.26E-01	-0.26	1.96E-01	-0.39	4.52E-02	0.39	4.56E-02
ENSCAFG0000001999	CMIP	darkgreen	VSMC_M4	0.02	9.25E-01	0.18	3.62E-01	-0.44	2.12E-02	0.62	6.17E-04	0.28	1.54E-01	0.15	4.49E-01	-0.48	1.10E-02	-0.48	1.19E-02	-0.18	3.64E-01	0.40	3.69E-02
ENSCAFG0000000359	BCL2L12	grey	VSMC_M1C	0.02	9.25E-01	0.41	3.40E-02	0.41	3.39E-02	-0.10	6.18E-01	-0.52	5.04E-03	0.08	6.83E-01	-0.30	1.22E-01	-0.01	9.77E-01	-0.39	4.54E-02	0.32	1.07E-01
ENSCAFG0000001435	CCDC96	grey	VSMC_M1C	0.02	9.26E-01	0.08	6.79E-01	-0.06	7.82E-01	0.00	9.98E-01	0.01	9.64E-01	-0.11	5.79E-01	0.07	7.37E-01	-0.08	6.81E-01	-0.12	5.51E-01	0.14	4.80E-01
ENSCAFG0000003120	ENSCAFG0000003120	grey	VSMC_M1C	0.02	9.26E-01	0.07	7.12E-01	0.26	1.84E-01	-0.30	1.30E-01	-0.30	1.27E-01	-0.11	5.88E-01	0.27	1.81E-01	0.25	2.13E-01	-0.17	4.11E-01	0.06	7.65E-01
ENSCAFG0000001716	NGB	grey	VSMC_M1C	0.02	9.26E-01	0.04	8.55E-01	0.11	5.87E-01	-0.23	2.49E-01	0.02	9.08E-01	-0.08	6.93E-01	0.27	1.71E-01	0.14	4.87E-01	-0.03	8.97E-01	-0.17	3.85E-01
ENSCAFG0000001280	MACO1	grey	VSMC_M1C	0.02	9.26E-01	-0.07	7.18E-01	0.00	9.92E-01	0.06	7.59E-01	0.11	5.73E-01	0.02	9.33E-01	-0.30	1.27E-01	-0.06	7.49E-01	0.16	4.37E-01	-0.23	2.41E-01
ENSCAFG0000003204	ENSCAFG0000003204	grey	VSMC_M1C	0.02	9.27E-01	0.18	3.75E-01	-0.08	6.93E-01	0.38	5.25E-02	-0.11	5.90E-01	0.34	8.13E-02	-0.36	6.41E-02	-0.28	1.58E-01	-0.19	3.44E-01	0.36	6.17E-02
ENSCAFG0000002977	ENSCAFG0000002977	grey	VSMC_M1C	0.02	9.27E-01	0.01	9.43E-01	0.20	3.08E-01	-0.19	3.43E-01	-0.21	3.00E-01	-0.03	9.01E-01	0.39	4.15E-02	0.15	4.49E-01	-0.06	7.75E-01	0.00	9.95E-01
ENSCAFG0000000782	YPEL4	grey	VSMC_M1C	0.02	9.27E-01	0.13	5.09E-01	-0.32	9.85E-02	0.31	1.20E-01	0.37	6.09E-02	-0.29	1.39E-01	0.01	9.63E-01	-0.31	1.10E-01	-0.12	5.44E-01	0.08	6.76E-01
ENSCAFG0000002336	CNDP2	cyan	VSMC_M2	0.02	9.28E-01	0.53	4.57E-03	0.59	1.30E-03	-0.21	2.83E-01	-0.57	1.73E-03	0.13	5.33E-01	0.02	9.37E-01	-0.07	7.30E-01	-0.42	2.85E-02	0.20	3.22E-01
ENSCAFG0000001999	IER5L	cyan	VSMC_M2	0.02	9.28E-01	0.59	1.06E-03	0.49	9.21E-03	-0.22	2.65E-01	-0.43	2.63E-02	0.01	9.44E-01	-0.49	1.02E-02	-0.06	7.56E-01	-0.46	1.68E-02	0.16	4.14E-01
ENSCAFG0000000037	UST	darkgreen	VSMC_M4	0.02	9.28E-01	-0.11	5.71E-01	-0.65	2.78E-04	0.78	1.99E-06	0.57	1.77E-03	-0.13	5.26E-01	-0.32	1.09E-01	-0.61	7.29E-04	0.19	3.55E-01	0.23	2.46E-01
ENSCAFG0000000090	ENSCAFG0000000090	grey	VSMC_M1C	0.02	9.28E-01	0.25	2.16E-01	-0.03	9.00E-01	0.15	4.46E-01	-0.02	9.04E-01	0.36	6.31E-02	-0.17	4.08E-01	-0.22	2.65E-01	-0.18	3.58E-01	0.26	1.83E-01
ENSCAFG0000001069	FGF19	violet	VSMC_M7	0.02	9.28E-01	-0.12	5.64E-01	0.12	5.63E-01	-0.19	3.49E-01	-0.13	5.22E-01	-0.04	8.45E-01	0.84	5.94E-08	0.20	3.08E-01	-0.01	9.76E-01	-0.03	8.69E-01
ENSCAFG0000002846	ENSCAFG0000002846	violet	VSMC_M7	0.02	9.28E-01	-0.12	5.64E-01	0.12	5.63E-01	-0.19	3.49E-01	-0.13	5.22E-01	-0.04	8.45E-01	0.84	5.94E-08	0.20	3.08E-01	-0.01	9.76E-01	-0.03	8.69E-01
ENSCAFG0000000270	ENSCAFG0000000270	grey	VSMC_M1C	0.02	9.28E-01	0.03	8.73E-01	-0.25	2.07E-01	0.24	2.27E-01	0.25	2.02E-01	0.06	7.55E-01	0.11	5.70E-01	-0.22	2.67E-01	-0.09	6.69E-01	0.07	7.45E-01
ENSCAFG0000001196	ENSCAFG0000001196	grey	VSMC_M1C	0.02	9.28E-01	-0.04	8.24E-01	-0.24	2.30E-01	0.22	2.79E-01	0.20	3.20E-01	-0.10	6.27E-01	0.01	9.47E-01	-0.13	5.27E-01	0.06	7.72E-01	0.14	4.91E-01
ENSCAFG0000000539	CMTM8	grey	VSMC_M1C	0.02	9.29E-01	0.23	2.48E-01	0.12	5.64E-01	-0.06	7.49E-01	-0.11	5.87E-01	-0.09	6.58E-01	0.38	5.20E-02	-0.07	7.22E-01	-0.22	2.77E-01	0.26	1.85E-01
ENSCAFG0000000635	METAP2	darkgrey	VSMC_M8	0.02	9.29E-01	-0.54	3.83E-03	0.21	2.84E-01	-0.73	1.70E-05	0.04	8.49E-01	-0.22	2.66E-01	0.33	9.05E-02	0.80	6.90E-07	0.45	1.71E-02	-0.83	8.04E-08
ENSCAFG0000002217	ENSCAFG0000002217	grey	VSMC_M1C	0.02	9.29E-01	0.27	1.74E-01	0.18	3.80E-01	-0.12	5.53E-01	-0.24	2.28E-01	-0.05	8.09E-01	0.13	5.34E-01	0.04	8.50E-01	-0.31	1.13E-01	0.18	3.64E-01
ENSCAFG0000000662	HOXC6	grey	VSMC_M1C	0.02	9.29E-01	-0.14	4.77E-01	0.49	8.80E-03	-0.65	2.61E-04	-0.29	1.39E-01	-0.03	8.98E-01	0.02	9.28E-01	0.51	6.51E-03	0.17	4.06E-01	-0.50	8.46E-03
ENSCAFG0000001783	GGNBP2	pink	VSMC_M5	0.02	9.29E-01	-0.64	2.96E-04	-0.61	7.67E-04	0.12	5.41E-01	0.68	8.71E-05	-0.21	3.04E-01	0.00	9.90E-01	0.15	4.49E-01	0.58	1.59E-03	-0.42	2.89E-02
ENSCAFG0000000401	ZNF438	grey	VSMC_M1C	0.02	9.29E-01	0.09	6.52E-01	-0.31	1.15E-01	0.28	1.56E-01	0.28	1.60E-01	-0.16	4.33E-01	0.08	7.00E-01	-0.30	1.29E-01	-0.09	6.45E-01	0.18	3.71E-01
ENSCAFG0000000100	SMPDL3A	grey	VSMC_M1C	0.02	9.29E-01	-0.23	2.40E-01	-0.03	8.80E-01	-0.17	4.11E-01	0.06	7.71E-01	-0.12	5.59E-01	0.21	2.93E-01	0.25	2.14E-01	0.13	5.07E-01	-0.16	4.23E-01
ENSCAFG0000001146	HOMEZ	grey	VSMC_M1C	0.02	9.30E-01	0.57	1.96E-03	0.11	5.75E-01	0.39	4.42E-02	-0.20	3.19E-01	-0.21	3.03E-01	-0.06	7.56E-01	-0.54	3.48E-03	-0.46	1.67E-02	0.63	4.64E-04
ENSCAFG0000000986	EHD4	darkolivegreen	VSMC_M9	0.02	9.30E-01	-0.72	2.75E-05	-0.44	2.03E-02	-0.02	9.05E-01	0.49	8.77E-03	0.05	8.02E-01	0.10	6.17E-01	0.25	2.11E-01	0.65	2.51E-04	-0.49	1.01E-02
ENSCAFG0000003196	ENSCAFG0000003196	grey	VSMC_M1C	0.02	9.30E-01	0.37	6.04E-02	0.16	4.37E-01	-0.05	8.22E-01	-0.21	2.82E-01	0.00	9.82E-01	-0.06	7.72E-01	-0.15	4.44E-01	-0.32	1.00E-01	0.26	1.90E-01
ENSCAFG0000000837	G3BP2	darkgreen	VSMC_M4	0.02	9.30E-01	-0.02	9.15E-01	-0.34	8.27E-02	0.56	2.27E-03	0.30	1.24E-01	0.05	8.05E-01	-0.10	6.35E-01	-0.48	1.22E-02	0.11	5.75E-01	0.14	4.75E-01
ENSCAFG0000000525	CDC20	darkgrey	VSMC_M8	0.02	9.30E-01	-0.45	1.80E-02	0.45	1.87E-02	-0.93	3.24E-12	-0.28	1.54E-01	0.10	6.24E-01	0.38	4.81E-02	0.97	3.14E-16	0.29	1.37E-01	-0.70	5.29E-05
ENSCAFG0000001087	ABCA9	grey	VSMC_M1C	0.02	9.31E-01	0.68	1.11E-04	-0.03	8.76E-01	0.50	8.10E-03	-0.09	6.62E-01	-0.15	4.70E-01	-0.26	1.89E-01	-0.61	7.54E-04	-0.60	8.40E-04	0.65	2.56E-04
ENSCAFG0000001529	SMTNL2	grey	VSMC_M1C	0.02	9.31E-01	0.53	4.41E-03	-0.03	8.65E-01	0.31	1.11E-01	-0.15	4.41E-01	-0.04	8.25E-01	0.05	8.01E-01	-0.35	7.30E-02	-0.55	3.11E-03	0.64	3.30E-04
ENSCAFG0000002313	ENSCAFG0000002313	grey	VSMC_M1C	0.02	9.31E-01	0.11	5.78E-01	0.18	3.57E-01	-0.17	4.03E-01	-0.15	4.44E-01	0.08	6.84E-01	0.02	9.13E-01	0.12	5.35E-01	-0.10	6.16E-01	0.02	9.16E-01
ENSCAFG0000000722	ENSCAFG0000000722	grey	VSMC_M1C	0.02	9.31E-01	0.39	4.67E-02	0.19	3.46E-01	-0.06	7.78E-01	-0.31	1.19E-01	0.20	3.19E-01	0.20	3.10E-01	-0.08	6.78E-01	-0.43	2.67E-02	0.45	1.73E-02
ENSCAFG0000001410	ADAM11	cyan	VSMC_M2	0.02	9.32E-01	0.43	2.68E-02	0.27	1.65E-01	0.27	1.77E-01	-0.31	1.10E-01	-0.33	8.84E-02	-0.11	5.71E-01	-0.37	5.75E-02	-0.29	1.45E-01	0.38	5.33E-02
ENSCAFG0000000888	PRDM8	turquoise	VSMC_M6	0.02	9.32E-01	0.06	7.67E-01	-0.15	4.43E-01	0.16	4.36E-01	0.07	7.46E-01	0.41	3.16E-02	0.31	1.14E-01	-0.11	5.72E-01	-0.12	5.49E-01	0.17	4.03E-01
ENSCAFG0000000417	C5AR2	grey	VSMC_M1C	0.02	9.32E-01	-0.20	3.09E-01	-0.27	1.65E-01	0.03	8.67E-01	0.33	8.99E-02	0.13	5.05E-01	0.12	5.64E-01	0.05	7.88E-01	0.15	4.60E-01	-0.20	3.19E-01
ENSCAFG0000003042	AIFM3	grey	VSMC_M1C	0.02	9.32E-01	0.03	8.70E-01	-0.26	1.88E-01	0.23	2.46E-01	0.26	1.95E-01	-0.25	1.99E-01	0.13	5.27E-01	-0.18	3.56E-01	-0.06	7.71E-01	0.11	5.97E-01
ENSCAFG0000001699	PGF	grey	VSMC_M1C	0.02	9.32E-01	0.25	2.02E-01	-0.06	7.76E-01	0.19	3.48E-01	-0.09	6.47E-01	0.20	3.16E-01	0.24	2.35E-01	-0.15	4.42E-01	-0.35	7.50E-02	0.39	4.17E-02
ENSCAFG0000003262	SRM	darkgrey	VSMC_M8	0.02	9.33E-01	-0.27	1.67E-01	0.52	5.65E-03	-0.81	2.94E-07	-0.43	2.46E-02	0.17	3.98E-01	0.04	8.38E-01	0.85	2.01E-08	0.14	4.88E-01	-0.52	5.77E-03
ENSCAFG0000000024	HNRNPAB	darkgrey	VSMC_M8	0.02	9.33E-01	-0.41	3.56E-02	0.38	4.86E-02	-0.77	2.46E-06	-0.33	8.79E-02	0.29	1.48E-01	0.31	1.16E-01	0.88	2.27E-09	0.21	2.85E-01	-0.50	7.47E-03
ENSCAFG0000000011	PAN2	grey	VSMC_M1C	0.02	9.33E-01	-0.27	1.73E-01	-0.08	7.05E-01	-0.31	1.20E-01	0.08	6.98E-01	0.12	5.61E-01	0.40	3.82E-02	0.36	6.85E-02	0.14	4.71E-01	-0.17	3.83E-01
ENSCAFG0000001213	ENSCAFG0000001213	grey	VSMC_M1C	0.02	9.33E-01	0.22	2.78E-01	0.49	9.23E-03	-0.20	3.20E-01	-0.42	2.77E-02	0.22	2.60E-01	-0.20	3.27E-01	0.03	8.98E-01	-0.15	4.68E-01	-0.05	7.89E-01
ENSCAFG0000002134	ENSCAFG0000002134	grey	VSMC_M1C	0.02	9.33E-01	-0.36	6.29E-02	-0.28	1.62E-01	-0.15	4.60E-01	0.42	2.74E-02	-0.11	5.92E-01	0.21	2.83E-01	0.20	3.26E-01	0.31	1.15E-01	-0.37	5.90E-02
ENSCAFG0000001350	DGKG	grey	VSMC_M1C	0.02	9.34E-01	0.44	2.09E-02	0.36	6.32E-02	-0.14	4.94E-01	-0.42	2.90E-02	0.11	5.84E-01	-0.05	8.11E-01	0.07	7.43E-01	-0.46	1.51E-02	0.26	1.91E-01

ENSCAFG0000001488	GDF15	grey	VSMC_M1C	0.02	9.34E-01	0.30	1.24E-01	0.39	4.24E-02	-0.14	5.02E-01	-0.42	2.77E-02	0.37	5.96E-02	-0.13	5.04E-01	-0.08	6.77E-01	-0.29	1.41E-01	0.22	2.72E-01
ENSCAFG0000003158	CLYBL	grey	VSMC_M1C	0.02	9.34E-01	0.12	5.59E-01	-0.31	1.17E-01	0.28	1.60E-01	0.14	4.85E-01	0.01	9.78E-01	0.12	5.68E-01	-0.21	3.05E-01	-0.15	4.59E-01	0.43	2.66E-02
ENSCAFG0000002294	ENSCAFG0000002294	grey	VSMC_M1C	0.02	9.34E-01	0.38	4.91E-02	0.12	5.55E-01	0.01	9.52E-01	-0.18	3.63E-01	-0.06	7.72E-01	-0.13	5.08E-01	-0.25	2.10E-01	-0.33	9.78E-02	0.28	1.50E-01
ENSCAFG0000000797	TEAD1	darkgreen	VSMC_M4	0.02	9.34E-01	0.17	3.86E-01	-0.66	2.00E-04	0.83	6.33E-08	0.50	7.96E-03	-0.02	9.37E-01	-0.47	1.40E-02	-0.71	3.41E-05	-0.10	6.16E-01	0.48	1.06E-02
ENSCAFG0000001460	TAF6	grey	VSMC_M1C	0.02	9.35E-01	0.30	1.32E-01	0.04	8.60E-01	0.07	7.11E-01	-0.10	6.09E-01	0.05	8.21E-01	-0.15	4.58E-01	-0.15	4.65E-01	-0.27	1.69E-01	0.34	8.53E-02
ENSCAFG0000002984	LASP1	cyan	VSMC_M2	0.02	9.35E-01	0.39	4.60E-02	0.61	7.75E-04	-0.32	1.05E-01	-0.59	1.10E-03	0.10	6.10E-01	-0.28	1.62E-01	0.09	6.61E-01	-0.29	1.36E-01	0.09	6.71E-01
ENSCAFG0000000475	ZNF575	grey	VSMC_M1C	0.02	9.35E-01	0.52	5.93E-03	0.26	1.93E-01	-0.11	6.02E-01	-0.32	1.00E-01	0.02	9.26E-01	0.04	8.33E-01	-0.09	6.44E-01	-0.47	1.23E-02	0.36	6.60E-02
ENSCAFG0000001478	PHC3	grey	VSMC_M1C	0.02	9.35E-01	0.08	7.02E-01	-0.37	5.60E-02	0.16	4.28E-01	0.33	9.63E-02	0.01	9.80E-01	-0.09	6.49E-01	-0.16	4.39E-01	-0.08	7.02E-01	0.17	3.99E-01
ENSCAFG0000001613	ENSCAFG0000001613	grey	VSMC_M1C	0.02	9.35E-01	-0.11	5.74E-01	-0.03	8.87E-01	-0.21	2.95E-01	0.15	4.46E-01	-0.26	1.99E-01	0.16	4.25E-01	0.13	5.07E-01	0.09	6.46E-01	-0.22	2.80E-01
ENSCAFG0000001066	ENSCAFG0000001066	darkolivegreen	VSMC_M9	0.02	9.35E-01	-0.51	6.70E-03	-0.04	8.60E-01	-0.36	6.62E-02	0.10	6.15E-01	0.21	2.84E-01	0.19	3.41E-01	0.39	4.31E-02	0.47	1.32E-02	-0.45	1.93E-02
ENSCAFG0000000620	ENSCAFG0000000620	grey	VSMC_M1C	0.02	9.35E-01	0.60	8.47E-04	0.07	7.14E-01	0.34	8.05E-02	-0.26	1.91E-01	0.21	2.96E-01	-0.03	8.67E-01	-0.51	7.17E-03	-0.55	2.95E-03	0.72	2.31E-05
ENSCAFG0000003027	ENSCAFG0000003027	grey	VSMC_M1C	0.02	9.35E-01	0.09	6.50E-01	0.17	4.01E-01	-0.15	4.46E-01	-0.21	2.91E-01	-0.08	6.75E-01	0.34	7.97E-02	0.11	5.83E-01	-0.10	6.21E-01	0.07	7.35E-01
ENSCAFG0000002011	ARHGAP29	darkgreen	VSMC_M4	0.02	9.35E-01	0.24	2.18E-01	-0.56	2.25E-03	0.76	3.90E-06	0.34	8.47E-02	0.08	6.86E-01	-0.37	5.58E-02	-0.62	6.02E-04	-0.25	2.04E-01	0.58	1.51E-03
ENSCAFG0000002000	VCAM1	darkgreen	VSMC_M4	0.02	9.35E-01	0.44	2.19E-02	-0.19	3.42E-01	0.62	5.41E-04	0.11	5.83E-01	-0.30	1.34E-01	-0.57	1.96E-03	-0.65	2.29E-04	-0.24	2.20E-01	0.47	1.24E-02
ENSCAFG0000001210	WASF2	grey	VSMC_M1C	0.02	9.36E-01	-0.10	6.20E-01	-0.20	3.27E-01	-0.19	3.39E-01	0.35	7.00E-02	-0.21	2.84E-01	-0.05	8.10E-01	0.12	5.48E-01	0.18	3.74E-01	-0.28	1.56E-01
ENSCAFG0000000082	RPS6KA2	cyan	VSMC_M2	0.02	9.36E-01	0.45	1.87E-02	0.69	8.04E-05	-0.34	8.53E-02	-0.73	1.33E-05	0.21	2.89E-01	0.28	1.53E-01	0.18	3.68E-01	-0.52	5.06E-03	0.29	1.47E-01
ENSCAFG0000000634	ENSCAFG0000000634	grey	VSMC_M1C	0.02	9.36E-01	-0.02	9.39E-01	0.40	3.80E-02	-0.31	1.15E-01	-0.29	1.37E-01	0.04	8.47E-01	-0.11	5.81E-01	0.31	1.12E-01	0.00	9.93E-01	-0.30	1.32E-01
ENSCAFG0000003199	HKR1	darkgreen	VSMC_M4	0.02	9.36E-01	0.02	9.27E-01	-0.51	6.62E-03	0.57	1.95E-03	0.35	7.28E-02	0.02	9.26E-01	-0.05	7.88E-01	-0.42	2.88E-02	-0.07	7.23E-01	0.42	2.76E-02
ENSCAFG0000000341	ST7	grey	VSMC_M1C	0.02	9.36E-01	-0.31	1.18E-01	0.10	6.13E-01	-0.39	4.27E-02	-0.01	9.59E-01	0.02	9.36E-01	0.01	9.74E-01	0.43	2.37E-02	0.35	7.75E-02	-0.41	3.33E-02
ENSCAFG0000001780	MOCOS	grey	VSMC_M1C	0.02	9.36E-01	0.36	6.72E-02	0.10	6.10E-01	0.21	2.88E-01	-0.01	9.80E-01	-0.40	4.11E-02	-0.13	5.11E-01	-0.33	9.00E-02	-0.19	3.37E-01	0.16	4.15E-01
ENSCAFG0000001816	TTC39C	yellow	VSMC_M3	0.02	9.36E-01	0.41	3.57E-02	0.78	2.01E-06	-0.51	7.03E-03	-0.66	1.82E-04	0.01	9.75E-01	-0.06	7.83E-01	0.23	2.52E-01	-0.33	9.06E-02	-0.02	9.02E-01
ENSCAFG0000000669	SLC46A3	grey	VSMC_M1C	0.02	9.36E-01	0.24	2.29E-01	0.25	2.14E-01	-0.29	1.36E-01	-0.22	2.63E-01	0.19	3.44E-01	-0.13	5.14E-01	0.08	6.88E-01	-0.15	4.47E-01	0.08	7.10E-01
ENSCAFG0000001120	SGMS2	grey	VSMC_M1C	0.02	9.36E-01	0.00	9.95E-01	-0.23	2.39E-01	0.05	8.12E-01	0.23	2.39E-01	0.12	5.45E-01	-0.27	1.66E-01	-0.08	7.04E-01	0.02	9.26E-01	-0.02	9.09E-01
ENSCAFG0000003186	ENSCAFG0000003186	turquoise	VSMC_M6	0.02	9.36E-01	0.24	2.22E-01	0.18	3.75E-01	0.24	2.35E-01	-0.30	1.31E-01	0.71	2.83E-05	0.01	9.60E-01	-0.25	2.11E-01	-0.26	1.90E-01	0.35	7.14E-02
ENSCAFG0000001873	VAPA	darkolivegreen	VSMC_M9	0.02	9.37E-01	-0.64	3.00E-04	-0.21	3.00E-01	-0.37	5.57E-02	0.32	9.98E-02	-0.03	8.65E-01	0.50	7.30E-03	0.48	1.09E-02	0.51	6.95E-03	-0.60	1.03E-03
ENSCAFG0000003040	ENSCAFG0000003040	grey	VSMC_M1C	0.02	9.37E-01	0.01	9.54E-01	-0.55	2.90E-03	0.45	1.75E-02	0.40	3.65E-02	-0.05	8.08E-01	0.04	8.27E-01	-0.26	1.97E-01	-0.09	6.62E-01	0.34	8.28E-02
ENSCAFG0000000756	ANKRD37	darkgreen	VSMC_M4	0.02	9.37E-01	-0.07	7.35E-01	-0.58	1.51E-03	0.63	4.61E-04	0.57	1.88E-03	-0.20	3.23E-01	-0.08	6.90E-01	-0.54	3.97E-03	0.17	4.01E-01	0.17	3.85E-01
ENSCAFG0000000299	COL9A2	darkgrey	VSMC_M8	0.02	9.37E-01	-0.47	1.31E-02	0.18	3.74E-01	-0.42	3.09E-02	-0.18	3.77E-01	0.33	8.96E-02	0.23	2.48E-01	0.57	2.01E-03	0.28	1.50E-01	-0.41	3.53E-02
ENSCAFG0000000161	DENND4C	darkolivegreen	VSMC_M9	0.02	9.38E-01	-0.51	5.98E-03	-0.56	2.16E-03	0.12	5.52E-01	0.62	6.20E-04	-0.08	6.81E-01	-0.16	4.28E-01	0.02	9.30E-01	0.56	2.61E-03	-0.31	1.21E-01
ENSCAFG0000001589	GHITM	pink	VSMC_M5	0.02	9.38E-01	-0.34	8.10E-02	-0.44	2.03E-02	0.30	1.25E-01	0.47	1.35E-02	0.16	4.16E-01	-0.04	8.36E-01	-0.07	7.36E-01	0.28	1.57E-01	-0.20	3.24E-01
ENSCAFG0000000789	HPGD	grey	VSMC_M1C	0.02	9.38E-01	0.64	3.70E-04	0.03	9.00E-01	0.55	2.94E-03	-0.20	3.25E-01	0.01	9.58E-01	-0.33	9.56E-02	-0.56	2.56E-03	-0.58	1.46E-03	0.72	2.47E-05
ENSCAFG0000000797	ENSCAFG0000000797	grey	VSMC_M1C	0.02	9.39E-01	0.35	6.93E-02	0.35	7.06E-02	0.06	7.60E-01	-0.30	1.29E-01	-0.08	6.87E-01	-0.12	5.55E-01	-0.21	2.86E-01	-0.28	1.64E-01	0.26	1.98E-01
ENSCAFG0000001231	TFAM	grey	VSMC_M1C	0.02	9.39E-01	-0.11	5.85E-01	-0.18	3.59E-01	0.03	8.74E-01	0.03	8.88E-01	0.22	2.64E-01	0.07	7.35E-01	0.15	4.41E-01	-0.09	6.64E-01	0.16	4.40E-01
ENSCAFG0000001883	RAP2C	darkgrey	VSMC_M8	0.02	9.39E-01	-0.22	2.80E-01	0.11	6.00E-01	-0.53	4.70E-03	0.04	8.53E-01	-0.04	8.40E-01	-0.19	3.55E-01	0.53	4.84E-03	0.19	3.48E-01	-0.45	1.82E-02
ENSCAFG0000000848	ENSCAFG0000000848	grey	VSMC_M1C	0.02	9.39E-01	-0.17	3.85E-01	-0.05	8.22E-01	-0.12	5.56E-01	0.14	5.01E-01	-0.16	4.20E-01	-0.22	2.64E-01	0.18	3.64E-01	0.15	4.47E-01	-0.28	1.55E-01
ENSCAFG0000001724	WDR83	pink	VSMC_M5	0.02	9.39E-01	-0.16	4.13E-01	-0.18	3.56E-01	0.03	8.68E-01	0.34	7.91E-02	-0.40	3.92E-02	-0.17	4.07E-01	0.07	7.27E-01	0.20	3.30E-01	-0.32	1.07E-01
ENSCAFG0000000239	EXOSC3	grey	VSMC_M1C	0.02	9.39E-01	-0.11	5.78E-01	0.09	6.69E-01	-0.15	4.63E-01	0.04	8.39E-01	-0.01	9.66E-01	-0.05	8.00E-01	0.15	4.58E-01	0.10	6.29E-01	-0.38	5.07E-02
ENSCAFG0000000556	IPO5	darkgrey	VSMC_M8	0.02	9.40E-01	-0.38	4.79E-02	0.15	4.61E-01	-0.60	8.90E-04	-0.03	8.73E-01	0.00	9.88E-01	-0.02	9.03E-01	0.70	4.28E-05	0.28	1.59E-01	-0.57	1.83E-03
ENSCAFG0000001422	PAPPA2	darkgreen	VSMC_M4	0.02	9.40E-01	0.31	1.10E-01	-0.43	2.62E-02	0.85	1.55E-08	0.31	1.14E-01	-0.13	5.04E-01	-0.38	5.33E-02	-0.75	6.31E-06	-0.25	2.00E-01	0.55	3.05E-03
ENSCAFG0000001712	GPATCH2L	darkgreen	VSMC_M4	0.02	9.40E-01	-0.03	8.74E-01	-0.44	2.12E-02	0.59	1.08E-03	0.48	1.07E-02	-0.17	4.11E-01	-0.22	2.74E-01	-0.53	4.44E-03	0.14	4.71E-01	0.04	8.44E-01
ENSCAFG0000000136	CBX7	grey	VSMC_M1C	0.02	9.40E-01	0.46	1.58E-02	0.03	8.95E-01	0.57	2.01E-03	0.00	9.94E-01	-0.17	3.94E-01	-0.28	1.50E-01	-0.70	4.37E-05	-0.28	1.51E-01	0.35	7.21E-02
ENSCAFG0000000694	ATP6V1H	grey	VSMC_M1C	0.02	9.40E-01	0.14	4.73E-01	0.03	8.73E-01	0.39	4.34E-02	-0.12	5.54E-01	0.21	2.88E-01	-0.18	3.80E-01	-0.39	4.33E-02	-0.09	6.65E-01	0.29	1.40E-01
ENSCAFG0000003054	FEN1	darkgrey	VSMC_M8	0.02	9.40E-01	-0.56	2.33E-03	0.23	2.40E-01	-0.70	5.68E-05	-0.16	4.21E-01	0.10	6.19E-01	0.27	1.70E-01	0.88	1.01E-09	0.37	5.52E-02	-0.59	1.32E-03
ENSCAFG0000001159	ARHGAP6	grey	VSMC_M1C	0.02	9.41E-01	0.35	7.28E-02	-0.25	2.07E-01	0.50	8.36E-03	0.05	7.95E-01	0.39	4.30E-02	0.07	7.47E-01	-0.49	1.02E-02	-0.35	7.68E-02	0.64	3.46E-04
ENSCAFG0000001923	ATP2B3	grey	VSMC_M1C	0.02	9.41E-01	-0.12	5.42E-01	0.00	9.95E-01	-0.07	7.11E-01	-0.03	8.67E-01	-0.08	6.89E-01	-0.08	6.85E-01	0.14	4.96E-01	0.13	5.14E-01	-0.07	7.28E-01
ENSCAFG0000002488	ENSCAFG0000002488	grey	VSMC_M1C	0.01	9.41E-01	-0.12	5.36E-01	-0.06	7.52E-01	-0.15	4.54E-01	0.10	6.13E-01	-0.03	8.87E-01	0.13	5.28E-01	0.21	2.92E-01	0.04	8.55E-01	-0.04	8.49E-01
ENSCAFG0000001046	MTR	grey	VSMC_M1C	0.01	9.41E-01	0.09	6.50E-01	0.23	2.54E-01	-0.26	1.94E-01	-0.34	8.14E-02	0.21	2.87E-01	0.43	2.49E-02	0.19	3.44E-01	-0.19	3.41E-01	0.28	1.58E-01
ENSCAFG0000000269	ENSCAFG0000000269	grey	VSMC_M1C	0.01	9.41E-01	0.24	2.20E-01	0.22	2.59E-01	-0.13	5.26E-01	-0.27	1.69E-01	-0.07	7.30E-01	0.31	1.10E-01	-0.04	8.62E-01	-0.25	2.03E-01	0.19	3.40E-01
ENSCAFG0000000781	ENSCAFG0000000781	grey	VSMC_M1C	0.01	9.41E-01	-0.13	5.16E-01	0.08	6.88E-01	-0.08	7.07E-01	-0.01	9.74E-01	0.00	9.84E-01	0.10	6.22E-01	0.06	7.48E-01	0.11	6.00E-01	-0.16	4.19E-01
ENSCAFG0000000454	DLG2	grey	VSMC_M1C	0.01	9.41E-01	0.12	5.36E-01	-0.30	1.34E-01	0.38	5.05E-02	0.18	3.57E-01	-0.12	5.55E-01	0.36	6.80E-02	-0.32	1.09E-01	-0.19	3.52E-01	0.40	4.00E-02
ENSCAFG0000000278	TRA2A	grey	VSMC_M1C	0.01	9.42E-01	-0.17	3.94E-01	-0.61	8.10E-04	0.38	5.20E-02	0.57	2.03E-03	-0.17	4.01E-01	0.28	1.62E-01	-0.24	2.21E-01	0.09	6.61E-01	0.12	5.52E-01
ENSCAFG0000003207	FNDC11	grey	VSMC_M1C	0.01	9.42E-01	0.15	4.48E-01	-0.23	2.48E-01	0.28	1.59E-01	0.06	7.66E-01	0.02	9.21E-01	0.07	7.39E-01	-0.35	7.28E-02	-0.13	5.34E-01	0.46	1.50E-02
ENSCAFG0000003192	C23H3orf58	grey	VSMC_M1C	0.01	9.42E-01	-0.45	1.80E-02	-0.20	3.15E-01	-0.43	2.71E-02	0.38	5.11E-02	-0.32	1.01E-01	0.05	8.17E-01	0.48	1.11E-02	0.45	1.98E-02	-0.58	1.48E-03
ENSCAFG0000001670	PCNX1	grey	VSMC_M1C	0.01	9.42E-01	0.13	5.06E-01	-0.36	6.36E-02	0.58	1.51E-03	0.18	3.65E-01	0.28	1.54E-01	-0.24	2.33E-01	-0.53	4.20E-03	-0.09	6.42E-01	0.50	7.85E-03
ENSCAFG0000003168	ENSCAFG0000003168	grey	VSMC_M1C	0.01	9.43E-01	0.02	9.27E-01	-0.01	9.46E-01	0.04	8.32E-01	-0.06	7.67E-01	-0.05	7.94E-01	0.15	4.59E-01	-0.07	7.22E-01	-0.07	7.34E-01	0.13	5.20E-01
ENSCAFG0000001771	SLC44A2	cyan	VSMC_M2	0.01	9.43E-01	0.74	8.54E-06	-0.05	8.20E-01	0.70	4.47E-05	-0.10	6.02E-01	-0.06	7.85E-01	-0.61	7.44E-04	-0.83	7.70E-08	-0.56	2.61E-03	0.75	5.65E-06
ENSCAFG0000000577	ENSCAFG0000000577	grey	VSMC_M1C	0.01	9.44E-01	0.54	3.29E-03	0.01	9.64E-01	0.51	6.02E-03	-0.04	8.58E-01	-0.29	1.44E-01	-0.52	5.41E-03	-0.66	1.86E-04	-0.33	9.32E-02	0.47	1.27E-02
ENSCAFG0000003041	ENSCAFG0000003041	grey	VSMC_M1C	0.01	9.44E-01	-0.27	1.74E-01	-0.05	8.08E-01	-0.17	3.90E-01	0.09	6.56E-01	-0.05	8.23E-01	0.07	7.40E-01	0.26	1.91E-01	0.21	3.03E-01	-0.22	2.80E-01
ENSCAFG0000000421	EFCAB14	grey	VSMC_M1C	0.01	9.44E-01	0.61	6.98E-04	-0.19	3.39E-01	0.61	6.50E-04	0.12	5.62E-01	-0.27	1.71E-01	-0.29	1.40E-01	-0.70	5.40E-05	-0.45	1.81E-02	0.58	1.39E-03
ENSCAFG0000000943	ENSCAFG0000000943	grey	VSMC_M1C	0.01	9.44E-01	-0.55	2.92E-03	-0.54	4.03E-03	0.02	9.10E-01	0.59	1.23E-03	-0.02	9.34E-01	0.09	6.68E-01	0.17	4.03E-01	0.47	1.37E-02	-0.37	5.61E-02
ENSCAFG0000001052	LGR6	grey	VSMC_M1C	0.01	9.45E-01	-0.02	9.10E-01	-0.52	5.42E-03	0.68	1.05E-04	0.37	5.72E-02	0.02	9.10E-01	0.21	2.84E-01	-0.53	4.58E-03	-0.02	9.21E-01	0.38	4.81E-02
ENSCAFG0000000973	NGF	darkgreen	VSMC_M4	0.01	9.45E-01	0.20	3.22E-01	-0.32	1.08E-01	0.72	2.18E-05	0.12	5.52E-01	0.16	4.29E-01	-0.10	6.21E-01	-0.58	1.52E-03	-0.26	1.96E-01	0.54	3.86E-03
ENSCAFG0000001159	MOCS3	yellow	VSMC_M3	0.01	9.45E-01	0.35	7.51E-02	0.73	1.36E-05	-0.29	1.38E-01	-0.74	1.00E-05	0.19	3.52E-01	-0.09	6.52E-01	0.08	6.80E-01	-0.31	1.20E-01	0.14	4.86E-01
ENSCAFG0000001832	KIF26A	turquoise	VSMC_M6	0.01	9.45E-01	-0.08	6.98E-01	0.03	8.83E-01	-0.08	6.97E-01	-0.08	6.93E-01	0.73	1.56E-05	-0.02	9.26E-01	0.15	4.59E-01	0.02	9.03E-01	-0.04	8.38E-01
ENSCAFG0000000201	ENSCAFG0000000201	darkgrey	VSMC_M8	0.01	9.45E-01	-0.56	2.34E-03	-0.08	6.86E-01	-0.51	6.10E-03	0.27	1.74E-01	-0.10	6.09E-01	-0.12	5.44E-01	0.63	4.61E-04	0.52	5.43E-03	-0.69	6.82E-05
ENSCAFG0000000909	KNSTRN	darkgrey	VSMC_M8	0.01	9.45E-01	-0.52	5.96E-03	0.35	7.53E-02	-0.81	2.72E-07	-0.13	5.31E-01	0.02	9.08E-01	0.33	9.44E-02	0.87	3.54E-09	0.38	5.05E-02	-0.82	1.20E-07
ENSCAFG0000000245	ENSCAFG0000000245	grey	VSMC_M1C	0.01	9.45E-01	0.17	4.05E-01	-0.03	8.67E-01	0.02	9.05E-01	-0.05	8.14E-01	0.30	1.25E-01	0.01	9.75E-01	-0.09	6.68E-01	-0.19	3.33E-01	0.22	2.70E-01
ENSCAFG0000000793	TRPC1	pink	VSMC_M5	0.01	9.45E-01	-0.28	1.58E-01	-0.66	2.08E-04	0.35	7.12E-02	0.70	4.88E-05	-0.12	5.65E-01	-0.11	5.85E-01	-0.28	1.55E-01	0.32	1.06E-01	-0.06	7.60E-01
ENSCAFG0000002310	OAS2	cyan	VSMC_M2	0.01	9.45E-01	0.82	2.17E-07	0.43	2.44E-02	0.15	4.63E-01	-0.52	5.78E-03	0.14	4.84E-01	-0.05	8.15E-01	-0.42	2.91E-02	-0.72	2.50E-05	0.62	5.08E-04
ENSCAFG0000001970	RNF126	grey	VSMC_M1C	0.01	9.45E-01	0.02	9.30E-01	0.20	3.17E-01	-0.05	8.18E-01	-0.28	1.55E-01	0.34	8.43E-02	-0.38	5.34E-02	0.05	7.95E-01	0.01	9.56E-01	0.06	7.61E-01
ENSCAFG0000000627	ATRN	pink	VSMC_M5	0.01	9.46E-01	-0.07	7.38E-01	-0.74	8.32E-06	0.79	9.00E-07	0.66	1.88E-04	-0.10	6.11E-01	-0.29	1.41E-01	-0.61	6.72E-04	0.12	5.67E-01	0.29	1.41E-01
ENSCAFG0000003218	ENSCAFG0000003218	grey	VSMC_M1C	0.01	9.46E-01	-0.14	4.92E-01	0.12	5.48E-01	-0.29	1.42E-01	-0.19	3.45E-01	0.36	6.82E-02	0.50	7.99E-03	0.34	8.72E-02	0.03	8.93E-01	-0.06	7.57E-01
ENSCAFG0000000512	ENTPD3	darkgreen	VSMC_M4	0.01	9.46E-01	0.13	5.04E-01	-0.65	2.64E-04	0.84	3.85E-08	0.57	1.73E-03	-0.15	4.53E-01	-0.56	2.58E-03	-0.73	1.63E-05	-0.02	9.20E-01	0.33	9.24E-02
ENSCAFG0000001862	C3	grey	VSMC_M1C	0.01	9.46E-01	0.59	1.28E-03	0.05	7.96E-01	0.31	1.16E-01	-0.19	3.51E-01	-0.06	7.60E-01	-0.18	3.57E-01	-0.45	2.00E-02	-0.54	4.02E-03	0.57	1.81E-03
ENSCAFG0000001216	EWSR1	darkgrey	VSMC_M8	0.01	9.46E-01	-0.43	2.64E-02	0.25	2.13E-01	-0.66	2.07E-04	-0.11	6.00E-01	0.09	6.47E-01	0.01	9.47E-01	0.75	6.26E-06	0.35	7.32E-02	-0.68	1.00E-04
ENSCAFG0000001682	UBTD2	grey	VSMC_M1C	0.01	9.47E-01	0.02	9.16E-01	-0.03	8.78E-01	-0.05	8.19E-01	0.18	3.67E-01	-0.34	7.91E-02	-0.11	5.83E-01	0.01	9.50E-01	0.07	7.30E-01	-0.22	2.68E-01
ENSCAFG0000000239	ENSCAFG0000000239	grey	VSMC_M1C	0.01	9.48E-01	0.46	1.54E-02	0.36	6.13E-02	-0.03	8.92E-01	-0.46	1.67E-02	0.04	8.59E-01	-0.21	2.91E-01	-0.08	6.82E-01	-0.45	1.92E-02	0.33	9.66E-02
ENSCAFG0000002442	ENSCAFG0000002442	grey	VSMC_M1C	0.01	9.49E-01	0.21	2.96E-01	0.07	7.42E-01	0.34	8.17E-02	-0.16	4.19E-01	0.09	6.55E-01	-0.10	6.18E-01	-0.33	9.49E-02	-0.19	3.32E-01	0.29	1.44E-01
ENSCAFG0000000114	PKD2L2	grey	VSMC_M1C	0.01	9.49E-01	0.15	4.60E-01	0.27	1.66E-01	-0.25	2.11E-01	-0.29	1.45E-01	0.06	7.49E-01	0.47	1.30E-02	0.15	4.50E-01	-0.17	3.91E-01	0.09	6.48E-01
ENSCAFG0000000706	BUB1	darkgrey	VSMC_M8	0.01	9.49E-01	-0.48	1.17E-02	0.35	7.47E-02	-0.88	2.12E-09	-0.23	2.47E-01	0.13	5.15E-01	0.47	1.31E-02	0.95	4.57E-14	0.28	1.54E-01	-0.62	5.84E-04
ENSCAFG0000002331	DUSP2	grey	VSMC_M1C	0.01	9.49E-01	0.31	1.11E-01	0.48	1.23E-02	-0.27	1.73E-01	-0.50	8.53E-03	0.23	2.58E-01	0.07	7.47E-01	0.12	5.40E-01	-0.27	1.80E-01	0.17	4.02E-01
ENSCAFG0000001696	LCP2	grey	VSMC_M1C	0.01	9.49E-01	-0.36	6.88E-02	-0.11	6.00E-01	-0.24	2.37E-01	0.16	4.26E-01	0.06	7.61E-01	0.23	2.58E-01	0.31	1.17E-01	0.25	2.15E-01	-0.23	2.41E-01
ENSCAFG0000001570	LRRN4CL	yellow	VSMC_M3	0.01	9.50E-01	0.38	4.77E-02	0.71	3.47E-05	-0.57	2.03E-03	-0.61	7.67E-04	-0.19	3.49E-01	0.26	1.95E-01	0.27	1.79E-01	-0.34	8.56E-02	0.00	9.84E-01
ENSCAFG0000001733	ENSCAFG0000001733	darkgreen	VSMC_M4	0.01	9.50E-01	0.22	2.77E-01	-0.27	1.69E-01	0.51	6.96E-03	0.05	7.88E-01	0.20	3.29E-01	0.01	9.42E-01	-0.42	2.78E-02	-0.30	1.28E-01	0.55	3.23E-03
ENSCAFG0000001224	NEFH	grey	VSMC_M1C	0.01	9.50E-01	0.18	3.69E-01	-0.01	9.50E-01	0.14	4.95E-01	-0.11	5.87E-01	0.24	2.24E-01	0.05	8.18E-01	-0.16	4.38E-01	-0.20	3.17E-01	0.29	1.40E-01
ENSCAFG0000000426	ENSCAFG0000000426	grey	VSMC_M1C	0.01	9.51E-01	-0.01	9.53E-01	0.11	5.97E-01	-0.22	2.80E-01	-0.09	6.72E-01	0.09	6.73E-01	0.01	9.66E-01	0.20	3.17E-01	-0.03	8.92E-01	-0.03	8.91E-01
ENSCAFG0000000623	ENSCAFG0000000623	darkgreen	VSMC_M4	0.01	9.51E-01	0.19	3.41E-01	-0.36	6.60E-02	0.63	4.54E-04	0.26	1.94E-01	-0.09	6.46E-01	0.01	9.47E-01	-0.56	2.45E-03	-0.22	2.72E-01	0.46	1.68E-02
ENSCAFG0000001581	ACLY	yellow	VSMC_M3	0.01	9.51E-01	-0.27	1.66E-01	0.41	3.17E-02	-0.85	2.41E-08	-0.19	3.38E-01	-0.03	8.72E-01	0.35	7.30E-02	0.71	2.82E-05	0.22	2.64E-01	-0.61	6.81E-04
ENSCAFG0000001389	ACBD4	cyan	VSMC_M2	0.01	9.51E-01	0.69	7.98E-05	0.14	4.73E-01	0.43	2.56E-02	-0.27	1.67E-01	-0.19	3.45E-01	-0.01	9.58E-01	-0.66	2.06E-04	-0.59	1.13E-03	0.72	2.34E-05
ENSCAFG0000001080	TMEM39E	grey	VSMC_M1C	0.01	9.51E-01	0.33	8.79E-02	0.40	3.86E-02	-0.24	2.33E-01	-0.43	2.54E-02	0.20	3.27E-01	0.25	2.05E-01	0.09	6.56E-01	-0.39	4.68E-02	0.19	3.51E-01
ENSCAFG0000001973	PLPP2	cyan	VSMC_M2	0.01	9.52E-01	0.82	1.43E-07	0.19	3.42E-01	0.51	6.11E-03	-0.33	8.90E-02	-0.02	9.41E-01	-0.49	9.13E-03	-0.69	7.56E-05	-0.69	6.40E-05	0.75	7.46E-06
ENSCAFG0000001880	SAFB	yellow	VSMC_M3	0.01	9.52E-01	0.19	3.41E-01	0.75	6.89E-06	-0.47	1.35E-02	-0.76	4.47E-06	0.16	4.36E-01	0.19	3.45E-01	0.36	6.28E-02	-0.29	1.39E-01	0.01	9.76E-01
ENSCAFG0000003083	PNRC1	darkgreen	VSMC_M4	0.01	9.52E-01	0.54	3.30E-03	-0.29	1.38E-01	0.81	3.33E-07	0.22	2.76E-01	-0.22	2.65E-01	-0.31	1.19E-01	-0.88	9.47E-10	-0.39	4.34E-02	0.62	5.07E-04
ENSCAFG0000000450	COG1	grey	VSMC_M1C	0.01	9.52E-01	0.22	2.62E-01	-0.19	3.44E-01	0.14	4.79E-01	0.29	1.48E-01	-0.19	3.46E-01	-0.11	5.70E-01	-0.22	2.80E-01	-0.14	4.88E-01	0.03	8.93E-01
ENSCAFG0000000570	MEMO1	pink	VSMC_M5	0.01	9.53E-01	-0.45	1.80E-02	-0.68	1.10E-04	0.12	5.62E-01	0.76	4.85E-06	-0.18	3.80E-01	-0.01	9.63E-01	-0.01	9.46E-01	0.46	1.63E-02	-0.35	7.79E-02
ENSCAFG0000003081	ENSCAFG0000003081	yellow	VSMC_M3	0.01	9.53E-01	-0.30	1.28E-01	0.29	1.36E-01	-0.67	1.27E-04	-0.04	8.30E-01	-0.12	5.48E-01	0.45	1.73E-02	0.58	1.59E-03	0.27	1.71E-01	-0.61	7.04E-04
ENSCAFG0000001045	RIPOR2	darkgreen	VSMC_M4	0.01	9.53E-01	0.23	2.47E-01	-0.50	8.18E-03	0.87	3.27E-09	0.34	8.18E-02	-0.14	4.79E-01	-0.06	7.66E-01	-0.73	1.50E-05	-0.19	3.36E-01	0.55	3.01E-03
ENSCAFG0000000297	HOXA13	grey	VSMC_M1C	0.01	9.53E-01	-0.22	2.74E-01	-0.49	9.17E-03	0.35	7.59E-02	0.49	9.90E-03	-0.01	9.72E-01	0.47	1.26E-02	-0.28	1.64E-01	0.21	3.02E-01	0.03	8.73E-01
ENSCAFG0000003193	ENSCAFG0000003193	grey	VSMC_M1C	0.01	9.53E-01	0.26	1.95E-01	0.21	3.04E-01	0.25	2.10E-01	-0.34	8.37E-02	0.26	1.98E-01	0.01	9.71E-01	-0.30	1.27E-01	-0.23	2.39E-01	0.42	2.78E-02
ENSCAFG0000002582	ENSCAFG0000002582	grey	VSMC_M1C	0.01	9.54E-01	0.12	5.49E-01	0.14	4.88E-01	-0.14	4.94E-01	-0.12	5.43E-01	-0.06	7.65E-01	0.12	5.43E-01	0.01	9.63E-01	-0.09	6.45E-01	0.08	6.75E-01
ENSCAFG0000001305	MAP3K15	grey	VSMC_M1C	0.01	9.54E-01	0.16	4.14E-01	-0.11	5.72E-01	0.24	2.22E-01	-0.06	7.76E-01	0.35	7.28E-02	0.08	7.07E-01	-0.23	2.43E-01	-0.24	2.37E-01	0.35	7.04E-02
ENSCAFG0000002048	ENSCAFG0000002048	grey	VSMC_M1C	0.01	9.54E-01	0.11	5.76E-01	-0.07	7.38E-01	0.25	2.08E-01	-0.01	9.45E-01	-0.08	7.04E-01	0.33	9.55E-02	-0.22	2.60E-01	-0.13	5.17E-01	0.25	2.06E-01
ENSCAFG0000002682	ENSCAFG0000002682	grey	VSMC_M1C	0.01	9.54E-01	-0.05	8.12E-01	0.13	5.25E-01	-0.27	1.78E-01	-0.15	4.47E-01	-0.20	3.24E-01	0.17	3.91E-01	0.29	1.43E-01	0.02	9.10E-01	0.01	9.58E-01
ENSCAFG0000000750	RGS9BP	violet	VSMC_M7	0.01	9.54E-01	-0.05	8.22E-01	0.13	5.07E-01	-0.17	3.93E-01	-0.11	5.91E-01	0.06	7.75E-01	0.62	5.19E-04	0.13	5.24E-01	0.01	9.68E-01	-0.02	9.39E-01
ENSCAFG0000000869	DHH	violet	VSMC_M7	0.01	9.54E-01	-0.05	8.22E-01	0.13	5.07E-01	-0.17	3.93E-01	-0.11	5.91E-01	0.06	7.75E-01	0.62	5.19E-04	0.13	5.24E-01	0.01	9.68E-01	-0.02	9.39E-01
ENSCAFG0000002261	ENSCAFG0000002261	violet	VSMC_M7	0.01	9.54E-01	-0.05	8.22E-01	0.13	5.07E-01	-0.17	3.93E-01	-0.11	5.91E-01	0.06	7.75E-01	0.62	5.19E-04	0.13	5.24E-01	0.01	9.68E-01	-0.02	9.39E-01
ENSCAFG0000003218	ENSCAFG0000003218	violet	VSMC_M7	0.01	9.54E-01	-0.05	8.22E-01	0.13	5.07E-01	-0.17	3.93E-01	-0.11	5.91E-01	0.06	7.75E-01	0.62	5.19E-04	0.13	5.24E-01	0.01	9.68E-01	-0.02	9.39E-01
ENSCAFG0000000871	KMT2D	grey	VSMC_M1C	0.01	9.55E-01	0.18	3.57E-01	-0.25	2.02E-01	0.47	1.33E-02	0.19	3.46E-01	-0.01	9.48E-01	-0.31	1.15E-01	-0.53	4.62E-03	-0.09	6.69E-01	0.34	7.92E-02
ENSCAFG0000001278	XPR1	grey	VSMC_M1C	0.01	9.55E-01	-0.46	1.48E-02	-0.44	2.20E-02	-0.10	6.09E-01	0.50	7.49E-03	0.11	5.77E-01	0.13	5.09E-01	0.23	2.47E-01	0.42	3.06E-02	-0.36	6.52E-02
ENSCAFG0000001818	ENSCAFG0000001818	darkgreen	VSMC_M4	0.01	9.55E-01	0.22	2.72E-01	-0.55	2.79E-03	0.88	2.04E-09	0.43	2.45E-02	-0.17	3.88E-01	-0.46	1.70E-02	-0.84	4.89E-08	-0.10	6.26E-01	0.48	1.11E-02
ENSCAFG0000001966	WFIKKN1	grey	VSMC_M1C	0.01	9.55E-01	-0.20	3.07E-01	0.09	6.58E-01	-0.03	8.74E-01	-0.11	5.93E-01	0.30	1.25E-01	0.28	1.60E-01	0.11	5.98E-01	0.18	3.80E-01	-0.12	5.38E-01
ENSCAFG0000000730	CWC27	darkgrey	VSMC_M8	0.01	9.55E-01	-0.48	1.08E-02	-0.05	8.09E-01	-0.45	1.82E-02	0.10	6.08E-01	-0.05	8.09E-01	0.14	4.84E-01	0.64	2.83E-04	0.28	1.59E-01	-0.47	1.25E-02
ENSCAFG0000001233	BICC1	grey	VSMC_M1C	0.01	9.56E-01	-0.27	1.71E-01	-0.12	5.64E-01	-0.34	8.38E-02	0.27	1.74E-01	-0.20	3.18E-01	0.55	2.66E-03	0.39	4.58E-02	0.20	3.20E-01	-0.39	4.29E-02
ENSCAFG0000000665	NOP56	darkgrey	VSMC_M8	0.01	9.56E-01	-0.27	1.68E-01	0.51	6.97E-03	-0.74	9.76E-06	-0.48	1.10E-02	0.25	2.09E-01	0.19	3.46E-01	0.77	2.19E-06	0.13	5.06E-01	-0.37	5.59E-02
ENSCAFG0000000485	TMEM185E	grey	VSMC_M1C	0.01	9.56E-01	0.00	9.84E-01	-0.19	3.55E-01	0.25	2.07E-01	0.18	3.56E-01	0.04	8.45E-01	0.29	1.43E-01	-0.28	1.54E-01	-0.04	8.47E-01	0.15	4.52E-01
ENSCAFG0000001997	RNPC3	cyan	VSMC_M2	0.01	9.56E-01	0.47	1.31E-02	-0.04	8.27E-01	0.33	9.04E-02	0.07	7.41E-01	-0.30	1.33E-01	-0.21	3.01E-01	-0.54	3.73E-03	-0.32	9.85E-02	0.34	8.52E-02

ENSCAFG0000000090	SEPT8	grey	VSMC_M10	0.01	9.56E-01	-0.23	2.54E-01	-0.15	4.58E-01	0.24	2.22E-01	0.11	5.87E-01	0.24	2.21E-01	0.30	1.23E-01	-0.15	4.59E-01	0.15	4.52E-01	0.03	8.63E-01
ENSCAFG0000001535	MPC2	grey	VSMC_M10	0.01	9.57E-01	0.18	3.76E-01	0.18	3.79E-01	-0.14	4.82E-01	-0.07	7.26E-01	-0.08	6.97E-01	-0.26	1.86E-01	0.02	9.07E-01	-0.10	6.34E-01	-0.12	5.49E-01
ENSCAFG0000002971	ENSCAFG0000002971	grey	VSMC_M10	0.01	9.57E-01	0.42	2.77E-02	0.11	5.77E-01	0.32	1.06E-01	-0.16	4.15E-01	0.11	5.96E-01	-0.12	5.60E-01	-0.46	1.52E-02	-0.36	6.61E-02	0.46	1.57E-02
ENSCAFG0000000524	ENSCAFG0000000524	grey	VSMC_M10	0.01	9.57E-01	-0.12	5.49E-01	-0.02	9.22E-01	-0.14	5.01E-01	0.02	9.14E-01	-0.09	6.45E-01	-0.03	8.80E-01	0.11	5.90E-01	0.07	7.19E-01	-0.09	6.67E-01
ENSCAFG0000002529	ENSCAFG0000002529	grey	VSMC_M10	0.01	9.58E-01	-0.03	8.91E-01	0.07	7.41E-01	-0.25	2.16E-01	-0.04	8.43E-01	0.04	8.35E-01	0.16	4.31E-01	0.24	2.34E-01	-0.01	9.67E-01	-0.04	8.37E-01
ENSCAFG0000003193	NRBF2	grey	VSMC_M10	0.01	9.58E-01	-0.43	2.64E-02	-0.20	3.09E-01	-0.08	6.75E-01	0.35	7.69E-02	-0.18	3.59E-01	-0.06	7.54E-01	0.11	5.99E-01	0.45	1.83E-02	-0.44	2.11E-02
ENSCAFG0000001501	ATP6V0A1	grey	VSMC_M10	0.01	9.58E-01	0.50	7.71E-03	0.18	3.82E-01	0.09	6.45E-01	-0.26	1.87E-01	-0.02	9.34E-01	-0.61	7.43E-04	-0.26	1.85E-01	-0.40	4.02E-02	0.36	6.82E-02
ENSCAFG0000001139	RASSF8	grey	VSMC_M10	0.01	9.58E-01	-0.14	4.91E-01	0.32	1.08E-01	-0.61	7.88E-04	-0.11	5.74E-01	-0.04	8.29E-01	-0.11	5.81E-01	0.53	4.80E-03	0.13	5.14E-01	-0.53	4.24E-03
ENSCAFG0000001168	TRUB1	grey	VSMC_M10	0.01	9.59E-01	0.25	2.00E-01	-0.28	1.53E-01	0.38	4.87E-02	0.15	4.47E-01	-0.04	8.33E-01	-0.19	3.36E-01	-0.28	1.61E-01	-0.27	1.70E-01	0.44	2.23E-02
ENSCAFG0000000626	MAB21L1	grey	VSMC_M10	0.01	9.59E-01	-0.33	9.58E-02	-0.09	6.52E-01	-0.25	2.00E-01	0.22	2.64E-01	0.06	7.65E-01	0.61	6.61E-04	0.30	1.28E-01	0.22	2.59E-01	-0.39	4.68E-02
ENSCAFG0000001008	CCT5	darkgrey	VSMC_M8	0.01	9.59E-01	-0.52	5.88E-03	0.35	7.73E-02	-0.83	9.90E-08	-0.21	2.83E-01	0.06	7.60E-01	0.16	4.34E-01	0.93	2.46E-12	0.37	6.07E-02	-0.72	1.97E-05
ENSCAFG0000001374	TAB3	grey	VSMC_M10	0.01	9.59E-01	0.05	8.18E-01	-0.26	1.87E-01	0.14	4.75E-01	0.36	6.50E-02	-0.34	8.02E-02	-0.44	2.26E-02	-0.21	2.87E-01	0.14	4.76E-01	-0.11	5.93E-01
ENSCAFG0000000748	RILPL1	grey	VSMC_M10	0.01	9.60E-01	0.20	3.27E-01	0.22	2.67E-01	0.11	5.86E-01	-0.12	5.65E-01	0.03	8.66E-01	-0.27	1.72E-01	-0.27	1.73E-01	-0.05	7.91E-01	0.02	9.26E-01
ENSCAFG0000001079	SLC9B1	grey	VSMC_M10	0.01	9.60E-01	-0.22	2.65E-01	0.27	1.74E-01	-0.51	6.49E-03	-0.22	2.63E-01	0.24	2.24E-01	0.15	4.44E-01	0.54	3.30E-03	0.11	5.71E-01	-0.34	8.58E-02
ENSCAFG0000000870	ENSCAFG0000000870	darkgrey	VSMC_M8	0.01	9.60E-01	-0.23	2.39E-01	0.58	1.49E-03	-0.70	4.09E-05	-0.42	2.99E-02	0.04	8.36E-01	0.16	4.40E-01	0.66	1.83E-04	0.20	3.20E-01	-0.58	1.47E-03
ENSCAFG0000000906	ZNF638	darkgreen	VSMC_M4	0.01	9.60E-01	0.09	6.58E-01	-0.33	8.90E-02	0.38	5.09E-02	0.21	2.85E-01	-0.05	8.06E-01	0.37	5.89E-02	-0.30	1.23E-01	-0.19	3.52E-01	0.40	3.80E-02
ENSCAFG0000000216	SPTLC1	grey	VSMC_M10	0.01	9.60E-01	-0.57	1.90E-03	-0.47	1.43E-02	0.12	5.43E-01	0.61	7.91E-04	-0.05	8.02E-01	0.01	9.64E-01	0.11	5.92E-01	0.51	6.32E-03	-0.53	4.10E-03
ENSCAFG0000001239	ENSCAFG0000001239	grey	VSMC_M10	0.01	9.61E-01	0.29	1.46E-01	0.18	3.68E-01	-0.20	3.07E-01	-0.26	1.97E-01	0.09	6.66E-01	0.06	7.68E-01	0.14	4.99E-01	-0.34	7.85E-02	0.19	3.34E-01
ENSCAFG0000001988	ZC3H18	darkgrey	VSMC_M8	0.01	9.61E-01	-0.33	9.14E-02	0.48	1.18E-02	-0.74	8.48E-06	-0.36	6.71E-02	0.19	3.40E-01	0.20	3.27E-01	0.81	3.51E-07	0.18	3.75E-01	-0.55	2.68E-03
ENSCAFG0000000283	CTNNAL1	grey	VSMC_M10	0.01	9.61E-01	-0.10	6.21E-01	-0.51	6.51E-03	0.49	9.89E-03	0.43	2.65E-02	0.37	6.12E-02	-0.64	2.93E-04	-0.32	1.03E-01	0.14	4.88E-01	0.08	6.78E-01
ENSCAFG0000003155	ERF	yellow	VSMC_M3	0.01	9.61E-01	0.15	4.47E-01	0.55	2.70E-03	-0.66	1.75E-04	-0.43	2.63E-02	0.22	2.81E-01	-0.14	4.86E-01	0.44	2.16E-02	-0.07	7.24E-01	-0.25	2.09E-01
ENSCAFG0000001824	FAM83G	grey	VSMC_M10	0.01	9.61E-01	0.70	5.41E-05	0.33	8.91E-02	0.24	2.36E-01	-0.54	3.65E-03	0.12	5.35E-01	-0.19	3.54E-01	-0.37	5.56E-02	-0.70	5.23E-05	0.73	1.42E-05
ENSCAFG0000000125	TNRC6B	grey	VSMC_M10	0.01	9.61E-01	-0.02	9.23E-01	0.47	1.29E-02	-0.56	2.19E-03	-0.39	4.37E-02	0.18	3.82E-01	0.52	5.46E-03	0.52	5.39E-03	-0.07	7.31E-01	-0.24	2.26E-01
ENSCAFG0000001103	NCL	darkgrey	VSMC_M8	0.01	9.61E-01	-0.47	1.32E-02	0.38	5.38E-02	-0.75	7.23E-06	-0.25	2.06E-01	0.11	5.81E-01	0.26	1.83E-01	0.88	1.36E-09	0.30	1.34E-01	-0.66	1.78E-04
ENSCAFG0000000113	ST13	grey	VSMC_M10	0.01	9.61E-01	-0.11	5.85E-01	0.25	2.07E-01	-0.36	6.68E-02	-0.06	7.51E-01	-0.10	6.21E-01	-0.27	1.66E-01	0.29	1.41E-01	0.18	3.72E-01	-0.47	1.29E-02
ENSCAFG0000001467	STIP1	grey	VSMC_M10	0.01	9.61E-01	-0.34	8.13E-02	0.21	2.89E-01	-0.41	3.43E-02	-0.34	8.65E-02	0.25	2.13E-01	0.34	8.11E-02	0.56	2.47E-03	0.14	4.79E-01	-0.10	6.18E-01
ENSCAFG0000001990	PRRC2B	darkgreen	VSMC_M4	0.01	9.61E-01	0.48	1.05E-02	-0.31	1.14E-01	0.83	9.92E-08	0.18	3.66E-01	-0.07	7.41E-01	-0.22	2.72E-01	-0.87	5.27E-09	-0.39	4.63E-02	0.69	5.75E-05
ENSCAFG0000001517	ZNF81	grey	VSMC_M10	0.01	9.62E-01	-0.39	4.54E-02	-0.40	4.11E-02	0.13	5.33E-01	0.58	1.43E-03	-0.48	1.11E-02	0.19	3.40E-01	-0.07	7.29E-01	0.41	3.40E-02	-0.36	6.22E-02
ENSCAFG0000001749	ARHGAP17	cyan	VSMC_M2	0.01	9.62E-01	0.55	2.82E-03	0.18	3.71E-01	0.17	4.06E-01	-0.21	2.89E-01	0.03	8.68E-01	0.06	7.63E-01	-0.43	2.56E-02	-0.45	1.72E-02	0.46	1.70E-02
ENSCAFG0000003040	TNFSF9	grey	VSMC_M10	0.01	9.62E-01	0.26	1.98E-01	0.05	8.17E-01	0.24	2.26E-01	-0.21	2.83E-01	0.15	4.59E-01	0.29	1.49E-01	-0.21	2.99E-01	-0.30	1.25E-01	0.46	1.51E-02
ENSCAFG0000001214	NPEPL1	grey	VSMC_M10	0.01	9.63E-01	-0.06	7.78E-01	0.03	8.83E-01	-0.03	8.78E-01	0.09	6.42E-01	-0.02	9.22E-01	0.20	3.18E-01	-0.08	6.93E-01	0.13	5.25E-01	-0.13	5.33E-01
ENSCAFG0000001292	DSCAML1	violet	VSMC_M7	0.01	9.63E-01	-0.07	7.24E-01	0.14	4.80E-01	-0.21	3.04E-01	-0.18	3.80E-01	-0.05	8.09E-01	0.77	2.20E-06	0.22	2.79E-01	-0.07	7.46E-01	0.00	9.91E-01
ENSCAFG0000000196	KRIT1	pink	VSMC_M5	0.01	9.63E-01	-0.36	6.87E-02	-0.61	8.26E-04	0.31	1.10E-01	0.71	3.28E-05	-0.31	1.15E-01	0.11	5.96E-01	-0.14	4.83E-01	0.33	8.88E-02	-0.29	1.36E-01
ENSCAFG0000001315	FZD9	grey	VSMC_M10	0.01	9.63E-01	0.01	9.78E-01	0.10	6.34E-01	0.04	8.50E-01	-0.25	2.06E-01	0.24	2.33E-01	0.39	4.19E-02	-0.04	8.37E-01	-0.05	7.86E-01	0.35	7.47E-02
ENSCAFG0000001914	UBN1	yellow	VSMC_M3	0.01	9.63E-01	0.07	7.44E-01	0.49	8.95E-03	-0.61	7.17E-04	-0.37	5.82E-02	0.08	7.01E-01	0.13	5.30E-01	0.40	3.65E-02	-0.01	9.50E-01	-0.27	1.70E-01
ENSCAFG0000001057	CHSY1	darkolivegreen	VSMC_M9	0.01	9.63E-01	-0.65	2.66E-04	-0.33	8.83E-02	-0.23	2.41E-01	0.46	1.55E-02	0.11	6.02E-01	-0.20	3.13E-01	0.41	3.46E-02	0.61	7.28E-04	-0.64	3.04E-04
ENSCAFG0000000242	SOSTDC1	grey	VSMC_M10	0.01	9.64E-01	-0.15	4.65E-01	0.00	9.91E-01	-0.25	2.15E-01	0.00	9.82E-01	0.01	9.74E-01	0.06	7.77E-01	0.19	3.46E-01	0.14	4.99E-01	-0.11	5.90E-01
ENSCAFG0000001286	RAPH1	darkgreen	VSMC_M4	0.01	9.64E-01	0.17	3.88E-01	-0.69	7.74E-05	0.86	8.02E-09	0.53	4.56E-03	-0.07	7.29E-01	-0.29	1.47E-01	-0.72	2.50E-05	-0.13	5.24E-01	0.47	1.27E-02
ENSCAFG0000000564	RAB2B	darkgreen	VSMC_M4	0.01	9.64E-01	0.28	1.50E-01	-0.31	1.11E-01	0.69	7.09E-05	0.14	4.86E-01	-0.06	7.77E-01	-0.31	1.14E-01	-0.62	4.93E-04	-0.23	2.59E-01	0.55	2.82E-03
ENSCAFG0000001302	CEP164	yellow	VSMC_M3	0.01	9.64E-01	0.19	3.44E-01	0.73	1.51E-05	-0.58	1.63E-03	-0.71	3.14E-05	0.07	7.19E-01	0.33	9.59E-02	0.36	6.34E-02	-0.23	2.40E-01	0.05	8.18E-01
ENSCAFG0000001248	CASTOR1	darkgreen	VSMC_M4	0.01	9.64E-01	0.25	2.01E-01	-0.55	2.69E-03	0.71	3.47E-05	0.49	9.17E-03	-0.14	4.98E-01	-0.20	3.26E-01	-0.69	6.22E-05	-0.14	4.96E-01	0.39	4.21E-02
ENSCAFG0000001996	MBTPS1	darkgreen	VSMC_M4	0.01	9.64E-01	0.38	5.19E-02	-0.29	1.43E-01	0.62	5.52E-04	0.27	1.79E-01	-0.17	3.92E-01	-0.69	5.78E-05	-0.69	6.42E-05	-0.16	4.29E-01	0.33	8.91E-02
ENSCAFG0000001933	HCFC1R1	yellow	VSMC_M3	0.01	9.64E-01	0.22	2.66E-01	0.67	1.16E-04	-0.55	3.06E-03	-0.58	1.63E-03	-0.19	3.45E-01	0.31	1.15E-01	0.34	8.14E-02	-0.23	2.51E-01	-0.11	5.80E-01
ENSCAFG0000000808	FAM208A	grey	VSMC_M10	0.01	9.64E-01	-0.14	4.81E-01	-0.56	2.34E-03	0.28	1.56E-01	0.56	2.14E-03	-0.27	1.79E-01	0.13	5.11E-01	-0.23	2.58E-01	0.10	6.08E-01	0.08	7.04E-01
ENSCAFG0000000424	ENSCAFG0000000424	grey	VSMC_M10	0.01	9.64E-01	0.48	1.21E-02	0.00	9.81E-01	0.45	1.89E-02	-0.22	2.73E-01	-0.01	9.57E-01	-0.10	6.17E-01	-0.46	1.70E-02	-0.44	2.07E-02	0.70	4.84E-05
ENSCAFG0000002365	ARPC4	darkgrey	VSMC_M8	0.01	9.64E-01	-0.15	4.68E-01	0.62	5.67E-04	-0.74	1.08E-05	-0.52	5.88E-03	0.09	6.59E-01	0.25	2.09E-01	0.65	2.69E-04	0.11	5.95E-01	-0.42	2.88E-02
ENSCAFG0000001432	ENSCAFG0000001432	grey	VSMC_M10	0.01	9.65E-01	0.04	8.58E-01	-0.04	8.50E-01	-0.05	7.97E-01	0.17	4.04E-01	-0.14	4.80E-01	-0.17	3.91E-01	-0.02	9.17E-01	0.00	9.94E-01	-0.17	3.98E-01
ENSCAFG0000003069	TMEM38A	darkgreen	VSMC_M4	0.01	9.65E-01	0.03	8.63E-01	-0.51	7.14E-03	0.69	7.00E-05	0.45	1.80E-02	0.15	4.62E-01	-0.16	4.38E-01	-0.63	4.75E-04	0.02	9.29E-01	0.23	2.51E-01
ENSCAFG0000001242	CCS	cyan	VSMC_M2	0.01	9.66E-01	0.64	3.53E-04	0.06	7.82E-01	0.39	4.18E-02	-0.18	3.59E-01	-0.05	8.01E-01	0.11	5.89E-01	-0.54	3.95E-03	-0.58	1.38E-03	0.69	6.24E-05
ENSCAFG0000003258	ENSCAFG0000003258	grey	VSMC_M10	0.01	9.66E-01	-0.01	9.77E-01	-0.43	2.54E-02	0.29	1.43E-01	0.31	1.11E-01	0.09	6.60E-01	0.08	7.06E-01	-0.17	3.85E-01	-0.06	7.85E-01	0.26	1.82E-01
ENSCAFG0000001881	SAFB2	yellow	VSMC_M3	0.01	9.66E-01	0.08	6.90E-01	0.67	1.19E-04	-0.53	4.66E-03	-0.71	3.41E-05	0.28	1.59E-01	0.13	5.09E-01	0.43	2.41E-02	-0.17	3.96E-01	-0.02	9.03E-01
ENSCAFG0000001842	TOM1L2	grey	VSMC_M10	0.01	9.66E-01	0.18	3.59E-01	-0.23	2.43E-01	0.55	2.69E-03	0.24	2.28E-01	0.06	7.76E-01	-0.38	4.87E-02	-0.60	8.50E-04	-0.01	9.69E-01	0.20	3.27E-01
ENSCAFG0000002997	TRIM62	turquoise	VSMC_M6	0.01	9.67E-01	-0.01	9.68E-01	0.02	9.23E-01	-0.11	5.98E-01	-0.11	5.80E-01	0.45	1.95E-02	-0.14	4.77E-01	0.13	5.20E-01	0.00	9.92E-01	0.03	8.76E-01
ENSCAFG0000002023	SH3GLB1	grey	VSMC_M10	0.01	9.67E-01	-0.45	1.74E-02	-0.62	5.45E-04	0.22	2.60E-01	0.63	4.90E-04	-0.12	5.43E-01	-0.20	3.17E-01	-0.01	9.58E-01	0.46	1.56E-02	-0.23	2.57E-01
ENSCAFG0000000713	NSMAF	grey	VSMC_M10	0.01	9.67E-01	0.37	5.41E-02	0.27	1.79E-01	-0.25	2.18E-01	-0.23	2.49E-01	-0.10	6.19E-01	0.24	2.31E-01	0.01	9.63E-01	-0.38	5.34E-02	0.17	3.87E-01
ENSCAFG0000001872	ENSCAFG0000001872	darkgrey	VSMC_M8	0.01	9.67E-01	-0.54	3.94E-03	0.30	1.30E-01	-0.88	2.29E-09	-0.16	4.38E-01	0.13	5.14E-01	0.34	8.34E-02	0.96	7.75E-16	0.36	6.25E-02	-0.70	5.26E-05
ENSCAFG0000002039	CES2	grey	VSMC_M10	0.01	9.68E-01	0.28	1.61E-01	-0.02	9.03E-01	0.50	7.81E-03	-0.15	4.63E-01	-0.01	9.42E-01	0.12	5.42E-01	-0.44	2.02E-02	-0.27	1.66E-01	0.57	1.71E-03
ENSCAFG0000000416	HMCES	grey	VSMC_M10	0.01	9.68E-01	0.32	1.05E-01	-0.06	7.75E-01	0.47	1.24E-02	0.08	7.02E-01	-0.10	6.10E-01	-0.05	7.92E-01	-0.52	5.45E-03	-0.22	2.63E-01	0.26	1.95E-01
ENSCAFG0000000237	ZAP70	darkgreen	VSMC_M4	0.01	9.68E-01	0.16	4.40E-01	-0.56	2.61E-03	0.88	1.48E-09	0.44	2.33E-02	-0.12	5.43E-01	-0.04	8.44E-01	-0.78	1.95E-06	-0.08	7.02E-01	0.50	8.36E-03
ENSCAFG0000002873	GEMIN8	cyan	VSMC_M2	0.01	9.68E-01	0.46	1.55E-02	0.42	2.83E-02	-0.03	8.99E-01	-0.47	1.39E-02	-0.09	6.51E-01	0.01	9.69E-01	-0.11	5.96E-01	-0.46	1.70E-02	0.31	1.11E-01
ENSCAFG0000000493	ENSCAFG0000000493	grey	VSMC_M10	0.01	9.69E-01	0.29	1.43E-01	0.21	2.83E-01	-0.12	5.65E-01	-0.25	2.01E-01	-0.02	9.27E-01	-0.12	5.66E-01	0.01	9.61E-01	-0.26	1.88E-01	0.16	4.23E-01
ENSCAFG0000001792	ENSCAFG0000001792	grey	VSMC_M10	0.01	9.69E-01	0.29	1.43E-01	0.21	2.83E-01	-0.12	5.65E-01	-0.25	2.01E-01	-0.02	9.27E-01	-0.12	5.66E-01	0.01	9.61E-01	-0.26	1.88E-01	0.16	4.23E-01
ENSCAFG0000002513	SLC6A19	grey	VSMC_M10	0.01	9.69E-01	0.29	1.43E-01	0.21	2.83E-01	-0.12	5.65E-01	-0.25	2.01E-01	-0.02	9.27E-01	-0.12	5.66E-01	0.01	9.61E-01	-0.26	1.88E-01	0.16	4.23E-01
ENSCAFG0000000007	ZCCHC2	grey	VSMC_M10	0.01	9.69E-01	0.00	9.95E-01	0.01	9.55E-01	-0.03	8.86E-01	0.01	9.66E-01	0.27	1.81E-01	-0.07	7.44E-01	-0.05	8.06E-01	0.04	8.55E-01	-0.03	8.90E-01
ENSCAFG0000000223	ENSCAFG0000000223	grey	VSMC_M10	0.01	9.69E-01	-0.03	8.77E-01	-0.36	6.66E-02	0.35	7.42E-02	0.29	1.41E-01	-0.16	4.19E-01	-0.11	5.79E-01	-0.21	3.04E-01	0.03	8.79E-01	0.18	3.73E-01
ENSCAFG0000001704	BATF	grey	VSMC_M10	0.01	9.69E-01	0.12	5.35E-01	0.42	2.94E-02	-0.32	1.06E-01	-0.42	2.75E-02	0.27	1.76E-01	0.06	7.56E-01	0.21	3.04E-01	-0.08	7.08E-01	0.02	9.21E-01
ENSCAFG0000000819	KIF13B	darkgreen	VSMC_M4	0.01	9.69E-01	0.51	6.78E-03	-0.33	9.31E-02	0.74	1.15E-05	0.19	3.54E-01	0.09	6.70E-01	-0.09	6.73E-01	-0.79	1.18E-06	-0.39	4.19E-02	0.70	5.08E-05
ENSCAFG0000001754	S100A13	grey	VSMC_M10	0.01	9.70E-01	-0.02	9.17E-01	0.35	7.25E-02	-0.15	4.46E-01	-0.22	2.60E-01	-0.29	1.48E-01	0.14	4.94E-01	0.07	7.37E-01	0.04	8.29E-01	-0.20	3.26E-01
ENSCAFG0000000797	ENSCAFG0000000797	grey	VSMC_M10	0.01	9.70E-01	-0.32	1.06E-01	-0.06	7.61E-01	-0.19	3.52E-01	0.07	7.11E-01	-0.10	6.36E-01	0.21	2.93E-01	0.31	1.14E-01	0.20	3.14E-01	-0.23	2.58E-01
ENSCAFG0000000156	BNC2	grey	VSMC_M10	0.01	9.70E-01	0.24	2.34E-01	-0.40	4.03E-02	0.42	3.12E-02	0.44	2.23E-02	-0.31	1.18E-01	-0.30	1.26E-01	-0.42	3.00E-02	-0.12	5.51E-01	0.12	5.46E-01
ENSCAFG0000001238	TRAPPC4	grey	VSMC_M10	0.01	9.71E-01	0.16	4.38E-01	0.22	2.76E-01	0.09	6.57E-01	-0.20	3.11E-01	0.08	6.87E-01	-0.10	6.22E-01	-0.16	4.18E-01	-0.18	3.56E-01	0.06	7.73E-01
ENSCAFG0000003068	ENSCAFG0000003068	grey	VSMC_M10	0.01	9.71E-01	0.34	7.99E-02	0.07	7.35E-01	0.13	5.04E-01	-0.18	3.78E-01	-0.04	8.31E-01	0.20	3.22E-01	-0.29	1.44E-01	-0.32	1.02E-01	0.38	5.36E-02
ENSCAFG0000001435	MEF2B	yellow	VSMC_M3	0.01	9.71E-01	0.52	5.35E-03	0.67	1.36E-04	-0.12	5.64E-01	-0.76	3.91E-06	0.16	4.24E-01	0.04	8.37E-01	-0.13	5.14E-01	-0.49	9.19E-03	0.42	2.86E-02
ENSCAFG0000000174	LARGE1	grey	VSMC_M10	0.01	9.71E-01	-0.08	6.88E-01	-0.13	5.18E-01	0.03	9.00E-01	0.24	2.23E-01	-0.10	6.16E-01	0.19	3.54E-01	-0.13	5.30E-01	0.17	3.92E-01	-0.12	5.44E-01
ENSCAFG0000001463	PNKD	grey	VSMC_M10	0.01	9.71E-01	-0.03	8.76E-01	0.16	4.27E-01	-0.23	2.58E-01	-0.20	3.17E-01	-0.07	7.43E-01	0.38	5.10E-02	0.29	1.42E-01	-0.06	7.52E-01	0.06	7.49E-01
ENSCAFG0000002483	RAB43	cyan	VSMC_M2	0.01	9.71E-01	0.38	4.90E-02	0.51	6.61E-03	-0.14	4.76E-01	-0.58	1.56E-03	0.17	4.07E-01	0.12	5.64E-01	-0.04	8.27E-01	-0.37	5.64E-02	0.37	5.70E-02
ENSCAFG0000002038	C5H16orf70	grey	VSMC_M10	0.01	9.71E-01	0.09	6.60E-01	-0.09	6.43E-01	0.02	9.08E-01	0.01	9.69E-01	0.17	3.98E-01	-0.13	5.26E-01	-0.01	9.48E-01	-0.08	6.88E-01	0.10	6.33E-01
ENSCAFG0000001094	ENSCAFG0000001094	grey	VSMC_M10	0.01	9.71E-01	0.12	5.55E-01	0.25	2.09E-01	-0.39	4.69E-02	-0.11	5.72E-01	0.07	7.29E-01	0.10	6.37E-01	0.26	1.89E-01	-0.13	5.15E-01	-0.24	2.28E-01
ENSCAFG0000003276	ENSCAFG0000003276	grey	VSMC_M10	0.01	9.71E-01	-0.13	5.31E-01	-0.05	8.15E-01	-0.09	6.39E-01	0.10	6.13E-01	-0.12	5.37E-01	-0.21	2.91E-01	0.14	4.86E-01	0.15	4.50E-01	-0.23	2.42E-01
ENSCAFG0000000673	ENSCAFG0000000673	grey	VSMC_M10	0.01	9.71E-01	-0.26	1.86E-01	-0.22	2.60E-01	-0.03	8.73E-01	0.32	9.91E-02	-0.27	1.70E-01	-0.09	6.62E-01	0.19	3.34E-01	0.17	3.84E-01	-0.35	7.54E-02
ENSCAFG0000001680	ZFYVE1	grey	VSMC_M10	0.01	9.72E-01	0.48	1.10E-02	-0.10	6.18E-01	0.49	8.87E-03	0.10	6.05E-01	-0.13	5.24E-01	-0.09	6.45E-01	-0.65	2.22E-04	-0.37	5.53E-02	0.44	2.31E-02
ENSCAFG0000000861	TROAP	darkgrey	VSMC_M8	0.01	9.72E-01	-0.49	9.55E-03	0.36	6.92E-02	-0.88	1.97E-09	-0.23	2.43E-01	0.16	4.34E-01	0.39	4.29E-02	0.95	6.09E-14	0.31	1.21E-01	-0.65	2.11E-04
ENSCAFG0000000045	THAP2	grey	VSMC_M10	0.01	9.72E-01	0.13	5.33E-01	-0.06	7.72E-01	0.05	8.06E-01	-0.10	6.10E-01	-0.14	4.74E-01	0.33	9.61E-02	-0.05	7.97E-01	-0.20	3.28E-01	0.36	6.46E-02
ENSCAFG0000001491	FAM227B	grey	VSMC_M10	0.01	9.72E-01	-0.25	2.11E-01	-0.12	5.48E-01	-0.09	6.70E-01	0.02	9.17E-01	-0.08	7.02E-01	0.23	2.39E-01	0.21	2.95E-01	0.18	3.81E-01	0.00	9.83E-01
ENSCAFG0000001367	DDX20	darkgrey	VSMC_M8	0.01	9.72E-01	-0.55	2.98E-03	-0.14	4.84E-01	-0.36	6.29E-02	0.16	4.32E-01	0.15	4.66E-01	0.32	1.09E-01	0.59	1.28E-03	0.35	7.29E-02	-0.40	3.73E-02
ENSCAFG0000001712	ZBTB7B	grey	VSMC_M10	0.01	9.73E-01	0.56	2.43E-03	0.25	2.00E-01	0.30	1.33E-01	-0.35	7.09E-02	-0.05	7.87E-01	-0.33	9.75E-02	-0.48	1.03E-02	-0.44	2.12E-02	0.50	7.67E-03
ENSCAFG0000000885	PPP2R2A	darkolivegreen	VSMC_M9	0.01	9.73E-01	-0.72	2.69E-05	-0.39	4.68E-02	-0.25	2.13E-01	0.47	1.28E-02	0.08	6.91E-01	0.05	8.19E-01	0.53	4.50E-03	0.57	1.75E-03	-0.60	8.55E-04
ENSCAFG0000000766	OGFOD2	grey	VSMC_M10	0.01	9.73E-01	0.46	1.68E-02	-0.01	9.50E-01	0.26	1.88E-01	-0.05	8.20E-01	0.09	6.73E-01	-0.04	8.24E-01	-0.37	6.04E-02	-0.41	3.49E-02	0.43	2.68E-02
ENSCAFG0000003148	ENSCAFG0000003148	grey	VSMC_M10	0.01	9.73E-01	0.14	4.85E-01	-0.07	7.37E-01	0.26	1.97E-01	0.02	9.14E-01	-0.06	7.85E-01	0.02	9.31E-01	-0.24	2.35E-01	-0.12	5.52E-01	0.23	2.44E-01
ENSCAFG0000000816	BTBD10	pink	VSMC_M5	0.01	9.74E-01	-0.39	4.57E-02	-0.75	5.71E-06	0.31	1.15E-01	0.72	2.70E-05	0.04	8.49E-01	-0.19	3.50E-01	-0.08	6.91E-01	0.32	1.05E-01	-0.18	3.76E-01
ENSCAFG0000000980	ENSCAFG0000000980	grey	VSMC_M10	0.01	9.74E-01	0.10	6.04E-01	-0.09	6.69E-01	0.01	9.51E-01	0.05	8.03E-01	-0.11	5.84E-01	-0.08	6.98E-01	-0.12	5.58E-01	-0.12	5.66E-01	0.14	4.72E-01
ENSCAFG0000000024	MAP7	grey	VSMC_M10	0.01	9.74E-01	0.24	2.36E-01	-0.11	5.93E-01	0.29	1.47E-01	-0.01	9.42E-01	-0.18	3.72E-01	-0.20	3.29E-01	-0.34	8.11E-02	-0.20	3.14E-01	0.34	8.04E-02
ENSCAFG0000000514	METTL23	grey	VSMC_M10	0.01	9.74E-01	0.36	6.91E-02	0.30	1.33E-01	0.16	4.37E-01	-0.52	5.92E-03	0.10	6.11E-01	0.20	3.26E-01	-0.17	4.00E-01	-0.39	4.27E-02	0.61	7.91E-04
ENSCAFG0000001139	NCF1	grey	VSMC_M10	0.01	9.74E-01	-0.22	2.73E-01	0.03	8.67E-01	-0.21	2.95E-01	-0.07	7.17E-01	0.43	2.64E-02	0.16	4.14E-01	0.27	1.70E-01	0.16	4.18E-01	-0.15	4.65E-01
ENSCAFG0000000024	PIP4K2C	grey	VSMC_M10	0.01	9.74E-01	0.64	3.36E-04	-0.12	5.61E-01	0.53	4.57E-03	0.08	6.91E-01	-0.23	2.45E-01	-0.47	1.44E-02	-0.72	2.45E-05	-0.42	2.78E-02	0.56	2.43E-03
ENSCAFG0000000875	CCL17	grey	VSMC_M10	0.01	9.75E-01	-0.37	5.54E-02	-0.06	7.82E-01	-0.39	4.38E-02	0.11	5.77E-01	0.25	2.10E-01	0.36	6.24E-02	0.47	1.40E-02	0.21	2.89E-01	-0.42	2.75E-02
ENSCAFG0000002121	ENSCAFG0000002121	grey	VSMC_M10	0.01	9.75E-01	0.12	5.59E-01	0.23	2.58E-01	-0.17	3.93E-01	-0.18	3.61E-01	-0.16	4.17E-01	0.21	2.85E-01	0.10	6.09E-01	-0.14	5.01E-01	0.00	9.91E-01
ENSCAFG0000001398	P3H2	grey	VSMC_M10	0.01	9.75E-01	-0.30	1.30E-01	0.10	6.05E-01	-0.33	9.32E-02	-0.12	5.43E-01	0.38	4.98E-02	0.50	7.91E-03	0.38	5.11E-02	0.15	4.65E-01	-0.18	3.66E-01
ENSCAFG0000001797	FRMPD3	darkgreen	VSMC_M4	0.01	9.75E-01	-0.17	4.03E-01	-0.61	6.47E-04	0.55	3.22E-03	0.48	1.21E-02	0.06	7.59E-01	-0.12	5.59E-01	-0.32	9.97E-02	0.07	7.47E-01	0.20	3.12E-01
ENSCAFG0000000253	PPM1B	grey	VSMC_M10	0.01	9.76E-01	-0.40	3.97E-02	-0.36	6.18E-02	0.17	4.04E-01	0.34	8.11E-02	-0.03	8.70E-01	0.10	6.05E-01	0.04	8.28E-01	0.36	6.13E-02	-0.16	4.29E-01
ENSCAFG0000001503	NOP2	darkgrey	VSMC_M8	0.01	9.76E-01	-0.37	5.40E-02	0.26	1.96E-01	-0.59	1.25E-03	-0.30	1.29E-01	0.34	8.37E-02	0.27	1.77E-01	0.77	2.82E-06	0.15	4.41E-01	-0.34	8.35E-02
ENSCAFG0000003100	SNX16	grey	VSMC_M10	0.01	9.76E-01	-0.33	9.69E-02	-0.56	2.45E-03	0.14	4.98E-01	0.55	2.88E-03	-0.07	7.24E-01	0.37	5.91E-02	0.00	9.92E-01	0.23	2.51E-01	-0.10	6.06E-01
ENSCAFG0000000922	WDR41	grey	VSMC_M10	0.01	9.76E-01	-0.13	5.31E-01	0.40	3.82E-02	-0.37	5.64E-02	-0.37	5.78E-02	0.14	4.90E-01	0.20	3.10E-01	0.32	1.09E-01	0.06	7.80E-01	-0.17	4.05E-01
ENSCAFG0000002215	ENSCAFG0000002215	grey	VSMC_M10	0.01	9.76E-01	0.28	1.59E-01	0.10	6.22E-01	0.06	7.76E-01	-0.17	3.88E-01	0.03	8.90E-01	0.29	1.44E-01	-0.13	5.22E-01	-0.27	1.77E-01	0.29	1.40E-01
ENSCAFG0000000249	DYNC2LI1	grey	VSMC_M10	0.01	9.76E-01	0.19	3.35E-01	-0.27	1.66E-01	0.43	2.38E-02	0.11	5.78E-01	-0.12	5.63E-01	0.00	9.98E-01	-0.38	5.14E-02	-0.24	2.22E-01	0.51	6.47E-03
ENSCAFG0000000951	YWHAB	darkgrey	VSMC_M8	0.01	9.77E-01	-0.37	5.85E-02	0.46	1.51E-02	-0.68	8.76E-05	-0.26	1.91E-01	0.05	8.15E-01	0.02	9.05E-01	0.69	7.75E-05	0.34	8.37E-02	-0.68	8.08E-05
ENSCAFG0000002863	ENSCAFG0000002863	turquoise	VSMC_M6	0.01	9.77E-01	-0.26	1.89E-01	-0.05	8.18E-01	-0.17	4.07E-01	-0.01	9.61E-01	0.49	1.02E-02	0.17	4.10E-01	0.28	1.54E-01	0.11	5.93E-01	-0.12	5.51E-01
ENSCAFG0000001079	TNNT2	darkgreen	VSMC_M4	0.01	9.77E-01	0.36	6.26E-02	-0.37	5.66E-02	0.87	5.05E-09	0.20	3.16E-01	-0.03	8.64E-01	-0.29	1.38E-01	-0.80	6.90E-07	-0.29	1.40E-01	0.61	8.07E-04
ENSCAFG0000002561	ENSCAFG0000002561	darkgrey	VSMC_M8	0.01	9.77E-01	-0.34	8.58E-02	0.48	1.05E-02	-0.70	4.29E-05	-0.35	7.60E-02	0.10	6.19E-01	0.28	1.56E-01	0.75	7.93E-06	0.17	3.96E-01	-0.56	2.38E-03
ENSCAFG0000001945	REXO1	cyan	VSMC_M2	0.01	9.78E-01	0.46	1.68E-02	0.53	4.76E-03	-0.09	6.54E-01	-0.62	6.20E-04	0.03	8.91E-01	-0.20	3.26E-01	-0.07	7.36E-01	-0.42	2.94E-02	0.40	4.00E-02
ENSCAFG0000001254	PLEKHA1	pink	VSMC_M5	0.01	9.78E-01	-0.26	1.91E-01	-0.50	7.75E-03	0.32	1.09E-01	0.67	1.43E-04	-0.27	1.69E-01	-0.15	4.64E-01	-0.30	1.25E-01	0.39	4.60E-02	-0.32	1.00E-01
ENSCAFG0000002879	ENSCAFG0000002879	grey	VSMC_M10	0.01	9.78E-01	-0.07	7.35E-01	-0.19	3.54E-01	0.06	7.85E-01	0.09	6.42E-01	0.13	5.15E-01	0.01	9.61E-01	0.04	8.45E-01	0.01	9.74E-01	0.15	4.66E-01
ENSCAFG0000000565	NMI	grey	VSMC_M10	0.01	9.78E-01	0.53	4.68E-03	0.04	8.53E-01	0.42	3.05E-02	-0.02	9.12E-01	-0.27	1.69E-01	-0.14	4.86E-01	-0.60	8.83E-04	-0.36	6.82E-02	0.38	5.09E-02
ENSCAFG0000001868	CPLANE1	pink	VSMC_M5	0.01	9.78E-01	-0.52	5.19E-03	-0.60	9.34E-04	0.20	3.24E-01	0.76	3.37E-06	-0.28	1.54E-01	-0.03	8.95E-01	-0.05	7.86E-01	0.53	4.08E-03	-0.46	1.47E-02
ENSCAFG0000001877	NTAN1	grey	VSMC_M10	0.01	9.78E-01	0.11	5.77E-01	0.07	7.33E-01	0.01	9.78E-01	-0.02	9.20E-01	-0.10	6.36E-01	-0.34	8.64E-02	-0.09	6.56E-01	0.03	8.98E-01	-0.03	8.88E-01
ENSCAFG0000001310	AP3B2	darkgreen	VSMC_M4	0.01	9.79E-01	0.11	5.93E-01	-0.44	2.18E-02	0.70	5.21E-05	0.30	1.23E-01	-0.05	7.94E-01	0.15	4.59E-01	-0.56	2.33E-03	-0.11	5.87E-01	0.44	2.19E-02

ENSCAFG0000000404	ARHGAP12	grey	VSMC_M1C	0.01	9.79E-01	0.32	1.03E-01	-0.14	4.90E-01	0.46	1.52E-02	0.08	6.98E-01	-0.26	1.90E-01	-0.37	5.92E-02	-0.44	2.19E-02	-0.22	2.65E-01	0.38	5.09E-02
ENSCAFG0000001844	TEDC1	yellow	VSMC_M3	0.01	9.79E-01	0.30	1.29E-01	0.43	2.65E-02	0.00	9.88E-01	-0.38	4.87E-02	0.10	6.32E-01	0.05	8.07E-01	-0.11	5.88E-01	-0.27	1.72E-01	0.14	4.85E-01
ENSCAFG0000000237	POLR1E	darkgrey	VSMC_M8	0.01	9.79E-01	-0.18	3.71E-01	0.32	1.03E-01	-0.52	5.64E-03	-0.31	1.17E-01	0.20	3.28E-01	0.22	2.62E-01	0.60	8.67E-04	0.02	9.10E-01	-0.26	1.87E-01
ENSCAFG0000000985	TRIM45	grey	VSMC_M1C	0.01	9.79E-01	0.54	3.83E-03	0.08	6.84E-01	0.53	4.32E-03	-0.27	1.74E-01	-0.01	9.79E-01	0.10	6.25E-01	-0.60	1.00E-03	-0.49	1.02E-02	0.77	2.38E-06
ENSCAFG0000000607	POSTN	violet	VSMC_M7	0.01	9.79E-01	-0.22	2.66E-01	0.25	2.14E-01	-0.55	2.82E-03	-0.16	4.33E-01	0.04	8.37E-01	0.81	2.97E-07	0.49	9.56E-03	0.12	5.62E-01	-0.26	1.92E-01
ENSCAFG0000001159	ENSCAFG0000001159	grey	VSMC_M1C	0.01	9.79E-01	0.44	2.09E-02	0.02	9.20E-01	0.21	2.94E-01	0.00	9.91E-01	-0.06	7.55E-01	-0.18	3.58E-01	-0.37	5.41E-02	-0.28	1.58E-01	0.36	6.12E-02
ENSCAFG0000000021	SLC2A12	grey	VSMC_M1C	0.01	9.80E-01	0.72	2.17E-05	0.13	5.32E-01	0.44	2.26E-02	-0.17	3.89E-01	-0.19	3.37E-01	-0.21	2.98E-01	-0.70	5.42E-05	-0.53	4.77E-03	0.63	4.28E-04
ENSCAFG0000000056	ENSCAFG0000000056	cyan	VSMC_M2	0.01	9.80E-01	0.53	4.87E-03	0.44	2.28E-02	0.04	8.33E-01	-0.62	5.43E-04	0.00	9.90E-01	0.04	8.54E-01	-0.21	2.88E-01	-0.53	4.71E-03	0.67	1.31E-04
ENSCAFG0000001920	PIP5K1C	grey	VSMC_M1C	0.01	9.80E-01	0.47	1.29E-02	0.19	3.48E-01	0.31	1.18E-01	-0.18	3.79E-01	0.15	4.68E-01	-0.32	1.09E-01	-0.50	7.90E-03	-0.32	1.00E-01	0.29	1.38E-01
ENSCAFG0000001175	TRAPPC2	grey	VSMC_M1C	0.01	9.80E-01	-0.05	8.04E-01	-0.28	1.62E-01	0.41	3.38E-02	0.30	1.27E-01	0.10	6.35E-01	-0.10	6.21E-01	-0.33	8.80E-02	0.11	5.99E-01	-0.03	8.77E-01
ENSCAFG0000001707	TTLL5	grey	VSMC_M1C	0.01	9.80E-01	-0.18	3.71E-01	-0.31	1.20E-01	0.04	8.35E-01	0.37	5.60E-02	-0.11	5.83E-01	0.44	2.25E-02	0.03	8.91E-01	0.17	4.07E-01	-0.10	6.20E-01
ENSCAFG0000003171	PHETA2	yellow	VSMC_M3	0.00	9.80E-01	0.30	1.24E-01	0.64	3.26E-04	-0.24	2.23E-01	-0.57	1.72E-03	-0.13	5.24E-01	-0.13	5.06E-01	0.07	7.43E-01	-0.21	2.82E-01	0.06	7.57E-01
ENSCAFG0000001466	SLC35F2	darkgrey	VSMC_M8	0.00	9.81E-01	-0.71	4.02E-05	0.12	5.66E-01	-0.70	4.06E-05	0.02	9.40E-01	0.13	5.21E-01	0.52	5.10E-03	0.83	6.59E-08	0.54	3.61E-03	-0.73	1.54E-05
ENSCAFG0000000458	RSU1	grey	VSMC_M1C	0.00	9.81E-01	0.31	1.20E-01	-0.24	2.21E-01	0.52	5.12E-03	0.18	3.61E-01	-0.28	1.61E-01	-0.29	1.47E-01	-0.62	6.31E-04	-0.11	5.83E-01	0.39	4.68E-02
ENSCAFG0000001991	FBXO31	grey	VSMC_M1C	0.00	9.81E-01	0.33	9.67E-02	0.19	3.53E-01	0.24	2.32E-01	-0.18	3.57E-01	-0.23	2.45E-01	-0.15	4.65E-01	-0.29	1.47E-01	-0.24	2.24E-01	0.23	2.43E-01
ENSCAFG0000000286	PTPN3	darkgreen	VSMC_M4	0.00	9.81E-01	0.48	1.14E-02	-0.11	5.69E-01	0.64	3.28E-04	-0.15	4.63E-01	0.22	2.62E-01	-0.33	9.25E-02	-0.63	4.64E-04	-0.44	2.27E-02	0.77	2.14E-06
ENSCAFG0000001182	TAGLN2	yellow	VSMC_M3	0.00	9.81E-01	0.11	5.85E-01	0.77	2.92E-06	-0.57	1.84E-03	-0.73	1.67E-05	0.07	7.25E-01	-0.10	6.16E-01	0.46	1.49E-02	-0.13	5.08E-01	-0.16	4.12E-01
ENSCAFG0000000273	CTPS1	darkgrey	VSMC_M8	0.00	9.81E-01	-0.38	5.13E-02	0.33	8.83E-02	-0.69	7.64E-05	-0.29	1.39E-01	0.30	1.34E-01	0.12	5.56E-01	0.81	3.50E-07	0.19	3.33E-01	-0.51	6.72E-03
ENSCAFG0000002312	ENSCAFG0000002312	grey	VSMC_M1C	0.00	9.82E-01	0.17	4.04E-01	0.42	2.91E-02	-0.24	2.19E-01	-0.47	1.24E-02	0.12	5.40E-01	0.21	2.88E-01	0.07	7.46E-01	-0.21	2.85E-01	0.18	3.76E-01
ENSCAFG0000003236	ENSCAFG0000003236	grey	VSMC_M1C	0.00	9.82E-01	-0.08	6.92E-01	-0.16	4.18E-01	0.13	5.03E-01	0.11	5.77E-01	0.01	9.66E-01	0.20	3.20E-01	-0.04	8.31E-01	-0.01	9.56E-01	0.09	6.55E-01
ENSCAFG0000000999	HPS6	cyan	VSMC_M2	0.00	9.82E-01	0.52	5.56E-03	0.21	3.02E-01	0.34	8.28E-02	-0.21	3.01E-01	-0.24	2.32E-01	-0.25	2.14E-01	-0.57	1.93E-03	-0.34	7.93E-02	0.47	1.32E-02
ENSCAFG0000001181	COL18A1	turquoise	VSMC_M6	0.00	9.82E-01	0.13	5.02E-01	0.28	1.59E-01	-0.14	4.92E-01	-0.40	3.95E-02	0.87	2.39E-09	0.21	2.82E-01	0.10	6.29E-01	-0.21	2.87E-01	0.22	2.68E-01
ENSCAFG0000002978	CDHR4	grey	VSMC_M1C	0.00	9.83E-01	0.17	3.85E-01	0.27	1.80E-01	-0.21	2.91E-01	-0.33	8.89E-02	-0.08	6.76E-01	0.20	3.21E-01	0.17	4.09E-01	-0.23	2.39E-01	0.12	5.62E-01
ENSCAFG0000000004	TMX3	darkgreen	VSMC_M4	0.00	9.83E-01	0.18	3.68E-01	-0.58	1.56E-03	0.75	5.91E-06	0.47	1.45E-02	-0.24	2.36E-01	-0.13	5.30E-01	-0.71	3.93E-05	-0.12	5.40E-01	0.48	1.10E-02
ENSCAFG0000000546	PFKP	grey	VSMC_M1C	0.00	9.83E-01	-0.01	9.77E-01	0.23	2.47E-01	-0.17	3.88E-01	-0.09	6.61E-01	-0.12	5.64E-01	-0.16	4.14E-01	0.07	7.47E-01	0.11	5.89E-01	-0.22	2.62E-01
ENSCAFG0000001779	ENSCAFG0000001779	darkgreen	VSMC_M4	0.00	9.83E-01	0.05	7.98E-01	-0.63	4.50E-04	0.73	1.29E-05	0.61	7.93E-04	-0.11	5.87E-01	-0.09	6.43E-01	-0.69	5.75E-05	0.06	7.67E-01	0.23	2.52E-01
ENSCAFG0000001735	XPO6	grey	VSMC_M1C	0.00	9.84E-01	-0.26	1.87E-01	-0.02	9.31E-01	-0.34	8.46E-02	-0.07	7.44E-01	0.42	2.78E-02	0.05	7.95E-01	0.49	1.01E-02	0.14	4.97E-01	-0.18	3.78E-01
ENSCAFG0000000587	NUP98	grey	VSMC_M1C	0.00	9.84E-01	0.11	5.75E-01	-0.36	6.35E-02	0.28	1.63E-01	0.22	2.74E-01	0.25	2.14E-01	0.10	6.20E-01	-0.18	3.65E-01	-0.17	4.00E-01	0.35	6.95E-02
ENSCAFG0000000574	THRB	darkgreen	VSMC_M4	0.00	9.84E-01	-0.10	6.24E-01	-0.62	5.36E-04	0.60	8.65E-04	0.54	3.56E-03	-0.18	3.63E-01	-0.21	2.88E-01	-0.45	1.85E-02	0.11	5.91E-01	0.19	3.35E-01
ENSCAFG0000001510	GABPB1	darkgrey	VSMC_M8	0.00	9.84E-01	-0.29	1.46E-01	0.03	9.00E-01	-0.50	7.63E-03	0.17	4.02E-01	-0.03	8.64E-01	0.10	6.15E-01	0.54	3.48E-03	0.22	2.61E-01	-0.58	1.45E-03
ENSCAFG0000000796	MLXIP	grey	VSMC_M1C	0.00	9.85E-01	0.18	3.63E-01	0.05	8.16E-01	0.24	2.26E-01	-0.11	5.86E-01	0.23	2.44E-01	-0.14	4.77E-01	-0.31	1.18E-01	-0.12	5.38E-01	0.35	7.76E-02
ENSCAFG0000000797	FAM167A	grey	VSMC_M1C	0.00	9.85E-01	0.23	2.57E-01	0.27	1.80E-01	-0.28	1.54E-01	-0.29	1.47E-01	0.22	2.78E-01	0.40	4.03E-02	0.19	3.48E-01	-0.24	2.29E-01	0.15	4.69E-01
ENSCAFG0000001503	CPSF4	grey	VSMC_M1C	0.00	9.85E-01	0.04	8.62E-01	0.41	3.35E-02	-0.27	1.68E-01	-0.30	1.26E-01	0.00	9.84E-01	-0.22	2.81E-01	0.18	3.82E-01	-0.01	9.58E-01	-0.30	1.30E-01
ENSCAFG0000001024	ENSCAFG0000001024	grey	VSMC_M1C	0.00	9.85E-01	-0.15	4.46E-01	-0.02	9.24E-01	-0.15	4.64E-01	0.12	5.53E-01	-0.09	6.44E-01	0.20	3.19E-01	0.11	5.72E-01	0.17	3.94E-01	-0.27	1.74E-01
ENSCAFG0000000297	PAPOLG	darkgreen	VSMC_M4	0.00	9.85E-01	-0.20	3.13E-01	-0.66	2.05E-04	0.66	1.91E-04	0.65	2.32E-04	-0.13	5.19E-01	-0.17	4.02E-01	-0.47	1.41E-02	0.20	3.21E-01	0.03	8.94E-01
ENSCAFG0000000994	STMND1	grey	VSMC_M1C	0.00	9.85E-01	-0.46	1.59E-02	-0.23	2.42E-01	-0.17	3.96E-01	0.28	1.59E-01	-0.03	8.84E-01	0.18	3.77E-01	0.29	1.41E-01	0.36	6.83E-02	-0.29	1.49E-01
ENSCAFG0000001617	ENSCAFG0000001617	grey	VSMC_M1C	0.00	9.85E-01	0.26	1.91E-01	-0.06	7.63E-01	0.16	4.23E-01	-0.02	9.30E-01	-0.13	5.15E-01	0.39	4.67E-02	-0.23	2.44E-01	-0.28	1.53E-01	0.41	3.54E-02
ENSCAFG0000001532	PLIN4	grey	VSMC_M1C	0.00	9.85E-01	-0.01	9.52E-01	0.04	8.60E-01	0.02	9.06E-01	-0.17	4.07E-01	0.24	2.37E-01	-0.07	7.43E-01	0.03	8.68E-01	0.01	9.72E-01	0.23	2.56E-01
ENSCAFG0000001325	NUF2	darkgrey	VSMC_M8	0.00	9.85E-01	-0.55	2.70E-03	0.30	1.26E-01	-0.85	2.44E-08	-0.11	5.81E-01	0.01	9.50E-01	0.37	5.73E-02	0.94	4.82E-13	0.39	4.49E-02	-0.78	1.41E-06
ENSCAFG0000000561	ARHGEF17	darkgreen	VSMC_M4	0.00	9.85E-01	0.15	4.46E-01	-0.40	4.10E-02	0.69	7.68E-05	0.39	4.55E-02	-0.27	1.77E-01	-0.33	9.31E-02	-0.68	9.37E-05	0.01	9.62E-01	0.26	1.94E-01
ENSCAFG0000002447	ENSCAFG0000002447	darkgreen	VSMC_M4	0.00	9.85E-01	-0.11	5.96E-01	-0.40	3.99E-02	0.43	2.71E-02	0.34	8.17E-02	-0.05	8.21E-01	-0.03	8.63E-01	-0.29	1.49E-01	0.13	5.03E-01	0.13	5.03E-01
ENSCAFG0000000979	VPS26C	grey	VSMC_M1C	0.00	9.86E-01	-0.12	5.38E-01	-0.24	2.32E-01	0.08	6.77E-01	0.20	3.25E-01	0.08	6.96E-01	0.29	1.48E-01	-0.06	7.59E-01	0.07	7.47E-01	0.05	8.17E-01
ENSCAFG0000001022	ENSCAFG0000001022	cyan	VSMC_M2	0.00	9.86E-01	0.52	5.00E-03	0.45	1.86E-02	0.02	9.13E-01	-0.49	1.02E-02	-0.16	4.24E-01	-0.07	7.41E-01	-0.25	2.01E-01	-0.43	2.62E-02	0.39	4.22E-02
ENSCAFG0000001875	RAB33A	grey	VSMC_M1C	0.00	9.86E-01	0.52	5.45E-03	0.15	4.63E-01	0.14	4.84E-01	-0.22	2.75E-01	-0.07	7.27E-01	0.06	7.67E-01	-0.27	1.67E-01	-0.44	2.17E-02	0.42	2.97E-02
ENSCAFG0000001442	SMARCAL1	darkgrey	VSMC_M8	0.00	9.86E-01	-0.08	6.79E-01	0.40	4.11E-02	-0.62	5.46E-04	-0.38	4.79E-02	0.03	8.81E-01	0.10	6.05E-01	0.60	1.04E-03	0.01	9.67E-01	-0.21	2.90E-01
ENSCAFG0000001351	BROX	grey	VSMC_M1C	0.00	9.86E-01	-0.43	2.51E-02	-0.60	9.44E-04	0.15	4.69E-01	0.64	3.59E-04	-0.10	6.03E-01	0.39	4.44E-02	0.01	9.76E-01	0.34	8.07E-02	-0.15	4.43E-01
ENSCAFG0000003273	SVIP	grey	VSMC_M1C	0.00	9.86E-01	0.29	1.45E-01	0.01	9.77E-01	0.46	1.57E-02	-0.15	4.42E-01	0.14	5.00E-01	-0.17	3.86E-01	-0.44	2.16E-02	-0.27	1.67E-01	0.48	1.07E-02
ENSCAFG0000000262	EPS8L1	grey	VSMC_M1C	0.00	9.86E-01	0.34	7.80E-02	-0.05	8.18E-01	0.24	2.25E-01	0.00	9.94E-01	-0.09	6.49E-01	-0.25	2.01E-01	-0.34	8.12E-02	-0.18	3.59E-01	0.34	7.84E-02
ENSCAFG0000000338	ADAM17	pink	VSMC_M5	0.00	9.87E-01	-0.04	8.62E-01	-0.48	1.05E-02	0.50	7.57E-03	0.52	5.03E-03	-0.03	8.71E-01	-0.16	4.37E-01	-0.45	1.86E-02	0.09	6.60E-01	0.03	8.67E-01
ENSCAFG0000001299	GRHL3	grey	VSMC_M1C	0.00	9.87E-01	-0.19	3.55E-01	-0.24	2.28E-01	0.05	8.08E-01	0.20	3.22E-01	-0.06	7.63E-01	0.34	8.08E-02	0.07	7.31E-01	0.07	7.11E-01	0.02	9.11E-01
ENSCAFG0000001709	YPEL3	grey	VSMC_M1C	0.00	9.87E-01	0.73	1.35E-05	-0.03	8.96E-01	0.55	3.16E-03	-0.09	6.38E-01	-0.06	7.68E-01	-0.21	3.01E-01	-0.76	4.33E-06	-0.56	2.45E-03	0.77	2.26E-06
ENSCAFG0000000481	LIPE	grey	VSMC_M1C	0.00	9.87E-01	0.60	9.29E-04	0.01	9.63E-01	0.61	7.43E-04	-0.17	3.99E-01	-0.03	8.78E-01	-0.53	4.65E-03	-0.68	9.66E-05	-0.46	1.48E-02	0.70	5.10E-05
ENSCAFG0000000908	PBRM1	grey	VSMC_M1C	0.00	9.88E-01	-0.35	7.14E-02	-0.60	1.03E-03	0.10	6.17E-01	0.55	2.80E-03	0.04	8.48E-01	0.20	3.09E-01	0.15	4.70E-01	0.21	2.83E-01	-0.15	4.65E-01
ENSCAFG0000000022	HBS1L	grey	VSMC_M1C	0.00	9.88E-01	-0.53	4.72E-03	-0.41	3.25E-02	0.06	7.48E-01	0.54	3.58E-03	-0.42	2.74E-02	0.16	4.34E-01	0.09	6.64E-01	0.50	8.40E-03	-0.43	2.44E-02
ENSCAFG0000000181	APTX	darkgrey	VSMC_M8	0.00	9.88E-01	-0.39	4.64E-02	0.31	1.17E-01	-0.52	5.83E-03	-0.29	1.49E-01	0.17	3.95E-01	0.29	1.41E-01	0.63	3.98E-04	0.21	2.90E-01	-0.39	4.32E-02
ENSCAFG0000001302	SMCO1	darkgreen	VSMC_M4	0.00	9.88E-01	0.14	4.82E-01	-0.29	1.38E-01	0.49	8.99E-03	0.18	3.80E-01	0.33	9.34E-02	0.08	6.79E-01	-0.41	3.61E-02	-0.12	5.44E-01	0.36	6.43E-02
ENSCAFG0000000004	ENSCAFG0000000004	grey	VSMC_M1C	0.00	9.88E-01	-0.03	8.66E-01	0.02	9.03E-01	0.01	9.53E-01	-0.07	7.35E-01	-0.05	8.11E-01	0.20	3.10E-01	0.09	6.49E-01	-0.06	7.82E-01	0.04	8.25E-01
ENSCAFG0000000315	ENSCAFG0000000315	grey	VSMC_M1C	0.00	9.89E-01	-0.08	7.09E-01	0.03	8.95E-01	0.13	5.32E-01	-0.10	6.12E-01	0.07	7.32E-01	0.21	2.90E-01	-0.09	6.66E-01	0.03	8.68E-01	0.11	5.71E-01
ENSCAFG0000001984	CELSR2	grey	VSMC_M1C	0.00	9.89E-01	0.71	2.97E-05	0.17	3.93E-01	0.42	2.99E-02	-0.40	3.84E-02	0.12	5.42E-01	-0.06	7.66E-01	-0.56	2.43E-03	-0.67	1.37E-04	0.88	1.25E-09
ENSCAFG0000001963	MTCP1	grey	VSMC_M1C	0.00	9.89E-01	0.39	4.72E-02	0.34	8.42E-02	-0.08	6.94E-01	-0.47	1.24E-02	0.22	2.61E-01	-0.03	8.99E-01	-0.03	8.81E-01	-0.39	4.24E-02	0.42	2.95E-02
ENSCAFG0000001015	MYH15	grey	VSMC_M1C	0.00	9.89E-01	-0.33	8.84E-02	-0.14	4.76E-01	-0.06	7.70E-01	0.09	6.47E-01	-0.10	6.32E-01	0.08	6.83E-01	0.22	2.74E-01	0.23	2.42E-01	-0.15	4.63E-01
ENSCAFG0000001956	RNF207	grey	VSMC_M1C	0.00	9.89E-01	0.34	7.81E-02	0.15	4.52E-01	-0.03	8.72E-01	-0.13	5.23E-01	-0.03	8.68E-01	-0.09	6.49E-01	-0.08	6.89E-01	-0.26	1.83E-01	0.19	3.54E-01
ENSCAFG0000002014	MTF2	grey	VSMC_M1C	0.00	9.89E-01	-0.27	1.65E-01	-0.24	2.21E-01	0.03	8.81E-01	0.07	7.29E-01	0.17	3.95E-01	0.15	4.48E-01	0.15	4.55E-01	0.18	3.59E-01	0.05	7.99E-01
ENSCAFG0000000709	FXYD1	darkgreen	VSMC_M4	0.00	9.89E-01	0.42	3.02E-02	-0.19	3.43E-01	0.71	3.98E-05	0.12	5.36E-01	-0.24	2.28E-01	-0.37	5.51E-02	-0.80	4.48E-07	-0.22	2.81E-01	0.49	9.41E-03
ENSCAFG0000001188	DNAJC8	darkgrey	VSMC_M8	0.00	9.90E-01	-0.49	1.02E-02	0.04	8.58E-01	-0.60	1.01E-03	0.16	4.22E-01	-0.27	1.66E-01	0.22	2.81E-01	0.68	9.64E-05	0.38	5.32E-02	-0.68	9.29E-05
ENSCAFG0000002364	REEP6	grey	VSMC_M1C	0.00	9.90E-01	0.19	3.37E-01	0.29	1.44E-01	-0.29	1.36E-01	-0.31	1.15E-01	0.00	9.99E-01	0.39	4.56E-02	0.20	3.22E-01	-0.20	3.16E-01	0.12	5.56E-01
ENSCAFG0000000476	CAD	darkgrey	VSMC_M8	0.00	9.90E-01	-0.03	8.99E-01	0.71	3.78E-05	-0.86	8.04E-09	-0.65	2.56E-04	0.22	2.77E-01	0.22	2.63E-01	0.75	6.37E-06	-0.08	7.02E-01	-0.30	1.32E-01
ENSCAFG0000002980	ENSCAFG0000002980	grey	VSMC_M1C	0.00	9.90E-01	0.40	3.75E-02	0.04	8.37E-01	0.03	8.86E-01	-0.09	6.54E-01	-0.02	9.39E-01	-0.11	5.72E-01	-0.20	3.27E-01	-0.30	1.22E-01	0.28	1.56E-01
ENSCAFG0000000641	PPP4R2	grey	VSMC_M1C	0.00	9.90E-01	-0.51	6.18E-03	-0.34	8.25E-02	0.07	7.32E-01	0.49	9.04E-03	-0.16	4.37E-01	-0.18	3.62E-01	0.10	6.13E-01	0.52	5.47E-03	-0.46	1.54E-02
ENSCAFG0000003142	PRPSAP1	darkgrey	VSMC_M8	0.00	9.90E-01	-0.58	1.64E-03	0.28	1.61E-01	-0.83	8.73E-08	-0.09	6.73E-01	-0.01	9.50E-01	0.46	1.58E-02	0.86	9.32E-09	0.47	1.44E-02	-0.77	2.40E-06
ENSCAFG0000002270	ENSCAFG0000002270	grey	VSMC_M1C	0.00	9.90E-01	-0.03	8.98E-01	-0.02	9.39E-01	-0.04	8.56E-01	-0.09	6.57E-01	-0.17	4.03E-01	0.06	7.51E-01	0.06	7.72E-01	0.01	9.46E-01	0.12	5.43E-01
ENSCAFG0000002451	ENSCAFG0000002451	grey	VSMC_M1C	0.00	9.90E-01	0.35	7.04E-02	0.34	8.61E-02	-0.08	6.74E-01	-0.48	1.22E-02	0.15	4.40E-01	-0.04	8.45E-01	0.03	8.76E-01	-0.39	4.29E-02	0.34	8.57E-02
ENSCAFG0000001276	COCH	grey	VSMC_M1C	0.00	9.90E-01	-0.05	7.98E-01	-0.38	5.04E-02	0.57	1.72E-03	0.35	7.40E-02	-0.08	6.94E-01	-0.16	4.39E-01	-0.38	5.04E-02	0.06	7.67E-01	0.06	7.68E-01
ENSCAFG0000000826	MND1	darkgrey	VSMC_M8	0.00	9.91E-01	-0.47	1.44E-02	0.06	7.52E-01	-0.69	7.84E-05	0.03	8.78E-01	0.16	4.37E-01	0.37	5.96E-02	0.79	8.66E-07	0.28	1.52E-01	-0.57	1.82E-03
ENSCAFG0000000677	C16H8orf48	grey	VSMC_M1C	0.00	9.91E-01	-0.25	2.05E-01	-0.30	1.30E-01	0.40	3.96E-02	0.37	5.87E-02	-0.24	2.38E-01	-0.13	5.11E-01	-0.21	3.04E-01	0.26	1.87E-01	-0.20	3.14E-01
ENSCAFG0000001482	SECISBP2L	darkgreen	VSMC_M4	0.00	9.92E-01	-0.14	4.84E-01	-0.51	6.63E-03	0.53	4.38E-03	0.49	9.58E-03	-0.10	6.37E-01	-0.05	7.96E-01	-0.42	2.98E-02	0.16	4.28E-01	0.11	5.96E-01
ENSCAFG0000000328	RSPO1	grey	VSMC_M1C	0.00	9.92E-01	0.37	6.07E-02	-0.04	8.48E-01	0.29	1.42E-01	-0.07	7.21E-01	-0.07	7.40E-01	-0.15	4.60E-01	-0.33	8.92E-02	-0.32	1.06E-01	0.39	4.71E-02
ENSCAFG0000001736	POU3F4	grey	VSMC_M1C	0.00	9.92E-01	0.37	6.07E-02	-0.04	8.48E-01	0.29	1.42E-01	-0.07	7.21E-01	-0.07	7.40E-01	-0.15	4.60E-01	-0.33	8.92E-02	-0.32	1.06E-01	0.39	4.71E-02
ENSCAFG0000001541	PRR19	grey	VSMC_M1C	0.00	9.92E-01	0.28	1.57E-01	-0.06	7.65E-01	0.27	1.76E-01	-0.07	7.27E-01	0.18	3.83E-01	-0.17	4.06E-01	-0.26	1.99E-01	-0.30	1.24E-01	0.37	5.61E-02
ENSCAFG0000000050	DDX39B	darkgrey	VSMC_M8	0.00	9.92E-01	-0.22	2.75E-01	0.40	3.80E-02	-0.66	1.84E-04	-0.38	5.36E-02	0.14	4.80E-01	0.19	3.39E-01	0.73	1.51E-05	0.06	7.74E-01	-0.33	9.06E-02
ENSCAFG0000000081	EIF3H	cyan	VSMC_M2	0.00	9.92E-01	0.51	6.99E-03	0.23	2.38E-01	0.05	8.19E-01	-0.30	1.34E-01	-0.07	7.23E-01	-0.16	4.12E-01	-0.23	2.44E-01	-0.44	2.28E-02	0.38	4.76E-02
ENSCAFG0000000935	ASNSD1	grey	VSMC_M1C	0.00	9.92E-01	0.15	4.63E-01	0.19	3.51E-01	0.04	8.30E-01	-0.30	1.22E-01	0.25	2.02E-01	0.29	1.40E-01	-0.04	8.51E-01	-0.18	3.70E-01	0.29	1.40E-01
ENSCAFG0000001024	PXN	grey	VSMC_M1C	0.00	9.92E-01	0.23	2.46E-01	0.60	9.99E-04	-0.45	1.77E-02	-0.65	2.77E-04	0.25	2.16E-01	-0.24	2.22E-01	0.36	6.31E-02	-0.24	2.32E-01	0.01	9.48E-01
ENSCAFG0000000362	USP38	grey	VSMC_M1C	0.00	9.92E-01	-0.70	4.87E-05	-0.52	5.90E-03	-0.20	3.26E-01	0.56	2.34E-03	0.04	8.35E-01	0.16	4.17E-01	0.42	3.12E-02	0.57	1.88E-03	-0.51	7.09E-03
ENSCAFG0000003039	NACC2	darkgreen	VSMC_M4	0.00	9.92E-01	0.51	6.74E-03	-0.26	1.87E-01	0.80	5.95E-07	0.10	6.26E-01	-0.16	4.22E-01	-0.50	8.03E-03	-0.82	1.22E-07	-0.35	7.78E-02	0.69	6.30E-05
ENSCAFG0000001185	SPC25	darkgrey	VSMC_M8	0.00	9.92E-01	-0.49	9.30E-03	0.42	3.12E-02	-0.87	2.60E-09	-0.30	1.22E-01	0.13	5.08E-01	0.47	1.43E-02	0.95	1.96E-14	0.30	1.33E-01	-0.62	5.94E-04
ENSCAFG0000001837	ENSCAFG0000001837	grey	VSMC_M1C	0.00	9.93E-01	0.22	2.66E-01	0.29	1.39E-01	-0.02	9.32E-01	-0.26	1.87E-01	0.11	5.99E-01	-0.14	4.72E-01	-0.08	7.10E-01	-0.13	5.22E-01	0.03	8.83E-01
ENSCAFG0000002580	ENSCAFG0000002580	grey	VSMC_M1C	0.00	9.93E-01	-0.32	1.03E-01	-0.06	7.50E-01	-0.22	2.81E-01	0.01	9.71E-01	0.20	3.13E-01	-0.04	8.25E-01	0.30	1.33E-01	0.23	2.40E-01	-0.20	3.17E-01
ENSCAFG0000001031	TENT4A	darkgrey	VSMC_M8	0.00	9.93E-01	-0.36	6.49E-02	0.35	7.34E-02	-0.70	4.36E-05	-0.24	2.30E-01	0.29	1.49E-01	0.31	1.10E-01	0.71	3.95E-05	0.23	2.51E-01	-0.51	7.20E-03
ENSCAFG0000000930	SCAF11	grey	VSMC_M1C	0.00	9.93E-01	-0.44	2.06E-02	-0.19	3.33E-01	-0.38	5.26E-02	0.33	9.53E-02	0.04	8.35E-01	-0.01	9.52E-01	0.50	7.36E-03	0.36	6.30E-02	-0.50	7.61E-03
ENSCAFG0000003062	LAYN	grey	VSMC_M1C	0.00	9.93E-01	-0.16	4.35E-01	0.18	3.81E-01	-0.29	1.40E-01	-0.22	2.70E-01	0.42	2.84E-02	0.11	5.99E-01	0.39	4.23E-02	0.04	8.41E-01	-0.12	5.50E-01
ENSCAFG0000001514	USP8	pink	VSMC_M5	0.00	9.93E-01	-0.37	5.69E-02	-0.86	8.60E-09	0.49	8.87E-03	0.86	8.48E-09	-0.20	3.15E-01	-0.27	1.76E-01	-0.26	1.85E-01	0.40	3.70E-02	-0.14	4.84E-01
ENSCAFG0000000560	ISM1	grey	VSMC_M1C	0.00	9.93E-01	0.56	2.54E-03	0.03	8.99E-01	0.31	1.16E-01	-0.03	8.71E-01	-0.29	1.37E-01	-0.13	5.14E-01	-0.41	3.25E-02	-0.48	1.22E-02	0.40	4.01E-02
ENSCAFG0000003026	MFAP4	darkgreen	VSMC_M4	0.00	9.93E-01	0.50	8.51E-03	-0.29	1.39E-01	0.82	2.25E-07	0.14	4.72E-01	-0.04	8.48E-01	-0.58	1.67E-03	-0.81	3.35E-07	-0.39	4.64E-02	0.60	8.39E-04
ENSCAFG0000001684	HOXB1	cyan	VSMC_M2	0.00	9.94E-01	0.42	3.02E-02	0.24	2.28E-01	-0.09	6.41E-01	-0.31	1.13E-01	-0.04	8.40E-01	-0.19	3.38E-01	-0.03	8.73E-01	-0.39	4.67E-02	0.26	1.96E-01
ENSCAFG0000001035	CD96	grey	VSMC_M1C	0.00	9.94E-01	-0.31	1.20E-01	-0.35	7.79E-02	0.13	5.20E-01	0.20	3.17E-01	0.35	7.65E-02	0.18	3.62E-01	0.09	6.67E-01	0.15	4.41E-01	0.07	7.21E-01
ENSCAFG0000001660	CCNB2	darkgrey	VSMC_M8	0.00	9.94E-01	-0.57	1.99E-03	0.35	7.05E-02	-0.90	1.99E-10	-0.20	3.27E-01	0.10	6.03E-01	0.38	5.26E-02	0.97	4.64E-16	0.41	3.58E-02	-0.75	7.75E-06
ENSCAFG0000001688	SSR2	grey	VSMC_M1C	0.00	9.94E-01	0.25	2.07E-01	0.61	7.69E-04	-0.39	4.61E-02	-0.57	1.92E-03	0.03	8.82E-01	-0.39	4.33E-02	0.23	2.39E-01	-0.20	3.21E-01	-0.11	6.01E-01
ENSCAFG0000000933	SV2C	violet	VSMC_M7	0.00	9.94E-01	-0.44	2.30E-02	0.06	7.67E-01	-0.46	1.62E-02	-0.03	9.00E-01	0.20	3.18E-01	0.66	1.99E-04	0.49	1.03E-02	0.29	1.45E-01	-0.33	8.98E-02
ENSCAFG0000001826	PNPLA6	grey	VSMC_M1C	0.00	9.94E-01	0.81	3.66E-07	0.05	8.12E-01	0.59	1.12E-03	-0.24	2.28E-01	-0.01	9.48E-01	-0.31	1.19E-01	-0.77	2.19E-06	-0.68	8.73E-05	0.87	3.79E-09
ENSCAFG0000000181	ZFAND5	grey	VSMC_M1C	0.00	9.94E-01	-0.32	1.06E-01	0.39	4.61E-02	-0.63	4.18E-04	-0.27	1.75E-01	0.10	6.37E-01	0.28	1.54E-01	0.53	4.51E-03	0.32	1.05E-01	-0.45	1.85E-02
ENSCAFG0000000171	LRRC19	cyan	VSMC_M2	0.00	9.94E-01	0.62	5.66E-04	0.06	7.59E-01	0.24	2.19E-01	-0.15	4.50E-01	-0.07	7.23E-01	-0.06	7.79E-01	-0.48	1.20E-02	-0.51	7.01E-03	0.57	1.92E-03
ENSCAFG0000003131	CAPNS1	grey	VSMC_M1C	0.00	9.94E-01	0.43	2.55E-02	0.38	5.32E-02	-0.26	1.84E-01	-0.40	3.68E-02	0.07	7.14E-01	-0.09	6.38E-01	0.02	9.38E-01	-0.34	8.22E-02	0.20	3.11E-01
ENSCAFG0000001539	VDAC2	darkolivegreen	VSMC_M9	0.00	9.95E-01	-0.78	1.75E-06	-0.18	3.69E-01	-0.34	8.45E-02	0.31	1.16E-01	0.09	6.42E-01	0.17	4.06E-01	0.58	1.56E-03	0.69	7.72E-05	-0.72	2.04E-05
ENSCAFG0000002475	ENSCAFG0000002475	turquoise	VSMC_M6	0.00	9.95E-01	0.12	5.53E-01	0.13	5.05E-01	-0.10	6.07E-01	-0.24	2.33E-01	0.54	3.37E-03	0.26	1.95E-01	0.08	6.84E-01	-0.16	4.23E-01	0.13	5.22E-01
ENSCAFG0000001342	CALY	grey	VSMC_M1C	0.00	9.95E-01	-0.12	5.47E-01	-0.06	7.78E-01	-0.04	8.47E-01	0.01	9.45E-01	-0.04	8.61E-01	0.20	3.29E-01	0.11	5.89E-01	0.09	6.54E-01	0.03	8.96E-01
ENSCAFG0000001939	TMPRSS9	grey	VSMC_M1C	0.00	9.95E-01	0.04	8.57E-01	0.49	9.78E-03	-0.23	2.43E-01	-0.50	7.60E-03	0.30	1.24E-01	-0.07	7.39E-01	0.16	4.39E-01	-0.05	8.19E-01	-0.01	9.64E-01
ENSCAFG0000001984	ACSF3	grey	VSMC_M1C	0.00	9.95E-01	0.26	1.90E-01	0.17	4.00E-01	-0.04	8.52E-01	-0.17	4.01E-01	0.07	7.22E-01	0.26	1.90E-01	-0.03	8.85E-01	-0.27	1.70E-01	0.17	4.09E-01
ENSCAFG0000000793	B3GNT4	grey	VSMC_M1C	0.00	9.95E-01	0.44	2.02E-02	0.15	4.55E-01	0.15	4.65E-01	-0.31	1.13E-01	0.33	9.65E-02	-0.18	3.79E-01	-0.20	3.05E-01	-0.47	1.44E-02	0.47	1.25E-02
ENSCAFG0000003144	FAM237B	grey	VSMC_M1C	0.00	9.95E-01	-0.20	3.21E-01	0.06	7.61E-01	-0.21	2.91E-01	-0.06	7.73E-01	-0.08	7.04E-01	0.09	6.73E-01	0.28	1.62E-01	0.14	4.74E-01	-0.15	4.58E-01
ENSCAFG0000000574	IDO2	grey	VSMC_M1C	0.00	9.95E-01	0.38	4.82E-02	-0.09	6.63E-01	0.34	8.28E-02	-0.11	5.86E-01	-0.03	8.89E-01	-0.01	9.72E-01	-0.31	1.15E-01	-0.41	3.30E-02	0.61	6.60E-04
ENSCAFG0000000943	E2F8	darkgrey	VSMC_M8	0.00	9.95E-01	-0.57	1.92E-03	0.26	1.98E-01	-0.85	2.74E-08	-0.12	5.44E-01	0.09	6.50E-01	0.36	6.32E-02	0.95	2.54E-14	0.38	4.95E-02	-0.72	2.06E-05
ENSCAFG0000000657	TEX26	grey	VSMC_M1C	0.00	9.96E-01	-0.11	5.77E-01	0.10	6.30E-01	-0.21	2.82E-01	-0.06	7.63E-01	-0.04	8.46E-01	0.16	4.35E-01	0.27	1.78E-01	0.02	9.26E-01	-0.10	6.25E-01
ENSCAFG0000003211	RCE1	yellow	VSMC_M3	0.00	9.96E-01	0.11	5.95E-01	0.67	1.47E-04	-0.49	9.99E-03	-0.55	2.83E-03	0.02	9.34E-01	0.09	6.68E-01	0.30	1.25E-01	-0.06	7.76E-01	-0.23	2.42E-01
ENSCAFG0000001709	FARSA	yellow	VSMC_M3	0.00	9.96E-01	-0.09	6.63E-01	0.71	3.28E-05	-0.71	3.32E-05	-0.72	1.90E-05	0.34	8.34E-02	0.27	1.80E-01	0.67	1.14E-04	-0.07	7.38E-01	-0.19	3.32E-01
ENSCAFG0000001484	SYNPO2L	darkgreen	VSMC_M4	0.00	9.96E-01	0.47	1.29E-02	-0.45	1.80E-02	0.94	7.10E-13	0.24	2.21E-01	-0.05	7.91E-01	-0.41	3.51E-02	-0.89	4.16E-10	-0.36	6.17E-02	0.74	9.32E-06

ENSCAFG0000000464	UROD	cyan	VSMC_M2	0.00	9.96E-01	0.76	3.42E-06	0.42	2.79E-02	0.25	1.99E-01	-0.53	4.44E-03	0.06	7.58E-01	-0.26	1.84E-01	-0.50	8.02E-03	-0.63	3.94E-04	0.60	8.99E-04
ENSCAFG0000001954	POLR2E	darkgrey	VSMC_M8	0.00	9.97E-01	-0.47	1.39E-02	0.33	9.01E-02	-0.77	2.78E-06	-0.22	2.72E-01	0.26	1.97E-01	0.14	4.81E-01	0.83	1.13E-07	0.36	6.20E-02	-0.65	2.42E-04
ENSCAFG0000000484	INHBB	darkgreen	VSMC_M4	0.00	9.97E-01	0.16	4.17E-01	-0.39	4.32E-02	0.74	9.27E-06	0.26	1.82E-01	-0.03	8.99E-01	0.16	4.14E-01	-0.70	4.62E-05	-0.15	4.63E-01	0.51	6.74E-03
ENSCAFG0000000226	FBXO9	darkolivegreen	VSMC_M9	0.00	9.97E-01	-0.52	5.02E-03	-0.26	1.87E-01	-0.17	3.97E-01	0.44	2.12E-02	-0.10	6.33E-01	-0.25	2.11E-01	0.27	1.75E-01	0.57	1.99E-03	-0.64	2.94E-04
ENSCAFG0000000865	KATNB1	grey	VSMC_M10	0.00	9.98E-01	0.19	3.43E-01	-0.16	4.32E-01	0.41	3.16E-02	0.05	8.19E-01	0.01	9.42E-01	0.13	5.07E-01	-0.44	2.18E-02	-0.12	5.34E-01	0.39	4.52E-02
ENSCAFG0000003191	OTOS	grey	VSMC_M10	0.00	9.98E-01	0.26	1.91E-01	0.15	4.56E-01	-0.09	6.52E-01	-0.17	4.07E-01	0.06	7.75E-01	0.04	8.33E-01	-0.03	8.98E-01	-0.22	2.73E-01	0.16	4.25E-01
ENSCAFG0000000637	C24H20orf194	darkgreen	VSMC_M4	0.00	9.98E-01	-0.02	9.28E-01	-0.63	4.37E-04	0.80	6.56E-07	0.55	3.18E-03	-0.08	6.76E-01	-0.15	4.57E-01	-0.65	2.79E-04	0.11	5.95E-01	0.28	1.53E-01
ENSCAFG0000000413	CCNA2	darkgrey	VSMC_M8	0.00	9.99E-01	-0.55	2.88E-03	0.34	8.07E-02	-0.90	1.98E-10	-0.20	3.08E-01	0.15	4.69E-01	0.39	4.26E-02	0.98	2.66E-20	0.38	5.07E-02	-0.72	2.14E-05
ENSCAFG0000001314	MAP3K20	darkgrey	VSMC_M8	0.00	9.99E-01	-0.42	2.94E-02	0.36	6.60E-02	-0.88	1.89E-09	-0.15	4.67E-01	0.06	7.54E-01	0.42	3.12E-02	0.86	1.03E-08	0.33	9.41E-02	-0.73	1.43E-05
ENSCAFG0000000578	ENSCAFG0000000578	grey	VSMC_M10	0.00	9.99E-01	0.65	2.28E-04	0.31	1.10E-01	0.21	2.97E-01	-0.52	5.64E-03	0.22	2.66E-01	0.27	1.75E-01	-0.38	4.93E-02	-0.66	1.61E-04	0.78	1.65E-06
ENSCAFG0000000448	RUBCNL	grey	VSMC_M10	0.00	1.00E+00	-0.05	8.03E-01	-0.15	4.43E-01	0.30	1.35E-01	0.08	6.85E-01	0.01	9.66E-01	0.12	5.65E-01	-0.26	1.94E-01	0.13	5.23E-01	0.22	2.63E-01
ENSCAFG0000001770	CLMN	grey	VSMC_M10	0.00	1.00E+00	-0.06	7.56E-01	-0.08	7.08E-01	-0.06	7.55E-01	0.09	6.73E-01	0.00	9.99E-01	0.03	8.68E-01	0.06	7.76E-01	0.03	8.91E-01	0.03	8.77E-01
ENSCAFG0000001274	PSMC5	darkgrey	VSMC_M8	0.00	1.00E+00	-0.50	7.96E-03	0.22	2.67E-01	-0.68	8.60E-05	-0.09	6.64E-01	0.05	7.94E-01	0.15	4.68E-01	0.80	5.35E-07	0.35	7.47E-02	-0.71	3.71E-05
ENSCAFG0000000997	ENSCAFG0000000997	grey	VSMC_M10	0.00	1.00E+00	-0.33	8.80E-02	-0.42	2.99E-02	0.11	5.78E-01	0.42	3.04E-02	-0.23	2.57E-01	-0.04	8.33E-01	-0.02	9.09E-01	0.35	7.05E-02	-0.10	6.03E-01
ENSCAFG0000000839	MRPS28	grey	VSMC_M10	0.00	1.00E+00	-0.36	6.61E-02	0.24	2.19E-01	-0.46	1.50E-02	-0.22	2.69E-01	0.18	3.65E-01	0.44	2.28E-02	0.53	4.40E-03	0.21	2.83E-01	-0.34	8.38E-02
ENSCAFG0000001832	ENSCAFG0000001832	grey	VSMC_M10	0.00	1.00E+00	-0.07	7.44E-01	0.10	6.24E-01	-0.29	1.37E-01	-0.16	4.22E-01	0.14	4.94E-01	0.25	2.13E-01	0.29	1.48E-01	-0.01	9.74E-01	-0.03	9.01E-01
ENSCAFG0000000255	CCDC106	yellow	VSMC_M3	0.00	1.00E+00	0.15	4.56E-01	0.60	9.42E-04	-0.57	1.98E-03	-0.46	1.67E-02	-0.11	5.90E-01	-0.01	9.45E-01	0.38	5.26E-02	-0.09	6.65E-01	-0.30	1.23E-01
ENSCAFG0000000608	ENSCAFG0000000608	grey	VSMC_M10	0.00	9.99E-01	0.05	8.10E-01	0.38	5.15E-02	-0.31	1.12E-01	-0.24	2.28E-01	-0.12	5.39E-01	0.04	8.41E-01	0.21	2.96E-01	-0.06	7.63E-01	-0.29	1.39E-01
ENSCAFG0000003155	ENSCAFG0000003155	grey	VSMC_M10	0.00	9.99E-01	0.13	5.30E-01	0.16	4.26E-01	0.23	2.53E-01	-0.24	2.32E-01	0.13	5.26E-01	0.12	5.63E-01	-0.19	3.55E-01	-0.13	5.14E-01	0.23	2.48E-01
ENSCAFG0000001132	ISCU	grey	VSMC_M10	0.00	9.99E-01	0.02	9.33E-01	0.24	2.23E-01	-0.03	9.01E-01	-0.12	5.59E-01	-0.07	7.47E-01	-0.03	8.68E-01	-0.08	6.77E-01	0.10	6.37E-01	-0.21	2.87E-01
ENSCAFG0000000888	MRAP	grey	VSMC_M10	0.00	9.98E-01	-0.08	7.03E-01	0.02	9.27E-01	-0.07	7.16E-01	-0.02	9.10E-01	-0.08	6.97E-01	-0.21	2.88E-01	0.10	6.22E-01	0.09	6.54E-01	-0.07	7.40E-01
ENSCAFG0000000358	PHF19	darkgrey	VSMC_M8	0.00	9.98E-01	-0.29	1.37E-01	0.52	5.83E-03	-0.92	1.60E-11	-0.36	6.17E-02	0.05	8.17E-01	0.33	9.60E-02	0.87	2.45E-09	0.19	3.50E-01	-0.56	2.18E-03
ENSCAFG0000000936	MAPK8IP1	cyan	VSMC_M2	0.00	9.98E-01	0.48	1.16E-02	0.27	1.68E-01	0.06	7.65E-01	-0.24	2.20E-01	0.02	9.19E-01	-0.21	2.87E-01	-0.24	2.28E-01	-0.29	1.42E-01	0.22	2.77E-01
ENSCAFG0000001136	KCNB1	grey	VSMC_M10	0.00	9.98E-01	0.14	4.99E-01	-0.29	1.35E-01	0.36	6.56E-02	0.16	4.22E-01	-0.34	8.48E-02	0.00	9.93E-01	-0.33	8.99E-02	-0.11	6.00E-01	0.35	7.39E-02
ENSCAFG0000001929	ENSCAFG0000001929	turquoise	VSMC_M6	0.00	9.98E-01	0.02	9.09E-01	0.09	6.60E-01	-0.19	3.49E-01	-0.10	6.08E-01	0.05	8.02E-01	0.31	1.21E-01	0.09	6.43E-01	-0.04	8.26E-01	0.05	7.99E-01
ENSCAFG0000001874	ENSCAFG0000001874	grey	VSMC_M10	0.00	9.97E-01	0.11	5.75E-01	0.11	5.94E-01	-0.10	6.06E-01	-0.18	3.59E-01	-0.03	8.87E-01	0.30	1.30E-01	-0.01	9.69E-01	-0.11	5.79E-01	0.15	4.64E-01
ENSCAFG0000001069	NAA50	darkgrey	VSMC_M8	0.00	9.97E-01	-0.67	1.24E-04	0.00	9.98E-01	-0.46	1.68E-02	0.11	5.69E-01	-0.02	9.07E-01	0.31	1.10E-01	0.69	7.10E-05	0.53	4.41E-03	-0.66	1.97E-04
ENSCAFG0000001928	ENSCAFG0000001928	grey	VSMC_M10	0.00	9.97E-01	-0.24	2.23E-01	0.01	9.41E-01	0.07	7.38E-01	-0.02	9.05E-01	0.07	7.44E-01	-0.07	7.23E-01	0.06	7.83E-01	0.17	3.85E-01	-0.06	7.75E-01
ENSCAFG0000001395	FSCB	violet	VSMC_M7	0.00	9.97E-01	-0.32	1.01E-01	-0.11	5.98E-01	-0.16	4.30E-01	0.13	5.25E-01	-0.08	6.93E-01	0.53	4.09E-03	0.23	2.57E-01	0.22	2.63E-01	-0.18	3.59E-01
ENSCAFG0000001492	MYOC	violet	VSMC_M7	0.00	9.97E-01	-0.32	1.01E-01	-0.11	5.98E-01	-0.16	4.30E-01	0.13	5.25E-01	-0.08	6.93E-01	0.53	4.09E-03	0.23	2.57E-01	0.22	2.63E-01	-0.18	3.59E-01
ENSCAFG0000001663	FBRS	grey	VSMC_M10	0.00	9.97E-01	0.25	2.05E-01	0.15	4.59E-01	0.04	8.49E-01	-0.27	1.77E-01	0.18	3.82E-01	0.11	5.86E-01	-0.16	4.16E-01	-0.24	2.29E-01	0.34	8.31E-02
ENSCAFG0000002990	ENSCAFG0000002990	grey	VSMC_M10	0.00	9.97E-01	0.28	1.60E-01	-0.13	5.26E-01	0.31	1.14E-01	0.09	6.67E-01	-0.08	7.00E-01	-0.16	4.23E-01	-0.30	1.24E-01	-0.25	2.12E-01	0.33	9.25E-02
ENSCAFG0000000225	PHF2	yellow	VSMC_M3	0.00	9.96E-01	0.30	1.30E-01	0.60	8.50E-04	-0.43	2.69E-02	-0.69	7.15E-05	0.17	4.02E-01	0.12	5.41E-01	0.32	1.01E-01	-0.41	3.52E-02	0.21	2.90E-01
ENSCAFG0000003208	ENSCAFG0000003208	darkolivegreen	VSMC_M9	0.00	9.96E-01	-0.68	8.21E-05	-0.28	1.52E-01	-0.38	5.28E-02	0.45	1.75E-02	-0.14	4.75E-01	0.36	6.79E-02	0.57	1.80E-03	0.57	2.06E-03	-0.76	4.45E-06
ENSCAFG0000001718	ENSCAFG0000001718	grey	VSMC_M10	0.00	9.96E-01	-0.02	9.20E-01	0.01	9.46E-01	-0.04	8.30E-01	-0.14	4.98E-01	0.27	1.74E-01	-0.18	3.74E-01	0.18	3.72E-01	-0.08	6.85E-01	0.12	5.62E-01
ENSCAFG0000002020	AP1G1	grey	VSMC_M10	0.00	9.95E-01	-0.52	4.98E-03	-0.70	4.16E-05	0.31	1.12E-01	0.59	1.08E-03	0.07	7.26E-01	0.00	9.97E-01	-0.03	8.87E-01	0.43	2.44E-02	-0.09	6.40E-01
ENSCAFG0000001035	ICE1	darkgrey	VSMC_M8	0.00	9.95E-01	-0.32	1.04E-01	-0.09	6.60E-01	-0.43	2.65E-02	0.12	5.64E-01	0.19	3.40E-01	0.12	5.46E-01	0.54	3.29E-03	0.21	2.86E-01	-0.33	9.02E-02
ENSCAFG0000001893	UHRF1	darkgrey	VSMC_M8	0.00	9.95E-01	-0.49	8.90E-03	0.39	4.62E-02	-0.87	4.83E-09	-0.29	1.37E-01	0.18	3.71E-01	0.39	4.32E-02	0.97	2.53E-16	0.31	1.17E-01	-0.62	6.08E-04
ENSCAFG0000003247	ENSCAFG0000003247	grey	VSMC_M10	0.00	9.95E-01	-0.30	1.33E-01	-0.04	8.31E-01	-0.16	4.14E-01	0.02	9.38E-01	-0.10	6.02E-01	-0.10	6.25E-01	0.23	2.47E-01	0.31	1.18E-01	-0.21	2.90E-01
ENSCAFG0000000318	SLC1A4	grey	VSMC_M10	0.00	9.95E-01	-0.15	4.46E-01	0.12	5.66E-01	-0.10	6.04E-01	-0.08	6.77E-01	-0.07	7.32E-01	0.34	8.00E-02	0.05	7.96E-01	0.19	3.44E-01	0.00	9.81E-01
ENSCAFG0000001185	SLAMF9	turquoise	VSMC_M6	0.00	9.95E-01	-0.31	1.16E-01	-0.12	5.47E-01	-0.15	4.61E-01	0.11	5.76E-01	0.49	9.44E-03	0.42	2.81E-02	0.29	1.48E-01	0.20	3.28E-01	-0.19	3.34E-01
ENSCAFG0000001730	GTF2A1	pink	VSMC_M5	0.00	9.95E-01	-0.34	7.92E-02	-0.61	7.70E-04	0.45	1.80E-02	0.67	1.29E-04	-0.27	1.74E-01	0.00	9.86E-01	-0.31	1.16E-01	0.39	4.54E-02	-0.09	6.69E-01
ENSCAFG0000000933	MYBL2	darkgrey	VSMC_M8	0.00	9.95E-01	-0.45	1.71E-02	0.47	1.44E-02	-0.94	2.27E-13	-0.33	9.28E-02	0.16	4.31E-01	0.34	8.62E-02	0.98	2.98E-18	0.30	1.25E-01	-0.68	1.05E-04
ENSCAFG0000001762	C20H19orf38	grey	VSMC_M10	0.00	9.94E-01	-0.04	8.50E-01	-0.06	7.74E-01	-0.07	7.30E-01	-0.01	9.53E-01	0.26	1.88E-01	0.26	1.88E-01	0.12	5.63E-01	-0.04	8.41E-01	0.08	6.94E-01
ENSCAFG0000001956	LCN15	darkgreen	VSMC_M4	0.00	9.94E-01	0.02	9.30E-01	-0.50	7.29E-03	0.68	9.89E-05	0.40	3.88E-02	-0.08	6.81E-01	-0.13	5.10E-01	-0.52	5.08E-03	0.03	8.71E-01	0.31	1.16E-01
ENSCAFG0000001533	CYP19A1	darkgreen	VSMC_M4	0.00	9.94E-01	0.33	9.62E-02	-0.34	8.46E-02	0.69	6.07E-05	0.17	4.02E-01	-0.12	5.60E-01	-0.12	5.42E-01	-0.61	6.43E-04	-0.30	1.29E-01	0.57	2.07E-03
ENSCAFG0000001082	TTC23	grey	VSMC_M10	0.00	9.94E-01	-0.35	7.15E-02	-0.15	4.47E-01	-0.33	9.05E-02	0.28	1.64E-01	-0.01	9.44E-01	0.22	2.74E-01	0.38	5.36E-02	0.27	1.65E-01	-0.48	1.12E-02
ENSCAFG0000000019	SEMA6A	grey	VSMC_M10	0.00	9.94E-01	0.20	3.25E-01	-0.22	2.65E-01	0.34	8.63E-02	0.13	5.03E-01	0.28	1.54E-01	-0.13	5.07E-01	-0.23	2.41E-01	-0.20	3.09E-01	0.30	1.27E-01
ENSCAFG0000001683	KIF4A	darkgrey	VSMC_M8	0.00	9.94E-01	-0.58	1.41E-03	0.18	3.69E-01	-0.82	1.68E-07	-0.01	9.78E-01	0.08	6.95E-01	0.38	5.24E-02	0.94	6.61E-13	0.42	2.96E-02	-0.77	2.18E-06
ENSCAFG0000001926	CCNL2	grey	VSMC_M10	0.00	9.94E-01	0.10	6.31E-01	0.29	1.47E-01	-0.24	2.19E-01	-0.38	4.75E-02	0.12	5.50E-01	0.03	8.81E-01	0.23	2.42E-01	-0.15	4.46E-01	0.15	4.70E-01
ENSCAFG0000001694	ENSCAFG0000001694	grey	VSMC_M10	0.00	9.93E-01	0.19	3.33E-01	0.04	8.38E-01	0.22	2.63E-01	-0.16	4.21E-01	0.06	7.50E-01	0.28	1.50E-01	-0.16	4.17E-01	-0.24	2.19E-01	0.36	6.84E-02
ENSCAFG0000000222	ENSCAFG0000000222	darkgreen	VSMC_M4	0.00	9.93E-01	0.09	6.40E-01	-0.42	2.84E-02	0.64	3.24E-04	0.29	1.48E-01	-0.08	6.98E-01	-0.15	4.49E-01	-0.50	7.42E-03	-0.08	6.96E-01	0.37	5.55E-02
ENSCAFG0000001734	COX11	grey	VSMC_M10	0.00	9.93E-01	0.11	5.84E-01	-0.12	5.43E-01	0.29	1.46E-01	-0.10	6.14E-01	0.13	5.26E-01	0.06	7.73E-01	-0.23	2.49E-01	-0.15	4.70E-01	0.51	6.66E-03
ENSCAFG0000000998	ENSCAFG0000000998	turquoise	VSMC_M6	0.00	9.93E-01	-0.09	6.53E-01	0.00	9.93E-01	-0.14	4.93E-01	-0.02	9.27E-01	-0.03	9.00E-01	-0.05	8.07E-01	0.07	7.12E-01	0.07	7.39E-01	-0.03	8.75E-01
ENSCAFG0000001322	DDX47	grey	VSMC_M10	0.00	9.93E-01	-0.53	4.44E-03	-0.48	1.20E-02	0.18	3.76E-01	0.49	9.09E-03	-0.06	7.84E-01	0.16	4.24E-01	0.12	5.44E-01	0.38	4.93E-02	-0.28	1.59E-01
ENSCAFG0000001582	DESI2	darkgrey	VSMC_M8	0.00	9.92E-01	-0.12	5.35E-01	0.47	1.30E-02	-0.75	6.65E-06	-0.38	5.14E-02	0.28	1.50E-01	0.21	2.94E-01	0.74	8.32E-06	0.03	8.84E-01	-0.39	4.70E-02
ENSCAFG0000002917	ENSCAFG0000002917	grey	VSMC_M10	0.00	9.92E-01	0.40	4.08E-02	-0.22	2.63E-01	0.55	2.78E-03	0.08	6.85E-01	-0.11	6.01E-01	-0.12	5.39E-01	-0.53	4.31E-03	-0.36	6.85E-02	0.52	5.39E-03
ENSCAFG0000000233	IGFBP7	darkgreen	VSMC_M4	0.00	9.92E-01	0.50	8.48E-03	-0.26	1.99E-01	0.79	8.06E-07	0.17	4.11E-01	-0.14	4.78E-01	-0.63	4.12E-04	-0.86	6.26E-09	-0.29	1.50E-01	0.55	2.93E-03
ENSCAFG0000000701	RASSF4	darkolivegreen	VSMC_M9	0.00	9.92E-01	-0.65	2.34E-04	-0.25	2.08E-01	-0.33	9.72E-02	0.32	1.03E-01	0.12	5.44E-01	0.34	8.62E-02	0.42	2.75E-02	0.56	2.29E-03	-0.52	5.41E-03
ENSCAFG0000003213	ENSCAFG0000003213	grey	VSMC_M10	0.00	9.92E-01	0.36	6.26E-02	0.09	6.51E-01	0.12	5.65E-01	-0.23	2.56E-01	0.24	2.31E-01	0.23	2.43E-01	-0.15	4.42E-01	-0.36	6.27E-02	0.46	1.48E-02
ENSCAFG0000001042	COL4A4	darkgreen	VSMC_M4	0.00	9.92E-01	0.29	1.37E-01	-0.19	3.45E-01	0.55	3.17E-03	0.07	7.21E-01	-0.10	6.24E-01	-0.21	2.93E-01	-0.50	8.52E-03	-0.24	2.20E-01	0.42	2.89E-02
ENSCAFG0000002304	ENSCAFG0000002304	darkgreen	VSMC_M4	0.00	9.92E-01	0.10	6.34E-01	-0.36	6.68E-02	0.51	6.18E-03	0.30	1.27E-01	-0.09	6.58E-01	-0.17	3.97E-01	-0.49	8.94E-03	-0.02	9.29E-01	0.29	1.39E-01
ENSCAFG0000002938	TNFRSF6B	grey	VSMC_M10	0.00	9.91E-01	0.37	5.89E-02	0.62	5.20E-04	-0.24	2.22E-01	-0.68	1.08E-04	0.10	6.14E-01	-0.05	7.94E-01	0.11	5.98E-01	-0.34	8.49E-02	0.28	1.55E-01
ENSCAFG0000001287	PDE6H	grey	VSMC_M10	0.00	9.91E-01	-0.21	2.95E-01	-0.02	9.39E-01	-0.20	3.14E-01	-0.01	9.59E-01	0.39	4.58E-02	0.31	1.13E-01	0.24	2.24E-01	0.15	4.58E-01	-0.06	7.64E-01
ENSCAFG0000001841	ENSCAFG0000001841	grey	VSMC_M10	0.00	9.91E-01	-0.15	4.68E-01	-0.12	5.45E-01	0.01	9.43E-01	0.10	6.20E-01	0.07	7.19E-01	0.32	1.03E-01	0.06	7.57E-01	0.04	8.26E-01	0.02	9.24E-01
ENSCAFG0000000161	IRF5	grey	VSMC_M10	0.00	9.91E-01	0.34	7.93E-02	0.18	3.64E-01	0.01	9.62E-01	-0.32	1.03E-01	0.13	5.30E-01	0.19	3.55E-01	-0.11	5.75E-01	-0.40	4.09E-02	0.41	3.31E-02
ENSCAFG0000001034	FAN1	grey	VSMC_M10	0.00	9.91E-01	0.18	3.65E-01	-0.46	1.60E-02	0.58	1.40E-03	0.38	5.22E-02	-0.01	9.60E-01	-0.04	8.54E-01	-0.56	2.66E-03	-0.17	3.99E-01	0.40	3.99E-02
ENSCAFG0000000220	S1PR3	grey	VSMC_M10	0.00	9.91E-01	-0.25	2.06E-01	0.06	7.55E-01	-0.29	1.39E-01	-0.11	5.82E-01	0.21	2.88E-01	0.64	3.48E-04	0.40	3.78E-02	0.08	6.76E-01	-0.09	6.64E-01
ENSCAFG0000001704	TRIM46	grey	VSMC_M10	0.00	9.91E-01	-0.13	5.29E-01	0.11	5.72E-01	-0.40	3.91E-02	-0.03	8.81E-01	0.12	5.50E-01	0.19	3.36E-01	0.32	9.97E-02	0.09	6.64E-01	-0.23	2.52E-01
ENSCAFG0000002875	BAG2	grey	VSMC_M10	0.00	9.91E-01	-0.18	3.56E-01	-0.08	6.90E-01	-0.17	3.86E-01	0.10	6.13E-01	0.12	5.37E-01	0.47	1.28E-02	0.13	5.20E-01	0.18	3.75E-01	-0.09	6.44E-01
ENSCAFG0000001908	RBFOX1	darkgreen	VSMC_M4	0.00	9.91E-01	0.35	7.16E-02	-0.26	1.84E-01	0.56	2.31E-03	0.12	5.66E-01	-0.12	5.54E-01	-0.26	1.92E-01	-0.50	7.59E-03	-0.26	1.94E-01	0.51	7.16E-03
ENSCAFG0000000840	TMBIM6	grey	VSMC_M10	0.00	9.90E-01	0.40	3.86E-02	-0.36	6.43E-02	0.60	8.31E-04	0.15	4.65E-01	0.13	5.29E-01	-0.26	1.94E-01	-0.61	7.40E-04	-0.31	1.14E-01	0.63	4.07E-04
ENSCAFG0000003272	ENSCAFG0000003272	yellow	VSMC_M3	0.00	9.90E-01	0.15	4.47E-01	0.70	4.79E-05	-0.68	1.02E-04	-0.60	1.04E-03	0.10	6.15E-01	-0.08	6.95E-01	0.49	1.02E-02	-0.16	4.28E-01	-0.23	2.43E-01
ENSCAFG0000000553	CEP295NL	grey	VSMC_M10	0.00	9.90E-01	0.19	3.34E-01	0.01	9.75E-01	-0.07	7.22E-01	0.03	8.81E-01	0.21	2.82E-01	0.10	6.32E-01	-0.07	7.45E-01	-0.13	5.03E-01	-0.01	9.77E-01
ENSCAFG0000000623	DCLK1	cyan	VSMC_M2	0.00	9.90E-01	0.58	1.56E-03	0.52	5.85E-03	-0.14	5.00E-01	-0.54	3.48E-03	0.06	7.75E-01	-0.28	1.52E-01	-0.15	4.50E-01	-0.46	1.46E-02	0.35	7.12E-02
ENSCAFG0000001624	PGM2	pink	VSMC_M5	0.00	9.90E-01	-0.45	1.88E-02	-0.21	3.00E-01	-0.31	1.18E-01	0.38	5.34E-02	-0.01	9.44E-01	-0.07	7.40E-01	0.40	3.99E-02	0.43	2.47E-02	-0.64	2.82E-04
ENSCAFG0000001889	CXXC1	grey	VSMC_M10	0.00	9.90E-01	0.19	3.42E-01	0.48	1.15E-02	-0.30	1.35E-01	-0.44	2.24E-02	0.15	4.67E-01	0.13	5.31E-01	0.21	2.91E-01	-0.24	2.25E-01	-0.02	9.14E-01
ENSCAFG0000000360	CNTRL	grey	VSMC_M10	0.00	9.90E-01	-0.16	4.14E-01	0.27	1.71E-01	-0.55	3.10E-03	-0.07	7.35E-01	-0.18	3.61E-01	0.25	2.01E-01	0.49	9.78E-03	0.11	5.78E-01	-0.46	1.47E-02
ENSCAFG0000002457	GPR182	grey	VSMC_M10	0.00	9.90E-01	0.11	5.85E-01	-0.08	7.07E-01	0.19	3.32E-01	-0.02	9.28E-01	-0.11	5.89E-01	-0.02	9.38E-01	-0.15	4.59E-01	-0.10	6.36E-01	0.22	2.81E-01
ENSCAFG0000002529	ENSCAFG0000002529	cyan	VSMC_M2	0.00	9.90E-01	0.47	1.23E-02	0.22	2.59E-01	0.08	7.08E-01	-0.33	9.35E-02	0.10	6.22E-01	0.07	7.12E-01	-0.17	3.85E-01	-0.50	7.90E-03	0.40	3.65E-02
ENSCAFG0000001064	IPO9	grey	VSMC_M10	0.00	9.89E-01	0.12	5.62E-01	0.23	2.57E-01	-0.24	2.21E-01	-0.26	1.95E-01	-0.01	9.77E-01	0.08	6.91E-01	0.29	1.44E-01	-0.21	2.99E-01	0.04	8.26E-01
ENSCAFG0000000274	ENSCAFG0000000274	grey	VSMC_M10	0.00	9.89E-01	0.33	9.43E-02	0.03	8.98E-01	0.21	3.00E-01	-0.07	7.19E-01	-0.07	7.19E-01	-0.39	4.20E-02	-0.20	3.21E-01	-0.28	1.54E-01	0.32	9.88E-02
ENSCAFG0000001587	CROCC	cyan	VSMC_M2	0.00	9.89E-01	0.63	4.21E-04	0.63	3.78E-04	-0.14	4.73E-01	-0.59	1.09E-03	-0.03	8.75E-01	-0.06	7.70E-01	-0.14	4.98E-01	-0.53	4.18E-03	0.33	9.52E-02
ENSCAFG000000024	COL23A1	darkgreen	VSMC_M4	0.00	9.89E-01	-0.14	4.86E-01	-0.44	2.09E-02	0.48	1.07E-02	0.35	7.59E-02	-0.11	5.90E-01	-0.17	4.02E-01	-0.29	1.41E-01	0.15	4.42E-01	0.13	5.05E-01
ENSCAFG0000001791	SEMA7A	darkolivegreen	VSMC_M9	0.00	9.89E-01	-0.54	3.41E-03	0.03	8.68E-01	-0.42	3.05E-02	0.03	8.81E-01	0.13	5.03E-01	-0.05	8.09E-01	0.54	3.83E-03	0.52	5.58E-03	-0.52	5.82E-03
ENSCAFG0000000965	DOP1B	grey	VSMC_M10	0.00	9.89E-01	-0.24	2.26E-01	-0.22	2.65E-01	-0.04	8.51E-01	0.31	1.21E-01	0.02	9.22E-01	0.55	2.83E-03	0.03	8.79E-01	0.24	2.28E-01	-0.19	3.43E-01
ENSCAFG0000002354	C9orf3	grey	VSMC_M10	0.00	9.89E-01	-0.25	2.08E-01	-0.26	1.90E-01	0.18	3.67E-01	0.44	2.03E-02	-0.16	4.35E-01	-0.03	8.85E-01	-0.18	3.65E-01	0.39	4.34E-02	-0.37	5.81E-02
ENSCAFG0000002955	ENSCAFG0000002955	grey	VSMC_M10	0.00	9.89E-01	0.56	2.53E-03	0.00	9.81E-01	0.54	3.84E-03	-0.18	3.61E-01	-0.10	6.20E-01	-0.50	7.77E-03	-0.56	2.28E-03	-0.47	1.33E-02	0.61	6.55E-04
ENSCAFG0000001033	ENSCAFG0000001033	grey	VSMC_M10	0.00	9.89E-01	-0.21	2.99E-01	-0.30	1.34E-01	0.14	4.90E-01	0.44	2.17E-02	-0.13	5.32E-01	-0.23	2.44E-01	-0.15	4.44E-01	0.35	7.75E-02	-0.34	8.00E-02
ENSCAFG0000001255	HTRA1	grey	VSMC_M10	0.00	9.88E-01	0.55	3.28E-03	-0.12	5.47E-01	0.65	2.21E-04	0.07	7.36E-01	-0.45	1.94E-02	-0.45	1.73E-02	-0.74	1.02E-05	-0.34	8.18E-02	0.54	3.66E-03
ENSCAFG0000000450	KLC3	grey	VSMC_M10	0.00	9.88E-01	0.22	2.63E-01	0.15	4.66E-01	-0.07	7.29E-01	-0.21	2.92E-01	-0.08	6.90E-01	-0.02	9.12E-01	0.02	9.10E-01	-0.21	2.92E-01	0.19	3.42E-01
ENSCAFG0000000998	NCOA5	darkgrey	VSMC_M8	0.00	9.88E-01	-0.22	2.65E-01	0.28	1.58E-01	-0.66	1.69E-04	-0.18	3.71E-01	-0.06	7.70E-01	0.23	2.42E-01	0.61	7.36E-04	0.15	4.68E-01	-0.44	2.02E-02
ENSCAFG0000001218	CTSZ	darkgreen	VSMC_M4	0.00	9.87E-01	-0.10	6.12E-01	-0.40	4.05E-02	0.60	8.83E-04	0.31	1.17E-01	-0.12	5.63E-01	-0.07	7.45E-01	-0.41	3.53E-02	0.11	5.73E-01	0.20	3.25E-01
ENSCAFG0000000669	CALCOCO1	grey	VSMC_M10	0.00	9.87E-01	0.69	6.93E-05	-0.02	9.33E-01	0.68	9.73E-05	-0.09	6.59E-01	-0.22	2.65E-01	-0.35	6.93E-02	-0.87	2.38E-09	-0.48	1.11E-02	0.72	2.00E-05
ENSCAFG0000000756	AMPD3	grey	VSMC_M10	0.00	9.87E-01	0.33	9.67E-02	-0.03	9.01E-01	0.40	3.97E-02	0.00	9.97E-01	0.17	4.10E-01	-0.40	3.62E-02	-0.48	1.09E-02	-0.22	2.66E-01	0.25	2.16E-01
ENSCAFG0000001795	COL5A3	violet	VSMC_M7	0.00	9.87E-01	-0.24	2.25E-01	0.27	1.74E-01	-0.40	3.66E-02	-0.21	2.92E-01	0.28	1.64E-01	0.75	6.48E-06	0.40	4.00E-02	0.10	6.07E-01	-0.27	1.81E-01
ENSCAFG0000001209	PGBD5	grey	VSMC_M10	0.00	9.87E-01	-0.12	5.44E-01	0.10	6.33E-01	-0.24	2.27E-01	-0.16	4.20E-01	0.27	1.71E-01	0.17	4.10E-01	0.28	1.62E-01	0.10	6.08E-01	-0.07	7.12E-01
ENSCAFG0000002498	FRZB	darkgreen	VSMC_M4	0.00	9.87E-01	0.28	1.52E-01	-0.48	1.08E-02	0.84	5.21E-08	0.32	1.04E-01	0.02	9.21E-01	-0.19	3.32E-01	-0.78	1.66E-06	-0.20	3.25E-01	0.58	1.56E-03
ENSCAFG0000002274	MT-ND6	grey	VSMC_M10	0.00	9.87E-01	0.16	4.35E-01	-0.03	8.91E-01	0.39	4.18E-02	-0.15	4.50E-01	0.18	3.67E-01	0.04	8.39E-01	-0.33	9.66E-02	-0.21	2.86E-01	0.44	2.32E-02
ENSCAFG0000001377	BCR	cyan	VSMC_M2	0.00	9.87E-01	0.57	1.95E-03	0.40	3.92E-02	0.07	7.46E-01	-0.49	9.20E-03	0.49	9.41E-03	0.01	9.69E-01	-0.26	1.90E-01	-0.49	8.73E-03	0.47	1.29E-02
ENSCAFG0000001801	SIN3A	grey	VSMC_M10	0.00	9.87E-01	-0.17	4.07E-01	0.18	3.65E-01	-0.44	2.29E-02	-0.04	8.38E-01	0.15	4.47E-01	0.10	6.25E-01	0.46	1.58E-02	0.13	5.02E-01	-0.40	3.91E-02
ENSCAFG0000000717	POLR1B	darkgrey	VSMC_M8	0.00	9.86E-01	-0.66	1.76E-04	0.02	9.02E-01	-0.56	2.53E-03	0.12	5.67E-01	0.16	4.18E-01	0.08	7.04E-01	0.70	4.13E-05	0.56	2.35E-03	-0.73	1.34E-05
ENSCAFG0000001316	EIF4ENIF1	grey	VSMC_M10	0.00	9.85E-01	0.40	3.88E-02	0.05	8.15E-01	0.25	2.07E-01	-0.25	2.17E-01	0.26	1.96E-01	-0.20	3.12E-01	-0.28	1.54E-01	-0.40	3.95E-02	0.55	3.18E-03
ENSCAFG0000000508	INSIG1	yellow	VSMC_M3	0.00	9.85E-01	0.05	8.14E-01	0.58	1.44E-03	-0.75	6.40E-06	-0.42	3.06E-02	-0.02	9.21E-01	0.41	3.37E-02	0.52	5.02E-03	-0.08	7.08E-01	-0.32	1.03E-01
ENSCAFG0000001291	HECTD1	pink	VSMC_M5	0.00	9.85E-01	-0.35	7.28E-02	-0.82	2.17E-07	0.61	7.48E-04	0.80	4.84E-07	-0.07	7.22E-01	-0.15	4.57E-01	-0.39	4.41E-02	0.35	7.69E-02	-0.03	8.74E-01
ENSCAFG0000000280	FPR2	grey	VSMC_M10	0.00	9.84E-01	0.45	1.75E-02	0.10	6.27E-01	0.16	4.40E-01	-0.20	3.08E-01	-0.06	7.66E-01	-0.18	3.63E-01	-0.25	2.03E-01	-0.41	3.51E-02	0.38	5.29E-02
ENSCAFG0000000300	SNX14	grey	VSMC_M10	0.00	9.84E-01	0.24	2.22E-01	-0.39	4.46E-02	0.56	2.33E-03	0.42	3.00E-02	-0.31	1.16E-01	-0.14	4.95E-01	-0.64	2.85E-04	-0.11	5.97E-01	0.28	1.57E-01
ENSCAFG0000002038	CBFB	darkgrey	VSMC_M8	0.00	9.84E-01	-0.27	1.71E-01	0.49	9.22E-03	-0.73	1.49E-05	-0.38	4.86E-02	0.01	9.78E-01	0.21	2.88E-01	0.71	2.97E-05	0.22	2.73E-01	-0.44	2.22E-02
ENSCAFG0000001877	ENSCAFG0000001877	grey	VSMC_M10	0.00	9.84E-01	0.35	7.68E-02	-0.08	6.80E-01	0.32	1.04E-01	-0.05	8.23E-01	0.26	1.93E-01	0.02	9.27E-01	-0.43	2.48E-02	-0.29	1.48E-01	0.49	9.09E-03
ENSCAFG0000001365	HMCN1	grey	VSMC_M10	0.00	9.83E-01	-0.37	5.90E-02	-0.33	9.71E-02	-0.17	3.86E-01	0.40	3.92E-02	0.04	8.47E-01	0.03	8.90E-01	0.24	2.22E-01	0.33	8.82E-02	-0.34	8.29E-02
ENSCAFG0000003014	LONRF1	grey	VSMC_M10	0.00	9.83E-01	-0.30	1.30E-01	-0.42	2.91E-02	0.13	5.29E-01	0.46	1.58E-02	-0.24	2.27E-01	0.20	3.10E-01	-0.04	8.58E-01	0.30	1.35E-01	-0.11	5.70E-01
ENSCAFG0000000232	NOL8	darkgrey	VSMC_M8	0.00	9.83E-01	-0.39	4.38E-02	0.32	9.85E-02	-0.75	7.06E-06	-0.20	3.12E-01	0.08	6.96E-01	0.26	1.92E-01	0.75	5.47E-06	0.24	2.23E-01	-0.54	3.67E-03
ENSCAFG0000001638	EIF2S1	darkgrey	VSMC_M8	0.00	9.83E-01	-0.78	2.05E-06	-0.17	3.98E-01	-0.47	1.45E-02	0.31	1.21E-01	-0.02	9.02E-01	0.20	3.26E-01	0.70	4.08E-05	0.64	2.88E-04	-0.78	1.38E-06
ENSCAFG0000001123	AXIN2	grey	VSMC_M10	0.00	9.82E-01	0.11	5.87E-01	-0.47	1.23E-02	0.34	8.34E-02	0.39	4.45E-02	-0.23	2.43E-01	0.13	5.06E-01	-0.27	1.79E-01	-0.10	6.08E-01	0.28	1.64E-01
ENSCAFG0000001617	CYB561A3	grey	VSMC_M10	0.00	9.82E-01	-0.42	3.10E-02	0.03	8.70E-01	-0.21	2.93E-01	0.03	8.93E-01	0.01	9.53E-01	0.37	5.63E-02	0.26	1.90E-01	0.35	7.47E-02	-0.29	1.49E-01
ENSCAFG0000001022	PRDM15	grey	VSMC_M10	0.00	9.82E-01	0.28	1.52E-01	-0.12	5.51E-01	0.31	1.17E-01	-0.12	5.37E-01	0.16	4.21E-01	0.18	3.76E-01	-0.27	1.66E-01	-0.36	6.18E-02	0.65	2.67E-04

ENSCAFG0000003121	ENSCAFG0000003121	grey	VSMC_M10	0.00	9.82E-01	0.64	3.58E-04	0.07	7.38E-01	0.62	5.36E-04	-0.23	2.41E-01	-0.10	6.26E-01	-0.43	2.50E-02	-0.70	5.69E-05	-0.53	4.68E-03	0.69	7.80E-05
ENSCAFG0000000605	EIF2AK2	grey	VSMC_M10	0.00	9.82E-01	0.16	4.28E-01	0.26	1.97E-01	-0.06	7.58E-01	-0.36	6.40E-02	0.09	6.38E-01	0.11	5.83E-01	0.06	7.60E-01	-0.24	2.34E-01	0.27	1.78E-01
ENSCAFG0000001646	ENSCAFG0000001646	darkgrey	VSMC_M8	0.00	9.81E-01	-0.61	7.16E-04	0.23	2.47E-01	-0.82	1.86E-07	-0.12	5.52E-01	0.18	3.79E-01	0.45	1.75E-02	0.95	1.74E-14	0.41	3.59E-02	-0.69	6.55E-05
ENSCAFG0000002307	ACYP2	darkgreen	VSMC_M4	0.00	9.81E-01	0.11	5.71E-01	-0.58	1.63E-03	0.72	2.76E-05	0.34	7.94E-02	0.13	5.31E-01	-0.11	5.87E-01	-0.56	2.42E-03	-0.10	6.25E-01	0.57	2.08E-03
ENSCAFG0000002961	LAMTOR2	grey	VSMC_M10	0.00	9.81E-01	0.22	2.63E-01	0.61	7.59E-04	-0.32	1.03E-01	-0.54	3.34E-03	0.05	7.91E-01	0.07	7.18E-01	0.17	3.96E-01	-0.21	2.85E-01	-0.07	7.17E-01
ENSCAFG0000000837	ESCO2	darkgrey	VSMC_M8	0.00	9.80E-01	-0.53	4.79E-03	0.31	1.13E-01	-0.88	9.83E-10	-0.16	4.16E-01	0.12	5.48E-01	0.40	3.94E-02	0.96	4.84E-16	0.36	6.33E-02	-0.71	3.04E-05
ENSCAFG0000000938	OSGEPL1	grey	VSMC_M10	-0.01	9.80E-01	0.49	9.75E-03	0.04	8.45E-01	0.51	6.96E-03	-0.23	2.47E-01	-0.17	4.07E-01	-0.14	4.88E-01	-0.58	1.52E-03	-0.40	3.87E-02	0.72	2.02E-05
ENSCAFG0000000909	ENSCAFG0000000909	grey	VSMC_M10	-0.01	9.80E-01	0.06	7.85E-01	-0.28	1.51E-01	0.47	1.27E-02	0.16	4.35E-01	-0.15	4.67E-01	0.08	6.86E-01	-0.34	8.56E-02	-0.04	8.36E-01	0.34	7.88E-02
ENSCAFG0000001047	SRPRA	grey	VSMC_M10	-0.01	9.80E-01	-0.57	2.07E-03	-0.17	3.91E-01	-0.13	5.34E-01	0.21	2.88E-01	0.10	6.13E-01	-0.19	3.50E-01	0.35	7.12E-02	0.49	8.71E-03	-0.45	1.80E-02
ENSCAFG0000000575	NR1D2	grey	VSMC_M10	-0.01	9.80E-01	-0.15	4.44E-01	-0.55	2.81E-03	0.31	1.16E-01	0.60	8.41E-04	-0.22	2.74E-01	0.25	2.10E-01	-0.18	3.59E-01	0.14	4.79E-01	0.00	9.96E-01
ENSCAFG0000001206	BMX	grey	VSMC_M10	-0.01	9.80E-01	0.36	6.15E-02	-0.13	5.24E-01	0.26	1.94E-01	0.00	9.97E-01	0.50	7.23E-03	-0.04	8.28E-01	-0.35	6.94E-02	-0.32	1.05E-01	0.41	3.56E-02
ENSCAFG0000001048	PDE9A	grey	VSMC_M10	-0.01	9.80E-01	0.33	8.98E-02	-0.30	1.32E-01	0.49	9.33E-03	0.11	5.95E-01	-0.09	6.40E-01	-0.11	5.77E-01	-0.52	5.22E-03	-0.23	2.46E-01	0.62	5.88E-04
ENSCAFG0000001286	MORC2	pink	VSMC_M5	-0.01	9.79E-01	0.02	9.17E-01	-0.36	6.46E-02	0.32	1.02E-01	0.46	1.47E-02	-0.40	3.99E-02	-0.12	5.64E-01	-0.32	1.09E-01	0.04	8.24E-01	0.00	9.89E-01
ENSCAFG0000001271	QSOX1	darkgreen	VSMC_M4	-0.01	9.79E-01	0.12	5.36E-01	-0.44	2.20E-02	0.73	1.85E-05	0.35	7.26E-02	-0.01	9.51E-01	-0.39	4.21E-02	-0.72	1.92E-05	0.00	9.89E-01	0.34	8.04E-02
ENSCAFG0000000515	UBE3C	grey	VSMC_M10	-0.01	9.78E-01	-0.14	4.80E-01	-0.46	1.59E-02	0.32	1.00E-01	0.40	3.91E-02	0.03	8.71E-01	0.32	9.98E-02	-0.20	3.26E-01	0.03	8.92E-01	0.14	4.94E-01
ENSCAFG0000001036	AZIN2	grey	VSMC_M10	-0.01	9.78E-01	0.18	3.70E-01	-0.16	4.23E-01	0.41	3.19E-02	-0.08	7.06E-01	0.12	5.41E-01	0.27	1.79E-01	-0.39	4.73E-02	-0.24	2.21E-01	0.62	5.07E-04
ENSCAFG0000002909	ENSCAFG0000002909	grey	VSMC_M10	-0.01	9.78E-01	0.40	4.05E-02	0.06	7.69E-01	0.14	4.95E-01	-0.09	6.58E-01	-0.08	7.05E-01	-0.23	2.39E-01	-0.33	9.62E-02	-0.27	1.80E-01	0.35	7.34E-02
ENSCAFG0000000852	P2RY14	turquoise	VSMC_M6	-0.01	9.77E-01	-0.11	5.72E-01	-0.26	1.94E-01	0.20	3.21E-01	0.15	4.44E-01	0.22	2.70E-01	-0.18	3.57E-01	-0.09	6.52E-01	0.02	9.19E-01	0.11	5.75E-01
ENSCAFG0000000974	NIPA2	darkgrey	VSMC_M8	-0.01	9.77E-01	-0.64	3.18E-04	0.12	5.54E-01	-0.69	8.01E-05	0.05	8.03E-01	0.09	6.54E-01	0.12	5.41E-01	0.78	1.53E-06	0.53	4.19E-03	-0.80	5.35E-07
ENSCAFG0000001115	ENSCAFG0000001115	grey	VSMC_M10	-0.01	9.77E-01	-0.22	2.71E-01	-0.19	3.39E-01	0.17	3.97E-01	0.12	5.67E-01	0.11	5.80E-01	-0.49	9.66E-03	-0.04	8.47E-01	0.24	2.19E-01	-0.06	7.78E-01
ENSCAFG0000001441	ASCC1	grey	VSMC_M10	-0.01	9.77E-01	-0.15	4.62E-01	0.11	5.93E-01	-0.36	6.58E-02	0.08	7.01E-01	-0.08	6.94E-01	-0.08	6.91E-01	0.29	1.44E-01	0.17	3.86E-01	-0.47	1.29E-02
ENSCAFG0000001459	TNFSF18	grey	VSMC_M10	-0.01	9.77E-01	-0.35	7.11E-02	-0.10	6.21E-01	-0.19	3.33E-01	0.12	5.41E-01	-0.15	4.52E-01	0.38	4.80E-02	0.26	1.95E-01	0.24	2.23E-01	-0.19	3.55E-01
ENSCAFG0000000036	SASH1	darkgreen	VSMC_M4	-0.01	9.76E-01	0.40	3.83E-02	-0.36	6.60E-02	0.78	1.87E-06	0.22	2.73E-01	-0.12	5.68E-01	-0.22	2.68E-01	-0.80	5.62E-07	-0.26	1.82E-01	0.64	3.04E-04
ENSCAFG0000001446	ENSCAFG0000001446	grey	VSMC_M10	-0.01	9.76E-01	-0.01	9.61E-01	-0.19	3.49E-01	-0.01	9.51E-01	0.20	3.20E-01	0.22	2.75E-01	-0.26	1.90E-01	-0.02	9.10E-01	0.06	7.64E-01	-0.12	5.38E-01
ENSCAFG0000001113	ARSD	grey	VSMC_M10	-0.01	9.76E-01	0.61	7.02E-04	-0.05	7.97E-01	0.65	2.27E-04	-0.12	5.38E-01	-0.03	8.88E-01	-0.07	7.24E-01	-0.80	4.34E-07	-0.49	9.46E-03	0.81	2.47E-07
ENSCAFG0000000574	HBEGF	grey	VSMC_M10	-0.01	9.76E-01	-0.15	4.68E-01	-0.42	2.77E-02	0.16	4.37E-01	0.29	1.39E-01	0.25	2.14E-01	0.33	9.15E-02	-0.09	6.56E-01	0.05	8.20E-01	0.20	3.07E-01
ENSCAFG0000000462	ENSCAFG0000000462	grey	VSMC_M10	-0.01	9.75E-01	-0.13	5.12E-01	-0.24	2.32E-01	0.01	9.52E-01	0.29	1.46E-01	0.13	5.26E-01	0.07	7.32E-01	0.07	7.31E-01	0.06	7.66E-01	-0.15	4.62E-01
ENSCAFG0000001159	TBC1D10C	grey	VSMC_M10	-0.01	9.75E-01	0.50	8.36E-03	0.11	5.96E-01	0.14	4.90E-01	-0.22	2.75E-01	-0.05	8.08E-01	0.30	1.29E-01	-0.31	1.19E-01	-0.47	1.35E-02	0.54	3.86E-03
ENSCAFG0000000744	TRIM23	darkgreen	VSMC_M4	-0.01	9.75E-01	-0.14	4.96E-01	-0.76	4.14E-06	0.65	2.66E-04	0.69	6.92E-05	0.03	8.88E-01	-0.11	5.71E-01	-0.51	7.11E-03	0.16	4.32E-01	0.14	4.97E-01
ENSCAFG0000000821	FHDC1	darkgrey	VSMC_M8	-0.01	9.75E-01	-0.46	1.51E-02	0.00	9.89E-01	-0.44	2.27E-02	0.01	9.75E-01	0.38	5.30E-02	0.63	4.66E-04	0.62	5.52E-04	0.24	2.18E-01	-0.36	6.79E-02
ENSCAFG0000001572	SNAPC1	pink	VSMC_M5	-0.01	9.75E-01	-0.59	1.17E-03	-0.59	1.09E-03	0.12	5.42E-01	0.72	2.43E-05	-0.16	4.19E-01	-0.08	7.05E-01	0.11	5.76E-01	0.54	3.78E-03	-0.53	4.53E-03
ENSCAFG0000000496	MKRN2	darkgreen	VSMC_M4	-0.01	9.75E-01	0.22	2.80E-01	-0.44	2.22E-02	0.63	3.93E-04	0.21	2.91E-01	0.06	7.49E-01	-0.05	8.13E-01	-0.52	5.44E-03	-0.25	2.17E-01	0.56	2.19E-03
ENSCAFG0000000800	ENSCAFG0000000800	violet	VSMC_M7	-0.01	9.74E-01	-0.05	8.19E-01	0.12	5.37E-01	-0.17	4.03E-01	-0.10	6.15E-01	0.08	6.84E-01	0.58	1.66E-03	0.12	5.43E-01	0.02	9.22E-01	-0.01	9.45E-01
ENSCAFG0000000924	DGKZ	yellow	VSMC_M3	-0.01	9.74E-01	0.12	5.52E-01	0.77	3.16E-06	-0.66	1.74E-04	-0.73	1.36E-05	0.12	5.37E-01	0.17	4.05E-01	0.46	1.59E-02	-0.12	5.45E-01	-0.08	6.90E-01
ENSCAFG0000001290	THEM5	grey	VSMC_M10	-0.01	9.74E-01	0.02	9.22E-01	-0.12	5.47E-01	0.16	4.15E-01	0.09	6.57E-01	-0.09	6.71E-01	-0.19	3.35E-01	-0.09	6.43E-01	-0.07	7.16E-01	0.13	5.30E-01
ENSCAFG0000000928	ACVR1	darkolivegreen	VSMC_M9	-0.01	9.74E-01	-0.45	1.77E-02	-0.43	2.46E-02	0.00	9.96E-01	0.53	4.23E-03	-0.07	7.29E-01	-0.18	3.62E-01	0.01	9.60E-01	0.52	5.91E-03	-0.37	5.49E-02
ENSCAFG0000001762	FAXDC2	darkgreen	VSMC_M4	-0.01	9.74E-01	0.55	2.99E-03	-0.34	8.19E-02	0.80	4.37E-07	0.24	2.23E-01	-0.16	4.22E-01	-0.40	3.79E-02	-0.92	6.51E-12	-0.35	7.40E-02	0.62	4.99E-04
ENSCAFG0000000834	CDKL2	darkgreen	VSMC_M4	-0.01	9.74E-01	0.18	3.66E-01	-0.46	1.46E-02	0.78	1.96E-06	0.38	5.24E-02	-0.33	9.44E-02	-0.23	2.38E-01	-0.73	1.76E-05	-0.05	8.16E-01	0.46	1.58E-02
ENSCAFG0000000397	ENSCAFG0000000397	grey	VSMC_M10	-0.01	9.73E-01	-0.01	9.72E-01	0.19	3.35E-01	-0.36	6.40E-02	-0.17	4.05E-01	-0.11	6.01E-01	0.39	4.70E-02	0.37	5.47E-02	-0.07	7.17E-01	-0.11	5.91E-01
ENSCAFG0000000621	POLE	darkgrey	VSMC_M8	-0.01	9.73E-01	-0.51	6.24E-03	0.31	1.16E-01	-0.86	8.28E-09	-0.21	2.97E-01	0.21	2.99E-01	0.32	1.06E-01	0.96	1.02E-15	0.34	7.98E-02	-0.65	2.21E-04
ENSCAFG0000001587	UCHL1	darkgrey	VSMC_M8	-0.01	9.73E-01	-0.51	6.59E-03	0.17	3.90E-01	-0.58	1.35E-03	-0.02	9.33E-01	0.02	9.24E-01	-0.03	8.92E-01	0.66	1.60E-04	0.47	1.31E-02	-0.71	4.02E-05
ENSCAFG0000000572	GINS4	darkgrey	VSMC_M8	-0.01	9.73E-01	-0.49	9.20E-03	0.37	6.03E-02	-0.87	2.60E-09	-0.24	2.28E-01	0.14	4.93E-01	0.35	7.17E-02	0.96	6.02E-16	0.33	9.55E-02	-0.68	1.05E-04
ENSCAFG0000001954	JPT2	darkgrey	VSMC_M8	-0.01	9.73E-01	-0.39	4.51E-02	0.11	5.81E-01	-0.45	1.73E-02	-0.18	3.73E-01	0.36	6.44E-02	0.20	3.23E-01	0.63	4.06E-04	0.21	2.88E-01	-0.28	1.59E-01
ENSCAFG0000000250	ABCG5	grey	VSMC_M10	-0.01	9.73E-01	0.19	3.31E-01	0.13	5.07E-01	-0.08	6.92E-01	-0.20	3.28E-01	0.01	9.75E-01	-0.01	9.58E-01	-0.05	8.13E-01	-0.21	2.96E-01	0.16	4.39E-01
ENSCAFG0000001698	YLPM1	grey	VSMC_M10	-0.01	9.72E-01	-0.36	6.35E-02	-0.53	4.51E-03	0.04	8.52E-01	0.61	6.57E-04	-0.16	4.40E-01	0.11	5.94E-01	0.10	6.34E-01	0.33	8.90E-02	-0.33	9.41E-02
ENSCAFG0000000095	B3GALT4	cyan	VSMC_M2	-0.01	9.72E-01	0.57	1.99E-03	0.46	1.62E-02	-0.10	6.30E-01	-0.52	5.69E-03	-0.12	5.54E-01	0.06	7.60E-01	-0.13	5.26E-01	-0.46	1.63E-02	0.41	3.23E-02
ENSCAFG0000000915	HDAC7	grey	VSMC_M10	-0.01	9.72E-01	0.69	5.95E-05	0.32	9.82E-02	0.25	2.16E-01	-0.35	7.46E-02	-0.19	3.31E-01	-0.31	1.18E-01	-0.47	1.33E-02	-0.54	3.65E-03	0.47	1.45E-02
ENSCAFG0000002397	ENSCAFG0000002397	grey	VSMC_M10	-0.01	9.72E-01	0.33	8.91E-02	0.19	3.42E-01	-0.12	5.64E-01	-0.23	2.49E-01	-0.04	8.38E-01	0.19	3.52E-01	0.01	9.60E-01	-0.33	9.13E-02	0.22	2.73E-01
ENSCAFG0000000771	ENSCAFG0000000771	grey	VSMC_M10	-0.01	9.71E-01	0.15	4.42E-01	0.04	8.31E-01	0.04	8.51E-01	-0.12	5.59E-01	-0.12	5.67E-01	0.00	9.83E-01	-0.13	5.17E-01	-0.12	5.63E-01	0.20	3.15E-01
ENSCAFG0000000033	FBXO30	grey	VSMC_M10	-0.01	9.71E-01	-0.39	4.65E-02	-0.03	8.97E-01	-0.40	3.71E-02	0.11	5.97E-01	0.15	4.61E-01	0.17	3.93E-01	0.41	3.38E-02	0.38	4.85E-02	-0.38	4.78E-02
ENSCAFG0000002966	ENSCAFG0000002966	darkgrey	VSMC_M8	-0.01	9.71E-01	-0.52	5.73E-03	-0.06	7.48E-01	-0.35	7.45E-02	0.11	5.93E-01	0.13	5.26E-01	0.25	2.01E-01	0.57	1.85E-03	0.38	4.96E-02	-0.45	2.00E-02
ENSCAFG0000001574	EVC	yellow	VSMC_M3	-0.01	9.71E-01	0.28	1.61E-01	0.76	3.58E-06	-0.56	2.65E-03	-0.75	6.02E-06	0.04	8.53E-01	0.31	1.13E-01	0.34	8.60E-02	-0.32	9.83E-02	0.08	6.84E-01
ENSCAFG0000000864	ATG10	grey	VSMC_M10	-0.01	9.71E-01	0.09	6.69E-01	-0.30	1.31E-01	0.20	3.23E-01	0.17	4.06E-01	0.17	4.06E-01	0.10	6.09E-01	-0.13	5.33E-01	-0.16	4.38E-01	0.23	2.48E-01
ENSCAFG0000000212	MRPS9	grey	VSMC_M10	-0.01	9.71E-01	-0.07	7.29E-01	0.36	6.89E-02	-0.34	7.95E-02	-0.32	9.86E-02	0.00	9.86E-01	0.15	4.62E-01	0.37	5.93E-02	-0.02	9.22E-01	-0.16	4.12E-01
ENSCAFG0000001963	FUNDC2	grey	VSMC_M10	-0.01	9.70E-01	-0.17	3.98E-01	-0.16	4.26E-01	0.12	5.40E-01	0.25	2.14E-01	-0.33	9.20E-02	-0.24	2.37E-01	-0.02	9.14E-01	0.24	2.26E-01	-0.26	1.86E-01
ENSCAFG0000002045	ENSCAFG0000002045	grey	VSMC_M10	-0.01	9.69E-01	0.39	4.60E-02	0.28	1.54E-01	-0.16	4.11E-01	-0.31	1.15E-01	0.03	8.77E-01	-0.05	8.10E-01	0.03	8.92E-01	-0.33	8.84E-02	0.22	2.78E-01
ENSCAFG0000003126	CYYR1	turquoise	VSMC_M6	-0.01	9.69E-01	-0.03	8.67E-01	0.11	5.88E-01	-0.19	3.50E-01	-0.17	3.95E-01	0.52	5.55E-03	-0.04	8.54E-01	0.23	2.50E-01	0.00	9.83E-01	-0.01	9.55E-01
ENSCAFG0000000734	ZMYM2	darkgreen	VSMC_M4	-0.01	9.69E-01	0.10	6.16E-01	-0.64	2.97E-04	0.81	3.15E-07	0.60	8.84E-04	-0.24	2.37E-01	-0.40	4.11E-02	-0.74	9.18E-06	0.00	9.95E-01	0.31	1.18E-01
ENSCAFG0000001876	SPEF2	grey	VSMC_M10	-0.01	9.69E-01	-0.25	2.00E-01	-0.01	9.61E-01	-0.21	3.02E-01	0.01	9.63E-01	-0.11	5.93E-01	0.07	7.11E-01	0.31	1.10E-01	0.17	3.99E-01	-0.19	3.51E-01
ENSCAFG0000000033	SHPRH	darkgreen	VSMC_M4	-0.01	9.69E-01	0.03	8.71E-01	-0.58	1.67E-03	0.60	1.01E-03	0.44	2.05E-02	-0.12	5.41E-01	0.12	5.39E-01	-0.49	8.71E-03	-0.08	7.01E-01	0.40	3.84E-02
ENSCAFG0000000931	CCDC148	pink	VSMC_M5	-0.01	9.69E-01	-0.36	6.52E-02	-0.37	5.47E-02	-0.08	6.83E-01	0.48	1.09E-02	-0.46	1.59E-02	-0.07	7.10E-01	0.20	3.16E-01	0.39	4.50E-02	-0.38	4.78E-02
ENSCAFG0000000087	TAF2	grey	VSMC_M10	-0.01	9.69E-01	-0.62	5.28E-04	-0.38	5.02E-02	0.04	8.49E-01	0.37	5.58E-02	0.00	9.93E-01	0.04	8.59E-01	0.25	2.02E-01	0.50	8.13E-03	-0.32	9.91E-02
ENSCAFG0000002397	ZSWIM5	grey	VSMC_M10	-0.01	9.69E-01	0.19	3.48E-01	-0.08	6.79E-01	0.28	1.61E-01	-0.02	9.37E-01	-0.10	6.13E-01	0.23	2.42E-01	-0.21	3.00E-01	-0.23	2.52E-01	0.34	7.85E-02
ENSCAFG0000001058	MARCKSL1	darkgreen	VSMC_M4	-0.01	9.69E-01	0.33	8.90E-02	-0.25	2.06E-01	0.77	3.17E-06	0.15	4.66E-01	0.00	9.94E-01	-0.62	5.09E-04	-0.75	7.59E-06	-0.20	3.11E-01	0.46	1.62E-02
ENSCAFG0000001370	ENSCAFG0000001370	grey	VSMC_M10	-0.01	9.68E-01	-0.07	7.41E-01	0.17	3.84E-01	-0.26	1.91E-01	-0.04	8.51E-01	0.02	9.21E-01	-0.12	5.59E-01	0.16	4.23E-01	0.13	5.23E-01	-0.33	9.00E-02
ENSCAFG0000001241	NSMCE4A	darkgrey	VSMC_M8	-0.01	9.68E-01	-0.50	7.97E-03	0.29	1.46E-01	-0.67	1.52E-04	-0.19	3.42E-01	0.12	5.57E-01	0.20	3.14E-01	0.80	5.36E-07	0.34	8.34E-02	-0.58	1.36E-03
ENSCAFG0000002966	CHN1	grey	VSMC_M10	-0.01	9.68E-01	-0.47	1.44E-02	-0.06	7.73E-01	-0.36	6.34E-02	0.20	3.09E-01	0.29	1.48E-01	0.02	9.15E-01	0.37	5.51E-02	0.44	2.21E-02	-0.58	1.44E-03
ENSCAFG0000000791	RFESD	grey	VSMC_M10	-0.01	9.68E-01	0.00	9.99E-01	-0.24	2.31E-01	0.25	2.13E-01	0.14	4.82E-01	0.23	2.49E-01	0.16	4.26E-01	-0.23	2.47E-01	-0.01	9.66E-01	0.26	1.87E-01
ENSCAFG0000001088	TPPP	cyan	VSMC_M2	-0.01	9.68E-01	0.49	9.92E-03	0.45	1.96E-02	0.00	9.86E-01	-0.56	2.59E-03	0.15	4.55E-01	-0.09	6.44E-01	-0.19	3.34E-01	-0.43	2.43E-02	0.45	1.73E-02
ENSCAFG0000001693	MSTO1	grey	VSMC_M10	-0.01	9.67E-01	0.23	2.39E-01	-0.31	1.22E-01	0.35	7.71E-02	0.26	1.90E-01	0.02	9.05E-01	-0.32	1.05E-01	-0.32	1.02E-01	-0.13	5.15E-01	0.24	2.21E-01
ENSCAFG0000000023	AHI1	darkolivegreen	VSMC_M9	-0.01	9.67E-01	-0.51	6.72E-03	-0.41	3.40E-02	-0.09	6.38E-01	0.46	1.49E-02	-0.01	9.75E-01	0.20	3.21E-01	0.21	2.94E-01	0.46	1.47E-02	-0.33	9.54E-02
ENSCAFG0000003021	DTD2	grey	VSMC_M10	-0.01	9.67E-01	-0.67	1.21E-04	-0.26	1.83E-01	-0.40	4.01E-02	0.43	2.43E-02	-0.05	7.87E-01	0.45	2.00E-02	0.58	1.62E-03	0.53	4.89E-03	-0.73	1.32E-05
ENSCAFG0000000308	AKIRIN2	grey	VSMC_M10	-0.01	9.67E-01	0.01	9.46E-01	0.01	9.73E-01	-0.11	5.78E-01	-0.09	6.40E-01	0.00	9.86E-01	0.08	6.79E-01	0.07	7.15E-01	0.02	9.21E-01	0.11	5.79E-01
ENSCAFG0000001398	CDCA5	darkgrey	VSMC_M8	-0.01	9.67E-01	-0.49	1.03E-02	0.26	1.95E-01	-0.75	5.88E-06	-0.23	2.54E-01	0.24	2.23E-01	0.54	4.03E-03	0.90	1.19E-10	0.26	1.84E-01	-0.50	7.68E-03
ENSCAFG0000000140	KCNJ4	grey	VSMC_M10	-0.01	9.66E-01	0.17	3.98E-01	-0.39	4.37E-02	0.52	5.16E-03	0.23	2.56E-01	0.24	2.25E-01	0.04	8.58E-01	-0.47	1.38E-02	-0.22	2.77E-01	0.40	3.80E-02
ENSCAFG0000001767	SETBP1	grey	VSMC_M10	-0.01	9.66E-01	0.45	1.89E-02	-0.11	6.02E-01	0.49	9.24E-03	0.07	7.41E-01	0.00	9.89E-01	-0.04	8.45E-01	-0.59	1.08E-03	-0.33	8.93E-02	0.44	2.07E-02
ENSCAFG0000000980	DYRK1A	darkolivegreen	VSMC_M9	-0.01	9.66E-01	-0.68	1.10E-04	-0.54	3.37E-03	-0.03	8.77E-01	0.65	2.26E-04	0.04	8.54E-01	-0.02	9.12E-01	0.20	3.11E-01	0.69	8.03E-05	-0.54	3.43E-03
ENSCAFG0000000555	ENSCAFG0000000555	yellow	VSMC_M3	-0.01	9.66E-01	0.36	6.48E-02	0.75	6.80E-06	-0.49	8.79E-03	-0.77	2.35E-06	-0.01	9.48E-01	0.27	1.79E-01	0.32	9.96E-02	-0.43	2.52E-02	0.11	5.69E-01
ENSCAFG0000001101	PLA1A	grey	VSMC_M10	-0.01	9.66E-01	0.33	9.11E-02	-0.19	3.32E-01	0.38	5.31E-02	0.06	7.85E-01	0.06	7.49E-01	-0.07	7.24E-01	-0.46	1.48E-02	-0.21	3.01E-01	0.50	8.40E-03
ENSCAFG0000000576	PDE6G	grey	VSMC_M10	-0.01	9.65E-01	0.17	4.00E-01	0.15	4.51E-01	-0.08	6.88E-01	-0.20	3.11E-01	-0.05	7.94E-01	0.19	3.44E-01	-0.11	5.72E-01	-0.16	4.27E-01	0.16	4.24E-01
ENSCAFG0000000681	TUSC3	darkgreen	VSMC_M4	-0.01	9.65E-01	-0.29	1.38E-01	-0.70	5.38E-05	0.59	1.16E-03	0.70	5.48E-05	-0.19	3.53E-01	-0.18	3.67E-01	-0.42	2.78E-02	0.36	6.85E-02	0.01	9.57E-01
ENSCAFG0000003078	ENSCAFG0000003078	grey	VSMC_M10	-0.01	9.65E-01	-0.57	2.05E-03	-0.38	5.29E-02	-0.20	3.20E-01	0.41	3.29E-02	-0.01	9.69E-01	0.11	5.93E-01	0.39	4.44E-02	0.47	1.40E-02	-0.41	3.49E-02
ENSCAFG0000001839	ATPAF2	grey	VSMC_M10	-0.01	9.64E-01	-0.24	2.35E-01	-0.02	9.14E-01	-0.35	7.73E-02	0.01	9.41E-01	0.19	3.35E-01	0.21	2.83E-01	0.33	9.30E-02	0.13	5.10E-01	-0.20	3.28E-01
ENSCAFG0000001945	NELFB	cyan	VSMC_M2	-0.01	9.64E-01	0.43	2.38E-02	0.59	1.20E-03	-0.22	2.79E-01	-0.73	1.71E-05	0.30	1.35E-01	0.40	3.81E-02	0.05	7.98E-01	-0.52	5.05E-03	0.43	2.58E-02
ENSCAFG0000002332	S100A12	grey	VSMC_M10	-0.01	9.64E-01	0.02	9.33E-01	-0.25	2.04E-01	0.24	2.30E-01	0.16	4.37E-01	-0.06	7.56E-01	0.39	4.35E-02	-0.23	2.51E-01	-0.05	7.92E-01	0.28	1.63E-01
ENSCAFG0000003051	TRAM1L1	grey	VSMC_M10	-0.01	9.63E-01	0.45	1.94E-02	0.14	4.75E-01	0.36	6.36E-02	-0.29	1.43E-01	0.05	7.94E-01	-0.50	8.16E-03	-0.46	1.58E-02	-0.40	3.91E-02	0.52	5.47E-03
ENSCAFG0000002036	TPPP3	darkgrey	VSMC_M8	-0.01	9.63E-01	-0.52	5.70E-03	0.04	8.24E-01	-0.52	5.09E-03	0.04	8.48E-01	0.09	6.50E-01	0.75	5.85E-06	0.60	8.65E-04	0.36	6.27E-02	-0.52	5.29E-03
ENSCAFG0000001103	ENSCAFG0000001103	turquoise	VSMC_M6	-0.01	9.63E-01	-0.04	8.41E-01	-0.12	5.65E-01	0.04	8.60E-01	0.02	9.38E-01	0.38	5.20E-02	0.01	9.71E-01	0.04	8.28E-01	-0.03	8.93E-01	0.10	6.21E-01
ENSCAFG0000001458	KDM6A	pink	VSMC_M5	-0.01	9.63E-01	-0.49	1.02E-02	-0.54	3.89E-03	0.09	6.63E-01	0.69	6.69E-05	-0.27	1.66E-01	-0.07	7.14E-01	0.04	8.35E-01	0.51	6.37E-03	-0.50	7.73E-03
ENSCAFG0000000835	ZC2HC1A	pink	VSMC_M5	-0.01	9.63E-01	-0.40	3.77E-02	-0.71	3.48E-05	0.33	8.87E-02	0.80	4.71E-07	-0.21	2.86E-01	-0.21	2.91E-01	-0.21	2.83E-01	0.47	1.25E-02	-0.31	1.18E-01
ENSCAFG0000000533	ENSCAFG0000000533	grey	VSMC_M10	-0.01	9.62E-01	0.34	8.36E-02	0.39	4.50E-02	-0.11	5.94E-01	-0.43	2.52E-02	-0.19	3.39E-01	-0.21	2.97E-01	-0.04	8.26E-01	-0.22	2.81E-01	0.17	4.03E-01
ENSCAFG0000001827	ENSCAFG0000001827	grey	VSMC_M10	-0.01	9.62E-01	-0.15	4.51E-01	-0.27	1.72E-01	0.24	2.27E-01	0.23	2.48E-01	-0.07	7.29E-01	-0.17	3.86E-01	-0.18	3.77E-01	0.25	2.18E-01	0.04	8.43E-01
ENSCAFG0000001973	ENSCAFG0000001973	grey	VSMC_M10	-0.01	9.62E-01	0.14	4.97E-01	-0.17	3.95E-01	0.16	4.37E-01	0.01	9.64E-01	0.10	6.31E-01	0.14	4.74E-01	-0.17	4.07E-01	-0.17	3.87E-01	0.43	2.50E-02
ENSCAFG0000001787	ARHGAP44	grey	VSMC_M10	-0.01	9.62E-01	-0.03	8.70E-01	-0.41	3.51E-02	0.29	1.35E-01	0.37	5.49E-02	-0.12	5.59E-01	0.15	4.54E-01	-0.25	2.14E-01	0.04	8.40E-01	0.15	4.56E-01
ENSCAFG0000001211	VAPB	darkolivegreen	VSMC_M9	-0.01	9.62E-01	-0.66	1.73E-04	-0.21	2.94E-01	-0.23	2.48E-01	0.41	3.17E-02	-0.04	8.33E-01	-0.17	3.88E-01	0.42	3.08E-02	0.67	1.33E-04	-0.78	1.39E-06
ENSCAFG0000002008	PTBP2	pink	VSMC_M5	-0.01	9.62E-01	-0.40	3.99E-02	-0.57	1.88E-03	0.36	6.86E-02	0.53	4.63E-03	0.06	7.82E-01	-0.25	2.09E-01	-0.12	5.64E-01	0.37	6.07E-02	-0.10	6.36E-01
ENSCAFG0000000273	ENSCAFG0000000273	grey	VSMC_M10	-0.01	9.61E-01	0.21	3.04E-01	0.26	1.84E-01	-0.18	3.66E-01	-0.33	9.53E-02	-0.02	9.30E-01	0.17	3.89E-01	0.10	6.36E-01	-0.21	2.87E-01	0.14	4.99E-01
ENSCAFG0000001661	IQGAP3	darkgrey	VSMC_M8	-0.01	9.61E-01	-0.38	5.07E-02	0.49	1.01E-02	-0.90	2.61E-10	-0.39	4.51E-02	0.16	4.12E-01	0.38	5.15E-02	0.92	1.20E-11	0.23	2.48E-01	-0.54	3.53E-03
ENSCAFG0000002936	ENSCAFG0000002936	grey	VSMC_M10	-0.01	9.61E-01	0.13	5.10E-01	0.43	2.70E-02	-0.17	3.96E-01	-0.48	1.18E-02	-0.01	9.78E-01	0.27	1.66E-01	0.06	7.77E-01	-0.13	5.03E-01	0.19	3.47E-01
ENSCAFG0000001852	PRAM1	grey	VSMC_M10	-0.01	9.61E-01	0.29	1.40E-01	0.31	1.21E-01	-0.10	6.18E-01	-0.25	2.08E-01	-0.13	5.08E-01	-0.28	1.64E-01	-0.10	6.19E-01	-0.14	4.95E-01	0.05	7.90E-01
ENSCAFG0000000518	FANCD2	darkgrey	VSMC_M8	-0.01	9.61E-01	-0.58	1.58E-03	0.19	3.45E-01	-0.77	2.14E-06	-0.11	5.86E-01	0.12	5.37E-01	0.43	2.52E-02	0.93	1.24E-12	0.37	5.62E-02	-0.61	8.23E-04
ENSCAFG0000000284	EPB41L4B	grey	VSMC_M10	-0.01	9.61E-01	0.33	9.78E-02	-0.13	5.15E-01	0.26	1.99E-01	-0.10	6.18E-01	0.11	5.76E-01	0.19	3.49E-01	-0.23	2.58E-01	-0.43	2.67E-02	0.58	1.49E-03
ENSCAFG0000001890	ZNF449	grey	VSMC_M10	-0.01	9.61E-01	0.38	5.10E-02	-0.05	8.15E-01	0.19	3.37E-01	-0.04	8.49E-01	-0.08	6.81E-01	0.13	5.14E-01	-0.20	3.25E-01	-0.41	3.59E-02	0.41	3.31E-02
ENSCAFG0000002864	ENSCAFG0000002864	grey	VSMC_M10	-0.01	9.61E-01	-0.01	9.80E-01	-0.24	2.37E-01	0.24	2.18E-01	0.08	6.97E-01	0.11	5.94E-01	0.20	3.25E-01	-0.10	6.22E-01	-0.04	8.47E-01	0.33	9.76E-02
ENSCAFG0000003242	PTPMT1	grey	VSMC_M10	-0.01	9.61E-01	0.26	1.82E-01	-0.12	5.41E-01	0.45	1.92E-02	0.04	8.52E-01	-0.18	3.82E-01	0.10	6.08E-01	-0.39	4.56E-02	-0.25	2.13E-01	0.38	4.99E-02
ENSCAFG0000000749	SGTB	pink	VSMC_M5	-0.01	9.61E-01	-0.31	1.12E-01	-0.17	3.91E-01	-0.10	6.27E-01	0.38	5.04E-02	-0.10	6.07E-01	-0.24	2.22E-01	0.16	4.39E-01	0.39	4.33E-02	-0.55	2.66E-03
ENSCAFG0000001290	CDK1	darkgrey	VSMC_M8	-0.01	9.61E-01	-0.59	1.09E-03	0.24	2.31E-01	-0.87	2.78E-09	-0.09	6.60E-01	0.13	5.29E-01	0.40	4.03E-02	0.96	2.86E-15	0.42	2.73E-02	-0.76	4.86E-06
ENSCAFG0000000716	ZSWIM6	grey	VSMC_M10	-0.01	9.60E-01	0.21	2.93E-01	-0.43	2.65E-02	0.58	1.59E-03	0.35	7.53E-02	-0.02	9.20E-01	-0.53	4.88E-03	-0.57	1.85E-03	-0.13	5.07E-01	0.34	7.98E-02
ENSCAFG0000000206	ADGRF1	grey	VSMC_M10	-0.01	9.60E-01	0.31	1.22E-01	0.20	3.27E-01	-0.09	6.66E-01	-0.28	1.52E-01	-0.03	8.99E-01	-0.17	3.97E-01	0.00	9.95E-01	-0.30	1.32E-01	0.20	3.18E-01
ENSCAFG0000002107	ENSCAFG0000002107	grey	VSMC_M10	-0.01	9.60E-01	0.17	4.09E-01	0.12	5.39E-01	-0.11	5.73E-01	-0.18	3.60E-01	-0.03	8.96E-01	0.27	1.67E-01	0.06	7.54E-01	-0.21	2.83E-01	0.13	5.04E-01
ENSCAFG0000002139	ENSCAFG0000002139	grey	VSMC_M10	-0.01	9.60E-01	-0.01	9.49E-01	0.17	3.90E-01	-0.20	3.05E-01	-0.25	2.01E-01	0.37	5.64E-02	0.26	1.87E-01	0.27	1.70E-01	-0.09	6.54E-01	0.02	9.14E-01
ENSCAFG0000003002	ENSCAFG0000003002	grey	VSMC_M10	-0.01	9.60E-01	-0.21	3.04E-01	-0.17	4.05E-01	0.06	7.53E-01	0.11	5.88E-01	-0.07	7.12E-01	0.19	3.45E-01	0.09	6.69E-01	0.07	7.38E-01	0.00	9.89E-01
ENSCAFG0000001865	NLK	grey	VSMC_M10	-0.01	9.60E-01	-0.13	5.07E-01	-0.50	8.30E-03	0.34	7.82E-02	0.31	1.15E-01	0.15	4.67E-01	-0.06	7.67E-01	-0.21	2.97E-01	0.09	6.49E-01	0.25	2.06E-01
ENSCAFG0000000156	DAAM2	grey	VSMC_M10	-0.01	9.60E-01	-0.33	9.65E-02	-0.24	2.21E-01	0.16	4.39E-01	0.21	2.97E-01	0.17	3.92E-01	0.11	5.86E-01	-0.08	6.99E-01	0.34	7.91E-02	-0.09	6.53E-01
ENSCAFG0000001246	UCN2	grey	VSMC_M10	-0.01	9.60E-01	0.27	1.68E-01	0.24	2.18E-01	-0.18	3.71E-01	-0.28	1.55E-01	-0.04	8.60E-01	0.22	2.61E-01	0.08	6.81E-01	-0.23	2.49E-01	0.17	4.03E-01
ENSCAFG0000000987	ITGB6	grey	VSMC_M10	-0.01	9.60E-01	0.10	6.26E-01	0.07	7.41E-01	-0.01	9.64E-01	-0.16	4.13E-01	0.24	2.22E-01	-0.01	9.42E-01	0.05	8.17E-01	-0.09	6.55E-01	0.17	4.04E-01

ENSCAFG0000001516	ENSCAFG0000001516	turquoise	VSMC_M6	-0.01	9.60E-01	0.06	7.80E-01	0.09	6.55E-01	-0.09	6.46E-01	-0.23	2.50E-01	0.42	2.75E-02	0.06	7.67E-01	0.11	5.83E-01	-0.12	5.65E-01	0.14	4.92E-01
ENSCAFG0000001270	BUB3	darkgrey	VSMC_M8	-0.01	9.60E-01	-0.61	7.86E-04	0.07	7.28E-01	-0.70	4.65E-05	0.10	6.13E-01	0.00	9.84E-01	0.24	2.28E-01	0.86	8.65E-09	0.46	1.70E-02	-0.80	5.45E-07
ENSCAFG0000001401	DLAT	darkgrey	VSMC_M8	-0.01	9.59E-01	-0.29	1.39E-01	0.20	3.28E-01	-0.65	2.61E-04	-0.01	9.48E-01	0.06	7.60E-01	0.29	1.38E-01	0.64	3.46E-04	0.18	3.63E-01	-0.59	1.11E-03
ENSCAFG0000000248	PLEKHH2	grey	VSMC_M10	-0.01	9.59E-01	0.07	7.26E-01	-0.30	1.28E-01	0.20	3.15E-01	0.22	2.64E-01	-0.01	9.42E-01	0.44	2.31E-02	-0.24	2.26E-01	-0.14	4.79E-01	0.28	1.56E-01
ENSCAFG0000001602	RFX7	pink	VSMC_M5	-0.01	9.59E-01	-0.42	2.90E-02	-0.83	6.61E-08	0.36	6.89E-02	0.82	1.89E-07	-0.15	4.68E-01	0.05	7.99E-01	-0.13	5.16E-01	0.38	5.29E-02	-0.12	5.62E-01
ENSCAFG0000002973	BRMS1L	pink	VSMC_M5	-0.01	9.58E-01	-0.59	1.13E-03	-0.49	8.79E-03	0.01	9.70E-01	0.57	1.90E-03	-0.12	5.62E-01	0.13	5.03E-01	0.23	2.48E-01	0.55	2.79E-03	-0.52	5.88E-03
ENSCAFG0000000445	NUDT15	grey	VSMC_M10	-0.01	9.58E-01	-0.22	2.72E-01	0.30	1.23E-01	-0.36	6.46E-02	-0.33	9.52E-02	0.26	1.82E-01	0.19	3.51E-01	0.40	3.79E-02	0.12	5.63E-01	-0.25	2.09E-01
ENSCAFG0000003010	TPC3	cyan	VSMC_M2	-0.01	9.58E-01	0.71	3.59E-05	-0.11	6.00E-01	0.67	1.25E-04	0.00	9.81E-01	-0.24	2.35E-01	-0.35	7.65E-02	-0.78	1.67E-06	-0.56	2.45E-03	0.67	1.21E-04
ENSCAFG0000001175	PLXNA2	darkgreen	VSMC_M4	-0.01	9.57E-01	0.38	5.10E-02	-0.40	3.75E-02	0.77	2.71E-06	0.29	1.44E-01	-0.04	8.40E-01	-0.46	1.68E-02	-0.72	2.26E-05	-0.23	2.57E-01	0.51	6.58E-03
ENSCAFG0000000733	CCDC73	turquoise	VSMC_M6	-0.01	9.57E-01	-0.23	2.49E-01	-0.10	6.19E-01	-0.07	7.39E-01	0.01	9.58E-01	0.67	1.39E-04	-0.09	6.43E-01	0.19	3.38E-01	0.13	5.29E-01	-0.08	6.74E-01
ENSCAFG0000000398	SPATA5	grey	VSMC_M10	-0.01	9.57E-01	-0.36	6.14E-02	-0.43	2.55E-02	0.42	2.74E-02	0.37	5.63E-02	-0.07	7.42E-01	0.06	7.71E-01	-0.19	3.51E-01	0.32	1.04E-01	-0.01	9.61E-01
ENSCAFG0000001566	ATAD1	darkolivegreen	VSMC_M9	-0.01	9.57E-01	-0.69	6.56E-05	-0.53	4.83E-03	0.13	5.24E-01	0.55	2.77E-03	-0.03	8.89E-01	-0.15	4.60E-01	0.15	4.46E-01	0.65	2.11E-04	-0.44	2.15E-02
ENSCAFG0000002884	TMEM141	grey	VSMC_M10	-0.01	9.56E-01	0.42	3.02E-02	0.13	5.29E-01	0.12	5.51E-01	-0.40	3.72E-02	0.34	7.80E-02	0.15	4.66E-01	-0.17	3.90E-01	-0.49	9.52E-03	0.69	7.45E-05
ENSCAFG0000002989	ENSCAFG0000002989	turquoise	VSMC_M6	-0.01	9.56E-01	0.00	9.87E-01	-0.31	1.21E-01	0.31	1.15E-01	0.17	4.07E-01	0.41	3.33E-02	-0.12	5.39E-01	-0.32	9.89E-02	-0.02	9.03E-01	0.23	2.56E-01
ENSCAFG0000000257	EDN2	darkgreen	VSMC_M4	-0.01	9.56E-01	-0.03	8.72E-01	-0.50	7.40E-03	0.63	4.05E-04	0.37	6.12E-02	0.32	1.02E-01	0.07	7.14E-01	-0.45	1.80E-02	0.01	9.65E-01	0.31	1.17E-01
ENSCAFG0000002371	MEX3D	cyan	VSMC_M2	-0.01	9.55E-01	0.48	1.20E-02	0.46	1.61E-02	-0.03	8.92E-01	-0.65	2.34E-04	0.30	1.23E-01	-0.09	6.60E-01	-0.11	5.93E-01	-0.49	9.81E-03	0.54	3.38E-03
ENSCAFG0000001015	BACE2	darkgreen	VSMC_M4	-0.01	9.55E-01	0.20	3.30E-01	-0.35	7.00E-02	0.58	1.66E-03	0.14	4.73E-01	0.31	1.21E-01	0.10	6.27E-01	-0.49	9.33E-03	-0.25	2.14E-01	0.57	1.86E-03
ENSCAFG0000000269	LENG1	grey	VSMC_M10	-0.01	9.55E-01	0.12	5.55E-01	0.56	2.37E-03	-0.43	2.40E-02	-0.62	6.31E-04	-0.06	7.59E-01	0.33	8.77E-02	0.31	1.22E-01	-0.16	4.32E-01	0.13	5.15E-01
ENSCAFG0000000187	TCP11L2	grey	VSMC_M10	-0.01	9.55E-01	0.09	6.64E-01	-0.50	7.49E-03	0.59	1.06E-03	0.59	1.25E-03	-0.24	2.37E-01	-0.30	1.24E-01	-0.62	6.37E-04	0.10	6.02E-01	0.05	7.90E-01
ENSCAFG0000000310	AQP1	grey	VSMC_M10	-0.01	9.55E-01	0.51	7.16E-03	0.21	2.98E-01	0.19	3.50E-01	-0.33	9.81E-02	-0.11	5.70E-01	0.31	1.17E-01	-0.25	2.12E-01	-0.51	7.12E-03	0.53	4.62E-03
ENSCAFG0000001546	CHRM1	grey	VSMC_M10	-0.01	9.55E-01	0.41	3.61E-02	0.02	9.12E-01	0.24	2.36E-01	-0.14	4.87E-01	-0.04	8.38E-01	0.00	1.00E+00	-0.27	1.77E-01	-0.39	4.23E-02	0.44	2.20E-02
ENSCAFG0000001734	TOM1L1	darkgreen	VSMC_M4	-0.01	9.55E-01	0.15	4.55E-01	-0.52	5.81E-03	0.78	1.52E-06	0.29	1.36E-01	0.27	1.66E-01	-0.29	1.49E-01	-0.64	3.10E-04	-0.15	4.51E-01	0.51	6.59E-03
ENSCAFG0000001045	SLC37A1	grey	VSMC_M10	-0.01	9.54E-01	0.39	4.54E-02	-0.19	3.38E-01	0.37	5.78E-02	0.16	4.32E-01	-0.14	4.72E-01	-0.16	4.17E-01	-0.53	4.24E-03	-0.28	1.64E-01	0.39	4.34E-02
ENSCAFG0000002979	EMC7	grey	VSMC_M10	-0.01	9.54E-01	-0.48	1.04E-02	-0.23	2.47E-01	0.12	5.53E-01	0.22	2.79E-01	-0.13	5.19E-01	-0.08	6.89E-01	0.10	6.06E-01	0.44	2.28E-02	-0.30	1.33E-01
ENSCAFG0000000455	ENSCAFG0000000455	grey	VSMC_M10	-0.01	9.54E-01	-0.34	7.82E-02	-0.19	3.49E-01	-0.13	5.15E-01	0.30	1.27E-01	-0.17	4.07E-01	-0.24	2.21E-01	0.19	3.44E-01	0.41	3.53E-02	-0.41	3.35E-02
ENSCAFG0000000671	RYK	grey	VSMC_M10	-0.01	9.53E-01	-0.10	6.24E-01	-0.13	5.24E-01	0.04	8.56E-01	0.14	4.83E-01	-0.07	7.41E-01	0.46	1.51E-02	0.04	8.25E-01	-0.01	9.64E-01	-0.06	7.67E-01
ENSCAFG0000001118	TM7SF3	darkgreen	VSMC_M4	-0.01	9.53E-01	0.19	3.45E-01	-0.60	8.76E-04	0.78	1.50E-06	0.57	2.14E-03	-0.22	2.61E-01	-0.35	7.37E-02	-0.73	1.71E-05	-0.07	7.41E-01	0.30	1.24E-01
ENSCAFG0000001753	CHTOP	grey	VSMC_M10	-0.01	9.53E-01	-0.14	5.00E-01	0.39	4.20E-02	-0.28	1.56E-01	-0.28	1.55E-01	-0.06	7.54E-01	-0.10	6.23E-01	0.28	1.64E-01	0.17	3.98E-01	-0.34	7.94E-02
ENSCAFG0000000612	PGAM5	darkgrey	VSMC_M8	-0.01	9.53E-01	-0.44	2.00E-02	0.30	1.25E-01	-0.76	5.01E-06	-0.27	1.77E-01	0.17	3.83E-01	0.47	1.42E-02	0.85	1.85E-08	0.27	1.68E-01	-0.45	1.73E-02
ENSCAFG0000001743	RAB3D	grey	VSMC_M10	-0.01	9.53E-01	0.66	1.71E-04	0.28	1.53E-01	0.21	2.85E-01	-0.39	4.17E-02	0.30	1.33E-01	-0.27	1.75E-01	-0.43	2.66E-02	-0.54	3.75E-03	0.55	2.68E-03
ENSCAFG0000000367	FZD8	darkgreen	VSMC_M4	-0.01	9.53E-01	0.24	2.19E-01	-0.58	1.40E-03	0.90	1.76E-10	0.41	3.23E-02	-0.07	7.19E-01	-0.14	4.71E-01	-0.78	1.37E-06	-0.20	3.24E-01	0.58	1.46E-03
ENSCAFG0000002481	ENSCAFG0000002481	grey	VSMC_M10	-0.01	9.53E-01	0.04	8.29E-01	0.08	6.97E-01	-0.13	5.10E-01	-0.13	5.32E-01	0.18	3.73E-01	0.13	5.30E-01	0.18	3.75E-01	-0.10	6.36E-01	0.06	7.73E-01
ENSCAFG0000002982	ENSCAFG0000002982	grey	VSMC_M10	-0.01	9.53E-01	0.44	2.17E-02	0.16	4.27E-01	-0.01	9.45E-01	-0.25	2.05E-01	-0.13	5.19E-01	-0.06	7.48E-01	-0.07	7.18E-01	-0.44	2.06E-02	0.36	6.19E-02
ENSCAFG0000001014	SLC45A3	cyan	VSMC_M2	-0.01	9.53E-01	0.53	4.63E-03	0.43	2.65E-02	0.06	7.71E-01	-0.44	2.25E-02	-0.08	6.87E-01	-0.45	1.94E-02	-0.25	2.18E-01	-0.39	4.65E-02	0.27	1.79E-01
ENSCAFG0000000918	CPOX	grey	VSMC_M10	-0.01	9.53E-01	0.02	9.32E-01	-0.21	3.01E-01	0.34	7.98E-02	0.23	2.54E-01	-0.02	9.32E-01	0.44	2.26E-02	-0.32	1.02E-01	-0.03	8.62E-01	0.15	4.42E-01
ENSCAFG0000001571	ENSCAFG0000001571	grey	VSMC_M10	-0.01	9.53E-01	-0.21	2.97E-01	0.07	7.10E-01	-0.43	2.55E-02	-0.02	9.15E-01	0.06	7.71E-01	0.23	2.55E-01	0.46	1.47E-02	0.16	4.30E-01	-0.33	9.24E-02
ENSCAFG0000003192	ENSCAFG0000003192	grey	VSMC_M10	-0.01	9.52E-01	0.12	5.62E-01	0.12	5.36E-01	-0.11	6.01E-01	-0.15	4.59E-01	0.03	8.69E-01	0.04	8.56E-01	0.05	8.01E-01	-0.13	5.06E-01	0.07	7.17E-01
ENSCAFG0000001879	C20H19orf70	grey	VSMC_M10	-0.01	9.52E-01	0.31	1.17E-01	0.14	4.77E-01	0.02	9.40E-01	-0.17	3.99E-01	0.04	8.46E-01	0.11	5.86E-01	-0.11	5.86E-01	-0.28	1.53E-01	0.13	5.13E-01
ENSCAFG0000001912	GLOD4	yellow	VSMC_M3	-0.01	9.52E-01	0.38	5.26E-02	0.46	1.68E-02	0.16	4.30E-01	-0.55	2.74E-03	0.06	7.72E-01	-0.07	7.41E-01	-0.19	3.37E-01	-0.37	5.79E-02	0.37	5.52E-02
ENSCAFG0000001513	VAMP1	grey	VSMC_M10	-0.01	9.52E-01	-0.20	3.26E-01	0.06	7.57E-01	-0.40	3.79E-02	-0.11	5.82E-01	0.19	3.53E-01	0.12	5.53E-01	0.47	1.42E-02	0.09	6.61E-01	-0.14	4.80E-01
ENSCAFG0000001881	RAI14	darkgrey	VSMC_M8	-0.01	9.51E-01	-0.31	1.13E-01	0.44	2.29E-02	-0.78	1.37E-06	-0.31	1.11E-01	0.18	3.79E-01	-0.07	7.44E-01	0.72	1.94E-05	0.26	1.84E-01	-0.57	1.86E-03
ENSCAFG0000001983	CPNE7	grey	VSMC_M10	-0.01	9.51E-01	0.47	1.38E-02	-0.25	2.01E-01	0.61	6.77E-04	0.10	6.35E-01	-0.19	3.51E-01	-0.23	2.52E-01	-0.64	3.24E-04	-0.36	6.36E-02	0.66	1.74E-04
ENSCAFG0000001046	NEURL1	grey	VSMC_M10	-0.01	9.50E-01	0.08	7.08E-01	0.05	8.04E-01	-0.18	3.70E-01	-0.01	9.64E-01	-0.11	5.97E-01	-0.13	5.07E-01	0.07	7.20E-01	-0.02	9.35E-01	-0.13	5.31E-01
ENSCAFG0000001208	TSSK4	grey	VSMC_M10	-0.01	9.50E-01	0.26	1.91E-01	0.44	2.20E-02	-0.37	5.97E-02	-0.51	6.41E-03	0.07	7.22E-01	0.18	3.81E-01	0.26	1.92E-01	-0.30	1.35E-01	0.17	3.93E-01
ENSCAFG0000001364	DUSP10	grey	VSMC_M10	-0.01	9.50E-01	-0.01	9.45E-01	-0.22	2.81E-01	0.28	1.59E-01	0.09	6.73E-01	0.25	2.00E-01	0.14	4.78E-01	-0.16	4.16E-01	-0.02	9.14E-01	0.25	2.03E-01
ENSCAFG0000001203	SPO11	grey	VSMC_M10	-0.01	9.50E-01	-0.09	6.51E-01	-0.22	2.70E-01	0.12	5.45E-01	0.20	3.14E-01	-0.14	4.96E-01	-0.09	6.57E-01	-0.18	3.57E-01	0.16	4.25E-01	0.03	8.80E-01
ENSCAFG0000001462	AAMP	yellow	VSMC_M3	-0.01	9.49E-01	0.44	2.13E-02	0.71	3.04E-05	-0.17	3.83E-01	-0.76	4.95E-06	0.09	6.37E-01	-0.02	9.08E-01	-0.01	9.69E-01	-0.42	2.92E-02	0.24	2.35E-01
ENSCAFG0000000924	ENSCAFG0000000924	turquoise	VSMC_M6	-0.01	9.49E-01	0.04	8.39E-01	0.16	4.36E-01	-0.01	9.65E-01	-0.21	2.81E-01	0.38	5.29E-02	0.19	3.41E-01	-0.09	6.47E-01	-0.03	8.65E-01	0.15	4.69E-01
ENSCAFG0000001410	SGPL1	yellow	VSMC_M3	-0.01	9.49E-01	0.16	4.23E-01	0.59	1.30E-03	-0.66	1.90E-04	-0.47	1.26E-02	0.14	4.81E-01	-0.12	5.53E-01	0.43	2.63E-02	-0.10	6.18E-01	-0.23	2.56E-01
ENSCAFG0000001458	TMEM106A	cyan	VSMC_M2	-0.01	9.48E-01	0.67	1.41E-04	0.14	5.01E-01	0.33	9.44E-02	-0.39	4.62E-02	0.12	5.42E-01	-0.09	6.54E-01	-0.43	2.41E-02	-0.62	5.32E-04	0.81	3.04E-07
ENSCAFG0000000355	TSKS	grey	VSMC_M10	-0.01	9.48E-01	0.17	4.03E-01	0.21	2.89E-01	-0.09	6.70E-01	-0.07	7.35E-01	-0.20	3.12E-01	-0.20	3.05E-01	0.02	9.34E-01	-0.08	6.96E-01	-0.19	3.30E-01
ENSCAFG0000001669	ANGPTL7	darkgreen	VSMC_M4	-0.01	9.48E-01	0.28	1.55E-01	-0.36	6.31E-02	0.66	1.68E-04	0.23	2.53E-01	-0.10	6.32E-01	-0.20	3.07E-01	-0.71	3.18E-05	-0.20	3.09E-01	0.48	1.07E-02
ENSCAFG0000002479	ENSCAFG0000002479	violet	VSMC_M7	-0.01	9.48E-01	-0.18	3.81E-01	0.02	9.06E-01	-0.14	4.89E-01	-0.06	7.56E-01	-0.05	8.06E-01	0.78	1.79E-06	0.19	3.41E-01	0.05	7.87E-01	-0.02	9.31E-01
ENSCAFG0000000347	LANCL2	grey	VSMC_M10	-0.01	9.48E-01	0.26	1.97E-01	0.52	5.70E-03	-0.21	2.84E-01	-0.64	3.58E-04	0.06	7.50E-01	0.07	7.17E-01	0.17	4.07E-01	-0.25	2.15E-01	0.27	1.73E-01
ENSCAFG0000000308	RNGTT	grey	VSMC_M10	-0.01	9.48E-01	-0.36	6.38E-02	-0.39	4.20E-02	0.03	8.94E-01	0.39	4.26E-02	0.10	6.32E-01	-0.03	8.66E-01	0.13	5.18E-01	0.33	9.32E-02	-0.24	2.34E-01
ENSCAFG0000002326	DPYSL5	grey	VSMC_M10	-0.01	9.48E-01	-0.04	8.43E-01	-0.08	6.78E-01	0.02	9.03E-01	0.10	6.27E-01	-0.05	8.17E-01	-0.12	5.65E-01	-0.08	6.87E-01	0.07	7.25E-01	0.03	8.64E-01
ENSCAFG0000000961	IFIT2	grey	VSMC_M10	-0.01	9.48E-01	0.42	2.88E-02	0.08	6.90E-01	0.12	5.51E-01	-0.19	3.45E-01	0.32	1.06E-01	-0.30	1.25E-01	-0.17	4.04E-01	-0.39	4.34E-02	0.39	4.66E-02
ENSCAFG0000000596	FOXO1	darkgreen	VSMC_M4	-0.01	9.48E-01	0.31	1.17E-01	-0.63	4.45E-04	0.90	1.55E-10	0.45	1.91E-02	0.01	9.55E-01	-0.39	4.71E-02	-0.82	1.99E-07	-0.22	2.74E-01	0.59	1.16E-03
ENSCAFG0000001717	ENSCAFG0000001717	grey	VSMC_M10	-0.01	9.47E-01	0.22	2.76E-01	-0.14	4.90E-01	0.22	2.73E-01	0.15	4.64E-01	-0.20	3.23E-01	-0.17	3.99E-01	-0.31	1.20E-01	-0.11	5.79E-01	0.20	3.06E-01
ENSCAFG0000002863	SMIM1	cyan	VSMC_M2	-0.01	9.47E-01	0.67	1.35E-04	0.04	8.38E-01	0.65	2.63E-04	-0.24	2.32E-01	0.01	9.56E-01	-0.43	2.66E-02	-0.71	2.80E-05	-0.57	1.87E-03	0.81	3.11E-07
ENSCAFG0000000752	KDM3A	pink	VSMC_M5	-0.01	9.46E-01	-0.23	2.47E-01	-0.52	5.22E-03	0.30	1.34E-01	0.64	3.59E-04	-0.23	2.44E-01	-0.07	7.32E-01	-0.23	2.45E-01	0.34	8.76E-02	-0.20	3.10E-01
ENSCAFG0000002929	ENSCAFG0000002929	grey	VSMC_M10	-0.01	9.46E-01	0.31	1.13E-01	0.26	1.90E-01	-0.05	8.15E-01	-0.41	3.49E-02	0.25	2.18E-01	0.38	5.10E-02	-0.01	9.47E-01	-0.36	6.82E-02	0.44	2.19E-02
ENSCAFG0000000029	AVIL	grey	VSMC_M10	-0.01	9.46E-01	0.15	4.47E-01	0.18	3.82E-01	-0.21	2.94E-01	-0.20	3.20E-01	-0.06	7.49E-01	0.17	4.08E-01	0.16	4.16E-01	-0.19	3.45E-01	0.08	6.95E-01
ENSCAFG0000001721	ABCB7	pink	VSMC_M5	-0.01	9.46E-01	-0.28	1.62E-01	-0.67	1.15E-04	0.24	2.27E-01	0.68	1.01E-04	-0.08	6.82E-01	-0.15	4.44E-01	-0.03	8.96E-01	0.25	2.02E-01	-0.14	5.02E-01
ENSCAFG0000001619	AP1S3	darkgreen	VSMC_M4	-0.01	9.46E-01	0.13	5.26E-01	-0.41	3.33E-02	0.66	1.99E-04	0.36	6.29E-02	-0.13	5.33E-01	-0.32	1.07E-01	-0.56	2.60E-03	-0.05	8.07E-01	0.29	1.46E-01
ENSCAFG0000000308	CCT4	darkgrey	VSMC_M8	-0.01	9.46E-01	-0.67	1.13E-04	0.08	7.03E-01	-0.64	3.15E-04	0.02	9.32E-01	0.24	2.30E-01	0.20	3.19E-01	0.85	2.49E-08	0.51	6.84E-03	-0.74	9.60E-06
ENSCAFG0000003233	KCNJ9	grey	VSMC_M10	-0.01	9.46E-01	0.08	6.95E-01	0.13	5.17E-01	-0.19	3.34E-01	-0.31	1.11E-01	0.19	3.35E-01	0.17	3.95E-01	0.24	2.35E-01	-0.19	3.37E-01	0.24	2.26E-01
ENSCAFG0000001755	TEX14	cyan	VSMC_M2	-0.01	9.46E-01	0.47	1.33E-02	0.32	1.06E-01	0.08	6.96E-01	-0.47	1.36E-02	0.26	1.87E-01	-0.01	9.74E-01	-0.23	2.53E-01	-0.41	3.44E-02	0.53	4.87E-03
ENSCAFG0000001105	ACP6	grey	VSMC_M10	-0.01	9.45E-01	0.42	2.96E-02	0.07	7.18E-01	0.27	1.70E-01	-0.29	1.48E-01	0.23	2.45E-01	0.20	3.10E-01	-0.31	1.17E-01	-0.47	1.31E-02	0.67	1.53E-04
ENSCAFG0000000291	EFEMP1	cyan	VSMC_M2	-0.01	9.45E-01	0.65	2.41E-04	-0.09	6.42E-01	0.61	7.83E-04	-0.02	9.29E-01	-0.05	7.86E-01	-0.23	2.54E-01	-0.68	1.11E-04	-0.56	2.33E-03	0.64	3.12E-04
ENSCAFG0000000492	ENSCAFG0000000492	grey	VSMC_M10	-0.01	9.45E-01	0.16	4.35E-01	0.02	9.02E-01	0.07	7.15E-01	-0.01	9.43E-01	-0.08	7.02E-01	-0.16	4.28E-01	-0.26	1.99E-01	-0.05	8.11E-01	0.15	4.67E-01
ENSCAFG0000001915	GABRE	grey	VSMC_M10	-0.01	9.45E-01	0.30	1.24E-01	0.14	4.85E-01	0.15	4.68E-01	-0.32	1.00E-01	0.04	8.28E-01	0.24	2.20E-01	-0.08	7.08E-01	-0.40	3.67E-02	0.48	1.13E-02
ENSCAFG0000000841	RASA1	darkolivegreen	VSMC_M9	-0.01	9.45E-01	-0.57	2.11E-03	-0.54	3.65E-03	0.05	8.20E-01	0.57	2.12E-03	0.11	5.88E-01	0.28	1.53E-01	0.16	4.29E-01	0.45	1.93E-02	-0.33	8.78E-02
ENSCAFG0000000753	ERBIN	pink	VSMC_M5	-0.01	9.44E-01	-0.17	3.97E-01	-0.69	6.56E-05	0.48	1.19E-02	0.74	9.63E-06	-0.20	3.10E-01	-0.03	8.71E-01	-0.38	5.23E-02	0.21	2.82E-01	0.00	9.84E-01
ENSCAFG0000000533	CAPN14	grey	VSMC_M10	-0.01	9.44E-01	0.14	4.74E-01	0.19	3.39E-01	-0.14	4.94E-01	-0.24	2.37E-01	-0.02	9.09E-01	-0.10	6.36E-01	0.09	6.65E-01	-0.16	4.16E-01	0.07	7.14E-01
ENSCAFG0000001698	DOCK2	grey	VSMC_M10	-0.01	9.44E-01	0.14	4.74E-01	0.19	3.39E-01	-0.14	4.94E-01	-0.24	2.37E-01	-0.02	9.09E-01	-0.10	6.36E-01	0.09	6.65E-01	-0.16	4.16E-01	0.07	7.14E-01
ENSCAFG0000001031	SRSF9	yellow	VSMC_M3	-0.01	9.44E-01	-0.12	5.50E-01	0.46	1.48E-02	-0.42	2.81E-02	-0.49	9.85E-03	0.10	6.18E-01	0.04	8.42E-01	0.47	1.40E-02	0.03	8.66E-01	-0.19	3.52E-01
ENSCAFG0000001295	NIPAL3	grey	VSMC_M10	-0.01	9.43E-01	0.61	8.18E-04	-0.06	7.53E-01	0.56	2.14E-03	-0.07	7.12E-01	0.02	9.40E-01	-0.02	9.40E-01	-0.68	8.62E-05	-0.55	3.25E-03	0.71	2.81E-05
ENSCAFG0000002424	ENSCAFG0000002424	grey	VSMC_M10	-0.01	9.43E-01	0.27	1.73E-01	-0.16	4.33E-01	0.39	4.39E-02	0.06	7.80E-01	-0.14	4.90E-01	-0.33	9.57E-02	-0.38	5.27E-02	-0.16	4.21E-01	0.39	4.59E-02
ENSCAFG0000000578	ANAPC15	grey	VSMC_M10	-0.01	9.43E-01	-0.47	1.31E-02	-0.07	7.39E-01	-0.28	1.62E-01	0.25	2.11E-01	-0.26	1.88E-01	0.00	9.82E-01	0.40	3.64E-02	0.42	2.96E-02	-0.67	1.46E-04
ENSCAFG0000001729	CEP128	darkgrey	VSMC_M8	-0.01	9.42E-01	-0.58	1.48E-03	0.10	6.09E-01	-0.69	6.55E-05	0.04	8.58E-01	0.03	8.67E-01	0.44	2.14E-02	0.81	2.28E-07	0.44	2.07E-02	-0.69	6.08E-05
ENSCAFG0000000842	IFT81	grey	VSMC_M10	-0.01	9.42E-01	-0.04	8.40E-01	-0.40	3.91E-02	0.18	3.70E-01	0.51	6.63E-03	-0.36	6.54E-02	0.03	8.88E-01	-0.17	3.91E-01	0.07	7.45E-01	-0.13	5.03E-01
ENSCAFG0000003009	ENSCAFG0000003009	grey	VSMC_M10	-0.01	9.42E-01	0.03	8.84E-01	0.08	6.77E-01	-0.04	8.35E-01	-0.16	4.22E-01	0.18	3.75E-01	-0.11	5.83E-01	0.00	9.99E-01	-0.03	8.69E-01	0.17	3.92E-01
ENSCAFG0000000502	SRP68	darkgrey	VSMC_M8	-0.01	9.42E-01	-0.51	6.27E-03	0.20	3.25E-01	-0.52	5.30E-03	-0.08	7.02E-01	0.03	8.84E-01	0.08	7.01E-01	0.67	1.33E-04	0.44	2.16E-02	-0.68	8.99E-05
ENSCAFG0000003065	ENSCAFG0000003065	grey	VSMC_M10	-0.01	9.41E-01	0.26	1.91E-01	0.21	3.00E-01	-0.12	5.52E-01	-0.29	1.38E-01	0.01	9.77E-01	0.22	2.80E-01	-0.06	7.59E-01	-0.25	2.07E-01	0.22	2.62E-01
ENSCAFG0000001594	ENSCAFG0000001594	grey	VSMC_M10	-0.01	9.41E-01	0.03	8.99E-01	0.36	6.59E-02	-0.61	6.67E-04	-0.29	1.40E-01	0.22	2.73E-01	0.25	2.06E-01	0.47	1.28E-02	-0.11	5.96E-01	-0.20	3.10E-01
ENSCAFG0000000504	AXL	darkgrey	VSMC_M8	-0.02	9.41E-01	-0.24	2.38E-01	0.62	5.14E-04	-0.84	3.61E-08	-0.50	8.63E-03	-0.04	8.45E-01	0.24	2.19E-01	0.80	7.25E-07	0.16	4.34E-01	-0.53	4.87E-03
ENSCAFG0000001074	PGAP1	grey	VSMC_M10	-0.02	9.41E-01	-0.50	7.24E-03	-0.44	2.09E-02	0.07	7.43E-01	0.49	9.48E-03	-0.01	9.74E-01	0.03	8.96E-01	0.19	3.40E-01	0.43	2.64E-02	-0.34	8.33E-02
ENSCAFG0000001792	TEKT3	grey	VSMC_M10	-0.02	9.40E-01	0.09	6.51E-01	0.01	9.41E-01	0.10	6.32E-01	-0.12	5.38E-01	-0.08	6.88E-01	0.47	1.26E-02	-0.07	7.33E-01	-0.18	3.76E-01	0.22	2.65E-01
ENSCAFG0000001580	WNK1	pink	VSMC_M5	-0.02	9.40E-01	-0.01	9.68E-01	-0.80	5.52E-07	0.76	4.77E-06	0.69	6.49E-05	0.00	9.92E-01	-0.28	1.61E-01	-0.62	5.56E-04	0.08	7.05E-01	0.32	1.07E-01
ENSCAFG0000003150	C15orf48	darkgreen	VSMC_M4	-0.02	9.40E-01	0.28	1.59E-01	-0.49	9.14E-03	0.88	9.96E-10	0.29	1.40E-01	0.07	7.46E-01	-0.26	1.97E-01	-0.78	1.82E-06	-0.22	2.68E-01	0.60	1.01E-03
ENSCAFG0000001395	FAH	grey	VSMC_M10	-0.02	9.40E-01	0.07	7.13E-01	-0.17	3.95E-01	0.52	5.45E-03	-0.05	8.13E-01	0.15	4.47E-01	-0.04	8.54E-01	-0.39	4.67E-02	-0.11	5.90E-01	0.49	1.02E-02
ENSCAFG0000000644	ULK1	grey	VSMC_M10	-0.02	9.40E-01	0.76	4.31E-06	0.06	7.48E-01	0.56	2.34E-03	-0.24	2.20E-01	0.06	7.70E-01	-0.44	2.16E-02	-0.75	6.10E-06	-0.58	1.45E-03	0.79	7.58E-07
ENSCAFG0000001602	SHROOM4	grey	VSMC_M10	-0.02	9.39E-01	0.25	2.07E-01	-0.13	5.31E-01	0.49	8.99E-03	-0.05	8.02E-01	0.12	5.50E-01	-0.41	3.57E-02	-0.42	3.10E-02	-0.23	2.49E-01	0.46	1.49E-02
ENSCAFG0000001004	PPRC1	darkgrey	VSMC_M8	-0.02	9.39E-01	-0.36	6.73E-02	0.34	7.81E-02	-0.75	6.47E-06	-0.31	1.20E-01	0.26	1.92E-01	0.04	8.54E-01	0.80	6.44E-07	0.22	2.62E-01	-0.51	6.09E-03
ENSCAFG0000001626	RELL1	grey	VSMC_M10	-0.02	9.39E-01	0.17	3.98E-01	0.20	3.28E-01	-0.20	3.27E-01	-0.19	3.53E-01	0.10	6.17E-01	0.26	1.96E-01	0.17	3.89E-01	-0.24	2.22E-01	0.03	8.94E-01
ENSCAFG0000000947	SERINC3	darkgreen	VSMC_M4	-0.02	9.39E-01	0.30	1.25E-01	-0.42	2.71E-02	0.76	4.38E-06	0.33	9.40E-02	-0.10	6.17E-01	-0.44	2.28E-02	-0.79	8.78E-07	-0.11	6.01E-01	0.48	1.16E-02
ENSCAFG0000000528	SYNGR2	darkgreen	VSMC_M4	-0.02	9.39E-01	0.57	2.07E-03	-0.18	3.73E-01	0.71	3.05E-05	0.03	9.01E-01	-0.14	4.96E-01	-0.39	4.58E-02	-0.80	6.39E-07	-0.36	6.15E-02	0.69	7.94E-05
ENSCAFG0000000417	DHX34	grey	VSMC_M10	-0.02	9.38E-01	0.52	5.94E-03	0.38	5.28E-02	0.15	4.44E-01	-0.52	5.37E-03	0.01	9.59E-01	-0.11	5.96E-01	-0.31	1.21E-01	-0.46	1.65E-02	0.62	5.71E-04
ENSCAFG0000001371	TPR	darkolivegreen	VSMC_M9	-0.02	9.37E-01	-0.61	8.04E-04	-0.47	1.44E-02	-0.18	3.60E-01	0.52	5.76E-03	-0.10	6.09E-01	0.10	6.36E-01	0.44	2.31E-02	0.49	1.01E-02	-0.45	1.74E-02
ENSCAFG0000001927	PLXNB3	darkgreen	VSMC_M4	-0.02	9.37E-01	0.49	1.02E-02	-0.21	2.93E-01	0.67	1.13E-04	0.17	4.03E-01	-0.23	2.54E-01	-0.66	1.83E-04	-0.79	1.08E-06	-0.25	2.10E-01	0.46	1.46E-02
ENSCAFG0000000198	TMEM151B	grey	VSMC_M10	-0.02	9.37E-01	0.60	8.51E-04	-0.07	7.11E-01	0.43	2.49E-02	-0.10	6.19E-01	0.26	1.95E-01	-0.12	5.50E-01	-0.53	4.42E-03	-0.52	5.61E-03	0.70	4.22E-05
ENSCAFG0000001676	PCGF3	grey	VSMC_M10	-0.02	9.37E-01	-0.07	7.41E-01	0.25	2.06E-01	-0.27	1.71E-01	-0.18	3.81E-01	0.18	3.58E-01	-0.34	8.62E-02	0.20	3.25E-01	0.14	4.89E-01	-0.23	2.39E-01
ENSCAFG0000003044	KCNA6	grey	VSMC_M10	-0.02	9.37E-01	-0.26	1.88E-01	-0.16	4.13E-01	-0.17	3.96E-01	0.21	3.00E-01	0.49	1.02E-02	0.29	1.44E-01	0.25	2.04E-01	0.16	4.12E-01	-0.29	1.37E-01
ENSCAFG0000002490	MAFB	grey	VSMC_M10	-0.02	9.36E-01	-0.14	4.97E-01	0.11	5.84E-01	-0.35	7.55E-02	0.04	8.52E-01	-0.01	9.49E-01	0.44	2.25E-02	0.28	1.57E-01	0.12	5.68E-01	-0.35	7.51E-02
ENSCAFG0000002332	RASSF9	grey	VSMC_M10	-0.02	9.36E-01	-0.46	1.64E-02	-0.42	2.82E-02	-0.09	6.39E-01	0.54	3.61E-03	-0.06	7.63E-01	0.22	2.73E-01	0.21	2.96E-01	0.41	3.18E-02	-0.46	1.47E-02
ENSCAFG0000000143	PLEC	yellow	VSMC_M3	-0.02	9.35E-01	0.23	2.50E-01	0.79	1.13E-06	-0.59	1.24E-03	-0.83	9.04E-08	0.24	2.22E-01	0.10	6.30E-01	0.45	1.87E-02	-0.30	1.30E-01	0.06	7.85E-01
ENSCAFG0000001700	AURKB	darkgrey	VSMC_M8	-0.02	9.35E-01	-0.38	5.12E-02	0.53	4.12E-03	-0.90	2.46E-10	-0.42	2.93E-02	0.16	4.33E-01	0.39	4.47E-02	0.92	1.49E-11	0.23	2.41E-01	-0.58	1.57E-03
ENSCAFG0000001774	AK7	darkgreen	VSMC_M4	-0.02	9.35E-01	0.24	2.30E-01	-0.43	2.60E-02	0.70	5.21E-05	0.28	1.65E-01	0.32	1.02E-01	-0.25	2.01E-01	-0.65	2.37E-04	-0.11	5.83E-01	0.49	1.00E-02
ENSCAFG0000001640	TMEM229B	grey	VSMC_M10	-0.02	9.35E-01	0.53	4.10E-03	-0.11	5.79E-01	0.45	1.82E-02	-0.08	7.08E-01	-0.17	4.09E-01	-0.19	3.50E-01	-0.47	1.26E-02	-0.49	9.39E-03	0.65	2.30E-04
ENSCAFG0000001088	PPA2	grey	VSMC_M10	-0.02	9.34E-01	-0.27	1.69E-01	0.05	7.88E-01	-0.44	2.25E-02	-0.03	8.73E-01	0.22	2.67E-01	0.38	5.16E-02	0.47	1.43E-02	0.12	5.64E-01	-0.28	1.64E-01
ENSCAFG0000000009	ERBB3	grey	VSMC_M10	-0.02	9.34E-01	0.08	6.82E-01	-0.02	9.34E-01	0.01	9.60E-01	-0.11	5.71E-01	0.06	7.84E-01	0.53	4.24E-03	0.03	8.90E-01	-0.19	3.46E-01	0.31	1.15E-01
ENSCAFG0000001116	UBA7	cyan	VSMC_M2	-0.02	9.34E-01	0.69	7.11E-05	0.46	1.54E-02	-0.08	7.04E-01	-0.54	3.65E-03	-0.04	8.32E-01	0.02	9.02E-01	-0.17	4.03E-01	-0.62	5.25E-04	0.52	5.69E-03
ENSCAFG0000000317	AGBL3	grey	VSMC_M10	-0.02	9.34E-01	0.16	4.17E-01	-0.31	1.17E-01	0.49	8.88E-03	0.16	4.27E-01	0.32	1.05E-01	-0.09	6.38E-01	-0.44	2.26E-02	-0.07	7.32E-01	0.43	2.63E-02
ENSCAFG0000003046	ENSCAFG0000003046	grey	VSMC_M10	-0.02	9.34E-01	0.19	3.36E-01	-0.16	4.35E-01	0.34	8.51E-02	0.02	9.26E-01	-0.01	9.48E-01	0.00	9.85E-01	-0.28	1.59E-01	-0.21	2.99E-01	0.35	7.41E-02
ENSCAFG0000000561	EIF4A3	darkgrey	VSMC_M8	-0.02	9.34E-01	-0.19	3.52E-01	0.63	4.24E-04	-0.81	3.42E-07	-0.55	2.88E-03	0.22	2.67E-01	0.14	5.00E-01	0.80	6.00E-07	0.10	6.06E-01	-0.47	1.23E-02

ENSCAFG0000000989	DTNBP1	grey	VSMC_M10	-0.02	9.34E-01	0.43	2.54E-02	0.34	7.87E-02	-0.15	4.50E-01	-0.28	1.50E-01	-0.22	2.60E-01	0.14	5.01E-01	-0.11	5.87E-01	-0.32	1.02E-01	0.15	4.47E-01
ENSCAFG0000001252	MUC13	grey	VSMC_M10	-0.02	9.34E-01	0.05	8.02E-01	-0.16	4.18E-01	0.23	2.45E-01	0.07	7.21E-01	-0.07	7.47E-01	-0.23	2.44E-01	-0.15	4.50E-01	-0.07	7.43E-01	0.19	3.40E-01
ENSCAFG0000001646	ALDH1A2	grey	VSMC_M10	-0.02	9.34E-01	0.07	7.29E-01	0.09	6.54E-01	-0.09	6.69E-01	-0.17	3.83E-01	0.34	8.68E-02	-0.15	4.68E-01	0.09	6.38E-01	-0.09	6.38E-01	0.07	7.20E-01
ENSCAFG0000001088	CEP72	darkgrey	VSMC_M8	-0.02	9.34E-01	-0.63	4.38E-04	0.03	8.82E-01	-0.64	2.92E-04	0.13	5.27E-01	0.13	5.11E-01	0.33	8.84E-02	0.78	1.51E-06	0.50	7.95E-03	-0.79	1.09E-06
ENSCAFG0000002962	SRSF7	darkgrey	VSMC_M8	-0.02	9.33E-01	-0.34	8.33E-02	0.19	3.34E-01	-0.41	3.36E-02	-0.08	7.08E-01	-0.08	7.01E-01	0.20	3.08E-01	0.56	2.57E-03	0.20	3.13E-01	-0.46	1.48E-02
ENSCAFG0000000655	PRKDC	grey	VSMC_M10	-0.02	9.33E-01	-0.24	2.24E-01	-0.10	6.10E-01	-0.26	1.90E-01	0.18	3.66E-01	-0.05	7.99E-01	0.17	3.94E-01	0.30	1.27E-01	0.17	3.93E-01	-0.30	1.25E-01
ENSCAFG0000003093	TMEM218	grey	VSMC_M10	-0.02	9.33E-01	0.49	1.01E-02	-0.03	8.74E-01	0.55	2.87E-03	-0.23	2.43E-01	0.09	6.72E-01	-0.11	5.94E-01	-0.60	1.06E-03	-0.43	2.39E-02	0.84	5.16E-08
ENSCAFG0000001418	ACSBG1	grey	VSMC_M10	-0.02	9.33E-01	0.44	2.31E-02	0.20	3.17E-01	-0.10	6.29E-01	-0.23	2.53E-01	-0.03	8.92E-01	-0.09	6.47E-01	-0.17	3.92E-01	-0.30	1.23E-01	0.32	1.06E-01
ENSCAFG0000001873	TXNDC2	grey	VSMC_M10	-0.02	9.32E-01	0.05	8.20E-01	-0.14	4.71E-01	0.08	7.03E-01	0.07	7.24E-01	-0.10	6.08E-01	0.02	9.27E-01	-0.10	6.21E-01	-0.04	8.50E-01	0.21	2.94E-01
ENSCAFG0000001277	USP21	grey	VSMC_M10	-0.02	9.32E-01	0.63	3.78E-04	0.25	2.16E-01	0.18	3.60E-01	-0.47	1.29E-02	0.06	7.83E-01	0.02	9.28E-01	-0.34	8.63E-02	-0.65	2.80E-04	0.79	9.43E-07
ENSCAFG0000000037	TAB2	pink	VSMC_M5	-0.02	9.32E-01	-0.24	2.38E-01	-0.86	1.28E-08	0.57	1.85E-03	0.87	5.44E-09	-0.12	5.50E-01	-0.13	5.28E-01	-0.40	4.05E-02	0.28	1.57E-01	-0.04	8.42E-01
ENSCAFG0000001962	CCDC78	darkgreen	VSMC_M4	-0.02	9.32E-01	0.46	1.63E-02	-0.12	5.51E-01	0.60	1.04E-03	-0.01	9.47E-01	-0.16	4.30E-01	-0.12	5.50E-01	-0.65	2.38E-04	-0.39	4.50E-02	0.66	1.98E-04
ENSCAFG0000001690	FGF18	grey	VSMC_M10	-0.02	9.32E-01	0.27	1.66E-01	-0.32	1.00E-01	0.49	9.22E-03	0.12	5.63E-01	0.03	8.80E-01	-0.09	6.55E-01	-0.45	1.88E-02	-0.28	1.56E-01	0.56	2.65E-03
ENSCAFG0000000753	ADM	darkgreen	VSMC_M4	-0.02	9.32E-01	0.33	9.78E-02	-0.26	1.87E-01	0.66	1.86E-04	0.14	4.93E-01	0.06	7.48E-01	-0.01	9.65E-01	-0.69	6.89E-05	-0.24	2.28E-01	0.52	5.47E-03
ENSCAFG0000000835	PKIA	grey	VSMC_M10	-0.02	9.32E-01	0.03	8.76E-01	-0.22	2.69E-01	0.26	1.98E-01	0.30	1.33E-01	-0.42	2.83E-02	-0.25	2.12E-01	-0.21	3.04E-01	0.03	8.79E-01	-0.04	8.42E-01
ENSCAFG0000003058	ENSCAFG0000003058	grey	VSMC_M10	-0.02	9.31E-01	-0.09	6.42E-01	0.15	4.51E-01	-0.08	6.94E-01	0.02	9.33E-01	-0.24	2.19E-01	0.24	2.29E-01	0.04	8.52E-01	0.15	4.58E-01	-0.32	1.02E-01
ENSCAFG0000002862	ENSCAFG0000002862	grey	VSMC_M10	-0.02	9.31E-01	-0.09	6.65E-01	0.27	1.79E-01	-0.50	7.77E-03	-0.31	1.18E-01	0.26	1.82E-01	0.10	6.27E-01	0.50	7.75E-03	0.04	8.40E-01	-0.11	5.79E-01
ENSCAFG0000001572	SLC4A3	grey	VSMC_M10	-0.02	9.31E-01	0.15	4.58E-01	-0.31	1.19E-01	0.37	5.40E-02	0.25	2.07E-01	-0.01	9.52E-01	-0.08	7.03E-01	-0.46	1.68E-02	-0.05	8.17E-01	0.31	1.13E-01
ENSCAFG0000000766	ARL6IP4	yellow	VSMC_M3	-0.02	9.30E-01	0.03	8.66E-01	0.55	2.99E-03	-0.26	1.85E-01	-0.55	3.03E-03	-0.04	8.40E-01	0.29	1.37E-01	0.20	3.09E-01	-0.07	7.27E-01	-0.03	8.94E-01
ENSCAFG0000000300	RLF	pink	VSMC_M5	-0.02	9.30E-01	-0.41	3.41E-02	-0.70	4.18E-05	0.47	1.34E-02	0.61	7.24E-04	0.02	9.34E-01	0.00	9.97E-01	-0.22	2.66E-01	0.34	8.20E-02	0.04	8.46E-01
ENSCAFG0000000553	PPME1	grey	VSMC_M10	-0.02	9.30E-01	-0.22	2.71E-01	-0.28	1.58E-01	0.22	2.77E-01	0.47	1.26E-02	-0.28	1.50E-01	-0.18	3.61E-01	-0.14	4.75E-01	0.32	1.05E-01	-0.37	5.96E-02
ENSCAFG0000001627	TECR	grey	VSMC_M10	-0.02	9.30E-01	0.54	3.72E-03	0.33	9.57E-02	-0.12	5.60E-01	-0.46	1.59E-02	0.17	3.96E-01	-0.08	7.05E-01	-0.07	7.42E-01	-0.48	1.18E-02	0.42	2.93E-02
ENSCAFG0000000140	PODXL	grey	VSMC_M10	-0.02	9.30E-01	0.57	1.84E-03	0.14	4.86E-01	0.30	1.25E-01	-0.32	9.82E-02	0.52	5.63E-03	-0.08	7.04E-01	-0.40	3.64E-02	-0.51	6.27E-03	0.68	9.28E-05
ENSCAFG0000001940	SPPL2B	grey	VSMC_M10	-0.02	9.29E-01	0.01	9.50E-01	0.39	4.59E-02	-0.48	1.20E-02	-0.42	2.87E-02	0.29	1.38E-01	0.30	1.32E-01	0.40	3.97E-02	-0.13	5.19E-01	-0.06	7.55E-01
ENSCAFG0000002893	IGSF8	yellow	VSMC_M3	-0.02	9.29E-01	0.53	4.37E-03	0.65	2.45E-04	-0.10	6.18E-01	-0.68	1.01E-04	-0.01	9.55E-01	-0.33	9.69E-02	-0.12	5.46E-01	-0.44	2.13E-02	0.31	1.13E-01
ENSCAFG0000000581	EFHB	grey	VSMC_M10	-0.02	9.28E-01	-0.32	9.90E-02	-0.07	7.21E-01	-0.15	4.65E-01	0.00	9.82E-01	0.41	3.41E-02	0.10	6.10E-01	0.27	1.80E-01	0.27	1.74E-01	-0.19	3.38E-01
ENSCAFG0000001948	TCF3	cyan	VSMC_M2	-0.02	9.28E-01	0.56	2.14E-03	0.40	3.96E-02	0.00	9.91E-01	-0.54	3.65E-03	0.16	4.32E-01	-0.09	6.56E-01	-0.14	4.83E-01	-0.54	4.01E-03	0.56	2.16E-03
ENSCAFG0000001998	PRRX2	grey	VSMC_M10	-0.02	9.27E-01	0.32	1.06E-01	0.16	4.37E-01	0.22	2.66E-01	-0.17	4.00E-01	0.05	8.17E-01	-0.07	7.42E-01	-0.27	1.68E-01	-0.22	2.80E-01	0.26	1.93E-01
ENSCAFG0000002861	TMPRSS11E	darkgreen	VSMC_M4	-0.02	9.27E-01	-0.03	8.98E-01	-0.38	5.00E-02	0.50	8.40E-03	0.29	1.38E-01	-0.11	5.81E-01	-0.21	2.88E-01	-0.33	9.03E-02	0.02	9.19E-01	0.22	2.71E-01
ENSCAFG0000000443	CHCHD4	darkgrey	VSMC_M8	-0.02	9.27E-01	-0.39	4.16E-02	0.20	3.13E-01	-0.52	5.15E-03	-0.13	5.13E-01	0.15	4.66E-01	0.17	3.97E-01	0.61	7.40E-04	0.28	1.58E-01	-0.51	7.08E-03
ENSCAFG0000003074	SRSF12	grey	VSMC_M10	-0.02	9.27E-01	0.10	6.08E-01	0.07	7.44E-01	0.20	3.14E-01	-0.10	6.28E-01	0.19	3.30E-01	-0.16	4.14E-01	-0.20	3.16E-01	0.02	9.17E-01	0.09	6.59E-01
ENSCAFG0000000840	AUP1	grey	VSMC_M10	-0.02	9.27E-01	0.00	9.86E-01	0.25	2.04E-01	-0.34	7.86E-02	-0.27	1.76E-01	0.20	3.06E-01	-0.16	4.26E-01	0.32	1.04E-01	-0.03	8.96E-01	-0.15	4.45E-01
ENSCAFG0000001753	SNAPIN	grey	VSMC_M10	-0.02	9.27E-01	0.40	3.91E-02	0.56	2.35E-03	0.01	9.66E-01	-0.72	2.28E-05	0.25	2.14E-01	-0.05	7.93E-01	-0.09	6.58E-01	-0.40	3.80E-02	0.44	2.14E-02
ENSCAFG0000001255	PRKD1	darkgreen	VSMC_M4	-0.02	9.27E-01	0.35	7.42E-02	-0.43	2.39E-02	0.70	5.00E-05	0.27	1.78E-01	-0.11	5.92E-01	-0.52	5.30E-03	-0.67	1.46E-04	-0.24	2.27E-01	0.58	1.67E-03
ENSCAFG0000001070	MXI1	darkgreen	VSMC_M4	-0.02	9.26E-01	0.13	5.28E-01	-0.67	1.29E-04	0.74	1.04E-05	0.65	2.57E-04	-0.18	3.60E-01	-0.17	4.02E-01	-0.72	2.12E-05	0.04	8.52E-01	0.31	1.19E-01
ENSCAFG0000000532	TGDS	grey	VSMC_M10	-0.02	9.26E-01	-0.58	1.37E-03	-0.35	6.93E-02	-0.11	6.01E-01	0.26	1.84E-01	0.16	4.35E-01	0.44	2.24E-02	0.37	5.59E-02	0.37	5.43E-02	-0.17	3.87E-01
ENSCAFG0000001731	TPM3	darkgrey	VSMC_M8	-0.02	9.26E-01	-0.45	1.95E-02	0.13	5.31E-01	-0.71	3.68E-05	-0.05	8.06E-01	0.17	4.10E-01	0.15	4.67E-01	0.82	1.50E-07	0.33	9.51E-02	-0.61	7.32E-04
ENSCAFG0000000333	ABCA13	grey	VSMC_M10	-0.02	9.26E-01	0.23	2.45E-01	-0.07	7.24E-01	0.29	1.47E-01	-0.05	8.18E-01	-0.07	7.17E-01	-0.13	5.17E-01	-0.27	1.65E-01	-0.23	2.53E-01	0.31	1.12E-01
ENSCAFG0000002966	ENSCAFG0000002966	grey	VSMC_M10	-0.02	9.26E-01	0.23	2.45E-01	-0.07	7.24E-01	0.29	1.47E-01	-0.05	8.18E-01	-0.07	7.17E-01	-0.13	5.17E-01	-0.27	1.65E-01	-0.23	2.53E-01	0.31	1.12E-01
ENSCAFG0000003028	ENSCAFG0000003028	darkgreen	VSMC_M4	-0.02	9.25E-01	0.26	1.91E-01	-0.42	3.01E-02	0.61	7.16E-04	0.20	3.06E-01	0.15	4.62E-01	0.17	3.89E-01	-0.58	1.41E-03	-0.24	2.22E-01	0.63	3.82E-04
ENSCAFG0000000282	GSDME	grey	VSMC_M10	-0.02	9.25E-01	0.38	4.85E-02	0.31	1.18E-01	0.12	5.44E-01	-0.33	9.60E-02	0.06	7.71E-01	-0.05	7.94E-01	-0.23	2.53E-01	-0.30	1.34E-01	0.23	2.49E-01
ENSCAFG0000001240	KHNYN	yellow	VSMC_M3	-0.02	9.25E-01	-0.07	7.43E-01	0.62	5.85E-04	-0.53	4.30E-03	-0.47	1.42E-02	-0.18	3.56E-01	0.02	9.22E-01	0.44	2.32E-02	0.05	8.16E-01	-0.39	4.71E-02
ENSCAFG0000000911	ABRAXAS1	grey	VSMC_M10	-0.02	9.25E-01	-0.28	1.54E-01	-0.44	2.20E-02	0.48	1.15E-02	0.39	4.42E-02	-0.24	2.37E-01	-0.13	5.15E-01	-0.27	1.74E-01	0.24	2.36E-01	0.04	8.53E-01
ENSCAFG0000002848	ENSCAFG0000002848	grey	VSMC_M10	-0.02	9.25E-01	-0.12	5.60E-01	0.07	7.38E-01	-0.10	6.11E-01	-0.20	3.28E-01	0.04	8.44E-01	0.43	2.40E-02	0.14	4.99E-01	0.02	9.23E-01	0.15	4.46E-01
ENSCAFG0000003112	ENSCAFG0000003112	grey	VSMC_M10	-0.02	9.25E-01	0.27	1.69E-01	-0.05	7.92E-01	0.19	3.34E-01	-0.11	5.76E-01	0.09	6.61E-01	-0.02	9.10E-01	-0.18	3.57E-01	-0.31	1.18E-01	0.43	2.59E-02
ENSCAFG0000000657	FAM19A4	grey	VSMC_M10	-0.02	9.25E-01	0.20	3.16E-01	0.30	1.29E-01	-0.23	2.55E-01	-0.40	3.73E-02	-0.17	3.83E-01	0.27	1.76E-01	0.15	4.47E-01	-0.22	2.73E-01	0.23	2.42E-01
ENSCAFG0000001224	ZNF831	grey	VSMC_M10	-0.02	9.25E-01	0.21	2.82E-01	-0.28	1.50E-01	0.47	1.41E-02	0.12	5.49E-01	0.04	8.36E-01	-0.01	9.76E-01	-0.35	7.50E-02	-0.22	2.78E-01	0.48	1.04E-02
ENSCAFG0000002604	ENSCAFG0000002604	grey	VSMC_M10	-0.02	9.25E-01	-0.13	5.28E-01	-0.03	8.67E-01	-0.08	6.85E-01	0.06	7.78E-01	0.03	8.76E-01	-0.02	9.26E-01	0.06	7.74E-01	0.13	5.14E-01	-0.04	8.39E-01
ENSCAFG0000000010	ENSCAFG0000000010	darkgrey	VSMC_M8	-0.02	9.25E-01	-0.63	4.91E-04	0.21	2.85E-01	-0.79	9.42E-07	-0.09	6.57E-01	0.09	6.65E-01	0.29	1.37E-01	0.94	3.77E-13	0.46	1.59E-02	-0.78	1.73E-06
ENSCAFG0000002314	PRSS48	darkgreen	VSMC_M4	-0.02	9.24E-01	0.19	3.34E-01	-0.39	4.25E-02	0.66	1.65E-04	0.25	2.06E-01	-0.09	6.59E-01	-0.16	4.30E-01	-0.60	8.32E-04	-0.17	4.00E-01	0.47	1.41E-02
ENSCAFG0000002624	ENSCAFG0000002624	grey	VSMC_M10	-0.02	9.24E-01	0.09	6.55E-01	0.00	1.00E+00	-0.04	8.61E-01	0.00	1.00E+00	-0.21	3.04E-01	0.28	1.58E-01	-0.01	9.57E-01	-0.11	5.72E-01	0.07	7.28E-01
ENSCAFG0000000788	PCSK1	grey	VSMC_M10	-0.02	9.24E-01	0.05	8.04E-01	-0.30	1.22E-01	0.41	3.15E-02	0.18	3.68E-01	0.21	3.02E-01	-0.06	7.81E-01	-0.33	9.78E-02	-0.01	9.42E-01	0.30	1.24E-01
ENSCAFG0000000354	NCDN	grey	VSMC_M10	-0.02	9.24E-01	0.25	2.13E-01	0.32	1.08E-01	0.11	5.72E-01	-0.47	1.38E-02	0.23	2.41E-01	-0.04	8.41E-01	-0.21	2.97E-01	-0.21	2.95E-01	0.41	3.32E-02
ENSCAFG0000000785	HAND2	grey	VSMC_M10	-0.02	9.24E-01	-0.21	2.83E-01	-0.37	5.63E-02	0.37	6.04E-02	0.32	1.09E-01	0.12	5.59E-01	0.14	4.85E-01	-0.30	1.23E-01	0.22	2.72E-01	0.05	7.95E-01
ENSCAFG0000001982	GNAT2	grey	VSMC_M10	-0.02	9.23E-01	0.37	5.61E-02	0.11	5.76E-01	0.02	9.14E-01	-0.15	4.59E-01	-0.06	7.75E-01	-0.12	5.48E-01	-0.26	1.82E-01	-0.29	1.50E-01	0.28	1.64E-01
ENSCAFG0000003075	ENSCAFG0000003075	grey	VSMC_M10	-0.02	9.23E-01	-0.16	4.34E-01	-0.06	7.63E-01	-0.17	3.88E-01	-0.01	9.80E-01	0.15	4.68E-01	0.37	5.63E-02	0.16	4.32E-01	0.14	4.92E-01	-0.02	9.39E-01
ENSCAFG0000001783	CCDC33	violet	VSMC_M7	-0.02	9.23E-01	-0.15	4.51E-01	0.09	6.54E-01	-0.19	3.41E-01	-0.12	5.36E-01	-0.03	8.91E-01	0.84	4.62E-08	0.23	2.53E-01	0.01	9.43E-01	-0.04	8.48E-01
ENSCAFG0000001546	TAS1R2	grey	VSMC_M10	-0.02	9.23E-01	0.54	3.68E-03	0.34	8.71E-02	0.17	3.84E-01	-0.36	6.26E-02	0.00	1.00E+00	-0.36	6.79E-02	-0.38	5.24E-02	-0.34	8.18E-02	0.40	3.78E-02
ENSCAFG0000001367	RUFY2	grey	VSMC_M10	-0.02	9.23E-01	0.15	4.45E-01	0.26	1.85E-01	-0.18	3.65E-01	-0.42	3.03E-02	0.09	6.40E-01	0.23	2.46E-01	0.20	3.15E-01	-0.25	2.16E-01	0.32	1.04E-01
ENSCAFG0000000360	GJB4	violet	VSMC_M7	-0.02	9.23E-01	-0.15	4.57E-01	0.13	5.17E-01	-0.24	2.35E-01	-0.16	4.24E-01	0.05	8.13E-01	0.87	3.80E-09	0.27	1.76E-01	0.02	9.23E-01	-0.04	8.30E-01
ENSCAFG0000000113	EPB41L2	grey	VSMC_M10	-0.02	9.22E-01	0.13	5.11E-01	0.36	6.90E-02	-0.41	3.43E-02	-0.27	1.81E-01	-0.21	2.91E-01	0.55	2.82E-03	0.30	1.32E-01	-0.18	3.69E-01	-0.10	6.19E-01
ENSCAFG0000000842	DQX1	grey	VSMC_M10	-0.02	9.22E-01	-0.03	8.99E-01	-0.02	9.20E-01	-0.20	3.12E-01	-0.01	9.49E-01	0.11	5.70E-01	0.09	6.54E-01	0.15	4.62E-01	0.03	8.95E-01	-0.05	7.89E-01
ENSCAFG0000002334	CALHM6	violet	VSMC_M7	-0.02	9.21E-01	-0.05	8.08E-01	0.03	8.66E-01	-0.22	2.76E-01	-0.12	5.44E-01	0.22	2.74E-01	0.59	1.34E-03	0.24	2.34E-01	-0.09	6.48E-01	0.05	7.97E-01
ENSCAFG0000001838	SNX18	yellow	VSMC_M3	-0.02	9.21E-01	0.40	3.77E-02	0.42	2.87E-02	-0.28	1.62E-01	-0.37	5.90E-02	0.05	8.00E-01	0.09	6.73E-01	0.01	9.79E-01	-0.32	1.09E-01	0.12	5.46E-01
ENSCAFG0000001705	PPIB	darkgrey	VSMC_M8	-0.02	9.21E-01	-0.06	7.62E-01	0.69	6.11E-05	-0.79	8.62E-07	-0.64	3.25E-04	0.04	8.48E-01	0.46	1.54E-02	0.71	2.92E-05	-0.06	7.51E-01	-0.29	1.40E-01
ENSCAFG0000000520	RBM17	darkgrey	VSMC_M8	-0.02	9.21E-01	-0.25	2.01E-01	0.48	1.04E-02	-0.75	7.93E-06	-0.43	2.64E-02	-0.02	9.10E-01	0.36	6.16E-02	0.79	1.10E-06	0.07	7.11E-01	-0.37	5.67E-02
ENSCAFG0000001823	MCOLN1	cyan	VSMC_M2	-0.02	9.21E-01	0.77	2.43E-06	0.38	5.30E-02	0.23	2.46E-01	-0.45	1.88E-02	0.02	9.02E-01	-0.39	4.43E-02	-0.54	3.85E-03	-0.57	1.72E-03	0.57	2.02E-03
ENSCAFG0000001936	ENSCAFG0000001936	yellow	VSMC_M3	-0.02	9.21E-01	0.32	1.03E-01	0.65	2.25E-04	-0.41	3.27E-02	-0.76	4.34E-06	0.18	3.57E-01	0.20	3.18E-01	0.28	1.52E-01	-0.40	4.03E-02	0.25	2.13E-01
ENSCAFG0000001053	TRMT10A	grey	VSMC_M10	-0.02	9.21E-01	-0.23	2.42E-01	-0.18	3.74E-01	-0.06	7.63E-01	0.28	1.62E-01	-0.39	4.36E-02	0.28	1.65E-01	0.07	7.40E-01	0.20	3.24E-01	-0.17	3.94E-01
ENSCAFG0000000905	CKAP5	darkgrey	VSMC_M8	-0.02	9.20E-01	-0.59	1.24E-03	-0.14	5.00E-01	-0.43	2.59E-02	0.11	5.75E-01	0.06	7.54E-01	0.46	1.46E-02	0.67	1.32E-04	0.36	6.45E-02	-0.35	7.37E-02
ENSCAFG0000002852	IFNLR1	grey	VSMC_M10	-0.02	9.20E-01	0.37	5.65E-02	-0.27	1.76E-01	0.32	9.84E-02	0.18	3.82E-01	-0.22	2.72E-01	-0.01	9.70E-01	-0.40	3.89E-02	-0.35	7.53E-02	0.41	3.35E-02
ENSCAFG0000001265	ENSCAFG0000001265	darkgreen	VSMC_M4	-0.02	9.20E-01	0.34	7.91E-02	-0.32	9.89E-02	0.69	7.70E-05	0.16	4.40E-01	-0.08	6.95E-01	-0.25	2.02E-01	-0.61	7.12E-04	-0.29	1.47E-01	0.55	2.79E-03
ENSCAFG0000000112	ENSCAFG0000000112	grey	VSMC_M10	-0.02	9.20E-01	-0.11	6.00E-01	-0.04	8.28E-01	-0.17	3.96E-01	0.00	9.95E-01	0.19	3.34E-01	0.05	7.87E-01	0.15	4.60E-01	0.08	6.82E-01	-0.02	9.07E-01
ENSCAFG0000000126	ADGRB1	darkgreen	VSMC_M4	-0.02	9.20E-01	0.33	9.37E-02	-0.48	1.07E-02	0.87	5.37E-09	0.30	1.33E-01	-0.06	7.63E-01	-0.32	9.85E-02	-0.81	2.37E-07	-0.24	2.28E-01	0.59	1.09E-03
ENSCAFG0000000716	SMIM15	grey	VSMC_M10	-0.02	9.19E-01	-0.04	8.41E-01	-0.07	7.22E-01	-0.10	6.26E-01	0.24	2.27E-01	-0.15	4.50E-01	-0.23	2.51E-01	0.04	8.35E-01	0.08	6.88E-01	-0.35	7.07E-02
ENSCAFG0000002483	ENSCAFG0000002483	grey	VSMC_M10	-0.02	9.19E-01	-0.14	4.88E-01	0.50	8.12E-03	-0.56	2.27E-03	-0.38	4.97E-02	0.04	8.32E-01	0.19	3.33E-01	0.53	4.52E-03	0.09	6.63E-01	-0.40	4.08E-02
ENSCAFG0000001522	MAP1S	cyan	VSMC_M2	-0.02	9.19E-01	0.52	5.38E-03	0.54	3.44E-03	-0.17	3.86E-01	-0.62	6.23E-04	0.20	3.16E-01	-0.43	2.59E-02	-0.01	9.50E-01	-0.40	3.83E-02	0.30	1.24E-01
ENSCAFG0000000432	RSBN1L	darkgreen	VSMC_M4	-0.02	9.19E-01	0.14	4.88E-01	-0.60	9.35E-04	0.77	2.55E-06	0.55	2.69E-03	-0.35	7.07E-02	-0.25	2.08E-01	-0.64	3.12E-04	-0.05	8.08E-01	0.32	1.03E-01
ENSCAFG0000002380	ENSCAFG0000002380	turquoise	VSMC_M6	-0.02	9.19E-01	-0.16	4.16E-01	-0.08	6.93E-01	-0.08	6.74E-01	0.03	8.96E-01	0.28	1.53E-01	-0.10	6.22E-01	0.12	5.66E-01	0.09	6.71E-01	-0.03	8.78E-01
ENSCAFG0000001648	ENSCAFG0000001648	darkgrey	VSMC_M8	-0.02	9.18E-01	-0.57	1.94E-03	0.33	9.39E-02	-0.88	1.51E-09	-0.16	4.37E-01	0.09	6.66E-01	0.38	5.35E-02	0.93	2.05E-12	0.44	2.30E-02	-0.78	1.62E-06
ENSCAFG0000000608	PCDHGC5	grey	VSMC_M10	-0.02	9.18E-01	0.57	1.96E-03	0.05	8.03E-01	0.25	2.00E-01	-0.03	8.69E-01	-0.17	3.94E-01	-0.11	5.91E-01	-0.50	7.67E-03	-0.41	3.38E-02	0.38	5.32E-02
ENSCAFG0000001747	CLN6	grey	VSMC_M10	-0.02	9.18E-01	0.65	2.28E-04	0.31	1.14E-01	0.05	7.95E-01	-0.37	5.52E-02	0.11	5.96E-01	-0.12	5.54E-01	-0.35	7.49E-02	-0.50	8.33E-03	0.45	1.76E-02
ENSCAFG0000000143	PICK1	grey	VSMC_M10	-0.02	9.18E-01	0.54	3.48E-03	-0.01	9.62E-01	0.50	8.65E-03	-0.14	4.93E-01	0.00	9.95E-01	-0.06	7.82E-01	-0.60	8.34E-04	-0.43	2.41E-02	0.69	6.58E-05
ENSCAFG0000000660	SMUG1	yellow	VSMC_M3	-0.02	9.18E-01	0.37	5.99E-02	0.79	8.82E-07	-0.41	3.44E-02	-0.83	9.27E-08	0.11	5.94E-01	-0.11	5.95E-01	0.21	2.85E-01	-0.36	6.59E-02	0.17	4.09E-01
ENSCAFG0000001891	SOCS1	grey	VSMC_M10	-0.02	9.18E-01	-0.12	5.47E-01	-0.13	5.16E-01	-0.11	5.80E-01	0.25	2.05E-01	-0.13	5.21E-01	0.22	2.74E-01	0.03	8.67E-01	0.15	4.52E-01	-0.27	1.80E-01
ENSCAFG0000001596	MTHFD1	darkgrey	VSMC_M8	-0.02	9.17E-01	-0.53	4.32E-03	0.30	1.25E-01	-0.84	3.03E-08	-0.19	3.36E-01	0.12	5.64E-01	0.41	3.27E-02	0.93	2.17E-12	0.35	7.74E-02	-0.67	1.13E-04
ENSCAFG0000001312	INSYN2	darkgreen	VSMC_M4	-0.02	9.17E-01	0.29	1.36E-01	-0.44	2.31E-02	0.76	3.62E-06	0.29	1.42E-01	-0.14	4.81E-01	-0.34	8.65E-02	-0.81	3.63E-07	-0.13	5.16E-01	0.54	3.69E-03
ENSCAFG0000001012	RAD54L2	grey	VSMC_M10	-0.02	9.17E-01	-0.12	5.45E-01	-0.43	2.38E-02	0.42	2.73E-02	0.32	1.08E-01	0.36	6.63E-02	-0.29	1.44E-01	-0.28	1.54E-01	0.15	4.46E-01	0.15	4.49E-01
ENSCAFG0000001778	PAPOLA	grey	VSMC_M10	-0.02	9.17E-01	-0.27	1.76E-01	-0.57	1.72E-03	0.10	6.13E-01	0.51	7.05E-03	0.23	2.52E-01	0.15	4.47E-01	0.09	6.66E-01	0.17	3.87E-01	-0.05	8.04E-01
ENSCAFG0000001177	GPR146	turquoise	VSMC_M6	-0.02	9.17E-01	0.04	8.46E-01	0.08	6.77E-01	-0.13	5.15E-01	-0.22	2.65E-01	0.63	4.87E-04	-0.10	6.29E-01	0.14	4.79E-01	-0.05	8.17E-01	0.11	5.94E-01
ENSCAFG0000001495	ENSCAFG0000001495	grey	VSMC_M10	-0.02	9.17E-01	-0.11	5.76E-01	-0.21	2.95E-01	0.08	6.82E-01	0.07	7.16E-01	0.28	1.59E-01	0.18	3.60E-01	-0.01	9.62E-01	0.00	9.81E-01	0.19	3.39E-01
ENSCAFG0000000951	HSD17B13	grey	VSMC_M10	-0.02	9.16E-01	0.12	5.65E-01	-0.23	2.42E-01	0.36	6.68E-02	0.05	8.03E-01	0.20	3.14E-01	0.19	3.34E-01	-0.30	1.35E-01	-0.13	5.28E-01	0.38	5.12E-02
ENSCAFG0000000587	ALYREF	darkgrey	VSMC_M8	-0.02	9.16E-01	-0.39	4.26E-02	0.46	1.70E-02	-0.67	1.22E-04	-0.40	4.07E-02	0.23	2.58E-01	0.32	1.07E-01	0.75	6.96E-06	0.29	1.45E-01	-0.49	8.80E-03
ENSCAFG0000002880	ANKRD66	grey	VSMC_M10	-0.02	9.16E-01	0.12	5.52E-01	-0.03	8.88E-01	-0.06	7.75E-01	0.01	9.69E-01	-0.08	6.92E-01	0.22	2.71E-01	-0.02	9.40E-01	-0.13	5.11E-01	0.15	4.47E-01
ENSCAFG0000001846	COPS3	darkgrey	VSMC_M8	-0.02	9.16E-01	-0.63	3.92E-04	-0.21	3.00E-01	-0.41	3.36E-02	0.27	1.74E-01	0.16	4.33E-01	0.14	4.71E-01	0.65	2.36E-04	0.50	8.63E-03	-0.60	1.02E-03
ENSCAFG0000001670	KIAA1191	darkgreen	VSMC_M4	-0.02	9.16E-01	0.04	8.28E-01	-0.68	1.05E-04	0.87	3.34E-09	0.57	1.82E-03	-0.12	5.62E-01	-0.45	1.80E-02	-0.78	1.32E-06	0.08	7.08E-01	0.35	7.40E-02
ENSCAFG0000001678	DNAH2	grey	VSMC_M10	-0.02	9.16E-01	-0.16	4.32E-01	0.10	6.37E-01	-0.15	4.44E-01	-0.13	5.06E-01	-0.08	7.00E-01	0.49	8.86E-03	0.23	2.51E-01	0.06	7.76E-01	-0.06	7.76E-01
ENSCAFG0000000073	LYRM7	grey	VSMC_M10	-0.02	9.16E-01	-0.44	2.26E-02	-0.04	8.55E-01	-0.25	2.03E-01	0.19	3.55E-01	-0.26	1.98E-01	0.13	5.29E-01	0.30	1.31E-01	0.38	5.19E-02	-0.55	2.99E-03
ENSCAFG0000002338	CCDC38	darkgreen	VSMC_M4	-0.02	9.16E-01	0.15	4.67E-01	-0.34	7.96E-02	0.55	2.68E-03	0.19	3.40E-01	0.12	5.63E-01	-0.23	2.59E-01	-0.41	3.37E-02	-0.15	4.52E-01	0.39	4.64E-02
ENSCAFG0000000106	ENSCAFG0000000106	grey	VSMC_M10	-0.02	9.15E-01	-0.19	3.39E-01	-0.31	1.21E-01	0.02	9.37E-01	0.35	7.23E-02	-0.05	8.12E-01	0.35	7.39E-02	0.10	6.15E-01	0.14	4.99E-01	-0.13	5.23E-01
ENSCAFG0000001374	ODR4	pink	VSMC_M5	-0.02	9.15E-01	-0.23	2.54E-01	-0.65	2.21E-04	0.56	2.45E-03	0.55	2.88E-03	-0.13	5.13E-01	-0.24	2.35E-01	-0.37	6.00E-02	0.24	2.20E-01	0.17	3.94E-01
ENSCAFG0000001443	PHB2	yellow	VSMC_M3	-0.02	9.15E-01	0.19	3.43E-01	0.85	2.01E-08	-0.70	4.47E-05	-0.83	9.85E-08	0.19	3.39E-01	0.19	3.45E-01	0.54	3.74E-03	-0.23	2.39E-01	-0.10	6.31E-01
ENSCAFG0000001215	LPGAT1	grey	VSMC_M10	-0.02	9.15E-01	-0.28	1.55E-01	-0.01	9.43E-01	-0.44	2.15E-02	0.21	2.93E-01	-0.33	9.35E-02	0.38	5.19E-02	0.37	5.66E-02	0.28	1.59E-01	-0.49	9.25E-03
ENSCAFG0000001254	HEG1	cyan	VSMC_M2	-0.02	9.14E-01	0.63	3.77E-04	-0.25	2.11E-01	0.71	3.72E-05	0.10	6.36E-01	-0.21	2.82E-01	-0.48	1.09E-02	-0.78	1.69E-06	-0.50	7.89E-03	0.70	4.13E-05
ENSCAFG0000000959	NELL2	grey	VSMC_M10	-0.02	9.14E-01	-0.29	1.37E-01	-0.08	7.10E-01	-0.14	5.01E-01	0.03	8.76E-01	-0.04	8.44E-01	-0.15	4.58E-01	0.26	1.91E-01	0.22	2.65E-01	-0.17	3.98E-01
ENSCAFG0000000141	KIF27	darkgrey	VSMC_M8	-0.02	9.14E-01	-0.40	3.85E-02	-0.06	7.61E-01	-0.46	1.46E-02	0.05	8.01E-01	0.14	4.98E-01	0.54	3.54E-03	0.62	5.58E-04	0.17	3.92E-01	-0.30	1.24E-01
ENSCAFG0000001947	NDOR1	cyan	VSMC_M2	-0.02	9.13E-01	0.45	1.80E-02	0.47	1.26E-02	-0.21	3.04E-01	-0.58	1.47E-03	0.24	2.25E-01	0.10	6.25E-01	0.03	8.70E-01	-0.49	9.35E-03	0.36	6.84E-02
ENSCAFG0000001792	UBL7	grey	VSMC_M10	-0.02	9.13E-01	0.62	5.23E-04	0.44	2.08E-02	0.17	3.96E-01	-0.64	3.49E-04	0.17	3.85E-01	0.01	9.58E-01	-0.36	6.49E-02	-0.57	1.80E-03	0.72	1.93E-05
ENSCAFG0000000014	CCDC68	grey	VSMC_M10	-0.02	9.13E-01	0.12	5.40E-01	-0.20	3.08E-01	0.24	2.26E-01	0.06	7.77E-01	0.26	1.98E-01	-0.07	7.14E-01	-0.17	3.91E-01	-0.12	5.62E-01	0.29	1.48E-01
ENSCAFG0000002038	ST6GALNAC3	darkgreen	VSMC_M4	-0.02	9.12E-01	-0.10	6.12E-01	-0.48	1.18E-02	0.54	3.49E-03	0.36	6.24E-02	-0.10	6.20E-01	-0.04	8.33E-01	-0.38	4.85E-02	0.10	6.19E-01	0.26	1.90E-01
ENSCAFG0000002010	ENG	grey	VSMC_M10	-0.02	9.12E-01	0.74	9.16E-06	0.23	2.58E-01	0.40	3.74E-02	-0.39	4.31E-02	0.34	8.67E-02	-0.51	7.17E-03	-0.59	1.21E-03	-0.60	9.43E-04	0.69	6.65E-05
ENSCAFG0000000563	KAT6A	pink	VSMC_M5	-0.02	9.12E-01	-0.23	2.53E-01	-0.74	1.07E-05	0.42	2.98E-02	0.78	1.40E-06	-0.33	9.19E-02	0.02	9.40E-01	-0.28	1.54E-01	0.24	2.31E-01	-0.08	7.06E-01
ENSCAFG0000001768	COG7	grey	VSMC_M10	-0.02	9.12E-01	0.27	1.69E-01	0.31	1.10E-01	-0.28	1.65E-01	-0.38	4.83E-02	0.10	6.23E-01	0.01	9.55E-01	0.19	3.45E-01	-0.25	2.17E-01	0.12	5.66E-01
ENSCAFG0000000407	TCTE3	grey	VSMC_M10	-0.02	9.12E-01	0.23	2.40E-01	0.17	4.09E-01	-0.11	5.88E-01	-0.16	4.27E-01	0.07	7.17E-01	0.07	7.16E-01	0.03	8.80E-01	-0.19	3.49E-01	0.13	5.11E-01
ENSCAFG0000000923	AMIGO2	grey	VSMC_M10	-0.02	9.12E-01	0.23	2.40E-01	0.17	4.09E-01	-0.11	5.88E-01	-0.16	4.27E-01	0.07	7.17E-01	0.07	7.16E-01	0.03	8.80E-01	-0.19	3.49E-01	0.13	5.11E-01
ENSCAFG0000001637	AADACL4	grey	VSMC_M10	-0.02	9.12E-01	0.23	2.40E-01	0.17	4.09E-01	-0.11	5.88E-01	-0.16	4.27E-01	0.07	7.17E-01	0.07	7.16E-01	0.03	8.80E-01	-0.19	3.49E-01	0.13	5.11E-01

ENSCAFG0000001712	LENEP	grey	VSMC_M10	-0.02	9.12E-01	0.23	2.40E-01	0.17	4.09E-01	-0.11	5.88E-01	-0.16	4.27E-01	0.07	7.17E-01	0.07	7.16E-01	0.03	8.80E-01	-0.19	3.49E-01	0.13	5.11E-01
ENSCAFG0000003039	GPR88	grey	VSMC_M10	-0.02	9.12E-01	0.23	2.40E-01	0.17	4.09E-01	-0.11	5.88E-01	-0.16	4.27E-01	0.07	7.17E-01	0.07	7.16E-01	0.03	8.80E-01	-0.19	3.49E-01	0.13	5.11E-01
ENSCAFG0000000564	SGSH	cyan	VSMC_M2	-0.02	9.12E-01	0.81	2.44E-07	0.54	3.65E-03	0.10	6.13E-01	-0.66	2.05E-04	0.10	6.25E-01	-0.05	7.95E-01	-0.39	4.18E-02	-0.72	2.09E-05	0.67	1.29E-04
ENSCAFG0000002926	ENSCAFG0000002926	grey	VSMC_M10	-0.02	9.12E-01	0.23	2.40E-01	0.17	4.09E-01	-0.11	5.88E-01	-0.16	4.27E-01	0.07	7.17E-01	0.07	7.16E-01	0.03	8.80E-01	-0.19	3.49E-01	0.13	5.11E-01
ENSCAFG0000002622	ENSCAFG0000002622	grey	VSMC_M10	-0.02	9.12E-01	-0.33	9.14E-02	0.30	1.28E-01	-0.49	9.70E-03	-0.18	3.76E-01	0.04	8.61E-01	0.33	8.96E-02	0.50	7.93E-03	0.26	1.98E-01	-0.50	8.05E-03
ENSCAFG0000002950	ENSCAFG0000002950	grey	VSMC_M10	-0.02	9.12E-01	0.04	8.43E-01	0.04	8.35E-01	-0.12	5.42E-01	-0.11	5.92E-01	0.16	4.13E-01	-0.13	5.30E-01	0.18	3.69E-01	-0.07	7.21E-01	0.06	7.58E-01
ENSCAFG0000001630	GPHN	grey	VSMC_M10	-0.02	9.12E-01	-0.32	1.06E-01	-0.47	1.44E-02	0.13	5.14E-01	0.54	3.90E-03	-0.36	6.45E-02	-0.02	9.04E-01	0.02	9.13E-01	0.33	9.43E-02	-0.23	2.50E-01
ENSCAFG0000000210	FHL2	grey	VSMC_M10	-0.02	9.11E-01	0.03	8.81E-01	-0.05	8.10E-01	0.43	2.43E-02	-0.10	6.16E-01	0.00	9.82E-01	-0.10	6.17E-01	-0.32	9.94E-02	-0.03	8.92E-01	0.27	1.70E-01
ENSCAFG0000003005	ECI1	grey	VSMC_M10	-0.02	9.10E-01	0.10	6.03E-01	0.40	3.85E-02	-0.16	4.16E-01	-0.39	4.24E-02	0.00	9.81E-01	-0.10	6.06E-01	0.13	5.34E-01	-0.08	7.06E-01	0.00	9.87E-01
ENSCAFG0000001807	DCTN4	pink	VSMC_M5	-0.02	9.10E-01	-0.38	4.97E-02	-0.78	1.98E-06	0.35	6.94E-02	0.78	1.66E-06	-0.12	5.56E-01	-0.22	2.77E-01	-0.13	5.30E-01	0.40	3.89E-02	-0.17	4.07E-01
ENSCAFG0000001652	PLOD1	darkgreen	VSMC_M4	-0.02	9.10E-01	0.34	7.87E-02	-0.07	7.15E-01	0.52	5.94E-03	0.05	8.17E-01	-0.24	2.37E-01	-0.56	2.29E-03	-0.57	1.85E-03	-0.15	4.48E-01	0.31	1.21E-01
ENSCAFG0000001838	METTL4	grey	VSMC_M10	-0.02	9.10E-01	-0.21	3.00E-01	-0.44	2.15E-02	-0.01	9.67E-01	0.47	1.43E-02	-0.15	4.43E-01	0.18	3.60E-01	0.06	7.62E-01	0.12	5.56E-01	-0.10	6.21E-01
ENSCAFG0000001369	PRG4	grey	VSMC_M10	-0.02	9.09E-01	0.35	7.14E-02	0.00	9.89E-01	0.29	1.41E-01	-0.16	4.15E-01	0.45	1.99E-02	-0.25	2.13E-01	-0.27	1.79E-01	-0.34	8.17E-02	0.42	2.98E-02
ENSCAFG0000000718	SKA3	darkgrey	VSMC_M8	-0.02	9.09E-01	-0.58	1.38E-03	0.26	1.95E-01	-0.79	8.77E-07	-0.10	6.13E-01	0.08	6.74E-01	0.28	1.51E-01	0.94	8.37E-13	0.43	2.48E-02	-0.80	5.98E-07
ENSCAFG0000000324	BSPRY	grey	VSMC_M10	-0.02	9.09E-01	-0.05	8.19E-01	0.01	9.55E-01	-0.18	3.70E-01	0.07	7.14E-01	-0.16	4.21E-01	-0.01	9.51E-01	0.10	6.11E-01	0.09	6.44E-01	-0.20	3.17E-01
ENSCAFG0000003072	PSMB9	cyan	VSMC_M2	-0.02	9.09E-01	0.58	1.46E-03	0.34	8.59E-02	0.14	4.90E-01	-0.43	2.35E-02	0.17	3.92E-01	-0.05	8.09E-01	-0.26	1.83E-01	-0.50	7.37E-03	0.47	1.34E-02
ENSCAFG0000000663	ENSCAFG0000000663	grey	VSMC_M10	-0.02	9.09E-01	0.72	2.20E-05	0.37	5.46E-02	0.26	1.89E-01	-0.53	4.06E-03	0.20	3.20E-01	-0.24	2.28E-01	-0.50	7.66E-03	-0.60	1.03E-03	0.69	5.96E-05
ENSCAFG0000001507	FAM184B	grey	VSMC_M10	-0.02	9.09E-01	-0.03	8.78E-01	0.22	2.63E-01	-0.34	8.49E-02	-0.26	1.96E-01	0.03	8.99E-01	0.47	1.26E-02	0.34	7.91E-02	-0.02	9.28E-01	0.00	9.91E-01
ENSCAFG0000001428	PYGM	grey	VSMC_M10	-0.02	9.09E-01	0.30	1.34E-01	-0.23	2.48E-01	0.45	1.96E-02	0.04	8.59E-01	0.12	5.58E-01	-0.21	3.00E-01	-0.43	2.70E-02	-0.28	1.64E-01	0.51	5.99E-03
ENSCAFG0000000906	EXOSC1	darkgrey	VSMC_M8	-0.02	9.09E-01	-0.49	9.28E-03	0.13	5.22E-01	-0.54	3.97E-03	0.07	7.31E-01	0.09	6.50E-01	0.19	3.47E-01	0.62	6.21E-04	0.42	2.87E-02	-0.73	1.37E-05
ENSCAFG0000001655	DCAF5	darkgreen	VSMC_M4	-0.02	9.08E-01	0.20	3.25E-01	-0.40	3.90E-02	0.64	3.07E-04	0.42	3.01E-02	-0.27	1.74E-01	-0.57	1.84E-03	-0.65	2.68E-04	0.02	9.03E-01	0.18	3.71E-01
ENSCAFG0000001275	UFC1	grey	VSMC_M10	-0.02	9.08E-01	0.14	4.88E-01	0.03	8.99E-01	0.26	1.85E-01	-0.06	7.82E-01	0.04	8.54E-01	0.02	9.29E-01	-0.30	1.27E-01	-0.11	5.89E-01	0.10	6.33E-01
ENSCAFG0000001103	GOLGA5	pink	VSMC_M5	-0.02	9.08E-01	-0.47	1.26E-02	-0.38	5.21E-02	-0.11	5.86E-01	0.61	7.22E-04	-0.35	7.64E-02	-0.08	6.84E-01	0.21	3.02E-01	0.54	3.76E-03	-0.68	8.84E-05
ENSCAFG0000002002	TRMT13	darkgrey	VSMC_M8	-0.02	9.08E-01	-0.30	1.24E-01	0.30	1.25E-01	-0.60	8.96E-04	-0.17	3.87E-01	-0.06	7.63E-01	0.34	8.25E-02	0.64	3.59E-04	0.20	3.19E-01	-0.53	4.68E-03
ENSCAFG0000001638	DEGS1	grey	VSMC_M10	-0.02	9.07E-01	-0.20	3.07E-01	-0.08	6.82E-01	-0.25	2.14E-01	0.17	3.83E-01	-0.11	5.91E-01	-0.20	3.25E-01	0.25	2.09E-01	0.28	1.56E-01	-0.38	5.14E-02
ENSCAFG0000003031	ENSCAFG0000003031	grey	VSMC_M10	-0.02	9.07E-01	0.03	8.98E-01	0.01	9.44E-01	-0.04	8.28E-01	0.02	9.16E-01	-0.01	9.74E-01	0.05	7.99E-01	-0.02	9.15E-01	0.03	8.72E-01	0.06	7.61E-01
ENSCAFG0000001548	TBC1D25	grey	VSMC_M10	-0.02	9.07E-01	0.15	4.44E-01	0.55	3.14E-03	-0.42	2.91E-02	-0.45	1.74E-02	0.05	7.98E-01	-0.06	7.82E-01	0.18	3.74E-01	-0.05	8.01E-01	-0.19	3.39E-01
ENSCAFG0000002897	CCDC85C	grey	VSMC_M10	-0.02	9.07E-01	0.08	7.00E-01	0.06	7.51E-01	-0.07	7.37E-01	-0.19	3.54E-01	0.24	2.26E-01	0.07	7.41E-01	0.00	9.84E-01	-0.07	7.37E-01	0.18	3.75E-01
ENSCAFG0000000180	TMEM263	darkgrey	VSMC_M8	-0.02	9.07E-01	-0.56	2.64E-03	0.08	6.96E-01	-0.47	1.35E-02	0.07	7.21E-01	-0.10	6.31E-01	0.16	4.37E-01	0.59	1.27E-03	0.48	1.04E-02	-0.67	1.14E-04
ENSCAFG0000002000	CDC14A	grey	VSMC_M10	-0.02	9.06E-01	0.04	8.40E-01	0.06	7.76E-01	0.00	9.93E-01	-0.14	4.72E-01	0.29	1.43E-01	0.49	9.26E-03	0.00	9.88E-01	-0.10	6.04E-01	0.21	2.92E-01
ENSCAFG0000001525	ATP1B1	grey	VSMC_M10	-0.02	9.06E-01	-0.33	9.35E-02	-0.04	8.53E-01	-0.15	4.68E-01	0.09	6.72E-01	-0.08	6.77E-01	-0.17	3.84E-01	0.31	1.13E-01	0.33	9.73E-02	-0.40	3.98E-02
ENSCAFG0000001628	TNK1	grey	VSMC_M10	-0.02	9.05E-01	0.31	1.18E-01	0.24	2.25E-01	0.16	4.17E-01	-0.41	3.55E-02	0.36	6.69E-02	0.06	7.65E-01	-0.24	2.31E-01	-0.28	1.56E-01	0.43	2.56E-02
ENSCAFG0000000395	ENSCAFG0000000395	grey	VSMC_M10	-0.02	9.05E-01	0.10	6.22E-01	0.12	5.45E-01	-0.11	5.72E-01	-0.14	4.78E-01	0.06	7.77E-01	0.07	7.16E-01	0.06	7.77E-01	-0.11	5.83E-01	0.07	7.47E-01
ENSCAFG0000001119	ENSCAFG0000001119	grey	VSMC_M10	-0.02	9.05E-01	0.10	6.22E-01	0.12	5.45E-01	-0.11	5.72E-01	-0.14	4.78E-01	0.06	7.77E-01	0.07	7.16E-01	0.06	7.77E-01	-0.11	5.83E-01	0.07	7.47E-01
ENSCAFG0000000207	ENSCAFG0000000207	grey	VSMC_M10	-0.02	9.05E-01	0.10	6.05E-01	-0.39	4.65E-02	0.48	1.12E-02	0.12	5.63E-01	0.35	7.25E-02	-0.07	7.30E-01	-0.35	7.48E-02	-0.19	3.34E-01	0.55	2.98E-03
ENSCAFG0000001307	ENSCAFG0000001307	grey	VSMC_M10	-0.02	9.05E-01	-0.10	6.15E-01	0.41	3.28E-02	-0.44	2.19E-02	-0.48	1.11E-02	-0.04	8.58E-01	0.48	1.14E-02	0.49	9.69E-03	-0.06	7.80E-01	-0.05	8.15E-01
ENSCAFG0000000497	ENSCAFG0000000497	grey	VSMC_M10	-0.02	9.04E-01	-0.19	3.45E-01	0.01	9.77E-01	-0.20	3.05E-01	-0.01	9.67E-01	0.00	9.82E-01	0.09	6.44E-01	0.29	1.46E-01	0.13	5.24E-01	-0.11	5.82E-01
ENSCAFG0000000237	ENSCAFG0000000237	grey	VSMC_M10	-0.02	9.04E-01	0.39	4.30E-02	0.21	2.98E-01	0.14	4.92E-01	-0.25	2.14E-01	-0.10	6.22E-01	0.05	8.16E-01	-0.24	2.24E-01	-0.35	7.34E-02	0.32	1.09E-01
ENSCAFG0000001769	ILF3	darkgrey	VSMC_M8	-0.02	9.04E-01	-0.32	1.05E-01	0.50	7.32E-03	-0.88	2.01E-09	-0.43	2.58E-02	0.15	4.63E-01	0.23	2.40E-01	0.90	9.68E-11	0.19	3.55E-01	-0.52	5.95E-03
ENSCAFG0000000373	C12H6orf203	grey	VSMC_M10	-0.02	9.04E-01	0.00	9.89E-01	0.36	6.85E-02	-0.36	6.14E-02	-0.39	4.16E-02	-0.02	9.08E-01	0.45	1.96E-02	0.33	9.45E-02	-0.10	6.37E-01	0.02	9.18E-01
ENSCAFG0000002947	CLIC2	cyan	VSMC_M2	-0.02	9.04E-01	0.63	4.91E-04	0.47	1.35E-02	0.03	8.90E-01	-0.52	5.55E-03	-0.05	8.18E-01	-0.33	9.57E-02	-0.21	3.00E-01	-0.50	7.63E-03	0.39	4.62E-02
ENSCAFG0000002887	ENSCAFG0000002887	cyan	VSMC_M2	-0.02	9.04E-01	0.69	6.10E-05	0.54	3.92E-03	-0.06	7.56E-01	-0.57	1.94E-03	-0.03	8.91E-01	0.04	8.26E-01	-0.29	1.41E-01	-0.53	4.50E-03	0.47	1.36E-02
ENSCAFG0000001646	ZFYVE26	darkgreen	VSMC_M4	-0.02	9.04E-01	0.37	5.65E-02	-0.28	1.58E-01	0.65	2.81E-04	0.13	5.31E-01	0.17	4.03E-01	-0.45	1.75E-02	-0.72	2.72E-05	-0.23	2.59E-01	0.55	2.96E-03
ENSCAFG0000000320	SERF2	grey	VSMC_M10	-0.02	9.04E-01	-0.01	9.78E-01	0.17	3.94E-01	0.05	8.18E-01	-0.19	3.54E-01	-0.08	6.88E-01	0.08	7.09E-01	-0.05	7.90E-01	0.00	1.00E+00	0.03	8.93E-01
ENSCAFG0000002338	DTX3	cyan	VSMC_M2	-0.02	9.03E-01	0.77	3.30E-06	0.33	9.67E-02	0.19	3.40E-01	-0.32	1.03E-01	-0.20	3.16E-01	-0.30	1.22E-01	-0.53	4.71E-03	-0.53	4.61E-03	0.48	1.17E-02
ENSCAFG0000001916	C5H1orf123	grey	VSMC_M10	-0.02	9.03E-01	0.41	3.30E-02	0.49	9.20E-03	-0.12	5.46E-01	-0.50	7.77E-03	-0.12	5.45E-01	0.28	1.54E-01	-0.11	5.97E-01	-0.36	6.32E-02	0.23	2.56E-01
ENSCAFG0000003052	C7H1orf74	grey	VSMC_M10	-0.02	9.03E-01	0.26	1.93E-01	0.38	5.12E-02	-0.06	7.73E-01	-0.41	3.47E-02	0.01	9.49E-01	-0.08	7.08E-01	-0.08	6.92E-01	-0.15	4.68E-01	0.14	4.94E-01
ENSCAFG0000001420	ENSCAFG0000001420	pink	VSMC_M5	-0.02	9.03E-01	0.02	9.19E-01	-0.79	8.10E-07	0.79	8.21E-07	0.71	3.35E-05	-0.19	3.50E-01	-0.22	2.70E-01	-0.65	2.34E-04	0.04	8.57E-01	0.31	1.19E-01
ENSCAFG0000001398	TEX12	grey	VSMC_M10	-0.02	9.03E-01	0.09	6.58E-01	0.02	9.19E-01	0.01	9.75E-01	-0.06	7.66E-01	0.05	8.06E-01	0.29	1.41E-01	-0.02	9.35E-01	-0.12	5.59E-01	0.15	4.43E-01
ENSCAFG0000001996	HSDL1	grey	VSMC_M10	-0.02	9.03E-01	0.56	2.55E-03	-0.16	4.15E-01	0.61	7.13E-04	0.02	9.29E-01	-0.22	2.62E-01	0.00	9.81E-01	-0.70	5.08E-05	-0.51	6.46E-03	0.68	8.99E-05
ENSCAFG0000001016	MX2	grey	VSMC_M10	-0.02	9.03E-01	0.32	1.06E-01	-0.06	7.64E-01	0.42	2.85E-02	-0.19	3.50E-01	0.40	4.00E-02	-0.21	2.97E-01	-0.37	6.00E-02	-0.38	5.19E-02	0.59	1.14E-03
ENSCAFG0000001828	NLE1	grey	VSMC_M10	-0.02	9.03E-01	-0.13	5.06E-01	0.13	5.13E-01	-0.44	2.24E-02	-0.02	9.30E-01	0.00	9.97E-01	-0.22	2.65E-01	0.40	3.72E-02	0.13	5.04E-01	-0.42	3.11E-02
ENSCAFG0000002859	ENSCAFG0000002859	grey	VSMC_M10	-0.02	9.02E-01	0.16	4.24E-01	0.10	6.22E-01	0.04	8.37E-01	-0.18	3.60E-01	0.03	8.96E-01	0.34	8.56E-02	-0.06	7.69E-01	-0.19	3.52E-01	0.21	2.85E-01
ENSCAFG0000001215	GABBR1	darkgreen	VSMC_M4	-0.02	9.02E-01	0.44	2.06E-02	-0.38	4.88E-02	0.80	5.95E-07	0.24	2.26E-01	-0.11	5.89E-01	-0.30	1.29E-01	-0.81	2.97E-07	-0.27	1.66E-01	0.63	4.34E-04
ENSCAFG0000003259	PTMA	darkgrey	VSMC_M8	-0.02	9.02E-01	-0.20	3.06E-01	0.58	1.54E-03	-0.88	1.80E-09	-0.43	2.50E-02	0.04	8.62E-01	0.30	1.25E-01	0.85	2.42E-08	0.12	5.67E-01	-0.55	2.92E-03
ENSCAFG0000000352	TRAPPC3	grey	VSMC_M10	-0.03	9.01E-01	0.19	3.48E-01	-0.24	2.38E-01	0.62	5.30E-04	0.12	5.58E-01	-0.18	3.57E-01	-0.25	2.00E-01	-0.60	9.73E-04	-0.07	7.31E-01	0.39	4.60E-02
ENSCAFG0000000536	CCDC13	grey	VSMC_M10	-0.03	9.01E-01	0.22	2.67E-01	0.03	8.81E-01	0.22	2.68E-01	-0.09	6.38E-01	-0.10	6.30E-01	0.04	8.44E-01	-0.20	3.23E-01	-0.23	2.54E-01	0.29	1.37E-01
ENSCAFG0000001231	ENSCAFG0000001231	grey	VSMC_M10	-0.03	9.01E-01	0.13	5.25E-01	0.39	4.64E-02	0.03	8.80E-01	-0.45	1.80E-02	0.01	9.77E-01	0.06	7.55E-01	-0.08	6.88E-01	-0.12	5.47E-01	0.17	3.91E-01
ENSCAFG0000001899	USP7	darkolivegreen	VSMC_M9	-0.03	9.01E-01	-0.65	2.62E-04	-0.46	1.63E-02	-0.24	2.22E-01	0.58	1.36E-03	0.02	9.38E-01	0.25	2.11E-01	0.49	9.04E-03	0.55	2.96E-03	-0.64	2.83E-04
ENSCAFG0000002165	ENSCAFG0000002165	grey	VSMC_M10	-0.03	9.01E-01	0.10	6.08E-01	0.24	2.26E-01	-0.23	2.49E-01	-0.26	1.85E-01	-0.03	8.63E-01	0.54	3.36E-03	0.18	3.82E-01	-0.16	4.39E-01	0.08	7.02E-01
ENSCAFG0000000650	TPP1	cyan	VSMC_M2	-0.03	9.00E-01	0.81	3.69E-07	0.30	1.32E-01	0.26	1.98E-01	-0.45	1.85E-02	0.02	9.31E-01	-0.28	1.59E-01	-0.55	3.27E-03	-0.66	1.75E-04	0.75	7.69E-06
ENSCAFG0000000766	ACSL1	yellow	VSMC_M3	-0.03	9.00E-01	0.06	7.73E-01	0.68	1.02E-04	-0.70	5.32E-05	-0.65	2.32E-04	0.10	6.22E-01	0.18	3.71E-01	0.56	2.51E-03	-0.07	7.14E-01	-0.13	5.29E-01
ENSCAFG0000002012	PTRH1	grey	VSMC_M10	-0.03	9.00E-01	0.17	3.89E-01	0.25	2.05E-01	-0.11	5.89E-01	-0.37	6.03E-02	0.25	2.15E-01	0.14	4.98E-01	0.14	4.96E-01	-0.29	1.44E-01	0.21	3.03E-01
ENSCAFG0000000527	CFAP61	grey	VSMC_M10	-0.03	9.00E-01	0.22	2.64E-01	0.03	8.88E-01	-0.01	9.55E-01	-0.11	5.83E-01	-0.02	9.28E-01	0.27	1.72E-01	-0.02	9.16E-01	-0.29	1.48E-01	0.26	1.96E-01
ENSCAFG0000000583	PCCA	darkgrey	VSMC_M8	-0.03	9.00E-01	-0.39	4.23E-02	0.13	5.27E-01	-0.53	4.79E-03	-0.07	7.10E-01	0.09	6.49E-01	0.46	1.69E-02	0.63	4.17E-04	0.25	2.04E-01	-0.41	3.50E-02
ENSCAFG0000000242	ZNF550	grey	VSMC_M10	-0.03	9.00E-01	0.02	9.41E-01	-0.31	1.22E-01	0.47	1.27E-02	0.06	7.62E-01	0.14	4.98E-01	0.13	5.27E-01	-0.31	1.20E-01	-0.09	6.38E-01	0.54	4.01E-03
ENSCAFG0000002003	KYAT1	grey	VSMC_M10	-0.03	9.00E-01	0.11	5.95E-01	-0.01	9.70E-01	0.07	7.32E-01	-0.04	8.39E-01	0.27	1.67E-01	-0.10	6.37E-01	-0.06	7.84E-01	-0.16	4.29E-01	0.09	6.47E-01
ENSCAFG0000001953	ENSCAFG0000001953	grey	VSMC_M10	-0.03	8.99E-01	0.16	4.36E-01	0.00	9.83E-01	0.21	2.90E-01	-0.08	7.03E-01	-0.05	8.03E-01	0.37	6.09E-02	-0.22	2.74E-01	-0.15	4.62E-01	0.26	1.98E-01
ENSCAFG0000002002	SASS6	darkgrey	VSMC_M8	-0.03	8.99E-01	-0.68	1.04E-04	0.05	7.90E-01	-0.72	2.48E-05	0.07	7.19E-01	0.02	9.07E-01	0.33	9.76E-02	0.91	2.59E-11	0.50	7.32E-03	-0.75	6.99E-06
ENSCAFG0000000222	ZNF169	darkgreen	VSMC_M4	-0.03	8.99E-01	0.05	8.17E-01	-0.56	2.42E-03	0.59	1.20E-03	0.43	2.70E-02	-0.10	6.34E-01	0.18	3.69E-01	-0.49	9.35E-03	-0.08	6.89E-01	0.40	3.92E-02
ENSCAFG0000002964	FXN	grey	VSMC_M10	-0.03	8.99E-01	0.16	4.12E-01	-0.03	8.85E-01	0.22	2.66E-01	-0.22	2.74E-01	0.42	2.86E-02	0.26	1.97E-01	-0.09	6.40E-01	-0.28	1.52E-01	0.51	6.25E-03
ENSCAFG0000000083	TAP1	grey	VSMC_M10	-0.03	8.99E-01	0.48	1.18E-02	-0.14	5.01E-01	0.47	1.30E-02	-0.02	9.23E-01	0.10	6.13E-01	-0.08	6.75E-01	-0.52	5.49E-03	-0.46	1.66E-02	0.54	3.87E-03
ENSCAFG0000002915	ENSCAFG0000002915	grey	VSMC_M10	-0.03	8.98E-01	-0.33	9.35E-02	-0.26	1.82E-01	0.01	9.53E-01	0.23	2.41E-01	-0.06	7.76E-01	0.07	7.46E-01	0.15	4.56E-01	0.34	8.53E-02	-0.21	3.05E-01
ENSCAFG0000000089	SULT4A1	grey	VSMC_M10	-0.03	8.98E-01	-0.14	4.74E-01	-0.22	2.68E-01	0.36	6.23E-02	0.13	5.13E-01	0.20	3.07E-01	-0.13	5.22E-01	-0.25	2.08E-01	0.19	3.35E-01	0.12	5.61E-01
ENSCAFG0000001389	MOGAT3	grey	VSMC_M10	-0.03	8.98E-01	0.00	9.99E-01	-0.15	4.66E-01	0.18	3.74E-01	0.06	7.75E-01	-0.10	6.34E-01	-0.10	6.29E-01	-0.16	4.18E-01	-0.04	8.25E-01	0.16	4.37E-01
ENSCAFG0000000933	TUBB2A	grey	VSMC_M10	-0.03	8.98E-01	-0.32	1.02E-01	0.13	5.25E-01	-0.20	3.09E-01	-0.17	4.03E-01	0.20	3.07E-01	0.25	2.14E-01	0.31	1.13E-01	0.30	1.26E-01	-0.17	4.11E-01
ENSCAFG0000001152	PTPRU	violet	VSMC_M7	-0.03	8.98E-01	-0.56	2.33E-03	-0.06	7.81E-01	-0.41	3.55E-02	0.10	6.32E-01	0.18	3.79E-01	0.73	1.77E-05	0.48	1.16E-02	0.42	2.85E-02	-0.40	3.87E-02
ENSCAFG0000002933	ENSCAFG0000002933	darkgrey	VSMC_M8	-0.03	8.98E-01	-0.41	3.54E-02	0.06	7.85E-01	-0.47	1.33E-02	-0.05	7.91E-01	0.21	2.90E-01	0.07	7.34E-01	0.64	2.90E-04	0.28	1.51E-01	-0.40	4.13E-02
ENSCAFG0000001008	ENSCAFG0000001008	grey	VSMC_M10	-0.03	8.98E-01	0.25	2.15E-01	0.36	6.28E-02	-0.04	8.52E-01	-0.35	7.55E-02	-0.29	1.45E-01	-0.01	9.45E-01	-0.03	8.77E-01	-0.17	4.00E-01	0.14	4.83E-01
ENSCAFG0000000691	STK33	grey	VSMC_M10	-0.03	8.96E-01	-0.28	1.64E-01	-0.43	2.62E-02	0.19	3.30E-01	0.36	6.80E-02	0.15	4.59E-01	0.33	9.70E-02	0.03	8.98E-01	0.18	3.65E-01	0.00	9.93E-01
ENSCAFG0000000088	DSCC1	darkgrey	VSMC_M8	-0.03	8.96E-01	-0.48	1.22E-02	0.23	2.55E-01	-0.52	5.60E-03	-0.23	2.43E-01	0.17	3.89E-01	0.63	3.99E-04	0.67	1.41E-04	0.26	1.97E-01	-0.33	8.96E-02
ENSCAFG0000002132	ENSCAFG0000002132	grey	VSMC_M10	-0.03	8.96E-01	0.21	2.96E-01	0.23	2.46E-01	-0.13	5.13E-01	-0.26	1.96E-01	-0.06	7.62E-01	0.29	1.43E-01	0.00	9.90E-01	-0.18	3.70E-01	0.16	4.15E-01
ENSCAFG0000001706	SLC50A1	grey	VSMC_M10	-0.03	8.96E-01	0.28	1.51E-01	-0.30	1.25E-01	0.32	1.04E-01	0.15	4.63E-01	0.09	6.46E-01	0.06	7.80E-01	-0.35	7.78E-02	-0.27	1.73E-01	0.45	1.78E-02
ENSCAFG0000002858	ENSCAFG0000002858	cyan	VSMC_M2	-0.03	8.96E-01	0.61	7.13E-04	-0.17	3.83E-01	0.58	1.48E-03	-0.04	8.41E-01	0.08	6.85E-01	-0.37	5.65E-02	-0.62	6.03E-04	-0.56	2.65E-03	0.73	1.45E-05
ENSCAFG0000002476	ENSCAFG0000002476	turquoise	VSMC_M6	-0.03	8.96E-01	0.32	1.06E-01	-0.17	3.87E-01	0.31	1.14E-01	0.01	9.74E-01	0.51	6.65E-03	-0.23	2.49E-01	-0.33	9.27E-02	-0.28	1.57E-01	0.37	5.95E-02
ENSCAFG0000000305	INIP	pink	VSMC_M5	-0.03	8.96E-01	0.00	9.84E-01	-0.42	3.10E-02	0.40	3.64E-02	0.34	7.82E-02	-0.13	5.26E-01	0.08	6.90E-01	-0.33	9.08E-02	-0.05	8.19E-01	0.16	4.13E-01
ENSCAFG0000000606	ENSCAFG0000000606	cyan	VSMC_M2	-0.03	8.96E-01	0.50	8.13E-03	0.48	1.12E-02	-0.01	9.71E-01	-0.61	7.90E-04	0.19	3.54E-01	0.00	9.81E-01	-0.14	4.79E-01	-0.49	8.98E-03	0.43	2.51E-02
ENSCAFG0000000007	ENSCAFG0000000007	grey	VSMC_M10	-0.03	8.96E-01	0.30	1.27E-01	0.14	4.86E-01	0.26	1.94E-01	-0.29	1.37E-01	0.23	2.39E-01	-0.16	4.22E-01	-0.32	1.00E-01	-0.22	2.61E-01	0.37	6.10E-02
ENSCAFG0000000922	RAD51	darkgrey	VSMC_M8	-0.03	8.96E-01	-0.51	6.17E-03	0.39	4.30E-02	-0.90	1.55E-10	-0.23	2.47E-01	0.05	7.86E-01	0.37	5.53E-02	0.95	2.76E-14	0.39	4.26E-02	-0.76	4.94E-06
ENSCAFG0000000445	CD3EAP	darkgrey	VSMC_M8	-0.03	8.95E-01	-0.48	1.21E-02	0.32	9.87E-02	-0.58	1.62E-03	-0.29	1.49E-01	0.12	5.40E-01	0.19	3.47E-01	0.70	4.52E-05	0.33	9.05E-02	-0.54	3.35E-03
ENSCAFG0000001581	SYNE2	grey	VSMC_M10	-0.03	8.95E-01	-0.64	2.98E-04	-0.25	2.05E-01	-0.35	7.53E-02	0.34	7.93E-02	0.07	7.37E-01	0.59	1.26E-03	0.55	3.11E-03	0.50	7.84E-03	-0.53	4.87E-03
ENSCAFG0000000676	ENSCAFG0000000676	grey	VSMC_M10	-0.03	8.95E-01	0.10	6.04E-01	0.11	5.91E-01	-0.13	5.31E-01	-0.17	3.88E-01	-0.02	9.14E-01	0.36	6.57E-02	0.10	6.29E-01	-0.17	3.90E-01	0.11	5.97E-01
ENSCAFG0000000325	RNASEH1	grey	VSMC_M10	-0.03	8.95E-01	-0.65	2.76E-04	-0.06	7.67E-01	-0.29	1.36E-01	0.15	4.54E-01	0.17	3.92E-01	0.22	2.81E-01	0.50	8.08E-03	0.52	5.94E-03	-0.67	1.28E-04
ENSCAFG0000000938	ORMDL1	grey	VSMC_M10	-0.03	8.95E-01	-0.33	8.79E-02	-0.06	7.62E-01	-0.34	8.09E-02	0.21	2.82E-01	0.08	6.92E-01	-0.06	7.72E-01	0.40	3.69E-02	0.33	9.80E-02	-0.59	1.09E-03
ENSCAFG0000000748	ENSCAFG0000000748	grey	VSMC_M10	-0.03	8.95E-01	0.24	2.38E-01	-0.09	6.45E-01	0.16	4.14E-01	0.04	8.49E-01	-0.13	5.32E-01	-0.11	5.96E-01	-0.26	1.95E-01	-0.16	4.23E-01	0.27	1.77E-01
ENSCAFG0000002946	SPAG8	violet	VSMC_M7	-0.03	8.95E-01	-0.17	4.00E-01	0.03	9.00E-01	-0.22	2.73E-01	-0.06	7.51E-01	-0.06	7.50E-01	0.75	6.15E-06	0.25	2.02E-01	0.03	8.75E-01	-0.05	7.93E-01
ENSCAFG0000001045	CALHM2	grey	VSMC_M10	-0.03	8.95E-01	0.24	2.32E-01	0.56	2.17E-03	-0.41	3.46E-02	-0.50	7.96E-03	0.05	8.07E-01	0.42	2.73E-02	0.18	3.78E-01	-0.23	2.41E-01	-0.02	9.09E-01
ENSCAFG0000000705	CHCHD7	grey	VSMC_M10	-0.03	8.95E-01	-0.20	3.13E-01	-0.03	9.00E-01	-0.01	9.75E-01	0.02	9.05E-01	0.27	1.65E-01	0.35	7.41E-02	0.10	6.08E-01	0.11	5.96E-01	-0.11	5.73E-01
ENSCAFG0000002678	ENSCAFG0000002678	grey	VSMC_M10	-0.03	8.94E-01	-0.02	9.29E-01	-0.16	4.25E-01	0.16	4.19E-01	-0.02	9.21E-01	0.30	1.34E-01	0.26	1.87E-01	-0.04	8.47E-01	-0.07	7.33E-01	0.28	1.64E-01
ENSCAFG0000002417	SPINK2	darkgreen	VSMC_M4	-0.03	8.94E-01	-0.11	5.73E-01	-0.53	4.49E-03	0.58	1.55E-03	0.59	1.35E-03	-0.23	2.39E-01	-0.37	5.53E-02	-0.39	4.23E-02	0.20	3.23E-01	-0.07	7.36E-01
ENSCAFG0000001678	DFFA	yellow	VSMC_M3	-0.03	8.94E-01	0.27	1.81E-01	0.51	6.68E-03	0.02	9.38E-01	-0.57	1.73E-03	0.01	9.51E-01	0.24	2.36E-01	-0.14	4.73E-01	-0.26	1.83E-01	0.29	1.36E-01
ENSCAFG0000001784	DNAH3	grey	VSMC_M10	-0.03	8.94E-01	-0.02	9.38E-01	-0.33	9.57E-02	0.27	1.74E-01	0.28	1.53E-01	-0.10	6.34E-01	0.25	2.05E-01	-0.28	1.62E-01	0.03	8.86E-01	0.20	3.05E-01
ENSCAFG0000001872	ENSCAFG0000001872	grey	VSMC_M10	-0.03	8.94E-01	0.06	7.54E-01	0.07	7.34E-01	-0.02	9.37E-01	-0.23	2.57E-01	0.36	6.90E-02	0.18	3.77E-01	0.03	8.81E-01	-0.10	6.33E-01	0.19	3.33E-01
ENSCAFG0000000858	CXCL10	turquoise	VSMC_M6	-0.03	8.94E-01	0.03	8.76E-01	-0.05	7.91E-01	0.05	7.95E-01	-0.08	6.77E-01	0.52	5.53E-03	0.07	7.31E-01	-0.07	7.36E-01	-0.09	6.63E-01	0.21	2.95E-01
ENSCAFG0000001581	ZNF316	yellow	VSMC_M3	-0.03	8.93E-01	0.25	2.09E-01	0.63	3.90E-04	-0.21	2.93E-01	-0.61	6.47E-04	0.12	5.66E-01	-0.03	8.90E-01	0.03	8.88E-01	-0.18	3.78E-01	0.03	8.96E-01
ENSCAFG0000001116	PAPSS1	yellow	VSMC_M3	-0.03	8.93E-01	0.15	4.54E-01	0.56	2.48E-03	-0.65	2.18E-04	-0.45	1.88E-02	-0.06	7.69E-01	0.21	2.91E-01	0.49	1.00E-02	-0.16	4.16E-01	-0.22	2.68E-01
ENSCAFG0000001787	STRA6	grey	VSMC_M10	-0.03	8.93E-01	-0.40	3.66E-02	-0.44	2.25E-02	0.25	2.11E-01	0.30	1.24E-01	0.28	1.57E-01	0.21	2.85E-01	0.03	8.87E-01	0.25	2.09E-01	0.02	9.30E-01
ENSCAFG0000001245	UBR3	pink	VSMC_M5	-0.03	8.93E-01	-0.57	1.75E-03	-0.72	2.16E-05	0.24	2.25E-01	0.68	8.10E-05	-0.05	8.05E-01	0.05	7.98E-01	0.03	8.75E-01	0.52	5.29E-03	-0.21	2.96E-01
ENSCAFG0000001976	DENND2D	grey	VSMC_M10	-0.03	8.92E-01	0.18	3.57E-01	0.16	4.36E-01	-0.12	5.47E-01	-0.15	4.41E-01	0.10	6.33E-01	0.11	5.75E-01	0.06	7.72E-01	-0.15	4.52E-01	0.11	5.96E-01
ENSCAFG0000000441	ENSCAFG0000000441	grey	VSMC_M10	-0.03	8.91E-01	-0.29	1.37E-01	-0.16	4.29E-01	-0.29	1.43E-01	0.32	1.09E-01	0.18	3.56E-01	0.01	9.43E-01	0.31	1.11E-01	0.29	1.48E-01	-0.48	1.14E-02
ENSCAFG0000000890	PTX3	darkgreen	VSMC_M4	-0.03	8.91E-01	0.35	7.52E-02	-0.32	9.96E-02	0.84	3.56E-08	0.14	4.79E-01	-0.05	8.20E-01	-0.28	1.63E-01	-0.74	1.03E-05	-0.30	1.32E-01	0.58	1.40E-03
ENSCAFG0000001846	FLCN	yellow	VSMC_M3	-0.03	8.91E-01	0.51	7.07E-03	0.56	2.25E-03	-0.33	9.69E-02	-0.50	8.00E-03	-0.02	9.22E-01	0.10	6.10E-01	-0.03	8.66E-01	-0.41	3.30E-02	0.17	3.96E-01
ENSCAFG0000002943	TIMM10B	grey	VSMC_M10	-0.03	8.91E-01	0.22	2.80E-01	0.44	2.03E-02	-0.07	7.18E-01	-0.60	8.77E-04	0.10	6.32E-01	-0.09	6.61E-01	0.04	8.60E-01	-0.22	2.60E-01	0.33	9.77E-02
ENSCAFG0000000600	PROSER1	grey	VSMC_M10	-0.03	8.91E-01	0.04	8.37E-01	-0.60	9.95E-04	0.52	5.90E-03	0.42	3.05E-02	0.12	5.40E-01	0.04	8.46E-01	-0.42	3.07E-02	-0.10	6.32E-01	0.42	2.72E-02
ENSCAFG0000001350	CASC4	darkgreen	VSMC_M4	-0.03	8.90E-01	0.13	5.04E-01	-0.53	4.67E-03	0.67	1.21E-04	0.44	2.22E-02	-0.21	3.02E-01	-0.19	3.36E-01	-0.58	1.53E-03	-0.10	6.16E-01	0.36	6.29E-02
ENSCAFG0000001412	CHCHD10	yellow	VSMC_M3	-0.03	8.90E-01	0.38	4.93E-02	0.70	4.61E-05	-0.37	5.69E-02	-0.68	1.02E-04	-0.08	6.96E-01	0.11	5.85E-01	0.08	6.99E-01	-0.31	1.12E-01	0.12	5.51E-01

ENSCAFG0000002627	ENSCAFG0000002627	grey	VSMC_M10	-0.03	8.90E-01	-0.01	9.47E-01	0.13	5.27E-01	-0.14	4.79E-01	-0.19	3.45E-01	-0.03	8.74E-01	0.47	1.27E-02	0.07	7.31E-01	-0.05	8.17E-01	0.07	7.40E-01
ENSCAFG0000001760	IREB2	turquoise	VSMC_M6	-0.03	8.89E-01	-0.21	2.88E-01	-0.19	3.36E-01	0.10	6.11E-01	0.08	6.85E-01	0.15	4.55E-01	-0.25	2.04E-01	0.01	9.50E-01	0.16	4.29E-01	0.10	6.31E-01
ENSCAFG0000001062	CATSPER3	grey	VSMC_M10	-0.03	8.89E-01	-0.01	9.75E-01	-0.03	9.00E-01	-0.11	5.77E-01	-0.09	6.38E-01	0.33	9.75E-02	0.29	1.40E-01	0.11	5.97E-01	-0.08	6.84E-01	0.12	5.49E-01
ENSCAFG0000003133	HOPX	darkgreen	VSMC_M4	-0.03	8.89E-01	0.03	8.73E-01	-0.52	5.01E-03	0.66	2.02E-04	0.41	3.42E-02	-0.09	6.55E-01	0.05	8.11E-01	-0.53	4.07E-03	0.00	1.00E+00	0.38	5.29E-02
ENSCAFG0000002260	REV1	grey	VSMC_M10	-0.03	8.89E-01	-0.22	2.63E-01	-0.35	7.67E-02	-0.13	5.09E-01	0.42	2.96E-02	-0.33	9.71E-02	0.11	5.90E-01	0.23	2.49E-01	0.18	3.63E-01	-0.26	1.82E-01
ENSCAFG0000001671	LEP	darkgreen	VSMC_M4	-0.03	8.89E-01	0.11	5.70E-01	-0.67	1.38E-04	0.93	2.72E-12	0.50	7.42E-03	-0.05	8.18E-01	-0.09	6.56E-01	-0.76	3.69E-06	-0.07	7.22E-01	0.50	7.40E-03
ENSCAFG0000001067	RSPO3	grey	VSMC_M10	-0.03	8.88E-01	0.13	5.05E-01	0.32	1.07E-01	-0.23	2.54E-01	-0.47	1.31E-02	0.36	6.66E-02	-0.07	7.44E-01	0.19	3.53E-01	-0.21	2.92E-01	0.20	3.16E-01
ENSCAFG0000001784	MRM1	darkgreen	VSMC_M4	-0.03	8.88E-01	-0.10	6.13E-01	-0.49	9.78E-03	0.51	6.43E-03	0.27	1.81E-01	0.13	5.15E-01	0.25	2.16E-01	-0.27	1.76E-01	-0.02	9.23E-01	0.38	4.82E-02
ENSCAFG0000000976	ACAD8	grey	VSMC_M10	-0.03	8.88E-01	-0.08	7.06E-01	-0.19	3.44E-01	0.06	7.75E-01	0.30	1.23E-01	-0.50	8.39E-03	-0.11	5.86E-01	-0.04	8.35E-01	0.17	3.94E-01	-0.24	2.23E-01
ENSCAFG0000001225	AP2M1	grey	VSMC_M10	-0.03	8.88E-01	0.32	1.03E-01	0.57	1.87E-03	-0.30	1.31E-01	-0.57	1.99E-03	0.21	3.05E-01	-0.35	6.95E-02	0.15	4.68E-01	-0.24	2.23E-01	0.01	9.49E-01
ENSCAFG0000000551	WDR37	darkgrey	VSMC_M8	-0.03	8.88E-01	-0.41	3.31E-02	0.13	5.30E-01	-0.50	8.55E-03	-0.06	7.81E-01	0.32	1.01E-01	0.01	9.41E-01	0.59	1.25E-03	0.34	8.64E-02	-0.57	1.79E-03
ENSCAFG0000001753	SEPT4	grey	VSMC_M10	-0.03	8.87E-01	0.11	5.74E-01	0.34	8.45E-02	-0.31	1.18E-01	-0.46	1.70E-02	0.20	3.17E-01	0.54	3.83E-03	0.24	2.31E-01	-0.24	2.33E-01	0.26	1.93E-01
ENSCAFG0000000966	SCD	yellow	VSMC_M3	-0.03	8.87E-01	-0.16	4.40E-01	0.40	3.68E-02	-0.78	1.87E-06	-0.20	3.16E-01	-0.03	8.81E-01	0.32	1.03E-01	0.58	1.38E-03	0.15	4.46E-01	-0.50	7.58E-03
ENSCAFG0000001213	NEK2	darkgrey	VSMC_M8	-0.03	8.87E-01	-0.59	1.10E-03	0.22	2.64E-01	-0.79	9.16E-07	-0.14	4.97E-01	0.14	4.80E-01	0.50	8.45E-03	0.93	4.51E-12	0.40	3.66E-02	-0.62	4.93E-04
ENSCAFG0000000329	ENSCAFG0000000329	grey	VSMC_M10	-0.03	8.87E-01	0.03	8.92E-01	-0.07	7.34E-01	0.06	7.63E-01	0.04	8.54E-01	-0.14	5.01E-01	0.22	2.78E-01	-0.14	4.78E-01	0.01	9.45E-01	0.19	3.52E-01
ENSCAFG0000002322	ENSCAFG0000002322	grey	VSMC_M10	-0.03	8.86E-01	0.12	5.64E-01	-0.13	5.31E-01	0.22	2.77E-01	-0.01	9.41E-01	0.41	3.22E-02	0.05	7.95E-01	-0.14	4.84E-01	-0.17	3.96E-01	0.31	1.21E-01
ENSCAFG0000001990	MED27	grey	VSMC_M10	-0.03	8.86E-01	-0.03	8.87E-01	-0.04	8.50E-01	0.16	4.33E-01	0.06	7.83E-01	-0.03	8.76E-01	-0.23	2.38E-01	-0.10	6.33E-01	0.08	7.07E-01	-0.06	7.58E-01
ENSCAFG0000002620	ENSCAFG0000002620	darkgrey	VSMC_M8	-0.03	8.86E-01	-0.13	5.04E-01	0.60	1.03E-03	-0.65	2.46E-04	-0.49	9.61E-03	0.13	5.28E-01	-0.08	7.07E-01	0.59	1.14E-03	0.14	4.94E-01	-0.46	1.69E-02
ENSCAFG0000001512	SCYL3	grey	VSMC_M10	-0.03	8.86E-01	0.29	1.47E-01	0.39	4.33E-02	0.09	6.44E-01	-0.50	8.57E-03	-0.01	9.80E-01	-0.14	4.74E-01	-0.21	2.90E-01	-0.19	3.55E-01	0.37	6.00E-02
ENSCAFG0000002876	ENSCAFG0000002876	grey	VSMC_M10	-0.03	8.86E-01	0.49	9.85E-03	0.20	3.30E-01	0.24	2.24E-01	-0.27	1.70E-01	0.14	5.01E-01	-0.26	1.87E-01	-0.30	1.30E-01	-0.43	2.50E-02	0.35	7.51E-02
ENSCAFG0000000327	ANTXR1	darkgreen	VSMC_M4	-0.03	8.86E-01	0.39	4.29E-02	-0.38	5.23E-02	0.71	3.36E-05	0.34	8.14E-02	-0.35	7.05E-02	-0.33	9.78E-02	-0.78	1.42E-06	-0.17	3.92E-01	0.45	1.97E-02
ENSCAFG0000000192	FAM122A	darkgreen	VSMC_M4	-0.03	8.86E-01	0.44	2.33E-02	-0.18	3.70E-01	0.65	2.51E-04	-0.04	8.39E-01	0.22	2.68E-01	-0.33	9.05E-02	-0.67	1.24E-04	-0.36	6.39E-02	0.67	1.53E-04
ENSCAFG0000000228	FGD3	grey	VSMC_M10	-0.03	8.86E-01	0.13	5.16E-01	0.11	5.72E-01	-0.08	6.82E-01	-0.19	3.52E-01	-0.07	7.39E-01	-0.03	9.00E-01	0.08	6.90E-01	-0.15	4.40E-01	0.11	5.96E-01
ENSCAFG0000000361	FAM131B	grey	VSMC_M10	-0.03	8.86E-01	0.44	2.01E-02	-0.11	5.87E-01	0.53	4.55E-03	0.11	5.70E-01	-0.39	4.55E-02	-0.36	6.19E-02	-0.68	1.10E-04	-0.18	3.72E-01	0.41	3.35E-02
ENSCAFG0000000876	SFMBT1	grey	VSMC_M10	-0.03	8.86E-01	-0.11	5.99E-01	0.01	9.75E-01	-0.21	2.93E-01	-0.16	4.13E-01	0.32	9.86E-02	0.38	5.11E-02	0.26	1.96E-01	-0.05	8.11E-01	0.16	4.39E-01
ENSCAFG0000001014	SLC39A10	darkolivegreen	VSMC_M9	-0.03	8.85E-01	-0.68	9.00E-05	-0.45	1.99E-02	-0.04	8.50E-01	0.50	7.39E-03	0.02	9.28E-01	0.52	5.29E-03	0.30	1.32E-01	0.58	1.48E-03	-0.44	2.06E-02
ENSCAFG0000000588	PGAP2	grey	VSMC_M10	-0.03	8.84E-01	0.30	1.24E-01	-0.17	3.89E-01	0.41	3.26E-02	-0.03	8.98E-01	0.19	3.35E-01	0.20	3.23E-01	-0.41	3.43E-02	-0.29	1.37E-01	0.53	4.11E-03
ENSCAFG0000000047	CDSN	darkgreen	VSMC_M4	-0.03	8.84E-01	0.14	4.95E-01	-0.48	1.21E-02	0.75	7.87E-06	0.37	5.66E-02	-0.08	6.76E-01	-0.21	3.01E-01	-0.67	1.29E-04	-0.04	8.52E-01	0.42	3.06E-02
ENSCAFG0000001525	VCL	grey	VSMC_M10	-0.03	8.84E-01	-0.08	6.99E-01	-0.12	5.62E-01	0.17	4.04E-01	0.11	5.93E-01	0.06	7.64E-01	-0.54	3.86E-03	-0.20	3.19E-01	0.23	2.59E-01	-0.02	9.38E-01
ENSCAFG0000001518	ENSCAFG0000001518	grey	VSMC_M10	-0.03	8.84E-01	0.51	6.61E-03	0.34	7.94E-02	-0.07	7.13E-01	-0.43	2.37E-02	0.08	6.83E-01	-0.03	8.66E-01	-0.13	5.25E-01	-0.45	1.86E-02	0.40	3.71E-02
ENSCAFG0000001289	ENSCAFG0000001289	grey	VSMC_M10	-0.03	8.84E-01	0.32	1.03E-01	-0.26	1.82E-01	0.55	2.75E-03	0.24	2.36E-01	-0.28	1.59E-01	0.01	9.66E-01	-0.60	9.78E-04	-0.21	2.89E-01	0.36	6.47E-02
ENSCAFG0000003201	RAB39A	grey	VSMC_M10	-0.03	8.84E-01	-0.28	1.61E-01	-0.30	1.25E-01	0.08	6.75E-01	0.27	1.81E-01	-0.08	6.80E-01	0.20	3.12E-01	0.05	8.00E-01	0.19	3.36E-01	0.01	9.65E-01
ENSCAFG0000002921	RBAK	pink	VSMC_M5	-0.03	8.83E-01	-0.60	9.66E-04	-0.69	7.62E-05	0.21	3.01E-01	0.78	1.74E-06	-0.26	1.97E-01	-0.04	8.34E-01	0.02	9.04E-01	0.54	3.55E-03	-0.43	2.49E-02
ENSCAFG0000000574	NAV3	grey	VSMC_M10	-0.03	8.83E-01	0.10	6.03E-01	-0.52	5.21E-03	0.61	8.24E-04	0.44	2.11E-02	-0.11	5.71E-01	-0.64	3.69E-04	-0.54	3.72E-03	0.00	9.86E-01	0.23	2.45E-01
ENSCAFG0000003082	GCSAM	grey	VSMC_M10	-0.03	8.83E-01	-0.03	8.99E-01	0.04	8.37E-01	-0.17	3.92E-01	-0.13	5.03E-01	0.32	1.08E-01	-0.22	2.81E-01	0.16	4.39E-01	0.00	9.95E-01	0.07	7.30E-01
ENSCAFG0000002145	ENSCAFG0000002145	grey	VSMC_M10	-0.03	8.83E-01	0.17	3.94E-01	0.09	6.53E-01	-0.16	4.18E-01	-0.09	6.39E-01	0.02	9.15E-01	0.13	5.24E-01	0.08	7.09E-01	-0.15	4.66E-01	0.10	6.11E-01
ENSCAFG0000002958	ENSCAFG0000002958	grey	VSMC_M10	-0.03	8.83E-01	-0.01	9.61E-01	0.05	8.21E-01	-0.15	4.43E-01	-0.05	8.17E-01	-0.11	6.02E-01	0.03	8.98E-01	0.13	5.07E-01	0.03	8.79E-01	0.06	7.61E-01
ENSCAFG0000000584	ARL6IP6	yellow	VSMC_M3	-0.03	8.83E-01	-0.24	2.29E-01	0.54	3.47E-03	-0.61	6.96E-04	-0.43	2.65E-02	-0.14	4.76E-01	0.28	1.51E-01	0.52	5.74E-03	0.22	2.61E-01	-0.37	5.53E-02
ENSCAFG0000000609	ANKRD10	grey	VSMC_M10	-0.03	8.83E-01	0.17	4.04E-01	-0.29	1.49E-01	0.40	3.85E-02	0.33	9.03E-02	-0.15	4.53E-01	-0.41	3.50E-02	-0.51	6.20E-03	0.04	8.57E-01	0.14	4.90E-01
ENSCAFG0000003239	FAM83H	darkgreen	VSMC_M4	-0.03	8.83E-01	0.22	2.73E-01	-0.26	1.98E-01	0.64	3.33E-04	0.19	3.45E-01	-0.17	4.02E-01	-0.50	7.73E-03	-0.66	1.77E-04	-0.06	7.70E-01	0.35	7.04E-02
ENSCAFG0000002378	ZBP1	violet	VSMC_M7	-0.03	8.83E-01	-0.16	4.31E-01	0.08	6.88E-01	-0.19	3.50E-01	-0.12	5.49E-01	-0.02	9.07E-01	0.82	1.53E-07	0.23	2.49E-01	0.02	9.22E-01	-0.04	8.46E-01
ENSCAFG0000001555	DKK1	darkolivegreen	VSMC_M9	-0.03	8.82E-01	-0.69	6.52E-05	0.01	9.54E-01	-0.47	1.31E-02	0.12	5.63E-01	-0.10	6.30E-01	0.19	3.48E-01	0.63	4.55E-04	0.62	6.38E-04	-0.71	3.98E-05
ENSCAFG0000001151	GTF2I	pink	VSMC_M5	-0.03	8.82E-01	0.09	6.41E-01	-0.59	1.25E-03	0.58	1.37E-03	0.49	9.28E-03	0.03	8.66E-01	0.18	3.73E-01	-0.51	6.43E-03	-0.09	6.45E-01	0.35	7.09E-02
ENSCAFG0000002143	ENSCAFG0000002143	grey	VSMC_M10	-0.03	8.82E-01	0.17	4.02E-01	0.28	1.54E-01	-0.22	2.59E-01	-0.35	7.42E-02	0.04	8.49E-01	0.22	2.80E-01	0.15	4.54E-01	-0.18	3.64E-01	0.11	5.71E-01
ENSCAFG0000001353	CCNYL1	grey	VSMC_M10	-0.03	8.81E-01	0.03	8.73E-01	-0.33	9.32E-02	0.54	3.83E-03	0.15	4.56E-01	0.31	1.14E-01	-0.07	7.31E-01	-0.38	4.74E-02	-0.01	9.52E-01	0.39	4.56E-02
ENSCAFG0000000990	OCA2	grey	VSMC_M10	-0.03	8.81E-01	0.73	1.31E-05	0.27	1.70E-01	0.36	6.69E-02	-0.46	1.54E-02	0.18	3.78E-01	-0.33	9.13E-02	-0.57	1.75E-03	-0.62	5.13E-04	0.76	4.72E-06
ENSCAFG0000000905	ENSCAFG0000000905	grey	VSMC_M10	-0.03	8.81E-01	0.03	8.94E-01	0.09	6.39E-01	-0.15	4.54E-01	-0.08	6.93E-01	0.01	9.53E-01	0.14	4.85E-01	0.08	6.93E-01	0.00	9.81E-01	0.04	8.26E-01
ENSCAFG0000001396	CD163	grey	VSMC_M10	-0.03	8.81E-01	0.02	9.36E-01	0.01	9.58E-01	-0.02	9.27E-01	-0.08	6.85E-01	0.00	9.80E-01	0.18	3.56E-01	0.06	7.80E-01	-0.06	7.52E-01	0.11	5.71E-01
ENSCAFG0000001597	NEDD4	darkgrey	VSMC_M8	-0.03	8.81E-01	-0.30	1.24E-01	0.29	1.44E-01	-0.81	4.03E-07	-0.13	5.33E-01	-0.06	7.75E-01	0.35	7.29E-02	0.71	2.94E-05	0.25	2.10E-01	-0.57	1.85E-03
ENSCAFG0000002595	ENSCAFG0000002595	grey	VSMC_M10	-0.03	8.81E-01	0.21	3.04E-01	0.25	1.99E-01	-0.20	3.27E-01	-0.34	8.25E-02	0.38	4.79E-02	0.16	4.14E-01	0.14	4.80E-01	-0.23	2.38E-01	0.15	4.56E-01
ENSCAFG0000001928	ENSCAFG0000001928	darkgreen	VSMC_M4	-0.03	8.81E-01	-0.18	3.60E-01	-0.37	5.50E-02	0.39	4.39E-02	0.27	1.77E-01	-0.10	6.13E-01	-0.10	6.18E-01	-0.18	3.81E-01	0.15	4.69E-01	0.10	6.26E-01
ENSCAFG0000001070	WASHC5	grey	VSMC_M10	-0.03	8.80E-01	0.40	3.68E-02	-0.06	7.78E-01	0.07	7.14E-01	0.07	7.29E-01	-0.14	4.77E-01	-0.21	2.82E-01	-0.31	1.22E-01	-0.24	2.19E-01	0.28	1.56E-01
ENSCAFG0000001306	ATF6	darkgreen	VSMC_M4	-0.03	8.80E-01	-0.24	2.20E-01	-0.79	8.97E-07	0.67	1.21E-04	0.66	1.85E-04	0.12	5.44E-01	-0.13	5.03E-01	-0.46	1.56E-02	0.23	2.42E-01	0.15	4.53E-01
ENSCAFG0000001009	ENSCAFG0000001009	grey	VSMC_M10	-0.03	8.80E-01	0.11	5.84E-01	0.22	2.71E-01	0.03	8.94E-01	-0.30	1.32E-01	0.19	3.51E-01	0.18	3.56E-01	0.00	9.80E-01	-0.18	3.70E-01	0.16	4.26E-01
ENSCAFG0000000260	RNF20	grey	VSMC_M10	-0.03	8.80E-01	0.05	8.09E-01	-0.28	1.53E-01	0.54	3.65E-03	0.19	3.32E-01	-0.28	1.63E-01	0.06	7.69E-01	-0.46	1.69E-02	-0.02	9.06E-01	0.28	1.62E-01
ENSCAFG0000001461	ENSCAFG0000001461	grey	VSMC_M10	-0.03	8.80E-01	0.26	1.94E-01	0.16	4.40E-01	-0.06	7.67E-01	-0.24	2.34E-01	0.00	9.98E-01	-0.15	4.67E-01	-0.02	9.29E-01	-0.23	2.57E-01	0.18	3.72E-01
ENSCAFG0000001329	IGF2BP2	grey	VSMC_M10	-0.03	8.80E-01	-0.52	5.54E-03	-0.12	5.39E-01	-0.34	8.36E-02	0.22	2.63E-01	0.09	6.42E-01	-0.32	1.06E-01	0.41	3.45E-02	0.52	5.07E-03	-0.53	4.05E-03
ENSCAFG0000001754	S100A14	grey	VSMC_M10	-0.03	8.80E-01	0.00	9.83E-01	-0.04	8.25E-01	0.00	9.98E-01	0.02	9.36E-01	0.04	8.30E-01	0.03	8.66E-01	0.03	8.75E-01	0.01	9.70E-01	0.07	7.15E-01
ENSCAFG0000000361	NOSIP	grey	VSMC_M10	-0.03	8.79E-01	0.11	5.88E-01	-0.28	1.56E-01	0.43	2.48E-02	0.23	2.54E-01	-0.21	2.88E-01	-0.04	8.26E-01	-0.48	1.19E-02	0.02	9.13E-01	0.26	1.84E-01
ENSCAFG0000001320	SCAP	grey	VSMC_M10	-0.03	8.79E-01	0.75	7.31E-06	0.32	1.02E-01	0.24	2.31E-01	-0.51	7.09E-03	0.14	4.98E-01	-0.23	2.47E-01	-0.45	1.92E-02	-0.65	2.33E-04	0.73	1.37E-05
ENSCAFG0000001452	DARS2	grey	VSMC_M10	-0.03	8.79E-01	-0.28	1.57E-01	-0.40	3.78E-02	0.33	9.18E-02	0.34	8.22E-02	0.11	6.01E-01	0.16	4.25E-01	-0.14	4.83E-01	0.22	2.80E-01	-0.04	8.30E-01
ENSCAFG0000001291	S100A11	darkgrey	VSMC_M8	-0.03	8.79E-01	-0.47	1.44E-02	0.31	1.11E-01	-0.70	4.72E-05	-0.18	3.76E-01	-0.02	9.04E-01	0.16	4.37E-01	0.70	4.26E-05	0.43	2.60E-02	-0.65	2.42E-04
ENSCAFG0000000327	CDCA8	darkgrey	VSMC_M8	-0.03	8.79E-01	-0.42	3.00E-02	0.45	1.81E-02	-0.92	9.23E-12	-0.32	1.00E-01	0.12	5.49E-01	0.42	2.82E-02	0.95	1.24E-14	0.27	1.79E-01	-0.64	2.96E-04
ENSCAFG0000000068	ABRA	darkgreen	VSMC_M4	-0.03	8.79E-01	0.00	9.96E-01	-0.53	4.22E-03	0.68	8.45E-05	0.40	3.81E-02	0.18	3.68E-01	-0.07	7.29E-01	-0.54	3.62E-03	0.05	8.08E-01	0.35	7.50E-02
ENSCAFG0000002488	SOX4	grey	VSMC_M10	-0.03	8.78E-01	0.61	7.66E-04	-0.11	5.85E-01	0.53	4.32E-03	-0.06	7.64E-01	-0.10	6.29E-01	-0.35	7.54E-02	-0.71	2.86E-05	-0.49	8.83E-03	0.72	2.58E-05
ENSCAFG0000003055	RWDD2A	grey	VSMC_M10	-0.03	8.78E-01	-0.03	8.95E-01	-0.19	3.31E-01	0.09	6.46E-01	0.30	1.31E-01	-0.41	3.17E-02	-0.09	6.60E-01	-0.16	4.32E-01	0.11	5.74E-01	-0.12	5.65E-01
ENSCAFG0000000401	GRIN2D	grey	VSMC_M10	-0.03	8.78E-01	-0.43	2.56E-02	-0.04	8.47E-01	-0.35	6.97E-02	-0.01	9.58E-01	0.15	4.67E-01	0.39	4.33E-02	0.38	5.32E-02	0.36	6.69E-02	-0.21	3.01E-01
ENSCAFG0000000605	RASSF2	darkgreen	VSMC_M4	-0.03	8.78E-01	0.30	1.35E-01	-0.51	6.49E-03	0.92	1.24E-11	0.29	1.42E-01	-0.03	8.87E-01	-0.13	5.28E-01	-0.78	1.58E-06	-0.26	1.93E-01	0.67	1.42E-04
ENSCAFG0000000222	TMEM14A	grey	VSMC_M10	-0.03	8.77E-01	0.00	9.82E-01	0.23	2.43E-01	-0.38	5.29E-02	-0.26	1.95E-01	-0.04	8.32E-01	0.37	5.57E-02	0.26	1.88E-01	-0.02	9.05E-01	-0.01	9.42E-01
ENSCAFG0000001909	ENSCAFG0000001909	grey	VSMC_M10	-0.03	8.77E-01	-0.14	4.76E-01	-0.17	3.92E-01	0.01	9.78E-01	0.15	4.47E-01	0.42	2.79E-02	0.51	6.11E-03	0.07	7.12E-01	0.06	7.55E-01	-0.08	6.99E-01
ENSCAFG0000000219	RNF149	darkgreen	VSMC_M4	-0.03	8.77E-01	0.25	2.17E-01	-0.36	6.28E-02	0.76	3.62E-06	0.15	4.68E-01	-0.04	8.44E-01	-0.06	7.65E-01	-0.68	9.99E-05	-0.18	3.63E-01	0.61	7.29E-04
ENSCAFG0000001780	BCL11B	grey	VSMC_M10	-0.03	8.77E-01	0.11	5.78E-01	-0.03	8.72E-01	0.10	6.22E-01	-0.06	7.83E-01	-0.15	4.63E-01	0.27	1.77E-01	-0.06	7.57E-01	-0.19	3.41E-01	0.25	2.05E-01
ENSCAFG0000001054	ENSCAFG0000001054	grey	VSMC_M10	-0.03	8.77E-01	0.29	1.36E-01	0.15	4.44E-01	-0.08	6.76E-01	-0.21	2.87E-01	0.07	7.17E-01	0.07	7.35E-01	-0.13	5.11E-01	-0.25	2.18E-01	0.24	2.35E-01
ENSCAFG0000001863	ENSCAFG0000001863	grey	VSMC_M10	-0.03	8.77E-01	0.34	8.56E-02	-0.20	3.27E-01	0.51	6.85E-03	0.06	7.70E-01	-0.08	7.01E-01	-0.27	1.68E-01	-0.48	1.18E-02	-0.27	1.67E-01	0.46	1.68E-02
ENSCAFG0000001893	CFAP53	grey	VSMC_M10	-0.03	8.77E-01	-0.09	6.50E-01	-0.38	4.93E-02	0.34	8.71E-02	0.28	1.63E-01	-0.12	5.39E-01	0.10	6.35E-01	-0.25	2.05E-01	0.09	6.40E-01	0.19	3.55E-01
ENSCAFG0000000930	TOMM70	darkgrey	VSMC_M8	-0.03	8.77E-01	-0.70	4.64E-05	-0.10	6.29E-01	-0.46	1.62E-02	0.18	3.57E-01	-0.01	9.49E-01	0.43	2.48E-02	0.67	1.37E-04	0.49	8.83E-03	-0.60	8.62E-04
ENSCAFG0000001937	HASPIN	darkgrey	VSMC_M8	-0.03	8.76E-01	-0.60	9.35E-04	0.34	8.66E-02	-0.82	1.54E-07	-0.21	3.00E-01	0.10	6.04E-01	0.41	3.32E-02	0.95	8.17E-14	0.43	2.70E-02	-0.74	1.17E-05
ENSCAFG0000001048	SPPL3	grey	VSMC_M10	-0.03	8.76E-01	0.48	1.21E-02	-0.21	3.00E-01	0.44	2.03E-02	0.03	8.82E-01	0.06	7.75E-01	-0.18	3.69E-01	-0.50	7.93E-03	-0.37	5.55E-02	0.61	6.50E-04
ENSCAFG0000000644	TRIM3	grey	VSMC_M10	-0.03	8.76E-01	0.42	3.04E-02	0.09	6.67E-01	0.17	4.00E-01	-0.22	2.75E-01	-0.14	4.76E-01	-0.22	2.81E-01	-0.29	1.37E-01	-0.30	1.23E-01	0.43	2.40E-02
ENSCAFG0000001980	EPS8L3	grey	VSMC_M10	-0.03	8.76E-01	-0.14	4.75E-01	0.02	9.38E-01	-0.17	3.91E-01	-0.11	5.78E-01	0.25	2.06E-01	0.13	5.29E-01	0.14	4.82E-01	0.12	5.66E-01	-0.05	8.22E-01
ENSCAFG0000003042	ENSCAFG0000003042	grey	VSMC_M10	-0.03	8.76E-01	0.01	9.72E-01	0.15	4.67E-01	-0.26	1.83E-01	-0.21	2.95E-01	0.35	7.71E-02	-0.05	8.08E-01	0.18	3.69E-01	0.04	8.40E-01	0.05	8.06E-01
ENSCAFG0000000806	ARHGEF3	darkgreen	VSMC_M4	-0.03	8.76E-01	0.60	9.93E-04	-0.26	1.93E-01	0.77	3.32E-06	0.04	8.38E-01	0.04	8.39E-01	-0.28	1.62E-01	-0.82	2.19E-07	-0.48	1.06E-02	0.77	2.69E-06
ENSCAFG0000001164	AP1AR	grey	VSMC_M10	-0.03	8.76E-01	-0.46	1.54E-02	-0.48	1.12E-02	0.16	4.31E-01	0.48	1.15E-02	0.20	3.23E-01	0.02	9.37E-01	0.07	7.33E-01	0.36	6.18E-02	-0.30	1.31E-01
ENSCAFG0000000712	HPN	darkgreen	VSMC_M4	-0.03	8.75E-01	-0.01	9.70E-01	-0.51	6.38E-03	0.71	2.86E-05	0.31	1.17E-01	0.16	4.22E-01	-0.11	5.94E-01	-0.50	7.44E-03	0.01	9.60E-01	0.37	5.60E-02
ENSCAFG0000001471	MACROD1	cyan	VSMC_M2	-0.03	8.75E-01	0.49	9.35E-03	0.35	7.34E-02	-0.03	8.66E-01	-0.30	1.28E-01	-0.10	6.08E-01	-0.02	9.11E-01	-0.25	2.08E-01	-0.33	9.25E-02	0.21	2.94E-01
ENSCAFG0000001692	PHB	grey	VSMC_M10	-0.03	8.75E-01	-0.15	4.57E-01	0.11	5.80E-01	-0.24	2.18E-01	-0.26	1.93E-01	0.33	9.46E-02	0.07	7.40E-01	0.39	4.33E-02	0.02	9.30E-01	-0.04	8.56E-01
ENSCAFG0000001315	GALE	darkgrey	VSMC_M8	-0.03	8.74E-01	-0.11	5.81E-01	0.54	3.91E-03	-0.59	1.20E-03	-0.60	9.76E-04	0.24	2.24E-01	0.09	6.41E-01	0.62	5.38E-04	-0.01	9.46E-01	-0.13	5.10E-01
ENSCAFG0000001433	EXPH5	darkgreen	VSMC_M4	-0.03	8.74E-01	0.27	1.78E-01	-0.57	1.86E-03	0.96	6.46E-15	0.35	7.63E-02	-0.05	8.15E-01	-0.21	2.99E-01	-0.83	9.35E-08	-0.21	2.82E-01	0.67	1.39E-04
ENSCAFG0000003234	ENSCAFG0000003234	grey	VSMC_M10	-0.03	8.74E-01	0.09	6.41E-01	0.09	6.43E-01	-0.21	2.86E-01	-0.12	5.66E-01	0.39	4.70E-02	0.01	9.44E-01	0.14	4.96E-01	-0.05	8.17E-01	-0.05	8.06E-01
ENSCAFG0000003041	STX3	cyan	VSMC_M2	-0.03	8.74E-01	0.62	6.02E-04	0.13	5.04E-01	0.20	3.24E-01	-0.23	2.48E-01	-0.15	4.66E-01	0.06	7.84E-01	-0.49	1.02E-02	-0.49	9.46E-03	0.59	1.21E-03
ENSCAFG0000000483	TRIM54	grey	VSMC_M10	-0.03	8.74E-01	0.34	7.80E-02	0.24	2.36E-01	-0.06	7.71E-01	-0.30	1.30E-01	0.01	9.60E-01	0.17	4.03E-01	-0.03	8.65E-01	-0.32	1.02E-01	0.27	1.71E-01
ENSCAFG0000000239	FAM178B	grey	VSMC_M10	-0.03	8.74E-01	-0.16	4.37E-01	-0.25	2.10E-01	0.21	2.89E-01	0.15	4.47E-01	-0.16	4.23E-01	0.27	1.81E-01	-0.11	5.80E-01	0.16	4.11E-01	0.15	4.55E-01
ENSCAFG0000000034	RUFY1	yellow	VSMC_M3	-0.03	8.74E-01	0.10	6.16E-01	0.62	5.30E-04	-0.59	1.08E-03	-0.49	8.69E-03	-0.13	5.22E-01	0.32	1.07E-01	0.36	6.46E-02	-0.05	8.03E-01	-0.18	3.71E-01
ENSCAFG0000000522	ANKRD16	darkgreen	VSMC_M4	-0.03	8.73E-01	0.45	1.88E-02	-0.20	3.20E-01	0.72	2.27E-05	0.03	8.79E-01	-0.08	6.93E-01	-0.03	8.74E-01	-0.76	3.62E-06	-0.30	1.27E-01	0.69	6.32E-05
ENSCAFG0000000640	SH3RF2	grey	VSMC_M10	-0.03	8.73E-01	0.68	8.77E-05	-0.01	9.56E-01	0.53	4.31E-03	-0.26	1.95E-01	0.20	3.25E-01	-0.28	1.51E-01	-0.57	1.88E-03	-0.64	2.88E-04	0.84	3.44E-08
ENSCAFG0000000294	HOXA4	grey	VSMC_M10	-0.03	8.73E-01	0.39	4.48E-02	0.25	2.02E-01	-0.17	3.97E-01	-0.42	2.95E-02	0.23	2.42E-01	-0.09	6.52E-01	0.06	7.53E-01	-0.44	2.29E-02	0.41	3.46E-02
ENSCAFG0000000311	GJA10	grey	VSMC_M10	-0.03	8.73E-01	0.12	5.36E-01	-0.21	2.92E-01	0.29	1.40E-01	0.09	6.56E-01	0.21	3.01E-01	0.16	4.32E-01	-0.24	2.20E-01	-0.11	5.85E-01	0.37	5.79E-02
ENSCAFG0000002661	ENSCAFG0000002661	grey	VSMC_M10	-0.03	8.73E-01	0.03	8.82E-01	0.56	2.42E-03	-0.46	1.59E-02	-0.41	3.31E-02	-0.16	4.35E-01	0.13	5.29E-01	0.23	2.44E-01	0.06	7.67E-01	-0.33	9.80E-02
ENSCAFG0000003149	BMP2	pink	VSMC_M5	-0.03	8.72E-01	-0.52	5.27E-03	-0.67	1.39E-04	0.31	1.13E-01	0.59	1.17E-03	0.09	6.59E-01	0.17	3.91E-01	-0.07	7.30E-01	0.39	4.55E-02	-0.12	5.56E-01
ENSCAFG0000002188	ENSCAFG0000002188	grey	VSMC_M10	-0.03	8.72E-01	0.08	6.98E-01	0.07	7.32E-01	-0.03	8.96E-01	-0.15	4.61E-01	-0.06	7.55E-01	0.05	7.86E-01	0.05	8.15E-01	-0.13	5.13E-01	0.16	4.12E-01
ENSCAFG0000000424	ARHGAP21	grey	VSMC_M10	-0.03	8.72E-01	-0.22	2.77E-01	-0.52	5.41E-03	0.27	1.67E-01	0.47	1.39E-02	-0.04	8.35E-01	0.48	1.21E-02	-0.09	6.69E-01	0.09	6.64E-01	0.04	8.47E-01
ENSCAFG0000003152	CROCC2	grey	VSMC_M10	-0.03	8.72E-01	0.13	5.05E-01	-0.04	8.44E-01	0.19	3.46E-01	-0.08	6.83E-01	-0.09	6.69E-01	0.44	2.02E-02	-0.25	2.10E-01	-0.17	3.99E-01	0.30	1.31E-01
ENSCAFG0000001019	RNF144B	grey	VSMC_M10	-0.03	8.72E-01	-0.08	6.90E-01	-0.12	5.60E-01	0.16	4.14E-01	0.06	7.82E-01	0.17	3.91E-01	0.03	8.86E-01	-0.12	5.48E-01	0.12	5.37E-01	0.14	4.76E-01
ENSCAFG0000001901	CPD	pink	VSMC_M5	-0.03	8.72E-01	-0.49	1.00E-02	-0.80	6.07E-07	0.30	1.27E-01	0.84	5.83E-08	-0.14	5.02E-01	0.03	8.81E-01	-0.11	5.96E-01	0.47	1.26E-02	-0.23	2.56E-01
ENSCAFG0000000936	HPS1	yellow	VSMC_M3	-0.03	8.72E-01	0.41	3.21E-02	0.65	2.48E-04	-0.42	2.98E-02	-0.63	4.46E-04	0.14	4.78E-01	-0.09	6.54E-01	0.15	4.67E-01	-0.32	1.05E-01	0.12	5.53E-01
ENSCAFG0000003273	ENSCAFG0000003273	grey	VSMC_M10	-0.03	8.71E-01	0.29	1.39E-01	0.02	9.28E-01	0.23	2.55E-01	-0.14	4.98E-01	-0.13	5.33E-01	-0.35	7.28E-02	-0.20	3.19E-01	-0.31	1.14E-01	0.35	7.19E-02
ENSCAFG0000000902	TMEM64	darkgreen	VSMC_M4	-0.03	8.71E-01	0.31	1.10E-01	-0.30	1.29E-01	0.72	2.05E-05	0.22	2.65E-01	-0.16	4.11E-01	-0.49	9.60E-03	-0.72	2.07E-05	-0.19	3.30E-01	0.42	3.12E-02
ENSCAFG0000000442	PPM1N	grey	VSMC_M10	-0.03	8.71E-01	0.29	1.43E-01	0.18	3.64E-01	-0.16	4.27E-01	-0.20	3.09E-01	-0.04	8.62E-01	0.17	3.86E-01	-0.05	7.97E-01	-0.22	2.69E-01	0.23	2.42E-01
ENSCAFG0000000686	FAHD2A	grey	VSMC_M10	-0.03	8.71E-01	0.28	1.51E-01	0.05	8.08E-01	0.16	4.20E-01	-0.20	3.25E-01	0.09	6.69E-01	0.14	4.87E-01	-0.22	2.71E-01	-0.31	1.11E-01	0.40	4.14E-02
ENSCAFG0000002945	ENSCAFG0000002945	turquoise	VSMC_M6	-0.03	8.71E-01	-0.07	7.41E-01	-0.06	7.77E-01	0.13	5.34E-01	-0.08	7.03E-01	0.30	1.23E-01	0.10	6.13E-01	0.03	9.01E-01	-0.02	9.31E-01	0.11	5.74E-01
ENSCAFG0000003030	ENSCAFG0000003030	grey	VSMC_M10	-0.03	8.71E-01	0.37	5.95E-02	0.06	7.63E-01	0.28	1.59E-01	-0.24	2.31E-01	0.06	7.53E-01	0.31	1.16E-01	-0.26	1.87E-01	-0.44	2.11E-02	0.54	3.30E-03
ENSCAFG0000002004	AGL	grey	VSMC_M10	-0.03	8.71E-01	-0.05	7.86E-01	-0.43	2.45E-02	0.25	2.00E-01	0.42	2.98E-02	-0.25	2.10E-01	0.04	8.44E-01	-0.27	1.71E-01	0.14	4.77E-01	0.12	5.66E-01
ENSCAFG0000000183	RUNDC3B	grey	VSMC_M10	-0.03	8.70E-01	0.43	2.52E-02	0.02	9.10E-01	0.26	1.97E-01	-0.14	5.01E-01	-0.04	8.38E-01	-0.16	4.16E-01	-0.34	8.60E-02	-0.35	7.24E-02	0.40	3.84E-02
ENSCAFG0000001830	PCP2	grey	VSMC_M10	-0.03	8.70E-01	0.43	2.52E-02	0.02	9.10E-01	0.26	1.97E-01	-0.14	5.01E-01	-0.04	8.38E-01	-0.16	4.16E-01	-0.34	8.60E-02	-0.35	7.24E-02	0.40	3.84E-02
ENSCAFG0000000467	AKAP11	pink	VSMC_M5	-0.03	8.70E-01	0.09	6.63E-01	-0.48	1.11E-02	0.33	8.79E-02	0.46	1.49E-02	-0.17	4.05E-01	-0.19	3.37E-01	-0.34	8.75E-02	0.02	9.31E-01	0.16	4.12E-01
ENSCAFG0000001255	SLC12A8	grey	VSMC_M10	-0.03	8.70E-01	0.42	2.84E-02	-0.18	3.81E-01	0.45	1.78E-02	0.01	9.63E-01	-0.13	5.09E-01	-0.11	5.78E-01	-0.45	1.87E-02	-0.37	6.05E-02	0.58	1.56E-03

ENSCAFG0000000899	OTULINL	pink	VSMC_M5	-0.03	8.70E-01	-0.54	3.37E-03	-0.66	1.55E-04	0.25	2.17E-01	0.77	3.32E-06	-0.15	4.51E-01	0.11	5.73E-01	-0.05	8.17E-01	0.52	5.98E-03	-0.42	3.10E-02
ENSCAFG0000000418	HEPHL1	grey	VSMC_M10	-0.03	8.70E-01	0.29	1.35E-01	-0.39	4.43E-02	0.56	2.63E-03	0.22	2.67E-01	0.19	3.53E-01	-0.24	2.27E-01	-0.62	6.20E-04	-0.22	2.61E-01	0.56	2.29E-03
ENSCAFG0000001909	AFF2	grey	VSMC_M10	-0.03	8.70E-01	-0.23	2.54E-01	-0.20	3.16E-01	-0.04	8.42E-01	0.05	8.21E-01	0.37	5.65E-02	0.16	4.29E-01	0.12	5.54E-01	0.17	4.00E-01	0.08	6.96E-01
ENSCAFG0000000478	GAB2	grey	VSMC_M10	-0.03	8.70E-01	0.47	1.26E-02	-0.09	6.57E-01	0.41	3.61E-02	-0.03	8.64E-01	0.06	7.59E-01	-0.59	1.23E-03	-0.56	2.53E-03	-0.32	1.02E-01	0.49	9.99E-03
ENSCAFG0000000105	MTSS1	grey	VSMC_M10	-0.03	8.70E-01	0.24	2.21E-01	0.47	1.27E-02	-0.23	2.50E-01	-0.54	3.81E-03	0.48	1.09E-02	0.16	4.16E-01	0.06	7.67E-01	-0.22	2.72E-01	0.19	3.30E-01
ENSCAFG0000000145	GCAT	darkgrey	VSMC_M8	-0.03	8.70E-01	-0.07	7.26E-01	0.45	1.81E-02	-0.72	2.49E-05	-0.43	2.39E-02	0.20	3.08E-01	0.39	4.64E-02	0.69	6.33E-05	-0.05	8.14E-01	-0.21	2.90E-01
ENSCAFG0000003137	ENSCAFG0000003137	grey	VSMC_M10	-0.03	8.70E-01	0.14	4.92E-01	0.17	3.96E-01	-0.14	4.87E-01	-0.29	1.45E-01	0.30	1.27E-01	0.13	5.22E-01	0.08	6.98E-01	-0.16	4.32E-01	0.19	3.46E-01
ENSCAFG0000000934	SLC40A1	grey	VSMC_M10	-0.03	8.69E-01	0.50	8.23E-03	0.12	5.58E-01	0.17	4.06E-01	-0.12	5.61E-01	-0.12	5.48E-01	0.01	9.65E-01	-0.29	1.49E-01	-0.43	2.66E-02	0.28	1.51E-01
ENSCAFG0000000873	ENSCAFG0000000873	grey	VSMC_M10	-0.03	8.69E-01	0.33	9.57E-02	0.19	3.35E-01	0.15	4.52E-01	-0.27	1.80E-01	0.00	9.98E-01	0.14	4.83E-01	-0.27	1.67E-01	-0.25	2.17E-01	0.36	6.63E-02
ENSCAFG0000000678	TMEM127	grey	VSMC_M10	-0.03	8.69E-01	0.56	2.59E-03	0.06	7.64E-01	0.47	1.32E-02	-0.27	1.75E-01	0.15	4.57E-01	-0.36	6.76E-02	-0.61	6.93E-04	-0.39	4.49E-02	0.65	2.23E-04
ENSCAFG0000001255	NCSTN	darkgreen	VSMC_M4	-0.03	8.69E-01	0.65	2.36E-04	-0.05	8.20E-01	0.65	2.43E-04	-0.16	4.39E-01	0.02	9.38E-01	-0.37	5.69E-02	-0.75	6.22E-06	-0.50	8.47E-03	0.77	2.28E-06
ENSCAFG0000002377	ENSCAFG0000002377	grey	VSMC_M10	-0.03	8.69E-01	0.22	2.63E-01	0.25	2.12E-01	-0.09	6.51E-01	-0.34	7.94E-02	-0.16	4.15E-01	-0.04	8.30E-01	0.10	6.32E-01	-0.26	1.99E-01	0.19	3.38E-01
ENSCAFG0000001377	ENSCAFG0000001377	grey	VSMC_M10	-0.03	8.69E-01	0.01	9.51E-01	-0.03	8.88E-01	-0.11	5.82E-01	-0.02	9.39E-01	-0.02	9.09E-01	0.12	5.48E-01	0.02	9.05E-01	-0.03	8.79E-01	0.05	8.00E-01
ENSCAFG0000000041	ENSCAFG0000000041	darkolivegreen	VSMC_M9	-0.03	8.69E-01	-0.80	7.07E-07	-0.36	6.64E-02	-0.35	7.10E-02	0.50	7.55E-03	0.02	9.31E-01	0.22	2.74E-01	0.57	1.89E-03	0.73	1.48E-05	-0.77	2.88E-06
ENSCAFG0000000589	HARS	grey	VSMC_M10	-0.03	8.68E-01	-0.59	1.27E-03	-0.08	6.89E-01	-0.28	1.64E-01	0.19	3.44E-01	0.00	1.00E+00	-0.05	8.03E-01	0.52	5.14E-03	0.50	7.74E-03	-0.68	8.38E-05
ENSCAFG0000002542	ENSCAFG0000002542	grey	VSMC_M10	-0.03	8.68E-01	0.27	1.71E-01	0.22	2.69E-01	-0.12	5.49E-01	-0.23	2.54E-01	-0.01	9.47E-01	-0.10	6.14E-01	0.02	9.19E-01	-0.21	2.88E-01	0.14	4.89E-01
ENSCAFG0000001692	ABI3	turquoise	VSMC_M6	-0.03	8.68E-01	-0.05	8.12E-01	0.05	7.87E-01	-0.08	6.77E-01	-0.07	7.15E-01	0.53	4.06E-03	0.05	7.97E-01	0.08	6.85E-01	0.06	7.58E-01	0.00	9.84E-01
ENSCAFG0000000340	MET	grey	VSMC_M10	-0.03	8.68E-01	0.15	4.59E-01	0.28	1.51E-01	-0.40	3.83E-02	-0.23	2.46E-01	-0.19	3.35E-01	0.01	9.46E-01	0.37	5.69E-02	-0.20	3.29E-01	-0.14	4.77E-01
ENSCAFG0000001167	ALPK1	grey	VSMC_M10	-0.03	8.68E-01	0.26	1.98E-01	-0.33	9.75E-02	0.60	8.90E-04	0.28	1.52E-01	-0.17	3.86E-01	0.12	5.54E-01	-0.62	6.28E-04	-0.19	3.43E-01	0.33	9.10E-02
ENSCAFG0000003120	ZCCHC10	grey	VSMC_M10	-0.03	8.68E-01	-0.28	1.53E-01	-0.19	3.40E-01	-0.04	8.31E-01	0.29	1.36E-01	-0.12	5.67E-01	0.03	8.86E-01	0.14	4.97E-01	0.23	2.42E-01	-0.32	1.02E-01
ENSCAFG0000001100	TCF7L2	grey	VSMC_M10	-0.03	8.67E-01	0.23	2.41E-01	-0.22	2.76E-01	0.24	2.35E-01	0.11	5.76E-01	0.03	8.91E-01	0.44	2.26E-02	-0.31	1.22E-01	-0.26	1.84E-01	0.41	3.58E-02
ENSCAFG0000001940	MRPL41	grey	VSMC_M10	-0.03	8.67E-01	0.22	2.80E-01	0.51	6.26E-03	-0.40	3.75E-02	-0.60	8.66E-04	0.17	4.06E-01	0.19	3.33E-01	0.24	2.30E-01	-0.23	2.39E-01	0.15	4.53E-01
ENSCAFG0000001097	ARMC9	grey	VSMC_M10	-0.03	8.66E-01	-0.17	3.88E-01	-0.51	7.11E-03	0.48	1.17E-02	0.32	1.08E-01	0.28	1.63E-01	0.29	1.37E-01	-0.31	1.22E-01	0.10	6.18E-01	0.30	1.24E-01
ENSCAFG0000000412	SESN3	grey	VSMC_M10	-0.03	8.66E-01	0.22	2.64E-01	-0.45	1.79E-02	0.58	1.47E-03	0.22	2.62E-01	0.11	5.96E-01	0.20	3.19E-01	-0.48	1.18E-02	-0.25	2.12E-01	0.57	2.10E-03
ENSCAFG0000000037	ENSCAFG0000000037	darkgreen	VSMC_M4	-0.03	8.66E-01	0.12	5.39E-01	-0.61	7.69E-04	0.79	8.85E-07	0.48	1.06E-02	-0.23	2.41E-01	-0.35	7.76E-02	-0.65	2.52E-04	-0.02	9.39E-01	0.38	4.90E-02
ENSCAFG0000001056	CFAP43	grey	VSMC_M10	-0.03	8.66E-01	0.01	9.74E-01	0.10	6.37E-01	-0.14	4.97E-01	-0.16	4.22E-01	-0.03	8.76E-01	0.32	1.09E-01	0.15	4.62E-01	-0.11	5.86E-01	0.04	8.37E-01
ENSCAFG0000002969	ENSCAFG0000002969	grey	VSMC_M10	-0.03	8.66E-01	0.01	9.74E-01	0.10	6.37E-01	-0.14	4.97E-01	-0.16	4.22E-01	-0.03	8.76E-01	0.32	1.09E-01	0.15	4.62E-01	-0.11	5.86E-01	0.04	8.37E-01
ENSCAFG0000002873	ENSCAFG0000002873	grey	VSMC_M10	-0.03	8.66E-01	0.18	3.63E-01	0.00	9.91E-01	0.15	4.50E-01	-0.19	3.48E-01	-0.18	3.74E-01	0.22	2.65E-01	-0.13	5.28E-01	-0.28	1.55E-01	0.39	4.25E-02
ENSCAFG0000001982	CDK10	grey	VSMC_M10	-0.03	8.65E-01	-0.01	9.68E-01	-0.28	1.54E-01	0.13	5.18E-01	0.31	1.15E-01	-0.22	2.67E-01	-0.07	7.32E-01	-0.12	5.46E-01	0.00	9.95E-01	-0.06	7.74E-01
ENSCAFG0000001927	CLUAP1	grey	VSMC_M10	-0.03	8.65E-01	-0.01	9.55E-01	-0.14	4.91E-01	0.17	3.84E-01	0.07	7.23E-01	-0.06	7.82E-01	0.36	6.86E-02	-0.18	3.70E-01	-0.06	7.82E-01	0.24	2.33E-01
ENSCAFG0000000829	FZD3	grey	VSMC_M10	-0.03	8.65E-01	-0.16	4.11E-01	-0.55	3.09E-03	0.46	1.67E-02	0.44	2.19E-02	0.13	5.31E-01	0.14	5.00E-01	-0.33	8.81E-02	0.09	6.62E-01	0.16	4.29E-01
ENSCAFG0000003249	ZNF785	grey	VSMC_M10	-0.03	8.65E-01	0.04	8.40E-01	-0.42	3.12E-02	0.43	2.67E-02	0.41	3.14E-02	-0.15	4.45E-01	-0.37	5.63E-02	-0.39	4.34E-02	0.13	5.30E-01	0.10	6.06E-01
ENSCAFG0000003262	ENSCAFG0000003262	grey	VSMC_M10	-0.03	8.64E-01	0.11	5.77E-01	-0.16	4.27E-01	0.39	4.52E-02	0.03	8.70E-01	0.05	8.14E-01	-0.23	2.47E-01	-0.31	1.19E-01	-0.08	6.76E-01	0.25	2.18E-01
ENSCAFG0000001214	PRC1	darkgrey	VSMC_M8	-0.03	8.64E-01	-0.58	1.51E-03	0.28	1.50E-01	-0.86	8.14E-09	-0.14	4.77E-01	0.07	7.37E-01	0.40	3.84E-02	0.97	1.75E-17	0.42	2.99E-02	-0.74	1.17E-05
ENSCAFG0000001542	KIAA0753	yellow	VSMC_M3	-0.03	8.64E-01	0.20	3.15E-01	0.59	1.16E-03	-0.34	8.40E-02	-0.63	4.24E-04	0.25	2.10E-01	-0.19	3.52E-01	0.20	3.20E-01	-0.20	3.21E-01	0.07	7.19E-01
ENSCAFG0000001254	ATRIP	grey	VSMC_M10	-0.03	8.64E-01	0.02	9.36E-01	0.28	1.62E-01	-0.52	5.24E-03	-0.32	1.07E-01	0.03	8.86E-01	0.05	7.99E-01	0.47	1.34E-02	-0.09	6.68E-01	-0.09	6.41E-01
ENSCAFG0000000660	VSTM4	grey	VSMC_M10	-0.03	8.64E-01	0.35	7.10E-02	0.20	3.20E-01	-0.16	4.39E-01	-0.24	2.23E-01	0.28	1.58E-01	-0.36	6.84E-02	-0.03	8.81E-01	-0.27	1.72E-01	0.15	4.52E-01
ENSCAFG0000000585	PPP1R27	cyan	VSMC_M2	-0.03	8.64E-01	0.45	1.81E-02	0.34	8.10E-02	-0.15	4.52E-01	-0.38	4.85E-02	-0.08	6.96E-01	0.09	6.40E-01	0.02	9.25E-01	-0.42	2.81E-02	0.35	7.79E-02
ENSCAFG0000002372	TSPYL1	turquoise	VSMC_M6	-0.03	8.63E-01	0.23	2.40E-01	0.46	1.57E-02	-0.24	2.20E-01	-0.51	6.07E-03	0.59	1.23E-03	0.07	7.31E-01	0.08	6.93E-01	-0.21	2.87E-01	0.13	5.17E-01
ENSCAFG0000002613	ENSCAFG0000002613	grey	VSMC_M10	-0.03	8.63E-01	0.31	1.12E-01	0.01	9.54E-01	0.25	2.06E-01	-0.15	4.68E-01	0.01	9.55E-01	0.24	2.34E-01	-0.28	1.63E-01	-0.29	1.44E-01	0.40	3.65E-02
ENSCAFG0000002443	GEMIN4	darkgrey	VSMC_M8	-0.03	8.63E-01	-0.14	4.75E-01	0.52	5.14E-03	-0.73	1.74E-05	-0.44	2.04E-02	0.13	5.24E-01	0.32	1.04E-01	0.71	2.95E-05	0.01	9.69E-01	-0.41	3.50E-02
ENSCAFG0000001177	C2CD5	grey	VSMC_M10	-0.03	8.63E-01	-0.12	5.56E-01	-0.05	8.08E-01	0.18	3.68E-01	-0.05	8.03E-01	-0.04	8.58E-01	0.29	1.48E-01	-0.10	6.26E-01	0.06	7.80E-01	0.19	3.35E-01
ENSCAFG0000002985	ZNF48	grey	VSMC_M10	-0.04	8.62E-01	0.46	1.64E-02	0.01	9.79E-01	0.36	6.45E-02	-0.06	7.57E-01	-0.04	8.40E-01	-0.05	7.90E-01	-0.51	6.68E-03	-0.36	6.66E-02	0.41	3.54E-02
ENSCAFG0000002333	C3H5orf30	grey	VSMC_M10	-0.04	8.62E-01	0.20	3.10E-01	0.38	5.21E-02	-0.22	2.64E-01	-0.47	1.30E-02	-0.03	8.80E-01	-0.11	5.81E-01	0.13	5.10E-01	-0.18	3.68E-01	0.19	3.46E-01
ENSCAFG0000003066	ENSCAFG0000003066	cyan	VSMC_M2	-0.04	8.62E-01	0.31	1.19E-01	0.40	4.14E-02	-0.16	4.33E-01	-0.42	3.10E-02	0.07	7.41E-01	0.08	6.97E-01	-0.04	8.24E-01	-0.27	1.76E-01	0.18	3.70E-01
ENSCAFG0000000611	ARHGEF7	grey	VSMC_M10	-0.04	8.62E-01	0.01	9.78E-01	0.02	9.39E-01	-0.02	9.06E-01	0.19	3.49E-01	-0.05	8.01E-01	-0.37	5.58E-02	-0.11	6.01E-01	0.17	3.84E-01	-0.35	7.16E-02
ENSCAFG0000000233	ENSCAFG0000000233	grey	VSMC_M10	-0.04	8.62E-01	-0.29	1.38E-01	-0.02	9.06E-01	-0.17	3.95E-01	0.00	9.97E-01	-0.05	7.91E-01	-0.03	8.92E-01	0.26	1.93E-01	0.30	1.26E-01	-0.20	3.10E-01
ENSCAFG0000000137	SCRIB	yellow	VSMC_M3	-0.04	8.61E-01	0.21	2.97E-01	0.75	7.46E-06	-0.61	7.94E-04	-0.74	8.78E-06	0.27	1.77E-01	0.32	1.09E-01	0.41	3.51E-02	-0.27	1.78E-01	-0.02	9.19E-01
ENSCAFG0000002501	C11H5orf24	grey	VSMC_M10	-0.04	8.61E-01	0.08	6.92E-01	0.08	6.76E-01	0.11	5.92E-01	-0.09	6.39E-01	0.01	9.80E-01	-0.59	1.27E-03	-0.14	4.90E-01	0.00	9.81E-01	0.07	7.32E-01
ENSCAFG0000000856	WDR54	grey	VSMC_M10	-0.04	8.61E-01	-0.06	7.81E-01	-0.18	3.72E-01	0.12	5.65E-01	-0.03	8.82E-01	0.18	3.59E-01	0.32	1.08E-01	-0.03	8.79E-01	-0.04	8.42E-01	0.38	5.23E-02
ENSCAFG0000003094	ENSCAFG0000003094	grey	VSMC_M10	-0.04	8.61E-01	-0.21	2.92E-01	0.08	7.04E-01	-0.22	2.79E-01	0.08	7.06E-01	-0.09	6.48E-01	0.13	5.30E-01	0.24	2.35E-01	0.22	2.61E-01	-0.43	2.38E-02
ENSCAFG0000000330	AAK1	grey	VSMC_M10	-0.04	8.61E-01	0.10	6.10E-01	-0.54	3.42E-03	0.55	2.93E-03	0.36	6.90E-02	0.13	5.25E-01	-0.26	1.91E-01	-0.46	1.68E-02	-0.06	7.60E-01	0.47	1.25E-02
ENSCAFG0000001050	UBE2T	darkgrey	VSMC_M8	-0.04	8.61E-01	-0.36	6.71E-02	0.48	1.22E-02	-0.83	6.93E-08	-0.41	3.24E-02	0.07	7.42E-01	0.52	5.04E-03	0.87	3.60E-09	0.20	3.25E-01	-0.48	1.20E-02
ENSCAFG0000000991	WDR3	darkgrey	VSMC_M8	-0.04	8.61E-01	-0.54	3.92E-03	0.23	2.40E-01	-0.56	2.14E-03	-0.21	2.90E-01	0.16	4.12E-01	0.25	2.10E-01	0.74	8.59E-06	0.33	8.85E-02	-0.52	5.73E-03
ENSCAFG0000003251	C15H1orf109	grey	VSMC_M10	-0.04	8.61E-01	-0.19	3.50E-01	0.42	2.91E-02	-0.47	1.38E-02	-0.36	6.52E-02	0.03	8.67E-01	0.29	1.39E-01	0.48	1.07E-02	0.09	6.43E-01	-0.30	1.24E-01
ENSCAFG0000000631	ENSCAFG0000000631	grey	VSMC_M10	-0.04	8.61E-01	-0.16	4.31E-01	-0.51	7.11E-03	0.47	1.40E-02	0.47	1.41E-02	0.07	7.42E-01	-0.21	2.94E-01	-0.40	3.68E-02	0.21	2.83E-01	0.06	7.82E-01
ENSCAFG0000001361	PRAME	turquoise	VSMC_M6	-0.04	8.61E-01	0.04	8.57E-01	0.07	7.30E-01	-0.19	3.35E-01	-0.19	3.47E-01	0.49	8.67E-03	-0.11	5.75E-01	0.20	3.09E-01	-0.04	8.24E-01	0.07	7.47E-01
ENSCAFG0000003253	ENSCAFG0000003253	grey	VSMC_M10	-0.04	8.60E-01	0.09	6.55E-01	-0.17	3.93E-01	0.25	2.15E-01	0.03	8.94E-01	0.12	5.45E-01	0.12	5.38E-01	-0.20	3.13E-01	-0.04	8.57E-01	0.31	1.11E-01
ENSCAFG0000002890	WDR83OS	grey	VSMC_M10	-0.04	8.60E-01	0.27	1.80E-01	0.58	1.39E-03	-0.35	7.21E-02	-0.53	4.22E-03	-0.07	7.19E-01	-0.04	8.52E-01	0.22	2.75E-01	-0.21	3.04E-01	-0.08	6.76E-01
ENSCAFG0000003266	SPCS2	grey	VSMC_M10	-0.04	8.60E-01	-0.52	5.26E-03	-0.37	5.51E-02	-0.21	2.94E-01	0.39	4.45E-02	0.10	6.32E-01	0.27	1.68E-01	0.43	2.34E-02	0.36	6.51E-02	-0.39	4.34E-02
ENSCAFG0000002548	ENSCAFG0000002548	grey	VSMC_M10	-0.04	8.59E-01	0.17	3.91E-01	0.30	1.32E-01	0.04	8.36E-01	-0.36	6.87E-02	0.08	6.86E-01	0.28	1.52E-01	-0.15	4.70E-01	-0.20	3.13E-01	0.28	1.62E-01
ENSCAFG0000002505	LAMB1	grey	VSMC_M10	-0.04	8.59E-01	0.04	8.31E-01	0.48	1.15E-02	-0.51	6.62E-03	-0.54	3.69E-03	0.52	5.62E-03	-0.03	8.90E-01	0.43	2.55E-02	-0.11	5.80E-01	-0.03	8.93E-01
ENSCAFG0000001099	LGMN	darkgreen	VSMC_M4	-0.04	8.59E-01	0.28	1.58E-01	-0.47	1.34E-02	0.73	1.31E-05	0.33	8.85E-02	-0.11	5.92E-01	-0.25	2.03E-01	-0.78	1.81E-06	-0.12	5.55E-01	0.53	4.28E-03
ENSCAFG0000000113	ITPR3	yellow	VSMC_M3	-0.04	8.59E-01	-0.14	4.86E-01	0.30	1.30E-01	-0.60	9.54E-04	-0.17	3.86E-01	-0.10	6.14E-01	0.37	5.65E-02	0.46	1.62E-02	0.15	4.51E-01	-0.34	8.62E-02
ENSCAFG0000001996	FNBP1	darkgreen	VSMC_M4	-0.04	8.59E-01	0.22	2.62E-01	-0.58	1.52E-03	0.91	7.67E-11	0.46	1.51E-02	-0.15	4.59E-01	-0.35	7.63E-02	-0.77	2.64E-06	-0.10	6.08E-01	0.44	2.11E-02
ENSCAFG0000000126	TSNARE1	grey	VSMC_M10	-0.04	8.58E-01	0.39	4.38E-02	0.09	6.56E-01	0.30	1.33E-01	-0.23	2.41E-01	-0.26	1.84E-01	0.16	4.14E-01	-0.37	5.47E-02	-0.38	4.79E-02	0.62	5.43E-04
ENSCAFG0000002432	ENSCAFG0000002432	grey	VSMC_M10	-0.04	8.58E-01	-0.02	9.08E-01	-0.13	5.19E-01	0.01	9.63E-01	0.24	2.28E-01	-0.28	1.62E-01	0.21	2.95E-01	-0.09	6.67E-01	0.09	6.42E-01	-0.16	4.28E-01
ENSCAFG0000002082	ENSCAFG0000002082	grey	VSMC_M10	-0.04	8.58E-01	0.21	2.82E-01	0.11	5.88E-01	-0.05	8.07E-01	-0.17	4.03E-01	0.01	9.58E-01	0.00	9.95E-01	-0.14	4.77E-01	-0.19	3.42E-01	0.19	3.45E-01
ENSCAFG0000002870	SCD5	darkgrey	VSMC_M8	-0.04	8.58E-01	-0.36	6.34E-02	0.00	9.94E-01	-0.52	5.51E-03	0.08	6.78E-01	-0.09	6.68E-01	0.30	1.28E-01	0.54	3.55E-03	0.29	1.48E-01	-0.42	2.76E-02
ENSCAFG0000002946	CDC34	yellow	VSMC_M3	-0.04	8.57E-01	0.17	4.02E-01	0.73	1.50E-05	-0.44	2.29E-02	-0.77	2.89E-06	0.14	4.85E-01	0.10	6.26E-01	0.33	9.14E-02	-0.21	2.96E-01	0.03	8.68E-01
ENSCAFG0000000185	DLK2	grey	VSMC_M10	-0.04	8.57E-01	0.05	8.15E-01	0.06	7.84E-01	-0.08	6.94E-01	-0.20	3.25E-01	0.28	1.57E-01	0.02	9.39E-01	0.04	8.24E-01	-0.01	9.54E-01	0.19	3.45E-01
ENSCAFG0000000199	CDC5L	darkgrey	VSMC_M8	-0.04	8.57E-01	-0.73	1.28E-05	-0.15	4.64E-01	-0.59	1.17E-03	0.31	1.21E-01	-0.06	7.68E-01	0.43	2.46E-02	0.77	2.28E-06	0.58	1.62E-03	-0.79	8.33E-07
ENSCAFG0000002003	TBC1D13	darkgreen	VSMC_M4	-0.04	8.57E-01	0.24	2.35E-01	-0.45	1.73E-02	0.70	5.51E-05	0.23	2.56E-01	0.12	5.50E-01	-0.06	7.74E-01	-0.57	2.00E-03	-0.22	2.60E-01	0.58	1.35E-03
ENSCAFG0000001947	ARHGEF16	grey	VSMC_M10	-0.04	8.57E-01	0.02	9.32E-01	0.06	7.50E-01	0.01	9.71E-01	-0.12	5.56E-01	0.02	9.07E-01	0.01	9.75E-01	0.06	7.71E-01	-0.04	8.29E-01	0.07	7.21E-01
ENSCAFG0000000974	UBE2C	darkgrey	VSMC_M8	-0.04	8.57E-01	-0.46	1.70E-02	0.42	3.11E-02	-0.91	7.93E-11	-0.32	1.01E-01	0.20	3.23E-01	0.39	4.41E-02	0.97	3.97E-16	0.29	1.39E-01	-0.62	5.21E-04
ENSCAFG0000000152	EXOSC4	grey	VSMC_M10	-0.04	8.57E-01	0.20	3.11E-01	0.55	3.23E-03	-0.16	4.39E-01	-0.62	5.97E-04	0.14	4.95E-01	-0.06	7.79E-01	0.11	5.83E-01	-0.19	3.50E-01	0.11	5.75E-01
ENSCAFG0000000889	URB1	grey	VSMC_M10	-0.04	8.57E-01	-0.42	3.08E-02	-0.33	9.73E-02	0.09	6.71E-01	0.24	2.36E-01	0.18	3.58E-01	0.09	6.60E-01	0.15	4.51E-01	0.27	1.72E-01	-0.15	4.60E-01
ENSCAFG0000002363	ZBTB33	grey	VSMC_M10	-0.04	8.57E-01	0.27	1.73E-01	-0.43	2.61E-02	0.55	2.80E-03	0.27	1.71E-01	-0.10	6.32E-01	-0.23	2.49E-01	-0.56	2.43E-03	-0.24	2.30E-01	0.53	4.30E-03
ENSCAFG0000001123	LYST	pink	VSMC_M5	-0.04	8.57E-01	-0.05	8.10E-01	-0.73	1.57E-05	0.62	5.29E-04	0.69	7.96E-05	-0.18	3.59E-01	-0.15	4.64E-01	-0.48	1.15E-02	0.10	6.28E-01	0.17	3.97E-01
ENSCAFG0000001407	TMEM42	grey	VSMC_M10	-0.04	8.57E-01	0.47	1.40E-02	0.46	1.60E-02	0.12	5.52E-01	-0.55	3.08E-03	-0.10	6.04E-01	0.05	8.17E-01	-0.20	3.15E-01	-0.42	2.73E-02	0.46	1.57E-02
ENSCAFG0000001679	BNIP1	grey	VSMC_M10	-0.04	8.56E-01	-0.33	9.46E-02	-0.41	3.23E-02	0.02	9.26E-01	0.49	9.35E-03	-0.16	4.20E-01	0.20	3.12E-01	0.05	7.95E-01	0.28	1.55E-01	-0.31	1.20E-01
ENSCAFG0000001047	CTTN	cyan	VSMC_M2	-0.04	8.56E-01	0.79	1.02E-06	0.64	3.45E-04	-0.04	8.53E-01	-0.67	1.40E-04	-0.13	5.32E-01	-0.16	4.34E-01	-0.29	1.42E-01	-0.63	4.11E-04	0.48	1.11E-02
ENSCAFG0000001824	SLC26A2	grey	VSMC_M10	-0.04	8.56E-01	-0.28	1.60E-01	-0.27	1.79E-01	-0.25	2.01E-01	0.36	6.13E-02	0.04	8.41E-01	0.13	5.12E-01	0.36	6.38E-02	0.25	2.02E-01	-0.43	2.57E-02
ENSCAFG0000001940	BRICD5	grey	VSMC_M10	-0.04	8.56E-01	-0.22	2.73E-01	-0.13	5.06E-01	0.03	8.92E-01	0.06	7.78E-01	-0.05	7.88E-01	-0.13	5.04E-01	0.12	5.44E-01	0.13	5.17E-01	-0.04	8.38E-01
ENSCAFG0000001019	RAB7B	grey	VSMC_M10	-0.04	8.55E-01	0.04	8.32E-01	-0.18	3.63E-01	0.22	2.59E-01	0.06	7.81E-01	0.06	7.56E-01	0.47	1.29E-02	-0.22	2.67E-01	-0.11	5.98E-01	0.32	1.05E-01
ENSCAFG0000000340	RRM2	darkgrey	VSMC_M8	-0.04	8.55E-01	-0.48	1.09E-02	0.39	4.17E-02	-0.90	2.99E-10	-0.31	1.14E-01	0.16	4.26E-01	0.47	1.35E-02	0.97	1.33E-16	0.31	1.10E-01	-0.60	1.01E-03
ENSCAFG0000000149	ENSCAFG0000000149	grey	VSMC_M10	-0.04	8.55E-01	0.07	7.44E-01	0.40	4.10E-02	-0.34	8.50E-02	-0.48	1.12E-02	0.21	2.82E-01	0.26	1.96E-01	0.27	1.72E-01	-0.17	3.84E-01	0.04	8.51E-01
ENSCAFG0000002944	ZNF688	grey	VSMC_M10	-0.04	8.55E-01	-0.01	9.55E-01	-0.40	4.09E-02	0.36	6.69E-02	0.31	1.19E-01	-0.27	1.68E-01	0.02	9.15E-01	-0.20	3.17E-01	0.01	9.63E-01	0.19	3.44E-01
ENSCAFG0000000086	ENPP2	turquoise	VSMC_M6	-0.04	8.55E-01	-0.06	7.61E-01	0.03	8.70E-01	-0.09	6.56E-01	-0.15	4.56E-01	0.38	4.92E-02	0.41	3.29E-02	0.13	5.29E-01	0.01	9.50E-01	0.08	6.97E-01
ENSCAFG0000000650	SIGIRR	violet	VSMC_M7	-0.04	8.55E-01	-0.05	7.95E-01	0.26	1.83E-01	-0.32	1.04E-01	-0.33	9.14E-02	0.10	6.22E-01	0.69	6.32E-05	0.27	1.71E-01	0.00	9.84E-01	0.05	8.11E-01
ENSCAFG0000001096	HTR2B	darkgreen	VSMC_M4	-0.04	8.55E-01	0.22	2.65E-01	-0.51	6.38E-03	0.86	7.21E-09	0.31	1.22E-01	0.17	3.91E-01	-0.30	1.27E-01	-0.73	1.87E-05	-0.17	3.97E-01	0.56	2.52E-03
ENSCAFG0000001773	ZMAT1	grey	VSMC_M10	-0.04	8.55E-01	0.24	2.21E-01	-0.25	2.00E-01	0.45	1.75E-02	0.07	7.39E-01	-0.14	4.82E-01	0.26	1.83E-01	-0.42	3.03E-02	-0.27	1.77E-01	0.52	5.09E-03
ENSCAFG0000001434	C1S	cyan	VSMC_M2	-0.04	8.55E-01	0.72	1.90E-05	-0.01	9.52E-01	0.57	1.85E-03	-0.15	4.58E-01	-0.05	8.05E-01	-0.35	7.12E-02	-0.70	4.23E-05	-0.62	6.30E-04	0.73	1.86E-05
ENSCAFG0000002799	ENSCAFG0000002799	turquoise	VSMC_M6	-0.04	8.54E-01	0.13	5.18E-01	0.17	4.03E-01	-0.19	3.46E-01	-0.20	3.26E-01	0.44	2.16E-02	0.00	9.89E-01	0.14	4.84E-01	-0.10	6.13E-01	0.08	6.87E-01
ENSCAFG0000000405	CABP5	grey	VSMC_M10	-0.04	8.54E-01	-0.39	4.34E-02	-0.17	4.00E-01	-0.26	1.91E-01	0.22	2.61E-01	0.03	9.01E-01	0.32	1.06E-01	0.37	5.65E-02	0.28	1.50E-01	-0.38	5.06E-02
ENSCAFG0000000971	DNAH1	grey	VSMC_M10	-0.04	8.53E-01	0.28	1.51E-01	-0.31	1.14E-01	0.60	9.36E-04	0.16	4.34E-01	-0.13	5.28E-01	-0.14	4.90E-01	-0.53	4.63E-03	-0.21	3.04E-01	0.54	3.84E-03
ENSCAFG0000001675	CPLX1	grey	VSMC_M10	-0.04	8.53E-01	0.10	6.12E-01	0.33	9.28E-02	-0.17	4.09E-01	-0.46	1.49E-02	0.19	3.31E-01	0.32	1.06E-01	0.15	4.41E-01	-0.21	2.90E-01	0.18	3.63E-01
ENSCAFG0000001031	METAP1	darkgrey	VSMC_M8	-0.04	8.52E-01	-0.76	3.63E-06	-0.30	1.24E-01	-0.26	1.86E-01	0.34	8.00E-02	0.04	8.58E-01	0.40	3.88E-02	0.59	1.35E-03	0.59	1.23E-03	-0.60	8.66E-04
ENSCAFG0000001930	RAP1GAP2	grey	VSMC_M10	-0.04	8.52E-01	0.37	6.11E-02	-0.26	1.93E-01	0.53	4.91E-03	0.13	5.13E-01	0.00	9.92E-01	-0.10	6.36E-01	-0.61	7.13E-04	-0.27	1.68E-01	0.56	2.54E-03
ENSCAFG0000000763	ACSS2	cyan	VSMC_M2	-0.04	8.52E-01	0.60	8.97E-04	0.29	1.47E-01	0.07	7.41E-01	-0.29	1.41E-01	-0.10	6.15E-01	0.11	5.81E-01	-0.33	9.29E-02	-0.48	1.22E-02	0.42	2.97E-02
ENSCAFG0000000504	EXOC7	cyan	VSMC_M2	-0.04	8.52E-01	0.66	1.58E-04	0.50	8.60E-03	0.05	8.03E-01	-0.52	5.71E-03	-0.23	2.43E-01	-0.24	2.33E-01	-0.39	4.61E-02	-0.46	1.51E-02	0.42	2.79E-02
ENSCAFG0000001403	ENSCAFG0000001403	grey	VSMC_M10	-0.04	8.52E-01	0.03	8.92E-01	-0.24	2.29E-01	0.27	1.72E-01	0.21	2.89E-01	-0.25	2.02E-01	-0.34	8.54E-02	-0.19	3.52E-01	0.09	6.68E-01	0.03	8.68E-01
ENSCAFG0000001787	GALNT1	grey	VSMC_M10	-0.04	8.52E-01	-0.01	9.60E-01	-0.15	4.57E-01	0.18	3.60E-01	0.13	5.05E-01	0.01	9.44E-01	-0.77	2.13E-06	-0.15	4.42E-01	0.13	5.34E-01	-0.06	7.73E-01
ENSCAFG0000002850	ENSCAFG0000002850	turquoise	VSMC_M6	-0.04	8.52E-01	0.02	9.35E-01	0.08	6.74E-01	-0.06	7.64E-01	-0.20	3.29E-01	0.38	4.92E-02	0.15	4.64E-01	0.07	7.15E-01	-0.02	9.04E-01	0.10	6.26E-01
ENSCAFG0000000659	MCM4	darkgrey	VSMC_M8	-0.04	8.52E-01	-0.48	1.19E-02	0.35	7.21E-02	-0.89	5.74E-10	-0.27	1.67E-01	0.16	4.32E-01	0.44	2.32E-02	0.97	1.38E-17	0.29	1.36E-01	-0.60	8.40E-04
ENSCAFG0000000213	IL18RAP	turquoise	VSMC_M6	-0.04	8.52E-01	-0.06	7.78E-01	0.07	7.20E-01	-0.13	5.23E-01	-0.09	6.55E-01	0.15	4.46E-01	0.22	2.61E-01	0.14	4.72E-01	0.03	8.76E-01	-0.02	9.16E-01
ENSCAFG0000000858	VCAN	darkolivegreen	VSMC_M9	-0.04	8.52E-01	-0.70	4.98E-05	-0.49	9.31E-03	-0.02	9.02E-01	0.62	5.40E-04	-0.26	1.95E-01	0.18	3.66E-01	0.22	2.63E-01	0.70	4.80E-05	-0.55	3.08E-03
ENSCAFG0000000068	ENSCAFG0000000068	grey	VSMC_M10	-0.04	8.51E-01	-0.07	7.15E-01	0.14	4.79E-01	-0.02	9.24E-01	-0.09	6.59E-01	-0.27	1.74E-01	0.03	8.68E-01	0.13	5.24E-01	0.03	8.93E-01	-0.13	5.33E-01
ENSCAFG0000001086	ENSCAFG0000001086	darkgrey	VSMC_M8	-0.04	8.51E-01	-0.63	4.75E-04	0.15	4.61E-01	-0.57	1.97E-03	-0.05	8.04E-01	0.06	7.79E-01	0.32	1.00E-01	0.70	5.39E-05	0.45	1.79E-02	-0.64	3.25E-04
ENSCAFG0000001708	EPN3	grey	VSMC_M10	-0.04	8.51E-01	0.35	7.57E-02	-0.05	7.88E-01	0.30	1.27E-01	-0.14	4.83E-01	0.34	8.13E-02	0.25	2.11E-01	-0.38	5.10E-02	-0.35	7.79E-02	0.53	4.68E-03
ENSCAFG0000000540	TEP1	cyan	VSMC_M2	-0.04	8.51E-01	0.66	1.94E-04	0.48	1.08E-02	0.15	4.48E-01	-0.48	1.09E-02	-0.11	5.72E-01	-0.16	4.40E-01	-0.41	3.16E-02	-0.50	7.68E-03	0.47	1.44E-02
ENSCAFG0000000449	GRIP2	turquoise	VSMC_M6	-0.04	8.51E-01	0.31	1.11E-01	0.14	4.76E-01	-0.05	8.10E-01	-0.25	2.05E-01	0.56	2.17E-03	-0.12	5.50E-01	-0.02	9.26E-01	-0.28	1.60E-01	0.23	2.38E-01
ENSCAFG0000000711	ENSCAFG0000000711	grey	VSMC_M10	-0.04	8.50E-01	0.09	6.39E-01	-0.14	4.73E-01	0.28	1.62E-01	-0.11	5.83E-01	0.44	2.22E-02	0.03	8.64E-01	-0.22	2.60E-01	-0.14	4.95E-01	0.48	1.16E-02
ENSCAFG0000000910	ZHX3	cyan	VSMC_M2	-0.04	8.50E-01	0.61	7.52E-04	0.22	2.75E-01	0.28	1.52E-01	-0.22	2.72E-01	-0.09	6.64E-01	-0.13	5.17E-01	-0.52	5.11E-03	-0.48	1.13E-02	0.42	3.06E-02
ENSCAFG0000001957	HES3	turquoise	VSMC_M6	-0.04	8.50E-01	-0.11	5.75E-01	0.05	7.90E-01	-0.10	6.24E-01	-0.16	4.21E-01	0.70	5.74E-05	0.40	4.00E-02	0.16	4.28E-01	0.04	8.54E-01	0.01	9.71E-01
ENSCAFG0000002615	ENSCAFG0000002615	turquoise	VSMC_M6	-0.04	8.50E-01	-0.11	5.75E-01	0.05	7.90E-01	-0.10	6.24E-01	-0.16	4.21E-01	0.70	5.74E-05	0.40	4.00E-02	0.16	4.28E-01	0.04	8.54E-01	0.01	9.71E-01

ENSCAFG0000000084	SLC22A4	grey	VSMC_M10	-0.04	8.50E-01	0.40	3.87E-02	0.35	7.78E-02	-0.05	8.15E-01	-0.38	4.77E-02	0.05	7.90E-01	0.17	3.99E-01	-0.13	5.16E-01	-0.40	3.91E-02	0.36	6.68E-02
ENSCAFG0000002037	EXOC3L1	turquoise	VSMC_M6	-0.04	8.50E-01	0.10	6.21E-01	-0.10	6.37E-01	0.25	2.18E-01	-0.06	7.67E-01	0.60	8.83E-04	-0.08	6.79E-01	-0.14	4.93E-01	-0.11	5.83E-01	0.26	1.94E-01
ENSCAFG0000000758	PATL1	cyan	VSMC_M2	-0.04	8.50E-01	0.25	2.12E-01	0.19	3.52E-01	-0.21	2.95E-01	-0.10	6.04E-01	-0.02	9.38E-01	-0.19	3.40E-01	-0.11	6.00E-01	-0.07	7.27E-01	0.01	9.46E-01
ENSCAFG0000001044	FNDC5	darkgreen	VSMC_M4	-0.04	8.50E-01	0.26	1.99E-01	-0.54	3.89E-03	0.76	4.18E-06	0.32	1.00E-01	0.02	9.28E-01	-0.21	2.89E-01	-0.69	6.92E-05	-0.23	2.52E-01	0.59	1.34E-03
ENSCAFG0000001014	BICDL1	grey	VSMC_M10	-0.04	8.50E-01	0.09	6.73E-01	-0.11	5.76E-01	0.22	2.62E-01	-0.01	9.80E-01	0.21	3.04E-01	0.29	1.39E-01	-0.14	5.00E-01	-0.16	4.34E-01	0.29	1.41E-01
ENSCAFG0000001938	GNG7	grey	VSMC_M10	-0.04	8.50E-01	0.26	1.92E-01	0.06	7.66E-01	0.19	3.44E-01	-0.28	1.62E-01	0.21	2.84E-01	0.20	3.10E-01	-0.21	3.02E-01	-0.34	8.61E-02	0.58	1.62E-03
ENSCAFG0000002015	ENSCAFG0000002015	grey	VSMC_M10	-0.04	8.50E-01	0.01	9.61E-01	-0.39	4.62E-02	0.19	3.45E-01	0.33	9.00E-02	0.06	7.83E-01	-0.01	9.78E-01	-0.17	3.95E-01	-0.02	9.27E-01	0.12	5.60E-01
ENSCAFG0000001946	RNF151	grey	VSMC_M10	-0.04	8.50E-01	0.04	8.59E-01	-0.22	2.79E-01	0.43	2.56E-02	0.04	8.44E-01	0.04	8.25E-01	0.08	6.96E-01	-0.31	1.15E-01	-0.11	5.91E-01	0.41	3.19E-02
ENSCAFG0000001756	RBBP6	darkolivegreen	VSMC_M9	-0.04	8.49E-01	-0.73	1.67E-05	-0.43	2.70E-02	-0.29	1.44E-01	0.53	4.69E-03	-0.06	7.55E-01	0.17	4.07E-01	0.52	5.67E-03	0.62	6.30E-04	-0.62	5.78E-04
ENSCAFG0000001326	MGMT	grey	VSMC_M10	-0.04	8.49E-01	0.29	1.45E-01	0.07	7.11E-01	0.45	2.00E-02	-0.19	3.46E-01	0.21	2.82E-01	-0.03	8.73E-01	-0.47	1.28E-02	-0.24	2.27E-01	0.45	1.89E-02
ENSCAFG0000001759	ADPRM	grey	VSMC_M10	-0.04	8.49E-01	0.32	1.01E-01	0.23	2.57E-01	-0.04	8.46E-01	-0.45	1.83E-02	0.32	1.05E-01	0.23	2.48E-01	-0.05	8.02E-01	-0.36	6.47E-02	0.55	2.89E-03
ENSCAFG0000001611	ENSCAFG0000001611	grey	VSMC_M10	-0.04	8.49E-01	0.21	2.88E-01	0.43	2.60E-02	-0.26	1.95E-01	-0.34	8.25E-02	-0.07	7.22E-01	-0.04	8.24E-01	0.06	7.72E-01	-0.17	3.83E-01	-0.10	6.11E-01
ENSCAFG0000002195	ENSCAFG0000002195	grey	VSMC_M10	-0.04	8.49E-01	0.36	6.21E-02	0.43	2.51E-02	-0.13	5.05E-01	-0.44	2.08E-02	0.14	4.97E-01	0.22	2.73E-01	0.00	9.91E-01	-0.38	5.30E-02	0.16	4.37E-01
ENSCAFG0000002903	ENSCAFG0000002903	grey	VSMC_M10	-0.04	8.49E-01	-0.32	1.04E-01	0.15	4.43E-01	-0.50	8.17E-03	-0.11	5.75E-01	0.38	5.22E-02	0.31	1.16E-01	0.53	4.41E-03	0.20	3.18E-01	-0.44	2.20E-02
ENSCAFG0000003066	ENSCAFG0000003066	grey	VSMC_M10	-0.04	8.48E-01	-0.02	9.32E-01	-0.18	3.82E-01	0.03	8.86E-01	0.01	9.45E-01	0.19	3.35E-01	0.02	9.34E-01	0.04	8.48E-01	-0.03	8.89E-01	0.18	3.80E-01
ENSCAFG0000000344	GPR63	darkgreen	VSMC_M4	-0.04	8.48E-01	0.25	2.01E-01	-0.48	1.15E-02	0.80	6.17E-07	0.26	1.99E-01	0.09	6.45E-01	-0.37	6.05E-02	-0.71	3.55E-05	-0.23	2.53E-01	0.59	1.31E-03
ENSCAFG0000002909	LYPD6	darkgreen	VSMC_M4	-0.04	8.48E-01	0.34	8.38E-02	-0.42	3.10E-02	0.78	1.27E-06	0.19	3.55E-01	0.25	2.09E-01	-0.18	3.67E-01	-0.71	3.66E-05	-0.28	1.51E-01	0.64	2.96E-04
ENSCAFG0000001290	ENSCAFG0000001290	grey	VSMC_M10	-0.04	8.47E-01	-0.10	6.12E-01	0.28	1.61E-01	-0.10	6.34E-01	-0.33	9.23E-02	0.10	6.17E-01	0.09	6.44E-01	0.14	4.86E-01	0.05	7.89E-01	0.01	9.47E-01
ENSCAFG0000002851	ENSCAFG0000002851	grey	VSMC_M10	-0.04	8.47E-01	0.04	8.46E-01	0.00	9.88E-01	-0.01	9.45E-01	-0.12	5.65E-01	-0.08	7.06E-01	-0.21	2.93E-01	0.00	9.93E-01	-0.08	6.81E-01	0.13	5.21E-01
ENSCAFG0000001132	EIF4E2	grey	VSMC_M10	-0.04	8.47E-01	-0.20	3.05E-01	0.15	4.47E-01	-0.35	6.98E-02	-0.10	6.36E-01	-0.23	2.47E-01	0.06	7.74E-01	0.38	4.91E-02	0.19	3.50E-01	-0.31	1.13E-01
ENSCAFG0000003163	ENSCAFG0000003163	grey	VSMC_M10	-0.04	8.47E-01	0.13	5.23E-01	0.26	1.92E-01	-0.26	1.94E-01	-0.31	1.16E-01	-0.01	9.51E-01	0.15	4.49E-01	0.23	2.50E-01	-0.17	4.05E-01	0.06	7.49E-01
ENSCAFG0000001071	TXLNA	darkgrey	VSMC_M8	-0.04	8.47E-01	-0.48	1.09E-02	0.08	7.02E-01	-0.70	4.78E-05	0.05	8.00E-01	0.05	7.95E-01	0.26	1.89E-01	0.77	2.36E-06	0.39	4.18E-02	-0.63	3.76E-04
ENSCAFG0000000427	RAD54L	darkgrey	VSMC_M8	-0.04	8.47E-01	-0.44	2.01E-02	0.42	2.99E-02	-0.89	3.93E-10	-0.31	1.11E-01	0.05	8.07E-01	0.41	3.54E-02	0.96	6.90E-15	0.27	1.66E-01	-0.63	4.00E-04
ENSCAFG0000002603	ENSCAFG0000002603	grey	VSMC_M10	-0.04	8.46E-01	-0.41	3.54E-02	-0.24	2.27E-01	-0.12	5.44E-01	0.24	2.35E-01	0.16	4.31E-01	0.06	7.67E-01	0.30	1.33E-01	0.27	1.70E-01	-0.29	1.45E-01
ENSCAFG0000000155	CCDC171	grey	VSMC_M10	-0.04	8.46E-01	-0.24	2.22E-01	0.17	4.07E-01	-0.46	1.50E-02	-0.20	3.26E-01	0.12	5.40E-01	0.56	2.47E-03	0.47	1.32E-02	0.09	6.69E-01	-0.22	2.78E-01
ENSCAFG0000001344	COLGALT2	darkgreen	VSMC_M4	-0.04	8.46E-01	0.23	2.46E-01	-0.51	7.10E-03	0.83	6.63E-08	0.35	7.22E-02	-0.06	7.70E-01	-0.10	6.11E-01	-0.74	9.42E-06	-0.18	3.65E-01	0.49	1.02E-02
ENSCAFG0000000070	ENSCAFG0000000070	darkgreen	VSMC_M4	-0.04	8.46E-01	0.57	1.96E-03	-0.08	7.00E-01	0.67	1.32E-04	-0.11	5.69E-01	-0.05	7.95E-01	-0.19	3.34E-01	-0.71	3.20E-05	-0.47	1.23E-02	0.72	2.11E-05
ENSCAFG0000003128	KCNJ8	grey	VSMC_M10	-0.04	8.46E-01	0.11	5.70E-01	0.19	3.41E-01	-0.29	1.45E-01	-0.30	1.26E-01	0.15	4.45E-01	0.47	1.44E-02	0.26	1.82E-01	-0.24	2.38E-01	0.19	3.55E-01
ENSCAFG0000000347	TSPAN12	grey	VSMC_M10	-0.04	8.45E-01	0.12	5.44E-01	-0.44	2.08E-02	0.50	7.46E-03	0.47	1.34E-02	0.01	9.46E-01	-0.11	5.91E-01	-0.55	2.84E-03	0.05	7.93E-01	0.14	4.88E-01
ENSCAFG0000001495	NSD2	darkgrey	VSMC_M8	-0.04	8.45E-01	-0.42	3.04E-02	0.44	2.25E-02	-0.91	7.06E-11	-0.33	8.99E-02	0.05	7.88E-01	0.42	2.75E-02	0.95	2.71E-14	0.27	1.77E-01	-0.60	8.99E-04
ENSCAFG0000000101	UBE2B	grey	VSMC_M10	-0.04	8.45E-01	0.43	2.39E-02	-0.13	5.05E-01	0.42	3.12E-02	0.04	8.36E-01	-0.26	1.85E-01	-0.31	1.10E-01	-0.52	5.59E-03	-0.28	1.54E-01	0.48	1.06E-02
ENSCAFG0000000424	NAPEPLD	grey	VSMC_M10	-0.04	8.45E-01	0.42	2.78E-02	0.11	5.92E-01	0.08	7.04E-01	-0.24	2.29E-01	0.14	4.97E-01	-0.42	2.84E-02	-0.19	3.34E-01	-0.35	7.40E-02	0.31	1.13E-01
ENSCAFG0000002957	KANK3	turquoise	VSMC_M6	-0.04	8.45E-01	-0.07	7.29E-01	0.04	8.34E-01	-0.14	4.87E-01	-0.13	5.10E-01	0.82	1.24E-07	0.06	7.82E-01	0.19	3.31E-01	0.03	8.86E-01	0.01	9.64E-01
ENSCAFG0000001663	EIF4A1	darkgrey	VSMC_M8	-0.04	8.45E-01	-0.45	1.80E-02	0.41	3.23E-02	-0.79	1.05E-06	-0.35	7.59E-02	0.20	3.14E-01	0.15	4.69E-01	0.90	2.37E-10	0.31	1.20E-01	-0.62	4.95E-04
ENSCAFG0000000576	SYT1	grey	VSMC_M10	-0.04	8.44E-01	0.00	1.00E+00	0.27	1.80E-01	-0.28	1.65E-01	-0.14	4.77E-01	0.08	6.98E-01	-0.17	4.10E-01	0.23	2.40E-01	0.06	7.48E-01	-0.25	2.05E-01
ENSCAFG0000002006	COQ4	grey	VSMC_M10	-0.04	8.44E-01	0.20	3.10E-01	0.43	2.51E-02	-0.24	2.18E-01	-0.42	2.90E-02	-0.07	7.10E-01	-0.10	6.20E-01	0.08	7.03E-01	-0.17	4.10E-01	0.06	7.65E-01
ENSCAFG0000001674	FAM155B	grey	VSMC_M10	-0.04	8.44E-01	0.27	1.68E-01	-0.19	3.48E-01	0.08	6.92E-01	0.16	4.15E-01	-0.06	7.47E-01	-0.19	3.41E-01	-0.20	3.09E-01	-0.24	2.25E-01	0.18	3.79E-01
ENSCAFG0000000929	NIT2	grey	VSMC_M10	-0.04	8.44E-01	0.24	2.32E-01	-0.28	1.57E-01	0.56	2.44E-03	0.15	4.61E-01	0.10	6.07E-01	0.11	5.85E-01	-0.56	2.60E-03	-0.19	3.41E-01	0.45	1.90E-02
ENSCAFG0000000908	PLCG1	cyan	VSMC_M2	-0.04	8.43E-01	0.55	3.22E-03	0.45	1.87E-02	-0.06	7.67E-01	-0.63	4.63E-04	0.41	3.22E-02	0.05	8.01E-01	-0.11	5.99E-01	-0.54	3.47E-03	0.55	3.06E-03
ENSCAFG0000000901	NEFM	grey	VSMC_M10	-0.04	8.42E-01	-0.14	5.00E-01	0.09	6.70E-01	-0.15	4.57E-01	-0.13	5.07E-01	-0.08	7.00E-01	0.40	3.95E-02	0.19	3.51E-01	0.12	5.61E-01	-0.06	7.75E-01
ENSCAFG0000001424	PDE1A	darkgreen	VSMC_M4	-0.04	8.42E-01	0.34	8.33E-02	-0.53	4.22E-03	0.89	3.44E-10	0.41	3.42E-02	-0.16	4.37E-01	-0.36	6.27E-02	-0.89	5.46E-10	-0.18	3.56E-01	0.55	2.92E-03
ENSCAFG0000000000	ENSCAFG0000000000	grey	VSMC_M10	-0.04	8.42E-01	-0.03	8.74E-01	-0.06	7.49E-01	0.03	8.87E-01	-0.06	7.61E-01	-0.15	4.42E-01	0.32	1.09E-01	0.06	7.70E-01	-0.06	7.85E-01	0.20	3.16E-01
ENSCAFG0000001212	TRAF5	grey	VSMC_M10	-0.04	8.42E-01	0.06	7.79E-01	0.11	5.71E-01	-0.09	6.60E-01	0.05	7.86E-01	-0.35	7.80E-02	-0.22	2.81E-01	-0.06	7.61E-01	0.14	5.02E-01	-0.23	2.49E-01
ENSCAFG0000001509	LAP3	darkgreen	VSMC_M4	-0.04	8.42E-01	0.45	1.75E-02	-0.12	5.37E-01	0.50	8.51E-03	0.03	8.97E-01	-0.28	1.52E-01	0.06	7.54E-01	-0.55	3.19E-03	-0.36	6.62E-02	0.47	1.40E-02
ENSCAFG0000000153	FREM1	turquoise	VSMC_M6	-0.04	8.42E-01	-0.41	3.17E-02	-0.05	7.87E-01	-0.29	1.41E-01	0.13	5.22E-01	0.24	2.36E-01	0.47	1.24E-02	0.33	8.86E-02	0.40	4.05E-02	-0.36	6.63E-02
ENSCAFG0000001871	RANBP3L	grey	VSMC_M10	-0.04	8.42E-01	-0.27	1.81E-01	-0.50	7.36E-03	0.43	2.50E-02	0.55	3.09E-03	-0.12	5.44E-01	-0.07	7.41E-01	-0.24	2.34E-01	0.25	2.15E-01	-0.11	5.71E-01
ENSCAFG0000002042	DEPDC1	darkgrey	VSMC_M8	-0.04	8.41E-01	-0.53	4.25E-03	0.35	6.95E-02	-0.85	1.43E-08	-0.26	1.87E-01	0.07	7.45E-01	0.40	3.61E-02	0.93	3.22E-12	0.37	5.56E-02	-0.63	4.55E-04
ENSCAFG0000000879	FKBP11	yellow	VSMC_M3	-0.04	8.41E-01	0.20	3.17E-01	0.70	4.51E-05	-0.62	5.44E-04	-0.66	1.79E-04	0.03	8.65E-01	-0.11	5.77E-01	0.49	9.04E-03	-0.22	2.79E-01	-0.18	3.73E-01
ENSCAFG0000000131	PPARD	yellow	VSMC_M3	-0.04	8.41E-01	-0.09	6.54E-01	0.32	9.98E-02	-0.63	4.83E-04	-0.18	3.79E-01	0.11	5.90E-01	0.08	6.88E-01	0.43	2.63E-02	0.12	5.53E-01	-0.38	5.37E-02
ENSCAFG0000001038	FAP	grey	VSMC_M10	-0.04	8.41E-01	-0.03	8.82E-01	0.27	1.69E-01	-0.44	2.21E-02	-0.13	5.14E-01	-0.28	1.52E-01	0.40	4.07E-02	0.31	1.17E-01	0.02	9.14E-01	-0.28	1.59E-01
ENSCAFG0000003078	ENSCAFG0000003078	grey	VSMC_M10	-0.04	8.41E-01	-0.02	9.37E-01	-0.30	1.34E-01	0.32	1.01E-01	0.12	5.61E-01	0.27	1.79E-01	0.12	5.56E-01	-0.15	4.47E-01	-0.06	7.53E-01	0.35	7.03E-02
ENSCAFG0000001412	C26H22orf15	grey	VSMC_M10	-0.04	8.40E-01	0.23	2.55E-01	-0.18	3.65E-01	0.32	1.09E-01	0.06	7.79E-01	-0.13	5.31E-01	-0.12	5.51E-01	-0.35	7.45E-02	-0.18	3.61E-01	0.39	4.61E-02
ENSCAFG0000002951	LDLRAP1	grey	VSMC_M10	-0.04	8.40E-01	0.35	7.70E-02	-0.08	6.80E-01	0.37	5.79E-02	0.00	9.96E-01	0.00	9.96E-01	-0.14	4.82E-01	-0.39	4.52E-02	-0.28	1.54E-01	0.34	8.11E-02
ENSCAFG0000000667	ERI1	darkolivegreen	VSMC_M9	-0.04	8.40E-01	-0.62	6.04E-04	-0.21	2.89E-01	-0.21	2.98E-01	0.19	3.52E-01	-0.04	8.60E-01	0.33	9.74E-02	0.48	1.21E-02	0.43	2.57E-02	-0.35	7.05E-02
ENSCAFG0000000571	E2F7	darkgrey	VSMC_M8	-0.04	8.40E-01	-0.55	2.91E-03	0.21	2.98E-01	-0.80	5.69E-07	-0.14	4.92E-01	0.20	3.22E-01	0.40	3.82E-02	0.94	3.07E-13	0.36	6.18E-02	-0.62	6.26E-04
ENSCAFG0000000874	CIAPIN1	grey	VSMC_M10	-0.04	8.40E-01	-0.14	4.73E-01	0.28	1.51E-01	-0.32	1.08E-01	-0.27	1.65E-01	0.26	1.91E-01	0.08	6.78E-01	0.34	8.13E-02	0.12	5.55E-01	-0.29	1.46E-01
ENSCAFG0000001739	KIAA0556	darkgreen	VSMC_M4	-0.04	8.40E-01	0.31	1.12E-01	-0.34	7.95E-02	0.75	7.46E-06	0.26	1.83E-01	-0.18	3.57E-01	-0.19	3.34E-01	-0.72	2.03E-05	-0.24	2.29E-01	0.46	1.67E-02
ENSCAFG0000000957	ZBED6	grey	VSMC_M10	-0.04	8.40E-01	-0.39	4.41E-02	-0.66	1.88E-04	0.20	3.21E-01	0.71	2.96E-05	-0.04	8.58E-01	-0.16	4.39E-01	0.02	9.12E-01	0.39	4.49E-02	-0.33	9.32E-02
ENSCAFG0000000482	DHTKD1	grey	VSMC_M10	-0.04	8.40E-01	-0.22	2.80E-01	0.10	6.11E-01	-0.49	9.08E-03	-0.15	4.64E-01	0.08	6.90E-01	0.46	1.61E-02	0.54	3.97E-03	0.09	6.55E-01	-0.12	5.56E-01
ENSCAFG0000000528	SS18L2	grey	VSMC_M10	-0.04	8.40E-01	-0.02	9.30E-01	0.33	9.37E-02	-0.42	3.10E-02	-0.27	1.78E-01	-0.08	6.94E-01	0.07	7.28E-01	0.36	6.66E-02	-0.01	9.48E-01	-0.28	1.63E-01
ENSCAFG0000001633	ENSCAFG0000001633	grey	VSMC_M10	-0.04	8.40E-01	-0.18	3.67E-01	-0.04	8.29E-01	-0.18	3.72E-01	0.01	9.61E-01	0.32	1.01E-01	-0.10	6.14E-01	0.29	1.46E-01	0.10	6.35E-01	-0.17	4.03E-01
ENSCAFG0000001648	STAC2	grey	VSMC_M10	-0.04	8.39E-01	0.38	4.94E-02	0.25	2.02E-01	0.13	5.03E-01	-0.35	7.20E-02	0.12	5.60E-01	-0.10	6.10E-01	-0.27	1.73E-01	-0.27	1.81E-01	0.37	5.72E-02
ENSCAFG0000000061	ZDHHC14	grey	VSMC_M10	-0.04	8.39E-01	0.10	6.18E-01	0.14	4.92E-01	-0.17	4.08E-01	-0.23	2.45E-01	0.17	3.99E-01	0.25	2.08E-01	0.15	4.65E-01	-0.15	4.61E-01	0.19	3.30E-01
ENSCAFG0000000671	FLT3	darkgreen	VSMC_M4	-0.04	8.39E-01	0.25	2.10E-01	-0.36	6.77E-02	0.55	2.76E-03	0.16	4.16E-01	0.03	8.85E-01	0.23	2.43E-01	-0.51	6.36E-03	-0.24	2.24E-01	0.56	2.16E-03
ENSCAFG0000002375	GMPPB	darkgrey	VSMC_M8	-0.04	8.39E-01	-0.40	4.05E-02	0.45	1.90E-02	-0.81	3.77E-07	-0.35	7.01E-02	0.09	6.65E-01	0.28	1.55E-01	0.81	3.04E-07	0.30	1.30E-01	-0.62	5.55E-04
ENSCAFG0000001142	LMNTD1	grey	VSMC_M10	-0.04	8.39E-01	0.22	2.70E-01	0.01	9.53E-01	0.11	5.99E-01	-0.15	4.63E-01	0.30	1.31E-01	-0.10	6.07E-01	-0.12	5.47E-01	-0.16	4.21E-01	0.30	1.24E-01
ENSCAFG0000002942	REPIN1	grey	VSMC_M10	-0.04	8.39E-01	0.47	1.35E-02	-0.32	1.03E-01	0.61	8.02E-04	0.19	3.54E-01	-0.14	5.00E-01	-0.36	6.13E-02	-0.64	3.04E-04	-0.37	5.88E-02	0.58	1.59E-03
ENSCAFG0000000392	MFSD4B	grey	VSMC_M10	-0.04	8.39E-01	-0.34	8.30E-02	-0.31	1.20E-01	0.19	3.52E-01	0.24	2.27E-01	0.19	3.54E-01	0.21	3.00E-01	-0.04	8.49E-01	0.36	6.44E-02	-0.05	8.20E-01
ENSCAFG0000001359	ENSCAFG0000001359	grey	VSMC_M10	-0.04	8.39E-01	0.10	6.18E-01	0.37	5.97E-02	-0.13	5.16E-01	-0.30	1.31E-01	-0.12	5.45E-01	0.14	4.92E-01	-0.08	6.98E-01	0.00	9.89E-01	-0.06	7.85E-01
ENSCAFG0000001783	CYP46A1	grey	VSMC_M10	-0.04	8.38E-01	0.45	1.92E-02	0.38	4.96E-02	-0.03	8.91E-01	-0.39	4.31E-02	-0.02	9.39E-01	-0.08	6.76E-01	-0.15	4.64E-01	-0.36	6.67E-02	0.19	3.50E-01
ENSCAFG0000001045	KDM5B	darkgreen	VSMC_M4	-0.04	8.38E-01	-0.05	8.17E-01	-0.46	1.48E-02	0.64	3.00E-04	0.40	4.07E-02	0.05	7.88E-01	-0.51	6.25E-03	-0.58	1.58E-03	0.19	3.55E-01	0.18	3.65E-01
ENSCAFG0000000834	RACGAP1	darkgrey	VSMC_M8	-0.04	8.38E-01	-0.47	1.28E-02	0.39	4.67E-02	-0.93	5.21E-12	-0.25	2.07E-01	0.09	6.53E-01	0.41	3.32E-02	0.97	5.02E-17	0.32	1.06E-01	-0.69	7.46E-05
ENSCAFG0000001653	ADGRA3	grey	VSMC_M10	-0.04	8.38E-01	-0.55	2.77E-03	-0.10	6.24E-01	-0.42	2.99E-02	0.09	6.39E-01	0.16	4.13E-01	0.16	4.13E-01	0.52	5.81E-03	0.48	1.12E-02	-0.39	4.40E-02
ENSCAFG0000003111	ENSCAFG0000003111	grey	VSMC_M10	-0.04	8.38E-01	-0.49	9.77E-03	-0.38	5.08E-02	-0.13	5.29E-01	0.43	2.57E-02	-0.05	8.16E-01	0.28	1.58E-01	0.30	1.22E-01	0.39	4.66E-02	-0.38	5.11E-02
ENSCAFG0000001095	PLCL1	darkgreen	VSMC_M4	-0.04	8.38E-01	0.06	7.72E-01	-0.51	6.23E-03	0.72	1.91E-05	0.43	2.70E-02	-0.15	4.51E-01	0.14	4.96E-01	-0.59	1.12E-03	0.01	9.65E-01	0.33	8.90E-02
ENSCAFG0000001210	PRDX3	grey	VSMC_M10	-0.04	8.38E-01	-0.38	5.18E-02	-0.32	1.08E-01	0.12	5.60E-01	0.23	2.48E-01	0.18	3.72E-01	-0.09	6.73E-01	0.14	4.71E-01	0.34	7.95E-02	-0.13	5.33E-01
ENSCAFG0000001739	SLC39A1	yellow	VSMC_M3	-0.04	8.37E-01	0.46	1.65E-02	0.82	1.24E-07	-0.44	2.15E-02	-0.90	1.45E-10	0.09	6.61E-01	0.14	4.72E-01	0.25	2.05E-01	-0.45	1.78E-02	0.26	1.87E-01
ENSCAFG0000001133	NEK7	darkolivegreen	VSMC_M9	-0.04	8.37E-01	-0.55	2.99E-03	-0.58	1.69E-03	0.21	3.05E-01	0.64	2.89E-04	-0.23	2.49E-01	-0.25	2.04E-01	0.03	8.63E-01	0.59	1.34E-03	-0.41	3.58E-02
ENSCAFG0000001003	BRWD1	grey	VSMC_M10	-0.04	8.37E-01	-0.52	5.20E-03	-0.22	2.76E-01	-0.25	2.05E-01	0.25	2.03E-01	0.06	7.69E-01	0.13	5.19E-01	0.36	6.57E-02	0.49	9.84E-03	-0.47	1.32E-02
ENSCAFG0000000212	CENPQ	darkgrey	VSMC_M8	-0.04	8.37E-01	-0.69	6.11E-05	0.03	8.99E-01	-0.54	3.63E-03	0.08	6.80E-01	0.13	5.23E-01	0.20	3.14E-01	0.79	1.16E-06	0.51	6.15E-03	-0.72	1.94E-05
ENSCAFG0000000877	EIF2AK4	grey	VSMC_M10	-0.04	8.37E-01	0.10	6.22E-01	-0.08	6.98E-01	0.20	3.16E-01	-0.05	8.00E-01	0.03	8.76E-01	0.14	5.01E-01	-0.12	5.41E-01	-0.16	4.30E-01	0.37	5.59E-02
ENSCAFG0000000986	BBX	pink	VSMC_M5	-0.04	8.37E-01	-0.04	8.34E-01	-0.69	8.06E-05	0.68	1.09E-04	0.61	6.45E-04	-0.06	7.48E-01	-0.18	3.74E-01	-0.60	9.51E-04	0.13	5.07E-01	0.22	2.74E-01
ENSCAFG0000000071	SLC27A6	grey	VSMC_M10	-0.04	8.37E-01	0.32	1.00E-01	-0.10	6.23E-01	0.37	5.59E-02	0.00	9.89E-01	-0.07	7.37E-01	-0.13	5.05E-01	-0.38	4.89E-02	-0.25	2.03E-01	0.39	4.29E-02
ENSCAFG0000000391	GTF3C6	grey	VSMC_M10	-0.04	8.37E-01	-0.02	9.32E-01	0.24	2.35E-01	-0.33	8.94E-02	-0.19	3.55E-01	-0.07	7.16E-01	0.27	1.77E-01	0.26	1.89E-01	0.01	9.71E-01	-0.18	3.77E-01
ENSCAFG0000001967	PLPPR3	darkgreen	VSMC_M4	-0.04	8.36E-01	0.45	1.90E-02	-0.41	3.29E-02	0.84	5.67E-08	0.22	2.80E-01	0.10	6.06E-01	-0.27	1.72E-01	-0.86	1.11E-08	-0.34	8.69E-02	0.72	1.94E-05
ENSCAFG0000001015	NHLRC1	grey	VSMC_M10	-0.04	8.36E-01	0.38	5.28E-02	0.29	1.45E-01	0.08	7.10E-01	-0.27	1.67E-01	0.03	8.71E-01	-0.03	8.90E-01	-0.29	1.42E-01	-0.28	1.52E-01	0.22	2.73E-01
ENSCAFG0000000442	NSUN6	grey	VSMC_M10	-0.04	8.36E-01	-0.14	5.01E-01	0.14	4.91E-01	-0.15	4.61E-01	-0.17	3.95E-01	-0.06	7.64E-01	0.20	3.18E-01	0.20	3.24E-01	0.06	7.78E-01	-0.04	8.48E-01
ENSCAFG0000000199	FANCG	grey	VSMC_M10	-0.04	8.36E-01	-0.30	1.23E-01	-0.10	6.03E-01	-0.14	4.89E-01	-0.01	9.45E-01	0.12	5.56E-01	0.29	1.47E-01	0.39	4.55E-02	0.11	5.98E-01	-0.06	7.70E-01
ENSCAFG0000001971	HCN2	darkgreen	VSMC_M4	-0.04	8.36E-01	0.42	2.97E-02	-0.17	3.93E-01	0.71	3.49E-05	0.02	9.30E-01	0.13	5.20E-01	-0.37	5.55E-02	-0.76	4.70E-06	-0.29	1.40E-01	0.58	1.61E-03
ENSCAFG0000001471	PSME3	grey	VSMC_M10	-0.04	8.36E-01	-0.40	3.95E-02	0.24	2.20E-01	-0.40	3.81E-02	-0.10	6.19E-01	-0.03	9.00E-01	0.16	4.40E-01	0.52	5.89E-03	0.31	1.17E-01	-0.59	1.18E-03
ENSCAFG0000002584	ENSCAFG0000002584	grey	VSMC_M10	-0.04	8.36E-01	0.04	8.56E-01	0.01	9.77E-01	0.04	8.46E-01	-0.13	5.32E-01	-0.12	5.40E-01	0.09	6.64E-01	0.05	8.05E-01	-0.10	6.35E-01	0.18	3.75E-01
ENSCAFG0000000192	RSPH9	cyan	VSMC_M2	-0.04	8.36E-01	0.33	9.21E-02	0.43	2.39E-02	0.01	9.41E-01	-0.57	1.94E-03	0.21	2.93E-01	-0.21	2.93E-01	-0.10	6.15E-01	-0.31	1.10E-01	0.36	6.64E-02
ENSCAFG0000000152	IFT27	yellow	VSMC_M3	-0.04	8.35E-01	-0.01	9.53E-01	0.70	4.35E-05	-0.61	6.77E-04	-0.64	3.18E-04	0.04	8.28E-01	0.27	1.65E-01	0.49	9.97E-03	-0.02	9.19E-01	-0.19	3.44E-01
ENSCAFG0000001879	DNAJC21	darkgrey	VSMC_M8	-0.04	8.35E-01	-0.31	1.15E-01	0.27	1.70E-01	-0.41	3.29E-02	-0.20	3.20E-01	-0.06	7.74E-01	0.09	6.61E-01	0.52	5.63E-03	0.24	2.21E-01	-0.36	6.89E-02
ENSCAFG0000001941	CASKIN1	grey	VSMC_M10	-0.04	8.35E-01	0.49	1.01E-02	0.26	1.95E-01	0.01	9.55E-01	-0.37	5.44E-02	-0.06	7.82E-01	0.04	8.33E-01	-0.14	4.85E-01	-0.43	2.36E-02	0.39	4.25E-02
ENSCAFG0000000840	P2RX7	grey	VSMC_M10	-0.04	8.34E-01	0.38	4.77E-02	-0.24	2.20E-01	0.57	2.03E-03	0.00	9.89E-01	0.18	3.74E-01	-0.12	5.63E-01	-0.55	3.25E-03	-0.39	4.58E-02	0.67	1.40E-04
ENSCAFG0000002491	ABCA8	grey	VSMC_M10	-0.04	8.34E-01	0.34	7.86E-02	0.00	9.90E-01	0.14	4.83E-01	-0.17	3.96E-01	0.13	5.32E-01	-0.28	1.50E-01	-0.18	3.75E-01	-0.39	4.18E-02	0.39	4.23E-02
ENSCAFG0000000195	IL11RA	grey	VSMC_M10	-0.04	8.34E-01	0.58	1.49E-03	0.25	2.17E-01	0.32	1.02E-01	-0.35	7.19E-02	-0.11	5.99E-01	-0.02	9.16E-01	-0.49	9.29E-03	-0.44	2.32E-02	0.57	1.88E-03
ENSCAFG0000000042	YEATS4	grey	VSMC_M10	-0.04	8.34E-01	0.00	9.85E-01	0.12	5.57E-01	-0.37	6.08E-02	-0.18	3.63E-01	0.20	3.18E-01	0.41	3.48E-02	0.31	1.21E-01	-0.06	7.66E-01	0.05	8.13E-01
ENSCAFG0000001667	RNF44	grey	VSMC_M10	-0.04	8.34E-01	0.37	5.43E-02	-0.07	7.34E-01	0.41	3.50E-02	-0.10	6.19E-01	0.00	9.90E-01	-0.32	1.02E-01	-0.54	3.47E-03	-0.23	2.40E-01	0.55	3.27E-03
ENSCAFG0000000045	ENSCAFG0000000045	grey	VSMC_M10	-0.04	8.33E-01	0.34	8.68E-02	0.29	1.47E-01	-0.28	1.57E-01	-0.38	5.26E-02	0.09	6.69E-01	-0.09	6.65E-01	0.10	6.12E-01	-0.32	1.07E-01	0.23	2.46E-01
ENSCAFG0000000968	DBNDD2	grey	VSMC_M10	-0.04	8.33E-01	-0.01	9.76E-01	-0.21	2.87E-01	0.44	2.13E-02	0.15	4.52E-01	-0.18	3.72E-01	0.12	5.65E-01	-0.41	3.29E-02	0.11	5.82E-01	0.20	3.25E-01
ENSCAFG0000000832	SEMA4F	grey	VSMC_M10	-0.04	8.33E-01	-0.41	3.24E-02	-0.11	5.93E-01	0.05	7.99E-01	0.11	5.98E-01	0.05	7.93E-01	0.14	4.85E-01	0.10	6.30E-01	0.40	4.12E-02	-0.19	3.54E-01
ENSCAFG0000002065	ENSCAFG0000002065	grey	VSMC_M10	-0.04	8.33E-01	0.04	8.50E-01	0.32	1.01E-01	-0.35	7.52E-02	-0.39	4.50E-02	0.35	7.55E-02	0.45	1.99E-02	0.25	2.16E-01	-0.12	5.63E-01	0.00	9.82E-01
ENSCAFG0000000078	PPARA	grey	VSMC_M10	-0.04	8.32E-01	0.11	5.96E-01	0.02	9.02E-01	0.10	6.22E-01	-0.16	4.17E-01	-0.12	5.61E-01	0.17	3.84E-01	-0.11	5.95E-01	-0.17	4.09E-01	0.25	2.18E-01
ENSCAFG0000003184	CGRRF1	grey	VSMC_M10	-0.04	8.32E-01	0.02	9.36E-01	-0.33	8.88E-02	0.33	9.52E-02	0.33	9.17E-02	-0.39	4.72E-02	-0.19	3.33E-01	-0.36	6.82E-02	0.13	5.28E-01	0.08	6.92E-01
ENSCAFG0000002157	ENSCAFG0000002157	grey	VSMC_M10	-0.04	8.32E-01	0.19	3.36E-01	-0.09	6.60E-01	0.25	2.14E-01	-0.07	7.34E-01	-0.10	6.27E-01	0.24	2.20E-01	-0.21	3.01E-01	-0.19	3.35E-01	0.36	6.67E-02
ENSCAFG0000001644	TSR2	grey	VSMC_M10	-0.04	8.32E-01	0.12	5.67E-01	0.31	1.22E-01	-0.07	7.43E-01	-0.47	1.33E-02	0.13	5.09E-01	0.09	6.46E-01	0.10	6.08E-01	-0.18	3.73E-01	0.25	2.17E-01
ENSCAFG0000000398	JCAD	pink	VSMC_M5	-0.04	8.32E-01	-0.47	1.38E-02	-0.69	7.17E-05	0.31	1.15E-01	0.67	1.54E-04	-0.04	8.31E-01	0.05	7.95E-01	-0.02	9.17E-01	0.36	6.13E-02	-0.18	3.65E-01
ENSCAFG0000001500	GORAB	grey	VSMC_M10	-0.04	8.32E-01	-0.40	3.87E-02	-0.26	1.84E-01	-0.12	5.46E-01	0.47	1.23E-02	-0.31	1.10E-01	0.17	3.96E-01	0.12	5.58E-01	0.47	1.35E-02	-0.56	2.40E-03
ENSCAFG0000001182	MN1	cyan	VSMC_M2	-0.04	8.32E-01	0.59	1.31E-03	0.17	4.09E-01	0.13	5.34E-01	-0.25	2.16E-01	-0.12	5.39E-01	-0.60	9.70E-04	-0.30	1.32E-01	-0.43	2.43E-02	0.39	4.27E-02
ENSCAFG0000001259	ENSCAFG0000001259	darkgrey	VSMC_M8	-0.04	8.32E-01	-0.57	1.96E-03	0.31	1.10E-01	-0.88	8.91E-10	-0.16	4.14E-01	0.07	7.22E-01	0.41	3.43E-02	0.97	2.60E-16	0.42	3.14E-02	-0.74	1.01E-05
ENSCAFG0000000861	DSN1	darkgrey	VSMC_M8	-0.04	8.32E-01	-0.49	8.94E-03	0.39	4.50E-02	-0.84	3.79E-08	-0.30	1.23E-01	0.19	3.36E-01	0.43	2.40E-02	0.96	7.18E-15	0.31	1.10E-01	-0.63	4.47E-04
ENSCAFG0000000196	CAPN11	grey	VSMC_M10	-0.04	8.32E-01	0.23	2.42E-01	0.21	2.97E-01	-0.17	4.01E-01	-0.21	2.82E-01	0.11	5.73E-01	0.14	5.01E-01	0.07	7.37E-01	-0.20	3.15E-01	0.14	4.88E-01
ENSCAFG0000002585	ENSCAFG0000002585	grey	VSMC_M10	-0.04	8.31E-01	-0.41	3.24E-02	-0.12	5.42E-01	-0.36	6.51E-02	0.22	2.75E-01	0.13	5.09E-01	0.30	1.28E-01	0.50	8.60E-03	0.31	1.16E-01	-0.54	3.32E-03
ENSCAFG0000001763	PPP4R4	grey	VSMC_M10	-0.04	8.31E-01	0.05	7.94E-01	-0.43	2.63E-02	0.55	3.25E-03	0.36	6.40E-02	-0.13	5.13E-01	-0.63	3.82E-04	-0.43	2.66E-02	0.04	8.31E-01	0.16	4.32E-01

ENSCAFG0000001261	KCNK2	grey	VSMC_M10	-0.04	8.31E-01	0.07	7.18E-01	0.03	8.64E-01	0.05	8.01E-01	-0.14	5.01E-01	-0.02	9.29E-01	0.54	3.77E-03	-0.04	8.41E-01	-0.15	4.69E-01	0.21	2.94E-01
ENSCAFG0000002369	ANGPTL5	darkgreen	VSMC_M4	-0.04	8.31E-01	0.01	9.62E-01	-0.34	7.99E-02	0.45	1.71E-02	0.26	1.91E-01	0.03	8.68E-01	0.23	2.42E-01	-0.36	6.41E-02	-0.01	9.64E-01	0.27	1.77E-01
ENSCAFG0000000386	DHDH	grey	VSMC_M10	-0.04	8.30E-01	0.21	2.95E-01	-0.08	6.83E-01	0.17	3.83E-01	0.05	7.93E-01	-0.16	4.21E-01	0.24	2.38E-01	-0.25	2.03E-01	-0.15	4.42E-01	0.24	2.26E-01
ENSCAFG0000001640	SEL1L3	grey	VSMC_M10	-0.04	8.30E-01	0.35	7.26E-02	0.25	2.12E-01	-0.04	8.61E-01	-0.34	8.00E-02	0.02	9.29E-01	0.12	5.59E-01	-0.03	8.83E-01	-0.41	3.23E-02	0.33	9.40E-02
ENSCAFG0000001134	ADAP1	grey	VSMC_M10	-0.04	8.29E-01	-0.07	7.24E-01	-0.25	2.18E-01	0.51	7.17E-03	0.11	5.88E-01	0.09	6.71E-01	-0.10	6.29E-01	-0.34	7.93E-02	0.07	7.27E-01	0.20	3.23E-01
ENSCAFG0000001139	KLHL25	grey	VSMC_M10	-0.04	8.29E-01	0.25	2.13E-01	-0.31	1.14E-01	0.53	4.57E-03	0.17	3.97E-01	0.11	5.78E-01	-0.24	2.36E-01	-0.52	5.05E-03	-0.13	5.07E-01	0.40	3.81E-02
ENSCAFG0000001544	ENSCAFG0000001544	grey	VSMC_M10	-0.04	8.29E-01	-0.06	7.76E-01	-0.04	8.51E-01	0.11	5.97E-01	-0.03	8.99E-01	-0.05	8.14E-01	0.20	3.24E-01	-0.10	6.15E-01	0.06	7.48E-01	0.04	8.58E-01
ENSCAFG0000001108	NDUFS8	cyan	VSMC_M2	-0.04	8.29E-01	0.50	7.70E-03	0.37	5.84E-02	0.12	5.67E-01	-0.60	1.01E-03	0.39	4.45E-02	-0.04	8.48E-01	-0.20	3.06E-01	-0.50	8.06E-03	0.57	1.71E-03
ENSCAFG0000001849	CRLF3	darkgreen	VSMC_M4	-0.04	8.29E-01	-0.05	8.04E-01	-0.64	2.83E-04	0.71	3.16E-05	0.57	2.08E-03	-0.11	5.92E-01	-0.31	1.20E-01	-0.52	5.30E-03	0.09	6.39E-01	0.20	3.20E-01
ENSCAFG0000003233	ENSCAFG0000003233	grey	VSMC_M10	-0.04	8.28E-01	0.45	1.94E-02	0.39	4.40E-02	-0.18	3.71E-01	-0.43	2.57E-02	0.14	4.73E-01	0.18	3.58E-01	-0.07	7.36E-01	-0.42	2.99E-02	0.29	1.39E-01
ENSCAFG0000000309	PM20D2	grey	VSMC_M10	-0.04	8.28E-01	-0.49	9.16E-03	-0.17	3.95E-01	0.01	9.44E-01	0.17	4.02E-01	0.14	4.95E-01	0.14	4.71E-01	0.17	3.89E-01	0.39	4.49E-02	-0.27	1.72E-01
ENSCAFG0000001638	GRK6	grey	VSMC_M10	-0.04	8.28E-01	-0.23	2.45E-01	0.39	4.41E-02	-0.48	1.18E-02	-0.40	3.72E-02	0.09	6.63E-01	0.03	8.76E-01	0.51	6.05E-03	0.17	3.90E-01	-0.26	1.99E-01
ENSCAFG0000001446	ZCWPW1	grey	VSMC_M10	-0.04	8.28E-01	0.01	9.56E-01	0.09	6.51E-01	-0.14	4.76E-01	-0.21	3.04E-01	0.30	1.32E-01	-0.16	4.21E-01	0.18	3.69E-01	-0.04	8.57E-01	0.07	7.21E-01
ENSCAFG0000002397	ENSCAFG0000002397	grey	VSMC_M10	-0.04	8.28E-01	0.12	5.42E-01	0.15	4.56E-01	-0.05	8.12E-01	-0.23	2.44E-01	-0.07	7.29E-01	0.27	1.71E-01	0.01	9.53E-01	-0.15	4.50E-01	0.17	3.92E-01
ENSCAFG0000001290	RUNX3	grey	VSMC_M10	-0.04	8.28E-01	0.07	7.42E-01	-0.27	1.71E-01	0.28	1.58E-01	0.22	2.73E-01	-0.18	3.81E-01	-0.27	1.73E-01	-0.31	1.11E-01	-0.01	9.71E-01	0.26	1.91E-01
ENSCAFG0000000805	MTMR9	grey	VSMC_M10	-0.04	8.27E-01	-0.22	2.73E-01	-0.05	7.89E-01	-0.17	3.83E-01	-0.01	9.54E-01	0.40	3.66E-02	0.41	3.36E-02	0.16	4.15E-01	0.16	4.13E-01	0.00	9.82E-01
ENSCAFG0000001330	ENSCAFG0000001330	grey	VSMC_M10	-0.04	8.27E-01	0.17	3.94E-01	0.16	4.32E-01	-0.11	5.96E-01	-0.21	2.86E-01	-0.08	6.83E-01	-0.05	7.99E-01	0.10	6.37E-01	-0.19	3.40E-01	0.12	5.60E-01
ENSCAFG0000000255	U2AF2	darkgrey	VSMC_M8	-0.04	8.27E-01	-0.08	6.95E-01	0.66	1.62E-04	-0.85	2.78E-08	-0.60	8.55E-04	0.21	3.01E-01	0.24	2.26E-01	0.78	1.64E-06	-0.02	9.11E-01	-0.38	5.14E-02
ENSCAFG0000000778	CAPG	yellow	VSMC_M3	-0.04	8.26E-01	-0.11	5.77E-01	0.67	1.31E-04	-0.84	5.20E-08	-0.53	4.77E-03	-0.03	8.99E-01	0.21	2.92E-01	0.65	2.62E-04	0.11	5.84E-01	-0.45	1.90E-02
ENSCAFG0000000538	MAP6	turquoise	VSMC_M6	-0.04	8.26E-01	0.16	4.40E-01	0.07	7.12E-01	-0.04	8.59E-01	-0.21	2.94E-01	0.65	2.18E-04	-0.08	6.82E-01	0.03	9.01E-01	-0.16	4.21E-01	0.16	4.32E-01
ENSCAFG0000002874	ENSCAFG0000002874	turquoise	VSMC_M6	-0.04	8.26E-01	0.16	4.40E-01	0.07	7.12E-01	-0.04	8.59E-01	-0.21	2.94E-01	0.65	2.18E-04	-0.08	6.82E-01	0.03	9.01E-01	-0.16	4.21E-01	0.16	4.32E-01
ENSCAFG0000000831	ASIC1	grey	VSMC_M10	-0.04	8.26E-01	0.37	5.81E-02	0.11	5.93E-01	-0.03	8.67E-01	-0.16	4.22E-01	0.07	7.34E-01	0.17	4.09E-01	-0.13	5.17E-01	-0.32	1.09E-01	0.26	1.88E-01
ENSCAFG0000001820	RIOK3	grey	VSMC_M10	-0.04	8.26E-01	-0.64	2.92E-04	-0.23	2.43E-01	-0.14	4.97E-01	0.24	2.33E-01	0.05	7.96E-01	0.22	2.60E-01	0.37	5.84E-02	0.54	3.84E-03	-0.38	4.78E-02
ENSCAFG0000000095	SNTB1	grey	VSMC_M10	-0.04	8.26E-01	0.53	4.32E-03	0.02	9.30E-01	0.28	1.62E-01	-0.20	3.13E-01	0.09	6.57E-01	-0.33	9.72E-02	-0.39	4.69E-02	-0.48	1.17E-02	0.59	1.30E-03
ENSCAFG0000001000	DUSP8	grey	VSMC_M10	-0.04	8.26E-01	0.21	2.87E-01	-0.10	6.16E-01	0.28	1.52E-01	-0.09	6.48E-01	0.05	8.06E-01	0.00	9.97E-01	-0.32	1.08E-01	-0.20	3.13E-01	0.47	1.23E-02
ENSCAFG0000001784	STOML1	darkgreen	VSMC_M4	-0.04	8.26E-01	0.28	1.53E-01	-0.35	7.51E-02	0.71	3.54E-05	0.09	6.73E-01	0.01	9.67E-01	-0.14	4.79E-01	-0.62	5.41E-04	-0.26	1.98E-01	0.69	7.46E-05
ENSCAFG0000001588	APBB2	darkgreen	VSMC_M4	-0.04	8.26E-01	0.00	9.93E-01	-0.62	5.05E-04	0.63	4.00E-04	0.48	1.17E-02	-0.03	8.92E-01	-0.15	4.67E-01	-0.44	2.11E-02	-0.02	9.28E-01	0.32	1.05E-01
ENSCAFG0000000831	ELP3	grey	VSMC_M10	-0.04	8.25E-01	-0.29	1.50E-01	-0.06	7.82E-01	-0.23	2.52E-01	0.16	4.24E-01	0.15	4.65E-01	-0.14	4.88E-01	0.26	1.99E-01	0.25	2.04E-01	-0.51	6.93E-03
ENSCAFG0000000940	LPCAT2	darkgreen	VSMC_M4	-0.04	8.25E-01	0.08	6.89E-01	-0.48	1.06E-02	0.64	3.37E-04	0.41	3.27E-02	-0.21	3.00E-01	-0.65	2.59E-04	-0.51	6.91E-03	0.02	9.27E-01	0.19	3.46E-01
ENSCAFG0000001311	RGSL1	grey	VSMC_M10	-0.04	8.25E-01	0.25	2.17E-01	-0.04	8.52E-01	0.23	2.41E-01	-0.04	8.39E-01	-0.04	8.59E-01	0.10	6.04E-01	-0.23	2.53E-01	-0.24	2.19E-01	0.36	6.65E-02
ENSCAFG0000003069	ZBED2	grey	VSMC_M10	-0.04	8.25E-01	-0.02	9.30E-01	0.23	2.49E-01	-0.29	1.49E-01	-0.26	1.93E-01	0.21	2.82E-01	-0.01	9.65E-01	0.23	2.53E-01	-0.07	7.42E-01	-0.04	8.35E-01
ENSCAFG0000002712	ENSCAFG0000002712	grey	VSMC_M10	-0.04	8.25E-01	-0.02	9.06E-01	0.13	5.17E-01	-0.12	5.55E-01	-0.19	3.36E-01	0.01	9.75E-01	0.29	1.49E-01	0.12	5.56E-01	-0.01	9.70E-01	0.02	9.10E-01
ENSCAFG0000001029	ENSCAFG0000001029	darkgreen	VSMC_M4	-0.04	8.25E-01	0.41	3.17E-02	-0.38	5.18E-02	0.89	5.62E-10	0.19	3.47E-01	-0.08	6.89E-01	-0.36	6.64E-02	-0.81	2.76E-07	-0.31	1.13E-01	0.64	3.63E-04
ENSCAFG0000000490	DIAPH3	darkgrey	VSMC_M8	-0.04	8.25E-01	-0.60	8.38E-04	0.28	1.55E-01	-0.86	7.77E-09	-0.16	4.25E-01	0.08	6.78E-01	0.44	2.13E-02	0.99	3.35E-21	0.43	2.42E-02	-0.73	1.28E-05
ENSCAFG0000000059	MARCH3	grey	VSMC_M10	-0.04	8.24E-01	0.00	9.82E-01	0.14	4.98E-01	0.00	9.83E-01	-0.20	3.11E-01	0.20	3.06E-01	-0.46	1.66E-02	0.03	8.84E-01	-0.02	9.16E-01	0.00	9.97E-01
ENSCAFG0000001955	RABL6	yellow	VSMC_M3	-0.04	8.24E-01	-0.08	6.79E-01	0.64	2.89E-04	-0.55	2.77E-03	-0.59	1.33E-03	0.01	9.43E-01	0.10	6.22E-01	0.48	1.15E-02	0.06	7.79E-01	-0.24	2.27E-01
ENSCAFG0000003153	INPP1	grey	VSMC_M10	-0.04	8.24E-01	0.62	5.04E-04	0.07	7.25E-01	0.38	4.83E-02	-0.28	1.51E-01	0.07	7.25E-01	0.13	5.20E-01	-0.53	4.08E-03	-0.61	7.45E-04	0.80	5.47E-07
ENSCAFG0000001651	CACNB1	grey	VSMC_M10	-0.04	8.24E-01	0.10	6.19E-01	-0.25	2.13E-01	0.35	7.36E-02	0.13	5.29E-01	-0.13	5.13E-01	0.06	7.48E-01	-0.35	7.08E-02	-0.06	7.55E-01	0.37	5.53E-02
ENSCAFG0000002001	CENPN	darkgrey	VSMC_M8	-0.04	8.24E-01	-0.62	5.13E-04	0.17	3.96E-01	-0.77	2.39E-06	0.01	9.51E-01	0.00	9.83E-01	0.32	1.07E-01	0.88	9.07E-10	0.51	6.30E-03	-0.82	1.32E-07
ENSCAFG0000001371	ENSCAFG0000001371	darkgrey	VSMC_M8	-0.05	8.24E-01	-0.29	1.46E-01	0.45	1.91E-02	-0.75	5.45E-06	-0.29	1.41E-01	0.04	8.38E-01	0.04	8.52E-01	0.73	1.67E-05	0.25	2.10E-01	-0.66	1.59E-04
ENSCAFG0000000973	SEMA4G	grey	VSMC_M10	-0.05	8.23E-01	0.44	2.32E-02	-0.13	5.27E-01	0.48	1.05E-02	-0.01	9.62E-01	0.02	9.39E-01	-0.32	1.09E-01	-0.54	3.71E-03	-0.29	1.39E-01	0.52	5.19E-03
ENSCAFG0000001561	GHDC	grey	VSMC_M10	-0.05	8.23E-01	0.59	1.24E-03	0.03	8.67E-01	0.40	4.06E-02	-0.21	2.95E-01	0.12	5.55E-01	0.00	9.96E-01	-0.56	2.32E-03	-0.54	3.88E-03	0.73	1.37E-05
ENSCAFG0000002429	ENSCAFG0000002429	grey	VSMC_M10	-0.05	8.23E-01	0.08	7.00E-01	0.08	7.09E-01	0.02	9.16E-01	-0.14	4.92E-01	0.01	9.64E-01	0.19	3.33E-01	0.00	9.93E-01	-0.07	7.43E-01	0.18	3.79E-01
ENSCAFG0000002974	ENSCAFG0000002974	grey	VSMC_M10	-0.05	8.23E-01	0.31	1.13E-01	0.21	2.82E-01	0.17	3.85E-01	-0.42	2.86E-02	0.24	2.21E-01	0.17	4.00E-01	-0.24	2.31E-01	-0.35	7.07E-02	0.55	2.86E-03
ENSCAFG0000000798	MCTP1	darkgreen	VSMC_M4	-0.05	8.22E-01	0.14	4.76E-01	-0.41	3.31E-02	0.68	9.40E-05	0.27	1.80E-01	-0.07	7.24E-01	0.02	9.19E-01	-0.56	2.23E-03	-0.12	5.49E-01	0.42	2.96E-02
ENSCAFG0000000756	IMMT	grey	VSMC_M10	-0.05	8.22E-01	0.03	8.94E-01	0.43	2.34E-02	-0.43	2.57E-02	-0.33	8.95E-02	-0.01	9.55E-01	0.36	6.61E-02	0.34	8.38E-02	-0.10	6.29E-01	-0.23	2.53E-01
ENSCAFG0000003274	ENSCAFG0000003274	grey	VSMC_M10	-0.05	8.22E-01	0.13	5.09E-01	0.45	1.74E-02	-0.34	7.84E-02	-0.26	1.91E-01	-0.21	2.96E-01	0.10	6.29E-01	0.16	4.37E-01	-0.03	8.62E-01	-0.30	1.30E-01
ENSCAFG0000001614	SYDE1	grey	VSMC_M10	-0.05	8.22E-01	0.11	5.89E-01	0.49	9.19E-03	-0.26	1.91E-01	-0.64	3.67E-04	0.31	1.14E-01	0.07	7.40E-01	0.23	2.54E-01	-0.19	3.34E-01	0.19	3.35E-01
ENSCAFG0000000353	ENSCAFG0000000353	grey	VSMC_M10	-0.05	8.21E-01	0.24	2.35E-01	-0.24	2.21E-01	0.57	2.09E-03	0.11	5.68E-01	0.13	5.17E-01	0.11	5.87E-01	-0.49	9.12E-03	-0.21	2.91E-01	0.37	5.98E-02
ENSCAFG0000000724	CYP7B1	grey	VSMC_M10	-0.05	8.21E-01	0.46	1.64E-02	0.11	5.80E-01	0.32	1.03E-01	-0.36	6.54E-02	0.17	3.84E-01	-0.26	1.89E-01	-0.33	9.81E-02	-0.44	2.17E-02	0.65	2.39E-04
ENSCAFG0000000582	APBB3	grey	VSMC_M10	-0.05	8.21E-01	0.33	9.27E-02	0.14	4.71E-01	0.30	1.33E-01	-0.36	6.80E-02	0.19	3.47E-01	0.14	4.77E-01	-0.33	9.25E-02	-0.33	9.76E-02	0.61	8.01E-04
ENSCAFG0000001667	SAT2	cyan	VSMC_M2	-0.05	8.21E-01	0.52	5.42E-03	0.35	7.53E-02	0.20	3.08E-01	-0.35	7.42E-02	-0.06	7.81E-01	-0.12	5.59E-01	-0.46	1.53E-02	-0.35	7.71E-02	0.39	4.57E-02
ENSCAFG0000000551	EOMES	grey	VSMC_M10	-0.05	8.21E-01	0.19	3.37E-01	0.15	4.70E-01	-0.11	5.90E-01	-0.17	3.89E-01	-0.01	9.54E-01	-0.01	9.49E-01	-0.07	7.35E-01	-0.14	4.81E-01	0.18	3.75E-01
ENSCAFG0000001406	PALD1	turquoise	VSMC_M6	-0.05	8.21E-01	0.12	5.66E-01	0.04	8.50E-01	0.02	9.31E-01	-0.19	3.38E-01	0.83	1.04E-07	-0.03	8.91E-01	0.03	8.68E-01	-0.13	5.22E-01	0.23	2.56E-01
ENSCAFG0000001749	TSPOAP1	yellow	VSMC_M3	-0.05	8.21E-01	0.11	5.92E-01	0.55	2.74E-03	-0.60	9.30E-04	-0.39	4.34E-02	-0.31	1.20E-01	0.12	5.62E-01	0.39	4.53E-02	-0.04	8.51E-01	-0.27	1.76E-01
ENSCAFG0000001787	ICAM5	grey	VSMC_M10	-0.05	8.21E-01	-0.27	1.70E-01	0.18	3.81E-01	-0.23	2.39E-01	-0.04	8.44E-01	-0.07	7.16E-01	-0.23	2.48E-01	0.24	2.23E-01	0.37	5.86E-02	-0.49	1.03E-02
ENSCAFG0000002920	ZNF354C	grey	VSMC_M10	-0.05	8.20E-01	0.06	7.60E-01	-0.56	2.41E-03	0.58	1.46E-03	0.31	1.16E-01	0.14	4.96E-01	0.04	8.32E-01	-0.42	2.89E-02	-0.13	5.05E-01	0.52	5.96E-03
ENSCAFG0000001541	ANO8	grey	VSMC_M10	-0.05	8.20E-01	0.03	8.98E-01	0.18	3.58E-01	-0.25	2.06E-01	-0.14	4.89E-01	0.02	9.16E-01	0.32	9.88E-02	0.16	4.36E-01	-0.02	9.28E-01	-0.01	9.54E-01
ENSCAFG0000001569	C20H19orf44	darkgreen	VSMC_M4	-0.05	8.20E-01	0.32	1.03E-01	-0.25	2.00E-01	0.66	1.68E-04	0.09	6.66E-01	-0.21	2.88E-01	-0.13	5.33E-01	-0.63	4.00E-04	-0.32	1.00E-01	0.57	1.81E-03
ENSCAFG0000001229	NDST3	darkgreen	VSMC_M4	-0.05	8.20E-01	0.04	8.27E-01	-0.38	5.17E-02	0.51	6.05E-03	0.28	1.56E-01	0.03	8.98E-01	-0.25	2.04E-01	-0.44	2.03E-02	-0.01	9.67E-01	0.28	1.61E-01
ENSCAFG0000001502	DLGAP5	darkgrey	VSMC_M8	-0.05	8.20E-01	-0.52	5.14E-03	0.33	9.28E-02	-0.90	1.43E-10	-0.20	3.14E-01	0.07	7.25E-01	0.46	1.63E-02	0.97	7.12E-17	0.37	5.87E-02	-0.69	6.56E-05
ENSCAFG0000000198	DMRT2	violet	VSMC_M7	-0.05	8.20E-01	-0.31	1.15E-01	0.01	9.45E-01	-0.33	9.66E-02	0.00	9.99E-01	0.03	8.89E-01	0.93	9.78E-13	0.35	7.30E-02	0.18	3.81E-01	-0.18	3.56E-01
ENSCAFG0000001422	C1R	cyan	VSMC_M2	-0.05	8.20E-01	0.71	3.61E-05	0.04	8.33E-01	0.48	1.18E-02	-0.20	3.15E-01	-0.05	7.91E-01	-0.25	2.00E-01	-0.61	7.81E-04	-0.61	7.43E-04	0.66	1.57E-04
ENSCAFG0000000908	ENSCAFG0000000908	grey	VSMC_M10	-0.05	8.20E-01	-0.05	8.22E-01	-0.01	9.79E-01	-0.21	2.84E-01	-0.10	6.11E-01	0.10	6.23E-01	0.24	2.21E-01	0.22	2.59E-01	0.00	9.99E-01	0.12	5.58E-01
ENSCAFG0000001434	SAP25	grey	VSMC_M10	-0.05	8.20E-01	-0.07	7.16E-01	0.02	9.35E-01	-0.13	5.18E-01	-0.02	9.04E-01	0.04	8.62E-01	0.04	8.57E-01	0.10	6.23E-01	0.07	7.26E-01	0.01	9.59E-01
ENSCAFG0000000909	COQ2	grey	VSMC_M10	-0.05	8.20E-01	0.18	3.72E-01	-0.39	4.18E-02	0.47	1.26E-02	0.14	4.93E-01	0.21	2.82E-01	0.15	4.68E-01	-0.45	1.86E-02	-0.19	3.42E-01	0.58	1.49E-03
ENSCAFG0000001789	COX10	grey	VSMC_M10	-0.05	8.20E-01	0.16	4.36E-01	0.35	7.30E-02	-0.39	4.15E-02	-0.43	2.45E-02	0.11	5.72E-01	0.14	4.87E-01	0.29	1.48E-01	-0.26	1.93E-01	0.10	6.08E-01
ENSCAFG0000000920	NR4A2	grey	VSMC_M10	-0.05	8.20E-01	-0.30	1.34E-01	-0.29	1.40E-01	-0.19	3.30E-01	0.36	6.39E-02	0.03	8.65E-01	-0.14	4.96E-01	0.27	1.70E-01	0.30	1.23E-01	-0.34	8.35E-02
ENSCAFG0000002756	ENSCAFG0000002756	grey	VSMC_M10	-0.05	8.19E-01	-0.17	4.04E-01	-0.06	7.51E-01	-0.02	9.22E-01	-0.02	9.25E-01	-0.08	7.08E-01	0.44	2.04E-02	0.03	8.68E-01	0.08	6.78E-01	0.07	7.27E-01
ENSCAFG0000000008	CDK2	darkgrey	VSMC_M8	-0.05	8.19E-01	-0.63	3.97E-04	0.02	9.20E-01	-0.64	3.26E-04	0.16	4.13E-01	-0.01	9.56E-01	0.18	3.63E-01	0.79	9.09E-07	0.52	4.93E-03	-0.84	3.05E-08
ENSCAFG0000001049	PPP1R12B	darkgreen	VSMC_M4	-0.05	8.18E-01	0.06	7.50E-01	-0.34	8.67E-02	0.63	3.89E-04	0.30	1.23E-01	-0.10	6.27E-01	-0.62	5.34E-04	-0.62	5.32E-04	0.12	5.43E-01	0.18	3.77E-01
ENSCAFG0000000309	CRHR2	grey	VSMC_M10	-0.05	8.18E-01	0.05	8.11E-01	0.15	4.40E-01	-0.13	5.19E-01	-0.18	3.58E-01	0.01	9.51E-01	-0.05	7.98E-01	0.10	6.22E-01	-0.04	8.32E-01	0.02	9.33E-01
ENSCAFG0000001894	ADGRG4	grey	VSMC_M10	-0.05	8.18E-01	0.05	8.11E-01	0.15	4.40E-01	-0.13	5.19E-01	-0.18	3.58E-01	0.01	9.51E-01	-0.05	7.98E-01	0.10	6.22E-01	-0.04	8.32E-01	0.02	9.33E-01
ENSCAFG0000000762	ENSCAFG0000000762	grey	VSMC_M10	-0.05	8.18E-01	-0.32	1.07E-01	-0.06	7.56E-01	-0.36	6.59E-02	0.06	7.71E-01	0.04	8.26E-01	0.31	1.19E-01	0.44	2.11E-02	0.18	3.82E-01	-0.33	9.43E-02
ENSCAFG0000000686	ZFAND4	darkgreen	VSMC_M4	-0.05	8.18E-01	-0.13	5.10E-01	-0.58	1.55E-03	0.57	1.88E-03	0.42	3.13E-02	-0.07	7.30E-01	-0.08	7.06E-01	-0.35	7.55E-02	0.06	7.57E-01	0.28	1.63E-01
ENSCAFG0000000672	RIMBP2	cyan	VSMC_M2	-0.05	8.17E-01	0.57	1.89E-03	0.19	3.49E-01	0.07	7.27E-01	-0.28	1.57E-01	-0.02	9.22E-01	-0.14	4.81E-01	-0.31	1.13E-01	-0.46	1.70E-02	0.45	1.97E-02
ENSCAFG0000001196	GCNT7	cyan	VSMC_M2	-0.05	8.17E-01	0.68	9.93E-05	-0.08	6.99E-01	0.71	3.02E-05	-0.09	6.61E-01	-0.12	5.51E-01	-0.52	5.30E-03	-0.81	2.92E-07	-0.50	8.50E-03	0.74	1.01E-05
ENSCAFG0000003155	ENSCAFG0000003155	grey	VSMC_M10	-0.05	8.17E-01	0.08	7.08E-01	0.14	4.91E-01	-0.15	4.69E-01	-0.21	2.94E-01	0.03	8.63E-01	0.11	5.78E-01	0.15	4.45E-01	-0.11	5.81E-01	0.08	7.03E-01
ENSCAFG0000000704	MOS	grey	VSMC_M10	-0.05	8.17E-01	-0.04	8.34E-01	-0.04	8.59E-01	0.02	9.03E-01	-0.01	9.67E-01	-0.07	7.36E-01	-0.08	7.00E-01	-0.02	9.20E-01	0.11	5.83E-01	0.05	7.90E-01
ENSCAFG0000002987	ENSCAFG0000002987	grey	VSMC_M10	-0.05	8.17E-01	0.24	2.30E-01	0.15	4.56E-01	-0.09	6.40E-01	-0.13	5.26E-01	0.07	7.11E-01	0.08	6.90E-01	-0.02	9.25E-01	-0.16	4.19E-01	0.14	4.92E-01
ENSCAFG0000001358	IVNS1ABP	grey	VSMC_M10	-0.05	8.17E-01	-0.02	9.41E-01	0.14	5.00E-01	-0.34	8.62E-02	-0.15	4.55E-01	0.12	5.53E-01	-0.04	8.59E-01	0.40	3.67E-02	-0.05	8.02E-01	-0.17	4.07E-01
ENSCAFG0000000991	ENSCAFG0000000991	grey	VSMC_M10	-0.05	8.16E-01	-0.17	3.86E-01	-0.07	7.35E-01	-0.17	3.87E-01	0.03	8.80E-01	-0.02	9.40E-01	0.25	2.03E-01	0.17	3.83E-01	0.11	5.93E-01	-0.07	7.24E-01
ENSCAFG0000001681	RHBG	grey	VSMC_M10	-0.05	8.16E-01	0.37	5.96E-02	0.42	2.87E-02	-0.08	6.95E-01	-0.49	8.88E-03	0.17	3.94E-01	0.02	9.04E-01	-0.08	7.03E-01	-0.30	1.31E-01	0.34	8.73E-02
ENSCAFG0000002851	FAM206A	pink	VSMC_M5	-0.05	8.16E-01	-0.08	7.04E-01	-0.58	1.52E-03	0.53	4.65E-03	0.44	2.01E-02	-0.24	2.36E-01	0.09	6.72E-01	-0.42	2.75E-02	0.08	6.85E-01	0.29	1.39E-01
ENSCAFG0000000848	MED12L	darkgreen	VSMC_M4	-0.05	8.16E-01	0.16	4.18E-01	-0.35	7.09E-02	0.50	8.64E-03	0.20	3.22E-01	-0.08	7.03E-01	0.07	7.42E-01	-0.54	3.65E-03	-0.13	5.12E-01	0.47	1.39E-02
ENSCAFG0000000593	CRLS1	grey	VSMC_M10	-0.05	8.15E-01	0.33	8.90E-02	-0.43	2.41E-02	0.54	3.53E-03	0.34	8.50E-02	-0.03	8.90E-01	-0.39	4.17E-02	-0.58	1.58E-03	-0.26	1.95E-01	0.39	4.33E-02
ENSCAFG0000003057	NNMT	grey	VSMC_M10	-0.05	8.15E-01	-0.31	1.12E-01	-0.10	6.29E-01	-0.23	2.56E-01	0.02	9.16E-01	0.56	2.65E-03	0.40	3.87E-02	0.34	8.29E-02	0.16	4.37E-01	-0.12	5.44E-01
ENSCAFG0000001292	NR1I3	grey	VSMC_M10	-0.05	8.15E-01	0.55	3.18E-03	0.06	7.61E-01	0.27	1.73E-01	-0.17	4.09E-01	-0.05	7.97E-01	-0.19	3.32E-01	-0.38	4.82E-02	-0.43	2.49E-02	0.47	1.26E-02
ENSCAFG0000001763	THSD4	turquoise	VSMC_M6	-0.05	8.15E-01	-0.02	9.07E-01	0.29	1.40E-01	-0.13	5.32E-01	-0.43	2.52E-02	0.80	5.52E-07	0.18	3.75E-01	0.17	3.90E-01	-0.04	8.49E-01	0.09	6.64E-01
ENSCAFG0000001171	DDX5	darkgrey	VSMC_M8	-0.05	8.14E-01	-0.25	2.14E-01	0.26	1.84E-01	-0.47	1.33E-02	-0.14	4.88E-01	-0.12	5.44E-01	-0.03	8.82E-01	0.53	4.52E-03	0.24	2.34E-01	-0.50	8.28E-03
ENSCAFG0000001918	BGN	darkgreen	VSMC_M4	-0.05	8.14E-01	0.53	4.53E-03	0.02	9.31E-01	0.44	2.07E-02	-0.01	9.71E-01	-0.27	1.77E-01	-0.55	2.67E-03	-0.63	3.78E-04	-0.26	1.90E-01	0.37	5.42E-02
ENSCAFG0000000753	DCDC1	grey	VSMC_M10	-0.05	8.14E-01	0.15	4.68E-01	0.05	8.08E-01	0.11	5.77E-01	-0.18	3.69E-01	-0.05	8.16E-01	0.13	5.32E-01	-0.10	6.05E-01	-0.17	4.04E-01	0.24	2.30E-01
ENSCAFG0000001676	NES	darkgrey	VSMC_M8	-0.05	8.14E-01	-0.63	3.89E-04	0.26	1.82E-01	-0.78	2.00E-06	-0.08	6.86E-01	0.03	8.77E-01	0.36	6.67E-02	0.81	2.61E-07	0.56	2.50E-03	-0.76	4.21E-06
ENSCAFG0000000343	CTTNBP2	grey	VSMC_M10	-0.05	8.13E-01	-0.14	4.97E-01	-0.26	1.88E-01	0.28	1.54E-01	0.08	6.88E-01	0.12	5.68E-01	0.34	7.92E-02	-0.13	5.15E-01	0.08	6.97E-01	0.30	1.27E-01
ENSCAFG0000001143	UBR1	pink	VSMC_M5	-0.05	8.12E-01	-0.31	1.11E-01	-0.86	7.34E-09	0.57	1.98E-03	0.82	1.27E-07	-0.15	4.53E-01	-0.01	9.59E-01	-0.32	1.08E-01	0.28	1.61E-01	-0.01	9.77E-01
ENSCAFG0000001167	OTUD7B	grey	VSMC_M10	-0.05	8.12E-01	0.04	8.26E-01	0.26	1.94E-01	-0.07	7.20E-01	-0.25	2.05E-01	0.26	1.99E-01	0.16	4.34E-01	-0.02	9.21E-01	-0.04	8.34E-01	0.02	9.25E-01
ENSCAFG0000002026	PTGS1	turquoise	VSMC_M6	-0.05	8.12E-01	0.15	4.61E-01	0.07	7.21E-01	-0.06	7.74E-01	-0.18	3.71E-01	0.62	5.22E-04	-0.07	7.43E-01	-0.05	7.97E-01	-0.13	5.10E-01	0.18	3.57E-01
ENSCAFG0000002012	ABCA4	darkgreen	VSMC_M4	-0.05	8.12E-01	0.32	1.02E-01	-0.32	1.02E-01	0.58	1.69E-03	0.09	6.54E-01	0.09	6.51E-01	-0.20	3.25E-01	-0.50	7.28E-03	-0.33	9.34E-02	0.58	1.43E-03
ENSCAFG0000000462	PRCP	darkgrey	VSMC_M8	-0.05	8.12E-01	-0.61	6.64E-04	0.08	6.79E-01	-0.67	1.24E-04	0.13	5.24E-01	0.09	6.61E-01	0.15	4.67E-01	0.74	9.19E-06	0.58	1.55E-03	-0.83	6.12E-08
ENSCAFG0000001840	JAG2	grey	VSMC_M10	-0.05	8.11E-01	0.32	9.96E-02	0.18	3.67E-01	0.11	5.79E-01	-0.43	2.45E-02	0.63	4.39E-04	-0.02	9.04E-01	-0.14	4.93E-01	-0.32	1.03E-01	0.57	1.98E-03
ENSCAFG0000001020	ID4	pink	VSMC_M5	-0.05	8.11E-01	-0.14	4.90E-01	-0.54	3.34E-03	0.39	4.23E-02	0.56	2.16E-03	0.04	8.34E-01	0.02	9.26E-01	-0.31	1.12E-01	0.19	3.51E-01	-0.05	8.20E-01
ENSCAFG0000002011	PDPR	grey	VSMC_M10	-0.05	8.11E-01	0.23	2.46E-01	0.21	2.91E-01	-0.10	6.12E-01	-0.24	2.26E-01	0.29	1.47E-01	0.04	8.35E-01	0.00	9.81E-01	-0.17	4.06E-01	0.13	5.15E-01
ENSCAFG0000001150	ENSCAFG0000001150	grey	VSMC_M10	-0.05	8.11E-01	0.07	7.29E-01	-0.02	9.29E-01	-0.03	8.68E-01	-0.06	7.52E-01	-0.22	2.80E-01	0.12	5.61E-01	-0.02	9.40E-01	-0.08	6.90E-01	0.22	2.67E-01
ENSCAFG0000001011	CIT	darkgrey	VSMC_M8	-0.05	8.11E-01	-0.46	1.61E-02	0.35	7.51E-02	-0.89	7.06E-10	-0.21	2.92E-01	0.07	7.28E-01	0.42	2.97E-02	0.93	1.08E-12	0.32	1.01E-01	-0.66	1.67E-04
ENSCAFG0000003095	ENSCAFG0000003095	grey	VSMC_M10	-0.05	8.11E-01	0.35	6.96E-02	0.17	4.10E-01	-0.04	8.62E-01	-0.25	2.13E-01	0.25	2.04E-01	-0.07	7.38E-01	-0.16	4.34E-01	-0.28	1.51E-01	0.30	1.34E-01
ENSCAFG0000001057	SELENOS	yellow	VSMC_M3	-0.05	8.11E-01	-0.17	4.07E-01	0.41	3.25E-02	-0.35	7.01E-02	-0.41	3.58E-02	0.04	8.61E-01	-0.12	5.38E-01	0.42	2.81E-02	0.14	4.82E-01	-0.29	1.37E-01
ENSCAFG0000000056	ARSA	cyan	VSMC_M2	-0.05	8.11E-01	0.85	2.04E-08	0.15	4.53E-01	0.53	4.60E-03	-0.31	1.17E-01	-0.03	8.66E-01	-0.32	9.91E-02	-0.79	9.30E-07	-0.65	2.60E-04	0.81	3.55E-07
ENSCAFG0000000067	ZBTB12	grey	VSMC_M10	-0.05	8.11E-01	0.17	3.94E-01	0.18	3.66E-01	-0.24	2.27E-01	-0.01	9.46E-01	-0.41	3.23E-02	0.14	4.84E-01	0.05	7.98E-01	-0.08	7.05E-01	-0.18	3.61E-01
ENSCAFG0000001320	ENSCAFG0000001320	grey	VSMC_M10	-0.05	8.10E-01	0.25	2.00E-01	-0.28	1.63E-01	0.46	1.57E-02	0.15	4.44E-01	-0.13	5.09E-01	-0.05	7.97E-01	-0.48	1.09E-02	-0.20	3.18E-01	0.39	4.66E-02
ENSCAFG0000001889	JUN	grey	VSMC_M10	-0.05	8.10E-01	0.12	5.57E-01	0.13	5.29E-01	0.26	1.87E-01	-0.25	2.08E-01	0.06	7.73E-01	0.29	1.49E-01	-0.27	1.72E-01	-0.17	3.96E-01	0.34	7.83E-02
ENSCAFG0000001612	ENSCAFG0000001612	grey	VSMC_M10	-0.05	8.10E-01	0.09	6.49E-01	0.20	3.25E-01	-0.21	2.84E-01	-0.26	1.91E-01	-0.06	7.53E-01	0.19	3.50E-01	0.14	4.74E-01	-0.13	5.31E-01	0.12	5.68E-01
ENSCAFG0000001392	MRPL49	yellow	VSMC_M3	-0.05	8.10E-01	-0.11	5.96E-01	0.54	3.32E-03	-0.59	1.12E-03	-0.42	2.87E-02	0.07	7.32E-01	0.02	9.07E-01	0.51	6.57E-03	0.10	6.10E-01	-0.49	9.12E-03
ENSCAFG0000002983	ENSCAFG0000002983	grey	VSMC_M10	-0.05	8.10E-01	0.17	3.95E-01	-0.20	3.25E-01	0.44	2.23E-02	0.07	7.32E-01	-0.06	7.75E-01	-0.10	6.21E-01	-0.39	4.66E-02	-0.14	4.96E-01	0.34	8.44E-02
ENSCAFG0000003251	ENSCAFG0000003251	grey	VSMC_M10	-0.05	8.10E-01	0.17	3.95E-01	-0.20	3.25E-01	0.44	2.23E-02	0.07	7.32E-01	-0.06	7.75E-01	-0.10	6.21E-01	-0.39	4.66E-02	-0.14	4.96E-01	0.34	8.44E-02
ENSCAFG0000000510	COQ8B	cyan	VSMC_M2	-0.05	8.10E-01	0.54	3.29E-03	0.34	7.92E-02	0.16	4.13E-01	-0.43	2.66E-02	-0.02	9.24E-01	0.06	7.49E-01	-0.29	1.45E-01	-0.49	9.45E-03	0.47	1.44E-02
ENSCAFG0000003109	ZNF79	darkgreen	VSMC_M4	-0.05	8.10E-01	0.11	5.86E-01	-0.49	9.76E-03	0.57	2.07E-03	0.34	8.48E-02	-0.12	5.43E-01	0.23	2.55E-01	-0.49	9.71E-03	-0.18	3.79E-01	0.43	2.34E-02

ENSCAFG0000000690	ANKRD55	grey	VSMC_M10	-0.05	8.10E-01	0.06	7.65E-01	0.15	4.66E-01	-0.25	2.17E-01	-0.27	1.80E-01	0.26	1.96E-01	0.41	3.45E-02	0.19	3.40E-01	-0.18	3.78E-01	0.14	4.86E-01
ENSCAFG0000001058	ENSCAFG0000001058	grey	VSMC_M10	-0.05	8.10E-01	0.44	2.15E-02	-0.13	5.16E-01	0.44	2.03E-02	0.01	9.43E-01	-0.06	7.85E-01	-0.21	2.89E-01	-0.52	5.34E-03	-0.31	1.13E-01	0.52	5.51E-03
ENSCAFG0000002074	ENSCAFG0000002074	grey	VSMC_M10	-0.05	8.10E-01	-0.13	5.17E-01	0.06	7.56E-01	-0.23	2.50E-01	-0.13	5.21E-01	-0.05	8.07E-01	0.09	6.63E-01	0.30	1.25E-01	0.05	7.94E-01	-0.06	7.56E-01
ENSCAFG0000001508	MED28	darkgrey	VSMC_M8	-0.05	8.09E-01	-0.54	3.74E-03	-0.03	8.84E-01	-0.45	1.93E-02	0.06	7.71E-01	0.21	2.89E-01	0.08	6.95E-01	0.68	8.63E-05	0.40	3.97E-02	-0.56	2.28E-03
ENSCAFG0000001867	ENSCAFG0000001867	grey	VSMC_M10	-0.05	8.09E-01	0.08	6.97E-01	0.07	7.12E-01	-0.16	4.36E-01	-0.08	6.97E-01	0.05	8.04E-01	0.32	1.02E-01	0.07	7.21E-01	-0.08	6.78E-01	0.11	5.99E-01
ENSCAFG0000001525	DPT	darkgrey	VSMC_M8	-0.05	8.09E-01	-0.22	2.63E-01	0.39	4.64E-02	-0.65	2.77E-04	-0.28	1.58E-01	-0.08	6.85E-01	0.72	1.98E-05	0.62	5.86E-04	0.11	5.83E-01	-0.39	4.15E-02
ENSCAFG0000000098	NDUFA6	grey	VSMC_M10	-0.05	8.09E-01	0.12	5.52E-01	0.44	2.04E-02	-0.17	4.02E-01	-0.56	2.29E-03	0.39	4.54E-02	0.06	7.63E-01	0.12	5.49E-01	-0.13	5.14E-01	0.15	4.52E-01
ENSCAFG0000000426	FBXL13	grey	VSMC_M10	-0.05	8.08E-01	-0.01	9.56E-01	-0.32	9.92E-02	0.31	1.15E-01	0.19	3.31E-01	-0.18	3.64E-01	0.25	2.14E-01	-0.29	1.45E-01	-0.01	9.73E-01	0.30	1.28E-01
ENSCAFG0000000783	SERPING1	cyan	VSMC_M2	-0.05	8.08E-01	0.73	1.51E-05	-0.06	7.69E-01	0.68	1.11E-04	-0.11	5.74E-01	-0.11	5.82E-01	-0.37	5.52E-02	-0.79	8.72E-07	-0.59	1.33E-03	0.76	4.52E-06
ENSCAFG0000001953	ATP5F1D	yellow	VSMC_M3	-0.05	8.08E-01	0.07	7.41E-01	0.73	1.51E-05	-0.56	2.17E-03	-0.63	4.85E-04	-0.04	8.34E-01	0.20	3.06E-01	0.42	3.04E-02	-0.06	7.78E-01	-0.27	1.68E-01
ENSCAFG0000000025	B4GALNT1	grey	VSMC_M10	-0.05	8.08E-01	0.19	3.45E-01	0.32	1.03E-01	-0.36	6.19E-02	-0.40	3.98E-02	-0.05	7.88E-01	0.13	5.23E-01	0.28	1.55E-01	-0.26	1.90E-01	0.15	4.68E-01
ENSCAFG0000000296	JOSD2	yellow	VSMC_M3	-0.05	8.08E-01	0.26	1.86E-01	0.63	4.54E-04	-0.30	1.30E-01	-0.59	1.23E-03	0.05	8.02E-01	0.11	5.71E-01	0.11	5.85E-01	-0.25	2.06E-01	-0.03	8.96E-01
ENSCAFG0000000107	ENSCAFG0000000107	grey	VSMC_M10	-0.05	8.07E-01	0.23	2.46E-01	0.02	9.07E-01	0.12	5.44E-01	-0.13	5.09E-01	0.01	9.78E-01	-0.12	5.48E-01	-0.12	5.52E-01	-0.24	2.34E-01	0.29	1.39E-01
ENSCAFG0000002115	ENSCAFG0000002115	grey	VSMC_M10	-0.05	8.07E-01	0.02	9.10E-01	0.06	7.57E-01	-0.27	1.69E-01	-0.05	7.94E-01	-0.15	4.62E-01	0.25	2.04E-01	0.15	4.60E-01	-0.01	9.46E-01	-0.07	7.22E-01
ENSCAFG0000000870	CKMT2	grey	VSMC_M10	-0.05	8.07E-01	0.25	2.10E-01	-0.05	8.03E-01	0.28	1.51E-01	-0.01	9.61E-01	-0.03	8.78E-01	-0.11	5.73E-01	-0.41	3.52E-02	-0.14	4.97E-01	0.33	9.33E-02
ENSCAFG0000001955	ARHGAP45	grey	VSMC_M10	-0.05	8.07E-01	-0.24	2.36E-01	-0.39	4.15E-02	0.22	2.79E-01	0.36	6.35E-02	-0.05	8.22E-01	-0.07	7.41E-01	-0.06	7.63E-01	0.25	2.09E-01	-0.11	5.77E-01
ENSCAFG0000001641	GNAI1	darkgreen	VSMC_M4	-0.05	8.07E-01	0.07	7.16E-01	-0.57	1.90E-03	0.80	5.88E-07	0.41	3.18E-02	0.13	5.32E-01	-0.07	7.21E-01	-0.72	2.53E-05	0.01	9.72E-01	0.47	1.32E-02
ENSCAFG0000003016	ENSCAFG0000003016	grey	VSMC_M10	-0.05	8.07E-01	-0.03	8.96E-01	0.07	7.23E-01	-0.31	1.14E-01	-0.09	6.38E-01	0.17	3.93E-01	0.38	5.05E-02	0.27	1.74E-01	-0.01	9.73E-01	-0.04	8.60E-01
ENSCAFG0000001753	KIF23	darkgrey	VSMC_M8	-0.05	8.06E-01	-0.58	1.50E-03	0.26	1.90E-01	-0.88	1.74E-09	-0.13	5.14E-01	0.11	5.97E-01	0.36	6.90E-02	0.97	3.92E-17	0.43	2.41E-02	-0.74	1.23E-05
ENSCAFG0000001669	TBKBP1	yellow	VSMC_M3	-0.05	8.06E-01	0.50	7.66E-03	0.30	1.35E-01	0.35	7.09E-02	-0.45	1.95E-02	0.01	9.50E-01	-0.35	7.59E-02	-0.49	9.04E-03	-0.37	5.62E-02	0.57	1.99E-03
ENSCAFG0000000224	ENSCAFG0000000224	grey	VSMC_M10	-0.05	8.06E-01	-0.06	7.64E-01	0.09	6.60E-01	-0.16	4.22E-01	0.06	7.64E-01	-0.41	3.38E-02	-0.33	8.93E-02	0.06	7.67E-01	0.21	2.99E-01	-0.33	8.89E-02
ENSCAFG0000000851	HVCN1	grey	VSMC_M10	-0.05	8.06E-01	0.56	2.62E-03	0.11	5.98E-01	0.47	1.36E-02	-0.18	3.56E-01	0.10	6.08E-01	-0.26	1.99E-01	-0.62	5.66E-04	-0.41	3.56E-02	0.51	5.98E-03
ENSCAFG0000002162	ENSCAFG0000002162	turquoise	VSMC_M6	-0.05	8.06E-01	-0.25	2.17E-01	-0.07	7.45E-01	-0.10	6.25E-01	-0.02	9.34E-01	0.33	9.57E-02	-0.12	5.45E-01	0.26	1.99E-01	0.11	5.85E-01	-0.08	6.92E-01
ENSCAFG0000001373	ITGB3	darkolivegreen	VSMC_M9	-0.05	8.06E-01	-0.42	2.94E-02	0.15	4.68E-01	-0.58	1.45E-03	-0.05	7.87E-01	0.31	1.11E-01	-0.07	7.39E-01	0.55	2.76E-03	0.45	1.77E-02	-0.55	3.15E-03
ENSCAFG0000001306	DOCK1	grey	VSMC_M10	-0.05	8.06E-01	0.38	4.88E-02	-0.04	8.45E-01	0.23	2.54E-01	0.03	8.85E-01	0.07	7.30E-01	-0.02	9.19E-01	-0.41	3.22E-02	-0.23	2.57E-01	0.30	1.30E-01
ENSCAFG0000000459	RELB	yellow	VSMC_M3	-0.05	8.06E-01	0.64	3.30E-04	0.54	3.71E-03	0.14	4.89E-01	-0.59	1.11E-03	0.05	8.10E-01	-0.48	1.07E-02	-0.36	6.38E-02	-0.46	1.59E-02	0.41	3.32E-02
ENSCAFG0000000519	ACER3	grey	VSMC_M10	-0.05	8.06E-01	0.09	6.47E-01	0.26	1.84E-01	-0.17	4.03E-01	-0.38	4.86E-02	0.13	5.30E-01	0.16	4.22E-01	0.21	2.88E-01	-0.14	4.86E-01	0.18	3.56E-01
ENSCAFG0000000380	NRDC	darkgrey	VSMC_M8	-0.05	8.05E-01	-0.63	4.73E-04	-0.04	8.57E-01	-0.55	2.77E-03	0.14	4.88E-01	0.27	1.73E-01	0.22	2.78E-01	0.72	2.15E-05	0.53	4.61E-03	-0.74	1.14E-05
ENSCAFG0000001519	PLAU	yellow	VSMC_M3	-0.05	8.05E-01	-0.01	9.80E-01	0.50	7.97E-03	-0.52	4.99E-03	-0.53	4.45E-03	0.31	1.15E-01	-0.10	6.35E-01	0.43	2.59E-02	-0.07	7.39E-01	-0.13	5.29E-01
ENSCAFG0000002546	TRIM65	grey	VSMC_M10	-0.05	8.04E-01	0.31	1.10E-01	0.14	4.96E-01	0.15	4.58E-01	-0.41	3.30E-02	0.27	1.72E-01	0.31	1.17E-01	-0.19	3.52E-01	-0.35	7.51E-02	0.67	1.25E-04
ENSCAFG0000001113	ENSCAFG0000001113	yellow	VSMC_M3	-0.05	8.04E-01	-0.07	7.29E-01	0.47	1.30E-02	-0.27	1.80E-01	-0.55	2.86E-03	0.27	1.68E-01	0.16	4.38E-01	0.29	1.42E-01	0.01	9.80E-01	-0.01	9.47E-01
ENSCAFG0000002485	KPNA4	darkolivegreen	VSMC_M9	-0.05	8.04E-01	-0.77	2.90E-06	-0.24	2.29E-01	-0.26	1.87E-01	0.33	9.55E-02	-0.02	9.09E-01	0.36	6.49E-02	0.46	1.65E-02	0.64	2.85E-04	-0.60	9.00E-04
ENSCAFG0000001454	SPSB2	grey	VSMC_M10	-0.05	8.04E-01	0.28	1.54E-01	0.09	6.63E-01	0.21	2.93E-01	-0.28	1.53E-01	0.16	4.19E-01	-0.12	5.45E-01	-0.18	3.65E-01	-0.29	1.40E-01	0.49	9.57E-03
ENSCAFG0000001041	ENSCAFG0000001041	darkgrey	VSMC_M8	-0.05	8.04E-01	-0.35	7.55E-02	0.42	2.98E-02	-0.85	1.36E-08	-0.32	1.09E-01	0.14	4.93E-01	0.34	8.72E-02	0.88	1.85E-09	0.21	2.90E-01	-0.57	1.87E-03
ENSCAFG0000001979	ALX3	grey	VSMC_M10	-0.05	8.04E-01	-0.49	9.67E-03	-0.18	3.65E-01	-0.18	3.74E-01	0.12	5.53E-01	0.45	1.94E-02	0.42	3.09E-02	0.34	7.83E-02	0.32	1.02E-01	-0.20	3.08E-01
ENSCAFG0000000732	KRT80	grey	VSMC_M10	-0.05	8.03E-01	0.24	2.22E-01	-0.11	5.99E-01	0.19	3.33E-01	-0.04	8.45E-01	0.11	6.02E-01	0.36	6.73E-02	-0.26	1.85E-01	-0.25	2.07E-01	0.45	1.75E-02
ENSCAFG0000000333	GPR85	grey	VSMC_M10	-0.05	8.03E-01	0.19	3.49E-01	0.12	5.49E-01	-0.04	8.26E-01	-0.19	3.49E-01	-0.03	8.91E-01	-0.10	6.34E-01	-0.14	4.79E-01	-0.15	4.63E-01	0.17	4.05E-01
ENSCAFG0000000884	FBXO41	grey	VSMC_M10	-0.05	8.03E-01	0.19	3.49E-01	0.12	5.49E-01	-0.04	8.26E-01	-0.19	3.49E-01	-0.03	8.91E-01	-0.10	6.34E-01	-0.14	4.79E-01	-0.15	4.63E-01	0.17	4.05E-01
ENSCAFG0000001242	CCDC87	grey	VSMC_M10	-0.05	8.03E-01	0.19	3.49E-01	0.12	5.49E-01	-0.04	8.26E-01	-0.19	3.49E-01	-0.03	8.91E-01	-0.10	6.34E-01	-0.14	4.79E-01	-0.15	4.63E-01	0.17	4.05E-01
ENSCAFG0000001121	PIK3CA	pink	VSMC_M5	-0.05	8.03E-01	-0.36	6.31E-02	-0.70	5.11E-05	0.26	1.84E-01	0.75	5.69E-06	-0.09	6.42E-01	-0.10	6.13E-01	-0.15	4.56E-01	0.43	2.51E-02	-0.20	3.06E-01
ENSCAFG0000000672	MAGI1	pink	VSMC_M5	-0.05	8.02E-01	-0.43	2.61E-02	-0.57	1.99E-03	0.11	5.99E-01	0.54	4.02E-03	0.04	8.32E-01	-0.14	4.72E-01	0.08	6.92E-01	0.42	3.13E-02	-0.26	1.83E-01
ENSCAFG0000000840	CLU	darkgreen	VSMC_M4	-0.05	8.02E-01	0.38	4.95E-02	-0.25	2.00E-01	0.61	6.85E-04	0.09	6.71E-01	0.18	3.71E-01	-0.44	2.21E-02	-0.57	1.73E-03	-0.33	9.36E-02	0.51	6.51E-03
ENSCAFG0000001492	RETREG3	grey	VSMC_M10	-0.05	8.01E-01	0.17	3.90E-01	0.05	7.88E-01	0.18	3.75E-01	-0.01	9.51E-01	-0.14	4.87E-01	-0.31	1.15E-01	-0.31	1.17E-01	-0.01	9.53E-01	0.03	8.67E-01
ENSCAFG0000000365	PREP	darkgreen	VSMC_M4	-0.05	8.00E-01	0.18	3.69E-01	-0.64	2.81E-04	0.96	7.61E-16	0.44	2.30E-02	-0.04	8.36E-01	-0.32	1.06E-01	-0.82	2.10E-07	-0.08	6.76E-01	0.58	1.51E-03
ENSCAFG0000001372	ENSCAFG0000001372	grey	VSMC_M10	-0.05	8.00E-01	0.03	8.66E-01	-0.03	9.01E-01	-0.12	5.55E-01	-0.10	6.14E-01	0.13	5.21E-01	0.21	2.93E-01	0.13	5.06E-01	-0.14	4.98E-01	0.20	3.26E-01
ENSCAFG0000001091	HEATR1	grey	VSMC_M10	-0.05	8.00E-01	-0.68	1.01E-04	-0.43	2.45E-02	-0.16	4.37E-01	0.49	9.93E-03	-0.07	7.12E-01	0.30	1.24E-01	0.41	3.22E-02	0.50	7.41E-03	-0.54	3.89E-03
ENSCAFG0000000365	CSMD2	yellow	VSMC_M3	-0.05	8.00E-01	0.02	9.28E-01	0.44	2.05E-02	-0.46	1.46E-02	-0.42	3.05E-02	0.11	5.89E-01	0.03	8.91E-01	0.30	1.30E-01	0.05	8.09E-01	-0.12	5.55E-01
ENSCAFG0000002959	ZNF527	grey	VSMC_M10	-0.05	8.00E-01	-0.10	6.31E-01	0.03	8.98E-01	0.07	7.36E-01	-0.08	7.01E-01	0.14	4.84E-01	0.24	2.22E-01	-0.11	5.87E-01	0.12	5.40E-01	0.08	6.91E-01
ENSCAFG0000002535	CREBZF	grey	VSMC_M10	-0.05	8.00E-01	0.45	1.78E-02	0.31	1.17E-01	0.23	2.51E-01	-0.48	1.13E-02	0.18	3.72E-01	0.02	9.34E-01	-0.31	1.21E-01	-0.46	1.46E-02	0.58	1.64E-03
ENSCAFG0000001330	DNAJC12	grey	VSMC_M10	-0.05	8.00E-01	-0.06	7.54E-01	-0.05	8.23E-01	-0.11	5.95E-01	-0.01	9.66E-01	-0.03	8.73E-01	0.12	5.57E-01	0.03	8.81E-01	0.02	9.20E-01	0.02	9.31E-01
ENSCAFG0000001867	SMARCA1	darkgreen	VSMC_M4	-0.05	7.99E-01	0.06	7.49E-01	-0.60	9.19E-04	0.89	3.58E-10	0.45	1.88E-02	-0.20	3.06E-01	-0.33	8.96E-02	-0.72	2.02E-05	0.02	9.22E-01	0.42	2.93E-02
ENSCAFG0000000340	CAV2	darkolivegreen	VSMC_M9	-0.05	7.99E-01	-0.67	1.23E-04	-0.28	1.58E-01	-0.37	5.64E-02	0.40	3.74E-02	0.15	4.61E-01	0.51	6.09E-03	0.56	2.34E-03	0.57	1.79E-03	-0.63	4.89E-04
ENSCAFG0000002574	ENSCAFG0000002574	grey	VSMC_M10	-0.05	7.99E-01	-0.04	8.34E-01	0.11	5.86E-01	-0.33	9.40E-02	-0.23	2.57E-01	0.15	4.65E-01	-0.02	9.04E-01	0.31	1.14E-01	-0.03	8.94E-01	0.09	6.66E-01
ENSCAFG0000002906	ENSCAFG0000002906	grey	VSMC_M10	-0.05	7.99E-01	-0.05	8.05E-01	0.12	5.42E-01	-0.31	1.12E-01	-0.23	2.43E-01	0.23	2.45E-01	-0.06	7.80E-01	0.38	5.22E-02	-0.02	9.06E-01	0.02	9.21E-01
ENSCAFG0000003019	TCF23	grey	VSMC_M10	-0.05	7.99E-01	0.29	1.36E-01	-0.02	9.15E-01	0.24	2.33E-01	-0.07	7.42E-01	0.01	9.54E-01	-0.02	9.27E-01	-0.26	1.95E-01	-0.24	2.36E-01	0.34	8.13E-02
ENSCAFG0000000729	ENSCAFG0000000729	grey	VSMC_M10	-0.05	7.98E-01	0.19	3.36E-01	-0.04	8.44E-01	0.21	2.93E-01	-0.06	7.55E-01	0.00	9.81E-01	-0.01	9.56E-01	-0.22	2.74E-01	-0.18	3.74E-01	0.28	1.57E-01
ENSCAFG0000001784	DHRS11	grey	VSMC_M10	-0.05	7.98E-01	-0.29	1.48E-01	-0.14	4.72E-01	-0.04	8.27E-01	0.18	3.58E-01	-0.07	7.32E-01	0.23	2.38E-01	0.12	5.39E-01	0.28	1.62E-01	-0.23	2.46E-01
ENSCAFG0000001300	ADH5	grey	VSMC_M10	-0.05	7.98E-01	0.55	3.06E-03	0.29	1.36E-01	-0.01	9.49E-01	-0.49	8.94E-03	0.15	4.58E-01	0.10	6.26E-01	-0.13	5.33E-01	-0.53	4.91E-03	0.62	6.16E-04
ENSCAFG0000000623	NDUFA12	grey	VSMC_M10	-0.05	7.98E-01	0.07	7.25E-01	0.25	2.06E-01	0.09	6.44E-01	-0.39	4.43E-02	-0.02	9.05E-01	0.26	1.99E-01	-0.01	9.53E-01	-0.17	4.09E-01	0.27	1.80E-01
ENSCAFG0000001903	HDGFL2	yellow	VSMC_M3	-0.05	7.98E-01	0.08	6.74E-01	0.72	2.20E-05	-0.52	5.57E-03	-0.61	6.52E-04	-0.07	7.21E-01	0.06	7.50E-01	0.41	3.28E-02	-0.08	6.89E-01	-0.23	2.46E-01
ENSCAFG0000003209	ENSCAFG0000003209	grey	VSMC_M10	-0.05	7.97E-01	-0.23	2.54E-01	-0.38	5.29E-02	0.34	7.86E-02	0.22	2.77E-01	0.41	3.31E-02	0.18	3.74E-01	-0.11	6.02E-01	0.12	5.57E-01	0.12	5.61E-01
ENSCAFG0000001720	BEST2	turquoise	VSMC_M6	-0.05	7.97E-01	-0.18	3.65E-01	-0.05	8.07E-01	-0.12	5.52E-01	-0.03	8.78E-01	-0.07	7.31E-01	0.07	7.46E-01	0.13	5.24E-01	0.12	5.59E-01	-0.04	8.30E-01
ENSCAFG0000002423	MLLT11	grey	VSMC_M10	-0.05	7.97E-01	-0.40	3.71E-02	0.34	8.20E-02	-0.56	2.32E-03	-0.24	2.38E-01	0.26	1.82E-01	0.02	9.20E-01	0.56	2.56E-03	0.41	3.35E-02	-0.55	3.14E-03
ENSCAFG0000001808	DSC3	grey	VSMC_M10	-0.05	7.97E-01	0.29	1.47E-01	-0.11	5.99E-01	0.30	1.29E-01	-0.12	5.36E-01	0.48	1.10E-02	-0.01	9.48E-01	-0.27	1.69E-01	-0.31	1.10E-01	0.50	8.03E-03
ENSCAFG0000001279	CELF3	grey	VSMC_M10	-0.05	7.96E-01	0.19	3.52E-01	-0.09	6.53E-01	0.23	2.51E-01	0.04	8.34E-01	-0.02	9.17E-01	-0.06	7.50E-01	-0.29	1.47E-01	-0.06	7.74E-01	0.29	1.46E-01
ENSCAFG0000001856	C4H5orf34	darkgrey	VSMC_M8	-0.05	7.96E-01	-0.43	2.58E-02	0.32	1.02E-01	-0.63	4.01E-04	-0.28	1.57E-01	0.03	8.73E-01	0.42	2.80E-02	0.74	8.87E-06	0.29	1.47E-01	-0.42	2.74E-02
ENSCAFG0000000892	OAS3	grey	VSMC_M10	-0.05	7.95E-01	0.22	2.71E-01	0.27	1.74E-01	-0.27	1.72E-01	-0.32	1.04E-01	0.23	2.42E-01	-0.29	1.45E-01	0.07	7.15E-01	-0.12	5.63E-01	0.08	6.78E-01
ENSCAFG0000001853	MAP2K3	cyan	VSMC_M2	-0.05	7.95E-01	0.35	7.56E-02	0.45	1.92E-02	-0.11	5.79E-01	-0.61	6.64E-04	0.37	5.53E-02	-0.19	3.45E-01	0.00	9.82E-01	-0.29	1.48E-01	0.35	7.07E-02
ENSCAFG0000000731	RRS1	yellow	VSMC_M3	-0.05	7.95E-01	0.02	9.40E-01	0.45	1.76E-02	-0.30	1.22E-01	-0.57	1.90E-03	0.15	4.70E-01	0.34	8.56E-02	0.35	7.32E-02	-0.15	4.46E-01	0.13	5.27E-01
ENSCAFG0000000827	LYSMD3	darkgreen	VSMC_M4	-0.05	7.95E-01	-0.08	6.97E-01	-0.53	4.24E-03	0.63	4.02E-04	0.29	1.40E-01	0.21	2.84E-01	-0.14	4.97E-01	-0.42	2.86E-02	0.04	8.52E-01	0.41	3.26E-02
ENSCAFG0000000598	ENSCAFG0000000598	grey	VSMC_M10	-0.05	7.95E-01	-0.18	3.75E-01	-0.48	1.08E-02	0.29	1.37E-01	0.42	3.12E-02	-0.35	7.73E-02	-0.25	2.00E-01	-0.18	3.81E-01	0.21	3.03E-01	0.09	6.60E-01
ENSCAFG0000000001	ATP9B	grey	VSMC_M10	-0.05	7.94E-01	0.29	1.36E-01	-0.10	6.03E-01	0.33	9.31E-02	-0.02	9.19E-01	0.06	7.59E-01	-0.54	3.44E-03	-0.37	5.96E-02	-0.19	3.52E-01	0.29	1.45E-01
ENSCAFG0000000902	DDX54	grey	VSMC_M10	-0.05	7.94E-01	0.53	4.76E-03	0.70	4.36E-05	-0.24	2.25E-01	-0.81	2.62E-07	0.20	3.21E-01	-0.08	6.94E-01	0.00	9.82E-01	-0.46	1.71E-02	0.39	4.29E-02
ENSCAFG0000000293	ENSCAFG0000000293	grey	VSMC_M10	-0.05	7.94E-01	0.12	5.37E-01	0.25	2.08E-01	-0.16	4.16E-01	-0.29	1.45E-01	0.01	9.73E-01	0.18	3.78E-01	-0.03	8.81E-01	-0.05	7.97E-01	0.08	7.00E-01
ENSCAFG0000001659	FBXO2	grey	VSMC_M10	-0.05	7.94E-01	0.20	3.19E-01	-0.03	9.00E-01	0.22	2.81E-01	0.09	6.42E-01	-0.26	1.96E-01	-0.38	5.25E-02	-0.37	5.71E-02	0.00	9.89E-01	0.05	8.15E-01
ENSCAFG0000001939	RNPS1	darkgrey	VSMC_M8	-0.05	7.94E-01	-0.49	8.81E-03	0.41	3.24E-02	-0.87	2.62E-09	-0.33	8.97E-02	0.14	4.82E-01	0.37	5.64E-02	0.95	1.24E-14	0.35	7.60E-02	-0.64	3.02E-04
ENSCAFG0000000136	APOBEC3Z3	grey	VSMC_M10	-0.05	7.93E-01	0.22	2.74E-01	-0.07	7.38E-01	0.12	5.53E-01	-0.03	8.78E-01	-0.09	6.65E-01	-0.05	8.02E-01	-0.24	2.23E-01	-0.18	3.66E-01	0.26	1.90E-01
ENSCAFG0000000525	TMC6	turquoise	VSMC_M6	-0.05	7.93E-01	0.07	7.40E-01	0.01	9.60E-01	0.19	3.31E-01	-0.22	2.76E-01	0.71	3.96E-05	0.18	3.65E-01	-0.16	4.34E-01	-0.10	6.10E-01	0.33	9.37E-02
ENSCAFG0000001562	KCNH4	grey	VSMC_M10	-0.05	7.93E-01	0.27	1.78E-01	-0.12	5.58E-01	0.34	8.00E-02	-0.02	9.35E-01	-0.07	7.14E-01	-0.13	5.22E-01	-0.47	1.45E-02	-0.21	2.96E-01	0.38	4.83E-02
ENSCAFG0000003076	ZNF835	grey	VSMC_M10	-0.05	7.93E-01	-0.24	2.22E-01	-0.16	4.25E-01	-0.08	6.87E-01	0.07	7.23E-01	0.17	3.84E-01	0.38	4.83E-02	0.12	5.45E-01	0.12	5.51E-01	0.01	9.57E-01
ENSCAFG0000002919	STMN1	darkgrey	VSMC_M8	-0.05	7.93E-01	-0.53	4.08E-03	0.36	6.76E-02	-0.90	1.22E-10	-0.24	2.20E-01	0.17	4.09E-01	0.43	2.41E-02	0.97	7.86E-18	0.38	5.09E-02	-0.70	5.59E-05
ENSCAFG0000001810	CD74	grey	VSMC_M10	-0.05	7.93E-01	0.27	1.71E-01	0.06	7.51E-01	0.06	7.75E-01	-0.17	3.83E-01	-0.09	6.48E-01	-0.02	9.36E-01	-0.07	7.44E-01	-0.33	9.54E-02	0.32	1.03E-01
ENSCAFG0000002157	ENSCAFG0000002157	turquoise	VSMC_M6	-0.05	7.93E-01	0.04	8.59E-01	0.15	4.49E-01	-0.24	2.28E-01	-0.26	1.92E-01	0.49	1.01E-02	0.37	6.04E-02	0.15	4.51E-01	-0.10	6.04E-01	0.12	5.38E-01
ENSCAFG0000003214	BLCAP	grey	VSMC_M10	-0.05	7.93E-01	0.53	4.20E-03	0.08	6.82E-01	0.45	1.76E-02	-0.34	7.91E-02	0.30	1.26E-01	-0.33	8.88E-02	-0.55	2.92E-03	-0.42	2.91E-02	0.70	5.37E-05
ENSCAFG0000001289	ENSCAFG0000001289	yellow	VSMC_M3	-0.05	7.93E-01	-0.15	4.45E-01	0.50	7.51E-03	-0.37	5.75E-02	-0.58	1.55E-03	0.15	4.62E-01	0.23	2.43E-01	0.47	1.34E-02	0.03	8.79E-01	-0.09	6.46E-01
ENSCAFG0000001524	AP5M1	grey	VSMC_M10	-0.05	7.93E-01	-0.34	8.66E-02	-0.43	2.45E-02	-0.08	7.02E-01	0.38	5.33E-02	0.08	6.94E-01	0.30	1.26E-01	0.24	2.29E-01	0.20	3.24E-01	-0.14	4.76E-01
ENSCAFG0000000561	SLC4A7	grey	VSMC_M10	-0.05	7.93E-01	-0.35	7.19E-02	-0.31	1.13E-01	-0.13	5.03E-01	0.44	2.29E-02	0.04	8.48E-01	0.05	8.17E-01	0.08	7.07E-01	0.45	1.97E-02	-0.37	5.90E-02
ENSCAFG0000000731	FGFBP3	grey	VSMC_M10	-0.05	7.93E-01	0.17	4.03E-01	0.05	8.08E-01	0.07	7.37E-01	-0.11	5.83E-01	-0.03	8.87E-01	-0.14	4.97E-01	-0.05	8.12E-01	-0.16	4.38E-01	0.20	3.07E-01
ENSCAFG0000000690	RIC3	darkgreen	VSMC_M4	-0.05	7.93E-01	0.09	6.54E-01	-0.50	8.10E-03	0.71	3.73E-05	0.29	1.42E-01	0.09	6.62E-01	-0.05	8.21E-01	-0.52	5.59E-03	-0.08	7.08E-01	0.49	9.66E-03
ENSCAFG0000003118	ENSCAFG0000003118	turquoise	VSMC_M6	-0.05	7.92E-01	-0.05	7.86E-01	0.06	7.71E-01	-0.01	9.66E-01	-0.20	3.09E-01	0.23	2.57E-01	0.30	1.30E-01	0.11	5.91E-01	-0.01	9.55E-01	0.10	6.10E-01
ENSCAFG0000003088	ENSCAFG0000003088	grey	VSMC_M10	-0.05	7.92E-01	0.06	7.70E-01	0.03	8.85E-01	-0.01	9.52E-01	-0.08	6.98E-01	-0.08	7.00E-01	-0.03	9.01E-01	0.01	9.53E-01	-0.09	6.70E-01	0.14	4.71E-01
ENSCAFG0000000622	ZNF703	darkgrey	VSMC_M8	-0.05	7.92E-01	-0.50	8.48E-03	-0.06	7.52E-01	-0.45	1.88E-02	0.10	6.19E-01	0.13	5.32E-01	0.22	2.65E-01	0.56	2.44E-03	0.42	2.87E-02	-0.47	1.38E-02
ENSCAFG0000001841	NKRF	grey	VSMC_M10	-0.05	7.92E-01	-0.69	6.13E-05	-0.35	7.38E-02	-0.16	4.20E-01	0.31	1.18E-01	0.39	4.36E-02	0.19	3.37E-01	0.46	1.63E-02	0.53	4.53E-03	-0.45	1.90E-02
ENSCAFG0000000723	ENSCAFG0000000723	grey	VSMC_M10	-0.05	7.92E-01	-0.15	4.54E-01	-0.39	4.30E-02	0.35	7.77E-02	0.20	3.23E-01	0.42	2.78E-02	0.03	8.81E-01	-0.14	4.84E-01	0.05	7.96E-01	0.20	3.13E-01
ENSCAFG0000000152	CYC1	darkgrey	VSMC_M8	-0.05	7.91E-01	-0.15	4.54E-01	0.40	3.69E-02	-0.65	2.11E-04	-0.45	1.85E-02	0.35	7.40E-02	0.19	3.39E-01	0.69	6.93E-05	0.01	9.50E-01	-0.28	1.57E-01
ENSCAFG0000001578	CEP170	grey	VSMC_M10	-0.05	7.91E-01	-0.37	5.71E-02	-0.70	4.26E-05	0.38	5.00E-02	0.60	8.51E-04	-0.15	4.42E-01	0.17	4.10E-01	-0.13	5.23E-01	0.24	2.32E-01	0.05	7.97E-01
ENSCAFG0000002589	ENSCAFG0000002589	grey	VSMC_M10	-0.05	7.91E-01	0.30	1.34E-01	0.16	4.26E-01	-0.06	7.57E-01	-0.27	1.79E-01	0.03	8.80E-01	-0.07	7.17E-01	-0.12	5.40E-01	-0.25	2.03E-01	0.25	2.08E-01
ENSCAFG0000001994	ENSCAFG0000001994	grey	VSMC_M10	-0.05	7.91E-01	0.30	1.26E-01	-0.05	8.14E-01	0.39	4.63E-02	-0.17	4.06E-01	0.21	2.94E-01	-0.03	8.97E-01	-0.32	1.09E-01	-0.34	8.36E-02	0.46	1.53E-02
ENSCAFG0000000387	PLK4	darkgrey	VSMC_M8	-0.05	7.91E-01	-0.60	1.03E-03	0.30	1.31E-01	-0.89	6.09E-10	-0.15	4.43E-01	0.09	6.44E-01	0.39	4.45E-02	0.98	1.33E-18	0.45	1.88E-02	-0.79	1.20E-06
ENSCAFG0000001068	SP110	grey	VSMC_M10	-0.05	7.91E-01	0.40	3.97E-02	-0.07	7.47E-01	0.21	2.82E-01	-0.07	7.44E-01	-0.03	8.79E-01	0.13	5.32E-01	-0.34	8.23E-02	-0.38	4.84E-02	0.50	8.11E-03
ENSCAFG0000000067	RIMS2	grey	VSMC_M10	-0.05	7.90E-01	-0.14	4.81E-01	-0.09	6.64E-01	0.00	9.95E-01	-0.11	5.97E-01	0.21	3.03E-01	0.18	3.76E-01	0.12	5.57E-01	0.06	7.48E-01	0.12	5.56E-01
ENSCAFG0000000092	DCBLD1	grey	VSMC_M10	-0.05	7.90E-01	0.27	1.79E-01	-0.28	1.56E-01	0.48	1.22E-02	0.04	8.44E-01	0.09	6.38E-01	-0.19	3.39E-01	-0.44	2.25E-02	-0.22	2.78E-01	0.59	1.26E-03
ENSCAFG0000000694	GPBP1	pink	VSMC_M5	-0.05	7.90E-01	-0.33	9.28E-02	-0.60	9.54E-04	0.14	4.85E-01	0.70	4.28E-05	-0.33	8.88E-02	-0.25	2.13E-01	-0.04	8.54E-01	0.38	5.29E-02	-0.35	7.09E-02
ENSCAFG0000000577	UBE2E1	pink	VSMC_M5	-0.05	7.90E-01	-0.61	7.08E-04	-0.69	7.55E-05	0.12	5.68E-01	0.74	1.12E-05	-0.23	2.53E-01	0.20	3.18E-01	0.11	5.97E-01	0.61	7.72E-04	-0.39	4.35E-02
ENSCAFG0000001643	MXD3	grey	VSMC_M10	-0.05	7.90E-01	-0.21	3.02E-01	0.01	9.77E-01	-0.27	1.72E-01	-0.14	4.96E-01	0.02	9.17E-01	0.46	1.50E-02	0.37	6.05E-02	0.08	6.87E-01	0.07	7.26E-01
ENSCAFG0000000409	EHD2	yellow	VSMC_M3	-0.05	7.90E-01	0.48	1.23E-02	0.81	2.40E-07	-0.37	5.91E-02	-0.90	1.24E-10	0.13	5.08E-01	0.06	7.57E-01	0.12	5.49E-01	-0.44	2.03E-02	0.34	8.26E-02
ENSCAFG0000003157	PRAP1	grey	VSMC_M10	-0.05	7.90E-01	0.35	7.51E-02	-0.02	9.05E-01	0.25	2.16E-01	-0.12	5.45E-01	0.16	4.14E-01	0.37	6.05E-02	-0.36	6.26E-02	-0.31	1.15E-01	0.49	9.72E-03
ENSCAFG0000001714	ESRRB	grey	VSMC_M10	-0.05	7.90E-01	0.42	2.87E-02	-0.01	9.51E-01	0.32	1.08E-01	-0.12	5.68E-01	-0.04	8.44E-01	-0.17	4.08E-01	-0.38	4.95E-02	-0.34	8.67E-02	0.42	2.72E-02
ENSCAFG0000002975	ENSCAFG0000002975	grey	VSMC_M10	-0.05	7.90E-01	-0.07	7.36E-01	-0.23	2.39E-01	0.25	2.12E-01	0.24	2.19E-01	-0.15	4.49E-01	-0.09	6.72E-01	-0.24	2.26E-01	0.11	5.82E-01	-0.02	9.37E-01
ENSCAFG0000001515	TNIK	darkolivegreen	VSMC_M9	-0.05	7.90E-01	-0.55	3.09E-03	-0.13	5.08E-01	-0.25	2.07E-01	0.11	5.99E-01	0.40	3.98E-02	0.08	6.74E-01	0.35	7.23E-02	0.48	1.05E-02	-0.35	7.45E-02
ENSCAFG0000001411	CCDC43	grey	VSMC_M10	-0.05	7.89E-01	-0.07	7.13E-01	0.15	4.70E-01	0.01	9.47E-01	-0.22	2.67E-01	0.16	4.22E-01	0.16	4.20E-01	0.05	7.90E-01	-0.03	9.00E-01	0.02	9.15E-01
ENSCAFG0000000648	ENSCAFG0000000648	grey	VSMC_M10	-0.05	7.89E-01	-0.08	6.80E-01	0.02	9.15E-01	-0.20	3.05E-01	-0.01	9.53E-01	-0.08	6.78E-01	0.27	1.68E-01	0.25	2.08E-01	0.00	9.82E-01	-0.02	9.31E-01
ENSCAFG0000001727	ENSCAFG0000001727	turquoise	VSMC_M6	-0.05	7.89E-01	0.03	9.00E-01	0.04	8.52E-01	-0.07	7.17E-01	-0.12	5.50E-01	0.59	1.17E-03	0.24	2.26E-01	0.08	7.02E-01	-0.04	8.61E-01	0.11	6.02E-01
ENSCAFG0000000458	CLASRP	grey	VSMC_M10	-0.05	7.89E-01	0.17	4.09E-01	-0.09	6.54E-01	0.19	3.35E-01	-0.19	3.53E-01	0.18	3.70E-01	-0.13	5.25E-01	-0.12	5.35E-01	-0.18	3.79E-01	0.49	9.57E-03
ENSCAFG0000003191	TMEM26	violet	VSMC_M7	-0.05	7.89E-01	-0.56	2.55E-03	-0.05	7.89E-01	-0.43	2.34E-02	0.14	4.73E-01	-0.03	8.71E-01	0.81	2.42E-07	0.55	2.76E-03	0.43	2.39E-02	-0.51	6.39E-03
ENSCAFG0000000039	RPP21	grey	VSMC_M10	-0.05	7.89E-01	-0.15	4.55E-01	0.05	7.92E-01	-0.11	5.70E-01	-0.03	8.89E-01	-0.25	2.05E-01	-0.04	8.48E-01	0.11	5.85E-01	0.24	2.37E-01	-0.17	3.97E-01

ENSCAFG0000001236	ENSCAFG0000001236	yellow	VSMC_M3	-0.05	7.89E-01	0.39	4.38E-02	0.61	7.05E-04	-0.42	3.07E-02	-0.71	3.73E-05	0.25	2.17E-01	0.30	1.23E-01	0.20	3.06E-01	-0.41	3.54E-02	0.24	2.30E-01
ENSCAFG0000001268	USF1	grey	VSMC_M10	-0.05	7.89E-01	0.26	1.82E-01	0.42	3.13E-02	-0.15	4.62E-01	-0.36	6.73E-02	0.16	4.39E-01	-0.01	9.42E-01	-0.04	8.46E-01	-0.17	3.99E-01	0.00	9.87E-01
ENSCAFG0000002872	FPGT	grey	VSMC_M10	-0.05	7.89E-01	0.08	6.99E-01	-0.26	1.96E-01	0.23	2.38E-01	0.26	1.98E-01	-0.22	2.74E-01	0.19	3.41E-01	-0.19	3.35E-01	-0.10	6.35E-01	0.09	6.58E-01
ENSCAFG0000001449	ENSCAFG0000001449	grey	VSMC_M10	-0.05	7.89E-01	-0.09	6.66E-01	-0.14	4.88E-01	0.16	4.17E-01	0.08	6.88E-01	-0.06	7.66E-01	-0.18	3.56E-01	-0.10	6.30E-01	0.15	4.58E-01	0.05	7.95E-01
ENSCAFG0000001100	CAMKV	grey	VSMC_M10	-0.05	7.88E-01	-0.05	7.93E-01	-0.01	9.50E-01	-0.15	4.61E-01	0.02	9.12E-01	0.02	9.25E-01	0.19	3.52E-01	0.15	4.58E-01	0.04	8.27E-01	-0.03	8.76E-01
ENSCAFG0000002398	ZNF24	grey	VSMC_M10	-0.05	7.88E-01	-0.02	9.34E-01	0.18	3.64E-01	0.00	9.93E-01	-0.21	2.98E-01	-0.01	9.59E-01	0.22	2.70E-01	-0.05	7.88E-01	0.01	9.53E-01	0.07	7.27E-01
ENSCAFG0000000293	HOXA1	yellow	VSMC_M3	-0.05	7.88E-01	0.16	4.21E-01	0.52	5.37E-03	-0.39	4.69E-02	-0.50	8.03E-03	-0.04	8.52E-01	0.02	9.28E-01	0.22	2.64E-01	-0.11	5.89E-01	0.01	9.52E-01
ENSCAFG0000002829	ENSCAFG0000002829	grey	VSMC_M10	-0.05	7.87E-01	0.39	4.24E-02	0.09	6.50E-01	0.12	5.39E-01	-0.26	1.82E-01	0.18	3.60E-01	-0.09	6.46E-01	-0.27	1.65E-01	-0.30	1.35E-01	0.46	1.63E-02
ENSCAFG0000003059	ENSCAFG0000003059	grey	VSMC_M10	-0.05	7.87E-01	-0.13	5.09E-01	0.11	5.93E-01	-0.16	4.35E-01	-0.17	4.09E-01	-0.03	8.75E-01	0.36	6.59E-02	0.20	3.19E-01	0.09	6.52E-01	-0.05	7.94E-01
ENSCAFG0000001383	MASP1	violet	VSMC_M7	-0.05	7.87E-01	-0.47	1.23E-02	-0.12	5.57E-01	-0.28	1.51E-01	0.10	6.29E-01	0.25	2.06E-01	0.81	2.38E-07	0.41	3.56E-02	0.30	1.34E-01	-0.27	1.76E-01
ENSCAFG0000000469	DCLRE1C	pink	VSMC_M5	-0.05	7.87E-01	-0.22	2.59E-01	-0.44	2.11E-02	0.31	1.12E-01	0.47	1.26E-02	0.19	3.33E-01	-0.03	8.85E-01	-0.16	4.26E-01	0.25	2.03E-01	-0.18	3.56E-01
ENSCAFG0000001291	CDC25A	darkgrey	VSMC_M8	-0.05	7.87E-01	-0.37	6.00E-02	0.46	1.70E-02	-0.74	9.07E-06	-0.45	1.86E-02	0.32	1.02E-01	0.28	1.54E-01	0.80	5.23E-07	0.24	2.22E-01	-0.46	1.63E-02
ENSCAFG0000001210	RCOR3	grey	VSMC_M10	-0.05	7.86E-01	0.14	4.78E-01	-0.28	1.63E-01	0.17	4.08E-01	0.20	3.10E-01	-0.12	5.36E-01	0.24	2.25E-01	-0.19	3.45E-01	-0.12	5.40E-01	0.26	1.87E-01
ENSCAFG0000000500	TAMM41	grey	VSMC_M10	-0.05	7.86E-01	0.52	5.09E-03	0.26	1.96E-01	-0.04	8.34E-01	-0.32	9.83E-02	-0.14	4.94E-01	0.08	7.03E-01	-0.19	3.37E-01	-0.46	1.64E-02	0.43	2.63E-02
ENSCAFG0000001546	TSPAN9	grey	VSMC_M10	-0.05	7.86E-01	0.21	2.95E-01	0.04	8.48E-01	0.29	1.45E-01	-0.28	1.60E-01	0.60	8.47E-04	0.17	4.05E-01	-0.31	1.18E-01	-0.21	3.04E-01	0.55	3.21E-03
ENSCAFG0000002003	VAT1L	grey	VSMC_M10	-0.05	7.86E-01	0.11	5.93E-01	0.13	5.22E-01	-0.08	6.94E-01	-0.22	2.76E-01	0.00	9.87E-01	-0.13	5.28E-01	0.06	7.65E-01	-0.12	5.36E-01	0.09	6.54E-01
ENSCAFG0000003126	ENSCAFG0000003126	grey	VSMC_M10	-0.05	7.85E-01	0.31	1.20E-01	-0.15	4.63E-01	0.43	2.63E-02	0.06	7.68E-01	0.00	9.94E-01	-0.08	6.80E-01	-0.48	1.13E-02	-0.19	3.52E-01	0.44	2.21E-02
ENSCAFG0000000119	PRXL2C	grey	VSMC_M10	-0.05	7.85E-01	0.39	4.18E-02	0.03	8.66E-01	0.45	1.71E-02	-0.07	7.40E-01	-0.15	4.52E-01	-0.04	8.29E-01	-0.59	1.22E-03	-0.25	2.04E-01	0.39	4.23E-02
ENSCAFG0000003038	ENSCAFG0000003038	grey	VSMC_M10	-0.05	7.85E-01	0.23	2.42E-01	-0.19	3.53E-01	0.30	1.27E-01	0.06	7.62E-01	-0.20	3.22E-01	-0.18	3.64E-01	-0.35	7.13E-02	-0.19	3.43E-01	0.41	3.48E-02
ENSCAFG0000001089	EI24	grey	VSMC_M10	-0.06	7.85E-01	-0.23	2.51E-01	0.21	2.90E-01	-0.38	4.94E-02	-0.25	2.05E-01	0.50	8.24E-03	0.31	1.10E-01	0.43	2.40E-02	0.12	5.43E-01	-0.22	2.67E-01
ENSCAFG0000002507	ENSCAFG0000002507	grey	VSMC_M10	-0.06	7.85E-01	-0.44	2.12E-02	-0.15	4.46E-01	-0.40	3.76E-02	0.24	2.37E-01	-0.06	7.74E-01	0.35	7.74E-02	0.48	1.04E-02	0.30	1.26E-01	-0.42	3.03E-02
ENSCAFG0000001528	GGT6	grey	VSMC_M10	-0.06	7.85E-01	-0.05	7.99E-01	-0.09	6.64E-01	-0.01	9.70E-01	0.04	8.41E-01	-0.04	8.40E-01	0.36	6.16E-02	0.01	9.48E-01	0.02	9.02E-01	0.07	7.45E-01
ENSCAFG0000001685	HOXB7	darkgrey	VSMC_M8	-0.06	7.85E-01	-0.34	7.81E-02	0.29	1.38E-01	-0.68	9.95E-05	-0.16	4.12E-01	0.13	5.09E-01	-0.15	4.60E-01	0.63	3.75E-04	0.35	7.36E-02	-0.60	8.37E-04
ENSCAFG0000000573	DOCK9	darkgrey	VSMC_M8	-0.06	7.85E-01	-0.30	1.30E-01	0.42	2.94E-02	-0.73	1.44E-05	-0.23	2.52E-01	0.10	6.12E-01	0.39	4.30E-02	0.64	3.55E-04	0.30	1.35E-01	-0.60	1.02E-03
ENSCAFG0000003002	FGL2	grey	VSMC_M10	-0.06	7.84E-01	-0.36	6.18E-02	0.06	7.69E-01	-0.31	1.19E-01	0.03	8.86E-01	-0.17	4.05E-01	0.39	4.55E-02	0.36	6.33E-02	0.38	5.26E-02	-0.38	5.16E-02
ENSCAFG0000000949	KLHL8	pink	VSMC_M5	-0.06	7.84E-01	0.02	9.25E-01	-0.66	2.04E-04	0.62	5.98E-04	0.56	2.15E-03	-0.13	5.03E-01	-0.01	9.61E-01	-0.54	3.45E-03	0.01	9.65E-01	0.32	1.04E-01
ENSCAFG0000002273	MT-ND4	pink	VSMC_M5	-0.06	7.84E-01	-0.18	3.58E-01	-0.67	1.31E-04	0.29	1.47E-01	0.66	1.72E-04	-0.19	3.51E-01	0.01	9.80E-01	-0.21	2.91E-01	0.21	2.82E-01	-0.03	8.81E-01
ENSCAFG0000001932	SDF4	grey	VSMC_M10	-0.06	7.84E-01	0.63	4.11E-04	0.18	3.60E-01	0.53	4.17E-03	-0.33	9.08E-02	-0.03	8.87E-01	-0.46	1.71E-02	-0.63	4.07E-04	-0.50	8.24E-03	0.59	1.24E-03
ENSCAFG0000000308	ENSCAFG0000000308	grey	VSMC_M10	-0.06	7.84E-01	-0.05	8.00E-01	0.05	7.89E-01	0.08	6.77E-01	0.00	9.90E-01	-0.05	7.91E-01	-0.33	9.04E-02	-0.03	8.82E-01	0.13	5.06E-01	-0.20	3.19E-01
ENSCAFG0000000867	LMBR1L	grey	VSMC_M10	-0.06	7.84E-01	0.11	5.92E-01	0.34	8.58E-02	-0.33	9.40E-02	-0.42	3.07E-02	0.41	3.17E-02	0.03	8.71E-01	0.20	3.17E-01	-0.11	5.99E-01	0.09	6.37E-01
ENSCAFG0000001134	ENSCAFG0000001134	grey	VSMC_M10	-0.06	7.84E-01	-0.52	5.22E-03	-0.21	2.97E-01	-0.23	2.59E-01	0.29	1.37E-01	0.05	7.96E-01	0.16	4.20E-01	0.37	5.40E-02	0.47	1.39E-02	-0.53	4.60E-03
ENSCAFG0000001789	ENSCAFG0000001789	darkgrey	VSMC_M8	-0.06	7.83E-01	-0.36	6.79E-02	0.42	2.94E-02	-0.84	3.01E-08	-0.38	5.10E-02	0.21	2.86E-01	0.52	5.35E-03	0.91	6.98E-11	0.16	4.15E-01	-0.47	1.32E-02
ENSCAFG0000002002	SLC35A3	pink	VSMC_M5	-0.06	7.83E-01	-0.47	1.42E-02	-0.69	7.28E-05	0.42	3.04E-02	0.75	8.20E-06	-0.31	1.21E-01	-0.02	9.26E-01	-0.21	2.85E-01	0.49	9.49E-03	-0.24	2.33E-01
ENSCAFG0000001816	CAMK2A	grey	VSMC_M10	-0.06	7.83E-01	0.36	6.39E-02	0.03	8.74E-01	0.21	2.94E-01	0.02	9.39E-01	-0.17	4.09E-01	0.03	8.73E-01	-0.45	1.80E-02	-0.19	3.55E-01	0.25	2.17E-01
ENSCAFG0000001652	INTS1	yellow	VSMC_M3	-0.06	7.83E-01	-0.22	2.61E-01	0.46	1.51E-02	-0.55	2.86E-03	-0.52	5.31E-03	0.41	3.55E-02	0.09	6.40E-01	0.62	6.22E-04	0.11	5.97E-01	-0.25	2.06E-01
ENSCAFG0000000809	CYP2C18	grey	VSMC_M10	-0.06	7.83E-01	-0.17	3.83E-01	0.05	8.04E-01	-0.29	1.36E-01	-0.13	5.18E-01	0.18	3.69E-01	-0.26	1.88E-01	0.33	8.79E-02	0.18	3.81E-01	-0.10	6.10E-01
ENSCAFG0000002836	ENSCAFG0000002836	darkgreen	VSMC_M4	-0.06	7.83E-01	-0.09	6.54E-01	-0.43	2.59E-02	0.55	3.27E-03	0.24	2.34E-01	0.32	1.00E-01	-0.09	6.66E-01	-0.31	1.13E-01	0.04	8.36E-01	0.26	1.82E-01
ENSCAFG0000000370	SHCBP1	darkgrey	VSMC_M8	-0.06	7.82E-01	-0.50	8.62E-03	0.40	3.70E-02	-0.92	1.42E-11	-0.27	1.81E-01	0.10	6.19E-01	0.40	3.79E-02	0.96	9.60E-16	0.35	7.38E-02	-0.70	5.26E-05
ENSCAFG0000001980	DEF8	grey	VSMC_M10	-0.06	7.82E-01	0.63	4.20E-04	0.07	7.15E-01	0.37	5.65E-02	-0.22	2.66E-01	-0.11	6.01E-01	-0.37	5.57E-02	-0.55	2.74E-03	-0.44	2.07E-02	0.68	1.01E-04
ENSCAFG0000001725	ENSCAFG0000001725	grey	VSMC_M10	-0.06	7.82E-01	-0.06	7.74E-01	0.24	2.36E-01	-0.23	2.44E-01	-0.28	1.52E-01	-0.06	7.75E-01	0.35	7.43E-02	0.29	1.46E-01	-0.03	8.64E-01	0.02	9.24E-01
ENSCAFG0000001466	AOC3	darkgreen	VSMC_M4	-0.06	7.82E-01	-0.12	5.43E-01	-0.47	1.29E-02	0.52	5.58E-03	0.30	1.25E-01	0.12	5.49E-01	-0.06	7.77E-01	-0.36	6.43E-02	0.10	6.28E-01	0.27	1.71E-01
ENSCAFG0000000033	HNRNPH1	darkgrey	VSMC_M8	-0.06	7.82E-01	-0.63	3.95E-04	0.24	2.19E-01	-0.80	4.23E-07	-0.13	5.30E-01	0.06	7.54E-01	0.34	8.32E-02	0.96	6.37E-15	0.47	1.26E-02	-0.77	2.74E-06
ENSCAFG0000002272	ENSCAFG0000002272	grey	VSMC_M10	-0.06	7.82E-01	0.27	1.70E-01	0.22	2.78E-01	0.01	9.68E-01	-0.30	1.32E-01	-0.11	5.91E-01	0.12	5.39E-01	-0.14	4.95E-01	-0.25	2.04E-01	0.32	9.98E-02
ENSCAFG0000000787	TRABD2A	grey	VSMC_M10	-0.06	7.82E-01	0.16	4.15E-01	0.23	2.38E-01	-0.26	1.95E-01	-0.27	1.76E-01	-0.06	7.62E-01	0.18	3.70E-01	0.09	6.71E-01	-0.05	7.90E-01	0.14	4.87E-01
ENSCAFG0000000736	EPB41L4A	darkgreen	VSMC_M4	-0.06	7.82E-01	0.12	5.55E-01	-0.67	1.19E-04	0.86	1.05E-08	0.58	1.67E-03	-0.12	5.56E-01	-0.21	2.86E-01	-0.72	2.10E-05	-0.01	9.58E-01	0.37	5.59E-02
ENSCAFG0000000632	MAK16	darkgrey	VSMC_M8	-0.06	7.81E-01	-0.58	1.45E-03	-0.02	9.21E-01	-0.51	6.03E-03	0.06	7.82E-01	0.00	9.96E-01	0.30	1.26E-01	0.73	1.76E-05	0.43	2.63E-02	-0.57	1.92E-03
ENSCAFG0000000842	ZNF704	grey	VSMC_M10	-0.06	7.81E-01	-0.29	1.44E-01	-0.25	2.01E-01	-0.15	4.44E-01	0.30	1.34E-01	0.09	6.71E-01	-0.08	6.90E-01	0.23	2.51E-01	0.27	1.74E-01	-0.31	1.10E-01
ENSCAFG0000001759	GDPD1	grey	VSMC_M10	-0.06	7.81E-01	0.33	9.37E-02	-0.22	2.73E-01	0.46	1.57E-02	-0.01	9.74E-01	0.05	7.94E-01	-0.10	6.08E-01	-0.45	1.90E-02	-0.30	1.26E-01	0.61	7.16E-04
ENSCAFG0000000644	EIF4E3	grey	VSMC_M10	-0.06	7.81E-01	0.18	3.69E-01	-0.34	8.69E-02	0.56	2.55E-03	0.22	2.64E-01	0.04	8.27E-01	0.10	6.09E-01	-0.54	3.56E-03	-0.14	4.73E-01	0.36	6.59E-02
ENSCAFG0000001873	MARF1	darkgreen	VSMC_M4	-0.06	7.80E-01	0.13	5.05E-01	-0.57	1.79E-03	0.78	1.38E-06	0.51	7.16E-03	-0.23	2.42E-01	-0.31	1.16E-01	-0.74	8.72E-06	-0.02	9.36E-01	0.36	6.20E-02
ENSCAFG0000001184	NRL	grey	VSMC_M10	-0.06	7.80E-01	-0.04	8.47E-01	-0.20	3.30E-01	0.10	6.05E-01	0.04	8.62E-01	0.18	3.66E-01	0.37	5.42E-02	0.06	7.71E-01	-0.03	8.76E-01	0.19	3.40E-01
ENSCAFG0000000893	ENSCAFG0000000893	grey	VSMC_M10	-0.06	7.80E-01	0.31	1.18E-01	0.32	1.08E-01	0.02	9.38E-01	-0.40	3.65E-02	0.05	8.00E-01	-0.19	3.34E-01	-0.09	6.55E-01	-0.22	2.60E-01	0.23	2.56E-01
ENSCAFG0000000139	HNRNPK	darkgrey	VSMC_M8	-0.06	7.80E-01	-0.60	1.04E-03	0.12	5.35E-01	-0.73	1.48E-05	-0.03	8.92E-01	0.09	6.42E-01	0.22	2.60E-01	0.88	1.19E-09	0.48	1.13E-02	-0.73	1.84E-05
ENSCAFG0000001572	OPN3	darkgreen	VSMC_M4	-0.06	7.80E-01	0.41	3.42E-02	-0.34	8.38E-02	0.78	1.99E-06	0.22	2.61E-01	-0.20	3.06E-01	-0.28	1.59E-01	-0.73	1.48E-05	-0.31	1.15E-01	0.52	5.42E-03
ENSCAFG0000001568	PIM2	grey	VSMC_M10	-0.06	7.80E-01	0.12	5.55E-01	-0.04	8.56E-01	0.06	7.83E-01	-0.13	5.24E-01	0.24	2.19E-01	-0.12	5.57E-01	0.00	9.89E-01	-0.15	4.45E-01	0.26	1.84E-01
ENSCAFG0000003274	ENSCAFG0000003274	grey	VSMC_M10	-0.06	7.80E-01	-0.37	5.45E-02	-0.23	2.56E-01	-0.09	6.40E-01	0.23	2.42E-01	-0.06	7.60E-01	0.20	3.22E-01	0.19	3.33E-01	0.30	1.26E-01	-0.21	2.84E-01
ENSCAFG0000003220	LYPD6B	grey	VSMC_M10	-0.06	7.79E-01	0.13	5.12E-01	0.12	5.54E-01	-0.12	5.54E-01	-0.15	4.63E-01	-0.02	9.13E-01	0.34	8.13E-02	0.08	6.83E-01	-0.16	4.30E-01	0.11	5.88E-01
ENSCAFG0000000071	ZBED4	grey	VSMC_M10	-0.06	7.79E-01	0.22	2.64E-01	0.11	5.89E-01	-0.02	9.35E-01	-0.35	7.37E-02	0.32	9.99E-02	-0.01	9.42E-01	0.04	8.54E-01	-0.33	9.75E-02	0.44	2.22E-02
ENSCAFG0000001859	ENSCAFG0000001859	violet	VSMC_M7	-0.06	7.79E-01	-0.36	6.55E-02	-0.04	8.41E-01	-0.25	2.04E-01	0.02	9.18E-01	0.02	9.28E-01	0.79	7.53E-07	0.34	8.03E-02	0.21	2.82E-01	-0.17	3.88E-01
ENSCAFG0000001960	PLEKHG5	darkgreen	VSMC_M4	-0.06	7.79E-01	0.45	1.73E-02	-0.26	1.88E-01	0.65	2.45E-04	0.18	3.73E-01	-0.17	4.01E-01	-0.58	1.39E-03	-0.69	6.01E-05	-0.23	2.43E-01	0.49	9.79E-03
ENSCAFG0000001443	MAOA	darkgreen	VSMC_M4	-0.06	7.79E-01	0.28	1.59E-01	-0.48	1.16E-02	0.78	1.77E-06	0.29	1.37E-01	0.01	9.78E-01	-0.50	7.58E-03	-0.67	1.16E-04	-0.20	3.18E-01	0.52	5.67E-03
ENSCAFG0000000266	MSH6	darkgrey	VSMC_M8	-0.06	7.79E-01	-0.64	3.52E-04	-0.24	2.20E-01	-0.33	9.00E-02	0.28	1.63E-01	0.09	6.41E-01	0.40	3.99E-02	0.59	1.13E-03	0.44	2.25E-02	-0.50	7.31E-03
ENSCAFG0000001599	DLG4	grey	VSMC_M10	-0.06	7.79E-01	0.22	2.62E-01	0.21	2.90E-01	0.14	4.86E-01	-0.28	1.52E-01	-0.14	4.95E-01	-0.06	7.65E-01	-0.16	4.19E-01	-0.16	4.23E-01	0.28	1.58E-01
ENSCAFG0000001422	CDH23	grey	VSMC_M10	-0.06	7.79E-01	0.04	8.62E-01	0.17	3.86E-01	-0.19	3.37E-01	-0.25	2.16E-01	-0.02	9.28E-01	0.23	2.54E-01	0.18	3.61E-01	-0.12	5.63E-01	0.04	8.25E-01
ENSCAFG0000000739	SLC25A46	darkolivegreen	VSMC_M9	-0.06	7.79E-01	-0.49	9.59E-03	-0.77	2.88E-06	0.52	5.48E-03	0.76	4.92E-06	-0.09	6.50E-01	-0.18	3.71E-01	-0.26	1.83E-01	0.51	7.14E-03	-0.16	4.30E-01
ENSCAFG0000001920	RILP	grey	VSMC_M10	-0.06	7.78E-01	0.23	2.49E-01	-0.07	7.22E-01	0.13	5.06E-01	0.11	5.97E-01	-0.02	9.18E-01	0.01	9.52E-01	-0.26	1.91E-01	-0.15	4.40E-01	0.13	5.05E-01
ENSCAFG0000000148	LGALS1	yellow	VSMC_M3	-0.06	7.78E-01	0.04	8.53E-01	0.70	5.53E-05	-0.77	3.26E-06	-0.70	4.11E-05	0.11	5.75E-01	0.38	5.12E-02	0.63	4.42E-04	-0.09	6.52E-01	-0.15	4.45E-01
ENSCAFG0000000831	ZFHX4	grey	VSMC_M10	-0.06	7.78E-01	-0.48	1.08E-02	-0.44	2.11E-02	0.12	5.64E-01	0.45	1.78E-02	-0.04	8.58E-01	0.53	4.17E-03	-0.03	8.92E-01	0.43	2.47E-02	-0.18	3.57E-01
ENSCAFG0000003120	RGS10	cyan	VSMC_M2	-0.06	7.77E-01	0.54	3.47E-03	0.66	1.99E-04	-0.26	1.88E-01	-0.72	2.67E-05	-0.03	8.92E-01	-0.22	2.60E-01	0.03	8.82E-01	-0.45	1.94E-02	0.33	9.08E-02
ENSCAFG0000000976	LZTS2	cyan	VSMC_M2	-0.06	7.77E-01	0.77	3.33E-06	0.58	1.60E-03	0.05	8.07E-01	-0.62	4.98E-04	-0.03	8.82E-01	-0.42	2.99E-02	-0.33	9.24E-02	-0.59	1.30E-03	0.48	1.18E-02
ENSCAFG0000002467	FBXO47	turquoise	VSMC_M6	-0.06	7.77E-01	-0.02	9.33E-01	0.07	7.26E-01	-0.15	4.50E-01	-0.12	5.50E-01	0.38	4.99E-02	0.12	5.39E-01	0.13	5.08E-01	0.02	9.13E-01	0.05	7.98E-01
ENSCAFG0000001388	ENSCAFG0000001388	grey	VSMC_M10	-0.06	7.77E-01	0.04	8.39E-01	0.42	2.93E-02	-0.42	2.97E-02	-0.35	6.99E-02	0.11	5.87E-01	0.46	1.69E-02	0.21	3.00E-01	-0.01	9.73E-01	-0.09	6.59E-01
ENSCAFG0000001525	SPNS3	grey	VSMC_M10	-0.06	7.77E-01	-0.36	6.20E-02	0.05	8.00E-01	-0.33	9.73E-02	-0.16	4.30E-01	0.54	3.88E-03	0.46	1.59E-02	0.39	4.55E-02	0.21	2.85E-01	-0.10	6.30E-01
ENSCAFG0000001266	CYBRD1	darkgreen	VSMC_M4	-0.06	7.76E-01	0.49	1.02E-02	-0.33	9.19E-02	0.76	3.70E-06	0.19	3.47E-01	-0.21	2.92E-01	-0.35	7.02E-02	-0.78	1.44E-06	-0.33	9.27E-02	0.61	7.46E-04
ENSCAFG0000001172	ENSCAFG0000001172	grey	VSMC_M10	-0.06	7.76E-01	0.26	1.85E-01	0.02	9.07E-01	0.06	7.48E-01	-0.07	7.38E-01	-0.03	8.79E-01	0.06	7.80E-01	-0.28	1.65E-01	-0.13	5.34E-01	0.27	1.73E-01
ENSCAFG0000001088	FMO5	grey	VSMC_M10	-0.06	7.76E-01	0.28	1.64E-01	-0.21	2.89E-01	0.48	1.08E-02	0.23	2.56E-01	-0.08	7.06E-01	-0.11	5.82E-01	-0.56	2.17E-03	-0.15	4.65E-01	0.19	3.46E-01
ENSCAFG0000000687	KMT2B	yellow	VSMC_M3	-0.06	7.76E-01	0.16	4.24E-01	0.74	8.66E-06	-0.45	1.78E-02	-0.76	4.01E-06	0.10	6.28E-01	-0.01	9.67E-01	0.28	1.56E-01	-0.11	5.70E-01	0.00	9.95E-01
ENSCAFG0000000857	ENSCAFG0000000857	grey	VSMC_M10	-0.06	7.76E-01	0.13	5.17E-01	-0.04	8.30E-01	0.03	8.73E-01	0.08	6.85E-01	-0.07	7.44E-01	-0.03	8.88E-01	-0.17	3.92E-01	-0.05	8.12E-01	0.03	8.82E-01
ENSCAFG0000001030	ENSCAFG0000001030	grey	VSMC_M10	-0.06	7.76E-01	0.17	3.93E-01	0.17	3.84E-01	-0.14	4.95E-01	-0.29	1.35E-01	0.42	3.08E-02	0.16	4.27E-01	0.11	6.00E-01	-0.15	4.43E-01	0.16	4.35E-01
ENSCAFG0000001729	TENT5D	grey	VSMC_M10	-0.06	7.75E-01	-0.07	7.18E-01	-0.01	9.62E-01	-0.06	7.64E-01	0.02	9.12E-01	-0.15	4.62E-01	0.23	2.51E-01	0.13	5.15E-01	-0.01	9.50E-01	-0.12	5.66E-01
ENSCAFG0000000976	PDZD7	darkgreen	VSMC_M4	-0.06	7.75E-01	0.02	9.06E-01	-0.44	2.31E-02	0.60	9.42E-04	0.28	1.63E-01	-0.10	6.18E-01	0.04	8.56E-01	-0.48	1.09E-02	-0.02	9.22E-01	0.36	6.53E-02
ENSCAFG0000000904	IQCD	grey	VSMC_M10	-0.06	7.75E-01	-0.62	5.00E-04	-0.47	1.26E-02	-0.02	9.09E-01	0.55	2.72E-03	-0.06	7.71E-01	0.37	5.83E-02	0.21	2.98E-01	0.55	3.10E-03	-0.48	1.04E-02
ENSCAFG0000001493	MED15	grey	VSMC_M10	-0.06	7.75E-01	0.33	9.39E-02	0.16	4.27E-01	-0.04	8.45E-01	-0.24	2.27E-01	0.10	6.21E-01	-0.11	5.97E-01	-0.15	4.51E-01	-0.20	3.11E-01	0.29	1.40E-01
ENSCAFG0000001145	LRMP	violet	VSMC_M7	-0.06	7.74E-01	-0.16	4.21E-01	0.10	6.31E-01	-0.22	2.77E-01	-0.13	5.16E-01	0.04	8.44E-01	0.86	1.11E-08	0.24	2.28E-01	0.04	8.33E-01	-0.04	8.49E-01
ENSCAFG0000001395	KIF18B	darkgrey	VSMC_M8	-0.06	7.74E-01	-0.51	7.14E-03	0.33	9.59E-02	-0.82	1.60E-07	-0.28	1.58E-01	0.19	3.42E-01	0.42	3.08E-02	0.92	6.53E-12	0.33	9.70E-02	-0.58	1.44E-03
ENSCAFG0000002914	FGF16	violet	VSMC_M7	-0.06	7.74E-01	-0.33	9.54E-02	-0.04	8.37E-01	-0.19	3.40E-01	0.01	9.56E-01	-0.07	7.32E-01	0.48	1.04E-02	0.28	1.62E-01	0.24	2.25E-01	-0.17	3.94E-01
ENSCAFG0000000835	LOXL3	darkgrey	VSMC_M8	-0.06	7.74E-01	-0.35	7.72E-02	0.22	2.63E-01	-0.64	2.90E-04	-0.07	7.11E-01	-0.13	5.32E-01	0.12	5.46E-01	0.54	3.86E-03	0.37	5.87E-02	-0.54	4.00E-03
ENSCAFG0000000863	USP16	grey	VSMC_M10	-0.06	7.74E-01	-0.53	4.92E-03	-0.50	8.42E-03	0.02	9.20E-01	0.48	1.08E-02	0.00	9.97E-01	0.19	3.47E-01	0.26	1.83E-01	0.41	3.61E-02	-0.29	1.45E-01
[truncated: 1,722,664 more chars]
