# Supplementary material for: Locational memory of macrovessel vascular cells is transcriptionally imprinted
Source: Sci Rep. 2023 Aug 10;13:13028. doi: 10.1038/s41598-023-38880-6 (PMC10415317; doi:10.1038/s41598-023-38880-6)
Supplement: Supplementary file 1 — Supplementary Figure 1. [file 41598_2023_38880_MOESM1_ESM.pdf]

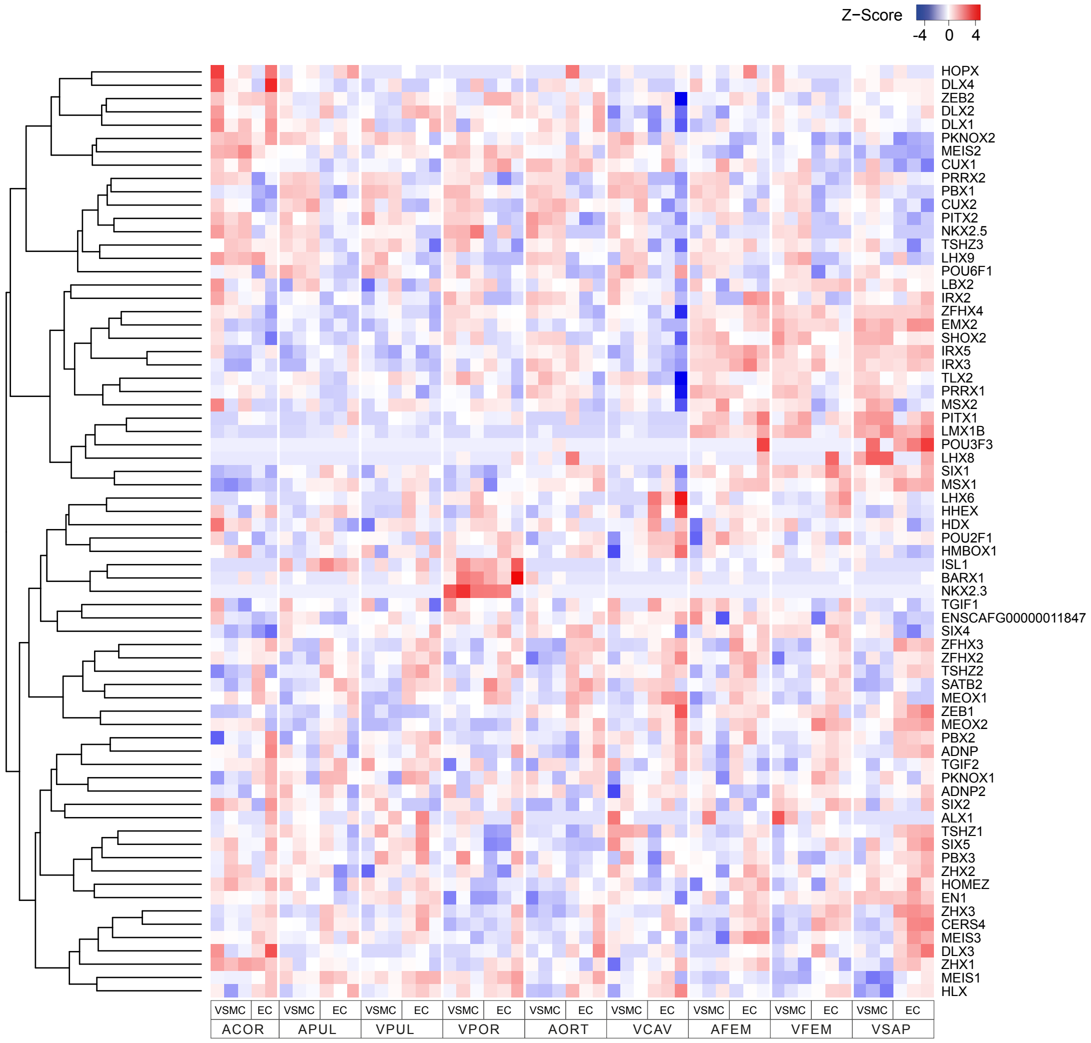

Supplemental Figure 1. Heatmap of genes with a homeobox domain (SMART SM00389, other than HOX) with expression in ECs and VSMCs.
ACOR, coronary artery; APUL, pulmonary artery; VPUL, pulmonary vein; VPOR, portal vein; VCAV, caval vein; AORT, aorta; AFEM, femoral artery; VFEM, femoral vein; VSAP, saphenous vein.
