# Supplementary material for: Locational memory of macrovessel vascular cells is transcriptionally imprinted
Source: Sci Rep. 2023 Aug 10;13:13028. doi: 10.1038/s41598-023-38880-6 (PMC10415317; doi:10.1038/s41598-023-38880-6)
Supplement: Supplementary file 8 — Supplementary Table 11. [file 41598_2023_38880_MOESM8_ESM.pdf]

Supplemental Table 11. Gene ontology terms for modules in the endothelial cell dataset.

category	over_represented_pvalue	under_represented_pvalue	numDEInCat	numInCat	term	ontology	p.adjust	EnsemblID	module	module_num
GO:0051276	6.33E-73	1	231	682	chromosome organization	BP	5.21E-69	RPA3,SHPRH,LATS1,NUP107,CCT2,FBXO5,MAP3K4,RAD21,DSCC1,KIFC1,ATAD2,XRCC6,SRPK1,RECQL4,MCM5,POT1,MTERF1,MCM3,TRIM28,ANP32B,MSH2,SMC2,SENP6,TTK,HNRNPA2B1,ZMPSTE24,CCT4,CDCA8,EZH2,ASCC3,DDX1,SMC6,GEN1,HDAC2,MCM2,CENPO,DNAJC2,RAD54L,SETDB2,CENPA,NASP,DCLRE1C,KIF2C,RSF1,MCM6,FANCD2,NCAPG2,CDC20,SUPT16H,CHD8,KAT6A,RIF1,NAP1L1,TOP2B,SGO1,RFC3,BRCA2,RRP8,CDC16,PRKDC,NCAPH,CCNB1,MCM4,ERCC6,ACTR6,ESPL1,BUB1,ANAPC1,TNKS2,RRS1,TDG,SMARCA5,CHD1,CENPH,XRN1,KNTC1,ATR,TERF1,FAM208A,HELLS,HLTF,RACGAP1,ESCO2,ANAPC7,DSN1,RBL1,MSH3,BUB1B,MIS18A,CCT7,NBN,TOP1,KNSTRN,ABRAXAS1,RAD51,DCLRE1B,ARID2,CCNE2,INO80,OIP5,NUSAP1,CHAF1B,CHD9,UBE2C,NCAPD3,RFC5,SMARCAD1,CCT5,CCT6A,NUCKS1,DEK,KIF18A,CUL3,NAP1L4,NAA50,SMC3,CENPE,TRIP13,CHD1L,SGO2,KIF14,ACIN1,ARID4B,GAR1,BPTF,ANP32E,HJURP,RFC2,RPA2,MCM3AP,SPO11,RAE1,NEK2,PRC1,NUP133,NSL1,BLM,CENPF,BUB3,HAT1,CDK1,SMARCC1,BAZ1B,DHX9,MKI67,NUF2,BAZ1A,SETD2,RFC4,DNA2,TPR,CDC27,KIF18B,CDCA5,FANCM,SMC4,XRCC5,MCM7,TACC3,HP1BP3,DLGAP5,NCAPD2,ZNF518B,ZWINT,SUV39H1,DDX11,EXO1,HNRNPU,INCENP,PDS5A,WAPL,SMARCE1,RFC1,TOP2A,CDC6,DCP2,SMC1A,MAD1L1,ASF1B,NCAPG,KPNB1,EXOSC10,CCT3,KIF4A,KIF22,SPDL1,AURKB,ATRX,PTTG1,ZWILCH,MBTD1,LCMT1,KIF23,CENPI,PLK1,BRIP1,G3BP1,ESCO1,TOP3A,NDC80,SMCHD1,STAG2,NUP155,NIPBL,AIFM1,SAFB,MYSM1,RMI2,CHAF1A,NDC1,UBN1,RPA1,HASPIN,EHMT1,CHTF18,CDT1,CENPN,CTCF,ERCC6L,HIST1H1A,HNRN	darkgrey	EC_M8
GO:0090304	6.91E-73	1	652	3916	nucleic acid metabolic process	BP	5.21E-69	CDK2,PAN2,NARS,ONECUT2,BCLAF1,CLK4,RPA3,SHPRH,HNRNPH1,PPIL4,NUP107,CCT2,MDC1,ZFC3H1,POP1,DDX39B,SRFBP1,ZNF608,SCAF8,UBR5,EIF3E,MAP3K4,RAD21,DSCC1,RRP7A,HSF2,ATAD2,XRCC6,DDX46,PHF5A,TNRC6B,FANCC,TUT7,SRPK1,UHRF2,RMI1,SRSF3,PPIL1,PSIP1,RECQL4,TONSL,RBM28,UBR2,IFT74,MCM5,POT1,PWP1,ZNF300,TOPORS,APTX,SMU1,POLR3B,XPO5,MTERF1,POLH,RFX3,CKS2,MCM3,REV1,NOL8,TRIM28,NCBP1,ZNF451,PHF3,TWISTNB,LRPPRC,PPIH,FOXJ3,ZNF189,MSH2,SP4,CDCA7L,PHIP,YTHDC1,HNRNPA2B1,PNPT1,PAPOLG,PUS10,ZMPSTE24,RLF,SYNCRIP,ZNF292,XPO1,PABPC4,CCT4,COMMD1,MDN1,CASP8AP2,ETAA1,TIA1,ZNF350,ITGB1BP1,THRAP3,TAF1B,EZH2,UFL1,NOL10,MMS22L,USP45,CCNC,CLSPN,ASCC3,ORC6,DDX1,NAA15,SMC6,ELF2,ORC1,GEN1,CDC40,ATAD2B,CDCA7,PUS7,HDAC2,MCM2,CCNA2,EXOSC9,MLLT10,DNAJC2,RAD54L,RNASEH2B,WDR33,SETDB2,SYMPK,ZC3H13,TRDMT1,PCF11,CNOT10,DCLRE1C,NIFK,NUP85,MCM10,ELF1,UPF2,MBD4,RSF1,DDX18,TSEN2,DIS3,CCNT2,PAXIP1,SNRPA,MCM6,DARS,FANCD2,XRN2,RBM17,NCAPG2,RBM26,WDR43,TK1,BIRC5,CRNKL1,EBNA1BP2,INTS10,PARP2,POLR3F,ORC4,GTPBP4,RAD18,EPC2,SUPT16H,CHD8,KAT6A,HNRNPL,METTL3,RIF1,TOP2B,E2F7,GINS4,PRPF40A,ALYREF,CKAP2,ZMAT2,CEP290,NSD3,CEBPZ,SUPT20H,TAF7,EXOSC8,POLE,NR2C1,RFC3,CUL4A,DDX51,MAK16,BRCA2,SNRPF,PPP4R2,LARS,TOPBP1,ELK3,AC091959.3,RRP8,TCERG1,MAPK8,PRKDC,CBX5,CCNB1,MCM4,ERCC6,NOP56,ZNF214,PAN3,MTREX,GPBP1,SP1,DHX37,UTP20,TCEA1,ZNF664,TGS1,BMS1,ZC3H8,POLR1B,DIMT1,PARPBP,TNKS2,RRS	darkgrey	EC_M8

GO:0006139	7.45E-66	1	673	4274	nucleobase-containing compound metabolic process	BP	3.75E-62	CDK2,PAN2,NARS,ONECUT2,BCLAF1,CLK4,RPA3,SHPRH,HNRNPH1,PPIL4,NUP107,CCT2,MDC1,ZFC3H1,POP1,DDX39B,SRFBP1,ZNF608,SCAF8,UBR5,EIF3E,MAP3K4,RAD21,DSCC1,RRP7A,HSF2,ATAD2,XRCC6,DDX46,PHF5A,TNRC6B,FANCC,TUT7,SRPK1,UHRF2,RMI1,SRSF3,PPIL1,PSIP1,RECQL4,TONSL,RBM28,UBR2,IFT74,MCM5,POT1,PWP1,ZNF300,TOPORS,APTX,SMU1,POLR3B,XPO5,MTERF1,POLH,RFX3,CKS2,MCM3,PPAT,PAICS,REV1,NOL8,TRIM28,NCBP1,ZNF451,PHF3,TWISTNB,LRPPRC,PPIH,FOXJ3,ZNF189,MSH2,SP4,CDCA7L,CTPS1,PHIP,YTHDC1,HNRNPA2B1,PNPT1,DCK,PAPOLG,PUS10,ZMPSTE24,RLF,SYNCRIP,ZNF292,XPO1,PABPC4,CCT4,COMMD1,MDN1,CASP8AP2,ETAA1,TIA1,ZNF350,ITGB1BP1,THRAP3,TAF1B,EZH2,UFL1,NOL10,MMS22L,USP45,CCNC,CLSPN,ASCC3,ORC6,DDX1,NAA15,SMC6,ELF2,ORC1,GEN1,CDC40,AMD1,ATAD2B,CDCA7,PUS7,HDAC2,MCM2,CCNA2,EXOSC9,MLLT10,DNAJC2,RAD54L,RNASEH2B,WDR33,SETDB2,SYMPK,ZC3H13,TRDMT1,PCF11,CNOT10,DCLRE1C,NIFK,NUP85,MCM10,CAD,ELF1,UPF2,MBD4,RSF1,DDX18,TSEN2,DIS3,CCNT2,PAXIP1,SNRPA,MCM6,DARS,FANCD2,XRN2,RBM17,NCAPG2,RBM26,WDR43,TK1,AFMID,BIRC5,CRNKL1,EBNA1BP2,INTS10,PARP2,POLR3F,ORC4,GTPBP4,RAD18,EPC2,SUPT16H,CHD8,KAT6A,HNRNPL,METTL3,RIF1,TOP2B,E2F7,GINS4,PRPF40A,ALYREF,CKAP2,ZMAT2,RRM1,CEP290,NSD3,CEBPZ,SUPT20H,TAF7,EXOSC8,POLE,NR2C1,RFC3,CUL4A,DDX51,MAK16,BRCA2,SNRPF,PPP4R2,LARS,TOPBP1,ELK3,AC091959.3,RRP8,TCERG1,MAPK8,PRKDC,CBX5,CCNB1,MCM4,SUCLG2,ERCC6,NOP56,ZNF214,PAN3,MTREX,GPBP1,SP1,DHX37,UTP20,TCEA1,ZNF664	darkgrey	EC_M8
GO:0046483	6.44E-65	1	681	4376	heterocycle metabolic process	BP	2.43E-61	CDK2,PAN2,NARS,ONECUT2,BCLAF1,CLK4,RPA3,SHPRH,HNRNPH1,PPIL4,MTHFD1L,NUP107,CCT2,MDC1,ZFC3H1,POP1,DDX39B,SRFBP1,ZNF608,SCAF8,UBR5,EIF3E,MAP3K4,RAD21,DSCC1,RRP7A,HSF2,ATAD2,XRCC6,DDX46,PHF5A,TNRC6B,FANCC,TUT7,SRPK1,UHRF2,RMI1,SRSF3,PPIL1,PSIP1,RECQL4,TONSL,RBM28,UBR2,IFT74,MCM5,POT1,PWP1,ZNF300,TOPORS,APTX,SMU1,POLR3B,XPO5,MTERF1,POLH,RFX3,CKS2,MCM3,PPAT,PAICS,REV1,NOL8,TRIM28,NCBP1,ZNF451,PHF3,TWISTNB,LRPPRC,PPIH,FOXJ3,ZNF189,MSH2,SP4,CDCA7L,CTPS1,PHIP,YTHDC1,HNRNPA2B1,PNPT1,DCK,PAPOLG,PUS10,ZMPSTE24,RLF,SYNCRIP,ZNF292,XPO1,PABPC4,CCT4,COMMD1,MDN1,CASP8AP2,ETAA1,TIA1,ZNF350,ITGB1BP1,THRAP3,TAF1B,EZH2,UFL1,NOL10,MMS22L,USP45,CCNC,CLSPN,ASCC3,ORC6,DDX1,NAA15,SMC6,ELF2,ORC1,GEN1,CDC40,AMD1,ATAD2B,DHFR,CDCA7,PUS7,HDAC2,MCM2,CCNA2,EXOSC9,MLLT10,DNAJC2,RAD54L,RNASEH2B,WDR33,SETDB2,SYMPK,ZC3H13,TRDMT1,PCF11,CNOT10,DCLRE1C,NIFK,NUP85,MCM10,CAD,ELF1,UPF2,MBD4,RSF1,DDX18,TSEN2,DIS3,CCNT2,PAXIP1,SNRPA,MCM6,DARS,FANCD2,XRN2,RBM17,NCAPG2,RBM26,WDR43,TK1,AFMID,BIRC5,CRNKL1,EBNA1BP2,INTS10,PARP2,POLR3F,ORC4,GTPBP4,RAD18,EPC2,SUPT16H,CHD8,KAT6A,HNRNPL,METTL3,RIF1,TOP2B,E2F7,GINS4,PRPF40A,ALYREF,CKAP2,ZMAT2,RRM1,CEP290,NSD3,CEBPZ,SUPT20H,TAF7,EXOSC8,POLE,NR2C1,RFC3,CUL4A,DDX51,MAK16,BRCA2,SNRPF,PPP4R2,LARS,TOPBP1,ELK3,AC091959.3,RRP8,TCERG1,MAPK8,PRKDC,CBX5,CCNB1,MCM4,SUCLG2,ERCC6,NOP56,ZNF214,PAN3,MTREX,GPBP1,SP1,DHX37,	darkgrey	EC_M8
GO:0007049	6.42E-64	1	333	1405	cell cycle	BP	1.94E-60	RTTN,CDK2,YTHDC2,RPA3,KATNA1,LATS1,NUP43,MDC1,DDX39B,FBXO43,FBXO5,GTSE1,RAD21,DSCC1,KIF3A,KIFC1,KIF20A,CDC25C,UHRF2,RMI1,PLEC,HAUS6,UBR2,DBF4,CENPQ,CKS2,MCM3,PPAT,MELK,FOXJ3,MSH2,SMC2,SENP6,PHIP,TTK,PNPT1,ZMPSTE24,XPO1,CASP8AP2,ETAA1,ANLN,CDCA8,EZH2,CUL1,CLSPN,CNTRL,CASP2,ORC1,GEN1,PLK4,RINT1,STIL,MCM2,CCNA2,CEP295,RAD54L,CHORDC1,RNASEH2B,SETDB2,MASTL,CENPA,NASP,CLASP2,DDIAS,KIF2C,PPM1G,BORA,CCNT2,PAXIP1,MCM6,FANCD2,NCAPG2,CDC20,BIRC5,GTPBP4,IPO5,METTL3,RIF1,TOP2B,E2F7,SGO1,PRPF40A,CKAP2,RRM1,TUBGCP3,POLE,CUL4A,BRCA2,TOPBP1,PSMD13,RRP8,CDC16,NEDD1,PRKDC,NCAPH,CCNB1,MCM4,WDR62,ERCC6,HAUS5,ESPL1,BUB1,TPX2,ANAPC1,KIF20B,SKA3,IPO7,RRS1,LSM14A,CENPJ,CDK5RAP1,KIF11,CENPH,KNTC1,ATR,CEP55,TERF1,HELLS,MND1,CETN3,RACGAP1,PBK,ESCO2,ANAPC7,LRRCC1,DSN1,USP16,RBL1,RPS6,MSH3,ALMS1,BUB1B,MIS18A,CCNL1,CDCA2,NBN,FAM83D,CKAP5,KNSTRN,ABRAXAS1,KNL1,RAD51,MYBL2,POC5,PKP4,CCNE2,E2F8,INO80,OIP5,NUSAP1,ZBED6,MDM4,CHAF1B,UBE2C,NCAPD3,HERC5,TAOK3,SMARCAD1,NOLC1,CIT,KIF18A,CUL3,NSUN2,GMNN,CHEK1,NAA50,SMC3,KHDRBS1,CENPE,TRIP13,TET2,PPP2R2D,CEP72,SGO2,HAUS2,KIF14,PRMT5,INTS13,AJUBA,TBCE,CFL1,ASPM,BCAT1,FANCI,ZBED9,RCC1,SPC25,HJURP,TICRR,RPA2,SPO11,RAE1,NEK2,PRC1,INTS7,DTL,NSL1,BLM,WDR12,ATRIP,CENPF,TXLNG,BUB3,AUNIP,CDKL5,CDK1,CDC25A,SEPT2,FOSL1,MKI67,NUF2,SLC16A1,SETD2,POLA1,DNA2,TPR,CDC27,CCAR1,KIF18B,CDCA5,FANCM,MIS18BP1,DBF4B,KIF15,IL12A,S	darkgrey	EC_M8

GO:0006725	2.08E-62	1	679	4410	cellular aromatic compound metabolic process	BP	5.24E-59	CDK2,PAN2,NARS,ONECUT2,BCLAF1,CLK4,RPA3,SHPRH,HNRNPH1,PPIL4,MTHFD1L,NUP107,CCT2,MDC1,ZFC3H1,POP1,DDX39B,SRFBP1,ZNF608,SCAF8,UBR5,EIF3E,MAP3K4,RAD21,DSCC1,RRP7A,HSF2,ATAD2,XRCC6,DDX46,PHF5A,TNRC6B,FANCC,TUT7,SRPK1,UHRF2,RMI1,SRSF3,PPIL1,PSIP1,RECQL4,TONSL,RBM28,UBR2,IFT74,MCM5,POT1,PWP1,ZNF300,TOPORS,APTX,SMU1,POLR3B,XPO5,MTERF1,POLH,RFX3,CKS2,MCM3,PPAT,PAICS,REV1,NOL8,TRIM28,NCBP1,ZNF451,PHF3,TWISTNB,LRPPRC,PPIH,FOXJ3,ZNF189,MSH2,SP4,CDCA7L,CTPS1,PHIP,YTHDC1,HNRNPA2B1,PNPT1,DCK,PAPOLG,PUS10,ZMPSTE24,RLF,SYNCRIP,ZNF292,XPO1,PABPC4,CCT4,COMMD1,MDN1,CASP8AP2,ETAA1,TIA1,ZNF350,ITGB1BP1,THRAP3,TAF1B,EZH2,UFL1,NOL10,MMS22L,USP45,CCNC,CLSPN,ASCC3,ORC6,DDX1,NAA15,SMC6,ELF2,ORC1,GEN1,CDC40,AMD1,ATAD2B,DHFR,CDCA7,PUS7,HDAC2,MCM2,CCNA2,EXOSC9,MLLT10,DNAJC2,RAD54L,RNASEH2B,WDR33,SETDB2,SYMPK,ZC3H13,TRDMT1,PCF11,CNOT10,DCLRE1C,NIFK,NUP85,MCM10,CAD,ELF1,UPF2,MBD4,RSF1,DDX18,TSEN2,DIS3,CCNT2,PAXIP1,SNRPA,MCM6,DARS,FANCD2,XRN2,RBM17,NCAPG2,RBM26,WDR43,TK1,AFMID,BIRC5,CRNKL1,EBNA1BP2,INTS10,PARP2,POLR3F,ORC4,GTPBP4,RAD18,EPC2,SUPT16H,CHD8,KAT6A,HNRNPL,METTL3,RIF1,TOP2B,E2F7,GINS4,PRPF40A,ALYREF,CKAP2,ZMAT2,RRM1,CEP290,NSD3,CEBPZ,SUPT20H,TAF7,EXOSC8,POLE,NR2C1,RFC3,CUL4A,DDX51,MAK16,BRCA2,SNRPF,PPP4R2,LARS,TOPBP1,ELK3,AC091959.3,RRP8,TCERG1,MAPK8,PRKDC,CBX5,CCNB1,MCM4,SUCLG2,ERCC6,NOP56,ZNF214,PAN3,MTREX,GPBP1,SP1,DHX37,	darkgrey	EC_M8
GO:0022402	3.51E-60	1	258	951	cell cycle process	BP	7.58E-57	RTTN,CDK2,YTHDC2,LATS1,MDC1,DDX39B,FBXO43,FBXO5,GTSE1,RAD21,DSCC1,KIF3A,KIFC1,KIF20A,CDC25C,RMI1,PLEC,HAUS6,UBR2,DBF4,CENPQ,CKS2,MCM3,PPAT,MELK,FOXJ3,MSH2,SMC2,SENP6,PHIP,TTK,ZMPSTE24,XPO1,ETAA1,ANLN,CDCA8,EZH2,CUL1,CLSPN,CASP2,ORC1,GEN1,PLK4,RINT1,STIL,MCM2,CCNA2,CEP295,RAD54L,CHORDC1,RNASEH2B,MASTL,CENPA,NASP,CLASP2,KIF2C,BORA,PAXIP1,MCM6,FANCD2,NCAPG2,CDC20,TOP2B,E2F7,SGO1,PRPF40A,CKAP2,TUBGCP3,POLE,CUL4A,BRCA2,TOPBP1,PSMD13,CDC16,PRKDC,NCAPH,CCNB1,MCM4,WDR62,ERCC6,HAUS5,ESPL1,BUB1,TPX2,ANAPC1,KIF20B,RRS1,LSM14A,CENPJ,KIF11,CENPH,KNTC1,ATR,CEP55,TERF1,MND1,CETN3,RACGAP1,ESCO2,ANAPC7,DSN1,USP16,RBL1,RPS6,MSH3,ALMS1,BUB1B,CDCA2,NBN,FAM83D,CKAP5,KNSTRN,ABRAXAS1,KNL1,RAD51,MYBL2,PKP4,CCNE2,E2F8,INO80,NUSAP1,UBE2C,NCAPD3,TAOK3,SMARCAD1,CIT,KIF18A,CUL3,NSUN2,GMNN,CHEK1,NAA50,SMC3,KHDRBS1,CENPE,TRIP13,PPP2R2D,CEP72,SGO2,HAUS2,KIF14,PRMT5,INTS13,TBCE,CFL1,ASPM,BCAT1,RCC1,SPC25,TICRR,RPA2,SPO11,RAE1,NEK2,PRC1,INTS7,DTL,NSL1,BLM,ATRIP,CENPF,TXLNG,BUB3,AUNIP,CDK1,CDC25A,SEPT2,MKI67,NUF2,SLC16A1,SETD2,POLA1,DNA2,TPR,CDC27,KIF18B,CDCA5,FANCM,DBF4B,KIF15,SMC4,NPAT,CDC45,BRCA1,CUL5,PSME3,CEP152,HAUS3,CDKN3,TACC3,DLGAP5,NCAPD2,TIPRL,RAD51AP1,ECT2,ZWINT,FOXM1,DDX11,HNRNPU,INCENP,PDS5A,WAPL,CCNB3,TOP2A,CDC6,SMC1A,MAD1L1,CCNB2,IQGAP3,NCAPG,KPNB1,KIF4A,KIF22,SPDL1,AURKB,EME1,TAF1,ATRX,PTTG1,TIPIN,ZWILCH,STXBP4,LCMT1,KIF23,TRIM37,P	darkgrey	EC_M8
GO:0034641	6.07E-60	1	717	4878	cellular nitrogen compound metabolic process	BP	1.15E-56	CDK2,PAN2,NARS,ONECUT2,BCLAF1,CLK4,RPA3,SHPRH,HNRNPH1,PPIL4,MTHFD1L,NUP107,CCT2,MDC1,ZFC3H1,POP1,DDX39B,SRFBP1,ZNF608,SCAF8,UBR5,EIF3E,MAP3K4,RAD21,DSCC1,MRPL13,RRP7A,HSF2,ATAD2,XRCC6,DDX46,PHF5A,TNRC6B,FANCC,TUT7,SRPK1,UHRF2,RMI1,SRSF3,PPIL1,PSIP1,RECQL4,TONSL,RBM28,UBR2,CARNMT1,IFT74,MCM5,POT1,PWP1,ZNF300,TOPORS,APTX,SMU1,POLR3B,XPO5,MTERF1,POLH,PUM3,RFX3,CKS2,MCM3,PPAT,PAICS,REV1,NOL8,TRIM28,NCBP1,ZNF451,PHF3,TWISTNB,LRPPRC,PPIH,FOXJ3,ZNF189,MSH2,SP4,CDCA7L,CTPS1,PHIP,YTHDC1,MTIF2,HNRNPA2B1,PNPT1,DCK,PAPOLG,PUS10,ZMPSTE24,RLF,SYNCRIP,ZNF292,XPO1,PABPC4,CCT4,COMMD1,MDN1,CASP8AP2,ETAA1,TIA1,ZNF350,ITGB1BP1,THRAP3,TAF1B,EZH2,UFL1,NOL10,MMS22L,USP45,MRPL32,CCNC,CLSPN,ASCC3,ORC6,DDX1,NAA15,SMC6,ELF2,ORC1,GEN1,WDR35,CDC40,AMD1,ATAD2B,DHFR,CDCA7,PUS7,HDAC2,MCM2,CCNA2,EXOSC9,MLLT10,DNAJC2,RAD54L,RNASEH2B,WDR33,SETDB2,SYMPK,ZC3H13,TRDMT1,PCF11,CNOT10,DCLRE1C,NIFK,NUP85,MRPS7,MCM10,CAD,ELF1,UPF2,MBD4,RSF1,DDX18,TSEN2,DIS3,CCNT2,PAXIP1,SNRPA,MCM6,DARS,FANCD2,XRN2,RBM17,NCAPG2,RBM26,WDR43,TK1,AFMID,BIRC5,CRNKL1,EBNA1BP2,INTS10,PARP2,POLR3F,ORC4,GTPBP4,RAD18,EPC2,SUPT16H,CHD8,KAT6A,HNRNPL,METTL3,RIF1,TOP2B,E2F7,GINS4,PRPF40A,ALYREF,CKAP2,ZMAT2,RRM1,CEP290,NSD3,CEBPZ,SUPT20H,TAF7,EXOSC8,POLE,NR2C1,RFC3,CUL4A,DDX51,MAK16,BRCA2,SNRPF,PPP4R2,LARS,LTA4H,TOPBP1,ELK3,AC091959.3,RRP8,TCERG1,MAPK8,PRKDC,CBX5,CCNB1,MCM4,S	darkgrey	EC_M8

GO:1901360	1.05E-56	1	682	4575	organic cyclic compound metabolic process	BP	1.76E-53	CDK2,PAN2,NARS,ONECUT2,BCLAF1,CLK4,RPA3,SHPRH,HNRNPH1,PPIL4,MTHFD1L,NUP107,CCT2,MDC1,ZFC3H1,POP1,DDX39B,SRFBP1,ZNF608,SCAF8,UBR5,EIF3E,MAP3K4,RAD21,DSCC1,RRP7A,HSF2,ATAD2,XRCC6,DDX46,PHF5A,TNRC6B,FANCC,TUT7,SRPK1,UHRF2,RMI1,SRSF3,PPIL1,PSIP1,RECQL4,TONSL,RBM28,UBR2,IFT74,MCM5,POT1,PWP1,ZNF300,TOPORS,APTX,SMU1,POLR3B,XPO5,MTERF1,POLH,RFX3,CKS2,MCM3,PPAT,PAICS,REV1,NOL8,TRIM28,NCBP1,ZNF451,PHF3,TWISTNB,LRPPRC,PPIH,FOXJ3,ZNF189,MSH2,SP4,CDCA7L,CTPS1,PHIP,YTHDC1,HNRNPA2B1,PNPT1,DCK,PAPOLG,PUS10,ZMPSTE24,RLF,SYNCRIP,ZNF292,XPO1,PABPC4,CCT4,COMMD1,MDN1,CASP8AP2,ETAA1,TIA1,ZNF350,ITGB1BP1,THRAP3,TAF1B,EZH2,UFL1,NOL10,MMS22L,USP45,CCNC,CLSPN,ASCC3,ORC6,DDX1,NAA15,SMC6,ELF2,ORC1,GEN1,CDC40,AMD1,ATAD2B,DHFR,CDCA7,PUS7,HDAC2,MCM2,CCNA2,EXOSC9,MLLT10,DNAJC2,RAD54L,RNASEH2B,WDR33,SETDB2,SYMPK,ZC3H13,TRDMT1,PCF11,CNOT10,DCLRE1C,NIFK,NUP85,MCM10,CAD,ELF1,UPF2,MBD4,RSF1,DDX18,TSEN2,DIS3,CCNT2,PAXIP1,SNRPA,MCM6,DARS,FANCD2,XRN2,RBM17,NCAPG2,RBM26,WDR43,TK1,AFMID,BIRC5,CRNKL1,EBNA1BP2,INTS10,PARP2,POLR3F,ORC4,GTPBP4,RAD18,EPC2,SUPT16H,CHD8,KAT6A,HNRNPL,METTL3,RIF1,TOP2B,E2F7,GINS4,PRPF40A,ALYREF,CKAP2,ZMAT2,RRM1,CEP290,NSD3,CEBPZ,SUPT20H,TAF7,EXOSC8,POLE,NR2C1,RFC3,CUL4A,DDX51,MAK16,BRCA2,SNRPF,PPP4R2,LARS,TOPBP1,ELK3,AC091959.3,RRP8,TCERG1,MAPK8,PRKDC,CBX5,CCNB1,MCM4,SUCLG2,ERCC6,NOP56,ZNF214,PAN3,MTREX,GPBP1,SP1,DHX37,	darkgrey	EC_M8
GO:0007059	2.70E-53	1	125	288	chromosome segregation	BP	4.07E-50	LATS1,NUP43,FBXO5,RAD21,DSCC1,KIFC1,SRPK1,RMI1,CENPQ,SMC2,TTK,CDCA8,SMC6,GEN1,SETDB2,KIF2C,FANCD2,NCAPG2,CDC20,BIRC5,RAD18,TOP2B,SGO1,CDC16,NCAPH,CCNB1,ESPL1,BUB1,ANAPC1,SKA3,RRS1,CENPH,KNTC1,TERF1,RACGAP1,ESCO2,ANAPC7,DSN1,BUB1B,MIS18A,CDCA2,FAM83D,TOP1,KNSTRN,ABRAXAS1,KNL1,CCNE2,INO80,OIP5,NUSAP1,UBE2C,NCAPD3,SMARCAD1,KIF18A,CUL3,NAA50,SMC3,CENPE,TRIP13,SGO2,KIF14,RCC1,SPC25,HJURP,SPO11,NEK2,PRC1,NSL1,CENPF,BUB3,MKI67,NUF2,TPR,CDC27,KIF18B,CDCA5,FANCM,SMC4,BRCA1,TACC3,DLGAP5,NCAPD2,ECT2,ZWINT,DDX11,HNRNPU,INCENP,PDS5A,WAPL,TOP2A,CDC6,SMC1A,MAD1L1,NCAPG,KPNB1,KIF4A,PMF1,KIF22,SPDL1,AURKB,EME1,CIAO2A,ATRX,PTTG1,ZWILCH,LCMT1,KIF23,SPC24,PLK1,BRIP1,RIOK3,ESCO1,TOP3A,NDC80,STAG2,NIPBL,RMI2,NDC1,HASPIN,CDT1,CENPN,CTCF,MAD2L1,CENPW,FEN1	darkgrey	EC_M8
GO:0006396	4.35E-53	1	232	950	RNA processing	BP	5.97E-50	PAN2,CLK4,HNRNPH1,ZFC3H1,POP1,DDX39B,SRFBP1,SCAF8,RRP7A,DDX46,PHF5A,TUT7,SRPK1,SRSF3,PPIL1,PSIP1,RBM28,PWP1,SMU1,NOL8,NCBP1,PPIH,YTHDC1,HNRNPA2B1,PNPT1,PAPOLG,PUS10,ZMPSTE24,SYNCRIP,PABPC4,MDN1,TIA1,THRAP3,NOL10,DDX1,CDC40,PUS7,EXOSC9,WDR33,SYMPK,ZC3H13,TRDMT1,PCF11,NIFK,DDX18,TSEN2,DIS3,SNRPA,XRN2,RBM17,RBM26,WDR43,CRNKL1,EBNA1BP2,INTS10,GTPBP4,HNRNPL,METTL3,PRPF40A,ALYREF,ZMAT2,EXOSC8,DDX51,MAK16,SNRPF,PPP4R2,AC091959.3,RRP8,TCERG1,PRKDC,CCNB1,NOP56,PAN3,MTREX,DHX37,UTP20,TGS1,BMS1,DIMT1,RRS1,WDR36,CDK5RAP1,PPWD1,SREK1,PDE12,SMN2,ZCCHC8,U2SURP,GCFC2,UTP15,RBM12,AQR,RBM39,RPS6,CCNL1,URB1,TENT2,ZNF638,VIRMA,SLC38A2,SCAF11,RIPK1,WDR75,PRPF4B,BCAS2,RIOK1,CWF19L1,WDR3,RBMS1,NOLC1,NSUN2,PDCD11,TRMT10A,ZBTB8OS,SNRPA1,U2AF1,RRP1B,RRP1,FP565260.1,QTRT2,KHDRBS1,SF3B1,RRP15,RBM5,CPSF2,RBM6,HEATR1,NCL,SNRNP40,PRMT5,DDX27,ACIN1,NOL11,GAR1,TARBP1,FXR1,NGDN,DDX5,INTS7,FASTKD1,PPIG,SSB,NOP58,WDR12,FTSJ3,WBP11,DHX9,HNRNPR,TRA2B,TSEN15,FASTKD2,HNRNPA3,IVNS1ABP,HNRNPH3,DDX20,DDX21,PNN,PRPF39,EFTUD2,CWC22,DGCR8,CWF19L2,NOP14,SUV39H1,LEO1,LYAR,HNRNPU,DDX39A,DHX15,LAS1L,SLTM,SRSF5,EXOSC10,RBM25,FUS,CLK2,LUC7L3,UTP18,THG1L,COIL,RBBP6,GEMIN5,INTS2,THUMPD1,RIOK3,THOC1,UTP6,HNRNPM,THOC2,UTP14A,BRIX1,SAFB,SAFB2,RSL1D1,ZC3H7A,DROSHA,RBMX,NSRP1,RBFOX1,FMR1,METTL16,SRRM2,NCBP3,RNPS1,DAZAP1,NOL9,PTBP1,EXOSC2,DNTTIP2,SF3B3,CMT	darkgrey	EC_M8

GO:0006259	2.52E-50	1	206	741	DNA metabolic process	BP	3.17E-47	CDK2,RPA3,SHPRH,CCT2,MDC1,DDX39B,UBR5,MAP3K4,RAD21,DSCC1,XRCC6,FANCC,UHRF2,RMI1,RECQL4,TONSL,UBR2,MCM5,POT1,APTX,POLH,MCM3,REV1,TRIM28,MSH2,HNRNPA2B1,ZMPSTE24,RLF,CCT4,COMMD1,ETAA1,EZH2,UFL1,MMS22L,USP45,CLSPN,ASCC3,ORC6,DDX1,SMC6,ORC1,GEN1,MCM2,DNAJC2,RAD54L,WDR33,DCLRE1C,MCM10,MBD4,PAXIP1,MCM6,FANCD2,XRN2,TK1,PARP2,ORC4,RAD18,EPC2,SUPT16H,RIF1,TOP2B,GINS4,ALYREF,POLE,RFC3,CUL4A,BRCA2,PPP4R2,TOPBP1,PRKDC,MCM4,ERCC6,PARPBP,TNKS2,PARP4,TDG,OTUD4,XRN1,ATR,TERF1,NOC3L,HELLS,MND1,ESCO2,MCRS1,MSH3,MIS18A,CCT7,NBN,TOP1,ABRAXAS1,RAD51,DCLRE1B,ARID2,CCNE2,INO80,CHAF1B,RFC5,TAOK3,SMARCAD1,CCT5,CCT6A,NUCKS1,DEK,UBE2T,CBSL,CHEK1,SMC3,TRIP13,TET2,CHD1L,TRAIP,PRMT5,GAR1,GTF2I,FANCI,ZGRF1,ZBED9,FANCB,TICRR,RFC2,RPA2,SPO11,ORC2,NEK2,DTL,BLM,ATRIP,CENPF,AUNIP,CDK1,DHX9,SETD2,POLA1,AP000944.2,RFC4,DNA2,DDX21,CDCA5,FANCM,C17orf53,APEX2,XRCC5,CDC45,BRCA1,MCM7,NSD2,WDHD1,RAD51AP1,BOD1L1,ANKLE1,FOXM1,FH,DDX11,EXO1,RAD52,HNRNPU,PDS5A,RFC1,TOP2A,CDC6,LIN9,DCP2,SMC1A,DCTPP1,EXOSC10,CCT3,FUS,KIF22,USP3,AURKB,EME1,ATRX,PTTG1,TIPIN,BRIP1,TOP3A,THOC1,TYMS,SMCHD1,CUL4B,NIPBL,AIFM1,TAOK1,RMI2,UHRF1,CHAF1A,RPA1,EHMT1,TP73,CHTF18,FANCA,CDT1,CTCF,PRIM2,HIST1H1A,HNRNPD,HMGB3,TIGAR,NAF1,RNF8,FEN1,PALB2,SFR1,CINP,RCHY1	darkgrey	EC_M8
GO:1903047	4.18E-48	1	181	608	mitotic cell cycle process	BP	4.85E-45	CDK2,LATS1,MDC1,FBXO43,FBXO5,GTSE1,RAD21,DSCC1,KIFC1,KIF20A,CDC25C,DBF4,CKS2,MCM3,PPAT,MELK,MSH2,SMC2,PHIP,TTK,ZMPSTE24,ANLN,CDCA8,EZH2,CUL1,CLSPN,CASP2,ORC1,GEN1,RINT1,STIL,MCM2,CCNA2,RNASEH2B,MASTL,CENPA,NASP,CLASP2,KIF2C,BORA,MCM6,FANCD2,NCAPG2,CDC20,E2F7,SGO1,CKAP2,POLE,CUL4A,BRCA2,TOPBP1,CDC16,PRKDC,NCAPH,CCNB1,MCM4,WDR62,ESPL1,BUB1,TPX2,ANAPC1,KIF20B,RRS1,LSM14A,KIF11,CENPH,KNTC1,CEP55,RACGAP1,ANAPC7,DSN1,USP16,RBL1,RPS6,BUB1B,CDCA2,NBN,CKAP5,KNSTRN,ABRAXAS1,RAD51,MYBL2,CCNE2,INO80,NUSAP1,UBE2C,NCAPD3,TAOK3,CIT,KIF18A,CUL3,CHEK1,NAA50,SMC3,KHDRBS1,CENPE,TRIP13,PPP2R2D,SGO2,KIF14,PRMT5,INTS13,TBCE,BCAT1,RCC1,SPC25,TICRR,RPA2,RAE1,NEK2,PRC1,DTL,NSL1,BLM,CENPF,BUB3,CDK1,CDC25A,MKI67,NUF2,SETD2,POLA1,DNA2,TPR,CDC27,KIF18B,CDCA5,DBF4B,KIF15,SMC4,NPAT,CDC45,BRCA1,CUL5,PSME3,CDKN3,TACC3,DLGAP5,NCAPD2,ECT2,ZWINT,FOXM1,HNRNPU,INCENP,PDS5A,CCNB3,CDC6,SMC1A,MAD1L1,CCNB2,IQGAP3,NCAPG,KPNB1,KIF4A,KIF22,SPDL1,AURKB,EME1,ATRX,PTTG1,TIPIN,ZWILCH,LCMT1,KIF23,PLK1,NDC80,CUL4B,STAG2,NIPBL,GSPT1,TAOK1,PKMYT1,HASPIN,CCNF,SAPCD2,CDT1,TMOD3,MAD2L1,STMN1,AURKA,DRG1	darkgrey	EC_M8
GO:0000278	1.94E-46	1	199	729	mitotic cell cycle	BP	2.09E-43	CDK2,YTHDC2,RPA3,LATS1,MDC1,FBXO43,FBXO5,GTSE1,RAD21,DSCC1,KIFC1,KIF20A,CDC25C,DBF4,CKS2,MCM3,PPAT,MELK,MSH2,SMC2,PHIP,TTK,ZMPSTE24,ANLN,CDCA8,EZH2,CUL1,CLSPN,CASP2,ORC1,GEN1,PLK4,RINT1,STIL,MCM2,CCNA2,RNASEH2B,SETDB2,MASTL,CENPA,NASP,CLASP2,KIF2C,BORA,MCM6,FANCD2,NCAPG2,CDC20,E2F7,SGO1,CKAP2,RRM1,TUBGCP3,POLE,CUL4A,BRCA2,TOPBP1,CDC16,PRKDC,NCAPH,CCNB1,MCM4,WDR62,ESPL1,BUB1,TPX2,ANAPC1,KIF20B,SKA3,RRS1,LSM14A,KIF11,CENPH,KNTC1,CEP55,RACGAP1,PBK,ANAPC7,DSN1,USP16,RBL1,RPS6,BUB1B,CDCA2,NBN,CKAP5,KNSTRN,ABRAXAS1,RAD51,MYBL2,CCNE2,E2F8,INO80,NUSAP1,UBE2C,NCAPD3,TAOK3,NOLC1,CIT,KIF18A,CUL3,CHEK1,NAA50,SMC3,KHDRBS1,CENPE,TRIP13,PPP2R2D,SGO2,KIF14,PRMT5,INTS13,TBCE,BCAT1,RCC1,SPC25,TICRR,RPA2,RAE1,NEK2,PRC1,DTL,NSL1,BLM,CENPF,BUB3,CDK1,CDC25A,MKI67,NUF2,SETD2,POLA1,DNA2,TPR,CDC27,KIF18B,CDCA5,DBF4B,KIF15,SMC4,NPAT,CDC45,BRCA1,CUL5,PSME3,CDKN3,WDHD1,TACC3,DLGAP5,NCAPD2,ECT2,ZWINT,FOXM1,HNRNPU,INCENP,PDS5A,WAPL,CCNB3,CDC6,SMC1A,MAD1L1,CCNB2,IQGAP3,NCAPG,KPNB1,KIF4A,KIF22,SPDL1,USP3,AURKB,EME1,ATRX,PTTG1,TIPIN,ZWILCH,LCMT1,KIF23,PLK1,AATF,NLE1,NDC80,CUL4B,STAG2,NIPBL,GSPT1,TAOK1,PKMYT1,HASPIN,CCNF,TP73,SAPCD2,CDT1,PKN2,TMOD3,MAD2L1,STMN1,AURKA,CENPW,DRG1	darkgrey	EC_M8

GO:0043170	7.17E-46	1	886	7034	macromolecule metabolic process	BP	7.22E-43	CDK2,PAN2,NARS,ONECUT2,MEX3C,BCLAF1,CLK4,RPA3,USP15,SHPRH,HNRNPH1,PPIL4,LATS1,NUP107,CCT2,MDC1,ZFC3H1,POP1,DDX39B,SRFBP1,FBXO43,FBXO5,CSNK1G3,ZNF608,SCAF8,UBR5,EIF3E,MAP3K4,RAD21,DSCC1,MRPL13,RRP7A,HSF2,ATAD2,XRCC6,DDX46,PHF5A,SLC35D2,CDC25C,TNRC6B,FANCC,TUT7,NAA35,MATR3,SRPK1,UHRF2,RMI1,SRSF3,PPIL1,PLEC,PSIP1,RECQL4,TONSL,RBM28,UBR2,IFT74,MCM5,POT1,PWP1,ZNF300,TOPORS,APTX,SMU1,DBF4,POLR3B,XPO5,MTERF1,POLH,AKAP9,PUM3,RFX3,IL1R1,CKS2,MCM3,C1GALT1,ICK,REV1,NOL8,MELK,TRIM28,NCBP1,ZNF451,ANP32B,PHF3,TWISTNB,LRPPRC,PPIH,FOXJ3,ZNF189,MSH2,FBXO11,SP4,SENP6,ASB3,CDCA7L,PHIP,YTHDC1,MTIF2,TTK,PPP4R3B,IBTK,HNRNPA2B1,PNPT1,PAPOLG,PUS10,ZMPSTE24,RLF,USP34,SYNCRIP,ZNF292,XPO1,PABPC4,CCT4,COMMD1,MDN1,CASP8AP2,DPY19L1,FKBP15,ETAA1,FBXO48,TIA1,ZNF350,ITGB1BP1,THRAP3,TAF1B,EZH2,UFL1,NOL10,CUL1,MMS22L,USP45,MRPL32,CCNC,CLSPN,ASCC3,CASP2,ORC6,DDX1,NAA15,SMC6,ELF2,ORC1,GEN1,AFG1L,WDR35,NRDC,CDC40,PLK4,ATAD2B,DHFR,CDCA7,PUS7,HDAC2,MCM2,CCNA2,EXOSC9,MLLT10,PMPCB,SAE1,CEP295,DNAJC2,RAD54L,CHORDC1,RNASEH2B,WDR33,SETDB2,SYMPK,MASTL,YME1L1,ZC3H13,NASP,COG3,TRDMT1,PCF11,CNOT10,DCLRE1C,NIFK,NUP85,MRPS7,MCM10,CAD,ELF1,ALG8,UPF2,MBD4,RSF1,PPM1G,DDX18,BORA,TSEN2,DIS3,CCNT2,PAXIP1,SNRPA,MCM6,DARS,FANCD2,XRN2,RBM17,ULK4,NCAPG2,RBM26,WDR43,CDC20,TK1,BIRC5,NKTR,CRNKL1,EBNA1BP2,INTS10,FNTA,PARP2,PITRM1,POLR3F,KAT14,ORC4,GTPBP4,RAD18,IP	darkgrey	EC_M8
GO:0006807	1.38E-43	1	899	7284	nitrogen compound metabolic process	BP	1.30E-40	CDK2,PAN2,NARS,ONECUT2,MEX3C,BCLAF1,CLK4,RPA3,USP15,SHPRH,HNRNPH1,PPIL4,LATS1,MTHFD1L,NUP107,CCT2,MDC1,ZFC3H1,POP1,DDX39B,SRFBP1,FBXO43,FBXO5,CSNK1G3,ZNF608,SCAF8,UBR5,EIF3E,MAP3K4,RAD21,DSCC1,MRPL13,RRP7A,HSF2,ATAD2,XRCC6,DDX46,PHF5A,SLC35D2,CDC25C,TNRC6B,FANCC,TUT7,NAA35,SRPK1,UHRF2,RMI1,SRSF3,PPIL1,PLEC,PSIP1,RECQL4,TONSL,RBM28,UBR2,CARNMT1,IFT74,MCM5,POT1,PWP1,ZNF300,TOPORS,APTX,SMU1,DBF4,POLR3B,XPO5,MTERF1,POLH,AKAP9,PUM3,RFX3,CKS2,MCM3,PPAT,C1GALT1,PAICS,ICK,REV1,NOL8,MELK,TRIM28,NCBP1,ZNF451,ANP32B,PHF3,TWISTNB,LRPPRC,PPIH,FOXJ3,ZNF189,MSH2,FBXO11,SP4,SENP6,ASB3,CDCA7L,CTPS1,PHIP,YTHDC1,MTIF2,TTK,PPP4R3B,IBTK,HNRNPA2B1,PNPT1,DCK,PAPOLG,PUS10,ZMPSTE24,RLF,USP34,SYNCRIP,ZNF292,XPO1,PABPC4,CCT4,COMMD1,MDN1,CASP8AP2,DPY19L1,FKBP15,ETAA1,FBXO48,TIA1,ZNF350,ITGB1BP1,THRAP3,TAF1B,EZH2,UFL1,NOL10,CUL1,MMS22L,USP45,MRPL32,CCNC,CLSPN,ASCC3,CASP2,ORC6,DDX1,NAA15,SMC6,ELF2,ORC1,GEN1,AFG1L,WDR35,NRDC,CDC40,PLK4,AMD1,ATAD2B,DHFR,CDCA7,PUS7,HDAC2,MCM2,CCNA2,EXOSC9,MLLT10,PMPCB,SAE1,CEP295,DNAJC2,RAD54L,CHORDC1,RNASEH2B,WDR33,SETDB2,SYMPK,MASTL,YME1L1,ZC3H13,COG3,TRDMT1,PCF11,CNOT10,DCLRE1C,NIFK,NUP85,MRPS7,MCM10,CAD,ELF1,ALG8,UPF2,MBD4,RSF1,PPM1G,DDX18,BORA,TSEN2,DIS3,CCNT2,PAXIP1,SNRPA,MCM6,DARS,FANCD2,XRN2,RBM17,ULK4,NCAPG2,RBM26,WDR43,CDC20,TK1,AFMID,BIRC5,NKTR,CRNKL1,EBNA1BP2,INTS10,FNTA,PARP2,PITRM1,POLR3F	darkgrey	EC_M8
GO:0000819	1.68E-43	1	86	168	sister chromatid segregation	BP	1.49E-40	LATS1,FBXO5,RAD21,DSCC1,KIFC1,SMC2,TTK,CDCA8,GEN1,KIF2C,NCAPG2,CDC20,TOP2B,SGO1,CDC16,NCAPH,CCNB1,ESPL1,BUB1,ANAPC1,RRS1,KNTC1,RACGAP1,ESCO2,ANAPC7,DSN1,BUB1B,KNSTRN,INO80,NUSAP1,UBE2C,NCAPD3,KIF18A,CUL3,NAA50,SMC3,CENPE,TRIP13,SGO2,KIF14,NEK2,PRC1,NSL1,CENPF,BUB3,NUF2,TPR,CDC27,KIF18B,CDCA5,SMC4,TACC3,DLGAP5,NCAPD2,ZWINT,DDX11,HNRNPU,INCENP,PDS5A,WAPL,TOP2A,CDC6,SMC1A,MAD1L1,NCAPG,KPNB1,KIF4A,KIF22,SPDL1,AURKB,ATRX,PTTG1,ZWILCH,LCMT1,KIF23,PLK1,ESCO1,NDC80,STAG2,NIPBL,RMI2,HASPIN,CDT1,CTCF,MAD2L1,FEN1	darkgrey	EC_M8
GO:0098813	1.18E-42	1	100	230	nuclear chromosome segregation	BP	9.93E-40	LATS1,FBXO5,RAD21,DSCC1,KIFC1,RMI1,CENPQ,SMC2,TTK,CDCA8,GEN1,KIF2C,FANCD2,NCAPG2,CDC20,TOP2B,SGO1,CDC16,NCAPH,CCNB1,ESPL1,BUB1,ANAPC1,RRS1,KNTC1,TERF1,RACGAP1,ESCO2,ANAPC7,DSN1,BUB1B,FAM83D,KNSTRN,ABRAXAS1,KNL1,CCNE2,INO80,NUSAP1,UBE2C,NCAPD3,KIF18A,CUL3,NAA50,SMC3,CENPE,TRIP13,SGO2,KIF14,SPO11,NEK2,PRC1,NSL1,CENPF,BUB3,NUF2,TPR,CDC27,KIF18B,CDCA5,FANCM,SMC4,TACC3,DLGAP5,NCAPD2,ECT2,ZWINT,DDX11,HNRNPU,INCENP,PDS5A,WAPL,TOP2A,CDC6,SMC1A,MAD1L1,NCAPG,KPNB1,KIF4A,KIF22,SPDL1,AURKB,EME1,ATRX,PTTG1,ZWILCH,LCMT1,KIF23,PLK1,BRIP1,ESCO1,NDC80,STAG2,NIPBL,RMI2,NDC1,HASPIN,CDT1,CTCF,MAD2L1,FEN1	darkgrey	EC_M8

GO:0006260	6.88E-41	1	99	230	DNA replication	BP	5.47E-38	CDK2,RPA3,FBXO5,DSCC1,RMI1,RECQL4,TONSL,MCM5,DBF4,POLH,MCM3,REV1,ZMPSTE24,ETAA1,MMS22L,ORC6,ORC1,GEN1,MCM2,CCNA2,DNAJC2,NASP,MCM10,MCM6,ORC4,GTPBP4,SUPT16H,NAP1L1,E2F7,GINS4,ALYREF,RRM1,POLE,RFC3,BRCA2,TOPBP1,MCM4,ATR,TERF1,NOC3L,ESCO2,NBN,TOP1,RAD51,CCNE2,E2F8,INO80,CHAF1B,RFC5,RBMS1,NUCKS1,GMNN,CHEK1,SMC3,TRAIP,ZGRF1,TICRR,RFC2,RPA2,ORC2,DTL,BLM,CDK1,ING5,DHX9,BAZ1A,POLA1,AP000944.2,RFC4,DNA2,DDX21,FANCM,DBF4B,CDC45,MCM7,WDHD1,BOD1L1,DDX11,PDS5A,WAPL,RFC1,CDC6,EME1,ATRX,TIPIN,RBBP6,ESCO1,THOC1,ATAD5,RMI2,CHAF1A,RPA1,CHTF18,CDT1,PRIM2,FAM111B,FEN1,RNF219,CINP	darkgrey	EC_M8
GO:0000280	3.19E-40	1	123	355	nuclear division	BP	2.41E-37	FBXO43,FBXO5,RAD21,DSCC1,KIFC1,CDC25C,RMI1,UBR2,CKS2,FOXJ3,SMC2,PHIP,TTK,ANLN,CDCA8,GEN1,RAD54L,CLASP2,KIF2C,BORA,FANCD2,NCAPG2,CDC20,TOP2B,SGO1,BRCA2,PSMD13,CDC16,NCAPH,CCNB1,ESPL1,BUB1,TPX2,ANAPC1,KIF20B,RRS1,LSM14A,KIF11,KNTC1,TERF1,MND1,RACGAP1,ANAPC7,DSN1,USP16,MSH3,BUB1B,CDCA2,KNSTRN,ABRAXAS1,RAD51,MYBL2,CCNE2,INO80,NUSAP1,UBE2C,NCAPD3,KIF18A,CUL3,CHEK1,NAA50,SMC3,CENPE,TRIP13,PPP2R2D,SGO2,KIF14,PRMT5,ASPM,RCC1,SPO11,NEK2,PRC1,NSL1,CENPF,BUB3,MKI67,NUF2,TPR,CDC27,KIF18B,CDCA5,FANCM,KIF15,SMC4,TACC3,DLGAP5,NCAPD2,RAD51AP1,ZWINT,HNRNPU,INCENP,PDS5A,TOP2A,CDC6,SMC1A,MAD1L1,CCNB2,NCAPG,KPNB1,KIF4A,KIF22,SPDL1,AURKB,EME1,ATRX,PTTG1,ZWILCH,LCMT1,KIF23,PLK1,BRIP1,NDC80,STAG2,NIPBL,NDC1,PKMYT1,HASPIN,FANCA,CDT1,MAD2L1,AURKA,DRG1	darkgrey	EC_M8
GO:0051301	1.55E-39	1	154	525	cell division	BP	1.12E-36	CDK2,KATNA1,LATS1,NUP43,FBXO5,RAD21,KIFC1,KIF20A,CDC25C,PLEC,HAUS6,CKS2,SMC2,ANLN,CDCA8,ITGB1BP1,CNTRL,ORC6,PLK4,CCNA2,SETDB2,MASTL,CENPA,CLASP2,KIF2C,BORA,CCNT2,NCAPG2,CDC20,BIRC5,E2F7,SGO1,PRPF40A,CKAP2,BRCA2,CDC16,NEDD1,NCAPH,CCNB1,HAUS5,ESPL1,BUB1,TPX2,ANAPC1,KIF20B,SKA3,CENPJ,KIF11,KNTC1,CEP55,TERF1,HELLS,CETN3,RACGAP1,ANAPC7,LRRCC1,DSN1,USP16,RBL1,BUB1B,MIS18A,SMYD5,CDCA2,FAM83D,CKAP5,TOP1,KNSTRN,KNL1,PKP4,CCNE2,E2F8,INO80,OIP5,NUSAP1,UBE2C,NCAPD3,CIT,CUL3,NSUN2,SMC3,CENPE,PPP2R2D,SGO2,HAUS2,KIF14,INTS13,ASPM,RCC1,SPC25,RAE1,NEK2,PRC1,TIAL1,NSL1,BLM,CENPF,BUB3,CDK1,CDC25A,SEPT2,NUF2,SETD2,TPR,CDC27,KIF18B,CDCA5,MIS18BP1,SMC4,CDCA3,HAUS3,TACC3,NCAPD2,ECT2,PIMREG,ZWINT,HNRNPU,INCENP,PDS5A,WAPL,CCNB3,TOP2A,CDC6,SMC1A,MAD1L1,CCNB2,NCAPG,KIF4A,PMF1,SPDL1,AURKB,PTTG1,TIPIN,ZWILCH,KIF23,SPC24,PLK1,BRIP1,AATF,NDC80,STAG2,CCNF,SAPCD2,CDT1,EVI5,IST1,PKN2,ERCC6L,MAD2L1,STMN1,ITGB3BP,AURKA,CENPW,RNF8,CINP	darkgrey	EC_M8
GO:0006281	4.03E-39	1	140	455	DNA repair	BP	2.77E-36	CDK2,RPA3,SHPRH,MDC1,UBR5,RAD21,XRCC6,FANCC,RMI1,RECQL4,TONSL,MCM5,POT1,APTX,POLH,MCM3,REV1,TRIM28,MSH2,ZMPSTE24,COMMD1,ETAA1,UFL1,MMS22L,USP45,CLSPN,ASCC3,DDX1,SMC6,GEN1,MCM2,RAD54L,WDR33,DCLRE1C,MBD4,PAXIP1,MCM6,FANCD2,PARP2,RAD18,EPC2,SUPT16H,RIF1,GINS4,POLE,RFC3,CUL4A,BRCA2,PPP4R2,TOPBP1,PRKDC,MCM4,ERCC6,PARPBP,PARP4,TDG,ATR,ESCO2,MCRS1,MSH3,NBN,ABRAXAS1,RAD51,DCLRE1B,ARID2,INO80,CHAF1B,RFC5,TAOK3,SMARCAD1,NUCKS1,DEK,UBE2T,CHEK1,SMC3,TRIP13,CHD1L,TRAIP,GTF2I,FANCI,FANCB,TICRR,RFC2,RPA2,SPO11,DTL,BLM,ATRIP,AUNIP,CDK1,DHX9,SETD2,POLA1,RFC4,DNA2,CDCA5,FANCM,C17orf53,APEX2,XRCC5,CDC45,BRCA1,MCM7,NSD2,WDHD1,RAD51AP1,BOD1L1,ANKLE1,FOXM1,FH,DDX11,EXO1,RAD52,PDS5A,RFC1,SMC1A,FUS,KIF22,USP3,EME1,ATRX,PTTG1,BRIP1,SMCHD1,CUL4B,NIPBL,TAOK1,RMI2,UHRF1,CHAF1A,RPA1,TP73,FANCA,TIGAR,RNF8,FEN1,PALB2,SFR1,CINP,RCHY1	darkgrey	EC_M8
GO:0000070	4.31E-39	1	75	143	mitotic sister chromatid segregation	BP	2.83E-36	FBXO5,RAD21,DSCC1,KIFC1,SMC2,TTK,CDCA8,GEN1,KIF2C,NCAPG2,CDC20,SGO1,CDC16,NCAPH,CCNB1,ESPL1,BUB1,ANAPC1,RRS1,KNTC1,RACGAP1,ANAPC7,DSN1,BUB1B,KNSTRN,INO80,NUSAP1,UBE2C,NCAPD3,KIF18A,CUL3,NAA50,SMC3,CENPE,TRIP13,SGO2,KIF14,NEK2,PRC1,NSL1,CENPF,BUB3,NUF2,TPR,CDC27,KIF18B,CDCA5,SMC4,TACC3,DLGAP5,NCAPD2,ZWINT,HNRNPU,INCENP,PDS5A,CDC6,SMC1A,MAD1L1,NCAPG,KPNB1,KIF4A,KIF22,SPDL1,AURKB,ATRX,PTTG1,ZWILCH,LCMT1,KIF23,PLK1,NDC80,NIPBL,HASPIN,CDT1,MAD2L1	darkgrey	EC_M8

GO:0140014	6.05E-39	1	99	244	mitotic nuclear division	BP	3.81E-36	FBXO43,FBXO5,RAD21,DSCC1,KIFC1,CDC25C,SMC2,PHIP,TTK,ANLN,CDCA8,GEN1,CLASP2,KIF2C,BORA,NCAPG2,CDC20,SGO1,CDC16,NCAPH,CCNB1,ESPL1,BUB1,TPX2,ANAPC1,KIF20B,RRS1,LSM14A,KIF11,KNTC1,RACGAP1,ANAPC7,DSN1,USP16,BUB1B,CDCA2,KNSTRN,ABRAXAS1,MYBL2,INO80,NUSAP1,UBE2C,NCAPD3,KIF18A,CUL3,CHEK1,NAA50,SMC3,CENPE,TRIP13,PPP2R2D,SGO2,KIF14,PRMT5,RCC1,NEK2,PRC1,NSL1,CENPF,BUB3,MKI67,NUF2,TPR,CDC27,KIF18B,CDCA5,KIF15,SMC4,TACC3,DLGAP5,NCAPD2,ZWINT,HNRNPU,INCENP,PDS5A,CDC6,SMC1A,MAD1L1,NCAPG,KPNB1,KIF4A,KIF22,SPDL1,AURKB,ATRX,PTTG1,ZWILCH,LCMT1,KIF23,PLK1,NDC80,STAG2,NIPBL,PKMYT1,HASPIN,CDT1,MAD2L1,AURKA,DRG1	darkgrey	EC_M8
GO:0006974	1.86E-38	1	180	699	cellular response to DNA damage stimulus	BP	1.12E-35	CDK2,BCLAF1,RPA3,SHPRH,MDC1,DDX39B,FBXO5,UBR5,GTSE1,RAD21,XRCC6,FANCC,RMI1,RECQL4,TONSL,MCM5,POT1,TOPORS,APTX,POLH,MCM3,REV1,TRIM28,MSH2,ZMPSTE24,COMMD1,ETAA1,UFL1,MMS22L,USP45,CLSPN,ASCC3,CASP2,DDX1,SMC6,GEN1,RINT1,MCM2,RAD54L,RNASEH2B,WDR33,MASTL,DDIAS,DCLRE1C,MCM10,MBD4,PAXIP1,MCM6,FANCD2,PARP2,RAD18,EPC2,SUPT16H,METTL3,RIF1,E2F7,GINS4,POLE,RFC3,CUL4A,BRCA2,PPP4R2,TOPBP1,PRKDC,CBX5,MCM4,ERCC6,MTREX,PARPBP,PARP4,TDG,TAF9,ATR,CDKN2AIP,ESCO2,MCRS1,USP16,MSH3,NBN,ABRAXAS1,RAD51,DCLRE1B,ARID2,INO80,EEF1E1,MDM4,CHAF1B,RFC5,TAOK3,SMARCAD1,NUCKS1,DEK,UBE2T,CHEK1,SMC3,TRIP13,CHD1L,TRAIP,GTF2I,FXR1,FANCI,DDX5,FANCB,TICRR,RFC2,RPA2,SPO11,INTS7,DTL,BLM,ATRIP,AUNIP,CDK1,BAZ1B,DHX9,SETD2,POLA1,RFC4,DNA2,CDCA5,FANCM,C17orf53,APEX2,XRCC5,CDC45,BRCA1,MCM7,NSD2,WDHD1,DCUN1D5,TIPRL,RAD51AP1,BOD1L1,ANKLE1,SUV39H1,FOXM1,FH,DDX11,EXO1,RAD52,PDS5A,NEDD4,RFC1,TOP2A,SMC1A,FUS,KIF22,USP3,EME1,TAF1,ATRX,PTTG1,TIPIN,STXBP4,RBBP6,PLK1,BRIP1,AATF,PSMD10,THOC1,SMCHD1,ATAD5,CUL4B,XIAP,NIPBL,TAOK1,RMI2,UHRF1,CHAF1A,FMR1,RPA1,TP73,FANCA,TIGAR,RNF8,FEN1,PALB2,SFR1,CINP,RCHY1	darkgrey	EC_M8
GO:0016070	1.32E-37	1	520	3480	RNA metabolic process	BP	7.68E-35	PAN2,NARS,ONECUT2,BCLAF1,CLK4,HNRNPH1,PPIL4,NUP107,ZFC3H1,POP1,DDX39B,SRFBP1,ZNF608,SCAF8,EIF3E,RAD21,RRP7A,HSF2,ATAD2,XRCC6,DDX46,PHF5A,TNRC6B,TUT7,SRPK1,SRSF3,PPIL1,PSIP1,RBM28,IFT74,PWP1,ZNF300,TOPORS,SMU1,POLR3B,XPO5,MTERF1,RFX3,CKS2,NOL8,TRIM28,NCBP1,ZNF451,PHF3,TWISTNB,LRPPRC,PPIH,FOXJ3,ZNF189,SP4,CDCA7L,PHIP,YTHDC1,HNRNPA2B1,PNPT1,PAPOLG,PUS10,ZMPSTE24,RLF,SYNCRIP,ZNF292,XPO1,PABPC4,COMMD1,MDN1,CASP8AP2,TIA1,ZNF350,ITGB1BP1,THRAP3,TAF1B,EZH2,UFL1,NOL10,CCNC,DDX1,NAA15,ELF2,CDC40,ATAD2B,CDCA7,PUS7,HDAC2,CCNA2,EXOSC9,MLLT10,DNAJC2,RNASEH2B,WDR33,SETDB2,SYMPK,ZC3H13,TRDMT1,PCF11,CNOT10,NIFK,NUP85,ELF1,UPF2,RSF1,DDX18,TSEN2,DIS3,CCNT2,PAXIP1,SNRPA,DARS,XRN2,RBM17,NCAPG2,RBM26,WDR43,BIRC5,CRNKL1,EBNA1BP2,INTS10,POLR3F,GTPBP4,EPC2,SUPT16H,CHD8,KAT6A,HNRNPL,METTL3,RIF1,E2F7,PRPF40A,ALYREF,CKAP2,ZMAT2,CEP290,NSD3,CEBPZ,SUPT20H,TAF7,EXOSC8,NR2C1,DDX51,MAK16,BRCA2,SNRPF,PPP4R2,LARS,ELK3,AC091959.3,RRP8,TCERG1,MAPK8,PRKDC,CBX5,CCNB1,ERCC6,NOP56,ZNF214,PAN3,MTREX,GPBP1,SP1,DHX37,UTP20,TCEA1,ZNF664,TGS1,BMS1,ZC3H8,POLR1B,DIMT1,RRS1,SGK3,BTAF1,ZNF143,WDR36,CENPJ,CDK5RAP1,PPWD1,FER,TDG,NCK1,SBNO1,SMARCA5,SREK1,RNF141,ZNF507,ABCE1,POLR1A,CHD1,ZFP91,TAF9,CGGBP1,XRN1,PDE12,SMN2,BDP1,TFCP2,SAP30,ZCCHC8,TTC37,U2SURP,GCFC2,UTP15,RBM12,HLTF,AQR,RBM39,ZBTB10,CNOT1,GABPA,MCRS1,USP16,PPID,RBL1,RPS6,CCNL1,URB1,SENP1,TENT2,ZNF638,VIR	darkgrey	EC_M8

GO:0044238	6.55E-37	1	911	7641	primary metabolic process	BP	3.67E-34	CDK2,PAN2,NARS,ONECUT2,MEX3C,BCLAF1,CLK4,RPA3,USP15,SHPRH,HNRNPH1,PPIL4,LATS1,NUP107,CCT2,MDC1,ZFC3H1,POP1,DDX39B,SRFBP1,FBXO43,FBXO5,CSNK1G3,ZNF608,SCAF8,UBR5,EIF3E,MAP3K4,RAD21,DSCC1,MRPL13,RRP7A,HSF2,ATAD2,XRCC6,DDX46,PHF5A,CDC25C,TNRC6B,FANCC,TUT7,NAA35,SRPK1,UHRF2,RMI1,SRSF3,PPIL1,PLEC,PSIP1,RECQL4,TONSL,RBM28,UBR2,CARNMT1,IFT74,MCM5,POT1,PWP1,ZNF300,TOPORS,APTX,SMU1,DBF4,POLR3B,XPO5,MTERF1,POLH,AKAP9,PUM3,RFX3,CKS2,MCM3,PPAT,C1GALT1,PAICS,ICK,REV1,NOL8,MELK,TRIM28,NCBP1,ZNF451,ANP32B,PHF3,TWISTNB,LRPPRC,PPIH,FOXJ3,ZNF189,MSH2,FBXO11,SP4,SENP6,ASB3,CDCA7L,CTPS1,PHIP,YTHDC1,MTIF2,TTK,PPP4R3B,IBTK,HNRNPA2B1,PNPT1,DCK,PAPOLG,PUS10,ZMPSTE24,RLF,USP34,SYNCRIP,ZNF292,XPO1,PABPC4,CCT4,COMMD1,MDN1,CASP8AP2,DPY19L1,FKBP15,ETAA1,FBXO48,TIA1,ZNF350,ITGB1BP1,THRAP3,TAF1B,EZH2,UFL1,NOL10,CUL1,MMS22L,USP45,FAM3C,MRPL32,CCNC,CLSPN,ASCC3,CASP2,ORC6,DDX1,NAA15,SMC6,ELF2,ORC1,GEN1,AFG1L,WDR35,NRDC,CDC40,PLK4,AMD1,ATAD2B,DHFR,CDCA7,PUS7,HDAC2,MCM2,CCNA2,EXOSC9,MLLT10,PMPCB,SAE1,CEP295,DNAJC2,RAD54L,CHORDC1,RNASEH2B,WDR33,PDSS1,SETDB2,SYMPK,MASTL,YME1L1,ZC3H13,COG3,TRDMT1,PCF11,CNOT10,DCLRE1C,NIFK,NUP85,MRPS7,MCM10,CAD,ELF1,ALG8,UPF2,MBD4,RSF1,PPM1G,DDX18,BORA,TSEN2,ACOX1,DIS3,CCNT2,PAXIP1,SNRPA,MCM6,DARS,FANCD2,XRN2,RBM17,ULK4,NCAPG2,RBM26,WDR43,CDC20,TK1,AFMID,BIRC5,NKTR,CRNKL1,EBNA1BP2,INTS10,FNTA,PARP2,PITRM1,P	darkgrey	EC_M8
GO:0022613	1.02E-36	1	118	376	ribonucleoprotein complex biogenesis	BP	5.49E-34	DDX39B,SRFBP1,EIF3E,RRP7A,SRPK1,PSIP1,PWP1,NOL8,NCBP1,YTHDC1,XPO1,MDN1,GNL2,NOL10,DDX1,EXOSC9,NIFK,MRPS7,DDX18,DIS3,XRN2,WDR43,CRNKL1,EBNA1BP2,GTPBP4,EXOSC8,CUL4A,DDX51,MAK16,SNRPF,RRP8,PRKDC,NOP56,DHX29,MTREX,DHX37,UTP20,TGS1,BMS1,DIMT1,RRS1,WDR36,ABCE1,TAF9,SMN2,ATR,GCFC2,UTP15,RPS6,URB1,SCAF11,WDR75,RIOK1,PAK1IP1,WDR3,HEATR3,NOLC1,PDCD11,RRP1B,RRP1,FP565260.1,SF3B1,RRP15,RBM5,HEATR1,PRMT5,DDX27,NOL11,GAR1,NGDN,EIF3A,URB2,NOP58,WDR12,FTSJ3,WBP11,DHX9,FASTKD2,DDX20,DDX21,PRPF39,RPL10L,NMD3,XRCC5,NOP14,SUV39H1,LYAR,EIF3B,LAS1L,SRSF5,EXOSC10,LUC7L3,UTP18,COIL,MRPL22,GEMIN5,AATF,RIOK3,EIF5,NLE1,UTP6,CUL4B,UTP14A,BRIX1,RSL1D1,DROSHA,METTL16,NOL9,EXOSC2,NOB1,RPF1,METTL18,GEMIN4,GEMIN6,NAF1,C1D,IMP4,TFB2M	darkgrey	EC_M8
GO:0006397	4.94E-36	1	125	407	mRNA processing	BP	2.57E-33	PAN2,HNRNPH1,DDX39B,SCAF8,DDX46,PHF5A,SRPK1,SRSF3,PPIL1,PSIP1,RBM28,SMU1,NCBP1,PPIH,YTHDC1,HNRNPA2B1,PNPT1,PAPOLG,SYNCRIP,TIA1,THRAP3,DDX1,CDC40,WDR33,SYMPK,ZC3H13,PCF11,TSEN2,SNRPA,XRN2,RBM17,RBM26,CRNKL1,HNRNPL,METTL3,PRPF40A,ALYREF,ZMAT2,SNRPF,PPP4R2,AC091959.3,TCERG1,CCNB1,PAN3,MTREX,TGS1,PPWD1,SREK1,PDE12,SMN2,ZCCHC8,GCFC2,AQR,RBM39,TENT2,VIRMA,SCAF11,PRPF4B,BCAS2,CWF19L1,PDCD11,SNRPA1,U2AF1,RRP1B,KHDRBS1,SF3B1,RBM5,CPSF2,RBM6,NCL,SNRNP40,PRMT5,ACIN1,FXR1,DDX5,WBP11,DHX9,HNRNPR,TRA2B,TSEN15,HNRNPA3,HNRNPH3,DDX20,PNN,PRPF39,EFTUD2,CWC22,CWF19L2,LEO1,HNRNPU,DDX39A,DHX15,SLTM,SRSF5,RBM25,LUC7L3,COIL,RBBP6,GEMIN5,THOC1,HNRNPM,THOC2,SAFB,SAFB2,RBMX,NSRP1,RBFOX1,FMR1,METTL16,SRRM2,NCBP3,RNPS1,DAZAP1,PTBP1,SF3B3,CMTR2,ZNF326,ZRANB2,SRSF11,RBM8A,SRRT,FASTKD5,GEMIN4,GEMIN6,SRSF7	darkgrey	EC_M8
GO:0071103	9.91E-36	1	86	200	DNA conformation change	BP	4.99E-33	SHPRH,DSCC1,XRCC6,SRPK1,RECQL4,MCM5,POT1,MTERF1,MCM3,ANP32B,SMC2,HNRNPA2B1,ASCC3,DDX1,MCM2,RAD54L,NASP,RSF1,MCM6,NCAPG2,CHD8,KAT6A,NAP1L1,TOP2B,RFC3,RRP8,NCAPH,CCNB1,MCM4,SMARCA5,CHD1,HELLS,MIS18A,NBN,TOP1,RAD51,OIP5,NUSAP1,CHAF1B,CHD9,NCAPD3,RFC5,SMARCAD1,NAP1L4,CHD1L,ACIN1,HJURP,RFC2,BLM,HAT1,CDK1,DHX9,RFC4,DNA2,CDCA5,FANCM,SMC4,XRCC5,MCM7,HP1BP3,NCAPD2,SUV39H1,DDX11,INCENP,WAPL,TOP2A,ASF1B,NCAPG,ATRX,PLK1,BRIP1,G3BP1,TOP3A,AIFM1,CHAF1A,UBN1,RPA1,CHTF18,CTCF,ERCC6L,HIST1H1A,HMGB3,ITGB3BP,HIST1H3H,CENPW,RNF8	darkgrey	EC_M8

GO:0048285	1.48E-35	1	125	401	organelle fission	BP	7.20E-33	MTFR2,FBXO43,FBXO5,RAD21,DSCC1,KIFC1,CDC25C,RMI1,UBR2,CKS2,FOXJ3,SMC2,PHIP,TTK,ANLN,CDCA8,GEN1,RAD54L,CLASP2,KIF2C,BORA,FANCD2,NCAPG2,CDC20,TOP2B,SGO1,BRCA2,PSMD13,CDC16,NCAPH,CCNB1,ESPL1,BUB1,TPX2,ANAPC1,KIF20B,RRS1,LSM14A,KIF11,KNTC1,TERF1,MND1,RACGAP1,ANAPC7,DSN1,USP16,MSH3,BUB1B,CDCA2,KNSTRN,ABRAXAS1,RAD51,MYBL2,CCNE2,INO80,NUSAP1,UBE2C,NCAPD3,KIF18A,CUL3,CHEK1,NAA50,SMC3,CENPE,TRIP13,PPP2R2D,SGO2,KIF14,PRMT5,ASPM,RCC1,SPO11,NEK2,PRC1,NSL1,CENPF,BUB3,MKI67,NUF2,TPR,CDC27,KIF18B,CDCA5,FANCM,KIF15,SMC4,TACC3,DLGAP5,NCAPD2,RAD51AP1,ZWINT,HNRNPU,INCENP,PDS5A,TOP2A,CDC6,SMC1A,MAD1L1,CCNB2,NCAPG,KPNB1,KIF4A,KIF22,SPDL1,AURKB,EME1,ATRX,PTTG1,ZWILCH,LCMT1,KIF23,PLK1,BRIP1,NDC80,STAG2,NIPBL,NDC1,PKMYT1,HASPIN,FANCA,CDT1,DHODH,MAD2L1,AURKA,DRG1	darkgrey	EC_M8
GO:0010564	5.11E-35	1	145	515	regulation of cell cycle process	BP	2.41E-32	CDK2,MDC1,DDX39B,FBXO43,FBXO5,GTSE1,RAD21,KIF20A,CDC25C,DBF4,MSH2,SENP6,PHIP,TTK,ZMPSTE24,XPO1,ETAA1,ANLN,EZH2,CLSPN,CASP2,ORC1,GEN1,PLK4,RINT1,STIL,CEP295,CHORDC1,RNASEH2B,BORA,PAXIP1,FANCD2,CDC20,E2F7,PRPF40A,CUL4A,BRCA2,TOPBP1,CDC16,PRKDC,CCNB1,ERCC6,ESPL1,BUB1,TPX2,ANAPC1,KIF20B,CENPJ,KIF11,KNTC1,ATR,TERF1,RACGAP1,ANAPC7,RBL1,ALMS1,BUB1B,CDCA2,NBN,FAM83D,KNSTRN,ABRAXAS1,PKP4,E2F8,INO80,NUSAP1,UBE2C,TAOK3,CIT,CUL3,NSUN2,CHEK1,CENPE,TRIP13,KIF14,PRMT5,RCC1,TICRR,RPA2,RAE1,NEK2,PRC1,INTS7,DTL,BLM,ATRIP,CENPF,TXLNG,BUB3,CDK1,CDC25A,MKI67,SETD2,DNA2,TPR,CDC27,CDCA5,DBF4B,KIF15,CDC45,BRCA1,PSME3,TACC3,DLGAP5,TIPRL,RAD51AP1,ECT2,ZWINT,DDX11,HNRNPU,INCENP,WAPL,CCNB3,CDC6,MAD1L1,SPDL1,AURKB,EME1,TAF1,ATRX,PTTG1,TIPIN,ZWILCH,STXBP4,LCMT1,KIF23,TRIM37,PLK1,BRIP1,THOC1,NDC80,ATAD5,CEP76,TAOK1,RMI2,PKMYT1,CCNF,CDT1,PKN2,CTCF,TMOD3,MAD2L1,AURKA,FEN1,DRG1	darkgrey	EC_M8
GO:0016071	1.79E-34	1	154	586	mRNA metabolic process	BP	8.19E-32	PAN2,HNRNPH1,DDX39B,SCAF8,EIF3E,DDX46,PHF5A,TNRC6B,TUT7,SRPK1,SRSF3,PPIL1,PSIP1,RBM28,SMU1,NCBP1,PPIH,YTHDC1,HNRNPA2B1,PNPT1,PAPOLG,SYNCRIP,PABPC4,TIA1,THRAP3,DDX1,CDC40,PUS7,EXOSC9,WDR33,SYMPK,ZC3H13,PCF11,CNOT10,UPF2,TSEN2,DIS3,SNRPA,XRN2,RBM17,RBM26,CRNKL1,HNRNPL,METTL3,PRPF40A,ALYREF,ZMAT2,EXOSC8,SNRPF,PPP4R2,AC091959.3,TCERG1,CCNB1,PAN3,MTREX,TGS1,PPWD1,SREK1,XRN1,PDE12,SMN2,TFCP2,ZCCHC8,TTC37,GCFC2,AQR,RBM39,CNOT1,TENT2,VIRMA,SCAF11,PRPF4B,BCAS2,CWF19L1,NSUN2,PDCD11,SNRPA1,U2AF1,RRP1B,KHDRBS1,SF3B1,RBM5,CPSF2,RBM6,NCL,SNRNP40,PRMT5,ACIN1,FXR1,DDX5,FASTKD1,SSB,WBP11,DHX9,HNRNPR,TRA2B,TSEN15,FASTKD2,HNRNPA3,HNRNPH3,DDX20,PNN,PRPF39,EFTUD2,CWC22,CWF19L2,CNOT9,LEO1,HNRNPU,DCP2,DDX39A,DHX15,SLTM,SRSF5,EXOSC10,RBM25,FUS,LUC7L3,COIL,RBBP6,GEMIN5,LARP1,TAF15,THOC1,UPF3B,HNRNPM,SERBP1,THOC2,SAFB,SAFB2,GSPT1,RBMX,NSRP1,RBFOX1,FMR1,METTL16,SRRM2,NCBP3,RNPS1,DAZAP1,PTBP1,EXOSC2,SF3B3,CMTR2,ZNF326,ZRANB2,SRSF11,RBM8A,SRRT,FASTKD5,GEMIN4,HNRNPD,GEMIN6,SRSF7	darkgrey	EC_M8
GO:0051726	3.54E-32	1	188	827	regulation of cell cycle	BP	1.57E-29	CDK2,YTHDC2,RPA3,LATS1,MDC1,DDX39B,FBXO43,FBXO5,GTSE1,RAD21,KIF20A,CDC25C,UHRF2,DBF4,CKS2,MSH2,SENP6,PHIP,TTK,PNPT1,ZMPSTE24,XPO1,ETAA1,ANLN,EZH2,CLSPN,CASP2,ORC1,GEN1,PLK4,RINT1,STIL,CCNA2,CEP295,CHORDC1,RNASEH2B,MASTL,PPM1G,BORA,CCNT2,PAXIP1,FANCD2,CDC20,BIRC5,GTPBP4,IPO5,METTL3,E2F7,PRPF40A,CUL4A,BRCA2,TOPBP1,RRP8,CDC16,PRKDC,CCNB1,ERCC6,ESPL1,BUB1,TPX2,ANAPC1,KIF20B,IPO7,CENPJ,CDK5RAP1,KIF11,KNTC1,ATR,TERF1,RACGAP1,ANAPC7,USP16,RBL1,ALMS1,BUB1B,CCNL1,CDCA2,NBN,FAM83D,KNSTRN,ABRAXAS1,PKP4,CCNE2,E2F8,INO80,NUSAP1,ZBED6,MDM4,UBE2C,HERC5,TAOK3,CIT,CUL3,NSUN2,GMNN,CHEK1,CENPE,TRIP13,KIF14,PRMT5,INTS13,ASPM,ZBED9,RCC1,TICRR,RPA2,RAE1,NEK2,PRC1,INTS7,DTL,BLM,WDR12,ATRIP,CENPF,TXLNG,BUB3,CDKL5,CDK1,CDC25A,FOSL1,MKI67,SETD2,DNA2,TPR,CDC27,CDCA5,DBF4B,KIF15,IL12A,CDC45,BRCA1,PSME3,CDKN3,TACC3,DLGAP5,TIPRL,RAD51AP1,ECT2,ZWINT,FOXM1,DDX11,HNRNPU,INCENP,WAPL,CCNB3,CDC6,LIN9,MAD1L1,SRSF5,CCNB2,YY1AP1,SPDL1,AURKB,EME1,TAF1,ATRX,PTTG1,TIPIN,ZWILCH,STXBP4,LCMT1,KIF23,TRIM37,PRR11,PLK1,BRIP1,AATF,PSMD10,NLE1,THOC1,NDC80,ATAD5,XIAP,CEP76,TAOK1,RMI2,PKMYT1,CCNF,TP73,CDT1,PKN2,CTCF,TMOD3,MAD2L1,AURKA,FEN1,DRG1	darkgrey	EC_M8

GO:0008380	5.21E-32	1	109	356	RNA splicing	BP	2.25E-29	CLK4,HNRNPH1,DDX39B,DDX46,PHF5A,SRPK1,SRSF3,PPIL1,PSIP1,RBM28,SMU1,NCBP1,PPIH,YTHDC1,HNRNPA2B1,SYNCRIP,TIA1,THRAP3,DDX1,CDC40,ZC3H13,TSEN2,SNRPA,RBM17,CRNKL1,HNRNPL,METTL3,PRPF40A,ALYREF,ZMAT2,SNRPF,PPP4R2,TCERG1,MTREX,TGS1,PPWD1,SREK1,SMN2,ZCCHC8,GCFC2,RBM12,AQR,RBM39,ZNF638,VIRMA,SLC38A2,SCAF11,PRPF4B,BCAS2,CWF19L1,ZBTB8OS,SNRPA1,U2AF1,RRP1B,KHDRBS1,SF3B1,RBM5,RBM6,NCL,SNRNP40,PRMT5,ACIN1,FXR1,DDX5,PPIG,WBP11,DHX9,HNRNPR,TRA2B,TSEN15,HNRNPA3,IVNS1ABP,HNRNPH3,DDX20,PNN,PRPF39,EFTUD2,CWC22,CWF19L2,HNRNPU,DDX39A,DHX15,SRSF5,RBM25,FUS,CLK2,LUC7L3,COIL,GEMIN5,THOC1,HNRNPM,THOC2,RBMX,NSRP1,RBFOX1,FMR1,METTL16,SRRM2,RNPS1,DAZAP1,PTBP1,SF3B3,ZNF326,ZRANB2,SRSF11,RBM8A,GEMIN4,GEMIN6,SRSF7	darkgrey	EC_M8
GO:0042254	6.70E-32	1	89	256	ribosome biogenesis	BP	2.81E-29	SRFBP1,RRP7A,PWP1,NOL8,XPO1,MDN1,GNL2,NOL10,EXOSC9,NIFK,MRPS7,DDX18,DIS3,XRN2,WDR43,EBNA1BP2,GTPBP4,EXOSC8,CUL4A,DDX51,MAK16,RRP8,PRKDC,NOP56,DHX29,MTREX,DHX37,UTP20,BMS1,DIMT1,RRS1,WDR36,ABCE1,UTP15,RPS6,URB1,WDR75,RIOK1,PAK1IP1,WDR3,HEATR3,NOLC1,PDCD11,RRP1B,RRP1,FP565260.1,RRP15,HEATR1,DDX27,NOL11,GAR1,NGDN,URB2,NOP58,WDR12,FTSJ3,WBP11,FASTKD2,DDX21,RPL10L,NMD3,XRCC5,NOP14,SUV39H1,LYAR,LAS1L,EXOSC10,UTP18,MRPL22,AATF,RIOK3,NLE1,UTP6,CUL4B,UTP14A,BRIX1,RSL1D1,DROSHA,METTL16,NOL9,EXOSC2,NOB1,RPF1,METTL18,GEMIN4,NAF1,C1D,IMP4,TFB2M	darkgrey	EC_M8
GO:0006261	2.88E-31	1	66	137	DNA-dependent DNA replication	BP	1.18E-28	FBXO5,DSCC1,RECQL4,TONSL,MCM5,DBF4,MCM3,ZMPSTE24,ETAA1,MMS22L,ORC6,ORC1,GEN1,MCM2,MCM10,MCM6,ORC4,E2F7,GINS4,ALYREF,POLE,RFC3,BRCA2,TOPBP1,MCM4,ATR,TERF1,NOC3L,NBN,RAD51,CCNE2,E2F8,INO80,RFC5,NUCKS1,GMNN,TRAIP,ZGRF1,TICRR,RFC2,ORC2,BLM,BAZ1A,POLA1,AP000944.2,RFC4,DNA2,DDX21,FANCM,DBF4B,CDC45,MCM7,WDHD1,BOD1L1,DDX11,RFC1,CDC6,EME1,ATRX,TIPIN,THOC1,RPA1,CHTF18,CDT1,PRIM2,FEN1	darkgrey	EC_M8
GO:0071704	2.96E-31	1	931	8083	organic substance metabolic process	BP	1.18E-28	CDK2,PAN2,NARS,ONECUT2,MEX3C,ME2,BCLAF1,CLK4,RPA3,USP15,SHPRH,HNRNPH1,PPIL4,LATS1,MTHFD1L,NUP107,CCT2,MDC1,ZFC3H1,POP1,DDX39B,SRFBP1,FBXO43,FBXO5,CSNK1G3,ZNF608,SCAF8,UBR5,EIF3E,MAP3K4,RAD21,DSCC1,MRPL13,RRP7A,HSF2,ATAD2,XRCC6,DDX46,PHF5A,SLC35D2,CDC25C,TNRC6B,FANCC,TUT7,NAA35,MATR3,SRPK1,UHRF2,RMI1,SRSF3,PPIL1,PLEC,PSIP1,RECQL4,TONSL,RBM28,UBR2,CARNMT1,IFT74,MCM5,POT1,PWP1,ZNF300,TOPORS,APTX,SMU1,DBF4,POLR3B,XPO5,MTERF1,POLH,AKAP9,PUM3,RFX3,IL1R1,CKS2,MCM3,PPAT,C1GALT1,PAICS,ICK,REV1,NOL8,MELK,TRIM28,NCBP1,ZNF451,ANP32B,PHF3,TWISTNB,LRPPRC,PPIH,FOXJ3,ZNF189,MSH2,FBXO11,SP4,SENP6,ASB3,CDCA7L,CTPS1,PHIP,YTHDC1,MTIF2,TTK,PPP4R3B,IBTK,HNRNPA2B1,PNPT1,DCK,PAPOLG,PUS10,ZMPSTE24,RLF,USP34,SYNCRIP,ZNF292,XPO1,PABPC4,CCT4,COMMD1,MDN1,CASP8AP2,DPY19L1,FKBP15,ETAA1,FBXO48,TIA1,ZNF350,ITGB1BP1,THRAP3,TAF1B,EZH2,UFL1,NOL10,CUL1,MMS22L,USP45,FAM3C,MRPL32,CCNC,CLSPN,ASCC3,CASP2,ORC6,DDX1,NAA15,SMC6,ELF2,ORC1,GEN1,AFG1L,WDR35,NRDC,CDC40,PLK4,AMD1,ATAD2B,DHFR,CDCA7,PUS7,HDAC2,MCM2,CCNA2,EXOSC9,MLLT10,PMPCB,SAE1,CEP295,DNAJC2,RAD54L,CHORDC1,RNASEH2B,WDR33,PDSS1,SETDB2,SYMPK,MASTL,YME1L1,ZC3H13,NASP,COG3,TRDMT1,PCF11,CNOT10,DCLRE1C,NIFK,NUP85,MRPS7,MCM10,CAD,ELF1,ALG8,UPF2,MBD4,RSF1,PPM1G,DDX18,BORA,TSEN2,ACOX1,DIS3,CCNT2,PAXIP1,SNRPA,MCM6,DARS,FANCD2,XRN2,RBM17,ULK4,NCAPG2,RBM26,WDR43,CDC20,TK1,AFMID,BIRC5,NKTR,CRNKL	darkgrey	EC_M8

GO:0044237	3.13E-31	1	916	7918	cellular metabolic process	BP	1.21E-28	CDK2,PAN2,NARS,ONECUT2,MEX3C,ME2,MTFR2,BCLAF1,CLK4,RPA3,USP15,SHPRH,HNRNPH1,PPIL4,LATS1,MTHFD1L,NUP107,CCT2,MDC1,ZFC3H1,POP1,DDX39B,SRFBP1,FBXO43,FBXO5,CSNK1G3,ZNF608,SCAF8,UBR5,EIF3E,MAP3K4,RAD21,DSCC1,MRPL13,RRP7A,HSF2,ATAD2,XRCC6,DDX46,PHF5A,SLC35D2,CDC25C,TNRC6B,FANCC,HSPA9,TUT7,NAA35,SRPK1,UHRF2,RMI1,SRSF3,PPIL1,PLEC,PSIP1,RECQL4,TONSL,RBM28,UBR2,CARNMT1,IFT74,MCM5,POT1,PWP1,ZNF300,TOPORS,APTX,SMU1,DBF4,POLR3B,XPO5,MTERF1,POLH,AKAP9,PUM3,RFX3,CKS2,MCM3,PPAT,C1GALT1,PAICS,ICK,REV1,NOL8,MELK,TRIM28,NCBP1,ZNF451,ANP32B,PHF3,TWISTNB,LRPPRC,STX17,PPIH,FOXJ3,ZNF189,MSH2,FBXO11,SP4,SENP6,ASB3,CDCA7L,CTPS1,PHIP,YTHDC1,MTIF2,TTK,PPP4R3B,IBTK,HNRNPA2B1,PNPT1,DCK,CYB5R4,PAPOLG,PUS10,ZMPSTE24,RLF,USP34,SYNCRIP,ZNF292,XPO1,PABPC4,CCT4,COMMD1,MDN1,CASP8AP2,DPY19L1,FKBP15,ETAA1,FBXO48,TIA1,ZNF350,ITGB1BP1,THRAP3,TAF1B,EZH2,UFL1,NOL10,CUL1,MMS22L,USP45,FAM3C,MRPL32,CCNC,CLSPN,ASCC3,CASP2,ORC6,DDX1,NAA15,SMC6,ELF2,ORC1,GEN1,AFG1L,WDR35,NRDC,CDC40,PLK4,AMD1,ATAD2B,DHFR,CDCA7,PUS7,HDAC2,MCM2,NT5DC1,CCNA2,EXOSC9,MLLT10,PMPCB,SAE1,CEP295,DNAJC2,RAD54L,CHORDC1,RNASEH2B,WDR33,PDSS1,SETDB2,SYMPK,MASTL,YME1L1,ZC3H13,NASP,COG3,TRDMT1,PCF11,CNOT10,DCLRE1C,NIFK,NUP85,MRPS7,MCM10,CAD,ELF1,ALG8,UPF2,MBD4,RSF1,PPM1G,DDX18,BORA,TSEN2,ACOX1,DIS3,CCNT2,PAXIP1,SNRPA,MCM6,DARS,FANCD2,XRN2,RBM17,ULK4,NCAPG2,RBM26,WDR43,CDC20,TK	darkgrey	EC_M8
GO:0010467	2.57E-30	1	596	4455	gene expression	BP	9.70E-28	CDK2,PAN2,NARS,ONECUT2,BCLAF1,CLK4,HNRNPH1,PPIL4,NUP107,ZFC3H1,POP1,DDX39B,SRFBP1,ZNF608,SCAF8,UBR5,EIF3E,MAP3K4,RAD21,MRPL13,RRP7A,HSF2,ATAD2,XRCC6,DDX46,PHF5A,TNRC6B,TUT7,NAA35,MATR3,SRPK1,SRSF3,PPIL1,PLEC,PSIP1,RBM28,IFT74,PWP1,ZNF300,TOPORS,SMU1,POLR3B,XPO5,MTERF1,PUM3,RFX3,IL1R1,CKS2,NOL8,TRIM28,NCBP1,ZNF451,PHF3,TWISTNB,LRPPRC,PPIH,FOXJ3,ZNF189,MSH2,SP4,CDCA7L,PHIP,YTHDC1,MTIF2,HNRNPA2B1,PNPT1,PAPOLG,PUS10,ZMPSTE24,RLF,SYNCRIP,ZNF292,XPO1,PABPC4,COMMD1,MDN1,CASP8AP2,TIA1,ZNF350,ITGB1BP1,THRAP3,TAF1B,EZH2,UFL1,NOL10,MRPL32,CCNC,ASCC3,CASP2,DDX1,NAA15,ELF2,WDR35,CDC40,ATAD2B,DHFR,CDCA7,PUS7,HDAC2,CCNA2,EXOSC9,MLLT10,PMPCB,DNAJC2,RNASEH2B,WDR33,SETDB2,SYMPK,YME1L1,ZC3H13,TRDMT1,PCF11,CNOT10,NIFK,NUP85,MRPS7,ELF1,UPF2,RSF1,DDX18,TSEN2,DIS3,CCNT2,PAXIP1,SNRPA,DARS,XRN2,RBM17,NCAPG2,RBM26,WDR43,BIRC5,CRNKL1,EBNA1BP2,INTS10,PITRM1,POLR3F,GTPBP4,EPC2,SUPT16H,CHD8,KAT6A,HNRNPL,METTL3,RIF1,E2F7,PRPF40A,ALYREF,CKAP2,ZMAT2,CEP290,NSD3,CEBPZ,SUPT20H,TAF7,EXOSC8,NR2C1,DDX51,MAK16,METAP2,BRCA2,SNRPF,PPP4R2,LARS,ELK3,AC091959.3,RRP8,TCERG1,MAPK8,PRKDC,CBX5,CCNB1,ERCC6,NOP56,NFKBID,ZNF214,DHX29,PAN3,MTREX,GPBP1,SP1,DHX37,UTP20,TCEA1,MRPL15,ZNF664,TGS1,MRPS5,CAPRIN1,BMS1,ZC3H8,POLR1B,DIMT1,RRS1,LSM14A,SGK3,BTAF1,ZNF143,WDR36,CENPJ,CDK5RAP1,PPWD1,FER,EIF2B1,TDG,NCK1,SBNO1,SMARCA5,MRPL35,SREK1,RNF141,PTCD3,ZNF507,ABCE1,POLR1A,C	darkgrey	EC_M8
GO:0034660	2.39E-29	1	114	412	ncRNA metabolic process	BP	8.82E-27	NARS,POP1,SRFBP1,RRP7A,TUT7,PWP1,XPO5,NOL8,HNRNPA2B1,PNPT1,PUS10,MDN1,NOL10,DDX1,PUS7,EXOSC9,TRDMT1,NIFK,DDX18,TSEN2,DIS3,DARS,XRN2,WDR43,EBNA1BP2,INTS10,GTPBP4,METTL3,EXOSC8,DDX51,MAK16,LARS,RRP8,PRKDC,NOP56,MTREX,DHX37,UTP20,BMS1,DIMT1,RRS1,WDR36,CDK5RAP1,XRN1,ZCCHC8,UTP15,RPS6,URB1,TENT2,WDR75,RIOK1,WDR3,NOLC1,NSUN2,PDCD11,YARS,TRMT10A,ZBTB8OS,RRP1B,RRP1,FP565260.1,NFKB1,QTRT2,RRP15,HEATR1,IARS2,DDX27,NOL11,GAR1,TARBP1,NGDN,INTS7,SSB,NOP58,WDR12,FTSJ3,WBP11,TSEN15,DDX21,DGCR8,DARS2,NOP14,SUV39H1,LYAR,MRPL44,SEPSECS,LAS1L,EXOSC10,RARS,DIS3L,UTP18,THG1L,INTS2,THUMPD1,RIOK3,UTP6,UTP14A,BRIX1,RSL1D1,DROSHA,METTL16,NOL9,EXOSC2,NOB1,RPF1,METTL18,SRRT,GEMIN4,INTS6,NAF1,C1D,DTD2,IMP4,TFB2M	darkgrey	EC_M8
GO:0016072	1.53E-28	1	75	206	rRNA metabolic process	BP	5.49E-26	SRFBP1,RRP7A,PWP1,NOL8,MDN1,NOL10,EXOSC9,NIFK,DDX18,DIS3,XRN2,WDR43,EBNA1BP2,GTPBP4,EXOSC8,DDX51,MAK16,RRP8,PRKDC,NOP56,MTREX,DHX37,UTP20,BMS1,DIMT1,RRS1,WDR36,XRN1,UTP15,RPS6,URB1,WDR75,RIOK1,WDR3,NOLC1,PDCD11,RRP1B,RRP1,FP565260.1,RRP15,HEATR1,DDX27,NOL11,GAR1,NGDN,NOP58,WDR12,FTSJ3,WBP11,DDX21,NOP14,SUV39H1,LYAR,MRPL44,LAS1L,EXOSC10,DIS3L,UTP18,RIOK3,UTP6,UTP14A,BRIX1,RSL1D1,DROSHA,METTL16,NOL9,EXOSC2,NOB1,RPF1,METTL18,GEMIN4,NAF1,C1D,IMP4,TFB2M	darkgrey	EC_M8

GO:0044260	1.81E-28	1	740	5916	cellular macromolecule metabolic process	BP	6.34E-26	CDK2,PAN2,NARS,ONECUT2,MEX3C,BCLAF1,CLK4,RPA3,USP15,SHPRH,PPIL4,LATS1,NUP107,CCT2,MDC1,POP1,DDX39B,FBXO43,FBXO5,CSNK1G3,ZNF608,SCAF8,UBR5,EIF3E,MAP3K4,RAD21,DSCC1,MRPL13,HSF2,ATAD2,XRCC6,PHF5A,CDC25C,TNRC6B,FANCC,TUT7,NAA35,SRPK1,UHRF2,RMI1,PPIL1,PLEC,PSIP1,RECQL4,TONSL,UBR2,IFT74,MCM5,POT1,PWP1,ZNF300,TOPORS,APTX,DBF4,POLR3B,MTERF1,POLH,AKAP9,PUM3,RFX3,CKS2,MCM3,C1GALT1,ICK,REV1,MELK,TRIM28,NCBP1,ZNF451,ANP32B,PHF3,TWISTNB,LRPPRC,PPIH,FOXJ3,ZNF189,MSH2,FBXO11,SP4,SENP6,ASB3,CDCA7L,PHIP,MTIF2,TTK,PPP4R3B,IBTK,HNRNPA2B1,PNPT1,ZMPSTE24,RLF,USP34,SYNCRIP,ZNF292,XPO1,PABPC4,CCT4,COMMD1,CASP8AP2,DPY19L1,FKBP15,ETAA1,FBXO48,TIA1,ZNF350,ITGB1BP1,THRAP3,TAF1B,EZH2,UFL1,CUL1,MMS22L,USP45,MRPL32,CCNC,CLSPN,ASCC3,CASP2,ORC6,DDX1,NAA15,SMC6,ELF2,ORC1,GEN1,AFG1L,WDR35,NRDC,PLK4,ATAD2B,DHFR,CDCA7,PUS7,HDAC2,MCM2,CCNA2,EXOSC9,MLLT10,PMPCB,SAE1,CEP295,DNAJC2,RAD54L,CHORDC1,RNASEH2B,WDR33,SETDB2,SYMPK,MASTL,YME1L1,ZC3H13,NASP,COG3,TRDMT1,PCF11,CNOT10,DCLRE1C,NIFK,NUP85,MRPS7,MCM10,CAD,ELF1,ALG8,UPF2,MBD4,RSF1,PPM1G,BORA,DIS3,CCNT2,PAXIP1,MCM6,DARS,FANCD2,XRN2,ULK4,NCAPG2,WDR43,CDC20,TK1,BIRC5,NKTR,FNTA,PARP2,POLR3F,KAT14,ORC4,GTPBP4,RAD18,IPO5,EPC2,SUPT16H,CHD8,KAT6A,METTL3,RIF1,NAP1L1,TOP2B,ZDHHC17,E2F7,GINS4,ALYREF,CKAP2,VPS36,RRM1,TPP2,CEP290,NSD3,CEBPZ,KITLG,SUPT20H,TAF7,EXOSC8,PRKD3,POLE,NR2C1,RFC3,CUL4A,METAP2,BRCA2,PPP4R2	darkgrey	EC_M8
GO:0044770	7.00E-28	1	117	426	cell cycle phase transition	BP	2.40E-25	CDK2,LATS1,MDC1,DDX39B,FBXO5,GTSE1,RAD21,CDC25C,DBF4,CKS2,PPAT,MELK,MSH2,TTK,ETAA1,ANLN,EZH2,CUL1,CLSPN,CASP2,ORC1,GEN1,RINT1,CCNA2,RNASEH2B,MASTL,NASP,CLASP2,PAXIP1,FANCD2,E2F7,POLE,CUL4A,TOPBP1,CDC16,PRKDC,CCNB1,ERCC6,ESPL1,BUB1,ANAPC1,KNTC1,ATR,ANAPC7,RBL1,RPS6,BUB1B,NBN,FAM83D,ABRAXAS1,CCNE2,INO80,UBE2C,TAOK3,CUL3,NSUN2,CHEK1,KHDRBS1,CENPE,TRIP13,PPP2R2D,KIF14,BCAT1,RCC1,TICRR,RPA2,INTS7,DTL,BLM,ATRIP,CENPF,BUB3,CDK1,CDC25A,DNA2,TPR,CDC27,CDCA5,DBF4B,NPAT,CDC45,BRCA1,CUL5,PSME3,CDKN3,TACC3,DLGAP5,TIPRL,ZWINT,FOXM1,CCNB3,CDC6,MAD1L1,CCNB2,IQGAP3,SPDL1,AURKB,EME1,TAF1,TIPIN,ZWILCH,STXBP4,LCMT1,PLK1,BRIP1,THOC1,NDC80,ATAD5,CUL4B,GSPT1,TAOK1,PKMYT1,CCNF,CDT1,TMOD3,MAD2L1,AURKA	darkgrey	EC_M8
GO:0000377	1.54E-27	1	84	255	RNA splicing, via transesterification reactions with bulged adenosine as nucleophile	BP	5.05E-25	HNRNPH1,DDX39B,DDX46,PHF5A,SRPK1,SRSF3,PPIL1,PSIP1,SMU1,NCBP1,PPIH,YTHDC1,HNRNPA2B1,SYNCRIP,TIA1,THRAP3,DDX1,CDC40,SNRPA,RBM17,CRNKL1,HNRNPL,METTL3,PRPF40A,ALYREF,ZMAT2,SNRPF,MTREX,TGS1,PPWD1,SMN2,ZCCHC8,GCFC2,AQR,RBM39,SCAF11,PRPF4B,BCAS2,CWF19L1,SNRPA1,U2AF1,KHDRBS1,SF3B1,RBM5,RBM6,NCL,SNRNP40,PRMT5,FXR1,DDX5,WBP11,DHX9,HNRNPR,TRA2B,HNRNPA3,HNRNPH3,DDX20,PNN,PRPF39,EFTUD2,CWC22,CWF19L2,HNRNPU,DDX39A,SRSF5,RBM25,LUC7L3,COIL,GEMIN5,HNRNPM,RBMX,NSRP1,RBFOX1,FMR1,METTL16,SRRM2,RNPS1,DAZAP1,PTBP1,SF3B3,RBM8A,GEMIN4,GEMIN6,SRSF7	darkgrey	EC_M8
GO:0000398	1.54E-27	1	84	255	mRNA splicing, via spliceosome	BP	5.05E-25	HNRNPH1,DDX39B,DDX46,PHF5A,SRPK1,SRSF3,PPIL1,PSIP1,SMU1,NCBP1,PPIH,YTHDC1,HNRNPA2B1,SYNCRIP,TIA1,THRAP3,DDX1,CDC40,SNRPA,RBM17,CRNKL1,HNRNPL,METTL3,PRPF40A,ALYREF,ZMAT2,SNRPF,MTREX,TGS1,PPWD1,SMN2,ZCCHC8,GCFC2,AQR,RBM39,SCAF11,PRPF4B,BCAS2,CWF19L1,SNRPA1,U2AF1,KHDRBS1,SF3B1,RBM5,RBM6,NCL,SNRNP40,PRMT5,FXR1,DDX5,WBP11,DHX9,HNRNPR,TRA2B,HNRNPA3,HNRNPH3,DDX20,PNN,PRPF39,EFTUD2,CWC22,CWF19L2,HNRNPU,DDX39A,SRSF5,RBM25,LUC7L3,COIL,GEMIN5,HNRNPM,RBMX,NSRP1,RBFOX1,FMR1,METTL16,SRRM2,RNPS1,DAZAP1,PTBP1,SF3B3,RBM8A,GEMIN4,GEMIN6,SRSF7	darkgrey	EC_M8
GO:0000375	4.78E-27	1	84	259	RNA splicing, via transesterification reactions	BP	1.54E-24	HNRNPH1,DDX39B,DDX46,PHF5A,SRPK1,SRSF3,PPIL1,PSIP1,SMU1,NCBP1,PPIH,YTHDC1,HNRNPA2B1,SYNCRIP,TIA1,THRAP3,DDX1,CDC40,SNRPA,RBM17,CRNKL1,HNRNPL,METTL3,PRPF40A,ALYREF,ZMAT2,SNRPF,MTREX,TGS1,PPWD1,SMN2,ZCCHC8,GCFC2,AQR,RBM39,SCAF11,PRPF4B,BCAS2,CWF19L1,SNRPA1,U2AF1,KHDRBS1,SF3B1,RBM5,RBM6,NCL,SNRNP40,PRMT5,FXR1,DDX5,WBP11,DHX9,HNRNPR,TRA2B,HNRNPA3,HNRNPH3,DDX20,PNN,PRPF39,EFTUD2,CWC22,CWF19L2,HNRNPU,DDX39A,SRSF5,RBM25,LUC7L3,COIL,GEMIN5,HNRNPM,RBMX,NSRP1,RBFOX1,FMR1,METTL16,SRRM2,RNPS1,DAZAP1,PTBP1,SF3B3,RBM8A,GEMIN4,GEMIN6,SRSF7	darkgrey	EC_M8

GO:0006364	8.18E-27	1	71	198	rRNA processing	BP	2.57E-24	SRFBP1,RRP7A,PWP1,NOL8,MDN1,NOL10,EXOSC9,NIFK,DDX18,DIS3,XRN2,WDR43,EBNA1BP2,GTPBP4,EXOSC8,DDX51,MAK16,RRP8,PRKDC,NOP56,MTREX,DHX37,UTP20,BMS1,DIMT1,RRS1,WDR36,UTP15,RPS6,URB1,WDR75,RIOK1,WDR3,NOLC1,PDCD11,RRP1B,RRP1,FP565260.1,RRP15,HEATR1,DDX27,NOL11,GAR1,NGDN,NOP58,WDR12,FTSJ3,WBP11,DDX21,NOP14,SUV39H1,LYAR,LAS1L,EXOSC10,UTP18,RIOK3,UTP6,UTP14A,BRIX1,RSL1D1,METTL16,NOL9,EXOSC2,NOB1,RPF1,METTL18,GEMIN4,NAF1,C1D,IMP4,TFB2M	darkgrey	EC_M8
GO:0034470	1.23E-26	1	98	345	ncRNA processing	BP	3.80E-24	POP1,SRFBP1,RRP7A,TUT7,PWP1,NOL8,HNRNPA2B1,PUS10,MDN1,NOL10,DDX1,PUS7,EXOSC9,TRDMT1,NIFK,DDX18,TSEN2,DIS3,XRN2,WDR43,EBNA1BP2,INTS10,GTPBP4,METTL3,EXOSC8,DDX51,MAK16,RRP8,PRKDC,NOP56,MTREX,DHX37,UTP20,BMS1,DIMT1,RRS1,WDR36,CDK5RAP1,ZCCHC8,UTP15,RPS6,URB1,WDR75,RIOK1,WDR3,NOLC1,NSUN2,PDCD11,TRMT10A,ZBTB8OS,RRP1B,RRP1,FP565260.1,QTRT2,RRP15,HEATR1,DDX27,NOL11,GAR1,TARBP1,NGDN,INTS7,SSB,NOP58,WDR12,FTSJ3,WBP11,TSEN15,DDX21,DGCR8,NOP14,SUV39H1,LYAR,LAS1L,EXOSC10,UTP18,THG1L,INTS2,THUMPD1,RIOK3,UTP6,UTP14A,BRIX1,RSL1D1,DROSHA,METTL16,NOL9,EXOSC2,NOB1,RPF1,METTL18,SRRT,GEMIN4,INTS6,NAF1,C1D,IMP4,TFB2M	darkgrey	EC_M8
GO:0033044	1.47E-26	1	68	168	regulation of chromosome organization	BP	4.44E-24	CCT2,FBXO5,MAP3K4,RAD21,POT1,TRIM28,SENP6,TTK,HNRNPA2B1,CCT4,GEN1,MCM2,SETDB2,CDC16,CCNB1,ESPL1,BUB1,ANAPC1,TNKS2,XRN1,KNTC1,ATR,TERF1,FAM208A,ANAPC7,BUB1B,CCT7,NBN,UBE2C,CCT5,CCT6A,CUL3,CENPE,TRIP13,NEK2,CENPF,BUB3,TPR,CDC27,CDCA5,XRCC5,TACC3,DLGAP5,ZWINT,DDX11,HNRNPU,WAPL,TOP2A,CDC6,DCP2,MAD1L1,EXOSC10,CCT3,SPDL1,AURKB,ATRX,PTTG1,ZWILCH,LCMT1,PLK1,NDC80,RMI2,CDT1,CTCF,HNRNPD,MAD2L1,NAF1,FEN1	darkgrey	EC_M8
GO:0034645	4.27E-25	1	491	3533	cellular macromolecule biosynthetic process	BP	1.26E-22	CDK2,PAN2,NARS,ONECUT2,BCLAF1,RPA3,PPIL4,NUP107,CCT2,DDX39B,FBXO5,ZNF608,SCAF8,EIF3E,MAP3K4,RAD21,DSCC1,MRPL13,HSF2,ATAD2,XRCC6,PHF5A,TNRC6B,TUT7,RMI1,PSIP1,RECQL4,TONSL,IFT74,MCM5,POT1,PWP1,ZNF300,TOPORS,DBF4,POLR3B,MTERF1,POLH,PUM3,RFX3,CKS2,MCM3,C1GALT1,REV1,TRIM28,NCBP1,ZNF451,PHF3,TWISTNB,LRPPRC,FOXJ3,ZNF189,SP4,CDCA7L,PHIP,MTIF2,HNRNPA2B1,PNPT1,ZMPSTE24,RLF,SYNCRIP,ZNF292,XPO1,PABPC4,CCT4,COMMD1,CASP8AP2,DPY19L1,ETAA1,TIA1,ZNF350,ITGB1BP1,THRAP3,TAF1B,EZH2,UFL1,MMS22L,MRPL32,CCNC,ASCC3,ORC6,DDX1,NAA15,ELF2,ORC1,GEN1,ATAD2B,DHFR,CDCA7,PUS7,HDAC2,MCM2,CCNA2,EXOSC9,MLLT10,DNAJC2,SETDB2,NASP,COG3,PCF11,CNOT10,NIFK,NUP85,MRPS7,MCM10,ELF1,ALG8,RSF1,DIS3,CCNT2,PAXIP1,MCM6,DARS,XRN2,NCAPG2,WDR43,TK1,BIRC5,POLR3F,ORC4,GTPBP4,EPC2,SUPT16H,CHD8,KAT6A,METTL3,RIF1,NAP1L1,ZDHHC17,E2F7,GINS4,ALYREF,CKAP2,RRM1,CEP290,NSD3,CEBPZ,SUPT20H,TAF7,EXOSC8,POLE,NR2C1,RFC3,BRCA2,LARS,TOPBP1,ELK3,RRP8,TCERG1,MAPK8,PRKDC,CBX5,MCM4,ERCC6,ZNF214,DHX29,PAN3,GPBP1,SP1,TCEA1,MRPL15,ZNF664,MRPS5,CAPRIN1,ZC3H8,POLR1B,TNKS2,LSM14A,SGK3,BTAF1,ZNF143,PARP4,CENPJ,CDK5RAP1,FER,EIF2B1,TDG,NCK1,SBNO1,ARFGEF1,SMARCA5,MRPL35,RNF141,PTCD3,ZNF507,ABCE1,POLR1A,CHD1,ZFP91,GALNT6,TAF9,CGGBP1,XRN1,PDE12,SMN2,BDP1,TFCP2,SAP30,ATR,TERF1,NOC3L,GCFC2,LARP4,UTP15,HLTF,ESCO2,ZBTB10,CNOT1,GABPA,MCRS1,LTN1,USP16,PPID,RBL1,RPS6,CCT7,CCNL1,NBN,SENP1,TOP1,RAD51,RIPK1,	darkgrey	EC_M8

GO:0009059	1.42E-24	1	493	3569	macromolecule biosynthetic process	BP	4.12E-22	CDK2,PAN2,NARS,ONECUT2,BCLAF1,RPA3,PPIL4,NUP107,CCT2,DDX39B,FBXO5,ZNF608,SCAF8,EIF3E,MAP3K4,RAD21,DSCC1,MRPL13,HSF2,ATAD2,XRCC6,PHF5A,SLC35D2,TNRC6B,TUT7,RMI1,PSIP1,RECQL4,TONSL,IFT74,MCM5,POT1,PWP1,ZNF300,TOPORS,DBF4,POLR3B,MTERF1,POLH,PUM3,RFX3,CKS2,MCM3,C1GALT1,REV1,TRIM28,NCBP1,ZNF451,PHF3,TWISTNB,LRPPRC,FOXJ3,ZNF189,SP4,CDCA7L,PHIP,MTIF2,HNRNPA2B1,PNPT1,ZMPSTE24,RLF,SYNCRIP,ZNF292,XPO1,PABPC4,CCT4,COMMD1,CASP8AP2,DPY19L1,ETAA1,TIA1,ZNF350,ITGB1BP1,THRAP3,TAF1B,EZH2,UFL1,MMS22L,MRPL32,CCNC,ASCC3,ORC6,DDX1,NAA15,ELF2,ORC1,GEN1,ATAD2B,DHFR,CDCA7,PUS7,HDAC2,MCM2,CCNA2,EXOSC9,MLLT10,DNAJC2,SETDB2,NASP,COG3,PCF11,CNOT10,NIFK,NUP85,MRPS7,MCM10,ELF1,ALG8,RSF1,DIS3,CCNT2,PAXIP1,MCM6,DARS,XRN2,NCAPG2,WDR43,TK1,BIRC5,POLR3F,ORC4,GTPBP4,EPC2,SUPT16H,CHD8,KAT6A,METTL3,RIF1,NAP1L1,ZDHHC17,E2F7,GINS4,ALYREF,CKAP2,RRM1,CEP290,NSD3,CEBPZ,SUPT20H,TAF7,EXOSC8,POLE,NR2C1,RFC3,BRCA2,LARS,TOPBP1,ELK3,RRP8,TCERG1,MAPK8,PRKDC,CBX5,MCM4,ERCC6,ZNF214,DHX29,PAN3,GPBP1,SP1,TCEA1,MRPL15,ZNF664,MRPS5,CAPRIN1,ZC3H8,POLR1B,TNKS2,LSM14A,SGK3,BTAF1,ZNF143,PARP4,CENPJ,CDK5RAP1,FER,EIF2B1,TDG,NCK1,SBNO1,ARFGEF1,SMARCA5,MRPL35,RNF141,PTCD3,ZNF507,ABCE1,POLR1A,CHD1,ZFP91,GALNT6,TAF9,CGGBP1,XRN1,PDE12,SMN2,BDP1,TFCP2,SAP30,ATR,TERF1,NOC3L,GCFC2,LARP4,UTP15,HLTF,ESCO2,ZBTB10,CNOT1,GABPA,MCRS1,LTN1,USP16,PPID,RBL1,RPS6,CCT7,CCNL1,NBN,SENP1,TOP1,RAD	darkgrey	EC_M8
GO:0008152	2.53E-24	1	939	8467	metabolic process	BP	7.21E-22	CDK2,PAN2,NARS,ONECUT2,MEX3C,ME2,MTFR2,BCLAF1,CLK4,RPA3,USP15,SHPRH,HNRNPH1,PPIL4,LATS1,MTHFD1L,NUP107,CCT2,MDC1,ZFC3H1,POP1,DDX39B,SRFBP1,FBXO43,FBXO5,CSNK1G3,ZNF608,SCAF8,UBR5,EIF3E,MAP3K4,RAD21,DSCC1,MRPL13,RRP7A,HSF2,ATAD2,XRCC6,DDX46,PHF5A,SLC35D2,CDC25C,TNRC6B,FANCC,HSPA9,TUT7,NAA35,MATR3,SRPK1,UHRF2,RMI1,SRSF3,PPIL1,PLEC,PSIP1,RECQL4,TONSL,RBM28,UBR2,CARNMT1,IFT74,MCM5,POT1,PWP1,ZNF300,TOPORS,APTX,SMU1,DBF4,POLR3B,XPO5,MTERF1,POLH,AKAP9,PUM3,RFX3,IL1R1,CKS2,MCM3,PPAT,C1GALT1,PAICS,ICK,REV1,NOL8,MELK,TRIM28,NCBP1,ZNF451,ANP32B,PHF3,TWISTNB,LRPPRC,STX17,PPIH,FOXJ3,ZNF189,MSH2,FBXO11,SP4,SENP6,ASB3,CDCA7L,CTPS1,PHIP,YTHDC1,MTIF2,TTK,PPP4R3B,IBTK,HNRNPA2B1,PNPT1,DCK,CYB5R4,PAPOLG,PUS10,ZMPSTE24,RLF,USP34,SYNCRIP,ZNF292,XPO1,PABPC4,CCT4,COMMD1,MDN1,CASP8AP2,DPY19L1,FKBP15,ETAA1,FBXO48,TIA1,ZNF350,ITGB1BP1,THRAP3,TAF1B,EZH2,UFL1,NOL10,CUL1,MMS22L,USP45,FAM3C,MRPL32,CCNC,CLSPN,ASCC3,CASP2,ORC6,DDX1,NAA15,SMC6,ELF2,ORC1,GEN1,AFG1L,WDR35,NRDC,CDC40,PLK4,AMD1,ATAD2B,DHFR,CDCA7,PUS7,HDAC2,MCM2,NT5DC1,CCNA2,EXOSC9,MLLT10,PMPCB,SAE1,CEP295,DNAJC2,RAD54L,CHORDC1,RNASEH2B,WDR33,PDSS1,SETDB2,SYMPK,MASTL,YME1L1,ZC3H13,NASP,COG3,TRDMT1,PCF11,CNOT10,DCLRE1C,NIFK,NUP85,MRPS7,MCM10,CAD,ELF1,ALG8,UPF2,MBD4,RSF1,PPM1G,DDX18,BORA,TSEN2,ACOX1,DIS3,CCNT2,PAXIP1,SNRPA,MCM6,DARS,FANCD2,XRN2,RBM17,ULK4,NCAPG2,RBM26,WDR43,	darkgrey	EC_M8
GO:0051983	3.13E-23	1	42	78	regulation of chromosome segregation	BP	8.75E-21	FBXO5,RAD21,TTK,SMC6,GEN1,KIF2C,RAD18,CDC16,CCNB1,ESPL1,BUB1,ANAPC1,KNTC1,ANAPC7,BUB1B,CDCA2,UBE2C,CUL3,CENPE,TRIP13,CENPF,BUB3,MKI67,TPR,CDC27,TACC3,DLGAP5,ZWINT,HNRNPU,WAPL,CDC6,MAD1L1,SPDL1,AURKB,PTTG1,ZWILCH,LCMT1,PLK1,NDC80,RMI2,CDT1,MAD2L1	darkgrey	EC_M8

GO:0006996	4.18E-23	1	425	2943	organelle organization	BP	1.15E-20	RTTN,CDK2,PAN2,ONECUT2,MTFR2,RPA3,SHPRH,KATNA1,LATS1,NUP107,CCT2,FBXO43,FBXO5,MAP3K4,RAD21,DSCC1,KIF3A,TBC1D32,RRP7A,KIFC1,ATAD2,XRCC6,KIF20A,CDC25C,HSPA9,CHCHD3,SRPK1,RMI1,PLEC,RECQL4,CEP78,HAUS6,UBR2,IFT74,MCM5,POT1,MTERF1,KIF24,AKAP9,GCC2,RFX3,CKS2,MCM3,ICK,TRIM28,ANP32B,STX17,FOXJ3,MSH2,SMC2,SENP6,PHIP,MTIF2,TTK,HNRNPA2B1,PNPT1,RUFY3,CEP162,ZMPSTE24,XPO1,CCT4,MDN1,NUP205,ANLN,CDCA8,ITGB1BP1,CAPZA2,EZH2,UFL1,ASCC3,ZMYM4,CNTRL,DDX1,SMC6,GEN1,AFG1L,WDR35,SCLT1,PLK4,STIL,HDAC2,MCM2,CENPO,CEP57,PMPCB,SAE1,CEP295,DNAJC2,RAD54L,CHORDC1,SETDB2,YME1L1,CENPA,NASP,COG3,CLASP2,DCLRE1C,MRPS7,KIF2C,RSF1,DIAPH3,BORA,MCM6,FANCD2,ULK4,NCAPG2,CDC20,PITRM1,HOOK3,SUPT16H,CHD8,KAT6A,RIF1,NAP1L1,TOP2B,SGO1,PRPF40A,CKAP2,VPS36,CEP290,TUBGCP3,DNAJC13,RFC3,BRCA2,PSMD13,RRP8,CDC16,MAPK8,PRKDC,NCAPH,CCNB1,MCM4,WDR62,ERCC6,DHX29,ACTR6,PAN3,GAS2L3,HAUS5,DHX37,ESPL1,BUB1,TPX2,ANAPC1,NDUFAF2,POLR1B,KIF20B,SKA3,TNKS2,RRS1,LSM14A,CENPJ,FER,TDG,NCK1,CEP89,KIF11,ARFGEF1,SMARCA5,CHD1,CENPH,XRN1,KNTC1,ATR,KATNBL1,TERF1,FAM208A,HELLS,CCDC66,LARP4,HK2,MND1,HLTF,CETN3,RACGAP1,ESCO2,CNOT1,ANAPC7,NUP54,DSN1,USP16,PPID,RBL1,MSH3,ALMS1,BUB1B,MIS18A,CCT7,CDCA2,NBN,CFAP298,CKAP5,TOP1,NUP93,KNSTRN,ABRAXAS1,KNL1,RAD51,DCLRE1B,TOMM70,ARID2,MYBL2,CCNE2,COL4A3BP,INO80,OIP5,NUSAP1,CHAF1B,CHD9,UBE2C,NCAPD3,RFC5,STMND1,SMARCAD1,BRWD1,NOLC1,CCT5,CIT,C	darkgrey	EC_M8
GO:1901987	8.60E-23	1	89	311	regulation of cell cycle phase transition	BP	2.32E-20	CDK2,MDC1,DDX39B,FBXO5,GTSE1,RAD21,CDC25C,DBF4,MSH2,TTK,ETAA1,ANLN,EZH2,CLSPN,CASP2,ORC1,GEN1,RINT1,RNASEH2B,PAXIP1,FANCD2,E2F7,CUL4A,TOPBP1,CDC16,PRKDC,CCNB1,ERCC6,ESPL1,BUB1,KNTC1,ATR,ANAPC7,RBL1,BUB1B,NBN,FAM83D,ABRAXAS1,INO80,UBE2C,TAOK3,CUL3,NSUN2,CHEK1,CENPE,TRIP13,KIF14,TICRR,RPA2,INTS7,DTL,BLM,ATRIP,CENPF,BUB3,CDK1,CDC25A,DNA2,TPR,CDC27,CDCA5,DBF4B,CDC45,BRCA1,PSME3,DLGAP5,TIPRL,ZWINT,CCNB3,CDC6,MAD1L1,SPDL1,AURKB,EME1,TAF1,TIPIN,ZWILCH,STXBP4,LCMT1,PLK1,BRIP1,THOC1,NDC80,ATAD5,TAOK1,CDT1,TMOD3,MAD2L1,AURKA	darkgrey	EC_M8
GO:0044772	1.24E-22	1	95	350	mitotic cell cycle phase transition	BP	3.29E-20	CDK2,LATS1,FBXO5,GTSE1,RAD21,CDC25C,DBF4,CKS2,PPAT,MELK,TTK,ANLN,EZH2,CUL1,CLSPN,CASP2,ORC1,GEN1,RINT1,CCNA2,RNASEH2B,MASTL,NASP,CLASP2,E2F7,POLE,CUL4A,TOPBP1,CDC16,PRKDC,CCNB1,ESPL1,BUB1,ANAPC1,KNTC1,ANAPC7,RBL1,RPS6,BUB1B,NBN,ABRAXAS1,CCNE2,INO80,UBE2C,TAOK3,CUL3,CHEK1,KHDRBS1,CENPE,TRIP13,PPP2R2D,KIF14,BCAT1,RCC1,TICRR,RPA2,DTL,BLM,CENPF,BUB3,CDK1,CDC25A,TPR,CDC27,CDCA5,DBF4B,NPAT,BRCA1,CUL5,PSME3,CDKN3,TACC3,DLGAP5,ZWINT,FOXM1,CCNB3,CDC6,MAD1L1,CCNB2,IQGAP3,SPDL1,AURKB,ZWILCH,LCMT1,PLK1,NDC80,CUL4B,GSPT1,TAOK1,PKMYT1,CCNF,CDT1,TMOD3,MAD2L1,AURKA	darkgrey	EC_M8
GO:0000075	2.24E-22	1	59	155	cell cycle checkpoint signaling	BP	5.84E-20	CDK2,MDC1,DDX39B,GTSE1,MSH2,TTK,ETAA1,CLSPN,CASP2,ORC1,GEN1,RINT1,RNASEH2B,FANCD2,CUL4A,TOPBP1,PRKDC,CCNB1,ERCC6,BUB1,KNTC1,ATR,BUB1B,NBN,ABRAXAS1,TAOK3,NSUN2,CHEK1,TRIP13,TICRR,RPA2,INTS7,DTL,BLM,ATRIP,CENPF,BUB3,CDK1,DNA2,TPR,CDC45,BRCA1,TIPRL,ZWINT,CDC6,MAD1L1,SPDL1,AURKB,EME1,TIPIN,ZWILCH,LCMT1,PLK1,BRIP1,THOC1,NDC80,TAOK1,CDT1,MAD2L1	darkgrey	EC_M8
GO:0010948	1.75E-21	1	74	237	negative regulation of cell cycle process	BP	4.49E-19	CDK2,MDC1,DDX39B,FBXO43,FBXO5,GTSE1,RAD21,MSH2,TTK,ETAA1,EZH2,CLSPN,CASP2,ORC1,GEN1,RINT1,RNASEH2B,FANCD2,E2F7,CUL4A,TOPBP1,PRKDC,CCNB1,ERCC6,ESPL1,BUB1,KNTC1,ATR,TERF1,RBL1,BUB1B,NBN,ABRAXAS1,E2F8,TAOK3,NSUN2,CHEK1,TRIP13,TICRR,RPA2,NEK2,INTS7,DTL,BLM,ATRIP,CENPF,BUB3,CDK1,DNA2,TPR,CDC45,BRCA1,TIPRL,ZWINT,WAPL,CDC6,MAD1L1,SPDL1,AURKB,EME1,ATRX,PTTG1,TIPIN,ZWILCH,LCMT1,TRIM37,PLK1,BRIP1,THOC1,NDC80,TAOK1,CCNF,CDT1,MAD2L1	darkgrey	EC_M8
GO:0006302	2.53E-21	1	72	225	double-strand break repair	BP	6.37E-19	RPA3,UBR5,RAD21,XRCC6,RMI1,RECQL4,TONSL,MCM5,POT1,APTX,MCM3,MSH2,MMS22L,DDX1,SMC6,GEN1,MCM2,RAD54L,DCLRE1C,MCM6,FANCD2,PARP2,RIF1,GINS4,BRCA2,PPP4R2,PRKDC,MCM4,ERCC6,PARPBP,ESCO2,NBN,ABRAXAS1,RAD51,DCLRE1B,ARID2,INO80,SMARCAD1,NUCKS1,DEK,CHEK1,TRIP13,FANCB,RPA2,SPO11,BLM,AUNIP,SETD2,POLA1,DNA2,CDCA5,FANCM,XRCC5,CDC45,BRCA1,MCM7,NSD2,RAD51AP1,FOXM1,DDX11,RAD52,FUS,EME1,BRIP1,SMCHD1,NIPBL,RMI2,RPA1,RNF8,FEN1,PALB2,SFR1	darkgrey	EC_M8
GO:0033045	5.09E-21	1	36	64	regulation of sister chromatid segregation	BP	1.26E-18	FBXO5,RAD21,TTK,GEN1,CDC16,CCNB1,ESPL1,BUB1,ANAPC1,KNTC1,ANAPC7,BUB1B,UBE2C,CUL3,CENPE,TRIP13,CENPF,BUB3,TPR,CDC27,TACC3,DLGAP5,ZWINT,HNRNPU,CDC6,MAD1L1,SPDL1,AURKB,PTTG1,ZWILCH,LCMT1,PLK1,NDC80,RMI2,CDT1,MAD2L1	darkgrey	EC_M8

GO:0006403	7.76E-21	1	60	171	RNA localization	BP	1.89E-18	XPOT,NUP43,NUP107,CCT2,DDX39B,SRSF3,XPO5,NCBP1,LRPPRC,YTHDC1,HNRNPA2B1,PNPT1,XPO1,CCT4,NUP205,NUP85,UPF2,ALYREF,NUP58,ATR,TERF1,CETN3,NUP160,NUP54,CCT7,CKAP5,NUP93,CCT5,CCT6A,NSUN2,KHDRBS1,MCM3AP,RAE1,NUP133,SSB,NOP58,DHX9,SETD2,HNRNPA3,TPR,HNRNPU,DCP2,DDX39A,EXOSC10,CCT3,NUP62CL,THOC1,UPF3B,THOC2,NUP155,RBFOX1,NDC1,FMR1,NCBP3,EXOSC2,RBM8A,SARNP,SRSF7,NAF1,NUP35	darkgrey	EC_M8
GO:0010965	8.99E-21	1	34	58	regulation of mitotic sister chromatid separation	BP	2.15E-18	FBXO5,RAD21,TTK,GEN1,CDC16,CCNB1,ESPL1,BUB1,ANAPC1,KNTC1,ANAPC7,BUB1B,UBE2C,CUL3,CENPE,TRIP13,CENPF,BUB3,TPR,CDC27,TACC3,DLGAP5,ZWINT,CDC6,MAD1L1,SPDL1,AURKB,PTTG1,ZWILCH,LCMT1,PLK1,NDC80,CDT1,MAD2L1	darkgrey	EC_M8
GO:0051321	1.86E-20	1	64	192	meiotic cell cycle	BP	4.39E-18	CDK2,YTHDC2,FBXO43,FBXO5,RAD21,CDC25C,RMI1,UBR2,CKS2,FOXJ3,SMC2,TTK,RAD54L,FANCD2,CDC20,METTL3,TOP2B,SGO1,TUBGCP3,BRCA2,PSMD13,ESPL1,BUB1,TERF1,MND1,MSH3,BUB1B,NBN,RAD51,CCNE2,NCAPD3,KIF18A,NSUN2,SMC3,TRIP13,SGO2,ASPM,SPO11,NEK2,BUB3,CDC25A,NUF2,FANCM,SMC4,NCAPD2,RAD51AP1,EXO1,INCENP,CCNB3,TOP2A,SMC1A,CCNB2,EME1,ATRX,PTTG1,PLK1,BRIP1,TOP3A,STAG2,NDC1,RPA1,PKMYT1,FANCA,AURKA	darkgrey	EC_M8
GO:0071824	2.17E-20	1	58	163	protein-DNA complex subunit organization	BP	5.04E-18	SHPRH,ANP32B,SENP6,TAF1B,MCM2,CENPO,CENPA,NASP,RSF1,SUPT16H,KAT6A,NAP1L1,TAF7,ACTR6,SMARCA5,CENPH,TAF9,BDP1,KNTC1,TERF1,HELLS,MIS18A,RAD51,ARID2,INO80,OIP5,CHAF1B,NAP1L4,GMNN,CENPE,BPTF,ANP32E,HJURP,MCM3AP,CENPF,HAT1,SMARCC1,SETD2,UBTF,CDC45,HP1BP3,DLGAP5,RAD52,SMARCE1,ASF1B,TAF1,ATRX,CENPI,CHAF1A,UBN1,CDT1,CENPN,CTCF,HIST1H1A,ITGB3BP,HIST1H3H,CENPW,RNF8	darkgrey	EC_M8
GO:0007051	2.34E-20	1	59	165	spindle organization	BP	5.34E-18	FBXO5,KIFC1,HAUS6,SENP6,TTK,STIL,CLASP2,BORA,CDC20,TUBGCP3,CCNB1,WDR62,HAUS5,ESPL1,TPX2,LSM14A,CENPJ,KIF11,CENPH,RACGAP1,CKAP5,KNSTRN,ABRAXAS1,MYBL2,INO80,SMC3,CENPE,CEP72,HAUS2,INTS13,TBCE,ASPM,RCC1,SPC25,RAE1,NEK2,PRC1,AUNIP,NUF2,TPR,KIF15,HAUS3,TACC3,DLGAP5,HNRNPU,INCENP,SMC1A,CCNB2,KPNB1,KIF4A,AURKB,ATRX,KIF23,PLK1,NDC80,STAG2,STMN1,AURKA,DRG1	darkgrey	EC_M8
GO:0051304	2.37E-20	1	41	85	chromosome separation	BP	5.34E-18	FBXO5,RAD21,RMI1,TTK,GEN1,TOP2B,CDC16,CCNB1,ESPL1,BUB1,ANAPC1,KNTC1,ANAPC7,BUB1B,UBE2C,SMARCAD1,CUL3,CENPE,TRIP13,CENPF,BUB3,TPR,CDC27,FANCM,TACC3,DLGAP5,ZWINT,TOP2A,CDC6,MAD1L1,SPDL1,AURKB,EME1,PTTG1,ZWILCH,LCMT1,PLK1,TOP3A,NDC80,CDT1,MAD2L1	darkgrey	EC_M8
GO:0007346	3.06E-20	1	93	360	regulation of mitotic cell cycle	BP	6.79E-18	CDK2,YTHDC2,RPA3,MDC1,FBXO43,FBXO5,GTSE1,RAD21,CDC25C,CKS2,MSH2,PHIP,TTK,ZMPSTE24,ANLN,EZH2,CLSPN,CASP2,ORC1,GEN1,RINT1,RNASEH2B,BORA,FANCD2,E2F7,CUL4A,BRCA2,TOPBP1,CDC16,PRKDC,CCNB1,ESPL1,BUB1,KIF20B,KNTC1,ANAPC7,RBL1,BUB1B,CDCA2,NBN,ABRAXAS1,INO80,NUSAP1,UBE2C,TAOK3,CUL3,CHEK1,CENPE,TRIP13,KIF14,PRMT5,INTS13,RCC1,TICRR,RPA2,NEK2,DTL,BLM,CENPF,BUB3,CDK1,CDC25A,MKI67,TPR,CDC27,CDCA5,DBF4B,BRCA1,PSME3,DLGAP5,ZWINT,HNRNPU,CCNB3,CDC6,MAD1L1,SPDL1,AURKB,EME1,TIPIN,ZWILCH,LCMT1,PLK1,AATF,NLE1,NDC80,TAOK1,PKMYT1,TP73,CDT1,PKN2,TMOD3,MAD2L1,AURKA	darkgrey	EC_M8
GO:0051306	3.49E-20	1	34	60	mitotic sister chromatid separation	BP	7.64E-18	FBXO5,RAD21,TTK,GEN1,CDC16,CCNB1,ESPL1,BUB1,ANAPC1,KNTC1,ANAPC7,BUB1B,UBE2C,CUL3,CENPE,TRIP13,CENPF,BUB3,TPR,CDC27,TACC3,DLGAP5,ZWINT,CDC6,MAD1L1,SPDL1,AURKB,PTTG1,ZWILCH,LCMT1,PLK1,NDC80,CDT1,MAD2L1	darkgrey	EC_M8
GO:0007091	6.69E-20	1	33	57	metaphase/anaphase transition of mitotic cell cycle	BP	1.44E-17	FBXO5,RAD21,TTK,GEN1,CDC16,CCNB1,ESPL1,BUB1,ANAPC1,KNTC1,ANAPC7,BUB1B,UBE2C,CUL3,CENPE,TRIP13,CENPF,BUB3,TPR,CDC27,TACC3,DLGAP5,ZWINT,CDC6,MAD1L1,SPDL1,AURKB,ZWILCH,LCMT1,PLK1,NDC80,CDT1,MAD2L1	darkgrey	EC_M8
GO:0034502	7.56E-20	1	41	86	protein localization to chromosome	BP	1.59E-17	CCT2,RAD21,POT1,CENPQ,MSH2,TTK,CCT4,EZH2,CENPA,BRCA2,TNKS2,KNTC1,ATR,TERF1,FAM208A,ESCO2,BUB1B,CCT7,KNL1,CCT5,CCT6A,RPA2,SPO11,BUB3,CDK1,SETD2,CDCA5,XRCC5,WAPL,CCT3,SPDL1,AURKB,ATRX,ZWILCH,PLK1,NDC80,NIPBL,RPA1,HASPIN,CDT1,CTCF	darkgrey	EC_M8
GO:0006310	7.59E-20	1	73	245	DNA recombination	BP	1.59E-17	RPA3,RAD21,XRCC6,RMI1,RECQL4,TONSL,UBR2,MCM5,MCM3,MSH2,MMS22L,SMC6,GEN1,MCM2,RAD54L,DCLRE1C,PAXIP1,MCM6,FANCD2,RIF1,TOP2B,GINS4,ALYREF,BRCA2,PPP4R2,PRKDC,MCM4,ERCC6,PARPBP,MND1,MCRS1,MSH3,NBN,RAD51,ARID2,INO80,SMARCAD1,NUCKS1,CHEK1,TRIP13,FANCB,RPA2,SPO11,BLM,AUNIP,SETD2,FANCM,C17orf53,APEX2,XRCC5,CDC45,BRCA1,MCM7,NSD2,RAD51AP1,ANKLE1,EXO1,RAD52,TOP2A,FUS,EME1,BRIP1,THOC1,SMCHD1,NIPBL,RMI2,RPA1,HIST1H1A,HMGB3,RNF8,FEN1,PALB2,SFR1	darkgrey	EC_M8

GO:0032392	1.51E-19	1	40	80	DNA geometric change	BP	3.12E-17	DSCC1,XRCC6,RECQL4,MCM5,POT1,MTERF1,MCM3,HNRNPA2B1,ASCC3,DDX1,MCM2,RAD54L,MCM6,CHD8,RFC3,MCM4,CHD1,NBN,RAD51,CHD9,RFC5,SMARCAD1,CHD1L,RFC2,BLM,DHX9,RFC4,DNA2,FANCM,XRCC5,MCM7,DDX11,TOP2A,ATRX,BRIP1,G3BP1,RPA1,CHTF18,ERCC6L,HMGB3	darkgrey	EC_M8
GO:1901988	3.17E-19	1	64	201	negative regulation of cell cycle phase transition	BP	6.46E-17	CDK2,MDC1,DDX39B,FBXO5,GTSE1,RAD21,MSH2,TTK,ETAA1,EZH2,CLSPN,CASP2,ORC1,GEN1,RINT1,RNASEH2B,FANCD2,E2F7,CUL4A,TOPBP1,PRKDC,CCNB1,ERCC6,BUB1,KNTC1,ATR,RBL1,BUB1B,NBN,ABRAXAS1,TAOK3,NSUN2,CHEK1,TRIP13,TICRR,RPA2,INTS7,DTL,BLM,ATRIP,CENPF,BUB3,CDK1,DNA2,TPR,CDC45,BRCA1,TIPRL,ZWINT,CDC6,MAD1L1,SPDL1,AURKB,EME1,TIPIN,ZWILCH,LCMT1,PLK1,BRIP1,THOC1,NDC80,TAOK1,CDT1,MAD2L1	darkgrey	EC_M8
GO:0051052	4.23E-19	1	81	297	regulation of DNA metabolic process	BP	8.51E-17	CCT2,DDX39B,UBR5,MAP3K4,DSCC1,POT1,POLH,TRIM28,MSH2,HNRNPA2B1,ZMPSTE24,RLF,CCT4,DNAJC2,PAXIP1,RIF1,ALYREF,RFC3,CUL4A,PPP4R2,PRKDC,ERCC6,PARPBP,TNKS2,PARP4,OTUD4,XRN1,ATR,TERF1,MSH3,MIS18A,CCT7,NBN,ABRAXAS1,RAD51,ARID2,RFC5,SMARCAD1,CCT5,CCT6A,NUCKS1,DEK,CHEK1,PRMT5,FANCB,TICRR,RFC2,RPA2,NEK2,BLM,AUNIP,DHX9,SETD2,RFC4,XRCC5,BRCA1,NSD2,RAD51AP1,ANKLE1,FOXM1,DDX11,RAD52,HNRNPU,DCP2,EXOSC10,CCT3,FUS,AURKB,ATRX,THOC1,SMCHD1,AIFM1,RMI2,CHTF18,CDT1,PRIM2,HIST1H1A,HNRNPD,TIGAR,NAF1,RNF8	darkgrey	EC_M8
GO:0044784	4.89E-19	1	33	60	metaphase/anaphase transition of cell cycle	BP	9.71E-17	FBXO5,RAD21,TTK,GEN1,CDC16,CCNB1,ESPL1,BUB1,ANAPC1,KNTC1,ANAPC7,BUB1B,UBE2C,CUL3,CENPE,TRIP13,CENPF,BUB3,TPR,CDC27,TACC3,DLGAP5,ZWINT,CDC6,MAD1L1,SPDL1,AURKB,ZWILCH,LCMT1,PLK1,NDC80,CDT1,MAD2L1	darkgrey	EC_M8
GO:0007088	7.74E-19	1	42	96	regulation of mitotic nuclear division	BP	1.52E-16	FBXO43,FBXO5,RAD21,CDC25C,PHIP,TTK,GEN1,BORA,CDC16,CCNB1,ESPL1,BUB1,KIF20B,KNTC1,ANAPC7,BUB1B,CDCA2,NUSAP1,CUL3,CHEK1,TRIP13,PRMT5,RCC1,NEK2,CENPF,BUB3,MKI67,TPR,CDC27,DLGAP5,ZWINT,MAD1L1,SPDL1,AURKB,ZWILCH,LCMT1,PLK1,NDC80,PKMYT1,CDT1,MAD2L1,AURKA	darkgrey	EC_M8
GO:1905818	1.12E-18	1	34	65	regulation of chromosome separation	BP	2.17E-16	FBXO5,RAD21,TTK,GEN1,CDC16,CCNB1,ESPL1,BUB1,ANAPC1,KNTC1,ANAPC7,BUB1B,UBE2C,CUL3,CENPE,TRIP13,CENPF,BUB3,TPR,CDC27,TACC3,DLGAP5,ZWINT,CDC6,MAD1L1,SPDL1,AURKB,PTTG1,ZWILCH,LCMT1,PLK1,NDC80,CDT1,MAD2L1	darkgrey	EC_M8
GO:0045786	1.93E-18	1	81	306	negative regulation of cell cycle	BP	3.68E-16	CDK2,YTHDC2,LATS1,MDC1,DDX39B,FBXO43,FBXO5,GTSE1,RAD21,MSH2,TTK,ETAA1,EZH2,CLSPN,CASP2,ORC1,GEN1,RINT1,RNASEH2B,FANCD2,IPO5,E2F7,CUL4A,TOPBP1,PRKDC,CCNB1,ERCC6,ESPL1,BUB1,IPO7,CDK5RAP1,KNTC1,ATR,TERF1,RBL1,BUB1B,NBN,ABRAXAS1,E2F8,TAOK3,NSUN2,GMNN,CHEK1,TRIP13,TICRR,RPA2,NEK2,INTS7,DTL,BLM,ATRIP,CENPF,BUB3,CDK1,DNA2,TPR,CDC45,BRCA1,TIPRL,ZWINT,WAPL,CDC6,MAD1L1,SPDL1,AURKB,EME1,ATRX,PTTG1,TIPIN,ZWILCH,LCMT1,TRIM37,PLK1,BRIP1,NLE1,THOC1,NDC80,TAOK1,CCNF,CDT1,MAD2L1	darkgrey	EC_M8
GO:0030071	2.51E-18	1	31	55	regulation of mitotic metaphase/anaphase transition	BP	4.74E-16	FBXO5,RAD21,TTK,GEN1,CDC16,CCNB1,ESPL1,BUB1,KNTC1,ANAPC7,BUB1B,UBE2C,CUL3,CENPE,TRIP13,CENPF,BUB3,TPR,CDC27,DLGAP5,ZWINT,CDC6,MAD1L1,SPDL1,AURKB,ZWILCH,LCMT1,PLK1,NDC80,CDT1,MAD2L1	darkgrey	EC_M8
GO:0019219	3.14E-18	1	397	2852	regulation of nucleobase-containing compound metabolic process	BP	5.85E-16	PAN2,ONECUT2,BCLAF1,CLK4,HNRNPH1,PPIL4,NUP107,CCT2,DDX39B,ZNF608,SCAF8,UBR5,MAP3K4,RAD21,DSCC1,HSF2,ATAD2,XRCC6,PHF5A,TNRC6B,TUT7,SRPK1,SRSF3,PSIP1,IFT74,POT1,PWP1,ZNF300,TOPORS,SMU1,MTERF1,POLH,RFX3,CKS2,TRIM28,NCBP1,ZNF451,LRPPRC,FOXJ3,ZNF189,MSH2,SP4,CDCA7L,PHIP,YTHDC1,HNRNPA2B1,PNPT1,ZMPSTE24,RLF,SYNCRIP,ZNF292,XPO1,PABPC4,CCT4,COMMD1,CASP8AP2,TIA1,ZNF350,ITGB1BP1,THRAP3,EZH2,UFL1,CCNC,DDX1,NAA15,ELF2,ATAD2B,CDCA7,HDAC2,CCNA2,EXOSC9,MLLT10,DNAJC2,SETDB2,TRDMT1,NUP85,ELF1,RSF1,DIS3,CCNT2,PAXIP1,WDR43,BIRC5,POLR3F,EPC2,SUPT16H,CHD8,KAT6A,HNRNPL,METTL3,RIF1,E2F7,ALYREF,CKAP2,CEP290,NSD3,CEBPZ,SUPT20H,TAF7,EXOSC8,NR2C1,RFC3,CUL4A,BRCA2,PPP4R2,ELK3,RRP8,TCERG1,MAPK8,PRKDC,CBX5,CCNB1,ERCC6,ZNF214,PAN3,GPBP1,SP1,TCEA1,ZNF664,ZC3H8,DIMT1,PARPBP,TNKS2,SGK3,BTAF1,ZNF143,PARP4,CENPJ,FER,TDG,NCK1,SBNO1,SMARCA5,RNF141,ZNF507,ABCE1,OTUD4,CHD1,ZFP91,TAF9,CGGBP1,XRN1,PDE12,TFCP2,SAP30,ATR,TERF1,GCFC2,UTP15,RBM12,HLTF,RBM39,ZBTB10,CNOT1,GABPA,MCRS1,USP16,PPID,RBL1,MSH3,MIS18A,CCT7,CCNL1,NBN,SENP1,TENT2,ABRAXAS1,RAD51,VIRMA,SLC38A2,RIPK1,WDR75,ARID2,MYBL2,E2F8,TRIM33,INO80,RIOK1,ZBED6,CHUK,ZBTB11,ZNF248,MGA,MDM4,HIVEP1,RFC5,SMARCAD1,BRWD1,NOLC1,CCT5,BMPR1B,UBE3A,CCT6A,PRDM10,NUCKS1,DEK,GCN1,CUL3,NSUN2,GMNN,CHEK1,H2AFZ,RRP1B,NFKB1,KHDRBS1,TRIP13,RBM5,TET2,TRIP11,HEATR1,NCL,TRAIP,ZNF184,PRMT5,AJUBA,GTF2IRD2,ZBTB41,ARID4B,NOL11,GT	darkgrey	EC_M8

GO:0044249	5.13E-18	1	536	4271	cellular biosynthetic process	BP	9.44E-16	CDK2,PAN2,NARS,ONECUT2,BCLAF1,RPA3,PPIL4,MTHFD1L,NUP107,CCT2,DDX39B,FBXO5,ZNF608,SCAF8,EIF3E,MAP3K4,RAD21,DSCC1,MRPL13,HSF2,ATAD2,XRCC6,PHF5A,SLC35D2,TNRC6B,TUT7,RMI1,PSIP1,RECQL4,TONSL,IFT74,MCM5,POT1,PWP1,ZNF300,TOPORS,DBF4,POLR3B,MTERF1,POLH,PUM3,RFX3,CKS2,MCM3,PPAT,C1GALT1,PAICS,REV1,TRIM28,NCBP1,ZNF451,PHF3,TWISTNB,LRPPRC,FOXJ3,ZNF189,SP4,CDCA7L,CTPS1,PHIP,MTIF2,PPP4R3B,HNRNPA2B1,PNPT1,DCK,ZMPSTE24,RLF,SYNCRIP,ZNF292,XPO1,PABPC4,CCT4,COMMD1,CASP8AP2,DPY19L1,ETAA1,TIA1,ZNF350,ITGB1BP1,THRAP3,TAF1B,EZH2,UFL1,MMS22L,FAM3C,MRPL32,CCNC,ASCC3,ORC6,DDX1,NAA15,ELF2,ORC1,GEN1,WDR35,AMD1,ATAD2B,DHFR,CDCA7,PUS7,HDAC2,MCM2,CCNA2,EXOSC9,MLLT10,DNAJC2,PDSS1,SETDB2,NASP,COG3,PCF11,CNOT10,NIFK,NUP85,MRPS7,MCM10,CAD,ELF1,ALG8,RSF1,ACOX1,DIS3,CCNT2,PAXIP1,MCM6,DARS,XRN2,NCAPG2,WDR43,TK1,AFMID,BIRC5,POLR3F,ORC4,GTPBP4,EPC2,SUPT16H,CHD8,KAT6A,METTL3,RIF1,NAP1L1,ZDHHC17,E2F7,GINS4,ALYREF,CKAP2,RRM1,CEP290,NSD3,CEBPZ,SUPT20H,TAF7,EXOSC8,PRKD3,POLE,NR2C1,RFC3,BRCA2,LARS,LTA4H,TOPBP1,ELK3,RRP8,TCERG1,MAPK8,PRKDC,CBX5,MCM4,ERCC6,ZNF214,DHX29,PAN3,GPBP1,SP1,TCEA1,MRPL15,ZNF664,MRPS5,CAPRIN1,ZC3H8,POLR1B,TNKS2,LSM14A,SGK3,BTAF1,ZNF143,PARP4,CENPJ,CDK5RAP1,FER,EIF2B1,TDG,NCK1,SBNO1,ARFGEF1,SMARCA5,MRPL35,RNF141,PTCD3,ZNF507,ABCE1,POLR1A,CHD1,ZFP91,GALNT6,TAF9,CGGBP1,XRN1,PDE12,SMN2,BDP1,TFCP2,SAP30,ATR,TERF1,NOC3L,GCFC2,LARP4,UTP15,HLT	darkgrey	EC_M8
GO:0006323	7.21E-18	1	46	119	DNA packaging	BP	1.31E-15	SHPRH,SRPK1,ANP32B,SMC2,MCM2,NASP,RSF1,NCAPG2,KAT6A,NAP1L1,RRP8,NCAPH,CCNB1,SMARCA5,HELLS,MIS18A,OIP5,NUSAP1,CHAF1B,NCAPD3,NAP1L4,ACIN1,HJURP,HAT1,CDK1,CDCA5,SMC4,HP1BP3,NCAPD2,SUV39H1,INCENP,WAPL,TOP2A,ASF1B,NCAPG,ATRX,PLK1,AIFM1,CHAF1A,UBN1,CTCF,HIST1H1A,ITGB3BP,HIST1H3H,CENPW,RNF8	darkgrey	EC_M8
GO:0140694	7.70E-18	1	81	305	non-membrane-bounded organelle assembly	BP	1.38E-15	RTTN,CDK2,PAN2,FBXO5,RRP7A,KIFC1,PLEC,HAUS6,SENP6,MDN1,ANLN,PLK4,STIL,CEP295,CENPA,CLASP2,MRPS7,CDC20,TUBGCP3,PRKDC,WDR62,DHX29,PAN3,HAUS5,DHX37,TPX2,RRS1,LSM14A,CENPJ,KIF11,CENPH,KNTC1,RACGAP1,CNOT1,ALMS1,ABRAXAS1,MYBL2,INO80,FP565260.1,SMC3,CENPE,CEP72,HAUS2,ASPM,RCC1,NEK2,PRC1,DDX6,CENPF,FASTKD2,TPR,KIF15,RPL10L,XRCC5,BRCA1,CEP152,HAUS3,PRRC2C,DLGAP5,HNRNPU,INCENP,SMC1A,CCNB2,KPNB1,KIF4A,AURKB,KIF23,TRIM37,MRPL22,PLK1,G3BP1,NLE1,STAG2,BRIX1,CEP76,CENPN,SASS6,TMOD3,AURKA,CENPW,DRG1	darkgrey	EC_M8
GO:0007093	8.01E-18	1	45	117	mitotic cell cycle checkpoint signaling	BP	1.42E-15	CDK2,MDC1,GTSE1,MSH2,TTK,CLSPN,CASP2,ORC1,GEN1,RINT1,FANCD2,TOPBP1,PRKDC,CCNB1,BUB1,KNTC1,BUB1B,NBN,ABRAXAS1,TAOK3,CHEK1,TRIP13,TICRR,RPA2,DTL,BLM,CENPF,BUB3,CDK1,TPR,BRCA1,ZWINT,CDC6,MAD1L1,SPDL1,AURKB,EME1,TIPIN,ZWILCH,LCMT1,PLK1,NDC80,TAOK1,CDT1,MAD2L1	darkgrey	EC_M8
GO:0032508	9.41E-18	1	37	75	DNA duplex unwinding	BP	1.65E-15	DSCC1,XRCC6,RECQL4,MCM5,POT1,MCM3,ASCC3,DDX1,MCM2,RAD54L,MCM6,CHD8,RFC3,MCM4,CHD1,NBN,RAD51,CHD9,RFC5,SMARCAD1,CHD1L,RFC2,BLM,DHX9,RFC4,DNA2,FANCM,XRCC5,MCM7,DDX11,TOP2A,ATRX,BRIP1,G3BP1,RPA1,CHTF18,ERCC6L	darkgrey	EC_M8
GO:1902099	1.67E-17	1	31	58	regulation of metaphase/anaphase transition of cell cycle	BP	2.89E-15	FBXO5,RAD21,TTK,GEN1,CDC16,CCNB1,ESPL1,BUB1,KNTC1,ANAPC7,BUB1B,UBE2C,CUL3,CENPE,TRIP13,CENPF,BUB3,TPR,CDC27,DLGAP5,ZWINT,CDC6,MAD1L1,SPDL1,AURKB,ZWILCH,LCMT1,PLK1,NDC80,CDT1,MAD2L1	darkgrey	EC_M8
GO:1902850	1.84E-17	1	49	133	microtubule cytoskeleton organization involved in mitosis	BP	3.17E-15	KIFC1,TTK,STIL,CENPA,CLASP2,BORA,CDC20,CCNB1,WDR62,ESPL1,TPX2,LSM14A,KIF11,CENPH,RACGAP1,CKAP5,ABRAXAS1,MYBL2,NUSAP1,SMC3,CENPE,INTS13,TBCE,RCC1,SPC25,RAE1,NEK2,PRC1,NUF2,SETD2,TPR,KIF15,TACC3,DLGAP5,HNRNPU,SMC1A,KPNB1,KIF4A,SPDL1,AURKB,KIF23,PLK1,NDC80,STAG2,SAPCD2,MAD2L1,STMN1,AURKA,DRG1	darkgrey	EC_M8
GO:0051783	2.22E-17	1	44	114	regulation of nuclear division	BP	3.76E-15	FBXO43,FBXO5,RAD21,CDC25C,PHIP,TTK,GEN1,BORA,CDC20,CDC16,CCNB1,ESPL1,BUB1,KIF20B,KNTC1,ANAPC7,BUB1B,CDCA2,NUSAP1,CUL3,CHEK1,TRIP13,PRMT5,RCC1,NEK2,CENPF,BUB3,MKI67,TPR,CDC27,DLGAP5,RAD51AP1,ZWINT,MAD1L1,SPDL1,AURKB,ZWILCH,LCMT1,PLK1,NDC80,PKMYT1,CDT1,MAD2L1,AURKA	darkgrey	EC_M8
GO:2001251	5.34E-17	1	36	77	negative regulation of chromosome organization	BP	8.97E-15	FBXO5,RAD21,POT1,TTK,GEN1,MCM2,CCNB1,ESPL1,BUB1,TNKS2,XRN1,KNTC1,TERF1,BUB1B,NBN,TRIP13,CENPF,BUB3,TPR,ZWINT,HNRNPU,WAPL,TOP2A,DCP2,MAD1L1,EXOSC10,SPDL1,AURKB,ATRX,PTTG1,ZWILCH,LCMT1,PLK1,NDC80,CDT1,MAD2L1	darkgrey	EC_M8

GO:0051171	7.45E-17	1	518	4082	regulation of nitrogen compound metabolic process	BP	1.24E-14	CDK2,PAN2,ONECUT2,BCLAF1,CLK4,HNRNPH1,PPIL4,LATS1,NUP107,CCT2,DDX39B,FBXO5,ZNF608,SCAF8,UBR5,EIF3E,MAP3K4,RAD21,DSCC1,MRPL13,HSF2,ATAD2,XRCC6,PHF5A,CDC25C,TNRC6B,TUT7,SRPK1,SRSF3,PLEC,PSIP1,IFT74,POT1,PWP1,ZNF300,TOPORS,SMU1,DBF4,MTERF1,POLH,AKAP9,PUM3,RFX3,CKS2,TRIM28,NCBP1,ZNF451,ANP32B,LRPPRC,FOXJ3,ZNF189,MSH2,SP4,CDCA7L,PHIP,YTHDC1,MTIF2,TTK,IBTK,HNRNPA2B1,PNPT1,ZMPSTE24,RLF,SYNCRIP,ZNF292,XPO1,PABPC4,CCT4,COMMD1,CASP8AP2,ETAA1,TIA1,ZNF350,ITGB1BP1,THRAP3,EZH2,UFL1,CCNC,CLSPN,ASCC3,CASP2,DDX1,NAA15,ELF2,WDR35,NRDC,ATAD2B,DHFR,CDCA7,PUS7,HDAC2,CCNA2,EXOSC9,MLLT10,SAE1,CEP295,DNAJC2,CHORDC1,SETDB2,SYMPK,MASTL,TRDMT1,CNOT10,NUP85,ELF1,RSF1,BORA,DIS3,CCNT2,PAXIP1,NCAPG2,WDR43,CDC20,BIRC5,FNTA,POLR3F,GTPBP4,IPO5,EPC2,SUPT16H,CHD8,KAT6A,HNRNPL,METTL3,RIF1,E2F7,ALYREF,CKAP2,CEP290,NSD3,CEBPZ,KITLG,SUPT20H,TAF7,EXOSC8,NR2C1,RFC3,CUL4A,BRCA2,PPP4R2,ELK3,RRP8,TCERG1,MAPK8,PRKDC,APAF1,CBX5,CCNB1,ERCC6,ZNF214,DHX29,PAN3,GPBP1,SP1,TCEA1,ZNF664,TPX2,CAPRIN1,ZC3H8,DIMT1,UBA2,IPO7,PARPBP,TNKS2,LSM14A,SGK3,BTAF1,ZNF143,PARP4,CENPJ,CDK5RAP1,FER,TDG,NCK1,IDE,SBNO1,ARFGEF1,SMARCA5,RNF141,PTCD3,ZNF507,ABCE1,OTUD4,CHD1,ZFP91,TAF9,CGGBP1,XRN1,PDE12,TFCP2,SAP30,ATR,TERF1,GCFC2,LARP4,USP25,UTP15,RBM12,HLTF,RBM39,PBK,ZBTB10,CNOT1,GABPA,MCRS1,SH2B3,LTN1,USP16,PPID,RBL1,MOB1A,MSH3,MIS18A,CCT7,CCNL1,CDCA2,NBN,FAM83D,SENP1,TENT2,ABRAXAS1,RAD5	darkgrey	EC_M8
GO:1901990	8.87E-17	1	66	237	regulation of mitotic cell cycle phase transition	BP	1.46E-14	CDK2,FBXO5,GTSE1,RAD21,CDC25C,TTK,ANLN,EZH2,CLSPN,CASP2,ORC1,GEN1,RINT1,RNASEH2B,E2F7,CUL4A,TOPBP1,CDC16,PRKDC,CCNB1,ESPL1,BUB1,KNTC1,ANAPC7,RBL1,BUB1B,NBN,ABRAXAS1,INO80,UBE2C,TAOK3,CUL3,CHEK1,CENPE,TRIP13,KIF14,TICRR,RPA2,DTL,BLM,CENPF,BUB3,CDK1,CDC25A,TPR,CDC27,CDCA5,DBF4B,BRCA1,PSME3,DLGAP5,ZWINT,CCNB3,CDC6,MAD1L1,SPDL1,AURKB,ZWILCH,LCMT1,PLK1,NDC80,TAOK1,CDT1,TMOD3,MAD2L1,AURKA	darkgrey	EC_M8
GO:1901576	1.32E-16	1	535	4324	organic substance biosynthetic process	BP	2.15E-14	CDK2,PAN2,NARS,ONECUT2,BCLAF1,RPA3,PPIL4,MTHFD1L,NUP107,CCT2,DDX39B,FBXO5,ZNF608,SCAF8,EIF3E,MAP3K4,RAD21,DSCC1,MRPL13,HSF2,ATAD2,XRCC6,PHF5A,SLC35D2,TNRC6B,TUT7,RMI1,PSIP1,RECQL4,TONSL,IFT74,MCM5,POT1,PWP1,ZNF300,TOPORS,DBF4,POLR3B,MTERF1,POLH,PUM3,RFX3,CKS2,MCM3,PPAT,C1GALT1,PAICS,REV1,TRIM28,NCBP1,ZNF451,PHF3,TWISTNB,LRPPRC,FOXJ3,ZNF189,SP4,CDCA7L,CTPS1,PHIP,MTIF2,PPP4R3B,HNRNPA2B1,PNPT1,DCK,ZMPSTE24,RLF,SYNCRIP,ZNF292,XPO1,PABPC4,CCT4,COMMD1,CASP8AP2,DPY19L1,ETAA1,TIA1,ZNF350,ITGB1BP1,THRAP3,TAF1B,EZH2,UFL1,MMS22L,FAM3C,MRPL32,CCNC,ASCC3,ORC6,DDX1,NAA15,ELF2,ORC1,GEN1,AMD1,ATAD2B,DHFR,CDCA7,PUS7,HDAC2,MCM2,CCNA2,EXOSC9,MLLT10,DNAJC2,PDSS1,SETDB2,NASP,COG3,PCF11,CNOT10,NIFK,NUP85,MRPS7,MCM10,CAD,ELF1,ALG8,RSF1,DIS3,CCNT2,PAXIP1,MCM6,DARS,XRN2,NCAPG2,WDR43,TK1,AFMID,BIRC5,POLR3F,ORC4,GTPBP4,EPC2,SUPT16H,CHD8,KAT6A,METTL3,RIF1,NAP1L1,ZDHHC17,E2F7,GINS4,ALYREF,CKAP2,RRM1,CEP290,NSD3,CEBPZ,SUPT20H,TAF7,EXOSC8,PRKD3,POLE,NR2C1,RFC3,BRCA2,LARS,LTA4H,TOPBP1,ELK3,RRP8,TCERG1,MAPK8,PRKDC,CBX5,MCM4,ERCC6,ZNF214,DHX29,PAN3,GPBP1,SP1,TCEA1,MRPL15,ZNF664,MRPS5,CAPRIN1,ZC3H8,POLR1B,TNKS2,LSM14A,SGK3,BTAF1,ZNF143,PARP4,CENPJ,CDK5RAP1,FER,EIF2B1,TDG,NCK1,SBNO1,ARFGEF1,SMARCA5,MRPL35,RNF141,PTCD3,ZNF507,ABCE1,POLR1A,CHD1,ZFP91,GK5,GALNT6,TAF9,CGGBP1,XRN1,PDE12,SMN2,BDP1,TFCP2,SAP30,ATR,TERF1,NOC3L,GCFC2,LARP4,UTP15,HLTF,ESCO2	darkgrey	EC_M8
GO:0051236	1.43E-16	1	48	138	establishment of RNA localization	BP	2.30E-14	XPOT,NUP43,NUP107,DDX39B,SRSF3,XPO5,NCBP1,LRPPRC,YTHDC1,HNRNPA2B1,PNPT1,XPO1,NUP205,NUP85,UPF2,ALYREF,NUP58,ATR,TERF1,CETN3,NUP160,NUP54,CKAP5,NUP93,NSUN2,KHDRBS1,MCM3AP,RAE1,NUP133,SSB,DHX9,SETD2,HNRNPA3,TPR,DDX39A,NUP62CL,THOC1,UPF3B,THOC2,NUP155,RBFOX1,NDC1,FMR1,NCBP3,RBM8A,SARNP,SRSF7,NUP35	darkgrey	EC_M8

GO:0000226	1.85E-16	1	112	522	microtubule cytoskeleton organization	BP	2.94E-14	RTTN,CDK2,KATNA1,FBXO5,KIF3A,KIFC1,KIF20A,HAUS6,KIF24,AKAP9,GCC2,SENP6,TTK,XPO1,GEN1,PLK4,STIL,CEP57,CEP295,CHORDC1,CENPA,CLASP2,KIF2C,BORA,ULK4,CDC20,HOOK3,SGO1,CKAP2,TUBGCP3,BRCA2,CCNB1,WDR62,GAS2L3,HAUS5,ESPL1,TPX2,SKA3,LSM14A,CENPJ,FER,KIF11,CENPH,CCDC66,CETN3,RACGAP1,ALMS1,CKAP5,KNSTRN,ABRAXAS1,MYBL2,INO80,NUSAP1,STMND1,KIF18A,SLK,CHEK1,SMC3,CENPE,CEP72,CAMSAP2,HAUS2,INTS13,TBCE,CFL1,ASPM,RCC1,SPC25,RAE1,NEK2,PRC1,AUNIP,CDK1,HDGFL3,NUF2,SLC16A1,SETD2,TPR,KIF18B,KIF15,BRCA1,CEP152,HAUS3,TACC3,DLGAP5,FKBP4,HNRNPU,INCENP,SMC1A,CCNB2,KPNB1,KIF4A,SPDL1,AURKB,ATRX,KIF23,TRIM37,PLK1,NDC80,STAG2,CEP76,TAOK1,CCNF,SAPCD2,GAS8,SASS6,ODF2,USP33,MAD2L1,STMN1,AURKA,DRG1	darkgrey	EC_M8
GO:0000725	2.17E-16	1	46	126	recombinational repair	BP	3.42E-14	RPA3,RAD21,RMI1,RECQL4,TONSL,MCM5,MCM3,MMS22L,SMC6,GEN1,MCM2,RAD54L,MCM6,RIF1,GINS4,BRCA2,PPP4R2,MCM4,ERCC6,PARPBP,NBN,RAD51,ARID2,INO80,NUCKS1,CHEK1,FANCB,RPA2,BLM,AUNIP,SETD2,FANCM,C17orf53,CDC45,BRCA1,MCM7,RAD51AP1,RAD52,FUS,SMCHD1,NIPBL,RMI2,RPA1,FEN1,PALB2,SFR1	darkgrey	EC_M8
GO:0060255	2.35E-16	1	555	4468	regulation of macromolecule metabolic process	BP	3.66E-14	CDK2,PAN2,ONECUT2,BCLAF1,CLK4,HNRNPH1,PPIL4,LATS1,NUP107,CCT2,DDX39B,FBXO5,ZNF608,SCAF8,UBR5,EIF3E,MAP3K4,RAD21,DSCC1,MRPL13,HSF2,ATAD2,XRCC6,PHF5A,CDC25C,TNRC6B,TUT7,MATR3,SRPK1,SRSF3,PLEC,PSIP1,IFT74,POT1,PWP1,ZNF300,TOPORS,SMU1,DBF4,POLR3B,XPO5,MTERF1,POLH,AKAP9,PUM3,RFX3,IL1R1,CKS2,TRIM28,NCBP1,ZNF451,ANP32B,LRPPRC,FOXJ3,ZNF189,MSH2,SP4,CDCA7L,PHIP,YTHDC1,MTIF2,TTK,IBTK,HNRNPA2B1,PNPT1,PUS10,ZMPSTE24,RLF,SYNCRIP,ZNF292,XPO1,PABPC4,CCT4,COMMD1,CASP8AP2,ETAA1,TIA1,ZNF350,ITGB1BP1,THRAP3,EZH2,UFL1,CCNC,CLSPN,ASCC3,CASP2,DDX1,NAA15,ELF2,WDR35,NRDC,ATAD2B,DHFR,CDCA7,PUS7,HDAC2,CCNA2,EXOSC9,MLLT10,SAE1,CEP295,DNAJC2,CHORDC1,RNASEH2B,SETDB2,SYMPK,MASTL,TRDMT1,CNOT10,NUP85,ELF1,UPF2,RSF1,BORA,DIS3,CCNT2,PAXIP1,XRN2,NCAPG2,WDR43,CDC20,BIRC5,FNTA,POLR3F,GTPBP4,IPO5,EPC2,SUPT16H,CHD8,KAT6A,HNRNPL,METTL3,RIF1,E2F7,ALYREF,CKAP2,CEP290,NSD3,CEBPZ,KITLG,SUPT20H,TAF7,EXOSC8,NR2C1,RFC3,CUL4A,BRCA2,PPP4R2,ELK3,RRP8,TCERG1,MAPK8,PRKDC,APAF1,CBX5,CCNB1,ERCC6,NFKBID,ZNF214,DHX29,PAN3,SCYL2,GPBP1,SP1,TCEA1,ZNF664,TPX2,CAPRIN1,ZC3H8,DIMT1,UBA2,IPO7,PARPBP,TNKS2,LSM14A,SGK3,BTAF1,ZNF143,PARP4,CENPJ,CDK5RAP1,FER,TDG,NCK1,IDE,SBNO1,ARFGEF1,SMARCA5,RNF141,PTCD3,ZNF507,ABCE1,OTUD4,CHD1,ZFP91,TAF9,CGGBP1,XRN1,PDE12,TFCP2,SAP30,ATR,TTC37,TERF1,MTMR9,FAM208A,HELLS,GCFC2,LARP4,USP25,UTP15,RBM12,HLTF,RBM39,PBK,ESCO2,ZBTB10,CNOT1,GABPA,MCRS1,SH2B3,LT	darkgrey	EC_M8
GO:0065004	2.70E-16	1	46	132	protein-DNA complex assembly	BP	4.15E-14	SHPRH,ANP32B,SENP6,TAF1B,MCM2,CENPO,CENPA,NASP,RSF1,KAT6A,NAP1L1,TAF7,SMARCA5,CENPH,TAF9,BDP1,KNTC1,TERF1,HELLS,MIS18A,RAD51,OIP5,CHAF1B,NAP1L4,GMNN,CENPE,HJURP,CENPF,HAT1,UBTF,CDC45,HP1BP3,DLGAP5,RAD52,ASF1B,TAF1,ATRX,CENPI,CHAF1A,UBN1,CDT1,CENPN,HIST1H1A,ITGB3BP,HIST1H3H,CENPW	darkgrey	EC_M8
GO:0044271	2.93E-16	1	444	3436	cellular nitrogen compound biosynthetic process	BP	4.47E-14	PAN2,NARS,ONECUT2,BCLAF1,PPIL4,MTHFD1L,NUP107,CCT2,DDX39B,ZNF608,SCAF8,EIF3E,MAP3K4,RAD21,DSCC1,MRPL13,HSF2,ATAD2,XRCC6,PHF5A,TNRC6B,TUT7,PSIP1,IFT74,POT1,PWP1,ZNF300,TOPORS,POLR3B,MTERF1,POLH,PUM3,RFX3,CKS2,PPAT,PAICS,REV1,TRIM28,NCBP1,ZNF451,PHF3,TWISTNB,LRPPRC,FOXJ3,ZNF189,SP4,CDCA7L,CTPS1,PHIP,MTIF2,HNRNPA2B1,PNPT1,DCK,ZMPSTE24,RLF,SYNCRIP,ZNF292,XPO1,PABPC4,CCT4,COMMD1,CASP8AP2,TIA1,ZNF350,ITGB1BP1,THRAP3,TAF1B,EZH2,UFL1,MRPL32,CCNC,ASCC3,DDX1,NAA15,ELF2,WDR35,AMD1,ATAD2B,DHFR,CDCA7,PUS7,HDAC2,CCNA2,EXOSC9,MLLT10,DNAJC2,SETDB2,PCF11,CNOT10,NIFK,NUP85,MRPS7,CAD,ELF1,RSF1,DIS3,CCNT2,PAXIP1,DARS,XRN2,NCAPG2,WDR43,TK1,AFMID,BIRC5,POLR3F,EPC2,SUPT16H,CHD8,KAT6A,METTL3,RIF1,E2F7,ALYREF,CKAP2,RRM1,CEP290,NSD3,CEBPZ,SUPT20H,TAF7,EXOSC8,POLE,NR2C1,RFC3,BRCA2,LARS,ELK3,RRP8,TCERG1,MAPK8,PRKDC,CBX5,ERCC6,ZNF214,DHX29,PAN3,GPBP1,SP1,TCEA1,MRPL15,ZNF664,MRPS5,CAPRIN1,ZC3H8,POLR1B,TNKS2,LSM14A,SGK3,BTAF1,ZNF143,PARP4,CENPJ,CDK5RAP1,FER,EIF2B1,TDG,NCK1,SBNO1,SMARCA5,MRPL35,RNF141,PTCD3,ZNF507,ABCE1,POLR1A,CHD1,ZFP91,TAF9,CGGBP1,XRN1,PDE12,SMN2,BDP1,TFCP2,SAP30,ATR,TERF1,GCFC2,LARP4,UTP15,HLTF,ZBTB10,CNOT1,GABPA,MCRS1,LTN1,USP16,PPID,RBL1,RPS6,GMPS,CCT7,CCNL1,SENP1,RIPK1,WDR75,ARID2,MYBL2,E2F8,TRIM33,INO80,ZBED6,EEF1E1,CHUK,ZBTB11,ZNF248,MGA,MDM4,HIVEP1,RFC5,BRWD1,NOLC1,CCT5,BMPR1B,UBE3A,CCT6A,PRDM10,NUCKS1,DEK,GCN1,CUL3,METAP1,	darkgrey	EC_M8

GO:0007052	3.65E-16	1	42	109	mitotic spindle organization	BP	5.51E-14	KIFC1,TTK,STIL,CLASP2,BORA,CDC20,CCNB1,WDR62,TPX2,LSM14A,KIF11,CENPH,RACGAP1,CKAP5,ABRAXAS1,MYBL2,SMC3,CENPE,INTS13,TBCE,RCC1,SPC25,RAE1,NEK2,PRC1,NUF2,TPR,KIF15,TACC3,DLGAP5,HNRNPU,SMC1A,KPNB1,KIF4A,AURKB,KIF23,PLK1,NDC80,STAG2,STMN1,AURKA,DRG1	darkgrey	EC_M8
GO:0000724	6.29E-16	1	45	124	double-strand break repair via homologous recombination	BP	9.40E-14	RPA3,RAD21,RMI1,RECQL4,TONSL,MCM5,MCM3,MMS22L,SMC6,GEN1,MCM2,RAD54L,MCM6,RIF1,GINS4,BRCA2,PPP4R2,MCM4,ERCC6,PARPBP,NBN,RAD51,ARID2,INO80,NUCKS1,CHEK1,FANCB,RPA2,BLM,AUNIP,SETD2,FANCM,CDC45,BRCA1,MCM7,RAD51AP1,RAD52,FUS,SMCHD1,NIPBL,RMI2,RPA1,FEN1,PALB2,SFR1	darkgrey	EC_M8
GO:2000112	9.68E-16	1	383	2822	regulation of cellular macromolecule biosynthetic process	BP	1.43E-13	PAN2,ONECUT2,BCLAF1,PPIL4,NUP107,CCT2,DDX39B,FBXO5,ZNF608,SCAF8,EIF3E,MAP3K4,RAD21,DSCC1,MRPL13,HSF2,ATAD2,XRCC6,PHF5A,TNRC6B,TUT7,PSIP1,IFT74,POT1,PWP1,ZNF300,TOPORS,DBF4,MTERF1,PUM3,RFX3,CKS2,TRIM28,NCBP1,ZNF451,LRPPRC,FOXJ3,ZNF189,SP4,CDCA7L,PHIP,MTIF2,HNRNPA2B1,PNPT1,ZMPSTE24,RLF,SYNCRIP,ZNF292,XPO1,CCT4,COMMD1,CASP8AP2,TIA1,ZNF350,ITGB1BP1,THRAP3,EZH2,UFL1,CCNC,ASCC3,DDX1,NAA15,ELF2,ATAD2B,DHFR,CDCA7,PUS7,HDAC2,CCNA2,EXOSC9,MLLT10,DNAJC2,SETDB2,CNOT10,NUP85,ELF1,RSF1,DIS3,CCNT2,PAXIP1,WDR43,BIRC5,POLR3F,GTPBP4,EPC2,SUPT16H,CHD8,KAT6A,METTL3,RIF1,E2F7,ALYREF,CKAP2,CEP290,NSD3,CEBPZ,SUPT20H,TAF7,EXOSC8,NR2C1,RFC3,BRCA2,ELK3,RRP8,TCERG1,MAPK8,PRKDC,CBX5,ERCC6,ZNF214,DHX29,PAN3,GPBP1,SP1,TCEA1,ZNF664,CAPRIN1,ZC3H8,TNKS2,LSM14A,SGK3,BTAF1,ZNF143,PARP4,CENPJ,CDK5RAP1,FER,TDG,NCK1,SBNO1,ARFGEF1,SMARCA5,RNF141,PTCD3,ZNF507,ABCE1,CHD1,ZFP91,TAF9,CGGBP1,XRN1,PDE12,TFCP2,SAP30,ATR,TERF1,GCFC2,LARP4,UTP15,HLTF,ESCO2,ZBTB10,CNOT1,GABPA,MCRS1,LTN1,USP16,PPID,RBL1,CCT7,CCNL1,NBN,SENP1,RIPK1,WDR75,ARID2,MYBL2,E2F8,TRIM33,INO80,ZBED6,CHUK,ZBTB11,ZNF248,MGA,MDM4,HIVEP1,RFC5,BRWD1,NOLC1,CCT5,BMPR1B,UBE3A,CCT6A,PRDM10,NUCKS1,DEK,GCN1,CUL3,METAP1,GMNN,CHEK1,H2AFZ,RRP1B,NFKB1,SMC3,KHDRBS1,TRIP13,TET2,TRIP11,HEATR1,NCL,TRAIP,ZNF184,PRMT5,AJUBA,GTF2IRD2,ZBTB41,ARID4B,NOL11,GTF2I,TARBP1,BPTF,FXR1,NGDN,DDX5,TICRR,RFC2,ORC2,NEK2,EIF2B5,EWSR1,TIAL1,	darkgrey	EC_M8
GO:0050657	1.11E-15	1	46	135	nucleic acid transport	BP	1.61E-13	XPOT,NUP43,NUP107,DDX39B,SRSF3,XPO5,NCBP1,LRPPRC,YTHDC1,HNRNPA2B1,PNPT1,XPO1,NUP205,NUP85,UPF2,ALYREF,NUP58,CETN3,NUP160,NUP54,CKAP5,NUP93,NSUN2,KHDRBS1,MCM3AP,RAE1,NUP133,SSB,DHX9,SETD2,HNRNPA3,TPR,DDX39A,NUP62CL,THOC1,UPF3B,THOC2,NUP155,RBFOX1,NDC1,FMR1,NCBP3,RBM8A,SARNP,SRSF7,NUP35	darkgrey	EC_M8
GO:0050658	1.11E-15	1	46	135	RNA transport	BP	1.61E-13	XPOT,NUP43,NUP107,DDX39B,SRSF3,XPO5,NCBP1,LRPPRC,YTHDC1,HNRNPA2B1,PNPT1,XPO1,NUP205,NUP85,UPF2,ALYREF,NUP58,CETN3,NUP160,NUP54,CKAP5,NUP93,NSUN2,KHDRBS1,MCM3AP,RAE1,NUP133,SSB,DHX9,SETD2,HNRNPA3,TPR,DDX39A,NUP62CL,THOC1,UPF3B,THOC2,NUP155,RBFOX1,NDC1,FMR1,NCBP3,RBM8A,SARNP,SRSF7,NUP35	darkgrey	EC_M8
GO:0010556	1.30E-15	1	385	2846	regulation of macromolecule biosynthetic process	BP	1.87E-13	PAN2,ONECUT2,BCLAF1,PPIL4,NUP107,CCT2,DDX39B,FBXO5,ZNF608,SCAF8,EIF3E,MAP3K4,RAD21,DSCC1,MRPL13,HSF2,ATAD2,XRCC6,PHF5A,TNRC6B,TUT7,PSIP1,IFT74,POT1,PWP1,ZNF300,TOPORS,DBF4,MTERF1,PUM3,RFX3,CKS2,TRIM28,NCBP1,ZNF451,LRPPRC,FOXJ3,ZNF189,SP4,CDCA7L,PHIP,MTIF2,HNRNPA2B1,PNPT1,ZMPSTE24,RLF,SYNCRIP,ZNF292,XPO1,CCT4,COMMD1,CASP8AP2,TIA1,ZNF350,ITGB1BP1,THRAP3,EZH2,UFL1,CCNC,ASCC3,DDX1,NAA15,ELF2,ATAD2B,DHFR,CDCA7,PUS7,HDAC2,CCNA2,EXOSC9,MLLT10,DNAJC2,SETDB2,CNOT10,NUP85,ELF1,RSF1,DIS3,CCNT2,PAXIP1,WDR43,BIRC5,POLR3F,GTPBP4,EPC2,SUPT16H,CHD8,KAT6A,METTL3,RIF1,E2F7,ALYREF,CKAP2,CEP290,NSD3,CEBPZ,SUPT20H,TAF7,EXOSC8,NR2C1,RFC3,BRCA2,ELK3,RRP8,TCERG1,MAPK8,PRKDC,CBX5,ERCC6,ZNF214,DHX29,PAN3,GPBP1,SP1,TCEA1,ZNF664,CAPRIN1,ZC3H8,TNKS2,LSM14A,SGK3,BTAF1,ZNF143,PARP4,CENPJ,CDK5RAP1,FER,TDG,NCK1,SBNO1,ARFGEF1,SMARCA5,RNF141,PTCD3,ZNF507,ABCE1,CHD1,ZFP91,TAF9,CGGBP1,XRN1,PDE12,TFCP2,SAP30,ATR,TERF1,GCFC2,LARP4,UTP15,HLTF,ESCO2,ZBTB10,CNOT1,GABPA,MCRS1,LTN1,USP16,PPID,RBL1,CCT7,CCNL1,NBN,SENP1,RIPK1,WDR75,ARID2,MYBL2,E2F8,TRIM33,INO80,ZBED6,CHUK,ZBTB11,ZNF248,MGA,MDM4,HIVEP1,RFC5,BRWD1,NOLC1,CCT5,BMPR1B,UBE3A,CCT6A,PRDM10,NUCKS1,DEK,GCN1,CUL3,METAP1,GMNN,CHEK1,H2AFZ,RRP1B,NFKB1,SMC3,KHDRBS1,TRIP13,TET2,TRIP11,HEATR1,NCL,TRAIP,ZNF184,PRMT5,AJUBA,GTF2IRD2,ZBTB41,ARID4B,NOL11,GTF2I,TARBP1,BPTF,FXR1,NGDN,DDX5,TICRR,RFC2,ORC2,NEK2,EIF2B5,EWSR1,TIAL1,	darkgrey	EC_M8

GO:0006275	1.35E-15	1	38	92	regulation of DNA replication	BP	1.92E-13	FBXO5,DSCC1,DBF4,ZMPSTE24,CCNA2,GTPBP4,E2F7,RFC3,BRCA2,ATR,TERF1,ESCO2,NBN,E2F8,INO80,RFC5,NUCKS1,GMNN,SMC3,TICRR,RFC2,BLM,CDK1,DHX9,RFC4,DNA2,DBF4B,PDS5A,WAPL,CDC6,ATRX,TIPIN,RBBP6,ESCO1,ATAD5,CHTF18,CDT1,RNF219	darkgrey	EC_M8
GO:0009058	1.43E-15	1	537	4393	biosynthetic process	BP	2.01E-13	CDK2,PAN2,NARS,ONECUT2,BCLAF1,RPA3,PPIL4,MTHFD1L,NUP107,CCT2,DDX39B,FBXO5,ZNF608,SCAF8,EIF3E,MAP3K4,RAD21,DSCC1,MRPL13,HSF2,ATAD2,XRCC6,PHF5A,SLC35D2,TNRC6B,TUT7,RMI1,PSIP1,RECQL4,TONSL,IFT74,MCM5,POT1,PWP1,ZNF300,TOPORS,DBF4,POLR3B,MTERF1,POLH,PUM3,RFX3,CKS2,MCM3,PPAT,C1GALT1,PAICS,REV1,TRIM28,NCBP1,ZNF451,PHF3,TWISTNB,LRPPRC,FOXJ3,ZNF189,SP4,CDCA7L,CTPS1,PHIP,MTIF2,PPP4R3B,HNRNPA2B1,PNPT1,DCK,ZMPSTE24,RLF,SYNCRIP,ZNF292,XPO1,PABPC4,CCT4,COMMD1,CASP8AP2,DPY19L1,ETAA1,TIA1,ZNF350,ITGB1BP1,THRAP3,TAF1B,EZH2,UFL1,MMS22L,FAM3C,MRPL32,CCNC,ASCC3,ORC6,DDX1,NAA15,ELF2,ORC1,GEN1,WDR35,AMD1,ATAD2B,DHFR,CDCA7,PUS7,HDAC2,MCM2,CCNA2,EXOSC9,MLLT10,DNAJC2,PDSS1,SETDB2,NASP,COG3,PCF11,CNOT10,NIFK,NUP85,MRPS7,MCM10,CAD,ELF1,ALG8,RSF1,ACOX1,DIS3,CCNT2,PAXIP1,MCM6,DARS,XRN2,NCAPG2,WDR43,TK1,AFMID,BIRC5,POLR3F,ORC4,GTPBP4,EPC2,SUPT16H,CHD8,KAT6A,METTL3,RIF1,NAP1L1,ZDHHC17,E2F7,GINS4,ALYREF,CKAP2,RRM1,CEP290,NSD3,CEBPZ,SUPT20H,TAF7,EXOSC8,PRKD3,POLE,NR2C1,RFC3,BRCA2,LARS,LTA4H,TOPBP1,ELK3,RRP8,TCERG1,MAPK8,PRKDC,CBX5,MCM4,ERCC6,ZNF214,DHX29,PAN3,GPBP1,SP1,TCEA1,MRPL15,ZNF664,MRPS5,CAPRIN1,ZC3H8,POLR1B,TNKS2,LSM14A,SGK3,BTAF1,ZNF143,PARP4,CENPJ,CDK5RAP1,FER,EIF2B1,TDG,NCK1,SBNO1,ARFGEF1,SMARCA5,MRPL35,RNF141,PTCD3,ZNF507,ABCE1,POLR1A,CHD1,ZFP91,GK5,GALNT6,TAF9,CGGBP1,XRN1,PDE12,SMN2,BDP1,TFCP2,SAP30,ATR,TERF1,NOC3L,GCFC2,LARP4,UTP15	darkgrey	EC_M8
GO:1903046	2.45E-15	1	49	153	meiotic cell cycle process	BP	3.43E-13	FBXO43,FBXO5,RAD21,CDC25C,RMI1,UBR2,CKS2,FOXJ3,SMC2,TTK,RAD54L,FANCD2,CDC20,TOP2B,SGO1,BRCA2,PSMD13,ESPL1,BUB1,TERF1,MND1,MSH3,BUB1B,RAD51,CCNE2,NCAPD3,KIF18A,NSUN2,TRIP13,SGO2,ASPM,SPO11,CDC25A,NUF2,FANCM,SMC4,NCAPD2,RAD51AP1,INCENP,TOP2A,CCNB2,EME1,ATRX,PTTG1,PLK1,BRIP1,NDC1,FANCA,AURKA	darkgrey	EC_M8
GO:0080090	3.35E-15	1	523	4206	regulation of primary metabolic process	BP	4.64E-13	CDK2,PAN2,ONECUT2,BCLAF1,CLK4,HNRNPH1,PPIL4,LATS1,NUP107,CCT2,DDX39B,FBXO5,ZNF608,SCAF8,UBR5,EIF3E,MAP3K4,RAD21,DSCC1,MRPL13,HSF2,ATAD2,XRCC6,PHF5A,CDC25C,TNRC6B,TUT7,SRPK1,SRSF3,PLEC,PSIP1,IFT74,POT1,PWP1,ZNF300,TOPORS,SMU1,DBF4,MTERF1,POLH,AKAP9,PUM3,RFX3,CKS2,TRIM28,NCBP1,ZNF451,ANP32B,LRPPRC,FOXJ3,ZNF189,MSH2,SP4,CDCA7L,PHIP,YTHDC1,MTIF2,TTK,PPP4R3B,IBTK,HNRNPA2B1,PNPT1,ZMPSTE24,RLF,SYNCRIP,ZNF292,XPO1,PABPC4,CCT4,COMMD1,CASP8AP2,ETAA1,TIA1,ZNF350,ITGB1BP1,THRAP3,EZH2,UFL1,FAM3C,CCNC,CLSPN,ASCC3,CASP2,DDX1,NAA15,ELF2,WDR35,NRDC,ATAD2B,DHFR,CDCA7,PUS7,HDAC2,CCNA2,EXOSC9,MLLT10,SAE1,CEP295,DNAJC2,CHORDC1,SETDB2,SYMPK,MASTL,TRDMT1,CNOT10,NUP85,ELF1,RSF1,BORA,DIS3,CCNT2,PAXIP1,NCAPG2,WDR43,CDC20,BIRC5,FNTA,POLR3F,GTPBP4,IPO5,EPC2,SUPT16H,CHD8,KAT6A,HNRNPL,METTL3,RIF1,E2F7,ALYREF,CKAP2,CEP290,NSD3,CEBPZ,KITLG,SUPT20H,TAF7,EXOSC8,NR2C1,RFC3,CUL4A,BRCA2,PPP4R2,ELK3,RRP8,TCERG1,MAPK8,PRKDC,APAF1,CBX5,CCNB1,ERCC6,ZNF214,DHX29,PAN3,GPBP1,SP1,TCEA1,ZNF664,TPX2,CAPRIN1,ZC3H8,DIMT1,UBA2,IPO7,PARPBP,TNKS2,LSM14A,SGK3,BTAF1,ZNF143,PARP4,CENPJ,CDK5RAP1,FER,TDG,NCK1,IDE,SBNO1,ARFGEF1,SMARCA5,RNF141,PTCD3,ZNF507,ABCE1,OTUD4,CHD1,ZFP91,TAF9,CGGBP1,XRN1,PDE12,TFCP2,SAP30,ATR,TERF1,MTMR9,GCFC2,LARP4,USP25,UTP15,RBM12,HLTF,RBM39,PBK,ZBTB10,CNOT1,GABPA,MCRS1,SH2B3,LTN1,USP16,PPID,RBL1,MOB1A,MSH3,MIS18A,CCT7,CCNL1,CDCA2,NBN,FAM83D,SENP1,	darkgrey	EC_M8
GO:0090068	3.50E-15	1	54	180	positive regulation of cell cycle process	BP	4.80E-13	DDX39B,FBXO5,RAD21,CDC25C,DBF4,PHIP,EZH2,GEN1,PLK4,CEP295,E2F7,CUL4A,CDC16,CCNB1,ESPL1,KIF20B,CENPJ,RACGAP1,ANAPC7,FAM83D,PKP4,E2F8,INO80,NUSAP1,UBE2C,CIT,CUL3,KIF14,DTL,CDK1,CDC25A,TPR,CDC27,CDCA5,DBF4B,DLGAP5,RAD51AP1,ECT2,DDX11,HNRNPU,CDC6,AURKB,ATRX,STXBP4,KIF23,THOC1,NDC80,ATAD5,CDT1,PKN2,TMOD3,MAD2L1,AURKA,FEN1	darkgrey	EC_M8

GO:0071840	4.76E-15	1	588	4827	cellular component organization or biogenesis	BP	6.48E-13	RTTN,CDK2,PAN2,ONECUT2,MTFR2,RPA3,SHPRH,KATNA1,LATS1,NUP107,CCT2,DDX39B,SRFBP1,FBXO43,FBXO5,SCAF8,EIF3E,EBAG9,MAP3K4,RAD21,DSCC1,KIF3A,TBC1D32,RRP7A,KIFC1,ATAD2,XRCC6,KIF20A,CDC25C,FANCC,HSPA9,CHCHD3,SRPK1,RMI1,PLEC,PSIP1,RECQL4,CEP78,HAUS6,UBR2,IFT74,MCM5,POT1,PWP1,TOPORS,MTERF1,KIF24,AKAP9,GCC2,RFX3,CKS2,MCM3,ICK,NOL8,TRIM28,NCBP1,ANP32B,STX17,PPIH,FOXJ3,MSH2,SMC2,SENP6,PHIP,YTHDC1,MTIF2,TTK,HNRNPA2B1,PNPT1,RUFY3,CEP162,ZMPSTE24,XPO1,PLEKHA8,CCT4,MDN1,NUP205,ANLN,CDCA8,GNL2,ITGB1BP1,TAF1B,CAPZA2,EZH2,UFL1,NOL10,ASCC3,ZMYM4,CNTRL,DDX1,SMC6,GEN1,AFG1L,WDR35,SCLT1,PLK4,DHFR,KIAA1109,STIL,HDAC2,MCM2,CENPO,CEP57,EXOSC9,PMPCB,SAE1,CEP295,DNAJC2,RAD54L,CHORDC1,SETDB2,YME1L1,CENPA,NASP,COG3,CLASP2,DCLRE1C,NIFK,NUP85,MRPS7,KIF2C,RSF1,DIAPH3,DDX18,BORA,DIS3,MCM6,DARS,FANCD2,XRN2,ULK4,NCAPG2,WDR43,CDC20,TK1,CRNKL1,EBNA1BP2,FNTA,PARP2,PITRM1,HOOK3,GTPBP4,SUPT16H,CHD8,KAT6A,RIF1,NAP1L1,TOP2B,ZDHHC17,SGO1,PRPF40A,CKAP2,VPS36,STRN,RRM1,CEP290,TAF7,EXOSC8,TUBGCP3,DNAJC13,RFC3,CUL4A,DDX51,MAK16,BRCA2,SNRPF,LARS,PSMD13,RRP8,CDC16,MAPK8,PRKDC,NCAPH,ARHGAP22,CCNB1,MCM4,WDR62,ERCC6,NOP56,DHX29,ACTR6,PAN3,SCYL2,MTREX,GAS2L3,HAUS5,DHX37,UTP20,TGS1,ESPL1,BUB1,TPX2,ANAPC1,CAPRIN1,BMS1,NDUFAF2,POLR1B,KIF20B,SKA3,DIMT1,TNKS2,RRS1,LSM14A,SGK3,WDR36,CENPJ,FER,TDG,NCK1,CEP89,KIF11,ARFGEF1,SMARCA5,ABCE1,CHD1,CENPH,TAF9,XRN1,SMN2,BDP1,KN	darkgrey	EC_M8
GO:0033047	5.72E-15	1	24	41	regulation of mitotic sister chromatid segregation	BP	7.71E-13	FBXO5,RAD21,TTK,GEN1,CCNB1,BUB1,KNTC1,BUB1B,TRIP13,CENPF,BUB3,TPR,ZWINT,HNRNPU,MAD1L1,SPDL1,AURKB,PTTG1,ZWILCH,LCMT1,PLK1,NDC80,CDT1,MAD2L1	darkgrey	EC_M8
GO:0033554	8.49E-15	1	235	1561	cellular response to stress	BP	1.13E-12	CDK2,BCLAF1,RPA3,SHPRH,MDC1,DDX39B,FBXO5,UBR5,MAP3K4,GTSE1,RAD21,XRCC6,FANCC,HSPA9,RMI1,PLEC,RECQL4,TONSL,MCM5,POT1,TOPORS,APTX,POLH,MCM3,MIOS,REV1,MELK,TRIM28,MSH2,PNPT1,ZMPSTE24,COMMD1,ETAA1,EZH2,UFL1,MMS22L,USP45,CLSPN,ASCC3,CASP2,DDX1,SMC6,GEN1,DHFR,RINT1,HDAC2,MCM2,CCNA2,DNAJC2,RAD54L,CHORDC1,RNASEH2B,WDR33,MASTL,DDIAS,DCLRE1C,MCM10,MBD4,PAXIP1,MCM6,FANCD2,ULK4,PARP2,RAD18,EPC2,SUPT16H,KAT6A,METTL3,RIF1,E2F7,GINS4,POLE,RFC3,CUL4A,BRCA2,PPP4R2,LARS,TOPBP1,RRP8,MAPK8,PRKDC,CBX5,CCNB1,MCM4,ERCC6,MTREX,PARPBP,PARP4,FER,TDG,NCK1,TAF9,ATR,CDKN2AIP,USP25,HK2,PBK,ESCO2,MCRS1,USP16,RBL1,MSH3,NBN,ABRAXAS1,RAD51,SLC38A2,DCLRE1B,RIPK1,ARID2,COL4A3BP,INO80,EEF1E1,CHUK,MDM4,CHAF1B,RFC5,TAOK3,SMARCAD1,NUCKS1,DEK,GCN1,CUL3,UBE2T,CBSL,CHEK1,NFKB1,SMC3,TRIP13,CHD1L,TRAIP,AJUBA,GTF2I,FXR1,FANCI,DDX5,FANCB,TICRR,RFC2,RPA2,SPO11,EIF2B5,INTS7,DTL,BLM,ATRIP,AUNIP,XPR1,CCDC47,CDK1,PDE8A,BAZ1B,DHX9,SETD2,POLA1,RFC4,DNA2,TPR,CDCA5,LANCL1,FANCM,C17orf53,APEX2,XRCC5,CDC45,BRCA1,MCM7,NSD2,LETM1,WDHD1,HP1BP3,DCUN1D5,TIPRL,RAD51AP1,BOD1L1,ANKLE1,ECT2,SUV39H1,FOXM1,EIF2AK1,FH,DDX11,DNAJC7,EXO1,RAD52,PDS5A,UBE2K,NEDD4,RFC1,TOP2A,SMC1A,DCTPP1,FUS,KIF22,USP3,EME1,TAF1,HACD3,ATRX,PTTG1,TIPIN,THG1L,STXBP4,RBBP6,PLK1,BRIP1,AATF,PSMD10,THOC1,SMCHD1,ATAD5,HNRNPM,CUL4B,XIAP,NIPBL,AIFM1,RSL1D1,TAOK1,RMI2,UHRF1,CHAF1A,FMR1,RPA1,TP73,FANCA,U	darkgrey	EC_M8
GO:0033046	1.10E-14	1	23	39	negative regulation of sister chromatid segregation	BP	1.43E-12	FBXO5,RAD21,TTK,GEN1,CCNB1,BUB1,KNTC1,BUB1B,TRIP13,CENPF,BUB3,TPR,ZWINT,MAD1L1,SPDL1,AURKB,PTTG1,ZWILCH,LCMT1,PLK1,NDC80,CDT1,MAD2L1	darkgrey	EC_M8
GO:0033048	1.10E-14	1	23	39	negative regulation of mitotic sister chromatid segregation	BP	1.43E-12	FBXO5,RAD21,TTK,GEN1,CCNB1,BUB1,KNTC1,BUB1B,TRIP13,CENPF,BUB3,TPR,ZWINT,MAD1L1,SPDL1,AURKB,PTTG1,ZWILCH,LCMT1,PLK1,NDC80,CDT1,MAD2L1	darkgrey	EC_M8
GO:2000816	1.10E-14	1	23	39	negative regulation of mitotic sister chromatid separation	BP	1.43E-12	FBXO5,RAD21,TTK,GEN1,CCNB1,BUB1,KNTC1,BUB1B,TRIP13,CENPF,BUB3,TPR,ZWINT,MAD1L1,SPDL1,AURKB,PTTG1,ZWILCH,LCMT1,PLK1,NDC80,CDT1,MAD2L1	darkgrey	EC_M8
GO:0000723	1.11E-14	1	42	118	telomere maintenance	BP	1.44E-12	RPA3,CCT2,MAP3K4,XRCC6,RECQL4,POT1,HNRNPA2B1,CCT4,SMC6,DCLRE1C,RIF1,BRCA2,PRKDC,TNKS2,XRN1,ATR,TERF1,CCT7,NBN,RAD51,DCLRE1B,CCNE2,CCT5,CCT6A,GAR1,RPA2,NEK2,BLM,DNA2,XRCC5,EXO1,HNRNPU,RFC1,DCP2,EXOSC10,CCT3,AURKB,ATRX,RPA1,HNRNPD,NAF1,FEN1	darkgrey	EC_M8

GO:0010608	1.38E-14	1	98	480	posttranscriptional regulation of gene expression	BP	1.77E-12	PAN2,DDX39B,EIF3E,MRPL13,TNRC6B,TUT7,MATR3,XPO5,PUM3,NCBP1,LRPPRC,YTHDC1,MTIF2,HNRNPA2B1,PNPT1,PUS10,ZMPSTE24,SYNCRIP,PABPC4,TIA1,THRAP3,ASCC3,DDX1,DHFR,PUS7,EXOSC9,TRDMT1,CNOT10,DIS3,METTL3,EXOSC8,PRKDC,DHX29,PAN3,CAPRIN1,LSM14A,CDK5RAP1,NCK1,PTCD3,ABCE1,XRN1,PDE12,LARP4,CNOT1,LTN1,USP16,TENT2,RIPK1,NOLC1,GCN1,METAP1,NSUN2,KHDRBS1,NCL,AJUBA,FXR1,NGDN,DDX5,EIF2B5,TIAL1,FASTKD1,DDX6,DHX9,HNRNPR,FASTKD2,TPR,DGCR8,CNOT9,EIF2AK1,HNRNPU,DCP2,EIF3B,SEPSECS,FUS,GEMIN5,LARP1,ILF3,TAF15,EIF5,TYMS,UPF3B,HNRNPM,SERBP1,GSPT1,ZC3H7A,DROSHA,FMR1,METTL16,EXOSC2,RBM8A,METTL18,SRRT,SARNP,FASTKD5,HNRNPD,NAF1,RNF139,PAIP2	darkgrey	EC_M8
GO:0045787	1.48E-14	1	64	243	positive regulation of cell cycle	BP	1.88E-12	YTHDC2,DDX39B,FBXO5,RAD21,CDC25C,DBF4,CKS2,PHIP,EZH2,GEN1,PLK4,CEP295,CCNT2,E2F7,CUL4A,BRCA2,CDC16,CCNB1,ESPL1,KIF20B,CENPJ,CDK5RAP1,RACGAP1,ANAPC7,FAM83D,PKP4,E2F8,INO80,NUSAP1,ZBED6,UBE2C,CIT,CUL3,CHEK1,KIF14,ZBED9,DTL,CDK1,CDC25A,FOSL1,TPR,CDC27,CDCA5,DBF4B,DLGAP5,RAD51AP1,ECT2,DDX11,HNRNPU,CDC6,AURKB,ATRX,STXBP4,KIF23,PSMD10,THOC1,NDC80,ATAD5,CDT1,PKN2,TMOD3,MAD2L1,AURKA,FEN1	darkgrey	EC_M8
GO:0031570	1.56E-14	1	40	111	DNA integrity checkpoint signaling	BP	1.96E-12	CDK2,MDC1,DDX39B,GTSE1,MSH2,ETAA1,CLSPN,CASP2,ORC1,RINT1,RNASEH2B,FANCD2,CUL4A,TOPBP1,PRKDC,ERCC6,ATR,NBN,ABRAXAS1,TAOK3,CHEK1,TICRR,RPA2,INTS7,DTL,BLM,ATRIP,CDK1,DNA2,CDC45,BRCA1,TIPRL,CDC6,EME1,TIPIN,PLK1,BRIP1,THOC1,TAOK1,CDT1	darkgrey	EC_M8
GO:0044085	1.75E-14	1	335	2454	cellular component biogenesis	BP	2.18E-12	RTTN,CDK2,PAN2,ONECUT2,SHPRH,LATS1,NUP107,CCT2,DDX39B,SRFBP1,FBXO5,EIF3E,RAD21,KIF3A,TBC1D32,RRP7A,KIFC1,FANCC,HSPA9,SRPK1,PLEC,PSIP1,HAUS6,IFT74,POT1,PWP1,KIF24,AKAP9,RFX3,ICK,NOL8,TRIM28,NCBP1,ANP32B,STX17,PPIH,SENP6,YTHDC1,PNPT1,CEP162,XPO1,MDN1,NUP205,ANLN,GNL2,ITGB1BP1,TAF1B,CAPZA2,NOL10,DDX1,WDR35,SCLT1,PLK4,STIL,MCM2,CENPO,CEP57,EXOSC9,CEP295,SETDB2,YME1L1,CENPA,NASP,CLASP2,NIFK,NUP85,MRPS7,RSF1,DIAPH3,DDX18,DIS3,DARS,XRN2,WDR43,CDC20,TK1,CRNKL1,EBNA1BP2,GTPBP4,KAT6A,NAP1L1,VPS36,STRN,RRM1,CEP290,TAF7,EXOSC8,TUBGCP3,CUL4A,DDX51,MAK16,SNRPF,RRP8,PRKDC,CCNB1,WDR62,NOP56,DHX29,PAN3,MTREX,HAUS5,DHX37,UTP20,TGS1,TPX2,BMS1,NDUFAF2,POLR1B,DIMT1,RRS1,LSM14A,WDR36,CENPJ,FER,NCK1,CEP89,KIF11,ARFGEF1,SMARCA5,ABCE1,CENPH,TAF9,SMN2,BDP1,KNTC1,ATR,TERF1,FAM208A,HELLS,CCDC66,GCFC2,UTP15,RACGAP1,CNOT1,USP16,PPID,RPS6,ALMS1,MIS18A,URB1,NBN,CFAP298,CKAP5,NUP93,ABRAXAS1,KNL1,RAD51,SCAF11,RIPK1,WDR75,MYBL2,PKP4,INO80,OIP5,RIOK1,STK4,MDM4,CHAF1B,PAK1IP1,UBE2C,WDR3,HEATR3,NOLC1,CUL3,NAP1L4,PDCD11,GMNN,SLK,RRP1B,RRP1,FP565260.1,KCTD3,SMC3,SF3B1,RRP15,CENPE,TRIP13,RBM5,CEP72,HEATR1,CAMSAP2,COPS7B,HAUS2,KIF14,PRMT5,AJUBA,TBCE,DDX27,ASPM,NOL11,GAR1,FXR1,NGDN,RCC1,HJURP,SPO11,EIF3A,URB2,NEK2,PRC1,ALS2,BLM,DDX6,NOP58,WDR12,CENPF,FTSJ3,CDKL5,HAT1,CDK1,WBP11,SEPT2,DHX9,HDGFL3,FASTKD2,DDX20,PIKFYVE,DNA2,TPR,DDX21,PRPF39,GJC1,KIF15,RPL10L,IF	darkgrey	EC_M8
GO:0031323	1.99E-14	1	538	4391	regulation of cellular metabolic process	BP	2.47E-12	CDK2,PAN2,ONECUT2,BCLAF1,CLK4,HNRNPH1,PPIL4,LATS1,NUP107,CCT2,DDX39B,FBXO5,ZNF608,SCAF8,UBR5,EIF3E,MAP3K4,RAD21,DSCC1,MRPL13,HSF2,ATAD2,XRCC6,PHF5A,CDC25C,TNRC6B,TUT7,SRPK1,SRSF3,PLEC,PSIP1,IFT74,POT1,PWP1,ZNF300,TOPORS,SMU1,DBF4,MTERF1,POLH,AKAP9,PUM3,RFX3,CKS2,TRIM28,NCBP1,ZNF451,ANP32B,LRPPRC,FOXJ3,ZNF189,MSH2,SP4,CDCA7L,PHIP,YTHDC1,MTIF2,TTK,PPP4R3B,IBTK,HNRNPA2B1,PNPT1,ZMPSTE24,RLF,SYNCRIP,ZNF292,XPO1,PABPC4,CCT4,COMMD1,CASP8AP2,ETAA1,TIA1,ZNF350,ITGB1BP1,THRAP3,EZH2,UFL1,FAM3C,CCNC,CLSPN,ASCC3,CASP2,DDX1,NAA15,ELF2,WDR35,NRDC,ATAD2B,DHFR,CDCA7,PUS7,HDAC2,CCNA2,EXOSC9,MLLT10,SAE1,CEP295,DNAJC2,CHORDC1,SETDB2,SYMPK,MASTL,TRDMT1,CNOT10,NUP85,ELF1,RSF1,BORA,DIS3,CCNT2,PAXIP1,NCAPG2,WDR43,CDC20,BIRC5,FNTA,POLR3F,GTPBP4,IPO5,EPC2,SUPT16H,CHD8,KAT6A,HNRNPL,METTL3,RIF1,E2F7,ALYREF,CKAP2,CEP290,NSD3,CEBPZ,KITLG,SUPT20H,TAF7,EXOSC8,NR2C1,RFC3,CUL4A,BRCA2,PPP4R2,ELK3,RRP8,TCERG1,MAPK8,PRKDC,APAF1,CBX5,CCNB1,ERCC6,ZNF214,DHX29,PAN3,SCYL2,GPBP1,SP1,TCEA1,ZNF664,TPX2,CAPRIN1,ZC3H8,DIMT1,UBA2,IPO7,PARPBP,TNKS2,LSM14A,SGK3,BTAF1,ZNF143,PARP4,CENPJ,CDK5RAP1,FER,TDG,NCK1,IDE,SBNO1,ARFGEF1,SMARCA5,RNF141,PTCD3,ZNF507,ABCE1,OTUD4,CHD1,ZFP91,TAF9,CGGBP1,XRN1,PDE12,TFCP2,SAP30,ATR,TERF1,MTMR9,GCFC2,LARP4,USP25,UTP15,RBM12,HK2,HLTF,RBM39,PBK,ESCO2,ZBTB10,CNOT1,GABPA,MCRS1,SH2B3,LTN1,USP16,PPID,RBL1,MOB1A,MSH3,MIS18A,CCT7,CCNL1,CDCA2,N	darkgrey	EC_M8

GO:0006270	2.85E-14	1	21	33	DNA replication initiation	BP	3.50E-12	MCM5,MCM3,ORC6,ORC1,MCM2,MCM10,MCM6,ORC4,TOPBP1,MCM4,NOC3L,NBN,CCNE2,TICRR,ORC2,POLA1,AP000944.2,CDC45,MCM7,CDC6,CDT1	darkgrey	EC_M8
GO:0031326	4.40E-14	1	389	2955	regulation of cellular biosynthetic process	BP	5.36E-12	PAN2,ONECUT2,BCLAF1,PPIL4,NUP107,CCT2,DDX39B,FBXO5,ZNF608,SCAF8,EIF3E,MAP3K4,RAD21,DSCC1,MRPL13,HSF2,ATAD2,XRCC6,PHF5A,TNRC6B,TUT7,PSIP1,IFT74,POT1,PWP1,ZNF300,TOPORS,DBF4,MTERF1,PUM3,RFX3,CKS2,TRIM28,NCBP1,ZNF451,LRPPRC,FOXJ3,ZNF189,SP4,CDCA7L,PHIP,MTIF2,PPP4R3B,HNRNPA2B1,PNPT1,ZMPSTE24,RLF,SYNCRIP,ZNF292,XPO1,CCT4,COMMD1,CASP8AP2,TIA1,ZNF350,ITGB1BP1,THRAP3,EZH2,UFL1,FAM3C,CCNC,ASCC3,DDX1,NAA15,ELF2,WDR35,ATAD2B,DHFR,CDCA7,PUS7,HDAC2,CCNA2,EXOSC9,MLLT10,DNAJC2,SETDB2,CNOT10,NUP85,ELF1,RSF1,DIS3,CCNT2,PAXIP1,WDR43,BIRC5,POLR3F,GTPBP4,EPC2,SUPT16H,CHD8,KAT6A,METTL3,RIF1,E2F7,ALYREF,CKAP2,CEP290,NSD3,CEBPZ,SUPT20H,TAF7,EXOSC8,NR2C1,RFC3,BRCA2,ELK3,RRP8,TCERG1,MAPK8,PRKDC,CBX5,ERCC6,ZNF214,DHX29,PAN3,GPBP1,SP1,TCEA1,ZNF664,CAPRIN1,ZC3H8,TNKS2,LSM14A,SGK3,BTAF1,ZNF143,PARP4,CENPJ,CDK5RAP1,FER,TDG,NCK1,SBNO1,ARFGEF1,SMARCA5,RNF141,PTCD3,ZNF507,ABCE1,CHD1,ZFP91,TAF9,CGGBP1,XRN1,PDE12,TFCP2,SAP30,ATR,TERF1,GCFC2,LARP4,UTP15,HLTF,ESCO2,ZBTB10,CNOT1,GABPA,MCRS1,LTN1,USP16,PPID,RBL1,CCT7,CCNL1,NBN,SENP1,RIPK1,WDR75,ARID2,MYBL2,E2F8,TRIM33,INO80,ZBED6,CHUK,ZBTB11,ZNF248,MGA,MDM4,HIVEP1,RFC5,BRWD1,NOLC1,CCT5,BMPR1B,UBE3A,CCT6A,PRDM10,NUCKS1,DEK,GCN1,CUL3,METAP1,GMNN,CHEK1,H2AFZ,RRP1B,NFKB1,SMC3,KHDRBS1,TRIP13,TET2,TRIP11,HEATR1,NCL,TRAIP,ZNF184,PRMT5,AJUBA,GTF2IRD2,ZBTB41,ARID4B,NOL11,GTF2I,TARBP1,BPTF,FXR1,NGDN,DDX5,TICRR,RFC2,O	darkgrey	EC_M8
GO:0051985	4.54E-14	1	23	41	negative regulation of chromosome segregation	BP	5.44E-12	FBXO5,RAD21,TTK,GEN1,CCNB1,BUB1,KNTC1,BUB1B,TRIP13,CENPF,BUB3,TPR,ZWINT,MAD1L1,SPDL1,AURKB,PTTG1,ZWILCH,LCMT1,PLK1,NDC80,CDT1,MAD2L1	darkgrey	EC_M8
GO:1905819	4.54E-14	1	23	41	negative regulation of chromosome separation	BP	5.44E-12	FBXO5,RAD21,TTK,GEN1,CCNB1,BUB1,KNTC1,BUB1B,TRIP13,CENPF,BUB3,TPR,ZWINT,MAD1L1,SPDL1,AURKB,PTTG1,ZWILCH,LCMT1,PLK1,NDC80,CDT1,MAD2L1	darkgrey	EC_M8
GO:1901991	6.50E-14	1	45	142	negative regulation of mitotic cell cycle phase transition	BP	7.73E-12	CDK2,FBXO5,GTSE1,RAD21,TTK,EZH2,CLSPN,CASP2,ORC1,GEN1,RINT1,E2F7,TOPBP1,PRKDC,CCNB1,BUB1,KNTC1,RBL1,BUB1B,NBN,ABRAXAS1,TAOK3,CHEK1,TRIP13,TICRR,RPA2,DTL,BLM,CENPF,BUB3,CDK1,TPR,BRCA1,ZWINT,CDC6,MAD1L1,SPDL1,AURKB,ZWILCH,LCMT1,PLK1,NDC80,TAOK1,CDT1,MAD2L1	darkgrey	EC_M8
GO:0032200	7.09E-14	1	43	130	telomere organization	BP	8.37E-12	RPA3,CCT2,MAP3K4,XRCC6,RECQL4,POT1,HNRNPA2B1,CCT4,SMC6,DCLRE1C,RIF1,BRCA2,PRKDC,TNKS2,XRN1,ATR,TERF1,CCT7,NBN,RAD51,DCLRE1B,CCNE2,CCT5,CCT6A,GAR1,RPA2,NEK2,BLM,DNA2,XRCC5,EXO1,HNRNPU,RFC1,DCP2,EXOSC10,CCT3,AURKB,ATRX,RPA1,HNRNPD,HIST1H3H,NAF1,FEN1	darkgrey	EC_M8
GO:0006325	7.76E-14	1	73	301	chromatin organization	BP	9.09E-12	SHPRH,ATAD2,SRPK1,TRIM28,ANP32B,EZH2,HDAC2,MCM2,DNAJC2,SETDB2,NASP,RSF1,SUPT16H,CHD8,KAT6A,NAP1L1,RRP8,ERCC6,ACTR6,TDG,SMARCA5,FAM208A,HELLS,HLTF,RBL1,MIS18A,TOP1,ABRAXAS1,ARID2,INO80,OIP5,CHAF1B,CHD9,SMARCAD1,NUCKS1,DEK,NAP1L4,CHD1L,ARID4B,BPTF,ANP32E,HJURP,MCM3AP,HAT1,SMARCC1,BAZ1B,MKI67,BAZ1A,SETD2,TPR,HP1BP3,ZNF518B,SUV39H1,DDX11,HNRNPU,SMARCE1,ASF1B,ATRX,MBTD1,SMCHD1,SAFB,MYSM1,CHAF1A,UBN1,EHMT1,CTCF,HIST1H1A,ITGB3BP,HIST1H3H,CENPW,HMGN4,RNF8,ZNF518A	darkgrey	EC_M8

GO:0002376	6.18E-16	1	279	1656	immune system process	BP	9.33E-12	TNFRSF11A,IFNGR1,TNFAIP3,TRIM39,LYZ,FLT4,GRAMD4,NOTCH4,TCF7,L3MBTL3,PTK2,C6orf106,DAPK1,ATF4,PDGFB,PIM1,MFNG,LRRC19,TEK,IREB2,DDX58,TEC,ADGRF5,KIT,TNFRSF21,KDR,NFIL3,SYK,SEMA4D,IL6,REL,PTBP3,CXCL8,BACH2,PELI1,RSAD2,TLR4,C5,IL15,TRPM4,RAB3B,ZBTB24,PUM2,PIK3CG,TAL1,GATA2,PRKD2,CST7,CTSC,LRCH1,SNRK,NECTIN2,GAB2,UNC13D,PPARG,GATA3,CXCR4,IRAK2,CD93,SEC14L1,EDNRB,IKBKB,JAG1,TMEM173,SFRP1,ASH2L,CD36,TMEM178A,ARID5A,MAP3K1,TCF15,EPHB1,PLK2,GAPT,RET,PDE4D,MERTK,CHD7,IL1A,IGF1,BPIFB3,HHEX,ITCH,ERBIN,SH3RF1,KIF13B,MEF2C,TLR2,IL7,TPD52,VEGFC,CXCL10,LMBR1L,ADGRG3,TIPARP,SERINC5,OAS3,NLRC5,THBS4,DYSF,PLCG1,RAPGEF3,DGKZ,STAB1,HIPK1,ANO6,DLL4,IFIT2,SOX13,IFIT1,TSPAN2,EDN1,STAT1,CD58,JAM3,SNCA,ALAS1,JARID2,NFKB2,MX2,MX1,DCAF1,ETS1,RNF19B,CD200,LCK,PPP3CA,HYAL2,ICOSLG,RIN3,SNAP23,PRKX,LYST,LAPTM5,HLX,NCF1,DYRK3,LRMP,ISG20,HCLS1,NFATC2,INPP5D,TIFA,CD34,PECAM1,CD86,BMX,FGR,LTB4R,FES,PLEKHA1,PRKD1,F11R,YTHDF1,MMP21,SRMS,ACE,PAK2,DLG1,SIPA1,APOD,ETV6,STYK1,MOV10,CREB1,NBEAL2,B2M,ST6GAL1,LTF,CCRL2,ADORA2A,MASP1,RTP4,CYBB,IL1RAP,ITGA4,DDX3X,SOS2,TBX1,MCU,TMEM106A,MECOM,BIRC3,SELE,SELP,PLVAP,CRACR2A,WAS,PRKCH,RAC1,HIF1A,ADSS,PLD2,RAB27A,BCL6B,EPHA2,ITPKB,TLR6,LBR,TNK1,LFNG,TNFRSF1B,ARG2,MTOR,TBKBP1,EFNB1,PSEN1,HOXB6,HOXB7,STK10,SEMA4A,HOXB8,PGF,EIF2B2,DAPK2,BATF,CHRNB2,CD19,S100A14,GPR68,DNM2,SCNN1B,YY1,LYL1,TNIP1,RCOR1,MMP28,CCL2,YES1,JAG2,PDE4B,JAK1,LGALS9,NOS2	turquoise	EC_M6
GO:0045841	8.26E-14	1	22	38	negative regulation of mitotic metaphase/anaphase transition	BP	9.59E-12	FBXO5,RAD21,TTK,GEN1,CCNB1,BUB1,KNTC1,BUB1B,TRIP13,CENPF,BUB3,TPR,ZWINT,MAD1L1,SPDL1,AURKB,ZWILCH,LCMT1,PLK1,NDC80,CDT1,MAD2L1	darkgrey	EC_M8
GO:0044786	8.64E-14	1	23	40	cell cycle DNA replication	BP	9.96E-12	FBXO5,DBF4,MCM3,ZMPSTE24,MCM2,MCM6,E2F7,BRCA2,MCM4,TERF1,RAD51,E2F8,INO80,GMNN,BLM,POLA1,DNA2,DBF4B,CDC45,ATRX,TIPIN,CDT1,FEN1	darkgrey	EC_M8
GO:0051179	7.13E-16	1	779	4586	localization	BP	1.08E-11	CYB5A,CD63,LMAN1,ATP8B1,LRP1,STX7,AP3S1,CDO1,AHI1,B4GALNT1,AGAP2,CANX,TBC1D9B,RAB21,RABL2A,IPCEF1,SLC12A2,AZIN1,ATP6V1C1,PLXNB2,FBN2,ANGPT1,FAM19A5,TCP1,PPARA,SLC22A4,NUP50,CALHM5,SLC39A7,NUS1,SLC35F1,HSPA4,SMDT1,SEC24A,CXCL14,FAM49B,MRTFA,KCNK9,PACSIN1,SLC45A4,PTP4A3,REEP2,ADGRB1,LY6E,PPARD,CTNNA1,SIL1,SUN2,CBY1,MKLN1,TMEM184B,UBQLN1,MDGA1,GGA1,SH3GL2,PLIN2,SLC39A4,ATP6V1F,MYH9,LEP,PLAA,PEX6,SLC30A9,SRF,VEGFA,DOCK8,VCP,SLC35B2,UNC13B,NCK2,CLOCK,DYNC1I1,EXOC1,CREB3,SRP72,REST,CNNM4,LMAN2L,DST,RAB23,SNX13,PTP4A1,NLRP5,P3H1,TGFBR1,RIMS1,SLC17A5,TMEM30A,ERLEC1,FAM126A,CENPC,RTN4,TPBG,SHANK1,COX18,EMC10,MFSD2A,SLC35A1,UBE2J1,AKIRIN1,ACTR2,SLC31A1,WHRN,PCYOX1,MDFIC,GLI3,ROCK2,INHBA,VPS41,BVES,RRAS,RCN3,STARD3NL,VPS35,MGARP,SLC7A11,ITGB1,PGRMC2,LIN7B,LDAH,MFSD4B,FKBP1B,SLC37A3,PLXNA1,FGF2,KIF5B,SEC61A1,NAPA,DNAJC1,RAB7A,PANX1,SLC36A4,COPG1,ATP7B,RB1,ABHD5,IWS1,ANO10,PICALM,MARK4,TPT1,MAPRE3,PDCD6IP,MEGF8,ATP6V0B,PCDH17,SNX17,PAK1,ACTR3,TGFB1,SRP68,AXL,RAB3GAP1,SCN5A,SEC13,CTNNB1,IL15RA,ESYT2,TRAK1,DGAT2,AKT2,GPD1L,SEC23B,OSBPL10,DZIP1,ZEB2,LGALS3BP,C1QTNF1,TBC1D16,SLC4A7,RAB2B,OSBPL8,SLC38A10,ACTG1,NAV3,NPLOC4,ACTN4,PPP1R12A,STAM2,LTBP1,FAM98A,STIM1,SPINT2,COG6,UFM1,ATP2C1,IRS2,PRNP,ATP2B1,DCN,HDAC3,RAB11FIP1,SIGLEC1,ZNF703,DCLK1,PCLO,STARD13,TMCO3,SEMA3C,CCKBR,DDRGK1,SMPD1,ILK,LEPROTL1,ARL6IP5,DPYSL3,APLP1,TUSC3,SNPH,W	darkgreen	EC_M4
GO:0007062	1.30E-13	1	25	47	sister chromatid cohesion	BP	1.48E-11	RAD21,DSCC1,CDC20,SGO1,ESPL1,BUB1,ESCO2,BUB1B,NAA50,SMC3,SGO2,CDCA5,DDX11,PDS5A,WAPL,SMC1A,KIF22,ATRX,PLK1,ESCO1,STAG2,NIPBL,HASPIN,CTCF,FEN1	darkgrey	EC_M8
GO:0051028	1.88E-13	1	38	109	mRNA transport	BP	2.13E-11	NUP43,NUP107,DDX39B,SRSF3,NCBP1,LRPPRC,YTHDC1,HNRNPA2B1,XPO1,NUP205,NUP85,UPF2,ALYREF,NUP58,CETN3,NUP160,NUP54,NUP93,NSUN2,MCM3AP,RAE1,NUP133,DHX9,SETD2,HNRNPA3,TPR,DDX39A,THOC1,UPF3B,THOC2,NUP155,NDC1,FMR1,NCBP3,RBM8A,SARNP,SRSF7,NUP35	darkgrey	EC_M8
GO:0045930	2.47E-13	1	52	186	negative regulation of mitotic cell cycle	BP	2.79E-11	CDK2,YTHDC2,MDC1,FBXO5,GTSE1,RAD21,MSH2,TTK,EZH2,CLSPN,CASP2,ORC1,GEN1,RINT1,FANCD2,E2F7,TOPBP1,PRKDC,CCNB1,BUB1,KNTC1,RBL1,BUB1B,NBN,ABRAXAS1,TAOK3,CHEK1,TRIP13,TICRR,RPA2,DTL,BLM,CENPF,BUB3,CDK1,TPR,BRCA1,ZWINT,CDC6,MAD1L1,SPDL1,AURKB,EME1,TIPIN,ZWILCH,LCMT1,PLK1,NLE1,NDC80,TAOK1,CDT1,MAD2L1	darkgrey	EC_M8

GO:0009889	2.78E-13	1	391	3010	regulation of biosynthetic process	BP	3.11E-11	PAN2,ONECUT2,BCLAF1,PPIL4,NUP107,CCT2,DDX39B,FBXO5,ZNF608,SCAF8,EIF3E,MAP3K4,RAD21,DSCC1,MRPL13,HSF2,ATAD2,XRCC6,PHF5A,TNRC6B,TUT7,PSIP1,IFT74,POT1,PWP1,ZNF300,TOPORS,DBF4,MTERF1,PUM3,RFX3,CKS2,TRIM28,NCBP1,ZNF451,LRPPRC,FOXJ3,ZNF189,SP4,CDCA7L,PHIP,MTIF2,PPP4R3B,HNRNPA2B1,PNPT1,ZMPSTE24,RLF,SYNCRIP,ZNF292,XPO1,CCT4,COMMD1,CASP8AP2,TIA1,ZNF350,ITGB1BP1,THRAP3,EZH2,UFL1,FAM3C,CCNC,ASCC3,DDX1,NAA15,ELF2,WDR35,ATAD2B,DHFR,CDCA7,PUS7,HDAC2,CCNA2,EXOSC9,MLLT10,DNAJC2,SETDB2,CNOT10,NUP85,ELF1,RSF1,DIS3,CCNT2,PAXIP1,WDR43,BIRC5,POLR3F,GTPBP4,EPC2,SUPT16H,CHD8,KAT6A,METTL3,RIF1,E2F7,ALYREF,CKAP2,CEP290,NSD3,CEBPZ,SUPT20H,TAF7,EXOSC8,NR2C1,RFC3,BRCA2,ELK3,RRP8,TCERG1,MAPK8,PRKDC,CBX5,ERCC6,ZNF214,DHX29,PAN3,GPBP1,SP1,TCEA1,ZNF664,CAPRIN1,ZC3H8,TNKS2,LSM14A,SGK3,BTAF1,ZNF143,PARP4,CENPJ,CDK5RAP1,FER,TDG,NCK1,SBNO1,ARFGEF1,SMARCA5,RNF141,PTCD3,ZNF507,ABCE1,CHD1,ZFP91,TAF9,CGGBP1,XRN1,PDE12,TFCP2,SAP30,ATR,TERF1,GCFC2,LARP4,UTP15,HLTF,ESCO2,ZBTB10,CNOT1,GABPA,MCRS1,LTN1,USP16,PPID,RBL1,CCT7,CCNL1,NBN,SENP1,RIPK1,WDR75,ARID2,MYBL2,E2F8,TRIM33,INO80,ZBED6,CHUK,ZBTB11,ZNF248,MGA,MDM4,HIVEP1,RFC5,BRWD1,NOLC1,CCT5,BMPR1B,UBE3A,CCT6A,PRDM10,NUCKS1,DEK,GCN1,CUL3,METAP1,GMNN,CHEK1,H2AFZ,RRP1B,NFKB1,SMC3,KHDRBS1,TRIP13,TET2,TRIP11,HEATR1,NCL,TRAIP,ZNF184,PRMT5,AJUBA,GTF2IRD2,ZBTB41,ARID4B,NOL11,GTF2I,TARBP1,BPTF,FXR1,NGDN,DDX5,TICRR,RFC2,O	darkgrey	EC_M8
GO:1902100	3.21E-13	1	22	40	negative regulation of metaphase/anaphase transition of cell cycle	BP	3.57E-11	FBXO5,RAD21,TTK,GEN1,CCNB1,BUB1,KNTC1,BUB1B,TRIP13,CENPF,BUB3,TPR,ZWINT,MAD1L1,SPDL1,AURKB,ZWILCH,LCMT1,PLK1,NDC80,CDT1,MAD2L1	darkgrey	EC_M8
GO:0140013	4.53E-13	1	43	139	meiotic nuclear division	BP	4.99E-11	FBXO43,FBXO5,RAD21,RMI1,UBR2,CKS2,FOXJ3,SMC2,TTK,RAD54L,FANCD2,CDC20,TOP2B,SGO1,BRCA2,PSMD13,ESPL1,BUB1,TERF1,MND1,MSH3,BUB1B,RAD51,CCNE2,KIF18A,TRIP13,SGO2,ASPM,SPO11,NUF2,FANCM,SMC4,RAD51AP1,INCENP,TOP2A,CCNB2,EME1,PTTG1,PLK1,BRIP1,NDC1,FANCA,AURKA	darkgrey	EC_M8
GO:0015931	5.16E-13	1	50	181	nucleobase-containing compound transport	BP	5.64E-11	XPOT,NUP43,NUP107,DDX39B,SLC35D2,SRSF3,XPO5,NCBP1,LRPPRC,YTHDC1,HNRNPA2B1,PNPT1,XPO1,NUP205,NUP85,UPF2,ALYREF,NUP58,CETN3,NUP160,NUP54,CKAP5,NUP93,RIPK1,NSUN2,KHDRBS1,MCM3AP,RAE1,NUP133,SSB,DHX9,SETD2,HNRNPA3,TPR,DDX39A,NUP62CL,THOC1,UPF3B,THOC2,NUP155,RBFOX1,NDC1,FMR1,NCBP3,RBM8A,SARNP,SLC25A32,SRSF7,SLC25A36,NUP35	darkgrey	EC_M8
GO:0034728	5.81E-13	1	35	97	nucleosome organization	BP	6.32E-11	SHPRH,ANP32B,MCM2,NASP,RSF1,SUPT16H,KAT6A,NAP1L1,ACTR6,SMARCA5,MIS18A,ARID2,INO80,OIP5,CHAF1B,NAP1L4,BPTF,ANP32E,HJURP,MCM3AP,HAT1,SMARCC1,SETD2,HP1BP3,SMARCE1,ASF1B,ATRX,CHAF1A,UBN1,CTCF,HIST1H1A,ITGB3BP,HIST1H3H,CENPW,RNF8	darkgrey	EC_M8
GO:0051225	6.97E-13	1	36	101	spindle assembly	BP	7.52E-11	FBXO5,KIFC1,HAUS6,SENP6,CLASP2,CDC20,TUBGCP3,HAUS5,TPX2,LSM14A,KIF11,RACGAP1,ABRAXAS1,MYBL2,INO80,SMC3,HAUS2,ASPM,RCC1,NEK2,PRC1,TPR,KIF15,HAUS3,HNRNPU,INCENP,SMC1A,CCNB2,KPNB1,KIF4A,AURKB,KIF23,PLK1,STAG2,AURKA,DRG1	darkgrey	EC_M8
GO:0071426	8.28E-13	1	28	66	ribonucleoprotein complex export from nucleus	BP	8.87E-11	XPOT,NUP107,DDX39B,SRSF3,NCBP1,YTHDC1,HNRNPA2B1,XPO1,NUP85,UPF2,ALYREF,RRS1,ABCE1,NUP160,NUP93,NSUN2,MCM3AP,RAE1,NUP133,SSB,SETD2,TPR,NMD3,DDX39A,THOC1,THOC2,NUP155,SARNP	darkgrey	EC_M8
GO:0006401	9.76E-13	1	60	237	RNA catabolic process	BP	1.04E-10	PAN2,POP1,EIF3E,TNRC6B,TUT7,NCBP1,LRPPRC,PNPT1,SYNCRIP,PABPC4,THRAP3,EXOSC9,RNASEH2B,TRDMT1,CNOT10,UPF2,DIS3,XRN2,METTL3,EXOSC8,PAN3,MTREX,XRN1,PDE12,ZCCHC8,TTC37,CNOT1,TENT2,NSUN2,FXR1,DDX5,FASTKD1,SSB,DHX9,HNRNPR,FASTKD2,DNA2,CNOT9,HNRNPU,MRPL44,DCP2,EXOSC10,FUS,DIS3L,LARP1,TAF15,UPF3B,HNRNPM,SERBP1,GSPT1,DROSHA,FMR1,METTL16,RNPS1,EXOSC2,RBM8A,FASTKD5,HNRNPD,NAF1,FEN1	darkgrey	EC_M8

GO:0006810	1.44E-14	1	583	3300	transport	BP	1.08E-10	CYB5A,CD63,LMAN1,ATP8B1,LRP1,STX7,AP3S1,CDO1,AHI1,AGAP2,CANX,TBC1D9B,RAB21,RABL2A,IPCEF1,SLC12A2,AZIN1,ATP6V1C1,ANGPT1,TCP1,PPARA,SLC22A4,NUP50,CALHM5,SLC39A7,NUS1,SLC35F1,HSPA4,SMDT1,SEC24A,KCNK9,PACSIN1,SLC45A4,REEP2,ADGRB1,LY6E,PPARD,SIL1,SUN2,MKLN1,TMEM184B,UBQLN1,GGA1,SH3GL2,PLIN2,SLC39A4,ATP6V1F,MYH9,LEP,PLAA,PEX6,SLC30A9,VEGFA,VCP,SLC35B2,UNC13B,CLOCK,DYNC1I1,EXOC1,SRP72,REST,CNNM4,LMAN2L,DST,RAB23,SNX13,NLRP5,P3H1,RIMS1,SLC17A5,TMEM30A,ERLEC1,SHANK1,COX18,MFSD2A,SLC35A1,UBE2J1,ACTR2,SLC31A1,PCYOX1,MDFIC,GLI3,INHBA,VPS41,BVES,RCN3,STARD3NL,VPS35,MGARP,SLC7A11,ITGB1,PGRMC2,LIN7B,MFSD4B,FKBP1B,SLC37A3,FGF2,KIF5B,SEC61A1,NAPA,DNAJC1,RAB7A,PANX1,SLC36A4,COPG1,ATP7B,IWS1,ANO10,PICALM,TPT1,PDCD6IP,ATP6V0B,PCDH17,SNX17,PAK1,TGFB1,SRP68,AXL,RAB3GAP1,SCN5A,SEC13,CTNNB1,IL15RA,ESYT2,TRAK1,AKT2,GPD1L,SEC23B,OSBPL10,LGALS3BP,C1QTNF1,TBC1D16,SLC4A7,RAB2B,OSBPL8,SLC38A10,ACTG1,NPLOC4,ACTN4,PPP1R12A,STAM2,STIM1,COG6,UFM1,ATP2C1,IRS2,PRNP,ATP2B1,HDAC3,RAB11FIP1,SIGLEC1,DCLK1,PCLO,TMCO3,CCKBR,SMPD1,LEPROTL1,ARL6IP5,APLP1,TUSC3,SNPH,WASHC2A,VPS37A,REM1,ARL1,CXCL12,MTMR6,FXYD1,TMEM87B,TM9SF4,RAB2A,SLC20A1,ANKRD1,REEP5,SNTA1,HSP90B1,TMED2,SLC25A4,COPB2,OSBP,VAMP8,SLMAP,ATP1B3,RIOK2,TRAM1,TRPC4AP,MAP1B,RYR3,ERGIC3,SORBS1,LOXL3,P2RX4,COPB1,SELENOT,ATP2A2,USO1,SDAD1,CACNA1D,APP,ATXN2,GLRB,SLC4A5,CCT8,CX3CL1,SLC33A1,ABCC8,CACNB3,	darkgreen	EC_M4
GO:0045839	1.11E-12	1	23	46	negative regulation of mitotic nuclear division	BP	1.17E-10	FBXO5,RAD21,TTK,GEN1,CCNB1,BUB1,KNTC1,BUB1B,CHEK1,TRIP13,CENPF,BUB3,TPR,ZWINT,MAD1L1,SPDL1,AURKB,ZWILCH,LCMT1,PLK1,NDC80,CDT1,MAD2L1	darkgrey	EC_M8
GO:0071166	1.20E-12	1	28	67	ribonucleoprotein complex localization	BP	1.25E-10	XPOT,NUP107,DDX39B,SRSF3,NCBP1,YTHDC1,HNRNPA2B1,XPO1,NUP85,UPF2,ALYREF,RRS1,ABCE1,NUP160,NUP93,NSUN2,MCM3AP,RAE1,NUP133,SSB,SETD2,TPR,NMD3,DDX39A,THOC1,THOC2,NUP155,SARNP	darkgrey	EC_M8
GO:0051784	1.97E-12	1	24	51	negative regulation of nuclear division	BP	2.05E-10	FBXO43,FBXO5,RAD21,TTK,GEN1,CCNB1,BUB1,KNTC1,BUB1B,CHEK1,TRIP13,CENPF,BUB3,TPR,ZWINT,MAD1L1,SPDL1,AURKB,ZWILCH,LCMT1,PLK1,NDC80,CDT1,MAD2L1	darkgrey	EC_M8
GO:0051234	5.00E-14	1	599	3429	establishment of localization	BP	2.52E-10	CYB5A,CD63,LMAN1,ATP8B1,LRP1,STX7,AP3S1,CDO1,AHI1,AGAP2,CANX,TBC1D9B,RAB21,RABL2A,IPCEF1,SLC12A2,AZIN1,ATP6V1C1,ANGPT1,TCP1,PPARA,SLC22A4,NUP50,CALHM5,SLC39A7,NUS1,SLC35F1,HSPA4,SMDT1,SEC24A,KCNK9,PACSIN1,SLC45A4,PTP4A3,REEP2,ADGRB1,LY6E,PPARD,SIL1,SUN2,MKLN1,TMEM184B,UBQLN1,GGA1,SH3GL2,PLIN2,SLC39A4,ATP6V1F,MYH9,LEP,PLAA,PEX6,SLC30A9,VEGFA,VCP,SLC35B2,UNC13B,CLOCK,DYNC1I1,EXOC1,SRP72,REST,CNNM4,LMAN2L,DST,RAB23,SNX13,NLRP5,P3H1,RIMS1,SLC17A5,TMEM30A,ERLEC1,CENPC,SHANK1,COX18,EMC10,MFSD2A,SLC35A1,UBE2J1,ACTR2,SLC31A1,WHRN,PCYOX1,MDFIC,GLI3,INHBA,VPS41,BVES,RCN3,STARD3NL,VPS35,MGARP,SLC7A11,ITGB1,PGRMC2,LIN7B,MFSD4B,FKBP1B,SLC37A3,FGF2,KIF5B,SEC61A1,NAPA,DNAJC1,RAB7A,PANX1,SLC36A4,COPG1,ATP7B,IWS1,ANO10,PICALM,TPT1,PDCD6IP,ATP6V0B,PCDH17,SNX17,PAK1,ACTR3,TGFB1,SRP68,AXL,RAB3GAP1,SCN5A,SEC13,CTNNB1,IL15RA,ESYT2,TRAK1,AKT2,GPD1L,SEC23B,OSBPL10,DZIP1,LGALS3BP,C1QTNF1,TBC1D16,SLC4A7,RAB2B,OSBPL8,SLC38A10,ACTG1,NPLOC4,ACTN4,PPP1R12A,STAM2,STIM1,COG6,UFM1,ATP2C1,IRS2,PRNP,ATP2B1,HDAC3,RAB11FIP1,SIGLEC1,DCLK1,PCLO,TMCO3,CCKBR,SMPD1,LEPROTL1,ARL6IP5,APLP1,TUSC3,SNPH,WASHC2A,VPS37A,REM1,ARL1,CXCL12,MTMR6,FXYD1,TMEM87B,TM9SF4,RAB2A,SLC20A1,ANKRD1,REEP5,SNTA1,HSP90B1,TMED2,SLC25A4,COPB2,OSBP,VAMP8,SLMAP,ATP1B3,RIOK2,TRAM1,TRPC4AP,MAP1B,EMC4,RYR3,ERGIC3,PINX1,SORBS1,LOXL3,P2RX4,COPB1,SELENOT,ATP2A2,USO1,SDAD1,CACNA1D,APP,AT	darkgreen	EC_M4
GO:0042770	2.61E-12	1	45	155	signal transduction in response to DNA damage	BP	2.70E-10	CDK2,MDC1,DDX39B,GTSE1,MSH2,ZMPSTE24,ETAA1,CLSPN,CASP2,RINT1,RNASEH2B,PAXIP1,FANCD2,E2F7,CUL4A,BRCA2,TOPBP1,PRKDC,ERCC6,ATR,NBN,ABRAXAS1,EEF1E1,MDM4,TAOK3,CHEK1,DDX5,TICRR,RPA2,INTS7,DTL,BLM,ATRIP,CDK1,BRCA1,TIPRL,FOXM1,EME1,ATRX,TIPIN,PLK1,BRIP1,PSMD10,THOC1,TAOK1	darkgrey	EC_M8
GO:0007094	3.16E-12	1	20	36	mitotic spindle assembly checkpoint signaling	BP	3.20E-10	TTK,GEN1,CCNB1,BUB1,KNTC1,BUB1B,TRIP13,CENPF,BUB3,TPR,ZWINT,MAD1L1,SPDL1,AURKB,ZWILCH,LCMT1,PLK1,NDC80,CDT1,MAD2L1	darkgrey	EC_M8
GO:0071173	3.16E-12	1	20	36	spindle assembly checkpoint signaling	BP	3.20E-10	TTK,GEN1,CCNB1,BUB1,KNTC1,BUB1B,TRIP13,CENPF,BUB3,TPR,ZWINT,MAD1L1,SPDL1,AURKB,ZWILCH,LCMT1,PLK1,NDC80,CDT1,MAD2L1	darkgrey	EC_M8
GO:0071174	3.16E-12	1	20	36	mitotic spindle checkpoint signaling	BP	3.20E-10	TTK,GEN1,CCNB1,BUB1,KNTC1,BUB1B,TRIP13,CENPF,BUB3,TPR,ZWINT,MAD1L1,SPDL1,AURKB,ZWILCH,LCMT1,PLK1,NDC80,CDT1,MAD2L1	darkgrey	EC_M8

GO:0051252	4.16E-12	1	349	2654	regulation of RNA metabolic process	BP	4.19E-10	PAN2,ONECUT2,BCLAF1,CLK4,HNRNPH1,PPIL4,NUP107,DDX39B,ZNF608,SCAF8,RAD21,HSF2,ATAD2,XRCC6,PHF5A,TNRC6B,TUT7,SRPK1,SRSF3,PSIP1,IFT74,PWP1,ZNF300,TOPORS,SMU1,MTERF1,RFX3,CKS2,TRIM28,NCBP1,ZNF451,LRPPRC,FOXJ3,ZNF189,SP4,CDCA7L,PHIP,YTHDC1,PNPT1,ZMPSTE24,RLF,SYNCRIP,ZNF292,XPO1,PABPC4,COMMD1,CASP8AP2,TIA1,ZNF350,ITGB1BP1,THRAP3,EZH2,UFL1,CCNC,DDX1,NAA15,ELF2,ATAD2B,CDCA7,HDAC2,CCNA2,EXOSC9,MLLT10,DNAJC2,SETDB2,TRDMT1,NUP85,ELF1,RSF1,DIS3,CCNT2,PAXIP1,WDR43,BIRC5,POLR3F,EPC2,SUPT16H,CHD8,KAT6A,HNRNPL,METTL3,RIF1,E2F7,ALYREF,CKAP2,CEP290,NSD3,CEBPZ,SUPT20H,TAF7,EXOSC8,NR2C1,BRCA2,ELK3,RRP8,TCERG1,MAPK8,PRKDC,CBX5,CCNB1,ERCC6,ZNF214,PAN3,GPBP1,SP1,TCEA1,ZNF664,ZC3H8,DIMT1,SGK3,BTAF1,ZNF143,CENPJ,FER,TDG,NCK1,SBNO1,SMARCA5,RNF141,ZNF507,ABCE1,CHD1,ZFP91,TAF9,CGGBP1,PDE12,TFCP2,SAP30,GCFC2,UTP15,RBM12,HLTF,RBM39,ZBTB10,CNOT1,GABPA,MCRS1,USP16,PPID,RBL1,CCNL1,SENP1,TENT2,VIRMA,SLC38A2,RIPK1,WDR75,ARID2,MYBL2,E2F8,TRIM33,INO80,RIOK1,ZBED6,CHUK,ZBTB11,ZNF248,MGA,MDM4,HIVEP1,BRWD1,NOLC1,BMPR1B,UBE3A,PRDM10,NUCKS1,DEK,GCN1,CUL3,NSUN2,GMNN,CHEK1,H2AFZ,RRP1B,NFKB1,KHDRBS1,TRIP13,RBM5,TET2,TRIP11,HEATR1,NCL,TRAIP,ZNF184,PRMT5,AJUBA,GTF2IRD2,ZBTB41,ARID4B,NOL11,GTF2I,TARBP1,BPTF,FXR1,DDX5,ORC2,EWSR1,TIAL1,FASTKD1,BLM,CENPF,EDRF1,PDE8A,ING5,BCLAF3,FOSL1,SMARCC1,BAZ1B,SP3,DHX9,HDGFL3,BAZ1A,HNRNPR,TRA2B,SETD2,ZFX,PSMA6,FASTKD2,HNRNPH3,DD	darkgrey	EC_M8
GO:1903311	4.92E-12	1	60	247	regulation of mRNA metabolic process	BP	4.92E-10	PAN2,TNRC6B,TUT7,SRPK1,SRSF3,SMU1,NCBP1,YTHDC1,PNPT1,SYNCRIP,PABPC4,TIA1,THRAP3,EXOSC9,DIS3,HNRNPL,METTL3,EXOSC8,CCNB1,PAN3,PDE12,RBM39,CNOT1,VIRMA,KHDRBS1,RBM5,NCL,FXR1,DDX5,FASTKD1,DHX9,HNRNPR,TRA2B,FASTKD2,CWC22,LEO1,HNRNPU,DCP2,SLTM,RBM25,FUS,LARP1,TAF15,HNRNPM,SERBP1,SAFB,SAFB2,RBMX,NSRP1,RBFOX1,FMR1,METTL16,RNPS1,DAZAP1,PTBP1,EXOSC2,RBM8A,FASTKD5,HNRNPD,SRSF7	darkgrey	EC_M8
GO:0006405	5.34E-12	1	28	69	RNA export from nucleus	BP	5.31E-10	XPOT,NUP107,DDX39B,SRSF3,XPO5,NCBP1,YTHDC1,HNRNPA2B1,NUP85,UPF2,ALYREF,NUP160,NUP93,NSUN2,KHDRBS1,MCM3AP,RAE1,NUP133,SSB,DHX9,SETD2,TPR,DDX39A,NUP62CL,THOC1,THOC2,NUP155,SARNP	darkgrey	EC_M8
GO:0044839	5.87E-12	1	40	132	cell cycle G2/M phase transition	BP	5.80E-10	CDK2,LATS1,FBXO5,RAD21,CDC25C,MELK,CLSPN,ORC1,RINT1,CCNA2,RNASEH2B,MASTL,PAXIP1,TOPBP1,CCNB1,NBN,ABRAXAS1,TAOK3,CHEK1,KHDRBS1,KIF14,TICRR,DTL,BLM,CENPF,CDK1,CDC25A,DBF4B,BRCA1,FOXM1,CCNB3,CDC6,CCNB2,AURKB,LCMT1,PLK1,ATAD5,TAOK1,PKMYT1,AURKA	darkgrey	EC_M8
GO:0071897	6.31E-12	1	44	150	DNA biosynthetic process	BP	6.19E-10	CCT2,DDX39B,MAP3K4,DSCC1,POT1,POLH,REV1,HNRNPA2B1,CCT4,DNAJC2,TK1,POLE,RFC3,TNKS2,PARP4,XRN1,ATR,TERF1,CCT7,RFC5,CCT5,CCT6A,GAR1,RFC2,NEK2,DTL,CENPF,POLA1,RFC4,C17orf53,XRCC5,HNRNPU,RFC1,LIN9,DCP2,EXOSC10,CCT3,AURKB,TYMS,RPA1,CHTF18,HNRNPD,NAF1,RCHY1	darkgrey	EC_M8
GO:0031577	6.53E-12	1	20	37	spindle checkpoint signaling	BP	6.36E-10	TTK,GEN1,CCNB1,BUB1,KNTC1,BUB1B,TRIP13,CENPF,BUB3,TPR,ZWINT,MAD1L1,SPDL1,AURKB,ZWILCH,LCMT1,PLK1,NDC80,CDT1,MAD2L1	darkgrey	EC_M8
GO:0006412	7.03E-12	1	97	548	translation	BP	6.80E-10	PAN2,NARS,DDX39B,EIF3E,MRPL13,TNRC6B,TUT7,PUM3,NCBP1,LRPPRC,MTIF2,PNPT1,SYNCRIP,PABPC4,TIA1,MRPL32,ASCC3,DDX1,DHFR,PUS7,EXOSC9,CNOT10,MRPS7,DIS3,DARS,METTL3,EXOSC8,LARS,PRKDC,DHX29,PAN3,MRPL15,MRPS5,CAPRIN1,LSM14A,CDK5RAP1,EIF2B1,NCK1,MRPL35,PTCD3,ABCE1,XRN1,PDE12,LARP4,CNOT1,LTN1,USP16,RPS6,EEF1E1,NOLC1,GCN1,METAP1,YARS,KHDRBS1,IARS2,NCL,FXR1,NGDN,EIF3A,EIF2B5,DDX6,DHX9,HNRNPR,FASTKD2,TPR,RPL10L,DARS2,CNOT9,RPL22L1,DHX33,EIF2AK1,MRPL44,DCP2,EIF3B,SEPSECS,EIF4A1,RARS,MRPS23,MRPL22,GEMIN5,LARP1,ILF3,EIF5,TYMS,UPF3B,GSPT1,FMR1,METTL16,EXOSC2,RBM8A,METTL18,SARNP,HNRNPD,RNF139,PAIP2,EIF1AX,DRG1	darkgrey	EC_M8
GO:0000077	8.70E-12	1	35	105	DNA damage checkpoint signaling	BP	8.36E-10	CDK2,MDC1,DDX39B,GTSE1,MSH2,ETAA1,CLSPN,CASP2,RINT1,RNASEH2B,FANCD2,CUL4A,TOPBP1,PRKDC,ERCC6,ATR,NBN,ABRAXAS1,TAOK3,CHEK1,TICRR,RPA2,INTS7,DTL,BLM,ATRIP,CDK1,BRCA1,TIPRL,EME1,TIPIN,PLK1,BRIP1,THOC1,TAOK1	darkgrey	EC_M8
GO:0033260	8.86E-12	1	20	36	nuclear DNA replication	BP	8.47E-10	DBF4,MCM3,ZMPSTE24,MCM2,MCM6,BRCA2,MCM4,TERF1,RAD51,INO80,GMNN,BLM,POLA1,DNA2,DBF4B,CDC45,ATRX,TIPIN,CDT1,FEN1	darkgrey	EC_M8

GO:0019222	9.44E-12	1	569	4858	regulation of metabolic process	BP	8.97E-10	CDK2,PAN2,ONECUT2,ME2,BCLAF1,CLK4,HNRNPH1,PPIL4,LATS1,NUP107,CCT2,DDX39B,FBXO5,ZNF608,SCAF8,UBR5,EIF3E,MAP3K4,RAD21,DSCC1,MRPL13,HSF2,ATAD2,XRCC6,PHF5A,CDC25C,TNRC6B,TUT7,MATR3,SRPK1,SRSF3,PLEC,PSIP1,IFT74,POT1,PWP1,ZNF300,TOPORS,SMU1,DBF4,POLR3B,XPO5,MTERF1,POLH,AKAP9,PUM3,RFX3,IL1R1,CKS2,TRIM28,NCBP1,ZNF451,ANP32B,LRPPRC,FOXJ3,ZNF189,MSH2,SP4,CDCA7L,PHIP,YTHDC1,MTIF2,TTK,PPP4R3B,IBTK,HNRNPA2B1,PNPT1,PUS10,ZMPSTE24,RLF,SYNCRIP,ZNF292,XPO1,PABPC4,CCT4,COMMD1,CASP8AP2,ETAA1,TIA1,ZNF350,ITGB1BP1,THRAP3,EZH2,UFL1,FAM3C,CCNC,CLSPN,ASCC3,CASP2,DDX1,NAA15,ELF2,WDR35,NRDC,ATAD2B,DHFR,CDCA7,PUS7,HDAC2,CCNA2,EXOSC9,MLLT10,SAE1,CEP295,DNAJC2,CHORDC1,RNASEH2B,SETDB2,SYMPK,MASTL,TRDMT1,CNOT10,NUP85,ELF1,UPF2,RSF1,BORA,DIS3,CCNT2,PAXIP1,XRN2,NCAPG2,WDR43,CDC20,BIRC5,FNTA,POLR3F,GTPBP4,IPO5,EPC2,SUPT16H,CHD8,KAT6A,HNRNPL,METTL3,RIF1,E2F7,ALYREF,CKAP2,CEP290,NSD3,CEBPZ,KITLG,SUPT20H,TAF7,EXOSC8,NR2C1,RFC3,CUL4A,BRCA2,PPP4R2,ELK3,RRP8,TCERG1,MAPK8,PRKDC,APAF1,CBX5,CCNB1,ERCC6,NFKBID,ZNF214,DHX29,PAN3,SCYL2,GPBP1,SP1,TCEA1,ZNF664,TPX2,CAPRIN1,ZC3H8,DIMT1,UBA2,IPO7,PARPBP,TNKS2,LSM14A,SGK3,BTAF1,ZNF143,PARP4,CENPJ,CDK5RAP1,FER,TDG,NCK1,IDE,SBNO1,ARFGEF1,SMARCA5,RNF141,PTCD3,ZNF507,ABCE1,OTUD4,CHD1,ZFP91,TAF9,CGGBP1,XRN1,PDE12,TFCP2,SAP30,ATR,TTC37,TERF1,MTMR9,FAM208A,HELLS,GCFC2,LARP4,USP25,UTP15,RBM12,HK2,HLTF,RBM39,PBK,ESCO2,ZBTB1	darkgrey	EC_M8
GO:0090329	1.04E-11	1	21	41	regulation of DNA-dependent DNA replication	BP	9.86E-10	FBXO5,DSCC1,DBF4,ZMPSTE24,E2F7,RFC3,BRCA2,NBN,E2F8,INO80,RFC5,GMNN,TICRR,RFC2,BLM,RFC4,DBF4B,ATRX,TIPIN,CHTF18,CDT1	darkgrey	EC_M8
GO:0051054	1.25E-11	1	47	176	positive regulation of DNA metabolic process	BP	1.17E-09	CCT2,DDX39B,MAP3K4,DSCC1,POT1,TRIM28,MSH2,HNRNPA2B1,CCT4,PAXIP1,RIF1,RFC3,PRKDC,ERCC6,TNKS2,OTUD4,ATR,CCT7,NBN,ABRAXAS1,RAD51,ARID2,RFC5,CCT5,CCT6A,FANCB,RFC2,NEK2,DHX9,RFC4,XRCC5,BRCA1,NSD2,RAD51AP1,FOXM1,DDX11,CCT3,FUS,AURKB,ATRX,SMCHD1,CHTF18,PRIM2,HNRNPD,TIGAR,NAF1,RNF8	darkgrey	EC_M8
GO:0034654	1.47E-11	1	364	2841	nucleobase-containing compound biosynthetic process	BP	1.37E-09	ONECUT2,BCLAF1,PPIL4,NUP107,CCT2,DDX39B,ZNF608,SCAF8,MAP3K4,RAD21,DSCC1,HSF2,ATAD2,XRCC6,PHF5A,PSIP1,IFT74,POT1,PWP1,ZNF300,TOPORS,POLR3B,MTERF1,POLH,RFX3,CKS2,PPAT,PAICS,REV1,TRIM28,ZNF451,PHF3,TWISTNB,FOXJ3,ZNF189,SP4,CDCA7L,CTPS1,PHIP,HNRNPA2B1,DCK,ZMPSTE24,RLF,ZNF292,XPO1,CCT4,COMMD1,CASP8AP2,ZNF350,ITGB1BP1,THRAP3,TAF1B,EZH2,UFL1,CCNC,DDX1,NAA15,ELF2,AMD1,ATAD2B,CDCA7,HDAC2,CCNA2,EXOSC9,MLLT10,DNAJC2,SETDB2,PCF11,NIFK,NUP85,CAD,ELF1,RSF1,CCNT2,PAXIP1,XRN2,NCAPG2,WDR43,TK1,AFMID,BIRC5,POLR3F,EPC2,SUPT16H,CHD8,KAT6A,RIF1,E2F7,ALYREF,CKAP2,RRM1,CEP290,NSD3,CEBPZ,SUPT20H,TAF7,POLE,NR2C1,RFC3,BRCA2,ELK3,RRP8,TCERG1,MAPK8,PRKDC,CBX5,ERCC6,ZNF214,GPBP1,SP1,TCEA1,ZNF664,ZC3H8,POLR1B,TNKS2,SGK3,BTAF1,ZNF143,PARP4,CENPJ,FER,TDG,NCK1,SBNO1,SMARCA5,RNF141,ZNF507,POLR1A,CHD1,ZFP91,TAF9,CGGBP1,XRN1,SMN2,BDP1,TFCP2,SAP30,ATR,TERF1,GCFC2,UTP15,HLTF,ZBTB10,CNOT1,GABPA,MCRS1,USP16,PPID,RBL1,GMPS,CCT7,CCNL1,SENP1,RIPK1,WDR75,ARID2,MYBL2,E2F8,TRIM33,INO80,ZBED6,CHUK,ZBTB11,ZNF248,MGA,MDM4,HIVEP1,RFC5,BRWD1,NOLC1,CCT5,BMPR1B,UBE3A,CCT6A,PRDM10,NUCKS1,DEK,GCN1,CUL3,GMNN,CHEK1,H2AFZ,RRP1B,NFKB1,KHDRBS1,TRIP13,TET2,TRIP11,HEATR1,ACSL5,NCL,TRAIP,PAPSS1,ZNF184,PRMT5,AJUBA,GTF2IRD2,ZBTB41,ARID4B,NOL11,GAR1,GTF2I,TARBP1,BPTF,DDX5,RFC2,ORC2,NEK2,EWSR1,TIAL1,DTL,BLM,UMPS,CENPF,EDRF1,PDE8A,ING5,BCLAF3,FOSL1,SMARCC1,BAZ1B,SP3,DHX9,HDGFL3,BAZ1A,SETD2,ZFX,P	darkgrey	EC_M8
GO:0061982	1.56E-11	1	33	99	meiosis I cell cycle process	BP	1.45E-09	FBXO5,RAD21,CDC25C,RMI1,UBR2,CKS2,FOXJ3,RAD54L,FANCD2,TOP2B,SGO1,BRCA2,PSMD13,ESPL1,TERF1,MND1,MSH3,RAD51,CCNE2,TRIP13,SGO2,SPO11,CDC25A,FANCM,RAD51AP1,TOP2A,CCNB2,EME1,PTTG1,PLK1,BRIP1,NDC1,AURKA	darkgrey	EC_M8
GO:0034508	1.74E-11	1	17	28	centromere complex assembly	BP	1.60E-09	SENP6,CENPO,CENPA,NASP,CENPH,KNTC1,HELLS,MIS18A,OIP5,CENPE,HJURP,CENPF,DLGAP5,CENPI,CENPN,ITGB3BP,CENPW	darkgrey	EC_M8

GO:0007165	2.74E-13	1	554	3919	signal transduction	BP	1.85E-09	TNFRSF11A,SEMA6A,LVRN,PDE7B,IFNGR1,TNFAIP3,TRIM39,PLEKHG1,AKAP12,FLT4,FZD6,CTHRC1,RIMS2,CDC42SE2,GRAMD4,CELSR1,NOTCH4,AFDN,TCF7,TPD52L1,MTSS1,RNF146,ARHGAP18,SGSM3,PTK2,DENND3,ANKS1A,DAPK1,ATF4,PDGFB,MAPK13,CSNK1E,PICK1,KDM4C,PIM1,CDC42EP1,MFNG,CSF2RB,MDFI,OSTF1,LRRC19,TEK,RIC8B,DDX58,PIP5K1B,FAM122A,TEC,SH3RF3,GNG11,ADGRF5,KIT,ADGRF1,TNFRSF21,KDR,CD2AP,SYK,GADD45G,SEMA4D,RNF149,SHB,RAPGEF5,IL6,REL,CHN2,CXCL8,KBTBD2,PELI1,RGS3,ARHGAP25,ELMO1,RSAD2,DOCK4,FIGNL1,CAV2,GRB10,LANCL2,TSPAN12,TLR4,GRIK2,TRAF1,C5,DLGAP3,INPP4B,IL15,FZD8,CCNY,CREM,TEAD2,SDCBP,TRPM4,NRP1,PUM2,RASIP1,PIK3CG,ARHGAP42,TAL1,MTMR2,MGLL,GATA2,ARHGAP21,PRKD2,CTSC,RAB38,CAB39L,GPR4,SNRK,FGD5,PLXND1,NECTIN2,PRCP,BCAM,ITGA8,NOS3,GAB2,LLGL2,ITGA9,RALB,ITGB4,ZFAND6,PPARG,TIMP4,GATA3,ACVR2B,CXCR4,LMBR1,IRAK2,SEC14L1,EDNRB,PPP1CB,NET1,RALGAPA2,NDFIP2,TMC8,TIE1,CYTH1,IKBKB,JAG1,P2RY6,P2RY2,NFKBIB,PDE2A,SFRP1,HBEGF,NR1D2,PLCB4,RASGRP3,FEZ2,NOTUM,FERMT1,RPL26,EFNB2,SH3BP5,ARHGEF7,SPEF1,ARAP3,ARHGAP26,P2RX2,CD36,RIC8A,SIRT3,RASSF7,MAGI1,PRAG1,SDCBP2,PRR5L,MAP3K1,SCARB1,EPHB1,PLK2,RET,PDE4D,MERTK,TNFRSF19,FAM3D,IL1A,IGF1,NR4A1,KIAA0355,EIF2AK3,ABHD6,HHEX,KDM3A,ITCH,ERBIN,UFSP2,SH3RF1,PITPNM2,PREX2,SMAD1,HIP1R,DCLK2,MCTP1,CHRM5,ARHGEF3,SOX7,KIF13B,ARHGEF28,MEF2C,TLR2,WWTR1,IL7,P2RX7,VEGFC,NDRG4,GUCY1A1,GUCY1B1,CXCL10,ANGPT2,SOGA1,MADD,LMBR1L,ADGRG3,DHH,KMT	turquoise	EC_M6
GO:0023052	3.67E-13	1	595	4258	signaling	BP	1.85E-09	TNFRSF11A,SEMA6A,LVRN,KIF5A,PDE7B,IFNGR1,TNFAIP3,STX11,TRIM39,PLEKHG1,AKAP12,FLT4,SNCAIP,FZD6,CTHRC1,RIMS2,CDC42SE2,GRAMD4,CELSR1,NOTCH4,AFDN,PACSIN2,TCF7,TRDN,TPD52L1,MTSS1,RNF146,ARHGAP18,SGSM3,PTK2,DENND3,ANKS1A,DAPK1,ATF4,PDGFB,MAPK13,CSNK1E,PICK1,KDM4C,PIM1,CDC42EP1,MFNG,CSF2RB,MDFI,OSTF1,LRRC19,TEK,RIC8B,DDX58,APBA1,PIP5K1B,FAM122A,TEC,SH3RF3,GNG11,ADGRF5,KIT,ADGRF1,TNFRSF21,KDR,CD2AP,SYK,GADD45G,SEMA4D,RNF149,ICA1,SHB,RAPGEF5,IL6,REL,PPT1,CHN2,CXCL8,KBTBD2,PELI1,RGS3,ARHGAP25,ELMO1,RSAD2,DOCK4,FIGNL1,CAV2,GRB10,LANCL2,TSPAN12,TLR4,GRIK2,TRAF1,C5,DLGAP3,GJA4,INPP4B,IL15,FZD8,CCNY,CREM,TEAD2,SDCBP,TRPM4,NRP1,RAB3B,PUM2,RASIP1,PIK3CG,ARHGAP42,TAL1,MTMR2,MGLL,GATA2,ARHGAP21,PRKD2,CTSC,RAB38,CAB39L,GPR4,SNRK,FGD5,PLXND1,NECTIN2,PRCP,BCAM,ITGA8,NOS3,GAB2,LLGL2,ITGA9,RALB,ITGB4,ZFAND6,PPARG,TIMP4,GATA3,ACVR2B,CXCR4,LMBR1,IRAK2,SEC14L1,EDNRB,PPP1CB,NET1,RALGAPA2,NDFIP2,TMC8,SPTBN4,TIE1,CYTH1,IKBKB,JAG1,P2RY6,P2RY2,FCHSD2,NFKBIB,PDE2A,SFRP1,HBEGF,NR1D2,PLCB4,RASGRP3,FEZ2,NOTUM,FERMT1,RPL26,EFNB2,SH3BP5,ARHGEF7,SPEF1,FCHSD1,ARAP3,PCDH1,ARHGAP26,P2RX2,NTN4,CD36,RIC8A,SIRT3,RASSF7,MAGI1,PRAG1,SDCBP2,PRR5L,MAP3K1,SCARB1,EPHB1,PLK2,RET,PDE4D,MERTK,TNFRSF19,CHD7,FAM3D,IL1A,IGF1,NR4A1,KIAA0355,EIF2AK3,ABHD6,HHEX,KDM3A,ITCH,ERBIN,UFSP2,SH3RF1,PITPNM2,PREX2,SMAD1,CLCN3,HIP1R,DCLK2,MCTP1,CHRM5,ARHGEF3,SOX7,KIF13B,ARHGEF28,MEF2C	turquoise	EC_M6
GO:0018130	2.10E-11	1	369	2900	heterocycle biosynthetic process	BP	1.92E-09	ONECUT2,BCLAF1,PPIL4,MTHFD1L,NUP107,CCT2,DDX39B,ZNF608,SCAF8,MAP3K4,RAD21,DSCC1,HSF2,ATAD2,XRCC6,PHF5A,PSIP1,IFT74,POT1,PWP1,ZNF300,TOPORS,POLR3B,MTERF1,POLH,RFX3,CKS2,PPAT,PAICS,REV1,TRIM28,ZNF451,PHF3,TWISTNB,FOXJ3,ZNF189,SP4,CDCA7L,CTPS1,PHIP,HNRNPA2B1,DCK,ZMPSTE24,RLF,ZNF292,XPO1,CCT4,COMMD1,CASP8AP2,ZNF350,ITGB1BP1,THRAP3,TAF1B,EZH2,UFL1,CCNC,DDX1,NAA15,ELF2,AMD1,ATAD2B,DHFR,CDCA7,HDAC2,CCNA2,EXOSC9,MLLT10,DNAJC2,SETDB2,PCF11,NIFK,NUP85,CAD,ELF1,RSF1,CCNT2,PAXIP1,XRN2,NCAPG2,WDR43,TK1,AFMID,BIRC5,POLR3F,EPC2,SUPT16H,CHD8,KAT6A,RIF1,E2F7,ALYREF,CKAP2,RRM1,CEP290,NSD3,CEBPZ,SUPT20H,TAF7,POLE,NR2C1,RFC3,BRCA2,ELK3,RRP8,TCERG1,MAPK8,PRKDC,CBX5,ERCC6,ZNF214,GPBP1,SP1,TCEA1,ZNF664,ZC3H8,POLR1B,TNKS2,SGK3,BTAF1,ZNF143,PARP4,CENPJ,FER,TDG,NCK1,SBNO1,SMARCA5,RNF141,ZNF507,POLR1A,CHD1,ZFP91,TAF9,CGGBP1,XRN1,SMN2,BDP1,TFCP2,SAP30,ATR,TERF1,GCFC2,UTP15,HLTF,ZBTB10,CNOT1,GABPA,MCRS1,USP16,PPID,RBL1,GMPS,CCT7,CCNL1,SENP1,RIPK1,WDR75,ARID2,MYBL2,E2F8,TRIM33,INO80,ZBED6,CHUK,ZBTB11,ZNF248,MGA,MDM4,HIVEP1,RFC5,BRWD1,NOLC1,CCT5,BMPR1B,UBE3A,CCT6A,PRDM10,NUCKS1,DEK,GCN1,CUL3,GMNN,CHEK1,H2AFZ,RRP1B,NFKB1,KHDRBS1,TRIP13,TET2,TRIP11,HEATR1,ACSL5,NCL,TRAIP,PAPSS1,ZNF184,PRMT5,AJUBA,GTF2IRD2,ZBTB41,ARID4B,NOL11,GAR1,GTF2I,TARBP1,BPTF,DDX5,RFC2,ORC2,NEK2,EWSR1,TIAL1,DTL,HMBS,FLVCR1,BLM,UMPS,CENPF,OAT,EDRF1,PDE8A,ING5,BCLAF3,FOSL1,SMARCC1,B	darkgrey	EC_M8

GO:1902749	2.19E-11	1	31	87	regulation of cell cycle G2/M phase transition	BP	1.99E-09	CDK2,FBXO5,RAD21,CDC25C,CLSPN,ORC1,RINT1,RNASEH2B,PAXIP1,TOPBP1,CCNB1,NBN,ABRAXAS1,TAOK3,CHEK1,KIF14,TICRR,DTL,BLM,CENPF,CDK1,CDC25A,DBF4B,BRCA1,CCNB3,CDC6,AURKB,PLK1,ATAD5,TAOK1,AURKA	darkgrey	EC_M8
GO:0022618	2.39E-11	1	43	163	ribonucleoprotein complex assembly	BP	2.16E-09	DDX39B,EIF3E,RRP7A,SRPK1,PSIP1,NCBP1,YTHDC1,MDN1,DDX1,MRPS7,CRNKL1,SNRPF,PRKDC,DHX29,TGS1,RRS1,TAF9,SMN2,ATR,GCFC2,SCAF11,FP565260.1,SF3B1,RBM5,PRMT5,EIF3A,DHX9,FASTKD2,DDX20,PRPF39,RPL10L,XRCC5,EIF3B,SRSF5,LUC7L3,COIL,GEMIN5,EIF5,NLE1,BRIX1,GEMIN4,GEMIN6,NAF1	darkgrey	EC_M8
GO:0045005	2.78E-11	1	21	41	DNA-dependent DNA replication maintenance of fidelity	BP	2.50E-09	TONSL,ETAA1,MMS22L,GEN1,ALYREF,POLE,BRCA2,ATR,RAD51,NUCKS1,TRAIP,ZGRF1,BLM,DNA2,FANCM,BOD1L1,DDX11,EME1,ATRX,TIPIN,THOC1	darkgrey	EC_M8
GO:0030261	2.79E-11	1	18	31	chromosome condensation	BP	2.50E-09	SMC2,NCAPG2,NCAPH,CCNB1,NUSAP1,NCAPD3,ACIN1,CDK1,CDCA5,SMC4,NCAPD2,INCENP,WAPL,TOP2A,NCAPG,PLK1,AIFM1,HIST1H1A	darkgrey	EC_M8
GO:0051168	3.27E-11	1	39	131	nuclear export	BP	2.90E-09	XPOT,NUP107,DDX39B,HSPA9,SRSF3,XPO5,NCBP1,ANP32B,YTHDC1,HNRNPA2B1,XPO1,NUP85,UPF2,ALYREF,XPO4,RRS1,ABCE1,NUP160,NUP93,NSUN2,KHDRBS1,CSE1L,MCM3AP,RAE1,NUP133,SSB,DHX9,SETD2,CTDSPL2,TPR,NMD3,ANKLE1,DDX39A,XPO6,NUP62CL,THOC1,THOC2,NUP155,SARNP	darkgrey	EC_M8
GO:0006417	3.78E-11	1	71	341	regulation of translation	BP	3.34E-09	PAN2,DDX39B,EIF3E,MRPL13,TNRC6B,TUT7,PUM3,NCBP1,LRPPRC,MTIF2,PNPT1,SYNCRIP,TIA1,ASCC3,DDX1,DHFR,PUS7,EXOSC9,CNOT10,DIS3,METTL3,EXOSC8,PRKDC,DHX29,PAN3,CAPRIN1,LSM14A,CDK5RAP1,NCK1,PTCD3,ABCE1,XRN1,PDE12,LARP4,CNOT1,LTN1,USP16,NOLC1,GCN1,METAP1,KHDRBS1,NCL,FXR1,NGDN,EIF2B5,DDX6,DHX9,HNRNPR,FASTKD2,TPR,CNOT9,EIF2AK1,DCP2,EIF3B,SEPSECS,GEMIN5,LARP1,ILF3,EIF5,TYMS,UPF3B,GSPT1,FMR1,METTL16,EXOSC2,RBM8A,METTL18,SARNP,HNRNPD,RNF139,PAIP2	darkgrey	EC_M8
GO:0019438	3.88E-11	1	368	2902	aromatic compound biosynthetic process	BP	3.41E-09	ONECUT2,BCLAF1,PPIL4,MTHFD1L,NUP107,CCT2,DDX39B,ZNF608,SCAF8,MAP3K4,RAD21,DSCC1,HSF2,ATAD2,XRCC6,PHF5A,PSIP1,IFT74,POT1,PWP1,ZNF300,TOPORS,POLR3B,MTERF1,POLH,RFX3,CKS2,PPAT,PAICS,REV1,TRIM28,ZNF451,PHF3,TWISTNB,FOXJ3,ZNF189,SP4,CDCA7L,CTPS1,PHIP,HNRNPA2B1,DCK,ZMPSTE24,RLF,ZNF292,XPO1,CCT4,COMMD1,CASP8AP2,ZNF350,ITGB1BP1,THRAP3,TAF1B,EZH2,UFL1,CCNC,DDX1,NAA15,ELF2,AMD1,ATAD2B,DHFR,CDCA7,HDAC2,CCNA2,EXOSC9,MLLT10,DNAJC2,SETDB2,PCF11,NIFK,NUP85,CAD,ELF1,RSF1,CCNT2,PAXIP1,XRN2,NCAPG2,WDR43,TK1,AFMID,BIRC5,POLR3F,EPC2,SUPT16H,CHD8,KAT6A,RIF1,E2F7,ALYREF,CKAP2,RRM1,CEP290,NSD3,CEBPZ,SUPT20H,TAF7,POLE,NR2C1,RFC3,BRCA2,ELK3,RRP8,TCERG1,MAPK8,PRKDC,CBX5,ERCC6,ZNF214,GPBP1,SP1,TCEA1,ZNF664,ZC3H8,POLR1B,TNKS2,SGK3,BTAF1,ZNF143,PARP4,CENPJ,FER,TDG,NCK1,SBNO1,SMARCA5,RNF141,ZNF507,POLR1A,CHD1,ZFP91,TAF9,CGGBP1,XRN1,SMN2,BDP1,TFCP2,SAP30,ATR,TERF1,GCFC2,UTP15,HLTF,ZBTB10,CNOT1,GABPA,MCRS1,USP16,PPID,RBL1,GMPS,CCT7,CCNL1,SENP1,RIPK1,WDR75,ARID2,MYBL2,E2F8,TRIM33,INO80,ZBED6,CHUK,ZBTB11,ZNF248,MGA,MDM4,HIVEP1,RFC5,BRWD1,NOLC1,CCT5,BMPR1B,UBE3A,CCT6A,PRDM10,NUCKS1,DEK,GCN1,CUL3,GMNN,CHEK1,H2AFZ,RRP1B,NFKB1,KHDRBS1,TRIP13,TET2,TRIP11,HEATR1,ACSL5,NCL,TRAIP,PAPSS1,ZNF184,PRMT5,AJUBA,GTF2IRD2,ZBTB41,ARID4B,NOL11,GAR1,GTF2I,TARBP1,BPTF,DDX5,RFC2,ORC2,NEK2,EWSR1,TIAL1,DTL,HMBS,FLVCR1,BLM,UMPS,CENPF,EDRF1,PDE8A,ING5,BCLAF3,FOSL1,SMARCC1,BAZ1	darkgrey	EC_M8
GO:0007098	4.17E-11	1	37	115	centrosome cycle	BP	3.64E-09	RTTN,CDK2,KIF3A,HAUS6,XPO1,GEN1,PLK4,STIL,CEP295,CHORDC1,SGO1,BRCA2,WDR62,HAUS5,CENPJ,KIF11,CETN3,ALMS1,CKAP5,CHEK1,CEP72,HAUS2,NEK2,CDK1,SLC16A1,BRCA1,CEP152,HAUS3,TRIM37,PLK1,NDC80,CEP76,CCNF,SASS6,ODF2,USP33,AURKA	darkgrey	EC_M8

GO:0010468	4.98E-11	1	423	3428	regulation of gene expression	BP	4.32E-09	CDK2,PAN2,ONECUT2,BCLAF1,CLK4,HNRNPH1,PPIL4,NUP107,DDX39B,ZNF608,SCAF8,UBR5,EIF3E,MAP3K4,RAD21,MRPL13,HSF2,ATAD2,XRCC6,PHF5A,TNRC6B,TUT7,MATR3,SRPK1,SRSF3,PSIP1,IFT74,PWP1,ZNF300,TOPORS,SMU1,POLR3B,XPO5,MTERF1,PUM3,RFX3,IL1R1,CKS2,TRIM28,NCBP1,ZNF451,LRPPRC,FOXJ3,ZNF189,MSH2,SP4,CDCA7L,PHIP,YTHDC1,MTIF2,HNRNPA2B1,PNPT1,PUS10,ZMPSTE24,RLF,SYNCRIP,ZNF292,XPO1,PABPC4,COMMD1,CASP8AP2,TIA1,ZNF350,ITGB1BP1,THRAP3,EZH2,UFL1,CCNC,ASCC3,DDX1,NAA15,ELF2,WDR35,ATAD2B,DHFR,CDCA7,PUS7,HDAC2,CCNA2,EXOSC9,MLLT10,DNAJC2,RNASEH2B,SETDB2,TRDMT1,CNOT10,NUP85,ELF1,UPF2,RSF1,DIS3,CCNT2,PAXIP1,XRN2,NCAPG2,WDR43,BIRC5,POLR3F,EPC2,SUPT16H,CHD8,KAT6A,HNRNPL,METTL3,RIF1,E2F7,ALYREF,CKAP2,CEP290,NSD3,CEBPZ,SUPT20H,TAF7,EXOSC8,NR2C1,BRCA2,ELK3,RRP8,TCERG1,MAPK8,PRKDC,CBX5,CCNB1,ERCC6,NFKBID,ZNF214,DHX29,PAN3,GPBP1,SP1,TCEA1,ZNF664,CAPRIN1,ZC3H8,DIMT1,LSM14A,SGK3,BTAF1,ZNF143,CENPJ,CDK5RAP1,FER,TDG,NCK1,SBNO1,SMARCA5,RNF141,PTCD3,ZNF507,ABCE1,CHD1,ZFP91,TAF9,CGGBP1,XRN1,PDE12,TFCP2,SAP30,TTC37,FAM208A,HELLS,GCFC2,LARP4,UTP15,RBM12,HLTF,RBM39,ZBTB10,CNOT1,GABPA,MCRS1,LTN1,USP16,PPID,RBL1,CCNL1,SMYD5,HNRNPDL,SENP1,TENT2,TOP1,SLC38A2,TOMM70,RIPK1,WDR75,ARID2,MYBL2,E2F8,TRIM33,INO80,RIOK1,ZBED6,CHUK,ZBTB11,ZNF248,MGA,MDM4,HIVEP1,BRWD1,NOLC1,BMPR1B,UBE3A,PRDM10,NUCKS1,DEK,GCN1,CUL3,METAP1,NSUN2,GMNN,CHEK1,TIMM17A,H2AFZ,RRP1B,NFKB1,KHDRBS1,TRIP13	darkgrey	EC_M8
GO:0043043	5.30E-11	1	97	566	peptide biosynthetic process	BP	4.57E-09	PAN2,NARS,DDX39B,EIF3E,MRPL13,TNRC6B,TUT7,PUM3,NCBP1,LRPPRC,MTIF2,PNPT1,SYNCRIP,PABPC4,TIA1,MRPL32,ASCC3,DDX1,DHFR,PUS7,EXOSC9,CNOT10,MRPS7,DIS3,DARS,METTL3,EXOSC8,LARS,PRKDC,DHX29,PAN3,MRPL15,MRPS5,CAPRIN1,LSM14A,CDK5RAP1,EIF2B1,NCK1,MRPL35,PTCD3,ABCE1,XRN1,PDE12,LARP4,CNOT1,LTN1,USP16,RPS6,EEF1E1,NOLC1,GCN1,METAP1,YARS,KHDRBS1,IARS2,NCL,FXR1,NGDN,EIF3A,EIF2B5,DDX6,DHX9,HNRNPR,FASTKD2,TPR,RPL10L,DARS2,CNOT9,RPL22L1,DHX33,EIF2AK1,MRPL44,DCP2,EIF3B,SEPSECS,EIF4A1,RARS,MRPS23,MRPL22,GEMIN5,LARP1,ILF3,EIF5,TYMS,UPF3B,GSPT1,FMR1,METTL16,EXOSC2,RBM8A,METTL18,SARNP,HNRNPD,RNF139,PAIP2,EIF1AX,DRG1	darkgrey	EC_M8
GO:0000086	5.68E-11	1	37	124	G2/M transition of mitotic cell cycle	BP	4.88E-09	CDK2,LATS1,FBXO5,RAD21,CDC25C,MELK,CLSPN,ORC1,RINT1,CCNA2,RNASEH2B,MASTL,TOPBP1,CCNB1,NBN,ABRAXAS1,TAOK3,CHEK1,KHDRBS1,KIF14,TICRR,DTL,BLM,CENPF,CDK1,CDC25A,DBF4B,BRCA1,FOXM1,CCNB3,CDC6,AURKB,LCMT1,PLK1,TAOK1,PKMYT1,AURKA	darkgrey	EC_M8
GO:0007017	5.72E-11	1	123	715	microtubule-based process	BP	4.88E-09	RTTN,CDK2,KATNA1,FBXO5,GTSE1,KIF3A,KIFC1,KIF20A,HAUS6,IFT74,KIF24,AKAP9,GCC2,RFX3,ICK,LRPPRC,SENP6,TTK,XPO1,GEN1,WDR35,PLK4,STIL,CEP57,CEP295,CHORDC1,CENPA,CLASP2,KIF2C,BORA,ULK4,CDC20,HOOK3,SGO1,CKAP2,TUBGCP3,BRCA2,CCNB1,WDR62,GAS2L3,HAUS5,ESPL1,TPX2,KIF20B,SKA3,LSM14A,CENPJ,FER,KIF11,CENPH,CCDC66,CETN3,RACGAP1,ALMS1,CFAP298,CKAP5,KNSTRN,ABRAXAS1,MYBL2,INO80,NUSAP1,STMND1,KIF18A,SLK,CHEK1,SMC3,SLC9B1,CENPE,CEP72,CAMSAP2,HAUS2,KIF14,INTS13,TBCE,CFL1,ASPM,RCC1,SPC25,RAE1,NEK2,PRC1,AUNIP,CDK1,HDGFL3,NUF2,SLC16A1,SETD2,TPR,KIF18B,KIF15,BRCA1,CEP152,HAUS3,TACC3,DLGAP5,FKBP4,HNRNPU,INCENP,SMC1A,CCNB2,KPNB1,KIF4A,KIF22,SPDL1,AURKB,ATRX,KIF23,TRIM37,PLK1,NDC80,STAG2,CEP76,TAOK1,CCNF,SAPCD2,GAS8,SASS6,ODF2,USP33,MAD2L1,STMN1,AURKA,DRG1	darkgrey	EC_M8
GO:0043484	7.05E-11	1	38	126	regulation of RNA splicing	BP	5.97E-09	CLK4,HNRNPH1,SRPK1,SRSF3,SMU1,NCBP1,YTHDC1,TIA1,THRAP3,HNRNPL,RBM12,RBM39,SLC38A2,RRP1B,KHDRBS1,RBM5,NCL,FXR1,DDX5,TRA2B,HNRNPH3,CWC22,HNRNPU,SRSF5,RBM25,FUS,CLK2,RBMX,NSRP1,RBFOX1,FMR1,METTL16,RNPS1,DAZAP1,PTBP1,ZNF326,RBM8A,SRSF7	darkgrey	EC_M8
GO:0031023	7.08E-11	1	39	127	microtubule organizing center organization	BP	5.97E-09	RTTN,CDK2,KIF3A,HAUS6,GCC2,XPO1,GEN1,PLK4,STIL,CEP295,CHORDC1,CLASP2,SGO1,BRCA2,WDR62,HAUS5,CENPJ,KIF11,CETN3,ALMS1,CKAP5,CHEK1,CEP72,HAUS2,NEK2,CDK1,SLC16A1,BRCA1,CEP152,HAUS3,TRIM37,PLK1,NDC80,CEP76,CCNF,SASS6,ODF2,USP33,AURKA	darkgrey	EC_M8
GO:0000460	7.65E-11	1	18	34	maturation of 5.8S rRNA	BP	6.41E-09	EXOSC9,EXOSC8,MAK16,PRKDC,MTREX,UTP20,RRS1,URB1,RRP15,WDR12,FTSJ3,NOP14,LAS1L,EXOSC10,NOL9,EXOSC2,RPF1,C1D	darkgrey	EC_M8
GO:0031297	8.25E-11	1	19	35	replication fork processing	BP	6.88E-09	TONSL,ETAA1,MMS22L,GEN1,ALYREF,BRCA2,ATR,RAD51,NUCKS1,TRAIP,ZGRF1,BLM,DNA2,FANCM,BOD1L1,DDX11,EME1,ATRX,THOC1	darkgrey	EC_M8
GO:0051303	9.73E-11	1	26	68	establishment of chromosome localization	BP	8.07E-09	KIFC1,CENPQ,CDCA8,KIF2C,CCNB1,RRS1,TERF1,FAM83D,KIF18A,CUL3,CENPE,KIF14,SPO11,CENPF,NUF2,CDCA5,DLGAP5,HNRNPU,INCENP,MAD1L1,KPNB1,KIF22,SPDL1,AURKB,NDC80,CDT1	darkgrey	EC_M8

GO:0071459	1.11E-10	1	15	23	protein localization to chromosome, centromeric region	BP	9.12E-09	CENPQ,TTK,CENPA,KNTC1,BUB1B,KNL1,BUB3,CDK1,SPDL1,AURKB,ZWILCH,NDC80,HASPIN,CDT1,CTCF	darkgrey	EC_M8
GO:0009987	1.21E-10	1	1153	11676	cellular process	BP	9.93E-09	RTTN,CDK2,PAN2,NARS,ONECUT2,C18orf54,MEX3C,ME2,YTHDC2,MTFR2,BCLAF1,CLK4,RPA3,USP15,SHPRH,HNRNPH1,XPOT,PPIL4,KATNA1,LATS1,NUP43,MTHFD1L,NUP107,CCT2,MDC1,ZFC3H1,TBC1D15,POP1,DDX39B,SRFBP1,FBXO43,FBXO5,CSNK1G3,ZNF608,SCAF8,UBR5,EIF3E,EBAG9,MAP3K4,GTSE1,RAD21,DSCC1,KIF3A,MRPL13,TBC1D32,RRP7A,HSF2,KIFC1,ATAD2,XRCC6,DDX46,PHF5A,AKAP7,KIF20A,SLC35D2,CDC25C,TNRC6B,FANCC,HSPA9,CHCHD3,TUT7,NAA35,SRPK1,UHRF2,RMI1,SRSF3,PPIL1,PLEC,TNPO3,PSIP1,RECQL4,CEP78,HAUS6,DENND4C,TONSL,RBM28,UBR2,CARNMT1,IFT74,MCM5,HMGXB4,POT1,PWP1,ZNF300,TOPORS,APTX,SMU1,DBF4,POLR3B,XPO5,MTERF1,KIF24,POLH,AKAP9,GCC2,PUM3,RFX3,ARHGEF39,CENPQ,IL1R1,CKS2,MCM3,PPAT,C1GALT1,PAICS,MIOS,ICK,REV1,NOL8,MELK,TRIM28,BZW2,NCBP1,ZNF451,ANP32B,PHF3,TWISTNB,LRPPRC,STX17,PPIH,FOXJ3,ZNF189,MSH2,SMC2,FBXO11,SP4,SENP6,ASB3,CDCA7L,CTPS1,PHIP,YTHDC1,MTIF2,TTK,PPP4R3B,IBTK,HNRNPA2B1,PNPT1,DOP1A,RUFY3,DCK,CYB5R4,PAPOLG,CEP162,PUS10,ZMPSTE24,RLF,USP34,SYNCRIP,ZNF292,XPO1,PLEKHA8,PABPC4,CCT4,COMMD1,MDN1,CASP8AP2,DPY19L1,FKBP15,NUP205,ETAA1,ANLN,FBXO48,CDCA8,GNL2,TIA1,ZNF350,ITGB1BP1,THRAP3,TAF1B,CAPZA2,EZH2,UFL1,NOL10,CUL1,MMS22L,USP45,FAM3C,MRPL32,CCNC,CLSPN,ASCC3,ZMYM4,CNTRL,CASP2,ORC6,DDX1,SHCBP1,NAA15,SMC6,ELF2,ORC1,GEN1,AFG1L,WDR35,SCLT1,NRDC,CDC40,PLK4,AMD1,BCAP29,ATAD2B,DHFR,CDCA7,SYPL1,KIAA1109,RINT1,STIL,PUS7,HDAC2,MCM2,NT5DC1,CENPO,CEP57,CCNA2,EXOSC9,MLLT10,PMPCB,	darkgrey	EC_M8
GO:0007127	1.26E-10	1	31	94	meiosis I	BP	1.02E-08	FBXO5,RAD21,RMI1,UBR2,CKS2,FOXJ3,RAD54L,FANCD2,TOP2B,SGO1,BRCA2,PSMD13,ESPL1,TERF1,MND1,MSH3,RAD51,CCNE2,TRIP13,SGO2,SPO11,FANCM,RAD51AP1,TOP2A,CCNB2,EME1,PTTG1,PLK1,BRIP1,NDC1,AURKA	darkgrey	EC_M8
GO:0006913	1.33E-10	1	58	257	nucleocytoplasmic transport	BP	1.07E-08	XPOT,NUP107,DDX39B,UBR5,HSPA9,SRSF3,TNPO3,XPO5,TRIM28,NCBP1,ANP32B,YTHDC1,HNRNPA2B1,XPO1,NUP205,NUP85,UPF2,IPO5,ALYREF,NUP58,XPO4,IPO11,IPO7,RRS1,ABCE1,TNPO1,NUP160,NUP54,NUP93,HEATR3,NOLC1,NSUN2,KHDRBS1,CSE1L,MCM3AP,RAE1,NUP133,SSB,CDK1,DHX9,SETD2,CTDSPL2,TPR,NMD3,ANKLE1,ECT2,NEDD4,DDX39A,KPNB1,XPO6,NUP62CL,THOC1,UPF3B,THOC2,NUP155,NDC1,SARNP,NUP35	darkgrey	EC_M8
GO:0051169	1.33E-10	1	58	257	nuclear transport	BP	1.07E-08	XPOT,NUP107,DDX39B,UBR5,HSPA9,SRSF3,TNPO3,XPO5,TRIM28,NCBP1,ANP32B,YTHDC1,HNRNPA2B1,XPO1,NUP205,NUP85,UPF2,IPO5,ALYREF,NUP58,XPO4,IPO11,IPO7,RRS1,ABCE1,TNPO1,NUP160,NUP54,NUP93,HEATR3,NOLC1,NSUN2,KHDRBS1,CSE1L,MCM3AP,RAE1,NUP133,SSB,CDK1,DHX9,SETD2,CTDSPL2,TPR,NMD3,ANKLE1,ECT2,NEDD4,DDX39A,KPNB1,XPO6,NUP62CL,THOC1,UPF3B,THOC2,NUP155,NDC1,SARNP,NUP35	darkgrey	EC_M8
GO:0010389	1.34E-10	1	29	82	regulation of G2/M transition of mitotic cell cycle	BP	1.07E-08	CDK2,FBXO5,RAD21,CDC25C,CLSPN,ORC1,RINT1,RNASEH2B,TOPBP1,CCNB1,NBN,ABRAXAS1,TAOK3,CHEK1,KIF14,TICRR,DTL,BLM,CENPF,CDK1,CDC25A,DBF4B,BRCA1,CCNB3,CDC6,AURKB,PLK1,TAOK1,AURKA	darkgrey	EC_M8
GO:0007154	2.88E-12	1	594	4300	cell communication	BP	1.09E-08	TNFRSF11A,SEMA6A,LVRN,KIF5A,PDE7B,IFNGR1,TNFAIP3,STX11,TRIM39,PLEKHG1,AKAP12,FLT4,SNCAIP,FZD6,CTHRC1,RIMS2,CDC42SE2,GRAMD4,CELSR1,NOTCH4,AFDN,PACSIN2,TCF7,TRDN,TPD52L1,MTSS1,RNF146,ARHGAP18,SGSM3,PTK2,DENND3,ANKS1A,DAPK1,ATF4,PDGFB,MAPK13,CSNK1E,PICK1,KDM4C,PIM1,CDC42EP1,MFNG,CSF2RB,MDFI,OSTF1,LRRC19,TEK,RIC8B,DDX58,APBA1,PIP5K1B,FAM122A,TEC,SH3RF3,GNG11,ADGRF5,KIT,ADGRF1,TNFRSF21,KDR,CD2AP,SYK,GADD45G,SEMA4D,RNF149,ICA1,SHB,RAPGEF5,IL6,REL,PPT1,CHN2,CXCL8,KBTBD2,PELI1,RGS3,ARHGAP25,ELMO1,RSAD2,DOCK4,FIGNL1,CAV2,GRB10,LANCL2,TSPAN12,TLR4,GRIK2,TRAF1,C5,DLGAP3,GJA4,INPP4B,IL15,FZD8,CCNY,CREM,TEAD2,SDCBP,TRPM4,NRP1,RAB3B,PUM2,RASIP1,PIK3CG,ARHGAP42,TAL1,MTMR2,MGLL,GATA2,ARHGAP21,PRKD2,CTSC,RAB38,CAB39L,GPR4,SNRK,FGD5,PLXND1,NECTIN2,PRCP,BCAM,ITGA8,NOS3,GAB2,LLGL2,ITGA9,RALB,ITGB4,ZFAND6,PPARG,TIMP4,GATA3,ACVR2B,CXCR4,LMBR1,IRAK2,SEC14L1,EDNRB,PPP1CB,NET1,RALGAPA2,NDFIP2,TMC8,SPTBN4,TIE1,CYTH1,IKBKB,JAG1,P2RY6,P2RY2,FCHSD2,NFKBIB,PDE2A,SFRP1,HBEGF,NR1D2,PLCB4,RASGRP3,FEZ2,NOTUM,FERMT1,RPL26,FREM2,EFNB2,SH3BP5,ARHGEF7,SPEF1,FCHSD1,ARAP3,PCDH1,ARHGAP26,P2RX2,NTN4,CD36,RIC8A,SIRT3,RASSF7,MAGI1,PRAG1,SDCBP2,PRR5L,MAP3K1,SCARB1,EPHB1,PLK2,RET,PDE4D,MERTK,TNFRSF19,CHD7,FAM3D,IL1A,IGF1,NR4A1,KIAA0355,EIF2AK3,ABHD6,HHEX,KDM3A,ITCH,ERBIN,UFSP2,SH3RF1,PITPNM2,PREX2,SMAD1,CLCN3,HIP1R,DCLK2,MCTP1,CHRM5,ARHGEF3,SOX7,KIF13B,ARHGEF2	turquoise	EC_M6

GO:0071826	1.38E-10	1	43	170	ribonucleoprotein complex subunit organization	BP	1.11E-08	DDX39B,EIF3E,RRP7A,SRPK1,PSIP1,NCBP1,YTHDC1,MDN1,DDX1,MRPS7,CRNKL1,SNRPF,PRKDC,DHX29,TGS1,RRS1,TAF9,SMN2,ATR,GCFC2,SCAF11,FP565260.1,SF3B1,RBM5,PRMT5,EIF3A,DHX9,FASTKD2,DDX20,PRPF39,RPL10L,XRCC5,EIF3B,SRSF5,LUC7L3,COIL,GEMIN5,EIF5,NLE1,BRIX1,GEMIN4,GEMIN6,NAF1	darkgrey	EC_M8
GO:0010033	3.63E-12	1	415	2273	response to organic substance	BP	1.37E-08	CD63,LRP1,AP3S1,CDO1,CANX,TAB2,RRM2B,SLC12A2,FBN2,ANGPT1,PPARA,TAF2,NUS1,HSPA4,FAM49B,PTP4A3,LY6E,PPARD,CTNNA1,UBQLN1,LGALS1,TXN2,SH3GL2,GNAQ,PLIN2,MTAP,LEP,CDKN2B,PLAA,RORB,KIAA1324L,SRF,VEGFA,DOCK8,VCP,RUNX2,RASL11B,UNC13B,COL1A2,NCK2,CLOCK,CREB3,ASNS,REST,ASPN,TGFBR1,ERP44,SOCS5,ERLEC1,ADAMTS3,UBE2J1,MAP3K7,ACTR2,KIDINS220,GLI3,ROCK2,INHBA,ZYX,RCN3,MYLK3,VPS35,MGARP,SLC7A11,ITGB1,PGRMC2,FKBP1B,FGF2,KIF5B,SEC61A1,SESN3,PANX1,ESD,GLB1,EIF2B3,TMUB1,GLI2,MEGF8,NUB1,PAK1,ACTR3,TGFB1,AXL,HTR5A,UBXN4,HNMT,CTNNB1,IL15RA,LBH,DGAT2,SERPINH1,AKT2,STT3B,PFKP,ZEB2,OSBPL8,RARB,ACTG1,NPLOC4,ACTN4,KAT2B,RNF121,LTBP1,TMTC4,CRIM1,SPRED3,SPINT2,UFM1,IRS2,PRNP,ATP2B1,LUM,DCN,SMAD9,ZNF703,UBA5,DDRGK1,SMPD1,ILK,LEPROTL1,HSPH1,ARL6IP5,DPYSL3,APLP1,CNOT7,RNF6,CXCL12,ANKRD1,HSP90B1,TMED2,IL20RB,MRAS,MAP1LC3A,CD180,FLNB,OSBP,SULF1,DKK3,TRAM1,ROBO1,MAP1B,RYR3,SORBS1,DOK1,P2RX4,HEY1,SELENOT,ATP2A2,USO1,MMP15,APP,GLRB,DHX36,RAPGEF2,CX3CL1,SLC33A1,ABCC8,PPP2R2A,PTPN11,ADCY6,HOMER1,OPA1,ARSB,AMFR,GTF2H1,ZFYVE27,PTPN22,ACVR1,CRY2,VPS18,CHAC1,MMP2,GDF6,MTDH,ADA,ATP2B4,SPP1,CPN1,PKD2,TANK,TOLLIP,PSMD14,BRSK2,MARCH6,PSPH,NDN,RAB7B,CHRNA7,EIF4E,DYNLL1,INA,ADIPOR1,KDM5B,NOTCH2,CDC73,DNM1L,GLRX2,TROVE2,SELENOS,HYAL1,SLC2A10,CCND1,CPT1A,TGFB2,ZBTB20,ZNF106,LRP5,EPRS,FEZ1,HTR2B,SATB2,GSK3B,CASP7,FSTL1,MMP14,DAG1,PIK3CA,HADH,LEF1,PRKCA,CMKLR1,HJ	darkgreen	EC_M4
GO:0008608	1.81E-10	1	17	30	attachment of spindle microtubules to kinetochore	BP	1.44E-08	KIF2C,SGO1,CCNB1,RACGAP1,KNSTRN,ABRAXAS1,KNL1,CENPE,NEK2,BUB3,NUF2,ECT2,HNRNPU,MAD1L1,AURKB,NDC80,CDT1	darkgrey	EC_M8
GO:0040012	4.90E-12	1	153	796	regulation of locomotion	BP	1.48E-08	SEMA6A,AKAP12,FLT4,AFDN,ARHGAP18,PTK2,PDGFB,PODXL,TEK,LIMCH1,DDX58,ATP8A1,KIT,KDR,SEMA4D,IL6,CXCL8,DOCK4,TLR4,TCAF2,C5,SDCBP,NRP1,PIK3CG,LAMA4,GATA2,PRKD2,NOVA2,LRCH1,PLXND1,PRCP,NOS3,DACH1,PPARG,GATA3,CXCR4,TIE1,JAG1,P2RY6,SFRP1,HBEGF,FERMT1,EFNB2,ARHGEF7,PRAG1,ADAMTS9,PRR5L,SCARB1,PLK2,RET,IGF1,ABHD6,MCTP1,MEF2C,VEGFC,NDRG4,CXCL10,ANGPT2,ADGRG3,THBS4,PLCG1,STC1,ANO6,DLL4,NEDD9,EDN1,JAM3,SNCA,SRGAP2C,ETS1,CD200,PPP3CA,HYAL2,SEMA3F,RIN3,ROBO4,PRKX,AP1AR,PECAM1,PLXNA2,FGR,MCAM,INPP5F,IGFBP3,FES,PROX1,PRKD1,LAMA5,YTHDF1,ARHGDIB,MAP3K3,ACE,ITGA6,DOCK1,LAMC2,APOD,ADORA2A,SEMA6D,ITGA4,IGFBP5,MCU,LDB2,SELE,SELP,PLVAP,FGF23,PPM1F,WAS,RAC1,HIF1A,RCC2,RHOJ,SYNE2,JUP,ADIPOR2,EPHA2,MMRN2,MCC,MTOR,STK10,SEMA4A,FGF18,ABI3,ITGA3,PGF,DAPK2,EFNA1,VASH1,S100A14,MMP28,AMOT,CCL2,LGALS9,STK26,SEMA6B,ARHGAP4,NOTCH1,PLCG2,S1PR1,ENG,DAB2IP,CARMIL2,RIPOR1,CDH5,MIR23A,ADGRG1,RELN,FGF16,STX3,MGAT3,BST2,BMP2,CBLL1	turquoise	EC_M6
GO:0051310	2.09E-10	1	23	55	metaphase plate congression	BP	1.65E-08	KIFC1,CENPQ,CDCA8,KIF2C,CCNB1,RRS1,FAM83D,KIF18A,CUL3,CENPE,KIF14,CENPF,NUF2,CDCA5,HNRNPU,INCENP,MAD1L1,KPNB1,KIF22,SPDL1,AURKB,NDC80,CDT1	darkgrey	EC_M8
GO:0050000	2.34E-10	1	26	70	chromosome localization	BP	1.84E-08	KIFC1,CENPQ,CDCA8,KIF2C,CCNB1,RRS1,TERF1,FAM83D,KIF18A,CUL3,CENPE,KIF14,SPO11,CENPF,NUF2,CDCA5,DLGAP5,HNRNPU,INCENP,MAD1L1,KPNB1,KIF22,SPDL1,AURKB,NDC80,CDT1	darkgrey	EC_M8

GO:0051270	9.53E-12	1	157	830	regulation of cellular component movement	BP	1.93E-08	SEMA6A,AKAP12,FLT4,AFDN,ARHGAP18,PTK2,PDGFB,PODXL,TEK,LIMCH1,DDX58,ATP8A1,KIT,KDR,SEMA4D,IL6,CXCL8,DOCK4,TLR4,TCAF2,C5,SDCBP,TRPM4,NRP1,PIK3CG,LAMA4,GATA2,PRKD2,NOVA2,LRCH1,PLXND1,PRCP,NOS3,DACH1,PPARG,GATA3,CXCR4,TIE1,JAG1,P2RY6,SFRP1,HBEGF,FERMT1,ARHGEF7,PRAG1,ADAMTS9,PRR5L,SCARB1,PLK2,RET,PDE4D,IGF1,ABHD6,MCTP1,MEF2C,VEGFC,NDRG4,CXCL10,ANGPT2,ADGRG3,THBS4,PLCG1,STC1,ANO6,DLL4,NEDD9,EDN1,JAM3,SRGAP2C,ETS1,CD200,PPP3CA,HYAL2,KCNJ2,SEMA3F,RIN3,ROBO4,PRKX,AP1AR,PECAM1,PLXNA2,FGR,MCAM,INPP5F,IGFBP3,FES,PROX1,PRKD1,LAMA5,YTHDF1,ARHGDIB,MAP3K3,ACE,ITGA6,DOCK1,LAMC2,DLG1,APOD,SEMA6D,ITGA4,IGFBP5,MCU,LDB2,SELE,SELP,PLVAP,FGF23,PPM1F,WAS,RAC1,HIF1A,RCC2,RHOJ,SYNE2,JUP,ADIPOR2,EPHA2,MMRN2,MCC,MTOR,STK10,SEMA4A,FGF18,ABI3,ITGA3,PGF,DAPK2,EFNA1,VASH1,ATP2A1,S100A14,DSG2,DSC2,MMP28,AMOT,CCL2,PDE4B,LGALS9,STK26,SEMA6B,ARHGAP4,NOTCH1,PLCG2,S1PR1,ENG,DAB2IP,CARMIL2,RIPOR1,CDH5,MIR23A,ADGRG1,RELN,FGF16,MGAT3,BST2,BMP2,CBLL1	turquoise	EC_M6
GO:0009605	9.91E-12	1	278	1770	response to external stimulus	BP	1.93E-08	TNFRSF11A,SEMA6A,KIF5A,IFNGR1,TNFAIP3,TRIM39,AKAP12,LYZ,TSPAN8,TRDN,PTK2,C6orf106,DAPK1,ATF4,PDGFB,MAPK13,PICK1,PIM1,CSF2RB,LRRC19,TEK,DDX58,KIT,KDR,SYK,SEMA4D,IL6,REL,CXCL8,PELI1,RSAD2,DOCK4,ODC1,TLR4,GRIK2,C5,IL15,NRCAM,NOCT,TRPM4,NRP1,PUM2,PIK3CG,MGLL,DNMT3A,PRKD2,CST7,NOVA2,CTSC,RAB38,GPR4,PLXND1,NECTIN2,NOS3,RALB,UNC13D,PPARG,TIMP4,GATA3,CXCR4,IRAK2,SEC14L1,EDNRB,PPP1CB,IKBKB,NFKBIB,PDE2A,TMEM173,SFRP1,HBEGF,NR1D2,FEZ2,EFNB2,CD36,ARID5A,MAP3K1,SCARB1,EPHB1,PLK2,RET,CHD7,IL1A,DNMT3B,IGF1,NR4A1,BPIFB3,KLKB1,EIF2AK3,ITCH,ERBIN,MMP24,MEF2C,TLR2,VEGFC,GPM6A,CXCL10,ANGPT2,SERINC5,OAS3,NLRC5,THBS4,TMEM150C,PLCG1,STC1,NR4A2,STAB1,ANO6,IFIT2,IFIT1,EDN1,STAT1,CD58,JAM3,SNCA,NUAK2,ANO3,NFKB2,MX2,MX1,ETS1,RNF19B,CD200,SIK1,HDAC1,LCK,HYAL2,ANO1,KCNJ2,SEMA3F,RIN3,ROBO4,LYST,NCF1,FABP4,PABPN1,ISG20,TIFA,CD34,CD86,PLXNA2,MN1,FGR,INPP5F,KALRN,FES,RBM4,PROX1,PRKD1,LAMA5,F11R,YTHDF1,IL10RA,SRMS,PAK2,LAMC2,SIPA1,STYK1,MOV10,CREB1,B2M,HMCN1,SSTR1,LTF,CCRL2,ADORA2A,MASP1,RTP4,SEMA6D,CYBB,IL1RAP,EPHB4,ITGA4,DDX3X,MCU,TMEM106A,PYGL,CALCRL,BIRC3,SELE,SELP,BAIAP2L1,FGF23,PPM1F,WAS,RAC1,HIF1A,JUP,RAB27A,ADIPOR2,BCL6B,EPHA2,TLR6,TNK1,TNFRSF1B,ARG2,ALDH1A2,MTOR,BNIP2,OPHN1,TBKBP1,EFNB1,ZFYVE1,SEMA4A,HOXB9,FGF18,GNGT2,PGF,DAPK2,BATF,EFNA1,RPS6KA5,S100A14,SCNN1B,CYP1A1,TNIP1,MMP28,AMOT,KIF26A,ADRB2,CCL2,YES1,PDE4B,JAK1,LGALS9,NOS2,UNC119,ELF4,IL7R,PI	turquoise	EC_M6
GO:0016477	1.02E-11	1	199	1120	cell migration	BP	1.93E-08	TNFRSF11A,SEMA6A,TNFAIP3,AKAP12,FLT4,CTHRC1,CELSR1,AFDN,PTK2,ANKS1A,PDGFB,PODXL,STRIP2,TEK,LIMCH1,DDX58,ATP8A1,KIT,KDR,CD2AP,SYK,SEMA4D,EFHC1,IL6,CXCL8,AVL9,ELMO1,DOCK4,GRB10,TLR4,TCAF2,C5,SDCBP,NRCAM,TRPM4,NRP1,PIK3CG,LAMA4,GATA2,PRKD2,LRCH1,PLXND1,PRCP,NOS3,ITGB4,DACH1,PPARG,GATA3,CXCR4,EDNRB,NET1,TIE1,JAG1,P2RY6,SFRP1,HBEGF,FERMT1,EFNB2,ARHGEF7,SPEF1,NTN4,RIC8A,PRAG1,ADAMTS9,PRR5L,SCARB1,EPHB1,PLK2,RET,MERTK,IGF1,NR4A1,ABHD6,SH3RF1,MCTP1,HEXB,MEF2C,VEGFC,NDRG4,GPM6A,CXCL10,ANGPT2,ADGRG3,THBS4,PLCG1,STC1,NR4A2,DGKZ,ANO6,DLL4,PIK3C2B,NEDD9,EDN1,JAM3,SRGAP2C,ETS1,CD200,LCK,PPP3CA,HYAL2,SEMA3F,RIN3,ROBO4,PRKX,LYST,NFATC2,CD34,PECAM1,PLEKHO1,PLXNA2,FGR,MCAM,IGFBP3,PROX1,PRKD1,VANGL2,LAMA5,F11R,ARHGDIB,MAP3K3,ACE,ITGA6,DOCK1,LAMC2,APOD,CLEC14A,CCRL2,SEMA6D,EPHB4,ITGA4,IGFBP5,TBX1,MCU,LDB2,SELE,SELP,PLVAP,FGF23,PPM1F,RAC1,HIF1A,RCC2,RHOJ,SYNE2,JUP,ADIPOR2,FSCN1,EPHA2,MMRN2,MCC,MTOR,OPHN1,EFNB1,PSEN1,STK10,SEMA4A,HOXB9,FGF18,ABI3,ITGA3,PGF,DAPK2,TAOK2,EFNA1,VASH1,S100A14,CDC42BPB,MMP28,AMOT,CCL2,YES1,PDE4B,LGALS9,STK26,MYO18A,SEMA6B,ARHGAP4,PLEKHG5,NOTCH1,PLCG2,S1PR1,ENG,LHX6,DAB2IP,CARMIL2,RIPOR1,CDH5,MIR23A,ADGRG1,SOX17,RELN,FGF16,SOX18,MGAT3,MSMP,BST2,BMP2,CBLL1,ARPC5L	turquoise	EC_M6

GO:0001525	1.38E-11	1	96	428	angiogenesis	BP	2.32E-08	SEMA6A,TNFAIP3,PTPRB,FLT4,NOTCH4,PTK2,TEK,KDR,SYK,THSD7A,SHB,IL6,CXCL8,TSPAN12,C5,NRCAM,NRP1,RASIP1,PIK3CG,TAL1,GATA2,PRKD2,GPR4,PLXND1,PRCP,NOS3,PPARG,CXCR4,TIE1,JAG1,SFRP1,EFNB2,ADAMTS9,EPHB1,PLK2,IL1A,NR4A1,EIF2AK3,HHEX,SMAD1,VEGFC,CXCL10,ANGPT2,THBS4,PPP1R16B,PLCG1,RAPGEF3,STAB1,HIPK1,DLL4,EDN1,STAT1,JAM3,ETS1,ROBO4,PRKX,CD34,MCAM,PRKD1,MAP3K3,APOLD1,APOD,CLEC14A,CYBB,EPHB4,TBX1,CALCRL,HSPG2,RAMP2,HIF1A,RHOJ,JUP,ADIPOR2,EPHA2,MMRN2,SEMA4A,FGF18,PGF,EFNA1,VASH1,NPR1,AMOT,CCL2,JAK1,LRG1,NRARP,EGFL7,NOTCH1,S1PR1,ENG,DAB2IP,CDH5,MIR23A,ADGRG1,SOX17,SOX18	turquoise	EC_M6
GO:0006406	4.37E-10	1	22	53	mRNA export from nucleus	BP	3.40E-08	NUP107,DDX39B,SRSF3,NCBP1,YTHDC1,HNRNPA2B1,NUP85,UPF2,ALYREF,NUP160,NUP93,NSUN2,MCM3AP,RAE1,NUP133,SETD2,TPR,DDX39A,THOC1,THOC2,NUP155,SARNP	darkgrey	EC_M8
GO:0071427	4.37E-10	1	22	53	mRNA-containing ribonucleoprotein complex export from nucleus	BP	3.40E-08	NUP107,DDX39B,SRSF3,NCBP1,YTHDC1,HNRNPA2B1,NUP85,UPF2,ALYREF,NUP160,NUP93,NSUN2,MCM3AP,RAE1,NUP133,SETD2,TPR,DDX39A,THOC1,THOC2,NUP155,SARNP	darkgrey	EC_M8
GO:2000145	2.51E-11	1	147	772	regulation of cell motility	BP	3.78E-08	SEMA6A,AKAP12,FLT4,AFDN,ARHGAP18,PTK2,PDGFB,PODXL,TEK,LIMCH1,DDX58,ATP8A1,KIT,KDR,SEMA4D,IL6,CXCL8,DOCK4,TLR4,TCAF2,C5,SDCBP,NRP1,PIK3CG,LAMA4,GATA2,PRKD2,LRCH1,PLXND1,PRCP,NOS3,DACH1,PPARG,GATA3,CXCR4,TIE1,JAG1,P2RY6,SFRP1,HBEGF,FERMT1,ARHGEF7,PRAG1,ADAMTS9,PRR5L,SCARB1,PLK2,RET,IGF1,ABHD6,MCTP1,MEF2C,VEGFC,NDRG4,CXCL10,ANGPT2,ADGRG3,THBS4,PLCG1,STC1,ANO6,DLL4,NEDD9,EDN1,JAM3,SRGAP2C,ETS1,CD200,PPP3CA,HYAL2,SEMA3F,RIN3,ROBO4,PRKX,AP1AR,PECAM1,PLXNA2,FGR,MCAM,INPP5F,IGFBP3,FES,PROX1,PRKD1,LAMA5,ARHGDIB,MAP3K3,ACE,ITGA6,DOCK1,LAMC2,APOD,SEMA6D,ITGA4,IGFBP5,MCU,LDB2,SELE,SELP,PLVAP,FGF23,PPM1F,WAS,RAC1,HIF1A,RCC2,RHOJ,SYNE2,JUP,ADIPOR2,EPHA2,MMRN2,MCC,MTOR,STK10,SEMA4A,FGF18,ABI3,ITGA3,PGF,DAPK2,EFNA1,VASH1,S100A14,MMP28,AMOT,CCL2,LGALS9,STK26,SEMA6B,ARHGAP4,NOTCH1,PLCG2,S1PR1,ENG,DAB2IP,CARMIL2,RIPOR1,CDH5,MIR23A,ADGRG1,RELN,FGF16,MGAT3,BST2,BMP2,CBLL1	turquoise	EC_M6
GO:0051383	4.93E-10	1	14	21	kinetochore organization	BP	3.81E-08	SMC2,SENP6,CENPA,CENPH,KNTC1,CENPE,CENPF,NUF2,SMC4,DLGAP5,NDC80,CDT1,CENPN,CENPW	darkgrey	EC_M8
GO:0043933	5.07E-10	1	193	1370	protein-containing complex subunit organization	BP	3.91E-08	SHPRH,LATS1,NUP107,CCT2,DDX39B,FBXO5,SCAF8,EIF3E,RRP7A,FANCC,SRPK1,PLEC,PSIP1,KIF24,AKAP9,NCBP1,ANP32B,STX17,PPIH,SENP6,YTHDC1,PNPT1,ZMPSTE24,MDN1,NUP205,ANLN,ITGB1BP1,TAF1B,CAPZA2,DDX1,MCM2,CENPO,CEP57,YME1L1,CENPA,NASP,CLASP2,NIFK,MRPS7,KIF2C,RSF1,DIAPH3,DARS,TK1,CRNKL1,SUPT16H,KAT6A,NAP1L1,CKAP2,RRM1,TAF7,TUBGCP3,SNRPF,PRKDC,CCNB1,DHX29,ACTR6,TGS1,TPX2,NDUFAF2,RRS1,CENPJ,FER,NCK1,ARFGEF1,SMARCA5,ABCE1,CENPH,TAF9,SMN2,BDP1,KNTC1,ATR,TERF1,HELLS,GCFC2,NUP54,USP16,PPID,MIS18A,CKAP5,NUP93,RAD51,SCAF11,RIPK1,ARID2,INO80,OIP5,STK4,MDM4,CHAF1B,UBE2C,STMND1,KIF18A,CUL3,NAP1L4,GMNN,FP565260.1,KCTD3,SF3B1,CENPE,RBM5,CAMSAP2,COPS7B,HAUS2,KIF14,PRMT5,AJUBA,TBCE,CFL1,BPTF,ANP32E,HJURP,MCM3AP,EIF3A,NUP133,ALS2,BLM,CENPF,HAT1,CDK1,SMARCC1,DHX9,HDGFL3,SETD2,FASTKD2,DDX20,TPR,KIF18B,PRPF39,RPL10L,UBTF,XRCC5,CDC45,LETM1,HP1BP3,DLGAP5,TRPM7,ECT2,DHX33,FKBP4,RAD52,UBE2K,SMARCE1,EIF3B,ASF1B,SRSF5,CCT3,FUS,TAF1,LUC7L3,ATRX,THG1L,COIL,LCMT1,CENPI,GEMIN5,PSMD10,RIOK3,EIF5,NLE1,RICTOR,AIFM1,BRIX1,AFG3L2,GSPT1,HPRT1,TAOK1,RBMX,LIPG,CHAF1A,NDC1,UBN1,TP73,FANCA,CDT1,CENPN,IST1,CTCF,CTH,HIST1H1A,GEMIN4,TMOD3,STMN1,ITGB3BP,HIST1H3H,GEMIN6,NAF1,CENPW,OTUD6B,RNF8,NUP35,DRG1	darkgrey	EC_M8

GO:0008104	1.30E-11	1	375	2028	protein localization	BP	3.92E-08	CD63,LMAN1,ATP8B1,LRP1,STX7,AP3S1,AHI1,AGAP2,CANX,TBC1D9B,RAB21,RABL2A,SLC12A2,FBN2,ANGPT1,TCP1,NUP50,HSPA4,SEC24A,PACSIN1,PTP4A3,REEP2,PPARD,CTNNA1,SIL1,SUN2,CBY1,GGA1,MYH9,LEP,PEX6,VCP,UNC13B,CLOCK,EXOC1,SRP72,REST,LMAN2L,RAB23,SNX13,P3H1,RIMS1,TMEM30A,ERLEC1,FAM126A,RTN4,TPBG,SHANK1,COX18,EMC10,UBE2J1,WHRN,MDFIC,GLI3,ROCK2,VPS41,RCN3,VPS35,MGARP,SLC7A11,ITGB1,LIN7B,FKBP1B,KIF5B,SEC61A1,NAPA,DNAJC1,RAB7A,COPG1,RB1,PICALM,MARK4,MAPRE3,PDCD6IP,SNX17,PAK1,TGFB1,SRP68,RAB3GAP1,SEC13,CTNNB1,TRAK1,AKT2,GPD1L,SEC23B,DZIP1,TBC1D16,RAB2B,OSBPL8,ACTG1,NPLOC4,ACTN4,STAM2,LTBP1,COG6,UFM1,ATP2C1,IRS2,PRNP,HDAC3,RAB11FIP1,DCLK1,PCLO,DDRGK1,ARL6IP5,WASHC2A,RIC3,VPS37A,ARL1,TM9SF4,RAB2A,ANKRD1,HSP90B1,TMED2,TP53INP2,COPB2,OSBP,TMEM150A,VAMP8,SLMAP,ATP1B3,TRAM1,EMC4,FAM160A1,PINX1,SORBS1,COPB1,SELENOT,USO1,TCTN1,SDAD1,GLRB,CCT8,RAPGEF2,TM9SF3,ABCC8,CACNB3,CPE,SSR3,PTPN11,EXOC6B,GNL3,PFKM,ARHGAP1,ATG13,AP3B1,ZFYVE27,TSG101,DKC1,CRY2,VPS18,AKTIP,BAP1,CHP1,ATP2B4,PKD2,TWF1,AP2A2,CBLB,TOLLIP,BRSK2,HPS6,VPS39,RAB7B,ARL3,DYNLL1,APBA2,PHLDB2,TJP1,SRPRA,CTTN,PKP2,DNM1L,SELENOS,KPNA6,SEC22B,CPT1A,TGFB2,FBLN5,LRP5,WIPI1,ZMAT3,GSK3B,MMP14,DAG1,HADH,CORO1C,DISP1,SHROOM2,PITPNM1,SGSM1,GOLGB1,HSPA8,LARP7,KPNA1,BMP7,IDH2,AP1B1,SEC22A,ADCY5,NEDD8,AP1S2,GOLT1B,AP2M1,MPP4,COPA,GORASP2,IQGAP1,SCFD1,KIF1A,SNX27,TOMM40L,TFRC,FZD9,SIDT2,SRP54,L	darkgreen	EC_M4
GO:2001020	5.38E-10	1	48	200	regulation of response to DNA damage stimulus	BP	4.12E-08	BCLAF1,DDX39B,FBXO5,UBR5,POT1,POLH,TRIM28,ZMPSTE24,ETAA1,RNASEH2B,DDIAS,PAXIP1,RIF1,CUL4A,PPP4R2,PRKDC,ERCC6,PARPBP,TAF9,ATR,ABRAXAS1,RAD51,ARID2,EEF1E1,DEK,CHEK1,FXR1,DDX5,FANCB,RPA2,AUNIP,DHX9,SETD2,BRCA1,NSD2,RAD51AP1,ANKLE1,FOXM1,DDX11,RAD52,FUS,PSMD10,THOC1,SMCHD1,RMI2,FMR1,TIGAR,RNF8	darkgrey	EC_M8
GO:0050684	5.63E-10	1	35	118	regulation of mRNA processing	BP	4.29E-08	SRPK1,SRSF3,SMU1,NCBP1,YTHDC1,TIA1,THRAP3,HNRNPL,CCNB1,RBM39,VIRMA,KHDRBS1,RBM5,NCL,FXR1,DDX5,DHX9,TRA2B,CWC22,LEO1,HNRNPU,SLTM,RBM25,SAFB,SAFB2,RBMX,NSRP1,RBFOX1,FMR1,METTL16,RNPS1,DAZAP1,PTBP1,RBM8A,SRSF7	darkgrey	EC_M8
GO:0006952	3.36E-11	1	166	949	defense response	BP	4.61E-08	TNFRSF11A,IFNGR1,TNFAIP3,TRIM39,LYZ,PTK2,C6orf106,DAPK1,MAPK13,LRRC19,TEK,DDX58,NFX1,KIT,SYK,IL6,REL,CXCL8,RSAD2,TLR4,C5,IL15,PUM2,PIK3CG,MGLL,CST7,CTSC,GPR4,NECTIN2,PRCP,PSG3,UNC13D,PPARG,GATA3,CXCR4,IRAK2,SEC14L1,EDNRB,IKBKB,PDE2A,TMEM173,NR1D2,CD36,ARID5A,PLK2,IL1A,IGF1,BPIFB3,KLKB1,ITCH,SMAD1,MEF2C,TLR2,CXCL10,SERINC5,OAS3,NLRC5,PLCG1,STAB1,ANO6,CHST1,IFIT2,IFIT1,TSPAN2,EDN1,STAT1,CD58,JAM3,SNCA,MX2,MX1,ETS1,RNF19B,CD200,LCK,HYAL2,ICOSLG,SNAP23,LYST,LAPTM5,NCF1,FABP4,ISG20,TIFA,FGR,ADCY1,LTB4R,FES,PRKD1,ZNF148,F11R,SRMS,PAK2,APOD,STYK1,MOV10,LTF,CCRL2,ADORA2A,MASP1,RTP4,CYBB,IL1RAP,DDX3X,TMEM106A,CALCRL,HSPG2,BIRC3,SELE,SELP,WAS,RAC1,HIF1A,LIPA,RAB27A,BCL6B,EPHA2,TLR6,TNK1,TNFRSF1B,ARG2,FBXL20,TBKBP1,ABI3,BATF,RPS6KA5,S100A14,GPR68,SCNN1B,TNIP1,CCL2,YES1,JAK1,LGALS9,NOS2,ELF4,IL7R,GIT1,ISG15,F8,NOTCH1,IRF8,PLCG2,STXBP1,LRSAM1,PTGS1,DAB2IP,CDH5,OAS1,VNN1,PMAIP1,TLR1,GBP5,RAB43,TRIM34,CD46,ACKR1,TRIM62,SERPINB9,MSMP,BST2,VAMP3,BMP2,IFIT3,RNF125,BMP6	turquoise	EC_M6
GO:0006955	3.74E-11	1	174	1012	immune response	BP	4.65E-08	TNFRSF11A,IFNGR1,TNFAIP3,TRIM39,LYZ,GRAMD4,TCF7,PTK2,C6orf106,DAPK1,MFNG,LRRC19,DDX58,TEC,KIT,TNFRSF21,NFIL3,SYK,SEMA4D,IL6,REL,CXCL8,BACH2,PELI1,RSAD2,TLR4,C5,IL15,TRPM4,PUM2,PIK3CG,GATA2,PRKD2,CST7,CTSC,NECTIN2,GAB2,UNC13D,PPARG,GATA3,CXCR4,IRAK2,SEC14L1,IKBKB,JAG1,TMEM173,CD36,ARID5A,MAP3K1,PLK2,GAPT,PDE4D,IL1A,BPIFB3,ITCH,ERBIN,MEF2C,TLR2,IL7,CXCL10,SERINC5,OAS3,NLRC5,DYSF,PLCG1,RAPGEF3,DGKZ,STAB1,IFIT2,IFIT1,EDN1,STAT1,CD58,JAM3,SNCA,NFKB2,MX2,MX1,ETS1,RNF19B,CD200,LCK,HYAL2,ICOSLG,SNAP23,LYST,LAPTM5,HLX,NCF1,ISG20,NFATC2,INPP5D,TIFA,CD86,BMX,FGR,LTB4R,FES,PLEKHA1,PRKD1,SRMS,PAK2,SIPA1,STYK1,MOV10,B2M,ST6GAL1,LTF,CCRL2,MASP1,RTP4,CYBB,IL1RAP,DDX3X,TMEM106A,BIRC3,SELP,CRACR2A,WAS,PRKCH,PLD2,RAB27A,BCL6B,EPHA2,TLR6,TNK1,LFNG,TNFRSF1B,ARG2,MTOR,TBKBP1,PSEN1,SEMA4A,EIF2B2,BATF,CD19,S100A14,SCNN1B,TNIP1,CCL2,YES1,PDE4B,JAK1,LGALS9,NOS2,ELF4,IL7R,ISG15,TRAF2,NOTCH1,IRF8,PLCG2,STXBP1,LRSAM1,DAB2IP,FYB1,OAS1,VNN1,PMAIP1,TLR1,GBP5,ERAP2,PKHD1L1,RAB43,TRIM34,CD46,TRIM62,FCER2,SERPINB9,BST2,VAMP3,IFIT3,RNF125,BMP6	turquoise	EC_M6

GO:0043207	4.56E-11	1	147	820	response to external biotic stimulus	BP	4.65E-08	TNFRSF11A,IFNGR1,TNFAIP3,TRIM39,AKAP12,LYZ,TRDN,PTK2,C6orf106,DAPK1,CSF2RB,DDX58,SYK,IL6,REL,CXCL8,PELI1,RSAD2,ODC1,TLR4,C5,IL15,NOCT,PUM2,PIK3CG,NECTIN2,NOS3,UNC13D,PPARG,TIMP4,GATA3,CXCR4,IRAK2,SEC14L1,EDNRB,IKBKB,NFKBIB,TMEM173,CD36,ARID5A,SCARB1,CHD7,IL1A,NR4A1,BPIFB3,ITCH,ERBIN,MEF2C,TLR2,GPM6A,CXCL10,SERINC5,OAS3,NLRC5,STAB1,IFIT2,IFIT1,EDN1,STAT1,CD58,SNCA,NFKB2,MX2,MX1,RNF19B,HDAC1,LCK,HYAL2,LYST,NCF1,FABP4,PABPN1,ISG20,TIFA,CD86,FGR,FES,PRKD1,IL10RA,SRMS,PAK2,STYK1,MOV10,B2M,HMCN1,LTF,MASP1,RTP4,CYBB,IL1RAP,DDX3X,TMEM106A,PYGL,BIRC3,SELE,SELP,BAIAP2L1,WAS,HIF1A,RAB27A,ADIPOR2,EPHA2,TLR6,TNK1,TNFRSF1B,ARG2,TBKBP1,BATF,S100A14,SCNN1B,CYP1A1,TNIP1,CCL2,YES1,PDE4B,JAK1,LGALS9,NOS2,ELF4,IL7R,GIT1,LRG1,ISG15,NOTCH1,IRF8,PLCG2,STXBP1,LRSAM1,DAB2IP,ADGRL2,OAS1,VNN1,PMAIP1,TLR1,GBP5,RAB43,TRIM34,CD46,TRIM62,SERPINB9,BST2,VAMP3,BMP2,C15orf48,IFIT3,RNF125,BMP6	turquoise	EC_M6
GO:0051707	4.56E-11	1	147	820	response to other organism	BP	4.65E-08	TNFRSF11A,IFNGR1,TNFAIP3,TRIM39,AKAP12,LYZ,TRDN,PTK2,C6orf106,DAPK1,CSF2RB,DDX58,SYK,IL6,REL,CXCL8,PELI1,RSAD2,ODC1,TLR4,C5,IL15,NOCT,PUM2,PIK3CG,NECTIN2,NOS3,UNC13D,PPARG,TIMP4,GATA3,CXCR4,IRAK2,SEC14L1,EDNRB,IKBKB,NFKBIB,TMEM173,CD36,ARID5A,SCARB1,CHD7,IL1A,NR4A1,BPIFB3,ITCH,ERBIN,MEF2C,TLR2,GPM6A,CXCL10,SERINC5,OAS3,NLRC5,STAB1,IFIT2,IFIT1,EDN1,STAT1,CD58,SNCA,NFKB2,MX2,MX1,RNF19B,HDAC1,LCK,HYAL2,LYST,NCF1,FABP4,PABPN1,ISG20,TIFA,CD86,FGR,FES,PRKD1,IL10RA,SRMS,PAK2,STYK1,MOV10,B2M,HMCN1,LTF,MASP1,RTP4,CYBB,IL1RAP,DDX3X,TMEM106A,PYGL,BIRC3,SELE,SELP,BAIAP2L1,WAS,HIF1A,RAB27A,ADIPOR2,EPHA2,TLR6,TNK1,TNFRSF1B,ARG2,TBKBP1,BATF,S100A14,SCNN1B,CYP1A1,TNIP1,CCL2,YES1,PDE4B,JAK1,LGALS9,NOS2,ELF4,IL7R,GIT1,LRG1,ISG15,NOTCH1,IRF8,PLCG2,STXBP1,LRSAM1,DAB2IP,ADGRL2,OAS1,VNN1,PMAIP1,TLR1,GBP5,RAB43,TRIM34,CD46,TRIM62,SERPINB9,BST2,VAMP3,BMP2,C15orf48,IFIT3,RNF125,BMP6	turquoise	EC_M6
GO:0030334	4.62E-11	1	141	735	regulation of cell migration	BP	4.65E-08	SEMA6A,AKAP12,FLT4,AFDN,PTK2,PDGFB,PODXL,TEK,LIMCH1,DDX58,ATP8A1,KIT,KDR,SEMA4D,IL6,CXCL8,DOCK4,TLR4,TCAF2,C5,SDCBP,NRP1,PIK3CG,LAMA4,GATA2,PRKD2,LRCH1,PLXND1,PRCP,NOS3,DACH1,PPARG,GATA3,CXCR4,TIE1,JAG1,P2RY6,SFRP1,HBEGF,FERMT1,ARHGEF7,ADAMTS9,PRR5L,SCARB1,PLK2,RET,IGF1,ABHD6,MCTP1,MEF2C,VEGFC,NDRG4,CXCL10,ANGPT2,ADGRG3,THBS4,PLCG1,STC1,ANO6,DLL4,NEDD9,EDN1,JAM3,SRGAP2C,ETS1,CD200,PPP3CA,HYAL2,SEMA3F,RIN3,ROBO4,PRKX,PECAM1,PLXNA2,FGR,MCAM,IGFBP3,PROX1,PRKD1,LAMA5,ARHGDIB,MAP3K3,ACE,ITGA6,DOCK1,LAMC2,APOD,SEMA6D,ITGA4,IGFBP5,MCU,LDB2,SELE,SELP,PLVAP,FGF23,PPM1F,RAC1,HIF1A,RCC2,RHOJ,SYNE2,JUP,ADIPOR2,EPHA2,MMRN2,MCC,MTOR,STK10,SEMA4A,FGF18,ABI3,ITGA3,PGF,DAPK2,EFNA1,VASH1,S100A14,MMP28,AMOT,CCL2,LGALS9,STK26,SEMA6B,ARHGAP4,NOTCH1,PLCG2,S1PR1,ENG,DAB2IP,CARMIL2,RIPOR1,CDH5,MIR23A,ADGRG1,RELN,FGF16,MGAT3,BST2,BMP2,CBLL1	turquoise	EC_M6
GO:0009607	5.13E-11	1	152	856	response to biotic stimulus	BP	4.84E-08	TNFRSF11A,IFNGR1,TNFAIP3,TRIM39,AKAP12,LYZ,TRDN,PTK2,C6orf106,DAPK1,CSF2RB,DDX58,SYK,IL6,REL,CXCL8,PELI1,RSAD2,ODC1,TLR4,C5,IL15,NOCT,PUM2,PIK3CG,NECTIN2,NOS3,UNC13D,DACH1,PPARG,TIMP4,GATA3,CXCR4,IRAK2,SEC14L1,EDNRB,IKBKB,NFKBIB,TMEM173,CD36,ARID5A,SCARB1,CHD7,IL1A,NR4A1,BPIFB3,EIF2AK3,ITCH,ERBIN,MEF2C,TLR2,GPM6A,CXCL10,SERINC5,OAS3,NLRC5,STAB1,IFIT2,IFIT1,EDN1,STAT1,CD58,SNCA,NFKB2,MX2,MX1,RNF19B,HDAC1,LCK,HYAL2,LYST,LAPTM5,NCF1,FABP4,PABPN1,ISG20,TIFA,CD86,FGR,FES,PRKD1,IL10RA,SRMS,PAK2,STYK1,MOV10,B2M,HMCN1,LTF,MASP1,RTP4,CYBB,IL1RAP,DDX3X,TMEM106A,PYGL,BIRC3,SELE,SELP,BAIAP2L1,WAS,HIF1A,RAB27A,ADIPOR2,EPHA2,BID,TLR6,TNK1,TNFRSF1B,ARG2,TBKBP1,ABI3,BATF,S100A14,SCNN1B,CYP1A1,TNIP1,CCL2,YES1,PDE4B,JAK1,LGALS9,NOS2,ELF4,IL7R,GIT1,LRG1,ISG15,NOTCH1,IRF8,PLCG2,STXBP1,LRSAM1,DAB2IP,ADGRL2,OAS1,VNN1,PMAIP1,TLR1,GBP5,RAB43,TRIM34,CD46,TRIM62,SERPINB9,BST2,VAMP3,BMP2,C15orf48,IFIT3,RNF125,BMP6	turquoise	EC_M6

GO:0006338	6.45E-10	1	45	175	chromatin remodeling	BP	4.89E-08	SHPRH,ANP32B,HDAC2,MCM2,NASP,RSF1,SUPT16H,CHD8,KAT6A,NAP1L1,ERCC6,ACTR6,SMARCA5,HELLS,MIS18A,TOP1,ARID2,INO80,OIP5,CHAF1B,SMARCAD1,NAP1L4,CHD1L,BPTF,ANP32E,HJURP,MCM3AP,HAT1,SMARCC1,BAZ1B,BAZ1A,SETD2,HP1BP3,SMARCE1,ASF1B,ATRX,MYSM1,CHAF1A,UBN1,CTCF,HIST1H1A,ITGB3BP,HIST1H3H,CENPW,RNF8	darkgrey	EC_M8
GO:0090307	7.56E-10	1	23	57	mitotic spindle assembly	BP	5.71E-08	KIFC1,CLASP2,CDC20,TPX2,LSM14A,KIF11,RACGAP1,ABRAXAS1,MYBL2,SMC3,NEK2,PRC1,TPR,KIF15,HNRNPU,SMC1A,KPNB1,KIF4A,AURKB,KIF23,PLK1,STAG2,DRG1	darkgrey	EC_M8
GO:0035556	7.56E-11	1	316	2063	intracellular signal transduction	BP	6.71E-08	TNFRSF11A,SEMA6A,PDE7B,TNFAIP3,TRIM39,PLEKHG1,AKAP12,FLT4,RIMS2,CDC42SE2,CELSR1,TPD52L1,ARHGAP18,SGSM3,PTK2,DENND3,DAPK1,ATF4,PDGFB,MAPK13,CDC42EP1,LRRC19,TEK,DDX58,PIP5K1B,FAM122A,TEC,SH3RF3,KIT,KDR,CD2AP,SYK,GADD45G,SEMA4D,RNF149,RAPGEF5,IL6,REL,CHN2,CXCL8,KBTBD2,PELI1,ARHGAP25,ELMO1,DOCK4,FIGNL1,CAV2,GRB10,TLR4,TRAF1,TEAD2,SDCBP,TRPM4,NRP1,PUM2,RASIP1,PIK3CG,ARHGAP42,ARHGAP21,PRKD2,RAB38,CAB39L,GPR4,SNRK,FGD5,NOS3,GAB2,RALB,ZFAND6,PPARG,GATA3,CXCR4,IRAK2,SEC14L1,EDNRB,PPP1CB,NET1,RALGAPA2,NDFIP2,CYTH1,IKBKB,P2RY6,PDE2A,SFRP1,HBEGF,PLCB4,RASGRP3,RPL26,SH3BP5,ARHGEF7,ARHGAP26,P2RX2,CD36,SIRT3,PRAG1,SDCBP2,PRR5L,MAP3K1,EPHB1,PLK2,RET,PDE4D,MERTK,TNFRSF19,IGF1,KIAA0355,EIF2AK3,ITCH,ERBIN,SH3RF1,PITPNM2,PREX2,SMAD1,DCLK2,MCTP1,ARHGEF3,ARHGEF28,MEF2C,TLR2,WWTR1,P2RX7,NDRG4,GUCY1A1,GUCY1B1,MADD,ADGRG3,OAS3,NLRC5,PPP1R16B,PLCB2,PLCG1,RAPGEF3,DGKZ,HIPK1,ZDHHC13,DNMBP,PIK3C2B,EDN1,STAT1,DYRK1A,DUSP8,NUAK2,NFKB2,PRDM15,ADCY2,SHANK2,PDE9A,SIK1,HDAC1,LCK,RASSF1,PPP3CA,HYAL2,MEF2A,SHOC2,MCTP2,HCRTR1,LAPTM5,NCF1,DYRK3,PABPN1,HCLS1,NFATC2,INPP5D,TIFA,CD86,ASB9,ARHGEF12,SH3BP4,BMX,FGR,CFLAR,MAP3K6,CASP10,INPP5F,ADCY1,KALRN,ADCY4,RPS6KA1,PROX1,PLEKHA1,PRKD1,F11R,ARHGDIB,MAP3K3,DOCK1,PAK2,CNKSR2,DUSP16,DLG1,ELL3,SIPA1,MAP3K11,USP28,ZW10,LTF,ARHGAP27,BCR,CCRL2,DDX3X,SOS2,RFXANK,IGFBP5,HOMER3,TBX1,MCU,TMEM106A,MECOM,ING4,WN	turquoise	EC_M6
GO:2001252	9.40E-10	1	26	72	positive regulation of chromosome organization	BP	7.06E-08	CCT2,MAP3K4,RAD21,POT1,TRIM28,HNRNPA2B1,CCT4,SETDB2,TNKS2,ATR,FAM208A,CCT7,NBN,CCT5,CCT6A,NEK2,TPR,XRCC5,DDX11,CCT3,AURKB,ATRX,CDT1,HNRNPD,NAF1,FEN1	darkgrey	EC_M8
GO:0044774	9.66E-10	1	26	74	mitotic DNA integrity checkpoint signaling	BP	7.22E-08	CDK2,MDC1,GTSE1,MSH2,CLSPN,CASP2,ORC1,RINT1,FANCD2,TOPBP1,PRKDC,NBN,ABRAXAS1,TAOK3,CHEK1,TICRR,RPA2,DTL,BLM,CDK1,BRCA1,CDC6,EME1,TIPIN,PLK1,TAOK1	darkgrey	EC_M8
GO:0065007	1.22E-10	1	1023	8375	biological regulation	BP	9.95E-08	PARD6G,ZNF516,TNFRSF11A,BAZ2A,TCF4,RDH16,TNFAIP8,DMXL1,SEMA6A,LVRN,ALDH8A1,PDE7B,IFNGR1,TNFAIP3,HECA,PHACTR2,PLAGL1,STX11,TBC1D30,TRIM39,PLEKHG1,AKAP12,LYZ,PTPRB,TSPAN8,LAPTM4B,CNOT6,FLT4,SNCAIP,MYCT1,FZD6,CTHRC1,RIMS2,OXR1,ZBED4,CDC42SE2,GRAMD4,CELSR1,QKI,NOTCH4,AFDN,PACSIN2,TCF20,TCF7,CDKL3,TRDN,TPD52L1,NCOA7,MTSS1,RNF146,ASAP1,ARHGAP18,L3MBTL3,KLHL3,SGSM3,PTK2,HSD17B3,DENND3,C6orf106,ANKS1A,DAPK1,ATF4,PDGFB,AGTPBP1,MAPK13,BRPF3,PODXL,CSNK1E,BAIAP2L2,PICK1,KDM4C,PIM1,TBC1D22B,CDC42EP1,MFNG,STRIP2,CSF2RB,NFYA,MDFI,OSTF1,LRRC19,TEK,LINGO2,IREB2,PRDM4,LIMCH1,RIC8B,DDX58,ATP8A1,NFX1,APBA1,DCAF12,TJP2,PIP5K1B,FAM122A,TEC,SH3RF3,GNG11,ADGRF5,KIT,ADGRF1,TNFRSF21,KDR,CD2AP,SRD5A3,NFIL3,SYK,GADD45G,SEMA4D,RNF149,EFHC1,RGP1,ICA1,SHB,TMOD1,TSPAN13,PPP6R1,RAPGEF5,IL6,IGF2BP3,NFE2L3,SNAP91,REL,PPT1,CREB5,PTBP3,CHN2,CXCL8,PPIE,BACH2,KBTBD2,VPS54,PELI1,UTP11,RGS3,ARHGAP25,ELMO1,RSAD2,DOCK4,ASAP2,FIGNL1,FOXP2,CAV2,KLF11,ODC1,GRB10,LANCL2,TSPAN12,NOBOX,TLR4,TCAF2,CPT1C,GRIK2,TRAF1,C5,DLGAP3,INPP4B,IL15,TBC1D9,FZD8,CCNY,CREM,TEAD2,SDCBP,MYCN,FAM49A,NRCAM,NOCT,TUT4,TRPM4,NRP1,KTI12,RAB3B,ZBTB24,PUM2,KIAA1147,RASIP1,BEND5,PIK3CG,ARHGAP42,SVIL,ITSN2,TAL1,LAMA4,PHF10,MTMR2,MGLL,EPC1,KDM4D,GATA2,DNMT3A,ARHGAP21,PRKD2,SAP130,KCNRG,CST7,NOVA2,CTSC,RAB38,CAB39L,GPR4,RCBTB2,LRCH1,SNRK,FGD5,PLXND1,NECTIN2,PRCP,BCAM,ITGA8,NOS3,GGA3,GAB2,LL	turquoise	EC_M6
GO:0001667	1.25E-10	1	82	360	ameboidal-type cell migration	BP	9.95E-08	SEMA6A,AKAP12,FLT4,PTK2,PDGFB,TEK,KIT,KDR,SEMA4D,NRP1,PIK3CG,GATA2,PRKD2,PLXND1,PRCP,NOS3,ITGB4,PPARG,GATA3,EDNRB,HBEGF,FERMT1,EFNB2,ARHGEF7,RIC8A,ADAMTS9,PRR5L,SCARB1,PLK2,RET,NR4A1,MEF2C,VEGFC,ANGPT2,PLCG1,STC1,DLL4,EDN1,ETS1,HYAL2,SEMA3F,PRKX,PROX1,PRKD1,LAMA5,ARHGDIB,MAP3K3,DOCK1,CLEC14A,SEMA6D,EPHB4,ITGA4,TBX1,PPM1F,RAC1,HIF1A,RCC2,RHOJ,JUP,ADIPOR2,EPHA2,MMRN2,MCC,MTOR,EFNB1,SEMA4A,FGF18,ITGA3,EFNA1,VASH1,AMOT,SEMA6B,ARHGAP4,PLEKHG5,NOTCH1,PLCG2,DAB2IP,CDH5,MIR23A,SOX17,FGF16,SOX18	turquoise	EC_M6

GO:0006333	1.44E-09	1	32	106	chromatin assembly or disassembly	BP	1.07E-07	SHPRH,ANP32B,MCM2,NASP,RSF1,SUPT16H,KAT6A,NAP1L1,RRP8,SMARCA5,HELLS,MIS18A,ARID2,OIP5,CHAF1B,NAP1L4,HJURP,HAT1,SMARCC1,BAZ1B,HP1BP3,SUV39H1,SMARCE1,ASF1B,ATRX,CHAF1A,UBN1,CTCF,HIST1H1A,ITGB3BP,HIST1H3H,CENPW	darkgrey	EC_M8
GO:1901362	1.49E-09	1	370	3012	organic cyclic compound biosynthetic process	BP	1.10E-07	ONECUT2,BCLAF1,PPIL4,MTHFD1L,NUP107,CCT2,DDX39B,ZNF608,SCAF8,MAP3K4,RAD21,DSCC1,HSF2,ATAD2,XRCC6,PHF5A,PSIP1,IFT74,POT1,PWP1,ZNF300,TOPORS,POLR3B,MTERF1,POLH,RFX3,CKS2,PPAT,PAICS,REV1,TRIM28,ZNF451,PHF3,TWISTNB,FOXJ3,ZNF189,SP4,CDCA7L,CTPS1,PHIP,HNRNPA2B1,DCK,ZMPSTE24,RLF,ZNF292,XPO1,CCT4,COMMD1,CASP8AP2,ZNF350,ITGB1BP1,THRAP3,TAF1B,EZH2,UFL1,CCNC,DDX1,NAA15,ELF2,AMD1,ATAD2B,DHFR,CDCA7,HDAC2,CCNA2,EXOSC9,MLLT10,DNAJC2,SETDB2,PCF11,NIFK,NUP85,CAD,ELF1,RSF1,CCNT2,PAXIP1,XRN2,NCAPG2,WDR43,TK1,AFMID,BIRC5,POLR3F,EPC2,SUPT16H,CHD8,KAT6A,RIF1,E2F7,ALYREF,CKAP2,RRM1,CEP290,NSD3,CEBPZ,SUPT20H,TAF7,POLE,NR2C1,RFC3,BRCA2,ELK3,RRP8,TCERG1,MAPK8,PRKDC,CBX5,ERCC6,ZNF214,GPBP1,SP1,TCEA1,ZNF664,ZC3H8,POLR1B,TNKS2,SGK3,BTAF1,ZNF143,PARP4,CENPJ,FER,TDG,NCK1,SBNO1,SMARCA5,RNF141,ZNF507,POLR1A,CHD1,ZFP91,TAF9,CGGBP1,XRN1,SMN2,BDP1,TFCP2,SAP30,ATR,TERF1,GCFC2,UTP15,HLTF,ZBTB10,CNOT1,GABPA,MCRS1,USP16,PPID,RBL1,GMPS,CCT7,CCNL1,SENP1,RIPK1,WDR75,ARID2,MYBL2,E2F8,TRIM33,INO80,ZBED6,CHUK,ZBTB11,ZNF248,MGA,MDM4,HIVEP1,RFC5,BRWD1,NOLC1,CCT5,BMPR1B,UBE3A,CCT6A,PRDM10,NUCKS1,DEK,GCN1,CUL3,GMNN,CHEK1,H2AFZ,RRP1B,NFKB1,KHDRBS1,TRIP13,TET2,TRIP11,HEATR1,ACSL5,NCL,TRAIP,PAPSS1,ZNF184,PRMT5,AJUBA,GTF2IRD2,ZBTB41,ARID4B,NOL11,GAR1,GTF2I,TARBP1,BPTF,DDX5,RFC2,ORC2,NEK2,EWSR1,TIAL1,DTL,HMBS,FLVCR1,BLM,UMPS,CENPF,OAT,EDRF1,PDE8A,ING5,BCLAF3,FOSL1,SMARCC1,B	darkgrey	EC_M8
GO:0042221	5.07E-11	1	506	2921	response to chemical	BP	1.15E-07	CYB5A,CD63,LRP1,AP3S1,CDO1,CANX,TAB2,IPCEF1,RRM2B,SLC12A2,PLXNB2,FBN2,ANGPT1,PPARA,TAF2,NUS1,HSPA4,CXCL14,FAM49B,PTP4A3,LY6E,PPARD,CTNNA1,UBQLN1,LGALS1,TXN2,SH3GL2,GNAQ,PLIN2,MTAP,LEP,CDKN2B,PLAA,RORB,KIAA1324L,SRF,VEGFA,DOCK8,VCP,RUNX2,RASL11B,UNC13B,COL1A2,NCK2,CLOCK,CREB3,ASNS,REST,ASPN,TGFBR1,ERP44,SOCS5,ERLEC1,TPBG,SHANK1,ADAMTS3,UBE2J1,MAP3K7,AKIRIN1,ACTR2,SLC31A1,KIDINS220,GLI3,ROCK2,INHBA,ZYX,RCN3,MYLK3,VPS35,MGARP,SLC7A11,ITGB1,PGRMC2,FKBP1B,PLXNA1,FGF2,SELENOW,KIF5B,SEC61A1,SESN3,PANX1,ATP7B,GAD2,ENTPD6,RB1,ESD,GLB1,EIF2B3,TMUB1,GLI2,MEGF8,NUB1,PAK1,ACTR3,TGFB1,SRP68,AXL,SCN5A,HTR5A,UBXN4,HNMT,CTNNB1,IL15RA,LBH,DGAT2,SERPINH1,AKT2,STT3B,PFKP,ZEB2,OSBPL8,RARB,ACTG1,NPLOC4,ACTN4,PPP1R12A,KAT2B,RNF121,LTBP1,TMTC4,CRIM1,STIM1,SPRED3,SPINT2,UFM1,LIG4,IRS2,PRNP,ATP2B1,LUM,DCN,SMAD9,ZNF703,SEMA3C,UBA5,DDRGK1,SMPD1,PPP2CB,ILK,LEPROTL1,HSPH1,ARL6IP5,DPYSL3,APLP1,RYK,CNOT7,RNF6,CXCL12,ANKRD1,HSP90B1,TMED2,IL20RB,PALLD,MRAS,MAP1LC3A,CD180,FLNB,OSBP,SULF1,DKK3,TRAM1,ROBO1,MAP1B,RYR3,SORBS1,SEMA4F,DOK1,P2RX4,HEY1,SELENOT,ATP2A2,USO1,MMP15,APP,GLRB,DHX36,RAPGEF2,RPN2,CX3CL1,SLC33A1,ABCC8,PPP2R2A,CACNB3,PTPN11,ADCY6,HOMER1,OPA1,ARSB,AMFR,GTF2H1,RAD54B,LOXL2,ZFYVE27,PTPN22,ACVR1,CRY2,VPS18,CHAC1,MMP2,GDF6,MTDH,ADA,BAP1,ATP2B4,SPP1,CPN1,PKD2,PLA2R1,HIF1AN,SEMA4G,TANK,TOLLIP,PSMD14,BRSK2,MARCH6,PSPH,NDN,RAB7B,CHRNA	darkgreen	EC_M4

GO:0045184	5.34E-11	1	271	1395	establishment of protein localization	BP	1.15E-07	CD63,LMAN1,LRP1,STX7,AP3S1,AGAP2,CANX,TBC1D9B,RAB21,RABL2A,SLC12A2,ANGPT1,TCP1,NUP50,HSPA4,SEC24A,PTP4A3,REEP2,PPARD,SIL1,GGA1,MYH9,LEP,PEX6,VCP,UNC13B,CLOCK,EXOC1,SRP72,REST,LMAN2L,RAB23,SNX13,P3H1,RIMS1,TMEM30A,ERLEC1,COX18,EMC10,UBE2J1,WHRN,MDFIC,GLI3,VPS41,RCN3,VPS35,MGARP,SLC7A11,LIN7B,FKBP1B,KIF5B,SEC61A1,NAPA,DNAJC1,RAB7A,COPG1,PDCD6IP,SNX17,PAK1,TGFB1,SRP68,RAB3GAP1,SEC13,TRAK1,AKT2,SEC23B,DZIP1,TBC1D16,RAB2B,NPLOC4,ACTN4,STAM2,COG6,UFM1,ATP2C1,IRS2,PRNP,HDAC3,RAB11FIP1,PCLO,ARL6IP5,WASHC2A,VPS37A,ARL1,TM9SF4,RAB2A,ANKRD1,HSP90B1,TMED2,COPB2,OSBP,VAMP8,TRAM1,EMC4,COPB1,SELENOT,USO1,SDAD1,CCT8,ABCC8,CACNB3,SSR3,PTPN11,EXOC6B,PFKM,ARHGAP1,ATG13,AP3B1,TSG101,DKC1,CRY2,VPS18,AKTIP,BAP1,CHP1,AP2A2,CBLB,BRSK2,VPS39,RAB7B,ARL3,DYNLL1,APBA2,PHLDB2,SRPRA,CTTN,DNM1L,SELENOS,KPNA6,SEC22B,CPT1A,TGFB2,LRP5,ZMAT3,GSK3B,HADH,CORO1C,DISP1,PITPNM1,SGSM1,HSPA8,KPNA1,IDH2,AP1B1,SEC22A,ADCY5,AP1S2,GOLT1B,AP2M1,COPA,GORASP2,SCFD1,KIF1A,SNX27,TOMM40L,TFRC,SIDT2,SRP54,LSG1,HSPB1,MAPT,SEC23A,AP1S1,SYVN1,SCAMP3,TANGO2,TFR2,C11orf65,PDCD10,COMP,CLTCL1,PRKCI,SMURF1,KTN1,EMC1,EXOC5,PTPRN,BLZF1,AP5M1,FMN2,ATAD1,STX18,KDELR2,ZDHHC4,PRAF2,B3GAT3,MYO5A,JAKMIP1,ERC1,HAP1,NLGN2,SNX8,LMAN2,MED1,SRCIN1,TSPAN17,STX1B,SNX11,SSR2,ARHGEF2,NGFR,LTBP2,COL1A1,TMED10,TGFB3,UBE2Q1,RAB11A,MYH10,SEL1L,TTC8,CALM1,SYTL4,TMED1,CLTC,VMP1,SCAMP5,ATG4A,	darkgreen	EC_M4
GO:2000278	1.70E-09	1	30	95	regulation of DNA biosynthetic process	BP	1.25E-07	CCT2,DDX39B,MAP3K4,DSCC1,POT1,HNRNPA2B1,CCT4,DNAJC2,RFC3,TNKS2,PARP4,XRN1,ATR,TERF1,CCT7,RFC5,CCT5,CCT6A,RFC2,NEK2,RFC4,XRCC5,HNRNPU,DCP2,EXOSC10,CCT3,AURKB,CHTF18,HNRNPD,NAF1	darkgrey	EC_M8
GO:0048024	1.71E-09	1	28	85	regulation of mRNA splicing, via spliceosome	BP	1.25E-07	SRPK1,SRSF3,SMU1,NCBP1,YTHDC1,TIA1,THRAP3,HNRNPL,RBM39,KHDRBS1,RBM5,NCL,FXR1,DDX5,TRA2B,CWC22,HNRNPU,RBM25,RBMX,NSRP1,RBFOX1,FMR1,METTL16,RNPS1,DAZAP1,PTBP1,RBM8A,SRSF7	darkgrey	EC_M8
GO:0007080	1.89E-09	1	19	43	mitotic metaphase plate congression	BP	1.38E-07	KIFC1,CDCA8,KIF2C,CCNB1,RRS1,KIF18A,CUL3,CENPE,KIF14,NUF2,CDCA5,HNRNPU,MAD1L1,KPNB1,KIF22,SPDL1,AURKB,NDC80,CDT1	darkgrey	EC_M8
GO:0051641	7.41E-11	1	412	2295	cellular localization	BP	1.40E-07	CD63,LMAN1,LRP1,STX7,AP3S1,AHI1,AGAP2,CANX,TBC1D9B,RAB21,RABL2A,ANGPT1,TCP1,NUP50,NUS1,HSPA4,SEC24A,PACSIN1,REEP2,PPARD,CTNNA1,SIL1,SUN2,GGA1,SH3GL2,MYH9,LEP,PLAA,PEX6,VCP,UNC13B,DYNC1I1,EXOC1,SRP72,LMAN2L,DST,RAB23,SNX13,NLRP5,RIMS1,TMEM30A,ERLEC1,FAM126A,CENPC,RTN4,TPBG,COX18,EMC10,UBE2J1,ACTR2,MDFIC,GLI3,ROCK2,VPS41,BVES,STARD3NL,VPS35,MGARP,SLC7A11,ITGB1,LIN7B,FKBP1B,FGF2,KIF5B,SEC61A1,NAPA,RAB7A,COPG1,ATP7B,RB1,IWS1,PICALM,MARK4,MAPRE3,PCDH17,SNX17,PAK1,ACTR3,TGFB1,SRP68,RAB3GAP1,SCN5A,SEC13,CTNNB1,ESYT2,TRAK1,AKT2,GPD1L,SEC23B,DZIP1,TBC1D16,OSBPL8,ACTG1,NPLOC4,ACTN4,PPP1R12A,FAM98A,UFM1,ATP2C1,PRNP,HDAC3,DCLK1,PCLO,DDRGK1,LEPROTL1,ARL6IP5,SNPH,WASHC2A,RIC3,VPS37A,ARL1,TMEM87B,TM9SF4,REEP5,SNTA1,HSP90B1,TMED2,TP53INP2,COPB2,OSBP,TMEM150A,VAMP8,SLMAP,ATP1B3,RIOK2,TRAM1,MAP1B,EMC4,RYR3,ERGIC3,PINX1,SORBS1,P2RX4,COPB1,SELENOT,ATP2A2,USO1,TCTN1,SDAD1,CACNA1D,APP,GLRB,DHX36,SLC4A5,CCT8,RAPGEF2,TM9SF3,CX3CL1,CACNB3,CPE,SSR3,PTPN11,EXOC6B,GNL3,OPA1,ARHGAP1,ATG13,AP3B1,ZFYVE27,TSG101,DKC1,CRY2,VPS18,PPP3CC,AKTIP,BAP1,CHP1,ATP2B4,PKD2,TWF1,AP2A2,CBLB,DNAH5,IFT57,TOLLIP,BRSK2,HPS6,VPS39,MAGEL2,RAB7B,ARL3,DYNLL1,SRPRA,CTTN,PKP2,DNM1L,SELENOS,KPNA6,SEC22B,CPT1A,FBLN5,ERGIC2,LRP5,FEZ1,HTR2B,WIPI1,GSK3B,MMP14,DAG1,CORO1C,ISCU,SHROOM2,SGSM1,GOLGB1,HSPA8,LARP7,ARV1,KPNA1,BMP7,AP1B1,SEC22A,ADCY5,VAPB,HYOU1,AP1S2,AP2	darkgreen	EC_M4
GO:0001944	1.99E-10	1	121	607	vasculature development	BP	1.50E-07	SEMA6A,TNFAIP3,PTPRB,FLT4,QKI,NOTCH4,PTK2,PDGFB,TEK,ADGRF5,KIT,KDR,SYK,THSD7A,SHB,IL6,CXCL8,TSPAN12,C5,GJA4,TEAD2,NRCAM,NRP1,RASIP1,PIK3CG,TAL1,EPC1,GATA2,PRKD2,GPR4,PLXND1,PRCP,NOS3,PPARG,ACVR2B,CXCR4,TIE1,JAG1,PDE2A,SFRP1,EFNB2,RIC8A,ADAMTS9,EPHB1,PLK2,CHD7,IL1A,NR4A1,EIF2AK3,HHEX,SMAD1,MEF2C,VEGFC,CXCL10,ANGPT2,TIPARP,THBS4,PPP1R16B,PLCG1,RAPGEF3,STAB1,HIPK1,DLL4,EDN1,STAT1,JAM3,ETS1,ROBO4,PRKX,SEC24B,NFATC2,CD34,PECAM1,MCAM,CFLAR,PROX1,PRKD1,MMP21,MAP3K3,APOLD1,APOD,CLEC14A,CYBB,EPHB4,TBX1,CALCRL,HSPG2,RAMP2,HIF1A,RHOJ,JUP,ADIPOR2,EPHA2,MMRN2,ALDH1A2,PSEN1,SEMA4A,FGF18,PGF,ARHGEF15,EFNA1,VASH1,NPR1,LYL1,AMOT,AP2B1,CCL2,JAK1,LRG1,NRARP,TMEM204,EGFL7,NOTCH1,S1PR1,ENG,DAB2IP,CDH5,MIR23A,ADGRG1,SOX17,SOX18	turquoise	EC_M6

GO:0034655	2.15E-09	1	66	335	nucleobase-containing compound catabolic process	BP	1.56E-07	PAN2,POP1,EIF3E,TNRC6B,TUT7,NCBP1,LRPPRC,PNPT1,SYNCRIP,PABPC4,THRAP3,EXOSC9,RNASEH2B,TRDMT1,CNOT10,UPF2,MBD4,DIS3,XRN2,METTL3,EXOSC8,PAN3,MTREX,TDG,XRN1,PDE12,ZCCHC8,TTC37,CNOT1,TENT2,NSUN2,FXR1,DDX5,FASTKD1,SSB,PDE8A,DHX9,HNRNPR,FASTKD2,DNA2,CNOT9,HNRNPU,MRPL44,DCP2,DCTPP1,EXOSC10,FUS,DIS3L,LARP1,TAF15,UPF3B,HNRNPM,SERBP1,AIFM1,GSPT1,HPRT1,DROSHA,FMR1,METTL16,RNPS1,EXOSC2,RBM8A,FASTKD5,HNRNPD,NAF1,FEN1	darkgrey	EC_M8
GO:0042273	2.75E-09	1	21	56	ribosomal large subunit biogenesis	BP	1.99E-07	MDN1,NIFK,DDX18,EBNA1BP2,GTPBP4,MAK16,RRS1,URB1,PAK1IP1,HEATR3,RRP15,WDR12,FTSJ3,FASTKD2,RPL10L,LAS1L,NLE1,BRIX1,RSL1D1,NOL9,RPF1	darkgrey	EC_M8
GO:0048731	1.30E-10	1	602	3556	system development	BP	2.14E-07	IL23A,ATP8B1,NAB2,LRP1,CDO1,AHI1,TAB2,RAB21,CDSN,RRM2B,SLC12A2,PLXNB2,FBN2,ANGPT1,FAM19A5,PPARA,HLA-DRA,COL11A2,NUS1,RSPO3,TRAPPC9,PACSIN1,ADGRB1,LY6E,PPARD,CTNNA1,TEAD3,SUN2,CBY1,MTCH1,LGALS1,MDGA1,SH3GL2,MYH9,LEP,CDKN2B,PLAA,LARGE1,RORB,KIAA1324L,SRF,VEGFA,RUNX2,COL1A2,C2orf40,NCK2,CLOCK,ASNS,PDCL3,REST,ASPN,CNNM4,RAB23,COL15A1,P3H1,TGFBR1,HIVEP3,SOCS5,RIMS1,TMEM30A,FAM126A,UBA6,RTN4,TPBG,SHANK1,LRRC4B,EMC10,MFSD2A,MYH14,CALD1,AKIRIN1,ACTR2,KIDINS220,WHRN,GLI3,NCDN,ROCK2,INHBA,BVES,RRAS,RCN3,MYLK3,VPS35,SLC7A11,OSTM1,PCDH18,ITGB1,PGRMC2,PLEKHA4,DBP,FKBP1B,PLXNA1,FGF2,SRPK2,KIF5B,SEC61A1,NAPA,RB1,ITM2B,EED,PICALM,MARK4,EIF2B3,PTCH2,TENM4,GLI2,MEGF8,SMARCD3,PCDH17,RHEB,SNX17,PAK1,TGFB1,AXL,RAB3GAP1,SCN5A,WDR48,HTR5A,HNMT,ACER3,CTNNB1,IL15RA,TRAK1,LBH,DGAT2,SERPINH1,AKT2,ZEB2,FARP1,RELT,SLC4A7,SLC38A10,RARB,ACTG1,NAV3,KAT2B,TACC1,CRIM1,STIM1,SPRED3,SPINT2,UFM1,LIG4,IRS2,ATP2B1,LUM,DCN,ZNF703,DCLK1,PCLO,STARD13,SEMA3C,CCKBR,UBA5,DDRGK1,SMPD1,ILK,DPYSL3,SPINK5,APLP1,RYK,PRICKLE2,SNPH,RNF6,CXCL12,IMPAD1,LGI4,ZSWIM6,KIF2A,SLC20A1,TMEM41B,ANKRD1,FAT1,ADAMTS6,SLC25A46,TMED2,PALLD,EIF2S2,MRAS,ZNF536,FLNB,ST8SIA4,EIF4G2,SULF1,CTNND1,DKK3,ROBO1,TRPC4AP,MAP1B,TEAD1,RDH10,DIP2B,SEMA4F,LOXL3,P2RX4,FAIM2,HEY1,RASA1,SELENOT,TLX2,CELF1,TCTN1,APP,GLRB,DHX36,SLC4A5,RAPGEF2,ACTG2,CX3CL1,ABCC8,CPE,PTPN11,P	darkgreen	EC_M4
GO:0007275	1.55E-10	1	639	3815	multicellular organism development	BP	2.14E-07	IL23A,ATP8B1,NAB2,LRP1,CDO1,AHI1,TAB2,RAB21,CDSN,RRM2B,SLC12A2,PLXNB2,FBN2,ANGPT1,FAM19A5,PPARA,HLA-DRA,COL11A2,ROS1,NUS1,RSPO3,TRAPPC9,PACSIN1,ADGRB1,LY6E,PPARD,CTNNA1,TEAD3,SUN2,CBY1,MTCH1,LGALS1,MDGA1,SH3GL2,MYH9,LEP,CDKN2B,PLAA,LARGE1,RORB,KIAA1324L,SRF,VEGFA,RUNX2,COL1A2,C2orf40,NCK2,CLOCK,ASNS,PDCL3,REST,ASPN,CNNM4,RAB23,NLRP5,COL15A1,P3H1,TGFBR1,HIVEP3,SOCS5,RIMS1,TMEM30A,FAM126A,UBA6,RTN4,TPBG,SHANK1,ADAMTS3,LRRC4B,EMC10,MFSD2A,MYH14,CALD1,AKIRIN1,ACTR2,KIDINS220,WHRN,GLI3,NCDN,ROCK2,INHBA,GREB1,BVES,RRAS,RCN3,MYLK3,VPS35,SLC7A11,OSTM1,PCDH18,ITGB1,PGRMC2,PLEKHA4,DBP,FKBP1B,PLXNA1,FGF2,SRPK2,KIF5B,SEC61A1,NAPA,RB1,ITM2B,EED,PICALM,MARK4,EIF2B3,PTCH2,TENM4,GLI2,MEGF8,SMARCD3,PCDH17,RHEB,SNX17,PAK1,TGFB1,AXL,RAB3GAP1,SCN5A,WDR48,HTR5A,HNMT,ACER3,CTNNB1,IL15RA,TRAK1,LBH,DGAT2,SERPINH1,AKT2,ZEB2,FARP1,RELT,SLC4A7,SLC38A10,RARB,ACTG1,NAV3,KAT2B,TACC1,CRIM1,MAFG,STIM1,SPRED3,SPINT2,UFM1,LIG4,IRS2,ATP2B1,LUM,DCN,ZNF703,DCLK1,PCLO,STARD13,SEMA3C,CCKBR,UBA5,DDRGK1,SMPD1,ILK,DPYSL3,SPINK5,APLP1,RYK,PRICKLE2,SNPH,RNF6,CXCL12,IMPAD1,LGI4,ZSWIM6,KIF2A,SLC20A1,TMEM41B,ANKRD1,FAT1,ADAMTS6,SLC25A46,TMED2,PALLD,EIF2S2,MRAS,ZNF536,FLNB,ST8SIA4,EIF4G2,SULF1,CTNND1,DKK3,ROBO1,TRPC4AP,MAP1B,TEAD1,CHST2,RDH10,DIP2B,SEMA4F,LOXL3,P2RX4,FAIM2,HEY1,RASA1,SELENOT,TLX2,CELF1,TCTN1,MMP15,APP,GLRB,DHX	darkgreen	EC_M4

GO:0033036	1.56E-10	1	419	2356	macromolecule localization	BP	2.14E-07	CD63,LMAN1,ATP8B1,LRP1,STX7,AP3S1,AHI1,B4GALNT1,AGAP2,CANX,TBC1D9B,RAB21,RABL2A,SLC12A2,FBN2,ANGPT1,TCP1,PPARA,NUP50,NUS1,HSPA4,SEC24A,PACSIN1,PTP4A3,REEP2,PPARD,CTNNA1,SIL1,SUN2,CBY1,GGA1,PLIN2,MYH9,LEP,PEX6,VCP,UNC13B,CLOCK,EXOC1,SRP72,REST,LMAN2L,RAB23,SNX13,P3H1,RIMS1,TMEM30A,ERLEC1,FAM126A,RTN4,TPBG,SHANK1,COX18,EMC10,MFSD2A,UBE2J1,WHRN,MDFIC,GLI3,ROCK2,INHBA,VPS41,RCN3,VPS35,MGARP,SLC7A11,ITGB1,LIN7B,LDAH,FKBP1B,KIF5B,SEC61A1,NAPA,DNAJC1,RAB7A,COPG1,RB1,ABHD5,IWS1,PICALM,MARK4,MAPRE3,PDCD6IP,SNX17,PAK1,TGFB1,SRP68,RAB3GAP1,SEC13,CTNNB1,ESYT2,TRAK1,DGAT2,AKT2,GPD1L,SEC23B,OSBPL10,DZIP1,C1QTNF1,TBC1D16,RAB2B,OSBPL8,ACTG1,NPLOC4,ACTN4,STAM2,LTBP1,COG6,UFM1,ATP2C1,IRS2,PRNP,HDAC3,RAB11FIP1,DCLK1,PCLO,DDRGK1,ARL6IP5,WASHC2A,RIC3,VPS37A,ARL1,TM9SF4,RAB2A,ANKRD1,HSP90B1,TMED2,TP53INP2,COPB2,OSBP,TMEM150A,VAMP8,SLMAP,ATP1B3,TRAM1,EMC4,FAM160A1,PINX1,SORBS1,P2RX4,COPB1,SELENOT,USO1,TCTN1,SDAD1,ATXN2,GLRB,DHX36,CCT8,RAPGEF2,TM9SF3,ABCC8,CACNB3,CPE,SSR3,PTPN11,EXOC6B,GNL3,PFKM,ARHGAP1,ATG13,AP3B1,ZFYVE27,TSG101,DKC1,CRY2,VPS18,AKTIP,BAP1,CHP1,ATP2B4,SPP1,PKD2,PLA2R1,TWF1,AP2A2,CBLB,TOLLIP,BRSK2,HPS6,VPS39,RAB7B,ARL3,DYNLL1,APBA2,PHLDB2,TJP1,KDM5B,SRPRA,CTTN,PKP2,DNM1L,SELENOS,KPNA6,SEC22B,CPT1A,TGFB2,FBLN5,LRP5,EPRS,WIPI1,ZMAT3,GSK3B,MMP14,DAG1,HADH,CORO1C,DISP1,SHROOM2,PITPNM1,SGSM1,GOLGB1,HSPA8,LARP7,PITPNB,AR	darkgreen	EC_M4
GO:0031497	3.36E-09	0.999999999	27	84	chromatin assembly	BP	2.42E-07	SHPRH,ANP32B,MCM2,NASP,RSF1,KAT6A,NAP1L1,RRP8,SMARCA5,HELLS,MIS18A,OIP5,CHAF1B,NAP1L4,HJURP,HAT1,HP1BP3,SUV39H1,ASF1B,ATRX,CHAF1A,UBN1,CTCF,HIST1H1A,ITGB3BP,HIST1H3H,CENPW	darkgrey	EC_M8
GO:0015031	2.19E-10	1	253	1301	protein transport	BP	2.60E-07	CD63,LMAN1,LRP1,STX7,AP3S1,AGAP2,CANX,TBC1D9B,RAB21,RABL2A,SLC12A2,ANGPT1,TCP1,NUP50,HSPA4,SEC24A,REEP2,PPARD,SIL1,GGA1,MYH9,LEP,PEX6,VCP,UNC13B,CLOCK,EXOC1,SRP72,REST,LMAN2L,RAB23,SNX13,P3H1,RIMS1,TMEM30A,ERLEC1,COX18,UBE2J1,MDFIC,GLI3,VPS41,RCN3,VPS35,MGARP,SLC7A11,LIN7B,FKBP1B,KIF5B,SEC61A1,NAPA,DNAJC1,RAB7A,COPG1,PDCD6IP,SNX17,PAK1,TGFB1,SRP68,SEC13,TRAK1,AKT2,SEC23B,TBC1D16,RAB2B,NPLOC4,ACTN4,STAM2,COG6,UFM1,ATP2C1,IRS2,PRNP,HDAC3,RAB11FIP1,PCLO,ARL6IP5,WASHC2A,VPS37A,ARL1,TM9SF4,RAB2A,ANKRD1,HSP90B1,TMED2,COPB2,OSBP,VAMP8,TRAM1,COPB1,SELENOT,USO1,SDAD1,ABCC8,CACNB3,SSR3,PTPN11,EXOC6B,PFKM,ARHGAP1,ATG13,AP3B1,TSG101,CRY2,VPS18,AKTIP,BAP1,CHP1,AP2A2,CBLB,BRSK2,VPS39,RAB7B,ARL3,DYNLL1,APBA2,SRPRA,CTTN,DNM1L,SELENOS,KPNA6,SEC22B,CPT1A,TGFB2,LRP5,ZMAT3,GSK3B,HADH,DISP1,PITPNM1,SGSM1,HSPA8,KPNA1,IDH2,AP1B1,SEC22A,ADCY5,AP1S2,GOLT1B,AP2M1,COPA,SCFD1,KIF1A,SNX27,TOMM40L,TFRC,SIDT2,SRP54,LSG1,HSPB1,SEC23A,AP1S1,SYVN1,SCAMP3,TANGO2,TFR2,C11orf65,PDCD10,COMP,CLTCL1,PRKCI,SMURF1,KTN1,EXOC5,PTPRN,BLZF1,AP5M1,FMN2,STX18,KDELR2,ZDHHC4,PRAF2,B3GAT3,MYO5A,JAKMIP1,ERC1,HAP1,NLGN2,SNX8,LMAN2,MED1,SRCIN1,STX1B,SNX11,SSR2,ARHGEF2,NGFR,LTBP2,COL1A1,TMED10,TGFB3,UBE2Q1,RAB11A,MYH10,SEL1L,TTC8,SYTL4,TMED1,CLTC,VMP1,SCAMP5,ATG4A,ACSL4,CD74,MIEF2,EVI5L,AKT1,MYO1D,SNX18,MYOM1,PRKAA1,ZDHHC9,RAB31,NAPG,RAB34,MYO5B,MYO1C,BCAP31,PAF	darkgreen	EC_M4

GO:0048856	2.34E-10	1	692	4193	anatomical structure development	BP	2.60E-07	IL23A,ATP8B1,NAB2,LRP1,CDO1,AHI1,TAB2,IER3,RAB21,CDSN,RRM2B,SLC12A2,PLXNB2,FBN2,ANGPT1,FAM19A5,PPARA,HLA-DRA,COL11A2,ROS1,AFF4,NUS1,RSPO3,FAM49B,MRTFA,TRAPPC9,PACSIN1,ADGRB1,LY6E,PPARD,CTNNA1,TEAD3,SUN2,CBY1,MKLN1,MTCH1,LGALS1,MDGA1,SH3GL2,FLNC,MYH9,LEP,CDKN2B,PLAA,TBCC,LARGE1,RORB,KIAA1324L,SRF,VEGFA,RUNX2,COL1A2,C2orf40,NCK2,CLOCK,ASNS,PDCL3,REST,ASPN,CNNM4,RAB23,NLRP5,COL15A1,P3H1,TGFBR1,HIVEP3,SOCS5,RIMS1,TMEM30A,PSME4,FAM126A,UBA6,RTN4,TPBG,SHANK1,ADAMTS3,LRRC4B,EMC10,MFSD2A,UBE2J1,MYH14,CALD1,AKIRIN1,ACTR2,GMCL1,IFRD1,KIDINS220,WHRN,GLI3,NCDN,ROCK2,INHBA,GREB1,BVES,RRAS,RCN3,MYLK3,DKKL1,VPS35,SLC7A11,OSTM1,PCDH18,ITGB1,PGRMC2,PLEKHA4,DBP,FKBP1B,PLXNA1,FGF2,SRPK2,KIF5B,SEC61A1,NAPA,PANX1,RB1,ITM2B,EED,PICALM,MARK4,TPT1,EIF2B3,PTCH2,TENM4,GLI2,MEGF8,SMARCD3,PCDH17,RHEB,SNX17,PAK1,TGFB1,AXL,RAB3GAP1,SCN5A,WDR48,HTR5A,HNMT,ACER3,CTNNB1,IL15RA,TRAK1,LBH,DGAT2,SERPINH1,AKT2,DZIP1,ZEB2,FARP1,RELT,SLC4A7,SLC38A10,RARB,ACTG1,NAV3,ACTN4,KAT2B,TACC1,CRIM1,MAFG,STIM1,SPRED3,SPINT2,UFM1,LIG4,ATP2C1,IRS2,ATP2B1,LUM,DCN,CDC42EP3,SMAD9,ZNF703,DCLK1,PCLO,STARD13,SEMA3C,CCKBR,UBA5,DDRGK1,SMPD1,ILK,DPYSL3,SPINK5,APLP1,RYK,PRICKLE2,SNPH,RNF6,AKIP1,CXCL12,IMPAD1,LGI4,ZSWIM6,KIF2A,SLC20A1,TMEM41B,ANKRD1,FAT1,ADAMTS6,SLC25A46,TMED2,PALLD,EIF2S2,MRAS,ZNF536,FLNB,ST8SIA4,EIF4G2,SULF1,CTNND1,DKK3,ROBO1,TRPC4	darkgreen	EC_M4
GO:0032501	2.41E-10	1	806	5003	multicellular organismal process	BP	2.60E-07	SERPINB2,LMAN1,IL23A,ATP8B1,NAB2,LRP1,CDO1,AHI1,B4GALNT1,TAB2,RAB21,CDSN,RRM2B,SLC12A2,PLXNB2,FBN2,ANGPT1,FAM19A5,PPARA,HLA-DRA,COL11A2,ROS1,AFF4,NUS1,RSPO3,FAM49B,TRAPPC9,PACSIN1,PTP4A3,REEP2,ADGRB1,LY6E,PPARD,CTNNA1,TEAD3,SUN2,CBY1,MTCH1,LGALS1,MDGA1,SH3GL2,GNAQ,MYH9,LEP,CDKN2B,PLAA,LARGE1,RORB,KIAA1324L,SRF,VEGFA,RUNX2,UNC13B,COL1A2,TPM2,C2orf40,NCK2,CLOCK,ASNS,PDCL3,REST,ASPN,CNNM4,RAB23,NLRP5,COL15A1,P3H1,TGFBR1,HIVEP3,SOCS5,RIMS1,TMEM30A,PSME4,FAM126A,UBA6,RTN4,TPBG,SHANK1,ADAMTS3,LRRC4B,EMC10,MFSD2A,UBE2J1,MAP3K7,MYH14,CALD1,AKIRIN1,ACTR2,GFPT1,GMCL1,KIDINS220,WHRN,GLI3,NCDN,ROCK2,INHBA,GREB1,BVES,RRAS,RCN3,MYLK3,DKKL1,VPS35,SLC7A11,OSTM1,PCDH18,ITGB1,PGRMC2,PLEKHA4,DBP,FKBP1B,PLXNA1,FGF2,SRPK2,KIF5B,SEC61A1,NAPA,PANX1,PSMC2,ABHD12,EML2,RB1,ITM2B,EED,PICALM,MARK4,TPT1,ZNF296,CRTAP,EIF2B3,PTCH2,TENM4,DGKH,GLI2,MEGF8,SMARCD3,PCDH17,RHEB,SNX17,PAK1,TGFB1,AXL,RAB3GAP1,SCN5A,WDR48,HTR5A,HNMT,ACER3,CTNNB1,IL15RA,TRAK1,LBH,DGAT2,SERPINH1,AKT2,GPD1L,DZIP1,ZEB2,C1QTNF1,FARP1,RELT,SLC4A7,SLC38A10,RARB,ACTG1,NAV3,NPLOC4,KAT2B,LTBP1,TMTC4,TACC1,CRIM1,MAFG,STIM1,SPRED3,SPINT2,UFM1,LIG4,IRS2,PRNP,ATP2B1,LUM,DCN,HDAC3,RAB11FIP1,ZNF703,DCLK1,ACPP,PCLO,STARD13,SEMA3C,CCKBR,CAVIN3,UBA5,DDRGK1,SMPD1,ILK,ARL6IP5,DPYSL3,KATNAL1,SPINK5,APLP1,RYK,PRICKLE2,TUSC3,SNPH,RNF6,CXCL12,IMPAD1,FXYD1,LGI4,ZSWIM6,KIF2A,SLC20A1,TME	darkgreen	EC_M4
GO:0034248	4.43E-09	1	73	395	regulation of cellular amide metabolic process	BP	3.17E-07	PAN2,DDX39B,EIF3E,MRPL13,TNRC6B,TUT7,PUM3,NCBP1,LRPPRC,MTIF2,PNPT1,SYNCRIP,TIA1,ASCC3,DDX1,DHFR,PUS7,EXOSC9,CNOT10,DIS3,METTL3,EXOSC8,PRKDC,DHX29,PAN3,SP1,CAPRIN1,LSM14A,CDK5RAP1,NCK1,PTCD3,ABCE1,XRN1,PDE12,LARP4,CNOT1,LTN1,USP16,NOLC1,GCN1,METAP1,KHDRBS1,NCL,FXR1,NGDN,EIF2B5,DDX6,DHX9,HNRNPR,FASTKD2,TPR,CNOT9,EIF2AK1,DCP2,EIF3B,SEPSECS,GEMIN5,LARP1,ILF3,EIF5,TYMS,UPF3B,GSPT1,FMR1,METTL16,EXOSC2,RBM8A,METTL18,SARNP,HNRNPD,TIGAR,RNF139,PAIP2	darkgrey	EC_M8
GO:0001568	4.87E-10	1	115	576	blood vessel development	BP	3.50E-07	SEMA6A,TNFAIP3,PTPRB,FLT4,QKI,NOTCH4,PTK2,PDGFB,TEK,ADGRF5,KDR,SYK,THSD7A,SHB,IL6,CXCL8,TSPAN12,C5,GJA4,TEAD2,NRCAM,NRP1,RASIP1,PIK3CG,TAL1,GATA2,PRKD2,GPR4,PLXND1,PRCP,NOS3,PPARG,ACVR2B,CXCR4,TIE1,JAG1,PDE2A,SFRP1,EFNB2,ADAMTS9,EPHB1,PLK2,CHD7,IL1A,NR4A1,EIF2AK3,HHEX,SMAD1,MEF2C,VEGFC,CXCL10,ANGPT2,TIPARP,THBS4,PPP1R16B,PLCG1,RAPGEF3,STAB1,HIPK1,DLL4,EDN1,STAT1,JAM3,ETS1,ROBO4,PRKX,SEC24B,CD34,PECAM1,MCAM,CFLAR,PROX1,PRKD1,MMP21,MAP3K3,APOLD1,APOD,CLEC14A,CYBB,EPHB4,TBX1,CALCRL,HSPG2,RAMP2,HIF1A,RHOJ,JUP,ADIPOR2,EPHA2,MMRN2,ALDH1A2,PSEN1,SEMA4A,FGF18,PGF,EFNA1,VASH1,NPR1,LYL1,AMOT,AP2B1,CCL2,JAK1,LRG1,NRARP,EGFL7,NOTCH1,S1PR1,ENG,DAB2IP,CDH5,MIR23A,ADGRG1,SOX17,SOX18	turquoise	EC_M6

GO:0046700	4.93E-09	1	68	357	heterocycle catabolic process	BP	3.51E-07	PAN2,POP1,EIF3E,TNRC6B,TUT7,NCBP1,LRPPRC,PNPT1,SYNCRIP,PABPC4,THRAP3,EXOSC9,RNASEH2B,TRDMT1,CNOT10,UPF2,MBD4,DIS3,XRN2,AFMID,METTL3,EXOSC8,PAN3,MTREX,TDG,XRN1,PDE12,ZCCHC8,TTC37,CNOT1,TENT2,NSUN2,TET2,FXR1,DDX5,FASTKD1,SSB,PDE8A,DHX9,HNRNPR,FASTKD2,DNA2,CNOT9,HNRNPU,MRPL44,DCP2,DCTPP1,EXOSC10,FUS,DIS3L,LARP1,TAF15,UPF3B,HNRNPM,SERBP1,AIFM1,GSPT1,HPRT1,DROSHA,FMR1,METTL16,RNPS1,EXOSC2,RBM8A,FASTKD5,HNRNPD,NAF1,FEN1	darkgrey	EC_M8
GO:0006402	5.43E-09	1	47	201	mRNA catabolic process	BP	3.85E-07	PAN2,EIF3E,TNRC6B,TUT7,NCBP1,PNPT1,SYNCRIP,PABPC4,THRAP3,EXOSC9,CNOT10,UPF2,DIS3,XRN2,METTL3,EXOSC8,PAN3,XRN1,PDE12,TTC37,CNOT1,TENT2,FXR1,DDX5,FASTKD1,SSB,DHX9,HNRNPR,FASTKD2,CNOT9,HNRNPU,DCP2,EXOSC10,FUS,LARP1,TAF15,UPF3B,HNRNPM,SERBP1,GSPT1,FMR1,METTL16,RNPS1,EXOSC2,RBM8A,FASTKD5,HNRNPD	darkgrey	EC_M8
GO:0048870	6.13E-10	1	206	1234	cell motility	BP	4.03E-07	TNFRSF11A,SEMA6A,TNFAIP3,AKAP12,FLT4,CTHRC1,CELSR1,AFDN,ARHGAP18,PTK2,ANKS1A,PDGFB,PODXL,STRIP2,TEK,LIMCH1,DDX58,ATP8A1,KIT,KDR,CD2AP,SYK,SEMA4D,EFHC1,IL6,CXCL8,AVL9,ELMO1,DOCK4,GRB10,TLR4,TCAF2,C5,SDCBP,NRCAM,TRPM4,NRP1,PIK3CG,LAMA4,GATA2,PRKD2,LRCH1,PLXND1,PRCP,NOS3,ITGB4,DACH1,PPARG,GATA3,CXCR4,EDNRB,NET1,TIE1,JAG1,P2RY6,SFRP1,HBEGF,FERMT1,EFNB2,ARHGEF7,SPEF1,NTN4,RIC8A,PRAG1,ADAMTS9,PRR5L,SCARB1,EPHB1,PLK2,RET,MERTK,IGF1,NR4A1,ABHD6,SH3RF1,MCTP1,HEXB,MEF2C,VEGFC,NDRG4,GPM6A,CXCL10,ANGPT2,ADGRG3,THBS4,PLCG1,STC1,NR4A2,DGKZ,ANO6,DLL4,PIK3C2B,NEDD9,EDN1,JAM3,SRGAP2C,ETS1,CD200,LCK,PPP3CA,HYAL2,SEMA3F,RIN3,ROBO4,PRKX,LYST,NFATC2,AP1AR,CD34,PECAM1,PLEKHO1,PLXNA2,FGR,MCAM,INPP5F,IGFBP3,FES,PROX1,PRKD1,VANGL2,LAMA5,F11R,ARHGDIB,MAP3K3,ACE,ITGA6,DOCK1,LAMC2,APOD,CLEC14A,CCRL2,SEMA6D,EPHB4,ITGA4,IGFBP5,TBX1,MCU,LDB2,SELE,SELP,PLVAP,FGF23,PPM1F,WAS,RAC1,HIF1A,RCC2,RHOJ,SYNE2,PLD2,JUP,ADIPOR2,FSCN1,EPHA2,MMRN2,MCC,MTOR,OPHN1,EFNB1,PSEN1,STK10,SEMA4A,HOXB9,FGF18,ABI3,ITGA3,PGF,DAPK2,TAOK2,EFNA1,VASH1,S100A14,CDC42BPB,MMP28,AMOT,CCL2,YES1,PDE4B,LGALS9,SPEF2,STK26,MYO18A,SEMA6B,ARHGAP4,PLEKHG5,NOTCH1,PLCG2,S1PR1,ENG,LHX6,DAB2IP,CARMIL2,RIPOR1,CDH5,MIR23A,ADGRG1,SOX17,RELN,FGF16,SOX18,MGAT3,MSMP,BST2,BMP2,CBLL1,ARPC5L	turquoise	EC_M6
GO:0051674	6.13E-10	1	206	1234	localization of cell	BP	4.03E-07	TNFRSF11A,SEMA6A,TNFAIP3,AKAP12,FLT4,CTHRC1,CELSR1,AFDN,ARHGAP18,PTK2,ANKS1A,PDGFB,PODXL,STRIP2,TEK,LIMCH1,DDX58,ATP8A1,KIT,KDR,CD2AP,SYK,SEMA4D,EFHC1,IL6,CXCL8,AVL9,ELMO1,DOCK4,GRB10,TLR4,TCAF2,C5,SDCBP,NRCAM,TRPM4,NRP1,PIK3CG,LAMA4,GATA2,PRKD2,LRCH1,PLXND1,PRCP,NOS3,ITGB4,DACH1,PPARG,GATA3,CXCR4,EDNRB,NET1,TIE1,JAG1,P2RY6,SFRP1,HBEGF,FERMT1,EFNB2,ARHGEF7,SPEF1,NTN4,RIC8A,PRAG1,ADAMTS9,PRR5L,SCARB1,EPHB1,PLK2,RET,MERTK,IGF1,NR4A1,ABHD6,SH3RF1,MCTP1,HEXB,MEF2C,VEGFC,NDRG4,GPM6A,CXCL10,ANGPT2,ADGRG3,THBS4,PLCG1,STC1,NR4A2,DGKZ,ANO6,DLL4,PIK3C2B,NEDD9,EDN1,JAM3,SRGAP2C,ETS1,CD200,LCK,PPP3CA,HYAL2,SEMA3F,RIN3,ROBO4,PRKX,LYST,NFATC2,AP1AR,CD34,PECAM1,PLEKHO1,PLXNA2,FGR,MCAM,INPP5F,IGFBP3,FES,PROX1,PRKD1,VANGL2,LAMA5,F11R,ARHGDIB,MAP3K3,ACE,ITGA6,DOCK1,LAMC2,APOD,CLEC14A,CCRL2,SEMA6D,EPHB4,ITGA4,IGFBP5,TBX1,MCU,LDB2,SELE,SELP,PLVAP,FGF23,PPM1F,WAS,RAC1,HIF1A,RCC2,RHOJ,SYNE2,PLD2,JUP,ADIPOR2,FSCN1,EPHA2,MMRN2,MCC,MTOR,OPHN1,EFNB1,PSEN1,STK10,SEMA4A,HOXB9,FGF18,ABI3,ITGA3,PGF,DAPK2,TAOK2,EFNA1,VASH1,S100A14,CDC42BPB,MMP28,AMOT,CCL2,YES1,PDE4B,LGALS9,SPEF2,STK26,MYO18A,SEMA6B,ARHGAP4,PLEKHG5,NOTCH1,PLCG2,S1PR1,ENG,LHX6,DAB2IP,CARMIL2,RIPOR1,CDH5,MIR23A,ADGRG1,SOX17,RELN,FGF16,SOX18,MGAT3,MSMP,BST2,BMP2,CBLL1,ARPC5L	turquoise	EC_M6

GO:0016192	4.41E-10	1	236	1192	vesicle-mediated transport	BP	4.44E-07	CD63,LMAN1,LRP1,STX7,AP3S1,AHI1,AGAP2,CANX,RAB21,ANGPT1,SEC24A,PACSIN1,ADGRB1,MKLN1,GGA1,SH3GL2,MYH9,LEP,PLAA,VEGFA,VCP,UNC13B,EXOC1,REST,LMAN2L,NLRP5,RIMS1,MFSD2A,VPS41,BVES,STARD3NL,VPS35,ITGB1,LIN7B,KIF5B,NAPA,RAB7A,COPG1,PICALM,PDCD6IP,PCDH17,SNX17,PAK1,AXL,RAB3GAP1,SEC13,CTNNB1,IL15RA,ESYT2,TRAK1,AKT2,SEC23B,LGALS3BP,RAB2B,ACTG1,COG6,ATP2C1,RAB11FIP1,SIGLEC1,DCLK1,PCLO,SMPD1,LEPROTL1,APLP1,SNPH,WASHC2A,ARL1,MTMR6,TMEM87B,TM9SF4,TMED2,COPB2,VAMP8,ERGIC3,LOXL3,COPB1,ATP2A2,USO1,APP,ATXN2,PTX3,EXOC6B,PIP4P2,ARHGAP1,LOXL2,AP3B1,ZFYVE27,TSG101,VPS18,PPP3CC,AKTIP,CHP1,TXNDC5,PLA2R1,LGI3,AP2A2,BRSK2,VPS39,MAGEL2,RAB7B,ARL3,ELMO2,CTTN,DNM1L,DNER,SEC22B,ERGIC2,LRP5,HTR2B,GOLGA5,WIPI1,GSK3B,PIK3CA,CORO1C,CFI,GRK3,HSPA8,PITPNB,AP1B1,SEC22A,VAPB,HYOU1,AP1S2,GOLT1B,AP2M1,COPA,SCFD1,KIF1A,SNX27,TMEM50A,TFRC,BACE1,MRC2,OLR1,SCYL1,SEC23A,AP1S1,SERPINE1,CD163,SCAMP3,TFR2,ITGAV,LRPAP1,HTT,KXD1,CLTCL1,PRKCI,RTN3,EXOC5,PLD1,BLZF1,AP5M1,CCZ1B,FMN2,ATAD1,STX18,KDELR2,MYO5A,ERC1,HAP1,SLC2A4,NLGN2,PDLIM7,STARD3,SNX8,LMAN2,MSN,SRCIN1,MYO1E,STX1B,SYT11,DOCK2,TMED10,EPN3,CACNA1G,RAB11A,MAP2K1,CLINT1,SOX30,CALM1,SYTL4,YIPF2,TMED1,CLTC,GRIA1,ICAM5,SCAMP5,GP2,ROCK1,MYO1D,SNX18,ITGA2,MARCH2,PRKAA1,RAB31,NAPG,RAB34,CLEC16A,MYO5B,YIPF1,MGRN1,SRL,BCAP31,SDF4,CSNK1G2,FLNA,ATP6AP1,VAMP7,GNAI3,GPR107,FNBP1,MBTPS1,CDH13,USP20,AP1G1,C16orf70,C	darkgreen	EC_M4
GO:0034622	7.10E-09	1	124	830	cellular protein-containing complex assembly	BP	5.01E-07	SHPRH,LATS1,NUP107,CCT2,DDX39B,FBXO5,EIF3E,RRP7A,SRPK1,PSIP1,AKAP9,NCBP1,ANP32B,SENP6,YTHDC1,MDN1,NUP205,ANLN,TAF1B,CAPZA2,DDX1,MCM2,CENPO,CENPA,NASP,CLASP2,MRPS7,RSF1,DIAPH3,CRNKL1,KAT6A,NAP1L1,TAF7,TUBGCP3,SNRPF,PRKDC,DHX29,TGS1,TPX2,NDUFAF2,RRS1,CENPJ,FER,NCK1,ARFGEF1,SMARCA5,CENPH,TAF9,SMN2,BDP1,KNTC1,ATR,TERF1,HELLS,GCFC2,MIS18A,CKAP5,NUP93,RAD51,SCAF11,OIP5,CHAF1B,UBE2C,NAP1L4,GMNN,FP565260.1,SF3B1,CENPE,RBM5,CAMSAP2,COPS7B,HAUS2,PRMT5,TBCE,HJURP,EIF3A,CENPF,HAT1,DHX9,HDGFL3,FASTKD2,DDX20,PRPF39,RPL10L,UBTF,XRCC5,CDC45,HP1BP3,DLGAP5,DHX33,FKBP4,RAD52,UBE2K,EIF3B,ASF1B,SRSF5,TAF1,LUC7L3,ATRX,COIL,CENPI,GEMIN5,PSMD10,EIF5,NLE1,RICTOR,AIFM1,BRIX1,CHAF1A,NDC1,UBN1,CDT1,CENPN,HIST1H1A,GEMIN4,TMOD3,STMN1,ITGB3BP,HIST1H3H,GEMIN6,NAF1,CENPW,OTUD6B,DRG1	darkgrey	EC_M8
GO:0044270	7.16E-09	1	68	361	cellular nitrogen compound catabolic process	BP	5.03E-07	PAN2,POP1,EIF3E,TNRC6B,TUT7,NCBP1,LRPPRC,PNPT1,SYNCRIP,PABPC4,THRAP3,EXOSC9,RNASEH2B,TRDMT1,CNOT10,UPF2,MBD4,DIS3,XRN2,AFMID,METTL3,EXOSC8,PAN3,MTREX,TDG,XRN1,PDE12,ZCCHC8,TTC37,CNOT1,TENT2,NSUN2,TET2,FXR1,DDX5,FASTKD1,SSB,PDE8A,DHX9,HNRNPR,FASTKD2,DNA2,CNOT9,HNRNPU,MRPL44,DCP2,DCTPP1,EXOSC10,FUS,DIS3L,LARP1,TAF15,UPF3B,HNRNPM,SERBP1,AIFM1,GSPT1,HPRT1,DROSHA,FMR1,METTL16,RNPS1,EXOSC2,RBM8A,FASTKD5,HNRNPD,NAF1,FEN1	darkgrey	EC_M8
GO:0048583	8.13E-10	1	402	2834	regulation of response to stimulus	BP	5.11E-07	TNFRSF11A,SEMA6A,TNFAIP3,TRIM39,PLEKHG1,AKAP12,TSPAN8,FLT4,FZD6,CTHRC1,RIMS2,OXR1,CDC42SE2,GRAMD4,TPD52L1,NCOA7,RNF146,ARHGAP18,SGSM3,PTK2,DENND3,C6orf106,ANKS1A,DAPK1,ATF4,PDGFB,MAPK13,CSNK1E,KDM4C,MFNG,MDFI,LRRC19,TEK,RIC8B,DDX58,PIP5K1B,TEC,SH3RF3,KIT,TNFRSF21,KDR,CD2AP,SYK,GADD45G,SEMA4D,RNF149,IL6,REL,CHN2,CXCL8,PELI1,RGS3,ARHGAP25,RSAD2,FIGNL1,CAV2,GRB10,LANCL2,TSPAN12,TLR4,TRAF1,C5,DLGAP3,IL15,CCNY,SDCBP,TRPM4,NRP1,PUM2,RASIP1,PIK3CG,ARHGAP42,MTMR2,MGLL,KDM4D,GATA2,ARHGAP21,PRKD2,CST7,NOVA2,CTSC,GPR4,FGD5,NECTIN2,PRCP,NOS3,GAB2,LLGL2,UNC13D,ZFAND6,PPARG,GATA3,ACVR2B,CXCR4,IRAK2,SEC14L1,EDNRB,NET1,RALGAPA2,NDFIP2,TMC8,CYTH1,IKBKB,JAG1,P2RY6,PDE2A,TMEM173,SFRP1,HBEGF,NR1D2,NOTUM,FERMT1,RPL26,EFNB2,SPEF1,ARHGAP26,P2RX2,CD36,SIRT3,PRAG1,PRR5L,MAP3K1,EPHB1,PLK2,RET,PDE4D,TNFRSF19,IL1A,IGF1,KLKB1,EIF2AK3,ABHD6,HHEX,ITCH,ERBIN,UFSP2,SH3RF1,PREX2,HIP1R,OCLN,MCTP1,ARHGEF3,SOX7,ARHGEF28,MEF2C,TLR2,WWTR1,IL7,P2RX7,VEGFC,NDRG4,GUCY1A1,CXCL10,ANGPT2,MADD,LMBR1L,ADGRG3,KMT2D,BMP2K,OAS3,NLRC5,THBS4,PPP1R16B,PLCG1,NR4A2,DGKZ,HIPK1,ANO6,DLL4,ZDHHC13,DNMBP,SOX13,IFIT1,ADAMTS20,EDN1,STAT1,DYRK1A,JAM3,SNCA,JARID2,DTNBP1,DUSP8,GAS2,PRDM15,ETS1,SHANK2,CD200,PDE9A,HDAC1,ITPRIP,LCK,PPP3CA,HYAL2,ICOSLG,GIT2,MEF2A,SEMA3F,SHOC2,RIN3,HCRTR1,LAPTM5,HLX,NCF1,DYRK3,USP13,FABP4,HCLS1,NFATC2,INPP5D,TIFA,CD34,CD86,MN1,ARHGEF12,SH3BP4,BMX,	turquoise	EC_M6

GO:0044419	8.91E-10	1	160	948	biological process involved in interspecies interaction between organisms	BP	5.38E-07	TNFRSF11A,IFNGR1,TNFAIP3,TRIM39,AKAP12,LYZ,TRDN,PTK2,C6orf106,DAPK1,CSF2RB,LRRC19,DDX58,SYK,IL6,REL,CXCL8,PELI1,RSAD2,CAV2,ODC1,TLR4,C5,IL15,NOCT,NRP1,PUM2,PIK3CG,NECTIN2,NOS3,UNC13D,PPARG,TIMP4,GATA3,CXCR4,IRAK2,SEC14L1,EDNRB,IKBKB,NFKBIB,TMEM173,EFNB2,CD36,ARID5A,SCARB1,CHD7,IL1A,NR4A1,BPIFB3,ITCH,ERBIN,MEF2C,TLR2,P2RX7,GPM6A,CXCL10,SERINC5,OAS3,NLRC5,TPCN1,STAB1,ANO6,IFIT2,IFIT1,EDN1,STAT1,CD58,SNCA,NFKB2,MX2,MX1,RNF19B,HDAC1,LCK,HYAL2,LYST,NCF1,FABP4,PABPN1,ISG20,TIFA,CD86,POU2F3,FGR,FES,PRKD1,F11R,IL10RA,SRMS,PAK2,STYK1,MOV10,B2M,HMCN1,LTF,MASP1,RTP4,CYBB,IL1RAP,DDX3X,THOC3,TMEM106A,PYGL,BIRC3,SELE,SELP,BAIAP2L1,WAS,HIF1A,RAB27A,ADIPOR2,EPHA2,TLR6,TNK1,TNFRSF1B,ARG2,TBKBP1,BATF,S100A14,SCNN1B,CYP1A1,TNIP1,CCL2,YES1,PDE4B,JAK1,LGALS9,NOS2,ELF4,IL7R,GIT1,LRG1,ISG15,NOTCH1,IRF8,PLCG2,STXBP1,LRSAM1,DAB2IP,ADGRL2,HAVCR1,OAS1,VNN1,PMAIP1,TLR1,GBP5,RAB43,TRIM34,CD46,TRIM62,FCER2,SERPINB9,TBC1D20,BST2,VAMP3,BMP2,C15orf48,IFIT3,RNF125,BMP6	turquoise	EC_M6
GO:0010833	7.96E-09	0.999999998	25	73	telomere maintenance via telomere lengthening	BP	5.56E-07	CCT2,MAP3K4,POT1,HNRNPA2B1,CCT4,TNKS2,XRN1,ATR,TERF1,CCT7,RAD51,DCLRE1B,CCT5,CCT6A,GAR1,NEK2,XRCC5,HNRNPU,RFC1,DCP2,EXOSC10,CCT3,AURKB,RPA1,NAF1	darkgrey	EC_M8
GO:0032879	7.27E-10	1	373	2074	regulation of localization	BP	6.49E-07	CD63,LMAN1,ATP8B1,LRP1,STX7,AHI1,RAB21,SLC12A2,AZIN1,PLXNB2,ANGPT1,FAM19A5,TCP1,PPARA,NUS1,FAM49B,PACSIN1,PTP4A3,REEP2,ADGRB1,PPARD,CTNNA1,SUN2,MKLN1,UBQLN1,SH3GL2,PLIN2,LEP,PLAA,SRF,VEGFA,DOCK8,VCP,UNC13B,CLOCK,EXOC1,CREB3,REST,RAB23,PTP4A1,NLRP5,P3H1,TGFBR1,RIMS1,TMEM30A,ERLEC1,RTN4,SHANK1,EMC10,UBE2J1,AKIRIN1,MDFIC,GLI3,ROCK2,INHBA,BVES,RRAS,VPS35,SLC7A11,ITGB1,FKBP1B,PLXNA1,FGF2,KIF5B,DNAJC1,RAB7A,RB1,ABHD5,IWS1,PICALM,MARK4,PDCD6IP,PCDH17,SNX17,PAK1,TGFB1,AXL,RAB3GAP1,SCN5A,CTNNB1,IL15RA,AKT2,GPD1L,C1QTNF1,RAB2B,OSBPL8,ACTG1,NAV3,ACTN4,PPP1R12A,STIM1,SPINT2,UFM1,ATP2C1,IRS2,PRNP,ATP2B1,DCN,HDAC3,RAB11FIP1,ZNF703,DCLK1,PCLO,STARD13,SEMA3C,DDRGK1,SMPD1,ILK,ARL6IP5,DPYSL3,RIC3,REM1,CXCL12,FXYD1,TM9SF4,KIF2A,ANKRD1,REEP5,SNTA1,TP53INP2,OSBP,SULF1,VAMP8,SLMAP,ATP1B3,RIOK2,ROBO1,MAP1B,CHST2,RYR3,PINX1,SORBS1,SEMA4F,P2RX4,PFN2,SELENOT,ATP2A2,CACNA1D,APP,ATXN2,DHX36,CCT8,RAPGEF2,CX3CL1,ABCC8,CACNB3,PTPN11,PTX3,GNL3,PIP4P2,PFKM,PGAM4,HOMER1,ARHGAP1,ARSB,ATG13,TSG101,ACVR1,DKC1,CRY2,VPS18,PPP3CC,ADA,BAP1,CHP1,ATP2B4,SPP1,PKD2,PLA2R1,LGI3,SEMA4G,NKD1,BRSK2,DOCK10,DYNLL1,ADIPOR1,AZIN2,PHLDB2,TJP1,KDM5B,CALHM1,PKP2,DNM1L,SELENOS,HYAL1,CPT1A,TGFB2,LRP5,EPRS,FEZ1,HTR2B,GSK3B,MMP14,DAG1,HADH,LEF1,CORO1C,ISCU,PRKCA,DISP1,CMKLR1,NTRK3,HSPA8,LARP7,SLAMF8,ARV1,BMP7,IDH2,ADCY5,AP2M1,OSM,CASQ1,PEA15,IQGAP1,SCFD1,CLIC4,S100A11,TFR	darkgreen	EC_M4
GO:0071705	7.30E-10	1	299	1609	nitrogen compound transport	BP	6.49E-07	CD63,LMAN1,ATP8B1,LRP1,STX7,AP3S1,AGAP2,CANX,TBC1D9B,RAB21,RABL2A,SLC12A2,AZIN1,ANGPT1,TCP1,SLC22A4,NUP50,HSPA4,SEC24A,REEP2,LY6E,PPARD,SIL1,GGA1,MYH9,LEP,PEX6,VCP,SLC35B2,UNC13B,CLOCK,EXOC1,SRP72,REST,LMAN2L,RAB23,SNX13,P3H1,RIMS1,SLC17A5,TMEM30A,ERLEC1,COX18,SLC35A1,UBE2J1,MDFIC,GLI3,VPS41,RCN3,VPS35,MGARP,SLC7A11,ITGB1,PGRMC2,LIN7B,FKBP1B,KIF5B,SEC61A1,NAPA,DNAJC1,RAB7A,SLC36A4,COPG1,IWS1,PDCD6IP,SNX17,PAK1,TGFB1,SRP68,RAB3GAP1,SEC13,TRAK1,AKT2,SEC23B,TBC1D16,RAB2B,SLC38A10,NPLOC4,ACTN4,STAM2,COG6,UFM1,ATP2C1,IRS2,PRNP,HDAC3,RAB11FIP1,PCLO,ARL6IP5,WASHC2A,VPS37A,ARL1,CXCL12,TM9SF4,RAB2A,ANKRD1,HSP90B1,TMED2,SLC25A4,COPB2,OSBP,VAMP8,TRAM1,COPB1,SELENOT,USO1,SDAD1,ATXN2,SLC33A1,ABCC8,CACNB3,SSR3,PTPN11,EXOC6B,PFKM,CDH17,ARHGAP1,ATG13,AP3B1,TSG101,CRY2,VPS18,SERINC3,ADA,AKTIP,BAP1,CHP1,AP2A2,CBLB,BRSK2,VPS39,RAB7B,ARL3,DYNLL1,AZIN2,APBA2,CALHM1,SRPRA,CTTN,SLC35A5,DNM1L,SELENOS,KPNA6,SEC22B,CPT1A,TGFB2,LRP5,ZMAT3,GSK3B,HADH,DISP1,PITPNM1,SGSM1,HSPA8,KPNA1,IDH2,AP1B1,SEC22A,ADCY5,AP1S2,GOLT1B,AP2M1,COPA,SCFD1,KIF1A,SNX27,TOMM40L,TFRC,SIDT2,SRP54,LSG1,HSPB1,LINC02210-CRHR1,SEC23A,AP1S1,SYVN1,SCAMP3,TANGO2,TFR2,C11orf65,PDCD10,UPF1,ADRA2C,COMP,CLTCL1,PRKCI,SMURF1,KTN1,EXOC5,PTPRN,BLZF1,AP5M1,SLC3A2,FMN2,STX18,KDELR2,ZDHHC4,SLC6A13,PRAF2,B3GAT3,MYO5A,JAKMIP1,ERC1,HAP1,SLC29A4,NLGN2,CLN8,SNX8,LMAN2,MED1,SRCIN1,SFXN1,STX1B,	darkgreen	EC_M4

GO:0090305	9.76E-09	1	47	213	nucleic acid phosphodiester bond hydrolysis	BP	6.79E-07	PAN2,POP1,APTX,NCBP1,PNPT1,DDX1,GEN1,EXOSC9,PCF11,DCLRE1C,TSEN2,DIS3,XRN2,EXOSC8,POLE,PAN3,UTP20,BMS1,RRS1,XRN1,PDE12,CNOT1,DCLRE1B,CPSF2,SPO11,TSEN15,DNA2,FANCM,APEX2,NOP14,ANKLE1,EXO1,N4BP2,MRPL44,DCP2,LAS1L,EXOSC10,EME1,DIS3L,G3BP1,REXO5,AIFM1,DROSHA,NOL9,EXOSC2,NOB1,FEN1	darkgrey	EC_M8
GO:0034613	8.29E-10	1	275	1451	cellular protein localization	BP	6.83E-07	CD63,LMAN1,LRP1,STX7,AP3S1,AHI1,TBC1D9B,RAB21,RABL2A,ANGPT1,TCP1,NUP50,HSPA4,SEC24A,PACSIN1,REEP2,CTNNA1,SIL1,SUN2,GGA1,LEP,PEX6,VCP,SRP72,RAB23,SNX13,RIMS1,TMEM30A,ERLEC1,FAM126A,RTN4,TPBG,COX18,EMC10,UBE2J1,MDFIC,GLI3,ROCK2,VPS41,VPS35,MGARP,SLC7A11,ITGB1,LIN7B,KIF5B,SEC61A1,NAPA,RAB7A,COPG1,RB1,PICALM,MARK4,MAPRE3,SNX17,PAK1,TGFB1,SRP68,RAB3GAP1,SEC13,CTNNB1,TRAK1,AKT2,GPD1L,SEC23B,DZIP1,TBC1D16,OSBPL8,NPLOC4,UFM1,ATP2C1,PRNP,HDAC3,DCLK1,DDRGK1,ARL6IP5,WASHC2A,RIC3,VPS37A,ARL1,TM9SF4,HSP90B1,TMED2,TP53INP2,COPB2,TMEM150A,VAMP8,SLMAP,ATP1B3,TRAM1,EMC4,PINX1,SORBS1,COPB1,USO1,TCTN1,GLRB,CCT8,RAPGEF2,TM9SF3,CACNB3,CPE,SSR3,PTPN11,EXOC6B,GNL3,ATG13,AP3B1,ZFYVE27,TSG101,DKC1,CRY2,VPS18,BAP1,CHP1,ATP2B4,TWF1,AP2A2,CBLB,TOLLIP,BRSK2,HPS6,VPS39,ARL3,SRPRA,CTTN,PKP2,DNM1L,SELENOS,KPNA6,FBLN5,WIPI1,GSK3B,MMP14,DAG1,SGSM1,GOLGB1,HSPA8,LARP7,KPNA1,BMP7,AP1B1,AP1S2,AP2M1,COPA,GORASP2,IQGAP1,SCFD1,KIF1A,SNX27,TOMM40L,TFRC,FZD9,SRP54,LRRC15,HSPB1,SCYL1,MAPT,SEC23A,AP1S1,SYVN1,C11orf65,GPC2,PDCD10,CLTCL1,PRKCI,SMURF1,TNIK,EMC1,EXOC5,BLZF1,VCL,KCNA1,TULP3,SIX4,OBSL1,ATAD1,STX18,KDELR2,ZDHHC4,B3GAT3,ROM1,MYO5A,HAP1,MAGED1,WIPI2,IQSEC2,NLGN2,ATP6V1D,SNX8,MED1,MSN,TSPAN17,STX1B,SNX11,SSR2,ARHGEF2,SYT11,NGFR,LTBP2,COL1A1,TMED10,RAB11A,SEL1L,TTC8,CALM1,SYTL4,TMED1,CLTC,ARMCX3,NPTN,CYP46A1,PIN1,CD74,MIEF2,ROCK1,EVI5L,AKT1,TMEM98,PRK	darkgreen	EC_M4
GO:0097435	8.59E-10	1	140	622	supramolecular fiber organization	BP	6.83E-07	B4GALT7,COL11A2,FAM49B,PACSIN1,CTNNA1,SRF,COL1A2,TPM2,NCK2,DST,NLRP5,TGFBR1,SHANK1,ADAMTS3,CALD1,ACTR2,ROCK2,ZYX,MYLK3,ITGB1,EML2,RB1,MAPRE3,CRTAP,PAK1,ACTR3,NCKAP5,CLIP4,SERPINH1,PLEKHG2,ACTG1,NAV3,LUM,CDC42EP3,ILK,DPYSL3,TTC17,CXCL12,KIF2A,ANKRD1,FAT1,HSP90B1,MAP1B,SORBS1,LOXL3,PFN2,RASA1,APP,CX3CL1,NEFM,LOXL2,CHP1,BMP1,TWF1,MAGEL2,CHRNA7,ARL3,RAP1GDS1,ELMO2,PHLDB2,TJP1,CTTN,PKP2,MARCKSL1,LMOD1,TGFB2,FBLN5,PRKAR1A,PIK3CA,CORO1C,SHROOM2,HSPA8,SPATC1L,SYNPO2,CASQ1,ITGB5,BACE1,ARPC5,COLGALT2,MAPT,PLOD3,NCKAP1,P4HA1,COMP,ARPC2,COL3A1,PRKCI,COL5A2,SYNPO2L,CAPZB,WDR1,FMN2,SIX4,OBSL1,TPM4,MYO5A,FKBP10,ENAH,CGNL1,TCAP,ACTN1,MYO1E,ARHGEF2,LTBP2,TPM1,COL1A1,TGFB3,RAB11A,GPR65,TTC8,DIAPH2,PDGFRB,PLS3,ROCK1,FLII,MYO1D,MPRIP,TUBB4A,TNFAIP1,MYO5B,DAPK3,MYO1C,PAFAH1B1,FLNA,PARK7,COL5A1,COL11A1,SPTAN1,FKBP1A,LOXL1,LOX,PDLIM1,CSRP1,MFAP4,TMSB4X,MZT1,DSTN,MAP1A,COTL1,CFL2	darkgreen	EC_M4
GO:0044773	1.01E-08	0.999999998	24	71	mitotic DNA damage checkpoint signaling	BP	7.01E-07	CDK2,MDC1,GTSE1,MSH2,CLSPN,CASP2,RINT1,FANCD2,TOPBP1,PRKDC,NBN,ABRAXAS1,TAOK3,CHEK1,TICRR,RPA2,DTL,BLM,CDK1,BRCA1,EME1,TIPIN,PLK1,TAOK1	darkgrey	EC_M8
GO:0032502	9.76E-10	1	743	4589	developmental process	BP	7.37E-07	CD63,IL23A,ATP8B1,NAB2,LRP1,CDO1,AHI1,B4GALNT1,CANX,TAB2,CAND1,IER3,RAB21,CDSN,RRM2B,SLC12A2,PLXNB2,FBN2,ANGPT1,FAM19A5,PPARA,HLA-DRA,COL11A2,ROS1,AFF4,NUS1,RSPO3,FAM49B,MRTFA,TRAPPC9,PACSIN1,ADGRB1,LY6E,PPARD,CTNNA1,TEAD3,SUN2,CBY1,MKLN1,MTCH1,LGALS1,MDGA1,SH3GL2,FLNC,MYH9,LEP,CDKN2B,PLAA,TBCC,LARGE1,RORB,KIAA1324L,SRF,NUAK1,VEGFA,RUNX2,UNC13B,COL1A2,C2orf40,NCK2,CLOCK,ASNS,PDCL3,REST,ASPN,CNNM4,RAB23,NLRP5,COL15A1,P3H1,TGFBR1,HIVEP3,SOCS5,RIMS1,TMEM30A,PSME4,FAM126A,UBA6,RTN4,TPBG,SHANK1,ADAMTS3,LRRC4B,EMC10,MFSD2A,UBE2J1,MYH14,CALD1,AKIRIN1,ACTR2,GMCL1,IFRD1,KIDINS220,WHRN,GLI3,NCDN,ROCK2,INHBA,GREB1,BVES,RRAS,RCN3,MYLK3,DKKL1,VPS35,SLC7A11,OSTM1,PCDH18,ITGB1,PGRMC2,PLEKHA4,DBP,FKBP1B,PLXNA1,FGF2,SRPK2,KIF5B,SEC61A1,NAPA,PANX1,PSMC2,RB1,ITM2B,ABHD5,EED,PICALM,MARK4,TPT1,ZNF296,CRTAP,EIF2B3,PTCH2,TENM4,GLI2,MEGF8,SMARCD3,PCDH17,RHEB,SNX17,PAK1,TGFB1,AXL,RAB3GAP1,SCN5A,WDR48,HTR5A,HNMT,ACER3,CTNNB1,IL15RA,TRAK1,LBH,DGAT2,SERPINH1,AKT2,DZIP1,ZEB2,FARP1,RELT,SLC4A7,OSBPL8,SLC38A10,RARB,ACTG1,NAV3,ACTN4,KAT2B,TACC1,CRIM1,MAFG,STIM1,SPRED3,SPINT2,UFM1,LIG4,ATP2C1,IRS2,PRNP,ATP2B1,LUM,DCN,CDC42EP3,SMAD9,ZNF703,DCLK1,PCLO,STARD13,SEMA3C,CCKBR,UBA5,DDRGK1,SMPD1,ILK,DPYSL3,KATNAL1,SPINK5,APLP1,RYK,PRICKLE2,SNPH,RNF6,AKIP1,CXCL12,IMPAD1,LGI4,ZSWIM6,KIF2A,SLC20A1,TMEM41B,ANKRD1,FAT1,ADAMTS6,SLC25A46,TMED2,PALLD,EIF2S2,	darkgreen	EC_M4

GO:0007154	1.03E-09	1	701	4300	cell communication	BP	7.41E-07	CD63,IL23A,LRP1,AP3S1,HBS1L,AHI1,ARHGEF25,TAB2,DYRK2,RAB21,RNF19A,RRM2B,SLC12A2,PLXNB2,FBN2,ANGPT1,FAM19A5,PPARA,ROS1,NUS1,RSPO3,CXCL14,FAM49B,GNG5,PTP4A3,ADGRB1,LY6E,SCUBE3,PPARD,CTNNA1,TEAD3,CBY1,MKLN1,UBQLN1,MTCH1,LGALS1,KLHDC10,TXN2,SH3GL2,GNAQ,SLC39A4,MYH9,ARHGAP39,LEP,CDKN2B,PLAA,RORB,BTBD11,KIAA1324L,SRF,BAG1,NUAK1,CDK14,VEGFA,DOCK8,VCP,SLC35B2,RUNX2,RASL11B,UNC13B,COL1A2,NCK2,CLOCK,EXOC1,CREB3,ASNS,REST,ASPN,INPP4A,DST,SNX13,COL15A1,TGFBR1,SOCS5,RIMS1,ERLEC1,RTN4,TPBG,SHANK1,ADAMTS3,EMC10,MFSD2A,GGCT,MAP3K7,MYH14,SH3BP5L,KIDINS220,MDFIC,GLI3,NCDN,ROCK2,INHBA,VPS41,RRAS,ZYX,RCN3,DKKL1,VPS35,CUL2,SLC7A11,ITGB1,PGRMC2,LIN7B,PLEKHA4,FKBP1B,PLXNA1,FGF2,SRPK2,KIF5B,NAPA,SESN3,RAB7A,PANX1,ARHGEF4,GAD2,APBB1IP,RB1,MARK4,TPT1,EIF2B3,PTCH2,TENM4,DGKH,KCTD21,GLI2,MEGF8,PCDH17,RHEB,SNX17,PAK1,TGFB1,AXL,RAB3GAP1,SCN5A,HTR5A,SEC13,CTNNB1,IL15RA,LBH,DGAT2,AKT2,DYNC1LI1,GPD1L,DZIP1,ZEB2,LGALS3BP,CANT1,C1QTNF1,FARP1,ARHGEF17,OSBPL8,CXXC5,CLPB,PSD2,RARB,NPLOC4,ACTN4,PPP1R12A,ANAPC15,STAM2,LTBP1,TMTC4,CRIM1,SPRED3,GPS1,UFM1,ATP2C1,IRS2,PRNP,CHFR,ATP2B1,DCN,CDC42EP3,SMAD9,HDAC3,RAB11FIP1,ZNF703,DCLK1,ACPP,PCLO,STARD13,KCTD16,SEMA3C,CCKBR,CAVIN3,UBA5,DDRGK1,SMPD1,PPP2CB,ILK,RBPMS,GPR84,LEPROTL1,ARL6IP5,APLP1,RYK,TMEM127,PRICKLE2,SNPH,CNOT7,RNF6,RIC3,TMEM9B,ST5,AKIP1,CXCL12,SLC20A1,ANKRD1,FAT1,PJA2,TMED2,IL20RB,MRAS,MAP1L	darkgreen	EC_M4
GO:0070727	1.16E-09	1	276	1462	cellular macromolecule localization	BP	7.86E-07	CD63,LMAN1,LRP1,STX7,AP3S1,AHI1,TBC1D9B,RAB21,RABL2A,ANGPT1,TCP1,NUP50,HSPA4,SEC24A,PACSIN1,REEP2,CTNNA1,SIL1,SUN2,GGA1,LEP,PEX6,VCP,SRP72,RAB23,SNX13,RIMS1,TMEM30A,ERLEC1,FAM126A,RTN4,TPBG,COX18,EMC10,UBE2J1,MDFIC,GLI3,ROCK2,VPS41,VPS35,MGARP,SLC7A11,ITGB1,LIN7B,KIF5B,SEC61A1,NAPA,RAB7A,COPG1,RB1,PICALM,MARK4,MAPRE3,SNX17,PAK1,TGFB1,SRP68,RAB3GAP1,SEC13,CTNNB1,TRAK1,AKT2,GPD1L,SEC23B,DZIP1,TBC1D16,OSBPL8,NPLOC4,UFM1,ATP2C1,PRNP,HDAC3,DCLK1,DDRGK1,ARL6IP5,WASHC2A,RIC3,VPS37A,ARL1,TM9SF4,HSP90B1,TMED2,TP53INP2,COPB2,TMEM150A,VAMP8,SLMAP,ATP1B3,TRAM1,EMC4,PINX1,SORBS1,COPB1,USO1,TCTN1,GLRB,DHX36,CCT8,RAPGEF2,TM9SF3,CACNB3,CPE,SSR3,PTPN11,EXOC6B,GNL3,ATG13,AP3B1,ZFYVE27,TSG101,DKC1,CRY2,VPS18,BAP1,CHP1,ATP2B4,TWF1,AP2A2,CBLB,TOLLIP,BRSK2,HPS6,VPS39,ARL3,SRPRA,CTTN,PKP2,DNM1L,SELENOS,KPNA6,FBLN5,WIPI1,GSK3B,MMP14,DAG1,SGSM1,GOLGB1,HSPA8,LARP7,KPNA1,BMP7,AP1B1,AP1S2,AP2M1,COPA,GORASP2,IQGAP1,SCFD1,KIF1A,SNX27,TOMM40L,TFRC,FZD9,SRP54,LRRC15,HSPB1,SCYL1,MAPT,SEC23A,AP1S1,SYVN1,C11orf65,GPC2,PDCD10,CLTCL1,PRKCI,SMURF1,TNIK,EMC1,EXOC5,BLZF1,VCL,KCNA1,TULP3,SIX4,OBSL1,ATAD1,STX18,KDELR2,ZDHHC4,B3GAT3,ROM1,MYO5A,HAP1,MAGED1,WIPI2,IQSEC2,NLGN2,ATP6V1D,SNX8,MED1,MSN,TSPAN17,STX1B,SNX11,SSR2,ARHGEF2,SYT11,NGFR,LTBP2,COL1A1,TMED10,RAB11A,SEL1L,TTC8,CALM1,SYTL4,TMED1,CLTC,ARMCX3,NPTN,CYP46A1,PIN1,CD74,MIEF2,ROCK1,EVI5L,AKT1,TME	darkgreen	EC_M4
GO:0030029	1.21E-09	1	147	662	actin filament-based process	BP	7.86E-07	LRP1,AGAP2,ABRACL,FAM49B,MRTFA,PACSIN1,CTNNA1,SUN2,MKLN1,FLNC,MYH9,SRF,TPM2,NCK2,PDCL3,NLRP5,TGFBR1,SHANK1,MYH14,CALD1,ACTR2,ROCK2,ZYX,MYLK3,ITGB1,PDCD6IP,PAK1,ACTR3,SCN5A,GPD1L,PLEKHG2,FARP1,ARHGEF17,ACTG1,ACTN4,ATP2C1,CDC42EP3,STARD13,CAVIN3,ILK,DPYSL3,TTC17,CXCL12,ANKRD1,FAT1,SNTA1,HSP90B1,PALLD,MRAS,FLNB,SORBS1,PFN2,RASA1,EPB41L1,ATP2A2,RTKN,SDAD1,CACNA1D,CX3CL1,TWF1,BRSK2,PDLIM5,MAGEL2,RAP1GDS1,ELMO2,INA,PHLDB2,TJP1,NOTCH2,CTTN,PKP2,MARCKSL1,LMOD1,PRKAR1A,PIK3CA,AUTS2,CORO1C,SHROOM2,KRAS,NTRK3,SPATC1L,SYNPO2,CASQ1,ITGB5,IQGAP1,FARP2,ARPC5,EHBP1L1,ALKBH4,NCKAP1,ARPC2,PRKCI,SYNPO2L,CAPZB,TNIK,DAAM1,WDR1,MYO9B,FMN2,SIX4,OBSL1,TPM4,MYO5A,CACNA1C,IQSEC2,ENAH,CGNL1,PDLIM7,TCAP,ACTN1,MYO1E,ARHGEF2,TPM1,DOCK2,TGFB3,CACNA1G,SHC1,MYH10,CNN1,GPR65,TTC8,DIAPH2,SETD3,PDGFRB,PLS3,ROCK1,FLII,MYO1D,MPRIP,TNFAIP1,MYO5B,DAPK3,MYO1C,PAFAH1B1,NPHP4,FLNA,SPTAN1,DMD,PDLIM1,CSRP1,TMSB4X,DSTN,HCN4,PRKG1,COTL1,CFL2,FGF10	darkgreen	EC_M4

GO:0050896	1.25E-09	1	919	5876	response to stimulus	BP	7.86E-07	CYB5A,SERPINB2,CD63,LMAN1,IL23A,LRP1,STX7,AP3S1,CDO1,HBS1L,AHI1,ARHGEF25,CANX,ENPP3,TAB2,DYRK2,IER3,RAB21,IPCEF1,RRM2B,SLC12A2,PLXNB2,FBN2,ANGPT1,FAM19A5,PPARA,HLA-DRA,RNASET2,TAF2,ROS1,NUS1,HSPA4,FER1L6,RSPO3,CXCL14,FAM49B,MRTFA,HABP4,GNG5,PTP4A3,ADGRB1,LY6E,SCUBE3,PPARD,CTNNA1,TEAD3,CBY1,MKLN1,C6orf89,UBQLN1,MTCH1,LGALS1,KLHDC10,TXN2,SH3GL2,GNAQ,PLIN2,SLC39A4,MYH9,ARHGAP39,MTAP,LEP,CDKN2B,PLAA,RORB,BTBD11,SLC30A9,KIAA1324L,SRF,BAG1,NUAK1,CDK14,VEGFA,DOCK8,VCP,SLC35B2,RUNX2,RASL11B,UNC13B,COL1A2,C2orf40,NCK2,CLOCK,EXOC1,CREB3,ASNS,PDCL3,REST,ASPN,INPP4A,CNNM4,DST,RAB23,SNX13,COL15A1,TGFBR1,ERP44,SOCS5,RIMS1,SLC17A5,LAIR1,ERLEC1,PSME4,COPS9,UBA6,RTN4,TPBG,SHANK1,ADAMTS3,EMC10,MFSD2A,GGCT,UBE2J1,MAP3K7,SH3BP5L,AKIRIN1,ACTR2,SLC31A1,KIDINS220,WHRN,MDFIC,GLI3,ROCK2,INHBA,VPS41,BVES,RRAS,ZYX,RCN3,MYLK3,DKKL1,VPS35,CUL2,MGARP,SLC7A11,ITGB1,PGRMC2,PLEKHA4,FKBP1B,PLXNA1,FGF2,SRPK2,SELENOW,KIF5B,SEC61A1,SESN3,RAB7A,PANX1,ARHGEF4,ATP7B,GAD2,APBB1IP,ENTPD6,ABHD12,RB1,ESD,MARK4,TPT1,GLB1,EIF2B3,PTCH2,TENM4,DGKH,TMUB1,KCTD21,GLI2,MEGF8,NUB1,RHEB,SNX17,PAK1,ACTR3,TGFB1,SRP68,AXL,RAB3GAP1,SCN5A,WDR48,HTR5A,UBXN4,SEC13,HNMT,ACER3,CTNNB1,IL15RA,LBH,DGAT2,SERPINH1,AKT2,DYNC1LI1,GPD1L,STT3B,DZIP1,PFKP,ZEB2,LGALS3BP,CANT1,C1QTNF1,ARHGEF17,OSBPL8,CXXC5,CLPB,PSD2,RARB,ACTG1,NPLOC4,ACTN4,PPP1R12A,ANAPC15,STAM2,KAT2B,RNF121,L	darkgreen	EC_M4
GO:0006334	1.30E-08	0.999999998	23	68	nucleosome assembly	BP	8.96E-07	SHPRH,ANP32B,MCM2,NASP,RSF1,KAT6A,NAP1L1,SMARCA5,MIS18A,OIP5,CHAF1B,NAP1L4,HJURP,HAT1,HP1BP3,ASF1B,ATRX,CHAF1A,UBN1,HIST1H1A,ITGB3BP,HIST1H3H,CENPW	darkgrey	EC_M8
GO:0034501	1.33E-08	0.999999999	12	19	protein localization to kinetochore	BP	9.09E-07	CENPQ,TTK,KNTC1,KNL1,BUB3,CDK1,SPDL1,AURKB,ZWILCH,NDC80,HASPIN,CDT1	darkgrey	EC_M8
GO:1903083	1.33E-08	0.999999999	12	19	protein localization to condensed chromosome	BP	9.09E-07	CENPQ,TTK,KNTC1,KNL1,BUB3,CDK1,SPDL1,AURKB,ZWILCH,NDC80,HASPIN,CDT1	darkgrey	EC_M8
GO:0048514	1.57E-09	1	103	508	blood vessel morphogenesis	BP	9.11E-07	SEMA6A,TNFAIP3,PTPRB,FLT4,QKI,NOTCH4,PTK2,TEK,ADGRF5,KDR,SYK,THSD7A,SHB,IL6,CXCL8,TSPAN12,C5,TEAD2,NRCAM,NRP1,RASIP1,PIK3CG,TAL1,GATA2,PRKD2,GPR4,PLXND1,PRCP,NOS3,PPARG,CXCR4,TIE1,JAG1,SFRP1,EFNB2,ADAMTS9,EPHB1,PLK2,CHD7,IL1A,NR4A1,EIF2AK3,HHEX,SMAD1,VEGFC,CXCL10,ANGPT2,TIPARP,THBS4,PPP1R16B,PLCG1,RAPGEF3,STAB1,HIPK1,DLL4,EDN1,STAT1,JAM3,ETS1,ROBO4,PRKX,SEC24B,CD34,MCAM,PROX1,PRKD1,MAP3K3,APOLD1,APOD,CLEC14A,CYBB,EPHB4,TBX1,CALCRL,HSPG2,RAMP2,HIF1A,RHOJ,JUP,ADIPOR2,EPHA2,MMRN2,SEMA4A,FGF18,PGF,EFNA1,VASH1,NPR1,AMOT,CCL2,JAK1,LRG1,NRARP,EGFL7,NOTCH1,S1PR1,ENG,DAB2IP,CDH5,MIR23A,ADGRG1,SOX17,SOX18	turquoise	EC_M6
GO:0009888	1.59E-09	1	266	1389	tissue development	BP	9.51E-07	NAB2,AHI1,CDSN,PLXNB2,FBN2,PPARA,COL11A2,ROS1,RSPO3,MRTFA,LY6E,PPARD,CBY1,LEP,KIAA1324L,SRF,VEGFA,RUNX2,COL1A2,CLOCK,ASPN,CNNM4,COL15A1,TGFBR1,HIVEP3,RTN4,MFSD2A,MYH14,AKIRIN1,WHRN,GLI3,ROCK2,INHBA,BVES,RCN3,MYLK3,SLC7A11,ITGB1,PGRMC2,PLEKHA4,PLXNA1,FGF2,RB1,TPT1,PTCH2,TENM4,GLI2,MEGF8,RHEB,PAK1,TGFB1,SCN5A,CTNNB1,LBH,DGAT2,SERPINH1,AKT2,SLC4A7,RARB,ACTG1,MAFG,STIM1,SPRED3,SPINT2,ATP2C1,ATP2B1,LUM,DCN,ZNF703,STARD13,SEMA3C,DDRGK1,ILK,SPINK5,RYK,PRICKLE2,IMPAD1,SLC20A1,ANKRD1,FAT1,TMED2,PALLD,FLNB,SULF1,ROBO1,TRPC4AP,RDH10,SEMA4F,LOXL3,HEY1,ATP2A2,TLX2,TCTN1,MMP15,SLC4A5,RAPGEF2,ACTG2,PTPN11,HOMER1,OPA1,LOXL2,AP3B1,TSG101,ACVR1,MMP2,GDF6,ADA,SPP1,BMP1,PKD2,RUNX1,SEMA4G,NKD1,IFT57,TOLLIP,BRSK2,PDLIM5,ADIPOR1,PHLDB2,TJP1,KDM5B,NOTCH2,PKP2,CDC73,DNER,CHSY1,HYAL1,EYA2,CCND1,CPT1A,SBDS,TGFB2,LRP5,HTR2B,SATB2,PRKAR1A,ITPK1,GSK3B,FSTL1,FAM20C,MMP14,DAG1,PIK3CA,LEF1,CORO1C,KRAS,TAGLN2,COL6A1,BMP7,ATF3,FGFR2,CASQ1,ITGB5,PHLDB1,IQGAP1,CLIC4,ARID5B,FZD9,TAGLN,SIDT2,OVOL1,PLOD3,SERPINE1,ATP6AP2,FZD2,FN1,MEOX1,PDCD10,ITGAV,COMP,FKBP8,COL5A2,SKIL,FGFR3,PRRX1,GORAB,SMURF1,TIMP1,EXOC5,VCL,DAAM1,WDR1,TULP3,SIX4,OBSL1,ACTA2,EVC,MAGED1,BMPR1A,TCAP,MED1,PPARGC1A,NPPB,MYLPF,MSN,MYO1E,TGFB1I1,NGFR,TPM1,THBS3,COL1A1,TGFB3,CACNA1G,PBXIP1,SPTLC2,MAP2K1,TMEM100,TTC8,SGCD,MYOCD,ANXA6,PIN1,FBXO22,LAMA3,PDGFRB,ROCK1,AKT1,ITGA2,MYOM1,P	darkgreen	EC_M4

GO:0023051	1.64E-09	1	440	2524	regulation of signaling	BP	9.51E-07	CD63,IL23A,LRP1,ARHGEF25,TAB2,DYRK2,RNF19A,RRM2B,SLC12A2,FBN2,ANGPT1,PPARA,ROS1,RSPO3,FAM49B,PTP4A3,ADGRB1,LY6E,PPARD,CTNNA1,CBY1,UBQLN1,MTCH1,LGALS1,KLHDC10,GNAQ,ARHGAP39,LEP,CDKN2B,KIAA1324L,SRF,NUAK1,CDK14,VEGFA,DOCK8,VCP,SLC35B2,RUNX2,RASL11B,UNC13B,NCK2,CLOCK,CREB3,REST,ASPN,SNX13,TGFBR1,SOCS5,RIMS1,RTN4,TPBG,SHANK1,ADAMTS3,EMC10,MAP3K7,MDFIC,GLI3,NCDN,ROCK2,INHBA,BVES,RRAS,RCN3,DKKL1,VPS35,SLC7A11,ITGB1,PLEKHA4,FKBP1B,FGF2,KIF5B,SESN3,RAB7A,ARHGEF4,RB1,TPT1,PTCH2,KCTD21,GLI2,MEGF8,PCDH17,RHEB,PAK1,TGFB1,AXL,RAB3GAP1,LMO7,SEC13,CTNNB1,LBH,DYNC1LI1,GPD1L,ZEB2,CANT1,C1QTNF1,TBC1D16,ARHGEF17,OSBPL8,CXXC5,PSD2,NPLOC4,ACTN4,ANAPC15,LTBP1,CRIM1,SPRED3,UFM1,ATP2C1,IRS2,PRNP,ATP2B1,DCN,HDAC3,RAB11FIP1,ZNF703,ACPP,STARD13,KCTD16,CAVIN3,UBA5,DDRGK1,SMPD1,PPP2CB,ILK,RBPMS,LEPROTL1,ARL6IP5,APLP1,RYK,TMEM127,CNOT7,RNF6,TMEM9B,ST5,AKIP1,CXCL12,SLC20A1,ANKRD1,PJA2,TMED2,CD180,ZNF536,OSBP,SULF1,VAMP8,GRK7,CTNND1,DKK3,ROBO1,WWC2,MAP1B,SORBS1,LOXL3,P2RX4,RNF13,FAIM2,HEY1,RASA1,SELENOT,ATP2A2,DLGAP4,TCTN1,APP,DHX36,RAPGEF2,MAPKAPK5,CX3CL1,ABCC8,CACNB3,PTPN11,RALGAPB,PFKM,HOMER1,OPA1,ARHGAP1,AMFR,PTPN22,TSG101,ACVR1,CRY2,VPS18,CHAC1,GDF6,SERINC3,MTDH,ADA,CHP1,ATP2B4,SPP1,PKD2,PLA2R1,HIF1AN,CBLB,NKD1,TANK,BRSK2,SLC39A10,RAB7B,CHRNA7,EIF4E,DYNLL1,ADIPOR1,KDM5B,NOTCH2,CTTN,CDC73,DNM1L,GLRX2,UCHL5,SELENOS,CHSY1,SLC2A10,EYA2,C	darkgreen	EC_M4
GO:0006954	1.73E-09	1	97	497	inflammatory response	BP	9.66E-07	TNFRSF11A,TNFAIP3,LYZ,MAPK13,LRRC19,TEK,NFX1,KIT,SYK,IL6,REL,CXCL8,TLR4,C5,IL15,PIK3CG,MGLL,CST7,CTSC,GPR4,PRCP,UNC13D,PPARG,GATA3,CXCR4,IRAK2,EDNRB,IKBKB,PDE2A,TMEM173,NR1D2,CD36,PLK2,IL1A,IGF1,KLKB1,ITCH,SMAD1,TLR2,CXCL10,PLCG1,STAB1,ANO6,CHST1,TSPAN2,JAM3,SNCA,ETS1,CD200,HYAL2,SNAP23,NCF1,FABP4,FGR,ADCY1,LTB4R,PRKD1,F11R,APOD,CCRL2,ADORA2A,CYBB,IL1RAP,CALCRL,HSPG2,BIRC3,SELE,SELP,RAC1,HIF1A,LIPA,BCL6B,EPHA2,TLR6,TNFRSF1B,RPS6KA5,GPR68,SCNN1B,TNIP1,CCL2,YES1,LGALS9,NOS2,GIT1,F8,NOTCH1,PLCG2,PTGS1,DAB2IP,CDH5,VNN1,TLR1,GBP5,ACKR1,MSMP,BMP2,BMP6	turquoise	EC_M6
GO:0043534	2.09E-09	1	35	108	blood vessel endothelial cell migration	BP	1.11E-06	PDGFB,KDR,NRP1,GATA2,PRKD2,PRCP,NOS3,PPARG,EFNB2,SCARB1,PLK2,NR4A1,MEF2C,VEGFC,ANGPT2,PLCG1,DLL4,ETS1,PRKD1,MAP3K3,CLEC14A,EPHB4,HIF1A,RHOJ,JUP,EPHA2,MMRN2,FGF18,EFNA1,VASH1,AMOT,NOTCH1,CDH5,MIR23A,SOX18	turquoise	EC_M6
GO:0009617	2.13E-09	1	76	362	response to bacterium	BP	1.11E-06	TNFRSF11A,TNFAIP3,AKAP12,LYZ,TRDN,CSF2RB,SYK,IL6,CXCL8,PELI1,TLR4,NOCT,NOS3,TIMP4,IRAK2,EDNRB,NFKBIB,CD36,SCARB1,CHD7,IL1A,NR4A1,ERBIN,MEF2C,TLR2,GPM6A,CXCL10,OAS3,NLRC5,STAB1,EDN1,SNCA,NFKB2,HDAC1,LYST,FABP4,PABPN1,CD86,FGR,PRKD1,IL10RA,B2M,HMCN1,LTF,PYGL,SELE,SELP,BAIAP2L1,ADIPOR2,EPHA2,TLR6,TNFRSF1B,ARG2,S100A14,SCNN1B,CYP1A1,TNIP1,CCL2,PDE4B,LGALS9,NOS2,IL7R,GIT1,LRG1,ISG15,NOTCH1,IRF8,PLCG2,DAB2IP,ADGRL2,OAS1,TLR1,SERPINB9,BMP2,C15orf48,BMP6	turquoise	EC_M6
GO:0023052	2.05E-09	1	693	4258	signaling	BP	1.15E-06	CD63,IL23A,LRP1,AP3S1,HBS1L,AHI1,ARHGEF25,TAB2,DYRK2,RAB21,RNF19A,RRM2B,SLC12A2,PLXNB2,FBN2,ANGPT1,FAM19A5,PPARA,ROS1,NUS1,RSPO3,CXCL14,FAM49B,GNG5,PTP4A3,ADGRB1,LY6E,SCUBE3,PPARD,CTNNA1,TEAD3,CBY1,MKLN1,UBQLN1,MTCH1,LGALS1,KLHDC10,SH3GL2,GNAQ,MYH9,ARHGAP39,LEP,CDKN2B,PLAA,RORB,BTBD11,KIAA1324L,SRF,BAG1,NUAK1,CDK14,VEGFA,DOCK8,VCP,SLC35B2,RUNX2,RASL11B,UNC13B,COL1A2,NCK2,CLOCK,EXOC1,CREB3,REST,ASPN,INPP4A,DST,SNX13,COL15A1,TGFBR1,SOCS5,RIMS1,ERLEC1,RTN4,TPBG,SHANK1,ADAMTS3,EMC10,GGCT,MAP3K7,MYH14,SH3BP5L,KIDINS220,MDFIC,GLI3,NCDN,ROCK2,INHBA,BVES,RRAS,ZYX,RCN3,DKKL1,VPS35,CUL2,SLC7A11,ITGB1,PGRMC2,LIN7B,PLEKHA4,FKBP1B,PLXNA1,FGF2,SRPK2,KIF5B,NAPA,SESN3,RAB7A,PANX1,ARHGEF4,GAD2,APBB1IP,RB1,MARK4,TPT1,EIF2B3,PTCH2,TENM4,DGKH,KCTD21,GLI2,MEGF8,PCDH17,RHEB,SNX17,PAK1,TGFB1,AXL,RAB3GAP1,SCN5A,LMO7,HTR5A,SEC13,CTNNB1,IL15RA,LBH,DGAT2,AKT2,DYNC1LI1,GPD1L,DZIP1,ZEB2,LGALS3BP,CANT1,C1QTNF1,TBC1D16,FARP1,ARHGEF17,OSBPL8,CXXC5,CLPB,PSD2,RARB,NPLOC4,ACTN4,PPP1R12A,ANAPC15,STAM2,LTBP1,TMTC4,CRIM1,SPRED3,GPS1,UFM1,ATP2C1,IRS2,PRNP,CHFR,ATP2B1,DCN,CDC42EP3,SMAD9,HDAC3,RAB11FIP1,ZNF703,DCLK1,ACPP,PCLO,STARD13,KCTD16,SEMA3C,CCKBR,CAVIN3,UBA5,DDRGK1,SMPD1,PPP2CB,ILK,RBPMS,GPR84,LEPROTL1,ARL6IP5,APLP1,RYK,TMEM127,PRICKLE2,SNPH,CNOT7,RNF6,RIC3,TMEM9B,ST5,AKIP1,CXCL12,SLC20A1,ANKRD1,FAT1,PJA2,TMED2,IL20RB,MRAS,MAST4,CD180,ZNF536,FLNB	darkgreen	EC_M4

GO:0045132	1.69E-08	0.999999997	24	73	meiotic chromosome segregation	BP	1.15E-06	RAD21,RMI1,SMC2,TTK,FANCD2,TOP2B,SGO1,ESPL1,BUB1,TERF1,BUB1B,CCNE2,TRIP13,SGO2,SPO11,NUF2,FANCM,SMC4,TOP2A,EME1,PTTG1,PLK1,BRIP1,NDC1	darkgrey	EC_M8
GO:0030036	2.18E-09	1	135	600	actin cytoskeleton organization	BP	1.18E-06	LRP1,AGAP2,FAM49B,MRTFA,PACSIN1,CTNNA1,MKLN1,FLNC,MYH9,SRF,TPM2,NCK2,PDCL3,NLRP5,TGFBR1,SHANK1,MYH14,CALD1,ACTR2,ROCK2,ZYX,MYLK3,ITGB1,PDCD6IP,PAK1,ACTR3,PLEKHG2,FARP1,ARHGEF17,ACTG1,ACTN4,ATP2C1,CDC42EP3,STARD13,CAVIN3,ILK,DPYSL3,TTC17,CXCL12,ANKRD1,FAT1,HSP90B1,PALLD,MRAS,FLNB,SORBS1,PFN2,RASA1,EPB41L1,RTKN,SDAD1,CX3CL1,TWF1,BRSK2,PDLIM5,MAGEL2,RAP1GDS1,ELMO2,INA,PHLDB2,TJP1,NOTCH2,CTTN,MARCKSL1,LMOD1,PRKAR1A,PIK3CA,AUTS2,CORO1C,SHROOM2,KRAS,NTRK3,SPATC1L,SYNPO2,CASQ1,ITGB5,IQGAP1,FARP2,ARPC5,EHBP1L1,ALKBH4,NCKAP1,ARPC2,PRKCI,SYNPO2L,CAPZB,TNIK,DAAM1,WDR1,FMN2,SIX4,OBSL1,TPM4,MYO5A,IQSEC2,ENAH,CGNL1,PDLIM7,TCAP,ACTN1,MYO1E,ARHGEF2,TPM1,DOCK2,TGFB3,SHC1,MYH10,CNN1,GPR65,TTC8,DIAPH2,SETD3,PDGFRB,PLS3,ROCK1,FLII,MYO1D,MPRIP,TNFAIP1,MYO5B,DAPK3,MYO1C,PAFAH1B1,NPHP4,FLNA,SPTAN1,DMD,PDLIM1,CSRP1,TMSB4X,DSTN,PRKG1,COTL1,CFL2,FGF10	darkgreen	EC_M4
GO:0043542	2.42E-09	0.999999999	51	195	endothelial cell migration	BP	1.22E-06	FLT4,PTK2,PDGFB,TEK,KDR,NRP1,PIK3CG,GATA2,PRKD2,PLXND1,PRCP,NOS3,PPARG,GATA3,EFNB2,ADAMTS9,SCARB1,PLK2,NR4A1,MEF2C,VEGFC,ANGPT2,PLCG1,STC1,DLL4,EDN1,ETS1,PRKX,PROX1,PRKD1,MAP3K3,CLEC14A,EPHB4,RAC1,HIF1A,RHOJ,JUP,EPHA2,MMRN2,SEMA4A,FGF18,EFNA1,VASH1,AMOT,PLEKHG5,NOTCH1,DAB2IP,CDH5,MIR23A,FGF16,SOX18	turquoise	EC_M6
GO:0001935	2.83E-09	0.999999999	38	123	endothelial cell proliferation	BP	1.38E-06	FLT4,PDGFB,TEK,KDR,CAV2,NRP1,GATA2,PRKD2,PPARG,TIE1,SCARB1,NR4A1,MEF2C,VEGFC,THBS4,PPP1R16B,PLCG1,DLL4,STAT1,PRKX,CD34,PROX1,PRKD1,DLG1,ITGA4,HIF1A,EPHA2,ALDH1A2,MTOR,PGF,VASH1,CCL2,LRG1,NRARP,EGFL7,MIR23A,BMP2,BMP6	turquoise	EC_M6
GO:0040011	3.45E-09	1	218	1349	locomotion	BP	1.62E-06	TNFRSF11A,SEMA6A,KIF5A,TNFAIP3,AKAP12,FLT4,CTHRC1,CELSR1,AFDN,ARHGAP18,PTK2,ANKS1A,PDGFB,PODXL,STRIP2,TEK,LIMCH1,DDX58,ATP8A1,KIT,KDR,CD2AP,SYK,SEMA4D,EFHC1,IL6,CXCL8,AVL9,ELMO1,DOCK4,GRB10,TLR4,TCAF2,C5,SDCBP,NRCAM,TRPM4,NRP1,PIK3CG,LAMA4,GATA2,PRKD2,NOVA2,LRCH1,PLXND1,PRCP,NOS3,ITGB4,DACH1,PPARG,GATA3,CXCR4,EDNRB,NET1,TIE1,JAG1,P2RY6,SFRP1,HBEGF,FEZ2,FERMT1,EFNB2,ARHGEF7,SPEF1,NTN4,RIC8A,PRAG1,ADAMTS9,PRR5L,SCARB1,EPHB1,PLK2,RET,MERTK,IGF1,NR4A1,ABHD6,SH3RF1,MCTP1,HEXB,MEF2C,VEGFC,NDRG4,GPM6A,CXCL10,ANGPT2,ADGRG3,THBS4,PLCG1,STC1,NR4A2,DGKZ,ANO6,DLL4,PIK3C2B,NEDD9,EDN1,JAM3,SNCA,SRGAP2C,ETS1,CD200,LCK,PPP3CA,HYAL2,SEMA3F,RIN3,ROBO4,PRKX,LYST,NFATC2,AP1AR,CD34,PECAM1,PLEKHO1,PLXNA2,FGR,MCAM,INPP5F,KALRN,IGFBP3,FES,PROX1,PRKD1,VANGL2,LAMA5,F11R,YTHDF1,ARHGDIB,MAP3K3,ACE,ITGA6,DOCK1,LAMC2,APOD,CREB1,CLEC14A,CCRL2,ADORA2A,SEMA6D,EPHB4,ITGA4,IGFBP5,TBX1,MCU,LDB2,SELE,SELP,PLVAP,FGF23,PPM1F,WAS,RAC1,HIF1A,RCC2,RHOJ,SYNE2,PLD2,JUP,ADIPOR2,FSCN1,EPHA2,MMRN2,MCC,MTOR,OPHN1,EFNB1,PSEN1,STK10,SEMA4A,HOXB9,FGF18,ABI3,ITGA3,PGF,DAPK2,TAOK2,EFNA1,VASH1,RPS6KA5,S100A14,CDC42BPB,MMP28,AMOT,CCL2,YES1,PDE4B,LGALS9,SPEF2,STK26,MYO18A,SEMA6B,ARHGAP4,PLEKHG5,NOTCH1,PLCG2,S1PR1,ENG,LHX6,DAB2IP,CARMIL2,RIPOR1,CDH5,MIR23A,ADGRG1,VASP,SOX17,RELN,FGF16,SOX18,STX3,MGAT3,MSMP,RALA,BST2,BMP2,CBLL1,ARPC5L	turquoise	EC_M6

GO:0051239	3.55E-09	1	291	1937	regulation of multicellular organismal process	BP	1.62E-06	ZNF516,TNFRSF11A,SEMA6A,IFNGR1,TNFAIP3,AKAP12,TSPAN8,LAPTM4B,FLT4,RIMS2,NOTCH4,TCF7,CDKL3,TRDN,PTK2,C6orf106,ATF4,PDGFB,MAPK13,PIM1,LRRC19,TEK,LINGO2,DDX58,KIT,TNFRSF21,KDR,CD2AP,SYK,GADD45G,SEMA4D,IL6,IGF2BP3,REL,CXCL8,PELI1,RSAD2,DOCK4,GRB10,TSPAN12,TLR4,C5,DLGAP3,IL15,SDCBP,MYCN,NOCT,TRPM4,NRP1,PIK3CG,ARHGAP42,TAL1,LAMA4,MTMR2,MGLL,GATA2,PRKD2,CST7,NOVA2,CTSC,GPR4,PLXND1,PRCP,NOS3,PPARG,GATA3,ACVR2B,CXCR4,EDNRB,SPTBN4,TIE1,IKBKB,JAG1,P2RY2,TMEM173,GPAT4,SFRP1,HBEGF,NOTUM,FERMT1,EFNB2,NTN4,CD36,TMEM178A,LMOD3,ADAMTS9,SCARB1,EPHB1,PLK2,RET,PDE4D,MERTK,CHD7,IL1A,IGF1,NR4A1,KLKB1,EIF2AK3,ABHD6,HHEX,KDM3A,ITCH,ERBIN,SH3RF1,SMAD1,HIP1R,PROCR,MEF2C,TLR2,WWTR1,IL7,P2RX7,VEGFC,GUCY1A1,CXCL10,ANGPT2,SFMBT1,BMP2K,OAS3,THBS4,PPP1R16B,PLCG1,STC1,RAPGEF3,KCNJ3,NR4A2,STAB1,HIPK1,ANO6,DLL4,SOX13,EDN1,STAT1,CD58,JARID2,MYLIP,ETS1,CD200,PDE9A,HDAC1,PPP3CA,HYAL2,ICOSLG,KCNJ2,MEF2A,SEMA3F,PRKX,LAPTM5,HLX,NCF1,FABP4,HCLS1,ZFHX2,INPP5D,EAF2,EPB41,CD34,CD86,PLXNA2,FGR,TRAK2,FES,PROX1,SS18L1,PRKD1,LAMA5,F11R,ARHGDIB,MAP3K3,ACE,DOCK1,APOLD1,DLG1,ELL3,APOD,CREB1,B2M,LTF,ADORA2A,SEMA6D,CYBB,IL1RAP,BCOR,ADAMTS7,DDX3X,SOS2,IGFBP5,HOMER3,ETV4,TMEM106A,CALCRL,HSPG2,WNK4,RAMP2,PROM1,FGF23,PPM1F,PRKCH,RAC1,HIF1A,RHOJ,JUP,ADIPOR2,BCL6B,EPHA2,ITPKB,MMRN2,SYT7,TLR6,MCC,LFNG,TNFRSF1B,RGS14,ARG2,MTOR,AR,PSEN1,HOXB7,SEMA4A,HOXB8,FGF18,ITGA3,PGF,B	turquoise	EC_M6
GO:0065008	3.94E-09	1	392	2770	regulation of biological quality	BP	1.75E-06	ZNF516,TNFRSF11A,RDH16,DMXL1,SEMA6A,LVRN,ALDH8A1,TNFAIP3,STX11,TRIM39,AKAP12,LYZ,TSPAN8,LAPTM4B,CNOT6,SNCAIP,MYCT1,FZD6,RIMS2,CDC42SE2,CDKL3,TRDN,ARHGAP18,L3MBTL3,KLHL3,PTK2,HSD17B3,ATF4,PDGFB,AGTPBP1,BAIAP2L2,PICK1,PIM1,CDC42EP1,STRIP2,LRRC19,TEK,LINGO2,IREB2,ATP8A1,APBA1,TJP2,TEC,ADGRF5,KIT,KDR,SRD5A3,SYK,GADD45G,SEMA4D,RNF149,ICA1,TMOD1,IL6,PPT1,PTBP3,VPS54,DOCK4,CAV2,GRB10,TLR4,CPT1C,GRIK2,IL15,FAM49A,NRCAM,NOCT,TUT4,TRPM4,NRP1,RAB3B,PUM2,RASIP1,PIK3CG,ARHGAP42,SVIL,TAL1,LAMA4,MTMR2,GATA2,NOVA2,RAB38,GPR4,FGD5,PLXND1,PRCP,NOS3,GGA3,PPARG,GATA3,ACVR2B,CXCR4,EDNRB,TMC8,SPTBN4,SETD5,P2RY6,P2RY2,FCHSD2,PDE2A,GPAT4,SFRP1,NR1D2,DND1,KCNK6,POC1B,ATP11A,FCHSD1,P2RX2,FGD6,SLC4A11,CD36,SIRT3,TMEM178A,LMOD3,PRAG1,MICU3,SCARB1,EPHB1,PLK2,GAPT,MALL,RET,PDE4D,MERTK,CHD7,FAM3D,IL1A,IGF1,NR4A1,KLKB1,ABHD6,KDM3A,ERBIN,SMAD1,CLCN3,HIP1R,OCLN,PROCR,MCTP1,HEXB,MEF2C,CP,TLR2,WWTR1,IL7,P2RX7,VEGFC,ENTPD1,GPM6A,GUCY1A1,CXCL10,SEPT11,TIPARP,USH1C,TPCN1,PLCG1,STC1,KCNJ3,DGKZ,SLC38A1,IQGAP2,ANO6,BAZ2B,DNMBP,IFIT1,NAV2,EDN1,STAT1,JAM3,SNCA,ALAS1,MMRN1,DTNBP1,MYLIP,GAS2,ANO3,PM20D1,ABCG1,ETS1,SHANK2,LPCAT1,MTTP,HDAC1,LCK,PPP3CA,HYAL2,ANO1,SLC39A8,KCNJ2,TESMIN,SLC12A7,SEMA3F,SCN3A,MCTP2,RIN3,HCRTR1,SNAP23,ESAM,LAPTM5,DYRK3,USP13,SLC9A8,FABP4,HCLS1,AP1AR,INPP5D,ATP9A,EPB41,CD34,PECAM1,PLEKHO1,PLXNA2,POLG,FGR,ADCY1,FES,TMOD4,PLEKHA1,PR	turquoise	EC_M6
GO:0045935	2.68E-08	1	203	1489	positive regulation of nucleobase-containing compound metabolic process	BP	1.81E-06	PAN2,ONECUT2,BCLAF1,CCT2,DDX39B,SCAF8,MAP3K4,DSCC1,HSF2,ATAD2,XRCC6,PHF5A,TNRC6B,TUT7,IFT74,POT1,PWP1,ZNF300,TOPORS,RFX3,TRIM28,NCBP1,ZNF451,FOXJ3,MSH2,PHIP,HNRNPA2B1,PNPT1,RLF,ZNF292,CCT4,ITGB1BP1,THRAP3,CCNC,NAA15,ELF2,ATAD2B,HDAC2,CCNA2,EXOSC9,MLLT10,DNAJC2,NUP85,ELF1,RSF1,DIS3,CCNT2,PAXIP1,WDR43,SUPT16H,CHD8,KAT6A,METTL3,RIF1,E2F7,ALYREF,CKAP2,CEP290,NSD3,CEBPZ,TAF7,EXOSC8,RFC3,BRCA2,ELK3,TCERG1,PRKDC,CCNB1,ERCC6,PAN3,GPBP1,SP1,TCEA1,ZC3H8,DIMT1,TNKS2,ZNF143,CENPJ,NCK1,SMARCA5,ZNF507,OTUD4,TAF9,PDE12,TFCP2,ATR,UTP15,HLTF,CNOT1,GABPA,MCRS1,USP16,CCT7,NBN,SENP1,ABRAXAS1,RAD51,SLC38A2,RIPK1,WDR75,ARID2,MYBL2,E2F8,INO80,RIOK1,CHUK,HIVEP1,RFC5,NOLC1,CCT5,BMPR1B,UBE3A,CCT6A,PRDM10,NUCKS1,GCN1,CHEK1,H2AFZ,RRP1B,NFKB1,TET2,TRIP11,HEATR1,NCL,NOL11,GTF2I,BPTF,DDX5,FANCB,RFC2,NEK2,BLM,EDRF1,ING5,BCLAF3,FOSL1,SMARCC1,SP3,DHX9,HNRNPR,TRA2B,RFC4,CCAR1,UBTF,NMD3,XRCC5,NPAT,BRCA1,CNOT9,NSD2,RAD51AP1,DCAF6,DHX33,TBL1XR1,LEO1,FOXM1,LYAR,DDX11,HNRNPU,SMARCE1,RFC1,TOP2A,DCP2,NSD1,SRSF5,MED6,ICE2,CCT3,PMF1,FUS,AURKB,ZNF609,OGT,ATRX,ZNF711,ILF2,TRIM37,ILF3,MED13,AATF,TAF15,THOC1,SMCHD1,NIPBL,MYSM1,UHRF1,RBMX,METTL16,TP73,DAZAP1,CHTF18,EXOSC2,CTCF,PRIM2,HNRNPD,DRAP1,ZNF770,TIGAR,NAF1,RNF8,ZNF782,ZFP62,SFR1	darkgrey	EC_M8

GO:0051049	3.59E-09	1	244	1276	regulation of transport	BP	1.87E-06	CD63,LMAN1,ATP8B1,LRP1,AHI1,RAB21,SLC12A2,AZIN1,ANGPT1,PPARA,NUS1,PACSIN1,REEP2,PPARD,MKLN1,UBQLN1,SH3GL2,LEP,PLAA,VEGFA,UNC13B,CLOCK,EXOC1,REST,RAB23,P3H1,RIMS1,TMEM30A,ERLEC1,SHANK1,UBE2J1,MDFIC,GLI3,INHBA,BVES,VPS35,SLC7A11,ITGB1,FKBP1B,KIF5B,DNAJC1,RAB7A,IWS1,PICALM,PDCD6IP,SNX17,PAK1,TGFB1,AXL,RAB3GAP1,SCN5A,CTNNB1,IL15RA,AKT2,GPD1L,C1QTNF1,RAB2B,OSBPL8,ACTG1,ACTN4,PPP1R12A,STIM1,UFM1,ATP2C1,IRS2,PRNP,ATP2B1,HDAC3,RAB11FIP1,PCLO,SMPD1,ARL6IP5,REM1,CXCL12,FXYD1,TM9SF4,ANKRD1,REEP5,SNTA1,OSBP,VAMP8,SLMAP,ATP1B3,RIOK2,MAP1B,SORBS1,P2RX4,SELENOT,ATP2A2,CACNA1D,APP,ATXN2,CX3CL1,ABCC8,CACNB3,PTPN11,PTX3,PIP4P2,PFKM,HOMER1,ARHGAP1,ATG13,TSG101,CRY2,VPS18,PPP3CC,ADA,BAP1,CHP1,ATP2B4,SPP1,PKD2,PLA2R1,LGI3,BRSK2,DYNLL1,AZIN2,KDM5B,CALHM1,PKP2,DNM1L,SELENOS,CPT1A,TGFB2,LRP5,EPRS,FEZ1,GSK3B,HADH,ISCU,DISP1,HSPA8,ARV1,IDH2,ADCY5,AP2M1,OSM,CASQ1,PEA15,SCFD1,CLIC4,BACE1,FZD9,SIDT2,LINC02210-CRHR1,SERPINE1,COMT,TFR2,C11orf65,PTPN6,KCNK4,PDCD10,ITGAV,ADRA2C,LRPAP1,HTT,CLTCL1,PRKCI,PLD1,KCNA1,CYP19A1,WDR1,ATAD1,STX18,B3GAT3,HAP1,CACNA1C,NLGN2,STIM2,LMAN2,PPARGC1A,NPPB,MSN,SRCIN1,STX1B,SYT11,KCNMB1,DOCK2,TMED10,TGFB3,CACNA1G,RAB11A,MYH10,MAP2K1,CALM1,SYTL4,VMP1,SCAMP5,TRPV2,ACSL4,CD74,RASL10B,PDGFRB,MIEF2,ROCK1,AKT1,ITGA2,MYOM1,PRKAA1,RAB31,SUPT6H,MYO1C,BCAP31,ABCA3,FLNA,KCNAB2,MIDN,ATP6AP1,SSTR5,VAMP7,PARK7,R	darkgreen	EC_M4
GO:2000573	2.92E-08	0.999999995	21	60	positive regulation of DNA biosynthetic process	BP	1.97E-06	CCT2,DDX39B,MAP3K4,DSCC1,POT1,CCT4,RFC3,TNKS2,ATR,CCT7,RFC5,CCT5,CCT6A,RFC2,NEK2,RFC4,XRCC5,CCT3,AURKB,CHTF18,NAF1	darkgrey	EC_M8
GO:0019439	3.01E-08	1	68	375	aromatic compound catabolic process	BP	2.02E-06	PAN2,POP1,EIF3E,TNRC6B,TUT7,NCBP1,LRPPRC,PNPT1,SYNCRIP,PABPC4,THRAP3,EXOSC9,RNASEH2B,TRDMT1,CNOT10,UPF2,MBD4,DIS3,XRN2,AFMID,METTL3,EXOSC8,PAN3,MTREX,TDG,XRN1,PDE12,ZCCHC8,TTC37,CNOT1,TENT2,NSUN2,TET2,FXR1,DDX5,FASTKD1,SSB,PDE8A,DHX9,HNRNPR,FASTKD2,DNA2,CNOT9,HNRNPU,MRPL44,DCP2,DCTPP1,EXOSC10,FUS,DIS3L,LARP1,TAF15,UPF3B,HNRNPM,SERBP1,AIFM1,GSPT1,HPRT1,DROSHA,FMR1,METTL16,RNPS1,EXOSC2,RBM8A,FASTKD5,HNRNPD,NAF1,FEN1	darkgrey	EC_M8
GO:0042274	3.19E-08	0.999999994	21	64	ribosomal small subunit biogenesis	BP	2.13E-06	SRFBP1,RRP7A,NOL10,MRPS7,PRKDC,DHX37,UTP20,BMS1,RRS1,RPS6,RIOK1,WDR3,FP565260.1,HEATR1,NOL11,NGDN,XRCC5,NOP14,RIOK3,UTP6,NOB1	darkgrey	EC_M8
GO:0022610	4.61E-09	1	210	1050	biological adhesion	BP	2.26E-06	CD63,IL23A,LRP1,CDSN,PLXNB2,ANGPT1,PPARA,HLA-DRA,FAM49B,ADGRB1,PPARD,CTNNA1,MKLN1,LGALS1,MDGA1,MYH9,LEP,SRF,NUAK1,VEGFA,DOCK8,NCK2,CHST10,DST,COL15A1,SOCS5,TPBG,LRRC4B,GLI3,ROCK2,BVES,RRAS,ZYX,SLC7A11,PCDH18,ITGB1,PLXNA1,APBB1IP,FBLN2,CGREF1,TENM4,GLI2,PCDH17,TGFB1,AXL,LMO7,CTNNB1,LGALS3BP,C1QTNF1,ACTG1,ACTN4,PPP1R12A,SPINT2,ATP2C1,PRNP,SIGLEC1,ZNF703,VEZT,ILK,HSPH1,SPINK5,APLP1,AKIP1,CXCL12,TM9SF4,FAT1,IL20RB,PALLD,CTNND1,ROBO1,CHST2,SORBS1,LOXL3,RASA1,ATP2A2,EDIL3,MYL9,APP,PLEKHA7,CX3CL1,PTPN11,CDH17,LOXL2,AP3B1,AMIGO2,PTPN22,ACVR1,ADA,SPP1,RUNX1,CBLB,PDLIM5,ELMO2,FAP,PHLDB2,TJP1,CTTN,PKP2,HYAL1,TGFB2,FBLN5,FEZ1,PRKAR1A,GSK3B,MMP14,DAG1,PIK3CA,LEF1,CORO1C,ANOS1,PRKCA,ACAN,HAPLN3,COL6A1,BMP7,COL6A3,ITGB5,IQGAP1,NECTIN4,S100A11,TFRC,FARP2,LRRC15,OLR1,HSPB1,COL26A1,SERPINE1,LPP,ITGA2B,TNR,FN1,PTPN6,IGFBP2,FBN1,ITGAV,COMP,ARPC2,AOC3,PKD1L1,COL3A1,VCL,GLDN,OBSL1,ROM1,NINJ2,NLGN2,PCDH7,ADGRL1,ACTN1,MSN,SRCIN1,FGFRL1,TGFB1I1,TLN2,TPM1,THBS3,COL1A1,SHC1,PBXIP1,MYH10,VMP1,NPTN,FAT2,ICAM5,ICAM4,CD74,LAMA3,ROCK1,AKT1,ITGA2,EGFLAM,PTPN2,CD40LG,MYO10,CD99L2,DAPK3,MXRA8,PLXNB3,TNFRSF18,PKD1,IRAK1,NPHP4,FLNA,PLXNA3,BSG,COL5A1,NTNG2,FIBCD1,CDH13,CERCAM,CFDP1,BCL10,NEXN,FSTL3,CD151,PCDHB2,PDLIM1,VSTM2L,CD164,COL6A2,CSRP1,THY1,HAS2,STXBP6,EDA,FGL2,MFAP4,DUSP22,PRKG1,COL8A2	darkgreen	EC_M4

GO:0007155	4.63E-09	1	209	1044	cell adhesion	BP	2.26E-06	CD63,IL23A,LRP1,CDSN,PLXNB2,ANGPT1,PPARA,HLA-DRA,FAM49B,ADGRB1,PPARD,CTNNA1,MKLN1,LGALS1,MDGA1,MYH9,LEP,SRF,NUAK1,VEGFA,DOCK8,NCK2,CHST10,DST,COL15A1,SOCS5,TPBG,LRRC4B,GLI3,ROCK2,BVES,RRAS,ZYX,SLC7A11,PCDH18,ITGB1,PLXNA1,APBB1IP,FBLN2,CGREF1,TENM4,GLI2,PCDH17,TGFB1,AXL,LMO7,CTNNB1,LGALS3BP,C1QTNF1,ACTG1,ACTN4,PPP1R12A,SPINT2,ATP2C1,PRNP,SIGLEC1,ZNF703,VEZT,ILK,HSPH1,SPINK5,APLP1,AKIP1,CXCL12,TM9SF4,FAT1,IL20RB,PALLD,CTNND1,ROBO1,CHST2,SORBS1,LOXL3,RASA1,ATP2A2,EDIL3,MYL9,APP,PLEKHA7,CX3CL1,PTPN11,CDH17,LOXL2,AP3B1,AMIGO2,PTPN22,ACVR1,ADA,SPP1,RUNX1,CBLB,PDLIM5,ELMO2,FAP,PHLDB2,TJP1,CTTN,PKP2,HYAL1,TGFB2,FBLN5,FEZ1,PRKAR1A,GSK3B,MMP14,DAG1,PIK3CA,LEF1,CORO1C,ANOS1,PRKCA,ACAN,HAPLN3,COL6A1,BMP7,COL6A3,ITGB5,IQGAP1,NECTIN4,S100A11,TFRC,FARP2,OLR1,HSPB1,COL26A1,SERPINE1,LPP,ITGA2B,TNR,FN1,PTPN6,IGFBP2,FBN1,ITGAV,COMP,ARPC2,AOC3,PKD1L1,COL3A1,VCL,GLDN,OBSL1,ROM1,NINJ2,NLGN2,PCDH7,ADGRL1,ACTN1,MSN,SRCIN1,FGFRL1,TGFB1I1,TLN2,TPM1,THBS3,COL1A1,SHC1,PBXIP1,MYH10,VMP1,NPTN,FAT2,ICAM5,ICAM4,CD74,LAMA3,ROCK1,AKT1,ITGA2,EGFLAM,PTPN2,CD40LG,MYO10,CD99L2,DAPK3,MXRA8,PLXNB3,TNFRSF18,PKD1,IRAK1,NPHP4,FLNA,PLXNA3,BSG,COL5A1,NTNG2,FIBCD1,CDH13,CERCAM,CFDP1,BCL10,NEXN,FSTL3,CD151,PCDHB2,PDLIM1,VSTM2L,CD164,COL6A2,CSRP1,THY1,HAS2,STXBP6,EDA,FGL2,MFAP4,DUSP22,PRKG1,COL8A2	darkgreen	EC_M4
GO:0048646	5.33E-09	1	155	877	anatomical structure formation involved in morphogenesis	BP	2.30E-06	SEMA6A,TNFAIP3,PTPRB,FLT4,FZD6,CTHRC1,CELSR1,NOTCH4,PTK2,AGTPBP1,PODXL,PIM1,MFNG,TEK,PGM5,KDR,SYK,THSD7A,SHB,TMOD1,IL6,CXCL8,TSPAN12,C5,TEAD2,NRCAM,NRP1,RASIP1,PIK3CG,TAL1,MTMR2,GATA2,PRKD2,GPR4,PLXND1,NECTIN2,PRCP,ITGA8,NOS3,ITGB4,UNC13D,PPARG,GATA3,ACVR2B,CXCR4,TIE1,JAG1,SFRP1,EFNB2,CD36,LMOD3,ADAMTS9,TCF15,EPHB1,PLK2,RET,IL1A,NR4A1,EIF2AK3,HHEX,SMAD1,SOX7,MEF2C,TLR2,VEGFC,CXCL10,ANGPT2,THBS4,PPP1R16B,PLCG1,RAPGEF3,STAB1,HIPK1,TANC1,DLL4,EDN1,STAT1,JAM3,ETS2,NFKB2,ETS1,HDAC1,MEF2A,ROBO4,NRAP,PRKX,SEC24B,NFATC2,CD34,PLEKHO1,PLXNA2,MCAM,CFLAR,PROX1,TMOD4,PRKD1,VANGL2,MAP3K3,APOLD1,APOD,NBEAL2,TET1,CLEC14A,CYBB,EPHB4,ITGA4,TBX1,CALCRL,HSPG2,RAMP2,PROM1,TMOD2,HIF1A,RHOJ,JUP,ADIPOR2,EPHA2,MMRN2,CECR2,MPP5,LFNG,ALDH1A2,AR,OPHN1,PSEN1,SEMA4A,FGF18,ITGA3,PGF,EFNA1,VASH1,NPR1,SMAD2,AMOT,CCL2,JAK1,LRG1,NRARP,EGFL7,NOTCH1,S1PR1,ENG,DAB2IP,CDH5,MIR23A,ADGRG1,VASP,SOX17,RELN,SOX18,DUSP5,TBC1D20,PERP,RALA,BMP2	turquoise	EC_M6
GO:0034661	3.49E-08	0.999999996	16	35	ncRNA catabolic process	BP	2.32E-06	POP1,PNPT1,EXOSC9,TRDMT1,DIS3,EXOSC8,MTREX,XRN1,ZCCHC8,TENT2,NSUN2,MRPL44,EXOSC10,DIS3L,DROSHA,EXOSC2	darkgrey	EC_M8
GO:0010646	5.02E-09	1	435	2513	regulation of cell communication	BP	2.35E-06	CD63,IL23A,LRP1,ARHGEF25,TAB2,DYRK2,RNF19A,RRM2B,SLC12A2,FBN2,ANGPT1,PPARA,ROS1,RSPO3,FAM49B,PTP4A3,ADGRB1,LY6E,PPARD,CTNNA1,CBY1,UBQLN1,MTCH1,LGALS1,KLHDC10,GNAQ,ARHGAP39,LEP,CDKN2B,KIAA1324L,SRF,NUAK1,CDK14,VEGFA,DOCK8,VCP,SLC35B2,RUNX2,RASL11B,UNC13B,NCK2,CLOCK,CREB3,REST,ASPN,SNX13,TGFBR1,SOCS5,RIMS1,RTN4,TPBG,SHANK1,ADAMTS3,EMC10,MAP3K7,MDFIC,GLI3,NCDN,ROCK2,INHBA,RRAS,RCN3,DKKL1,VPS35,SLC7A11,ITGB1,PLEKHA4,FKBP1B,FGF2,KIF5B,SESN3,RAB7A,ARHGEF4,RB1,TPT1,PTCH2,KCTD21,GLI2,MEGF8,PCDH17,RHEB,PAK1,TGFB1,AXL,RAB3GAP1,SEC13,CTNNB1,LBH,DYNC1LI1,GPD1L,ZEB2,CANT1,C1QTNF1,ARHGEF17,OSBPL8,CXXC5,PSD2,NPLOC4,ACTN4,ANAPC15,LTBP1,CRIM1,SPRED3,UFM1,ATP2C1,IRS2,PRNP,DCN,HDAC3,RAB11FIP1,ZNF703,ACPP,STARD13,KCTD16,CAVIN3,UBA5,DDRGK1,SMPD1,PPP2CB,ILK,RBPMS,LEPROTL1,ARL6IP5,APLP1,RYK,TMEM127,CNOT7,RNF6,TMEM9B,ST5,AKIP1,CXCL12,SLC20A1,ANKRD1,PJA2,TMED2,CD180,ZNF536,OSBP,SULF1,VAMP8,GRK7,CTNND1,DKK3,ROBO1,WWC2,MAP1B,SORBS1,LOXL3,P2RX4,RNF13,FAIM2,HEY1,RASA1,SELENOT,ATP2A2,DLGAP4,TCTN1,APP,DHX36,RAPGEF2,MAPKAPK5,CX3CL1,ABCC8,CACNB3,PTPN11,RALGAPB,PFKM,HOMER1,OPA1,ARHGAP1,AMFR,PTPN22,TSG101,ACVR1,CRY2,VPS18,CHAC1,GDF6,SERINC3,MTDH,ADA,CHP1,ATP2B4,SPP1,PKD2,PLA2R1,HIF1AN,CBLB,NKD1,TANK,BRSK2,SLC39A10,RAB7B,CHRNA7,EIF4E,DYNLL1,ADIPOR1,KDM5B,NOTCH2,CTTN,CDC73,DNM1L,GLRX2,UCHL5,SELENOS,CHSY1,SLC2A10,EYA2,CAPRIN2,CPT1A,TGFB2,LRP5,	darkgreen	EC_M4

GO:0071702	5.13E-09	1	338	1891	organic substance transport	BP	2.35E-06	CD63,LMAN1,ATP8B1,LRP1,STX7,AP3S1,AGAP2,CANX,TBC1D9B,RAB21,RABL2A,SLC12A2,AZIN1,ANGPT1,TCP1,PPARA,SLC22A4,NUP50,NUS1,HSPA4,SEC24A,SLC45A4,REEP2,LY6E,PPARD,SIL1,GGA1,PLIN2,MYH9,LEP,PEX6,VCP,SLC35B2,UNC13B,CLOCK,EXOC1,SRP72,REST,LMAN2L,RAB23,SNX13,P3H1,RIMS1,SLC17A5,TMEM30A,ERLEC1,COX18,MFSD2A,SLC35A1,UBE2J1,MDFIC,GLI3,INHBA,VPS41,RCN3,VPS35,MGARP,SLC7A11,ITGB1,PGRMC2,LIN7B,MFSD4B,FKBP1B,SLC37A3,KIF5B,SEC61A1,NAPA,DNAJC1,RAB7A,SLC36A4,COPG1,IWS1,PDCD6IP,SNX17,PAK1,TGFB1,SRP68,RAB3GAP1,SEC13,ESYT2,TRAK1,AKT2,SEC23B,OSBPL10,C1QTNF1,TBC1D16,SLC4A7,RAB2B,OSBPL8,SLC38A10,NPLOC4,ACTN4,STAM2,COG6,UFM1,ATP2C1,IRS2,PRNP,HDAC3,RAB11FIP1,PCLO,ARL6IP5,WASHC2A,VPS37A,ARL1,CXCL12,TM9SF4,RAB2A,ANKRD1,HSP90B1,TMED2,SLC25A4,COPB2,OSBP,VAMP8,TRAM1,SORBS1,P2RX4,COPB1,SELENOT,USO1,SDAD1,ATXN2,SLC4A5,SLC33A1,ABCC8,CACNB3,SSR3,PTPN11,EXOC6B,PFKM,SLC26A7,CDH17,ARHGAP1,ATG13,AP3B1,TSG101,CRY2,VPS18,SERINC3,ADA,AKTIP,BAP1,CHP1,SPP1,PLA2R1,AP2A2,CBLB,BRSK2,VPS39,RAB7B,ARL3,DYNLL1,AZIN2,APBA2,KDM5B,CALHM1,SRPRA,CTTN,SLC35A5,DNM1L,SELENOS,SLC2A10,KPNA6,SEC22B,CPT1A,TGFB2,LRP5,EPRS,ZMAT3,GSK3B,HADH,DISP1,PITPNM1,SGSM1,HSPA8,PITPNB,ARV1,KPNA1,IDH2,AP1B1,SEC22A,ADCY5,AP1S2,PRELID3B,GOLT1B,AP2M1,FABP2,PEA15,COPA,OSBPL2,SCFD1,KIF1A,SNX27,OSBP2,TOMM40L,HDLBP,TFRC,SIDT2,SRP54,LSG1,HSPB1,LINC02210-CRHR1,SEC23A,AP1S1,SYVN1,SCAMP3,TANGO2,TFR	darkgreen	EC_M4
GO:0002682	6.15E-09	1	153	915	regulation of immune system process	BP	2.58E-06	TNFAIP3,GRAMD4,TCF7,PTK2,C6orf106,LRRC19,DDX58,TEC,ADGRF5,KIT,TNFRSF21,SYK,IL6,CXCL8,PELI1,RSAD2,TLR4,C5,IL15,TRPM4,PUM2,TAL1,GATA2,PRKD2,CST7,CTSC,LRCH1,NECTIN2,GAB2,UNC13D,PPARG,GATA3,IRAK2,SEC14L1,IKBKB,JAG1,TMEM173,SFRP1,CD36,TMEM178A,MAP3K1,PLK2,PDE4D,MERTK,IL1A,IGF1,ITCH,ERBIN,SH3RF1,MEF2C,TLR2,IL7,VEGFC,CXCL10,OAS3,NLRC5,THBS4,PLCG1,DGKZ,ANO6,SOX13,IFIT1,EDN1,STAT1,JAM3,SNCA,ETS1,CD200,LCK,ICOSLG,RIN3,LAPTM5,HLX,NCF1,HCLS1,NFATC2,INPP5D,TIFA,CD86,BMX,FGR,FES,PLEKHA1,YTHDF1,PAK2,DLG1,APOD,CREB1,B2M,LTF,ADORA2A,MASP1,ITGA4,DDX3X,SOS2,MCU,BIRC3,SELE,SELP,PLVAP,WAS,PRKCH,RAC1,HIF1A,PLD2,BCL6B,ITPKB,TLR6,TNFRSF1B,ARG2,MTOR,EFNB1,PSEN1,STK10,HOXB8,PGF,EIF2B2,DAPK2,CHRNB2,CD19,S100A14,GPR68,TNIP1,RCOR1,MMP28,CCL2,YES1,PDE4B,LGALS9,NOS2,IL7R,MYO18A,ISG15,AP3D1,NRARP,TRAF2,PLCG2,STXBP1,DAB2IP,FYB1,HAVCR1,OAS1,VNN1,TLR1,GBP5,CD46,CA2,TRIM62,FCER2,SERPINB9,HIST1H4D,BST2,RNF125	turquoise	EC_M6
GO:0048513	6.46E-09	1	437	2529	animal organ development	BP	2.87E-06	IL23A,ATP8B1,NAB2,LRP1,CDO1,AHI1,TAB2,CDSN,RRM2B,PLXNB2,FBN2,ANGPT1,PPARA,HLA-DRA,COL11A2,RSPO3,TRAPPC9,ADGRB1,LY6E,PPARD,CTNNA1,TEAD3,SUN2,CBY1,LGALS1,MDGA1,MYH9,LEP,CDKN2B,LARGE1,RORB,SRF,VEGFA,RUNX2,COL1A2,CLOCK,ASNS,REST,ASPN,CNNM4,RAB23,P3H1,TGFBR1,HIVEP3,SOCS5,UBA6,RTN4,MFSD2A,MYH14,AKIRIN1,WHRN,GLI3,ROCK2,INHBA,BVES,RRAS,RCN3,MYLK3,SLC7A11,OSTM1,PCDH18,ITGB1,PGRMC2,PLEKHA4,DBP,PLXNA1,FGF2,KIF5B,SEC61A1,NAPA,RB1,EED,PICALM,EIF2B3,PTCH2,TENM4,GLI2,MEGF8,SMARCD3,RHEB,SNX17,PAK1,TGFB1,AXL,RAB3GAP1,SCN5A,WDR48,HTR5A,HNMT,CTNNB1,IL15RA,LBH,DGAT2,SERPINH1,AKT2,RELT,SLC4A7,SLC38A10,RARB,KAT2B,TACC1,STIM1,SPRED3,SPINT2,UFM1,LIG4,IRS2,ATP2B1,LUM,DCN,ZNF703,DCLK1,SEMA3C,CCKBR,UBA5,DDRGK1,ILK,SPINK5,APLP1,RYK,PRICKLE2,SNPH,CXCL12,IMPAD1,ZSWIM6,SLC20A1,ANKRD1,FAT1,ADAMTS6,TMED2,PALLD,EIF2S2,MRAS,FLNB,EIF4G2,SULF1,CTNND1,DKK3,ROBO1,TRPC4AP,TEAD1,RDH10,SEMA4F,LOXL3,FAIM2,HEY1,SELENOT,TLX2,CELF1,TCTN1,APP,DHX36,SLC4A5,RAPGEF2,ACTG2,CPE,PTPN11,PTX3,HOMER1,OPA1,CDH17,LOXL2,AP3B1,AMIGO2,PTPN22,TSG101,ACVR1,PPP3CC,MMP2,GDF6,ADA,ATP2B4,SPP1,BMP1,PKD2,RUNX1,CSDE1,TMEM14C,SEMA4G,NKD1,DNAH5,MAN1A2,IFT57,PSMG1,PDLIM5,RAB7B,ARL3,DOCK10,EIF4E,DYNLL1,INA,ADIPOR1,PHLDB2,KDM5B,NOTCH2,PKP2,CDC73,DNER,UCHL5,CHSY1,HYAL1,SLC2A10,CCND1,CPT1A,SBDS,TGFB2,LRP5,FEZ1,HTR2B,SATB2,PRKAR1A,GSK3B,CASP7,FAM20C,MMP14,DAG1,PIK3CA,LEF1,CORO1	darkgreen	EC_M4

GO:0032501	7.53E-09	1	653	5003	multicellular organismal process	BP	3.07E-06	ZNF516,TNFRSF11A,TCF4,SEMA6A,LVRN,N4BP3,KIF5A,IFNGR1,TNFAIP3,HECA,AKAP12,LYZ,PTPRB,TSPAN8,LAPTM4B,FLT4,SYNJ2,FZD6,CTHRC1,RIMS2,OXR1,CELSR1,QKI,NOTCH4,ASF1A,TCF7,CDKL3,TRDN,MTSS1,ASAP1,L3MBTL3,KLHL3,PTK2,HSD17B3,C6orf106,ANKS1A,ATF4,PDGFB,AGTPBP1,MAPK13,PODXL,CSNK1E,PICK1,KDM4C,MICALL1,PIM1,MFNG,CSF2RB,MDFI,OSTF1,LRRC19,TEK,LINGO2,IREB2,DDX58,ATP8A1,APBA1,TJP2,TEC,ADGRF5,KIT,ADGRF1,TNFRSF21,KDR,CD2AP,NFIL3,SYK,GADD45G,SEMA4D,EFHC1,THSD7A,SHB,TMOD1,RAPGEF5,IL6,IGF2BP3,REL,PPT1,PTBP3,CXCL8,VPS54,PELI1,UTP11,RSAD2,DOCK4,FIGNL1,FOXP2,CAV2,GRB10,TSPAN12,NOBOX,TLR4,GRIK2,C5,DLGAP3,GJA4,IL15,FZD8,CREM,TEAD2,SDCBP,MYCN,NRCAM,NOCT,TUT4,TRPM4,NRP1,ZBTB24,IZUMO1,RASIP1,SPATA6,PIK3CG,ARHGAP42,SVIL,ITSN2,TAL1,LAMA4,PHF10,MTMR2,MGLL,EPC1,GATA2,DNMT3A,HS6ST1,PRKD2,CST7,NOVA2,CTSC,GPR4,SNRK,PLXND1,NECTIN2,PRCP,ITGA8,NOS3,PSG3,GAB2,ITGB4,DACH1,PPARG,TIMP4,GATA3,ACVR2B,MYO7A,CXCR4,LMBR1,GZF1,EDNRB,PPP1CB,SPTBN4,TIE1,GDPD5,SLCO2B1,SETD5,IKBKB,JAG1,P2RY2,PDE2A,TMEM173,GPAT4,SFRP1,HBEGF,NR1D2,DND1,FEZ2,NOTUM,FERMT1,KCNK6,FREM2,EFNB2,GOLGA3,ANKLE2,POC1B,ARHGEF7,ASH2L,SPEF1,ATP11A,PCDH1,PCDH12,ARHGAP26,P2RX2,NTN4,CD36,RIC8A,TMEM178A,LMOD3,PRAG1,ADAMTS9,SDCBP2,TCF15,SCARB1,EPHB1,PLK2,MALL,RET,PDE4D,MERTK,TNFRSF19,CHD7,IL1A,DNMT3B,IGF1,NR4A1,KLKB1,EIF2AK3,ABHD6,HHEX,KDM3A,ITCH,ERBIN,SH3RF1,PREX2,SMAD1,CLCN3,HIP1R,POU6F1,MARVELD2,OCLN,STOX2,	turquoise	EC_M6
GO:0007004	4.79E-08	0.999999991	22	63	telomere maintenance via telomerase	BP	3.17E-06	CCT2,MAP3K4,POT1,CCT4,TNKS2,XRN1,ATR,TERF1,CCT7,CCT5,CCT6A,GAR1,NEK2,XRCC5,HNRNPU,RFC1,DCP2,EXOSC10,CCT3,AURKB,RPA1,NAF1	darkgrey	EC_M8
GO:0022616	4.88E-08	0.999999997	12	20	DNA strand elongation	BP	3.22E-06	POT1,MCM3,POLE,RFC3,MCM4,NBN,DCLRE1B,NUCKS1,POLA1,RFC4,DNA2,MCM7	darkgrey	EC_M8
GO:0002181	2.36E-10	1	39	111	cytoplasmic translation	BP	3.56E-06	EIF3H,RPS18,RPL3,EIF3L,EIF3D,RPL8,RPS5,RPL28,RPS9,RPS7,RPS11,RPL18,RPS8,UNK,RPS3,RPS16,RPL15,EIF3K,EIF3F,EIF4B,RPS3A,RPLP2,EIF4H,RPS21,RPL35A,RPL11,YBX3,RPL27,UBA52,RPL7A,EIF3G,RPL23A,EEF2,DPH1,RPL13,RPS15,RPS19,RPL23,RPS20	cyan	EC_M2
GO:0031503	5.58E-08	0.999999981	43	186	protein-containing complex localization	BP	3.66E-06	XPOT,NUP107,DDX39B,SRSF3,IFT74,AKAP9,MIOS,ICK,NCBP1,YTHDC1,HNRNPA2B1,XPO1,WDR35,GRIP2,NUP85,UPF2,BIRC5,ALYREF,ANKS1B,RRS1,ABCE1,ATR,TERF1,NUP160,NUP93,NSUN2,CEP72,MCM3AP,RAE1,NUP133,SSB,SETD2,TPR,NMD3,RAB8A,HNRNPU,DDX39A,THOC1,THOC2,NUP155,NDC1,HASPIN,SARNP	darkgrey	EC_M8
GO:0065009	1.03E-08	1	331	2307	regulation of molecular function	BP	3.99E-06	TNFRSF11A,TNFAIP8,TNFAIP3,PHACTR2,TBC1D30,PLEKHG1,PTPRB,FLT4,FZD6,CTHRC1,GRAMD4,AFDN,TRDN,TPD52L1,ASAP1,ARHGAP18,SGSM3,PTK2,DENND3,C6orf106,DAPK1,PDGFB,CSNK1E,PIM1,TBC1D22B,MFNG,MDFI,TEK,RIC8B,DDX58,FAM122A,KIT,ADGRF1,KDR,SYK,SEMA4D,RGP1,PPP6R1,RAPGEF5,IL6,PPT1,CHN2,PELI1,RGS3,ARHGAP25,ELMO1,DOCK4,ASAP2,CAV2,TLR4,TCAF2,TRAF1,C5,DLGAP3,TBC1D9,CCNY,NRP1,KIAA1147,RASIP1,PIK3CG,ARHGAP42,ITSN2,TAL1,KDM4D,ARHGAP21,PRKD2,KCNRG,CST7,CAB39L,RCBTB2,LRCH1,FGD5,PLXND1,NOS3,LLGL2,RALB,USP6NL,PPARG,TIMP4,GATA3,TAF3,TBC1D4,ITIH5,IRAK2,EDNRB,NET1,RALGAPA2,NDFIP2,TMC8,TIE1,CYTH1,IKBKB,P2RY6,NFKBIB,TMEM173,SFRP1,HBEGF,RASGRP3,SH3BP5,ANKLE2,ARHGEF7,ARAP3,ARHGAP26,FGD6,CD36,RIC8A,SIRT3,SCARB1,EPHB1,PLK2,RET,PDE4D,MERTK,IL1A,IGF1,NR4A1,EIF2AK3,HHEX,ITCH,ERBIN,SH3RF1,PREX2,HIP1R,MMP24,ARHGEF3,SOX7,HMBOX1,ARHGEF28,MEF2C,TLR2,WWTR1,IL7,VEGFC,CXCL10,MADD,ADGRG3,FAM192A,BMP2K,SERINC5,OAS3,NLRC5,PPP1R16B,PLCB2,RAPGEF3,NR4A2,DGKZ,IQGAP2,CCNJL,DNMBP,IFIT2,IFIT1,NEDD9,EDN1,SNCA,DTNBP1,PM20D1,CD200,SIK1,HDAC1,ITPRIP,LCK,PPP3CA,HYAL2,HECW2,GIT2,SHOC2,RIN3,ADPRH,LAPTM5,NCF1,FABP4,PABPN1,HCLS1,EPB41,CD86,PLXNA2,ARHGEF12,SH3BP4,FGR,CFLAR,CASP10,ADCY1,KALRN,IGFBP3,PPP2R5A,RPS6KA1,PROX1,PRKD1,TOR1AIP1,F11R,OAZ3,ARHGDIB,ACE,ITGA6,DOCK1,PAK2,CST6,DLG1,SIPA1,MAP3K11,RNF2,B2M,CTTNBP2NL,EIF4A2,ALS2CL,LTF,ARHGAP27,BCR,ADORA2A,IL1RAP,EPHB4,ITGA4,DDX3X,SOS2,AGFG2	turquoise	EC_M6

GO:0022610	1.03E-08	1	179	1050	biological adhesion	BP	3.99E-06	SEMA6A,CELSR1,NOTCH4,AFDN,PARVG,MTSS1,PTK2,PDGFB,PODXL,CDC42EP1,TEK,LIMCH1,APBA1,TJP2,PGM5,KIT,TNFRSF21,KDR,CD2AP,CNTNAP3,SYK,SEMA4D,IL6,CXCL8,PELI1,IL15,NRCAM,NRP1,IZUMO1,PIK3CG,LAMA4,PRKD2,GPR4,PLXND1,NECTIN2,BCAM,ITGA8,ITGA9,ITGB4,UNC13D,GATA3,CXCR4,CD93,PPP1CB,CYTH1,JAG1,SFRP1,FERMT1,THSD1,FREM2,EFNB2,ARHGEF7,PCDH1,PCDH12,NTN4,CD36,RIC8A,MAGI1,ADAMTS9,SCARB1,EPHB1,RET,MERTK,IL1A,IGF1,ITCH,ERBIN,LYVE1,MMP24,IL7,ENTPD1,ANGPT2,THBS4,CYTIP,STAB1,SPARCL1,SOX13,NEDD9,CD58,JAM3,MMRN1,ETS1,CD200,LCK,PPP3CA,SLC39A8,ICOSLG,LSAMP,ROBO4,ESAM,PRKX,LAPTM5,HLX,CLDN34,CDH24,AP1AR,CD34,CADM3,PECAM1,CD86,PLXNA2,ICAM2,BMX,MCAM,FES,LAMA5,F11R,ARHGDIB,ITGA6,DOCK1,MELTF,LAMC2,DLG1,SIPA1,APOD,B2M,HMCN1,LTF,BCR,TMEM47,ADORA2A,IL1RAP,EPHB4,ITGA4,SELE,SELP,VWF,BAIAP2L1,PPM1F,RAC1,RCC2,JUP,EPHA2,ITPKB,ARG2,PEAR1,EFNB1,PSEN1,STK10,ITGA3,TAOK2,EFNA1,PCDH11X,DNM2,TNIP1,COL4A6,DSG2,DSC2,DSC3,CCL2,YES1,JAG2,JAK1,LGALS9,IL7R,SLITRK2,AP3D1,NRARP,EGFL7,NOTCH1,PIEZO1,S1PR1,CNN3,ENG,STXBP1,NFAT5,CDH5,PDZD2,VNN1,ADGRG1,CD46,RELN,STX3,PERP,VAMP3,BMP2,SCARF1,CBLL1,BMP6	turquoise	EC_M6
GO:0050794	1.06E-08	1	929	7581	regulation of cellular process	BP	3.99E-06	PARD6G,ZNF516,TNFRSF11A,BAZ2A,TCF4,TNFAIP8,SEMA6A,LVRN,PDE7B,IFNGR1,TNFAIP3,HECA,PLAGL1,TBC1D30,TRIM39,PLEKHG1,AKAP12,PTPRB,LAPTM4B,CNOT6,FLT4,SNCAIP,FZD6,CTHRC1,RIMS2,OXR1,ZBED4,CDC42SE2,GRAMD4,CELSR1,QKI,NOTCH4,AFDN,PACSIN2,TCF20,TCF7,CDKL3,TRDN,TPD52L1,NCOA7,MTSS1,RNF146,ASAP1,ARHGAP18,L3MBTL3,SGSM3,PTK2,DENND3,C6orf106,ANKS1A,DAPK1,ATF4,PDGFB,AGTPBP1,MAPK13,BRPF3,PODXL,CSNK1E,BAIAP2L2,PICK1,KDM4C,PIM1,CDC42EP1,MFNG,CSF2RB,NFYA,MDFI,OSTF1,LRRC19,TEK,LINGO2,IREB2,PRDM4,LIMCH1,RIC8B,DDX58,ATP8A1,NFX1,APBA1,DCAF12,PIP5K1B,FAM122A,TEC,SH3RF3,GNG11,ADGRF5,KIT,ADGRF1,TNFRSF21,KDR,CD2AP,NFIL3,SYK,GADD45G,SEMA4D,RNF149,EFHC1,RGP1,ICA1,SHB,TMOD1,TSPAN13,PPP6R1,RAPGEF5,IL6,IGF2BP3,NFE2L3,SNAP91,REL,PPT1,CREB5,PTBP3,CHN2,CXCL8,PPIE,BACH2,KBTBD2,PELI1,UTP11,RGS3,ARHGAP25,ELMO1,RSAD2,DOCK4,FIGNL1,FOXP2,CAV2,KLF11,GRB10,LANCL2,TSPAN12,NOBOX,TLR4,TCAF2,GRIK2,TRAF1,C5,DLGAP3,INPP4B,IL15,FZD8,CCNY,CREM,TEAD2,SDCBP,MYCN,FAM49A,NRCAM,NOCT,TUT4,TRPM4,NRP1,KTI12,RAB3B,ZBTB24,PUM2,RASIP1,BEND5,PIK3CG,ARHGAP42,SVIL,ITSN2,TAL1,LAMA4,PHF10,MTMR2,MGLL,EPC1,KDM4D,GATA2,DNMT3A,ARHGAP21,PRKD2,SAP130,KCNRG,CST7,NOVA2,CTSC,RAB38,CAB39L,GPR4,LRCH1,SNRK,FGD5,PLXND1,NECTIN2,PRCP,BCAM,ITGA8,NOS3,GGA3,GAB2,LLGL2,ITGA9,RALB,USP6NL,ITGB4,UNC13D,ZFAND6,DACH1,PPARG,TIMP4,GATA3,TAF3,ACVR2B,TBC1D4,ITIH5,CXCR4,LMBR1,IRAK2,GZF1,SEC14L1,EDNRB,PPP1CB,NET1,RAL	turquoise	EC_M6
GO:0032989	9.97E-09	1	135	609	cellular component morphogenesis	BP	4.30E-06	LRP1,RAB21,PLXNB2,PACSIN1,ADGRB1,SH3GL2,PLAA,SRF,VEGFA,RIMS1,RTN4,TPBG,SHANK1,MFSD2A,MYH14,ACTR2,KIDINS220,GLI3,MYLK3,ITGB1,PLXNA1,KIF5B,PICALM,TENM4,GLI2,MEGF8,PAK1,CTNNB1,TRAK1,FARP1,ACTG1,DCLK1,SEMA3C,ILK,RYK,RNF6,CXCL12,ANKRD1,PALLD,EIF4G2,ROBO1,MAP1B,DIP2B,SEMA4F,TLX2,TCTN1,APP,DHX36,RAPGEF2,PTPN11,OPA1,ZFYVE27,SPP1,SEMA4G,BRSK2,PDLIM5,NDN,CHRNA7,DOCK10,PHLDB2,NOTCH2,CTTN,DNM1L,LMOD1,CAPRIN2,FEZ1,PRKAR1A,GSK3B,DAG1,AUTS2,CORO1C,ANOS1,BMP7,FGFR2,CASQ1,PHLDB1,IQGAP1,KIF1A,TANC2,MAPT,TNR,FN1,NCKAP1,SKIL,SYNPO2L,SMURF1,NBL1,TNIK,VCL,WDR1,SIX4,OBSL1,CRMP1,NOTCH3,ENAH,TCAP,SRCIN1,NES,NGFR,TPM1,RAB11A,MAP2K1,SOX30,TTC8,DICER1,NPTN,ISLR2,TRPV2,LAMA3,PDGFRB,ROCK1,FLII,PLXNB3,PAFAH1B1,NTN3,PLXNA3,RERE,BSG,NTNG2,BARHL2,NEXN,DPYSL5,CCDC136,SPINK2,VSTM2L,GDNF,CSRP1,THY1,CHN1,ARTN,YWHAH,SDC2,MAP1A,OLFM1,CFL2	darkgreen	EC_M4
GO:0017148	6.83E-08	0.999999979	38	158	negative regulation of translation	BP	4.47E-06	PAN2,EIF3E,MRPL13,TNRC6B,TUT7,PNPT1,SYNCRIP,TIA1,DHFR,PUS7,EXOSC9,CNOT10,DIS3,METTL3,EXOSC8,PAN3,CAPRIN1,LSM14A,XRN1,PDE12,CNOT1,NCL,FXR1,DDX6,HNRNPR,TPR,CNOT9,EIF2AK1,DCP2,LARP1,ILF3,TYMS,FMR1,METTL16,EXOSC2,HNRNPD,RNF139,PAIP2	darkgrey	EC_M8

GO:0009719	1.12E-08	1	231	1202	response to endogenous stimulus	BP	4.70E-06	LRP1,AP3S1,CDO1,FBN2,PPARA,PPARD,CTNNA1,TXN2,SH3GL2,LEP,CDKN2B,KIAA1324L,SRF,RUNX2,RASL11B,COL1A2,CLOCK,ASNS,REST,ASPN,TGFBR1,SOCS5,MAP3K7,ACTR2,KIDINS220,GLI3,ROCK2,INHBA,ZYX,VPS35,MGARP,ITGB1,PGRMC2,FGF2,SESN3,GLB1,EIF2B3,MEGF8,PAK1,TGFB1,HTR5A,HNMT,CTNNB1,LBH,AKT2,ZEB2,OSBPL8,RARB,KAT2B,LTBP1,CRIM1,SPRED3,SPINT2,UFM1,IRS2,PRNP,ATP2B1,SMAD9,ZNF703,UBA5,DDRGK1,ILK,LEPROTL1,APLP1,RNF6,CXCL12,ANKRD1,HSP90B1,SULF1,DKK3,MAP1B,RYR3,SORBS1,P2RX4,HEY1,USO1,APP,RAPGEF2,SLC33A1,ABCC8,PTPN11,ADCY6,OPA1,ARSB,GTF2H1,PTPN22,ACVR1,CRY2,VPS18,MMP2,GDF6,ATP2B4,SPP1,CPN1,PKD2,EIF4E,ADIPOR1,KDM5B,NOTCH2,SELENOS,HYAL1,SLC2A10,CCND1,TGFB2,ZNF106,LRP5,EPRS,HTR2B,GSK3B,CASP7,FSTL1,MMP14,DAG1,PIK3CA,HADH,LEF1,HJV,KRAS,NTRK3,COL6A1,BMP7,ADCY5,WDTC1,FGFR2,ITGB5,HTRA1,NCSTN,SYAP1,IQGAP1,STRAP,RBBP7,STRN3,UROS,BACE1,FAM89B,LINC02210-CRHR1,MAPT,PLOD3,EIF4EBP2,COMT,IGFBP2,FBN1,COMP,PKD1L1,FKBP8,COL3A1,PRKCI,COL5A2,SKIL,FGFR3,QDPR,SMURF1,NBL1,TIMP1,PTPRN,GREM2,MYO5A,BMPR1A,SLC2A4,MED1,PPARGC1A,RNF111,FGFRL1,CPEB4,ARHGEF2,TGFB1I1,NGFR,LTBP2,COL1A1,TGFB3,SHC1,SOX30,TMEM100,CALM1,SMARCA4,PKM,NPTN,MYOCD,PIN1,CHRDL1,PDGFRB,ROCK1,AKT1,ITGA2,LPIN2,PRKAA1,NLK,RAB31,GNAL,PTPN2,LDOC1,MYO1C,GNB1,VASN,ABCA3,FLNA,SSTR5,PARK7,HCN2,BSG,AHCYL1,GNAI3,CDH13,AGL,GLG1,BCAR3,SLC2A8,MTSS1L,HSPA5,DYNC1LI2,FSTL3,FKBP1A,SERPINF1,TMEM38B,PTPN12,LOX,CACYBP,TIMP3,HA	darkgreen	EC_M4
GO:0006278	7.53E-08	0.999999985	22	64	RNA-dependent DNA biosynthetic process	BP	4.90E-06	CCT2,MAP3K4,POT1,CCT4,TNKS2,XRN1,ATR,TERF1,CCT7,CCT5,CCT6A,GAR1,NEK2,XRCC5,HNRNPU,RFC1,DCP2,EXOSC10,CCT3,AURKB,RPA1,NAF1	darkgrey	EC_M8
GO:0051649	1.32E-08	1	304	1679	establishment of localization in cell	BP	5.39E-06	CD63,LMAN1,LRP1,STX7,AP3S1,AGAP2,CANX,TBC1D9B,RAB21,RABL2A,ANGPT1,NUP50,NUS1,HSPA4,SEC24A,PACSIN1,REEP2,PPARD,SIL1,SUN2,GGA1,SH3GL2,MYH9,LEP,PLAA,PEX6,VCP,UNC13B,DYNC1I1,SRP72,LMAN2L,DST,RAB23,SNX13,NLRP5,RIMS1,TMEM30A,ERLEC1,CENPC,COX18,UBE2J1,ACTR2,MDFIC,GLI3,VPS41,BVES,STARD3NL,VPS35,MGARP,ITGB1,LIN7B,FKBP1B,FGF2,KIF5B,SEC61A1,NAPA,RAB7A,COPG1,IWS1,PICALM,PCDH17,SNX17,PAK1,ACTR3,TGFB1,SRP68,RAB3GAP1,SCN5A,SEC13,CTNNB1,TRAK1,AKT2,SEC23B,TBC1D16,ACTG1,NPLOC4,ACTN4,PPP1R12A,UFM1,PRNP,HDAC3,DCLK1,PCLO,LEPROTL1,SNPH,WASHC2A,VPS37A,ARL1,TMEM87B,TM9SF4,REEP5,SNTA1,HSP90B1,TMED2,COPB2,OSBP,VAMP8,RIOK2,TRAM1,MAP1B,RYR3,ERGIC3,PINX1,P2RX4,COPB1,SELENOT,ATP2A2,USO1,SDAD1,CACNA1D,APP,CX3CL1,CACNB3,SSR3,PTPN11,EXOC6B,OPA1,ARHGAP1,ATG13,AP3B1,TSG101,CRY2,VPS18,PPP3CC,AKTIP,BAP1,CHP1,ATP2B4,PKD2,AP2A2,CBLB,DNAH5,IFT57,BRSK2,VPS39,MAGEL2,RAB7B,ARL3,DYNLL1,SRPRA,CTTN,DNM1L,SELENOS,KPNA6,SEC22B,CPT1A,ERGIC2,LRP5,FEZ1,HTR2B,WIPI1,GSK3B,CORO1C,ISCU,SHROOM2,SGSM1,HSPA8,ARV1,KPNA1,AP1B1,SEC22A,ADCY5,VAPB,HYOU1,AP1S2,AP2M1,CASQ1,COPA,OSBPL2,SCFD1,DYNC1I2,KIF1A,SNX27,TMEM50A,TOMM40L,TANC2,BACE1,SIDT2,SRP54,LSG1,HSPB1,MAPT,SEC23A,AP1S1,SYVN1,TFR2,C11orf65,PTPN6,PDCD10,UPF1,HTT,CLTCL1,ERO1A,PRKCI,SMURF1,PTPRN,PLD1,AP5M1,ACTR10,WDR1,FMN2,STX18,KDELR2,ZDHHC4,SLC6A13,B3GAT3,MYO5A,ERC1,HAP1,CACNA1C,SLC29A4,SLC2A4,NLGN2,CLN8,STARD3,SNX8,	darkgreen	EC_M4
GO:0032879	1.46E-08	1	304	2074	regulation of localization	BP	5.39E-06	PARD6G,TNFRSF11A,SEMA6A,AKAP12,FLT4,SNCAIP,RIMS2,AFDN,PACSIN2,TRDN,ARHGAP18,PTK2,C6orf106,DAPK1,PDGFB,PODXL,CSNK1E,PICK1,PIM1,TEK,LIMCH1,DDX58,ATP8A1,APBA1,KIT,KDR,CD2AP,SYK,SEMA4D,ICA1,TSPAN13,IL6,SNAP91,PPT1,CXCL8,RSAD2,DOCK4,GRB10,TLR4,TCAF2,C5,IL15,SDCBP,TRPM4,NRP1,RAB3B,PIK3CG,LAMA4,MTMR2,GATA2,PRKD2,KCNRG,NOVA2,LRCH1,PLXND1,PRCP,NOS3,GAB2,LLGL2,RALB,UNC13D,DACH1,PPARG,GATA3,TBC1D4,CXCR4,EDNRB,NDFIP2,SPTBN4,TIE1,JAG1,P2RY6,P2RY2,SFRP1,HBEGF,FERMT1,KCNK6,ARHGEF7,P2RX2,CD36,SIRT3,PRAG1,ADAMTS9,PRR5L,SCARB1,PLK2,RET,PDE4D,MERTK,CHD7,FAM3D,IL1A,IGF1,EIF2AK3,ABHD6,HIP1R,OCLN,DCLK2,MCTP1,MEF2C,TLR2,WWTR1,P2RX7,VEGFC,NDRG4,CXCL10,ANGPT2,ADGRG3,BMP2K,THBS4,DYSF,TPCN1,PLCG1,STC1,RAPGEF3,KCNJ3,ANO6,DLL4,CLIC6,NEDD9,EDN1,JAM3,KCNJ15,EHD4,SNCA,DTNBP1,PM20D1,MX2,SRGAP2C,ABCG1,ETS1,CD200,SIK1,HDAC1,LCK,PPP3CA,HYAL2,ANO1,HECW2,KCNJ2,MEF2A,SEMA3F,SCN3A,MCTP2,RIN3,ROBO4,PRKX,SEC24B,HCLS1,AP1AR,ATP9A,EPB41,PECAM1,PLXNA2,FGR,MCAM,INPP5F,ADCY1,IGFBP3,FES,PPP2R5A,RBM4,PROX1,PRKD1,LAMA5,YTHDF1,OAZ3,ARHGDIB,MAP3K3,ACE,ITGA6,LIMK2,DOCK1,LAMC2,DLG1,APOD,KCNK7,CREB1,B2M,CTTNBP2NL,OSBPL6,ADORA2A,SEMA6D,CYBB,ITGA4,IGFBP5,HOMER3,MCU,WNK4,DNM3,CDK16,LDB2,SELE,SELP,PLVAP,FGF23,CRACR2A,PPM1F,WAS,TMEM30B,PRKCH,RAC1,HIF1A,RCC2,RHOJ,SYNE2,JUP,RAB27A,ADIPOR2,EPHA2,MMRN2,SNCG,SYT7,TBC1D1,RSC1A1,MCC,TNFRSF1B,FBXL20,MTOR,AR,OPHN1,PSEN1	turquoise	EC_M6

GO:0035239	1.57E-08	1	128	699	tube morphogenesis	BP	5.63E-06	SEMA6A,TNFAIP3,PTPRB,FLT4,FZD6,CTHRC1,CELSR1,QKI,NOTCH4,MTSS1,KLHL3,PTK2,PODXL,TEK,ADGRF5,KDR,SYK,THSD7A,SHB,IL6,CXCL8,TSPAN12,C5,TEAD2,MYCN,NRCAM,NRP1,RASIP1,PIK3CG,TAL1,GATA2,PRKD2,GPR4,PLXND1,PRCP,NOS3,PPARG,GATA3,CXCR4,GZF1,TIE1,JAG1,SFRP1,EFNB2,ADAMTS9,EPHB1,PLK2,RET,CHD7,IL1A,NR4A1,EIF2AK3,HHEX,SMAD1,MEF2C,VEGFC,NDRG4,CXCL10,ANGPT2,TIPARP,THBS4,PPP1R16B,PLCG1,RAPGEF3,STAB1,HIPK1,DLL4,EDN1,STAT1,JAM3,ETS1,ROBO4,PRKX,HLX,SEC24B,CD34,MCAM,PROX1,PRKD1,VANGL2,LAMA5,MAP3K3,APOLD1,DLG1,APOD,CLEC14A,CYBB,EPHB4,TBX1,CALCRL,HSPG2,WNK4,RAMP2,HIF1A,RHOJ,JUP,ADIPOR2,EPHA2,MMRN2,CECR2,AR,PSEN1,HOXB7,SEMA4A,FGF18,PGF,EFNA1,VASH1,NPR1,SMAD2,AMOT,CCL2,JAK1,LRG1,NRARP,EGFL7,NOTCH1,S1PR1,ENG,DAB2IP,CDH5,MIR23A,ADGRG1,VASP,SOX17,SOX18,RALA,BMP2	turquoise	EC_M6
GO:0045765	1.72E-08	1	55	229	regulation of angiogenesis	BP	6.06E-06	SEMA6A,TNFAIP3,TEK,KDR,IL6,CXCL8,TSPAN12,C5,NRP1,GATA2,PRKD2,GPR4,PLXND1,NOS3,PPARG,CXCR4,TIE1,SFRP1,ADAMTS9,PLK2,IL1A,HHEX,SMAD1,VEGFC,CXCL10,ANGPT2,THBS4,PPP1R16B,PLCG1,RAPGEF3,STAB1,HIPK1,STAT1,ETS1,CD34,PRKD1,CYBB,HSPG2,RAMP2,HIF1A,RHOJ,JUP,EPHA2,SEMA4A,FGF18,PGF,EFNA1,VASH1,NPR1,AMOT,JAK1,LRG1,ENG,DAB2IP,CDH5	turquoise	EC_M6
GO:0006282	9.63E-08	0.999999974	31	118	regulation of DNA repair	BP	6.24E-06	UBR5,POT1,POLH,TRIM28,RIF1,CUL4A,PPP4R2,PRKDC,ERCC6,PARPBP,ABRAXAS1,RAD51,ARID2,DEK,CHEK1,FANCB,RPA2,AUNIP,DHX9,SETD2,BRCA1,NSD2,RAD51AP1,FOXM1,DDX11,RAD52,FUS,SMCHD1,RMI2,TIGAR,RNF8	darkgrey	EC_M8
GO:0007155	1.86E-08	1	177	1044	cell adhesion	BP	6.39E-06	SEMA6A,CELSR1,NOTCH4,AFDN,PARVG,MTSS1,PTK2,PDGFB,PODXL,CDC42EP1,TEK,LIMCH1,APBA1,TJP2,PGM5,KIT,TNFRSF21,KDR,CD2AP,CNTNAP3,SYK,SEMA4D,IL6,CXCL8,PELI1,IL15,NRCAM,NRP1,IZUMO1,PIK3CG,LAMA4,PRKD2,GPR4,PLXND1,NECTIN2,BCAM,ITGA8,ITGA9,ITGB4,UNC13D,GATA3,CXCR4,CD93,PPP1CB,CYTH1,JAG1,SFRP1,FERMT1,THSD1,FREM2,EFNB2,ARHGEF7,PCDH1,PCDH12,NTN4,CD36,RIC8A,MAGI1,ADAMTS9,EPHB1,RET,MERTK,IL1A,IGF1,ITCH,ERBIN,LYVE1,MMP24,IL7,ENTPD1,ANGPT2,THBS4,CYTIP,STAB1,SPARCL1,SOX13,NEDD9,CD58,JAM3,MMRN1,ETS1,CD200,LCK,PPP3CA,SLC39A8,ICOSLG,LSAMP,ROBO4,ESAM,PRKX,LAPTM5,HLX,CLDN34,CDH24,AP1AR,CD34,CADM3,PECAM1,CD86,PLXNA2,ICAM2,BMX,MCAM,FES,LAMA5,F11R,ARHGDIB,ITGA6,DOCK1,MELTF,LAMC2,DLG1,SIPA1,APOD,B2M,HMCN1,BCR,TMEM47,ADORA2A,IL1RAP,EPHB4,ITGA4,SELE,SELP,VWF,BAIAP2L1,PPM1F,RAC1,RCC2,JUP,EPHA2,ITPKB,ARG2,PEAR1,EFNB1,PSEN1,STK10,ITGA3,TAOK2,EFNA1,PCDH11X,DNM2,TNIP1,COL4A6,DSG2,DSC2,DSC3,CCL2,YES1,JAG2,JAK1,LGALS9,IL7R,SLITRK2,AP3D1,NRARP,EGFL7,NOTCH1,PIEZO1,S1PR1,CNN3,ENG,STXBP1,NFAT5,CDH5,PDZD2,VNN1,ADGRG1,CD46,RELN,STX3,PERP,VAMP3,BMP2,SCARF1,CBLL1,BMP6	turquoise	EC_M6
GO:0006518	9.99E-08	1	101	688	peptide metabolic process	BP	6.45E-06	PAN2,NARS,DDX39B,EIF3E,MRPL13,TNRC6B,TUT7,CARNMT1,PUM3,NCBP1,LRPPRC,MTIF2,PNPT1,SYNCRIP,PABPC4,TIA1,MRPL32,ASCC3,DDX1,DHFR,PUS7,EXOSC9,CNOT10,MRPS7,DIS3,DARS,METTL3,EXOSC8,LARS,LTA4H,PRKDC,DHX29,PAN3,SP1,MRPL15,MRPS5,CAPRIN1,LSM14A,CDK5RAP1,EIF2B1,NCK1,IDE,MRPL35,PTCD3,ABCE1,XRN1,PDE12,LARP4,CNOT1,LTN1,USP16,RPS6,EEF1E1,NOLC1,GCN1,METAP1,YARS,KHDRBS1,IARS2,NCL,FXR1,NGDN,EIF3A,EIF2B5,DDX6,DHX9,HNRNPR,FASTKD2,TPR,RPL10L,DARS2,CNOT9,RPL22L1,DHX33,EIF2AK1,MRPL44,DCP2,EIF3B,SEPSECS,EIF4A1,RARS,MRPS23,MRPL22,GEMIN5,LARP1,ILF3,EIF5,TYMS,UPF3B,GSPT1,FMR1,METTL16,EXOSC2,RBM8A,METTL18,SARNP,HNRNPD,RNF139,PAIP2,EIF1AX,DRG1	darkgrey	EC_M8
GO:0034976	1.66E-08	1	61	222	response to endoplasmic reticulum stress	BP	6.59E-06	CANX,UBQLN1,VCP,NCK2,CREB3,ERP44,ERLEC1,UBE2J1,RCN3,TMUB1,UBXN4,STT3B,NPLOC4,RNF121,TMTC4,UFM1,TMTC3,UBA5,DDRGK1,PPP2CB,HSP90B1,TMED2,ATP2A2,OPA1,AMFR,CHAC1,SERINC3,BRSK2,MARCH6,SELENOS,CCND1,GSK3B,VAPB,HYOU1,ATF3,GORASP2,UBE4A,ATF6,PDIA3,RHBDD2,TMCO1,EDEM3,SYVN1,DNAJC10,DNAJB12,ERO1A,JKAMP,EIF2S1,SEL1L,SCAMP5,USP14,PTPN2,BCAP31,TMEM259,PARK7,MBTPS1,HSPA5,FBXO27,DNAJB9,UBE2G2,DERL2	darkgreen	EC_M4
GO:0032210	1.03E-07	0.999999983	19	50	regulation of telomere maintenance via telomerase	BP	6.63E-06	CCT2,MAP3K4,POT1,CCT4,TNKS2,XRN1,ATR,TERF1,CCT7,CCT5,CCT6A,NEK2,XRCC5,HNRNPU,DCP2,EXOSC10,CCT3,AURKB,NAF1	darkgrey	EC_M8

GO:0032774	1.08E-07	1	311	2534	RNA biosynthetic process	BP	6.88E-06	ONECUT2,BCLAF1,PPIL4,NUP107,DDX39B,ZNF608,SCAF8,RAD21,HSF2,ATAD2,XRCC6,PHF5A,PSIP1,IFT74,PWP1,ZNF300,TOPORS,POLR3B,MTERF1,RFX3,CKS2,TRIM28,ZNF451,PHF3,TWISTNB,FOXJ3,ZNF189,SP4,CDCA7L,PHIP,ZMPSTE24,RLF,ZNF292,XPO1,COMMD1,CASP8AP2,ZNF350,ITGB1BP1,THRAP3,TAF1B,EZH2,UFL1,CCNC,DDX1,NAA15,ELF2,ATAD2B,CDCA7,HDAC2,CCNA2,EXOSC9,MLLT10,DNAJC2,SETDB2,PCF11,NIFK,NUP85,ELF1,RSF1,CCNT2,PAXIP1,XRN2,NCAPG2,WDR43,BIRC5,POLR3F,EPC2,SUPT16H,CHD8,KAT6A,RIF1,E2F7,ALYREF,CKAP2,CEP290,NSD3,CEBPZ,SUPT20H,TAF7,NR2C1,BRCA2,ELK3,RRP8,TCERG1,MAPK8,PRKDC,CBX5,ERCC6,ZNF214,GPBP1,SP1,TCEA1,ZNF664,ZC3H8,POLR1B,SGK3,BTAF1,ZNF143,CENPJ,FER,TDG,NCK1,SBNO1,SMARCA5,RNF141,ZNF507,POLR1A,CHD1,ZFP91,TAF9,CGGBP1,SMN2,BDP1,TFCP2,SAP30,GCFC2,UTP15,HLTF,ZBTB10,CNOT1,GABPA,MCRS1,USP16,PPID,RBL1,CCNL1,SENP1,RIPK1,WDR75,ARID2,MYBL2,E2F8,TRIM33,INO80,ZBED6,CHUK,ZBTB11,ZNF248,MGA,MDM4,HIVEP1,BRWD1,NOLC1,BMPR1B,UBE3A,PRDM10,NUCKS1,DEK,GCN1,CUL3,GMNN,CHEK1,H2AFZ,RRP1B,NFKB1,KHDRBS1,TRIP13,TET2,TRIP11,HEATR1,NCL,TRAIP,ZNF184,PRMT5,AJUBA,GTF2IRD2,ZBTB41,ARID4B,NOL11,GTF2I,TARBP1,BPTF,DDX5,ORC2,EWSR1,TIAL1,BLM,CENPF,EDRF1,PDE8A,ING5,BCLAF3,FOSL1,SMARCC1,BAZ1B,SP3,DHX9,HDGFL3,BAZ1A,SETD2,ZFX,PSMA6,POLA1,IVNS1ABP,TAF1A,DDX20,AP000944.2,TPR,CCAR1,DDX21,UBTF,NMD3,XRCC5,NPAT,BRCA1,CNOT9,NSD2,HP1BP3,DCAF6,ZNF518B,DHX33,TBL1XR1,SUV39H1,POLR3A,LEO1,FOXM1,LYAR,DDX11,FAM220A,HNR	darkgrey	EC_M8
GO:0044265	1.09E-07	1	136	947	cellular macromolecule catabolic process	BP	6.95E-06	CDK2,PAN2,USP15,LATS1,POP1,EIF3E,TNRC6B,TUT7,UBR2,TOPORS,NCBP1,LRPPRC,FBXO11,PNPT1,ZMPSTE24,USP34,SYNCRIP,XPO1,PABPC4,COMMD1,FBXO48,THRAP3,UFL1,CUL1,USP45,ASCC3,AFG1L,EXOSC9,RNASEH2B,YME1L1,TRDMT1,CNOT10,UPF2,DIS3,XRN2,CDC20,METTL3,VPS36,EXOSC8,CUL4A,PSMD13,CDC16,PAN3,MTREX,ANAPC1,IDE,TAF9,XRN1,PDE12,ZCCHC8,TTC37,USP25,PBK,CNOT1,ANAPC7,LTN1,USP16,TENT2,RIPK1,UBE2C,OGA,UBE3A,CUL3,NSUN2,KIF14,UBR1,PSMD12,FXR1,DDX5,DTL,FASTKD1,SSB,UBR3,PSMD4,CCDC47,SMARCC1,DHX9,HNRNPR,PSMA6,FASTKD2,DNA2,CDC27,COP1,CUL5,PSME3,CNOT9,TBL1XR1,HNRNPU,UBE2K,NEDD4,MRPL44,DCP2,EXOSC10,FUS,USP3,TAF1,CLPX,OGT,RLIM,DIS3L,RBBP6,LARP1,PLK1,APPBP2,PSMD10,TAF15,MIB1,UPF3B,COPS3,HNRNPM,CUL4B,SERBP1,AIFM1,GSPT1,UHRF1,DROSHA,FMR1,METTL16,CCNF,RNPS1,PSMA5,EXOSC2,GLMN,USP33,RBM8A,FASTKD5,HNRNPD,AURKA,NAF1,RNF139,RNF8,FEN1,GID4,UBXN2A,PSMD7,RCHY1	darkgrey	EC_M8
GO:0010631	2.11E-08	1	60	261	epithelial cell migration	BP	7.09E-06	FLT4,PTK2,PDGFB,TEK,KIT,KDR,NRP1,PIK3CG,GATA2,PRKD2,PLXND1,PRCP,NOS3,PPARG,GATA3,HBEGF,FERMT1,EFNB2,ADAMTS9,SCARB1,PLK2,NR4A1,MEF2C,VEGFC,ANGPT2,PLCG1,STC1,DLL4,EDN1,ETS1,PRKX,PROX1,PRKD1,MAP3K3,DOCK1,CLEC14A,EPHB4,PPM1F,RAC1,HIF1A,RHOJ,JUP,EPHA2,MMRN2,MCC,MTOR,SEMA4A,FGF18,ITGA3,EFNA1,VASH1,AMOT,PLEKHG5,NOTCH1,PLCG2,DAB2IP,CDH5,MIR23A,FGF16,SOX18	turquoise	EC_M6
GO:0007166	2.21E-08	1	286	1935	cell surface receptor signaling pathway	BP	7.26E-06	TNFRSF11A,SEMA6A,IFNGR1,TNFAIP3,FLT4,FZD6,CTHRC1,RIMS2,CELSR1,NOTCH4,TCF7,MTSS1,RNF146,PTK2,ANKS1A,PDGFB,CSNK1E,MFNG,CSF2RB,MDFI,TEK,TEC,ADGRF5,KIT,ADGRF1,TNFRSF21,KDR,SYK,SEMA4D,IL6,CXCL8,PELI1,CAV2,GRB10,TSPAN12,TLR4,GRIK2,TRAF1,C5,IL15,FZD8,CCNY,SDCBP,TRPM4,NRP1,TAL1,MTMR2,GATA2,PRKD2,PLXND1,NECTIN2,ITGA8,NOS3,GAB2,LLGL2,ITGA9,ITGB4,PPARG,TIMP4,GATA3,ACVR2B,CXCR4,IRAK2,EDNRB,TMC8,TIE1,IKBKB,JAG1,P2RY6,P2RY2,SFRP1,HBEGF,NOTUM,FERMT1,EFNB2,ARHGEF7,SPEF1,P2RX2,CD36,MAGI1,PRAG1,MAP3K1,SCARB1,EPHB1,RET,PDE4D,MERTK,TNFRSF19,IL1A,IGF1,EIF2AK3,HHEX,ERBIN,SH3RF1,SMAD1,HIP1R,SOX7,ARHGEF28,MEF2C,TLR2,WWTR1,IL7,P2RX7,VEGFC,NDRG4,CXCL10,ANGPT2,SOGA1,MADD,LMBR1L,ADGRG3,DHH,BMP2K,TIPARP,OAS3,NLRC5,PLCG1,NR4A2,DGKZ,HIPK1,ANO6,DLL4,SOX13,NEDD9,EDN1,STAT1,SNCA,GAS2,MX1,PRDM15,HDAC1,ITPRIP,LCK,PPP3CA,HYAL2,ICOSLG,SEMA3F,SHOC2,BCL9,LAPTM5,NCF1,HCLS1,NFATC2,INPP5D,PECAM1,CD86,PLXNA2,HHAT,BMX,FGR,CFLAR,INPP5F,KALRN,IGFBP3,FES,PLEKHA1,PRKD1,VANGL2,LAMA5,IL10RA,SRMS,ITGA6,DOCK1,PAK2,INSYN2,PTPRE,SENP2,PTCHD1,APOD,STYK1,CREB1,ADGRA1,SSTR1,CLEC14A,LTF,BCR,CCRL2,ADORA2A,SEMA6D,TSPAN15,IL1RAP,EPHB4,ITGA4,DDX3X,IGFBP5,HOMER3,CALCRL,CDA,BIRC3,PLVAP,BAIAP2L1,FGF23,LMTK2,PRKCH,RAC1,HIF1A,RCC2,PLD2,TSPAN14,JUP,ADIPOR2,EPHA2,ITPKB,MMRN2,BID,MCC,TNK1,CHRNB1,MPP5,LFNG,TNFRSF1B,RGS14,AR,EFNB1,PSEN1,SEMA4A,FBXW11,FGF18,ITGA3,PGF,EIF2B2,EFNA1,CHRNB2,CD1	turquoise	EC_M6

GO:0030154	2.34E-08	1	415	2966	cell differentiation	BP	7.53E-06	ZNF516,TNFRSF11A,TCF4,SEMA6A,KIF5A,IFNGR1,FLT4,CTHRC1,RIMS2,CELSR1,QKI,NOTCH4,AFDN,PARVG,ASF1A,TCF7,CDKL3,MTSS1,ASAP1,L3MBTL3,PTK2,ANKS1A,ATF4,PDGFB,AGTPBP1,PODXL,CSNK1E,PICK1,KDM4C,MICALL1,PIM1,MFNG,MDFI,TEK,IREB2,TJP2,PGM5,ADGRF5,KIT,ADGRF1,TNFRSF21,KDR,NFIL3,SYK,GADD45G,SEMA4D,THSD7A,SHB,TMOD1,IL6,PPT1,PTBP3,RSAD2,FIGNL1,CAV2,NOBOX,TLR4,IL15,FZD8,CREM,TEAD2,SDCBP,MYCN,NRCAM,NOCT,TUT4,TRPM4,NRP1,ZBTB24,RASIP1,SPATA6,ITSN2,TAL1,MTMR2,EPC1,GATA2,DNMT3A,HS6ST1,NOVA2,RAB38,GPR4,SNRK,PLXND1,NECTIN2,ITGA8,GAB2,ITGB4,UNC13D,PPARG,GATA3,ACVR2B,MYO7A,CXCR4,EDNRB,SPTBN4,TIE1,IKBKB,JAG1,PDE2A,GPAT4,SFRP1,NR1D2,DND1,FEZ2,EFNB2,ARHGEF7,ATP11A,PCDH12,P2RX2,NTN4,SLC4A11,CD36,TMEM178A,LMOD3,SYNE4,PRAG1,TCF15,EPHB1,PLK2,RET,PDE4D,MERTK,CHD7,IL1A,DNMT3B,IGF1,NR4A1,EIF2AK3,HHEX,KDM3A,ITCH,SH3RF1,PREX2,SMAD1,MARVELD2,DCLK2,MMP24,SOX7,KIF13B,TMEM120B,ARHGEF28,ENC1,HEXB,MEF2C,TLR2,WWTR1,IL7,TPD52,VEGFC,NDRG4,GPM6A,CXCL10,ANGPT2,TLL2,LMBR1L,ADGRG3,DHH,KMT2D,SFMBT1,TIPARP,TLL1,USH1C,PPP1R16B,DYSF,STC1,RAPGEF3,ANKRD2,NR4A2,HIPK1,TANC1,DLL4,CDYL,SOX13,TSPAN2,ADAMTS20,NEDD9,NAV2,EDN1,STAT1,JAM3,ALAS1,JARID2,DTNBP1,MYLIP,ERG,ETS2,SLC45A3,NFKB2,DCAF1,SRGAP2C,ABCG1,ETS1,SIK1,HDAC1,LCK,PPP3CA,HYAL2,HECW2,TESMIN,MEF2A,SEMA3F,ROBO4,BCL9,NRAP,PRKX,HLX,SEC24B,DYRK3,USP13,FABP4,HCLS1,NFATC2,AP1AR,ZFHX2,INPP5D,JHY,CD34,PECAM1,PLEKHO1,CD86,PLXNA2,F	turquoise	EC_M6
GO:1901342	2.49E-08	1	55	232	regulation of vasculature development	BP	7.80E-06	SEMA6A,TNFAIP3,TEK,KDR,IL6,CXCL8,TSPAN12,C5,NRP1,GATA2,PRKD2,GPR4,PLXND1,NOS3,PPARG,CXCR4,TIE1,SFRP1,ADAMTS9,PLK2,IL1A,HHEX,SMAD1,VEGFC,CXCL10,ANGPT2,THBS4,PPP1R16B,PLCG1,RAPGEF3,STAB1,HIPK1,STAT1,ETS1,CD34,PRKD1,CYBB,HSPG2,RAMP2,HIF1A,RHOJ,JUP,EPHA2,SEMA4A,FGF18,PGF,EFNA1,VASH1,NPR1,AMOT,JAK1,LRG1,ENG,DAB2IP,CDH5	turquoise	EC_M6
GO:0090132	2.53E-08	1	60	262	epithelium migration	BP	7.80E-06	FLT4,PTK2,PDGFB,TEK,KIT,KDR,NRP1,PIK3CG,GATA2,PRKD2,PLXND1,PRCP,NOS3,PPARG,GATA3,HBEGF,FERMT1,EFNB2,ADAMTS9,SCARB1,PLK2,NR4A1,MEF2C,VEGFC,ANGPT2,PLCG1,STC1,DLL4,EDN1,ETS1,PRKX,PROX1,PRKD1,MAP3K3,DOCK1,CLEC14A,EPHB4,PPM1F,RAC1,HIF1A,RHOJ,JUP,EPHA2,MMRN2,MCC,MTOR,SEMA4A,FGF18,ITGA3,EFNA1,VASH1,AMOT,PLEKHG5,NOTCH1,PLCG2,DAB2IP,CDH5,MIR23A,FGF16,SOX18	turquoise	EC_M6
GO:0009653	2.02E-08	1	364	2050	anatomical structure morphogenesis	BP	7.80E-06	ATP8B1,NAB2,LRP1,AHI1,IER3,RAB21,CDSN,SLC12A2,PLXNB2,FBN2,ANGPT1,FAM19A5,PPARA,NUS1,RSPO3,FAM49B,MRTFA,PACSIN1,ADGRB1,LY6E,CTNNA1,MKLN1,SH3GL2,MYH9,LEP,PLAA,TBCC,RORB,SRF,VEGFA,RUNX2,COL1A2,PDCL3,ASPN,CNNM4,RAB23,COL15A1,TGFBR1,RIMS1,RTN4,TPBG,SHANK1,EMC10,MFSD2A,MYH14,CALD1,ACTR2,KIDINS220,WHRN,GLI3,ROCK2,INHBA,BVES,RRAS,MYLK3,DKKL1,VPS35,ITGB1,PLEKHA4,PLXNA1,FGF2,SRPK2,KIF5B,RB1,PICALM,TENM4,GLI2,MEGF8,SMARCD3,PAK1,TGFB1,AXL,RAB3GAP1,SCN5A,WDR48,CTNNB1,TRAK1,SERPINH1,FARP1,RELT,RARB,ACTG1,ACTN4,STIM1,SPINT2,DCN,CDC42EP3,SMAD9,DCLK1,STARD13,SEMA3C,ILK,SPINK5,APLP1,RYK,PRICKLE2,RNF6,AKIP1,CXCL12,IMPAD1,ANKRD1,FAT1,TMED2,PALLD,FLNB,EIF4G2,SULF1,DKK3,ROBO1,MAP1B,RDH10,DIP2B,SEMA4F,FAIM2,HEY1,RASA1,TLX2,TCTN1,MMP15,APP,DHX36,RAPGEF2,ACTG2,CX3CL1,ABCC8,CPE,PTPN11,OPA1,LOXL2,AP3B1,ZFYVE27,ACVR1,MMP2,MTDH,ADA,ATP2B4,SPP1,BMP1,PKD2,RUNX1,HIF1AN,SEMA4G,NKD1,IFT57,BRSK2,PDLIM5,NDN,CHRNA7,DOCK10,FAP,PHLDB2,TJP1,KDM5B,NOTCH2,CTTN,PKP2,CDC73,DNM1L,UCHL5,CHSY1,HYAL1,LMOD1,EYA2,CAPRIN2,TGFB2,LRP5,ADGRB2,FEZ1,HTR2B,SATB2,PRKAR1A,GSK3B,FAM20C,MMP14,DAG1,PIK3CA,AUTS2,LEF1,CORO1C,ANOS1,PRKCA,SHROOM2,KRAS,TCTA,COL6A1,BMP7,NEDD8,FGFR2,VASH2,CASQ1,ITGB5,PHLDB1,HTRA1,IQGAP1,SCFD1,KIF1A,CLIC4,TFRC,ARID5B,TANC2,SIDT2,HSPB1,MAPT,PLOD3,SERPINE1,SGPL1,ATP6AP2,FZD2,TNR,FN1,NCKAP1,C11orf65,PTPN6,MEOX1,FBN1,PDCD10,ITGAV,COMP,ARPC2,CLT	darkgreen	EC_M4
GO:0003158	2.73E-08	0.999999993	34	107	endothelium development	BP	8.12E-06	NOTCH4,AFDN,TJP2,KDR,GJA4,NRP1,PRKD2,ACVR2B,CXCR4,TIE1,IKBKB,JAG1,PDE2A,PDE4D,MARVELD2,CXCL10,PPP1R16B,STC1,RAPGEF3,DLL4,ROBO4,CD34,PECAM1,PROX1,F11R,APOLD1,HOXB5,NOTCH1,RAPGEF1,S1PR1,CDH5,SOX17,SOX18,BMP6	turquoise	EC_M6

GO:0050793	2.74E-08	1	278	1852	regulation of developmental process	BP	8.12E-06	TCF4,SEMA6A,TNFAIP3,FLT4,FZD6,CTHRC1,RIMS2,CDC42SE2,CELSR1,NOTCH4,TCF7,CDKL3,ASAP1,ARHGAP18,PTK2,ATF4,PDGFB,CSNK1E,KDM4C,PIM1,CDC42EP1,STRIP2,TEK,LINGO2,KIT,TNFRSF21,KDR,SYK,SEMA4D,IL6,PTBP3,CXCL8,TSPAN12,TLR4,C5,IL15,TEAD2,SDCBP,MYCN,NRCAM,NOCT,TRPM4,NRP1,ITSN2,TAL1,LAMA4,MTMR2,GATA2,PRKD2,CST7,CTSC,GPR4,FGD5,PLXND1,NOS3,UNC13D,PPARG,GATA3,ACVR2B,CXCR4,EDNRB,SPTBN4,TIE1,IKBKB,JAG1,GPAT4,SFRP1,NR1D2,NOTUM,FERMT1,EFNB2,ARHGEF7,SPEF1,ATP11A,FGD6,NTN4,SLC4A11,CD36,TMEM178A,LMOD3,PRAG1,ADAMTS9,TCF15,EPHB1,PLK2,RET,CHD7,IL1A,DNMT3B,IGF1,EIF2AK3,HHEX,KDM3A,ITCH,SH3RF1,SMAD1,KIF13B,HEXB,MEF2C,TLR2,WWTR1,IL7,P2RX7,VEGFC,CXCL10,ANGPT2,TLL2,SFMBT1,BMP2K,THBS4,PPP1R16B,PLCG1,RAPGEF3,ANKRD2,STAB1,HIPK1,ANO6,DLL4,DNMBP,SOX13,ADAMTS20,NEDD9,EDN1,STAT1,JARID2,GAS2,SLC45A3,SRGAP2C,ABCG1,ETS1,SIK1,HDAC1,PPP3CA,HECW2,SEMA3F,ZDHHC6,PRKX,HLX,SEC24B,HCLS1,NFATC2,AP1AR,ZFHX2,INPP5D,EAF2,EPB41,JHY,CD34,PLEKHO1,CD86,PLXNA2,FGR,CFLAR,INPP5F,TRAK2,IGFBP3,FES,RBM4,RPS6KA1,PROX1,SS18L1,PRKD1,VANGL2,LAMA5,F11R,MAP3K3,DOCK1,PAK2,MELTF,APOLD1,DLG1,ELL3,MOV10,CREB1,B2M,LTF,SEMA6D,CYBB,IL1RAP,BCOR,ADAMTS7,SOS2,IGFBP5,ETV4,TBX1,MCU,HSPG2,RAMP2,DNM3,PROM1,FGF23,PRKCH,RAC1,HIF1A,RCC2,RHOJ,JUP,BCL6B,EPHA2,ITPKB,LFNG,TNFRSF1B,RGS14,ARG2,FGD1,ZC4H2,SMOC1,MTOR,AR,CASZ1,PSEN1,HOXB7,SEMA4A,HOXB8,FGF18,ABI3,PGF,TAOK2,ARHGEF15,EFNA1,VASH1,CHRNB2,NAP1L2,ZD	turquoise	EC_M6
GO:0000470	1.28E-07	0.99999999	12	23	maturation of LSU-rRNA	BP	8.14E-06	NIFK,DDX18,GTPBP4,MAK16,URB1,RRP15,WDR12,FTSJ3,LAS1L,RSL1D1,NOL9,RPF1	darkgrey	EC_M8
GO:0032206	1.32E-07	0.999999979	18	48	positive regulation of telomere maintenance	BP	8.34E-06	CCT2,MAP3K4,POT1,HNRNPA2B1,CCT4,TNKS2,ATR,CCT7,NBN,CCT5,CCT6A,NEK2,XRCC5,CCT3,AURKB,ATRX,HNRNPD,NAF1	darkgrey	EC_M8
GO:0048869	3.15E-08	1	422	3033	cellular developmental process	BP	9.14E-06	ZNF516,TNFRSF11A,TCF4,SEMA6A,KIF5A,IFNGR1,FLT4,SNX2,CTHRC1,RIMS2,CELSR1,QKI,NOTCH4,AFDN,PARVG,PACSIN2,ASF1A,TCF7,CDKL3,MTSS1,ASAP1,L3MBTL3,PTK2,ANKS1A,ATF4,PDGFB,AGTPBP1,PODXL,CSNK1E,PICK1,KDM4C,MICALL1,PIM1,MFNG,MDFI,TEK,IREB2,TJP2,PGM5,ADGRF5,KIT,ADGRF1,TNFRSF21,KDR,NFIL3,SYK,GADD45G,SEMA4D,THSD7A,SHB,TMOD1,IL6,PPT1,PTBP3,RSAD2,FIGNL1,CAV2,NOBOX,TLR4,IL15,FZD8,CREM,TEAD2,SDCBP,MYCN,NRCAM,NOCT,TUT4,TRPM4,NRP1,ZBTB24,RASIP1,SPATA6,ITSN2,TAL1,MTMR2,EPC1,GATA2,DNMT3A,HS6ST1,NOVA2,RAB38,GPR4,SNRK,PLXND1,NECTIN2,ITGA8,GAB2,ITGB4,UNC13D,PPARG,GATA3,ACVR2B,MYO7A,CXCR4,EDNRB,SPTBN4,TIE1,IKBKB,JAG1,PDE2A,GPAT4,SFRP1,NR1D2,DND1,FEZ2,EFNB2,ARHGEF7,ATP11A,PCDH12,P2RX2,NTN4,SLC4A11,CD36,TMEM178A,LMOD3,SYNE4,PRAG1,TCF15,EPHB1,PLK2,RET,PDE4D,MERTK,CHD7,IL1A,DNMT3B,IGF1,NR4A1,EIF2AK3,HHEX,KDM3A,ITCH,SH3RF1,PREX2,SMAD1,MARVELD2,DCLK2,MMP24,SOX7,KIF13B,TMEM120B,ARHGEF28,ENC1,HEXB,MEF2C,TLR2,WWTR1,IL7,TPD52,VEGFC,NDRG4,GPM6A,CXCL10,ANGPT2,TLL2,LMBR1L,ADGRG3,DHH,KMT2D,SFMBT1,TIPARP,TLL1,USH1C,PPP1R16B,DYSF,STC1,RAPGEF3,ANKRD2,NR4A2,HIPK1,TANC1,DLL4,CDYL,SOX13,TSPAN2,ADAMTS20,NEDD9,NAV2,EDN1,STAT1,JAM3,ALAS1,JARID2,DTNBP1,MYLIP,ERG,ETS2,SLC45A3,NFKB2,DCAF1,SRGAP2C,ABCG1,ETS1,SIK1,HDAC1,LCK,PPP3CA,HYAL2,HECW2,TESMIN,MEF2A,SEMA3F,ROBO4,BCL9,NRAP,PRKX,HLX,SEC24B,DYRK3,USP13,FABP4,HCLS1,NFATC2,AP1AR,ZFHX2,INPP5D,JHY,CD34,PECAM1,PLEKH	turquoise	EC_M6
GO:0006886	2.52E-08	1	152	739	intracellular protein transport	BP	9.50E-06	LMAN1,STX7,AP3S1,TBC1D9B,RAB21,RABL2A,ANGPT1,NUP50,HSPA4,SEC24A,REEP2,SIL1,GGA1,LEP,PEX6,VCP,SRP72,RAB23,SNX13,RIMS1,TMEM30A,ERLEC1,COX18,UBE2J1,MDFIC,GLI3,VPS41,VPS35,MGARP,KIF5B,SEC61A1,NAPA,RAB7A,COPG1,SNX17,PAK1,TGFB1,SRP68,SEC13,TRAK1,AKT2,SEC23B,TBC1D16,NPLOC4,UFM1,PRNP,HDAC3,VPS37A,ARL1,TM9SF4,HSP90B1,TMED2,COPB2,TRAM1,COPB1,USO1,CACNB3,SSR3,PTPN11,EXOC6B,ATG13,AP3B1,CRY2,VPS18,BAP1,CHP1,AP2A2,CBLB,BRSK2,VPS39,SRPRA,CTTN,SELENOS,KPNA6,GSK3B,SGSM1,HSPA8,KPNA1,AP1B1,AP1S2,AP2M1,COPA,SCFD1,KIF1A,SNX27,TOMM40L,SRP54,HSPB1,SEC23A,AP1S1,SYVN1,C11orf65,PDCD10,CLTCL1,PRKCI,SMURF1,STX18,ZDHHC4,B3GAT3,HAP1,SNX8,MED1,STX1B,SNX11,SSR2,ARHGEF2,NGFR,LTBP2,TMED10,RAB11A,SEL1L,SYTL4,TMED1,CLTC,CD74,MIEF2,EVI5L,AKT1,PRKAA1,ZDHHC9,RAB31,NAPG,MYO1C,BCAP31,PKD1,FLNA,PARK7,AHCYL1,HSPA5,AP1G1,WLS,RANBP2,KPNA4,SPCS3,RAB6A,SNX10,ARF4,VPS26B,CDKN1A,TXN,ARFRP1,PTTG1IP,YWHAH,TIMM22,LEPROT,TMED3,ZPR1,GRPEL2,UBE2G2,TOMM7,MAP1A,DERL2	darkgreen	EC_M4

GO:0016043	1.54E-07	1	527	4652	cellular component organization	BP	9.66E-06	RTTN,CDK2,PAN2,ONECUT2,MTFR2,RPA3,SHPRH,KATNA1,LATS1,NUP107,CCT2,DDX39B,FBXO43,FBXO5,SCAF8,EIF3E,EBAG9,MAP3K4,RAD21,DSCC1,KIF3A,TBC1D32,RRP7A,KIFC1,ATAD2,XRCC6,KIF20A,CDC25C,FANCC,HSPA9,CHCHD3,SRPK1,RMI1,PLEC,PSIP1,RECQL4,CEP78,HAUS6,UBR2,IFT74,MCM5,POT1,TOPORS,MTERF1,KIF24,AKAP9,GCC2,RFX3,CKS2,MCM3,ICK,TRIM28,NCBP1,ANP32B,STX17,PPIH,FOXJ3,MSH2,SMC2,SENP6,PHIP,YTHDC1,MTIF2,TTK,HNRNPA2B1,PNPT1,RUFY3,CEP162,ZMPSTE24,XPO1,PLEKHA8,CCT4,MDN1,NUP205,ANLN,CDCA8,ITGB1BP1,TAF1B,CAPZA2,EZH2,UFL1,ASCC3,ZMYM4,CNTRL,DDX1,SMC6,GEN1,AFG1L,WDR35,SCLT1,PLK4,DHFR,KIAA1109,STIL,HDAC2,MCM2,CENPO,CEP57,EXOSC9,PMPCB,SAE1,CEP295,DNAJC2,RAD54L,CHORDC1,SETDB2,YME1L1,CENPA,NASP,COG3,CLASP2,DCLRE1C,NIFK,NUP85,MRPS7,KIF2C,RSF1,DIAPH3,BORA,MCM6,DARS,FANCD2,ULK4,NCAPG2,CDC20,TK1,CRNKL1,FNTA,PARP2,PITRM1,HOOK3,SUPT16H,CHD8,KAT6A,RIF1,NAP1L1,TOP2B,ZDHHC17,SGO1,PRPF40A,CKAP2,VPS36,STRN,RRM1,CEP290,TAF7,TUBGCP3,DNAJC13,RFC3,BRCA2,SNRPF,LARS,PSMD13,RRP8,CDC16,MAPK8,PRKDC,NCAPH,ARHGAP22,CCNB1,MCM4,WDR62,ERCC6,DHX29,ACTR6,PAN3,SCYL2,GAS2L3,HAUS5,DHX37,TGS1,ESPL1,BUB1,TPX2,ANAPC1,CAPRIN1,NDUFAF2,POLR1B,KIF20B,SKA3,TNKS2,RRS1,LSM14A,SGK3,CENPJ,FER,TDG,NCK1,CEP89,KIF11,ARFGEF1,SMARCA5,ABCE1,CHD1,CENPH,TAF9,XRN1,SMN2,BDP1,KNTC1,ATR,CEP55,CDKN2AIP,KATNBL1,TERF1,FAM208A,HELLS,CCDC66,GCFC2,LARP4,HK2,MND1,HLTF,CETN3,RACGAP1,ESCO2,CNOT1,ANAPC7,NUP54,DSN1,	darkgrey	EC_M8
GO:0051298	1.56E-07	0.999999969	22	64	centrosome duplication	BP	9.77E-06	RTTN,CDK2,XPO1,GEN1,PLK4,STIL,CEP295,CHORDC1,BRCA2,WDR62,CENPJ,ALMS1,CKAP5,CEP72,BRCA1,CEP152,TRIM37,NDC80,CEP76,CCNF,SASS6,USP33	darkgrey	EC_M8
GO:1904358	1.63E-07	0.99999998	15	35	positive regulation of telomere maintenance via telomere lengthening	BP	1.02E-05	CCT2,MAP3K4,POT1,HNRNPA2B1,CCT4,TNKS2,ATR,CCT7,CCT5,CCT6A,NEK2,XRCC5,CCT3,AURKB,NAF1	darkgrey	EC_M8
GO:0032101	3.58E-08	1	114	646	regulation of response to external stimulus	BP	1.02E-05	TNFRSF11A,SEMA6A,TNFAIP3,TSPAN8,PTK2,C6orf106,PDGFB,MAPK13,LRRC19,TEK,DDX58,KDR,SYK,SEMA4D,IL6,CXCL8,TLR4,C5,IL15,NRP1,PUM2,PIK3CG,MGLL,PRKD2,CST7,NOVA2,CTSC,GPR4,NECTIN2,NOS3,PPARG,GATA3,CXCR4,SEC14L1,EDNRB,IKBKB,PDE2A,TMEM173,NR1D2,EFNB2,CD36,PLK2,IGF1,KLKB1,ITCH,TLR2,VEGFC,CXCL10,ANGPT2,OAS3,NLRC5,THBS4,PLCG1,ANO6,IFIT1,EDN1,STAT1,JAM3,SNCA,ETS1,CD200,HYAL2,SEMA3F,RIN3,NCF1,FABP4,CD34,MN1,FGR,INPP5F,PRKD1,YTHDF1,PAK2,LTF,ADORA2A,SEMA6D,MCU,CALCRL,BIRC3,SELE,PPM1F,RAC1,BCL6B,TNFRSF1B,ARG2,MTOR,SEMA4A,FGF18,PGF,DAPK2,S100A14,TNIP1,MMP28,CCL2,YES1,LGALS9,GIT1,SEMA6B,ISG15,NOTCH1,PLCG2,S1PR1,LRSAM1,CDH5,OAS1,GBP5,TRIM24,FGF16,TRIM62,SERPINB9,STX3,SCARF1,RNF125,BMP6	turquoise	EC_M6
GO:0070192	1.68E-07	0.999999971	19	54	chromosome organization involved in meiotic cell cycle	BP	1.04E-05	RAD21,SMC2,FANCD2,SGO1,BUB1,TERF1,BUB1B,RAD51,CCNE2,NCAPD3,TRIP13,SGO2,SPO11,SMC4,NCAPD2,INCENP,ATRX,BRIP1,NDC1	darkgrey	EC_M8
GO:0009653	3.82E-08	1	307	2050	anatomical structure morphogenesis	BP	1.07E-05	SEMA6A,KIF5A,TNFAIP3,PTPRB,FLT4,SNX2,FZD6,CTHRC1,RIMS2,CDC42SE2,CELSR1,QKI,NOTCH4,PARVG,PACSIN2,CDKL3,MTSS1,ARHGAP18,KLHL3,PTK2,ATF4,AGTPBP1,PODXL,PIM1,CDC42EP1,MFNG,STRIP2,MDFI,TEK,PGM5,ADGRF5,KIT,KDR,SYK,SEMA4D,THSD7A,SHB,TMOD1,IL6,IGF2BP3,PTBP3,CXCL8,TSPAN12,C5,TEAD2,MYCN,NRCAM,NRP1,RASIP1,PIK3CG,ITSN2,TAL1,MTMR2,GATA2,PRKD2,NOVA2,GPR4,FGD5,PLXND1,NECTIN2,PRCP,ITGA8,NOS3,ITGB4,UNC13D,PPARG,GATA3,ACVR2B,MYO7A,CXCR4,LMBR1,GZF1,SPTBN4,TIE1,JAG1,SFRP1,HBEGF,FEZ2,FERMT1,FREM2,EFNB2,ARHGEF7,SPEF1,FGD6,NTN4,CD36,RIC8A,LMOD3,SYNE4,PRAG1,ADAMTS9,TCF15,EPHB1,PLK2,RET,MERTK,CHD7,IL1A,NR4A1,EIF2AK3,HHEX,LYVE1,PREX2,SMAD1,SOX7,KIF13B,ARHGEF28,HEXB,MEF2C,TLR2,WWTR1,IL7,TPD52,VEGFC,NDRG4,GPM6A,CXCL10,ANGPT2,TIPARP,THBS4,USH1C,PPP1R16B,PLCG1,STC1,RAPGEF3,NR4A2,STAB1,HIPK1,TANC1,DLL4,DNMBP,SOX13,NEDD9,EDN1,STAT1,JAM3,DTNBP1,ETS2,GAS2,NFKB2,ETS1,HDAC1,PPP3CA,HYAL2,HECW2,MEF2A,SEMA3F,ZDHHC6,ROBO4,NRAP,PRKX,HLX,CDH24,SEC24B,ABLIM1,NFATC2,AP1AR,EPB41,JHY,CD34,PLEKHO1,PLXNA2,FGR,MCAM,CFLAR,TRAK2,ADCY1,KALRN,FES,RPS6KA1,PROX1,TMOD4,PLEKHA1,SS18L1,PRKD1,VANGL2,LAMA5,F11R,YTHDF1,MAP3K3,MLXIPL,ITGA6,DOCK1,PAK2,CST6,MELTF,APOLD1,LAMC2,DLG1,APOD,HOXD10,MOV10,RNF2,CREB1,NBEAL2,TET1,CLEC14A,LTF,ADORA2A,SEMA6D,CYBB,BCOR,EPHB4,ITGA4,IGFBP5,TBX1,MCU,CALCRL,HSPG2,WNK4,RAMP2,DNM3,MMP13,PROM1,FGF23,LMTK2,TMOD2,RAC1,HIF1A,LIPA,RCC2,RHOJ,JUP,	turquoise	EC_M6

GO:0090130	4.22E-08	1	60	266	tissue migration	BP	1.16E-05	FLT4,PTK2,PDGFB,TEK,KIT,KDR,NRP1,PIK3CG,GATA2,PRKD2,PLXND1,PRCP,NOS3,PPARG,GATA3,HBEGF,FERMT1,EFNB2,ADAMTS9,SCARB1,PLK2,NR4A1,MEF2C,VEGFC,ANGPT2,PLCG1,STC1,DLL4,EDN1,ETS1,PRKX,PROX1,PRKD1,MAP3K3,DOCK1,CLEC14A,EPHB4,PPM1F,RAC1,HIF1A,RHOJ,JUP,EPHA2,MMRN2,MCC,MTOR,SEMA4A,FGF18,ITGA3,EFNA1,VASH1,AMOT,PLEKHG5,NOTCH1,PLCG2,DAB2IP,CDH5,MIR23A,FGF16,SOX18	turquoise	EC_M6
GO:1901361	1.91E-07	1	68	395	organic cyclic compound catabolic process	BP	1.18E-05	PAN2,POP1,EIF3E,TNRC6B,TUT7,NCBP1,LRPPRC,PNPT1,SYNCRIP,PABPC4,THRAP3,EXOSC9,RNASEH2B,TRDMT1,CNOT10,UPF2,MBD4,DIS3,XRN2,AFMID,METTL3,EXOSC8,PAN3,MTREX,TDG,XRN1,PDE12,ZCCHC8,TTC37,CNOT1,TENT2,NSUN2,TET2,FXR1,DDX5,FASTKD1,SSB,PDE8A,DHX9,HNRNPR,FASTKD2,DNA2,CNOT9,HNRNPU,MRPL44,DCP2,DCTPP1,EXOSC10,FUS,DIS3L,LARP1,TAF15,UPF3B,HNRNPM,SERBP1,AIFM1,GSPT1,HPRT1,DROSHA,FMR1,METTL16,RNPS1,EXOSC2,RBM8A,FASTKD5,HNRNPD,NAF1,FEN1	darkgrey	EC_M8
GO:1904356	2.00E-07	0.999999963	20	57	regulation of telomere maintenance via telomere lengthening	BP	1.23E-05	CCT2,MAP3K4,POT1,HNRNPA2B1,CCT4,TNKS2,XRN1,ATR,TERF1,CCT7,CCT5,CCT6A,NEK2,XRCC5,HNRNPU,DCP2,EXOSC10,CCT3,AURKB,NAF1	darkgrey	EC_M8
GO:0071310	3.56E-08	1	318	1784	cellular response to organic substance	BP	1.31E-05	CD63,LRP1,AP3S1,SLC12A2,FBN2,ANGPT1,PPARA,NUS1,PTP4A3,PPARD,CTNNA1,LGALS1,SH3GL2,LEP,CDKN2B,PLAA,RORB,KIAA1324L,SRF,VEGFA,DOCK8,VCP,RUNX2,RASL11B,UNC13B,COL1A2,NCK2,CLOCK,CREB3,ASNS,REST,ASPN,TGFBR1,SOCS5,ERLEC1,ADAMTS3,MAP3K7,ACTR2,KIDINS220,ROCK2,INHBA,ZYX,MYLK3,VPS35,MGARP,PGRMC2,FKBP1B,FGF2,KIF5B,SESN3,GLI2,MEGF8,PAK1,ACTR3,TGFB1,AXL,HTR5A,CTNNB1,IL15RA,LBH,DGAT2,AKT2,PFKP,ZEB2,OSBPL8,RARB,ACTG1,ACTN4,KAT2B,LTBP1,TMTC4,CRIM1,SPRED3,SPINT2,UFM1,IRS2,PRNP,ATP2B1,DCN,SMAD9,ZNF703,UBA5,DDRGK1,ILK,LEPROTL1,ARL6IP5,DPYSL3,APLP1,CNOT7,RNF6,CXCL12,ANKRD1,HSP90B1,TMED2,IL20RB,MRAS,CD180,FLNB,OSBP,SULF1,DKK3,ROBO1,MAP1B,RYR3,SORBS1,DOK1,P2RX4,HEY1,SELENOT,USO1,APP,RAPGEF2,CX3CL1,SLC33A1,ABCC8,PTPN11,ADCY6,OPA1,AMFR,GTF2H1,ZFYVE27,PTPN22,ACVR1,CRY2,VPS18,MMP2,GDF6,MTDH,ATP2B4,SPP1,PKD2,TANK,TOLLIP,BRSK2,NDN,RAB7B,EIF4E,DYNLL1,INA,ADIPOR1,KDM5B,NOTCH2,CDC73,DNM1L,TROVE2,SELENOS,HYAL1,SLC2A10,CCND1,CPT1A,TGFB2,ZBTB20,ZNF106,LRP5,EPRS,FEZ1,HTR2B,SATB2,GSK3B,CASP7,FSTL1,DAG1,PIK3CA,LEF1,PRKCA,CMKLR1,HJV,KRAS,NTRK3,HSPA8,COL6A1,BMP7,ADCY5,VAPB,WDTC1,ATF3,FGFR2,OSM,ITGB5,HTRA1,NCSTN,SYAP1,IQGAP1,STRAP,STRN3,UROS,TFRC,ARID5B,ATF6,BACE1,SIDT2,PDIA3,RHBDD2,FAM89B,HSPB1,LINC02210-CRHR1,MAPT,PLOD3,SERPINE1,EIF4EBP2,DNAJB12,PTPN6,KCNK4,IGFBP2,ZC3H15,FBN1,PDCD10,UPF1,COMP,STIP1,FKBP8,ERO1A,COL3A1,PRKCI,COL5A2,S	darkgreen	EC_M4
GO:0043604	2.22E-07	1	98	672	amide biosynthetic process	BP	1.36E-05	PAN2,NARS,DDX39B,EIF3E,MRPL13,TNRC6B,TUT7,PUM3,NCBP1,LRPPRC,MTIF2,PNPT1,SYNCRIP,PABPC4,TIA1,MRPL32,ASCC3,DDX1,DHFR,PUS7,EXOSC9,CNOT10,MRPS7,DIS3,DARS,METTL3,EXOSC8,LARS,PRKDC,DHX29,PAN3,MRPL15,MRPS5,CAPRIN1,LSM14A,CDK5RAP1,EIF2B1,NCK1,MRPL35,PTCD3,ABCE1,XRN1,PDE12,LARP4,CNOT1,LTN1,USP16,RPS6,EEF1E1,NOLC1,GCN1,METAP1,YARS,KHDRBS1,ACSL5,IARS2,NCL,FXR1,NGDN,EIF3A,EIF2B5,DDX6,DHX9,HNRNPR,FASTKD2,TPR,RPL10L,DARS2,CNOT9,RPL22L1,DHX33,EIF2AK1,MRPL44,DCP2,EIF3B,SEPSECS,EIF4A1,RARS,MRPS23,MRPL22,GEMIN5,LARP1,ILF3,EIF5,TYMS,UPF3B,GSPT1,FMR1,METTL16,EXOSC2,RBM8A,METTL18,SARNP,HNRNPD,RNF139,PAIP2,EIF1AX,DRG1	darkgrey	EC_M8
GO:0022603	5.89E-08	1	135	761	regulation of anatomical structure morphogenesis	BP	1.59E-05	SEMA6A,TNFAIP3,FLT4,FZD6,CTHRC1,RIMS2,CDC42SE2,CELSR1,CDKL3,ARHGAP18,PTK2,CDC42EP1,STRIP2,TEK,KIT,KDR,SEMA4D,IL6,CXCL8,TSPAN12,C5,NRP1,ITSN2,GATA2,PRKD2,GPR4,FGD5,PLXND1,NOS3,UNC13D,PPARG,GATA3,CXCR4,TIE1,SFRP1,ARHGEF7,SPEF1,FGD6,NTN4,PRAG1,ADAMTS9,PLK2,RET,IL1A,HHEX,SMAD1,KIF13B,HEXB,MEF2C,VEGFC,CXCL10,ANGPT2,THBS4,PPP1R16B,PLCG1,RAPGEF3,STAB1,HIPK1,DNMBP,NEDD9,EDN1,STAT1,GAS2,ETS1,PPP3CA,HECW2,SEMA3F,ZDHHC6,SEC24B,AP1AR,EPB41,JHY,CD34,PLEKHO1,PLXNA2,FGR,TRAK2,FES,SS18L1,PRKD1,VANGL2,F11R,DOCK1,PAK2,MELTF,DLG1,MOV10,SEMA6D,CYBB,TBX1,MCU,HSPG2,RAMP2,DNM3,RAC1,HIF1A,RCC2,RHOJ,JUP,EPHA2,LFNG,FGD1,AR,PSEN1,HOXB7,SEMA4A,FGF18,ABI3,PGF,TAOK2,EFNA1,VASH1,CHRNB2,ZDHHC15,ADCK1,NPR1,DNM2,AMOT,INF2,CCL2,JAK1,SPIRE1,SEMA6B,LRG1,ARHGAP4,NOTCH1,PALMD,ENG,DAB2IP,CDH5,RELN,SHANK3,TGIF2,RALA,BMP2	turquoise	EC_M6

GO:0098655	4.44E-08	1	132	623	cation transmembrane transport	BP	1.60E-05	CYB5A,CD63,SLC12A2,ATP6V1C1,SLC22A4,CALHM5,SLC39A7,SMDT1,KCNK9,UBQLN1,SLC39A4,ATP6V1F,SLC30A9,CNNM4,SLC17A5,SHANK1,SLC31A1,ITGB1,FKBP1B,FGF2,KIF5B,SEC61A1,PANX1,SLC36A4,ATP7B,ANO10,ATP6V0B,SCN5A,GPD1L,SLC4A7,ACTN4,STIM1,ATP2C1,PRNP,ATP2B1,TMCO3,TUSC3,REM1,FXYD1,SLC20A1,SNTA1,SLC25A4,SLMAP,ATP1B3,TRPC4AP,RYR3,P2RX4,ATP2A2,CACNA1D,APP,SLC4A5,CX3CL1,SLC33A1,ABCC8,CACNB3,HOMER1,OPA1,CHP1,ATP2B4,PKD2,NIPA1,ATP6V1B2,SLC39A10,CHRNA7,MRS2,CNNM2,CALHM1,SLC2A10,HTR2B,ISCU,SHROOM2,CASQ1,TMCO1,LINC02210-CRHR1,PTPN6,KCNK4,ITGAV,HTT,PKD1L1,ERO1A,KCNA1,SLC3A2,ASIC4,SLC6A13,CHRNE,HAP1,CACNA1C,SLC29A4,NLGN2,STIM2,ATP6V1D,PPARGC1A,SFXN1,RHBG,COX6A2,SLC7A3,KCNMB1,CACNA1G,TMEM63C,CALM1,VMP1,SLC39A6,ANXA6,TRPV2,AKT1,SCN1D,PKD1,FLNA,KCNAB2,ATP6AP1,PARK7,HCN2,JPH3,SLC30A7,ATP6V0D1,MIR24-1,DMD,SURF1,FKBP1A,CBARP,SLC36A1,TMEM38B,THY1,TMSB4X,TMEM38A,YWHAH,SLC39A3,NIPAL1,HCN4,ATP6V0E1,GEM,KCNMB4	darkgreen	EC_M4
GO:0098609	5.09E-08	1	128	592	cell-cell adhesion	BP	1.79E-05	IL23A,CDSN,PLXNB2,PPARA,HLA-DRA,FAM49B,CTNNA1,LGALS1,MDGA1,MYH9,LEP,SRF,VEGFA,DOCK8,NCK2,SOCS5,LRRC4B,GLI3,BVES,SLC7A11,PCDH18,ITGB1,TENM4,GLI2,PCDH17,TGFB1,CTNNB1,C1QTNF1,ACTG1,SPINT2,ATP2C1,PRNP,SIGLEC1,ZNF703,VEZT,ILK,HSPH1,CXCL12,FAT1,IL20RB,PALLD,CTNND1,ROBO1,CHST2,LOXL3,MYL9,PLEKHA7,CX3CL1,PTPN11,CDH17,AP3B1,AMIGO2,PTPN22,ACVR1,ADA,RUNX1,CBLB,PDLIM5,ELMO2,TJP1,PKP2,TGFB2,PRKAR1A,PIK3CA,LEF1,PRKCA,BMP7,ITGB5,NECTIN4,S100A11,TFRC,OLR1,HSPB1,LPP,ITGA2B,TNR,FN1,PTPN6,IGFBP2,ITGAV,COMP,PKD1L1,VCL,GLDN,OBSL1,NINJ2,NLGN2,PCDH7,ADGRL1,MSN,FGFRL1,TLN2,SHC1,VMP1,NPTN,FAT2,ICAM5,ICAM4,CD74,LAMA3,ROCK1,AKT1,PTPN2,CD40LG,MYO10,PLXNB3,PKD1,IRAK1,NPHP4,FLNA,BSG,NTNG2,CDH13,CERCAM,BCL10,NEXN,FSTL3,PCDHB2,PDLIM1,VSTM2L,CD164,CSRP1,THY1,HAS2,STXBP6,FGL2,PRKG1,COL8A2	darkgreen	EC_M4
GO:0032212	2.92E-07	0.999999965	14	32	positive regulation of telomere maintenance via telomerase	BP	1.79E-05	CCT2,MAP3K4,POT1,CCT4,TNKS2,ATR,CCT7,CCT5,CCT6A,NEK2,XRCC5,CCT3,AURKB,NAF1	darkgrey	EC_M8
GO:0007399	5.32E-08	1	333	1867	nervous system development	BP	1.82E-05	ATP8B1,NAB2,LRP1,AHI1,RAB21,PLXNB2,TRAPPC9,PACSIN1,ADGRB1,PPARD,CTNNA1,TEAD3,SUN2,MTCH1,LGALS1,MDGA1,SH3GL2,LEP,PLAA,RORB,KIAA1324L,SRF,VEGFA,RUNX2,C2orf40,NCK2,REST,P3H1,TGFBR1,RIMS1,TMEM30A,FAM126A,UBA6,RTN4,TPBG,SHANK1,LRRC4B,MFSD2A,ACTR2,KIDINS220,WHRN,GLI3,NCDN,INHBA,VPS35,SLC7A11,PCDH18,ITGB1,FKBP1B,PLXNA1,FGF2,KIF5B,NAPA,RB1,ITM2B,EED,PICALM,MARK4,EIF2B3,TENM4,GLI2,MEGF8,SMARCD3,PCDH17,RHEB,PAK1,TGFB1,AXL,RAB3GAP1,SCN5A,HTR5A,HNMT,ACER3,CTNNB1,TRAK1,AKT2,ZEB2,FARP1,SLC4A7,RARB,NAV3,TACC1,CRIM1,SPINT2,UFM1,LIG4,IRS2,ATP2B1,DCLK1,PCLO,SEMA3C,SMPD1,ILK,DPYSL3,SPINK5,APLP1,RYK,SNPH,RNF6,CXCL12,LGI4,ZSWIM6,KIF2A,TMEM41B,ANKRD1,SLC25A46,TMED2,PALLD,ZNF536,ST8SIA4,EIF4G2,SULF1,CTNND1,ROBO1,MAP1B,DIP2B,SEMA4F,LOXL3,P2RX4,FAIM2,HEY1,TLX2,CELF1,TCTN1,APP,GLRB,DHX36,RAPGEF2,CX3CL1,ABCC8,PTPN11,ADCY6,OPA1,ARSB,AMIGO2,ZFYVE27,CRTAC1,TUBB2A,PPP3CC,CHAC1,ATP2B4,SPP1,PKD2,RUNX1,TWF1,SEMA4G,NKD1,DNAH5,GPRIN3,IFT57,BRSK2,PSMG1,PDLIM5,NDN,CHRNA7,ARL3,DOCK10,EIF4E,DYNLL1,INA,APBA2,TDP2,NOTCH2,CTTN,DNM1L,DNER,UCHL5,MARCKSL1,CAPRIN2,TGFB2,ADGRB2,FEZ1,SATB2,ITPK1,GSK3B,MMP14,DAG1,AUTS2,LEF1,ANOS1,SHROOM2,KRAS,PITPNM1,NTRK3,ACAN,HAPLN3,GFRA1,BMP7,IDH2,FGFR2,VASH2,NCSTN,IQGAP1,STRAP,KIF1A,MACO1,FARP2,TANC2,FZD9,RPS6KA3,PAFAH1B2,ARPC5,SCYL1,MAPT,PLOD3,C1QL1,ATP6AP2,FZD2,MDGA2,TNR,FN1,NCKAP1,GPC2,ADRA2C,FKBP8,ESS2,COL3A1,PRKCI,SKIL,PR	darkgreen	EC_M4

GO:0065003	3.06E-07	1	161	1210	protein-containing complex assembly	BP	1.86E-05	SHPRH,LATS1,NUP107,CCT2,DDX39B,FBXO5,EIF3E,RRP7A,FANCC,SRPK1,PSIP1,AKAP9,NCBP1,ANP32B,PPIH,SENP6,YTHDC1,PNPT1,MDN1,NUP205,ANLN,ITGB1BP1,TAF1B,CAPZA2,DDX1,MCM2,CENPO,CEP57,YME1L1,CENPA,NASP,CLASP2,NIFK,MRPS7,RSF1,DIAPH3,DARS,TK1,CRNKL1,KAT6A,NAP1L1,RRM1,TAF7,TUBGCP3,SNRPF,PRKDC,CCNB1,DHX29,TGS1,TPX2,NDUFAF2,RRS1,CENPJ,FER,NCK1,ARFGEF1,SMARCA5,CENPH,TAF9,SMN2,BDP1,KNTC1,ATR,TERF1,HELLS,GCFC2,USP16,PPID,MIS18A,CKAP5,NUP93,RAD51,SCAF11,RIPK1,OIP5,STK4,MDM4,CHAF1B,UBE2C,CUL3,NAP1L4,GMNN,FP565260.1,KCTD3,SF3B1,CENPE,RBM5,CAMSAP2,COPS7B,HAUS2,KIF14,PRMT5,AJUBA,TBCE,HJURP,EIF3A,ALS2,BLM,CENPF,HAT1,CDK1,DHX9,HDGFL3,FASTKD2,DDX20,PRPF39,RPL10L,UBTF,XRCC5,CDC45,LETM1,HP1BP3,DLGAP5,TRPM7,ECT2,DHX33,FKBP4,RAD52,UBE2K,EIF3B,ASF1B,SRSF5,CCT3,FUS,TAF1,LUC7L3,ATRX,THG1L,COIL,LCMT1,CENPI,GEMIN5,PSMD10,RIOK3,EIF5,NLE1,RICTOR,AIFM1,BRIX1,AFG3L2,HPRT1,RBMX,CHAF1A,NDC1,UBN1,TP73,FANCA,CDT1,CENPN,CTH,HIST1H1A,GEMIN4,TMOD3,STMN1,ITGB3BP,HIST1H3H,GEMIN6,NAF1,CENPW,OTUD6B,DRG1	darkgrey	EC_M8
GO:0007064	3.09E-07	0.999999974	12	22	mitotic sister chromatid cohesion	BP	1.87E-05	RAD21,DSCC1,CDC20,SGO1,NAA50,SMC3,CDCA5,PDS5A,SMC1A,ATRX,NIPBL,HASPIN	darkgrey	EC_M8
GO:0070925	3.10E-07	1	108	705	organelle assembly	BP	1.87E-05	RTTN,CDK2,PAN2,ONECUT2,FBXO5,KIF3A,TBC1D32,RRP7A,KIFC1,PLEC,HAUS6,IFT74,KIF24,RFX3,ICK,STX17,SENP6,CEP162,MDN1,ANLN,WDR35,SCLT1,PLK4,STIL,CEP295,CENPA,CLASP2,MRPS7,CDC20,VPS36,CEP290,TUBGCP3,PRKDC,WDR62,DHX29,PAN3,HAUS5,DHX37,TPX2,RRS1,LSM14A,CENPJ,CEP89,KIF11,CENPH,KNTC1,CCDC66,RACGAP1,CNOT1,ALMS1,CFAP298,ABRAXAS1,KNL1,MYBL2,INO80,FP565260.1,SMC3,CENPE,CEP72,HAUS2,ASPM,RCC1,NEK2,PRC1,DDX6,CENPF,CDKL5,SEPT2,FASTKD2,PIKFYVE,TPR,KIF15,RPL10L,IFT80,XRCC5,BRCA1,CEP152,RP2,HAUS3,PRRC2C,DLGAP5,KIAA0586,RAB8A,HNRNPU,INCENP,SMC1A,CCNB2,KPNB1,KIF4A,AURKB,KIF23,TRIM37,MRPL22,PLK1,G3BP1,NLE1,STAG2,BRIX1,CEP76,GAS8,CENPN,SASS6,IST1,TMOD3,AURKA,CENPW,UBXN2A,DRG1	darkgrey	EC_M8
GO:0043487	3.32E-07	0.999999894	35	148	regulation of RNA stability	BP	2.00E-05	PAN2,TNRC6B,TUT7,PNPT1,SYNCRIP,PABPC4,THRAP3,EXOSC9,TRDMT1,DIS3,METTL3,EXOSC8,PAN3,PDE12,CNOT1,TENT2,NSUN2,FXR1,FASTKD1,DHX9,HNRNPR,FASTKD2,HNRNPU,DCP2,FUS,LARP1,TAF15,HNRNPM,SERBP1,FMR1,METTL16,EXOSC2,FASTKD5,HNRNPD,NAF1	darkgrey	EC_M8
GO:0000079	3.36E-07	0.999999923	23	80	regulation of cyclin-dependent protein serine/threonine kinase activity	BP	2.01E-05	LATS1,CDC25C,CKS2,CCNA2,CCNT2,GTPBP4,IPO5,CCNB1,IPO7,CDK5RAP1,CCNL1,CCNE2,HERC5,BLM,CDC25A,CDKN3,CCNB3,CDC6,CCNB2,PLK1,PSMD10,PKMYT1,CCNF	darkgrey	EC_M8
GO:0010594	8.23E-08	0.999999973	41	157	regulation of endothelial cell migration	BP	2.14E-05	FLT4,PTK2,PDGFB,TEK,KDR,NRP1,PIK3CG,GATA2,PRKD2,PRCP,NOS3,PPARG,GATA3,ADAMTS9,SCARB1,PLK2,MEF2C,VEGFC,ANGPT2,PLCG1,STC1,DLL4,EDN1,ETS1,PROX1,PRKD1,MAP3K3,RAC1,HIF1A,RHOJ,JUP,EPHA2,MMRN2,SEMA4A,FGF18,EFNA1,VASH1,NOTCH1,DAB2IP,MIR23A,FGF16	turquoise	EC_M6
GO:0001936	8.31E-08	0.999999977	33	111	regulation of endothelial cell proliferation	BP	2.14E-05	FLT4,PDGFB,TEK,KDR,CAV2,NRP1,GATA2,PRKD2,PPARG,TIE1,NR4A1,MEF2C,VEGFC,THBS4,PPP1R16B,PLCG1,DLL4,STAT1,PROX1,PRKD1,ITGA4,HIF1A,ALDH1A2,MTOR,PGF,VASH1,CCL2,LRG1,NRARP,EGFL7,MIR23A,BMP2,BMP6	turquoise	EC_M6
GO:0098542	8.36E-08	1	102	574	defense response to other organism	BP	2.14E-05	IFNGR1,TNFAIP3,TRIM39,LYZ,PTK2,C6orf106,DAPK1,DDX58,SYK,IL6,REL,RSAD2,TLR4,C5,IL15,PUM2,PIK3CG,NECTIN2,UNC13D,PPARG,GATA3,SEC14L1,IKBKB,TMEM173,CD36,ARID5A,BPIFB3,ITCH,TLR2,CXCL10,SERINC5,OAS3,NLRC5,STAB1,IFIT2,IFIT1,EDN1,STAT1,CD58,SNCA,MX2,MX1,RNF19B,LCK,HYAL2,LYST,NCF1,ISG20,TIFA,FGR,FES,PRKD1,SRMS,PAK2,STYK1,MOV10,LTF,MASP1,RTP4,CYBB,IL1RAP,DDX3X,TMEM106A,BIRC3,SELP,WAS,RAB27A,EPHA2,TLR6,TNK1,ARG2,TBKBP1,BATF,S100A14,SCNN1B,CCL2,YES1,JAK1,LGALS9,NOS2,ELF4,IL7R,ISG15,IRF8,PLCG2,STXBP1,LRSAM1,DAB2IP,OAS1,VNN1,PMAIP1,TLR1,GBP5,RAB43,TRIM34,CD46,TRIM62,SERPINB9,BST2,VAMP3,IFIT3,RNF125	turquoise	EC_M6
GO:1901989	3.60E-07	0.999999911	25	91	positive regulation of cell cycle phase transition	BP	2.15E-05	DDX39B,FBXO5,CDC25C,EZH2,CUL4A,CDC16,CCNB1,ESPL1,ANAPC7,FAM83D,UBE2C,CUL3,DTL,CDK1,CDC25A,CDC27,CDCA5,DBF4B,DLGAP5,CDC6,STXBP4,THOC1,ATAD5,CDT1,TMOD3	darkgrey	EC_M8
GO:0032204	3.66E-07	0.999999917	23	74	regulation of telomere maintenance	BP	2.17E-05	CCT2,MAP3K4,POT1,HNRNPA2B1,CCT4,TNKS2,XRN1,ATR,TERF1,CCT7,NBN,CCT5,CCT6A,NEK2,XRCC5,HNRNPU,DCP2,EXOSC10,CCT3,AURKB,ATRX,HNRNPD,NAF1	darkgrey	EC_M8

GO:0035295	8.85E-08	1	145	849	tube development	BP	2.23E-05	SEMA6A,TNFAIP3,HECA,PTPRB,FLT4,FZD6,CTHRC1,CELSR1,QKI,NOTCH4,MTSS1,KLHL3,PTK2,PODXL,TEK,ADGRF5,KIT,KDR,SYK,THSD7A,SHB,IL6,CXCL8,TSPAN12,C5,TEAD2,MYCN,NRCAM,NRP1,RASIP1,PIK3CG,TAL1,GATA2,PRKD2,GPR4,PLXND1,PRCP,NOS3,PPARG,GATA3,CXCR4,GZF1,EDNRB,TIE1,JAG1,SFRP1,EFNB2,ADAMTS9,EPHB1,PLK2,RET,CHD7,IL1A,NR4A1,EIF2AK3,HHEX,SMAD1,MEF2C,WWTR1,VEGFC,NDRG4,CXCL10,ANGPT2,TIPARP,THBS4,PPP1R16B,PLCG1,RAPGEF3,STAB1,HIPK1,DLL4,EDN1,STAT1,JAM3,ETS1,DPPA4,ROBO4,PRKX,HLX,SEC24B,CD34,PLXNA2,MCAM,PROX1,PRKD1,VANGL2,LAMA5,MAP3K3,APOLD1,DLG1,APOD,CREB1,CLEC14A,CYBB,EPHB4,IGFBP5,TBX1,CALCRL,HSPG2,WNK4,RAMP2,PROM1,HIF1A,LIPA,RHOJ,JUP,ADIPOR2,EPHA2,MMRN2,CECR2,ARG2,ALDH1A2,AR,PSEN1,HOXB7,SEMA4A,FGF18,ITGA3,PGF,EFNA1,VASH1,NPR1,SMAD2,CYP1A1,AMOT,CCL2,JAK1,LRG1,NRARP,EGFL7,NOTCH1,S1PR1,ENG,DAB2IP,CDH5,MIR23A,ATXN1L,ADGRG1,VASP,SOX17,SOX18,TBC1D20,RALA,BMP2,PIFO	turquoise	EC_M6
GO:0007166	6.69E-08	1	340	1935	cell surface receptor signaling pathway	BP	2.24E-05	CD63,IL23A,LRP1,AP3S1,AHI1,DYRK2,PLXNB2,FBN2,ANGPT1,PPARA,ROS1,NUS1,RSPO3,PTP4A3,ADGRB1,LY6E,CTNNA1,CBY1,GNAQ,MYH9,LEP,CDKN2B,BTBD11,KIAA1324L,BAG1,CDK14,VEGFA,VCP,RUNX2,RASL11B,UNC13B,COL1A2,NCK2,ASPN,DST,TGFBR1,SOCS5,RIMS1,RTN4,TPBG,SHANK1,ADAMTS3,MAP3K7,KIDINS220,MDFIC,GLI3,INHBA,ZYX,DKKL1,VPS35,ITGB1,PLEKHA4,PLXNA1,FGF2,SESN3,RAB7A,RB1,EIF2B3,PTCH2,KCTD21,GLI2,MEGF8,PAK1,TGFB1,AXL,RAB3GAP1,CTNNB1,IL15RA,AKT2,DZIP1,ZEB2,OSBPL8,ACTN4,LTBP1,CRIM1,SPRED3,IRS2,PRNP,DCN,SMAD9,ZNF703,ACPP,SEMA3C,CCKBR,ILK,RBPMS,LEPROTL1,RYK,PRICKLE2,CNOT7,CXCL12,IL20RB,CD180,OSBP,SULF1,CTNND1,DKK3,ROBO1,SORBS1,SEMA4F,DOK1,LOXL3,P2RX4,FAIM2,HEY1,RASA1,TCTN1,P2RY14,APP,GLRB,RAPGEF2,CX3CL1,SLC33A1,CACNB3,CPE,PTPN11,HOMER1,OPA1,CDH17,AMFR,ZFYVE27,PTPN22,TSG101,ACVR1,CHAC1,MMP2,GDF6,MTDH,ADA,PKD2,HIF1AN,SEMA4G,CBLB,NKD1,BRD7,IFT57,TOLLIP,DOK2,NDN,SLC39A10,CHRNA7,ARL3,ADIPOR1,TDP2,NOTCH2,CTTN,CDC73,DNER,TROVE2,UCHL5,CHSY1,SLC2A10,EYA2,CCND1,CAPRIN2,TGFB2,ZNF106,LRP5,ADGRB2,ARMC9,GSK3B,FSTL1,MMP14,PIK3CA,LEF1,PRKCA,ADAP1,CMKLR1,HJV,KRAS,NTRK3,GFRA1,KPNA1,BMP7,ADCY5,BICC1,ATF3,FGFR2,OSM,PEA15,ITGB5,HTRA1,NCSTN,IQGAP1,STRAP,MUC20,FARP2,ARID5B,FZD9,PDIA3,FAM89B,HSPB1,LINC02210-CRHR1,SERPINE1,EIF4EBP2,SGPL1,ADAM11,ATP6AP2,FZD2,ITGA2B,FN1,MPP2,PTPN6,IGFBP2,ZC3H15,GPC2,FBN1,PDCD10,ITGAV,COMP,HTT,FKBP8,COL3A1,PRKCI,SKIL,FGFR3,PRRX1,GORAB,SMURF1,NBL1,TI	darkgreen	EC_M4
GO:0033365	3.80E-07	1	110	737	protein localization to organelle	BP	2.25E-05	LATS1,NUP107,CCT2,UBR5,RAD21,TBC1D32,TNPO3,POT1,TOPORS,GCC2,CENPQ,NOL8,TRIM28,MSH2,TTK,XPO1,CCT4,TIA1,EZH2,DDX1,WDR35,BCAP29,STIL,PMPCB,SAE1,CENPA,COG3,NUP85,PITRM1,HOOK3,IPO5,VPS36,BRCA2,NEDD1,MAPK8,NUP58,KIF20B,IPO11,IPO7,TNKS2,RRS1,POLR1A,KNTC1,ATR,TERF1,TNPO1,FAM208A,CCDC66,HK2,ESCO2,MCRS1,NUP54,BUB1B,CCT7,FAM83D,NUP93,KNL1,TOMM70,MORC3,HEATR3,NOLC1,CCT5,CCT6A,TIMM17A,CEP72,INTS13,CSE1L,RPA2,SPO11,NUP133,SSB,BUB3,CCDC47,CDK1,DHX9,SETD2,PIKFYVE,TPR,CDCA5,IFT80,NMD3,GRPEL1,XRCC5,ECT2,HNRNPU,WAPL,NEDD4,KPNB1,CCT3,SPDL1,AURKB,ATRX,ZWILCH,TIMM8A,PLK1,NUP62CL,NDC80,NUP155,NIPBL,AIFM1,RPA1,HASPIN,RER1,PMPCA,GAS8,CDT1,CTCF,ANKRD13C,AURKA,NUP35	darkgrey	EC_M8

GO:0009966	9.37E-08	1	319	2237	regulation of signal transduction	BP	2.32E-05	TNFRSF11A,SEMA6A,TNFAIP3,TRIM39,PLEKHG1,AKAP12,FLT4,FZD6,CTHRC1,RIMS2,CDC42SE2,GRAMD4,TPD52L1,RNF146,ARHGAP18,SGSM3,PTK2,DENND3,ANKS1A,DAPK1,PDGFB,CSNK1E,KDM4C,MFNG,MDFI,LRRC19,TEK,RIC8B,PIP5K1B,SH3RF3,KIT,KDR,CD2AP,SYK,GADD45G,SEMA4D,RNF149,IL6,REL,CHN2,CXCL8,PELI1,RGS3,ARHGAP25,RSAD2,FIGNL1,CAV2,GRB10,LANCL2,TSPAN12,TLR4,TRAF1,DLGAP3,CCNY,SDCBP,TRPM4,NRP1,PUM2,RASIP1,PIK3CG,ARHGAP42,MTMR2,MGLL,GATA2,ARHGAP21,PRKD2,CTSC,GPR4,FGD5,NECTIN2,PRCP,NOS3,LLGL2,ZFAND6,PPARG,GATA3,ACVR2B,CXCR4,IRAK2,SEC14L1,NET1,RALGAPA2,NDFIP2,TMC8,CYTH1,IKBKB,JAG1,P2RY6,PDE2A,SFRP1,HBEGF,NOTUM,FERMT1,RPL26,SPEF1,ARHGAP26,P2RX2,CD36,SIRT3,PRAG1,PRR5L,EPHB1,PLK2,RET,PDE4D,TNFRSF19,IL1A,IGF1,EIF2AK3,ABHD6,HHEX,ITCH,ERBIN,UFSP2,SH3RF1,PREX2,HIP1R,ARHGEF3,SOX7,ARHGEF28,MEF2C,TLR2,WWTR1,IL7,P2RX7,VEGFC,NDRG4,GUCY1A1,MADD,LMBR1L,ADGRG3,KMT2D,BMP2K,OAS3,NLRC5,PPP1R16B,NR4A2,DGKZ,HIPK1,DLL4,ZDHHC13,DNMBP,SOX13,ADAMTS20,EDN1,STAT1,DYRK1A,SNCA,DTNBP1,DUSP8,GAS2,PRDM15,SHANK2,HDAC1,ITPRIP,LCK,HYAL2,GIT2,SHOC2,HCRTR1,LAPTM5,NCF1,DYRK3,HCLS1,INPP5D,TIFA,CD86,MN1,ARHGEF12,SH3BP4,FGR,CFLAR,CASP10,INPP5F,KALRN,IGFBP3,PROX1,PLEKHA1,PRKD1,F11R,IL10RA,ARHGDIB,MAP3K3,SRMS,ITGA6,PAK2,PTPRE,CNKSR2,SENP2,DUSP16,DLG1,ELL3,SIPA1,APOD,MAP3K11,LTF,ARHGAP27,BCR,ADORA2A,TSPAN15,DDX3X,SOS2,IGFBP5,HOMER3,TBX1,TMEM106A,MECOM,RAMP2,BIRC3,SELP,VWF,TNFAIP8L3,BAIA	turquoise	EC_M6
GO:0048584	9.61E-08	1	222	1488	positive regulation of response to stimulus	BP	2.34E-05	TNFRSF11A,TNFAIP3,TRIM39,AKAP12,FLT4,RIMS2,TPD52L1,RNF146,PTK2,PDGFB,MAPK13,CSNK1E,MFNG,LRRC19,TEK,DDX58,TEC,SH3RF3,KIT,TNFRSF21,KDR,SYK,GADD45G,SEMA4D,IL6,REL,CXCL8,PELI1,RSAD2,CAV2,GRB10,LANCL2,TLR4,TRAF1,C5,IL15,SDCBP,TRPM4,NRP1,PUM2,PIK3CG,KDM4D,GATA2,PRKD2,CTSC,GPR4,NECTIN2,NOS3,GAB2,PPARG,GATA3,ACVR2B,CXCR4,NET1,NDFIP2,IKBKB,JAG1,P2RY6,PDE2A,TMEM173,SFRP1,HBEGF,FERMT1,RPL26,P2RX2,CD36,PRAG1,PRR5L,PLK2,RET,PDE4D,TNFRSF19,IGF1,HHEX,ERBIN,SH3RF1,HIP1R,OCLN,ARHGEF3,MEF2C,TLR2,IL7,VEGFC,NDRG4,GUCY1A1,CXCL10,MADD,KMT2D,BMP2K,NLRC5,THBS4,PLCG1,DGKZ,ANO6,DLL4,ZDHHC13,ADAMTS20,EDN1,SNCA,PRDM15,ETS1,LCK,PPP3CA,HYAL2,ICOSLG,SHOC2,HCRTR1,LAPTM5,HLX,NCF1,USP13,FABP4,HCLS1,NFATC2,INPP5D,TIFA,CD86,MN1,BMX,FGR,CFLAR,CASP10,PROX1,PLEKHA1,PRKD1,F11R,IL10RA,MAP3K3,PAK2,MAP3K11,B2M,LTF,ADORA2A,MASP1,DDX3X,SOS2,IGFBP5,TBX1,MCU,TMEM106A,BIRC3,SELP,VWF,TNFAIP8L3,FGF23,CRACR2A,PPM1F,WAS,TMOD2,PRKCH,RAC1,HIF1A,PLD2,TSPAN14,JUP,ITPKB,BID,TLR6,RELL1,ARG2,MTOR,AR,PSEN1,FGF18,ABI3,PGF,EIF2B2,DAPK2,TAOK2,EFNA1,CD19,PIK3R5,NPR1,S100A14,SMAD2,TNIP1,ADRB2,CCL2,YES1,JAG2,PDE4B,LGALS9,IL7R,SPIRE1,LRG1,TEDC2,NRARP,TRAF2,PLEKHG5,NOTCH1,PLCG2,S1PR1,ENG,STXBP1,LRSAM1,NFAT5,DAB2IP,CDH5,MIR23A,FYB1,PMAIP1,TLR1,ADGRG1,GBP5,TRIM34,CD46,RELN,ALKAL1,LYPD6,FGF16,SHANK3,LPAR6,RGS4,TRIM62,FCER2,STX3,PDCL,BST2,BMP2,SCARF1,NMNAT1,BMP6	turquoise	EC_M6
GO:0050789	9.98E-08	1	955	7907	regulation of biological process	BP	2.39E-05	PARD6G,ZNF516,TNFRSF11A,BAZ2A,TCF4,TNFAIP8,SEMA6A,LVRN,PDE7B,IFNGR1,TNFAIP3,HECA,PLAGL1,TBC1D30,TRIM39,PLEKHG1,AKAP12,PTPRB,TSPAN8,LAPTM4B,CNOT6,FLT4,SNCAIP,FZD6,CTHRC1,RIMS2,OXR1,ZBED4,CDC42SE2,GRAMD4,CELSR1,QKI,NOTCH4,AFDN,PACSIN2,TCF20,TCF7,CDKL3,TRDN,TPD52L1,NCOA7,MTSS1,RNF146,ASAP1,ARHGAP18,L3MBTL3,SGSM3,PTK2,DENND3,C6orf106,ANKS1A,DAPK1,ATF4,PDGFB,AGTPBP1,MAPK13,BRPF3,PODXL,CSNK1E,BAIAP2L2,PICK1,KDM4C,PIM1,CDC42EP1,MFNG,STRIP2,CSF2RB,NFYA,MDFI,OSTF1,LRRC19,TEK,LINGO2,IREB2,PRDM4,LIMCH1,RIC8B,DDX58,ATP8A1,NFX1,APBA1,DCAF12,PIP5K1B,FAM122A,TEC,SH3RF3,GNG11,ADGRF5,KIT,ADGRF1,TNFRSF21,KDR,CD2AP,NFIL3,SYK,GADD45G,SEMA4D,RNF149,EFHC1,RGP1,ICA1,SHB,TMOD1,TSPAN13,PPP6R1,RAPGEF5,IL6,IGF2BP3,NFE2L3,SNAP91,REL,PPT1,CREB5,PTBP3,CHN2,CXCL8,PPIE,BACH2,KBTBD2,VPS54,PELI1,UTP11,RGS3,ARHGAP25,ELMO1,RSAD2,DOCK4,FIGNL1,FOXP2,CAV2,KLF11,ODC1,GRB10,LANCL2,TSPAN12,NOBOX,TLR4,TCAF2,GRIK2,TRAF1,C5,DLGAP3,INPP4B,IL15,FZD8,CCNY,CREM,TEAD2,SDCBP,MYCN,FAM49A,NRCAM,NOCT,TUT4,TRPM4,NRP1,KTI12,RAB3B,ZBTB24,PUM2,RASIP1,BEND5,PIK3CG,ARHGAP42,SVIL,ITSN2,TAL1,LAMA4,PHF10,MTMR2,MGLL,EPC1,KDM4D,GATA2,DNMT3A,ARHGAP21,PRKD2,SAP130,KCNRG,CST7,NOVA2,CTSC,RAB38,CAB39L,GPR4,LRCH1,SNRK,FGD5,PLXND1,NECTIN2,PRCP,BCAM,ITGA8,NOS3,GGA3,GAB2,LLGL2,ITGA9,RALB,USP6NL,ITGB4,UNC13D,ZFAND6,DACH1,PPARG,TIMP4,GATA3,TAF3,ACVR2B,TBC1D4,ITIH5,CXCR4,LMBR1,IRAK2,GZF1,SEC14L	turquoise	EC_M6
GO:0000018	4.32E-07	0.999999896	24	83	regulation of DNA recombination	BP	2.55E-05	MSH2,PAXIP1,RIF1,ALYREF,PPP4R2,ERCC6,PARPBP,MSH3,RAD51,ARID2,SMARCAD1,CHEK1,FANCB,RPA2,BLM,SETD2,NSD2,RAD51AP1,ANKLE1,FUS,THOC1,SMCHD1,RMI2,HIST1H1A	darkgrey	EC_M8

GO:0035825	4.49E-07	0.999999926	17	46	homologous recombination	BP	2.64E-05	RAD21,RMI1,UBR2,RAD54L,FANCD2,TOP2B,MND1,MSH3,RAD51,NUCKS1,TRIP13,SPO11,FANCM,RAD51AP1,TOP2A,EME1,BRIP1	darkgrey	EC_M8
GO:0051173	4.68E-07	1	285	2331	positive regulation of nitrogen compound metabolic process	BP	2.74E-05	PAN2,ONECUT2,BCLAF1,LATS1,CCT2,DDX39B,SCAF8,EIF3E,MAP3K4,DSCC1,HSF2,ATAD2,XRCC6,PHF5A,TNRC6B,TUT7,IFT74,POT1,PWP1,ZNF300,TOPORS,DBF4,AKAP9,RFX3,CKS2,TRIM28,NCBP1,ZNF451,ANP32B,FOXJ3,MSH2,PHIP,TTK,HNRNPA2B1,PNPT1,RLF,ZNF292,CCT4,COMMD1,CASP8AP2,ETAA1,ITGB1BP1,THRAP3,EZH2,CCNC,CLSPN,CASP2,NAA15,ELF2,WDR35,NRDC,ATAD2B,HDAC2,CCNA2,EXOSC9,MLLT10,SAE1,CEP295,DNAJC2,SYMPK,NUP85,ELF1,RSF1,BORA,DIS3,CCNT2,PAXIP1,WDR43,CDC20,FNTA,SUPT16H,CHD8,KAT6A,METTL3,RIF1,E2F7,ALYREF,CKAP2,CEP290,NSD3,CEBPZ,KITLG,TAF7,EXOSC8,RFC3,BRCA2,ELK3,TCERG1,MAPK8,PRKDC,APAF1,CCNB1,ERCC6,DHX29,PAN3,GPBP1,SP1,TCEA1,TPX2,ZC3H8,DIMT1,UBA2,TNKS2,ZNF143,CENPJ,CDK5RAP1,NCK1,IDE,ARFGEF1,SMARCA5,ZNF507,OTUD4,ZFP91,TAF9,PDE12,TFCP2,ATR,LARP4,UTP15,HLTF,CNOT1,GABPA,MCRS1,USP16,MOB1A,CCT7,CDCA2,NBN,SENP1,ABRAXAS1,RAD51,SLC38A2,RIPK1,WDR75,ARID2,MYBL2,E2F8,INO80,RIOK1,CHUK,STK4,UBE2C,HIVEP1,RFC5,TAOK3,NOLC1,CCT5,BMPR1B,UBE3A,CCT6A,PRDM10,NUCKS1,GCN1,CUL3,CHEK1,TIMM17A,H2AFZ,RRP1B,NFKB1,KHDRBS1,CENPE,TET2,TRIP11,HEATR1,NCL,KIF14,AJUBA,NOL11,GTF2I,BPTF,FXR1,FANCI,DDX5,FANCB,RFC2,NEK2,EIF2B5,DTL,ALS2,BLM,EDRF1,PDE8A,ING5,BCLAF3,FOSL1,SMARCC1,SP3,DHX9,HNRNPR,TRA2B,FASTKD2,RFC4,CCAR1,FANCM,DBF4B,IL12A,UBTF,COP1,NMD3,XRCC5,NPAT,BRCA1,PSME3,CNOT9,NSD2,DCUN1D5,RAD51AP1,DCAF6,ECT2,DHX33,TBL1XR1,LEO1,FOXM1,LYAR,DDX11,HNRNPU,INCENP,UBE2K,NEDD4,SMARCE1,RFC1,TOP2A,CDC6,DCP2,	darkgrey	EC_M8
GO:0006928	1.21E-07	1	239	1565	movement of cell or subcellular component	BP	2.85E-05	TNFRSF11A,SEMA6A,KIF5A,TNFAIP3,AKAP12,FLT4,CTHRC1,CELSR1,AFDN,ARHGAP18,PTK2,ANKS1A,PDGFB,AGTPBP1,PODXL,STRIP2,TEK,LIMCH1,DDX58,ATP8A1,APBA1,KIT,KDR,CD2AP,SYK,SEMA4D,EFHC1,IL6,CXCL8,AVL9,ELMO1,DOCK4,GRB10,TLR4,TCAF2,C5,SDCBP,NRCAM,TRPM4,NRP1,PIK3CG,LAMA4,TCTE3,GATA2,KIF3C,ARHGAP21,PRKD2,NOVA2,LRCH1,PLXND1,PRCP,NOS3,ITGB4,DACH1,PPARG,GATA3,MYO7A,CXCR4,EDNRB,NET1,TIE1,JAG1,P2RY6,SFRP1,HBEGF,FEZ2,FERMT1,EFNB2,ARHGEF7,SPEF1,NTN4,RIC8A,PRAG1,ADAMTS9,PRR5L,SCARB1,EPHB1,PLK2,DNAH10,RET,PDE4D,MERTK,IGF1,NR4A1,ABHD6,SH3RF1,MCTP1,KIF13B,HEXB,MEF2C,VEGFC,NDRG4,GPM6A,CXCL10,ANGPT2,ADGRG3,THBS4,PLCG1,STC1,KCNJ3,NR4A2,DGKZ,ANO6,DLL4,PIK3C2B,NEDD9,EDN1,JAM3,DTNBP1,SRGAP2C,ETS1,CD200,LCK,PPP3CA,HYAL2,KCNJ2,SEMA3F,RIN3,ROBO4,PRKX,LYST,NFATC2,AP1AR,JHY,CD34,PECAM1,PLEKHO1,PLXNA2,FGR,MCAM,INPP5F,TRAK2,KALRN,IGFBP3,FES,PROX1,PRKD1,VANGL2,LAMA5,F11R,YTHDF1,ARHGDIB,MAP3K3,ACE,ITGA6,DOCK1,LAMC2,DLG1,APOD,CLEC14A,CCRL2,SEMA6D,EPHB4,ITGA4,IGFBP5,TBX1,MCU,LDB2,SELE,SELP,PLVAP,FGF23,PPM1F,WAS,RAC1,HIF1A,RCC2,RHOJ,SYNE2,PLD2,JUP,ADIPOR2,FSCN1,EPHA2,MMRN2,MCC,MTOR,OPHN1,EFNB1,PSEN1,STK10,SEMA4A,HOXB9,FBXW11,FGF18,ABI3,ITGA3,PGF,DAPK2,TAOK2,EFNA1,VASH1,ATP2A1,RPS6KA5,S100A14,CCDC88C,DSG2,DSC2,CDC42BPB,MMP28,AMOT,CCL2,YES1,PDE4B,LGALS9,SPEF2,STK26,MYO18A,SEMA6B,BICDL2,ARHGAP4,AP3D1,PLEKHG5,NOTCH1,PLCG2,S1PR1,ENG,FNBP1L,TERF2,LHX6,DAB2IP,CARMIL	turquoise	EC_M6
GO:0000904	9.15E-08	1	123	562	cell morphogenesis involved in differentiation	BP	3.00E-05	LRP1,RAB21,PLXNB2,ADGRB1,MYH9,SRF,VEGFA,COL15A1,RTN4,TPBG,SHANK1,MFSD2A,ACTR2,KIDINS220,WHRN,GLI3,BVES,ITGB1,PLXNA1,KIF5B,RB1,PICALM,GLI2,MEGF8,PAK1,AXL,CTNNB1,TRAK1,FARP1,ACTN4,SPINT2,DCLK1,SEMA3C,ILK,RYK,RNF6,AKIP1,CXCL12,FAT1,PALLD,FLNB,EIF4G2,ROBO1,MAP1B,DIP2B,SEMA4F,TLX2,TCTN1,APP,DHX36,RAPGEF2,PTPN11,OPA1,ZFYVE27,SPP1,SEMA4G,BRSK2,PDLIM5,NDN,CHRNA7,DOCK10,NOTCH2,CTTN,DNM1L,CAPRIN2,FEZ1,GSK3B,DAG1,AUTS2,LEF1,CORO1C,ANOS1,BMP7,FGFR2,KIF1A,CLIC4,TANC2,MAPT,PLOD3,TNR,FN1,PTPN6,ITGAV,ARPC2,SKIL,TNIK,VCL,WDR1,OBSL1,CRMP1,NOTCH3,ENAH,ACTN1,SRCIN1,NGFR,RAB11A,MAP2K1,TTC8,NPTN,ISLR2,TRPV2,LAMA3,PLXNB3,PAFAH1B1,NTN3,FLNA,PLXNA3,RERE,BSG,NTNG2,BARHL2,NEXN,DPYSL5,VSTM2L,GDNF,THY1,HAS2,CHN1,ARTN,YWHAH,SDC2,MAP1A,OLFM1	darkgreen	EC_M4
GO:0010972	5.28E-07	0.999999906	18	51	negative regulation of G2/M transition of mitotic cell cycle	BP	3.08E-05	RAD21,CLSPN,ORC1,RINT1,TOPBP1,NBN,ABRAXAS1,TAOK3,CHEK1,TICRR,DTL,BLM,CDK1,BRCA1,CDC6,AURKB,PLK1,TAOK1	darkgrey	EC_M8

GO:0051241	1.40E-07	1	126	743	negative regulation of multicellular organismal process	BP	3.25E-05	SEMA6A,IFNGR1,TNFAIP3,TSPAN8,LAPTM4B,CDKL3,C6orf106,ATF4,PDGFB,TEK,TNFRSF21,CD2AP,SEMA4D,IL6,REL,DOCK4,TLR4,IL15,MYCN,TRPM4,NRP1,PIK3CG,ARHGAP42,LAMA4,MTMR2,GATA2,NOVA2,CTSC,GPR4,NOS3,PPARG,GATA3,ACVR2B,SPTBN4,TIE1,SFRP1,FERMT1,TMEM178A,ADAMTS9,PLK2,PDE4D,MERTK,IGF1,KLKB1,EIF2AK3,ABHD6,HHEX,ITCH,ERBIN,PROCR,MEF2C,TLR2,P2RX7,GUCY1A1,CXCL10,ANGPT2,SFMBT1,OAS3,THBS4,STC1,STAB1,DLL4,EDN1,STAT1,JARID2,MYLIP,CD200,PPP3CA,SEMA3F,LAPTM5,HLX,INPP5D,EAF2,CD34,TRAK2,ARHGDIB,APOD,B2M,LTF,SEMA6D,BCOR,ADAMTS7,IGFBP5,HOMER3,CALCRL,HSPG2,WNK4,FGF23,RAC1,HIF1A,JUP,EPHA2,MMRN2,TLR6,MCC,TNFRSF1B,ARG2,MTOR,PSEN1,SEMA4A,EFNA1,VASH1,NPR1,RPS6KA5,GPR68,TLE3,YY1,AMOT,ADRB2,RAI1,PDE4B,LGALS9,GIT1,SEMA6B,RFLNB,ARHGAP4,NRARP,NOTCH1,DAB2IP,OAS1,SOX17,RGS4,TRIM62,BST2,BMP2,RNF125	turquoise	EC_M6
GO:0022008	1.01E-07	1	239	1272	neurogenesis	BP	3.26E-05	ATP8B1,NAB2,LRP1,AHI1,RAB21,PLXNB2,TRAPPC9,PACSIN1,ADGRB1,CTNNA1,TEAD3,SUN2,MTCH1,LGALS1,MDGA1,SH3GL2,LEP,PLAA,RORB,SRF,VEGFA,RUNX2,NCK2,REST,P3H1,TGFBR1,RIMS1,TMEM30A,UBA6,RTN4,TPBG,SHANK1,MFSD2A,ACTR2,KIDINS220,WHRN,GLI3,NCDN,INHBA,ITGB1,FKBP1B,PLXNA1,FGF2,KIF5B,NAPA,RB1,PICALM,EIF2B3,TENM4,GLI2,MEGF8,SMARCD3,RHEB,PAK1,TGFB1,AXL,CTNNB1,TRAK1,AKT2,FARP1,SLC4A7,RARB,NAV3,LIG4,DCLK1,SEMA3C,ILK,DPYSL3,SPINK5,RYK,SNPH,RNF6,CXCL12,LGI4,ZSWIM6,ANKRD1,SLC25A46,PALLD,ZNF536,EIF4G2,ROBO1,MAP1B,DIP2B,SEMA4F,P2RX4,FAIM2,HEY1,TLX2,TCTN1,APP,DHX36,RAPGEF2,CX3CL1,ABCC8,PTPN11,ADCY6,OPA1,ARSB,ZFYVE27,CRTAC1,TUBB2A,CHAC1,SPP1,RUNX1,TWF1,SEMA4G,NKD1,GPRIN3,BRSK2,PDLIM5,NDN,CHRNA7,ARL3,DOCK10,EIF4E,TDP2,NOTCH2,CTTN,DNM1L,DNER,CAPRIN2,TGFB2,FEZ1,SATB2,GSK3B,MMP14,DAG1,AUTS2,LEF1,ANOS1,KRAS,NTRK3,BMP7,IDH2,FGFR2,VASH2,NCSTN,IQGAP1,STRAP,KIF1A,FARP2,TANC2,FZD9,SCYL1,MAPT,C1QL1,FZD2,MDGA2,TNR,FN1,NCKAP1,GPC2,ADRA2C,COL3A1,PRKCI,SKIL,PRRX1,SMURF1,NBL1,TNIK,VCL,KCNA1,GLDN,WDR1,TULP3,SIX4,OBSL1,ROM1,CRMP1,HAP1,PRTG,UGDH,BMPR1A,NOTCH3,ENAH,CLN8,MED1,SRCIN1,STX1B,ARHGEF2,NGFR,RAB11A,MAP2K1,TTC8,DICER1,CLMN,NPTN,ISLR2,EML1,PIN1,TRPV2,ACSL4,LAMA3,ROCK1,AKT1,TMEM98,ROGDI,DAPK3,MXRA8,PLXNB3,PAFAH1B1,NTN3,NPHP4,FLNA,PLXNA3,RERE,BSG,NTNG2,BARHL2,HSPA5,LMO4,NEXN,LHX8,DPYSL5,DMD,SERPINF1,BNIP3,VXN,FRY,VSTM2L,ITM2C,UBE2V2,ARF4,LBX1,GDNF,THY1,BLO	darkgreen	EC_M4
GO:0000380	5.83E-07	0.999999875	21	66	alternative mRNA splicing, via spliceosome	BP	3.39E-05	SMU1,YTHDC1,THRAP3,RBM17,HNRNPL,KHDRBS1,RBM5,FXR1,DDX5,DHX9,TRA2B,HNRNPU,RBM25,HNRNPM,RBMX,NSRP1,RBFOX1,FMR1,RNPS1,PTBP1,RBM8A	darkgrey	EC_M8
GO:0000727	6.14E-07	0.99999998	8	11	double-strand break repair via break-induced replication	BP	3.55E-05	MCM5,MCM3,MCM2,MCM6,GINS4,MCM4,CDC45,MCM7	darkgrey	EC_M8
GO:0046112	6.40E-07	0.999999956	10	18	nucleobase biosynthetic process	BP	3.69E-05	PPAT,PAICS,CTPS1,CAD,GMPS,UMPS,PRPS1,HPRT1,DHODH,GART	darkgrey	EC_M8
GO:0051960	1.20E-07	1	83	344	regulation of nervous system development	BP	3.78E-05	LRP1,RAB21,PLXNB2,ADGRB1,CTNNA1,KIAA1324L,SRF,VEGFA,REST,RTN4,TPBG,LRRC4B,ACTR2,GLI3,PLXNA1,TENM4,MEGF8,SMARCD3,RHEB,PAK1,TGFB1,CTNNB1,TRAK1,RARB,LIG4,SEMA3C,ILK,RYK,RNF6,CXCL12,LGI4,EIF4G2,ROBO1,MAP1B,DIP2B,SEMA4F,HEY1,TLX2,DHX36,RAPGEF2,CX3CL1,ABCC8,OPA1,AMIGO2,ZFYVE27,SPP1,SEMA4G,DNM1L,CAPRIN2,DAG1,NTRK3,BMP7,IDH2,MAPT,TNR,FN1,PRKCI,SKIL,OBSL1,HAP1,PRTG,BMPR1A,NLGN2,RAB11A,MAP2K1,DICER1,NPTN,ISLR2,TRPV2,AKT1,TMEM98,PLXNB3,PAFAH1B1,PLXNA3,SERPINF1,LRRC24,UBE2V2,THY1,YWHAH,BHLHE41,ZPR1,WNT2,LRRTM2	darkgreen	EC_M4

GO:0006351	6.64E-07	0.999999724	306	2521	transcription, DNA-templated	BP	3.81E-05	ONECUT2,BCLAF1,PPIL4,NUP107,DDX39B,ZNF608,SCAF8,RAD21,HSF2,ATAD2,XRCC6,PHF5A,PSIP1,IFT74,PWP1,ZNF300,TOPORS,POLR3B,MTERF1,RFX3,CKS2,TRIM28,ZNF451,PHF3,TWISTNB,FOXJ3,ZNF189,SP4,CDCA7L,PHIP,ZMPSTE24,RLF,ZNF292,XPO1,COMMD1,CASP8AP2,ZNF350,ITGB1BP1,THRAP3,TAF1B,EZH2,UFL1,CCNC,DDX1,NAA15,ELF2,ATAD2B,CDCA7,HDAC2,CCNA2,EXOSC9,MLLT10,DNAJC2,SETDB2,PCF11,NIFK,NUP85,ELF1,RSF1,CCNT2,PAXIP1,XRN2,NCAPG2,WDR43,BIRC5,POLR3F,EPC2,SUPT16H,CHD8,KAT6A,RIF1,E2F7,ALYREF,CKAP2,CEP290,NSD3,CEBPZ,SUPT20H,TAF7,NR2C1,BRCA2,ELK3,RRP8,TCERG1,MAPK8,PRKDC,CBX5,ERCC6,ZNF214,GPBP1,SP1,TCEA1,ZNF664,ZC3H8,POLR1B,SGK3,BTAF1,ZNF143,CENPJ,FER,TDG,NCK1,SBNO1,SMARCA5,RNF141,ZNF507,POLR1A,CHD1,ZFP91,TAF9,CGGBP1,SMN2,BDP1,TFCP2,SAP30,GCFC2,UTP15,HLTF,ZBTB10,CNOT1,GABPA,MCRS1,USP16,PPID,RBL1,CCNL1,SENP1,RIPK1,WDR75,ARID2,MYBL2,E2F8,TRIM33,INO80,ZBED6,CHUK,ZBTB11,ZNF248,MGA,MDM4,HIVEP1,BRWD1,NOLC1,BMPR1B,UBE3A,PRDM10,NUCKS1,DEK,GCN1,CUL3,GMNN,CHEK1,H2AFZ,RRP1B,NFKB1,KHDRBS1,TRIP13,TET2,TRIP11,HEATR1,NCL,TRAIP,ZNF184,PRMT5,AJUBA,GTF2IRD2,ZBTB41,ARID4B,NOL11,GTF2I,TARBP1,BPTF,DDX5,ORC2,EWSR1,TIAL1,BLM,CENPF,EDRF1,PDE8A,ING5,BCLAF3,FOSL1,SMARCC1,BAZ1B,SP3,DHX9,HDGFL3,BAZ1A,SETD2,ZFX,PSMA6,IVNS1ABP,TAF1A,DDX20,TPR,CCAR1,DDX21,UBTF,XRCC5,NPAT,BRCA1,CNOT9,NSD2,HP1BP3,DCAF6,ZNF518B,DHX33,TBL1XR1,SUV39H1,POLR3A,LEO1,FOXM1,LYAR,DDX11,FAM220A,HNRNPU,NEDD4,SMARCE1,RF	darkgrey	EC_M8
GO:0030335	1.67E-07	1	85	443	positive regulation of cell migration	BP	3.82E-05	SEMA6A,AKAP12,FLT4,PTK2,PDGFB,PODXL,TEK,ATP8A1,KIT,KDR,SEMA4D,IL6,CXCL8,DOCK4,TLR4,TCAF2,SDCBP,NRP1,PIK3CG,GATA2,PRKD2,NOS3,GATA3,CXCR4,P2RY6,HBEGF,FERMT1,ARHGEF7,SCARB1,PLK2,RET,IGF1,VEGFC,CXCL10,THBS4,PLCG1,ANO6,NEDD9,EDN1,JAM3,SRGAP2C,ETS1,PPP3CA,SEMA3F,PECAM1,FGR,MCAM,PROX1,PRKD1,MAP3K3,ITGA6,DOCK1,LAMC2,SEMA6D,ITGA4,IGFBP5,MCU,SELP,PLVAP,PPM1F,RAC1,HIF1A,RHOJ,SYNE2,MTOR,SEMA4A,FGF18,ITGA3,PGF,DAPK2,S100A14,LGALS9,SEMA6B,NOTCH1,PLCG2,S1PR1,DAB2IP,CARMIL2,RIPOR1,CDH5,MIR23A,RELN,FGF16,BMP2,CBLL1	turquoise	EC_M6
GO:0007095	6.78E-07	0.999999913	14	32	mitotic G2 DNA damage checkpoint signaling	BP	3.86E-05	CLSPN,RINT1,TOPBP1,NBN,ABRAXAS1,TAOK3,CHEK1,TICRR,DTL,BLM,CDK1,BRCA1,PLK1,TAOK1	darkgrey	EC_M8
GO:1904029	6.78E-07	0.999999838	23	83	regulation of cyclin-dependent protein kinase activity	BP	3.86E-05	LATS1,CDC25C,CKS2,CCNA2,CCNT2,GTPBP4,IPO5,CCNB1,IPO7,CDK5RAP1,CCNL1,CCNE2,HERC5,BLM,CDC25A,CDKN3,CCNB3,CDC6,CCNB2,PLK1,PSMD10,PKMYT1,CCNF	darkgrey	EC_M8
GO:0097659	6.85E-07	0.999999716	306	2522	nucleic acid-templated transcription	BP	3.89E-05	ONECUT2,BCLAF1,PPIL4,NUP107,DDX39B,ZNF608,SCAF8,RAD21,HSF2,ATAD2,XRCC6,PHF5A,PSIP1,IFT74,PWP1,ZNF300,TOPORS,POLR3B,MTERF1,RFX3,CKS2,TRIM28,ZNF451,PHF3,TWISTNB,FOXJ3,ZNF189,SP4,CDCA7L,PHIP,ZMPSTE24,RLF,ZNF292,XPO1,COMMD1,CASP8AP2,ZNF350,ITGB1BP1,THRAP3,TAF1B,EZH2,UFL1,CCNC,DDX1,NAA15,ELF2,ATAD2B,CDCA7,HDAC2,CCNA2,EXOSC9,MLLT10,DNAJC2,SETDB2,PCF11,NIFK,NUP85,ELF1,RSF1,CCNT2,PAXIP1,XRN2,NCAPG2,WDR43,BIRC5,POLR3F,EPC2,SUPT16H,CHD8,KAT6A,RIF1,E2F7,ALYREF,CKAP2,CEP290,NSD3,CEBPZ,SUPT20H,TAF7,NR2C1,BRCA2,ELK3,RRP8,TCERG1,MAPK8,PRKDC,CBX5,ERCC6,ZNF214,GPBP1,SP1,TCEA1,ZNF664,ZC3H8,POLR1B,SGK3,BTAF1,ZNF143,CENPJ,FER,TDG,NCK1,SBNO1,SMARCA5,RNF141,ZNF507,POLR1A,CHD1,ZFP91,TAF9,CGGBP1,SMN2,BDP1,TFCP2,SAP30,GCFC2,UTP15,HLTF,ZBTB10,CNOT1,GABPA,MCRS1,USP16,PPID,RBL1,CCNL1,SENP1,RIPK1,WDR75,ARID2,MYBL2,E2F8,TRIM33,INO80,ZBED6,CHUK,ZBTB11,ZNF248,MGA,MDM4,HIVEP1,BRWD1,NOLC1,BMPR1B,UBE3A,PRDM10,NUCKS1,DEK,GCN1,CUL3,GMNN,CHEK1,H2AFZ,RRP1B,NFKB1,KHDRBS1,TRIP13,TET2,TRIP11,HEATR1,NCL,TRAIP,ZNF184,PRMT5,AJUBA,GTF2IRD2,ZBTB41,ARID4B,NOL11,GTF2I,TARBP1,BPTF,DDX5,ORC2,EWSR1,TIAL1,BLM,CENPF,EDRF1,PDE8A,ING5,BCLAF3,FOSL1,SMARCC1,BAZ1B,SP3,DHX9,HDGFL3,BAZ1A,SETD2,ZFX,PSMA6,IVNS1ABP,TAF1A,DDX20,TPR,CCAR1,DDX21,UBTF,XRCC5,NPAT,BRCA1,CNOT9,NSD2,HP1BP3,DCAF6,ZNF518B,DHX33,TBL1XR1,SUV39H1,POLR3A,LEO1,FOXM1,LYAR,DDX11,FAM220A,HNRNPU,NEDD4,SMARCE1,RF	darkgrey	EC_M8

GO:0048667	1.31E-07	1	102	446	cell morphogenesis involved in neuron differentiation	BP	4.00E-05	LRP1,RAB21,PLXNB2,ADGRB1,SRF,VEGFA,RTN4,TPBG,SHANK1,MFSD2A,ACTR2,KIDINS220,WHRN,GLI3,ITGB1,PLXNA1,KIF5B,RB1,PICALM,GLI2,MEGF8,PAK1,TRAK1,FARP1,DCLK1,SEMA3C,ILK,RYK,RNF6,CXCL12,PALLD,EIF4G2,ROBO1,MAP1B,DIP2B,SEMA4F,TLX2,TCTN1,APP,DHX36,RAPGEF2,PTPN11,OPA1,ZFYVE27,SPP1,SEMA4G,BRSK2,PDLIM5,NDN,CHRNA7,DOCK10,NOTCH2,CTTN,DNM1L,CAPRIN2,FEZ1,GSK3B,DAG1,AUTS2,ANOS1,BMP7,FGFR2,KIF1A,TANC2,MAPT,TNR,FN1,SKIL,TNIK,VCL,OBSL1,CRMP1,NOTCH3,ENAH,SRCIN1,NGFR,RAB11A,MAP2K1,TTC8,NPTN,ISLR2,TRPV2,LAMA3,PLXNB3,PAFAH1B1,NTN3,PLXNA3,RERE,BSG,NTNG2,BARHL2,NEXN,DPYSL5,VSTM2L,GDNF,THY1,CHN1,ARTN,YWHAH,SDC2,MAP1A,OLFM1	darkgreen	EC_M4
GO:0030199	1.32E-07	0.999999973	24	56	collagen fibril organization	BP	4.00E-05	COL11A2,COL1A2,TGFBR1,ADAMTS3,RB1,CRTAP,SERPINH1,LUM,LOXL3,LOXL2,BMP1,TGFB2,COLGALT2,PLOD3,P4HA1,COMP,COL3A1,COL5A2,FKBP10,COL1A1,COL5A1,COL11A1,LOXL1,LOX	darkgreen	EC_M4
GO:1902750	7.14E-07	0.999999869	18	52	negative regulation of cell cycle G2/M phase transition	BP	4.04E-05	RAD21,CLSPN,ORC1,RINT1,TOPBP1,NBN,ABRAXAS1,TAOK3,CHEK1,TICRR,DTL,BLM,CDK1,BRCA1,CDC6,AURKB,PLK1,TAOK1	darkgrey	EC_M8
GO:0051716	1.84E-07	1	641	5042	cellular response to stimulus	BP	4.14E-05	TNFRSF11A,SEMA6A,LVRN,PDE7B,IFNGR1,TNFAIP3,TRIM39,PLEKHG1,AKAP12,FLT4,FZD6,CTHRC1,RIMS2,OXR1,CDC42SE2,GRAMD4,CELSR1,NOTCH4,AFDN,ASF1A,TCF7,TPD52L1,NCOA7,MTSS1,RNF146,ARHGAP18,SGSM3,PTK2,DENND3,ANKS1A,DAPK1,ATF4,PDGFB,MAPK13,CSNK1E,PICK1,KDM4C,MICALL1,PIM1,CDC42EP1,MFNG,CSF2RB,MDFI,OSTF1,LRRC19,TEK,RIC8B,DDX58,PIP5K1B,FAM122A,TEC,SH3RF3,GNG11,ADGRF5,KIT,ADGRF1,TNFRSF21,KDR,CD2AP,NFIL3,SYK,GADD45G,SEMA4D,RNF149,SHB,RAPGEF5,IL6,REL,CHN2,CXCL8,KBTBD2,PELI1,RGS3,ARHGAP25,ELMO1,RSAD2,DOCK4,FIGNL1,CAV2,KLF11,GRB10,LANCL2,TSPAN12,TLR4,GRIK2,TRAF1,C5,DLGAP3,INPP4B,IL15,FZD8,CCNY,CREM,TEAD2,SDCBP,TRPM4,NRP1,PUM2,RASIP1,PIK3CG,ARHGAP42,TAL1,MTMR2,MGLL,KDM4D,GATA2,DNMT3A,ARHGAP21,PRKD2,CTSC,RAB38,CAB39L,GPR4,LRCH1,SNRK,FGD5,PLXND1,NECTIN2,PRCP,BCAM,ITGA8,NOS3,GAB2,LLGL2,ITGA9,RALB,ITGB4,ZFAND6,PPARG,TIMP4,GATA3,ACVR2B,TBC1D4,CXCR4,LMBR1,IRAK2,SEC14L1,EDNRB,PPP1CB,NET1,RALGAPA2,NDFIP2,TMC8,TIE1,CYTH1,IKBKB,SEL1L2,JAG1,P2RY6,P2RY2,NFKBIB,PDE2A,TMEM173,SFRP1,HBEGF,NR1D2,PLCB4,RASGRP3,FEZ2,NOTUM,FERMT1,RPL26,EFNB2,SH3BP5,ARHGEF7,ASH2L,SPEF1,ARAP3,ARHGAP26,P2RX2,SLC4A11,CD36,RIC8A,SIRT3,RASSF7,ARID5A,MAGI1,PRAG1,SDCBP2,PRR5L,MAP3K1,SCARB1,EPHB1,PLK2,RET,PDE4D,MERTK,TNFRSF19,GRAMD1A,FAM3D,IL1A,DNMT3B,IGF1,NR4A1,KIAA0355,EIF2AK3,ABHD6,HHEX,KDM3A,ITCH,ERBIN,UFSP2,SH3RF1,PITPNM2,PREX2,SMAD1,ZBTB38,HIP1R,OCLN,DCLK2,MCTP1,CHRM5,A	turquoise	EC_M6
GO:0098662	1.46E-07	1	116	541	inorganic cation transmembrane transport	BP	4.31E-05	CYB5A,CD63,SLC12A2,ATP6V1C1,SLC39A7,SMDT1,KCNK9,UBQLN1,SLC39A4,ATP6V1F,CNNM4,SLC17A5,SLC31A1,ITGB1,FKBP1B,FGF2,KIF5B,SEC61A1,PANX1,ATP7B,ATP6V0B,SCN5A,GPD1L,SLC4A7,ACTN4,STIM1,ATP2C1,PRNP,ATP2B1,TMCO3,TUSC3,REM1,FXYD1,SLC20A1,SNTA1,SLC25A4,SLMAP,ATP1B3,TRPC4AP,RYR3,P2RX4,ATP2A2,CACNA1D,SLC4A5,CX3CL1,SLC33A1,ABCC8,CACNB3,OPA1,CHP1,ATP2B4,PKD2,NIPA1,ATP6V1B2,SLC39A10,CHRNA7,MRS2,CNNM2,CALHM1,SLC2A10,HTR2B,ISCU,SHROOM2,CASQ1,TMCO1,LINC02210-CRHR1,PTPN6,KCNK4,ITGAV,HTT,PKD1L1,ERO1A,KCNA1,SLC3A2,ASIC4,SLC6A13,HAP1,CACNA1C,STIM2,ATP6V1D,RHBG,COX6A2,KCNMB1,CACNA1G,CALM1,VMP1,SLC39A6,ANXA6,TRPV2,AKT1,SCNN1D,PKD1,FLNA,KCNAB2,ATP6AP1,PARK7,HCN2,JPH3,ATP6V0D1,MIR24-1,DMD,SURF1,FKBP1A,CBARP,SLC36A1,TMEM38B,THY1,TMSB4X,TMEM38A,YWHAH,SLC39A3,NIPAL1,HCN4,ATP6V0E1,GEM,KCNMB4	darkgreen	EC_M4
GO:0030433	1.50E-07	0.999999962	29	78	ubiquitin-dependent ERAD pathway	BP	4.37E-05	CANX,UBQLN1,VCP,ERLEC1,UBE2J1,TMUB1,UBXN4,STT3B,NPLOC4,RNF121,HSP90B1,AMFR,MARCH6,SELENOS,UBE4A,RHBDD2,EDEM3,SYVN1,DNAJC10,DNAJB12,JKAMP,SEL1L,USP14,BCAP31,HSPA5,FBXO27,DNAJB9,UBE2G2,DERL2	darkgreen	EC_M4
GO:0070198	7.82E-07	0.999999908	13	29	protein localization to chromosome, telomeric region	BP	4.41E-05	CCT2,POT1,CCT4,BRCA2,TNKS2,ATR,TERF1,CCT7,CCT5,CCT6A,XRCC5,CCT3,ATRX	darkgrey	EC_M8

GO:0030097	2.00E-07	1	109	618	hemopoiesis	BP	4.44E-05	TNFRSF11A,FLT4,NOTCH4,TCF7,L3MBTL3,ATF4,PIM1,MFNG,TEK,IREB2,ADGRF5,KIT,KDR,NFIL3,SYK,IL6,PTBP3,RSAD2,TLR4,IL15,ZBTB24,TAL1,GATA2,SNRK,GAB2,PPARG,GATA3,JAG1,SFRP1,ASH2L,TMEM178A,TCF15,MERTK,CHD7,IL1A,HHEX,ITCH,SH3RF1,MEF2C,TLR2,IL7,TPD52,LMBR1L,ADGRG3,TIPARP,HIPK1,DLL4,SOX13,TSPAN2,STAT1,JAM3,ALAS1,DCAF1,ETS1,LCK,HYAL2,PRKX,HLX,DYRK3,HCLS1,INPP5D,CD34,CD86,FES,MMP21,ACE,ETV6,CREB1,NBEAL2,B2M,LTF,ITGA4,SOS2,MECOM,CRACR2A,HIF1A,EPHA2,ITPKB,LBR,LFNG,MTOR,PSEN1,HOXB7,SEMA4A,HOXB8,BATF,CD19,GPR68,YY1,LYL1,RCOR1,JAG2,LGALS9,IL7R,ISG15,AP3D1,NRARP,NOTCH1,IRF8,PLCG2,VNN1,SLC8A3,ARID4A,ATXN1L,CD46,CA2,CDK13,HIST1H4D,FLI1	turquoise	EC_M6
GO:0007264	2.11E-07	1	87	437	small GTPase mediated signal transduction	BP	4.61E-05	PLEKHG1,CDC42SE2,CELSR1,ARHGAP18,SGSM3,DENND3,CDC42EP1,CD2AP,RAPGEF5,CHN2,ARHGAP25,ELMO1,DOCK4,SDCBP,NRP1,RASIP1,PIK3CG,ARHGAP42,ARHGAP21,RAB38,GPR4,FGD5,RALB,NET1,RALGAPA2,CYTH1,RASGRP3,ARHGAP26,PRAG1,PLK2,IGF1,KIAA0355,ERBIN,PREX2,ARHGEF3,ARHGEF28,MADD,RAPGEF3,DNMBP,RASSF1,SHOC2,ARHGEF12,KALRN,PRKD1,F11R,ARHGDIB,DOCK1,SIPA1,USP28,ARHGAP27,BCR,SOS2,RFXANK,WAS,RAC1,RHOJ,PLD2,ITPKB,FGD1,ARHGEF9,ARHGAP23,OPHN1,STARD8,RIT1,ITGA3,ARHGEF15,DOCK6,CCDC88C,DNM2,AMOT,ARHGAP28,SH2D3A,ARHGAP4,PLEKHG5,NOTCH1,RAPGEF1,SH2D3C,DAB2IP,RIPOR1,ADGRG1,RELN,RAB18,GRAP,LPAR6,ARHGEF1,RALA,RAB39A	turquoise	EC_M6
GO:0043062	1.69E-07	1	65	251	extracellular structure organization	BP	4.81E-05	LRP1,COL11A2,COL1A2,COL15A1,TGFBR1,ADAMTS3,ITGB1,FBLN2,RB1,CRTAP,TGFB1,SERPINH1,SPINT2,LUM,MMP17,SPINK5,APLP1,ADAMTS6,SULF1,LOXL3,MMP15,APP,PTX3,LOXL2,MMP2,BMP1,RUNX1,FAP,PHLDB2,SLC2A10,TGFB2,FBLN5,MMP14,DAG1,PHLDB1,QSOX1,PRDX4,COLGALT2,PLOD3,TNR,P4HA1,COMP,ERO1A,COL3A1,COL5A2,FKBP10,MYO1E,COL1A1,PBXIP1,TEX14,EGFLAM,MMP23B,VWA1,COL5A1,NTNG2,POMT1,COL11A1,LOXL1,SERPINB5,ADAMTS5,TMEM38B,LOX,HAS2,MFAP4,COL8A2	darkgreen	EC_M4
GO:0007165	1.76E-07	1	628	3919	signal transduction	BP	4.92E-05	CD63,IL23A,LRP1,AP3S1,HBS1L,AHI1,ARHGEF25,TAB2,DYRK2,RAB21,RRM2B,PLXNB2,FBN2,ANGPT1,FAM19A5,PPARA,ROS1,NUS1,RSPO3,CXCL14,FAM49B,GNG5,PTP4A3,ADGRB1,LY6E,SCUBE3,PPARD,CTNNA1,TEAD3,CBY1,MKLN1,UBQLN1,MTCH1,LGALS1,KLHDC10,SH3GL2,GNAQ,MYH9,ARHGAP39,LEP,CDKN2B,PLAA,RORB,BTBD11,KIAA1324L,BAG1,NUAK1,CDK14,VEGFA,DOCK8,VCP,SLC35B2,RUNX2,RASL11B,UNC13B,COL1A2,NCK2,CLOCK,EXOC1,CREB3,ASPN,INPP4A,DST,SNX13,COL15A1,TGFBR1,SOCS5,RIMS1,ERLEC1,RTN4,TPBG,SHANK1,ADAMTS3,EMC10,GGCT,MAP3K7,SH3BP5L,KIDINS220,MDFIC,GLI3,ROCK2,INHBA,RRAS,ZYX,RCN3,DKKL1,VPS35,CUL2,ITGB1,PGRMC2,PLEKHA4,FKBP1B,PLXNA1,FGF2,SRPK2,SESN3,RAB7A,ARHGEF4,APBB1IP,RB1,MARK4,TPT1,EIF2B3,PTCH2,TENM4,DGKH,KCTD21,GLI2,MEGF8,RHEB,SNX17,PAK1,TGFB1,AXL,RAB3GAP1,HTR5A,SEC13,CTNNB1,IL15RA,LBH,DGAT2,AKT2,DYNC1LI1,GPD1L,DZIP1,ZEB2,LGALS3BP,CANT1,C1QTNF1,ARHGEF17,OSBPL8,CXXC5,CLPB,PSD2,RARB,NPLOC4,ACTN4,PPP1R12A,ANAPC15,STAM2,LTBP1,TMTC4,CRIM1,SPRED3,GPS1,UFM1,ATP2C1,IRS2,PRNP,CHFR,DCN,CDC42EP3,SMAD9,HDAC3,ZNF703,DCLK1,ACPP,PCLO,STARD13,KCTD16,SEMA3C,CCKBR,CAVIN3,UBA5,DDRGK1,SMPD1,PPP2CB,ILK,RBPMS,GPR84,LEPROTL1,ARL6IP5,APLP1,RYK,TMEM127,PRICKLE2,CNOT7,RNF6,TMEM9B,ST5,AKIP1,CXCL12,SLC20A1,ANKRD1,PJA2,TMED2,IL20RB,MRAS,MAST4,CD180,ZNF536,FLNB,OSBP,MRVI1,SULF1,GRK7,CTNND1,LNPEP,DKK3,ROBO1,WWC2,TEAD1,SORBS1,SEMA4F,CDKL2,DOK1,LOXL3,P2RX4,RNF13,FAIM2,HEY1,RASA1,ATP2A2,USO1,DLG	darkgreen	EC_M4

GO:0050896	2.33E-07	1	730	5876	response to stimulus	BP	5.00E-05	ZNF516,TNFRSF11A,SEMA6A,LVRN,KIF5A,PDE7B,IFNGR1,TNFAIP3,TRIM39,PLEKHG1,AKAP12,LYZ,TSPAN8,FLT4,FZD6,CTHRC1,RIMS2,OXR1,CDC42SE2,GRAMD4,CELSR1,NOTCH4,AFDN,ASF1A,TCF7,TRDN,TPD52L1,NCOA7,MTSS1,RNF146,ARHGAP18,SGSM3,PTK2,DENND3,C6orf106,ANKS1A,DAPK1,ATF4,PDGFB,MAPK13,CSNK1E,PICK1,KDM4C,MICALL1,PIM1,CDC42EP1,MFNG,CSF2RB,MDFI,OSTF1,LRRC19,TEK,RIC8B,DDX58,NFX1,PIP5K1B,FAM122A,TEC,DNAJB5,SH3RF3,GNG11,ADGRF5,KIT,ADGRF1,TNFRSF21,KDR,CD2AP,NFIL3,SYK,GADD45G,SEMA4D,RNF149,SHB,RAPGEF5,IL6,REL,PPT1,CHN2,CXCL8,BACH2,KBTBD2,PELI1,RGS3,ARHGAP25,ELMO1,RSAD2,DOCK4,FIGNL1,FOXP2,CAV2,KLF11,ODC1,GRB10,LANCL2,TSPAN12,TLR4,GRIK2,TRAF1,C5,DLGAP3,GJA4,INPP4B,IL15,FZD8,CCNY,CREM,TEAD2,SDCBP,NRCAM,NOCT,TRPM4,NRP1,PUM2,RASIP1,PIK3CG,ARHGAP42,TAL1,MTMR2,MGLL,KDM4D,GATA2,DNMT3A,MSRB2,ARHGAP21,PRKD2,CST7,NOVA2,CTSC,RAB38,CAB39L,GPR4,LRCH1,SNRK,FGD5,PLXND1,NECTIN2,PRCP,BCAM,ITGA8,NOS3,PSG3,GAB2,LLGL2,ITGA9,RALB,ITGB4,UNC13D,ZFAND6,DACH1,PPARG,TIMP4,GATA3,ACVR2B,TBC1D4,CXCR4,LMBR1,IRAK2,SEC14L1,EDNRB,PPP1CB,NET1,RALGAPA2,NDFIP2,TMC8,TIE1,CYTH1,IKBKB,SEL1L2,JAG1,P2RY6,P2RY2,NFKBIB,PDE2A,TMEM173,SFRP1,HBEGF,NR1D2,PLCB4,RASGRP3,FEZ2,NOTUM,FERMT1,RPL26,EFNB2,SH3BP5,ARHGEF7,ASH2L,SPEF1,ARAP3,ARHGAP26,P2RX2,SLC4A11,CD36,RIC8A,SIRT3,RASSF7,ARID5A,MAGI1,PRAG1,SDCBP2,PRR5L,MAP3K1,SCARB1,EPHB1,PLK2,GAPT,RET,PDE4D,MERTK,TNFRSF19,GRAMD1A,CHD7,FAM3D,IL1A,DNMT	turquoise	EC_M6
GO:0048534	2.35E-07	1	113	649	hematopoietic or lymphoid organ development	BP	5.00E-05	TNFRSF11A,FLT4,NOTCH4,TCF7,L3MBTL3,ATF4,PIM1,MFNG,TEK,IREB2,ADGRF5,KIT,KDR,NFIL3,SYK,IL6,PTBP3,RSAD2,TLR4,IL15,ZBTB24,TAL1,GATA2,SNRK,GAB2,PPARG,GATA3,JAG1,SFRP1,ASH2L,TMEM178A,TCF15,RET,MERTK,CHD7,IL1A,HHEX,ITCH,SH3RF1,MEF2C,TLR2,IL7,TPD52,LMBR1L,ADGRG3,TIPARP,HIPK1,DLL4,SOX13,TSPAN2,STAT1,JAM3,ALAS1,JARID2,NFKB2,DCAF1,ETS1,LCK,HYAL2,PRKX,HLX,DYRK3,HCLS1,INPP5D,CD34,CD86,FES,MMP21,ACE,ETV6,CREB1,NBEAL2,B2M,LTF,ITGA4,SOS2,TBX1,MECOM,CRACR2A,HIF1A,EPHA2,ITPKB,LBR,LFNG,MTOR,PSEN1,HOXB7,SEMA4A,HOXB8,BATF,CD19,GPR68,YY1,LYL1,RCOR1,JAG2,LGALS9,IL7R,ISG15,AP3D1,NRARP,NOTCH1,IRF8,PLCG2,VNN1,SLC8A3,ARID4A,ATXN1L,CD46,CA2,CDK13,HIST1H4D,FLI1	turquoise	EC_M6
GO:0043535	2.63E-07	0.999999933	27	87	regulation of blood vessel endothelial cell migration	BP	5.51E-05	PDGFB,KDR,NRP1,GATA2,PRKD2,PRCP,NOS3,PPARG,PLK2,MEF2C,VEGFC,ANGPT2,PLCG1,DLL4,ETS1,PRKD1,MAP3K3,HIF1A,RHOJ,JUP,EPHA2,MMRN2,FGF18,EFNA1,VASH1,NOTCH1,MIR23A	turquoise	EC_M6
GO:0048856	2.67E-07	1	551	4193	anatomical structure development	BP	5.52E-05	ZNF516,TNFRSF11A,TCF4,SEMA6A,N4BP3,KIF5A,IFNGR1,TNFAIP3,HECA,PTPRB,FLT4,SNX2,SYNJ2,FZD6,CTHRC1,RIMS2,CDC42SE2,CELSR1,QKI,NOTCH4,AFDN,PARVG,PACSIN2,ASF1A,TCF7,CDKL3,MTSS1,ASAP1,ARHGAP18,L3MBTL3,KLHL3,PTK2,HSD17B3,ANKS1A,ATF4,PDGFB,AGTPBP1,PODXL,CSNK1E,PICK1,KDM4C,MICALL1,PIM1,CDC42EP1,MFNG,STRIP2,MDFI,TEK,LINGO2,IREB2,APBA1,TJP2,PGM5,TEC,ADGRF5,KIT,ADGRF1,TNFRSF21,KDR,NFIL3,SYK,SEMA4D,EFHC1,THSD7A,SHB,TMOD1,RAPGEF5,IL6,IGF2BP3,PPT1,PTBP3,CXCL8,UTP11,RSAD2,FOXP2,CAV2,TSPAN12,NOBOX,TLR4,C5,GJA4,IL15,FZD8,TEAD2,SDCBP,MYCN,NRCAM,NOCT,TUT4,TRPM4,NRP1,ZBTB24,RASIP1,PIK3CG,SVIL,ITSN2,TAL1,LAMA4,PHF10,MTMR2,EPC1,GATA2,DNMT3A,HS6ST1,PRKD2,CST7,NOVA2,CTSC,GPR4,SNRK,FGD5,PLXND1,NECTIN2,PRCP,ITGA8,NOS3,GAB2,ITGB4,UNC13D,DACH1,PPARG,TIMP4,GATA3,ACVR2B,MYO7A,CXCR4,LMBR1,GZF1,EDNRB,SPTBN4,TIE1,GDPD5,SETD5,IKBKB,JAG1,PDE2A,GPAT4,SFRP1,HBEGF,NR1D2,DND1,FEZ2,NOTUM,FERMT1,FREM2,EFNB2,ANKLE2,ARHGEF7,ASH2L,SPEF1,ATP11A,PCDH1,PCDH12,ARHGAP26,P2RX2,FGD6,NTN4,CD36,RIC8A,TMEM178A,LMOD3,SYNE4,PRAG1,ADAMTS9,SDCBP2,TCF15,EPHB1,PLK2,MALL,RET,PDE4D,MERTK,TNFRSF19,CHD7,IL1A,DNMT3B,IGF1,NR4A1,EIF2AK3,HHEX,KDM3A,ITCH,LYVE1,SH3RF1,PREX2,SMAD1,HIP1R,POU6F1,MARVELD2,STOX2,DCLK2,MMP24,SOX7,KIF13B,ARHGEF28,ENC1,HEXB,MEF2C,SMARCD1,TLR2,WWTR1,IL7,P2RX7,TPD52,VEGFC,NDRG4,GPM6A,CXCL10,ANGPT2,TLL2,LMBR1L,ADGRG3,DHH,KMT2D,SFMBT1,PLLP,BMP2K,TIPARP,TLL1,SERINC5,	turquoise	EC_M6
GO:0007178	2.09E-07	1	69	272	transmembrane receptor protein serine/threonine kinase signaling pathway	BP	5.75E-05	LRP1,FBN2,PPARA,CDKN2B,BTBD11,KIAA1324L,RUNX2,RASL11B,COL1A2,ASPN,TGFBR1,MAP3K7,INHBA,ZYX,MEGF8,TGFB1,ZEB2,LTBP1,CRIM1,SPRED3,SMAD9,ZNF703,ILK,RBPMS,SULF1,DKK3,SLC33A1,ACVR1,GDF6,NOTCH2,SLC2A10,TGFB2,FSTL1,LEF1,HJV,BMP7,ITGB5,HTRA1,STRAP,FAM89B,FBN1,COMP,FKBP8,COL3A1,SKIL,SMURF1,NBL1,GREM2,BMPR1A,RNF111,TGFB1I1,LTBP2,TGFB3,TMEM100,MYOCD,PIN1,CHRDL1,NOMO1,NLK,VASN,GLG1,HSPA5,FSTL3,FKBP1A,LOX,DUSP22,PMEPA1,CHST11,FGF10	darkgreen	EC_M4

GO:0009124	1.03E-06	0.999999855	14	37	nucleoside monophosphate biosynthetic process	BP	5.78E-05	PPAT,PAICS,DCK,CAD,TK1,GMPS,UMPS,PFAS,PRPS1,TYMS,HPRT1,DHODH,RFK,GART	darkgrey	EC_M8
GO:0045321	2.88E-07	1	102	586	leukocyte activation	BP	5.87E-05	IFNGR1,TNFAIP3,TCF7,MFNG,ADGRF5,KIT,TNFRSF21,NFIL3,SYK,IL6,CXCL8,PELI1,RSAD2,TLR4,IL15,PIK3CG,GATA2,CST7,CTSC,NECTIN2,GAB2,UNC13D,GATA3,CD93,SFRP1,EPHB1,GAPT,MERTK,CHD7,IL1A,IGF1,HHEX,ITCH,SH3RF1,KIF13B,MEF2C,TLR2,IL7,TPD52,LMBR1L,ADGRG3,DYSF,DLL4,SOX13,EDN1,SNCA,DCAF1,CD200,LCK,PPP3CA,HYAL2,ICOSLG,SNAP23,LAPTM5,HLX,NFATC2,INPP5D,CD86,FGR,FES,DLG1,B2M,ADORA2A,ITGA4,SOS2,TMEM106A,CRACR2A,WAS,RAB27A,ITPKB,TLR6,LFNG,TNFRSF1B,ARG2,MTOR,EFNB1,PSEN1,SEMA4A,BATF,CHRNB2,CD19,SCNN1B,YY1,LYL1,CCL2,YES1,JAG2,LGALS9,ELF4,IL7R,MYO18A,AP3D1,NRARP,IRF8,PLCG2,STXBP1,HAVCR1,VNN1,TLR1,CD46,AKAP17A,BST2	turquoise	EC_M6
GO:0044818	1.08E-06	0.999999822	16	43	mitotic G2/M transition checkpoint	BP	6.06E-05	CLSPN,ORC1,RINT1,TOPBP1,NBN,ABRAXAS1,TAOK3,CHEK1,TICRR,DTL,BLM,CDK1,BRCA1,CDC6,PLK1,TAOK1	darkgrey	EC_M8
GO:0050790	3.01E-07	1	262	1823	regulation of catalytic activity	BP	6.07E-05	TNFRSF11A,TNFAIP8,TNFAIP3,PHACTR2,TBC1D30,PLEKHG1,PTPRB,FLT4,GRAMD4,AFDN,TPD52L1,ASAP1,ARHGAP18,SGSM3,PTK2,DENND3,DAPK1,PDGFB,TBC1D22B,TEK,RIC8B,FAM122A,KIT,KDR,SYK,SEMA4D,RGP1,PPP6R1,RAPGEF5,PPT1,CHN2,RGS3,ARHGAP25,ELMO1,DOCK4,ASAP2,CAV2,TLR4,C5,TBC1D9,CCNY,NRP1,KIAA1147,RASIP1,PIK3CG,ARHGAP42,ITSN2,TAL1,ARHGAP21,PRKD2,CST7,CAB39L,RCBTB2,LRCH1,FGD5,PLXND1,NOS3,LLGL2,RALB,USP6NL,PPARG,TIMP4,TBC1D4,ITIH5,EDNRB,NET1,RALGAPA2,TIE1,CYTH1,IKBKB,P2RY6,SFRP1,HBEGF,RASGRP3,SH3BP5,ANKLE2,ARHGEF7,ARAP3,ARHGAP26,FGD6,RIC8A,SIRT3,SCARB1,EPHB1,RET,MERTK,IL1A,IGF1,NR4A1,EIF2AK3,HHEX,SH3RF1,PREX2,HIP1R,MMP24,ARHGEF3,SOX7,HMBOX1,ARHGEF28,MEF2C,WWTR1,IL7,VEGFC,CXCL10,MADD,BMP2K,SERINC5,OAS3,NLRC5,PPP1R16B,PLCB2,RAPGEF3,NR4A2,DGKZ,IQGAP2,CCNJL,DNMBP,IFIT1,NEDD9,EDN1,SNCA,DTNBP1,CD200,HDAC1,ITPRIP,LCK,HYAL2,GIT2,SHOC2,RIN3,ADPRH,LAPTM5,NCF1,FABP4,PABPN1,CD86,PLXNA2,ARHGEF12,SH3BP4,FGR,CFLAR,CASP10,KALRN,IGFBP3,PPP2R5A,RPS6KA1,PROX1,PRKD1,TOR1AIP1,F11R,OAZ3,ARHGDIB,ACE,ITGA6,DOCK1,PAK2,CST6,DLG1,SIPA1,MAP3K11,EIF4A2,ALS2CL,LTF,ARHGAP27,BCR,ADORA2A,EPHB4,DDX3X,SOS2,AGFG2,RUNDC1,ELMOD1,BIRC3,LDB2,SELE,TNFAIP8L3,FGF23,CPEB2,LMTK2,PPM1F,WAS,HIF1A,RCC2,KDM5A,EPHA2,BID,TLR6,TBC1D1,RGS14,FGD1,ARHGEF9,ARHGAP23,MTOR,BNIP2,OPHN1,STARD8,PSEN1,PAPLN,FGF18,EIF2B2,TAOK2,ARHGEF15,EFNA1,RABEP2,CD19,PIK3R5,CALML4,DOCK6,CCDC88C,TBC1D8B,PPP2R5C,AMOT,ARHG	turquoise	EC_M6
GO:0048699	2.31E-07	1	222	1177	generation of neurons	BP	6.23E-05	ATP8B1,LRP1,AHI1,RAB21,PLXNB2,TRAPPC9,PACSIN1,ADGRB1,CTNNA1,TEAD3,MTCH1,LGALS1,MDGA1,SH3GL2,LEP,PLAA,RORB,SRF,VEGFA,RUNX2,NCK2,REST,P3H1,TGFBR1,RIMS1,TMEM30A,UBA6,RTN4,TPBG,SHANK1,MFSD2A,ACTR2,KIDINS220,WHRN,GLI3,NCDN,INHBA,ITGB1,FKBP1B,PLXNA1,KIF5B,NAPA,RB1,PICALM,TENM4,GLI2,MEGF8,SMARCD3,RHEB,PAK1,TGFB1,AXL,CTNNB1,TRAK1,FARP1,SLC4A7,LIG4,DCLK1,SEMA3C,ILK,DPYSL3,SPINK5,RYK,SNPH,RNF6,CXCL12,LGI4,ZSWIM6,ANKRD1,SLC25A46,PALLD,ZNF536,EIF4G2,ROBO1,MAP1B,DIP2B,SEMA4F,FAIM2,HEY1,TLX2,TCTN1,APP,DHX36,RAPGEF2,CX3CL1,ABCC8,PTPN11,ADCY6,OPA1,ARSB,ZFYVE27,CRTAC1,TUBB2A,SPP1,RUNX1,TWF1,SEMA4G,NKD1,GPRIN3,BRSK2,PDLIM5,NDN,CHRNA7,ARL3,DOCK10,EIF4E,TDP2,NOTCH2,CTTN,DNM1L,DNER,CAPRIN2,TGFB2,FEZ1,SATB2,GSK3B,DAG1,AUTS2,LEF1,ANOS1,NTRK3,BMP7,IDH2,FGFR2,VASH2,IQGAP1,STRAP,KIF1A,FARP2,TANC2,FZD9,SCYL1,MAPT,C1QL1,FZD2,MDGA2,TNR,FN1,NCKAP1,GPC2,ADRA2C,COL3A1,PRKCI,SKIL,PRRX1,SMURF1,NBL1,TNIK,VCL,KCNA1,GLDN,TULP3,SIX4,OBSL1,ROM1,CRMP1,HAP1,PRTG,UGDH,BMPR1A,NOTCH3,ENAH,CLN8,MED1,SRCIN1,STX1B,ARHGEF2,NGFR,RAB11A,MAP2K1,TTC8,DICER1,CLMN,NPTN,ISLR2,EML1,PIN1,TRPV2,ACSL4,LAMA3,ROCK1,AKT1,TMEM98,DAPK3,PLXNB3,PAFAH1B1,NTN3,NPHP4,FLNA,PLXNA3,RERE,BSG,NTNG2,BARHL2,HSPA5,LMO4,NEXN,LHX8,DPYSL5,DMD,SERPINF1,VXN,FRY,VSTM2L,ITM2C,UBE2V2,ARF4,LBX1,GDNF,THY1,BLOC1S2,CHN1,EFHD1,ARTN,YWHAH,BHLHE41,ZPR1,SDC2,PRKG1,MAP1A,OLFM1,WNT2	darkgreen	EC_M4
GO:0032465	1.13E-06	0.999999736	22	72	regulation of cytokinesis	BP	6.29E-05	KIF20A,PLK4,E2F7,PRPF40A,BRCA2,KIF20B,RACGAP1,PKP4,E2F8,CIT,CUL3,KIF14,PRC1,SETD2,ECT2,INCENP,CDC6,AURKB,KIF23,PLK1,PKN2,AURKA	darkgrey	EC_M8

GO:0003008	2.41E-07	1	230	1240	system process	BP	6.35E-05	ATP8B1,LRP1,RRM2B,SLC12A2,ANGPT1,PPARA,HLA-DRA,COL11A2,PTP4A3,REEP2,PPARD,SH3GL2,LEP,RORB,SRF,VEGFA,UNC13B,COL1A2,TPM2,CNNM4,RIMS1,UBA6,TPBG,SHANK1,MFSD2A,MYH14,CALD1,ACTR2,WHRN,ROCK2,INHBA,BVES,MYLK3,SLC7A11,ITGB1,FKBP1B,EML2,PICALM,TENM4,PAK1,TGFB1,RAB3GAP1,SCN5A,GPD1L,C1QTNF1,TMTC4,PRNP,ATP2B1,LUM,RAB11FIP1,ACPP,CCKBR,UBA5,ARL6IP5,TUSC3,CXCL12,FXYD1,SNTA1,PJA2,SULF1,SLMAP,GRK7,LNPEP,RDH10,P2RX4,ATP2A2,DLGAP4,CACNA1D,MYL9,APP,GLRB,SLC4A5,ABCC8,CACNB3,PTPN11,ADCY6,SLC26A7,HOMER1,OPA1,AMFR,AP3B1,CRY2,ADA,GLS,ATP2B4,TWF1,PDLIM5,NDN,CHRNA7,RAP1GDS1,TJP1,CALHM1,CTTN,PKP2,PPP1R12B,DNM1L,LMOD1,SLC2A10,TGFB2,LRP5,HTR2B,GSK3B,DAG1,PIK3CA,LEF1,PRKCA,KRAS,RLBP1,CRYBA4,ADCY5,FABP2,CASQ1,COPA,NCSTN,STRAP,TFRC,ATF6,BACE1,FZD9,GLRX3,OLR1,LINC02210-CRHR1,MAPT,PLOD3,C1QL1,EIF4EBP2,ATP6AP2,FZD2,COMT,TNR,MPP2,KCNK4,ADRA2C,COMP,HTT,ECE1,KCNA1,WDR1,ATAD1,ACTA2,SLC6A13,ROM1,TPM4,CHRNE,JAKMIP1,CACNA1C,SLC29A4,HSPB7,SLC2A4,NLGN2,CLN8,TCAP,MAGED2,PPARGC1A,NPPB,MYO1E,STX1B,SLC7A3,SYT11,KCNMB1,TPM1,SGCA,COL1A1,CACNA1G,TMEM63C,ADRA1B,CNN1,TMEM100,CLN6,TTC8,CALM1,SGCD,DICER1,GRIA1,NPTN,SETD3,MYOCD,BEGAIN,ANXA6,DTNA,PIN1,TRPV2,ADORA2B,RASL10B,ROCK1,AKT1,TMEM98,ITGA2,MYOM1,GNAL,NPR3,MYO5B,DAPK3,PITPNA,GNB1,PAFAH1B1,SCNN1D,FLNA,ENO1,JPH3,HSBP1,COL11A1,AARS,LHX8,MIR24-	darkgreen	EC_M4
GO:0071495	2.47E-07	1	195	1018	cellular response to endogenous stimulus	BP	6.35E-05	LRP1,AP3S1,FBN2,PPARA,PPARD,CTNNA1,SH3GL2,LEP,CDKN2B,KIAA1324L,RUNX2,RASL11B,COL1A2,CLOCK,ASNS,REST,ASPN,TGFBR1,MAP3K7,ACTR2,KIDINS220,ROCK2,INHBA,ZYX,VPS35,MGARP,PGRMC2,FGF2,SESN3,MEGF8,PAK1,TGFB1,HTR5A,CTNNB1,LBH,AKT2,ZEB2,OSBPL8,RARB,KAT2B,LTBP1,CRIM1,SPRED3,SPINT2,UFM1,IRS2,PRNP,ATP2B1,SMAD9,ZNF703,UBA5,DDRGK1,ILK,LEPROTL1,APLP1,RNF6,ANKRD1,HSP90B1,SULF1,DKK3,MAP1B,RYR3,SORBS1,P2RX4,HEY1,USO1,APP,RAPGEF2,SLC33A1,PTPN11,ADCY6,OPA1,GTF2H1,PTPN22,ACVR1,CRY2,VPS18,MMP2,GDF6,ATP2B4,PKD2,EIF4E,ADIPOR1,KDM5B,NOTCH2,SELENOS,HYAL1,SLC2A10,TGFB2,ZNF106,EPRS,HTR2B,GSK3B,CASP7,FSTL1,PIK3CA,LEF1,HJV,NTRK3,COL6A1,BMP7,ADCY5,WDTC1,FGFR2,ITGB5,HTRA1,NCSTN,SYAP1,IQGAP1,STRAP,STRN3,UROS,BACE1,FAM89B,LINC02210-CRHR1,MAPT,PLOD3,EIF4EBP2,IGFBP2,FBN1,COMP,FKBP8,COL3A1,PRKCI,COL5A2,SKIL,FGFR3,SMURF1,NBL1,GREM2,MYO5A,BMPR1A,SLC2A4,MED1,PPARGC1A,RNF111,FGFRL1,CPEB4,ARHGEF2,TGFB1I1,NGFR,LTBP2,COL1A1,TGFB3,SHC1,TMEM100,SMARCA4,PKM,NPTN,MYOCD,PIN1,CHRDL1,PDGFRB,ROCK1,AKT1,LPIN2,PRKAA1,NLK,RAB31,GNAL,PTPN2,LDOC1,MYO1C,GNB1,VASN,FLNA,SSTR5,PARK7,HCN2,AHCYL1,GNAI3,GLG1,BCAR3,SLC2A8,MTSS1L,HSPA5,DYNC1LI2,FSTL3,FKBP1A,SERPINF1,TMEM38B,PTPN12,LOX,HAS2,TMEM38A,DUSP22,YWHAH,PMEPA1,LEPROT,PIK3C2A,ZPR1,HCN4,CHST11,WNT2,FGF10	darkgreen	EC_M4
GO:0048812	2.50E-07	1	111	503	neuron projection morphogenesis	BP	6.35E-05	LRP1,RAB21,PLXNB2,PACSIN1,ADGRB1,SH3GL2,PLAA,SRF,VEGFA,RIMS1,RTN4,TPBG,SHANK1,MFSD2A,ACTR2,KIDINS220,GLI3,ITGB1,PLXNA1,KIF5B,PICALM,GLI2,MEGF8,PAK1,CTNNB1,TRAK1,FARP1,DCLK1,SEMA3C,ILK,RYK,RNF6,CXCL12,PALLD,EIF4G2,ROBO1,MAP1B,DIP2B,SEMA4F,TLX2,TCTN1,APP,DHX36,RAPGEF2,PTPN11,OPA1,ZFYVE27,SPP1,SEMA4G,BRSK2,PDLIM5,NDN,CHRNA7,DOCK10,NOTCH2,CTTN,DNM1L,CAPRIN2,FEZ1,GSK3B,DAG1,AUTS2,ANOS1,BMP7,FGFR2,IQGAP1,KIF1A,TANC2,MAPT,TNR,FN1,NCKAP1,SKIL,SMURF1,NBL1,TNIK,VCL,OBSL1,CRMP1,NOTCH3,ENAH,SRCIN1,NGFR,RAB11A,MAP2K1,TTC8,DICER1,NPTN,ISLR2,TRPV2,LAMA3,ROCK1,PLXNB3,PAFAH1B1,NTN3,PLXNA3,RERE,BSG,NTNG2,BARHL2,NEXN,DPYSL5,VSTM2L,GDNF,THY1,CHN1,ARTN,YWHAH,SDC2,MAP1A,OLFM1	darkgreen	EC_M4

GO:0046907	2.52E-07	1	231	1261	intracellular transport	BP	6.35E-05	CD63,LMAN1,STX7,AP3S1,AGAP2,TBC1D9B,RAB21,RABL2A,ANGPT1,NUP50,NUS1,HSPA4,SEC24A,REEP2,SIL1,SUN2,GGA1,LEP,PEX6,VCP,DYNC1I1,SRP72,LMAN2L,DST,RAB23,SNX13,RIMS1,TMEM30A,ERLEC1,COX18,UBE2J1,ACTR2,MDFIC,GLI3,VPS41,BVES,VPS35,MGARP,KIF5B,SEC61A1,NAPA,RAB7A,COPG1,IWS1,PICALM,SNX17,PAK1,TGFB1,SRP68,SEC13,TRAK1,AKT2,SEC23B,TBC1D16,NPLOC4,ACTN4,PPP1R12A,UFM1,PRNP,HDAC3,DCLK1,LEPROTL1,WASHC2A,VPS37A,ARL1,TMEM87B,TM9SF4,REEP5,HSP90B1,TMED2,COPB2,OSBP,RIOK2,TRAM1,MAP1B,ERGIC3,COPB1,ATP2A2,USO1,SDAD1,APP,CACNB3,SSR3,PTPN11,EXOC6B,OPA1,ARHGAP1,ATG13,AP3B1,TSG101,CRY2,VPS18,AKTIP,BAP1,CHP1,AP2A2,CBLB,IFT57,BRSK2,VPS39,MAGEL2,RAB7B,ARL3,SRPRA,CTTN,DNM1L,SELENOS,KPNA6,SEC22B,CPT1A,ERGIC2,FEZ1,WIPI1,GSK3B,CORO1C,SGSM1,HSPA8,ARV1,KPNA1,AP1B1,SEC22A,VAPB,HYOU1,AP1S2,AP2M1,COPA,OSBPL2,SCFD1,DYNC1I2,KIF1A,SNX27,TMEM50A,TOMM40L,TANC2,SRP54,LSG1,HSPB1,MAPT,SEC23A,AP1S1,SYVN1,C11orf65,PDCD10,UPF1,HTT,CLTCL1,PRKCI,SMURF1,AP5M1,ACTR10,FMN2,STX18,KDELR2,ZDHHC4,B3GAT3,MYO5A,ERC1,HAP1,SNX8,LMAN2,MED1,MSN,MYO1E,STX1B,SNX11,SSR2,ARHGEF2,NGFR,LTBP2,TMED10,IFT43,RAB11A,MAP2K1,SEL1L,SYTL4,TMED1,CLTC,ARMCX3,CLMN,CD74,MIEF2,EVI5L,AKT1,MYO1D,SNX18,PRKAA1,ZDHHC9,RAB31,NAPG,SUPT6H,CLEC16A,MYO5B,MYO10,MYO1C,MGRN1,SRL,BCAP31,PAFAH1B1,PKD1,FLNA,VAMP7,PARK7,AHCYL1,HSBP1,HSPA5,AP1G1,WLS,RANBP2,KPNA4,SPCS3,RAB6A,SNX10,ARF4,TMEM167A,BLOC1S2,VPS26B,C	darkgreen	EC_M4
GO:0000381	1.16E-06	0.999999779	18	52	regulation of alternative mRNA splicing, via spliceosome	BP	6.43E-05	SMU1,YTHDC1,THRAP3,HNRNPL,KHDRBS1,RBM5,FXR1,DDX5,TRA2B,HNRNPU,RBM25,RBMX,NSRP1,RBFOX1,FMR1,RNPS1,PTBP1,RBM8A	darkgrey	EC_M8
GO:0030490	1.19E-06	0.999999799	16	46	maturation of SSU-rRNA	BP	6.58E-05	SRFBP1,NOL10,DHX37,UTP20,BMS1,RRS1,RIOK1,WDR3,FP565260.1,HEATR1,NOL11,NGDN,NOP14,RIOK3,UTP6,NOB1	darkgrey	EC_M8
GO:1901976	1.20E-06	0.99999985	13	31	regulation of cell cycle checkpoint	BP	6.59E-05	DDX39B,ETAA1,GEN1,RNASEH2B,CUL4A,CCNB1,RPA2,TPR,LCMT1,THOC1,NDC80,CDT1,MAD2L1	darkgrey	EC_M8
GO:0045087	3.47E-07	1	84	459	innate immune response	BP	6.89E-05	IFNGR1,TNFAIP3,TRIM39,PTK2,C6orf106,DAPK1,DDX58,SYK,REL,RSAD2,TLR4,C5,PIK3CG,NECTIN2,UNC13D,PPARG,GATA3,SEC14L1,IKBKB,TMEM173,ARID5A,BPIFB3,ITCH,TLR2,CXCL10,SERINC5,OAS3,NLRC5,IFIT2,IFIT1,EDN1,STAT1,CD58,SNCA,MX2,MX1,RNF19B,LCK,LYST,NCF1,ISG20,TIFA,FGR,FES,PRKD1,SRMS,PAK2,STYK1,LTF,MASP1,CYBB,IL1RAP,DDX3X,TMEM106A,BIRC3,WAS,RAB27A,TLR6,TNK1,ARG2,TBKBP1,CCL2,YES1,JAK1,LGALS9,NOS2,ELF4,ISG15,IRF8,PLCG2,STXBP1,DAB2IP,OAS1,VNN1,TLR1,GBP5,RAB43,TRIM34,CD46,TRIM62,SERPINB9,BST2,VAMP3,IFIT3	turquoise	EC_M6
GO:0006812	2.82E-07	1	157	796	cation transport	BP	6.98E-05	CYB5A,CD63,SLC12A2,ATP6V1C1,SLC22A4,CALHM5,SLC39A7,SMDT1,KCNK9,UBQLN1,SLC39A4,ATP6V1F,SLC30A9,CNNM4,SLC17A5,SHANK1,SLC31A1,ITGB1,PGRMC2,MFSD4B,FKBP1B,FGF2,KIF5B,SEC61A1,PANX1,SLC36A4,ATP7B,ANO10,TPT1,ATP6V0B,SCN5A,CTNNB1,GPD1L,SLC4A7,SLC38A10,ACTN4,STIM1,ATP2C1,PRNP,ATP2B1,TMCO3,TUSC3,REM1,CXCL12,FXYD1,SLC20A1,SNTA1,SLC25A4,VAMP8,SLMAP,ATP1B3,TRPC4AP,RYR3,P2RX4,ATP2A2,CACNA1D,APP,SLC4A5,CX3CL1,SLC33A1,ABCC8,CACNB3,HOMER1,OPA1,ARHGAP1,SERINC3,ADA,CHP1,ATP2B4,PKD2,NIPA1,ATP6V1B2,SLC39A10,CHRNA7,MRS2,CNNM2,CALHM1,PKP2,DNM1L,SLC2A10,HTR2B,ISCU,SHROOM2,CASQ1,TFRC,TMCO1,LINC02210-CRHR1,TFR2,PTPN6,KCNK4,ITGAV,HTT,PKD1L1,ERO1A,KCNA1,SLC3A2,ASIC4,SLC6A13,CHRNE,HAP1,CACNA1C,SLC29A4,NLGN2,STIM2,ATP6V1D,MAGED2,PPARGC1A,SFXN1,RHBG,COX6A2,SLC7A3,SYT11,KCNMB1,CACNA1G,TMEM63C,CALM1,CLTC,VMP1,SLC44A2,SLC39A6,ANXA6,TRPV2,PDGFRB,AKT1,SCNN1D,PKD1,FLNA,KCNAB2,ATP6AP1,VAMP7,PARK7,HCN2,AHCYL1,JPH3,SLC30A7,ATP6V0D1,MIR24-1,DMD,SURF1,FKBP1A,CBARP,SLC36A1,TMEM38B,SLC22A17,NMUR1,GDNF,THY1,TMSB4X,TMEM38A,YWHAH,SARAF,SLC39A3,NIPAL1,HCN4,ATP6V0E1,GEM,KCNMB4	darkgreen	EC_M4

GO:0010243	2.88E-07	1	157	793	response to organonitrogen compound	BP	7.01E-05	LRP1,AP3S1,CDO1,CANX,RRM2B,PPARA,CTNNA1,UBQLN1,LEP,VCP,COL1A2,ASNS,SOCS5,ERLEC1,UBE2J1,ACTR2,ROCK2,RCN3,VPS35,MGARP,ITGB1,SESN3,PANX1,EIF2B3,TMUB1,PAK1,HTR5A,UBXN4,HNMT,CTNNB1,AKT2,STT3B,OSBPL8,NPLOC4,KAT2B,RNF121,IRS2,PRNP,ATP2B1,SMPD1,LEPROTL1,APLP1,CXCL12,HSP90B1,MAP1LC3A,MAP1B,RYR3,SORBS1,P2RX4,USO1,APP,GLRB,RAPGEF2,ABCC8,PPP2R2A,PTPN11,ADCY6,HOMER1,OPA1,AMFR,PTPN22,CRY2,MMP2,ADA,ATP2B4,PKD2,BRSK2,MARCH6,CHRNA7,ADIPOR1,SELENOS,CCND1,CPT1A,ZNF106,LRP5,EPRS,HTR2B,GSK3B,CASP7,DAG1,PIK3CA,HADH,COL6A1,BMP7,ADCY5,WDTC1,PEA15,NCSTN,SYAP1,IQGAP1,UBE4A,UROS,BACE1,RHBDD2,EDEM3,LINC02210-CRHR1,SYVN1,EIF4EBP2,DNAJC10,DNAJB12,FBN1,PRKCI,COL5A2,QDPR,TIMP1,PTPRN,JKAMP,MYO5A,SLC2A4,PPARGC1A,CPEB4,ARHGEF2,NGFR,COL1A1,TMED10,SHC1,SEL1L,CALM1,PKM,ROCK1,USP14,AKT1,ITGA2,LPIN2,PRKAA1,RAB31,GNAL,PTPN2,LDOC1,MYO1C,GNB1,BCAP31,FLNA,TMEM259,HCN2,BSG,AHCYL1,GNAI3,CDH13,BCAR3,SLC2A8,HSPA5,RANBP2,SERPINF1,FBXO27,TMEM38B,PEMT,CACYBP,CDKN1A,DNAJB9,NNMT,TMEM38A,LEPROT,PIK3C2A,UBE2G2,HCN4,DERL2	darkgreen	EC_M4
GO:0050920	3.81E-07	0.999999865	41	167	regulation of chemotaxis	BP	7.46E-05	SEMA6A,PTK2,PDGFB,KDR,SEMA4D,IL6,CXCL8,C5,NRP1,PRKD2,NOVA2,CXCR4,EFNB2,VEGFC,CXCL10,ANGPT2,THBS4,ANO6,EDN1,JAM3,SEMA3F,RIN3,PRKD1,YTHDF1,SEMA6D,MCU,PPM1F,RAC1,SEMA4A,FGF18,PGF,DAPK2,S100A14,MMP28,CCL2,LGALS9,SEMA6B,NOTCH1,S1PR1,FGF16,STX3	turquoise	EC_M6
GO:0007131	1.37E-06	0.999999769	16	44	reciprocal meiotic recombination	BP	7.48E-05	RAD21,RMI1,UBR2,RAD54L,FANCD2,TOP2B,MND1,MSH3,RAD51,TRIP13,SPO11,FANCM,RAD51AP1,TOP2A,EME1,BRIP1	darkgrey	EC_M8
GO:0140527	1.37E-06	0.999999769	16	44	reciprocal homologous recombination	BP	7.48E-05	RAD21,RMI1,UBR2,RAD54L,FANCD2,TOP2B,MND1,MSH3,RAD51,TRIP13,SPO11,FANCM,RAD51AP1,TOP2A,EME1,BRIP1	darkgrey	EC_M8
GO:1902969	1.38E-06	0.999999924	9	14	mitotic DNA replication	BP	7.54E-05	MCM3,ZMPSTE24,MCM2,MCM6,BRCA2,MCM4,RAD51,POLA1,CDC45	darkgrey	EC_M8
GO:0030198	3.16E-07	1	64	250	extracellular matrix organization	BP	7.57E-05	LRP1,COL11A2,COL1A2,COL15A1,TGFBR1,ADAMTS3,ITGB1,FBLN2,RB1,CRTAP,TGFB1,SERPINH1,SPINT2,LUM,MMP17,SPINK5,APLP1,ADAMTS6,SULF1,LOXL3,MMP15,APP,PTX3,LOXL2,MMP2,BMP1,RUNX1,FAP,PHLDB2,SLC2A10,TGFB2,FBLN5,MMP14,DAG1,PHLDB1,QSOX1,PRDX4,COLGALT2,PLOD3,TNR,P4HA1,COMP,ERO1A,COL3A1,COL5A2,FKBP10,MYO1E,COL1A1,PBXIP1,EGFLAM,MMP23B,VWA1,COL5A1,NTNG2,POMT1,COL11A1,LOXL1,SERPINB5,ADAMTS5,TMEM38B,LOX,HAS2,MFAP4,COL8A2	darkgreen	EC_M4
GO:0072359	3.25E-07	1	176	902	circulatory system development	BP	7.66E-05	LRP1,AHI1,TAB2,SLC12A2,ANGPT1,FAM19A5,PPARA,NUS1,RSPO3,ADGRB1,LY6E,PPARD,CBY1,MYH9,LEP,SRF,VEGFA,COL1A2,PDCL3,REST,COL15A1,TGFBR1,RTN4,EMC10,CALD1,GLI3,ROCK2,BVES,RRAS,MYLK3,ITGB1,FGF2,SRPK2,RB1,TENM4,GLI2,MEGF8,SMARCD3,SNX17,PAK1,TGFB1,SCN5A,CTNNB1,RARB,ACTG1,KAT2B,STIM1,DCN,STARD13,SEMA3C,ILK,SPINK5,ANKRD1,ADAMTS6,TMED2,EIF4G2,SULF1,ROBO1,HEY1,RASA1,DHX36,RAPGEF2,CX3CL1,ABCC8,CPE,PTPN11,LOXL2,ACVR1,MMP2,MTDH,ATP2B4,PKD2,RUNX1,HIF1AN,DNAH5,IFT57,PDLIM5,CHRNA7,FAP,TJP1,NOTCH2,PKP2,HYAL1,SLC2A10,TGFB2,LRP5,ADGRB2,HTR2B,PRKAR1A,CASP7,MMP14,DAG1,PIK3CA,LEF1,PRKCA,NTRK3,BMP7,AP1B1,BICC1,FGFR2,VASH2,CLIC4,HSPB1,SERPINE1,SGPL1,FZD2,FN1,FBN1,PDCD10,ITGAV,COMP,COL3A1,SYNPO2L,ECE1,PRRX1,MNAT1,OBSL1,ACTA2,ROM1,AKT3,FKBP10,CACNA1C,BMPR1A,HSPB7,PDLIM7,TCAP,MED1,NPPB,MYO1E,FGFRL1,NGFR,TPM1,COL1A1,CACNA1G,SHC1,MAP2K1,TMEM100,SGCD,PKM,MYOCD,ANGPTL6,PIN1,PDGFRB,ROCK1,AKT1,MYDGF,NXN,PKD1,FLNA,BSG,SYPL2,SARS,COL5A1,CDH13,COL11A1,F3,LMO4,MIR24-1,FKBP1A,LOXL1,SERPINF1,LOX,PDLIM1,CACYBP,LBX1,THY1,HAS2,CDKN1A,HOPX,PIK3C2A,HCN4,OLFM1,WNT2,COL8A2,FGF10,MBD2	darkgreen	EC_M4
GO:0045943	1.48E-06	0.999999861	11	22	positive regulation of transcription by RNA polymerase I	BP	8.02E-05	PWP1,WDR43,UTP15,WDR75,HEATR1,NCL,NOL11,UBTF,DHX33,LYAR,DDX11	darkgrey	EC_M8
GO:2000779	1.48E-06	0.999999643	22	76	regulation of double-strand break repair	BP	8.02E-05	UBR5,POT1,RIF1,PPP4R2,PRKDC,ERCC6,PARPBP,RAD51,ARID2,DEK,CHEK1,FANCB,RPA2,AUNIP,SETD2,NSD2,RAD51AP1,FOXM1,DDX11,FUS,SMCHD1,RMI2	darkgrey	EC_M8
GO:0000910	1.53E-06	0.99999949	33	141	cytokinesis	BP	8.27E-05	KIF20A,PLEC,ANLN,PLK4,CENPA,E2F7,PRPF40A,CKAP2,BRCA2,ESPL1,KIF20B,CEP55,RACGAP1,PKP4,E2F8,NUSAP1,CIT,CUL3,KIF14,PRC1,SEPT2,SETD2,ECT2,INCENP,CDC6,KIF4A,AURKB,KIF23,PLK1,IST1,PKN2,STMN1,AURKA	darkgrey	EC_M8

GO:0034080	1.56E-06	0.999999981	6	7	CENP-A containing nucleosome assembly	BP	8.33E-05	NASP,MIS18A,OIP5,HJURP,ITGB3BP,CENPW	darkgrey	EC_M8
GO:0061641	1.56E-06	0.999999981	6	7	CENP-A containing chromatin organization	BP	8.33E-05	NASP,MIS18A,OIP5,HJURP,ITGB3BP,CENPW	darkgrey	EC_M8
GO:0045446	4.30E-07	0.999999883	29	91	endothelial cell differentiation	BP	8.33E-05	NOTCH4,AFDN,TJP2,KDR,NRP1,ACVR2B,CXCR4,TIE1,IKBKB,JAG1,PDE2A,PDE4D,MARVELD2,PPP1R16B,STC1,RAPGEF3,ROBO4,PECAM1,PROX1,F11R,APOLD1,HOXB5,NOTCH1,RAPGEF1,S1PR1,CDH5,SOX17,SOX18,BMP6	turquoise	EC_M6
GO:0045229	3.65E-07	1	64	251	external encapsulating structure organization	BP	8.49E-05	LRP1,COL11A2,COL1A2,COL15A1,TGFBR1,ADAMTS3,ITGB1,FBLN2,RB1,CRTAP,TGFB1,SERPINH1,SPINT2,LUM,MMP17,SPINK5,APLP1,ADAMTS6,SULF1,LOXL3,MMP15,APP,PTX3,LOXL2,MMP2,BMP1,RUNX1,FAP,PHLDB2,SLC2A10,TGFB2,FBLN5,MMP14,DAG1,PHLDB1,QSOX1,PRDX4,COLGALT2,PLOD3,TNR,P4HA1,COMP,ERO1A,COL3A1,COL5A2,FKBP10,MYO1E,COL1A1,PBXIP1,EGFLAM,MMP23B,VWA1,COL5A1,NTNG2,POMT1,COL11A1,LOXL1,SERPINB5,ADAMTS5,TMEM38B,LOX,HAS2,MFAP4,COL8A2	darkgreen	EC_M4
GO:0030516	3.73E-07	0.999999899	30	83	regulation of axon extension	BP	8.54E-05	LRP1,RAB21,SRF,VEGFA,RTN4,MEGF8,PAK1,SEMA3C,ILK,RYK,RNF6,CXCL12,EIF4G2,MAP1B,DIP2B,SEMA4F,ZFYVE27,SEMA4G,CTTN,GSK3B,MAPT,TNR,FN1,RAB11A,ISLR2,TRPV2,PAFAH1B1,PLXNA3,BARHL2,OLFM1	darkgreen	EC_M4
GO:0031055	1.66E-06	0.999999948	7	10	chromatin remodeling at centromere	BP	8.86E-05	NASP,HELLS,MIS18A,OIP5,HJURP,ITGB3BP,CENPW	darkgrey	EC_M8
GO:0001775	4.72E-07	1	116	692	cell activation	BP	9.03E-05	IFNGR1,TNFAIP3,AKAP12,FZD6,TCF7,PDGFB,MFNG,TEC,ADGRF5,KIT,TNFRSF21,NFIL3,SYK,IL6,CXCL8,PELI1,RSAD2,TLR4,IL15,PIK3CG,GATA2,CST7,CTSC,NECTIN2,NOS3,GAB2,UNC13D,GATA3,CD93,SFRP1,EPHB1,GAPT,MERTK,CHD7,IL1A,IGF1,HHEX,ITCH,SH3RF1,KIF13B,MEF2C,TLR2,IL7,TPD52,CXCL10,LMBR1L,ADGRG3,DYSF,DGKZ,DLL4,SOX13,EDN1,SNCA,NFKB2,DCAF1,CD200,LCK,PPP3CA,HYAL2,ICOSLG,SNAP23,LAPTM5,HLX,NFATC2,INPP5D,CD86,FGR,FES,RPS6KA1,F11R,APOLD1,DLG1,B2M,ADORA2A,ITGA4,SOS2,TMEM106A,SELP,VWF,CRACR2A,WAS,RAB27A,ITPKB,TLR6,LFNG,TNFRSF1B,ARG2,PEAR1,MTOR,EFNB1,PSEN1,SEMA4A,BATF,CHRNB2,CD19,SCNN1B,YY1,LYL1,CCL2,YES1,JAG2,LGALS9,ELF4,IL7R,MYO18A,AP3D1,NRARP,IRF8,PLCG2,STXBP1,HAVCR1,VNN1,TLR1,CD46,AKAP17A,BST2	turquoise	EC_M6
GO:0006986	4.23E-07	0.999999861	38	124	response to unfolded protein	BP	9.53E-05	HSPA4,VCP,NCK2,CREB3,ERP44,ERLEC1,UBXN4,SERPINH1,STT3B,TMTC4,DDRGK1,HSPH1,TMED2,TRAM1,AMFR,CHAC1,SELENOS,CCND1,HSPA8,VAPB,ATF3,ATF6,RHBDD2,EDEM3,HSPB1,COMP,ERO1A,JKAMP,HSPB7,EIF2S1,HSPB3,PTPN2,MBTPS1,HSPA5,HSPB2,DNAJB9,HSPA13,DERL2	darkgreen	EC_M4
GO:0048858	4.39E-07	1	113	520	cell projection morphogenesis	BP	9.74E-05	LRP1,RAB21,PLXNB2,PACSIN1,ADGRB1,SH3GL2,PLAA,SRF,VEGFA,RIMS1,RTN4,TPBG,SHANK1,MFSD2A,ACTR2,KIDINS220,GLI3,ITGB1,PLXNA1,KIF5B,PICALM,GLI2,MEGF8,PAK1,CTNNB1,TRAK1,FARP1,DCLK1,SEMA3C,ILK,RYK,RNF6,CXCL12,PALLD,EIF4G2,ROBO1,MAP1B,DIP2B,SEMA4F,TLX2,TCTN1,APP,DHX36,RAPGEF2,PTPN11,OPA1,ZFYVE27,SPP1,SEMA4G,BRSK2,PDLIM5,NDN,CHRNA7,DOCK10,NOTCH2,CTTN,DNM1L,CAPRIN2,FEZ1,GSK3B,DAG1,AUTS2,CORO1C,ANOS1,BMP7,FGFR2,IQGAP1,KIF1A,TANC2,MAPT,TNR,FN1,NCKAP1,SKIL,SMURF1,NBL1,TNIK,VCL,OBSL1,CRMP1,NOTCH3,ENAH,SRCIN1,NES,NGFR,RAB11A,MAP2K1,TTC8,DICER1,NPTN,ISLR2,TRPV2,LAMA3,ROCK1,PLXNB3,PAFAH1B1,NTN3,PLXNA3,RERE,BSG,NTNG2,BARHL2,NEXN,DPYSL5,VSTM2L,GDNF,THY1,CHN1,ARTN,YWHAH,SDC2,MAP1A,OLFM1	darkgreen	EC_M4
GO:0030155	5.40E-07	1	98	541	regulation of cell adhesion	BP	1.00E-04	SEMA6A,NOTCH4,AFDN,PTK2,PDGFB,PODXL,TEK,LIMCH1,TNFRSF21,KDR,SYK,SEMA4D,IL6,CXCL8,PELI1,IL15,NRP1,PIK3CG,LAMA4,PRKD2,GPR4,PLXND1,UNC13D,GATA3,CXCR4,PPP1CB,CYTH1,JAG1,SFRP1,FERMT1,ARHGEF7,CD36,MAGI1,RET,IL1A,IGF1,ITCH,IL7,ANGPT2,CYTIP,SOX13,NEDD9,JAM3,ETS1,LCK,PPP3CA,ICOSLG,PRKX,LAPTM5,HLX,AP1AR,CD86,PLXNA2,FES,LAMA5,F11R,ARHGDIB,ITGA6,DOCK1,MELTF,DLG1,SIPA1,APOD,B2M,ADORA2A,ITGA4,SELE,SELP,PPM1F,RAC1,RCC2,JUP,EPHA2,ITPKB,ARG2,EFNB1,ITGA3,EFNA1,DNM2,CCL2,YES1,JAG2,JAK1,LGALS9,IL7R,AP3D1,NRARP,NOTCH1,PIEZO1,S1PR1,NFAT5,VNN1,ADGRG1,CD46,STX3,BMP2,CBLL1,BMP6	turquoise	EC_M6

GO:0010632	5.41E-07	0.999999845	48	208	regulation of epithelial cell migration	BP	1.00E-04	FLT4,PTK2,PDGFB,TEK,KDR,NRP1,PIK3CG,GATA2,PRKD2,PRCP,NOS3,PPARG,GATA3,HBEGF,ADAMTS9,SCARB1,PLK2,MEF2C,VEGFC,ANGPT2,PLCG1,STC1,DLL4,EDN1,ETS1,PROX1,PRKD1,MAP3K3,DOCK1,PPM1F,RAC1,HIF1A,RHOJ,JUP,EPHA2,MMRN2,MCC,MTOR,SEMA4A,FGF18,ITGA3,EFNA1,VASH1,NOTCH1,PLCG2,DAB2IP,MIR23A,FGF16	turquoise	EC_M6
GO:0040017	5.43E-07	1	86	462	positive regulation of locomotion	BP	1.00E-04	SEMA6A,AKAP12,FLT4,PTK2,PDGFB,PODXL,TEK,ATP8A1,KIT,KDR,SEMA4D,IL6,CXCL8,DOCK4,TLR4,TCAF2,SDCBP,NRP1,PIK3CG,GATA2,PRKD2,NOS3,GATA3,CXCR4,P2RY6,HBEGF,FERMT1,ARHGEF7,SCARB1,PLK2,RET,IGF1,VEGFC,CXCL10,THBS4,PLCG1,ANO6,NEDD9,EDN1,JAM3,SRGAP2C,ETS1,PPP3CA,SEMA3F,PECAM1,FGR,MCAM,PROX1,PRKD1,MAP3K3,ITGA6,DOCK1,LAMC2,SEMA6D,ITGA4,IGFBP5,MCU,SELP,PLVAP,PPM1F,RAC1,HIF1A,RHOJ,SYNE2,MTOR,SEMA4A,FGF18,ITGA3,PGF,DAPK2,S100A14,LGALS9,SEMA6B,NOTCH1,PLCG2,S1PR1,DAB2IP,CARMIL2,RIPOR1,CDH5,MIR23A,RELN,FGF16,STX3,BMP2,CBLL1	turquoise	EC_M6
GO:0048666	4.58E-07	1	172	877	neuron development	BP	1.00E-04	ATP8B1,LRP1,AHI1,RAB21,PLXNB2,PACSIN1,ADGRB1,CTNNA1,MTCH1,LGALS1,SH3GL2,LEP,PLAA,RORB,SRF,VEGFA,NCK2,P3H1,RIMS1,TMEM30A,UBA6,RTN4,TPBG,SHANK1,MFSD2A,ACTR2,KIDINS220,WHRN,GLI3,NCDN,ITGB1,FKBP1B,PLXNA1,KIF5B,RB1,PICALM,TENM4,GLI2,MEGF8,PAK1,CTNNB1,TRAK1,FARP1,SLC4A7,DCLK1,SEMA3C,ILK,DPYSL3,RYK,RNF6,CXCL12,LGI4,ANKRD1,SLC25A46,PALLD,EIF4G2,ROBO1,MAP1B,DIP2B,SEMA4F,TLX2,TCTN1,APP,DHX36,RAPGEF2,CX3CL1,PTPN11,ADCY6,OPA1,ARSB,ZFYVE27,CRTAC1,SPP1,RUNX1,TWF1,SEMA4G,GPRIN3,BRSK2,PDLIM5,NDN,CHRNA7,ARL3,DOCK10,TDP2,NOTCH2,CTTN,DNM1L,CAPRIN2,TGFB2,FEZ1,GSK3B,DAG1,AUTS2,ANOS1,NTRK3,BMP7,FGFR2,VASH2,IQGAP1,KIF1A,FARP2,TANC2,SCYL1,MAPT,C1QL1,FZD2,TNR,FN1,NCKAP1,GPC2,PRKCI,SKIL,PRRX1,SMURF1,NBL1,TNIK,VCL,GLDN,OBSL1,ROM1,CRMP1,UGDH,NOTCH3,ENAH,SRCIN1,STX1B,NGFR,RAB11A,MAP2K1,TTC8,DICER1,CLMN,NPTN,ISLR2,TRPV2,ACSL4,LAMA3,ROCK1,AKT1,PLXNB3,PAFAH1B1,NTN3,NPHP4,FLNA,PLXNA3,RERE,BSG,NTNG2,BARHL2,HSPA5,NEXN,LHX8,DPYSL5,DMD,SERPINF1,FRY,VSTM2L,ITM2C,UBE2V2,ARF4,GDNF,THY1,BLOC1S2,CHN1,EFHD1,ARTN,YWHAH,ZPR1,SDC2,PRKG1,MAP1A,OLFM1	darkgreen	EC_M4
GO:0000076	1.93E-06	0.99999988	9	15	DNA replication checkpoint signaling	BP	1.02E-04	CLSPN,ORC1,TOPBP1,TICRR,DNA2,CDC45,CDC6,TIPIN,CDT1	darkgrey	EC_M8
GO:0006360	1.99E-06	0.999999625	17	50	transcription by RNA polymerase I	BP	1.05E-04	PWP1,ZMPSTE24,TAF1B,WDR43,ERCC6,POLR1A,UTP15,WDR75,HEATR1,NCL,NOL11,TAF1A,UBTF,DHX33,LYAR,DDX11,TAF1	darkgrey	EC_M8
GO:0051272	6.71E-07	1	86	466	positive regulation of cellular component movement	BP	1.22E-04	SEMA6A,AKAP12,FLT4,PTK2,PDGFB,PODXL,TEK,ATP8A1,KIT,KDR,SEMA4D,IL6,CXCL8,DOCK4,TLR4,TCAF2,SDCBP,NRP1,PIK3CG,GATA2,PRKD2,NOS3,GATA3,CXCR4,P2RY6,HBEGF,FERMT1,ARHGEF7,SCARB1,PLK2,RET,IGF1,VEGFC,CXCL10,THBS4,PLCG1,ANO6,NEDD9,EDN1,JAM3,SRGAP2C,ETS1,PPP3CA,SEMA3F,PECAM1,FGR,MCAM,PROX1,PRKD1,MAP3K3,ITGA6,DOCK1,LAMC2,SEMA6D,ITGA4,IGFBP5,MCU,SELP,PLVAP,PPM1F,RAC1,HIF1A,RHOJ,SYNE2,MTOR,SEMA4A,FGF18,ITGA3,PGF,DAPK2,ATP2A1,S100A14,LGALS9,SEMA6B,NOTCH1,PLCG2,S1PR1,DAB2IP,CARMIL2,RIPOR1,CDH5,MIR23A,RELN,FGF16,BMP2,CBLL1	turquoise	EC_M6
GO:0010639	2.32E-06	0.999999073	55	297	negative regulation of organelle organization	BP	1.23E-04	FBXO43,FBXO5,RAD21,POT1,TTK,CAPZA2,GEN1,MCM2,CLASP2,CKAP2,CCNB1,ESPL1,BUB1,TPX2,TNKS2,ARFGEF1,XRN1,KNTC1,TERF1,BUB1B,NBN,CHEK1,TRIP13,CAMSAP2,CENPF,BUB3,HDGFL3,TPR,BRCA1,ZWINT,FKBP4,HNRNPU,WAPL,TOP2A,DCP2,PRELID1,MAD1L1,EXOSC10,SPDL1,AURKB,ATRX,PTTG1,ZWILCH,LCMT1,TRIM37,PLK1,PSMD10,NDC80,TAOK1,CCNF,CDT1,TMOD3,MAD2L1,STMN1,TIGAR	darkgrey	EC_M8
GO:0051053	2.34E-06	0.999999351	25	94	negative regulation of DNA metabolic process	BP	1.23E-04	UBR5,POT1,TRIM28,MSH2,DNAJC2,RIF1,ERCC6,PARPBP,TNKS2,XRN1,TERF1,MSH3,NBN,FANCB,BLM,AUNIP,XRCC5,ANKLE1,HNRNPU,DCP2,EXOSC10,THOC1,SMCHD1,RMI2,HIST1H1A	darkgrey	EC_M8

GO:0120039	5.75E-07	1	112	517	plasma membrane bounded cell projection morphogenesis	BP	1.24E-04	LRP1,RAB21,PLXNB2,PACSIN1,ADGRB1,SH3GL2,PLAA,SRF,VEGFA,RIMS1,RTN4,TPBG,SHANK1,MFSD2A,ACTR2,KIDINS220,GLI3,ITGB1,PLXNA1,KIF5B,PICALM,GLI2,MEGF8,PAK1,CTNNB1,TRAK1,FARP1,DCLK1,SEMA3C,ILK,RYK,RNF6,CXCL12,PALLD,EIF4G2,ROBO1,MAP1B,DIP2B,SEMA4F,TLX2,TCTN1,APP,DHX36,RAPGEF2,PTPN11,OPA1,ZFYVE27,SPP1,SEMA4G,BRSK2,PDLIM5,NDN,CHRNA7,DOCK10,NOTCH2,CTTN,DNM1L,CAPRIN2,FEZ1,GSK3B,DAG1,AUTS2,CORO1C,ANOS1,BMP7,FGFR2,IQGAP1,KIF1A,TANC2,MAPT,TNR,FN1,NCKAP1,SKIL,SMURF1,NBL1,TNIK,VCL,OBSL1,CRMP1,NOTCH3,ENAH,SRCIN1,NGFR,RAB11A,MAP2K1,TTC8,DICER1,NPTN,ISLR2,TRPV2,LAMA3,ROCK1,PLXNB3,PAFAH1B1,NTN3,PLXNA3,RERE,BSG,NTNG2,BARHL2,NEXN,DPYSL5,VSTM2L,GDNF,THY1,CHN1,ARTN,YWHAH,SDC2,MAP1A,OLFM1	darkgreen	EC_M4
GO:0048675	5.88E-07	0.999999816	35	106	axon extension	BP	1.25E-04	LRP1,RAB21,SRF,VEGFA,RTN4,ITGB1,MEGF8,PAK1,DCLK1,SEMA3C,ILK,RYK,RNF6,CXCL12,EIF4G2,MAP1B,DIP2B,SEMA4F,ZFYVE27,SEMA4G,NDN,CTTN,GSK3B,AUTS2,MAPT,TNR,FN1,VCL,RAB11A,ISLR2,TRPV2,PAFAH1B1,PLXNA3,BARHL2,OLFM1	darkgreen	EC_M4
GO:0042476	5.99E-07	0.999999823	32	95	odontogenesis	BP	1.26E-04	PPARA,CTNNA1,RUNX2,COL1A2,ASPN,CNNM4,GLI3,INHBA,GLI2,SCN5A,CTNNB1,RELT,STIM1,TGFB2,FAM20C,LEF1,BMP7,FGFR2,HTRA1,SERPINE1,BMPR1A,NGFR,COL1A1,TGFB3,ROGDI,BSG,ANKRD11,LHX8,ADAMTS5,SNX10,EDA,FGF10	darkgreen	EC_M4
GO:1902531	7.11E-07	0.999999717	199	1309	regulation of intracellular signal transduction	BP	1.28E-04	TNFRSF11A,SEMA6A,TNFAIP3,TRIM39,PLEKHG1,AKAP12,FLT4,CDC42SE2,TPD52L1,ARHGAP18,SGSM3,PTK2,DENND3,PDGFB,LRRC19,TEK,PIP5K1B,SH3RF3,KIT,KDR,CD2AP,SYK,GADD45G,SEMA4D,RNF149,IL6,REL,CHN2,PELI1,ARHGAP25,FIGNL1,CAV2,TLR4,TRAF1,SDCBP,NRP1,PUM2,RASIP1,PIK3CG,ARHGAP42,ARHGAP21,PRKD2,GPR4,FGD5,ZFAND6,PPARG,GATA3,SEC14L1,NET1,RALGAPA2,NDFIP2,CYTH1,IKBKB,P2RY6,PDE2A,SFRP1,HBEGF,RPL26,ARHGAP26,P2RX2,CD36,SIRT3,PRAG1,PRR5L,EPHB1,PLK2,RET,PDE4D,TNFRSF19,IGF1,EIF2AK3,ITCH,ERBIN,SH3RF1,PREX2,ARHGEF3,ARHGEF28,TLR2,P2RX7,NDRG4,GUCY1A1,MADD,ADGRG3,PPP1R16B,ZDHHC13,DNMBP,EDN1,STAT1,DYRK1A,DUSP8,PRDM15,SHANK2,HDAC1,LCK,HYAL2,SHOC2,HCRTR1,LAPTM5,NCF1,DYRK3,HCLS1,TIFA,CD86,ARHGEF12,SH3BP4,FGR,CFLAR,CASP10,INPP5F,KALRN,PROX1,PLEKHA1,PRKD1,F11R,ARHGDIB,MAP3K3,PAK2,DUSP16,DLG1,ELL3,SIPA1,MAP3K11,LTF,ARHGAP27,BCR,DDX3X,SOS2,IGFBP5,HOMER3,TBX1,TMEM106A,MECOM,BIRC3,SELP,VWF,TNFAIP8L3,FGF23,CRACR2A,RAC1,HIF1A,EPHA2,ITPKB,BID,TLR6,RELL1,RGS14,FGD1,ARHGEF9,ARHGAP23,MTOR,AR,OPHN1,STARD8,PSEN1,FGF18,ITGA3,DAPK2,TAOK2,ARHGEF15,EFNA1,PIK3R5,NPR1,DNM2,TNIP1,AMOT,MARK3,ADRB2,CCL2,ARHGAP28,LGALS9,USP7,TNFAIP8L1,ARHGAP4,TRAF2,PLEKHG5,NOTCH1,PLCG2,ENG,RFWD3,NFAT5,DAB2IP,MIR23A,VNN1,PMAIP1,ADGRG1,TRIM24,TRIM34,RELN,ALKAL1,LPAR6,TRIM62,ARHGEF1,DUSP5,BST2,BMP2,RNF125,DUSP7,RNF152,NMNAT1	turquoise	EC_M6
GO:0002764	7.41E-07	0.99999977	58	278	immune response-regulating signaling pathway	BP	1.32E-04	TNFAIP3,GRAMD4,PTK2,LRRC19,DDX58,TEC,KIT,TNFRSF21,SYK,PELI1,RSAD2,TLR4,PUM2,PRKD2,NECTIN2,GATA3,IRAK2,SEC14L1,IKBKB,CD36,MAP3K1,PDE4D,ITCH,ERBIN,MEF2C,TLR2,OAS3,PLCG1,DGKZ,LCK,ICOSLG,LAPTM5,NFATC2,INPP5D,TIFA,BMX,FGR,PLEKHA1,PAK2,LTF,DDX3X,BIRC3,PRKCH,PLD2,TLR6,PSEN1,EIF2B2,CD19,S100A14,TNIP1,YES1,PDE4B,PLCG2,DAB2IP,FYB1,OAS1,TLR1,RNF125	turquoise	EC_M6

GO:0023051	7.58E-07	0.999999681	351	2524	regulation of signaling	BP	1.32E-04	TNFRSF11A,SEMA6A,TNFAIP3,TRIM39,PLEKHG1,AKAP12,FLT4,SNCAIP,FZD6,CTHRC1,RIMS2,CDC42SE2,GRAMD4,PACSIN2,TPD52L1,RNF146,ARHGAP18,SGSM3,PTK2,DENND3,ANKS1A,DAPK1,ATF4,PDGFB,CSNK1E,PICK1,KDM4C,MFNG,MDFI,LRRC19,TEK,RIC8B,APBA1,PIP5K1B,SH3RF3,KIT,KDR,CD2AP,SYK,GADD45G,SEMA4D,RNF149,ICA1,IL6,REL,CHN2,CXCL8,PELI1,RGS3,ARHGAP25,RSAD2,FIGNL1,CAV2,GRB10,LANCL2,TSPAN12,TLR4,GRIK2,TRAF1,DLGAP3,CCNY,SDCBP,TRPM4,NRP1,PUM2,RASIP1,PIK3CG,ARHGAP42,MTMR2,MGLL,GATA2,ARHGAP21,PRKD2,CTSC,GPR4,FGD5,NECTIN2,PRCP,NOS3,LLGL2,ZFAND6,PPARG,GATA3,ACVR2B,CXCR4,IRAK2,SEC14L1,NET1,RALGAPA2,NDFIP2,TMC8,CYTH1,IKBKB,JAG1,P2RY6,PDE2A,SFRP1,HBEGF,PLCB4,NOTUM,FERMT1,RPL26,SPEF1,ARHGAP26,P2RX2,CD36,SIRT3,PRAG1,PRR5L,EPHB1,PLK2,RET,PDE4D,TNFRSF19,CHD7,FAM3D,IL1A,IGF1,EIF2AK3,ABHD6,HHEX,ITCH,ERBIN,UFSP2,SH3RF1,PREX2,HIP1R,MCTP1,ARHGEF3,SOX7,ARHGEF28,MEF2C,TLR2,WWTR1,IL7,P2RX7,VEGFC,NDRG4,GUCY1A1,MADD,LMBR1L,ADGRG3,KMT2D,BMP2K,OAS3,NLRC5,PPP1R16B,PLCG1,NR4A2,DGKZ,HIPK1,DLL4,ZDHHC13,DNMBP,SOX13,ADAMTS20,EDN1,STAT1,DYRK1A,SNCA,DTNBP1,DUSP8,GAS2,PRDM15,ABCG1,SHANK2,HDAC1,ITPRIP,LCK,PPP3CA,HYAL2,ANO1,GIT2,SHOC2,MCTP2,HCRTR1,LAPTM5,NCF1,DYRK3,HCLS1,AP1AR,INPP5D,TIFA,CD86,MN1,ARHGEF12,SH3BP4,FGR,CFLAR,CASP10,INPP5F,ADCY1,KALRN,IGFBP3,PROX1,PLEKHA1,PRKD1,F11R,YTHDF1,IL10RA,ARHGDIB,MAP3K3,SRMS,ITGA6,PAK2,PTPRE,CNKSR2,SENP2,DUSP16,DLG1,ELL3,SIPA1,APOD,MAP3K11,CREB1,	turquoise	EC_M6
GO:2000147	7.59E-07	0.999999738	85	455	positive regulation of cell motility	BP	1.32E-04	SEMA6A,AKAP12,FLT4,PTK2,PDGFB,PODXL,TEK,ATP8A1,KIT,KDR,SEMA4D,IL6,CXCL8,DOCK4,TLR4,TCAF2,SDCBP,NRP1,PIK3CG,GATA2,PRKD2,NOS3,GATA3,CXCR4,P2RY6,HBEGF,FERMT1,ARHGEF7,SCARB1,PLK2,RET,IGF1,VEGFC,CXCL10,THBS4,PLCG1,ANO6,NEDD9,EDN1,JAM3,SRGAP2C,ETS1,PPP3CA,SEMA3F,PECAM1,FGR,MCAM,PROX1,PRKD1,MAP3K3,ITGA6,DOCK1,LAMC2,SEMA6D,ITGA4,IGFBP5,MCU,SELP,PLVAP,PPM1F,RAC1,HIF1A,RHOJ,SYNE2,MTOR,SEMA4A,FGF18,ITGA3,PGF,DAPK2,S100A14,LGALS9,SEMA6B,NOTCH1,PLCG2,S1PR1,DAB2IP,CARMIL2,RIPOR1,CDH5,MIR23A,RELN,FGF16,BMP2,CBLL1	turquoise	EC_M6
GO:0070200	2.61E-06	0.999999822	9	16	establishment of protein localization to telomere	BP	1.37E-04	CCT2,POT1,CCT4,BRCA2,TERF1,CCT7,CCT5,CCT6A,CCT3	darkgrey	EC_M8
GO:0001938	8.31E-07	0.99999979	25	78	positive regulation of endothelial cell proliferation	BP	1.43E-04	FLT4,PDGFB,TEK,KDR,CAV2,NRP1,GATA2,PRKD2,NR4A1,VEGFC,THBS4,PPP1R16B,PLCG1,PROX1,PRKD1,ITGA4,HIF1A,MTOR,PGF,LRG1,NRARP,EGFL7,MIR23A,BMP2,BMP6	turquoise	EC_M6
GO:0090501	2.80E-06	0.999999114	29	124	RNA phosphodiester bond hydrolysis	BP	1.46E-04	PAN2,POP1,NCBP1,PNPT1,EXOSC9,PCF11,TSEN2,DIS3,XRN2,EXOSC8,PAN3,UTP20,BMS1,RRS1,XRN1,PDE12,CNOT1,CPSF2,NOP14,EXO1,MRPL44,DCP2,EXOSC10,DIS3L,DROSHA,NOL9,EXOSC2,NOB1,FEN1	darkgrey	EC_M8
GO:0051315	2.86E-06	0.999999865	8	12	attachment of mitotic spindle microtubules to kinetochore	BP	1.49E-04	KIF2C,CENPE,NUF2,HNRNPU,MAD1L1,AURKB,NDC80,CDT1	darkgrey	EC_M8
GO:0070887	7.36E-07	0.999999688	377	2230	cellular response to chemical stimulus	BP	1.52E-04	CD63,LRP1,AP3S1,IPCEF1,SLC12A2,FBN2,ANGPT1,PPARA,NUS1,CXCL14,PTP4A3,LY6E,PPARD,CTNNA1,UBQLN1,LGALS1,SH3GL2,GNAQ,LEP,CDKN2B,PLAA,RORB,KIAA1324L,SRF,VEGFA,DOCK8,VCP,RUNX2,RASL11B,UNC13B,COL1A2,NCK2,CLOCK,CREB3,ASNS,REST,ASPN,TGFBR1,SOCS5,ERLEC1,TPBG,ADAMTS3,MAP3K7,AKIRIN1,ACTR2,SLC31A1,KIDINS220,ROCK2,INHBA,ZYX,MYLK3,VPS35,MGARP,SLC7A11,PGRMC2,FKBP1B,FGF2,SELENOW,KIF5B,SESN3,RB1,ESD,GLI2,MEGF8,PAK1,ACTR3,TGFB1,AXL,SCN5A,HTR5A,CTNNB1,IL15RA,LBH,DGAT2,AKT2,PFKP,ZEB2,OSBPL8,RARB,ACTG1,ACTN4,PPP1R12A,KAT2B,LTBP1,TMTC4,CRIM1,SPRED3,SPINT2,UFM1,LIG4,IRS2,PRNP,ATP2B1,DCN,SMAD9,ZNF703,UBA5,DDRGK1,SMPD1,ILK,LEPROTL1,ARL6IP5,DPYSL3,APLP1,RYK,CNOT7,RNF6,CXCL12,ANKRD1,HSP90B1,TMED2,IL20RB,MRAS,MAP1LC3A,CD180,FLNB,OSBP,SULF1,DKK3,ROBO1,MAP1B,RYR3,SORBS1,DOK1,P2RX4,HEY1,SELENOT,ATP2A2,USO1,APP,DHX36,RAPGEF2,CX3CL1,SLC33A1,ABCC8,CACNB3,PTPN11,ADCY6,OPA1,AMFR,GTF2H1,ZFYVE27,PTPN22,ACVR1,CRY2,VPS18,MMP2,GDF6,MTDH,ATP2B4,SPP1,PKD2,PLA2R1,HIF1AN,TANK,TOLLIP,BRSK2,NDN,RAB7B,CHRNA7,EIF4E,ELMO2,DYNLL1,INA,ADIPOR1,KDM5B,NOTCH2,CDC73,DNM1L,GLRX2,TROVE2,SELENOS,HYAL1,SLC2A10,CCND1,CPT1A,SBDS,TGFB2,ZBTB20,ZNF106,FBLN5,LRP5,EPRS,FEZ1,HTR2B,SATB2,GSK3B,CASP7,FSTL1,DAG1,PIK3CA,LEF1,PRKCA,CMKLR1,HJV,KRAS,NTRK3,HSPA8,SLAMF8,FAM162A,COL6A1,BMP7,ADCY5,VAPB,HYOU1,WDTC1,PYROXD1,ATF3,FGFR2,OSM,ITGB5,HTRA1,NCSTN,SYAP1,IQGAP1,STRAP,STRN3,UROS,CLIC4,TFRC,ARI	darkgreen	EC_M4

GO:0034220	7.63E-07	0.999999707	151	765	ion transmembrane transport	BP	1.56E-04	CYB5A,CD63,ATP8B1,SLC12A2,ATP6V1C1,SLC22A4,CALHM5,SLC39A7,SMDT1,KCNK9,UBQLN1,SLC39A4,ATP6V1F,SLC30A9,SLC35B2,CNNM4,SLC17A5,SHANK1,SLC35A1,SLC31A1,PCYOX1,SLC7A11,ITGB1,FKBP1B,FGF2,KIF5B,SEC61A1,PANX1,SLC36A4,ATP7B,ANO10,ATP6V0B,SCN5A,GPD1L,SLC4A7,ACTN4,STIM1,ATP2C1,PRNP,ATP2B1,TMCO3,ARL6IP5,TUSC3,REM1,FXYD1,SLC20A1,SNTA1,SLC25A4,SLMAP,ATP1B3,TRPC4AP,RYR3,P2RX4,ATP2A2,CACNA1D,APP,GLRB,SLC4A5,CX3CL1,SLC33A1,ABCC8,CACNB3,SLC26A7,HOMER1,OPA1,CHP1,ATP2B4,PKD2,NIPA1,ATP6V1B2,SLC39A10,CHRNA7,MRS2,CNNM2,CALHM1,SLC2A10,HTR2B,ISCU,SHROOM2,CASQ1,CLIC4,TMCO1,LINC02210-CRHR1,PTPN6,KCNK4,ITGAV,HTT,PKD1L1,ERO1A,KCNA1,VDAC2,SLC3A2,WDR1,ASIC4,SLC6A13,PRAF2,CHRNE,HAP1,CACNA1C,SLC29A4,NLGN2,CLN8,STIM2,ATP6V1D,PPARGC1A,SFXN1,RHBG,COX6A2,SLC7A3,SLC35B1,KCNMB1,CACNA1G,TMEM63C,CALM1,VMP1,GRIA1,SLC39A6,ANXA6,TRPV2,AKT1,ATP11C,SCN1D,PKD1,FLNA,KCNAB2,ATP6AP1,PARK7,HCN2,AHCYL1,JPH3,SLC30A7,SLC35A3,ATP6V0D1,MIR24-1,DMD,SURF1,FKBP1A,CBARP,SLC36A1,TMEM38B,THY1,TMSB4X,TMEM38A,YWHAH,TMEM109,SLC39A3,NIPAL1,HCN4,ATP6V0E1,GEM,KCNMB4	darkgreen	EC_M4
GO:0034249	3.03E-06	0.999998859	38	183	negative regulation of cellular amide metabolic process	BP	1.57E-04	PAN2,EIF3E,MRPL13,TNRC6B,TUT7,PNPT1,SYNCRIP,TIA1,DHFR,PUS7,EXOSC9,CNOT10,DIS3,METTL3,EXOSC8,PAN3,CAPRIN1,LSM14A,XRN1,PDE12,CNOT1,NCL,FXR1,DDX6,HNRNPR,TPR,CNOT9,EIF2AK1,DCP2,LARP1,ILF3,TYMS,FMR1,METTL16,EXOSC2,HNRNPD,RNF139,PAIP2	darkgrey	EC_M8
GO:0050767	7.80E-07	0.999999746	70	285	regulation of neurogenesis	BP	1.57E-04	LRP1,RAB21,PLXNB2,CTNNA1,SRF,VEGFA,REST,RTN4,ACTR2,GLI3,PLXNA1,TENM4,MEGF8,SMARCD3,RHEB,PAK1,TGFB1,CTNNB1,TRAK1,LIG4,SEMA3C,ILK,RYK,RNF6,CXCL12,EIF4G2,ROBO1,MAP1B,DIP2B,SEMA4F,HEY1,TLX2,DHX36,RAPGEF2,CX3CL1,ABCC8,OPA1,ZFYVE27,SPP1,SEMA4G,DNM1L,CAPRIN2,DAG1,NTRK3,BMP7,IDH2,MAPT,TNR,FN1,PRKCI,SKIL,OBSL1,HAP1,PRTG,BMPR1A,RAB11A,MAP2K1,DICER1,NPTN,ISLR2,TRPV2,TMEM98,PLXNB3,PAFAH1B1,PLXNA3,SERPINF1,THY1,YWHAH,BHLHE41,WNT2	darkgreen	EC_M4
GO:0000466	3.09E-06	0.99999967	11	24	maturation of 5.8S rRNA from tricistronic rRNA transcript (SSU-rRNA, 5.8S rRNA, LSU-rRNA)	BP	1.60E-04	EXOSC9,EXOSC8,UTP20,RRS1,URB1,WDR12,FTSJ3,NOP14,EXOSC10,NOL9,EXOSC2	darkgrey	EC_M8
GO:0006928	8.24E-07	0.999999659	279	1565	movement of cell or subcellular component	BP	1.64E-04	CD63,LRP1,AP3S1,RAB21,SLC12A2,PLXNB2,ANGPT1,FAM19A5,NUS1,CXCL14,FAM49B,MRTFA,PTP4A3,ADGRB1,PPARD,CTNNA1,SUN2,MDGA1,MYH9,LEP,PLAA,SRF,VEGFA,DOCK8,NCK2,DYNC1I1,CREB3,DST,PTP4A1,TGFBR1,RTN4,TPBG,EMC10,MYH14,AKIRIN1,ACTR2,GLI3,ROCK2,BVES,RRAS,MGARP,CFAP100,ITGB1,PLXNA1,FGF2,KIF5B,COPG1,GLI2,MEGF8,PAK1,ACTR3,TGFB1,AXL,SCN5A,CTNNB1,TRAK1,AKT2,DYNC1LI1,GPD1L,ZEB2,OSBPL8,ACTG1,NAV3,ACTN4,SPINT2,IRS2,DCN,ZNF703,DCLK1,STARD13,SEMA3C,DDRGK1,ILK,DPYSL3,RYK,CXCL12,KIF2A,FAT1,SNTA1,PALLD,SULF1,ROBO1,WWC2,MAP1B,CHST2,SEMA4F,P2RX4,PFN2,ATP2A2,CACNA1D,APP,RAPGEF2,CX3CL1,ABCC8,PTPN11,PGAM4,OPA1,ARSB,LOXL2,AP3B1,ACVR1,TUBB2A,ADA,ATP2B4,DNAH1,SEMA4G,NKD1,DNAH5,IFT57,NDN,ARL3,DOCK10,ELMO2,ADIPOR1,FAP,PHLDB2,TJP1,NOTCH2,CTTN,PKP2,DNM1L,DNER,HYAL1,SBDS,TGFB2,LRP5,FEZ1,HTR2B,SATB2,FSTL1,MMP14,DAG1,PIK3CA,AUTS2,SDC3,LEF1,DNAAF5,CORO1C,ANOS1,PRKCA,CMKLR1,SHROOM2,NTRK3,WWC3,HSPA8,SLAMF8,BMP7,IDH2,ITGB5,IQGAP1,DYNC1I2,KIF1A,CLIC4,S100A11,ARID5B,SRP54,ARPC5,LRRC15,FAM89B,HSPB1,MAPT,SERPINE1,SGPL1,ITGA2B,DNAJA4,TNR,S100P,FN1,NCKAP1,GPC2,PDCD10,ITGAV,ARPC2,HTT,COL3A1,PRKCI,NBL1,TIMP1,KTN1,PLD1,VCL,ACTR10,WDR1,MYO9B,FMN2,SIX4,CRMP1,MYO5A,AKT3,HAP1,CACNA1C,BMPR1A,NOTCH3,ENAH,PPARGC1A,ACTN1,MSN,SRCIN1,MYO1E,ARHGEF2,NGFR,TPM1,COL1A1,IFT43,CACNA1G,PBXIP1,RAB11A,MYH10,MAP2K1,TTC8,ARMCX3,NPTN,MYOCD,FAT2,ANXA6,PIN1,GP2,CD74,LAMA3,PDGFRB,ROCK	darkgreen	EC_M4
GO:0060326	9.71E-07	0.999999651	46	206	cell chemotaxis	BP	1.65E-04	TNFRSF11A,PTK2,PDGFB,KIT,KDR,SYK,IL6,CXCL8,DOCK4,C5,TRPM4,NRP1,PIK3CG,PRKD2,CXCR4,EDNRB,HBEGF,EPHB1,NR4A1,VEGFC,CXCL10,THBS4,ANO6,EDN1,JAM3,RIN3,LYST,PRKD1,CCRL2,MCU,RAC1,EPHA2,HOXB9,FGF18,PGF,DAPK2,S100A14,MMP28,CCL2,PDE4B,LGALS9,PLEKHG5,NOTCH1,S1PR1,FGF16,MSMP	turquoise	EC_M6
GO:0006999	3.34E-06	0.999999839	8	12	nuclear pore organization	BP	1.72E-04	NUP107,NUP205,NUP54,NUP93,NUP133,TPR,NDC1,NUP35	darkgrey	EC_M8

GO:0016043	9.61E-07	0.99999944	728	4652	cellular component organization	BP	1.84E-04	CD63,LMAN1,ATP8B1,LRP1,STX7,TRIM36,B4GALT7,AHI1,AGAP2,CAND1,RAB21,RNF19A,SLC12A2,PLXNB2,ANGPT1,TCP1,PPARA,HLA-DRA,COL11A2,ROS1,HSPA4,SEC24A,FER1L6,FAM49B,MRTFA,HABP4,PACSIN1,REEP2,ADGRB1,PPARD,CTNNA1,GOLM1,SUN2,CBY1,MKLN1,UBQLN1,MTCH1,LGALS1,MDGA1,SH3GL2,FLNC,MYH9,ARHGAP39,PLAA,TBCC,PEX6,SRF,VEGFA,VCP,UNC13B,COL1A2,TPM2,NCK2,PDCL3,REST,TXNDC9,LMAN2L,DST,RAB23,NLRP5,COL15A1,P3H1,TGFBR1,RIMS1,TMEM30A,PSME4,CENPC,UBA6,RTN4,TPBG,SHANK1,ADAMTS3,COX18,LRRC4B,EMC10,MFSD2A,GGCT,MYH14,CALD1,AKIRIN1,ACTR2,KIDINS220,WHRN,GLI3,NCDN,ROCK2,INHBA,VPS41,ZYX,STARD3NL,MYLK3,VPS35,MGARP,SLC7A11,CFAP100,ITGB1,FKBP1B,PLXNA1,SRPK2,KIF5B,SEC61A1,NAPA,RAB7A,ARHGEF4,FBLN2,EML2,RB1,PICALM,MARK4,NUFIP1,MAPRE3,CRTAP,PTCH2,PDCD6IP,TENM4,KCTD21,GLI2,MEGF8,SMARCD3,PCDH17,SNX17,PAK1,ACTR3,NCKAP5,TGFB1,AXL,RAB3GAP1,SEC13,CTNNB1,ESYT2,TRAK1,CLIP4,SERPINH1,AKT2,DYNC1LI1,SEC23B,DZIP1,ZEB2,PLEKHG2,DNAJB13,FARP1,ARHGEF17,OSBPL8,ACTG1,NAV3,NPLOC4,ACTN4,PPP1R12A,ANAPC15,STAM2,KAT2B,FAM98A,TACC1,SPINT2,UFM1,LIG4,ATP2C1,PRNP,CHFR,LUM,DCN,CDC42EP3,HDAC3,ZNF703,DCLK1,ACPP,PCLO,STARD13,KCTD16,SEMA3C,CAVIN3,UBA5,DDRGK1,SMPD1,PPP2CB,ILK,DCTN6,MMP17,DPYSL3,KATNAL1,SPINK5,APLP1,RYK,TTC17,CIAO1,TMEM127,CNOT7,RNF6,RIC3,VPS37A,ARL1,CXCL12,TM9SF4,SACS,RAB2A,KIF2A,CHPT1,TMEM41B,ANKRD1,FAT1,REEP5,ADAMTS6,SLC25A46,SNTA1,HSP90B1,TMED2,PA	darkgreen	EC_M4
GO:0098660	9.71E-07	0.999999641	119	574	inorganic ion transmembrane transport	BP	1.84E-04	CYB5A,CD63,SLC12A2,ATP6V1C1,SLC39A7,SMDT1,KCNK9,UBQLN1,SLC39A4,ATP6V1F,CNNM4,SLC17A5,SLC31A1,PCYOX1,ITGB1,FKBP1B,FGF2,KIF5B,SEC61A1,PANX1,ATP7B,ATP6V0B,SCN5A,GPD1L,SLC4A7,ACTN4,STIM1,ATP2C1,PRNP,ATP2B1,TMCO3,TUSC3,REM1,FXYD1,SLC20A1,SNTA1,SLC25A4,SLMAP,ATP1B3,TRPC4AP,RYR3,P2RX4,ATP2A2,CACNA1D,GLRB,SLC4A5,CX3CL1,SLC33A1,ABCC8,CACNB3,SLC26A7,OPA1,CHP1,ATP2B4,PKD2,NIPA1,ATP6V1B2,SLC39A10,CHRNA7,MRS2,CNNM2,CALHM1,SLC2A10,HTR2B,ISCU,SHROOM2,CASQ1,TMCO1,LINC02210-CRHR1,PTPN6,KCNK4,ITGAV,HTT,PKD1L1,ERO1A,KCNA1,SLC3A2,ASIC4,SLC6A13,HAP1,CACNA1C,STIM2,ATP6V1D,RHBG,COX6A2,KCNMB1,CACNA1G,CALM1,VMP1,SLC39A6,ANXA6,TRPV2,AKT1,SCNN1D,PKD1,FLNA,KCNAB2,ATP6AP1,PARK7,HCN2,JPH3,ATP6V0D1,MIR24-1,DMD,SURF1,FKBP1A,CBARP,SLC36A1,TMEM38B,THY1,TMSB4X,TMEM38A,YWHAH,SLC39A3,NIPAL1,HCN4,ATP6V0E1,GEM,KCNMB4	darkgreen	EC_M4
GO:0060341	9.75E-07	0.999999631	136	675	regulation of cellular localization	BP	1.84E-04	LMAN1,LRP1,STX7,RAB21,ANGPT1,TCP1,NUS1,REEP2,PPARD,CTNNA1,LEP,VCP,RAB23,TMEM30A,ERLEC1,RTN4,UBE2J1,MDFIC,GLI3,ROCK2,BVES,SLC7A11,ITGB1,FKBP1B,KIF5B,RB1,IWS1,PICALM,MARK4,PCDH17,PAK1,TGFB1,CTNNB1,AKT2,GPD1L,PPP1R12A,UFM1,ATP2C1,PRNP,HDAC3,DCLK1,DDRGK1,RIC3,TM9SF4,REEP5,TP53INP2,OSBP,VAMP8,RIOK2,MAP1B,PINX1,SORBS1,P2RX4,DHX36,CCT8,CX3CL1,CACNB3,PTPN11,GNL3,ARHGAP1,ATG13,TSG101,DKC1,BAP1,CHP1,ATP2B4,PKD2,BRSK2,DYNLL1,LRP5,FEZ1,GSK3B,MMP14,DAG1,ISCU,LARP7,ARV1,ADCY5,AP2M1,CASQ1,IQGAP1,SCFD1,TFRC,FZD9,SIDT2,LRRC15,MAPT,C11orf65,PTPN6,GPC2,PDCD10,HTT,PRKCI,STX18,B3GAT3,HAP1,CACNA1C,MAGED1,IQSEC2,NLGN2,MSN,SYT11,NGFR,RAB11A,MAP2K1,CALM1,CLTC,NPTN,PIN1,MIEF2,AKT1,TMEM98,PRKAA1,SUPT6H,MYO1C,BCAP31,TAX1BP3,FLNA,KCNAB2,VAMP7,PARK7,JPH3,DMD,FKBP1A,TMEM38B,THY1,TXN,TMEM38A,LEPROT,ZPR1,ORMDL3,UBE2G2,TOMM7,MESD,MAP1A,DERL2	darkgreen	EC_M4

GO:0048468	9.77E-07	0.999999595	283	1587	cell development	BP	1.84E-04	ATP8B1,LRP1,AHI1,RAB21,CDSN,PLXNB2,PPARA,ROS1,AFF4,PACSIN1,ADGRB1,CTNNA1,MTCH1,LGALS1,SH3GL2,FLNC,MYH9,LEP,PLAA,RORB,SRF,VEGFA,RUNX2,NCK2,CLOCK,REST,COL15A1,P3H1,RIMS1,TMEM30A,PSME4,UBA6,RTN4,TPBG,SHANK1,MFSD2A,UBE2J1,ACTR2,GMCL1,KIDINS220,WHRN,GLI3,NCDN,ROCK2,BVES,MYLK3,ITGB1,FKBP1B,PLXNA1,FGF2,KIF5B,PANX1,RB1,PICALM,EIF2B3,TENM4,GLI2,MEGF8,SMARCD3,RHEB,PAK1,TGFB1,AXL,CTNNB1,TRAK1,SERPINH1,AKT2,DZIP1,FARP1,SLC4A7,RARB,ACTG1,ACTN4,SPINT2,LIG4,DCLK1,SEMA3C,ILK,DPYSL3,RYK,RNF6,AKIP1,CXCL12,IMPAD1,LGI4,ANKRD1,FAT1,SLC25A46,PALLD,FLNB,EIF4G2,SULF1,ROBO1,MAP1B,RDH10,DIP2B,SEMA4F,HEY1,ATP2A2,TLX2,CELF1,TCTN1,APP,DHX36,SLC4A5,RAPGEF2,CX3CL1,ABCC8,PTPN11,PTX3,ADCY6,HOMER1,OPA1,ARSB,ZFYVE27,CRTAC1,ACVR1,SPP1,RUNX1,TWF1,DNAH1,SEMA4G,GPRIN3,BRSK2,PDLIM5,NDN,CHRNA7,ARL3,DOCK10,DYNLL1,TJP1,TDP2,NOTCH2,CTTN,DNM1L,DNER,CHSY1,LMOD1,CAPRIN2,TGFB2,LRP5,FEZ1,HTR2B,SATB2,PRKAR1A,GSK3B,DAG1,AUTS2,LEF1,CORO1C,ANOS1,KRAS,NTRK3,SLAMF8,BMP7,IDH2,FGFR2,VASH2,CASQ1,NCSTN,IQGAP1,KIF1A,OSBP2,CLIC4,FARP2,ARID5B,TANC2,SIDT2,SCYL1,MAPT,PLOD3,C1QL1,FZD2,TNR,FN1,NCKAP1,PTPN6,GPC2,FBN1,ITGAV,COMP,ARPC2,PRKCI,SKIL,SYNPO2L,PRRX1,SMURF1,NBL1,TNIK,VCL,GLDN,WDR1,FMN2,SIX4,OBSL1,ACTA2,ROM1,CRMP1,HAP1,PRTG,UGDH,BMPR1A,NOTCH3,ENAH,TCAP,MED1,ACTN1,MSN,SRCIN1,MYO1E,STX1B,NGFR,TPM1,RAB11A,MAP2K1,SOX30,TTC8,DIAPH2,DICER1,CLMN,NPTN,ISLR2,ANXA6,PIN1,TRPV2,ACSL	darkgreen	EC_M4
GO:0010646	1.11E-06	0.999999383	349	2513	regulation of cell communication	BP	1.86E-04	TNFRSF11A,SEMA6A,TNFAIP3,TRIM39,PLEKHG1,AKAP12,FLT4,SNCAIP,FZD6,CTHRC1,RIMS2,CDC42SE2,GRAMD4,PACSIN2,TRDN,TPD52L1,RNF146,ARHGAP18,SGSM3,PTK2,DENND3,ANKS1A,DAPK1,ATF4,PDGFB,CSNK1E,PICK1,KDM4C,MFNG,MDFI,LRRC19,TEK,RIC8B,APBA1,PIP5K1B,SH3RF3,KIT,KDR,CD2AP,SYK,GADD45G,SEMA4D,RNF149,ICA1,IL6,REL,CHN2,CXCL8,PELI1,RGS3,ARHGAP25,RSAD2,FIGNL1,CAV2,GRB10,LANCL2,TSPAN12,TLR4,GRIK2,TRAF1,DLGAP3,CCNY,SDCBP,TRPM4,NRP1,PUM2,RASIP1,PIK3CG,ARHGAP42,MTMR2,MGLL,GATA2,ARHGAP21,PRKD2,CTSC,GPR4,FGD5,NECTIN2,PRCP,NOS3,LLGL2,ZFAND6,PPARG,GATA3,ACVR2B,CXCR4,IRAK2,SEC14L1,NET1,RALGAPA2,NDFIP2,TMC8,CYTH1,IKBKB,JAG1,P2RY6,PDE2A,SFRP1,HBEGF,PLCB4,NOTUM,FERMT1,RPL26,SPEF1,ARHGAP26,P2RX2,CD36,SIRT3,PRAG1,PRR5L,EPHB1,PLK2,RET,PDE4D,TNFRSF19,CHD7,FAM3D,IL1A,IGF1,EIF2AK3,ABHD6,HHEX,ITCH,ERBIN,UFSP2,SH3RF1,PREX2,HIP1R,MCTP1,ARHGEF3,SOX7,ARHGEF28,MEF2C,TLR2,WWTR1,IL7,P2RX7,VEGFC,NDRG4,GUCY1A1,MADD,LMBR1L,ADGRG3,KMT2D,BMP2K,OAS3,NLRC5,PPP1R16B,PLCG1,NR4A2,DGKZ,HIPK1,DLL4,ZDHHC13,DNMBP,SOX13,ADAMTS20,EDN1,STAT1,DYRK1A,SNCA,DTNBP1,DUSP8,GAS2,PRDM15,ABCG1,SHANK2,HDAC1,ITPRIP,LCK,PPP3CA,HYAL2,ANO1,GIT2,SHOC2,MCTP2,HCRTR1,LAPTM5,NCF1,DYRK3,HCLS1,INPP5D,TIFA,CD86,MN1,ARHGEF12,SH3BP4,FGR,CFLAR,CASP10,INPP5F,ADCY1,KALRN,IGFBP3,PROX1,PLEKHA1,PRKD1,F11R,YTHDF1,IL10RA,ARHGDIB,MAP3K3,SRMS,ITGA6,PAK2,PTPRE,CNKSR2,SENP2,DUSP16,DLG1,ELL3,SIPA1,APOD,MAP3K11,CREB1,	turquoise	EC_M6
GO:0033043	3.80E-06	0.999997889	134	950	regulation of organelle organization	BP	1.95E-04	PAN2,LATS1,CCT2,FBXO43,FBXO5,MAP3K4,RAD21,CDC25C,POT1,AKAP9,TRIM28,SENP6,PHIP,TTK,HNRNPA2B1,CCT4,ITGB1BP1,CAPZA2,CNTRL,GEN1,WDR35,PLK4,STIL,MCM2,CEP295,SETDB2,CLASP2,BORA,CDC20,CKAP2,CDC16,MAPK8,CCNB1,PAN3,ESPL1,BUB1,TPX2,ANAPC1,KIF20B,SKA3,TNKS2,CENPJ,FER,NCK1,ARFGEF1,XRN1,KNTC1,ATR,KATNBL1,TERF1,FAM208A,CNOT1,ANAPC7,ALMS1,BUB1B,CCT7,CDCA2,NBN,CKAP5,NUSAP1,UBE2C,STMND1,CCT5,CCT6A,KIF18A,CUL3,CHEK1,CENPE,TRIP13,CAMSAP2,PRMT5,RCC1,C2CD5,RAE1,NEK2,CENPF,BUB3,CDKL5,HDGFL3,MKI67,PIKFYVE,TPR,CDC27,ABL2,CDCA5,KIF15,XRCC5,BRCA1,TACC3,DLGAP5,RAD51AP1,ECT2,ZWINT,FKBP4,DDX11,HNRNPU,WAPL,TOP2A,CDC6,DCP2,PRELID1,MAD1L1,IQGAP3,EXOSC10,CCT3,SPDL1,AURKB,ATRX,PTTG1,ZWILCH,LCMT1,TRIM37,PLK1,G3BP1,PSMD10,NDC80,RICTOR,CEP76,TAOK1,RMI2,PKMYT1,CCNF,CDT1,DHODH,CTCF,TMOD3,HNRNPD,MAD2L1,STMN1,AURKA,TIGAR,NAF1,FEN1,DRG1	darkgrey	EC_M8
GO:0000245	4.00E-06	0.999999233	16	51	spliceosomal complex assembly	BP	2.05E-04	DDX39B,SRPK1,PSIP1,NCBP1,YTHDC1,DDX1,CRNKL1,SMN2,GCFC2,SCAF11,SF3B1,RBM5,PRPF39,SRSF5,LUC7L3,GEMIN6	darkgrey	EC_M8
GO:0043486	4.01E-06	0.999999696	9	17	histone exchange	BP	2.05E-04	NASP,ACTR6,MIS18A,OIP5,ANP32E,HJURP,ITGB3BP,CENPW,RNF8	darkgrey	EC_M8
GO:0000956	4.32E-06	0.999998713	26	102	nuclear-transcribed mRNA catabolic process	BP	2.20E-04	PAN2,EIF3E,TNRC6B,TUT7,NCBP1,THRAP3,EXOSC9,UPF2,DIS3,XRN2,EXOSC8,PAN3,XRN1,PDE12,TTC37,CNOT1,TENT2,DDX5,SSB,DCP2,EXOSC10,UPF3B,GSPT1,RNPS1,EXOSC2,RBM8A	darkgrey	EC_M8

GO:0001816	1.38E-06	0.999999513	90	504	cytokine production	BP	2.29E-04	IFNGR1,TNFAIP3,AKAP12,LAPTM4B,FLT4,C6orf106,ATF4,MAPK13,LRRC19,DDX58,KIT,TNFRSF21,CD2AP,SYK,IL6,IGF2BP3,REL,PELI1,RSAD2,TLR4,C5,IL15,TRPM4,PIK3CG,PRKD2,GATA3,TMEM173,FERMT1,CD36,PDE4D,MERTK,IL1A,IGF1,EIF2AK3,ITCH,ERBIN,TLR2,IL7,P2RX7,OAS3,STAT1,CD58,CD200,HDAC1,HYAL2,ICOSLG,LAPTM5,NFATC2,INPP5D,CD34,CD86,FGR,F11R,APOD,CREB1,B2M,LTF,CYBB,IL1RAP,DDX3X,HOMER3,TMEM106A,RAC1,HIF1A,BCL6B,EPHA2,TLR6,TNFRSF1B,ARG2,PSEN1,BATF,RPS6KA5,YY1,PDE4B,LGALS9,NOS2,GIT1,ISG15,TRAF2,IRF8,PLCG2,NFAT5,OAS1,TLR1,ADGRG1,GBP5,CD46,ACKR1,BST2,RNF125	turquoise	EC_M6
GO:0036503	1.30E-06	0.999999597	32	99	ERAD pathway	BP	2.43E-04	CANX,UBQLN1,VCP,ERLEC1,UBE2J1,RCN3,TMUB1,UBXN4,STT3B,NPLOC4,RNF121,HSP90B1,AMFR,BRSK2,MARCH6,SELENOS,UBE4A,RHBDD2,EDEM3,SYVN1,DNAJC10,DNAJB12,JKAMP,SEL1L,USP14,BCAP31,TMEM259,HSPA5,FBXO27,DNAJB9,UBE2G2,DERL2	darkgreen	EC_M4
GO:0043488	4.87E-06	0.999998342	31	137	regulation of mRNA stability	BP	2.47E-04	PAN2,TNRC6B,TUT7,PNPT1,SYNCRIP,PABPC4,THRAP3,EXOSC9,DIS3,METTL3,EXOSC8,PAN3,PDE12,CNOT1,FXR1,FASTKD1,DHX9,HNRNPR,FASTKD2,HNRNPU,DCP2,FUS,LARP1,TAF15,HNRNPM,SERBP1,FMR1,METTL16,EXOSC2,FASTKD5,HNRNPD	darkgrey	EC_M8
GO:1901698	1.35E-06	0.999999474	163	845	response to nitrogen compound	BP	2.48E-04	LRP1,AP3S1,CDO1,CANX,RRM2B,PPARA,LY6E,CTNNA1,UBQLN1,GNAQ,LEP,VCP,COL1A2,ASNS,SOCS5,ERLEC1,UBE2J1,ACTR2,ROCK2,RCN3,VPS35,MGARP,ITGB1,SESN3,PANX1,EIF2B3,TMUB1,PAK1,HTR5A,UBXN4,HNMT,CTNNB1,AKT2,STT3B,OSBPL8,NPLOC4,KAT2B,RNF121,IRS2,PRNP,ATP2B1,SMPD1,LEPROTL1,APLP1,CXCL12,HSP90B1,MAP1LC3A,MAP1B,RYR3,SORBS1,P2RX4,USO1,APP,GLRB,DHX36,RAPGEF2,ABCC8,PPP2R2A,PTPN11,ADCY6,HOMER1,OPA1,AMFR,PTPN22,CRY2,MMP2,ADA,ATP2B4,PKD2,BRSK2,MARCH6,CHRNA7,ADIPOR1,SELENOS,CCND1,CPT1A,ZNF106,LRP5,EPRS,HTR2B,GSK3B,CASP7,DAG1,PIK3CA,HADH,COL6A1,BMP7,ADCY5,WDTC1,PEA15,NCSTN,SYAP1,IQGAP1,UBE4A,UROS,BACE1,RHBDD2,EDEM3,LINC02210-CRHR1,SYVN1,EIF4EBP2,DNAJC10,DNAJB12,FBN1,PRKCI,COL5A2,QDPR,TIMP1,PTPRN,SLC3A2,JKAMP,MYO5A,SLC2A4,PPARGC1A,CPEB4,ARHGEF2,NGFR,COL1A1,TMED10,SHC1,SEL1L,CALM1,PKM,GRIA1,ROCK1,USP14,AKT1,ITGA2,LPIN2,PRKAA1,RAB31,GNAL,PTPN2,LDOC1,MYO1C,GNB1,BCAP31,FLNA,TMEM259,HCN2,BSG,AHCYL1,GNAI3,CDH13,BCAR3,SLC2A8,HSPA5,RANBP2,SERPINF1,FBXO27,TMEM38B,PEMT,CACYBP,CDKN1A,DNAJB9,TXN,NNMT,TMEM38A,LEPROT,PIK3C2A,UBE2G2,HCN4,DERL2	darkgreen	EC_M4
GO:0051382	5.07E-06	0.999999622	9	16	kinetochore assembly	BP	2.56E-04	SENP6,CENPA,CENPH,KNTC1,CENPE,CENPF,DLGAP5,CENPN,CENPW	darkgrey	EC_M8
GO:2001022	5.25E-06	0.999998501	24	95	positive regulation of response to DNA damage stimulus	BP	2.64E-04	BCLAF1,TRIM28,PAXIP1,RIF1,PRKDC,ERCC6,ATR,ABRAXAS1,ARID2,EEF1E1,FXR1,DDX5,FANCB,DHX9,BRCA1,RAD51AP1,ANKLE1,FOXM1,DDX11,FUS,SMCHD1,FMR1,TIGAR,RNF8	darkgrey	EC_M8
GO:0016075	5.26E-06	0.999999588	9	17	rRNA catabolic process	BP	2.64E-04	EXOSC9,DIS3,EXOSC8,XRN1,MRPL44,EXOSC10,DIS3L,DROSHA,EXOSC2	darkgrey	EC_M8
GO:0008156	5.28E-06	0.999999401	11	24	negative regulation of DNA replication	BP	2.64E-04	FBXO5,GTPBP4,BRCA2,ATR,TERF1,GMNN,BLM,PDS5A,WAPL,CDC6,TIPIN	darkgrey	EC_M8
GO:0002237	1.62E-06	0.999999387	50	236	response to molecule of bacterial origin	BP	2.65E-04	TNFRSF11A,TNFAIP3,AKAP12,CSF2RB,IL6,CXCL8,PELI1,TLR4,NOCT,NOS3,TIMP4,IRAK2,EDNRB,NFKBIB,CD36,SCARB1,IL1A,NR4A1,ERBIN,MEF2C,TLR2,CXCL10,EDN1,SNCA,NFKB2,HDAC1,PABPN1,CD86,IL10RA,B2M,LTF,SELE,SELP,ADIPOR2,TLR6,TNFRSF1B,S100A14,CYP1A1,TNIP1,CCL2,PDE4B,LGALS9,NOS2,GIT1,NOTCH1,IRF8,PLCG2,DAB2IP,TLR1,BMP6	turquoise	EC_M6
GO:0034097	1.70E-06	0.999999374	109	659	response to cytokine	BP	2.76E-04	TNFRSF11A,IFNGR1,TNFAIP3,AKAP12,TCF7,DAPK1,PDGFB,MAPK13,CSF2RB,DDX58,KIT,TNFRSF21,NFIL3,SYK,IL6,REL,CXCL8,TLR4,TRAF1,IL15,LRCH1,ZFAND6,PPARG,TIMP4,GATA3,CXCR4,IRAK2,IKBKB,PDE2A,TMEM173,SFRP1,TNFRSF19,IL1A,KDM3A,ERBIN,OCLN,GPD1,TLR2,IL7,VEGFC,CXCL10,OAS3,NLRC5,ST3GAL6,HIPK1,IFIT2,IFIT1,EDN1,STAT1,CD58,SNCA,JARID2,NFKB2,MX2,MX1,ETS1,HDAC1,HYAL2,LAPTM5,FABP4,HCLS1,IL10RA,CREB1,EIF4A2,SSTR1,CCRL2,IL1RAP,ADAMTS7,ITGA4,BIRC3,SELE,SELP,PLVAP,FGF23,WAS,HIF1A,ADIPOR2,TNFRSF1B,ALDH1A2,RPS6KA5,YY1,CCL2,IL13RA1,JAK1,LGALS9,NOS2,IL7R,ISG15,TRAF2,TNFRSF25,NOTCH1,IRF8,STXBP1,MTF2,NFAT5,DAB2IP,MIR99A,AFF3,OAS1,GBP5,RAB43,SOX17,ACKR1,COMMD7,BST2,VAMP3,XAF1,IFIT3,RNF125	turquoise	EC_M6

GO:0090092	1.52E-06	0.999999362	52	193	regulation of transmembrane receptor protein serine/threonine kinase signaling pathway	BP	2.77E-04	LRP1,FBN2,PPARA,CDKN2B,KIAA1324L,RASL11B,ASPN,TGFBR1,INHBA,TGFB1,ZEB2,LTBP1,CRIM1,SPRED3,ZNF703,ILK,RBPMS,SULF1,DKK3,ACVR1,GDF6,NOTCH2,SLC2A10,TGFB2,FSTL1,BMP7,HTRA1,STRAP,FAM89B,FBN1,FKBP8,SKIL,SMURF1,NBL1,GREM2,BMPR1A,RNF111,TGFB1I1,TGFB3,MYOCD,PIN1,CHRDL1,NOMO1,VASN,GLG1,HSPA5,FSTL3,FKBP1A,LOX,PMEPA1,CHST11,FGF10	darkgreen	EC_M4
GO:0007076	5.89E-06	0.999999552	9	16	mitotic chromosome condensation	BP	2.94E-04	SMC2,NCAPH,NUSAP1,NCAPD3,CDCA5,SMC4,NCAPD2,NCAPG,PLK1	darkgrey	EC_M8
GO:1904872	6.02E-06	0.999999502	9	18	regulation of telomerase RNA localization to Cajal body	BP	2.99E-04	CCT2,CCT4,CCT7,CCT5,CCT6A,DCP2,EXOSC10,CCT3,NAF1	darkgrey	EC_M8
GO:0040029	6.06E-06	0.999998321	23	86	regulation of gene expression, epigenetic	BP	3.00E-04	TRIM28,YTHDC1,EZH2,SETDB2,METTL3,RRP8,TDG,SMARCA5,CGGBP1,FAM208A,HELLS,SMYD5,CHEK1,TPR,BRCA1,SUV39H1,HNRNPU,EXOSC10,RLIM,SMCHD1,LRIF1,GLMN,CTCF	darkgrey	EC_M8
GO:0031145	6.50E-06	0.999999456	9	18	anaphase-promoting complex-dependent catabolic process	BP	3.21E-04	CDK2,CDC20,CDC16,ANAPC1,ANAPC7,UBE2C,CUL3,CDC27,PLK1	darkgrey	EC_M8
GO:0051988	6.55E-06	0.999999746	7	10	regulation of attachment of spindle microtubules to kinetochore	BP	3.22E-04	CCNB1,RACGAP1,KNSTRN,NEK2,ECT2,HNRNPU,AURKB	darkgrey	EC_M8
GO:0030182	1.86E-06	0.999999019	201	1067	neuron differentiation	BP	3.34E-04	ATP8B1,LRP1,AHI1,RAB21,PLXNB2,TRAPPC9,PACSIN1,ADGRB1,CTNNA1,MTCH1,LGALS1,MDGA1,SH3GL2,LEP,PLAA,RORB,SRF,VEGFA,RUNX2,NCK2,REST,P3H1,TGFBR1,RIMS1,TMEM30A,UBA6,RTN4,TPBG,SHANK1,MFSD2A,ACTR2,KIDINS220,WHRN,GLI3,NCDN,INHBA,ITGB1,FKBP1B,PLXNA1,KIF5B,NAPA,RB1,PICALM,TENM4,GLI2,MEGF8,PAK1,CTNNB1,TRAK1,FARP1,SLC4A7,DCLK1,SEMA3C,ILK,DPYSL3,SPINK5,RYK,SNPH,RNF6,CXCL12,LGI4,ZSWIM6,ANKRD1,SLC25A46,PALLD,ZNF536,EIF4G2,ROBO1,MAP1B,DIP2B,SEMA4F,FAIM2,HEY1,TLX2,TCTN1,APP,DHX36,RAPGEF2,CX3CL1,PTPN11,ADCY6,OPA1,ARSB,ZFYVE27,CRTAC1,SPP1,RUNX1,TWF1,SEMA4G,NKD1,GPRIN3,BRSK2,PDLIM5,NDN,CHRNA7,ARL3,DOCK10,EIF4E,TDP2,NOTCH2,CTTN,DNM1L,CAPRIN2,TGFB2,FEZ1,SATB2,GSK3B,DAG1,AUTS2,ANOS1,NTRK3,BMP7,FGFR2,VASH2,IQGAP1,STRAP,KIF1A,FARP2,TANC2,SCYL1,MAPT,C1QL1,FZD2,MDGA2,TNR,FN1,NCKAP1,GPC2,ADRA2C,PRKCI,SKIL,PRRX1,SMURF1,NBL1,TNIK,VCL,GLDN,TULP3,OBSL1,ROM1,CRMP1,UGDH,NOTCH3,ENAH,CLN8,MED1,SRCIN1,STX1B,ARHGEF2,NGFR,RAB11A,MAP2K1,TTC8,DICER1,CLMN,NPTN,ISLR2,PIN1,TRPV2,ACSL4,LAMA3,ROCK1,AKT1,DAPK3,PLXNB3,PAFAH1B1,NTN3,NPHP4,FLNA,PLXNA3,RERE,BSG,NTNG2,BARHL2,HSPA5,LMO4,NEXN,LHX8,DPYSL5,DMD,SERPINF1,VXN,FRY,VSTM2L,ITM2C,UBE2V2,ARF4,LBX1,GDNF,THY1,BLOC1S2,CHN1,EFHD1,ARTN,YWHAH,ZPR1,SDC2,PRKG1,MAP1A,OLFM1,WNT2	darkgreen	EC_M4
GO:0006935	2.12E-06	0.999999058	80	426	chemotaxis	BP	3.40E-04	TNFRSF11A,SEMA6A,KIF5A,PTK2,PDGFB,KIT,KDR,SYK,SEMA4D,IL6,CXCL8,DOCK4,C5,NRCAM,TRPM4,NRP1,PIK3CG,PRKD2,NOVA2,PLXND1,GATA3,CXCR4,EDNRB,HBEGF,FEZ2,EFNB2,EPHB1,RET,NR4A1,VEGFC,CXCL10,ANGPT2,THBS4,ANO6,EDN1,JAM3,SEMA3F,RIN3,ROBO4,LYST,PLXNA2,KALRN,FES,PRKD1,LAMA5,YTHDF1,LAMC2,CREB1,CCRL2,SEMA6D,EPHB4,MCU,PPM1F,RAC1,EPHA2,OPHN1,EFNB1,SEMA4A,HOXB9,FGF18,PGF,DAPK2,EFNA1,RPS6KA5,S100A14,MMP28,AMOT,CCL2,PDE4B,LGALS9,SEMA6B,PLEKHG5,NOTCH1,S1PR1,VASP,RELN,FGF16,STX3,MSMP,RALA	turquoise	EC_M6

GO:0032502	2.18E-06	0.999998598	590	4589	developmental process	BP	3.46E-04	ZNF516,TNFRSF11A,TCF4,SEMA6A,N4BP3,KIF5A,IFNGR1,TNFAIP3,HECA,PTPRB,TSPAN8,FLT4,SNX2,SYNJ2,FZD6,CTHRC1,RIMS2,CDC42SE2,CELSR1,QKI,NOTCH4,AFDN,PARVG,PACSIN2,ASF1A,TCF7,CDKL3,MTSS1,ASAP1,ARHGAP18,L3MBTL3,KLHL3,PTK2,HSD17B3,ANKS1A,ATF4,PDGFB,AGTPBP1,PODXL,CSNK1E,PICK1,KDM4C,MICALL1,PIM1,CDC42EP1,MFNG,STRIP2,MDFI,TEK,LINGO2,IREB2,APBA1,TJP2,PGM5,TEC,ADGRF5,KIT,ADGRF1,TNFRSF21,KDR,NFIL3,SYK,GADD45G,SEMA4D,EFHC1,THSD7A,SHB,TMOD1,RAPGEF5,IL6,IGF2BP3,PPT1,PTBP3,CXCL8,UTP11,RSAD2,FIGNL1,FOXP2,CAV2,TSPAN12,NOBOX,TLR4,C5,GJA4,IL15,FZD8,CREM,TEAD2,SDCBP,MYCN,NRCAM,NOCT,TUT4,TRPM4,NRP1,ZBTB24,RASIP1,SPATA6,PIK3CG,SVIL,ITSN2,TAL1,LAMA4,PHF10,MTMR2,EPC1,GATA2,DNMT3A,HS6ST1,PRKD2,CST7,NOVA2,CTSC,RAB38,GPR4,SNRK,FGD5,PLXND1,NECTIN2,PRCP,ITGA8,NOS3,GAB2,ITGB4,UNC13D,DACH1,PPARG,TIMP4,GATA3,ACVR2B,MYO7A,CXCR4,LMBR1,GZF1,EDNRB,SPTBN4,TIE1,GDPD5,SETD5,IKBKB,JAG1,PDE2A,GPAT4,SFRP1,HBEGF,NR1D2,DND1,FEZ2,NOTUM,FERMT1,FREM2,EFNB2,GOLGA3,ANKLE2,ARHGEF7,ASH2L,SPEF1,ATP11A,PCDH1,PCDH12,ARHGAP26,P2RX2,FGD6,NTN4,SLC4A11,CD36,RIC8A,SIRT3,TMEM178A,LMOD3,SYNE4,PRAG1,ADAMTS9,SDCBP2,TCF15,EPHB1,PLK2,MALL,RET,PDE4D,MERTK,TNFRSF19,CHD7,IL1A,DNMT3B,IGF1,NR4A1,EIF2AK3,HHEX,KDM3A,ITCH,LYVE1,SH3RF1,PREX2,SMAD1,CLCN3,HIP1R,POU6F1,MARVELD2,STOX2,DCLK2,MMP24,SOX7,KIF13B,TMEM120B,ARHGEF28,ENC1,HEXB,MEF2C,SMARCD1,TLR2,WWTR1,IL7,P2RX7,TPD52,VEGFC,NDRG4,GPM6A,CX	turquoise	EC_M6
GO:2000026	2.28E-06	0.999998829	161	1014	regulation of multicellular organismal development	BP	3.58E-04	SEMA6A,TNFAIP3,NOTCH4,TCF7,CDKL3,PIM1,TEK,LINGO2,KIT,TNFRSF21,KDR,SYK,SEMA4D,IL6,CXCL8,TSPAN12,TLR4,C5,IL15,MYCN,NOCT,TRPM4,NRP1,TAL1,LAMA4,MTMR2,GATA2,PRKD2,CST7,CTSC,GPR4,PLXND1,NOS3,PPARG,GATA3,ACVR2B,CXCR4,TIE1,IKBKB,JAG1,SFRP1,NOTUM,FERMT1,TMEM178A,ADAMTS9,EPHB1,PLK2,CHD7,IL1A,IGF1,EIF2AK3,HHEX,SH3RF1,SMAD1,MEF2C,TLR2,WWTR1,IL7,P2RX7,VEGFC,CXCL10,ANGPT2,BMP2K,THBS4,PPP1R16B,PLCG1,RAPGEF3,STAB1,HIPK1,ANO6,DLL4,SOX13,EDN1,STAT1,JARID2,ETS1,HDAC1,PPP3CA,SEMA3F,PRKX,HLX,HCLS1,INPP5D,CD34,CD86,PLXNA2,TRAK2,FES,PROX1,SS18L1,PRKD1,LAMA5,F11R,APOLD1,DLG1,ELL3,CREB1,B2M,LTF,SEMA6D,CYBB,IL1RAP,BCOR,ADAMTS7,SOS2,ETV4,HSPG2,RAMP2,PROM1,FGF23,PRKCH,HIF1A,RHOJ,JUP,EPHA2,ITPKB,TNFRSF1B,RGS14,MTOR,PSEN1,SEMA4A,HOXB8,FGF18,PGF,EFNA1,VASH1,NPR1,GPR68,YY1,RCOR1,AMOT,ADRB2,JAK1,LGALS9,IL7R,SEMA6B,LRG1,SLITRK2,RFLNB,ISG15,ARHGAP4,AP3D1,NRARP,NOTCH1,S1PR1,ENG,DAB2IP,ADGRL2,CDH5,VNN1,CD46,CA2,SOX17,RELN,SHANK3,MAFF,RGS4,TGIF2,HIST1H4D,BMP2,BMP6	turquoise	EC_M6
GO:0032990	2.03E-06	0.999999024	114	536	cell part morphogenesis	BP	3.61E-04	LRP1,RAB21,PLXNB2,PACSIN1,ADGRB1,SH3GL2,PLAA,SRF,VEGFA,RIMS1,RTN4,TPBG,SHANK1,MFSD2A,MYH14,ACTR2,KIDINS220,GLI3,ITGB1,PLXNA1,KIF5B,PICALM,GLI2,MEGF8,PAK1,CTNNB1,TRAK1,FARP1,DCLK1,SEMA3C,ILK,RYK,RNF6,CXCL12,PALLD,EIF4G2,ROBO1,MAP1B,DIP2B,SEMA4F,TLX2,TCTN1,APP,DHX36,RAPGEF2,PTPN11,OPA1,ZFYVE27,SPP1,SEMA4G,BRSK2,PDLIM5,NDN,CHRNA7,DOCK10,NOTCH2,CTTN,DNM1L,CAPRIN2,FEZ1,GSK3B,DAG1,AUTS2,CORO1C,ANOS1,BMP7,FGFR2,IQGAP1,KIF1A,TANC2,MAPT,TNR,FN1,NCKAP1,SKIL,SMURF1,NBL1,TNIK,VCL,OBSL1,CRMP1,NOTCH3,ENAH,SRCIN1,NES,NGFR,RAB11A,MAP2K1,TTC8,DICER1,NPTN,ISLR2,TRPV2,LAMA3,ROCK1,PLXNB3,PAFAH1B1,NTN3,PLXNA3,RERE,BSG,NTNG2,BARHL2,NEXN,DPYSL5,VSTM2L,GDNF,THY1,CHN1,ARTN,YWHAH,SDC2,MAP1A,OLFM1	darkgreen	EC_M4
GO:0009112	7.48E-06	0.999998949	12	31	nucleobase metabolic process	BP	3.67E-04	PPAT,PAICS,CTPS1,CAD,RRM1,GMPS,UMPS,PRPS1,TYMS,HPRT1,DHODH,GART	darkgrey	EC_M8
GO:0070203	7.57E-06	0.999999657	7	11	regulation of establishment of protein localization to telomere	BP	3.70E-04	CCT2,CCT4,TERF1,CCT7,CCT5,CCT6A,CCT3	darkgrey	EC_M8
GO:0042330	2.38E-06	0.999998939	80	427	taxis	BP	3.70E-04	TNFRSF11A,SEMA6A,KIF5A,PTK2,PDGFB,KIT,KDR,SYK,SEMA4D,IL6,CXCL8,DOCK4,C5,NRCAM,TRPM4,NRP1,PIK3CG,PRKD2,NOVA2,PLXND1,GATA3,CXCR4,EDNRB,HBEGF,FEZ2,EFNB2,EPHB1,RET,NR4A1,VEGFC,CXCL10,ANGPT2,THBS4,ANO6,EDN1,JAM3,SEMA3F,RIN3,ROBO4,LYST,PLXNA2,KALRN,FES,PRKD1,LAMA5,YTHDF1,LAMC2,CREB1,CCRL2,SEMA6D,EPHB4,MCU,PPM1F,RAC1,EPHA2,OPHN1,EFNB1,SEMA4A,HOXB9,FGF18,PGF,DAPK2,EFNA1,RPS6KA5,S100A14,MMP28,AMOT,CCL2,PDE4B,LGALS9,SEMA6B,PLEKHG5,NOTCH1,S1PR1,VASP,RELN,FGF16,STX3,MSMP,RALA	turquoise	EC_M6

GO:1900262	7.76E-06	0.999999803	6	8	regulation of DNA-directed DNA polymerase activity	BP	3.77E-04	DSCC1,RFC3,RFC5,RFC2,RFC4,CHTF18	darkgrey	EC_M8
GO:1900264	7.76E-06	0.999999803	6	8	positive regulation of DNA-directed DNA polymerase activity	BP	3.77E-04	DSCC1,RFC3,RFC5,RFC2,RFC4,CHTF18	darkgrey	EC_M8
GO:0016570	7.97E-06	0.999996184	67	399	histone modification	BP	3.86E-04	CDK2,USP15,UBR5,UBR2,PWP1,ZNF451,ZMPSTE24,RLF,EZH2,UFL1,HDAC2,CCNA2,SETDB2,PAXIP1,NCAPG2,PARP2,KAT14,EPC2,KAT6A,RIF1,NSD3,TAF7,BRCA2,MAPK8,CCNB1,TAF9,MCRS1,USP16,SMYD5,SMARCAD1,CHEK1,NAA50,TET2,PRMT5,MCM3AP,HAT1,CDK1,ING5,BAZ1B,SETD2,BRCA1,NSD2,TBL1XR1,SUV39H1,LEO1,INCENP,NSD1,USP3,AURKB,TAF1,OGT,ATRX,TRIM37,SUZ12,CUL4B,MIER1,NIPBL,MYSM1,UHRF1,FMR1,HASPIN,EHMT1,CTCF,AC118549.1,AURKA,BRMS1L,RNF8	darkgrey	EC_M8
GO:1901576	5.88E-08	1	564	4324	organic substance biosynthetic process	BP	3.90E-04	NFATC1,ZNF236,TSHZ1,ORMDL2,PYM1,DGKA,SMARCC2,STAT6,ATG12,PIP4K2C,MAP3K5,PEX7,CDK4,CITED2,MAML1,SQSTM1,IRAK3,HELB,GFPT2,OSR2,CHKB,ALDH7A1,HSPA1B,EHMT2,ZBTB12,HDAC10,ALG12,ATF6B,AGPAT1,EIF3H,DSE,RXRB,TSPO,HSD17B8,RING1,RPS18,B3GALT4,CYB5R3,ZHX2,ZBTB22,PMM1,MYC,TOB2,SMAD5,EP300,IBA57,KDM3B,ZNF76,EEF1D,RPL3,DDX17,EIF3L,NFIB,GPAA1,EIF3D,HSF1,CCND3,TRERF1,CDKN2A,RPL8,HMOX1,WASL,KLF9,POLR1C,PIGO,PLPP6,JAK2,HINT2,ZBTB45,RPS5,ZNF135,ZNF667,ALG2,ZNF865,RPL28,TTC7A,RPS9,EEF1A1,MALSU1,SLC44A1,EFEMP1,UGP2,ZNF692,RPS7,ANXA4,AKNA,MED25,IL4I1,COQ3,ING3,ARHGEF5,AP2A1,IRF3,LPIN1,RPS11,FOXO3,KLF15,SPHK2,RPL18,NOP53,ABTB1,MAML2,BICRA,KMT2E,BBS7,MEIS3,FKRP,TRIM13,ACSS1,IRF2BP1,SIX5,FOSB,ZNF746,NR2C2,ZNF862,TSC22D1,SERP2,FDXR,UROD,SEPHS1,RPS8,ZNF575,MIF4GD,NAA16,MLH1,GTF3C2,UNK,ZNF574,GALNT11,WBP2,KMT2C,MYD88,TAMM41,TADA3,CSGALNACT1,RPS3,TEP1,BRPF1,PNP,DYRK1B,EID2,ZNF219,RPS16,MED29,SAMD4B,MRPS12,SIRT2,RPTOR,RPL15,APBB3,EIF3K,NUP98,GALNT15,TRIM21,CYP1B1,GNPDA1,HGF,APBB1,TAF10,PTDSS2,DEAF1,HOXC4,CLIP3,NCOA4,LIN37,TARBP2,KMT2B,EIF3F,APIP,RBCK1,RARG,SDR16C5,ZNF740,EIF4B,LMO2,POFUT1,THNSL2,NECAB3,CBR4,URI1,PIK3R1,ACSS2,NCOA2,ATF1,TNKS1BP1,RPS3A,POLE4,KDM2B,CHDH,ZFHX4,CAMKK2,SCAND1,EIF2A,SETD6,MCPH1,SSBP2,FNIP2,COQ9,DGUOK,PRKAG1,ARL2BP,SHOX2,NEK4,SPR,ZHX3,VDR,MMS19,GPAT3,LPIN3,CHST14,CPOX,FOXF2,F2RL1,SORBS3,ORMDL1,HNF4A,TP53INP1,FARS2,PIDD1,RPL	cyan	EC_M2
GO:0044249	7.75E-08	1	557	4271	cellular biosynthetic process	BP	3.90E-04	NFATC1,ZNF236,TSHZ1,ORMDL2,PYM1,DGKA,SMARCC2,STAT6,ATG12,PIP4K2C,MAP3K5,PEX7,CDK4,CITED2,MAML1,SQSTM1,IRAK3,HELB,GFPT2,OSR2,CHKB,ALDH7A1,HSPA1B,EHMT2,ZBTB12,HDAC10,ALG12,ATF6B,AGPAT1,EIF3H,DSE,RXRB,TSPO,HSD17B8,RING1,RPS18,B3GALT4,CYB5R3,ZHX2,ZBTB22,PMM1,MYC,TOB2,SMAD5,EP300,IBA57,KDM3B,ZNF76,EEF1D,RPL3,DDX17,EIF3L,MPST,NFIB,GPAA1,EIF3D,HSF1,CCND3,TRERF1,CDKN2A,RPL8,HMOX1,WASL,KLF9,POLR1C,PIGO,JAK2,ZBTB45,RPS5,ZNF135,ZNF667,ALG2,ZNF865,RPL28,TTC7A,RPS9,EEF1A1,MALSU1,SLC44A1,EFEMP1,UGP2,ZNF692,RPS7,ANXA4,AKNA,MED25,IL4I1,COQ3,ING3,ARHGEF5,IRF3,LPIN1,RPS11,FOXO3,KLF15,SPHK2,RPL18,NOP53,ABTB1,MAML2,BICRA,KMT2E,BBS7,MEIS3,FKRP,TRIM13,ACSS1,IRF2BP1,SIX5,FOSB,ZNF746,NR2C2,ZNF862,TSC22D1,SERP2,FDXR,UROD,SEPHS1,RPS8,ZNF575,MIF4GD,NAA16,MLH1,GTF3C2,UNK,ZNF574,GALNT11,WBP2,KMT2C,MYD88,TAMM41,TADA3,CSGALNACT1,RPS3,TEP1,BRPF1,PNP,DYRK1B,EID2,ZNF219,RPS16,MED29,SAMD4B,MRPS12,SIRT2,RPTOR,RPL15,APBB3,EIF3K,NUP98,GALNT15,TRIM21,CYP1B1,GNPDA1,HGF,APBB1,TAF10,PTDSS2,DEAF1,HOXC4,CLIP3,NCOA4,LIN37,TARBP2,KMT2B,EIF3F,APIP,RBCK1,RARG,SDR16C5,ZNF740,EIF4B,LMO2,POFUT1,THNSL2,NECAB3,CBR4,URI1,PIK3R1,ACSS2,NCOA2,ATF1,TNKS1BP1,RPS3A,POLE4,KDM2B,CHDH,ZFHX4,CAMKK2,SCAND1,EIF2A,SETD6,MCPH1,SSBP2,FNIP2,COQ9,DGUOK,PRKAG1,ARL2BP,SHOX2,NEK4,SPR,ZHX3,VDR,MMS19,GPAT3,LPIN3,CHST14,CPOX,FOXF2,F2RL1,SORBS3,ORMDL1,HNF4A,TP53INP1,FARS2,PIDD1,RPLP2,ERLIN1,HR,PIG	cyan	EC_M2

GO:0048523	2.58E-06	0.999998257	476	3647	negative regulation of cellular process	BP	3.98E-04	TNFAIP8,SEMA6A,TNFAIP3,HECA,PLAGL1,TBC1D30,TRIM39,PTPRB,LAPTM4B,CNOT6,FLT4,FZD6,CTHRC1,OXR1,GRAMD4,NOTCH4,AFDN,PACSIN2,TCF7,CDKL3,TRDN,NCOA7,MTSS1,L3MBTL3,PTK2,C6orf106,DAPK1,ATF4,PDGFB,AGTPBP1,PODXL,PICK1,PIM1,MDFI,TEK,IREB2,LIMCH1,NFX1,FAM122A,ADGRF5,KIT,TNFRSF21,KDR,CD2AP,NFIL3,SEMA4D,RNF149,RGP1,TMOD1,IL6,IGF2BP3,NFE2L3,REL,PPT1,PTBP3,CXCL8,BACH2,PELI1,RGS3,ARHGAP25,RSAD2,FIGNL1,FOXP2,CAV2,KLF11,GRB10,LANCL2,TLR4,TCAF2,C5,IL15,CREM,TEAD2,SDCBP,MYCN,NOCT,TUT4,TRPM4,NRP1,RASIP1,BEND5,PIK3CG,ARHGAP42,SVIL,TAL1,PHF10,MTMR2,EPC1,KDM4D,GATA2,DNMT3A,SAP130,KCNRG,CST7,CTSC,LRCH1,PLXND1,NOS3,GGA3,ZFAND6,DACH1,PPARG,TIMP4,GATA3,TAF3,ACVR2B,TBC1D4,ITIH5,GZF1,SEC14L1,EDNRB,TMC8,SPTBN4,TIE1,IKBKB,JAG1,P2RY6,PDE2A,SFRP1,HBEGF,NR1D2,DND1,FEZ2,NOTUM,FERMT1,RPL26,EFNB2,SH3BP5,ANKLE2,ARHGEF7,SPEF1,CD36,SIRT3,TMEM178A,LMOD3,ARID5A,PRAG1,ADAMTS9,PRR5L,TCF15,EPHB1,PLK2,PDE4D,MERTK,CHD7,FAM3D,IL1A,DNMT3B,IGF1,NR4A1,EIF2AK3,ABHD6,HHEX,KDM3A,ITCH,ERBIN,SH3RF1,SMAD1,ZBTB38,HIP1R,POU6F1,OCLN,DCLK2,MCTP1,SOX7,HMBOX1,ENC1,MEF2C,TLR2,WWTR1,IL7,P2RX7,VEGFC,NDRG4,CXCL10,ANGPT2,SOGA1,LMBR1L,ADGRG3,SFMBT1,FAM192A,SCAF4,OAS3,NLRC5,PPP1R16B,DYSF,TPCN1,PLCG1,STC1,RAPGEF3,ANKRD2,NR4A2,DGKZ,TP53I11,DLL4,CDYL,CEP97,SOX13,ADAMTS20,YAF2,EDN1,STAT1,DYRK1A,JAM3,SNCA,JARID2,DTNBP1,MYLIP,ETS2,DUSP8,NUAK2,PM20D1,DCAF1,RNF144B,PRDM15,SRGAP2C,ABCG1,ETS	turquoise	EC_M6
GO:0034724	8.26E-06	0.999998979	11	26	DNA replication-independent nucleosome organization	BP	3.98E-04	NASP,SUPT16H,MIS18A,OIP5,HJURP,HAT1,ASF1B,ATRX,UBN1,ITGB3BP,CENPW	darkgrey	EC_M8
GO:0048519	2.65E-06	0.999998205	512	3961	negative regulation of biological process	BP	4.04E-04	BAZ2A,TNFAIP8,SEMA6A,IFNGR1,TNFAIP3,HECA,PLAGL1,TBC1D30,TRIM39,PTPRB,TSPAN8,LAPTM4B,CNOT6,FLT4,FZD6,CTHRC1,OXR1,GRAMD4,NOTCH4,AFDN,PACSIN2,TCF7,CDKL3,TRDN,NCOA7,MTSS1,ASAP1,L3MBTL3,PTK2,C6orf106,DAPK1,ATF4,PDGFB,AGTPBP1,PODXL,PICK1,PIM1,MDFI,TEK,IREB2,LIMCH1,NFX1,FAM122A,ADGRF5,KIT,TNFRSF21,KDR,CD2AP,NFIL3,SYK,SEMA4D,RNF149,RGP1,TMOD1,IL6,IGF2BP3,NFE2L3,REL,PPT1,PTBP3,CXCL8,BACH2,PELI1,RGS3,ARHGAP25,RSAD2,DOCK4,FIGNL1,FOXP2,CAV2,KLF11,GRB10,LANCL2,TLR4,TCAF2,C5,IL15,CREM,TEAD2,SDCBP,MYCN,NOCT,TUT4,TRPM4,NRP1,PUM2,RASIP1,BEND5,PIK3CG,ARHGAP42,SVIL,TAL1,LAMA4,PHF10,MTMR2,EPC1,KDM4D,GATA2,DNMT3A,SAP130,KCNRG,CST7,NOVA2,CTSC,GPR4,LRCH1,PLXND1,NOS3,GGA3,ZFAND6,DACH1,PPARG,TIMP4,GATA3,TAF3,ACVR2B,TBC1D4,ITIH5,GZF1,SEC14L1,EDNRB,NDFIP2,TMC8,SPTBN4,TIE1,IKBKB,JAG1,P2RY6,PDE2A,SFRP1,HBEGF,NR1D2,DND1,FEZ2,NOTUM,FERMT1,RPL26,EFNB2,SH3BP5,ANKLE2,ARHGEF7,SPEF1,CD36,SIRT3,TMEM178A,LMOD3,ARID5A,PRAG1,ADAMTS9,PRR5L,TCF15,EPHB1,PLK2,PDE4D,MERTK,CHD7,FAM3D,IL1A,DNMT3B,IGF1,NR4A1,KLKB1,EIF2AK3,ABHD6,HHEX,KDM3A,ITCH,ERBIN,SH3RF1,SMAD1,ZBTB38,HIP1R,POU6F1,OCLN,DCLK2,PROCR,MCTP1,SOX7,HMBOX1,ENC1,MEF2C,TLR2,WWTR1,IL7,P2RX7,VEGFC,NDRG4,GUCY1A1,CXCL10,ANGPT2,TLL2,SOGA1,LMBR1L,ADGRG3,SFMBT1,FAM192A,SCAF4,TIPARP,OAS3,NLRC5,THBS4,PPP1R16B,DYSF,TPCN1,PLCG1,STC1,RAPGEF3,ANKRD2,NR4A2,DGKZ,STAB1,TP53I11,DLL4,CDYL,CEP97,SOX13,IFIT1,ADAMTS20,YAF2,	turquoise	EC_M6
GO:0007267	2.54E-06	0.999998499	211	1146	cell-cell signaling	BP	4.45E-04	LRP1,RNF19A,SLC12A2,RSPO3,CXCL14,ADGRB1,LY6E,PPARD,CBY1,GNAQ,LEP,SRF,CDK14,VCP,UNC13B,CLOCK,REST,RIMS1,TPBG,SHANK1,MDFIC,GLI3,NCDN,INHBA,ZYX,DKKL1,VPS35,SLC7A11,ITGB1,LIN7B,PLEKHA4,FKBP1B,FGF2,KIF5B,NAPA,PANX1,GAD2,GLI2,PCDH17,TGFB1,RAB3GAP1,SCN5A,HTR5A,CTNNB1,ZEB2,C1QTNF1,FARP1,IRS2,PRNP,RAB11FIP1,ZNF703,PCLO,ILK,RYK,PRICKLE2,SNPH,RIC3,CXCL12,FAT1,OSBP,SULF1,VAMP8,CTNND1,LNPEP,DKK3,KCMF1,PCSK1,MAP1B,SEMA4F,P2RX4,SELENOT,ATP2A2,DLGAP4,CACNA1D,APP,GLRB,RAPGEF2,CX3CL1,ABCC8,CACNB3,CPE,PTPN11,PFKM,HOMER1,AMFR,CRY2,VPS18,GLS,SPP1,PKD2,NKD1,BRD7,BRSK2,CHRNA7,EIF4E,DYNLL1,APBA2,KDM5B,PKP2,CDC73,DNM1L,CCND1,CAPRIN2,CPT1A,LRP5,HTR2B,GSK3B,DAG1,HADH,LEF1,KRAS,KPNA1,ADCY5,BICC1,FGFR2,OSM,NCSTN,SYAP1,BACE1,FZD9,RPS6KA3,SIDT2,LINC02210-CRHR1,MAPT,EIF4EBP2,ATP6AP2,FZD2,TNR,TFR2,MPP2,IGFBP2,ADRA2C,FGFR3,SMURF1,TNIK,PTPRN,KCNA1,CYP19A1,DAAM1,ATAD1,OTUD5,CHRNE,ERC1,HAP1,CACNA1C,IQSEC2,NLGN2,STX1B,TGFB1I1,SYT11,KCNMB1,COL1A1,MYCBPAP,CACNA1G,PBXIP1,UBE2Q1,RAB11A,ADRA1B,SOX30,CALM1,SYTL4,SMARCA4,TMED1,GSKIP,GRIA1,NPTN,CYP46A1,BEGAIN,DTNA,PIN1,ACSL4,RASL10B,AKT1,PRKAA1,NLK,ABR,NXN,DAPK3,GNB1,PAFAH1B1,TAX1BP3,PKD1,CSNK1G2,TPRG1L,NPHP4,FLNA,MIDN,SSTR5,VAMP7,PARK7,HCN2,NTNG2,JPH3,WLS,CCL16,PCDHB2,IL36G,GDNF,THY1,EDA,DACT3,INSYN1,YWHAH,HCN4,MESD,KCNMB4,MAP1A,WNT2,LRRTM2,FGF10,MBD2	darkgreen	EC_M4

GO:0008150	9.75E-06	0.999996521	1172	12183	biological_process	BP	4.69E-04	RTTN,CDK2,PAN2,NARS,ONECUT2,C18orf54,MEX3C,ME2,YTHDC2,MTFR2,BCLAF1,CLK4,RPA3,USP15,SHPRH,HNRNPH1,XPOT,PPIL4,KATNA1,LATS1,NUP43,MTHFD1L,NUP107,CCT2,MDC1,ZFC3H1,TBC1D15,POP1,DDX39B,SRFBP1,FBXO43,FBXO5,CSNK1G3,ZNF608,SCAF8,UBR5,VWA7,EIF3E,NUDCD1,EBAG9,MAP3K4,GTSE1,RAD21,DSCC1,KIF3A,MRPL13,TBC1D32,RRP7A,HSF2,KIFC1,ATAD2,XRCC6,DDX46,PHF5A,AKAP7,KIF20A,SLC35D2,CDC25C,TNRC6B,FANCC,HSPA9,CHCHD3,TUT7,NAA35,MATR3,SRPK1,UHRF2,RMI1,SRSF3,PPIL1,PLEC,TNPO3,PSIP1,RECQL4,CEP78,HAUS6,DENND4C,TONSL,MRPS10,RBM28,UBR2,CARNMT1,IFT74,MCM5,HMGXB4,POT1,PWP1,ZNF300,TOPORS,APTX,SMU1,DBF4,POLR3B,XPO5,MTERF1,KIF24,POLH,AKAP9,GCC2,PUM3,RFX3,ARHGEF39,CENPQ,IL1R1,CKS2,MCM3,PPAT,C1GALT1,PAICS,MIOS,ICK,REV1,NOL8,MELK,TRIM28,BZW2,NCBP1,ZNF451,ANP32B,PHF3,TWISTNB,LRPPRC,STX17,PPIH,FOXJ3,ZNF189,MSH2,SMC2,FBXO11,SP4,SENP6,ASB3,CDCA7L,CTPS1,FSD1L,PHIP,YTHDC1,MTIF2,TTK,PPP4R3B,IBTK,HNRNPA2B1,PNPT1,DOP1A,RUFY3,DCK,CYB5R4,PAPOLG,CEP162,PUS10,ZMPSTE24,RLF,USP34,SYNCRIP,ZNF292,XPO1,PLEKHA8,SNX30,PABPC4,CCT4,COMMD1,MDN1,CASP8AP2,DPY19L1,FKBP15,NUP205,ETAA1,ANLN,FBXO48,CDCA8,GNL2,TIA1,ZNF350,ITGB1BP1,THRAP3,TAF1B,CAPZA2,EZH2,UFL1,NOL10,CUL1,MMS22L,USP45,FAM3C,MRPL32,CCNC,CLSPN,ASCC3,ZMYM4,CNTRL,CASP2,ORC6,DDX1,SHCBP1,NAA15,SMC6,ELF2,ORC1,GEN1,AFG1L,WDR35,SCLT1,NRDC,CDC40,PLK4,AMD1,BCAP29,ATAD2B,DHFR,CDCA7,SYPL1,KIAA1109,RINT1,STIL,PUS7,HDAC2,MCM2,NT5	darkgrey	EC_M8
GO:0016458	9.98E-06	0.999996238	34	169	gene silencing	BP	4.78E-04	CDK2,TNRC6B,TUT7,XPO5,TRIM28,NCBP1,HNRNPA2B1,PUS10,ZMPSTE24,SETDB2,CNOT10,METTL3,RRP8,SMARCA5,FAM208A,HELLS,CNOT1,RIPK1,AJUBA,FXR1,DDX5,TIAL1,DDX6,DHX9,TPR,DGCR8,CNOT9,SUV39H1,SMCHD1,ZC3H7A,DROSHA,FMR1,SRRT,HIST1H3H	darkgrey	EC_M8
GO:0001817	3.22E-06	0.999998517	87	492	regulation of cytokine production	BP	4.87E-04	IFNGR1,TNFAIP3,AKAP12,LAPTM4B,FLT4,C6orf106,ATF4,MAPK13,LRRC19,DDX58,KIT,TNFRSF21,CD2AP,SYK,IL6,IGF2BP3,REL,PELI1,RSAD2,TLR4,C5,IL15,TRPM4,PIK3CG,PRKD2,GATA3,TMEM173,FERMT1,CD36,PDE4D,MERTK,IL1A,IGF1,EIF2AK3,ITCH,ERBIN,TLR2,IL7,P2RX7,OAS3,STAT1,CD58,CD200,HDAC1,HYAL2,ICOSLG,LAPTM5,INPP5D,CD34,CD86,FGR,F11R,APOD,CREB1,B2M,LTF,CYBB,IL1RAP,DDX3X,HOMER3,TMEM106A,RAC1,HIF1A,BCL6B,EPHA2,TLR6,TNFRSF1B,ARG2,PSEN1,BATF,RPS6KA5,YY1,PDE4B,LGALS9,NOS2,GIT1,ISG15,TRAF2,IRF8,PLCG2,OAS1,TLR1,GBP5,CD46,ACKR1,BST2,RNF125	turquoise	EC_M6
GO:0010212	1.02E-05	0.999996432	30	137	response to ionizing radiation	BP	4.89E-04	XRCC6,MSH2,ZMPSTE24,RAD54L,DCLRE1C,PAXIP1,FANCD2,RRM1,BRCA2,TOPBP1,PRKDC,ERCC6,ATR,ABRAXAS1,RAD51,INO80,NUCKS1,TICRR,INTS7,BLM,COP1,XRCC5,BRCA1,RAD51AP1,ECT2,CLK2,NIPBL,TIGAR,RNF8,LZIC	darkgrey	EC_M8
GO:0090670	1.05E-05	0.999999039	9	19	RNA localization to Cajal body	BP	4.94E-04	CCT2,CCT4,CCT7,CCT5,CCT6A,DCP2,EXOSC10,CCT3,NAF1	darkgrey	EC_M8
GO:0090671	1.05E-05	0.999999039	9	19	telomerase RNA localization to Cajal body	BP	4.94E-04	CCT2,CCT4,CCT7,CCT5,CCT6A,DCP2,EXOSC10,CCT3,NAF1	darkgrey	EC_M8
GO:0090672	1.05E-05	0.999999039	9	19	telomerase RNA localization	BP	4.94E-04	CCT2,CCT4,CCT7,CCT5,CCT6A,DCP2,EXOSC10,CCT3,NAF1	darkgrey	EC_M8
GO:0090685	1.05E-05	0.999999039	9	19	RNA localization to nucleus	BP	4.94E-04	CCT2,CCT4,CCT7,CCT5,CCT6A,DCP2,EXOSC10,CCT3,NAF1	darkgrey	EC_M8
GO:0061061	2.86E-06	0.999998637	106	504	muscle structure development	BP	4.96E-04	PPARA,MRTFA,ADGRB1,LY6E,PPARD,CBY1,LGALS1,FLNC,MYH9,LARGE1,SRF,VEGFA,REST,TGFBR1,HIVEP3,MYH14,AKIRIN1,IFRD1,BVES,MYLK3,ITGB1,RB1,SMARCD3,PAK1,TGFB1,CTNNB1,RARB,ACTG1,ACTN4,DCN,ILK,ANKRD1,MRAS,FLNB,HEY1,ATP2A2,HOMER1,ACVR1,HIF1AN,NKD1,PDLIM5,PKP2,DNER,LMOD1,TGFB2,PRKAR1A,CASP7,MMP14,DAG1,LEF1,DISP1,KRAS,COL6A3,ATF3,FGFR2,CASQ1,ARID5B,TAGLN,FZD2,COMP,SYNPO2L,WDR1,SIX4,OBSL1,EVC,BMPR1A,PDLIM7,TCAP,MED1,ACTN1,MYLPF,FGFRL1,TPM1,SGCA,ITGB1BP2,SGCD,MORF4L2,SETD3,MYOCD,PIN1,FBXO22,PDGFRB,FLII,AKT1,MYOM1,PRKAA1,SUPT6H,SYPL2,COL11A1,MIR24-1,DMD,FKBP1A,ADAMTS5,LOX,PDLIM1,IGSF8,CD164,CACYBP,LBX1,CSRP1,SRPK3,BHLHE41,HOPX,WNT2,CFL2,FGF10	darkgreen	EC_M4

GO:0044093	3.33E-06	0.999998071	176	1151	positive regulation of molecular function	BP	4.98E-04	TNFRSF11A,TBC1D30,FLT4,CTHRC1,GRAMD4,AFDN,TRDN,TPD52L1,ASAP1,SGSM3,PTK2,DAPK1,PDGFB,TBC1D22B,MFNG,TEK,DDX58,KIT,ADGRF1,KDR,SYK,SEMA4D,RGP1,IL6,CHN2,ARHGAP25,ASAP2,CAV2,TLR4,TRAF1,TBC1D9,CCNY,NRP1,PIK3CG,ARHGAP42,TAL1,KDM4D,PRKD2,CAB39L,PLXND1,NOS3,RALB,USP6NL,PPARG,GATA3,TBC1D4,IRAK2,NET1,RALGAPA2,TIE1,IKBKB,P2RY6,TMEM173,SFRP1,HBEGF,ARHGEF7,ARAP3,CD36,SIRT3,SCARB1,EPHB1,PLK2,RET,MERTK,IGF1,EIF2AK3,HIP1R,SOX7,HMBOX1,MEF2C,TLR2,VEGFC,MADD,SERINC5,PLCB2,RAPGEF3,NR4A2,DGKZ,NEDD9,EDN1,SNCA,CD200,HDAC1,LCK,PPP3CA,ADPRH,LAPTM5,NCF1,PABPN1,HCLS1,EPB41,CD86,FGR,CFLAR,CASP10,ADCY1,PROX1,PRKD1,TOR1AIP1,F11R,ACE,ITGA6,PAK2,DLG1,SIPA1,MAP3K11,B2M,LTF,ARHGAP27,BCR,IL1RAP,EPHB4,DDX3X,RUNDC1,ELMOD1,WNK4,RAMP2,SELE,TNFAIP8L3,FGF23,CRACR2A,LMTK2,PPM1F,PRKCH,HIF1A,RCC2,JUP,EPHA2,BID,TLR6,TBC1D1,RGS14,MTOR,AR,PSEN1,FGF18,TAOK2,ARHGEF15,EFNA1,ATP2A1,CD19,PIK3R5,RPS6KA5,DNM2,TBC1D8B,MARK3,ADRB2,CCL2,LGALS9,NOS2,UNC119,SGSM2,TRAF2,RAPGEF1,PLCG2,S1PR1,TBC1D13,TERF2,DAB2IP,PRSS22,PMAIP1,TRIM34,RELN,ALKAL1,SHANK3,MMD,RGS4,TRIM62,FCER2,RGS16,TBC1D20,PERP,DAZAP2,COPS8,BMP2,PIFO	turquoise	EC_M6
GO:0010557	1.07E-05	0.999993016	185	1433	positive regulation of macromolecule biosynthetic process	BP	5.02E-04	ONECUT2,BCLAF1,CCT2,DDX39B,SCAF8,EIF3E,MAP3K4,DSCC1,HSF2,ATAD2,XRCC6,PHF5A,IFT74,POT1,PWP1,ZNF300,TOPORS,DBF4,RFX3,TRIM28,ZNF451,FOXJ3,PHIP,RLF,ZNF292,CCT4,ITGB1BP1,THRAP3,CCNC,NAA15,ELF2,ATAD2B,HDAC2,CCNA2,EXOSC9,MLLT10,DNAJC2,NUP85,ELF1,RSF1,CCNT2,PAXIP1,WDR43,SUPT16H,CHD8,KAT6A,METTL3,E2F7,ALYREF,CKAP2,CEP290,NSD3,CEBPZ,TAF7,RFC3,BRCA2,ELK3,TCERG1,PRKDC,ERCC6,DHX29,GPBP1,SP1,TCEA1,ZC3H8,TNKS2,ZNF143,CENPJ,CDK5RAP1,NCK1,ARFGEF1,SMARCA5,ZNF507,TAF9,TFCP2,ATR,LARP4,UTP15,HLTF,GABPA,MCRS1,USP16,CCT7,SENP1,RIPK1,WDR75,MYBL2,E2F8,INO80,CHUK,HIVEP1,RFC5,NOLC1,CCT5,BMPR1B,UBE3A,CCT6A,PRDM10,NUCKS1,GCN1,CHEK1,H2AFZ,RRP1B,NFKB1,KHDRBS1,TET2,TRIP11,HEATR1,NCL,NOL11,GTF2I,BPTF,FXR1,RFC2,NEK2,EIF2B5,BLM,EDRF1,CDK1,ING5,BCLAF3,FOSL1,SMARCC1,SP3,DHX9,FASTKD2,RFC4,DNA2,CCAR1,DBF4B,UBTF,NMD3,XRCC5,NPAT,BRCA1,CNOT9,DCAF6,DHX33,TBL1XR1,LEO1,FOXM1,LYAR,DDX11,HNRNPU,SMARCE1,RFC1,TOP2A,NSD1,MED6,ICE2,CCT3,PMF1,FUS,AURKB,ZNF609,OGT,ATRX,ZNF711,ILF2,TRIM37,LARP1,ILF3,MED13,AATF,TAF15,THOC1,UPF3B,ATAD5,NIPBL,MYSM1,UHRF1,RBMX,FMR1,TP73,CHTF18,CDT1,CTCF,PRIM2,HNRNPD,DRAP1,ZNF770,NAF1,ZNF782,ZFP62,SFR1	darkgrey	EC_M8
GO:0060284	3.05E-06	0.999998614	86	384	regulation of cell development	BP	5.19E-04	LRP1,RAB21,PLXNB2,CTNNA1,SRF,VEGFA,CLOCK,REST,RTN4,ACTR2,GLI3,ROCK2,PLXNA1,TENM4,MEGF8,SMARCD3,RHEB,PAK1,TGFB1,CTNNB1,TRAK1,ACTN4,LIG4,SEMA3C,ILK,RYK,RNF6,CXCL12,EIF4G2,ROBO1,MAP1B,DIP2B,SEMA4F,HEY1,TLX2,DHX36,RAPGEF2,CX3CL1,ABCC8,OPA1,ZFYVE27,SPP1,SEMA4G,NOTCH2,DNM1L,CAPRIN2,GSK3B,DAG1,CORO1C,NTRK3,SLAMF8,BMP7,IDH2,MAPT,TNR,FN1,FBN1,ARPC2,PRKCI,SKIL,VCL,OBSL1,HAP1,PRTG,BMPR1A,RAB11A,MAP2K1,DICER1,NPTN,ISLR2,TRPV2,FBXO22,ROCK1,AKT1,TMEM98,PLXNB3,PAFAH1B1,FLNA,ATP6AP1,PLXNA3,SERPINF1,THY1,HAS2,YWHAH,BHLHE41,WNT2	darkgreen	EC_M4
GO:0061387	3.06E-06	0.999999031	31	95	regulation of extent of cell growth	BP	5.19E-04	LRP1,RAB21,SRF,VEGFA,RTN4,MEGF8,PAK1,SEMA3C,ILK,RYK,RNF6,CXCL12,EIF4G2,MAP1B,DIP2B,SEMA4F,ZFYVE27,SPP1,SEMA4G,CTTN,GSK3B,MAPT,TNR,FN1,RAB11A,ISLR2,TRPV2,PAFAH1B1,PLXNA3,BARHL2,OLFM1	darkgreen	EC_M4
GO:0006457	3.10E-06	0.999998766	44	166	protein folding	BP	5.19E-04	LMAN1,CANX,TCP1,SIL1,TBCC,BAG1,PDCL3,TXNDC9,LMAN2L,P3H1,ERP44,PDIA6,FKBP1B,DNAJC1,CRTAP,DNAJB13,HSPH1,RIC3,PDRG1,SACS,HSP90B1,CCT8,HSPA8,QSOX1,ATF6,PDIA3,PRDX4,HSPB1,DNAJA4,DNAJC10,DNAJB12,FKBP2,FKBP8,ERO1A,NPPB,CD74,HSPA5,RANBP2,FKBP1A,TBCA,TTC1,HSPA13,GRPEL2,MESD	darkgreen	EC_M4

GO:0048589	3.13E-06	0.999998507	106	501	developmental growth	BP	5.19E-04	LRP1,RAB21,PPARA,LY6E,PPARD,SH3GL2,LEP,PLAA,LARGE1,SRF,VEGFA,TGFBR1,RIMS1,RTN4,MFSD2A,AKIRIN1,GLI3,ITGB1,PLXNA1,FGF2,TENM4,GLI2,MEGF8,PAK1,WDR48,CTNNB1,RARB,DCLK1,SEMA3C,ILK,RYK,RNF6,CXCL12,TMED2,EIF4G2,WWC2,MAP1B,RDH10,DIP2B,SEMA4F,CELF1,APP,PTPN11,PTX3,ZFYVE27,SPP1,RUNX1,SEMA4G,NKD1,PDLIM5,NDN,KDM5B,NOTCH2,CTTN,SBDS,TGFB2,PRKAR1A,GSK3B,DAG1,PIK3CA,AUTS2,WWC3,KPNA1,WDTC1,FGFR2,IQGAP1,CLIC4,ARID5B,FZD9,MAPT,SGPL1,TNR,FN1,COMP,FKBP8,FGFR3,SMURF1,VCL,SIX4,EVC,NINJ2,BMPR1A,MED1,SGCA,THBS3,RAB11A,TTC8,ISLR2,ANXA6,PIN1,TRPV2,PDGFRB,AKT1,PAFAH1B1,PLXNA3,BARHL2,MIR24-1,TMEM38B,PTPN12,CDKN1A,HOPX,ZPR1,CHST11,OLFM1,WNT2,FGF10	darkgreen	EC_M4
GO:0016078	1.12E-05	0.99999946	7	11	tRNA catabolic process	BP	5.24E-04	POP1,EXOSC9,TRDMT1,EXOSC8,NSUN2,EXOSC10,EXOSC2	darkgrey	EC_M8
GO:0007099	1.15E-05	0.999997987	14	37	centriole replication	BP	5.37E-04	RTTN,CDK2,PLK4,STIL,CEP295,WDR62,CENPJ,ALMS1,CEP72,BRCA1,CEP152,TRIM37,CEP76,SASS6	darkgrey	EC_M8
GO:0007507	3.36E-06	0.999998253	102	474	heart development	BP	5.52E-04	AHI1,TAB2,PPARA,LY6E,PPARD,CBY1,SRF,VEGFA,REST,TGFBR1,RTN4,GLI3,ROCK2,BVES,MYLK3,ITGB1,FGF2,RB1,TENM4,GLI2,MEGF8,SMARCD3,SNX17,PAK1,TGFB1,SCN5A,CTNNB1,RARB,KAT2B,SEMA3C,ILK,ANKRD1,ADAMTS6,TMED2,EIF4G2,ROBO1,HEY1,DHX36,CPE,PTPN11,ACVR1,PKD2,RUNX1,DNAH5,IFT57,PDLIM5,NOTCH2,PKP2,TGFB2,HTR2B,PRKAR1A,CASP7,NTRK3,BMP7,AP1B1,BICC1,FGFR2,FZD2,FN1,FBN1,COL3A1,SYNPO2L,ECE1,MNAT1,OBSL1,CACNA1C,BMPR1A,HSPB7,PDLIM7,TCAP,MED1,NPPB,FGFRL1,TPM1,CACNA1G,SHC1,MAP2K1,TMEM100,SGCD,MYOCD,PIN1,PDGFRB,ROCK1,PKD1,FLNA,SYPL2,COL5A1,COL11A1,LMO4,MIR24-1,FKBP1A,LOX,PDLIM1,CACYBP,LBX1,HAS2,CDKN1A,HOPX,HCN4,OLFM1,WNT2,MBD2	darkgreen	EC_M4
GO:0006356	1.19E-05	0.999998239	12	31	regulation of transcription by RNA polymerase I	BP	5.56E-04	PWP1,ZMPSTE24,WDR43,UTP15,WDR75,HEATR1,NCL,NOL11,UBTF,DHX33,LYAR,DDX11	darkgrey	EC_M8
GO:0051239	3.43E-06	0.999997732	331	1937	regulation of multicellular organismal process	BP	5.57E-04	SERPINB2,IL23A,LRP1,AHI1,RAB21,SLC12A2,PLXNB2,FBN2,ANGPT1,FAM19A5,PPARA,HLA-DRA,NUS1,FAM49B,ADGRB1,PPARD,CTNNA1,LEP,KIAA1324L,SRF,VEGFA,RUNX2,UNC13B,CLOCK,PDCL3,REST,ASPN,NLRP5,TGFBR1,SOCS5,RIMS1,RTN4,TPBG,SHANK1,LRRC4B,EMC10,MFSD2A,UBE2J1,MAP3K7,ACTR2,GLI3,ROCK2,INHBA,BVES,RRAS,ITGB1,FKBP1B,PLXNA1,FGF2,PANX1,RB1,PTCH2,TENM4,GLI2,MEGF8,SMARCD3,RHEB,PAK1,TGFB1,AXL,SCN5A,CTNNB1,IL15RA,TRAK1,LBH,GPD1L,C1QTNF1,RARB,NAV3,NPLOC4,LTBP1,MAFG,STIM1,SPRED3,LIG4,PRNP,ATP2B1,LUM,DCN,HDAC3,RAB11FIP1,ZNF703,ACPP,STARD13,SEMA3C,ILK,SPINK5,RYK,RNF6,CXCL12,FXYD1,LGI4,SNTA1,IL20RB,EIF4G2,SULF1,VAMP8,LNPEP,ROBO1,WWC2,MAP1B,DIP2B,SEMA4F,LOXL3,P2RX4,PFN2,HEY1,ATP2A2,TLX2,CELF1,DLGAP4,CACNA1D,MYL9,APP,DHX36,RAPGEF2,CX3CL1,ABCC8,CACNB3,PTPN11,HOMER1,OPA1,ARSB,LOXL2,AP3B1,AMIGO2,ZFYVE27,PTPN22,ACVR1,CRY2,GDF6,POLR3D,MTDH,ADA,GLS,BAP1,ATP2B4,SPP1,CHID1,BMP1,RUNX1,PLA2R1,TWF1,SEMA4G,CBLB,RAB7B,CHRNA7,ADIPOR1,FAP,PHLDB2,TJP1,KDM5B,NOTCH2,CTTN,PKP2,PPP1R12B,CDC73,DNM1L,SELENOS,CHSY1,TRIP12,HYAL1,SLC2A10,CCND1,CAPRIN2,KPNA6,TGFB2,PRKAB2,ADGRB2,HTR2B,GSK3B,MMP14,DAG1,PIK3CA,HADH,LEF1,CORO1C,PRKCA,TLR5,CMKLR1,NTRK3,WWC3,TCTA,SLAMF8,BMP7,IDH2,PPP1R11,FGFR2,VASH2,ASB1,OSM,CASQ1,TP53BP1,PHLDB1,TFRC,FZD9,GLRX3,PRDX4,HSPB1,LINC02210-CRHR1,MAPT,SERPINE1,SGPL1,ATP6AP2,DNAJA4,COMT,TNR,FN1,PTPN6,GPR137,FBN1,PDCD10,ITGAV,A	darkgreen	EC_M4
GO:0002684	3.77E-06	0.999998071	92	542	positive regulation of immune system process	BP	5.58E-04	PTK2,DDX58,TEC,KIT,TNFRSF21,SYK,IL6,CXCL8,PELI1,RSAD2,TLR4,C5,IL15,GATA2,PRKD2,CTSC,NECTIN2,GAB2,UNC13D,GATA3,IKBKB,TMEM173,CD36,PDE4D,IL1A,IGF1,ITCH,MEF2C,IL7,VEGFC,CXCL10,NLRC5,THBS4,PLCG1,DGKZ,ANO6,SOX13,EDN1,JAM3,LCK,ICOSLG,LAPTM5,HLX,HCLS1,NFATC2,INPP5D,CD86,BMX,FGR,FES,PLEKHA1,PAK2,CREB1,B2M,MASP1,ITGA4,MCU,SELP,PLVAP,PRKCH,RAC1,PLD2,ITPKB,TLR6,EFNB1,PSEN1,PGF,EIF2B2,DAPK2,CHRNB2,CD19,S100A14,GPR68,CCL2,YES1,PDE4B,LGALS9,NOS2,IL7R,MYO18A,AP3D1,TRAF2,PLCG2,STXBP1,FYB1,HAVCR1,VNN1,GBP5,CD46,CA2,TRIM62,FCER2	turquoise	EC_M6

GO:0009966	3.55E-06	0.999997625	376	2237	regulation of signal transduction	BP	5.68E-04	CD63,IL23A,LRP1,ARHGEF25,TAB2,DYRK2,RRM2B,FBN2,ANGPT1,PPARA,ROS1,RSPO3,FAM49B,PTP4A3,LY6E,PPARD,CTNNA1,CBY1,UBQLN1,MTCH1,LGALS1,KLHDC10,GNAQ,ARHGAP39,LEP,CDKN2B,KIAA1324L,NUAK1,CDK14,VEGFA,DOCK8,VCP,SLC35B2,RUNX2,RASL11B,UNC13B,NCK2,CLOCK,CREB3,ASPN,SNX13,TGFBR1,SOCS5,RIMS1,RTN4,TPBG,SHANK1,ADAMTS3,EMC10,MAP3K7,MDFIC,GLI3,ROCK2,INHBA,RRAS,RCN3,DKKL1,VPS35,ITGB1,PLEKHA4,FGF2,SESN3,RAB7A,ARHGEF4,RB1,TPT1,PTCH2,KCTD21,GLI2,MEGF8,RHEB,PAK1,TGFB1,AXL,SEC13,CTNNB1,LBH,DYNC1LI1,GPD1L,ZEB2,CANT1,C1QTNF1,ARHGEF17,OSBPL8,CXXC5,PSD2,NPLOC4,ACTN4,ANAPC15,LTBP1,CRIM1,SPRED3,UFM1,ATP2C1,PRNP,DCN,HDAC3,ZNF703,ACPP,STARD13,KCTD16,CAVIN3,UBA5,DDRGK1,SMPD1,PPP2CB,ILK,RBPMS,LEPROTL1,ARL6IP5,APLP1,RYK,TMEM127,CNOT7,RNF6,TMEM9B,ST5,AKIP1,CXCL12,SLC20A1,ANKRD1,PJA2,TMED2,CD180,ZNF536,SULF1,GRK7,CTNND1,DKK3,ROBO1,WWC2,SORBS1,LOXL3,P2RX4,RNF13,FAIM2,HEY1,RASA1,DLGAP4,TCTN1,APP,DHX36,RAPGEF2,MAPKAPK5,CX3CL1,CACNB3,PTPN11,RALGAPB,HOMER1,OPA1,ARHGAP1,AMFR,PTPN22,TSG101,ACVR1,CRY2,VPS18,CHAC1,GDF6,SERINC3,MTDH,ADA,CHP1,ATP2B4,PKD2,PLA2R1,HIF1AN,CBLB,NKD1,TANK,SLC39A10,RAB7B,CHRNA7,ADIPOR1,NOTCH2,CTTN,CDC73,DNM1L,GLRX2,UCHL5,SELENOS,CHSY1,SLC2A10,EYA2,CAPRIN2,TGFB2,KCTD10,ARHGAP31,HTR2B,ARMC9,GSK3B,FSTL1,MMP14,DAG1,PIK3CA,AUTS2,LEF1,PRKCA,TLR5,CMKLR1,KRAS,NTRK3,WWC3,GRK3,KPNA1,SPATC1L,BMP7,HYOU1,GOLT1B,BICC1,ATF3,FGFR2,OSM,PEA15,	darkgreen	EC_M4
GO:0019725	3.59E-06	0.999998216	128	638	cellular homeostasis	BP	5.68E-04	LRP1,SLC12A2,SLC39A7,SMDT1,PPARD,LGALS1,TXN2,SLC39A4,PLAA,LARGE1,SLC30A9,SRF,UNC13B,ERP44,RTN4,SLC31A1,FKBP1B,FGF2,KIF5B,RAB7A,ATP7B,TPT1,DGAT2,C1QTNF1,SLC4A7,TMTC4,MAFG,STIM1,ATP2C1,IRS2,PRNP,ATP2B1,CCKBR,RIC3,CXCL12,TM9SF4,HSP90B1,OSBP,C19orf12,ATP1B3,RYR3,P2RX4,SELENOT,ATP2A2,APP,SLC4A5,CX3CL1,ABCC8,CACNB3,PFKM,OPA1,AP3B1,CHP1,ATP2B4,PKD2,BRSK2,SLC39A10,CHRNA7,RAP1GDS1,DYNLL1,GLRX2,SELENOS,SLC2A10,ZBTB20,LRP5,HTR2B,PIK3CA,ISCU,CMKLR1,HJV,SLAMF8,ADCY5,VAPB,WDTC1,CASQ1,CLIC4,TFRC,FZD9,SIDT2,GLRX3,PRDX4,TMCO1,ATP6AP2,TFR2,PTPN6,ITGAV,HTT,ERO1A,PTPRN,HAP1,CACNA1C,STIM2,PPARGC1A,NGFR,ADRA1B,GPR65,CLN6,CALM1,SMARCA4,GRIA1,NPTN,SLC39A6,ANXA6,TRPV2,PRKAA1,GNB1,BCAP31,PKD1,ATP6AP1,SYPL2,JPH3,SLC30A7,ATP6V0D1,DMD,FKBP1A,SERPINF1,BNIP3,TMEM38B,SLC22A17,LOX,THY1,TXN,TMEM38A,CCR6,ORMDL3,ATP6V0E1,MAP1A,CFL2	darkgreen	EC_M4
GO:0032880	3.61E-06	0.999997997	137	693	regulation of protein localization	BP	5.68E-04	LMAN1,LRP1,STX7,SLC12A2,ANGPT1,TCP1,PTP4A3,PPARD,CTNNA1,LEP,VCP,UNC13B,CLOCK,EXOC1,REST,RAB23,P3H1,TMEM30A,ERLEC1,RTN4,UBE2J1,MDFIC,GLI3,ROCK2,SLC7A11,ITGB1,FKBP1B,KIF5B,DNAJC1,RB1,PICALM,MARK4,PAK1,TGFB1,CTNNB1,AKT2,GPD1L,UFM1,ATP2C1,IRS2,PRNP,HDAC3,RAB11FIP1,DCLK1,DDRGK1,RIC3,TM9SF4,ANKRD1,TP53INP2,OSBP,VAMP8,PINX1,SORBS1,CCT8,ABCC8,CACNB3,PTPN11,GNL3,PFKM,ATG13,TSG101,DKC1,BAP1,CHP1,ATP2B4,BRSK2,DYNLL1,DNM1L,CPT1A,TGFB2,LRP5,GSK3B,MMP14,DAG1,HADH,DISP1,HSPA8,LARP7,IDH2,ADCY5,AP2M1,IQGAP1,SCFD1,TFRC,FZD9,SIDT2,LRRC15,MAPT,C11orf65,GPC2,PDCD10,PRKCI,VCL,SIX4,B3GAT3,MAGED1,IQSEC2,NLGN2,MSN,SRCIN1,SYT11,NGFR,TMED10,TGFB3,RAB11A,MYH10,TTC8,SYTL4,CLTC,NPTN,PIN1,ACSL4,MIEF2,AKT1,TMEM98,MYOM1,PRKAA1,MYO1C,BCAP31,TAX1BP3,FLNA,KCNAB2,MIDN,SSTR5,PARK7,RHBDF1,WLS,FKBP1A,TXN,LEPROT,ZPR1,ORMDL3,UBE2G2,TOMM7,MESD,MAP1A,DERL2	darkgreen	EC_M4
GO:0042255	1.23E-05	0.999997715	14	45	ribosome assembly	BP	5.72E-04	RRP7A,MDN1,MRPS7,PRKDC,DHX29,DHX37,RRS1,FP565260.1,FASTKD2,RPL10L,XRCC5,MRPL22,NLE1,BRIX1	darkgrey	EC_M8

GO:0003008	3.93E-06	0.999997693	189	1240	system process	BP	5.76E-04	LVRN,AKAP12,FLT4,RIMS2,TRDN,KLHL3,PDGFB,AGTPBP1,TEK,IREB2,ATP8A1,TJP2,ADGRF5,KIT,ADGRF1,TNFRSF21,TMOD1,PPT1,VPS54,DOCK4,TLR4,GRIK2,DLGAP3,IL15,TRPM4,PIK3CG,ARHGAP42,MTMR2,MGLL,CST7,CTSC,GPR4,PRCP,NOS3,PPARG,GATA3,MYO7A,CXCR4,EDNRB,SPTBN4,SLCO2B1,SETD5,P2RY2,PDE2A,HBEGF,KCNK6,P2RX2,CD36,RIC8A,LMOD3,TCF15,SCARB1,EPHB1,PLK2,PDE4D,CHD7,IGF1,NR4A1,EIF2AK3,HIP1R,MARVELD2,OCLN,MMP24,CHRM5,HEXB,MEF2C,TLR2,WWTR1,P2RX7,VEGFC,NDRG4,GUCY1A1,GUCY1B1,CXCL10,TMEM150C,USH1C,PLCB2,STC1,KCNJ3,ANKRD2,SLC38A1,TANC1,DLL4,IMPG2,DOP1B,NAV2,EDN1,STAT1,JAM3,SNCA,JARID2,SHANK2,PDE9A,PPP3CA,HYAL2,ANO1,KCNJ2,MEF2A,SYNM,SCN3A,PABPN1,ABLIM1,ZFHX2,EPB41,JHY,CD34,MCAM,CFLAR,INPP5F,ADCY1,LTB4R,TMOD4,F11R,YTHDF1,ACE,MYOM3,INSYN2,DLG1,PTCHD1,HOXD10,CREB1,B2M,HMCN1,ADORA2A,RTP4,CCDC50,IGFBP5,TBX1,CALCRL,WNK4,RAMP2,PLVAP,TMOD2,HIF1A,APBB2,JUP,SHROOM4,CHRNB1,TNFRSF1B,RGS14,ARG2,MTOR,AR,PSEN1,HOXB8,ITGA3,CHRNB2,ATP2A1,PIAS1,NPR1,SCNN1B,YY1,TBC1D8B,DSG2,DSC2,ADRB2,CCL2,YES1,JAG2,PDE4B,NOS2,UNC119,PIEZO2,GIT1,NOTCH1,S1PR1,NFAT5,PTGS1,CDH5,MIR23A,SLC8A3,ATXN1L,ERAP2,PKHD1L1,RELN,KCNH2,GRAP,SCN2B,KCNK5,SHANK3,RGS4,DRAM2,RGS16,MGAT3,PDCL,CACNG5,BMP6,KCNG2,FLI1	turquoise	EC_M6
GO:0044843	1.24E-05	0.999995108	36	180	cell cycle G1/S phase transition	BP	5.76E-04	CDK2,LATS1,GTSE1,DBF4,PPAT,EZH2,CUL1,CASP2,CCNA2,NASP,E2F7,POLE,CUL4A,PRKDC,RBL1,RPS6,FAM83D,CCNE2,INO80,CUL3,KHDRBS1,KIF14,BCAT1,RCC1,RPA2,CDC25A,NPAT,CUL5,PSME3,CDKN3,CDC6,IQGAP3,TAF1,STXBP4,CUL4B,GSPT1	darkgrey	EC_M8
GO:1990138	3.83E-06	0.999998509	42	147	neuron projection extension	BP	5.97E-04	LRP1,RAB21,SH3GL2,PLAA,SRF,VEGFA,RIMS1,RTN4,ITGB1,PLXNA1,MEGF8,PAK1,CTNNB1,DCLK1,SEMA3C,ILK,RYK,RNF6,CXCL12,EIF4G2,MAP1B,DIP2B,SEMA4F,ZFYVE27,SEMA4G,NDN,CTTN,GSK3B,AUTS2,IQGAP1,MAPT,TNR,FN1,SMURF1,VCL,RAB11A,ISLR2,TRPV2,PAFAH1B1,PLXNA3,BARHL2,OLFM1	darkgreen	EC_M4
GO:2000249	4.24E-06	0.999999342	14	34	regulation of actin cytoskeleton reorganization	BP	6.15E-04	BAIAP2L2,TEK,CD2AP,NRP1,RAPGEF3,IQGAP2,ESAM,HCLS1,FES,F11R,ARHGDIB,BAIAP2L1,ABI3,GMFG	turquoise	EC_M6
GO:0006936	4.12E-06	0.999998284	62	256	muscle contraction	BP	6.31E-04	SRF,TPM2,MYH14,CALD1,ROCK2,FKBP1B,SCN5A,GPD1L,ATP2B1,FXYD1,SNTA1,SULF1,SLMAP,P2RX4,ATP2A2,CACNA1D,MYL9,HOMER1,ADA,ATP2B4,RAP1GDS1,CTTN,PKP2,PPP1R12B,LMOD1,HTR2B,PIK3CA,CASQ1,ADRA2C,COMP,KCNA1,ACTA2,TPM4,CHRNE,CACNA1C,TCAP,TPM1,SGCA,CACNA1G,ADRA1B,CNN1,CALM1,SETD3,MYOCD,ANXA6,DTNA,ROCK1,ITGA2,MYOM1,DAPK3,FLNA,ENO1,HSBP1,DMD,TMEM38B,NMUR1,GDNF,TMEM38A,PIK3C2A,ORMDL3,HCN4,PRKG1	darkgreen	EC_M4
GO:0007167	4.14E-06	0.999997666	148	752	enzyme linked receptor protein signaling pathway	BP	6.31E-04	CD63,LRP1,AP3S1,AHI1,FBN2,ANGPT1,PPARA,ROS1,NUS1,PTP4A3,LEP,CDKN2B,BTBD11,KIAA1324L,VEGFA,RUNX2,RASL11B,COL1A2,NCK2,ASPN,TGFBR1,SOCS5,RTN4,ADAMTS3,MAP3K7,INHBA,ZYX,FGF2,SESN3,RAB7A,MEGF8,PAK1,TGFB1,AXL,CTNNB1,AKT2,ZEB2,OSBPL8,LTBP1,CRIM1,SPRED3,IRS2,DCN,SMAD9,ZNF703,ILK,RBPMS,LEPROTL1,RYK,SULF1,DKK3,ROBO1,SORBS1,DOK1,RASA1,RAPGEF2,SLC33A1,PTPN11,OPA1,ZFYVE27,TSG101,ACVR1,MMP2,GDF6,CBLB,DOK2,NDN,ADIPOR1,NOTCH2,SLC2A10,TGFB2,ZNF106,GSK3B,FSTL1,PIK3CA,LEF1,HJV,NTRK3,GFRA1,BMP7,FGFR2,ITGB5,HTRA1,IQGAP1,STRAP,MUC20,ARID5B,FAM89B,HSPB1,EIF4EBP2,SGPL1,IGFBP2,FBN1,COMP,FKBP8,COL3A1,SKIL,FGFR3,SMURF1,NBL1,GREM2,HAP1,BMPR1A,SLC2A4,NPPB,RNF111,MYO1E,FGFRL1,TGFB1I1,NGFR,LTBP2,COL1A1,TGFB3,SHC1,TMEM100,NPTN,MYOCD,PIN1,CHRDL1,SS18,NOMO1,PDGFRB,AKT1,PRKAA1,OSMR,NLK,PTPN2,MYO1C,VASN,RHBDF1,CDH13,F3,GLG1,BCAR3,SLC2A8,HSPA5,FSTL3,FKBP1A,PTPN12,LOX,ARF4,CHN1,DUSP22,PMEPA1,LEPROT,PIK3C2A,CHST11,FGF10	darkgreen	EC_M4
GO:0006271	1.38E-05	0.99999903	8	14	DNA strand elongation involved in DNA replication	BP	6.39E-04	MCM3,POLE,RFC3,MCM4,POLA1,RFC4,DNA2,MCM7	darkgrey	EC_M8

GO:0009058	1.80E-07	1	568	4393	biosynthetic process	BP	6.81E-04	NFATC1,ZNF236,TSHZ1,ORMDL2,PYM1,DGKA,SMARCC2,STAT6,ATG12,PIP4K2C,MAP3K5,PEX7,CDK4,CITED2,MAML1,SQSTM1,IRAK3,HELB,GFPT2,OSR2,CHKB,ALDH7A1,HSPA1B,EHMT2,ZBTB12,HDAC10,ALG12,ATF6B,AGPAT1,EIF3H,DSE,RXRB,TSPO,HSD17B8,RING1,RPS18,B3GALT4,CYB5R3,ZHX2,ZBTB22,PMM1,MYC,TOB2,SMAD5,EP300,IBA57,KDM3B,ZNF76,EEF1D,RPL3,DDX17,EIF3L,MPST,NFIB,GPAA1,EIF3D,HSF1,CCND3,TRERF1,CDKN2A,RPL8,HMOX1,WASL,KLF9,POLR1C,PIGO,PLPP6,JAK2,HINT2,ZBTB45,RPS5,ZNF135,ZNF667,ALG2,ZNF865,RPL28,TTC7A,RPS9,EEF1A1,MALSU1,SLC44A1,EFEMP1,UGP2,ZNF692,RPS7,ANXA4,AKNA,MED25,IL4I1,COQ3,ING3,ARHGEF5,AP2A1,IRF3,LPIN1,RPS11,FOXO3,KLF15,SPHK2,RPL18,NOP53,ABTB1,MAML2,BICRA,KMT2E,BBS7,MEIS3,FKRP,TRIM13,ACSS1,IRF2BP1,SIX5,FOSB,ZNF746,NR2C2,ZNF862,TSC22D1,SERP2,FDXR,UROD,SEPHS1,RPS8,ZNF575,MIF4GD,NAA16,MLH1,GTF3C2,UNK,ZNF574,GALNT11,WBP2,KMT2C,MYD88,TAMM41,CYGB,TADA3,CSGALNACT1,RPS3,TEP1,BRPF1,PNP,DYRK1B,EID2,ZNF219,RPS16,MED29,SAMD4B,MRPS12,SIRT2,RPTOR,RPL15,APBB3,EIF3K,NUP98,GALNT15,TRIM21,CYP1B1,GNPDA1,HGF,APBB1,TAF10,PTDSS2,DEAF1,HOXC4,CLIP3,NCOA4,LIN37,TARBP2,KMT2B,EIF3F,APIP,RBCK1,RARG,SDR16C5,ZNF740,EIF4B,LMO2,POFUT1,THNSL2,NECAB3,CBR4,URI1,PIK3R1,ACSS2,NCOA2,ATF1,TNKS1BP1,RPS3A,POLE4,KDM2B,CHDH,ZFHX4,CAMKK2,SCAND1,EIF2A,SETD6,MCPH1,SSBP2,FNIP2,COQ9,DGUOK,PRKAG1,ARL2BP,SHOX2,NEK4,SPR,ZHX3,VDR,MMS19,GPAT3,LPIN3,CHST14,CPOX,FOXF2,F2RL1,SORBS3,ORMDL1,HNF4A,TP53INP1,FARS2,PI	cyan	EC_M2
GO:0061013	1.48E-05	0.999994605	31	144	regulation of mRNA catabolic process	BP	6.83E-04	PAN2,TNRC6B,TUT7,PNPT1,SYNCRIP,PABPC4,THRAP3,EXOSC9,DIS3,METTL3,EXOSC8,PAN3,PDE12,CNOT1,FXR1,FASTKD1,DHX9,HNRNPR,FASTKD2,HNRNPU,DCP2,FUS,LARP1,TAF15,HNRNPM,SERBP1,FMR1,METTL16,EXOSC2,FASTKD5,HNRNPD	darkgrey	EC_M8
GO:0046903	4.70E-06	0.999997396	133	673	secretion	BP	7.03E-04	LRP1,CDO1,CANX,RAB21,SLC12A2,SLC22A4,LY6E,PPARD,MYH9,LEP,VEGFA,UNC13B,CLOCK,EXOC1,REST,NLRP5,P3H1,RIMS1,INHBA,VPS41,RCN3,LIN7B,FKBP1B,KIF5B,NAPA,DNAJC1,RAB7A,COPG1,PDCD6IP,PAK1,TGFB1,AXL,RAB3GAP1,C1QTNF1,RAB2B,IRS2,RAB11FIP1,PCLO,CCKBR,SNPH,CXCL12,ANKRD1,OSBP,VAMP8,P2RX4,SELENOT,ATP2A2,SLC4A5,ABCC8,PTPN11,EXOC6B,PFKM,SLC26A7,TSG101,CRY2,VPS18,ADA,SPP1,PLA2R1,LGI3,BRSK2,DYNLL1,KDM5B,DNM1L,CCND1,CPT1A,TGFB2,FBLN5,LRP5,GSK3B,HADH,DISP1,IDH2,ADCY5,OSM,COPA,BACE1,SIDT2,LINC02210-CRHR1,TANGO2,COMT,TFR2,ADRA2C,COMP,PRKCI,EXOC5,PTPRN,CYP19A1,HAP1,NLGN2,MED1,NPPB,SRCIN1,STX1B,SYT11,LTBP2,TMED10,TGFB3,CACNA1G,UBE2Q1,RAB11A,MYH10,SEL1L,CALM1,SYTL4,SCAMP5,ACSL4,CD74,RASL10B,MYOM1,PRKAA1,RAB31,NPR3,PAFAH1B1,SDF4,MIDN,ATP6AP1,SSTR5,VAMP7,PARK7,RHBDF1,AP1G1,WLS,CBARP,TMEM38B,SNX10,GDNF,TMEM167A,STXBP6,PIK3C2A,KCNMB4,FGF10,EXOC3L4	darkgreen	EC_M4
GO:0042475	4.73E-06	0.999998784	23	63	odontogenesis of dentin-containing tooth	BP	7.03E-04	PPARA,CTNNA1,RUNX2,CNNM4,GLI3,GLI2,SCN5A,CTNNB1,RELT,STIM1,FAM20C,LEF1,BMP7,HTRA1,SERPINE1,BMPR1A,NGFR,ROGDI,BSG,ANKRD11,LHX8,EDA,FGF10	darkgreen	EC_M4
GO:0051050	4.75E-06	0.999997374	131	660	positive regulation of transport	BP	7.03E-04	CD63,LRP1,AHI1,RAB21,SLC12A2,AZIN1,ANGPT1,PPARD,LEP,PLAA,VEGFA,UNC13B,EXOC1,TMEM30A,GLI3,INHBA,ITGB1,KIF5B,RAB7A,PDCD6IP,PAK1,TGFB1,AXL,RAB3GAP1,SCN5A,IL15RA,AKT2,GPD1L,C1QTNF1,RAB2B,OSBPL8,ACTN4,STIM1,ATP2C1,IRS2,PRNP,ATP2B1,HDAC3,SMPD1,CXCL12,FXYD1,TM9SF4,ANKRD1,OSBP,VAMP8,ATP1B3,RIOK2,SORBS1,P2RX4,SELENOT,CACNA1D,CX3CL1,ABCC8,CACNB3,PTPN11,PTX3,PFKM,HOMER1,ATG13,TSG101,PPP3CC,BAP1,CHP1,SPP1,PKD2,PLA2R1,DYNLL1,AZIN2,PKP2,DNM1L,TGFB2,FEZ1,GSK3B,AP2M1,CASQ1,SERPINE1,TFR2,PDCD10,HTT,CLTCL1,PRKCI,KCNA1,CYP19A1,ATAD1,STX18,B3GAT3,HAP1,NLGN2,STIM2,LMAN2,NPPB,MSN,STX1B,KCNMB1,DOCK2,TMED10,TGFB3,CACNA1G,MYH10,CALM1,SYTL4,VMP1,SCAMP5,TRPV2,ACSL4,RASL10B,PDGFRB,MIEF2,AKT1,ITGA2,MYOM1,PRKAA1,RAB31,MYO1C,BCAP31,ABCA3,FLNA,ATP6AP1,VAMP7,PARK7,AHCYL1,AP1G1,WLS,DMD,CD151,GDNF,THY1,TMSB4X,LEPROT,ZPR1,TOMM7	darkgreen	EC_M4
GO:0051984	1.57E-05	0.999999196	7	11	positive regulation of chromosome segregation	BP	7.22E-04	SMC6,RAD18,CCNB1,HNRNPU,CDC6,AURKB,CDT1	darkgrey	EC_M8

GO:2001141	1.60E-05	0.9999887	288	2416	regulation of RNA biosynthetic process	BP	7.33E-04	ONECUT2,BCLAF1,PPIL4,NUP107,DDX39B,ZNF608,SCAF8,RAD21,HSF2,ATAD2,XRCC6,PHF5A,PSIP1,IFT74,PWP1,ZNF300,TOPORS,MTERF1,RFX3,CKS2,TRIM28,ZNF451,FOXJ3,ZNF189,SP4,CDCA7L,PHIP,ZMPSTE24,RLF,ZNF292,XPO1,COMMD1,CASP8AP2,ZNF350,ITGB1BP1,THRAP3,EZH2,UFL1,CCNC,DDX1,NAA15,ELF2,ATAD2B,CDCA7,HDAC2,CCNA2,EXOSC9,MLLT10,DNAJC2,SETDB2,NUP85,ELF1,RSF1,CCNT2,PAXIP1,WDR43,BIRC5,POLR3F,EPC2,SUPT16H,CHD8,KAT6A,RIF1,E2F7,ALYREF,CKAP2,CEP290,NSD3,CEBPZ,SUPT20H,TAF7,NR2C1,BRCA2,ELK3,RRP8,TCERG1,MAPK8,PRKDC,CBX5,ERCC6,ZNF214,GPBP1,SP1,TCEA1,ZNF664,ZC3H8,SGK3,BTAF1,ZNF143,CENPJ,FER,TDG,NCK1,SBNO1,SMARCA5,RNF141,ZNF507,CHD1,ZFP91,TAF9,CGGBP1,TFCP2,SAP30,GCFC2,UTP15,HLTF,ZBTB10,CNOT1,GABPA,MCRS1,USP16,PPID,RBL1,CCNL1,SENP1,RIPK1,WDR75,ARID2,MYBL2,E2F8,TRIM33,INO80,ZBED6,CHUK,ZBTB11,ZNF248,MGA,MDM4,HIVEP1,BRWD1,NOLC1,BMPR1B,UBE3A,PRDM10,NUCKS1,DEK,GCN1,CUL3,GMNN,CHEK1,H2AFZ,RRP1B,NFKB1,KHDRBS1,TRIP13,TET2,TRIP11,HEATR1,NCL,TRAIP,ZNF184,PRMT5,AJUBA,GTF2IRD2,ZBTB41,ARID4B,NOL11,GTF2I,TARBP1,BPTF,DDX5,ORC2,EWSR1,TIAL1,BLM,CENPF,EDRF1,PDE8A,ING5,BCLAF3,FOSL1,SMARCC1,BAZ1B,SP3,DHX9,HDGFL3,BAZ1A,SETD2,ZFX,PSMA6,DDX20,TPR,CCAR1,UBTF,NMD3,XRCC5,NPAT,BRCA1,CNOT9,NSD2,HP1BP3,DCAF6,ZNF518B,DHX33,TBL1XR1,SUV39H1,LEO1,FOXM1,LYAR,DDX11,FAM220A,HNRNPU,NEDD4,SMARCE1,RFX7,RFC1,TOP2A,LIN9,NSD1,SLTM,MED6,ICE2,PMF1,FUS,YY1AP1,USP3,AURKB,ZNF609,TAF1,OGT,RLIM,DENND4A,ATRX,MB	darkgrey	EC_M8
GO:0032496	5.18E-06	0.999997862	47	225	response to lipopolysaccharide	BP	7.44E-04	TNFRSF11A,TNFAIP3,AKAP12,CSF2RB,IL6,CXCL8,PELI1,TLR4,NOCT,NOS3,TIMP4,IRAK2,EDNRB,NFKBIB,CD36,SCARB1,IL1A,NR4A1,ERBIN,MEF2C,TLR2,CXCL10,EDN1,SNCA,NFKB2,HDAC1,PABPN1,CD86,IL10RA,LTF,SELE,SELP,ADIPOR2,TNFRSF1B,S100A14,CYP1A1,TNIP1,CCL2,PDE4B,LGALS9,NOS2,GIT1,NOTCH1,IRF8,PLCG2,DAB2IP,BMP6	turquoise	EC_M6
GO:0050776	5.22E-06	0.999997309	92	538	regulation of immune response	BP	7.44E-04	TNFAIP3,GRAMD4,PTK2,C6orf106,LRRC19,DDX58,TEC,KIT,TNFRSF21,SYK,IL6,PELI1,RSAD2,TLR4,C5,IL15,TRPM4,PUM2,GATA2,PRKD2,NECTIN2,GAB2,UNC13D,PPARG,GATA3,IRAK2,SEC14L1,IKBKB,TMEM173,CD36,MAP3K1,PLK2,PDE4D,ITCH,ERBIN,MEF2C,TLR2,OAS3,NLRC5,PLCG1,DGKZ,IFIT1,STAT1,CD200,LCK,ICOSLG,LAPTM5,HLX,NCF1,NFATC2,INPP5D,TIFA,CD86,BMX,FGR,FES,PLEKHA1,PAK2,B2M,LTF,MASP1,DDX3X,BIRC3,WAS,PRKCH,PLD2,TLR6,TNFRSF1B,ARG2,PSEN1,EIF2B2,CD19,S100A14,TNIP1,YES1,PDE4B,LGALS9,IL7R,ISG15,TRAF2,PLCG2,STXBP1,DAB2IP,FYB1,OAS1,TLR1,GBP5,CD46,TRIM62,FCER2,SERPINB9,RNF125	turquoise	EC_M6
GO:0061448	5.29E-06	0.999997759	52	203	connective tissue development	BP	7.76E-04	COL11A2,PPARD,LEP,SRF,RUNX2,TGFBR1,GLI3,PGRMC2,FGF2,GLI2,TGFB1,CTNNB1,DGAT2,SERPINH1,RARB,LUM,DDRGK1,IMPAD1,SULF1,PTPN11,LOXL2,GDF6,BMP1,RUNX1,CHSY1,HYAL1,LRP5,SATB2,PIK3CA,BMP7,ARID5B,COMP,FGFR3,PRRX1,TIMP1,ACTA2,EVC,BMPR1A,PPARGC1A,THBS3,COL1A1,PBXIP1,SPTLC2,ANXA6,PDGFRB,PKD1,COL5A1,COL11A1,GLG1,LOX,OTOR,CHST11	darkgreen	EC_M4
GO:0090503	1.73E-05	0.999996727	14	42	RNA phosphodiester bond hydrolysis, exonucleolytic	BP	7.91E-04	PAN2,PNPT1,EXOSC9,DIS3,XRN2,EXOSC8,PAN3,XRN1,PDE12,CNOT1,DCP2,EXOSC10,DIS3L,EXOSC2	darkgrey	EC_M8
GO:0044267	1.77E-05	0.999986913	413	3725	cellular protein metabolic process	BP	8.03E-04	CDK2,PAN2,NARS,MEX3C,CLK4,USP15,SHPRH,PPIL4,LATS1,DDX39B,FBXO43,FBXO5,CSNK1G3,UBR5,EIF3E,MAP3K4,DSCC1,MRPL13,XRCC6,CDC25C,TNRC6B,TUT7,NAA35,SRPK1,UHRF2,PPIL1,PLEC,UBR2,PWP1,TOPORS,DBF4,AKAP9,PUM3,CKS2,C1GALT1,ICK,MELK,TRIM28,NCBP1,ZNF451,ANP32B,LRPPRC,PPIH,FBXO11,SENP6,ASB3,PHIP,MTIF2,TTK,PPP4R3B,IBTK,PNPT1,ZMPSTE24,RLF,USP34,SYNCRIP,XPO1,PABPC4,COMMD1,CASP8AP2,DPY19L1,FKBP15,ETAA1,FBXO48,TIA1,ITGB1BP1,EZH2,UFL1,CUL1,USP45,MRPL32,CLSPN,ASCC3,CASP2,DDX1,NAA15,AFG1L,WDR35,NRDC,PLK4,DHFR,PUS7,HDAC2,CCNA2,EXOSC9,PMPCB,SAE1,CEP295,CHORDC1,SETDB2,SYMPK,MASTL,YME1L1,COG3,CNOT10,MRPS7,CAD,ALG8,PPM1G,BORA,DIS3,CCNT2,PAXIP1,DARS,ULK4,NCAPG2,CDC20,BIRC5,NKTR,FNTA,PARP2,KAT14,GTPBP4,RAD18,IPO5,EPC2,KAT6A,METTL3,RIF1,ZDHHC17,VPS36,TPP2,NSD3,KITLG,TAF7,EXOSC8,PRKD3,CUL4A,METAP2,BRCA2,PPP4R2,LARS,LTA4H,PSMD13,CDC16,MAPK8,PRKDC,APAF1,CCNB1,ERCC6,DHX29,PAN3,SCYL2,LNX2,MRPL15,MRPS5,BUB1,TPX2,ANAPC1,CAPRIN1,UBA2,IPO7,TNKS2,LSM14A,SGK3,PARP4,CDK5RAP1,PPWD1,FER,EIF2B1,NCK1,IDE,ARFGEF1,MRPL35,RNF141,PTCD3,ABCE1,OTUD4,ZFP91,GALNT6,TAF9,XRN1,PDE12,ATR,LARP4,USP25,HLTF,PBK,ESCO2,CNOT1,ANAPC7,MCRS1,SH2B3,LTN1,USP16,PPID,MOB1A,RPS6,NAA25,CPNE3,BUB1B,CCNL1,SMYD5,CDCA2,NBN,SENP1,TOP1,ABRAXAS1,RAD51,RIPK1,PRPF4B,CCNE2,TRIM33,RIOK1,EEF1E1,CHUK,STK4,MORC3,UBE2C,GXYLT1,HERC5,TAOK3,OGA,SMARCAD1,NOLC1,BMPR1B,UBE3A,CIT,GCN1,CUL3,METAP1,ALG10,YARS,UBE2	darkgrey	EC_M8

GO:0000003	1.77E-05	0.999988891	137	1031	reproduction	BP	8.03E-04	CDK2,YTHDC2,NUP107,CCT2,FBXO43,FBXO5,MAP3K4,RAD21,HSF2,KIFC1,CDC25C,FANCC,TUT7,SRPK1,RMI1,UBR2,CKS2,PPAT,TRIM28,FOXJ3,MSH2,SMC2,YTHDC1,TTK,CCT4,CASP2,PLK4,HDAC2,CEP57,RAD54L,WDR33,NASP,CAD,ACOX1,FANCD2,XRN2,CDC20,METTL3,TOP2B,E2F7,SGO1,RRM1,KITLG,TUBGCP3,BRCA2,PSMD13,PRKDC,CCNB1,DHX37,ESPL1,BUB1,POLR1B,TERF1,MND1,RACGAP1,RPS6,MSH3,BUB1B,CCT7,NBN,KNL1,RAD51,SLC38A2,CCNE2,E2F8,STK4,NCAPD3,CCT5,BMPR1B,UBE3A,KIF18A,NSUN2,SNRPA1,SMC3,KHDRBS1,SLC9B1,TRIP13,SGO2,INTS13,ASPM,BPTF,SPO11,NEK2,EIF2B5,TIAL1,UMPS,DDX6,BUB3,CDK1,CDC25A,SEPT2,FOSL1,SMARCC1,SP3,NUF2,SETD2,ZFX,DDX20,FANCM,RPL10L,C17orf53,SMC4,CNOT9,NCAPD2,RAD51AP1,EXO1,INCENP,CCNB3,TOP2A,LIN9,SMC1A,ASF1B,CCNB2,CCT3,EME1,ATRX,PTTG1,CENPI,PLK1,BRIP1,TOP3A,STAG2,NIPBL,SAFB2,NDC1,RPA1,PKMYT1,CCNF,DAZAP1,GAS8,FANCA,ODF2,DHODH,HIST1H1A,AURKA,RNF8,PAIP2	darkgrey	EC_M8
GO:0071345	5.76E-06	0.99999699	96	574	cellular response to cytokine stimulus	BP	8.13E-04	TNFRSF11A,IFNGR1,TNFAIP3,AKAP12,TCF7,DAPK1,PDGFB,MAPK13,CSF2RB,KIT,TNFRSF21,NFIL3,SYK,IL6,CXCL8,TLR4,TRAF1,IL15,LRCH1,ZFAND6,PPARG,GATA3,CXCR4,IRAK2,IKBKB,PDE2A,TMEM173,SFRP1,TNFRSF19,IL1A,KDM3A,ERBIN,OCLN,GPD1,TLR2,IL7,VEGFC,CXCL10,OAS3,NLRC5,ST3GAL6,HIPK1,IFIT2,IFIT1,EDN1,STAT1,CD58,JARID2,MX1,HDAC1,HYAL2,LAPTM5,FABP4,HCLS1,IL10RA,CREB1,EIF4A2,SSTR1,CCRL2,IL1RAP,ADAMTS7,ITGA4,BIRC3,PLVAP,FGF23,WAS,HIF1A,ADIPOR2,TNFRSF1B,RPS6KA5,YY1,CCL2,IL13RA1,JAK1,LGALS9,NOS2,IL7R,ISG15,TRAF2,TNFRSF25,NOTCH1,IRF8,STXBP1,MTF2,NFAT5,DAB2IP,MIR99A,OAS1,GBP5,RAB43,SOX17,ACKR1,COMMD7,VAMP3,IFIT3,RNF125	turquoise	EC_M6
GO:0034394	5.70E-06	0.999998573	22	59	protein localization to cell surface	BP	8.28E-04	ANGPT1,GGA1,PICALM,CTNNB1,GPD1L,RIC3,TM9SF4,AP3B1,FBLN5,SMURF1,VCL,RAB11A,AKT1,TAX1BP3,FLNA,KCNAB2,PIGK,FCN1,LEPROT,MESD,MAP1A,FGF10	darkgreen	EC_M4
GO:0072657	5.81E-06	0.99999697	98	466	protein localization to membrane	BP	8.36E-04	LRP1,STX7,HSPA4,PACSIN1,REEP2,SIL1,GGA1,SRP72,FAM126A,TPBG,COX18,EMC10,ROCK2,VPS35,SLC7A11,ITGB1,LIN7B,KIF5B,SEC61A1,RAB7A,PICALM,PAK1,TGFB1,SRP68,RAB3GAP1,AKT2,ATP2C1,PRNP,ARL6IP5,VPS37A,TM9SF4,TMED2,TMEM150A,VAMP8,SLMAP,ATP1B3,TRAM1,EMC4,SORBS1,GLRB,RAPGEF2,TM9SF3,CACNB3,CPE,SSR3,AP3B1,ZFYVE27,CHP1,ATP2B4,HPS6,ARL3,SRPRA,PKP2,MMP14,DAG1,AP2M1,GORASP2,FZD9,SRP54,LRRC15,SEC23A,GPC2,PRKCI,SMURF1,TNIK,EMC1,EXOC5,BLZF1,ATAD1,ZDHHC4,MYO5A,IQSEC2,NLGN2,TSPAN17,STX1B,SSR2,RAB11A,TTC8,CALM1,CLTC,CYP46A1,MIEF2,ROCK1,AKT1,ZDHHC9,RAB31,RAB34,MYO1C,FLNA,BSG,HSPA5,C16orf70,THY1,LHFPL4,EMC7,ARFRP1,TIMM22,MESD	darkgreen	EC_M4
GO:0032467	1.88E-05	0.999997413	11	27	positive regulation of cytokinesis	BP	8.48E-04	KIF20B,RACGAP1,PKP4,CIT,CUL3,KIF14,ECT2,CDC6,AURKB,KIF23,PKN2	darkgrey	EC_M8
GO:0070202	1.88E-05	0.999998914	7	12	regulation of establishment of protein localization to chromosome	BP	8.48E-04	CCT2,CCT4,TERF1,CCT7,CCT5,CCT6A,CCT3	darkgrey	EC_M8
GO:1901992	1.94E-05	0.999994944	19	73	positive regulation of mitotic cell cycle phase transition	BP	8.70E-04	FBXO5,CDC25C,CUL4A,CDC16,CCNB1,ESPL1,ANAPC7,UBE2C,CUL3,DTL,CDK1,CDC25A,CDC27,CDCA5,DBF4B,DLGAP5,CDC6,CDT1,TMOD3	darkgrey	EC_M8

GO:0010604	1.96E-05	0.999985848	304	2586	positive regulation of macromolecule metabolic process	BP	8.78E-04	PAN2,ONECUT2,BCLAF1,LATS1,CCT2,DDX39B,SCAF8,UBR5,EIF3E,MAP3K4,DSCC1,HSF2,ATAD2,XRCC6,PHF5A,TNRC6B,TUT7,IFT74,POT1,PWP1,ZNF300,TOPORS,DBF4,POLR3B,AKAP9,RFX3,IL1R1,CKS2,TRIM28,NCBP1,ZNF451,ANP32B,FOXJ3,MSH2,PHIP,TTK,HNRNPA2B1,PNPT1,ZMPSTE24,RLF,SYNCRIP,ZNF292,CCT4,COMMD1,CASP8AP2,ETAA1,ITGB1BP1,THRAP3,EZH2,CCNC,CLSPN,CASP2,DDX1,NAA15,ELF2,WDR35,NRDC,ATAD2B,HDAC2,CCNA2,EXOSC9,MLLT10,SAE1,CEP295,DNAJC2,SYMPK,NUP85,ELF1,RSF1,BORA,DIS3,CCNT2,PAXIP1,WDR43,CDC20,FNTA,POLR3F,SUPT16H,CHD8,KAT6A,METTL3,RIF1,E2F7,ALYREF,CKAP2,CEP290,NSD3,CEBPZ,KITLG,TAF7,EXOSC8,RFC3,BRCA2,ELK3,TCERG1,MAPK8,PRKDC,APAF1,CCNB1,ERCC6,DHX29,PAN3,SCYL2,GPBP1,SP1,TCEA1,TPX2,ZC3H8,DIMT1,UBA2,TNKS2,ZNF143,CENPJ,CDK5RAP1,NCK1,IDE,ARFGEF1,SMARCA5,ZNF507,OTUD4,ZFP91,TAF9,PDE12,TFCP2,ATR,LARP4,UTP15,HLTF,CNOT1,GABPA,MCRS1,USP16,MOB1A,CCT7,CDCA2,NBN,SENP1,ABRAXAS1,RAD51,SLC38A2,TOMM70,RIPK1,WDR75,ARID2,MYBL2,E2F8,INO80,RIOK1,CHUK,STK4,UBE2C,HIVEP1,RFC5,TAOK3,NOLC1,CCT5,BMPR1B,UBE3A,CCT6A,PRDM10,NUCKS1,GCN1,CUL3,CHEK1,TIMM17A,H2AFZ,RRP1B,NFKB1,KHDRBS1,CENPE,TET2,TRIP11,HEATR1,NCL,KIF14,AJUBA,NOL11,GTF2I,BPTF,FXR1,FANCI,DDX5,FANCB,RFC2,NEK2,EIF2B5,DTL,ALS2,BLM,EDRF1,CDK1,PDE8A,ING5,BCLAF3,FOSL1,SMARCC1,SP3,DHX9,HNRNPR,TRA2B,SETD2,FASTKD2,RFC4,DNA2,CCAR1,DDX21,FANCM,DBF4B,IL12A,UBTF,COP1,NMD3,XRCC5,NPAT,BRCA1,PSME3,CNOT9,NSD2,DCUN1D5,RAD51AP1,DCAF6,ECT2,DHX33,TBL1X	darkgrey	EC_M8
GO:0007063	2.00E-05	0.999998064	9	18	regulation of sister chromatid cohesion	BP	8.91E-04	RAD21,ESPL1,BUB1,CDCA5,DDX11,WAPL,ATRX,CTCF,FEN1	darkgrey	EC_M8
GO:0055082	6.37E-06	0.99999655	115	564	cellular chemical homeostasis	BP	9.08E-04	LRP1,SLC12A2,SLC39A7,SMDT1,PPARD,LGALS1,SLC39A4,SLC30A9,SRF,UNC13B,RTN4,SLC31A1,FKBP1B,FGF2,KIF5B,RAB7A,ATP7B,TPT1,DGAT2,C1QTNF1,SLC4A7,TMTC4,MAFG,STIM1,ATP2C1,IRS2,PRNP,ATP2B1,CCKBR,RIC3,CXCL12,TM9SF4,HSP90B1,OSBP,C19orf12,ATP1B3,RYR3,P2RX4,SELENOT,ATP2A2,APP,SLC4A5,CX3CL1,ABCC8,CACNB3,OPA1,AP3B1,CHP1,ATP2B4,PKD2,BRSK2,SLC39A10,CHRNA7,RAP1GDS1,DYNLL1,ZBTB20,LRP5,HTR2B,PIK3CA,ISCU,CMKLR1,HJV,SLAMF8,ADCY5,VAPB,WDTC1,CASQ1,CLIC4,TFRC,FZD9,SIDT2,GLRX3,TMCO1,ATP6AP2,TFR2,PTPN6,ITGAV,HTT,ERO1A,PTPRN,HAP1,CACNA1C,STIM2,PPARGC1A,NGFR,ADRA1B,GPR65,CLN6,CALM1,SMARCA4,GRIA1,NPTN,SLC39A6,ANXA6,TRPV2,PRKAA1,GNB1,BCAP31,PKD1,ATP6AP1,SYPL2,JPH3,SLC30A7,ATP6V0D1,DMD,FKBP1A,SERPINF1,BNIP3,TMEM38B,SLC22A17,THY1,TMEM38A,CCR6,ORMDL3,ATP6V0E1	darkgreen	EC_M4
GO:2000105	2.12E-05	0.99999886	7	11	positive regulation of DNA-dependent DNA replication	BP	9.46E-04	DBF4,E2F7,E2F8,INO80,DBF4B,ATRX,CDT1	darkgrey	EC_M8
GO:0036297	2.15E-05	0.999996203	13	37	interstrand cross-link repair	BP	9.56E-04	FANCC,DCLRE1C,FANCD2,ATR,RAD51,DCLRE1B,NUCKS1,FANCB,FANCM,C17orf53,RAD51AP1,FANCA,RNF8	darkgrey	EC_M8
GO:0009156	2.18E-05	0.999996836	11	30	ribonucleoside monophosphate biosynthetic process	BP	9.66E-04	PPAT,PAICS,CAD,GMPS,UMPS,PFAS,PRPS1,HPRT1,DHODH,RFK,GART	darkgrey	EC_M8
GO:0048646	6.87E-06	0.999995815	167	877	anatomical structure formation involved in morphogenesis	BP	9.69E-04	ATP8B1,AHI1,SLC12A2,PLXNB2,FBN2,ANGPT1,FAM19A5,PPARA,NUS1,RSPO3,ADGRB1,MYH9,LEP,SRF,VEGFA,PDCL3,CNNM4,COL15A1,TGFBR1,RTN4,EMC10,CALD1,WHRN,GLI3,ROCK2,INHBA,RRAS,MYLK3,ITGB1,FGF2,SRPK2,TENM4,GLI2,SMARCD3,TGFB1,CTNNB1,RELT,ACTG1,STIM1,SPINT2,DCN,STARD13,SEMA3C,ILK,SPINK5,ANKRD1,TMED2,SULF1,ROBO1,RDH10,FAIM2,HEY1,TLX2,TCTN1,MMP15,CX3CL1,ABCC8,PTPN11,OPA1,LOXL2,ACVR1,MMP2,MTDH,ADA,ATP2B4,RUNX1,NKD1,IFT57,CHRNA7,FAP,PHLDB2,TJP1,NOTCH2,CDC73,HYAL1,LMOD1,EYA2,TGFB2,ADGRB2,PRKAR1A,FAM20C,MMP14,DAG1,PIK3CA,LEF1,PRKCA,TCTA,COL6A1,BMP7,FGFR2,VASH2,CASQ1,ITGB5,PHLDB1,HTRA1,HSPB1,SERPINE1,FZD2,FN1,PTPN6,MEOX1,PDCD10,ITGAV,COL5A2,SKIL,SYNPO2L,WDR1,TULP3,SIX4,OBSL1,AKT3,BMPR1A,TCAP,MED1,ACTN1,NPPB,NGFR,TPM1,THBS3,COL1A1,CHAD,SHC1,MAP2K1,SOX30,TMEM100,PKM,DICER1,ANGPTL6,LAMA3,PDGFRB,ROCK1,FLII,AKT1,ITGA2,SUPT6H,MYDGF,PAFAH1B1,FLNA,BSG,SARS,COL5A1,HSBP1,CDH13,COL11A1,F3,LMO4,BCL10,WLS,MIR24-1,DUSP2,FKBP1A,CCDC136,SPINK2,SERPINF1,ADAMTS5,GDNF,CSRP1,THY1,EDA,DUSP4,HOPX,PIK3C2A,OLFM1,WNT2,COL8A2,CFL2,FGF10	darkgreen	EC_M4

GO:0070199	2.20E-05	0.999997282	10	24	establishment of protein localization to chromosome	BP	9.70E-04	CCT2,POT1,CCT4,BRCA2,TERF1,CCT7,CCT5,CCT6A,CCT3,NIPBL	darkgrey	EC_M8
GO:0035966	7.10E-06	0.999997248	39	143	response to topologically incorrect protein	BP	9.93E-04	HSPA4,VCP,NCK2,CREB3,ERP44,ERLEC1,UBXN4,SERPINH1,STT3B,TMTC4,DDRGK1,HSPH1,TMED2,TRAM1,AMFR,CHAC1,SELENOS,CCND1,HSPA8,VAPB,ATF3,ATF6,RHBDD2,EDEM3,HSPB1,DNAJB12,COMP,ERO1A,JKAMP,HSPB7,EIF2S1,HSPB3,PTPN2,MBTPS1,HSPA5,HSPB2,DNAJB9,HSPA13,DERL2	darkgreen	EC_M4
GO:0002520	7.13E-06	0.999996116	114	694	immune system development	BP	9.97E-04	TNFRSF11A,TNFAIP3,FLT4,NOTCH4,TCF7,L3MBTL3,ATF4,PIM1,MFNG,TEK,IREB2,ADGRF5,KIT,KDR,NFIL3,SYK,IL6,PTBP3,RSAD2,TLR4,IL15,ZBTB24,TAL1,GATA2,SNRK,GAB2,PPARG,GATA3,JAG1,SFRP1,ASH2L,TMEM178A,TCF15,RET,MERTK,CHD7,IL1A,HHEX,ITCH,SH3RF1,MEF2C,TLR2,IL7,TPD52,LMBR1L,ADGRG3,TIPARP,HIPK1,DLL4,SOX13,TSPAN2,STAT1,JAM3,ALAS1,JARID2,NFKB2,DCAF1,ETS1,LCK,HYAL2,PRKX,HLX,DYRK3,HCLS1,INPP5D,CD34,CD86,FES,MMP21,ACE,ETV6,CREB1,NBEAL2,B2M,LTF,ITGA4,SOS2,TBX1,MECOM,CRACR2A,HIF1A,EPHA2,ITPKB,LBR,LFNG,MTOR,PSEN1,HOXB7,SEMA4A,HOXB8,BATF,CD19,GPR68,YY1,LYL1,RCOR1,JAG2,LGALS9,IL7R,ISG15,AP3D1,NRARP,NOTCH1,IRF8,PLCG2,VNN1,SLC8A3,ARID4A,ATXN1L,CD46,CA2,CDK13,HIST1H4D,FLI1	turquoise	EC_M6
GO:0007179	7.30E-06	0.999997031	43	156	transforming growth factor beta receptor signaling pathway	BP	1.01E-03	FBN2,PPARA,CDKN2B,RASL11B,COL1A2,ASPN,TGFBR1,MAP3K7,ZYX,TGFB1,ZEB2,LTBP1,SPRED3,SMAD9,ZNF703,DKK3,ACVR1,SLC2A10,TGFB2,ITGB5,HTRA1,STRAP,FAM89B,FBN1,COL3A1,SKIL,SMURF1,BMPR1A,RNF111,TGFB1I1,LTBP2,TGFB3,MYOCD,PIN1,NLK,VASN,GLG1,HSPA5,FKBP1A,LOX,DUSP22,PMEPA1,CHST11	darkgreen	EC_M4
GO:0071363	8.13E-06	0.999995406	112	545	cellular response to growth factor stimulus	BP	1.12E-03	CD63,FBN2,PPARA,NUS1,PTP4A3,SH3GL2,CDKN2B,KIAA1324L,VEGFA,RUNX2,RASL11B,COL1A2,ASPN,TGFBR1,ADAMTS3,MAP3K7,KIDINS220,ZYX,FGF2,MEGF8,TGFB1,CTNNB1,ZEB2,LTBP1,CRIM1,SPRED3,SPINT2,DCN,SMAD9,ZNF703,ILK,ANKRD1,SULF1,DKK3,ROBO1,MAP1B,APP,RAPGEF2,SLC33A1,PTPN11,ZFYVE27,ACVR1,GDF6,ATP2B4,NDN,KDM5B,NOTCH2,HYAL1,SLC2A10,TGFB2,FEZ1,FSTL1,PIK3CA,LEF1,HJV,NTRK3,BMP7,FGFR2,ITGB5,HTRA1,SYAP1,IQGAP1,STRAP,FAM89B,HSPB1,MAPT,FBN1,COMP,FKBP8,COL3A1,SKIL,FGFR3,SMURF1,NBL1,GREM2,HAP1,BMPR1A,MED1,PPARGC1A,RNF111,FGFRL1,TGFB1I1,NGFR,LTBP2,COL1A1,TGFB3,SHC1,TMEM100,NPTN,MYOCD,PIN1,CHRDL1,PDGFRB,AKT1,NLK,MYO1C,VASN,GLG1,MTSS1L,HSPA5,DYNC1LI2,FSTL3,FKBP1A,PTPN12,LOX,HAS2,DUSP22,PMEPA1,ZPR1,CHST11,WNT2,FGF10	darkgreen	EC_M4
GO:0022414	2.54E-05	0.999983828	136	1029	reproductive process	BP	1.12E-03	CDK2,YTHDC2,NUP107,CCT2,FBXO43,FBXO5,MAP3K4,RAD21,HSF2,KIFC1,CDC25C,FANCC,TUT7,SRPK1,RMI1,UBR2,CKS2,PPAT,TRIM28,FOXJ3,MSH2,SMC2,YTHDC1,TTK,CCT4,CASP2,PLK4,HDAC2,CEP57,RAD54L,WDR33,NASP,CAD,ACOX1,FANCD2,XRN2,CDC20,METTL3,TOP2B,E2F7,SGO1,RRM1,KITLG,TUBGCP3,BRCA2,PSMD13,PRKDC,CCNB1,DHX37,ESPL1,BUB1,POLR1B,TERF1,MND1,RACGAP1,RPS6,MSH3,BUB1B,CCT7,NBN,KNL1,RAD51,SLC38A2,CCNE2,E2F8,STK4,NCAPD3,CCT5,BMPR1B,UBE3A,KIF18A,NSUN2,SNRPA1,SMC3,KHDRBS1,SLC9B1,TRIP13,SGO2,INTS13,ASPM,BPTF,SPO11,NEK2,EIF2B5,TIAL1,UMPS,DDX6,BUB3,CDK1,CDC25A,SEPT2,FOSL1,SMARCC1,SP3,NUF2,SETD2,ZFX,DDX20,FANCM,RPL10L,C17orf53,SMC4,CNOT9,NCAPD2,RAD51AP1,EXO1,INCENP,CCNB3,TOP2A,SMC1A,ASF1B,CCNB2,CCT3,EME1,ATRX,PTTG1,CENPI,PLK1,BRIP1,TOP3A,STAG2,NIPBL,SAFB2,NDC1,RPA1,PKMYT1,CCNF,DAZAP1,GAS8,FANCA,ODF2,DHODH,HIST1H1A,AURKA,RNF8,PAIP2	darkgrey	EC_M8
GO:0048771	8.10E-06	0.999997138	32	129	tissue remodeling	BP	1.12E-03	TNFRSF11A,TNFAIP3,FLT4,CTHRC1,SYK,IL6,IL15,NOS3,ACVR2B,TIE1,JAG1,SFRP1,CHD7,IL1A,MEF2C,IL7,P2RX7,THBS4,DLL4,PPP3CA,INPP5D,ACE,IGFBP5,RAC1,HIF1A,LIPA,EPHA2,SYT7,RAB3D,ADRB2,S1PR1,CA2	turquoise	EC_M6
GO:0031346	8.44E-06	0.999995957	70	302	positive regulation of cell projection organization	BP	1.15E-03	LRP1,RAB21,PLXNB2,PACSIN1,SRF,VEGFA,P3H1,TGFBR1,TMEM30A,AKIRIN1,ACTR2,KIDINS220,FKBP1B,PLXNA1,MARK4,MEGF8,PAK1,ACTR3,TRAK1,DZIP1,FAM98A,CDC42EP3,ILK,DPYSL3,CXCL12,ANKRD1,EIF4G2,ROBO1,MAP1B,DHX36,RAPGEF2,CX3CL1,OPA1,ARSB,ZFYVE27,TWF1,DNM1L,CAPRIN2,FEZ1,PIK3CA,AUTS2,CORO1C,NTRK3,BMP7,IQGAP1,MAPT,FN1,NCKAP1,GPC2,ARPC2,HTT,PRKCI,SKIL,OBSL1,HAP1,TGFB3,RAB11A,MAP2K1,NPTN,ISLR2,TRPV2,ITGA2,PLXNB3,PAFAH1B1,FLNA,PLXNA3,DEF8,HSPA5,SERPINF1,UBE2V2	darkgreen	EC_M4

GO:0010569	2.62E-05	0.999994841	14	43	regulation of double-strand break repair via homologous recombination	BP	1.15E-03	RIF1,PPP4R2,ERCC6,PARPBP,RAD51,ARID2,CHEK1,FANCB,RPA2,SETD2,RAD51AP1,FUS,SMCHD1,RMI2	darkgrey	EC_M8
GO:0006355	2.68E-05	0.999980715	286	2411	regulation of transcription, DNA-templated	BP	1.17E-03	ONECUT2,BCLAF1,PPIL4,NUP107,DDX39B,ZNF608,SCAF8,RAD21,HSF2,ATAD2,XRCC6,PHF5A,PSIP1,IFT74,PWP1,ZNF300,TOPORS,MTERF1,RFX3,CKS2,TRIM28,ZNF451,FOXJ3,ZNF189,SP4,CDCA7L,PHIP,ZMPSTE24,RLF,ZNF292,XPO1,COMMD1,CASP8AP2,ZNF350,ITGB1BP1,THRAP3,EZH2,UFL1,CCNC,DDX1,NAA15,ELF2,ATAD2B,CDCA7,HDAC2,CCNA2,EXOSC9,MLLT10,DNAJC2,SETDB2,NUP85,ELF1,RSF1,CCNT2,PAXIP1,WDR43,BIRC5,POLR3F,EPC2,SUPT16H,CHD8,KAT6A,RIF1,E2F7,ALYREF,CKAP2,CEP290,NSD3,CEBPZ,SUPT20H,TAF7,NR2C1,BRCA2,ELK3,RRP8,TCERG1,MAPK8,PRKDC,CBX5,ERCC6,ZNF214,GPBP1,SP1,TCEA1,ZNF664,ZC3H8,SGK3,BTAF1,ZNF143,CENPJ,FER,TDG,NCK1,SBNO1,SMARCA5,RNF141,ZNF507,CHD1,ZFP91,TAF9,CGGBP1,TFCP2,SAP30,GCFC2,UTP15,HLTF,ZBTB10,CNOT1,GABPA,MCRS1,USP16,PPID,RBL1,CCNL1,SENP1,RIPK1,WDR75,ARID2,MYBL2,E2F8,TRIM33,INO80,ZBED6,CHUK,ZBTB11,ZNF248,MGA,MDM4,HIVEP1,BRWD1,NOLC1,BMPR1B,UBE3A,PRDM10,NUCKS1,DEK,GCN1,CUL3,GMNN,CHEK1,H2AFZ,RRP1B,NFKB1,KHDRBS1,TRIP13,TET2,TRIP11,HEATR1,NCL,TRAIP,ZNF184,PRMT5,AJUBA,GTF2IRD2,ZBTB41,ARID4B,NOL11,GTF2I,TARBP1,BPTF,DDX5,ORC2,EWSR1,TIAL1,BLM,CENPF,EDRF1,PDE8A,ING5,BCLAF3,FOSL1,SMARCC1,BAZ1B,SP3,DHX9,HDGFL3,BAZ1A,SETD2,ZFX,PSMA6,DDX20,TPR,CCAR1,UBTF,XRCC5,NPAT,BRCA1,CNOT9,NSD2,HP1BP3,DCAF6,ZNF518B,DHX33,TBL1XR1,SUV39H1,LEO1,FOXM1,LYAR,DDX11,FAM220A,HNRNPU,NEDD4,SMARCE1,RFX7,RFC1,TOP2A,LIN9,NSD1,SLTM,MED6,ICE2,PMF1,FUS,YY1AP1,USP3,AURKB,ZNF609,TAF1,OGT,RLIM,DENND4A,ATRX,MBTD1,ZNF	darkgrey	EC_M8
GO:0098534	2.73E-05	0.999994689	14	40	centriole assembly	BP	1.19E-03	RTTN,CDK2,PLK4,STIL,CEP295,WDR62,CENPJ,ALMS1,CEP72,BRCA1,CEP152,TRIM37,CEP76,SASS6	darkgrey	EC_M8
GO:1903506	2.75E-05	0.999980161	286	2412	regulation of nucleic acid-templated transcription	BP	1.20E-03	ONECUT2,BCLAF1,PPIL4,NUP107,DDX39B,ZNF608,SCAF8,RAD21,HSF2,ATAD2,XRCC6,PHF5A,PSIP1,IFT74,PWP1,ZNF300,TOPORS,MTERF1,RFX3,CKS2,TRIM28,ZNF451,FOXJ3,ZNF189,SP4,CDCA7L,PHIP,ZMPSTE24,RLF,ZNF292,XPO1,COMMD1,CASP8AP2,ZNF350,ITGB1BP1,THRAP3,EZH2,UFL1,CCNC,DDX1,NAA15,ELF2,ATAD2B,CDCA7,HDAC2,CCNA2,EXOSC9,MLLT10,DNAJC2,SETDB2,NUP85,ELF1,RSF1,CCNT2,PAXIP1,WDR43,BIRC5,POLR3F,EPC2,SUPT16H,CHD8,KAT6A,RIF1,E2F7,ALYREF,CKAP2,CEP290,NSD3,CEBPZ,SUPT20H,TAF7,NR2C1,BRCA2,ELK3,RRP8,TCERG1,MAPK8,PRKDC,CBX5,ERCC6,ZNF214,GPBP1,SP1,TCEA1,ZNF664,ZC3H8,SGK3,BTAF1,ZNF143,CENPJ,FER,TDG,NCK1,SBNO1,SMARCA5,RNF141,ZNF507,CHD1,ZFP91,TAF9,CGGBP1,TFCP2,SAP30,GCFC2,UTP15,HLTF,ZBTB10,CNOT1,GABPA,MCRS1,USP16,PPID,RBL1,CCNL1,SENP1,RIPK1,WDR75,ARID2,MYBL2,E2F8,TRIM33,INO80,ZBED6,CHUK,ZBTB11,ZNF248,MGA,MDM4,HIVEP1,BRWD1,NOLC1,BMPR1B,UBE3A,PRDM10,NUCKS1,DEK,GCN1,CUL3,GMNN,CHEK1,H2AFZ,RRP1B,NFKB1,KHDRBS1,TRIP13,TET2,TRIP11,HEATR1,NCL,TRAIP,ZNF184,PRMT5,AJUBA,GTF2IRD2,ZBTB41,ARID4B,NOL11,GTF2I,TARBP1,BPTF,DDX5,ORC2,EWSR1,TIAL1,BLM,CENPF,EDRF1,PDE8A,ING5,BCLAF3,FOSL1,SMARCC1,BAZ1B,SP3,DHX9,HDGFL3,BAZ1A,SETD2,ZFX,PSMA6,DDX20,TPR,CCAR1,UBTF,XRCC5,NPAT,BRCA1,CNOT9,NSD2,HP1BP3,DCAF6,ZNF518B,DHX33,TBL1XR1,SUV39H1,LEO1,FOXM1,LYAR,DDX11,FAM220A,HNRNPU,NEDD4,SMARCE1,RFX7,RFC1,TOP2A,LIN9,NSD1,SLTM,MED6,ICE2,PMF1,FUS,YY1AP1,USP3,AURKB,ZNF609,TAF1,OGT,RLIM,DENND4A,ATRX,MBTD1,ZNF	darkgrey	EC_M8
GO:0070601	2.81E-05	0.999998879	6	9	centromeric sister chromatid cohesion	BP	1.22E-03	SGO1,BUB1,BUB1B,NAA50,SGO2,CTCF	darkgrey	EC_M8
GO:0060968	2.86E-05	0.999993064	17	59	regulation of gene silencing	BP	1.24E-03	CDK2,TNRC6B,XPO5,TRIM28,ZMPSTE24,SETDB2,FAM208A,RIPK1,AJUBA,FXR1,DDX5,TIAL1,DDX6,DHX9,TPR,FMR1,HIST1H3H	darkgrey	EC_M8
GO:0009048	2.87E-05	0.999997756	8	14	dosage compensation by inactivation of X chromosome	BP	1.24E-03	YTHDC1,METTL3,BRCA1,HNRNPU,EXOSC10,RLIM,SMCHD1,LRIF1	darkgrey	EC_M8
GO:0010975	9.59E-06	0.999994964	83	376	regulation of neuron projection development	BP	1.29E-03	LRP1,RAB21,PLXNB2,PACSIN1,LGALS1,SRF,VEGFA,P3H1,TMEM30A,RTN4,MFSD2A,ACTR2,KIDINS220,FKBP1B,PLXNA1,MEGF8,PAK1,TRAK1,SEMA3C,ILK,DPYSL3,RYK,RNF6,CXCL12,ANKRD1,EIF4G2,ROBO1,MAP1B,DIP2B,SEMA4F,TLX2,DHX36,RAPGEF2,CX3CL1,ADCY6,OPA1,ARSB,ZFYVE27,SPP1,TWF1,SEMA4G,BRSK2,PDLIM5,DNM1L,CAPRIN2,FEZ1,GSK3B,NTRK3,BMP7,IQGAP1,KIF1A,TANC2,MAPT,TNR,FN1,GPC2,PRKCI,SKIL,PRRX1,TNIK,OBSL1,CRMP1,SRCIN1,STX1B,RAB11A,MAP2K1,NPTN,ISLR2,TRPV2,AKT1,PLXNB3,PAFAH1B1,FLNA,PLXNA3,NTNG2,HSPA5,SERPINF1,ITM2C,UBE2V2,THY1,CHN1,YWHAH,SDC2	darkgreen	EC_M4

GO:0051962	9.68E-06	0.999995621	54	216	positive regulation of nervous system development	BP	1.29E-03	LRP1,RAB21,PLXNB2,ADGRB1,SRF,VEGFA,TPBG,LRRC4B,ACTR2,GLI3,PLXNA1,TENM4,MEGF8,SMARCD3,RHEB,PAK1,TGFB1,CTNNB1,TRAK1,LIG4,ILK,CXCL12,EIF4G2,ROBO1,MAP1B,DHX36,CX3CL1,OPA1,AMIGO2,ZFYVE27,DNM1L,CAPRIN2,DAG1,MAPT,FN1,PRKCI,SKIL,OBSL1,HAP1,NLGN2,RAB11A,MAP2K1,DICER1,NPTN,ISLR2,TRPV2,PLXNB3,PAFAH1B1,PLXNA3,SERPINF1,LRRC24,UBE2V2,WNT2,LRRTM2	darkgreen	EC_M4
GO:0061564	9.71E-06	0.999995075	84	380	axon development	BP	1.29E-03	LRP1,RAB21,PLXNB2,ADGRB1,CTNNA1,SRF,VEGFA,RTN4,GLI3,ITGB1,FKBP1B,PLXNA1,KIF5B,PICALM,GLI2,MEGF8,PAK1,TRAK1,DCLK1,SEMA3C,ILK,RYK,RNF6,CXCL12,SLC25A46,PALLD,EIF4G2,ROBO1,MAP1B,DIP2B,SEMA4F,TCTN1,APP,PTPN11,ZFYVE27,CRTAC1,SPP1,SEMA4G,BRSK2,NDN,NOTCH2,CTTN,FEZ1,GSK3B,DAG1,AUTS2,ANOS1,BMP7,FGFR2,VASH2,MAPT,TNR,FN1,SKIL,VCL,CRMP1,NOTCH3,ENAH,NGFR,RAB11A,MAP2K1,TTC8,NPTN,ISLR2,TRPV2,LAMA3,PLXNB3,PAFAH1B1,NTN3,FLNA,PLXNA3,BSG,NTNG2,BARHL2,NEXN,DPYSL5,VSTM2L,GDNF,THY1,CHN1,ARTN,ZPR1,MAP1A,OLFM1	darkgreen	EC_M4
GO:0051716	1.01E-05	0.999992195	775	5042	cellular response to stimulus	BP	1.32E-03	CD63,IL23A,LRP1,AP3S1,HBS1L,AHI1,ARHGEF25,CANX,TAB2,DYRK2,IER3,RAB21,IPCEF1,RRM2B,SLC12A2,PLXNB2,FBN2,ANGPT1,FAM19A5,PPARA,ROS1,NUS1,RSPO3,CXCL14,FAM49B,HABP4,GNG5,PTP4A3,ADGRB1,LY6E,SCUBE3,PPARD,CTNNA1,TEAD3,CBY1,MKLN1,UBQLN1,MTCH1,LGALS1,KLHDC10,TXN2,SH3GL2,GNAQ,SLC39A4,MYH9,ARHGAP39,LEP,CDKN2B,PLAA,RORB,BTBD11,SLC30A9,KIAA1324L,SRF,BAG1,NUAK1,CDK14,VEGFA,DOCK8,VCP,SLC35B2,RUNX2,RASL11B,UNC13B,COL1A2,C2orf40,NCK2,CLOCK,EXOC1,CREB3,ASNS,PDCL3,REST,ASPN,INPP4A,DST,SNX13,COL15A1,TGFBR1,ERP44,SOCS5,RIMS1,ERLEC1,PSME4,COPS9,UBA6,RTN4,TPBG,SHANK1,ADAMTS3,EMC10,MFSD2A,GGCT,UBE2J1,MAP3K7,SH3BP5L,AKIRIN1,ACTR2,SLC31A1,KIDINS220,MDFIC,GLI3,ROCK2,INHBA,VPS41,RRAS,ZYX,RCN3,MYLK3,DKKL1,VPS35,CUL2,MGARP,SLC7A11,ITGB1,PGRMC2,PLEKHA4,FKBP1B,PLXNA1,FGF2,SRPK2,SELENOW,KIF5B,SESN3,RAB7A,ARHGEF4,APBB1IP,RB1,ESD,MARK4,TPT1,EIF2B3,PTCH2,TENM4,DGKH,TMUB1,KCTD21,GLI2,MEGF8,RHEB,SNX17,PAK1,ACTR3,TGFB1,AXL,RAB3GAP1,SCN5A,WDR48,HTR5A,UBXN4,SEC13,CTNNB1,IL15RA,LBH,DGAT2,AKT2,DYNC1LI1,GPD1L,STT3B,DZIP1,PFKP,ZEB2,LGALS3BP,CANT1,C1QTNF1,ARHGEF17,OSBPL8,CXXC5,CLPB,PSD2,RARB,ACTG1,NPLOC4,ACTN4,PPP1R12A,ANAPC15,STAM2,KAT2B,RNF121,LTBP1,TMTC4,CRIM1,SPRED3,GPS1,SPINT2,UFM1,LIG4,ATP2C1,IRS2,TMTC3,PRNP,CHFR,ATP2B1,DCN,CDC42EP3,SMAD9,HDAC3,ZNF703,DCLK1,ACPP,PCLO,STARD13,KCTD16,SEMA3C,CCKBR,CAVIN3,UBA5,DDRGK1,SMPD1,PPP2CB,ILK,RBPMS,GPR84,LEPROTL1,	darkgreen	EC_M4
GO:0050772	1.01E-05	0.999997154	24	68	positive regulation of axonogenesis	BP	1.32E-03	LRP1,PLXNB2,SRF,VEGFA,PLXNA1,MEGF8,PAK1,TRAK1,ILK,CXCL12,EIF4G2,ROBO1,MAP1B,ZFYVE27,MAPT,FN1,SKIL,RAB11A,MAP2K1,ISLR2,TRPV2,PLXNB3,PAFAH1B1,PLXNA3	darkgreen	EC_M4
GO:0006268	3.07E-05	0.999997449	8	15	DNA unwinding involved in DNA replication	BP	1.32E-03	RECQL4,MCM2,MCM6,MCM4,RAD51,BLM,MCM7,RPA1	darkgrey	EC_M8
GO:0000902	1.04E-05	0.999993531	155	799	cell morphogenesis	BP	1.34E-03	LRP1,RAB21,PLXNB2,PACSIN1,ADGRB1,MKLN1,SH3GL2,MYH9,PLAA,TBCC,SRF,VEGFA,COL15A1,RIMS1,RTN4,TPBG,SHANK1,MFSD2A,MYH14,ACTR2,KIDINS220,WHRN,GLI3,BVES,ITGB1,PLXNA1,KIF5B,RB1,PICALM,GLI2,MEGF8,PAK1,AXL,CTNNB1,TRAK1,FARP1,ACTN4,SPINT2,CDC42EP3,DCLK1,SEMA3C,ILK,RYK,RNF6,AKIP1,CXCL12,FAT1,PALLD,FLNB,EIF4G2,ROBO1,MAP1B,DIP2B,SEMA4F,RASA1,TLX2,TCTN1,APP,DHX36,RAPGEF2,PTPN11,OPA1,AP3B1,ZFYVE27,SPP1,SEMA4G,BRSK2,PDLIM5,NDN,CHRNA7,DOCK10,NOTCH2,CTTN,DNM1L,CAPRIN2,TGFB2,FEZ1,GSK3B,DAG1,AUTS2,LEF1,CORO1C,ANOS1,BMP7,FGFR2,IQGAP1,SCFD1,KIF1A,CLIC4,TANC2,SIDT2,MAPT,PLOD3,TNR,FN1,NCKAP1,PTPN6,ITGAV,ARPC2,SKIL,SMURF1,NBL1,CAPZB,TNIK,VCL,WDR1,OBSL1,CRMP1,NOTCH3,ENAH,MED1,ACTN1,MSN,SRCIN1,NES,ARHGEF2,NGFR,TPM1,RAB11A,MYH10,MAP2K1,TTC8,DICER1,NPTN,ISLR2,TRPV2,SS18,LAMA3,ROCK1,MYO10,DAPK3,PLXNB3,PAFAH1B1,NTN3,FLNA,PLXNA3,RERE,BSG,NTNG2,CFDP1,BARHL2,NEXN,DPYSL5,FRY,VSTM2L,GDNF,THY1,HAS2,CHN1,ARTN,YWHAH,SDC2,SLC39A3,MAP1A,OLFM1	darkgreen	EC_M4
GO:0001945	9.95E-06	0.99999868	12	25	lymph vessel development	BP	1.35E-03	FLT4,SYK,ACVR2B,TIE1,VEGFC,PROX1,CLEC14A,TBX1,EPHA2,VASH1,TMEM204,SOX18	turquoise	EC_M6

GO:0043085	9.95E-06	0.999993925	138	875	positive regulation of catalytic activity	BP	1.35E-03	TNFRSF11A,TBC1D30,FLT4,GRAMD4,AFDN,TPD52L1,ASAP1,SGSM3,PTK2,DAPK1,PDGFB,TBC1D22B,TEK,KIT,KDR,SYK,SEMA4D,RGP1,CHN2,ARHGAP25,ASAP2,CAV2,TLR4,TBC1D9,CCNY,PIK3CG,ARHGAP42,TAL1,PRKD2,CAB39L,NOS3,RALB,USP6NL,PPARG,TBC1D4,NET1,RALGAPA2,TIE1,P2RY6,SFRP1,HBEGF,ARHGEF7,ARAP3,SIRT3,SCARB1,EPHB1,RET,MERTK,IGF1,EIF2AK3,HIP1R,SOX7,HMBOX1,MEF2C,VEGFC,MADD,SERINC5,PLCB2,RAPGEF3,NR4A2,DGKZ,NEDD9,EDN1,SNCA,CD200,LCK,ADPRH,LAPTM5,NCF1,PABPN1,CD86,FGR,CFLAR,CASP10,PROX1,PRKD1,TOR1AIP1,F11R,ACE,ITGA6,PAK2,DLG1,SIPA1,MAP3K11,LTF,ARHGAP27,BCR,EPHB4,DDX3X,RUNDC1,ELMOD1,SELE,TNFAIP8L3,FGF23,LMTK2,PPM1F,HIF1A,RCC2,EPHA2,BID,TLR6,TBC1D1,RGS14,MTOR,PSEN1,FGF18,TAOK2,ARHGEF15,EFNA1,CD19,PIK3R5,TBC1D8B,ADRB2,CCL2,LGALS9,NOS2,UNC119,SGSM2,TRAF2,RAPGEF1,S1PR1,TBC1D13,TERF2,DAB2IP,PRSS22,PMAIP1,RELN,ALKAL1,MMD,RGS4,FCER2,RGS16,TBC1D20,PERP,DAZAP2,COPS8,BMP2,PIFO	turquoise	EC_M6
GO:1903313	3.16E-05	0.999989756	24	105	positive regulation of mRNA metabolic process	BP	1.35E-03	PAN2,TNRC6B,TUT7,NCBP1,PNPT1,THRAP3,EXOSC9,DIS3,METTL3,EXOSC8,CCNB1,PAN3,PDE12,CNOT1,NCL,HNRNPR,TRA2B,LEO1,DCP2,RBMX,METTL16,DAZAP1,EXOSC2,HNRNPD	darkgrey	EC_M8
GO:0120035	1.08E-05	0.99999388	108	524	regulation of plasma membrane bounded cell projection organization	BP	1.38E-03	ATP8B1,LRP1,RAB21,PLXNB2,PACSIN1,LGALS1,SRF,VEGFA,P3H1,TGFBR1,TMEM30A,RTN4,MFSD2A,AKIRIN1,ACTR2,KIDINS220,VPS35,FKBP1B,PLXNA1,MARK4,MEGF8,PAK1,ACTR3,TRAK1,DZIP1,FAM98A,PRNP,CDC42EP3,SEMA3C,ILK,DPYSL3,RYK,RNF6,CXCL12,ANKRD1,EIF4G2,ROBO1,MAP1B,DIP2B,SEMA4F,PFN2,TLX2,DHX36,RAPGEF2,CX3CL1,ADCY6,HOMER1,OPA1,ARSB,ZFYVE27,SPP1,TWF1,SEMA4G,BRSK2,PDLIM5,DNM1L,CAPRIN2,FEZ1,GSK3B,PIK3CA,AUTS2,CORO1C,NTRK3,BMP7,IQGAP1,KIF1A,TANC2,MAPT,TNR,FN1,NCKAP1,GPC2,ARPC2,HTT,PRKCI,SKIL,PRRX1,CAPZB,TNIK,PLD1,OBSL1,CRMP1,HAP1,SRCIN1,STX1B,TGFB3,RAB11A,MAP2K1,NPTN,ISLR2,TRPV2,EVI5L,AKT1,MYO10,PLXNB3,PAFAH1B1,FLNA,PLXNA3,DEF8,NTNG2,HSPA5,SERPINF1,ITM2C,UBE2V2,THY1,CHN1,YWHAH,SDC2	darkgreen	EC_M4
GO:0023056	1.09E-05	0.999992666	224	1258	positive regulation of signaling	BP	1.38E-03	CD63,IL23A,LRP1,TAB2,ANGPT1,RSPO3,PTP4A3,PPARD,CTNNA1,LGALS1,KLHDC10,LEP,CDKN2B,KIAA1324L,VEGFA,VCP,SLC35B2,NCK2,TGFBR1,RIMS1,RTN4,TPBG,SHANK1,ADAMTS3,EMC10,MAP3K7,ROCK2,INHBA,BVES,VPS35,ITGB1,PLEKHA4,FGF2,KIF5B,RHEB,PAK1,TGFB1,AXL,RAB3GAP1,SEC13,CTNNB1,DYNC1LI1,ZEB2,CANT1,C1QTNF1,OSBPL8,CXXC5,ACTN4,ATP2C1,IRS2,PRNP,DCN,HDAC3,ACPP,CAVIN3,ILK,RBPMS,ARL6IP5,RYK,TMEM9B,ST5,SLC20A1,ANKRD1,PJA2,CD180,OSBP,SULF1,VAMP8,ROBO1,SORBS1,LOXL3,P2RX4,RNF13,SELENOT,APP,DHX36,RAPGEF2,CX3CL1,CACNB3,PTPN11,PFKM,HOMER1,OPA1,PTPN22,ACVR1,GDF6,SERINC3,MTDH,ADA,SPP1,PKD2,PLA2R1,NKD1,SLC39A10,CHRNA7,DYNLL1,ADIPOR1,NOTCH2,CDC73,DNM1L,UCHL5,CHSY1,SLC2A10,CAPRIN2,TGFB2,HTR2B,ARMC9,GSK3B,PIK3CA,AUTS2,LEF1,PRKCA,TLR5,KRAS,NTRK3,SPATC1L,BMP7,GOLT1B,ATF3,FGFR2,OSM,PEA15,NCSTN,IQGAP1,TFRC,ATF6,FZD9,PDIA3,ATP6AP2,TNR,TFR2,FN1,MPP2,MAOA,PTPN6,GPR137,PDCD10,ITGAV,ADRA2C,HTT,FKBP8,COL3A1,PRKCI,SKIL,SYNPO2L,ECE1,FGFR3,PRRX1,GORAB,TNIK,CYP19A1,OTUD5,EVC,AKT3,HAP1,MAGED1,BMPR1A,SLC2A4,NLGN2,MED1,RNF111,STX1B,TGFB1I1,NGFR,COL1A1,TGFB3,SHC1,PBXIP1,ADRA1B,MAP2K1,GPR65,TSPAN6,SMARCA4,SLC44A2,GSKIP,NPTN,CYP46A1,MYOCD,PIN1,ACSL4,CD74,RASL10B,PDGFRB,ROCK1,ITGA2,CRLF3,RAB34,PTPN2,CLEC16A,MYDGF,DAPK3,MYO1C,BCAP31,PAFAH1B1,CSNK1G2,IRAK1,FLNA,ATP6AP1,IKBKG,VAMP7,PARK7,CDH13,F3,BCAR3,BCL10,WLS,MIR24-1,CCL16,FKBP1A,AGO3,ITM2C,C18orf32,TIMP3,EDA,	darkgreen	EC_M4
GO:0030001	1.16E-05	0.999993128	118	587	metal ion transport	BP	1.47E-03	CD63,SLC12A2,SLC22A4,SLC39A7,SMDT1,KCNK9,UBQLN1,SLC39A4,SLC30A9,CNNM4,SLC31A1,ITGB1,PGRMC2,MFSD4B,FKBP1B,FGF2,KIF5B,SEC61A1,PANX1,ATP7B,TPT1,SCN5A,CTNNB1,GPD1L,SLC4A7,SLC38A10,ACTN4,STIM1,ATP2C1,PRNP,ATP2B1,TUSC3,REM1,CXCL12,FXYD1,SLC20A1,SNTA1,SLMAP,ATP1B3,TRPC4AP,RYR3,P2RX4,ATP2A2,CACNA1D,SLC4A5,CX3CL1,ABCC8,CACNB3,HOMER1,OPA1,ARHGAP1,CHP1,ATP2B4,PKD2,NIPA1,SLC39A10,CHRNA7,MRS2,CNNM2,CALHM1,PKP2,DNM1L,HTR2B,ISCU,SHROOM2,CASQ1,TFRC,TMCO1,LINC02210-CRHR1,TFR2,PTPN6,KCNK4,ITGAV,HTT,PKD1L1,ERO1A,KCNA1,SLC3A2,ASIC4,SLC6A13,HAP1,CACNA1C,STIM2,MAGED2,KCNMB1,CACNA1G,CALM1,CLTC,VMP1,SLC39A6,ANXA6,TRPV2,PDGFRB,AKT1,SCNN1D,PKD1,FLNA,KCNAB2,HCN2,AHCYL1,JPH3,SLC30A7,MIR24-1,DMD,FKBP1A,CBARP,TMEM38B,SLC22A17,NMUR1,THY1,TMEM38A,YWHAH,SARAF,SLC39A3,NIPAL1,HCN4,GEM,KCNMB4	darkgreen	EC_M4

GO:0003012	1.20E-05	0.999993882	74	332	muscle system process	BP	1.49E-03	PPARA,LEP,SRF,TPM2,MYH14,CALD1,ROCK2,FKBP1B,PAK1,SCN5A,GPD1L,ATP2B1,FXYD1,SNTA1,SULF1,SLMAP,P2RX4,ATP2A2,CACNA1D,MYL9,ABCC8,HOMER1,ADA,ATP2B4,TWF1,PDLIM5,RAP1GDS1,CTTN,PKP2,PPP1R12B,LMOD1,HTR2B,DAG1,PIK3CA,PRKCA,CASQ1,GLRX3,ADRA2C,COMP,KCNA1,ACTA2,TPM4,CHRNE,CACNA1C,TCAP,PPARGC1A,TPM1,SGCA,CACNA1G,ADRA1B,CNN1,CALM1,SETD3,MYOCD,ANXA6,DTNA,PIN1,ROCK1,ITGA2,MYOM1,DAPK3,FLNA,ENO1,HSBP1,MIR24-1,DMD,TMEM38B,NMUR1,GDNF,TMEM38A,PIK3C2A,ORMDL3,HCN4,PRKG1	darkgreen	EC_M4
GO:0006336	3.53E-05	0.999995298	10	25	DNA replication-independent nucleosome assembly	BP	1.51E-03	NASP,MIS18A,OIP5,HJURP,HAT1,ASF1B,ATRX,UBN1,ITGB3BP,CENPW	darkgrey	EC_M8
GO:0000082	3.56E-05	0.999986073	32	160	G1/S transition of mitotic cell cycle	BP	1.52E-03	CDK2,LATS1,GTSE1,DBF4,PPAT,EZH2,CUL1,CASP2,NASP,E2F7,POLE,CUL4A,PRKDC,RBL1,RPS6,CCNE2,INO80,CUL3,KHDRBS1,KIF14,BCAT1,RCC1,RPA2,CDC25A,NPAT,CUL5,PSME3,CDKN3,CDC6,IQGAP3,CUL4B,GSPT1	darkgrey	EC_M8
GO:0007409	1.23E-05	0.999993647	77	341	axonogenesis	BP	1.52E-03	LRP1,RAB21,PLXNB2,ADGRB1,SRF,VEGFA,RTN4,GLI3,ITGB1,PLXNA1,KIF5B,PICALM,GLI2,MEGF8,PAK1,TRAK1,DCLK1,SEMA3C,ILK,RYK,RNF6,CXCL12,PALLD,EIF4G2,ROBO1,MAP1B,DIP2B,SEMA4F,TCTN1,APP,PTPN11,ZFYVE27,SPP1,SEMA4G,BRSK2,NDN,NOTCH2,CTTN,FEZ1,GSK3B,DAG1,AUTS2,ANOS1,BMP7,FGFR2,MAPT,TNR,FN1,SKIL,VCL,CRMP1,NOTCH3,ENAH,NGFR,RAB11A,MAP2K1,TTC8,NPTN,ISLR2,TRPV2,LAMA3,PLXNB3,PAFAH1B1,NTN3,PLXNA3,BSG,NTNG2,BARHL2,NEXN,DPYSL5,VSTM2L,GDNF,THY1,CHN1,ARTN,MAP1A,OLFM1	darkgreen	EC_M4
GO:0060560	1.26E-05	0.999994404	51	200	developmental growth involved in morphogenesis	BP	1.54E-03	LRP1,RAB21,SH3GL2,PLAA,SRF,VEGFA,RIMS1,RTN4,ITGB1,PLXNA1,MEGF8,PAK1,CTNNB1,DCLK1,SEMA3C,ILK,RYK,RNF6,CXCL12,EIF4G2,MAP1B,RDH10,DIP2B,SEMA4F,APP,ZFYVE27,SPP1,SEMA4G,NKD1,NDN,KDM5B,CTTN,GSK3B,AUTS2,FGFR2,IQGAP1,MAPT,TNR,FN1,SMURF1,VCL,SIX4,MED1,RAB11A,ISLR2,TRPV2,PAFAH1B1,PLXNA3,BARHL2,OLFM1,FGF10	darkgreen	EC_M4
GO:0010629	3.68E-05	0.999977603	106	766	negative regulation of gene expression	BP	1.56E-03	CDK2,PAN2,EIF3E,MRPL13,TNRC6B,TUT7,XPO5,TRIM28,NCBP1,HNRNPA2B1,PNPT1,PUS10,ZMPSTE24,SYNCRIP,PABPC4,TIA1,THRAP3,EZH2,WDR35,DHFR,PUS7,EXOSC9,RNASEH2B,SETDB2,CNOT10,UPF2,DIS3,XRN2,METTL3,EXOSC8,RRP8,PRKDC,CCNB1,PAN3,CAPRIN1,LSM14A,SMARCA5,XRN1,PDE12,TTC37,FAM208A,HELLS,CNOT1,RBL1,SMYD5,TENT2,RIPK1,NFKB1,NCL,TRAIP,AJUBA,FXR1,DDX5,TIAL1,FASTKD1,SSB,DDX6,CDK1,DHX9,HNRNPR,FASTKD2,TPR,DGCR8,IL12A,CNOT9,SUV39H1,EIF2AK1,HNRNPU,DCP2,IQGAP3,EXOSC10,FUS,TAF1,LARP1,ILF3,BRIP1,CSK,TAF15,THOC1,TYMS,SMCHD1,UPF3B,SUZ12,HNRNPM,SERBP1,XIAP,GSPT1,ZC3H7A,DROSHA,RBMX,FMR1,METTL16,RNPS1,PTBP1,EXOSC2,CTCF,RBM8A,SRRT,FASTKD5,HNRNPD,AURKA,HIST1H3H,SRSF7,RNF139,ERP29,PAIP2	darkgrey	EC_M8
GO:0051256	3.95E-05	0.999999335	5	6	mitotic spindle midzone assembly	BP	1.67E-03	RACGAP1,PRC1,KIF4A,AURKB,KIF23	darkgrey	EC_M8
GO:0010927	1.39E-05	0.999995482	28	87	cellular component assembly involved in morphogenesis	BP	1.68E-03	SRF,MYLK3,ITGB1,TENM4,ACTG1,ILK,ANKRD1,PHLDB2,LMOD1,PRKAR1A,DAG1,CASQ1,PHLDB1,SYNPO2L,WDR1,SIX4,OBSL1,TCAP,TPM1,SOX30,DICER1,PDGFRB,FLII,PAFAH1B1,CCDC136,SPINK2,CSRP1,CFL2	darkgreen	EC_M4

GO:0055085	1.40E-05	0.99999077	190	1047	transmembrane transport	BP	1.68E-03	CYB5A,CD63,ATP8B1,SLC12A2,AZIN1,ATP6V1C1,SLC22A4,CALHM5,SLC39A7,SLC35F1,SMDT1,KCNK9,PPARD,UBQLN1,SLC39A4,ATP6V1F,LEP,PEX6,SLC30A9,SLC35B2,CNNM4,SLC17A5,TMEM30A,SHANK1,MFSD2A,SLC35A1,SLC31A1,PCYOX1,SLC7A11,ITGB1,MFSD4B,FKBP1B,FGF2,KIF5B,SEC61A1,PANX1,SLC36A4,ATP7B,ANO10,ATP6V0B,SCN5A,AKT2,GPD1L,SLC4A7,OSBPL8,SLC38A10,ACTN4,STIM1,ATP2C1,IRS2,PRNP,ATP2B1,TMCO3,ARL6IP5,TUSC3,REM1,FXYD1,SLC20A1,SNTA1,SLC25A4,SLMAP,ATP1B3,TRAM1,TRPC4AP,RYR3,SORBS1,P2RX4,ATP2A2,CACNA1D,APP,GLRB,SLC4A5,CX3CL1,SLC33A1,ABCC8,CACNB3,PTPN11,SLC26A7,HOMER1,OPA1,CDH17,CHP1,ATP2B4,PKD2,NIPA1,ATP6V1B2,SLC39A10,CHRNA7,MRS2,CNNM2,AZIN2,CALHM1,SLC35A5,SELENOS,SLC2A10,CPT1A,HTR2B,ISCU,SHROOM2,CASQ1,PEA15,TOMM40L,CLIC4,SRP54,TMCO1,LINC02210-CRHR1,SLC25A16,PTPN6,KCNK4,ITGAV,HTT,PKD1L1,CLTCL1,ERO1A,PRKCI,KCNA1,VDAC2,SLC3A2,WDR1,ASIC4,SLC6A13,PRAF2,CHRNE,HAP1,CACNA1C,SLC29A4,SLC2A4,NLGN2,CLN8,STIM2,ATP6V1D,PPARGC1A,SFXN1,RHBG,SLC25A44,COX6A2,SLC7A3,SLC35B1,KCNMB1,TMED10,CACNA1G,TMEM63C,CALM1,VMP1,SLC44A2,GRIA1,SLC39A6,ANXA6,TRPV2,AKT1,ATP11C,SCNN1D,ABCA3,PKD1,FLNA,KCNAB2,ATP6AP1,PARK7,HCN2,SLC2A6,AHCYL1,JPH3,SLC30A7,SLC35A3,SLC2A8,HSPA5,ATP6V0D1,MIR24-1,DMD,SURF1,FKBP1A,CBARP,SLC36A1,TMEM38B,SLC22A17,THY1,TMSB4X,TMEM38A,YWHAH,TMEM109,TIMM22,GRPEL2,SLC39A3,NIPAL1,HCN4,TOMM7,ATP6V0E1,SLC35C1,GEM,KCNMB4	darkgreen	EC_M4
GO:1903828	1.41E-05	0.999995197	30	99	negative regulation of cellular protein localization	BP	1.68E-03	LMAN1,ANGPT1,CTNNA1,RAB23,ERLEC1,UBE2J1,MDFIC,ROCK2,PICALM,TGFB1,UFM1,DCLK1,TP53INP2,CHP1,GSK3B,AP2M1,FZD9,LRRC15,MAPT,C11orf65,MAGED1,CLTC,TMEM98,TAX1BP3,PARK7,TXN,LEPROT,UBE2G2,MAP1A,DERL2	darkgreen	EC_M4
GO:0051302	4.02E-05	0.999985063	29	138	regulation of cell division	BP	1.70E-03	KIF20A,ITGB1BP1,ORC6,PLK4,E2F7,PRPF40A,BRCA2,KIF20B,RACGAP1,RBL1,SMYD5,PKP4,E2F8,CIT,CUL3,KIF14,ASPM,PRC1,BLM,SETD2,KIF18B,ECT2,INCENP,CDC6,AURKB,KIF23,PLK1,PKN2,AURKA	darkgrey	EC_M8
GO:0030029	1.28E-05	0.999992487	113	662	actin filament-based process	BP	1.72E-03	PHACTR2,CELSR1,PARVG,PACSIN2,MTSS1,ARHGAP18,BAIAP2L2,PICK1,CDC42EP1,TEK,LIMCH1,PGM5,KIT,CD2AP,THSD7A,TMOD1,ARHGAP25,ELMO1,SDCBP,FAM49A,TRPM4,NRP1,SVIL,MSRB2,FGD5,LLGL2,MYO7A,SPTBN4,IKBKB,FCHSD2,SFRP1,FCHSD1,ARHGAP26,FGD6,LMOD3,PDE4D,HIP1R,FHDC1,USH1C,STC1,RAPGEF3,KCNJ3,IQGAP2,NEDD9,EDN1,JAM3,DTNBP1,NUAK2,GAS2,KCNJ2,MEF2A,NRAP,ESAM,HCLS1,AP1AR,EPB41,CFLAR,FES,PROX1,TMOD4,F11R,ARHGDIB,LIMK2,PAK2,DLG1,MCU,SELE,BAIAP2L1,PPM1F,WAS,TMOD2,RAC1,RHOJ,SYNE2,JUP,FSCN1,SHROOM4,MICAL3,FGD1,MTOR,OPHN1,STARD8,ABI3,TAOK2,ARHGEF15,ATP2A1,CCDC88C,DSG2,DSC2,CDC42BPB,AMOT,ABLIM3,INF2,PDE4B,ARHGAP28,SPIRE1,MYO18A,RFLNB,S1PR1,CNN3,FNBP1L,CARMIL2,VASP,KCNH2,GMFG,SCN2B,KANK3,SHANK3,RGS4,GBA2,RALA,BST2,ARPC5L	turquoise	EC_M6
GO:0070848	1.48E-05	0.999991257	115	570	response to growth factor	BP	1.76E-03	CD63,FBN2,PPARA,NUS1,PTP4A3,SH3GL2,CDKN2B,KIAA1324L,VEGFA,RUNX2,RASL11B,COL1A2,ASPN,TGFBR1,ADAMTS3,MAP3K7,KIDINS220,ROCK2,ZYX,FGF2,MEGF8,TGFB1,CTNNB1,ZEB2,LTBP1,CRIM1,SPRED3,SPINT2,LUM,DCN,SMAD9,ZNF703,ILK,ANKRD1,SULF1,DKK3,ROBO1,MAP1B,APP,RAPGEF2,SLC33A1,PTPN11,ZFYVE27,ACVR1,GDF6,ATP2B4,NDN,KDM5B,NOTCH2,HYAL1,SLC2A10,TGFB2,FEZ1,FSTL1,PIK3CA,LEF1,HJV,NTRK3,BMP7,FGFR2,ITGB5,HTRA1,SYAP1,IQGAP1,STRAP,FAM89B,HSPB1,MAPT,FBN1,COMP,FKBP8,COL3A1,SKIL,FGFR3,SMURF1,NBL1,GREM2,HAP1,BMPR1A,MED1,PPARGC1A,RNF111,FGFRL1,TGFB1I1,NGFR,LTBP2,COL1A1,TGFB3,SHC1,TMEM100,NPTN,MYOCD,PIN1,CHRDL1,PDGFRB,ROCK1,AKT1,NLK,MYO1C,VASN,GLG1,MTSS1L,HSPA5,DYNC1LI2,FSTL3,FKBP1A,PTPN12,LOX,HAS2,DUSP22,PMEPA1,ZPR1,CHST11,WNT2,FGF10	darkgreen	EC_M4
GO:0051046	1.51E-05	0.999991775	91	434	regulation of secretion	BP	1.77E-03	LRP1,RAB21,SLC12A2,PPARD,LEP,UNC13B,CLOCK,EXOC1,REST,P3H1,RIMS1,INHBA,FKBP1B,KIF5B,DNAJC1,RAB7A,PDCD6IP,TGFB1,RAB3GAP1,C1QTNF1,RAB2B,IRS2,RAB11FIP1,PCLO,CXCL12,ANKRD1,OSBP,VAMP8,P2RX4,SELENOT,ATP2A2,ABCC8,PTPN11,PFKM,TSG101,CRY2,VPS18,ADA,SPP1,PLA2R1,LGI3,BRSK2,DYNLL1,KDM5B,DNM1L,CPT1A,TGFB2,LRP5,GSK3B,HADH,DISP1,IDH2,ADCY5,OSM,BACE1,SIDT2,LINC02210-CRHR1,COMT,TFR2,ADRA2C,CYP19A1,HAP1,NLGN2,NPPB,SRCIN1,STX1B,SYT11,TMED10,TGFB3,CACNA1G,MYH10,CALM1,SYTL4,SCAMP5,ACSL4,CD74,RASL10B,MYOM1,PRKAA1,MIDN,ATP6AP1,SSTR5,VAMP7,RHBDF1,AP1G1,WLS,CBARP,GDNF,STXBP6,KCNMB4,FGF10	darkgreen	EC_M4

GO:0010647	1.51E-05	0.999989658	222	1251	positive regulation of cell communication	BP	1.77E-03	CD63,IL23A,LRP1,TAB2,ANGPT1,RSPO3,PTP4A3,PPARD,CTNNA1,LGALS1,KLHDC10,LEP,CDKN2B,KIAA1324L,VEGFA,VCP,SLC35B2,NCK2,TGFBR1,RIMS1,RTN4,TPBG,SHANK1,ADAMTS3,EMC10,MAP3K7,ROCK2,INHBA,VPS35,ITGB1,PLEKHA4,FGF2,KIF5B,RHEB,PAK1,TGFB1,AXL,RAB3GAP1,SEC13,CTNNB1,DYNC1LI1,ZEB2,CANT1,C1QTNF1,OSBPL8,CXXC5,ACTN4,ATP2C1,IRS2,PRNP,DCN,HDAC3,ACPP,CAVIN3,ILK,RBPMS,ARL6IP5,RYK,TMEM9B,ST5,SLC20A1,ANKRD1,PJA2,CD180,OSBP,SULF1,VAMP8,ROBO1,SORBS1,LOXL3,P2RX4,RNF13,SELENOT,APP,DHX36,RAPGEF2,CX3CL1,CACNB3,PTPN11,PFKM,HOMER1,OPA1,PTPN22,ACVR1,GDF6,SERINC3,MTDH,ADA,SPP1,PKD2,PLA2R1,NKD1,SLC39A10,CHRNA7,DYNLL1,ADIPOR1,NOTCH2,CDC73,DNM1L,UCHL5,CHSY1,SLC2A10,CAPRIN2,TGFB2,HTR2B,ARMC9,GSK3B,PIK3CA,AUTS2,LEF1,PRKCA,TLR5,KRAS,NTRK3,SPATC1L,BMP7,GOLT1B,ATF3,FGFR2,OSM,PEA15,NCSTN,IQGAP1,TFRC,ATF6,FZD9,PDIA3,ATP6AP2,TNR,TFR2,FN1,MPP2,MAOA,PTPN6,GPR137,PDCD10,ITGAV,ADRA2C,HTT,FKBP8,COL3A1,PRKCI,SKIL,SYNPO2L,FGFR3,PRRX1,GORAB,TNIK,CYP19A1,OTUD5,EVC,AKT3,HAP1,MAGED1,BMPR1A,SLC2A4,NLGN2,MED1,RNF111,STX1B,TGFB1I1,NGFR,COL1A1,TGFB3,SHC1,PBXIP1,ADRA1B,MAP2K1,GPR65,TSPAN6,SMARCA4,SLC44A2,GSKIP,NPTN,CYP46A1,MYOCD,PIN1,ACSL4,CD74,RASL10B,PDGFRB,ROCK1,ITGA2,CRLF3,RAB34,PTPN2,CLEC16A,MYDGF,DAPK3,MYO1C,BCAP31,PAFAH1B1,CSNK1G2,IRAK1,FLNA,ATP6AP1,IKBKG,VAMP7,PARK7,CDH13,F3,BCAR3,BCL10,WLS,MIR24-1,CCL16,FKBP1A,AGO3,ITM2C,C18orf32,TIMP3,EDA,	darkgreen	EC_M4
GO:0033262	4.20E-05	0.999997233	7	12	regulation of nuclear cell cycle DNA replication	BP	1.77E-03	DBF4,ZMPSTE24,INO80,DBF4B,ATRX,TIPIN,CDT1	darkgrey	EC_M8
GO:0045842	4.24E-05	0.999997206	7	12	positive regulation of mitotic metaphase/anaphase transition	BP	1.78E-03	CDC16,ESPL1,ANAPC7,CUL3,CDC27,DLGAP5,CDT1	darkgrey	EC_M8
GO:1901970	4.24E-05	0.999997206	7	12	positive regulation of mitotic sister chromatid separation	BP	1.78E-03	CDC16,ESPL1,ANAPC7,CUL3,CDC27,DLGAP5,CDT1	darkgrey	EC_M8
GO:0045773	1.58E-05	0.999996892	16	37	positive regulation of axon extension	BP	1.83E-03	LRP1,SRF,VEGFA,MEGF8,PAK1,ILK,CXCL12,EIF4G2,MAP1B,ZFYVE27,MAPT,FN1,RAB11A,ISLR2,TRPV2,PAFAH1B1	darkgreen	EC_M4
GO:0010605	4.49E-05	0.999967763	249	2090	negative regulation of macromolecule metabolic process	BP	1.88E-03	CDK2,PAN2,BCLAF1,LATS1,FBXO5,ZNF608,SCAF8,UBR5,EIF3E,MRPL13,XRCC6,TNRC6B,TUT7,PLEC,POT1,PWP1,XPO5,RFX3,TRIM28,NCBP1,ZNF451,LRPPRC,MSH2,IBTK,HNRNPA2B1,PNPT1,PUS10,ZMPSTE24,SYNCRIP,XPO1,PABPC4,CASP8AP2,TIA1,ZNF350,ITGB1BP1,THRAP3,EZH2,UFL1,WDR35,DHFR,PUS7,HDAC2,EXOSC9,DNAJC2,CHORDC1,RNASEH2B,SETDB2,MASTL,TRDMT1,CNOT10,UPF2,RSF1,DIS3,XRN2,NCAPG2,BIRC5,GTPBP4,IPO5,CHD8,KAT6A,METTL3,RIF1,E2F7,TAF7,EXOSC8,NR2C1,BRCA2,ELK3,RRP8,TCERG1,PRKDC,CBX5,CCNB1,ERCC6,PAN3,CAPRIN1,ZC3H8,IPO7,PARPBP,TNKS2,LSM14A,BTAF1,CDK5RAP1,TDG,NCK1,IDE,SMARCA5,ABCE1,TAF9,CGGBP1,XRN1,PDE12,SAP30,ATR,TTC37,TERF1,FAM208A,HELLS,GCFC2,USP25,PBK,CNOT1,GABPA,SH2B3,PPID,RBL1,MSH3,SMYD5,NBN,TENT2,RIPK1,E2F8,TRIM33,ZBED6,ZNF248,MDM4,HIVEP1,CUL3,NSUN2,GMNN,NFKB1,KHDRBS1,NCL,TRAIP,PRMT5,AJUBA,BPTF,FXR1,DDX5,FANCB,ORC2,TIAL1,FASTKD1,BLM,SSB,DDX6,CENPF,AUNIP,CDK1,SMARCC1,SP3,DHX9,HNRNPR,FASTKD2,IVNS1ABP,DDX20,TPR,CCAR1,DGCR8,IL12A,XRCC5,NPAT,BRCA1,TFPI,CNOT9,NSD2,TIPRL,BOD1L1,ANKLE1,TBL1XR1,SUV39H1,FOXM1,EIF2AK1,LYAR,FAM220A,HNRNPU,PDS5A,WAPL,NEDD4,SMARCE1,RFC1,CDC6,DCP2,NSD1,IQGAP3,EXOSC10,FUS,USP3,AURKB,TAF1,OGT,RLIM,ATRX,PTTG1,SPAG9,TIPIN,MBTD1,TRIM37,LARP1,PLK1,ILF3,BRIP1,AATF,CSK,PSMD10,TAF15,NLE1,THOC1,TYMS,SMCHD1,UPF3B,ZNF3,SUZ12,HNRNPM,SERBP1,MIER1,XIAP,NIPBL,GSPT1,PHF6,ZC3H7A,RMI2,UHRF1,DROSHA,RBMX,FMR1,METTL16,EHMT1,RNPS1,PTBP1,EXOSC2,SF3B3,GLMN,SCAI,CT	darkgrey	EC_M8
GO:0031123	4.66E-05	0.999985361	22	94	RNA 3'-end processing	BP	1.94E-03	SCAF8,TUT7,NCBP1,YTHDC1,PNPT1,PAPOLG,EXOSC9,WDR33,SYMPK,PCF11,EXOSC8,CCNB1,TENT2,VIRMA,CPSF2,INTS7,SSB,LEO1,EXOSC10,INTS2,EXOSC2,INTS6	darkgrey	EC_M8
GO:0051056	1.46E-05	0.999993056	56	259	regulation of small GTPase mediated signal transduction	BP	1.95E-03	PLEKHG1,CDC42SE2,ARHGAP18,SGSM3,DENND3,CD2AP,CHN2,ARHGAP25,NRP1,RASIP1,PIK3CG,ARHGAP42,ARHGAP21,GPR4,FGD5,NET1,RALGAPA2,CYTH1,ARHGAP26,PRAG1,IGF1,ERBIN,PREX2,ARHGEF3,ARHGEF28,MADD,DNMBP,SHOC2,ARHGEF12,KALRN,F11R,ARHGDIB,SIPA1,ARHGAP27,BCR,SOS2,RAC1,ITPKB,FGD1,ARHGEF9,ARHGAP23,OPHN1,STARD8,ITGA3,ARHGEF15,DNM2,AMOT,ARHGAP28,ARHGAP4,PLEKHG5,NOTCH1,DAB2IP,ADGRG1,RELN,LPAR6,ARHGEF1	turquoise	EC_M6

GO:0043087	1.54E-05	0.999992514	59	285	regulation of GTPase activity	BP	2.03E-03	TBC1D30,AFDN,ASAP1,SGSM3,PTK2,TBC1D22B,SEMA4D,RGP1,CHN2,ARHGAP25,ASAP2,CAV2,TBC1D9,RASIP1,ARHGAP42,LRCH1,FGD5,PLXND1,USP6NL,TBC1D4,NET1,RALGAPA2,SFRP1,RASGRP3,ARHGEF7,ARAP3,FGD6,RAPGEF3,IQGAP2,NEDD9,ADPRH,PLXNA2,SH3BP4,F11R,ITGA6,SIPA1,ARHGAP27,BCR,RUNDC1,ELMOD1,CPEB2,RCC2,EPHA2,TBC1D1,RGS14,FGD1,MTOR,ARHGEF15,TBC1D8B,AMOT,CCL2,SGSM2,RAPGEF1,S1PR1,TBC1D13,DAB2IP,RGS4,RGS16,TBC1D20	turquoise	EC_M6
GO:0051668	1.78E-05	0.99998962	108	537	localization within membrane	BP	2.05E-03	LRP1,STX7,HSPA4,PACSIN1,REEP2,SIL1,GGA1,EXOC1,SRP72,FAM126A,TPBG,COX18,EMC10,ROCK2,BVES,VPS35,SLC7A11,ITGB1,LIN7B,KIF5B,SEC61A1,RAB7A,PICALM,SNX17,PAK1,TGFB1,SRP68,RAB3GAP1,AKT2,ATP2C1,PRNP,ARL6IP5,VPS37A,TM9SF4,TMED2,TMEM150A,VAMP8,SLMAP,ATP1B3,TRAM1,EMC4,SORBS1,GLRB,RAPGEF2,TM9SF3,CACNB3,CPE,SSR3,EXOC6B,ARHGAP1,AP3B1,ZFYVE27,CHP1,ATP2B4,HPS6,VPS39,ARL3,SRPRA,PKP2,MMP14,DAG1,AP2M1,GORASP2,SNX27,FZD9,SRP54,LRRC15,SEC23A,GPC2,PRKCI,SMURF1,TNIK,EMC1,EXOC5,BLZF1,ATAD1,ZDHHC4,MYO5A,IQSEC2,NLGN2,TSPAN17,STX1B,SSR2,DOCK2,RAB11A,TTC8,CALM1,CLTC,CYP46A1,MIEF2,ROCK1,AKT1,ZDHHC9,RAB31,RAB34,MYO1C,FLNA,BSG,CDH13,HSPA5,C16orf70,THY1,LHFPL4,STXBP6,EMC7,ARFRP1,TIMM22,MESD	darkgreen	EC_M4
GO:0002274	1.59E-05	0.999993822	35	156	myeloid leukocyte activation	BP	2.09E-03	IFNGR1,ADGRF5,KIT,SYK,IL6,CXCL8,TLR4,IL15,PIK3CG,GATA2,CST7,CTSC,NECTIN2,GAB2,UNC13D,CD93,TLR2,DYSF,SNCA,CD200,HYAL2,SNAP23,FGR,FES,TMEM106A,TLR6,PSEN1,BATF,SCNN1B,LGALS9,MYO18A,PLCG2,STXBP1,HAVCR1,TLR1	turquoise	EC_M6
GO:0018108	1.61E-05	0.99999234	57	287	peptidyl-tyrosine phosphorylation	BP	2.09E-03	FLT4,PTK2,PDGFB,TEK,TEC,KIT,KDR,SYK,SEMA4D,IL6,IL15,NRP1,TIE1,SFRP1,HBEGF,SH3BP5,CD36,EPHB1,RET,MERTK,IGF1,IL7,THBS4,HIPK1,NEDD9,DYRK1A,EHD4,HDAC1,LCK,HYAL2,NCF1,DYRK3,HCLS1,PECAM1,BMX,FGR,INPP5F,FES,SRMS,ACE,PAK2,STYK1,BCR,EPHB4,EPHA2,TNK1,MTOR,PSEN1,EFNA1,YES1,JAK1,UNC119,MATK,PLCG2,RELN,ALKAL1,BMP6	turquoise	EC_M6
GO:0000469	5.07E-05	0.999992837	10	26	cleavage involved in rRNA processing	BP	2.11E-03	EXOSC9,EXOSC8,UTP20,BMS1,RRS1,NOP14,EXOSC10,NOL9,EXOSC2,NOB1	darkgrey	EC_M8
GO:0072507	1.87E-05	0.99999037	72	323	divalent inorganic cation homeostasis	BP	2.14E-03	LRP1,SLC39A7,SMDT1,SLC39A4,SLC30A9,CNNM4,FKBP1B,FGF2,ATP7B,TPT1,C1QTNF1,TMTC4,STIM1,ATP2C1,PRNP,ATP2B1,CCKBR,RIC3,CXCL12,HSP90B1,C19orf12,RYR3,P2RX4,SELENOT,ATP2A2,APP,CX3CL1,CACNB3,AP3B1,ATP2B4,PKD2,SLC39A10,CHRNA7,RAP1GDS1,CNNM2,HTR2B,CMKLR1,ADCY5,VAPB,CASQ1,FZD9,TMCO1,PTPN6,ITGAV,HTT,ERO1A,KCNA1,HAP1,CACNA1C,STIM2,ADRA1B,GPR65,CALM1,GRIA1,NPTN,SLC39A6,ANXA6,TRPV2,GNB1,BCAP31,PKD1,SYPL2,JPH3,SLC30A7,DMD,FKBP1A,BNIP3,TMEM38B,SNX10,THY1,TMEM38A,CCR6	darkgreen	EC_M4
GO:1904851	5.23E-05	0.99999734	6	10	positive regulation of establishment of protein localization to telomere	BP	2.17E-03	CCT2,CCT4,CCT7,CCT5,CCT6A,CCT3	darkgrey	EC_M8
GO:0006811	1.93E-05	0.999987106	186	1026	ion transport	BP	2.19E-03	CYB5A,CD63,ATP8B1,SLC12A2,ATP6V1C1,SLC22A4,CALHM5,SLC39A7,SMDT1,KCNK9,UBQLN1,SLC39A4,ATP6V1F,LEP,SLC30A9,SLC35B2,CNNM4,SLC17A5,SHANK1,MFSD2A,SLC35A1,SLC31A1,PCYOX1,SLC7A11,ITGB1,PGRMC2,MFSD4B,FKBP1B,FGF2,KIF5B,SEC61A1,PANX1,SLC36A4,ATP7B,ANO10,TPT1,ATP6V0B,RAB3GAP1,SCN5A,CTNNB1,GPD1L,SLC4A7,SLC38A10,ACTN4,STIM1,ATP2C1,PRNP,ATP2B1,TMCO3,ARL6IP5,TUSC3,REM1,CXCL12,FXYD1,SLC20A1,SNTA1,SLC25A4,VAMP8,SLMAP,ATP1B3,TRPC4AP,RYR3,P2RX4,ATP2A2,CACNA1D,APP,GLRB,SLC4A5,CX3CL1,SLC33A1,ABCC8,CACNB3,SLC26A7,HOMER1,OPA1,ARHGAP1,CRY2,SERINC3,ADA,CHP1,ATP2B4,PKD2,PLA2R1,NIPA1,ATP6V1B2,SLC39A10,CHRNA7,MRS2,CNNM2,CALHM1,PKP2,DNM1L,SLC2A10,HTR2B,ISCU,SHROOM2,CASQ1,TOMM40L,CLIC4,TFRC,TMCO1,LINC02210-CRHR1,TFR2,PTPN6,KCNK4,ITGAV,HTT,PKD1L1,ERO1A,KCNA1,VDAC2,SLC3A2,WDR1,ASIC4,SLC6A13,PRAF2,CHRNE,HAP1,CACNA1C,SLC29A4,NLGN2,CLN8,STIM2,ATP6V1D,MAGED2,PPARGC1A,SFXN1,RHBG,COX6A2,SLC7A3,SYT11,SLC35B1,KCNMB1,CACNA1G,TMEM63C,CALM1,CLTC,VMP1,SLC44A2,GRIA1,SLC39A6,ANXA6,TRPV2,PDGFRB,AKT1,PRKAA1,ATP11C,SCNN1D,PKD1,FLNA,KCNAB2,ATP6AP1,VAMP7,PARK7,HCN2,SLC2A6,AHCYL1,CLCC1,JPH3,SLC30A7,SLC35A3,SLC2A8,ATP6V0D1,MIR24-1,DMD,SURF1,FKBP1A,CBARP,SLC36A1,TMEM38B,SLC22A17,NMUR1,PLEKHA3,GDNF,THY1,TMSB4X,TMEM38A,YWHAH,TMEM109,SARAF,SLC39A3,NIPAL1,HCN4,ATP6V0E1,GEM,KCNMB4	darkgreen	EC_M4

GO:0001704	1.98E-05	0.999993267	29	93	formation of primary germ layer	BP	2.23E-03	SRF,INHBA,ITGB1,CTNNB1,TLX2,MMP15,ACVR1,MMP2,CDC73,EYA2,PRKAR1A,MMP14,LEF1,COL6A1,BMP7,FGFR2,ITGB5,FN1,ITGAV,COL5A2,BMPR1A,LAMA3,ITGA2,COL5A1,HSBP1,COL11A1,WLS,DUSP2,DUSP4	darkgreen	EC_M4
GO:0032940	2.03E-05	0.999987774	114	574	secretion by cell	BP	2.27E-03	LRP1,CANX,RAB21,SLC12A2,LY6E,PPARD,MYH9,LEP,UNC13B,CLOCK,EXOC1,REST,NLRP5,P3H1,RIMS1,INHBA,VPS41,RCN3,LIN7B,FKBP1B,KIF5B,NAPA,DNAJC1,RAB7A,COPG1,PDCD6IP,PAK1,TGFB1,AXL,RAB3GAP1,C1QTNF1,RAB2B,IRS2,RAB11FIP1,PCLO,SNPH,CXCL12,ANKRD1,OSBP,VAMP8,P2RX4,SELENOT,ATP2A2,ABCC8,PTPN11,EXOC6B,PFKM,TSG101,CRY2,VPS18,SPP1,LGI3,BRSK2,DYNLL1,KDM5B,DNM1L,CPT1A,TGFB2,LRP5,GSK3B,HADH,DISP1,IDH2,ADCY5,OSM,BACE1,SIDT2,LINC02210-CRHR1,TANGO2,TFR2,ADRA2C,COMP,EXOC5,PTPRN,CYP19A1,HAP1,NLGN2,SRCIN1,STX1B,SYT11,LTBP2,TMED10,TGFB3,CACNA1G,UBE2Q1,RAB11A,MYH10,SEL1L,CALM1,SYTL4,SCAMP5,ACSL4,RASL10B,MYOM1,RAB31,PAFAH1B1,SDF4,MIDN,ATP6AP1,SSTR5,VAMP7,PARK7,RHBDF1,AP1G1,WLS,CBARP,TMEM38B,GDNF,TMEM167A,STXBP6,PIK3C2A,KCNMB4,FGF10,EXOC3L4	darkgreen	EC_M4
GO:0051049	1.81E-05	0.999987873	187	1276	regulation of transport	BP	2.34E-03	TNFRSF11A,SNCAIP,RIMS2,PACSIN2,TRDN,DAPK1,PDGFB,PICK1,PIM1,ATP8A1,APBA1,CD2AP,SYK,ICA1,TSPAN13,IL6,SNAP91,PPT1,RSAD2,GRB10,TLR4,TCAF2,IL15,SDCBP,TRPM4,NRP1,RAB3B,PIK3CG,MTMR2,GATA2,KCNRG,GAB2,LLGL2,UNC13D,PPARG,TBC1D4,CXCR4,EDNRB,NDFIP2,SPTBN4,P2RY6,P2RY2,SFRP1,FERMT1,KCNK6,P2RX2,CD36,SIRT3,PRR5L,SCARB1,PDE4D,MERTK,CHD7,FAM3D,IL1A,IGF1,HIP1R,OCLN,MCTP1,MEF2C,TLR2,P2RX7,VEGFC,NDRG4,CXCL10,BMP2K,DYSF,TPCN1,PLCG1,STC1,RAPGEF3,KCNJ3,ANO6,CLIC6,EDN1,KCNJ15,EHD4,SNCA,DTNBP1,PM20D1,MX2,ABCG1,CD200,SIK1,HDAC1,PPP3CA,HYAL2,ANO1,HECW2,KCNJ2,MEF2A,SCN3A,MCTP2,RIN3,SEC24B,HCLS1,ATP9A,EPB41,FGR,INPP5F,ADCY1,FES,RBM4,PRKD1,OAZ3,DLG1,APOD,KCNK7,CREB1,B2M,CTTNBP2NL,OSBPL6,ADORA2A,CYBB,HOMER3,MCU,WNK4,DNM3,CDK16,SELE,FGF23,CRACR2A,PPM1F,TMEM30B,RAC1,HIF1A,JUP,RAB27A,ADIPOR2,SNCG,SYT7,RSC1A1,TNFRSF1B,FBXL20,MTOR,OPHN1,PSEN1,SLC51B,CHRNB2,ATP2A1,CD19,RAB3D,NPR1,GPR68,DNM2,AP2B1,ABLIM3,SH3TC2,ADRB2,CCL2,YES1,KCNJ18,PDE4B,LGALS9,NOS2,UNC119,MYO18A,GIT1,USP7,RPH3AL,SLC43A2,NOTCH1,NUP214,PLCG2,STXBP1,LRSAM1,RIPOR1,PLA2G4E,CA2,RELN,KCNH2,SCN2B,KCNK5,SHANK3,RGS4,KCNA6,TBC1D20,RALA,BST2,VAMP3,BMP2,CACNG5,CBLL1,FXYD6,BMP6,KCNG2,GSG1L	turquoise	EC_M6
GO:0006413	5.71E-05	0.999981706	22	99	translational initiation	BP	2.36E-03	EIF3E,NCBP1,MTIF2,DDX1,METTL3,DHX29,EIF2B1,NCK1,ABCE1,KHDRBS1,EIF3A,EIF2B5,TPR,DHX33,EIF2AK1,EIF3B,EIF4A1,LARP1,EIF5,FMR1,PAIP2,EIF1AX	darkgrey	EC_M8
GO:0009113	5.74E-05	0.999997026	6	10	purine nucleobase biosynthetic process	BP	2.37E-03	PPAT,PAICS,GMPS,PRPS1,HPRT1,GART	darkgrey	EC_M8
GO:0031325	5.91E-05	0.999956432	290	2483	positive regulation of cellular metabolic process	BP	2.43E-03	PAN2,ONECUT2,BCLAF1,LATS1,CCT2,DDX39B,SCAF8,EIF3E,MAP3K4,DSCC1,HSF2,ATAD2,XRCC6,PHF5A,TNRC6B,TUT7,IFT74,POT1,PWP1,ZNF300,TOPORS,DBF4,AKAP9,RFX3,CKS2,TRIM28,NCBP1,ZNF451,ANP32B,FOXJ3,MSH2,PHIP,TTK,PPP4R3B,HNRNPA2B1,PNPT1,RLF,ZNF292,CCT4,COMMD1,CASP8AP2,ETAA1,ITGB1BP1,THRAP3,EZH2,UFL1,CCNC,CLSPN,CASP2,NAA15,ELF2,WDR35,NRDC,ATAD2B,HDAC2,CCNA2,EXOSC9,MLLT10,SAE1,CEP295,DNAJC2,SYMPK,NUP85,ELF1,RSF1,BORA,DIS3,CCNT2,PAXIP1,WDR43,CDC20,FNTA,SUPT16H,CHD8,KAT6A,METTL3,RIF1,E2F7,ALYREF,CKAP2,CEP290,NSD3,CEBPZ,KITLG,TAF7,EXOSC8,RFC3,BRCA2,ELK3,TCERG1,PRKDC,APAF1,CCNB1,ERCC6,DHX29,PAN3,SCYL2,GPBP1,SP1,TCEA1,TPX2,ZC3H8,DIMT1,UBA2,TNKS2,ZNF143,CENPJ,CDK5RAP1,NCK1,ARFGEF1,SMARCA5,ZNF507,OTUD4,ZFP91,TAF9,PDE12,TFCP2,ATR,MTMR9,LARP4,UTP15,HLTF,CNOT1,GABPA,MCRS1,USP16,MOB1A,CCT7,CDCA2,NBN,SENP1,ABRAXAS1,RAD51,SLC38A2,RIPK1,WDR75,ARID2,MYBL2,E2F8,INO80,RIOK1,CHUK,STK4,UBE2C,HIVEP1,RFC5,TAOK3,NOLC1,CCT5,BMPR1B,UBE3A,CCT6A,PRDM10,NUCKS1,GCN1,CUL3,CHEK1,TIMM17A,H2AFZ,RRP1B,NFKB1,KHDRBS1,CENPE,TET2,TRIP11,HEATR1,NCL,KIF14,AJUBA,NOL11,GTF2I,BPTF,FXR1,FANCI,DDX5,FANCB,RFC2,NEK2,EIF2B5,ALS2,BLM,EDRF1,CDK1,PDE8A,ING5,BCLAF3,FOSL1,SMARCC1,SP3,DHX9,HNRNPR,TRA2B,FASTKD2,RFC4,DNA2,CCAR1,FANCM,DBF4B,IL12A,UBTF,COP1,NMD3,XRCC5,NPAT,BRCA1,PSME3,CNOT9,NSD2,DCUN1D5,RAD51AP1,DCAF6,ECT2,DHX33,TBL1XR1,LEO1,FOXM1,LYAR,DDX11,HNRNPU,INCENP,UBE2K,SMARCE1,RFC	darkgrey	EC_M8

GO:0050770	2.19E-05	0.999991301	37	132	regulation of axonogenesis	BP	2.43E-03	LRP1,PLXNB2,SRF,VEGFA,RTN4,PLXNA1,MEGF8,PAK1,TRAK1,SEMA3C,ILK,RYK,RNF6,CXCL12,EIF4G2,ROBO1,MAP1B,DIP2B,SEMA4F,ZFYVE27,SPP1,SEMA4G,BRSK2,GSK3B,MAPT,TNR,FN1,SKIL,RAB11A,MAP2K1,ISLR2,TRPV2,PLXNB3,PAFAH1B1,PLXNA3,THY1,CHN1	darkgreen	EC_M4
GO:0001894	1.92E-05	0.999991821	41	189	tissue homeostasis	BP	2.46E-03	TNFRSF11A,TNFAIP3,LYZ,TJP2,ADGRF5,SYK,IL6,VPS54,TLR4,GATA2,NOS3,POC1B,CLCN3,OCLN,WWTR1,IL7,P2RX7,USH1C,JAM3,LPCAT1,SLC39A8,ESAM,INPP5D,CD34,PECAM1,F11R,B2M,LTF,LDB2,PROM1,RAC1,LIPA,APBB2,RAB3D,ADRB2,NOTCH1,S1PR1,CDH5,OAS1,CA2,DRAM2	turquoise	EC_M6
GO:0031344	2.24E-05	0.999986791	109	538	regulation of cell projection organization	BP	2.47E-03	ATP8B1,LRP1,RAB21,PLXNB2,PACSIN1,LGALS1,SRF,VEGFA,P3H1,TGFBR1,TMEM30A,RTN4,MFSD2A,AKIRIN1,ACTR2,KIDINS220,VPS35,FKBP1B,PLXNA1,MARK4,MEGF8,PAK1,ACTR3,TRAK1,DZIP1,FAM98A,PRNP,CDC42EP3,SEMA3C,ILK,DPYSL3,RYK,RNF6,CXCL12,ANKRD1,EIF4G2,ROBO1,MAP1B,DIP2B,SEMA4F,PFN2,TLX2,DHX36,RAPGEF2,CX3CL1,ADCY6,HOMER1,OPA1,ARSB,ZFYVE27,SPP1,TWF1,SEMA4G,BRSK2,PDLIM5,DNM1L,CAPRIN2,FEZ1,GSK3B,PIK3CA,AUTS2,CORO1C,NTRK3,BMP7,IQGAP1,KIF1A,TANC2,MAPT,TNR,FN1,NCKAP1,GPC2,ARPC2,HTT,PRKCI,SKIL,PRRX1,CAPZB,TNIK,PLD1,OBSL1,CRMP1,HAP1,SRCIN1,STX1B,TGFB3,RAB11A,MAP2K1,NPTN,ISLR2,TRPV2,EVI5L,AKT1,ITGA2,MYO10,PLXNB3,PAFAH1B1,FLNA,PLXNA3,DEF8,NTNG2,HSPA5,SERPINF1,ITM2C,UBE2V2,THY1,CHN1,YWHAH,SDC2	darkgreen	EC_M4
GO:2000234	6.27E-05	0.999996699	6	10	positive regulation of rRNA processing	BP	2.57E-03	WDR43,DIMT1,UTP15,WDR75,RIOK1,HEATR1	darkgrey	EC_M8
GO:0031175	2.36E-05	0.999984801	150	784	neuron projection development	BP	2.58E-03	LRP1,RAB21,PLXNB2,PACSIN1,ADGRB1,CTNNA1,LGALS1,SH3GL2,PLAA,SRF,VEGFA,NCK2,P3H1,RIMS1,TMEM30A,UBA6,RTN4,TPBG,SHANK1,MFSD2A,ACTR2,KIDINS220,WHRN,GLI3,NCDN,ITGB1,FKBP1B,PLXNA1,KIF5B,RB1,PICALM,GLI2,MEGF8,PAK1,CTNNB1,TRAK1,FARP1,DCLK1,SEMA3C,ILK,DPYSL3,RYK,RNF6,CXCL12,ANKRD1,SLC25A46,PALLD,EIF4G2,ROBO1,MAP1B,DIP2B,SEMA4F,TLX2,TCTN1,APP,DHX36,RAPGEF2,CX3CL1,PTPN11,ADCY6,OPA1,ARSB,ZFYVE27,CRTAC1,SPP1,TWF1,SEMA4G,GPRIN3,BRSK2,PDLIM5,NDN,CHRNA7,DOCK10,NOTCH2,CTTN,DNM1L,CAPRIN2,FEZ1,GSK3B,DAG1,AUTS2,ANOS1,NTRK3,BMP7,FGFR2,VASH2,IQGAP1,KIF1A,TANC2,MAPT,TNR,FN1,NCKAP1,GPC2,PRKCI,SKIL,PRRX1,SMURF1,NBL1,TNIK,VCL,OBSL1,CRMP1,NOTCH3,ENAH,SRCIN1,STX1B,NGFR,RAB11A,MAP2K1,TTC8,DICER1,CLMN,NPTN,ISLR2,TRPV2,ACSL4,LAMA3,ROCK1,AKT1,PLXNB3,PAFAH1B1,NTN3,FLNA,PLXNA3,RERE,BSG,NTNG2,BARHL2,HSPA5,NEXN,DPYSL5,SERPINF1,FRY,VSTM2L,ITM2C,UBE2V2,ARF4,GDNF,THY1,BLOC1S2,CHN1,EFHD1,ARTN,YWHAH,ZPR1,SDC2,PRKG1,MAP1A,OLFM1	darkgreen	EC_M4
GO:0048010	2.06E-05	0.999995348	17	48	vascular endothelial growth factor receptor signaling pathway	BP	2.61E-03	FLT4,PTK2,KDR,GRB10,NRP1,PRKD2,HHEX,VEGFC,PRKD1,PAK2,CLEC14A,HIF1A,MMRN2,FGF18,PGF,TMEM204,DAB2IP	turquoise	EC_M6
GO:0006909	2.10E-05	0.99999149	37	163	phagocytosis	BP	2.64E-03	CDC42SE2,PTK2,SYK,ARHGAP25,ELMO1,TLR4,IL15,GATA2,UNC13D,MYO7A,CD93,P2RY6,CD36,SCARB1,MERTK,CLCN3,TLR2,DYSF,ANO6,LYST,PECAM1,FGR,DOCK1,ADORA2A,RAC1,PLD2,RAB27A,SYT7,PEAR1,DNM2,CCL2,YES1,MYO18A,IRF8,PLCG2,HAVCR1,RAB39A	turquoise	EC_M6
GO:0018212	2.13E-05	0.999989573	57	290	peptidyl-tyrosine modification	BP	2.66E-03	FLT4,PTK2,PDGFB,TEK,TEC,KIT,KDR,SYK,SEMA4D,IL6,IL15,NRP1,TIE1,SFRP1,HBEGF,SH3BP5,CD36,EPHB1,RET,MERTK,IGF1,IL7,THBS4,HIPK1,NEDD9,DYRK1A,EHD4,HDAC1,LCK,HYAL2,NCF1,DYRK3,HCLS1,PECAM1,BMX,FGR,INPP5F,FES,SRMS,ACE,PAK2,STYK1,BCR,EPHB4,EPHA2,TNK1,MTOR,PSEN1,EFNA1,YES1,JAK1,UNC119,MATK,PLCG2,RELN,ALKAL1,BMP6	turquoise	EC_M6
GO:0016572	6.60E-05	0.999987956	12	34	histone phosphorylation	BP	2.70E-03	CDK2,ZMPSTE24,CCNA2,CCNB1,CHEK1,CDK1,BAZ1B,INCENP,AURKB,FMR1,HASPIN,AURKA	darkgrey	EC_M8
GO:0032963	2.52E-05	0.999992096	25	78	collagen metabolic process	BP	2.73E-03	PPARD,COL1A2,P3H1,ADAMTS3,RCN3,ITGB1,TGFB1,SERPINH1,MMP17,MMP15,MMP2,RUNX1,FAP,MMP14,MRC2,PLOD3,SUCO,COL1A1,TGFB3,PDGFRB,ITGA2,MMP23B,COL5A1,MFAP4,P3H3	darkgreen	EC_M4

GO:0065008	2.53E-05	0.999980618	448	2770	regulation of biological quality	BP	2.73E-03	SERPINB2,LMAN1,ATP8B1,HSD17B6,LRP1,CDO1,RAB21,SLC12A2,PLXNB2,ANGPT1,TCP1,PPARA,SLC22A4,SLC39A7,NUS1,SMDT1,FAM49B,KCNK9,PTP4A3,ADGRB1,PPARD,MKLN1,LGALS1,MDGA1,TXN2,SH3GL2,GNAQ,SLC39A4,MYH9,LEP,PLAA,LARGE1,PEX6,SLC30A9,SRF,BAG1,VEGFA,VCP,UNC13B,COL1A2,NCK2,CLOCK,PDCL3,REST,CNNM4,P3H1,ERP44,ISOC2,RIMS1,TMEM30A,RTN4,TPBG,SHANK1,LRRC4B,MFSD2A,MYH14,ACTR2,SLC31A1,WHRN,NCDN,ROCK2,INHBA,BVES,RCN3,MYLK3,VPS35,SLC7A11,ITGB1,LIN7B,FKBP1B,PLXNA1,FGF2,KIF5B,NAPA,SESN3,RAB7A,ATP7B,GAD2,RB1,ABHD5,PICALM,TPT1,CRTAP,PDCD6IP,DGKH,MEGF8,RHEB,PAK1,ACTR3,TGFB1,AXL,RAB3GAP1,SCN5A,WDR48,HNMT,CTNNB1,DGAT2,AKT2,GPD1L,PLEKHG2,C1QTNF1,FARP1,SLC4A7,ACTG1,TMTC4,MAFG,STIM1,ATP2C1,IRS2,PRNP,CHFR,ATP2B1,DCN,CDC42EP3,HDAC3,RAB11FIP1,PCLO,SEMA3C,CCKBR,UBA5,DDRGK1,ILK,ARL6IP5,RYK,SNPH,CNOT7,RNF6,RIC3,LMO1,CXCL12,FXYD1,TM9SF4,SNTA1,HSP90B1,IL20RB,TP53INP2,SLC25A4,OSBP,C19orf12,EIF4G2,VAMP8,SLMAP,ATP1B3,CTNND1,LNPEP,DKK3,PCSK1,MAP1B,RDH10,DIP2B,RYR3,PINX1,SEMA4F,P2RX4,PFN2,RASA1,SELENOT,ATP2A2,CELF1,CACNA1D,MYL9,APP,GLRB,DHX36,SLC4A5,CCT8,RAPGEF2,CX3CL1,ABCC8,HECTD4,CACNB3,PTPN11,ADCY6,PFKM,HOMER1,OPA1,AP3B1,AMIGO2,ZFYVE27,DKC1,CRY2,VPS18,ADA,GLS,BAP1,CHP1,ATP2B4,SPP1,PKD2,TWF1,TMEM14C,SEMA4G,TENT5C,TENT4B,BRSK2,HPS6,PDLIM5,NDN,MAGEL2,SLC39A10,CHRNA7,RAP1GDS1,DOCK10,CNNM2,DYNLL1,ADIPOR1,FAP,TJP1,KDM5B,CTTN,PKP2,CDC73,DNM1L,GLRX2,SELENOS,L	darkgreen	EC_M4
GO:0050769	2.67E-05	0.999988027	46	181	positive regulation of neurogenesis	BP	2.86E-03	LRP1,RAB21,PLXNB2,SRF,VEGFA,ACTR2,GLI3,PLXNA1,TENM4,MEGF8,SMARCD3,RHEB,PAK1,TGFB1,CTNNB1,TRAK1,LIG4,ILK,CXCL12,EIF4G2,ROBO1,MAP1B,DHX36,CX3CL1,OPA1,ZFYVE27,DNM1L,CAPRIN2,DAG1,MAPT,FN1,PRKCI,SKIL,OBSL1,HAP1,RAB11A,MAP2K1,DICER1,NPTN,ISLR2,TRPV2,PLXNB3,PAFAH1B1,PLXNA3,SERPINF1,WNT2	darkgreen	EC_M4
GO:0044255	9.47E-07	0.999999646	125	743	cellular lipid metabolic process	BP	2.86E-03	ZADH2,ORMDL2,DGKA,PIP4K2C,PEX7,CHKB,ABHD16A,ALG12,CERK,AGPAT1,HSD17B8,B3GALT4,GPAA1,PIGO,PLPP6,ALG2,TTC7A,SLC44A1,ETFB,PTGR1,LPIN1,SPHK2,ACSS1,LIPE,ECHDC3,ACAA1,TAMM41,CYGB,PIP4P1,SIRT2,LAMTOR1,HACL1,GPCPD1,PLPP5,DDHD2,CYP1B1,PTDSS2,SDR16C5,THNSL2,CBR4,PIK3R1,ACSS2,RETSAT,HTRA2,PRKAG1,PLBD2,GPAT3,LPIN3,ORMDL1,ERLIN1,PIGT,PLA2G4B,ACOT8,LONP2,SRD5A1,OSBPL5,IRS1,ACADS,GPAM,CHKA,ABHD4,GPX1,MTMR11,PIGM,ABCB11,CERS2,PER2,ALG3,SCAP,EHHADH,POR,IDH1,ACADL,SOAT1,PECR,PHB2,ABHD12B,SMPD4,PLA2G16,TNFRSF1A,ABHD8,FADS3,CTDNEP1,ILVBL,EPHX1,TECR,PLSCR3,PIGH,PIP4K2B,CRABP2,ACOT2,ABCD4,GBA,DPM3,GCDH,AC138894.1,SCPEP1,SLC27A3,MTMR4,HEXA,ACSM3,ACACA,SREBF1,CERS4,MPPE1,DHRS13,ACAA2,ALG1,MTMR1,CPT2,PNPLA7,TAZ,LMF1,PIGQ,INPP5E,PLPPR3,GSTM4,PTGES2,VAC14,LCAT,ABCA2,PLPP3,RDH14,SCP2,DNAJC19	cyan	EC_M2
GO:0031328	7.25E-05	0.999949357	187	1503	positive regulation of cellular biosynthetic process	BP	2.96E-03	ONECUT2,BCLAF1,CCT2,DDX39B,SCAF8,EIF3E,MAP3K4,DSCC1,HSF2,ATAD2,XRCC6,PHF5A,IFT74,POT1,PWP1,ZNF300,TOPORS,DBF4,RFX3,TRIM28,ZNF451,FOXJ3,PHIP,PPP4R3B,RLF,ZNF292,CCT4,ITGB1BP1,THRAP3,CCNC,NAA15,ELF2,ATAD2B,HDAC2,CCNA2,EXOSC9,MLLT10,DNAJC2,NUP85,ELF1,RSF1,CCNT2,PAXIP1,WDR43,SUPT16H,CHD8,KAT6A,METTL3,E2F7,ALYREF,CKAP2,CEP290,NSD3,CEBPZ,TAF7,RFC3,BRCA2,ELK3,TCERG1,PRKDC,ERCC6,DHX29,GPBP1,SP1,TCEA1,ZC3H8,TNKS2,ZNF143,CENPJ,CDK5RAP1,NCK1,ARFGEF1,SMARCA5,ZNF507,TAF9,TFCP2,ATR,LARP4,UTP15,HLTF,GABPA,MCRS1,USP16,CCT7,SENP1,RIPK1,WDR75,MYBL2,E2F8,INO80,CHUK,HIVEP1,RFC5,NOLC1,CCT5,BMPR1B,UBE3A,CCT6A,PRDM10,NUCKS1,GCN1,CHEK1,H2AFZ,RRP1B,NFKB1,KHDRBS1,TET2,TRIP11,HEATR1,NCL,AJUBA,NOL11,GTF2I,BPTF,FXR1,RFC2,NEK2,EIF2B5,BLM,EDRF1,CDK1,ING5,BCLAF3,FOSL1,SMARCC1,SP3,DHX9,FASTKD2,RFC4,DNA2,CCAR1,DBF4B,UBTF,NMD3,XRCC5,NPAT,BRCA1,CNOT9,DCAF6,DHX33,TBL1XR1,LEO1,FOXM1,LYAR,DDX11,HNRNPU,SMARCE1,RFC1,TOP2A,NSD1,MED6,ICE2,CCT3,PMF1,FUS,AURKB,ZNF609,OGT,ATRX,ZNF711,ILF2,TRIM37,LARP1,ILF3,MED13,AATF,TAF15,THOC1,UPF3B,ATAD5,NIPBL,MYSM1,UHRF1,RBMX,FMR1,TP73,CHTF18,CDT1,CTCF,PRIM2,HNRNPD,DRAP1,ZNF770,NAF1,ZNF782,ZFP62,SFR1	darkgrey	EC_M8

GO:0009887	2.80E-05	0.999981907	149	784	animal organ morphogenesis	BP	2.98E-03	NAB2,AHI1,CDSN,FBN2,PPARA,RSPO3,LY6E,CTNNA1,RORB,SRF,VEGFA,RUNX2,COL1A2,ASPN,CNNM4,RAB23,TGFBR1,RTN4,MFSD2A,WHRN,GLI3,INHBA,PLEKHA4,PLXNA1,FGF2,GLI2,MEGF8,SMARCD3,TGFB1,SCN5A,WDR48,CTNNB1,SERPINH1,RELT,RARB,STIM1,DCN,SEMA3C,ILK,APLP1,RYK,PRICKLE2,IMPAD1,ANKRD1,FAT1,TMED2,SULF1,ROBO1,RDH10,HEY1,ACTG2,CPE,AP3B1,ACVR1,MMP2,BMP1,PKD2,NKD1,IFT57,KDM5B,NOTCH2,PKP2,UCHL5,CHSY1,HYAL1,TGFB2,LRP5,HTR2B,SATB2,FAM20C,MMP14,DAG1,LEF1,SHROOM2,KRAS,BMP7,FGFR2,HTRA1,ARID5B,PLOD3,SERPINE1,SGPL1,FZD2,FBN1,COMP,PRKCI,COL5A2,SYNPO2L,FGFR3,PRRX1,SMURF1,NBL1,DAAM1,TULP3,SIX4,GREM2,OBSL1,ACTA2,ROM1,AKT3,MAGED1,BMPR1A,TCAP,MED1,MSN,FGFRL1,NGFR,TPM1,THBS3,COL1A1,CHAD,TGFB3,MAP2K1,TMEM100,TTC8,ANXA6,LAMA3,PDGFRB,ITGA2,EGFLAM,ROGDI,PAFAH1B1,NTN3,PKD1,FLNA,BSG,ANKRD11,COL5A1,NTNG2,COL11A1,GLG1,BCAR3,LHX8,FKBP1A,SERPINB5,ADAMTS5,SNX10,LBX1,GDNF,THY1,HAS2,EDA,OTOR,SLC39A3,CHST11,OLFM1,WNT2,COL8A2,FGF10	darkgreen	EC_M4
GO:1903530	2.85E-05	0.99998417	84	399	regulation of secretion by cell	BP	3.01E-03	LRP1,RAB21,SLC12A2,PPARD,LEP,UNC13B,CLOCK,EXOC1,REST,P3H1,RIMS1,INHBA,FKBP1B,KIF5B,DNAJC1,RAB7A,PDCD6IP,TGFB1,RAB3GAP1,C1QTNF1,RAB2B,IRS2,RAB11FIP1,PCLO,CXCL12,ANKRD1,OSBP,VAMP8,P2RX4,SELENOT,ATP2A2,ABCC8,PTPN11,PFKM,TSG101,CRY2,VPS18,SPP1,LGI3,BRSK2,DYNLL1,KDM5B,DNM1L,CPT1A,TGFB2,LRP5,GSK3B,HADH,DISP1,IDH2,ADCY5,OSM,BACE1,SIDT2,LINC02210-CRHR1,TFR2,ADRA2C,CYP19A1,HAP1,NLGN2,SRCIN1,STX1B,SYT11,TMED10,TGFB3,CACNA1G,MYH10,CALM1,SYTL4,SCAMP5,ACSL4,RASL10B,MYOM1,MIDN,ATP6AP1,SSTR5,VAMP7,RHBDF1,AP1G1,WLS,CBARP,GDNF,STXBP6,KCNMB4	darkgreen	EC_M4
GO:0035987	2.87E-05	0.999994429	15	34	endodermal cell differentiation	BP	3.01E-03	INHBA,CTNNB1,MMP15,MMP2,CDC73,MMP14,COL6A1,ITGB5,FN1,ITGAV,COL5A2,LAMA3,COL5A1,HSBP1,COL11A1	darkgreen	EC_M4
GO:0051255	7.55E-05	0.999995885	6	10	spindle midzone assembly	BP	3.06E-03	RACGAP1,PRC1,INCENP,KIF4A,AURKB,KIF23	darkgrey	EC_M8
GO:1904814	7.55E-05	0.999993836	7	14	regulation of protein localization to chromosome, telomeric region	BP	3.06E-03	CCT2,CCT4,TERF1,CCT7,CCT5,CCT6A,CCT3	darkgrey	EC_M8
GO:1902101	7.56E-05	0.999994135	7	13	positive regulation of metaphase/anaphase transition of cell cycle	BP	3.06E-03	CDC16,ESPL1,ANAPC7,CUL3,CDC27,DLGAP5,CDT1	darkgrey	EC_M8
GO:0022603	2.96E-05	0.999980967	145	761	regulation of anatomical structure morphogenesis	BP	3.08E-03	LRP1,AHI1,RAB21,SLC12A2,PLXNB2,FAM19A5,RSPO3,FAM49B,ADGRB1,MKLN1,MYH9,LEP,PLAA,SRF,VEGFA,RUNX2,PDCL3,RIMS1,RTN4,EMC10,MFSD2A,MYH14,ACTR2,ROCK2,BVES,RRAS,VPS35,ITGB1,PLEKHA4,PLXNA1,FGF2,TENM4,MEGF8,PAK1,TGFB1,CTNNB1,TRAK1,ACTN4,STIM1,DCN,CDC42EP3,SEMA3C,ILK,SPINK5,RYK,PRICKLE2,RNF6,CXCL12,EIF4G2,SULF1,ROBO1,MAP1B,DIP2B,SEMA4F,RASA1,TLX2,DHX36,RAPGEF2,ABCC8,OPA1,ZFYVE27,MTDH,ADA,ATP2B4,SPP1,RUNX1,SEMA4G,NKD1,BRSK2,PDLIM5,CHRNA7,PHLDB2,TJP1,DNM1L,HYAL1,CAPRIN2,TGFB2,ADGRB2,GSK3B,DAG1,CORO1C,PRKCA,BMP7,FGFR2,VASH2,PHLDB1,KIF1A,TFRC,TANC2,HSPB1,MAPT,SERPINE1,FZD2,TNR,FN1,C11orf65,ARPC2,SKIL,SMURF1,CAPZB,TNIK,DAAM1,WDR1,SIX4,OBSL1,AKT3,MAGED1,BMPR1A,PPARGC1A,NPPB,MSN,SRCIN1,NGFR,TPM1,RAB11A,MYH10,MAP2K1,PKM,ISLR2,TRPV2,MIEF2,ROCK1,OMA1,MYDGF,MYO10,DAPK3,PLXNB3,PAFAH1B1,FLNA,PLXNA3,SARS,NTNG2,CFDP1,F3,MIR24-1,SERPINF1,BNIP3,GDNF,THY1,HAS2,CHN1,YWHAH,SDC2,WNT2,FGF10	darkgreen	EC_M4
GO:0044057	3.03E-05	0.999983159	84	398	regulation of system process	BP	3.12E-03	PPARA,LEP,SRF,UNC13B,RIMS1,SHANK1,ROCK2,INHBA,BVES,FKBP1B,TENM4,PAK1,SCN5A,GPD1L,C1QTNF1,ATP2B1,ACPP,FXYD1,SNTA1,P2RX4,ATP2A2,DLGAP4,CACNA1D,MYL9,APP,ABCC8,CACNB3,PTPN11,HOMER1,CRY2,ADA,GLS,ATP2B4,TWF1,CHRNA7,CTTN,PKP2,PPP1R12B,TGFB2,DAG1,PRKCA,CASQ1,GLRX3,LINC02210-CRHR1,COMT,TNR,ADRA2C,ECE1,KCNA1,CACNA1C,HSPB7,NLGN2,PPARGC1A,NPPB,STX1B,TPM1,SGCA,CACNA1G,ADRA1B,CNN1,TMEM100,CALM1,DICER1,SETD3,MYOCD,BEGAIN,ANXA6,PIN1,ROCK1,AKT1,TMEM98,ITGA2,DAPK3,FLNA,ENO1,DMD,TMEM38B,TMEM38A,HOPX,ORMDL3,HCN4,PRKG1,KCNMB4,FGF10	darkgreen	EC_M4

GO:0034330	3.04E-05	0.999981734	110	541	cell junction organization	BP	3.12E-03	LRP1,PLXNB2,ADGRB1,CTNNA1,MDGA1,ARHGAP39,SRF,VEGFA,VCP,UNC13B,REST,DST,TGFBR1,TPBG,SHANK1,LRRC4B,ACTR2,WHRN,ROCK2,VPS35,SLC7A11,PDCD6IP,PCDH17,TGFB1,CTNNB1,FARP1,ACTG1,PRNP,ZNF703,PCLO,RYK,SNTA1,CTNND1,MAP1B,SORBS1,APP,GLRB,PLEKHA7,DHX36,RAPGEF2,CX3CL1,ABCC8,CACNB3,PTPN11,HOMER1,OPA1,AMIGO2,MTDH,PDLIM5,CHRNA7,DOCK10,INA,PHLDB2,TJP1,CTTN,PKP2,DNM1L,DNER,CAPRIN2,TGFB2,MMP14,DAG1,CORO1C,PRKCA,NTRK3,HSPA8,IQGAP1,KIF1A,TANC2,FZD9,MAPT,C1QL1,TNR,FN1,DOK7,PRKCI,VCL,WDR1,SIX4,OBSL1,ERC1,NLGN2,ADGRL1,ACTN1,SRCIN1,TLN2,TGFB3,MYH10,VMP1,NPTN,PIN1,ROCK1,ITGA2,DAPK3,MYO1C,PAFAH1B1,SDF4,NPHP4,FLNA,NTNG2,PCDHB2,LRRC24,UBE2V2,ARF4,GDNF,THY1,LHFPL4,DUSP22,MESD,LRRTM2	darkgreen	EC_M4
GO:1904874	7.86E-05	0.999993277	7	15	positive regulation of telomerase RNA localization to Cajal body	BP	3.17E-03	CCT2,CCT4,CCT7,CCT5,CCT6A,CCT3,NAF1	darkgrey	EC_M8
GO:0006997	7.90E-05	0.999972006	25	112	nucleus organization	BP	3.18E-03	NUP107,SRPK1,PLEC,ZMPSTE24,NUP205,RRP8,POLR1B,SMARCA5,NUP54,NUP93,NOLC1,CHEK1,RCC1,NUP133,CDK1,TPR,SUV39H1,DDX11,PLK1,SERBP1,NUP155,NDC1,RNF8,UBXN2A,NUP35	darkgrey	EC_M8
GO:0048878	3.16E-05	0.999979277	155	832	chemical homeostasis	BP	3.22E-03	LRP1,SLC12A2,SLC39A7,NUS1,SMDT1,PPARD,LGALS1,SLC39A4,LEP,SLC30A9,SRF,VEGFA,UNC13B,CNNM4,RTN4,SLC31A1,RCN3,SLC7A11,FKBP1B,FGF2,KIF5B,SESN3,RAB7A,ATP7B,ABHD5,PICALM,TPT1,SCN5A,DGAT2,GPD1L,C1QTNF1,SLC4A7,TMTC4,MAFG,STIM1,ATP2C1,IRS2,PRNP,ATP2B1,DCN,CCKBR,RIC3,CXCL12,TM9SF4,HSP90B1,OSBP,C19orf12,SLMAP,ATP1B3,RYR3,P2RX4,SELENOT,ATP2A2,APP,SLC4A5,CX3CL1,ABCC8,HECTD4,CACNB3,PTPN11,ADCY6,PFKM,HOMER1,OPA1,AP3B1,CRY2,GLS,CHP1,ATP2B4,PKD2,TENT4B,BRSK2,SLC39A10,CHRNA7,RAP1GDS1,CNNM2,DYNLL1,ADIPOR1,ZBTB20,LRP5,HTR2B,PIK3CA,ISCU,CMKLR1,HJV,SLAMF8,ADCY5,VAPB,WDTC1,CASQ1,CLIC4,TFRC,FZD9,SIDT2,GLRX3,TMCO1,ATP6AP2,COMT,TFR2,PTPN6,ACOX3,ITGAV,HTT,ERO1A,PTPRN,KCNA1,FBXL5,HAP1,CACNA1C,SLC2A4,CLN8,STIM2,PPARGC1A,NPPB,NGFR,CACNA1G,ADRA1B,GPR65,CLN6,CALM1,SMARCA4,GRIA1,NPTN,SLC39A6,ANXA6,TRPV2,AKT1,PRKAA1,PTPN2,MYO5B,GNB1,BCAP31,ABCA3,PKD1,ATP6AP1,SSTR5,PARK7,SYPL2,JPH3,SLC30A7,ATP6V0D1,DMD,FKBP1A,SERPINF1,BNIP3,TMEM38B,SLC22A17,SNX10,THY1,HAS2,TMEM38A,CCR6,ORMDL3,HCN4,ATP6V0E1	darkgreen	EC_M4
GO:0031334	2.61E-05	0.999988632	42	199	positive regulation of protein-containing complex assembly	BP	3.24E-03	ASAP1,BAIAP2L2,PICK1,CDC42EP1,SYK,TLR4,RASIP1,TAL1,FCHSD2,FERMT1,FCHSD1,CD36,LMOD3,HIP1R,OCLN,IQGAP2,SNCA,AP1AR,FES,DLG1,CREB1,DDX3X,MMP1,BAIAP2L1,WAS,RAC1,FSCN1,BID,TLR6,MTOR,SPIRE1,GIT1,ISG15,PIEZO1,PLCG2,DAB2IP,CARMIL2,CDH5,GBP5,VASP,GMFG,ARPC5L	turquoise	EC_M6
GO:0072359	2.67E-05	0.999982831	143	902	circulatory system development	BP	3.26E-03	SEMA6A,TNFAIP3,PTPRB,FLT4,QKI,NOTCH4,PTK2,PDGFB,PIM1,TEK,ADGRF5,KIT,KDR,SYK,THSD7A,SHB,IL6,CXCL8,TSPAN12,C5,GJA4,TEAD2,NRCAM,NRP1,RASIP1,PIK3CG,TAL1,EPC1,GATA2,PRKD2,GPR4,PLXND1,PRCP,NOS3,PPARG,GATA3,ACVR2B,CXCR4,TIE1,JAG1,PDE2A,SFRP1,FREM2,EFNB2,RIC8A,ADAMTS9,EPHB1,PLK2,CHD7,IL1A,IGF1,NR4A1,EIF2AK3,HHEX,SMAD1,POU6F1,MEF2C,VEGFC,NDRG4,CXCL10,ANGPT2,TIPARP,THBS4,PPP1R16B,PLCG1,RAPGEF3,STAB1,HIPK1,DLL4,EDN1,STAT1,JAM3,JARID2,ETS1,SIK1,MEF2A,ROBO4,NRAP,PRKX,SEC24B,NFATC2,CD34,PECAM1,MCAM,CFLAR,PROX1,PRKD1,VANGL2,MMP21,MAP3K3,APOLD1,APOD,CREB1,CLEC14A,CYBB,BCOR,EPHB4,TBX1,CALCRL,HSPG2,RAMP2,HIF1A,RHOJ,JUP,ADIPOR2,EPHA2,MMRN2,TNFRSF1B,ALDH1A2,MTOR,PSEN1,SEMA4A,FGF18,ITGA3,PGF,ARHGEF15,EFNA1,VASH1,NPR1,SMAD2,YY1,LYL1,DSG2,AMOT,AP2B1,CCL2,JAK1,LRG1,NRARP,TMEM204,EGFL7,NOTCH1,S1PR1,ENG,DAB2IP,CDH5,MIR23A,ADGRG1,SOX17,SOX18,RGS4,BMP2,PIFO	turquoise	EC_M6

GO:0051094	2.68E-05	0.999982496	151	982	positive regulation of developmental process	BP	3.26E-03	TCF4,CTHRC1,RIMS2,CDKL3,PDGFB,CSNK1E,KDM4C,PIM1,TEK,LINGO2,KIT,KDR,SYK,SEMA4D,IL6,CXCL8,C5,IL15,SDCBP,NRCAM,NOCT,TRPM4,NRP1,ITSN2,TAL1,GATA2,PRKD2,PLXND1,NOS3,UNC13D,PPARG,GATA3,ACVR2B,CXCR4,SPTBN4,TIE1,JAG1,SFRP1,EFNB2,ARHGEF7,ATP11A,CD36,LMOD3,TCF15,EPHB1,RET,CHD7,IL1A,DNMT3B,IGF1,SMAD1,MEF2C,TLR2,WWTR1,IL7,P2RX7,VEGFC,ANGPT2,PLCG1,RAPGEF3,HIPK1,ANO6,SOX13,ADAMTS20,NEDD9,EDN1,STAT1,ETS1,HDAC1,ZDHHC6,HLX,HCLS1,NFATC2,INPP5D,CD34,CD86,PLXNA2,IGFBP3,FES,RBM4,RPS6KA1,PROX1,SS18L1,PRKD1,F11R,DOCK1,ELL3,CREB1,B2M,LTF,CYBB,IL1RAP,ETV4,TBX1,MCU,RAMP2,PROM1,PRKCH,RAC1,HIF1A,JUP,ITPKB,TNFRSF1B,RGS14,ARG2,ZC4H2,MTOR,AR,PSEN1,HOXB7,SEMA4A,FGF18,ABI3,PGF,NAP1L2,ZDHHC15,PIAS1,GPR68,SMAD2,DNM2,RCOR1,AMOT,ADRB2,JAK1,LGALS9,IL7R,MRTFB,SPIRE1,LRG1,SLITRK2,ISG15,AP3D1,NOTCH1,PIEZO1,ENG,DAB2IP,ADGRL2,CDH5,VNN1,CD46,CA2,SOX17,RELN,DIO3,SHANK3,MMD,TGIF2,RALA,BMP2,SCARF1,BMP6	turquoise	EC_M6
GO:0007549	8.31E-05	0.999991621	8	16	dosage compensation	BP	3.34E-03	YTHDC1,METTL3,BRCA1,HNRNPU,EXOSC10,RLIM,SMCHD1,LRIF1	darkgrey	EC_M8
GO:0001819	2.84E-05	0.99998595	57	304	positive regulation of cytokine production	BP	3.43E-03	IFNGR1,AKAP12,FLT4,ATF4,MAPK13,DDX58,KIT,SYK,IL6,PELI1,RSAD2,TLR4,C5,IL15,PIK3CG,PRKD2,GATA3,TMEM173,FERMT1,CD36,PDE4D,IL1A,EIF2AK3,TLR2,IL7,P2RX7,OAS3,STAT1,CD58,CD200,HDAC1,HYAL2,LAPTM5,CD34,CD86,FGR,CREB1,B2M,CYBB,IL1RAP,DDX3X,TMEM106A,HIF1A,TLR6,PSEN1,BATF,PDE4B,LGALS9,NOS2,ISG15,TRAF2,IRF8,PLCG2,OAS1,TLR1,GBP5,CD46	turquoise	EC_M6
GO:0030512	3.42E-05	0.999990119	22	64	negative regulation of transforming growth factor beta receptor signaling pathway	BP	3.46E-03	FBN2,PPARA,RASL11B,ASPN,LTBP1,SPRED3,SLC2A10,HTRA1,STRAP,FAM89B,FBN1,SKIL,SMURF1,TGFB1I1,TGFB3,PIN1,VASN,GLG1,HSPA5,FKBP1A,PMEPA1,CHST11	darkgreen	EC_M4
GO:0072503	3.47E-05	0.999981649	68	306	cellular divalent inorganic cation homeostasis	BP	3.48E-03	LRP1,SLC39A7,SMDT1,SLC39A4,SLC30A9,FKBP1B,FGF2,ATP7B,TPT1,C1QTNF1,TMTC4,STIM1,ATP2C1,PRNP,ATP2B1,CCKBR,RIC3,CXCL12,HSP90B1,C19orf12,RYR3,P2RX4,SELENOT,ATP2A2,APP,CX3CL1,CACNB3,AP3B1,ATP2B4,PKD2,SLC39A10,CHRNA7,RAP1GDS1,HTR2B,CMKLR1,ADCY5,VAPB,CASQ1,FZD9,TMCO1,PTPN6,ITGAV,HTT,ERO1A,HAP1,CACNA1C,STIM2,ADRA1B,GPR65,CALM1,GRIA1,NPTN,SLC39A6,ANXA6,TRPV2,GNB1,BCAP31,PKD1,SYPL2,JPH3,SLC30A7,DMD,FKBP1A,BNIP3,TMEM38B,THY1,TMEM38A,CCR6	darkgreen	EC_M4
GO:0016477	3.49E-05	0.999975849	201	1120	cell migration	BP	3.48E-03	CD63,LRP1,SLC12A2,PLXNB2,ANGPT1,FAM19A5,NUS1,CXCL14,FAM49B,MRTFA,PTP4A3,ADGRB1,PPARD,CTNNA1,SUN2,MDGA1,MYH9,LEP,PLAA,SRF,VEGFA,DOCK8,NCK2,CREB3,PTP4A1,TGFBR1,RTN4,TPBG,EMC10,AKIRIN1,GLI3,ROCK2,BVES,RRAS,ITGB1,PLXNA1,FGF2,MEGF8,PAK1,TGFB1,AXL,CTNNB1,AKT2,ZEB2,OSBPL8,ACTG1,NAV3,ACTN4,IRS2,DCN,ZNF703,DCLK1,STARD13,SEMA3C,DDRGK1,ILK,DPYSL3,CXCL12,KIF2A,FAT1,PALLD,SULF1,ROBO1,WWC2,MAP1B,CHST2,SEMA4F,P2RX4,PFN2,APP,RAPGEF2,CX3CL1,ABCC8,PTPN11,ARSB,LOXL2,ACVR1,TUBB2A,ADA,ATP2B4,SEMA4G,NKD1,NDN,DOCK10,ELMO2,ADIPOR1,FAP,PHLDB2,TJP1,CTTN,DNM1L,DNER,HYAL1,SBDS,TGFB2,LRP5,HTR2B,SATB2,FSTL1,MMP14,DAG1,PIK3CA,AUTS2,SDC3,LEF1,CORO1C,PRKCA,CMKLR1,SHROOM2,NTRK3,WWC3,SLAMF8,BMP7,IDH2,ITGB5,IQGAP1,CLIC4,S100A11,ARID5B,SRP54,ARPC5,LRRC15,FAM89B,HSPB1,SERPINE1,SGPL1,ITGA2B,DNAJA4,TNR,S100P,FN1,NCKAP1,GPC2,PDCD10,ITGAV,COL3A1,PRKCI,NBL1,TIMP1,VCL,WDR1,SIX4,AKT3,BMPR1A,PPARGC1A,MSN,SRCIN1,ARHGEF2,NGFR,TPM1,COL1A1,PBXIP1,RAB11A,MYOCD,FAT2,ANXA6,GP2,CD74,LAMA3,PDGFRB,ROCK1,AKT1,ITGA2,TNFAIP1,DAPK3,MYO1C,PLXNB3,PAFAH1B1,TNFRSF18,FLNA,PLXNA3,RERE,RHBDF1,BSG,COL5A1,NTNG2,CDH13,F3,BARHL2,HSPA5,LMO4,NEXN,MIR24-1,CCL16,CD151,SERPINF1,LOX,PDLIM1,ARF4,GDNF,THY1,HAS2,ARTN,TMSB4X,GLIPR2,CCR6,DUSP22,PIK3C2A,SDC2,PRKG1,FGF10	darkgreen	EC_M4
GO:0090101	3.51E-05	0.999987305	30	101	negative regulation of transmembrane receptor protein serine/threonine kinase signaling pathway	BP	3.48E-03	LRP1,FBN2,PPARA,RASL11B,ASPN,LTBP1,CRIM1,SPRED3,ACVR1,SLC2A10,HTRA1,STRAP,FAM89B,FBN1,SKIL,SMURF1,NBL1,GREM2,TGFB1I1,TGFB3,PIN1,CHRDL1,NOMO1,VASN,GLG1,HSPA5,FSTL3,FKBP1A,PMEPA1,CHST11	darkgreen	EC_M4

GO:0030003	3.53E-05	0.999979723	90	434	cellular cation homeostasis	BP	3.48E-03	LRP1,SLC12A2,SLC39A7,SMDT1,SLC39A4,SLC30A9,SLC31A1,FKBP1B,FGF2,RAB7A,ATP7B,TPT1,C1QTNF1,SLC4A7,TMTC4,MAFG,STIM1,ATP2C1,PRNP,ATP2B1,CCKBR,RIC3,CXCL12,TM9SF4,HSP90B1,C19orf12,ATP1B3,RYR3,P2RX4,SELENOT,ATP2A2,APP,SLC4A5,CX3CL1,CACNB3,AP3B1,CHP1,ATP2B4,PKD2,SLC39A10,CHRNA7,RAP1GDS1,HTR2B,ISCU,CMKLR1,HJV,SLAMF8,ADCY5,VAPB,CASQ1,CLIC4,TFRC,FZD9,GLRX3,TMCO1,ATP6AP2,TFR2,PTPN6,ITGAV,HTT,ERO1A,HAP1,CACNA1C,STIM2,ADRA1B,GPR65,CLN6,CALM1,GRIA1,NPTN,SLC39A6,ANXA6,TRPV2,GNB1,BCAP31,PKD1,ATP6AP1,SYPL2,JPH3,SLC30A7,ATP6V0D1,DMD,FKBP1A,BNIP3,TMEM38B,SLC22A17,THY1,TMEM38A,CCR6,ATP6V0E1	darkgreen	EC_M4
GO:0045597	2.91E-05	0.9999826	105	639	positive regulation of cell differentiation	BP	3.49E-03	TCF4,CTHRC1,CDKL3,CSNK1E,KDM4C,PIM1,KIT,KDR,SYK,SEMA4D,IL6,IL15,SDCBP,NRCAM,NOCT,TRPM4,NRP1,TAL1,GATA2,PLXND1,UNC13D,PPARG,GATA3,ACVR2B,CXCR4,JAG1,SFRP1,EFNB2,ARHGEF7,ATP11A,CD36,LMOD3,TCF15,RET,DNMT3B,IGF1,SMAD1,MEF2C,TLR2,WWTR1,IL7,VEGFC,SOX13,ADAMTS20,NEDD9,EDN1,STAT1,ETS1,HDAC1,HLX,HCLS1,INPP5D,CD34,CD86,PLXNA2,IGFBP3,FES,RBM4,RPS6KA1,PROX1,SS18L1,PRKD1,F11R,DOCK1,ELL3,CREB1,LTF,ETV4,TBX1,RAMP2,PROM1,PRKCH,RAC1,HIF1A,ITPKB,TNFRSF1B,RGS14,ZC4H2,MTOR,AR,FGF18,NAP1L2,PIAS1,GPR68,SMAD2,DNM2,RCOR1,LGALS9,IL7R,ISG15,AP3D1,NOTCH1,PIEZO1,ENG,CDH5,VNN1,CD46,CA2,SOX17,RELN,SHANK3,MMD,TGIF2,BMP2,BMP6	turquoise	EC_M6
GO:0000463	8.78E-05	0.999992652	7	14	maturation of LSU-rRNA from tricistronic rRNA transcript (SSU-rRNA, 5.8S rRNA, LSU-rRNA)	BP	3.50E-03	NIFK,DDX18,GTPBP4,URB1,WDR12,FTSJ3,NOL9	darkgrey	EC_M8
GO:0009057	8.79E-05	0.999941343	141	1102	macromolecule catabolic process	BP	3.50E-03	CDK2,PAN2,USP15,LATS1,POP1,EIF3E,TNRC6B,TUT7,UBR2,TOPORS,NCBP1,LRPPRC,FBXO11,PNPT1,ZMPSTE24,USP34,SYNCRIP,XPO1,PABPC4,COMMD1,FBXO48,THRAP3,UFL1,CUL1,USP45,ASCC3,AFG1L,NRDC,EXOSC9,RNASEH2B,YME1L1,TRDMT1,CNOT10,UPF2,DIS3,XRN2,CDC20,METTL3,VPS36,EXOSC8,CUL4A,PSMD13,CDC16,PAN3,MTREX,ANAPC1,IDE,TAF9,XRN1,PDE12,ZCCHC8,TTC37,USP25,PBK,CNOT1,ANAPC7,LTN1,USP16,FAM83D,TENT2,RIPK1,MDM4,UBE2C,OGA,UBE3A,CUL3,NSUN2,KIF14,UBR1,PSMD12,FXR1,DDX5,DTL,FASTKD1,SSB,UBR3,PSMD4,CCDC47,SMARCC1,DHX9,HNRNPR,PSMA6,FASTKD2,DNA2,CDC27,COP1,CUL5,PSME3,CNOT9,TBL1XR1,HNRNPU,UBE2K,NEDD4,MRPL44,DCP2,EXOSC10,FUS,USP3,TAF1,CLPX,OGT,RLIM,DIS3L,RBBP6,LARP1,PLK1,APPBP2,PSMD10,TAF15,MIB1,UPF3B,COPS3,HNRNPM,CUL4B,SERBP1,AIFM1,GSPT1,UHRF1,DROSHA,FMR1,METTL16,CCNF,RNPS1,PSMA5,EXOSC2,SF3B3,GLMN,USP33,RBM8A,FASTKD5,HNRNPD,MAD2L1,AURKA,NAF1,RNF139,RNF8,FEN1,GID4,UBXN2A,PSMD7,RCHY1	darkgrey	EC_M8
GO:0022607	8.79E-05	0.999935329	262	2230	cellular component assembly	BP	3.50E-03	RTTN,CDK2,PAN2,ONECUT2,SHPRH,LATS1,NUP107,CCT2,DDX39B,FBXO5,EIF3E,RAD21,KIF3A,TBC1D32,RRP7A,KIFC1,FANCC,HSPA9,SRPK1,PLEC,PSIP1,HAUS6,IFT74,POT1,KIF24,AKAP9,RFX3,ICK,NCBP1,ANP32B,STX17,PPIH,SENP6,YTHDC1,PNPT1,CEP162,MDN1,NUP205,ANLN,ITGB1BP1,TAF1B,CAPZA2,DDX1,WDR35,SCLT1,PLK4,STIL,MCM2,CENPO,CEP57,CEP295,YME1L1,CENPA,NASP,CLASP2,NIFK,NUP85,MRPS7,RSF1,DIAPH3,DARS,CDC20,TK1,CRNKL1,KAT6A,NAP1L1,VPS36,STRN,RRM1,CEP290,TAF7,TUBGCP3,SNRPF,RRP8,PRKDC,CCNB1,WDR62,DHX29,PAN3,HAUS5,DHX37,TGS1,TPX2,NDUFAF2,RRS1,LSM14A,CENPJ,FER,NCK1,CEP89,KIF11,ARFGEF1,SMARCA5,CENPH,TAF9,SMN2,BDP1,KNTC1,ATR,TERF1,HELLS,CCDC66,GCFC2,RACGAP1,CNOT1,USP16,PPID,ALMS1,MIS18A,NBN,CFAP298,CKAP5,NUP93,ABRAXAS1,KNL1,RAD51,SCAF11,RIPK1,MYBL2,PKP4,INO80,OIP5,STK4,MDM4,CHAF1B,UBE2C,CUL3,NAP1L4,GMNN,SLK,FP565260.1,KCTD3,SMC3,SF3B1,CENPE,TRIP13,RBM5,CEP72,CAMSAP2,COPS7B,HAUS2,KIF14,PRMT5,AJUBA,TBCE,ASPM,FXR1,RCC1,HJURP,SPO11,EIF3A,NEK2,PRC1,ALS2,BLM,DDX6,CENPF,CDKL5,HAT1,CDK1,SEPT2,DHX9,HDGFL3,FASTKD2,DDX20,PIKFYVE,DNA2,TPR,PRPF39,GJC1,KIF15,RPL10L,IFT80,UBTF,XRCC5,CDC45,BRCA1,CEP152,RP2,HAUS3,PRRC2C,LETM1,HP1BP3,DLGAP5,TRPM7,KIAA0586,ECT2,DHX33,SUV39H1,FKBP4,EXO1,RAB8A,RAD52,HNRNPU,INCENP,UBE2K,SMC1A,EIF3B,ASF1B,SRSF5,CCNB2,KPNB1,CCT3,KIF4A,FUS,AURKB,CIAO2A,TAF1,LUC7L3,ATRX,THG1L,COIL,LCMT1,KIF23,TRIM37,CENPI,MRPL22,GEMIN5,MYO9A,PLK1,BRIP1,G3BP1,PSMD10,RIOK3,EIF5,NLE1,STAG2	darkgrey	EC_M8

GO:1903827	3.60E-05	0.99997909	93	454	regulation of cellular protein localization	BP	3.53E-03	LMAN1,LRP1,STX7,ANGPT1,TCP1,CTNNA1,LEP,VCP,RAB23,TMEM30A,ERLEC1,RTN4,UBE2J1,MDFIC,GLI3,ROCK2,SLC7A11,ITGB1,KIF5B,RB1,PICALM,MARK4,PAK1,TGFB1,CTNNB1,AKT2,GPD1L,UFM1,ATP2C1,PRNP,HDAC3,DCLK1,DDRGK1,RIC3,TM9SF4,TP53INP2,VAMP8,PINX1,SORBS1,CCT8,CACNB3,PTPN11,GNL3,ATG13,TSG101,DKC1,BAP1,CHP1,ATP2B4,BRSK2,GSK3B,MMP14,DAG1,LARP7,AP2M1,IQGAP1,TFRC,FZD9,LRRC15,MAPT,C11orf65,GPC2,PDCD10,PRKCI,B3GAT3,MAGED1,IQSEC2,MSN,SYT11,NGFR,RAB11A,CLTC,NPTN,PIN1,MIEF2,AKT1,TMEM98,PRKAA1,MYO1C,BCAP31,TAX1BP3,FLNA,KCNAB2,PARK7,TXN,LEPROT,ZPR1,ORMDL3,UBE2G2,TOMM7,MESD,MAP1A,DERL2	darkgreen	EC_M4
GO:0042060	3.68E-05	0.999980143	72	329	wound healing	BP	3.59E-03	SERPINB2,LMAN1,SLC12A2,FAM19A5,PPARA,MRTFA,PPARD,C6orf89,GNAQ,MYH9,SRF,VEGFA,DST,TGFBR1,GLI3,SLC7A11,ITGB1,FGF2,DGKH,PAK1,TGFB1,AXL,C1QTNF1,ACTG1,DCN,SMPD1,ILK,MYL9,CX3CL1,ABCC8,AP3B1,HPS6,FAP,PHLDB2,NOTCH2,TGFB2,ITPK1,DAG1,PIK3CA,PRKCA,FGFR2,HSPB1,SERPINE1,ITGA2B,FN1,PTPN6,PDCD10,ADRA2C,COMP,COL3A1,TIMP1,VCL,FKBP10,SYT11,TPM1,COL1A1,TGFB3,SYTL4,ANXA6,PDGFRB,ITGA2,CD40LG,FLNA,COL5A1,F3,CD151,LOX,CD59,CSRP1,CDKN1A,PRKG1,FGF10	darkgreen	EC_M4
GO:0032970	3.07E-05	0.999984048	64	337	regulation of actin filament-based process	BP	3.65E-03	CELSR1,MTSS1,ARHGAP18,BAIAP2L2,PICK1,CDC42EP1,TEK,LIMCH1,CD2AP,TMOD1,FAM49A,TRPM4,NRP1,SVIL,SPTBN4,FCHSD2,SFRP1,FCHSD1,LMOD3,PDE4D,HIP1R,STC1,RAPGEF3,IQGAP2,EDN1,JAM3,KCNJ2,ESAM,HCLS1,AP1AR,FES,PROX1,TMOD4,F11R,ARHGDIB,PAK2,DLG1,BAIAP2L1,PPM1F,WAS,TMOD2,RAC1,JUP,FSCN1,MTOR,ABI3,TAOK2,ARHGEF15,ATP2A1,DSG2,DSC2,PDE4B,ARHGAP28,SPIRE1,S1PR1,CARMIL2,VASP,GMFG,KANK3,SHANK3,RGS4,GBA2,BST2,ARPC5L	turquoise	EC_M6
GO:0001946	3.11E-05	0.999997046	9	16	lymphangiogenesis	BP	3.67E-03	FLT4,ACVR2B,TIE1,VEGFC,PROX1,CLEC14A,EPHA2,VASH1,SOX18	turquoise	EC_M6
GO:0040011	3.85E-05	0.999972611	236	1349	locomotion	BP	3.72E-03	CD63,LRP1,SLC12A2,PLXNB2,ANGPT1,FAM19A5,NUS1,CXCL14,FAM49B,MRTFA,PTP4A3,ADGRB1,PPARD,CTNNA1,SUN2,MDGA1,MYH9,LEP,PLAA,SRF,VEGFA,DOCK8,NCK2,CREB3,DST,PTP4A1,TGFBR1,RTN4,TPBG,EMC10,AKIRIN1,GLI3,ROCK2,BVES,RRAS,ITGB1,PLXNA1,FGF2,KIF5B,GLI2,MEGF8,PAK1,ACTR3,TGFB1,AXL,CTNNB1,AKT2,ZEB2,OSBPL8,ACTG1,NAV3,ACTN4,SPINT2,IRS2,DCN,ZNF703,DCLK1,STARD13,SEMA3C,DDRGK1,ILK,DPYSL3,RYK,CXCL12,KIF2A,FAT1,PALLD,SULF1,ROBO1,WWC2,MAP1B,CHST2,SEMA4F,P2RX4,PFN2,APP,RAPGEF2,CX3CL1,ABCC8,PTPN11,PGAM4,ARSB,LOXL2,ACVR1,TUBB2A,ADA,ATP2B4,DNAH1,SEMA4G,NKD1,DNAH5,NDN,DOCK10,ELMO2,ADIPOR1,FAP,PHLDB2,TJP1,NOTCH2,CTTN,DNM1L,DNER,HYAL1,SBDS,TGFB2,LRP5,FEZ1,HTR2B,SATB2,FSTL1,MMP14,DAG1,PIK3CA,AUTS2,SDC3,LEF1,CORO1C,ANOS1,PRKCA,CMKLR1,SHROOM2,NTRK3,WWC3,SLAMF8,BMP7,IDH2,ITGB5,IQGAP1,MACO1,CLIC4,S100A11,ARID5B,SRP54,ARPC5,LRRC15,FAM89B,HSPB1,SERPINE1,SGPL1,ITGA2B,DNAJA4,TNR,S100P,FN1,NCKAP1,GPC2,PDCD10,ITGAV,ARPC2,COL3A1,PRKCI,NBL1,TIMP1,PLD1,VCL,WDR1,SIX4,CRMP1,AKT3,BMPR1A,NOTCH3,ENAH,PPARGC1A,MSN,SRCIN1,ARHGEF2,NGFR,TPM1,DOCK2,COL1A1,PBXIP1,RAB11A,MAP2K1,CLN6,TTC8,NPTN,MYOCD,FAT2,ANXA6,PIN1,GP2,CD74,LAMA3,PDGFRB,ROCK1,USP14,AKT1,ITGA2,TNFAIP1,PTPN2,DAPK3,MYO1C,PLXNB3,PAFAH1B1,TNFRSF18,NTN3,NPHP4,FLNA,PLXNA3,RERE,RHBDF1,BSG,COL5A1,NTNG2,JPH3,CDH13,F3,BARHL2,HSPA5,LMO4,NEXN,MIR24-1,CCL16,DPYSL5,CD151,SERPINF1,LOX,PDLIM1,VST	darkgreen	EC_M4

GO:0009967	4.02E-05	0.999972143	200	1127	positive regulation of signal transduction	BP	3.87E-03	CD63,IL23A,LRP1,TAB2,ANGPT1,RSPO3,PTP4A3,PPARD,CTNNA1,LGALS1,KLHDC10,LEP,CDKN2B,KIAA1324L,VEGFA,VCP,SLC35B2,NCK2,TGFBR1,RIMS1,RTN4,TPBG,SHANK1,ADAMTS3,EMC10,MAP3K7,ROCK2,INHBA,VPS35,ITGB1,PLEKHA4,FGF2,RHEB,PAK1,TGFB1,AXL,SEC13,CTNNB1,DYNC1LI1,ZEB2,CANT1,C1QTNF1,OSBPL8,CXXC5,ACTN4,ATP2C1,DCN,HDAC3,ACPP,CAVIN3,ILK,RBPMS,ARL6IP5,RYK,TMEM9B,ST5,SLC20A1,ANKRD1,PJA2,CD180,SULF1,ROBO1,SORBS1,LOXL3,P2RX4,RNF13,APP,DHX36,RAPGEF2,CX3CL1,CACNB3,PTPN11,HOMER1,OPA1,PTPN22,ACVR1,GDF6,SERINC3,MTDH,ADA,PKD2,PLA2R1,NKD1,SLC39A10,CHRNA7,ADIPOR1,NOTCH2,CDC73,DNM1L,UCHL5,CHSY1,SLC2A10,CAPRIN2,TGFB2,HTR2B,ARMC9,PIK3CA,AUTS2,LEF1,PRKCA,TLR5,KRAS,NTRK3,SPATC1L,BMP7,GOLT1B,ATF3,FGFR2,OSM,PEA15,IQGAP1,TFRC,ATF6,FZD9,PDIA3,ATP6AP2,FN1,MAOA,PTPN6,GPR137,PDCD10,ITGAV,ADRA2C,HTT,FKBP8,COL3A1,PRKCI,SKIL,SYNPO2L,FGFR3,PRRX1,GORAB,TNIK,OTUD5,EVC,AKT3,HAP1,MAGED1,BMPR1A,SLC2A4,NLGN2,MED1,RNF111,STX1B,TGFB1I1,NGFR,COL1A1,TGFB3,SHC1,PBXIP1,ADRA1B,MAP2K1,GPR65,TSPAN6,SMARCA4,SLC44A2,GSKIP,NPTN,MYOCD,PIN1,CD74,PDGFRB,ROCK1,CRLF3,RAB34,PTPN2,CLEC16A,MYDGF,DAPK3,MYO1C,BCAP31,PAFAH1B1,CSNK1G2,IRAK1,FLNA,ATP6AP1,IKBKG,PARK7,CDH13,F3,BCAR3,BCL10,WLS,MIR24-1,CCL16,FKBP1A,AGO3,ITM2C,C18orf32,TIMP3,EDA,TXN,NACC2,GLIPR2,DUSP22,TSLP,PLAGL2,OCIAD2,FGF10,MBD2	darkgreen	EC_M4
GO:0060537	4.05E-05	0.999978358	71	323	muscle tissue development	BP	3.87E-03	PPARA,LY6E,CBY1,SRF,VEGFA,TGFBR1,HIVEP3,MYH14,AKIRIN1,BVES,MYLK3,ITGB1,FGF2,RB1,TENM4,PAK1,TGFB1,SCN5A,CTNNB1,RARB,DCN,SEMA3C,ANKRD1,FLNB,HEY1,HOMER1,ACVR1,PKD2,RUNX1,PDLIM5,NOTCH2,PKP2,DNER,EYA2,TGFB2,PRKAR1A,LEF1,BMP7,ATF3,FGFR2,CASQ1,SIX4,OBSL1,BMPR1A,TCAP,MED1,PPARGC1A,NPPB,MYLPF,TPM1,CACNA1G,SGCD,MYOCD,PIN1,FBXO22,PDGFRB,MYOM1,PRKAA1,COL11A1,MIR24-1,DMD,FKBP1A,LOX,IGSF8,CACYBP,CSRP1,SRPK3,HOPX,HCN4,WNT2,CFL2	darkgreen	EC_M4
GO:0001706	4.14E-05	0.999990373	17	43	endoderm formation	BP	3.93E-03	INHBA,CTNNB1,MMP15,MMP2,CDC73,MMP14,COL6A1,ITGB5,FN1,ITGAV,COL5A2,LAMA3,COL5A1,HSBP1,COL11A1,DUSP2,DUSP4	darkgreen	EC_M4
GO:0010032	9.95E-05	0.999997939	5	6	meiotic chromosome condensation	BP	3.95E-03	SMC2,NCAPD3,SMC4,NCAPD2,INCENP	darkgrey	EC_M8
GO:0032784	1.00E-04	0.999976814	14	49	regulation of DNA-templated transcription, elongation	BP	3.98E-03	DDX39B,SCAF8,EZH2,CCNT2,WDR43,SUPT16H,ALYREF,TCERG1,ERCC6,LEO1,HNRNPU,THOC1,ZNF326,RNF8	darkgrey	EC_M8
GO:2000113	1.04E-04	0.999929779	147	1144	negative regulation of cellular macromolecule biosynthetic process	BP	4.11E-03	PAN2,BCLAF1,FBXO5,ZNF608,SCAF8,EIF3E,MRPL13,XRCC6,TNRC6B,TUT7,POT1,RFX3,TRIM28,ZNF451,PNPT1,SYNCRIP,XPO1,CASP8AP2,TIA1,ZNF350,EZH2,DHFR,PUS7,HDAC2,EXOSC9,DNAJC2,SETDB2,CNOT10,RSF1,DIS3,BIRC5,GTPBP4,CHD8,KAT6A,METTL3,RIF1,E2F7,TAF7,EXOSC8,NR2C1,BRCA2,ELK3,TCERG1,CBX5,PAN3,CAPRIN1,ZC3H8,LSM14A,BTAF1,TDG,NCK1,CGGBP1,XRN1,PDE12,SAP30,ATR,TERF1,GCFC2,CNOT1,GABPA,PPID,RBL1,E2F8,TRIM33,ZBED6,ZNF248,MDM4,HIVEP1,CUL3,GMNN,NFKB1,KHDRBS1,NCL,PRMT5,AJUBA,BPTF,FXR1,DDX5,ORC2,BLM,DDX6,CENPF,SP3,HNRNPR,DDX20,TPR,CCAR1,XRCC5,NPAT,BRCA1,CNOT9,NSD2,TBL1XR1,SUV39H1,FOXM1,EIF2AK1,LYAR,FAM220A,HNRNPU,PDS5A,WAPL,NEDD4,SMARCE1,RFC1,CDC6,DCP2,NSD1,EXOSC10,USP3,AURKB,TAF1,RLIM,ATRX,TIPIN,MBTD1,TRIM37,LARP1,PLK1,ILF3,AATF,PSMD10,TYMS,ZNF3,MIER1,NIPBL,PHF6,UHRF1,FMR1,METTL16,EHMT1,EXOSC2,SCAI,CTCF,DEPDC1,HNRNPD,ZNF684,DRAP1,ZNF614,RBAK,BRMS1L,RNF139,C1D,RNF8,ZNF558,IKZF5,PAIP2,ZNF529	darkgrey	EC_M8
GO:0022904	1.90E-06	0.999999418	30	99	respiratory electron transport chain	BP	4.16E-03	UQCRQ,NDUFB2,UQCRH,NDUFB6,NDUFC2,NDUFA2,COX7A1,UQCRFS1,COQ9,DGUOK,NDUFV1,NDUFS2,AIFM2,UQCC3,NDUFB7,GBA,NDUFA7,UQCR11,NDUFS7,TAZ,MT-ND1,MT-ND2,MT-CO1,MT-CO2,MT-CO3,MT-ND3,MT-ND4L,MT-ND4,MT-ND5,MT-CYB	cyan	EC_M2
GO:0042773	2.34E-06	0.999999335	27	83	ATP synthesis coupled electron transport	BP	4.16E-03	UQCRQ,NDUFB2,UQCRH,NDUFB6,NDUFC2,NDUFA2,COX7A1,COQ9,DGUOK,NDUFV1,NDUFS2,UQCC3,NDUFB7,NDUFA7,UQCR11,NDUFS7,TAZ,MT-ND1,MT-ND2,MT-CO1,MT-CO2,MT-CO3,MT-ND3,MT-ND4L,MT-ND4,MT-ND5,MT-CYB	cyan	EC_M2
GO:0042775	2.34E-06	0.999999335	27	83	mitochondrial ATP synthesis coupled electron transport	BP	4.16E-03	UQCRQ,NDUFB2,UQCRH,NDUFB6,NDUFC2,NDUFA2,COX7A1,COQ9,DGUOK,NDUFV1,NDUFS2,UQCC3,NDUFB7,NDUFA7,UQCR11,NDUFS7,TAZ,MT-ND1,MT-ND2,MT-CO1,MT-CO2,MT-CO3,MT-ND3,MT-ND4L,MT-ND4,MT-ND5,MT-CYB	cyan	EC_M2

GO:0006629	2.48E-06	0.999998728	155	982	lipid metabolic process	BP	4.16E-03	ZADH2,ORMDL2,DGKA,PIP4K2C,PEX7,CDK4,CHKB,ABHD16A,ALG12,CERK,AGPAT1,TSPO,HSD17B8,B3GALT4,CYB5R3,GPAA1,PIGO,PDGFRA,PLPP6,HINT2,ALG2,TTC7A,SLC44A1,ETFB,PTGR1,LPIN1,SPHK2,TPRA1,ACSS1,FDXR,LIPE,PAFAH1B3,ECHDC3,ACAA1,TAMM41,CYGB,PIP4P1,SIRT2,LAMTOR1,HACL1,GPCPD1,PLPP5,DDHD2,CYP1B1,PTDSS2,SDR16C5,THNSL2,CBR4,PIK3R1,ACSS2,RETSAT,HTRA2,PRKAG1,PLBD2,GPAT3,LPIN3,ORMDL1,HNF4A,ERLIN1,PIGT,PLA2G4B,ACOT8,LONP2,SRD5A1,OSBPL5,CYB5R1,IRS1,ACADS,GPAM,FMO5,CHKA,ABHD4,LRP10,GPX1,MTMR11,PIGM,ABCB11,PC,CERS2,PER2,ALG3,CLCN2,SCAP,EHHADH,POR,IDH1,ACADL,SOAT1,PECR,PHB2,ABHD12B,DDHD1,CYP27A1,SMPD4,PLA2G16,TNFRSF1A,ABHD8,WASHC1,FADS3,CTDNEP1,ILVBL,GPS2,EPHX1,THRA,TECR,PLSCR3,PIGH,PIP4K2B,CRABP2,OSBPL7,HSD3B7,ACOT2,ABCD4,GBA,ERG28,DPM3,GCDH,AC138894.1,SCPEP1,SLC27A3,MTMR4,HEXA,ACSM3,ACACA,SREBF1,CERS4,LEPR,MPPE1,DHRS13,ACAA2,ALG1,MTMR1,CPT2,TRPV1,PNPLA7,TAZ,LMF1,PIGQ,INPP5E,PLPPR3,GSTM4,RXRA,VAV3,PTGES2,VAC14,LCAT,ABCA2,BSCL2,PLPP3,EPHX3,C1QTNF2,RDH14,STUB1,SCP2,DNAJC19	cyan	EC_M2
GO:0014706	4.41E-05	0.999976483	68	307	striated muscle tissue development	BP	4.17E-03	PPARA,LY6E,CBY1,SRF,VEGFA,TGFBR1,HIVEP3,MYH14,AKIRIN1,BVES,MYLK3,ITGB1,FGF2,RB1,TENM4,PAK1,TGFB1,SCN5A,CTNNB1,RARB,DCN,SEMA3C,ANKRD1,FLNB,HEY1,HOMER1,ACVR1,RUNX1,PDLIM5,NOTCH2,PKP2,DNER,EYA2,TGFB2,PRKAR1A,LEF1,BMP7,ATF3,FGFR2,CASQ1,SIX4,OBSL1,BMPR1A,TCAP,MED1,NPPB,MYLPF,TPM1,CACNA1G,SGCD,MYOCD,PIN1,FBXO22,PDGFRB,MYOM1,PRKAA1,COL11A1,MIR24-1,DMD,FKBP1A,LOX,IGSF8,CACYBP,SRPK3,HOPX,HCN4,WNT2,CFL2	darkgreen	EC_M4
GO:0017015	4.45E-05	0.999983661	30	102	regulation of transforming growth factor beta receptor signaling pathway	BP	4.17E-03	FBN2,PPARA,CDKN2B,RASL11B,ASPN,TGFB1,ZEB2,LTBP1,SPRED3,ZNF703,DKK3,SLC2A10,HTRA1,STRAP,FAM89B,FBN1,SKIL,SMURF1,RNF111,TGFB1I1,TGFB3,MYOCD,PIN1,VASN,GLG1,HSPA5,FKBP1A,LOX,PMEPA1,CHST11	darkgreen	EC_M4
GO:0048193	4.48E-05	0.999977396	59	259	Golgi vesicle transport	BP	4.18E-03	LMAN1,AP3S1,SEC24A,GGA1,VCP,EXOC1,LMAN2L,NAPA,COPG1,SEC13,SEC23B,COG6,ATP2C1,ARL1,TMED2,COPB2,ERGIC3,COPB1,USO1,EXOC6B,RAB7B,ARL3,SEC22B,ERGIC2,GOLGA5,WIPI1,PITPNB,SEC22A,VAPB,HYOU1,COPA,SCFD1,SCYL1,SEC23A,SCAMP3,HTT,PRKCI,EXOC5,BLZF1,STX18,KDELR2,MYO5A,SNX8,LMAN2,TMED10,TMED1,RAB31,NAPG,RAB34,BCAP31,VAMP7,AP1G1,C16orf70,RAB6A,ARF4,STXBP6,ARFRP1,GABARAPL2,TMED3	darkgreen	EC_M4
GO:0009100	4.61E-05	0.999975647	66	299	glycoprotein metabolic process	BP	4.24E-03	B4GALT7,NUS1,PPARD,LEP,LARGE1,CHST10,ERP44,UBE2J1,GFPT1,RPN1,POMGNT1,ITM2B,CHPF2,ST6GALNAC2,CTNNB1,TRAK1,STT3B,CANT1,TMTC4,KDELC1,TMTC3,DCN,TUSC3,IMPAD1,ST8SIA4,SULF1,B3GNT4,TET3,RPN2,PTX3,AMFR,DPY19L4,CHP1,MAN1A2,MARCH6,CHSY1,HYAL1,SLC2A10,B4GALT4,B3GALT1,GALNT2,MAN2A2,NCSTN,EDEM3,PLOD3,SYVN1,DDOST,B3GAT3,UGDH,CHST12,GALNT16,XYLT2,GALNT10,GALNT1,MPI,EGFLAM,PARK7,POMT1,COL11A1,DPM2,FBXO27,ITM2C,DPM1,SLC35C1,CHST11,HS2ST1	darkgreen	EC_M4
GO:0071692	4.61E-05	0.999976279	61	271	protein localization to extracellular region	BP	4.24E-03	LRP1,CANX,SLC12A2,FBN2,PPARD,LEP,UNC13B,CLOCK,EXOC1,REST,P3H1,RCN3,FKBP1B,KIF5B,DNAJC1,COPG1,TGFB1,LTBP1,IRS2,RAB11FIP1,PCLO,ANKRD1,OSBP,SELENOT,ABCC8,PTPN11,PFKM,BRSK2,DYNLL1,DNM1L,CPT1A,TGFB2,LRP5,HADH,DISP1,IDH2,ADCY5,SIDT2,TANGO2,FBN1,COMP,NBL1,PTPRN,NLGN2,SRCIN1,LTBP2,TMED10,TGFB3,UBE2Q1,MYH10,SEL1L,SYTL4,ACSL4,MYOM1,PAFAH1B1,MIDN,SSTR5,PARK7,RHBDF1,WLS,TMEM167A	darkgreen	EC_M4
GO:0051216	4.65E-05	0.999979903	40	154	cartilage development	BP	4.26E-03	COL11A2,LEP,SRF,RUNX2,TGFBR1,GLI3,FGF2,GLI2,TGFB1,CTNNB1,SERPINH1,RARB,LUM,DDRGK1,IMPAD1,SULF1,PTPN11,LOXL2,GDF6,BMP1,RUNX1,CHSY1,HYAL1,SATB2,BMP7,COMP,FGFR3,PRRX1,TIMP1,EVC,BMPR1A,THBS3,COL1A1,PBXIP1,ANXA6,PKD1,COL11A1,GLG1,OTOR,CHST11	darkgreen	EC_M4
GO:0034504	1.08E-04	0.99994873	42	244	protein localization to nucleus	BP	4.27E-03	LATS1,NUP107,CCT2,UBR5,TNPO3,TOPORS,NOL8,TRIM28,XPO1,CCT4,NUP85,IPO5,NUP58,IPO11,IPO7,RRS1,POLR1A,TNPO1,MCRS1,NUP54,CCT7,NUP93,MORC3,HEATR3,NOLC1,CCT5,CCT6A,INTS13,CSE1L,NUP133,CDK1,PIKFYVE,TPR,NMD3,ECT2,HNRNPU,KPNB1,CCT3,PLK1,NUP62CL,NUP155,NUP35	darkgrey	EC_M8
GO:0048588	4.74E-05	0.999977726	48	195	developmental cell growth	BP	4.31E-03	LRP1,RAB21,PPARA,SH3GL2,PLAA,SRF,VEGFA,RIMS1,RTN4,ITGB1,PLXNA1,MEGF8,PAK1,CTNNB1,DCLK1,SEMA3C,ILK,RYK,RNF6,CXCL12,EIF4G2,MAP1B,DIP2B,SEMA4F,APP,ZFYVE27,SPP1,SEMA4G,PDLIM5,NDN,CTTN,GSK3B,AUTS2,IQGAP1,MAPT,TNR,FN1,SMURF1,VCL,RAB11A,ISLR2,PIN1,TRPV2,PAFAH1B1,PLXNA3,BARHL2,MIR24-1,OLFM1	darkgreen	EC_M4

GO:0044271	2.86E-06	0.999998068	451	3436	cellular nitrogen compound biosynthetic process	BP	4.32E-03	NFATC1,ZNF236,TSHZ1,ORMDL2,PYM1,SMARCC2,STAT6,MAP3K5,CDK4,CITED2,MAML1,SQSTM1,IRAK3,HELB,GFPT2,OSR2,ALDH7A1,HSPA1B,EHMT2,ZBTB12,HDAC10,ATF6B,EIF3H,RXRB,TSPO,RING1,RPS18,B3GALT4,CYB5R3,ZHX2,ZBTB22,PMM1,MYC,TOB2,SMAD5,EP300,IBA57,KDM3B,ZNF76,EEF1D,RPL3,DDX17,EIF3L,NFIB,EIF3D,HSF1,CCND3,TRERF1,CDKN2A,RPL8,HMOX1,WASL,KLF9,POLR1C,JAK2,ZBTB45,RPS5,ZNF135,ZNF667,ZNF865,RPL28,RPS9,EEF1A1,MALSU1,EFEMP1,ZNF692,RPS7,ANXA4,AKNA,MED25,IL4I1,ING3,ARHGEF5,IRF3,LPIN1,RPS11,FOXO3,KLF15,SPHK2,RPL18,NOP53,ABTB1,MAML2,BICRA,KMT2E,BBS7,MEIS3,TRIM13,ACSS1,IRF2BP1,SIX5,FOSB,ZNF746,NR2C2,ZNF862,TSC22D1,UROD,RPS8,ZNF575,MIF4GD,NAA16,MLH1,GTF3C2,UNK,ZNF574,WBP2,KMT2C,MYD88,TADA3,RPS3,TEP1,BRPF1,PNP,DYRK1B,EID2,ZNF219,RPS16,MED29,SAMD4B,MRPS12,SIRT2,RPTOR,RPL15,APBB3,EIF3K,NUP98,TRIM21,CYP1B1,GNPDA1,HGF,APBB1,TAF10,DEAF1,HOXC4,NCOA4,LIN37,TARBP2,KMT2B,EIF3F,RBCK1,RARG,SDR16C5,ZNF740,EIF4B,LMO2,POFUT1,URI1,PIK3R1,ACSS2,NCOA2,ATF1,TNKS1BP1,RPS3A,POLE4,KDM2B,CHDH,ZFHX4,CAMKK2,SCAND1,EIF2A,SETD6,MCPH1,SSBP2,FNIP2,DGUOK,ARL2BP,SHOX2,NEK4,SPR,ZHX3,VDR,MMS19,LPIN3,CPOX,FOXF2,F2RL1,SORBS3,ORMDL1,HNF4A,TP53INP1,FARS2,PIDD1,RPLP2,HR,PCIF1,TRIM45,CD47,NPM3,WARS2,PARP3,FASTKD3,ELK4,LGR4,ICE1,MRPL36,SP140,TCF7L2,DIS3L2,PUM1,TXNIP,APEH,AIP,SOX5,ADNP,RPS6KB2,ZNF217,QARS,IMPDH2,DTX3L,DALRD3,PARP14,EIF4H,ECM1,RCOR3,ARNT,PRMT2,CERS2,RIPK3,CTPS2,GABPB2,PER2,TWIST2,VPS72,	cyan	EC_M2
GO:0048518	3.76E-05	0.999970781	568	4533	positive regulation of biological process	BP	4.40E-03	ZNF516,TNFRSF11A,TCF4,TNFAIP8,SEMA6A,IFNGR1,TNFAIP3,PLAGL1,TRIM39,AKAP12,CNOT6,FLT4,CTHRC1,RIMS2,ZBED4,GRAMD4,QKI,NOTCH4,AFDN,TCF20,CDKL3,TRDN,TPD52L1,NCOA7,MTSS1,RNF146,ASAP1,SGSM3,PTK2,DAPK1,ATF4,PDGFB,AGTPBP1,MAPK13,BRPF3,PODXL,CSNK1E,BAIAP2L2,PICK1,KDM4C,PIM1,CDC42EP1,MFNG,NFYA,LRRC19,TEK,LINGO2,PRDM4,LIMCH1,DDX58,ATP8A1,FAM122A,TEC,SH3RF3,ADGRF5,KIT,TNFRSF21,KDR,CD2AP,NFIL3,SYK,GADD45G,SEMA4D,IL6,REL,PPT1,CREB5,CXCL8,PPIE,PELI1,UTP11,RSAD2,DOCK4,CAV2,KLF11,GRB10,LANCL2,TLR4,TCAF2,GRIK2,TRAF1,C5,IL15,CCNY,TEAD2,SDCBP,MYCN,NRCAM,NOCT,TUT4,TRPM4,NRP1,RAB3B,PUM2,RASIP1,PIK3CG,SVIL,ITSN2,TAL1,PHF10,MTMR2,EPC1,KDM4D,GATA2,DNMT3A,PRKD2,CST7,CTSC,RAB38,CAB39L,GPR4,PLXND1,NECTIN2,NOS3,GGA3,GAB2,RALB,UNC13D,PPARG,GATA3,ACVR2B,CXCR4,EDNRB,NET1,NDFIP2,SPTBN4,TIE1,IKBKB,JAG1,P2RY6,P2RY2,FCHSD2,NFKBIB,PDE2A,TMEM173,SFRP1,HBEGF,NR1D2,DND1,FERMT1,RPL26,EFNB2,ANKLE2,ARHGEF7,ASH2L,ATP11A,FCHSD1,P2RX2,CD36,SIRT3,LMOD3,MAGI1,PRAG1,PRR5L,TCF15,SCARB1,EPHB1,PLK2,RET,PDE4D,MERTK,TNFRSF19,CHD7,IL1A,DNMT3B,IGF1,NR4A1,KLKB1,EIF2AK3,ABHD6,CSPP1,HHEX,KDM3A,ITCH,ERBIN,SH3RF1,SMAD1,ZBTB38,CLCN3,HIP1R,OCLN,STOX2,ARHGEF3,SOX7,HMBOX1,GPD1,ENC1,HEXB,MEF2C,SMARCD1,TLR2,WWTR1,IL7,P2RX7,VEGFC,NDRG4,GPM6A,GUCY1A1,CXCL10,ANGPT2,MADD,KMT2D,BMP2K,TIPARP,OAS3,NLRC5,THBS4,PPP1R16B,TPCN1,PLCG1,STC1,RAPGEF3,NR4A2,DGKZ,IQGAP2,HIPK1,TOX2,ANO6,DLL4,ZDHHC1	turquoise	EC_M6
GO:0071559	4.94E-05	0.999976533	50	205	response to transforming growth factor beta	BP	4.47E-03	FBN2,PPARA,CDKN2B,RASL11B,COL1A2,ASPN,TGFBR1,MAP3K7,ROCK2,ZYX,TGFB1,ZEB2,LTBP1,SPRED3,SMAD9,ZNF703,ANKRD1,DKK3,ACVR1,SLC2A10,TGFB2,FGFR2,ITGB5,HTRA1,STRAP,FAM89B,FBN1,COL3A1,SKIL,SMURF1,BMPR1A,PPARGC1A,RNF111,TGFB1I1,LTBP2,COL1A1,TGFB3,MYOCD,PIN1,ROCK1,NLK,VASN,GLG1,HSPA5,FKBP1A,LOX,DUSP22,PMEPA1,CHST11,WNT2	darkgreen	EC_M4
GO:0050673	3.88E-05	0.999980173	59	316	epithelial cell proliferation	BP	4.51E-03	TNFAIP3,FLT4,MTSS1,PDGFB,TEK,KIT,KDR,IL6,CAV2,NRP1,GATA2,PRKD2,PPARG,GATA3,EDNRB,TIE1,SFRP1,FERMT1,SCARB1,IGF1,NR4A1,MEF2C,VEGFC,THBS4,PPP1R16B,PLCG1,DLL4,STAT1,PRKX,EAF2,CD34,CFLAR,TGM1,RPS6KA1,PROX1,PRKD1,DLG1,B2M,ITGA4,IGFBP5,TBX1,HIF1A,EPHA2,MCC,ALDH1A2,MTOR,AR,PSEN1,PGF,VASH1,CCL2,LRG1,NRARP,EGFL7,NOTCH1,DAB2IP,MIR23A,BMP2,BMP6	turquoise	EC_M6
GO:0018126	5.08E-05	0.999991783	12	26	protein hydroxylation	BP	4.57E-03	P4HA2,P3H1,CRTAP,PLOD2,HIF1AN,EGLN3,PRDX4,PLOD3,P4HA1,ERO1A,FKBP10,P3H3	darkgreen	EC_M4
GO:0019646	3.43E-06	0.999999063	25	75	aerobic electron transport chain	BP	4.70E-03	UQCRQ,NDUFB2,UQCRH,NDUFB6,NDUFC2,NDUFA2,COX7A1,COQ9,NDUFV1,NDUFS2,UQCC3,NDUFB7,NDUFA7,UQCR11,NDUFS7,MT-ND1,MT-ND2,MT-CO1,MT-CO2,MT-CO3,MT-ND3,MT-ND4L,MT-ND4,MT-ND5,MT-CYB	cyan	EC_M2
GO:1904869	1.22E-04	0.99999205	6	11	regulation of protein localization to Cajal body	BP	4.79E-03	CCT2,CCT4,CCT7,CCT5,CCT6A,CCT3	darkgrey	EC_M8
GO:1904871	1.22E-04	0.99999205	6	11	positive regulation of protein localization to Cajal body	BP	4.79E-03	CCT2,CCT4,CCT7,CCT5,CCT6A,CCT3	darkgrey	EC_M8

GO:0043547	4.49E-05	0.999979631	44	202	positive regulation of GTPase activity	BP	5.18E-03	TBC1D30,AFDN,ASAP1,SGSM3,TBC1D22B,SEMA4D,RGP1,CHN2,ARHGAP25,ASAP2,CAV2,TBC1D9,ARHGAP42,USP6NL,TBC1D4,NET1,RALGAPA2,SFRP1,ARHGEF7,ARAP3,RAPGEF3,NEDD9,ADPRH,F11R,ITGA6,SIPA1,ARHGAP27,BCR,RUNDC1,ELMOD1,RCC2,EPHA2,TBC1D1,RGS14,ARHGEF15,TBC1D8B,CCL2,SGSM2,RAPGEF1,S1PR1,TBC1D13,RGS4,RGS16,TBC1D20	turquoise	EC_M6
GO:0140352	5.81E-05	0.999963334	119	618	export from cell	BP	5.19E-03	LRP1,CANX,RAB21,SLC12A2,LY6E,PPARD,MYH9,LEP,UNC13B,CLOCK,EXOC1,REST,NLRP5,P3H1,RIMS1,INHBA,VPS41,RCN3,LIN7B,FKBP1B,KIF5B,NAPA,DNAJC1,RAB7A,COPG1,ATP7B,PDCD6IP,PAK1,TGFB1,AXL,RAB3GAP1,C1QTNF1,RAB2B,IRS2,ATP2B1,RAB11FIP1,PCLO,SNPH,CXCL12,FXYD1,ANKRD1,OSBP,VAMP8,ATP1B3,P2RX4,SELENOT,ATP2A2,ABCC8,PTPN11,EXOC6B,PFKM,TSG101,CRY2,VPS18,SPP1,LGI3,BRSK2,DYNLL1,KDM5B,DNM1L,CPT1A,TGFB2,LRP5,GSK3B,HADH,DISP1,IDH2,ADCY5,OSM,BACE1,SIDT2,LINC02210-CRHR1,TANGO2,TFR2,ADRA2C,COMP,EXOC5,PTPRN,CYP19A1,HAP1,SLC29A4,NLGN2,SRCIN1,STX1B,SYT11,LTBP2,TMED10,TGFB3,CACNA1G,UBE2Q1,RAB11A,MYH10,SEL1L,CALM1,SYTL4,SCAMP5,ACSL4,RASL10B,MYOM1,RAB31,PAFAH1B1,SDF4,MIDN,ATP6AP1,SSTR5,VAMP7,PARK7,RHBDF1,AP1G1,WLS,CBARP,TMEM38B,GDNF,TMEM167A,STXBP6,PIK3C2A,KCNMB4,FGF10,EXOC3L4	darkgreen	EC_M4
GO:0010558	1.34E-04	0.999908986	147	1150	negative regulation of macromolecule biosynthetic process	BP	5.23E-03	PAN2,BCLAF1,FBXO5,ZNF608,SCAF8,EIF3E,MRPL13,XRCC6,TNRC6B,TUT7,POT1,RFX3,TRIM28,ZNF451,PNPT1,SYNCRIP,XPO1,CASP8AP2,TIA1,ZNF350,EZH2,DHFR,PUS7,HDAC2,EXOSC9,DNAJC2,SETDB2,CNOT10,RSF1,DIS3,BIRC5,GTPBP4,CHD8,KAT6A,METTL3,RIF1,E2F7,TAF7,EXOSC8,NR2C1,BRCA2,ELK3,TCERG1,CBX5,PAN3,CAPRIN1,ZC3H8,LSM14A,BTAF1,TDG,NCK1,CGGBP1,XRN1,PDE12,SAP30,ATR,TERF1,GCFC2,CNOT1,GABPA,PPID,RBL1,E2F8,TRIM33,ZBED6,ZNF248,MDM4,HIVEP1,CUL3,GMNN,NFKB1,KHDRBS1,NCL,PRMT5,AJUBA,BPTF,FXR1,DDX5,ORC2,BLM,DDX6,CENPF,SP3,HNRNPR,DDX20,TPR,CCAR1,XRCC5,NPAT,BRCA1,CNOT9,NSD2,TBL1XR1,SUV39H1,FOXM1,EIF2AK1,LYAR,FAM220A,HNRNPU,PDS5A,WAPL,NEDD4,SMARCE1,RFC1,CDC6,DCP2,NSD1,EXOSC10,USP3,AURKB,TAF1,RLIM,ATRX,TIPIN,MBTD1,TRIM37,LARP1,PLK1,ILF3,AATF,PSMD10,TYMS,ZNF3,MIER1,NIPBL,PHF6,UHRF1,FMR1,METTL16,EHMT1,EXOSC2,SCAI,CTCF,DEPDC1,HNRNPD,ZNF684,DRAP1,ZNF614,RBAK,BRMS1L,RNF139,C1D,RNF8,ZNF558,IKZF5,PAIP2,ZNF529	darkgrey	EC_M8
GO:0007369	5.90E-05	0.999975216	37	138	gastrulation	BP	5.24E-03	SRF,INHBA,ITGB1,TENM4,MEGF8,CTNNB1,TLX2,MMP15,ACVR1,MMP2,PHLDB2,CDC73,EYA2,LRP5,PRKAR1A,MMP14,DAG1,LEF1,COL6A1,BMP7,FGFR2,ITGB5,PHLDB1,FN1,ITGAV,COL5A2,UGDH,BMPR1A,LAMA3,ITGA2,COL5A1,HSBP1,COL11A1,WLS,DUSP2,DUSP4,ARFRP1	darkgreen	EC_M4
GO:0000413	1.34E-04	0.999977284	10	32	protein peptidyl-prolyl isomerization	BP	5.24E-03	PPIL4,PPIL1,PPIH,FKBP15,NKTR,PPWD1,PPID,PPIG,AIPL1,FKBP4	darkgrey	EC_M8
GO:0010562	4.73E-05	0.999970786	109	669	positive regulation of phosphorus metabolic process	BP	5.37E-03	TNFRSF11A,FLT4,TPD52L1,PTK2,PDGFB,TEK,LIMCH1,TEC,ADGRF5,KIT,KDR,SYK,SEMA4D,IL6,TLR4,IL15,CCNY,SDCBP,NRP1,PIK3CG,TAL1,PRKD2,RAB38,CAB39L,NOS3,RALB,PPARG,EDNRB,TIE1,P2RY6,HBEGF,ANKLE2,CD36,PRR5L,EPHB1,RET,MERTK,IGF1,GPD1,MEF2C,P2RX7,VEGFC,MADD,THBS4,PPP1R16B,RAPGEF3,DGKZ,NEDD9,EDN1,EHD4,SNCA,HDAC1,NCF1,HCLS1,PECAM1,CD86,FGR,PPP2R5A,PROX1,PRKD1,MLXIPL,ACE,ITGA6,LIMK2,PAK2,DLG1,MAP3K11,LTF,EPHB4,DDX3X,TBX1,TNFAIP8L3,FGF23,LMTK2,RAC1,HIF1A,ADIPOR2,EPHA2,TLR6,MTOR,AR,PSEN1,FGF18,PGF,TAOK2,EFNA1,CD19,PIK3R5,RPS6KA5,ADRB2,YES1,SH2D3A,NOS2,UNC119,TRAF2,PLCG2,ENG,SH2D3C,DAB2IP,CDH5,RELN,ALKAL1,FGF16,MMD,DAZAP2,COPS8,BMP2,PIFO,BMP6	turquoise	EC_M6
GO:0045937	4.73E-05	0.999970786	109	669	positive regulation of phosphate metabolic process	BP	5.37E-03	TNFRSF11A,FLT4,TPD52L1,PTK2,PDGFB,TEK,LIMCH1,TEC,ADGRF5,KIT,KDR,SYK,SEMA4D,IL6,TLR4,IL15,CCNY,SDCBP,NRP1,PIK3CG,TAL1,PRKD2,RAB38,CAB39L,NOS3,RALB,PPARG,EDNRB,TIE1,P2RY6,HBEGF,ANKLE2,CD36,PRR5L,EPHB1,RET,MERTK,IGF1,GPD1,MEF2C,P2RX7,VEGFC,MADD,THBS4,PPP1R16B,RAPGEF3,DGKZ,NEDD9,EDN1,EHD4,SNCA,HDAC1,NCF1,HCLS1,PECAM1,CD86,FGR,PPP2R5A,PROX1,PRKD1,MLXIPL,ACE,ITGA6,LIMK2,PAK2,DLG1,MAP3K11,LTF,EPHB4,DDX3X,TBX1,TNFAIP8L3,FGF23,LMTK2,RAC1,HIF1A,ADIPOR2,EPHA2,TLR6,MTOR,AR,PSEN1,FGF18,PGF,TAOK2,EFNA1,CD19,PIK3R5,RPS6KA5,ADRB2,YES1,SH2D3A,NOS2,UNC119,TRAF2,PLCG2,ENG,SH2D3C,DAB2IP,CDH5,RELN,ALKAL1,FGF16,MMD,DAZAP2,COPS8,BMP2,PIFO,BMP6	turquoise	EC_M6
GO:0071168	1.39E-04	0.999977509	10	27	protein localization to chromatin	BP	5.39E-03	RAD21,MSH2,EZH2,FAM208A,ESCO2,SETD2,CDCA5,WAPL,PLK1,NIPBL	darkgrey	EC_M8

GO:0046605	1.39E-04	0.999969427	13	42	regulation of centrosome cycle	BP	5.39E-03	XPO1,GEN1,PLK4,STIL,CEP295,CHORDC1,CENPJ,ALMS1,BRCA1,TRIM37,CEP76,CCNF,AURKA	darkgrey	EC_M8
GO:1901566	4.30E-06	0.999997319	200	1317	organonitrogen compound biosynthetic process	BP	5.41E-03	ORMDL2,PYM1,ATG12,CDK4,GFPT2,CHKB,ALDH7A1,ALG12,EIF3H,DSE,TSPO,RPS18,B3GALT4,PMM1,IBA57,EEF1D,RPL3,EIF3L,GPAA1,EIF3D,RPL8,PIGO,RPS5,ALG2,RPL28,RPS9,EEF1A1,MALSU1,SLC44A1,RPS7,AP2A1,RPS11,FOXO3,SPHK2,RPL18,ABTB1,FKRP,ACSS1,SERP2,UROD,SEPHS1,RPS8,MIF4GD,MLH1,UNK,GALNT11,CSGALNACT1,RPS3,PNP,RPS16,SAMD4B,MRPS12,RPL15,EIF3K,GALNT15,PTDSS2,CLIP3,TARBP2,EIF3F,APIP,EIF4B,POFUT1,NECAB3,ACSS2,RPS3A,CHDH,EIF2A,DGUOK,SPR,CHST14,CPOX,ORMDL1,FARS2,RPLP2,PIGT,ACOT8,PCIF1,WARS2,FASTKD3,MRPL36,CHKA,TCF7L2,DIS3L2,PUM1,APEH,RPS6KB2,QARS,PIGM,IMPDH2,GCNT7,DALRD3,EIF4H,CERS2,CTPS2,PER2,ALG3,ZDHHC24,RPS21,RPL35A,PPOX,DNAJC30,GLUL,RPL11,YBX3,XXYLT1,NFE2L2,SOAT1,MRPL49,MTHFS,EXOSC7,PCBD1,ZDHHC8,ATIC,KDELC2,RPL27,UBA52,SMPD4,AC022400.6,UQCC3,WDR45,RARA,ILVBL,TECR,PIGH,ZFP36L1,MTHFR,MRI1,MRPL10,PNPO,PER1,GBA,KRTCAP2,DPM3,GCDH,FLAD1,AC138894.1,PARP16,TNRC6A,LARP6,RPL7A,HEXA,EEF2K,MOCOS,MRPL4,EIF3G,ACACA,CERS4,NDUFA7,RPL23A,PARN,MPPE1,MRPL37,ALG1,NAGPA,EEF2,DPH1,MRPL41,MRPS34,GAMT,ATP5F1D,TAZ,PIGQ,INPP5E,MRPL28,RPL13,MRPS2,APRT,ZDHHC12,DPYD,KARS,FPGS,LCAT,ZDHHC1,B3GNT9,TK2,MT-CO2,MT-ATP6,ABCA2,RPS15,AGO4,RACK1,FUT4,RPS19,PLPP3,MRPL34,RPL23,HS3ST3B1,EEF1B2,RPUSD3,MRPL57,RPS20,PID1,POMGNT2,ZFP36L2,PDF,PDXK,NUDT2,UPP1,EXOSC5,MAP6D1	cyan	EC_M2
GO:0090257	6.19E-05	0.999971572	44	179	regulation of muscle system process	BP	5.47E-03	PPARA,SRF,ROCK2,FKBP1B,PAK1,SCN5A,ATP2B1,P2RX4,ATP2A2,MYL9,ABCC8,ADA,ATP2B4,TWF1,CTTN,PKP2,PPP1R12B,DAG1,PRKCA,CASQ1,GLRX3,ADRA2C,KCNA1,CACNA1C,PPARGC1A,TPM1,SGCA,ADRA1B,CNN1,CALM1,SETD3,MYOCD,ANXA6,PIN1,ROCK1,ITGA2,DAPK3,ENO1,DMD,TMEM38B,TMEM38A,ORMDL3,HCN4,PRKG1	darkgreen	EC_M4
GO:0051130	6.27E-05	0.999957752	160	870	positive regulation of cellular component organization	BP	5.51E-03	CD63,LMAN1,LRP1,AHI1,CAND1,RAB21,PLXNB2,ANGPT1,TCP1,PACSIN1,ADGRB1,SH3GL2,SRF,VEGFA,VCP,UNC13B,NCK2,P3H1,TGFBR1,TMEM30A,TPBG,LRRC4B,AKIRIN1,ACTR2,KIDINS220,ROCK2,MYLK3,VPS35,MGARP,FKBP1B,PLXNA1,KIF5B,RB1,MARK4,PDCD6IP,MEGF8,PAK1,ACTR3,TGFB1,AXL,RAB3GAP1,CTNNB1,TRAK1,AKT2,DZIP1,NAV3,FAM98A,LIG4,DCN,CDC42EP3,SMPD1,ILK,DPYSL3,CXCL12,ANKRD1,SLC25A4,OSBP,EIF4G2,ROBO1,MAP1B,PFN2,APP,DHX36,CCT8,RAPGEF2,MAPKAPK5,CX3CL1,ABCC8,PTPN11,GNL3,OPA1,CDH17,ARSB,AMIGO2,ZFYVE27,TSG101,DKC1,PPP3CC,RUNX1,TWF1,MAGEL2,ARL3,PHLDB2,CTTN,DNM1L,HYAL1,LMOD1,CAPRIN2,TGFB2,LRP5,FEZ1,GSK3B,DAG1,PIK3CA,AUTS2,CORO1C,NEK7,NTRK3,FAM162A,BMP7,AP2M1,SYNPO2,PHLDB1,IQGAP1,TFRC,ARPC5,MAPT,SERPINE1,TFR2,FN1,NCKAP1,GPC2,ARPC2,HTT,PRKCI,SKIL,SYNPO2L,WDR1,OBSL1,ATAD1,STX18,HAP1,NLGN2,ANAPC4,ADGRL1,PPARGC1A,MSN,NES,TPM1,TGFB3,RAB11A,MAP2K1,GPR65,NPTN,ISLR2,TRPV2,PDGFRB,MIEF2,ROCK1,ITGA2,RAB31,MYO1C,PLXNB3,PAFAH1B1,ABCA3,NPHP4,FLNA,PLXNA3,PARK7,DEF8,HSPA5,CD151,SERPINF1,BNIP3,LRRC24,UBE2V2,THY1,DSTN,CFL2,LRRTM2	darkgreen	EC_M4
GO:0032268	1.42E-04	0.999896901	215	1819	regulation of cellular protein metabolic process	BP	5.51E-03	CDK2,PAN2,PPIL4,LATS1,DDX39B,FBXO5,UBR5,EIF3E,MAP3K4,MRPL13,XRCC6,CDC25C,TNRC6B,TUT7,PLEC,PWP1,TOPORS,DBF4,AKAP9,PUM3,CKS2,NCBP1,ZNF451,ANP32B,LRPPRC,PHIP,MTIF2,TTK,IBTK,PNPT1,ZMPSTE24,SYNCRIP,XPO1,COMMD1,CASP8AP2,ETAA1,TIA1,ITGB1BP1,EZH2,UFL1,CLSPN,ASCC3,CASP2,DDX1,WDR35,NRDC,DHFR,PUS7,HDAC2,CCNA2,EXOSC9,SAE1,CEP295,CHORDC1,SYMPK,MASTL,CNOT10,BORA,DIS3,CCNT2,PAXIP1,NCAPG2,CDC20,BIRC5,FNTA,GTPBP4,IPO5,METTL3,RIF1,NSD3,KITLG,TAF7,EXOSC8,MAPK8,PRKDC,APAF1,CCNB1,ERCC6,DHX29,PAN3,TPX2,CAPRIN1,UBA2,IPO7,LSM14A,CDK5RAP1,FER,NCK1,IDE,ARFGEF1,PTCD3,ABCE1,OTUD4,ZFP91,TAF9,XRN1,PDE12,LARP4,USP25,PBK,CNOT1,SH2B3,LTN1,USP16,MOB1A,CCNL1,CDCA2,NBN,SENP1,RAD51,RIPK1,CCNE2,STK4,UBE2C,HERC5,TAOK3,NOLC1,BMPR1B,UBE3A,GCN1,CUL3,METAP1,CHEK1,TIMM17A,NFKB1,KHDRBS1,CENPE,NCL,KIF14,AJUBA,FXR1,FANCI,NGDN,EIF2B5,ALS2,BLM,DDX6,CDC25A,PDE8A,SMARCC1,DHX9,HNRNPR,FASTKD2,IVNS1ABP,TPR,FANCM,DBF4B,IL12A,COP1,XRCC5,BRCA1,PSME3,TFPI,CNOT9,CDKN3,DCUN1D5,TIPRL,BOD1L1,ECT2,EIF2AK1,INCENP,UBE2K,CCNB3,CDC6,DCP2,EIF3B,PRELID1,SEPSECS,NSD1,CCNB2,IQGAP3,TAF1,CLPX,OGT,ATRX,PTTG1,SPAG9,GEMIN5,LARP1,PLK1,ILF3,CSK,PSMD10,EIF5,NLE1,TYMS,UPF3B,SUZ12,XIAP,RICTOR,NIPBL,AIFM1,GSPT1,TAOK1,UHRF1,FMR1,METTL16,PKMYT1,CCNF,PTBP1,EXOSC2,GLMN,IST1,CTCF,RBM8A,METTL18,SARNP,HNRNPD,MAD2L1,AURKA,RNF139,ERP29,PAIP2,UBXN2A,RCHY1	darkgrey	EC_M8

GO:0008015	4.91E-05	0.999973596	67	369	blood circulation	BP	5.54E-03	LVRN,AKAP12,TRDN,PDGFB,TEK,TJP2,DOCK4,TRPM4,PIK3CG,ARHGAP42,GPR4,PRCP,NOS3,PPARG,CXCR4,EDNRB,SPTBN4,P2RY2,PDE2A,HBEGF,KCNK6,P2RX2,PDE4D,CHD7,OCLN,VEGFC,GUCY1A1,GUCY1B1,CXCL10,STC1,KCNJ3,DLL4,NAV2,EDN1,STAT1,KCNJ2,MEF2A,CD34,F11R,ACE,DLG1,ADORA2A,RAMP2,PLVAP,JUP,MTOR,AR,ATP2A1,NPR1,SCNN1B,DSG2,DSC2,ADRB2,YES1,PDE4B,NOS2,PTGS1,CDH5,MIR23A,SLC8A3,ERAP2,KCNH2,SCN2B,RGS4,BMP6,KCNG2,FLI1	turquoise	EC_M6
GO:0045214	6.41E-05	0.999987185	14	33	sarcomere organization	BP	5.60E-03	SRF,MYLK3,ITGB1,ACTG1,ANKRD1,PRKAR1A,CASQ1,SYNPO2L,WDR1,SIX4,TCAP,TPM1,CSRP1,CFL2	darkgreen	EC_M4
GO:0006399	1.47E-04	0.999940354	29	162	tRNA metabolic process	BP	5.66E-03	NARS,POP1,PUS10,DDX1,PUS7,EXOSC9,TRDMT1,TSEN2,DARS,EXOSC8,LARS,CDK5RAP1,NSUN2,YARS,TRMT10A,ZBTB8OS,QTRT2,IARS2,TARBP1,SSB,TSEN15,DARS2,SEPSECS,EXOSC10,RARS,THG1L,THUMPD1,EXOSC2,DTD2	darkgrey	EC_M8
GO:1903844	6.52E-05	0.999975431	30	104	regulation of cellular response to transforming growth factor beta stimulus	BP	5.66E-03	FBN2,PPARA,CDKN2B,RASL11B,ASPN,TGFB1,ZEB2,LTBP1,SPRED3,ZNF703,DKK3,SLC2A10,HTRA1,STRAP,FAM89B,FBN1,SKIL,SMURF1,RNF111,TGFB1I1,TGFB3,MYOCD,PIN1,VASN,GLG1,HSPA5,FKBP1A,LOX,PMEPA1,CHST11	darkgreen	EC_M4
GO:0060148	1.49E-04	0.999975383	10	28	positive regulation of posttranscriptional gene silencing	BP	5.72E-03	TNRC6B,XPO5,RIPK1,AJUBA,FXR1,DDX5,TIAL1,DDX6,DHX9,FMR1	darkgrey	EC_M8
GO:0034329	5.15E-05	0.999972952	63	318	cell junction assembly	BP	5.76E-03	AFDN,PTK2,TEK,LINGO2,LIMCH1,ADGRF1,KDR,SEMA4D,GJA4,SDCBP,NRCAM,NRP1,PLXND1,ITGB4,SETD5,IKBKB,SFRP1,THSD1,EFNB2,ARHGEF7,EPHB1,MARVELD2,OCLN,MEF2C,TLR2,GPM6A,HIPK1,JAM3,SNCA,SHANK2,ESAM,CLDN34,CDH24,PECAM1,F11R,ACE,ITGA6,PAK2,DLG1,APOD,BCR,IL1RAP,RAMP2,DNM3,PPM1F,PRKCH,RAC1,RCC2,JUP,FSCN1,EPHA2,LGI2,OPHN1,SEMA4A,ABI3,TAOK2,CHRNB2,PATJ,SLITRK2,RAPGEF1,ADGRL2,CDH5,SHANK3	turquoise	EC_M6
GO:0002250	5.24E-05	0.999975125	47	244	adaptive immune response	BP	5.82E-03	TNFRSF11A,TNFAIP3,TEC,TNFRSF21,SYK,IL6,BACH2,RSAD2,TLR4,C5,TRPM4,PIK3CG,PRKD2,CTSC,NECTIN2,UNC13D,GATA3,JAG1,GAPT,MEF2C,RAPGEF3,JAM3,NFKB2,RNF19B,ICOSLG,HLX,INPP5D,CD86,BMX,SIPA1,B2M,CRACR2A,WAS,RAB27A,TNFRSF1B,ARG2,MTOR,SEMA4A,BATF,CD19,IL7R,TRAF2,NOTCH1,ERAP2,CD46,FCER2,RNF125	turquoise	EC_M6
GO:0090287	6.78E-05	0.999966154	54	230	regulation of cellular response to growth factor stimulus	BP	5.85E-03	CD63,FBN2,PPARA,PTP4A3,CDKN2B,KIAA1324L,RUNX2,RASL11B,ASPN,ADAMTS3,FGF2,TGFB1,CTNNB1,ZEB2,LTBP1,CRIM1,SPRED3,DCN,ZNF703,ILK,SULF1,DKK3,ROBO1,ATP2B4,NOTCH2,SLC2A10,FSTL1,HTRA1,STRAP,FAM89B,FBN1,FKBP8,SKIL,SMURF1,NBL1,GREM2,RNF111,TGFB1I1,NGFR,TGFB3,NPTN,MYOCD,PIN1,CHRDL1,MYO1C,VASN,GLG1,HSPA5,FSTL3,FKBP1A,LOX,PMEPA1,CHST11,FGF10	darkgreen	EC_M4
GO:0090231	1.54E-04	0.99998522	7	15	regulation of spindle checkpoint	BP	5.90E-03	GEN1,CCNB1,TPR,LCMT1,NDC80,CDT1,MAD2L1	darkgrey	EC_M8
GO:0090266	1.54E-04	0.99998522	7	15	regulation of mitotic cell cycle spindle assembly checkpoint	BP	5.90E-03	GEN1,CCNB1,TPR,LCMT1,NDC80,CDT1,MAD2L1	darkgrey	EC_M8
GO:1903504	1.54E-04	0.99998522	7	15	regulation of mitotic spindle checkpoint	BP	5.90E-03	GEN1,CCNB1,TPR,LCMT1,NDC80,CDT1,MAD2L1	darkgrey	EC_M8
GO:0040007	6.90E-05	0.999954679	139	742	growth	BP	5.92E-03	LRP1,RAB21,PPARA,ROS1,LY6E,PPARD,SH3GL2,LEP,PLAA,LARGE1,SRF,VEGFA,TGFBR1,SOCS5,RIMS1,RTN4,MFSD2A,AKIRIN1,GLI3,INHBA,ITGB1,PLXNA1,FGF2,RB1,PTCH2,TENM4,KCTD21,GLI2,MEGF8,PAK1,TGFB1,WDR48,CTNNB1,RARB,DCLK1,SEMA3C,ILK,RYK,RNF6,CXCL12,CHPT1,TMED2,EIF4G2,WWC2,MAP1B,RDH10,DIP2B,SEMA4F,CELF1,APP,PTPN11,PTX3,ZFYVE27,TSG101,BAP1,SPP1,RUNX1,SEMA4G,NKD1,PDLIM5,NDN,ADIPOR1,KDM5B,NOTCH2,CTTN,CDC73,HYAL1,CAPRIN2,SBDS,TGFB2,FBLN5,PRKAR1A,ZMAT3,GSK3B,MMP14,DAG1,PIK3CA,AUTS2,WWC3,KPNA1,WDTC1,FGFR2,OSM,IQGAP1,RBBP7,CLIC4,ARID5B,FZD9,RPS6KA3,MAPT,SGPL1,TNR,FN1,IGFBP2,COMP,FKBP8,FGFR3,SMURF1,VCL,SIX4,EVC,NINJ2,BMPR1A,TSPYL2,MED1,NPPB,SGCA,THBS3,SHC1,RAB11A,TAF9B,TTC8,SMARCA4,MORF4L2,MYOCD,ISLR2,ANXA6,PIN1,TRPV2,PDGFRB,AKT1,CRLF3,PAFAH1B1,PLXNA3,ENO1,BARHL2,MIR24-1,TMEM38B,PTPN12,CDKN1A,DACT3,NME6,HOPX,ZPR1,CHST11,OLFM1,DERL2,WNT2,FGF10	darkgreen	EC_M4

GO:0010647	5.65E-05	0.999961015	181	1251	positive regulation of cell communication	BP	6.18E-03	TNFRSF11A,TNFAIP3,TRIM39,AKAP12,FLT4,RIMS2,TRDN,TPD52L1,RNF146,PTK2,PDGFB,CSNK1E,MFNG,LRRC19,TEK,SH3RF3,KIT,KDR,SYK,GADD45G,SEMA4D,IL6,REL,PELI1,RSAD2,CAV2,GRB10,LANCL2,TLR4,GRIK2,TRAF1,SDCBP,TRPM4,NRP1,PUM2,PIK3CG,PRKD2,CTSC,GPR4,NECTIN2,NOS3,PPARG,GATA3,ACVR2B,CXCR4,NET1,NDFIP2,IKBKB,JAG1,P2RY6,SFRP1,HBEGF,FERMT1,RPL26,P2RX2,CD36,SIRT3,PRAG1,PRR5L,PLK2,RET,TNFRSF19,IGF1,HHEX,ERBIN,SH3RF1,HIP1R,ARHGEF3,TLR2,IL7,NDRG4,GUCY1A1,MADD,KMT2D,BMP2K,NLRC5,DLL4,ZDHHC13,ADAMTS20,EDN1,SNCA,DTNBP1,PRDM15,ABCG1,SHANK2,LCK,HYAL2,ANO1,SHOC2,HCRTR1,LAPTM5,NCF1,HCLS1,TIFA,CD86,MN1,FGR,CFLAR,CASP10,ADCY1,PROX1,PRKD1,F11R,YTHDF1,IL10RA,MAP3K3,PAK2,MAP3K11,CREB1,LTF,ADORA2A,DDX3X,SOS2,IGFBP5,TBX1,MCU,TMEM106A,BIRC3,SELP,VWF,TNFAIP8L3,FGF23,CRACR2A,TMOD2,PRKCH,RAC1,HIF1A,TSPAN14,JUP,ITPKB,BID,TLR6,RELL1,RGS14,MTOR,AR,PSEN1,FGF18,TAOK2,EFNA1,PIK3R5,NPR1,GPR68,SMAD2,ADRB2,CCL2,JAG2,LGALS9,IL7R,LRG1,TEDC2,NRARP,TRAF2,PLEKHG5,NOTCH1,PLCG2,ENG,STXBP1,NFAT5,DAB2IP,CDH5,MIR23A,PMAIP1,SLC8A3,TLR1,ADGRG1,TRIM34,RELN,ALKAL1,LYPD6,SHANK3,LPAR6,RGS4,TRIM62,STX3,PDCL,BST2,BMP2,CACNG5,NMNAT1,BMP6	turquoise	EC_M6
GO:0023056	5.68E-05	0.999960808	182	1258	positive regulation of signaling	BP	6.18E-03	TNFRSF11A,TNFAIP3,TRIM39,AKAP12,FLT4,RIMS2,TPD52L1,RNF146,PTK2,PDGFB,CSNK1E,MFNG,LRRC19,TEK,SH3RF3,KIT,KDR,SYK,GADD45G,SEMA4D,IL6,REL,PELI1,RSAD2,CAV2,GRB10,LANCL2,TLR4,GRIK2,TRAF1,SDCBP,TRPM4,NRP1,PUM2,PIK3CG,PRKD2,CTSC,GPR4,NECTIN2,NOS3,PPARG,GATA3,ACVR2B,CXCR4,NET1,NDFIP2,IKBKB,JAG1,P2RY6,SFRP1,HBEGF,FERMT1,RPL26,P2RX2,CD36,SIRT3,PRAG1,PRR5L,PLK2,RET,TNFRSF19,IGF1,HHEX,ERBIN,SH3RF1,HIP1R,ARHGEF3,TLR2,IL7,NDRG4,GUCY1A1,MADD,KMT2D,BMP2K,NLRC5,DLL4,ZDHHC13,ADAMTS20,EDN1,SNCA,DTNBP1,PRDM15,ABCG1,SHANK2,LCK,HYAL2,ANO1,SHOC2,HCRTR1,LAPTM5,NCF1,HCLS1,TIFA,CD86,MN1,FGR,CFLAR,CASP10,INPP5F,ADCY1,PROX1,PRKD1,F11R,YTHDF1,IL10RA,MAP3K3,PAK2,MAP3K11,CREB1,LTF,ADORA2A,DDX3X,SOS2,IGFBP5,TBX1,MCU,TMEM106A,BIRC3,SELP,VWF,TNFAIP8L3,FGF23,CRACR2A,TMOD2,PRKCH,RAC1,HIF1A,TSPAN14,JUP,ITPKB,BID,TLR6,RELL1,RGS14,MTOR,AR,PSEN1,FGF18,TAOK2,EFNA1,PIK3R5,NPR1,GPR68,SMAD2,ADRB2,CCL2,JAG2,LGALS9,IL7R,LRG1,TEDC2,NRARP,TRAF2,PLEKHG5,NOTCH1,PLCG2,ENG,STXBP1,NFAT5,DAB2IP,CDH5,MIR23A,PMAIP1,SLC8A3,TLR1,ADGRG1,TRIM34,RELN,ALKAL1,LYPD6,SHANK3,LPAR6,RGS4,TRIM62,STX3,PDCL,BST2,VAMP3,BMP2,CACNG5,NMNAT1,BMP6	turquoise	EC_M6
GO:0040013	5.69E-05	0.999972067	51	261	negative regulation of locomotion	BP	6.18E-03	SEMA6A,AFDN,LIMCH1,SEMA4D,C5,NRP1,LRCH1,DACH1,PPARG,GATA3,TIE1,JAG1,SFRP1,ADAMTS9,ABHD6,MCTP1,MEF2C,NDRG4,ANGPT2,STC1,DLL4,SRGAP2C,CD200,HYAL2,SEMA3F,RIN3,AP1AR,IGFBP3,ARHGDIB,APOD,ADORA2A,SEMA6D,IGFBP5,WAS,RAC1,JUP,ADIPOR2,MMRN2,MCC,SEMA4A,VASH1,MMP28,CCL2,STK26,SEMA6B,ARHGAP4,NOTCH1,ENG,DAB2IP,ADGRG1,BST2	turquoise	EC_M6
GO:0051240	5.77E-05	0.999961175	157	1050	positive regulation of multicellular organismal process	BP	6.22E-03	ZNF516,TNFRSF11A,IFNGR1,AKAP12,FLT4,RIMS2,CDKL3,ATF4,PDGFB,MAPK13,PIM1,TEK,LINGO2,DDX58,KIT,KDR,SYK,GADD45G,SEMA4D,IL6,CXCL8,PELI1,RSAD2,GRB10,TLR4,C5,IL15,SDCBP,TRPM4,NRP1,PIK3CG,GATA2,PRKD2,CST7,PLXND1,NOS3,GATA3,ACVR2B,CXCR4,EDNRB,SPTBN4,TIE1,JAG1,P2RY2,TMEM173,FERMT1,EFNB2,CD36,LMOD3,EPHB1,RET,PDE4D,CHD7,IL1A,IGF1,EIF2AK3,KDM3A,ITCH,SMAD1,MEF2C,TLR2,WWTR1,IL7,P2RX7,VEGFC,ANGPT2,OAS3,PLCG1,RAPGEF3,HIPK1,ANO6,SOX13,EDN1,STAT1,CD58,ETS1,CD200,PDE9A,HDAC1,PPP3CA,HYAL2,MEF2A,LAPTM5,HLX,FABP4,HCLS1,INPP5D,CD34,CD86,PLXNA2,FGR,FES,PROX1,SS18L1,PRKD1,ELL3,CREB1,B2M,LTF,ADORA2A,CYBB,IL1RAP,DDX3X,ETV4,TMEM106A,RAMP2,PRKCH,HIF1A,JUP,ADIPOR2,ITPKB,TLR6,TNFRSF1B,RGS14,MTOR,AR,PSEN1,HOXB7,SEMA4A,FGF18,PGF,BATF,ATP2A1,NPR1,GPR68,SMAD2,AMOT,ADRB2,PDE4B,JAK1,LGALS9,NOS2,IL7R,MRTFB,LRG1,SLITRK2,ISG15,AP3D1,TRAF2,NOTCH1,IRF8,PLCG2,ENG,ADGRL2,CDH5,OAS1,VNN1,TLR1,GBP5,CD46,CA2,RELN,DIO3,SHANK3,RGS4,BMP2,BMP6	turquoise	EC_M6
GO:0000022	1.69E-04	0.999993933	5	7	mitotic spindle elongation	BP	6.44E-03	RACGAP1,PRC1,KIF4A,AURKB,KIF23	darkgrey	EC_M8
GO:0051231	1.69E-04	0.999993933	5	7	spindle elongation	BP	6.44E-03	RACGAP1,PRC1,KIF4A,AURKB,KIF23	darkgrey	EC_M8
GO:0061640	1.72E-04	0.999944052	20	87	cytoskeleton-dependent cytokinesis	BP	6.51E-03	KIF20A,PLEC,ANLN,CENPA,CKAP2,ESPL1,KIF20B,CEP55,RACGAP1,NUSAP1,CIT,SEPT2,ECT2,INCENP,KIF4A,AURKB,KIF23,PLK1,IST1,STMN1	darkgrey	EC_M8

GO:0048731	6.12E-05	0.999952497	464	3556	system development	BP	6.56E-03	ZNF516,TNFRSF11A,TCF4,SEMA6A,N4BP3,KIF5A,IFNGR1,TNFAIP3,HECA,PTPRB,FLT4,SYNJ2,FZD6,CTHRC1,RIMS2,CELSR1,QKI,NOTCH4,TCF7,CDKL3,MTSS1,ASAP1,L3MBTL3,KLHL3,PTK2,HSD17B3,ANKS1A,ATF4,PDGFB,AGTPBP1,PODXL,CSNK1E,PICK1,KDM4C,MICALL1,PIM1,MFNG,MDFI,TEK,LINGO2,IREB2,APBA1,ADGRF5,KIT,ADGRF1,TNFRSF21,KDR,NFIL3,SYK,SEMA4D,EFHC1,THSD7A,SHB,TMOD1,RAPGEF5,IL6,IGF2BP3,PPT1,PTBP3,CXCL8,UTP11,RSAD2,FOXP2,CAV2,TSPAN12,TLR4,C5,GJA4,IL15,FZD8,TEAD2,SDCBP,MYCN,NRCAM,TRPM4,NRP1,ZBTB24,RASIP1,PIK3CG,SVIL,ITSN2,TAL1,PHF10,MTMR2,EPC1,GATA2,DNMT3A,HS6ST1,PRKD2,CST7,NOVA2,CTSC,GPR4,SNRK,PLXND1,PRCP,ITGA8,NOS3,GAB2,ITGB4,PPARG,TIMP4,GATA3,ACVR2B,MYO7A,CXCR4,GZF1,EDNRB,SPTBN4,TIE1,GDPD5,SETD5,JAG1,PDE2A,GPAT4,SFRP1,HBEGF,NR1D2,FEZ2,NOTUM,FERMT1,FREM2,EFNB2,ANKLE2,ARHGEF7,ASH2L,SPEF1,PCDH1,PCDH12,ARHGAP26,P2RX2,NTN4,RIC8A,TMEM178A,LMOD3,PRAG1,ADAMTS9,SDCBP2,TCF15,EPHB1,PLK2,MALL,RET,MERTK,TNFRSF19,CHD7,IL1A,DNMT3B,IGF1,NR4A1,EIF2AK3,HHEX,ITCH,SH3RF1,PREX2,SMAD1,HIP1R,POU6F1,STOX2,DCLK2,MMP24,KIF13B,ENC1,HEXB,MEF2C,SMARCD1,TLR2,WWTR1,IL7,P2RX7,TPD52,VEGFC,NDRG4,GPM6A,CXCL10,ANGPT2,TLL2,LMBR1L,ADGRG3,DHH,SFMBT1,PLLP,BMP2K,TIPARP,TLL1,SERINC5,THBS4,USH1C,PPP1R16B,PLCG1,STC1,RAPGEF3,ANKRD2,NR4A2,STAB1,HIPK1,ANO6,DLL4,SOX13,TSPAN2,NAV2,EDN1,STAT1,DYRK1A,JAM3,IGSF3,SNCA,ALAS1,JARID2,DTNBP1,MYLIP,ETS2,GAS2,SLC45A3,NFKB2,DCAF1,SRGAP2C,ETS1,DPPA4,SHANK2,LPCAT1	turquoise	EC_M6
GO:0051345	6.18E-05	0.999965042	76	440	positive regulation of hydrolase activity	BP	6.57E-03	TBC1D30,GRAMD4,AFDN,ASAP1,SGSM3,DAPK1,TBC1D22B,KIT,SYK,SEMA4D,RGP1,CHN2,ARHGAP25,ASAP2,CAV2,TBC1D9,ARHGAP42,PRKD2,USP6NL,PPARG,TBC1D4,NET1,RALGAPA2,P2RY6,SFRP1,ARHGEF7,ARAP3,EIF2AK3,HIP1R,SOX7,MEF2C,PLCB2,RAPGEF3,NEDD9,SNCA,CD200,LCK,ADPRH,LAPTM5,CD86,CFLAR,CASP10,PRKD1,TOR1AIP1,F11R,ITGA6,SIPA1,ARHGAP27,BCR,DDX3X,RUNDC1,ELMOD1,SELE,PPM1F,RCC2,EPHA2,BID,TBC1D1,RGS14,MTOR,ARHGEF15,EFNA1,TBC1D8B,CCL2,LGALS9,SGSM2,RAPGEF1,S1PR1,TBC1D13,PRSS22,PMAIP1,RGS4,RGS16,TBC1D20,PERP,BMP2	turquoise	EC_M6
GO:0007019	1.76E-04	0.999960649	13	40	microtubule depolymerization	BP	6.64E-03	KIF24,CLASP2,KIF2C,CKAP2,TPX2,CKAP5,STMND1,KIF18A,CAMSAP2,HDGFL3,KIF18B,TAOK1,STMN1	darkgrey	EC_M8
GO:0051445	1.76E-04	0.999966054	11	35	regulation of meiotic cell cycle	BP	6.64E-03	YTHDC2,FBXO43,FBXO5,CDC25C,TTK,CDC20,METTL3,TRIP13,ASPM,CDC25A,RAD51AP1	darkgrey	EC_M8
GO:0090066	7.84E-05	0.999954097	86	418	regulation of anatomical structure size	BP	6.69E-03	LRP1,RAB21,SLC12A2,FAM49B,PPARD,LEP,SRF,VEGFA,NCK2,RTN4,ACTR2,ROCK2,PICALM,MEGF8,PAK1,ACTR3,PLEKHG2,ATP2B1,CDC42EP3,SEMA3C,ILK,RYK,RNF6,CXCL12,SNTA1,EIF4G2,MAP1B,DIP2B,SEMA4F,PFN2,RASA1,ADCY6,ZFYVE27,SPP1,TWF1,SEMA4G,MAGEL2,RAP1GDS1,CTTN,LMOD1,HTR2B,GSK3B,PIK3CA,WDTC1,AP2M1,ARPC5,MAPT,PLOD3,TNR,FN1,NCKAP1,ADRA2C,COMP,ARPC2,ECE1,CAPZB,WDR1,ACTA2,AKT3,CLN8,NPPB,MSN,KCNMB1,RAB11A,ADRA1B,ISLR2,TRPV2,PEX11G,ROCK1,FLII,AKT1,NPR3,MYO1C,PAFAH1B1,PLXNA3,SPTAN1,BARHL2,TMSB4X,DSTN,TMEM123,PIK3C2A,PRKG1,KCNMB4,OLFM1,COTL1,CFL2	darkgreen	EC_M4
GO:0030970	7.98E-05	0.999986204	12	27	retrograde protein transport, ER to cytosol	BP	6.73E-03	VCP,ERLEC1,UBE2J1,NPLOC4,HSP90B1,BRSK2,SELENOS,SYVN1,SEL1L,BCAP31,UBE2G2,DERL2	darkgreen	EC_M4
GO:1903513	7.98E-05	0.999986204	12	27	endoplasmic reticulum to cytosol transport	BP	6.73E-03	VCP,ERLEC1,UBE2J1,NPLOC4,HSP90B1,BRSK2,SELENOS,SYVN1,SEL1L,BCAP31,UBE2G2,DERL2	darkgreen	EC_M4
GO:0019896	8.12E-05	0.999991178	9	16	axonal transport of mitochondrion	BP	6.74E-03	MGARP,TRAK1,OPA1,FEZ1,MAPT,ACTR10,HAP1,ARMCX3,HSBP1	darkgreen	EC_M4

GO:0048870	8.12E-05	0.999941861	216	1234	cell motility	BP	6.74E-03	CD63,LRP1,SLC12A2,PLXNB2,ANGPT1,FAM19A5,NUS1,CXCL14,FAM49B,MRTFA,PTP4A3,ADGRB1,PPARD,CTNNA1,SUN2,MDGA1,MYH9,LEP,PLAA,SRF,VEGFA,DOCK8,NCK2,CREB3,DST,PTP4A1,TGFBR1,RTN4,TPBG,EMC10,AKIRIN1,GLI3,ROCK2,BVES,RRAS,ITGB1,PLXNA1,FGF2,MEGF8,PAK1,ACTR3,TGFB1,AXL,CTNNB1,AKT2,ZEB2,OSBPL8,ACTG1,NAV3,ACTN4,SPINT2,IRS2,DCN,ZNF703,DCLK1,STARD13,SEMA3C,DDRGK1,ILK,DPYSL3,CXCL12,KIF2A,FAT1,PALLD,SULF1,ROBO1,WWC2,MAP1B,CHST2,SEMA4F,P2RX4,PFN2,APP,RAPGEF2,CX3CL1,ABCC8,PTPN11,PGAM4,ARSB,LOXL2,ACVR1,TUBB2A,ADA,ATP2B4,DNAH1,SEMA4G,NKD1,DNAH5,NDN,DOCK10,ELMO2,ADIPOR1,FAP,PHLDB2,TJP1,CTTN,DNM1L,DNER,HYAL1,SBDS,TGFB2,LRP5,HTR2B,SATB2,FSTL1,MMP14,DAG1,PIK3CA,AUTS2,SDC3,LEF1,CORO1C,PRKCA,CMKLR1,SHROOM2,NTRK3,WWC3,SLAMF8,BMP7,IDH2,ITGB5,IQGAP1,CLIC4,S100A11,ARID5B,SRP54,ARPC5,LRRC15,FAM89B,HSPB1,SERPINE1,SGPL1,ITGA2B,DNAJA4,TNR,S100P,FN1,NCKAP1,GPC2,PDCD10,ITGAV,ARPC2,COL3A1,PRKCI,NBL1,TIMP1,PLD1,VCL,WDR1,SIX4,AKT3,BMPR1A,ENAH,PPARGC1A,MSN,SRCIN1,ARHGEF2,NGFR,TPM1,COL1A1,PBXIP1,RAB11A,MAP2K1,MYOCD,FAT2,ANXA6,PIN1,GP2,CD74,LAMA3,PDGFRB,ROCK1,AKT1,ITGA2,TNFAIP1,DAPK3,MYO1C,PLXNB3,PAFAH1B1,TNFRSF18,NPHP4,FLNA,PLXNA3,RERE,RHBDF1,BSG,COL5A1,NTNG2,CDH13,F3,BARHL2,HSPA5,LMO4,NEXN,MIR24-1,CCL16,CD151,SERPINF1,LOX,PDLIM1,IGSF8,ARF4,GDNF,THY1,HAS2,ARTN,TMSB4X,DSTN,GLIPR2,CCR6,DUSP22,PIK3C2A,SDC2,PRKG1,CFL2,FGF10	darkgreen	EC_M4
GO:0051674	8.12E-05	0.999941861	216	1234	localization of cell	BP	6.74E-03	CD63,LRP1,SLC12A2,PLXNB2,ANGPT1,FAM19A5,NUS1,CXCL14,FAM49B,MRTFA,PTP4A3,ADGRB1,PPARD,CTNNA1,SUN2,MDGA1,MYH9,LEP,PLAA,SRF,VEGFA,DOCK8,NCK2,CREB3,DST,PTP4A1,TGFBR1,RTN4,TPBG,EMC10,AKIRIN1,GLI3,ROCK2,BVES,RRAS,ITGB1,PLXNA1,FGF2,MEGF8,PAK1,ACTR3,TGFB1,AXL,CTNNB1,AKT2,ZEB2,OSBPL8,ACTG1,NAV3,ACTN4,SPINT2,IRS2,DCN,ZNF703,DCLK1,STARD13,SEMA3C,DDRGK1,ILK,DPYSL3,CXCL12,KIF2A,FAT1,PALLD,SULF1,ROBO1,WWC2,MAP1B,CHST2,SEMA4F,P2RX4,PFN2,APP,RAPGEF2,CX3CL1,ABCC8,PTPN11,PGAM4,ARSB,LOXL2,ACVR1,TUBB2A,ADA,ATP2B4,DNAH1,SEMA4G,NKD1,DNAH5,NDN,DOCK10,ELMO2,ADIPOR1,FAP,PHLDB2,TJP1,CTTN,DNM1L,DNER,HYAL1,SBDS,TGFB2,LRP5,HTR2B,SATB2,FSTL1,MMP14,DAG1,PIK3CA,AUTS2,SDC3,LEF1,CORO1C,PRKCA,CMKLR1,SHROOM2,NTRK3,WWC3,SLAMF8,BMP7,IDH2,ITGB5,IQGAP1,CLIC4,S100A11,ARID5B,SRP54,ARPC5,LRRC15,FAM89B,HSPB1,SERPINE1,SGPL1,ITGA2B,DNAJA4,TNR,S100P,FN1,NCKAP1,GPC2,PDCD10,ITGAV,ARPC2,COL3A1,PRKCI,NBL1,TIMP1,PLD1,VCL,WDR1,SIX4,AKT3,BMPR1A,ENAH,PPARGC1A,MSN,SRCIN1,ARHGEF2,NGFR,TPM1,COL1A1,PBXIP1,RAB11A,MAP2K1,MYOCD,FAT2,ANXA6,PIN1,GP2,CD74,LAMA3,PDGFRB,ROCK1,AKT1,ITGA2,TNFAIP1,DAPK3,MYO1C,PLXNB3,PAFAH1B1,TNFRSF18,NPHP4,FLNA,PLXNA3,RERE,RHBDF1,BSG,COL5A1,NTNG2,CDH13,F3,BARHL2,HSPA5,LMO4,NEXN,MIR24-1,CCL16,CD151,SERPINF1,LOX,PDLIM1,IGSF8,ARF4,GDNF,THY1,HAS2,ARTN,TMSB4X,DSTN,GLIPR2,CCR6,DUSP22,PIK3C2A,SDC2,PRKG1,CFL2,FGF10	darkgreen	EC_M4
GO:0016073	1.85E-04	0.999964142	11	35	snRNA metabolic process	BP	6.96E-03	EXOSC9,INTS10,EXOSC8,MTREX,ZCCHC8,INTS7,EXOSC10,INTS2,METTL16,EXOSC2,INTS6	darkgrey	EC_M8
GO:0071840	8.44E-05	0.999931726	735	4827	cellular component organization or biogenesis	BP	6.96E-03	CD63,LMAN1,ATP8B1,LRP1,STX7,TRIM36,B4GALT7,AHI1,AGAP2,CAND1,RAB21,RNF19A,SLC12A2,PLXNB2,ANGPT1,TCP1,PPARA,HLA-DRA,COL11A2,ROS1,HSPA4,SERINC1,SEC24A,FER1L6,FAM49B,MRTFA,HABP4,PACSIN1,REEP2,ADGRB1,PPARD,CTNNA1,GOLM1,SUN2,CBY1,MKLN1,UBQLN1,MTCH1,LGALS1,MDGA1,SH3GL2,FLNC,MYH9,ARHGAP39,PLAA,TBCC,PEX6,SRF,VEGFA,VCP,UNC13B,COL1A2,TPM2,NCK2,PDCL3,REST,TXNDC9,LMAN2L,DST,RAB23,NLRP5,COL15A1,P3H1,TGFBR1,RIMS1,TMEM30A,PSME4,CENPC,UBA6,RTN4,TPBG,SHANK1,ADAMTS3,COX18,LRRC4B,EMC10,MFSD2A,GGCT,MYH14,CALD1,AKIRIN1,ACTR2,KIDINS220,WHRN,GLI3,NCDN,ROCK2,INHBA,VPS41,ZYX,STARD3NL,MYLK3,VPS35,MGARP,SLC7A11,CFAP100,ITGB1,FKBP1B,PLXNA1,SRPK2,KIF5B,SEC61A1,NAPA,RAB7A,ARHGEF4,FBLN2,EML2,RB1,PICALM,MARK4,NUFIP1,MAPRE3,CRTAP,PTCH2,PDCD6IP,TENM4,KCTD21,GLI2,MEGF8,SMARCD3,PCDH17,SNX17,PAK1,ACTR3,NCKAP5,TGFB1,AXL,RAB3GAP1,SEC13,CTNNB1,ESYT2,TRAK1,CLIP4,SERPINH1,AKT2,DYNC1LI1,SEC23B,DZIP1,ZEB2,PLEKHG2,DNAJB13,FARP1,ARHGEF17,OSBPL8,ACTG1,NAV3,NPLOC4,ACTN4,PPP1R12A,ANAPC15,STAM2,KAT2B,FAM98A,TACC1,SPINT2,UFM1,LIG4,ATP2C1,PRNP,CHFR,NUDT16,LUM,DCN,CDC42EP3,HDAC3,ZNF703,DCLK1,ACPP,PCLO,STARD13,KCTD16,SEMA3C,CAVIN3,UBA5,DDRGK1,SMPD1,PPP2CB,ILK,DCTN6,MMP17,DPYSL3,KATNAL1,SPINK5,ERI1,APLP1,RYK,TTC17,CIAO1,TMEM127,GTF3A,CNOT7,RNF6,RIC3,VPS37A,ARL1,CXCL12,TM9SF4,SACS,RAB2A,KIF2A,CHPT1,TMEM41B,ANKRD1,FAT1,REEP5,ADAMTS6,SLC25	darkgreen	EC_M4

GO:0051336	6.71E-05	0.999957338	117	754	regulation of hydrolase activity	BP	7.01E-03	TNFAIP8,TBC1D30,GRAMD4,AFDN,ASAP1,SGSM3,PTK2,DAPK1,TBC1D22B,KIT,SYK,SEMA4D,RGP1,PPP6R1,PPT1,CHN2,ARHGAP25,ASAP2,CAV2,C5,TBC1D9,RASIP1,ARHGAP42,PRKD2,CST7,LRCH1,FGD5,PLXND1,USP6NL,PPARG,TIMP4,TBC1D4,ITIH5,NET1,RALGAPA2,IKBKB,P2RY6,SFRP1,RASGRP3,ARHGEF7,ARAP3,FGD6,NR4A1,EIF2AK3,SH3RF1,HIP1R,SOX7,MEF2C,OAS3,PLCB2,RAPGEF3,IQGAP2,NEDD9,SNCA,CD200,HDAC1,LCK,ADPRH,LAPTM5,CD86,PLXNA2,SH3BP4,CFLAR,CASP10,RPS6KA1,PRKD1,TOR1AIP1,F11R,ITGA6,PAK2,CST6,SIPA1,LTF,ARHGAP27,BCR,ADORA2A,DDX3X,RUNDC1,ELMOD1,BIRC3,SELE,CPEB2,PPM1F,RCC2,KDM5A,EPHA2,BID,TBC1D1,RGS14,FGD1,MTOR,PAPLN,ARHGEF15,EFNA1,TBC1D8B,AMOT,CCL2,LGALS9,SGSM2,RAPGEF1,S1PR1,TBC1D13,CNN3,TERF2,DAB2IP,OAS1,PRSS22,PMAIP1,RGS4,SERPINB9,RGS16,PSMB9,TBC1D20,PERP,BST2,BMP2,PPP1R2	turquoise	EC_M6
GO:0098609	6.73E-05	0.999958991	99	592	cell-cell adhesion	BP	7.01E-03	CELSR1,NOTCH4,AFDN,PTK2,PODXL,CDC42EP1,TJP2,KIT,TNFRSF21,CD2AP,SYK,SEMA4D,IL6,PELI1,IL15,NRCAM,IZUMO1,PIK3CG,NECTIN2,ITGA8,GATA3,CD93,JAG1,PCDH1,PCDH12,RIC8A,MAGI1,RET,IL1A,IGF1,ITCH,MMP24,IL7,THBS4,SPARCL1,SOX13,CD58,JAM3,ETS1,CD200,LCK,SLC39A8,ICOSLG,ROBO4,ESAM,LAPTM5,HLX,CDH24,CD34,CADM3,PECAM1,CD86,ICAM2,LAMA5,F11R,ITGA6,DLG1,B2M,HMCN1,TMEM47,ADORA2A,IL1RAP,ITGA4,SELE,SELP,BAIAP2L1,PPM1F,JUP,ITPKB,ARG2,PEAR1,EFNB1,PSEN1,STK10,PCDH11X,TNIP1,DSG2,DSC2,DSC3,CCL2,YES1,JAK1,LGALS9,IL7R,SLITRK2,AP3D1,NRARP,NOTCH1,PIEZO1,CNN3,STXBP1,NFAT5,CDH5,VNN1,CD46,PERP,BMP2,CBLL1,BMP6	turquoise	EC_M6
GO:0007186	6.73E-05	0.999961439	78	458	G protein-coupled receptor signaling pathway	BP	7.01E-03	AKAP12,FZD6,CELSR1,PICK1,RIC8B,GNG11,ADGRF5,ADGRF1,CXCL8,RGS3,C5,FZD8,PIK3CG,MGLL,GPR4,PPARG,CXCR4,EDNRB,P2RY6,P2RY2,PDE2A,PLCB4,RIC8A,PDE4D,ABHD6,PREX2,CHRM5,CXCL10,ADGRG3,PLCB2,DGKZ,IQGAP2,EDN1,SNCA,ADCY2,ANO1,GIT2,HCRTR1,GPR146,ARHGEF12,ADCY1,LTB4R,ADCY4,ADGRA1,SSTR1,CCRL2,ADORA2A,HOMER3,CALCRL,RAMP2,TMOD2,RAC1,RGS14,GNGT2,PIK3R5,NPR1,GPR68,DNM2,ADRB2,CCL2,PDE4B,GIT1,S1PR1,ADGRL2,ADGRL4,ADGRE3,ADGRG1,GPR182,CA2,ACKR1,LPAR6,RGS4,ARHGEF1,RGS16,PDCL,GNG4,GNG2,GPR162	turquoise	EC_M6
GO:0070201	8.64E-05	0.999948727	88	436	regulation of establishment of protein localization	BP	7.06E-03	LMAN1,LRP1,SLC12A2,ANGPT1,TCP1,PTP4A3,PPARD,LEP,UNC13B,CLOCK,EXOC1,REST,RAB23,P3H1,TMEM30A,ERLEC1,UBE2J1,MDFIC,GLI3,SLC7A11,FKBP1B,KIF5B,DNAJC1,PAK1,TGFB1,AKT2,UFM1,ATP2C1,IRS2,PRNP,HDAC3,RAB11FIP1,TM9SF4,ANKRD1,OSBP,CCT8,ABCC8,CACNB3,PTPN11,PFKM,ATG13,TSG101,DKC1,BAP1,CHP1,BRSK2,DYNLL1,DNM1L,CPT1A,TGFB2,LRP5,GSK3B,HADH,DISP1,HSPA8,IDH2,ADCY5,SCFD1,SIDT2,MAPT,C11orf65,PDCD10,B3GAT3,NLGN2,SRCIN1,TMED10,TGFB3,RAB11A,MYH10,SYTL4,ACSL4,MIEF2,MYOM1,PRKAA1,MYO1C,BCAP31,FLNA,MIDN,SSTR5,PARK7,RHBDF1,WLS,TXN,LEPROT,ZPR1,UBE2G2,TOMM7,DERL2	darkgreen	EC_M4
GO:0006873	8.65E-05	0.999948401	90	444	cellular ion homeostasis	BP	7.06E-03	LRP1,SLC12A2,SLC39A7,SMDT1,SLC39A4,SLC30A9,SLC31A1,FKBP1B,FGF2,RAB7A,ATP7B,TPT1,C1QTNF1,SLC4A7,TMTC4,MAFG,STIM1,ATP2C1,PRNP,ATP2B1,CCKBR,RIC3,CXCL12,TM9SF4,HSP90B1,C19orf12,ATP1B3,RYR3,P2RX4,SELENOT,ATP2A2,APP,SLC4A5,CX3CL1,CACNB3,AP3B1,CHP1,ATP2B4,PKD2,SLC39A10,CHRNA7,RAP1GDS1,HTR2B,ISCU,CMKLR1,HJV,SLAMF8,ADCY5,VAPB,CASQ1,CLIC4,TFRC,FZD9,GLRX3,TMCO1,ATP6AP2,TFR2,PTPN6,ITGAV,HTT,ERO1A,HAP1,CACNA1C,STIM2,ADRA1B,GPR65,CLN6,CALM1,GRIA1,NPTN,SLC39A6,ANXA6,TRPV2,GNB1,BCAP31,PKD1,ATP6AP1,SYPL2,JPH3,SLC30A7,ATP6V0D1,DMD,FKBP1A,BNIP3,TMEM38B,SLC22A17,THY1,TMEM38A,CCR6,ATP6V0E1	darkgreen	EC_M4
GO:0045931	1.88E-04	0.999935913	21	97	positive regulation of mitotic cell cycle	BP	7.06E-03	FBXO5,CDC25C,CUL4A,BRCA2,CDC16,CCNB1,ESPL1,ANAPC7,UBE2C,CUL3,DTL,CDK1,CDC25A,CDC27,CDCA5,DBF4B,DLGAP5,CDC6,CDT1,PKN2,TMOD3	darkgrey	EC_M8
GO:0070588	8.82E-05	0.999956592	50	211	calcium ion transmembrane transport	BP	7.10E-03	SMDT1,UBQLN1,FKBP1B,FGF2,SEC61A1,PANX1,STIM1,ATP2C1,PRNP,ATP2B1,REM1,TRPC4AP,RYR3,P2RX4,ATP2A2,CACNA1D,CX3CL1,CACNB3,OPA1,ATP2B4,PKD2,CHRNA7,CALHM1,HTR2B,CASQ1,TMCO1,LINC02210-CRHR1,PTPN6,ITGAV,HTT,PKD1L1,ERO1A,HAP1,CACNA1C,STIM2,CACNA1G,CALM1,VMP1,ANXA6,TRPV2,AKT1,PKD1,JPH3,DMD,FKBP1A,CBARP,TMEM38B,THY1,TMEM38A,GEM	darkgreen	EC_M4

GO:0071560	8.84E-05	0.99995735	48	199	cellular response to transforming growth factor beta stimulus	BP	7.10E-03	FBN2,PPARA,CDKN2B,RASL11B,COL1A2,ASPN,TGFBR1,MAP3K7,ZYX,TGFB1,ZEB2,LTBP1,SPRED3,SMAD9,ZNF703,ANKRD1,DKK3,ACVR1,SLC2A10,TGFB2,FGFR2,ITGB5,HTRA1,STRAP,FAM89B,FBN1,COL3A1,SKIL,SMURF1,BMPR1A,PPARGC1A,RNF111,TGFB1I1,LTBP2,COL1A1,TGFB3,MYOCD,PIN1,NLK,VASN,GLG1,HSPA5,FKBP1A,LOX,DUSP22,PMEPA1,CHST11,WNT2	darkgreen	EC_M4
GO:0010171	8.86E-05	0.999980099	15	38	body morphogenesis	BP	7.10E-03	RRAS,RAB3GAP1,PTPN11,MMP2,PHLDB2,DAG1,LEF1,PHLDB1,ARID5B,SGPL1,ATP6AP2,GREM2,COL1A1,TGFB3,ANKRD11	darkgreen	EC_M4
GO:0050801	8.89E-05	0.999944702	106	542	ion homeostasis	BP	7.10E-03	LRP1,SLC12A2,SLC39A7,SMDT1,SLC39A4,SLC30A9,CNNM4,SLC31A1,SLC7A11,FKBP1B,FGF2,RAB7A,ATP7B,PICALM,TPT1,SCN5A,GPD1L,C1QTNF1,SLC4A7,TMTC4,MAFG,STIM1,ATP2C1,PRNP,ATP2B1,DCN,CCKBR,RIC3,CXCL12,TM9SF4,HSP90B1,C19orf12,SLMAP,ATP1B3,RYR3,P2RX4,SELENOT,ATP2A2,APP,SLC4A5,CX3CL1,CACNB3,AP3B1,GLS,CHP1,ATP2B4,PKD2,SLC39A10,CHRNA7,RAP1GDS1,CNNM2,HTR2B,ISCU,CMKLR1,HJV,SLAMF8,ADCY5,VAPB,CASQ1,CLIC4,TFRC,FZD9,GLRX3,TMCO1,ATP6AP2,COMT,TFR2,PTPN6,ITGAV,HTT,ERO1A,KCNA1,FBXL5,HAP1,CACNA1C,STIM2,NPPB,CACNA1G,ADRA1B,GPR65,CLN6,CALM1,GRIA1,NPTN,SLC39A6,ANXA6,TRPV2,GNB1,BCAP31,PKD1,ATP6AP1,SYPL2,JPH3,SLC30A7,ATP6V0D1,DMD,FKBP1A,BNIP3,TMEM38B,SLC22A17,SNX10,THY1,TMEM38A,CCR6,HCN4,ATP6V0E1	darkgreen	EC_M4
GO:0055080	9.25E-05	0.999943354	98	494	cation homeostasis	BP	7.32E-03	LRP1,SLC12A2,SLC39A7,SMDT1,SLC39A4,SLC30A9,CNNM4,SLC31A1,FKBP1B,FGF2,RAB7A,ATP7B,PICALM,TPT1,C1QTNF1,SLC4A7,TMTC4,MAFG,STIM1,ATP2C1,PRNP,ATP2B1,CCKBR,RIC3,CXCL12,TM9SF4,HSP90B1,C19orf12,ATP1B3,RYR3,P2RX4,SELENOT,ATP2A2,APP,SLC4A5,CX3CL1,CACNB3,AP3B1,CHP1,ATP2B4,PKD2,SLC39A10,CHRNA7,RAP1GDS1,CNNM2,HTR2B,ISCU,CMKLR1,HJV,SLAMF8,ADCY5,VAPB,CASQ1,CLIC4,TFRC,FZD9,GLRX3,TMCO1,ATP6AP2,COMT,TFR2,PTPN6,ITGAV,HTT,ERO1A,KCNA1,FBXL5,HAP1,CACNA1C,STIM2,NPPB,ADRA1B,GPR65,CLN6,CALM1,GRIA1,NPTN,SLC39A6,ANXA6,TRPV2,GNB1,BCAP31,PKD1,ATP6AP1,SYPL2,JPH3,SLC30A7,ATP6V0D1,DMD,FKBP1A,BNIP3,TMEM38B,SLC22A17,SNX10,THY1,TMEM38A,CCR6,ATP6V0E1	darkgreen	EC_M4
GO:0001501	9.26E-05	0.999945467	85	413	skeletal system development	BP	7.32E-03	NAB2,FBN2,COL11A2,LEP,SRF,RUNX2,COL1A2,RAB23,P3H1,TGFBR1,GLI3,FGF2,GLI2,MEGF8,TGFB1,WDR48,CTNNB1,SERPINH1,SLC38A10,RARB,LUM,DDRGK1,RYK,IMPAD1,SULF1,RDH10,PTPN11,LOXL2,MMP2,GDF6,BMP1,RUNX1,NOTCH2,CHSY1,HYAL1,SLC2A10,SBDS,TGFB2,LRP5,SATB2,MMP14,CMKLR1,ACAN,HAPLN3,BMP7,FGFR2,ARID5B,RPS6KA3,SGPL1,PTPN6,FBN1,COMP,COL3A1,COL5A2,HSD17B1,FGFR3,PRRX1,TIMP1,TULP3,SIX4,EVC,BMPR1A,MED1,FGFRL1,THBS3,COL1A1,CHAD,TGFB3,PBXIP1,ANXA6,PLS3,NPR3,PAFAH1B1,PKD1,ATP6AP1,ANKRD11,COL11A1,GLG1,TMEM38B,LOX,SNX10,HAS2,OTOR,SLC39A3,CHST11	darkgreen	EC_M4
GO:0009611	9.36E-05	0.999944656	86	422	response to wounding	BP	7.36E-03	SERPINB2,LMAN1,LRP1,SLC12A2,FAM19A5,PPARA,MRTFA,PPARD,CTNNA1,C6orf89,LGALS1,TXN2,GNAQ,MYH9,SRF,VEGFA,DST,TGFBR1,GLI3,SLC7A11,ITGB1,FKBP1B,FGF2,DGKH,PAK1,TGFB1,AXL,C1QTNF1,ACTG1,DCN,SMPD1,ILK,DPYSL3,MAP1B,P2RX4,MYL9,CX3CL1,ABCC8,AP3B1,SPP1,HPS6,FAP,PHLDB2,NOTCH2,TGFB2,ITPK1,DAG1,PIK3CA,PRKCA,NTRK3,FGFR2,HSPB1,SERPINE1,ITGA2B,TNR,FN1,PTPN6,PDCD10,ADRA2C,COMP,COL3A1,TIMP1,VCL,FKBP10,SYT11,TPM1,COL1A1,TGFB3,MAP2K1,SYTL4,ANXA6,PDGFRB,ITGA2,CD40LG,FLNA,COL5A1,F3,CD151,BNIP3,LOX,CD59,ARF4,CSRP1,CDKN1A,PRKG1,FGF10	darkgreen	EC_M4
GO:0045910	1.98E-04	0.999961532	11	34	negative regulation of DNA recombination	BP	7.42E-03	MSH2,RIF1,PARPBP,MSH3,FANCB,BLM,ANKLE1,THOC1,SMCHD1,RMI2,HIST1H1A	darkgrey	EC_M8
GO:0006353	2.01E-04	0.999974691	8	19	DNA-templated transcription, termination	BP	7.52E-03	SCAF8,MTERF1,ZMPSTE24,PCF11,XRN2,SMN2,PRMT5,DHX9	darkgrey	EC_M8
GO:0006875	1.01E-04	0.999941747	79	381	cellular metal ion homeostasis	BP	7.87E-03	LRP1,SLC12A2,SLC39A7,SMDT1,SLC39A4,SLC30A9,SLC31A1,FKBP1B,FGF2,ATP7B,TPT1,C1QTNF1,TMTC4,STIM1,ATP2C1,PRNP,ATP2B1,CCKBR,RIC3,CXCL12,HSP90B1,C19orf12,ATP1B3,RYR3,P2RX4,SELENOT,ATP2A2,APP,CX3CL1,CACNB3,AP3B1,ATP2B4,PKD2,SLC39A10,CHRNA7,RAP1GDS1,HTR2B,ISCU,CMKLR1,HJV,ADCY5,VAPB,CASQ1,TFRC,FZD9,GLRX3,TMCO1,TFR2,PTPN6,ITGAV,HTT,ERO1A,HAP1,CACNA1C,STIM2,ADRA1B,GPR65,CALM1,GRIA1,NPTN,SLC39A6,ANXA6,TRPV2,GNB1,BCAP31,PKD1,ATP6AP1,SYPL2,JPH3,SLC30A7,ATP6V0D1,DMD,FKBP1A,BNIP3,TMEM38B,SLC22A17,THY1,TMEM38A,CCR6	darkgreen	EC_M4

GO:0000165	7.66E-05	0.999953671	94	579	MAPK cascade	BP	7.92E-03	TNFRSF11A,SEMA6A,AKAP12,FLT4,TPD52L1,PDGFB,MAPK13,TEK,SH3RF3,KIT,KDR,SYK,GADD45G,RNF149,IL6,CAV2,GRB10,TLR4,TRAF1,SDCBP,NRP1,PIK3CG,PRKD2,PPARG,PPP1CB,IKBKB,P2RY6,SFRP1,RASGRP3,CD36,SIRT3,MAP3K1,EPHB1,RET,TNFRSF19,IGF1,ITCH,SH3RF1,SMAD1,MEF2C,P2RX7,NDRG4,MADD,EDN1,DUSP8,PRDM15,HYAL2,MEF2A,HCRTR1,LAPTM5,NCF1,PABPN1,CFLAR,MAP3K6,MAP3K3,PAK2,DUSP16,DLG1,MAP3K11,TBX1,TMEM106A,MECOM,TNFAIP8L3,PLVAP,FGF23,CRACR2A,EPHA2,ITPKB,TLR6,RELL1,RGS14,AR,PSEN1,FGF18,TAOK2,EFNA1,PIK3R5,TNIP1,ADRB2,CCL2,SH2D3A,LGALS9,TRAF2,NOTCH1,PLCG2,SH2D3C,DAB2IP,MIR23A,ALKAL1,SHANK3,DUSP5,BMP2,DUSP7,NMNAT1	turquoise	EC_M6
GO:0045143	2.15E-04	0.999949156	13	47	homologous chromosome segregation	BP	8.03E-03	RAD21,FANCD2,SGO1,ESPL1,TERF1,CCNE2,TRIP13,SGO2,SPO11,PTTG1,PLK1,BRIP1,NDC1	darkgrey	EC_M8
GO:0009891	2.18E-04	0.999843381	187	1533	positive regulation of biosynthetic process	BP	8.12E-03	ONECUT2,BCLAF1,CCT2,DDX39B,SCAF8,EIF3E,MAP3K4,DSCC1,HSF2,ATAD2,XRCC6,PHF5A,IFT74,POT1,PWP1,ZNF300,TOPORS,DBF4,RFX3,TRIM28,ZNF451,FOXJ3,PHIP,PPP4R3B,RLF,ZNF292,CCT4,ITGB1BP1,THRAP3,CCNC,NAA15,ELF2,ATAD2B,HDAC2,CCNA2,EXOSC9,MLLT10,DNAJC2,NUP85,ELF1,RSF1,CCNT2,PAXIP1,WDR43,SUPT16H,CHD8,KAT6A,METTL3,E2F7,ALYREF,CKAP2,CEP290,NSD3,CEBPZ,TAF7,RFC3,BRCA2,ELK3,TCERG1,PRKDC,ERCC6,DHX29,GPBP1,SP1,TCEA1,ZC3H8,TNKS2,ZNF143,CENPJ,CDK5RAP1,NCK1,ARFGEF1,SMARCA5,ZNF507,TAF9,TFCP2,ATR,LARP4,UTP15,HLTF,GABPA,MCRS1,USP16,CCT7,SENP1,RIPK1,WDR75,MYBL2,E2F8,INO80,CHUK,HIVEP1,RFC5,NOLC1,CCT5,BMPR1B,UBE3A,CCT6A,PRDM10,NUCKS1,GCN1,CHEK1,H2AFZ,RRP1B,NFKB1,KHDRBS1,TET2,TRIP11,HEATR1,NCL,AJUBA,NOL11,GTF2I,BPTF,FXR1,RFC2,NEK2,EIF2B5,BLM,EDRF1,CDK1,ING5,BCLAF3,FOSL1,SMARCC1,SP3,DHX9,FASTKD2,RFC4,DNA2,CCAR1,DBF4B,UBTF,NMD3,XRCC5,NPAT,BRCA1,CNOT9,DCAF6,DHX33,TBL1XR1,LEO1,FOXM1,LYAR,DDX11,HNRNPU,SMARCE1,RFC1,TOP2A,NSD1,MED6,ICE2,CCT3,PMF1,FUS,AURKB,ZNF609,OGT,ATRX,ZNF711,ILF2,TRIM37,LARP1,ILF3,MED13,AATF,TAF15,THOC1,UPF3B,ATAD5,NIPBL,MYSM1,UHRF1,RBMX,FMR1,TP73,CHTF18,CDT1,CTCF,PRIM2,HNRNPD,DRAP1,ZNF770,NAF1,ZNF782,ZFP62,SFR1	darkgrey	EC_M8
GO:0051336	1.05E-04	0.999930077	139	754	regulation of hydrolase activity	BP	8.18E-03	SERPINB2,LRP1,TBC1D9B,PLXNB2,C6orf89,MTCH1,GNAQ,PLAA,NUAK1,VEGFA,DOCK8,VCP,TPM2,REST,SNX13,PSME4,CYCS,ROCK2,BVES,RCN3,ITGB1,FKBP1B,PLXNA1,FGF2,NAPA,ABHD5,PICALM,RAB3GAP1,SERPINH1,AKT2,TBC1D16,PPP1R12A,CRIM1,SPINT2,PRNP,DDRGK1,ARL6IP5,SPINK5,ARL1,HSP90B1,TMED2,ATP1B3,ROBO1,PFN2,RASA1,APP,RAPGEF2,CX3CL1,PTX3,RALGAPB,CRY2,CHP1,PLA2R1,CBLB,IFT57,TANK,PSMD14,BRSK2,SLC39A10,RAP1GDS1,DOCK10,UCHL5,TGFB2,PPP6R3,HTR2B,GSK3B,CORO1C,ANOS1,ADAP1,NTRK3,SGSM1,FAM162A,PPP1R11,COL6A3,FGFR2,NCSTN,ADRM1,IQGAP1,RPS6KA3,EGLN3,MAPT,SERPINE1,RASAL2,DNAJC10,PCOLCE,FN1,ZC3H15,HTT,FGFR3,TIMP1,PPP1R36,EIF2S1,NGFR,TPM1,TMED10,PBXIP1,GPR65,TTC8,CALM1,UACA,PPP4R4,PIN1,PDGFRB,ROCK1,USP14,EVI5L,AKT1,SNX18,ITGA2,OCRL,ABR,BCAP31,PLXNB3,PAFAH1B1,TAX1BP3,PLXNA3,PARK7,GPSM1,F3,ARHGAP29,BCAR3,MTSS1L,BCL10,MIR24-1,CCL16,FKBP1A,SPINK2,SERPINF1,SERPINB5,A2M,NMUR1,ARF4,TIMP3,THY1,TOR1AIP2,CHN1,GABARAPL2,PRKG1,FGF10	darkgreen	EC_M4
GO:0000212	2.23E-04	0.999983691	6	11	meiotic spindle organization	BP	8.28E-03	FBXO5,ESPL1,ASPM,CCNB2,ATRX,AURKA	darkgrey	EC_M8
GO:1903405	2.25E-04	0.999982452	6	12	protein localization to nuclear body	BP	8.32E-03	CCT2,CCT4,CCT7,CCT5,CCT6A,CCT3	darkgrey	EC_M8
GO:1904867	2.25E-04	0.999982452	6	12	protein localization to Cajal body	BP	8.32E-03	CCT2,CCT4,CCT7,CCT5,CCT6A,CCT3	darkgrey	EC_M8

GO:0048522	8.18E-05	0.999935721	529	4212	positive regulation of cellular process	BP	8.36E-03	ZNF516,TNFRSF11A,TCF4,TNFAIP8,SEMA6A,IFNGR1,TNFAIP3,PLAGL1,TRIM39,AKAP12,CNOT6,FLT4,CTHRC1,RIMS2,ZBED4,GRAMD4,NOTCH4,AFDN,TCF20,CDKL3,TRDN,TPD52L1,NCOA7,MTSS1,RNF146,ASAP1,PTK2,DAPK1,ATF4,PDGFB,AGTPBP1,BRPF3,PODXL,CSNK1E,BAIAP2L2,PICK1,KDM4C,PIM1,CDC42EP1,MFNG,NFYA,LRRC19,TEK,LINGO2,PRDM4,LIMCH1,DDX58,ATP8A1,FAM122A,TEC,SH3RF3,ADGRF5,KIT,KDR,CD2AP,NFIL3,SYK,GADD45G,SEMA4D,IL6,REL,PPT1,CREB5,CXCL8,PELI1,UTP11,RSAD2,DOCK4,CAV2,KLF11,GRB10,LANCL2,TLR4,TCAF2,GRIK2,TRAF1,IL15,CCNY,TEAD2,SDCBP,MYCN,NRCAM,NOCT,TUT4,TRPM4,NRP1,PUM2,RASIP1,PIK3CG,SVIL,ITSN2,TAL1,PHF10,MTMR2,EPC1,KDM4D,GATA2,DNMT3A,PRKD2,CST7,CTSC,RAB38,CAB39L,GPR4,PLXND1,NECTIN2,NOS3,GAB2,RALB,UNC13D,PPARG,GATA3,ACVR2B,CXCR4,EDNRB,NET1,NDFIP2,TIE1,IKBKB,JAG1,P2RY6,FCHSD2,NFKBIB,TMEM173,SFRP1,HBEGF,NR1D2,DND1,FERMT1,RPL26,EFNB2,ANKLE2,ARHGEF7,ASH2L,ATP11A,FCHSD1,P2RX2,CD36,SIRT3,LMOD3,MAGI1,PRAG1,PRR5L,TCF15,SCARB1,EPHB1,PLK2,RET,MERTK,TNFRSF19,CHD7,IL1A,DNMT3B,IGF1,NR4A1,KLKB1,EIF2AK3,CSPP1,HHEX,KDM3A,ITCH,ERBIN,SH3RF1,SMAD1,ZBTB38,CLCN3,HIP1R,OCLN,STOX2,ARHGEF3,SOX7,HMBOX1,GPD1,ENC1,HEXB,MEF2C,SMARCD1,TLR2,WWTR1,IL7,P2RX7,VEGFC,NDRG4,GPM6A,GUCY1A1,CXCL10,MADD,KMT2D,BMP2K,NLRC5,THBS4,PPP1R16B,TPCN1,PLCG1,RAPGEF3,NR4A2,DGKZ,IQGAP2,HIPK1,TOX2,ANO6,DLL4,ZDHHC13,IFIT2,SOX13,ADAMTS20,NEDD9,YAF2,EDN1,STAT1,DYRK1A,JAM3,EHD4,SNCA,JARID2,DTNBP1,ERG,ETS2,SLC45A3,N	turquoise	EC_M6
GO:0032103	8.19E-05	0.999958574	52	286	positive regulation of response to external stimulus	BP	8.36E-03	TNFRSF11A,PTK2,PDGFB,MAPK13,KDR,SYK,IL6,CXCL8,TLR4,IL15,NRP1,PUM2,PIK3CG,PRKD2,CTSC,GPR4,NECTIN2,CXCR4,IKBKB,PDE2A,TMEM173,TLR2,VEGFC,CXCL10,NLRC5,THBS4,ANO6,EDN1,SNCA,ETS1,HYAL2,FABP4,PRKD1,PAK2,MCU,PPM1F,RAC1,FGF18,PGF,DAPK2,S100A14,TNIP1,LGALS9,PLCG2,S1PR1,LRSAM1,GBP5,FGF16,TRIM62,STX3,SCARF1,BMP6	turquoise	EC_M6
GO:0072331	2.29E-04	0.999907952	27	139	signal transduction by p53 class mediator	BP	8.44E-03	GTSE1,TOPORS,MSH2,ZMPSTE24,CASP2,PAXIP1,KAT6A,E2F7,BRCA2,RRP8,RRS1,TAF9,ATR,NBN,EEF1E1,MDM4,PAK1IP1,CHEK1,PRMT5,DDX5,FOXM1,AURKB,TAF1,ATRX,PSMD10,TP73,AURKA	darkgrey	EC_M8
GO:0007405	1.09E-04	0.999973342	16	42	neuroblast proliferation	BP	8.45E-03	PLXNB2,CTNNA1,TEAD3,VEGFA,GLI3,SMARCD3,CTNNB1,CX3CL1,LEF1,FGFR2,FZD9,KCNA1,ARHGEF2,EML1,PAFAH1B1,ARTN	darkgreen	EC_M4
GO:0051254	2.32E-04	0.999836207	169	1357	positive regulation of RNA metabolic process	BP	8.51E-03	PAN2,ONECUT2,BCLAF1,DDX39B,SCAF8,HSF2,ATAD2,XRCC6,PHF5A,TNRC6B,TUT7,IFT74,PWP1,ZNF300,TOPORS,RFX3,TRIM28,NCBP1,ZNF451,FOXJ3,PHIP,PNPT1,RLF,ZNF292,ITGB1BP1,THRAP3,CCNC,NAA15,ELF2,ATAD2B,HDAC2,CCNA2,EXOSC9,MLLT10,DNAJC2,NUP85,ELF1,RSF1,DIS3,CCNT2,PAXIP1,WDR43,SUPT16H,CHD8,KAT6A,METTL3,E2F7,ALYREF,CKAP2,CEP290,NSD3,CEBPZ,TAF7,EXOSC8,BRCA2,ELK3,TCERG1,PRKDC,CCNB1,ERCC6,PAN3,GPBP1,SP1,TCEA1,ZC3H8,DIMT1,ZNF143,CENPJ,NCK1,SMARCA5,ZNF507,TAF9,PDE12,TFCP2,UTP15,HLTF,CNOT1,GABPA,MCRS1,USP16,SENP1,SLC38A2,RIPK1,WDR75,MYBL2,E2F8,INO80,RIOK1,CHUK,HIVEP1,NOLC1,BMPR1B,UBE3A,PRDM10,NUCKS1,GCN1,CHEK1,H2AFZ,RRP1B,NFKB1,TET2,TRIP11,HEATR1,NCL,NOL11,GTF2I,BPTF,DDX5,BLM,EDRF1,ING5,BCLAF3,FOSL1,SMARCC1,SP3,DHX9,HNRNPR,TRA2B,CCAR1,UBTF,NMD3,NPAT,BRCA1,CNOT9,DCAF6,DHX33,TBL1XR1,LEO1,FOXM1,LYAR,DDX11,HNRNPU,SMARCE1,RFC1,TOP2A,DCP2,NSD1,SRSF5,MED6,ICE2,PMF1,FUS,ZNF609,OGT,ATRX,ZNF711,ILF2,TRIM37,ILF3,MED13,AATF,TAF15,THOC1,NIPBL,MYSM1,UHRF1,RBMX,METTL16,TP73,DAZAP1,EXOSC2,CTCF,PRIM2,HNRNPD,DRAP1,ZNF770,ZNF782,ZFP62,SFR1	darkgrey	EC_M8

GO:0010648	1.12E-04	0.999921271	185	1047	negative regulation of cell communication	BP	8.62E-03	LRP1,DYRK2,RRM2B,FBN2,PPARA,FAM49B,CTNNA1,CBY1,LEP,SRF,VCP,RUNX2,RASL11B,NCK2,CLOCK,CREB3,REST,ASPN,SNX13,TGFBR1,SOCS5,TPBG,GLI3,INHBA,DKKL1,ITGB1,FKBP1B,FGF2,SESN3,RAB7A,RB1,TPT1,PTCH2,KCTD21,GLI2,MEGF8,PCDH17,CTNNB1,LBH,GPD1L,NPLOC4,LTBP1,CRIM1,SPRED3,PRNP,DCN,HDAC3,RAB11FIP1,CAVIN3,DDRGK1,SMPD1,PPP2CB,LEPROTL1,APLP1,TMEM127,CNOT7,CXCL12,ZNF536,SULF1,CTNND1,DKK3,ROBO1,WWC2,FAIM2,HEY1,RASA1,MAPKAPK5,CX3CL1,ABCC8,PTPN11,OPA1,AMFR,PTPN22,TSG101,ACVR1,CRY2,VPS18,CHAC1,ADA,CHP1,ATP2B4,HIF1AN,CBLB,NKD1,TANK,RAB7B,ADIPOR1,CTTN,SELENOS,SLC2A10,EYA2,TGFB2,KCTD10,HTR2B,GSK3B,MMP14,DAG1,HADH,WWC3,GRK3,BMP7,HYOU1,BICC1,ATF3,OSM,PEA15,HTRA1,STRAP,STRN3,FZD9,FAM89B,HSPB1,MAPT,SERPINE1,SYVN1,TNR,PTPN6,IGFBP2,FBN1,ITGAV,LRPAP1,HTT,SKIL,SMURF1,NBL1,VDAC2,TULP3,GREM2,ATAD1,OTUD5,GHITM,CGNL1,ARHGEF2,TGFB1I1,SYT11,NGFR,TGFB3,TAF9B,SOX30,TSPAN6,SYTL4,SMARCA4,UACA,GRIA1,MYOCD,PIN1,CD74,CHRDL1,NOMO1,AKT1,PRKAA1,NLK,TNFAIP1,PTPN2,NXN,MGRN1,VASN,PAFAH1B1,TAX1BP3,NPHP4,MIDN,PARK7,ENO1,GNAI3,USP10,GLG1,HSPA5,MIR24-1,FSTL3,DUSP2,FKBP1A,PTPN12,GDNF,TIMP3,THY1,DNAJB9,DACT3,DUSP4,TMSB4X,PTTG1IP,PMEPA1,LEPROT,SLC35C1,CHST11,FGF10	darkgreen	EC_M4
GO:0051046	8.52E-05	0.999951509	74	434	regulation of secretion	BP	8.63E-03	TNFRSF11A,SNCAIP,RIMS2,PICK1,APBA1,CD2AP,SYK,ICA1,IL6,RSAD2,TLR4,SDCBP,TRPM4,RAB3B,GATA2,GAB2,LLGL2,UNC13D,EDNRB,P2RY2,SFRP1,SIRT3,CHD7,FAM3D,IL1A,IGF1,HIP1R,MCTP1,MEF2C,TLR2,VEGFC,STC1,EDN1,SNCA,DTNBP1,ABCG1,CD200,HDAC1,PPP3CA,ANO1,MCTP2,ATP9A,FGR,ADCY1,FES,CREB1,ADORA2A,MCU,WNK4,CDK16,FGF23,RAC1,HIF1A,RAB27A,SNCG,SYT7,RSC1A1,TNFRSF1B,FBXL20,CHRNB2,RAB3D,NPR1,GPR68,LGALS9,NOS2,MYO18A,GIT1,RPH3AL,NOTCH1,STXBP1,RALA,VAMP3,BMP2,BMP6	turquoise	EC_M6
GO:0010824	2.38E-04	0.999948105	12	39	regulation of centrosome duplication	BP	8.73E-03	XPO1,GEN1,PLK4,STIL,CEP295,CHORDC1,CENPJ,ALMS1,BRCA1,TRIM37,CEP76,CCNF	darkgrey	EC_M8
GO:1904816	2.43E-04	0.999980763	6	12	positive regulation of protein localization to chromosome, telomeric region	BP	8.90E-03	CCT2,CCT4,CCT7,CCT5,CCT6A,CCT3	darkgrey	EC_M8
GO:0061024	1.17E-04	0.999925405	114	599	membrane organization	BP	8.94E-03	ATP8B1,STX7,HSPA4,SEC24A,FER1L6,PACSIN1,REEP2,ADGRB1,SUN2,MTCH1,SH3GL2,MYH9,RIMS1,TMEM30A,RTN4,COX18,EMC10,MFSD2A,VPS41,SLC7A11,KIF5B,NAPA,RAB7A,PICALM,PDCD6IP,SEC13,AKT2,SEC23B,OSBPL8,ATP2C1,SMPD1,SLC25A46,TMED2,SLC25A4,OSBP,VAMP8,TRAM1,EMC4,ATP2A2,USO1,GLRB,OPA1,AP3B1,LPCAT2,CHP1,VPS39,RAB7B,AZIN2,DNM1L,EYA2,SEC22B,TGFB2,GSK3B,CORO1C,PRKCA,PITPNM1,HSPA8,PITPNB,ARV1,VAPB,PRELID3B,AP2M1,OSBPL2,FZD9,ALKBH4,SEC23A,PRKCI,SMURF1,EMC1,GLDN,VDAC2,STX18,GHITM,SLC2A4,NLGN2,CLN8,STARD3,STX1B,SYT11,DOCK2,TMED10,SOX30,CALM1,SYTL4,ANXA6,MIEF2,AKT1,SNX18,ITGA2,OCRL,RAB31,NAPG,RAB34,OMA1,ATP11C,PITPNA,PAFAH1B1,ABCA3,VAMP7,GNAI3,SYPL2,COL5A1,MTSS1L,BNIP3,PLEKHA3,GDNF,TOR1AIP2,BLOC1S2,LHFPL4,STXBP6,EMC7,TIMM22,PIK3C2A,MESD	darkgreen	EC_M4

GO:0023057	1.18E-04	0.999916847	185	1048	negative regulation of signaling	BP	9.00E-03	LRP1,DYRK2,RRM2B,FBN2,PPARA,FAM49B,CTNNA1,CBY1,LEP,SRF,VCP,RUNX2,RASL11B,NCK2,CLOCK,CREB3,REST,ASPN,SNX13,TGFBR1,SOCS5,TPBG,GLI3,INHBA,DKKL1,ITGB1,FKBP1B,FGF2,SESN3,RAB7A,RB1,TPT1,PTCH2,KCTD21,GLI2,MEGF8,PCDH17,CTNNB1,LBH,GPD1L,NPLOC4,LTBP1,CRIM1,SPRED3,PRNP,DCN,HDAC3,RAB11FIP1,CAVIN3,DDRGK1,SMPD1,PPP2CB,LEPROTL1,APLP1,TMEM127,CNOT7,CXCL12,ZNF536,SULF1,CTNND1,DKK3,ROBO1,WWC2,FAIM2,HEY1,RASA1,MAPKAPK5,CX3CL1,ABCC8,PTPN11,OPA1,AMFR,PTPN22,TSG101,ACVR1,CRY2,VPS18,CHAC1,ADA,CHP1,ATP2B4,HIF1AN,CBLB,NKD1,TANK,RAB7B,ADIPOR1,CTTN,SELENOS,SLC2A10,EYA2,TGFB2,KCTD10,HTR2B,GSK3B,MMP14,DAG1,HADH,WWC3,GRK3,BMP7,HYOU1,BICC1,ATF3,OSM,PEA15,HTRA1,STRAP,STRN3,FZD9,FAM89B,HSPB1,MAPT,SERPINE1,SYVN1,TNR,PTPN6,IGFBP2,FBN1,ITGAV,LRPAP1,HTT,SKIL,SMURF1,NBL1,VDAC2,TULP3,GREM2,ATAD1,OTUD5,GHITM,CGNL1,ARHGEF2,TGFB1I1,SYT11,NGFR,TGFB3,TAF9B,SOX30,TSPAN6,SYTL4,SMARCA4,UACA,GRIA1,MYOCD,PIN1,CD74,CHRDL1,NOMO1,AKT1,PRKAA1,NLK,TNFAIP1,PTPN2,NXN,MGRN1,VASN,PAFAH1B1,TAX1BP3,NPHP4,MIDN,PARK7,ENO1,GNAI3,USP10,GLG1,HSPA5,MIR24-1,FSTL3,DUSP2,FKBP1A,PTPN12,GDNF,TIMP3,THY1,DNAJB9,DACT3,DUSP4,TMSB4X,PTTG1IP,PMEPA1,LEPROT,SLC35C1,CHST11,FGF10	darkgreen	EC_M4
GO:0006914	1.21E-04	0.999926853	90	450	autophagy	BP	9.13E-03	TAB2,ATP6V1C1,UBQLN1,LEP,PLAA,VCP,EXOC1,RAB23,MAP3K7,VPS41,VPS35,SESN3,RAB7A,ATP6V0B,RHEB,RAB3GAP1,MTMR14,STAM2,UFM1,DCN,UBA5,DDRGK1,VPS37A,TMEM41B,MAP1LC3A,TP53INP2,SLC25A4,C19orf12,EIF4G2,VAMP8,ATP2A2,ARSB,ATG13,PTPN22,TSG101,VPS18,MTDH,TOLLIP,VPS39,ATP6V1B2,CTTN,DNM1L,SEC22B,CISD2,FEZ1,HTR2B,WIPI1,GSK3B,PIK3CA,HSPA8,SYNPO2,SCFD1,QSOX1,ATF6,PAFAH1B2,HSPB1,MAPT,GPR137,HTT,SMURF1,HAP1,WIPI2,ATP6V1D,PPARGC1A,SYT11,SPTLC2,CALM1,CLTC,VMP1,ATG4A,ROCK1,AKT1,PRKAA1,CLEC16A,DAPK3,IKBKG,FUNDC2,PARK7,GPSM1,GNAI3,USP10,ATP6V0D1,BNIP3,VPS26B,CAPNS1,GABARAPL2,PIK3C2A,ORMDL3,TOMM7,ATP6V0E1	darkgreen	EC_M4
GO:0061919	1.21E-04	0.999926853	90	450	process utilizing autophagic mechanism	BP	9.13E-03	TAB2,ATP6V1C1,UBQLN1,LEP,PLAA,VCP,EXOC1,RAB23,MAP3K7,VPS41,VPS35,SESN3,RAB7A,ATP6V0B,RHEB,RAB3GAP1,MTMR14,STAM2,UFM1,DCN,UBA5,DDRGK1,VPS37A,TMEM41B,MAP1LC3A,TP53INP2,SLC25A4,C19orf12,EIF4G2,VAMP8,ATP2A2,ARSB,ATG13,PTPN22,TSG101,VPS18,MTDH,TOLLIP,VPS39,ATP6V1B2,CTTN,DNM1L,SEC22B,CISD2,FEZ1,HTR2B,WIPI1,GSK3B,PIK3CA,HSPA8,SYNPO2,SCFD1,QSOX1,ATF6,PAFAH1B2,HSPB1,MAPT,GPR137,HTT,SMURF1,HAP1,WIPI2,ATP6V1D,PPARGC1A,SYT11,SPTLC2,CALM1,CLTC,VMP1,ATG4A,ROCK1,AKT1,PRKAA1,CLEC16A,DAPK3,IKBKG,FUNDC2,PARK7,GPSM1,GNAI3,USP10,ATP6V0D1,BNIP3,VPS26B,CAPNS1,GABARAPL2,PIK3C2A,ORMDL3,TOMM7,ATP6V0E1	darkgreen	EC_M4
GO:0021700	9.30E-05	0.999956756	42	204	developmental maturation	BP	9.36E-03	L3MBTL3,ANKS1A,PICK1,SEMA4D,PTBP3,IL15,NRCAM,TUT4,TAL1,GATA2,RAB38,PPARG,GATA3,EDNRB,SPTBN4,GPAT4,FERMT1,NTN4,RET,IGF1,CLCN3,NR4A2,LTF,HIF1A,ALDH1A2,MTOR,ARHGEF15,ZDHHC15,LYL1,B4GALT6,RFLNB,AP3D1,S1PR1,STXBP1,LHX6,DAB2IP,CDH5,RELN,SOX18,RAB32,BMP2,SCARF1	turquoise	EC_M6
GO:0006888	1.26E-04	0.999951251	29	106	endoplasmic reticulum to Golgi vesicle-mediated transport	BP	9.40E-03	LMAN1,SEC24A,VCP,LMAN2L,COPG1,SEC13,SEC23B,TMED2,COPB2,ERGIC3,COPB1,USO1,SEC22B,ERGIC2,SEC22A,VAPB,HYOU1,COPA,SCFD1,SEC23A,STX18,KDELR2,LMAN2,TMED10,TMED1,BCAP31,VAMP7,ARF4,TMED3	darkgreen	EC_M4
GO:0040013	1.26E-04	0.99993342	58	261	negative regulation of locomotion	BP	9.40E-03	LRP1,FAM19A5,ADGRB1,PPARD,CTNNA1,SRF,RRAS,FGF2,TGFB1,OSBPL8,NAV3,SPINT2,DCN,STARD13,SEMA3C,ILK,DPYSL3,RYK,CXCL12,SULF1,ROBO1,SEMA4F,PFN2,CX3CL1,ABCC8,ADA,ATP2B4,SEMA4G,ADIPOR1,PHLDB2,DAG1,CORO1C,SLAMF8,IDH2,CLIC4,SERPINE1,DNAJA4,PDCD10,COL3A1,NBL1,TIMP1,VCL,BMPR1A,PPARGC1A,NGFR,TPM1,MYOCD,PIN1,CD74,AKT1,PTPN2,PLXNB3,PLXNA3,MIR24-1,SERPINF1,THY1,DUSP22,PRKG1	darkgreen	EC_M4
GO:0030239	1.28E-04	0.999964718	18	51	myofibril assembly	BP	9.49E-03	SRF,MYLK3,ITGB1,ACTG1,ANKRD1,LMOD1,PRKAR1A,CASQ1,SYNPO2L,WDR1,SIX4,OBSL1,TCAP,TPM1,PDGFRB,FLII,CSRP1,CFL2	darkgreen	EC_M4
GO:0002521	9.51E-05	0.999947258	67	383	leukocyte differentiation	BP	9.51E-03	TNFRSF11A,TCF7,L3MBTL3,MFNG,IREB2,KIT,NFIL3,SYK,IL6,RSAD2,TLR4,IL15,TAL1,GATA2,GAB2,PPARG,GATA3,SFRP1,TMEM178A,MERTK,CHD7,IL1A,HHEX,SH3RF1,TLR2,IL7,TPD52,LMBR1L,ADGRG3,DLL4,SOX13,TSPAN2,DCAF1,LCK,HLX,HCLS1,INPP5D,CD86,FES,CREB1,B2M,LTF,ITGA4,SOS2,CRACR2A,EPHA2,ITPKB,LBR,LFNG,MTOR,PSEN1,SEMA4A,BATF,CD19,GPR68,YY1,LYL1,JAG2,LGALS9,IL7R,AP3D1,NRARP,IRF8,PLCG2,VNN1,CD46,CA2	turquoise	EC_M6

GO:0045739	2.62E-04	0.999924381	16	66	positive regulation of DNA repair	BP	9.54E-03	TRIM28,RIF1,PRKDC,ERCC6,ABRAXAS1,ARID2,FANCB,DHX9,BRCA1,RAD51AP1,FOXM1,DDX11,FUS,SMCHD1,TIGAR,RNF8	darkgrey	EC_M8
GO:0043633	2.62E-04	0.999987336	5	8	polyadenylation-dependent RNA catabolic process	BP	9.54E-03	PNPT1,EXOSC9,EXOSC8,EXOSC10,EXOSC2	darkgrey	EC_M8
GO:0000281	2.64E-04	0.999924247	16	63	mitotic cytokinesis	BP	9.56E-03	KIF20A,ANLN,CENPA,CKAP2,ESPL1,KIF20B,CEP55,RACGAP1,NUSAP1,CIT,ECT2,INCENP,KIF4A,KIF23,PLK1,STMN1	darkgrey	EC_M8
GO:1905820	2.64E-04	0.9999723	7	15	positive regulation of chromosome separation	BP	9.56E-03	CDC16,ESPL1,ANAPC7,CUL3,CDC27,DLGAP5,CDT1	darkgrey	EC_M8
GO:0032102	9.64E-05	0.999951709	50	266	negative regulation of response to external stimulus	BP	9.57E-03	SEMA6A,TNFAIP3,TSPAN8,C6orf106,PDGFB,TEK,SYK,SEMA4D,C5,NRP1,CST7,NOS3,PPARG,GATA3,SEC14L1,NR1D2,PLK2,IGF1,KLKB1,ITCH,ANGPT2,OAS3,NLRC5,PLCG1,EDN1,CD200,SEMA3F,RIN3,CD34,FGR,INPP5F,LTF,ADORA2A,SEMA6D,CALCRL,TNFRSF1B,ARG2,SEMA4A,MMP28,CCL2,YES1,LGALS9,GIT1,SEMA6B,ISG15,NOTCH1,CDH5,OAS1,SERPINB9,RNF125	turquoise	EC_M6
GO:0006446	2.66E-04	0.999923152	16	66	regulation of translational initiation	BP	9.59E-03	EIF3E,NCBP1,MTIF2,DDX1,METTL3,DHX29,NCK1,KHDRBS1,EIF2B5,TPR,EIF2AK1,EIF3B,LARP1,EIF5,FMR1,PAIP2	darkgrey	EC_M8
GO:2000232	2.66E-04	0.99997112	7	16	regulation of rRNA processing	BP	9.59E-03	WDR43,DIMT1,UTP15,WDR75,RIOK1,HEATR1,METTL18	darkgrey	EC_M8
GO:0031327	2.68E-04	0.999813401	150	1198	negative regulation of cellular biosynthetic process	BP	9.63E-03	PAN2,BCLAF1,FBXO5,ZNF608,SCAF8,EIF3E,MRPL13,XRCC6,TNRC6B,TUT7,POT1,RFX3,TRIM28,ZNF451,PNPT1,SYNCRIP,XPO1,CASP8AP2,TIA1,ZNF350,EZH2,FAM3C,WDR35,DHFR,PUS7,HDAC2,EXOSC9,DNAJC2,SETDB2,CNOT10,RSF1,DIS3,BIRC5,GTPBP4,CHD8,KAT6A,METTL3,RIF1,E2F7,TAF7,EXOSC8,NR2C1,BRCA2,ELK3,TCERG1,CBX5,PAN3,CAPRIN1,ZC3H8,LSM14A,BTAF1,TDG,NCK1,CGGBP1,XRN1,PDE12,SAP30,ATR,TERF1,GCFC2,CNOT1,GABPA,PPID,RBL1,E2F8,TRIM33,ZBED6,ZNF248,MDM4,HIVEP1,CUL3,GMNN,NFKB1,KHDRBS1,NCL,PRMT5,AJUBA,BPTF,FXR1,DDX5,ORC2,BLM,DDX6,CENPF,SP3,HNRNPR,DDX20,TPR,CCAR1,XRCC5,NPAT,BRCA1,CNOT9,NSD2,TBL1XR1,SUV39H1,FOXM1,EIF2AK1,LYAR,FAM220A,HNRNPU,PDS5A,WAPL,NEDD4,SMARCE1,RFC1,CDC6,DCP2,NSD1,EXOSC10,CLK2,USP3,AURKB,TAF1,RLIM,ATRX,TIPIN,MBTD1,TRIM37,LARP1,PLK1,ILF3,AATF,PSMD10,TYMS,ZNF3,MIER1,NIPBL,PHF6,UHRF1,FMR1,METTL16,EHMT1,EXOSC2,SCAI,CTCF,DEPDC1,HNRNPD,ZNF684,DRAP1,ZNF614,RBAK,BRMS1L,RNF139,C1D,RNF8,ZNF558,IKZF5,PAIP2,ZNF529	darkgrey	EC_M8
GO:0046649	9.77E-05	0.999943246	78	470	lymphocyte activation	BP	9.64E-03	TNFAIP3,TCF7,MFNG,KIT,TNFRSF21,NFIL3,SYK,IL6,PELI1,RSAD2,TLR4,IL15,PIK3CG,UNC13D,GATA3,SFRP1,EPHB1,GAPT,MERTK,CHD7,IL1A,IGF1,HHEX,ITCH,SH3RF1,KIF13B,MEF2C,IL7,TPD52,LMBR1L,ADGRG3,DLL4,SOX13,DCAF1,LCK,PPP3CA,ICOSLG,LAPTM5,HLX,NFATC2,INPP5D,CD86,FGR,DLG1,B2M,ADORA2A,ITGA4,SOS2,CRACR2A,WAS,RAB27A,ITPKB,LFNG,TNFRSF1B,ARG2,MTOR,EFNB1,PSEN1,SEMA4A,BATF,CHRNB2,CD19,YY1,LYL1,CCL2,YES1,JAG2,LGALS9,ELF4,IL7R,AP3D1,NRARP,IRF8,PLCG2,VNN1,CD46,AKAP17A,BST2	turquoise	EC_M6
GO:0044806	2.70E-04	0.999989229	5	7	G-quadruplex DNA unwinding	BP	9.69E-03	HNRNPA2B1,BLM,DHX9,DNA2,DDX11	darkgrey	EC_M8
GO:0043536	9.93E-05	0.999974638	16	53	positive regulation of blood vessel endothelial cell migration	BP	9.74E-03	PDGFB,KDR,NRP1,GATA2,PRKD2,NOS3,PLK2,VEGFC,PLCG1,ETS1,PRKD1,MAP3K3,HIF1A,RHOJ,FGF18,MIR23A	turquoise	EC_M6
GO:0006898	1.01E-04	0.999953957	40	192	receptor-mediated endocytosis	BP	9.80E-03	PICK1,MICALL1,CD2AP,SYK,SNAP91,PPT1,CXCL8,CAV2,SDCBP,ITSN2,MTMR2,FCHSD2,CD36,HIP1R,LMBR1L,BMP2K,STAB1,SNCA,RIN3,INPP5F,B2M,ARHGAP27,ITGA4,CALCRL,HSPG2,RAMP2,DNM3,SELE,FCHO1,RAC1,OPHN1,DNM2,AP2B1,ADRB2,UNC119,PLCG2,FNBP1L,CACNG5,SCARF1,GSG1L	turquoise	EC_M6

GO:0048583	1.33E-04	0.999895371	450	2834	regulation of response to stimulus	BP	9.81E-03	SERPINB2,CD63,IL23A,LRP1,STX7,ARHGEF25,ENPP3,TAB2,DYRK2,IER3,RRM2B,SLC12A2,FBN2,ANGPT1,FAM19A5,PPARA,HLA-DRA,ROS1,RSPO3,FAM49B,PTP4A3,LY6E,PPARD,CTNNA1,CBY1,UBQLN1,MTCH1,LGALS1,KLHDC10,GNAQ,ARHGAP39,LEP,CDKN2B,KIAA1324L,NUAK1,CDK14,VEGFA,DOCK8,VCP,SLC35B2,RUNX2,RASL11B,UNC13B,NCK2,CLOCK,CREB3,REST,ASPN,SNX13,TGFBR1,SOCS5,RIMS1,RTN4,TPBG,SHANK1,ADAMTS3,EMC10,MAP3K7,AKIRIN1,ACTR2,MDFIC,GLI3,ROCK2,INHBA,RRAS,ZYX,RCN3,DKKL1,VPS35,SLC7A11,ITGB1,PLEKHA4,FKBP1B,FGF2,SESN3,RAB7A,ARHGEF4,ABHD12,RB1,TPT1,EIF2B3,PTCH2,KCTD21,GLI2,MEGF8,RHEB,PAK1,TGFB1,AXL,SCN5A,WDR48,SEC13,CTNNB1,LBH,DYNC1LI1,GPD1L,ZEB2,CANT1,C1QTNF1,ARHGEF17,OSBPL8,CXXC5,CLPB,PSD2,ACTG1,NPLOC4,ACTN4,ANAPC15,LTBP1,CRIM1,SPRED3,UFM1,ATP2C1,PRNP,ATP2B1,DCN,HDAC3,ZNF703,ACPP,STARD13,KCTD16,SEMA3C,CAVIN3,UBA5,DDRGK1,SMPD1,PPP2CB,ILK,RBPMS,LEPROTL1,ARL6IP5,SPINK5,APLP1,RYK,TMEM127,CNOT7,RNF6,TMEM9B,ST5,AKIP1,CXCL12,SLC20A1,ANKRD1,PJA2,TMED2,IL20RB,CD180,ZNF536,SULF1,VAMP8,GRK7,CTNND1,DKK3,ROBO1,WWC2,SORBS1,SEMA4F,LOXL3,P2RX4,RNF13,FAIM2,HEY1,RASA1,ATP2A2,USO1,CELF1,DLGAP4,TCTN1,APP,DHX36,RAPGEF2,MAPKAPK5,CX3CL1,ABCC8,CACNB3,PTPN11,RALGAPB,HOMER1,OPA1,ARHGAP1,AMFR,AP3B1,PTPN22,TSG101,ACVR1,CRY2,VPS18,CHAC1,GDF6,POLR3D,SERINC3,MTDH,ADA,BAP1,CHP1,ATP2B4,SPP1,PKD2,PLA2R1,TWF1,HIF1AN,SEMA4G,CBLB,NKD1,TANK,SLC39A10,RAB7B,CHRNA7,ADIPOR1,FAP,	darkgreen	EC_M4
GO:0032786	2.85E-04	0.999954146	9	25	positive regulation of DNA-templated transcription, elongation	BP	1.02E-02	DDX39B,SCAF8,CCNT2,SUPT16H,ALYREF,TCERG1,ERCC6,LEO1,THOC1	darkgrey	EC_M8
GO:1902533	1.06E-04	0.999932532	112	731	positive regulation of intracellular signal transduction	BP	1.02E-02	TNFRSF11A,AKAP12,FLT4,TPD52L1,PTK2,PDGFB,LRRC19,TEK,SH3RF3,KIT,KDR,SYK,GADD45G,SEMA4D,IL6,REL,PELI1,CAV2,TLR4,TRAF1,SDCBP,NRP1,PUM2,PIK3CG,PRKD2,GPR4,GATA3,NET1,NDFIP2,IKBKB,P2RY6,HBEGF,RPL26,P2RX2,CD36,PRAG1,PRR5L,PLK2,RET,TNFRSF19,IGF1,ERBIN,SH3RF1,ARHGEF3,TLR2,NDRG4,GUCY1A1,MADD,ZDHHC13,EDN1,LCK,SHOC2,HCRTR1,LAPTM5,NCF1,HCLS1,TIFA,CD86,FGR,CFLAR,CASP10,PROX1,PRKD1,F11R,MAP3K3,MAP3K11,LTF,DDX3X,SOS2,IGFBP5,TBX1,TMEM106A,BIRC3,SELP,VWF,TNFAIP8L3,FGF23,CRACR2A,RAC1,ITPKB,BID,TLR6,RELL1,MTOR,AR,PSEN1,FGF18,TAOK2,EFNA1,PIK3R5,NPR1,ADRB2,CCL2,LGALS9,TRAF2,PLEKHG5,NOTCH1,PLCG2,ENG,NFAT5,DAB2IP,MIR23A,PMAIP1,ADGRG1,TRIM34,RELN,ALKAL1,LPAR6,TRIM62,BST2,BMP2,NMNAT1	turquoise	EC_M6
GO:0051094	1.39E-04	0.999902689	175	982	positive regulation of developmental process	BP	1.02E-02	IL23A,LRP1,AHI1,RAB21,PLXNB2,FBN2,HLA-DRA,PACSIN1,ADGRB1,PPARD,LEP,PLAA,KIAA1324L,SRF,VEGFA,RUNX2,PDCL3,REST,NLRP5,TGFBR1,SOCS5,RIMS1,RTN4,TPBG,SHANK1,LRRC4B,EMC10,AKIRIN1,ACTR2,GLI3,INHBA,RRAS,MYLK3,DKKL1,VPS35,ITGB1,FKBP1B,PLXNA1,FGF2,RB1,PTCH2,TENM4,GLI2,MEGF8,SMARCD3,RHEB,PAK1,TGFB1,AXL,CTNNB1,IL15RA,TRAK1,LBH,ZEB2,STIM1,LIG4,ATP2B1,DCN,ZNF703,ILK,CXCL12,EIF4G2,ROBO1,MAP1B,CELF1,DHX36,RAPGEF2,CX3CL1,OPA1,LOXL2,AP3B1,AMIGO2,ZFYVE27,ACVR1,GDF6,MTDH,ADA,BMP1,RUNX1,HIF1AN,RAB7B,CHRNA7,PHLDB2,TJP1,FNDC5,KDM5B,NOTCH2,DNM1L,HYAL1,CCND1,CAPRIN2,TGFB2,LRP5,FEZ1,MMP14,DAG1,LEF1,CORO1C,PRKCA,CMKLR1,KRAS,BMP7,FGFR2,VASH2,TP53BP1,PHLDB1,SYAP1,IQGAP1,TFRC,FZD9,RPS6KA3,HSPB1,MAPT,SERPINE1,FN1,ADRA2C,ARPC2,SUCO,PRKCI,SKIL,SMURF1,NBL1,SIX4,OBSL1,AKT3,HAP1,MAGED1,BMPR1A,NLGN2,PDLIM7,MED1,ADGRL1,PPARGC1A,ARHGEF2,TGFB1I1,NGFR,COL1A1,TGFB3,RAB11A,MAP2K1,TMEM100,PKM,DICER1,MORF4L2,NPTN,SETD3,MYOCD,ISLR2,PIN1,TRPV2,CD74,MIEF2,AKT1,PRKAA1,MYDGF,PLXNB3,PAFAH1B1,FLNA,ATP6AP1,PLXNA3,F3,SERPINF1,BNIP3,LRRC24,UBE2V2,GDNF,HAS2,GLIPR2,ZNF322,FAM210B,HOPX,OLFM1,WNT2,LRRTM2,FGF10	darkgreen	EC_M4
GO:0060055	1.07E-04	0.999983923	10	24	angiogenesis involved in wound healing	BP	1.02E-02	TNFAIP3,KDR,GATA2,GPR4,PRCP,CXCR4,ETS1,CD34,MCAM,ADIPOR2	turquoise	EC_M6
GO:0061028	1.08E-04	0.999976351	14	38	establishment of endothelial barrier	BP	1.02E-02	AFDN,TJP2,IKBKB,PDE2A,PDE4D,MARVELD2,PPP1R16B,RAPGEF3,ROBO4,PECAM1,F11R,RAPGEF1,CDH5,SOX18	turquoise	EC_M6
GO:0010595	1.08E-04	0.99996122	25	105	positive regulation of endothelial cell migration	BP	1.02E-02	FLT4,PDGFB,TEK,KDR,NRP1,PIK3CG,GATA2,PRKD2,NOS3,GATA3,SCARB1,PLK2,VEGFC,PLCG1,EDN1,ETS1,PROX1,PRKD1,MAP3K3,RAC1,HIF1A,RHOJ,FGF18,MIR23A,FGF16	turquoise	EC_M6

GO:0051972	2.90E-04	0.999934516	12	42	regulation of telomerase activity	BP	1.03E-02	CCT2,MAP3K4,POT1,HNRNPA2B1,CCT4,PARP4,TERF1,NEK2,XRCC5,AURKB,HNRNPD,NAF1	darkgrey	EC_M8
GO:0051223	1.43E-04	0.999915094	83	412	regulation of protein transport	BP	1.05E-02	LMAN1,LRP1,SLC12A2,ANGPT1,PPARD,LEP,UNC13B,CLOCK,EXOC1,REST,RAB23,P3H1,TMEM30A,ERLEC1,UBE2J1,MDFIC,GLI3,SLC7A11,FKBP1B,KIF5B,DNAJC1,PAK1,TGFB1,AKT2,UFM1,ATP2C1,IRS2,PRNP,HDAC3,RAB11FIP1,TM9SF4,ANKRD1,OSBP,ABCC8,CACNB3,PTPN11,PFKM,ATG13,TSG101,BAP1,CHP1,BRSK2,DYNLL1,DNM1L,CPT1A,TGFB2,LRP5,GSK3B,HADH,DISP1,HSPA8,IDH2,ADCY5,SCFD1,SIDT2,C11orf65,PDCD10,B3GAT3,NLGN2,SRCIN1,TMED10,TGFB3,RAB11A,MYH10,SYTL4,ACSL4,MIEF2,MYOM1,PRKAA1,MYO1C,BCAP31,FLNA,MIDN,SSTR5,PARK7,RHBDF1,WLS,TXN,LEPROT,ZPR1,UBE2G2,TOMM7,DERL2	darkgreen	EC_M4
GO:1904062	1.44E-04	0.999925367	54	241	regulation of cation transmembrane transport	BP	1.05E-02	CD63,UBQLN1,SHANK1,ITGB1,FKBP1B,KIF5B,SCN5A,GPD1L,ACTN4,STIM1,PRNP,REM1,FXYD1,SNTA1,SLMAP,ATP1B3,CACNA1D,APP,CX3CL1,ABCC8,CACNB3,HOMER1,CHP1,ATP2B4,PKD2,ISCU,CASQ1,LINC02210-CRHR1,PTPN6,HTT,KCNA1,HAP1,CACNA1C,NLGN2,STIM2,PPARGC1A,KCNMB1,CALM1,VMP1,AKT1,FLNA,KCNAB2,PARK7,JPH3,MIR24-1,DMD,FKBP1A,CBARP,TMEM38B,THY1,TMSB4X,TMEM38A,YWHAH,GEM	darkgreen	EC_M4
GO:0001568	1.47E-04	0.999906005	111	576	blood vessel development	BP	1.07E-02	LRP1,SLC12A2,ANGPT1,FAM19A5,NUS1,RSPO3,ADGRB1,MYH9,LEP,SRF,VEGFA,COL1A2,PDCL3,COL15A1,TGFBR1,EMC10,CALD1,GLI3,ROCK2,RRAS,ITGB1,FGF2,SRPK2,MEGF8,SNX17,TGFB1,CTNNB1,ACTG1,STIM1,DCN,STARD13,SEMA3C,SPINK5,ADAMTS6,TMED2,SULF1,ROBO1,HEY1,RASA1,RAPGEF2,CX3CL1,ABCC8,LOXL2,ACVR1,MMP2,MTDH,ATP2B4,PKD2,RUNX1,HIF1AN,CHRNA7,FAP,TJP1,NOTCH2,HYAL1,SLC2A10,TGFB2,LRP5,ADGRB2,MMP14,DAG1,PIK3CA,LEF1,PRKCA,BMP7,FGFR2,VASH2,HSPB1,SERPINE1,SGPL1,FN1,PDCD10,ITGAV,COMP,PRRX1,ACTA2,AKT3,FKBP10,BMPR1A,MED1,NPPB,MYO1E,NGFR,COL1A1,SHC1,MAP2K1,TMEM100,PKM,MYOCD,ANGPTL6,PDGFRB,ROCK1,AKT1,MYDGF,PKD1,FLNA,BSG,SARS,COL5A1,CDH13,F3,MIR24-1,LOXL1,SERPINF1,LOX,THY1,HAS2,PIK3C2A,WNT2,COL8A2,FGF10	darkgreen	EC_M4
GO:0048468	1.13E-04	0.99991785	228	1587	cell development	BP	1.07E-02	SEMA6A,KIF5A,IFNGR1,CTHRC1,RIMS2,QKI,NOTCH4,AFDN,PARVG,CDKL3,ASAP1,L3MBTL3,PTK2,ANKS1A,ATF4,PDGFB,AGTPBP1,PODXL,PICK1,MICALL1,TEK,TJP2,PGM5,ADGRF5,KIT,ADGRF1,TNFRSF21,SEMA4D,TMOD1,IL6,PPT1,PTBP3,CAV2,NOBOX,TLR4,IL15,MYCN,NRCAM,TUT4,NRP1,ITSN2,TAL1,MTMR2,GATA2,HS6ST1,NOVA2,GPR4,PLXND1,NECTIN2,ITGA8,ITGB4,UNC13D,PPARG,GATA3,MYO7A,CXCR4,EDNRB,SPTBN4,IKBKB,JAG1,PDE2A,GPAT4,SFRP1,DND1,FEZ2,EFNB2,ARHGEF7,PCDH12,P2RX2,NTN4,LMOD3,PRAG1,EPHB1,PLK2,RET,PDE4D,MERTK,CHD7,IL1A,IGF1,EIF2AK3,KDM3A,PREX2,MARVELD2,DCLK2,KIF13B,ENC1,HEXB,MEF2C,TLR2,VEGFC,NDRG4,GPM6A,ANGPT2,DHH,KMT2D,USH1C,PPP1R16B,DYSF,STC1,RAPGEF3,NR4A2,DLL4,CDYL,TSPAN2,NEDD9,EDN1,JAM3,ALAS1,DTNBP1,MYLIP,SRGAP2C,HDAC1,PPP3CA,HECW2,MEF2A,SEMA3F,ROBO4,NRAP,SEC24B,NFATC2,AP1AR,PECAM1,PLXNA2,CFLAR,INPP5F,TRAK2,ADCY1,KALRN,FES,PROX1,TMOD4,SS18L1,PRKD1,LAMA5,F11R,YTHDF1,ITGA6,DOCK1,PAK2,MELTF,LAMC2,ELL3,APOD,HOXD10,MOV10,RNF2,CREB1,NBEAL2,B2M,LTF,ADORA2A,SEMA6D,EPHB4,ITGA4,TBX1,RAMP2,DNM3,CDK16,LMTK2,TMOD2,PRKCH,RAC1,HIF1A,RCC2,EPHA2,CECR2,CHRNB1,MPP5,TNFRSF1B,RGS14,ALDH1A2,MTOR,AR,OPHN1,EFNB1,PSEN1,SEMA4A,FBXW11,FGF18,ABI3,ITGA3,EIF2B2,TAOK2,EFNA1,CHRNB2,ZDHHC15,CCDC42,RPS6KA5,GPR68,CCDC88C,DNM2,YY1,B4GALT6,SH3TC2,SPEF2,GIT1,SEMA6B,SLITRK2,RFLNB,ATCAY,ARHGAP4,NOTCH1,RAPGEF1,ENG,STXBP1,STRBP,LHX6,DAB2IP,CDH5,MIR23A,ARID4A,VASP,SOX17,RELN,ALKAL1,SOX18,DIO3,SHAN	turquoise	EC_M6
GO:0034085	3.04E-04	0.999987548	5	7	establishment of sister chromatid cohesion	BP	1.08E-02	NAA50,CDCA5,DDX11,WAPL,NIPBL	darkgrey	EC_M8

GO:2000026	1.52E-04	0.99989299	180	1014	regulation of multicellular organismal development	BP	1.10E-02	IL23A,LRP1,AHI1,RAB21,SLC12A2,PLXNB2,FBN2,FAM19A5,PPARA,HLA-DRA,ADGRB1,PPARD,CTNNA1,LEP,KIAA1324L,SRF,VEGFA,RUNX2,PDCL3,REST,ASPN,NLRP5,TGFBR1,SOCS5,RTN4,TPBG,LRRC4B,EMC10,ACTR2,GLI3,ROCK2,INHBA,RRAS,ITGB1,PLXNA1,FGF2,RB1,PTCH2,TENM4,GLI2,MEGF8,SMARCD3,RHEB,PAK1,TGFB1,AXL,CTNNB1,IL15RA,TRAK1,RARB,MAFG,STIM1,SPRED3,LIG4,ATP2B1,DCN,SEMA3C,ILK,SPINK5,RYK,RNF6,CXCL12,LGI4,EIF4G2,SULF1,ROBO1,MAP1B,DIP2B,SEMA4F,LOXL3,HEY1,TLX2,DHX36,RAPGEF2,CX3CL1,ABCC8,PTPN11,OPA1,LOXL2,AP3B1,AMIGO2,ZFYVE27,ACVR1,GDF6,MTDH,ADA,ATP2B4,SPP1,BMP1,RUNX1,SEMA4G,RAB7B,CHRNA7,PHLDB2,TJP1,NOTCH2,CDC73,DNM1L,TRIP12,HYAL1,CCND1,CAPRIN2,TGFB2,ADGRB2,MMP14,DAG1,LEF1,PRKCA,NTRK3,TCTA,SLAMF8,BMP7,IDH2,FGFR2,VASH2,PHLDB1,FZD9,HSPB1,MAPT,SERPINE1,TNR,FN1,PTPN6,GPR137,FBN1,COMP,PRKCI,SKIL,VCL,SIX4,OBSL1,AKT3,HAP1,PRTG,BMPR1A,NLGN2,MED1,NPPB,NGFR,TGFB3,RAB11A,MAP2K1,TMEM100,PKM,DICER1,NPTN,ISLR2,PIN1,TRPV2,CD74,LAMA3,ROCK1,AKT1,TMEM98,PTPN2,MYDGF,PLXNB3,PAFAH1B1,ATP6AP1,PLXNA3,SARS,F3,GLG1,MIR24-1,PRMT6,FSTL3,SERPINF1,LOX,LRRC24,UBE2V2,GDNF,THY1,FGL2,FAM210B,YWHAH,BHLHE41,HOPX,ZPR1,WNT2,LRRTM2	darkgreen	EC_M4
GO:1903532	1.52E-04	0.999925295	46	196	positive regulation of secretion by cell	BP	1.10E-02	LRP1,SLC12A2,PPARD,LEP,UNC13B,EXOC1,INHBA,KIF5B,RAB7A,PDCD6IP,TGFB1,RAB3GAP1,C1QTNF1,RAB2B,IRS2,CXCL12,ANKRD1,OSBP,VAMP8,P2RX4,SELENOT,PTPN11,PFKM,TSG101,SPP1,DYNLL1,DNM1L,TGFB2,TFR2,CYP19A1,NLGN2,STX1B,TMED10,TGFB3,CACNA1G,MYH10,SYTL4,SCAMP5,ACSL4,RASL10B,MYOM1,ATP6AP1,VAMP7,AP1G1,WLS,GDNF	darkgreen	EC_M4
GO:0045056	1.56E-04	0.999980512	9	17	transcytosis	BP	1.12E-02	LRP1,MFSD2A,VPS35,PICALM,USO1,LRPAP1,RAB11A,CLTC,GP2	darkgreen	EC_M4
GO:0009967	1.21E-04	0.999916633	163	1127	positive regulation of signal transduction	BP	1.13E-02	TNFRSF11A,TNFAIP3,TRIM39,AKAP12,FLT4,RIMS2,TPD52L1,RNF146,PTK2,PDGFB,CSNK1E,MFNG,LRRC19,TEK,SH3RF3,KIT,KDR,SYK,GADD45G,SEMA4D,IL6,REL,PELI1,RSAD2,CAV2,GRB10,LANCL2,TLR4,TRAF1,SDCBP,TRPM4,NRP1,PUM2,PIK3CG,PRKD2,CTSC,GPR4,NECTIN2,NOS3,PPARG,GATA3,ACVR2B,CXCR4,NET1,NDFIP2,IKBKB,JAG1,P2RY6,SFRP1,HBEGF,FERMT1,RPL26,P2RX2,CD36,PRAG1,PRR5L,PLK2,RET,TNFRSF19,IGF1,HHEX,ERBIN,SH3RF1,HIP1R,ARHGEF3,TLR2,IL7,NDRG4,GUCY1A1,MADD,KMT2D,BMP2K,NLRC5,DLL4,ZDHHC13,ADAMTS20,EDN1,PRDM15,LCK,HYAL2,SHOC2,HCRTR1,LAPTM5,NCF1,HCLS1,TIFA,CD86,MN1,FGR,CFLAR,CASP10,PROX1,PRKD1,F11R,IL10RA,MAP3K3,PAK2,MAP3K11,LTF,ADORA2A,DDX3X,SOS2,IGFBP5,TBX1,TMEM106A,BIRC3,SELP,VWF,TNFAIP8L3,FGF23,CRACR2A,TMOD2,PRKCH,RAC1,HIF1A,TSPAN14,JUP,ITPKB,BID,TLR6,RELL1,MTOR,AR,PSEN1,FGF18,TAOK2,EFNA1,PIK3R5,NPR1,SMAD2,ADRB2,CCL2,JAG2,LGALS9,IL7R,LRG1,TEDC2,NRARP,TRAF2,PLEKHG5,NOTCH1,PLCG2,ENG,NFAT5,DAB2IP,CDH5,MIR23A,PMAIP1,TLR1,ADGRG1,TRIM34,RELN,ALKAL1,LYPD6,SHANK3,LPAR6,RGS4,TRIM62,PDCL,BST2,BMP2,NMNAT1,BMP6	turquoise	EC_M6
GO:0045766	1.22E-04	0.999950899	30	133	positive regulation of angiogenesis	BP	1.13E-02	TEK,KDR,CXCL8,C5,NRP1,GATA2,PRKD2,NOS3,CXCR4,TIE1,IL1A,SMAD1,VEGFC,ANGPT2,PLCG1,RAPGEF3,HIPK1,ETS1,CD34,PRKD1,CYBB,RAMP2,HIF1A,JUP,FGF18,PGF,JAK1,LRG1,ENG,CDH5	turquoise	EC_M6
GO:1904018	1.22E-04	0.999950899	30	133	positive regulation of vasculature development	BP	1.13E-02	TEK,KDR,CXCL8,C5,NRP1,GATA2,PRKD2,NOS3,CXCR4,TIE1,IL1A,SMAD1,VEGFC,ANGPT2,PLCG1,RAPGEF3,HIPK1,ETS1,CD34,PRKD1,CYBB,RAMP2,HIF1A,JUP,FGF18,PGF,JAK1,LRG1,ENG,CDH5	turquoise	EC_M6
GO:0050921	1.24E-04	0.999954655	25	109	positive regulation of chemotaxis	BP	1.13E-02	PTK2,PDGFB,KDR,IL6,CXCL8,NRP1,PRKD2,CXCR4,VEGFC,CXCL10,THBS4,ANO6,EDN1,PRKD1,MCU,PPM1F,RAC1,FGF18,PGF,DAPK2,S100A14,LGALS9,S1PR1,FGF16,STX3	turquoise	EC_M6
GO:0044087	1.25E-04	0.999917952	125	801	regulation of cellular component biogenesis	BP	1.13E-02	TBC1D30,CNOT6,SNCAIP,MTSS1,ASAP1,ARHGAP18,PTK2,PODXL,BAIAP2L2,PICK1,CDC42EP1,TEK,LINGO2,LIMCH1,KIT,KDR,SYK,SEMA4D,TMOD1,TLR4,SDCBP,FAM49A,NRP1,RASIP1,SVIL,TAL1,RALB,TMC8,SPTBN4,TIE1,SETD5,IKBKB,FCHSD2,SFRP1,FEZ2,FERMT1,ARHGEF7,FCHSD1,CD36,SIRT3,LMOD3,EPHB1,PLK2,HIP1R,OCLN,MEF2C,TLR2,P2RX7,GPM6A,PPP1R16B,RAPGEF3,IQGAP2,CEP97,DYRK1A,JAM3,SNCA,SRGAP2C,KIAA1551,ADPRH,ESAM,HCLS1,AP1AR,FES,TMOD4,F11R,ACE,LIMK2,PAK2,DLG1,APOD,CREB1,IL1RAP,DDX3X,DNM3,MMP1,LDB2,SELP,BAIAP2L1,SAMD8,PPM1F,WAS,TMOD2,PRKCH,RAC1,RCC2,FSCN1,EPHA2,BID,TLR6,MTOR,SEMA4A,ABI3,ARHGEF15,CHRNB2,RABEP2,DNM2,ARHGAP28,SPIRE1,GIT1,SLITRK2,MTM1,ISG15,HCFC1,TRAF2,NOTCH1,PIEZO1,RAPGEF1,PLCG2,S1PR1,STXBP1,LRSAM1,FNBP1L,TERF2,DAB2IP,ADGRL2,CARMIL2,CDH5,GBP5,VASP,GMFG,KANK3,SHANK3,GBA2,RALA,ARPC5L	turquoise	EC_M6

GO:0050900	1.25E-04	0.999938365	46	245	leukocyte migration	BP	1.13E-02	TNFRSF11A,PTK2,PDGFB,KIT,SYK,IL6,CXCL8,C5,TRPM4,PIK3CG,LRCH1,GATA3,CXCR4,EDNRB,RET,VEGFC,CXCL10,THBS4,ANO6,EDN1,JAM3,CD200,LCK,RIN3,LYST,CD34,PECAM1,F11R,APOD,ITGA4,MCU,SELE,SELP,PLVAP,RAC1,STK10,PGF,DAPK2,S100A14,MMP28,CCL2,YES1,PDE4B,LGALS9,S1PR1,MSMP	turquoise	EC_M6
GO:0048017	1.26E-04	0.99994787	32	137	inositol lipid-mediated signaling	BP	1.13E-02	PTK2,PDGFB,TEK,PIP5K1B,KIT,KDR,SEMA4D,KBTBD2,PIK3CG,GAB2,GATA3,PLCB4,PRR5L,IGF1,PITPNM2,PREX2,PPP1R16B,PLCB2,PIK3C2B,EDN1,NCF1,HCLS1,FGR,INPP5F,PLEKHA1,SELP,TNFAIP8L3,PLD2,PEAR1,PIK3R5,DAB2IP,RELN	turquoise	EC_M6
GO:0043114	1.26E-04	0.999971447	14	40	regulation of vascular permeability	BP	1.13E-02	AKAP12,TEK,TJP2,GPR4,PDE2A,OCLN,ADORA2A,RAMP2,PLVAP,NPR1,YES1,CDH5,MIR23A,BMP6	turquoise	EC_M6
GO:0032682	1.26E-04	0.999986423	8	16	negative regulation of chemokine production	BP	1.13E-02	IL6,ERBIN,OAS3,APOD,EPHA2,ARG2,LGALS9,OAS1	turquoise	EC_M6
GO:0007010	1.62E-04	0.999883343	206	1177	cytoskeleton organization	BP	1.15E-02	LRP1,TRIM36,AGAP2,RNF19A,FAM49B,MRTFA,PACSIN1,CTNNA1,SUN2,MKLN1,FLNC,MYH9,SRF,VCP,TPM2,NCK2,PDCL3,TXNDC9,DST,NLRP5,TGFBR1,SHANK1,MYH14,CALD1,ACTR2,ROCK2,ZYX,MYLK3,CFAP100,ITGB1,EML2,MARK4,MAPRE3,PDCD6IP,PAK1,ACTR3,NCKAP5,CTNNB1,CLIP4,DYNC1LI1,PLEKHG2,DNAJB13,FARP1,ARHGEF17,ACTG1,NAV3,ACTN4,PPP1R12A,KAT2B,TACC1,ATP2C1,CDC42EP3,HDAC3,PCLO,STARD13,CAVIN3,ILK,DCTN6,DPYSL3,KATNAL1,TTC17,CXCL12,KIF2A,ANKRD1,FAT1,HSP90B1,PALLD,MRAS,MAST4,FLNB,MAP1B,SORBS1,PFN2,RASA1,EPB41L1,RTKN,SDAD1,CX3CL1,NEFM,TUBB2A,CHP1,PKD2,TWF1,DNAH1,DNAH5,BRSK2,PDLIM5,MAGEL2,ARL3,RAP1GDS1,ELMO2,INA,PHLDB2,TJP1,NOTCH2,CTTN,PKP2,MARCKSL1,LMOD1,SBDS,PRKAR1A,GSK3B,DAG1,PIK3CA,AUTS2,DNAAF5,CORO1C,NEK7,SHROOM2,KRAS,NTRK3,SPATC1L,SYNPO2,CCDC6,CASQ1,ITGB5,PHLDB1,IQGAP1,ABRAXAS2,CLIC4,FARP2,ARPC5,EHBP1L1,MAPT,ALKBH4,NCKAP1,ARPC2,HTT,PRKCI,SYNPO2L,CAPZB,TNIK,TUBA4A,DAAM1,WDR1,FMN2,SIX4,OBSL1,TPM4,CRMP1,MYO5A,CCSER2,IQSEC2,ENAH,CGNL1,CLN8,PDLIM7,TCAP,ACTN1,MSN,MYO1E,NES,ARHGEF2,TLN2,TPM1,DOCK2,TGFB3,SHC1,RAB11A,MYH10,CNN1,GPR65,TTC8,DIAPH2,LRRC49,CLTC,SETD3,EML1,SS18,PDGFRB,PLS3,ROCK1,FLII,MYO1D,MPRIP,PRKAA1,TUBB4A,TNFAIP1,MYO5B,DAPK3,MYO1C,PAFAH1B1,PKD1,NPHP4,FLNA,PLXNA3,SPTAN1,NEXN,DYNC1LI2,DMD,MAP7D1,PDLIM1,CSRP1,SKA2,THY1,TMSB4X,MZT1,DSTN,TUBA1C,ZPR1,RMDN1,PRKG1,MAP1A,COTL1,CFL2,FGF10	darkgreen	EC_M4
GO:0060572	1.64E-04	0.99998325	8	14	morphogenesis of an epithelial bud	BP	1.16E-02	GLI2,CTNNB1,SULF1,RDH10,BMP7,FGFR2,WNT2,FGF10	darkgreen	EC_M4
GO:0055002	1.67E-04	0.999952576	18	52	striated muscle cell development	BP	1.18E-02	SRF,MYLK3,ITGB1,ACTG1,ANKRD1,LMOD1,PRKAR1A,CASQ1,SYNPO2L,WDR1,SIX4,OBSL1,TCAP,TPM1,PDGFRB,FLII,CSRP1,CFL2	darkgreen	EC_M4
GO:0006189	3.35E-04	0.999993347	4	5	'de novo' IMP biosynthetic process	BP	1.19E-02	PPAT,PAICS,PFAS,GART	darkgrey	EC_M8
GO:0098771	1.75E-04	0.999890577	98	502	inorganic ion homeostasis	BP	1.22E-02	LRP1,SLC12A2,SLC39A7,SMDT1,SLC39A4,SLC30A9,CNNM4,SLC31A1,FKBP1B,FGF2,RAB7A,ATP7B,PICALM,TPT1,C1QTNF1,SLC4A7,TMTC4,MAFG,STIM1,ATP2C1,PRNP,ATP2B1,CCKBR,RIC3,CXCL12,TM9SF4,HSP90B1,C19orf12,ATP1B3,RYR3,P2RX4,SELENOT,ATP2A2,APP,SLC4A5,CX3CL1,CACNB3,AP3B1,CHP1,ATP2B4,PKD2,SLC39A10,CHRNA7,RAP1GDS1,CNNM2,HTR2B,ISCU,CMKLR1,HJV,SLAMF8,ADCY5,VAPB,CASQ1,CLIC4,TFRC,FZD9,GLRX3,TMCO1,ATP6AP2,COMT,TFR2,PTPN6,ITGAV,HTT,ERO1A,KCNA1,FBXL5,HAP1,CACNA1C,STIM2,NPPB,ADRA1B,GPR65,CLN6,CALM1,GRIA1,NPTN,SLC39A6,ANXA6,TRPV2,GNB1,BCAP31,PKD1,ATP6AP1,SYPL2,JPH3,SLC30A7,ATP6V0D1,DMD,FKBP1A,BNIP3,TMEM38B,SLC22A17,SNX10,THY1,TMEM38A,CCR6,ATP6V0E1	darkgreen	EC_M4
GO:0016236	1.76E-04	0.999905662	58	265	macroautophagy	BP	1.22E-02	ATP6V1C1,UBQLN1,PLAA,VCP,EXOC1,RAB23,MAP3K7,VPS41,VPS35,SESN3,RAB7A,ATP6V0B,RHEB,RAB3GAP1,MTMR14,STAM2,UFM1,DCN,UBA5,DDRGK1,VPS37A,TMEM41B,MAP1LC3A,TP53INP2,SLC25A4,VAMP8,ATP2A2,ATG13,TSG101,VPS39,ATP6V1B2,SEC22B,FEZ1,WIPI1,PIK3CA,SYNPO2,SCFD1,QSOX1,PAFAH1B2,HTT,SMURF1,WIPI2,ATP6V1D,SPTLC2,CALM1,VMP1,AKT1,CLEC16A,IKBKG,GPSM1,GNAI3,ATP6V0D1,BNIP3,VPS26B,CAPNS1,GABARAPL2,TOMM7,ATP6V0E1	darkgreen	EC_M4

GO:0051128	1.76E-04	0.999865534	310	1877	regulation of cellular component organization	BP	1.22E-02	CD63,LMAN1,ATP8B1,LRP1,TRIM36,AHI1,CAND1,RAB21,PLXNB2,ANGPT1,TCP1,PPARA,ROS1,FAM49B,PACSIN1,ADGRB1,PPARD,MKLN1,LGALS1,MDGA1,SH3GL2,MYH9,PLAA,SRF,VEGFA,VCP,UNC13B,NCK2,P3H1,TGFBR1,RIMS1,TMEM30A,RTN4,TPBG,SHANK1,LRRC4B,MFSD2A,AKIRIN1,ACTR2,KIDINS220,ROCK2,INHBA,MYLK3,VPS35,MGARP,SLC7A11,FKBP1B,PLXNA1,KIF5B,NAPA,RAB7A,EML2,RB1,PICALM,MARK4,MAPRE3,PTCH2,PDCD6IP,MEGF8,SNX17,PAK1,ACTR3,TGFB1,AXL,RAB3GAP1,CTNNB1,TRAK1,AKT2,DYNC1LI1,DZIP1,ZEB2,PLEKHG2,FARP1,ACTG1,NAV3,ANAPC15,KAT2B,FAM98A,LIG4,PRNP,CHFR,DCN,CDC42EP3,SEMA3C,SMPD1,ILK,DPYSL3,RYK,RNF6,CXCL12,SACS,CHPT1,ANKRD1,SLC25A4,OSBP,EIF4G2,RIOK2,ROBO1,MAP1B,DIP2B,PINX1,ANKRA2,SEMA4F,PFN2,RASA1,TLX2,APP,ATXN2,DHX36,CCT8,RAPGEF2,MAPKAPK5,CX3CL1,ABCC8,PTPN11,ADCY6,GNL3,HOMER1,OPA1,CDH17,ARSB,AMIGO2,ZFYVE27,TSG101,DKC1,PPP3CC,BAP1,SPP1,RUNX1,TWF1,SEMA4G,TENT4B,BRSK2,PDLIM5,MAGEL2,CHRNA7,ARL3,ADIPOR1,FAP,PHLDB2,TJP1,NOTCH2,CTTN,CDC73,DNM1L,HYAL1,LMOD1,SLC2A10,CAPRIN2,SEC22B,TGFB2,FBLN5,LRP5,FEZ1,PRKAR1A,GSK3B,MMP14,DAG1,PIK3CA,AUTS2,CORO1C,NEK7,NTRK3,HSPA8,FAM162A,BMP7,AP2M1,SYNPO2,PHLDB1,IQGAP1,SCFD1,RBBP7,KIF1A,CLIC4,TFRC,FARP2,TANC2,FZD9,RPS6KA3,ARPC5,MAPT,ALKBH4,SERPINE1,DNAJA4,TNR,TFR2,FN1,NCKAP1,C11orf65,GPC2,PDCD10,UPF1,ITGAV,LRPAP1,ARPC2,HTT,PRKCI,SKIL,SYNPO2L,PRRX1,SMURF1,CAPZB,TNIK,PLD1,VCL,VDAC2,WDR1,SIX4,MNAT1,OBSL1,ATAD1,STX18,CRMP1,GHITM,HAP1,TSPYL2,SLC2A	darkgreen	EC_M4
GO:0042327	1.39E-04	0.999913051	99	612	positive regulation of phosphorylation	BP	1.24E-02	TNFRSF11A,FLT4,TPD52L1,PTK2,PDGFB,TEK,LIMCH1,TEC,KIT,KDR,SYK,SEMA4D,IL6,TLR4,IL15,CCNY,SDCBP,NRP1,PIK3CG,TAL1,PRKD2,CAB39L,RALB,PPARG,EDNRB,TIE1,HBEGF,CD36,PRR5L,EPHB1,RET,MERTK,IGF1,GPD1,P2RX7,VEGFC,MADD,THBS4,RAPGEF3,DGKZ,NEDD9,EDN1,EHD4,SNCA,HDAC1,NCF1,HCLS1,PECAM1,CD86,FGR,PROX1,PRKD1,MLXIPL,ACE,ITGA6,LIMK2,PAK2,DLG1,MAP3K11,LTF,EPHB4,DDX3X,TBX1,TNFAIP8L3,FGF23,LMTK2,RAC1,HIF1A,ADIPOR2,EPHA2,TLR6,MTOR,AR,PSEN1,FGF18,PGF,TAOK2,EFNA1,CD19,PIK3R5,RPS6KA5,ADRB2,YES1,SH2D3A,UNC119,TRAF2,PLCG2,ENG,SH2D3C,DAB2IP,RELN,ALKAL1,FGF16,MMD,DAZAP2,COPS8,BMP2,PIFO,BMP6	turquoise	EC_M6
GO:0006468	1.40E-04	0.999901131	178	1223	protein phosphorylation	BP	1.24E-02	TNFRSF11A,TNFAIP3,PTPRB,FLT4,CDKL3,TPD52L1,PTK2,DAPK1,PDGFB,MAPK13,CSNK1E,PICK1,PIM1,TEK,LIMCH1,TEC,KIT,KDR,SYK,SEMA4D,IL6,TLR4,IL15,CCNY,SDCBP,NRP1,RASIP1,PIK3CG,TAL1,KDM4D,PRKD2,CAB39L,SNRK,RALB,PPARG,ACVR2B,IRAK2,EDNRB,SPTBN4,TIE1,IKBKB,SFRP1,HBEGF,SH3BP5,CD36,CDK17,PRAG1,PRR5L,MAP3K1,EPHB1,PLK2,RET,PDE4D,MERTK,IGF1,EIF2AK3,HHEX,SMAD1,OCLN,DCLK2,TLR2,WWTR1,IL7,VEGFC,MADD,BMP2K,THBS4,RAPGEF3,HIPK1,CCNJL,NEDD9,EDN1,DYRK1A,EHD4,SNCA,DTNBP1,ERG,NUAK2,CDK18,DCAF1,SIK1,HDAC1,LCK,HYAL2,PRKX,NCF1,DYRK3,FABP4,HCLS1,PECAM1,CD86,BMX,FGR,MAP3K6,INPP5F,KALRN,IGFBP3,FES,RPS6KA1,PROX1,PRKD1,PDIK1L,MAP3K3,SRMS,MLXIPL,ACE,LIMK2,PAK2,DLG1,MAP3K11,STYK1,PDK3,CREB1,LTF,BCR,ADORA2A,EPHB4,DDX3X,TBX1,WNK4,CDK16,FGF23,LMTK2,PPM1F,PRKCH,RAC1,ADIPOR2,EPHA2,ITPKB,TLR6,TNK1,RGS14,MTOR,PSEN1,STK10,FGF18,RPS6KL1,PGF,NEK9,DAPK2,TAOK2,EFNA1,ADCK1,SBK1,PIK3R5,NPR1,RPS6KA5,CCDC88C,SMAD2,CDC42BPB,MARK3,ADRB2,CCL2,YES1,SH2D3A,JAK1,UNC119,STK26,MATK,TRAF2,PLCG2,ENG,SH2D3C,DAB2IP,CDH5,SLC8A3,TRIM24,RELN,ALKAL1,GMFG,FGF16,CDK13,MMD,DAZAP2,COPS8,BMP2,DUSP7,BMP6	turquoise	EC_M6
GO:0048638	1.81E-04	0.999903159	57	257	regulation of developmental growth	BP	1.26E-02	LRP1,RAB21,PPARA,PPARD,LEP,PLAA,SRF,VEGFA,TGFBR1,RIMS1,RTN4,MFSD2A,FGF2,MEGF8,PAK1,SEMA3C,ILK,RYK,RNF6,CXCL12,EIF4G2,WWC2,MAP1B,DIP2B,SEMA4F,CELF1,APP,ZFYVE27,SPP1,RUNX1,SEMA4G,NKD1,CTTN,GSK3B,PIK3CA,WWC3,FGFR2,MAPT,SGPL1,TNR,FN1,FGFR3,SMURF1,SIX4,BMPR1A,RAB11A,ISLR2,PIN1,TRPV2,AKT1,PAFAH1B1,PLXNA3,BARHL2,CDKN1A,HOPX,OLFM1,WNT2	darkgreen	EC_M4
GO:0030968	1.83E-04	0.999940295	21	69	endoplasmic reticulum unfolded protein response	BP	1.26E-02	VCP,NCK2,CREB3,ERLEC1,TMTC4,DDRGK1,TMED2,AMFR,SELENOS,CCND1,VAPB,ATF3,ATF6,RHBDD2,ERO1A,EIF2S1,PTPN2,MBTPS1,HSPA5,DNAJB9,DERL2	darkgreen	EC_M4

GO:0060548	1.90E-04	0.999871721	137	754	negative regulation of cell death	BP	1.30E-02	SERPINB2,LRP1,AHI1,AGAP2,IER3,CDSN,RRM2B,ANGPT1,PPARA,PPARD,CTNNA1,LEP,BAG1,VEGFA,DOCK8,CREB3,ASNS,REST,TGFBR1,TAX1BP1,GLI3,VPS35,SLC7A11,ITGB1,FGF2,RB1,TPT1,GLI2,AXL,CTNNB1,AKT2,UFM1,LIG4,IRS2,PRNP,HDAC3,DDRGK1,ILK,CXCL12,HSP90B1,SLC25A4,FAIM2,RASA1,CX3CL1,OPA1,AMIGO2,ACVR1,MTDH,ADA,TXNDC5,SLC39A10,TJP1,NOTCH2,CTTN,CDC73,SELENOS,EYA2,HTR2B,GSK3B,INPP5A,FSTL1,PIK3CA,LEF1,PRKCA,KRAS,NTRK3,BMP7,HYOU1,FGFR2,PEA15,TFRC,FZD9,RPS6KA3,HSPB1,SERPINE1,SYVN1,PDCD10,ITGAV,LRPAP1,COMP,HTT,FKBP8,PRKCI,ARAF,TIMP1,VDAC2,FMN2,SIX4,MNAT1,ERC1,GHITM,CLN8,MED1,PPARGC1A,CPEB4,NES,ARHGEF2,TGFB3,SHC1,TAF9B,MYOCD,PIN1,CD74,PDGFRB,AKT1,PRKAA1,CD40LG,MYDGF,TNFRSF18,FLNA,IKBKG,TMEM259,PARK7,ENO1,GNAI3,CFDP1,AARS,HSPA5,BCL10,MIR24-1,SERPINF1,BNIP3,VSTM2L,UBE2V2,CACYBP,ARF4,GDNF,GLO1,CDKN1A,TXN,PTTG1IP,TSLP,TMEM109,ZPR1,ORMDL3,CHST11,FGF10	darkgreen	EC_M4
GO:0048729	1.90E-04	0.999881086	97	490	tissue morphogenesis	BP	1.30E-02	AHI1,PLXNB2,RSPO3,MRTFA,LY6E,SRF,VEGFA,TGFBR1,GLI3,INHBA,ITGB1,PLEKHA4,PLXNA1,FGF2,GLI2,MEGF8,PAK1,TGFB1,CTNNB1,ACTG1,SPINT2,STARD13,SEMA3C,ILK,RYK,PRICKLE2,ANKRD1,FAT1,TMED2,SULF1,ROBO1,RDH10,HEY1,TLX2,TCTN1,ACTG2,OPA1,ACVR1,PKD2,NKD1,IFT57,BRSK2,PHLDB2,KDM5B,NOTCH2,PKP2,EYA2,TGFB2,LRP5,PRKAR1A,MMP14,DAG1,LEF1,KRAS,BMP7,FGFR2,CLIC4,PLOD3,FZD2,PDCD10,GORAB,SMURF1,EXOC5,VCL,DAAM1,WDR1,TULP3,SIX4,ACTA2,MAGED1,BMPR1A,TCAP,MED1,MSN,TGFB1I1,NGFR,TPM1,TMEM100,TTC8,LAMA3,ITGA2,PAFAH1B1,PKD1,FLNA,COL5A1,COL11A1,LMO4,BCL10,WLS,FKBP1A,CD151,SERPINB5,LBX1,GDNF,ARTN,WNT2,FGF10	darkgreen	EC_M4
GO:0007015	1.92E-04	0.999887179	77	373	actin filament organization	BP	1.30E-02	FAM49B,PACSIN1,CTNNA1,SRF,TPM2,NCK2,NLRP5,TGFBR1,SHANK1,CALD1,ACTR2,ROCK2,ZYX,PAK1,ACTR3,PLEKHG2,ACTG1,CDC42EP3,DPYSL3,TTC17,CXCL12,FAT1,HSP90B1,SORBS1,PFN2,RASA1,CX3CL1,TWF1,MAGEL2,ELMO2,PHLDB2,TJP1,CTTN,MARCKSL1,LMOD1,PIK3CA,CORO1C,SHROOM2,SPATC1L,SYNPO2,ITGB5,ARPC5,NCKAP1,ARPC2,PRKCI,SYNPO2L,CAPZB,WDR1,FMN2,TPM4,MYO5A,ENAH,CGNL1,TCAP,ACTN1,MYO1E,ARHGEF2,TPM1,TGFB3,GPR65,TTC8,DIAPH2,PLS3,ROCK1,FLII,MYO1D,MPRIP,TNFAIP1,MYO5B,MYO1C,FLNA,SPTAN1,PDLIM1,TMSB4X,DSTN,COTL1,CFL2	darkgreen	EC_M4
GO:0009611	1.50E-04	0.999913752	72	422	response to wounding	BP	1.31E-02	TNFAIP3,TSPAN8,FZD6,NOTCH4,PTK2,PDGFB,TEC,KDR,SYK,IL6,TLR4,NRP1,PIK3CG,GATA2,GPR4,PRCP,NOS3,ITGA9,ITGB4,CXCR4,HBEGF,FERMT1,CD36,SCARB1,MERTK,IL1A,IGF1,KLKB1,HHEX,LYVE1,OCLN,PROCR,ENTPD1,PLLP,DYSF,DGKZ,ANO6,EDN1,JAM3,MMRN1,DTNBP1,ETS1,LCK,PPP3CA,CD34,MCAM,CFLAR,INPP5F,F11R,APOD,ADORA2A,SELP,VWF,WAS,RAC1,HIF1A,RAB27A,ADIPOR2,SYT7,PEAR1,MTOR,VASH1,SMAD2,CYP1A1,GIT1,F8,PLCG2,STXBP1,CARMIL2,RIPOR1,SCARF1,NMNAT1	turquoise	EC_M6
GO:0051179	1.53E-04	0.999877815	568	4586	localization	BP	1.34E-02	PARD6G,TNFRSF11A,SEMA6A,KIF5A,TNFAIP3,STX11,TBC1D30,AKAP12,LAPTM4B,FLT4,SNCAIP,SNX2,SNX24,ZDHHC14,SYNJ2,SERAC1,CTHRC1,RIMS2,CDC42SE2,CELSR1,QKI,AFDN,PACSIN2,TRDN,FAM91A1,ARHGAP18,KLHL3,SGSM3,PTK2,FAM53C,DENND3,C6orf106,TSNARE1,ANKS1A,DAPK1,PDGFB,AGTPBP1,PODXL,CSNK1E,PICK1,MICALL1,PIM1,TBC1D22B,STRIP2,MDFI,TEK,IREB2,LIMCH1,DDX58,ATP8A1,APBA1,TJP2,UBAP1,KIT,KDR,CD2AP,SYK,SEMA4D,EFHC1,TRAM2,RGP1,ICA1,TSPAN13,SLC3A1,IL6,IGF2BP3,SNAP91,PPT1,CXCL8,BACH2,AVL9,VPS54,CEP68,ARHGAP25,ELMO1,RSAD2,DOCK4,CAV2,GRB10,TLR4,TCAF2,GRIK2,C5,GJA4,IL15,TBC1D9,SDCBP,NRCAM,TRPM4,NRP1,RAB3B,OSBPL9,ANKRD50,PIK3CG,ITSN2,LAMA4,MTMR2,GATA2,KIF3C,ARHGAP21,PRKD2,KCNRG,NOVA2,RAB38,LRCH1,EXOC3L2,TMCC1,PLXND1,NECTIN2,PRCP,NOS3,GGA3,GAB2,LLGL2,RALB,USP6NL,ITGB4,UNC13D,ZFAND6,DACH1,PPARG,GATA3,TAF3,TBC1D4,MYO7A,CXCR4,CD93,SEC14L1,EDNRB,NET1,NDFIP2,TMC8,SPTBN4,TIE1,SLCO2B1,CYTH1,IKBKB,JAG1,P2RY6,P2RY2,FCHSD2,NFKBIB,GPAT4,SFRP1,HBEGF,FERMT1,KCNK6,CDS2,EFNB2,ARHGEF7,SPEF1,ATP11A,ARAP3,P2RX2,NTN4,SLC4A11,CD36,RIC8A,SIRT3,EML4,SLCO2A1,PRAG1,ADAMTS9,SDCBP2,PRR5L,MICU3,SCARB1,EPHB1,PLK2,RET,PDE4D,MERTK,GRAMD1A,CHD7,FAM3D,IL1A,IGF1,NR4A1,EIF2AK3,ABHD6,HHEX,EXOC6,ERBIN,SH3RF1,PITPNM2,CLCN3,SLCO5A1,HIP1R,OCLN,DCLK2,MCTP1,CHRM5,KIF13B,HEXB,MEF2C,CP,TLR2,WWTR1,P2RX7,TPD52,VEGFC,NDRG4,GPM6A,CXCL10,ANGPT2,SEPT11,TMEM144,LMBR1L,ADGRG3,PLLP,BMP2K,SERINC5,THBS4,TME	turquoise	EC_M6

GO:0060249	1.57E-04	0.999924348	43	218	anatomical structure homeostasis	BP	1.35E-02	TNFRSF11A,TNFAIP3,LYZ,TJP2,ADGRF5,SYK,IL6,VPS54,TLR4,GATA2,NOS3,POC1B,CLCN3,OCLN,WWTR1,IL7,P2RX7,USH1C,JAM3,LPCAT1,SLC39A8,ESAM,INPP5D,CD34,PECAM1,F11R,B2M,LTF,LDB2,PROM1,RAC1,HIF1A,LIPA,APBB2,RAB3D,ADRB2,MTM1,NOTCH1,S1PR1,CDH5,OAS1,CA2,DRAM2	turquoise	EC_M6
GO:0043254	1.57E-04	0.999912975	63	361	regulation of protein-containing complex assembly	BP	1.35E-02	ASAP1,ARHGAP18,BAIAP2L2,PICK1,CDC42EP1,SYK,TMOD1,TLR4,FAM49A,RASIP1,SVIL,TAL1,RALB,TMC8,SPTBN4,FCHSD2,FERMT1,ARHGEF7,FCHSD1,CD36,LMOD3,HIP1R,OCLN,IQGAP2,DYRK1A,JAM3,SNCA,ESAM,HCLS1,AP1AR,FES,TMOD4,DLG1,CREB1,DDX3X,MMP1,SELP,BAIAP2L1,WAS,TMOD2,RAC1,FSCN1,BID,TLR6,MTOR,ARHGAP28,SPIRE1,GIT1,ISG15,HCFC1,TRAF2,PIEZO1,PLCG2,STXBP1,DAB2IP,CARMIL2,CDH5,GBP5,VASP,GMFG,KANK3,GBA2,ARPC5L	turquoise	EC_M6
GO:0031032	2.01E-04	0.999904052	42	172	actomyosin structure organization	BP	1.36E-02	MYH9,SRF,TGFBR1,MYH14,ROCK2,ZYX,MYLK3,ITGB1,PDCD6IP,PAK1,ACTG1,ANKRD1,SORBS1,PFN2,EPB41L1,RTKN,PHLDB2,TJP1,LMOD1,PRKAR1A,CASQ1,ITGB5,ALKBH4,SYNPO2L,WDR1,SIX4,OBSL1,CGNL1,TCAP,TPM1,TGFB3,MYH10,CNN1,GPR65,TTC8,PDGFRB,ROCK1,FLII,TNFAIP1,PDLIM1,CSRP1,CFL2	darkgreen	EC_M4
GO:0098930	2.03E-04	0.99993836	19	58	axonal transport	BP	1.37E-02	AP3S1,RAB21,DST,MGARP,KIF5B,TRAK1,OPA1,AP3B1,FEZ1,KIF1A,HSPB1,MAPT,ACTR10,HAP1,ARMCX3,PAFAH1B1,HSBP1,BLOC1S2,MAP1A	darkgreen	EC_M4
GO:0055065	2.07E-04	0.999873783	87	438	metal ion homeostasis	BP	1.39E-02	LRP1,SLC12A2,SLC39A7,SMDT1,SLC39A4,SLC30A9,CNNM4,SLC31A1,FKBP1B,FGF2,ATP7B,PICALM,TPT1,C1QTNF1,TMTC4,STIM1,ATP2C1,PRNP,ATP2B1,CCKBR,RIC3,CXCL12,HSP90B1,C19orf12,ATP1B3,RYR3,P2RX4,SELENOT,ATP2A2,APP,CX3CL1,CACNB3,AP3B1,ATP2B4,PKD2,SLC39A10,CHRNA7,RAP1GDS1,CNNM2,HTR2B,ISCU,CMKLR1,HJV,ADCY5,VAPB,CASQ1,TFRC,FZD9,GLRX3,TMCO1,COMT,TFR2,PTPN6,ITGAV,HTT,ERO1A,KCNA1,FBXL5,HAP1,CACNA1C,STIM2,NPPB,ADRA1B,GPR65,CALM1,GRIA1,NPTN,SLC39A6,ANXA6,TRPV2,GNB1,BCAP31,PKD1,ATP6AP1,SYPL2,JPH3,SLC30A7,ATP6V0D1,DMD,FKBP1A,BNIP3,TMEM38B,SLC22A17,SNX10,THY1,TMEM38A,CCR6	darkgreen	EC_M4
GO:0009314	3.93E-04	0.999782624	54	359	response to radiation	BP	1.39E-02	MAP3K4,XRCC6,POLH,REV1,MSH2,ZMPSTE24,RAD54L,DCLRE1C,PAXIP1,FANCD2,METTL3,RRM1,BRCA2,TOPBP1,MAPK8,PRKDC,ERCC6,ATR,CCDC66,PBK,PPID,TENT2,ABRAXAS1,RAD51,INO80,NUCKS1,CHEK1,RGS9,TICRR,INTS7,DTL,BLM,CDC25A,IL12A,COP1,XRCC5,BRCA1,RAD51AP1,ECT2,AIPL1,NEDD4,SMC1A,CLK2,AURKB,TAF1,TIPIN,COPS3,CUL4B,NIPBL,FMR1,TIGAR,RNF8,LZIC,FEN1	darkgrey	EC_M8
GO:0120192	1.63E-04	0.999958898	15	48	tight junction assembly	BP	1.40E-02	AFDN,IKBKB,MARVELD2,OCLN,ESAM,CLDN34,PECAM1,F11R,PAK2,DLG1,RAMP2,PRKCH,EPHA2,PATJ,CDH5	turquoise	EC_M6
GO:0050679	1.64E-04	0.999929241	33	154	positive regulation of epithelial cell proliferation	BP	1.40E-02	TNFAIP3,FLT4,PDGFB,TEK,KDR,CAV2,NRP1,GATA2,PRKD2,SFRP1,IGF1,NR4A1,VEGFC,THBS4,PPP1R16B,PLCG1,CFLAR,TGM1,PROX1,PRKD1,ITGA4,TBX1,HIF1A,MTOR,AR,PGF,LRG1,NRARP,EGFL7,NOTCH1,MIR23A,BMP2,BMP6	turquoise	EC_M6
GO:1902369	3.98E-04	0.999886213	15	60	negative regulation of RNA catabolic process	BP	1.41E-02	LRPPRC,SYNCRIP,THRAP3,TRDMT1,TENT2,NSUN2,DHX9,HNRNPU,FUS,LARP1,TAF15,FMR1,METTL16,HNRNPD,NAF1	darkgrey	EC_M8
GO:1903530	1.67E-04	0.999904926	68	399	regulation of secretion by cell	BP	1.41E-02	TNFRSF11A,SNCAIP,RIMS2,PICK1,APBA1,CD2AP,SYK,ICA1,IL6,RSAD2,TLR4,SDCBP,TRPM4,RAB3B,GATA2,GAB2,LLGL2,UNC13D,SFRP1,SIRT3,CHD7,FAM3D,IL1A,IGF1,MCTP1,MEF2C,TLR2,VEGFC,EDN1,SNCA,DTNBP1,ABCG1,CD200,HDAC1,PPP3CA,ANO1,MCTP2,ATP9A,FGR,ADCY1,FES,CREB1,ADORA2A,MCU,CDK16,FGF23,RAC1,HIF1A,RAB27A,SNCG,SYT7,RSC1A1,TNFRSF1B,FBXL20,CHRNB2,RAB3D,GPR68,LGALS9,NOS2,MYO18A,GIT1,RPH3AL,NOTCH1,STXBP1,RALA,VAMP3,BMP2,BMP6	turquoise	EC_M6
GO:0050808	2.16E-04	0.999875734	70	329	synapse organization	BP	1.44E-02	PLXNB2,ADGRB1,MDGA1,ARHGAP39,VCP,UNC13B,REST,TPBG,SHANK1,LRRC4B,ACTR2,VPS35,SLC7A11,PCDH17,CTNNB1,FARP1,ACTG1,PRNP,PCLO,RYK,SNTA1,MAP1B,APP,GLRB,DHX36,CX3CL1,CACNB3,HOMER1,OPA1,AMIGO2,PDLIM5,CHRNA7,DOCK10,INA,CTTN,DNM1L,DNER,CAPRIN2,DAG1,NTRK3,HSPA8,KIF1A,TANC2,FZD9,MAPT,C1QL1,TNR,DOK7,SIX4,OBSL1,ERC1,NLGN2,ADGRL1,ACTN1,SRCIN1,MYH10,NPTN,PIN1,PAFAH1B1,SDF4,FLNA,NTNG2,PCDHB2,LRRC24,UBE2V2,ARF4,GDNF,LHFPL4,MESD,LRRTM2	darkgreen	EC_M4
GO:0006874	2.17E-04	0.999880375	61	283	cellular calcium ion homeostasis	BP	1.44E-02	LRP1,SMDT1,FKBP1B,FGF2,ATP7B,TPT1,C1QTNF1,TMTC4,STIM1,ATP2C1,PRNP,ATP2B1,CCKBR,RIC3,CXCL12,HSP90B1,C19orf12,RYR3,P2RX4,SELENOT,ATP2A2,APP,CX3CL1,CACNB3,ATP2B4,PKD2,CHRNA7,RAP1GDS1,HTR2B,CMKLR1,ADCY5,VAPB,CASQ1,FZD9,TMCO1,PTPN6,ITGAV,HTT,ERO1A,HAP1,CACNA1C,STIM2,ADRA1B,GPR65,CALM1,GRIA1,NPTN,ANXA6,TRPV2,GNB1,BCAP31,PKD1,SYPL2,JPH3,DMD,FKBP1A,BNIP3,TMEM38B,THY1,TMEM38A,CCR6	darkgreen	EC_M4

GO:0051047	2.29E-04	0.999883829	48	211	positive regulation of secretion	BP	1.51E-02	LRP1,SLC12A2,PPARD,LEP,UNC13B,EXOC1,INHBA,KIF5B,RAB7A,PDCD6IP,TGFB1,RAB3GAP1,C1QTNF1,RAB2B,IRS2,CXCL12,ANKRD1,OSBP,VAMP8,P2RX4,SELENOT,PTPN11,PFKM,TSG101,SPP1,PLA2R1,DYNLL1,DNM1L,TGFB2,TFR2,CYP19A1,NLGN2,NPPB,STX1B,TMED10,TGFB3,CACNA1G,MYH10,SYTL4,SCAMP5,ACSL4,RASL10B,MYOM1,ATP6AP1,VAMP7,AP1G1,WLS,GDNF	darkgreen	EC_M4
GO:0043066	2.32E-04	0.999845887	122	662	negative regulation of apoptotic process	BP	1.53E-02	SERPINB2,LRP1,AHI1,AGAP2,IER3,RRM2B,ANGPT1,PPARA,PPARD,CTNNA1,LEP,BAG1,VEGFA,DOCK8,CREB3,ASNS,TGFBR1,TAX1BP1,GLI3,ITGB1,RB1,TPT1,GLI2,AXL,CTNNB1,AKT2,UFM1,LIG4,IRS2,PRNP,HDAC3,DDRGK1,ILK,CXCL12,HSP90B1,FAIM2,RASA1,CX3CL1,OPA1,AMIGO2,ACVR1,MTDH,ADA,TXNDC5,SLC39A10,TJP1,NOTCH2,CTTN,CDC73,SELENOS,EYA2,HTR2B,GSK3B,FSTL1,PIK3CA,LEF1,PRKCA,KRAS,BMP7,HYOU1,FGFR2,PEA15,TFRC,FZD9,RPS6KA3,HSPB1,SERPINE1,SYVN1,PDCD10,ITGAV,COMP,HTT,FKBP8,PRKCI,ARAF,TIMP1,VDAC2,FMN2,SIX4,MNAT1,ERC1,GHITM,CLN8,MED1,PPARGC1A,CPEB4,NES,ARHGEF2,TGFB3,SHC1,TAF9B,MYOCD,PIN1,CD74,PDGFRB,AKT1,PRKAA1,CD40LG,MYDGF,TNFRSF18,FLNA,PARK7,ENO1,GNAI3,CFDP1,AARS,HSPA5,BCL10,MIR24-1,BNIP3,VSTM2L,UBE2V2,ARF4,GDNF,GLO1,CDKN1A,PTTG1IP,TSLP,ZPR1,ORMDL3,CHST11,FGF10	darkgreen	EC_M4
GO:0099173	2.36E-04	0.999894928	35	137	postsynapse organization	BP	1.55E-02	ARHGAP39,SHANK1,LRRC4B,ACTR2,VPS35,SLC7A11,FARP1,ACTG1,PRNP,GLRB,DHX36,HOMER1,OPA1,PDLIM5,CHRNA7,DOCK10,INA,CTTN,DNM1L,CAPRIN2,NTRK3,HSPA8,KIF1A,TANC2,FZD9,NLGN2,ACTN1,SRCIN1,PAFAH1B1,NTNG2,ARF4,GDNF,LHFPL4,MESD,LRRTM2	darkgreen	EC_M4
GO:0010634	1.86E-04	0.999921733	31	143	positive regulation of epithelial cell migration	BP	1.57E-02	FLT4,PDGFB,TEK,KDR,NRP1,PIK3CG,GATA2,PRKD2,NOS3,GATA3,HBEGF,SCARB1,PLK2,VEGFC,PLCG1,EDN1,ETS1,PROX1,PRKD1,MAP3K3,DOCK1,PPM1F,RAC1,HIF1A,RHOJ,MTOR,FGF18,ITGA3,PLCG2,MIR23A,FGF16	turquoise	EC_M6
GO:0048738	2.41E-04	0.999881559	44	186	cardiac muscle tissue development	BP	1.57E-02	PPARA,LY6E,CBY1,SRF,VEGFA,TGFBR1,BVES,MYLK3,ITGB1,FGF2,TENM4,PAK1,TGFB1,SCN5A,RARB,ANKRD1,HEY1,ACVR1,RUNX1,PDLIM5,NOTCH2,PKP2,TGFB2,PRKAR1A,BMP7,FGFR2,OBSL1,BMPR1A,TCAP,MED1,NPPB,TPM1,CACNA1G,SGCD,MYOCD,PIN1,PDGFRB,COL11A1,MIR24-1,FKBP1A,CACYBP,HOPX,HCN4,WNT2	darkgreen	EC_M4
GO:0045934	4.54E-04	0.999683496	140	1115	negative regulation of nucleobase-containing compound metabolic process	BP	1.60E-02	BCLAF1,ZNF608,SCAF8,UBR5,XRCC6,POT1,RFX3,TRIM28,ZNF451,LRPPRC,MSH2,ZMPSTE24,SYNCRIP,XPO1,CASP8AP2,ZNF350,THRAP3,EZH2,HDAC2,DNAJC2,SETDB2,TRDMT1,RSF1,BIRC5,CHD8,KAT6A,RIF1,E2F7,TAF7,NR2C1,ELK3,TCERG1,CBX5,ERCC6,ZC3H8,PARPBP,TNKS2,BTAF1,TDG,NCK1,ABCE1,CGGBP1,XRN1,SAP30,TERF1,GCFC2,CNOT1,GABPA,PPID,RBL1,MSH3,NBN,TENT2,E2F8,TRIM33,ZBED6,ZNF248,MDM4,HIVEP1,CUL3,NSUN2,GMNN,NFKB1,KHDRBS1,PRMT5,AJUBA,BPTF,DDX5,FANCB,ORC2,BLM,CENPF,AUNIP,SP3,DHX9,DDX20,TPR,CCAR1,XRCC5,NPAT,BRCA1,NSD2,ANKLE1,TBL1XR1,SUV39H1,FOXM1,LYAR,FAM220A,HNRNPU,NEDD4,SMARCE1,RFC1,DCP2,NSD1,EXOSC10,FUS,USP3,AURKB,TAF1,RLIM,ATRX,MBTD1,TRIM37,LARP1,PLK1,ILF3,PSMD10,TAF15,THOC1,SMCHD1,ZNF3,MIER1,NIPBL,PHF6,RMI2,UHRF1,RBMX,FMR1,METTL16,EHMT1,RNPS1,PTBP1,SCAI,CTCF,DEPDC1,HIST1H1A,HNRNPD,ZNF684,DRAP1,ZNF614,RBAK,TIGAR,SRSF7,NAF1,BRMS1L,C1D,RNF8,ZNF558,IKZF5,ZNF529	darkgrey	EC_M8
GO:0043414	4.58E-04	0.999767683	41	254	macromolecule methylation	BP	1.61E-02	PWP1,TRIM28,FBXO11,ZMPSTE24,RLF,EZH2,SETDB2,ZC3H13,TRDMT1,PAXIP1,METTL3,RIF1,NSD3,TGS1,DIMT1,HELLS,MIS18A,SMYD5,VIRMA,NSUN2,TRMT10A,TET2,PRMT5,TARBP1,FTSJ3,SETD2,BRCA1,NSD2,SUV39H1,NSD1,OGT,ATRX,LCMT1,SUZ12,GSPT1,METTL16,EHMT1,CMTR2,CTCF,METTL18,TFB2M	darkgrey	EC_M8
GO:0045071	1.93E-04	0.999959947	12	35	negative regulation of viral genome replication	BP	1.62E-02	RSAD2,OAS3,IFIT1,MX1,KIAA1551,ISG20,PROX1,LTF,TNIP1,ISG15,OAS1,BST2	turquoise	EC_M6
GO:0010959	2.51E-04	0.999863733	57	263	regulation of metal ion transport	BP	1.63E-02	CD63,UBQLN1,ITGB1,FKBP1B,KIF5B,SCN5A,CTNNB1,GPD1L,ACTN4,STIM1,PRNP,ATP2B1,REM1,CXCL12,FXYD1,SNTA1,SLMAP,ATP1B3,P2RX4,CACNA1D,CX3CL1,ABCC8,CACNB3,HOMER1,CHP1,ATP2B4,PKD2,PKP2,ISCU,CASQ1,LINC02210-CRHR1,PTPN6,HTT,KCNA1,HAP1,CACNA1C,STIM2,KCNMB1,CALM1,VMP1,TRPV2,PDGFRB,AKT1,FLNA,KCNAB2,AHCYL1,JPH3,MIR24-1,DMD,FKBP1A,CBARP,TMEM38B,THY1,TMEM38A,YWHAH,SARAF,GEM	darkgreen	EC_M4
GO:0090317	2.52E-04	0.999943717	13	34	negative regulation of intracellular protein transport	BP	1.63E-02	LMAN1,ANGPT1,RAB23,ERLEC1,UBE2J1,MDFIC,UFM1,CHP1,C11orf65,PARK7,TXN,UBE2G2,DERL2	darkgreen	EC_M4

GO:1900227	1.96E-04	0.999988652	6	9	positive regulation of NLRP3 inflammasome complex assembly	BP	1.63E-02	TLR4,CD36,DDX3X,TLR6,PLCG2,GBP5	turquoise	EC_M6
GO:0008284	1.97E-04	0.999873771	104	681	positive regulation of cell population proliferation	BP	1.63E-02	TNFRSF11A,TNFAIP3,CNOT6,FLT4,CTHRC1,PTK2,PDGFB,KDM4C,PIM1,TEK,KIT,KDR,SYK,IL6,PELI1,CAV2,TLR4,IL15,SDCBP,MYCN,TRPM4,NRP1,GATA2,PRKD2,GAB2,EDNRB,P2RY6,SFRP1,HBEGF,EFNB2,ASH2L,IL1A,IGF1,NR4A1,MEF2C,WWTR1,IL7,VEGFC,CXCL10,KMT2D,THBS4,PPP1R16B,PLCG1,HIPK1,DLL4,EDN1,STAT1,ETS1,SHANK2,HDAC1,ICOSLG,HLX,HCLS1,NFATC2,CD86,CFLAR,TGM1,PROX1,PRKD1,MAP3K3,MLXIPL,LAMC2,DLG1,ELL3,TET1,LTF,ARNT2,ITGA4,IGFBP5,TBX1,CALCRL,FGF23,PRKCH,HIF1A,ALDH1A2,MTOR,AR,EFNB1,FGF18,PGF,CHRNB2,CNOT8,IL13RA1,LGALS9,IL7R,LRG1,MATK,DOT1L,NRARP,EGFL7,NOTCH1,SLC25A33,S1PR1,MIR23A,ATXN1L,ADGRG1,CD46,FGF16,CDK13,STX3,E2F3,BST2,BMP2,BMP6	turquoise	EC_M6
GO:0008088	2.54E-04	0.999915361	21	68	axo-dendritic transport	BP	1.64E-02	AP3S1,RAB21,DST,MGARP,KIF5B,TRAK1,APP,OPA1,AP3B1,FEZ1,HSPA8,KIF1A,HSPB1,MAPT,ACTR10,HAP1,ARMCX3,PAFAH1B1,HSBP1,BLOC1S2,MAP1A	darkgreen	EC_M4
GO:0030595	1.99E-04	0.999915784	31	150	leukocyte chemotaxis	BP	1.64E-02	TNFRSF11A,PTK2,PDGFB,KIT,SYK,IL6,CXCL8,C5,TRPM4,PIK3CG,CXCR4,EDNRB,VEGFC,CXCL10,THBS4,ANO6,EDN1,JAM3,RIN3,LYST,MCU,RAC1,PGF,DAPK2,S100A14,MMP28,CCL2,PDE4B,LGALS9,S1PR1,MSMP	turquoise	EC_M6
GO:0032886	4.67E-04	0.999775549	36	202	regulation of microtubule-based process	BP	1.64E-02	AKAP9,SENP6,XPO1,GEN1,PLK4,STIL,CEP295,CHORDC1,CLASP2,BORA,CKAP2,TPX2,SKA3,CENPJ,ALMS1,CFAP298,CKAP5,STMND1,KIF18A,CAMSAP2,RAE1,HDGFL3,TPR,KIF15,BRCA1,TACC3,FKBP4,HNRNPU,TRIM37,PLK1,CEP76,TAOK1,CCNF,STMN1,AURKA,DRG1	darkgrey	EC_M8
GO:0006298	4.77E-04	0.99991577	9	27	mismatch repair	BP	1.67E-02	RPA3,MSH2,TDG,MSH3,RPA2,SETD2,EXO1,RPA1,TP73	darkgrey	EC_M8
GO:0045740	4.78E-04	0.99989512	11	36	positive regulation of DNA replication	BP	1.67E-02	DBF4,E2F7,E2F8,INO80,CDK1,DHX9,DNA2,DBF4B,ATRX,ATAD5,CDT1	darkgrey	EC_M8
GO:2000146	2.61E-04	0.999862663	52	234	negative regulation of cell motility	BP	1.68E-02	LRP1,FAM19A5,ADGRB1,PPARD,CTNNA1,SRF,RRAS,FGF2,TGFB1,OSBPL8,NAV3,SPINT2,DCN,STARD13,ILK,DPYSL3,CXCL12,SULF1,ROBO1,PFN2,CX3CL1,ABCC8,ADA,ATP2B4,ADIPOR1,PHLDB2,DAG1,CORO1C,SLAMF8,IDH2,CLIC4,SERPINE1,DNAJA4,PDCD10,COL3A1,NBL1,TIMP1,VCL,BMPR1A,PPARGC1A,NGFR,TPM1,MYOCD,PIN1,CD74,AKT1,PLXNB3,MIR24-1,SERPINF1,THY1,DUSP22,PRKG1	darkgreen	EC_M4
GO:0048863	2.04E-04	0.999907319	36	171	stem cell differentiation	BP	1.68E-02	SEMA6A,KIT,SEMA4D,TEAD2,NRP1,TAL1,EDNRB,JAG1,SFRP1,SLC4A11,TCF15,RET,KDM3A,ITCH,MEF2C,GPM6A,LMBR1L,EDN1,JARID2,SEMA3F,LAMA5,ACE,ELL3,SEMA6D,TBX1,HIF1A,ALDH1A2,EFNB1,SEMA4A,BATF,SEMA6B,NOTCH1,MTF2,SOX17,CDK13,SOX18	turquoise	EC_M6
GO:0070830	2.06E-04	0.999949736	14	44	bicellular tight junction assembly	BP	1.68E-02	AFDN,IKBKB,MARVELD2,OCLN,ESAM,CLDN34,PECAM1,F11R,PAK2,DLG1,RAMP2,PRKCH,EPHA2,CDH5	turquoise	EC_M6
GO:0007292	4.84E-04	0.9998214	21	101	female gamete generation	BP	1.69E-02	YTHDC2,FBXO5,TUT7,YTHDC1,TTK,METTL3,BRCA2,CCNB1,RPS6,BMPR1B,TRIP13,ASPM,SPO11,FOSL1,ZFX,DDX20,C17orf53,TOP2A,CCNB2,PLK1,AURKA	darkgrey	EC_M8
GO:0070997	2.65E-04	0.999852459	61	288	neuron death	BP	1.70E-02	LRP1,AGAP2,ANGPT1,PPARA,REST,VPS35,SLC7A11,SRPK2,RB1,PICALM,AXL,CTNNB1,AKT2,TMTC4,LIG4,PRNP,ILK,ARL6IP5,FAIM2,RASA1,APP,CX3CL1,CHP1,CHRNA7,TGFB2,GSK3B,INPP5A,CASP7,PIK3CA,KRAS,FAM162A,HYOU1,NCSTN,BACE1,FZD9,EGLN3,MAPT,PRKCI,SIX4,EIF2S1,PPARGC1A,CPEB4,NES,NGFR,TGFB3,PIN1,AKT1,IKBKG,TMEM259,PARK7,AARS,HSPA5,NAE1,SERPINF1,BNIP3,ATN1,VSTM2L,UBE2V2,GDNF,SIGMAR1,ZPR1	darkgreen	EC_M4
GO:0055074	2.68E-04	0.999850001	62	291	calcium ion homeostasis	BP	1.70E-02	LRP1,SMDT1,FKBP1B,FGF2,ATP7B,TPT1,C1QTNF1,TMTC4,STIM1,ATP2C1,PRNP,ATP2B1,CCKBR,RIC3,CXCL12,HSP90B1,C19orf12,RYR3,P2RX4,SELENOT,ATP2A2,APP,CX3CL1,CACNB3,ATP2B4,PKD2,CHRNA7,RAP1GDS1,HTR2B,CMKLR1,ADCY5,VAPB,CASQ1,FZD9,TMCO1,PTPN6,ITGAV,HTT,ERO1A,HAP1,CACNA1C,STIM2,ADRA1B,GPR65,CALM1,GRIA1,NPTN,ANXA6,TRPV2,GNB1,BCAP31,PKD1,SYPL2,JPH3,DMD,FKBP1A,BNIP3,TMEM38B,SNX10,THY1,TMEM38A,CCR6	darkgreen	EC_M4
GO:0019511	2.69E-04	0.999968824	8	15	peptidyl-proline hydroxylation	BP	1.70E-02	P4HA2,P3H1,CRTAP,EGLN3,PRDX4,P4HA1,ERO1A,P3H3	darkgreen	EC_M4
GO:0007029	2.71E-04	0.999903163	23	80	endoplasmic reticulum organization	BP	1.71E-02	LMAN1,REEP2,LMAN2L,RTN4,EMC10,SEC61A1,RAB3GAP1,ESYT2,REEP5,TMED2,TRAM1,EMC4,ZFYVE27,VAPB,CASQ1,RTN3,EMC1,STX18,LMAN2,TMEM38B,TOR1AIP2,EMC7,TMEM38A	darkgreen	EC_M4

GO:2000587	2.77E-04	1	4	4	negative regulation of platelet-derived growth factor receptor-beta signaling pathway	BP	1.74E-02	LRP1,MYOCD,PTPN2,PTPN12	darkgreen	EC_M4
GO:0051051	2.79E-04	0.99984088	65	314	negative regulation of transport	BP	1.75E-02	LMAN1,ANGPT1,PACSIN1,UBQLN1,LEP,REST,RAB23,ERLEC1,UBE2J1,MDFIC,INHBA,VPS35,FKBP1B,RAB7A,PICALM,AKT2,UFM1,IRS2,RAB11FIP1,ARL6IP5,REM1,VAMP8,MAP1B,ATXN2,ABCC8,CACNB3,PTPN11,PIP4P2,ARHGAP1,CRY2,ADA,CHP1,PKD2,PLA2R1,SELENOS,HADH,ISCU,IDH2,OSM,PEA15,LINC02210-CRHR1,COMT,C11orf65,ITGAV,ADRA2C,LRPAP1,SRCIN1,STX1B,SYT11,CALM1,SYTL4,SCAMP5,CD74,AKT1,MIDN,PARK7,RHBDF1,MIR24-1,CBARP,STXBP6,TXN,UBE2G2,GEM,DERL2,LRRTM2	darkgreen	EC_M4
GO:0055001	2.84E-04	0.999868733	37	150	muscle cell development	BP	1.77E-02	PPARA,FLNC,SRF,VEGFA,BVES,MYLK3,ITGB1,PAK1,ACTG1,ACTN4,ANKRD1,ATP2A2,HOMER1,PDLIM5,DNER,LMOD1,PRKAR1A,CASQ1,COMP,SYNPO2L,WDR1,SIX4,OBSL1,BMPR1A,TCAP,ACTN1,TPM1,PIN1,FBXO22,PDGFRB,FLII,SYPL2,MIR24-1,DMD,LOX,CSRP1,CFL2	darkgreen	EC_M4
GO:0032259	5.12E-04	0.999729803	45	290	methylation	BP	1.77E-02	CARNMT1,PWP1,TRIM28,FBXO11,ZMPSTE24,RLF,EZH2,SETDB2,ZC3H13,TRDMT1,PAXIP1,METTL3,RIF1,NSD3,RRP8,TGS1,DIMT1,HELLS,MIS18A,SMYD5,VIRMA,PRDM10,NSUN2,TRMT10A,TET2,PRMT5,TARBP1,FTSJ3,SETD2,BRCA1,NSD2,SUV39H1,NSD1,OGT,ATRX,LCMT1,TYMS,SUZ12,GSPT1,METTL16,EHMT1,CMTR2,CTCF,METTL18,TFB2M	darkgrey	EC_M8
GO:0031053	5.12E-04	0.999953141	6	12	primary miRNA processing	BP	1.77E-02	HNRNPA2B1,PUS10,METTL3,DGCR8,DROSHA,SRRT	darkgrey	EC_M8
GO:0045785	2.22E-04	0.999880478	55	306	positive regulation of cell adhesion	BP	1.80E-02	AFDN,PDGFB,PODXL,TEK,KDR,SYK,IL6,IL15,NRP1,PRKD2,UNC13D,GATA3,SFRP1,FERMT1,ARHGEF7,CD36,MAGI1,RET,IL1A,IGF1,IL7,SOX13,NEDD9,ETS1,LCK,PPP3CA,ICOSLG,HLX,CD86,F11R,ITGA6,DOCK1,B2M,ITGA4,SELE,SELP,PPM1F,RAC1,JUP,ITPKB,EFNB1,ITGA3,DNM2,CCL2,YES1,JAK1,LGALS9,IL7R,AP3D1,PIEZO1,NFAT5,VNN1,ADGRG1,CD46,STX3	turquoise	EC_M6
GO:0007275	2.24E-04	0.999821879	488	3815	multicellular organism development	BP	1.81E-02	ZNF516,TNFRSF11A,TCF4,SEMA6A,N4BP3,KIF5A,IFNGR1,TNFAIP3,HECA,PTPRB,FLT4,SYNJ2,FZD6,CTHRC1,RIMS2,CELSR1,QKI,NOTCH4,TCF7,CDKL3,MTSS1,ASAP1,L3MBTL3,KLHL3,PTK2,HSD17B3,ANKS1A,ATF4,PDGFB,AGTPBP1,PODXL,CSNK1E,PICK1,KDM4C,MICALL1,PIM1,MFNG,MDFI,TEK,LINGO2,IREB2,APBA1,ADGRF5,KIT,ADGRF1,TNFRSF21,KDR,NFIL3,SYK,SEMA4D,EFHC1,THSD7A,SHB,TMOD1,RAPGEF5,IL6,IGF2BP3,PPT1,PTBP3,CXCL8,UTP11,RSAD2,FOXP2,CAV2,TSPAN12,TLR4,C5,GJA4,IL15,FZD8,TEAD2,SDCBP,MYCN,NRCAM,NOCT,TRPM4,NRP1,ZBTB24,RASIP1,PIK3CG,SVIL,ITSN2,TAL1,LAMA4,PHF10,MTMR2,EPC1,GATA2,DNMT3A,HS6ST1,PRKD2,CST7,NOVA2,CTSC,GPR4,SNRK,PLXND1,PRCP,ITGA8,NOS3,GAB2,ITGB4,DACH1,PPARG,TIMP4,GATA3,ACVR2B,MYO7A,CXCR4,LMBR1,GZF1,EDNRB,SPTBN4,TIE1,GDPD5,SETD5,IKBKB,JAG1,PDE2A,GPAT4,SFRP1,HBEGF,NR1D2,FEZ2,NOTUM,FERMT1,FREM2,EFNB2,ANKLE2,ARHGEF7,ASH2L,SPEF1,ATP11A,PCDH1,PCDH12,ARHGAP26,P2RX2,NTN4,RIC8A,TMEM178A,LMOD3,PRAG1,ADAMTS9,SDCBP2,TCF15,EPHB1,PLK2,MALL,RET,MERTK,TNFRSF19,CHD7,IL1A,DNMT3B,IGF1,NR4A1,EIF2AK3,HHEX,ITCH,SH3RF1,PREX2,SMAD1,HIP1R,POU6F1,STOX2,DCLK2,MMP24,SOX7,KIF13B,ENC1,HEXB,MEF2C,SMARCD1,TLR2,WWTR1,IL7,P2RX7,TPD52,VEGFC,NDRG4,GPM6A,CXCL10,ANGPT2,TLL2,LMBR1L,ADGRG3,DHH,SFMBT1,PLLP,BMP2K,TIPARP,TLL1,SERINC5,THBS4,USH1C,PPP1R16B,PLCG1,STC1,RAPGEF3,ANKRD2,NR4A2,STAB1,HIPK1,ANO6,DLL4,SOX13,DOP1B,TSPAN2,NAV2,EDN1,STAT1,DYRK1A,JAM3,IGSF3,SNCA,ALAS1,JARID2,DTNBP1,MYLIP,ERG,ETS2,GAS2,	turquoise	EC_M6
GO:0048871	2.28E-04	0.999870321	65	378	multicellular organismal homeostasis	BP	1.83E-02	ZNF516,TNFRSF11A,TNFAIP3,LYZ,ATF4,TJP2,ADGRF5,SYK,GADD45G,IL6,VPS54,GRB10,TLR4,IL15,LAMA4,GATA2,NOVA2,PRCP,NOS3,ACVR2B,CXCR4,EDNRB,NR1D2,POC1B,CD36,IL1A,ABHD6,KDM3A,CLCN3,OCLN,WWTR1,IL7,P2RX7,USH1C,JAM3,PM20D1,LPCAT1,HYAL2,SLC39A8,ESAM,FABP4,INPP5D,CD34,PECAM1,F11R,MLXIPL,B2M,LTF,LDB2,PROM1,RAC1,LIPA,APBB2,ADIPOR2,RAB3D,TLE3,SCNN1B,ADRB2,NOTCH1,S1PR1,CDH5,OAS1,CA2,DRAM2,BMP6	turquoise	EC_M6

GO:0016049	2.98E-04	0.999819453	80	399	cell growth	BP	1.85E-02	LRP1,RAB21,PPARA,ROS1,PPARD,SH3GL2,PLAA,SRF,VEGFA,TGFBR1,RIMS1,RTN4,MFSD2A,INHBA,ITGB1,PLXNA1,RB1,PTCH2,MEGF8,PAK1,TGFB1,CTNNB1,DCLK1,SEMA3C,ILK,RYK,RNF6,CXCL12,CHPT1,EIF4G2,MAP1B,DIP2B,SEMA4F,APP,ZFYVE27,TSG101,BAP1,SPP1,SEMA4G,PDLIM5,NDN,ADIPOR1,CTTN,CDC73,HYAL1,CAPRIN2,TGFB2,FBLN5,GSK3B,MMP14,AUTS2,IQGAP1,RBBP7,RPS6KA3,MAPT,TNR,FN1,SMURF1,VCL,TSPYL2,NPPB,RAB11A,TAF9B,SMARCA4,MYOCD,ISLR2,PIN1,TRPV2,AKT1,CRLF3,PAFAH1B1,PLXNA3,ENO1,BARHL2,MIR24-1,CDKN1A,DACT3,NME6,OLFM1,DERL2	darkgreen	EC_M4
GO:0051271	2.99E-04	0.99984036	53	241	negative regulation of cellular component movement	BP	1.85E-02	LRP1,FAM19A5,ADGRB1,PPARD,CTNNA1,SRF,RRAS,FGF2,TGFB1,OSBPL8,NAV3,SPINT2,DCN,STARD13,ILK,DPYSL3,CXCL12,SULF1,ROBO1,PFN2,CX3CL1,ABCC8,ADA,ATP2B4,ADIPOR1,PHLDB2,DAG1,CORO1C,SLAMF8,IDH2,CLIC4,SERPINE1,DNAJA4,PDCD10,COL3A1,NBL1,TIMP1,VCL,BMPR1A,PPARGC1A,ACTN1,NGFR,TPM1,MYOCD,PIN1,CD74,AKT1,PLXNB3,MIR24-1,SERPINF1,THY1,DUSP22,PRKG1	darkgreen	EC_M4
GO:1902292	5.41E-04	0.999987732	4	5	cell cycle DNA replication initiation	BP	1.86E-02	MCM3,MCM2,MCM4,POLA1	darkgrey	EC_M8
GO:1902315	5.41E-04	0.999987732	4	5	nuclear cell cycle DNA replication initiation	BP	1.86E-02	MCM3,MCM2,MCM4,POLA1	darkgrey	EC_M8
GO:1902975	5.41E-04	0.999987732	4	5	mitotic DNA replication initiation	BP	1.86E-02	MCM3,MCM2,MCM4,POLA1	darkgrey	EC_M8
GO:0007265	2.34E-04	0.999873849	55	296	Ras protein signal transduction	BP	1.87E-02	CDC42SE2,CELSR1,SGSM3,DENND3,CDC42EP1,ELMO1,SDCBP,NRP1,RASIP1,PIK3CG,ARHGAP42,GPR4,RALB,NET1,CYTH1,RASGRP3,PRAG1,PLK2,IGF1,KIAA0355,ERBIN,ARHGEF3,ARHGEF28,MADD,RAPGEF3,RASSF1,SHOC2,ARHGEF12,PRKD1,F11R,ARHGDIB,USP28,BCR,RFXANK,WAS,RAC1,RHOJ,ITPKB,OPHN1,STARD8,RIT1,ITGA3,DNM2,ARHGAP4,PLEKHG5,NOTCH1,RAPGEF1,DAB2IP,RIPOR1,ADGRG1,GRAP,LPAR6,ARHGEF1,RALA,RAB39A	turquoise	EC_M6
GO:0007266	2.37E-04	0.999906169	27	116	Rho protein signal transduction	BP	1.87E-02	CDC42SE2,CELSR1,CDC42EP1,NRP1,RASIP1,ARHGAP42,GPR4,NET1,PRAG1,ARHGEF3,ARHGEF28,ARHGEF12,F11R,ARHGDIB,BCR,WAS,RAC1,RHOJ,OPHN1,STARD8,ITGA3,ARHGAP4,PLEKHG5,RIPOR1,ADGRG1,LPAR6,ARHGEF1	turquoise	EC_M6
GO:0051767	2.37E-04	0.99997093	8	17	nitric-oxide synthase biosynthetic process	BP	1.87E-02	AKAP12,KDR,TLR4,TLR2,EDN1,STAT1,CCL2,FCER2	turquoise	EC_M6
GO:0051769	2.37E-04	0.99997093	8	17	regulation of nitric-oxide synthase biosynthetic process	BP	1.87E-02	AKAP12,KDR,TLR4,TLR2,EDN1,STAT1,CCL2,FCER2	turquoise	EC_M6
GO:0051246	5.46E-04	0.999589749	224	1953	regulation of protein metabolic process	BP	1.87E-02	CDK2,PAN2,PPIL4,LATS1,DDX39B,FBXO5,UBR5,EIF3E,MAP3K4,MRPL13,XRCC6,CDC25C,TNRC6B,TUT7,PLEC,PWP1,TOPORS,DBF4,AKAP9,PUM3,CKS2,NCBP1,ZNF451,ANP32B,LRPPRC,PHIP,MTIF2,TTK,IBTK,PNPT1,ZMPSTE24,SYNCRIP,XPO1,COMMD1,CASP8AP2,ETAA1,TIA1,ITGB1BP1,EZH2,UFL1,CLSPN,ASCC3,CASP2,DDX1,WDR35,NRDC,DHFR,PUS7,HDAC2,CCNA2,EXOSC9,SAE1,CEP295,CHORDC1,SYMPK,MASTL,CNOT10,BORA,DIS3,CCNT2,PAXIP1,NCAPG2,CDC20,BIRC5,FNTA,GTPBP4,IPO5,METTL3,RIF1,NSD3,KITLG,TAF7,EXOSC8,CUL4A,MAPK8,PRKDC,APAF1,CCNB1,ERCC6,DHX29,PAN3,SP1,TPX2,CAPRIN1,UBA2,IPO7,LSM14A,CDK5RAP1,FER,NCK1,IDE,ARFGEF1,PTCD3,ABCE1,OTUD4,ZFP91,TAF9,XRN1,PDE12,LARP4,USP25,PBK,CNOT1,SH2B3,LTN1,USP16,MOB1A,CCNL1,CDCA2,NBN,FAM83D,SENP1,RAD51,RIPK1,CCNE2,STK4,MDM4,UBE2C,HERC5,TAOK3,NOLC1,BMPR1B,UBE3A,GCN1,CUL3,METAP1,CHEK1,TIMM17A,NFKB1,KHDRBS1,CENPE,NCL,KIF14,AJUBA,FXR1,FANCI,NGDN,EIF2B5,DTL,ALS2,BLM,DDX6,CDC25A,PDE8A,SMARCC1,DHX9,HNRNPR,FASTKD2,IVNS1ABP,TPR,FANCM,DBF4B,IL12A,COP1,XRCC5,BRCA1,PSME3,TFPI,CNOT9,CDKN3,DCUN1D5,TIPRL,BOD1L1,ECT2,EIF2AK1,INCENP,UBE2K,NEDD4,CCNB3,CDC6,DCP2,EIF3B,PRELID1,SEPSECS,NSD1,CCNB2,IQGAP3,TAF1,CLPX,OGT,ATRX,PTTG1,SPAG9,GEMIN5,LARP1,PLK1,ILF3,AATF,CSK,PSMD10,EIF5,NLE1,TYMS,UPF3B,SUZ12,XIAP,RICTOR,NIPBL,AIFM1,GSPT1,TAOK1,UHRF1,LIPG,FMR1,METTL16,PKMYT1,CCNF,PTBP1,EXOSC2,SF3B3,GLMN,IST1,CTCF,RBM8A,METTL18,SARNP,HNRNPD,MAD2L1,AURKA,RNF139,ERP29,PAIP2,UBXN2A,RCHY1	darkgrey	EC_M8
GO:0048477	5.47E-04	0.999837992	15	62	oogenesis	BP	1.87E-02	YTHDC2,FBXO5,TUT7,YTHDC1,METTL3,BRCA2,CCNB1,RPS6,BMPR1B,TRIP13,ASPM,SPO11,ZFX,DDX20,AURKA	darkgrey	EC_M8

GO:0050865	2.40E-04	0.999863622	64	385	regulation of cell activation	BP	1.88E-02	TNFAIP3,TCF7,PDGFB,TEC,ADGRF5,TNFRSF21,SYK,IL6,PELI1,TLR4,IL15,GATA2,CST7,CTSC,NECTIN2,NOS3,GAB2,UNC13D,GATA3,SFRP1,MERTK,IL1A,IGF1,ITCH,SH3RF1,MEF2C,IL7,SOX13,SNCA,CD200,LCK,ICOSLG,LAPTM5,HLX,NFATC2,INPP5D,CD86,FGR,FES,RPS6KA1,F11R,DLG1,B2M,ADORA2A,SOS2,SELP,ITPKB,TLR6,TNFRSF1B,ARG2,EFNB1,CHRNB2,CD19,CCL2,YES1,LGALS9,IL7R,MYO18A,AP3D1,NRARP,STXBP1,HAVCR1,VNN1,CD46	turquoise	EC_M6
GO:1990173	5.52E-04	0.999944352	6	14	protein localization to nucleoplasm	BP	1.88E-02	CCT2,CCT4,CCT7,CCT5,CCT6A,CCT3	darkgrey	EC_M8
GO:0071029	5.57E-04	0.999980888	4	6	nuclear ncRNA surveillance	BP	1.88E-02	EXOSC9,EXOSC8,EXOSC10,EXOSC2	darkgrey	EC_M8
GO:0071035	5.57E-04	0.999980888	4	6	nuclear polyadenylation-dependent rRNA catabolic process	BP	1.88E-02	EXOSC9,EXOSC8,EXOSC10,EXOSC2	darkgrey	EC_M8
GO:0071038	5.57E-04	0.999980888	4	6	nuclear polyadenylation-dependent tRNA catabolic process	BP	1.88E-02	EXOSC9,EXOSC8,EXOSC10,EXOSC2	darkgrey	EC_M8
GO:0071046	5.57E-04	0.999980888	4	6	nuclear polyadenylation-dependent ncRNA catabolic process	BP	1.88E-02	EXOSC9,EXOSC8,EXOSC10,EXOSC2	darkgrey	EC_M8
GO:0106354	5.57E-04	0.999980888	4	6	tRNA surveillance	BP	1.88E-02	EXOSC9,EXOSC8,EXOSC10,EXOSC2	darkgrey	EC_M8
GO:0032527	3.05E-04	0.999919884	15	43	protein exit from endoplasmic reticulum	BP	1.88E-02	VCP,TMEM30A,ERLEC1,UBE2J1,SEC13,NPLOC4,TM9SF4,HSP90B1,BRSK2,SELENOS,SYVN1,SEL1L,BCAP31,UBE2G2,DERL2	darkgreen	EC_M4
GO:0030154	3.07E-04	0.999753593	468	2966	cell differentiation	BP	1.88E-02	CD63,IL23A,ATP8B1,NAB2,LRP1,AHI1,CAND1,RAB21,CDSN,PLXNB2,FBN2,ANGPT1,PPARA,HLA-DRA,ROS1,AFF4,NUS1,MRTFA,TRAPPC9,PACSIN1,ADGRB1,PPARD,CTNNA1,TEAD3,SUN2,CBY1,MTCH1,LGALS1,MDGA1,SH3GL2,FLNC,MYH9,LEP,CDKN2B,PLAA,RORB,SRF,VEGFA,RUNX2,NCK2,CLOCK,REST,COL15A1,P3H1,TGFBR1,HIVEP3,SOCS5,RIMS1,TMEM30A,PSME4,UBA6,RTN4,TPBG,SHANK1,MFSD2A,UBE2J1,AKIRIN1,ACTR2,GMCL1,IFRD1,KIDINS220,WHRN,GLI3,NCDN,ROCK2,INHBA,BVES,RRAS,MYLK3,DKKL1,SLC7A11,OSTM1,ITGB1,FKBP1B,PLXNA1,FGF2,SRPK2,KIF5B,NAPA,PANX1,PSMC2,RB1,ABHD5,PICALM,EIF2B3,PTCH2,TENM4,GLI2,MEGF8,SMARCD3,RHEB,PAK1,TGFB1,AXL,CTNNB1,IL15RA,TRAK1,LBH,SERPINH1,AKT2,DZIP1,ZEB2,FARP1,SLC4A7,OSBPL8,RARB,ACTG1,NAV3,ACTN4,CRIM1,MAFG,SPRED3,SPINT2,LIG4,SMAD9,ZNF703,DCLK1,SEMA3C,UBA5,ILK,DPYSL3,SPINK5,RYK,SNPH,RNF6,AKIP1,CXCL12,IMPAD1,LGI4,ZSWIM6,KIF2A,ANKRD1,FAT1,SLC25A46,PALLD,TP53INP2,ZNF536,FLNB,EIF4G2,SULF1,ROBO1,MAP1B,RDH10,DIP2B,SEMA4F,LOXL3,P2RX4,FAIM2,HEY1,RASA1,ATP2A2,TLX2,CELF1,TCTN1,MMP15,APP,DHX36,SLC4A5,RAPGEF2,CX3CL1,ABCC8,PTPN11,PTX3,ADCY6,HOMER1,RUNX1T1,OPA1,CDH17,ARSB,LOXL2,AP3B1,ZFYVE27,PTPN22,CRTAC1,TSG101,ACVR1,TUBB2A,CHAC1,MMP2,GDF6,ADA,SPP1,BMP1,RUNX1,TWF1,TMEM14C,HIF1AN,DNAH1,SEMA4G,NKD1,GPRIN3,TOLLIP,BRSK2,HPS6,PDLIM5,NDN,RAB7B,CHRNA7,ARL3,DOCK10,EIF4E,DYNLL1,INA,ADIPOR1,PHLDB2,TJP1,TDP2,FNDC5,KDM5B,NOTCH2,CTTN,PKP2,CDC73,DNM1L,DNER,GLRX2,CHSY1,LMOD1,EYA2,CCND	darkgreen	EC_M4
GO:0007169	2.42E-04	0.999851618	84	499	transmembrane receptor protein tyrosine kinase signaling pathway	BP	1.89E-02	SEMA6A,FLT4,MTSS1,PTK2,ANKS1A,PDGFB,TEK,KIT,KDR,SYK,CAV2,GRB10,TSPAN12,NRP1,TAL1,PRKD2,GAB2,GATA3,TIE1,HBEGF,EFNB2,ARHGEF7,EPHB1,RET,MERTK,IGF1,EIF2AK3,HHEX,ERBIN,HIP1R,ARHGEF28,VEGFC,NDRG4,ANGPT2,SOGA1,TIPARP,PLCG1,NEDD9,SNCA,LCK,SHOC2,NCF1,FGR,KALRN,IGFBP3,FES,PLEKHA1,PRKD1,SRMS,PAK2,PTPRE,APOD,STYK1,CLEC14A,BCR,EPHB4,IGFBP5,BAIAP2L1,FGF23,LMTK2,RAC1,HIF1A,EPHA2,MMRN2,TNK1,RGS14,AR,EFNB1,PSEN1,FGF18,PGF,EFNA1,RPS6KA5,COL4A6,COL4A5,SH3TC2,ADRB2,YES1,GIT1,TMEM204,RAPGEF1,DAB2IP,FGF16,BMP2	turquoise	EC_M6

GO:0045595	2.44E-04	0.999828003	167	1153	regulation of cell differentiation	BP	1.89E-02	TCF4,SEMA6A,CTHRC1,NOTCH4,TCF7,CDKL3,PTK2,ATF4,PDGFB,CSNK1E,KDM4C,PIM1,KIT,TNFRSF21,KDR,SYK,SEMA4D,IL6,PTBP3,TLR4,IL15,TEAD2,SDCBP,MYCN,NRCAM,NOCT,TRPM4,NRP1,TAL1,GATA2,PLXND1,UNC13D,PPARG,GATA3,ACVR2B,CXCR4,EDNRB,IKBKB,JAG1,SFRP1,NR1D2,EFNB2,ARHGEF7,ATP11A,SLC4A11,CD36,TMEM178A,LMOD3,TCF15,EPHB1,RET,CHD7,DNMT3B,IGF1,KDM3A,ITCH,SH3RF1,SMAD1,MEF2C,TLR2,WWTR1,IL7,VEGFC,CXCL10,ANKRD2,DLL4,SOX13,ADAMTS20,NEDD9,EDN1,STAT1,SLC45A3,ABCG1,ETS1,SIK1,HDAC1,PPP3CA,SEMA3F,PRKX,HLX,HCLS1,NFATC2,AP1AR,ZFHX2,INPP5D,CD34,CD86,PLXNA2,TRAK2,IGFBP3,FES,RBM4,RPS6KA1,PROX1,SS18L1,PRKD1,F11R,DOCK1,MELTF,APOLD1,ELL3,CREB1,B2M,LTF,SEMA6D,ADAMTS7,SOS2,IGFBP5,ETV4,TBX1,RAMP2,PROM1,FGF23,PRKCH,RAC1,HIF1A,RCC2,BCL6B,ITPKB,TNFRSF1B,RGS14,ZC4H2,SMOC1,MTOR,AR,CASZ1,PSEN1,SEMA4A,HOXB8,FGF18,EFNA1,NAP1L2,PIAS1,GPR68,SMAD2,DNM2,YY1,HMG20A,RCOR1,LGALS9,IL7R,SEMA6B,RFLNB,ISG15,ARHGAP4,AP3D1,NRARP,NOTCH1,PIEZO1,ENG,DAB2IP,CDH5,VNN1,CD46,CA2,SOX17,RELN,CDK13,SHANK3,MMD,MAFF,RGS4,TRIM62,TGIF2,HIST1H4D,BMP2,BMP6	turquoise	EC_M6
GO:0009306	3.13E-04	0.999828153	57	266	protein secretion	BP	1.91E-02	LRP1,CANX,SLC12A2,PPARD,LEP,UNC13B,CLOCK,EXOC1,REST,P3H1,RCN3,FKBP1B,KIF5B,DNAJC1,COPG1,TGFB1,IRS2,RAB11FIP1,PCLO,ANKRD1,OSBP,SELENOT,ABCC8,PTPN11,PFKM,BRSK2,DYNLL1,DNM1L,CPT1A,TGFB2,LRP5,HADH,DISP1,IDH2,ADCY5,SIDT2,TANGO2,COMP,PTPRN,NLGN2,SRCIN1,LTBP2,TMED10,TGFB3,UBE2Q1,MYH10,SEL1L,SYTL4,ACSL4,MYOM1,PAFAH1B1,MIDN,SSTR5,PARK7,RHBDF1,WLS,TMEM167A	darkgreen	EC_M4
GO:0035592	3.13E-04	0.999828153	57	266	establishment of protein localization to extracellular region	BP	1.91E-02	LRP1,CANX,SLC12A2,PPARD,LEP,UNC13B,CLOCK,EXOC1,REST,P3H1,RCN3,FKBP1B,KIF5B,DNAJC1,COPG1,TGFB1,IRS2,RAB11FIP1,PCLO,ANKRD1,OSBP,SELENOT,ABCC8,PTPN11,PFKM,BRSK2,DYNLL1,DNM1L,CPT1A,TGFB2,LRP5,HADH,DISP1,IDH2,ADCY5,SIDT2,TANGO2,COMP,PTPRN,NLGN2,SRCIN1,LTBP2,TMED10,TGFB3,UBE2Q1,MYH10,SEL1L,SYTL4,ACSL4,MYOM1,PAFAH1B1,MIDN,SSTR5,PARK7,RHBDF1,WLS,TMEM167A	darkgreen	EC_M4
GO:2000637	5.75E-04	0.999895659	9	27	positive regulation of gene silencing by miRNA	BP	1.94E-02	TNRC6B,RIPK1,AJUBA,FXR1,DDX5,TIAL1,DDX6,DHX9,FMR1	darkgrey	EC_M8
GO:0045840	5.82E-04	0.999880811	10	32	positive regulation of mitotic nuclear division	BP	1.96E-02	PHIP,CDC16,ESPL1,ANAPC7,NUSAP1,CUL3,CDC27,DLGAP5,CDT1,AURKA	darkgrey	EC_M8
GO:0071219	2.55E-04	0.99989558	28	133	cellular response to molecule of bacterial origin	BP	1.96E-02	TNFAIP3,IL6,CXCL8,TLR4,IRAK2,EDNRB,NFKBIB,CD36,SCARB1,IL1A,MEF2C,TLR2,CXCL10,PABPN1,CD86,LTF,TLR6,TNFRSF1B,TNIP1,CCL2,PDE4B,NOS2,GIT1,IRF8,PLCG2,DAB2IP,TLR1,BMP6	turquoise	EC_M6
GO:0051402	3.24E-04	0.999836147	45	198	neuron apoptotic process	BP	1.96E-02	LRP1,AGAP2,ANGPT1,SRPK2,RB1,AXL,CTNNB1,AKT2,TMTC4,LIG4,PRNP,ILK,FAIM2,RASA1,APP,CX3CL1,TGFB2,CASP7,PIK3CA,KRAS,FAM162A,HYOU1,NCSTN,BACE1,FZD9,EGLN3,PRKCI,SIX4,PPARGC1A,CPEB4,NES,NGFR,TGFB3,PIN1,PARK7,AARS,HSPA5,NAE1,BNIP3,ATN1,VSTM2L,UBE2V2,GDNF,SIGMAR1,ZPR1	darkgreen	EC_M4
GO:0006939	3.25E-04	0.999878958	24	84	smooth muscle contraction	BP	1.97E-02	SRF,ROCK2,FKBP1B,ATP2B1,SULF1,ADA,ATP2B4,RAP1GDS1,CTTN,HTR2B,ADRA2C,COMP,ACTA2,CNN1,SETD3,MYOCD,ROCK1,ITGA2,DAPK3,NMUR1,GDNF,PIK3C2A,ORMDL3,PRKG1	darkgreen	EC_M4
GO:0046599	5.93E-04	0.999910017	8	20	regulation of centriole replication	BP	1.98E-02	PLK4,STIL,CEP295,CENPJ,ALMS1,BRCA1,TRIM37,CEP76	darkgrey	EC_M8
GO:0000462	5.93E-04	0.999876577	10	34	maturation of SSU-rRNA from tricistronic rRNA transcript (SSU-rRNA, 5.8S rRNA, LSU-rRNA)	BP	1.98E-02	NOL10,DHX37,UTP20,BMS1,RRS1,FP565260.1,HEATR1,NGDN,NOP14,UTP6	darkgrey	EC_M8
GO:0006284	5.95E-04	0.999863903	11	39	base-excision repair	BP	1.98E-02	RPA3,MBD4,PARP2,POLE,ERCC6,TDG,RPA2,DNA2,APEX2,RPA1,FEN1	darkgrey	EC_M8
GO:0006937	3.35E-04	0.999855908	31	121	regulation of muscle contraction	BP	2.02E-02	SRF,FKBP1B,SCN5A,ATP2B1,P2RX4,ATP2A2,MYL9,ADA,CTTN,PKP2,PPP1R12B,CASQ1,ADRA2C,KCNA1,CACNA1C,TPM1,ADRA1B,CNN1,CALM1,SETD3,MYOCD,ANXA6,ITGA2,DAPK3,ENO1,DMD,TMEM38B,TMEM38A,ORMDL3,HCN4,PRKG1	darkgreen	EC_M4
GO:1903829	3.36E-04	0.999819417	53	241	positive regulation of cellular protein localization	BP	2.02E-02	LRP1,TCP1,LEP,RTN4,GLI3,ROCK2,ITGB1,KIF5B,MARK4,PAK1,TGFB1,AKT2,GPD1L,ATP2C1,PRNP,HDAC3,DDRGK1,RIC3,TM9SF4,PINX1,SORBS1,CCT8,CACNB3,GNL3,ATG13,TSG101,DKC1,BAP1,CHP1,ATP2B4,GSK3B,LARP7,IQGAP1,TFRC,MAPT,PRKCI,MSN,SYT11,NGFR,RAB11A,NPTN,MIEF2,AKT1,PRKAA1,MYO1C,FLNA,PARK7,LEPROT,ZPR1,ORMDL3,TOMM7,MESD,MAP1A	darkgreen	EC_M4

GO:0009059	1.75E-05	0.999986782	459	3569	macromolecule biosynthetic process	BP	2.03E-02	NFATC1,ZNF236,TSHZ1,ORMDL2,PYM1,SMARCC2,STAT6,ATG12,MAP3K5,CDK4,CITED2,MAML1,SQSTM1,IRAK3,HELB,GFPT2,OSR2,HSPA1B,EHMT2,ZBTB12,HDAC10,ALG12,ATF6B,EIF3H,DSE,RXRB,RING1,RPS18,B3GALT4,ZHX2,ZBTB22,PMM1,MYC,TOB2,SMAD5,EP300,KDM3B,ZNF76,EEF1D,RPL3,DDX17,EIF3L,NFIB,GPAA1,EIF3D,HSF1,CCND3,TRERF1,CDKN2A,RPL8,HMOX1,WASL,KLF9,POLR1C,PIGO,JAK2,ZBTB45,RPS5,ZNF135,ZNF667,ALG2,ZNF865,RPL28,RPS9,EEF1A1,MALSU1,EFEMP1,UGP2,ZNF692,RPS7,ANXA4,AKNA,MED25,IL4I1,ING3,ARHGEF5,AP2A1,IRF3,LPIN1,RPS11,FOXO3,KLF15,SPHK2,RPL18,NOP53,ABTB1,MAML2,BICRA,KMT2E,BBS7,MEIS3,FKRP,TRIM13,IRF2BP1,SIX5,FOSB,ZNF746,NR2C2,ZNF862,TSC22D1,SERP2,RPS8,ZNF575,MIF4GD,NAA16,MLH1,GTF3C2,UNK,ZNF574,GALNT11,WBP2,KMT2C,MYD88,TADA3,CSGALNACT1,RPS3,TEP1,BRPF1,DYRK1B,EID2,ZNF219,RPS16,MED29,SAMD4B,MRPS12,SIRT2,RPTOR,RPL15,APBB3,EIF3K,NUP98,GALNT15,TRIM21,CYP1B1,HGF,APBB1,TAF10,DEAF1,HOXC4,CLIP3,NCOA4,LIN37,TARBP2,KMT2B,EIF3F,RBCK1,RARG,SDR16C5,ZNF740,EIF4B,LMO2,POFUT1,NECAB3,URI1,PIK3R1,NCOA2,ATF1,TNKS1BP1,RPS3A,POLE4,KDM2B,ZFHX4,CAMKK2,SCAND1,EIF2A,SETD6,MCPH1,SSBP2,FNIP2,ARL2BP,SHOX2,NEK4,ZHX3,VDR,MMS19,LPIN3,CHST14,FOXF2,F2RL1,SORBS3,ORMDL1,HNF4A,TP53INP1,FARS2,PIDD1,RPLP2,HR,PIGT,ACOT8,PCIF1,TRIM45,NPM3,WARS2,PARP3,FASTKD3,ELK4,LGR4,ICE1,IRS1,MRPL36,SP140,TCF7L2,DIS3L2,PUM1,TXNIP,APEH,AIP,SOX5,ADNP,RPS6KB2,ZNF217,QARS,PIGM,DTX3L,GCNT7,DALRD3,PARP14,EIF4H,ECM1,RCOR3,ARNT,PRMT	cyan	EC_M2
GO:0009890	6.19E-04	0.999559084	150	1222	negative regulation of biosynthetic process	BP	2.06E-02	PAN2,BCLAF1,FBXO5,ZNF608,SCAF8,EIF3E,MRPL13,XRCC6,TNRC6B,TUT7,POT1,RFX3,TRIM28,ZNF451,PNPT1,SYNCRIP,XPO1,CASP8AP2,TIA1,ZNF350,EZH2,FAM3C,WDR35,DHFR,PUS7,HDAC2,EXOSC9,DNAJC2,SETDB2,CNOT10,RSF1,DIS3,BIRC5,GTPBP4,CHD8,KAT6A,METTL3,RIF1,E2F7,TAF7,EXOSC8,NR2C1,BRCA2,ELK3,TCERG1,CBX5,PAN3,CAPRIN1,ZC3H8,LSM14A,BTAF1,TDG,NCK1,CGGBP1,XRN1,PDE12,SAP30,ATR,TERF1,GCFC2,CNOT1,GABPA,PPID,RBL1,E2F8,TRIM33,ZBED6,ZNF248,MDM4,HIVEP1,CUL3,GMNN,NFKB1,KHDRBS1,NCL,PRMT5,AJUBA,BPTF,FXR1,DDX5,ORC2,BLM,DDX6,CENPF,SP3,HNRNPR,DDX20,TPR,CCAR1,XRCC5,NPAT,BRCA1,CNOT9,NSD2,TBL1XR1,SUV39H1,FOXM1,EIF2AK1,LYAR,FAM220A,HNRNPU,PDS5A,WAPL,NEDD4,SMARCE1,RFC1,CDC6,DCP2,NSD1,EXOSC10,CLK2,USP3,AURKB,TAF1,RLIM,ATRX,TIPIN,MBTD1,TRIM37,LARP1,PLK1,ILF3,AATF,PSMD10,TYMS,ZNF3,MIER1,NIPBL,PHF6,UHRF1,FMR1,METTL16,EHMT1,EXOSC2,SCAI,CTCF,DEPDC1,HNRNPD,ZNF684,DRAP1,ZNF614,RBAK,BRMS1L,RNF139,C1D,RNF8,ZNF558,IKZF5,PAIP2,ZNF529	darkgrey	EC_M8
GO:0043666	3.49E-04	0.999902101	16	47	regulation of phosphoprotein phosphatase activity	BP	2.08E-02	NUAK1,ROCK2,FKBP1B,PPP1R12A,HSP90B1,CRY2,SLC39A10,PPP6R3,GSK3B,PPP1R11,HTT,CALM1,PPP4R4,PDGFRB,ROCK1,FKBP1A	darkgreen	EC_M4
GO:0043069	3.50E-04	0.999764258	124	681	negative regulation of programmed cell death	BP	2.08E-02	SERPINB2,LRP1,AHI1,AGAP2,IER3,CDSN,RRM2B,ANGPT1,PPARA,PPARD,CTNNA1,LEP,BAG1,VEGFA,DOCK8,CREB3,ASNS,TGFBR1,TAX1BP1,GLI3,ITGB1,RB1,TPT1,GLI2,AXL,CTNNB1,AKT2,UFM1,LIG4,IRS2,PRNP,HDAC3,DDRGK1,ILK,CXCL12,HSP90B1,SLC25A4,FAIM2,RASA1,CX3CL1,OPA1,AMIGO2,ACVR1,MTDH,ADA,TXNDC5,SLC39A10,TJP1,NOTCH2,CTTN,CDC73,SELENOS,EYA2,HTR2B,GSK3B,FSTL1,PIK3CA,LEF1,PRKCA,KRAS,BMP7,HYOU1,FGFR2,PEA15,TFRC,FZD9,RPS6KA3,HSPB1,SERPINE1,SYVN1,PDCD10,ITGAV,COMP,HTT,FKBP8,PRKCI,ARAF,TIMP1,VDAC2,FMN2,SIX4,MNAT1,ERC1,GHITM,CLN8,MED1,PPARGC1A,CPEB4,NES,ARHGEF2,TGFB3,SHC1,TAF9B,MYOCD,PIN1,CD74,PDGFRB,AKT1,PRKAA1,CD40LG,MYDGF,TNFRSF18,FLNA,PARK7,ENO1,GNAI3,CFDP1,AARS,HSPA5,BCL10,MIR24-1,BNIP3,VSTM2L,UBE2V2,ARF4,GDNF,GLO1,CDKN1A,PTTG1IP,TSLP,ZPR1,ORMDL3,CHST11,FGF10	darkgreen	EC_M4
GO:0006119	1.96E-05	0.999992815	32	119	oxidative phosphorylation	BP	2.11E-02	UQCRQ,NDUFB2,UQCRH,NDUFB6,NDUFC2,NDUFA2,COX7A1,COQ9,DGUOK,NDUFV1,NDUFS2,DNAJC30,CHCHD10,UQCC3,NDUFB7,NUPR1,NDUFA7,UQCR11,NDUFS7,ATP5F1D,TAZ,MT-ND1,MT-ND2,MT-CO1,MT-CO2,MT-ATP6,MT-CO3,MT-ND3,MT-ND4L,MT-ND4,MT-ND5,MT-CYB	cyan	EC_M2
GO:0030705	3.67E-04	0.999820233	41	173	cytoskeleton-dependent intracellular transport	BP	2.17E-02	AP3S1,RAB21,SUN2,DYNC1I1,DST,MGARP,KIF5B,COPG1,TRAK1,ACTN4,MAP1B,APP,OPA1,AP3B1,IFT57,ARL3,FEZ1,HSPA8,DYNC1I2,KIF1A,TANC2,HSPB1,MAPT,HTT,ACTR10,MYO5A,HAP1,MYO1E,IFT43,ARMCX3,MYO1D,MYO5B,MYO10,MYO1C,PAFAH1B1,VAMP7,HSBP1,RAB6A,BLOC1S2,TUBA1C,MAP1A	darkgreen	EC_M4

GO:0032885	3.71E-04	0.999920429	12	30	regulation of polysaccharide biosynthetic process	BP	2.19E-02	DYRK2,TGFB1,AKT2,IRS2,SORBS1,SELENOS,GSK3B,PPP1R3D,PPP1R3F,CLTC,AKT1,HAS2	darkgreen	EC_M4
GO:0031640	2.89E-04	0.999960785	8	20	killing of cells of other organism	BP	2.22E-02	LYZ,SYK,P2RX7,LTF,SCNN1B,NOS2,FCER2,SERPINB9	turquoise	EC_M6
GO:0034645	2.25E-05	0.999982944	454	3533	cellular macromolecule biosynthetic process	BP	2.26E-02	NFATC1,ZNF236,TSHZ1,PYM1,SMARCC2,STAT6,ATG12,MAP3K5,CDK4,CITED2,MAML1,SQSTM1,IRAK3,HELB,GFPT2,OSR2,HSPA1B,EHMT2,ZBTB12,HDAC10,ALG12,ATF6B,EIF3H,DSE,RXRB,RING1,RPS18,B3GALT4,ZHX2,ZBTB22,PMM1,MYC,TOB2,SMAD5,EP300,KDM3B,ZNF76,EEF1D,RPL3,DDX17,EIF3L,NFIB,GPAA1,EIF3D,HSF1,CCND3,TRERF1,CDKN2A,RPL8,HMOX1,WASL,KLF9,POLR1C,PIGO,JAK2,ZBTB45,RPS5,ZNF135,ZNF667,ALG2,ZNF865,RPL28,RPS9,EEF1A1,MALSU1,EFEMP1,UGP2,ZNF692,RPS7,ANXA4,AKNA,MED25,IL4I1,ING3,ARHGEF5,IRF3,LPIN1,RPS11,FOXO3,KLF15,RPL18,NOP53,ABTB1,MAML2,BICRA,KMT2E,BBS7,MEIS3,FKRP,TRIM13,IRF2BP1,SIX5,FOSB,ZNF746,NR2C2,ZNF862,TSC22D1,SERP2,RPS8,ZNF575,MIF4GD,NAA16,MLH1,GTF3C2,UNK,ZNF574,GALNT11,WBP2,KMT2C,MYD88,TADA3,CSGALNACT1,RPS3,TEP1,BRPF1,DYRK1B,EID2,ZNF219,RPS16,MED29,SAMD4B,MRPS12,SIRT2,RPTOR,RPL15,APBB3,EIF3K,NUP98,GALNT15,TRIM21,CYP1B1,HGF,APBB1,TAF10,DEAF1,HOXC4,CLIP3,NCOA4,LIN37,TARBP2,KMT2B,EIF3F,RBCK1,RARG,SDR16C5,ZNF740,EIF4B,LMO2,POFUT1,NECAB3,URI1,PIK3R1,NCOA2,ATF1,TNKS1BP1,RPS3A,POLE4,KDM2B,ZFHX4,CAMKK2,SCAND1,EIF2A,SETD6,MCPH1,SSBP2,FNIP2,ARL2BP,SHOX2,NEK4,ZHX3,VDR,MMS19,LPIN3,CHST14,FOXF2,F2RL1,SORBS3,HNF4A,TP53INP1,FARS2,PIDD1,RPLP2,HR,PIGT,ACOT8,PCIF1,TRIM45,NPM3,WARS2,PARP3,FASTKD3,ELK4,LGR4,ICE1,IRS1,MRPL36,SP140,TCF7L2,DIS3L2,PUM1,TXNIP,APEH,AIP,SOX5,ADNP,RPS6KB2,ZNF217,QARS,PIGM,DTX3L,GCNT7,DALRD3,PARP14,EIF4H,ECM1,RCOR3,ARNT,PRMT2,RIPK3,GABPB2,PER2,ALG3,TWIST2,	cyan	EC_M2
GO:0051240	3.86E-04	0.999720722	182	1050	positive regulation of multicellular organismal process	BP	2.27E-02	IL23A,LRP1,RAB21,PLXNB2,FBN2,HLA-DRA,FAM49B,ADGRB1,LEP,SRF,VEGFA,RUNX2,UNC13B,PDCL3,REST,NLRP5,TGFBR1,SOCS5,RIMS1,RTN4,TPBG,LRRC4B,EMC10,MAP3K7,ACTR2,GLI3,ROCK2,INHBA,RRAS,ITGB1,PLXNA1,FGF2,PANX1,RB1,TENM4,GLI2,MEGF8,SMARCD3,RHEB,PAK1,TGFB1,AXL,CTNNB1,IL15RA,TRAK1,LBH,C1QTNF1,STIM1,LIG4,ATP2B1,LUM,HDAC3,ZNF703,ILK,CXCL12,IL20RB,EIF4G2,SULF1,VAMP8,ROBO1,MAP1B,ATP2A2,CELF1,APP,DHX36,CX3CL1,ABCC8,PTPN11,OPA1,LOXL2,AP3B1,AMIGO2,ZFYVE27,PTPN22,ACVR1,GDF6,POLR3D,MTDH,ADA,BMP1,RUNX1,PLA2R1,TWF1,CBLB,RAB7B,CHRNA7,ADIPOR1,TJP1,KDM5B,NOTCH2,CTTN,DNM1L,HYAL1,CCND1,CAPRIN2,KPNA6,TGFB2,PRKAB2,HTR2B,MMP14,DAG1,HADH,LEF1,PRKCA,TLR5,CMKLR1,BMP7,FGFR2,VASH2,OSM,TP53BP1,TFRC,FZD9,HSPB1,MAPT,SERPINE1,ATP6AP2,TNR,FN1,ADRA2C,LRPAP1,PRKCI,SKIL,SIX4,OBSL1,AKT3,HAP1,MAGED1,BMPR1A,NLGN2,MED1,PPARGC1A,NPPB,ARHGEF2,TGFB1I1,NGFR,TPM1,COL1A1,TMED10,TGFB3,RAB11A,ADRA1B,MAP2K1,PKM,DICER1,NPTN,MYOCD,ISLR2,PIN1,SCAMP5,TRPV2,CD74,ROCK1,AKT1,ITGA2,PRKAA1,OMA1,CD40LG,MYDGF,PLXNB3,PAFAH1B1,IRAK1,ATP6AP1,PLXNA3,VAMP7,PARK7,ENO1,F3,BCL10,SERPINF1,LRRC24,PEMT,UBE2V2,GDNF,FCN1,GLIPR2,ZNF322,TSLP,OLFM1,WNT2,LRRTM2,FGF10	darkgreen	EC_M4
GO:0001824	6.90E-04	0.999760372	18	86	blastocyst development	BP	2.28E-02	LATS1,RRP7A,NASP,NCAPG2,GINS4,BRCA2,CNOT1,GABPA,NBN,ZBED6,CUL3,CHEK1,NEK2,SP3,ASF1B,NLE1,PHF6,PALB2	darkgrey	EC_M8
GO:0072711	6.98E-04	0.999952593	5	9	cellular response to hydroxyurea	BP	2.30E-02	RAD51,BLM,DDX11,ATRX,FMR1	darkgrey	EC_M8

GO:0042592	3.97E-04	0.999704934	219	1295	homeostatic process	BP	2.32E-02	LRP1,SLC12A2,ANGPT1,SLC39A7,NUS1,SMDT1,PPARD,LGALS1,TXN2,SH3GL2,SLC39A4,LEP,PLAA,LARGE1,SLC30A9,SRF,VEGFA,UNC13B,CNNM4,ERP44,RTN4,MFSD2A,SLC31A1,WHRN,NCDN,INHBA,RCN3,SLC7A11,ITGB1,FKBP1B,FGF2,KIF5B,SESN3,RAB7A,ATP7B,RB1,ABHD5,PICALM,TPT1,RHEB,AXL,SCN5A,WDR48,CTNNB1,DGAT2,GPD1L,C1QTNF1,SLC4A7,ACTG1,TMTC4,MAFG,STIM1,ATP2C1,IRS2,PRNP,ATP2B1,DCN,HDAC3,CCKBR,UBA5,RIC3,LMO1,CXCL12,TM9SF4,HSP90B1,IL20RB,TP53INP2,OSBP,C19orf12,SLMAP,ATP1B3,LNPEP,RYR3,P2RX4,SELENOT,ATP2A2,APP,SLC4A5,CX3CL1,ABCC8,HECTD4,CACNB3,PTPN11,ADCY6,PFKM,HOMER1,OPA1,AP3B1,CRY2,ADA,GLS,BAP1,CHP1,ATP2B4,SPP1,PKD2,TMEM14C,TENT4B,BRSK2,NDN,SLC39A10,CHRNA7,RAP1GDS1,DOCK10,CNNM2,DYNLL1,ADIPOR1,TJP1,GLRX2,SELENOS,SLC2A10,ZBTB20,PRKAB2,LRP5,HTR2B,FSTL1,PIK3CA,HADH,ISCU,PRKCA,CMKLR1,HJV,KRAS,SLAMF8,ADCY5,VAPB,WDTC1,CASQ1,NCSTN,STRAP,CLIC4,TFRC,FZD9,SIDT2,GLRX3,PRDX4,TMCO1,HSPB1,ATP6AP2,COMT,TFR2,PTPN6,GPR137,ACOX3,ITGAV,HTT,ERO1A,SKIL,PTPRN,VCL,KCNA1,FBXL5,AKT3,HAP1,CACNA1C,SLC2A4,CLN8,STIM2,MED1,PPARGC1A,NPPB,SLC25A44,NGFR,DNASE2,CACNA1G,ADRA1B,GPR65,CLN6,CALM1,SMARCA4,GRIA1,NPTN,SLC39A6,ANXA6,TRPV2,CD74,AKT1,PRKAA1,PTPN2,NPR3,OMA1,MYO5B,GNB1,BCAP31,ABCA3,PKD1,NPHP4,ATP6AP1,SSTR5,PARK7,DEF8,SYPL2,JPH3,SLC30A7,BCL10,ATP6V0D1,DMD,FKBP1A,SERPINF1,BNIP3,ADAMTS5,TMEM38B,SLC22A17,LOX,PEMT,SNX10,THY1,HAS2,TXN,TMEM38A,FAM210B,CCR6,OCIAD2,ORMDL3,SLC39A3,HCN4,AT	darkgreen	EC_M4
GO:0008361	4.03E-04	0.999807881	38	157	regulation of cell size	BP	2.35E-02	LRP1,RAB21,SLC12A2,SRF,VEGFA,RTN4,MEGF8,PAK1,SEMA3C,ILK,RYK,RNF6,CXCL12,EIF4G2,MAP1B,DIP2B,SEMA4F,ZFYVE27,SPP1,SEMA4G,CTTN,GSK3B,WDTC1,MAPT,TNR,FN1,AKT3,CLN8,MSN,RAB11A,ISLR2,TRPV2,AKT1,PAFAH1B1,PLXNA3,BARHL2,TMEM123,OLFM1	darkgreen	EC_M4
GO:0040001	7.16E-04	0.999850025	10	31	establishment of mitotic spindle localization	BP	2.36E-02	CENPA,CLASP2,ESPL1,NUSAP1,KPNB1,SPDL1,PLK1,NDC80,SAPCD2,MAD2L1	darkgrey	EC_M8
GO:0043270	4.12E-04	0.999794452	42	182	positive regulation of ion transport	BP	2.39E-02	SLC12A2,LEP,ITGB1,KIF5B,RAB3GAP1,SCN5A,GPD1L,ACTN4,STIM1,ATP2B1,CXCL12,FXYD1,VAMP8,ATP1B3,P2RX4,CACNA1D,CX3CL1,ABCC8,CACNB3,HOMER1,CHP1,PKD2,PLA2R1,PKP2,CASQ1,HTT,KCNA1,HAP1,STIM2,KCNMB1,CALM1,VMP1,TRPV2,PDGFRB,AKT1,FLNA,VAMP7,AHCYL1,DMD,GDNF,THY1,TMSB4X	darkgreen	EC_M4
GO:0048705	4.18E-04	0.999789817	43	186	skeletal system morphogenesis	BP	2.42E-02	NAB2,FBN2,RUNX2,RAB23,TGFBR1,GLI3,MEGF8,WDR48,CTNNB1,SERPINH1,RARB,RYK,IMPAD1,RDH10,MMP2,BMP1,CHSY1,HYAL1,LRP5,SATB2,MMP14,BMP7,FGFR2,ARID5B,SGPL1,COMP,FGFR3,PRRX1,TULP3,SIX4,THBS3,COL1A1,CHAD,TGFB3,ANXA6,PKD1,ANKRD11,COL11A1,GLG1,HAS2,OTOR,SLC39A3,CHST11	darkgreen	EC_M4
GO:0031532	3.18E-04	0.999882354	23	94	actin cytoskeleton reorganization	BP	2.43E-02	PARVG,BAIAP2L2,TEK,KIT,CD2AP,THSD7A,NRP1,RAPGEF3,IQGAP2,NEDD9,DTNBP1,ESAM,HCLS1,FES,F11R,ARHGDIB,MCU,BAIAP2L1,ABI3,CDC42BPB,S1PR1,GMFG,RALA	turquoise	EC_M6
GO:0072396	7.48E-04	0.999954054	5	8	response to cell cycle checkpoint signaling	BP	2.44E-02	ZMPSTE24,APAF1,SMC1A,EME1,TIGAR	darkgrey	EC_M8
GO:0072402	7.48E-04	0.999954054	5	8	response to DNA integrity checkpoint signaling	BP	2.44E-02	ZMPSTE24,APAF1,SMC1A,EME1,TIGAR	darkgrey	EC_M8
GO:0072423	7.48E-04	0.999954054	5	8	response to DNA damage checkpoint signaling	BP	2.44E-02	ZMPSTE24,APAF1,SMC1A,EME1,TIGAR	darkgrey	EC_M8
GO:0046321	4.38E-04	0.99997027	6	9	positive regulation of fatty acid oxidation	BP	2.53E-02	PPARA,PPARD,AKT2,IRS2,CPT1A,PPARGC1A	darkgreen	EC_M4
GO:0034330	3.33E-04	0.999789411	92	541	cell junction organization	BP	2.53E-02	AFDN,MTSS1,PTK2,PICK1,TEK,LINGO2,LIMCH1,TJP2,ADGRF1,KDR,SEMA4D,DLGAP3,GJA4,SDCBP,NRCAM,NRP1,MTMR2,PLXND1,ITGB4,SETD5,IKBKB,SFRP1,THSD1,EFNB2,ARHGEF7,P2RX2,EPHB1,MARVELD2,OCLN,MEF2C,TLR2,GPM6A,SEPT11,HIPK1,TANC1,SPARCL1,JAM3,SNCA,SHANK2,SEMA3F,ESAM,CLDN34,CDH24,PECAM1,F11R,IL10RA,ACE,ITGA6,PAK2,CNKSR2,DLG1,APOD,BCR,IL1RAP,RAMP2,DNM3,PPM1F,PRKCH,RAC1,RCC2,APBB2,JUP,FSCN1,EPHA2,SNCG,CHRNB1,LGI2,ZC4H2,OPHN1,PSEN1,SEMA4A,ABI3,ITGA3,TAOK2,ARHGEF15,EFNA1,CHRNB2,ZDHHC15,COL4A5,DSG2,PATJ,SLITRK2,RAPGEF1,DAB2IP,ADGRL2,CDH5,SLC8A3,RELN,ABHD17C,SHANK3,PERP,BMP6	turquoise	EC_M6
GO:0002040	3.39E-04	0.999864754	26	114	sprouting angiogenesis	BP	2.56E-02	SEMA6A,FLT4,TEK,KDR,NRP1,GATA2,EFNB2,ADAMTS9,PLK2,NR4A1,SMAD1,VEGFC,PPP1R16B,DLL4,MAP3K3,CLEC14A,EPHB4,RAMP2,RHOJ,EPHA2,MMRN2,PGF,JAK1,NRARP,NOTCH1,MIR23A	turquoise	EC_M6

GO:0007267	3.49E-04	0.999751905	165	1146	cell-cell signaling	BP	2.62E-02	TNFRSF11A,KIF5A,PDE7B,TNFAIP3,STX11,AKAP12,SNCAIP,FZD6,CTHRC1,RIMS2,CELSR1,AFDN,PACSIN2,TCF7,RNF146,ATF4,PDGFB,CSNK1E,PICK1,MDFI,TEK,APBA1,KIT,SYK,ICA1,IL6,PPT1,GRB10,GRIK2,DLGAP3,GJA4,IL15,FZD8,CCNY,SDCBP,TRPM4,NRP1,RAB3B,MTMR2,GATA3,FCHSD2,SFRP1,PLCB4,NOTUM,FERMT1,EFNB2,SPEF1,FCHSD1,PCDH1,P2RX2,SIRT3,EPHB1,PLK2,MERTK,CHD7,FAM3D,NR4A1,ABHD6,HHEX,CLCN3,MCTP1,CHRM5,SOX7,MEF2C,TLR2,WWTR1,IL7,P2RX7,GUCY1A1,GUCY1B1,CXCL10,LMBR1L,DHH,PLCG1,KCNJ3,NR4A2,DGKZ,STAB1,SOX13,EDN1,SNCA,DTNBP1,PRDM15,ABCG1,SHANK2,HDAC1,PPP3CA,ANO1,SLC12A7,MCTP2,HCRTR1,BCL9,SNAP23,CD34,ADCY1,RPS6KA1,VANGL2,YTHDF1,INSYN2,SENP2,DLG1,PTCHD1,CREB1,BCR,ADORA2A,IL1RAP,DDX3X,MCU,CDK16,SNAP29,FGF23,TMOD2,RAC1,HIF1A,JUP,SNCG,SYT7,MCC,CHRNB1,TNFRSF1B,RGS14,FBXL20,MTOR,AR,OPHN1,EFNB1,PSEN1,FBXW11,FGF18,ITGA3,PGF,EFNA1,CHRNB2,GPR68,CCDC88C,SMAD2,TLE3,ADRB2,CCL2,NOS2,UNC119,GIT1,NRARP,NOTCH1,PLCG2,STXBP1,DAB2IP,EXOC3L1,ZBTB33,BCL7B,SLC8A3,ADGRG1,SOX17,RELN,LYPD6,FGF16,GRAP,SCN2B,SHANK3,RGS4,STX3,BMP2,CACNG5,SRD5A2,BMP6	turquoise	EC_M6
GO:0043269	3.55E-04	0.999787143	73	444	regulation of ion transport	BP	2.65E-02	TNFRSF11A,TRDN,DAPK1,PDGFB,SYK,TSPAN13,TCAF2,RAB3B,PIK3CG,KCNRG,CXCR4,SPTBN4,P2RY6,KCNK6,P2RX2,PDE4D,CHD7,MEF2C,P2RX7,CXCL10,TPCN1,PLCG1,STC1,KCNJ3,ANO6,CLIC6,EDN1,KCNJ15,SNCA,DTNBP1,PM20D1,SIK1,HECW2,KCNJ2,SCN3A,EPB41,PRKD1,DLG1,KCNK7,B2M,ADORA2A,CYBB,HOMER3,WNK4,FGF23,CRACR2A,SNCG,SYT7,MTOR,PSEN1,CHRNB2,ATP2A1,CD19,DNM2,ADRB2,CCL2,KCNJ18,PDE4B,SLC43A2,PLCG2,STXBP1,CA2,RELN,KCNH2,SCN2B,KCNK5,SHANK3,RGS4,KCNA6,CACNG5,FXYD6,KCNG2,GSG1L	turquoise	EC_M6
GO:0032956	3.60E-04	0.999802097	55	304	regulation of actin cytoskeleton organization	BP	2.65E-02	CELSR1,MTSS1,ARHGAP18,BAIAP2L2,PICK1,CDC42EP1,TEK,LIMCH1,CD2AP,TMOD1,FAM49A,NRP1,SVIL,SPTBN4,FCHSD2,SFRP1,FCHSD1,LMOD3,HIP1R,RAPGEF3,IQGAP2,EDN1,JAM3,ESAM,HCLS1,AP1AR,FES,PROX1,TMOD4,F11R,ARHGDIB,PAK2,DLG1,BAIAP2L1,PPM1F,WAS,TMOD2,RAC1,FSCN1,MTOR,ABI3,TAOK2,ARHGEF15,ARHGAP28,SPIRE1,S1PR1,CARMIL2,VASP,GMFG,KANK3,SHANK3,RGS4,GBA2,BST2,ARPC5L	turquoise	EC_M6
GO:0009888	3.60E-04	0.999736467	197	1389	tissue development	BP	2.65E-02	ZNF516,SEMA6A,FZD6,CTHRC1,CELSR1,NOTCH4,AFDN,MTSS1,KLHL3,ATF4,PDGFB,PODXL,PIM1,TJP2,PGM5,KDR,SEMA4D,TMOD1,IL6,CAV2,GJA4,TEAD2,SDCBP,MYCN,TRPM4,NRP1,RASIP1,SVIL,TAL1,PRKD2,GPR4,PLXND1,ITGA8,NOS3,ITGB4,PPARG,GATA3,ACVR2B,MYO7A,CXCR4,GZF1,EDNRB,TIE1,IKBKB,JAG1,PDE2A,GPAT4,SFRP1,HBEGF,NR1D2,NOTUM,FERMT1,FREM2,EFNB2,SPEF1,P2RX2,NTN4,LMOD3,SYNE4,ADAMTS9,TCF15,EPHB1,RET,PDE4D,TNFRSF19,CHD7,IGF1,NR4A1,EIF2AK3,SMAD1,MARVELD2,SOX7,MEF2C,WWTR1,P2RX7,VEGFC,NDRG4,CXCL10,TLL2,BMP2K,TIPARP,USH1C,PPP1R16B,STC1,RAPGEF3,ANO6,DLL4,EDN1,STAT1,JARID2,ETS2,DPPA4,SIK1,HDAC1,PPP3CA,HYAL2,MEF2A,SEMA3F,ROBO4,BCL9,NRAP,PRKX,HLX,SEC24B,JHY,CD34,PECAM1,PLXNA2,POU2F3,BMX,FGR,CFLAR,TGM1,PROX1,VANGL2,LAMA5,F11R,ITGA6,CST6,APOLD1,LAMC2,DLG1,ELL3,HOXD10,CREB1,LTF,BCR,SEMA6D,BCOR,ADAMTS7,ITGA4,IGFBP5,ETV4,TBX1,HSPG2,WNK4,MMP13,LDB2,PROM1,FGF23,WAS,PRKCH,RAC1,HIF1A,ZBTB18,EPHA2,CECR2,MPP5,LFNG,ARG2,ALDH1A2,MTOR,AR,OPHN1,EFNB1,PSEN1,HOXB5,HOXB7,SEMA4A,FGF18,ITGA3,EFNA1,CCDC88C,SMAD2,YY1,CYP1A1,DSG2,ADRB2,JAG2,MRTFB,SEMA6B,RFLNB,ISG15,NRARP,NOTCH1,RAPGEF1,S1PR1,SLC25A25,CNN3,ENG,DAB2IP,CARMIL2,CDH5,MIR23A,ARID4A,VASP,SOX17,SOX18,MAFF,RGS4,TRIM62,DUSP5,TBC1D20,RALA,BMP2,PIFO,BMP6	turquoise	EC_M6
GO:2000146	3.61E-04	0.999815765	44	234	negative regulation of cell motility	BP	2.65E-02	AFDN,LIMCH1,C5,LRCH1,DACH1,PPARG,GATA3,TIE1,JAG1,SFRP1,ADAMTS9,ABHD6,MCTP1,MEF2C,NDRG4,ANGPT2,STC1,DLL4,SRGAP2C,CD200,HYAL2,RIN3,AP1AR,IGFBP3,ARHGDIB,APOD,SEMA6D,IGFBP5,WAS,RAC1,JUP,ADIPOR2,MMRN2,MCC,VASH1,MMP28,CCL2,STK26,ARHGAP4,NOTCH1,ENG,DAB2IP,ADGRG1,BST2	turquoise	EC_M6
GO:0002443	3.64E-04	0.999819222	41	221	leukocyte mediated immunity	BP	2.65E-02	DDX58,KIT,SYK,IL6,RSAD2,TLR4,C5,TRPM4,PIK3CG,GATA2,CTSC,NECTIN2,GAB2,UNC13D,GATA3,JAG1,GAPT,RNF19B,SNAP23,LYST,INPP5D,FGR,FES,ACE,B2M,WAS,RAB27A,TNFRSF1B,BATF,CD19,SCNN1B,LGALS9,NOS2,IL7R,TRAF2,PLCG2,STXBP1,CD46,FCER2,SERPINB9,BST2	turquoise	EC_M6

GO:0030036	3.64E-04	0.999766325	97	600	actin cytoskeleton organization	BP	2.65E-02	PHACTR2,CELSR1,PARVG,PACSIN2,MTSS1,ARHGAP18,BAIAP2L2,PICK1,CDC42EP1,TEK,LIMCH1,PGM5,KIT,CD2AP,THSD7A,TMOD1,ARHGAP25,ELMO1,SDCBP,FAM49A,NRP1,SVIL,MSRB2,FGD5,LLGL2,MYO7A,SPTBN4,IKBKB,FCHSD2,SFRP1,FCHSD1,ARHGAP26,FGD6,LMOD3,HIP1R,FHDC1,USH1C,RAPGEF3,IQGAP2,NEDD9,EDN1,JAM3,DTNBP1,NUAK2,GAS2,MEF2A,NRAP,ESAM,HCLS1,AP1AR,EPB41,CFLAR,FES,PROX1,TMOD4,F11R,ARHGDIB,LIMK2,PAK2,DLG1,MCU,BAIAP2L1,PPM1F,WAS,TMOD2,RAC1,RHOJ,FSCN1,SHROOM4,MICAL3,FGD1,MTOR,OPHN1,STARD8,ABI3,TAOK2,ARHGEF15,CDC42BPB,AMOT,ABLIM3,INF2,ARHGAP28,SPIRE1,MYO18A,RFLNB,S1PR1,CNN3,CARMIL2,VASP,GMFG,KANK3,SHANK3,RGS4,GBA2,RALA,BST2,ARPC5L	turquoise	EC_M6
GO:1900115	4.74E-04	0.999967352	6	9	extracellular regulation of signal transduction	BP	2.71E-02	FBN2,LTBP1,FBN1,LRPAP1,NBL1,GREM2	darkgreen	EC_M4
GO:1900116	4.74E-04	0.999967352	6	9	extracellular negative regulation of signal transduction	BP	2.71E-02	FBN2,LTBP1,FBN1,LRPAP1,NBL1,GREM2	darkgreen	EC_M4
GO:0008589	4.77E-04	0.999831375	21	71	regulation of smoothened signaling pathway	BP	2.72E-02	CTNNA1,VCP,RUNX2,GLI3,RB1,PTCH2,KCTD21,GLI2,MEGF8,TCTN1,UCHL5,CHSY1,ARMC9,FGFR2,PRRX1,GORAB,TULP3,EVC,RAB34,MGRN1,FGF10	darkgreen	EC_M4
GO:0009187	3.76E-04	0.999916853	12	33	cyclic nucleotide metabolic process	BP	2.73E-02	PDE7B,PDE2A,PDE4D,GUCY1A1,GUCY1B1,ADCY2,PDE9A,ADCY1,ADCY4,EPHA2,NPR1,PDE4B	turquoise	EC_M6
GO:0035195	8.39E-04	0.999682119	20	107	gene silencing by miRNA	BP	2.74E-02	TNRC6B,TUT7,XPO5,HNRNPA2B1,PUS10,ZMPSTE24,METTL3,CNOT1,RIPK1,AJUBA,FXR1,DDX5,TIAL1,DDX6,DHX9,DGCR8,ZC3H7A,DROSHA,FMR1,SRRT	darkgrey	EC_M8
GO:1903557	3.91E-04	0.999869938	19	72	positive regulation of tumor necrosis factor superfamily cytokine production	BP	2.83E-02	IFNGR1,AKAP12,DDX58,SYK,IL6,TLR4,CD36,IL1A,TLR2,OAS3,HDAC1,CD86,CYBB,TMEM106A,PSEN1,LGALS9,PLCG2,OAS1,TLR1	turquoise	EC_M6
GO:0033314	8.71E-04	0.999937642	5	9	mitotic DNA replication checkpoint signaling	BP	2.84E-02	CLSPN,ORC1,TOPBP1,TICRR,CDC6	darkgrey	EC_M8
GO:0048015	3.98E-04	0.99982784	30	133	phosphatidylinositol-mediated signaling	BP	2.86E-02	PTK2,PDGFB,TEK,PIP5K1B,KIT,KDR,SEMA4D,KBTBD2,PIK3CG,GAB2,GATA3,PLCB4,PRR5L,IGF1,PITPNM2,PREX2,PPP1R16B,PLCB2,PIK3C2B,EDN1,NCF1,HCLS1,FGR,INPP5F,PLEKHA1,SELP,PEAR1,PIK3R5,DAB2IP,RELN	turquoise	EC_M6
GO:0051648	5.05E-04	0.9997583	37	154	vesicle localization	BP	2.87E-02	AP3S1,DYNC1I1,STARD3NL,KIF5B,PICALM,PCDH17,CTNNB1,TRAK1,ACTN4,PCLO,TMED2,USO1,EXOC6B,AP3B1,CHP1,BRSK2,WIPI1,SHROOM2,KIF1A,TANC2,HTT,MYO5A,HAP1,SLC2A4,NLGN2,STARD3,MYO1E,SYT11,TMED10,RAB11A,MYO1D,MYO5B,MYO1C,PAFAH1B1,IKBKG,VAMP7,BLOC1S2	darkgreen	EC_M4
GO:0006354	8.85E-04	0.999696815	17	80	DNA-templated transcription, elongation	BP	2.87E-02	DDX39B,SCAF8,EZH2,CCNT2,WDR43,SUPT16H,ALYREF,TCERG1,ERCC6,CHEK1,SETD2,LEO1,HNRNPU,THOC1,TEFM,ZNF326,RNF8	darkgrey	EC_M8
GO:0000912	8.98E-04	0.999964806	4	6	assembly of actomyosin apparatus involved in cytokinesis	BP	2.90E-02	PLEC,ANLN,RACGAP1,ECT2	darkgrey	EC_M8
GO:0000915	8.98E-04	0.999964806	4	6	actomyosin contractile ring assembly	BP	2.90E-02	PLEC,ANLN,RACGAP1,ECT2	darkgrey	EC_M8
GO:0015988	3.28E-05	1	5	5	energy coupled proton transmembrane transport, against electrochemical gradient	BP	2.92E-02	NDUFS7,MT-CO1,MT-ND4,MT-ND5,MT-CYB	cyan	EC_M2
GO:0015990	3.28E-05	1	5	5	electron transport coupled proton transport	BP	2.92E-02	NDUFS7,MT-CO1,MT-ND4,MT-ND5,MT-CYB	cyan	EC_M2

GO:0035295	5.19E-04	0.999633041	151	849	tube development	BP	2.93E-02	LRP1,AHI1,SLC12A2,PLXNB2,ANGPT1,FAM19A5,NUS1,RSPO3,ADGRB1,MYH9,LEP,SRF,VEGFA,PDCL3,COL15A1,TGFBR1,EMC10,CALD1,GLI3,ROCK2,RRAS,RCN3,SLC7A11,ITGB1,FGF2,SRPK2,RB1,GLI2,MEGF8,PAK1,TGFB1,WDR48,CTNNB1,RARB,ACTG1,STIM1,SPINT2,DCN,STARD13,SEMA3C,CCKBR,ILK,SPINK5,TMED2,SULF1,ROBO1,RDH10,LOXL3,HEY1,RASA1,TCTN1,RAPGEF2,CX3CL1,ABCC8,OPA1,LOXL2,AP3B1,ACVR1,MMP2,MTDH,ADA,ATP2B4,PKD2,RUNX1,HIF1AN,MAN1A2,IFT57,CHRNA7,EIF4E,FAP,TJP1,KDM5B,NOTCH2,HYAL1,TGFB2,LRP5,ADGRB2,ITPK1,MMP14,DAG1,PIK3CA,LEF1,PRKCA,KRAS,CLMP,BMP7,FGFR2,VASH2,CLIC4,HSPB1,PLOD3,SERPINE1,SGPL1,ATP6AP2,FZD2,FN1,PTPN6,PDCD10,ITGAV,COMP,FKBP8,PRRX1,GORAB,TULP3,SIX4,AKT3,FKBP10,MAGED1,BMPR1A,TCAP,MED1,NPPB,MYO1E,NGFR,TGFB3,SHC1,MAP2K1,TMEM100,TTC8,PKM,MYOCD,ANGPTL6,PDGFRB,ROCK1,AKT1,MYDGF,ABCA3,PKD1,FLNA,BSG,SARS,CDH13,F3,LMO4,BCL10,MIR24-1,FSTL3,SERPINF1,TMEM38B,LOX,LBX1,GDNF,THY1,HAS2,CDKN1A,EDA,HOPX,PIK3C2A,WNT2,COL8A2,FGF10	darkgreen	EC_M4
GO:0001944	5.20E-04	0.999651842	113	607	vasculature development	BP	2.93E-02	LRP1,SLC12A2,ANGPT1,FAM19A5,NUS1,RSPO3,ADGRB1,MYH9,LEP,SRF,VEGFA,COL1A2,PDCL3,COL15A1,TGFBR1,EMC10,CALD1,GLI3,ROCK2,RRAS,ITGB1,FGF2,SRPK2,MEGF8,SNX17,TGFB1,CTNNB1,ACTG1,STIM1,DCN,STARD13,SEMA3C,SPINK5,ADAMTS6,TMED2,SULF1,ROBO1,HEY1,RASA1,RAPGEF2,CX3CL1,ABCC8,LOXL2,ACVR1,MMP2,MTDH,ATP2B4,PKD2,RUNX1,HIF1AN,CHRNA7,FAP,TJP1,NOTCH2,HYAL1,SLC2A10,TGFB2,LRP5,ADGRB2,MMP14,DAG1,PIK3CA,LEF1,PRKCA,BMP7,FGFR2,VASH2,CLIC4,HSPB1,SERPINE1,SGPL1,FN1,PDCD10,ITGAV,COMP,PRRX1,ACTA2,ROM1,AKT3,FKBP10,BMPR1A,MED1,NPPB,MYO1E,NGFR,COL1A1,SHC1,MAP2K1,TMEM100,PKM,MYOCD,ANGPTL6,PDGFRB,ROCK1,AKT1,MYDGF,PKD1,FLNA,BSG,SARS,COL5A1,CDH13,F3,MIR24-1,LOXL1,SERPINF1,LOX,THY1,HAS2,PIK3C2A,WNT2,COL8A2,FGF10	darkgreen	EC_M4
GO:0002768	4.10E-04	0.999815501	32	158	immune response-regulating cell surface receptor signaling pathway	BP	2.93E-02	PTK2,TEC,KIT,TNFRSF21,SYK,PRKD2,NECTIN2,GATA3,IKBKB,MAP3K1,PDE4D,MEF2C,PLCG1,DGKZ,LCK,ICOSLG,LAPTM5,NFATC2,INPP5D,BMX,FGR,PLEKHA1,PAK2,PRKCH,PLD2,PSEN1,EIF2B2,CD19,YES1,PDE4B,PLCG2,FYB1	turquoise	EC_M6
GO:0036303	4.12E-04	0.999934208	9	21	lymph vessel morphogenesis	BP	2.94E-02	FLT4,ACVR2B,TIE1,VEGFC,PROX1,CLEC14A,EPHA2,VASH1,SOX18	turquoise	EC_M6
GO:0001885	4.16E-04	0.999878595	16	53	endothelial cell development	BP	2.94E-02	NOTCH4,AFDN,TJP2,IKBKB,PDE2A,PDE4D,MARVELD2,PPP1R16B,STC1,RAPGEF3,ROBO4,PECAM1,F11R,RAPGEF1,CDH5,SOX18	turquoise	EC_M6
GO:1904019	4.17E-04	0.999850554	21	86	epithelial cell apoptotic process	BP	2.94E-02	TNFAIP3,TEK,KDR,IL6,PIK3CG,GATA2,DNMT3A,GATA3,PLCG1,HIPK1,ANO6,HDAC1,CFLAR,ITGA4,RAMP2,BID,MTOR,CCL2,JAG2,DAB2IP,CDH5	turquoise	EC_M6
GO:0042552	4.26E-04	0.999830705	25	109	myelination	BP	2.95E-02	QKI,TNFRSF21,MTMR2,CST7,CTSC,ITGB4,CXCR4,MALL,EIF2AK3,HEXB,TLR2,DHH,PLLP,SERINC5,TSPAN2,JAM3,DLG1,MPP5,TNFRSF1B,MTOR,EIF2B2,B4GALT6,SH3TC2,SLC8A3,ZNF24	turquoise	EC_M6
GO:0051607	4.26E-04	0.999801851	34	174	defense response to virus	BP	2.95E-02	TNFAIP3,C6orf106,DDX58,IL6,RSAD2,IL15,PUM2,UNC13D,SEC14L1,TMEM173,ITCH,TLR2,CXCL10,SERINC5,OAS3,NLRC5,IFIT2,IFIT1,STAT1,MX2,MX1,HYAL2,LYST,ISG20,MOV10,RTP4,BIRC3,ISG15,OAS1,PMAIP1,TRIM34,BST2,IFIT3,RNF125	turquoise	EC_M6
GO:0140546	4.26E-04	0.999801851	34	174	defense response to symbiont	BP	2.95E-02	TNFAIP3,C6orf106,DDX58,IL6,RSAD2,IL15,PUM2,UNC13D,SEC14L1,TMEM173,ITCH,TLR2,CXCL10,SERINC5,OAS3,NLRC5,IFIT2,IFIT1,STAT1,MX2,MX1,HYAL2,LYST,ISG20,MOV10,RTP4,BIRC3,ISG15,OAS1,PMAIP1,TRIM34,BST2,IFIT3,RNF125	turquoise	EC_M6
GO:0050727	4.27E-04	0.999781807	43	239	regulation of inflammatory response	BP	2.95E-02	TNFRSF11A,TNFAIP3,MAPK13,LRRC19,TEK,SYK,IL6,TLR4,IL15,PIK3CG,MGLL,CST7,CTSC,GPR4,PPARG,GATA3,EDNRB,PDE2A,TMEM173,NR1D2,PLK2,IGF1,KLKB1,TLR2,PLCG1,SNCA,ETS1,CD200,HYAL2,NCF1,FABP4,FGR,ADORA2A,CALCRL,BIRC3,SELE,BCL6B,TNFRSF1B,TNIP1,YES1,GIT1,PLCG2,CDH5	turquoise	EC_M6
GO:0030336	5.28E-04	0.99971749	49	223	negative regulation of cell migration	BP	2.97E-02	LRP1,FAM19A5,ADGRB1,PPARD,SRF,RRAS,FGF2,TGFB1,OSBPL8,NAV3,DCN,STARD13,ILK,DPYSL3,CXCL12,SULF1,ROBO1,PFN2,CX3CL1,ABCC8,ADA,ATP2B4,ADIPOR1,PHLDB2,DAG1,CORO1C,SLAMF8,IDH2,CLIC4,SERPINE1,DNAJA4,PDCD10,COL3A1,NBL1,TIMP1,VCL,BMPR1A,PPARGC1A,NGFR,TPM1,MYOCD,CD74,AKT1,PLXNB3,MIR24-1,SERPINF1,THY1,DUSP22,PRKG1	darkgreen	EC_M4

GO:1901564	3.54E-05	0.999972395	601	4789	organonitrogen compound metabolic process	BP	2.97E-02	ORMDL2,PYM1,ATG12,MAP3K5,OS9,CDK4,CTDSP2,ATP23,ADAMTS2,MAML1,SQSTM1,SASH1,IRAK3,GFPT2,BAG6,CHKB,ALDH7A1,ABHD16A,PPP6R2,SNX9,HSPA1B,EHMT2,HDAC10,SELENOO,ALG12,CERK,RNF5,EIF3H,RPS6KA2,DEPTOR,DSE,TSPO,RING1,RPS18,B3GALT4,JADE2,PMM1,MYC,SMAD5,EP300,IBA57,CDC14C,KDM3B,EEF1D,TAB1,RPL3,JOSD1,NRBP2,STK38,EIF3L,OPLAH,MPST,GPAA1,SHARPIN,EIF3D,FOXRED2,HSF1,FBXL6,SLC52A2,VPS28,CCND3,PPP1R16A,TRERF1,CDKN2A,RPL8,HMOX1,CUL7,CUL9,DNPH1,PIGO,PDGFRA,JAK2,HINT2,RECK,RNF38,RPS5,ANKRD39,ALG2,RPL28,RPS9,EEF1A1,CD109,GPNMB,MALSU1,STAP1,SLC44A1,EFEMP1,RPS7,ANTXR1,STK40,IL4I1,AKT1S1,ING3,ARHGEF5,AP2A1,RPS11,GSN,PARD3,FOXO3,KLF15,ADCK2,SPHK2,RPL18,SPRY1,NOP53,ABTB1,KMT2E,BBS7,HMCES,FKRP,LRRC41,TRIM13,ACSS1,IRF2BP1,QPCTL,SERP2,UROD,FASTK,SEPHS1,RPS8,XRCC1,MIF4GD,AGAP3,NAA16,MLH1,RNF220,LIPE,UNK,GALNT11,WBP2,TRIM47,KMT2C,DMAC2,BCKDHA,EGLN2,NUMBL,LTBP4,BABAM2,TADA3,CSGALNACT1,RPS3,BRPF1,PNP,DYRK1B,TGFBR2,RPS16,TIMP2,ENGASE,SAMD4B,XDH,MRPS12,SIRT2,RPTOR,RPL15,LRTOMT,HGS,UBAC2,ASPSCR1,EIF3K,NUP98,GALNT15,FGFR1,TRIM21,RNF24,GNPDA1,NDFIP1,SPRY4,HGF,APBB1,TAF10,PTDSS2,SMUG1,CLIP3,PCBP2,PSMF1,TARBP2,KMT2B,EIF3F,APIP,RBCK1,SDR16C5,TNS2,EIF4B,PTPRG,POFUT1,GGH,LATS2,KCTD6,PEPD,THNSL2,NECAB3,RMND5A,PIK3R1,ACSS2,GGCX,CAST,FBXO8,PCOLCE2,NEIL2,TNKS1BP1,RPS3A,SH3D19,ARRDC3,KDM2B,CHDH,CAMKK2,AGA,HTRA2,EIF2A,SETD6,PPP1CC,PDGFC,SPRED1,CCNG2,FNIP2,DGUOK,PRK	cyan	EC_M2
GO:0048869	5.31E-04	0.999569615	475	3033	cellular developmental process	BP	2.97E-02	CD63,IL23A,ATP8B1,NAB2,LRP1,AHI1,CAND1,RAB21,CDSN,PLXNB2,FBN2,ANGPT1,PPARA,HLA-DRA,ROS1,AFF4,NUS1,MRTFA,TRAPPC9,PACSIN1,ADGRB1,PPARD,CTNNA1,TEAD3,SUN2,CBY1,MTCH1,LGALS1,MDGA1,SH3GL2,FLNC,MYH9,LEP,CDKN2B,PLAA,RORB,SRF,NUAK1,VEGFA,RUNX2,C2orf40,NCK2,CLOCK,REST,COL15A1,P3H1,TGFBR1,HIVEP3,SOCS5,RIMS1,TMEM30A,PSME4,UBA6,RTN4,TPBG,SHANK1,MFSD2A,UBE2J1,MYH14,AKIRIN1,ACTR2,GMCL1,IFRD1,KIDINS220,WHRN,GLI3,NCDN,ROCK2,INHBA,BVES,RRAS,MYLK3,DKKL1,SLC7A11,OSTM1,ITGB1,FKBP1B,PLXNA1,FGF2,SRPK2,KIF5B,NAPA,PANX1,PSMC2,RB1,ABHD5,PICALM,EIF2B3,PTCH2,TENM4,GLI2,MEGF8,SMARCD3,RHEB,PAK1,TGFB1,AXL,CTNNB1,IL15RA,TRAK1,LBH,SERPINH1,AKT2,DZIP1,ZEB2,FARP1,SLC4A7,OSBPL8,RARB,ACTG1,NAV3,ACTN4,CRIM1,MAFG,SPRED3,SPINT2,LIG4,SMAD9,ZNF703,DCLK1,SEMA3C,UBA5,ILK,DPYSL3,SPINK5,RYK,SNPH,RNF6,AKIP1,CXCL12,IMPAD1,LGI4,ZSWIM6,KIF2A,ANKRD1,FAT1,SLC25A46,PALLD,TP53INP2,ZNF536,FLNB,EIF4G2,SULF1,ROBO1,MAP1B,RDH10,DIP2B,SEMA4F,LOXL3,P2RX4,FAIM2,HEY1,RASA1,ATP2A2,TLX2,CELF1,TCTN1,MMP15,APP,DHX36,SLC4A5,RAPGEF2,MAPKAPK5,CX3CL1,ABCC8,PTPN11,PTX3,ADCY6,HOMER1,RUNX1T1,OPA1,CDH17,ARSB,LOXL2,AP3B1,ZFYVE27,PTPN22,CRTAC1,TSG101,ACVR1,TUBB2A,CHAC1,MMP2,GDF6,ADA,SPP1,BMP1,RUNX1,PLA2R1,TWF1,TMEM14C,HIF1AN,DNAH1,SEMA4G,NKD1,GPRIN3,TOLLIP,BRSK2,HPS6,PDLIM5,NDN,RAB7B,CHRNA7,ARL3,DOCK10,EIF4E,DYNLL1,INA,ADIPOR1,PHLDB2,TJP1,TDP2,FNDC5,KDM5B,NOTCH2,CTTN,PKP2,CDC73,DN	darkgreen	EC_M4
GO:0030500	4.34E-04	0.999865141	17	62	regulation of bone mineralization	BP	2.98E-02	TRPM4,ACVR2B,NOTUM,MEF2C,P2RX7,BMP2K,ANO6,LTF,BCOR,FGF23,HIF1A,ADRB2,RFLNB,ISG15,S1PR1,BMP2,BMP6	turquoise	EC_M6
GO:0032602	4.37E-04	0.999863607	17	64	chemokine production	BP	2.98E-02	SYK,IL6,TLR4,C5,ERBIN,TLR2,IL7,OAS3,HDAC1,APOD,DDX3X,HIF1A,EPHA2,ARG2,LGALS9,OAS1,ACKR1	turquoise	EC_M6
GO:0032642	4.37E-04	0.999863607	17	64	regulation of chemokine production	BP	2.98E-02	SYK,IL6,TLR4,C5,ERBIN,TLR2,IL7,OAS3,HDAC1,APOD,DDX3X,HIF1A,EPHA2,ARG2,LGALS9,OAS1,ACKR1	turquoise	EC_M6
GO:0051770	4.53E-04	0.99994968	7	14	positive regulation of nitric-oxide synthase biosynthetic process	BP	3.08E-02	AKAP12,KDR,TLR4,TLR2,STAT1,CCL2,FCER2	turquoise	EC_M6
GO:0051785	9.57E-04	0.999765221	11	41	positive regulation of nuclear division	BP	3.09E-02	PHIP,CDC16,ESPL1,ANAPC7,NUSAP1,CUL3,CDC27,DLGAP5,RAD51AP1,CDT1,AURKA	darkgrey	EC_M8
GO:0051057	4.60E-04	0.999863598	16	55	positive regulation of small GTPase mediated signal transduction	BP	3.11E-02	PIK3CG,GPR4,NET1,PRAG1,IGF1,ERBIN,ARHGEF3,SHOC2,F11R,SOS2,RAC1,ITPKB,NOTCH1,ADGRG1,RELN,LPAR6	turquoise	EC_M6
GO:1905953	5.59E-04	0.999860328	13	37	negative regulation of lipid localization	BP	3.11E-02	PPARA,PPARD,LEP,ABHD5,AKT2,OSBPL8,IRS2,PTPN11,CRY2,PLA2R1,ITGAV,AKT1,PTPN2	darkgreen	EC_M4

GO:0009892	9.69E-04	0.999251979	255	2254	negative regulation of metabolic process	BP	3.12E-02	CDK2,PAN2,BCLAF1,LATS1,FBXO5,ZNF608,SCAF8,UBR5,EIF3E,MRPL13,XRCC6,TNRC6B,TUT7,PLEC,POT1,PWP1,XPO5,RFX3,TRIM28,NCBP1,ZNF451,LRPPRC,MSH2,IBTK,HNRNPA2B1,PNPT1,PUS10,ZMPSTE24,SYNCRIP,XPO1,PABPC4,CASP8AP2,TIA1,ZNF350,ITGB1BP1,THRAP3,EZH2,UFL1,FAM3C,WDR35,NRDC,DHFR,PUS7,HDAC2,EXOSC9,DNAJC2,CHORDC1,RNASEH2B,SETDB2,MASTL,TRDMT1,CNOT10,UPF2,RSF1,DIS3,XRN2,NCAPG2,BIRC5,GTPBP4,IPO5,CHD8,KAT6A,METTL3,RIF1,E2F7,TAF7,EXOSC8,NR2C1,BRCA2,ELK3,RRP8,TCERG1,PRKDC,CBX5,CCNB1,ERCC6,PAN3,CAPRIN1,ZC3H8,IPO7,PARPBP,TNKS2,LSM14A,BTAF1,CDK5RAP1,TDG,NCK1,IDE,SMARCA5,ABCE1,TAF9,CGGBP1,XRN1,PDE12,SAP30,ATR,TTC37,TERF1,MTMR9,FAM208A,HELLS,GCFC2,USP25,HK2,PBK,CNOT1,GABPA,SH2B3,PPID,RBL1,MSH3,SMYD5,NBN,TENT2,RIPK1,E2F8,TRIM33,ZBED6,ZNF248,MDM4,HIVEP1,CUL3,NSUN2,GMNN,NFKB1,KHDRBS1,NCL,TRAIP,PRMT5,AJUBA,BPTF,FXR1,DDX5,FANCB,ORC2,TIAL1,FASTKD1,BLM,SSB,DDX6,CENPF,AUNIP,CDK1,SMARCC1,SP3,DHX9,HNRNPR,FASTKD2,IVNS1ABP,DDX20,TPR,CCAR1,DGCR8,IL12A,XRCC5,NPAT,BRCA1,TFPI,CNOT9,NSD2,TIPRL,BOD1L1,ANKLE1,TBL1XR1,SUV39H1,FOXM1,EIF2AK1,LYAR,FAM220A,HNRNPU,PDS5A,WAPL,NEDD4,SMARCE1,RFC1,CDC6,DCP2,NSD1,IQGAP3,EXOSC10,FUS,CLK2,USP3,AURKB,TAF1,OGT,RLIM,ATRX,PTTG1,SPAG9,TIPIN,MBTD1,TRIM37,LARP1,PLK1,ILF3,BRIP1,AATF,CSK,PSMD10,TAF15,NLE1,THOC1,TYMS,SMCHD1,UPF3B,ZNF3,SUZ12,HNRNPM,SERBP1,MIER1,XIAP,NIPBL,GSPT1,PHF6,ZC3H7A,RMI2,UHRF1,DROSHA,RBMX,FMR1,METTL16,EHMT1,RNPS1,PT	darkgrey	EC_M8
GO:0001667	5.68E-04	0.99965712	72	360	ameboidal-type cell migration	BP	3.15E-02	ANGPT1,NUS1,PTP4A3,ADGRB1,PPARD,MYH9,SRF,VEGFA,TGFBR1,RTN4,TPBG,EMC10,ROCK2,RRAS,ITGB1,FGF2,MEGF8,PAK1,TGFB1,ZEB2,DCN,STARD13,SEMA3C,ILK,ROBO1,SEMA4F,P2RX4,PFN2,PTPN11,ARSB,LOXL2,ACVR1,ATP2B4,SEMA4G,ADIPOR1,FAP,HYAL1,TGFB2,LRP5,HTR2B,FSTL1,PIK3CA,CORO1C,PRKCA,BMP7,ARID5B,HSPB1,SGPL1,DNAJA4,S100P,FN1,PDCD10,TIMP1,AKT3,RAB11A,FAT2,ANXA6,ROCK1,AKT1,ITGA2,PAFAH1B1,CDH13,MIR24-1,SERPINF1,PDLIM1,GDNF,HAS2,TMSB4X,GLIPR2,CCR6,PIK3C2A,FGF10	darkgreen	EC_M4
GO:0030509	5.69E-04	0.999750925	30	116	BMP signaling pathway	BP	3.15E-02	KIAA1324L,RUNX2,MEGF8,TGFB1,CRIM1,SMAD9,ILK,SULF1,SLC33A1,ACVR1,GDF6,NOTCH2,TGFB2,FSTL1,LEF1,HJV,BMP7,HTRA1,FBN1,COMP,FKBP8,SKIL,SMURF1,NBL1,GREM2,BMPR1A,TGFB3,TMEM100,CHRDL1,FSTL3	darkgreen	EC_M4
GO:0090306	9.84E-04	0.999960436	4	6	meiotic spindle assembly	BP	3.16E-02	FBXO5,ASPM,CCNB2,AURKA	darkgrey	EC_M8
GO:0043603	9.85E-04	0.999328661	107	897	cellular amide metabolic process	BP	3.16E-02	PAN2,NARS,DDX39B,EIF3E,MRPL13,TNRC6B,TUT7,CARNMT1,PUM3,NCBP1,LRPPRC,MTIF2,PNPT1,SYNCRIP,PABPC4,TIA1,MRPL32,ASCC3,DDX1,DHFR,PUS7,EXOSC9,CNOT10,MRPS7,DIS3,DARS,METTL3,EXOSC8,LARS,LTA4H,PRKDC,SUCLG2,DHX29,PAN3,SP1,MRPL15,MRPS5,CAPRIN1,LSM14A,CDK5RAP1,EIF2B1,NCK1,IDE,MRPL35,PTCD3,ABCE1,XRN1,PDE12,LARP4,CNOT1,LTN1,USP16,RPS6,COL4A3BP,EEF1E1,NOLC1,GCN1,METAP1,YARS,KHDRBS1,ACSL5,IARS2,NCL,FXR1,NGDN,MCCC1,EIF3A,EIF2B5,DDX6,DHX9,HNRNPR,FASTKD2,TPR,RPL10L,DARS2,CNOT9,RPL22L1,DHX33,EIF2AK1,MRPL44,DCP2,EIF3B,SEPSECS,EIF4A1,RARS,MRPS23,MRPL22,GEMIN5,LARP1,ILF3,EIF5,TYMS,UPF3B,GSPT1,FMR1,METTL16,EXOSC2,RBM8A,METTL18,SARNP,HNRNPD,SLC25A32,TIGAR,RNF139,PAIP2,EIF1AX,DRG1	darkgrey	EC_M8
GO:0043489	9.98E-04	0.999716712	13	52	RNA stabilization	BP	3.19E-02	SYNCRIP,THRAP3,TRDMT1,TENT2,NSUN2,DHX9,HNRNPU,FUS,LARP1,TAF15,METTL16,HNRNPD,NAF1	darkgrey	EC_M8
GO:0010765	5.81E-04	0.999878834	11	27	positive regulation of sodium ion transport	BP	3.20E-02	KIF5B,SCN5A,GPD1L,ACTN4,FXYD1,ATP1B3,CHP1,PKP2,AKT1,AHCYL1,DMD	darkgreen	EC_M4
GO:0000972	1.01E-03	0.999959211	4	6	transcription-dependent tethering of RNA polymerase II gene DNA at nuclear periphery	BP	3.22E-02	NUP107,RAE1,NUP133,NUP155	darkgrey	EC_M8
GO:0006890	5.86E-04	0.99983382	15	45	retrograde vesicle-mediated transport, Golgi to endoplasmic reticulum	BP	3.22E-02	COPB2,ERGIC3,SEC22B,ERGIC2,PITPNB,COPA,SCFD1,SCYL1,HTT,STX18,KDELR2,LMAN2,TMED10,RAB6A,ARF4	darkgreen	EC_M4
GO:0045070	1.01E-03	0.999798825	9	28	positive regulation of viral genome replication	BP	3.22E-02	SRPK1,PPIH,TOP2B,PDE12,PPID,TOP2A,HACD3,LARP1,PKN2	darkgrey	EC_M8

GO:0031047	1.01E-03	0.999578679	23	129	gene silencing by RNA	BP	3.22E-02	TNRC6B,TUT7,XPO5,NCBP1,HNRNPA2B1,PUS10,ZMPSTE24,CNOT10,METTL3,CNOT1,RIPK1,AJUBA,FXR1,DDX5,TIAL1,DDX6,DHX9,DGCR8,CNOT9,ZC3H7A,DROSHA,FMR1,SRRT	darkgrey	EC_M8
GO:0006303	1.02E-03	0.999692541	14	59	double-strand break repair via nonhomologous end joining	BP	3.23E-02	XRCC6,POT1,DCLRE1C,RIF1,PRKDC,ERCC6,DCLRE1B,DEK,AUNIP,POLA1,XRCC5,NSD2,SMCHD1,RNF8	darkgrey	EC_M8
GO:1902992	5.98E-04	0.999886435	10	24	negative regulation of amyloid precursor protein catabolic process	BP	3.27E-02	RTN4,PICALM,PRNP,CHRNA7,RTN3,HAP1,TMED10,PIN1,ROCK1,MIR24-1	darkgreen	EC_M4
GO:0032387	6.02E-04	0.999828158	15	46	negative regulation of intracellular transport	BP	3.28E-02	LMAN1,ANGPT1,RAB23,ERLEC1,UBE2J1,MDFIC,UFM1,MAP1B,ARHGAP1,CHP1,C11orf65,PARK7,TXN,UBE2G2,DERL2	darkgreen	EC_M4
GO:0040012	6.05E-04	0.999575208	142	796	regulation of locomotion	BP	3.28E-02	LRP1,PLXNB2,ANGPT1,FAM19A5,NUS1,FAM49B,ADGRB1,PPARD,CTNNA1,SUN2,PLAA,SRF,VEGFA,DOCK8,CREB3,PTP4A1,TGFBR1,RTN4,TPBG,EMC10,AKIRIN1,ROCK2,BVES,RRAS,ITGB1,PLXNA1,FGF2,MEGF8,PAK1,TGFB1,AKT2,OSBPL8,ACTG1,NAV3,ACTN4,SPINT2,IRS2,DCN,ZNF703,STARD13,SEMA3C,DDRGK1,ILK,DPYSL3,RYK,CXCL12,KIF2A,SULF1,ROBO1,CHST2,SEMA4F,P2RX4,PFN2,APP,RAPGEF2,CX3CL1,ABCC8,PGAM4,ARSB,ACVR1,ADA,ATP2B4,SEMA4G,NKD1,DOCK10,ADIPOR1,PHLDB2,TJP1,DNM1L,HYAL1,TGFB2,MMP14,DAG1,LEF1,CORO1C,PRKCA,CMKLR1,NTRK3,SLAMF8,BMP7,IDH2,IQGAP1,CLIC4,S100A11,LRRC15,FAM89B,HSPB1,SERPINE1,ITGA2B,DNAJA4,FN1,PDCD10,ITGAV,COL3A1,NBL1,TIMP1,VCL,AKT3,BMPR1A,PPARGC1A,MSN,SRCIN1,ARHGEF2,NGFR,TPM1,COL1A1,RAB11A,MYOCD,PIN1,CD74,LAMA3,PDGFRB,ROCK1,USP14,AKT1,ITGA2,PTPN2,DAPK3,MYO1C,PLXNB3,TNFRSF18,FLNA,PLXNA3,NTNG2,CDH13,F3,HSPA5,LMO4,NEXN,MIR24-1,CD151,SERPINF1,THY1,HAS2,ARTN,TMSB4X,GLIPR2,CCR6,DUSP22,PIK3C2A,PRKG1,FGF10	darkgreen	EC_M4
GO:0070339	4.93E-04	0.999989099	4	5	response to bacterial lipopeptide	BP	3.29E-02	CD36,TLR2,TLR6,TLR1	turquoise	EC_M6
GO:0071220	4.93E-04	0.999989099	4	5	cellular response to bacterial lipoprotein	BP	3.29E-02	CD36,TLR2,TLR6,TLR1	turquoise	EC_M6
GO:0071221	4.93E-04	0.999989099	4	5	cellular response to bacterial lipopeptide	BP	3.29E-02	CD36,TLR2,TLR6,TLR1	turquoise	EC_M6
GO:0043570	1.05E-03	0.999956819	4	6	maintenance of DNA repeat elements	BP	3.33E-02	MSH2,ZMPSTE24,MSH3,RMI2	darkgrey	EC_M8
GO:0009127	1.06E-03	0.999841596	7	20	purine nucleoside monophosphate biosynthetic process	BP	3.33E-02	PPAT,PAICS,DCK,GMPS,PFAS,HPRT1,GART	darkgrey	EC_M8
GO:0007272	5.05E-04	0.99979669	25	110	ensheathment of neurons	BP	3.34E-02	QKI,TNFRSF21,MTMR2,CST7,CTSC,ITGB4,CXCR4,MALL,EIF2AK3,HEXB,TLR2,DHH,PLLP,SERINC5,TSPAN2,JAM3,DLG1,MPP5,TNFRSF1B,MTOR,EIF2B2,B4GALT6,SH3TC2,SLC8A3,ZNF24	turquoise	EC_M6
GO:0008366	5.05E-04	0.99979669	25	110	axon ensheathment	BP	3.34E-02	QKI,TNFRSF21,MTMR2,CST7,CTSC,ITGB4,CXCR4,MALL,EIF2AK3,HEXB,TLR2,DHH,PLLP,SERINC5,TSPAN2,JAM3,DLG1,MPP5,TNFRSF1B,MTOR,EIF2B2,B4GALT6,SH3TC2,SLC8A3,ZNF24	turquoise	EC_M6
GO:0002694	5.07E-04	0.999710591	58	352	regulation of leukocyte activation	BP	3.34E-02	TNFAIP3,TCF7,ADGRF5,TNFRSF21,SYK,IL6,PELI1,TLR4,IL15,GATA2,CST7,CTSC,NECTIN2,GAB2,UNC13D,GATA3,SFRP1,MERTK,IL1A,IGF1,ITCH,SH3RF1,MEF2C,IL7,SOX13,SNCA,CD200,LCK,ICOSLG,LAPTM5,HLX,NFATC2,INPP5D,CD86,FGR,FES,DLG1,B2M,ADORA2A,SOS2,ITPKB,TLR6,TNFRSF1B,ARG2,EFNB1,CHRNB2,CD19,CCL2,YES1,LGALS9,IL7R,MYO18A,AP3D1,NRARP,STXBP1,HAVCR1,VNN1,CD46	turquoise	EC_M6
GO:0070167	5.12E-04	0.999825098	19	74	regulation of biomineral tissue development	BP	3.35E-02	TRPM4,NOS3,ACVR2B,NOTUM,MEF2C,P2RX7,BMP2K,ANO6,LTF,BCOR,FGF23,HIF1A,ADRB2,RFLNB,ISG15,NOTCH1,S1PR1,BMP2,BMP6	turquoise	EC_M6
GO:0008360	5.13E-04	0.99977702	29	134	regulation of cell shape	BP	3.35E-02	CDC42SE2,ARHGAP18,PTK2,CDC42EP1,STRIP2,KIT,KDR,SEMA4D,FGD5,PLXND1,FGD6,PRAG1,HEXB,DNMBP,GAS2,EPB41,PLEKHO1,PLXNA2,FGR,FES,F11R,DLG1,RAC1,RHOJ,FGD1,SEMA4A,TAOK2,CCL2,PALMD	turquoise	EC_M6
GO:0016525	5.18E-04	0.999816385	20	82	negative regulation of angiogenesis	BP	3.35E-02	SEMA6A,TEK,GPR4,PPARG,TIE1,ADAMTS9,PLK2,HHEX,CXCL10,ANGPT2,THBS4,STAB1,STAT1,HSPG2,EPHA2,SEMA4A,VASH1,NPR1,AMOT,DAB2IP	turquoise	EC_M6

GO:0048872	5.21E-04	0.999733175	42	229	homeostasis of number of cells	BP	3.35E-02	TNFAIP3,MYCT1,L3MBTL3,LRRC19,IREB2,ADGRF5,KIT,IL6,PTBP3,VPS54,TAL1,GATA2,NOS3,GATA3,GAPT,MERTK,MEF2C,IL7,STAT1,JAM3,ALAS1,ETS1,DYRK3,HCLS1,INPP5D,B2M,SOS2,HIF1A,LIPA,ITPKB,HOXB6,SCNN1B,RCOR1,PDE4B,LGALS9,IL7R,ISG15,NOTCH1,PMAIP1,ARID4A,ADGRG1,EMCN	turquoise	EC_M6
GO:0030098	5.23E-04	0.999721551	47	259	lymphocyte differentiation	BP	3.35E-02	TCF7,MFNG,KIT,NFIL3,SYK,IL6,RSAD2,IL15,GATA3,SFRP1,MERTK,CHD7,IL1A,HHEX,SH3RF1,IL7,TPD52,LMBR1L,ADGRG3,DLL4,SOX13,DCAF1,LCK,HLX,INPP5D,CD86,B2M,ITGA4,SOS2,CRACR2A,ITPKB,LFNG,MTOR,SEMA4A,BATF,CD19,YY1,LYL1,JAG2,LGALS9,IL7R,AP3D1,NRARP,IRF8,PLCG2,VNN1,CD46	turquoise	EC_M6
GO:0072577	5.25E-04	0.999865548	13	43	endothelial cell apoptotic process	BP	3.35E-02	TNFAIP3,TEK,KDR,GATA2,GATA3,PLCG1,HIPK1,ANO6,ITGA4,RAMP2,CCL2,DAB2IP,CDH5	turquoise	EC_M6
GO:0002429	5.28E-04	0.999764845	30	148	immune response-activating cell surface receptor signaling pathway	BP	3.35E-02	PTK2,TEC,TNFRSF21,SYK,PRKD2,NECTIN2,GATA3,IKBKB,PDE4D,MEF2C,PLCG1,DGKZ,LCK,ICOSLG,LAPTM5,NFATC2,INPP5D,BMX,FGR,PLEKHA1,PAK2,PRKCH,PLD2,PSEN1,EIF2B2,CD19,YES1,PDE4B,PLCG2,FYB1	turquoise	EC_M6
GO:0002757	5.28E-04	0.999764845	30	148	immune response-activating signal transduction	BP	3.35E-02	PTK2,TEC,TNFRSF21,SYK,PRKD2,NECTIN2,GATA3,IKBKB,PDE4D,MEF2C,PLCG1,DGKZ,LCK,ICOSLG,LAPTM5,NFATC2,INPP5D,BMX,FGR,PLEKHA1,PAK2,PRKCH,PLD2,PSEN1,EIF2B2,CD19,YES1,PDE4B,PLCG2,FYB1	turquoise	EC_M6
GO:0071216	5.29E-04	0.999760938	31	157	cellular response to biotic stimulus	BP	3.35E-02	TNFAIP3,SYK,IL6,CXCL8,TLR4,IRAK2,EDNRB,NFKBIB,CD36,SCARB1,IL1A,EIF2AK3,MEF2C,TLR2,CXCL10,PABPN1,CD86,LTF,BID,TLR6,TNFRSF1B,TNIP1,CCL2,PDE4B,NOS2,GIT1,IRF8,PLCG2,DAB2IP,TLR1,BMP6	turquoise	EC_M6
GO:0050678	5.31E-04	0.999712521	49	275	regulation of epithelial cell proliferation	BP	3.36E-02	TNFAIP3,FLT4,MTSS1,PDGFB,TEK,KDR,CAV2,NRP1,GATA2,PRKD2,PPARG,GATA3,EDNRB,TIE1,SFRP1,IGF1,NR4A1,MEF2C,VEGFC,THBS4,PPP1R16B,PLCG1,DLL4,STAT1,EAF2,CFLAR,TGM1,PROX1,PRKD1,DLG1,B2M,ITGA4,TBX1,HIF1A,MCC,ALDH1A2,MTOR,AR,PGF,VASH1,CCL2,LRG1,NRARP,EGFL7,NOTCH1,DAB2IP,MIR23A,BMP2,BMP6	turquoise	EC_M6
GO:0006091	4.23E-05	0.99997681	72	392	generation of precursor metabolites and energy	BP	3.36E-02	GFPT2,UQCRQ,MYC,EP300,ETFB,UGP2,STK40,NDUFB2,SPHK2,NOP53,UQCRH,ACSS1,NDUFB6,FDXR,KHK,NDUFC2,GAA,NDUFA2,GNPDA1,COX7A1,UQCRFS1,ACSS2,PPP1CC,COQ9,DGUOK,PRKAG1,SRD5A1,IRS1,NDUFV1,OXA1L,ARNT,PER2,HDAC4,MTFR1L,NDUFS2,DNAJC30,POR,IDH1,AIFM2,CHCHD10,PHB2,PGLS,UQCC3,FOXK1,NDUFB7,GBA,NUPR1,INSR,NDUFA7,OXCT1,LEPR,NDOR1,UQCR11,NDUFS7,ATP5F1D,TAZ,JMJD8,CYBA,MT-ND1,MT-ND2,MT-CO1,MT-CO2,MT-ATP6,MT-CO3,MT-ND3,MT-ND4L,MT-ND4,MT-ND5,MT-CYB,ZBTB7A,PID1,RPIA	cyan	EC_M2
GO:0006188	1.07E-03	0.999913093	5	10	IMP biosynthetic process	BP	3.36E-02	PPAT,PAICS,PFAS,HPRT1,GART	darkgrey	EC_M8
GO:0050685	1.07E-03	0.999808668	8	25	positive regulation of mRNA processing	BP	3.36E-02	NCBP1,THRAP3,CCNB1,NCL,TRA2B,LEO1,RBMX,DAZAP1	darkgrey	EC_M8
GO:0018205	1.07E-03	0.999402859	47	313	peptidyl-lysine modification	BP	3.36E-02	UHRF2,PWP1,TOPORS,TRIM28,ZNF451,SENP6,ZMPSTE24,RLF,EZH2,HDAC2,SAE1,SETDB2,PAXIP1,KAT14,EPC2,KAT6A,RIF1,NSD3,TAF7,BRCA2,UBA2,TAF9,MCRS1,SMYD5,SENP1,CHEK1,NAA50,TET2,MCM3AP,HAT1,ING5,SETD2,BRCA1,NSD2,SUV39H1,NSD1,TAF1,OGT,ATRX,ESCO1,SUZ12,EHMT1,CTCF,AC118549.1,CTH,METTL18,SENP7	darkgrey	EC_M8
GO:0003012	5.40E-04	0.999693121	57	332	muscle system process	BP	3.40E-02	TRDN,KIT,TMOD1,DOCK4,IL15,TRPM4,PIK3CG,ARHGAP42,NOS3,CXCR4,EDNRB,P2RX2,LMOD3,PDE4D,IGF1,NR4A1,GUCY1A1,STC1,KCNJ3,ANKRD2,EDN1,JARID2,PDE9A,PPP3CA,KCNJ2,MEF2A,SYNM,PABPN1,CFLAR,INPP5F,LTB4R,TMOD4,MYOM3,DLG1,IGFBP5,CALCRL,TMOD2,APBB2,JUP,CHRNB1,TNFRSF1B,ARG2,MTOR,CHRNB2,ATP2A1,SCNN1B,YY1,DSG2,DSC2,ADRB2,PDE4B,NOTCH1,MIR23A,SLC8A3,KCNH2,SCN2B,RGS4	turquoise	EC_M6
GO:0009123	1.09E-03	0.999665372	14	65	nucleoside monophosphate metabolic process	BP	3.40E-02	PPAT,PAICS,DCK,CAD,TK1,GMPS,UMPS,PFAS,PRPS1,TYMS,HPRT1,DHODH,RFK,GART	darkgrey	EC_M8
GO:0016584	1.09E-03	0.999954734	4	6	nucleosome positioning	BP	3.41E-02	RSF1,SMARCA5,CTCF,HIST1H1A	darkgrey	EC_M8
GO:0043255	6.36E-04	0.999761929	22	78	regulation of carbohydrate biosynthetic process	BP	3.44E-02	DYRK2,PPARA,LEP,TGFB1,DGAT2,AKT2,KAT2B,IRS2,SORBS1,SELENOS,GSK3B,PPP1R3D,PPP1R3F,PPARGC1A,CLTC,AKT1,PTPN2,PGP,FAM3A,RANBP2,HAS2,NNMT	darkgreen	EC_M4
GO:1904238	5.51E-04	0.999968435	5	8	pericyte cell differentiation	BP	3.45E-02	PDGFB,GPR4,CD34,EPHA2,NOTCH1	turquoise	EC_M6
GO:0030833	5.59E-04	0.999750669	30	145	regulation of actin filament polymerization	BP	3.48E-02	ARHGAP18,BAIAP2L2,PICK1,CDC42EP1,TMOD1,FAM49A,SVIL,SPTBN4,FCHSD2,FCHSD1,LMOD3,HIP1R,IQGAP2,ESAM,HCLS1,AP1AR,TMOD4,DLG1,BAIAP2L1,WAS,TMOD2,MTOR,ARHGAP28,SPIRE1,CARMIL2,VASP,GMFG,KANK3,GBA2,ARPC5L	turquoise	EC_M6

GO:0071356	5.60E-04	0.999742099	32	163	cellular response to tumor necrosis factor	BP	3.48E-02	TNFRSF11A,TNFAIP3,AKAP12,TNFRSF21,SYK,CXCL8,TRAF1,ZFAND6,GATA3,IKBKB,SFRP1,TNFRSF19,ERBIN,OCLN,GPD1,HIPK1,EDN1,STAT1,CD58,HDAC1,HYAL2,LAPTM5,FABP4,ADAMTS7,BIRC3,PLVAP,TNFRSF1B,CCL2,TRAF2,TNFRSF25,DAB2IP,COMMD7	turquoise	EC_M6
GO:0051172	1.12E-03	0.999154907	205	1770	negative regulation of nitrogen compound metabolic process	BP	3.48E-02	PAN2,BCLAF1,LATS1,FBXO5,ZNF608,SCAF8,UBR5,EIF3E,MRPL13,XRCC6,TNRC6B,TUT7,PLEC,POT1,PWP1,RFX3,TRIM28,ZNF451,LRPPRC,MSH2,IBTK,PNPT1,ZMPSTE24,SYNCRIP,XPO1,CASP8AP2,TIA1,ZNF350,ITGB1BP1,THRAP3,EZH2,UFL1,WDR35,DHFR,PUS7,HDAC2,EXOSC9,DNAJC2,CHORDC1,SETDB2,MASTL,TRDMT1,CNOT10,RSF1,DIS3,NCAPG2,BIRC5,GTPBP4,IPO5,CHD8,KAT6A,METTL3,RIF1,E2F7,TAF7,EXOSC8,NR2C1,ELK3,TCERG1,PRKDC,CBX5,CCNB1,ERCC6,PAN3,CAPRIN1,ZC3H8,IPO7,PARPBP,TNKS2,LSM14A,BTAF1,CDK5RAP1,TDG,NCK1,IDE,ABCE1,TAF9,CGGBP1,XRN1,PDE12,SAP30,TERF1,GCFC2,USP25,PBK,CNOT1,GABPA,SH2B3,PPID,RBL1,MSH3,NBN,TENT2,E2F8,TRIM33,ZBED6,ZNF248,MDM4,HIVEP1,CUL3,NSUN2,GMNN,NFKB1,KHDRBS1,NCL,PRMT5,AJUBA,BPTF,FXR1,DDX5,FANCB,ORC2,BLM,DDX6,CENPF,AUNIP,SMARCC1,SP3,DHX9,HNRNPR,IVNS1ABP,DDX20,TPR,CCAR1,XRCC5,NPAT,BRCA1,TFPI,CNOT9,NSD2,TIPRL,BOD1L1,ANKLE1,TBL1XR1,SUV39H1,FOXM1,EIF2AK1,LYAR,FAM220A,HNRNPU,NEDD4,SMARCE1,RFC1,DCP2,NSD1,EXOSC10,FUS,USP3,AURKB,TAF1,OGT,RLIM,ATRX,PTTG1,SPAG9,MBTD1,TRIM37,LARP1,PLK1,ILF3,AATF,PSMD10,TAF15,NLE1,THOC1,TYMS,SMCHD1,ZNF3,SUZ12,MIER1,XIAP,NIPBL,PHF6,RMI2,UHRF1,RBMX,FMR1,METTL16,EHMT1,RNPS1,PTBP1,EXOSC2,SF3B3,GLMN,SCAI,CTCF,DEPDC1,HIST1H1A,HNRNPD,ZNF684,MAD2L1,DRAP1,ZNF614,RBAK,TIGAR,SRSF7,NAF1,BRMS1L,RNF139,C1D,RNF8,ZNF558,IKZF5,PAIP2,ZNF529	darkgrey	EC_M8
GO:0050764	5.67E-04	0.999826304	16	59	regulation of phagocytosis	BP	3.51E-02	SYK,IL15,GATA2,CD36,SCARB1,MERTK,TLR2,DYSF,ANO6,FGR,RAB27A,SYT7,DNM2,CCL2,MYO18A,PLCG2	turquoise	EC_M6
GO:0040016	1.13E-03	0.999952609	4	6	embryonic cleavage	BP	3.52E-02	TOP1,CUL3,TOP2A,AATF	darkgrey	EC_M8
GO:0035767	5.73E-04	0.999899359	9	24	endothelial cell chemotaxis	BP	3.53E-02	KDR,NRP1,PRKD2,NR4A1,PRKD1,FGF18,PLEKHG5,NOTCH1,FGF16	turquoise	EC_M6
GO:0006029	6.57E-04	0.999768385	20	67	proteoglycan metabolic process	BP	3.53E-02	B4GALT7,PPARD,CHST10,CHPF2,CTNNB1,CANT1,DCN,IMPAD1,SULF1,CHSY1,HYAL1,SLC2A10,B3GAT3,UGDH,CHST12,XYLT2,EGFLAM,COL11A1,CHST11,HS2ST1	darkgreen	EC_M4
GO:0010256	6.58E-04	0.999586749	82	422	endomembrane system organization	BP	3.53E-02	LMAN1,ATP8B1,FER1L6,PACSIN1,REEP2,SUN2,SH3GL2,MYH9,LMAN2L,RTN4,EMC10,SEC61A1,RAB7A,PDCD6IP,RAB3GAP1,ESYT2,AKT2,NPLOC4,STAM2,SMPD1,TMEM127,VPS37A,ARL1,RAB2A,REEP5,TMED2,OSBP,TRAM1,EMC4,ATP2A2,USO1,AP3B1,ZFYVE27,TSG101,VPS18,AKTIP,SEC22B,TGFB2,GOLGA5,CORO1C,PRKCA,GOLGB1,ARV1,VAPB,CASQ1,OSBPL2,GORASP2,TANGO2,PDCD10,HTT,RTN3,EMC1,PTPRN,BLZF1,OBSL1,STX18,LMAN2,STX1B,SYT11,TMED10,RAB11A,MAP2K1,SOX30,SYTL4,TMED1,VMP1,ANXA6,AKT1,PAFAH1B1,VAMP7,SYPL2,COL5A1,MTSS1L,CCDC136,SPINK2,TMEM38B,SNX10,TOR1AIP2,BLOC1S2,EMC7,TMEM38A,TMED3	darkgreen	EC_M4
GO:0071346	5.78E-04	0.99979863	19	80	cellular response to interferon-gamma	BP	3.55E-02	IFNGR1,DAPK1,TLR4,PPARG,TLR2,NLRC5,EDN1,STAT1,CD58,WAS,CCL2,JAK1,LGALS9,NOS2,IRF8,STXBP1,GBP5,RAB43,VAMP3	turquoise	EC_M6
GO:2000181	5.81E-04	0.99979175	20	83	negative regulation of blood vessel morphogenesis	BP	3.55E-02	SEMA6A,TEK,GPR4,PPARG,TIE1,ADAMTS9,PLK2,HHEX,CXCL10,ANGPT2,THBS4,STAB1,STAT1,HSPG2,EPHA2,SEMA4A,VASH1,NPR1,AMOT,DAB2IP	turquoise	EC_M6
GO:0010921	6.65E-04	0.999764694	20	68	regulation of phosphatase activity	BP	3.56E-02	NUAK1,ROCK2,FKBP1B,PPP1R12A,HSP90B1,CRY2,CHP1,SLC39A10,TGFB2,PPP6R3,GSK3B,PPP1R11,HTT,PPP1R36,CALM1,PPP4R4,PDGFRB,ROCK1,ITGA2,FKBP1A	darkgreen	EC_M4
GO:0001325	1.16E-03	0.999864515	6	14	formation of extrachromosomal circular DNA	BP	3.57E-02	TERF1,NBN,BLM,DNA2,XRCC5,EXO1	darkgrey	EC_M8
GO:0090656	1.16E-03	0.999864515	6	14	t-circle formation	BP	3.57E-02	TERF1,NBN,BLM,DNA2,XRCC5,EXO1	darkgrey	EC_M8
GO:0090737	1.16E-03	0.999864515	6	14	telomere maintenance via telomere trimming	BP	3.57E-02	TERF1,NBN,BLM,DNA2,XRCC5,EXO1	darkgrey	EC_M8
GO:0110149	5.94E-04	0.999793972	19	75	regulation of biomineralization	BP	3.62E-02	TRPM4,NOS3,ACVR2B,NOTUM,MEF2C,P2RX7,BMP2K,ANO6,LTF,BCOR,FGF23,HIF1A,ADRB2,RFLNB,ISG15,NOTCH1,S1PR1,BMP2,BMP6	turquoise	EC_M6

GO:0006816	6.80E-04	0.999609134	59	282	calcium ion transport	BP	3.62E-02	SMDT1,UBQLN1,FKBP1B,FGF2,SEC61A1,PANX1,TPT1,CTNNB1,STIM1,ATP2C1,PRNP,ATP2B1,REM1,CXCL12,TRPC4AP,RYR3,P2RX4,ATP2A2,CACNA1D,CX3CL1,CACNB3,HOMER1,OPA1,ATP2B4,PKD2,CHRNA7,CALHM1,DNM1L,HTR2B,CASQ1,TMCO1,LINC02210-CRHR1,PTPN6,ITGAV,HTT,PKD1L1,ERO1A,SLC3A2,HAP1,CACNA1C,STIM2,CACNA1G,CALM1,VMP1,ANXA6,TRPV2,PDGFRB,AKT1,PKD1,JPH3,DMD,FKBP1A,CBARP,TMEM38B,NMUR1,THY1,TMEM38A,SARAF,GEM	darkgreen	EC_M4
GO:0072384	6.81E-04	0.99974367	22	78	organelle transport along microtubule	BP	3.62E-02	AP3S1,SUN2,DYNC1I1,MGARP,KIF5B,COPG1,TRAK1,MAP1B,OPA1,AP3B1,FEZ1,KIF1A,MAPT,HTT,ACTR10,HAP1,ARMCX3,PAFAH1B1,VAMP7,HSBP1,RAB6A,BLOC1S2	darkgreen	EC_M4
GO:0060840	6.86E-04	0.999735715	23	81	artery development	BP	3.63E-02	LRP1,LEP,SRF,VEGFA,TGFBR1,GLI3,SNX17,ADAMTS6,ROBO1,HEY1,PKD2,SLC2A10,TGFB2,COMP,PRRX1,AKT3,FKBP10,BMPR1A,NGFR,MYOCD,PDGFRB,LOXL1,LOX	darkgreen	EC_M4
GO:0007224	6.89E-04	0.999693638	30	119	smoothened signaling pathway	BP	3.64E-02	DYRK2,CTNNA1,VCP,RUNX2,GLI3,RB1,PTCH2,KCTD21,GLI2,MEGF8,DZIP1,TCTN1,IFT57,ARL3,TROVE2,UCHL5,CHSY1,ARMC9,FGFR2,GPC2,FKBP8,PRRX1,GORAB,TULP3,EVC2,EVC,RAB34,MGRN1,TCTN2,FGF10	darkgreen	EC_M4
GO:0051235	6.92E-04	0.999615063	53	248	maintenance of location	BP	3.64E-02	B4GALNT1,FBN2,PPARA,PPARD,SUN2,PLIN2,LEP,LDAH,FKBP1B,FGF2,ATP7B,ABHD5,DGAT2,DZIP1,OSBPL8,LTBP1,HSP90B1,RYR3,CX3CL1,CRY2,PKD2,TWF1,CPT1A,HTR2B,CASQ1,PTPN6,FBN1,ITGAV,HTT,ERO1A,NBL1,KDELR2,HAP1,CACNA1C,CALM1,TMEM159,ANXA6,AKT1,PTPN2,PAFAH1B1,PKD1,FLNA,PARK7,JPH3,SLC30A7,HSPA5,DMD,FKBP1A,TMEM38B,THY1,TXN,TMSB4X,TMEM38A	darkgreen	EC_M4
GO:0014912	6.97E-04	0.999864897	10	24	negative regulation of smooth muscle cell migration	BP	3.65E-02	LRP1,FAM19A5,PPARD,ILK,SERPINE1,BMPR1A,PPARGC1A,TPM1,MYOCD,PRKG1	darkgreen	EC_M4
GO:0090224	1.19E-03	0.999726676	10	34	regulation of spindle organization	BP	3.67E-02	SENP6,BORA,TPX2,RAE1,TPR,KIF15,TACC3,HNRNPU,PLK1,DRG1	darkgrey	EC_M8
GO:0030030	7.04E-04	0.999472961	209	1226	cell projection organization	BP	3.67E-02	ATP8B1,LRP1,AHI1,RAB21,PLXNB2,PACSIN1,ADGRB1,CTNNA1,CBY1,LGALS1,SH3GL2,MYH9,PLAA,SRF,VEGFA,NCK2,RAB23,P3H1,TGFBR1,RIMS1,TMEM30A,UBA6,RTN4,TPBG,SHANK1,MFSD2A,AKIRIN1,ACTR2,KIDINS220,WHRN,GLI3,NCDN,VPS35,CFAP100,ITGB1,FKBP1B,PLXNA1,KIF5B,ARHGEF4,RB1,PICALM,MARK4,GLI2,MEGF8,PAK1,ACTR3,CTNNB1,TRAK1,DZIP1,DNAJB13,FARP1,FAM98A,PRNP,CDC42EP3,DCLK1,SEMA3C,ILK,DPYSL3,RYK,TTC17,RNF6,CXCL12,ANKRD1,SLC25A46,PALLD,EIF4G2,ROBO1,MAP1B,DIP2B,SEMA4F,PFN2,TLX2,TCTN1,APP,DHX36,RAPGEF2,CX3CL1,PTPN11,ADCY6,HOMER1,OPA1,ARSB,ZFYVE27,CRTAC1,SPP1,PKD2,TWF1,DNAH1,SEMA4G,DNAH5,GPRIN3,IFT57,BRSK2,PDLIM5,NDN,CHRNA7,ARL3,DOCK10,DYNLL1,NOTCH2,CTTN,DNM1L,TROVE2,CAPRIN2,FEZ1,ARMC9,GSK3B,DAG1,PIK3CA,AUTS2,DNAAF5,CORO1C,ANOS1,NTRK3,BMP7,FGFR2,VASH2,IQGAP1,KIF1A,TANC2,ARPC5,MAPT,TNR,FN1,NCKAP1,GPC2,ARPC2,HTT,PRKCI,SKIL,PRRX1,GORAB,SMURF1,NBL1,CAPZB,TNIK,EXOC5,PLD1,VCL,GLDN,OBSL1,CRMP1,HAP1,NOTCH3,ENAH,ATP6V1D,SRCIN1,NES,STX1B,NGFR,TPM1,TGFB3,IFT43,RAB11A,MAP2K1,TTC8,LRRC49,DICER1,CLMN,AK7,NPTN,ISLR2,TRPV2,ACSL4,LAMA3,ROCK1,EVI5L,AKT1,ITGA2,OCRL,RAB34,MYO10,PLXNB3,PAFAH1B1,NTN3,FLNA,PLXNA3,RERE,BSG,DEF8,NTNG2,CDH13,MTSS1L,BARHL2,HSPA5,NEXN,ATP6V0D1,DPYSL5,TCTN2,SERPINF1,FRY,VSTM2L,ITM2C,UBE2V2,SNX10,ARF4,GDNF,THY1,BLOC1S2,CHN1,EFHD1,ARTN,CC2D2B,YWHAH,ZPR1,SDC2,PRKG1,MAP1A,OLFM1	darkgreen	EC_M4
GO:0060348	7.05E-04	0.999644571	40	172	bone development	BP	3.67E-02	NAB2,LEP,SRF,RUNX2,RAB23,P3H1,GLI3,MEGF8,SERPINH1,SLC38A10,RARB,IMPAD1,SULF1,PTPN11,NOTCH2,CHSY1,SBDS,LRP5,MMP14,FGFR2,PTPN6,FBN1,COMP,HSD17B1,FGFR3,TULP3,MED1,THBS3,COL1A1,CHAD,TGFB3,ANXA6,PLS3,PAFAH1B1,ATP6AP1,GLG1,TMEM38B,LOX,SNX10,HAS2	darkgreen	EC_M4
GO:0071222	6.08E-04	0.999744564	26	126	cellular response to lipopolysaccharide	BP	3.68E-02	TNFAIP3,IL6,CXCL8,TLR4,IRAK2,EDNRB,NFKBIB,CD36,SCARB1,IL1A,MEF2C,TLR2,CXCL10,PABPN1,CD86,LTF,TNFRSF1B,TNIP1,CCL2,PDE4B,NOS2,GIT1,IRF8,PLCG2,DAB2IP,BMP6	turquoise	EC_M6
GO:0050851	6.11E-04	0.999749073	25	117	antigen receptor-mediated signaling pathway	BP	3.68E-02	TEC,TNFRSF21,SYK,PRKD2,NECTIN2,GATA3,IKBKB,PDE4D,MEF2C,PLCG1,DGKZ,LCK,ICOSLG,LAPTM5,NFATC2,INPP5D,BMX,PLEKHA1,PRKCH,PSEN1,EIF2B2,CD19,PDE4B,PLCG2,FYB1	turquoise	EC_M6
GO:0042060	6.12E-04	0.999650659	57	329	wound healing	BP	3.68E-02	TNFAIP3,TSPAN8,FZD6,NOTCH4,PTK2,PDGFB,TEC,KDR,SYK,IL6,TLR4,PIK3CG,GATA2,GPR4,PRCP,NOS3,ITGA9,CXCR4,HBEGF,FERMT1,CD36,SCARB1,MERTK,IL1A,IGF1,KLKB1,OCLN,PROCR,ENTPD1,DYSF,DGKZ,ANO6,EDN1,MMRN1,DTNBP1,ETS1,LCK,PPP3CA,CD34,MCAM,CFLAR,F11R,ADORA2A,SELP,VWF,WAS,HIF1A,RAB27A,ADIPOR2,SYT7,PEAR1,MTOR,SMAD2,F8,PLCG2,STXBP1,CARMIL2	turquoise	EC_M6

GO:0034643	7.17E-04	0.999860522	10	24	establishment of mitochondrion localization, microtubule-mediated	BP	3.71E-02	MGARP,TRAK1,MAP1B,OPA1,FEZ1,MAPT,ACTR10,HAP1,ARMCX3,HSBP1	darkgreen	EC_M4
GO:0047497	7.17E-04	0.999860522	10	24	mitochondrion transport along microtubule	BP	3.71E-02	MGARP,TRAK1,MAP1B,OPA1,FEZ1,MAPT,ACTR10,HAP1,ARMCX3,HSBP1	darkgreen	EC_M4
GO:0007135	1.22E-03	0.999937489	4	7	meiosis II	BP	3.71E-02	SGO1,BUB1,BUB1B,SGO2	darkgrey	EC_M8
GO:0045144	1.22E-03	0.999937489	4	7	meiotic sister chromatid segregation	BP	3.71E-02	SGO1,BUB1,BUB1B,SGO2	darkgrey	EC_M8
GO:0051177	1.22E-03	0.999937489	4	7	meiotic sister chromatid cohesion	BP	3.71E-02	SGO1,BUB1,BUB1B,SGO2	darkgrey	EC_M8
GO:0061983	1.22E-03	0.999937489	4	7	meiosis II cell cycle process	BP	3.71E-02	SGO1,BUB1,BUB1B,SGO2	darkgrey	EC_M8
GO:0032760	6.20E-04	0.99979213	18	69	positive regulation of tumor necrosis factor production	BP	3.71E-02	IFNGR1,AKAP12,DDX58,SYK,IL6,TLR4,CD36,IL1A,TLR2,OAS3,HDAC1,CYBB,TMEM106A,PSEN1,LGALS9,PLCG2,OAS1,TLR1	turquoise	EC_M6
GO:0060147	1.22E-03	0.999690702	11	41	regulation of posttranscriptional gene silencing	BP	3.72E-02	TNRC6B,XPO5,ZMPSTE24,RIPK1,AJUBA,FXR1,DDX5,TIAL1,DDX6,DHX9,FMR1	darkgrey	EC_M8
GO:0035850	6.23E-04	0.999838737	13	41	epithelial cell differentiation involved in kidney development	BP	3.72E-02	MTSS1,PDGFB,PODXL,GPR4,GATA3,JAG1,MEF2C,WWTR1,STAT1,PRKX,CD34,PROM1,NOTCH1	turquoise	EC_M6
GO:1901652	7.25E-04	0.999554333	74	375	response to peptide	BP	3.72E-02	LRP1,AP3S1,CDO1,PPARA,LEP,ROCK2,MGARP,SESN3,EIF2B3,PAK1,AKT2,OSBPL8,KAT2B,IRS2,PRNP,ATP2B1,LEPROTL1,CXCL12,MAP1B,SORBS1,USO1,APP,ABCC8,PTPN11,ADCY6,OPA1,PTPN22,CRY2,MMP2,CHRNA7,ADIPOR1,SELENOS,ZNF106,LRP5,EPRS,GSK3B,DAG1,PIK3CA,HADH,BMP7,WDTC1,SYAP1,IQGAP1,BACE1,LINC02210-CRHR1,EIF4EBP2,FBN1,PRKCI,QDPR,TIMP1,PTPRN,MYO5A,SLC2A4,ARHGEF2,NGFR,COL1A1,SHC1,PKM,ROCK1,AKT1,LPIN2,PRKAA1,RAB31,PTPN2,LDOC1,MYO1C,BSG,AHCYL1,BCAR3,SLC2A8,SERPINF1,CACYBP,LEPROT,PIK3C2A	darkgreen	EC_M4
GO:0043434	7.25E-04	0.999573433	63	308	response to peptide hormone	BP	3.72E-02	AP3S1,CDO1,PPARA,LEP,ROCK2,MGARP,SESN3,EIF2B3,PAK1,AKT2,OSBPL8,KAT2B,IRS2,ATP2B1,LEPROTL1,CXCL12,MAP1B,SORBS1,USO1,ABCC8,PTPN11,ADCY6,OPA1,CRY2,ADIPOR1,SELENOS,ZNF106,LRP5,EPRS,GSK3B,DAG1,PIK3CA,HADH,BMP7,WDTC1,SYAP1,IQGAP1,LINC02210-CRHR1,EIF4EBP2,FBN1,PRKCI,QDPR,TIMP1,PTPRN,MYO5A,SLC2A4,COL1A1,SHC1,PKM,ROCK1,AKT1,LPIN2,PRKAA1,RAB31,PTPN2,MYO1C,BSG,AHCYL1,BCAR3,SLC2A8,CACYBP,LEPROT,PIK3C2A	darkgreen	EC_M4
GO:0042742	6.29E-04	0.999751343	23	109	defense response to bacterium	BP	3.74E-02	LYZ,SYK,IL6,TLR4,CD36,TLR2,OAS3,STAB1,LYST,FGR,PRKD1,LTF,SELP,EPHA2,TLR6,ARG2,S100A14,SCNN1B,NOS2,IL7R,ISG15,IRF8,OAS1	turquoise	EC_M6
GO:0044528	1.23E-03	0.999947212	4	6	regulation of mitochondrial mRNA stability	BP	3.74E-02	PDE12,FASTKD1,FASTKD2,FASTKD5	darkgrey	EC_M8
GO:0007417	7.40E-04	0.999476875	142	795	central nervous system development	BP	3.78E-02	LRP1,AHI1,PLXNB2,TRAPPC9,SUN2,MDGA1,SH3GL2,LEP,SRF,C2orf40,UBA6,RTN4,MFSD2A,WHRN,GLI3,INHBA,SLC7A11,PCDH18,ITGB1,FGF2,KIF5B,NAPA,EED,EIF2B3,TENM4,GLI2,RHEB,PAK1,TGFB1,AXL,RAB3GAP1,SCN5A,HTR5A,HNMT,CTNNB1,RARB,TACC1,UFM1,LIG4,IRS2,ATP2B1,DCLK1,SPINK5,APLP1,RYK,SNPH,CXCL12,ZSWIM6,CTNND1,ROBO1,LOXL3,FAIM2,CELF1,TCTN1,APP,RAPGEF2,PTPN11,ARSB,AMIGO2,PPP3CC,ATP2B4,PKD2,DNAH5,PSMG1,NDN,DYNLL1,INA,DNER,UCHL5,MARCKSL1,FEZ1,SATB2,GSK3B,DAG1,LEF1,SHROOM2,KRAS,PITPNM1,NTRK3,ACAN,HAPLN3,BMP7,FGFR2,NCSTN,KIF1A,MACO1,RPS6KA3,PAFAH1B2,ARPC5,SCYL1,MAPT,ATP6AP2,MDGA2,TNR,NCKAP1,COL3A1,KCNA1,WDR1,TULP3,MNAT1,AKT3,HAP1,BMPR1A,CLN8,MED1,PPARGC1A,NES,NGFR,MAP2K1,TTC8,CALM1,EML1,AKT1,TMEM98,MYO1D,ROGDI,MXRA8,PAFAH1B1,SDF4,PKD1,FLNA,PLXNA3,RERE,GNAI3,SYPL2,AARS,HSPA5,LMO4,ATP6V0D1,LHX8,WLS,BNIP3,ATN1,ARF4,LBX1,GDNF,YWHAH,ZPR1,PRKG1,WNT2,NPAS1,FGF10	darkgreen	EC_M4

GO:0120036	7.43E-04	0.999444917	204	1193	plasma membrane bounded cell projection organization	BP	3.78E-02	ATP8B1,LRP1,AHI1,RAB21,PLXNB2,PACSIN1,ADGRB1,CTNNA1,LGALS1,SH3GL2,MYH9,PLAA,SRF,VEGFA,NCK2,RAB23,P3H1,TGFBR1,RIMS1,TMEM30A,UBA6,RTN4,TPBG,SHANK1,MFSD2A,AKIRIN1,ACTR2,KIDINS220,WHRN,GLI3,NCDN,VPS35,CFAP100,ITGB1,FKBP1B,PLXNA1,KIF5B,ARHGEF4,RB1,PICALM,MARK4,GLI2,MEGF8,PAK1,ACTR3,CTNNB1,TRAK1,DZIP1,DNAJB13,FARP1,FAM98A,PRNP,CDC42EP3,DCLK1,SEMA3C,ILK,DPYSL3,RYK,TTC17,RNF6,CXCL12,ANKRD1,SLC25A46,PALLD,EIF4G2,ROBO1,MAP1B,DIP2B,SEMA4F,PFN2,TLX2,TCTN1,APP,DHX36,RAPGEF2,CX3CL1,PTPN11,ADCY6,HOMER1,OPA1,ARSB,ZFYVE27,CRTAC1,SPP1,PKD2,TWF1,DNAH1,SEMA4G,DNAH5,GPRIN3,IFT57,BRSK2,PDLIM5,NDN,CHRNA7,ARL3,DOCK10,DYNLL1,NOTCH2,CTTN,DNM1L,TROVE2,CAPRIN2,FEZ1,ARMC9,GSK3B,DAG1,PIK3CA,AUTS2,DNAAF5,CORO1C,ANOS1,NTRK3,BMP7,FGFR2,VASH2,IQGAP1,KIF1A,TANC2,ARPC5,MAPT,TNR,FN1,NCKAP1,GPC2,ARPC2,HTT,PRKCI,SKIL,PRRX1,GORAB,SMURF1,NBL1,CAPZB,TNIK,EXOC5,PLD1,VCL,GLDN,OBSL1,CRMP1,HAP1,NOTCH3,ENAH,ATP6V1D,SRCIN1,STX1B,NGFR,TPM1,TGFB3,IFT43,RAB11A,MAP2K1,TTC8,LRRC49,DICER1,CLMN,NPTN,ISLR2,TRPV2,ACSL4,LAMA3,ROCK1,EVI5L,AKT1,OCRL,MYO10,PLXNB3,PAFAH1B1,NTN3,FLNA,PLXNA3,RERE,BSG,DEF8,NTNG2,CDH13,MTSS1L,BARHL2,HSPA5,NEXN,ATP6V0D1,DPYSL5,TCTN2,SERPINF1,FRY,VSTM2L,ITM2C,UBE2V2,SNX10,ARF4,GDNF,THY1,BLOC1S2,CHN1,EFHD1,ARTN,CC2D2B,YWHAH,ZPR1,SDC2,PRKG1,MAP1A,OLFM1	darkgreen	EC_M4
GO:0034620	7.43E-04	0.999709558	23	86	cellular response to unfolded protein	BP	3.78E-02	VCP,NCK2,CREB3,ERLEC1,TMTC4,DDRGK1,TMED2,AMFR,SELENOS,CCND1,HSPA8,VAPB,ATF3,ATF6,RHBDD2,ERO1A,EIF2S1,PTPN2,MBTPS1,HSPA5,DNAJB9,HSPA13,DERL2	darkgreen	EC_M4
GO:0031347	6.44E-04	0.999617881	64	397	regulation of defense response	BP	3.79E-02	TNFRSF11A,TNFAIP3,C6orf106,MAPK13,LRRC19,TEK,DDX58,SYK,IL6,TLR4,IL15,PIK3CG,MGLL,CST7,CTSC,GPR4,NECTIN2,PPARG,GATA3,SEC14L1,EDNRB,IKBKB,PDE2A,TMEM173,NR1D2,PLK2,IGF1,KLKB1,ITCH,MEF2C,TLR2,OAS3,NLRC5,PLCG1,IFIT1,STAT1,SNCA,ETS1,CD200,HYAL2,NCF1,FABP4,FGR,PAK2,ADORA2A,CALCRL,BIRC3,SELE,BCL6B,TNFRSF1B,ARG2,TNIP1,YES1,LGALS9,GIT1,ISG15,PLCG2,LRSAM1,CDH5,OAS1,GBP5,TRIM62,SERPINB9,RNF125	turquoise	EC_M6
GO:0071695	6.45E-04	0.99969745	33	163	anatomical structure maturation	BP	3.79E-02	L3MBTL3,ANKS1A,PICK1,SEMA4D,PTBP3,IL15,NRCAM,TUT4,TAL1,GATA2,PPARG,GATA3,EDNRB,SPTBN4,GPAT4,FERMT1,NTN4,RET,IGF1,NR4A2,LTF,HIF1A,ALDH1A2,MTOR,LYL1,B4GALT6,RFLNB,S1PR1,LHX6,CDH5,SOX18,BMP2,SCARF1	turquoise	EC_M6
GO:0033005	6.45E-04	0.999920847	7	15	positive regulation of mast cell activation	BP	3.79E-02	SYK,GATA2,NECTIN2,GAB2,FGR,STXBP1,HAVCR1	turquoise	EC_M6
GO:0150093	7.52E-04	0.999961979	5	7	amyloid-beta clearance by transcytosis	BP	3.80E-02	LRP1,PICALM,LRPAP1,RAB11A,CLTC	darkgreen	EC_M4
GO:0001558	7.52E-04	0.999547381	68	339	regulation of cell growth	BP	3.80E-02	LRP1,RAB21,PPARA,ROS1,PPARD,PLAA,SRF,VEGFA,TGFBR1,RIMS1,RTN4,MFSD2A,INHBA,RB1,PTCH2,MEGF8,PAK1,TGFB1,SEMA3C,ILK,RYK,RNF6,CXCL12,CHPT1,EIF4G2,MAP1B,DIP2B,SEMA4F,ZFYVE27,TSG101,BAP1,SPP1,SEMA4G,ADIPOR1,CTTN,CDC73,HYAL1,CAPRIN2,TGFB2,FBLN5,GSK3B,MMP14,RBBP7,RPS6KA3,MAPT,TNR,FN1,SMURF1,TSPYL2,NPPB,RAB11A,TAF9B,SMARCA4,MYOCD,ISLR2,PIN1,TRPV2,AKT1,CRLF3,PAFAH1B1,PLXNA3,ENO1,BARHL2,CDKN1A,DACT3,NME6,OLFM1,DERL2	darkgreen	EC_M4
GO:0051640	7.61E-04	0.999513597	86	447	organelle localization	BP	3.83E-02	STX7,AP3S1,SUN2,MYH9,UNC13B,DYNC1I1,NLRP5,CENPC,ACTR2,BVES,STARD3NL,MGARP,ITGB1,KIF5B,COPG1,PICALM,PCDH17,ACTR3,CTNNB1,ESYT2,TRAK1,ACTN4,FAM98A,PCLO,SNPH,TMED2,VAMP8,RIOK2,MAP1B,PINX1,ATP2A2,USO1,SDAD1,SLC4A5,EXOC6B,OPA1,AP3B1,VPS18,CHP1,BRSK2,HPS6,DNM1L,FEZ1,WIPI1,SHROOM2,VAPB,KIF1A,TANC2,LSG1,MAPT,PDCD10,HTT,KXD1,ACTR10,FMN2,MYO5A,HAP1,SLC2A4,NLGN2,STARD3,MYO1E,STX1B,SYT11,TMED10,RAB11A,CALM1,SYTL4,VMP1,ARMCX3,CLMN,MYO1D,RAB34,MYO5B,MYO1C,PAFAH1B1,ATP6AP1,IKBKG,VAMP7,AHCYL1,DEF8,HSBP1,RAB6A,BLOC1S2,RMDN1,GEM,FGF10	darkgreen	EC_M4
GO:0060323	7.81E-04	0.999828955	11	28	head morphogenesis	BP	3.91E-02	RRAS,RAB3GAP1,PTPN11,MMP2,LEF1,ARID5B,SGPL1,ATP6AP2,COL1A1,TGFB3,ANKRD11	darkgreen	EC_M4
GO:0042692	7.81E-04	0.999541	62	303	muscle cell differentiation	BP	3.91E-02	PPARA,MRTFA,ADGRB1,CBY1,FLNC,MYH9,SRF,VEGFA,BVES,MYLK3,ITGB1,RB1,SMARCD3,PAK1,TGFB1,CTNNB1,RARB,ACTG1,ACTN4,ANKRD1,HEY1,ATP2A2,HOMER1,ACVR1,PDLIM5,DNER,LMOD1,PRKAR1A,CASP7,MMP14,KRAS,FGFR2,CASQ1,COMP,SYNPO2L,WDR1,SIX4,OBSL1,BMPR1A,TCAP,ACTN1,TPM1,MORF4L2,SETD3,MYOCD,PIN1,FBXO22,PDGFRB,FLII,AKT1,SUPT6H,SYPL2,MIR24-1,DMD,ADAMTS5,LOX,CACYBP,CSRP1,BHLHE41,HOPX,CFL2,FGF10	darkgreen	EC_M4

GO:0032271	6.71E-04	0.999665172	38	199	regulation of protein polymerization	BP	3.92E-02	ARHGAP18,BAIAP2L2,PICK1,CDC42EP1,TMOD1,FAM49A,SVIL,SPTBN4,FCHSD2,ARHGEF7,FCHSD1,LMOD3,HIP1R,OCLN,IQGAP2,DYRK1A,SNCA,ESAM,HCLS1,AP1AR,FES,TMOD4,DLG1,BAIAP2L1,WAS,TMOD2,RAC1,MTOR,ARHGAP28,SPIRE1,GIT1,CARMIL2,CDH5,VASP,GMFG,KANK3,GBA2,ARPC5L	turquoise	EC_M6
GO:0016192	6.72E-04	0.999511721	169	1192	vesicle-mediated transport	BP	3.92E-02	KIF5A,STX11,LAPTM4B,SNX2,SYNJ2,RIMS2,CDC42SE2,PACSIN2,FAM91A1,SGSM3,PTK2,DENND3,TSNARE1,CSNK1E,PICK1,MICALL1,APBA1,KIT,CD2AP,SYK,RGP1,SNAP91,PPT1,CXCL8,VPS54,ARHGAP25,ELMO1,CAV2,TLR4,IL15,SDCBP,NRP1,RAB3B,ANKRD50,PIK3CG,ITSN2,MTMR2,GATA2,RAB38,EXOC3L2,TMCC1,GGA3,GAB2,LLGL2,RALB,USP6NL,UNC13D,TBC1D4,MYO7A,CD93,SPTBN4,CYTH1,P2RY6,FCHSD2,ARAP3,CD36,ADAMTS9,SCARB1,MERTK,EXOC6,CLCN3,HIP1R,MCTP1,TLR2,NDRG4,LMBR1L,BMP2K,DYSF,TPCN1,AP4B1,STAB1,ANO6,DOP1B,EHD4,SNCA,DTNBP1,PPP3CA,RIN3,TINAGL1,SNAP23,LYST,LAPTM5,SEC24B,LRMP,AP1AR,ATP9A,PECAM1,SH3BP4,FGR,INPP5F,TRAK2,ADCY1,KALRN,FES,PRKD1,RNF215,IL10RA,DOCK1,B2M,ZW10,ARHGAP27,ADORA2A,NECAP1,ITGA4,CALCRL,HSPG2,RAMP2,DNM3,CDK16,SELE,SNAP29,FCHO1,LMTK2,WAS,RAC1,RHOJ,PLD2,RAB27A,SNCG,SYT7,CECR2,AP1S3,RSC1A1,MICAL3,FBXL20,PEAR1,OPHN1,PSEN1,RABEP2,RAB3D,DNM2,TBC1D8B,AP2B1,ADRB2,CCL2,YES1,RAB11FIP4,LGALS9,UNC119,SPIRE1,MYO18A,GIT1,USP7,MTM1,RPH3AL,SGSM2,AP3D1,NOTCH1,CACFD1,IRF8,PLCG2,STXBP1,LRSAM1,FNBP1L,EXOC3L1,RASSF9,HAVCR1,RAB43,PLA2G4E,RAB32,STX3,TBC1D20,RALA,VAMP3,CACNG5,SCARF1,CBLL1,RAB39A,GSG1L	turquoise	EC_M6
GO:1901343	6.75E-04	0.999754454	20	84	negative regulation of vasculature development	BP	3.92E-02	SEMA6A,TEK,GPR4,PPARG,TIE1,ADAMTS9,PLK2,HHEX,CXCL10,ANGPT2,THBS4,STAB1,STAT1,HSPG2,EPHA2,SEMA4A,VASH1,NPR1,AMOT,DAB2IP	turquoise	EC_M6
GO:0051271	6.78E-04	0.999642325	44	241	negative regulation of cellular component movement	BP	3.92E-02	AFDN,LIMCH1,C5,LRCH1,DACH1,PPARG,GATA3,TIE1,JAG1,SFRP1,ADAMTS9,ABHD6,MCTP1,MEF2C,NDRG4,ANGPT2,STC1,DLL4,SRGAP2C,CD200,HYAL2,RIN3,AP1AR,IGFBP3,ARHGDIB,APOD,SEMA6D,IGFBP5,WAS,RAC1,JUP,ADIPOR2,MMRN2,MCC,VASH1,MMP28,CCL2,STK26,ARHGAP4,NOTCH1,ENG,DAB2IP,ADGRG1,BST2	turquoise	EC_M6
GO:0010720	7.91E-04	0.999565033	50	231	positive regulation of cell development	BP	3.94E-02	LRP1,RAB21,PLXNB2,SRF,VEGFA,ACTR2,GLI3,PLXNA1,TENM4,MEGF8,SMARCD3,RHEB,PAK1,TGFB1,CTNNB1,TRAK1,LIG4,ILK,CXCL12,EIF4G2,ROBO1,MAP1B,DHX36,CX3CL1,OPA1,ZFYVE27,DNM1L,CAPRIN2,DAG1,MAPT,FN1,ARPC2,PRKCI,SKIL,OBSL1,HAP1,RAB11A,MAP2K1,DICER1,NPTN,ISLR2,TRPV2,PLXNB3,PAFAH1B1,FLNA,ATP6AP1,PLXNA3,SERPINF1,HAS2,WNT2	darkgreen	EC_M4
GO:0060541	7.95E-04	0.999604475	38	163	respiratory system development	BP	3.95E-02	SRF,VEGFA,GLI3,RCN3,SLC7A11,FGF2,GLI2,CTNNB1,RDH10,LOXL3,AP3B1,ADA,MAN1A2,EIF4E,MMP14,DAG1,LEF1,DISP1,KRAS,FGFR2,PLOD3,TULP3,SIX4,BMPR1A,FGFRL1,TGFB3,MAP2K1,MYOCD,PDGFRB,ABCA3,PKD1,FSTL3,TMEM38B,LOX,EDA,HOPX,WNT2,FGF10	darkgreen	EC_M4
GO:0071027	1.31E-03	0.999882614	5	11	nuclear RNA surveillance	BP	3.95E-02	EXOSC9,EXOSC8,XRN1,EXOSC10,EXOSC2	darkgrey	EC_M8
GO:0071028	1.31E-03	0.999882614	5	11	nuclear mRNA surveillance	BP	3.95E-02	EXOSC9,EXOSC8,XRN1,EXOSC10,EXOSC2	darkgrey	EC_M8
GO:0018208	8.00E-04	0.999762734	15	48	peptidyl-proline modification	BP	3.96E-02	P4HA2,P3H1,FKBP1B,CRTAP,EGLN3,PRDX4,FKBP2,P4HA1,FKBP8,ERO1A,FKBP10,PIN1,RANBP2,FKBP1A,P3H3	darkgreen	EC_M4
GO:0072710	1.34E-03	0.999885375	5	10	response to hydroxyurea	BP	4.05E-02	RAD51,BLM,DDX11,ATRX,FMR1	darkgrey	EC_M8
GO:0001837	8.22E-04	0.99961867	32	131	epithelial to mesenchymal transition	BP	4.06E-02	TGFBR1,RTN4,ROCK2,TGFB1,CTNNB1,SPRED3,ZNF703,LOXL3,HEY1,LOXL2,ACVR1,ADIPOR1,PHLDB2,TGFB2,GSK3B,DAG1,LEF1,BMP7,FGFR2,PHLDB1,TGFB1I1,COL1A1,TGFB3,TMEM100,ROCK1,VASN,FLNA,HAS2,DACT3,GLIPR2,OLFM1,WNT2	darkgreen	EC_M4
GO:0010571	1.35E-03	0.999940647	4	6	positive regulation of nuclear cell cycle DNA replication	BP	4.07E-02	DBF4,INO80,DBF4B,ATRX	darkgrey	EC_M8
GO:0032733	7.13E-04	0.999869007	9	25	positive regulation of interleukin-10 production	BP	4.11E-02	SYK,IL6,TLR4,TLR2,CD34,LGALS9,ISG15,PLCG2,CD46	turquoise	EC_M6
GO:2001257	8.37E-04	0.999628157	29	116	regulation of cation channel activity	BP	4.12E-02	UBQLN1,SHANK1,ITGB1,FKBP1B,KIF5B,STIM1,REM1,SLMAP,APP,ABCC8,CACNB3,HOMER1,PKD2,CASQ1,LINC02210-CRHR1,HTT,KCNA1,HAP1,NLGN2,STIM2,PPARGC1A,CALM1,JPH3,MIR24-1,DMD,FKBP1A,CBARP,TMSB4X,GEM	darkgreen	EC_M4

GO:0009615	7.25E-04	0.999615329	44	253	response to virus	BP	4.15E-02	IFNGR1,TNFAIP3,C6orf106,DDX58,IL6,RSAD2,ODC1,IL15,PUM2,UNC13D,GATA3,CXCR4,SEC14L1,IKBKB,TMEM173,ITCH,TLR2,CXCL10,SERINC5,OAS3,NLRC5,IFIT2,IFIT1,STAT1,MX2,MX1,HYAL2,LYST,ISG20,FGR,MOV10,RTP4,DDX3X,BIRC3,HIF1A,CYP1A1,LGALS9,ISG15,OAS1,PMAIP1,TRIM34,BST2,IFIT3,RNF125	turquoise	EC_M6
GO:0003013	7.25E-04	0.999554524	72	437	circulatory system process	BP	4.15E-02	LVRN,AKAP12,TRDN,PDGFB,TEK,ATP8A1,TJP2,DOCK4,TRPM4,PIK3CG,ARHGAP42,GPR4,PRCP,NOS3,PPARG,CXCR4,EDNRB,SPTBN4,SLCO2B1,P2RY2,PDE2A,HBEGF,KCNK6,P2RX2,CD36,PDE4D,CHD7,OCLN,WWTR1,VEGFC,GUCY1A1,GUCY1B1,CXCL10,STC1,KCNJ3,SLC38A1,DLL4,NAV2,EDN1,STAT1,KCNJ2,MEF2A,CD34,F11R,ACE,DLG1,ADORA2A,RAMP2,PLVAP,JUP,MTOR,AR,ATP2A1,NPR1,SCNN1B,DSG2,DSC2,ADRB2,YES1,PDE4B,NOS2,PTGS1,CDH5,MIR23A,SLC8A3,ERAP2,KCNH2,SCN2B,RGS4,BMP6,KCNG2,FLI1	turquoise	EC_M6
GO:0006613	8.57E-04	0.999847545	9	21	cotranslational protein targeting to membrane	BP	4.19E-02	SIL1,SRP72,SEC61A1,SRP68,TRAM1,SSR3,SRPRA,SRP54,SSR2	darkgreen	EC_M4
GO:0034975	8.58E-04	0.999924479	6	10	protein folding in endoplasmic reticulum	BP	4.19E-02	CANX,HSP90B1,PDIA3,DNAJC10,ERO1A,HSPA5	darkgreen	EC_M4
GO:0048585	8.66E-04	0.999349427	206	1229	negative regulation of response to stimulus	BP	4.21E-02	SERPINB2,LRP1,ENPP3,DYRK2,RRM2B,SLC12A2,FBN2,FAM19A5,PPARA,FAM49B,PPARD,CTNNA1,CBY1,LEP,VCP,RUNX2,RASL11B,NCK2,CLOCK,CREB3,ASPN,SNX13,TGFBR1,SOCS5,TPBG,GLI3,DKKL1,VPS35,ITGB1,FKBP1B,FGF2,SESN3,RAB7A,RB1,TPT1,PTCH2,KCTD21,GLI2,MEGF8,CTNNB1,LBH,GPD1L,C1QTNF1,NPLOC4,LTBP1,CRIM1,SPRED3,PRNP,DCN,HDAC3,SEMA3C,CAVIN3,DDRGK1,SMPD1,PPP2CB,LEPROTL1,SPINK5,APLP1,RYK,TMEM127,CNOT7,CXCL12,IL20RB,ZNF536,SULF1,CTNND1,DKK3,ROBO1,WWC2,SEMA4F,LOXL3,FAIM2,HEY1,RASA1,MAPKAPK5,CX3CL1,ABCC8,CACNB3,OPA1,AMFR,PTPN22,TSG101,ACVR1,CRY2,VPS18,CHAC1,ADA,CHP1,ATP2B4,SPP1,HIF1AN,SEMA4G,CBLB,NKD1,TANK,RAB7B,ADIPOR1,FAP,PHLDB2,CTTN,SELENOS,TRIP12,SLC2A10,EYA2,TGFB2,KCTD10,HTR2B,GSK3B,MMP14,DAG1,WWC3,POLQ,GRK3,SLAMF8,BMP7,HYOU1,BICC1,ATF3,PEA15,TP53BP1,HTRA1,STRAP,STRN3,BACE1,FZD9,FAM89B,HSPB1,SERPINE1,SYVN1,TNR,PTPN6,IGFBP2,FBN1,ITGAV,LRPAP1,HTT,COL3A1,SKIL,SMURF1,NBL1,VDAC2,TULP3,GREM2,OTUD5,AKT3,GHITM,CGNL1,ARHGEF2,TGFB1I1,SYT11,NGFR,TGFB3,TAF9B,SOX30,TSPAN6,SMARCA4,UACA,MYOCD,PIN1,CD74,CHRDL1,NOMO1,USP14,AKT1,PRKAA1,NLK,TNFAIP1,PTPN2,NXN,MGRN1,VASN,PAFAH1B1,TAX1BP3,NPHP4,PLXNA3,FAM3A,PARK7,ENO1,GNAI3,USP10,GLG1,HSPA5,MIR24-1,FSTL3,DUSP2,FKBP1A,SERPINF1,PTPN12,A2M,CD59,GDNF,TIMP3,THY1,DNAJB9,FGL2,TXN,DACT3,DUSP4,TMSB4X,PTTG1IP,PMEPA1,LEPROT,SLC35C1,PRKG1,CHST11,FGF10	darkgreen	EC_M4
GO:0006508	8.67E-04	0.999344942	214	1295	proteolysis	BP	4.21E-02	SERPINB2,SEC11C,LRP1,CANX,RNF19A,UBQLN1,MTCH1,KLHDC10,MYH9,PLAA,VEGFA,VCP,C2orf40,CLOCK,PDCL3,REST,SOCS5,ERLEC1,PSME4,UBA6,CYCS,ADAMTS3,MINDY4,UBE2J1,PCYOX1,GLI3,ROCK2,RCN3,PREP,CUL2,DNAJC1,PSMC2,GSAP,PRSS23,PICALM,PDCD6IP,TMUB1,KCTD21,NUB1,TGFB1,WDR48,UBXN4,SPOPL,CTNNB1,SERPINH1,STT3B,NPLOC4,RNF121,CRIM1,PSMD8,GPS1,SPINT2,TMTC3,PRNP,CHFR,NUDT16,DDRGK1,PPP2CB,MMP17,ARL6IP5,UBA3,CPXM1,SPINK5,PSMD6,RNF6,VPS37A,ADAMTS6,HSP90B1,TP53INP2,LNPEP,PCSK1,ROBO1,TRPC4AP,ELOC,RNF13,MMP15,APP,CPE,AMFR,TSG101,CHAC1,MMP2,BAP1,CPN1,BMP1,RUNX1,TTC3,IFT57,TANK,TOLLIP,PSMD14,BRSK2,MARCH6,FAP,TMPRSS7,TM4SF20,UCHL5,SELENOS,TRIP12,PSMD1,UBE3B,KCTD10,AMZ2,GSK3B,CASP7,MMP14,DAG1,ANOS1,HJV,CFI,ACAN,FAM162A,NEDD8,PPP1R11,COL6A3,VASH2,HTRA1,NCSTN,PSMD2,ADRM1,ABRAXAS2,UBE4A,SEC11A,TFRC,BACE1,PCSK7,RPS6KA3,EGLN3,PDIA3,RHBDD2,EDEM3,OLR1,MAPT,SERPINE1,SYVN1,ADAM11,ATP6AP2,DNAJC10,TFR2,PCOLCE,FN1,DNAJB12,COMP,USP48,PGPEP1,ECE1,LAP3,ARAF,SMURF1,TIMP1,FBXL5,JKAMP,OTUD5,ASRGL1,CLN8,ANAPC4,RNF111,TGFB1I1,NGFR,SPOP,FBXL22,TMED10,SEL1L,CLN6,UACA,ARIH1,ATG4A,FBXO22,ROCK1,PCYOX1L,USP14,AKT1,TMEM98,PSMD11,UBE2A,TNFAIP1,OMA1,MMP23B,BCAP31,PKD1,TMEM259,PARK7,ENO1,RHBDF1,USP10,MBTPS1,USP20,F3,GLG1,HSPA5,BCL10,MIR24-1,CNDP2,C9orf3,MINDY3,SPINK2,SERPINF1,FBXO27,SERPINB5,ADAMTS5,SPCS3,A2M,UBE2V2,TIMP3,PIG	darkgreen	EC_M4
GO:0045667	7.41E-04	0.999698376	24	109	regulation of osteoblast differentiation	BP	4.22E-02	CTHRC1,PTK2,ATF4,SEMA4D,IL6,NOCT,TRPM4,PPARG,ACVR2B,JAG1,SFRP1,IGF1,SMAD1,MEF2C,WWTR1,VEGFC,PRKD1,LTF,IGFBP5,FGF23,SMOC1,NOTCH1,BMP2,BMP6	turquoise	EC_M6
GO:0002903	8.75E-04	0.999922754	6	10	negative regulation of B cell apoptotic process	BP	4.23E-02	IRS2,ADA,SLC39A10,CD74,BCL10,ORMDL3	darkgreen	EC_M4

GO:0061042	7.49E-04	0.99990265	7	16	vascular wound healing	BP	4.25E-02	TNFAIP3,KDR,GATA2,CXCR4,CD34,MCAM,ADIPOR2	turquoise	EC_M6
GO:2000781	1.42E-03	0.99966436	10	36	positive regulation of double-strand break repair	BP	4.26E-02	RIF1,PRKDC,ERCC6,ARID2,FANCB,RAD51AP1,FOXM1,DDX11,FUS,SMCHD1	darkgrey	EC_M8
GO:0012501	8.83E-04	0.999322582	241	1474	programmed cell death	BP	4.26E-02	SERPINB2,LRP1,AHI1,AGAP2,DYRK2,IER3,CDSN,RRM2B,ANGPT1,TCP1,PPARA,PPARD,CTNNA1,UBQLN1,MTCH1,LGALS1,PRUNE2,LEP,BAG1,VEGFA,DOCK8,VCP,UNC13B,NCK2,CREB3,ASNS,PDCL3,REST,TGFBR1,RTN4,CYCS,TAX1BP1,GGCT,MAP3K7,GLI3,INHBA,DKKL1,VPS35,CUL2,SLC7A11,ITGB1,FGF2,SRPK2,RB1,MARK4,TPT1,PDCD6IP,GLI2,PAK1,TGFB1,AXL,ACER3,CTNNB1,AKT2,RELT,RARB,ACTN4,TMTC4,UFM1,LIG4,IRS2,PRNP,HDAC3,DDRGK1,SMPD1,PPP2CB,ILK,ARL6IP5,PPP2R2B,APLP1,CXCL12,ANKRD1,HSP90B1,SLC25A4,C19orf12,SULF1,EMC4,P2RX4,FAIM2,RASA1,RTKN,APP,RAPGEF2,CX3CL1,OPA1,AMIGO2,ACVR1,CHAC1,GDF6,SERINC3,MTDH,ADA,AKTIP,TXNDC5,PLA2R1,IFT57,BRSK2,SLC39A10,ELMO2,DYNLL1,FAP,TJP1,NOTCH2,CTTN,CDC73,DNM1L,GLRX2,SELENOS,EYA2,CLPTM1L,TGFB2,LRP5,HTR2B,ITPK1,ZMAT3,GSK3B,CASP7,FSTL1,PIK3CA,LEF1,PRKCA,KRAS,FAM162A,KPNA1,BMP7,HYOU1,ATF3,FGFR2,PEA15,NCSTN,TFRC,ATF6,BACE1,FZD9,RPS6KA3,EGLN3,PDIA3,HSPB1,SERPINE1,SYVN1,SGPL1,ZNF385B,DNAJC10,NCKAP1,PTPN6,PDCD10,ITGAV,COMP,HTT,FKBP8,ERO1A,PRKCI,SKIL,FGFR3,RTN3,ARAF,TIMP1,EXOC5,VDAC2,FMN2,SIX4,SGMS1,MNAT1,ERC1,GHITM,MAGED1,CLN8,EIF2S1,MED1,PPARGC1A,ACTN1,CPEB4,NES,PNMA1,ARHGEF2,NGFR,TGFB3,DNASE2,SHC1,TAF9B,GPR65,UACA,PKM,DICER1,GSKIP,MYOCD,ANXA6,PIN1,CD74,PDGFRB,ROCK1,AKT1,PRKAA1,TNFAIP1,TM2D1,PTPN2,OMA1,CD40LG,MYDGF,DAPK3,GNB1,BCAP31,TNFRSF18,FLNA,IKBKG,PARK7,ENO1,AHCYL1,GNAI3,CFDP1,AARS,HSPA5,BCL10,NAE1,MIR24-1,MINDY3,BNIP3,ATN1,LOX,VSTM2L,ITM2C,UBE2V2	darkgreen	EC_M4
GO:0071478	1.42E-03	0.999357002	26	150	cellular response to radiation	BP	4.26E-02	XRCC6,POLH,ZMPSTE24,METTL3,ATR,PBK,PPID,TENT2,RAD51,INO80,NUCKS1,CHEK1,RGS9,INTS7,BLM,CDC25A,XRCC5,RAD51AP1,ECT2,AIPL1,NEDD4,AURKB,TAF1,CUL4B,NIPBL,FMR1	darkgrey	EC_M8
GO:2000001	1.43E-03	0.999820607	6	15	regulation of DNA damage checkpoint	BP	4.27E-02	DDX39B,ETAA1,RNASEH2B,CUL4A,RPA2,THOC1	darkgrey	EC_M8
GO:0006206	1.43E-03	0.999820493	6	15	pyrimidine nucleobase metabolic process	BP	4.27E-02	CTPS1,CAD,RRM1,UMPS,TYMS,DHODH	darkgrey	EC_M8
GO:0030041	7.60E-04	0.9996436	32	160	actin filament polymerization	BP	4.28E-02	ARHGAP18,BAIAP2L2,PICK1,CDC42EP1,TMOD1,FAM49A,SVIL,MSRB2,SPTBN4,FCHSD2,FCHSD1,LMOD3,HIP1R,IQGAP2,ESAM,HCLS1,AP1AR,TMOD4,DLG1,BAIAP2L1,WAS,TMOD2,RAC1,MTOR,ARHGAP28,SPIRE1,CARMIL2,VASP,GMFG,KANK3,GBA2,ARPC5L	turquoise	EC_M6
GO:0034341	7.67E-04	0.999708001	21	95	response to interferon-gamma	BP	4.28E-02	IFNGR1,DAPK1,TLR4,PPARG,TLR2,NLRC5,EDN1,STAT1,CD58,SNCA,WAS,CCL2,JAK1,LGALS9,NOS2,IRF8,STXBP1,GBP5,RAB43,BST2,VAMP3	turquoise	EC_M6
GO:0071605	7.67E-04	0.999928395	6	11	monocyte chemotactic protein-1 production	BP	4.28E-02	SYK,ERBIN,OAS3,APOD,LGALS9,OAS1	turquoise	EC_M6
GO:0071637	7.67E-04	0.999928395	6	11	regulation of monocyte chemotactic protein-1 production	BP	4.28E-02	SYK,ERBIN,OAS3,APOD,LGALS9,OAS1	turquoise	EC_M6
GO:0071706	7.74E-04	0.999677129	25	115	tumor necrosis factor superfamily cytokine production	BP	4.28E-02	IFNGR1,TNFAIP3,AKAP12,C6orf106,DDX58,SYK,IL6,TLR4,CD36,IL1A,IGF1,TLR2,OAS3,HDAC1,CD34,CD86,LTF,CYBB,TMEM106A,ARG2,PSEN1,LGALS9,PLCG2,OAS1,TLR1	turquoise	EC_M6
GO:1903555	7.74E-04	0.999677129	25	115	regulation of tumor necrosis factor superfamily cytokine production	BP	4.28E-02	IFNGR1,TNFAIP3,AKAP12,C6orf106,DDX58,SYK,IL6,TLR4,CD36,IL1A,IGF1,TLR2,OAS3,HDAC1,CD34,CD86,LTF,CYBB,TMEM106A,ARG2,PSEN1,LGALS9,PLCG2,OAS1,TLR1	turquoise	EC_M6
GO:0070309	7.75E-04	0.99998054	4	5	lens fiber cell morphogenesis	BP	4.28E-02	ATF4,PROX1,EPHA2,TBC1D20	turquoise	EC_M6

GO:0010628	7.79E-04	0.999466149	116	803	positive regulation of gene expression	BP	4.28E-02	IFNGR1,AKAP12,FLT4,RIMS2,QKI,AFDN,ATF4,PDGFB,MAPK13,KDM4C,DDX58,KIT,NFIL3,SYK,IL6,CXCL8,PELI1,RSAD2,TLR4,C5,IL15,MYCN,NOCT,PIK3CG,GATA2,PRKD2,NOS3,PPARG,GATA3,PDE2A,TMEM173,DND1,FERMT1,RPL26,CD36,RET,PDE4D,IL1A,DNMT3B,IGF1,KLKB1,EIF2AK3,SMAD1,OCLN,MEF2C,SMARCD1,TLR2,IL7,P2RX7,OAS3,DLL4,STAT1,CD58,DYRK1A,DTNBP1,ETS1,CD200,HDAC1,PPP3CA,HYAL2,MEF2A,LAPTM5,NFATC2,CD34,CD86,FGR,ARID1A,YTHDF1,MELTF,CREB1,B2M,TET1,CYBB,IL1RAP,DDX3X,TMEM106A,RAMP2,FGF23,PPM1F,HIF1A,RAB27A,TLR6,ALDH1A2,MTOR,AR,PSEN1,FGF18,ITGA3,BATF,SMAD2,YY1,PDE4B,LGALS9,NOS2,IL7R,ISG15,HCFC1,TRAF2,NOTCH1,IRF8,PLCG2,ENG,NFAT5,TERF2,FUBP1,CDH5,OAS1,TLR1,GBP5,TRIM24,CD46,SOX17,FGF16,SERPINB9,BMP2,BMP6	turquoise	EC_M6
GO:0097529	7.79E-04	0.999654171	28	142	myeloid leukocyte migration	BP	4.28E-02	TNFRSF11A,PTK2,PDGFB,KIT,SYK,IL6,CXCL8,C5,PIK3CG,EDNRB,VEGFC,CXCL10,THBS4,ANO6,EDN1,JAM3,CD200,RIN3,PECAM1,MCU,RAC1,PGF,DAPK2,S100A14,MMP28,CCL2,PDE4B,MSMP	turquoise	EC_M6
GO:0010165	1.44E-03	0.999695371	9	29	response to X-ray	BP	4.29E-02	XRCC6,MSH2,BRCA2,ERCC6,RAD51,NUCKS1,BLM,XRCC5,NIPBL	darkgrey	EC_M8
GO:0045597	8.98E-04	0.999384904	116	639	positive regulation of cell differentiation	BP	4.32E-02	IL23A,LRP1,AHI1,RAB21,PLXNB2,FBN2,HLA-DRA,PPARD,SRF,VEGFA,RUNX2,REST,TGFBR1,SOCS5,AKIRIN1,ACTR2,GLI3,INHBA,RRAS,MYLK3,DKKL1,PLXNA1,FGF2,RB1,PTCH2,TENM4,GLI2,MEGF8,SMARCD3,RHEB,PAK1,TGFB1,AXL,CTNNB1,IL15RA,TRAK1,ZEB2,LIG4,ZNF703,ILK,CXCL12,EIF4G2,ROBO1,MAP1B,DHX36,RAPGEF2,CX3CL1,OPA1,LOXL2,AP3B1,ZFYVE27,ACVR1,GDF6,ADA,RUNX1,HIF1AN,RAB7B,FNDC5,NOTCH2,DNM1L,CAPRIN2,TGFB2,LRP5,FEZ1,MMP14,DAG1,LEF1,PRKCA,CMKLR1,BMP7,SYAP1,RPS6KA3,MAPT,SERPINE1,FN1,ADRA2C,ARPC2,SUCO,PRKCI,SKIL,NBL1,OBSL1,HAP1,BMPR1A,PDLIM7,MED1,ARHGEF2,TGFB1I1,COL1A1,TGFB3,RAB11A,MAP2K1,TMEM100,DICER1,MORF4L2,NPTN,SETD3,MYOCD,ISLR2,PIN1,TRPV2,CD74,AKT1,PLXNB3,PAFAH1B1,FLNA,ATP6AP1,PLXNA3,SERPINF1,GDNF,HAS2,GLIPR2,FAM210B,HOPX,OLFM1,WNT2	darkgreen	EC_M4
GO:0034612	7.94E-04	0.999615975	34	181	response to tumor necrosis factor	BP	4.34E-02	TNFRSF11A,TNFAIP3,AKAP12,TNFRSF21,SYK,CXCL8,TRAF1,ZFAND6,GATA3,IKBKB,SFRP1,TNFRSF19,ERBIN,OCLN,GPD1,HIPK1,EDN1,STAT1,CD58,HDAC1,HYAL2,LAPTM5,FABP4,ADAMTS7,BIRC3,SELE,PLVAP,TNFRSF1B,CCL2,TRAF2,TNFRSF25,DAB2IP,AFF3,COMMD7	turquoise	EC_M6
GO:0060966	1.46E-03	0.999619	11	42	regulation of gene silencing by RNA	BP	4.35E-02	TNRC6B,XPO5,ZMPSTE24,RIPK1,AJUBA,FXR1,DDX5,TIAL1,DDX6,DHX9,FMR1	darkgrey	EC_M8
GO:0090150	9.18E-04	0.999507912	45	210	establishment of protein localization to membrane	BP	4.37E-02	HSPA4,REEP2,SIL1,GGA1,SRP72,COX18,EMC10,VPS35,SEC61A1,RAB7A,PAK1,SRP68,RAB3GAP1,AKT2,ATP2C1,PRNP,VPS37A,TRAM1,EMC4,CACNB3,SSR3,AP3B1,CHP1,SRPRA,GORASP2,SRP54,PRKCI,EMC1,BLZF1,ATAD1,ZDHHC4,STX1B,SSR2,RAB11A,CALM1,MIEF2,ZDHHC9,RAB31,RAB34,MYO1C,HSPA5,C16orf70,EMC7,ARFRP1,TIMM22	darkgreen	EC_M4
GO:0051345	9.20E-04	0.999410432	84	440	positive regulation of hydrolase activity	BP	4.37E-02	TBC1D9B,C6orf89,MTCH1,GNAQ,PLAA,VEGFA,DOCK8,VCP,REST,SNX13,PSME4,CYCS,ROCK2,RCN3,ITGB1,FGF2,NAPA,ABHD5,PICALM,RAB3GAP1,AKT2,TBC1D16,PPP1R12A,DDRGK1,ARL6IP5,ARL1,ATP1B3,ROBO1,PFN2,RASA1,APP,RAPGEF2,CX3CL1,RALGAPB,IFT57,TANK,PSMD14,SLC39A10,RAP1GDS1,DOCK10,HTR2B,GSK3B,CORO1C,ADAP1,NTRK3,SGSM1,FAM162A,FGFR2,NCSTN,ADRM1,EGLN3,MAPT,DNAJC10,PCOLCE,FN1,ZC3H15,FGFR3,NGFR,TPM1,GPR65,CALM1,UACA,PIN1,PDGFRB,EVI5L,AKT1,SNX18,ITGA2,ABR,BCAP31,TAX1BP3,F3,ARHGAP29,BCAR3,MTSS1L,BCL10,CCL16,NMUR1,ARF4,THY1,TOR1AIP2,CHN1,GABARAPL2,FGF10	darkgreen	EC_M4
GO:0072659	9.21E-04	0.999486522	51	239	protein localization to plasma membrane	BP	4.37E-02	LRP1,STX7,PACSIN1,GGA1,FAM126A,TPBG,ROCK2,VPS35,ITGB1,KIF5B,RAB7A,PICALM,TGFB1,AKT2,ATP2C1,PRNP,ARL6IP5,TMED2,TMEM150A,VAMP8,SLMAP,ATP1B3,SORBS1,RAPGEF2,CACNB3,ZFYVE27,ATP2B4,PKP2,MMP14,AP2M1,GORASP2,LRRC15,SEC23A,PRKCI,SMURF1,TNIK,EXOC5,BLZF1,MYO5A,TSPAN17,RAB11A,TTC8,CLTC,ROCK1,AKT1,RAB31,RAB34,FLNA,BSG,C16orf70,ARFRP1	darkgreen	EC_M4

GO:0009725	9.21E-04	0.999366528	118	652	response to hormone	BP	4.37E-02	AP3S1,CDO1,PPARA,PPARD,CTNNA1,TXN2,LEP,SRF,CLOCK,ASNS,REST,GLI3,ROCK2,INHBA,MGARP,PGRMC2,SESN3,GLB1,EIF2B3,PAK1,TGFB1,HNMT,LBH,AKT2,OSBPL8,RARB,KAT2B,UFM1,IRS2,ATP2B1,UBA5,DDRGK1,LEPROTL1,RNF6,CXCL12,MAP1B,SORBS1,HEY1,USO1,ABCC8,PTPN11,ADCY6,OPA1,ARSB,GTF2H1,CRY2,VPS18,SPP1,CPN1,EIF4E,ADIPOR1,SELENOS,CCND1,TGFB2,ZNF106,LRP5,EPRS,GSK3B,MMP14,DAG1,PIK3CA,HADH,KRAS,NTRK3,BMP7,WDTC1,SYAP1,IQGAP1,RBBP7,STRN3,LINC02210-CRHR1,PLOD3,EIF4EBP2,COMT,IGFBP2,FBN1,PRKCI,QDPR,TIMP1,PTPRN,MYO5A,SLC2A4,MED1,PPARGC1A,COL1A1,TGFB3,SHC1,SOX30,CALM1,SMARCA4,PKM,PDGFRB,ROCK1,AKT1,ITGA2,LPIN2,PRKAA1,RAB31,PTPN2,MYO1C,GNB1,ABCA3,SSTR5,PARK7,BSG,AHCYL1,AGL,BCAR3,SLC2A8,SERPINF1,LOX,CACYBP,TIMP3,CDKN1A,YWHAH,PMEPA1,LEPROT,PIK3C2A	darkgreen	EC_M4
GO:0120193	8.04E-04	0.99975685	15	54	tight junction organization	BP	4.38E-02	AFDN,IKBKB,MARVELD2,OCLN,ESAM,CLDN34,PECAM1,F11R,PAK2,DLG1,RAMP2,PRKCH,EPHA2,PATJ,CDH5	turquoise	EC_M6
GO:0060627	8.19E-04	0.999505452	66	403	regulation of vesicle-mediated transport	BP	4.42E-02	RIMS2,PACSIN2,PICK1,APBA1,CD2AP,SYK,SNAP91,PPT1,IL15,SDCBP,NRP1,RAB3B,MTMR2,GATA2,GAB2,UNC13D,TBC1D4,CD36,SCARB1,MERTK,HIP1R,MCTP1,TLR2,NDRG4,BMP2K,DYSF,ANO6,EHD4,SNCA,DTNBP1,PPP3CA,RIN3,ATP9A,FGR,INPP5F,ADCY1,FES,PRKD1,B2M,ADORA2A,SELE,RAC1,RAB27A,SYT7,RSC1A1,FBXL20,OPHN1,RAB3D,DNM2,AP2B1,CCL2,LGALS9,UNC119,MYO18A,GIT1,USP7,RPH3AL,NOTCH1,PLCG2,STXBP1,LRSAM1,PLA2G4E,TBC1D20,RALA,CBLL1,GSG1L	turquoise	EC_M6
GO:0030947	8.20E-04	0.999850161	9	23	regulation of vascular endothelial growth factor receptor signaling pathway	BP	4.42E-02	GRB10,PRKD2,HHEX,VEGFC,HIF1A,MMRN2,FGF18,TMEM204,DAB2IP	turquoise	EC_M6
GO:0045069	8.20E-04	0.999737469	16	62	regulation of viral genome replication	BP	4.42E-02	CXCL8,PPIE,RSAD2,OAS3,IFIT1,MX1,KIAA1551,ISG20,PROX1,LTF,DDX3X,TNIP1,ISG15,NOTCH1,OAS1,BST2	turquoise	EC_M6
GO:0050654	9.38E-04	0.999789361	11	28	chondroitin sulfate proteoglycan metabolic process	BP	4.44E-02	CHPF2,DCN,IMPAD1,CHSY1,HYAL1,B3GAT3,UGDH,CHST12,XYLT2,EGFLAM,CHST11	darkgreen	EC_M4
GO:0007000	1.50E-03	0.999814731	6	14	nucleolus organization	BP	4.46E-02	RRP8,POLR1B,SMARCA5,NOLC1,SUV39H1,DDX11	darkgrey	EC_M8
GO:0006378	1.51E-03	0.999609978	11	40	mRNA polyadenylation	BP	4.46E-02	SCAF8,YTHDC1,PNPT1,PAPOLG,WDR33,SYMPK,PCF11,TENT2,VIRMA,CPSF2,LEO1	darkgrey	EC_M8
GO:0006915	9.59E-04	0.999265349	235	1436	apoptotic process	BP	4.53E-02	SERPINB2,LRP1,AHI1,AGAP2,DYRK2,IER3,RRM2B,ANGPT1,TCP1,PPARA,PPARD,CTNNA1,UBQLN1,MTCH1,LGALS1,PRUNE2,LEP,BAG1,VEGFA,DOCK8,VCP,UNC13B,NCK2,CREB3,ASNS,PDCL3,REST,TGFBR1,RTN4,CYCS,TAX1BP1,GGCT,MAP3K7,GLI3,INHBA,DKKL1,VPS35,CUL2,SLC7A11,ITGB1,SRPK2,RB1,TPT1,PDCD6IP,GLI2,PAK1,TGFB1,AXL,CTNNB1,AKT2,RELT,RARB,ACTN4,TMTC4,UFM1,LIG4,IRS2,PRNP,HDAC3,DDRGK1,SMPD1,PPP2CB,ILK,ARL6IP5,PPP2R2B,APLP1,CXCL12,ANKRD1,HSP90B1,SLC25A4,C19orf12,SULF1,EMC4,P2RX4,FAIM2,RASA1,RTKN,APP,RAPGEF2,CX3CL1,OPA1,AMIGO2,ACVR1,CHAC1,GDF6,SERINC3,MTDH,ADA,AKTIP,TXNDC5,PLA2R1,IFT57,BRSK2,SLC39A10,ELMO2,DYNLL1,FAP,TJP1,NOTCH2,CTTN,CDC73,DNM1L,GLRX2,SELENOS,EYA2,CLPTM1L,TGFB2,LRP5,HTR2B,ZMAT3,GSK3B,CASP7,FSTL1,PIK3CA,LEF1,PRKCA,KRAS,FAM162A,KPNA1,BMP7,HYOU1,ATF3,FGFR2,PEA15,NCSTN,TFRC,ATF6,BACE1,FZD9,RPS6KA3,EGLN3,PDIA3,HSPB1,SERPINE1,SYVN1,SGPL1,ZNF385B,DNAJC10,NCKAP1,PTPN6,PDCD10,ITGAV,COMP,HTT,FKBP8,ERO1A,PRKCI,SKIL,FGFR3,RTN3,ARAF,TIMP1,EXOC5,VDAC2,FMN2,SIX4,SGMS1,MNAT1,ERC1,GHITM,MAGED1,CLN8,EIF2S1,MED1,PPARGC1A,ACTN1,CPEB4,NES,PNMA1,ARHGEF2,NGFR,TGFB3,DNASE2,SHC1,TAF9B,GPR65,UACA,DICER1,GSKIP,MYOCD,ANXA6,PIN1,CD74,PDGFRB,ROCK1,AKT1,PRKAA1,TNFAIP1,TM2D1,PTPN2,OMA1,CD40LG,MYDGF,DAPK3,GNB1,BCAP31,TNFRSF18,FLNA,IKBKG,PARK7,ENO1,AHCYL1,GNAI3,CFDP1,AARS,HSPA5,BCL10,NAE1,MIR24-1,MINDY3,BNIP3,ATN1,LOX,VSTM2L,ITM2C,UBE2V2	darkgreen	EC_M4
GO:0035023	8.48E-04	0.999707328	18	69	regulation of Rho protein signal transduction	BP	4.56E-02	CDC42SE2,NRP1,RASIP1,ARHGAP42,GPR4,NET1,PRAG1,ARHGEF3,ARHGEF28,F11R,ARHGDIB,BCR,RAC1,OPHN1,STARD8,ITGA3,ADGRG1,LPAR6	turquoise	EC_M6
GO:1901215	9.73E-04	0.999514401	37	161	negative regulation of neuron death	BP	4.57E-02	LRP1,AGAP2,ANGPT1,PPARA,REST,VPS35,SLC7A11,AXL,CTNNB1,LIG4,ILK,FAIM2,RASA1,CX3CL1,GSK3B,INPP5A,PIK3CA,KRAS,HYOU1,FZD9,PRKCI,SIX4,PPARGC1A,CPEB4,NES,TGFB3,PIN1,AKT1,IKBKG,TMEM259,PARK7,AARS,SERPINF1,VSTM2L,UBE2V2,GDNF,ZPR1	darkgreen	EC_M4

GO:0050918	9.74E-04	0.999706387	15	47	positive chemotaxis	BP	4.57E-02	LRP1,ANGPT1,VEGFA,FGF2,CXCL12,CX3CL1,NTRK3,ITGA2,PLXNB3,CDH13,F3,CCL16,GDNF,ARTN,FGF10	darkgreen	EC_M4
GO:0009893	1.55E-03	0.998768727	311	2815	positive regulation of metabolic process	BP	4.58E-02	PAN2,ONECUT2,BCLAF1,LATS1,CCT2,DDX39B,SCAF8,UBR5,EIF3E,MAP3K4,DSCC1,HSF2,ATAD2,XRCC6,PHF5A,TNRC6B,TUT7,IFT74,POT1,PWP1,ZNF300,TOPORS,DBF4,POLR3B,AKAP9,RFX3,IL1R1,CKS2,TRIM28,NCBP1,ZNF451,ANP32B,FOXJ3,MSH2,PHIP,TTK,PPP4R3B,HNRNPA2B1,PNPT1,ZMPSTE24,RLF,SYNCRIP,ZNF292,CCT4,COMMD1,CASP8AP2,ETAA1,ITGB1BP1,THRAP3,EZH2,UFL1,CCNC,CLSPN,CASP2,DDX1,NAA15,ELF2,WDR35,NRDC,ATAD2B,HDAC2,CCNA2,EXOSC9,MLLT10,SAE1,CEP295,DNAJC2,SYMPK,NUP85,ELF1,RSF1,BORA,DIS3,CCNT2,PAXIP1,WDR43,CDC20,FNTA,POLR3F,SUPT16H,CHD8,KAT6A,METTL3,RIF1,E2F7,ALYREF,CKAP2,CEP290,NSD3,CEBPZ,KITLG,TAF7,EXOSC8,RFC3,BRCA2,ELK3,TCERG1,MAPK8,PRKDC,APAF1,CCNB1,ERCC6,DHX29,PAN3,SCYL2,GPBP1,SP1,TCEA1,TPX2,ZC3H8,DIMT1,UBA2,TNKS2,ZNF143,CENPJ,CDK5RAP1,NCK1,IDE,ARFGEF1,SMARCA5,ZNF507,OTUD4,ZFP91,TAF9,PDE12,TFCP2,ATR,MTMR9,LARP4,UTP15,HLTF,CNOT1,GABPA,MCRS1,USP16,MOB1A,ALMS1,CCT7,CDCA2,NBN,SENP1,ABRAXAS1,RAD51,SLC38A2,TOMM70,RIPK1,WDR75,ARID2,MYBL2,E2F8,INO80,RIOK1,CHUK,STK4,UBE2C,HIVEP1,RFC5,TAOK3,NOLC1,CCT5,BMPR1B,UBE3A,CCT6A,PRDM10,NUCKS1,GCN1,CUL3,CHEK1,TIMM17A,H2AFZ,RRP1B,NFKB1,KHDRBS1,CENPE,TET2,TRIP11,HEATR1,NCL,KIF14,AJUBA,NOL11,GTF2I,BPTF,FXR1,FANCI,DDX5,FANCB,RFC2,NEK2,EIF2B5,DTL,ALS2,BLM,EDRF1,CDK1,PDE8A,ING5,BCLAF3,FOSL1,SMARCC1,SP3,DHX9,HNRNPR,TRA2B,SETD2,FASTKD2,RFC4,DNA2,CCAR1,DDX21,FANCM,DBF4B,IL12A,UBTF,COP1,NMD3,XRCC5,NPAT,BRCA1,PSME3,CNOT9,NSD2,DCUN1D5,RAD	darkgrey	EC_M8
GO:0032239	1.55E-03	0.999807411	6	14	regulation of nucleobase-containing compound transport	BP	4.58E-02	RIPK1,NSUN2,KHDRBS1,DHX9,SETD2,TPR	darkgrey	EC_M8
GO:0032881	9.80E-04	0.999756864	12	33	regulation of polysaccharide metabolic process	BP	4.58E-02	DYRK2,TGFB1,AKT2,IRS2,SORBS1,SELENOS,GSK3B,PPP1R3D,PPP1R3F,CLTC,AKT1,HAS2	darkgreen	EC_M4
GO:2001032	1.56E-03	0.999704332	8	25	regulation of double-strand break repair via nonhomologous end joining	BP	4.59E-02	POT1,RIF1,PRKDC,ERCC6,DEK,AUNIP,NSD2,SMCHD1	darkgrey	EC_M8
GO:0009968	9.94E-04	0.999272961	167	973	negative regulation of signal transduction	BP	4.63E-02	LRP1,DYRK2,RRM2B,FBN2,PPARA,FAM49B,CTNNA1,CBY1,VCP,RUNX2,RASL11B,NCK2,CLOCK,CREB3,ASPN,SNX13,TGFBR1,SOCS5,TPBG,GLI3,DKKL1,ITGB1,FGF2,SESN3,RAB7A,RB1,TPT1,PTCH2,KCTD21,GLI2,MEGF8,CTNNB1,LBH,GPD1L,NPLOC4,LTBP1,CRIM1,SPRED3,PRNP,DCN,HDAC3,CAVIN3,DDRGK1,SMPD1,PPP2CB,LEPROTL1,APLP1,TMEM127,CNOT7,CXCL12,ZNF536,SULF1,CTNND1,DKK3,ROBO1,WWC2,FAIM2,HEY1,RASA1,MAPKAPK5,CX3CL1,OPA1,AMFR,PTPN22,TSG101,ACVR1,CRY2,VPS18,CHAC1,ADA,CHP1,ATP2B4,HIF1AN,CBLB,NKD1,TANK,RAB7B,ADIPOR1,CTTN,SELENOS,SLC2A10,EYA2,TGFB2,KCTD10,HTR2B,GSK3B,MMP14,DAG1,WWC3,GRK3,BMP7,HYOU1,BICC1,ATF3,PEA15,HTRA1,STRAP,STRN3,FZD9,FAM89B,HSPB1,SERPINE1,SYVN1,PTPN6,IGFBP2,FBN1,ITGAV,LRPAP1,HTT,SKIL,SMURF1,NBL1,VDAC2,TULP3,GREM2,OTUD5,GHITM,CGNL1,ARHGEF2,TGFB1I1,NGFR,TGFB3,TAF9B,SOX30,TSPAN6,SMARCA4,UACA,MYOCD,PIN1,CD74,CHRDL1,NOMO1,AKT1,PRKAA1,NLK,TNFAIP1,PTPN2,NXN,MGRN1,VASN,PAFAH1B1,TAX1BP3,NPHP4,PARK7,ENO1,GNAI3,USP10,GLG1,HSPA5,MIR24-1,FSTL3,DUSP2,FKBP1A,PTPN12,GDNF,TIMP3,THY1,DNAJB9,DACT3,DUSP4,TMSB4X,PTTG1IP,PMEPA1,LEPROT,SLC35C1,CHST11,FGF10	darkgreen	EC_M4
GO:0009411	1.60E-03	0.999313392	23	127	response to UV	BP	4.69E-02	MAP3K4,POLH,REV1,MSH2,METTL3,BRCA2,MAPK8,ERCC6,ATR,PBK,PPID,INO80,CHEK1,DTL,CDC25A,IL12A,NEDD4,AURKB,TAF1,TIPIN,CUL4B,FMR1,FEN1	darkgrey	EC_M8
GO:0005975	1.02E-03	0.999342349	86	458	carbohydrate metabolic process	BP	4.72E-02	B4GALT7,B4GALNT1,DYRK2,MIOX,PPARA,PPARD,LEP,GALT,CHST10,NANS,SLC35A1,GLB1,TGFB1,RBKS,DGAT2,AKT2,GPD1L,PFKP,C1QTNF1,KAT2B,IRS2,IMPAD1,ST8SIA4,B3GNT4,CHST2,GYG1,SORBS1,APP,HECTD4,PFKM,PGAM4,CHID1,MAN1A2,ADIPOR1,SELENOS,HYAL1,CPT1A,ZBTB20,LRP5,B4GALT4,ITPK1,GYG2,GSK3B,INPP5A,SIAE,PIK3CA,B3GALT1,IDH2,WDTC1,MAN2A2,PPP1R3D,ATF3,EDEM3,MOGAT3,TPI1,SLC3A2,B3GAT3,PPP1R3F,UGDH,SLC2A4,CHST12,PPARGC1A,CLTC,PKM,MPI,AKT1,PRKAA1,OCRL,PTPN2,OMA1,PGP,MIDN,GNPTG,FAM3A,ENO1,SLC2A6,POMT1,AGL,SLC2A8,RANBP2,HAS2,GLO1,PPP1R1A,NNMT,CHST11,HS2ST1	darkgreen	EC_M4

GO:0030334	1.02E-03	0.999283643	131	735	regulation of cell migration	BP	4.72E-02	LRP1,PLXNB2,ANGPT1,FAM19A5,NUS1,FAM49B,ADGRB1,PPARD,SUN2,PLAA,SRF,VEGFA,DOCK8,CREB3,PTP4A1,TGFBR1,RTN4,EMC10,AKIRIN1,ROCK2,RRAS,ITGB1,PLXNA1,FGF2,PAK1,TGFB1,AKT2,OSBPL8,ACTG1,NAV3,ACTN4,IRS2,DCN,ZNF703,STARD13,SEMA3C,DDRGK1,ILK,DPYSL3,CXCL12,KIF2A,SULF1,ROBO1,CHST2,SEMA4F,P2RX4,PFN2,APP,RAPGEF2,CX3CL1,ABCC8,ARSB,ACVR1,ADA,ATP2B4,SEMA4G,NKD1,DOCK10,ADIPOR1,PHLDB2,TJP1,DNM1L,HYAL1,TGFB2,MMP14,DAG1,LEF1,CORO1C,PRKCA,CMKLR1,NTRK3,SLAMF8,BMP7,IDH2,IQGAP1,CLIC4,S100A11,LRRC15,FAM89B,HSPB1,SERPINE1,ITGA2B,DNAJA4,FN1,PDCD10,ITGAV,COL3A1,NBL1,TIMP1,VCL,AKT3,BMPR1A,PPARGC1A,MSN,SRCIN1,ARHGEF2,NGFR,TPM1,COL1A1,RAB11A,MYOCD,CD74,LAMA3,PDGFRB,ROCK1,AKT1,ITGA2,DAPK3,MYO1C,PLXNB3,TNFRSF18,FLNA,PLXNA3,NTNG2,CDH13,F3,HSPA5,LMO4,NEXN,MIR24-1,CD151,SERPINF1,THY1,HAS2,TMSB4X,GLIPR2,CCR6,DUSP22,PIK3C2A,PRKG1,FGF10	darkgreen	EC_M4
GO:0006473	1.61E-03	0.999230385	29	168	protein acetylation	BP	4.72E-02	DSCC1,NAA35,ZNF451,ZMPSTE24,NAA15,HDAC2,CEP295,PAXIP1,KAT14,EPC2,KAT6A,TAF7,BRCA2,TAF9,ESCO2,MCRS1,NAA25,CHEK1,NAA50,MCM3AP,HAT1,ING5,BRCA1,TAF1,OGT,ESCO1,TAOK1,CTCF,AC118549.1	darkgrey	EC_M8
GO:0002064	8.90E-04	0.999563889	35	173	epithelial cell development	BP	4.76E-02	NOTCH4,AFDN,ATF4,PDGFB,PODXL,TJP2,TMOD1,GPR4,MYO7A,CXCR4,IKBKB,JAG1,PDE2A,GPAT4,PDE4D,MARVELD2,PPP1R16B,STC1,RAPGEF3,ROBO4,SEC24B,PECAM1,PROX1,F11R,HIF1A,EPHA2,AR,CCDC88C,NOTCH1,RAPGEF1,CDH5,ARID4A,SOX18,TBC1D20,BMP6	turquoise	EC_M6
GO:0070527	1.04E-03	0.999670947	16	51	platelet aggregation	BP	4.78E-02	MYH9,SLC7A11,C1QTNF1,ACTG1,ILK,MYL9,PRKCA,HSPB1,ITGA2B,FN1,PTPN6,COMP,VCL,FLNA,CSRP1,PRKG1	darkgreen	EC_M4
GO:0090130	1.04E-03	0.999402877	55	266	tissue migration	BP	4.78E-02	ANGPT1,NUS1,PTP4A3,ADGRB1,PPARD,MYH9,SRF,VEGFA,TGFBR1,RTN4,EMC10,ROCK2,RRAS,ITGB1,FGF2,TGFB1,ZEB2,DCN,STARD13,ROBO1,P2RX4,PFN2,ACTG2,PTPN11,ARSB,LOXL2,ATP2B4,ADIPOR1,FAP,HYAL1,TGFB2,FSTL1,PIK3CA,CORO1C,PRKCA,HSPB1,DNAJA4,S100P,PDCD10,ACTA2,AKT3,RAB11A,FAT2,ROCK1,AKT1,ITGA2,CDH13,MIR24-1,SERPINF1,HAS2,TMSB4X,GLIPR2,CCR6,PIK3C2A,FGF10	darkgreen	EC_M4
GO:0007159	8.97E-04	0.99952988	41	234	leukocyte cell-cell adhesion	BP	4.79E-02	TNFRSF21,SYK,SEMA4D,IL6,PELI1,IL15,GATA3,IL1A,IGF1,ITCH,IL7,SOX13,ETS1,LCK,SLC39A8,ICOSLG,LAPTM5,HLX,PECAM1,CD86,F11R,DLG1,B2M,ADORA2A,ITGA4,SELE,SELP,ITPKB,ARG2,EFNB1,STK10,TNIP1,CCL2,YES1,LGALS9,IL7R,AP3D1,NRARP,NFAT5,VNN1,CD46	turquoise	EC_M6
GO:0008219	1.07E-03	0.99917265	257	1587	cell death	BP	4.89E-02	SERPINB2,LRP1,AHI1,AGAP2,DYRK2,IER3,CDSN,RRM2B,ANGPT1,TCP1,PPARA,PPARD,CTNNA1,UBQLN1,MTCH1,LGALS1,PRUNE2,LEP,BAG1,VEGFA,DOCK8,VCP,UNC13B,NCK2,CREB3,ASNS,PDCL3,REST,TGFBR1,RTN4,CYCS,TAX1BP1,GGCT,MAP3K7,GLI3,INHBA,DKKL1,VPS35,CUL2,SLC7A11,ITGB1,FGF2,SRPK2,RB1,PICALM,MARK4,TPT1,PDCD6IP,GLI2,PAK1,TGFB1,AXL,ACER3,CTNNB1,AKT2,RELT,RARB,ACTN4,TMTC4,UFM1,LIG4,IRS2,PRNP,HDAC3,DDRGK1,SMPD1,PPP2CB,ILK,ARL6IP5,PPP2R2B,APLP1,CXCL12,ANKRD1,HSP90B1,SLC25A4,C19orf12,EIF4G2,SULF1,EMC4,P2RX4,FAIM2,RASA1,CELF1,RTKN,APP,RAPGEF2,CX3CL1,OPA1,AMIGO2,ACVR1,CHAC1,GDF6,SERINC3,MTDH,ADA,AKTIP,CHP1,TXNDC5,PLA2R1,IFT57,BRSK2,SLC39A10,CHRNA7,ELMO2,DYNLL1,FAP,TJP1,NOTCH2,CTTN,CDC73,DNM1L,GLRX2,SELENOS,EYA2,CLPTM1L,TGFB2,LRP5,HTR2B,ITPK1,ZMAT3,GSK3B,INPP5A,CASP7,FSTL1,PIK3CA,LEF1,PRKCA,KRAS,NTRK3,FAM162A,KPNA1,BMP7,HYOU1,ATF3,FGFR2,PEA15,NCSTN,TFRC,ATF6,BACE1,FZD9,RPS6KA3,EGLN3,PDIA3,OLR1,HSPB1,MAPT,SERPINE1,SYVN1,SGPL1,ZNF385B,DNAJC10,NCKAP1,PTPN6,PDCD10,ITGAV,LRPAP1,COMP,HTT,FKBP8,ERO1A,PRKCI,SKIL,FGFR3,RTN3,ARAF,TIMP1,EXOC5,VDAC2,FMN2,SIX4,SGMS1,MNAT1,SLC6A13,ERC1,GHITM,MAGED1,CLN8,EIF2S1,MED1,PPARGC1A,ACTN1,CPEB4,NES,PNMA1,ARHGEF2,NGFR,TGFB3,DNASE2,SHC1,TAF9B,GPR65,UACA,PKM,DICER1,GSKIP,MYOCD,ANXA6,PIN1,CD74,PDGFRB,ROCK1,AKT1,PRKAA1,TNFAIP1,TM2D1,PTPN2,OMA1,CD40LG,MYDGF,DAPK3,GNB1,BCAP31,TNFRSF18,FLNA,IKBKG,TMEM259,PARK7,ENO1,AHCYL1,GNAI3,CFDP1,A	darkgreen	EC_M4
GO:0035194	1.68E-03	0.999322008	20	112	post-transcriptional gene silencing by RNA	BP	4.91E-02	TNRC6B,TUT7,XPO5,HNRNPA2B1,PUS10,ZMPSTE24,METTL3,CNOT1,RIPK1,AJUBA,FXR1,DDX5,TIAL1,DDX6,DHX9,DGCR8,ZC3H7A,DROSHA,FMR1,SRRT	darkgrey	EC_M8

GO:0006897	1.08E-03	0.99930246	85	444	endocytosis	BP	4.94E-02	CD63,LRP1,AHI1,CANX,RAB21,ANGPT1,PACSIN1,ADGRB1,MKLN1,SH3GL2,MYH9,VEGFA,ITGB1,RAB7A,PICALM,SNX17,AXL,ESYT2,LGALS3BP,ACTG1,SIGLEC1,SMPD1,APLP1,MTMR6,LOXL3,APP,ATXN2,PTX3,LOXL2,TSG101,PPP3CC,PLA2R1,AP2A2,CTTN,DNM1L,DNER,LRP5,HTR2B,CFI,GRK3,AP2M1,TFRC,MRC2,OLR1,AP1S1,SERPINE1,CD163,TFR2,ITGAV,LRPAP1,CLTCL1,ATAD1,SLC2A4,NLGN2,PDLIM7,MYO1E,STX1B,SYT11,DOCK2,EPN3,CLINT1,CALM1,CLTC,GRIA1,SCAMP5,ROCK1,SNX18,ITGA2,MARCH2,RAB31,RAB34,SRL,CSNK1G2,VAMP7,GPR107,FNBP1,CDH13,USP20,AP1G1,CD151,SNX10,FCN1,OCIAD2,PIK3C2A,LRRTM2	darkgreen	EC_M4
GO:0042127	9.35E-04	0.999315354	167	1230	regulation of cell population proliferation	BP	4.95E-02	TNFRSF11A,TNFAIP3,CNOT6,FLT4,CTHRC1,MTSS1,PTK2,PDGFB,AGTPBP1,KDM4C,PIM1,TEK,KIT,TNFRSF21,KDR,SYK,IL6,CXCL8,PELI1,CAV2,KLF11,TLR4,IL15,SDCBP,MYCN,TRPM4,NRP1,TAL1,GATA2,PRKD2,NOS3,GAB2,DACH1,PPARG,GATA3,EDNRB,TIE1,JAG1,P2RY6,SFRP1,HBEGF,FERMT1,EFNB2,ASH2L,EPHB1,IL1A,IGF1,NR4A1,HHEX,ITCH,SH3RF1,SMAD1,SOX7,MEF2C,TLR2,WWTR1,IL7,VEGFC,NDRG4,CXCL10,KMT2D,THBS4,PPP1R16B,PLCG1,HIPK1,TP53I11,DLL4,EDN1,STAT1,JARID2,ETS1,SHANK2,CD200,HDAC1,ICOSLG,HLX,HCLS1,NFATC2,INPP5D,EAF2,CD86,MN1,WDR6,SH3BP4,CFLAR,TGM1,IGFBP3,FES,PROX1,PRKD1,LAMA5,MAP3K3,MLXIPL,ACE,LAMC2,DLG1,ELL3,APOD,CREB1,B2M,TET1,SSTR1,LTF,ADORA2A,ARNT2,P3H2,ITGA4,SOS2,IGFBP5,TBX1,CALCRL,ING4,FGF23,PRKCH,HIF1A,JUP,BCL6B,BID,MCC,TNFRSF1B,ARG2,ALDH1A2,MTOR,AR,EFNB1,FGF18,PGF,VASH1,CHRNB2,PIAS1,NPR1,SMAD2,CNOT8,PPP2R5C,CCL2,IL13RA1,JAG2,LGALS9,NOS2,IL7R,LRG1,MATK,DOT1L,NRARP,EGFL7,NOTCH1,SLC25A33,S1PR1,PTGS1,DAB2IP,CDH5,MIR23A,ATXN1L,ADGRG1,TRIM24,CD46,SOX17,FGF16,CDK13,SOX18,STX3,E2F3,COPS8,BST2,BMP2,IFIT3,BMP6	turquoise	EC_M6
GO:0007492	1.09E-03	0.999622959	18	59	endoderm development	BP	4.97E-02	INHBA,CTNNB1,MMP15,MMP2,CDC73,MMP14,COL6A1,ITGB5,FN1,ITGAV,COL5A2,BMPR1A,LAMA3,COL5A1,HSBP1,COL11A1,DUSP2,DUSP4	darkgreen	EC_M4
GO:0032964	1.09E-03	0.999703299	13	38	collagen biosynthetic process	BP	4.97E-02	PPARD,ADAMTS3,RCN3,TGFB1,SERPINH1,RUNX1,SUCO,COL1A1,TGFB3,PDGFRB,ITGA2,COL5A1,P3H3	darkgreen	EC_M4
GO:0046777	9.42E-04	0.999512233	40	204	protein autophosphorylation	BP	4.98E-02	FLT4,PTK2,DAPK1,PDGFB,PIM1,TEK,TEC,KIT,KDR,SYK,PRKD2,EPHB1,EIF2AK3,VEGFC,DYRK1A,SIK1,PRKX,BMX,FGR,FES,PRKD1,MAP3K3,SRMS,ACE,PAK2,MAP3K11,BCR,EPHB4,DDX3X,LMTK2,TNK1,MTOR,STK10,DAPK2,TAOK2,MARK3,YES1,STK26,ENG,TRIM24	turquoise	EC_M6
GO:0007517	1.10E-03	0.999369961	54	259	muscle organ development	BP	4.98E-02	ADGRB1,LY6E,LARGE1,TGFBR1,HIVEP3,MYH14,AKIRIN1,BVES,RB1,TGFB1,CTNNB1,DCN,ANKRD1,MRAS,FLNB,HOMER1,PKP2,DNER,TGFB2,DAG1,LEF1,DISP1,COL6A3,ATF3,FGFR2,CASQ1,ARID5B,TAGLN,FZD2,SIX4,EVC,BMPR1A,TCAP,MED1,MYLPF,FGFRL1,TPM1,SGCA,ITGB1BP2,SGCD,MYOCD,FBXO22,MYOM1,PRKAA1,COL11A1,DMD,FKBP1A,LOX,IGSF8,CD164,LBX1,SRPK3,WNT2,CFL2	darkgreen	EC_M4
GO:0050817	9.52E-04	0.999546004	32	166	coagulation	BP	4.99E-02	TSPAN8,FZD6,PDGFB,TEC,SYK,IL6,TLR4,PIK3CG,NOS3,CD36,MERTK,KLKB1,PROCR,ENTPD1,DGKZ,ANO6,EDN1,MMRN1,DTNBP1,LCK,CD34,F11R,ADORA2A,SELP,VWF,WAS,RAB27A,PEAR1,PSEN1,F8,PLCG2,STXBP1	turquoise	EC_M6
GO:0032755	9.53E-04	0.999690333	16	62	positive regulation of interleukin-6 production	BP	4.99E-02	MAPK13,DDX58,SYK,IL6,TLR4,CD36,IL1A,TLR2,HYAL2,IL1RAP,TMEM106A,TLR6,LGALS9,NOS2,PLCG2,TLR1	turquoise	EC_M6
GO:0007599	9.56E-04	0.999544155	32	166	hemostasis	BP	4.99E-02	TSPAN8,FZD6,PDGFB,TEC,SYK,IL6,TLR4,PIK3CG,NOS3,CD36,MERTK,KLKB1,PROCR,ENTPD1,DGKZ,ANO6,EDN1,MMRN1,DTNBP1,LCK,CD34,F11R,ADORA2A,SELP,VWF,WAS,RAB27A,PEAR1,F8,PLCG2,STXBP1,FLI1	turquoise	EC_M6
GO:0006289	1.72E-03	0.999505378	12	50	nucleotide-excision repair	BP	5.03E-02	RPA3,FANCC,COMMD1,USP45,POLE,CUL4A,BRCA2,ERCC6,RPA2,RAD52,BRIP1,RPA1	darkgrey	EC_M8
GO:0002252	9.69E-04	0.999427825	59	360	immune effector process	BP	5.05E-02	PTK2,MFNG,DDX58,KIT,SYK,IL6,RSAD2,TLR4,C5,TRPM4,PIK3CG,GATA2,CTSC,NECTIN2,GAB2,UNC13D,GATA3,JAG1,CD36,GAPT,DYSF,RNF19B,SNAP23,LYST,LAPTM5,HLX,NCF1,INPP5D,CD86,FGR,FES,ACE,B2M,MASP1,CRACR2A,WAS,PLD2,RAB27A,LFNG,TNFRSF1B,MTOR,PSEN1,SEMA4A,BATF,CD19,SCNN1B,YES1,LGALS9,NOS2,IL7R,MYO18A,TRAF2,IRF8,PLCG2,STXBP1,CD46,FCER2,SERPINB9,BST2	turquoise	EC_M6
GO:0043634	1.75E-03	0.999900451	4	7	polyadenylation-dependent ncRNA catabolic process	BP	5.10E-02	EXOSC9,EXOSC8,EXOSC10,EXOSC2	darkgrey	EC_M8
GO:0032835	9.86E-04	0.999697169	15	52	glomerulus development	BP	5.10E-02	MTSS1,PDGFB,PODXL,TEK,GPR4,JAG1,RET,MEF2C,WWTR1,ANGPT2,CD34,PECAM1,CFLAR,PROM1,NOTCH1	turquoise	EC_M6

GO:0030336	9.87E-04	0.999481505	41	223	negative regulation of cell migration	BP	5.10E-02	AFDN,LIMCH1,C5,LRCH1,DACH1,PPARG,TIE1,JAG1,SFRP1,ADAMTS9,ABHD6,MCTP1,MEF2C,NDRG4,ANGPT2,STC1,DLL4,SRGAP2C,CD200,HYAL2,RIN3,IGFBP3,ARHGDIB,APOD,SEMA6D,IGFBP5,RAC1,JUP,ADIPOR2,MMRN2,MCC,VASH1,MMP28,CCL2,STK26,ARHGAP4,NOTCH1,ENG,DAB2IP,ADGRG1,BST2	turquoise	EC_M6
GO:0071772	1.14E-03	0.999469443	31	126	response to BMP	BP	5.11E-02	KIAA1324L,RUNX2,MEGF8,TGFB1,CRIM1,SPINT2,SMAD9,ILK,SULF1,SLC33A1,ACVR1,GDF6,NOTCH2,TGFB2,FSTL1,LEF1,HJV,BMP7,HTRA1,FBN1,COMP,FKBP8,SKIL,SMURF1,NBL1,GREM2,BMPR1A,TGFB3,TMEM100,CHRDL1,FSTL3	darkgreen	EC_M4
GO:0071773	1.14E-03	0.999469443	31	126	cellular response to BMP stimulus	BP	5.11E-02	KIAA1324L,RUNX2,MEGF8,TGFB1,CRIM1,SPINT2,SMAD9,ILK,SULF1,SLC33A1,ACVR1,GDF6,NOTCH2,TGFB2,FSTL1,LEF1,HJV,BMP7,HTRA1,FBN1,COMP,FKBP8,SKIL,SMURF1,NBL1,GREM2,BMPR1A,TGFB3,TMEM100,CHRDL1,FSTL3	darkgreen	EC_M4
GO:0048514	1.14E-03	0.999245761	95	508	blood vessel morphogenesis	BP	5.11E-02	LRP1,SLC12A2,ANGPT1,FAM19A5,NUS1,RSPO3,ADGRB1,MYH9,LEP,SRF,VEGFA,PDCL3,COL15A1,TGFBR1,EMC10,CALD1,ROCK2,RRAS,ITGB1,FGF2,SRPK2,TGFB1,CTNNB1,ACTG1,STIM1,DCN,STARD13,SPINK5,SULF1,ROBO1,HEY1,RASA1,RAPGEF2,CX3CL1,ABCC8,LOXL2,ACVR1,MMP2,MTDH,ATP2B4,PKD2,RUNX1,HIF1AN,CHRNA7,FAP,TJP1,NOTCH2,HYAL1,TGFB2,LRP5,ADGRB2,MMP14,DAG1,PIK3CA,LEF1,PRKCA,FGFR2,VASH2,HSPB1,SERPINE1,SGPL1,FN1,PDCD10,ITGAV,COMP,PRRX1,AKT3,FKBP10,BMPR1A,MED1,NPPB,MYO1E,NGFR,SHC1,TMEM100,PKM,MYOCD,ANGPTL6,PDGFRB,ROCK1,AKT1,MYDGF,FLNA,BSG,SARS,CDH13,F3,MIR24-1,SERPINF1,LOX,THY1,HAS2,PIK3C2A,COL8A2,FGF10	darkgreen	EC_M4
GO:0002448	1.00E-03	0.999740505	12	40	mast cell mediated immunity	BP	5.15E-02	KIT,SYK,PIK3CG,GATA2,GAB2,UNC13D,SNAP23,FGR,FES,LGALS9,STXBP1,SERPINB9	turquoise	EC_M6
GO:0051241	1.16E-03	0.999181009	131	743	negative regulation of multicellular organismal process	BP	5.19E-02	SERPINB2,IL23A,SLC12A2,ANGPT1,FAM19A5,PPARA,ADGRB1,CTNNA1,LEP,KIAA1324L,SRF,REST,ASPN,TGFBR1,SOCS5,RTN4,GLI3,ROCK2,INHBA,FKBP1B,FGF2,RB1,GLI2,RHEB,PAK1,TGFB1,AXL,CTNNB1,C1QTNF1,RARB,NAV3,NPLOC4,SPRED3,PRNP,ATP2B1,DCN,RAB11FIP1,STARD13,SEMA3C,SPINK5,RYK,RNF6,IL20RB,SULF1,LNPEP,ROBO1,WWC2,DIP2B,SEMA4F,LOXL3,P2RX4,PFN2,HEY1,ATP2A2,TLX2,RAPGEF2,CX3CL1,ABCC8,CACNB3,PTPN11,LOXL2,PTPN22,CRY2,ADA,ATP2B4,SPP1,CHID1,RUNX1,SEMA4G,CHRNA7,ADIPOR1,FAP,SELENOS,CHSY1,SLC2A10,TGFB2,ADGRB2,GSK3B,LEF1,CORO1C,CMKLR1,NTRK3,WWC3,TCTA,SLAMF8,BMP7,IDH2,PPP1R11,ASB1,GLRX3,PRDX4,SERPINE1,DNAJA4,COMT,TNR,FN1,PTPN6,GPR137,FBN1,PDCD10,ADRA2C,LRPAP1,TIMP1,OTUD5,PRTG,BMPR1A,NPPB,SYT11,NGFR,TGFB3,DICER1,NPTN,MYOCD,CD74,ROCK1,TMEM98,PTPN2,NPR3,VASN,PLXNA3,SARS,MIR24-1,FSTL3,SERPINF1,ADAMTS5,THY1,FGL2,DACT3,TMSB4X,YWHAH,PRKG1	darkgreen	EC_M4
GO:0032623	1.02E-03	0.999731529	12	43	interleukin-2 production	BP	5.19E-02	TNFAIP3,PRKD2,GATA3,PDE4D,IL1A,LAPTM5,CD34,CD86,HOMER3,PDE4B,TRAF2,PLCG2	turquoise	EC_M6
GO:0032663	1.02E-03	0.999731529	12	43	regulation of interleukin-2 production	BP	5.19E-02	TNFAIP3,PRKD2,GATA3,PDE4D,IL1A,LAPTM5,CD34,CD86,HOMER3,PDE4B,TRAF2,PLCG2	turquoise	EC_M6
GO:0050731	1.02E-03	0.999540505	28	136	positive regulation of peptidyl-tyrosine phosphorylation	BP	5.19E-02	PDGFB,TEC,KIT,SYK,SEMA4D,IL6,IL15,NRP1,HBEGF,CD36,IGF1,THBS4,NEDD9,EHD4,HDAC1,NCF1,HCLS1,PECAM1,ACE,PAK2,MTOR,EFNA1,YES1,UNC119,PLCG2,RELN,ALKAL1,BMP6	turquoise	EC_M6
GO:0051149	1.02E-03	0.999694608	14	53	positive regulation of muscle cell differentiation	BP	5.19E-02	KIT,EFNB2,ATP11A,LMOD3,IGF1,MEF2C,EDN1,RBM4,PROX1,TBX1,MTOR,PIAS1,PIEZO1,ENG	turquoise	EC_M6
GO:0045794	1.03E-03	0.999972127	4	5	negative regulation of cell volume	BP	5.19E-02	CLCN3,P2RX7,ANO6,SHANK3	turquoise	EC_M6
GO:0014065	1.03E-03	0.999562202	25	111	phosphatidylinositol 3-kinase signaling	BP	5.20E-02	PTK2,PDGFB,TEK,PIP5K1B,KIT,KDR,SEMA4D,KBTBD2,PIK3CG,GATA3,PRR5L,IGF1,PREX2,PPP1R16B,PIK3C2B,EDN1,NCF1,HCLS1,FGR,PLEKHA1,SELP,PEAR1,PIK3R5,DAB2IP,RELN	turquoise	EC_M6
GO:1903131	1.04E-03	0.999412062	51	297	mononuclear cell differentiation	BP	5.22E-02	TCF7,MFNG,KIT,NFIL3,SYK,IL6,RSAD2,IL15,PPARG,GATA3,SFRP1,MERTK,CHD7,IL1A,HHEX,SH3RF1,IL7,TPD52,LMBR1L,ADGRG3,DLL4,SOX13,DCAF1,LCK,HLX,INPP5D,CD86,FES,B2M,ITGA4,SOS2,CRACR2A,ITPKB,LFNG,MTOR,PSEN1,SEMA4A,BATF,CD19,GPR68,YY1,LYL1,JAG2,LGALS9,IL7R,AP3D1,NRARP,IRF8,PLCG2,VNN1,CD46	turquoise	EC_M6
GO:0042116	1.04E-03	0.999643951	17	69	macrophage activation	BP	5.22E-02	IFNGR1,ADGRF5,SYK,IL6,TLR4,CST7,CTSC,CD93,TLR2,DYSF,SNCA,CD200,TMEM106A,TLR6,MYO18A,PLCG2,TLR1	turquoise	EC_M6

GO:0043408	1.05E-03	0.999331491	78	502	regulation of MAPK cascade	BP	5.25E-02	TNFRSF11A,SEMA6A,AKAP12,FLT4,TPD52L1,PDGFB,TEK,SH3RF3,KIT,KDR,SYK,GADD45G,RNF149,IL6,CAV2,TLR4,TRAF1,SDCBP,NRP1,PIK3CG,PRKD2,PPARG,P2RY6,SFRP1,CD36,SIRT3,EPHB1,RET,TNFRSF19,IGF1,ITCH,SH3RF1,P2RX7,NDRG4,MADD,EDN1,DUSP8,PRDM15,HYAL2,HCRTR1,LAPTM5,NCF1,CFLAR,MAP3K3,PAK2,DUSP16,DLG1,MAP3K11,TBX1,TMEM106A,MECOM,TNFAIP8L3,FGF23,CRACR2A,EPHA2,TLR6,RELL1,RGS14,AR,PSEN1,FGF18,TAOK2,EFNA1,PIK3R5,TNIP1,ADRB2,CCL2,LGALS9,TRAF2,NOTCH1,PLCG2,DAB2IP,MIR23A,ALKAL1,DUSP5,BMP2,DUSP7,NMNAT1	turquoise	EC_M6
GO:0032640	1.06E-03	0.999557295	24	111	tumor necrosis factor production	BP	5.25E-02	IFNGR1,TNFAIP3,AKAP12,C6orf106,DDX58,SYK,IL6,TLR4,CD36,IL1A,IGF1,TLR2,OAS3,HDAC1,CD34,LTF,CYBB,TMEM106A,ARG2,PSEN1,LGALS9,PLCG2,OAS1,TLR1	turquoise	EC_M6
GO:0032680	1.06E-03	0.999557295	24	111	regulation of tumor necrosis factor production	BP	5.25E-02	IFNGR1,TNFAIP3,AKAP12,C6orf106,DDX58,SYK,IL6,TLR4,CD36,IL1A,IGF1,TLR2,OAS3,HDAC1,CD34,LTF,CYBB,TMEM106A,ARG2,PSEN1,LGALS9,PLCG2,OAS1,TLR1	turquoise	EC_M6
GO:1902991	1.18E-03	0.999674512	13	39	regulation of amyloid precursor protein catabolic process	BP	5.25E-02	RTN4,ROCK2,GSAP,PICALM,PRNP,CHRNA7,RTN3,HAP1,TMED10,PIN1,ROCK1,MIR24-1,FKBP1A	darkgreen	EC_M4
GO:0061351	1.18E-03	0.999471011	28	111	neural precursor cell proliferation	BP	5.25E-02	PLXNB2,CTNNA1,TEAD3,VEGFA,GLI3,FGF2,GLI2,SMARCD3,CTNNB1,RARB,SPINT2,ILK,RYK,CX3CL1,PSMG1,LEF1,FGFR2,KIF1A,FZD9,KCNA1,NES,ARHGEF2,EML1,PAFAH1B1,FLNA,RERE,ARTN,WNT2	darkgreen	EC_M4
GO:0016241	1.18E-03	0.999444843	31	128	regulation of macroautophagy	BP	5.25E-02	ATP6V1C1,UBQLN1,EXOC1,MAP3K7,VPS35,SESN3,ATP6V0B,RHEB,RAB3GAP1,DCN,SLC25A4,ATP6V1B2,SEC22B,FEZ1,PIK3CA,SCFD1,QSOX1,PAFAH1B2,HTT,ATP6V1D,SPTLC2,AKT1,CLEC16A,IKBKG,GPSM1,GNAI3,ATP6V0D1,BNIP3,VPS26B,CAPNS1,ATP6V0E1	darkgreen	EC_M4
GO:0030282	1.07E-03	0.999572668	22	97	bone mineralization	BP	5.29E-02	ATF4,TRPM4,ACVR2B,NOTUM,IGF1,EIF2AK3,MEF2C,P2RX7,BMP2K,ANO6,FGR,LTF,BCOR,MMP13,FGF23,HIF1A,ADRB2,RFLNB,ISG15,S1PR1,BMP2,BMP6	turquoise	EC_M6
GO:0001654	1.20E-03	0.999295595	58	280	eye development	BP	5.31E-02	AHI1,FBN2,RORB,SRF,VEGFA,TGFBR1,MFSD2A,GLI3,INHBA,SLC7A11,FGF2,SMARCD3,RAB3GAP1,CTNNB1,RARB,SPRED3,ATP2B1,FAT1,RDH10,SLC4A5,OPA1,ATP2B4,NKD1,KDM5B,NOTCH2,TGFB2,LRP5,SHROOM2,CRYBA4,BMP7,CLIC4,ATF6,FBN1,FKBP8,PRKCI,COL5A2,SKIL,TULP3,OBSL1,ROM1,CACNA1C,CLN8,MED1,NES,TTC8,SMARCA4,CHRDL1,PDGFRB,MYOM1,GNB1,NPHP4,COL5A1,BCAR3,SERPINF1,THY1,WNT2,COL8A2,FGF10	darkgreen	EC_M4
GO:0045069	1.83E-03	0.999406929	14	62	regulation of viral genome replication	BP	5.32E-02	SRPK1,TRIM28,PPIH,TOP2B,IFIT5,PDE12,FAM208A,PPID,DDX5,TOP2A,HACD3,LARP1,ILF3,PKN2	darkgrey	EC_M8
GO:0060325	1.21E-03	0.999743154	10	25	face morphogenesis	BP	5.32E-02	RRAS,RAB3GAP1,PTPN11,MMP2,LEF1,ARID5B,SGPL1,COL1A1,TGFB3,ANKRD11	darkgreen	EC_M4
GO:0060485	1.21E-03	0.999318782	50	235	mesenchyme development	BP	5.32E-02	TGFBR1,RTN4,ROCK2,TGFB1,CTNNB1,SPRED3,ZNF703,SEMA3C,ROBO1,RDH10,SEMA4F,LOXL3,HEY1,ACTG2,LOXL2,ACVR1,PKD2,SEMA4G,ADIPOR1,PHLDB2,TGFB2,HTR2B,GSK3B,DAG1,LEF1,CORO1C,BMP7,FGFR2,PHLDB1,FN1,MEOX1,SIX4,ACTA2,BMPR1A,TGFB1I1,COL1A1,TGFB3,TMEM100,ANXA6,PDGFRB,ROCK1,VASN,FLNA,GDNF,HAS2,DACT3,GLIPR2,OLFM1,WNT2,FGF10	darkgreen	EC_M4
GO:0050793	1.21E-03	0.999041276	299	1852	regulation of developmental process	BP	5.32E-02	IL23A,NAB2,LRP1,AHI1,RAB21,SLC12A2,PLXNB2,FBN2,FAM19A5,PPARA,HLA-DRA,RSPO3,FAM49B,PACSIN1,ADGRB1,PPARD,CTNNA1,MKLN1,MYH9,LEP,PLAA,RORB,KIAA1324L,SRF,NUAK1,VEGFA,RUNX2,CLOCK,PDCL3,REST,ASPN,NLRP5,TGFBR1,SOCS5,RIMS1,RTN4,TPBG,SHANK1,LRRC4B,EMC10,MFSD2A,MYH14,AKIRIN1,ACTR2,GLI3,ROCK2,INHBA,BVES,RRAS,MYLK3,DKKL1,VPS35,ITGB1,PLEKHA4,FKBP1B,PLXNA1,FGF2,RB1,TPT1,PTCH2,TENM4,GLI2,MEGF8,SMARCD3,RHEB,PAK1,TGFB1,AXL,CTNNB1,IL15RA,TRAK1,LBH,ZEB2,RARB,ACTN4,CRIM1,MAFG,STIM1,SPRED3,LIG4,ATP2B1,DCN,CDC42EP3,ZNF703,SEMA3C,ILK,SPINK5,RYK,PRICKLE2,RNF6,CXCL12,LGI4,ZNF536,EIF4G2,SULF1,ROBO1,WWC2,MAP1B,DIP2B,SEMA4F,LOXL3,HEY1,RASA1,TLX2,CELF1,APP,DHX36,RAPGEF2,CX3CL1,ABCC8,PTPN11,RUNX1T1,OPA1,LOXL2,AP3B1,AMIGO2,ZFYVE27,ACVR1,GDF6,MTDH,ADA,ATP2B4,SPP1,BMP1,RUNX1,HIF1AN,SEMA4G,NKD1,BRSK2,PDLIM5,RAB7B,CHRNA7,EIF4E,ADIPOR1,PHLDB2,TJP1,FNDC5,KDM5B,NOTCH2,CTTN,PKP2,CDC73,DNM1L,TRIP12,HYAL1,CCND1,CAPRIN2,TGFB2,LRP5,ADGRB2,FEZ1,GSK3B,MMP14,DAG1,PIK3CA,LEF1,CORO1C,PRKCA,CMKLR1,KRAS,NTRK3,WWC3,TCTA,SLAMF8,BMP7,IDH2,FGFR2,VASH2,OSM,TP53BP1,PHLDB1,SYAP1,IQGAP1,KIF1A,TFRC,TANC2,FZD9,RPS6KA3,HSPB1,MAPT,SERPINE1,SGPL1,FZD2,TNR,FN1,C11orf65,PTPN6,GPR137,FBN1,ITGAV,ADRA2C,COMP,ARPC2,SUCO,PRKCI,COL5A2,SKIL,FGFR3,PRRX1,SMURF1,NBL1,CAPZB,TNIK,VCL,DAAM1,WDR1,SIX4,OBSL1,AKT3,HAP1,PRTG,MAGED1,BMPR1A,NOTCH3,NLGN2,PDLIM7,CDK12,MED1,ADGRL	darkgreen	EC_M4
GO:0035272	1.22E-03	0.999686779	12	34	exocrine system development	BP	5.34E-02	PLXNA1,TGFB1,SEMA3C,TGFB2,DAG1,BMP7,FGFR2,SRP54,TGFB3,WLS,EDA,FGF10	darkgreen	EC_M4

GO:0002253	1.09E-03	0.999455256	35	191	activation of immune response	BP	5.38E-02	PTK2,TEC,TNFRSF21,SYK,TLR4,C5,PRKD2,NECTIN2,GATA3,IKBKB,TMEM173,PDE4D,MEF2C,PLCG1,DGKZ,LCK,ICOSLG,LAPTM5,NFATC2,INPP5D,BMX,FGR,PLEKHA1,PAK2,MASP1,PRKCH,PLD2,PSEN1,EIF2B2,CD19,YES1,PDE4B,PLCG2,FYB1,CD46	turquoise	EC_M6
GO:0018206	1.86E-03	0.999817978	5	11	peptidyl-methionine modification	BP	5.41E-02	NAA35,NAA15,METAP2,NAA25,METAP1	darkgrey	EC_M8
GO:0000959	1.87E-03	0.999494458	11	44	mitochondrial RNA metabolic process	BP	5.42E-02	MTERF1,LRPPRC,PNPT1,CDK5RAP1,PDE12,FASTKD1,FASTKD2,DARS2,TEFM,FASTKD5,TFB2M	darkgrey	EC_M8
GO:0046579	1.11E-03	0.999671644	14	49	positive regulation of Ras protein signal transduction	BP	5.44E-02	PIK3CG,GPR4,NET1,PRAG1,IGF1,ERBIN,ARHGEF3,SHOC2,F11R,RAC1,ITPKB,NOTCH1,ADGRG1,LPAR6	turquoise	EC_M6
GO:0046784	1.90E-03	0.999877156	4	8	viral mRNA export from host cell nucleus	BP	5.47E-02	DDX39B,ALYREF,THOC1,THOC2	darkgrey	EC_M8
GO:0043631	1.90E-03	0.999489533	11	41	RNA polyadenylation	BP	5.47E-02	SCAF8,YTHDC1,PNPT1,PAPOLG,WDR33,SYMPK,PCF11,TENT2,VIRMA,CPSF2,LEO1	darkgrey	EC_M8
GO:0009438	1.26E-03	0.999963771	4	5	methylglyoxal metabolic process	BP	5.48E-02	TPI1,HAGH,PARK7,GLO1	darkgreen	EC_M4
GO:0042110	1.12E-03	0.999356666	52	310	T cell activation	BP	5.49E-02	TCF7,KIT,TNFRSF21,SYK,IL6,PELI1,RSAD2,IL15,PIK3CG,GATA3,CHD7,IL1A,IGF1,ITCH,SH3RF1,KIF13B,IL7,LMBR1L,DLL4,SOX13,LCK,PPP3CA,ICOSLG,LAPTM5,HLX,CD86,DLG1,B2M,ADORA2A,SOS2,CRACR2A,WAS,RAB27A,ITPKB,LFNG,TNFRSF1B,ARG2,MTOR,EFNB1,PSEN1,SEMA4A,BATF,CCL2,YES1,JAG2,LGALS9,ELF4,IL7R,AP3D1,NRARP,VNN1,CD46	turquoise	EC_M6
GO:0061437	1.14E-03	0.999776286	9	25	renal system vasculature development	BP	5.50E-02	PDGFB,TEK,NRP1,GPR4,ANGPT2,CD34,PECAM1,CFLAR,NOTCH1	turquoise	EC_M6
GO:0061440	1.14E-03	0.999776286	9	25	kidney vasculature development	BP	5.50E-02	PDGFB,TEK,NRP1,GPR4,ANGPT2,CD34,PECAM1,CFLAR,NOTCH1	turquoise	EC_M6
GO:0044837	1.92E-03	0.999887787	4	7	actomyosin contractile ring organization	BP	5.51E-02	PLEC,ANLN,RACGAP1,ECT2	darkgrey	EC_M8
GO:0009101	1.27E-03	0.999276323	51	245	glycoprotein biosynthetic process	BP	5.53E-02	B4GALT7,NUS1,LARGE1,CHST10,UBE2J1,GFPT1,RPN1,POMGNT1,ITM2B,CHPF2,ST6GALNAC2,CTNNB1,TRAK1,STT3B,CANT1,TMTC4,KDELC1,TMTC3,TUSC3,ST8SIA4,B3GNT4,TET3,RPN2,DPY19L4,CHP1,MAN1A2,CHSY1,SLC2A10,B4GALT4,B3GALT1,GALNT2,MAN2A2,NCSTN,EDEM3,PLOD3,DDOST,B3GAT3,UGDH,CHST12,GALNT16,XYLT2,GALNT10,GALNT1,MPI,POMT1,DPM2,ITM2C,DPM1,SLC35C1,CHST11,HS2ST1	darkgreen	EC_M4
GO:0032535	1.28E-03	0.999221904	65	325	regulation of cellular component size	BP	5.53E-02	LRP1,RAB21,SLC12A2,FAM49B,SRF,VEGFA,NCK2,RTN4,ACTR2,PICALM,MEGF8,PAK1,ACTR3,PLEKHG2,CDC42EP3,SEMA3C,ILK,RYK,RNF6,CXCL12,EIF4G2,MAP1B,DIP2B,SEMA4F,PFN2,RASA1,ZFYVE27,SPP1,TWF1,SEMA4G,MAGEL2,CTTN,LMOD1,GSK3B,PIK3CA,WDTC1,AP2M1,ARPC5,MAPT,TNR,FN1,NCKAP1,ARPC2,CAPZB,WDR1,AKT3,CLN8,MSN,RAB11A,ISLR2,TRPV2,PEX11G,FLII,AKT1,MYO1C,PAFAH1B1,PLXNA3,SPTAN1,BARHL2,TMSB4X,DSTN,TMEM123,OLFM1,COTL1,CFL2	darkgreen	EC_M4
GO:0030155	1.28E-03	0.999138922	100	541	regulation of cell adhesion	BP	5.53E-02	IL23A,LRP1,CDSN,PLXNB2,ANGPT1,PPARA,HLA-DRA,FAM49B,LGALS1,MDGA1,LEP,SRF,NUAK1,VEGFA,DOCK8,NCK2,SOCS5,GLI3,ROCK2,RRAS,PLXNA1,APBB1IP,FBLN2,GLI2,TGFB1,LMO7,C1QTNF1,ACTG1,ACTN4,PPP1R12A,SPINT2,PRNP,ZNF703,ILK,HSPH1,SPINK5,CXCL12,IL20RB,CHST2,LOXL3,RASA1,EDIL3,CX3CL1,PTPN11,AP3B1,PTPN22,ADA,RUNX1,CBLB,PHLDB2,TJP1,HYAL1,TGFB2,PRKAR1A,GSK3B,MMP14,DAG1,PIK3CA,LEF1,CORO1C,PRKCA,BMP7,IQGAP1,TFRC,COL26A1,SERPINE1,TNR,FN1,PTPN6,IGFBP2,ITGAV,ARPC2,VCL,TPM1,COL1A1,CD74,LAMA3,ROCK1,AKT1,ITGA2,EGFLAM,PTPN2,CD40LG,MYO10,DAPK3,PLXNB3,TNFRSF18,PKD1,IRAK1,FLNA,PLXNA3,CDH13,BCL10,FSTL3,CD164,THY1,HAS2,FGL2,DUSP22,PRKG1	darkgreen	EC_M4
GO:0070085	1.28E-03	0.999316619	42	194	glycosylation	BP	5.53E-02	B4GALT7,B4GALNT1,NUS1,LARGE1,NANS,UBE2J1,GFPT1,RPN1,POMGNT1,ST6GALNAC2,TRAK1,STT3B,TMTC4,COG6,KDELC1,TMTC3,TUSC3,ST8SIA4,B3GNT4,TET3,RPN2,DPY19L4,CHP1,MAN1A2,B4GALT4,B3GALT1,GALNT2,MAN2A2,PIGZ,EDEM3,PLOD3,DDOST,B3GAT3,GALNT16,GALNT10,GALNT1,MPI,POMT1,DPM2,DPM1,PIGB,SLC35C1	darkgreen	EC_M4
GO:0090232	1.94E-03	0.999886697	4	7	positive regulation of spindle checkpoint	BP	5.54E-02	GEN1,TPR,NDC80,MAD2L1	darkgrey	EC_M8

GO:0090267	1.94E-03	0.999886697	4	7	positive regulation of mitotic cell cycle spindle assembly checkpoint	BP	5.54E-02	GEN1,TPR,NDC80,MAD2L1	darkgrey	EC_M8
GO:0031348	1.15E-03	0.999467485	29	149	negative regulation of defense response	BP	5.55E-02	TNFAIP3,C6orf106,TEK,SYK,CST7,PPARG,GATA3,SEC14L1,NR1D2,PLK2,IGF1,ITCH,OAS3,NLRC5,PLCG1,CD200,FGR,ADORA2A,CALCRL,TNFRSF1B,ARG2,YES1,LGALS9,GIT1,ISG15,CDH5,OAS1,SERPINB9,RNF125	turquoise	EC_M6
GO:0080009	1.95E-03	0.999733527	6	16	mRNA methylation	BP	5.56E-02	ZC3H13,METTL3,VIRMA,NSUN2,METTL16,CMTR2	darkgrey	EC_M8
GO:0002456	1.16E-03	0.999649454	14	54	T cell mediated immunity	BP	5.56E-02	IL6,RSAD2,TRPM4,CTSC,NECTIN2,GATA3,JAG1,B2M,WAS,RAB27A,TNFRSF1B,IL7R,TRAF2,CD46	turquoise	EC_M6
GO:0045003	1.99E-03	0.999882943	4	7	double-strand break repair via synthesis-dependent strand annealing	BP	5.66E-02	RAD54L,RAD51,FANCM,RAD52	darkgrey	EC_M8
GO:0048026	2.00E-03	0.99971899	6	17	positive regulation of mRNA splicing, via spliceosome	BP	5.69E-02	NCBP1,THRAP3,NCL,TRA2B,RBMX,DAZAP1	darkgrey	EC_M8
GO:0006335	2.01E-03	0.999722517	6	16	DNA replication-dependent nucleosome assembly	BP	5.69E-02	NASP,CHAF1B,HAT1,ASF1B,CHAF1A,HIST1H3H	darkgrey	EC_M8
GO:0034723	2.01E-03	0.999722517	6	16	DNA replication-dependent nucleosome organization	BP	5.69E-02	NASP,CHAF1B,HAT1,ASF1B,CHAF1A,HIST1H3H	darkgrey	EC_M8
GO:0044089	1.19E-03	0.999253865	72	441	positive regulation of cellular component biogenesis	BP	5.70E-02	CNOT6,MTSS1,ASAP1,BAIAP2L2,PICK1,CDC42EP1,TEK,LINGO2,LIMCH1,KIT,KDR,SYK,SEMA4D,TLR4,SDCBP,NRP1,RASIP1,TAL1,RALB,FCHSD2,SFRP1,FERMT1,FCHSD1,CD36,LMOD3,EPHB1,HIP1R,OCLN,P2RX7,GPM6A,RAPGEF3,IQGAP2,SNCA,KIAA1551,AP1AR,FES,DLG1,CREB1,IL1RAP,DDX3X,DNM3,MMP1,LDB2,BAIAP2L1,PPM1F,WAS,RAC1,FSCN1,EPHA2,BID,TLR6,MTOR,SEMA4A,ARHGEF15,DNM2,SPIRE1,GIT1,SLITRK2,ISG15,PIEZO1,PLCG2,LRSAM1,FNBP1L,DAB2IP,ADGRL2,CARMIL2,CDH5,GBP5,VASP,GMFG,RALA,ARPC5L	turquoise	EC_M6
GO:0070661	1.21E-03	0.999384349	36	201	leukocyte proliferation	BP	5.71E-02	TNFAIP3,PTK2,KIT,TNFRSF21,SYK,IL6,PELI1,TLR4,IL15,PIK3CG,GAPT,IL1A,IGF1,HHEX,ITCH,SH3RF1,MEF2C,IL7,LMBR1L,ICOSLG,NFATC2,INPP5D,CD86,ACE,DLG1,SOS2,TNFRSF1B,ARG2,EFNB1,CHRNB2,CD19,LGALS9,ELF4,IL7R,CD46,BST2	turquoise	EC_M6
GO:0044546	1.21E-03	0.999868041	6	12	NLRP3 inflammasome complex assembly	BP	5.71E-02	TLR4,CD36,DDX3X,TLR6,PLCG2,GBP5	turquoise	EC_M6
GO:0140632	1.21E-03	0.999868041	6	12	inflammasome complex assembly	BP	5.71E-02	TLR4,CD36,DDX3X,TLR6,PLCG2,GBP5	turquoise	EC_M6
GO:1900225	1.21E-03	0.999868041	6	12	regulation of NLRP3 inflammasome complex assembly	BP	5.71E-02	TLR4,CD36,DDX3X,TLR6,PLCG2,GBP5	turquoise	EC_M6
GO:0002221	1.21E-03	0.999454552	27	134	pattern recognition receptor signaling pathway	BP	5.71E-02	TNFAIP3,GRAMD4,LRRC19,DDX58,PELI1,RSAD2,TLR4,PUM2,IRAK2,SEC14L1,CD36,ITCH,ERBIN,TLR2,OAS3,TIFA,LTF,DDX3X,BIRC3,TLR6,S100A14,TNIP1,PLCG2,DAB2IP,OAS1,TLR1,RNF125	turquoise	EC_M6
GO:0033003	1.21E-03	0.999726882	10	31	regulation of mast cell activation	BP	5.71E-02	SYK,GATA2,NECTIN2,GAB2,UNC13D,FGR,FES,LGALS9,STXBP1,HAVCR1	turquoise	EC_M6

GO:0042592	1.22E-03	0.999093775	177	1295	homeostatic process	BP	5.72E-02	ZNF516,TNFRSF11A,DMXL1,TNFAIP3,LYZ,MYCT1,TRDN,L3MBTL3,KLHL3,ATF4,LRRC19,IREB2,TJP2,ADGRF5,KIT,KDR,SYK,GADD45G,IL6,PPT1,PTBP3,VPS54,CAV2,GRB10,TLR4,IL15,TRPM4,PIK3CG,TAL1,LAMA4,GATA2,NOVA2,RAB38,GPR4,PRCP,NOS3,PPARG,GATA3,ACVR2B,CXCR4,EDNRB,TMC8,P2RY6,P2RY2,NR1D2,POC1B,P2RX2,SLC4A11,CD36,TMEM178A,MICU3,SCARB1,GAPT,MALL,PDE4D,MERTK,CHD7,IL1A,ABHD6,KDM3A,SMAD1,CLCN3,OCLN,HEXB,MEF2C,CP,WWTR1,IL7,P2RX7,CXCL10,USH1C,TPCN1,PLCG1,STC1,ANO6,IFIT1,EDN1,STAT1,JAM3,SNCA,ALAS1,MYLIP,PM20D1,ABCG1,ETS1,LPCAT1,MTTP,LCK,PPP3CA,HYAL2,ANO1,SLC39A8,KCNJ2,TESMIN,SLC12A7,HCRTR1,ESAM,DYRK3,SLC9A8,FABP4,HCLS1,INPP5D,CD34,PECAM1,POLG,PRKD1,F11R,MLXIPL,MELTF,PDK3,B2M,LTF,CCRL2,ADORA2A,DDX3X,SOS2,IGFBP5,MCU,PYGL,SLC9A7,WNK4,CDK16,LDB2,PROM1,FGF23,RAC1,HIF1A,LIPA,APBB2,ADIPOR2,ITPKB,HEPH,PSEN1,HOXB6,ADCK1,ATP2A1,CD19,RAB3D,NPR1,S100A14,GPR68,TLE3,SCNN1B,RCOR1,ADRB2,CCL2,PDE4B,LGALS9,NOS2,IL7R,GIT1,MTM1,HMOX2,ISG15,AP3D1,NOTCH1,PLCG2,S1PR1,CDH5,OAS1,PMAIP1,SLC8A3,ARID4A,LRRC8D,ADGRG1,TRIM24,CA2,KCNH2,SCN2B,SHANK3,LPAR6,DRAM2,TBC1D20,RAB39A,BMP6,RBP1,EMCN	turquoise	EC_M6
GO:0006220	2.08E-03	0.999467908	10	42	pyrimidine nucleotide metabolic process	BP	5.86E-02	CTPS1,DCK,CAD,MBD4,TDG,UMPS,DCTPP1,PRPS1,TYMS,DHODH	darkgrey	EC_M8
GO:0051709	1.25E-03	0.999902172	5	9	regulation of killing of cells of other organism	BP	5.87E-02	SYK,P2RX7,NOS2,FCER2,SERPINB9	turquoise	EC_M6
GO:0071025	2.09E-03	0.999781519	5	12	RNA surveillance	BP	5.88E-02	EXOSC9,EXOSC8,XRN1,EXOSC10,EXOSC2	darkgrey	EC_M8
GO:0034205	1.37E-03	0.999591754	14	44	amyloid-beta formation	BP	5.88E-02	RTN4,ROCK2,GSAP,PICALM,PRNP,CHRNA7,NCSTN,BACE1,RTN3,HAP1,TMED10,PIN1,ROCK1,MIR24-1	darkgreen	EC_M4
GO:0051782	2.10E-03	0.999714472	6	15	negative regulation of cell division	BP	5.91E-02	ORC6,E2F7,E2F8,ASPM,BLM,AURKB	darkgrey	EC_M8
GO:1902003	1.38E-03	0.999637062	12	35	regulation of amyloid-beta formation	BP	5.92E-02	RTN4,ROCK2,GSAP,PICALM,PRNP,CHRNA7,RTN3,HAP1,TMED10,PIN1,ROCK1,MIR24-1	darkgreen	EC_M4
GO:0043632	2.13E-03	0.998615157	74	561	modification-dependent macromolecule catabolic process	BP	5.96E-02	CDK2,USP15,LATS1,TUT7,UBR2,TOPORS,FBXO11,PNPT1,ZMPSTE24,USP34,XPO1,COMMD1,FBXO48,UFL1,CUL1,USP45,ASCC3,EXOSC9,CDC20,VPS36,EXOSC8,CUL4A,PSMD13,CDC16,ANAPC1,TAF9,USP25,PBK,ANAPC7,LTN1,USP16,UBE2C,UBE3A,CUL3,KIF14,UBR1,PSMD12,DTL,UBR3,PSMD4,CCDC47,SMARCC1,PSMA6,CDC27,COP1,CUL5,TBL1XR1,UBE2K,NEDD4,EXOSC10,USP3,TAF1,OGT,RLIM,RBBP6,PLK1,APPBP2,PSMD10,MIB1,COPS3,CUL4B,UHRF1,CCNF,PSMA5,EXOSC2,GLMN,USP33,AURKA,RNF139,RNF8,GID4,UBXN2A,PSMD7,RCHY1	darkgrey	EC_M8
GO:0000902	1.29E-03	0.999095461	123	799	cell morphogenesis	BP	6.00E-02	SEMA6A,KIF5A,SNX2,RIMS2,CDC42SE2,NOTCH4,PARVG,PACSIN2,CDKL3,ARHGAP18,PTK2,ATF4,CDC42EP1,STRIP2,TEK,KIT,KDR,SEMA4D,NRCAM,NRP1,ITSN2,TAL1,NOVA2,FGD5,PLXND1,ITGA8,UNC13D,GATA3,MYO7A,CXCR4,SPTBN4,FEZ2,EFNB2,ARHGEF7,FGD6,NTN4,PRAG1,EPHB1,RET,MERTK,PREX2,KIF13B,ARHGEF28,HEXB,MEF2C,GPM6A,STC1,NR4A2,DNMBP,NEDD9,DTNBP1,GAS2,PPP3CA,HECW2,MEF2A,SEMA3F,ROBO4,CDH24,SEC24B,AP1AR,EPB41,PLEKHO1,PLXNA2,FGR,TRAK2,ADCY1,KALRN,FES,PROX1,SS18L1,LAMA5,F11R,YTHDF1,DOCK1,PAK2,MELTF,LAMC2,DLG1,MOV10,CREB1,NBEAL2,ADORA2A,SEMA6D,EPHB4,ITGA4,DNM3,LMTK2,RAC1,LIPA,RCC2,RHOJ,EPHA2,FGD1,AR,OPHN1,EFNB1,PSEN1,SEMA4A,ABI3,TAOK2,EFNA1,CHRNB2,ZDHHC15,RPS6KA5,CCDC88C,DNM2,B4GALT6,CCL2,IL7R,SEMA6B,SLITRK2,ARHGAP4,NOTCH1,PALMD,STXBP1,DAB2IP,CDH5,VASP,SOX17,RELN,SHANK3,TBC1D20,VAMP3	turquoise	EC_M6
GO:0032940	1.30E-03	0.999144274	87	574	secretion by cell	BP	6.04E-02	TNFRSF11A,STX11,SNCAIP,RIMS2,PICK1,APBA1,KIT,CD2AP,SYK,ICA1,IL6,PPT1,RSAD2,TLR4,SDCBP,TRPM4,RAB3B,PIK3CG,GATA2,EXOC3L2,GAB2,LLGL2,RALB,UNC13D,GATA3,SFRP1,SIRT3,MERTK,CHD7,FAM3D,IL1A,IGF1,NR4A1,EXOC6,MCTP1,MEF2C,TLR2,VEGFC,EDN1,SNCA,DTNBP1,ABCG1,CD200,MTTP,HDAC1,PPP3CA,ANO1,MCTP2,SNAP23,ATP9A,FGR,ADCY1,FES,CREB1,ADORA2A,MCU,CDK16,SNAP29,FGF23,RAC1,HIF1A,RAB27A,SNCG,SYT7,RSC1A1,MICAL3,TNFRSF1B,FBXL20,PSEN1,CHRNB2,RAB3D,GPR68,SMAD2,LGALS9,NOS2,MYO18A,GIT1,RPH3AL,NOTCH1,ENG,STXBP1,EXOC3L1,STX3,RALA,VAMP3,BMP2,BMP6	turquoise	EC_M6

GO:0046034	8.05E-05	0.999961555	46	219	ATP metabolic process	BP	6.08E-02	HSPA1B,TSPO,UQCRQ,EP300,NDUFB2,SPHK2,UQCRH,NDUFB6,NDUFC2,NDUFA2,COX7A1,COQ9,DGUOK,PRKAG1,NDUFV1,ARNT,HDAC4,NDUFS2,DNAJC30,FIS1,CHCHD10,UQCC3,FOXK1,NDUFB7,NUPR1,INSR,NDUFA7,UQCR11,NDUFS7,ATP5F1D,TAZ,JMJD8,MT-ND1,MT-ND2,MT-CO1,MT-CO2,MT-ATP6,MT-CO3,MT-ND3,MT-ND4L,MT-ND4,MT-ND5,MT-CYB,ZBTB7A,PID1,NUDT2	cyan	EC_M2
GO:1901386	1.42E-03	0.999848908	6	11	negative regulation of voltage-gated calcium channel activity	BP	6.09E-02	REM1,CACNB3,LINC02210-CRHR1,CALM1,CBARP,GEM	darkgreen	EC_M4
GO:0150063	1.44E-03	0.999144574	58	282	visual system development	BP	6.15E-02	AHI1,FBN2,RORB,SRF,VEGFA,TGFBR1,MFSD2A,GLI3,INHBA,SLC7A11,FGF2,SMARCD3,RAB3GAP1,CTNNB1,RARB,SPRED3,ATP2B1,FAT1,RDH10,SLC4A5,OPA1,ATP2B4,NKD1,KDM5B,NOTCH2,TGFB2,LRP5,SHROOM2,CRYBA4,BMP7,CLIC4,ATF6,FBN1,FKBP8,PRKCI,COL5A2,SKIL,TULP3,OBSL1,ROM1,CACNA1C,CLN8,MED1,NES,TTC8,SMARCA4,CHRDL1,PDGFRB,MYOM1,GNB1,NPHP4,COL5A1,BCAR3,SERPINF1,THY1,WNT2,COL8A2,FGF10	darkgreen	EC_M4
GO:1904950	1.45E-03	0.99937408	25	100	negative regulation of establishment of protein localization	BP	6.16E-02	LMAN1,ANGPT1,REST,RAB23,ERLEC1,UBE2J1,MDFIC,FKBP1B,UFM1,RAB11FIP1,ABCC8,PTPN11,CHP1,HADH,IDH2,MAPT,C11orf65,SRCIN1,SYTL4,MIDN,PARK7,RHBDF1,TXN,UBE2G2,DERL2	darkgreen	EC_M4
GO:0030330	2.21E-03	0.999265139	14	63	DNA damage response, signal transduction by p53 class mediator	BP	6.18E-02	GTSE1,ZMPSTE24,CASP2,PAXIP1,E2F7,BRCA2,ATR,NBN,EEF1E1,MDM4,DDX5,FOXM1,ATRX,PSMD10	darkgrey	EC_M8
GO:0032613	1.34E-03	0.999691678	10	32	interleukin-10 production	BP	6.19E-02	TNFRSF21,SYK,IL6,TLR4,TLR2,CD34,LGALS9,ISG15,PLCG2,CD46	turquoise	EC_M6
GO:0032653	1.34E-03	0.999691678	10	32	regulation of interleukin-10 production	BP	6.19E-02	TNFRSF21,SYK,IL6,TLR4,TLR2,CD34,LGALS9,ISG15,PLCG2,CD46	turquoise	EC_M6
GO:0043297	1.35E-03	0.999587446	14	52	apical junction assembly	BP	6.20E-02	AFDN,IKBKB,MARVELD2,OCLN,ESAM,CLDN34,PECAM1,F11R,PAK2,DLG1,RAMP2,PRKCH,EPHA2,CDH5	turquoise	EC_M6
GO:0061077	1.46E-03	0.999512197	16	55	chaperone-mediated protein folding	BP	6.20E-02	BAG1,PDCL3,P3H1,FKBP1B,CRTAP,DNAJB13,HSPH1,HSPA8,HSPB1,DNAJB12,FKBP2,ERO1A,CD74,HSPA5,FKBP1A,HSPA13	darkgreen	EC_M4
GO:0072008	1.36E-03	0.999939844	4	6	glomerular mesangial cell differentiation	BP	6.24E-02	PDGFB,GPR4,CD34,NOTCH1	turquoise	EC_M6
GO:0060236	2.25E-03	0.999483012	9	31	regulation of mitotic spindle organization	BP	6.26E-02	BORA,TPX2,RAE1,TPR,KIF15,TACC3,HNRNPU,PLK1,DRG1	darkgrey	EC_M8
GO:0007189	1.38E-03	0.999482117	19	80	adenylate cyclase-activating G protein-coupled receptor signaling pathway	BP	6.28E-02	ADGRF1,GPR4,PDE4D,CXCL10,ADGRG3,ADCY2,ADCY1,ADCY4,ADORA2A,CALCRL,RAMP2,ADRB2,PDE4B,S1PR1,ADGRL2,ADGRL4,ADGRE3,ADGRG1,GNG2	turquoise	EC_M6
GO:0050975	1.50E-03	0.999954628	4	5	sensory perception of touch	BP	6.30E-02	P2RX4,CACNB3,KCNK4,KCNA1	darkgreen	EC_M4
GO:0007435	1.50E-03	0.999667217	10	26	salivary gland morphogenesis	BP	6.30E-02	PLXNA1,TGFB1,SEMA3C,TGFB2,DAG1,BMP7,FGFR2,TGFB3,EDA,FGF10	darkgreen	EC_M4
GO:0034762	1.50E-03	0.999040563	76	396	regulation of transmembrane transport	BP	6.30E-02	CD63,AZIN1,UBQLN1,LEP,SHANK1,ITGB1,FKBP1B,KIF5B,SCN5A,AKT2,GPD1L,OSBPL8,ACTN4,STIM1,IRS2,PRNP,ARL6IP5,REM1,FXYD1,SNTA1,SLMAP,ATP1B3,SORBS1,CACNA1D,APP,CX3CL1,ABCC8,CACNB3,PTPN11,HOMER1,CHP1,ATP2B4,PKD2,AZIN2,CALHM1,SELENOS,ISCU,CASQ1,PEA15,CLIC4,LINC02210-CRHR1,PTPN6,KCNK4,HTT,CLTCL1,PRKCI,KCNA1,WDR1,HAP1,CACNA1C,NLGN2,STIM2,PPARGC1A,KCNMB1,CACNA1G,CALM1,VMP1,AKT1,FLNA,KCNAB2,PARK7,HCN2,AHCYL1,JPH3,MIR24-1,DMD,FKBP1A,CBARP,TMEM38B,THY1,TMSB4X,TMEM38A,YWHAH,TMEM109,HCN4,GEM	darkgreen	EC_M4
GO:0043010	1.51E-03	0.999138214	51	242	camera-type eye development	BP	6.30E-02	AHI1,FBN2,RORB,SRF,VEGFA,TGFBR1,MFSD2A,GLI3,INHBA,SLC7A11,FGF2,SMARCD3,RAB3GAP1,CTNNB1,SPRED3,ATP2B1,FAT1,RDH10,SLC4A5,OPA1,ATP2B4,KDM5B,NOTCH2,TGFB2,LRP5,SHROOM2,CRYBA4,BMP7,CLIC4,FBN1,FKBP8,SKIL,TULP3,OBSL1,ROM1,CACNA1C,CLN8,MED1,NES,TTC8,SMARCA4,PDGFRB,MYOM1,GNB1,NPHP4,BCAR3,SERPINF1,THY1,WNT2,COL8A2,FGF10	darkgreen	EC_M4
GO:0085029	1.51E-03	0.999572336	13	39	extracellular matrix assembly	BP	6.30E-02	COL1A2,TGFB1,PHLDB2,FBLN5,DAG1,PHLDB1,QSOX1,PLOD3,FKBP10,NTNG2,LOX,HAS2,MFAP4	darkgreen	EC_M4
GO:0022409	1.39E-03	0.999312433	33	176	positive regulation of cell-cell adhesion	BP	6.31E-02	AFDN,PODXL,SYK,IL6,IL15,GATA3,MAGI1,IL1A,IGF1,IL7,SOX13,ETS1,LCK,ICOSLG,HLX,CD86,F11R,B2M,ITGA4,SELE,SELP,ITPKB,EFNB1,CCL2,YES1,JAK1,LGALS9,IL7R,AP3D1,PIEZO1,NFAT5,VNN1,CD46	turquoise	EC_M6

GO:0016233	2.28E-03	0.999420837	10	37	telomere capping	BP	6.32E-02	MAP3K4,POT1,DCLRE1C,PRKDC,TNKS2,NBN,DCLRE1B,NEK2,AURKB,HNRNPD	darkgrey	EC_M8
GO:1902751	2.28E-03	0.999531391	8	27	positive regulation of cell cycle G2/M phase transition	BP	6.32E-02	FBXO5,CDC25C,CCNB1,DTL,CDK1,CDC25A,DBF4B,ATAD5	darkgrey	EC_M8
GO:0045576	1.40E-03	0.999592479	13	48	mast cell activation	BP	6.33E-02	KIT,SYK,PIK3CG,GATA2,NECTIN2,GAB2,UNC13D,SNAP23,FGR,FES,LGALS9,STXBP1,HAVCR1	turquoise	EC_M6
GO:0043410	1.41E-03	0.999159004	58	356	positive regulation of MAPK cascade	BP	6.36E-02	TNFRSF11A,AKAP12,FLT4,TPD52L1,PDGFB,TEK,SH3RF3,KIT,KDR,SYK,GADD45G,IL6,CAV2,TLR4,TRAF1,SDCBP,NRP1,PIK3CG,PRKD2,P2RY6,CD36,RET,TNFRSF19,IGF1,SH3RF1,NDRG4,MADD,EDN1,HCRTR1,LAPTM5,NCF1,CFLAR,MAP3K3,MAP3K11,TBX1,TMEM106A,TNFAIP8L3,FGF23,CRACR2A,TLR6,RELL1,AR,PSEN1,FGF18,TAOK2,EFNA1,PIK3R5,ADRB2,CCL2,LGALS9,TRAF2,NOTCH1,PLCG2,DAB2IP,MIR23A,ALKAL1,BMP2,NMNAT1	turquoise	EC_M6
GO:0030900	1.53E-03	0.999090349	58	282	forebrain development	BP	6.38E-02	LRP1,TRAPPC9,SUN2,MDGA1,SRF,UBA6,RTN4,MFSD2A,GLI3,INHBA,SLC7A11,FGF2,KIF5B,EIF2B3,GLI2,AXL,RAB3GAP1,SCN5A,HTR5A,CTNNB1,RARB,TACC1,DCLK1,APLP1,RYK,CXCL12,ZSWIM6,ROBO1,CELF1,TCTN1,APP,RAPGEF2,ATP2B4,DNAH5,UCHL5,FEZ1,SATB2,GSK3B,LEF1,KRAS,FGFR2,KIF1A,ARPC5,TNR,COL3A1,KCNA1,HAP1,BMPR1A,PPARGC1A,TTC8,MYO1D,PAFAH1B1,FLNA,PLXNA3,LHX8,BNIP3,PRKG1,FGF10	darkgreen	EC_M4
GO:0046822	2.31E-03	0.99909567	18	94	regulation of nucleocytoplasmic transport	BP	6.39E-02	UBR5,TRIM28,ANP32B,XPO1,IPO5,NUP58,XPO4,NUP54,NOLC1,NSUN2,KHDRBS1,CDK1,DHX9,SETD2,CTDSPL2,TPR,ECT2,NEDD4	darkgrey	EC_M8
GO:0031589	1.55E-03	0.999069627	60	294	cell-substrate adhesion	BP	6.44E-02	CD63,LRP1,ANGPT1,PPARD,MKLN1,LGALS1,SRF,VEGFA,ROCK2,BVES,RRAS,ZYX,ITGB1,FBLN2,AXL,CTNNB1,ACTG1,ACTN4,SIGLEC1,ILK,AKIP1,SORBS1,RASA1,EDIL3,CX3CL1,PTPN11,PHLDB2,CTTN,FBLN5,GSK3B,MMP14,DAG1,CORO1C,ITGB5,IQGAP1,COL26A1,SERPINE1,ITGA2B,FN1,ITGAV,ARPC2,COL3A1,VCL,ACTN1,SRCIN1,THBS3,COL1A1,FAT2,ROCK1,ITGA2,EGFLAM,DAPK3,PKD1,FLNA,NTNG2,CDH13,THY1,HAS2,EDA,DUSP22	darkgreen	EC_M4
GO:1903608	2.34E-03	0.999855422	4	7	protein localization to cytoplasmic stress granule	BP	6.45E-02	TIA1,DDX1,SSB,DHX9	darkgrey	EC_M8
GO:0016441	2.34E-03	0.999024447	20	115	posttranscriptional gene silencing	BP	6.45E-02	TNRC6B,TUT7,XPO5,HNRNPA2B1,PUS10,ZMPSTE24,METTL3,CNOT1,RIPK1,AJUBA,FXR1,DDX5,TIAL1,DDX6,DHX9,DGCR8,ZC3H7A,DROSHA,FMR1,SRRT	darkgrey	EC_M8
GO:0060571	1.58E-03	0.999730517	8	18	morphogenesis of an epithelial fold	BP	6.52E-02	GLI2,CTNNB1,SULF1,RDH10,BMP7,FGFR2,WNT2,FGF10	darkgreen	EC_M4
GO:0034765	1.58E-03	0.999034665	64	323	regulation of ion transmembrane transport	BP	6.52E-02	CD63,UBQLN1,SHANK1,ITGB1,FKBP1B,KIF5B,SCN5A,GPD1L,ACTN4,STIM1,PRNP,ARL6IP5,REM1,FXYD1,SNTA1,SLMAP,ATP1B3,CACNA1D,APP,CX3CL1,ABCC8,CACNB3,HOMER1,CHP1,ATP2B4,PKD2,CALHM1,ISCU,CASQ1,CLIC4,LINC02210-CRHR1,PTPN6,KCNK4,HTT,KCNA1,WDR1,HAP1,CACNA1C,NLGN2,STIM2,PPARGC1A,KCNMB1,CACNA1G,CALM1,VMP1,AKT1,FLNA,KCNAB2,PARK7,HCN2,AHCYL1,JPH3,MIR24-1,DMD,FKBP1A,CBARP,TMEM38B,THY1,TMSB4X,TMEM38A,YWHAH,TMEM109,HCN4,GEM	darkgreen	EC_M4
GO:0006304	2.39E-03	0.999094446	17	84	DNA modification	BP	6.57E-02	TRIM28,ZMPSTE24,RLF,EZH2,ASCC3,MBD4,PARP2,TDG,OTUD4,HELLS,MIS18A,TET2,PRMT5,BRCA1,ATRX,EHMT1,CTCF	darkgrey	EC_M8
GO:0000271	1.60E-03	0.99944313	17	58	polysaccharide biosynthetic process	BP	6.59E-02	DYRK2,TGFB1,AKT2,IRS2,B3GNT4,GYG1,SORBS1,SELENOS,GYG2,GSK3B,PPP1R3D,PPP1R3F,CLTC,AKT1,AGL,HAS2,HS2ST1	darkgreen	EC_M4
GO:0045944	1.47E-03	0.998955634	130	904	positive regulation of transcription by RNA polymerase II	BP	6.60E-02	TCF4,PLAGL1,ZBED4,NOTCH4,TCF20,NCOA7,ATF4,PDGFB,PRDM4,DDX58,IL6,REL,TLR4,TEAD2,MYCN,TAL1,PHF10,EPC1,GATA2,PRKD2,PPARG,GATA3,IKBKB,JAG1,TMEM173,NR1D2,TCF15,CHD7,IL1A,IGF1,NR4A1,HHEX,KDM3A,SMAD1,ZBTB38,STOX2,HEXB,MEF2C,TLR2,WWTR1,CXCL10,KMT2D,NLRC5,NR4A2,TOX2,EDN1,STAT1,ERG,ETS2,NFKB2,PRDM15,ETS1,HDAC1,PPP3CA,HYAL2,MEF2A,BCL9,HCLS1,NFATC2,EAF2,GMEB1,POU2F3,RPS6KA1,PROX1,SS18L1,PRKD1,ZNF148,HELZ2,MLXIPL,ITGA6,ELL3,ETV6,HOXD10,CREB1,TET1,ARNT2,DDX3X,RFXANK,ETV4,TBX1,MECOM,LDB2,POU2F1,WAS,HIF1A,ZBTB18,APBB2,JUP,AR,CASZ1,HOXB5,HOXB7,HOXB9,BATF,RPS6KA5,SMAD2,YY1,ARID3B,TNIP1,ZNF521,ABLIM3,ADRB2,YES1,ELF4,MRTFB,DOT1L,AP3D1,HCFC1,NOTCH1,MED16,IRF8,S1PR1,ENG,MTF2,NFAT5,DAB2IP,ARID4A,ZNF24,SOX17,ZNF850,CDK13,SOX18,MAFF,ZNF229,E2F3,BMP2,GLIS3,ZNF71,BMP6,FLI1	turquoise	EC_M6

GO:0030855	1.47E-03	0.999079411	69	430	epithelial cell differentiation	BP	6.60E-02	NOTCH4,AFDN,MTSS1,ATF4,PDGFB,PODXL,TJP2,KDR,TMOD1,MYCN,NRP1,GPR4,PPARG,GATA3,ACVR2B,MYO7A,CXCR4,TIE1,IKBKB,JAG1,PDE2A,GPAT4,SYNE4,TCF15,PDE4D,MARVELD2,MEF2C,WWTR1,USH1C,PPP1R16B,STC1,RAPGEF3,STAT1,HDAC1,ROBO4,PRKX,SEC24B,CD34,PECAM1,TGM1,PROX1,F11R,APOLD1,CREB1,BCR,ETV4,TBX1,PROM1,PRKCH,HIF1A,EPHA2,AR,OPHN1,HOXB5,CCDC88C,CYP1A1,JAG2,NOTCH1,RAPGEF1,S1PR1,CNN3,CDH5,ARID4A,SOX17,SOX18,MAFF,TBC1D20,BMP2,BMP6	turquoise	EC_M6
GO:0050708	1.61E-03	0.999123511	43	200	regulation of protein secretion	BP	6.63E-02	LRP1,SLC12A2,PPARD,LEP,UNC13B,CLOCK,EXOC1,REST,P3H1,FKBP1B,KIF5B,DNAJC1,TGFB1,IRS2,RAB11FIP1,ANKRD1,OSBP,ABCC8,PTPN11,PFKM,BRSK2,DYNLL1,DNM1L,CPT1A,TGFB2,LRP5,HADH,DISP1,IDH2,ADCY5,SIDT2,NLGN2,SRCIN1,TMED10,TGFB3,MYH10,SYTL4,ACSL4,MYOM1,MIDN,SSTR5,RHBDF1,WLS	darkgreen	EC_M4
GO:0030203	1.62E-03	0.999311355	24	93	glycosaminoglycan metabolic process	BP	6.63E-02	B4GALT7,ANGPT1,FGF2,GLB1,CHPF2,TGFB1,DCN,IMPAD1,B3GNT4,CHST2,CHSY1,HYAL1,B3GAT3,UGDH,CHST12,XYLT2,CLN6,CLTC,PDGFRB,AKT1,EGFLAM,HAS2,CHST11,HS2ST1	darkgreen	EC_M4
GO:0006144	2.43E-03	0.999644751	6	17	purine nucleobase metabolic process	BP	6.68E-02	PPAT,PAICS,GMPS,PRPS1,HPRT1,GART	darkgrey	EC_M8
GO:0018057	1.64E-03	0.999949146	4	5	peptidyl-lysine oxidation	BP	6.69E-02	LOXL3,LOXL2,LOXL1,LOX	darkgreen	EC_M4
GO:0014070	1.64E-03	0.998836373	124	703	response to organic cyclic compound	BP	6.71E-02	CDO1,PPARA,TAF2,PPARD,CTNNA1,LGALS1,TXN2,PLIN2,MTAP,LEP,CLOCK,ASNS,REST,TGFBR1,ACTR2,ROCK2,INHBA,VPS35,MGARP,SLC7A11,PGRMC2,FKBP1B,PANX1,GLB1,GLI2,PAK1,TGFB1,HTR5A,HNMT,CTNNB1,LBH,UFM1,ATP2B1,LUM,ZNF703,UBA5,DDRGK1,SMPD1,ARL6IP5,APLP1,RNF6,ANKRD1,HSP90B1,MAP1LC3A,MAP1B,RYR3,P2RX4,HEY1,MMP15,APP,DHX36,RAPGEF2,PPP2R2A,ADCY6,HOMER1,OPA1,CRY2,VPS18,ADA,ATP2B4,SPP1,CPN1,PKD2,PSPH,EIF4E,DNM1L,CCND1,CPT1A,TGFB2,HTR2B,GSK3B,CASP7,MMP14,DAG1,KRAS,NTRK3,BMP7,ADCY5,NEDD8,PEA15,NCSTN,RBBP7,STRN3,COMT,IGFBP2,PTPRN,CYP19A1,SLC3A2,MED1,PPARGC1A,MSN,KCNMB1,COL1A1,TGFB3,SOX30,CALM1,SMARCA4,PDGFRB,RBBP8,AKT1,ITGA2,PRKAA1,GNAL,GNB1,ABCA3,FLNA,SSTR5,PARK7,HCN2,BSG,GNAI3,AGL,HSPA5,SERPINF1,TMEM38B,LOX,CDKN1A,TMEM38A,FAM210B,YWHAH,PMEPA1,HCN4,FGF10,MBD2	darkgreen	EC_M4
GO:0050778	1.50E-03	0.999135986	50	306	positive regulation of immune response	BP	6.73E-02	PTK2,TEC,TNFRSF21,SYK,IL6,RSAD2,TLR4,C5,IL15,GATA2,PRKD2,NECTIN2,GAB2,GATA3,IKBKB,TMEM173,PDE4D,MEF2C,NLRC5,PLCG1,DGKZ,LCK,ICOSLG,LAPTM5,HLX,NFATC2,INPP5D,CD86,BMX,FGR,PLEKHA1,PAK2,B2M,MASP1,PRKCH,PLD2,PSEN1,EIF2B2,CD19,YES1,PDE4B,LGALS9,TRAF2,PLCG2,STXBP1,FYB1,GBP5,CD46,TRIM62,FCER2	turquoise	EC_M6
GO:0006606	2.48E-03	0.998891277	23	129	protein import into nucleus	BP	6.78E-02	NUP107,UBR5,TNPO3,TRIM28,NUP85,IPO5,NUP58,IPO11,IPO7,TNPO1,NUP54,NUP93,HEATR3,NOLC1,CSE1L,NUP133,CDK1,TPR,ECT2,KPNB1,NUP62CL,NUP155,NUP35	darkgrey	EC_M8
GO:0031124	2.48E-03	0.999207471	13	56	mRNA 3'-end processing	BP	6.78E-02	SCAF8,NCBP1,YTHDC1,PNPT1,PAPOLG,WDR33,SYMPK,PCF11,CCNB1,TENT2,VIRMA,CPSF2,LEO1	darkgrey	EC_M8
GO:0006265	2.50E-03	0.999842261	4	7	DNA topological change	BP	6.82E-02	TOP2B,TOP1,TOP2A,TOP3A	darkgrey	EC_M8
GO:0031324	2.51E-03	0.998056917	215	1896	negative regulation of cellular metabolic process	BP	6.82E-02	PAN2,BCLAF1,LATS1,FBXO5,ZNF608,SCAF8,UBR5,EIF3E,MRPL13,XRCC6,TNRC6B,TUT7,PLEC,POT1,PWP1,RFX3,TRIM28,ZNF451,LRPPRC,MSH2,IBTK,PNPT1,ZMPSTE24,SYNCRIP,XPO1,CASP8AP2,TIA1,ZNF350,ITGB1BP1,THRAP3,EZH2,UFL1,FAM3C,WDR35,DHFR,PUS7,HDAC2,EXOSC9,DNAJC2,CHORDC1,SETDB2,MASTL,TRDMT1,CNOT10,RSF1,DIS3,NCAPG2,BIRC5,GTPBP4,IPO5,CHD8,KAT6A,METTL3,RIF1,E2F7,TAF7,EXOSC8,NR2C1,BRCA2,ELK3,TCERG1,PRKDC,CBX5,CCNB1,ERCC6,PAN3,CAPRIN1,ZC3H8,IPO7,PARPBP,TNKS2,LSM14A,BTAF1,CDK5RAP1,TDG,NCK1,IDE,ABCE1,TAF9,CGGBP1,XRN1,PDE12,SAP30,ATR,TERF1,MTMR9,GCFC2,USP25,HK2,PBK,CNOT1,GABPA,SH2B3,PPID,RBL1,MSH3,NBN,TENT2,E2F8,TRIM33,ZBED6,ZNF248,MDM4,HIVEP1,CUL3,NSUN2,GMNN,NFKB1,KHDRBS1,NCL,PRMT5,AJUBA,BPTF,FXR1,DDX5,FANCB,ORC2,BLM,DDX6,CENPF,AUNIP,SMARCC1,SP3,DHX9,HNRNPR,IVNS1ABP,DDX20,TPR,CCAR1,XRCC5,NPAT,BRCA1,TFPI,CNOT9,NSD2,TIPRL,BOD1L1,ANKLE1,TBL1XR1,SUV39H1,FOXM1,EIF2AK1,LYAR,FAM220A,HNRNPU,PDS5A,WAPL,NEDD4,SMARCE1,RFC1,CDC6,DCP2,NSD1,EXOSC10,FUS,CLK2,USP3,AURKB,TAF1,OGT,RLIM,ATRX,PTTG1,SPAG9,TIPIN,MBTD1,TRIM37,LARP1,PLK1,ILF3,AATF,CSK,PSMD10,TAF15,NLE1,THOC1,TYMS,SMCHD1,ZNF3,SUZ12,MIER1,XIAP,NIPBL,PHF6,RMI2,UHRF1,RBMX,FMR1,METTL16,EHMT1,RNPS1,PTBP1,EXOSC2,GLMN,SCAI,CTCF,DEPDC1,HIST1H1A,HNRNPD,ZNF684,MAD2L1,DRAP1,ZNF614,RBAK,TIGAR,SRSF7,NAF1,BRMS1L,RNF139,C1D,RNF8,ZNF558,IKZF5,PAIP2,ZNF529	darkgrey	EC_M8

GO:0048762	1.68E-03	0.999083691	43	198	mesenchymal cell differentiation	BP	6.84E-02	TGFBR1,RTN4,ROCK2,TGFB1,CTNNB1,SPRED3,ZNF703,SEMA3C,RDH10,SEMA4F,LOXL3,HEY1,LOXL2,ACVR1,SEMA4G,ADIPOR1,PHLDB2,TGFB2,HTR2B,GSK3B,DAG1,LEF1,CORO1C,BMP7,FGFR2,PHLDB1,FN1,BMPR1A,TGFB1I1,COL1A1,TGFB3,TMEM100,ANXA6,ROCK1,VASN,FLNA,GDNF,HAS2,DACT3,GLIPR2,OLFM1,WNT2,FGF10	darkgreen	EC_M4
GO:0022900	9.60E-05	0.999959137	35	149	electron transport chain	BP	6.84E-02	UQCRQ,ETFB,NDUFB2,UQCRH,NDUFB6,NDUFC2,NDUFA2,COX7A1,UQCRFS1,COQ9,DGUOK,SRD5A1,NDUFV1,NDUFS2,POR,AIFM2,UQCC3,NDUFB7,GBA,NDUFA7,NDOR1,UQCR11,NDUFS7,TAZ,CYBA,MT-ND1,MT-ND2,MT-CO1,MT-CO2,MT-CO3,MT-ND3,MT-ND4L,MT-ND4,MT-ND5,MT-CYB	cyan	EC_M2
GO:0006412	9.97E-05	0.999939547	92	548	translation	BP	6.84E-02	PYM1,CDK4,EIF3H,RPS18,EEF1D,RPL3,EIF3L,EIF3D,RPL8,RPS5,RPL28,RPS9,EEF1A1,MALSU1,RPS7,RPS11,FOXO3,RPL18,ABTB1,RPS8,MIF4GD,MLH1,UNK,RPS3,RPS16,SAMD4B,MRPS12,RPL15,EIF3K,TARBP2,EIF3F,EIF4B,RPS3A,EIF2A,FARS2,RPLP2,PCIF1,WARS2,FASTKD3,MRPL36,DIS3L2,PUM1,APEH,RPS6KB2,QARS,DALRD3,EIF4H,PER2,RPS21,RPL35A,RPL11,YBX3,MRPL49,EXOSC7,RPL27,UBA52,RARA,ZFP36L1,MRPL10,PER1,TNRC6A,LARP6,RPL7A,EEF2K,MRPL4,EIF3G,NDUFA7,RPL23A,PARN,MRPL37,EEF2,DPH1,MRPL41,MRPS34,INPP5E,MRPL28,RPL13,MRPS2,KARS,RPS15,AGO4,RACK1,RPS19,MRPL34,RPL23,EEF1B2,RPUSD3,MRPL57,RPS20,ZFP36L2,PDF,EXOSC5	cyan	EC_M2
GO:0031109	2.52E-03	0.998924492	21	109	microtubule polymerization or depolymerization	BP	6.85E-02	FBXO5,KIF24,AKAP9,CLASP2,KIF2C,CKAP2,TUBGCP3,TPX2,SKA3,CENPJ,CKAP5,STMND1,KIF18A,CAMSAP2,HAUS2,HDGFL3,KIF18B,FKBP4,TAOK1,STMN1,DRG1	darkgrey	EC_M8
GO:0090435	2.53E-03	0.999839863	4	7	protein localization to nuclear envelope	BP	6.87E-02	NUP54,INTS13,PLK1,NUP155	darkgrey	EC_M8
GO:0060322	1.70E-03	0.998816451	113	624	head development	BP	6.90E-02	LRP1,AHI1,PLXNB2,TRAPPC9,SUN2,MDGA1,SRF,UBA6,RTN4,MFSD2A,WHRN,GLI3,INHBA,RRAS,SLC7A11,PCDH18,ITGB1,FGF2,KIF5B,NAPA,EIF2B3,GLI2,PAK1,AXL,RAB3GAP1,SCN5A,HTR5A,HNMT,CTNNB1,RARB,TACC1,UFM1,IRS2,ATP2B1,DCLK1,APLP1,RYK,SNPH,CXCL12,ZSWIM6,CTNND1,ROBO1,FAIM2,CELF1,TCTN1,APP,RAPGEF2,PTPN11,AMIGO2,PPP3CC,MMP2,ATP2B4,DNAH5,PSMG1,DYNLL1,INA,UCHL5,FEZ1,SATB2,GSK3B,MMP14,LEF1,SHROOM2,KRAS,PITPNM1,BMP7,FGFR2,NCSTN,KIF1A,MACO1,ARID5B,PAFAH1B2,ARPC5,SGPL1,ATP6AP2,TNR,COL3A1,KCNA1,TULP3,MNAT1,AKT3,HAP1,BMPR1A,MED1,PPARGC1A,NES,COL1A1,TGFB3,MAP2K1,TTC8,CALM1,EML1,MYO1D,ROGDI,PAFAH1B1,SDF4,FLNA,PLXNA3,RERE,GNAI3,SYPL2,ANKRD11,AARS,HSPA5,ATP6V0D1,LHX8,WLS,BNIP3,ARF4,YWHAH,PRKG1,WNT2,FGF10	darkgreen	EC_M4
GO:0045814	2.56E-03	0.9992791	11	43	negative regulation of gene expression, epigenetic	BP	6.94E-02	TRIM28,EZH2,SETDB2,RRP8,SMARCA5,FAM208A,HELLS,SMYD5,TPR,SUV39H1,SMCHD1	darkgrey	EC_M8
GO:0007596	1.56E-03	0.999238191	31	164	blood coagulation	BP	6.99E-02	TSPAN8,FZD6,PDGFB,TEC,SYK,IL6,TLR4,PIK3CG,NOS3,CD36,MERTK,KLKB1,PROCR,ENTPD1,DGKZ,ANO6,EDN1,MMRN1,DTNBP1,LCK,CD34,F11R,ADORA2A,SELP,VWF,WAS,RAB27A,PEAR1,F8,PLCG2,STXBP1	turquoise	EC_M6
GO:0034762	1.57E-03	0.999031978	64	396	regulation of transmembrane transport	BP	6.99E-02	TRDN,DAPK1,PIM1,TSPAN13,GRB10,TCAF2,PIK3CG,KCNRG,P2RY6,KCNK6,PDE4D,CHD7,IGF1,OCLN,MEF2C,P2RX7,CXCL10,TPCN1,PLCG1,KCNJ3,ANO6,CLIC6,EDN1,KCNJ15,SNCA,PM20D1,HECW2,KCNJ2,MEF2A,SCN3A,PRKD1,OAZ3,DLG1,KCNK7,CTTNBP2NL,CYBB,WNK4,CRACR2A,ADIPOR2,RSC1A1,MTOR,PSEN1,ATP2A1,CD19,DNM2,ADRB2,CCL2,YES1,KCNJ18,PDE4B,SLC43A2,PLCG2,CA2,RELN,KCNH2,SCN2B,KCNK5,SHANK3,RGS4,KCNA6,CACNG5,FXYD6,KCNG2,GSG1L	turquoise	EC_M6
GO:0051224	1.74E-03	0.999251591	24	96	negative regulation of protein transport	BP	7.04E-02	LMAN1,ANGPT1,REST,RAB23,ERLEC1,UBE2J1,MDFIC,FKBP1B,UFM1,RAB11FIP1,ABCC8,PTPN11,CHP1,HADH,IDH2,C11orf65,SRCIN1,SYTL4,MIDN,PARK7,RHBDF1,TXN,UBE2G2,DERL2	darkgreen	EC_M4
GO:0006006	1.74E-03	0.999114729	35	155	glucose metabolic process	BP	7.04E-02	DYRK2,PPARA,PPARD,LEP,DGAT2,AKT2,PFKP,C1QTNF1,KAT2B,IRS2,SORBS1,HECTD4,PFKM,ADIPOR1,SELENOS,CPT1A,LRP5,GSK3B,PIK3CA,WDTC1,PPP1R3D,ATF3,TPI1,PPP1R3F,PPARGC1A,AKT1,PRKAA1,PTPN2,OMA1,PGP,MIDN,FAM3A,ENO1,RANBP2,NNMT	darkgreen	EC_M4

GO:0050877	1.77E-03	0.998750641	121	678	nervous system process	BP	7.12E-02	ATP8B1,HLA-DRA,COL11A2,REEP2,RORB,SRF,UNC13B,CNNM4,RIMS1,UBA6,TPBG,SHANK1,MFSD2A,MYH14,ACTR2,WHRN,ROCK2,SLC7A11,ITGB1,FKBP1B,EML2,PICALM,TENM4,RAB3GAP1,SCN5A,TMTC4,PRNP,LUM,ACPP,UBA5,ARL6IP5,TUSC3,CXCL12,PJA2,GRK7,RDH10,P2RX4,ATP2A2,DLGAP4,CACNA1D,APP,GLRB,ABCC8,CACNB3,HOMER1,OPA1,AMFR,GLS,NDN,CHRNA7,CALHM1,GSK3B,DAG1,LEF1,KRAS,RLBP1,CRYBA4,ADCY5,CASQ1,NCSTN,ATF6,BACE1,FZD9,MAPT,C1QL1,EIF4EBP2,FZD2,COMT,TNR,MPP2,KCNK4,ADRA2C,COMP,HTT,KCNA1,WDR1,ATAD1,ROM1,CHRNE,JAKMIP1,SLC2A4,NLGN2,CLN8,TCAP,STX1B,SYT11,COL1A1,CACNA1G,TMEM100,CLN6,TTC8,DICER1,GRIA1,NPTN,BEGAIN,TRPV2,AKT1,TMEM98,ITGA2,GNAL,PITPNA,GNB1,PAFAH1B1,SCNN1D,JPH3,COL11A1,AARS,LHX8,DMD,SERPINF1,LRRN4,ARF4,TIMP3,LHFPL4,OTOR,OR5AN1,OR6N2,INSYN1,KCNMB4,MAP1A,NPAS1	darkgreen	EC_M4
GO:2000145	1.78E-03	0.998716846	135	772	regulation of cell motility	BP	7.16E-02	LRP1,PLXNB2,ANGPT1,FAM19A5,NUS1,FAM49B,ADGRB1,PPARD,CTNNA1,SUN2,PLAA,SRF,VEGFA,DOCK8,CREB3,PTP4A1,TGFBR1,RTN4,EMC10,AKIRIN1,ROCK2,RRAS,ITGB1,PLXNA1,FGF2,PAK1,TGFB1,AKT2,OSBPL8,ACTG1,NAV3,ACTN4,SPINT2,IRS2,DCN,ZNF703,STARD13,SEMA3C,DDRGK1,ILK,DPYSL3,CXCL12,KIF2A,SULF1,ROBO1,CHST2,SEMA4F,P2RX4,PFN2,APP,RAPGEF2,CX3CL1,ABCC8,PGAM4,ARSB,ACVR1,ADA,ATP2B4,SEMA4G,NKD1,DOCK10,ADIPOR1,PHLDB2,TJP1,DNM1L,HYAL1,TGFB2,MMP14,DAG1,LEF1,CORO1C,PRKCA,CMKLR1,NTRK3,SLAMF8,BMP7,IDH2,IQGAP1,CLIC4,S100A11,LRRC15,FAM89B,HSPB1,SERPINE1,ITGA2B,DNAJA4,FN1,PDCD10,ITGAV,COL3A1,NBL1,TIMP1,VCL,AKT3,BMPR1A,PPARGC1A,MSN,SRCIN1,ARHGEF2,NGFR,TPM1,COL1A1,RAB11A,MYOCD,PIN1,CD74,LAMA3,PDGFRB,ROCK1,AKT1,ITGA2,DAPK3,MYO1C,PLXNB3,TNFRSF18,FLNA,PLXNA3,NTNG2,CDH13,F3,HSPA5,LMO4,NEXN,MIR24-1,CD151,SERPINF1,THY1,HAS2,TMSB4X,GLIPR2,CCR6,DUSP22,PIK3C2A,PRKG1,FGF10	darkgreen	EC_M4
GO:0110053	1.62E-03	0.999110913	43	237	regulation of actin filament organization	BP	7.19E-02	MTSS1,ARHGAP18,BAIAP2L2,PICK1,CDC42EP1,LIMCH1,TMOD1,FAM49A,NRP1,SVIL,SPTBN4,FCHSD2,SFRP1,FCHSD1,LMOD3,HIP1R,RAPGEF3,IQGAP2,ESAM,HCLS1,AP1AR,TMOD4,F11R,PAK2,DLG1,BAIAP2L1,PPM1F,WAS,TMOD2,RAC1,MTOR,ARHGEF15,ARHGAP28,SPIRE1,S1PR1,CARMIL2,VASP,GMFG,KANK3,SHANK3,RGS4,GBA2,ARPC5L	turquoise	EC_M6
GO:0008593	1.63E-03	0.999396674	18	76	regulation of Notch signaling pathway	BP	7.20E-02	MFNG,KIT,GATA2,NOS3,LLGL2,JAG1,PRAG1,BMP2K,DLL4,GAS2,TSPAN15,TSPAN14,LFNG,JAG2,NRARP,EGFL7,NOTCH1,CD46	turquoise	EC_M6
GO:1901137	1.81E-03	0.998794126	90	494	carbohydrate derivative biosynthetic process	BP	7.23E-02	B4GALT7,B4GALNT1,RRM2B,ANGPT1,PPARA,ACSL6,NUS1,MTAP,LARGE1,VCP,SLC35B2,CHST10,NANS,TMEM246,UBE2J1,GFPT1,CMPK1,RPN1,POMGNT1,ITM2B,CHPF2,TGFB1,ST6GALNAC2,CTNNB1,TRAK1,ST3B,CANT1,TMTC4,KDELC1,TMTC3,TUSC3,ST8SIA4,B3GNT4,CHST2,TET3,RPN2,ADCY6,DPY19L4,ADA,CHP1,PIGP,MAN1A2,CHSY1,HYAL1,SLC2A10,B4GALT4,TYW5,B3GALT1,ADCY5,HACD2,GALNT2,MAN2A2,NCSTN,PIGZ,EDEM3,SLC25A16,PLOD3,TPI1,DDOST,B3GAT3,UGDH,SLC2A4,CHST12,PPARGC1A,NPPB,GALNT16,XYLT2,GALNT10,CLTC,GALNT1,MPI,PIGL,ACSL4,PDGFRB,PRKAA1,ACOT7,ENO1,POMT1,DPM2,HACD1,ITM2C,HAS2,PIGK,DPM1,TMSB4X,NME6,PIGB,SLC35C1,CHST11,HS2ST1	darkgreen	EC_M4
GO:2000586	1.82E-03	0.999861517	5	8	regulation of platelet-derived growth factor receptor-beta signaling pathway	BP	7.28E-02	LRP1,MYOCD,PTPN2,PTPN12,LOX	darkgreen	EC_M4
GO:0051090	1.66E-03	0.999021046	54	328	regulation of DNA-binding transcription factor activity	BP	7.31E-02	TNFRSF11A,TNFAIP3,FZD6,PIM1,MDFI,DDX58,KIT,ADGRF1,SYK,IL6,PELI1,TLR4,TRAF1,PRKD2,PPARG,TAF3,IRAK2,IKBKB,NFKBIB,TMEM173,CD36,ITCH,ERBIN,TLR2,ADGRG3,NLRC5,EDN1,CD200,SIK1,PPP3CA,HCLS1,CFLAR,ADCY1,PROX1,PRKD1,RNF2,LTF,IL1RAP,PRKCH,KDM5A,JUP,TLR6,AR,RPS6KA5,LGALS9,USP7,TRAF2,PLCG2,DAB2IP,TRIM34,RELN,TRIM62,COMMD7,BMP2	turquoise	EC_M6
GO:0086004	1.67E-03	0.999614193	10	29	regulation of cardiac muscle cell contraction	BP	7.33E-02	TRPM4,PDE4D,STC1,KCNJ2,DLG1,JUP,ATP2A1,DSG2,DSC2,PDE4B	turquoise	EC_M6
GO:0002703	1.68E-03	0.999225905	27	136	regulation of leukocyte mediated immunity	BP	7.33E-02	DDX58,KIT,SYK,IL6,RSAD2,TLR4,TRPM4,GATA2,NECTIN2,GAB2,UNC13D,GATA3,FGR,FES,B2M,WAS,TNFRSF1B,LGALS9,NOS2,IL7R,TRAF2,PLCG2,STXBP1,CD46,FCER2,SERPINB9,BST2	turquoise	EC_M6
GO:0003160	1.68E-03	0.999921184	4	6	endocardium morphogenesis	BP	7.33E-02	PROX1,NOTCH1,SOX17,SOX18	turquoise	EC_M6

GO:0046903	1.68E-03	0.998851862	99	673	secretion	BP	7.33E-02	TNFRSF11A,STX11,SNCAIP,RIMS2,PICK1,APBA1,KIT,CD2AP,SYK,ICA1,IL6,PPT1,RSAD2,TLR4,SDCBP,TRPM4,RAB3B,PIK3CG,GATA2,EXOC3L2,GAB2,LLGL2,RALB,UNC13D,GATA3,EDNRB,P2RY2,GPAT4,SFRP1,SIRT3,MERTK,CHD7,FAM3D,IL1A,IGF1,NR4A1,EXOC6,HIP1R,MCTP1,CHRM5,MEF2C,TLR2,TPD52,VEGFC,STC1,EDN1,SNCA,DTNBP1,ABCG1,CD200,MTTP,HDAC1,PPP3CA,ANO1,MCTP2,SNAP23,ATP9A,FGR,ADCY1,FES,ACE,CREB1,ADORA2A,MCU,WNK4,CDK16,SNAP29,FGF23,RAC1,HIF1A,RAB27A,SNCG,SYT7,RSC1A1,MICAL3,TNFRSF1B,FBXL20,PSEN1,SLC51B,CHRNB2,RAB3D,NPR1,GPR68,SMAD2,SCNN1B,LGALS9,NOS2,MYO18A,GIT1,RPH3AL,NOTCH1,ENG,STXBP1,EXOC3L1,STX3,RALA,VAMP3,BMP2,BMP6	turquoise	EC_M6
GO:0006312	2.72E-03	0.99950961	7	21	mitotic recombination	BP	7.34E-02	SMC6,GEN1,BRCA2,MSH3,RAD51,ANKLE1,RAD52	darkgrey	EC_M8
GO:0060964	2.73E-03	0.9992818	10	39	regulation of gene silencing by miRNA	BP	7.35E-02	TNRC6B,ZMPSTE24,RIPK1,AJUBA,FXR1,DDX5,TIAL1,DDX6,DHX9,FMR1	darkgrey	EC_M8
GO:0009451	2.73E-03	0.998763165	23	141	RNA modification	BP	7.35E-02	PUS10,PUS7,ZC3H13,TRDMT1,METTL3,TGS1,DIMT1,CDK5RAP1,VIRMA,PUS7L,NSUN2,TRMT10A,QTRT2,GAR1,TARBP1,SSB,FTSJ3,THG1L,THUMPD1,METTL16,CMTR2,NAF1,TFB2M	darkgrey	EC_M8
GO:0051307	2.74E-03	0.99950558	7	21	meiotic chromosome separation	BP	7.35E-02	RMI1,TTK,TOP2B,ESPL1,FANCM,TOP2A,EME1	darkgrey	EC_M8
GO:0048584	1.86E-03	0.998551055	241	1488	positive regulation of response to stimulus	BP	7.40E-02	CD63,IL23A,LRP1,STX7,TAB2,ANGPT1,HLA-DRA,RSPO3,FAM49B,PTP4A3,PPARD,CTNNA1,UBQLN1,LGALS1,KLHDC10,LEP,CDKN2B,KIAA1324L,VEGFA,VCP,SLC35B2,NCK2,CLOCK,CREB3,TGFBR1,SOCS5,RIMS1,RTN4,TPBG,SHANK1,ADAMTS3,EMC10,MAP3K7,AKIRIN1,ACTR2,ROCK2,INHBA,VPS35,ITGB1,PLEKHA4,FKBP1B,FGF2,EIF2B3,MEGF8,RHEB,PAK1,TGFB1,AXL,WDR48,SEC13,CTNNB1,DYNC1LI1,ZEB2,CANT1,C1QTNF1,OSBPL8,CXXC5,ACTG1,ACTN4,ATP2C1,PRNP,DCN,HDAC3,ACPP,CAVIN3,ILK,RBPMS,ARL6IP5,RYK,TMEM9B,ST5,CXCL12,SLC20A1,ANKRD1,PJA2,CD180,SULF1,VAMP8,ROBO1,SORBS1,LOXL3,P2RX4,RNF13,APP,DHX36,RAPGEF2,CX3CL1,CACNB3,PTPN11,HOMER1,OPA1,PTPN22,ACVR1,GDF6,POLR3D,SERINC3,MTDH,ADA,PKD2,PLA2R1,CBLB,NKD1,SLC39A10,CHRNA7,ADIPOR1,NOTCH2,CDC73,DNM1L,UCHL5,CHSY1,SLC2A10,EYA2,CAPRIN2,TGFB2,HTR2B,ARMC9,PIK3CA,AUTS2,LEF1,PRKCA,TLR5,CMKLR1,KRAS,NTRK3,CFI,SPATC1L,BMP7,GOLT1B,ATF3,FGFR2,OSM,PEA15,TP53BP1,IQGAP1,TFRC,ATF6,FZD9,PDIA3,HSPB1,SERPINE1,ATP6AP2,FN1,MAOA,PTPN6,GPR137,PDCD10,ITGAV,ADRA2C,HTT,FKBP8,COL3A1,PRKCI,SKIL,SYNPO2L,FGFR3,PRRX1,GORAB,TNIK,FMN2,OTUD5,EVC,AKT3,HAP1,MAGED1,BMPR1A,SLC2A4,NLGN2,MED1,RNF111,STX1B,TGFB1I1,NGFR,COL1A1,TGFB3,SHC1,PBXIP1,ADRA1B,MAP2K1,GPR65,TSPAN6,SMARCA4,SLC44A2,GSKIP,NPTN,MYOCD,PIN1,CD74,PDGFRB,ROCK1,ITGA2,CRLF3,C6,OSMR,RAB34,PTPN2,CLEC16A,MYDGF,DAPK3,MYO1C,BCAP31,PAFAH1B1,CSNK1G2,IRAK1,FLNA,ATP6AP1,IKBKG,VAMP7,TMEM259,PARK7,CDH13,F3,BCAR3,AP1G1,BCL1	darkgreen	EC_M4
GO:0015980	1.15E-04	0.999941532	51	259	energy derivation by oxidation of organic compounds	BP	7.41E-02	GFPT2,UQCRQ,MYC,UGP2,STK40,NDUFB2,NOP53,UQCRH,NDUFB6,KHK,NDUFC2,GAA,NDUFA2,COX7A1,UQCRFS1,PPP1CC,COQ9,DGUOK,IRS1,NDUFV1,OXA1L,PER2,MTFR1L,NDUFS2,DNAJC30,IDH1,AIFM2,CHCHD10,UQCC3,NDUFB7,GBA,NUPR1,INSR,NDUFA7,LEPR,UQCR11,NDUFS7,ATP5F1D,TAZ,MT-ND1,MT-ND2,MT-CO1,MT-CO2,MT-ATP6,MT-CO3,MT-ND3,MT-ND4L,MT-ND4,MT-ND5,MT-CYB,PID1	cyan	EC_M2
GO:0006120	1.18E-04	0.999970356	16	46	mitochondrial electron transport, NADH to ubiquinone	BP	7.41E-02	NDUFB2,NDUFB6,NDUFC2,NDUFA2,COQ9,NDUFV1,NDUFS2,NDUFB7,NDUFA7,NDUFS7,MT-ND1,MT-ND2,MT-ND3,MT-ND4L,MT-ND4,MT-ND5	cyan	EC_M2
GO:0071694	1.88E-03	0.999939251	4	5	maintenance of protein location in extracellular region	BP	7.46E-02	FBN2,LTBP1,FBN1,NBL1	darkgreen	EC_M4
GO:0001833	2.79E-03	0.999700245	5	11	inner cell mass cell proliferation	BP	7.46E-02	NCAPG2,GINS4,BRCA2,CHEK1,PALB2	darkgrey	EC_M8
GO:0032490	1.73E-03	0.999853957	5	9	detection of molecule of bacterial origin	BP	7.50E-02	TLR4,SCARB1,TLR2,TLR6,TLR1	turquoise	EC_M6
GO:0051047	1.74E-03	0.999080542	38	211	positive regulation of secretion	BP	7.50E-02	TNFRSF11A,CD2AP,SYK,TLR4,SDCBP,TRPM4,GATA2,GAB2,UNC13D,EDNRB,P2RY2,SIRT3,IL1A,IGF1,TLR2,VEGFC,EDN1,SNCA,DTNBP1,ABCG1,ANO1,FGR,CREB1,ADORA2A,MCU,RAC1,HIF1A,RAB27A,SYT7,CHRNB2,RAB3D,NPR1,GPR68,MYO18A,RPH3AL,STXBP1,BMP2,BMP6	turquoise	EC_M6
GO:0032493	1.74E-03	0.999917773	4	6	response to bacterial lipoprotein	BP	7.50E-02	CD36,TLR2,TLR6,TLR1	turquoise	EC_M6
GO:0051653	2.84E-03	0.99913528	12	49	spindle localization	BP	7.58E-02	CENPA,CLASP2,ESPL1,NUSAP1,CFL1,ASPM,KPNB1,SPDL1,PLK1,NDC80,SAPCD2,MAD2L1	darkgrey	EC_M8

GO:0007043	1.77E-03	0.999259772	22	99	cell-cell junction assembly	BP	7.62E-02	AFDN,GJA4,IKBKB,MARVELD2,OCLN,HIPK1,JAM3,ESAM,CLDN34,CDH24,PECAM1,F11R,ACE,PAK2,DLG1,RAMP2,PRKCH,JUP,FSCN1,EPHA2,PATJ,CDH5	turquoise	EC_M6
GO:0010631	1.93E-03	0.998867572	53	261	epithelial cell migration	BP	7.63E-02	ANGPT1,NUS1,PTP4A3,ADGRB1,PPARD,MYH9,SRF,VEGFA,TGFBR1,RTN4,EMC10,ROCK2,RRAS,ITGB1,FGF2,TGFB1,ZEB2,DCN,STARD13,ROBO1,P2RX4,PFN2,PTPN11,ARSB,LOXL2,ATP2B4,ADIPOR1,FAP,HYAL1,TGFB2,FSTL1,PIK3CA,CORO1C,PRKCA,HSPB1,DNAJA4,S100P,PDCD10,AKT3,RAB11A,FAT2,ROCK1,AKT1,ITGA2,CDH13,MIR24-1,SERPINF1,HAS2,TMSB4X,GLIPR2,CCR6,PIK3C2A,FGF10	darkgreen	EC_M4
GO:0032412	1.93E-03	0.998971364	39	178	regulation of ion transmembrane transporter activity	BP	7.63E-02	UBQLN1,SHANK1,ITGB1,FKBP1B,KIF5B,GPD1L,ACTN4,STIM1,PRNP,REM1,FXYD1,SLMAP,ATP1B3,CACNA1D,APP,ABCC8,CACNB3,HOMER1,CHP1,PKD2,CASQ1,LINC02210-CRHR1,HTT,KCNA1,HAP1,NLGN2,STIM2,PPARGC1A,CALM1,VMP1,AHCYL1,JPH3,MIR24-1,DMD,FKBP1A,CBARP,TMSB4X,YWHAH,GEM	darkgreen	EC_M4
GO:0035239	1.94E-03	0.998619104	124	699	tube morphogenesis	BP	7.63E-02	LRP1,AHI1,SLC12A2,PLXNB2,ANGPT1,FAM19A5,NUS1,RSPO3,ADGRB1,MYH9,LEP,SRF,VEGFA,PDCL3,COL15A1,TGFBR1,EMC10,CALD1,GLI3,ROCK2,RRAS,ITGB1,FGF2,SRPK2,GLI2,MEGF8,PAK1,TGFB1,CTNNB1,ACTG1,STIM1,SPINT2,DCN,STARD13,ILK,SPINK5,TMED2,SULF1,ROBO1,RDH10,HEY1,RASA1,TCTN1,RAPGEF2,CX3CL1,ABCC8,OPA1,LOXL2,ACVR1,MMP2,MTDH,ATP2B4,PKD2,RUNX1,HIF1AN,IFT57,CHRNA7,FAP,TJP1,KDM5B,NOTCH2,HYAL1,TGFB2,LRP5,ADGRB2,MMP14,DAG1,PIK3CA,LEF1,PRKCA,KRAS,BMP7,FGFR2,VASH2,CLIC4,HSPB1,SERPINE1,SGPL1,FZD2,FN1,PDCD10,ITGAV,COMP,PRRX1,TULP3,SIX4,AKT3,FKBP10,MAGED1,BMPR1A,TCAP,MED1,NPPB,MYO1E,NGFR,SHC1,TMEM100,PKM,MYOCD,ANGPTL6,PDGFRB,ROCK1,AKT1,MYDGF,PKD1,FLNA,BSG,SARS,CDH13,F3,LMO4,BCL10,MIR24-1,SERPINF1,LOX,LBX1,GDNF,THY1,HAS2,EDA,PIK3C2A,WNT2,COL8A2,FGF10	darkgreen	EC_M4
GO:0050922	1.79E-03	0.999465237	13	46	negative regulation of chemotaxis	BP	7.68E-02	SEMA6A,SEMA4D,C5,NRP1,ANGPT2,SEMA3F,RIN3,SEMA6D,SEMA4A,MMP28,CCL2,SEMA6B,NOTCH1	turquoise	EC_M6
GO:0043269	1.96E-03	0.998708789	83	444	regulation of ion transport	BP	7.69E-02	CD63,ATP8B1,SLC12A2,UBQLN1,LEP,SHANK1,ITGB1,FKBP1B,KIF5B,RAB3GAP1,SCN5A,CTNNB1,GPD1L,ACTN4,STIM1,PRNP,ATP2B1,ARL6IP5,REM1,CXCL12,FXYD1,SNTA1,VAMP8,SLMAP,ATP1B3,P2RX4,CACNA1D,APP,CX3CL1,ABCC8,CACNB3,HOMER1,CRY2,CHP1,ATP2B4,PKD2,PLA2R1,CALHM1,PKP2,ISCU,CASQ1,CLIC4,LINC02210-CRHR1,PTPN6,KCNK4,HTT,KCNA1,WDR1,HAP1,CACNA1C,NLGN2,STIM2,PPARGC1A,SYT11,KCNMB1,CACNA1G,CALM1,VMP1,TRPV2,PDGFRB,AKT1,PRKAA1,FLNA,KCNAB2,VAMP7,PARK7,HCN2,AHCYL1,JPH3,MIR24-1,DMD,FKBP1A,CBARP,TMEM38B,GDNF,THY1,TMSB4X,TMEM38A,YWHAH,TMEM109,SARAF,HCN4,GEM	darkgreen	EC_M4
GO:0008283	1.80E-03	0.998635069	191	1442	cell population proliferation	BP	7.70E-02	TNFRSF11A,TNFAIP3,CNOT6,FLT4,FZD6,CTHRC1,MTSS1,PTK2,PDGFB,AGTPBP1,KDM4C,PIM1,TEK,KIT,TNFRSF21,KDR,SYK,IL6,CXCL8,PELI1,FIGNL1,CAV2,KLF11,TLR4,IL15,SDCBP,MYCN,TRPM4,NRP1,PIK3CG,TAL1,GATA2,PRKD2,NOS3,GAB2,DACH1,PPARG,GATA3,EDNRB,TIE1,JAG1,P2RY6,SFRP1,HBEGF,FERMT1,EFNB2,POC1B,ASH2L,SDCBP2,SCARB1,EPHB1,GAPT,IL1A,IGF1,NR4A1,HHEX,ITCH,SH3RF1,SMAD1,SOX7,MEF2C,TLR2,WWTR1,IL7,VEGFC,NDRG4,CXCL10,LMBR1L,KMT2D,THBS4,PPP1R16B,PLCG1,STC1,HIPK1,TP53I11,DLL4,EDN1,STAT1,JARID2,ETS1,SHANK2,CD200,HDAC1,ICOSLG,PRKX,HLX,USP13,HCLS1,NFATC2,INPP5D,EAF2,CD34,CD86,MN1,WDR6,SH3BP4,CFLAR,TGM1,IGFBP3,FES,RPS6KA1,PROX1,PRKD1,LAMA5,MAP3K3,MLXIPL,ACE,LAMC2,DLG1,ELL3,APOD,ETV6,CREB1,B2M,USP28,TET1,SSTR1,LTF,ADORA2A,ARNT2,P3H2,ITGA4,SOS2,IGFBP5,TBX1,MECOM,CALCRL,ING4,FGF23,PRKCH,RAC1,HIF1A,LIPA,JUP,BCL6B,EPHA2,ITPKB,BID,MCC,TNFRSF1B,ARG2,ALDH1A2,MTOR,AR,EFNB1,PSEN1,FGF18,PGF,VASH1,CHRNB2,CD19,PIAS1,NPR1,SMAD2,CNOT8,CYP1A1,PPP2R5C,CCL2,IL13RA1,JAG2,LGALS9,NOS2,ELF4,IL7R,LRG1,MATK,DOT1L,NRARP,EGFL7,NOTCH1,SLC25A33,S1PR1,PTGS1,DAB2IP,CDH5,MIR23A,ATXN1L,ADGRG1,TRIM24,CD46,SOX17,FGF16,CDK13,SOX18,STX3,E2F3,COPS8,BST2,BMP2,IFIT3,BMP6	turquoise	EC_M6
GO:0061157	2.89E-03	0.99892202	16	79	mRNA destabilization	BP	7.70E-02	PAN2,TNRC6B,TUT7,PNPT1,EXOSC9,DIS3,METTL3,EXOSC8,PAN3,PDE12,CNOT1,HNRNPR,DCP2,METTL16,EXOSC2,HNRNPD	darkgrey	EC_M8
GO:0030204	1.98E-03	0.999543069	10	26	chondroitin sulfate metabolic process	BP	7.74E-02	CHPF2,DCN,IMPAD1,CHSY1,HYAL1,UGDH,CHST12,XYLT2,EGFLAM,CHST11	darkgreen	EC_M4

GO:1900181	2.00E-03	0.999533838	10	27	negative regulation of protein localization to nucleus	BP	7.74E-02	ANGPT1,CTNNA1,RAB23,MDFIC,UFM1,DCLK1,CHP1,GSK3B,MAGED1,TMEM98	darkgreen	EC_M4
GO:0007160	2.00E-03	0.998908903	42	192	cell-matrix adhesion	BP	7.74E-02	CD63,LRP1,MKLN1,SRF,VEGFA,ROCK2,RRAS,ZYX,ITGB1,CTNNB1,ACTG1,SIGLEC1,ILK,SORBS1,RASA1,CX3CL1,PTPN11,PHLDB2,CTTN,FBLN5,GSK3B,MMP14,DAG1,CORO1C,ITGB5,IQGAP1,SERPINE1,ITGA2B,FN1,ITGAV,COL3A1,VCL,ACTN1,THBS3,ROCK1,ITGA2,DAPK3,PKD1,CDH13,THY1,EDA,DUSP22	darkgreen	EC_M4
GO:0014812	2.00E-03	0.999189249	21	79	muscle cell migration	BP	7.74E-02	LRP1,FAM19A5,PPARD,AKIRIN1,PLXNA1,PAK1,ILK,IQGAP1,S100A11,ARPC5,SERPINE1,SIX4,BMPR1A,PPARGC1A,TPM1,MYOCD,PDGFRB,ROCK1,ITGA2,HAS2,PRKG1	darkgreen	EC_M4
GO:0071417	2.00E-03	0.998666367	87	466	cellular response to organonitrogen compound	BP	7.74E-02	LRP1,AP3S1,CTNNA1,LEP,COL1A2,ACTR2,ROCK2,VPS35,MGARP,SESN3,PAK1,HTR5A,CTNNB1,AKT2,OSBPL8,KAT2B,IRS2,PRNP,ATP2B1,LEPROTL1,APLP1,HSP90B1,MAP1B,RYR3,SORBS1,P2RX4,USO1,APP,RAPGEF2,PTPN11,ADCY6,OPA1,PTPN22,MMP2,ATP2B4,PKD2,ADIPOR1,SELENOS,ZNF106,EPRS,HTR2B,GSK3B,CASP7,PIK3CA,COL6A1,ADCY5,WDTC1,NCSTN,SYAP1,UROS,BACE1,LINC02210-CRHR1,EIF4EBP2,FBN1,PRKCI,COL5A2,MYO5A,SLC2A4,PPARGC1A,CPEB4,ARHGEF2,NGFR,COL1A1,SHC1,PKM,ROCK1,AKT1,LPIN2,PRKAA1,RAB31,GNAL,PTPN2,LDOC1,MYO1C,GNB1,FLNA,HCN2,AHCYL1,GNAI3,BCAR3,SLC2A8,HSPA5,TMEM38B,TMEM38A,LEPROT,PIK3C2A,HCN4	darkgreen	EC_M4
GO:0048880	2.00E-03	0.998791331	58	286	sensory system development	BP	7.74E-02	AHI1,FBN2,RORB,SRF,VEGFA,TGFBR1,MFSD2A,GLI3,INHBA,SLC7A11,FGF2,SMARCD3,RAB3GAP1,CTNNB1,RARB,SPRED3,ATP2B1,FAT1,RDH10,SLC4A5,OPA1,ATP2B4,NKD1,KDM5B,NOTCH2,TGFB2,LRP5,SHROOM2,CRYBA4,BMP7,CLIC4,ATF6,FBN1,FKBP8,PRKCI,COL5A2,SKIL,TULP3,OBSL1,ROM1,CACNA1C,CLN8,MED1,NES,TTC8,SMARCA4,CHRDL1,PDGFRB,MYOM1,GNB1,NPHP4,COL5A1,BCAR3,SERPINF1,THY1,WNT2,COL8A2,FGF10	darkgreen	EC_M4
GO:0072527	2.93E-03	0.999033109	13	63	pyrimidine-containing compound metabolic process	BP	7.81E-02	CTPS1,DCK,CAD,MBD4,TK1,RRM1,TDG,TET2,UMPS,DCTPP1,PRPS1,TYMS,DHODH	darkgrey	EC_M8
GO:0045947	2.94E-03	0.999562283	6	16	negative regulation of translational initiation	BP	7.81E-02	EIF3E,TPR,EIF2AK1,LARP1,FMR1,PAIP2	darkgrey	EC_M8
GO:0046883	2.05E-03	0.998915993	38	174	regulation of hormone secretion	BP	7.87E-02	LRP1,PPARD,LEP,CLOCK,REST,INHBA,FKBP1B,KIF5B,C1QTNF1,IRS2,RAB11FIP1,OSBP,VAMP8,SELENOT,ABCC8,PTPN11,PFKM,CRY2,SPP1,BRSK2,DYNLL1,KDM5B,CPT1A,LRP5,HADH,ADCY5,OSM,SIDT2,LINC02210-CRHR1,TFR2,CYP19A1,NLGN2,SYTL4,ACSL4,RASL10B,MIDN,SSTR5,VAMP7	darkgreen	EC_M4
GO:1990414	3.01E-03	0.999800195	4	7	replication-born double-strand break repair via sister chromatid exchange	BP	7.97E-02	RAD21,RAD51,FANCB,NIPBL	darkgrey	EC_M8
GO:0016358	2.09E-03	0.998839752	44	204	dendrite development	BP	7.99E-02	RAB21,PACSIN1,NCK2,UBA6,TPBG,SHANK1,MFSD2A,ACTR2,KIDINS220,ITGB1,PICALM,TRAK1,FARP1,DCLK1,ILK,EIF4G2,MAP1B,TLX2,APP,DHX36,RAPGEF2,OPA1,PDLIM5,CHRNA7,DOCK10,DNM1L,CAPRIN2,GSK3B,BMP7,IQGAP1,KIF1A,TANC2,TNIK,OBSL1,SRCIN1,ACSL4,PAFAH1B1,NTN3,RERE,ARF4,YWHAH,SDC2,PRKG1,MAP1A	darkgreen	EC_M4

GO:0019538	3.03E-03	0.997510087	429	4113	protein metabolic process	BP	8.01E-02	CDK2,PAN2,NARS,MEX3C,CLK4,USP15,SHPRH,PPIL4,LATS1,DDX39B,FBXO43,FBXO5,CSNK1G3,UBR5,EIF3E,MAP3K4,DSCC1,MRPL13,XRCC6,CDC25C,TNRC6B,TUT7,NAA35,SRPK1,UHRF2,PPIL1,PLEC,UBR2,PWP1,TOPORS,DBF4,AKAP9,PUM3,CKS2,C1GALT1,ICK,MELK,TRIM28,NCBP1,ZNF451,ANP32B,LRPPRC,PPIH,FBXO11,SENP6,ASB3,PHIP,MTIF2,TTK,PPP4R3B,IBTK,PNPT1,ZMPSTE24,RLF,USP34,SYNCRIP,XPO1,PABPC4,COMMD1,CASP8AP2,DPY19L1,FKBP15,ETAA1,FBXO48,TIA1,ITGB1BP1,EZH2,UFL1,CUL1,USP45,MRPL32,CLSPN,ASCC3,CASP2,DDX1,NAA15,AFG1L,WDR35,NRDC,PLK4,DHFR,PUS7,HDAC2,CCNA2,EXOSC9,PMPCB,SAE1,CEP295,CHORDC1,SETDB2,SYMPK,MASTL,YME1L1,COG3,CNOT10,MRPS7,CAD,ALG8,PPM1G,BORA,DIS3,CCNT2,PAXIP1,DARS,ULK4,NCAPG2,CDC20,BIRC5,NKTR,FNTA,PARP2,PITRM1,KAT14,GTPBP4,RAD18,IPO5,EPC2,KAT6A,METTL3,RIF1,ZDHHC17,VPS36,TPP2,NSD3,KITLG,TAF7,EXOSC8,PRKD3,CUL4A,METAP2,BRCA2,PPP4R2,LARS,LTA4H,PSMD13,CDC16,MAPK8,PRKDC,APAF1,CCNB1,ERCC6,DHX29,PAN3,SCYL2,LNX2,SP1,MRPL15,ESPL1,MRPS5,BUB1,TPX2,ANAPC1,CAPRIN1,UBA2,IPO7,TNKS2,LSM14A,SGK3,PARP4,CDK5RAP1,PPWD1,FER,EIF2B1,NCK1,IDE,ARFGEF1,MRPL35,RNF141,PTCD3,ABCE1,OTUD4,ZFP91,GALNT6,TAF9,XRN1,PDE12,ATR,LARP4,USP25,HLTF,PBK,ESCO2,CNOT1,ANAPC7,MCRS1,SH2B3,LTN1,USP16,PPID,MOB1A,RPS6,NAA25,CPNE3,BUB1B,CCNL1,SMYD5,CDCA2,NBN,FAM83D,SENP1,TOP1,ABRAXAS1,RAD51,RIPK1,PRPF4B,CCNE2,TRIM33,RIOK1,EEF1E1,CHUK,STK4,MORC3,MDM4,UBE2C,GXYLT1,HERC5,TAOK3,OGA,SMARCAD1,NOLC1,BMPR1B,UBE3A,CIT,G	darkgrey	EC_M8
GO:0140352	1.88E-03	0.998732121	92	618	export from cell	BP	8.01E-02	TNFRSF11A,STX11,SNCAIP,RIMS2,PICK1,APBA1,KIT,CD2AP,SYK,ICA1,IL6,PPT1,RSAD2,TLR4,SDCBP,TRPM4,RAB3B,PIK3CG,GATA2,EXOC3L2,GAB2,LLGL2,RALB,UNC13D,GATA3,SFRP1,SIRT3,MERTK,CHD7,FAM3D,IL1A,IGF1,NR4A1,EXOC6,MCTP1,MEF2C,TLR2,VEGFC,ANO6,EDN1,SNCA,DTNBP1,ABCG1,CD200,MTTP,HDAC1,PPP3CA,ANO1,MCTP2,SNAP23,ATP9A,FGR,ADCY1,FES,DLG1,CREB1,ADORA2A,MCU,CDK16,SNAP29,FGF23,RAC1,HIF1A,RAB27A,SNCG,SYT7,RSC1A1,MICAL3,TNFRSF1B,FBXL20,PSEN1,CHRNB2,RAB3D,GPR68,SMAD2,LGALS9,NOS2,MYO18A,GIT1,RPH3AL,NOTCH1,ENG,STXBP1,EXOC3L1,SLC8A3,KCNH2,KCNK5,STX3,RALA,VAMP3,BMP2,BMP6	turquoise	EC_M6
GO:0007431	2.10E-03	0.999506452	10	27	salivary gland development	BP	8.02E-02	PLXNA1,TGFB1,SEMA3C,TGFB2,DAG1,BMP7,FGFR2,TGFB3,EDA,FGF10	darkgreen	EC_M4
GO:0000028	3.04E-03	0.999622989	5	15	ribosomal small subunit assembly	BP	8.02E-02	RRP7A,MRPS7,PRKDC,FP565260.1,XRCC5	darkgrey	EC_M8
GO:0017156	2.12E-03	0.999335635	14	44	calcium-ion regulated exocytosis	BP	8.03E-02	MYH9,UNC13B,REST,NLRP5,RIMS1,RAB3GAP1,ATP2A2,STX1B,SYT11,CACNA1G,SCAMP5,SDF4,VAMP7,CBARP	darkgreen	EC_M4
GO:0090132	2.12E-03	0.998750166	53	262	epithelium migration	BP	8.03E-02	ANGPT1,NUS1,PTP4A3,ADGRB1,PPARD,MYH9,SRF,VEGFA,TGFBR1,RTN4,EMC10,ROCK2,RRAS,ITGB1,FGF2,TGFB1,ZEB2,DCN,STARD13,ROBO1,P2RX4,PFN2,PTPN11,ARSB,LOXL2,ATP2B4,ADIPOR1,FAP,HYAL1,TGFB2,FSTL1,PIK3CA,CORO1C,PRKCA,HSPB1,DNAJA4,S100P,PDCD10,AKT3,RAB11A,FAT2,ROCK1,AKT1,ITGA2,CDH13,MIR24-1,SERPINF1,HAS2,TMSB4X,GLIPR2,CCR6,PIK3C2A,FGF10	darkgreen	EC_M4
GO:0034770	3.06E-03	0.999663051	5	11	histone H4-K20 methylation	BP	8.05E-02	PWP1,SMYD5,BRCA1,NSD2,NSD1	darkgrey	EC_M8
GO:0051654	2.13E-03	0.99949795	10	27	establishment of mitochondrion localization	BP	8.06E-02	MGARP,TRAK1,MAP1B,OPA1,FEZ1,MAPT,ACTR10,HAP1,ARMCX3,HSBP1	darkgreen	EC_M4
GO:0006301	3.08E-03	0.999334669	8	27	postreplication repair	BP	8.08E-02	POLH,REV1,MSH2,WDR33,RAD18,DTL,BRCA1,RCHY1	darkgrey	EC_M8

GO:0006366	3.12E-03	0.997570035	213	1862	transcription by RNA polymerase II	BP	8.18E-02	ONECUT2,BCLAF1,PPIL4,ZNF608,SCAF8,RAD21,HSF2,ATAD2,XRCC6,PSIP1,IFT74,ZNF300,RFX3,CKS2,TRIM28,ZNF451,FOXJ3,ZNF189,SP4,PHIP,RLF,ZNF292,XPO1,ZNF350,ITGB1BP1,THRAP3,EZH2,CCNC,ELF2,ATAD2B,HDAC2,EXOSC9,MLLT10,PCF11,ELF1,RSF1,CCNT2,PAXIP1,NCAPG2,WDR43,EPC2,SUPT16H,CHD8,KAT6A,RIF1,E2F7,CKAP2,CEBPZ,SUPT20H,TAF7,NR2C1,ELK3,TCERG1,PRKDC,CBX5,ERCC6,ZNF214,SP1,TCEA1,ZNF664,ZC3H8,ZNF143,TDG,NCK1,SMARCA5,ZNF507,CHD1,ZFP91,TAF9,CGGBP1,TFCP2,SAP30,HLTF,ZBTB10,CNOT1,GABPA,MCRS1,USP16,PPID,RBL1,CCNL1,SENP1,RIPK1,MYBL2,E2F8,TRIM33,INO80,ZBED6,CHUK,ZBTB11,ZNF248,MGA,MDM4,HIVEP1,BRWD1,BMPR1B,UBE3A,PRDM10,NUCKS1,DEK,GCN1,CUL3,CHEK1,H2AFZ,RRP1B,NFKB1,KHDRBS1,TRIP13,TET2,TRIP11,NCL,ZNF184,AJUBA,GTF2IRD2,ZBTB41,ARID4B,GTF2I,TARBP1,BPTF,DDX5,ORC2,TIAL1,BCLAF3,FOSL1,SMARCC1,BAZ1B,SP3,DHX9,HDGFL3,BAZ1A,SETD2,ZFX,TAF1A,DDX20,TPR,DDX21,NPAT,BRCA1,NSD2,DCAF6,ZNF518B,TBL1XR1,SUV39H1,LEO1,FOXM1,LYAR,FAM220A,HNRNPU,NEDD4,SMARCE1,RFX7,RFC1,TOP2A,LIN9,NSD1,SLTM,MED6,ICE2,PMF1,FUS,USP3,AURKB,ZNF609,TAF1,OGT,RLIM,ATRX,GTF2A1,ZKSCAN2,TRIM37,PLK1,MED13,BRIP1,ZNF699,AATF,PSMD10,ZNF3,MIER1,NIPBL,ZNF280C,SAFB,SAFB2,PHF6,MYSM1,UHRF1,RBMX,EHMT1,TP73,CTCF,ZNF684,DRAP1,ZFP30,HMGB3,ZNF22,ZNF614,RBAK,ZNF770,FOXN2,BRMS1L,ZFP14,RNF8,ZNF558,ZNF624,ZNF782,IKZF5,ZFP62,ZNF302,ZNF34,ZNF529,ZNF146,ZNF518A,ZNF420,ZSCAN16	darkgrey	EC_M8
GO:0005978	2.20E-03	0.999383619	12	36	glycogen biosynthetic process	BP	8.28E-02	DYRK2,AKT2,IRS2,GYG1,SORBS1,SELENOS,GYG2,GSK3B,PPP1R3D,PPP1R3F,AKT1,AGL	darkgreen	EC_M4
GO:0009250	2.20E-03	0.999383619	12	36	glucan biosynthetic process	BP	8.28E-02	DYRK2,AKT2,IRS2,GYG1,SORBS1,SELENOS,GYG2,GSK3B,PPP1R3D,PPP1R3F,AKT1,AGL	darkgreen	EC_M4
GO:0050792	1.96E-03	0.999131031	24	119	regulation of viral process	BP	8.35E-02	CXCL8,PPIE,RSAD2,CAV2,NECTIN2,CXCR4,OAS3,IFIT1,STAT1,MX1,KIAA1551,ISG20,PROX1,LTF,DDX3X,TNIP1,LGALS9,ISG15,NOTCH1,OAS1,TRIM34,TRIM62,TBC1D20,BST2	turquoise	EC_M6
GO:0050877	1.97E-03	0.998642386	101	678	nervous system process	BP	8.37E-02	RIMS2,AGTPBP1,ATP8A1,KIT,ADGRF1,TNFRSF21,PPT1,VPS54,GRIK2,DLGAP3,MTMR2,MGLL,CST7,CTSC,NOS3,MYO7A,CXCR4,EDNRB,SPTBN4,SETD5,P2RX2,CD36,RIC8A,TCF15,EPHB1,PLK2,CHD7,NR4A1,EIF2AK3,MARVELD2,MMP24,CHRM5,HEXB,MEF2C,TLR2,P2RX7,NDRG4,TMEM150C,USH1C,PLCB2,TANC1,DLL4,IMPG2,DOP1B,NAV2,EDN1,JAM3,SNCA,SHANK2,PPP3CA,ANO1,KCNJ2,SYNM,SCN3A,ABLIM1,ZFHX2,JHY,ADCY1,YTHDF1,INSYN2,PTCHD1,HOXD10,CREB1,B2M,HMCN1,ADORA2A,RTP4,CCDC50,TBX1,TMOD2,HIF1A,SHROOM4,CHRNB1,TNFRSF1B,RGS14,MTOR,PSEN1,HOXB8,ITGA3,CHRNB2,ATP2A1,PIAS1,SCNN1B,ADRB2,CCL2,UNC119,PIEZO2,GIT1,S1PR1,SLC8A3,ATXN1L,PKHD1L1,RELN,GRAP,SHANK3,RGS4,DRAM2,RGS16,MGAT3,PDCL,CACNG5	turquoise	EC_M6
GO:1905603	2.23E-03	0.999821826	5	8	regulation of blood-brain barrier permeability	BP	8.38E-02	ANGPT1,SH3GL2,VEGFA,ABCC8,TJP1	darkgreen	EC_M4
GO:0050982	2.24E-03	0.999330698	13	40	detection of mechanical stimulus	BP	8.38E-02	WHRN,CTNNB1,CXCL12,CACNB3,PKD2,BACE1,KCNK4,PKD1L1,KCNA1,TCAP,ITGA2,PKD1,COL11A1	darkgreen	EC_M4
GO:0048813	2.24E-03	0.998911478	30	126	dendrite morphogenesis	BP	8.39E-02	RAB21,TPBG,SHANK1,ACTR2,KIDINS220,ITGB1,PICALM,TRAK1,FARP1,DCLK1,ILK,TLX2,DHX36,RAPGEF2,OPA1,PDLIM5,CHRNA7,DOCK10,DNM1L,CAPRIN2,GSK3B,KIF1A,TANC2,TNIK,OBSL1,SRCIN1,PAFAH1B1,RERE,YWHAH,SDC2	darkgreen	EC_M4
GO:1990778	2.26E-03	0.998639579	56	279	protein localization to cell periphery	BP	8.42E-02	LRP1,STX7,PACSIN1,GGA1,FAM126A,TPBG,ROCK2,VPS35,ITGB1,LIN7B,KIF5B,RAB7A,PICALM,TGFB1,AKT2,ATP2C1,PRNP,ARL6IP5,TMED2,TMEM150A,VAMP8,SLMAP,ATP1B3,SORBS1,RAPGEF2,CACNB3,ZFYVE27,ATP2B4,ARL3,PKP2,MMP14,DAG1,AP2M1,GORASP2,LRRC15,SEC23A,PRKCI,SMURF1,TNIK,EXOC5,BLZF1,MYO5A,IQSEC2,TSPAN17,STX1B,RAB11A,TTC8,CLTC,ROCK1,AKT1,RAB31,RAB34,FLNA,BSG,C16orf70,ARFRP1	darkgreen	EC_M4
GO:0071375	2.26E-03	0.998715083	46	219	cellular response to peptide hormone stimulus	BP	8.42E-02	AP3S1,LEP,ROCK2,MGARP,SESN3,PAK1,AKT2,OSBPL8,KAT2B,IRS2,ATP2B1,LEPROTL1,MAP1B,SORBS1,USO1,PTPN11,ADCY6,OPA1,ADIPOR1,SELENOS,ZNF106,EPRS,GSK3B,PIK3CA,WDTC1,SYAP1,LINC02210-CRHR1,EIF4EBP2,FBN1,PRKCI,MYO5A,SLC2A4,SHC1,PKM,ROCK1,AKT1,LPIN2,PRKAA1,RAB31,PTPN2,MYO1C,AHCYL1,BCAR3,SLC2A8,LEPROT,PIK3C2A	darkgreen	EC_M4
GO:0002042	2.00E-03	0.999353433	14	55	cell migration involved in sprouting angiogenesis	BP	8.45E-02	KDR,NRP1,GATA2,EFNB2,PLK2,NR4A1,DLL4,MAP3K3,CLEC14A,EPHB4,RHOJ,MMRN2,NOTCH1,MIR23A	turquoise	EC_M6

GO:0099537	2.00E-03	0.998700176	76	477	trans-synaptic signaling	BP	8.45E-02	KIF5A,PDE7B,STX11,AKAP12,SNCAIP,RIMS2,PACSIN2,ATF4,PICK1,APBA1,KIT,PPT1,GRIK2,DLGAP3,SDCBP,RAB3B,MTMR2,FCHSD2,PLCB4,FCHSD1,P2RX2,EPHB1,PLK2,NR4A1,ABHD6,CLCN3,MCTP1,CHRM5,MEF2C,P2RX7,GUCY1A1,GUCY1B1,PLCG1,DGKZ,EDN1,SNCA,DTNBP1,SHANK2,PPP3CA,SLC12A7,MCTP2,HCRTR1,SNAP23,ADCY1,RPS6KA1,YTHDF1,INSYN2,DLG1,PTCHD1,CREB1,BCR,ADORA2A,IL1RAP,SNAP29,TMOD2,SNCG,SYT7,CHRNB1,RGS14,FBXL20,MTOR,OPHN1,PSEN1,CHRNB2,ADRB2,CCL2,UNC119,GIT1,STXBP1,SLC8A3,RELN,SCN2B,SHANK3,RGS4,STX3,CACNG5	turquoise	EC_M6
GO:0010216	3.24E-03	0.999780475	4	7	maintenance of DNA methylation	BP	8.47E-02	UHRF2,HELLS,UHRF1,CTCF	darkgrey	EC_M8
GO:0051170	3.25E-03	0.99851307	23	132	import into nucleus	BP	8.50E-02	NUP107,UBR5,TNPO3,TRIM28,NUP85,IPO5,NUP58,IPO11,IPO7,TNPO1,NUP54,NUP93,HEATR3,NOLC1,CSE1L,NUP133,CDK1,TPR,ECT2,KPNB1,NUP62CL,NUP155,NUP35	darkgrey	EC_M8
GO:0044803	2.03E-03	0.999899795	4	6	multi-organism membrane organization	BP	8.53E-02	NECTIN2,FCHSD2,FCHSD1,HYAL2	turquoise	EC_M6
GO:0032869	2.31E-03	0.998793439	36	161	cellular response to insulin stimulus	BP	8.57E-02	AP3S1,LEP,SESN3,PAK1,AKT2,OSBPL8,KAT2B,IRS2,ATP2B1,SORBS1,USO1,PTPN11,OPA1,ADIPOR1,SELENOS,ZNF106,EPRS,GSK3B,PIK3CA,WDTC1,SYAP1,EIF4EBP2,PRKCI,MYO5A,SLC2A4,SHC1,PKM,AKT1,LPIN2,PRKAA1,RAB31,PTPN2,MYO1C,BCAR3,SLC2A8,PIK3C2A	darkgreen	EC_M4
GO:0001755	2.05E-03	0.99937669	13	47	neural crest cell migration	BP	8.59E-02	SEMA6A,SEMA4D,NRP1,EDNRB,RET,SEMA3F,LAMA5,SEMA6D,TBX1,HIF1A,EFNB1,SEMA4A,SEMA6B	turquoise	EC_M6
GO:0010718	2.32E-03	0.999260457	14	45	positive regulation of epithelial to mesenchymal transition	BP	8.59E-02	TGFBR1,TGFB1,CTNNB1,ZNF703,LOXL2,ACVR1,TGFB2,LEF1,BMP7,TGFB1I1,COL1A1,TGFB3,GLIPR2,OLFM1	darkgreen	EC_M4
GO:0007568	2.33E-03	0.998630356	51	252	aging	BP	8.61E-02	LRP1,CANX,SLC12A2,CTNNA1,LEP,CDKN2B,SRF,NUAK1,C2orf40,FGF2,PICALM,PRNP,ATP2B1,DCN,ILK,APP,RPN2,MAPKAPK5,CX3CL1,OPA1,AMFR,RAD54B,LOXL2,ADA,PLA2R1,GLRX2,CISD2,CASP7,DAG1,KRAS,SERPINE1,IGFBP2,COMP,ERO1A,TIMP1,AKT3,BMPR1A,CLN8,EIF2S1,PPARGC1A,KCNMB1,TGFB3,MAP2K1,PDGFRB,AKT1,IRAK1,PRMT6,SERPINF1,CACYBP,CDKN1A,MBD2	darkgreen	EC_M4
GO:0035581	2.34E-03	0.999811317	5	8	sequestering of extracellular ligand from receptor	BP	8.61E-02	FBN2,LTBP1,FBN1,NBL1,GREM2	darkgreen	EC_M4
GO:1904888	2.35E-03	0.999178934	16	54	cranial skeletal system development	BP	8.61E-02	RUNX2,RAB23,TGFBR1,GLI3,MEGF8,CTNNB1,RDH10,TGFB2,MMP14,FGFR2,PRRX1,TULP3,SIX4,TGFB3,SLC39A3,CHST11	darkgreen	EC_M4
GO:0005979	2.37E-03	0.99948992	9	23	regulation of glycogen biosynthetic process	BP	8.61E-02	DYRK2,AKT2,IRS2,SORBS1,SELENOS,GSK3B,PPP1R3D,PPP1R3F,AKT1	darkgreen	EC_M4
GO:0010962	2.37E-03	0.99948992	9	23	regulation of glucan biosynthetic process	BP	8.61E-02	DYRK2,AKT2,IRS2,SORBS1,SELENOS,GSK3B,PPP1R3D,PPP1R3F,AKT1	darkgreen	EC_M4
GO:0010970	2.38E-03	0.998810936	32	139	transport along microtubule	BP	8.61E-02	AP3S1,RAB21,SUN2,DYNC1I1,DST,MGARP,KIF5B,COPG1,TRAK1,MAP1B,APP,OPA1,AP3B1,IFT57,ARL3,FEZ1,HSPA8,DYNC1I2,KIF1A,HSPB1,MAPT,HTT,ACTR10,HAP1,IFT43,ARMCX3,PAFAH1B1,VAMP7,HSBP1,RAB6A,BLOC1S2,MAP1A	darkgreen	EC_M4
GO:0022898	2.38E-03	0.99870526	40	186	regulation of transmembrane transporter activity	BP	8.61E-02	UBQLN1,SHANK1,ITGB1,FKBP1B,KIF5B,GPD1L,ACTN4,STIM1,PRNP,REM1,FXYD1,SLMAP,ATP1B3,CACNA1D,APP,ABCC8,CACNB3,HOMER1,CHP1,PKD2,CASQ1,LINC02210-CRHR1,HTT,KCNA1,HAP1,NLGN2,STIM2,PPARGC1A,CALM1,VMP1,PARK7,AHCYL1,JPH3,MIR24-1,DMD,FKBP1A,CBARP,TMSB4X,YWHAH,GEM	darkgreen	EC_M4
GO:0010717	2.38E-03	0.999017389	21	80	regulation of epithelial to mesenchymal transition	BP	8.61E-02	TGFBR1,TGFB1,CTNNB1,SPRED3,ZNF703,LOXL2,ACVR1,ADIPOR1,PHLDB2,TGFB2,DAG1,LEF1,BMP7,PHLDB1,TGFB1I1,COL1A1,TGFB3,VASN,DACT3,GLIPR2,OLFM1	darkgreen	EC_M4
GO:2000351	2.06E-03	0.999446774	11	39	regulation of endothelial cell apoptotic process	BP	8.61E-02	TNFAIP3,TEK,KDR,GATA2,GATA3,PLCG1,ANO6,ITGA4,RAMP2,CCL2,CDH5	turquoise	EC_M6
GO:0032781	2.41E-03	0.999538177	8	20	positive regulation of ATPase activity	BP	8.64E-02	NAPA,ATP1B3,PFN2,DNAJC10,TPM1,TOR1AIP2,GABARAPL2,FGF10	darkgreen	EC_M4
GO:0051146	2.41E-03	0.998626159	46	220	striated muscle cell differentiation	BP	8.64E-02	PPARA,ADGRB1,CBY1,MYH9,SRF,VEGFA,BVES,MYLK3,ITGB1,RB1,PAK1,TGFB1,RARB,ACTG1,ANKRD1,HOMER1,ACVR1,PDLIM5,DNER,LMOD1,PRKAR1A,CASP7,MMP14,KRAS,CASQ1,SYNPO2L,WDR1,SIX4,OBSL1,BMPR1A,TCAP,TPM1,MORF4L2,MYOCD,PIN1,FBXO22,PDGFRB,FLII,AKT1,MIR24-1,ADAMTS5,CACYBP,CSRP1,BHLHE41,HOPX,CFL2	darkgreen	EC_M4

GO:0014910	2.42E-03	0.999116921	17	60	regulation of smooth muscle cell migration	BP	8.64E-02	LRP1,FAM19A5,PPARD,PLXNA1,PAK1,ILK,IQGAP1,S100A11,SERPINE1,BMPR1A,PPARGC1A,TPM1,MYOCD,PDGFRB,ITGA2,HAS2,PRKG1	darkgreen	EC_M4
GO:0051093	2.43E-03	0.998270009	119	676	negative regulation of developmental process	BP	8.68E-02	SLC12A2,FAM19A5,PPARA,ADGRB1,PPARD,CTNNA1,LEP,RORB,KIAA1324L,VEGFA,REST,ASPN,TGFBR1,SOCS5,RTN4,AKIRIN1,GLI3,ROCK2,INHBA,ITGB1,RB1,TPT1,GLI2,PAK1,TGFB1,CTNNB1,LBH,RARB,ACTN4,CRIM1,SPRED3,DCN,SEMA3C,SPINK5,RYK,RNF6,ZNF536,SULF1,WWC2,DIP2B,SEMA4F,LOXL3,HEY1,TLX2,APP,RAPGEF2,ABCC8,PTPN11,RUNX1T1,LOXL2,ATP2B4,SPP1,RUNX1,SEMA4G,NKD1,EIF4E,ADIPOR1,PKP2,CDC73,DNM1L,CCND1,TGFB2,LRP5,ADGRB2,GSK3B,LEF1,CORO1C,KRAS,NTRK3,WWC3,TCTA,BMP7,IDH2,TFRC,MAPT,SERPINE1,TNR,C11orf65,GPR137,FBN1,ITGAV,COL5A2,SKIL,FGFR3,SIX4,AKT3,PRTG,BMPR1A,CDK12,MED1,PPARGC1A,NPPB,TGFB1I1,NGFR,CHAD,DICER1,MYOCD,CD74,ROCK1,TMEM98,PTPN2,OMA1,VASN,PLXNA3,SARS,COL5A1,MIR24-1,FSTL3,SERPINF1,BNIP3,LBX1,THY1,CDKN1A,FGL2,DACT3,YWHAH,BHLHE41,HOPX,FGF10	darkgreen	EC_M4
GO:0009168	3.33E-03	0.99947182	6	18	purine ribonucleoside monophosphate biosynthetic process	BP	8.69E-02	PPAT,PAICS,GMPS,PFAS,HPRT1,GART	darkgrey	EC_M8
GO:0030323	2.44E-03	0.998760357	33	145	respiratory tube development	BP	8.69E-02	SRF,VEGFA,GLI3,RCN3,SLC7A11,FGF2,GLI2,CTNNB1,RDH10,LOXL3,AP3B1,ADA,MAN1A2,EIF4E,MMP14,DAG1,KRAS,FGFR2,PLOD3,TULP3,BMPR1A,TGFB3,MAP2K1,MYOCD,PDGFRB,ABCA3,PKD1,FSTL3,TMEM38B,LOX,HOPX,WNT2,FGF10	darkgreen	EC_M4
GO:0051270	2.46E-03	0.998197195	143	830	regulation of cellular component movement	BP	8.73E-02	LRP1,PLXNB2,ANGPT1,FAM19A5,NUS1,FAM49B,ADGRB1,PPARD,CTNNA1,SUN2,PLAA,SRF,VEGFA,DOCK8,CREB3,PTP4A1,TGFBR1,RTN4,EMC10,AKIRIN1,ROCK2,RRAS,ITGB1,PLXNA1,FGF2,PAK1,TGFB1,SCN5A,AKT2,OSBPL8,ACTG1,NAV3,ACTN4,SPINT2,IRS2,DCN,ZNF703,STARD13,SEMA3C,DDRGK1,ILK,DPYSL3,CXCL12,KIF2A,SULF1,ROBO1,CHST2,SEMA4F,P2RX4,PFN2,ATP2A2,APP,RAPGEF2,CX3CL1,ABCC8,PGAM4,ARSB,ACVR1,ADA,ATP2B4,SEMA4G,NKD1,DOCK10,ADIPOR1,PHLDB2,TJP1,PKP2,DNM1L,HYAL1,TGFB2,FEZ1,MMP14,DAG1,LEF1,CORO1C,PRKCA,CMKLR1,NTRK3,SLAMF8,BMP7,IDH2,IQGAP1,CLIC4,S100A11,LRRC15,FAM89B,HSPB1,SERPINE1,ITGA2B,DNAJA4,FN1,PDCD10,ITGAV,COL3A1,NBL1,TIMP1,VCL,AKT3,HAP1,CACNA1C,BMPR1A,PPARGC1A,ACTN1,MSN,SRCIN1,ARHGEF2,NGFR,TPM1,COL1A1,RAB11A,MYOCD,PIN1,CD74,LAMA3,PDGFRB,ROCK1,AKT1,ITGA2,DAPK3,MYO1C,PLXNB3,TNFRSF18,FLNA,PLXNA3,NTNG2,CDH13,F3,HSPA5,LMO4,NEXN,MIR24-1,CD151,SERPINF1,THY1,HAS2,TMSB4X,GLIPR2,CCR6,DUSP22,PIK3C2A,HCN4,PRKG1,FGF10	darkgreen	EC_M4
GO:0061383	2.46E-03	0.999254488	13	40	trabecula morphogenesis	BP	8.73E-02	FBN2,SRF,TGFBR1,HEY1,MMP2,TGFB2,BMP7,BMPR1A,MED1,THBS3,COL1A1,CHAD,FKBP1A	darkgreen	EC_M4
GO:0010923	2.47E-03	0.999345091	11	32	negative regulation of phosphatase activity	BP	8.74E-02	ROCK2,FKBP1B,CRY2,CHP1,TGFB2,GSK3B,PPP1R11,PPP1R36,PPP4R4,ROCK1,FKBP1A	darkgreen	EC_M4
GO:0000731	3.39E-03	0.999262265	8	26	DNA synthesis involved in DNA repair	BP	8.82E-02	POLH,REV1,POLE,RFC3,DTL,POLA1,C17orf53,RCHY1	darkgrey	EC_M8
GO:0000729	3.41E-03	0.999475832	6	16	DNA double-strand break processing	BP	8.85E-02	SMARCAD1,SPO11,BLM,DNA2,RAD52,BRIP1	darkgrey	EC_M8

GO:0009893	2.13E-03	0.998277383	353	2815	positive regulation of metabolic process	BP	8.88E-02	ZNF516,TNFRSF11A,TCF4,IFNGR1,TNFAIP3,PLAGL1,AKAP12,CNOT6,FLT4,RIMS2,ZBED4,GRAMD4,QKI,NOTCH4,AFDN,TCF20,TPD52L1,NCOA7,SGSM3,PTK2,DAPK1,ATF4,PDGFB,AGTPBP1,MAPK13,BRPF3,CSNK1E,PICK1,KDM4C,PIM1,NFYA,TEK,PRDM4,LIMCH1,DDX58,FAM122A,TEC,ADGRF5,KIT,KDR,NFIL3,SYK,GADD45G,SEMA4D,IL6,REL,CREB5,CXCL8,PELI1,RSAD2,GRB10,TLR4,C5,IL15,CCNY,TEAD2,SDCBP,MYCN,NOCT,TUT4,NRP1,PIK3CG,TAL1,PHF10,EPC1,KDM4D,GATA2,PRKD2,CTSC,RAB38,CAB39L,NOS3,GGA3,RALB,PPARG,GATA3,CXCR4,EDNRB,NDFIP2,TIE1,IKBKB,JAG1,P2RY6,NFKBIB,PDE2A,TMEM173,SFRP1,HBEGF,NR1D2,DND1,FERMT1,RPL26,ANKLE2,CD36,SIRT3,PRR5L,TCF15,SCARB1,EPHB1,PLK2,RET,PDE4D,MERTK,CHD7,IL1A,DNMT3B,IGF1,NR4A1,KLKB1,EIF2AK3,ABHD6,HHEX,KDM3A,ITCH,SMAD1,ZBTB38,CLCN3,HIP1R,OCLN,STOX2,SOX7,HMBOX1,GPD1,HEXB,MEF2C,SMARCD1,TLR2,WWTR1,IL7,P2RX7,VEGFC,CXCL10,MADD,KMT2D,TIPARP,OAS3,NLRC5,THBS4,PPP1R16B,TPCN1,RAPGEF3,NR4A2,DGKZ,TOX2,DLL4,NEDD9,YAF2,EDN1,STAT1,CD58,DYRK1A,EHD4,SNCA,JARID2,DTNBP1,MYLIP,ERG,ETS2,SLC45A3,NFKB2,RNF144B,PRDM15,ABCG1,ETS1,RNF19B,CD200,LPCAT1,HDAC1,LCK,PPP3CA,HYAL2,HECW2,MEF2A,BCL9,LAPTM5,NCF1,USP13,FABP4,HCLS1,NFATC2,EAF2,GMEB1,CD34,PECAM1,CD86,ASB9,POU2F3,SH3BP4,FGR,CFLAR,CASP10,INPP5F,PPP2R5A,ARID1A,RPS6KA1,PROX1,SS18L1,PRKD1,ZNF148,YTHDF1,OAZ3,HELZ2,MLXIPL,ACE,ITGA6,LIMK2,PAK2,MELTF,SENP2,DLG1,ELL3,ETV6,MAP3K11,HOXD10,MOV10,CREB1,B2M,TET1,LTF,ARNT2,CYBB,IL1RAP,EPHB4,DDX3X,RFXANK,ETV4	turquoise	EC_M6
GO:0086046	2.53E-03	0.99991079	4	5	membrane depolarization during SA node cell action potential	BP	8.91E-02	SCN5A,CACNA1D,CACNA1G,HCN4	darkgreen	EC_M4
GO:0040008	2.53E-03	0.998272468	94	518	regulation of growth	BP	8.91E-02	LRP1,RAB21,PPARA,ROS1,PPARD,LEP,PLAA,SRF,VEGFA,TGFBR1,SOCS5,RIMS1,RTN4,MFSD2A,INHBA,FGF2,RB1,PTCH2,KCTD21,MEGF8,PAK1,TGFB1,SEMA3C,ILK,RYK,RNF6,CXCL12,CHPT1,EIF4G2,WWC2,MAP1B,DIP2B,SEMA4F,CELF1,APP,ZFYVE27,TSG101,BAP1,SPP1,RUNX1,SEMA4G,NKD1,NDN,ADIPOR1,NOTCH2,CTTN,CDC73,HYAL1,CAPRIN2,TGFB2,FBLN5,ZMAT3,GSK3B,MMP14,PIK3CA,WWC3,FGFR2,OSM,RBBP7,RPS6KA3,MAPT,SGPL1,TNR,FN1,IGFBP2,FGFR3,SMURF1,SIX4,BMPR1A,TSPYL2,NPPB,SHC1,RAB11A,TAF9B,SMARCA4,MORF4L2,MYOCD,ISLR2,PIN1,TRPV2,AKT1,CRLF3,PAFAH1B1,PLXNA3,ENO1,BARHL2,CDKN1A,DACT3,NME6,HOPX,ZPR1,OLFM1,DERL2,WNT2	darkgreen	EC_M4
GO:0009452	3.46E-03	0.999760506	4	7	7-methylguanosine RNA capping	BP	8.95E-02	NCBP1,TGS1,NCBP3,CMTR2	darkgrey	EC_M8
GO:0036260	3.46E-03	0.999760506	4	7	RNA capping	BP	8.95E-02	NCBP1,TGS1,NCBP3,CMTR2	darkgrey	EC_M8
GO:0044417	3.49E-03	0.999711295	4	9	translocation of molecules into host	BP	9.01E-02	DDX39B,ALYREF,THOC1,THOC2	darkgrey	EC_M8
GO:0043303	2.18E-03	0.999411139	11	38	mast cell degranulation	BP	9.06E-02	KIT,SYK,PIK3CG,GATA2,GAB2,UNC13D,SNAP23,FGR,FES,LGALS9,STXBP1	turquoise	EC_M6
GO:0030099	2.19E-03	0.998723201	49	293	myeloid cell differentiation	BP	9.06E-02	TNFRSF11A,L3MBTL3,IREB2,ADGRF5,KIT,PTBP3,TLR4,IL15,TAL1,GATA2,SNRK,GAB2,PPARG,GATA3,JAG1,SFRP1,TMEM178A,MEF2C,TLR2,TSPAN2,STAT1,ALAS1,ETS1,PRKX,DYRK3,HCLS1,INPP5D,FES,CREB1,NBEAL2,B2M,LTF,HIF1A,EPHA2,ITPKB,LBR,MTOR,PSEN1,HOXB7,HOXB8,BATF,GPR68,RCOR1,ISG15,IRF8,ARID4A,CA2,HIST1H4D,FLI1	turquoise	EC_M6

GO:0016310	2.19E-03	0.998326596	195	1440	phosphorylation	BP	9.06E-02	TNFRSF11A,TNFAIP3,PTPRB,FLT4,CDKL3,TPD52L1,PTK2,DAPK1,PDGFB,MAPK13,CSNK1E,PICK1,PIM1,TEK,LIMCH1,PIP5K1B,TEC,KIT,KDR,SYK,SEMA4D,IL6,GRB10,TLR4,IL15,CCNY,SDCBP,NRP1,RASIP1,PIK3CG,TAL1,KDM4D,PRKD2,CAB39L,SNRK,RALB,PPARG,ACVR2B,IRAK2,EDNRB,SPTBN4,TIE1,IKBKB,SFRP1,HBEGF,SH3BP5,ANKLE2,CD36,CDK17,PRAG1,PRR5L,MAP3K1,EPHB1,PLK2,RET,PDE4D,MERTK,IGF1,EIF2AK3,HHEX,SMAD1,OCLN,DCLK2,GPD1,TLR2,WWTR1,IL7,P2RX7,VEGFC,MADD,BMP2K,NLRC5,THBS4,RAPGEF3,DGKZ,HIPK1,CCNJL,PIK3C2B,NEDD9,EDN1,DYRK1A,EHD4,SNCA,DTNBP1,ERG,NUAK2,CDK18,DCAF1,SIK1,HDAC1,LCK,HYAL2,PRKX,NCF1,DYRK3,FABP4,HCLS1,PECAM1,CD86,BMX,FGR,MAP3K6,INPP5F,KALRN,IGFBP3,FES,PPP2R5A,RPS6KA1,PROX1,PRKD1,PDIK1L,MAP3K3,SRMS,MLXIPL,ACE,ITGA6,LIMK2,PAK2,DLG1,MAP3K11,STYK1,PDK3,CREB1,LTF,BCR,ADORA2A,EPHB4,DDX3X,TBX1,WNK4,CDK16,LDB2,TNFAIP8L3,FGF23,LMTK2,PPM1F,PRKCH,RAC1,HIF1A,ADIPOR2,EPHA2,ITPKB,TLR6,TNK1,RGS14,MTOR,AR,PSEN1,STK10,FGF18,RPS6KL1,PGF,NEK9,DAPK2,TAOK2,EFNA1,ADCK1,CD19,SBK1,PIK3R5,NPR1,RPS6KA5,CCDC88C,SMAD2,CDC42BPB,MARK3,ADRB2,CCL2,YES1,SH2D3A,JAK1,UNC119,STK26,GIT1,MATK,TRAF2,PLCG2,ENG,SH2D3C,DAB2IP,CDH5,SLC8A3,TRIM24,RELN,ALKAL1,GMFG,FGF16,CDK13,MMD,DAZAP2,COPS8,BMP2,DUSP7,PIFO,BMP6	turquoise	EC_M6
GO:0050931	2.20E-03	0.999507686	9	28	pigment cell differentiation	BP	9.07E-02	KIT,RAB38,EDNRB,MEF2C,ADAMTS20,USP13,RAB27A,AP3D1,RAB32	turquoise	EC_M6
GO:0006614	2.60E-03	0.999575715	7	16	SRP-dependent cotranslational protein targeting to membrane	BP	9.12E-02	SRP72,SEC61A1,SRP68,TRAM1,SSR3,SRPRA,SRP54	darkgreen	EC_M4
GO:0001906	2.23E-03	0.999168216	17	78	cell killing	BP	9.15E-02	LYZ,SYK,CTSC,NECTIN2,UNC13D,P2RX7,RNF19B,LYST,B2M,LTF,RAB27A,SCNN1B,LGALS9,NOS2,IL7R,FCER2,SERPINB9	turquoise	EC_M6
GO:0019220	2.26E-03	0.998335309	149	1074	regulation of phosphate metabolic process	BP	9.23E-02	TNFRSF11A,TNFAIP3,PTPRB,FLT4,TPD52L1,PTK2,PDGFB,TEK,LIMCH1,TEC,ADGRF5,KIT,KDR,SYK,SEMA4D,PPP6R1,IL6,GRB10,TLR4,IL15,CCNY,SDCBP,NRP1,RASIP1,PIK3CG,TAL1,MTMR2,KDM4D,PRKD2,RAB38,CAB39L,NOS3,RALB,PPARG,EDNRB,SPTBN4,TIE1,IKBKB,P2RY6,SFRP1,HBEGF,SH3BP5,ANKLE2,CD36,PRR5L,SCARB1,EPHB1,RET,PDE4D,MERTK,IGF1,EIF2AK3,HHEX,OCLN,CHRM5,GPD1,MEF2C,WWTR1,IL7,P2RX7,VEGFC,MADD,NLRC5,THBS4,PPP1R16B,RAPGEF3,DGKZ,CCNJL,NEDD9,EDN1,EHD4,SNCA,DTNBP1,LPCAT1,HDAC1,HYAL2,NCF1,FABP4,HCLS1,PECAM1,CD86,FGR,INPP5F,IGFBP3,PPP2R5A,PROX1,PRKD1,MLXIPL,ACE,ITGA6,LIMK2,PAK2,DLG1,SMG7,MAP3K11,PDK3,LTF,ADORA2A,EPHB4,DDX3X,TBX1,CDA,LDB2,TNFAIP8L3,FGF23,LMTK2,PPM1F,RAC1,HIF1A,ADIPOR2,EPHA2,ITPKB,TLR6,RGS14,MTOR,AR,PSEN1,FGF18,PGF,TAOK2,EFNA1,CD19,PIK3R5,RPS6KA5,CCDC88C,ADRB2,YES1,SH2D3A,NOS2,UNC119,GIT1,TRAF2,PLCG2,ENG,SH2D3C,DAB2IP,CDH5,SLC8A3,RELN,ALKAL1,FGF16,MMD,DAZAP2,COPS8,BMP2,DUSP7,PIFO,BMP6,PPP1R2	turquoise	EC_M6
GO:0051174	2.26E-03	0.998335309	149	1074	regulation of phosphorus metabolic process	BP	9.23E-02	TNFRSF11A,TNFAIP3,PTPRB,FLT4,TPD52L1,PTK2,PDGFB,TEK,LIMCH1,TEC,ADGRF5,KIT,KDR,SYK,SEMA4D,PPP6R1,IL6,GRB10,TLR4,IL15,CCNY,SDCBP,NRP1,RASIP1,PIK3CG,TAL1,MTMR2,KDM4D,PRKD2,RAB38,CAB39L,NOS3,RALB,PPARG,EDNRB,SPTBN4,TIE1,IKBKB,P2RY6,SFRP1,HBEGF,SH3BP5,ANKLE2,CD36,PRR5L,SCARB1,EPHB1,RET,PDE4D,MERTK,IGF1,EIF2AK3,HHEX,OCLN,CHRM5,GPD1,MEF2C,WWTR1,IL7,P2RX7,VEGFC,MADD,NLRC5,THBS4,PPP1R16B,RAPGEF3,DGKZ,CCNJL,NEDD9,EDN1,EHD4,SNCA,DTNBP1,LPCAT1,HDAC1,HYAL2,NCF1,FABP4,HCLS1,PECAM1,CD86,FGR,INPP5F,IGFBP3,PPP2R5A,PROX1,PRKD1,MLXIPL,ACE,ITGA6,LIMK2,PAK2,DLG1,SMG7,MAP3K11,PDK3,LTF,ADORA2A,EPHB4,DDX3X,TBX1,CDA,LDB2,TNFAIP8L3,FGF23,LMTK2,PPM1F,RAC1,HIF1A,ADIPOR2,EPHA2,ITPKB,TLR6,RGS14,MTOR,AR,PSEN1,FGF18,PGF,TAOK2,EFNA1,CD19,PIK3R5,RPS6KA5,CCDC88C,ADRB2,YES1,SH2D3A,NOS2,UNC119,GIT1,TRAF2,PLCG2,ENG,SH2D3C,DAB2IP,CDH5,SLC8A3,RELN,ALKAL1,FGF16,MMD,DAZAP2,COPS8,BMP2,DUSP7,PIFO,BMP6,PPP1R2	turquoise	EC_M6
GO:0044764	2.28E-03	0.999703668	6	13	multi-organism cellular process	BP	9.28E-02	CAV2,NECTIN2,FCHSD2,FCHSD1,TPCN1,HYAL2	turquoise	EC_M6

GO:0007268	2.29E-03	0.998512	75	471	chemical synaptic transmission	BP	9.28E-02	KIF5A,PDE7B,STX11,AKAP12,SNCAIP,RIMS2,PACSIN2,ATF4,PICK1,APBA1,KIT,PPT1,GRIK2,DLGAP3,SDCBP,RAB3B,MTMR2,FCHSD2,PLCB4,FCHSD1,P2RX2,EPHB1,PLK2,NR4A1,ABHD6,CLCN3,MCTP1,CHRM5,MEF2C,P2RX7,GUCY1A1,GUCY1B1,PLCG1,DGKZ,EDN1,SNCA,DTNBP1,SHANK2,PPP3CA,SLC12A7,MCTP2,HCRTR1,SNAP23,ADCY1,RPS6KA1,YTHDF1,INSYN2,DLG1,PTCHD1,CREB1,BCR,ADORA2A,SNAP29,TMOD2,SNCG,SYT7,CHRNB1,RGS14,FBXL20,MTOR,OPHN1,PSEN1,CHRNB2,ADRB2,CCL2,UNC119,GIT1,STXBP1,SLC8A3,RELN,SCN2B,SHANK3,RGS4,STX3,CACNG5	turquoise	EC_M6
GO:0098916	2.29E-03	0.998512	75	471	anterograde trans-synaptic signaling	BP	9.28E-02	KIF5A,PDE7B,STX11,AKAP12,SNCAIP,RIMS2,PACSIN2,ATF4,PICK1,APBA1,KIT,PPT1,GRIK2,DLGAP3,SDCBP,RAB3B,MTMR2,FCHSD2,PLCB4,FCHSD1,P2RX2,EPHB1,PLK2,NR4A1,ABHD6,CLCN3,MCTP1,CHRM5,MEF2C,P2RX7,GUCY1A1,GUCY1B1,PLCG1,DGKZ,EDN1,SNCA,DTNBP1,SHANK2,PPP3CA,SLC12A7,MCTP2,HCRTR1,SNAP23,ADCY1,RPS6KA1,YTHDF1,INSYN2,DLG1,PTCHD1,CREB1,BCR,ADORA2A,SNAP29,TMOD2,SNCG,SYT7,CHRNB1,RGS14,FBXL20,MTOR,OPHN1,PSEN1,CHRNB2,ADRB2,CCL2,UNC119,GIT1,STXBP1,SLC8A3,RELN,SCN2B,SHANK3,RGS4,STX3,CACNG5	turquoise	EC_M6
GO:0051293	3.63E-03	0.998927897	11	44	establishment of spindle localization	BP	9.34E-02	CENPA,CLASP2,ESPL1,NUSAP1,CFL1,KPNB1,SPDL1,PLK1,NDC80,SAPCD2,MAD2L1	darkgrey	EC_M8
GO:0000027	3.63E-03	0.99941301	6	18	ribosomal large subunit assembly	BP	9.34E-02	MDN1,RRS1,FASTKD2,RPL10L,NLE1,BRIX1	darkgrey	EC_M8
GO:0000387	3.64E-03	0.999070098	9	38	spliceosomal snRNP assembly	BP	9.34E-02	SNRPF,TGS1,SMN2,PRMT5,DDX20,COIL,GEMIN5,GEMIN4,GEMIN6	darkgrey	EC_M8
GO:1903039	2.33E-03	0.998896508	27	143	positive regulation of leukocyte cell-cell adhesion	BP	9.42E-02	SYK,IL6,IL15,GATA3,IL1A,IGF1,IL7,SOX13,ETS1,LCK,ICOSLG,HLX,CD86,B2M,ITGA4,SELE,SELP,ITPKB,EFNB1,CCL2,YES1,LGALS9,IL7R,AP3D1,NFAT5,VNN1,CD46	turquoise	EC_M6
GO:0010907	2.70E-03	0.999275056	11	32	positive regulation of glucose metabolic process	BP	9.43E-02	DYRK2,PPARA,DGAT2,AKT2,KAT2B,IRS2,SORBS1,PPARGC1A,AKT1,PTPN2,NNMT	darkgreen	EC_M4
GO:0008090	2.72E-03	0.999478811	8	19	retrograde axonal transport	BP	9.45E-02	DST,MGARP,KIF5B,KIF1A,ACTR10,HAP1,PAFAH1B1,MAP1A	darkgreen	EC_M4
GO:0006458	2.73E-03	0.999261271	11	33	'de novo' protein folding	BP	9.48E-02	BAG1,FKBP1B,DNAJB13,HSPH1,HSPA8,DNAJB12,ERO1A,CD74,HSPA5,FKBP1A,HSPA13	darkgreen	EC_M4
GO:0006204	1.58E-04	1	4	4	IMP catabolic process	BP	9.52E-02	PNP,XDH,NT5C2,NT5C	cyan	EC_M2
GO:0032386	2.76E-03	0.99831531	57	290	regulation of intracellular transport	BP	9.53E-02	LMAN1,RAB21,ANGPT1,NUS1,REEP2,LEP,RAB23,TMEM30A,ERLEC1,UBE2J1,MDFIC,GLI3,BVES,KIF5B,IWS1,PAK1,TGFB1,AKT2,PPP1R12A,UFM1,PRNP,HDAC3,TM9SF4,REEP5,RIOK2,MAP1B,CACNB3,PTPN11,ARHGAP1,ATG13,BAP1,CHP1,BRSK2,FEZ1,GSK3B,ARV1,SCFD1,C11orf65,PDCD10,STX18,B3GAT3,HAP1,MSN,MAP2K1,MIEF2,PRKAA1,SUPT6H,MYO1C,BCAP31,FLNA,PARK7,TXN,LEPROT,ZPR1,UBE2G2,TOMM7,DERL2	darkgreen	EC_M4
GO:1901653	2.76E-03	0.998337231	54	270	cellular response to peptide	BP	9.53E-02	LRP1,AP3S1,LEP,ROCK2,MGARP,SESN3,PAK1,AKT2,OSBPL8,KAT2B,IRS2,PRNP,ATP2B1,LEPROTL1,MAP1B,SORBS1,USO1,APP,PTPN11,ADCY6,OPA1,PTPN22,ADIPOR1,SELENOS,ZNF106,EPRS,GSK3B,PIK3CA,WDTC1,SYAP1,BACE1,LINC02210-CRHR1,EIF4EBP2,FBN1,PRKCI,MYO5A,SLC2A4,ARHGEF2,NGFR,SHC1,PKM,ROCK1,AKT1,LPIN2,PRKAA1,RAB31,PTPN2,LDOC1,MYO1C,AHCYL1,BCAR3,SLC2A8,LEPROT,PIK3C2A	darkgreen	EC_M4
GO:0051961	2.77E-03	0.998714989	26	106	negative regulation of nervous system development	BP	9.53E-02	CTNNA1,KIAA1324L,REST,RTN4,CTNNB1,SEMA3C,RYK,RNF6,DIP2B,SEMA4F,TLX2,RAPGEF2,ABCC8,SPP1,SEMA4G,NTRK3,BMP7,IDH2,TNR,PRTG,BMPR1A,DICER1,TMEM98,PLXNA3,THY1,YWHAH	darkgreen	EC_M4
GO:0009161	3.73E-03	0.99887097	11	51	ribonucleoside monophosphate metabolic process	BP	9.54E-02	PPAT,PAICS,CAD,GMPS,UMPS,PFAS,PRPS1,HPRT1,DHODH,RFK,GART	darkgrey	EC_M8
GO:0042987	2.79E-03	0.999039237	15	52	amyloid precursor protein catabolic process	BP	9.58E-02	RTN4,ROCK2,GSAP,PICALM,PRNP,CHRNA7,NCSTN,BACE1,RTN3,HAP1,TMED10,PIN1,ROCK1,MIR24-1,FKBP1A	darkgreen	EC_M4

GO:0065009	2.82E-03	0.997694804	359	2307	regulation of molecular function	BP	9.65E-02	SERPINB2,IL23A,NAB2,LRP1,ARHGEF25,AGAP2,TAB2,TBC1D9B,CAND1,PRRC1,DENND6B,AZIN1,PLXNB2,ANGPT1,TCP1,PPARA,ROS1,NUS1,SERINC1,TRAPPC9,HABP4,LY6E,SIL1,C6orf89,UBQLN1,MTCH1,GNAQ,ARHGAP39,LEP,CDKN2B,PLAA,SRF,BAG1,NUAK1,VEGFA,DOCK8,VCP,TPM2,NCK2,CLOCK,REST,SNX13,TGFBR1,SOCS5,RIMS1,PSME4,CYCS,SHANK1,TAX1BP1,MAP3K7,SH3BP5L,KIDINS220,ROCK2,BVES,RCN3,DKKL1,ITGB1,FKBP1B,PLXNA1,FGF2,KIF5B,NAPA,DNAJC1,ARHGEF4,RB1,ABHD5,EED,PICALM,MAPRE3,EIF2B3,SMARCD3,PAK1,TGFB1,AXL,RAB3GAP1,CTNNB1,SERPINH1,AKT2,GPD1L,SEC23B,DZIP1,ZEB2,PLEKHG2,TBC1D16,FARP1,ARHGEF17,OSBPL8,PSD2,ACTN4,PPP1R12A,KAT2B,CRIM1,STIM1,GPS1,SPINT2,IRS2,PRNP,STARD13,CCKBR,DDRGK1,SMPD1,PPP2CB,ILK,MMP17,HSPH1,ARL6IP5,PPP2R2B,SPINK5,RYK,PSMD6,ST5,REM1,ARL1,FXYD1,HSP90B1,TMED2,SLMAP,ATP1B3,DKK3,ROBO1,PINX1,PFN2,HEY1,RASA1,ATP2A2,CCNJ,DLGAP4,RTKN,CACNA1D,MMP15,APP,RAPGEF2,MAPKAPK5,CX3CL1,ABCC8,PPP2R2A,CACNB3,PTX3,RALGAPB,HOMER1,ARHGAP1,AMFR,GTF2H1,AP3B1,PTPN22,TSG101,DKC1,CRY2,MTDH,AKTIP,CHP1,ATP2B4,PKD2,PLA2R1,DBNDD2,CBLB,IFT57,TANK,PSMD14,BRSK2,NDN,SLC39A10,RAB7B,CHRNA7,RAP1GDS1,DOCK10,DYNLL1,AZIN2,PPP1R12B,DNM1L,UCHL5,CCND1,CAPRIN2,TGFB2,PPP6R3,PSMD1,PRKAB2,LRP5,ARHGAP31,HTR2B,PRKAR1A,GSK3B,DAG1,PIK3CA,LEF1,CORO1C,ANOS1,ISCU,NEK7,ADAP1,CMKLR1,KRAS,NTRK3,SGSM1,SLAMF8,FAM162A,SPATC1L,BMP7,PPP1R11,WDTC1,COL6A3,FGFR2,CASQ1,TP53BP1,NCSTN,SYAP1,PSMD2,ADRM1,IQGAP1,TFRC,FAR	darkgreen	EC_M4
GO:0035633	2.84E-03	0.999233199	11	32	maintenance of blood-brain barrier	BP	9.70E-02	SLC12A2,ANGPT1,SH3GL2,VEGFA,MFSD2A,ITGB1,ACTG1,ABCC8,TJP1,VCL,DMD	darkgreen	EC_M4
GO:1905168	3.81E-03	0.999524257	5	13	positive regulation of double-strand break repair via homologous recombination	BP	9.72E-02	ERCC6,ARID2,FANCB,RAD51AP1,FUS	darkgrey	EC_M8
GO:0060429	2.41E-03	0.998283743	118	810	epithelium development	BP	9.73E-02	FZD6,CTHRC1,CELSR1,NOTCH4,AFDN,MTSS1,KLHL3,ATF4,PDGFB,PODXL,TJP2,KDR,TMOD1,GJA4,TEAD2,MYCN,NRP1,RASIP1,PRKD2,GPR4,PLXND1,PPARG,GATA3,ACVR2B,MYO7A,CXCR4,GZF1,TIE1,IKBKB,JAG1,PDE2A,GPAT4,SFRP1,HBEGF,FERMT1,FREM2,SPEF1,NTN4,SYNE4,TCF15,RET,PDE4D,TNFRSF19,CHD7,SMAD1,MARVELD2,MEF2C,WWTR1,VEGFC,NDRG4,CXCL10,USH1C,PPP1R16B,STC1,RAPGEF3,DLL4,EDN1,STAT1,HDAC1,ROBO4,PRKX,SEC24B,JHY,CD34,PECAM1,PLXNA2,TGM1,PROX1,VANGL2,LAMA5,F11R,APOLD1,DLG1,CREB1,BCR,IGFBP5,ETV4,TBX1,WNK4,LDB2,PROM1,PRKCH,RAC1,HIF1A,EPHA2,CECR2,MPP5,LFNG,ARG2,ALDH1A2,MTOR,AR,OPHN1,PSEN1,HOXB5,HOXB7,CCDC88C,SMAD2,CYP1A1,JAG2,NRARP,NOTCH1,RAPGEF1,S1PR1,CNN3,ENG,CARMIL2,CDH5,ARID4A,VASP,SOX17,SOX18,MAFF,TBC1D20,RALA,BMP2,PIFO,BMP6	turquoise	EC_M6
GO:0019856	3.83E-03	0.999696146	4	8	pyrimidine nucleobase biosynthetic process	BP	9.76E-02	CTPS1,CAD,UMPS,DHODH	darkgrey	EC_M8
GO:0002688	2.43E-03	0.999023221	19	92	regulation of leukocyte chemotaxis	BP	9.78E-02	PTK2,IL6,CXCL8,C5,VEGFC,CXCL10,THBS4,ANO6,EDN1,JAM3,RIN3,MCU,RAC1,PGF,DAPK2,S100A14,MMP28,CCL2,LGALS9	turquoise	EC_M6
GO:0035268	2.87E-03	0.999432898	8	20	protein mannosylation	BP	9.80E-02	NUS1,LARGE1,TMTC4,TMTC3,DPY19L4,POMT1,DPM2,DPM1	darkgreen	EC_M4
GO:1904152	2.88E-03	0.999623995	6	12	regulation of retrograde protein transport, ER to cytosol	BP	9.80E-02	ERLEC1,UBE2J1,BRSK2,BCAP31,UBE2G2,DERL2	darkgreen	EC_M4
GO:0140353	2.90E-03	0.999278049	10	28	lipid export from cell	BP	9.84E-02	LEP,INHBA,C1QTNF1,P2RX4,PTPN11,CRY2,SPP1,KDM5B,LINC02210-CRHR1,CYP19A1	darkgreen	EC_M4
GO:0048013	2.45E-03	0.999242217	13	45	ephrin receptor signaling pathway	BP	9.85E-02	PTK2,ANKS1A,EFNB2,ARHGEF7,EPHB1,ARHGEF28,KALRN,EPHB4,EPHA2,EFNB1,EFNA1,YES1,GIT1	turquoise	EC_M6
GO:0001763	2.91E-03	0.998468621	35	157	morphogenesis of a branching structure	BP	9.87E-02	RSPO3,SRF,VEGFA,RTN4,GLI3,PLXNA1,FGF2,GLI2,PAK1,CTNNB1,SPINT2,SEMA3C,ILK,TMED2,SULF1,RDH10,ACVR1,PKD2,KDM5B,LRP5,MMP14,DAG1,LEF1,KRAS,BMP7,FGFR2,CLIC4,SIX4,MAGED1,MED1,PKD1,RERE,GDNF,WNT2,FGF10	darkgreen	EC_M4
GO:0072006	2.47E-03	0.998872667	25	118	nephron development	BP	9.88E-02	MTSS1,KLHL3,PDGFB,PODXL,TEK,GPR4,GATA3,GZF1,JAG1,RET,MEF2C,WWTR1,ANGPT2,STAT1,CD34,PECAM1,CFLAR,LAMA5,DLG1,WNK4,PROM1,HOXB7,ITGA3,NOTCH1,BMP2	turquoise	EC_M6

GO:0033674	2.48E-03	0.998455933	61	373	positive regulation of kinase activity	BP	9.91E-02	TNFRSF11A,FLT4,TPD52L1,PTK2,PDGFB,TEK,KIT,KDR,SYK,TLR4,CCNY,PIK3CG,TAL1,CAB39L,RALB,TIE1,HBEGF,EPHB1,RET,MERTK,IGF1,VEGFC,MADD,DGKZ,NEDD9,EDN1,SNCA,NCF1,CD86,FGR,PROX1,PRKD1,ACE,PAK2,DLG1,MAP3K11,LTF,EPHB4,DDX3X,TNFAIP8L3,LMTK2,EPHA2,TLR6,MTOR,PSEN1,FGF18,TAOK2,EFNA1,CD19,PIK3R5,ADRB2,UNC119,TRAF2,DAB2IP,RELN,ALKAL1,MMD,DAZAP2,COPS8,BMP2,PIFO	turquoise	EC_M6
GO:0034109	2.95E-03	0.998822964	19	71	homotypic cell-cell adhesion	BP	9.92E-02	LGALS1,MYH9,SLC7A11,C1QTNF1,ACTG1,ZNF703,ILK,MYL9,PKP2,PRKCA,HSPB1,ITGA2B,FN1,PTPN6,COMP,VCL,FLNA,CSRP1,PRKG1	darkgreen	EC_M4
GO:0030324	2.98E-03	0.998483331	32	141	lung development	BP	9.99E-02	SRF,VEGFA,GLI3,RCN3,SLC7A11,FGF2,GLI2,CTNNB1,RDH10,LOXL3,AP3B1,ADA,MAN1A2,EIF4E,MMP14,DAG1,KRAS,FGFR2,PLOD3,BMPR1A,TGFB3,MAP2K1,MYOCD,PDGFRB,ABCA3,PKD1,FSTL3,TMEM38B,LOX,HOPX,WNT2,FGF10	darkgreen	EC_M4
GO:1901135	2.98E-03	0.997817859	134	791	carbohydrate derivative metabolic process	BP	9.99E-02	B4GALT7,B4GALNT1,RRM2B,ANGPT1,PPARA,ACSL6,NUS1,PPARD,MTAP,LEP,LARGE1,GALT,VCP,SLC35B2,CHST10,RAB23,NANS,ERP44,TMEM246,UBE2J1,GFPT1,FGF2,CMPK1,RPN1,POMGNT1,ITM2B,GLB1,CHPF2,TGFB1,ST6GALNAC2,RBKS,CTNNB1,TRAK1,DGAT2,GPD1L,STT3B,PFKP,CANT1,TMTC4,SUMF1,KDELC1,TMTC3,NUDT16,DCN,ACPP,SMPD1,TUSC3,IMPAD1,ST8SIA4,SULF1,B3GNT4,CHST2,FAR1,APP,TET3,RPN2,PTX3,ADCY6,PFKM,PGAM4,OPA1,ENTPD4,AMFR,DPY19L4,ADA,CHP1,NUDT18,PIGP,MAN1A2,MARCH6,CHSY1,HYAL1,SLC2A10,ZBTB20,B4GALT4,TYW5,B3GALT1,TPST2,CMAS,ADCY5,HACD2,GALNT2,MAN2A2,NCSTN,PIGZ,EDEM3,SLC25A16,PLOD3,SYVN1,TPI1,DDOST,B3GAT3,UGDH,SLC2A4,CHST12,PPARGC1A,NPPB,GALNT16,XYLT2,CLN6,GALNT10,CLTC,PKM,GALNT1,MPI,PIGL,ACSL4,PDGFRB,AKT1,NT5M,PRKAA1,EGFLAM,ACOT7,PARK7,ENO1,SLC2A6,GNAI3,POMT1,COL11A1,SLC35A3,DPM2,HACD1,FBXO27,ITM2C,PEMT,HAS2,PIGK,DPM1,TMSB4X,NME6,PIGB,SLC35C1,CHST11,HS2ST1	darkgreen	EC_M4
GO:1905114	3.01E-03	0.997986594	83	448	cell surface receptor signaling pathway involved in cell-cell signaling	BP	1.00E-01	LRP1,RSPO3,CBY1,GNAQ,CDK14,VCP,UNC13B,RIMS1,TPBG,SHANK1,MDFIC,GLI3,DKKL1,VPS35,PLEKHA4,FGF2,GLI2,TGFB1,RAB3GAP1,CTNNB1,ZEB2,ZNF703,ILK,RYK,PRICKLE2,SULF1,CTNND1,DKK3,P2RX4,APP,GLRB,CACNB3,CPE,AMFR,PKD2,NKD1,BRD7,CHRNA7,CDC73,CCND1,CAPRIN2,LRP5,GSK3B,LEF1,KPNA1,BICC1,FGFR2,FZD9,ATP6AP2,FZD2,MPP2,IGFBP2,SMURF1,TNIK,DAAM1,OTUD5,CHRNE,NLGN2,STX1B,TGFB1I1,COL1A1,PBXIP1,SOX30,SMARCA4,GSKIP,PIN1,AKT1,PRKAA1,NLK,NXN,DAPK3,TAX1BP3,PKD1,CSNK1G2,NPHP4,WLS,EDA,DACT3,INSYN1,MESD,WNT2,FGF10,MBD2	darkgreen	EC_M4
GO:0000291	3.96E-03	0.999349194	6	18	nuclear-transcribed mRNA catabolic process, exonucleolytic	BP	1.01E-01	EXOSC9,DIS3,EXOSC8,TTC37,DCP2,EXOSC2	darkgrey	EC_M8
GO:0070663	2.53E-03	0.998774688	28	153	regulation of leukocyte proliferation	BP	1.01E-01	TNFAIP3,PTK2,TNFRSF21,SYK,IL6,PELI1,TLR4,IL15,IL1A,IGF1,HHEX,ITCH,SH3RF1,MEF2C,IL7,ICOSLG,NFATC2,INPP5D,CD86,DLG1,SOS2,TNFRSF1B,ARG2,EFNB1,CHRNB2,LGALS9,CD46,BST2	turquoise	EC_M6
GO:0071044	3.97E-03	0.999534639	5	11	histone mRNA catabolic process	BP	1.01E-01	XRN1,TENT2,SSB,DCP2,EXOSC10	darkgrey	EC_M8
GO:0008542	2.54E-03	0.999366191	10	31	visual learning	BP	1.01E-01	KIT,RIC8A,NDRG4,TANC1,CREB1,HIF1A,RGS14,MTOR,CHRNB2,PIAS1	turquoise	EC_M6
GO:0008334	3.99E-03	0.999365487	6	16	histone mRNA metabolic process	BP	1.01E-01	XRN1,TENT2,CPSF2,SSB,DCP2,EXOSC10	darkgrey	EC_M8
GO:0051973	4.01E-03	0.999076662	8	30	positive regulation of telomerase activity	BP	1.01E-01	CCT2,MAP3K4,POT1,CCT4,NEK2,XRCC5,AURKB,NAF1	darkgrey	EC_M8
GO:0045722	3.06E-03	0.999596349	6	12	positive regulation of gluconeogenesis	BP	1.02E-01	PPARA,DGAT2,KAT2B,PPARGC1A,PTPN2,NNMT	darkgreen	EC_M4
GO:0150076	2.59E-03	0.99947108	8	24	neuroinflammatory response	BP	1.03E-01	IL6,CST7,CTSC,IGF1,CD200,ADCY1,TNFRSF1B,PLCG2	turquoise	EC_M6
GO:0002183	4.08E-03	0.999057629	8	30	cytoplasmic translational initiation	BP	1.03E-01	EIF3E,METTL3,DHX29,NCK1,EIF3A,EIF3B,EIF4A1,EIF5	darkgrey	EC_M8
GO:0072012	2.60E-03	0.999474843	8	23	glomerulus vasculature development	BP	1.03E-01	PDGFB,TEK,GPR4,ANGPT2,CD34,PECAM1,CFLAR,NOTCH1	turquoise	EC_M6
GO:0002279	2.60E-03	0.999278146	11	39	mast cell activation involved in immune response	BP	1.03E-01	KIT,SYK,PIK3CG,GATA2,GAB2,UNC13D,SNAP23,FGR,FES,LGALS9,STXBP1	turquoise	EC_M6

GO:0099518	3.09E-03	0.998801688	18	65	vesicle cytoskeletal trafficking	BP	1.03E-01	AP3S1,DYNC1I1,KIF5B,TRAK1,ACTN4,AP3B1,KIF1A,TANC2,HTT,MYO5A,HAP1,MYO1E,MYO1D,MYO5B,MYO1C,PAFAH1B1,VAMP7,BLOC1S2	darkgreen	EC_M4
GO:0072160	2.62E-03	0.999649581	6	13	nephron tubule epithelial cell differentiation	BP	1.03E-01	MTSS1,GATA3,MEF2C,WWTR1,STAT1,PROM1	turquoise	EC_M6
GO:0008217	2.63E-03	0.998804435	24	122	regulation of blood pressure	BP	1.03E-01	LVRN,PDGFB,ARHGAP42,PRCP,NOS3,PPARG,EDNRB,KCNK6,P2RX2,PDE4D,VEGFC,GUCY1A1,NAV2,EDN1,CD34,F11R,ACE,RAMP2,AR,NPR1,ADRB2,NOS2,PTGS1,ERAP2	turquoise	EC_M6
GO:0045595	3.12E-03	0.997599815	191	1153	regulation of cell differentiation	BP	1.04E-01	IL23A,LRP1,AHI1,RAB21,PLXNB2,FBN2,PPARA,HLA-DRA,PPARD,CTNNA1,LEP,RORB,SRF,VEGFA,RUNX2,CLOCK,REST,TGFBR1,SOCS5,RTN4,AKIRIN1,ACTR2,GLI3,ROCK2,INHBA,RRAS,MYLK3,DKKL1,ITGB1,PLXNA1,FGF2,RB1,PTCH2,TENM4,GLI2,MEGF8,SMARCD3,RHEB,PAK1,TGFB1,AXL,CTNNB1,IL15RA,TRAK1,LBH,ZEB2,RARB,ACTN4,CRIM1,MAFG,SPRED3,LIG4,ZNF703,SEMA3C,ILK,SPINK5,RYK,RNF6,CXCL12,ZNF536,EIF4G2,ROBO1,MAP1B,DIP2B,SEMA4F,LOXL3,HEY1,TLX2,APP,DHX36,RAPGEF2,CX3CL1,ABCC8,PTPN11,RUNX1T1,OPA1,LOXL2,AP3B1,ZFYVE27,ACVR1,GDF6,ADA,SPP1,RUNX1,HIF1AN,SEMA4G,RAB7B,EIF4E,ADIPOR1,PHLDB2,FNDC5,NOTCH2,PKP2,CDC73,DNM1L,CCND1,CAPRIN2,TGFB2,LRP5,FEZ1,GSK3B,MMP14,DAG1,LEF1,CORO1C,PRKCA,CMKLR1,KRAS,NTRK3,TCTA,SLAMF8,BMP7,IDH2,FGFR2,OSM,PHLDB1,SYAP1,RPS6KA3,MAPT,SERPINE1,TNR,FN1,PTPN6,GPR137,FBN1,ITGAV,ADRA2C,ARPC2,SUCO,PRKCI,COL5A2,SKIL,NBL1,VCL,WDR1,SIX4,OBSL1,HAP1,PRTG,BMPR1A,PDLIM7,CDK12,MED1,ARHGEF2,TGFB1I1,COL1A1,TGFB3,RAB11A,MAP2K1,TMEM100,DICER1,MORF4L2,NPTN,SETD3,MYOCD,ISLR2,PIN1,TRPV2,CD74,FBXO22,ROCK1,AKT1,TMEM98,SUPT6H,PTPN2,VASN,PLXNB3,PAFAH1B1,FLNA,ATP6AP1,PLXNA3,COL5A1,GLG1,PRMT6,FSTL3,SERPINF1,LOX,LBX1,GDNF,THY1,HAS2,FGL2,DACT3,GLIPR2,FAM210B,YWHAH,BHLHE41,HOPX,OLFM1,WNT2,FGF10	darkgreen	EC_M4
GO:0050927	3.13E-03	0.999373668	8	20	positive regulation of positive chemotaxis	BP	1.04E-01	VEGFA,CXCL12,NTRK3,ITGA2,CDH13,F3,ARTN,FGF10	darkgreen	EC_M4
GO:0035025	2.66E-03	0.99946635	8	22	positive regulation of Rho protein signal transduction	BP	1.04E-01	GPR4,NET1,PRAG1,ARHGEF3,F11R,RAC1,ADGRG1,LPAR6	turquoise	EC_M6
GO:0032793	2.66E-03	0.999552522	7	17	positive regulation of CREB transcription factor activity	BP	1.04E-01	ADGRF1,PRKD2,CD200,ADCY1,PRKD1,RPS6KA5,RELN	turquoise	EC_M6
GO:0002460	2.66E-03	0.998650087	31	173	adaptive immune response based on somatic recombination of immune receptors built from immunoglobulin superfamily domains	BP	1.04E-01	TNFAIP3,IL6,BACH2,RSAD2,TLR4,C5,TRPM4,CTSC,NECTIN2,UNC13D,GATA3,JAG1,GAPT,MEF2C,NFKB2,HLX,INPP5D,B2M,CRACR2A,WAS,RAB27A,TNFRSF1B,MTOR,SEMA4A,BATF,CD19,IL7R,TRAF2,NOTCH1,CD46,FCER2	turquoise	EC_M6
GO:0008344	2.67E-03	0.999105202	14	56	adult locomotory behavior	BP	1.04E-01	OXR1,AGTPBP1,TMOD1,PPT1,SPTBN4,CHD7,PREX2,CLCN3,NR4A2,SNCA,INPP5F,HOXD10,SNCG,HOXB8	turquoise	EC_M6

GO:0006810	2.68E-03	0.997797827	406	3300	transport	BP	1.04E-01	TNFRSF11A,KIF5A,STX11,TBC1D30,LAPTM4B,SNCAIP,SNX2,SNX24,ZDHHC14,SYNJ2,SERAC1,RIMS2,CDC42SE2,QKI,PACSIN2,TRDN,FAM91A1,KLHL3,SGSM3,PTK2,FAM53C,DENND3,TSNARE1,DAPK1,PDGFB,AGTPBP1,CSNK1E,PICK1,MICALL1,PIM1,TBC1D22B,IREB2,ATP8A1,APBA1,TJP2,UBAP1,KIT,CD2AP,SYK,TRAM2,RGP1,ICA1,TSPAN13,SLC3A1,IL6,IGF2BP3,SNAP91,PPT1,CXCL8,BACH2,VPS54,ARHGAP25,ELMO1,RSAD2,CAV2,GRB10,TLR4,TCAF2,GRIK2,GJA4,IL15,TBC1D9,SDCBP,TRPM4,NRP1,RAB3B,OSBPL9,ANKRD50,PIK3CG,ITSN2,MTMR2,GATA2,KIF3C,ARHGAP21,KCNRG,RAB38,EXOC3L2,TMCC1,GGA3,GAB2,LLGL2,RALB,USP6NL,UNC13D,ZFAND6,PPARG,GATA3,TBC1D4,MYO7A,CXCR4,CD93,SEC14L1,EDNRB,NDFIP2,TMC8,SPTBN4,SLCO2B1,CYTH1,P2RY6,P2RY2,FCHSD2,GPAT4,SFRP1,FERMT1,KCNK6,ATP11A,ARAP3,P2RX2,SLC4A11,CD36,SIRT3,SLCO2A1,ADAMTS9,SDCBP2,PRR5L,MICU3,SCARB1,PDE4D,MERTK,GRAMD1A,CHD7,FAM3D,IL1A,IGF1,NR4A1,HHEX,EXOC6,ERBIN,PITPNM2,CLCN3,SLCO5A1,HIP1R,OCLN,MCTP1,CHRM5,KIF13B,MEF2C,CP,TLR2,P2RX7,TPD52,VEGFC,NDRG4,GPM6A,CXCL10,TMEM144,LMBR1L,PLLP,BMP2K,SERINC5,TMEM150C,MTX3,DYSF,TPCN1,PLCG1,STC1,RAPGEF3,KCNJ3,AP4B1,STAB1,SLC38A1,ANO6,ZDHHC13,CLIC6,ZC3H11A,IFIT1,DOP1B,EDN1,KCNJ15,EHD4,SNCA,DTNBP1,ANO5,MFSD4A,SLC45A3,ANO3,PM20D1,MX2,ABCG1,CD200,MTTP,SIK1,HDAC1,LCK,PPP3CA,HYAL2,ANO1,SLC16A14,HECW2,SLC39A8,KCNJ2,MEF2A,SLC12A7,SCN3A,RFTN2,MCTP2,RIN3,ZDHHC6,TINAGL1,ADPRH,SNAP23,SLC7A7,LYST,LAPTM5,NCF1,SEC24B,LRMP,SLC9A8,FABP4,PABPN1,HCLS1,AP	turquoise	EC_M6
GO:0098581	2.68E-03	0.999729034	5	10	detection of external biotic stimulus	BP	1.04E-01	TLR4,SCARB1,TLR2,TLR6,TLR1	turquoise	EC_M6
GO:0016556	4.13E-03	0.999168049	7	24	mRNA modification	BP	1.04E-01	PUS7,ZC3H13,METTL3,VIRMA,NSUN2,METTL16,CMTR2	darkgrey	EC_M8
GO:1905049	3.15E-03	0.999693045	5	9	negative regulation of metallopeptidase activity	BP	1.04E-01	LRP1,PICALM,TIMP1,MIR24-1,TIMP3	darkgreen	EC_M4
GO:0034103	2.72E-03	0.999087936	14	57	regulation of tissue remodeling	BP	1.04E-01	TNFRSF11A,TNFAIP3,FLT4,SYK,IL6,IL15,SFRP1,P2RX7,THBS4,PPP3CA,INPP5D,SYT7,S1PR1,CA2	turquoise	EC_M6
GO:0002683	2.72E-03	0.998412829	47	290	negative regulation of immune system process	BP	1.04E-01	TNFAIP3,C6orf106,ADGRF5,TNFRSF21,SYK,PELI1,TLR4,C5,GATA2,CST7,LRCH1,PPARG,SEC14L1,SFRP1,TMEM178A,PLK2,MERTK,ITCH,OAS3,NLRC5,PLCG1,DGKZ,CD200,RIN3,LAPTM5,HLX,INPP5D,CD86,FGR,DLG1,APOD,LTF,ADORA2A,ARG2,GPR68,MMP28,CCL2,YES1,LGALS9,IL7R,ISG15,NRARP,OAS1,CD46,SERPINB9,BST2,RNF125	turquoise	EC_M6
GO:0016051	3.18E-03	0.998333926	34	155	carbohydrate biosynthetic process	BP	1.05E-01	DYRK2,PPARA,LEP,CHST10,NANS,TGFB1,DGAT2,AKT2,KAT2B,IRS2,B3GNT4,GYG1,SORBS1,SELENOS,GYG2,GSK3B,B3GALT1,PPP1R3D,ATF3,TPI1,PPP1R3F,CHST12,PPARGC1A,CLTC,AKT1,PTPN2,PGP,FAM3A,AGL,RANBP2,HAS2,NNMT,CHST11,HS2ST1	darkgreen	EC_M4
GO:1903510	3.20E-03	0.998712538	19	71	mucopolysaccharide metabolic process	BP	1.05E-01	ANGPT1,FGF2,GLB1,CHPF2,TGFB1,DCN,IMPAD1,B3GNT4,CHST2,CHSY1,HYAL1,UGDH,CHST12,XYLT2,CLTC,AKT1,EGFLAM,HAS2,CHST11	darkgreen	EC_M4
GO:0030838	2.74E-03	0.998923319	18	82	positive regulation of actin filament polymerization	BP	1.05E-01	BAIAP2L2,PICK1,CDC42EP1,FCHSD2,FCHSD1,LMOD3,HIP1R,IQGAP2,AP1AR,DLG1,BAIAP2L1,WAS,MTOR,SPIRE1,CARMIL2,VASP,GMFG,ARPC5L	turquoise	EC_M6
GO:0050790	3.22E-03	0.997411062	288	1823	regulation of catalytic activity	BP	1.05E-01	SERPINB2,IL23A,NAB2,LRP1,ARHGEF25,AGAP2,TAB2,TBC1D9B,CAND1,PRRC1,DENND6B,AZIN1,PLXNB2,ANGPT1,TCP1,ROS1,NUS1,SERINC1,SIL1,C6orf89,MTCH1,GNAQ,ARHGAP39,LEP,CDKN2B,PLAA,BAG1,NUAK1,VEGFA,DOCK8,VCP,TPM2,NCK2,REST,SNX13,SOCS5,RIMS1,PSME4,CYCS,MAP3K7,SH3BP5L,KIDINS220,ROCK2,BVES,RCN3,ITGB1,FKBP1B,PLXNA1,FGF2,NAPA,DNAJC1,ARHGEF4,RB1,ABHD5,EED,PICALM,MAPRE3,EIF2B3,PAK1,TGFB1,AXL,RAB3GAP1,CTNNB1,SERPINH1,AKT2,SEC23B,ZEB2,PLEKHG2,TBC1D16,FARP1,ARHGEF17,OSBPL8,PSD2,PPP1R12A,KAT2B,CRIM1,STIM1,GPS1,SPINT2,IRS2,PRNP,STARD13,CCKBR,DDRGK1,SMPD1,ILK,MMP17,HSPH1,ARL6IP5,PPP2R2B,SPINK5,RYK,PSMD6,ST5,ARL1,HSP90B1,TMED2,ATP1B3,ROBO1,PINX1,PFN2,RASA1,CCNJ,RTKN,CACNA1D,MMP15,APP,RAPGEF2,MAPKAPK5,CX3CL1,PPP2R2A,PTX3,RALGAPB,ARHGAP1,GTF2H1,AP3B1,PTPN22,TSG101,DKC1,CRY2,CHP1,ATP2B4,PKD2,PLA2R1,DBNDD2,CBLB,IFT57,TANK,PSMD14,BRSK2,SLC39A10,CHRNA7,RAP1GDS1,DOCK10,DYNLL1,AZIN2,PPP1R12B,DNM1L,UCHL5,CCND1,TGFB2,PPP6R3,PSMD1,PRKAB2,LRP5,ARHGAP31,HTR2B,PRKAR1A,GSK3B,DAG1,PIK3CA,CORO1C,ANOS1,ISCU,NEK7,ADAP1,KRAS,NTRK3,SGSM1,SLAMF8,FAM162A,SPATC1L,BMP7,PPP1R11,WDTC1,COL6A3,FGFR2,NCSTN,SYAP1,PSMD2,ADRM1,IQGAP1,FARP2,RPS6KA3,EGLN3,HSPB1,LINC02210-CRHR1,MAPT,SEC23A,SERPINE1,RASAL2,DNAJC10,PCOLCE,FN1,PTPN6,ZC3H15,PDCD10,ADRA2C,DOK7,AOC3,HTT,FGFR3,DDOST,TIMP1,MYO9B,MNAT1,B3GAT3,PPP2R5E,PPP1R3F,PPP1R36,CACNA1C,MAGE	darkgreen	EC_M4

GO:0072007	2.77E-03	0.999820643	4	7	mesangial cell differentiation	BP	1.06E-01	PDGFB,GPR4,CD34,NOTCH1	turquoise	EC_M6
GO:0001934	2.79E-03	0.998134383	82	543	positive regulation of protein phosphorylation	BP	1.06E-01	TNFRSF11A,FLT4,TPD52L1,PTK2,PDGFB,TEK,LIMCH1,TEC,KIT,KDR,SYK,SEMA4D,IL6,TLR4,IL15,CCNY,SDCBP,NRP1,PIK3CG,TAL1,PRKD2,CAB39L,RALB,PPARG,EDNRB,HBEGF,CD36,PRR5L,RET,IGF1,VEGFC,MADD,THBS4,RAPGEF3,NEDD9,EDN1,EHD4,SNCA,HDAC1,NCF1,HCLS1,PECAM1,CD86,PROX1,PRKD1,ACE,LIMK2,PAK2,DLG1,MAP3K11,LTF,DDX3X,TBX1,FGF23,RAC1,ADIPOR2,TLR6,MTOR,PSEN1,FGF18,PGF,TAOK2,EFNA1,PIK3R5,RPS6KA5,ADRB2,YES1,SH2D3A,UNC119,TRAF2,PLCG2,ENG,SH2D3C,DAB2IP,RELN,ALKAL1,FGF16,MMD,DAZAP2,COPS8,BMP2,BMP6	turquoise	EC_M6
GO:0061138	3.27E-03	0.998303253	33	147	morphogenesis of a branching epithelium	BP	1.06E-01	RSPO3,SRF,VEGFA,GLI3,PLXNA1,FGF2,GLI2,PAK1,CTNNB1,SPINT2,SEMA3C,ILK,TMED2,SULF1,RDH10,ACVR1,PKD2,KDM5B,LRP5,MMP14,DAG1,LEF1,KRAS,BMP7,FGFR2,CLIC4,SIX4,MAGED1,MED1,PKD1,GDNF,WNT2,FGF10	darkgreen	EC_M4
GO:0050768	3.28E-03	0.998482606	25	102	negative regulation of neurogenesis	BP	1.06E-01	CTNNA1,REST,RTN4,CTNNB1,SEMA3C,RYK,RNF6,DIP2B,SEMA4F,TLX2,RAPGEF2,ABCC8,SPP1,SEMA4G,NTRK3,BMP7,IDH2,TNR,PRTG,BMPR1A,DICER1,TMEM98,PLXNA3,THY1,YWHAH	darkgreen	EC_M4
GO:1901019	3.28E-03	0.998713881	18	67	regulation of calcium ion transmembrane transporter activity	BP	1.06E-01	UBQLN1,FKBP1B,STIM1,REM1,CACNB3,PKD2,CASQ1,LINC02210-CRHR1,HTT,HAP1,STIM2,CALM1,VMP1,JPH3,DMD,FKBP1A,CBARP,GEM	darkgreen	EC_M4
GO:0043112	2.80E-03	0.998677	26	131	receptor metabolic process	BP	1.06E-01	PICK1,SYK,CXCL8,SDCBP,MTMR2,CD36,ITCH,SNCA,MYLIP,RIN3,LAPTM5,AP1AR,INPP5F,CALCRL,RAMP2,DNM3,SELE,LMTK2,OPHN1,PSEN1,DNM2,AP2B1,PLCG2,VAMP3,CACNG5,GSG1L	turquoise	EC_M6
GO:0006820	2.81E-03	0.998311331	52	319	anion transport	BP	1.06E-01	TNFRSF11A,APBA1,SYK,SLC3A1,TCAF2,LLGL2,SLCO2B1,P2RY6,SLC4A11,CD36,SLCO2A1,CLCN3,SLCO5A1,SERINC5,STC1,SLC38A1,ANO6,CLIC6,EDN1,SNCA,ANO5,ANO3,MTTP,ANO1,SLC16A14,SLC39A8,SLC12A7,SLC7A7,SLC9A8,SLC16A9,SLCO4A1,ACE,ADORA2A,SLC9A7,WNK4,FGF23,SLC2A9,SLC16A13,MTOR,SLC26A1,PSEN1,SLC51B,CA4,NOS2,SLC43A2,SLC25A25,STXBP1,LRRC8C,LRRC8D,CA2,SLC25A21,RGS4	turquoise	EC_M6
GO:0001822	2.81E-03	0.998392776	44	248	kidney development	BP	1.06E-01	MTSS1,KLHL3,PDGFB,PODXL,TEK,NRP1,GPR4,PLXND1,ITGA8,GATA3,GZF1,JAG1,SFRP1,FREM2,RET,SMAD1,MEF2C,WWTR1,ANGPT2,TIPARP,STAT1,HYAL2,PRKX,CD34,PECAM1,CFLAR,PROX1,VANGL2,LAMA5,ACE,DLG1,WNK4,PROM1,ARG2,ALDH1A2,HOXB7,ITGA3,SMAD2,AP2B1,NOTCH1,CA2,SOX17,BMP2,BMP6	turquoise	EC_M6
GO:0014033	2.83E-03	0.99888914	18	77	neural crest cell differentiation	BP	1.07E-01	SEMA6A,SEMA4D,NRP1,EDNRB,JAG1,SFRP1,RET,MEF2C,EDN1,SEMA3F,LAMA5,SEMA6D,TBX1,HIF1A,ALDH1A2,EFNB1,SEMA4A,SEMA6B	turquoise	EC_M6
GO:0033002	3.31E-03	0.998207764	37	174	muscle cell proliferation	BP	1.07E-01	ANGPT1,FAM19A5,PPARD,TGFBR1,AKIRIN1,FGF2,TENM4,PAK1,TGFB1,CTNNB1,ILK,CX3CL1,PTPN11,MMP2,RUNX1,TGFB2,PRKAR1A,KPNA1,FGFR2,COMT,MNAT1,BMPR1A,PPARGC1A,TPM1,TGFB3,CNN1,MYOCD,PDGFRB,AKT1,ITGA2,NPR3,IRAK1,GNAI3,CDH13,CDKN1A,PRKG1,WNT2	darkgreen	EC_M4
GO:1903170	3.32E-03	0.999148101	10	29	negative regulation of calcium ion transmembrane transport	BP	1.07E-01	UBQLN1,FKBP1B,REM1,CACNB3,PKD2,LINC02210-CRHR1,CALM1,AKT1,CBARP,GEM	darkgreen	EC_M4
GO:0050779	4.28E-03	0.99832667	16	82	RNA destabilization	BP	1.07E-01	PAN2,TNRC6B,TUT7,PNPT1,EXOSC9,DIS3,METTL3,EXOSC8,PAN3,PDE12,CNOT1,HNRNPR,DCP2,METTL16,EXOSC2,HNRNPD	darkgrey	EC_M8
GO:0070647	4.31E-03	0.996950684	99	806	protein modification by small protein conjugation or removal	BP	1.07E-01	MEX3C,USP15,SHPRH,FBXO43,FBXO5,UBR5,UHRF2,UBR2,TOPORS,TRIM28,ZNF451,FBXO11,SENP6,ASB3,USP34,COMMD1,UFL1,CUL1,USP45,SAE1,PAXIP1,CDC20,GTPBP4,RAD18,TPP2,CUL4A,CDC16,LNX2,ANAPC1,UBA2,TNKS2,RNF141,OTUD4,ZFP91,USP25,HLTF,ANAPC7,LTN1,USP16,SENP1,ABRAXAS1,TRIM33,UBE2C,HERC5,UBE3A,CUL3,UBE2T,ASB7,TRAIP,COPS7B,UBR1,FANCI,DTL,UBR3,G2E3,CDK1,IVNS1ABP,CDC27,FANCM,LRR1,COP1,BRCA1,CUL5,CDCA3,DCUN1D5,DCAF6,LEO1,UBE2K,NEDD4,MED6,USP3,TAF1,OGT,RLIM,RBBP6,TRIM37,PLK1,APPBP2,PSMD10,MIB1,COPS3,CUL4B,XIAP,MYSM1,UHRF1,BLMH,CCNF,GLMN,USP33,MAD2L1,SENP7,OTUD6B,RNF139,RNF8,TRIM59,GID4,UBXN2A,RNF219,RCHY1	darkgrey	EC_M8
GO:0046824	4.31E-03	0.998596212	12	54	positive regulation of nucleocytoplasmic transport	BP	1.07E-01	UBR5,TRIM28,ANP32B,IPO5,XPO4,KHDRBS1,CDK1,DHX9,CTDSPL2,TPR,ECT2,NEDD4	darkgrey	EC_M8
GO:0000459	4.31E-03	0.999620691	4	9	exonucleolytic trimming involved in rRNA processing	BP	1.07E-01	EXOSC9,EXOSC8,EXOSC10,EXOSC2	darkgrey	EC_M8

GO:0000467	4.31E-03	0.999620691	4	9	exonucleolytic trimming to generate mature 3'-end of 5.8S rRNA from tricistronic rRNA transcript (SSU-rRNA, 5.8S rRNA, LSU-rRNA)	BP	1.07E-01	EXOSC9,EXOSC8,EXOSC10,EXOSC2	darkgrey	EC_M8
GO:0006935	3.35E-03	0.997768643	79	426	chemotaxis	BP	1.08E-01	LRP1,SLC12A2,PLXNB2,ANGPT1,CXCL14,FAM49B,VEGFA,CREB3,TPBG,AKIRIN1,GLI3,PLXNA1,FGF2,KIF5B,GLI2,MEGF8,TGFB1,SEMA3C,RYK,CXCL12,PALLD,ROBO1,SEMA4F,P2RX4,APP,CX3CL1,SEMA4G,ELMO2,NOTCH2,DNM1L,SBDS,TGFB2,FEZ1,DAG1,LEF1,ANOS1,CMKLR1,NTRK3,SLAMF8,BMP7,MACO1,SRP54,HSPB1,SERPINE1,TNR,ITGAV,NBL1,PLD1,CRMP1,NOTCH3,ENAH,NGFR,DOCK2,MAP2K1,TTC8,NPTN,CD74,LAMA3,PDGFRB,USP14,ITGA2,PTPN2,PLXNB3,NTN3,PLXNA3,BSG,CDH13,F3,NEXN,CCL16,DPYSL5,LOX,VSTM2L,GDNF,CHN1,ARTN,TMSB4X,CCR6,FGF10	darkgreen	EC_M4
GO:1903169	3.36E-03	0.998414047	26	110	regulation of calcium ion transmembrane transport	BP	1.08E-01	UBQLN1,FKBP1B,STIM1,PRNP,REM1,CX3CL1,CACNB3,PKD2,CASQ1,LINC02210-CRHR1,PTPN6,HTT,HAP1,CACNA1C,STIM2,CALM1,VMP1,AKT1,JPH3,DMD,FKBP1A,CBARP,TMEM38B,THY1,TMEM38A,GEM	darkgreen	EC_M4
GO:0016050	3.37E-03	0.99798082	51	255	vesicle organization	BP	1.08E-01	STX7,SEC24A,UNC13B,VPS41,KIF5B,RAB7A,PICALM,PDCD6IP,SEC13,AKT2,SEC23B,ZEB2,STAM2,TMEM127,VPS37A,TMED2,OSBP,VAMP8,USO1,AP3B1,TSG101,VPS18,AKTIP,CHP1,VPS39,RAB7B,SEC22B,CORO1C,SHROOM2,VAPB,AP2M1,SEC23A,PRKCI,PTPRN,SLC2A4,STX1B,SNX11,SYT11,TMED10,RAB11A,SOX30,RAB34,PAFAH1B1,VAMP7,GNAI3,AP1G1,CCDC136,SPINK2,SNX10,BLOC1S2,STXBP6	darkgreen	EC_M4
GO:0060349	3.38E-03	0.998597521	20	76	bone morphogenesis	BP	1.08E-01	NAB2,RUNX2,RAB23,GLI3,MEGF8,SERPINH1,RARB,IMPAD1,CHSY1,LRP5,MMP14,FGFR2,COMP,FGFR3,THBS3,COL1A1,TGFB3,ANXA6,GLG1,HAS2	darkgreen	EC_M4
GO:0002262	2.88E-03	0.998633623	26	134	myeloid cell homeostasis	BP	1.08E-01	L3MBTL3,IREB2,ADGRF5,KIT,IL6,PTBP3,TAL1,GATA2,GATA3,MERTK,STAT1,JAM3,ALAS1,ETS1,DYRK3,HCLS1,INPP5D,B2M,HIF1A,ITPKB,HOXB6,SCNN1B,RCOR1,PDE4B,ISG15,ARID4A	turquoise	EC_M6
GO:0032535	2.89E-03	0.998245176	54	325	regulation of cellular component size	BP	1.08E-01	SEMA6A,CDKL3,ARHGAP18,BAIAP2L2,PICK1,CDC42EP1,SEMA4D,TMOD1,FAM49A,NRCAM,NRP1,RAB3B,SVIL,SPTBN4,FCHSD2,FCHSD1,LMOD3,RET,CLCN3,HIP1R,P2RX7,USH1C,IQGAP2,ANO6,EDN1,SLC12A7,SEMA3F,RIN3,ESAM,HCLS1,AP1AR,TMOD4,DLG1,CREB1,SEMA6D,BAIAP2L1,WAS,TMOD2,RAC1,MTOR,SEMA4A,DNM2,ARHGAP28,IL7R,SPIRE1,SEMA6B,ARHGAP4,CARMIL2,VASP,GMFG,KANK3,SHANK3,GBA2,ARPC5L	turquoise	EC_M6
GO:1903409	2.89E-03	0.999121142	12	47	reactive oxygen species biosynthetic process	BP	1.08E-01	TLR4,CD36,CLCN3,SNCA,NCF1,CFLAR,RAB27A,TLR6,ARG2,CYP1A1,SLC25A33,PLCG2	turquoise	EC_M6
GO:2000251	2.90E-03	0.999568708	6	16	positive regulation of actin cytoskeleton reorganization	BP	1.08E-01	BAIAP2L2,TEK,NRP1,HCLS1,FES,BAIAP2L1	turquoise	EC_M6
GO:0080135	4.36E-03	0.99710019	71	553	regulation of cellular response to stress	BP	1.08E-01	BCLAF1,DDX39B,FBXO5,UBR5,MAP3K4,POT1,POLH,TRIM28,PNPT1,ZMPSTE24,ETAA1,UFL1,DHFR,DNAJC2,CHORDC1,RNASEH2B,DDIAS,PAXIP1,ULK4,RIF1,CUL4A,PPP4R2,PRKDC,ERCC6,PARPBP,NCK1,TAF9,ATR,USP25,PBK,RBL1,ABRAXAS1,RAD51,SLC38A2,RIPK1,ARID2,EEF1E1,TAOK3,DEK,CHEK1,AJUBA,FXR1,DDX5,FANCB,RPA2,AUNIP,PDE8A,DHX9,SETD2,LANCL1,BRCA1,NSD2,LETM1,RAD51AP1,ANKLE1,FOXM1,EIF2AK1,DDX11,DNAJC7,RAD52,FUS,PSMD10,THOC1,SMCHD1,RSL1D1,TAOK1,RMI2,FMR1,TIGAR,ERP29,RNF8	darkgrey	EC_M8
GO:0035904	3.41E-03	0.99880637	15	50	aorta development	BP	1.09E-01	LRP1,LEP,SRF,SNX17,ADAMTS6,ROBO1,HEY1,PKD2,TGFB2,FKBP10,BMPR1A,NGFR,PDGFRB,LOXL1,LOX	darkgreen	EC_M4
GO:1900180	3.42E-03	0.998356147	27	116	regulation of protein localization to nucleus	BP	1.09E-01	ANGPT1,TCP1,CTNNA1,LEP,RAB23,MDFIC,GLI3,TGFB1,UFM1,HDAC3,DCLK1,PINX1,CCT8,DKC1,CHP1,GSK3B,LARP7,TFRC,MAGED1,NGFR,PIN1,AKT1,TMEM98,FLNA,PARK7,ZPR1,ORMDL3	darkgreen	EC_M4
GO:0033692	3.42E-03	0.998796361	15	52	cellular polysaccharide biosynthetic process	BP	1.09E-01	DYRK2,AKT2,IRS2,B3GNT4,GYG1,SORBS1,SELENOS,GYG2,GSK3B,PPP1R3D,PPP1R3F,AKT1,AGL,HAS2,HS2ST1	darkgreen	EC_M4
GO:0006022	3.43E-03	0.998438067	24	98	aminoglycan metabolic process	BP	1.09E-01	B4GALT7,ANGPT1,FGF2,GLB1,CHPF2,TGFB1,DCN,IMPAD1,B3GNT4,CHST2,CHSY1,HYAL1,B3GAT3,UGDH,CHST12,XYLT2,CLN6,CLTC,PDGFRB,AKT1,EGFLAM,HAS2,CHST11,HS2ST1	darkgreen	EC_M4

GO:0019318	3.44E-03	0.998101611	39	185	hexose metabolic process	BP	1.09E-01	DYRK2,PPARA,PPARD,LEP,GALT,GLB1,DGAT2,AKT2,PFKP,C1QTNF1,KAT2B,IRS2,SORBS1,HECTD4,PFKM,ADIPOR1,SELENOS,CPT1A,LRP5,GSK3B,PIK3CA,WDTC1,MAN2A2,PPP1R3D,ATF3,TPI1,PPP1R3F,PPARGC1A,MPI,AKT1,PRKAA1,PTPN2,OMA1,PGP,MIDN,FAM3A,ENO1,RANBP2,NNMT	darkgreen	EC_M4
GO:0008064	2.94E-03	0.998522122	30	159	regulation of actin polymerization or depolymerization	BP	1.09E-01	ARHGAP18,BAIAP2L2,PICK1,CDC42EP1,TMOD1,FAM49A,SVIL,SPTBN4,FCHSD2,FCHSD1,LMOD3,HIP1R,IQGAP2,ESAM,HCLS1,AP1AR,TMOD4,DLG1,BAIAP2L1,WAS,TMOD2,MTOR,ARHGAP28,SPIRE1,CARMIL2,VASP,GMFG,KANK3,GBA2,ARPC5L	turquoise	EC_M6
GO:0014902	2.94E-03	0.998802924	19	86	myotube differentiation	BP	1.09E-01	CAV2,ATP11A,P2RX2,LMOD3,IGF1,MEF2C,CXCL10,TANC1,SIK1,PPP3CA,BCL9,NFATC2,PLEKHO1,CFLAR,MYEF2,TBX1,MTOR,NOTCH1,PIEZO1	turquoise	EC_M6
GO:0031050	4.42E-03	0.99864858	11	46	dsRNA processing	BP	1.09E-01	TUT7,HNRNPA2B1,PUS10,ZMPSTE24,METTL3,RIPK1,DDX5,DGCR8,ZC3H7A,DROSHA,SRRT	darkgrey	EC_M8
GO:0035196	4.42E-03	0.99864858	11	46	production of miRNAs involved in gene silencing by miRNA	BP	1.09E-01	TUT7,HNRNPA2B1,PUS10,ZMPSTE24,METTL3,RIPK1,DDX5,DGCR8,ZC3H7A,DROSHA,SRRT	darkgrey	EC_M8
GO:0070918	4.42E-03	0.99864858	11	46	production of small RNA involved in gene silencing by RNA	BP	1.09E-01	TUT7,HNRNPA2B1,PUS10,ZMPSTE24,METTL3,RIPK1,DDX5,DGCR8,ZC3H7A,DROSHA,SRRT	darkgrey	EC_M8
GO:0043928	4.43E-03	0.999426535	5	13	exonucleolytic catabolism of deadenylated mRNA	BP	1.09E-01	EXOSC9,DIS3,EXOSC8,DCP2,EXOSC2	darkgrey	EC_M8
GO:0003007	3.48E-03	0.998031121	42	197	heart morphogenesis	BP	1.10E-01	AHI1,LY6E,SRF,VEGFA,TGFBR1,RTN4,MEGF8,SMARCD3,TGFB1,CTNNB1,SEMA3C,ILK,ANKRD1,TMED2,ROBO1,HEY1,CPE,ACVR1,PKD2,IFT57,NOTCH2,PKP2,TGFB2,HTR2B,BMP7,FGFR2,FZD2,SYNPO2L,BMPR1A,TCAP,MED1,FGFRL1,TPM1,TMEM100,FLNA,COL5A1,COL11A1,FKBP1A,LBX1,HAS2,OLFM1,WNT2	darkgreen	EC_M4
GO:0045911	4.46E-03	0.998830949	9	36	positive regulation of DNA recombination	BP	1.10E-01	MSH2,PAXIP1,RIF1,ERCC6,ARID2,FANCB,NSD2,RAD51AP1,FUS	darkgrey	EC_M8
GO:0061014	4.48E-03	0.99824296	16	82	positive regulation of mRNA catabolic process	BP	1.10E-01	PAN2,TNRC6B,TUT7,PNPT1,EXOSC9,DIS3,METTL3,EXOSC8,PAN3,PDE12,CNOT1,HNRNPR,DCP2,METTL16,EXOSC2,HNRNPD	darkgrey	EC_M8
GO:0014909	3.50E-03	0.998619721	18	67	smooth muscle cell migration	BP	1.10E-01	LRP1,FAM19A5,PPARD,PLXNA1,PAK1,ILK,IQGAP1,S100A11,ARPC5,SERPINE1,BMPR1A,PPARGC1A,TPM1,MYOCD,PDGFRB,ITGA2,HAS2,PRKG1	darkgreen	EC_M4
GO:0043043	1.91E-04	0.999880965	93	566	peptide biosynthetic process	BP	1.11E-01	PYM1,CDK4,EIF3H,RPS18,EEF1D,RPL3,EIF3L,EIF3D,RPL8,RPS5,RPL28,RPS9,EEF1A1,MALSU1,RPS7,RPS11,FOXO3,RPL18,ABTB1,RPS8,MIF4GD,MLH1,UNK,RPS3,RPS16,SAMD4B,MRPS12,RPL15,EIF3K,TARBP2,EIF3F,EIF4B,RPS3A,EIF2A,FARS2,RPLP2,PCIF1,WARS2,FASTKD3,MRPL36,DIS3L2,PUM1,APEH,RPS6KB2,QARS,DALRD3,EIF4H,PER2,RPS21,RPL35A,RPL11,YBX3,NFE2L2,MRPL49,EXOSC7,RPL27,UBA52,RARA,ZFP36L1,MRPL10,PER1,TNRC6A,LARP6,RPL7A,EEF2K,MRPL4,EIF3G,NDUFA7,RPL23A,PARN,MRPL37,EEF2,DPH1,MRPL41,MRPS34,INPP5E,MRPL28,RPL13,MRPS2,KARS,RPS15,AGO4,RACK1,RPS19,MRPL34,RPL23,EEF1B2,RPUSD3,MRPL57,RPS20,ZFP36L2,PDF,EXOSC5	cyan	EC_M2
GO:0051128	3.01E-03	0.997621571	247	1877	regulation of cellular component organization	BP	1.12E-01	SEMA6A,TBC1D30,LAPTM4B,CNOT6,SNCAIP,RIMS2,CELSR1,PACSIN2,CDKL3,MTSS1,ASAP1,ARHGAP18,PTK2,PDGFB,PODXL,BAIAP2L2,PICK1,CDC42EP1,TEK,LINGO2,LIMCH1,ATP8A1,FAM122A,KIT,KDR,CD2AP,SYK,SEMA4D,TMOD1,IL6,SNAP91,PPT1,CAV2,TLR4,SDCBP,FAM49A,NRCAM,NRP1,RASIP1,SVIL,ITSN2,TAL1,MTMR2,GATA2,PLXND1,RALB,USP6NL,PPARG,GATA3,TBC1D4,CXCR4,NET1,TMC8,SPTBN4,TIE1,SETD5,IKBKB,FCHSD2,PDE2A,SFRP1,HBEGF,FEZ2,FERMT1,EFNB2,ARHGEF7,SPEF1,FCHSD1,CD36,RASSF7,LMOD3,PRAG1,TCF15,EPHB1,PLK2,RET,IL1A,IGF1,ITCH,HIP1R,OCLN,MCTP1,KIF13B,HMBOX1,ENC1,MEF2C,TLR2,P2RX7,NDRG4,GPM6A,CXCL10,SEPT11,BMP2K,SCAF4,USH1C,PPP1R16B,RAPGEF3,IQGAP2,ANO6,CEP97,EDN1,DYRK1A,JAM3,EHD4,SNCA,MYLIP,SRGAP2C,ABCG1,ETS1,RASSF1,KIAA1551,PPP3CA,HYAL2,HECW2,SEMA3F,RIN3,ZDHHC6,ADPRH,ESAM,USP30,HCLS1,NFATC2,AP1AR,EAF2,PLEKHO1,PLXNA2,SH3BP4,CFLAR,INPP5F,TRAK2,FES,RPS6KA1,PROX1,TMOD4,SS18L1,PRKD1,F11R,IL10RA,ARHGDIB,ACE,ITGA6,LIMK2,PAK2,MELTF,DLG1,SIPA1,APOD,MOV10,CREB1,B2M,ZW10,TET1,SEMA6D,IL1RAP,DDX3X,IGFBP5,MCU,DNM3,CDA,MMP1,SELE,SELP,BAIAP2L1,PPM1F,WAS,TMOD2,PRKCH,RAC1,HIF1A,RCC2,ADIPOR2,FSCN1,EPHA2,BID,TLR6,TNFRSF1B,MTOR,AR,OPHN1,PSEN1,SEMA4A,ABI3,ITGA3,TAOK2,ARHGEF15,EFNA1,CHRNB2,ZDHHC15,ADCK1,RABEP2,NPR1,DNM2,YY1,AP2B1,KIF26A,INF2,ADRB2,ARHGAP28,UNC119,PATJ,SPIRE1,GIT1,USP7,SEMA6B,SLITRK2,MTM1,ISG15,ARHGAP4,HCFC1,TRAF2,NOTCH1,SLC25A33,PIEZO1,RAPGEF1,PLCG2,S1PR1,STXBP1,LRSAM1,FNBP1L,T	turquoise	EC_M6

GO:0046849	3.03E-03	0.998878307	16	69	bone remodeling	BP	1.12E-01	TNFRSF11A,TNFAIP3,CTHRC1,SYK,IL6,SFRP1,IL7,P2RX7,INPP5D,RAC1,EPHA2,SYT7,RAB3D,ADRB2,S1PR1,CA2	turquoise	EC_M6
GO:0070482	3.57E-03	0.997812154	55	280	response to oxygen levels	BP	1.12E-01	PPARA,PPARD,UBQLN1,TXN2,LEP,SRF,VEGFA,REST,ROCK2,MGARP,PAK1,CXCL12,TM9SF4,ANKRD1,HSP90B1,PLOD2,CACNB3,OPA1,LOXL2,MMP2,ADA,HIF1AN,CHRNA7,DNM1L,TGFB2,MMP14,FAM162A,BMP7,HYOU1,SCFD1,EGLN3,ERO1A,FMN2,SLC2A4,PPARGC1A,CPEB4,KCNMB1,COL1A1,TGFB3,MYOCD,PIN1,PDGFRB,AKT1,ITGA2,PRKAA1,GNB1,VASN,IRAK1,ATP6AP1,ENO1,SLC2A8,ATP6V0D1,BNIP3,PDLIM1,CDKN1A	darkgreen	EC_M4
GO:0051093	3.05E-03	0.997873334	99	676	negative regulation of developmental process	BP	1.13E-01	SEMA6A,NOTCH4,CDKL3,ASAP1,PDGFB,TEK,SEMA4D,IL6,TLR4,MYCN,NOCT,TRPM4,NRP1,GATA2,GPR4,NOS3,PPARG,GATA3,EDNRB,TIE1,JAG1,SFRP1,FERMT1,TMEM178A,ADAMTS9,TCF15,EPHB1,PLK2,IGF1,HHEX,SMAD1,TLR2,WWTR1,VEGFC,CXCL10,ANGPT2,TLL2,SFMBT1,THBS4,RAPGEF3,ANKRD2,STAB1,STAT1,JARID2,SRGAP2C,ABCG1,PPP3CA,SEMA3F,HLX,NFATC2,AP1AR,INPP5D,EAF2,CFLAR,INPP5F,TRAK2,MAP3K3,MELTF,B2M,LTF,SEMA6D,BCOR,ADAMTS7,IGFBP5,TBX1,HSPG2,DNM3,FGF23,HIF1A,RCC2,EPHA2,ITPKB,PSEN1,SEMA4A,HOXB8,ARHGEF15,EFNA1,VASH1,ADCK1,NPR1,GPR68,YY1,HMG20A,AMOT,ADRB2,RAI1,SEMA6B,RFLNB,ARHGAP4,NRARP,NOTCH1,TERF2,DAB2IP,SOX17,CDK13,RGS4,TRIM62,HIST1H4D,BMP2	turquoise	EC_M6
GO:1903426	3.06E-03	0.999187826	10	37	regulation of reactive oxygen species biosynthetic process	BP	1.13E-01	TLR4,CD36,CLCN3,SNCA,CFLAR,RAB27A,TLR6,ARG2,SLC25A33,PLCG2	turquoise	EC_M6
GO:0042330	3.60E-03	0.997594909	79	427	taxis	BP	1.13E-01	LRP1,SLC12A2,PLXNB2,ANGPT1,CXCL14,FAM49B,VEGFA,CREB3,TPBG,AKIRIN1,GLI3,PLXNA1,FGF2,KIF5B,GLI2,MEGF8,TGFB1,SEMA3C,RYK,CXCL12,PALLD,ROBO1,SEMA4F,P2RX4,APP,CX3CL1,SEMA4G,ELMO2,NOTCH2,DNM1L,SBDS,TGFB2,FEZ1,DAG1,LEF1,ANOS1,CMKLR1,NTRK3,SLAMF8,BMP7,MACO1,SRP54,HSPB1,SERPINE1,TNR,ITGAV,NBL1,PLD1,CRMP1,NOTCH3,ENAH,NGFR,DOCK2,MAP2K1,TTC8,NPTN,CD74,LAMA3,PDGFRB,USP14,ITGA2,PTPN2,PLXNB3,NTN3,PLXNA3,BSG,CDH13,F3,NEXN,CCL16,DPYSL5,LOX,VSTM2L,GDNF,CHN1,ARTN,TMSB4X,CCR6,FGF10	darkgreen	EC_M4
GO:0010721	3.60E-03	0.998187397	30	130	negative regulation of cell development	BP	1.13E-01	CTNNA1,VEGFA,REST,RTN4,CTNNB1,ACTN4,SEMA3C,RYK,RNF6,DIP2B,SEMA4F,TLX2,RAPGEF2,ABCC8,SPP1,SEMA4G,GSK3B,CORO1C,NTRK3,BMP7,IDH2,TNR,FBN1,PRTG,BMPR1A,DICER1,TMEM98,PLXNA3,THY1,YWHAH	darkgreen	EC_M4
GO:0060343	3.60E-03	0.999260721	8	20	trabecula formation	BP	1.13E-01	FBN2,SRF,HEY1,MMP2,THBS3,COL1A1,CHAD,FKBP1A	darkgreen	EC_M4
GO:0006811	3.07E-03	0.997733181	140	1026	ion transport	BP	1.13E-01	TNFRSF11A,TRDN,KLHL3,DAPK1,PDGFB,IREB2,ATP8A1,APBA1,SYK,TSPAN13,SLC3A1,TCAF2,GRIK2,GJA4,TRPM4,RAB3B,PIK3CG,KCNRG,LLGL2,CXCR4,SEC14L1,NDFIP2,TMC8,SPTBN4,SLCO2B1,P2RY6,KCNK6,P2RX2,SLC4A11,CD36,SLCO2A1,MICU3,PDE4D,CHD7,CLCN3,SLCO5A1,CHRM5,MEF2C,CP,P2RX7,GPM6A,CXCL10,PLLP,SERINC5,TMEM150C,TPCN1,PLCG1,STC1,KCNJ3,SLC38A1,ANO6,ZDHHC13,CLIC6,EDN1,KCNJ15,SNCA,DTNBP1,ANO5,ANO3,PM20D1,MTTP,SIK1,LCK,PPP3CA,ANO1,SLC16A14,HECW2,SLC39A8,KCNJ2,SLC12A7,SCN3A,SNAP23,SLC7A7,SLC9A8,EPB41,SLC16A9,PRKD1,SLCO4A1,ACE,MELTF,DLG1,KCNK7,B2M,LTF,ADORA2A,CYBB,HOMER3,MCU,CALCRL,SLC9A7,WNK4,RAMP2,FGF23,CRACR2A,LMTK2,SLC2A9,SLC16A13,SNCG,SYT7,CHRNB1,HEPH,MTOR,SLC26A1,PSEN1,SLC51B,KCNN3,CHRNB2,ATP2A1,CD19,DNM2,SCNN1B,CA4,TMC7,ADRB2,CCL2,KCNJ18,PDE4B,NOS2,PIEZO2,SLC43A2,PIEZO1,PLCG2,SLC25A25,STXBP1,LRRC8C,SLC8A3,LRRC8D,CA2,RELN,KCNH2,SLC25A21,SCN2B,KCNK5,SHANK3,RGS4,KCNA6,CACNG5,FXYD6,KCNG2,GSG1L	turquoise	EC_M6
GO:0051208	3.63E-03	0.998406331	22	87	sequestering of calcium ion	BP	1.13E-01	FKBP1B,FGF2,ATP7B,HSP90B1,RYR3,CX3CL1,PKD2,HTR2B,CASQ1,PTPN6,HTT,ERO1A,HAP1,CACNA1C,CALM1,ANXA6,JPH3,DMD,FKBP1A,TMEM38B,THY1,TMEM38A	darkgreen	EC_M4
GO:0045292	4.62E-03	0.999051608	7	24	mRNA cis splicing, via spliceosome	BP	1.14E-01	PSIP1,NCBP1,RBM17,PRPF40A,PRPF4B,WBP11,PRPF39	darkgrey	EC_M8
GO:0019079	4.63E-03	0.998053173	18	99	viral genome replication	BP	1.14E-01	YTHDC2,SRPK1,TRIM28,PPIH,TOP2B,IFIT5,PDE12,FAM208A,PPID,NUCKS1,DEK,DDX5,TOP2A,HACD3,LARP1,ILF3,FMR1,PKN2	darkgrey	EC_M8
GO:0046831	4.68E-03	0.999405757	5	12	regulation of RNA export from nucleus	BP	1.15E-01	NSUN2,KHDRBS1,DHX9,SETD2,TPR	darkgrey	EC_M8
GO:1903115	3.13E-03	0.999187902	10	32	regulation of actin filament-based movement	BP	1.15E-01	TRPM4,PDE4D,STC1,KCNJ2,DLG1,JUP,ATP2A1,DSG2,DSC2,PDE4B	turquoise	EC_M6

GO:0010604	3.14E-03	0.997457762	325	2586	positive regulation of macromolecule metabolic process	BP	1.15E-01	ZNF516,TNFRSF11A,TCF4,IFNGR1,TNFAIP3,PLAGL1,AKAP12,CNOT6,FLT4,RIMS2,ZBED4,GRAMD4,QKI,NOTCH4,AFDN,TCF20,TPD52L1,NCOA7,SGSM3,PTK2,DAPK1,ATF4,PDGFB,AGTPBP1,MAPK13,BRPF3,CSNK1E,PICK1,KDM4C,PIM1,NFYA,TEK,PRDM4,LIMCH1,DDX58,FAM122A,TEC,KIT,KDR,NFIL3,SYK,SEMA4D,IL6,REL,CREB5,CXCL8,PELI1,RSAD2,TLR4,C5,IL15,CCNY,TEAD2,SDCBP,MYCN,NOCT,TUT4,NRP1,PIK3CG,TAL1,PHF10,EPC1,KDM4D,GATA2,PRKD2,CTSC,CAB39L,NOS3,GGA3,RALB,PPARG,GATA3,EDNRB,NDFIP2,IKBKB,JAG1,NFKBIB,PDE2A,TMEM173,SFRP1,HBEGF,NR1D2,DND1,FERMT1,RPL26,ANKLE2,CD36,PRR5L,TCF15,PLK2,RET,PDE4D,CHD7,IL1A,DNMT3B,IGF1,NR4A1,KLKB1,EIF2AK3,HHEX,KDM3A,ITCH,SMAD1,ZBTB38,HIP1R,OCLN,STOX2,SOX7,HMBOX1,HEXB,MEF2C,SMARCD1,TLR2,WWTR1,IL7,P2RX7,VEGFC,CXCL10,MADD,KMT2D,TIPARP,OAS3,NLRC5,THBS4,PPP1R16B,RAPGEF3,NR4A2,TOX2,DLL4,NEDD9,YAF2,EDN1,STAT1,CD58,DYRK1A,EHD4,SNCA,JARID2,DTNBP1,MYLIP,ERG,ETS2,NFKB2,RNF144B,PRDM15,ABCG1,ETS1,RNF19B,CD200,LPCAT1,HDAC1,LCK,PPP3CA,HYAL2,HECW2,MEF2A,BCL9,LAPTM5,NCF1,USP13,HCLS1,NFATC2,EAF2,GMEB1,CD34,PECAM1,CD86,ASB9,POU2F3,FGR,CFLAR,CASP10,INPP5F,PPP2R5A,ARID1A,RPS6KA1,PROX1,SS18L1,PRKD1,ZNF148,YTHDF1,OAZ3,HELZ2,MLXIPL,ACE,ITGA6,LIMK2,PAK2,MELTF,SENP2,DLG1,ELL3,ETV6,MAP3K11,HOXD10,MOV10,CREB1,B2M,TET1,LTF,ARNT2,CYBB,IL1RAP,DDX3X,RFXANK,ETV4,TBX1,TMEM106A,MECOM,ING4,RAMP2,BIRC3,LDB2,SELE,FGF23,PPM1F,POU2F1,WAS,RAC1,HIF1A,KDM5A,ZBTB18,APBB2,JUP,RAB27A,ADIPOR2,BI	turquoise	EC_M6
GO:0050867	3.15E-03	0.99826054	38	226	positive regulation of cell activation	BP	1.15E-01	SYK,IL6,PELI1,TLR4,IL15,GATA2,CTSC,NECTIN2,GAB2,GATA3,IL1A,IGF1,MEF2C,IL7,SOX13,LCK,ICOSLG,HLX,NFATC2,INPP5D,CD86,FGR,RPS6KA1,B2M,SELP,ITPKB,TLR6,EFNB1,CHRNB2,CCL2,YES1,LGALS9,IL7R,AP3D1,STXBP1,HAVCR1,VNN1,CD46	turquoise	EC_M6
GO:1903532	3.15E-03	0.998311646	35	196	positive regulation of secretion by cell	BP	1.15E-01	TNFRSF11A,CD2AP,SYK,TLR4,SDCBP,TRPM4,GATA2,GAB2,UNC13D,SIRT3,IL1A,IGF1,TLR2,VEGFC,EDN1,SNCA,DTNBP1,ABCG1,ANO1,FGR,CREB1,ADORA2A,MCU,RAC1,HIF1A,RAB27A,SYT7,CHRNB2,RAB3D,GPR68,MYO18A,RPH3AL,STXBP1,BMP2,BMP6	turquoise	EC_M6
GO:2000104	4.69E-03	0.999383943	5	13	negative regulation of DNA-dependent DNA replication	BP	1.15E-01	FBXO5,BRCA2,GMNN,BLM,TIPIN	darkgrey	EC_M8
GO:0022407	3.18E-03	0.998119409	48	293	regulation of cell-cell adhesion	BP	1.15E-01	NOTCH4,AFDN,PTK2,PODXL,TNFRSF21,SYK,IL6,PELI1,IL15,GATA3,JAG1,MAGI1,IL1A,IGF1,ITCH,IL7,SOX13,ETS1,LCK,ICOSLG,LAPTM5,HLX,CD86,F11R,DLG1,B2M,ADORA2A,ITGA4,SELE,SELP,PPM1F,ITPKB,ARG2,EFNB1,CCL2,YES1,JAK1,LGALS9,IL7R,AP3D1,NRARP,NOTCH1,PIEZO1,NFAT5,VNN1,CD46,BMP2,BMP6	turquoise	EC_M6
GO:1901020	3.72E-03	0.999118925	9	25	negative regulation of calcium ion transmembrane transporter activity	BP	1.16E-01	UBQLN1,FKBP1B,REM1,CACNB3,PKD2,LINC02210-CRHR1,CALM1,CBARP,GEM	darkgreen	EC_M4
GO:0035810	3.21E-03	0.999643187	5	11	positive regulation of urine volume	BP	1.16E-01	EDNRB,EDN1,HYAL2,ADORA2A,NPR1	turquoise	EC_M6
GO:0030032	3.21E-03	0.998853307	15	61	lamellipodium assembly	BP	1.16E-01	KIT,ARHGEF7,SPEF1,OCLN,ABLIM1,WAS,RAC1,FSCN1,EPHA2,MTOR,ABI3,DNM2,ABLIM3,S1PR1,CARMIL2	turquoise	EC_M6
GO:0042551	3.23E-03	0.999082749	11	38	neuron maturation	BP	1.16E-01	ANKS1A,PICK1,NRCAM,EDNRB,SPTBN4,NTN4,RET,NR4A2,MTOR,B4GALT6,SCARF1	turquoise	EC_M6

GO:0048513	3.23E-03	0.997381804	323	2529	animal organ development	BP	1.16E-01	ZNF516,TNFRSF11A,SEMA6A,TNFAIP3,HECA,FLT4,SYNJ2,FZD6,CTHRC1,CELSR1,NOTCH4,TCF7,MTSS1,L3MBTL3,KLHL3,PTK2,HSD17B3,ATF4,PDGFB,AGTPBP1,PODXL,CSNK1E,PIM1,MFNG,MDFI,TEK,IREB2,ADGRF5,KIT,KDR,NFIL3,SYK,SEMA4D,EFHC1,TMOD1,IL6,PPT1,PTBP3,CXCL8,RSAD2,FOXP2,CAV2,TSPAN12,TLR4,IL15,TEAD2,SDCBP,MYCN,TRPM4,NRP1,ZBTB24,SVIL,TAL1,GATA2,GPR4,SNRK,PLXND1,ITGA8,NOS3,GAB2,ITGB4,PPARG,GATA3,ACVR2B,MYO7A,CXCR4,GZF1,EDNRB,TIE1,JAG1,PDE2A,GPAT4,SFRP1,HBEGF,NR1D2,NOTUM,FERMT1,FREM2,EFNB2,ASH2L,SPEF1,PCDH12,P2RX2,NTN4,TMEM178A,LMOD3,ADAMTS9,TCF15,EPHB1,RET,MERTK,TNFRSF19,CHD7,IL1A,IGF1,NR4A1,EIF2AK3,HHEX,ITCH,SH3RF1,SMAD1,POU6F1,STOX2,DCLK2,MEF2C,TLR2,WWTR1,IL7,P2RX7,TPD52,VEGFC,NDRG4,GPM6A,CXCL10,ANGPT2,TLL2,LMBR1L,ADGRG3,DHH,SFMBT1,BMP2K,TIPARP,USH1C,STC1,ANKRD2,NR4A2,HIPK1,ANO6,DLL4,SOX13,TSPAN2,EDN1,STAT1,JAM3,IGSF3,ALAS1,JARID2,GAS2,NFKB2,DCAF1,SRGAP2C,ETS1,DPPA4,LPCAT1,SIK1,HDAC1,LCK,PPP3CA,HYAL2,MEF2A,SEMA3F,BCL9,NRAP,PRKX,HLX,SEC24B,DYRK3,ABLIM1,HCLS1,ZFHX2,INPP5D,EAF2,JHY,CD34,PECAM1,CD86,PLXNA2,B3GNT5,FGR,CFLAR,TGM1,ADCY1,FES,RPS6KA1,PROX1,PLEKHA1,VANGL2,ZNF148,LAMA5,MMP21,ACE,ITGA6,LIMK2,LAMC2,DLG1,ELL3,PTCHD1,APOD,ETV6,HOXD10,CREB1,NBEAL2,B2M,SSTR1,LTF,BCR,ARNT2,SEMA6D,BCOR,ADAMTS7,EPHB4,ITGA4,SOS2,IGFBP5,ETV4,TBX1,MECOM,CALCRL,HSPG2,WNK4,RAMP2,MMP13,LDB2,PROM1,FGF23,CRACR2A,TEAD4,PRKCH,RAC1,HIF1A,LIPA,RHOJ,KDM5A,ZBTB18,JUP,ADIP	turquoise	EC_M6
GO:0034446	3.23E-03	0.998640406	20	89	substrate adhesion-dependent cell spreading	BP	1.16E-01	PARVG,PTK2,TEK,NRP1,ITGA8,UNC13D,ARHGEF7,NTN4,MERTK,NEDD9,AP1AR,LAMA5,DOCK1,MELTF,ITGA4,RAC1,RCC2,EFNA1,DNM2,VAMP3	turquoise	EC_M6
GO:0033120	4.75E-03	0.998871324	8	30	positive regulation of RNA splicing	BP	1.16E-01	NCBP1,THRAP3,SLC38A2,NCL,TRA2B,SRSF5,RBMX,DAZAP1	darkgrey	EC_M8
GO:1901566	4.76E-03	0.996414997	145	1317	organonitrogen compound biosynthetic process	BP	1.16E-01	PAN2,NARS,MTHFD1L,DDX39B,EIF3E,MRPL13,SLC35D2,TNRC6B,TUT7,PUM3,PPAT,C1GALT1,PAICS,NCBP1,LRPPRC,CTPS1,MTIF2,PNPT1,DCK,SYNCRIP,PABPC4,DPY19L1,TIA1,MRPL32,ASCC3,DDX1,AMD1,DHFR,PUS7,EXOSC9,COG3,CNOT10,MRPS7,CAD,ALG8,DIS3,DARS,AFMID,METTL3,ZDHHC17,EXOSC8,PRKD3,LARS,PRKDC,DHX29,PAN3,MRPL15,MRPS5,CAPRIN1,LSM14A,CDK5RAP1,EIF2B1,NCK1,ARFGEF1,MRPL35,PTCD3,ABCE1,GALNT6,XRN1,PDE12,LARP4,CNOT1,LTN1,USP16,RPS6,GMPS,ENOPH1,ASNSD1,EEF1E1,GXYLT1,OGA,NOLC1,BMPR1B,GCN1,METAP1,ALG10,YARS,CBSL,NFKB1,KHDRBS1,STT3A,TET2,TRIP11,ACSL5,IARS2,NCL,PAPSS1,BCAT1,FXR1,NGDN,EIF3A,EIF2B5,HMBS,FLVCR1,UMPS,DDX6,OAT,DHX9,HNRNPR,FASTKD2,TPR,RPL10L,DARS2,CNOT9,RPL22L1,DHX33,EIF2AK1,MRPL44,DCP2,EIF3B,SEPSECS,EIF4A1,PFAS,RARS,OGT,HACD3,MRPS23,MRPL22,GEMIN5,LARP1,ILF3,AATF,PRPS1,EIF5,TYMS,UPF3B,GSPT1,HPRT1,FMR1,METTL16,EXOSC2,DHODH,ACADM,CTH,MT-ATP8,RBM8A,METTL18,SARNP,HNRNPD,RNF139,RFK,GART,PAIP2,EIF1AX,DRG1	darkgrey	EC_M8
GO:0098657	3.75E-03	0.99801481	34	155	import into cell	BP	1.16E-01	SLC12A2,SLC22A4,KCNK9,SLC39A4,SLC31A1,SLC7A11,ITGB1,SLC36A4,AKT2,IRS2,PRNP,ARL6IP5,ATP1B3,TSG101,ATP2B4,SLC39A10,SLC2A10,EPRS,ISCU,TFR2,SLC3A2,SLC6A13,SLC29A4,CLN8,SLC7A3,SLC39A6,TRPV2,AKT1,PARK7,HCN2,MIR24-1,SLC36A1,GDNF,HCN4	darkgreen	EC_M4
GO:0001525	3.76E-03	0.997478884	79	428	angiogenesis	BP	1.16E-01	SLC12A2,ANGPT1,FAM19A5,NUS1,RSPO3,ADGRB1,MYH9,LEP,SRF,VEGFA,PDCL3,COL15A1,TGFBR1,EMC10,CALD1,ROCK2,RRAS,ITGB1,FGF2,SRPK2,CTNNB1,ACTG1,STIM1,DCN,STARD13,SPINK5,SULF1,ROBO1,HEY1,CX3CL1,ABCC8,LOXL2,ACVR1,MMP2,MTDH,ATP2B4,RUNX1,CHRNA7,FAP,TJP1,NOTCH2,HYAL1,TGFB2,ADGRB2,MMP14,DAG1,PIK3CA,LEF1,PRKCA,FGFR2,VASH2,HSPB1,SERPINE1,FN1,PDCD10,ITGAV,AKT3,MED1,NPPB,NGFR,SHC1,TMEM100,PKM,ANGPTL6,PDGFRB,ROCK1,AKT1,MYDGF,FLNA,BSG,SARS,CDH13,F3,MIR24-1,SERPINF1,THY1,PIK3C2A,COL8A2,FGF10	darkgreen	EC_M4
GO:0032757	3.27E-03	0.999057476	11	41	positive regulation of interleukin-8 production	BP	1.17E-01	DDX58,SYK,IL6,TLR4,PRKD2,TLR2,CD58,HYAL2,LGALS9,NOS2,TLR1	turquoise	EC_M6
GO:0006891	3.80E-03	0.999005269	10	29	intra-Golgi vesicle-mediated transport	BP	1.17E-01	NAPA,COPG1,COG6,COPB2,COPB1,GOLGA5,COPA,NAPG,RAB6A,GABARAPL2	darkgreen	EC_M4
GO:0002444	3.30E-03	0.998763427	16	70	myeloid leukocyte mediated immunity	BP	1.18E-01	DDX58,KIT,SYK,IL6,PIK3CG,GATA2,GAB2,UNC13D,SNAP23,FGR,FES,ACE,SCNN1B,LGALS9,STXBP1,SERPINB9	turquoise	EC_M6

GO:0090630	3.32E-03	0.998569122	21	93	activation of GTPase activity	BP	1.18E-01	TBC1D30,SGSM3,TBC1D22B,ARHGAP25,TBC1D9,ARHGAP42,USP6NL,TBC1D4,RALGAPA2,NEDD9,ADPRH,SIPA1,BCR,RUNDC1,RCC2,EPHA2,TBC1D1,ARHGEF15,TBC1D8B,SGSM2,TBC1D13	turquoise	EC_M6
GO:0030832	3.32E-03	0.99831504	30	160	regulation of actin filament length	BP	1.18E-01	ARHGAP18,BAIAP2L2,PICK1,CDC42EP1,TMOD1,FAM49A,SVIL,SPTBN4,FCHSD2,FCHSD1,LMOD3,HIP1R,IQGAP2,ESAM,HCLS1,AP1AR,TMOD4,DLG1,BAIAP2L1,WAS,TMOD2,MTOR,ARHGAP28,SPIRE1,CARMIL2,VASP,GMFG,KANK3,GBA2,ARPC5L	turquoise	EC_M6
GO:0090066	3.32E-03	0.997862831	66	418	regulation of anatomical structure size	BP	1.18E-01	SEMA6A,CDKL3,ARHGAP18,BAIAP2L2,PICK1,CDC42EP1,SEMA4D,TMOD1,DOCK4,FAM49A,NRCAM,TRPM4,NRP1,RAB3B,ARHGAP42,SVIL,NOS3,EDNRB,SPTBN4,P2RY2,FCHSD2,FCHSD1,LMOD3,RET,CLCN3,HIP1R,P2RX7,GUCY1A1,USH1C,IQGAP2,ANO6,EDN1,SLC12A7,SEMA3F,RIN3,ESAM,HCLS1,AP1AR,TMOD4,ACE,DLG1,CREB1,ADORA2A,SEMA6D,BAIAP2L1,WAS,TMOD2,RAC1,MTOR,SEMA4A,NPR1,DNM2,SCNN1B,ADRB2,ARHGAP28,IL7R,SPIRE1,SEMA6B,ARHGAP4,CARMIL2,VASP,GMFG,KANK3,SHANK3,GBA2,ARPC5L	turquoise	EC_M6
GO:1900182	4.87E-03	0.998136946	15	76	positive regulation of protein localization to nucleus	BP	1.19E-01	CCT2,UBR5,TRIM28,CCT4,IPO5,MCRS1,CCT7,CCT5,CCT6A,CDK1,TPR,NMD3,ECT2,CCT3,PLK1	darkgrey	EC_M8
GO:0007229	3.86E-03	0.998258588	23	92	integrin-mediated signaling pathway	BP	1.19E-01	CD63,CTNNA1,MYH9,DST,ZYX,ITGB1,ILK,LOXL3,PTPN11,CDH17,SLC2A10,ITGB5,ADAM11,ITGA2B,FN1,ITGAV,COL3A1,TIMP1,LAMA3,ITGA2,CD40LG,FLNA,THY1	darkgreen	EC_M4
GO:0099536	3.88E-03	0.997328873	90	494	synaptic signaling	BP	1.19E-01	RNF19A,SLC12A2,ADGRB1,LY6E,GNAQ,SRF,UNC13B,RIMS1,TPBG,SHANK1,NCDN,SLC7A11,ITGB1,LIN7B,KIF5B,NAPA,GAD2,PCDH17,RAB3GAP1,HTR5A,CTNNB1,FARP1,PRNP,PCLO,SNPH,RIC3,KCMF1,MAP1B,P2RX4,ATP2A2,DLGAP4,APP,GLRB,RAPGEF2,CX3CL1,CACNB3,HOMER1,VPS18,GLS,CHRNA7,EIF4E,APBA2,DNM1L,HTR2B,GSK3B,DAG1,KRAS,KPNA1,NCSTN,SYAP1,BACE1,RPS6KA3,MAPT,EIF4EBP2,TNR,MPP2,KCNA1,ATAD1,CHRNE,ERC1,HAP1,IQSEC2,NLGN2,STX1B,SYT11,KCNMB1,MYCBPAP,CACNA1G,RAB11A,CALM1,GRIA1,NPTN,CYP46A1,BEGAIN,DTNA,AKT1,ABR,GNB1,PAFAH1B1,TPRG1L,PARK7,NTNG2,JPH3,PCDHB2,GDNF,INSYN1,YWHAH,KCNMB4,MAP1A,LRRTM2	darkgreen	EC_M4
GO:0051650	3.89E-03	0.997981059	32	143	establishment of vesicle localization	BP	1.19E-01	AP3S1,DYNC1I1,STARD3NL,KIF5B,PICALM,CTNNB1,TRAK1,ACTN4,TMED2,EXOC6B,AP3B1,CHP1,WIPI1,SHROOM2,KIF1A,TANC2,HTT,MYO5A,HAP1,SLC2A4,STARD3,MYO1E,SYT11,TMED10,RAB11A,MYO1D,MYO5B,MYO1C,PAFAH1B1,IKBKG,VAMP7,BLOC1S2	darkgreen	EC_M4
GO:0010941	3.91E-03	0.996964521	198	1223	regulation of cell death	BP	1.19E-01	SERPINB2,LRP1,AHI1,AGAP2,IER3,CDSN,RRM2B,ANGPT1,TCP1,PPARA,PPARD,CTNNA1,UBQLN1,MTCH1,LGALS1,LEP,BAG1,VEGFA,DOCK8,VCP,UNC13B,NCK2,CREB3,ASNS,REST,TGFBR1,RTN4,TAX1BP1,GLI3,INHBA,DKKL1,VPS35,SLC7A11,ITGB1,FGF2,SRPK2,RB1,PICALM,MARK4,TPT1,GLI2,AXL,ACER3,CTNNB1,AKT2,RARB,ACTN4,UFM1,LIG4,IRS2,PRNP,HDAC3,DDRGK1,SMPD1,ILK,ARL6IP5,CXCL12,ANKRD1,HSP90B1,SLC25A4,FAIM2,RASA1,CELF1,RTKN,RAPGEF2,CX3CL1,OPA1,AMIGO2,ACVR1,SERINC3,MTDH,ADA,CHP1,TXNDC5,PLA2R1,IFT57,SLC39A10,CHRNA7,FAP,TJP1,NOTCH2,CTTN,CDC73,DNM1L,SELENOS,EYA2,TGFB2,LRP5,HTR2B,ZMAT3,GSK3B,INPP5A,FSTL1,PIK3CA,LEF1,PRKCA,KRAS,NTRK3,FAM162A,KPNA1,BMP7,HYOU1,ATF3,FGFR2,PEA15,TFRC,ATF6,BACE1,FZD9,RPS6KA3,EGLN3,PDIA3,HSPB1,MAPT,SERPINE1,SYVN1,PTPN6,PDCD10,ITGAV,LRPAP1,COMP,HTT,FKBP8,PRKCI,SKIL,ARAF,TIMP1,VDAC2,FMN2,SIX4,SGMS1,MNAT1,SLC6A13,ERC1,GHITM,MAGED1,CLN8,EIF2S1,MED1,PPARGC1A,ACTN1,CPEB4,NES,PNMA1,ARHGEF2,NGFR,TGFB3,SHC1,TAF9B,MYOCD,PIN1,CD74,PDGFRB,AKT1,PRKAA1,PTPN2,OMA1,CD40LG,MYDGF,DAPK3,BCAP31,TNFRSF18,FLNA,IKBKG,TMEM259,PARK7,ENO1,GNAI3,CFDP1,AARS,HSPA5,BCL10,NAE1,MIR24-1,SERPINF1,BNIP3,LOX,VSTM2L,ITM2C,UBE2V2,CACYBP,ARF4,GDNF,TIMP3,GLO1,CDKN1A,SIGMAR1,TXN,NACC2,PTTG1IP,TSLP,PLAGL2,TMEM109,ZPR1,ORMDL3,CHST11,OLFM1,FGF10	darkgreen	EC_M4
GO:0060401	3.92E-03	0.998040994	29	126	cytosolic calcium ion transport	BP	1.19E-01	SMDT1,FKBP1B,FGF2,PRNP,ATP2B1,RYR3,P2RX4,ATP2A2,CX3CL1,CACNB3,OPA1,ATP2B4,PKD2,HTR2B,CASQ1,PTPN6,HTT,ERO1A,HAP1,CACNA1C,CALM1,TRPV2,AKT1,JPH3,DMD,FKBP1A,TMEM38B,THY1,TMEM38A	darkgreen	EC_M4
GO:0016180	4.93E-03	0.998973343	7	24	snRNA processing	BP	1.20E-01	EXOSC9,INTS10,EXOSC8,INTS7,INTS2,EXOSC2,INTS6	darkgrey	EC_M8
GO:1904035	3.39E-03	0.998772056	15	65	regulation of epithelial cell apoptotic process	BP	1.20E-01	TNFAIP3,TEK,KDR,IL6,GATA2,GATA3,PLCG1,ANO6,HDAC1,CFLAR,ITGA4,RAMP2,MTOR,CCL2,CDH5	turquoise	EC_M6

GO:0034637	3.95E-03	0.998471223	17	63	cellular carbohydrate biosynthetic process	BP	1.20E-01	DYRK2,LEP,AKT2,IRS2,B3GNT4,GYG1,SORBS1,SELENOS,GYG2,GSK3B,PPP1R3D,PPP1R3F,AKT1,PGP,AGL,HAS2,HS2ST1	darkgreen	EC_M4
GO:0046856	3.41E-03	0.999183056	9	28	phosphatidylinositol dephosphorylation	BP	1.20E-01	SYNJ2,INPP4B,MTMR2,CHRM5,MTMR10,INPP5D,INPP5F,MTMR12,MTM1	turquoise	EC_M6
GO:0044093	3.96E-03	0.996941049	189	1151	positive regulation of molecular function	BP	1.20E-01	IL23A,NAB2,LRP1,AGAP2,TAB2,TBC1D9B,PRRC1,ANGPT1,TCP1,ROS1,NUS1,SERINC1,TRAPPC9,C6orf89,MTCH1,GNAQ,LEP,PLAA,SRF,VEGFA,DOCK8,VCP,CLOCK,REST,SNX13,PSME4,CYCS,MAP3K7,ROCK2,RCN3,ITGB1,FGF2,KIF5B,NAPA,RB1,ABHD5,PICALM,MAPRE3,PAK1,TGFB1,AXL,RAB3GAP1,CTNNB1,AKT2,TBC1D16,OSBPL8,ACTN4,PPP1R12A,STIM1,PRNP,DDRGK1,PPP2CB,ILK,ARL6IP5,RYK,ARL1,ATP1B3,ROBO1,PINX1,PFN2,RASA1,CACNA1D,APP,RAPGEF2,MAPKAPK5,CX3CL1,ABCC8,CACNB3,RALGAPB,AMFR,DKC1,MTDH,AKTIP,CHP1,ATP2B4,PKD2,IFT57,TANK,PSMD14,SLC39A10,RAB7B,CHRNA7,RAP1GDS1,DOCK10,AZIN2,CCND1,CAPRIN2,TGFB2,LRP5,HTR2B,GSK3B,DAG1,PIK3CA,CORO1C,ISCU,NEK7,ADAP1,KRAS,NTRK3,SGSM1,FAM162A,SPATC1L,FGFR2,CASQ1,TP53BP1,NCSTN,SYAP1,ADRM1,IQGAP1,TFRC,ARID5B,EGLN3,MAPT,FZD2,DNAJC10,PCOLCE,FN1,ZC3H15,PDCD10,ADRA2C,DOK7,HTT,PRKCI,FGFR3,KCNA1,MNAT1,B3GAT3,ERC1,HAP1,CACNA1C,MAGED1,BMPR1A,STIM2,EIF2S1,PPARGC1A,SRCIN1,ARHGEF2,NGFR,TPM1,EIF3C,MAP2K1,GPR65,CALM1,SMARCA4,UACA,VMP1,MYOCD,PIN1,CD74,PDGFRB,EVI5L,AKT1,SNX18,ITGA2,NPR3,CD40LG,ABR,BCAP31,TAX1BP3,PKD1,IRAK1,IKBKG,PARK7,GPSM1,GNAI3,F3,ARHGAP29,BCAR3,MTSS1L,LMO4,BCL10,RANBP2,CCL16,DMD,FKBP1A,NMUR1,ARF4,GDNF,THY1,TOR1AIP2,CHN1,CDKN1A,EDA,TXN,TMSB4X,GABARAPL2,WNT2,FGF10,MBD2	darkgreen	EC_M4
GO:0071624	3.42E-03	0.999355658	7	21	positive regulation of granulocyte chemotaxis	BP	1.20E-01	CXCL8,THBS4,EDN1,MCU,RAC1,DAPK2,S100A14	turquoise	EC_M6
GO:0070555	3.43E-03	0.998539171	20	98	response to interleukin-1	BP	1.20E-01	TNFRSF11A,AKAP12,MAPK13,IL6,CXCL8,IRAK2,IKBKB,SFRP1,EDN1,SNCA,ETS1,HYAL2,ADAMTS7,SELE,HIF1A,RPS6KA5,YY1,CCL2,LGALS9,DAB2IP	turquoise	EC_M6
GO:1903037	3.45E-03	0.998135775	35	206	regulation of leukocyte cell-cell adhesion	BP	1.21E-01	TNFRSF21,SYK,IL6,PELI1,IL15,GATA3,IL1A,IGF1,ITCH,IL7,SOX13,ETS1,LCK,ICOSLG,LAPTM5,HLX,CD86,DLG1,B2M,ADORA2A,ITGA4,SELE,SELP,ITPKB,ARG2,EFNB1,CCL2,YES1,LGALS9,IL7R,AP3D1,NRARP,NFAT5,VNN1,CD46	turquoise	EC_M6
GO:0061005	3.46E-03	0.998869991	13	49	cell differentiation involved in kidney development	BP	1.21E-01	MTSS1,PDGFB,PODXL,GPR4,GATA3,JAG1,MEF2C,WWTR1,STAT1,PRKX,CD34,PROM1,NOTCH1	turquoise	EC_M6
GO:0008209	3.48E-03	0.999334595	7	22	androgen metabolic process	BP	1.21E-01	HSD17B3,SRD5A3,SCARB1,TIPARP,PLEKHA1,SRD5A2,AKR1D1	turquoise	EC_M6
GO:0031214	3.49E-03	0.99835133	25	126	biomineral tissue development	BP	1.21E-01	ATF4,TRPM4,NOS3,ACVR2B,NOTUM,IGF1,EIF2AK3,MEF2C,P2RX7,BMP2K,ANO6,FGR,LTF,BCOR,TBX1,MMP13,FGF23,HIF1A,ADRB2,RFLNB,ISG15,NOTCH1,S1PR1,BMP2,BMP6	turquoise	EC_M6
GO:0048524	5.01E-03	0.998334177	12	55	positive regulation of viral process	BP	1.22E-01	SRPK1,PPIH,RSF1,TOP2B,PDE12,PPID,DHX9,TOP2A,HACD3,LARP1,FMR1,PKN2	darkgrey	EC_M8
GO:0006388	5.02E-03	0.999515241	4	10	tRNA splicing, via endonucleolytic cleavage and ligation	BP	1.22E-01	DDX1,TSEN2,ZBTB8OS,TSEN15	darkgrey	EC_M8
GO:0010452	5.04E-03	0.999374015	5	11	histone H3-K36 methylation	BP	1.22E-01	PAXIP1,NSD3,SETD2,NSD2,NSD1	darkgrey	EC_M8
GO:0051694	3.53E-03	0.99975407	4	7	pointed-end actin filament capping	BP	1.22E-01	TMOD1,LMOD3,TMOD4,TMOD2	turquoise	EC_M6
GO:0042092	3.54E-03	0.999223461	8	26	type 2 immune response	BP	1.22E-01	IL6,RSAD2,GATA3,HLX,CD86,BCL6B,ARG2,BATF	turquoise	EC_M6
GO:1902903	3.55E-03	0.997820095	54	327	regulation of supramolecular fiber organization	BP	1.23E-01	MTSS1,ARHGAP18,BAIAP2L2,PICK1,CDC42EP1,LIMCH1,TMOD1,FAM49A,NRP1,SVIL,SPTBN4,FCHSD2,SFRP1,ARHGEF7,SPEF1,FCHSD1,LMOD3,HIP1R,OCLN,RAPGEF3,IQGAP2,EDN1,DYRK1A,SNCA,ESAM,HCLS1,AP1AR,FES,PROX1,TMOD4,F11R,PAK2,DLG1,BAIAP2L1,PPM1F,WAS,TMOD2,RAC1,MTOR,PSEN1,ARHGEF15,ARHGAP28,SPIRE1,GIT1,S1PR1,CARMIL2,CDH5,VASP,GMFG,KANK3,SHANK3,RGS4,GBA2,ARPC5L	turquoise	EC_M6
GO:2000116	3.56E-03	0.998198608	29	167	regulation of cysteine-type endopeptidase activity	BP	1.23E-01	TNFAIP8,GRAMD4,DAPK1,SYK,CST7,PPARG,NR4A1,EIF2AK3,SH3RF1,HIP1R,SOX7,SNCA,LCK,LAPTM5,CFLAR,CASP10,RPS6KA1,PAK2,LTF,ADORA2A,DDX3X,BIRC3,PPM1F,BID,LGALS9,PMAIP1,SERPINB9,PSMB9,PERP	turquoise	EC_M6

GO:0031507	5.10E-03	0.998655854	9	33	heterochromatin assembly	BP	1.23E-01	TRIM28,SETDB2,RRP8,SMARCA5,FAM208A,HELLS,TPR,SUV39H1,SMCHD1	darkgrey	EC_M8
GO:0018208	5.13E-03	0.998479757	10	48	peptidyl-proline modification	BP	1.23E-01	PPIL4,PPIL1,PPIH,FKBP15,NKTR,PPWD1,PPID,PPIG,AIPL1,FKBP4	darkgrey	EC_M8
GO:0032479	3.60E-03	0.998590581	17	75	regulation of type I interferon production	BP	1.24E-01	C6orf106,DDX58,SYK,REL,TLR4,TMEM173,ITCH,TLR2,OAS3,STAT1,DDX3X,YY1,ISG15,IRF8,PLCG2,OAS1,RNF125	turquoise	EC_M6
GO:0032606	3.60E-03	0.998590581	17	75	type I interferon production	BP	1.24E-01	C6orf106,DDX58,SYK,REL,TLR4,TMEM173,ITCH,TLR2,OAS3,STAT1,DDX3X,YY1,ISG15,IRF8,PLCG2,OAS1,RNF125	turquoise	EC_M6
GO:0062207	3.61E-03	0.998583508	17	77	regulation of pattern recognition receptor signaling pathway	BP	1.24E-01	TNFAIP3,GRAMD4,PELI1,RSAD2,TLR4,PUM2,SEC14L1,CD36,ERBIN,TLR2,LTF,DDX3X,BIRC3,TLR6,DAB2IP,TLR1,RNF125	turquoise	EC_M6
GO:0043304	3.64E-03	0.999210761	8	24	regulation of mast cell degranulation	BP	1.24E-01	SYK,GATA2,GAB2,UNC13D,FGR,FES,LGALS9,STXBP1	turquoise	EC_M6
GO:0050852	3.66E-03	0.998515918	18	85	T cell receptor signaling pathway	BP	1.25E-01	TEC,TNFRSF21,PRKD2,NECTIN2,GATA3,IKBKB,PDE4D,PLCG1,DGKZ,LCK,ICOSLG,LAPTM5,INPP5D,PSEN1,EIF2B2,PDE4B,PLCG2,FYB1	turquoise	EC_M6
GO:0035924	3.66E-03	0.998670913	15	62	cellular response to vascular endothelial growth factor stimulus	BP	1.25E-01	SEMA6A,FLT4,KDR,TSPAN12,NRP1,PRKD2,NR4A1,VEGFC,DLL4,PRKD1,RAMP2,PGF,NOTCH1,DAB2IP,MIR23A	turquoise	EC_M6
GO:0010506	4.11E-03	0.997483407	53	268	regulation of autophagy	BP	1.25E-01	TAB2,ATP6V1C1,UBQLN1,LEP,EXOC1,MAP3K7,VPS35,SESN3,ATP6V0B,RHEB,RAB3GAP1,DCN,TP53INP2,SLC25A4,EIF4G2,PTPN22,MTDH,ATP6V1B2,CTTN,DNM1L,SEC22B,CISD2,FEZ1,HTR2B,GSK3B,PIK3CA,SCFD1,QSOX1,ATF6,PAFAH1B2,HSPB1,MAPT,GPR137,HTT,ATP6V1D,SPTLC2,ROCK1,AKT1,PRKAA1,CLEC16A,DAPK3,IKBKG,PARK7,GPSM1,GNAI3,USP10,ATP6V0D1,BNIP3,VPS26B,CAPNS1,PIK3C2A,ORMDL3,ATP6V0E1	darkgreen	EC_M4
GO:0045785	4.14E-03	0.997394023	59	306	positive regulation of cell adhesion	BP	1.25E-01	IL23A,ANGPT1,HLA-DRA,FAM49B,LGALS1,LEP,VEGFA,DOCK8,NCK2,SOCS5,GLI3,RRAS,APBB1IP,FBLN2,GLI2,ILK,HSPH1,CXCL12,CHST2,EDIL3,CX3CL1,PTPN11,AP3B1,PTPN22,ADA,RUNX1,TJP1,HYAL1,TGFB2,GSK3B,DAG1,PIK3CA,LEF1,PRKCA,BMP7,IQGAP1,TFRC,COL26A1,FN1,PTPN6,IGFBP2,ITGAV,ARPC2,TPM1,CD74,ROCK1,AKT1,ITGA2,EGFLAM,CD40LG,MYO10,TNFRSF18,IRAK1,FLNA,CDH13,BCL10,FSTL3,THY1,HAS2	darkgreen	EC_M4
GO:0035967	4.17E-03	0.998059854	24	103	cellular response to topologically incorrect protein	BP	1.26E-01	VCP,NCK2,CREB3,ERLEC1,TMTC4,DDRGK1,TMED2,AMFR,SELENOS,CCND1,HSPA8,VAPB,ATF3,ATF6,RHBDD2,DNAJB12,ERO1A,EIF2S1,PTPN2,MBTPS1,HSPA5,DNAJB9,HSPA13,DERL2	darkgreen	EC_M4
GO:0018198	3.74E-03	0.998891993	11	43	peptidyl-cysteine modification	BP	1.26E-01	ZDHHC14,OXR1,NCOA7,ZDHHC6,HHAT,ZDHHC18,ACE,ZDHHC15,RAB3D,NOS2,ZDHHC22	turquoise	EC_M6
GO:0014031	3.74E-03	0.998534494	17	73	mesenchymal cell development	BP	1.26E-01	SEMA6A,SEMA4D,NRP1,EDNRB,JAG1,RET,EDN1,SEMA3F,LAMA5,SEMA6D,TBX1,HIF1A,ALDH1A2,EFNB1,SEMA4A,SEMA6B,NOTCH1	turquoise	EC_M6
GO:0051129	3.75E-03	0.997442941	85	569	negative regulation of cellular component organization	BP	1.26E-01	SEMA6A,TBC1D30,PACSIN2,CDKL3,PICK1,SEMA4D,TMOD1,SDCBP,NRP1,SVIL,MTMR2,PPARG,TBC1D4,TMC8,SPTBN4,IKBKB,FEZ2,EFNB2,ARHGEF7,SPEF1,LMOD3,PRAG1,IGF1,HIP1R,MCTP1,TLR2,CXCL10,SCAF4,RAPGEF3,CEP97,DYRK1A,JAM3,SNCA,MYLIP,SRGAP2C,PPP3CA,SEMA3F,RIN3,USP30,CFLAR,INPP5F,TRAK2,TMOD4,PRKD1,F11R,ACE,LIMK2,PAK2,APOD,B2M,ZW10,TET1,SEMA6D,DDX3X,DNM3,WAS,TMOD2,RAC1,RCC2,TNFRSF1B,AR,PSEN1,SEMA4A,ABI3,ITGA3,ARHGEF15,EFNA1,ADCK1,DNM2,ARHGAP28,UNC119,SEMA6B,MTM1,ARHGAP4,NOTCH1,S1PR1,STXBP1,LRSAM1,TERF2,CARMIL2,CDH5,GMFG,KANK3,SHANK3,BMP6	turquoise	EC_M6
GO:0023061	4.24E-03	0.997293344	62	325	signal release	BP	1.28E-01	LRP1,PPARD,LEP,UNC13B,CLOCK,REST,RIMS1,INHBA,LIN7B,FKBP1B,KIF5B,NAPA,RAB3GAP1,C1QTNF1,IRS2,RAB11FIP1,PCLO,SNPH,CXCL12,OSBP,VAMP8,P2RX4,SELENOT,ATP2A2,ABCC8,PTPN11,PFKM,CRY2,VPS18,SPP1,BRSK2,DYNLL1,KDM5B,DNM1L,CPT1A,LRP5,GSK3B,HADH,ADCY5,OSM,BACE1,SIDT2,LINC02210-CRHR1,TFR2,ADRA2C,PTPRN,CYP19A1,NLGN2,STX1B,SYT11,UBE2Q1,CALM1,SYTL4,ACSL4,RASL10B,MIDN,SSTR5,VAMP7,PARK7,WLS,GDNF,KCNMB4	darkgreen	EC_M4
GO:2001258	4.25E-03	0.998862295	10	30	negative regulation of cation channel activity	BP	1.28E-01	UBQLN1,FKBP1B,REM1,CACNB3,PKD2,LINC02210-CRHR1,CALM1,MIR24-1,CBARP,GEM	darkgreen	EC_M4
GO:0045879	4.26E-03	0.998866145	10	29	negative regulation of smoothened signaling pathway	BP	1.28E-01	VCP,RUNX2,GLI3,RB1,PTCH2,KCTD21,GLI2,MEGF8,TULP3,MGRN1	darkgreen	EC_M4

GO:0110148	3.82E-03	0.998181734	25	127	biomineralization	BP	1.28E-01	ATF4,TRPM4,NOS3,ACVR2B,NOTUM,IGF1,EIF2AK3,MEF2C,P2RX7,BMP2K,ANO6,FGR,LTF,BCOR,TBX1,MMP13,FGF23,HIF1A,ADRB2,RFLNB,ISG15,NOTCH1,S1PR1,BMP2,BMP6	turquoise	EC_M6
GO:0044070	3.82E-03	0.998665476	14	57	regulation of anion transport	BP	1.28E-01	TNFRSF11A,SYK,TCAF2,STC1,EDN1,SNCA,ADORA2A,FGF23,MTOR,PSEN1,SLC43A2,STXBP1,CA2,RGS4	turquoise	EC_M6
GO:0007411	4.28E-03	0.997604984	39	181	axon guidance	BP	1.28E-01	LRP1,PLXNB2,VEGFA,GLI3,PLXNA1,KIF5B,GLI2,MEGF8,SEMA3C,RYK,CXCL12,PALLD,ROBO1,SEMA4F,APP,SEMA4G,NOTCH2,FEZ1,DAG1,ANOS1,BMP7,TNR,CRMP1,NOTCH3,ENAH,NGFR,TTC8,NPTN,LAMA3,PLXNB3,NTN3,PLXNA3,BSG,NEXN,DPYSL5,VSTM2L,GDNF,CHN1,ARTN	darkgreen	EC_M4
GO:0032616	3.89E-03	0.999546675	5	11	interleukin-13 production	BP	1.30E-01	TNFRSF21,GATA3,IL1RAP,ARG2,LGALS9	turquoise	EC_M6
GO:0032656	3.89E-03	0.999546675	5	11	regulation of interleukin-13 production	BP	1.30E-01	TNFRSF21,GATA3,IL1RAP,ARG2,LGALS9	turquoise	EC_M6
GO:0090148	4.35E-03	0.999541654	5	9	membrane fission	BP	1.30E-01	SH3GL2,SLC25A46,DNM1L,CORO1C,MIEF2	darkgreen	EC_M4
GO:0070307	3.92E-03	0.999541413	5	11	lens fiber cell development	BP	1.31E-01	ATF4,TMOD1,PROX1,EPHA2,TBC1D20	turquoise	EC_M6
GO:0062013	4.39E-03	0.997986585	23	96	positive regulation of small molecule metabolic process	BP	1.31E-01	DYRK2,PPARA,PPARD,VCP,DGAT2,AKT2,KAT2B,IRS2,RDH10,SORBS1,APP,CPT1A,ZBTB20,COMT,PPARGC1A,CD74,AKT1,PRKAA1,PTPN2,PARK7,ENO1,TMSB4X,NNMT	darkgreen	EC_M4
GO:2000780	5.45E-03	0.998682186	8	29	negative regulation of double-strand break repair	BP	1.31E-01	UBR5,RIF1,ERCC6,PARPBP,FANCB,AUNIP,SMCHD1,RMI2	darkgrey	EC_M8
GO:0032000	4.43E-03	0.999724511	4	6	positive regulation of fatty acid beta-oxidation	BP	1.32E-01	PPARA,AKT2,IRS2,CPT1A	darkgreen	EC_M4
GO:1901701	4.43E-03	0.99666659	149	887	cellular response to oxygen-containing compound	BP	1.32E-01	LRP1,AP3S1,LY6E,PPARD,CTNNA1,LGALS1,GNAQ,LEP,PLAA,RORB,SRF,VCP,UNC13B,COL1A2,ACTR2,ROCK2,INHBA,VPS35,MGARP,FKBP1B,KIF5B,SESN3,ESD,PAK1,TGFB1,AXL,HTR5A,CTNNB1,DGAT2,AKT2,OSBPL8,KAT2B,IRS2,PRNP,ATP2B1,ZNF703,LEPROTL1,APLP1,ANKRD1,HSP90B1,MAP1LC3A,CD180,OSBP,MAP1B,RYR3,SORBS1,P2RX4,SELENOT,USO1,APP,DHX36,RAPGEF2,CX3CL1,PTPN11,ADCY6,OPA1,PTPN22,MMP2,MTDH,ATP2B4,SPP1,PKD2,BRSK2,CHRNA7,EIF4E,DYNLL1,ADIPOR1,CDC73,DNM1L,GLRX2,SELENOS,CPT1A,ZBTB20,ZNF106,FBLN5,LRP5,EPRS,HTR2B,GSK3B,DAG1,PIK3CA,PRKCA,NTRK3,COL6A1,BMP7,ADCY5,WDTC1,FGFR2,NCSTN,SYAP1,BACE1,SIDT2,LINC02210-CRHR1,MAPT,SERPINE1,EIF4EBP2,KCNK4,FBN1,PDCD10,UPF1,PRKCI,COL5A2,PTPRN,SGMS1,MYO5A,SLC2A4,MED1,PPARGC1A,MSN,CPEB4,ARHGEF2,NGFR,KCNMB1,TPM1,COL1A1,SHC1,SMARCA4,PKM,ROCK1,AKT1,ITGA2,LPIN2,PRKAA1,RAB31,GNAL,PTPN2,LDOC1,MYO1C,GNB1,IRAK1,FLNA,PARK7,HCN2,AHCYL1,GNAI3,BCAR3,SLC2A8,HSPA5,BCL10,SERPINF1,BNIP3,IL36G,SIGMAR1,TXN,FAM210B,LEPROT,PIK3C2A,HCN4,WNT2	darkgreen	EC_M4
GO:0090257	3.98E-03	0.997902716	32	179	regulation of muscle system process	BP	1.32E-01	KIT,DOCK4,TRPM4,PIK3CG,ARHGAP42,NOS3,PDE4D,IGF1,NR4A1,GUCY1A1,STC1,EDN1,JARID2,PDE9A,PPP3CA,KCNJ2,MEF2A,DLG1,IGFBP5,CALCRL,JUP,TNFRSF1B,MTOR,ATP2A1,YY1,DSG2,DSC2,ADRB2,PDE4B,NOTCH1,SLC8A3,RGS4	turquoise	EC_M6
GO:0050730	3.99E-03	0.997851997	34	193	regulation of peptidyl-tyrosine phosphorylation	BP	1.32E-01	PDGFB,TEC,KIT,SYK,SEMA4D,IL6,IL15,NRP1,SFRP1,HBEGF,SH3BP5,CD36,IGF1,IL7,THBS4,NEDD9,EHD4,HDAC1,HYAL2,NCF1,HCLS1,PECAM1,INPP5F,ACE,PAK2,MTOR,PSEN1,EFNA1,YES1,UNC119,PLCG2,RELN,ALKAL1,BMP6	turquoise	EC_M6
GO:0048008	4.47E-03	0.998447051	14	48	platelet-derived growth factor receptor signaling pathway	BP	1.32E-01	LRP1,VEGFA,PTPN11,CBLB,ARID5B,SGPL1,MYO1E,MYOCD,PDGFRB,PTPN2,F3,PTPN12,LOX,PIK3C2A	darkgreen	EC_M4
GO:2001021	5.55E-03	0.997924805	14	72	negative regulation of response to DNA damage stimulus	BP	1.33E-01	DDX39B,FBXO5,UBR5,DDIAS,RIF1,ERCC6,PARPBP,TAF9,FANCB,AUNIP,PSMD10,THOC1,SMCHD1,RMI2	darkgrey	EC_M8
GO:0060346	4.51E-03	0.999519635	5	9	bone trabecula formation	BP	1.33E-01	FBN2,MMP2,THBS3,COL1A1,CHAD	darkgreen	EC_M4
GO:0046716	4.52E-03	0.999322314	6	13	muscle cell cellular homeostasis	BP	1.33E-01	LARGE1,SRF,PFKM,DMD,LOX,CFL2	darkgreen	EC_M4

GO:0048523	4.52E-03	0.996163975	548	3647	negative regulation of cellular process	BP	1.33E-01	SERPINB2,LMAN1,ATP8B1,NAB2,LRP1,B4GALT7,AHI1,AGAP2,ENPP3,TAB2,DYRK2,IER3,CDSN,RRM2B,PLXNB2,FBN2,ANGPT1,FAM19A5,PPARA,FAM49B,PACSIN1,ADGRB1,PPARD,CTNNA1,CBY1,UBQLN1,LGALS1,SH3GL2,GNAQ,MYH9,LEP,CDKN2B,PLAA,RORB,SRF,BAG1,VEGFA,DOCK8,VCP,RUNX2,RASL11B,NCK2,CLOCK,CREB3,ASNS,PDCL3,REST,ASPN,RAB23,SNX13,P3H1,TGFBR1,SOCS5,ERLEC1,COPS9,RTN4,TPBG,SHANK1,TAX1BP1,MFSD2A,UBE2J1,SH3BP5L,AKIRIN1,THAP5,MDFIC,TES,GLI3,ROCK2,INHBA,RRAS,DKKL1,VPS35,SLC7A11,ITGB1,FKBP1B,PLXNA1,FGF2,SESN3,DNAJC1,RAB7A,EML2,ZNF398,RB1,ITM2B,EED,PICALM,TPT1,ZNF296,CRTAP,CGREF1,PTCH2,KCTD21,GLI2,MEGF8,PCDH17,PAK1,TGFB1,AXL,SFMBT2,SPOPL,CTNNB1,LBH,DGAT2,SERPINH1,AKT2,DYNC1LI1,GPD1L,ZEB2,C1QTNF1,OSBPL8,CXXC5,RARB,NAV3,NPLOC4,ACTN4,ANAPC15,KAT2B,LTBP1,CRIM1,SPRED3,SPINT2,UFM1,LIG4,IRS2,PRNP,CHFR,NUDT16,DCN,HDAC3,RAB11FIP1,ZNF703,DCLK1,STARD13,SEMA3C,CAVIN3,DDRGK1,SMPD1,PPP2CB,ILK,LEPROTL1,ARL6IP5,UBA3,DPYSL3,SPINK5,APLP1,RYK,TMEM127,CNOT7,RNF6,LMO1,REM1,CXCL12,FXYD1,SACS,ANKRD1,SNTA1,HSP90B1,TMED2,IL20RB,TP53INP2,SLC25A4,ZNF536,EIF4G2,SULF1,VAMP8,CTNND1,DKK3,ROBO1,WWC2,MAP1B,DIP2B,RYR3,PINX1,SEMA4F,LOXL3,FAIM2,PFN2,BCDIN3D,HEY1,RASA1,TLX2,CELF1,APP,ATXN2,DHX36,RAPGEF2,MAPKAPK5,CX3CL1,ABCC8,CACNB3,PTPN11,PTX3,ADCY6,PIP4P2,RUNX1T1,OPA1,ARHGAP1,AMFR,LOXL2,AMIGO2,PTPN22,TSG101,ACVR1,DKC1,CRY2,VPS18,CHAC1,MTDH,ADA,BAP1,CHP1,ATP2B4,SPP1,TXNDC5,PKD2,RUNX1,TWF1,DBAKAP12,TNFRSF21,PRKCH,BID,CCL2,TRAF2	darkgreen	EC_M4
GO:0034349	4.03E-03	0.999372661	6	15	glial cell apoptotic process	BP	1.33E-01		turquoise	EC_M6
GO:0060717	5.57E-03	0.999507775	4	8	chorion development	BP	1.33E-01	MAP3K4,PAXIP1,E2F7,E2F8	darkgrey	EC_M8
GO:0010971	5.61E-03	0.998793264	7	25	positive regulation of G2/M transition of mitotic cell cycle	BP	1.34E-01	FBXO5,CDC25C,CCNB1,DTL,CDK1,CDC25A,DBF4B	darkgrey	EC_M8
GO:0043603	2.40E-04	0.999836417	136	897	cellular amide metabolic process	BP	1.34E-01	ORMDL2,PYM1,CDK4,CERK,EIF3H,RPS18,B3GALT4,EEF1D,RPL3,EIF3L,OPLAH,EIF3D,RPL8,DNPH1,RPS5,RPL28,RPS9,EEF1A1,MALSU1,RPS7,RPS11,FOXO3,SPHK2,RPL18,ABTB1,ACSS1,RPS8,MIF4GD,MLH1,UNK,RPS3,PNP,RPS16,SAMD4B,XDH,MRPS12,RPL15,EIF3K,GNPDA1,TARBP2,EIF3F,EIF4B,ACSS2,RPS3A,HTRA2,EIF2A,SPCS1,ORMDL1,FARS2,RPLP2,ACOT8,PCIF1,WARS2,FASTKD3,NT5C2,MRPL36,PCSK6,GPAM,ABHD4,DIS3L2,PUM1,APEH,GPX1,RPS6KB2,QARS,DALRD3,ANPEP,EIF4H,CERS2,PER2,RPS21,RPL35A,RPL11,YBX3,NFE2L2,IDH1,MRPL49,MTHFS,EXOSC7,CTSH,RPL27,UBA52,SMPD4,PLA2G16,RARA,TECR,ZFP36L1,MRPL10,ACOT2,PER1,GBA,GCDH,TNRC6A,LARP6,RPL7A,HEXA,EEF2K,MRPL4,EIF3G,ACSM3,ACACA,CERS4,NDUFA7,SLC46A1,RPL23A,PARN,MRPL37,EEF2,DPH1,MRPL41,MRPS34,INPP5E,MRPL28,GSTM4,RPL13,MRPS2,KARS,PTGES2,FPGS,ABCA2,RPS15,AGO4,RACK1,NT5C,RPS19,PLPP3,MRPL34,RPL23,EEF1B2,RPUSD3,MRPL57,RPS20,ZFP36L2,PDF,PSENEN,EXOSC5	cyan	EC_M2
GO:0070828	5.65E-03	0.998337641	10	40	heterochromatin organization	BP	1.35E-01	TRIM28,SETDB2,RRP8,SMARCA5,FAM208A,HELLS,TPR,HP1BP3,SUV39H1,SMCHD1	darkgrey	EC_M8
GO:0000184	5.65E-03	0.998476493	9	34	nuclear-transcribed mRNA catabolic process, nonsense-mediated decay	BP	1.35E-01	EIF3E,NCBP1,UPF2,DCP2,EXOSC10,UPF3B,GSPT1,RNPS1,RBM8A	darkgrey	EC_M8
GO:0017038	5.69E-03	0.997082094	27	174	protein import	BP	1.35E-01	NUP107,UBR5,TNPO3,TRIM28,NUP85,IPO5,NUP58,IPO11,IPO7,TNPO1,NUP54,NUP93,TOMM70,HEATR3,NOLC1,TIMM17A,CSE1L,NUP133,CDK1,TPR,GRPEL1,ECT2,KPNB1,NUP62CL,NUP155,AIFM1,NUP35	darkgrey	EC_M8
GO:0044728	5.69E-03	0.997878654	14	67	DNA methylation or demethylation	BP	1.35E-01	TRIM28,ZMPSTE24,RLF,EZH2,TDG,OTUD4,HELLS,MIS18A,TET2,PRMT5,BRCA1,ATRX,EHMT1,CTCF	darkgrey	EC_M8
GO:1903900	4.09E-03	0.998185545	21	104	regulation of viral life cycle	BP	1.35E-01	CXCL8,PPIE,RSAD2,CAV2,NECTIN2,OAS3,IFIT1,MX1,KIAA1551,ISG20,PROX1,LTF,DDX3X,TNIP1,LGALS9,ISG15,NOTCH1,OAS1,TRIM34,TRIM62,BST2	turquoise	EC_M6
GO:0050926	4.60E-03	0.99899935	8	21	regulation of positive chemotaxis	BP	1.35E-01	VEGFA,CXCL12,NTRK3,ITGA2,CDH13,F3,ARTN,FGF10	darkgreen	EC_M4

GO:0043067	4.61E-03	0.996439497	182	1119	regulation of programmed cell death	BP	1.35E-01	SERPINB2,LRP1,AHI1,AGAP2,IER3,CDSN,RRM2B,ANGPT1,TCP1,PPARA,PPARD,CTNNA1,UBQLN1,MTCH1,LGALS1,LEP,BAG1,VEGFA,DOCK8,VCP,UNC13B,NCK2,CREB3,ASNS,REST,TGFBR1,RTN4,TAX1BP1,GLI3,INHBA,DKKL1,SLC7A11,ITGB1,FGF2,SRPK2,RB1,MARK4,TPT1,GLI2,AXL,ACER3,CTNNB1,AKT2,RARB,ACTN4,UFM1,LIG4,IRS2,PRNP,HDAC3,DDRGK1,SMPD1,ILK,ARL6IP5,CXCL12,ANKRD1,HSP90B1,SLC25A4,FAIM2,RASA1,RTKN,RAPGEF2,CX3CL1,OPA1,AMIGO2,ACVR1,SERINC3,MTDH,ADA,TXNDC5,PLA2R1,IFT57,SLC39A10,FAP,TJP1,NOTCH2,CTTN,CDC73,DNM1L,SELENOS,EYA2,TGFB2,LRP5,HTR2B,ZMAT3,GSK3B,FSTL1,PIK3CA,LEF1,PRKCA,KRAS,FAM162A,KPNA1,BMP7,HYOU1,ATF3,FGFR2,PEA15,TFRC,ATF6,BACE1,FZD9,RPS6KA3,EGLN3,PDIA3,HSPB1,SERPINE1,SYVN1,PTPN6,PDCD10,ITGAV,COMP,HTT,FKBP8,PRKCI,SKIL,ARAF,TIMP1,VDAC2,FMN2,SIX4,SGMS1,MNAT1,ERC1,GHITM,MAGED1,CLN8,EIF2S1,MED1,PPARGC1A,ACTN1,CPEB4,NES,PNMA1,ARHGEF2,NGFR,TGFB3,SHC1,TAF9B,MYOCD,PIN1,CD74,PDGFRB,AKT1,PRKAA1,PTPN2,OMA1,CD40LG,MYDGF,DAPK3,BCAP31,TNFRSF18,FLNA,PARK7,ENO1,GNAI3,CFDP1,AARS,HSPA5,BCL10,NAE1,MIR24-1,BNIP3,LOX,VSTM2L,ITM2C,UBE2V2,ARF4,GDNF,TIMP3,GLO1,CDKN1A,SIGMAR1,NACC2,PTTG1IP,TSLP,PLAGL2,ZPR1,ORMDL3,CHST11,OLFM1,FGF10	darkgreen	EC_M4
GO:0010888	4.63E-03	0.999135348	7	17	negative regulation of lipid storage	BP	1.36E-01	PPARA,PPARD,LEP,ABHD5,OSBPL8,ITGAV,PTPN2	darkgreen	EC_M4
GO:0034472	5.75E-03	0.998963013	6	19	snRNA 3'-end processing	BP	1.36E-01	EXOSC9,EXOSC8,INTS7,INTS2,EXOSC2,INTS6	darkgrey	EC_M8
GO:0008154	4.15E-03	0.997783605	33	183	actin polymerization or depolymerization	BP	1.37E-01	ARHGAP18,BAIAP2L2,PICK1,CDC42EP1,TMOD1,FAM49A,SVIL,MSRB2,SPTBN4,FCHSD2,FCHSD1,LMOD3,HIP1R,IQGAP2,ESAM,HCLS1,AP1AR,TMOD4,DLG1,BAIAP2L1,WAS,TMOD2,RAC1,MICAL3,MTOR,ARHGAP28,SPIRE1,CARMIL2,VASP,GMFG,KANK3,GBA2,ARPC5L	turquoise	EC_M6
GO:0022607	4.70E-03	0.996132167	345	2230	cellular component assembly	BP	1.37E-01	ATP8B1,LRP1,STX7,AHI1,CAND1,PLXNB2,ANGPT1,TCP1,HLA-DRA,HSPA4,FAM49B,ADGRB1,CTNNA1,CBY1,UBQLN1,MDGA1,SH3GL2,TBCC,SRF,VEGFA,VCP,UNC13B,COL1A2,NCK2,DST,RAB23,TGFBR1,RIMS1,CENPC,RTN4,TPBG,SHANK1,COX18,LRRC4B,MFSD2A,CALD1,AKIRIN1,ACTR2,ROCK2,ZYX,MYLK3,VPS35,CFAP100,ITGB1,SRPK2,NAPA,RAB7A,ARHGEF4,EML2,RB1,PICALM,MARK4,NUFIP1,MAPRE3,PDCD6IP,TENM4,KCTD21,PCDH17,PAK1,ACTR3,TGFB1,RAB3GAP1,CTNNB1,DZIP1,PLEKHG2,DNAJB13,FARP1,ACTG1,NAV3,STAM2,KAT2B,FAM98A,PRNP,CDC42EP3,HDAC3,ZNF703,PCLO,KCTD16,ILK,DPYSL3,RYK,TTC17,CIAO1,CNOT7,RIC3,VPS37A,CXCL12,TM9SF4,SACS,KIF2A,TMEM41B,ANKRD1,HSP90B1,TMED2,EIF2S2,MAP1LC3A,TP53INP2,VAMP8,CTNND1,MAP1B,TEAD1,RYR3,ANKRA2,SORBS1,PFN2,RASA1,ATP2A2,CELF1,TCTN1,RTKN,APP,ATXN2,PLEKHA7,CCT8,RAPGEF2,CX3CL1,PTPN11,NEFM,HOMER1,OPA1,CDH17,ATG13,AMIGO2,ZFYVE27,TSG101,MTDH,GLS,CHP1,PKD2,CSDE1,TWF1,DNAH1,DNAH5,IFT57,PSMG1,PDLIM5,MAGEL2,RAB7B,ARL3,RAP1GDS1,DYNLL1,PHLDB2,TJP1,CALHM1,CTTN,PKP2,CDC73,DNM1L,DNER,TROVE2,HYAL1,LMOD1,SEC22B,SBDS,FBLN5,EPRS,KCTD10,FEZ1,ARMC9,PRKAR1A,WIPI1,GSK3B,MMP14,DAG1,PIK3CA,AUTS2,DNAAF5,CORO1C,ISCU,PRKCA,NEK7,DISP1,NTRK3,POLQ,HSPA8,PRPF3,BMP7,NDUFB3,ZRSR2,AP2M1,SYNPO2,CASQ1,ITGB5,TP53BP1,PHLDB1,OSBPL2,GORASP2,ADRM1,IQGAP1,SCFD1,STRAP,QSOX1,ABRAXAS2,TFRC,FARP2,GLRX3,SRP54,ARPC5,EIF3J,MAPT,PLOD3,DNAJA4,FN1,NCKAP1,MPP2,DNAJB12,PDCD	darkgreen	EC_M4
GO:0097485	4.70E-03	0.997351091	39	182	neuron projection guidance	BP	1.37E-01	LRP1,PLXNB2,VEGFA,GLI3,PLXNA1,KIF5B,GLI2,MEGF8,SEMA3C,RYK,CXCL12,PALLD,ROBO1,SEMA4F,APP,SEMA4G,NOTCH2,FEZ1,DAG1,ANOS1,BMP7,TNR,CRMP1,NOTCH3,ENAH,NGFR,TTC8,NPTN,LAMA3,PLXNB3,NTN3,PLXNA3,BSG,NEXN,DPYSL5,VSTM2L,GDNF,CHN1,ARTN	darkgreen	EC_M4
GO:0009060	2.62E-04	0.999879971	36	161	aerobic respiration	BP	1.37E-01	UQCRQ,NDUFB2,NOP53,UQCRH,NDUFB6,NDUFC2,NDUFA2,COX7A1,COQ9,DGUOK,NDUFV1,OXA1L,MTFR1L,NDUFS2,DNAJC30,IDH1,CHCHD10,UQCC3,NDUFB7,NUPR1,NDUFA7,UQCR11,NDUFS7,ATP5F1D,TAZ,MT-ND1,MT-ND2,MT-CO1,MT-CO2,MT-ATP6,MT-CO3,MT-ND3,MT-ND4L,MT-ND4,MT-ND5,MT-CYB	cyan	EC_M2
GO:0032787	2.64E-04	0.999846084	69	404	monocarboxylic acid metabolic process	BP	1.37E-01	ZADH2,HSD17B8,EP300,ETFB,PTGR1,IL4I1,LPIN1,ACSS1,ECHDC3,ACAA1,CYGB,HYI,HACL1,CYP1B1,THNSL2,CBR4,ACSS2,PRKAG1,LPIN3,ERLIN1,PLA2G4B,ACOT8,LONP2,ALDH5A1,IRS1,ACADS,GPAM,GPX1,ABCB11,PC,ARNT,PER2,HDAC4,SCAP,EHHADH,POR,IDH1,ACADL,MTHFS,PECR,CYP27A1,TNFRSF1A,FADS3,FOXK1,ILVBL,EPHX1,TECR,CRABP2,OSBPL7,HSD3B7,ACOT2,ABCD4,GCDH,NUPR1,SCPEP1,SLC27A3,ACSM3,ACACA,INSR,SREBF1,ACAA2,CPT2,GAMT,JMJD8,GSTM4,PTGES2,LDHD,ZBTB7A,SCP2	cyan	EC_M2

GO:0032943	4.19E-03	0.997803194	31	179	mononuclear cell proliferation	BP	1.38E-01	TNFRSF21,SYK,IL6,PELI1,TLR4,IL15,PIK3CG,GAPT,IL1A,IGF1,ITCH,SH3RF1,MEF2C,IL7,LMBR1L,ICOSLG,NFATC2,INPP5D,CD86,ACE,DLG1,SOS2,TNFRSF1B,ARG2,EFNB1,CHRNB2,CD19,LGALS9,ELF4,IL7R,CD46	turquoise	EC_M6
GO:0002685	4.21E-03	0.997901919	27	152	regulation of leukocyte migration	BP	1.38E-01	PTK2,IL6,CXCL8,C5,LRCH1,VEGFC,CXCL10,THBS4,ANO6,EDN1,JAM3,CD200,RIN3,APOD,ITGA4,MCU,SELE,SELP,PLVAP,RAC1,STK10,PGF,DAPK2,S100A14,MMP28,CCL2,LGALS9	turquoise	EC_M6
GO:1904996	4.22E-03	0.999337251	6	15	positive regulation of leukocyte adhesion to vascular endothelial cell	BP	1.38E-01	IL6,ETS1,ITGA4,SELE,SELP,NFAT5	turquoise	EC_M6
GO:0048469	4.23E-03	0.998039146	23	116	cell maturation	BP	1.38E-01	L3MBTL3,ANKS1A,PICK1,PTBP3,IL15,NRCAM,TUT4,TAL1,GATA2,PPARG,GATA3,EDNRB,SPTBN4,GPAT4,NTN4,RET,NR4A2,HIF1A,MTOR,B4GALT6,LHX6,SOX18,SCARF1	turquoise	EC_M6
GO:0060562	4.24E-03	0.997483677	46	269	epithelial tube morphogenesis	BP	1.38E-01	FZD6,CTHRC1,CELSR1,NOTCH4,MTSS1,KLHL3,PODXL,KDR,TEAD2,MYCN,NRP1,RASIP1,PRKD2,PLXND1,GATA3,CXCR4,GZF1,TIE1,SFRP1,RET,MEF2C,NDRG4,CXCL10,DLL4,EDN1,PRKX,SEC24B,PROX1,VANGL2,LAMA5,DLG1,WNK4,HIF1A,EPHA2,CECR2,AR,PSEN1,HOXB7,NRARP,NOTCH1,ENG,VASP,SOX17,SOX18,RALA,BMP2	turquoise	EC_M6
GO:0042982	4.76E-03	0.998113737	17	65	amyloid precursor protein metabolic process	BP	1.39E-01	RTN4,ROCK2,GSAP,ITM2B,PICALM,PRNP,CHRNA7,NCSTN,BACE1,RTN3,HAP1,TMED10,PIN1,ROCK1,MIR24-1,FKBP1A,ITM2C	darkgreen	EC_M4
GO:1901700	4.78E-03	0.996265011	200	1234	response to oxygen-containing compound	BP	1.39E-01	LRP1,AP3S1,CDO1,TAB2,PPARA,LY6E,PPARD,CTNNA1,LGALS1,TXN2,GNAQ,MTAP,LEP,PLAA,RORB,SRF,VCP,UNC13B,COL1A2,ASNS,TGFBR1,ACTR2,ROCK2,INHBA,VPS35,MGARP,FKBP1B,KIF5B,SESN3,PANX1,ESD,GLB1,EIF2B3,PAK1,TGFB1,AXL,HTR5A,HNMT,CTNNB1,DGAT2,AKT2,OSBPL8,KAT2B,IRS2,PRNP,ATP2B1,DCN,ZNF703,SMPD1,PPP2CB,LEPROTL1,APLP1,CXCL12,ANKRD1,HSP90B1,MAP1LC3A,CD180,OSBP,MAP1B,RYR3,SORBS1,P2RX4,SELENOT,ATP2A2,USO1,MMP15,APP,GLRB,DHX36,RAPGEF2,CX3CL1,ABCC8,PTPN11,ADCY6,HOMER1,OPA1,PTPN22,CRY2,MMP2,MTDH,ADA,ATP2B4,SPP1,PKD2,PSMD14,BRSK2,PSPH,CHRNA7,EIF4E,DYNLL1,ADIPOR1,CDC73,DNM1L,GLRX2,SELENOS,HYAL1,CCND1,CPT1A,TGFB2,ZBTB20,ZNF106,FBLN5,LRP5,EPRS,HTR2B,GSK3B,DAG1,PIK3CA,HADH,PRKCA,NTRK3,COL6A1,BMP7,ADCY5,WDTC1,FGFR2,NCSTN,SYAP1,IQGAP1,STRN3,BACE1,RPS6KA3,SIDT2,EDEM3,LINC02210-CRHR1,MAPT,SERPINE1,EIF4EBP2,COMT,KCNK4,IGFBP2,FBN1,PDCD10,UPF1,PRKCI,COL5A2,QDPR,TIMP1,PTPRN,CYP19A1,SGMS1,OTUD5,MYO5A,SLC2A4,MED1,PPARGC1A,MSN,CPEB4,ARHGEF2,NGFR,KCNMB1,TPM1,COL1A1,TGFB3,SHC1,CALM1,SMARCA4,PKM,PDGFRB,RBBP8,ROCK1,AKT1,ITGA2,LPIN2,PRKAA1,RAB31,GNAL,PTPN2,LDOC1,MYO1C,GNB1,SDF4,IRAK1,FLNA,PARK7,HCN2,BSG,AHCYL1,GNAI3,BCAR3,SLC2A8,HSPA5,BCL10,LOXL1,SERPINF1,BNIP3,PEMT,CACYBP,COL6A2,IL36G,CDKN1A,SIGMAR1,TXN,FAM210B,LEPROT,PIK3C2A,HCN4,WNT2,FGF10,MBD2	darkgreen	EC_M4
GO:0006814	4.79E-03	0.997391437	35	161	sodium ion transport	BP	1.39E-01	SLC12A2,SLC22A4,CNNM4,MFSD4B,KIF5B,SCN5A,GPD1L,SLC4A7,SLC38A10,ACTN4,FXYD1,SLC20A1,SNTA1,SLMAP,ATP1B3,P2RX4,SLC4A5,CHP1,ATP2B4,PKD2,PKP2,SHROOM2,SLC3A2,ASIC4,SLC6A13,MAGED2,CACNA1G,AKT1,SCNN1D,HCN2,AHCYL1,MIR24-1,DMD,YWHAH,HCN4	darkgreen	EC_M4
GO:0035304	4.79E-03	0.997984363	19	74	regulation of protein dephosphorylation	BP	1.39E-01	NUAK1,ROCK2,FKBP1B,TGFB1,PPP1R12A,SMPD1,HSP90B1,CRY2,SLC39A10,PPP6R3,GSK3B,PPP1R11,HTT,CALM1,PPP4R4,PIN1,PDGFRB,ROCK1,FKBP1A	darkgreen	EC_M4
GO:0106027	4.81E-03	0.997928788	20	78	neuron projection organization	BP	1.39E-01	SHANK1,ACTR2,VPS35,PRNP,APP,DHX36,HOMER1,OPA1,PDLIM5,CHRNA7,DOCK10,CTTN,DNM1L,CAPRIN2,GSK3B,KIF1A,TANC2,SRCIN1,PAFAH1B1,MAP1A	darkgreen	EC_M4
GO:1900180	5.89E-03	0.997328143	20	116	regulation of protein localization to nucleus	BP	1.39E-01	LATS1,CCT2,UBR5,TRIM28,CCT4,IPO5,NUP58,POLR1A,MCRS1,NUP54,CCT7,NOLC1,CCT5,CCT6A,CDK1,TPR,NMD3,ECT2,CCT3,PLK1	darkgrey	EC_M8
GO:0051865	5.89E-03	0.997887833	13	62	protein autoubiquitination	BP	1.39E-01	UHRF2,RAD18,RNF141,LTN1,UBE3A,CUL3,UBE2T,BRCA1,TAF1,TRIM37,UHRF1,RNF8,RNF219	darkgrey	EC_M8
GO:0030011	4.84E-03	0.999088485	7	17	maintenance of cell polarity	BP	1.40E-01	DST,LIN7B,PDCD6IP,GSK3B,ARPC5,WDR1,PDLIM1	darkgreen	EC_M4
GO:0046879	4.84E-03	0.997199075	42	206	hormone secretion	BP	1.40E-01	LRP1,PPARD,LEP,CLOCK,REST,INHBA,FKBP1B,KIF5B,C1QTNF1,IRS2,RAB11FIP1,PCLO,OSBP,VAMP8,SELENOT,ABCC8,PTPN11,PFKM,CRY2,SPP1,BRSK2,DYNLL1,KDM5B,CPT1A,LRP5,HADH,ADCY5,OSM,SIDT2,LINC02210-CRHR1,TFR2,PTPRN,CYP19A1,NLGN2,UBE2Q1,SYTL4,ACSL4,RASL10B,MIDN,SSTR5,VAMP7,PARK7	darkgreen	EC_M4

GO:0034329	4.85E-03	0.996888003	62	318	cell junction assembly	BP	1.40E-01	LRP1,PLXNB2,ADGRB1,CTNNA1,MDGA1,SRF,VEGFA,DST,TPBG,LRRC4B,ROCK2,VPS35,PDCD6IP,PCDH17,CTNNB1,FARP1,ACTG1,ZNF703,PCLO,RYK,CTNND1,MAP1B,SORBS1,APP,RAPGEF2,PTPN11,AMIGO2,MTDH,PDLIM5,PHLDB2,TJP1,CTTN,PKP2,DNER,MMP14,CORO1C,PRKCA,NTRK3,IQGAP1,FN1,VCL,WDR1,SIX4,OBSL1,NLGN2,ACTN1,TLN2,VMP1,NPTN,ROCK1,ITGA2,DAPK3,MYO1C,NPHP4,NTNG2,PCDHB2,LRRC24,UBE2V2,THY1,LHFPL4,DUSP22,LRRTM2	darkgreen	EC_M4
GO:0008643	4.86E-03	0.997630407	26	112	carbohydrate transport	BP	1.40E-01	SLC45A4,PPARD,LEP,SLC17A5,MFSD2A,SLC35A1,MFSD4B,SLC37A3,AKT2,OSBPL8,IRS2,SORBS1,PTPN11,SLC35A5,SELENOS,SLC2A10,PEA15,CLTCL1,PRKCI,SLC2A4,SLC35B1,AKT1,SLC2A6,SLC35A3,SLC2A8,SLC35C1	darkgreen	EC_M4
GO:0061820	5.92E-03	0.999467091	4	8	telomeric D-loop disassembly	BP	1.40E-01	RECQL4,POT1,TERF1,BLM	darkgrey	EC_M8
GO:2000273	4.30E-03	0.998804122	10	36	positive regulation of signaling receptor activity	BP	1.40E-01	NRP1,HBEGF,EDN1,HDAC1,NCF1,HIF1A,ADRB2,CCL2,RELN,SHANK3	turquoise	EC_M6
GO:0034765	4.31E-03	0.99734893	52	323	regulation of ion transmembrane transport	BP	1.40E-01	TRDN,DAPK1,TSPAN13,TCAF2,PIK3CG,KCNRG,P2RY6,KCNK6,PDE4D,CHD7,MEF2C,P2RX7,CXCL10,TPCN1,PLCG1,KCNJ3,ANO6,CLIC6,KCNJ15,SNCA,PM20D1,HECW2,KCNJ2,SCN3A,PRKD1,DLG1,KCNK7,CYBB,WNK4,CRACR2A,MTOR,PSEN1,ATP2A1,CD19,DNM2,ADRB2,CCL2,KCNJ18,PDE4B,SLC43A2,PLCG2,RELN,KCNH2,SCN2B,KCNK5,SHANK3,RGS4,KCNA6,CACNG5,FXYD6,KCNG2,GSG1L	turquoise	EC_M6
GO:0031125	5.96E-03	0.999395212	4	10	rRNA 3'-end processing	BP	1.40E-01	EXOSC9,EXOSC8,EXOSC10,EXOSC2	darkgrey	EC_M8
GO:0031100	5.96E-03	0.99795796	12	58	animal organ regeneration	BP	1.40E-01	PPAT,PNPT1,EZH2,WDR35,CCNA2,CAD,UPF2,PRMT5,CDK1,SRSF5,TYMS,AURKA	darkgrey	EC_M8
GO:0009790	4.90E-03	0.996323146	143	841	embryo development	BP	1.41E-01	AHI1,PLXNB2,FBN2,ANGPT1,RSPO3,LY6E,TEAD3,MYH9,SRF,VEGFA,RUNX2,NLRP5,TGFBR1,RTN4,ADAMTS3,KIDINS220,WHRN,GLI3,ROCK2,INHBA,ITGB1,FGF2,TENM4,GLI2,MEGF8,TGFB1,WDR48,CTNNB1,RARB,MAFG,SPINT2,LIG4,SEMA3C,IMPAD1,TMED2,EIF2S2,SULF1,TEAD1,RDH10,LOXL3,HEY1,TLX2,CELF1,TCTN1,MMP15,DHX36,TET3,OPA1,ACVR1,MMP2,ADA,PKD2,NKD1,TENT5C,IFT57,PSPH,PHLDB2,NOTCH2,CDC73,TRIP12,HYAL1,SLC2A10,EYA2,SBDS,TGFB2,LRP5,HTR2B,SATB2,PRKAR1A,ITPK1,MMP14,DAG1,LEF1,COL6A1,BMP7,WDTC1,FGFR2,VASH2,ITGB5,PHLDB1,RAI2,TANC2,PLOD3,ATP6AP2,FZD2,FN1,MEOX1,FBN1,ITGAV,FKBP8,COL5A2,SKIL,ECE1,PRRX1,GORAB,TULP3,SIX4,GREM2,FKBP10,UGDH,CACNA1C,BMPR1A,TCAP,MED1,MYO1E,NES,TGFB1I1,COL1A1,TGFB3,MAP2K1,TMEM100,PPP4R4,ACSL4,LAMA3,RBBP8,ROCK1,AKT1,UBE2A,ITGA2,C6,SUPT6H,NXN,PAFAH1B1,PKD1,COL5A1,HSBP1,COL11A1,LMO4,BCL10,WLS,DUSP2,PEMT,LBX1,GDNF,DUSP4,ARFRP1,HOPX,ZPR1,SLC39A3,CHST11,WNT2,FGF10,MBD2	darkgreen	EC_M4
GO:0071447	4.34E-03	0.999678777	4	7	cellular response to hydroperoxide	BP	1.41E-01	OXR1,DAPK1,CD36,PRKD1	turquoise	EC_M6
GO:0033280	4.35E-03	0.99890059	9	30	response to vitamin D	BP	1.41E-01	PIM1,IL15,SFRP1,CXCL10,STC1,MN1,FES,FGF23,TRIM24	turquoise	EC_M6
GO:0099565	4.36E-03	0.998264293	17	74	chemical synaptic transmission, postsynaptic	BP	1.41E-01	RIMS2,GRIK2,MTMR2,P2RX2,MEF2C,P2RX7,SNCA,PPP3CA,INSYN2,ADORA2A,CHRNB1,CHRNB2,ADRB2,SLC8A3,RELN,SHANK3,RGS4	turquoise	EC_M6
GO:1990830	6.02E-03	0.99764024	15	77	cellular response to leukemia inhibitory factor	BP	1.41E-01	SRSF3,WDR35,RIF1,MRPL15,SMARCA5,HELLS,HK2,MYBL2,NCL,XRCC5,HNRNPU,CTH,SRSF7,UBXN2A,NUP35	darkgrey	EC_M8
GO:0051085	4.96E-03	0.998759111	9	26	chaperone cofactor-dependent protein refolding	BP	1.42E-01	BAG1,DNAJB13,HSPH1,HSPA8,DNAJB12,ERO1A,CD74,HSPA5,HSPA13	darkgreen	EC_M4
GO:0048678	4.96E-03	0.998023616	17	65	response to axon injury	BP	1.42E-01	LRP1,CTNNA1,LGALS1,TXN2,FKBP1B,FGF2,DPYSL3,MAP1B,P2RX4,SPP1,DAG1,NTRK3,TNR,MAP2K1,FLNA,BNIP3,ARF4	darkgreen	EC_M4
GO:0051292	6.14E-03	0.999441307	4	8	nuclear pore complex assembly	BP	1.44E-01	NUP107,NUP205,NUP93,NDC1	darkgrey	EC_M8
GO:1902235	5.07E-03	0.998726519	9	26	regulation of endoplasmic reticulum stress-induced intrinsic apoptotic signaling pathway	BP	1.44E-01	NCK2,CREB3,OPA1,SERINC3,SELENOS,HYOU1,SYVN1,PTPN2,PARK7	darkgreen	EC_M4

GO:0007420	5.07E-03	0.996378189	104	587	brain development	BP	1.44E-01	LRP1,AHI1,PLXNB2,TRAPPC9,SUN2,MDGA1,SRF,UBA6,RTN4,MFSD2A,WHRN,GLI3,INHBA,SLC7A11,PCDH18,ITGB1,FGF2,KIF5B,NAPA,EIF2B3,GLI2,PAK1,AXL,RAB3GAP1,SCN5A,HTR5A,HNMT,CTNNB1,RARB,TACC1,UFM1,IRS2,ATP2B1,DCLK1,APLP1,RYK,SNPH,CXCL12,ZSWIM6,CTNND1,ROBO1,FAIM2,CELF1,TCTN1,APP,RAPGEF2,PTPN11,AMIGO2,PPP3CC,ATP2B4,DNAH5,PSMG1,DYNLL1,INA,UCHL5,FEZ1,SATB2,GSK3B,LEF1,SHROOM2,KRAS,PITPNM1,BMP7,FGFR2,NCSTN,KIF1A,MACO1,PAFAH1B2,ARPC5,TNR,COL3A1,KCNA1,TULP3,MNAT1,AKT3,HAP1,BMPR1A,MED1,PPARGC1A,NES,MAP2K1,TTC8,CALM1,EML1,MYO1D,ROGDI,PAFAH1B1,SDF4,FLNA,PLXNA3,RERE,GNAI3,SYPL2,AARS,HSPA5,ATP6V0D1,LHX8,WLS,BNIP3,ARF4,YWHAH,PRKG1,WNT2,FGF10	darkgreen	EC_M4
GO:0019932	4.49E-03	0.997470646	38	218	second-messenger-mediated signaling	BP	1.45E-01	PDE7B,RIMS2,KDR,SYK,CXCL8,TRPM4,NOS3,CXCR4,EDNRB,PDE2A,P2RX2,CD36,PDE4D,IGF1,EIF2AK3,MCTP1,P2RX7,GUCY1A1,GUCY1B1,PLCG1,RAPGEF3,EDN1,ADCY2,PDE9A,PPP3CA,MCTP2,NFATC2,ADCY1,CCRL2,HOMER3,MCU,SELE,SELP,MTOR,NPR1,NOS2,PLCG2,NFAT5	turquoise	EC_M6
GO:0033006	4.53E-03	0.998971622	8	25	regulation of mast cell activation involved in immune response	BP	1.45E-01	SYK,GATA2,GAB2,UNC13D,FGR,FES,LGALS9,STXBP1	turquoise	EC_M6
GO:0030258	2.90E-04	0.999867218	35	171	lipid modification	BP	1.46E-01	DGKA,CERK,PLPP6,ETFB,SPHK2,ACAA1,CYGB,PIP4P1,LAMTOR1,HACL1,PLPP5,ACOT8,LONP2,IRS1,ACADS,MTMR11,ABCB11,EHHADH,POR,ACADL,SOAT1,ILVBL,ABCD4,GBA,GCDH,MTMR4,ACAA2,MTMR1,CPT2,INPP5E,PLPPR3,LCAT,ABCA2,PLPP3,SCP2	cyan	EC_M2
GO:0043412	6.24E-03	0.994876689	325	3029	macromolecule modification	BP	1.46E-01	CDK2,MEX3C,CLK4,USP15,SHPRH,PPIL4,LATS1,FBXO43,FBXO5,CSNK1G3,UBR5,MAP3K4,DSCC1,XRCC6,CDC25C,NAA35,SRPK1,UHRF2,PPIL1,PLEC,UBR2,PWP1,TOPORS,DBF4,AKAP9,PUM3,CKS2,C1GALT1,ICK,MELK,TRIM28,ZNF451,PPIH,FBXO11,SENP6,ASB3,PHIP,TTK,PPP4R3B,IBTK,PUS10,ZMPSTE24,RLF,USP34,COMMD1,DPY19L1,FKBP15,ETAA1,ITGB1BP1,EZH2,UFL1,CUL1,USP45,CLSPN,ASCC3,NAA15,PLK4,PUS7,HDAC2,CCNA2,SAE1,CEP295,CHORDC1,SETDB2,SYMPK,MASTL,ZC3H13,COG3,TRDMT1,CAD,ALG8,MBD4,PPM1G,BORA,CCNT2,PAXIP1,ULK4,NCAPG2,CDC20,BIRC5,NKTR,FNTA,PARP2,KAT14,GTPBP4,RAD18,IPO5,EPC2,KAT6A,METTL3,RIF1,ZDHHC17,TPP2,NSD3,KITLG,TAF7,PRKD3,CUL4A,METAP2,BRCA2,PPP4R2,CDC16,MAPK8,PRKDC,CCNB1,ERCC6,PAN3,SCYL2,LNX2,TGS1,BUB1,TPX2,ANAPC1,DIMT1,UBA2,IPO7,TNKS2,SGK3,PARP4,CDK5RAP1,PPWD1,FER,TDG,NCK1,ARFGEF1,RNF141,OTUD4,ZFP91,GALNT6,TAF9,ATR,HELLS,USP25,HLTF,PBK,ESCO2,ANAPC7,MCRS1,SH2B3,LTN1,USP16,PPID,MOB1A,NAA25,CPNE3,BUB1B,MIS18A,CCNL1,SMYD5,CDCA2,NBN,SENP1,TOP1,ABRAXAS1,RAD51,VIRMA,RIPK1,PRPF4B,CCNE2,TRIM33,RIOK1,CHUK,STK4,MORC3,PUS7L,UBE2C,GXYLT1,HERC5,TAOK3,OGA,SMARCAD1,BMPR1B,UBE3A,CIT,CUL3,METAP1,NSUN2,ALG10,UBE2T,SLK,TRMT10A,CHEK1,ASB7,NAA50,QTRT2,STT3A,PTP4A2,CENPE,TET2,TRIP11,PPP2R2D,TRAIP,COPS7B,KIF14,PRMT5,AJUBA,UBR1,GAR1,TARBP1,FXR1,FANCI,MCM3AP,NEK2,DTL,HMBS,ALS2,PPIG,BLM,SSB,UBR3,G2E3,FTSJ3,CDKL5,HAT1,CDK1,CDC25A,PDE8A,ING5,BAZ1B,SETD2,CTDSPL2,IVNS1ABP,PIKFYVE,CDC27,ABL2,FANCM,DBF4B	darkgrey	EC_M8
GO:0050435	5.15E-03	0.998165493	14	50	amyloid-beta metabolic process	BP	1.46E-01	RTN4,ROCK2,GSAP,PICALM,PRNP,CHRNA7,NCSTN,BACE1,RTN3,HAP1,TMED10,PIN1,ROCK1,MIR24-1	darkgreen	EC_M4
GO:2001233	5.17E-03	0.996760354	55	292	regulation of apoptotic signaling pathway	BP	1.46E-01	RRM2B,CTNNA1,UBQLN1,NCK2,CREB3,TGFBR1,INHBA,RB1,TPT1,CTNNB1,CXCL12,FAIM2,CX3CL1,OPA1,ACVR1,SERINC3,CTTN,DNM1L,SELENOS,EYA2,GSK3B,HYOU1,ATF3,PEA15,FZD9,PDIA3,HSPB1,SERPINE1,SYVN1,ITGAV,HTT,SKIL,VDAC2,SGMS1,GHITM,MAGED1,ARHGEF2,NGFR,TAF9B,CD74,AKT1,PTPN2,DAPK3,BCAP31,PARK7,ENO1,GNAI3,BCL10,ITM2C,GDNF,TIMP3,NACC2,PTTG1IP,PLAGL2,FGF10	darkgreen	EC_M4
GO:0035162	4.60E-03	0.999109731	7	19	embryonic hemopoiesis	BP	1.47E-01	ATF4,KIT,KDR,TAL1,GATA2,GATA3,HIF1A	turquoise	EC_M6
GO:0099536	4.63E-03	0.996885005	76	494	synaptic signaling	BP	1.48E-01	KIF5A,PDE7B,STX11,AKAP12,SNCAIP,RIMS2,PACSIN2,ATF4,PICK1,APBA1,KIT,PPT1,GRIK2,DLGAP3,SDCBP,RAB3B,MTMR2,FCHSD2,PLCB4,FCHSD1,P2RX2,EPHB1,PLK2,NR4A1,ABHD6,CLCN3,MCTP1,CHRM5,MEF2C,P2RX7,GUCY1A1,GUCY1B1,PLCG1,DGKZ,EDN1,SNCA,DTNBP1,SHANK2,PPP3CA,SLC12A7,MCTP2,HCRTR1,SNAP23,ADCY1,RPS6KA1,YTHDF1,INSYN2,DLG1,PTCHD1,CREB1,BCR,ADORA2A,IL1RAP,SNAP29,TMOD2,SNCG,SYT7,CHRNB1,RGS14,FBXL20,MTOR,OPHN1,PSEN1,CHRNB2,ADRB2,CCL2,UNC119,GIT1,STXBP1,SLC8A3,RELN,SCN2B,SHANK3,RGS4,STX3,CACNG5	turquoise	EC_M6
GO:0048066	4.63E-03	0.998802406	9	32	developmental pigmentation	BP	1.48E-01	KIT,RAB38,EDNRB,MEF2C,ADAMTS20,USP13,RAB27A,AP3D1,RAB32	turquoise	EC_M6

GO:0048525	4.64E-03	0.99832904	14	61	negative regulation of viral process	BP	1.48E-01	RSAD2,OAS3,IFIT1,STAT1,MX1,KIAA1551,ISG20,PROX1,LTF,TNIP1,ISG15,OAS1,TRIM62,BST2	turquoise	EC_M6
GO:0007009	4.65E-03	0.997967331	20	94	plasma membrane organization	BP	1.48E-01	PACSIN2,MTSS1,ASAP1,BAIAP2L2,MICALL1,CAV2,IZUMO1,CXCR4,TIE1,P2RX7,DYSF,ANO6,ANO3,BAIAP2L1,SYT7,MPP5,AR,DNM2,SH3TC2,FNBP1L	turquoise	EC_M6
GO:0002224	4.65E-03	0.998010218	19	92	toll-like receptor signaling pathway	BP	1.48E-01	TNFAIP3,GRAMD4,LRRC19,PELI1,RSAD2,TLR4,IRAK2,CD36,TLR2,LTF,DDX3X,BIRC3,TLR6,S100A14,TNIP1,PLCG2,DAB2IP,OAS1,TLR1	turquoise	EC_M6
GO:0030168	5.23E-03	0.997568082	23	96	platelet activation	BP	1.48E-01	MYH9,SRF,SLC7A11,DGKH,AXL,C1QTNF1,ACTG1,ILK,MYL9,PIK3CA,PRKCA,HSPB1,ITGA2B,FN1,PTPN6,ADRA2C,COMP,COL3A1,VCL,CD40LG,FLNA,CSRP1,PRKG1	darkgreen	EC_M4
GO:0008347	5.23E-03	0.99832352	12	40	glial cell migration	BP	1.48E-01	LRP1,SUN2,RTN4,GLI3,CTNNB1,P2RX4,CX3CL1,NDN,TGFB2,MMP14,IDH2,PAFAH1B1	darkgreen	EC_M4
GO:1901699	5.25E-03	0.996336713	91	508	cellular response to nitrogen compound	BP	1.48E-01	LRP1,AP3S1,LY6E,CTNNA1,GNAQ,LEP,COL1A2,ACTR2,ROCK2,VPS35,MGARP,SESN3,PAK1,HTR5A,CTNNB1,AKT2,OSBPL8,KAT2B,IRS2,PRNP,ATP2B1,LEPROTL1,APLP1,HSP90B1,MAP1B,RYR3,SORBS1,P2RX4,USO1,APP,RAPGEF2,PTPN11,ADCY6,OPA1,PTPN22,MMP2,ATP2B4,PKD2,CHRNA7,ADIPOR1,SELENOS,ZNF106,EPRS,HTR2B,GSK3B,CASP7,PIK3CA,COL6A1,ADCY5,WDTC1,NCSTN,SYAP1,UROS,BACE1,LINC02210-CRHR1,EIF4EBP2,FBN1,PRKCI,COL5A2,MYO5A,SLC2A4,PPARGC1A,CPEB4,ARHGEF2,NGFR,COL1A1,SHC1,PKM,GRIA1,ROCK1,AKT1,LPIN2,PRKAA1,RAB31,GNAL,PTPN2,LDOC1,MYO1C,GNB1,FLNA,HCN2,AHCYL1,GNAI3,BCAR3,SLC2A8,HSPA5,TMEM38B,TMEM38A,LEPROT,PIK3C2A,HCN4	darkgreen	EC_M4
GO:0098739	5.27E-03	0.997334253	28	123	import across plasma membrane	BP	1.48E-01	SLC12A2,SLC22A4,KCNK9,SLC39A4,SLC31A1,SLC7A11,ITGB1,SLC36A4,AKT2,IRS2,PRNP,ARL6IP5,ATP1B3,ATP2B4,SLC39A10,SLC2A10,ISCU,SLC3A2,SLC6A13,CLN8,SLC7A3,SLC39A6,TRPV2,AKT1,HCN2,MIR24-1,SLC36A1,HCN4	darkgreen	EC_M4
GO:0060393	5.28E-03	0.998212212	13	44	regulation of pathway-restricted SMAD protein phosphorylation	BP	1.48E-01	LRP1,TGFBR1,INHBA,TGFB1,RBPMS,ACVR1,GDF6,TGFB2,BMP7,BMPR1A,TGFB3,PIN1,PMEPA1	darkgreen	EC_M4
GO:1902533	5.28E-03	0.996108994	124	731	positive regulation of intracellular signal transduction	BP	1.48E-01	IL23A,LRP1,TAB2,ANGPT1,PTP4A3,PPARD,LGALS1,KLHDC10,LEP,VEGFA,SLC35B2,NCK2,TGFBR1,RTN4,TPBG,EMC10,MAP3K7,ROCK2,ITGB1,FGF2,RHEB,PAK1,TGFB1,AXL,SEC13,CTNNB1,DYNC1LI1,CANT1,C1QTNF1,OSBPL8,CXXC5,ACTN4,ATP2C1,DCN,HDAC3,CAVIN3,ILK,ARL6IP5,RYK,TMEM9B,ST5,SLC20A1,ANKRD1,PJA2,ROBO1,P2RX4,RNF13,APP,DHX36,RAPGEF2,CX3CL1,PTPN11,PTPN22,GDF6,SERINC3,MTDH,ADA,PLA2R1,CHRNA7,NOTCH2,DNM1L,TGFB2,HTR2B,PIK3CA,AUTS2,PRKCA,KRAS,NTRK3,SPATC1L,GOLT1B,FGFR2,OSM,IQGAP1,TFRC,FN1,PTPN6,GPR137,PDCD10,ITGAV,ADRA2C,COL3A1,SKIL,SYNPO2L,FGFR3,TNIK,OTUD5,AKT3,MAGED1,SHC1,ADRA1B,MAP2K1,GPR65,TSPAN6,SLC44A2,NPTN,CD74,PDGFRB,ROCK1,PTPN2,CLEC16A,MYDGF,BCAP31,IRAK1,FLNA,ATP6AP1,IKBKG,PARK7,CDH13,F3,BCAR3,BCL10,WLS,MIR24-1,CCL16,FKBP1A,AGO3,C18orf32,EDA,TXN,NACC2,GLIPR2,DUSP22,PLAGL2,FGF10	darkgreen	EC_M4
GO:1903053	4.70E-03	0.998563383	11	43	regulation of extracellular matrix organization	BP	1.49E-01	IL6,TIE1,TCF15,ETS1,CFLAR,MELTF,FSCN1,TNFRSF1B,NOTCH1,CARMIL2,BMP2	turquoise	EC_M6
GO:0033601	5.35E-03	0.999648113	4	6	positive regulation of mammary gland epithelial cell proliferation	BP	1.50E-01	RTN4,ZNF703,KDM5B,CCND1	darkgreen	EC_M4
GO:0043604	3.08E-04	0.999798962	106	672	amide biosynthetic process	BP	1.50E-01	ORMDL2,PYM1,CDK4,EIF3H,RPS18,B3GALT4,EEF1D,RPL3,EIF3L,EIF3D,RPL8,RPS5,RPL28,RPS9,EEF1A1,MALSU1,RPS7,RPS11,FOXO3,SPHK2,RPL18,ABTB1,ACSS1,RPS8,MIF4GD,MLH1,UNK,RPS3,RPS16,SAMD4B,MRPS12,RPL15,EIF3K,TARBP2,EIF3F,EIF4B,ACSS2,RPS3A,EIF2A,ORMDL1,FARS2,RPLP2,PCIF1,WARS2,FASTKD3,MRPL36,DIS3L2,PUM1,APEH,RPS6KB2,QARS,DALRD3,EIF4H,CERS2,PER2,RPS21,RPL35A,RPL11,YBX3,NFE2L2,MRPL49,EXOSC7,RPL27,UBA52,SMPD4,RARA,TECR,ZFP36L1,MRPL10,PER1,GBA,GCDH,TNRC6A,LARP6,RPL7A,EEF2K,MRPL4,EIF3G,ACACA,CERS4,NDUFA7,RPL23A,PARN,MRPL37,EEF2,DPH1,MRPL41,MRPS34,INPP5E,MRPL28,RPL13,MRPS2,KARS,RPS15,AGO4,RACK1,RPS19,MRPL34,RPL23,EEF1B2,RPUSD3,MRPL57,RPS20,ZFP36L2,PDF,EXOSC5	cyan	EC_M2
GO:0051896	5.39E-03	0.997266307	28	125	regulation of protein kinase B signaling	BP	1.50E-01	ANGPT1,PPARA,LEP,TGFBR1,RTN4,TPBG,RRAS,RCN3,ITGB1,FGF2,SESN3,TGFB1,AXL,C1QTNF1,OSBPL8,CAVIN3,ILK,P2RX4,CX3CL1,MTDH,DAG1,PIK3CA,NTRK3,OSM,MYDGF,PARK7,F3,TXN	darkgreen	EC_M4

GO:0031623	4.80E-03	0.997999013	18	85	receptor internalization	BP	1.51E-01	PICK1,SYK,CXCL8,SDCBP,MTMR2,CD36,SNCA,RIN3,CALCRL,RAMP2,DNM3,SELE,OPHN1,DNM2,AP2B1,PLCG2,CACNG5,GSG1L	turquoise	EC_M6
GO:0010976	5.44E-03	0.997135052	31	140	positive regulation of neuron projection development	BP	1.52E-01	LRP1,PLXNB2,PACSIN1,P3H1,TMEM30A,ACTR2,KIDINS220,FKBP1B,DPYSL3,ANKRD1,DHX36,RAPGEF2,CX3CL1,OPA1,ARSB,TWF1,DNM1L,CAPRIN2,FEZ1,NTRK3,BMP7,IQGAP1,GPC2,PRKCI,NPTN,PLXNB3,PAFAH1B1,FLNA,HSPA5,SERPINF1,UBE2V2	darkgreen	EC_M4
GO:0001832	6.61E-03	0.998786963	6	18	blastocyst growth	BP	1.53E-01	NCAPG2,GINS4,BRCA2,NBN,CHEK1,PALB2	darkgrey	EC_M8
GO:0033993	4.87E-03	0.996573857	94	657	response to lipid	BP	1.53E-01	TNFRSF11A,TNFAIP3,AKAP12,KDM4C,PIM1,CSF2RB,SYK,IL6,CXCL8,PELI1,FOXP2,LANCL2,TLR4,IL15,TEAD2,NOCT,GATA2,DNMT3A,NOS3,PPARG,TIMP4,IRAK2,EDNRB,TIE1,NFKBIB,SFRP1,CD36,SCARB1,RET,GRAMD1A,IL1A,DNMT3B,NR4A1,KDM3A,ERBIN,UFSP2,MEF2C,SMARCD1,TLR2,CXCL10,DHH,KMT2D,STC1,EDN1,SNCA,NFKB2,ADCY2,ABCG1,ETS1,HDAC1,PABPN1,CD86,MN1,CFLAR,ADCY1,FES,ARID1A,IL10RA,PDK3,CREB1,SSTR1,LTF,ARNT2,CYBB,TBX1,RAMP2,SELE,SELP,FGF23,ADIPOR2,BID,TNFRSF1B,ALDH1A2,AR,S100A14,SMAD2,SCNN1B,CYP1A1,TNIP1,CCL2,YES1,PDE4B,LGALS9,NOS2,GIT1,NOTCH1,IRF8,PLCG2,ENG,STXBP1,DAB2IP,TRIM24,CA2,BMP6	turquoise	EC_M6
GO:0032735	4.87E-03	0.99886898	8	26	positive regulation of interleukin-12 production	BP	1.53E-01	SYK,TLR4,CD36,TLR2,LAPTM5,LGALS9,IRF8,PLCG2	turquoise	EC_M6
GO:0034113	4.89E-03	0.998614084	10	36	heterotypic cell-cell adhesion	BP	1.54E-01	IZUMO1,CD58,JAM3,CD200,LCK,ITGA4,JUP,DSG2,DSC2,PERP	turquoise	EC_M6
GO:0035507	5.53E-03	0.999632797	4	6	regulation of myosin-light-chain-phosphatase activity	BP	1.54E-01	NUAK1,ROCK2,PPP1R12A,ROCK1	darkgreen	EC_M4
GO:0048659	5.54E-03	0.997180154	28	128	smooth muscle cell proliferation	BP	1.54E-01	FAM19A5,PPARD,FGF2,PAK1,TGFB1,CTNNB1,ILK,CX3CL1,PTPN11,MMP2,FGFR2,COMT,MNAT1,BMPR1A,PPARGC1A,TPM1,TGFB3,CNN1,MYOCD,PDGFRB,AKT1,ITGA2,NPR3,IRAK1,GNAI3,CDH13,CDKN1A,PRKG1	darkgreen	EC_M4
GO:0060079	4.91E-03	0.998081773	16	69	excitatory postsynaptic potential	BP	1.54E-01	RIMS2,GRIK2,MTMR2,P2RX2,MEF2C,P2RX7,SNCA,PPP3CA,ADORA2A,CHRNB1,CHRNB2,ADRB2,SLC8A3,RELN,SHANK3,RGS4	turquoise	EC_M6
GO:0006702	4.93E-03	0.999579981	4	8	androgen biosynthetic process	BP	1.54E-01	HSD17B3,SRD5A3,SCARB1,SRD5A2	turquoise	EC_M6
GO:1990823	6.66E-03	0.997354496	15	78	response to leukemia inhibitory factor	BP	1.54E-01	SRSF3,WDR35,RIF1,MRPL15,SMARCA5,HELLS,HK2,MYBL2,NCL,XRCC5,HNRNPU,CTH,SRSF7,UBXN2A,NUP35	darkgrey	EC_M8
GO:1901978	6.68E-03	0.999332908	4	9	positive regulation of cell cycle checkpoint	BP	1.54E-01	GEN1,TPR,NDC80,MAD2L1	darkgrey	EC_M8
GO:1902430	5.57E-03	0.998729728	8	22	negative regulation of amyloid-beta formation	BP	1.54E-01	RTN4,PRNP,CHRNA7,RTN3,HAP1,PIN1,ROCK1,MIR24-1	darkgreen	EC_M4
GO:0071639	4.95E-03	0.999619445	4	7	positive regulation of monocyte chemotactic protein-1 production	BP	1.54E-01	SYK,OAS3,LGALS9,OAS1	turquoise	EC_M6
GO:0034427	6.70E-03	0.999295443	4	10	nuclear-transcribed mRNA catabolic process, exonucleolytic, 3'-5'	BP	1.55E-01	EXOSC9,EXOSC8,TTC37,EXOSC2	darkgrey	EC_M8
GO:0014032	5.00E-03	0.998043191	16	69	neural crest cell development	BP	1.56E-01	SEMA6A,SEMA4D,NRP1,EDNRB,JAG1,RET,EDN1,SEMA3F,LAMA5,SEMA6D,TBX1,HIF1A,ALDH1A2,EFNB1,SEMA4A,SEMA6B	turquoise	EC_M6
GO:0032722	5.04E-03	0.998348351	12	48	positive regulation of chemokine production	BP	1.57E-01	SYK,IL6,TLR4,C5,TLR2,IL7,OAS3,HDAC1,DDX3X,HIF1A,LGALS9,OAS1	turquoise	EC_M6
GO:0060249	5.67E-03	0.996640438	44	218	anatomical structure homeostasis	BP	1.57E-01	SLC12A2,ANGPT1,SH3GL2,LARGE1,SRF,VEGFA,MFSD2A,WHRN,NCDN,RCN3,ITGB1,RB1,CTNNB1,ACTG1,ATP2B1,IL20RB,TP53INP2,P2RX4,CX3CL1,ABCC8,PTPN11,PFKM,HOMER1,SPP1,TJP1,PRKCA,KRAS,STRAP,TFRC,HSPB1,GPR137,VCL,AKT3,CLN8,PRKAA1,ABCA3,NPHP4,ATP6AP1,DEF8,DMD,LOX,SNX10,MAP1A,CFL2	darkgreen	EC_M4
GO:0030217	5.07E-03	0.99728258	32	179	T cell differentiation	BP	1.57E-01	TCF7,KIT,SYK,IL6,RSAD2,IL15,GATA3,CHD7,IL1A,SH3RF1,IL7,LMBR1L,DLL4,SOX13,LCK,HLX,CD86,B2M,SOS2,CRACR2A,ITPKB,LFNG,MTOR,SEMA4A,BATF,JAG2,LGALS9,IL7R,AP3D1,NRARP,VNN1,CD46	turquoise	EC_M6

GO:0048729	5.11E-03	0.99654319	76	490	tissue morphogenesis	BP	1.57E-01	FZD6,CTHRC1,CELSR1,NOTCH4,MTSS1,KLHL3,PODXL,KDR,TEAD2,MYCN,NRP1,RASIP1,TAL1,PRKD2,PLXND1,ITGA8,NOS3,ITGB4,GATA3,CXCR4,GZF1,TIE1,JAG1,SFRP1,HBEGF,FERMT1,FREM2,SPEF1,NTN4,SYNE4,TCF15,RET,CHD7,SMAD1,MEF2C,VEGFC,NDRG4,CXCL10,STC1,DLL4,EDN1,STAT1,PRKX,SEC24B,JHY,PROX1,VANGL2,LAMA5,DLG1,IGFBP5,TBX1,WNK4,RAC1,HIF1A,EPHA2,CECR2,MPP5,ALDH1A2,MTOR,AR,OPHN1,PSEN1,HOXB7,ITGA3,SMAD2,JAG2,NRARP,NOTCH1,S1PR1,ENG,CARMIL2,VASP,SOX17,SOX18,RALA,BMP2	turquoise	EC_M6
GO:0035909	5.12E-03	0.998689165	9	28	aorta morphogenesis	BP	1.57E-01	JAG1,ADAMTS9,CHD7,DLL4,SEC24B,PROX1,TBX1,NOTCH1,ENG	turquoise	EC_M6
GO:0002696	5.12E-03	0.997130875	36	218	positive regulation of leukocyte activation	BP	1.57E-01	SYK,IL6,PELI1,TLR4,IL15,GATA2,CTSC,NECTIN2,GAB2,GATA3,IL1A,IGF1,MEF2C,IL7,SOX13,LCK,ICOSLG,HLX,NFATC2,INPP5D,CD86,FGR,B2M,ITPKB,TLR6,EFNB1,CHRNB2,CCL2,YES1,LGALS9,IL7R,AP3D1,STXBP1,HAVCR1,VNN1,CD46	turquoise	EC_M6
GO:0072001	5.13E-03	0.996962119	44	256	renal system development	BP	1.57E-01	MTSS1,KLHL3,PDGFB,PODXL,TEK,NRP1,GPR4,PLXND1,ITGA8,GATA3,GZF1,JAG1,SFRP1,FREM2,RET,SMAD1,MEF2C,WWTR1,ANGPT2,TIPARP,STAT1,HYAL2,PRKX,CD34,PECAM1,CFLAR,PROX1,VANGL2,LAMA5,ACE,DLG1,WNK4,PROM1,ARG2,ALDH1A2,HOXB7,ITGA3,SMAD2,AP2B1,NOTCH1,CA2,SOX17,BMP2,BMP6	turquoise	EC_M6
GO:0051050	5.13E-03	0.996378236	95	660	positive regulation of transport	BP	1.57E-01	TNFRSF11A,TRDN,PDGFB,PICK1,ATP8A1,CD2AP,SYK,PPT1,TLR4,TCAF2,IL15,SDCBP,TRPM4,RAB3B,MTMR2,GATA2,GAB2,UNC13D,PPARG,EDNRB,P2RY6,P2RY2,P2RX2,CD36,SIRT3,PRR5L,MERTK,IL1A,IGF1,HIP1R,OCLN,TLR2,P2RX7,VEGFC,CXCL10,PLCG1,STC1,RAPGEF3,ANO6,EDN1,SNCA,DTNBP1,ABCG1,PPP3CA,HYAL2,ANO1,KCNJ2,MEF2A,HCLS1,FGR,PRKD1,OAZ3,DLG1,CREB1,B2M,ADORA2A,MCU,WNK4,DNM3,SELE,CRACR2A,TMEM30B,RAC1,HIF1A,JUP,RAB27A,ADIPOR2,SYT7,PSEN1,SLC51B,CHRNB2,ATP2A1,CD19,RAB3D,NPR1,GPR68,DNM2,AP2B1,ABLIM3,ADRB2,CCL2,MYO18A,RPH3AL,PLCG2,STXBP1,RIPOR1,PLA2G4E,CA2,RELN,KCNH2,SHANK3,TBC1D20,BMP2,CBLL1,BMP6	turquoise	EC_M6
GO:0022604	5.13E-03	0.996940168	45	261	regulation of cell morphogenesis	BP	1.57E-01	RIMS2,CDC42SE2,CDKL3,ARHGAP18,PTK2,CDC42EP1,STRIP2,KIT,KDR,SEMA4D,NRP1,ITSN2,FGD5,PLXND1,UNC13D,CXCR4,ARHGEF7,FGD6,PRAG1,HEXB,DNMBP,NEDD9,GAS2,AP1AR,EPB41,PLEKHO1,PLXNA2,FGR,FES,SS18L1,F11R,DOCK1,MELTF,DLG1,MOV10,RAC1,RCC2,RHOJ,FGD1,SEMA4A,TAOK2,DNM2,CCL2,PALMD,RELN	turquoise	EC_M6
GO:0033008	5.17E-03	0.999353152	5	11	positive regulation of mast cell activation involved in immune response	BP	1.58E-01	SYK,GATA2,GAB2,FGR,STXBP1	turquoise	EC_M6
GO:0043306	5.17E-03	0.999353152	5	11	positive regulation of mast cell degranulation	BP	1.58E-01	SYK,GATA2,GAB2,FGR,STXBP1	turquoise	EC_M6
GO:0097502	5.76E-03	0.998374085	10	31	mannosylation	BP	1.59E-01	NUS1,LARGE1,TMTC4,TMTC3,DPY19L4,PIGZ,POMT1,DPM2,DPM1,PIGB	darkgreen	EC_M4
GO:0002062	5.77E-03	0.997342073	22	91	chondrocyte differentiation	BP	1.59E-01	RUNX2,TGFBR1,GLI3,GLI2,TGFB1,CTNNB1,SERPINH1,RARB,IMPAD1,SULF1,PTPN11,LOXL2,GDF6,RUNX1,CHSY1,COMP,FGFR3,BMPR1A,ANXA6,COL11A1,GLG1,CHST11	darkgreen	EC_M4
GO:0019221	5.24E-03	0.996821457	47	306	cytokine-mediated signaling pathway	BP	1.59E-01	TNFRSF11A,IFNGR1,TNFAIP3,PDGFB,CSF2RB,KIT,SYK,IL6,CXCL8,TRAF1,IL15,PPARG,CXCR4,IRAK2,IKBKB,TNFRSF19,IL1A,IL7,CXCL10,OAS3,NLRC5,HIPK1,EDN1,STAT1,MX1,LAPTM5,IL10RA,CCRL2,IL1RAP,BIRC3,PLVAP,HIF1A,ADIPOR2,TNFRSF1B,RPS6KA5,CCL2,IL13RA1,JAK1,IL7R,ISG15,TRAF2,TNFRSF25,NOTCH1,MIR99A,OAS1,ACKR1,COMMD7	turquoise	EC_M6
GO:0001569	5.24E-03	0.99877865	8	25	branching involved in blood vessel morphogenesis	BP	1.59E-01	NOTCH4,KDR,NRP1,PLXND1,DLL4,EDN1,NRARP,ENG	turquoise	EC_M6

GO:0042981	5.81E-03	0.995497407	177	1092	regulation of apoptotic process	BP	1.60E-01	SERPINB2,LRP1,AHI1,AGAP2,IER3,RRM2B,ANGPT1,TCP1,PPARA,PPARD,CTNNA1,UBQLN1,MTCH1,LGALS1,LEP,BAG1,VEGFA,DOCK8,VCP,UNC13B,NCK2,CREB3,ASNS,REST,TGFBR1,RTN4,TAX1BP1,GLI3,INHBA,DKKL1,SLC7A11,ITGB1,SRPK2,RB1,TPT1,GLI2,AXL,CTNNB1,AKT2,RARB,ACTN4,UFM1,LIG4,IRS2,PRNP,HDAC3,DDRGK1,SMPD1,ILK,ARL6IP5,CXCL12,ANKRD1,HSP90B1,FAIM2,RASA1,RTKN,RAPGEF2,CX3CL1,OPA1,AMIGO2,ACVR1,SERINC3,MTDH,ADA,TXNDC5,PLA2R1,IFT57,SLC39A10,FAP,TJP1,NOTCH2,CTTN,CDC73,DNM1L,SELENOS,EYA2,TGFB2,LRP5,HTR2B,ZMAT3,GSK3B,FSTL1,PIK3CA,LEF1,PRKCA,KRAS,FAM162A,KPNA1,BMP7,HYOU1,ATF3,FGFR2,PEA15,TFRC,ATF6,BACE1,FZD9,RPS6KA3,EGLN3,PDIA3,HSPB1,SERPINE1,SYVN1,PTPN6,PDCD10,ITGAV,COMP,HTT,FKBP8,PRKCI,SKIL,ARAF,TIMP1,VDAC2,FMN2,SIX4,SGMS1,MNAT1,ERC1,GHITM,MAGED1,CLN8,EIF2S1,MED1,PPARGC1A,ACTN1,CPEB4,NES,PNMA1,ARHGEF2,NGFR,TGFB3,SHC1,TAF9B,MYOCD,PIN1,CD74,PDGFRB,AKT1,PRKAA1,PTPN2,OMA1,CD40LG,MYDGF,DAPK3,BCAP31,TNFRSF18,FLNA,PARK7,ENO1,GNAI3,CFDP1,AARS,HSPA5,BCL10,NAE1,MIR24-1,BNIP3,LOX,VSTM2L,ITM2C,UBE2V2,ARF4,GDNF,TIMP3,GLO1,CDKN1A,SIGMAR1,NACC2,PTTG1IP,TSLP,PLAGL2,ZPR1,ORMDL3,CHST11,OLFM1,FGF10	darkgreen	EC_M4
GO:0006950	6.99E-03	0.994293086	290	2715	response to stress	BP	1.60E-01	CDK2,BCLAF1,RPA3,USP15,SHPRH,MDC1,DDX39B,FBXO5,UBR5,MAP3K4,GTSE1,RAD21,XRCC6,FANCC,HSPA9,MATR3,SRPK1,RMI1,PLEC,PSIP1,RECQL4,TONSL,MCM5,POT1,TOPORS,APTX,POLR3B,POLH,IL1R1,MCM3,MIOS,REV1,MELK,TRIM28,MSH2,PNPT1,ZMPSTE24,SYNCRIP,PABPC4,COMMD1,ETAA1,EZH2,UFL1,MMS22L,USP45,CLSPN,ASCC3,CASP2,DDX1,SMC6,GEN1,DHFR,RINT1,HDAC2,MCM2,CCNA2,DNAJC2,RAD54L,CHORDC1,RNASEH2B,WDR33,MASTL,CLASP2,DDIAS,DCLRE1C,MCM10,CAD,MBD4,PAXIP1,MCM6,FANCD2,ULK4,PARP2,POLR3F,RAD18,EPC2,SUPT16H,KAT6A,METTL3,RIF1,E2F7,GINS4,POLE,RFC3,CUL4A,BRCA2,PPP4R2,LARS,TOPBP1,ELK3,RRP8,MAPK8,PRKDC,APAF1,CBX5,CCNB1,MCM4,ERCC6,NFKBID,MTREX,SP1,IFIT5,IPO7,PARPBP,LSM14A,PARP4,FER,EIF2B1,TDG,NCK1,ARFGEF1,OTUD4,TAF9,PDE12,ATR,CDKN2AIP,USP25,HK2,PBK,ESCO2,MCRS1,SH2B3,USP16,RBL1,MSH3,NBN,ABRAXAS1,RAD51,SLC38A2,DCLRE1B,TOMM70,RIPK1,ARID2,COL4A3BP,INO80,EEF1E1,CHUK,MDM4,CHAF1B,HERC5,RFC5,TAOK3,SMARCAD1,NOLC1,BMPR1B,UBE3A,NUCKS1,DEK,GCN1,CUL3,METAP1,UBE2T,CBSL,CHEK1,NFKB1,SMC3,TRIP13,CHD1L,TRAIP,FGFR1OP2,AJUBA,GTF2I,BPTF,FXR1,FANCI,DDX5,DGKD,FANCB,TICRR,RFC2,RPA2,SPO11,EIF2B5,TIAL1,INTS7,DTL,ALS2,BLM,ATRIP,AUNIP,XPR1,CCDC47,CDK1,PDE8A,FOSL1,BAZ1B,DHX9,TRA2B,SETD2,PSMA6,POLA1,RFC4,DNA2,TPR,DDX21,CDCA5,LANCL1,FANCM,IL12A,C17orf53,APEX2,XRCC5,CDC45,BRCA1,MCM7,TFPI,NSD2,LETM1,WDHD1,HP1BP3,DCUN1D5,TIPRL,RAD51AP1,BOD1L1,ANKLE1,ECT2,SUV39H1,POLR3A,FOXM1,EIF2AK1,LYAR,FH,DDX1	darkgrey	EC_M8
GO:0002275	5.30E-03	0.998057012	14	62	myeloid cell activation involved in immune response	BP	1.61E-01	KIT,SYK,PIK3CG,GATA2,GAB2,UNC13D,DYSF,SNAP23,FGR,FES,SCNN1B,LGALS9,PLCG2,STXBP1	turquoise	EC_M6
GO:0007143	7.06E-03	0.99841497	7	25	female meiotic nuclear division	BP	1.61E-01	FBXO5,TTK,TRIP13,TOP2A,CCNB2,PLK1,AURKA	darkgrey	EC_M8
GO:0040017	5.88E-03	0.995941455	83	462	positive regulation of locomotion	BP	1.61E-01	LRP1,ANGPT1,NUS1,SUN2,PLAA,VEGFA,DOCK8,CREB3,PTP4A1,TGFBR1,RTN4,TPBG,EMC10,AKIRIN1,ROCK2,BVES,RRAS,ITGB1,FGF2,MEGF8,PAK1,TGFB1,AKT2,ACTG1,ACTN4,IRS2,ZNF703,SEMA3C,DDRGK1,ILK,CXCL12,SEMA4F,P2RX4,APP,RAPGEF2,CX3CL1,PGAM4,ACVR1,SEMA4G,TJP1,DNM1L,HYAL1,TGFB2,MMP14,LEF1,PRKCA,CMKLR1,NTRK3,BMP7,IQGAP1,S100A11,LRRC15,FAM89B,HSPB1,SERPINE1,ITGA2B,FN1,PDCD10,ITGAV,AKT3,ARHGEF2,COL1A1,RAB11A,CD74,PDGFRB,AKT1,ITGA2,DAPK3,MYO1C,TNFRSF18,FLNA,CDH13,F3,HSPA5,CD151,THY1,HAS2,ARTN,TMSB4X,GLIPR2,CCR6,PIK3C2A,FGF10	darkgreen	EC_M4
GO:0045453	5.37E-03	0.99821716	12	50	bone resorption	BP	1.62E-01	TNFRSF11A,TNFAIP3,SYK,IL6,IL7,P2RX7,INPP5D,RAC1,RAB3D,ADRB2,S1PR1,CA2	turquoise	EC_M6
GO:0006541	7.15E-03	0.998629864	6	20	glutamine metabolic process	BP	1.63E-01	PPAT,CTPS1,CAD,GMPS,ASNSD1,PFAS	darkgrey	EC_M8
GO:0070873	5.95E-03	0.998466528	9	26	regulation of glycogen metabolic process	BP	1.63E-01	DYRK2,AKT2,IRS2,SORBS1,SELENOS,GSK3B,PPP1R3D,PPP1R3F,AKT1	darkgreen	EC_M4

GO:0006897	5.43E-03	0.996388586	69	444	endocytosis	BP	1.64E-01	SNX2,SYNJ2,PACSIN2,CSNK1E,PICK1,MICALL1,CD2AP,SYK,SNAP91,PPT1,CXCL8,ARHGAP25,ELMO1,CAV2,SDCBP,PIK3CG,ITSN2,MTMR2,GATA2,FCHSD2,CD36,SCARB1,CLCN3,HIP1R,MCTP1,LMBR1L,BMP2K,TPCN1,STAB1,ANO6,EHD4,SNCA,PPP3CA,RIN3,ATP9A,SH3BP4,INPP5F,PRKD1,IL10RA,DOCK1,B2M,ARHGAP27,NECAP1,ITGA4,CALCRL,HSPG2,RAMP2,DNM3,SELE,FCHO1,RAC1,RHOJ,SNCG,PEAR1,OPHN1,RABEP2,DNM2,AP2B1,ADRB2,UNC119,MYO18A,PLCG2,LRSAM1,FNBP1L,HAVCR1,CACNG5,SCARF1,CBLL1,GSG1L	turquoise	EC_M6
GO:0002699	5.43E-03	0.997282939	26	142	positive regulation of immune effector process	BP	1.64E-01	DDX58,KIT,SYK,IL6,RSAD2,TLR4,GATA2,NECTIN2,GAB2,UNC13D,GATA3,CD36,LAPTM5,HLX,CD86,FGR,FES,B2M,LGALS9,NOS2,MYO18A,TRAF2,PLCG2,STXBP1,CD46,FCER2	turquoise	EC_M6
GO:0018401	5.99E-03	0.99926226	5	10	peptidyl-proline hydroxylation to 4-hydroxy-L-proline	BP	1.64E-01	P4HA2,EGLN3,PRDX4,P4HA1,ERO1A	darkgreen	EC_M4
GO:1901214	6.00E-03	0.996323283	49	251	regulation of neuron death	BP	1.64E-01	LRP1,AGAP2,ANGPT1,PPARA,REST,VPS35,SLC7A11,SRPK2,PICALM,AXL,CTNNB1,LIG4,PRNP,ILK,FAIM2,RASA1,CX3CL1,CHP1,CHRNA7,TGFB2,GSK3B,INPP5A,PIK3CA,KRAS,HYOU1,BACE1,FZD9,EGLN3,MAPT,PRKCI,SIX4,EIF2S1,PPARGC1A,CPEB4,NES,TGFB3,PIN1,AKT1,IKBKG,TMEM259,PARK7,AARS,NAE1,SERPINF1,VSTM2L,UBE2V2,GDNF,SIGMAR1,ZPR1	darkgreen	EC_M4
GO:0090100	6.02E-03	0.997279757	21	85	positive regulation of transmembrane receptor protein serine/threonine kinase signaling pathway	BP	1.64E-01	CDKN2B,KIAA1324L,TGFBR1,INHBA,TGFB1,ZEB2,ILK,RBPMS,SULF1,ACVR1,GDF6,NOTCH2,SLC2A10,TGFB2,BMP7,FKBP8,BMPR1A,RNF111,TGFB1I1,TGFB3,MYOCD	darkgreen	EC_M4
GO:0048598	6.03E-03	0.995821	85	469	embryonic morphogenesis	BP	1.64E-01	AHI1,PLXNB2,FBN2,RSPO3,SRF,RUNX2,TGFBR1,WHRN,GLI3,ROCK2,INHBA,ITGB1,FGF2,TENM4,GLI2,MEGF8,TGFB1,CTNNB1,RARB,SPINT2,IMPAD1,TMED2,SULF1,RDH10,TLX2,TCTN1,MMP15,OPA1,ACVR1,MMP2,PKD2,IFT57,PHLDB2,NOTCH2,CDC73,HYAL1,EYA2,TGFB2,LRP5,HTR2B,SATB2,PRKAR1A,MMP14,DAG1,LEF1,COL6A1,BMP7,FGFR2,ITGB5,PHLDB1,FZD2,FN1,FBN1,ITGAV,COL5A2,ECE1,PRRX1,TULP3,SIX4,GREM2,UGDH,CACNA1C,BMPR1A,TCAP,MED1,TGFB1I1,TGFB3,LAMA3,ROCK1,ITGA2,COL5A1,HSBP1,COL11A1,LMO4,BCL10,WLS,DUSP2,LBX1,GDNF,DUSP4,ARFRP1,SLC39A3,CHST11,WNT2,FGF10	darkgreen	EC_M4
GO:0051048	6.06E-03	0.997031683	25	110	negative regulation of secretion	BP	1.65E-01	LEP,REST,INHBA,FKBP1B,RAB7A,RAB11FIP1,VAMP8,ABCC8,PTPN11,CRY2,ADA,PLA2R1,HADH,IDH2,OSM,COMT,ADRA2C,SRCIN1,SYT11,SYTL4,CD74,MIDN,RHBDF1,CBARP,STXBP6	darkgreen	EC_M4
GO:0033500	6.07E-03	0.996540813	38	184	carbohydrate homeostasis	BP	1.65E-01	LRP1,PPARD,LGALS1,LEP,SRF,UNC13B,FKBP1B,KIF5B,SESN3,IRS2,OSBP,SELENOT,ABCC8,HECTD4,PTPN11,PFKM,OPA1,CRY2,TENT4B,BRSK2,DYNLL1,ADIPOR1,ZBTB20,LRP5,PIK3CA,ADCY5,SIDT2,PTPRN,SLC2A4,PPARGC1A,NGFR,SMARCA4,AKT1,PRKAA1,PTPN2,SSTR5,PARK7,SERPINF1	darkgreen	EC_M4
GO:0002009	6.08E-03	0.995873146	76	412	morphogenesis of an epithelium	BP	1.65E-01	AHI1,PLXNB2,RSPO3,MRTFA,SRF,VEGFA,GLI3,PLEKHA4,PLXNA1,FGF2,GLI2,MEGF8,PAK1,TGFB1,CTNNB1,ACTG1,SPINT2,STARD13,SEMA3C,ILK,RYK,PRICKLE2,FAT1,TMED2,SULF1,RDH10,TCTN1,OPA1,ACVR1,PKD2,NKD1,IFT57,BRSK2,PHLDB2,KDM5B,NOTCH2,TGFB2,LRP5,MMP14,DAG1,LEF1,KRAS,BMP7,FGFR2,CLIC4,PLOD3,FZD2,PDCD10,GORAB,SMURF1,EXOC5,VCL,DAAM1,WDR1,TULP3,SIX4,MAGED1,TCAP,MED1,MSN,TGFB1I1,NGFR,TTC8,LAMA3,PAFAH1B1,PKD1,FLNA,COL5A1,LMO4,BCL10,CD151,SERPINB5,LBX1,GDNF,WNT2,FGF10	darkgreen	EC_M4
GO:0098543	5.47E-03	0.99956663	4	7	detection of other organism	BP	1.65E-01	TLR4,TLR2,TLR6,TLR1	turquoise	EC_M6
GO:0000266	6.11E-03	0.998000164	12	40	mitochondrial fission	BP	1.65E-01	FAM49B,VPS35,DCN,SLC25A46,OPA1,AP3B1,DNM1L,MAPT,C11orf65,PPARGC1A,MIEF2,BNIP3	darkgreen	EC_M4
GO:1902570	7.32E-03	0.998899516	5	14	protein localization to nucleolus	BP	1.66E-01	NOL8,RRS1,POLR1A,MCRS1,NMD3	darkgrey	EC_M8
GO:0001666	6.19E-03	0.996219699	48	244	response to hypoxia	BP	1.67E-01	PPARA,PPARD,UBQLN1,TXN2,LEP,SRF,VEGFA,REST,ROCK2,MGARP,PAK1,CXCL12,TM9SF4,ANKRD1,HSP90B1,PLOD2,OPA1,LOXL2,MMP2,ADA,HIF1AN,CHRNA7,DNM1L,TGFB2,MMP14,FAM162A,BMP7,HYOU1,SCFD1,EGLN3,ERO1A,FMN2,SLC2A4,PPARGC1A,KCNMB1,TGFB3,MYOCD,PIN1,AKT1,ITGA2,PRKAA1,GNB1,VASN,IRAK1,ENO1,SLC2A8,BNIP3,PDLIM1	darkgreen	EC_M4
GO:0032637	5.57E-03	0.998033449	13	57	interleukin-8 production	BP	1.67E-01	LRRC19,DDX58,SYK,IL6,TLR4,PRKD2,TLR2,CD58,HYAL2,TLR6,LGALS9,NOS2,TLR1	turquoise	EC_M6
GO:0030534	5.59E-03	0.997568679	19	90	adult behavior	BP	1.67E-01	OXR1,AGTPBP1,TMOD1,PPT1,SPTBN4,CHD7,PREX2,CLCN3,NR4A2,SNCA,SHANK2,ZFHX2,INPP5F,HOXD10,SNCG,CHRNB1,HOXB8,CHRNB2,SHANK3	turquoise	EC_M6

GO:0060075	5.61E-03	0.999552959	4	7	regulation of resting membrane potential	BP	1.67E-01	KCNK6,KCNJ2,PSEN1,KCNK5	turquoise	EC_M6
GO:0150077	5.63E-03	0.998821516	7	22	regulation of neuroinflammatory response	BP	1.67E-01	IL6,CST7,CTSC,IGF1,CD200,TNFRSF1B,PLCG2	turquoise	EC_M6
GO:0002720	5.67E-03	0.998229749	11	42	positive regulation of cytokine production involved in immune response	BP	1.67E-01	DDX58,KIT,IL6,RSAD2,TLR4,GATA3,CD36,LAPTM5,B2M,TRAF2,PLCG2	turquoise	EC_M6
GO:0002369	5.67E-03	0.998811985	7	22	T cell cytokine production	BP	1.67E-01	IL6,RSAD2,TRPM4,GATA3,B2M,TNFRSF1B,TRAF2	turquoise	EC_M6
GO:0002724	5.67E-03	0.998811985	7	22	regulation of T cell cytokine production	BP	1.67E-01	IL6,RSAD2,TRPM4,GATA3,B2M,TNFRSF1B,TRAF2	turquoise	EC_M6
GO:0014821	5.67E-03	0.999243422	5	12	phasic smooth muscle contraction	BP	1.67E-01	KIT,EDNRB,P2RX2,EDN1,DLG1	turquoise	EC_M6
GO:0061000	5.69E-03	0.999271206	5	11	negative regulation of dendritic spine development	BP	1.67E-01	ASAP1,PLK2,SRGAP2C,DNM3,EFNA1	turquoise	EC_M6
GO:0042325	5.69E-03	0.99574877	133	967	regulation of phosphorylation	BP	1.67E-01	TNFRSF11A,TNFAIP3,PTPRB,FLT4,TPD52L1,PTK2,PDGFB,TEK,LIMCH1,TEC,KIT,KDR,SYK,SEMA4D,IL6,GRB10,TLR4,IL15,CCNY,SDCBP,NRP1,RASIP1,PIK3CG,TAL1,KDM4D,PRKD2,CAB39L,RALB,PPARG,EDNRB,SPTBN4,TIE1,IKBKB,SFRP1,HBEGF,SH3BP5,ANKLE2,CD36,PRR5L,EPHB1,RET,PDE4D,MERTK,IGF1,EIF2AK3,HHEX,OCLN,GPD1,WWTR1,IL7,P2RX7,VEGFC,MADD,NLRC5,THBS4,RAPGEF3,DGKZ,CCNJL,NEDD9,EDN1,EHD4,SNCA,DTNBP1,HDAC1,HYAL2,NCF1,FABP4,HCLS1,PECAM1,CD86,FGR,INPP5F,IGFBP3,PPP2R5A,PROX1,PRKD1,MLXIPL,ACE,ITGA6,LIMK2,PAK2,DLG1,MAP3K11,LTF,ADORA2A,EPHB4,DDX3X,TBX1,LDB2,TNFAIP8L3,FGF23,LMTK2,PPM1F,RAC1,HIF1A,ADIPOR2,EPHA2,ITPKB,TLR6,RGS14,MTOR,AR,PSEN1,FGF18,PGF,TAOK2,EFNA1,CD19,PIK3R5,RPS6KA5,CCDC88C,ADRB2,YES1,SH2D3A,UNC119,GIT1,TRAF2,PLCG2,ENG,SH2D3C,DAB2IP,CDH5,SLC8A3,RELN,ALKAL1,FGF16,MMD,DAZAP2,COPS8,BMP2,DUSP7,PIFO,BMP6	turquoise	EC_M6
GO:0007229	5.69E-03	0.997468614	20	92	integrin-mediated signaling pathway	BP	1.67E-01	PTK2,TEC,SYK,NRP1,ITGA8,ITGA9,ITGB4,FERMT1,ERBIN,NEDD9,FGR,PRKD1,LAMA5,ITGA6,DOCK1,ITGA4,RCC2,ITGA3,ISG15,FYB1	turquoise	EC_M6
GO:0071305	5.70E-03	0.998831913	7	20	cellular response to vitamin D	BP	1.67E-01	PIM1,IL15,SFRP1,MN1,FES,FGF23,TRIM24	turquoise	EC_M6
GO:0021522	6.25E-03	0.998544218	8	22	spinal cord motor neuron differentiation	BP	1.68E-01	GLI3,GLI2,TCTN1,SCYL1,MDGA2,CLN8,LMO4,LBX1	darkgreen	EC_M4
GO:0031573	7.44E-03	0.998853649	5	15	mitotic intra-S DNA damage checkpoint signaling	BP	1.69E-01	MDC1,MSH2,FANCD2,EME1,TIPIN	darkgrey	EC_M8
GO:0043524	6.35E-03	0.99682618	26	115	negative regulation of neuron apoptotic process	BP	1.71E-01	LRP1,AGAP2,ANGPT1,AXL,LIG4,ILK,FAIM2,RASA1,CX3CL1,PIK3CA,KRAS,HYOU1,FZD9,PRKCI,SIX4,PPARGC1A,CPEB4,NES,TGFB3,PIN1,PARK7,AARS,VSTM2L,UBE2V2,GDNF,ZPR1	darkgreen	EC_M4
GO:0002009	5.82E-03	0.996166426	65	412	morphogenesis of an epithelium	BP	1.71E-01	FZD6,CTHRC1,CELSR1,NOTCH4,MTSS1,KLHL3,PODXL,KDR,TEAD2,MYCN,NRP1,RASIP1,PRKD2,PLXND1,GATA3,CXCR4,GZF1,TIE1,JAG1,SFRP1,HBEGF,FERMT1,FREM2,SPEF1,NTN4,SYNE4,TCF15,RET,MEF2C,VEGFC,NDRG4,CXCL10,DLL4,EDN1,STAT1,PRKX,SEC24B,JHY,PROX1,VANGL2,LAMA5,DLG1,IGFBP5,WNK4,RAC1,HIF1A,EPHA2,CECR2,MPP5,ALDH1A2,MTOR,AR,OPHN1,PSEN1,HOXB7,JAG2,NRARP,NOTCH1,ENG,CARMIL2,VASP,SOX17,SOX18,RALA,BMP2	turquoise	EC_M6
GO:0001702	6.37E-03	0.998733243	7	17	gastrulation with mouth forming second	BP	1.71E-01	SRF,TENM4,MEGF8,CTNNB1,ACVR1,LRP5,UGDH	darkgreen	EC_M4
GO:0006959	5.89E-03	0.997613095	16	84	humoral immune response	BP	1.72E-01	LYZ,TNFRSF21,IL6,CXCL8,C5,GATA3,MEF2C,IL7,CXCL10,ST6GAL1,LTF,MASP1,CCL2,NOTCH1,CD46,FCER2	turquoise	EC_M6
GO:1903428	5.89E-03	0.998962094	6	17	positive regulation of reactive oxygen species biosynthetic process	BP	1.72E-01	TLR4,CD36,CLCN3,RAB27A,TLR6,PLCG2	turquoise	EC_M6

GO:0002551	5.89E-03	0.999206663	5	12	mast cell chemotaxis	BP	1.72E-01	KIT,VEGFC,RIN3,RAC1,PGF	turquoise	EC_M6
GO:0051775	6.45E-03	0.998918543	6	14	response to redox state	BP	1.73E-01	CLOCK,SLC7A11,FKBP1B,GLRX2,SELENOS,VASN	darkgreen	EC_M4
GO:1902680	7.68E-03	0.994130801	146	1246	positive regulation of RNA biosynthetic process	BP	1.73E-01	ONECUT2,BCLAF1,DDX39B,SCAF8,HSF2,ATAD2,XRCC6,PHF5A,IFT74,PWP1,ZNF300,TOPORS,RFX3,TRIM28,ZNF451,FOXJ3,PHIP,RLF,ZNF292,ITGB1BP1,THRAP3,CCNC,NAA15,ELF2,ATAD2B,HDAC2,CCNA2,EXOSC9,MLLT10,DNAJC2,NUP85,ELF1,RSF1,CCNT2,PAXIP1,WDR43,SUPT16H,CHD8,KAT6A,E2F7,ALYREF,CKAP2,CEP290,NSD3,CEBPZ,TAF7,BRCA2,ELK3,TCERG1,PRKDC,ERCC6,GPBP1,SP1,TCEA1,ZC3H8,ZNF143,CENPJ,NCK1,SMARCA5,ZNF507,TAF9,TFCP2,UTP15,HLTF,GABPA,MCRS1,USP16,SENP1,RIPK1,WDR75,MYBL2,E2F8,INO80,CHUK,HIVEP1,NOLC1,BMPR1B,UBE3A,PRDM10,NUCKS1,GCN1,CHEK1,H2AFZ,RRP1B,NFKB1,TET2,TRIP11,HEATR1,NCL,NOL11,GTF2I,BPTF,BLM,EDRF1,ING5,BCLAF3,FOSL1,SMARCC1,SP3,DHX9,CCAR1,UBTF,NMD3,NPAT,BRCA1,CNOT9,DCAF6,DHX33,TBL1XR1,LEO1,FOXM1,LYAR,DDX11,HNRNPU,SMARCE1,RFC1,TOP2A,NSD1,MED6,ICE2,PMF1,FUS,ZNF609,OGT,ATRX,ZNF711,ILF2,TRIM37,ILF3,MED13,AATF,TAF15,THOC1,NIPBL,MYSM1,UHRF1,RBMX,TP73,CTCF,PRIM2,HNRNPD,DRAP1,ZNF770,ZNF782,ZFP62,SFR1	darkgrey	EC_M8
GO:0042113	5.95E-03	0.996730317	33	193	B cell activation	BP	1.73E-01	TNFAIP3,MFNG,KIT,TNFRSF21,SYK,IL6,PELI1,TLR4,SFRP1,GAPT,HHEX,MEF2C,IL7,TPD52,ADGRG3,DCAF1,ICOSLG,LAPTM5,NFATC2,INPP5D,CD86,ITGA4,LFNG,BATF,CHRNB2,CD19,YY1,LYL1,IL7R,IRF8,PLCG2,AKAP17A,BST2	turquoise	EC_M6
GO:0006525	5.97E-03	0.998945929	6	17	arginine metabolic process	BP	1.73E-01	NOS3,SLC39A8,SLC7A7,FAH,ARG2,NOS2	turquoise	EC_M6
GO:0072594	7.73E-03	0.995185254	47	355	establishment of protein localization to organelle	BP	1.74E-01	NUP107,CCT2,UBR5,TNPO3,POT1,GCC2,TRIM28,CCT4,PMPCB,SAE1,NUP85,PITRM1,IPO5,VPS36,BRCA2,MAPK8,NUP58,IPO11,IPO7,TERF1,TNPO1,HK2,NUP54,CCT7,NUP93,TOMM70,HEATR3,NOLC1,CCT5,CCT6A,TIMM17A,CSE1L,NUP133,CDK1,TPR,GRPEL1,ECT2,NEDD4,KPNB1,CCT3,TIMM8A,NUP62CL,NUP155,NIPBL,AIFM1,PMPCA,NUP35	darkgrey	EC_M8
GO:0051091	6.00E-03	0.996641882	35	204	positive regulation of DNA-binding transcription factor activity	BP	1.74E-01	TNFRSF11A,DDX58,KIT,ADGRF1,IL6,TLR4,TRAF1,PRKD2,PPARG,IRAK2,IKBKB,TMEM173,CD36,TLR2,EDN1,CD200,PPP3CA,HCLS1,CFLAR,ADCY1,PRKD1,LTF,IL1RAP,PRKCH,JUP,TLR6,AR,RPS6KA5,LGALS9,TRAF2,PLCG2,TRIM34,RELN,TRIM62,BMP2	turquoise	EC_M6
GO:0000904	6.00E-03	0.995807426	87	562	cell morphogenesis involved in differentiation	BP	1.74E-01	SEMA6A,KIF5A,NOTCH4,PARVG,CDKL3,PTK2,ATF4,TEK,SEMA4D,NRCAM,NRP1,TAL1,NOVA2,PLXND1,ITGA8,UNC13D,GATA3,MYO7A,SPTBN4,FEZ2,EFNB2,ARHGEF7,NTN4,EPHB1,RET,MERTK,PREX2,KIF13B,MEF2C,STC1,NR4A2,NEDD9,DTNBP1,PPP3CA,HECW2,MEF2A,SEMA3F,ROBO4,SEC24B,AP1AR,PLXNA2,TRAK2,ADCY1,KALRN,PROX1,SS18L1,LAMA5,YTHDF1,DOCK1,PAK2,MELTF,LAMC2,CREB1,NBEAL2,SEMA6D,EPHB4,ITGA4,DNM3,LMTK2,RAC1,RCC2,EPHA2,AR,OPHN1,EFNB1,PSEN1,SEMA4A,ABI3,TAOK2,EFNA1,CHRNB2,ZDHHC15,RPS6KA5,CCDC88C,DNM2,B4GALT6,SEMA6B,SLITRK2,ARHGAP4,NOTCH1,STXBP1,VASP,SOX17,RELN,SHANK3,TBC1D20,VAMP3	turquoise	EC_M6
GO:0072073	6.04E-03	0.997113191	23	115	kidney epithelium development	BP	1.74E-01	MTSS1,KLHL3,PODXL,GATA3,GZF1,JAG1,SFRP1,RET,SMAD1,MEF2C,WWTR1,STAT1,CD34,PECAM1,LAMA5,DLG1,WNK4,PROM1,ARG2,HOXB7,SMAD2,NOTCH1,BMP2	turquoise	EC_M6
GO:0090091	6.05E-03	0.999451409	4	8	positive regulation of extracellular matrix disassembly	BP	1.74E-01	IL6,MELTF,FSCN1,CARMIL2	turquoise	EC_M6
GO:0051147	6.07E-03	0.997139134	22	115	regulation of muscle cell differentiation	BP	1.74E-01	PDGFB,KIT,EFNB2,ATP11A,LMOD3,IGF1,SMAD1,MEF2C,EDN1,SIK1,NFATC2,RBM4,PROX1,TBX1,MTOR,PIAS1,YY1,NOTCH1,PIEZO1,ENG,RGS4,BMP2	turquoise	EC_M6
GO:0007632	6.08E-03	0.998228054	10	35	visual behavior	BP	1.74E-01	KIT,RIC8A,NDRG4,TANC1,CREB1,HIF1A,RGS14,MTOR,CHRNB2,PIAS1	turquoise	EC_M6
GO:0051095	7.77E-03	0.99914685	4	10	regulation of helicase activity	BP	1.74E-01	POT1,MSH2,MCM2,MSH3	darkgrey	EC_M8
GO:1904951	6.52E-03	0.995962618	50	257	positive regulation of establishment of protein localization	BP	1.74E-01	LRP1,TCP1,PTP4A3,PPARD,LEP,UNC13B,EXOC1,TMEM30A,GLI3,KIF5B,PAK1,TGFB1,AKT2,ATP2C1,IRS2,PRNP,HDAC3,TM9SF4,ANKRD1,OSBP,CCT8,CACNB3,PFKM,ATG13,TSG101,DKC1,BAP1,CHP1,DYNLL1,DNM1L,TGFB2,GSK3B,PDCD10,B3GAT3,NLGN2,TMED10,TGFB3,MYH10,SYTL4,ACSL4,MIEF2,MYOM1,PRKAA1,MYO1C,BCAP31,FLNA,WLS,LEPROT,ZPR1,TOMM7	darkgreen	EC_M4
GO:0032743	6.11E-03	0.998699278	7	22	positive regulation of interleukin-2 production	BP	1.75E-01	PRKD2,PDE4D,IL1A,CD86,PDE4B,TRAF2,PLCG2	turquoise	EC_M6

GO:0060836	6.14E-03	0.999496953	4	7	lymphatic endothelial cell differentiation	BP	1.75E-01	ACVR2B,TIE1,PROX1,SOX18	turquoise	EC_M6
GO:0045821	6.14E-03	0.99895271	6	15	positive regulation of glycolytic process	BP	1.75E-01	IGF1,GPD1,P2RX7,MLXIPL,HIF1A,PSEN1	turquoise	EC_M6
GO:0007269	6.15E-03	0.997107051	22	110	neurotransmitter secretion	BP	1.75E-01	STX11,SNCAIP,RIMS2,APBA1,PPT1,NR4A1,MCTP1,MEF2C,SNCA,DTNBP1,MCTP2,SNAP23,ADCY1,ADORA2A,SNAP29,SNCG,SYT7,FBXL20,PSEN1,GIT1,STXBP1,STX3	turquoise	EC_M6
GO:0099643	6.15E-03	0.997107051	22	110	signal release from synapse	BP	1.75E-01	STX11,SNCAIP,RIMS2,APBA1,PPT1,NR4A1,MCTP1,MEF2C,SNCA,DTNBP1,MCTP2,SNAP23,ADCY1,ADORA2A,SNAP29,SNCG,SYT7,FBXL20,PSEN1,GIT1,STXBP1,STX3	turquoise	EC_M6
GO:0046320	6.57E-03	0.998279763	9	26	regulation of fatty acid oxidation	BP	1.75E-01	PPARA,PPARD,MFSD2A,DGAT2,AKT2,IRS2,CPT1A,PPARGC1A,AKT1	darkgreen	EC_M4
GO:0045738	7.89E-03	0.99794356	8	31	negative regulation of DNA repair	BP	1.76E-01	UBR5,RIF1,ERCC6,PARPBP,FANCB,AUNIP,SMCHD1,RMI2	darkgrey	EC_M8
GO:0031642	6.29E-03	0.999423966	4	8	negative regulation of myelination	BP	1.78E-01	TNFRSF21,MTMR2,CTSC,EIF2AK3	turquoise	EC_M6
GO:0048713	6.32E-03	0.998454158	8	27	regulation of oligodendrocyte differentiation	BP	1.78E-01	TNFRSF21,CXCR4,TLR2,SLC45A3,HDAC1,TNFRSF1B,MTOR,NOTCH1	turquoise	EC_M6
GO:0033197	6.72E-03	0.999148628	5	10	response to vitamin E	BP	1.79E-01	LEP,FKBP1B,ADA,CCND1,COL1A1	darkgreen	EC_M4
GO:0070665	6.35E-03	0.997252234	18	93	positive regulation of leukocyte proliferation	BP	1.79E-01	PTK2,SYK,IL6,PELI1,TLR4,IL15,IL1A,IGF1,MEF2C,IL7,ICOSLG,NFATC2,CD86,EFNB1,CHRNB2,LGALS9,CD46,BST2	turquoise	EC_M6
GO:0051155	6.40E-03	0.998105059	10	37	positive regulation of striated muscle cell differentiation	BP	1.80E-01	EFNB2,ATP11A,LMOD3,IGF1,MEF2C,EDN1,PROX1,TBX1,MTOR,PIEZO1	turquoise	EC_M6
GO:0006796	6.40E-03	0.994806019	270	2150	phosphate-containing compound metabolic process	BP	1.80E-01	TNFRSF11A,PDE7B,TNFAIP3,PTPRB,FLT4,SYNJ2,SERAC1,CDKL3,TPD52L1,HDDC2,PTK2,DAPK1,PDGFB,MAPK13,CSNK1E,PICK1,PIM1,TEK,LIMCH1,TJP2,PIP5K1B,TEC,ADGRF5,KIT,KDR,SRD5A3,SYK,SEMA4D,PPP6R1,PIGF,IL6,PPT1,GRB10,TLR4,INPP4B,IL15,CCNY,SDCBP,NOCT,NRP1,RASIP1,PIK3CG,TAL1,MTMR2,KDM4D,PRKD2,RAB38,CAB39L,SNRK,NOS3,RALB,PTPN4,PPARG,ACVR2B,IRAK2,EDNRB,PPP1CB,SPTBN4,TIE1,PGS1,IKBKB,P2RY6,PDE2A,GPAT4,SFRP1,HBEGF,CDS2,SH3BP5,ANKLE2,CD36,CDK17,PRAG1,PRR5L,MAP3K1,SCARB1,EPHB1,PLK2,RET,PDE4D,MERTK,ELOVL7,IGF1,EIF2AK3,ABHD6,HHEX,PITPNM2,SMAD1,AADAT,OCLN,DCLK2,CHRM5,GPD1,HEXB,MEF2C,TLR2,WWTR1,IL7,P2RX7,VEGFC,ENTPD1,GUCY1A1,GUCY1B1,MADD,ACOT12,BMP2K,SERINC5,NLRC5,THBS4,PPP1R16B,PLCB2,PLCG1,RAPGEF3,DGKZ,HIPK1,CCNJL,AMPD1,PIK3C2B,NEDD9,EDN1,DYRK1A,EHD4,SNCA,DTNBP1,ERG,DUSP8,NUAK2,CDK18,DCAF1,ADCY2,MTMR10,PDE9A,LPCAT1,SIK1,HDAC1,LCK,PPP3CA,HYAL2,PLA1A,PRKX,NCF1,DYRK3,FABP4,HCLS1,INPP5D,PECAM1,CD86,BMX,FGR,MAP3K6,INPP5F,ADCY1,KALRN,ADCY4,IGFBP3,FES,PPP2R5A,RPS6KA1,PROX1,PRKD1,PDIK1L,MAP3K3,SRMS,MLXIPL,ACE,ITGA6,LIMK2,PAK2,PTPRE,DUSP16,DLG1,SMG7,MAP3K11,STYK1,PDK3,CREB1,CTTNBP2NL,LTF,BCR,ADORA2A,PALD1,EPHB4,DDX3X,TBX1,PYGL,WNK4,CDK16,CDA,LDB2,TNFAIP8L3,FGF23,SAMD8,LMTK2,PPM1F,PRKCH,RAC1,HIF1A,ADSS,PLD2,ADIPOR2,EPHA2,ITPKB,TLR6,PGM2,TNK1,RGS14,MTOR,AR,SLC26A1,PSEN1,STK10,FBXW11,FGF18,RPS6KL1,PGF,NEK9,DAPK2,TAOK2,EFNA1,GDPD3,ADCK1,CD19,SBK1,PIK3R5,NPR1,RPS6K	turquoise	EC_M6
GO:0050863	6.43E-03	0.996408334	34	206	regulation of T cell activation	BP	1.80E-01	TCF7,TNFRSF21,SYK,IL6,PELI1,IL15,GATA3,IL1A,IGF1,ITCH,SH3RF1,IL7,SOX13,LCK,ICOSLG,LAPTM5,HLX,CD86,DLG1,B2M,ADORA2A,SOS2,ITPKB,TNFRSF1B,ARG2,EFNB1,CCL2,YES1,LGALS9,IL7R,AP3D1,NRARP,VNN1,CD46	turquoise	EC_M6
GO:0072583	6.43E-03	0.997834921	12	46	clathrin-dependent endocytosis	BP	1.80E-01	SNAP91,ITSN2,FCHSD2,HIP1R,BMP2K,INPP5F,ITGA4,FCHO1,DNM2,AP2B1,UNC119,FNBP1L	turquoise	EC_M6
GO:0035791	6.79E-03	0.998848096	6	14	platelet-derived growth factor receptor-beta signaling pathway	BP	1.80E-01	LRP1,MYOCD,PDGFRB,PTPN2,PTPN12,LOX	darkgreen	EC_M4
GO:0048660	6.80E-03	0.996526152	27	124	regulation of smooth muscle cell proliferation	BP	1.80E-01	FAM19A5,PPARD,FGF2,PAK1,TGFB1,CTNNB1,ILK,CX3CL1,MMP2,FGFR2,COMT,MNAT1,BMPR1A,PPARGC1A,TPM1,TGFB3,CNN1,MYOCD,PDGFRB,AKT1,ITGA2,NPR3,IRAK1,GNAI3,CDH13,CDKN1A,PRKG1	darkgreen	EC_M4
GO:0060601	6.82E-03	0.999132363	5	10	lateral sprouting from an epithelium	BP	1.80E-01	GLI2,SULF1,BMP7,FGFR2,FGF10	darkgreen	EC_M4

GO:0007162	6.82E-03	0.995936873	43	215	negative regulation of cell adhesion	BP	1.80E-01	LRP1,CDSN,PLXNB2,ANGPT1,PPARA,LGALS1,VEGFA,SOCS5,GLI3,PLXNA1,TGFB1,C1QTNF1,ACTN4,SPINT2,PRNP,ZNF703,CXCL12,IL20RB,LOXL3,RASA1,CX3CL1,PTPN11,PTPN22,RUNX1,CBLB,PHLDB2,PRKAR1A,MMP14,CORO1C,SERPINE1,TNR,PTPN6,COL1A1,CD74,AKT1,PTPN2,PLXNB3,PLXNA3,CDH13,CD164,FGL2,DUSP22,PRKG1	darkgreen	EC_M4
GO:0032891	6.83E-03	0.998838368	6	14	negative regulation of organic acid transport	BP	1.80E-01	LEP,AKT2,IRS2,ARL6IP5,PLA2R1,AKT1	darkgreen	EC_M4
GO:0005996	6.84E-03	0.996007692	40	199	monosaccharide metabolic process	BP	1.80E-01	DYRK2,PPARA,PPARD,LEP,GALT,GLB1,RBKS,DGAT2,AKT2,PFKP,C1QTNF1,KAT2B,IRS2,SORBS1,HECTD4,PFKM,ADIPOR1,SELENOS,CPT1A,LRP5,GSK3B,PIK3CA,WDTC1,MAN2A2,PPP1R3D,ATF3,TPI1,PPP1R3F,PPARGC1A,MPI,AKT1,PRKAA1,PTPN2,OMA1,PGP,MIDN,FAM3A,ENO1,RANBP2,NNMT	darkgreen	EC_M4
GO:0046651	6.45E-03	0.996543554	30	177	lymphocyte proliferation	BP	1.80E-01	TNFRSF21,SYK,IL6,PELI1,TLR4,IL15,PIK3CG,GAPT,IL1A,IGF1,ITCH,SH3RF1,MEF2C,IL7,LMBR1L,ICOSLG,NFATC2,INPP5D,CD86,DLG1,SOS2,TNFRSF1B,ARG2,EFNB1,CHRNB2,CD19,LGALS9,ELF4,IL7R,CD46	turquoise	EC_M6
GO:0045986	6.47E-03	0.999109168	5	12	negative regulation of smooth muscle contraction	BP	1.80E-01	DOCK4,ARHGAP42,GUCY1A1,CALCRL,ADRB2	turquoise	EC_M6
GO:0000054	8.10E-03	0.999099867	4	10	ribosomal subunit export from nucleus	BP	1.81E-01	XPO1,RRS1,ABCE1,NMD3	darkgrey	EC_M8
GO:0033750	8.10E-03	0.999099867	4	10	ribosome localization	BP	1.81E-01	XPO1,RRS1,ABCE1,NMD3	darkgrey	EC_M8
GO:0031214	6.89E-03	0.996437557	28	126	biomineral tissue development	BP	1.81E-01	FBN2,PPARA,LEP,COL1A2,ASPN,CNNM4,ROCK2,TGFB1,STIM1,ATP2B1,SLC20A1,HEY1,ACVR1,SPP1,SBDS,FAM20C,BMP7,FGFR2,FZD9,COMP,FGFR3,BMPR1A,COL1A1,TGFB3,ROCK1,TMEM38B,LOX,SNX10	darkgreen	EC_M4
GO:0034764	6.97E-03	0.996172423	33	155	positive regulation of transmembrane transport	BP	1.83E-01	AZIN1,ITGB1,KIF5B,AKT2,OSBPL8,ACTN4,STIM1,IRS2,FXYD1,ATP1B3,SORBS1,CX3CL1,ABCC8,CACNB3,PTPN11,CHP1,PKD2,AZIN2,CASQ1,HTT,CLTCL1,PRKCI,KCNA1,HAP1,STIM2,KCNMB1,CALM1,VMP1,AKT1,FLNA,DMD,THY1,TMSB4X	darkgreen	EC_M4
GO:0014870	7.00E-03	0.99940313	4	7	response to muscle inactivity	BP	1.83E-01	SCN5A,DAG1,CASQ1,SGCA	darkgreen	EC_M4
GO:0014877	7.00E-03	0.99940313	4	7	response to muscle inactivity involved in regulation of muscle adaptation	BP	1.83E-01	SCN5A,DAG1,CASQ1,SGCA	darkgreen	EC_M4
GO:0014894	7.00E-03	0.99940313	4	7	response to denervation involved in regulation of muscle adaptation	BP	1.83E-01	SCN5A,DAG1,CASQ1,SGCA	darkgreen	EC_M4
GO:0032409	7.01E-03	0.995901519	40	198	regulation of transporter activity	BP	1.83E-01	UBQLN1,SHANK1,ITGB1,FKBP1B,KIF5B,GPD1L,ACTN4,STIM1,PRNP,REM1,FXYD1,SLMAP,ATP1B3,CACNA1D,APP,ABCC8,CACNB3,HOMER1,CHP1,PKD2,CASQ1,LINC02210-CRHR1,HTT,KCNA1,HAP1,NLGN2,STIM2,PPARGC1A,CALM1,VMP1,PARK7,AHCYL1,JPH3,MIR24-1,DMD,FKBP1A,CBARP,TMSB4X,YWHAH,GEM	darkgreen	EC_M4
GO:1900078	7.04E-03	0.998324912	8	22	positive regulation of cellular response to insulin stimulus	BP	1.83E-01	LEP,PAK1,OSBPL8,SORBS1,PTPN11,OPA1,ADIPOR1,MYO1C	darkgreen	EC_M4
GO:0036293	7.05E-03	0.995614967	50	258	response to decreased oxygen levels	BP	1.83E-01	PPARA,PPARD,UBQLN1,TXN2,LEP,SRF,VEGFA,REST,ROCK2,MGARP,PAK1,CXCL12,TM9SF4,ANKRD1,HSP90B1,PLOD2,CACNB3,OPA1,LOXL2,MMP2,ADA,HIF1AN,CHRNA7,DNM1L,TGFB2,MMP14,FAM162A,BMP7,HYOU1,SCFD1,EGLN3,ERO1A,FMN2,SLC2A4,PPARGC1A,CPEB4,KCNMB1,TGFB3,MYOCD,PIN1,AKT1,ITGA2,PRKAA1,GNB1,VASN,IRAK1,ENO1,SLC2A8,BNIP3,PDLIM1	darkgreen	EC_M4
GO:2001044	7.09E-03	0.998785999	6	14	regulation of integrin-mediated signaling pathway	BP	1.83E-01	CD63,CTNNA1,LOXL3,SLC2A10,TIMP1,FLNA	darkgreen	EC_M4
GO:0002028	7.09E-03	0.997061776	17	66	regulation of sodium ion transport	BP	1.83E-01	KIF5B,SCN5A,GPD1L,ACTN4,FXYD1,SNTA1,SLMAP,ATP1B3,P2RX4,CHP1,ATP2B4,PKP2,AKT1,AHCYL1,MIR24-1,DMD,YWHAH	darkgreen	EC_M4
GO:0060674	7.11E-03	0.998111104	9	26	placenta blood vessel development	BP	1.84E-01	TMED2,HEY1,PKD2,NOTCH2,VASH2,MAP2K1,AKT1,PKD1,WNT2	darkgreen	EC_M4

GO:0000255	3.90E-04	0.999983086	5	7	allantoin metabolic process	BP	1.84E-01	DNPH1,PNP,XDH,NT5C2,NT5C	cyan	EC_M2
GO:0000966	8.27E-03	0.998347537	6	21	RNA 5'-end processing	BP	1.84E-01	POP1,PNPT1,UTP20,SSB,NOP14,THG1L	darkgrey	EC_M8
GO:0060324	7.15E-03	0.997607341	12	40	face development	BP	1.84E-01	SRF,RRAS,RAB3GAP1,PTPN11,MMP2,LEF1,ARID5B,SGPL1,COL1A1,TGFB3,MAP2K1,ANKRD11	darkgreen	EC_M4
GO:0045666	6.63E-03	0.9975043	14	61	positive regulation of neuron differentiation	BP	1.84E-01	TCF4,CSNK1E,KDM4C,NRCAM,GATA2,DNMT3B,MEF2C,PROX1,ZC4H2,NAP1L2,MMD,TGIF2,BMP2,BMP6	turquoise	EC_M6
GO:0034982	8.33E-03	0.999031765	4	11	mitochondrial protein processing	BP	1.85E-01	PMPCB,YME1L1,AFG3L2,PMPCA	darkgrey	EC_M8
GO:0022417	7.20E-03	0.999380518	4	7	protein maturation by protein folding	BP	1.85E-01	FKBP1B,PRDX4,ERO1A,FKBP1A	darkgreen	EC_M4
GO:0001556	8.36E-03	0.998363816	6	19	oocyte maturation	BP	1.86E-01	FBXO5,TUT7,BRCA2,CCNB1,TRIP13,AURKA	darkgrey	EC_M8
GO:0006479	8.41E-03	0.99573886	24	148	protein methylation	BP	1.86E-01	PWP1,FBXO11,RLF,EZH2,SETDB2,PAXIP1,RIF1,NSD3,SMYD5,TET2,PRMT5,SETD2,BRCA1,NSD2,SUV39H1,NSD1,OGT,ATRX,LCMT1,SUZ12,GSPT1,EHMT1,CTCF,METTL18	darkgrey	EC_M8
GO:0008213	8.41E-03	0.99573886	24	148	protein alkylation	BP	1.86E-01	PWP1,FBXO11,RLF,EZH2,SETDB2,PAXIP1,RIF1,NSD3,SMYD5,TET2,PRMT5,SETD2,BRCA1,NSD2,SUV39H1,NSD1,OGT,ATRX,LCMT1,SUZ12,GSPT1,EHMT1,CTCF,METTL18	darkgrey	EC_M8
GO:0034968	8.41E-03	0.996239912	18	98	histone lysine methylation	BP	1.86E-01	PWP1,RLF,EZH2,SETDB2,PAXIP1,RIF1,NSD3,SMYD5,TET2,SETD2,BRCA1,NSD2,SUV39H1,NSD1,OGT,ATRX,SUZ12,EHMT1	darkgrey	EC_M8
GO:0048701	7.27E-03	0.997560819	12	40	embryonic cranial skeleton morphogenesis	BP	1.86E-01	RUNX2,TGFBR1,GLI3,RDH10,MMP14,FGFR2,PRRX1,TULP3,SIX4,TGFB3,SLC39A3,CHST11	darkgreen	EC_M4
GO:0006486	7.28E-03	0.995829435	37	182	protein glycosylation	BP	1.86E-01	B4GALT7,NUS1,LARGE1,UBE2J1,GFPT1,RPN1,POMGNT1,ST6GALNAC2,TRAK1,STT3B,TMTC4,KDELC1,TMTC3,TUSC3,ST8SIA4,B3GNT4,TET3,RPN2,DPY19L4,CHP1,MAN1A2,B4GALT4,B3GALT1,GALNT2,MAN2A2,EDEM3,PLOD3,DDOST,B3GAT3,GALNT16,GALNT10,GALNT1,MPI,POMT1,DPM2,DPM1,SLC35C1	darkgreen	EC_M4
GO:0043413	7.28E-03	0.995829435	37	182	macromolecule glycosylation	BP	1.86E-01	B4GALT7,NUS1,LARGE1,UBE2J1,GFPT1,RPN1,POMGNT1,ST6GALNAC2,TRAK1,STT3B,TMTC4,KDELC1,TMTC3,TUSC3,ST8SIA4,B3GNT4,TET3,RPN2,DPY19L4,CHP1,MAN1A2,B4GALT4,B3GALT1,GALNT2,MAN2A2,EDEM3,PLOD3,DDOST,B3GAT3,GALNT16,GALNT10,GALNT1,MPI,POMT1,DPM2,DPM1,SLC35C1	darkgreen	EC_M4
GO:1903867	8.46E-03	0.999092912	4	9	extraembryonic membrane development	BP	1.87E-01	MAP3K4,PAXIP1,E2F7,E2F8	darkgrey	EC_M8
GO:0000394	8.48E-03	0.999008137	4	11	RNA splicing, via endonucleolytic cleavage and ligation	BP	1.87E-01	DDX1,TSEN2,ZBTB8OS,TSEN15	darkgrey	EC_M8
GO:1902905	6.77E-03	0.996293532	32	179	positive regulation of supramolecular fiber organization	BP	1.87E-01	MTSS1,BAIAP2L2,PICK1,CDC42EP1,LIMCH1,NRP1,FCHSD2,SFRP1,FCHSD1,LMOD3,HIP1R,OCLN,RAPGEF3,IQGAP2,EDN1,AP1AR,FES,PROX1,DLG1,BAIAP2L1,PPM1F,WAS,RAC1,MTOR,PSEN1,ARHGEF15,SPIRE1,GIT1,CARMIL2,VASP,GMFG,ARPC5L	turquoise	EC_M6
GO:0046058	6.77E-03	0.998587584	7	19	cAMP metabolic process	BP	1.87E-01	PDE7B,PDE4D,ADCY2,ADCY1,ADCY4,EPHA2,PDE4B	turquoise	EC_M6
GO:0090050	6.77E-03	0.99851683	7	23	positive regulation of cell migration involved in sprouting angiogenesis	BP	1.87E-01	KDR,NRP1,GATA2,PLK2,MAP3K3,RHOJ,MIR23A	turquoise	EC_M6
GO:0051495	6.80E-03	0.996194651	34	194	positive regulation of cytoskeleton organization	BP	1.88E-01	MTSS1,BAIAP2L2,PICK1,CDC42EP1,TEK,LIMCH1,NRP1,FCHSD2,SFRP1,FCHSD1,LMOD3,HIP1R,OCLN,P2RX7,RAPGEF3,IQGAP2,EDN1,HCLS1,AP1AR,FES,PROX1,DLG1,BAIAP2L1,PPM1F,WAS,RAC1,MTOR,ARHGEF15,SPIRE1,GIT1,CARMIL2,VASP,GMFG,ARPC5L	turquoise	EC_M6
GO:0071674	6.83E-03	0.996677387	23	126	mononuclear cell migration	BP	1.88E-01	TNFRSF11A,PTK2,PDGFB,IL6,TRPM4,PIK3CG,LRCH1,GATA3,CXCR4,RET,CXCL10,ANO6,CD200,PECAM1,F11R,APOD,ITGA4,STK10,S100A14,CCL2,LGALS9,S1PR1,MSMP	turquoise	EC_M6
GO:2000192	7.37E-03	0.99936136	4	7	negative regulation of fatty acid transport	BP	1.88E-01	AKT2,IRS2,PLA2R1,AKT1	darkgreen	EC_M4
GO:0002902	7.38E-03	0.998723303	6	14	regulation of B cell apoptotic process	BP	1.88E-01	IRS2,ADA,SLC39A10,CD74,BCL10,ORMDL3	darkgreen	EC_M4

GO:0007015	6.88E-03	0.995526854	59	373	actin filament organization	BP	1.89E-01	MTSS1,ARHGAP18,BAIAP2L2,PICK1,CDC42EP1,LIMCH1,CD2AP,TMOD1,ARHGAP25,ELMO1,FAM49A,NRP1,SVIL,MSRB2,MYO7A,SPTBN4,FCHSD2,SFRP1,FCHSD1,LMOD3,HIP1R,FHDC1,USH1C,RAPGEF3,IQGAP2,NEDD9,GAS2,NRAP,ESAM,HCLS1,AP1AR,PROX1,TMOD4,F11R,PAK2,DLG1,MCU,BAIAP2L1,PPM1F,WAS,TMOD2,RAC1,FSCN1,SHROOM4,MICAL3,MTOR,ARHGEF15,ARHGAP28,SPIRE1,RFLNB,S1PR1,CARMIL2,VASP,GMFG,KANK3,SHANK3,RGS4,GBA2,ARPC5L	turquoise	EC_M6
GO:0006936	6.90E-03	0.995883033	42	256	muscle contraction	BP	1.89E-01	TRDN,KIT,TMOD1,DOCK4,TRPM4,PIK3CG,ARHGAP42,CXCR4,EDNRB,P2RX2,LMOD3,PDE4D,NR4A1,GUCY1A1,STC1,KCNJ3,ANKRD2,EDN1,KCNJ2,SYNM,PABPN1,LTB4R,TMOD4,MYOM3,DLG1,CALCRL,TMOD2,APBB2,JUP,CHRNB1,ARG2,MTOR,CHRNB2,ATP2A1,SCNN1B,DSG2,DSC2,ADRB2,PDE4B,SLC8A3,KCNH2,SCN2B	turquoise	EC_M6
GO:0001655	6.98E-03	0.995708376	47	283	urogenital system development	BP	1.91E-01	MTSS1,KLHL3,PDGFB,PODXL,TEK,NRP1,GATA2,GPR4,PLXND1,ITGA8,GATA3,GZF1,JAG1,SFRP1,FREM2,RET,SMAD1,MEF2C,WWTR1,ANGPT2,TIPARP,STAT1,HYAL2,PRKX,EAF2,CD34,PECAM1,CFLAR,PROX1,VANGL2,LAMA5,ACE,DLG1,WNK4,PROM1,ARG2,ALDH1A2,AR,HOXB7,ITGA3,SMAD2,AP2B1,NOTCH1,CA2,SOX17,BMP2,BMP6	turquoise	EC_M6
GO:0034754	6.99E-03	0.997123938	16	79	cellular hormone metabolic process	BP	1.91E-01	RDH16,ALDH8A1,HSD17B3,SRD5A3,SCARB1,TIPARP,PLEKHA1,ALDH1A2,SCNN1B,DHRS11,CYP1A1,BMP2,SRD5A2,BMP6,AKR1D1,RBP1	turquoise	EC_M6
GO:0055008	7.52E-03	0.997194652	14	50	cardiac muscle tissue morphogenesis	BP	1.92E-01	LY6E,TGFBR1,TGFB1,ANKRD1,PKP2,TGFB2,FGFR2,BMPR1A,TCAP,MED1,TPM1,COL11A1,FKBP1A,WNT2	darkgreen	EC_M4
GO:0048799	7.06E-03	0.998267524	8	25	animal organ maturation	BP	1.92E-01	SEMA4D,GATA3,RET,IGF1,LTF,ALDH1A2,RFLNB,BMP2	turquoise	EC_M6
GO:0051897	7.55E-03	0.996500458	21	88	positive regulation of protein kinase B signaling	BP	1.92E-01	ANGPT1,LEP,TGFBR1,RTN4,TPBG,ITGB1,FGF2,TGFB1,AXL,C1QTNF1,OSBPL8,ILK,P2RX4,CX3CL1,MTDH,PIK3CA,OSM,MYDGF,PARK7,F3,TXN	darkgreen	EC_M4
GO:0006357	8.79E-03	0.993003451	201	1784	regulation of transcription by RNA polymerase II	BP	1.93E-01	ONECUT2,BCLAF1,PPIL4,ZNF608,SCAF8,RAD21,HSF2,ATAD2,XRCC6,PSIP1,IFT74,ZNF300,RFX3,CKS2,TRIM28,ZNF451,FOXJ3,ZNF189,SP4,PHIP,RLF,ZNF292,XPO1,ZNF350,ITGB1BP1,THRAP3,EZH2,CCNC,ELF2,ATAD2B,HDAC2,EXOSC9,MLLT10,ELF1,RSF1,CCNT2,PAXIP1,EPC2,SUPT16H,CHD8,KAT6A,RIF1,E2F7,CKAP2,CEBPZ,SUPT20H,TAF7,NR2C1,ELK3,TCERG1,PRKDC,CBX5,ERCC6,ZNF214,SP1,TCEA1,ZNF664,ZC3H8,ZNF143,TDG,NCK1,SMARCA5,ZNF507,CHD1,ZFP91,TAF9,CGGBP1,TFCP2,SAP30,HLTF,ZBTB10,CNOT1,GABPA,MCRS1,USP16,PPID,RBL1,CCNL1,SENP1,RIPK1,MYBL2,E2F8,TRIM33,INO80,ZBED6,CHUK,ZBTB11,ZNF248,MGA,MDM4,HIVEP1,BRWD1,BMPR1B,UBE3A,PRDM10,NUCKS1,DEK,GCN1,CUL3,CHEK1,H2AFZ,RRP1B,NFKB1,KHDRBS1,TET2,NCL,ZNF184,AJUBA,GTF2IRD2,ZBTB41,ARID4B,GTF2I,TARBP1,BPTF,DDX5,ORC2,TIAL1,BCLAF3,FOSL1,SMARCC1,BAZ1B,SP3,DHX9,HDGFL3,BAZ1A,ZFX,DDX20,TPR,NPAT,BRCA1,NSD2,DCAF6,ZNF518B,TBL1XR1,SUV39H1,LEO1,FOXM1,LYAR,FAM220A,HNRNPU,NEDD4,SMARCE1,RFX7,RFC1,TOP2A,LIN9,NSD1,SLTM,MED6,FUS,USP3,AURKB,ZNF609,TAF1,OGT,RLIM,ATRX,ZKSCAN2,TRIM37,PLK1,MED13,BRIP1,ZNF699,AATF,PSMD10,ZNF3,MIER1,NIPBL,ZNF280C,SAFB,SAFB2,PHF6,MYSM1,UHRF1,RBMX,EHMT1,TP73,CTCF,ZNF684,DRAP1,ZFP30,HMGB3,ZNF22,ZNF614,RBAK,ZNF770,FOXN2,BRMS1L,ZFP14,ZNF558,ZNF624,ZNF782,IKZF5,ZFP62,ZNF302,ZNF34,ZNF529,ZNF146,ZNF518A,ZNF420,ZSCAN16	darkgrey	EC_M8
GO:0018022	8.81E-03	0.995938347	19	108	peptidyl-lysine methylation	BP	1.93E-01	PWP1,RLF,EZH2,SETDB2,PAXIP1,RIF1,NSD3,SMYD5,TET2,SETD2,BRCA1,NSD2,SUV39H1,NSD1,OGT,ATRX,SUZ12,EHMT1,METTL18	darkgrey	EC_M8
GO:0089718	7.61E-03	0.997757489	10	31	amino acid import across plasma membrane	BP	1.93E-01	SLC22A4,SLC7A11,ITGB1,SLC36A4,ARL6IP5,SLC3A2,SLC6A13,CLN8,SLC7A3,SLC36A1	darkgreen	EC_M4
GO:0046823	7.61E-03	0.99837385	7	19	negative regulation of nucleocytoplasmic transport	BP	1.93E-01	ANGPT1,RAB23,MDFIC,UFM1,CHP1,PARK7,TXN	darkgreen	EC_M4
GO:0048645	7.63E-03	0.997020397	15	56	animal organ formation	BP	1.93E-01	GLI3,FGF2,GLI2,SMARCD3,CTNNB1,SULF1,ROBO1,RDH10,BMP7,FGFR2,BMPR1A,MAP2K1,GDNF,WNT2,FGF10	darkgreen	EC_M4
GO:0110148	7.67E-03	0.995993818	28	127	biomineralization	BP	1.94E-01	FBN2,PPARA,LEP,COL1A2,ASPN,CNNM4,ROCK2,TGFB1,STIM1,ATP2B1,SLC20A1,HEY1,ACVR1,SPP1,SBDS,FAM20C,BMP7,FGFR2,FZD9,COMP,FGFR3,BMPR1A,COL1A1,TGFB3,ROCK1,TMEM38B,LOX,SNX10	darkgreen	EC_M4
GO:0035265	7.70E-03	0.995922722	29	134	organ growth	BP	1.94E-01	PPARA,LY6E,LEP,TGFBR1,FGF2,TENM4,PAK1,RARB,WWC2,PTPN11,RUNX1,PDLIM5,TGFB2,PRKAR1A,WWC3,FGFR2,COMP,FGFR3,EVC,BMPR1A,THBS3,ANXA6,PIN1,PDGFRB,AKT1,MIR24-1,TMEM38B,WNT2,FGF10	darkgreen	EC_M4
GO:0007144	8.91E-03	0.998981189	4	10	female meiosis I	BP	1.95E-01	FBXO5,TRIP13,CCNB2,AURKA	darkgrey	EC_M8

GO:0032201	8.94E-03	0.999024976	4	9	telomere maintenance via semi-conservative replication	BP	1.95E-01	TERF1,BLM,DNA2,FEN1	darkgrey	EC_M8
GO:0030278	7.24E-03	0.99676734	19	91	regulation of ossification	BP	1.96E-01	TRPM4,ACVR2B,SFRP1,NOTUM,MEF2C,P2RX7,BMP2K,ANO6,LTF,BCOR,FGF23,HIF1A,ADRB2,RFLNB,ISG15,NOTCH1,S1PR1,BMP2,BMP6	turquoise	EC_M6
GO:1905952	7.82E-03	0.996015062	26	118	regulation of lipid localization	BP	1.97E-01	LRP1,PPARA,NUS1,PPARD,PLIN2,LEP,TMEM30A,ABHD5,AKT2,C1QTNF1,OSBPL8,IRS2,P2RX4,PTPN11,CRY2,SPP1,PLA2R1,KDM5B,EPRS,ARV1,LINC02210-CRHR1,ITGAV,CYP19A1,AKT1,PTPN2,ABCA3	darkgreen	EC_M4
GO:0001778	7.87E-03	0.998962226	5	10	plasma membrane repair	BP	1.98E-01	MYH9,SMPD1,SYT11,SYTL4,ANXA6	darkgreen	EC_M4
GO:0043161	7.89E-03	0.994714077	67	368	proteasome-mediated ubiquitin-dependent protein catabolic process	BP	1.98E-01	CANX,RNF19A,UBQLN1,KLHDC10,PLAA,VCP,C2orf40,CLOCK,SOCS5,ERLEC1,UBE2J1,CUL2,PSMC2,TMUB1,NUB1,UBXN4,SPOPL,CTNNB1,STT3B,NPLOC4,RNF121,PSMD8,CHFR,DDRGK1,PPP2CB,PSMD6,HSP90B1,TRPC4AP,AMFR,PSMD14,MARCH6,UCHL5,SELENOS,PSMD1,KCTD10,GSK3B,PSMD2,UBE4A,RHBDD2,EDEM3,SYVN1,DNAJC10,DNAJB12,ARAF,SMURF1,FBXL5,JKAMP,ANAPC4,SPOP,FBXL22,SEL1L,ARIH1,FBXO22,USP14,AKT1,UBE2A,TNFAIP1,BCAP31,PARK7,HSPA5,FBXO27,UBE2V2,DNAJB9,DDA1,UBE2G2,MAP1A,DERL2	darkgreen	EC_M4
GO:0007596	7.94E-03	0.995547599	34	164	blood coagulation	BP	1.99E-01	SERPINB2,LMAN1,GNAQ,MYH9,SRF,SLC7A11,DGKH,AXL,C1QTNF1,ACTG1,ILK,MYL9,AP3B1,HPS6,FAP,ITPK1,PIK3CA,PRKCA,HSPB1,SERPINE1,ITGA2B,FN1,PTPN6,ADRA2C,COMP,COL3A1,VCL,ITGA2,CD40LG,FLNA,F3,CD59,CSRP1,PRKG1	darkgreen	EC_M4
GO:0043090	7.95E-03	0.997454668	11	36	amino acid import	BP	1.99E-01	SLC22A4,SLC7A11,ITGB1,SLC36A4,ARL6IP5,SLC3A2,SLC6A13,CLN8,SFXN1,SLC7A3,SLC36A1	darkgreen	EC_M4
GO:1901838	9.13E-03	0.998947934	4	10	positive regulation of transcription of nucleolar large rRNA by RNA polymerase I	BP	1.99E-01	PWP1,NCL,NOL11,DDX11	darkgrey	EC_M8
GO:0043085	7.98E-03	0.993908158	145	875	positive regulation of catalytic activity	BP	1.99E-01	IL23A,NAB2,AGAP2,TAB2,TBC1D9B,PRRC1,ANGPT1,TCP1,ROS1,NUS1,SERINC1,C6orf89,MTCH1,GNAQ,LEP,PLAA,VEGFA,DOCK8,VCP,REST,SNX13,PSME4,CYCS,MAP3K7,ROCK2,RCN3,ITGB1,FGF2,NAPA,ABHD5,PICALM,MAPRE3,PAK1,TGFB1,AXL,RAB3GAP1,CTNNB1,AKT2,TBC1D16,OSBPL8,PPP1R12A,STIM1,PRNP,DDRGK1,ILK,ARL6IP5,RYK,ARL1,ATP1B3,ROBO1,PFN2,RASA1,CACNA1D,APP,RAPGEF2,MAPKAPK5,CX3CL1,RALGAPB,DKC1,ATP2B4,PKD2,IFT57,TANK,PSMD14,SLC39A10,CHRNA7,RAP1GDS1,DOCK10,AZIN2,CCND1,TGFB2,HTR2B,GSK3B,DAG1,PIK3CA,CORO1C,ISCU,NEK7,ADAP1,KRAS,NTRK3,SGSM1,FAM162A,SPATC1L,FGFR2,NCSTN,SYAP1,ADRM1,IQGAP1,EGLN3,MAPT,DNAJC10,PCOLCE,FN1,ZC3H15,PDCD10,ADRA2C,DOK7,FGFR3,MNAT1,B3GAT3,CACNA1C,MAGED1,BMPR1A,SRCIN1,NGFR,TPM1,MAP2K1,GPR65,CALM1,UACA,PIN1,CD74,PDGFRB,EVI5L,AKT1,SNX18,ITGA2,NPR3,ABR,BCAP31,TAX1BP3,PKD1,IRAK1,PARK7,GNAI3,F3,ARHGAP29,BCAR3,MTSS1L,LMO4,BCL10,RANBP2,CCL16,NMUR1,ARF4,GDNF,THY1,TOR1AIP2,CHN1,CDKN1A,TXN,TMSB4X,GABARAPL2,FGF10	darkgreen	EC_M4
GO:0097581	7.37E-03	0.996878367	17	78	lamellipodium organization	BP	2.00E-01	SNX2,KIT,ARHGEF7,SPEF1,OCLN,ABLIM1,PLEKHO1,WAS,RAC1,FSCN1,EPHA2,MTOR,ABI3,DNM2,ABLIM3,S1PR1,CARMIL2	turquoise	EC_M6
GO:0046851	7.39E-03	0.998914242	5	13	negative regulation of bone remodeling	BP	2.00E-01	TNFAIP3,IL6,SFRP1,P2RX7,INPP5D	turquoise	EC_M6
GO:0000712	9.23E-03	0.998529663	5	14	resolution of meiotic recombination intermediates	BP	2.00E-01	RMI1,TOP2B,FANCM,TOP2A,EME1	darkgrey	EC_M8
GO:0050728	7.47E-03	0.996719258	18	91	negative regulation of inflammatory response	BP	2.01E-01	TNFAIP3,TEK,SYK,CST7,PPARG,GATA3,NR1D2,PLK2,IGF1,PLCG1,CD200,FGR,ADORA2A,CALCRL,TNFRSF1B,YES1,GIT1,CDH5	turquoise	EC_M6
GO:0050766	7.48E-03	0.997721329	10	38	positive regulation of phagocytosis	BP	2.01E-01	IL15,GATA2,CD36,MERTK,ANO6,RAB27A,DNM2,CCL2,MYO18A,PLCG2	turquoise	EC_M6

GO:1901701	7.48E-03	0.994423929	122	887	cellular response to oxygen-containing compound	BP	2.01E-01	TNFAIP3,OXR1,PTK2,DAPK1,ATF4,PDGFB,MAPK13,PIM1,IL6,CXCL8,CAV2,KLF11,GRB10,LANCL2,TLR4,IL15,TEAD2,TRPM4,PIK3CG,DNMT3A,NOS3,PPARG,TBC1D4,IRAK2,EDNRB,NET1,P2RY6,P2RY2,NFKBIB,PDE2A,SFRP1,CD36,SCARB1,RET,GRAMD1A,IL1A,DNMT3B,IGF1,NR4A1,CHRM5,GPD1,MEF2C,SMARCD1,TLR2,P2RX7,GUCY1B1,CXCL10,SOGA1,STC1,RAPGEF3,NR4A2,EDN1,STAT1,SNCA,DTNBP1,ADCY2,ABCG1,ETS1,PPP3CA,ANO1,NCF1,PABPN1,CD86,POLG,MN1,SH3BP4,CFLAR,ADCY1,FES,PLEKHA1,PRKD1,MLXIPL,PTPRE,SIPA1,PDK3,CREB1,SSTR1,LTF,CYBB,ITGA4,IGFBP5,TBX1,CALCRL,CDK16,BAIAP2L1,FGF23,CPEB2,RAC1,HIF1A,JUP,ADIPOR2,TLR6,TNFRSF1B,ALDH1A2,MTOR,AR,PSEN1,CHRNB2,GPR68,DNM2,SCNN1B,TNIP1,COL4A6,ADRB2,CCL2,YES1,PDE4B,NOS2,STK26,GIT1,TRAF2,SLC25A33,RAPGEF1,IRF8,PLCG2,DAB2IP,SLC8A3,TRIM24,CA2,RGS4,GNG2,BMP6	turquoise	EC_M6
GO:0045727	9.27E-03	0.995787431	18	109	positive regulation of translation	BP	2.01E-01	DDX39B,EIF3E,METTL3,PRKDC,DHX29,CDK5RAP1,NCK1,LARP4,USP16,KHDRBS1,FXR1,EIF2B5,DHX9,FASTKD2,LARP1,UPF3B,FMR1,HNRNPD	darkgrey	EC_M8
GO:0021537	8.09E-03	0.995305617	38	184	telencephalon development	BP	2.01E-01	LRP1,TRAPPC9,SUN2,MDGA1,SRF,UBA6,RTN4,MFSD2A,GLI3,INHBA,SLC7A11,KIF5B,EIF2B3,SCN5A,HTR5A,CTNNB1,RARB,TACC1,RYK,CXCL12,ZSWIM6,ROBO1,CELF1,TCTN1,ATP2B4,DNAH5,UCHL5,FEZ1,GSK3B,LEF1,TNR,COL3A1,KCNA1,TTC8,PAFAH1B1,FLNA,PLXNA3,BNIP3	darkgreen	EC_M4
GO:0048259	7.52E-03	0.99671503	18	85	regulation of receptor-mediated endocytosis	BP	2.02E-01	PICK1,CD2AP,SYK,SNAP91,PPT1,SDCBP,MTMR2,HIP1R,BMP2K,RIN3,B2M,SELE,RAC1,OPHN1,DNM2,UNC119,PLCG2,GSG1L	turquoise	EC_M6
GO:0007611	8.18E-03	0.995249604	38	183	learning or memory	BP	2.03E-01	SRF,UBA6,TPBG,SHANK1,ACTR2,SLC7A11,ITGB1,PICALM,PRNP,ARL6IP5,PJA2,APP,ABCC8,AMFR,CHRNA7,KRAS,NCSTN,FZD9,MAPT,C1QL1,EIF4EBP2,COMT,TNR,KCNK4,HTT,ATAD1,SLC2A4,CLN8,SYT11,GRIA1,NPTN,PAFAH1B1,JPH3,LHX8,SERPINF1,LRRN4,ARF4,MAP1A	darkgreen	EC_M4
GO:0009914	8.22E-03	0.995076399	42	212	hormone transport	BP	2.03E-01	LRP1,PPARD,LEP,CLOCK,REST,INHBA,FKBP1B,KIF5B,C1QTNF1,IRS2,RAB11FIP1,PCLO,OSBP,VAMP8,SELENOT,ABCC8,PTPN11,PFKM,CRY2,SPP1,BRSK2,DYNLL1,KDM5B,CPT1A,LRP5,HADH,ADCY5,OSM,SIDT2,LINC02210-CRHR1,TFR2,PTPRN,CYP19A1,NLGN2,UBE2Q1,SYTL4,ACSL4,RASL10B,MIDN,SSTR5,VAMP7,PARK7	darkgreen	EC_M4
GO:0006024	8.24E-03	0.996528385	17	66	glycosaminoglycan biosynthetic process	BP	2.04E-01	B4GALT7,ANGPT1,CHPF2,TGFB1,B3GNT4,CHST2,CHSY1,HYAL1,B3GAT3,UGDH,CHST12,XYLT2,CLTC,PDGFRB,HAS2,CHST11,HS2ST1	darkgreen	EC_M4
GO:0048754	8.26E-03	0.995721732	27	121	branching morphogenesis of an epithelial tube	BP	2.04E-01	SRF,VEGFA,GLI3,FGF2,GLI2,PAK1,CTNNB1,ILK,RDH10,ACVR1,PKD2,KDM5B,LRP5,MMP14,DAG1,LEF1,KRAS,BMP7,FGFR2,CLIC4,SIX4,MAGED1,MED1,PKD1,GDNF,WNT2,FGF10	darkgreen	EC_M4
GO:0043589	8.26E-03	0.998896308	5	10	skin morphogenesis	BP	2.04E-01	CDSN,SRF,COL1A2,COL1A1,ITGA2	darkgreen	EC_M4
GO:0030307	8.30E-03	0.995576616	29	134	positive regulation of cell growth	BP	2.04E-01	LRP1,PLAA,SRF,VEGFA,TGFBR1,RIMS1,MFSD2A,MEGF8,PAK1,ILK,CXCL12,EIF4G2,MAP1B,ZFYVE27,HYAL1,TGFB2,MMP14,RPS6KA3,MAPT,FN1,SMURF1,RAB11A,TAF9B,ISLR2,PIN1,TRPV2,AKT1,PAFAH1B1,DERL2	darkgreen	EC_M4
GO:0008286	8.32E-03	0.99603097	22	93	insulin receptor signaling pathway	BP	2.04E-01	AP3S1,LEP,SESN3,PAK1,AKT2,OSBPL8,IRS2,SORBS1,PTPN11,OPA1,ADIPOR1,ZNF106,GSK3B,PIK3CA,EIF4EBP2,SHC1,AKT1,PRKAA1,PTPN2,BCAR3,SLC2A8,PIK3C2A	darkgreen	EC_M4
GO:0048644	8.37E-03	0.996575826	16	61	muscle organ morphogenesis	BP	2.05E-01	LY6E,TGFBR1,TGFB1,ANKRD1,PKP2,TGFB2,FGFR2,ARID5B,FZD2,BMPR1A,TCAP,MED1,TPM1,COL11A1,FKBP1A,WNT2	darkgreen	EC_M4
GO:1903312	9.49E-03	0.996204543	14	74	negative regulation of mRNA metabolic process	BP	2.06E-01	SYNCRIP,THRAP3,DHX9,HNRNPU,FUS,LARP1,TAF15,RBMX,FMR1,METTL16,RNPS1,PTBP1,HNRNPD,SRSF7	darkgrey	EC_M8
GO:0030048	8.43E-03	0.995902269	23	97	actin filament-based movement	BP	2.06E-01	SUN2,MYH9,MYH14,SCN5A,GPD1L,ACTN4,SNTA1,ATP2A2,CACNA1D,PKP2,PIK3CA,MYO9B,MYO5A,CACNA1C,MYO1E,TPM1,CACNA1G,MYH10,MYO1D,MYO5B,MYO1C,FLNA,HCN4	darkgreen	EC_M4
GO:0140374	7.75E-03	0.998817462	5	14	antiviral innate immune response	BP	2.08E-01	DDX58,CXCL10,IFIT1,MX1,OAS1	turquoise	EC_M6
GO:0042692	7.78E-03	0.995151993	48	303	muscle cell differentiation	BP	2.08E-01	QKI,ASF1A,PDGFB,PGM5,KIT,TMOD1,CAV2,EPC1,EDNRB,EFNB2,ATP11A,P2RX2,LMOD3,IGF1,SMAD1,MEF2C,CXCL10,DYSF,TANC1,EDN1,SIK1,PPP3CA,MEF2A,BCL9,NRAP,NFATC2,PLEKHO1,CFLAR,RBM4,PROX1,TMOD4,MYEF2,IGFBP5,TBX1,RAMP2,TMOD2,CHRNB1,MTOR,PIAS1,YY1,MRTFB,TMEM204,NOTCH1,PIEZO1,ENG,MIR23A,RGS4,BMP2	turquoise	EC_M6

GO:0050890	8.51E-03	0.994860238	43	214	cognition	BP	2.08E-01	HLA-DRA,SRF,UBA6,TPBG,SHANK1,MFSD2A,ACTR2,SLC7A11,ITGB1,PICALM,PRNP,ARL6IP5,TUSC3,PJA2,APP,ABCC8,AMFR,CHRNA7,KRAS,NCSTN,FZD9,MAPT,C1QL1,EIF4EBP2,COMT,TNR,KCNK4,HTT,ATAD1,JAKMIP1,SLC2A4,CLN8,SYT11,TTC8,GRIA1,NPTN,PAFAH1B1,JPH3,LHX8,SERPINF1,LRRN4,ARF4,MAP1A	darkgreen	EC_M4
GO:2000048	7.84E-03	0.999233082	4	8	negative regulation of cell-cell adhesion mediated by cadherin	BP	2.09E-01	NOTCH4,PPM1F,NOTCH1,BMP6	turquoise	EC_M6
GO:0003341	1.39E-05	0.999994668	76	103	cilium movement	BP	2.09E-01	TTLL1,UBE2B,CATSPER3,KIF27,VPS13A,CFAP69,RSPH9,DNAJA1,DNAH11,INPP5B,ARMC2,ARMC4,APOB,CCDC114,ADCY3,ENKUR,DRC1,RSPH6A,SLC9A3R1,CFAP61,TTLL3,TPPP2,MKKS,NEK10,CEP131,YIF1B,DNHD1,CFAP54,TMF1,ING2,CFAP20,CCDC65,PLTP,DPCD,ROPN1L,DNAH7,CFAP43,ZMYND10,SLC9B2,CFAP46,CFAP45,TSSK4,IQCG,CELF3,PLA2G3,CATSPER2,SORD,TTC12,CABYR,DNAAF2,SPAG16,CFAP70,RGN,STK36,ADCY10,TTC25,DNAAF4,MNS1,TTLL6,ASH1L,KIAA0556,CCDC151,BBS4,TEKT3,CAMSAP3,WDR78,CATSPERD,CFAP53,CELSR2,DNAAF1,HYDIN,WDR63,ATP1A4,CCSAP,MEIG1,SPA17	grey	EC_M10
GO:0045834	7.86E-03	0.996281611	21	109	positive regulation of lipid metabolic process	BP	2.09E-01	PTK2,PDGFB,TEK,ADGRF5,KIT,RAB38,PPARG,SIRT3,SCARB1,ABHD6,DGKZ,SLC45A3,ABCG1,FGR,PRKD1,MLXIPL,CREB1,TNFAIP8L3,MTOR,CD19,BMP6	turquoise	EC_M6
GO:0046330	7.88E-03	0.996846671	15	68	positive regulation of JNK cascade	BP	2.09E-01	FLT4,TPD52L1,SH3RF3,GADD45G,TLR4,TRAF1,SDCBP,TNFRSF19,SH3RF1,NCF1,MAP3K11,CRACR2A,TAOK2,TRAF2,DAB2IP	turquoise	EC_M6
GO:0048864	7.90E-03	0.996735751	16	72	stem cell development	BP	2.10E-01	SEMA6A,SEMA4D,NRP1,EDNRB,JAG1,RET,EDN1,SEMA3F,LAMA5,SEMA6D,TBX1,HIF1A,ALDH1A2,EFNB1,SEMA4A,SEMA6B	turquoise	EC_M6
GO:0032515	8.60E-03	0.997855025	8	23	negative regulation of phosphoprotein phosphatase activity	BP	2.10E-01	ROCK2,FKBP1B,CRY2,GSK3B,PPP1R11,PPP4R4,ROCK1,FKBP1A	darkgreen	EC_M4
GO:0097421	9.70E-03	0.997636234	7	28	liver regeneration	BP	2.10E-01	PNPT1,EZH2,WDR35,PRMT5,SRSF5,TYMS,AURKA	darkgrey	EC_M8
GO:0099504	8.63E-03	0.995339217	30	138	synaptic vesicle cycle	BP	2.10E-01	CANX,PACSIN1,SH3GL2,PLAA,UNC13B,RIMS1,NAPA,PICALM,PCDH17,RAB3GAP1,CTNNB1,ACTG1,PCLO,SNPH,ATP2A2,AP3B1,VPS18,PPP3CC,BRSK2,DNM1L,GSK3B,AP2M1,BACE1,PLD1,SLC2A4,NLGN2,STX1B,SYT11,CALM1,ROCK1	darkgreen	EC_M4
GO:0050650	8.64E-03	0.998137298	7	18	chondroitin sulfate proteoglycan biosynthetic process	BP	2.10E-01	CHPF2,CHSY1,B3GAT3,UGDH,CHST12,XYLT2,CHST11	darkgreen	EC_M4
GO:0035725	8.67E-03	0.995492888	27	120	sodium ion transmembrane transport	BP	2.10E-01	SLC12A2,CNNM4,KIF5B,SCN5A,GPD1L,SLC4A7,ACTN4,FXYD1,SLC20A1,SNTA1,SLMAP,ATP1B3,SLC4A5,CHP1,ATP2B4,PKD2,SHROOM2,SLC3A2,ASIC4,SLC6A13,CACNA1G,SCNN1D,HCN2,MIR24-1,DMD,YWHAH,HCN4	darkgreen	EC_M4
GO:1901888	8.72E-03	0.995038126	35	165	regulation of cell junction assembly	BP	2.11E-01	LRP1,ADGRB1,MDGA1,VEGFA,TPBG,LRRC4B,ROCK2,VPS35,FARP1,ACTG1,APP,RAPGEF2,PTPN11,AMIGO2,PDLIM5,PHLDB2,TJP1,MMP14,CORO1C,NTRK3,IQGAP1,VCL,SIX4,NLGN2,ROCK1,DAPK3,MYO1C,NPHP4,NTNG2,LRRC24,UBE2V2,THY1,LHFPL4,DUSP22,LRRTM2	darkgreen	EC_M4
GO:0032211	9.80E-03	0.998039361	6	18	negative regulation of telomere maintenance via telomerase	BP	2.12E-01	POT1,XRN1,TERF1,HNRNPU,DCP2,EXOSC10	darkgrey	EC_M8

GO:0070887	8.02E-03	0.993438158	279	2230	cellular response to chemical stimulus	BP	2.12E-01	TNFRSF11A,SEMA6A,IFNGR1,TNFAIP3,AKAP12,FLT4,OXR1,TCF7,NCOA7,PTK2,DAPK1,ATF4,PDGFB,MAPK13,PICK1,KDM4C,MICALL1,PIM1,CSF2RB,DDX58,KIT,TNFRSF21,KDR,NFIL3,SYK,RNF149,IL6,CXCL8,DOCK4,CAV2,KLF11,GRB10,LANCL2,TSPAN12,TLR4,TRAF1,C5,IL15,TEAD2,SDCBP,TRPM4,NRP1,PIK3CG,DNMT3A,PRKD2,LRCH1,PRCP,NOS3,RALB,ZFAND6,PPARG,GATA3,ACVR2B,TBC1D4,CXCR4,IRAK2,EDNRB,NET1,IKBKB,P2RY6,P2RY2,NFKBIB,PDE2A,TMEM173,SFRP1,HBEGF,NR1D2,FERMT1,P2RX2,SLC4A11,CD36,ARID5A,PRR5L,SCARB1,EPHB1,RET,TNFRSF19,GRAMD1A,IL1A,DNMT3B,IGF1,NR4A1,EIF2AK3,HHEX,KDM3A,ERBIN,UFSP2,SMAD1,OCLN,CHRM5,GPD1,MEF2C,SMARCD1,TLR2,IL7,P2RX7,VEGFC,GUCY1B1,CXCL10,ANGPT2,SOGA1,KMT2D,TIPARP,OAS3,NLRC5,THBS4,PLCG1,STC1,RAPGEF3,ANKRD2,ST3GAL6,NR4A2,HIPK1,ANO6,DLL4,IFIT2,IFIT1,EDN1,STAT1,CD58,JAM3,EHD4,SNCA,JARID2,DTNBP1,MX1,ADCY2,ABCG1,ETS1,HDAC1,PPP3CA,HYAL2,ANO1,MEF2A,SHOC2,RIN3,LYST,LAPTM5,NCF1,FABP4,PABPN1,HCLS1,CD34,CD86,POLG,MN1,TGIF1,SH3BP4,CFLAR,ADCY1,FES,ARID1A,PLEKHA1,PRKD1,IL10RA,MLXIPL,ITGA6,PAK2,PTPRE,SIPA1,PDK3,CREB1,B2M,EIF4A2,SSTR1,LTF,CCRL2,CYBB,IL1RAP,ADAMTS7,ITGA4,DDX3X,IGFBP5,TBX1,MCU,CALCRL,RAMP2,CDK16,BIRC3,PLVAP,BAIAP2L1,FGF23,CPEB2,PPM1F,WAS,RAC1,HIF1A,JUP,ADIPOR2,EPHA2,ITPKB,MMRN2,SYT7,TLR6,DDI2,MPP5,TNFRSF1B,ALDH1A2,MTOR,AR,PSEN1,HOXB9,FGF18,ITGA3,PGF,DAPK2,CHRNB2,RPS6KA5,S100A14,GPR68,SMAD2,DNM2,SCNN1B,YY1,CYP1A1,TNIP1,COL4A6,MMP28,FAM83G,ADRB2,CCL2,IL13RA1,YES1,PDE4B,J	turquoise	EC_M6
GO:0006793	8.04E-03	0.993437734	271	2169	phosphorus metabolic process	BP	2.12E-01	TNFRSF11A,PDE7B,TNFAIP3,PTPRB,FLT4,SYNJ2,SERAC1,CDKL3,TPD52L1,HDDC2,PTK2,DAPK1,PDGFB,MAPK13,CSNK1E,PICK1,PIM1,TEK,LIMCH1,TJP2,PIP5K1B,TEC,ADGRF5,KIT,KDR,SRD5A3,SYK,SEMA4D,PPP6R1,PIGF,IL6,PPT1,GRB10,TLR4,INPP4B,IL15,CCNY,SDCBP,NOCT,NRP1,RASIP1,PIK3CG,TAL1,MTMR2,KDM4D,PRKD2,RAB38,CAB39L,SNRK,NOS3,RALB,PTPN4,PPARG,ACVR2B,IRAK2,EDNRB,PPP1CB,SPTBN4,TIE1,PGS1,IKBKB,P2RY6,PDE2A,GPAT4,SFRP1,HBEGF,CDS2,SH3BP5,ANKLE2,CD36,CDK17,PRAG1,PRR5L,MAP3K1,SCARB1,EPHB1,PLK2,RET,PDE4D,MERTK,ELOVL7,IGF1,EIF2AK3,ABHD6,HHEX,PITPNM2,SMAD1,AADAT,OCLN,DCLK2,CHRM5,GPD1,HEXB,MEF2C,TLR2,WWTR1,IL7,P2RX7,VEGFC,ENTPD1,GUCY1A1,GUCY1B1,MADD,ACOT12,BMP2K,SERINC5,NLRC5,THBS4,PPP1R16B,PLCB2,PLCG1,RAPGEF3,DGKZ,HIPK1,CCNJL,AMPD1,PIK3C2B,NEDD9,EDN1,DYRK1A,EHD4,SNCA,DTNBP1,ERG,DUSP8,NUAK2,CDK18,DCAF1,ADCY2,MTMR10,PDE9A,LPCAT1,SIK1,HDAC1,LCK,PPP3CA,HYAL2,PLA1A,PRKX,NCF1,DYRK3,FABP4,HCLS1,INPP5D,PECAM1,CD86,BMX,FGR,MAP3K6,INPP5F,ADCY1,KALRN,ADCY4,IGFBP3,FES,PPP2R5A,RPS6KA1,PROX1,PRKD1,PDIK1L,MAP3K3,MGAT1,SRMS,MLXIPL,ACE,ITGA6,LIMK2,PAK2,PTPRE,DUSP16,DLG1,SMG7,MAP3K11,STYK1,PDK3,CREB1,CTTNBP2NL,LTF,BCR,ADORA2A,PALD1,EPHB4,DDX3X,TBX1,PYGL,WNK4,CDK16,CDA,LDB2,TNFAIP8L3,FGF23,SAMD8,LMTK2,PPM1F,PRKCH,RAC1,HIF1A,ADSS,PLD2,ADIPOR2,EPHA2,ITPKB,TLR6,PGM2,TNK1,RGS14,MTOR,AR,SLC26A1,PSEN1,STK10,FBXW11,FGF18,RPS6KL1,PGF,NEK9,DAPK2,TAOK2,EFNA1,GDPD3,ADCK1,CD19,SBK1,PIK3R5,NPR	turquoise	EC_M6
GO:0032635	8.06E-03	0.996345736	19	96	interleukin-6 production	BP	2.12E-01	TNFAIP3,MAPK13,DDX58,SYK,IL6,TLR4,CD36,IL1A,TLR2,CD200,HYAL2,INPP5D,IL1RAP,TMEM106A,TLR6,LGALS9,NOS2,PLCG2,TLR1	turquoise	EC_M6
GO:0032675	8.06E-03	0.996345736	19	96	regulation of interleukin-6 production	BP	2.12E-01	TNFAIP3,MAPK13,DDX58,SYK,IL6,TLR4,CD36,IL1A,TLR2,CD200,HYAL2,INPP5D,IL1RAP,TMEM106A,TLR6,LGALS9,NOS2,PLCG2,TLR1	turquoise	EC_M6
GO:0044771	9.86E-03	0.998837248	4	10	meiotic cell cycle phase transition	BP	2.12E-01	CDC25C,TTK,CDC25A,CCNB2	darkgrey	EC_M8
GO:1901673	9.89E-03	0.998362933	5	15	regulation of mitotic spindle assembly	BP	2.13E-01	TPR,KIF15,HNRNPU,PLK1,DRG1	darkgrey	EC_M8
GO:0043491	8.84E-03	0.995154282	31	147	protein kinase B signaling	BP	2.13E-01	ANGPT1,PPARA,LEP,TGFBR1,RTN4,TPBG,RRAS,RCN3,ITGB1,FGF2,SESN3,TGFB1,AXL,C1QTNF1,OSBPL8,CAVIN3,ILK,P2RX4,CX3CL1,MTDH,DAG1,PIK3CA,NTRK3,OSM,TMEM100,AKT1,MYDGF,PARK7,F3,LOX,TXN	darkgreen	EC_M4
GO:0035845	8.87E-03	0.999187645	4	7	photoreceptor cell outer segment organization	BP	2.13E-01	AHI1,MFSD2A,ROM1,NPHP4	darkgreen	EC_M4
GO:0006644	8.88E-03	0.994195427	58	314	phospholipid metabolic process	BP	2.13E-01	NUS1,SERINC1,PPARD,PLAA,INPP4A,TMEM246,SOCS5,FAM126A,MFSD2A,FGF2,SELENOI,ABHD12,ABHD5,DGKH,OSBPL10,MTMR14,OSBPL8,SMPD1,IMPAD1,MTMR6,CHPT1,TMEM150A,FAR1,PIP4P2,LPCAT2,SERINC3,CHP1,PIGP,AGPAT3,HTR2B,INPP5A,PIK3CA,PITPNM1,ETNK1,PIGZ,PCYT1B,PLD1,SGMS1,CLN8,SPTLC2,SLC44A2,PIGL,PDGFRB,PNPLA6,OCRL,PAFAH1B1,ABCA3,PGP,DPM2,PLA2G15,TMEM38B,PEMT,PIGK,DPM1,PIGB,PIK3C2A,ORMDL3,CDIPT	darkgreen	EC_M4
GO:0060415	8.89E-03	0.996465951	15	56	muscle tissue morphogenesis	BP	2.13E-01	LY6E,TGFBR1,TGFB1,ANKRD1,PKP2,TGFB2,FGFR2,FZD2,BMPR1A,TCAP,MED1,TPM1,COL11A1,FKBP1A,WNT2	darkgreen	EC_M4

GO:0043299	8.13E-03	0.997134878	12	51	leukocyte degranulation	BP	2.14E-01	KIT,SYK,PIK3CG,GATA2,GAB2,UNC13D,SNAP23,FGR,FES,RAB27A,LGALS9,STXBP1	turquoise	EC_M6
GO:0007188	8.18E-03	0.995897958	24	129	adenylate cyclase-modulating G protein-coupled receptor signaling pathway	BP	2.14E-01	AKAP12,ADGRF1,GPR4,PDE2A,RIC8A,PDE4D,CHRM5,CXCL10,ADGRG3,EDN1,ADCY2,ADCY1,ADCY4,ADORA2A,CALCRL,RAMP2,ADRB2,PDE4B,S1PR1,ADGRL2,ADGRL4,ADGRE3,ADGRG1,GNG2	turquoise	EC_M6
GO:0048730	8.98E-03	0.997742464	8	23	epidermis morphogenesis	BP	2.14E-01	GLI2,CTNNB1,TGFB2,FGFR2,PLOD3,GORAB,NGFR,FGF10	darkgreen	EC_M4
GO:0097581	9.00E-03	0.995945734	19	78	lamellipodium organization	BP	2.15E-01	NCK2,AKIRIN1,ACTR2,ITGB1,ARHGEF4,ACTR3,TWF1,CTTN,PIK3CA,AUTS2,CORO1C,ARPC5,NCKAP1,ARPC2,CAPZB,VCL,PLXNB3,CDH13,MTSS1L	darkgreen	EC_M4
GO:1905048	9.05E-03	0.998317356	6	15	regulation of metallopeptidase activity	BP	2.16E-01	LRP1,PICALM,DDRGK1,TIMP1,MIR24-1,TIMP3	darkgreen	EC_M4
GO:0070861	9.07E-03	0.997715565	8	23	regulation of protein exit from endoplasmic reticulum	BP	2.16E-01	TMEM30A,ERLEC1,UBE2J1,TM9SF4,BRSK2,BCAP31,UBE2G2,DERL2	darkgreen	EC_M4
GO:0099537	9.10E-03	0.99357372	85	477	trans-synaptic signaling	BP	2.16E-01	RNF19A,SLC12A2,ADGRB1,SRF,UNC13B,RIMS1,TPBG,SHANK1,NCDN,SLC7A11,ITGB1,LIN7B,KIF5B,NAPA,GAD2,PCDH17,RAB3GAP1,HTR5A,CTNNB1,FARP1,PRNP,PCLO,SNPH,RIC3,MAP1B,P2RX4,ATP2A2,DLGAP4,APP,GLRB,RAPGEF2,CX3CL1,CACNB3,HOMER1,VPS18,GLS,CHRNA7,EIF4E,APBA2,DNM1L,HTR2B,GSK3B,DAG1,KRAS,NCSTN,SYAP1,BACE1,RPS6KA3,MAPT,EIF4EBP2,TNR,MPP2,KCNA1,ATAD1,CHRNE,ERC1,HAP1,IQSEC2,NLGN2,STX1B,SYT11,KCNMB1,MYCBPAP,CACNA1G,RAB11A,CALM1,GRIA1,NPTN,CYP46A1,BEGAIN,DTNA,AKT1,ABR,PAFAH1B1,TPRG1L,PARK7,NTNG2,JPH3,PCDHB2,GDNF,INSYN1,YWHAH,KCNMB4,MAP1A,LRRTM2	darkgreen	EC_M4
GO:0051234	8.29E-03	0.99302346	414	3429	establishment of localization	BP	2.16E-01	TNFRSF11A,KIF5A,TNFAIP3,STX11,TBC1D30,LAPTM4B,SNCAIP,SNX2,SNX24,ZDHHC14,SYNJ2,SERAC1,RIMS2,CDC42SE2,QKI,AFDN,PACSIN2,TRDN,FAM91A1,KLHL3,SGSM3,PTK2,FAM53C,DENND3,TSNARE1,DAPK1,PDGFB,AGTPBP1,CSNK1E,PICK1,MICALL1,PIM1,TBC1D22B,IREB2,ATP8A1,APBA1,TJP2,UBAP1,KIT,CD2AP,SYK,TRAM2,RGP1,ICA1,TSPAN13,SLC3A1,IL6,IGF2BP3,SNAP91,PPT1,CXCL8,BACH2,VPS54,ARHGAP25,ELMO1,RSAD2,CAV2,GRB10,TLR4,TCAF2,GRIK2,GJA4,IL15,TBC1D9,SDCBP,TRPM4,NRP1,RAB3B,OSBPL9,ANKRD50,PIK3CG,ITSN2,MTMR2,GATA2,KIF3C,ARHGAP21,KCNRG,RAB38,EXOC3L2,TMCC1,NECTIN2,GGA3,GAB2,LLGL2,RALB,USP6NL,UNC13D,ZFAND6,PPARG,GATA3,TBC1D4,MYO7A,CXCR4,CD93,SEC14L1,EDNRB,NDFIP2,TMC8,SPTBN4,SLCO2B1,CYTH1,P2RY6,P2RY2,FCHSD2,GPAT4,SFRP1,FERMT1,KCNK6,ATP11A,ARAP3,P2RX2,SLC4A11,CD36,SIRT3,EML4,SLCO2A1,ADAMTS9,SDCBP2,PRR5L,MICU3,SCARB1,PDE4D,MERTK,GRAMD1A,CHD7,FAM3D,IL1A,IGF1,NR4A1,HHEX,EXOC6,ERBIN,PITPNM2,CLCN3,SLCO5A1,HIP1R,OCLN,MCTP1,CHRM5,KIF13B,MEF2C,CP,TLR2,P2RX7,TPD52,VEGFC,NDRG4,GPM6A,CXCL10,TMEM144,LMBR1L,PLLP,BMP2K,SERINC5,TMEM150C,MTX3,DYSF,TPCN1,PLCG1,STC1,RAPGEF3,KCNJ3,AP4B1,STAB1,SLC38A1,ANO6,ZDHHC13,CLIC6,ZC3H11A,IFIT1,DOP1B,EDN1,KCNJ15,EHD4,SNCA,DTNBP1,ANO5,MFSD4A,SLC45A3,ANO3,PM20D1,MX2,ABCG1,CD200,MTTP,SIK1,HDAC1,LCK,PPP3CA,HYAL2,ANO1,SLC16A14,HECW2,SLC39A8,KCNJ2,MEF2A,SLC12A7,SCN3A,RFTN2,MCTP2,RIN3,ZDHHC6,TINAGL1,ADPRH,SNAP23,SLC7A7,LYST,LAPTM5,NCF1,SEC24B,LRMP,SLC	turquoise	EC_M6
GO:0032273	8.31E-03	0.995969509	22	116	positive regulation of protein polymerization	BP	2.16E-01	BAIAP2L2,PICK1,CDC42EP1,FCHSD2,FCHSD1,LMOD3,HIP1R,OCLN,IQGAP2,AP1AR,FES,DLG1,BAIAP2L1,WAS,RAC1,MTOR,SPIRE1,GIT1,CARMIL2,VASP,GMFG,ARPC5L	turquoise	EC_M6
GO:0060429	9.13E-03	0.9930591	136	810	epithelium development	BP	2.16E-01	AHI1,CDSN,PLXNB2,ROS1,RSPO3,MRTFA,SRF,VEGFA,CLOCK,COL15A1,RTN4,MFSD2A,WHRN,GLI3,ROCK2,INHBA,RCN3,SLC7A11,PLEKHA4,PLXNA1,FGF2,PTCH2,GLI2,MEGF8,RHEB,PAK1,TGFB1,CTNNB1,LBH,AKT2,SLC4A7,RARB,ACTG1,MAFG,SPRED3,SPINT2,ZNF703,STARD13,SEMA3C,ILK,SPINK5,RYK,PRICKLE2,FAT1,TMED2,PALLD,FLNB,SULF1,ROBO1,TRPC4AP,RDH10,LOXL3,HEY1,TCTN1,SLC4A5,RAPGEF2,OPA1,AP3B1,TSG101,ACVR1,PKD2,NKD1,IFT57,TOLLIP,BRSK2,PHLDB2,TJP1,KDM5B,NOTCH2,CCND1,CPT1A,TGFB2,LRP5,ITPK1,GSK3B,FSTL1,FAM20C,MMP14,DAG1,LEF1,KRAS,TAGLN2,BMP7,FGFR2,IQGAP1,CLIC4,TAGLN,SIDT2,OVOL1,PLOD3,SERPINE1,ATP6AP2,FZD2,MEOX1,PDCD10,FKBP8,SKIL,GORAB,SMURF1,EXOC5,VCL,DAAM1,WDR1,TULP3,SIX4,ACTA2,MAGED1,BMPR1A,TCAP,MED1,MSN,MYO1E,TGFB1I1,NGFR,MAP2K1,TMEM100,TTC8,LAMA3,ROCK1,AKT1,ITGA2,PAFAH1B1,PKD1,FLNA,COL5A1,LMO4,BCL10,CD151,SERPINB5,TMEM38B,LBX1,GDNF,CDKN1A,EDA,WNT2,FGF10	darkgreen	EC_M4

GO:0033157	9.17E-03	0.994578629	39	196	regulation of intracellular protein transport	BP	2.17E-01	LMAN1,ANGPT1,LEP,RAB23,TMEM30A,ERLEC1,UBE2J1,MDFIC,GLI3,KIF5B,PAK1,TGFB1,AKT2,UFM1,PRNP,HDAC3,TM9SF4,CACNB3,PTPN11,ATG13,BAP1,CHP1,BRSK2,GSK3B,C11orf65,PDCD10,B3GAT3,MIEF2,PRKAA1,MYO1C,BCAP31,FLNA,PARK7,TXN,LEPROT,ZPR1,UBE2G2,TOMM7,DERL2	darkgreen	EC_M4
GO:1902531	9.20E-03	0.992636379	209	1309	regulation of intracellular signal transduction	BP	2.17E-01	IL23A,LRP1,ARHGEF25,TAB2,DYRK2,RRM2B,ANGPT1,PPARA,ROS1,FAM49B,PTP4A3,PPARD,UBQLN1,LGALS1,KLHDC10,ARHGAP39,LEP,NUAK1,VEGFA,DOCK8,SLC35B2,NCK2,CREB3,TGFBR1,RTN4,TPBG,EMC10,MAP3K7,MDFIC,ROCK2,RRAS,RCN3,ITGB1,FGF2,SESN3,ARHGEF4,TPT1,RHEB,PAK1,TGFB1,AXL,SEC13,CTNNB1,LBH,DYNC1LI1,GPD1L,CANT1,C1QTNF1,ARHGEF17,OSBPL8,CXXC5,PSD2,NPLOC4,ACTN4,ANAPC15,SPRED3,ATP2C1,PRNP,DCN,HDAC3,STARD13,CAVIN3,SMPD1,PPP2CB,ILK,ARL6IP5,RYK,TMEM127,TMEM9B,ST5,AKIP1,CXCL12,SLC20A1,ANKRD1,PJA2,ROBO1,WWC2,P2RX4,RNF13,RASA1,APP,DHX36,RAPGEF2,MAPKAPK5,CX3CL1,PTPN11,RALGAPB,OPA1,ARHGAP1,PTPN22,GDF6,SERINC3,MTDH,ADA,CHP1,ATP2B4,PLA2R1,TANK,CHRNA7,ADIPOR1,NOTCH2,DNM1L,SELENOS,TGFB2,KCTD10,ARHGAP31,HTR2B,GSK3B,DAG1,PIK3CA,AUTS2,PRKCA,CMKLR1,KRAS,NTRK3,WWC3,SPATC1L,BMP7,HYOU1,GOLT1B,ATF3,FGFR2,OSM,IQGAP1,TFRC,HSPB1,MAPT,SYVN1,ATP6AP2,FN1,PTPN6,GPR137,PDCD10,ITGAV,ADRA2C,HTT,COL3A1,SKIL,SYNPO2L,FGFR3,ARAF,TNIK,VDAC2,MYO9B,SGMS1,OTUD5,AKT3,MAGED1,IQSEC2,CGNL1,ARHGEF2,DOCK2,SHC1,TAF9B,ADRA1B,MAP2K1,GPR65,TMEM100,CALM1,TSPAN6,UACA,SLC44A2,NPTN,PIN1,CD74,PDGFRB,ROCK1,AKT1,PRKAA1,TNFAIP1,PTPN2,CLEC16A,MYDGF,ABR,MGRN1,BCAP31,PAFAH1B1,IRAK1,FLNA,ATP6AP1,IKBKG,PARK7,ENO1,USP10,CDH13,F3,ARHGAP29,BCAR3,BCL10,WLS,MIR24-1,PRMT6,CCL16,DUSP2,FKBP1A,AGO3,C18orf32,TIMP3,CHN1,EDA,TXN,DUSP4,NACC2,TMSB4X,GLIPR2,DUSP22,PTTG1IP,PLAGL2,FGF10	darkgreen	EC_M4
GO:0008277	8.38E-03	0.996183952	19	96	regulation of G protein-coupled receptor signaling pathway	BP	2.18E-01	RIC8B,CXCL8,RGS3,MGLL,ABHD6,EDN1,SNCA,GIT2,RAMP2,TMOD2,RGS14,DNM2,ADRB2,PDE4B,GIT1,RGS4,RGS16,PDCL,GNG4	turquoise	EC_M6
GO:0045913	9.24E-03	0.996300409	15	57	positive regulation of carbohydrate metabolic process	BP	2.18E-01	DYRK2,PPARA,DGAT2,AKT2,KAT2B,IRS2,SORBS1,APP,ZBTB20,PPARGC1A,AKT1,PRKAA1,PTPN2,HAS2,NNMT	darkgreen	EC_M4
GO:0016055	9.27E-03	0.993717848	68	373	Wnt signaling pathway	BP	2.18E-01	LRP1,RSPO3,CBY1,GNAQ,CDK14,VCP,TPBG,MDFIC,GLI3,DKKL1,VPS35,PLEKHA4,FGF2,TGFB1,CTNNB1,ZEB2,ZNF703,ILK,RYK,PRICKLE2,SULF1,CTNND1,DKK3,APP,CPE,AMFR,PKD2,NKD1,BRD7,CDC73,CCND1,CAPRIN2,LRP5,GSK3B,LEF1,KPNA1,BICC1,FGFR2,FZD9,ATP6AP2,FZD2,IGFBP2,SMURF1,TNIK,DAAM1,OTUD5,TGFB1I1,COL1A1,PBXIP1,SOX30,SMARCA4,GSKIP,PIN1,PRKAA1,NLK,NXN,DAPK3,TAX1BP3,PKD1,CSNK1G2,NPHP4,WLS,EDA,DACT3,MESD,WNT2,FGF10,MBD2	darkgreen	EC_M4
GO:0051017	9.29E-03	0.994946849	30	139	actin filament bundle assembly	BP	2.18E-01	SRF,TGFBR1,SHANK1,CALD1,ROCK2,ZYX,PAK1,ACTG1,DPYSL3,HSP90B1,SORBS1,PFN2,CX3CL1,PHLDB2,TJP1,SYNPO2,ITGB5,SYNPO2L,FMN2,CGNL1,ACTN1,TPM1,TGFB3,GPR65,TTC8,PLS3,ROCK1,TNFAIP1,FLNA,PDLIM1	darkgreen	EC_M4
GO:0060711	9.29E-03	0.996941656	11	37	labyrinthine layer development	BP	2.18E-01	RSPO3,SPINT2,TMED2,HEY1,LEF1,BMP7,FGFR2,VASH2,MAP2K1,AKT1,WNT2	darkgreen	EC_M4
GO:0016240	9.32E-03	0.999133008	4	7	autophagosome membrane docking	BP	2.18E-01	VAMP8,ATP2A2,CALM1,VMP1	darkgreen	EC_M4
GO:2001171	9.34E-03	0.998642693	5	11	positive regulation of ATP biosynthetic process	BP	2.18E-01	PPARA,VCP,PPARGC1A,ENO1,TMSB4X	darkgreen	EC_M4
GO:0001818	8.44E-03	0.995282501	32	195	negative regulation of cytokine production	BP	2.19E-01	TNFAIP3,LAPTM4B,C6orf106,TNFRSF21,CD2AP,IL6,REL,TLR4,GATA3,MERTK,IGF1,ITCH,ERBIN,OAS3,CD200,LAPTM5,INPP5D,CD34,APOD,LTF,HOMER3,RAC1,EPHA2,TLR6,ARG2,RPS6KA5,YY1,LGALS9,GIT1,OAS1,BST2,RNF125	turquoise	EC_M6
GO:0046850	8.45E-03	0.997377549	10	38	regulation of bone remodeling	BP	2.19E-01	TNFRSF11A,TNFAIP3,SYK,IL6,SFRP1,P2RX7,INPP5D,SYT7,S1PR1,CA2	turquoise	EC_M6
GO:1904036	8.47E-03	0.997562031	9	34	negative regulation of epithelial cell apoptotic process	BP	2.19E-01	TNFAIP3,TEK,KDR,GATA2,GATA3,CFLAR,RAMP2,MTOR,CDH5	turquoise	EC_M6
GO:0035590	8.48E-03	0.998334901	6	19	purinergic nucleotide receptor signaling pathway	BP	2.19E-01	P2RY6,P2RY2,P2RX2,P2RX7,ANO6,ADORA2A	turquoise	EC_M6

GO:0035815	8.49E-03	0.999088638	4	9	positive regulation of renal sodium excretion	BP	2.19E-01	EDNRB,EDN1,ADORA2A,NPR1	turquoise	EC_M6
GO:0048585	8.51E-03	0.993389584	161	1229	negative regulation of response to stimulus	BP	2.19E-01	SEMA6A,TNFAIP3,TRIM39,TSPAN8,FZD6,CTHRC1,OXR1,GRAMD4,NCOA7,C6orf106,PDGFB,MDFI,TEK,CD2AP,SYK,SEMA4D,RNF149,IL6,CXCL8,RGS3,ARHGAP25,FIGNL1,CAV2,GRB10,TLR4,C5,NRP1,RASIP1,ARHGAP42,MTMR2,GATA2,CST7,NOS3,PPARG,GATA3,SEC14L1,PDE2A,SFRP1,NR1D2,NOTUM,FERMT1,SIRT3,PRR5L,PLK2,PDE4D,IL1A,IGF1,KLKB1,HHEX,ITCH,ERBIN,SH3RF1,MCTP1,WWTR1,IL7,P2RX7,NDRG4,ANGPT2,LMBR1L,ADGRG3,OAS3,NLRC5,PLCG1,NR4A2,DGKZ,DLL4,SOX13,EDN1,STAT1,DYRK1A,SNCA,DUSP8,PRDM15,SHANK2,CD200,HDAC1,ITPRIP,HYAL2,SEMA3F,RIN3,LAPTM5,HLX,DYRK3,INPP5D,CD34,SH3BP4,FGR,CFLAR,INPP5F,IGFBP3,PLEKHA1,MAP3K3,SRMS,ITGA6,PTPRE,DUSP16,DLG1,ELL3,APOD,LTF,ADORA2A,SEMA6D,TSPAN15,DDX3X,IGFBP5,HOMER3,MECOM,CALCRL,HIF1A,EPHA2,MMRN2,BID,TLR6,MCC,LFNG,TNFRSF1B,RGS14,ARG2,MTOR,AR,PSEN1,SEMA4A,ITGA3,EFNA1,CCDC88C,TLE3,DNM2,TNIP1,MMP28,MARK3,KIF26A,ADRB2,CCL2,YES1,PDE4B,LGALS9,IL7R,BFAR,GIT1,TNFAIP8L1,SEMA6B,ISG15,NRARP,EGFL7,NOTCH1,TERF2,DAB2IP,CDH5,MIR99A,OAS1,VNN1,CD46,SOX17,RGS4,SERPINB9,RGS16,DUSP5,BMP2,RNF125,DUSP7,RNF152	turquoise	EC_M6
GO:0032611	8.53E-03	0.996970825	12	52	interleukin-1 beta production	BP	2.19E-01	TNFAIP3,IL6,TLR4,CD36,IGF1,P2RX7,TMEM106A,TLR6,ARG2,LGALS9,GIT1,GBP5	turquoise	EC_M6
GO:0032651	8.53E-03	0.996970825	12	52	regulation of interleukin-1 beta production	BP	2.19E-01	TNFAIP3,IL6,TLR4,CD36,IGF1,P2RX7,TMEM106A,TLR6,ARG2,LGALS9,GIT1,GBP5	turquoise	EC_M6
GO:0150003	9.37E-03	0.999127219	4	7	regulation of spontaneous synaptic transmission	BP	2.19E-01	SLC12A2,ITGB1,APP,STX1B	darkgreen	EC_M4
GO:0042593	9.38E-03	0.994530834	37	183	glucose homeostasis	BP	2.19E-01	LRP1,PPARD,LGALS1,LEP,SRF,UNC13B,FKBP1B,KIF5B,SESN3,IRS2,OSBP,SELENOT,ABCC8,HECTD4,PTPN11,PFKM,OPA1,CRY2,BRSK2,DYNLL1,ADIPOR1,ZBTB20,LRP5,PIK3CA,ADCY5,SIDT2,PTPRN,SLC2A4,PPARGC1A,NGFR,SMARCA4,AKT1,PRKAA1,PTPN2,SSTR5,PARK7,SERPINF1	darkgreen	EC_M4
GO:0050671	8.59E-03	0.996375562	16	82	positive regulation of lymphocyte proliferation	BP	2.20E-01	SYK,IL6,PELI1,TLR4,IL15,IL1A,IGF1,MEF2C,IL7,ICOSLG,NFATC2,CD86,EFNB1,CHRNB2,LGALS9,CD46	turquoise	EC_M6
GO:2001236	8.62E-03	0.995790895	22	121	regulation of extrinsic apoptotic signaling pathway	BP	2.20E-01	TNFAIP3,TRAF1,NRP1,NOS3,TMC8,SFRP1,RET,IL1A,IGF1,SH3RF1,IL7,MADD,ITPRIP,HYAL2,CFLAR,ITGA6,PAK2,DDX3X,BID,AR,TRAF2,PMAIP1	turquoise	EC_M6
GO:0043122	8.62E-03	0.995127998	33	197	regulation of I-kappaB kinase/NF-kappaB signaling	BP	2.20E-01	TNFAIP3,TRIM39,REL,PELI1,TLR4,TRAF1,ZFAND6,NDFIP2,IKBKB,CD36,PLK2,TNFRSF19,ZDHHC13,STAT1,HDAC1,TIFA,CFLAR,CASP10,PRKD1,MAP3K3,LTF,TMEM106A,BIRC3,TLR6,TNIP1,LGALS9,TRAF2,PLEKHG5,PLCG2,DAB2IP,TRIM34,TRIM62,BST2	turquoise	EC_M6
GO:0007399	8.63E-03	0.993005386	246	1867	nervous system development	BP	2.20E-01	TCF4,SEMA6A,N4BP3,KIF5A,IFNGR1,SYNJ2,FZD6,CTHRC1,RIMS2,CELSR1,QKI,CDKL3,ASAP1,PTK2,ANKS1A,AGTPBP1,CSNK1E,PICK1,KDM4C,MICALL1,LINGO2,APBA1,KIT,ADGRF1,TNFRSF21,SEMA4D,EFHC1,RAPGEF5,IL6,IGF2BP3,PPT1,UTP11,FOXP2,TLR4,FZD8,TEAD2,SDCBP,MYCN,NRCAM,NRP1,ITSN2,TAL1,PHF10,MTMR2,GATA2,DNMT3A,HS6ST1,CST7,NOVA2,CTSC,PLXND1,ITGA8,ITGB4,TIMP4,GATA3,MYO7A,CXCR4,EDNRB,SPTBN4,GDPD5,SETD5,JAG1,SFRP1,FEZ2,EFNB2,ANKLE2,ARHGEF7,PCDH1,PCDH12,ARHGAP26,NTN4,PRAG1,SDCBP2,EPHB1,PLK2,MALL,RET,MERTK,CHD7,DNMT3B,EIF2AK3,SH3RF1,PREX2,SMAD1,POU6F1,DCLK2,MMP24,KIF13B,ENC1,HEXB,MEF2C,SMARCD1,TLR2,VEGFC,NDRG4,GPM6A,DHH,PLLP,SERINC5,USH1C,NR4A2,HIPK1,DLL4,SOX13,TSPAN2,NAV2,DYRK1A,JAM3,SNCA,JARID2,DTNBP1,MYLIP,SLC45A3,SRGAP2C,ETS1,SHANK2,HDAC1,PPP3CA,HECW2,MEF2A,SEMA3F,LSAMP,ROBO4,HLX,SEC24B,ZFHX2,JHY,PLXNA2,B3GNT5,CFLAR,INPP5F,TRAK2,ADCY1,KALRN,FES,ARID1A,PROX1,SS18L1,PRKD1,VANGL2,ZNF148,LAMA5,YTHDF1,ITGA6,PAK2,LAMC2,DLG1,ELL3,PTCHD1,APOD,ETV6,HOXD10,MOV10,CREB1,B2M,SSTR1,ADORA2A,ARNT2,SEMA6D,MYEF2,IL1RAP,EPHB4,ITGA4,TBX1,HSPG2,DNM3,CDK16,LDB2,PROM1,LMTK2,TMOD2,PRKCH,RAC1,HIF1A,EPHA2,SHROOM4,CECR2,BID,MPP5,TNFRSF1B,RGS14,ALDH1A2,LGI2,MAFK,ZC4H2,MTOR,OPHN1,EFNB1,CASZ1,PSEN1,SEMA4A,HOXB8,FBXW11,ABI3,ITGA3,EIF2B2,TAOK2,ARHGEF15,EFNA1,CHRNB2,NAP1L2,ZDHHC15,RPS6KA5,DNM2,B4GALT6,HMG20A,ZNF521,KIF26A,SH3TC2,CCL2,JAG2,UNC119,SPEF2,GIT1,SEMA6B,SLITRK2	turquoise	EC_M6

GO:0051283	9.47E-03	0.995610746	20	83	negative regulation of sequestering of calcium ion	BP	2.20E-01	FKBP1B,FGF2,RYR3,CX3CL1,PKD2,HTR2B,CASQ1,PTPN6,HTT,ERO1A,HAP1,CACNA1C,CALM1,ANXA6,JPH3,DMD,FKBP1A,TMEM38B,THY1,TMEM38A	darkgreen	EC_M4
GO:0002366	8.66E-03	0.995208057	31	181	leukocyte activation involved in immune response	BP	2.20E-01	MFNG,KIT,SYK,IL6,TLR4,PIK3CG,GATA2,GAB2,UNC13D,GATA3,GAPT,DYSF,SNAP23,HLX,CD86,FGR,FES,CRACR2A,RAB27A,LFNG,MTOR,PSEN1,SEMA4A,BATF,CD19,SCNN1B,LGALS9,IRF8,PLCG2,STXBP1,CD46	turquoise	EC_M6
GO:0006493	9.49E-03	0.995798977	18	74	protein O-linked glycosylation	BP	2.20E-01	LARGE1,POMGNT1,ST6GALNAC2,TRAK1,TMTC4,KDELC1,TMTC3,B3GNT4,TET3,GALNT2,PLOD3,GALNT16,GALNT10,GALNT1,POMT1,DPM2,DPM1,SLC35C1	darkgreen	EC_M4
GO:0006686	9.51E-03	0.999109591	4	7	sphingomyelin biosynthetic process	BP	2.20E-01	SGMS1,SPTLC2,PEMT,ORMDL3	darkgreen	EC_M4
GO:0033138	9.53E-03	0.995782365	18	74	positive regulation of peptidyl-serine phosphorylation	BP	2.20E-01	ANGPT1,VEGFA,PAK1,TGFB1,PFN2,APP,ATP2B4,CAPRIN2,PIK3CA,NTRK3,OSM,TFRC,PDCD10,ARAF,AKT1,PARK7,BCAR3,TXN	darkgreen	EC_M4
GO:0051249	8.70E-03	0.994650252	44	287	regulation of lymphocyte activation	BP	2.20E-01	TNFAIP3,TCF7,TNFRSF21,SYK,IL6,PELI1,TLR4,IL15,GATA3,SFRP1,MERTK,IL1A,IGF1,ITCH,SH3RF1,MEF2C,IL7,SOX13,LCK,ICOSLG,LAPTM5,HLX,NFATC2,INPP5D,CD86,FGR,DLG1,B2M,ADORA2A,SOS2,ITPKB,TNFRSF1B,ARG2,EFNB1,CHRNB2,CD19,CCL2,YES1,LGALS9,IL7R,AP3D1,NRARP,VNN1,CD46	turquoise	EC_M6
GO:0050817	9.58E-03	0.994552498	34	166	coagulation	BP	2.21E-01	SERPINB2,LMAN1,GNAQ,MYH9,SRF,SLC7A11,DGKH,AXL,C1QTNF1,ACTG1,ILK,MYL9,AP3B1,HPS6,FAP,ITPK1,PIK3CA,PRKCA,HSPB1,SERPINE1,ITGA2B,FN1,PTPN6,ADRA2C,COMP,COL3A1,VCL,ITGA2,CD40LG,FLNA,F3,CD59,CSRP1,PRKG1	darkgreen	EC_M4
GO:0010862	9.59E-03	0.997051141	10	32	positive regulation of pathway-restricted SMAD protein phosphorylation	BP	2.21E-01	TGFBR1,INHBA,TGFB1,RBPMS,ACVR1,GDF6,TGFB2,BMP7,BMPR1A,TGFB3	darkgreen	EC_M4
GO:0007599	9.60E-03	0.99453777	34	166	hemostasis	BP	2.21E-01	SERPINB2,LMAN1,GNAQ,MYH9,SRF,SLC7A11,DGKH,AXL,C1QTNF1,ACTG1,ILK,MYL9,AP3B1,HPS6,FAP,ITPK1,PIK3CA,PRKCA,HSPB1,SERPINE1,ITGA2B,FN1,PTPN6,ADRA2C,COMP,COL3A1,VCL,ITGA2,CD40LG,FLNA,F3,CD59,CSRP1,PRKG1	darkgreen	EC_M4
GO:0009595	8.75E-03	0.998318712	6	17	detection of biotic stimulus	BP	2.21E-01	TLR4,DACH1,SCARB1,TLR2,TLR6,TLR1	turquoise	EC_M6
GO:0097709	9.75E-03	0.998566532	5	11	connective tissue replacement	BP	2.23E-01	ROCK2,TGFB1,RUNX1,TIMP1,ROCK1	darkgreen	EC_M4
GO:1990456	9.76E-03	0.999080054	4	7	mitochondrion-endoplasmic reticulum membrane tethering	BP	2.23E-01	ATP2A2,CALM1,VMP1,AHCYL1	darkgreen	EC_M4
GO:0007204	9.76E-03	0.994210297	39	194	positive regulation of cytosolic calcium ion concentration	BP	2.23E-01	LRP1,FKBP1B,FGF2,C1QTNF1,PRNP,CCKBR,RIC3,RYR3,P2RX4,SELENOT,CX3CL1,CACNB3,ATP2B4,PKD2,HTR2B,CMKLR1,ADCY5,CASQ1,PTPN6,ITGAV,HTT,ERO1A,HAP1,CACNA1C,ADRA1B,GPR65,CALM1,NPTN,TRPV2,GNB1,BCAP31,PKD1,JPH3,DMD,FKBP1A,TMEM38B,THY1,TMEM38A,CCR6	darkgreen	EC_M4
GO:0048639	9.76E-03	0.994731489	29	134	positive regulation of developmental growth	BP	2.23E-01	LRP1,PPARD,LEP,PLAA,SRF,VEGFA,RIMS1,FGF2,MEGF8,PAK1,ILK,CXCL12,EIF4G2,MAP1B,CELF1,ZFYVE27,FGFR2,MAPT,FN1,SMURF1,BMPR1A,RAB11A,ISLR2,PIN1,TRPV2,AKT1,PAFAH1B1,HOPX,WNT2	darkgreen	EC_M4
GO:0032868	9.77E-03	0.994117816	41	206	response to insulin	BP	2.23E-01	AP3S1,PPARA,LEP,SESN3,PAK1,AKT2,OSBPL8,KAT2B,IRS2,ATP2B1,SORBS1,USO1,ABCC8,PTPN11,OPA1,CRY2,ADIPOR1,SELENOS,ZNF106,EPRS,GSK3B,PIK3CA,HADH,WDTC1,SYAP1,EIF4EBP2,PRKCI,PTPRN,MYO5A,SLC2A4,SHC1,PKM,AKT1,LPIN2,PRKAA1,RAB31,PTPN2,MYO1C,BCAR3,SLC2A8,PIK3C2A	darkgreen	EC_M4
GO:0050670	8.85E-03	0.995514698	24	137	regulation of lymphocyte proliferation	BP	2.23E-01	TNFRSF21,SYK,IL6,PELI1,TLR4,IL15,IL1A,IGF1,ITCH,SH3RF1,MEF2C,IL7,ICOSLG,NFATC2,INPP5D,CD86,DLG1,SOS2,TNFRSF1B,ARG2,EFNB1,CHRNB2,LGALS9,CD46	turquoise	EC_M6
GO:0016445	1.05E-02	0.99630315	11	54	somatic diversification of immunoglobulins	BP	2.24E-01	MSH2,PAXIP1,RIF1,PRKDC,MSH3,NBN,MCM3AP,NSD2,EXO1,THOC1,RNF8	darkgrey	EC_M8
GO:0051924	9.87E-03	0.994319125	35	172	regulation of calcium ion transport	BP	2.25E-01	UBQLN1,FKBP1B,CTNNB1,STIM1,PRNP,ATP2B1,REM1,CXCL12,P2RX4,CACNA1D,CX3CL1,CACNB3,HOMER1,PKD2,CASQ1,LINC02210-CRHR1,PTPN6,HTT,HAP1,CACNA1C,STIM2,CALM1,VMP1,TRPV2,PDGFRB,AKT1,JPH3,DMD,FKBP1A,CBARP,TMEM38B,THY1,TMEM38A,SARAF,GEM	darkgreen	EC_M4

GO:0198738	9.88E-03	0.993285116	68	374	cell-cell signaling by wnt	BP	2.25E-01	LRP1,RSPO3,CBY1,GNAQ,CDK14,VCP,TPBG,MDFIC,GLI3,DKKL1,VPS35,PLEKHA4,FGF2,TGFB1,CTNNB1,ZEB2,ZNF703,ILK,RYK,PRICKLE2,SULF1,CTNND1,DKK3,APP,CPE,AMFR,PKD2,NKD1,BRD7,CDC73,CCND1,CAPRIN2,LRP5,GSK3B,LEF1,KPNA1,BICC1,FGFR2,FZD9,ATP6AP2,FZD2,IGFBP2,SMURF1,TNIK,DAAM1,OTUD5,TGFB1I1,COL1A1,PBXIP1,SOX30,SMARCA4,GSKIP,PIN1,PRKAA1,NLK,NXN,DAPK3,TAX1BP3,PKD1,CSNK1G2,NPHP4,WLS,EDA,DACT3,MESD,WNT2,FGF10,MBD2	darkgreen	EC_M4
GO:0033365	9.91E-03	0.992545875	123	737	protein localization to organelle	BP	2.25E-01	LMAN1,ANGPT1,TCP1,NUP50,HSPA4,CTNNA1,SUN2,GGA1,LEP,PEX6,VCP,SRP72,RAB23,TMEM30A,RTN4,COX18,EMC10,MDFIC,GLI3,ROCK2,VPS41,VPS35,MGARP,SEC61A1,RAB7A,RB1,MARK4,MAPRE3,TGFB1,SRP68,RAB3GAP1,SEC13,DZIP1,OSBPL8,UFM1,HDAC3,DCLK1,DDRGK1,WASHC2A,VPS37A,ARL1,TRAM1,EMC4,PINX1,TCTN1,CCT8,SSR3,GNL3,ATG13,AP3B1,TSG101,DKC1,CRY2,BAP1,CHP1,CBLB,TOLLIP,ARL3,SRPRA,DNM1L,KPNA6,GSK3B,DAG1,GOLGB1,HSPA8,LARP7,KPNA1,BMP7,TOMM40L,TFRC,SRP54,MAPT,C11orf65,SMURF1,EMC1,TULP3,SIX4,OBSL1,KDELR2,ROM1,MAGED1,IQSEC2,ATP6V1D,MED1,MSN,TSPAN17,SYT11,NGFR,COL1A1,TMED10,RAB11A,CALM1,PIN1,AKT1,TMEM98,PRKAA1,BCAP31,NPHP4,FLNA,PARK7,HSPA5,RANBP2,TCTN2,KPNA4,SPCS3,RAB6A,SNX10,ARF4,TOR1AIP2,EMC7,CDKN1A,TXN,CC2D2B,ARFRP1,PTTG1IP,GABARAPL2,TIMM22,LEPROT,ZPR1,GRPEL2,ORMDL3,TOMM7,MAP1A	darkgreen	EC_M4
GO:0010906	9.91E-03	0.995176971	22	96	regulation of glucose metabolic process	BP	2.25E-01	DYRK2,PPARA,LEP,DGAT2,AKT2,C1QTNF1,KAT2B,IRS2,SORBS1,ADIPOR1,SELENOS,GSK3B,PPP1R3D,PPP1R3F,PPARGC1A,AKT1,PTPN2,PGP,MIDN,FAM3A,RANBP2,NNMT	darkgreen	EC_M4
GO:0022412	1.06E-02	0.993802926	35	247	cellular process involved in reproduction in multicellular organism	BP	2.25E-01	YTHDC2,FBXO5,FANCC,TUT7,SRPK1,UBR2,FOXJ3,MSH2,YTHDC1,TTK,CEP57,METTL3,BRCA2,CCNB1,RPS6,KNL1,BMPR1B,KIF18A,NSUN2,TRIP13,ASPM,SPO11,TIAL1,DDX6,FOSL1,ZFX,DDX20,TOP2A,CCNB2,PLK1,BRIP1,FANCA,ODF2,AURKA,RNF8	darkgrey	EC_M8
GO:0048599	1.06E-02	0.997079733	8	32	oocyte development	BP	2.25E-01	YTHDC2,FBXO5,TUT7,BRCA2,CCNB1,TRIP13,ZFX,AURKA	darkgrey	EC_M8
GO:0060389	9.98E-03	0.996292544	13	47	pathway-restricted SMAD protein phosphorylation	BP	2.26E-01	LRP1,TGFBR1,INHBA,TGFB1,RBPMS,ACVR1,GDF6,TGFB2,BMP7,BMPR1A,TGFB3,PIN1,PMEPA1	darkgreen	EC_M4
GO:1901362	4.94E-04	0.999607881	380	3012	organic cyclic compound biosynthetic process	BP	2.26E-01	NFATC1,ZNF236,TSHZ1,SMARCC2,STAT6,MAP3K5,CDK4,CITED2,MAML1,SQSTM1,IRAK3,HELB,GFPT2,OSR2,HSPA1B,EHMT2,ZBTB12,HDAC10,ATF6B,RXRB,TSPO,HSD17B8,RING1,CYB5R3,ZHX2,ZBTB22,PMM1,MYC,TOB2,SMAD5,EP300,IBA57,KDM3B,ZNF76,DDX17,NFIB,HSF1,CCND3,TRERF1,CDKN2A,HMOX1,WASL,KLF9,POLR1C,PLPP6,JAK2,HINT2,ZBTB45,ZNF135,ZNF667,ZNF865,EFEMP1,ZNF692,ANXA4,AKNA,MED25,IL4I1,ING3,ARHGEF5,IRF3,LPIN1,FOXO3,KLF15,SPHK2,NOP53,MAML2,BICRA,KMT2E,BBS7,MEIS3,TRIM13,ACSS1,IRF2BP1,SIX5,FOSB,ZNF746,NR2C2,ZNF862,TSC22D1,FDXR,UROD,ZNF575,NAA16,GTF3C2,ZNF574,WBP2,KMT2C,MYD88,TADA3,RPS3,TEP1,BRPF1,PNP,DYRK1B,EID2,ZNF219,MED29,SIRT2,RPTOR,APBB3,NUP98,TRIM21,CYP1B1,GNPDA1,HGF,APBB1,TAF10,DEAF1,HOXC4,NCOA4,LIN37,KMT2B,RBCK1,RARG,SDR16C5,ZNF740,LMO2,POFUT1,URI1,PIK3R1,ACSS2,NCOA2,ATF1,TNKS1BP1,POLE4,KDM2B,ZFHX4,CAMKK2,SCAND1,SETD6,MCPH1,SSBP2,FNIP2,DGUOK,ARL2BP,SHOX2,NEK4,SPR,ZHX3,VDR,MMS19,LPIN3,CPOX,FOXF2,F2RL1,SORBS3,HNF4A,TP53INP1,PIDD1,ERLIN1,HR,ACOT8,TRIM45,NPM3,PARP3,ELK4,LGR4,SRD5A1,CYB5R1,ICE1,SP140,TCF7L2,TXNIP,AIP,SOX5,ADNP,ZNF217,QARS,IMPDH2,DTX3L,PARP14,ABCB11,ECM1,RCOR3,ARNT,PRMT2,RIPK3,CTPS2,GABPB2,PER2,TWIST2,VPS72,HDAC4,USF1,SMARCD2,POGZ,CLCN2,USP21,PPOX,DNAJC30,PATZ1,RNASEL,CCDC85B,MAGEF1,SCAP,CDKN1B,TCEA3,KAT5,LRP6,YBX3,NFKBIA,POR,NFE2L2,POLR2J2,PHC1,HEXIM2,MTHFS,BATF2,PCBD1,CHCHD10,MEN1,ATIC,PHB2,TSC22D4,SAV1,TAF6,CTDSP1,EID1,SSBP4,RNF25,FGF7,NELF	cyan	EC_M2
GO:0050920	1.00E-02	0.99428077	34	167	regulation of chemotaxis	BP	2.26E-01	FAM49B,VEGFA,CREB3,TPBG,AKIRIN1,FGF2,MEGF8,TGFB1,SEMA3C,RYK,CXCL12,ROBO1,SEMA4F,P2RX4,SEMA4G,DNM1L,CMKLR1,NTRK3,SLAMF8,HSPB1,SERPINE1,NBL1,CD74,PDGFRB,USP14,ITGA2,PTPN2,PLXNA3,CDH13,F3,ARTN,TMSB4X,CCR6,FGF10	darkgreen	EC_M4
GO:0051480	1.00E-02	0.993906876	42	212	regulation of cytosolic calcium ion concentration	BP	2.26E-01	LRP1,FKBP1B,FGF2,C1QTNF1,PRNP,ATP2B1,CCKBR,RIC3,RYR3,P2RX4,SELENOT,CX3CL1,CACNB3,ATP2B4,PKD2,HTR2B,CMKLR1,ADCY5,CASQ1,FZD9,PTPN6,ITGAV,HTT,ERO1A,HAP1,CACNA1C,ADRA1B,GPR65,CALM1,GRIA1,NPTN,TRPV2,GNB1,BCAP31,PKD1,JPH3,DMD,FKBP1A,TMEM38B,THY1,TMEM38A,CCR6	darkgreen	EC_M4
GO:0001508	1.00E-02	0.995028347	23	99	action potential	BP	2.26E-01	GNAQ,MYH14,FKBP1B,SCN5A,GPD1L,SNTA1,SLMAP,P2RX4,ATP2A2,CACNA1D,CACNB3,PKP2,KCNA1,CACNA1C,CACNA1G,CALM1,FLNA,KCNAB2,HCN2,DMD,YWHAH,HCN4,KCNMB4	darkgreen	EC_M4

GO:0008015	1.01E-02	0.993138193	67	369	blood circulation	BP	2.28E-01	ANGPT1,PPARA,PTP4A3,PPARD,SH3GL2,LEP,VEGFA,COL1A2,ROCK2,BVES,MYLK3,FKBP1B,TGFB1,SCN5A,GPD1L,ATP2B1,CXCL12,FXYD1,SNTA1,LNPEP,P2RX4,ATP2A2,CACNA1D,SLC4A5,ABCC8,ADCY6,ADA,ATP2B4,RAP1GDS1,TJP1,PKP2,DNM1L,TGFB2,LRP5,HTR2B,PIK3CA,GLRX3,OLR1,PLOD3,ATP6AP2,ADRA2C,COMP,ECE1,ACTA2,CACNA1C,HSPB7,TCAP,NPPB,KCNMB1,TPM1,CACNA1G,ADRA1B,CALM1,SGCD,RASL10B,ROCK1,AKT1,NPR3,FLNA,DMD,TMEM38B,TMEM38A,HOPX,PIK3C2A,HCN4,PRKG1,KCNMB4	darkgreen	EC_M4
GO:1903960	9.04E-03	0.999076528	4	8	negative regulation of anion transmembrane transport	BP	2.28E-01	TCAF2,MTOR,SLC43A2,RGS4	turquoise	EC_M6
GO:0097531	9.04E-03	0.998601182	5	13	mast cell migration	BP	2.28E-01	KIT,VEGFC,RIN3,RAC1,PGF	turquoise	EC_M6
GO:2001026	9.06E-03	0.998217853	6	18	regulation of endothelial cell chemotaxis	BP	2.28E-01	KDR,PRKD2,PRKD1,FGF18,NOTCH1,FGF16	turquoise	EC_M6
GO:0016048	9.10E-03	0.998237891	6	17	detection of temperature stimulus	BP	2.28E-01	GRIK2,CXCR4,EPHB1,MMP24,ANO3,ANO1	turquoise	EC_M6
GO:0010591	9.12E-03	0.997341219	9	34	regulation of lamellipodium assembly	BP	2.28E-01	OCLN,WAS,RAC1,FSCN1,EPHA2,MTOR,ABI3,DNM2,CARMIL2	turquoise	EC_M6
GO:0090049	9.19E-03	0.997078203	10	41	regulation of cell migration involved in sprouting angiogenesis	BP	2.30E-01	KDR,NRP1,GATA2,PLK2,DLL4,MAP3K3,RHOJ,MMRN2,NOTCH1,MIR23A	turquoise	EC_M6
GO:0008654	1.02E-02	0.993728427	43	222	phospholipid biosynthetic process	BP	2.30E-01	NUS1,SERINC1,PPARD,INPP4A,TMEM246,SOCS5,FAM126A,MFSD2A,FGF2,SELENOI,ABHD5,DGKH,MTMR14,IMPAD1,MTMR6,CHPT1,TMEM150A,FAR1,LPCAT2,CHP1,PIGP,AGPAT3,HTR2B,PIK3CA,PITPNM1,ETNK1,PIGZ,PCYT1B,PLD1,SGMS1,SPTLC2,SLC44A2,PIGL,OCRL,DPM2,TMEM38B,PEMT,PIGK,DPM1,PIGB,PIK3C2A,ORMDL3,CDIPT	darkgreen	EC_M4
GO:1990700	1.08E-02	0.998748001	4	9	nucleolar chromatin organization	BP	2.30E-01	RRP8,SMARCA5,SUV39H1,DDX11	darkgrey	EC_M8
GO:1904357	1.08E-02	0.997371106	7	24	negative regulation of telomere maintenance via telomere lengthening	BP	2.30E-01	POT1,TNKS2,XRN1,TERF1,HNRNPU,DCP2,EXOSC10	darkgrey	EC_M8
GO:0099175	1.03E-02	0.99540253	18	73	regulation of postsynapse organization	BP	2.31E-01	LRRC4B,ACTR2,VPS35,PRNP,DHX36,HOMER1,OPA1,PDLIM5,DNM1L,CAPRIN2,NTRK3,HSPA8,KIF1A,TANC2,SRCIN1,PAFAH1B1,ARF4,LRRTM2	darkgreen	EC_M4
GO:0050679	1.03E-02	0.99421037	32	154	positive regulation of epithelial cell proliferation	BP	2.31E-01	VEGFA,PDCL3,TGFBR1,RTN4,EMC10,FGF2,SCN5A,WDR48,CTNNB1,ZNF703,CXCL12,KDM5B,NOTCH2,HYAL1,CCND1,HTR2B,PRKCA,FGFR2,VASH2,HTRA1,PTPRN,AKT3,BMPR1A,MED1,AKT1,MYDGF,PLXNB3,CDH13,F3,HAS2,WNT2,FGF10	darkgreen	EC_M4
GO:1905897	1.03E-02	0.995501452	17	70	regulation of response to endoplasmic reticulum stress	BP	2.31E-01	UBQLN1,NCK2,CREB3,DDRGK1,OPA1,SERINC3,SELENOS,HYOU1,ATF6,SYVN1,USP14,PTPN2,BCAP31,TMEM259,PARK7,HSPA5,DNAJB9	darkgreen	EC_M4
GO:0050746	1.04E-02	0.998452298	5	11	regulation of lipoprotein metabolic process	BP	2.31E-01	LEP,RAB3GAP1,DGAT2,ITGAV,PEMT	darkgreen	EC_M4
GO:0003018	1.04E-02	0.993758767	40	200	vascular process in circulatory system	BP	2.32E-01	LRP1,SLC12A2,ANGPT1,PTP4A3,PPARD,SH3GL2,LEP,VEGFA,MFSD2A,ROCK2,MYLK3,TGFB1,ATP2B1,SNTA1,ABCC8,ADCY6,ATP2B4,RAP1GDS1,TJP1,SLC2A10,HTR2B,TFRC,PLOD3,ADRA2C,COMP,ECE1,ACTA2,SLC6A13,SLC29A4,SLC2A4,NPPB,SLC7A3,KCNMB1,ADRA1B,ROCK1,AKT1,NPR3,PIK3C2A,PRKG1,KCNMB4	darkgreen	EC_M4
GO:1900024	9.29E-03	0.996862017	11	44	regulation of substrate adhesion-dependent cell spreading	BP	2.32E-01	PTK2,NRP1,UNC13D,ARHGEF7,NEDD9,AP1AR,DOCK1,MELTF,RAC1,RCC2,DNM2	turquoise	EC_M6
GO:0001510	1.09E-02	0.995695631	13	73	RNA methylation	BP	2.32E-01	ZC3H13,TRDMT1,METTL3,TGS1,DIMT1,VIRMA,NSUN2,TRMT10A,TARBP1,FTSJ3,METTL16,CMTR2,TFB2M	darkgrey	EC_M8
GO:0044062	9.38E-03	0.998499518	5	14	regulation of excretion	BP	2.33E-01	EDNRB,STC1,EDN1,ADORA2A,NPR1	turquoise	EC_M6
GO:0007249	9.39E-03	0.994516821	36	222	I-kappaB kinase/NF-kappaB signaling	BP	2.33E-01	TNFAIP3,TRIM39,REL,PELI1,TLR4,TRAF1,ZFAND6,IRAK2,NDFIP2,IKBKB,CD36,PLK2,TNFRSF19,TLR2,ZDHHC13,STAT1,HDAC1,TIFA,CFLAR,CASP10,PRKD1,MAP3K3,LTF,TMEM106A,BIRC3,TLR6,TBKBP1,TNIP1,LGALS9,TRAF2,PLEKHG5,PLCG2,DAB2IP,TRIM34,TRIM62,BST2	turquoise	EC_M6

GO:0008610	1.05E-02	0.992412089	93	544	lipid biosynthetic process	BP	2.33E-01	HSD17B6,B4GALNT1,PPARA,ACSL6,NUS1,SERINC1,PPARD,LEP,LARGE1,REST,INPP4A,TMEM246,SOCS5,FAM126A,MFSD2A,DKKL1,FGF2,SELENOI,ABHD5,ABHD1,DGKH,ACER3,DGAT2,MTMR14,SMPD1,IMPAD1,MTMR6,CHPT1,OSBP,ST8SIA4,TMEM150A,DKK3,RDH10,FAR1,CERS5,SORBS1,LPCAT2,CHP1,PIGP,AGPAT3,ZBTB20,PRKAB2,HTR2B,PIK3CA,PITPNM1,ETNK1,B3GALT1,ARV1,HACD2,WDTC1,OSBPL2,PIGZ,PCYT1B,MOGAT3,SGPL1,HSD17B1,PLD1,CYP19A1,SGMS1,ACBD3,HSD17B10,CLN8,STARD3,MED1,PPARGC1A,SPTLC2,SLC44A2,CYP46A1,PIGL,ACSL4,CD74,AKT1,LPIN2,PRKAA1,OCRL,ABCA3,CSNK1G2,ACOT7,FAM3A,MBTPS1,DPM2,PLA2G15,HACD1,TMEM38B,PEMT,AMACR,PIGK,DPM1,PIGB,TMEM189,PIK3C2A,ORMDL3,CDIPT	darkgreen	EC_M4
GO:0051279	1.05E-02	0.995717045	15	58	regulation of release of sequestered calcium ion into cytosol	BP	2.33E-01	FKBP1B,CX3CL1,PKD2,CASQ1,PTPN6,HTT,HAP1,CACNA1C,CALM1,JPH3,DMD,FKBP1A,TMEM38B,THY1,TMEM38A	darkgreen	EC_M4
GO:0090502	1.11E-02	0.995845576	12	62	RNA phosphodiester bond hydrolysis, endonucleolytic	BP	2.34E-01	POP1,TSEN2,UTP20,BMS1,RRS1,NOP14,EXO1,MRPL44,DROSHA,NOL9,NOB1,FEN1	darkgrey	EC_M8
GO:0002562	1.11E-02	0.996066928	11	54	somatic diversification of immune receptors via germline recombination within a single locus	BP	2.34E-01	MSH2,DCLRE1C,PAXIP1,RIF1,PRKDC,MSH3,NBN,NSD2,EXO1,THOC1,RNF8	darkgrey	EC_M8
GO:0016444	1.11E-02	0.996066928	11	54	somatic cell DNA recombination	BP	2.34E-01	MSH2,DCLRE1C,PAXIP1,RIF1,PRKDC,MSH3,NBN,NSD2,EXO1,THOC1,RNF8	darkgrey	EC_M8
GO:0043543	1.11E-02	0.993758016	30	202	protein acylation	BP	2.34E-01	DSCC1,NAA35,ZNF451,ZMPSTE24,NAA15,HDAC2,CEP295,PAXIP1,KAT14,EPC2,KAT6A,ZDHHC17,TAF7,BRCA2,TAF9,ESCO2,MCRS1,NAA25,CHEK1,NAA50,MCM3AP,HAT1,ING5,BRCA1,TAF1,OGT,ESCO1,TAOK1,CTCF,AC118549.1	darkgrey	EC_M8
GO:0050860	1.06E-02	0.997952789	6	15	negative regulation of T cell receptor signaling pathway	BP	2.35E-01	PRNP,PTPN22,CBLB,PTPN6,PTPN2,THY1	darkgreen	EC_M4
GO:0046887	1.06E-02	0.994997899	20	86	positive regulation of hormone secretion	BP	2.35E-01	LRP1,PPARD,LEP,INHBA,KIF5B,C1QTNF1,IRS2,OSBP,VAMP8,SELENOT,PTPN11,PFKM,SPP1,DYNLL1,TFR2,CYP19A1,NLGN2,ACSL4,RASL10B,VAMP7	darkgreen	EC_M4
GO:0048662	1.06E-02	0.99618152	12	44	negative regulation of smooth muscle cell proliferation	BP	2.35E-01	FAM19A5,PPARD,ILK,COMT,PPARGC1A,TPM1,TGFB3,CNN1,MYOCD,NPR3,CDKN1A,PRKG1	darkgreen	EC_M4
GO:0002718	9.49E-03	0.996255432	14	63	regulation of cytokine production involved in immune response	BP	2.35E-01	DDX58,KIT,IL6,RSAD2,TLR4,TRPM4,GATA3,CD36,LAPTM5,B2M,TNFRSF1B,TRAF2,PLCG2,BST2	turquoise	EC_M6
GO:0046578	9.50E-03	0.994785219	30	161	regulation of Ras protein signal transduction	BP	2.35E-01	CDC42SE2,SGSM3,DENND3,NRP1,RASIP1,PIK3CG,ARHGAP42,GPR4,NET1,CYTH1,PRAG1,IGF1,ERBIN,ARHGEF3,ARHGEF28,MADD,SHOC2,F11R,ARHGDIB,BCR,RAC1,ITPKB,OPHN1,STARD8,ITGA3,DNM2,NOTCH1,DAB2IP,ADGRG1,LPAR6	turquoise	EC_M6

GO:0071310	9.58E-03	0.992260622	228	1784	cellular response to organic substance	BP	2.36E-01	TNFRSF11A,SEMA6A,IFNGR1,TNFAIP3,AKAP12,FLT4,TCF7,PTK2,DAPK1,ATF4,PDGFB,MAPK13,KDM4C,MICALL1,PIM1,CSF2RB,DDX58,KIT,TNFRSF21,KDR,NFIL3,SYK,IL6,CXCL8,CAV2,KLF11,GRB10,LANCL2,TSPAN12,TLR4,TRAF1,IL15,TEAD2,SDCBP,TRPM4,NRP1,PIK3CG,DNMT3A,PRKD2,LRCH1,PRCP,RALB,ZFAND6,PPARG,GATA3,ACVR2B,TBC1D4,CXCR4,IRAK2,EDNRB,IKBKB,P2RY6,P2RY2,NFKBIB,PDE2A,TMEM173,SFRP1,NR1D2,FERMT1,CD36,ARID5A,SCARB1,RET,TNFRSF19,GRAMD1A,IL1A,DNMT3B,IGF1,NR4A1,EIF2AK3,HHEX,KDM3A,ERBIN,UFSP2,SMAD1,OCLN,CHRM5,GPD1,MEF2C,SMARCD1,TLR2,IL7,P2RX7,VEGFC,CXCL10,ANGPT2,SOGA1,KMT2D,TIPARP,OAS3,NLRC5,PLCG1,STC1,RAPGEF3,ST3GAL6,NR4A2,HIPK1,DLL4,IFIT2,IFIT1,EDN1,STAT1,CD58,EHD4,SNCA,JARID2,DTNBP1,MX1,ADCY2,ABCG1,HDAC1,PPP3CA,HYAL2,ANO1,SHOC2,LAPTM5,FABP4,PABPN1,HCLS1,CD86,POLG,MN1,TGIF1,SH3BP4,CFLAR,ADCY1,FES,ARID1A,PRKD1,IL10RA,MLXIPL,ITGA6,PAK2,PTPRE,PDK3,CREB1,EIF4A2,SSTR1,LTF,CCRL2,CYBB,IL1RAP,ADAMTS7,ITGA4,IGFBP5,TBX1,CALCRL,RAMP2,CDK16,BIRC3,PLVAP,BAIAP2L1,FGF23,CPEB2,WAS,RAC1,HIF1A,JUP,ADIPOR2,MMRN2,TLR6,MPP5,TNFRSF1B,ALDH1A2,MTOR,AR,PSEN1,FGF18,ITGA3,PGF,RPS6KA5,GPR68,SMAD2,DNM2,SCNN1B,YY1,CYP1A1,TNIP1,COL4A6,FAM83G,ADRB2,CCL2,IL13RA1,YES1,PDE4B,JAK1,LGALS9,NOS2,IL7R,BFAR,GIT1,LRG1,ISG15,HCFC1,TRAF2,TMEM204,TNFRSF25,NOTCH1,SLC25A33,RAPGEF1,IRF8,PLCG2,ENG,STXBP1,MTF2,NFAT5,DAB2IP,CDH5,MIR23A,MIR99A,OAS1,SLC8A3,GBP5,TRIM24,RAB43,CA2,SOX17,ACKR1,FGF16,RGS4,SERPINB9,COMMD7,	turquoise	EC_M6
GO:0098911	9.61E-03	0.998536777	5	12	regulation of ventricular cardiac muscle cell action potential	BP	2.36E-01	TRPM4,DLG1,JUP,DSG2,DSC2	turquoise	EC_M6
GO:0032944	9.64E-03	0.995071632	24	138	regulation of mononuclear cell proliferation	BP	2.36E-01	TNFRSF21,SYK,IL6,PELI1,TLR4,IL15,IL1A,IGF1,ITCH,SH3RF1,MEF2C,IL7,ICOSLG,NFATC2,INPP5D,CD86,DLG1,SOS2,TNFRSF1B,ARG2,EFNB1,CHRNB2,LGALS9,CD46	turquoise	EC_M6
GO:0007411	9.64E-03	0.994529692	33	181	axon guidance	BP	2.36E-01	SEMA6A,KIF5A,PTK2,SEMA4D,NRCAM,NRP1,NOVA2,PLXND1,GATA3,FEZ2,EFNB2,EPHB1,RET,SEMA3F,ROBO4,PLXNA2,KALRN,LAMA5,YTHDF1,LAMC2,SEMA6D,EPHB4,RAC1,EPHA2,OPHN1,EFNB1,SEMA4A,EFNA1,RPS6KA5,SEMA6B,NOTCH1,VASP,RELN	turquoise	EC_M6
GO:0032946	9.65E-03	0.995870633	16	83	positive regulation of mononuclear cell proliferation	BP	2.36E-01	SYK,IL6,PELI1,TLR4,IL15,IL1A,IGF1,MEF2C,IL7,ICOSLG,NFATC2,CD86,EFNB1,CHRNB2,LGALS9,CD46	turquoise	EC_M6
GO:0009887	9.65E-03	0.992838484	111	784	animal organ morphogenesis	BP	2.36E-01	SEMA6A,TNFAIP3,FZD6,CTHRC1,CELSR1,KLHL3,PTK2,ATF4,AGTPBP1,PIM1,MDFI,TEK,SYK,IL6,TSPAN12,TEAD2,MYCN,NRP1,GATA2,PLXND1,NOS3,ITGB4,GATA3,MYO7A,GZF1,JAG1,SFRP1,SPEF1,NTN4,TCF15,EPHB1,CHD7,MEF2C,WWTR1,IL7,VEGFC,NDRG4,TIPARP,USH1C,STC1,HIPK1,DLL4,EDN1,STAT1,HDAC1,HYAL2,PRKX,HLX,SEC24B,ABLIM1,JHY,CD34,FGR,CFLAR,RPS6KA1,PROX1,PLEKHA1,VANGL2,LAMA5,ITGA6,LAMC2,DLG1,HOXD10,LTF,BCOR,EPHB4,IGFBP5,TBX1,HSPG2,WNK4,MMP13,PROM1,FGF23,RAC1,HIF1A,EPHA2,LFNG,FGD1,ALDH1A2,MTOR,AR,PSEN1,HOXB5,HOXB6,HOXB7,HOXB8,HOXB9,FBXW11,FGF18,EFNA1,SMAD2,TLE3,YY1,CCL2,JAG2,SPEF2,RFLNB,NOTCH1,S1PR1,ENG,CA2,SOX17,FGF16,SOX18,DIO3,SHANK3,TBC1D20,PERP,BMP2,BMP6,FLI1	turquoise	EC_M6
GO:0033209	9.68E-03	0.995996612	15	75	tumor necrosis factor-mediated signaling pathway	BP	2.36E-01	TNFRSF11A,TNFAIP3,SYK,TRAF1,IKBKB,TNFRSF19,HIPK1,STAT1,LAPTM5,BIRC3,PLVAP,TNFRSF1B,TRAF2,TNFRSF25,COMMD7	turquoise	EC_M6
GO:0046631	9.69E-03	0.995520863	19	97	alpha-beta T cell activation	BP	2.36E-01	SYK,IL6,RSAD2,IL15,GATA3,ITCH,SH3RF1,HLX,CD86,ADORA2A,CRACR2A,ITPKB,ARG2,MTOR,SEMA4A,BATF,LGALS9,ELF4,AP3D1	turquoise	EC_M6
GO:0010457	1.12E-02	0.998116392	5	14	centriole-centriole cohesion	BP	2.36E-01	RTTN,KIF3A,SGO1,NEK2,ODF2	darkgrey	EC_M8
GO:0006221	1.13E-02	0.997532042	6	24	pyrimidine nucleotide biosynthetic process	BP	2.36E-01	CTPS1,CAD,UMPS,PRPS1,TYMS,DHODH	darkgrey	EC_M8
GO:0002200	1.13E-02	0.99576467	12	61	somatic diversification of immune receptors	BP	2.36E-01	MSH2,DCLRE1C,PAXIP1,RIF1,PRKDC,MSH3,NBN,MCM3AP,NSD2,EXO1,THOC1,RNF8	darkgrey	EC_M8
GO:0046040	1.13E-02	0.998031658	5	16	IMP metabolic process	BP	2.36E-01	PPAT,PAICS,PFAS,HPRT1,GART	darkgrey	EC_M8

GO:0030182	9.72E-03	0.992497153	149	1067	neuron differentiation	BP	2.36E-01	TCF4,SEMA6A,KIF5A,CTHRC1,RIMS2,CDKL3,ASAP1,PTK2,ANKS1A,AGTPBP1,CSNK1E,PICK1,KDM4C,MICALL1,ADGRF1,TNFRSF21,SEMA4D,IL6,PPT1,FZD8,MYCN,NRCAM,NRP1,ITSN2,TAL1,MTMR2,GATA2,DNMT3A,HS6ST1,NOVA2,PLXND1,GATA3,MYO7A,CXCR4,EDNRB,SPTBN4,JAG1,SFRP1,FEZ2,EFNB2,PCDH12,NTN4,PRAG1,EPHB1,PLK2,RET,DNMT3B,PREX2,DCLK2,KIF13B,ENC1,MEF2C,NDRG4,GPM6A,USH1C,NR4A2,HIPK1,DLL4,TSPAN2,JAM3,DTNBP1,MYLIP,SRGAP2C,HDAC1,PPP3CA,HECW2,MEF2A,SEMA3F,ROBO4,SEC24B,ZFHX2,PLXNA2,CFLAR,INPP5F,TRAK2,ADCY1,KALRN,FES,PROX1,SS18L1,PRKD1,LAMA5,YTHDF1,ITGA6,PAK2,LAMC2,APOD,HOXD10,MOV10,CREB1,B2M,ADORA2A,SEMA6D,MYEF2,EPHB4,ITGA4,DNM3,CDK16,PROM1,LMTK2,RAC1,HIF1A,EPHA2,CECR2,MPP5,ALDH1A2,ZC4H2,MTOR,OPHN1,EFNB1,CASZ1,PSEN1,SEMA4A,ABI3,ITGA3,TAOK2,EFNA1,CHRNB2,NAP1L2,ZDHHC15,RPS6KA5,DNM2,B4GALT6,HMG20A,ZNF521,JAG2,GIT1,SEMA6B,SLITRK2,ATCAY,ARHGAP4,NOTCH1,RAPGEF1,S1PR1,STXBP1,LHX6,DAB2IP,VASP,RELN,ALKAL1,DIO3,SHANK3,MMD,GBA2,STX3,TGIF2,BMP2,SCARF1,BMP6	turquoise	EC_M6
GO:0051282	1.08E-02	0.994934636	20	84	regulation of sequestering of calcium ion	BP	2.37E-01	FKBP1B,FGF2,RYR3,CX3CL1,PKD2,HTR2B,CASQ1,PTPN6,HTT,ERO1A,HAP1,CACNA1C,CALM1,ANXA6,JPH3,DMD,FKBP1A,TMEM38B,THY1,TMEM38A	darkgreen	EC_M4
GO:0046628	1.08E-02	0.997530425	7	19	positive regulation of insulin receptor signaling pathway	BP	2.37E-01	LEP,PAK1,OSBPL8,SORBS1,PTPN11,OPA1,ADIPOR1	darkgreen	EC_M4
GO:1902893	1.08E-02	0.995942621	13	47	regulation of pri-miRNA transcription by RNA polymerase II	BP	2.37E-01	PPARA,MRTFA,PPARD,SRF,REST,FGF2,TGFB1,TEAD1,GNL3,TGFB2,BMPR1A,NGFR,SMARCA4	darkgreen	EC_M4
GO:0014743	9.79E-03	0.996456666	12	52	regulation of muscle hypertrophy	BP	2.38E-01	IGF1,EDN1,JARID2,PDE9A,PPP3CA,MEF2A,IGFBP5,TNFRSF1B,MTOR,YY1,NOTCH1,RGS4	turquoise	EC_M6
GO:1904706	1.09E-02	0.997459424	7	20	negative regulation of vascular associated smooth muscle cell proliferation	BP	2.38E-01	FAM19A5,TPM1,TGFB3,CNN1,MYOCD,CDKN1A,PRKG1	darkgreen	EC_M4
GO:0000478	1.15E-02	0.998030835	5	15	endonucleolytic cleavage involved in rRNA processing	BP	2.39E-01	UTP20,BMS1,RRS1,NOP14,NOL9	darkgrey	EC_M8
GO:0000479	1.15E-02	0.998030835	5	15	endonucleolytic cleavage of tricistronic rRNA transcript (SSU-rRNA, 5.8S rRNA, LSU-rRNA)	BP	2.39E-01	UTP20,BMS1,RRS1,NOP14,NOL9	darkgrey	EC_M8
GO:0003208	1.10E-02	0.995522313	15	57	cardiac ventricle morphogenesis	BP	2.39E-01	LY6E,TGFBR1,SMARCD3,TGFB1,SEMA3C,HEY1,CPE,PKP2,TGFB2,FGFR2,BMPR1A,MED1,TPM1,COL11A1,FKBP1A	darkgreen	EC_M4
GO:0097553	1.10E-02	0.994601458	22	95	calcium ion transmembrane import into cytosol	BP	2.39E-01	FKBP1B,FGF2,PRNP,RYR3,CX3CL1,ATP2B4,PKD2,HTR2B,CASQ1,PTPN6,HTT,ERO1A,HAP1,CACNA1C,CALM1,TRPV2,JPH3,DMD,FKBP1A,TMEM38B,THY1,TMEM38A	darkgreen	EC_M4
GO:0043954	1.10E-02	0.995854886	13	47	cellular component maintenance	BP	2.39E-01	WHRN,VPS35,PRNP,PCLO,PLEKHA7,HOMER1,TJP1,CTTN,C1QL1,ERC1,NLGN2,MYOCD,SDF4	darkgreen	EC_M4
GO:0033628	9.89E-03	0.996832646	10	40	regulation of cell adhesion mediated by integrin	BP	2.40E-01	PTK2,PODXL,SYK,PIK3CG,FERMT1,RET,JAM3,EPHA2,EFNA1,PIEZO1	turquoise	EC_M6
GO:0045926	9.91E-03	0.99434929	33	195	negative regulation of growth	BP	2.40E-01	SEMA6A,CDKL3,SEMA4D,PPT1,NRP1,SFRP1,TLL2,JARID2,HYAL2,SEMA3F,EAF2,SH3BP4,SIPA1,SEMA6D,DDX3X,IGFBP5,ING4,CDA,HIF1A,ADIPOR2,SEMA4A,NPR1,YY1,ADRB2,RAI1,SEMA6B,ARHGAP4,NOTCH1,SOX17,OSGIN2,RGS4,BST2,GNG4	turquoise	EC_M6
GO:0034101	9.95E-03	0.995180214	21	111	erythrocyte homeostasis	BP	2.40E-01	L3MBTL3,IREB2,ADGRF5,KIT,PTBP3,TAL1,GATA2,GATA3,STAT1,ALAS1,ETS1,DYRK3,HCLS1,INPP5D,B2M,HIF1A,HOXB6,SCNN1B,RCOR1,ISG15,ARID4A	turquoise	EC_M6
GO:0071526	9.98E-03	0.996822009	10	38	semaphorin-plexin signaling pathway	BP	2.40E-01	SEMA6A,SEMA4D,NRP1,PLXND1,SEMA3F,PLXNA2,SEMA6D,RAC1,SEMA4A,SEMA6B	turquoise	EC_M6
GO:0034315	9.99E-03	0.997993051	6	18	regulation of Arp2/3 complex-mediated actin nucleation	BP	2.40E-01	PICK1,FCHSD2,HIP1R,AP1AR,WAS,GMFG	turquoise	EC_M6

GO:0006930	1.00E-02	0.998942597	4	8	substrate-dependent cell migration, cell extension	BP	2.40E-01	CD2AP,SDCBP,NRP1,OPHN1	turquoise	EC_M6
GO:0097091	1.11E-02	0.998311614	5	11	synaptic vesicle clustering	BP	2.41E-01	PCDH17,CTNNB1,PCLO,BRSK2,NLGN2	darkgreen	EC_M4
GO:0042461	1.11E-02	0.996763854	9	28	photoreceptor cell development	BP	2.41E-01	AHI1,RORB,VEGFA,MFSD2A,ARL3,PRKCI,ROM1,NPHP4,THY1	darkgreen	EC_M4
GO:0045091	1.17E-02	0.998545025	4	10	regulation of single stranded viral RNA replication via double stranded DNA intermediate	BP	2.43E-01	TRIM28,TOP2B,FAM208A,TOP2A	darkgrey	EC_M8
GO:0060317	1.12E-02	0.996726754	9	28	cardiac epithelial to mesenchymal transition	BP	2.43E-01	TGFBR1,RTN4,HEY1,ACVR1,TGFB2,TMEM100,HAS2,OLFM1,WNT2	darkgreen	EC_M4
GO:0043467	1.12E-02	0.994356548	23	104	regulation of generation of precursor metabolites and energy	BP	2.43E-01	DYRK2,PPARA,VCP,AKT2,IRS2,CAVIN3,SORBS1,APP,SELENOS,ZBTB20,GSK3B,PIK3CA,ISCU,PPP1R3D,GHITM,PPP1R3F,PPARGC1A,AKT1,PRKAA1,PARK7,SLC2A6,BNIP3,COX7A2	darkgreen	EC_M4
GO:0050961	1.02E-02	0.998382612	5	13	detection of temperature stimulus involved in sensory perception	BP	2.43E-01	GRIK2,CXCR4,EPHB1,MMP24,ANO1	turquoise	EC_M6
GO:0006023	1.13E-02	0.995068004	17	68	aminoglycan biosynthetic process	BP	2.43E-01	B4GALT7,ANGPT1,CHPF2,TGFB1,B3GNT4,CHST2,CHSY1,HYAL1,B3GAT3,UGDH,CHST12,XYLT2,CLTC,PDGFRB,HAS2,CHST11,HS2ST1	darkgreen	EC_M4
GO:0032092	1.02E-02	0.995762077	15	73	positive regulation of protein binding	BP	2.44E-01	CTHRC1,MFNG,NRP1,PLXND1,RALB,TMEM173,PLK2,HIP1R,EPB41,ACE,B2M,RAMP2,PSEN1,MARK3,BMP2	turquoise	EC_M6
GO:0010764	1.03E-02	0.998831602	4	9	negative regulation of fibroblast migration	BP	2.45E-01	HYAL2,RAC1,ADIPOR2,ARHGAP4	turquoise	EC_M6
GO:0017157	1.03E-02	0.994567928	26	147	regulation of exocytosis	BP	2.45E-01	RIMS2,APBA1,SYK,SDCBP,RAB3B,GATA2,GAB2,UNC13D,SNCA,DTNBP1,ATP9A,FGR,ADCY1,FES,ADORA2A,RAB27A,SYT7,RSC1A1,FBXL20,RAB3D,LGALS9,GIT1,RPH3AL,NOTCH1,STXBP1,RALA	turquoise	EC_M6
GO:0044057	1.03E-02	0.99312292	60	398	regulation of system process	BP	2.45E-01	RIMS2,TRDN,PDGFB,KIT,TNFRSF21,DOCK4,DLGAP3,TRPM4,PIK3CG,ARHGAP42,MTMR2,MGLL,CST7,CTSC,NOS3,EDNRB,SPTBN4,HBEGF,PDE4D,IGF1,NR4A1,EIF2AK3,HIP1R,GUCY1A1,STC1,KCNJ3,EDN1,JARID2,PDE9A,PPP3CA,KCNJ2,MEF2A,ZFHX2,EPB41,ACE,DLG1,ADORA2A,IGFBP5,CALCRL,WNK4,JUP,TNFRSF1B,MTOR,ATP2A1,NPR1,YY1,DSG2,DSC2,ADRB2,PDE4B,NOTCH1,SLC8A3,RELN,KCNH2,SCN2B,SHANK3,RGS4,CACNG5,BMP6,KCNG2	turquoise	EC_M6
GO:0002573	1.03E-02	0.994475009	27	154	myeloid leukocyte differentiation	BP	2.45E-01	TNFRSF11A,L3MBTL3,IREB2,KIT,TLR4,IL15,TAL1,GATA2,GAB2,PPARG,GATA3,SFRP1,TMEM178A,TLR2,TSPAN2,HCLS1,INPP5D,FES,CREB1,LTF,EPHA2,LBR,MTOR,PSEN1,BATF,GPR68,CA2	turquoise	EC_M6
GO:0000288	1.19E-02	0.995749352	11	53	nuclear-transcribed mRNA catabolic process, deadenylation-dependent decay	BP	2.46E-01	PAN2,TNRC6B,TUT7,EXOSC9,DIS3,EXOSC8,PAN3,PDE12,CNOT1,DCP2,EXOSC2	darkgrey	EC_M8
GO:0043628	1.19E-02	0.996582387	8	36	ncRNA 3'-end processing	BP	2.46E-01	EXOSC9,EXOSC8,INTS7,SSB,EXOSC10,INTS2,EXOSC2,INTS6	darkgrey	EC_M8
GO:0097485	1.04E-02	0.994081899	33	182	neuron projection guidance	BP	2.46E-01	SEMA6A,KIF5A,PTK2,SEMA4D,NRCAM,NRP1,NOVA2,PLXND1,GATA3,FEZ2,EFNB2,EPHB1,RET,SEMA3F,ROBO4,PLXNA2,KALRN,LAMA5,YTHDF1,LAMC2,SEMA6D,EPHB4,RAC1,EPHA2,OPHN1,EFNB1,SEMA4A,EFNA1,RPS6KA5,SEMA6B,NOTCH1,VASP,RELN	turquoise	EC_M6
GO:0061572	1.14E-02	0.993679641	30	141	actin filament bundle organization	BP	2.46E-01	SRF,TGFBR1,SHANK1,CALD1,ROCK2,ZYX,PAK1,ACTG1,DPYSL3,HSP90B1,SORBS1,PFN2,CX3CL1,PHLDB2,TJP1,SYNPO2,ITGB5,SYNPO2L,FMN2,CGNL1,ACTN1,TPM1,TGFB3,GPR65,TTC8,PLS3,ROCK1,TNFAIP1,FLNA,PDLIM1	darkgreen	EC_M4
GO:0001522	1.20E-02	0.99782802	5	18	pseudouridine synthesis	BP	2.46E-01	PUS10,PUS7,PUS7L,GAR1,NAF1	darkgrey	EC_M8
GO:0007569	1.20E-02	0.994546659	17	98	cell aging	BP	2.47E-01	CDK2,FBXO5,PNPT1,ZMPSTE24,SMC6,KAT6A,BRCA2,MAPK8,PRKDC,ATR,RBL1,EEF1E1,MORC3,CHEK1,CDK1,FOXM1,RSL1D1	darkgrey	EC_M8
GO:0002367	1.05E-02	0.995818349	14	64	cytokine production involved in immune response	BP	2.47E-01	DDX58,KIT,IL6,RSAD2,TLR4,TRPM4,GATA3,CD36,LAPTM5,B2M,TNFRSF1B,TRAF2,PLCG2,BST2	turquoise	EC_M6

GO:0035459	1.15E-02	0.997327627	7	19	vesicle cargo loading	BP	2.48E-01	SEC24A,PICALM,SEC13,SEC23B,TMED2,SEC23A,TMED10	darkgreen	EC_M4
GO:0061081	1.05E-02	0.997903334	6	17	positive regulation of myeloid leukocyte cytokine production involved in immune response	BP	2.48E-01	DDX58,KIT,TLR4,CD36,LAPTM5,PLCG2	turquoise	EC_M6
GO:0016973	1.21E-02	0.997823043	5	17	poly(A)+ mRNA export from nucleus	BP	2.48E-01	NUP93,MCM3AP,NUP133,THOC2,SARNP	darkgrey	EC_M8
GO:0031644	1.06E-02	0.994967664	20	100	regulation of nervous system process	BP	2.49E-01	RIMS2,TNFRSF21,DLGAP3,MTMR2,MGLL,CST7,CTSC,NOS3,EDNRB,EIF2AK3,EDN1,ZFHX2,TNFRSF1B,MTOR,ADRB2,SLC8A3,RELN,SHANK3,RGS4,CACNG5	turquoise	EC_M6
GO:0071391	1.06E-02	0.998255599	5	14	cellular response to estrogen stimulus	BP	2.49E-01	SFRP1,ARID5A,AR,TRIM24,SERPINB9	turquoise	EC_M6
GO:0001774	1.06E-02	0.997116182	8	29	microglial cell activation	BP	2.49E-01	IFNGR1,IL6,CST7,CTSC,TLR2,SNCA,TLR6,TLR1	turquoise	EC_M6
GO:0030279	1.06E-02	0.997166418	8	26	negative regulation of ossification	BP	2.50E-01	TRPM4,SFRP1,MEF2C,BCOR,FGF23,HIF1A,RFLNB,NOTCH1	turquoise	EC_M6
GO:0035455	1.06E-02	0.998283879	5	13	response to interferon-alpha	BP	2.50E-01	GATA3,IFIT2,MX2,BST2,IFIT3	turquoise	EC_M6
GO:0006643	1.16E-02	0.993344492	33	164	membrane lipid metabolic process	BP	2.50E-01	B4GALNT1,SERINC1,LARGE1,TMEM246,GLB1,ACER3,SUMF1,SMPD1,ST8SIA4,CERS5,SERINC3,PIGP,B4GALT4,B3GALT1,ARV1,HACD2,PIGZ,SGPL1,SGMS1,CLN8,SPTLC2,CLN6,PIGL,PRKAA1,CSNK1G2,DPM2,PLA2G15,HACD1,PEMT,PIGK,DPM1,PIGB,ORMDL3	darkgreen	EC_M4
GO:0070509	1.16E-02	0.995199469	15	58	calcium ion import	BP	2.50E-01	SMDT1,CTNNB1,PRNP,CXCL12,ATP2A2,CACNA1D,HOMER1,OPA1,ATP2B4,PKD2,CACNA1C,CACNA1G,TRPV2,PDGFRB,AKT1	darkgreen	EC_M4
GO:1904355	1.22E-02	0.997829156	5	16	positive regulation of telomere capping	BP	2.50E-01	MAP3K4,TNKS2,NEK2,AURKB,HNRNPD	darkgrey	EC_M8
GO:0051781	1.22E-02	0.995345598	12	63	positive regulation of cell division	BP	2.50E-01	ITGB1BP1,KIF20B,RACGAP1,PKP4,CIT,CUL3,KIF14,ECT2,CDC6,AURKB,KIF23,PKN2	darkgrey	EC_M8
GO:0045893	1.22E-02	0.990499617	144	1244	positive regulation of transcription, DNA-templated	BP	2.50E-01	ONECUT2,BCLAF1,DDX39B,SCAF8,HSF2,ATAD2,XRCC6,PHF5A,IFT74,PWP1,ZNF300,TOPORS,RFX3,TRIM28,ZNF451,FOXJ3,PHIP,RLF,ZNF292,ITGB1BP1,THRAP3,CCNC,NAA15,ELF2,ATAD2B,HDAC2,CCNA2,EXOSC9,MLLT10,DNAJC2,NUP85,ELF1,RSF1,CCNT2,PAXIP1,WDR43,SUPT16H,CHD8,KAT6A,E2F7,ALYREF,CKAP2,CEP290,NSD3,CEBPZ,TAF7,BRCA2,ELK3,TCERG1,PRKDC,ERCC6,GPBP1,SP1,TCEA1,ZC3H8,ZNF143,CENPJ,NCK1,SMARCA5,ZNF507,TAF9,TFCP2,UTP15,HLTF,GABPA,MCRS1,USP16,SENP1,RIPK1,WDR75,MYBL2,E2F8,INO80,CHUK,HIVEP1,NOLC1,BMPR1B,UBE3A,PRDM10,NUCKS1,GCN1,CHEK1,H2AFZ,RRP1B,NFKB1,TET2,TRIP11,HEATR1,NCL,NOL11,GTF2I,BPTF,BLM,EDRF1,ING5,BCLAF3,FOSL1,SMARCC1,SP3,DHX9,CCAR1,UBTF,NPAT,BRCA1,CNOT9,DCAF6,DHX33,TBL1XR1,LEO1,FOXM1,LYAR,DDX11,HNRNPU,SMARCE1,RFC1,TOP2A,NSD1,MED6,ICE2,PMF1,FUS,ZNF609,OGT,ATRX,ZNF711,ILF2,TRIM37,ILF3,MED13,AATF,TAF15,THOC1,NIPBL,MYSM1,UHRF1,RBMX,TP73,CTCF,HNRNPD,DRAP1,ZNF770,ZNF782,ZFP62,SFR1	darkgrey	EC_M8
GO:1903508	1.22E-02	0.990499617	144	1244	positive regulation of nucleic acid-templated transcription	BP	2.50E-01	ONECUT2,BCLAF1,DDX39B,SCAF8,HSF2,ATAD2,XRCC6,PHF5A,IFT74,PWP1,ZNF300,TOPORS,RFX3,TRIM28,ZNF451,FOXJ3,PHIP,RLF,ZNF292,ITGB1BP1,THRAP3,CCNC,NAA15,ELF2,ATAD2B,HDAC2,CCNA2,EXOSC9,MLLT10,DNAJC2,NUP85,ELF1,RSF1,CCNT2,PAXIP1,WDR43,SUPT16H,CHD8,KAT6A,E2F7,ALYREF,CKAP2,CEP290,NSD3,CEBPZ,TAF7,BRCA2,ELK3,TCERG1,PRKDC,ERCC6,GPBP1,SP1,TCEA1,ZC3H8,ZNF143,CENPJ,NCK1,SMARCA5,ZNF507,TAF9,TFCP2,UTP15,HLTF,GABPA,MCRS1,USP16,SENP1,RIPK1,WDR75,MYBL2,E2F8,INO80,CHUK,HIVEP1,NOLC1,BMPR1B,UBE3A,PRDM10,NUCKS1,GCN1,CHEK1,H2AFZ,RRP1B,NFKB1,TET2,TRIP11,HEATR1,NCL,NOL11,GTF2I,BPTF,BLM,EDRF1,ING5,BCLAF3,FOSL1,SMARCC1,SP3,DHX9,CCAR1,UBTF,NPAT,BRCA1,CNOT9,DCAF6,DHX33,TBL1XR1,LEO1,FOXM1,LYAR,DDX11,HNRNPU,SMARCE1,RFC1,TOP2A,NSD1,MED6,ICE2,PMF1,FUS,ZNF609,OGT,ATRX,ZNF711,ILF2,TRIM37,ILF3,MED13,AATF,TAF15,THOC1,NIPBL,MYSM1,UHRF1,RBMX,TP73,CTCF,HNRNPD,DRAP1,ZNF770,ZNF782,ZFP62,SFR1	darkgrey	EC_M8

GO:0016447	1.23E-02	0.995852988	10	48	somatic recombination of immunoglobulin gene segments	BP	2.50E-01	MSH2,PAXIP1,RIF1,PRKDC,MSH3,NBN,NSD2,EXO1,THOC1,RNF8	darkgrey	EC_M8
GO:0016571	1.23E-02	0.994010904	20	118	histone methylation	BP	2.50E-01	PWP1,RLF,EZH2,SETDB2,PAXIP1,RIF1,NSD3,SMYD5,TET2,PRMT5,SETD2,BRCA1,NSD2,SUV39H1,NSD1,OGT,ATRX,SUZ12,EHMT1,CTCF	darkgrey	EC_M8
GO:0061180	1.17E-02	0.995186713	15	58	mammary gland epithelium development	BP	2.50E-01	RTN4,FGF2,GLI2,LBH,AKT2,ZNF703,ROBO1,KDM5B,CCND1,LRP5,FGFR2,MED1,AKT1,WNT2,FGF10	darkgreen	EC_M4
GO:0048814	1.17E-02	0.995191994	15	57	regulation of dendrite morphogenesis	BP	2.50E-01	RAB21,ACTR2,ILK,TLX2,DHX36,RAPGEF2,OPA1,DNM1L,CAPRIN2,GSK3B,TNIK,OBSL1,PAFAH1B1,YWHAH,SDC2	darkgreen	EC_M4
GO:0006662	1.17E-02	0.997235054	7	20	glycerol ether metabolic process	BP	2.50E-01	TXN2,CHPT1,FAR1,LPCAT2,PAFAH1B1,TXN,TMEM189	darkgreen	EC_M4
GO:0003156	1.17E-02	0.996868634	8	24	regulation of animal organ formation	BP	2.50E-01	FGF2,CTNNB1,SULF1,ROBO1,BMP7,GDNF,WNT2,FGF10	darkgreen	EC_M4
GO:0060538	1.18E-02	0.993623669	28	132	skeletal muscle organ development	BP	2.50E-01	LARGE1,HIVEP3,MYH14,AKIRIN1,BVES,RB1,TGFB1,CTNNB1,DCN,ANKRD1,FLNB,HOMER1,DNER,DAG1,DISP1,ATF3,CASQ1,SIX4,MYLPF,FGFRL1,MYOCD,FBXO22,MYOM1,PRKAA1,DMD,IGSF8,SRPK3,CFL2	darkgreen	EC_M4
GO:0050803	1.18E-02	0.993141746	35	170	regulation of synapse structure or activity	BP	2.50E-01	ADGRB1,MDGA1,VCP,TPBG,LRRC4B,ACTR2,VPS35,SLC7A11,FARP1,PRNP,APP,DHX36,HOMER1,OPA1,AMIGO2,PDLIM5,DNM1L,CAPRIN2,DAG1,NTRK3,HSPA8,KIF1A,TANC2,SIX4,NLGN2,ADGRL1,SRCIN1,STX1B,PAFAH1B1,NTNG2,LRRC24,UBE2V2,ARF4,LHFPL4,LRRTM2	darkgreen	EC_M4
GO:0010712	1.18E-02	0.996210978	10	34	regulation of collagen metabolic process	BP	2.50E-01	PPARD,ITGB1,TGFB1,RUNX1,FAP,SUCO,TGFB3,PDGFRB,ITGA2,MFAP4	darkgreen	EC_M4
GO:0010591	1.18E-02	0.996208935	10	34	regulation of lamellipodium assembly	BP	2.50E-01	AKIRIN1,ACTR2,ACTR3,TWF1,PIK3CA,AUTS2,NCKAP1,ARPC2,CAPZB,PLXNB3	darkgreen	EC_M4
GO:1903573	1.18E-02	0.995936423	11	39	negative regulation of response to endoplasmic reticulum stress	BP	2.50E-01	NCK2,CREB3,DDRGK1,OPA1,SELENOS,HYOU1,SYVN1,USP14,PARK7,HSPA5,DNAJB9	darkgreen	EC_M4
GO:0030335	1.18E-02	0.991669919	78	443	positive regulation of cell migration	BP	2.50E-01	LRP1,ANGPT1,NUS1,SUN2,PLAA,VEGFA,DOCK8,CREB3,PTP4A1,TGFBR1,RTN4,EMC10,AKIRIN1,ROCK2,RRAS,ITGB1,FGF2,PAK1,TGFB1,AKT2,ACTG1,ACTN4,IRS2,ZNF703,SEMA3C,DDRGK1,ILK,CXCL12,SEMA4F,P2RX4,APP,RAPGEF2,CX3CL1,ACVR1,SEMA4G,TJP1,DNM1L,HYAL1,TGFB2,MMP14,LEF1,PRKCA,CMKLR1,NTRK3,BMP7,IQGAP1,S100A11,LRRC15,FAM89B,HSPB1,SERPINE1,ITGA2B,FN1,PDCD10,ITGAV,AKT3,ARHGEF2,COL1A1,RAB11A,CD74,PDGFRB,AKT1,ITGA2,DAPK3,MYO1C,TNFRSF18,FLNA,CDH13,F3,HSPA5,CD151,THY1,HAS2,TMSB4X,GLIPR2,CCR6,PIK3C2A,FGF10	darkgreen	EC_M4
GO:0046209	1.07E-02	0.995877153	13	60	nitric oxide metabolic process	BP	2.50E-01	TLR4,NOS3,CD36,TLR2,EDN1,CD34,TMEM106A,RAC1,TLR6,ARG2,MTOR,DNM2,NOS2	turquoise	EC_M6
GO:0048662	1.07E-02	0.996478737	10	44	negative regulation of smooth muscle cell proliferation	BP	2.50E-01	TNFAIP3,IL15,PPARG,MEF2C,NDRG4,IGFBP3,APOD,IGFBP5,NPR1,BMP2	turquoise	EC_M6
GO:2000311	1.07E-02	0.997808374	6	18	regulation of AMPA receptor activity	BP	2.50E-01	MEF2C,ADRB2,RELN,SHANK3,CACNG5,GSG1L	turquoise	EC_M6
GO:0048762	1.07E-02	0.993782574	34	198	mesenchymal cell differentiation	BP	2.50E-01	SEMA6A,NOTCH4,SEMA4D,IL6,SDCBP,NRP1,GATA3,EDNRB,JAG1,SFRP1,RET,MEF2C,WWTR1,EDN1,STAT1,SEMA3F,LAMA5,ELL3,SEMA6D,TBX1,HIF1A,ALDH1A2,MTOR,EFNB1,SEMA4A,EFNA1,SMAD2,SEMA6B,RFLNB,NOTCH1,ENG,DAB2IP,TRIM62,BMP2	turquoise	EC_M6
GO:0099504	1.08E-02	0.994296208	26	138	synaptic vesicle cycle	BP	2.50E-01	STX11,SYNJ2,RIMS2,APBA1,SNAP91,RAB3B,ITSN2,CLCN3,SNCA,DTNBP1,SNAP23,ADCY1,ADORA2A,DNM3,SNAP29,PLD2,SNCG,SYT7,FBXL20,OPHN1,PSEN1,DNM2,GIT1,AP3D1,STXBP1,STX3	turquoise	EC_M6
GO:0060402	1.19E-02	0.993888692	24	107	calcium ion transport into cytosol	BP	2.51E-01	FKBP1B,FGF2,PRNP,RYR3,P2RX4,CX3CL1,CACNB3,ATP2B4,PKD2,HTR2B,CASQ1,PTPN6,HTT,ERO1A,HAP1,CACNA1C,CALM1,TRPV2,JPH3,DMD,FKBP1A,TMEM38B,THY1,TMEM38A	darkgreen	EC_M4
GO:0003206	1.19E-02	0.993990624	23	101	cardiac chamber morphogenesis	BP	2.51E-01	LY6E,SRF,TGFBR1,SMARCD3,TGFB1,SEMA3C,ROBO1,HEY1,CPE,ACVR1,NOTCH2,PKP2,TGFB2,BMP7,FGFR2,FZD2,BMPR1A,MED1,FGFRL1,TPM1,COL11A1,FKBP1A,WNT2	darkgreen	EC_M4
GO:0072599	1.20E-02	0.996150226	10	34	establishment of protein localization to endoplasmic reticulum	BP	2.51E-01	SRP72,SEC61A1,SRP68,RAB3GAP1,TRAM1,SSR3,SRPRA,SRP54,HSPA5,SPCS3	darkgreen	EC_M4

GO:0045948	1.24E-02	0.997279582	6	22	positive regulation of translational initiation	BP	2.51E-01	METTL3,DHX29,NCK1,KHDRBS1,EIF2B5,LARP1	darkgrey	EC_M8
GO:0007252	1.09E-02	0.997803368	6	17	I-kappaB phosphorylation	BP	2.51E-01	TLR4,IKBKB,TLR2,TRAF2,PLCG2,DAB2IP	turquoise	EC_M6
GO:0008283	1.20E-02	0.990265164	226	1442	cell population proliferation	BP	2.51E-01	IL23A,LRP1,B4GALT7,ENPP3,PLXNB2,ANGPT1,FAM19A5,ADGRB1,PPARD,CTNNA1,TEAD3,C6orf89,LEP,CDKN2B,SRF,NUAK1,VEGFA,DOCK8,RUNX2,C2orf40,NCK2,PDCL3,REST,P3H1,TGFBR1,COPS9,RTN4,TPBG,EMC10,AKIRIN1,TES,GLI3,INHBA,SLC7A11,ITGB1,FKBP1B,FGF2,SRPK2,RB1,CGREF1,TENM4,GLI2,SMARCD3,PAK1,TGFB1,SCN5A,WDR48,ACER3,CTNNB1,LBH,AKT2,RARB,KAT2B,FAM98A,TACC1,MAFG,SPINT2,LIG4,IRS2,PRNP,ZNF703,CCKBR,DDRGK1,ILK,RYK,CIAO1,TMEM127,CNOT7,LMO1,CXCL12,LGI4,IL20RB,EIF2S2,CD180,SULF1,ROBO1,PINX1,CELF1,APP,FAM98B,RAPGEF2,CX3CL1,ABCC8,PTPN11,GNL3,LOXL2,PTPN22,TSG101,MMP2,ADA,BAP1,PKD2,RUNX1,CBLB,BRD7,IFT57,PSMG1,NDN,SLC39A10,CHRNA7,FAP,TJP1,KDM5B,NOTCH2,CDC73,MARCKSL1,HYAL1,CCND1,SBDS,TGFB2,LRP5,HTR2B,PRKAR1A,MMP14,LEF1,PRKCA,KRAS,NTRK3,GOLGB1,KPNA1,BMP7,IDH2,ATF3,FGFR2,VASH2,OSM,HTRA1,NCSTN,KIF1A,S100A11,TFRC,FZD9,EGLN3,SIDT2,OVOL1,PRDX4,COMT,FN1,PTPN6,IGFBP2,PDCD10,ITGAV,COMP,PRKCI,FGFR3,PRRX1,TIMP1,PTPRN,KCNA1,SIX4,MNAT1,AKT3,MAGED1,BMPR1A,MED1,PPARGC1A,MSN,FGFRL1,NES,ARHGEF2,TGFB1I1,NGFR,TPM1,DOCK2,TGFB3,SHC1,PBXIP1,MAP2K1,CNN1,DICER1,CLMN,MYOCD,EML1,CD74,PDGFRB,AKT1,UBE2A,ITGA2,PRKAA1,OSMR,PTPN2,NPR3,CD40LG,MYDGF,LDOC1,ROGDI,GNB1,PLXNB3,PAFAH1B1,TAX1BP3,BRICD5,IRAK1,FLNA,SSTR5,RERE,RHBDF1,GNAI3,CDH13,CFDP1,F3,MIR24-1,CD151,SERPINF1,SERPINB5,AGO3,PEMT,CD164,LBX1,GDNF,HAS2,CDKN1A,ARTN,NACC2,DUSP22,TSLP,YPEL5,CAPNS1,P3H3,ZPR1,PRKG1,CHST11,DERL2,W	darkgreen	EC_M4
GO:0035051	1.20E-02	0.993657764	26	119	cardiocyte differentiation	BP	2.51E-01	PPARA,CBY1,SRF,VEGFA,REST,BVES,MYLK3,ITGB1,TENM4,PAK1,TGFB1,RARB,SEMA3C,DHX36,ACVR1,PDLIM5,TGFB2,BMP7,OBSL1,BMPR1A,TCAP,MYOCD,PIN1,PDGFRB,MIR24-1,CACYBP	darkgreen	EC_M4
GO:0048592	1.20E-02	0.993651609	26	118	eye morphogenesis	BP	2.51E-01	AHI1,FBN2,RORB,VEGFA,MFSD2A,GLI3,FGF2,CTNNB1,RARB,FAT1,NKD1,NOTCH2,LRP5,SHROOM2,BMP7,FBN1,PRKCI,COL5A2,OBSL1,ROM1,TTC8,COL5A1,BCAR3,THY1,WNT2,COL8A2	darkgreen	EC_M4
GO:0061458	1.20E-02	0.991922101	61	333	reproductive system development	BP	2.51E-01	RSPO3,PPARD,CTNNA1,LEP,VEGFA,TGFBR1,GLI3,INHBA,GLI2,AXL,WDR48,CTNNB1,SPINT2,DCN,TMED2,EIF2S2,SULF1,RDH10,HEY1,PTPN11,PTX3,ADA,SPP1,PKD2,CSDE1,KDM5B,NOTCH2,CCND1,TGFB2,MMP14,LEF1,BMP7,FGFR2,VASH2,ASB1,HTRA1,ARID5B,PRDX4,PCYT1B,SGPL1,PTPN6,PTPRN,CYP19A1,SIX4,MED1,UBE2Q1,MAP2K1,MYOCD,PDGFRB,AKT1,PLAC1,PKD1,FLNA,BSG,HSPA5,LHX8,FSTL3,SERPINF1,SERPINB5,WNT2,FGF10	darkgreen	EC_M4
GO:0016567	1.24E-02	0.991130563	80	656	protein ubiquitination	BP	2.52E-01	MEX3C,SHPRH,FBXO43,FBXO5,UBR5,UHRF2,UBR2,TOPORS,TRIM28,FBXO11,ASB3,COMMD1,UFL1,CUL1,SAE1,PAXIP1,CDC20,GTPBP4,RAD18,TPP2,CUL4A,CDC16,LNX2,ANAPC1,TNKS2,RNF141,ZFP91,HLTF,ANAPC7,LTN1,TRIM33,UBE2C,HERC5,UBE3A,CUL3,UBE2T,ASB7,TRAIP,UBR1,FANCI,DTL,UBR3,G2E3,IVNS1ABP,CDC27,FANCM,LRR1,COP1,BRCA1,CUL5,CDCA3,DCUN1D5,DCAF6,LEO1,UBE2K,NEDD4,MED6,TAF1,OGT,RLIM,RBBP6,TRIM37,PLK1,APPBP2,PSMD10,MIB1,CUL4B,XIAP,UHRF1,BLMH,CCNF,GLMN,MAD2L1,RNF139,RNF8,TRIM59,GID4,UBXN2A,RNF219,RCHY1	darkgrey	EC_M8
GO:0099003	1.10E-02	0.994047197	28	151	vesicle-mediated transport in synapse	BP	2.52E-01	STX11,SYNJ2,RIMS2,APBA1,SNAP91,RAB3B,ITSN2,CLCN3,SNCA,DTNBP1,SNAP23,ADCY1,ADORA2A,DNM3,SNAP29,PLD2,SNCG,SYT7,FBXL20,OPHN1,PSEN1,DNM2,AP2B1,GIT1,AP3D1,STXBP1,STX3,GSG1L	turquoise	EC_M6
GO:0030949	1.10E-02	0.998806895	4	8	positive regulation of vascular endothelial growth factor receptor signaling pathway	BP	2.53E-01	GRB10,PRKD2,HIF1A,FGF18	turquoise	EC_M6
GO:0045932	1.10E-02	0.997739329	6	18	negative regulation of muscle contraction	BP	2.53E-01	DOCK4,PIK3CG,ARHGAP42,GUCY1A1,CALCRL,ADRB2	turquoise	EC_M6
GO:0051153	1.10E-02	0.995379076	15	72	regulation of striated muscle cell differentiation	BP	2.53E-01	EFNB2,ATP11A,LMOD3,IGF1,MEF2C,EDN1,SIK1,PROX1,TBX1,MTOR,YY1,NOTCH1,PIEZO1,RGS4,BMP2	turquoise	EC_M6
GO:1903421	1.21E-02	0.996739543	8	24	regulation of synaptic vesicle recycling	BP	2.53E-01	PLAA,PPP3CC,DNM1L,AP2M1,STX1B,SYT11,CALM1,ROCK1	darkgreen	EC_M4

GO:0045776	1.11E-02	0.997290205	7	24	negative regulation of blood pressure	BP	2.54E-01	ARHGAP42,PRCP,NOS3,KCNK6,VEGFC,ADRB2,NOS2	turquoise	EC_M6
GO:0090218	1.11E-02	0.996700909	9	31	positive regulation of lipid kinase activity	BP	2.54E-01	PTK2,PDGFB,TEK,KIT,DGKZ,FGR,PRKD1,TNFAIP8L3,CD19	turquoise	EC_M6
GO:0044237	5.74E-04	0.99953254	938	7918	cellular metabolic process	BP	2.55E-01	NFATC1,ZNF236,TSHZ1,ZADH2,ORMDL2,PYM1,DGKA,SUOX,SMARCC2,STAT6,ATG12,PIP4K2C,MAP3K5,PEX7,OS9,CDK4,CITED2,CTDSP2,ATP23,MAML1,SQSTM1,SASH1,IRAK3,HELB,GFPT2,OSR2,BAG6,CHKB,ALDH7A1,ABHD16A,PPP6R2,MSH5,SNX9,HSPA1B,EHMT2,ZBTB12,HDAC10,SELENOO,ALG12,CERK,ATF6B,AGPAT1,RNF5,EIF3H,RPS6KA2,DEPTOR,DSE,RXRB,TSPO,UQCRQ,HSD17B8,RING1,RPS18,B3GALT4,CYB5R3,ZHX2,ZBTB22,JADE2,PMM1,MYC,TOB2,SMAD5,EP300,IBA57,CDC14C,ERCC6L2,KDM3B,ZC3H3,ZNF76,FANCE,EEF1D,TAB1,RPL3,JOSD1,DDX17,NRBP2,STK38,EIF3L,OPLAH,MPST,NFIB,GPAA1,SHARPIN,EIF3D,FOXRED2,HSF1,FBXL6,SLC52A2,CPSF1,VPS28,CCND3,PPP1R16A,TRERF1,CDKN2A,RPL8,HMOX1,WASL,CUL7,CUL9,DNPH1,KLF9,POLR1C,PIGO,PDGFRA,PLPP6,RCL1,JAK2,HINT2,RECK,RNF38,ZBTB45,RPS5,ZNF135,ANKRD39,TRMO,ZNF667,ALG2,ZNF865,RPL28,TTC7A,RPS9,EEF1A1,CD109,GPNMB,MALSU1,STAP1,SLC44A1,ETFB,EFEMP1,PTGR1,UGP2,ZNF692,CYREN,RPS7,ANTXR1,ANXA4,AKNA,LSM10,STK40,MED25,IL4I1,TBC1D17,COQ3,AKT1S1,ING3,ARHGEF5,IRF3,LPIN1,RPS11,GSN,PARD3,FOXO3,SESN1,KLF15,NDUFB2,ADCK2,SPHK2,RPL18,SPRY1,NOP53,ABTB1,MAML2,BICRA,ZC3HAV1,KMT2E,BBS7,MEIS3,HMCES,FKRP,UQCRH,LRRC41,TRIM13,ACSS1,IRF2BP1,SIX5,QPCTL,FOSB,ZNF746,NDUFB6,NR2C2,ZNF862,TSC22D1,SERP2,FDXR,UROD,KHK,SMG9,FASTK,SEPHS1,RPS8,XRCC1,ZNF575,MIF4GD,AGAP3,NAA16,MLH1,RNF220,LIPE,NDUFC2,ECHDC3,GTF3C2,UNK,ZNF574,GALNT11,WBP2,TRIM47,KMT2C,ACAA1,MYD88,CELF2,DMAC2,TAMM41,BCKDHA,EGLN2,CYGB,HYI,BABAM2,ANKRD16,	cyan	EC_M2
GO:0097190	1.23E-02	0.991285863	81	471	apoptotic signaling pathway	BP	2.55E-01	DYRK2,RRM2B,PPARD,CTNNA1,UBQLN1,NCK2,CREB3,TGFBR1,GGCT,INHBA,CUL2,RB1,TPT1,TGFB1,CTNNB1,ARL6IP5,CXCL12,P2RX4,FAIM2,CX3CL1,OPA1,ACVR1,CHAC1,SERINC3,BRSK2,CTTN,DNM1L,SELENOS,EYA2,TGFB2,GSK3B,PRKCA,FAM162A,HYOU1,ATF3,PEA15,FZD9,PDIA3,HSPB1,SERPINE1,SYVN1,SGPL1,ZNF385B,DNAJC10,PDCD10,ITGAV,HTT,ERO1A,SKIL,FGFR3,VDAC2,SGMS1,GHITM,MAGED1,ARHGEF2,NGFR,TAF9B,UACA,GSKIP,ANXA6,CD74,AKT1,TM2D1,PTPN2,DAPK3,BCAP31,PARK7,ENO1,GNAI3,BCL10,BNIP3,ITM2C,GDNF,TIMP3,BLOC1S2,CDKN1A,NACC2,PTTG1IP,PLAGL2,TMEM109,FGF10	darkgreen	EC_M4
GO:0023061	1.12E-02	0.992768404	50	325	signal release	BP	2.56E-01	TNFRSF11A,STX11,SNCAIP,RIMS2,PICK1,APBA1,SYK,ICA1,IL6,PPT1,TRPM4,GATA3,SFRP1,SIRT3,CHD7,FAM3D,NR4A1,MCTP1,MEF2C,EDN1,SNCA,DTNBP1,ABCG1,HDAC1,PPP3CA,ANO1,MCTP2,SNAP23,ADCY1,CREB1,ADORA2A,MCU,CDK16,SNAP29,FGF23,RAC1,HIF1A,SNCG,SYT7,FBXL20,PSEN1,CHRNB2,GPR68,SMAD2,NOS2,GIT1,STXBP1,EXOC3L1,STX3,BMP6	turquoise	EC_M6
GO:0002730	1.13E-02	0.998214349	5	12	regulation of dendritic cell cytokine production	BP	2.56E-01	DDX58,KIT,TLR4,PLCG2,BST2	turquoise	EC_M6
GO:0030100	1.13E-02	0.993704947	30	174	regulation of endocytosis	BP	2.56E-01	PACSIN2,PICK1,CD2AP,SYK,SNAP91,PPT1,SDCBP,MTMR2,GATA2,CD36,HIP1R,MCTP1,BMP2K,ANO6,EHD4,SNCA,PPP3CA,RIN3,PRKD1,B2M,SELE,RAC1,OPHN1,DNM2,AP2B1,UNC119,PLCG2,LRSAM1,CBLL1,GSG1L	turquoise	EC_M6
GO:0050853	1.13E-02	0.996297479	10	41	B cell receptor signaling pathway	BP	2.56E-01	TEC,SYK,MEF2C,LCK,NFATC2,BMX,PLEKHA1,PRKCH,CD19,PLCG2	turquoise	EC_M6
GO:0150079	1.13E-02	0.998674482	4	9	negative regulation of neuroinflammatory response	BP	2.56E-01	CST7,IGF1,CD200,TNFRSF1B	turquoise	EC_M6
GO:0071900	1.27E-02	0.992113118	41	300	regulation of protein serine/threonine kinase activity	BP	2.56E-01	LATS1,MAP3K4,CDC25C,DBF4,CKS2,ETAA1,EZH2,CCNA2,CHORDC1,CCNT2,GTPBP4,IPO5,KITLG,CCNB1,IPO7,CDK5RAP1,SH2B3,CCNL1,CCNE2,STK4,HERC5,TAOK3,BMPR1B,AJUBA,ALS2,BLM,CDC25A,DBF4B,CDKN3,INCENP,CCNB3,CDC6,CCNB2,PLK1,CSK,PSMD10,RICTOR,TAOK1,PKMYT1,CCNF,ERP29	darkgrey	EC_M8
GO:0031998	1.23E-02	0.997542063	6	15	regulation of fatty acid beta-oxidation	BP	2.56E-01	PPARA,MFSD2A,AKT2,IRS2,CPT1A,AKT1	darkgreen	EC_M4
GO:0043523	1.24E-02	0.992830585	34	169	regulation of neuron apoptotic process	BP	2.57E-01	LRP1,AGAP2,ANGPT1,SRPK2,AXL,CTNNB1,LIG4,PRNP,ILK,FAIM2,RASA1,CX3CL1,TGFB2,PIK3CA,KRAS,HYOU1,BACE1,FZD9,EGLN3,PRKCI,SIX4,PPARGC1A,CPEB4,NES,TGFB3,PIN1,PARK7,AARS,NAE1,VSTM2L,UBE2V2,GDNF,SIGMAR1,ZPR1	darkgreen	EC_M4

GO:0002690	1.14E-02	0.995368757	14	71	positive regulation of leukocyte chemotaxis	BP	2.57E-01	PTK2,IL6,CXCL8,VEGFC,CXCL10,THBS4,ANO6,EDN1,MCU,RAC1,PGF,DAPK2,S100A14,LGALS9	turquoise	EC_M6
GO:0001701	1.25E-02	0.99170892	58	314	in utero embryonic development	BP	2.58E-01	ANGPT1,RSPO3,LY6E,MYH9,SRF,VEGFA,TGFBR1,RTN4,ADAMTS3,KIDINS220,GLI3,ITGB1,GLI2,CTNNB1,MAFG,SPINT2,LIG4,TMED2,EIF2S2,RDH10,HEY1,TCTN1,ACVR1,ADA,PKD2,TENT5C,PSPH,NOTCH2,SBDS,LEF1,BMP7,WDTC1,FGFR2,VASH2,TANC2,PLOD3,SKIL,FKBP10,BMPR1A,MED1,MYO1E,TGFB3,MAP2K1,TMEM100,PPP4R4,RBBP8,AKT1,UBE2A,C6,SUPT6H,NXN,PKD1,PEMT,HOPX,ZPR1,SLC39A3,CHST11,WNT2	darkgreen	EC_M4
GO:0051222	1.25E-02	0.992103423	46	241	positive regulation of protein transport	BP	2.59E-01	LRP1,PPARD,LEP,UNC13B,EXOC1,TMEM30A,GLI3,KIF5B,PAK1,TGFB1,AKT2,ATP2C1,IRS2,PRNP,HDAC3,TM9SF4,ANKRD1,OSBP,CACNB3,PFKM,ATG13,TSG101,BAP1,CHP1,DYNLL1,DNM1L,TGFB2,GSK3B,PDCD10,B3GAT3,NLGN2,TMED10,TGFB3,MYH10,SYTL4,ACSL4,MIEF2,MYOM1,PRKAA1,MYO1C,BCAP31,FLNA,WLS,LEPROT,ZPR1,TOMM7	darkgreen	EC_M4
GO:0007156	1.26E-02	0.993881046	21	88	homophilic cell adhesion via plasma membrane adhesion molecules	BP	2.59E-01	PLXNB2,PCDH18,ITGB1,PCDH17,FAT1,PALLD,ROBO1,CDH17,AMIGO2,NECTIN4,OBSL1,PCDH7,NPTN,FAT2,PLXNB3,PKD1,BSG,CDH13,NEXN,PCDHB2,VSTM2L	darkgreen	EC_M4
GO:0051656	1.26E-02	0.991505756	61	334	establishment of organelle localization	BP	2.60E-01	AP3S1,SUN2,MYH9,DYNC1I1,NLRP5,CENPC,ACTR2,STARD3NL,MGARP,ITGB1,KIF5B,COPG1,PICALM,ACTR3,CTNNB1,TRAK1,ACTN4,TMED2,VAMP8,RIOK2,MAP1B,PINX1,SDAD1,EXOC6B,OPA1,AP3B1,CHP1,FEZ1,WIPI1,SHROOM2,KIF1A,TANC2,LSG1,MAPT,PDCD10,HTT,ACTR10,FMN2,MYO5A,HAP1,SLC2A4,STARD3,MYO1E,SYT11,TMED10,RAB11A,ARMCX3,CLMN,MYO1D,MYO5B,MYO1C,PAFAH1B1,ATP6AP1,IKBKG,VAMP7,HSBP1,RAB6A,BLOC1S2,RMDN1,GEM,FGF10	darkgreen	EC_M4
GO:0070972	1.27E-02	0.994691624	15	60	protein localization to endoplasmic reticulum	BP	2.61E-01	SRP72,RTN4,SEC61A1,SRP68,RAB3GAP1,DDRGK1,TRAM1,SSR3,SRPRA,SRP54,KDELR2,BCAP31,HSPA5,SPCS3,GABARAPL2	darkgreen	EC_M4
GO:0098868	1.27E-02	0.9965611	8	24	bone growth	BP	2.61E-01	LEP,RARB,FGFR2,COMP,FGFR3,EVC,THBS3,ANXA6	darkgreen	EC_M4
GO:0008306	1.27E-02	0.994882191	14	53	associative learning	BP	2.62E-01	SRF,TPBG,SHANK1,ACTR2,SLC7A11,ITGB1,APP,ABCC8,KRAS,TNR,CLN8,NPTN,LRRN4,MAP1A	darkgreen	EC_M4
GO:0045216	1.16E-02	0.993746313	27	146	cell-cell junction organization	BP	2.62E-01	AFDN,MTSS1,TJP2,GJA4,IKBKB,MARVELD2,OCLN,HIPK1,JAM3,ESAM,CLDN34,CDH24,PECAM1,F11R,ACE,PAK2,DLG1,RAMP2,PRKCH,JUP,FSCN1,EPHA2,DSG2,PATJ,CDH5,PERP,BMP6	turquoise	EC_M6
GO:0071482	1.30E-02	0.994182161	16	94	cellular response to light stimulus	BP	2.62E-01	POLH,METTL3,ATR,PBK,PPID,TENT2,INO80,CHEK1,RGS9,CDC25A,AIPL1,NEDD4,AURKB,TAF1,CUL4B,FMR1	darkgrey	EC_M8
GO:0050807	1.28E-02	0.99257753	34	165	regulation of synapse organization	BP	2.62E-01	ADGRB1,MDGA1,VCP,TPBG,LRRC4B,ACTR2,VPS35,SLC7A11,FARP1,PRNP,APP,DHX36,HOMER1,OPA1,AMIGO2,PDLIM5,DNM1L,CAPRIN2,DAG1,NTRK3,HSPA8,KIF1A,TANC2,SIX4,NLGN2,ADGRL1,SRCIN1,PAFAH1B1,NTNG2,LRRC24,UBE2V2,ARF4,LHFPL4,LRRTM2	darkgreen	EC_M4
GO:0071634	1.17E-02	0.996477072	9	33	regulation of transforming growth factor beta production	BP	2.62E-01	LAPTM4B,CD2AP,FERMT1,CD200,CD34,CREB1,HIF1A,LGALS9,CD46	turquoise	EC_M6
GO:0014854	1.28E-02	0.998539778	4	8	response to inactivity	BP	2.63E-01	SCN5A,DAG1,CASQ1,SGCA	darkgreen	EC_M4
GO:0061614	1.29E-02	0.995036583	13	48	pri-miRNA transcription by RNA polymerase II	BP	2.63E-01	PPARA,MRTFA,PPARD,SRF,REST,FGF2,TGFB1,TEAD1,GNL3,TGFB2,BMPR1A,NGFR,SMARCA4	darkgreen	EC_M4
GO:0009130	1.31E-02	0.998202843	4	12	pyrimidine nucleoside monophosphate biosynthetic process	BP	2.63E-01	CAD,UMPS,TYMS,DHODH	darkgrey	EC_M8
GO:0006984	1.29E-02	0.995245474	12	44	ER-nucleus signaling pathway	BP	2.63E-01	NCK2,TMED2,ATP2A2,AMFR,SELENOS,GSK3B,ATF6,TMCO1,EIF2S1,PTPN2,MBTPS1,HSPA5	darkgreen	EC_M4
GO:0002755	1.18E-02	0.997551704	6	18	MyD88-dependent toll-like receptor signaling pathway	BP	2.64E-01	TLR4,IRAK2,TLR2,TLR6,TNIP1,TLR1	turquoise	EC_M6
GO:0060907	1.18E-02	0.998601477	4	9	positive regulation of macrophage cytokine production	BP	2.64E-01	TLR4,CD36,LAPTM5,PLCG2	turquoise	EC_M6

GO:0071636	1.18E-02	0.997505789	6	19	positive regulation of transforming growth factor beta production	BP	2.64E-01	FERMT1,CD200,CD34,CREB1,LGALS9,CD46	turquoise	EC_M6
GO:0002263	1.18E-02	0.993296741	31	185	cell activation involved in immune response	BP	2.64E-01	MFNG,KIT,SYK,IL6,TLR4,PIK3CG,GATA2,GAB2,UNC13D,GATA3,GAPT,DYSF,SNAP23,HLX,CD86,FGR,FES,CRACR2A,RAB27A,LFNG,MTOR,PSEN1,SEMA4A,BATF,CD19,SCNN1B,LGALS9,IRF8,PLCG2,STXBP1,CD46	turquoise	EC_M6
GO:0046847	1.18E-02	0.995413938	13	56	filopodium assembly	BP	2.64E-01	NRP1,FGD5,ITGB4,SPEF1,FGD6,GPM6A,PPP1R16B,SRGAP2C,DNM3,FSCN1,FGD1,FNBP1L,RALA	turquoise	EC_M6
GO:0071428	1.32E-02	0.998237904	4	11	rRNA-containing ribonucleoprotein complex export from nucleus	BP	2.65E-01	XPO1,RRS1,ABCE1,NMD3	darkgrey	EC_M8
GO:0007031	6.16E-04	0.999864247	11	32	peroxisome organization	BP	2.65E-01	PEX7,HACL1,ACOT8,LONP2,FIS1,PLA2G16,ABCD4,RAB8B,PEX10,PEX11A,SCP2	cyan	EC_M2
GO:0045333	6.35E-04	0.999681964	39	191	cellular respiration	BP	2.65E-01	UQCRQ,NDUFB2,NOP53,UQCRH,NDUFB6,NDUFC2,NDUFA2,COX7A1,UQCRFS1,COQ9,DGUOK,NDUFV1,OXA1L,MTFR1L,NDUFS2,DNAJC30,IDH1,AIFM2,CHCHD10,UQCC3,NDUFB7,GBA,NUPR1,NDUFA7,UQCR11,NDUFS7,ATP5F1D,TAZ,MT-ND1,MT-ND2,MT-CO1,MT-CO2,MT-ATP6,MT-CO3,MT-ND3,MT-ND4L,MT-ND4,MT-ND5,MT-CYB	cyan	EC_M2
GO:0019695	6.55E-04	0.999984303	4	5	choline metabolic process	BP	2.65E-01	ALDH7A1,SLC44A1,CHDH,CHKA	cyan	EC_M2
GO:0034599	6.66E-04	0.999651541	43	234	cellular response to oxidative stress	BP	2.65E-01	STAT6,MAP3K5,HSPA1B,ERCC6L2,HSF1,HMOX1,PDGFRA,JAK2,FOXO3,AGAP3,RPS3,SIRT2,CYP1B1,HGF,CAMKK2,HTRA2,TP53INP1,GPX1,MCL1,ARNT,RIPK3,CCS,MGMT,NFE2L2,AIFM2,PRODH,TMEM161A,ANKZF1,HDAC6,ATP13A2,FOS,KEAP1,SIN3A,PPARGC1B,SLC25A24,NOL3,RACK1,SRXN1,ZNF580,ADPRHL2,NUDT2,PDGFD,MPV17	cyan	EC_M2
GO:0006305	1.33E-02	0.995183797	11	53	DNA alkylation	BP	2.65E-01	TRIM28,ZMPSTE24,RLF,EZH2,HELLS,MIS18A,PRMT5,BRCA1,ATRX,EHMT1,CTCF	darkgrey	EC_M8
GO:0006306	1.33E-02	0.995183797	11	53	DNA methylation	BP	2.65E-01	TRIM28,ZMPSTE24,RLF,EZH2,HELLS,MIS18A,PRMT5,BRCA1,ATRX,EHMT1,CTCF	darkgrey	EC_M8
GO:0006450	1.33E-02	0.997556259	5	17	regulation of translational fidelity	BP	2.65E-01	DNAJC2,LARS,CDK5RAP1,IARS2,DTD2	darkgrey	EC_M8
GO:2001057	1.19E-02	0.995338384	13	61	reactive nitrogen species metabolic process	BP	2.65E-01	TLR4,NOS3,CD36,TLR2,EDN1,CD34,TMEM106A,RAC1,TLR6,ARG2,MTOR,DNM2,NOS2	turquoise	EC_M6
GO:0006911	1.19E-02	0.996085463	10	39	phagocytosis, engulfment	BP	2.65E-01	ARHGAP25,ELMO1,GATA2,CD36,CLCN3,ANO6,DOCK1,RAC1,PLCG2,HAVCR1	turquoise	EC_M6
GO:0032205	1.33E-02	0.996184472	8	31	negative regulation of telomere maintenance	BP	2.65E-01	POT1,TNKS2,XRN1,TERF1,NBN,HNRNPU,DCP2,EXOSC10	darkgrey	EC_M8
GO:2000352	1.20E-02	0.99704264	7	24	negative regulation of endothelial cell apoptotic process	BP	2.66E-01	TNFAIP3,TEK,KDR,GATA2,GATA3,RAMP2,CDH5	turquoise	EC_M6
GO:0045747	1.20E-02	0.996369375	9	33	positive regulation of Notch signaling pathway	BP	2.66E-01	MFNG,KIT,NOS3,JAG1,BMP2K,DLL4,TSPAN14,JAG2,NOTCH1	turquoise	EC_M6
GO:0009214	1.20E-02	0.998055507	5	12	cyclic nucleotide catabolic process	BP	2.67E-01	PDE7B,PDE2A,PDE4D,PDE9A,PDE4B	turquoise	EC_M6
GO:0032677	1.21E-02	0.995456923	12	56	regulation of interleukin-8 production	BP	2.68E-01	DDX58,SYK,IL6,TLR4,PRKD2,TLR2,CD58,HYAL2,TLR6,LGALS9,NOS2,TLR1	turquoise	EC_M6
GO:0042026	1.32E-02	0.997277403	6	16	protein refolding	BP	2.68E-01	FKBP1B,HSPA8,DNAJA4,HSPA5,FKBP1A,HSPA13	darkgreen	EC_M4
GO:0042308	1.32E-02	0.997822924	5	12	negative regulation of protein import into nucleus	BP	2.68E-01	ANGPT1,RAB23,MDFIC,UFM1,CHP1	darkgreen	EC_M4
GO:1904590	1.32E-02	0.997822924	5	12	negative regulation of protein import	BP	2.68E-01	ANGPT1,RAB23,MDFIC,UFM1,CHP1	darkgreen	EC_M4

GO:0044087	1.32E-02	0.989866706	133	801	regulation of cellular component biogenesis	BP	2.68E-01	ATP8B1,LRP1,CAND1,FAM49B,ADGRB1,MDGA1,SRF,VEGFA,VCP,UNC13B,NCK2,REST,TGFBR1,TPBG,SHANK1,LRRC4B,AKIRIN1,ACTR2,ROCK2,VPS35,NAPA,RAB7A,EML2,RB1,MARK4,MAPRE3,PDCD6IP,PAK1,ACTR3,TGFB1,RAB3GAP1,DZIP1,PLEKHG2,FARP1,ACTG1,NAV3,KAT2B,FAM98A,NUDT16,CDC42EP3,DPYSL3,SACS,MAP1B,ANKRA2,PFN2,RASA1,APP,RAPGEF2,CX3CL1,PTPN11,CDH17,AMIGO2,TSG101,TWF1,PDLIM5,MAGEL2,ARL3,PHLDB2,TJP1,CTTN,HYAL1,LMOD1,SEC22B,FEZ1,GSK3B,MMP14,DAG1,PIK3CA,AUTS2,CORO1C,PRKCA,NTRK3,HSPA8,BMP7,SYNPO2,PHLDB1,IQGAP1,SCFD1,TFRC,FARP2,ARPC5,MAPT,DNAJA4,NCKAP1,ARPC2,HTT,SYNPO2L,CAPZB,PLD1,VCL,VDAC2,SIX4,STX18,HAP1,CGNL1,NLGN2,MSN,STX1B,SYT11,TPM1,TGFB3,GPR65,TTC8,ROCK1,FLII,EVI5L,PRKAA1,TUBB4A,MYO10,DAPK3,MYO1C,PLXNB3,ABCA3,NPHP4,FLNA,PARK7,DEF8,NTNG2,SPTAN1,HSPA5,LMO4,LRRC24,UBE2V2,THY1,HAS2,LHFPL4,STXBP6,TMSB4X,DUSP22,PMEPA1,ORMDL3,COTL1,LRRTM2	darkgreen	EC_M4
GO:0036465	1.32E-02	0.994263254	16	64	synaptic vesicle recycling	BP	2.68E-01	CANX,PACSIN1,SH3GL2,PLAA,PICALM,ACTG1,AP3B1,PPP3CC,DNM1L,AP2M1,SLC2A4,NLGN2,STX1B,SYT11,CALM1,ROCK1	darkgreen	EC_M4
GO:0007268	1.32E-02	0.99053324	83	471	chemical synaptic transmission	BP	2.68E-01	RNF19A,SLC12A2,ADGRB1,SRF,UNC13B,RIMS1,TPBG,SHANK1,NCDN,SLC7A11,ITGB1,LIN7B,KIF5B,NAPA,GAD2,PCDH17,RAB3GAP1,HTR5A,CTNNB1,PRNP,PCLO,SNPH,RIC3,MAP1B,P2RX4,ATP2A2,DLGAP4,APP,GLRB,RAPGEF2,CX3CL1,CACNB3,HOMER1,VPS18,GLS,CHRNA7,EIF4E,APBA2,DNM1L,HTR2B,GSK3B,KRAS,NCSTN,SYAP1,BACE1,RPS6KA3,MAPT,EIF4EBP2,TNR,MPP2,KCNA1,ATAD1,CHRNE,ERC1,HAP1,IQSEC2,NLGN2,STX1B,SYT11,KCNMB1,MYCBPAP,CACNA1G,RAB11A,CALM1,GRIA1,NPTN,CYP46A1,BEGAIN,DTNA,AKT1,ABR,PAFAH1B1,TPRG1L,PARK7,NTNG2,JPH3,PCDHB2,GDNF,INSYN1,YWHAH,KCNMB4,MAP1A,LRRTM2	darkgreen	EC_M4
GO:0098916	1.32E-02	0.99053324	83	471	anterograde trans-synaptic signaling	BP	2.68E-01	RNF19A,SLC12A2,ADGRB1,SRF,UNC13B,RIMS1,TPBG,SHANK1,NCDN,SLC7A11,ITGB1,LIN7B,KIF5B,NAPA,GAD2,PCDH17,RAB3GAP1,HTR5A,CTNNB1,PRNP,PCLO,SNPH,RIC3,MAP1B,P2RX4,ATP2A2,DLGAP4,APP,GLRB,RAPGEF2,CX3CL1,CACNB3,HOMER1,VPS18,GLS,CHRNA7,EIF4E,APBA2,DNM1L,HTR2B,GSK3B,KRAS,NCSTN,SYAP1,BACE1,RPS6KA3,MAPT,EIF4EBP2,TNR,MPP2,KCNA1,ATAD1,CHRNE,ERC1,HAP1,IQSEC2,NLGN2,STX1B,SYT11,KCNMB1,MYCBPAP,CACNA1G,RAB11A,CALM1,GRIA1,NPTN,CYP46A1,BEGAIN,DTNA,AKT1,ABR,PAFAH1B1,TPRG1L,PARK7,NTNG2,JPH3,PCDHB2,GDNF,INSYN1,YWHAH,KCNMB4,MAP1A,LRRTM2	darkgreen	EC_M4
GO:0002244	1.21E-02	0.994255266	19	96	hematopoietic progenitor cell differentiation	BP	2.68E-01	FLT4,KIT,KDR,ZBTB24,TAL1,GATA3,SFRP1,TCF15,ITCH,LMBR1L,JAM3,HYAL2,MMP21,ACE,SOS2,PSEN1,BATF,NOTCH1,SLC8A3	turquoise	EC_M6
GO:0019058	1.22E-02	0.992659023	38	239	viral life cycle	BP	2.68E-01	CXCL8,PPIE,RSAD2,CAV2,NRP1,NECTIN2,USP6NL,CXCR4,EFNB2,SCARB1,ITCH,OAS3,TPCN1,IFIT1,MX1,KIAA1551,HYAL2,ISG20,CD86,PROX1,F11R,LTF,DDX3X,EPHA2,TNIP1,CCL2,LGALS9,ISG15,NOTCH1,LRSAM1,HAVCR1,OAS1,RAB43,TRIM34,CD46,TRIM62,TBC1D20,BST2	turquoise	EC_M6
GO:0007588	1.22E-02	0.996247029	9	36	excretion	BP	2.68E-01	EDNRB,STC1,EDN1,ADORA2A,CHRNB2,NPR1,SCNN1B,NFAT5,KCNK5	turquoise	EC_M6
GO:0032506	1.35E-02	0.996056992	8	35	cytokinetic process	BP	2.69E-01	KIF20A,PLEC,ANLN,KIF20B,CEP55,RACGAP1,ECT2,AURKB	darkgrey	EC_M8
GO:0071347	1.22E-02	0.994787646	15	75	cellular response to interleukin-1	BP	2.69E-01	AKAP12,MAPK13,IL6,CXCL8,IRAK2,IKBKB,SFRP1,EDN1,HYAL2,ADAMTS7,HIF1A,RPS6KA5,YY1,CCL2,DAB2IP	turquoise	EC_M6
GO:0045047	1.33E-02	0.995938911	9	30	protein targeting to ER	BP	2.70E-01	SRP72,SEC61A1,SRP68,TRAM1,SSR3,SRPRA,SRP54,HSPA5,SPCS3	darkgreen	EC_M4
GO:0044331	1.23E-02	0.996996831	7	22	cell-cell adhesion mediated by cadherin	BP	2.70E-01	NOTCH4,AFDN,MMP24,PPM1F,NOTCH1,CDH5,BMP6	turquoise	EC_M6

GO:0009628	1.34E-02	0.98960689	145	891	response to abiotic stimulus	BP	2.70E-01	SLC12A2,PPARA,HABP4,PPARD,UBQLN1,TXN2,GNAQ,LEP,SRF,VEGFA,VCP,CLOCK,ASNS,PDCL3,REST,COPS9,WHRN,ROCK2,MGARP,SLC7A11,ITGB1,ABHD12,EIF2B3,PAK1,HNMT,CTNNB1,AKT2,CLPB,LIG4,ATP2B1,DCN,SMPD1,IKBIP,CXCL12,TM9SF4,ANKRD1,HSP90B1,GRK7,MAP1B,PLOD2,ATP2A2,APP,DHX36,ABCC8,CACNB3,PTPN11,OPA1,ARSB,RAD54B,LOXL2,CRY2,MMP2,ADA,CHP1,ATP2B4,PKD2,HIF1AN,TANK,PSPH,CHRNA7,DNM1L,GLRX2,TROVE2,HYAL1,CCND1,TGFB2,LRP5,HTR2B,GSK3B,CASP7,MMP14,DAG1,PIK3CA,TLR5,KRAS,PITPNM1,FAM162A,BMP7,HYOU1,CASQ1,TP53BP1,SCFD1,RBBP7,BACE1,EGLN3,MAPT,DNAJA4,KCNK4,IGFBP2,HTT,PKD1L1,ERO1A,COL3A1,TIMP1,KCNA1,FMN2,ROM1,SLC2A4,TCAP,EIF2S1,PPARGC1A,CPEB4,ARHGEF2,TGFB1I1,NGFR,KCNMB1,FBXL22,COL1A1,TGFB3,GPR65,NPTN,MYOCD,PIN1,TRPV2,PDGFRB,AKT1,UBE2A,ITGA2,PRKAA1,GNB1,VASN,SDF4,PKD1,IRAK1,NPHP4,ATP6AP1,ENO1,HSBP1,COL11A1,SLC2A8,HSPA5,BCL10,ATP6V0D1,DMD,SERPINF1,BNIP3,LRRN4,PDLIM1,CDKN1A,EFHD1,TXN,MFAP4,TMEM109,MBD2,GTF2H5	darkgreen	EC_M4
GO:0034104	1.24E-02	0.997835915	5	15	negative regulation of tissue remodeling	BP	2.70E-01	TNFAIP3,IL6,SFRP1,P2RX7,INPP5D	turquoise	EC_M6
GO:0001780	1.24E-02	0.997927592	5	13	neutrophil homeostasis	BP	2.70E-01	IL6,MERTK,JAM3,ITPKB,PDE4B	turquoise	EC_M6
GO:0090023	1.24E-02	0.997318163	6	20	positive regulation of neutrophil chemotaxis	BP	2.71E-01	CXCL8,THBS4,EDN1,MCU,RAC1,DAPK2	turquoise	EC_M6
GO:0006513	1.37E-02	0.994737176	12	61	protein monoubiquitination	BP	2.71E-01	TOPORS,CUL1,RAD18,CUL3,UBE2T,DTL,FANCM,LEO1,TRIM37,CUL4B,UHRF1,RNF219	darkgrey	EC_M8
GO:0099515	1.35E-02	0.996761983	7	19	actin filament-based transport	BP	2.72E-01	SUN2,ACTN4,MYO5A,MYO1E,MYO1D,MYO5B,MYO1C	darkgreen	EC_M4
GO:2000107	1.35E-02	0.995566405	10	34	negative regulation of leukocyte apoptotic process	BP	2.72E-01	DOCK8,AXL,IRS2,CXCL12,ADA,SLC39A10,SELENOS,CD74,BCL10,ORMDL3	darkgreen	EC_M4
GO:0006109	1.35E-02	0.992481999	29	139	regulation of carbohydrate metabolic process	BP	2.72E-01	DYRK2,PPARA,LEP,TGFB1,DGAT2,AKT2,C1QTNF1,KAT2B,IRS2,SORBS1,APP,ADIPOR1,SELENOS,ZBTB20,GSK3B,PPP1R3D,PPP1R3F,PPARGC1A,CLTC,AKT1,PRKAA1,PTPN2,PGP,MIDN,FAM3A,SLC2A6,RANBP2,HAS2,NNMT	darkgreen	EC_M4
GO:0022008	1.25E-02	0.990111447	173	1272	neurogenesis	BP	2.72E-01	TCF4,SEMA6A,KIF5A,IFNGR1,CTHRC1,RIMS2,CELSR1,CDKL3,ASAP1,PTK2,ANKS1A,AGTPBP1,CSNK1E,PICK1,KDM4C,MICALL1,KIT,ADGRF1,TNFRSF21,SEMA4D,IL6,PPT1,TLR4,FZD8,MYCN,NRCAM,NRP1,ITSN2,TAL1,MTMR2,GATA2,DNMT3A,HS6ST1,NOVA2,PLXND1,ITGB4,GATA3,MYO7A,CXCR4,EDNRB,SPTBN4,JAG1,SFRP1,FEZ2,EFNB2,PCDH12,NTN4,PRAG1,EPHB1,PLK2,RET,CHD7,DNMT3B,SH3RF1,PREX2,DCLK2,MMP24,KIF13B,ENC1,HEXB,MEF2C,TLR2,VEGFC,NDRG4,GPM6A,USH1C,NR4A2,HIPK1,DLL4,SOX13,TSPAN2,NAV2,JAM3,DTNBP1,MYLIP,SLC45A3,SRGAP2C,HDAC1,PPP3CA,HECW2,MEF2A,SEMA3F,ROBO4,SEC24B,ZFHX2,PLXNA2,CFLAR,INPP5F,TRAK2,ADCY1,KALRN,FES,PROX1,SS18L1,PRKD1,LAMA5,YTHDF1,ITGA6,PAK2,LAMC2,ELL3,APOD,ETV6,HOXD10,MOV10,CREB1,B2M,ADORA2A,SEMA6D,MYEF2,EPHB4,ITGA4,DNM3,CDK16,PROM1,LMTK2,PRKCH,RAC1,HIF1A,EPHA2,CECR2,MPP5,TNFRSF1B,RGS14,ALDH1A2,ZC4H2,MTOR,OPHN1,EFNB1,CASZ1,PSEN1,SEMA4A,ABI3,ITGA3,EIF2B2,TAOK2,EFNA1,CHRNB2,NAP1L2,ZDHHC15,RPS6KA5,DNM2,B4GALT6,HMG20A,ZNF521,SH3TC2,CCL2,JAG2,GIT1,SEMA6B,SLITRK2,ATCAY,ARHGAP4,NOTCH1,RAPGEF1,S1PR1,STXBP1,LHX6,DAB2IP,SLC8A3,ADGRG1,VASP,RELN,ALKAL1,DIO3,SHANK3,MMD,GBA2,STX3,TGIF2,BMP2,SCARF1,BMP6	turquoise	EC_M6
GO:0008089	1.36E-02	0.994958482	12	44	anterograde axonal transport	BP	2.72E-01	AP3S1,RAB21,MGARP,KIF5B,TRAK1,AP3B1,FEZ1,KIF1A,HSPB1,HAP1,BLOC1S2,MAP1A	darkgreen	EC_M4

GO:0048522	1.36E-02	0.988171831	620	4212	positive regulation of cellular process	BP	2.72E-01	CD63,LMAN1,IL23A,NAB2,LRP1,STX7,AHI1,AGAP2,TAB2,CAND1,DYRK2,RAB21,RNF19A,PRRC1,SLC12A2,AZIN1,PLXNB2,FBN2,ANGPT1,TCP1,PPARA,HLA-DRA,ROS1,NUS1,RSPO3,FAM49B,MRTFA,HABP4,PACSIN1,PTP4A3,ADGRB1,PPARD,CTNNA1,TEAD3,SUN2,C6orf89,UBQLN1,MTCH1,LGALS1,KLHDC10,SH3GL2,MYH9,LEP,CDKN2B,PLAA,RORB,SLC30A9,KIAA1324L,SRF,VEGFA,DOCK8,VCP,SLC35B2,RUNX2,UNC13B,NCK2,CLOCK,EXOC1,CREB3,ASNS,PDCL3,REST,PTP4A1,P3H1,TGFBR1,HIVEP3,SOCS5,RIMS1,TMEM30A,PSME4,COPS9,RTN4,CYCS,TPBG,SHANK1,ADAMTS3,LRRC4B,EMC10,MFSD2A,MAP3K7,AKIRIN1,ACTR2,KIDINS220,MDFIC,GLI3,ROCK2,INHBA,BVES,RRAS,RCN3,MYLK3,DKKL1,VPS35,MGARP,ITGB1,PLEKHA4,DBP,FKBP1B,PLXNA1,FGF2,SRPK2,KIF5B,SESN3,RAB7A,PSMC2,GSAP,APBB1IP,FBLN2,ZNF398,RB1,ABHD5,PICALM,MARK4,NUFIP1,ZNF296,MAPRE3,PTCH2,PDCD6IP,TENM4,GLI2,MEGF8,SMARCD3,NUB1,RHEB,PAK1,ACTR3,TGFB1,AXL,RAB3GAP1,SCN5A,LMO7,WDR48,SEC13,ACER3,CTNNB1,IL15RA,TRAK1,LBH,DGAT2,AKT2,DYNC1LI1,GPD1L,DZIP1,ZEB2,CANT1,C1QTNF1,RAB2B,OSBPL8,CXXC5,RARB,ACTG1,NAV3,ACTN4,PPP1R12A,KAT2B,FAM98A,TACC1,MAFG,STIM1,EAF1,LIG4,ATP2C1,IRS2,TMTC3,PRNP,CHFR,NUDT16,LUM,DCN,CDC42EP3,SMAD9,HDAC3,ZNF703,ACPP,SEMA3C,CCKBR,CAVIN3,DDRGK1,SMPD1,ILK,RBPMS,HSPH1,ARL6IP5,DPYSL3,RYK,CIAO1,CNOT7,RNF6,RIC3,LMO1,TMEM9B,ST5,CXCL12,FXYD1,TM9SF4,SLC20A1,ANKRD1,PJA2,TP53INP2,CD180,SLC25A4,OSBP,EIF4G2,SULF1,VAMP8,ATP1B3,RIOK2,ROBO1,MAP1B,TEAD1,CHST2,RDH10,DIP2B,PINX1,SORBS1,SEMA4F,	darkgreen	EC_M4
GO:0086012	1.36E-02	0.996729568	7	19	membrane depolarization during cardiac muscle cell action potential	BP	2.72E-01	SCN5A,SLMAP,CACNA1D,CACNA1C,CACNA1G,HCN2,HCN4	darkgreen	EC_M4
GO:0021761	1.36E-02	0.993725385	18	75	limbic system development	BP	2.72E-01	SRF,UBA6,MFSD2A,GLI3,KIF5B,EIF2B3,RAB3GAP1,HTR5A,ATP2B4,FEZ1,GSK3B,LEF1,FGFR2,ARPC5,KCNA1,HAP1,PAFAH1B1,PLXNA3	darkgreen	EC_M4
GO:0010640	1.36E-02	0.996676038	7	20	regulation of platelet-derived growth factor receptor signaling pathway	BP	2.72E-01	LRP1,CBLB,MYOCD,PTPN2,F3,PTPN12,LOX	darkgreen	EC_M4
GO:0035269	1.37E-02	0.997151589	6	16	protein O-linked mannosylation	BP	2.72E-01	LARGE1,TMTC4,TMTC3,POMT1,DPM2,DPM1	darkgreen	EC_M4
GO:0014874	1.37E-02	0.997711357	5	12	response to stimulus involved in regulation of muscle adaptation	BP	2.73E-01	SCN5A,DAG1,CASQ1,PPARGC1A,SGCA	darkgreen	EC_M4
GO:0086015	1.37E-02	0.998559586	4	7	SA node cell action potential	BP	2.73E-01	SCN5A,CACNA1D,CACNA1G,HCN4	darkgreen	EC_M4
GO:0086018	1.37E-02	0.998559586	4	7	SA node cell to atrial cardiac muscle cell signaling	BP	2.73E-01	SCN5A,CACNA1D,CACNA1G,HCN4	darkgreen	EC_M4
GO:2000074	1.37E-02	0.998403709	4	8	regulation of type B pancreatic cell development	BP	2.73E-01	CLOCK,RHEB,GSK3B,AKT1	darkgreen	EC_M4
GO:0090657	1.38E-02	0.99819273	4	10	telomeric loop disassembly	BP	2.73E-01	RECQL4,POT1,TERF1,BLM	darkgrey	EC_M8
GO:0009994	1.38E-02	0.995952084	8	34	oocyte differentiation	BP	2.73E-01	YTHDC2,FBXO5,TUT7,BRCA2,CCNB1,TRIP13,ZFX,AURKA	darkgrey	EC_M8
GO:0061024	1.26E-02	0.990977308	83	599	membrane organization	BP	2.74E-01	STX11,LAPTM4B,SYNJ2,PACSIN2,TRDN,MTSS1,ASAP1,TSNARE1,BAIAP2L2,PICK1,MICALL1,ATP8A1,TRAM2,SNAP91,PPT1,ARHGAP25,ELMO1,CAV2,NRCAM,IZUMO1,GATA2,TMCC1,NECTIN2,TBC1D4,CXCR4,SPTBN4,TIE1,FCHSD2,ANKLE2,ATP11A,FCHSD1,CD36,GRAMD1A,PITPNM2,CLCN3,HIP1R,P2RX7,MTX3,DYSF,ANO6,SNCA,ANO3,ABCG1,MTTP,HYAL2,SNAP23,LAPTM5,SEC24B,LRMP,ATP9A,TOR1AIP1,DOCK1,DLG1,RTP4,ATP8B4,SNAP29,TNFAIP8L3,BAIAP2L1,TMEM30B,RAC1,SYT7,BID,CHRNB1,MPP5,AR,ADCK1,DNM2,SH3TC2,SPIRE1,MYO18A,AP3D1,PLCG2,STXBP1,FNBP1L,HAVCR1,PMAIP1,RELN,SHANK3,GBA2,STX3,TBC1D20,VAMP3,RAB39A	turquoise	EC_M6
GO:0002709	1.26E-02	0.995762411	10	43	regulation of T cell mediated immunity	BP	2.74E-01	IL6,RSAD2,TRPM4,NECTIN2,GATA3,B2M,WAS,TNFRSF1B,IL7R,TRAF2	turquoise	EC_M6
GO:0060425	1.38E-02	0.995125028	11	39	lung morphogenesis	BP	2.74E-01	SRF,CTNNB1,RDH10,AP3B1,DAG1,KRAS,FGFR2,PLOD3,MAP2K1,WNT2,FGF10	darkgreen	EC_M4

GO:0006650	1.38E-02	0.991111912	48	257	glycerophospholipid metabolic process	BP	2.74E-01	SERINC1,INPP4A,TMEM246,SOCS5,FAM126A,MFSD2A,FGF2,SELENOI,ABHD12,ABHD5,DGKH,OSBPL10,MTMR14,OSBPL8,IMPAD1,MTMR6,CHPT1,TMEM150A,FAR1,PIP4P2,LPCAT2,SERINC3,PIGP,AGPAT3,HTR2B,INPP5A,PIK3CA,PITPNM1,ETNK1,PIGZ,PCYT1B,PLD1,SLC44A2,PIGL,PDGFRB,PNPLA6,OCRL,PAFAH1B1,ABCA3,PGP,DPM2,PLA2G15,PEMT,PIGK,DPM1,PIGB,PIK3C2A,CDIPT	darkgreen	EC_M4
GO:0001764	1.38E-02	0.992689523	25	113	neuron migration	BP	2.74E-01	PLXNB2,MDGA1,PLAA,SRF,VEGFA,AXL,CTNNB1,DCLK1,CXCL12,MAP1B,RAPGEF2,CX3CL1,TUBB2A,NDN,DNER,SATB2,AUTS2,NTRK3,COL3A1,ARHGEF2,PAFAH1B1,FLNA,NTNG2,BARHL2,PRKG1	darkgreen	EC_M4
GO:0043567	1.27E-02	0.997814589	5	14	regulation of insulin-like growth factor receptor signaling pathway	BP	2.74E-01	IGF1,IGFBP3,IGFBP5,AR,BMP2	turquoise	EC_M6
GO:0090169	1.39E-02	0.996880084	6	21	regulation of spindle assembly	BP	2.75E-01	SENP6,TPR,KIF15,HNRNPU,PLK1,DRG1	darkgrey	EC_M8
GO:0019941	1.39E-02	0.99027503	68	550	modification-dependent protein catabolic process	BP	2.75E-01	CDK2,USP15,LATS1,UBR2,TOPORS,FBXO11,ZMPSTE24,USP34,XPO1,COMMD1,FBXO48,UFL1,CUL1,USP45,ASCC3,CDC20,VPS36,CUL4A,PSMD13,CDC16,ANAPC1,TAF9,USP25,PBK,ANAPC7,LTN1,USP16,UBE2C,UBE3A,CUL3,KIF14,UBR1,PSMD12,DTL,UBR3,PSMD4,CCDC47,SMARCC1,PSMA6,CDC27,COP1,CUL5,TBL1XR1,UBE2K,NEDD4,USP3,TAF1,OGT,RLIM,RBBP6,PLK1,APPBP2,PSMD10,MIB1,COPS3,CUL4B,UHRF1,CCNF,PSMA5,GLMN,USP33,AURKA,RNF139,RNF8,GID4,UBXN2A,PSMD7,RCHY1	darkgrey	EC_M8
GO:0070059	1.40E-02	0.994525388	13	50	intrinsic apoptotic signaling pathway in response to endoplasmic reticulum stress	BP	2.75E-01	NCK2,CREB3,OPA1,CHAC1,SERINC3,BRSK2,SELENOS,HYOU1,SYVN1,DNAJC10,ERO1A,PTPN2,PARK7	darkgreen	EC_M4
GO:0071481	1.40E-02	0.998164341	4	10	cellular response to X-ray	BP	2.76E-01	XRCC6,NUCKS1,XRCC5,NIPBL	darkgrey	EC_M8
GO:0099590	1.27E-02	0.997847532	5	13	neurotransmitter receptor internalization	BP	2.76E-01	DNM3,OPHN1,AP2B1,CACNG5,GSG1L	turquoise	EC_M6
GO:0007159	1.40E-02	0.991182951	44	234	leukocyte cell-cell adhesion	BP	2.76E-01	IL23A,PPARA,HLA-DRA,FAM49B,LGALS1,LEP,DOCK8,NCK2,SOCS5,GLI3,ITGB1,GLI2,PRNP,HSPH1,CXCL12,IL20RB,CHST2,LOXL3,PTPN11,AP3B1,PTPN22,ADA,RUNX1,CBLB,PRKAR1A,PIK3CA,LEF1,BMP7,TFRC,OLR1,PTPN6,IGFBP2,MSN,CD74,ROCK1,AKT1,PTPN2,CD40LG,IRAK1,CERCAM,BCL10,THY1,HAS2,FGL2	darkgreen	EC_M4
GO:0034121	1.28E-02	0.995182858	12	54	regulation of toll-like receptor signaling pathway	BP	2.76E-01	TNFAIP3,GRAMD4,PELI1,RSAD2,CD36,TLR2,LTF,DDX3X,BIRC3,TLR6,DAB2IP,TLR1	turquoise	EC_M6
GO:0006112	1.40E-02	0.993854363	16	65	energy reserve metabolic process	BP	2.76E-01	DYRK2,LEP,GFPT1,AKT2,IRS2,GYG1,SORBS1,PFKM,SELENOS,GYG2,GSK3B,PPP1R3D,PPP1R3F,AKT1,AGL,PPP1R1A	darkgreen	EC_M4
GO:0007423	1.41E-02	0.990115236	74	415	sensory organ development	BP	2.76E-01	ATP8B1,AHI1,FBN2,RORB,SRF,VEGFA,TGFBR1,MFSD2A,WHRN,GLI3,INHBA,SLC7A11,FGF2,GLI2,SMARCD3,RAB3GAP1,CTNNB1,SLC4A7,RARB,SPRED3,ATP2B1,FAT1,RDH10,SLC4A5,PTPN11,OPA1,ATP2B4,NKD1,KDM5B,NOTCH2,TGFB2,LRP5,LEF1,SHROOM2,NTRK3,CRYBA4,BMP7,FGFR2,CLIC4,ATF6,FZD2,FBN1,FKBP8,PRKCI,COL5A2,SKIL,ECE1,PRRX1,TULP3,SIX4,OBSL1,ROM1,CACNA1C,CLN8,TCAP,MED1,NES,TGFB3,TTC8,SMARCA4,CHRDL1,PDGFRB,MYOM1,GNB1,PAFAH1B1,NPHP4,COL5A1,COL11A1,BCAR3,SERPINF1,THY1,WNT2,COL8A2,FGF10	darkgreen	EC_M4
GO:2000008	1.41E-02	0.99533534	10	34	regulation of protein localization to cell surface	BP	2.76E-01	PICALM,CTNNB1,GPD1L,RIC3,TM9SF4,AKT1,TAX1BP3,KCNAB2,LEPROT,MAP1A	darkgreen	EC_M4
GO:0051084	1.41E-02	0.995656011	9	30	'de novo' posttranslational protein folding	BP	2.77E-01	BAG1,DNAJB13,HSPH1,HSPA8,DNAJB12,ERO1A,CD74,HSPA5,HSPA13	darkgreen	EC_M4
GO:0098742	1.43E-02	0.991798908	32	152	cell-cell adhesion via plasma-membrane adhesion molecules	BP	2.79E-01	PLXNB2,MDGA1,LRRC4B,PCDH18,ITGB1,TENM4,PCDH17,ATP2C1,FAT1,PALLD,ROBO1,CX3CL1,CDH17,AMIGO2,ACVR1,TGFB2,NECTIN4,OBSL1,PCDH7,ADGRL1,FGFRL1,NPTN,FAT2,PLXNB3,PKD1,BSG,NTNG2,CDH13,NEXN,PCDHB2,VSTM2L,CD164	darkgreen	EC_M4
GO:0070168	1.30E-02	0.996797582	7	22	negative regulation of biomineral tissue development	BP	2.79E-01	TRPM4,NOS3,BCOR,FGF23,HIF1A,RFLNB,NOTCH1	turquoise	EC_M6

GO:0110150	1.30E-02	0.996797582	7	22	negative regulation of biomineralization	BP	2.79E-01	TRPM4,NOS3,BCOR,FGF23,HIF1A,RFLNB,NOTCH1	turquoise	EC_M6
GO:0045981	1.30E-02	0.996330559	8	29	positive regulation of nucleotide metabolic process	BP	2.79E-01	NOS3,IGF1,GPD1,P2RX7,MLXIPL,HIF1A,PSEN1,NOS2	turquoise	EC_M6
GO:1900544	1.30E-02	0.996330559	8	29	positive regulation of purine nucleotide metabolic process	BP	2.79E-01	NOS3,IGF1,GPD1,P2RX7,MLXIPL,HIF1A,PSEN1,NOS2	turquoise	EC_M6
GO:0045165	1.30E-02	0.992639634	30	172	cell fate commitment	BP	2.79E-01	NOTCH4,IL6,NRP1,TAL1,GATA2,PPARG,GATA3,JAG1,SFRP1,SMAD1,MEF2C,IL7,DHH,DLL4,SOX13,ETS2,PROX1,HOXD10,TBX1,MTOR,AR,PSEN1,BATF,SMAD2,ZNF521,JAG2,NOTCH1,SOX17,SOX18,BMP2	turquoise	EC_M6
GO:0022406	1.43E-02	0.993339537	18	76	membrane docking	BP	2.80E-01	STX7,UNC13B,BVES,RAB7A,ESYT2,SNPH,VAMP8,ATP2A2,USO1,VPS18,CHP1,VAPB,MSN,STX1B,CALM1,VMP1,ROCK1,AHCYL1	darkgreen	EC_M4
GO:0017185	1.44E-02	0.998306616	4	8	peptidyl-lysine hydroxylation	BP	2.80E-01	PLOD2,PLOD3,FKBP10,P3H3	darkgreen	EC_M4
GO:0001973	1.44E-02	0.998306153	4	8	G protein-coupled adenosine receptor signaling pathway	BP	2.80E-01	ACPP,ADA,ADCY5,ADORA2B	darkgreen	EC_M4
GO:0035588	1.44E-02	0.998306153	4	8	G protein-coupled purinergic receptor signaling pathway	BP	2.80E-01	ACPP,ADA,ADCY5,ADORA2B	darkgreen	EC_M4
GO:0006611	1.42E-02	0.995052269	10	49	protein export from nucleus	BP	2.80E-01	HSPA9,XPO5,ANP32B,XPO1,XPO4,CSE1L,CTDSPL2,TPR,ANKLE1,XPO6	darkgrey	EC_M8
GO:0051347	1.31E-02	0.991007314	65	441	positive regulation of transferase activity	BP	2.80E-01	TNFRSF11A,FLT4,TPD52L1,PTK2,PDGFB,TEK,KIT,KDR,SYK,TLR4,CCNY,PIK3CG,TAL1,CAB39L,RALB,TIE1,HBEGF,EPHB1,RET,MERTK,IGF1,HMBOX1,VEGFC,MADD,SERINC5,DGKZ,NEDD9,EDN1,SNCA,CD200,NCF1,PABPN1,CD86,FGR,PROX1,PRKD1,ACE,PAK2,DLG1,MAP3K11,LTF,EPHB4,DDX3X,TNFAIP8L3,LMTK2,EPHA2,TLR6,MTOR,PSEN1,FGF18,TAOK2,EFNA1,CD19,PIK3R5,ADRB2,UNC119,TRAF2,DAB2IP,RELN,ALKAL1,MMD,DAZAP2,COPS8,BMP2,PIFO	turquoise	EC_M6
GO:0072009	1.31E-02	0.993897477	18	89	nephron epithelium development	BP	2.80E-01	MTSS1,KLHL3,PODXL,GATA3,GZF1,JAG1,MEF2C,WWTR1,STAT1,CD34,PECAM1,LAMA5,DLG1,WNK4,PROM1,HOXB7,NOTCH1,BMP2	turquoise	EC_M6
GO:0071479	1.43E-02	0.994437979	12	64	cellular response to ionizing radiation	BP	2.81E-01	XRCC6,ZMPSTE24,ATR,RAD51,INO80,NUCKS1,INTS7,BLM,XRCC5,RAD51AP1,ECT2,NIPBL	darkgrey	EC_M8
GO:0006457	1.44E-02	0.992404819	23	166	protein folding	BP	2.81E-01	CCT2,HSPA9,PPIL1,PPIH,CCT4,DNAJC2,CHORDC1,NKTR,PPID,CCT7,CCT5,CCT6A,TBCE,PPIG,CCDC47,GRPEL1,RP2,FKBP4,DNAJC7,CCT3,CLPX,DNAJC21,ERP29	darkgrey	EC_M8
GO:0001701	1.44E-02	0.990880712	43	314	in utero embryonic development	BP	2.81E-01	LATS1,RRP7A,TRIM28,MSH2,YTHDC1,PLK4,STIL,RNASEH2B,NASP,NCAPG2,CHD8,E2F7,GINS4,CUL4A,BRCA2,CCNB1,CNOT1,GABPA,NBN,E2F8,COL4A3BP,ZBED6,STK4,CUL3,NSUN2,CHEK1,BPTF,NEK2,ALS2,FLVCR1,FOSL1,SP3,SETD2,NPAT,ASF1B,CCNB2,RBBP6,MIB1,NLE1,COPS3,PHF6,NSRP1,PALB2	darkgrey	EC_M8
GO:0032481	1.44E-02	0.994988518	10	48	positive regulation of type I interferon production	BP	2.81E-01	POLR3B,POLR3F,TOMM70,CHUK,DHX9,SETD2,DHX33,POLR3A,G3BP1,RIOK3	darkgrey	EC_M8
GO:1904666	1.44E-02	0.997218017	5	19	regulation of ubiquitin protein ligase activity	BP	2.81E-01	FBXO5,CDC20,UBE2C,PLK1,MAD2L1	darkgrey	EC_M8
GO:0021510	1.45E-02	0.993436574	17	70	spinal cord development	BP	2.82E-01	MDGA1,GLI3,EED,GLI2,LOXL3,TCTN1,PKD2,SCYL1,MDGA2,TULP3,CLN8,AKT1,PKD1,LMO4,LBX1,GDNF,ZPR1	darkgreen	EC_M4
GO:0010522	1.46E-02	0.993394717	17	71	regulation of calcium ion transport into cytosol	BP	2.83E-01	FKBP1B,PRNP,P2RX4,CX3CL1,PKD2,CASQ1,PTPN6,HTT,HAP1,CACNA1C,CALM1,JPH3,DMD,FKBP1A,TMEM38B,THY1,TMEM38A	darkgreen	EC_M4

GO:0048608	1.46E-02	0.990127371	60	330	reproductive structure development	BP	2.83E-01	RSPO3,PPARD,CTNNA1,LEP,VEGFA,TGFBR1,GLI3,INHBA,GLI2,AXL,WDR48,CTNNB1,SPINT2,DCN,TMED2,EIF2S2,SULF1,RDH10,HEY1,PTPN11,PTX3,ADA,SPP1,PKD2,CSDE1,KDM5B,NOTCH2,CCND1,TGFB2,MMP14,LEF1,BMP7,FGFR2,VASH2,ASB1,HTRA1,ARID5B,PRDX4,PCYT1B,SGPL1,PTPN6,PTPRN,CYP19A1,SIX4,MED1,MAP2K1,MYOCD,PDGFRB,AKT1,PLAC1,PKD1,FLNA,BSG,HSPA5,LHX8,FSTL3,SERPINF1,SERPINB5,WNT2,FGF10	darkgreen	EC_M4
GO:0060513	1.46E-02	0.998263881	4	8	prostatic bud formation	BP	2.84E-01	GLI2,SULF1,BMP7,FGF10	darkgreen	EC_M4
GO:2000628	1.48E-02	0.998033804	4	10	regulation of miRNA metabolic process	BP	2.85E-01	PNPT1,TENT2,NFKB1,DROSHA	darkgrey	EC_M8
GO:0019081	1.48E-02	0.997235462	5	16	viral translation	BP	2.86E-01	EIF3A,SSB,DHX9,EIF3B,PTBP1	darkgrey	EC_M8
GO:0050996	1.48E-02	0.997473867	5	12	positive regulation of lipid catabolic process	BP	2.87E-01	PPARA,ABHD5,AKT2,IRS2,CPT1A	darkgreen	EC_M4
GO:0031069	1.48E-02	0.996317251	7	20	hair follicle morphogenesis	BP	2.87E-01	GLI2,CTNNB1,TGFB2,FGFR2,GORAB,NGFR,FGF10	darkgreen	EC_M4
GO:0015780	1.49E-02	0.997464061	5	12	nucleotide-sugar transmembrane transport	BP	2.87E-01	SLC35A1,SLC35A5,SLC35B1,SLC35A3,SLC35C1	darkgreen	EC_M4
GO:0002053	1.50E-02	0.996280604	7	20	positive regulation of mesenchymal cell proliferation	BP	2.87E-01	CTNNB1,IRS2,LRP5,FGFR2,PRRX1,BMPR1A,WNT2	darkgreen	EC_M4
GO:0009056	1.50E-02	0.987448912	304	2000	catabolic process	BP	2.87E-01	HSD17B6,LRP1,CDO1,AGAP2,CANX,ENPP3,TAB2,RNF19A,MIOX,AZIN1,ATP6V1C1,SKIV2L,PPARA,RNASET2,ADGRB1,PPARD,UBQLN1,GGA1,KLHDC10,LEP,PLAA,ALDH1L2,GALT,VCP,C2orf40,CLOCK,EXOC1,PDCL3,RAB23,SOCS5,ERLEC1,PSME4,UBA6,ADAMTS3,MFSD2A,UBE2J1,MAP3K7,RNASEH1,PCYOX1,ROCK2,VPS41,RCN3,VPS35,CUL2,ITGB1,LDAH,FGF2,SESN3,RAB7A,PSMC2,GAD2,ENTPD6,ABHD12,ABHD5,ESD,GLB1,ABHD1,PDCD6IP,TMUB1,KCTD21,ATP6V0B,NUB1,RHEB,SNX17,TGFB1,RAB3GAP1,UBXN4,HNMT,SPOPL,RBKS,ACER3,CTNNB1,AKT2,GPD1L,STT3B,MTMR14,PFKP,NPLOC4,STAM2,RNF121,PSMD8,UFM1,IRS2,TMTC3,CHFR,NUDT16,DCN,UBA5,DDRGK1,SMPD1,PPP2CB,MMP17,PSMD6,CNOT7,RNF6,VPS37A,IMPAD1,TMEM41B,HSP90B1,MAP1LC3A,TP53INP2,SLC25A4,C19orf12,EIF4G2,TMEM150A,VAMP8,LNPEP,TRPC4AP,ELOC,RNF13,ATP2A2,CELF1,MMP15,APP,DHX36,TET3,PFKM,PGAM4,ENTPD4,ARSB,AMFR,ATG13,PTPN22,TSG101,DKC1,VPS18,CHAC1,MMP2,MTDH,ADA,GLS,BAP1,ATP2B4,SPP1,CPN1,CSDE1,NUDT18,TTC3,CBLB,NKD1,TENT5C,TENT4B,TOLLIP,PSMD14,BRSK2,VPS39,MARCH6,ATP6V1B2,AZIN2,FAP,CTTN,DNM1L,UCHL5,SELENOS,TRIP12,HYAL1,SEC22B,CPT1A,CISD2,ZBTB20,PSMD1,UBE3B,LRP5,KCTD10,FEZ1,HTR2B,WIPI1,GSK3B,INPP5A,MMP14,PIK3CA,HADH,PRKCA,HSPA8,NEDD8,PPP1R11,PPP1R3D,SYNPO2,PSMD2,ADRM1,SCFD1,QSOX1,UBE4A,ATF6,SIDT2,PAFAH1B2,MRC2,PRDX4,RHBDD2,EDEM3,HSPB1,MAPT,SYVN1,SGPL1,COMT,DNAJC10,MAOA,DNAJB12,GPR137,TPI1,ACOX3,UPF1,HTT,USP48,ECE1,SAMD4A,QDPR,ARAF,SMURF1,TIMP1,PLD1,FBXL5,CYP19A1,JKAMP,F	darkgreen	EC_M4
GO:0048771	1.50E-02	0.991794429	27	129	tissue remodeling	BP	2.87E-01	IL23A,RSPO3,LEP,NCDN,ROCK2,TGFB1,AXL,CTNNB1,SEMA3C,MMP2,SPP1,RUNX1,NOTCH2,LRP5,MMP14,PRKCA,TFRC,GPR137,SUCO,TIMP1,TGFB3,ROCK1,FLNA,ATP6AP1,DEF8,SNX10,FGF10	darkgreen	EC_M4
GO:0060627	1.50E-02	0.989466545	71	403	regulation of vesicle-mediated transport	BP	2.88E-01	CD63,LRP1,AHI1,RAB21,ANGPT1,PACSIN1,MKLN1,SH3GL2,VEGFA,REST,BVES,KIF5B,RAB7A,PICALM,PDCD6IP,SNX17,AXL,RAB3GAP1,IL15RA,AKT2,RAB2B,ATP2C1,PCLO,SMPD1,VAMP8,ATP2A2,ATXN2,PTX3,PIP4P2,ARHGAP1,TSG101,VPS18,PPP3CC,LGI3,DNM1L,GSK3B,AP2M1,SCFD1,BACE1,SERPINE1,TFR2,ITGAV,LRPAP1,PLD1,ATAD1,STX18,HAP1,LMAN2,MSN,STX1B,SYT11,DOCK2,CACNA1G,RAB11A,MAP2K1,CALM1,SYTL4,SCAMP5,ROCK1,ITGA2,PRKAA1,RAB31,ATP6AP1,VAMP7,MBTPS1,CDH13,AP1G1,CD151,CBARP,STXBP6,LRRTM2	darkgreen	EC_M4
GO:0006511	1.50E-02	0.989520586	67	542	ubiquitin-dependent protein catabolic process	BP	2.89E-01	CDK2,USP15,LATS1,UBR2,TOPORS,FBXO11,USP34,XPO1,COMMD1,FBXO48,UFL1,CUL1,USP45,ASCC3,CDC20,VPS36,CUL4A,PSMD13,CDC16,ANAPC1,TAF9,USP25,PBK,ANAPC7,LTN1,USP16,UBE2C,UBE3A,CUL3,KIF14,UBR1,PSMD12,DTL,UBR3,PSMD4,CCDC47,SMARCC1,PSMA6,CDC27,COP1,CUL5,TBL1XR1,UBE2K,NEDD4,USP3,TAF1,OGT,RLIM,RBBP6,PLK1,APPBP2,PSMD10,MIB1,COPS3,CUL4B,UHRF1,CCNF,PSMA5,GLMN,USP33,AURKA,RNF139,RNF8,GID4,UBXN2A,PSMD7,RCHY1	darkgrey	EC_M8
GO:0046068	1.36E-02	0.997665728	5	13	cGMP metabolic process	BP	2.90E-01	PDE2A,GUCY1A1,GUCY1B1,PDE9A,NPR1	turquoise	EC_M6
GO:0097061	1.52E-02	0.993088224	17	70	dendritic spine organization	BP	2.91E-01	SHANK1,ACTR2,VPS35,PRNP,DHX36,HOMER1,OPA1,PDLIM5,CHRNA7,DOCK10,CTTN,DNM1L,CAPRIN2,KIF1A,TANC2,SRCIN1,PAFAH1B1	darkgreen	EC_M4

GO:0002726	1.37E-02	0.997540489	5	15	positive regulation of T cell cytokine production	BP	2.91E-01	IL6,RSAD2,GATA3,B2M,TRAF2	turquoise	EC_M6
GO:0010596	1.37E-02	0.99504649	11	48	negative regulation of endothelial cell migration	BP	2.91E-01	PPARG,ADAMTS9,MEF2C,ANGPT2,STC1,DLL4,JUP,MMRN2,VASH1,NOTCH1,DAB2IP	turquoise	EC_M6
GO:0032612	1.37E-02	0.994509747	13	62	interleukin-1 production	BP	2.91E-01	TNFAIP3,IL6,TLR4,CD36,IGF1,P2RX7,HDAC1,TMEM106A,TLR6,ARG2,LGALS9,GIT1,GBP5	turquoise	EC_M6
GO:0032652	1.37E-02	0.994509747	13	62	regulation of interleukin-1 production	BP	2.91E-01	TNFAIP3,IL6,TLR4,CD36,IGF1,P2RX7,HDAC1,TMEM106A,TLR6,ARG2,LGALS9,GIT1,GBP5	turquoise	EC_M6
GO:0032368	1.52E-02	0.99238346	21	95	regulation of lipid transport	BP	2.91E-01	LRP1,PPARA,NUS1,LEP,TMEM30A,AKT2,C1QTNF1,IRS2,P2RX4,PTPN11,CRY2,SPP1,PLA2R1,KDM5B,EPRS,ARV1,LINC02210-CRHR1,ITGAV,CYP19A1,AKT1,ABCA3	darkgreen	EC_M4
GO:0036294	1.53E-02	0.991628956	27	129	cellular response to decreased oxygen levels	BP	2.91E-01	PPARD,UBQLN1,VEGFA,ROCK2,MGARP,ANKRD1,CACNB3,OPA1,HIF1AN,DNM1L,FAM162A,BMP7,HYOU1,EGLN3,ERO1A,FMN2,SLC2A4,PPARGC1A,CPEB4,KCNMB1,AKT1,PRKAA1,GNB1,VASN,IRAK1,ENO1,BNIP3	darkgreen	EC_M4
GO:0006836	1.38E-02	0.992631402	25	139	neurotransmitter transport	BP	2.92E-01	STX11,SNCAIP,RIMS2,APBA1,ICA1,PPT1,RAB3B,NR4A1,MCTP1,MEF2C,SLC38A1,SNCA,DTNBP1,MCTP2,SNAP23,ADCY1,ADORA2A,SNAP29,SNCG,SYT7,FBXL20,PSEN1,GIT1,STXBP1,STX3	turquoise	EC_M6
GO:0032870	7.53E-04	0.999538726	70	450	cellular response to hormone stimulus	BP	2.92E-01	STAT6,PIP4K2C,CDK4,RXRB,EP300,DDX17,HSF1,CCND3,TRERF1,KLF9,JAK2,LPIN1,FOXO3,FOSB,ECHDC3,WBP2,EGLN2,TADA3,NCOA4,RARG,TNS2,LMO2,LATS2,URI1,PIK3R1,NCOA2,BBS2,LPIN3,LGR4,SRD5A1,IRS1,PRMT2,HDAC4,USF1,CAPN10,POR,NFE2L2,CTSH,MEN1,PHB2,STAT5A,HDAC6,PELP1,RARA,THRA,CASP9,ZFP36L1,AGTRAP,PIP4K2B,RAB8B,PER1,FOS,ZBTB7B,KANK2,EEF2K,ACACA,INSR,PPARGC1B,SREBF1,LEPR,TFAP4,NSMF,RXRA,APRT,CYBA,PTGFR,MT-ND3,ZBTB7A,PID1,ZFP36L2	cyan	EC_M2
GO:0016042	7.73E-04	0.999596384	42	225	lipid catabolic process	BP	2.92E-01	CDK4,ABHD16A,HINT2,ETFB,LPIN1,LIPE,PAFAH1B3,ACAA1,SIRT2,HACL1,GPCPD1,DDHD2,CYP1B1,PLBD2,LPIN3,PLA2G4B,ACOT8,LONP2,SRD5A1,IRS1,ACADS,ABHD4,ABCB11,EHHADH,IDH1,ACADL,ABHD12B,DDHD1,CYP27A1,SMPD4,PLA2G16,ILVBL,THRA,ABCD4,GBA,GCDH,HEXA,ACAA2,CPT2,PNPLA7,BSCL2,SCP2	cyan	EC_M2
GO:1904491	1.54E-02	0.99814563	4	8	protein localization to ciliary transition zone	BP	2.93E-01	TCTN1,NPHP4,TCTN2,CC2D2B	darkgreen	EC_M4
GO:0009408	1.54E-02	0.992596438	19	84	response to heat	BP	2.93E-01	VCP,PDCL3,EIF2B3,CLPB,CXCL12,ATP2A2,DHX36,GSK3B,CASQ1,RBBP7,MAPT,DNAJA4,EIF2S1,TGFB1I1,TRPV2,AKT1,IRAK1,HSBP1,CDKN1A	darkgreen	EC_M4
GO:0035150	1.55E-02	0.992087892	22	100	regulation of tube size	BP	2.94E-01	PPARD,LEP,ROCK2,ATP2B1,SNTA1,ADCY6,RAP1GDS1,HTR2B,PLOD3,ADRA2C,COMP,ECE1,ACTA2,NPPB,KCNMB1,ADRA1B,ROCK1,AKT1,NPR3,PIK3C2A,PRKG1,KCNMB4	darkgreen	EC_M4
GO:0035296	1.55E-02	0.992087892	22	100	regulation of tube diameter	BP	2.94E-01	PPARD,LEP,ROCK2,ATP2B1,SNTA1,ADCY6,RAP1GDS1,HTR2B,PLOD3,ADRA2C,COMP,ECE1,ACTA2,NPPB,KCNMB1,ADRA1B,ROCK1,AKT1,NPR3,PIK3C2A,PRKG1,KCNMB4	darkgreen	EC_M4
GO:0097746	1.55E-02	0.992087892	22	100	blood vessel diameter maintenance	BP	2.94E-01	PPARD,LEP,ROCK2,ATP2B1,SNTA1,ADCY6,RAP1GDS1,HTR2B,PLOD3,ADRA2C,COMP,ECE1,ACTA2,NPPB,KCNMB1,ADRA1B,ROCK1,AKT1,NPR3,PIK3C2A,PRKG1,KCNMB4	darkgreen	EC_M4
GO:0030166	1.56E-02	0.993809129	13	49	proteoglycan biosynthetic process	BP	2.94E-01	B4GALT7,CHST10,CHPF2,CTNNB1,CANT1,CHSY1,SLC2A10,B3GAT3,UGDH,CHST12,XYLT2,CHST11,HS2ST1	darkgreen	EC_M4
GO:0018242	1.56E-02	0.998113711	4	8	protein O-linked glycosylation via serine	BP	2.94E-01	KDELC1,GALNT2,GALNT16,GALNT1	darkgreen	EC_M4
GO:1903830	1.56E-02	0.996641967	6	16	magnesium ion transmembrane transport	BP	2.94E-01	CNNM4,TUSC3,NIPA1,MRS2,CNNM2,NIPAL1	darkgreen	EC_M4
GO:0018158	1.56E-02	0.99730095	5	12	protein oxidation	BP	2.94E-01	SUMF1,LOXL3,LOXL2,LOXL1,LOX	darkgreen	EC_M4
GO:1903706	1.40E-02	0.99134682	40	258	regulation of hemopoiesis	BP	2.95E-01	TCF7,SYK,TLR4,IL15,TAL1,GATA2,GATA3,JAG1,SFRP1,TMEM178A,SH3RF1,MEF2C,IL7,SOX13,STAT1,ETS1,HLX,HCLS1,INPP5D,CD86,FES,CREB1,B2M,LTF,SOS2,HIF1A,ITPKB,MTOR,HOXB8,GPR68,RCOR1,LGALS9,IL7R,ISG15,AP3D1,NRARP,VNN1,CD46,CA2,HIST1H4D	turquoise	EC_M6
GO:0031663	1.40E-02	0.995197649	10	44	lipopolysaccharide-mediated signaling pathway	BP	2.95E-01	TNFAIP3,TLR4,IRAK2,CD36,SCARB1,TLR2,LTF,CCL2,PLCG2,BMP6	turquoise	EC_M6
GO:2000169	1.57E-02	0.99809957	4	8	regulation of peptidyl-cysteine S-nitrosylation	BP	2.95E-01	SNTA1,ATP2B4,DMD,TXN	darkgreen	EC_M4

GO:0022411	1.57E-02	0.989076537	67	375	cellular component disassembly	BP	2.95E-01	LRP1,UBQLN1,VCP,TGFBR1,KIF5B,NAPA,SMARCD3,NCKAP5,TGFB1,NAV3,UFM1,UBA5,DDRGK1,KIF2A,MAP1LC3A,SLC25A4,VAMP8,MAP1B,ATP2A2,MMP15,CX3CL1,ABCC8,ATG13,TSG101,MMP2,TWF1,FAP,CTTN,DNM1L,LMOD1,CISD2,WIPI1,GSK3B,MMP14,PIK3CA,PRKCA,HSPA8,UPF1,HTT,CAPZB,WDR1,WIPI2,PPARGC1A,NES,ARHGEF2,TGFB3,DNASE2,PBXIP1,CALM1,SMARCA4,VMP1,DICER1,FLII,CLEC16A,PAFAH1B1,FUNDC2,PARK7,SPTAN1,BNIP3,ADAMTS5,DSTN,GABARAPL2,TOMM7,BLCAP,MAP1A,DENR,CFL2	darkgreen	EC_M4
GO:2000147	1.57E-02	0.988724066	79	455	positive regulation of cell motility	BP	2.95E-01	LRP1,ANGPT1,NUS1,SUN2,PLAA,VEGFA,DOCK8,CREB3,PTP4A1,TGFBR1,RTN4,EMC10,AKIRIN1,ROCK2,RRAS,ITGB1,FGF2,PAK1,TGFB1,AKT2,ACTG1,ACTN4,IRS2,ZNF703,SEMA3C,DDRGK1,ILK,CXCL12,SEMA4F,P2RX4,APP,RAPGEF2,CX3CL1,PGAM4,ACVR1,SEMA4G,TJP1,DNM1L,HYAL1,TGFB2,MMP14,LEF1,PRKCA,CMKLR1,NTRK3,BMP7,IQGAP1,S100A11,LRRC15,FAM89B,HSPB1,SERPINE1,ITGA2B,FN1,PDCD10,ITGAV,AKT3,ARHGEF2,COL1A1,RAB11A,CD74,PDGFRB,AKT1,ITGA2,DAPK3,MYO1C,TNFRSF18,FLNA,CDH13,F3,HSPA5,CD151,THY1,HAS2,TMSB4X,GLIPR2,CCR6,PIK3C2A,FGF10	darkgreen	EC_M4
GO:1901339	1.57E-02	0.998090978	4	8	regulation of store-operated calcium channel activity	BP	2.95E-01	UBQLN1,STIM1,CASQ1,STIM2	darkgreen	EC_M4
GO:0010944	1.41E-02	0.998150641	4	10	negative regulation of transcription by competitive promoter binding	BP	2.96E-01	DACH1,HHEX,KDM2A,CREB1	turquoise	EC_M6
GO:0043281	1.41E-02	0.992382848	25	153	regulation of cysteine-type endopeptidase activity involved in apoptotic process	BP	2.96E-01	TNFAIP8,GRAMD4,DAPK1,SYK,PPARG,NR4A1,EIF2AK3,SH3RF1,HIP1R,SOX7,SNCA,LCK,LAPTM5,CFLAR,CASP10,RPS6KA1,PAK2,ADORA2A,DDX3X,BIRC3,PPM1F,BID,LGALS9,PMAIP1,SERPINB9	turquoise	EC_M6
GO:0007626	1.42E-02	0.992522551	24	132	locomotory behavior	BP	2.96E-01	OXR1,AGTPBP1,APBA1,TMOD1,PPT1,TAL1,SPTBN4,CHD7,PREX2,CLCN3,HEXB,NR4A2,NAV2,SNCA,CIART,INPP5F,HOXD10,ADORA2A,SNCG,MTOR,HOXB8,CHRNB2,GIT1,RELN	turquoise	EC_M6
GO:0009190	1.42E-02	0.996965625	6	17	cyclic nucleotide biosynthetic process	BP	2.96E-01	GUCY1A1,GUCY1B1,ADCY2,ADCY1,ADCY4,NPR1	turquoise	EC_M6
GO:0052652	1.42E-02	0.996965625	6	17	cyclic purine nucleotide metabolic process	BP	2.96E-01	GUCY1A1,GUCY1B1,ADCY2,ADCY1,ADCY4,NPR1	turquoise	EC_M6
GO:0018196	1.58E-02	0.995982668	7	21	peptidyl-asparagine modification	BP	2.96E-01	UBE2J1,RPN1,STT3B,TUSC3,RPN2,HIF1AN,DDOST	darkgreen	EC_M4
GO:0140212	1.58E-02	0.998076066	4	8	regulation of long-chain fatty acid import into cell	BP	2.96E-01	AKT2,IRS2,EPRS,AKT1	darkgreen	EC_M4
GO:0035809	1.42E-02	0.997410743	5	15	regulation of urine volume	BP	2.97E-01	EDNRB,EDN1,HYAL2,ADORA2A,NPR1	turquoise	EC_M6
GO:1902305	1.60E-02	0.993617609	13	50	regulation of sodium ion transmembrane transport	BP	2.98E-01	KIF5B,SCN5A,GPD1L,ACTN4,FXYD1,SNTA1,SLMAP,ATP1B3,CHP1,ATP2B4,MIR24-1,DMD,YWHAH	darkgreen	EC_M4
GO:1905475	1.60E-02	0.990877509	30	147	regulation of protein localization to membrane	BP	2.98E-01	LRP1,STX7,SLC7A11,ITGB1,KIF5B,PICALM,PAK1,TGFB1,AKT2,ATP2C1,PRNP,VAMP8,SORBS1,CACNB3,CHP1,ATP2B4,MMP14,DAG1,AP2M1,FZD9,LRRC15,GPC2,PRKCI,IQSEC2,RAB11A,CLTC,MIEF2,AKT1,MYO1C,MESD	darkgreen	EC_M4
GO:0008333	1.60E-02	0.993586832	13	50	endosome to lysosome transport	BP	2.98E-01	VCP,VPS41,RAB7A,TRAK1,TSG101,VPS18,AKTIP,VPS39,RAB7B,SNX27,CLEC16A,MGRN1,VAMP7	darkgreen	EC_M4
GO:0002921	1.61E-02	0.998039384	4	8	negative regulation of humoral immune response	BP	2.98E-01	SPINK5,PTPN6,A2M,CD59	darkgreen	EC_M4
GO:2001235	1.61E-02	0.991892426	21	98	positive regulation of apoptotic signaling pathway	BP	2.98E-01	CTNNA1,NCK2,TGFBR1,INHBA,SERINC3,DNM1L,ATF3,PEA15,PDIA3,SKIL,MAGED1,NGFR,PTPN2,DAPK3,BCAP31,PARK7,BCL10,ITM2C,TIMP3,NACC2,PLAGL2	darkgreen	EC_M4

GO:0010498	1.61E-02	0.988550153	75	434	proteasomal protein catabolic process	BP	2.98E-01	CANX,RNF19A,UBQLN1,KLHDC10,PLAA,VCP,C2orf40,CLOCK,SOCS5,ERLEC1,PSME4,UBE2J1,RCN3,CUL2,PSMC2,TMUB1,NUB1,UBXN4,SPOPL,CTNNB1,STT3B,NPLOC4,RNF121,PSMD8,TMTC3,CHFR,DDRGK1,PPP2CB,PSMD6,HSP90B1,TRPC4AP,AMFR,PSMD14,BRSK2,MARCH6,UCHL5,SELENOS,PSMD1,KCTD10,GSK3B,PSMD2,UBE4A,RHBDD2,EDEM3,SYVN1,DNAJC10,DNAJB12,ARAF,SMURF1,FBXL5,JKAMP,ANAPC4,SPOP,FBXL22,SEL1L,ARIH1,FBXO22,USP14,AKT1,UBE2A,TNFAIP1,BCAP31,PKD1,TMEM259,PARK7,RHBDF1,HSPA5,FBXO27,UBE2V2,DNAJB9,DDA1,GABARAPL2,UBE2G2,MAP1A,DERL2	darkgreen	EC_M4
GO:0006111	1.62E-02	0.994133659	11	40	regulation of gluconeogenesis	BP	3.00E-01	PPARA,LEP,DGAT2,KAT2B,SELENOS,PPARGC1A,PTPN2,PGP,FAM3A,RANBP2,NNMT	darkgreen	EC_M4
GO:2001234	1.63E-02	0.990182178	36	187	negative regulation of apoptotic signaling pathway	BP	3.01E-01	RRM2B,CTNNA1,CREB3,TGFBR1,RB1,TPT1,CTNNB1,CXCL12,FAIM2,CX3CL1,OPA1,ACVR1,CTTN,SELENOS,EYA2,GSK3B,HYOU1,PEA15,FZD9,HSPB1,SERPINE1,SYVN1,ITGAV,HTT,VDAC2,GHITM,ARHGEF2,TAF9B,CD74,AKT1,PARK7,ENO1,GNAI3,GDNF,PTTG1IP,FGF10	darkgreen	EC_M4
GO:1904153	1.63E-02	0.998002011	4	8	negative regulation of retrograde protein transport, ER to cytosol	BP	3.01E-01	ERLEC1,UBE2J1,UBE2G2,DERL2	darkgreen	EC_M4
GO:0038093	1.45E-02	0.995094167	10	39	Fc receptor signaling pathway	BP	3.01E-01	PTK2,KIT,SYK,IKBKB,MAP3K1,PLCG1,FGR,PLD2,YES1,PLCG2	turquoise	EC_M6
GO:0002371	1.45E-02	0.997467354	5	13	dendritic cell cytokine production	BP	3.01E-01	DDX58,KIT,TLR4,PLCG2,BST2	turquoise	EC_M6
GO:0010676	1.64E-02	0.994057193	11	40	positive regulation of cellular carbohydrate metabolic process	BP	3.02E-01	DYRK2,PPARA,DGAT2,AKT2,KAT2B,IRS2,SORBS1,PPARGC1A,AKT1,PTPN2,NNMT	darkgreen	EC_M4
GO:1903039	1.64E-02	0.990709402	29	143	positive regulation of leukocyte cell-cell adhesion	BP	3.02E-01	IL23A,HLA-DRA,FAM49B,LGALS1,LEP,DOCK8,NCK2,SOCS5,GLI3,GLI2,HSPH1,CHST2,PTPN11,AP3B1,PTPN22,ADA,RUNX1,PIK3CA,LEF1,TFRC,PTPN6,IGFBP2,CD74,AKT1,CD40LG,IRAK1,BCL10,THY1,HAS2	darkgreen	EC_M4
GO:0006684	1.65E-02	0.997111023	5	12	sphingomyelin metabolic process	BP	3.03E-01	SMPD1,SGMS1,SPTLC2,PEMT,ORMDL3	darkgreen	EC_M4
GO:0051251	1.65E-02	0.990003145	36	186	positive regulation of lymphocyte activation	BP	3.03E-01	IL23A,HLA-DRA,FAM49B,LGALS1,LEP,DOCK8,NCK2,SOCS5,GLI3,GLI2,TGFB1,AXL,IL15RA,IRS2,HSPH1,PTPN11,AP3B1,PTPN22,ADA,RUNX1,SLC39A10,MMP14,PIK3CA,LEF1,TP53BP1,TFRC,PTPN6,IGFBP2,CD74,AKT1,CD40LG,AP1G1,BCL10,THY1,CDKN1A,FGF10	darkgreen	EC_M4
GO:0046467	1.66E-02	0.991057833	25	120	membrane lipid biosynthetic process	BP	3.03E-01	B4GALNT1,LARGE1,TMEM246,ACER3,SMPD1,ST8SIA4,CERS5,PIGP,B3GALT1,HACD2,PIGZ,SGPL1,SGMS1,CLN8,SPTLC2,PIGL,PRKAA1,CSNK1G2,DPM2,HACD1,PEMT,PIGK,DPM1,PIGB,ORMDL3	darkgreen	EC_M4
GO:0006607	1.66E-02	0.997085815	5	12	NLS-bearing protein import into nucleus	BP	3.03E-01	CBLB,KPNA6,KPNA1,RANBP2,KPNA4	darkgreen	EC_M4
GO:0030032	1.66E-02	0.992797307	15	61	lamellipodium assembly	BP	3.03E-01	NCK2,AKIRIN1,ACTR2,ITGB1,ARHGEF4,ACTR3,TWF1,PIK3CA,AUTS2,NCKAP1,ARPC2,CAPZB,VCL,PLXNB3,CDH13	darkgreen	EC_M4
GO:0035305	1.67E-02	0.993942135	11	40	negative regulation of dephosphorylation	BP	3.03E-01	ROCK2,FKBP1B,CRY2,CHP1,TGFB2,GSK3B,PPP1R11,PPP1R36,PPP4R4,ROCK1,FKBP1A	darkgreen	EC_M4
GO:0048311	1.67E-02	0.99636373	6	16	mitochondrion distribution	BP	3.03E-01	TRAK1,SLC4A5,OPA1,DNM1L,MAPT,HAP1	darkgreen	EC_M4

GO:0048519	1.67E-02	0.985438806	583	3961	negative regulation of biological process	BP	3.03E-01	SERPINB2,LMAN1,IL23A,ATP8B1,NAB2,LRP1,B4GALT7,AHI1,AGAP2,ENPP3,TAB2,DYRK2,IER3,CDSN,RRM2B,SLC12A2,AZIN1,PLXNB2,SKIV2L,FBN2,ANGPT1,FAM19A5,PPARA,FAM49B,PACSIN1,ADGRB1,LY6E,PPARD,CTNNA1,CBY1,UBQLN1,LGALS1,SH3GL2,GNAQ,MYH9,LEP,CDKN2B,PLAA,RORB,KIAA1324L,SRF,BAG1,VEGFA,DOCK8,VCP,RUNX2,RASL11B,NCK2,CLOCK,CREB3,ASNS,PDCL3,REST,ASPN,RAB23,SNX13,NLRP5,P3H1,TGFBR1,SOCS5,ERLEC1,COPS9,RTN4,TPBG,SHANK1,TAX1BP1,MFSD2A,UBE2J1,SH3BP5L,AKIRIN1,THAP5,MDFIC,TES,GLI3,ROCK2,INHBA,RRAS,DKKL1,VPS35,SLC7A11,ITGB1,FKBP1B,PLXNA1,FGF2,SRPK2,SESN3,DNAJC1,RAB7A,EML2,ZNF398,RB1,ITM2B,ABHD5,EED,PICALM,TPT1,ZNF296,CRTAP,CGREF1,PTCH2,KCTD21,GLI2,MEGF8,PCDH17,RHEB,PAK1,TGFB1,AXL,SFMBT2,SPOPL,CTNNB1,LBH,DGAT2,SERPINH1,AKT2,DYNC1LI1,GPD1L,ZEB2,C1QTNF1,OSBPL8,CXXC5,RARB,NAV3,NPLOC4,ACTN4,ANAPC15,KAT2B,LTBP1,CRIM1,SPRED3,SPINT2,UFM1,LIG4,IRS2,PRNP,CHFR,NUDT16,ATP2B1,DCN,HDAC3,RAB11FIP1,ZNF703,DCLK1,STARD13,SEMA3C,CAVIN3,DDRGK1,SMPD1,PPP2CB,ILK,LEPROTL1,ARL6IP5,UBA3,DPYSL3,SPINK5,ERI1,APLP1,RYK,TMEM127,CNOT7,RNF6,LMO1,REM1,CXCL12,FXYD1,SACS,ANKRD1,SNTA1,HSP90B1,TMED2,IL20RB,TP53INP2,SLC25A4,ZNF536,EIF4G2,SULF1,VAMP8,CTNND1,LNPEP,DKK3,ROBO1,WWC2,MAP1B,DIP2B,RYR3,PINX1,SEMA4F,LOXL3,P2RX4,FAIM2,PFN2,BCDIN3D,HEY1,RASA1,ATP2A2,TLX2,CELF1,APP,ATXN2,DHX36,RAPGEF2,MAPKAPK5,CX3CL1,ABCC8,CACNB3,PTPN11,PTX3,ADCY6,PIP4P2,RUNX1T1,OPA1,ARHGAP1,AMFR,LOXL2,AMIGO2,PTPN22,T	darkgreen	EC_M4
GO:0034350	1.47E-02	0.998138002	4	9	regulation of glial cell apoptotic process	BP	3.04E-01	AKAP12,PRKCH,CCL2,TRAF2	turquoise	EC_M6
GO:0071453	1.68E-02	0.990502866	29	142	cellular response to oxygen levels	BP	3.04E-01	PPARD,UBQLN1,VEGFA,ROCK2,MGARP,ANKRD1,CACNB3,OPA1,HIF1AN,DNM1L,FAM162A,BMP7,HYOU1,EGLN3,ERO1A,FMN2,SLC2A4,PPARGC1A,CPEB4,KCNMB1,AKT1,PRKAA1,GNB1,VASN,IRAK1,ATP6AP1,ENO1,ATP6V0D1,BNIP3	darkgreen	EC_M4
GO:0019233	1.47E-02	0.994040257	13	63	sensory perception of pain	BP	3.04E-01	MGLL,CXCR4,EDNRB,EPHB1,MMP24,P2RX7,EDN1,ANO1,ZFHX2,MTOR,HOXB8,CHRNB2,CCL2	turquoise	EC_M6
GO:0009159	8.27E-04	0.999921529	6	11	deoxyribonucleoside monophosphate catabolic process	BP	3.05E-01	DNPH1,PNP,XDH,DPYD,NT5C,UPP1	cyan	EC_M2
GO:0010635	1.69E-02	0.997017449	5	12	regulation of mitochondrial fusion	BP	3.05E-01	OPA1,DNM1L,TFRC,OMA1,BNIP3	darkgreen	EC_M4
GO:0030220	1.69E-02	0.996288005	6	16	platelet formation	BP	3.05E-01	MYH9,SRF,PTPN11,PTPN6,WDR1,ACTN1	darkgreen	EC_M4
GO:0036344	1.69E-02	0.996288005	6	16	platelet morphogenesis	BP	3.05E-01	MYH9,SRF,PTPN11,PTPN6,WDR1,ACTN1	darkgreen	EC_M4
GO:0010942	1.48E-02	0.989742763	65	470	positive regulation of cell death	BP	3.05E-01	TNFAIP8,TRIM39,AKAP12,TPD52L1,DAPK1,ATF4,SYK,GADD45G,IL6,UTP11,KLF11,TLR4,MYCN,DNMT3A,CTSC,PPARG,NET1,SFRP1,RPL26,EFNB2,ARHGEF7,CD36,RET,NR4A1,HIP1R,MEF2C,PLCG1,ANO6,IFIT2,SNCA,SIK1,HDAC1,LCK,HYAL2,LAPTM5,INPP5D,IGFBP3,ITGA6,PAK2,MAP3K11,CREB1,ADORA2A,ITGA4,DDX3X,ING4,BID,TLR6,TNFRSF1B,ALDH1A2,MTOR,PSEN1,DAPK2,DNM2,PPP1R13B,CCL2,LGALS9,NOS2,TRAF2,NOTCH1,DAB2IP,MIR23A,PMAIP1,FCER2,E2F3,BMP2	turquoise	EC_M6
GO:0019941	1.70E-02	0.987435852	93	550	modification-dependent protein catabolic process	BP	3.05E-01	CANX,RNF19A,UBQLN1,KLHDC10,PLAA,VCP,C2orf40,CLOCK,PDCL3,SOCS5,ERLEC1,UBA6,UBE2J1,PCYOX1,CUL2,PSMC2,TMUB1,KCTD21,NUB1,UBXN4,SPOPL,CTNNB1,STT3B,NPLOC4,RNF121,PSMD8,CHFR,DDRGK1,PPP2CB,PSMD6,RNF6,VPS37A,HSP90B1,TP53INP2,TRPC4AP,ELOC,RNF13,AMFR,TSG101,BAP1,TTC3,TOLLIP,PSMD14,MARCH6,UCHL5,SELENOS,TRIP12,PSMD1,UBE3B,KCTD10,GSK3B,NEDD8,PPP1R11,PSMD2,ADRM1,UBE4A,RHBDD2,EDEM3,SYVN1,DNAJC10,DNAJB12,USP48,ARAF,SMURF1,FBXL5,JKAMP,ANAPC4,RNF111,TGFB1I1,SPOP,FBXL22,SEL1L,ARIH1,FBXO22,PCYOX1L,USP14,AKT1,PSMD11,UBE2A,TNFAIP1,BCAP31,PARK7,USP10,USP20,HSPA5,FBXO27,UBE2V2,DNAJB9,UBE2D2,DDA1,UBE2G2,MAP1A,DERL2	darkgreen	EC_M4
GO:0051209	1.70E-02	0.991747388	19	82	release of sequestered calcium ion into cytosol	BP	3.05E-01	FKBP1B,FGF2,RYR3,CX3CL1,PKD2,HTR2B,CASQ1,PTPN6,HTT,ERO1A,HAP1,CACNA1C,CALM1,JPH3,DMD,FKBP1A,TMEM38B,THY1,TMEM38A	darkgreen	EC_M4
GO:0005976	1.70E-02	0.991726098	19	83	polysaccharide metabolic process	BP	3.05E-01	DYRK2,TGFB1,AKT2,IRS2,B3GNT4,GYG1,SORBS1,PFKM,SELENOS,GYG2,GSK3B,PPP1R3D,PPP1R3F,CLTC,AKT1,AGL,HAS2,PPP1R1A,HS2ST1	darkgreen	EC_M4
GO:0055010	1.71E-02	0.993798171	11	39	ventricular cardiac muscle tissue morphogenesis	BP	3.05E-01	LY6E,TGFBR1,TGFB1,PKP2,TGFB2,FGFR2,BMPR1A,MED1,TPM1,COL11A1,FKBP1A	darkgreen	EC_M4

GO:1905214	1.60E-02	0.997669133	4	12	regulation of RNA binding	BP	3.06E-01	EIF3E,NCBP1,NUCKS1,FMR1	darkgrey	EC_M8
GO:0019934	1.49E-02	0.996681563	6	19	cGMP-mediated signaling	BP	3.07E-01	EDNRB,PDE2A,CD36,GUCY1B1,PDE9A,NPR1	turquoise	EC_M6
GO:0090136	1.73E-02	0.996179878	6	16	epithelial cell-cell adhesion	BP	3.08E-01	CTNNA1,SRF,BVES,PLEKHA7,ITGB5,VCL	darkgreen	EC_M4
GO:0031099	1.73E-02	0.990033279	30	150	regeneration	BP	3.08E-01	LRP1,PPARD,CTNNA1,LARGE1,AKIRIN1,GLI3,FKBP1B,AXL,CXCL12,MAP1B,SPP1,RUNX1,CCND1,CPT1A,DAG1,KPNA1,FZD9,TNR,PRRX1,NINJ2,MED1,SGCA,MAP2K1,FLNA,PTPN12,THY1,CDKN1A,NNMT,HOPX,FGF10	darkgreen	EC_M4
GO:0032481	1.50E-02	0.994498672	11	48	positive regulation of type I interferon production	BP	3.09E-01	DDX58,SYK,TLR4,TMEM173,TLR2,OAS3,STAT1,DDX3X,ISG15,PLCG2,OAS1	turquoise	EC_M6
GO:0039692	1.62E-02	0.997700431	4	11	single stranded viral RNA replication via double stranded DNA intermediate	BP	3.09E-01	TRIM28,TOP2B,FAM208A,TOP2A	darkgrey	EC_M8
GO:0003176	1.51E-02	0.995220452	9	34	aortic valve development	BP	3.09E-01	NOS3,GATA3,TIE1,JAG1,DLL4,TNFRSF1B,EFNA1,NOTCH1,BMP2	turquoise	EC_M6
GO:0023052	2.05E-05	0.999989508	86	4258	signaling	BP	3.10E-01	DDR1,PRRT1,LRRC4,BRSK1,RIMS3,TBX18,AQP1,ANKRD6,EPHB6,NAMPT,INHBB,GRIK5,SNAP25,PDE6G,PTPRA,IRF7,IL37,CLU,NDRG3,TTI1,LBP,GNAO1,MAPK8IP1,FMOD,NFASC,ESRRG,STAU1,ARHGAP6,OPRD1,SORL1,TEX2,IFI6,SEMA4B,CIDEB,ANXA9,MYOZ2,SNX4,PDE6H,FCER1G,NR1I3,NOS1AP,CALY,FZD5,PTH1R,DUOX1,STOX1,DIXDC1,LPAR2,CPZ,RND2,GPBAR1,CNTNAP1,PRKAG3,ATP1B1,PORCN,PCSK1N,SLC4A3,PADI2,PLEK2,FGFR4,ATP1B2,TOM1L1,EPOR,DRP2,GAS2L2,NF1,PTGER4,AGRN,PRDM16,FOXL1,OSGIN1,VCAM1,CNTNAP4,CDH3,CCR10,EPS8L2,GATA4,DUSP26,TRPM2,APCDD1L,HPCAL4,RTN4RL2,CCDC3,RHOU,HEY2,RGS18	magenta	EC_M13
GO:0043462	1.75E-02	0.993937688	10	37	regulation of ATPase activity	BP	3.10E-01	TPM2,NAPA,ATP1B3,PFN2,BRSK2,DNAJC10,TPM1,TOR1AIP2,GABARAPL2,FGF10	darkgreen	EC_M4
GO:0099003	1.76E-02	0.989815533	31	151	vesicle-mediated transport in synapse	BP	3.11E-01	CANX,PACSIN1,SH3GL2,PLAA,UNC13B,RIMS1,NAPA,PICALM,PCDH17,RAB3GAP1,CTNNB1,ACTG1,PCLO,SNPH,ATP2A2,AP3B1,VPS18,PPP3CC,BRSK2,DNM1L,GSK3B,AP2M1,BACE1,PLD1,SLC2A4,NLGN2,STX1B,SYT11,RAB11A,CALM1,ROCK1	darkgreen	EC_M4
GO:0046633	1.52E-02	0.996021982	7	25	alpha-beta T cell proliferation	BP	3.11E-01	SYK,IL15,ITCH,SH3RF1,ARG2,LGALS9,ELF4	turquoise	EC_M6
GO:0042221	1.52E-02	0.98713611	353	2921	response to chemical	BP	3.11E-01	TNFRSF11A,SEMA6A,KIF5A,IFNGR1,TNFAIP3,AKAP12,FLT4,OXR1,TCF7,NCOA7,PTK2,DAPK1,ATF4,PDGFB,MAPK13,PICK1,KDM4C,MICALL1,PIM1,CSF2RB,TEK,DDX58,DNAJB5,KIT,TNFRSF21,KDR,NFIL3,SYK,SEMA4D,RNF149,IL6,REL,CXCL8,KBTBD2,PELI1,DOCK4,FOXP2,CAV2,KLF11,GRB10,LANCL2,TSPAN12,TLR4,TRAF1,C5,IL15,TEAD2,SDCBP,NRCAM,NOCT,TRPM4,NRP1,PIK3CG,GATA2,DNMT3A,PRKD2,NOVA2,CTSC,LRCH1,PLXND1,PRCP,NOS3,RALB,ZFAND6,PPARG,TIMP4,GATA3,ACVR2B,TBC1D4,CXCR4,IRAK2,EDNRB,NET1,TIE1,IKBKB,SEL1L2,JAG1,P2RY6,P2RY2,NFKBIB,PDE2A,TMEM173,SFRP1,HBEGF,NR1D2,FEZ2,FERMT1,EFNB2,ASH2L,P2RX2,SLC4A11,CD36,ARID5A,PRR5L,SCARB1,EPHB1,RET,TNFRSF19,GRAMD1A,CHD7,IL1A,DNMT3B,IGF1,NR4A1,EIF2AK3,HHEX,KDM3A,ERBIN,UFSP2,SMAD1,OCLN,CHRM5,GPD1,MEF2C,SMARCD1,TLR2,IL7,P2RX7,VEGFC,GUCY1B1,CXCL10,ANGPT2,SOGA1,DHH,KMT2D,TIPARP,OAS3,NLRC5,THBS4,PLCG1,STC1,RAPGEF3,ANKRD2,ST3GAL6,NR4A2,HIPK1,ANO6,DLL4,AMPD1,IFIT2,IFIT1,EDN1,STAT1,CD58,JAM3,EHD4,SNCA,JARID2,DTNBP1,RBBP5,NFKB2,MX2,MX1,ADCY2,ABCG1,ETS1,MTTP,HDAC1,LCK,PPP3CA,HYAL2,ANO1,TESMIN,MEF2A,SEMA3F,SHOC2,RFTN2,RIN3,ROBO4,LYST,LAPTM5,NCF1,USP13,FABP4,PABPN1,HCLS1,NFATC2,CD34,CD86,PLXNA2,POLG,MN1,TGIF1,SH3BP4,CFLAR,ADCY1,KALRN,FES,ARID1A,RBM4,PLEKHA1,PRKD1,LAMA5,YTHDF1,IL10RA,MLXIPL,ITGA6,PAK2,PTPRE,LAMC2,SIPA1,APOD,PDK3,CREB1,B2M,EIF4A2,SSTR1,LTF,CCRL2,ADORA2A,RTP4,ARNT2,SEMA6D,CYBB,IL1RAP,ADAMTS7,EPHB4,ITGA4,DDX3X,IGFBP5,TBX1,MCU,CALCRL,RAMP2,CDK16,	turquoise	EC_M6
GO:0002687	1.53E-02	0.99271418	18	101	positive regulation of leukocyte migration	BP	3.11E-01	PTK2,IL6,CXCL8,VEGFC,CXCL10,THBS4,ANO6,EDN1,JAM3,ITGA4,MCU,SELP,PLVAP,RAC1,PGF,DAPK2,S100A14,LGALS9	turquoise	EC_M6
GO:0051966	1.53E-02	0.994754927	10	40	regulation of synaptic transmission, glutamatergic	BP	3.11E-01	MEF2C,DGKZ,ADORA2A,OPHN1,PSEN1,CCL2,STXBP1,RELN,SHANK3,CACNG5	turquoise	EC_M6

GO:2000696	1.53E-02	0.997222764	5	14	regulation of epithelial cell differentiation involved in kidney development	BP	3.11E-01	GATA3,WWTR1,STAT1,PRKX,PROM1	turquoise	EC_M6
GO:0003014	1.53E-02	0.993298507	15	74	renal system process	BP	3.11E-01	KLHL3,PDGFB,ADGRF5,EDNRB,STC1,EDN1,HYAL2,CD34,MCAM,ADORA2A,WNK4,ITGA3,CHRNB2,NPR1,TBC1D8B	turquoise	EC_M6
GO:0043252	1.54E-02	0.99802353	4	9	sodium-independent organic anion transport	BP	3.11E-01	SLCO2B1,SLCO2A1,SLCO5A1,SLCO4A1	turquoise	EC_M6
GO:0014812	1.54E-02	0.99305181	16	79	muscle cell migration	BP	3.11E-01	PDGFB,DOCK4,GRB10,TLR4,NRP1,NET1,P2RY6,IGF1,MEF2C,NDRG4,THBS4,PLEKHO1,IGFBP3,ACE,SEMA6D,IGFBP5	turquoise	EC_M6
GO:0009125	8.80E-04	0.999885815	7	15	nucleoside monophosphate catabolic process	BP	3.12E-01	DNPH1,PNP,XDH,NT5C2,DPYD,NT5C,UPP1	cyan	EC_M2
GO:0043543	8.88E-04	0.999552704	37	202	protein acylation	BP	3.12E-01	BAG6,JADE2,EP300,HINT2,ING3,KLF15,SPHK2,NAA16,WBP2,TADA3,BRPF1,APBB1,TAF10,CLIP3,SDR16C5,ZDHHC24,GLUL,KAT5,POR,ZDHHC8,EID1,KAT6B,MED24,CTBP1,KAT8,KAT7,PER1,SGF29,NUPR1,CRTC2,SIN3A,CREBBP,WDR5,GTF3C4,ZDHHC12,ZDHHC1,MAP6D1	cyan	EC_M2
GO:0021515	1.77E-02	0.994347959	9	30	cell differentiation in spinal cord	BP	3.12E-01	MDGA1,GLI3,GLI2,TCTN1,SCYL1,MDGA2,CLN8,LMO4,LBX1	darkgreen	EC_M4
GO:0045725	1.77E-02	0.99683532	5	12	positive regulation of glycogen biosynthetic process	BP	3.12E-01	DYRK2,AKT2,IRS2,SORBS1,AKT1	darkgreen	EC_M4
GO:0021953	1.55E-02	0.992009111	22	116	central nervous system neuron differentiation	BP	3.12E-01	AGTPBP1,NRP1,TAL1,GATA2,NOVA2,SPTBN4,EPHB1,DCLK2,NR4A2,DLL4,PROX1,HOXD10,B2M,MPP5,ZC4H2,OPHN1,PSEN1,CHRNB2,B4GALT6,LHX6,SHANK3,GBA2	turquoise	EC_M6
GO:0070979	1.64E-02	0.996058456	6	25	protein K11-linked ubiquitination	BP	3.13E-01	CDC16,ANAPC1,ANAPC7,UBE2C,UBE2T,CDC27	darkgrey	EC_M8
GO:0007154	5.66E-05	0.999970013	85	4300	cell communication	BP	3.13E-01	DDR1,PRRT1,LRRC4,BRSK1,RIMS3,TBX18,AQP1,ANKRD6,EPHB6,NAMPT,INHBB,GRIK5,SNAP25,PDE6G,PTPRA,IRF7,IL37,CLU,NDRG3,TTI1,LBP,GNAO1,MAPK8IP1,FMOD,NFASC,ESRRG,STAU1,ARHGAP6,OPRD1,SORL1,TEX2,IFI6,SEMA4B,CIDEB,ANXA9,MYOZ2,SNX4,PDE6H,FCER1G,NR1I3,NOS1AP,CALY,FZD5,PTH1R,DUOX1,STOX1,DIXDC1,LPAR2,CPZ,RND2,GPBAR1,CNTNAP1,PRKAG3,ATP1B1,PORCN,PCSK1N,PADI2,PLEK2,FGFR4,ATP1B2,TOM1L1,EPOR,DRP2,GAS2L2,NF1,PTGER4,AGRN,PRDM16,FOXL1,OSGIN1,VCAM1,CNTNAP4,CDH3,CCR10,EPS8L2,GATA4,DUSP26,TRPM2,APCDD1L,HPCAL4,RTN4RL2,CCDC3,RHOU,HEY2,RGS18	magenta	EC_M13
GO:0065008	6.22E-05	0.999968692	61	2770	regulation of biological quality	BP	3.13E-01	DDR1,PRRT1,BRSK1,RIMS3,TBX18,AQP1,DDO,NAMPT,SLC30A3,INHBB,GRIK5,CYP2S1,SNAP25,SLC4A9,ACOXL,CHMP4B,SLC35G1,CLU,SHROOM3,SLIT1,CPQ,ESRRG,PRSS56,STAU1,OPRD1,IFI6,SEMA4B,SLC12A8,SNX4,FCER1G,NOS1AP,CALY,PTH1R,DUOX1,STOX1,CPS1,LPAR2,RND2,HGFAC,CNTNAP1,ATP1B1,PORCN,PCSK1N,SLC4A3,FGFR4,ATP1B2,SLC16A2,RNF128,GAS2L2,NF1,C7,PTGER4,AGRN,PRDM16,CDH3,MAFB,CCR10,GATA4,TRPM2,RHOU,HEY2	magenta	EC_M13
GO:0099111	1.78E-02	0.989448528	33	165	microtubule-based transport	BP	3.13E-01	AP3S1,RAB21,SUN2,DYNC1I1,DST,MGARP,KIF5B,COPG1,TRAK1,MAP1B,APP,OPA1,AP3B1,DNAH5,IFT57,ARL3,FEZ1,HSPA8,DYNC1I2,KIF1A,HSPB1,MAPT,HTT,ACTR10,HAP1,IFT43,ARMCX3,PAFAH1B1,VAMP7,HSBP1,RAB6A,BLOC1S2,MAP1A	darkgreen	EC_M4
GO:0043627	1.56E-02	0.993905651	12	57	response to estrogen	BP	3.14E-01	TEK,GATA3,SFRP1,ASH2L,ARID5A,DHH,KMT2D,RBBP5,AR,TRIM24,CA2,SERPINB9	turquoise	EC_M6
GO:0051493	1.56E-02	0.989065347	68	457	regulation of cytoskeleton organization	BP	3.14E-01	CELSR1,MTSS1,ARHGAP18,PTK2,BAIAP2L2,PICK1,CDC42EP1,TEK,LIMCH1,CD2AP,TMOD1,FAM49A,NRP1,SVIL,SPTBN4,FCHSD2,SFRP1,ARHGEF7,SPEF1,FCHSD1,RASSF7,LMOD3,HIP1R,OCLN,P2RX7,RAPGEF3,IQGAP2,CEP97,EDN1,DYRK1A,JAM3,SNCA,RASSF1,ESAM,HCLS1,AP1AR,FES,PROX1,TMOD4,F11R,ARHGDIB,PAK2,DLG1,BAIAP2L1,PPM1F,WAS,TMOD2,RAC1,FSCN1,MTOR,ABI3,TAOK2,ARHGEF15,ARHGAP28,PATJ,SPIRE1,GIT1,S1PR1,CARMIL2,CDH5,VASP,GMFG,KANK3,SHANK3,RGS4,GBA2,BST2,ARPC5L	turquoise	EC_M6

GO:0050673	1.80E-02	0.987824503	57	316	epithelial cell proliferation	BP	3.14E-01	PPARD,C6orf89,LEP,CDKN2B,VEGFA,PDCL3,TGFBR1,RTN4,EMC10,FGF2,RB1,TGFB1,SCN5A,WDR48,CTNNB1,ZNF703,CXCL12,SULF1,ROBO1,LOXL2,IFT57,FAP,KDM5B,NOTCH2,CDC73,HYAL1,CCND1,TGFB2,HTR2B,MMP14,PRKCA,FGFR2,VASH2,HTRA1,NCSTN,SIDT2,PDCD10,PTPRN,SIX4,AKT3,MAGED1,BMPR1A,MED1,NGFR,MAP2K1,AKT1,MYDGF,PLXNB3,CDH13,F3,MIR24-1,SERPINF1,SERPINB5,HAS2,WNT2,COL8A2,FGF10	darkgreen	EC_M4
GO:0010762	1.57E-02	0.994953171	9	35	regulation of fibroblast migration	BP	3.14E-01	AKAP12,PTK2,ARHGEF7,PRR5L,HYAL2,RAC1,RCC2,ADIPOR2,ARHGAP4	turquoise	EC_M6
GO:0072109	1.57E-02	0.997068927	5	15	glomerular mesangium development	BP	3.14E-01	PDGFB,GPR4,CD34,CFLAR,NOTCH1	turquoise	EC_M6
GO:0043030	1.57E-02	0.994888538	9	38	regulation of macrophage activation	BP	3.14E-01	ADGRF5,IL6,TLR4,CST7,CTSC,SNCA,CD200,TLR6,MYO18A	turquoise	EC_M6
GO:0030502	1.58E-02	0.997174308	5	13	negative regulation of bone mineralization	BP	3.15E-01	TRPM4,BCOR,FGF23,HIF1A,RFLNB	turquoise	EC_M6
GO:0050921	1.81E-02	0.99046166	23	109	positive regulation of chemotaxis	BP	3.16E-01	VEGFA,CREB3,TPBG,AKIRIN1,FGF2,MEGF8,TGFB1,CXCL12,P2RX4,DNM1L,CMKLR1,NTRK3,HSPB1,SERPINE1,CD74,PDGFRB,ITGA2,CDH13,F3,ARTN,TMSB4X,CCR6,FGF10	darkgreen	EC_M4
GO:0015711	1.59E-02	0.990358519	36	228	organic anion transport	BP	3.16E-01	TNFRSF11A,APBA1,SYK,SLC3A1,LLGL2,SLCO2B1,SLC4A11,CD36,SLCO2A1,SLCO5A1,SERINC5,SLC38A1,EDN1,SNCA,MTTP,SLC16A14,SLC39A8,SLC7A7,SLC16A9,SLCO4A1,ACE,ADORA2A,SLC2A9,SLC16A13,SLC26A1,PSEN1,SLC51B,CA4,NOS2,SLC43A2,SLC25A25,STXBP1,LRRC8C,LRRC8D,SLC25A21,RGS4	turquoise	EC_M6
GO:0008219	1.59E-02	0.987142557	197	1587	cell death	BP	3.16E-01	TNFAIP8,SEMA6A,TNFAIP3,PLAGL1,TRIM39,AKAP12,FLT4,SNCAIP,OXR1,GRAMD4,TPD52L1,NCOA7,PTK2,DAPK1,ATF4,PIM1,TEK,SH3RF3,KIT,TNFRSF21,KDR,SYK,GADD45G,SEMA4D,SHB,IL6,REL,PPT1,PELI1,UTP11,ELMO1,FIGNL1,KLF11,TLR4,TRAF1,TEAD2,MYCN,NRP1,PIK3CG,GATA2,DNMT3A,PRKD2,CTSC,NOS3,RALB,ZFAND6,PPARG,GATA3,CXCR4,EDNRB,NET1,TMC8,SFRP1,RPL26,EFNB2,ANKLE2,ARHGEF7,SPEF1,CD36,RASSF7,EPHB1,PLK2,RET,MERTK,TNFRSF19,IL1A,IGF1,NR4A1,EIF2AK3,ITCH,SH3RF1,HIP1R,MEF2C,TLR2,IL7,P2RX7,CXCL10,MADD,LMBR1L,PLCG1,NR4A2,HIPK1,ANO6,IFIT2,ADAMTS20,EDN1,STAT1,SNCA,NUAK2,GAS2,PM20D1,MX1,RNF144B,CD200,SIK1,HDAC1,ITPRIP,LCK,HYAL2,MEF2A,LAPTM5,NCF1,DYRK3,HCLS1,INPP5D,EAF2,CD34,BMX,CFLAR,CASP10,IGFBP3,RPS6KA1,PRKD1,DDX42,ITGA6,DOCK1,PAK2,ELL3,ETV6,MAP3K11,CREB1,TRIM69,USP28,LTF,ADORA2A,ARNT2,ITGA4,DDX3X,TBX1,PYGL,MECOM,ING4,RAMP2,BIRC3,TNFAIP8L3,PPM1F,USP42,PRKCH,HIF1A,EPHA2,ITPKB,SNCG,CECR2,BID,TLR6,TNFRSF1B,ARG2,ALDH1A2,MTOR,AR,BNIP2,PSEN1,DAPK2,TAOK2,EFNA1,ATP2A1,PIAS1,S100A14,DNM2,ATG4D,AP2B1,PPP1R13B,CCL2,JAG2,LGALS9,NOS2,IL7R,BFAR,STK26,MYO18A,TNFAIP8L1,ATCAY,TRAF2,TNFRSF25,NOTCH1,PLCG2,STXBP1,DAB2IP,DUS2,CDH5,MIR23A,VNN1,BCL7B,PMAIP1,TRIM24,DIO3,FCER2,DRAM2,SERPINB9,PERP,E2F3,BMP2,XAF1,IFIT3,RNF152,NMNAT1,EVA1A	turquoise	EC_M6
GO:0045669	1.59E-02	0.993785354	12	56	positive regulation of osteoblast differentiation	BP	3.16E-01	CTHRC1,IL6,ACVR2B,JAG1,IGF1,SMAD1,MEF2C,WWTR1,PRKD1,LTF,BMP2,BMP6	turquoise	EC_M6
GO:2001028	1.59E-02	0.997015963	5	15	positive regulation of endothelial cell chemotaxis	BP	3.16E-01	KDR,PRKD2,PRKD1,FGF18,FGF16	turquoise	EC_M6
GO:0033555	1.60E-02	0.99444088	10	42	multicellular organismal response to stress	BP	3.17E-01	GJA4,EDNRB,P2RX2,RET,MEF2C,THBS4,NR4A2,PPP3CA,FBXL20,RELN	turquoise	EC_M6

GO:0010629	1.61E-02	0.987957503	103	766	negative regulation of gene expression	BP	3.18E-01	BAZ2A,TNFAIP3,LAPTM4B,CNOT6,C6orf106,DAPK1,ATF4,PDGFB,IREB2,TNFRSF21,KDR,CD2AP,IL6,IGF2BP3,REL,PTBP3,CXCL8,TLR4,MYCN,NOCT,TUT4,PUM2,EPC1,GATA2,DNMT3A,CST7,PPARG,GATA3,NDFIP2,SFRP1,DND1,FERMT1,CD36,PRR5L,MERTK,IGF1,EIF2AK3,ITCH,ERBIN,SMAD1,OCLN,ENC1,MEF2C,TIPARP,OAS3,DLL4,EDN1,DYRK1A,CD200,HDAC1,KIAA1551,PPP3CA,LAPTM5,INPP5D,CD34,TGIF1,PARP15,RBM4,ZNF148,YTHDF1,HELZ2,ACE,SMG7,APOD,MOV10,CREB1,TET1,LTF,MYEF2,DDX3X,IGFBP5,HOMER3,CPEB2,RAC1,HIF1A,ADIPOR2,EPHA2,CASC3,TLR6,ARG2,MTOR,PSEN1,RPS6KA5,SMAD2,CNOT8,YY1,RCOR1,LGALS9,NOS2,GIT1,TRAF2,NOTCH1,ENG,TERF2,MIR23A,MIR99A,OAS1,CD46,MIR1307,HIST1H3F,BST2,BMP2,RNF125	turquoise	EC_M6
GO:0045010	1.61E-02	0.994359461	10	44	actin nucleation	BP	3.18E-01	PICK1,FCHSD2,LMOD3,HIP1R,IQGAP2,AP1AR,WAS,SPIRE1,GMFG,ARPC5L	turquoise	EC_M6
GO:0045050	1.82E-02	0.997523002	4	9	protein insertion into ER membrane by stop-transfer membrane-anchor sequence	BP	3.19E-01	EMC10,EMC4,EMC1,EMC7	darkgreen	EC_M4
GO:0071326	1.83E-02	0.990207274	24	113	cellular response to monosaccharide stimulus	BP	3.19E-01	LRP1,PPARD,LGALS1,LEP,SRF,UNC13B,FKBP1B,KIF5B,IRS2,OSBP,SELENOT,OPA1,BRSK2,DYNLL1,ZBTB20,LRP5,PIK3CA,ADCY5,SIDT2,PTPRN,PPARGC1A,SMARCA4,PRKAA1,SERPINF1	darkgreen	EC_M4
GO:0006352	1.69E-02	0.992011173	17	105	DNA-templated transcription, initiation	BP	3.19E-01	BCLAF1,ZNF451,TWISTNB,TAF1B,RSF1,PAXIP1,TAF7,ERCC6,SMARCA5,TAF9,BDP1,FOSL1,UBTF,TAF1,GTF2A1,MED13,TFB2M	darkgrey	EC_M8
GO:0050966	1.84E-02	0.996682091	5	12	detection of mechanical stimulus involved in sensory perception of pain	BP	3.19E-01	CXCL12,CACNB3,BACE1,KCNA1,ITGA2	darkgreen	EC_M4
GO:0010575	1.62E-02	0.995778964	7	23	positive regulation of vascular endothelial growth factor production	BP	3.19E-01	FLT4,ATF4,IL6,C5,IL1A,EIF2AK3,HIF1A	turquoise	EC_M6
GO:0010543	1.62E-02	0.995206004	8	30	regulation of platelet activation	BP	3.19E-01	PDGFB,TEC,SYK,IL6,TLR4,NOS3,F11R,SELP	turquoise	EC_M6
GO:0006596	1.86E-02	0.9965404	5	13	polyamine biosynthetic process	BP	3.21E-01	AZIN1,AZIN2,SMS,AGMAT,NNMT	darkgreen	EC_M4
GO:0030206	1.86E-02	0.995844291	6	16	chondroitin sulfate biosynthetic process	BP	3.21E-01	CHPF2,CHSY1,UGDH,CHST12,XYLT2,CHST11	darkgreen	EC_M4
GO:2001242	1.87E-02	0.989537681	27	135	regulation of intrinsic apoptotic signaling pathway	BP	3.21E-01	RRM2B,UBQLN1,NCK2,CREB3,TPT1,CXCL12,OPA1,SERINC3,DNM1L,SELENOS,HYOU1,HSPB1,SYVN1,SKIL,VDAC2,SGMS1,ARHGEF2,TAF9B,CD74,AKT1,PTPN2,BCAP31,PARK7,ENO1,NACC2,PTTG1IP,PLAGL2	darkgreen	EC_M4
GO:0035303	1.87E-02	0.990127481	23	106	regulation of dephosphorylation	BP	3.21E-01	NUAK1,ROCK2,FKBP1B,TGFB1,PPP1R12A,SMPD1,HSP90B1,CRY2,CHP1,SLC39A10,TGFB2,PPP6R3,GSK3B,PPP1R11,HTT,PPP1R36,CALM1,PPP4R4,PIN1,PDGFRB,ROCK1,ITGA2,FKBP1A	darkgreen	EC_M4
GO:0030177	1.87E-02	0.989685883	26	124	positive regulation of Wnt signaling pathway	BP	3.21E-01	RSPO3,VCP,PLEKHA4,FGF2,TGFB1,ZEB2,ILK,SULF1,NKD1,CDC73,CAPRIN2,LEF1,FGFR2,FZD9,ATP6AP2,COL1A1,PBXIP1,SMARCA4,GSKIP,PIN1,DAPK3,CSNK1G2,WLS,EDA,FGF10,MBD2	darkgreen	EC_M4
GO:0060041	1.64E-02	0.991671495	21	108	retina development in camera-type eye	BP	3.21E-01	AGTPBP1,TSPAN12,NRP1,ACVR2B,RET,MERTK,CHD7,GPM6A,USH1C,HIPK1,DLL4,LPCAT1,PROX1,PROM1,HIF1A,RHOJ,PSEN1,ARHGEF15,TTLL5,DIO3,TGIF2	turquoise	EC_M6
GO:0048265	1.64E-02	0.996224939	6	20	response to pain	BP	3.21E-01	GJA4,EDNRB,P2RX2,RET,THBS4,RELN	turquoise	EC_M6
GO:0099024	1.64E-02	0.993906342	11	47	plasma membrane invagination	BP	3.22E-01	ARHGAP25,ELMO1,GATA2,CD36,CLCN3,ANO6,DOCK1,RAC1,SPIRE1,PLCG2,HAVCR1	turquoise	EC_M6
GO:0006612	1.88E-02	0.990363312	21	97	protein targeting to membrane	BP	3.22E-01	SIL1,SRP72,SEC61A1,PAK1,SRP68,AKT2,PRNP,VPS37A,TRAM1,CACNB3,SSR3,CHP1,SRPRA,SRP54,PRKCI,ZDHHC4,SSR2,MIEF2,ZDHHC9,MYO1C,HSPA5	darkgreen	EC_M4
GO:0008209	1.89E-02	0.994963271	7	22	androgen metabolic process	BP	3.22E-01	HSD17B6,SPP1,SGPL1,CYP19A1,HSD17B10,MED1,PPARGC1A	darkgreen	EC_M4
GO:0090594	1.65E-02	0.997721876	4	10	inflammatory response to wounding	BP	3.22E-01	TLR4,IL1A,HIF1A,GIT1	turquoise	EC_M6

GO:0001570	1.65E-02	0.992738609	15	71	vasculogenesis	BP	3.22E-01	QKI,KDR,TEAD2,RASIP1,TIE1,TIPARP,CD34,RAMP2,EPHA2,AMOT,EGFL7,NOTCH1,ENG,SOX17,SOX18	turquoise	EC_M6
GO:0045779	1.66E-02	0.99762967	4	11	negative regulation of bone resorption	BP	3.23E-01	TNFAIP3,IL6,P2RX7,INPP5D	turquoise	EC_M6
GO:0032753	1.66E-02	0.996863292	5	15	positive regulation of interleukin-4 production	BP	3.23E-01	SYK,GATA3,CD86,IL1RAP,LGALS9	turquoise	EC_M6
GO:0015833	1.90E-02	0.988389359	36	186	peptide transport	BP	3.23E-01	LRP1,PPARD,LEP,CLOCK,REST,SLC7A11,FKBP1B,KIF5B,IRS2,PCLO,OSBP,SELENOT,ABCC8,PTPN11,PFKM,CDH17,BRSK2,DYNLL1,CPT1A,LRP5,HADH,DISP1,ADCY5,SIDT2,LINC02210-CRHR1,TFR2,PTPRN,NLGN2,UBE2Q1,SYTL4,ACSL4,CD74,RASL10B,MIDN,SSTR5,PARK7	darkgreen	EC_M4
GO:0006940	1.90E-02	0.992525614	12	46	regulation of smooth muscle contraction	BP	3.23E-01	SRF,ATP2B1,ADA,CTTN,ADRA2C,CNN1,SETD3,MYOCD,ITGA2,DAPK3,ORMDL3,PRKG1	darkgreen	EC_M4
GO:0090022	1.66E-02	0.995506453	7	27	regulation of neutrophil chemotaxis	BP	3.23E-01	CXCL8,THBS4,EDN1,JAM3,MCU,RAC1,DAPK2	turquoise	EC_M6
GO:0002697	1.67E-02	0.990068654	33	206	regulation of immune effector process	BP	3.24E-01	DDX58,KIT,SYK,IL6,RSAD2,TLR4,TRPM4,GATA2,NECTIN2,GAB2,UNC13D,GATA3,CD36,LAPTM5,HLX,NCF1,CD86,FGR,FES,B2M,WAS,TNFRSF1B,LGALS9,NOS2,IL7R,MYO18A,TRAF2,PLCG2,STXBP1,CD46,FCER2,SERPINB9,BST2	turquoise	EC_M6
GO:0050856	1.92E-02	0.993746535	9	31	regulation of T cell receptor signaling pathway	BP	3.24E-01	PRNP,PTPN22,ADA,CBLB,PTPN6,PTPN2,IKBKG,BCL10,THY1	darkgreen	EC_M4
GO:0051968	1.67E-02	0.996174235	6	19	positive regulation of synaptic transmission, glutamatergic	BP	3.24E-01	ADORA2A,CCL2,STXBP1,RELN,SHANK3,CACNG5	turquoise	EC_M6
GO:0003013	1.92E-02	0.986193121	76	437	circulatory system process	BP	3.24E-01	LRP1,SLC12A2,ANGPT1,PPARA,PTP4A3,PPARD,SH3GL2,LEP,VEGFA,COL1A2,MFSD2A,ROCK2,BVES,MYLK3,FKBP1B,TGFB1,SCN5A,GPD1L,ATP2B1,CXCL12,FXYD1,SNTA1,LNPEP,P2RX4,ATP2A2,CACNA1D,SLC4A5,ABCC8,ADCY6,ADA,ATP2B4,RAP1GDS1,TJP1,PKP2,DNM1L,SLC2A10,TGFB2,LRP5,HTR2B,PIK3CA,TFRC,GLRX3,OLR1,PLOD3,ATP6AP2,ADRA2C,COMP,ECE1,ACTA2,SLC6A13,CACNA1C,SLC29A4,HSPB7,SLC2A4,TCAP,NPPB,SLC7A3,KCNMB1,TPM1,CACNA1G,ADRA1B,CALM1,SGCD,RASL10B,ROCK1,AKT1,NPR3,FLNA,DMD,TMEM38B,TMEM38A,HOPX,PIK3C2A,HCN4,PRKG1,KCNMB4	darkgreen	EC_M4
GO:0031644	1.93E-02	0.989958784	22	100	regulation of nervous system process	BP	3.24E-01	UNC13B,RIMS1,SHANK1,ROCK2,TENM4,ACPP,DLGAP4,APP,CACNB3,HOMER1,CHRNA7,DAG1,COMT,TNR,ADRA2C,NLGN2,STX1B,TMEM100,DICER1,BEGAIN,TMEM98,ITGA2	darkgreen	EC_M4
GO:0051646	1.93E-02	0.992418288	12	46	mitochondrion localization	BP	3.24E-01	MGARP,TRAK1,MAP1B,SLC4A5,OPA1,DNM1L,FEZ1,MAPT,ACTR10,HAP1,ARMCX3,HSBP1	darkgreen	EC_M4
GO:0051272	1.93E-02	0.985991881	80	466	positive regulation of cellular component movement	BP	3.24E-01	LRP1,ANGPT1,NUS1,SUN2,PLAA,VEGFA,DOCK8,CREB3,PTP4A1,TGFBR1,RTN4,EMC10,AKIRIN1,ROCK2,RRAS,ITGB1,FGF2,PAK1,TGFB1,AKT2,ACTG1,ACTN4,IRS2,ZNF703,SEMA3C,DDRGK1,ILK,CXCL12,SEMA4F,P2RX4,APP,RAPGEF2,CX3CL1,PGAM4,ACVR1,SEMA4G,TJP1,DNM1L,HYAL1,TGFB2,FEZ1,MMP14,LEF1,PRKCA,CMKLR1,NTRK3,BMP7,IQGAP1,S100A11,LRRC15,FAM89B,HSPB1,SERPINE1,ITGA2B,FN1,PDCD10,ITGAV,AKT3,ARHGEF2,COL1A1,RAB11A,CD74,PDGFRB,AKT1,ITGA2,DAPK3,MYO1C,TNFRSF18,FLNA,CDH13,F3,HSPA5,CD151,THY1,HAS2,TMSB4X,GLIPR2,CCR6,PIK3C2A,FGF10	darkgreen	EC_M4
GO:0031623	1.93E-02	0.990463095	19	85	receptor internalization	BP	3.24E-01	CD63,LRP1,AHI1,ANGPT1,MKLN1,VEGFA,ITGB1,PICALM,ATXN2,HTR2B,GRK3,AP2M1,TFRC,LRPAP1,ATAD1,CLTC,GRIA1,RAB31,LRRTM2	darkgreen	EC_M4
GO:0061008	1.72E-02	0.991447104	19	119	hepaticobiliary system development	BP	3.25E-01	ONECUT2,DDX39B,PNPT1,ZMPSTE24,EZH2,WDR35,CAD,UPF2,E2F7,E2F8,CUL3,PRMT5,SP3,SRSF5,TYMS,NIPBL,ACADM,HNRNPD,AURKA	darkgrey	EC_M8
GO:0061099	1.95E-02	0.994177046	8	26	negative regulation of protein tyrosine kinase activity	BP	3.26E-01	SOCS5,SH3BP5L,TSG101,CBLB,SRCIN1,PTPN2,THY1,DUSP22	darkgreen	EC_M4
GO:0075522	1.73E-02	0.997490679	4	11	IRES-dependent viral translational initiation	BP	3.26E-01	EIF3A,SSB,EIF3B,PTBP1	darkgrey	EC_M8

GO:0010941	1.69E-02	0.986635986	154	1223	regulation of cell death	BP	3.27E-01	TNFAIP8,TNFAIP3,TRIM39,AKAP12,FLT4,OXR1,TPD52L1,NCOA7,PTK2,DAPK1,ATF4,PIM1,TEK,KIT,KDR,SYK,GADD45G,SEMA4D,IL6,REL,PPT1,PELI1,UTP11,FIGNL1,KLF11,TLR4,TRAF1,TEAD2,MYCN,NRP1,PIK3CG,GATA2,DNMT3A,CTSC,NOS3,ZFAND6,PPARG,GATA3,CXCR4,EDNRB,NET1,TMC8,SFRP1,RPL26,EFNB2,ANKLE2,ARHGEF7,SPEF1,CD36,EPHB1,PLK2,RET,MERTK,IL1A,IGF1,NR4A1,EIF2AK3,ITCH,SH3RF1,HIP1R,MEF2C,IL7,CXCL10,MADD,PLCG1,NR4A2,ANO6,IFIT2,ADAMTS20,EDN1,STAT1,SNCA,NUAK2,PM20D1,RNF144B,CD200,SIK1,HDAC1,ITPRIP,LCK,HYAL2,LAPTM5,DYRK3,HCLS1,INPP5D,CD34,CFLAR,CASP10,IGFBP3,RPS6KA1,PRKD1,DDX42,ITGA6,PAK2,ELL3,MAP3K11,CREB1,LTF,ADORA2A,ARNT2,ITGA4,DDX3X,TBX1,MECOM,ING4,RAMP2,BIRC3,TNFAIP8L3,USP42,PRKCH,HIF1A,ITPKB,SNCG,BID,TLR6,TNFRSF1B,ARG2,ALDH1A2,MTOR,AR,BNIP2,PSEN1,DAPK2,EFNA1,PIAS1,DNM2,AP2B1,PPP1R13B,CCL2,LGALS9,NOS2,IL7R,BFAR,STK26,MYO18A,TNFAIP8L1,TRAF2,TNFRSF25,NOTCH1,PLCG2,STXBP1,DAB2IP,DUS2,CDH5,MIR23A,VNN1,PMAIP1,TRIM24,FCER2,SERPINB9,E2F3,BMP2,IFIT3,NMNAT1	turquoise	EC_M6
GO:1902903	1.96E-02	0.986532107	59	327	regulation of supramolecular fiber organization	BP	3.27E-01	FAM49B,NCK2,TGFBR1,SHANK1,ACTR2,ROCK2,MYLK3,EML2,RB1,MAPRE3,PAK1,ACTR3,PLEKHG2,ACTG1,NAV3,CDC42EP3,CXCL12,MAP1B,PFN2,RASA1,APP,CX3CL1,TWF1,MAGEL2,CHRNA7,ARL3,PHLDB2,TJP1,CTTN,LMOD1,PIK3CA,HSPA8,SYNPO2,ARPC5,MAPT,NCKAP1,ARPC2,SYNPO2L,CAPZB,WDR1,CGNL1,ARHGEF2,TPM1,TGFB3,GPR65,TTC8,ROCK1,FLII,TUBB4A,DAPK3,MYO1C,FLNA,PARK7,SPTAN1,TMSB4X,DSTN,MAP1A,COTL1,CFL2	darkgreen	EC_M4
GO:0045454	1.96E-02	0.993534928	9	32	cell redox homeostasis	BP	3.27E-01	TXN2,ERP44,SELENOT,GLRX2,SELENOS,SLC2A10,PRDX4,ERO1A,TXN	darkgreen	EC_M4
GO:0050870	1.70E-02	0.991115891	22	130	positive regulation of T cell activation	BP	3.28E-01	SYK,IL6,IL15,GATA3,IL1A,IGF1,IL7,SOX13,LCK,ICOSLG,HLX,CD86,B2M,ITPKB,EFNB1,CCL2,YES1,LGALS9,IL7R,AP3D1,VNN1,CD46	turquoise	EC_M6
GO:0070424	1.70E-02	0.997636116	4	10	regulation of nucleotide-binding oligomerization domain containing signaling pathway	BP	3.28E-01	TNFAIP3,TLR4,ERBIN,BIRC3	turquoise	EC_M6
GO:0034243	1.74E-02	0.995203374	7	29	regulation of transcription elongation from RNA polymerase II promoter	BP	3.28E-01	EZH2,WDR43,SUPT16H,TCERG1,LEO1,HNRNPU,RNF8	darkgrey	EC_M8
GO:2000117	1.71E-02	0.993227807	12	60	negative regulation of cysteine-type endopeptidase activity	BP	3.28E-01	TNFAIP8,CST7,NR4A1,SH3RF1,SNCA,RPS6KA1,PAK2,LTF,ADORA2A,DDX3X,BIRC3,SERPINB9	turquoise	EC_M6
GO:0030501	1.71E-02	0.99439954	9	37	positive regulation of bone mineralization	BP	3.28E-01	ACVR2B,MEF2C,P2RX7,ANO6,LTF,ADRB2,ISG15,BMP2,BMP6	turquoise	EC_M6
GO:0080134	1.71E-02	0.986758405	126	970	regulation of response to stress	BP	3.29E-01	TNFRSF11A,TNFAIP3,TSPAN8,FLT4,OXR1,TPD52L1,NCOA7,PTK2,C6orf106,ATF4,PDGFB,MAPK13,LRRC19,TEK,DDX58,SH3RF3,SYK,GADD45G,IL6,FIGNL1,TLR4,TRAF1,IL15,SDCBP,PIK3CG,MGLL,KDM4D,CST7,CTSC,GPR4,NECTIN2,NOS3,PPARG,GATA3,CXCR4,SEC14L1,EDNRB,IKBKB,PDE2A,TMEM173,SFRP1,HBEGF,NR1D2,FERMT1,RPL26,CD36,EPHB1,PLK2,TNFRSF19,IGF1,KLKB1,EIF2AK3,ITCH,SH3RF1,OCLN,MCTP1,MEF2C,TLR2,OAS3,NLRC5,PLCG1,ANO6,IFIT1,EDN1,STAT1,DYRK1A,SNCA,ETS1,CD200,PPP3CA,HYAL2,NCF1,DYRK3,USP13,FABP4,CD34,FGR,INPP5F,MAP3K3,PAK2,DLG1,ELL3,MAP3K11,B2M,ADORA2A,MECOM,CALCRL,BIRC3,SELE,CRACR2A,WAS,HIF1A,BCL6B,BID,TLR6,RELL1,TNFRSF1B,ARG2,MTOR,ABI3,TAOK2,VASH1,TNIP1,YES1,LGALS9,BFAR,STK26,SPIRE1,GIT1,ISG15,TRAF2,PLCG2,RFWD3,LRSAM1,TERF2,DAB2IP,CDH5,OAS1,VNN1,PMAIP1,GBP5,TRIM62,SERPINB9,BMP2,SCARF1,RNF125	turquoise	EC_M6
GO:0072594	1.98E-02	0.986247289	62	355	establishment of protein localization to organelle	BP	3.29E-01	LMAN1,ANGPT1,TCP1,NUP50,HSPA4,LEP,PEX6,SRP72,RAB23,COX18,MDFIC,GLI3,VPS41,MGARP,SEC61A1,RAB7A,TGFB1,SRP68,RAB3GAP1,SEC13,UFM1,HDAC3,VPS37A,TRAM1,CCT8,SSR3,ATG13,AP3B1,TSG101,DKC1,CRY2,BAP1,CHP1,CBLB,SRPRA,KPNA6,HSPA8,KPNA1,TOMM40L,SRP54,MAPT,C11orf65,SMURF1,MED1,TSPAN17,RAB11A,CALM1,AKT1,PRKAA1,BCAP31,FLNA,HSPA5,RANBP2,KPNA4,SPCS3,CDKN1A,PTTG1IP,TIMM22,LEPROT,ZPR1,GRPEL2,TOMM7	darkgreen	EC_M4

GO:0071313	1.98E-02	0.997411697	4	8	cellular response to caffeine	BP	3.29E-01	RYR3,PPARGC1A,TMEM38B,TMEM38A	darkgreen	EC_M4
GO:0042246	1.99E-02	0.991758165	13	53	tissue regeneration	BP	3.30E-01	PPARD,LARGE1,AKIRIN1,RUNX1,DAG1,KPNA1,FZD9,NINJ2,SGCA,PTPN12,CDKN1A,HOPX,FGF10	darkgreen	EC_M4
GO:2000242	1.99E-02	0.992498942	11	42	negative regulation of reproductive process	BP	3.30E-01	MYH9,NLRP5,CHFR,SULF1,ADA,PRKAR1A,BMP7,OVOL1,PRDX4,TIMP1,SERPINF1	darkgreen	EC_M4
GO:0033623	1.72E-02	0.996771763	5	14	regulation of integrin activation	BP	3.30E-01	RASIP1,FERMT1,JAM3,SELP,PIEZO1	turquoise	EC_M6
GO:0038084	1.73E-02	0.993938547	10	41	vascular endothelial growth factor signaling pathway	BP	3.30E-01	SEMA6A,FLT4,KDR,TSPAN12,NRP1,PRKD2,VEGFC,PRKD1,PGF,DAB2IP	turquoise	EC_M6
GO:0022407	1.99E-02	0.986591068	53	293	regulation of cell-cell adhesion	BP	3.30E-01	IL23A,CDSN,PPARA,HLA-DRA,FAM49B,LGALS1,MDGA1,LEP,VEGFA,DOCK8,NCK2,SOCS5,GLI3,GLI2,TGFB1,C1QTNF1,SPINT2,PRNP,ZNF703,HSPH1,CXCL12,IL20RB,CHST2,LOXL3,CX3CL1,PTPN11,AP3B1,PTPN22,ADA,RUNX1,CBLB,TJP1,PRKAR1A,PIK3CA,LEF1,PRKCA,BMP7,TFRC,TNR,PTPN6,IGFBP2,CD74,AKT1,PTPN2,CD40LG,MYO10,IRAK1,BCL10,FSTL3,THY1,HAS2,FGL2,PRKG1	darkgreen	EC_M4
GO:2001236	2.00E-02	0.989028665	25	121	regulation of extrinsic apoptotic signaling pathway	BP	3.30E-01	CTNNA1,TGFBR1,INHBA,FAIM2,CX3CL1,ACVR1,CTTN,EYA2,GSK3B,ATF3,PEA15,PDIA3,SERPINE1,ITGAV,HTT,SKIL,ARHGEF2,AKT1,DAPK3,PARK7,BCL10,ITM2C,GDNF,TIMP3,FGF10	darkgreen	EC_M4
GO:0042790	1.76E-02	0.996481588	5	18	nucleolar large rRNA transcription by RNA polymerase I	BP	3.30E-01	PWP1,TAF1B,NCL,NOL11,DDX11	darkgrey	EC_M8
GO:0031401	1.73E-02	0.986972873	102	747	positive regulation of protein modification process	BP	3.31E-01	TNFRSF11A,FLT4,TPD52L1,PTK2,PDGFB,TEK,LIMCH1,TEC,KIT,KDR,SYK,SEMA4D,IL6,PELI1,TLR4,IL15,CCNY,SDCBP,NRP1,PIK3CG,TAL1,PRKD2,CAB39L,RALB,PPARG,GATA3,EDNRB,NDFIP2,HBEGF,ANKLE2,CD36,PRR5L,RET,DNMT3B,IGF1,VEGFC,MADD,THBS4,PPP1R16B,RAPGEF3,NEDD9,EDN1,DYRK1A,EHD4,SNCA,JARID2,HDAC1,LAPTM5,NCF1,HCLS1,PECAM1,CD86,PPP2R5A,PROX1,PRKD1,ACE,LIMK2,PAK2,SENP2,DLG1,MAP3K11,TET1,LTF,DDX3X,TBX1,BIRC3,FGF23,RAC1,ADIPOR2,TLR6,MTOR,RNF40,PSEN1,FGF18,PGF,TAOK2,EFNA1,SLC51B,NAP1L2,PIK3R5,PIAS1,RPS6KA5,TNIP1,ADRB2,YES1,SH2D3A,UNC119,TRAF2,PLCG2,ENG,SH2D3C,MTF2,DAB2IP,CDH5,RELN,ALKAL1,FGF16,MMD,DAZAP2,COPS8,BMP2,BMP6	turquoise	EC_M6
GO:0051130	1.73E-02	0.986697049	119	870	positive regulation of cellular component organization	BP	3.31E-01	CNOT6,CDKL3,MTSS1,ASAP1,PDGFB,BAIAP2L2,PICK1,CDC42EP1,TEK,LINGO2,LIMCH1,ATP8A1,KIT,KDR,SYK,SEMA4D,IL6,PPT1,TLR4,SDCBP,NRP1,RASIP1,TAL1,GATA2,PLXND1,RALB,FCHSD2,SFRP1,FERMT1,ARHGEF7,FCHSD1,CD36,LMOD3,EPHB1,RET,IL1A,IGF1,HIP1R,OCLN,HMBOX1,ENC1,P2RX7,NDRG4,GPM6A,RAPGEF3,IQGAP2,ANO6,EDN1,SNCA,ABCG1,KIAA1551,PPP3CA,ZDHHC6,HCLS1,NFATC2,AP1AR,PLXNA2,CFLAR,FES,PROX1,SS18L1,PRKD1,ITGA6,MELTF,DLG1,CREB1,B2M,IL1RAP,DDX3X,MCU,DNM3,MMP1,SELE,BAIAP2L1,PPM1F,WAS,RAC1,HIF1A,FSCN1,EPHA2,BID,TLR6,MTOR,AR,PSEN1,SEMA4A,ITGA3,ARHGEF15,ZDHHC15,ADCK1,DNM2,AP2B1,ADRB2,SPIRE1,GIT1,SLITRK2,ISG15,PIEZO1,RAPGEF1,PLCG2,LRSAM1,FNBP1L,TERF2,DAB2IP,ADGRL2,CARMIL2,CDH5,PMAIP1,GBP5,VASP,RELN,ALKAL1,GMFG,SHANK3,RALA,BMP2,SCARF1,CBLL1,ARPC5L	turquoise	EC_M6
GO:0070374	1.74E-02	0.990563065	24	145	positive regulation of ERK1 and ERK2 cascade	BP	3.32E-01	TNFRSF11A,AKAP12,FLT4,PDGFB,TEK,KDR,TLR4,NRP1,PRKD2,P2RY6,CD36,IGF1,NDRG4,HCRTR1,CFLAR,TNFAIP8L3,FGF23,FGF18,CCL2,LGALS9,NOTCH1,MIR23A,ALKAL1,BMP2	turquoise	EC_M6
GO:0086003	1.74E-02	0.992846543	13	57	cardiac muscle cell contraction	BP	3.32E-01	TRPM4,PDE4D,STC1,KCNJ3,KCNJ2,DLG1,JUP,ATP2A1,DSG2,DSC2,PDE4B,KCNH2,SCN2B	turquoise	EC_M6
GO:0009162	9.67E-04	0.999844078	8	19	deoxyribonucleoside monophosphate metabolic process	BP	3.32E-01	DNPH1,PNP,XDH,DGUOK,DPYD,TK2,NT5C,UPP1	cyan	EC_M2
GO:0061430	2.03E-02	0.996238394	5	12	bone trabecula morphogenesis	BP	3.33E-01	FBN2,MMP2,THBS3,COL1A1,CHAD	darkgreen	EC_M4
GO:1902236	2.03E-02	0.995273514	6	17	negative regulation of endoplasmic reticulum stress-induced intrinsic apoptotic signaling pathway	BP	3.33E-01	CREB3,OPA1,SELENOS,HYOU1,SYVN1,PARK7	darkgreen	EC_M4

GO:0006887	2.03E-02	0.986548798	49	266	exocytosis	BP	3.33E-01	RAB21,MYH9,UNC13B,EXOC1,REST,NLRP5,RIMS1,VPS41,LIN7B,NAPA,RAB7A,PDCD6IP,PAK1,RAB3GAP1,RAB2B,RAB11FIP1,PCLO,SNPH,VAMP8,ATP2A2,EXOC6B,TSG101,VPS18,LGI3,BRSK2,DNM1L,GSK3B,BACE1,EXOC5,HAP1,SRCIN1,STX1B,SYT11,TMED10,CACNA1G,RAB11A,CALM1,SYTL4,SCAMP5,RAB31,SDF4,ATP6AP1,VAMP7,AP1G1,CBARP,TMEM167A,STXBP6,PIK3C2A,EXOC3L4	darkgreen	EC_M4
GO:0014841	2.04E-02	0.996219244	5	12	skeletal muscle satellite cell proliferation	BP	3.33E-01	ANGPT1,PPARD,AKIRIN1,FGF2,KPNA1	darkgreen	EC_M4
GO:0014856	2.04E-02	0.996219244	5	12	skeletal muscle cell proliferation	BP	3.33E-01	ANGPT1,PPARD,AKIRIN1,FGF2,KPNA1	darkgreen	EC_M4
GO:1902373	1.78E-02	0.99358728	10	49	negative regulation of mRNA catabolic process	BP	3.34E-01	SYNCRIP,THRAP3,DHX9,HNRNPU,FUS,LARP1,TAF15,FMR1,METTL16,HNRNPD	darkgrey	EC_M8
GO:0052547	2.05E-02	0.986223239	52	294	regulation of peptidase activity	BP	3.34E-01	SERPINB2,LRP1,MTCH1,VEGFA,VCP,REST,PSME4,CYCS,ROCK2,RCN3,PICALM,SERPINH1,CRIM1,SPINT2,PRNP,DDRGK1,ARL6IP5,SPINK5,ROBO1,APP,IFT57,TANK,PSMD14,UCHL5,ANOS1,FAM162A,COL6A3,NCSTN,ADRM1,RPS6KA3,EGLN3,MAPT,SERPINE1,PCOLCE,FN1,TIMP1,NGFR,TMED10,UACA,ROCK1,USP14,AKT1,BCAP31,PARK7,F3,BCL10,MIR24-1,SPINK2,SERPINF1,SERPINB5,A2M,TIMP3	darkgreen	EC_M4
GO:0032890	2.05E-02	0.991827092	12	47	regulation of organic acid transport	BP	3.34E-01	SLC12A2,PPARA,LEP,ITGB1,RAB3GAP1,AKT2,IRS2,ARL6IP5,P2RX4,PLA2R1,EPRS,AKT1	darkgreen	EC_M4
GO:0043632	2.05E-02	0.984674172	94	561	modification-dependent macromolecule catabolic process	BP	3.34E-01	CANX,RNF19A,UBQLN1,KLHDC10,PLAA,VCP,C2orf40,CLOCK,PDCL3,SOCS5,ERLEC1,UBA6,UBE2J1,PCYOX1,CUL2,PSMC2,TMUB1,KCTD21,NUB1,UBXN4,SPOPL,CTNNB1,STT3B,NPLOC4,RNF121,PSMD8,CHFR,DDRGK1,PPP2CB,PSMD6,RNF6,VPS37A,HSP90B1,TP53INP2,TRPC4AP,ELOC,RNF13,AMFR,TSG101,BAP1,TTC3,TENT4B,TOLLIP,PSMD14,MARCH6,UCHL5,SELENOS,TRIP12,PSMD1,UBE3B,KCTD10,GSK3B,NEDD8,PPP1R11,PSMD2,ADRM1,UBE4A,RHBDD2,EDEM3,SYVN1,DNAJC10,DNAJB12,USP48,ARAF,SMURF1,FBXL5,JKAMP,ANAPC4,RNF111,TGFB1I1,SPOP,FBXL22,SEL1L,ARIH1,FBXO22,PCYOX1L,USP14,AKT1,PSMD11,UBE2A,TNFAIP1,BCAP31,PARK7,USP10,USP20,HSPA5,FBXO27,UBE2V2,DNAJB9,UBE2D2,DDA1,UBE2G2,MAP1A,DERL2	darkgreen	EC_M4
GO:0071604	1.76E-02	0.994235549	9	35	transforming growth factor beta production	BP	3.34E-01	LAPTM4B,CD2AP,FERMT1,CD200,CD34,CREB1,HIF1A,LGALS9,CD46	turquoise	EC_M6
GO:0098810	2.06E-02	0.995177277	6	17	neurotransmitter reuptake	BP	3.35E-01	ITGB1,SLC6A13,SLC29A4,CLN8,PARK7,GDNF	darkgreen	EC_M4
GO:0046916	2.07E-02	0.989714251	19	86	cellular transition metal ion homeostasis	BP	3.35E-01	SLC39A7,SLC39A4,SLC30A9,SLC31A1,ATP7B,PRNP,APP,AP3B1,SLC39A10,ISCU,HJV,TFRC,GLRX3,TFR2,SLC39A6,ATP6AP1,SLC30A7,ATP6V0D1,SLC22A17	darkgreen	EC_M4
GO:0005977	2.08E-02	0.991029785	14	57	glycogen metabolic process	BP	3.35E-01	DYRK2,AKT2,IRS2,GYG1,SORBS1,PFKM,SELENOS,GYG2,GSK3B,PPP1R3D,PPP1R3F,AKT1,AGL,PPP1R1A	darkgreen	EC_M4
GO:0043588	2.08E-02	0.987422977	34	171	skin development	BP	3.35E-01	CDSN,SRF,COL1A2,ROCK2,INHBA,PTCH2,GLI2,WDR48,CTNNB1,SPINK5,PALLD,FLNB,TRPC4AP,AP3B1,TSG101,SLC2A10,TGFB2,FGFR2,CLIC4,OVOL1,COMP,COL3A1,COL5A2,GORAB,MED1,NGFR,COL1A1,MAP2K1,ROCK1,ITGA2,PKD1,COL5A1,EDA,FGF10	darkgreen	EC_M4
GO:0090140	2.09E-02	0.993088976	9	31	regulation of mitochondrial fission	BP	3.36E-01	FAM49B,VPS35,DCN,DNM1L,MAPT,C11orf65,PPARGC1A,MIEF2,BNIP3	darkgreen	EC_M4
GO:0043270	1.78E-02	0.989679316	30	182	positive regulation of ion transport	BP	3.37E-01	TNFRSF11A,TRDN,PDGFB,RAB3B,P2RY6,P2RX2,P2RX7,CXCL10,PLCG1,STC1,ANO6,EDN1,SNCA,KCNJ2,DLG1,ADORA2A,WNK4,CRACR2A,PSEN1,CHRNB2,ATP2A1,CD19,DNM2,ADRB2,CCL2,PLCG2,STXBP1,RELN,KCNH2,SHANK3	turquoise	EC_M6
GO:0030574	2.11E-02	0.993012642	9	31	collagen catabolic process	BP	3.37E-01	ADAMTS3,ITGB1,MMP17,MMP15,MMP2,FAP,MMP14,MRC2,MMP23B	darkgreen	EC_M4
GO:0007212	2.11E-02	0.99300549	9	31	dopamine receptor signaling pathway	BP	3.37E-01	VPS35,ADCY6,GSK3B,ADCY5,NCSTN,GNAL,GNB1,FLNA,GNAI3	darkgreen	EC_M4

GO:0045893	1.79E-02	0.985742589	162	1244	positive regulation of transcription, DNA-templated	BP	3.38E-01	ZNF516,TCF4,PLAGL1,CNOT6,ZBED4,NOTCH4,TCF20,NCOA7,ATF4,PDGFB,PIM1,NFYA,PRDM4,DDX58,NFIL3,IL6,REL,CREB5,TLR4,TEAD2,MYCN,TAL1,PHF10,EPC1,GATA2,PRKD2,PPARG,GATA3,IKBKB,JAG1,NFKBIB,TMEM173,SFRP1,NR1D2,TCF15,RET,CHD7,IL1A,IGF1,NR4A1,EIF2AK3,HHEX,KDM3A,SMAD1,ZBTB38,STOX2,SOX7,HMBOX1,HEXB,MEF2C,SMARCD1,TLR2,WWTR1,CXCL10,KMT2D,NLRC5,NR4A2,TOX2,YAF2,EDN1,STAT1,DYRK1A,ERG,ETS2,NFKB2,PRDM15,ETS1,HDAC1,PPP3CA,HYAL2,MEF2A,BCL9,NCF1,HCLS1,NFATC2,EAF2,GMEB1,CD86,POU2F3,ARID1A,RPS6KA1,PROX1,SS18L1,PRKD1,ZNF148,HELZ2,MLXIPL,ITGA6,ELL3,ETV6,HOXD10,CREB1,TET1,ARNT2,DDX3X,RFXANK,ETV4,TBX1,MECOM,ING4,LDB2,FGF23,POU2F1,WAS,HIF1A,KDM5A,ZBTB18,APBB2,JUP,MTOR,AR,CASZ1,PSEN1,HOXB5,HOXB7,HOXB9,FBXW11,BATF,PIAS1,RPS6KA5,SMAD2,DNM2,YY1,ARID3B,LYL1,TNIP1,ZNF521,ABLIM3,ADRB2,YES1,RAI1,ELF4,MRTFB,DOT1L,AP3D1,HCFC1,NOTCH1,MED16,IRF8,S1PR1,ENG,MTF2,NFAT5,DAB2IP,ARID4A,ZNF24,TRIM24,SOX17,KCNH2,ZNF850,ANKRD49,CDK13,SOX18,MAFF,TRIM62,ZNF229,E2F3,BMP2,GLIS3,ZNF71,BMP6,FLI1	turquoise	EC_M6
GO:1903508	1.79E-02	0.985742589	162	1244	positive regulation of nucleic acid-templated transcription	BP	3.38E-01	ZNF516,TCF4,PLAGL1,CNOT6,ZBED4,NOTCH4,TCF20,NCOA7,ATF4,PDGFB,PIM1,NFYA,PRDM4,DDX58,NFIL3,IL6,REL,CREB5,TLR4,TEAD2,MYCN,TAL1,PHF10,EPC1,GATA2,PRKD2,PPARG,GATA3,IKBKB,JAG1,NFKBIB,TMEM173,SFRP1,NR1D2,TCF15,RET,CHD7,IL1A,IGF1,NR4A1,EIF2AK3,HHEX,KDM3A,SMAD1,ZBTB38,STOX2,SOX7,HMBOX1,HEXB,MEF2C,SMARCD1,TLR2,WWTR1,CXCL10,KMT2D,NLRC5,NR4A2,TOX2,YAF2,EDN1,STAT1,DYRK1A,ERG,ETS2,NFKB2,PRDM15,ETS1,HDAC1,PPP3CA,HYAL2,MEF2A,BCL9,NCF1,HCLS1,NFATC2,EAF2,GMEB1,CD86,POU2F3,ARID1A,RPS6KA1,PROX1,SS18L1,PRKD1,ZNF148,HELZ2,MLXIPL,ITGA6,ELL3,ETV6,HOXD10,CREB1,TET1,ARNT2,DDX3X,RFXANK,ETV4,TBX1,MECOM,ING4,LDB2,FGF23,POU2F1,WAS,HIF1A,KDM5A,ZBTB18,APBB2,JUP,MTOR,AR,CASZ1,PSEN1,HOXB5,HOXB7,HOXB9,FBXW11,BATF,PIAS1,RPS6KA5,SMAD2,DNM2,YY1,ARID3B,LYL1,TNIP1,ZNF521,ABLIM3,ADRB2,YES1,RAI1,ELF4,MRTFB,DOT1L,AP3D1,HCFC1,NOTCH1,MED16,IRF8,S1PR1,ENG,MTF2,NFAT5,DAB2IP,ARID4A,ZNF24,TRIM24,SOX17,KCNH2,ZNF850,ANKRD49,CDK13,SOX18,MAFF,TRIM62,ZNF229,E2F3,BMP2,GLIS3,ZNF71,BMP6,FLI1	turquoise	EC_M6
GO:0060078	1.79E-02	0.991558466	17	85	regulation of postsynaptic membrane potential	BP	3.38E-01	RIMS2,GRIK2,MTMR2,P2RX2,MEF2C,P2RX7,SNCA,PPP3CA,INSYN2,ADORA2A,CHRNB1,CHRNB2,ADRB2,SLC8A3,RELN,SHANK3,RGS4	turquoise	EC_M6
GO:0030183	1.80E-02	0.991284526	18	96	B cell differentiation	BP	3.39E-01	MFNG,KIT,SYK,IL6,SFRP1,HHEX,IL7,TPD52,ADGRG3,DCAF1,INPP5D,ITGA4,LFNG,CD19,YY1,LYL1,IRF8,PLCG2	turquoise	EC_M6
GO:0002830	1.81E-02	0.99734691	4	11	positive regulation of type 2 immune response	BP	3.39E-01	IL6,RSAD2,GATA3,CD86	turquoise	EC_M6
GO:0070252	1.81E-02	0.991967744	15	70	actin-mediated cell contraction	BP	3.39E-01	LIMCH1,TRPM4,PDE4D,STC1,KCNJ3,KCNJ2,DLG1,JUP,ATP2A1,CCDC88C,DSG2,DSC2,PDE4B,KCNH2,SCN2B	turquoise	EC_M6
GO:0061050	1.81E-02	0.99643856	5	16	regulation of cell growth involved in cardiac muscle cell development	BP	3.39E-01	IGF1,EDN1,MTOR,YY1,RGS4	turquoise	EC_M6
GO:0019079	1.81E-02	0.991175998	18	99	viral genome replication	BP	3.39E-01	CXCL8,PPIE,RSAD2,OAS3,IFIT1,MX1,KIAA1551,ISG20,PROX1,LTF,DDX3X,TNIP1,CCL2,ISG15,NOTCH1,OAS1,TBC1D20,BST2	turquoise	EC_M6
GO:0042752	1.82E-02	0.991407514	17	89	regulation of circadian rhythm	BP	3.39E-01	CSNK1E,NOCT,PPARG,PPP1CB,NR1D2,SIK1,KDM2A,ADCY1,RBM4,PROX1,CREB1,ADORA2A,MTOR,FBXW11,CHRNB2,USP7,PASD1	turquoise	EC_M6
GO:0006527	1.82E-02	0.997417964	4	10	arginine catabolic process	BP	3.39E-01	NOS3,FAH,ARG2,NOS2	turquoise	EC_M6
GO:0008306	1.83E-02	0.992744154	12	53	associative learning	BP	3.39E-01	KIT,PPT1,RIC8A,NDRG4,TANC1,CREB1,HIF1A,RGS14,MTOR,CHRNB2,PIAS1,RELN	turquoise	EC_M6
GO:0060982	1.83E-02	0.997513026	4	9	coronary artery morphogenesis	BP	3.39E-01	NRP1,SEC24B,TBX1,NOTCH1	turquoise	EC_M6
GO:1904994	1.84E-02	0.995611672	6	21	regulation of leukocyte adhesion to vascular endothelial cell	BP	3.39E-01	IL6,ETS1,ITGA4,SELE,SELP,NFAT5	turquoise	EC_M6
GO:0070372	1.84E-02	0.988952139	33	211	regulation of ERK1 and ERK2 cascade	BP	3.39E-01	TNFRSF11A,SEMA6A,AKAP12,FLT4,PDGFB,TEK,KDR,SYK,TLR4,NRP1,PRKD2,P2RY6,CD36,SIRT3,EPHB1,IGF1,NDRG4,HCRTR1,CFLAR,DLG1,TNFAIP8L3,FGF23,EPHA2,RGS14,FGF18,TNIP1,CCL2,LGALS9,NOTCH1,DAB2IP,MIR23A,ALKAL1,BMP2	turquoise	EC_M6

GO:0050926	1.85E-02	0.9955681	6	21	regulation of positive chemotaxis	BP	3.39E-01	KDR,CXCL8,VEGFC,ANGPT2,PGF,S1PR1	turquoise	EC_M6
GO:0006140	1.85E-02	0.992582641	12	57	regulation of nucleotide metabolic process	BP	3.39E-01	NOS3,IGF1,GPD1,P2RX7,SNCA,MLXIPL,PDK3,CDA,HIF1A,PSEN1,NOS2,GIT1	turquoise	EC_M6
GO:0032608	1.86E-02	0.993346563	10	44	interferon-beta production	BP	3.39E-01	DDX58,REL,TLR4,TMEM173,TLR2,OAS3,DDX3X,YY1,ISG15,OAS1	turquoise	EC_M6
GO:0032648	1.86E-02	0.993346563	10	44	regulation of interferon-beta production	BP	3.39E-01	DDX58,REL,TLR4,TMEM173,TLR2,OAS3,DDX3X,YY1,ISG15,OAS1	turquoise	EC_M6
GO:0060485	1.86E-02	0.988425923	38	235	mesenchyme development	BP	3.40E-01	SEMA6A,NOTCH4,SEMA4D,IL6,TEAD2,SDCBP,NRP1,NOS3,GATA3,EDNRB,JAG1,SFRP1,TCF15,RET,MEF2C,WWTR1,EDN1,STAT1,DPPA4,SEMA3F,LAMA5,ELL3,SEMA6D,TBX1,HIF1A,ALDH1A2,MTOR,EFNB1,SEMA4A,EFNA1,SMAD2,SEMA6B,RFLNB,NOTCH1,ENG,DAB2IP,TRIM62,BMP2	turquoise	EC_M6
GO:2001238	2.13E-02	0.992342218	10	37	positive regulation of extrinsic apoptotic signaling pathway	BP	3.40E-01	CTNNA1,INHBA,ATF3,PEA15,PDIA3,SKIL,DAPK3,BCL10,ITM2C,TIMP3	darkgreen	EC_M4
GO:0003170	1.87E-02	0.992262641	13	58	heart valve development	BP	3.40E-01	NOS3,GATA3,TIE1,JAG1,PDE2A,ADAMTS9,MEF2C,DLL4,TNFRSF1B,MTOR,EFNA1,NOTCH1,BMP2	turquoise	EC_M6
GO:0038084	2.14E-02	0.991878461	11	41	vascular endothelial growth factor signaling pathway	BP	3.40E-01	CD63,NUS1,PTP4A3,VEGFA,ADAMTS3,DCN,ROBO1,PIK3CA,HSPB1,PDGFRB,MYO1C	darkgreen	EC_M4
GO:0007612	1.87E-02	0.990508988	20	104	learning	BP	3.41E-01	ATP8A1,KIT,PPT1,RIC8A,TLR2,NDRG4,TANC1,SHANK2,YTHDF1,CREB1,HIF1A,RGS14,MTOR,CHRNB2,PIAS1,GIT1,SLC8A3,ATXN1L,RELN,SHANK3	turquoise	EC_M6
GO:1902680	1.88E-02	0.985044948	162	1246	positive regulation of RNA biosynthetic process	BP	3.41E-01	ZNF516,TCF4,PLAGL1,CNOT6,ZBED4,NOTCH4,TCF20,NCOA7,ATF4,PDGFB,PIM1,NFYA,PRDM4,DDX58,NFIL3,IL6,REL,CREB5,TLR4,TEAD2,MYCN,TAL1,PHF10,EPC1,GATA2,PRKD2,PPARG,GATA3,IKBKB,JAG1,NFKBIB,TMEM173,SFRP1,NR1D2,TCF15,RET,CHD7,IL1A,IGF1,NR4A1,EIF2AK3,HHEX,KDM3A,SMAD1,ZBTB38,STOX2,SOX7,HMBOX1,HEXB,MEF2C,SMARCD1,TLR2,WWTR1,CXCL10,KMT2D,NLRC5,NR4A2,TOX2,YAF2,EDN1,STAT1,DYRK1A,ERG,ETS2,NFKB2,PRDM15,ETS1,HDAC1,PPP3CA,HYAL2,MEF2A,BCL9,NCF1,HCLS1,NFATC2,EAF2,GMEB1,CD86,POU2F3,ARID1A,RPS6KA1,PROX1,SS18L1,PRKD1,ZNF148,HELZ2,MLXIPL,ITGA6,ELL3,ETV6,HOXD10,CREB1,TET1,ARNT2,DDX3X,RFXANK,ETV4,TBX1,MECOM,ING4,LDB2,FGF23,POU2F1,WAS,HIF1A,KDM5A,ZBTB18,APBB2,JUP,MTOR,AR,CASZ1,PSEN1,HOXB5,HOXB7,HOXB9,FBXW11,BATF,PIAS1,RPS6KA5,SMAD2,DNM2,YY1,ARID3B,LYL1,TNIP1,ZNF521,ABLIM3,ADRB2,YES1,RAI1,ELF4,MRTFB,DOT1L,AP3D1,HCFC1,NOTCH1,MED16,IRF8,S1PR1,ENG,MTF2,NFAT5,DAB2IP,ARID4A,ZNF24,TRIM24,SOX17,KCNH2,ZNF850,ANKRD49,CDK13,SOX18,MAFF,TRIM62,ZNF229,E2F3,BMP2,GLIS3,ZNF71,BMP6,FLI1	turquoise	EC_M6
GO:0030593	1.88E-02	0.992445885	12	60	neutrophil chemotaxis	BP	3.41E-01	SYK,CXCL8,PIK3CG,CXCL10,THBS4,EDN1,JAM3,MCU,RAC1,DAPK2,CCL2,PDE4B	turquoise	EC_M6
GO:0007252	2.15E-02	0.994937828	6	17	I-kappaB phosphorylation	BP	3.41E-01	MAP3K7,DDRGK1,CX3CL1,PRDX4,ERC1,AKT1	darkgreen	EC_M4
GO:0099054	2.15E-02	0.99233105	10	35	presynapse assembly	BP	3.41E-01	MDGA1,LRRC4B,VPS35,PCDH17,FARP1,PCLO,APP,NTRK3,NLGN2,NTNG2	darkgreen	EC_M4
GO:0042447	2.16E-02	0.996914733	4	9	hormone catabolic process	BP	3.41E-01	HSD17B6,SPP1,ECE1,CYP19A1	darkgreen	EC_M4
GO:0050974	2.16E-02	0.993424248	8	26	detection of mechanical stimulus involved in sensory perception	BP	3.41E-01	WHRN,CXCL12,CACNB3,BACE1,KCNK4,KCNA1,ITGA2,COL11A1	darkgreen	EC_M4
GO:1900242	2.16E-02	0.99489369	6	17	regulation of synaptic vesicle endocytosis	BP	3.41E-01	PPP3CC,DNM1L,AP2M1,SYT11,CALM1,ROCK1	darkgreen	EC_M4

GO:0006511	2.18E-02	0.983809516	91	542	ubiquitin-dependent protein catabolic process	BP	3.42E-01	CANX,RNF19A,UBQLN1,KLHDC10,PLAA,VCP,C2orf40,CLOCK,PDCL3,SOCS5,ERLEC1,UBA6,UBE2J1,CUL2,PSMC2,TMUB1,KCTD21,NUB1,UBXN4,SPOPL,CTNNB1,STT3B,NPLOC4,RNF121,PSMD8,CHFR,DDRGK1,PPP2CB,PSMD6,RNF6,VPS37A,HSP90B1,TP53INP2,TRPC4AP,ELOC,RNF13,AMFR,TSG101,BAP1,TTC3,TOLLIP,PSMD14,MARCH6,UCHL5,SELENOS,TRIP12,PSMD1,UBE3B,KCTD10,GSK3B,NEDD8,PPP1R11,PSMD2,ADRM1,UBE4A,RHBDD2,EDEM3,SYVN1,DNAJC10,DNAJB12,USP48,ARAF,SMURF1,FBXL5,JKAMP,ANAPC4,RNF111,TGFB1I1,SPOP,FBXL22,SEL1L,ARIH1,FBXO22,USP14,AKT1,PSMD11,UBE2A,TNFAIP1,BCAP31,PARK7,USP10,USP20,HSPA5,FBXO27,UBE2V2,DNAJB9,UBE2D2,DDA1,UBE2G2,MAP1A,DERL2	darkgreen	EC_M4
GO:0035904	1.89E-02	0.992505092	12	50	aorta development	BP	3.43E-01	PLXND1,JAG1,PDE2A,ADAMTS9,CHD7,DLL4,SEC24B,PROX1,TBX1,AP2B1,NOTCH1,ENG	turquoise	EC_M6
GO:0042492	1.90E-02	0.997394911	4	9	gamma-delta T cell differentiation	BP	3.43E-01	TCF7,SYK,SOX13,JAG2	turquoise	EC_M6
GO:0048699	1.90E-02	0.984896037	160	1177	generation of neurons	BP	3.43E-01	TCF4,SEMA6A,KIF5A,CTHRC1,RIMS2,CELSR1,CDKL3,ASAP1,PTK2,ANKS1A,AGTPBP1,CSNK1E,PICK1,KDM4C,MICALL1,KIT,ADGRF1,TNFRSF21,SEMA4D,IL6,PPT1,FZD8,MYCN,NRCAM,NRP1,ITSN2,TAL1,MTMR2,GATA2,DNMT3A,HS6ST1,NOVA2,PLXND1,GATA3,MYO7A,CXCR4,EDNRB,SPTBN4,JAG1,SFRP1,FEZ2,EFNB2,PCDH12,NTN4,PRAG1,EPHB1,PLK2,RET,CHD7,DNMT3B,SH3RF1,PREX2,DCLK2,KIF13B,ENC1,MEF2C,TLR2,VEGFC,NDRG4,GPM6A,USH1C,NR4A2,HIPK1,DLL4,TSPAN2,JAM3,DTNBP1,MYLIP,SRGAP2C,HDAC1,PPP3CA,HECW2,MEF2A,SEMA3F,ROBO4,SEC24B,ZFHX2,PLXNA2,CFLAR,INPP5F,TRAK2,ADCY1,KALRN,FES,PROX1,SS18L1,PRKD1,LAMA5,YTHDF1,ITGA6,PAK2,LAMC2,ELL3,APOD,HOXD10,MOV10,CREB1,B2M,ADORA2A,SEMA6D,MYEF2,EPHB4,ITGA4,DNM3,CDK16,PROM1,LMTK2,PRKCH,RAC1,HIF1A,EPHA2,CECR2,MPP5,TNFRSF1B,RGS14,ALDH1A2,ZC4H2,MTOR,OPHN1,EFNB1,CASZ1,PSEN1,SEMA4A,ABI3,ITGA3,TAOK2,EFNA1,CHRNB2,NAP1L2,ZDHHC15,RPS6KA5,DNM2,B4GALT6,HMG20A,ZNF521,JAG2,GIT1,SEMA6B,SLITRK2,ATCAY,ARHGAP4,NOTCH1,RAPGEF1,S1PR1,STXBP1,LHX6,DAB2IP,ADGRG1,VASP,RELN,ALKAL1,DIO3,SHANK3,MMD,GBA2,STX3,TGIF2,BMP2,SCARF1,BMP6	turquoise	EC_M6
GO:0050777	1.90E-02	0.989964465	22	128	negative regulation of immune response	BP	3.43E-01	TNFAIP3,C6orf106,SYK,PPARG,SEC14L1,PLK2,ITCH,OAS3,NLRC5,PLCG1,HLX,INPP5D,FGR,ARG2,YES1,LGALS9,IL7R,ISG15,OAS1,CD46,SERPINB9,RNF125	turquoise	EC_M6
GO:0060395	2.18E-02	0.990201456	15	62	SMAD protein signal transduction	BP	3.43E-01	LRP1,BTBD11,TGFBR1,INHBA,TGFB1,SMAD9,RBPMS,SLC33A1,GDF6,SLC2A10,TGFB2,BMP7,FAM89B,BMPR1A,TGFB3	darkgreen	EC_M4
GO:1901857	2.19E-02	0.996856142	4	9	positive regulation of cellular respiration	BP	3.44E-01	VCP,ISCU,PPARGC1A,PARK7	darkgreen	EC_M4
GO:0034504	2.19E-02	0.985717066	45	244	protein localization to nucleus	BP	3.44E-01	ANGPT1,TCP1,NUP50,CTNNA1,SUN2,LEP,RAB23,MDFIC,GLI3,TGFB1,SEC13,OSBPL8,UFM1,HDAC3,DCLK1,PINX1,CCT8,DKC1,CRY2,CHP1,CBLB,KPNA6,GSK3B,LARP7,KPNA1,BMP7,TFRC,SIX4,MAGED1,MED1,NGFR,COL1A1,PIN1,AKT1,TMEM98,FLNA,PARK7,RANBP2,KPNA4,TOR1AIP2,CDKN1A,TXN,PTTG1IP,ZPR1,ORMDL3	darkgreen	EC_M4
GO:0042129	1.91E-02	0.990649322	18	102	regulation of T cell proliferation	BP	3.44E-01	TNFRSF21,SYK,IL6,PELI1,IL15,IL1A,IGF1,ITCH,SH3RF1,ICOSLG,CD86,DLG1,SOS2,TNFRSF1B,ARG2,EFNB1,LGALS9,CD46	turquoise	EC_M6
GO:0015693	2.19E-02	0.994804769	6	17	magnesium ion transport	BP	3.44E-01	CNNM4,TUSC3,NIPA1,MRS2,CNNM2,NIPAL1	darkgreen	EC_M4
GO:0043280	1.91E-02	0.990841723	17	96	positive regulation of cysteine-type endopeptidase activity involved in apoptotic process	BP	3.44E-01	GRAMD4,DAPK1,SYK,PPARG,EIF2AK3,HIP1R,SOX7,SNCA,LCK,LAPTM5,CFLAR,CASP10,DDX3X,PPM1F,BID,LGALS9,PMAIP1	turquoise	EC_M6
GO:0003205	2.20E-02	0.987392573	28	135	cardiac chamber development	BP	3.44E-01	LY6E,SRF,TGFBR1,SMARCD3,SNX17,TGFB1,SCN5A,SEMA3C,ADAMTS6,ROBO1,HEY1,CPE,ACVR1,NOTCH2,PKP2,TGFB2,BMP7,FGFR2,FZD2,BMPR1A,MED1,FGFRL1,TPM1,MYOCD,COL11A1,LMO4,FKBP1A,WNT2	darkgreen	EC_M4
GO:0002407	1.92E-02	0.996222085	5	15	dendritic cell chemotaxis	BP	3.44E-01	TRPM4,PIK3CG,CXCR4,ANO6,LGALS9	turquoise	EC_M6
GO:0051258	1.93E-02	0.987895751	39	250	protein polymerization	BP	3.44E-01	ARHGAP18,BAIAP2L2,PICK1,CDC42EP1,TMOD1,FAM49A,SVIL,MSRB2,SPTBN4,FCHSD2,ARHGEF7,FCHSD1,LMOD3,HIP1R,OCLN,IQGAP2,DYRK1A,SNCA,ESAM,HCLS1,AP1AR,FES,TMOD4,DLG1,BAIAP2L1,WAS,TMOD2,RAC1,MTOR,ARHGAP28,SPIRE1,GIT1,CARMIL2,CDH5,VASP,GMFG,KANK3,GBA2,ARPC5L	turquoise	EC_M6

GO:0034314	1.93E-02	0.994032267	8	33	Arp2/3 complex-mediated actin nucleation	BP	3.44E-01	PICK1,FCHSD2,HIP1R,IQGAP2,AP1AR,WAS,GMFG,ARPC5L	turquoise	EC_M6
GO:0051051	1.93E-02	0.987376769	46	314	negative regulation of transport	BP	3.44E-01	PACSIN2,TRDN,RSAD2,GRB10,TCAF2,SDCBP,MTMR2,KCNRG,TBC1D4,NDFIP2,SFRP1,FERMT1,CD36,FAM3D,MCTP1,TLR2,DYSF,STC1,EDN1,SNCA,CD200,HDAC1,PPP3CA,HECW2,RIN3,ATP9A,PRKD1,OAZ3,APOD,CTTNBP2NL,WNK4,FGF23,PPM1F,RAC1,RSC1A1,TNFRSF1B,MTOR,LGALS9,UNC119,SLC43A2,NOTCH1,LRSAM1,KCNH2,RGS4,BST2,VAMP3	turquoise	EC_M6
GO:0007623	1.94E-02	0.989010959	27	160	circadian rhythm	BP	3.46E-01	TNFRSF11A,ATF4,CSNK1E,NFIL3,NOCT,PPARG,PPP1CB,NR1D2,DYRK1A,MTTP,SIK1,HDAC1,KDM2A,CIART,ADCY1,RBM4,PROX1,CREB1,ADORA2A,KDM5A,MTOR,FBXW11,CHRNB2,RAI1,NOS2,USP7,PASD1	turquoise	EC_M6
GO:0010324	1.94E-02	0.992234003	12	54	membrane invagination	BP	3.46E-01	ARHGAP25,ELMO1,GATA2,CD36,CLCN3,ANO6,DOCK1,RAC1,SPIRE1,PLCG2,FNBP1L,HAVCR1	turquoise	EC_M6
GO:0070507	1.85E-02	0.990211816	22	135	regulation of microtubule cytoskeleton organization	BP	3.46E-01	AKAP9,SENP6,CLASP2,BORA,CKAP2,TPX2,SKA3,CKAP5,STMND1,KIF18A,CAMSAP2,RAE1,HDGFL3,TPR,KIF15,TACC3,FKBP4,HNRNPU,PLK1,TAOK1,STMN1,DRG1	darkgrey	EC_M8
GO:0008152	1.07E-03	0.999120661	995	8467	metabolic process	BP	3.46E-01	NFATC1,ZNF236,TSHZ1,ZADH2,ORMDL2,PYM1,DGKA,SUOX,SMARCC2,STAT6,ATG12,PIP4K2C,MAP3K5,PEX7,OS9,CDK4,CITED2,CTDSP2,ATP23,ADAMTS2,MAML1,SQSTM1,SASH1,IRAK3,HELB,GFPT2,OSR2,BAG6,CHKB,ALDH7A1,ABHD16A,PPP6R2,MSH5,SNX9,HSPA1B,EHMT2,ZBTB12,HDAC10,SELENOO,ALG12,CERK,ATF6B,AGPAT1,RNF5,EIF3H,RPS6KA2,DEPTOR,DSE,RXRB,TSPO,UQCRQ,HSD17B8,RING1,RPS18,B3GALT4,CYB5R3,ZHX2,ZBTB22,JADE2,PMM1,MYC,TOB2,SMAD5,EP300,IBA57,CDC14C,ERCC6L2,KDM3B,ZC3H3,GSDMD,ZNF76,FANCE,EEF1D,TAB1,RPL3,JOSD1,DDX17,NRBP2,STK38,EIF3L,OPLAH,MPST,NFIB,GPAA1,SHARPIN,EIF3D,FOXRED2,HSF1,FBXL6,SLC52A2,CPSF1,VPS28,CCND3,PPP1R16A,TRERF1,CDKN2A,RPL8,HMOX1,WASL,CUL7,CUL9,DNPH1,KLF9,POLR1C,PIGO,PDGFRA,PLPP6,RCL1,JAK2,HINT2,RECK,RNF38,ZBTB45,RPS5,ZNF135,ANKRD39,TDRD7,TRMO,ZNF667,ALG2,ZNF865,SSC5D,RPL28,TTC7A,RPS9,EEF1A1,CD109,GPNMB,MALSU1,STAP1,SLC44A1,EPB41L4B,ETFB,EFEMP1,SVEP1,LPAR1,PTGR1,UGP2,ZNF692,CYREN,RPS7,ANTXR1,ANXA4,AKNA,LSM10,STK40,MED25,IL4I1,TBC1D17,COQ3,AKT1S1,ING3,ARHGEF5,AP2A1,IRF3,LPIN1,RPS11,GSN,PARD3,FOXO3,SESN1,KLF15,DHDH,NDUFB2,ADCK2,SPHK2,RPL18,SPRY1,TPRA1,NOP53,ABTB1,MAML2,BICRA,ZC3HAV1,KMT2E,BBS7,MEIS3,HMCES,FKRP,UQCRH,LRRC41,LURAP1,TRIM13,ACSS1,IRF2BP1,SIX5,QPCTL,FOSB,ZNF746,NDUFB6,NR2C2,ZNF862,TSC22D1,SERP2,FDXR,UROD,KHK,SMG9,FASTK,SEPHS1,RPS8,XRCC1,ZNF575,MIF4GD,AGAP3,NAA16,MLH1,RNF220,LIPE,NDUFC2,PAFAH1B3,ECHDC3,GTF3C2,UNK,ZNF574,GALNT11,WBP2,TRI	cyan	EC_M2
GO:0006631	1.09E-03	0.999393512	48	271	fatty acid metabolic process	BP	3.46E-01	ZADH2,HSD17B8,ETFB,PTGR1,LPIN1,ACSS1,ECHDC3,ACAA1,CYGB,HACL1,CYP1B1,THNSL2,CBR4,ACSS2,PRKAG1,LPIN3,ERLIN1,PLA2G4B,ACOT8,LONP2,IRS1,ACADS,GPAM,GPX1,ABCB11,PER2,SCAP,EHHADH,POR,ACADL,PECR,TNFRSF1A,FADS3,ILVBL,EPHX1,TECR,ACOT2,ABCD4,GCDH,SLC27A3,ACSM3,ACACA,SREBF1,ACAA2,CPT2,GSTM4,PTGES2,SCP2	cyan	EC_M2
GO:0006355	1.09E-03	0.999139957	305	2411	regulation of transcription, DNA-templated	BP	3.46E-01	NFATC1,ZNF236,TSHZ1,SMARCC2,STAT6,MAP3K5,CDK4,CITED2,MAML1,SQSTM1,IRAK3,OSR2,HSPA1B,EHMT2,ZBTB12,HDAC10,ATF6B,RXRB,RING1,ZHX2,ZBTB22,MYC,TOB2,SMAD5,EP300,KDM3B,ZNF76,DDX17,NFIB,HSF1,CCND3,TRERF1,CDKN2A,HMOX1,WASL,KLF9,JAK2,ZBTB45,ZNF135,ZNF667,ZNF865,EFEMP1,ZNF692,ANXA4,AKNA,MED25,IL4I1,ING3,ARHGEF5,IRF3,LPIN1,FOXO3,KLF15,NOP53,MAML2,BICRA,KMT2E,BBS7,MEIS3,TRIM13,IRF2BP1,SIX5,FOSB,ZNF746,NR2C2,ZNF862,TSC22D1,ZNF575,NAA16,ZNF574,WBP2,KMT2C,MYD88,TADA3,RPS3,BRPF1,DYRK1B,EID2,ZNF219,MED29,SIRT2,RPTOR,APBB3,NUP98,TRIM21,CYP1B1,HGF,APBB1,DEAF1,HOXC4,NCOA4,LIN37,KMT2B,RBCK1,RARG,SDR16C5,ZNF740,LMO2,POFUT1,URI1,PIK3R1,NCOA2,ATF1,KDM2B,ZFHX4,CAMKK2,SCAND1,SETD6,MCPH1,SSBP2,FNIP2,ARL2BP,SHOX2,NEK4,ZHX3,VDR,MMS19,LPIN3,FOXF2,F2RL1,SORBS3,HNF4A,TP53INP1,PIDD1,HR,TRIM45,ELK4,LGR4,ICE1,SP140,TCF7L2,TXNIP,AIP,SOX5,ADNP,ZNF217,QARS,DTX3L,PARP14,ECM1,RCOR3,ARNT,PRMT2,RIPK3,GABPB2,PER2,TWIST2,VPS72,HDAC4,USF1,SMARCD2,POGZ,USP21,PATZ1,RNASEL,CCDC85B,MAGEF1,CDKN1B,TCEA3,KAT5,LRP6,YBX3,NFKBIA,NFE2L2,PHC1,HEXIM2,BATF2,PCBD1,CHCHD10,MEN1,PHB2,TSC22D4,SAV1,TAF6,CTDSP1,EID1,SSBP4,RNF25,FGF7,NELFA,ZSCAN25,ZNF394,TNFRSF1A,STK16,STAT5A,KAT6B,CREG1,NR2F6,HDAC6,PQBP1,CAMTA2,TFE3,PELP1,ZNF12,WIZ,ZBTB25,RARA,FOXK1,AC090517.4,ZBTB17,GPS2,THRA,KLF3,MED24,PRDM2,ARHGEF11,SPIN4,UIMC1,CC2D1A,ZNF771,MLLT6,CTBP1,CRABP2,ZNF646,CREBRF,CDK5RAP3,GLMP,KAT8,CHD	cyan	EC_M2

GO:0018394	1.12E-03	0.99949187	28	143	peptidyl-lysine acetylation	BP	3.46E-01	BAG6,JADE2,EP300,HINT2,ING3,KLF15,SPHK2,WBP2,TADA3,BRPF1,APBB1,TAF10,SDR16C5,KAT5,POR,EID1,KAT6B,MED24,CTBP1,KAT8,KAT7,PER1,SGF29,CRTC2,SIN3A,CREBBP,WDR5,GTF3C4	cyan	EC_M2
GO:1903506	1.12E-03	0.999113334	305	2412	regulation of nucleic acid-templated transcription	BP	3.46E-01	NFATC1,ZNF236,TSHZ1,SMARCC2,STAT6,MAP3K5,CDK4,CITED2,MAML1,SQSTM1,IRAK3,OSR2,HSPA1B,EHMT2,ZBTB12,HDAC10,ATF6B,RXRB,RING1,ZHX2,ZBTB22,MYC,TOB2,SMAD5,EP300,KDM3B,ZNF76,DDX17,NFIB,HSF1,CCND3,TRERF1,CDKN2A,HMOX1,WASL,KLF9,JAK2,ZBTB45,ZNF135,ZNF667,ZNF865,EFEMP1,ZNF692,ANXA4,AKNA,MED25,IL4I1,ING3,ARHGEF5,IRF3,LPIN1,FOXO3,KLF15,NOP53,MAML2,BICRA,KMT2E,BBS7,MEIS3,TRIM13,IRF2BP1,SIX5,FOSB,ZNF746,NR2C2,ZNF862,TSC22D1,ZNF575,NAA16,ZNF574,WBP2,KMT2C,MYD88,TADA3,RPS3,BRPF1,DYRK1B,EID2,ZNF219,MED29,SIRT2,RPTOR,APBB3,NUP98,TRIM21,CYP1B1,HGF,APBB1,DEAF1,HOXC4,NCOA4,LIN37,KMT2B,RBCK1,RARG,SDR16C5,ZNF740,LMO2,POFUT1,URI1,PIK3R1,NCOA2,ATF1,KDM2B,ZFHX4,CAMKK2,SCAND1,SETD6,MCPH1,SSBP2,FNIP2,ARL2BP,SHOX2,NEK4,ZHX3,VDR,MMS19,LPIN3,FOXF2,F2RL1,SORBS3,HNF4A,TP53INP1,PIDD1,HR,TRIM45,ELK4,LGR4,ICE1,SP140,TCF7L2,TXNIP,AIP,SOX5,ADNP,ZNF217,QARS,DTX3L,PARP14,ECM1,RCOR3,ARNT,PRMT2,RIPK3,GABPB2,PER2,TWIST2,VPS72,HDAC4,USF1,SMARCD2,POGZ,USP21,PATZ1,RNASEL,CCDC85B,MAGEF1,CDKN1B,TCEA3,KAT5,LRP6,YBX3,NFKBIA,NFE2L2,PHC1,HEXIM2,BATF2,PCBD1,CHCHD10,MEN1,PHB2,TSC22D4,SAV1,TAF6,CTDSP1,EID1,SSBP4,RNF25,FGF7,NELFA,ZSCAN25,ZNF394,TNFRSF1A,STK16,STAT5A,KAT6B,CREG1,NR2F6,HDAC6,PQBP1,CAMTA2,TFE3,PELP1,ZNF12,WIZ,ZBTB25,RARA,FOXK1,AC090517.4,ZBTB17,GPS2,THRA,KLF3,MED24,PRDM2,ARHGEF11,SPIN4,UIMC1,CC2D1A,ZNF771,MLLT6,CTBP1,CRABP2,ZNF646,CREBRF,CDK5RAP3,GLMP,KAT8,CHD	cyan	EC_M2
GO:0030318	1.94E-02	0.995345274	6	20	melanocyte differentiation	BP	3.46E-01	KIT,EDNRB,MEF2C,ADAMTS20,USP13,RAB27A	turquoise	EC_M6
GO:0045124	1.95E-02	0.993981495	8	32	regulation of bone resorption	BP	3.46E-01	TNFRSF11A,TNFAIP3,SYK,IL6,P2RX7,INPP5D,S1PR1,CA2	turquoise	EC_M6
GO:0046530	2.21E-02	0.991548113	11	41	photoreceptor cell differentiation	BP	3.46E-01	AHI1,RORB,VEGFA,MFSD2A,NKD1,ARL3,PRKCI,ROM1,TTC8,NPHP4,THY1	darkgreen	EC_M4
GO:0048660	1.95E-02	0.989818247	21	124	regulation of smooth muscle cell proliferation	BP	3.46E-01	TNFAIP3,PDGFB,IL6,TLR4,IL15,PPARG,P2RY6,HBEGF,IGF1,MEF2C,NDRG4,EDN1,STAT1,IGFBP3,APOD,IGFBP5,CALCRL,MTOR,NPR1,S1PR1,BMP2	turquoise	EC_M6
GO:0060037	2.22E-02	0.993920765	7	21	pharyngeal system development	BP	3.47E-01	TGFBR1,ACVR1,TGFB2,BMP7,ECE1,SIX4,BMPR1A	darkgreen	EC_M4
GO:0015872	1.96E-02	0.994488137	7	28	dopamine transport	BP	3.47E-01	RAB3B,CHRM5,SNCA,DTNBP1,SNCG,SYT7,CHRNB2	turquoise	EC_M6
GO:0032369	2.23E-02	0.99377774	7	23	negative regulation of lipid transport	BP	3.47E-01	AKT2,IRS2,PTPN11,CRY2,PLA2R1,ITGAV,AKT1	darkgreen	EC_M4
GO:0044281	1.15E-03	0.99914325	182	1301	small molecule metabolic process	BP	3.47E-01	ZADH2,PEX7,GFPT2,HSD17B8,CYB5R3,PMM1,EP300,MPST,SLC52A2,DNPH1,PLPP6,ETFB,PTGR1,UGP2,ZNF692,IL4I1,COQ3,LPIN1,DHDH,SPHK2,ACSS1,FDXR,KHK,SEPHS1,LIPE,ECHDC3,ACAA1,BCKDHA,CYGB,HYI,CSGALNACT1,PIP4P1,PNP,GAA,XDH,HACL1,CYP1B1,GNPDA1,SMUG1,APIP,SDR16C5,POFUT1,PEPD,THNSL2,CBR4,ACSS2,NCOA2,RETSAT,NEIL2,COQ9,DGUOK,PRKAG1,SPR,LPIN3,HNF4A,FARS2,ERLIN1,PLA2G4B,ACOT8,WARS2,LONP2,SRD5A1,OSBPL5,NT5C2,ALDH5A1,IRS1,ACADS,CARMIL1,MMAB,GPAM,FMO5,CHKA,GPX1,QARS,IMPDH2,DALRD3,ABCB11,PC,ARNT,SLC37A4,CTPS2,PER2,HDAC4,USF1,CLCN2,DNAJC30,GLUL,SCAP,EHHADH,FUOM,POR,IDH1,ACADL,SOAT1,MTHFS,AIFM2,PCBD1,PRODH,ATIC,PECR,G6PC3,GALK2,NAT8L,CYP27A1,PLA2G16,TNFRSF1A,STAT5A,UQCC3,FADS3,COQ8A,FOXK1,ILVBL,EPHX1,TECR,BRAT1,MTHFR,UBIAD1,MRI1,CRABP2,OSBPL7,HSD3B7,NAXE,PNPO,ACOT2,ALDH6A1,ABCD4,GBA,GCDH,FLAD1,NUPR1,AC138894.1,PARP16,CRTC2,SCPEP1,SLC27A3,ACSM3,ACACA,INSR,SREBF1,OXCT1,LEPR,SLC46A1,DHRS13,FGGY,ACAA2,CPT2,ASPA,GAMT,ATP5F1D,TAZ,JMJD8,INPP5E,GSTM4,WDR5,RXRA,APRT,DPYD,KARS,PTGES2,LDHD,FPGS,FUK,LCAT,TK2,MT-CO2,MT-ATP6,ABCA2,SULT1C4,ZBTB7A,FUT4,NT5C,FPGT,PLPP3,EPHX3,PID1,RDH14,PDXK,CLYBL,NUDT2,UPP1,SCP2,RPIA	cyan	EC_M2
GO:0120255	2.23E-02	0.994686596	6	17	olefinic compound biosynthetic process	BP	3.47E-01	REST,DKKL1,DKK3,HSD17B1,STARD3,PPARGC1A	darkgreen	EC_M4
GO:0048714	1.98E-02	0.996003186	5	16	positive regulation of oligodendrocyte differentiation	BP	3.49E-01	CXCR4,TLR2,HDAC1,TNFRSF1B,MTOR	turquoise	EC_M6

GO:0048843	1.99E-02	0.994544657	7	24	negative regulation of axon extension involved in axon guidance	BP	3.49E-01	SEMA6A,SEMA4D,NRP1,SEMA3F,SEMA6D,SEMA4A,SEMA6B	turquoise	EC_M6
GO:0010715	1.99E-02	0.995993198	5	16	regulation of extracellular matrix disassembly	BP	3.49E-01	IL6,ETS1,MELTF,FSCN1,CARMIL2	turquoise	EC_M6
GO:1901620	2.25E-02	0.996739815	4	9	regulation of smoothened signaling pathway involved in dorsal/ventral neural tube patterning	BP	3.49E-01	GLI3,GLI2,GORAB,TULP3	darkgreen	EC_M4
GO:0022409	2.25E-02	0.986273203	34	176	positive regulation of cell-cell adhesion	BP	3.50E-01	IL23A,HLA-DRA,FAM49B,LGALS1,LEP,DOCK8,NCK2,SOCS5,GLI3,GLI2,HSPH1,CHST2,CX3CL1,PTPN11,AP3B1,PTPN22,ADA,RUNX1,TJP1,PIK3CA,LEF1,BMP7,TFRC,PTPN6,IGFBP2,CD74,AKT1,CD40LG,MYO10,IRAK1,BCL10,FSTL3,THY1,HAS2	darkgreen	EC_M4
GO:0043552	2.00E-02	0.993911414	8	28	positive regulation of phosphatidylinositol 3-kinase activity	BP	3.51E-01	PTK2,PDGFB,TEK,KIT,FGR,PRKD1,TNFAIP8L3,CD19	turquoise	EC_M6
GO:0045064	2.01E-02	0.996953155	4	11	T-helper 2 cell differentiation	BP	3.51E-01	GATA3,HLX,CD86,BATF	turquoise	EC_M6
GO:2000027	2.27E-02	0.98773849	23	108	regulation of animal organ morphogenesis	BP	3.51E-01	AHI1,RSPO3,VEGFA,RUNX2,PLEKHA4,FGF2,CTNNB1,RYK,PRICKLE2,SULF1,ROBO1,NKD1,BMP7,FZD2,SMURF1,DAAM1,SIX4,MAGED1,BMPR1A,NGFR,GDNF,WNT2,FGF10	darkgreen	EC_M4
GO:0009792	2.28E-02	0.983001862	91	537	embryo development ending in birth or egg hatching	BP	3.51E-01	PLXNB2,ANGPT1,RSPO3,LY6E,MYH9,SRF,VEGFA,RUNX2,TGFBR1,RTN4,ADAMTS3,KIDINS220,GLI3,ITGB1,FGF2,GLI2,MEGF8,TGFB1,CTNNB1,MAFG,SPINT2,LIG4,SEMA3C,TMED2,EIF2S2,SULF1,RDH10,HEY1,CELF1,TCTN1,OPA1,ACVR1,ADA,PKD2,NKD1,TENT5C,IFT57,PSPH,NOTCH2,HYAL1,SLC2A10,SBDS,TGFB2,SATB2,ITPK1,MMP14,LEF1,BMP7,WDTC1,FGFR2,VASH2,RAI2,TANC2,PLOD3,ATP6AP2,FZD2,MEOX1,FKBP8,SKIL,ECE1,PRRX1,GORAB,TULP3,SIX4,FKBP10,BMPR1A,TCAP,MED1,MYO1E,COL1A1,TGFB3,MAP2K1,TMEM100,PPP4R4,ACSL4,RBBP8,AKT1,UBE2A,C6,SUPT6H,NXN,PKD1,COL11A1,LMO4,BCL10,PEMT,HOPX,ZPR1,SLC39A3,CHST11,WNT2	darkgreen	EC_M4
GO:0034505	2.29E-02	0.994518203	6	17	tooth mineralization	BP	3.52E-01	PPARA,ASPN,CNNM4,STIM1,FAM20C,COL1A1	darkgreen	EC_M4
GO:0045927	2.29E-02	0.985600528	38	199	positive regulation of growth	BP	3.52E-01	LRP1,PPARD,LEP,PLAA,SRF,VEGFA,TGFBR1,RIMS1,MFSD2A,FGF2,MEGF8,PAK1,ILK,CXCL12,EIF4G2,MAP1B,CELF1,ZFYVE27,HYAL1,TGFB2,MMP14,FGFR2,RPS6KA3,MAPT,FN1,SMURF1,BMPR1A,RAB11A,TAF9B,ISLR2,PIN1,TRPV2,AKT1,PAFAH1B1,HOPX,ZPR1,DERL2,WNT2	darkgreen	EC_M4
GO:0060688	2.30E-02	0.991151475	11	42	regulation of morphogenesis of a branching structure	BP	3.52E-01	VEGFA,RTN4,CTNNB1,SULF1,BMP7,FGFR2,SIX4,MAGED1,GDNF,WNT2,FGF10	darkgreen	EC_M4
GO:0008037	2.31E-02	0.987924708	21	97	cell recognition	BP	3.52E-01	TCP1,ADGRB1,DOCK8,NCK2,RTN4,MEGF8,PALLD,ROBO1,APP,CCT8,PTX3,CRTAC1,NDN,VDAC2,MSN,DOCK2,NPTN,BSG,NEXN,VSTM2L,FCN1	darkgreen	EC_M4
GO:0045017	2.31E-02	0.985265525	40	214	glycerolipid biosynthetic process	BP	3.52E-01	INPP4A,TMEM246,SOCS5,FAM126A,MFSD2A,FGF2,SELENOI,ABHD5,DGKH,DGAT2,MTMR14,IMPAD1,MTMR6,CHPT1,TMEM150A,FAR1,LPCAT2,PIGP,AGPAT3,HTR2B,PIK3CA,PITPNM1,ETNK1,PIGZ,PCYT1B,MOGAT3,PLD1,SLC44A2,PIGL,ACSL4,LPIN2,OCRL,DPM2,PLA2G15,PEMT,PIGK,DPM1,PIGB,PIK3C2A,CDIPT	darkgreen	EC_M4
GO:0032770	2.31E-02	0.992795397	8	27	positive regulation of monooxygenase activity	BP	3.52E-01	NUS1,HTR2B,KRAS,CALM1,AKT1,NPR3,PARK7,GDNF	darkgreen	EC_M4
GO:0010573	2.02E-02	0.993785336	8	30	vascular endothelial growth factor production	BP	3.52E-01	FLT4,ATF4,IL6,C5,IL1A,EIF2AK3,HIF1A,ADGRG1	turquoise	EC_M6
GO:1903531	2.33E-02	0.987783996	21	99	negative regulation of secretion by cell	BP	3.54E-01	LEP,REST,INHBA,FKBP1B,RAB7A,RAB11FIP1,VAMP8,ABCC8,PTPN11,CRY2,HADH,IDH2,OSM,ADRA2C,SRCIN1,SYT11,SYTL4,MIDN,RHBDF1,CBARP,STXBP6	darkgreen	EC_M4

GO:0018193	2.03E-02	0.984167266	130	997	peptidyl-amino acid modification	BP	3.54E-01	FLT4,ZDHHC14,OXR1,NCOA7,PTK2,PDGFB,AGTPBP1,MAPK13,BRPF3,CSNK1E,TEK,PRDM4,TEC,KIT,KDR,SYK,SEMA4D,IL6,PPIE,IL15,TRPM4,NRP1,EPC1,KDM4D,GATA2,PRKD2,GATA3,SPTBN4,TIE1,SETD5,IKBKB,SFRP1,HBEGF,SH3BP5,ASH2L,CD36,SIRT3,EOGT,EPHB1,PLK2,RET,PDE4D,MERTK,DNMT3B,IGF1,EIF2AK3,KDM3A,DCLK2,IL7,KMT2D,KANSL2,THBS4,RAPGEF3,HIPK1,NEDD9,DYRK1A,EHD4,SNCA,JARID2,RBBP5,DCAF1,HDAC1,LCK,HYAL2,ZDHHC6,PRKX,NCF1,DYRK3,HCLS1,PECAM1,HHAT,BMX,FGR,INPP5F,ZDHHC18,FES,RPS6KA1,PRKD1,MGAT1,SRMS,MLXIPL,ACE,PAK2,SENP2,STYK1,PDK3,ST6GAL1,KANSL1,BCR,P3H2,BCOR,EPHB4,MECOM,ING4,JADE3,LMTK2,PPM1F,PRKCH,EPHA2,TNK1,MTOR,PSEN1,EFNA1,TTLL5,NAP1L2,ZDHHC15,SBK1,RAB3D,PIAS1,RPS6KA5,HMG20A,CDC42BPB,MARK3,YES1,SH2D3A,JAK1,NOS2,UNC119,MATK,DOT1L,HCFC1,PLCG2,DPH5,SH2D3C,MTF2,ARID4A,RELN,ALKAL1,BMP6,ZDHHC22	turquoise	EC_M6
GO:1901700	2.03E-02	0.983791035	158	1234	response to oxygen-containing compound	BP	3.54E-01	TNFRSF11A,TNFAIP3,AKAP12,OXR1,PTK2,DAPK1,ATF4,PDGFB,MAPK13,PIM1,CSF2RB,TEK,IL6,CXCL8,KBTBD2,PELI1,FOXP2,CAV2,KLF11,GRB10,LANCL2,TLR4,IL15,TEAD2,NOCT,TRPM4,PIK3CG,DNMT3A,NOS3,PPARG,TIMP4,GATA3,TBC1D4,IRAK2,EDNRB,NET1,TIE1,JAG1,P2RY6,P2RY2,NFKBIB,PDE2A,SFRP1,P2RX2,CD36,SCARB1,RET,GRAMD1A,IL1A,DNMT3B,IGF1,NR4A1,HHEX,ERBIN,CHRM5,GPD1,MEF2C,SMARCD1,TLR2,P2RX7,GUCY1B1,CXCL10,ANGPT2,SOGA1,DHH,STC1,RAPGEF3,NR4A2,EDN1,STAT1,SNCA,DTNBP1,NFKB2,ADCY2,ABCG1,ETS1,HDAC1,PPP3CA,HYAL2,ANO1,NCF1,PABPN1,CD86,POLG,MN1,SH3BP4,CFLAR,ADCY1,FES,PLEKHA1,PRKD1,IL10RA,MLXIPL,PTPRE,SIPA1,APOD,PDK3,CREB1,SSTR1,LTF,ARNT2,CYBB,ITGA4,IGFBP5,TBX1,CALCRL,RAMP2,CDK16,MMP13,SELE,SELP,BAIAP2L1,FGF23,CPEB2,RAC1,HIF1A,JUP,ADIPOR2,BID,TLR6,TNFRSF1B,ALDH1A2,MTOR,AR,RNF40,PSEN1,EIF2B2,CHRNB2,S100A14,GPR68,SMAD2,DNM2,SCNN1B,CYP1A1,TNIP1,COL4A6,ADRB2,CCL2,YES1,PDE4B,LGALS9,NOS2,STK26,GIT1,TRAF2,NOTCH1,SLC25A33,RAPGEF1,IRF8,PLCG2,STXBP1,DAB2IP,SLC8A3,TRIM24,CA2,RGS4,GNG2,BMP6	turquoise	EC_M6
GO:0015844	2.04E-02	0.9925181	10	48	monoamine transport	BP	3.55E-01	PICK1,SYK,RAB3B,CHRM5,SNCA,DTNBP1,ADORA2A,SNCG,SYT7,CHRNB2	turquoise	EC_M6
GO:1904064	2.34E-02	0.987525864	22	103	positive regulation of cation transmembrane transport	BP	3.55E-01	KIF5B,ACTN4,STIM1,FXYD1,ATP1B3,CX3CL1,ABCC8,CACNB3,CHP1,PKD2,CASQ1,HTT,KCNA1,HAP1,STIM2,KCNMB1,CALM1,VMP1,FLNA,DMD,THY1,TMSB4X	darkgreen	EC_M4
GO:0043966	1.23E-03	0.999625141	14	54	histone H3 acetylation	BP	3.55E-01	JADE2,EP300,SPHK2,WBP2,TADA3,BRPF1,TAF10,KAT6B,KAT7,PER1,SGF29,CRTC2,SIN3A,WDR5	cyan	EC_M2
GO:2001141	1.26E-03	0.999005566	305	2416	regulation of RNA biosynthetic process	BP	3.55E-01	NFATC1,ZNF236,TSHZ1,SMARCC2,STAT6,MAP3K5,CDK4,CITED2,MAML1,SQSTM1,IRAK3,OSR2,HSPA1B,EHMT2,ZBTB12,HDAC10,ATF6B,RXRB,RING1,ZHX2,ZBTB22,MYC,TOB2,SMAD5,EP300,KDM3B,ZNF76,DDX17,NFIB,HSF1,CCND3,TRERF1,CDKN2A,HMOX1,WASL,KLF9,JAK2,ZBTB45,ZNF135,ZNF667,ZNF865,EFEMP1,ZNF692,ANXA4,AKNA,MED25,IL4I1,ING3,ARHGEF5,IRF3,LPIN1,FOXO3,KLF15,NOP53,MAML2,BICRA,KMT2E,BBS7,MEIS3,TRIM13,IRF2BP1,SIX5,FOSB,ZNF746,NR2C2,ZNF862,TSC22D1,ZNF575,NAA16,ZNF574,WBP2,KMT2C,MYD88,TADA3,RPS3,BRPF1,DYRK1B,EID2,ZNF219,MED29,SIRT2,RPTOR,APBB3,NUP98,TRIM21,CYP1B1,HGF,APBB1,DEAF1,HOXC4,NCOA4,LIN37,KMT2B,RBCK1,RARG,SDR16C5,ZNF740,LMO2,POFUT1,URI1,PIK3R1,NCOA2,ATF1,KDM2B,ZFHX4,CAMKK2,SCAND1,SETD6,MCPH1,SSBP2,FNIP2,ARL2BP,SHOX2,NEK4,ZHX3,VDR,MMS19,LPIN3,FOXF2,F2RL1,SORBS3,HNF4A,TP53INP1,PIDD1,HR,TRIM45,ELK4,LGR4,ICE1,SP140,TCF7L2,TXNIP,AIP,SOX5,ADNP,ZNF217,QARS,DTX3L,PARP14,ECM1,RCOR3,ARNT,PRMT2,RIPK3,GABPB2,PER2,TWIST2,VPS72,HDAC4,USF1,SMARCD2,POGZ,USP21,PATZ1,RNASEL,CCDC85B,MAGEF1,CDKN1B,TCEA3,KAT5,LRP6,YBX3,NFKBIA,NFE2L2,PHC1,HEXIM2,BATF2,PCBD1,CHCHD10,MEN1,PHB2,TSC22D4,SAV1,TAF6,CTDSP1,EID1,SSBP4,RNF25,FGF7,NELFA,ZSCAN25,ZNF394,TNFRSF1A,STK16,STAT5A,KAT6B,CREG1,NR2F6,HDAC6,PQBP1,CAMTA2,TFE3,PELP1,ZNF12,WIZ,ZBTB25,RARA,FOXK1,AC090517.4,ZBTB17,GPS2,THRA,KLF3,MED24,PRDM2,ARHGEF11,SPIN4,UIMC1,CC2D1A,ZNF771,MLLT6,CTBP1,CRABP2,ZNF646,CREBRF,CDK5RAP3,GLMP,KAT8,CHD	cyan	EC_M2
GO:0006991	1.29E-03	0.99981002	7	17	response to sterol depletion	BP	3.55E-01	PIP4P1,EIF2A,ERLIN1,SCAP,ARHGEF10L,ZBTB7B,SREBF1	cyan	EC_M2

GO:0044260	1.29E-03	0.998925172	705	5916	cellular macromolecule metabolic process	BP	3.55E-01	NFATC1,ZNF236,TSHZ1,PYM1,SMARCC2,STAT6,ATG12,MAP3K5,OS9,CDK4,CITED2,CTDSP2,ATP23,MAML1,SQSTM1,SASH1,IRAK3,HELB,GFPT2,OSR2,BAG6,PPP6R2,MSH5,SNX9,HSPA1B,EHMT2,ZBTB12,HDAC10,SELENOO,ALG12,ATF6B,RNF5,EIF3H,RPS6KA2,DEPTOR,DSE,RXRB,TSPO,RING1,RPS18,B3GALT4,ZHX2,ZBTB22,JADE2,PMM1,MYC,TOB2,SMAD5,EP300,CDC14C,ERCC6L2,KDM3B,ZNF76,FANCE,EEF1D,TAB1,RPL3,JOSD1,DDX17,NRBP2,STK38,EIF3L,NFIB,GPAA1,SHARPIN,EIF3D,FOXRED2,HSF1,FBXL6,VPS28,CCND3,PPP1R16A,TRERF1,CDKN2A,RPL8,HMOX1,WASL,CUL7,CUL9,KLF9,POLR1C,PIGO,PDGFRA,JAK2,HINT2,RECK,RNF38,ZBTB45,RPS5,ZNF135,ANKRD39,TRMO,ZNF667,ALG2,ZNF865,RPL28,RPS9,EEF1A1,CD109,GPNMB,MALSU1,STAP1,EFEMP1,UGP2,ZNF692,CYREN,RPS7,ANTXR1,ANXA4,AKNA,STK40,MED25,IL4I1,AKT1S1,ING3,ARHGEF5,IRF3,LPIN1,RPS11,GSN,PARD3,FOXO3,KLF15,ADCK2,SPHK2,RPL18,SPRY1,NOP53,ABTB1,MAML2,BICRA,ZC3HAV1,KMT2E,BBS7,MEIS3,HMCES,FKRP,LRRC41,TRIM13,IRF2BP1,SIX5,QPCTL,FOSB,ZNF746,NR2C2,ZNF862,TSC22D1,SERP2,KHK,SMG9,FASTK,SEPHS1,RPS8,XRCC1,ZNF575,MIF4GD,AGAP3,NAA16,MLH1,RNF220,LIPE,GTF3C2,UNK,ZNF574,GALNT11,WBP2,TRIM47,KMT2C,MYD88,DMAC2,EGLN2,BABAM2,TADA3,CSGALNACT1,RPS3,TEP1,BRPF1,DYRK1B,EID2,TGFBR2,ZNF219,RPS16,TIMP2,MED29,ENGASE,SAMD4B,GAA,XDH,MRPS12,SIRT2,ARAP1,RPTOR,FAAP100,RPL15,LAMTOR1,HGS,UBAC2,APBB3,ASPSCR1,TBC1D5,EIF3K,NUP98,GALNT15,FGFR1,TRIM21,RNF24,CYP1B1,NDFIP1,SPRY4,HGF,APBB1,TAF10,DEAF1,SMUG1,HOXC4,CLIP3,NCOA4,PCBP2,LIN3	cyan	EC_M2
GO:0044242	1.30E-03	0.999369856	32	164	cellular lipid catabolic process	BP	3.55E-01	ABHD16A,ETFB,LPIN1,LIPE,ACAA1,SIRT2,HACL1,GPCPD1,DDHD2,CYP1B1,PLBD2,LPIN3,PLA2G4B,ACOT8,LONP2,IRS1,ACADS,ABCB11,EHHADH,IDH1,ACADL,ABHD12B,SMPD4,ILVBL,ABCD4,GBA,GCDH,HEXA,ACAA2,CPT2,PNPLA7,SCP2	cyan	EC_M2
GO:0033108	1.32E-03	0.999482001	21	83	mitochondrial respiratory chain complex assembly	BP	3.55E-01	NDUFB2,NDUFB6,NDUFC2,DMAC2,NDUFA2,SDHAF1,UQCRFS1,FASTKD3,OXA1L,NDUFS2,UQCC3,NDUFB7,SMIM20,NDUFS7,TAZ,MT-ND1,MT-ND2,MT-ND4,MT-ND5,NDUFAF8,COX14	cyan	EC_M2
GO:0032924	2.36E-02	0.992011732	9	31	activin receptor signaling pathway	BP	3.56E-01	TGFBR1,INHBA,ACVR1,GDF6,HJV,NOMO1,FSTL3,FKBP1A,FGF10	darkgreen	EC_M4
GO:0007610	2.36E-02	0.983059995	71	406	behavior	BP	3.56E-01	AHI1,PPARA,LEP,SRF,UBA6,TPBG,SHANK1,MFSD2A,MYH14,ACTR2,GLI3,SLC7A11,ITGB1,ABHD12,PICALM,PCDH17,MAFG,PRNP,ARL6IP5,CXCL12,LGI4,PJA2,P2RX4,APP,GLRB,ABCC8,HOMER1,AMFR,ADA,GLS,CHRNA7,EIF4E,CPT1A,HTR2B,KRAS,ADCY5,NCSTN,FZD9,MAPT,C1QL1,EIF4EBP2,COMT,TNR,KCNK4,HTT,ASIC4,ATAD1,SLC2A4,NLGN2,CLN8,SYT11,UBE2Q1,CLN6,GRIA1,NPTN,PRKAA1,VWA1,PAFAH1B1,NPHP4,PARK7,JPH3,LHX8,SERPINF1,LRRN4,ARF4,GDNF,ORMDL3,MAP1A,DERL2,NPAS1,MBD2	darkgreen	EC_M4
GO:1900221	2.36E-02	0.995302206	5	13	regulation of amyloid-beta clearance	BP	3.56E-01	LRP1,SRF,LRPAP1,MYOCD,ROCK1	darkgreen	EC_M4
GO:0030326	2.36E-02	0.987623284	21	96	embryonic limb morphogenesis	BP	3.56E-01	FBN2,RUNX2,GLI3,GLI2,MEGF8,CTNNB1,RARB,IMPAD1,RDH10,NOTCH2,TGFB2,LRP5,LEF1,BMP7,ECE1,PRRX1,TULP3,CACNA1C,BMPR1A,MED1,CHST11	darkgreen	EC_M4
GO:0035113	2.36E-02	0.987623284	21	96	embryonic appendage morphogenesis	BP	3.56E-01	FBN2,RUNX2,GLI3,GLI2,MEGF8,CTNNB1,RARB,IMPAD1,RDH10,NOTCH2,TGFB2,LRP5,LEF1,BMP7,ECE1,PRRX1,TULP3,CACNA1C,BMPR1A,MED1,CHST11	darkgreen	EC_M4
GO:0006658	2.36E-02	0.993377036	7	22	phosphatidylserine metabolic process	BP	3.56E-01	SERINC1,MFSD2A,ABHD12,OSBPL10,OSBPL8,SERINC3,PLA2G15	darkgreen	EC_M4
GO:0035930	2.37E-02	0.99650287	4	9	corticosteroid hormone secretion	BP	3.56E-01	C1QTNF1,PTPN11,CRY2,LINC02210-CRHR1	darkgreen	EC_M4
GO:2000846	2.37E-02	0.99650287	4	9	regulation of corticosteroid hormone secretion	BP	3.56E-01	C1QTNF1,PTPN11,CRY2,LINC02210-CRHR1	darkgreen	EC_M4
GO:0035929	2.37E-02	0.995271803	5	13	steroid hormone secretion	BP	3.56E-01	INHBA,C1QTNF1,PTPN11,CRY2,LINC02210-CRHR1	darkgreen	EC_M4
GO:1903305	2.05E-02	0.989924526	18	95	regulation of regulated secretory pathway	BP	3.57E-01	APBA1,SYK,GATA2,GAB2,UNC13D,DTNBP1,FGR,ADCY1,FES,ADORA2A,RAB27A,SYT7,FBXL20,RAB3D,LGALS9,GIT1,NOTCH1,STXBP1	turquoise	EC_M6
GO:0034405	2.38E-02	0.991928084	9	31	response to fluid shear stress	BP	3.57E-01	SOCS5,HDAC3,P2RX4,PKD2,TGFB3,PDGFRB,AKT1,PKD1,HAS2	darkgreen	EC_M4
GO:0032874	2.06E-02	0.98987722	18	97	positive regulation of stress-activated MAPK cascade	BP	3.57E-01	FLT4,TPD52L1,SH3RF3,GADD45G,TLR4,TRAF1,SDCBP,TNFRSF19,SH3RF1,NCF1,MAP3K3,MAP3K11,CRACR2A,RELL1,TAOK2,TRAF2,DAB2IP,BMP2	turquoise	EC_M6

GO:0051092	2.06E-02	0.989232823	21	119	positive regulation of NF-kappaB transcription factor activity	BP	3.57E-01	TNFRSF11A,TLR4,TRAF1,PRKD2,IRAK2,IKBKB,CD36,TLR2,CFLAR,PRKD1,LTF,IL1RAP,PRKCH,TLR6,AR,RPS6KA5,LGALS9,TRAF2,PLCG2,TRIM34,TRIM62	turquoise	EC_M6
GO:0033135	2.39E-02	0.987227409	22	104	regulation of peptidyl-serine phosphorylation	BP	3.57E-01	ANGPT1,VEGFA,NCK2,PAK1,TGFB1,GPD1L,PFN2,APP,ATP2B4,CAPRIN2,PIK3CA,NTRK3,OSM,TFRC,PDCD10,ARAF,AKT1,PRKAA1,PARK7,BCAR3,DMD,TXN	darkgreen	EC_M4
GO:0006073	2.39E-02	0.989444437	14	58	cellular glucan metabolic process	BP	3.57E-01	DYRK2,AKT2,IRS2,GYG1,SORBS1,PFKM,SELENOS,GYG2,GSK3B,PPP1R3D,PPP1R3F,AKT1,AGL,PPP1R1A	darkgreen	EC_M4
GO:0044042	2.39E-02	0.989444437	14	58	glucan metabolic process	BP	3.57E-01	DYRK2,AKT2,IRS2,GYG1,SORBS1,PFKM,SELENOS,GYG2,GSK3B,PPP1R3D,PPP1R3F,AKT1,AGL,PPP1R1A	darkgreen	EC_M4
GO:0003161	2.40E-02	0.991862284	9	31	cardiac conduction system development	BP	3.58E-01	BVES,SCN5A,HEY1,NOTCH2,BMPR1A,NPPB,CACNA1G,HOPX,HCN4	darkgreen	EC_M4
GO:0086070	2.41E-02	0.996658915	4	8	SA node cell to atrial cardiac muscle cell communication	BP	3.59E-01	SCN5A,CACNA1D,CACNA1G,HCN4	darkgreen	EC_M4
GO:2000272	2.41E-02	0.9917627	9	32	negative regulation of signaling receptor activity	BP	3.59E-01	PPARA,LY6E,SOCS5,DKKL1,DKK3,TSG101,CBLB,LRPAP1,PPARGC1A	darkgreen	EC_M4
GO:0090279	2.41E-02	0.992422825	8	27	regulation of calcium ion import	BP	3.59E-01	CTNNB1,PRNP,CXCL12,HOMER1,PKD2,TRPV2,PDGFRB,AKT1	darkgreen	EC_M4
GO:0031646	2.42E-02	0.992459412	8	26	positive regulation of nervous system process	BP	3.59E-01	UNC13B,RIMS1,TENM4,DAG1,TNR,NLGN2,DICER1,ITGA2	darkgreen	EC_M4
GO:0003231	2.42E-02	0.987055298	22	101	cardiac ventricle development	BP	3.60E-01	LY6E,TGFBR1,SMARCD3,TGFB1,SCN5A,SEMA3C,ROBO1,HEY1,CPE,ACVR1,PKP2,TGFB2,FGFR2,FZD2,BMPR1A,MED1,FGFRL1,TPM1,MYOCD,COL11A1,LMO4,FKBP1A	darkgreen	EC_M4
GO:0018345	2.08E-02	0.994080332	7	28	protein palmitoylation	BP	3.60E-01	ZDHHC14,ZDHHC13,ZDHHC6,HHAT,ZDHHC18,ZDHHC15,ZDHHC22	turquoise	EC_M6
GO:0043537	2.08E-02	0.993449273	8	33	negative regulation of blood vessel endothelial cell migration	BP	3.60E-01	PPARG,MEF2C,ANGPT2,DLL4,JUP,MMRN2,VASH1,NOTCH1	turquoise	EC_M6
GO:0010611	2.08E-02	0.991923604	11	51	regulation of cardiac muscle hypertrophy	BP	3.60E-01	IGF1,EDN1,JARID2,PDE9A,PPP3CA,MEF2A,TNFRSF1B,MTOR,YY1,NOTCH1,RGS4	turquoise	EC_M6
GO:0097094	2.43E-02	0.995125655	5	13	craniofacial suture morphogenesis	BP	3.61E-01	RAB23,GLI3,MEGF8,MMP14,TGFB3	darkgreen	EC_M4
GO:0060218	2.09E-02	0.994053374	7	28	hematopoietic stem cell differentiation	BP	3.61E-01	TAL1,SFRP1,TCF15,ITCH,LMBR1L,ACE,BATF	turquoise	EC_M6
GO:0002828	2.09E-02	0.99480542	6	22	regulation of type 2 immune response	BP	3.61E-01	IL6,RSAD2,GATA3,HLX,CD86,ARG2	turquoise	EC_M6
GO:1902307	2.44E-02	0.994071168	6	17	positive regulation of sodium ion transmembrane transport	BP	3.61E-01	KIF5B,ACTN4,FXYD1,ATP1B3,CHP1,DMD	darkgreen	EC_M4
GO:0071333	2.44E-02	0.986717685	23	110	cellular response to glucose stimulus	BP	3.61E-01	LRP1,PPARD,LGALS1,SRF,UNC13B,FKBP1B,KIF5B,IRS2,OSBP,SELENOT,OPA1,BRSK2,DYNLL1,ZBTB20,LRP5,PIK3CA,ADCY5,SIDT2,PTPRN,PPARGC1A,SMARCA4,PRKAA1,SERPINF1	darkgreen	EC_M4
GO:0010823	2.44E-02	0.990477355	11	43	negative regulation of mitochondrion organization	BP	3.61E-01	OPA1,DNM1L,TFRC,FZD9,MAPT,C11orf65,GHITM,PPARGC1A,AKT1,OMA1,BNIP3	darkgreen	EC_M4
GO:0031453	1.95E-02	0.997060298	4	11	positive regulation of heterochromatin assembly	BP	3.62E-01	TRIM28,SETDB2,FAM208A,TPR	darkgrey	EC_M8
GO:0120263	1.95E-02	0.997060298	4	11	positive regulation of heterochromatin organization	BP	3.62E-01	TRIM28,SETDB2,FAM208A,TPR	darkgrey	EC_M8
GO:1901796	1.95E-02	0.991357805	14	83	regulation of signal transduction by p53 class mediator	BP	3.62E-01	ZMPSTE24,KAT6A,RRS1,TAF9,ATR,EEF1E1,PAK1IP1,CHEK1,PRMT5,DDX5,AURKB,TAF1,PSMD10,AURKA	darkgrey	EC_M8

GO:0007044	2.46E-02	0.987321083	20	89	cell-substrate junction assembly	BP	3.63E-01	LRP1,VEGFA,DST,ROCK2,ACTG1,SORBS1,PTPN11,PHLDB2,CTTN,MMP14,CORO1C,IQGAP1,FN1,VCL,ACTN1,ROCK1,ITGA2,DAPK3,THY1,DUSP22	darkgreen	EC_M4
GO:0048483	2.12E-02	0.992791734	9	37	autonomic nervous system development	BP	3.63E-01	NRP1,GATA3,EDNRB,RET,NAV2,SEMA3F,HLX,TBX1,KIF26A	turquoise	EC_M6
GO:0043981	2.12E-02	0.99573138	5	15	histone H4-K5 acetylation	BP	3.63E-01	KANSL2,KANSL1,ING4,JADE3,HCFC1	turquoise	EC_M6
GO:0043982	2.12E-02	0.99573138	5	15	histone H4-K8 acetylation	BP	3.63E-01	KANSL2,KANSL1,ING4,JADE3,HCFC1	turquoise	EC_M6
GO:0070875	2.46E-02	0.995049374	5	13	positive regulation of glycogen metabolic process	BP	3.63E-01	DYRK2,AKT2,IRS2,SORBS1,AKT1	darkgreen	EC_M4
GO:0051651	2.47E-02	0.985103358	32	163	maintenance of location in cell	BP	3.64E-01	SUN2,FKBP1B,FGF2,ATP7B,HSP90B1,RYR3,CX3CL1,PKD2,TWF1,HTR2B,CASQ1,PTPN6,HTT,ERO1A,KDELR2,HAP1,CACNA1C,CALM1,ANXA6,AKT1,PAFAH1B1,PARK7,JPH3,SLC30A7,HSPA5,DMD,FKBP1A,TMEM38B,THY1,TXN,TMSB4X,TMEM38A	darkgreen	EC_M4
GO:0050870	2.47E-02	0.98597966	26	130	positive regulation of T cell activation	BP	3.64E-01	IL23A,HLA-DRA,FAM49B,LGALS1,LEP,DOCK8,NCK2,SOCS5,GLI3,GLI2,HSPH1,PTPN11,AP3B1,PTPN22,ADA,RUNX1,PIK3CA,LEF1,TFRC,PTPN6,IGFBP2,CD74,AKT1,CD40LG,BCL10,THY1	darkgreen	EC_M4
GO:0006887	2.12E-02	0.986378473	41	266	exocytosis	BP	3.64E-01	STX11,RIMS2,APBA1,KIT,SYK,SDCBP,RAB3B,PIK3CG,GATA2,EXOC3L2,GAB2,LLGL2,RALB,UNC13D,EXOC6,SNCA,DTNBP1,SNAP23,ATP9A,FGR,ADCY1,FES,ADORA2A,CDK16,SNAP29,RAB27A,SYT7,RSC1A1,MICAL3,FBXL20,PSEN1,RAB3D,LGALS9,GIT1,RPH3AL,NOTCH1,STXBP1,EXOC3L1,STX3,RALA,VAMP3	turquoise	EC_M6
GO:0048488	2.48E-02	0.989421662	13	52	synaptic vesicle endocytosis	BP	3.64E-01	CANX,PACSIN1,SH3GL2,PICALM,ACTG1,PPP3CC,DNM1L,AP2M1,SLC2A4,NLGN2,SYT11,CALM1,ROCK1	darkgreen	EC_M4
GO:0140238	2.48E-02	0.989421662	13	52	presynaptic endocytosis	BP	3.64E-01	CANX,PACSIN1,SH3GL2,PICALM,ACTG1,PPP3CC,DNM1L,AP2M1,SLC2A4,NLGN2,SYT11,CALM1,ROCK1	darkgreen	EC_M4
GO:0003416	2.49E-02	0.99295986	7	22	endochondral bone growth	BP	3.65E-01	RARB,FGFR2,COMP,FGFR3,EVC,THBS3,ANXA6	darkgreen	EC_M4
GO:1901607	2.49E-02	0.989761538	12	49	alpha-amino acid biosynthetic process	BP	3.65E-01	PSAT1,MTAP,ASNS,PLOD2,ALDH18A1,SERINC3,GLS,ATP2B4,PSPH,PLOD3,PARK7,ADI1	darkgreen	EC_M4
GO:0031325	2.14E-02	0.981951832	303	2483	positive regulation of cellular metabolic process	BP	3.66E-01	ZNF516,TNFRSF11A,TCF4,IFNGR1,TNFAIP3,PLAGL1,CNOT6,FLT4,ZBED4,GRAMD4,NOTCH4,TCF20,TPD52L1,NCOA7,PTK2,DAPK1,ATF4,PDGFB,AGTPBP1,BRPF3,CSNK1E,PICK1,PIM1,NFYA,TEK,PRDM4,LIMCH1,DDX58,FAM122A,TEC,ADGRF5,KIT,KDR,NFIL3,SYK,SEMA4D,IL6,REL,CREB5,CXCL8,PELI1,TLR4,IL15,CCNY,TEAD2,SDCBP,MYCN,NOCT,TUT4,NRP1,PIK3CG,TAL1,PHF10,EPC1,KDM4D,GATA2,PRKD2,CTSC,RAB38,CAB39L,NOS3,RALB,PPARG,GATA3,EDNRB,NDFIP2,TIE1,IKBKB,JAG1,P2RY6,NFKBIB,TMEM173,SFRP1,HBEGF,NR1D2,DND1,RPL26,ANKLE2,CD36,SIRT3,PRR5L,TCF15,SCARB1,EPHB1,PLK2,RET,MERTK,CHD7,IL1A,DNMT3B,IGF1,NR4A1,KLKB1,EIF2AK3,HHEX,KDM3A,ITCH,SMAD1,ZBTB38,CLCN3,HIP1R,STOX2,SOX7,HMBOX1,GPD1,HEXB,MEF2C,SMARCD1,TLR2,WWTR1,P2RX7,VEGFC,CXCL10,MADD,KMT2D,NLRC5,THBS4,PPP1R16B,TPCN1,RAPGEF3,NR4A2,DGKZ,TOX2,NEDD9,YAF2,EDN1,STAT1,DYRK1A,EHD4,SNCA,JARID2,ERG,ETS2,SLC45A3,NFKB2,RNF144B,PRDM15,ABCG1,ETS1,RNF19B,HDAC1,LCK,PPP3CA,HYAL2,MEF2A,BCL9,LAPTM5,NCF1,USP13,HCLS1,NFATC2,EAF2,GMEB1,PECAM1,CD86,POU2F3,SH3BP4,FGR,CFLAR,CASP10,INPP5F,PPP2R5A,ARID1A,RPS6KA1,PROX1,SS18L1,PRKD1,ZNF148,YTHDF1,HELZ2,MLXIPL,ACE,ITGA6,LIMK2,PAK2,MELTF,SENP2,DLG1,ELL3,ETV6,MAP3K11,HOXD10,MOV10,CREB1,TET1,LTF,ARNT2,EPHB4,DDX3X,RFXANK,ETV4,TBX1,MECOM,ING4,BIRC3,LDB2,SELE,TNFAIP8L3,FGF23,LMTK2,PPM1F,POU2F1,WAS,RAC1,HIF1A,KDM5A,ZBTB18,APBB2,JUP,RAB27A,ADIPOR2,EPHA2,BID,TLR6,TNFRSF1B,MTOR,AR,RNF40,CASZ1,PSEN1,HOXB5,HOXB7,HOXB9,FBXW11,FGF18	turquoise	EC_M6
GO:0009612	2.50E-02	0.984651403	34	176	response to mechanical stimulus	BP	3.66E-01	HABP4,ASNS,WHRN,ABHD12,CTNNB1,DCN,CXCL12,ANKRD1,MAP1B,CACNB3,PTPN11,PKD2,PSPH,MMP14,DAG1,PIK3CA,TLR5,BACE1,KCNK4,IGFBP2,HTT,PKD1L1,COL3A1,KCNA1,TCAP,COL1A1,AKT1,ITGA2,PKD1,COL11A1,BCL10,DMD,BNIP3,MBD2	darkgreen	EC_M4
GO:2000644	2.14E-02	0.996795367	4	10	regulation of receptor catabolic process	BP	3.66E-01	MTMR2,ITCH,MYLIP,LAPTM5	turquoise	EC_M6
GO:0070229	2.51E-02	0.992888718	7	22	negative regulation of lymphocyte apoptotic process	BP	3.67E-01	DOCK8,IRS2,ADA,SLC39A10,CD74,BCL10,ORMDL3	darkgreen	EC_M4

GO:0009894	1.41E-03	0.999014204	115	806	regulation of catabolic process	BP	3.68E-01	PIP4K2C,CDK4,IRAK3,BAG6,SNX9,HSPA1B,EHMT2,RNF5,EIF3H,TSPO,EP300,NRBP2,HSF1,VPS28,HMOX1,EEF1A1,RPS7,FOXO3,SESN1,NOP53,ZC3HAV1,BBS7,TRIM13,FASTK,MLH1,MYD88,EGLN2,TIMP2,SAMD4B,SIRT2,RPTOR,HGS,TRIM21,NDFIP1,HGF,PSMF1,PLEKHF1,SH3D19,CAMKK2,HTRA2,PRKAG1,FOXF2,RNFT2,TP53INP1,LONP2,FASTKD3,IRS1,TPCN2,DIS3L2,PUM1,GPX1,AC013489.1,USP19,DTX3L,ABCB11,MCL1,RHBDD3,ARNT,HDAC4,RNASEL,MAGEF1,RPL11,CDKN1B,KAT5,LRIG2,YBX3,NFE2L2,IDH1,CAPN1,EXOSC7,VSIR,PHB2,HDAC6,UBXN1,WASHC1,ATP13A2,GABARAP,FOXK1,THRA,ZFP36L1,PIP4K2B,OSBPL7,CDK5RAP3,KAT8,GBA,NUPR1,AC138894.1,MTMR4,TNRC6A,KEAP1,SNX33,INSR,SREBF1,LEPR,PARN,RILP,FZR1,DVL1,JMJD8,AXIN1,ZER1,ABCA2,BSCL2,ZBTB7A,RACK1,TRIM65,TENT5B,CST3,RNFT1,RPL23,TRIB2,HSPB8,ZFP36L2,STUB1,EXOSC5	cyan	EC_M2
GO:0061098	2.16E-02	0.992071849	10	44	positive regulation of protein tyrosine kinase activity	BP	3.69E-01	PDGFB,HBEGF,NEDD9,NCF1,ACE,PAK2,EFNA1,UNC119,RELN,ALKAL1	turquoise	EC_M6
GO:2001214	2.52E-02	0.996202138	4	9	positive regulation of vasculogenesis	BP	3.69E-01	RRAS,RAPGEF2,HIF1AN,TMEM100	darkgreen	EC_M4
GO:0044264	2.52E-02	0.987725773	17	75	cellular polysaccharide metabolic process	BP	3.69E-01	DYRK2,AKT2,IRS2,B3GNT4,GYG1,SORBS1,PFKM,SELENOS,GYG2,GSK3B,PPP1R3D,PPP1R3F,AKT1,AGL,HAS2,PPP1R1A,HS2ST1	darkgreen	EC_M4
GO:0045596	2.53E-02	0.981326817	83	489	negative regulation of cell differentiation	BP	3.69E-01	PPARA,PPARD,CTNNA1,RORB,VEGFA,REST,TGFBR1,SOCS5,RTN4,AKIRIN1,GLI3,INHBA,ITGB1,RB1,GLI2,PAK1,TGFB1,CTNNB1,LBH,RARB,ACTN4,CRIM1,SPRED3,SEMA3C,RYK,RNF6,ZNF536,DIP2B,SEMA4F,LOXL3,HEY1,TLX2,APP,RAPGEF2,ABCC8,PTPN11,RUNX1T1,SPP1,RUNX1,SEMA4G,EIF4E,ADIPOR1,PKP2,CDC73,CCND1,TGFB2,LRP5,GSK3B,CORO1C,KRAS,NTRK3,TCTA,BMP7,IDH2,TNR,GPR137,FBN1,ITGAV,COL5A2,SKIL,SIX4,PRTG,BMPR1A,CDK12,MED1,TGFB1I1,DICER1,MYOCD,CD74,TMEM98,PTPN2,VASN,PLXNA3,COL5A1,FSTL3,LBX1,THY1,FGL2,DACT3,YWHAH,BHLHE41,HOPX,FGF10	darkgreen	EC_M4
GO:0031345	2.16E-02	0.98777657	26	150	negative regulation of cell projection organization	BP	3.69E-01	SEMA6A,TBC1D30,CDKL3,SEMA4D,NRP1,EFNB2,PRAG1,CEP97,MYLIP,SRGAP2C,PPP3CA,SEMA3F,INPP5F,TRAK2,LIMK2,B2M,SEMA6D,DNM3,PSEN1,SEMA4A,ABI3,ITGA3,EFNA1,DNM2,SEMA6B,ARHGAP4	turquoise	EC_M6
GO:0007167	2.17E-02	0.983518262	105	752	enzyme linked receptor protein signaling pathway	BP	3.69E-01	SEMA6A,FLT4,MTSS1,PTK2,ANKS1A,PDGFB,TEK,KIT,KDR,SYK,CAV2,GRB10,TSPAN12,SDCBP,NRP1,TAL1,PRKD2,GAB2,PPARG,GATA3,ACVR2B,TIE1,SFRP1,HBEGF,FERMT1,EFNB2,ARHGEF7,EPHB1,RET,MERTK,IGF1,EIF2AK3,HHEX,ERBIN,SMAD1,HIP1R,ARHGEF28,WWTR1,VEGFC,NDRG4,ANGPT2,SOGA1,TIPARP,PLCG1,NEDD9,SNCA,LCK,SHOC2,NCF1,FGR,KALRN,IGFBP3,FES,PLEKHA1,PRKD1,SRMS,PAK2,PTPRE,APOD,STYK1,CREB1,CLEC14A,BCR,EPHB4,IGFBP5,BAIAP2L1,FGF23,LMTK2,RAC1,HIF1A,EPHA2,MMRN2,TNK1,MPP5,RGS14,AR,EFNB1,PSEN1,FGF18,ITGA3,PGF,EFNA1,NPR1,RPS6KA5,SMAD2,DNM2,COL4A6,COL4A5,FAM83G,SH3TC2,ADRB2,IL13RA1,YES1,GIT1,LRG1,TMEM204,NOTCH1,RAPGEF1,ENG,DAB2IP,CDH5,FGF16,TGIF2,BMP2,BMP6	turquoise	EC_M6
GO:0046486	2.53E-02	0.982529596	56	317	glycerolipid metabolic process	BP	3.69E-01	SLC22A4,SERINC1,INPP4A,TMEM246,SOCS5,FAM126A,MFSD2A,FGF2,SELENOI,ABHD12,ABHD5,DGKH,DGAT2,OSBPL10,MTMR14,OSBPL8,IMPAD1,MTMR6,CHPT1,TMEM150A,FAR1,PTPN11,PIP4P2,LPCAT2,SERINC3,PIGP,AGPAT3,CPT1A,HTR2B,INPP5A,PIK3CA,PITPNM1,ETNK1,PIGZ,PCYT1B,MOGAT3,PLD1,SEL1L,SLC44A2,PIGL,ACSL4,PDGFRB,PNPLA6,LPIN2,OCRL,PAFAH1B1,ABCA3,PGP,DPM2,PLA2G15,PEMT,PIGK,DPM1,PIGB,PIK3C2A,CDIPT	darkgreen	EC_M4
GO:2001212	2.53E-02	0.994862186	5	13	regulation of vasculogenesis	BP	3.69E-01	RRAS,HEY1,RAPGEF2,HIF1AN,TMEM100	darkgreen	EC_M4
GO:0010637	2.54E-02	0.996173448	4	9	negative regulation of mitochondrial fusion	BP	3.70E-01	DNM1L,TFRC,OMA1,BNIP3	darkgreen	EC_M4
GO:0010092	2.54E-02	0.991226319	9	32	specification of animal organ identity	BP	3.70E-01	GLI3,FGF2,SMARCD3,CTNNB1,ROBO1,FGFR2,GDNF,WNT2,FGF10	darkgreen	EC_M4
GO:0030111	2.54E-02	0.982842719	50	277	regulation of Wnt signaling pathway	BP	3.70E-01	LRP1,RSPO3,CBY1,GNAQ,CDK14,VCP,TPBG,MDFIC,GLI3,DKKL1,PLEKHA4,FGF2,TGFB1,ZEB2,ZNF703,ILK,SULF1,CTNND1,DKK3,APP,AMFR,NKD1,CDC73,CAPRIN2,GSK3B,LEF1,KPNA1,BICC1,FGFR2,FZD9,ATP6AP2,IGFBP2,OTUD5,COL1A1,PBXIP1,SOX30,SMARCA4,GSKIP,PIN1,NLK,NXN,DAPK3,TAX1BP3,CSNK1G2,NPHP4,WLS,EDA,DACT3,FGF10,MBD2	darkgreen	EC_M4

GO:0051248	2.55E-02	0.97998458	130	813	negative regulation of protein metabolic process	BP	3.70E-01	SERPINB2,LRP1,AGAP2,AZIN1,ANGPT1,ADGRB1,SH3GL2,GNAQ,CDKN2B,PLAA,VEGFA,NCK2,PDCL3,P3H1,SOCS5,COPS9,RTN4,SH3BP5L,ROCK2,FKBP1B,DNAJC1,RB1,ITM2B,PICALM,CRTAP,TGFB1,SPOPL,CTNNB1,SERPINH1,GPD1L,KAT2B,CRIM1,SPINT2,PRNP,DDRGK1,SMPD1,ILK,SPINK5,CNOT7,FXYD1,SNTA1,TMED2,PINX1,CELF1,APP,DHX36,PTX3,PTPN22,TSG101,CRY2,CHAC1,CHP1,ATP2B4,DBNDD2,CBLB,TENT4B,CHRNA7,EIF4E,AZIN2,TM4SF20,UCHL5,TRIP12,SLC2A10,CAPRIN2,LRP5,EPRS,PRKAR1A,GSK3B,CORO1C,ANOS1,NTRK3,BMP7,PPP1R11,COL6A3,RPS6KA3,HSPB1,MAPT,SERPINE1,EIF4EBP2,DNAJC10,PTPN6,UPF1,ITGAV,FKBP8,SAMD4A,RTN3,TIMP1,FMN2,HAP1,CLN8,EIF2S1,PPARGC1A,SRCIN1,CPEB4,TMED10,CALM1,PPP4R4,GSKIP,MYOCD,PIN1,ROCK1,USP14,AKT1,TMEM98,PRKAA1,SUPT6H,PTPN2,NXN,DAPK3,FLNA,PARK7,GLG1,MIR24-1,PRMT6,DMD,FKBP1A,SPINK2,SERPINF1,SERPINB5,AGO3,A2M,FRY,ITM2C,TIMP3,THY1,CDKN1A,DUSP22,PMEPA1,GABARAPL2,MAP1A	darkgreen	EC_M4
GO:0016525	2.55E-02	0.98726671	18	82	negative regulation of angiogenesis	BP	3.70E-01	SLC12A2,FAM19A5,ADGRB1,ROCK2,CTNNB1,DCN,SPINK5,SULF1,ABCC8,ATP2B4,TGFB2,ADGRB2,SERPINE1,NPPB,ROCK1,SARS,MIR24-1,SERPINF1	darkgreen	EC_M4
GO:0007169	2.56E-02	0.981007084	85	499	transmembrane receptor protein tyrosine kinase signaling pathway	BP	3.71E-01	CD63,LRP1,AP3S1,AHI1,ANGPT1,ROS1,NUS1,PTP4A3,LEP,VEGFA,RUNX2,NCK2,SOCS5,RTN4,ADAMTS3,FGF2,SESN3,RAB7A,PAK1,TGFB1,AXL,CTNNB1,AKT2,OSBPL8,CRIM1,IRS2,DCN,LEPROTL1,RYK,SULF1,ROBO1,SORBS1,DOK1,RASA1,RAPGEF2,PTPN11,OPA1,ZFYVE27,TSG101,MMP2,CBLB,DOK2,NDN,ADIPOR1,ZNF106,GSK3B,PIK3CA,NTRK3,GFRA1,FGFR2,IQGAP1,MUC20,ARID5B,HSPB1,EIF4EBP2,SGPL1,IGFBP2,FGFR3,HAP1,SLC2A4,MYO1E,FGFRL1,NGFR,COL1A1,SHC1,NPTN,MYOCD,SS18,PDGFRB,AKT1,PRKAA1,PTPN2,MYO1C,RHBDF1,CDH13,F3,BCAR3,SLC2A8,PTPN12,LOX,ARF4,CHN1,LEPROT,PIK3C2A,FGF10	darkgreen	EC_M4
GO:0032446	2.01E-02	0.985246956	83	703	protein modification by small protein conjugation	BP	3.71E-01	MEX3C,SHPRH,FBXO43,FBXO5,UBR5,UHRF2,UBR2,TOPORS,TRIM28,ZNF451,FBXO11,SENP6,ASB3,COMMD1,UFL1,CUL1,SAE1,PAXIP1,CDC20,GTPBP4,RAD18,TPP2,CUL4A,CDC16,LNX2,ANAPC1,UBA2,TNKS2,RNF141,ZFP91,HLTF,ANAPC7,LTN1,TRIM33,UBE2C,HERC5,UBE3A,CUL3,UBE2T,ASB7,TRAIP,UBR1,FANCI,DTL,UBR3,G2E3,IVNS1ABP,CDC27,FANCM,LRR1,COP1,BRCA1,CUL5,CDCA3,DCUN1D5,DCAF6,LEO1,UBE2K,NEDD4,MED6,TAF1,OGT,RLIM,RBBP6,TRIM37,PLK1,APPBP2,PSMD10,MIB1,CUL4B,XIAP,UHRF1,BLMH,CCNF,GLMN,MAD2L1,RNF139,RNF8,TRIM59,GID4,UBXN2A,RNF219,RCHY1	darkgrey	EC_M8
GO:0051129	2.56E-02	0.980640459	95	569	negative regulation of cellular component organization	BP	3.71E-01	LRP1,FAM49B,PACSIN1,LGALS1,VEGFA,RTN4,SHANK1,ROCK2,RAB7A,EML2,PICALM,TGFB1,DYNC1LI1,NAV3,ANAPC15,KAT2B,PRNP,SEMA3C,DPYSL3,RYK,RNF6,SACS,MAP1B,DIP2B,PINX1,SEMA4F,PFN2,TLX2,ATXN2,RAPGEF2,ADCY6,OPA1,SPP1,TWF1,SEMA4G,TENT4B,FAP,PHLDB2,TJP1,DNM1L,LMOD1,SEC22B,FEZ1,PRKAR1A,GSK3B,MMP14,PIK3CA,CORO1C,HSPA8,BMP7,SCFD1,TFRC,FZD9,MAPT,DNAJA4,TNR,C11orf65,ITGAV,LRPAP1,CAPZB,VDAC2,MNAT1,CRMP1,GHITM,CGNL1,PPARGC1A,STX1B,ARHGEF2,SYT11,TEX14,SCAMP5,ROCK1,FLII,EVI5L,AKT1,TUBB4A,OMA1,CLEC16A,PLXNB3,PAFAH1B1,FLNA,PLXNA3,PARK7,SPTAN1,HSPA5,LMO4,BNIP3,ITM2C,THY1,TMSB4X,NME6,DUSP22,YWHAH,PMEPA1,LRRTM2	darkgreen	EC_M4
GO:0031061	2.18E-02	0.994685529	6	19	negative regulation of histone methylation	BP	3.72E-01	KDM4D,DNMT3B,KDM3A,JARID2,BCOR,MTF2	turquoise	EC_M6
GO:0062208	2.18E-02	0.993061605	8	33	positive regulation of pattern recognition receptor signaling pathway	BP	3.72E-01	PELI1,RSAD2,TLR4,PUM2,TLR2,LTF,DDX3X,TLR1	turquoise	EC_M6
GO:0007610	2.19E-02	0.984740752	60	406	behavior	BP	3.72E-01	OXR1,AGTPBP1,ATP8A1,APBA1,KIT,ADGRF1,TMOD1,PPT1,FOXP2,TAL1,DACH1,SPTBN4,NR1D2,P2RX2,RIC8A,TCF15,PLK2,CHD7,PREX2,CLCN3,HEXB,MEF2C,TLR2,NDRG4,THBS4,NR4A2,TANC1,NAV2,SNCA,SHANK2,HCRTR1,ZFHX2,CIART,INPP5F,ADCY1,YTHDF1,PTCHD1,HOXD10,CREB1,B2M,ADORA2A,TBX1,TMOD2,HIF1A,SNCG,CHRNB1,RGS14,FBXL20,MTOR,PSEN1,HOXB8,ITGA3,CHRNB2,PIAS1,GIT1,STRBP,SLC8A3,ATXN1L,RELN,SHANK3	turquoise	EC_M6
GO:2001056	2.19E-02	0.98907343	18	105	positive regulation of cysteine-type endopeptidase activity	BP	3.72E-01	GRAMD4,DAPK1,SYK,PPARG,EIF2AK3,HIP1R,SOX7,SNCA,LCK,LAPTM5,CFLAR,CASP10,DDX3X,PPM1F,BID,LGALS9,PMAIP1,PERP	turquoise	EC_M6

GO:0051145	2.19E-02	0.991004982	12	57	smooth muscle cell differentiation	BP	3.72E-01	PDGFB,KIT,EPC1,EDNRB,MEF2C,NFATC2,RAMP2,PIAS1,MRTFB,TMEM204,NOTCH1,ENG	turquoise	EC_M6
GO:0051606	2.57E-02	0.985162247	27	135	detection of stimulus	BP	3.72E-01	GNAQ,WHRN,PAK1,CTNNB1,STIM1,CXCL12,GRK7,CACNB3,PKD2,FAP,PITPNM1,BACE1,KCNK4,PKD1L1,KCNA1,ROM1,TCAP,NGFR,KCNMB1,TGFB3,CALM1,ITGA2,PKD1,COL11A1,OR5AN1,OR6N2,KCNMB4	darkgreen	EC_M4
GO:0051349	2.57E-02	0.9947588	5	13	positive regulation of lyase activity	BP	3.72E-01	STIM1,CACNA1D,ISCU,CACNA1C,PARK7	darkgreen	EC_M4
GO:0048844	2.58E-02	0.988531902	14	57	artery morphogenesis	BP	3.72E-01	LRP1,SRF,VEGFA,TGFBR1,HEY1,PKD2,TGFB2,COMP,PRRX1,AKT3,FKBP10,BMPR1A,MYOCD,PDGFRB	darkgreen	EC_M4
GO:0046888	2.59E-02	0.989295233	12	48	negative regulation of hormone secretion	BP	3.73E-01	LEP,REST,INHBA,FKBP1B,RAB11FIP1,ABCC8,PTPN11,CRY2,HADH,OSM,SYTL4,MIDN	darkgreen	EC_M4
GO:0045059	2.59E-02	0.996064644	4	9	positive thymic T cell selection	BP	3.73E-01	SRF,DOCK2,CD74,PTPN2	darkgreen	EC_M4
GO:0045601	2.21E-02	0.993110821	8	29	regulation of endothelial cell differentiation	BP	3.74E-01	NOTCH4,IKBKB,JAG1,F11R,APOLD1,NOTCH1,CDH5,BMP6	turquoise	EC_M6
GO:0070862	2.61E-02	0.996032244	4	9	negative regulation of protein exit from endoplasmic reticulum	BP	3.75E-01	ERLEC1,UBE2J1,UBE2G2,DERL2	darkgreen	EC_M4
GO:0001894	2.61E-02	0.983631801	36	189	tissue homeostasis	BP	3.76E-01	SLC12A2,ANGPT1,SH3GL2,SRF,VEGFA,MFSD2A,WHRN,NCDN,RCN3,ITGB1,RB1,CTNNB1,ACTG1,IL20RB,TP53INP2,P2RX4,ABCC8,PTPN11,HOMER1,SPP1,TJP1,PRKCA,KRAS,STRAP,TFRC,HSPB1,GPR137,VCL,AKT3,CLN8,ABCA3,NPHP4,ATP6AP1,DEF8,DMD,SNX10	darkgreen	EC_M4
GO:0051347	2.04E-02	0.985973934	56	441	positive regulation of transferase activity	BP	3.76E-01	CCT2,MAP3K4,DSCC1,XRCC6,POT1,TOPORS,DBF4,CKS2,HNRNPA2B1,CCT4,ETAA1,ITGB1BP1,EZH2,CLSPN,BORA,CCNT2,CDC20,KITLG,RFC3,CCNB1,ERCC6,TPX2,CDK5RAP1,ZFP91,NBN,STK4,UBE2C,RFC5,TAOK3,BMPR1B,GCN1,CENPE,KIF14,AJUBA,RFC2,NEK2,ALS2,RFC4,DBF4B,XRCC5,DCUN1D5,ECT2,INCENP,CDC6,AURKB,PLK1,CSK,PSMD10,RICTOR,TAOK1,CHTF18,PRIM2,HNRNPD,TIGAR,NAF1,ERP29	darkgrey	EC_M8
GO:0060292	2.23E-02	0.994480964	6	20	long-term synaptic depression	BP	3.77E-01	PICK1,PLK2,ABHD6,SHANK2,STXBP1,SHANK3	turquoise	EC_M6
GO:0018394	2.05E-02	0.989030156	22	143	peptidyl-lysine acetylation	BP	3.77E-01	ZNF451,ZMPSTE24,HDAC2,PAXIP1,KAT14,EPC2,KAT6A,TAF7,BRCA2,TAF9,MCRS1,CHEK1,NAA50,MCM3AP,HAT1,ING5,BRCA1,TAF1,OGT,ESCO1,CTCF,AC118549.1	darkgrey	EC_M8
GO:0006950	2.24E-02	0.980956761	324	2715	response to stress	BP	3.78E-01	ZNF516,TNFRSF11A,IFNGR1,TNFAIP3,TRIM39,LYZ,TSPAN8,FLT4,FZD6,OXR1,NOTCH4,ASF1A,TPD52L1,NCOA7,MTSS1,PTK2,C6orf106,DAPK1,ATF4,PDGFB,MAPK13,CSNK1E,PICK1,LRRC19,TEK,DDX58,NFX1,TEC,DNAJB5,SH3RF3,KIT,KDR,SYK,GADD45G,IL6,REL,CXCL8,RSAD2,FIGNL1,TLR4,TRAF1,C5,GJA4,IL15,SDCBP,NRP1,PUM2,PIK3CG,MGLL,KDM4D,GATA2,DNMT3A,MSRB2,CST7,CTSC,GPR4,NECTIN2,PRCP,NOS3,PSG3,ITGA9,RALB,ITGB4,UNC13D,PPARG,GATA3,CXCR4,IRAK2,SEC14L1,EDNRB,NET1,IKBKB,SEL1L2,PDE2A,TMEM173,SFRP1,HBEGF,NR1D2,FERMT1,RPL26,ASH2L,P2RX2,SLC4A11,CD36,ARID5A,PRR5L,SCARB1,EPHB1,PLK2,RET,MERTK,TNFRSF19,IL1A,DNMT3B,IGF1,BPIFB3,KLKB1,EIF2AK3,HHEX,ITCH,LYVE1,SH3RF1,SMAD1,ZBTB38,OCLN,PROCR,MCTP1,MEF2C,TLR2,P2RX7,VEGFC,ENTPD1,CXCL10,ANGPT2,PLLP,SERINC5,OAS3,NLRC5,THBS4,ACTR5,DYSF,PLCG1,STC1,ANKRD2,NR4A2,DGKZ,STAB1,HIPK1,ANO6,CHST1,IFIT2,IFIT1,TSPAN2,EDN1,STAT1,CD58,DYRK1A,JAM3,SNCA,MMRN1,DTNBP1,RBBP5,NUAK2,MX2,MX1,ETS1,RNF19B,CD200,SIK1,HDAC1,LCK,PPP3CA,HYAL2,ANO1,ICOSLG,SNAP23,LYST,LAPTM5,NCF1,DYRK3,USP13,FABP4,ISG20,NFATC2,TIFA,CD34,POLG,KDM2A,FGR,MCAM,CFLAR,MAP3K6,INPP5F,ADCY1,LTB4R,FES,RBM4,RPS6KA1,PLEKHA1,PRKD1,ZNF148,F11R,MAP3K3,SRMS,PAK2,APOLD1,DLG1,ELL3,SIPA1,APOD,MAP3K11,STYK1,MOV10,PDK3,CREB1,B2M,USP28,SSTR1,LTF,CCRL2,ADORA2A,MASP1,RTP4,ARNT2,CYBB,MYEF2,IL1RAP,DDX3X,TMEM106A,MECOM,CALCRL,ING4,HSPG2,RAMP2,BIRC3,SELE,SELP,VWF,CPEB2,CRACR2A,WAS,RAC1,HIF1A,LIPA,RAB27A,ADIPOR2,BCL6B,	turquoise	EC_M6
GO:0098781	2.06E-02	0.992362996	10	53	ncRNA transcription	BP	3.78E-01	PWP1,TAF1B,NIFK,ZC3H8,POLR1B,ZNF143,NCL,NOL11,DDX11,ICE2	darkgrey	EC_M8

GO:0042127	2.64E-02	0.97832918	191	1230	regulation of cell population proliferation	BP	3.79E-01	IL23A,B4GALT7,ENPP3,ANGPT1,FAM19A5,ADGRB1,PPARD,CTNNA1,LEP,CDKN2B,SRF,NUAK1,VEGFA,RUNX2,C2orf40,NCK2,PDCL3,REST,P3H1,TGFBR1,COPS9,RTN4,TPBG,EMC10,AKIRIN1,TES,GLI3,INHBA,SLC7A11,ITGB1,FGF2,SRPK2,RB1,CGREF1,GLI2,SMARCD3,PAK1,TGFB1,SCN5A,WDR48,ACER3,CTNNB1,LBH,AKT2,RARB,KAT2B,FAM98A,MAFG,SPINT2,LIG4,IRS2,PRNP,ZNF703,CCKBR,DDRGK1,ILK,CIAO1,TMEM127,CNOT7,LMO1,CXCL12,IL20RB,SULF1,ROBO1,PINX1,CELF1,APP,FAM98B,RAPGEF2,CX3CL1,ABCC8,GNL3,PTPN22,TSG101,MMP2,ADA,BAP1,PKD2,RUNX1,CBLB,BRD7,IFT57,NDN,SLC39A10,CHRNA7,FAP,TJP1,KDM5B,NOTCH2,CDC73,MARCKSL1,HYAL1,CCND1,TGFB2,LRP5,HTR2B,PRKAR1A,LEF1,PRKCA,KRAS,NTRK3,BMP7,IDH2,ATF3,FGFR2,VASH2,OSM,HTRA1,S100A11,TFRC,FZD9,EGLN3,OVOL1,PRDX4,COMT,FN1,PTPN6,IGFBP2,PDCD10,ITGAV,COMP,PRKCI,FGFR3,PRRX1,TIMP1,PTPRN,SIX4,MNAT1,AKT3,MAGED1,BMPR1A,MED1,PPARGC1A,FGFRL1,NES,ARHGEF2,TGFB1I1,NGFR,TPM1,TGFB3,SHC1,PBXIP1,MAP2K1,CNN1,DICER1,CLMN,MYOCD,CD74,PDGFRB,AKT1,UBE2A,ITGA2,PRKAA1,OSMR,PTPN2,NPR3,CD40LG,MYDGF,LDOC1,ROGDI,PLXNB3,TAX1BP3,BRICD5,IRAK1,FLNA,SSTR5,GNAI3,CDH13,CFDP1,F3,MIR24-1,SERPINF1,SERPINB5,AGO3,PEMT,CD164,LBX1,GDNF,HAS2,CDKN1A,NACC2,DUSP22,TSLP,CAPNS1,P3H3,PRKG1,CHST11,DERL2,WNT2,FGF10,MBD2	darkgreen	EC_M4
GO:0021543	2.65E-02	0.984895094	26	127	pallium development	BP	3.79E-01	LRP1,TRAPPC9,SUN2,MDGA1,SRF,UBA6,RTN4,MFSD2A,GLI3,KIF5B,EIF2B3,HTR5A,CTNNB1,TACC1,ROBO1,CELF1,ATP2B4,FEZ1,GSK3B,LEF1,COL3A1,KCNA1,PAFAH1B1,FLNA,PLXNA3,BNIP3	darkgreen	EC_M4
GO:0001975	2.26E-02	0.994228416	6	23	response to amphetamine	BP	3.81E-01	NR4A1,NR4A2,HDAC1,PPP3CA,ADORA2A,RGS4	turquoise	EC_M6
GO:0046474	2.67E-02	0.983365454	35	185	glycerophospholipid biosynthetic process	BP	3.82E-01	INPP4A,TMEM246,SOCS5,FAM126A,MFSD2A,FGF2,SELENOI,ABHD5,DGKH,MTMR14,IMPAD1,MTMR6,CHPT1,TMEM150A,FAR1,LPCAT2,PIGP,AGPAT3,HTR2B,PIK3CA,PITPNM1,ETNK1,PIGZ,PCYT1B,PLD1,SLC44A2,PIGL,OCRL,DPM2,PEMT,PIGK,DPM1,PIGB,PIK3C2A,CDIPT	darkgreen	EC_M4
GO:0010817	2.67E-02	0.981353473	58	331	regulation of hormone levels	BP	3.83E-01	HSD17B6,LRP1,PPARD,LEP,CLOCK,REST,INHBA,FKBP1B,KIF5B,DGAT2,C1QTNF1,IRS2,RAB11FIP1,PCLO,OSBP,VAMP8,DKK3,PCSK1,RDH10,SELENOT,ABCC8,PTPN11,PFKM,CRY2,SPP1,BRSK2,DYNLL1,KDM5B,CPT1A,LRP5,HADH,ADCY5,OSM,PCSK7,SIDT2,LINC02210-CRHR1,SGPL1,ATP6AP2,COMT,TFR2,ECE1,HSD17B1,PTPRN,CYP19A1,HSD17B10,NLGN2,STARD3,MED1,PPARGC1A,UBE2Q1,SYTL4,CYP46A1,ACSL4,RASL10B,MIDN,SSTR5,VAMP7,PARK7	darkgreen	EC_M4
GO:0002790	2.68E-02	0.983588289	33	172	peptide secretion	BP	3.83E-01	LRP1,PPARD,LEP,CLOCK,REST,FKBP1B,KIF5B,IRS2,PCLO,OSBP,SELENOT,ABCC8,PTPN11,PFKM,BRSK2,DYNLL1,CPT1A,LRP5,HADH,ADCY5,SIDT2,LINC02210-CRHR1,TFR2,PTPRN,NLGN2,UBE2Q1,SYTL4,ACSL4,CD74,RASL10B,MIDN,SSTR5,PARK7	darkgreen	EC_M4
GO:0050878	2.28E-02	0.985308526	41	273	regulation of body fluid levels	BP	3.83E-01	TSPAN8,FZD6,PDGFB,TEC,SYK,IL6,TLR4,PIK3CG,NOS3,EDNRB,P2RY2,GPAT4,CD36,MERTK,KLKB1,PROCR,ENTPD1,DGKZ,ANO6,EDN1,MMRN1,DTNBP1,LCK,HYAL2,CD34,F11R,CREB1,ADORA2A,WNK4,SELP,VWF,WAS,HIF1A,RAB27A,PEAR1,NPR1,SCNN1B,F8,PLCG2,STXBP1,FLI1	turquoise	EC_M6
GO:0007614	2.68E-02	0.995883286	4	9	short-term memory	BP	3.83E-01	CHRNA7,COMT,SLC2A4,SERPINF1	darkgreen	EC_M4
GO:0044848	2.69E-02	0.995876161	4	9	biological phase	BP	3.83E-01	CTNNB1,SPINK5,SYCP2L,TGFB2	darkgreen	EC_M4
GO:0030050	2.69E-02	0.993302591	6	17	vesicle transport along actin filament	BP	3.83E-01	ACTN4,MYO5A,MYO1E,MYO1D,MYO5B,MYO1C	darkgreen	EC_M4
GO:0099174	2.69E-02	0.992250696	7	22	regulation of presynapse organization	BP	3.83E-01	MDGA1,LRRC4B,VPS35,FARP1,APP,NTRK3,NTNG2	darkgreen	EC_M4
GO:1905606	2.69E-02	0.992250696	7	22	regulation of presynapse assembly	BP	3.83E-01	MDGA1,LRRC4B,VPS35,FARP1,APP,NTRK3,NTNG2	darkgreen	EC_M4
GO:0007007	2.09E-02	0.993868054	7	35	inner mitochondrial membrane organization	BP	3.83E-01	HSPA9,CHCHD3,TOMM70,LETM1,TIMM8A,AFG3L2,DNAJC11	darkgrey	EC_M8
GO:0035886	2.29E-02	0.993424858	7	26	vascular associated smooth muscle cell differentiation	BP	3.84E-01	PDGFB,KIT,EPC1,NFATC2,RAMP2,NOTCH1,ENG	turquoise	EC_M6

GO:0044267	1.50E-03	0.998769067	460	3725	cellular protein metabolic process	BP	3.84E-01	PYM1,ATG12,MAP3K5,OS9,CDK4,CTDSP2,ATP23,MAML1,SQSTM1,SASH1,IRAK3,GFPT2,BAG6,PPP6R2,SNX9,HSPA1B,EHMT2,HDAC10,SELENOO,ALG12,RNF5,EIF3H,RPS6KA2,DEPTOR,TSPO,RING1,RPS18,B3GALT4,JADE2,PMM1,MYC,SMAD5,EP300,CDC14C,KDM3B,EEF1D,TAB1,RPL3,JOSD1,NRBP2,STK38,EIF3L,GPAA1,SHARPIN,EIF3D,FOXRED2,HSF1,FBXL6,VPS28,CCND3,PPP1R16A,TRERF1,CDKN2A,RPL8,CUL7,CUL9,PIGO,PDGFRA,JAK2,HINT2,RECK,RNF38,RPS5,ANKRD39,ALG2,RPL28,RPS9,EEF1A1,CD109,GPNMB,MALSU1,STAP1,EFEMP1,RPS7,ANTXR1,STK40,AKT1S1,ING3,ARHGEF5,RPS11,GSN,PARD3,FOXO3,KLF15,ADCK2,SPHK2,RPL18,SPRY1,NOP53,ABTB1,KMT2E,BBS7,HMCES,FKRP,LRRC41,TRIM13,IRF2BP1,QPCTL,SERP2,FASTK,SEPHS1,RPS8,XRCC1,MIF4GD,AGAP3,NAA16,MLH1,RNF220,LIPE,UNK,GALNT11,WBP2,TRIM47,KMT2C,DMAC2,EGLN2,BABAM2,TADA3,RPS3,BRPF1,DYRK1B,TGFBR2,RPS16,TIMP2,ENGASE,SAMD4B,XDH,MRPS12,SIRT2,RPTOR,RPL15,HGS,UBAC2,ASPSCR1,EIF3K,GALNT15,FGFR1,TRIM21,RNF24,NDFIP1,SPRY4,HGF,APBB1,TAF10,CLIP3,PCBP2,PSMF1,TARBP2,KMT2B,EIF3F,RBCK1,SDR16C5,TNS2,EIF4B,PTPRG,POFUT1,LATS2,KCTD6,RMND5A,PIK3R1,GGCX,CAST,FBXO8,PCOLCE2,TNKS1BP1,RPS3A,SH3D19,ARRDC3,KDM2B,CAMKK2,AGA,HTRA2,EIF2A,SETD6,PPP1CC,PDGFC,SPRED1,CCNG2,FNIP2,DGUOK,PRKAG1,ARL2BP,TIAM1,NEK4,SPCS1,PROS1,TMEM67,FOXF2,RNFT2,FARS2,PIDD1,RPLP2,ERLIN1,HR,PIGT,NGF,HERC6,PCIF1,WDR82,TRIM45,PPM1M,FBXW4,WARS2,LONP2,PARP3,FASTKD3,ELK4,IFIH1,MRPL36,CSTB,TCIRG1,UBA7,DIS3L2,PUM1,APEH,GPX1,AC013489.1,AIP,USP	cyan	EC_M2
GO:0045621	2.29E-02	0.989736233	14	72	positive regulation of lymphocyte differentiation	BP	3.85E-01	SYK,IL15,GATA3,IL7,SOX13,HLX,INPP5D,CD86,ITPKB,LGALS9,IL7R,AP3D1,VNN1,CD46	turquoise	EC_M6
GO:0050905	2.71E-02	0.985345649	22	103	neuromuscular process	BP	3.85E-01	SHANK1,MYH14,UBA5,ATP2A2,APP,GLRB,HOMER1,ADCY5,CASQ1,BACE1,TNR,COMP,KCNA1,NLGN2,CLN8,TCAP,PAFAH1B1,JPH3,AARS,DMD,MAP1A,NPAS1	darkgreen	EC_M4
GO:1903307	2.30E-02	0.992042271	9	37	positive regulation of regulated secretory pathway	BP	3.85E-01	SYK,GATA2,GAB2,UNC13D,FGR,RAB27A,SYT7,RAB3D,STXBP1	turquoise	EC_M6
GO:0038083	2.31E-02	0.994314233	6	19	peptidyl-tyrosine autophosphorylation	BP	3.85E-01	TEC,KDR,DYRK1A,BMX,SRMS,ACE	turquoise	EC_M6
GO:0060445	2.72E-02	0.993120623	6	18	branching involved in salivary gland morphogenesis	BP	3.86E-01	PLXNA1,SEMA3C,DAG1,BMP7,FGFR2,FGF10	darkgreen	EC_M4
GO:0006607	2.11E-02	0.996655966	4	12	NLS-bearing protein import into nucleus	BP	3.86E-01	IPO5,NUP54,KPNB1,NUP35	darkgrey	EC_M8
GO:0043300	2.32E-02	0.992590884	8	32	regulation of leukocyte degranulation	BP	3.87E-01	SYK,GATA2,GAB2,UNC13D,FGR,FES,LGALS9,STXBP1	turquoise	EC_M6
GO:0045664	2.32E-02	0.98693528	25	145	regulation of neuron differentiation	BP	3.87E-01	TCF4,CSNK1E,KDM4C,MYCN,NRCAM,GATA2,EDNRB,JAG1,SFRP1,RET,DNMT3B,MEF2C,ZFHX2,PROX1,B2M,ZC4H2,MTOR,CASZ1,NAP1L2,HMG20A,NOTCH1,MMD,TGIF2,BMP2,BMP6	turquoise	EC_M6
GO:0060612	2.73E-02	0.990446845	9	32	adipose tissue development	BP	3.87E-01	PPARD,LEP,PGRMC2,DGAT2,LRP5,PIK3CA,ARID5B,PPARGC1A,SPTLC2	darkgreen	EC_M4
GO:0010592	2.74E-02	0.992016122	7	23	positive regulation of lamellipodium assembly	BP	3.88E-01	AKIRIN1,ACTR2,ACTR3,PIK3CA,AUTS2,NCKAP1,ARPC2	darkgreen	EC_M4
GO:0009266	2.75E-02	0.98404283	27	137	response to temperature stimulus	BP	3.89E-01	VCP,PDCL3,EIF2B3,CLPB,ATP2B1,CXCL12,ATP2A2,DHX36,GLRX2,HTR2B,GSK3B,CASQ1,RBBP7,MAPT,DNAJA4,KCNK4,ERO1A,EIF2S1,PPARGC1A,TGFB1I1,NGFR,TRPV2,AKT1,PRKAA1,IRAK1,HSBP1,CDKN1A	darkgreen	EC_M4
GO:0050878	2.75E-02	0.981414573	49	273	regulation of body fluid levels	BP	3.89E-01	SERPINB2,LMAN1,CDO1,SLC22A4,GNAQ,MYH9,SRF,VEGFA,SLC7A11,DGKH,AXL,C1QTNF1,ACTG1,ILK,VAMP8,MYL9,SLC4A5,ADCY6,AP3B1,ADA,HPS6,FAP,CCND1,ITPK1,PIK3CA,PRKCA,COPA,HSPB1,SERPINE1,ITGA2B,FN1,PTPN6,ADRA2C,COMP,COL3A1,VCL,MED1,NPPB,ITGA2,NPR3,CD40LG,MYO5B,FLNA,F3,CD59,CSRP1,HAS2,PRKG1,FGF10	darkgreen	EC_M4

GO:0032870	2.75E-02	0.979792799	77	450	cellular response to hormone stimulus	BP	3.89E-01	AP3S1,PPARA,PPARD,LEP,CLOCK,ASNS,REST,ROCK2,INHBA,MGARP,PGRMC2,SESN3,PAK1,LBH,AKT2,OSBPL8,RARB,KAT2B,UFM1,IRS2,ATP2B1,UBA5,DDRGK1,LEPROTL1,RNF6,MAP1B,SORBS1,HEY1,USO1,PTPN11,ADCY6,OPA1,GTF2H1,CRY2,VPS18,EIF4E,ADIPOR1,SELENOS,ZNF106,EPRS,GSK3B,PIK3CA,BMP7,WDTC1,SYAP1,STRN3,LINC02210-CRHR1,PLOD3,EIF4EBP2,IGFBP2,FBN1,PRKCI,MYO5A,SLC2A4,MED1,PPARGC1A,SHC1,SMARCA4,PKM,ROCK1,AKT1,LPIN2,PRKAA1,RAB31,PTPN2,MYO1C,GNB1,SSTR5,PARK7,AHCYL1,BCAR3,SLC2A8,SERPINF1,YWHAH,PMEPA1,LEPROT,PIK3C2A	darkgreen	EC_M4
GO:0001503	2.75E-02	0.980762809	58	328	ossification	BP	3.89E-01	NAB2,FBN2,LEP,RORB,RUNX2,COL1A2,REST,ASPN,GLI3,FGF2,PSMC2,GLI2,TGFB1,CTNNB1,CRIM1,ATP2B1,ILK,IMPAD1,TP53INP2,DHX36,PTPN11,ACVR1,MMP2,SPP1,BMP1,RUNX1,CHSY1,SBDS,TGFB2,LRP5,SATB2,MMP14,LEF1,COL6A1,BMP7,FGFR2,FZD9,MRC2,COMP,SUCO,COL5A2,FGFR3,TPM4,BMPR1A,PDLIM7,THBS3,COL1A1,TGFB3,CLTC,CHRDL1,AKT1,ATP6AP1,COL11A1,FSTL3,TMEM38B,LOX,SNX10,MESD	darkgreen	EC_M4
GO:0003157	2.34E-02	0.996279031	4	11	endocardium development	BP	3.89E-01	PROX1,NOTCH1,SOX17,SOX18	turquoise	EC_M6
GO:0071622	2.34E-02	0.991789231	9	41	regulation of granulocyte chemotaxis	BP	3.89E-01	PTK2,CXCL8,THBS4,EDN1,JAM3,MCU,RAC1,DAPK2,S100A14	turquoise	EC_M6
GO:2000191	2.77E-02	0.991917346	7	23	regulation of fatty acid transport	BP	3.89E-01	PPARA,AKT2,IRS2,P2RX4,PLA2R1,EPRS,AKT1	darkgreen	EC_M4
GO:0050858	2.77E-02	0.992961784	6	18	negative regulation of antigen receptor-mediated signaling pathway	BP	3.89E-01	PRNP,PTPN22,CBLB,PTPN6,PTPN2,THY1	darkgreen	EC_M4
GO:0035107	2.77E-02	0.984522553	24	115	appendage morphogenesis	BP	3.89E-01	FBN2,RUNX2,GLI3,GLI2,MEGF8,CTNNB1,RARB,SEMA3C,IMPAD1,RDH10,NOTCH2,TGFB2,LRP5,LEF1,BMP7,FGFR2,ECE1,PRRX1,TULP3,CACNA1C,BMPR1A,MED1,CHST11,FGF10	darkgreen	EC_M4
GO:0035108	2.77E-02	0.984522553	24	115	limb morphogenesis	BP	3.89E-01	FBN2,RUNX2,GLI3,GLI2,MEGF8,CTNNB1,RARB,SEMA3C,IMPAD1,RDH10,NOTCH2,TGFB2,LRP5,LEF1,BMP7,FGFR2,ECE1,PRRX1,TULP3,CACNA1C,BMPR1A,MED1,CHST11,FGF10	darkgreen	EC_M4
GO:0051338	2.13E-02	0.984258553	84	706	regulation of transferase activity	BP	3.90E-01	LATS1,CCT2,FBXO5,MAP3K4,DSCC1,XRCC6,CDC25C,PLEC,POT1,TOPORS,DBF4,CKS2,IBTK,HNRNPA2B1,CCT4,ETAA1,ITGB1BP1,EZH2,CLSPN,CCNA2,CHORDC1,BORA,CCNT2,NCAPG2,CDC20,GTPBP4,IPO5,KITLG,TAF7,RFC3,CCNB1,ERCC6,TPX2,IPO7,PARP4,CDK5RAP1,ZFP91,TERF1,SH2B3,RBL1,CCNL1,NBN,CCNE2,STK4,UBE2C,HERC5,RFC5,TAOK3,BMPR1B,GCN1,CENPE,KIF14,AJUBA,RFC2,NEK2,ALS2,BLM,CDC25A,RFC4,DBF4B,XRCC5,CDKN3,DCUN1D5,ECT2,HNRNPU,INCENP,CCNB3,CDC6,CCNB2,AURKB,PLK1,CSK,PSMD10,RICTOR,TAOK1,PKMYT1,CCNF,CHTF18,PRIM2,HNRNPD,MAD2L1,TIGAR,NAF1,ERP29	darkgrey	EC_M8
GO:0033572	2.78E-02	0.995686419	4	9	transferrin transport	BP	3.90E-01	ARHGAP1,TFRC,TFR2,CLTC	darkgreen	EC_M4
GO:0010881	2.79E-02	0.992903159	6	18	regulation of cardiac muscle contraction by regulation of the release of sequestered calcium ion	BP	3.90E-01	FKBP1B,CACNA1C,CALM1,DMD,TMEM38B,TMEM38A	darkgreen	EC_M4
GO:1903037	2.79E-02	0.982187447	38	206	regulation of leukocyte cell-cell adhesion	BP	3.90E-01	IL23A,PPARA,HLA-DRA,FAM49B,LGALS1,LEP,DOCK8,NCK2,SOCS5,GLI3,GLI2,PRNP,HSPH1,CXCL12,IL20RB,CHST2,LOXL3,PTPN11,AP3B1,PTPN22,ADA,RUNX1,CBLB,PRKAR1A,PIK3CA,LEF1,TFRC,PTPN6,IGFBP2,CD74,AKT1,PTPN2,CD40LG,IRAK1,BCL10,THY1,HAS2,FGL2	darkgreen	EC_M4
GO:0061384	2.79E-02	0.990992534	8	27	heart trabecula morphogenesis	BP	3.90E-01	SRF,TGFBR1,HEY1,TGFB2,BMP7,BMPR1A,MED1,FKBP1A	darkgreen	EC_M4
GO:0010172	2.79E-02	0.995664593	4	9	embryonic body morphogenesis	BP	3.90E-01	PHLDB2,DAG1,PHLDB1,GREM2	darkgreen	EC_M4
GO:0006882	2.79E-02	0.991828319	7	23	cellular zinc ion homeostasis	BP	3.90E-01	SLC39A7,SLC39A4,SLC30A9,AP3B1,SLC39A10,SLC39A6,SLC30A7	darkgreen	EC_M4
GO:0003229	2.80E-02	0.988342773	12	47	ventricular cardiac muscle tissue development	BP	3.91E-01	LY6E,TGFBR1,TGFB1,PKP2,TGFB2,FGFR2,BMPR1A,MED1,TPM1,COL11A1,FKBP1A,HOPX	darkgreen	EC_M4

GO:0018193	2.14E-02	0.983513254	114	997	peptidyl-amino acid modification	BP	3.91E-01	CDK2,CLK4,PPIL4,LATS1,CSNK1G3,NAA35,UHRF2,PPIL1,PWP1,TOPORS,AKAP9,MELK,TRIM28,ZNF451,PPIH,FBXO11,SENP6,TTK,IBTK,ZMPSTE24,RLF,DPY19L1,FKBP15,EZH2,CLSPN,NAA15,HDAC2,SAE1,SETDB2,MASTL,CAD,ALG8,PAXIP1,NCAPG2,NKTR,PARP2,KAT14,EPC2,KAT6A,RIF1,ZDHHC17,NSD3,KITLG,TAF7,METAP2,BRCA2,MAPK8,PRKDC,CCNB1,ERCC6,UBA2,SGK3,PPWD1,FER,NCK1,TAF9,ATR,PBK,MCRS1,SH2B3,PPID,NAA25,SMYD5,SENP1,TOP1,RIPK1,CHUK,STK4,MORC3,CIT,METAP1,CHEK1,NAA50,STT3A,TET2,PRMT5,MCM3AP,HMBS,PPIG,HAT1,CDK1,ING5,BAZ1B,SETD2,PIKFYVE,ABL2,IL12A,BRCA1,CNOT9,NSD2,AIPL1,SUV39H1,FKBP4,UBE2K,NSD1,CLK2,AURKB,TAF1,OGT,ATRX,PLK1,CSK,ESCO1,SUZ12,RICTOR,HASPIN,EHMT1,PKN2,CTCF,AC118549.1,CTH,METTL18,AURKA,SENP7	darkgrey	EC_M8
GO:0010882	2.80E-02	0.991781466	7	23	regulation of cardiac muscle contraction by calcium ion signaling	BP	3.91E-01	FKBP1B,ATP2A2,CACNA1C,CALM1,DMD,TMEM38B,TMEM38A	darkgreen	EC_M4
GO:2000181	2.80E-02	0.985835149	18	83	negative regulation of blood vessel morphogenesis	BP	3.91E-01	SLC12A2,FAM19A5,ADGRB1,ROCK2,CTNNB1,DCN,SPINK5,SULF1,ABCC8,ATP2B4,TGFB2,ADGRB2,SERPINE1,NPPB,ROCK1,SARS,MIR24-1,SERPINF1	darkgreen	EC_M4
GO:0048255	2.14E-02	0.992548924	9	43	mRNA stabilization	BP	3.91E-01	SYNCRIP,THRAP3,DHX9,HNRNPU,FUS,LARP1,TAF15,METTL16,HNRNPD	darkgrey	EC_M8
GO:0002791	2.81E-02	0.983479543	28	142	regulation of peptide secretion	BP	3.91E-01	LRP1,PPARD,LEP,CLOCK,REST,FKBP1B,KIF5B,IRS2,OSBP,SELENOT,ABCC8,PTPN11,PFKM,BRSK2,DYNLL1,CPT1A,LRP5,HADH,ADCY5,SIDT2,TFR2,NLGN2,SYTL4,ACSL4,CD74,RASL10B,MIDN,SSTR5	darkgreen	EC_M4
GO:0072080	2.36E-02	0.989108638	15	75	nephron tubule development	BP	3.92E-01	MTSS1,KLHL3,GATA3,GZF1,JAG1,MEF2C,WWTR1,STAT1,LAMA5,DLG1,WNK4,PROM1,HOXB7,NOTCH1,BMP2	turquoise	EC_M6
GO:0043502	2.82E-02	0.986439674	16	71	regulation of muscle adaptation	BP	3.92E-01	PPARA,ROCK2,PAK1,SCN5A,P2RX4,ATP2A2,ATP2B4,TWF1,DAG1,PRKCA,CASQ1,GLRX3,PPARGC1A,SGCA,PIN1,ROCK1	darkgreen	EC_M4
GO:0008038	2.84E-02	0.988677657	11	42	neuron recognition	BP	3.95E-01	RTN4,MEGF8,PALLD,ROBO1,APP,CRTAC1,NDN,NPTN,BSG,NEXN,VSTM2L	darkgreen	EC_M4
GO:1902036	2.17E-02	0.996446247	4	13	regulation of hematopoietic stem cell differentiation	BP	3.96E-01	HSPA9,PUS7,METTL3,PRKDC	darkgrey	EC_M8
GO:0006950	2.85E-02	0.975287205	402	2715	response to stress	BP	3.96E-01	SERPINB2,LMAN1,IL23A,LRP1,CDO1,CANX,ENPP3,DYRK2,IER3,IPCEF1,RRM2B,SLC12A2,FAM19A5,PPARA,RNASET2,HSPA4,MRTFA,ADGRB1,PPARD,CTNNA1,C6orf89,UBQLN1,LGALS1,KLHDC10,TXN2,GNAQ,SLC39A4,MYH9,LEP,CDKN2B,PLAA,SLC30A9,SRF,NUAK1,VEGFA,VCP,C2orf40,NCK2,CLOCK,EXOC1,CREB3,ASNS,PDCL3,REST,DST,RAB23,TGFBR1,ERP44,SOCS5,ERLEC1,PSME4,UBA6,EMC10,MFSD2A,UBE2J1,MAP3K7,ACTR2,MDFIC,GLI3,ROCK2,INHBA,VPS41,BVES,ZYX,RCN3,MYLK3,VPS35,MGARP,SLC7A11,ITGB1,FKBP1B,FGF2,SRPK2,KIF5B,SEC61A1,SESN3,PANX1,ABHD12,RB1,TPT1,EIF2B3,DGKH,TMUB1,NUB1,PAK1,ACTR3,TGFB1,AXL,WDR48,UBXN4,HNMT,ACER3,CTNNB1,SERPINH1,STT3B,LGALS3BP,C1QTNF1,CLPB,ACTG1,NPLOC4,RNF121,TMTC4,GPS1,UFM1,LIG4,TMTC3,PRNP,ATP2B1,DCN,HDAC3,SIGLEC1,UBA5,DDRGK1,SMPD1,PPP2CB,ILK,RBPMS,HSPH1,ARL6IP5,DPYSL3,SPINK5,CNOT7,CXCL12,TM9SF4,ANKRD1,PJA2,HSP90B1,TMED2,IL20RB,MAP1LC3A,CD180,FLNB,C19orf12,VAMP8,TRAM1,MAP1B,CHST2,PLOD2,ACTR8,LOXL3,P2RX4,RNF13,FAIM2,ATP2A2,CELF1,MYL9,APP,DHX36,MAPKAPK5,CX3CL1,ABCC8,PTPN11,PTX3,OPA1,AMFR,GTF2H1,RAD54B,LOXL2,AP3B1,PTPN22,ACVR1,CHAC1,MMP2,GDF6,POLR3D,SERINC3,ADA,BAP1,ATP2B4,SPP1,CHID1,PKD2,PLA2R1,HIF1AN,TANK,TOLLIP,PSMD14,BRSK2,HPS6,MARCH6,RAB7B,CHRNA7,EIF4E,FAP,PHLDB2,TDP2,NOTCH2,DNM1L,GLRX2,UCHL5,SELENOS,TRIP12,HYAL1,EYA2,CCND1,KPNA6,TGFB2,FBLN5,EPRS,WIPI1,ITPK1,ZMAT3,GSK3B,MMP14,DAG1,PIK3CA,PRKCA,TLR5,CMKLR1,KRAS,NTRK3,CFI,POLQ,HSPA8,SLAMF8,FAM162A,BMP7,VAPB,HY	darkgreen	EC_M4
GO:0070570	2.86E-02	0.99066195	8	28	regulation of neuron projection regeneration	BP	3.97E-01	LRP1,FKBP1B,SPP1,TNR,PRRX1,MAP2K1,FLNA,THY1	darkgreen	EC_M4

GO:0030162	2.86E-02	0.978515404	88	538	regulation of proteolysis	BP	3.97E-01	SERPINB2,LRP1,RNF19A,UBQLN1,MTCH1,MYH9,VEGFA,VCP,PDCL3,REST,SOCS5,PSME4,CYCS,ROCK2,RCN3,DNAJC1,PSMC2,GSAP,PICALM,NUB1,WDR48,SPOPL,SERPINH1,CRIM1,SPINT2,TMTC3,PRNP,CHFR,DDRGK1,ARL6IP5,SPINK5,ROBO1,APP,CHAC1,RUNX1,IFT57,TANK,PSMD14,TM4SF20,UCHL5,GSK3B,MMP14,ANOS1,FAM162A,NEDD8,COL6A3,NCSTN,ADRM1,RPS6KA3,EGLN3,MAPT,SERPINE1,PCOLCE,FN1,ARAF,SMURF1,TIMP1,CLN8,NGFR,SPOP,TMED10,CLN6,UACA,ARIH1,FBXO22,ROCK1,USP14,AKT1,TMEM98,BCAP31,PKD1,TMEM259,PARK7,ENO1,RHBDF1,F3,GLG1,BCL10,MIR24-1,SPINK2,SERPINF1,SERPINB5,A2M,UBE2V2,TIMP3,DDA1,GABARAPL2,MAP1A	darkgreen	EC_M4
GO:0031649	2.39E-02	0.996289113	4	10	heat generation	BP	3.97E-01	TNFRSF11A,EDNRB,IL1A,ADRB2	turquoise	EC_M6
GO:0030048	2.40E-02	0.987785769	19	97	actin filament-based movement	BP	3.97E-01	LIMCH1,TRPM4,MYO7A,PDE4D,STC1,KCNJ3,KCNJ2,DLG1,WAS,SYNE2,JUP,ATP2A1,CCDC88C,DSG2,DSC2,PDE4B,FNBP1L,KCNH2,SCN2B	turquoise	EC_M6
GO:0010288	2.87E-02	0.992633612	6	18	response to lead ion	BP	3.98E-01	PPP2CB,MAP1LC3A,APP,BACE1,MAPT,QDPR	darkgreen	EC_M4
GO:0043086	2.87E-02	0.978306632	91	555	negative regulation of catalytic activity	BP	3.98E-01	SERPINB2,LRP1,CAND1,GNAQ,CDKN2B,VEGFA,SOCS5,SH3BP5L,ROCK2,FKBP1B,RB1,PICALM,SERPINH1,PPP1R12A,KAT2B,CRIM1,GPS1,SPINT2,IRS2,PRNP,SMPD1,ILK,SPINK5,TMED2,PINX1,RTKN,APP,PTX3,PTPN22,TSG101,CRY2,CHP1,ATP2B4,PLA2R1,DBNDD2,CBLB,DYNLL1,PPP1R12B,UCHL5,TGFB2,LRP5,PRKAR1A,GSK3B,CORO1C,ANOS1,BMP7,PPP1R11,WDTC1,COL6A3,IQGAP1,RPS6KA3,HSPB1,MAPT,SERPINE1,PTPN6,AOC3,TIMP1,MNAT1,PPP1R36,SRCIN1,NES,TMED10,TTC8,PPP4R4,GSKIP,MYOCD,ROCK1,USP14,AKT1,PTPN2,NPR3,FAM122B,PLXNB3,MIDN,PARK7,GPSM1,GNAI3,MIR24-1,FKBP1A,SPINK2,SERPINF1,SERPINB5,A2M,FRY,TIMP3,PPP1R14A,THY1,PPP1R1A,CDKN1A,DUSP22,ORMDL3	darkgreen	EC_M4
GO:0001707	2.88E-02	0.987436291	13	53	mesoderm formation	BP	3.98E-01	SRF,INHBA,ITGB1,TLX2,ACVR1,EYA2,PRKAR1A,LEF1,BMP7,FGFR2,BMPR1A,ITGA2,WLS	darkgreen	EC_M4
GO:0062012	2.88E-02	0.980907132	44	243	regulation of small molecule metabolic process	BP	3.98E-01	DYRK2,PPARA,PPARD,LEP,VCP,REST,MFSD2A,DKKL1,SLC7A11,DGAT2,AKT2,C1QTNF1,KAT2B,IRS2,DKK3,RDH10,SORBS1,APP,ATP2B4,ADIPOR1,SELENOS,CPT1A,ZBTB20,GSK3B,ARV1,WDTC1,PPP1R3D,COMT,PPP1R3F,PPARGC1A,CD74,AKT1,PRKAA1,PTPN2,PGP,MIDN,FAM3A,PARK7,ENO1,SLC2A6,MBTPS1,RANBP2,TMSB4X,NNMT	darkgreen	EC_M4
GO:0006518	1.58E-03	0.998912818	104	688	peptide metabolic process	BP	3.98E-01	PYM1,CDK4,EIF3H,RPS18,EEF1D,RPL3,EIF3L,OPLAH,EIF3D,RPL8,RPS5,RPL28,RPS9,EEF1A1,MALSU1,RPS7,RPS11,FOXO3,RPL18,ABTB1,RPS8,MIF4GD,MLH1,UNK,RPS3,RPS16,SAMD4B,MRPS12,RPL15,EIF3K,TARBP2,EIF3F,EIF4B,RPS3A,EIF2A,SPCS1,FARS2,RPLP2,PCIF1,WARS2,FASTKD3,MRPL36,PCSK6,DIS3L2,PUM1,APEH,GPX1,RPS6KB2,QARS,DALRD3,ANPEP,EIF4H,PER2,RPS21,RPL35A,RPL11,YBX3,NFE2L2,IDH1,MRPL49,EXOSC7,CTSH,RPL27,UBA52,RARA,ZFP36L1,MRPL10,PER1,TNRC6A,LARP6,RPL7A,EEF2K,MRPL4,EIF3G,NDUFA7,RPL23A,PARN,MRPL37,EEF2,DPH1,MRPL41,MRPS34,INPP5E,MRPL28,GSTM4,RPL13,MRPS2,KARS,PTGES2,ABCA2,RPS15,AGO4,RACK1,RPS19,MRPL34,RPL23,EEF1B2,RPUSD3,MRPL57,RPS20,ZFP36L2,PDF,PSENEN,EXOSC5	cyan	EC_M2
GO:0045945	2.20E-02	0.996559752	4	11	positive regulation of transcription by RNA polymerase III	BP	3.99E-01	CHD8,ZC3H8,ZNF143,ICE2	darkgrey	EC_M8
GO:0045055	2.42E-02	0.985871562	28	167	regulated exocytosis	BP	4.00E-01	STX11,RIMS2,APBA1,KIT,SYK,PIK3CG,GATA2,GAB2,UNC13D,SNCA,DTNBP1,SNAP23,FGR,ADCY1,FES,ADORA2A,SNAP29,RAB27A,SYT7,FBXL20,PSEN1,RAB3D,LGALS9,GIT1,NOTCH1,STXBP1,STX3,VAMP3	turquoise	EC_M6
GO:0035813	2.42E-02	0.995995756	4	12	regulation of renal sodium excretion	BP	4.00E-01	EDNRB,EDN1,ADORA2A,NPR1	turquoise	EC_M6
GO:0048873	2.42E-02	0.993777914	6	22	homeostasis of number of cells within a tissue	BP	4.00E-01	VPS54,GATA2,NOS3,IL7,LIPA,NOTCH1	turquoise	EC_M6
GO:0003073	2.43E-02	0.989835976	12	60	regulation of systemic arterial blood pressure	BP	4.01E-01	PDGFB,ARHGAP42,PRCP,NOS3,KCNK6,P2RX2,PDE4D,NAV2,EDN1,ACE,AR,ADRB2	turquoise	EC_M6
GO:0098727	2.21E-02	0.988954629	18	108	maintenance of cell number	BP	4.01E-01	FANCC,EZH2,ZC3H13,WDR43,HOOK3,METTL3,RIF1,CUL4A,CNOT1,SMC3,ASPM,DDX6,MED28,LEO1,SMC1A,MED6,NIPBL,SRRT	darkgrey	EC_M8
GO:0001825	2.22E-02	0.99351461	7	31	blastocyst formation	BP	4.01E-01	LATS1,RRP7A,CNOT1,GABPA,CUL3,SP3,NLE1	darkgrey	EC_M8

GO:2000042	2.22E-02	0.99533219	5	17	negative regulation of double-strand break repair via homologous recombination	BP	4.01E-01	RIF1,PARPBP,FANCB,SMCHD1,RMI2	darkgrey	EC_M8
GO:0006359	2.23E-02	0.995308184	5	17	regulation of transcription by RNA polymerase III	BP	4.02E-01	POLR3F,CHD8,ZC3H8,ZNF143,ICE2	darkgrey	EC_M8
GO:0060713	2.91E-02	0.992500114	6	18	labyrinthine layer morphogenesis	BP	4.02E-01	RSPO3,SPINT2,TMED2,LEF1,BMP7,FGFR2	darkgreen	EC_M4
GO:0044257	2.92E-02	0.977392332	111	685	cellular protein catabolic process	BP	4.02E-01	LRP1,CANX,RNF19A,UBQLN1,KLHDC10,PLAA,VCP,C2orf40,CLOCK,PDCL3,SOCS5,ERLEC1,PSME4,UBA6,UBE2J1,PCYOX1,RCN3,VPS35,CUL2,RAB7A,PSMC2,PDCD6IP,TMUB1,KCTD21,NUB1,UBXN4,SPOPL,CTNNB1,STT3B,NPLOC4,RNF121,PSMD8,TMTC3,CHFR,DDRGK1,PPP2CB,PSMD6,RNF6,VPS37A,HSP90B1,TP53INP2,TRPC4AP,ELOC,RNF13,AMFR,TSG101,BAP1,TTC3,TOLLIP,PSMD14,BRSK2,MARCH6,FAP,UCHL5,SELENOS,TRIP12,PSMD1,UBE3B,KCTD10,GSK3B,NEDD8,PPP1R11,PSMD2,ADRM1,UBE4A,RHBDD2,EDEM3,SYVN1,DNAJC10,DNAJB12,USP48,ARAF,SMURF1,FBXL5,JKAMP,CLN8,ANAPC4,MSN,RNF111,TGFB1I1,SPOP,FBXL22,SEL1L,ARIH1,FBXO22,PCYOX1L,USP14,AKT1,PSMD11,UBE2A,TNFAIP1,OMA1,BCAP31,PKD1,TMEM259,PARK7,RHBDF1,USP10,USP20,HSPA5,FBXO27,BNIP3,UBE2V2,DNAJB9,PTTG1IP,UBE2D2,DDA1,GABARAPL2,UBE2G2,MAP1A,DERL2	darkgreen	EC_M4
GO:0045058	2.45E-02	0.992045805	8	33	T cell selection	BP	4.02E-01	SYK,IL6,IL15,GATA3,ITPKB,MTOR,BATF,JAG2	turquoise	EC_M6
GO:0032728	2.45E-02	0.992035738	8	33	positive regulation of interferon-beta production	BP	4.02E-01	DDX58,TLR4,TMEM173,TLR2,OAS3,DDX3X,ISG15,OAS1	turquoise	EC_M6
GO:0035630	2.46E-02	0.9960202	4	11	bone mineralization involved in bone maturation	BP	4.03E-01	IGF1,LTF,RFLNB,BMP2	turquoise	EC_M6
GO:1902895	2.94E-02	0.988868408	10	37	positive regulation of pri-miRNA transcription by RNA polymerase II	BP	4.04E-01	MRTFA,SRF,FGF2,TGFB1,TEAD1,GNL3,TGFB2,BMPR1A,NGFR,SMARCA4	darkgreen	EC_M4
GO:0030810	2.94E-02	0.993643734	5	14	positive regulation of nucleotide biosynthetic process	BP	4.04E-01	PPARA,VCP,PPARGC1A,ENO1,TMSB4X	darkgreen	EC_M4
GO:1900373	2.94E-02	0.993643734	5	14	positive regulation of purine nucleotide biosynthetic process	BP	4.04E-01	PPARA,VCP,PPARGC1A,ENO1,TMSB4X	darkgreen	EC_M4
GO:0051603	2.95E-02	0.977317002	104	639	proteolysis involved in cellular protein catabolic process	BP	4.05E-01	CANX,RNF19A,UBQLN1,KLHDC10,PLAA,VCP,C2orf40,CLOCK,PDCL3,SOCS5,ERLEC1,PSME4,UBA6,UBE2J1,PCYOX1,RCN3,CUL2,PSMC2,PDCD6IP,TMUB1,KCTD21,NUB1,UBXN4,SPOPL,CTNNB1,STT3B,NPLOC4,RNF121,PSMD8,TMTC3,CHFR,DDRGK1,PPP2CB,PSMD6,RNF6,VPS37A,HSP90B1,TP53INP2,TRPC4AP,ELOC,RNF13,AMFR,TSG101,BAP1,TTC3,TOLLIP,PSMD14,BRSK2,MARCH6,FAP,UCHL5,SELENOS,TRIP12,PSMD1,UBE3B,KCTD10,GSK3B,NEDD8,PPP1R11,PSMD2,ADRM1,UBE4A,RHBDD2,EDEM3,SYVN1,DNAJC10,DNAJB12,USP48,ARAF,SMURF1,FBXL5,JKAMP,ANAPC4,RNF111,TGFB1I1,SPOP,FBXL22,SEL1L,ARIH1,FBXO22,PCYOX1L,USP14,AKT1,PSMD11,UBE2A,TNFAIP1,OMA1,BCAP31,PKD1,TMEM259,PARK7,RHBDF1,USP10,USP20,HSPA5,FBXO27,UBE2V2,DNAJB9,UBE2D2,DDA1,GABARAPL2,UBE2G2,MAP1A,DERL2	darkgreen	EC_M4
GO:0071331	2.95E-02	0.983594462	23	112	cellular response to hexose stimulus	BP	4.05E-01	LRP1,PPARD,LGALS1,SRF,UNC13B,FKBP1B,KIF5B,IRS2,OSBP,SELENOT,OPA1,BRSK2,DYNLL1,ZBTB20,LRP5,PIK3CA,ADCY5,SIDT2,PTPRN,PPARGC1A,SMARCA4,PRKAA1,SERPINF1	darkgreen	EC_M4
GO:0007030	2.96E-02	0.983113147	25	122	Golgi organization	BP	4.06E-01	LMAN1,ATP8B1,LMAN2L,NPLOC4,ARL1,RAB2A,TMED2,USO1,SEC22B,GOLGA5,GOLGB1,GORASP2,TANGO2,PDCD10,HTT,BLZF1,OBSL1,STX18,LMAN2,TMED10,MAP2K1,TMED1,VMP1,VAMP7,TMED3	darkgreen	EC_M4
GO:0086001	2.97E-02	0.986505261	14	58	cardiac muscle cell action potential	BP	4.07E-01	SCN5A,GPD1L,SNTA1,SLMAP,ATP2A2,CACNA1D,PKP2,CACNA1C,CACNA1G,CALM1,FLNA,HCN2,DMD,HCN4	darkgreen	EC_M4

GO:0070304	2.49E-02	0.987462057	18	99	positive regulation of stress-activated protein kinase signaling cascade	BP	4.07E-01	FLT4,TPD52L1,SH3RF3,GADD45G,TLR4,TRAF1,SDCBP,TNFRSF19,SH3RF1,NCF1,MAP3K3,MAP3K11,CRACR2A,RELL1,TAOK2,TRAF2,DAB2IP,BMP2	turquoise	EC_M6
GO:0070374	2.98E-02	0.982366305	28	145	positive regulation of ERK1 and ERK2 cascade	BP	4.08E-01	LRP1,ANGPT1,TPBG,FGF2,TGFB1,CAVIN3,ST5,APP,RAPGEF2,CX3CL1,PTPN11,PTPN22,CHRNA7,NOTCH2,HTR2B,PRKCA,FGFR2,FGFR3,SHC1,MAP2K1,NPTN,CD74,PDGFRB,ATP6AP1,MIR24-1,CCL16,GLIPR2,FGF10	darkgreen	EC_M4
GO:0045807	2.98E-02	0.984847351	18	82	positive regulation of endocytosis	BP	4.08E-01	CD63,LRP1,AHI1,RAB21,ANGPT1,VEGFA,AXL,SMPD1,TSG101,PPP3CC,DNM1L,AP2M1,SERPINE1,TFR2,ATAD1,ITGA2,RAB31,CD151	darkgreen	EC_M4
GO:0045216	2.99E-02	0.982145837	29	146	cell-cell junction organization	BP	4.08E-01	CTNNA1,SRF,VEGFA,TGFBR1,WHRN,ROCK2,PDCD6IP,TGFB1,CTNNB1,ACTG1,ZNF703,CTNND1,PLEKHA7,ABCC8,MTDH,TJP1,PKP2,TGFB2,PRKCA,PRKCI,VCL,WDR1,NLGN2,TLN2,TGFB3,ROCK1,MYO1C,NPHP4,FLNA	darkgreen	EC_M4
GO:0002063	2.99E-02	0.990210387	8	27	chondrocyte development	BP	4.08E-01	RUNX2,SERPINH1,IMPAD1,SULF1,CHSY1,COMP,COL11A1,CHST11	darkgreen	EC_M4
GO:0120009	2.99E-02	0.98857815	10	38	intermembrane lipid transfer	BP	4.08E-01	OSBPL8,OSBP,PITPNM1,PITPNB,PRELID3B,OSBPL2,STARD3,PITPNA,ABCA3,PLEKHA3	darkgreen	EC_M4
GO:0001505	2.50E-02	0.985825487	25	147	regulation of neurotransmitter levels	BP	4.09E-01	STX11,SNCAIP,RIMS2,AGTPBP1,APBA1,PPT1,RAB3B,NR4A1,MCTP1,MEF2C,SLC38A1,SNCA,DTNBP1,MCTP2,SNAP23,ADCY1,ADORA2A,SNAP29,SNCG,SYT7,FBXL20,PSEN1,GIT1,STXBP1,STX3	turquoise	EC_M6
GO:0046328	2.51E-02	0.987110624	19	104	regulation of JNK cascade	BP	4.09E-01	FLT4,TPD52L1,SH3RF3,GADD45G,TLR4,TRAF1,SDCBP,SFRP1,EPHB1,TNFRSF19,ITCH,SH3RF1,NCF1,MAP3K11,MECOM,CRACR2A,TAOK2,TRAF2,DAB2IP	turquoise	EC_M6
GO:1903008	3.01E-02	0.983769863	21	101	organelle disassembly	BP	4.10E-01	VCP,KIF5B,UFM1,UBA5,DDRGK1,MAP1LC3A,SLC25A4,ATG13,CTTN,DNM1L,CISD2,WIPI1,HTT,WIPI2,PPARGC1A,FUNDC2,PARK7,BNIP3,GABARAPL2,TOMM7,DENR	darkgreen	EC_M4
GO:0009303	2.28E-02	0.993244993	7	33	rRNA transcription	BP	4.10E-01	PWP1,TAF1B,NIFK,POLR1B,NCL,NOL11,DDX11	darkgrey	EC_M8
GO:0031396	2.29E-02	0.987022911	25	180	regulation of protein ubiquitination	BP	4.10E-01	FBXO5,UBR5,TOPORS,COMMD1,UFL1,PAXIP1,CDC20,GTPBP4,UBE2C,UBE3A,CUL3,FANCI,IVNS1ABP,FANCM,BRCA1,DCUN1D5,TAF1,OGT,PLK1,PSMD10,XIAP,GLMN,MAD2L1,UBXN2A,RCHY1	darkgrey	EC_M8
GO:0021932	3.02E-02	0.995187461	4	9	hindbrain radial glia guided cell migration	BP	4.11E-01	ITGB1,LEF1,FLNA,RERE	darkgreen	EC_M4
GO:0060441	3.03E-02	0.990978177	7	23	epithelial tube branching involved in lung morphogenesis	BP	4.12E-01	CTNNB1,RDH10,DAG1,KRAS,FGFR2,WNT2,FGF10	darkgreen	EC_M4
GO:1902041	2.53E-02	0.990987078	9	42	regulation of extrinsic apoptotic signaling pathway via death domain receptors	BP	4.12E-01	TNFAIP3,NOS3,TMC8,SFRP1,MADD,ITPRIP,CFLAR,DDX3X,PMAIP1	turquoise	EC_M6
GO:0099172	3.03E-02	0.988450173	10	37	presynapse organization	BP	4.12E-01	MDGA1,LRRC4B,VPS35,PCDH17,FARP1,PCLO,APP,NTRK3,NLGN2,NTNG2	darkgreen	EC_M4
GO:0010633	2.54E-02	0.989339301	12	59	negative regulation of epithelial cell migration	BP	4.13E-01	PPARG,ADAMTS9,MEF2C,ANGPT2,STC1,DLL4,JUP,MMRN2,MCC,VASH1,NOTCH1,DAB2IP	turquoise	EC_M6
GO:0032731	2.54E-02	0.991695715	8	33	positive regulation of interleukin-1 beta production	BP	4.13E-01	IL6,TLR4,CD36,P2RX7,TMEM106A,TLR6,LGALS9,GBP5	turquoise	EC_M6

GO:0051173	2.54E-02	0.978543444	284	2331	positive regulation of nitrogen compound metabolic process	BP	4.13E-01	ZNF516,TNFRSF11A,TCF4,IFNGR1,TNFAIP3,PLAGL1,CNOT6,FLT4,ZBED4,GRAMD4,NOTCH4,TCF20,TPD52L1,NCOA7,SGSM3,PTK2,DAPK1,ATF4,PDGFB,AGTPBP1,CSNK1E,PIM1,NFYA,TEK,PRDM4,LIMCH1,DDX58,FAM122A,TEC,KIT,KDR,NFIL3,SYK,SEMA4D,IL6,REL,CREB5,PELI1,TLR4,IL15,CCNY,TEAD2,SDCBP,MYCN,NOCT,TUT4,NRP1,PIK3CG,TAL1,PHF10,EPC1,KDM4D,GATA2,PRKD2,CTSC,RAB38,CAB39L,NOS3,GGA3,RALB,PPARG,GATA3,EDNRB,NDFIP2,IKBKB,JAG1,NFKBIB,TMEM173,SFRP1,HBEGF,NR1D2,DND1,RPL26,ANKLE2,CD36,SIRT3,PRR5L,TCF15,PLK2,RET,CHD7,IL1A,DNMT3B,IGF1,NR4A1,KLKB1,EIF2AK3,HHEX,KDM3A,ITCH,SMAD1,ZBTB38,HIP1R,STOX2,SOX7,HMBOX1,GPD1,HEXB,MEF2C,SMARCD1,TLR2,WWTR1,P2RX7,VEGFC,CXCL10,MADD,KMT2D,TIPARP,NLRC5,THBS4,PPP1R16B,RAPGEF3,NR4A2,TOX2,NEDD9,YAF2,EDN1,STAT1,DYRK1A,EHD4,SNCA,JARID2,MYLIP,ERG,ETS2,NFKB2,RNF144B,PRDM15,ABCG1,ETS1,RNF19B,LPCAT1,HDAC1,LCK,PPP3CA,HYAL2,HECW2,MEF2A,BCL9,LAPTM5,NCF1,USP13,HCLS1,NFATC2,EAF2,GMEB1,PECAM1,CD86,ASB9,POU2F3,CFLAR,CASP10,PPP2R5A,ARID1A,RPS6KA1,PROX1,SS18L1,PRKD1,ZNF148,YTHDF1,OAZ3,HELZ2,MLXIPL,ACE,ITGA6,LIMK2,PAK2,MELTF,SENP2,DLG1,ELL3,ETV6,MAP3K11,HOXD10,MOV10,CREB1,TET1,LTF,ARNT2,DDX3X,RFXANK,ETV4,TBX1,TMEM106A,MECOM,ING4,BIRC3,LDB2,FGF23,PPM1F,POU2F1,WAS,RAC1,HIF1A,KDM5A,ZBTB18,APBB2,JUP,ADIPOR2,BID,TLR6,TNFRSF1B,MTOR,AR,RNF40,CASZ1,PSEN1,HOXB5,HOXB7,HOXB9,FBXW11,FGF18,PGF,TAOK2,BATF,EFNA1,SLC51B,NAP1L2,PIK3R5,PIAS1,RPS6KA5,SMAD2,CNOT8,DNM2,YY1,A	turquoise	EC_M6
GO:0042098	2.55E-02	0.986305769	21	127	T cell proliferation	BP	4.13E-01	TNFRSF21,SYK,IL6,PELI1,IL15,PIK3CG,IL1A,IGF1,ITCH,SH3RF1,LMBR1L,ICOSLG,CD86,DLG1,SOS2,TNFRSF1B,ARG2,EFNB1,LGALS9,ELF4,CD46	turquoise	EC_M6
GO:0060759	2.55E-02	0.986284295	21	128	regulation of response to cytokine stimulus	BP	4.13E-01	TNFAIP3,DDX58,SYK,IL6,TLR4,PPARG,CXCR4,IRAK2,IKBKB,TLR2,IL7,OAS3,NLRC5,HIPK1,EDN1,LAPTM5,HIF1A,ISG15,TRAF2,MIR99A,OAS1	turquoise	EC_M6
GO:0003085	2.56E-02	0.995795109	4	11	negative regulation of systemic arterial blood pressure	BP	4.14E-01	ARHGAP42,PRCP,KCNK6,ADRB2	turquoise	EC_M6
GO:0033630	2.56E-02	0.994469151	5	16	positive regulation of cell adhesion mediated by integrin	BP	4.14E-01	PODXL,SYK,FERMT1,RET,PIEZO1	turquoise	EC_M6
GO:0072574	2.56E-02	0.994387414	5	17	hepatocyte proliferation	BP	4.14E-01	TNFAIP3,IL6,CFLAR,RPS6KA1,PROX1	turquoise	EC_M6
GO:0072575	2.56E-02	0.994387414	5	17	epithelial cell proliferation involved in liver morphogenesis	BP	4.14E-01	TNFAIP3,IL6,CFLAR,RPS6KA1,PROX1	turquoise	EC_M6
GO:0035633	2.57E-02	0.991595874	8	32	maintenance of blood-brain barrier	BP	4.15E-01	TJP2,IL6,OCLN,JAM3,ESAM,PECAM1,F11R,CDH5	turquoise	EC_M6
GO:0140029	2.58E-02	0.988699067	13	66	exocytic process	BP	4.15E-01	STX11,SYK,RAB3B,RALB,EXOC6,SNCA,SNAP23,SNAP29,PSEN1,RAB3D,STXBP1,STX3,VAMP3	turquoise	EC_M6
GO:0002753	2.58E-02	0.989629577	11	53	cytoplasmic pattern recognition receptor signaling pathway	BP	4.15E-01	TNFAIP3,DDX58,TLR4,PUM2,SEC14L1,ITCH,ERBIN,OAS3,TIFA,BIRC3,RNF125	turquoise	EC_M6
GO:0060142	2.58E-02	0.994339972	5	17	regulation of syncytium formation by plasma membrane fusion	BP	4.15E-01	CXCL10,RAPGEF3,NFATC2,PLEKHO1,CFLAR	turquoise	EC_M6
GO:0001909	2.59E-02	0.989054216	12	61	leukocyte mediated cytotoxicity	BP	4.16E-01	CTSC,NECTIN2,UNC13D,RNF19B,LYST,B2M,RAB27A,SCNN1B,LGALS9,NOS2,IL7R,SERPINB9	turquoise	EC_M6
GO:0051253	2.33E-02	0.981940701	118	1032	negative regulation of RNA metabolic process	BP	4.16E-01	BCLAF1,ZNF608,SCAF8,XRCC6,RFX3,TRIM28,ZNF451,LRPPRC,ZMPSTE24,SYNCRIP,XPO1,CASP8AP2,ZNF350,THRAP3,EZH2,HDAC2,SETDB2,TRDMT1,RSF1,BIRC5,CHD8,KAT6A,RIF1,E2F7,TAF7,NR2C1,ELK3,TCERG1,CBX5,ZC3H8,BTAF1,TDG,NCK1,ABCE1,CGGBP1,SAP30,GCFC2,CNOT1,GABPA,PPID,RBL1,TENT2,E2F8,TRIM33,ZBED6,ZNF248,MDM4,HIVEP1,CUL3,NSUN2,GMNN,NFKB1,KHDRBS1,PRMT5,AJUBA,BPTF,DDX5,ORC2,CENPF,SP3,DHX9,DDX20,TPR,CCAR1,XRCC5,NPAT,BRCA1,NSD2,TBL1XR1,SUV39H1,FOXM1,LYAR,FAM220A,HNRNPU,NEDD4,SMARCE1,RFC1,NSD1,FUS,USP3,AURKB,TAF1,RLIM,ATRX,MBTD1,TRIM37,LARP1,PLK1,ILF3,PSMD10,TAF15,ZNF3,MIER1,NIPBL,PHF6,UHRF1,RBMX,FMR1,METTL16,EHMT1,RNPS1,PTBP1,SCAI,CTCF,DEPDC1,HNRNPD,ZNF684,DRAP1,ZNF614,RBAK,SRSF7,NAF1,BRMS1L,C1D,RNF8,ZNF558,IKZF5,ZNF529	darkgrey	EC_M8

GO:0007339	2.33E-02	0.994873447	5	20	binding of sperm to zona pellucida	BP	4.16E-01	CCT2,CCT4,CCT7,CCT5,CCT3	darkgrey	EC_M8
GO:0140053	2.33E-02	0.989040472	15	101	mitochondrial gene expression	BP	4.16E-01	MTERF1,LRPPRC,MTIF2,PNPT1,MRPS7,CDK5RAP1,PTCD3,IARS2,FASTKD1,FASTKD2,DARS2,MRPL44,TEFM,FASTKD5,TFB2M	darkgrey	EC_M8
GO:0071621	2.60E-02	0.98812231	14	76	granulocyte chemotaxis	BP	4.16E-01	PTK2,SYK,CXCL8,PIK3CG,CXCL10,THBS4,EDN1,JAM3,MCU,RAC1,DAPK2,S100A14,CCL2,PDE4B	turquoise	EC_M6
GO:0001678	3.08E-02	0.982353602	25	124	cellular glucose homeostasis	BP	4.18E-01	LRP1,PPARD,LGALS1,SRF,UNC13B,FKBP1B,KIF5B,IRS2,OSBP,SELENOT,ABCC8,OPA1,BRSK2,DYNLL1,ZBTB20,LRP5,PIK3CA,ADCY5,SIDT2,PTPRN,PPARGC1A,NGFR,SMARCA4,PRKAA1,SERPINF1	darkgreen	EC_M4
GO:0045923	3.08E-02	0.99078255	7	23	positive regulation of fatty acid metabolic process	BP	4.18E-01	PPARA,PPARD,AKT2,IRS2,CPT1A,PPARGC1A,CD74	darkgreen	EC_M4
GO:0042446	2.62E-02	0.98998436	10	47	hormone biosynthetic process	BP	4.18E-01	HSD17B3,SRD5A3,SCARB1,HIF1A,DHRS11,CHST9,DIO3,BMP2,SRD5A2,BMP6	turquoise	EC_M6
GO:0046942	2.63E-02	0.984514602	28	173	carboxylic acid transport	BP	4.19E-01	TNFRSF11A,APBA1,SYK,SLC3A1,LLGL2,SLCO2B1,CD36,SLCO2A1,SERINC5,SLC38A1,EDN1,SNCA,SLC16A14,SLC7A7,SLC16A9,ACE,ADORA2A,SLC16A13,SLC26A1,PSEN1,SLC51B,NOS2,SLC43A2,STXBP1,LRRC8C,LRRC8D,SLC25A21,RGS4	turquoise	EC_M6
GO:1904353	2.36E-02	0.993891905	6	24	regulation of telomere capping	BP	4.19E-01	MAP3K4,TNKS2,NBN,NEK2,AURKB,HNRNPD	darkgrey	EC_M8
GO:0048659	2.63E-02	0.985794851	21	128	smooth muscle cell proliferation	BP	4.20E-01	TNFAIP3,PDGFB,IL6,TLR4,IL15,PPARG,P2RY6,HBEGF,IGF1,MEF2C,NDRG4,EDN1,STAT1,IGFBP3,APOD,IGFBP5,CALCRL,MTOR,NPR1,S1PR1,BMP2	turquoise	EC_M6
GO:0002831	2.65E-02	0.983512926	34	224	regulation of response to biotic stimulus	BP	4.20E-01	TNFAIP3,C6orf106,DDX58,SYK,TLR4,IL15,PUM2,NECTIN2,PPARG,SEC14L1,IKBKB,TMEM173,CD36,ITCH,OAS3,NLRC5,IFIT1,STAT1,NCF1,FGR,PAK2,LTF,BIRC3,ARG2,LGALS9,ISG15,PLCG2,LRSAM1,OAS1,GBP5,TRIM62,SERPINB9,RNF125,BMP6	turquoise	EC_M6
GO:0120178	2.65E-02	0.992022163	7	29	steroid hormone biosynthetic process	BP	4.20E-01	HSD17B3,SRD5A3,SCARB1,DHRS11,BMP2,SRD5A2,BMP6	turquoise	EC_M6
GO:0048518	3.10E-02	0.972526398	658	4533	positive regulation of biological process	BP	4.20E-01	CD63,LMAN1,IL23A,NAB2,LRP1,STX7,AHI1,AGAP2,ENPP3,TAB2,CAND1,DYRK2,RAB21,RNF19A,PRRC1,SLC12A2,AZIN1,PLXNB2,FBN2,ANGPT1,TCP1,PPARA,HLA-DRA,ROS1,NUS1,RSPO3,FAM49B,MRTFA,HABP4,PACSIN1,PTP4A3,ADGRB1,PPARD,CTNNA1,TEAD3,SUN2,C6orf89,UBQLN1,MTCH1,LGALS1,GGA1,KLHDC10,SH3GL2,PLIN2,MYH9,LEP,CDKN2B,PLAA,RORB,SLC30A9,KIAA1324L,SRF,VEGFA,DOCK8,VCP,SLC35B2,RUNX2,UNC13B,NCK2,CLOCK,EXOC1,CREB3,ASNS,PDCL3,REST,PTP4A1,NLRP5,P3H1,TGFBR1,HIVEP3,SOCS5,RIMS1,TMEM30A,PSME4,COPS9,RTN4,CYCS,TPBG,SHANK1,ADAMTS3,LRRC4B,EMC10,MFSD2A,MAP3K7,AKIRIN1,ACTR2,KIDINS220,WHRN,MDFIC,GLI3,ROCK2,INHBA,BVES,RRAS,RCN3,MYLK3,DKKL1,VPS35,MGARP,ITGB1,PLEKHA4,DBP,FKBP1B,PLXNA1,FGF2,SRPK2,KIF5B,SESN3,RAB7A,PANX1,PSMC2,GSAP,APBB1IP,FBLN2,ZNF398,RB1,ABHD5,PICALM,MARK4,NUFIP1,ZNF296,MAPRE3,EIF2B3,PTCH2,PDCD6IP,TENM4,GLI2,MEGF8,SMARCD3,NUB1,RHEB,PAK1,ACTR3,TGFB1,AXL,RAB3GAP1,SCN5A,LMO7,WDR48,SEC13,ACER3,CTNNB1,IL15RA,TRAK1,LBH,DGAT2,AKT2,DYNC1LI1,GPD1L,DZIP1,ZEB2,CANT1,C1QTNF1,RAB2B,OSBPL8,CXXC5,RARB,ACTG1,NAV3,ACTN4,PPP1R12A,KAT2B,FAM98A,TACC1,MAFG,STIM1,EAF1,LIG4,ATP2C1,IRS2,TMTC3,PRNP,CHFR,NUDT16,ATP2B1,LUM,DCN,CDC42EP3,SMAD9,HDAC3,ZNF703,ACPP,SEMA3C,CCKBR,CAVIN3,DDRGK1,SMPD1,ILK,RBPMS,HSPH1,ARL6IP5,DPYSL3,RYK,CIAO1,CNOT7,RNF6,RIC3,LMO1,TMEM9B,ST5,CXCL12,FXYD1,TM9SF4,SLC20A1,ANKRD1,PJA2,TMED2,IL20RB,TP53INP2,CD180,SLC25	darkgreen	EC_M4
GO:0021885	3.11E-02	0.98739584	11	43	forebrain cell migration	BP	4.21E-01	SUN2,MDGA1,SRF,RTN4,GLI3,AXL,CTNNB1,CXCL12,ROBO1,TNR,PAFAH1B1	darkgreen	EC_M4
GO:0043500	2.66E-02	0.987085938	16	88	muscle adaptation	BP	4.21E-01	IL15,NOS3,IGF1,EDN1,JARID2,PDE9A,PPP3CA,MEF2A,CFLAR,INPP5F,IGFBP5,TNFRSF1B,MTOR,YY1,NOTCH1,RGS4	turquoise	EC_M6
GO:0048736	3.12E-02	0.981505045	28	140	appendage development	BP	4.21E-01	FBN2,RUNX2,GLI3,SLC7A11,GLI2,MEGF8,CTNNB1,RARB,KAT2B,SEMA3C,IMPAD1,RDH10,NOTCH2,TGFB2,LRP5,LEF1,BMP7,FGFR2,COMP,ECE1,PRRX1,TULP3,CACNA1C,BMPR1A,MED1,SLC39A3,CHST11,FGF10	darkgreen	EC_M4
GO:0060173	3.12E-02	0.981505045	28	140	limb development	BP	4.21E-01	FBN2,RUNX2,GLI3,SLC7A11,GLI2,MEGF8,CTNNB1,RARB,KAT2B,SEMA3C,IMPAD1,RDH10,NOTCH2,TGFB2,LRP5,LEF1,BMP7,FGFR2,COMP,ECE1,PRRX1,TULP3,CACNA1C,BMPR1A,MED1,SLC39A3,CHST11,FGF10	darkgreen	EC_M4
GO:0048484	2.67E-02	0.995567864	4	11	enteric nervous system development	BP	4.22E-01	EDNRB,RET,HLX,KIF26A	turquoise	EC_M6
GO:0086091	2.67E-02	0.991302834	8	30	regulation of heart rate by cardiac conduction	BP	4.22E-01	TRPM4,KCNJ3,KCNJ2,JUP,DSG2,DSC2,KCNH2,SCN2B	turquoise	EC_M6

GO:1902547	3.13E-02	0.990609478	7	23	regulation of cellular response to vascular endothelial growth factor stimulus	BP	4.22E-01	CD63,PTP4A3,ADAMTS3,DCN,ROBO1,ATP2B4,MYO1C	darkgreen	EC_M4
GO:1901343	3.13E-02	0.983971723	18	84	negative regulation of vasculature development	BP	4.22E-01	SLC12A2,FAM19A5,ADGRB1,ROCK2,CTNNB1,DCN,SPINK5,SULF1,ABCC8,ATP2B4,TGFB2,ADGRB2,SERPINE1,NPPB,ROCK1,SARS,MIR24-1,SERPINF1	darkgreen	EC_M4
GO:0007010	2.38E-02	0.981197895	137	1177	cytoskeleton organization	BP	4.23E-01	RTTN,CDK2,KATNA1,LATS1,FBXO5,KIF3A,KIFC1,KIF20A,PLEC,HAUS6,KIF24,AKAP9,GCC2,SENP6,PHIP,TTK,RUFY3,XPO1,ANLN,ITGB1BP1,CAPZA2,ZMYM4,CNTRL,GEN1,PLK4,STIL,CEP57,CEP295,CHORDC1,CENPA,CLASP2,KIF2C,DIAPH3,BORA,ULK4,CDC20,HOOK3,SGO1,PRPF40A,CKAP2,TUBGCP3,BRCA2,CCNB1,WDR62,GAS2L3,HAUS5,ESPL1,TPX2,SKA3,LSM14A,CENPJ,FER,NCK1,KIF11,ARFGEF1,CENPH,KATNBL1,CCDC66,LARP4,CETN3,RACGAP1,ALMS1,CKAP5,KNSTRN,ABRAXAS1,MYBL2,INO80,NUSAP1,STMND1,BRWD1,CIT,KIF18A,CUL3,SLK,CHEK1,SMC3,CENPE,CEP72,CAMSAP2,HAUS2,INTS13,AJUBA,TBCE,CFL1,ASPM,RCC1,SPC25,RAE1,NEK2,PRC1,AUNIP,CDK1,HDGFL3,NUF2,SLC16A1,SETD2,TPR,ABL2,KIF18B,KIF15,BRCA1,CEP152,HAUS3,TACC3,DLGAP5,TRPM7,ECT2,FKBP4,HNRNPU,INCENP,SMC1A,CCNB2,IQGAP3,KPNB1,KIF4A,SPDL1,AURKB,ATRX,KIF23,TRIM37,PLK1,NDC80,STAG2,RICTOR,CEP76,TAOK1,CCNF,SAPCD2,GAS8,SASS6,ODF2,USP33,TMOD3,MAD2L1,STMN1,AURKA,DRG1	darkgrey	EC_M8
GO:0010458	2.39E-02	0.993715863	6	26	exit from mitosis	BP	4.24E-01	ANLN,CLASP2,KNTC1,UBE2C,PPP2R2D,CDCA5	darkgrey	EC_M8
GO:0021766	3.15E-02	0.985550549	14	59	hippocampus development	BP	4.24E-01	SRF,UBA6,MFSD2A,GLI3,KIF5B,EIF2B3,HTR5A,ATP2B4,FEZ1,GSK3B,LEF1,KCNA1,PAFAH1B1,PLXNA3	darkgreen	EC_M4
GO:0048704	3.16E-02	0.983829982	18	83	embryonic skeletal system morphogenesis	BP	4.25E-01	RUNX2,TGFBR1,GLI3,MEGF8,CTNNB1,RDH10,HYAL1,SATB2,MMP14,BMP7,FGFR2,PRRX1,TULP3,SIX4,TGFB3,COL11A1,SLC39A3,CHST11	darkgreen	EC_M4
GO:0006071	3.16E-02	0.993037417	5	14	glycerol metabolic process	BP	4.25E-01	LEP,DGAT2,MOGAT3,TPI1,PGP	darkgreen	EC_M4
GO:0060644	3.16E-02	0.993033381	5	14	mammary gland epithelial cell differentiation	BP	4.25E-01	FGF2,LBH,AKT2,ZNF703,AKT1	darkgreen	EC_M4
GO:0044248	3.17E-02	0.973122363	265	1755	cellular catabolic process	BP	4.26E-01	HSD17B6,LRP1,CDO1,CANX,ENPP3,TAB2,RNF19A,MIOX,ATP6V1C1,SKIV2L,PPARA,RNASET2,PPARD,UBQLN1,KLHDC10,LEP,PLAA,ALDH1L2,GALT,VCP,C2orf40,CLOCK,EXOC1,PDCL3,RAB23,SOCS5,ERLEC1,PSME4,UBA6,MFSD2A,UBE2J1,MAP3K7,RNASEH1,PCYOX1,ROCK2,VPS41,RCN3,VPS35,CUL2,SESN3,RAB7A,PSMC2,GAD2,ENTPD6,ABHD12,ABHD5,ESD,GLB1,ABHD1,PDCD6IP,TMUB1,KCTD21,ATP6V0B,NUB1,RHEB,TGFB1,RAB3GAP1,UBXN4,HNMT,SPOPL,ACER3,CTNNB1,AKT2,STT3B,MTMR14,NPLOC4,STAM2,RNF121,PSMD8,UFM1,IRS2,TMTC3,CHFR,NUDT16,DCN,UBA5,DDRGK1,SMPD1,PPP2CB,PSMD6,CNOT7,RNF6,VPS37A,TMEM41B,HSP90B1,MAP1LC3A,TP53INP2,SLC25A4,C19orf12,EIF4G2,VAMP8,LNPEP,TRPC4AP,ELOC,RNF13,ATP2A2,CELF1,APP,DHX36,TET3,PFKM,ENTPD4,ARSB,AMFR,ATG13,PTPN22,TSG101,DKC1,VPS18,CHAC1,MTDH,ADA,GLS,BAP1,ATP2B4,CPN1,CSDE1,NUDT18,TTC3,TENT5C,TENT4B,TOLLIP,PSMD14,BRSK2,VPS39,MARCH6,ATP6V1B2,FAP,CTTN,DNM1L,UCHL5,SELENOS,TRIP12,HYAL1,SEC22B,CPT1A,CISD2,ZBTB20,PSMD1,UBE3B,KCTD10,FEZ1,HTR2B,WIPI1,GSK3B,PIK3CA,HADH,PRKCA,HSPA8,NEDD8,PPP1R11,PPP1R3D,SYNPO2,PSMD2,ADRM1,SCFD1,QSOX1,UBE4A,ATF6,SIDT2,PAFAH1B2,PRDX4,RHBDD2,EDEM3,HSPB1,MAPT,SYVN1,SGPL1,COMT,DNAJC10,MAOA,DNAJB12,GPR137,TPI1,ACOX3,UPF1,HTT,USP48,ECE1,SAMD4A,QDPR,ARAF,SMURF1,TIMP1,FBXL5,JKAMP,ASRGL1,HAP1,WIPI2,HSD17B10,CLN8,ATP6V1D,ANAPC4,PPARGC1A,MSN,RNF111,SLC25A44,TGFB1I1,SYT11,SPOP,FBXL22,DNASE2,SPTLC2,SEL1L,CLN6,CALM1,CLTC,VMP1,DICER1,ARIH1,ATG4A,FBXO22,PNP	darkgreen	EC_M4
GO:0021954	2.70E-02	0.988155227	13	61	central nervous system neuron development	BP	4.26E-01	AGTPBP1,NRP1,GATA2,NOVA2,SPTBN4,EPHB1,DCLK2,NR4A2,MPP5,CHRNB2,B4GALT6,LHX6,GBA2	turquoise	EC_M6
GO:0006665	3.19E-02	0.981881499	24	120	sphingolipid metabolic process	BP	4.26E-01	B4GALNT1,SERINC1,LARGE1,GLB1,ACER3,SUMF1,SMPD1,ST8SIA4,CERS5,SERINC3,B3GALT1,ARV1,HACD2,SGPL1,SGMS1,CLN8,SPTLC2,CLN6,PRKAA1,CSNK1G2,PLA2G15,HACD1,PEMT,ORMDL3	darkgreen	EC_M4
GO:0030100	3.19E-02	0.980142181	33	174	regulation of endocytosis	BP	4.26E-01	CD63,LRP1,AHI1,RAB21,ANGPT1,PACSIN1,MKLN1,SH3GL2,VEGFA,PICALM,SNX17,AXL,SMPD1,ATXN2,TSG101,PPP3CC,DNM1L,AP2M1,SERPINE1,TFR2,ITGAV,LRPAP1,ATAD1,STX1B,SYT11,CALM1,SCAMP5,ROCK1,ITGA2,RAB31,CDH13,CD151,LRRTM2	darkgreen	EC_M4
GO:0071456	3.19E-02	0.981887502	24	119	cellular response to hypoxia	BP	4.26E-01	PPARD,UBQLN1,VEGFA,ROCK2,MGARP,ANKRD1,OPA1,HIF1AN,FAM162A,BMP7,HYOU1,EGLN3,ERO1A,FMN2,SLC2A4,PPARGC1A,KCNMB1,AKT1,PRKAA1,GNB1,VASN,IRAK1,ENO1,BNIP3	darkgreen	EC_M4

GO:0000963	2.41E-02	0.994693063	5	19	mitochondrial RNA processing	BP	4.27E-01	PNPT1,CDK5RAP1,FASTKD1,FASTKD2,FASTKD5	darkgrey	EC_M8
GO:0030517	2.71E-02	0.990384414	9	36	negative regulation of axon extension	BP	4.27E-01	SEMA6A,CDKL3,SEMA4D,NRP1,SEMA3F,SEMA6D,SEMA4A,SEMA6B,ARHGAP4	turquoise	EC_M6
GO:0050714	3.20E-02	0.982636855	21	100	positive regulation of protein secretion	BP	4.27E-01	LRP1,PPARD,UNC13B,EXOC1,KIF5B,TGFB1,IRS2,ANKRD1,OSBP,PFKM,DYNLL1,DNM1L,TGFB2,NLGN2,TMED10,TGFB3,MYH10,SYTL4,ACSL4,MYOM1,WLS	darkgreen	EC_M4
GO:0007612	3.20E-02	0.982338803	22	104	learning	BP	4.28E-01	SRF,UBA6,TPBG,SHANK1,ACTR2,SLC7A11,ITGB1,APP,ABCC8,KRAS,C1QL1,COMT,TNR,HTT,ATAD1,CLN8,SYT11,NPTN,JPH3,LRRN4,ARF4,MAP1A	darkgreen	EC_M4
GO:0034767	2.72E-02	0.985585915	20	114	positive regulation of ion transmembrane transport	BP	4.28E-01	TRDN,P2RY6,P2RX7,CXCL10,PLCG1,ANO6,SNCA,KCNJ2,WNK4,CRACR2A,PSEN1,ATP2A1,CD19,DNM2,ADRB2,CCL2,PLCG2,RELN,KCNH2,SHANK3	turquoise	EC_M6
GO:0061061	2.72E-02	0.980278256	70	504	muscle structure development	BP	4.28E-01	QKI,ASF1A,PDGFB,PGM5,KIT,TMOD1,CAV2,SVIL,EPC1,EDNRB,JAG1,HBEGF,NR1D2,EFNB2,ATP11A,P2RX2,LMOD3,TCF15,EPHB1,CHD7,IGF1,NR4A1,SMAD1,POU6F1,MEF2C,CXCL10,TLL2,SFMBT1,DYSF,ANKRD2,TANC1,DLL4,EDN1,SIK1,PPP3CA,MEF2A,BCL9,NRAP,HLX,NFATC2,PLEKHO1,CFLAR,IGFBP3,RBM4,PROX1,TMOD4,LAMA5,HOXD10,CREB1,MYEF2,IGFBP5,TBX1,RAMP2,TEAD4,TMOD2,ZBTB18,CHRNB1,MTOR,PIAS1,YY1,MRTFB,TMEM204,NOTCH1,PIEZO1,S1PR1,ENG,MIR23A,MAFF,RGS4,BMP2	turquoise	EC_M6
GO:0071526	3.22E-02	0.987581592	10	38	semaphorin-plexin signaling pathway	BP	4.28E-01	PLXNB2,PLXNA1,SEMA3C,SEMA4F,SEMA4G,FARP2,PLXNB3,FLNA,PLXNA3,GDNF	darkgreen	EC_M4
GO:0001952	3.22E-02	0.982255315	22	105	regulation of cell-matrix adhesion	BP	4.28E-01	LRP1,VEGFA,ROCK2,RRAS,ACTG1,ILK,RASA1,CX3CL1,PTPN11,PHLDB2,GSK3B,MMP14,DAG1,CORO1C,IQGAP1,SERPINE1,VCL,ROCK1,DAPK3,CDH13,THY1,DUSP22	darkgreen	EC_M4
GO:0030072	3.22E-02	0.980103377	32	168	peptide hormone secretion	BP	4.28E-01	LRP1,PPARD,LEP,CLOCK,REST,FKBP1B,KIF5B,IRS2,PCLO,OSBP,SELENOT,ABCC8,PTPN11,PFKM,BRSK2,DYNLL1,CPT1A,LRP5,HADH,ADCY5,SIDT2,LINC02210-CRHR1,TFR2,PTPRN,NLGN2,UBE2Q1,SYTL4,ACSL4,RASL10B,MIDN,SSTR5,PARK7	darkgreen	EC_M4
GO:0051926	3.22E-02	0.987517045	10	39	negative regulation of calcium ion transport	BP	4.28E-01	UBQLN1,FKBP1B,REM1,CACNB3,PKD2,LINC02210-CRHR1,CALM1,AKT1,CBARP,GEM	darkgreen	EC_M4
GO:0051952	2.73E-02	0.988885303	11	55	regulation of amine transport	BP	4.29E-01	RAB3B,SNCA,DTNBP1,ADORA2A,SNCG,SYT7,PSEN1,CHRNB2,SLC43A2,STXBP1,RGS4	turquoise	EC_M6
GO:0046323	3.23E-02	0.985597697	13	54	glucose import	BP	4.29E-01	LEP,AKT2,OSBPL8,IRS2,SORBS1,PTPN11,SELENOS,SLC2A10,PEA15,CLTCL1,PRKCI,SLC2A4,AKT1	darkgreen	EC_M4
GO:0050773	3.23E-02	0.983058183	19	87	regulation of dendrite development	BP	4.29E-01	RAB21,PACSIN1,MFSD2A,ACTR2,ILK,TLX2,DHX36,RAPGEF2,OPA1,DNM1L,CAPRIN2,GSK3B,BMP7,IQGAP1,TNIK,OBSL1,PAFAH1B1,YWHAH,SDC2	darkgreen	EC_M4
GO:0018904	3.24E-02	0.990121906	7	24	ether metabolic process	BP	4.29E-01	TXN2,CHPT1,FAR1,LPCAT2,PAFAH1B1,TXN,TMEM189	darkgreen	EC_M4
GO:0006898	3.24E-02	0.979332916	36	192	receptor-mediated endocytosis	BP	4.29E-01	CD63,LRP1,AHI1,CANX,RAB21,ANGPT1,MKLN1,SH3GL2,VEGFA,ITGB1,PICALM,SNX17,SIGLEC1,ATXN2,PLA2R1,AP2A2,CTTN,HTR2B,GRK3,AP2M1,TFRC,AP1S1,SERPINE1,TFR2,ITGAV,LRPAP1,CLTCL1,ATAD1,PDLIM7,SYT11,CLTC,GRIA1,RAB31,GPR107,AP1G1,LRRTM2	darkgreen	EC_M4
GO:0050678	3.25E-02	0.977814426	49	275	regulation of epithelial cell proliferation	BP	4.30E-01	PPARD,LEP,CDKN2B,VEGFA,PDCL3,TGFBR1,RTN4,EMC10,FGF2,RB1,TGFB1,SCN5A,WDR48,CTNNB1,ZNF703,CXCL12,SULF1,ROBO1,IFT57,KDM5B,NOTCH2,CDC73,HYAL1,CCND1,TGFB2,HTR2B,PRKCA,FGFR2,VASH2,HTRA1,PDCD10,PTPRN,SIX4,AKT3,MAGED1,BMPR1A,MED1,NGFR,AKT1,MYDGF,PLXNB3,CDH13,F3,MIR24-1,SERPINF1,SERPINB5,HAS2,WNT2,FGF10	darkgreen	EC_M4
GO:0001974	2.74E-02	0.990857253	8	34	blood vessel remodeling	BP	4.31E-01	FLT4,NOS3,ACVR2B,JAG1,CHD7,MEF2C,DLL4,ACE	turquoise	EC_M6
GO:0072111	2.75E-02	0.99387954	5	17	cell proliferation involved in kidney development	BP	4.31E-01	PDGFB,GATA3,STAT1,CFLAR,BMP2	turquoise	EC_M6
GO:1905314	2.75E-02	0.990168963	9	37	semi-lunar valve development	BP	4.31E-01	NOS3,GATA3,TIE1,JAG1,DLL4,TNFRSF1B,EFNA1,NOTCH1,BMP2	turquoise	EC_M6
GO:0048041	3.27E-02	0.983210102	18	81	focal adhesion assembly	BP	4.32E-01	LRP1,VEGFA,ROCK2,ACTG1,SORBS1,PTPN11,PHLDB2,CTTN,MMP14,CORO1C,IQGAP1,VCL,ACTN1,ROCK1,ITGA2,DAPK3,THY1,DUSP22	darkgreen	EC_M4
GO:1901888	2.77E-02	0.983470835	29	165	regulation of cell junction assembly	BP	4.33E-01	PTK2,TEK,LINGO2,LIMCH1,KDR,SEMA4D,NRP1,SETD5,IKBKB,SFRP1,EPHB1,MEF2C,TLR2,SNCA,F11R,ACE,APOD,IL1RAP,PPM1F,PRKCH,RAC1,RCC2,EPHA2,SEMA4A,ABI3,CHRNB2,SLITRK2,RAPGEF1,ADGRL2	turquoise	EC_M6
GO:0010721	2.77E-02	0.984574771	23	130	negative regulation of cell development	BP	4.33E-01	SEMA6A,CDKL3,SEMA4D,IL6,MYCN,NRP1,EDNRB,IGF1,PPP3CA,SEMA3F,AP1AR,TRAK2,MELTF,B2M,LTF,SEMA6D,RCC2,PSEN1,SEMA4A,SEMA6B,RFLNB,ARHGAP4,NOTCH1	turquoise	EC_M6

GO:0035821	2.77E-02	0.987231374	14	75	modulation of process of other organism	BP	4.33E-01	LRRC19,SYK,CAV2,P2RX7,ANO6,HDAC1,HYAL2,POU2F3,LTF,TNIP1,NOS2,FCER2,SERPINB9,TBC1D20	turquoise	EC_M6
GO:0009605	3.29E-02	0.972120724	268	1770	response to external stimulus	BP	4.33E-01	SERPINB2,CD63,IL23A,LRP1,ENPP3,TAB2,SLC12A2,PLXNB2,ANGPT1,FAM19A5,PPARA,RNASET2,FER1L6,CXCL14,FAM49B,HABP4,ADGRB1,PPARD,TXN2,GNAQ,SLC39A4,LEP,CDKN2B,PLAA,SRF,NUAK1,VEGFA,CLOCK,EXOC1,CREB3,ASNS,SOCS5,SLC17A5,TPBG,SHANK1,MFSD2A,MAP3K7,AKIRIN1,ACTR2,WHRN,GLI3,VPS41,ZYX,VPS35,FKBP1B,PLXNA1,FGF2,SRPK2,KIF5B,SEC61A1,SESN3,ABHD12,RB1,TPT1,GLI2,MEGF8,NUB1,PAK1,ACTR3,TGFB1,AXL,CTNNB1,C1QTNF1,CLPB,ACTG1,NPLOC4,ATP2B1,DCN,DCLK1,SEMA3C,SMPD1,SPINK5,RYK,CNOT7,CXCL12,ANKRD1,PJA2,IL20RB,PALLD,MAP1LC3A,CD180,FLNB,VAMP8,GRK7,ROBO1,MAP1B,SEMA4F,P2RX4,ATP2A2,CELF1,APP,GLRB,DHX36,CX3CL1,ABCC8,CACNB3,PTPN11,PTX3,OPA1,ARSB,PTPN22,CRY2,POLR3D,SERINC3,MTDH,ADA,BAP1,SPP1,CHID1,PKD2,SEMA4G,TOLLIP,BRSK2,PSPH,RAB7B,ELMO2,FAP,PHLDB2,NOTCH2,CDC73,DNM1L,GLRX2,SELENOS,CHSY1,HYAL1,CCND1,CPT1A,SBDS,TGFB2,EPRS,FEZ1,WIPI1,ARNTL2,MMP14,DAG1,PIK3CA,LEF1,ANOS1,PRKCA,TLR5,CMKLR1,SHROOM2,PITPNM1,NTRK3,CFI,HSPA8,SLAMF8,BMP7,PPP1R11,ATF3,FGFR2,OSM,HTRA1,MACO1,BACE1,RPS6KA3,SRP54,HSPB1,AP1S1,SERPINE1,COMT,TNR,PTPN6,KCNK4,IGFBP2,UPF1,ITGAV,HTT,PKD1L1,COL3A1,NBL1,PLD1,KCNA1,SGMS1,OTUD5,ACTA2,ROM1,CRMP1,WIPI2,NOTCH3,ENAH,TCAP,EIF2S1,MED1,PPARGC1A,CPEB4,ARHGEF2,SYT11,NGFR,DOCK2,FBXL22,COL1A1,SHC1,MAP2K1,TTC8,TSPAN6,NPTN,MYOCD,GP2,ACSL4,CD74,LAMA3,PDGFRB,USP14,AKT1,ITGA2,C6,PRKAA1,OSMR,PTPN2,OMA1,CLEC16A,LDOC1,DAPK3,MYO1C,PLXNB3,NTN3,PKD1,IRAK1,FLNA,PLXNA	darkgreen	EC_M4
GO:0048284	3.29E-02	0.981257957	24	120	organelle fusion	BP	4.33E-01	STX7,VPS41,KIF5B,RAB7A,AKT2,VAMP8,USO1,OPA1,VPS18,CHP1,VPS39,RAB7B,DNM1L,SEC22B,TFRC,STX1B,SYT11,SOX30,RAB34,OMA1,VAMP7,GNAI3,BNIP3,STXBP6	darkgreen	EC_M4
GO:0070831	3.30E-02	0.992641193	5	14	basement membrane assembly	BP	4.34E-01	PHLDB2,DAG1,PHLDB1,PLOD3,NTNG2	darkgreen	EC_M4
GO:0090087	3.30E-02	0.980276487	28	144	regulation of peptide transport	BP	4.34E-01	LRP1,PPARD,LEP,CLOCK,REST,FKBP1B,KIF5B,IRS2,OSBP,SELENOT,ABCC8,PTPN11,PFKM,BRSK2,DYNLL1,CPT1A,LRP5,HADH,ADCY5,SIDT2,TFR2,NLGN2,SYTL4,ACSL4,CD74,RASL10B,MIDN,SSTR5	darkgreen	EC_M4
GO:1902743	2.80E-02	0.989232454	10	45	regulation of lamellipodium organization	BP	4.36E-01	ARHGEF7,OCLN,WAS,RAC1,FSCN1,EPHA2,MTOR,ABI3,DNM2,CARMIL2	turquoise	EC_M6
GO:0034142	2.81E-02	0.991398441	7	30	toll-like receptor 4 signaling pathway	BP	4.37E-01	TNFAIP3,PELI1,TLR4,LTF,S100A14,DAB2IP,OAS1	turquoise	EC_M6
GO:0086005	2.81E-02	0.991576503	7	26	ventricular cardiac muscle cell action potential	BP	4.37E-01	TRPM4,KCNJ3,DLG1,JUP,DSG2,DSC2,KCNH2	turquoise	EC_M6
GO:0048499	2.81E-02	0.992450222	6	23	synaptic vesicle membrane organization	BP	4.37E-01	STX11,SNAP23,SNAP29,AP3D1,STXBP1,STX3	turquoise	EC_M6
GO:0010638	2.48E-02	0.982874514	54	428	positive regulation of organelle organization	BP	4.38E-01	PAN2,CCT2,MAP3K4,RAD21,POT1,AKAP9,TRIM28,PHIP,HNRNPA2B1,CCT4,ITGB1BP1,WDR35,PLK4,CEP295,SETDB2,CDC16,MAPK8,PAN3,ESPL1,TNKS2,CENPJ,FER,NCK1,ATR,KATNBL1,FAM208A,CNOT1,ANAPC7,CCT7,NBN,CKAP5,NUSAP1,CCT5,CCT6A,CUL3,C2CD5,NEK2,TPR,CDC27,XRCC5,DLGAP5,RAD51AP1,DDX11,CCT3,AURKB,ATRX,G3BP1,RICTOR,CDT1,HNRNPD,AURKA,NAF1,FEN1,DRG1	darkgrey	EC_M8
GO:0045582	2.83E-02	0.987896497	12	60	positive regulation of T cell differentiation	BP	4.38E-01	SYK,GATA3,IL7,SOX13,HLX,CD86,ITPKB,LGALS9,IL7R,AP3D1,VNN1,CD46	turquoise	EC_M6
GO:0097035	2.83E-02	0.989130311	10	43	regulation of membrane lipid distribution	BP	4.38E-01	ATP8A1,ATP11A,P2RX7,ANO6,ANO3,ABCG1,ATP9A,ATP8B4,TMEM30B,GBA2	turquoise	EC_M6
GO:0009582	3.34E-02	0.982780773	18	83	detection of abiotic stimulus	BP	4.39E-01	GNAQ,WHRN,CTNNB1,CXCL12,GRK7,CACNB3,PKD2,PITPNM1,BACE1,KCNK4,PKD1L1,KCNA1,ROM1,TCAP,NGFR,ITGA2,PKD1,COL11A1	darkgreen	EC_M4
GO:0051346	3.34E-02	0.977733075	43	242	negative regulation of hydrolase activity	BP	4.39E-01	SERPINB2,LRP1,VEGFA,ROCK2,FKBP1B,PICALM,SERPINH1,CRIM1,SPINT2,PRNP,SPINK5,TMED2,APP,PTX3,CRY2,CHP1,PLA2R1,UCHL5,TGFB2,GSK3B,ANOS1,PPP1R11,COL6A3,RPS6KA3,SERPINE1,TIMP1,PPP1R36,TMED10,TTC8,PPP4R4,ROCK1,USP14,AKT1,PLXNB3,PARK7,GPSM1,MIR24-1,FKBP1A,SPINK2,SERPINF1,SERPINB5,A2M,TIMP3	darkgreen	EC_M4
GO:0120178	3.36E-02	0.98860698	8	29	steroid hormone biosynthetic process	BP	4.40E-01	HSD17B6,REST,DKK3,HSD17B1,CYP19A1,STARD3,MED1,PPARGC1A	darkgreen	EC_M4

GO:0001916	3.37E-02	0.994163598	4	10	positive regulation of T cell mediated cytotoxicity	BP	4.41E-01	IL23A,STX7,HLA-DRA,FAM49B	darkgreen	EC_M4
GO:0150105	2.87E-02	0.992449399	6	20	protein localization to cell-cell junction	BP	4.42E-01	TJP2,PECAM1,F11R,PAK2,JAK1,CDH5	turquoise	EC_M6
GO:1902107	2.88E-02	0.984912728	19	110	positive regulation of leukocyte differentiation	BP	4.42E-01	SYK,IL15,GATA3,IL7,SOX13,HLX,HCLS1,INPP5D,CD86,FES,CREB1,ITPKB,GPR68,LGALS9,IL7R,AP3D1,VNN1,CD46,CA2	turquoise	EC_M6
GO:1903708	2.88E-02	0.984912728	19	110	positive regulation of hemopoiesis	BP	4.42E-01	SYK,IL15,GATA3,IL7,SOX13,HLX,HCLS1,INPP5D,CD86,FES,CREB1,ITPKB,GPR68,LGALS9,IL7R,AP3D1,VNN1,CD46,CA2	turquoise	EC_M6
GO:0003298	2.88E-02	0.992161922	6	24	physiological muscle hypertrophy	BP	4.42E-01	IGF1,EDN1,MTOR,YY1,MIR23A,RGS4	turquoise	EC_M6
GO:0003301	2.88E-02	0.992161922	6	24	physiological cardiac muscle hypertrophy	BP	4.42E-01	IGF1,EDN1,MTOR,YY1,MIR23A,RGS4	turquoise	EC_M6
GO:0061049	2.88E-02	0.992161922	6	24	cell growth involved in cardiac muscle cell development	BP	4.42E-01	IGF1,EDN1,MTOR,YY1,MIR23A,RGS4	turquoise	EC_M6
GO:0044030	2.51E-02	0.99447555	5	18	regulation of DNA methylation	BP	4.42E-01	ZMPSTE24,RLF,MIS18A,PRMT5,BRCA1	darkgrey	EC_M8
GO:0035590	3.38E-02	0.990863	6	19	purinergic nucleotide receptor signaling pathway	BP	4.42E-01	ACPP,P2RX4,P2RY14,ADA,ADCY5,ADORA2B	darkgreen	EC_M4
GO:0001889	2.52E-02	0.987158908	18	116	liver development	BP	4.43E-01	ONECUT2,DDX39B,PNPT1,ZMPSTE24,EZH2,WDR35,CAD,UPF2,E2F7,E2F8,CUL3,PRMT5,SP3,SRSF5,TYMS,ACADM,HNRNPD,AURKA	darkgrey	EC_M8
GO:0051608	3.40E-02	0.994090789	4	10	histamine transport	BP	4.44E-01	VAMP8,ADA,SLC29A4,VAMP7	darkgreen	EC_M4
GO:0034644	2.54E-02	0.988778697	13	77	cellular response to UV	BP	4.45E-01	POLH,METTL3,ATR,PBK,PPID,INO80,CHEK1,CDC25A,NEDD4,AURKB,TAF1,CUL4B,FMR1	darkgrey	EC_M8
GO:0098884	2.91E-02	0.995193885	4	10	postsynaptic neurotransmitter receptor internalization	BP	4.45E-01	DNM3,OPHN1,AP2B1,GSG1L	turquoise	EC_M6
GO:0140239	2.91E-02	0.995193885	4	10	postsynaptic endocytosis	BP	4.45E-01	DNM3,OPHN1,AP2B1,GSG1L	turquoise	EC_M6
GO:0032732	2.91E-02	0.989384905	9	40	positive regulation of interleukin-1 production	BP	4.45E-01	IL6,TLR4,CD36,P2RX7,HDAC1,TMEM106A,TLR6,LGALS9,GBP5	turquoise	EC_M6
GO:0034250	2.55E-02	0.986722578	19	130	positive regulation of cellular amide metabolic process	BP	4.46E-01	DDX39B,EIF3E,METTL3,PRKDC,DHX29,SP1,CDK5RAP1,NCK1,LARP4,USP16,KHDRBS1,FXR1,EIF2B5,DHX9,FASTKD2,LARP1,UPF3B,FMR1,HNRNPD	darkgrey	EC_M8
GO:0098801	2.92E-02	0.992079678	6	23	regulation of renal system process	BP	4.46E-01	PDGFB,EDNRB,STC1,EDN1,ADORA2A,NPR1	turquoise	EC_M6
GO:0097305	1.80E-03	0.999100691	32	174	response to alcohol	BP	4.46E-01	CDK4,EHMT2,TSPO,KLF9,FOXO3,SPHK2,FOSB,TGFBR2,RPL15,GGH,TP53INP1,EPS8,MGMT,LRP6,RPL27,RARA,OSBPL7,FOS,ADAM15,ACACA,SREBF1,OXCT1,EEF2,TBXA2R,CYBA,TGFBR3,PTGFR,MT-ND4,MT-CYB,ABCA2,SETDB1,EEF1B2	cyan	EC_M2
GO:0050848	3.43E-02	0.984549516	13	55	regulation of calcium-mediated signaling	BP	4.47E-01	DYRK2,PRNP,P2RX4,ADA,CHP1,ATP2B4,GSK3B,CMKLR1,MAPT,HTT,TMEM100,CALM1,CDH13	darkgreen	EC_M4
GO:0006900	3.44E-02	0.984529416	13	55	vesicle budding from membrane	BP	4.47E-01	SEC24A,RAB7A,PICALM,SEC13,SEC23B,TMED2,AP3B1,VAPB,AP2M1,SEC23A,PRKCI,SLC2A4,TMED10	darkgreen	EC_M4
GO:0018209	3.44E-02	0.976470967	48	267	peptidyl-serine modification	BP	4.48E-01	DYRK2,ANGPT1,VEGFA,NCK2,TGFBR1,ROCK2,SRPK2,PAK1,TGFB1,AKT2,GPD1L,KDELC1,DCN,DCLK1,ILK,MAST4,PFN2,APP,MAPKAPK5,ATP2B4,BRSK2,CAPRIN2,GSK3B,PIK3CA,PRKCA,NTRK3,GALNT2,OSM,TFRC,RPS6KA3,PDCD10,PRKCI,ARAF,AKT3,GALNT16,GALNT1,ROCK1,AKT1,PRKAA1,NLK,EGFLAM,PNCK,PKD1,CSNK1G2,PARK7,BCAR3,DMD,TXN	darkgreen	EC_M4
GO:0045780	3.46E-02	0.992181452	5	14	positive regulation of bone resorption	BP	4.49E-01	SPP1,PRKCA,TFRC,ATP6AP1,DEF8	darkgreen	EC_M4

GO:0031326	1.95E-03	0.998421456	366	2955	regulation of cellular biosynthetic process	BP	4.49E-01	NFATC1,ZNF236,TSHZ1,ORMDL2,PYM1,SMARCC2,STAT6,MAP3K5,CDK4,CITED2,MAML1,SQSTM1,IRAK3,OSR2,HSPA1B,EHMT2,ZBTB12,HDAC10,ATF6B,EIF3H,RXRB,TSPO,RING1,ZHX2,ZBTB22,MYC,TOB2,SMAD5,EP300,KDM3B,ZNF76,DDX17,NFIB,EIF3D,HSF1,CCND3,TRERF1,CDKN2A,HMOX1,WASL,KLF9,JAK2,ZBTB45,ZNF135,ZNF667,ZNF865,RPS9,MALSU1,EFEMP1,ZNF692,ANXA4,AKNA,MED25,IL4I1,COQ3,ING3,ARHGEF5,IRF3,LPIN1,FOXO3,KLF15,SPHK2,NOP53,MAML2,BICRA,KMT2E,BBS7,MEIS3,TRIM13,IRF2BP1,SIX5,FOSB,ZNF746,NR2C2,ZNF862,TSC22D1,ZNF575,MIF4GD,NAA16,MLH1,UNK,ZNF574,WBP2,KMT2C,MYD88,TADA3,RPS3,BRPF1,DYRK1B,EID2,ZNF219,MED29,SAMD4B,SIRT2,RPTOR,APBB3,EIF3K,NUP98,TRIM21,CYP1B1,HGF,APBB1,DEAF1,HOXC4,NCOA4,LIN37,TARBP2,KMT2B,RBCK1,RARG,SDR16C5,ZNF740,EIF4B,LMO2,POFUT1,NECAB3,URI1,PIK3R1,NCOA2,ATF1,KDM2B,ZFHX4,CAMKK2,SCAND1,EIF2A,SETD6,MCPH1,SSBP2,FNIP2,ARL2BP,SHOX2,NEK4,ZHX3,VDR,MMS19,LPIN3,FOXF2,F2RL1,SORBS3,ORMDL1,HNF4A,TP53INP1,PIDD1,ERLIN1,HR,ACOT8,PCIF1,TRIM45,CD47,PARP3,FASTKD3,ELK4,LGR4,ICE1,IRS1,SP140,TCF7L2,DIS3L2,PUM1,TXNIP,AIP,SOX5,ADNP,RPS6KB2,ZNF217,QARS,DTX3L,PARP14,EIF4H,ECM1,RCOR3,ARNT,PRMT2,RIPK3,GABPB2,PER2,TWIST2,VPS72,HDAC4,USF1,SMARCD2,POGZ,CLCN2,USP21,DNAJC30,PATZ1,RNASEL,CCDC85B,MAGEF1,SCAP,CDKN1B,TCEA3,KAT5,LRP6,YBX3,NFKBIA,POR,NFE2L2,IDH1,PHC1,HEXIM2,ACADL,SOAT1,BATF2,EXOSC7,PCBD1,CHCHD10,MEN1,PHB2,TSC22D4,SAV1,TAF6,CTDSP1,EID1,SSBP4,RNF25,FGF7,NELFA,ZSCAN25,ZNF394,TNFR	cyan	EC_M2
GO:0019538	1.96E-03	0.998380405	503	4113	protein metabolic process	BP	4.49E-01	PYM1,ATG12,MAP3K5,OS9,CDK4,CTDSP2,ATP23,ADAMTS2,MAML1,SQSTM1,SASH1,IRAK3,GFPT2,BAG6,PPP6R2,SNX9,HSPA1B,EHMT2,HDAC10,SELENOO,ALG12,RNF5,EIF3H,RPS6KA2,DEPTOR,DSE,TSPO,RING1,RPS18,B3GALT4,JADE2,PMM1,MYC,SMAD5,EP300,CDC14C,KDM3B,EEF1D,TAB1,RPL3,JOSD1,NRBP2,STK38,EIF3L,GPAA1,SHARPIN,EIF3D,FOXRED2,HSF1,FBXL6,VPS28,CCND3,PPP1R16A,TRERF1,CDKN2A,RPL8,CUL7,CUL9,PIGO,PDGFRA,JAK2,HINT2,RECK,RNF38,RPS5,ANKRD39,ALG2,RPL28,RPS9,EEF1A1,CD109,GPNMB,MALSU1,STAP1,EFEMP1,RPS7,ANTXR1,STK40,AKT1S1,ING3,ARHGEF5,RPS11,GSN,PARD3,FOXO3,KLF15,ADCK2,SPHK2,RPL18,SPRY1,NOP53,ABTB1,KMT2E,BBS7,HMCES,FKRP,LRRC41,TRIM13,IRF2BP1,QPCTL,SERP2,FASTK,SEPHS1,RPS8,XRCC1,MIF4GD,AGAP3,NAA16,MLH1,RNF220,LIPE,UNK,GALNT11,WBP2,TRIM47,KMT2C,DMAC2,EGLN2,NUMBL,LTBP4,BABAM2,TADA3,CSGALNACT1,RPS3,BRPF1,DYRK1B,TGFBR2,RPS16,TIMP2,ENGASE,SAMD4B,XDH,MRPS12,SIRT2,RPTOR,RPL15,HGS,UBAC2,ASPSCR1,EIF3K,NUP98,GALNT15,FGFR1,TRIM21,RNF24,NDFIP1,SPRY4,HGF,APBB1,TAF10,CLIP3,PCBP2,PSMF1,TARBP2,KMT2B,EIF3F,RBCK1,SDR16C5,TNS2,EIF4B,PTPRG,POFUT1,GGH,LATS2,KCTD6,PEPD,NECAB3,RMND5A,PIK3R1,GGCX,CAST,FBXO8,PCOLCE2,TNKS1BP1,RPS3A,SH3D19,ARRDC3,KDM2B,CAMKK2,AGA,HTRA2,EIF2A,SETD6,PPP1CC,PDGFC,SPRED1,CCNG2,FNIP2,DGUOK,PRKAG1,ARL2BP,TIAM1,NEK4,SPCS1,PROS1,TMEM67,MMS19,CHST14,FOXF2,RNFT2,FARS2,PIDD1,RPLP2,ERLIN1,HR,PIGT,NGF,ACOT8,HERC6,PCIF1,WDR82,TRIM45,PPM1M,FBXW4,WARS2,LONP2,PARP3,GANC,F	cyan	EC_M2
GO:0009725	1.97E-03	0.998658105	94	652	response to hormone	BP	4.49E-01	STAT6,PIP4K2C,CDK4,CITED2,RXRB,TSPO,EP300,DDX17,HSF1,CCND3,TRERF1,HMOX1,KLF9,JAK2,LPIN1,FOXO3,KLF15,FOSB,KHK,ECHDC3,WBP2,EGLN2,TADA3,TGFBR2,TIMP2,NCOA4,RARG,TNS2,LMO2,GGH,LATS2,URI1,PIK3R1,NCOA2,BBS2,MMS19,LPIN3,LGR4,SRD5A1,IRS1,TXNIP,PRMT2,HDAC4,USF1,CAPN10,SCAP,CDKN1B,LRP6,POR,NFE2L2,IDH1,CTSH,MEN1,PHB2,RPL27,ARPC1B,STAT5A,HDAC6,PELP1,RARA,THRA,CASP9,ZFP36L1,AGTRAP,PIP4K2B,RAB8B,PER1,GBA,FOS,ZBTB7B,KANK2,TRIM25,EEF2K,ACACA,INSR,PPARGC1B,SREBF1,OXCT1,LEPR,TFAP4,TRPV1,NSMF,RXRA,APRT,CYBA,TGFBR3,PTGFR,MT-ND3,MT-CYB,ABCA2,JUND,ZBTB7A,PID1,ZFP36L2	cyan	EC_M2
GO:1903578	1.99E-03	0.999298338	16	66	regulation of ATP metabolic process	BP	4.49E-01	TSPO,EP300,SPHK2,COX7A1,PRKAG1,ARNT,HDAC4,DNAJC30,FIS1,NUPR1,INSR,TAZ,JMJD8,MT-CO2,ZBTB7A,PID1	cyan	EC_M2
GO:0018393	2.00E-03	0.999080508	26	134	internal peptidyl-lysine acetylation	BP	4.49E-01	BAG6,JADE2,EP300,ING3,SPHK2,WBP2,TADA3,BRPF1,APBB1,TAF10,SDR16C5,KAT5,POR,EID1,KAT6B,MED24,CTBP1,KAT8,KAT7,PER1,SGF29,CRTC2,SIN3A,CREBBP,WDR5,GTF3C4	cyan	EC_M2
GO:0006650	2.00E-03	0.998875778	44	257	glycerophospholipid metabolic process	BP	4.49E-01	DGKA,PIP4K2C,CHKB,ABHD16A,AGPAT1,GPAA1,PIGO,TTC7A,SLC44A1,LPIN1,TAMM41,PIP4P1,GPCPD1,PTDSS2,PIK3R1,GPAT3,PIGT,PLA2G4B,OSBPL5,GPAM,CHKA,ABHD4,MTMR11,PIGM,PHB2,ABHD12B,SMPD4,PLA2G16,ABHD8,PLSCR3,PIGH,PIP4K2B,DPM3,AC138894.1,MTMR4,MPPE1,MTMR1,PNPLA7,TAZ,PIGQ,INPP5E,VAC14,LCAT,DNAJC19	cyan	EC_M2
GO:0009158	2.10E-03	0.999732059	6	13	ribonucleoside monophosphate catabolic process	BP	4.49E-01	PNP,XDH,NT5C2,DPYD,NT5C,UPP1	cyan	EC_M2

GO:0010257	2.16E-03	0.999275832	15	53	NADH dehydrogenase complex assembly	BP	4.49E-01	NDUFB2,NDUFB6,NDUFC2,DMAC2,NDUFA2,OXA1L,NDUFS2,NDUFB7,NDUFS7,TAZ,MT-ND1,MT-ND2,MT-ND4,MT-ND5,NDUFAF8	cyan	EC_M2
GO:0032981	2.16E-03	0.999275832	15	53	mitochondrial respiratory chain complex I assembly	BP	4.49E-01	NDUFB2,NDUFB6,NDUFC2,DMAC2,NDUFA2,OXA1L,NDUFS2,NDUFB7,NDUFS7,TAZ,MT-ND1,MT-ND2,MT-ND4,MT-ND5,NDUFAF8	cyan	EC_M2
GO:0071704	2.16E-03	0.998201576	948	8083	organic substance metabolic process	BP	4.49E-01	NFATC1,ZNF236,TSHZ1,ZADH2,ORMDL2,PYM1,DGKA,SMARCC2,STAT6,ATG12,PIP4K2C,MAP3K5,PEX7,OS9,CDK4,CITED2,CTDSP2,ATP23,ADAMTS2,MAML1,SQSTM1,SASH1,IRAK3,HELB,GFPT2,OSR2,BAG6,CHKB,ALDH7A1,ABHD16A,PPP6R2,MSH5,SNX9,HSPA1B,EHMT2,ZBTB12,HDAC10,SELENOO,ALG12,CERK,ATF6B,AGPAT1,RNF5,EIF3H,RPS6KA2,DEPTOR,DSE,RXRB,TSPO,HSD17B8,RING1,RPS18,B3GALT4,CYB5R3,ZHX2,ZBTB22,JADE2,PMM1,MYC,TOB2,SMAD5,EP300,IBA57,CDC14C,ERCC6L2,KDM3B,ZC3H3,GSDMD,ZNF76,FANCE,EEF1D,TAB1,RPL3,JOSD1,DDX17,NRBP2,STK38,EIF3L,OPLAH,MPST,NFIB,GPAA1,SHARPIN,EIF3D,FOXRED2,HSF1,FBXL6,SLC52A2,CPSF1,VPS28,CCND3,PPP1R16A,TRERF1,CDKN2A,RPL8,HMOX1,WASL,CUL7,CUL9,DNPH1,KLF9,POLR1C,PIGO,PDGFRA,PLPP6,RCL1,JAK2,HINT2,RECK,RNF38,ZBTB45,RPS5,ZNF135,ANKRD39,TDRD7,TRMO,ZNF667,ALG2,ZNF865,SSC5D,RPL28,TTC7A,RPS9,EEF1A1,CD109,GPNMB,MALSU1,STAP1,SLC44A1,EPB41L4B,ETFB,EFEMP1,SVEP1,PTGR1,UGP2,ZNF692,CYREN,RPS7,ANTXR1,ANXA4,AKNA,LSM10,STK40,MED25,IL4I1,COQ3,AKT1S1,ING3,ARHGEF5,AP2A1,IRF3,LPIN1,RPS11,GSN,PARD3,FOXO3,KLF15,DHDH,ADCK2,SPHK2,RPL18,SPRY1,TPRA1,NOP53,ABTB1,MAML2,BICRA,ZC3HAV1,KMT2E,BBS7,MEIS3,HMCES,FKRP,LRRC41,LURAP1,TRIM13,ACSS1,IRF2BP1,SIX5,QPCTL,FOSB,ZNF746,NR2C2,ZNF862,TSC22D1,SERP2,FDXR,UROD,KHK,SMG9,FASTK,SEPHS1,RPS8,XRCC1,ZNF575,MIF4GD,AGAP3,NAA16,MLH1,RNF220,LIPE,PAFAH1B3,ECHDC3,GTF3C2,UNK,ZNF574,GALNT11,WBP2,TRIM47,KMT2C,ACAA1,MYD88,CELF2,DMAC2,TAMM41,BCKDHA,EGLN2,CY	cyan	EC_M2
GO:0009170	2.17E-03	0.99980691	5	9	purine deoxyribonucleoside monophosphate metabolic process	BP	4.49E-01	DNPH1,PNP,XDH,DGUOK,NT5C	cyan	EC_M2
GO:0018130	2.20E-03	0.998212796	360	2900	heterocycle biosynthetic process	BP	4.49E-01	NFATC1,ZNF236,TSHZ1,SMARCC2,STAT6,MAP3K5,CDK4,CITED2,MAML1,SQSTM1,IRAK3,HELB,GFPT2,OSR2,HSPA1B,EHMT2,ZBTB12,HDAC10,ATF6B,RXRB,TSPO,RING1,ZHX2,ZBTB22,PMM1,MYC,TOB2,SMAD5,EP300,IBA57,KDM3B,ZNF76,DDX17,NFIB,HSF1,CCND3,TRERF1,CDKN2A,HMOX1,WASL,KLF9,POLR1C,JAK2,ZBTB45,ZNF135,ZNF667,ZNF865,EFEMP1,ZNF692,ANXA4,AKNA,MED25,IL4I1,ING3,ARHGEF5,IRF3,LPIN1,FOXO3,KLF15,SPHK2,NOP53,MAML2,BICRA,KMT2E,BBS7,MEIS3,TRIM13,ACSS1,IRF2BP1,SIX5,FOSB,ZNF746,NR2C2,ZNF862,TSC22D1,UROD,ZNF575,NAA16,GTF3C2,ZNF574,WBP2,KMT2C,MYD88,TADA3,RPS3,TEP1,BRPF1,PNP,DYRK1B,EID2,ZNF219,MED29,SIRT2,RPTOR,APBB3,NUP98,TRIM21,CYP1B1,GNPDA1,HGF,APBB1,TAF10,DEAF1,HOXC4,NCOA4,LIN37,KMT2B,RBCK1,RARG,SDR16C5,ZNF740,LMO2,POFUT1,URI1,PIK3R1,ACSS2,NCOA2,ATF1,TNKS1BP1,POLE4,KDM2B,ZFHX4,CAMKK2,SCAND1,SETD6,MCPH1,SSBP2,FNIP2,DGUOK,ARL2BP,SHOX2,NEK4,SPR,ZHX3,VDR,MMS19,LPIN3,CPOX,FOXF2,F2RL1,SORBS3,HNF4A,TP53INP1,PIDD1,HR,TRIM45,NPM3,PARP3,ELK4,LGR4,ICE1,SP140,TCF7L2,TXNIP,AIP,SOX5,ADNP,ZNF217,QARS,IMPDH2,DTX3L,PARP14,ECM1,RCOR3,ARNT,PRMT2,RIPK3,CTPS2,GABPB2,PER2,TWIST2,VPS72,HDAC4,USF1,SMARCD2,POGZ,USP21,PPOX,DNAJC30,PATZ1,RNASEL,CCDC85B,MAGEF1,CDKN1B,TCEA3,KAT5,LRP6,YBX3,NFKBIA,NFE2L2,POLR2J2,PHC1,HEXIM2,MTHFS,BATF2,PCBD1,CHCHD10,MEN1,ATIC,PHB2,TSC22D4,SAV1,TAF6,CTDSP1,EID1,SSBP4,RNF25,FGF7,NELFA,ZSCAN25,ZNF394,TNFRSF1A,STK16,STAT5A,KAT6B,CREG1,NR2F6,HDAC6,PQBP1,CAMTA2,UQC	cyan	EC_M2
GO:0006941	2.95E-02	0.983697882	22	128	striated muscle contraction	BP	4.49E-01	TRPM4,PIK3CG,CXCR4,LMOD3,PDE4D,NR4A1,STC1,KCNJ3,KCNJ2,SYNM,DLG1,JUP,CHRNB1,ARG2,MTOR,ATP2A1,DSG2,DSC2,PDE4B,SLC8A3,KCNH2,SCN2B	turquoise	EC_M6

GO:0019438	2.23E-03	0.99818677	360	2902	aromatic compound biosynthetic process	BP	4.49E-01	NFATC1,ZNF236,TSHZ1,SMARCC2,STAT6,MAP3K5,CDK4,CITED2,MAML1,SQSTM1,IRAK3,HELB,GFPT2,OSR2,HSPA1B,EHMT2,ZBTB12,HDAC10,ATF6B,RXRB,TSPO,RING1,ZHX2,ZBTB22,PMM1,MYC,TOB2,SMAD5,EP300,IBA57,KDM3B,ZNF76,DDX17,NFIB,HSF1,CCND3,TRERF1,CDKN2A,HMOX1,WASL,KLF9,POLR1C,JAK2,ZBTB45,ZNF135,ZNF667,ZNF865,EFEMP1,ZNF692,ANXA4,AKNA,MED25,IL4I1,ING3,ARHGEF5,IRF3,LPIN1,FOXO3,KLF15,SPHK2,NOP53,MAML2,BICRA,KMT2E,BBS7,MEIS3,TRIM13,ACSS1,IRF2BP1,SIX5,FOSB,ZNF746,NR2C2,ZNF862,TSC22D1,UROD,ZNF575,NAA16,GTF3C2,ZNF574,WBP2,KMT2C,MYD88,TADA3,RPS3,TEP1,BRPF1,PNP,DYRK1B,EID2,ZNF219,MED29,SIRT2,RPTOR,APBB3,NUP98,TRIM21,CYP1B1,GNPDA1,HGF,APBB1,TAF10,DEAF1,HOXC4,NCOA4,LIN37,KMT2B,RBCK1,RARG,SDR16C5,ZNF740,LMO2,POFUT1,URI1,PIK3R1,ACSS2,NCOA2,ATF1,TNKS1BP1,POLE4,KDM2B,ZFHX4,CAMKK2,SCAND1,SETD6,MCPH1,SSBP2,FNIP2,DGUOK,ARL2BP,SHOX2,NEK4,SPR,ZHX3,VDR,MMS19,LPIN3,CPOX,FOXF2,F2RL1,SORBS3,HNF4A,TP53INP1,PIDD1,HR,TRIM45,NPM3,PARP3,ELK4,LGR4,ICE1,SP140,TCF7L2,TXNIP,AIP,SOX5,ADNP,ZNF217,QARS,IMPDH2,DTX3L,PARP14,ECM1,RCOR3,ARNT,PRMT2,RIPK3,CTPS2,GABPB2,PER2,TWIST2,VPS72,HDAC4,USF1,SMARCD2,POGZ,USP21,PPOX,DNAJC30,PATZ1,RNASEL,CCDC85B,MAGEF1,CDKN1B,TCEA3,KAT5,LRP6,YBX3,NFKBIA,NFE2L2,POLR2J2,PHC1,HEXIM2,MTHFS,BATF2,PCBD1,CHCHD10,MEN1,ATIC,PHB2,TSC22D4,SAV1,TAF6,CTDSP1,EID1,SSBP4,RNF25,FGF7,NELFA,ZSCAN25,ZNF394,TNFRSF1A,STK16,STAT5A,KAT6B,CREG1,NR2F6,HDAC6,PQBP1,CAMTA2,UQC	cyan	EC_M2
GO:0032989	2.96E-02	0.977734741	88	609	cellular component morphogenesis	BP	4.50E-01	SEMA6A,KIF5A,SNX2,RIMS2,PACSIN2,CDKL3,PTK2,PGM5,SEMA4D,TMOD1,NRCAM,NRP1,ITSN2,MTMR2,NOVA2,PLXND1,NECTIN2,ITGB4,GATA3,CXCR4,SPTBN4,FEZ2,EFNB2,ARHGEF7,LMOD3,EPHB1,RET,PREX2,KIF13B,TLR2,GPM6A,NR4A2,EDN1,DTNBP1,PPP3CA,HECW2,MEF2A,SEMA3F,ROBO4,NRAP,PLEKHO1,PLXNA2,CFLAR,TRAK2,ADCY1,KALRN,PROX1,TMOD4,SS18L1,LAMA5,YTHDF1,PAK2,LAMC2,MOV10,CREB1,ADORA2A,SEMA6D,EPHB4,ITGA4,DNM3,LMTK2,TMOD2,RAC1,EPHA2,MPP5,OPHN1,EFNB1,PSEN1,SEMA4A,ABI3,TAOK2,EFNA1,CHRNB2,ZDHHC15,RPS6KA5,DNM2,B4GALT6,SEMA6B,SLITRK2,MTM1,ARHGAP4,NOTCH1,STXBP1,DAB2IP,VASP,RELN,SHANK3,TBC1D20	turquoise	EC_M6
GO:0061326	2.96E-02	0.985892706	15	77	renal tubule development	BP	4.50E-01	MTSS1,KLHL3,GATA3,GZF1,JAG1,MEF2C,WWTR1,STAT1,LAMA5,DLG1,WNK4,PROM1,HOXB7,NOTCH1,BMP2	turquoise	EC_M6
GO:0048709	2.96E-02	0.986725978	13	67	oligodendrocyte differentiation	BP	4.50E-01	TNFRSF21,CXCR4,TLR2,SOX13,TSPAN2,SLC45A3,HDAC1,TNFRSF1B,MTOR,EIF2B2,B4GALT6,NOTCH1,SLC8A3	turquoise	EC_M6
GO:0051590	2.97E-02	0.993252307	5	17	positive regulation of neurotransmitter transport	BP	4.50E-01	RAB3B,SNCA,DTNBP1,ADORA2A,STXBP1	turquoise	EC_M6
GO:0006595	3.47E-02	0.9919963	5	15	polyamine metabolic process	BP	4.50E-01	AZIN1,AZIN2,SMS,AGMAT,NNMT	darkgreen	EC_M4
GO:0045685	2.97E-02	0.987742552	11	54	regulation of glial cell differentiation	BP	4.51E-01	TNFRSF21,IL6,MYCN,CXCR4,TLR2,SLC45A3,HDAC1,TNFRSF1B,MTOR,NOTCH1,BMP2	turquoise	EC_M6
GO:1905114	2.98E-02	0.978600482	64	448	cell surface receptor signaling pathway involved in cell-cell signaling	BP	4.51E-01	TNFAIP3,FZD6,CTHRC1,RIMS2,CELSR1,TCF7,RNF146,CSNK1E,MDFI,GRB10,GRIK2,FZD8,CCNY,TRPM4,MTMR2,GATA3,SFRP1,NOTUM,FERMT1,SPEF1,P2RX2,HHEX,SOX7,MEF2C,TLR2,WWTR1,P2RX7,LMBR1L,NR4A2,SOX13,SNCA,PRDM15,HDAC1,PPP3CA,BCL9,VANGL2,INSYN2,SENP2,ADORA2A,DDX3X,RAC1,JUP,MCC,CHRNB1,PSEN1,FBXW11,ITGA3,CHRNB2,CCDC88C,TLE3,ADRB2,NRARP,NOTCH1,PLCG2,DAB2IP,ZBTB33,BCL7B,SLC8A3,SOX17,RELN,LYPD6,SHANK3,RGS4,BMP2	turquoise	EC_M6
GO:0071396	2.99E-02	0.978786011	60	422	cellular response to lipid	BP	4.52E-01	TNFAIP3,KDM4C,PIM1,SYK,IL6,CXCL8,LANCL2,TLR4,IL15,TEAD2,IRAK2,EDNRB,NFKBIB,SFRP1,CD36,SCARB1,RET,GRAMD1A,IL1A,DNMT3B,KDM3A,UFSP2,MEF2C,SMARCD1,TLR2,CXCL10,KMT2D,STC1,EDN1,ADCY2,HDAC1,PABPN1,CD86,MN1,CFLAR,ADCY1,FES,ARID1A,PDK3,CREB1,SSTR1,LTF,TBX1,FGF23,ADIPOR2,TNFRSF1B,ALDH1A2,AR,SCNN1B,TNIP1,CCL2,YES1,PDE4B,NOS2,GIT1,IRF8,PLCG2,DAB2IP,TRIM24,BMP6	turquoise	EC_M6
GO:0043388	3.49E-02	0.986254711	10	40	positive regulation of DNA binding	BP	4.52E-01	RB1,TGFB1,CTNNB1,DDRGK1,PINX1,CALM1,MYOCD,ITGA2,PARK7,TXN	darkgreen	EC_M4
GO:0090382	3.00E-02	0.991831858	6	23	phagosome maturation	BP	4.52E-01	RAB38,MYO7A,SYT7,RAB43,RAB32,RAB39A	turquoise	EC_M6
GO:0097503	3.01E-02	0.993048156	5	18	sialylation	BP	4.52E-01	ST3GAL5,ST3GAL6,ST6GAL1,ST6GALNAC3,ST8SIA6	turquoise	EC_M6

GO:0031589	3.01E-02	0.979723149	46	294	cell-substrate adhesion	BP	4.52E-01	PARVG,PTK2,PDGFB,TEK,LIMCH1,KDR,NRP1,BCAM,ITGA8,ITGB4,UNC13D,JAG1,SFRP1,FERMT1,THSD1,ARHGEF7,NTN4,CD36,ADAMTS9,EPHB1,MERTK,LYVE1,ANGPT2,NEDD9,JAM3,PRKX,AP1AR,CD34,LAMA5,ITGA6,DOCK1,MELTF,APOD,BCR,ITGA4,VWF,PPM1F,RAC1,RCC2,JUP,ITGA3,TAOK2,EFNA1,DNM2,NOTCH1,VAMP3	turquoise	EC_M6
GO:1902105	3.02E-02	0.981570655	30	193	regulation of leukocyte differentiation	BP	4.52E-01	TCF7,SYK,TLR4,IL15,TAL1,GATA2,GATA3,SFRP1,TMEM178A,SH3RF1,IL7,SOX13,HLX,HCLS1,INPP5D,CD86,FES,CREB1,LTF,SOS2,ITPKB,MTOR,GPR68,LGALS9,IL7R,AP3D1,NRARP,VNN1,CD46,CA2	turquoise	EC_M6
GO:0010574	3.03E-02	0.99072151	7	27	regulation of vascular endothelial growth factor production	BP	4.52E-01	FLT4,ATF4,IL6,C5,IL1A,EIF2AK3,HIF1A	turquoise	EC_M6
GO:0031329	2.28E-03	0.998428791	99	689	regulation of cellular catabolic process	BP	4.52E-01	PIP4K2C,BAG6,SNX9,HSPA1B,EHMT2,RNF5,EIF3H,TSPO,EP300,NRBP2,HSF1,HMOX1,EEF1A1,RPS7,FOXO3,SESN1,NOP53,ZC3HAV1,BBS7,TRIM13,FASTK,MLH1,MYD88,TIMP2,SAMD4B,SIRT2,RPTOR,TRIM21,HGF,PSMF1,PLEKHF1,SH3D19,CAMKK2,HTRA2,FOXF2,RNFT2,TP53INP1,LONP2,FASTKD3,IRS1,TPCN2,DIS3L2,PUM1,GPX1,AC013489.1,USP19,DTX3L,ABCB11,MCL1,ARNT,RNASEL,MAGEF1,RPL11,KAT5,LRIG2,YBX3,NFE2L2,IDH1,CAPN1,EXOSC7,PHB2,HDAC6,UBXN1,WASHC1,ATP13A2,GABARAP,FOXK1,ZFP36L1,PIP4K2B,OSBPL7,CDK5RAP3,KAT8,GBA,NUPR1,AC138894.1,MTMR4,TNRC6A,KEAP1,SNX33,INSR,SREBF1,LEPR,PARN,FZR1,DVL1,AXIN1,ZER1,ABCA2,RACK1,TRIM65,TENT5B,CST3,RNFT1,RPL23,TRIB2,HSPB8,ZFP36L2,STUB1,EXOSC5	cyan	EC_M2
GO:0001932	3.03E-02	0.976375952	111	845	regulation of protein phosphorylation	BP	4.53E-01	TNFRSF11A,TNFAIP3,PTPRB,FLT4,TPD52L1,PTK2,PDGFB,TEK,LIMCH1,TEC,KIT,KDR,SYK,SEMA4D,IL6,TLR4,IL15,CCNY,SDCBP,NRP1,RASIP1,PIK3CG,TAL1,KDM4D,PRKD2,CAB39L,RALB,PPARG,EDNRB,SPTBN4,SFRP1,HBEGF,SH3BP5,CD36,PRR5L,RET,PDE4D,IGF1,EIF2AK3,HHEX,OCLN,WWTR1,IL7,VEGFC,MADD,THBS4,RAPGEF3,CCNJL,NEDD9,EDN1,EHD4,SNCA,DTNBP1,HDAC1,HYAL2,NCF1,FABP4,HCLS1,PECAM1,CD86,FGR,INPP5F,IGFBP3,PROX1,PRKD1,MLXIPL,ACE,LIMK2,PAK2,DLG1,MAP3K11,LTF,ADORA2A,DDX3X,TBX1,FGF23,PPM1F,RAC1,ADIPOR2,ITPKB,TLR6,RGS14,MTOR,PSEN1,FGF18,PGF,TAOK2,EFNA1,PIK3R5,RPS6KA5,CCDC88C,ADRB2,YES1,SH2D3A,UNC119,TRAF2,PLCG2,ENG,SH2D3C,DAB2IP,CDH5,SLC8A3,RELN,ALKAL1,FGF16,MMD,DAZAP2,COPS8,BMP2,DUSP7,BMP6	turquoise	EC_M6
GO:2001237	3.50E-02	0.982222453	17	79	negative regulation of extrinsic apoptotic signaling pathway	BP	4.53E-01	CTNNA1,TGFBR1,FAIM2,CX3CL1,ACVR1,CTTN,EYA2,GSK3B,PEA15,SERPINE1,ITGAV,HTT,ARHGEF2,AKT1,PARK7,GDNF,FGF10	darkgreen	EC_M4
GO:1902624	3.04E-02	0.991629402	6	24	positive regulation of neutrophil migration	BP	4.53E-01	CXCL8,THBS4,EDN1,MCU,RAC1,DAPK2	turquoise	EC_M6
GO:1902895	3.05E-02	0.98891945	9	37	positive regulation of pri-miRNA transcription by RNA polymerase II	BP	4.53E-01	PDGFB,GATA2,PPARG,GATA3,SMAD1,ETS1,HIF1A,MRTFB,BMP2	turquoise	EC_M6
GO:0060055	3.50E-02	0.989132925	7	24	angiogenesis involved in wound healing	BP	4.53E-01	SLC12A2,FAM19A5,SRF,VEGFA,CX3CL1,DAG1,SERPINE1	darkgreen	EC_M4
GO:0051588	3.05E-02	0.985786408	14	73	regulation of neurotransmitter transport	BP	4.53E-01	SNCAIP,APBA1,RAB3B,MCTP1,MEF2C,SNCA,DTNBP1,MCTP2,ADCY1,ADORA2A,SNCG,FBXL20,GIT1,STXBP1	turquoise	EC_M6
GO:0034123	3.06E-02	0.991635694	6	23	positive regulation of toll-like receptor signaling pathway	BP	4.53E-01	PELI1,RSAD2,TLR2,LTF,DDX3X,TLR1	turquoise	EC_M6
GO:0002449	3.06E-02	0.982253652	25	159	lymphocyte mediated immunity	BP	4.53E-01	IL6,RSAD2,C5,TRPM4,CTSC,NECTIN2,UNC13D,GATA3,JAG1,GAPT,RNF19B,LYST,INPP5D,B2M,WAS,RAB27A,TNFRSF1B,BATF,CD19,LGALS9,IL7R,TRAF2,CD46,FCER2,SERPINB9	turquoise	EC_M6
GO:0070169	3.07E-02	0.988665184	9	41	positive regulation of biomineral tissue development	BP	4.53E-01	ACVR2B,MEF2C,P2RX7,ANO6,LTF,ADRB2,ISG15,BMP2,BMP6	turquoise	EC_M6
GO:0045806	3.07E-02	0.98866093	9	41	negative regulation of endocytosis	BP	4.53E-01	PACSIN2,SDCBP,MTMR2,MCTP1,RIN3,PRKD1,RAC1,UNC119,LRSAM1	turquoise	EC_M6

GO:0050951	3.07E-02	0.992933344	5	17	sensory perception of temperature stimulus	BP	4.53E-01	GRIK2,CXCR4,EPHB1,MMP24,ANO1	turquoise	EC_M6
GO:0010959	3.08E-02	0.979942459	39	263	regulation of metal ion transport	BP	4.53E-01	TRDN,PDGFB,TSPAN13,PIK3CG,KCNRG,CXCR4,SPTBN4,P2RY6,P2RX2,PDE4D,CHD7,P2RX7,CXCL10,PLCG1,STC1,ANO6,SNCA,SIK1,HECW2,KCNJ2,EPB41,PRKD1,DLG1,B2M,ADORA2A,HOMER3,WNK4,CRACR2A,ATP2A1,CD19,DNM2,ADRB2,CCL2,PDE4B,PLCG2,KCNH2,SCN2B,RGS4,FXYD6	turquoise	EC_M6
GO:0035249	3.08E-02	0.986730785	12	57	synaptic transmission, glutamatergic	BP	4.53E-01	GRIK2,CLCN3,MEF2C,DGKZ,ADORA2A,OPHN1,PSEN1,CCL2,STXBP1,RELN,SHANK3,CACNG5	turquoise	EC_M6
GO:0016032	3.08E-02	0.979095567	47	325	viral process	BP	4.53E-01	MGAT4A,CXCL8,PPIE,RSAD2,CAV2,NRP1,NECTIN2,USP6NL,CXCR4,EFNB2,SCARB1,ITCH,OAS3,TPCN1,ANO6,IFIT1,STAT1,MX1,HDAC1,KIAA1551,HYAL2,ISG20,CD86,POU2F3,PROX1,F11R,MGAT1,ST6GAL1,LTF,DDX3X,EPHA2,TNIP1,CCL2,LGALS9,ISG15,HCFC1,NOTCH1,LRSAM1,ST6GALNAC3,HAVCR1,OAS1,RAB43,TRIM34,CD46,TRIM62,TBC1D20,BST2	turquoise	EC_M6
GO:0006812	3.08E-02	0.976202793	104	796	cation transport	BP	4.53E-01	TRDN,KLHL3,DAPK1,PDGFB,IREB2,ATP8A1,SYK,TSPAN13,GJA4,TRPM4,RAB3B,PIK3CG,KCNRG,CXCR4,SEC14L1,NDFIP2,SPTBN4,P2RY6,KCNK6,P2RX2,SLC4A11,MICU3,PDE4D,CHD7,CLCN3,CHRM5,MEF2C,CP,P2RX7,GPM6A,CXCL10,SERINC5,TPCN1,PLCG1,STC1,KCNJ3,SLC38A1,ANO6,ZDHHC13,EDN1,KCNJ15,SNCA,DTNBP1,PM20D1,SIK1,LCK,PPP3CA,ANO1,HECW2,SLC39A8,KCNJ2,SLC12A7,SCN3A,SNAP23,SLC9A8,EPB41,PRKD1,MELTF,DLG1,KCNK7,B2M,LTF,ADORA2A,HOMER3,MCU,CALCRL,SLC9A7,WNK4,RAMP2,CRACR2A,LMTK2,SLC2A9,SNCG,SYT7,CHRNB1,HEPH,PSEN1,KCNN3,CHRNB2,ATP2A1,CD19,DNM2,SCNN1B,ADRB2,CCL2,KCNJ18,PDE4B,PIEZO2,SLC43A2,PIEZO1,PLCG2,SLC25A25,SLC8A3,RELN,KCNH2,SCN2B,KCNK5,SHANK3,RGS4,KCNA6,CACNG5,FXYD6,KCNG2,GSG1L	turquoise	EC_M6
GO:0051247	3.09E-02	0.975231209	144	1140	positive regulation of protein metabolic process	BP	4.54E-01	TNFRSF11A,IFNGR1,TNFAIP3,FLT4,GRAMD4,TPD52L1,SGSM3,PTK2,DAPK1,PDGFB,AGTPBP1,CSNK1E,TEK,LIMCH1,DDX58,FAM122A,TEC,KIT,KDR,SYK,SEMA4D,IL6,PELI1,TLR4,IL15,CCNY,SDCBP,NRP1,PIK3CG,TAL1,PRKD2,CTSC,CAB39L,GGA3,RALB,PPARG,GATA3,EDNRB,NDFIP2,HBEGF,RPL26,ANKLE2,CD36,PRR5L,PLK2,RET,DNMT3B,IGF1,KLKB1,EIF2AK3,ITCH,HIP1R,SOX7,VEGFC,MADD,TIPARP,THBS4,PPP1R16B,RAPGEF3,NEDD9,EDN1,DYRK1A,EHD4,SNCA,JARID2,MYLIP,RNF144B,ABCG1,RNF19B,LPCAT1,HDAC1,LCK,HECW2,LAPTM5,NCF1,USP13,HCLS1,PECAM1,CD86,ASB9,CFLAR,CASP10,PPP2R5A,PROX1,PRKD1,YTHDF1,OAZ3,ACE,LIMK2,PAK2,MELTF,SENP2,DLG1,MAP3K11,TET1,LTF,DDX3X,TBX1,BIRC3,FGF23,PPM1F,RAC1,ADIPOR2,BID,TLR6,TNFRSF1B,MTOR,RNF40,PSEN1,FBXW11,FGF18,PGF,TAOK2,EFNA1,SLC51B,NAP1L2,PIK3R5,PIAS1,RPS6KA5,TNIP1,ADRB2,YES1,SH2D3A,LGALS9,UNC119,TRAF2,PLCG2,ENG,SH2D3C,MTF2,DAB2IP,CDH5,PRSS22,PMAIP1,SOX17,RELN,ALKAL1,FGF16,MMD,PERP,DAZAP2,COPS8,BMP2,BMP6	turquoise	EC_M6
GO:0061647	3.09E-02	0.988685215	9	38	histone H3-K9 modification	BP	4.54E-01	KDM4D,GATA3,SETD5,DNMT3B,KDM3A,JARID2,MECOM,NAP1L2,ARID4A	turquoise	EC_M6
GO:0048009	3.09E-02	0.990478387	7	27	insulin-like growth factor receptor signaling pathway	BP	4.54E-01	GRB10,IGF1,EIF2AK3,IGFBP3,IGFBP5,AR,BMP2	turquoise	EC_M6
GO:0019935	3.10E-02	0.986602594	12	57	cyclic-nucleotide-mediated signaling	BP	4.55E-01	PDE7B,RIMS2,EDNRB,PDE2A,CD36,PDE4D,GUCY1B1,RAPGEF3,ADCY2,PDE9A,ADCY1,NPR1	turquoise	EC_M6
GO:0060390	3.52E-02	0.988015579	8	28	regulation of SMAD protein signal transduction	BP	4.55E-01	LRP1,TGFBR1,TGFB1,RBPMS,SLC2A10,FAM89B,BMPR1A,TGFB3	darkgreen	EC_M4
GO:0048562	3.11E-02	0.980225567	35	225	embryonic organ morphogenesis	BP	4.55E-01	FZD6,CTHRC1,MDFI,TEAD2,MYCN,GATA2,GATA3,MYO7A,CHD7,MEF2C,NDRG4,USH1C,HIPK1,EDN1,HLX,SEC24B,PROX1,VANGL2,DLG1,HOXD10,TBX1,HIF1A,EPHA2,PSEN1,HOXB5,HOXB6,HOXB7,HOXB8,HOXB9,EFNA1,SMAD2,NOTCH1,ENG,SOX17,SOX18	turquoise	EC_M6
GO:0001967	3.53E-02	0.993789587	4	10	suckling behavior	BP	4.56E-01	APP,GLS,UBE2Q1,DERL2	darkgreen	EC_M4
GO:0002832	3.13E-02	0.985343119	14	77	negative regulation of response to biotic stimulus	BP	4.56E-01	TNFAIP3,C6orf106,PPARG,SEC14L1,ITCH,OAS3,NLRC5,LTF,ARG2,LGALS9,ISG15,OAS1,SERPINB9,RNF125	turquoise	EC_M6

GO:1903055	3.13E-02	0.991393692	6	23	positive regulation of extracellular matrix organization	BP	4.56E-01	IL6,CFLAR,MELTF,FSCN1,CARMIL2,BMP2	turquoise	EC_M6
GO:0072148	3.13E-02	0.994526112	4	11	epithelial cell fate commitment	BP	4.56E-01	NRP1,PROX1,JAG2,NOTCH1	turquoise	EC_M6
GO:0032634	3.13E-02	0.994366351	4	12	interleukin-5 production	BP	4.56E-01	TNFRSF21,GATA3,PDE4D,IL1RAP	turquoise	EC_M6
GO:0032674	3.13E-02	0.994366351	4	12	regulation of interleukin-5 production	BP	4.56E-01	TNFRSF21,GATA3,PDE4D,IL1RAP	turquoise	EC_M6
GO:0072576	3.14E-02	0.992655769	5	18	liver morphogenesis	BP	4.56E-01	TNFAIP3,IL6,CFLAR,RPS6KA1,PROX1	turquoise	EC_M6
GO:0021904	3.55E-02	0.99028616	6	19	dorsal/ventral neural tube patterning	BP	4.56E-01	GLI3,GLI2,TCTN1,FKBP8,GORAB,TULP3	darkgreen	EC_M4
GO:0006575	3.55E-02	0.97906317	26	136	cellular modified amino acid metabolic process	BP	4.56E-01	SLC22A4,SERINC1,ALDH1L2,MFSD2A,PCYOX1,SLC7A11,ABHD12,MMACHC,OSBPL10,OSBPL8,ARL6IP5,PLOD2,MTHFD2,CHAC1,SERINC3,GLRX2,CPT1A,PLOD3,MED1,PCYOX1L,HAGH,TMLHE,PARK7,PLA2G15,PEMT,GLO1	darkgreen	EC_M4
GO:0003272	3.55E-02	0.989033605	7	23	endocardial cushion formation	BP	4.56E-01	TGFBR1,ROBO1,ACVR1,TGFB2,BMP7,BMPR1A,TMEM100	darkgreen	EC_M4
GO:0006929	3.15E-02	0.991539964	6	20	substrate-dependent cell migration	BP	4.57E-01	ANKS1A,CD2AP,SDCBP,NRP1,VEGFC,OPHN1	turquoise	EC_M6
GO:0070371	3.15E-02	0.980053365	34	227	ERK1 and ERK2 cascade	BP	4.57E-01	TNFRSF11A,SEMA6A,AKAP12,FLT4,PDGFB,TEK,KDR,SYK,GRB10,TLR4,NRP1,PRKD2,P2RY6,CD36,SIRT3,EPHB1,IGF1,NDRG4,HCRTR1,CFLAR,DLG1,TNFAIP8L3,FGF23,EPHA2,RGS14,FGF18,TNIP1,CCL2,LGALS9,NOTCH1,DAB2IP,MIR23A,ALKAL1,BMP2	turquoise	EC_M6
GO:0007274	3.16E-02	0.991439719	6	21	neuromuscular synaptic transmission	BP	4.57E-01	FCHSD2,FCHSD1,P2RX2,CHRNB1,CHRNB2,STXBP1	turquoise	EC_M6
GO:0046839	3.16E-02	0.988310351	9	40	phospholipid dephosphorylation	BP	4.57E-01	SYNJ2,INPP4B,MTMR2,CHRM5,MTMR10,INPP5D,INPP5F,MTMR12,MTM1	turquoise	EC_M6
GO:0045229	3.17E-02	0.979316704	39	251	external encapsulating structure organization	BP	4.58E-01	SERAC1,IL6,CAV2,TIE1,FERMT1,NTN4,RIC8A,ADAMTS9,TCF15,MMP24,TLL2,TLL1,ADAMTS20,GAS2,NFKB2,ETS1,SLC39A8,CFLAR,TGM1,MMP21,MELTF,ADAMTS7,RAMP2,MMP13,MMP1,FSCN1,TNFRSF1B,SMOC1,PAPLN,THSD4,COL4A6,COL4A5,MMP28,NOTCH1,ENG,GFOD2,CARMIL2,ATXN1L,BMP2	turquoise	EC_M6
GO:0051000	3.58E-02	0.990195692	6	19	positive regulation of nitric-oxide synthase activity	BP	4.58E-01	NUS1,HTR2B,KRAS,CALM1,AKT1,NPR3	darkgreen	EC_M4
GO:0046324	3.58E-02	0.98511967	11	44	regulation of glucose import	BP	4.58E-01	LEP,AKT2,OSBPL8,IRS2,SORBS1,PTPN11,SELENOS,PEA15,CLTCL1,PRKCI,AKT1	darkgreen	EC_M4
GO:0072132	3.58E-02	0.985083075	11	44	mesenchyme morphogenesis	BP	4.58E-01	TGFBR1,ROBO1,HEY1,ACTG2,ACVR1,TGFB2,LEF1,BMP7,ACTA2,BMPR1A,TMEM100	darkgreen	EC_M4
GO:0070278	3.17E-02	0.99426971	4	12	extracellular matrix constituent secretion	BP	4.58E-01	TNFRSF1B,NOTCH1,ENG,BMP2	turquoise	EC_M6
GO:1904407	3.18E-02	0.99007978	7	28	positive regulation of nitric oxide metabolic process	BP	4.59E-01	TLR4,CD36,EDN1,TMEM106A,TLR6,MTOR,DNM2	turquoise	EC_M6
GO:0032501	1.22E-04	0.999933291	94	5003	multicellular organismal process	BP	4.59E-01	DDR1,PRRT1,TRPS1,LRRC6,LRRC4,TRPM3,MLIP,COL9A1,BRSK1,CABS1,TBX18,AQP1,ANKRD6,EPHB6,DDO,SLC22A16,NAMPT,INHBB,GRIK5,SNAP25,PDE6G,ZIC5,IRF7,IL37,CHMP4B,CLU,NDRG3,SHROOM3,LBP,SLIT1,GNAO1,NFASC,AGFG1,ESRRG,RBM20,PRSS56,RNF114,OPRD1,SORL1,SEMA4B,MYOZ2,TREH,SNX4,PDE6H,FCER1G,NOS1AP,FZD5,PTH1R,DUOX1,STOX1,GGT2,CPS1,DIXDC1,ANKH,RND2,HGFAC,CNTNAP1,PRKAG3,ATP1B1,SPEG,CPAMD8,PCSK1N,SLC4A3,PADI2,HSPA2,LDB3,FGFR4,ATP1B2,SLC16A2,LCTL,EPOR,DRP2,RNF128,PLD4,NF1,PTGER4,AGRN,PRDM16,PTGDS,FOXL1,VCAM1,CDH3,TCFL5,MAFB,EPS8L2,ISL2,GATA4,TRPM2,GGT5,TCF23,HPCAL4,RTN4RL2,DLX4,HEY2	magenta	EC_M13
GO:0048167	3.60E-02	0.978308167	28	143	regulation of synaptic plasticity	BP	4.60E-01	ADGRB1,SRF,RIMS1,NCDN,RAB3GAP1,PRNP,MAP1B,APP,RAPGEF2,CX3CL1,CHRNA7,GSK3B,KRAS,NCSTN,SYAP1,MAPT,EIF4EBP2,TNR,MPP2,ERC1,RAB11A,GRIA1,NPTN,CYP46A1,JPH3,YWHAH,MAP1A,LRRTM2	darkgreen	EC_M4

GO:0045669	3.61E-02	0.983595481	13	56	positive regulation of osteoblast differentiation	BP	4.60E-01	FBN2,RUNX2,GLI3,FGF2,CTNNB1,ILK,ACVR1,LRP5,BMP7,SUCO,BMPR1A,PDLIM7,ATP6AP1	darkgreen	EC_M4
GO:0070206	3.61E-02	0.993606287	4	10	protein trimerization	BP	4.60E-01	COL1A2,DISP1,CD74,SIGMAR1	darkgreen	EC_M4
GO:0042100	3.20E-02	0.986605582	11	56	B cell proliferation	BP	4.61E-01	TNFRSF21,PELI1,TLR4,GAPT,MEF2C,IL7,NFATC2,INPP5D,CHRNB2,CD19,IL7R	turquoise	EC_M6
GO:0045860	3.21E-02	0.978359867	45	305	positive regulation of protein kinase activity	BP	4.62E-01	TNFRSF11A,TPD52L1,PTK2,PDGFB,KIT,SYK,TLR4,CCNY,PIK3CG,TAL1,CAB39L,RALB,HBEGF,IGF1,VEGFC,MADD,NEDD9,EDN1,SNCA,NCF1,CD86,PROX1,ACE,PAK2,DLG1,MAP3K11,LTF,DDX3X,TLR6,MTOR,PSEN1,FGF18,TAOK2,EFNA1,PIK3R5,ADRB2,UNC119,TRAF2,DAB2IP,RELN,ALKAL1,MMD,DAZAP2,COPS8,BMP2	turquoise	EC_M6
GO:0035456	3.23E-02	0.992316811	5	19	response to interferon-beta	BP	4.62E-01	TMEM173,STAT1,OAS1,BST2,XAF1	turquoise	EC_M6
GO:0050890	3.23E-02	0.979581181	34	214	cognition	BP	4.62E-01	ATP8A1,KIT,ADGRF1,PPT1,SETD5,RIC8A,PLK2,CHD7,MEF2C,TLR2,NDRG4,TANC1,DOP1B,SHANK2,ADCY1,YTHDF1,PTCHD1,CREB1,B2M,TMOD2,HIF1A,SHROOM4,RGS14,MTOR,PSEN1,ITGA3,CHRNB2,PIAS1,GIT1,SLC8A3,ATXN1L,RELN,SHANK3,MGAT3	turquoise	EC_M6
GO:0014819	3.23E-02	0.994295288	4	11	regulation of skeletal muscle contraction	BP	4.62E-01	NR4A1,KCNJ2,ATP2A1,SLC8A3	turquoise	EC_M6
GO:2001141	3.24E-02	0.972364561	294	2416	regulation of RNA biosynthetic process	BP	4.63E-01	ZNF516,TNFRSF11A,BAZ2A,TCF4,TNFAIP3,PLAGL1,CNOT6,FZD6,ZBED4,NOTCH4,TCF20,TCF7,NCOA7,L3MBTL3,ATF4,PDGFB,KDM4C,PIM1,NFYA,MDFI,PRDM4,DDX58,NFX1,KIT,ADGRF1,NFIL3,SYK,SEMA4D,IL6,NFE2L3,REL,CREB5,PPIE,BACH2,PELI1,FOXP2,KLF11,LANCL2,NOBOX,TLR4,TRAF1,CREM,TEAD2,SDCBP,MYCN,KTI12,ZBTB24,BEND5,TAL1,PHF10,EPC1,GATA2,DNMT3A,PRKD2,SAP130,DACH1,PPARG,GATA3,TAF3,ACVR2B,IRAK2,GZF1,EDNRB,SETD5,IKBKB,JAG1,NFKBIB,PDE2A,TMEM173,SFRP1,NR1D2,CD36,CDK17,ARID5A,TCF15,RET,CHD7,IL1A,DNMT3B,IGF1,NR4A1,EIF2AK3,HHEX,KDM3A,ITCH,ERBIN,SMAD1,ZBTB38,POU6F1,STOX2,SOX7,HMBOX1,HEXB,MEF2C,SMARCD1,TLR2,WWTR1,ZNF704,CXCL10,ADGRG3,KMT2D,SFMBT1,SCAF4,NLRC5,ACTR5,ANKRD2,NR4A2,TOX2,DLL4,CDYL,BAZ2B,SOX13,YAF2,EDN1,STAT1,DYRK1A,SNCA,JARID2,ERG,ETS2,CDK18,NFKB2,DCAF1,PRDM15,ETS1,CD200,SIK1,HDAC1,PPP3CA,HYAL2,SLC39A8,TESMIN,MEF2A,BCL9,HLX,NCF1,USP13,FABP4,HCLS1,NFATC2,ZFHX2,EAF2,GMEB1,CD86,KDM2A,CIART,MN1,TGIF1,PARP15,POU2F3,ZSCAN29,CFLAR,TRAK2,ADCY1,ARID1A,RPS6KA1,PROX1,SS18L1,PRKD1,ZNF148,ZSCAN2,HELZ2,MLXIPL,ITGA6,DLG1,ELL3,ETV6,HOXD10,RNF2,CREB1,EIF4A2,TET1,LTF,ADORA2A,ARNT2,MYEF2,IL1RAP,BCOR,DDX3X,RFXANK,ETV4,TBX1,MECOM,ING4,CDK16,SPINDOC,LDB2,FGF23,PPM1F,POU2F1,WAS,TEAD4,PRKCH,HIF1A,KDM5A,ZBTB18,APBB2,JUP,BCL6B,KDM5C,TLR6,RSC1A1,TNFRSF1B,RGS14,MAFK,SPIN2A,MTOR,AR,CASZ1,PSEN1,CBX1,HOXB5,HOXB6,HOXB7,HOXB8,HOXB9,ZNF410,FBXW11,BATF,KRBA2,EFNA1,PIAS1,RPS6KA5,SM	turquoise	EC_M6
GO:0007219	3.25E-02	0.981582218	23	138	Notch signaling pathway	BP	4.63E-01	NOTCH4,MFNG,KIT,GATA2,NOS3,LLGL2,TIMP4,JAG1,PRAG1,BMP2K,DLL4,GAS2,TSPAN15,TSPAN14,LFNG,PSEN1,JAG2,NRARP,EGFL7,NOTCH1,CD46,PERP,BMP2	turquoise	EC_M6
GO:0034333	3.25E-02	0.994244412	4	11	adherens junction assembly	BP	4.63E-01	HIPK1,JAM3,PAK2,RAMP2	turquoise	EC_M6
GO:0006809	3.25E-02	0.986378351	11	55	nitric oxide biosynthetic process	BP	4.63E-01	TLR4,NOS3,CD36,EDN1,CD34,RAC1,TLR6,ARG2,MTOR,DNM2,NOS2	turquoise	EC_M6
GO:0072182	3.26E-02	0.99421799	4	11	regulation of nephron tubule epithelial cell differentiation	BP	4.63E-01	GATA3,WWTR1,STAT1,PROM1	turquoise	EC_M6
GO:0002886	3.27E-02	0.987819141	9	41	regulation of myeloid leukocyte mediated immunity	BP	4.63E-01	DDX58,SYK,GATA2,GAB2,UNC13D,FGR,FES,LGALS9,STXBP1	turquoise	EC_M6
GO:0035812	3.27E-02	0.993921484	4	13	renal sodium excretion	BP	4.63E-01	EDNRB,EDN1,ADORA2A,NPR1	turquoise	EC_M6
GO:0048511	3.27E-02	0.979137918	35	223	rhythmic process	BP	4.63E-01	TNFRSF11A,ATF4,CSNK1E,NFYA,NFIL3,CREM,NOCT,PPARG,TIMP4,PPP1CB,NR1D2,DYRK1A,GAS2,NFKB2,ETS1,MTTP,SIK1,HDAC1,KDM2A,CIART,ADCY1,RBM4,PROX1,PLEKHA1,CREB1,ADORA2A,KDM5A,KDM5C,MTOR,FBXW11,CHRNB2,RAI1,NOS2,USP7,PASD1	turquoise	EC_M6
GO:0046928	3.28E-02	0.985115748	13	66	regulation of neurotransmitter secretion	BP	4.63E-01	SNCAIP,APBA1,MCTP1,MEF2C,SNCA,DTNBP1,MCTP2,ADCY1,ADORA2A,SNCG,FBXL20,GIT1,STXBP1	turquoise	EC_M6

GO:0097623	3.28E-02	0.994183826	4	11	potassium ion export across plasma membrane	BP	4.63E-01	ANO6,DLG1,KCNH2,KCNK5	turquoise	EC_M6
GO:0010717	3.28E-02	0.984097823	15	80	regulation of epithelial to mesenchymal transition	BP	4.63E-01	IL6,SDCBP,GATA3,JAG1,SFRP1,WWTR1,ELL3,MTOR,EFNA1,SMAD2,NOTCH1,ENG,DAB2IP,TRIM62,BMP2	turquoise	EC_M6
GO:0045737	2.66E-02	0.992825784	6	26	positive regulation of cyclin-dependent protein serine/threonine kinase activity	BP	4.64E-01	CKS2,CCNT2,CCNB1,CDK5RAP1,CDC6,PSMD10	darkgrey	EC_M8
GO:0043009	3.65E-02	0.972343437	87	522	chordate embryonic development	BP	4.65E-01	PLXNB2,ANGPT1,RSPO3,LY6E,MYH9,SRF,VEGFA,RUNX2,TGFBR1,RTN4,ADAMTS3,KIDINS220,GLI3,ITGB1,GLI2,MEGF8,TGFB1,CTNNB1,MAFG,SPINT2,LIG4,SEMA3C,TMED2,EIF2S2,SULF1,RDH10,HEY1,TCTN1,OPA1,ACVR1,ADA,PKD2,NKD1,TENT5C,IFT57,PSPH,NOTCH2,HYAL1,SLC2A10,SBDS,TGFB2,SATB2,ITPK1,MMP14,LEF1,BMP7,WDTC1,FGFR2,VASH2,TANC2,PLOD3,ATP6AP2,FZD2,MEOX1,FKBP8,SKIL,ECE1,PRRX1,GORAB,TULP3,SIX4,FKBP10,BMPR1A,TCAP,MED1,MYO1E,COL1A1,TGFB3,MAP2K1,TMEM100,PPP4R4,RBBP8,AKT1,UBE2A,C6,SUPT6H,NXN,PKD1,COL11A1,LMO4,BCL10,PEMT,HOPX,ZPR1,SLC39A3,CHST11,WNT2	darkgreen	EC_M4
GO:0010831	3.29E-02	0.994144773	4	11	positive regulation of myotube differentiation	BP	4.65E-01	ATP11A,TBX1,MTOR,PIEZO1	turquoise	EC_M6
GO:0033363	3.65E-02	0.987400033	8	29	secretory granule organization	BP	4.65E-01	OSBP,AP3B1,PTPRN,SOX30,PAFAH1B1,CCDC136,SPINK2,BLOC1S2	darkgreen	EC_M4
GO:0007281	2.67E-02	0.984681265	25	177	germ cell development	BP	4.65E-01	YTHDC2,FBXO5,FANCC,TUT7,SRPK1,MSH2,YTHDC1,CEP57,METTL3,BRCA2,CCNB1,RPS6,KNL1,BMPR1B,NSUN2,TRIP13,ASPM,SPO11,TIAL1,ZFX,DDX20,BRIP1,ODF2,AURKA,RNF8	darkgrey	EC_M8
GO:0097696	3.30E-02	0.982750811	18	105	receptor signaling pathway via STAT	BP	4.65E-01	KIT,IL6,IL15,PPARG,RET,IGF1,STAT1,HDAC1,HCLS1,INPP5F,IL10RA,PIAS1,CCL2,JAK1,IL7R,DOT1L,NOTCH1,MIR99A	turquoise	EC_M6
GO:1904062	3.30E-02	0.978732801	36	241	regulation of cation transmembrane transport	BP	4.65E-01	TRDN,DAPK1,TSPAN13,PIK3CG,KCNRG,P2RY6,PDE4D,CHD7,MEF2C,CXCL10,PLCG1,ANO6,SNCA,PM20D1,HECW2,KCNJ2,PRKD1,DLG1,WNK4,CRACR2A,ATP2A1,CD19,DNM2,ADRB2,CCL2,PDE4B,SLC43A2,PLCG2,RELN,KCNH2,SCN2B,SHANK3,RGS4,CACNG5,FXYD6,GSG1L	turquoise	EC_M6
GO:0048545	3.66E-02	0.975077368	46	258	response to steroid hormone	BP	4.66E-01	CDO1,PPARA,PPARD,CLOCK,REST,MGARP,PGRMC2,GLB1,PAK1,TGFB1,HNMT,LBH,UFM1,ATP2B1,UBA5,DDRGK1,RNF6,HEY1,CRY2,VPS18,SPP1,CPN1,EIF4E,CCND1,TGFB2,KRAS,NTRK3,BMP7,RBBP7,STRN3,IGFBP2,MED1,COL1A1,TGFB3,SOX30,CALM1,SMARCA4,ABCA3,SSTR5,PARK7,AGL,SERPINF1,LOX,CDKN1A,YWHAH,PMEPA1	darkgreen	EC_M4
GO:0001783	3.66E-02	0.989897044	6	19	B cell apoptotic process	BP	4.66E-01	IRS2,ADA,SLC39A10,CD74,BCL10,ORMDL3	darkgreen	EC_M4
GO:0034767	3.67E-02	0.979126464	23	114	positive regulation of ion transmembrane transport	BP	4.66E-01	ITGB1,KIF5B,ACTN4,STIM1,FXYD1,ATP1B3,CX3CL1,ABCC8,CACNB3,CHP1,PKD2,CASQ1,HTT,KCNA1,HAP1,STIM2,KCNMB1,CALM1,VMP1,FLNA,DMD,THY1,TMSB4X	darkgreen	EC_M4
GO:0072678	3.31E-02	0.987570191	9	42	T cell migration	BP	4.66E-01	PIK3CG,LRCH1,CXCL10,CD200,F11R,APOD,ITGA4,CCL2,S1PR1	turquoise	EC_M6

GO:0006996	3.69E-02	0.967701832	434	2943	organelle organization	BP	4.68E-01	LMAN1,ATP8B1,LRP1,STX7,TRIM36,AHI1,AGAP2,RNF19A,TCP1,HSPA4,SEC24A,FAM49B,MRTFA,HABP4,PACSIN1,REEP2,CTNNA1,GOLM1,SUN2,MKLN1,UBQLN1,FLNC,MYH9,PEX6,SRF,VCP,UNC13B,TPM2,NCK2,PDCL3,TXNDC9,LMAN2L,DST,RAB23,NLRP5,TGFBR1,PSME4,CENPC,RTN4,SHANK1,COX18,LRRC4B,EMC10,GGCT,MYH14,CALD1,ACTR2,ROCK2,VPS41,ZYX,MYLK3,VPS35,MGARP,CFAP100,ITGB1,SRPK2,KIF5B,SEC61A1,RAB7A,EML2,RB1,PICALM,MARK4,MAPRE3,PDCD6IP,SMARCD3,PAK1,ACTR3,NCKAP5,RAB3GAP1,SEC13,CTNNB1,ESYT2,TRAK1,CLIP4,AKT2,DYNC1LI1,SEC23B,DZIP1,ZEB2,PLEKHG2,DNAJB13,FARP1,ARHGEF17,ACTG1,NAV3,NPLOC4,ACTN4,PPP1R12A,ANAPC15,STAM2,KAT2B,TACC1,UFM1,LIG4,ATP2C1,CHFR,DCN,CDC42EP3,HDAC3,ACPP,PCLO,STARD13,CAVIN3,UBA5,DDRGK1,PPP2CB,ILK,DCTN6,DPYSL3,KATNAL1,TTC17,TMEM127,CNOT7,VPS37A,ARL1,CXCL12,RAB2A,KIF2A,TMEM41B,ANKRD1,FAT1,REEP5,SLC25A46,HSP90B1,TMED2,PALLD,MRAS,MAP1LC3A,TP53INP2,MAST4,SLC25A4,FLNB,OSBP,VAMP8,RIOK2,TRAM1,MAP1B,EMC4,PINX1,ACTR8,SORBS1,PFN2,RASA1,EPB41L1,ATP2A2,USO1,TCTN1,RTKN,SDAD1,ATXN2,DHX36,SLC4A5,CCT8,MAPKAPK5,CX3CL1,GNL3,NEFM,OPA1,ARSB,ATG13,RAD54B,LOXL2,AP3B1,ZFYVE27,TSG101,DKC1,TUBB2A,VPS18,AKTIP,BAP1,CHP1,PKD2,CSDE1,TWF1,DNAH1,SYCP2L,DNAH5,IFT57,TENT4B,BRSK2,HPS6,VPS39,PDLIM5,MAGEL2,RAB7B,ARL3,RAP1GDS1,ELMO2,DYNLL1,INA,PHLDB2,TJP1,KDM5B,NOTCH2,CTTN,PKP2,DNM1L,TROVE2,MARCKSL1,LMOD1,EYA2,SEC22B,CISD2,SBDS,LRP5,FEZ1,SATB2,ARMC9,PRKAR1A,GOLGA5,WIPI1,GSK3B,DAG1,TBC1D30,CEP97,LIMK2,DNM2	darkgreen	EC_M4
GO:1902018	3.34E-02	0.994032391	4	11	negative regulation of cilium assembly	BP	4.69E-01		turquoise	EC_M6
GO:0033363	3.34E-02	0.989427064	7	29	secretory granule organization	BP	4.69E-01	RAB38,NECTIN2,DTNBP1,ABCG1,LYST,CREB1,TBC1D20	turquoise	EC_M6
GO:0050804	3.35E-02	0.97707572	48	317	modulation of chemical synaptic transmission	BP	4.69E-01	AKAP12,SNCAIP,RIMS2,PACSIN2,ATF4,PICK1,APBA1,KIT,GRIK2,DLGAP3,MTMR2,PLCB4,EPHB1,PLK2,ABHD6,MCTP1,MEF2C,PLCG1,DGKZ,EDN1,SNCA,DTNBP1,SHANK2,PPP3CA,MCTP2,ADCY1,YTHDF1,CREB1,BCR,ADORA2A,SNCG,SYT7,RGS14,FBXL20,MTOR,OPHN1,PSEN1,CHRNB2,ADRB2,CCL2,GIT1,STXBP1,SLC8A3,RELN,SHANK3,RGS4,STX3,CACNG5	turquoise	EC_M6
GO:1903203	3.35E-02	0.990630546	6	23	regulation of oxidative stress-induced neuron death	BP	4.69E-01	OXR1,NCOA7,ATF4,TLR4,HIF1A,TLR6	turquoise	EC_M6
GO:2000047	3.35E-02	0.992213578	5	16	regulation of cell-cell adhesion mediated by cadherin	BP	4.69E-01	NOTCH4,AFDN,PPM1F,NOTCH1,BMP6	turquoise	EC_M6
GO:0003179	3.36E-02	0.985987702	11	50	heart valve morphogenesis	BP	4.69E-01	NOS3,GATA3,TIE1,JAG1,ADAMTS9,MEF2C,DLL4,MTOR,EFNA1,NOTCH1,BMP2	turquoise	EC_M6
GO:0030308	3.37E-02	0.980322198	25	153	negative regulation of cell growth	BP	4.69E-01	SEMA6A,CDKL3,SEMA4D,PPT1,NRP1,SFRP1,HYAL2,SEMA3F,EAF2,SH3BP4,SIPA1,SEMA6D,DDX3X,CDA,ADIPOR2,SEMA4A,NPR1,YY1,SEMA6B,ARHGAP4,SOX17,OSGIN2,RGS4,BST2,GNG4	turquoise	EC_M6
GO:0002335	3.37E-02	0.99056145	6	23	mature B cell differentiation	BP	4.69E-01	MFNG,IL6,LFNG,CD19,IRF8,PLCG2	turquoise	EC_M6
GO:0043161	2.71E-02	0.981765935	46	368	proteasome-mediated ubiquitin-dependent protein catabolic process	BP	4.69E-01	CDK2,UBR2,TOPORS,XPO1,COMMD1,FBXO48,UFL1,CUL1,ASCC3,CDC20,CUL4A,CDC16,ANAPC1,TAF9,PBK,ANAPC7,LTN1,UBE2C,CUL3,KIF14,UBR1,PSMD12,UBR3,PSMD4,CCDC47,SMARCC1,CDC27,COP1,CUL5,TBL1XR1,UBE2K,NEDD4,TAF1,OGT,PLK1,APPBP2,PSMD10,CUL4B,CCNF,PSMA5,GLMN,AURKA,GID4,UBXN2A,PSMD7,RCHY1	darkgrey	EC_M8
GO:0034471	2.71E-02	0.993784815	5	20	ncRNA 5'-end processing	BP	4.69E-01	POP1,UTP20,SSB,NOP14,THG1L	darkgrey	EC_M8
GO:0009581	3.71E-02	0.980588156	18	84	detection of external stimulus	BP	4.69E-01	GNAQ,WHRN,CTNNB1,CXCL12,GRK7,CACNB3,PKD2,PITPNM1,BACE1,KCNK4,PKD1L1,KCNA1,ROM1,TCAP,NGFR,ITGA2,PKD1,COL11A1	darkgreen	EC_M4
GO:2001169	3.71E-02	0.99126488	5	15	regulation of ATP biosynthetic process	BP	4.69E-01	PPARA,VCP,PPARGC1A,ENO1,TMSB4X	darkgreen	EC_M4
GO:0010517	3.72E-02	0.983700834	12	50	regulation of phospholipase activity	BP	4.69E-01	LRP1,GNAQ,PLAA,FGF2,ARL1,PLA2R1,HTR2B,FGFR2,FGFR3,PDGFRB,NMUR1,ARF4	darkgreen	EC_M4
GO:0005513	3.72E-02	0.99335652	4	10	detection of calcium ion	BP	4.69E-01	STIM1,KCNMB1,CALM1,KCNMB4	darkgreen	EC_M4
GO:0086069	3.38E-02	0.993934089	4	11	bundle of His cell to Purkinje myocyte communication	BP	4.69E-01	TRPM4,JUP,DSG2,DSC2	turquoise	EC_M6
GO:1901739	3.39E-02	0.993616983	4	13	regulation of myoblast fusion	BP	4.70E-01	CXCL10,NFATC2,PLEKHO1,CFLAR	turquoise	EC_M6

GO:0043068	3.40E-02	0.975851622	57	422	positive regulation of programmed cell death	BP	4.71E-01	TNFAIP8,TRIM39,AKAP12,TPD52L1,DAPK1,ATF4,SYK,GADD45G,IL6,UTP11,KLF11,CTSC,PPARG,NET1,SFRP1,RPL26,ARHGEF7,RET,NR4A1,HIP1R,MEF2C,PLCG1,ANO6,IFIT2,SNCA,SIK1,HDAC1,LCK,HYAL2,LAPTM5,INPP5D,IGFBP3,ITGA6,PAK2,MAP3K11,CREB1,ADORA2A,ITGA4,DDX3X,ING4,BID,TNFRSF1B,ALDH1A2,PSEN1,DAPK2,DNM2,CCL2,LGALS9,NOS2,TRAF2,NOTCH1,DAB2IP,MIR23A,PMAIP1,FCER2,E2F3,BMP2	turquoise	EC_M6
GO:0010952	3.74E-02	0.977621341	27	142	positive regulation of peptidase activity	BP	4.71E-01	MTCH1,VCP,REST,PSME4,CYCS,ROCK2,RCN3,PICALM,DDRGK1,ARL6IP5,ROBO1,APP,IFT57,TANK,PSMD14,FAM162A,NCSTN,ADRM1,EGLN3,MAPT,PCOLCE,FN1,NGFR,UACA,BCAP31,F3,BCL10	darkgreen	EC_M4
GO:1902743	3.74E-02	0.984285977	11	45	regulation of lamellipodium organization	BP	4.71E-01	AKIRIN1,ACTR2,ACTR3,TWF1,PIK3CA,AUTS2,CORO1C,NCKAP1,ARPC2,CAPZB,PLXNB3	darkgreen	EC_M4
GO:2001256	3.76E-02	0.993258922	4	10	regulation of store-operated calcium entry	BP	4.72E-01	STIM1,HOMER1,CASQ1,SARAF	darkgreen	EC_M4
GO:0045834	3.76E-02	0.978857124	22	109	positive regulation of lipid metabolic process	BP	4.72E-01	AGAP2,PPARA,PPARD,MFSD2A,FGF2,ABHD5,TGFB1,DGAT2,AKT2,IRS2,RDH10,SORBS1,CHP1,CPT1A,ZBTB20,HTR2B,FGFR3,PPARGC1A,CD74,PDGFRB,AKT1,PRKAA1	darkgreen	EC_M4
GO:0034331	3.76E-02	0.987025227	8	28	cell junction maintenance	BP	4.72E-01	WHRN,PCLO,PLEKHA7,TJP1,C1QL1,ERC1,NLGN2,SDF4	darkgreen	EC_M4
GO:0007173	3.76E-02	0.979879646	19	90	epidermal growth factor receptor signaling pathway	BP	4.72E-01	NCK2,SOCS5,RAB7A,TGFB1,PTPN11,TSG101,CBLB,PIK3CA,IQGAP1,HAP1,SHC1,AKT1,PTPN2,RHBDF1,CDH13,BCAR3,PTPN12,ARF4,PIK3C2A	darkgreen	EC_M4
GO:0031645	3.42E-02	0.993680894	4	12	negative regulation of nervous system process	BP	4.73E-01	TNFRSF21,MTMR2,CTSC,EIF2AK3	turquoise	EC_M6
GO:0072528	2.75E-02	0.992436324	6	28	pyrimidine-containing compound biosynthetic process	BP	4.74E-01	CTPS1,CAD,UMPS,PRPS1,TYMS,DHODH	darkgrey	EC_M8
GO:0030163	3.78E-02	0.969783617	129	815	protein catabolic process	BP	4.75E-01	LRP1,AGAP2,CANX,RNF19A,AZIN1,ADGRB1,UBQLN1,GGA1,KLHDC10,PLAA,VCP,C2orf40,CLOCK,PDCL3,SOCS5,ERLEC1,PSME4,UBA6,UBE2J1,PCYOX1,RCN3,VPS35,CUL2,RAB7A,PSMC2,ABHD12,PDCD6IP,TMUB1,KCTD21,NUB1,UBXN4,SPOPL,CTNNB1,STT3B,NPLOC4,RNF121,PSMD8,TMTC3,CHFR,DDRGK1,PPP2CB,PSMD6,RNF6,VPS37A,HSP90B1,TP53INP2,TRPC4AP,ELOC,RNF13,AMFR,TSG101,BAP1,TTC3,CBLB,NKD1,TOLLIP,PSMD14,BRSK2,MARCH6,AZIN2,FAP,UCHL5,SELENOS,TRIP12,SEC22B,PSMD1,UBE3B,KCTD10,GSK3B,HSPA8,NEDD8,PPP1R11,SYNPO2,PSMD2,ADRM1,UBE4A,RHBDD2,EDEM3,SYVN1,DNAJC10,DNAJB12,USP48,ARAF,SMURF1,TIMP1,FBXL5,JKAMP,FMN2,CLN8,ANAPC4,MSN,RNF111,TGFB1I1,SPOP,FBXL22,SEL1L,CLN6,ARIH1,PIN1,FBXO22,ROCK1,PCYOX1L,USP14,AKT1,PSMD11,UBE2A,TNFAIP1,OMA1,BCAP31,PKD1,FLNA,TMEM259,PARK7,RHBDF1,USP10,USP20,HSPA5,FBXO27,BNIP3,UBE2V2,TIMP3,DNAJB9,PTTG1IP,UBE2D2,DDA1,GABARAPL2,UBE2G2,MAP1A,DERL2	darkgreen	EC_M4
GO:0030218	3.44E-02	0.981959839	18	102	erythrocyte differentiation	BP	4.75E-01	L3MBTL3,ADGRF5,KIT,PTBP3,TAL1,GATA2,GATA3,STAT1,ALAS1,ETS1,DYRK3,HCLS1,INPP5D,B2M,HIF1A,RCOR1,ISG15,ARID4A	turquoise	EC_M6
GO:0050714	3.44E-02	0.981956243	18	100	positive regulation of protein secretion	BP	4.75E-01	CD2AP,TLR4,TRPM4,SIRT3,IL1A,IGF1,TLR2,VEGFC,ABCG1,ANO1,ADORA2A,MCU,RAC1,HIF1A,GPR68,MYO18A,RPH3AL,BMP6	turquoise	EC_M6
GO:0050801	3.44E-02	0.974481611	74	542	ion homeostasis	BP	4.75E-01	DMXL1,TRDN,KLHL3,IREB2,KDR,PPT1,CAV2,TRPM4,PIK3CG,GATA2,RAB38,GPR4,CXCR4,EDNRB,TMC8,P2RY6,P2RY2,P2RX2,SLC4A11,CD36,TMEM178A,MICU3,PDE4D,CHD7,IL1A,CLCN3,HEXB,CP,P2RX7,CXCL10,TPCN1,PLCG1,STC1,EDN1,SNCA,LCK,HYAL2,SLC39A8,KCNJ2,TESMIN,SLC12A7,HCRTR1,SLC9A8,PRKD1,MELTF,B2M,LTF,CCRL2,ADORA2A,MCU,SLC9A7,WNK4,FGF23,HIF1A,HEPH,PSEN1,ATP2A1,CD19,NPR1,S100A14,SCNN1B,HMOX2,AP3D1,PLCG2,S1PR1,CDH5,SLC8A3,TRIM24,CA2,KCNH2,SCN2B,LPAR6,RAB39A,BMP6	turquoise	EC_M6
GO:1903580	3.81E-02	0.986740655	8	29	positive regulation of ATP metabolic process	BP	4.75E-01	PPARA,VCP,APP,ZBTB20,PPARGC1A,PRKAA1,ENO1,TMSB4X	darkgreen	EC_M4
GO:0006829	3.81E-02	0.987946122	7	24	zinc ion transport	BP	4.75E-01	SLC39A7,SLC39A4,SLC30A9,SLC39A10,SLC39A6,SLC30A7,SLC39A3	darkgreen	EC_M4
GO:0016322	3.82E-02	0.993116998	4	10	neuron remodeling	BP	4.75E-01	APP,CX3CL1,FARP2,C1QL1	darkgreen	EC_M4

GO:0045981	3.82E-02	0.986711302	8	29	positive regulation of nucleotide metabolic process	BP	4.75E-01	PPARA,VCP,APP,ZBTB20,PPARGC1A,PRKAA1,ENO1,TMSB4X	darkgreen	EC_M4
GO:1900544	3.82E-02	0.986711302	8	29	positive regulation of purine nucleotide metabolic process	BP	4.75E-01	PPARA,VCP,APP,ZBTB20,PPARGC1A,PRKAA1,ENO1,TMSB4X	darkgreen	EC_M4
GO:0051893	3.82E-02	0.981921119	14	60	regulation of focal adhesion assembly	BP	4.75E-01	LRP1,VEGFA,ROCK2,ACTG1,PTPN11,PHLDB2,MMP14,CORO1C,IQGAP1,VCL,ROCK1,DAPK3,THY1,DUSP22	darkgreen	EC_M4
GO:0090109	3.82E-02	0.981921119	14	60	regulation of cell-substrate junction assembly	BP	4.75E-01	LRP1,VEGFA,ROCK2,ACTG1,PTPN11,PHLDB2,MMP14,CORO1C,IQGAP1,VCL,ROCK1,DAPK3,THY1,DUSP22	darkgreen	EC_M4
GO:0034103	3.83E-02	0.982437113	13	57	regulation of tissue remodeling	BP	4.75E-01	IL23A,LEP,ROCK2,TGFB1,SPP1,RUNX1,PRKCA,TFRC,GPR137,SUCO,ROCK1,ATP6AP1,DEF8	darkgreen	EC_M4
GO:0099177	3.45E-02	0.976293311	48	318	regulation of trans-synaptic signaling	BP	4.76E-01	AKAP12,SNCAIP,RIMS2,PACSIN2,ATF4,PICK1,APBA1,KIT,GRIK2,DLGAP3,MTMR2,PLCB4,EPHB1,PLK2,ABHD6,MCTP1,MEF2C,PLCG1,DGKZ,EDN1,SNCA,DTNBP1,SHANK2,PPP3CA,MCTP2,ADCY1,YTHDF1,CREB1,BCR,ADORA2A,SNCG,SYT7,RGS14,FBXL20,MTOR,OPHN1,PSEN1,CHRNB2,ADRB2,CCL2,GIT1,STXBP1,SLC8A3,RELN,SHANK3,RGS4,STX3,CACNG5	turquoise	EC_M6
GO:0042158	2.43E-03	0.999030912	19	84	lipoprotein biosynthetic process	BP	4.76E-01	ATG12,GPAA1,PIGO,CLIP3,PIGT,PIGM,ZDHHC24,GLUL,ZDHHC8,WDR45,PIGH,GBA,DPM3,MPPE1,PIGQ,ZDHHC12,LCAT,ZDHHC1,MAP6D1	cyan	EC_M2
GO:0006475	2.49E-03	0.998831071	26	136	internal protein amino acid acetylation	BP	4.76E-01	BAG6,JADE2,EP300,ING3,SPHK2,WBP2,TADA3,BRPF1,APBB1,TAF10,SDR16C5,KAT5,POR,EID1,KAT6B,MED24,CTBP1,KAT8,KAT7,PER1,SGF29,CRTC2,SIN3A,CREBBP,WDR5,GTF3C4	cyan	EC_M2
GO:0080090	2.51E-03	0.997910043	507	4206	regulation of primary metabolic process	BP	4.76E-01	NFATC1,ZNF236,TSHZ1,ORMDL2,PYM1,SMARCC2,STAT6,PIP4K2C,MAP3K5,CDK4,CITED2,CTDSP2,MAML1,SQSTM1,SASH1,IRAK3,HELB,OSR2,BAG6,PPP6R2,SNX9,HSPA1B,EHMT2,ZBTB12,HDAC10,ATF6B,EIF3H,RPS6KA2,DEPTOR,RXRB,TSPO,RING1,ZHX2,ZBTB22,MYC,TOB2,SMAD5,EP300,KDM3B,ZC3H3,ZNF76,TAB1,DDX17,STK38,NFIB,EIF3D,HSF1,VPS28,CCND3,PPP1R16A,TRERF1,CDKN2A,HMOX1,WASL,KLF9,PDGFRA,JAK2,HINT2,RECK,ZBTB45,ZNF135,ZNF667,ZNF865,RPS9,EEF1A1,CD109,GPNMB,MALSU1,STAP1,EFEMP1,ZNF692,CYREN,RPS7,ANTXR1,ANXA4,AKNA,MED25,IL4I1,AKT1S1,ING3,ARHGEF5,AP2A1,IRF3,LPIN1,GSN,PARD3,FOXO3,KLF15,SPHK2,SPRY1,NOP53,MAML2,BICRA,ZC3HAV1,KMT2E,BBS7,MEIS3,HMCES,TRIM13,IRF2BP1,SIX5,FOSB,ZNF746,NR2C2,ZNF862,TSC22D1,KHK,FASTK,XRCC1,ZNF575,MIF4GD,NAA16,MLH1,UNK,ZNF574,WBP2,KMT2C,MYD88,CELF2,EGLN2,LTBP4,BABAM2,TADA3,RPS3,BRPF1,DYRK1B,EID2,TGFBR2,ZNF219,TIMP2,MED29,SAMD4B,XDH,SIRT2,RPTOR,LAMTOR1,HGS,APBB3,ASPSCR1,EIF3K,NUP98,FGFR1,TRIM21,CYP1B1,NDFIP1,SPRY4,HGF,APBB1,DEAF1,HOXC4,CLIP3,NCOA4,PCBP2,LIN37,SLC15A4,PSMF1,TARBP2,KMT2B,RBCK1,RARG,SDR16C5,ZNF740,EIF4B,LMO2,POFUT1,LATS2,NECAB3,URI1,PIK3R1,NCOA2,CAST,PCOLCE2,ATF1,TNKS1BP1,SH3D19,ARRDC3,KDM2B,ZFHX4,CAMKK2,SCAND1,HTRA2,EIF2A,SETD6,MCPH1,PDGFC,SPRED1,SSBP2,CCNG2,FNIP2,PRKAG1,ARL2BP,TIAM1,SHOX2,NEK4,PROS1,ZHX3,VDR,RBM12B,MMS19,LPIN3,FOXF2,F2RL1,RNFT2,SORBS3,ORMDL1,HNF4A,TP53INP1,PIDD1,ERLIN1,HR,NGF,ACOT8,PCIF1,TRIM45,LONP2,PARP3,FAS	cyan	EC_M2
GO:0009172	2.57E-03	0.999863729	4	6	purine deoxyribonucleoside monophosphate catabolic process	BP	4.76E-01	DNPH1,PNP,XDH,NT5C	cyan	EC_M2

GO:0010468	2.59E-03	0.997870572	418	3428	regulation of gene expression	BP	4.76E-01	NFATC1,ZNF236,TSHZ1,PYM1,SMARCC2,STAT6,MAP3K5,CDK4,CITED2,MAML1,SQSTM1,IRAK3,OSR2,HSPA1B,EHMT2,ZBTB12,HDAC10,ATF6B,AGPAT1,EIF3H,RPS6KA2,RXRB,TSPO,RING1,ZHX2,ZBTB22,MYC,TOB2,SMAD5,EP300,KDM3B,GSDMD,ZNF76,DDX17,NFIB,EIF3D,HSF1,CCND3,TRERF1,CDKN2A,HMOX1,WASL,KLF9,PDGFRA,JAK2,ZBTB45,ZNF135,TDRD7,ZNF667,ZNF865,SSC5D,RPS9,GPNMB,MALSU1,STAP1,EPB41L4B,EFEMP1,ZNF692,RPS7,ANXA4,AKNA,STK40,MED25,IL4I1,ING3,ARHGEF5,IRF3,LPIN1,GSN,FOXO3,KLF15,SPHK2,NOP53,MAML2,BICRA,ZC3HAV1,KMT2E,BBS7,MEIS3,HMCES,LURAP1,TRIM13,IRF2BP1,SIX5,FOSB,ZNF746,NR2C2,ZNF862,TSC22D1,SMG9,FASTK,ZNF575,MIF4GD,NAA16,MLH1,UNK,ZNF574,WBP2,KMT2C,MYD88,CELF2,TADA3,RPS3,BRPF1,PNP,DYRK1B,EID2,TGFBR2,RBMS3,ZNF219,MED29,SAMD4B,XDH,SIRT2,RPTOR,HGS,APBB3,EIF3K,NUP98,TRIM21,CYP1B1,NDFIP1,HGF,APBB1,TAF10,DEAF1,HOXC4,NCOA4,PCBP2,LIN37,SLC15A4,TARBP2,KMT2B,RBCK1,RARG,SDR16C5,ZNF740,EIF4B,LMO2,POFUT1,URI1,PIK3R1,NCOA2,CAST,ATF1,KDM2B,ZFHX4,CAMKK2,SCAND1,EIF2A,SETD6,PPP1CC,MCPH1,SSBP2,FNIP2,PRKAG1,ARL2BP,SHOX2,NEK4,ZHX3,VDR,RBM12B,MMS19,LPIN3,FOXF2,F2RL1,SORBS3,HNF4A,TP53INP1,AFF1,PIDD1,NFKBIZ,HR,NGF,PCIF1,TRIM45,CD47,PARP3,FASTKD3,ELK4,LGR4,RBM15B,ICE1,IFIH1,SP140,GPAM,SULF2,TCIRG1,TCF7L2,DIS3L2,PUM1,C14orf93,TXNIP,GPX1,AIP,SOX5,ADNP,RPS6KB2,SF3B4,ANK2,ZNF217,QARS,DTX3L,PARP14,EIF4H,ECM1,RNF26,PC,RCOR3,ARNT,PRMT2,RIPK3,GABPB2,PER2,TWIST2,VPS72,HEG1,HDAC4,USF1,ANK3,SMARCD2,POGZ,USP	cyan	EC_M2
GO:0008610	2.59E-03	0.99827079	82	544	lipid biosynthetic process	BP	4.76E-01	ORMDL2,DGKA,PIP4K2C,PEX7,CDK4,CHKB,AGPAT1,TSPO,HSD17B8,B3GALT4,CYB5R3,GPAA1,PIGO,PLPP6,HINT2,TTC7A,SLC44A1,LPIN1,SPHK2,ACSS1,FDXR,TAMM41,PTDSS2,THNSL2,CBR4,PIK3R1,ACSS2,PRKAG1,GPAT3,LPIN3,ORMDL1,ERLIN1,PIGT,ACOT8,SRD5A1,CYB5R1,GPAM,CHKA,ABHD4,PIGM,ABCB11,CERS2,CLCN2,SCAP,POR,IDH1,ACADL,PECR,CYP27A1,SMPD4,PLA2G16,ABHD8,FADS3,CTDNEP1,TECR,PLSCR3,PIGH,PIP4K2B,OSBPL7,HSD3B7,GBA,ERG28,DPM3,AC138894.1,MTMR4,ACSM3,ACACA,SREBF1,CERS4,MPPE1,ACAA2,MTMR1,TAZ,PIGQ,INPP5E,GSTM4,PTGES2,VAC14,LCAT,ABCA2,PLPP3,SCP2	cyan	EC_M2
GO:0098732	2.63E-03	0.998881846	21	102	macromolecule deacylation	BP	4.76E-01	ABHD16A,HDAC10,EP300,TRERF1,SPHK2,TADA3,SIRT2,SDR16C5,SPRED1,ELK4,RCOR3,PER2,HDAC4,ABHD12B,HDAC6,CTBP1,PER1,ZBTB7B,SIN3A,SREBF1,ABHD17A	cyan	EC_M2
GO:0062197	2.67E-03	0.998443876	47	282	cellular response to chemical stress	BP	4.76E-01	STAT6,MAP3K5,HSPA1B,TSPO,ERCC6L2,HSF1,HMOX1,PDGFRA,JAK2,FOXO3,AGAP3,RPS3,SIRT2,CYP1B1,HGF,CAMKK2,HTRA2,TP53INP1,GPX1,MCL1,ARNT,RIPK3,CCS,MGMT,YBX3,NFE2L2,AIFM2,PRODH,TMEM161A,ANKZF1,HDAC6,ATP13A2,ZFP36L1,FOS,AC138894.1,KEAP1,SIN3A,PPARGC1B,SLC25A24,NOL3,RACK1,SRXN1,ZNF580,ADPRHL2,NUDT2,PDGFD,MPV17	cyan	EC_M2
GO:0006122	2.70E-03	0.999747329	5	9	mitochondrial electron transport, ubiquinol to cytochrome c	BP	4.76E-01	UQCRQ,UQCRH,UQCC3,UQCR11,MT-CYB	cyan	EC_M2
GO:0044238	2.71E-03	0.997728747	898	7641	primary metabolic process	BP	4.76E-01	NFATC1,ZNF236,TSHZ1,ZADH2,ORMDL2,PYM1,DGKA,SMARCC2,STAT6,ATG12,PIP4K2C,MAP3K5,PEX7,OS9,CDK4,CITED2,CTDSP2,ATP23,ADAMTS2,MAML1,SQSTM1,SASH1,IRAK3,HELB,GFPT2,OSR2,BAG6,CHKB,ABHD16A,PPP6R2,MSH5,SNX9,HSPA1B,EHMT2,ZBTB12,HDAC10,SELENOO,ALG12,CERK,ATF6B,AGPAT1,RNF5,EIF3H,RPS6KA2,DEPTOR,DSE,RXRB,TSPO,HSD17B8,RING1,RPS18,B3GALT4,CYB5R3,ZHX2,ZBTB22,JADE2,PMM1,MYC,TOB2,SMAD5,EP300,CDC14C,ERCC6L2,KDM3B,ZC3H3,ZNF76,FANCE,EEF1D,TAB1,RPL3,JOSD1,DDX17,NRBP2,STK38,EIF3L,MPST,NFIB,GPAA1,SHARPIN,EIF3D,FOXRED2,HSF1,FBXL6,CPSF1,VPS28,CCND3,PPP1R16A,TRERF1,CDKN2A,RPL8,HMOX1,WASL,CUL7,CUL9,DNPH1,KLF9,POLR1C,PIGO,PDGFRA,PLPP6,RCL1,JAK2,HINT2,RECK,RNF38,ZBTB45,RPS5,ZNF135,ANKRD39,TRMO,ZNF667,ALG2,ZNF865,RPL28,TTC7A,RPS9,EEF1A1,CD109,GPNMB,MALSU1,STAP1,SLC44A1,ETFB,EFEMP1,PTGR1,UGP2,ZNF692,CYREN,RPS7,ANTXR1,ANXA4,AKNA,LSM10,STK40,MED25,IL4I1,COQ3,AKT1S1,ING3,ARHGEF5,AP2A1,IRF3,LPIN1,RPS11,GSN,PARD3,FOXO3,KLF15,DHDH,ADCK2,SPHK2,RPL18,SPRY1,TPRA1,NOP53,ABTB1,MAML2,BICRA,ZC3HAV1,KMT2E,BBS7,MEIS3,HMCES,FKRP,LRRC41,TRIM13,ACSS1,IRF2BP1,SIX5,QPCTL,FOSB,ZNF746,NR2C2,ZNF862,TSC22D1,SERP2,FDXR,KHK,SMG9,FASTK,SEPHS1,RPS8,XRCC1,ZNF575,MIF4GD,AGAP3,NAA16,MLH1,RNF220,LIPE,PAFAH1B3,ECHDC3,GTF3C2,UNK,ZNF574,GALNT11,WBP2,TRIM47,KMT2C,ACAA1,MYD88,CELF2,DMAC2,TAMM41,BCKDHA,EGLN2,CYGB,NUMBL,LTBP4,BABAM2,ANKRD16,TADA3,CSGALNACT1,RPS3,TEP1,BRPF1,PIP4P1,PNP,D	cyan	EC_M2

GO:0055080	3.46E-02	0.974701731	68	494	cation homeostasis	BP	4.76E-01	DMXL1,TRDN,IREB2,PPT1,CAV2,TRPM4,PIK3CG,GATA2,RAB38,GPR4,CXCR4,EDNRB,TMC8,P2RY6,P2RX2,SLC4A11,CD36,TMEM178A,MICU3,PDE4D,CHD7,IL1A,CLCN3,HEXB,CP,P2RX7,CXCL10,TPCN1,PLCG1,STC1,EDN1,SNCA,LCK,HYAL2,SLC39A8,KCNJ2,TESMIN,SLC12A7,HCRTR1,SLC9A8,PRKD1,MELTF,B2M,LTF,CCRL2,ADORA2A,MCU,SLC9A7,HIF1A,HEPH,PSEN1,ATP2A1,CD19,NPR1,S100A14,SCNN1B,HMOX2,AP3D1,PLCG2,S1PR1,CDH5,SLC8A3,TRIM24,CA2,KCNH2,LPAR6,RAB39A,BMP6	turquoise	EC_M6
GO:0110151	3.47E-02	0.986872086	9	42	positive regulation of biomineralization	BP	4.77E-01	ACVR2B,MEF2C,P2RX7,ANO6,LTF,ADRB2,ISG15,BMP2,BMP6	turquoise	EC_M6
GO:0010876	3.85E-02	0.972862202	54	311	lipid localization	BP	4.77E-01	ATP8B1,LRP1,B4GALNT1,PPARA,NUS1,PPARD,PLIN2,LEP,TMEM30A,MFSD2A,INHBA,LDAH,ABHD5,ESYT2,DGAT2,AKT2,OSBPL10,C1QTNF1,OSBPL8,IRS2,OSBP,P2RX4,PTPN11,CRY2,SPP1,PLA2R1,KDM5B,CPT1A,EPRS,PITPNM1,PITPNB,ARV1,PRELID3B,FABP2,OSBPL2,OSBP2,HDLBP,LINC02210-CRHR1,ITGAV,CYP19A1,CLN8,STARD3,TMEM159,STOML1,ACSL4,AKT1,PRKAA1,PTPN2,ATP11C,PITPNA,ABCA3,VAMP7,PLEKHA3,SIGMAR1	darkgreen	EC_M4
GO:0110053	3.87E-02	0.973972579	43	237	regulation of actin filament organization	BP	4.77E-01	FAM49B,NCK2,TGFBR1,SHANK1,ACTR2,ROCK2,PAK1,ACTR3,PLEKHG2,ACTG1,CDC42EP3,CXCL12,PFN2,RASA1,CX3CL1,TWF1,MAGEL2,PHLDB2,TJP1,CTTN,LMOD1,PIK3CA,SYNPO2,ARPC5,NCKAP1,ARPC2,SYNPO2L,CAPZB,WDR1,CGNL1,TPM1,TGFB3,GPR65,TTC8,ROCK1,FLII,MYO1C,FLNA,SPTAN1,TMSB4X,DSTN,COTL1,CFL2	darkgreen	EC_M4
GO:0008611	3.87E-02	0.992992441	4	10	ether lipid biosynthetic process	BP	4.77E-01	CHPT1,FAR1,LPCAT2,TMEM189	darkgreen	EC_M4
GO:0046504	3.87E-02	0.992992441	4	10	glycerol ether biosynthetic process	BP	4.77E-01	CHPT1,FAR1,LPCAT2,TMEM189	darkgreen	EC_M4
GO:0097384	3.87E-02	0.992992441	4	10	cellular lipid biosynthetic process	BP	4.77E-01	CHPT1,FAR1,LPCAT2,TMEM189	darkgreen	EC_M4
GO:1901503	3.87E-02	0.992992441	4	10	ether biosynthetic process	BP	4.77E-01	CHPT1,FAR1,LPCAT2,TMEM189	darkgreen	EC_M4
GO:0010880	3.87E-02	0.987726203	7	24	regulation of release of sequestered calcium ion into cytosol by sarcoplasmic reticulum	BP	4.77E-01	FKBP1B,CASQ1,CACNA1C,CALM1,DMD,TMEM38B,TMEM38A	darkgreen	EC_M4
GO:0048332	3.87E-02	0.982263729	13	55	mesoderm morphogenesis	BP	4.77E-01	SRF,INHBA,ITGB1,TLX2,ACVR1,EYA2,PRKAR1A,LEF1,BMP7,FGFR2,BMPR1A,ITGA2,WLS	darkgreen	EC_M4
GO:0017014	3.49E-02	0.99351003	4	12	protein nitrosylation	BP	4.78E-01	OXR1,NCOA7,ACE,NOS2	turquoise	EC_M6
GO:0018119	3.49E-02	0.99351003	4	12	peptidyl-cysteine S-nitrosylation	BP	4.78E-01	OXR1,NCOA7,ACE,NOS2	turquoise	EC_M6
GO:0010332	2.78E-02	0.989139941	10	53	response to gamma radiation	BP	4.78E-01	XRCC6,ZMPSTE24,FANCD2,BRCA2,PRKDC,ERCC6,ATR,RAD51,XRCC5,TIGAR	darkgrey	EC_M8
GO:2000179	3.88E-02	0.984443301	10	39	positive regulation of neural precursor cell proliferation	BP	4.78E-01	VEGFA,GLI3,FGF2,GLI2,SMARCD3,CTNNB1,CX3CL1,FZD9,NES,FLNA	darkgreen	EC_M4
GO:0000455	2.76E-03	0.999851171	4	6	enzyme-directed rRNA pseudouridine synthesis	BP	4.78E-01	RPUSD2,TSR3,RPUSD1,RPUSD3	cyan	EC_M2
GO:0071322	3.90E-02	0.977382631	24	121	cellular response to carbohydrate stimulus	BP	4.79E-01	LRP1,PPARD,LGALS1,LEP,SRF,UNC13B,FKBP1B,KIF5B,IRS2,OSBP,SELENOT,OPA1,BRSK2,DYNLL1,ZBTB20,LRP5,PIK3CA,ADCY5,SIDT2,PTPRN,PPARGC1A,SMARCA4,PRKAA1,SERPINF1	darkgreen	EC_M4
GO:0048791	3.90E-02	0.990882702	5	14	calcium ion-regulated exocytosis of neurotransmitter	BP	4.79E-01	UNC13B,RIMS1,RAB3GAP1,ATP2A2,STX1B	darkgreen	EC_M4
GO:0042391	3.90E-02	0.972595472	53	302	regulation of membrane potential	BP	4.79E-01	KCNK9,GNAQ,VCP,UNC13B,RIMS1,SHANK1,MYH14,BVES,FKBP1B,KIF5B,RAB3GAP1,SCN5A,AKT2,GPD1L,DCN,ARL6IP5,FXYD1,SNTA1,SLMAP,ATP1B3,P2RX4,ATP2A2,CACNA1D,APP,GLRB,CACNB3,CHRNA7,PKP2,GSK3B,FZD9,MAPT,MPP2,KCNK4,KCNA1,WDR1,CHRNE,CACNA1C,NLGN2,STX1B,CACNA1G,CALM1,GRIA1,AKT1,FLNA,KCNAB2,PARK7,HCN2,DMD,BNIP3,INSYN1,YWHAH,HCN4,KCNMB4	darkgreen	EC_M4
GO:0009395	3.51E-02	0.98762327	8	36	phospholipid catabolic process	BP	4.79E-01	SCARB1,ABHD6,PLCG1,INPP5F,GDPD3,GDPD1,PLCG2,PLA2G4E	turquoise	EC_M6

GO:0019216	3.52E-02	0.977044771	37	251	regulation of lipid metabolic process	BP	4.79E-01	PTK2,PDGFB,TEK,ADGRF5,KIT,PIK3CG,MTMR2,RAB38,PPARG,NR1D2,SIRT3,SCARB1,ABHD6,CHRM5,DHH,DGKZ,SNCA,SLC45A3,ABCG1,LPCAT1,SIK1,FGR,PPP2R5A,PROX1,PRKD1,MLXIPL,APOD,PDK3,CREB1,TNFAIP8L3,SAMD8,MTOR,CD19,PLCG2,DAB2IP,BMP2,BMP6	turquoise	EC_M6
GO:0061515	3.52E-02	0.984433161	12	59	myeloid cell development	BP	4.79E-01	L3MBTL3,ADGRF5,KIT,PTBP3,TAL1,TLR2,TSPAN2,ALAS1,LTF,GPR68,ARID4A,FLI1	turquoise	EC_M6
GO:0014910	3.53E-02	0.984388765	12	60	regulation of smooth muscle cell migration	BP	4.80E-01	PDGFB,DOCK4,TLR4,NRP1,P2RY6,IGF1,MEF2C,NDRG4,IGFBP3,ACE,SEMA6D,IGFBP5	turquoise	EC_M6
GO:0042953	3.53E-02	0.993398922	4	12	lipoprotein transport	BP	4.80E-01	PPARG,CD36,MTTP,UNC119	turquoise	EC_M6
GO:0072017	3.54E-02	0.993780624	4	10	distal tubule development	BP	4.80E-01	KLHL3,JAG1,WNK4,NOTCH1	turquoise	EC_M6
GO:0046632	3.54E-02	0.983165915	14	74	alpha-beta T cell differentiation	BP	4.80E-01	SYK,IL6,RSAD2,GATA3,SH3RF1,HLX,CD86,CRACR2A,ITPKB,MTOR,SEMA4A,BATF,LGALS9,AP3D1	turquoise	EC_M6
GO:1903506	3.54E-02	0.96972658	293	2412	regulation of nucleic acid-templated transcription	BP	4.80E-01	ZNF516,TNFRSF11A,BAZ2A,TCF4,TNFAIP3,PLAGL1,CNOT6,FZD6,ZBED4,NOTCH4,TCF20,TCF7,NCOA7,L3MBTL3,ATF4,PDGFB,KDM4C,PIM1,NFYA,MDFI,PRDM4,DDX58,NFX1,KIT,ADGRF1,NFIL3,SYK,SEMA4D,IL6,NFE2L3,REL,CREB5,PPIE,BACH2,PELI1,FOXP2,KLF11,LANCL2,NOBOX,TLR4,TRAF1,CREM,TEAD2,SDCBP,MYCN,KTI12,ZBTB24,BEND5,TAL1,PHF10,EPC1,GATA2,DNMT3A,PRKD2,SAP130,DACH1,PPARG,GATA3,TAF3,ACVR2B,IRAK2,GZF1,EDNRB,SETD5,IKBKB,JAG1,NFKBIB,PDE2A,TMEM173,SFRP1,NR1D2,CD36,CDK17,ARID5A,TCF15,RET,CHD7,IL1A,DNMT3B,IGF1,NR4A1,EIF2AK3,HHEX,KDM3A,ITCH,ERBIN,SMAD1,ZBTB38,POU6F1,STOX2,SOX7,HMBOX1,HEXB,MEF2C,SMARCD1,TLR2,WWTR1,ZNF704,CXCL10,ADGRG3,KMT2D,SFMBT1,SCAF4,NLRC5,ACTR5,ANKRD2,NR4A2,TOX2,DLL4,CDYL,BAZ2B,SOX13,YAF2,EDN1,STAT1,DYRK1A,SNCA,JARID2,ERG,ETS2,CDK18,NFKB2,DCAF1,PRDM15,ETS1,CD200,SIK1,HDAC1,PPP3CA,HYAL2,SLC39A8,TESMIN,MEF2A,BCL9,HLX,NCF1,USP13,FABP4,HCLS1,NFATC2,ZFHX2,EAF2,GMEB1,CD86,KDM2A,CIART,MN1,TGIF1,PARP15,POU2F3,ZSCAN29,CFLAR,TRAK2,ADCY1,ARID1A,RPS6KA1,PROX1,SS18L1,PRKD1,ZNF148,ZSCAN2,HELZ2,MLXIPL,ITGA6,DLG1,ELL3,ETV6,HOXD10,RNF2,CREB1,EIF4A2,TET1,LTF,ADORA2A,ARNT2,MYEF2,IL1RAP,BCOR,DDX3X,RFXANK,ETV4,TBX1,MECOM,ING4,CDK16,SPINDOC,LDB2,FGF23,PPM1F,POU2F1,WAS,TEAD4,PRKCH,HIF1A,KDM5A,ZBTB18,APBB2,JUP,BCL6B,KDM5C,TLR6,RSC1A1,RGS14,MAFK,SPIN2A,MTOR,AR,CASZ1,PSEN1,CBX1,HOXB5,HOXB6,HOXB7,HOXB8,HOXB9,ZNF410,FBXW11,BATF,KRBA2,EFNA1,PIAS1,RPS6KA5,SMAD2,TLE3,D	turquoise	EC_M6
GO:0045668	3.54E-02	0.986577165	9	41	negative regulation of osteoblast differentiation	BP	4.80E-01	SEMA4D,NOCT,TRPM4,PPARG,SFRP1,VEGFC,IGFBP5,FGF23,NOTCH1	turquoise	EC_M6
GO:0002027	3.91E-02	0.980306133	16	73	regulation of heart rate	BP	4.80E-01	BVES,FKBP1B,SCN5A,GPD1L,SNTA1,ATP2A2,CACNA1D,ADA,PKP2,CACNA1C,TPM1,CACNA1G,ADRA1B,CALM1,DMD,HCN4	darkgreen	EC_M4
GO:1903599	3.91E-02	0.992880724	4	10	positive regulation of autophagy of mitochondrion	BP	4.80E-01	SLC25A4,HTT,PARK7,BNIP3	darkgreen	EC_M4
GO:0002548	3.56E-02	0.987282848	8	40	monocyte chemotaxis	BP	4.80E-01	TNFRSF11A,PDGFB,IL6,CXCL10,ANO6,S100A14,CCL2,MSMP	turquoise	EC_M6
GO:0010524	3.56E-02	0.98736864	8	37	positive regulation of calcium ion transport into cytosol	BP	4.80E-01	TRDN,P2RY6,P2RX2,P2RX7,CXCL10,PLCG1,SNCA,CD19	turquoise	EC_M6
GO:0032272	3.57E-02	0.983580517	13	66	negative regulation of protein polymerization	BP	4.80E-01	TMOD1,SVIL,SPTBN4,ARHGEF7,LMOD3,HIP1R,DYRK1A,SNCA,TMOD4,TMOD2,CARMIL2,CDH5,KANK3	turquoise	EC_M6
GO:0001771	3.93E-02	0.992853629	4	10	immunological synapse formation	BP	4.80E-01	DOCK8,NCK2,MSN,DOCK2	darkgreen	EC_M4
GO:0055007	3.93E-02	0.978500605	20	96	cardiac muscle cell differentiation	BP	4.80E-01	PPARA,CBY1,SRF,VEGFA,BVES,MYLK3,ITGB1,PAK1,TGFB1,RARB,ACVR1,PDLIM5,OBSL1,BMPR1A,TCAP,MYOCD,PIN1,PDGFRB,MIR24-1,CACYBP	darkgreen	EC_M4
GO:0006517	3.94E-02	0.987447181	7	24	protein deglycosylation	BP	4.81E-01	AMFR,MAN1A2,MARCH6,MAN2A2,EDEM3,SYVN1,PARK7	darkgreen	EC_M4
GO:1901203	3.95E-02	0.992801965	4	10	positive regulation of extracellular matrix assembly	BP	4.81E-01	TGFB1,PHLDB2,DAG1,PHLDB1	darkgreen	EC_M4
GO:1903423	3.95E-02	0.992800025	4	10	positive regulation of synaptic vesicle recycling	BP	4.81E-01	PLAA,PPP3CC,DNM1L,AP2M1	darkgreen	EC_M4

GO:0002698	3.95E-02	0.981174951	14	62	negative regulation of immune effector process	BP	4.81E-01	ENPP3,ANGPT1,SOCS5,TGFB1,SPINK5,IL20RB,LOXL3,TGFB2,SLAMF8,PTPN6,TGFB3,A2M,CD59,FGL2	darkgreen	EC_M4
GO:0030038	3.95E-02	0.978389088	20	95	contractile actin filament bundle assembly	BP	4.81E-01	SRF,TGFBR1,ROCK2,ZYX,PAK1,ACTG1,SORBS1,PFN2,PHLDB2,TJP1,ITGB5,SYNPO2L,CGNL1,TPM1,TGFB3,GPR65,TTC8,ROCK1,TNFAIP1,PDLIM1	darkgreen	EC_M4
GO:0043149	3.95E-02	0.978389088	20	95	stress fiber assembly	BP	4.81E-01	SRF,TGFBR1,ROCK2,ZYX,PAK1,ACTG1,SORBS1,PFN2,PHLDB2,TJP1,ITGB5,SYNPO2L,CGNL1,TPM1,TGFB3,GPR65,TTC8,ROCK1,TNFAIP1,PDLIM1	darkgreen	EC_M4
GO:0016573	2.81E-02	0.98497856	20	131	histone acetylation	BP	4.81E-01	ZNF451,ZMPSTE24,PAXIP1,KAT14,EPC2,KAT6A,TAF7,BRCA2,TAF9,MCRS1,CHEK1,NAA50,MCM3AP,HAT1,ING5,BRCA1,TAF1,OGT,CTCF,AC118549.1	darkgrey	EC_M8
GO:0010586	2.81E-02	0.992459681	6	23	miRNA metabolic process	BP	4.81E-01	TUT7,XPO5,PNPT1,TENT2,NFKB1,DROSHA	darkgrey	EC_M8
GO:2000241	2.81E-02	0.986122803	16	105	regulation of reproductive process	BP	4.81E-01	YTHDC2,FBXO43,FBXO5,CDC25C,TTK,HDAC2,CDC20,METTL3,DHX37,STK4,TRIP13,INTS13,ASPM,CDC25A,RAD51AP1,AURKA	darkgrey	EC_M8
GO:1905269	2.82E-02	0.995104059	4	12	positive regulation of chromatin organization	BP	4.81E-01	TRIM28,SETDB2,FAM208A,TPR	darkgrey	EC_M8
GO:0007141	2.82E-02	0.993474489	5	20	male meiosis I	BP	4.81E-01	UBR2,FOXJ3,BRCA2,TRIP13,SPO11	darkgrey	EC_M8
GO:0050729	3.59E-02	0.981866456	16	93	positive regulation of inflammatory response	BP	4.82E-01	TNFRSF11A,MAPK13,IL6,TLR4,IL15,PIK3CG,CTSC,GPR4,PDE2A,TLR2,SNCA,ETS1,HYAL2,FABP4,TNIP1,PLCG2	turquoise	EC_M6
GO:0010951	3.96E-02	0.976360139	26	137	negative regulation of endopeptidase activity	BP	4.82E-01	SERPINB2,VEGFA,PICALM,SERPINH1,CRIM1,SPINT2,PRNP,SPINK5,APP,UCHL5,ANOS1,COL6A3,RPS6KA3,SERPINE1,TIMP1,TMED10,ROCK1,USP14,AKT1,PARK7,MIR24-1,SPINK2,SERPINF1,SERPINB5,A2M,TIMP3	darkgreen	EC_M4
GO:0030522	2.81E-03	0.998475572	37	217	intracellular receptor signaling pathway	BP	4.82E-01	CITED2,HSPA1B,RXRB,EP300,DDX17,TRERF1,JAK2,IRF3,NOP53,NR2C2,WBP2,EGLN2,TADA3,NCOA4,SLC15A4,RARG,VDR,HNF4A,IFIH1,PUM1,ARNT,PRMT2,NFKBIA,PHB2,NR2F6,HDAC6,RARA,GPS2,THRA,PER1,KANK2,PPARGC1B,SREBF1,RXRA,ZBTB7A,ZCCHC3,AHRR	cyan	EC_M2
GO:0001503	3.60E-02	0.975221349	48	328	ossification	BP	4.83E-01	TNFRSF11A,CTHRC1,ASF1A,PTK2,ATF4,OSTF1,TEK,SEMA4D,IL6,FIGNL1,NOCT,TRPM4,PPARG,ACVR2B,JAG1,SFRP1,NOTUM,IGF1,EIF2AK3,SMAD1,MEF2C,WWTR1,P2RX7,VEGFC,DHH,BMP2K,STC1,ANO6,MN1,FGR,PRKD1,LTF,BCOR,IGFBP5,MMP13,FGF23,HIF1A,EPHA2,SMOC1,FGF18,ADRB2,GIT1,RFLNB,ISG15,NOTCH1,S1PR1,BMP2,BMP6	turquoise	EC_M6
GO:0031365	2.83E-02	0.992291608	6	25	N-terminal protein amino acid modification	BP	4.83E-01	NAA35,NAA15,METAP2,NAA25,METAP1,NAA50	darkgrey	EC_M8
GO:0002526	3.61E-02	0.984567755	11	58	acute inflammatory response	BP	4.83E-01	TNFRSF11A,IL6,PIK3CG,PRCP,GATA3,EDNRB,IL1A,KLKB1,ANO6,F8,VNN1	turquoise	EC_M6
GO:0050708	3.61E-02	0.977465661	31	200	regulation of protein secretion	BP	4.83E-01	PICK1,CD2AP,ICA1,IL6,RSAD2,TLR4,TRPM4,LLGL2,SFRP1,SIRT3,FAM3D,IL1A,IGF1,TLR2,VEGFC,ABCG1,CD200,HDAC1,PPP3CA,ANO1,ADORA2A,MCU,CDK16,RAC1,HIF1A,SYT7,GPR68,NOS2,MYO18A,RPH3AL,BMP6	turquoise	EC_M6
GO:0010592	3.61E-02	0.989719043	6	23	positive regulation of lamellipodium assembly	BP	4.83E-01	OCLN,RAC1,FSCN1,MTOR,DNM2,CARMIL2	turquoise	EC_M6
GO:2000402	3.61E-02	0.993198198	4	12	negative regulation of lymphocyte migration	BP	4.83E-01	LRCH1,CD200,APOD,CCL2	turquoise	EC_M6
GO:0032411	3.99E-02	0.978934932	18	85	positive regulation of transporter activity	BP	4.84E-01	KIF5B,ACTN4,STIM1,ATP1B3,ABCC8,CACNB3,CHP1,PKD2,CASQ1,HTT,KCNA1,HAP1,STIM2,CALM1,VMP1,PARK7,DMD,TMSB4X	darkgreen	EC_M4
GO:0008210	4.00E-02	0.990397503	5	15	estrogen metabolic process	BP	4.85E-01	SGPL1,COMT,HSD17B1,CYP19A1,HSD17B10	darkgreen	EC_M4
GO:0043116	3.63E-02	0.991378269	5	16	negative regulation of vascular permeability	BP	4.85E-01	AKAP12,PDE2A,ADORA2A,RAMP2,MIR23A	turquoise	EC_M6
GO:0045670	3.65E-02	0.986088218	9	42	regulation of osteoclast differentiation	BP	4.86E-01	TLR4,SFRP1,TMEM178A,INPP5D,CREB1,LTF,MTOR,GPR68,CA2	turquoise	EC_M6
GO:0015837	3.65E-02	0.984356353	11	58	amine transport	BP	4.86E-01	RAB3B,SNCA,DTNBP1,ADORA2A,SNCG,SYT7,PSEN1,CHRNB2,SLC43A2,STXBP1,RGS4	turquoise	EC_M6
GO:0060760	3.66E-02	0.98514726	10	49	positive regulation of response to cytokine stimulus	BP	4.86E-01	DDX58,TLR4,CXCR4,TLR2,IL7,NLRC5,EDN1,LAPTM5,HIF1A,TRAF2	turquoise	EC_M6

GO:0050965	3.66E-02	0.993079415	4	12	detection of temperature stimulus involved in sensory perception of pain	BP	4.86E-01	CXCR4,EPHB1,MMP24,ANO1	turquoise	EC_M6
GO:0035907	4.01E-02	0.992642908	4	10	dorsal aorta development	BP	4.86E-01	SRF,HEY1,BMPR1A,NGFR	darkgreen	EC_M4
GO:0071732	2.86E-02	0.994904094	4	13	cellular response to nitric oxide	BP	4.86E-01	CDK2,CCNA2,AIFM1,HNRNPD	darkgrey	EC_M8
GO:0090276	4.02E-02	0.975750228	27	140	regulation of peptide hormone secretion	BP	4.87E-01	LRP1,PPARD,LEP,CLOCK,REST,FKBP1B,KIF5B,IRS2,OSBP,SELENOT,ABCC8,PTPN11,PFKM,BRSK2,DYNLL1,CPT1A,LRP5,HADH,ADCY5,SIDT2,TFR2,NLGN2,SYTL4,ACSL4,RASL10B,MIDN,SSTR5	darkgreen	EC_M4
GO:1903320	2.86E-02	0.982857225	28	211	regulation of protein modification by small protein conjugation or removal	BP	4.87E-01	FBXO5,UBR5,TOPORS,COMMD1,UFL1,SAE1,PAXIP1,CDC20,GTPBP4,UBA2,OTUD4,UBE2C,UBE3A,CUL3,FANCI,IVNS1ABP,FANCM,BRCA1,DCUN1D5,TAF1,OGT,PLK1,PSMD10,XIAP,GLMN,MAD2L1,UBXN2A,RCHY1	darkgrey	EC_M8
GO:0043302	3.67E-02	0.991137671	5	17	positive regulation of leukocyte degranulation	BP	4.87E-01	SYK,GATA2,GAB2,FGR,STXBP1	turquoise	EC_M6
GO:0002695	3.68E-02	0.980056963	19	118	negative regulation of leukocyte activation	BP	4.88E-01	TNFAIP3,ADGRF5,TNFRSF21,PELI1,CST7,SFRP1,MERTK,ITCH,CD200,LAPTM5,HLX,INPP5D,CD86,FGR,DLG1,ADORA2A,ARG2,LGALS9,NRARP	turquoise	EC_M6
GO:0050829	3.69E-02	0.987939123	7	32	defense response to Gram-negative bacterium	BP	4.88E-01	LYZ,IL6,TLR4,PRKD1,LTF,SELP,NOS2	turquoise	EC_M6
GO:0016079	3.69E-02	0.981869409	15	79	synaptic vesicle exocytosis	BP	4.88E-01	STX11,RIMS2,APBA1,SNCA,DTNBP1,SNAP23,ADCY1,ADORA2A,SNAP29,SYT7,FBXL20,PSEN1,GIT1,STXBP1,STX3	turquoise	EC_M6
GO:0098586	3.69E-02	0.983514945	12	63	cellular response to virus	BP	4.88E-01	DDX58,IL6,PUM2,SEC14L1,CXCL10,OAS3,DDX3X,BIRC3,HIF1A,LGALS9,OAS1,RNF125	turquoise	EC_M6
GO:0051339	4.03E-02	0.985800029	8	29	regulation of lyase activity	BP	4.88E-01	STIM1,CACNA1D,ISCU,LINC02210-CRHR1,CACNA1C,NPR3,PARK7,GNAI3	darkgreen	EC_M4
GO:0098814	4.04E-02	0.992578836	4	10	spontaneous synaptic transmission	BP	4.88E-01	SLC12A2,ITGB1,APP,STX1B	darkgreen	EC_M4
GO:0018279	4.05E-02	0.988439426	6	20	protein N-linked glycosylation via asparagine	BP	4.88E-01	UBE2J1,RPN1,STT3B,TUSC3,RPN2,DDOST	darkgreen	EC_M4
GO:0070296	4.05E-02	0.985731909	8	29	sarcoplasmic reticulum calcium ion transport	BP	4.88E-01	FKBP1B,ATP2A2,CASQ1,CACNA1C,CALM1,DMD,TMEM38B,TMEM38A	darkgreen	EC_M4
GO:0006869	4.07E-02	0.971865635	48	273	lipid transport	BP	4.90E-01	ATP8B1,LRP1,PPARA,NUS1,PPARD,PLIN2,LEP,TMEM30A,MFSD2A,INHBA,ESYT2,AKT2,OSBPL10,C1QTNF1,OSBPL8,IRS2,OSBP,P2RX4,PTPN11,CRY2,SPP1,PLA2R1,KDM5B,CPT1A,EPRS,PITPNM1,PITPNB,ARV1,PRELID3B,FABP2,OSBPL2,OSBP2,HDLBP,LINC02210-CRHR1,ITGAV,CYP19A1,CLN8,STARD3,STOML1,ACSL4,AKT1,PRKAA1,ATP11C,PITPNA,ABCA3,VAMP7,PLEKHA3,SIGMAR1	darkgreen	EC_M4
GO:0006706	4.07E-02	0.988355024	6	20	steroid catabolic process	BP	4.90E-01	HSD17B6,SNX17,SPP1,CYP19A1,CYP46A1,YWHAH	darkgreen	EC_M4
GO:0001767	4.09E-02	0.992464575	4	10	establishment of lymphocyte polarity	BP	4.91E-01	MYH9,DOCK8,SNX27,DOCK2	darkgreen	EC_M4
GO:0014909	3.72E-02	0.982770717	13	67	smooth muscle cell migration	BP	4.91E-01	PDGFB,DOCK4,GRB10,TLR4,NRP1,P2RY6,IGF1,MEF2C,NDRG4,IGFBP3,ACE,SEMA6D,IGFBP5	turquoise	EC_M6
GO:0070372	4.10E-02	0.973002543	38	211	regulation of ERK1 and ERK2 cascade	BP	4.92E-01	LRP1,ANGPT1,ROS1,TPBG,RRAS,FGF2,TGFB1,SPRED3,CAVIN3,ST5,APP,RAPGEF2,CX3CL1,PTPN11,PTPN22,CHRNA7,NOTCH2,HTR2B,PRKCA,ATF3,FGFR2,FN1,PTPN6,FGFR3,SHC1,MAP2K1,NPTN,PIN1,CD74,PDGFRB,PTPN2,ATP6AP1,MIR24-1,CCL16,TIMP3,DUSP4,GLIPR2,FGF10	darkgreen	EC_M4
GO:2001028	4.10E-02	0.990093391	5	15	positive regulation of endothelial cell chemotaxis	BP	4.92E-01	VEGFA,FGF2,P2RX4,HSPB1,TMSB4X	darkgreen	EC_M4

GO:0048666	3.73E-02	0.970459749	120	877	neuron development	BP	4.92E-01	SEMA6A,KIF5A,CTHRC1,RIMS2,CDKL3,ASAP1,PTK2,ANKS1A,AGTPBP1,PICK1,MICALL1,ADGRF1,TNFRSF21,SEMA4D,IL6,PPT1,NRCAM,NRP1,ITSN2,MTMR2,GATA2,HS6ST1,NOVA2,PLXND1,GATA3,MYO7A,CXCR4,EDNRB,SPTBN4,SFRP1,FEZ2,EFNB2,PCDH12,NTN4,PRAG1,EPHB1,PLK2,RET,PREX2,DCLK2,KIF13B,ENC1,MEF2C,NDRG4,GPM6A,USH1C,NR4A2,TSPAN2,JAM3,DTNBP1,MYLIP,SRGAP2C,PPP3CA,HECW2,MEF2A,SEMA3F,ROBO4,SEC24B,PLXNA2,CFLAR,INPP5F,TRAK2,ADCY1,KALRN,FES,SS18L1,PRKD1,LAMA5,YTHDF1,ITGA6,PAK2,LAMC2,APOD,HOXD10,MOV10,CREB1,B2M,ADORA2A,SEMA6D,EPHB4,ITGA4,DNM3,CDK16,LMTK2,RAC1,EPHA2,CECR2,MPP5,MTOR,OPHN1,EFNB1,PSEN1,SEMA4A,ABI3,ITGA3,TAOK2,EFNA1,CHRNB2,ZDHHC15,RPS6KA5,DNM2,B4GALT6,GIT1,SEMA6B,SLITRK2,ATCAY,ARHGAP4,NOTCH1,RAPGEF1,STXBP1,LHX6,DAB2IP,VASP,RELN,ALKAL1,DIO3,SHANK3,GBA2,STX3,SCARF1	turquoise	EC_M6
GO:0033002	3.73E-02	0.977446071	27	174	muscle cell proliferation	BP	4.92E-01	TNFAIP3,PDGFB,PIM1,IL6,TLR4,IL15,PPARG,P2RY6,HBEGF,EPHB1,IGF1,SMAD1,MEF2C,NDRG4,EDN1,STAT1,JARID2,CFLAR,IGFBP3,APOD,IGFBP5,CALCRL,MTOR,NPR1,NOTCH1,S1PR1,BMP2	turquoise	EC_M6
GO:0046629	3.74E-02	0.993062453	4	11	gamma-delta T cell activation	BP	4.92E-01	TCF7,SYK,SOX13,JAG2	turquoise	EC_M6
GO:0045580	3.74E-02	0.980543949	17	98	regulation of T cell differentiation	BP	4.92E-01	TCF7,SYK,IL15,GATA3,SH3RF1,IL7,SOX13,HLX,CD86,SOS2,ITPKB,LGALS9,IL7R,AP3D1,NRARP,VNN1,CD46	turquoise	EC_M6
GO:0019722	3.75E-02	0.978115787	24	147	calcium-mediated signaling	BP	4.93E-01	KDR,SYK,CXCL8,TRPM4,CXCR4,EDNRB,P2RX2,IGF1,EIF2AK3,MCTP1,P2RX7,PLCG1,EDN1,PPP3CA,MCTP2,NFATC2,CCRL2,HOMER3,MCU,SELE,SELP,MTOR,PLCG2,NFAT5	turquoise	EC_M6
GO:0051251	3.78E-02	0.976910612	28	186	positive regulation of lymphocyte activation	BP	4.94E-01	SYK,IL6,PELI1,TLR4,IL15,GATA3,IL1A,IGF1,MEF2C,IL7,SOX13,LCK,ICOSLG,HLX,NFATC2,INPP5D,CD86,B2M,ITPKB,EFNB1,CHRNB2,CCL2,YES1,LGALS9,IL7R,AP3D1,VNN1,CD46	turquoise	EC_M6
GO:1902894	4.12E-02	0.99001285	5	15	negative regulation of pri-miRNA transcription by RNA polymerase II	BP	4.95E-01	PPARA,PPARD,SRF,REST,TGFB1	darkgreen	EC_M4
GO:0021872	4.13E-02	0.984207168	9	35	forebrain generation of neurons	BP	4.95E-01	GLI3,INHBA,ZSWIM6,RAPGEF2,SATB2,LEF1,FGFR2,PLXNA3,LHX8	darkgreen	EC_M4
GO:0002696	4.14E-02	0.972524102	39	218	positive regulation of leukocyte activation	BP	4.96E-01	IL23A,HLA-DRA,FAM49B,LGALS1,LEP,DOCK8,NCK2,SOCS5,GLI3,GLI2,TGFB1,AXL,IL15RA,IRS2,HSPH1,VAMP8,PTPN11,AP3B1,PTPN22,ADA,RUNX1,SLC39A10,MMP14,PIK3CA,LEF1,TP53BP1,TFRC,PTPN6,IGFBP2,CD74,AKT1,CD40LG,VAMP7,AP1G1,BCL10,THY1,CDKN1A,TSLP,FGF10	darkgreen	EC_M4
GO:0030316	3.80E-02	0.98233071	13	68	osteoclast differentiation	BP	4.97E-01	TNFRSF11A,IREB2,TLR4,GAB2,SFRP1,TMEM178A,INPP5D,CREB1,LTF,EPHA2,MTOR,GPR68,CA2	turquoise	EC_M6
GO:0061564	3.80E-02	0.972886193	57	380	axon development	BP	4.97E-01	SEMA6A,KIF5A,CDKL3,PTK2,TNFRSF21,SEMA4D,NRCAM,NRP1,NOVA2,PLXND1,GATA3,SPTBN4,FEZ2,EFNB2,EPHB1,RET,KIF13B,NR4A2,TSPAN2,JAM3,SEMA3F,ROBO4,PLXNA2,INPP5F,TRAK2,ADCY1,KALRN,LAMA5,YTHDF1,PAK2,LAMC2,APOD,CREB1,SEMA6D,EPHB4,ITGA4,LMTK2,RAC1,EPHA2,OPHN1,EFNB1,PSEN1,SEMA4A,TAOK2,EFNA1,CHRNB2,RPS6KA5,DNM2,B4GALT6,SEMA6B,SLITRK2,ARHGAP4,NOTCH1,STXBP1,VASP,RELN,SCARF1	turquoise	EC_M6
GO:0045639	3.81E-02	0.981646405	14	77	positive regulation of myeloid cell differentiation	BP	4.97E-01	TAL1,GATA2,JAG1,STAT1,ETS1,HCLS1,INPP5D,FES,CREB1,HIF1A,GPR68,RCOR1,ISG15,CA2	turquoise	EC_M6
GO:0002313	3.81E-02	0.99059508	5	18	mature B cell differentiation involved in immune response	BP	4.97E-01	MFNG,IL6,LFNG,IRF8,PLCG2	turquoise	EC_M6
GO:0097242	4.16E-02	0.985273522	8	29	amyloid-beta clearance	BP	4.97E-01	LRP1,SRF,PICALM,LRPAP1,RAB11A,CLTC,MYOCD,ROCK1	darkgreen	EC_M4
GO:0097205	3.83E-02	0.989022064	6	22	renal filtration	BP	4.98E-01	PDGFB,ADGRF5,CD34,MCAM,ITGA3,TBC1D8B	turquoise	EC_M6
GO:0002862	3.83E-02	0.990648462	5	17	negative regulation of inflammatory response to antigenic stimulus	BP	4.98E-01	SYK,PLK2,PLCG1,FGR,YES1	turquoise	EC_M6
GO:0060033	4.18E-02	0.992237252	4	10	anatomical structure regression	BP	4.99E-01	GLI2,LRP5,LEF1,BMPR1A	darkgreen	EC_M4

GO:1904705	4.18E-02	0.981198002	12	53	regulation of vascular associated smooth muscle cell proliferation	BP	4.99E-01	FAM19A5,FGF2,PAK1,MMP2,BMPR1A,TPM1,TGFB3,CNN1,MYOCD,GNAI3,CDKN1A,PRKG1	darkgreen	EC_M4
GO:0009952	3.85E-02	0.977438124	24	150	anterior/posterior pattern specification	BP	5.00E-01	ACVR2B,SFRP1,TCF15,HHEX,HIPK1,ETS2,PLXNA2,HOXD10,RNF2,TBX1,LFNG,ALDH1A2,PSEN1,HOXB5,HOXB6,HOXB7,HOXB8,HOXB9,SMAD2,YY1,NRARP,NOTCH1,SOX17,BMP2	turquoise	EC_M6
GO:0051703	3.85E-02	0.986368442	8	32	biological process involved in intraspecies interaction between organisms	BP	5.00E-01	SHANK2,PTCHD1,LTF,TBX1,MTOR,CHRNB2,ATXN1L,SHANK3	turquoise	EC_M6
GO:0043501	3.86E-02	0.990569331	5	17	skeletal muscle adaptation	BP	5.00E-01	IL15,PPP3CA,CFLAR,IGFBP5,MTOR	turquoise	EC_M6
GO:0051928	4.20E-02	0.977685735	18	86	positive regulation of calcium ion transport	BP	5.00E-01	STIM1,ATP2B1,CXCL12,P2RX4,CACNA1D,CX3CL1,CACNB3,HOMER1,PKD2,CASQ1,HTT,HAP1,STIM2,CALM1,VMP1,TRPV2,PDGFRB,THY1	darkgreen	EC_M4
GO:0046622	3.87E-02	0.985094486	9	41	positive regulation of organ growth	BP	5.01E-01	PIM1,IGF1,MEF2C,IL7,EDN1,HLX,PROX1,MTOR,NOTCH1	turquoise	EC_M6
GO:1904064	3.88E-02	0.979336115	18	103	positive regulation of cation transmembrane transport	BP	5.01E-01	TRDN,P2RY6,CXCL10,PLCG1,ANO6,SNCA,KCNJ2,WNK4,CRACR2A,ATP2A1,CD19,DNM2,ADRB2,CCL2,PLCG2,RELN,KCNH2,SHANK3	turquoise	EC_M6
GO:0039529	3.88E-02	0.988658881	6	24	RIG-I signaling pathway	BP	5.01E-01	DDX58,PUM2,SEC14L1,OAS3,BIRC3,RNF125	turquoise	EC_M6
GO:1900542	3.90E-02	0.983128306	11	56	regulation of purine nucleotide metabolic process	BP	5.01E-01	NOS3,IGF1,GPD1,P2RX7,SNCA,MLXIPL,PDK3,HIF1A,PSEN1,NOS2,GIT1	turquoise	EC_M6
GO:0014066	3.91E-02	0.980105448	16	86	regulation of phosphatidylinositol 3-kinase signaling	BP	5.02E-01	PTK2,PDGFB,TEK,PIP5K1B,KIT,KDR,SEMA4D,PRR5L,IGF1,PPP1R16B,NCF1,HCLS1,FGR,SELP,DAB2IP,RELN	turquoise	EC_M6
GO:0050818	3.92E-02	0.983773929	10	54	regulation of coagulation	BP	5.02E-01	TSPAN8,PDGFB,NOS3,CD36,KLKB1,PROCR,ANO6,EDN1,CD34,PSEN1	turquoise	EC_M6
GO:1903507	3.92E-02	0.968830151	122	945	negative regulation of nucleic acid-templated transcription	BP	5.02E-01	PLAGL1,NOTCH4,TCF7,L3MBTL3,ATF4,PDGFB,MDFI,NFX1,NFIL3,SEMA4D,NFE2L3,BACH2,FOXP2,KLF11,LANCL2,CREM,SDCBP,BEND5,TAL1,PHF10,EPC1,GATA2,DNMT3A,SAP130,DACH1,PPARG,GATA3,TAF3,ACVR2B,GZF1,EDNRB,PDE2A,SFRP1,NR1D2,CD36,ARID5A,DNMT3B,HHEX,ZBTB38,POU6F1,SOX7,HMBOX1,MEF2C,WWTR1,SFMBT1,SCAF4,ANKRD2,NR4A2,DLL4,CDYL,SOX13,YAF2,EDN1,STAT1,SNCA,JARID2,ETS2,DCAF1,HDAC1,MEF2A,FABP4,HCLS1,NFATC2,KDM2A,CIART,TGIF1,PARP15,PROX1,ZNF148,MLXIPL,DLG1,ETV6,RNF2,CREB1,EIF4A2,BCOR,MECOM,ING4,SPINDOC,LDB2,PPM1F,POU2F1,ZBTB18,APBB2,BCL6B,KDM5C,MAFK,AR,PSEN1,CBX1,HOXB8,FBXW11,EFNA1,PIAS1,RPS6KA5,SMAD2,TLE3,YY1,HMG20A,RCOR1,HCFC1,NRARP,NOTCH1,IRF8,MTF2,ZBTB34,DAB2IP,ZBTB33,ARID4A,ZNF24,ATXN1L,TRIM24,SOX18,PASD1,COMMD7,TGIF2,BMP2,GLIS3,ZNF10,CBX2,BMP6,ZNF266	turquoise	EC_M6
GO:0043248	4.21E-02	0.991883484	4	11	proteasome assembly	BP	5.02E-01	PSMG1,ADRM1,PSMD11,PSMG4	darkgreen	EC_M4
GO:0042886	4.21E-02	0.972172275	38	210	amide transport	BP	5.02E-01	LRP1,PPARD,LEP,CLOCK,REST,SLC7A11,FKBP1B,KIF5B,IRS2,PCLO,OSBP,SELENOT,SLC33A1,ABCC8,PTPN11,PFKM,CDH17,BRSK2,DYNLL1,CPT1A,LRP5,HADH,DISP1,ADCY5,SIDT2,LINC02210-CRHR1,TFR2,PTPRN,NLGN2,UBE2Q1,SYTL4,ACSL4,CD74,RASL10B,MIDN,SSTR5,PARK7,PLEKHA3	darkgreen	EC_M4
GO:0055003	4.22E-02	0.98990927	5	14	cardiac myofibril assembly	BP	5.02E-01	SRF,MYLK3,OBSL1,TCAP,PDGFRB	darkgreen	EC_M4
GO:0050830	3.93E-02	0.986894096	7	33	defense response to Gram-positive bacterium	BP	5.02E-01	LYZ,IL6,CD36,TLR2,FGR,EPHA2,IL7R	turquoise	EC_M6
GO:0033627	3.93E-02	0.982300807	12	60	cell adhesion mediated by integrin	BP	5.02E-01	PTK2,PODXL,SYK,PIK3CG,ITGB4,FERMT1,RET,JAM3,ITGA4,EPHA2,EFNA1,PIEZO1	turquoise	EC_M6
GO:0002467	3.93E-02	0.992386615	4	12	germinal center formation	BP	5.02E-01	TNFAIP3,UNC13D,MEF2C,NFKB2	turquoise	EC_M6
GO:0044262	4.24E-02	0.971552222	41	229	cellular carbohydrate metabolic process	BP	5.04E-01	DYRK2,MIOX,PPARA,LEP,GLB1,RBKS,DGAT2,AKT2,C1QTNF1,KAT2B,IRS2,IMPAD1,B3GNT4,GYG1,SORBS1,PFKM,ADIPOR1,SELENOS,ITPK1,GYG2,GSK3B,INPP5A,IDH2,PPP1R3D,MOGAT3,TPI1,PPP1R3F,PPARGC1A,AKT1,OCRL,PTPN2,PGP,MIDN,GNPTG,FAM3A,AGL,RANBP2,HAS2,PPP1R1A,NNMT,HS2ST1	darkgreen	EC_M4

GO:0007096	2.98E-02	0.994438986	4	15	regulation of exit from mitosis	BP	5.05E-01	ANLN,KNTC1,UBE2C,CDCA5	darkgrey	EC_M8
GO:0047496	4.26E-02	0.981726671	11	45	vesicle transport along microtubule	BP	5.06E-01	AP3S1,DYNC1I1,KIF5B,TRAK1,AP3B1,KIF1A,HTT,HAP1,PAFAH1B1,VAMP7,BLOC1S2	darkgreen	EC_M4
GO:0014842	4.27E-02	0.992015351	4	10	regulation of skeletal muscle satellite cell proliferation	BP	5.06E-01	ANGPT1,PPARD,AKIRIN1,FGF2	darkgreen	EC_M4
GO:0014857	4.27E-02	0.992015351	4	10	regulation of skeletal muscle cell proliferation	BP	5.06E-01	ANGPT1,PPARD,AKIRIN1,FGF2	darkgreen	EC_M4
GO:0060291	3.98E-02	0.982034562	12	62	long-term synaptic potentiation	BP	5.07E-01	PLK2,SNCA,SHANK2,ADCY1,YTHDF1,CREB1,ADORA2A,RGS14,SLC8A3,RELN,SHANK3,STX3	turquoise	EC_M6
GO:0006942	3.98E-02	0.98133812	13	68	regulation of striated muscle contraction	BP	5.07E-01	TRPM4,PIK3CG,PDE4D,NR4A1,STC1,KCNJ2,DLG1,JUP,ATP2A1,DSG2,DSC2,PDE4B,SLC8A3	turquoise	EC_M6
GO:0002792	4.30E-02	0.983436646	9	35	negative regulation of peptide secretion	BP	5.09E-01	LEP,REST,FKBP1B,ABCC8,PTPN11,HADH,SYTL4,CD74,MIDN	darkgreen	EC_M4
GO:0010810	4.30E-02	0.972533017	33	175	regulation of cell-substrate adhesion	BP	5.09E-01	LRP1,LGALS1,VEGFA,ROCK2,RRAS,FBLN2,ACTG1,ACTN4,ILK,RASA1,EDIL3,CX3CL1,PTPN11,PHLDB2,GSK3B,MMP14,DAG1,CORO1C,IQGAP1,COL26A1,SERPINE1,FN1,ARPC2,VCL,COL1A1,ROCK1,EGFLAM,DAPK3,FLNA,CDH13,THY1,HAS2,DUSP22	darkgreen	EC_M4
GO:0048841	4.30E-02	0.984660424	8	29	regulation of axon extension involved in axon guidance	BP	5.09E-01	VEGFA,MEGF8,SEMA3C,RYK,CXCL12,SEMA4F,SEMA4G,PLXNA3	darkgreen	EC_M4
GO:1902679	4.00E-02	0.968125272	122	946	negative regulation of RNA biosynthetic process	BP	5.09E-01	PLAGL1,NOTCH4,TCF7,L3MBTL3,ATF4,PDGFB,MDFI,NFX1,NFIL3,SEMA4D,NFE2L3,BACH2,FOXP2,KLF11,LANCL2,CREM,SDCBP,BEND5,TAL1,PHF10,EPC1,GATA2,DNMT3A,SAP130,DACH1,PPARG,GATA3,TAF3,ACVR2B,GZF1,EDNRB,PDE2A,SFRP1,NR1D2,CD36,ARID5A,DNMT3B,HHEX,ZBTB38,POU6F1,SOX7,HMBOX1,MEF2C,WWTR1,SFMBT1,SCAF4,ANKRD2,NR4A2,DLL4,CDYL,SOX13,YAF2,EDN1,STAT1,SNCA,JARID2,ETS2,DCAF1,HDAC1,MEF2A,FABP4,HCLS1,NFATC2,KDM2A,CIART,TGIF1,PARP15,PROX1,ZNF148,MLXIPL,DLG1,ETV6,RNF2,CREB1,EIF4A2,BCOR,MECOM,ING4,SPINDOC,LDB2,PPM1F,POU2F1,ZBTB18,APBB2,BCL6B,KDM5C,MAFK,AR,PSEN1,CBX1,HOXB8,FBXW11,EFNA1,PIAS1,RPS6KA5,SMAD2,TLE3,YY1,HMG20A,RCOR1,HCFC1,NRARP,NOTCH1,IRF8,MTF2,ZBTB34,DAB2IP,ZBTB33,ARID4A,ZNF24,ATXN1L,TRIM24,SOX18,PASD1,COMMD7,TGIF2,BMP2,GLIS3,ZNF10,CBX2,BMP6,ZNF266	turquoise	EC_M6
GO:0010817	4.00E-02	0.972294309	47	331	regulation of hormone levels	BP	5.09E-01	RDH16,ALDH8A1,RIMS2,HSD17B3,PICK1,SRD5A3,ICA1,IL6,TRPM4,GATA3,SFRP1,SIRT3,SCARB1,CHD7,FAM3D,TIPARP,EDN1,ABCG1,HDAC1,PPP3CA,ANO1,SNAP23,PLEKHA1,SLCO4A1,ACE,CREB1,MCU,CDK16,FGF23,RAC1,HIF1A,SYT7,ALDH1A2,GPR68,SMAD2,SCN N1B,DHRS11,CYP1A1,CHST9,NOS2,EXOC3L1,DIO3,BMP2,SRD5A2,BMP6,AKR1D1,RBP1	turquoise	EC_M6
GO:0050792	3.02E-02	0.984299088	18	119	regulation of viral process	BP	5.09E-01	SRPK1,TRIM28,PPIH,RSF1,TOP2B,IFIT5,PDE12,FAM208A,PPID,DDX5,DHX9,TOP2A,HACD3,LARP1,ILF3,FMR1,PKN2,TRIM59	darkgrey	EC_M8
GO:0046785	3.02E-02	0.986344801	13	74	microtubule polymerization	BP	5.09E-01	FBXO5,AKAP9,CLASP2,TUBGCP3,TPX2,CENPJ,CKAP5,CAMSAP2,HAUS2,HDGFL3,FKBP4,STMN1,DRG1	darkgrey	EC_M8
GO:1901532	3.02E-02	0.991660594	6	25	regulation of hematopoietic progenitor cell differentiation	BP	5.09E-01	HSPA9,PUS7,METTL3,KITLG,PRKDC,ANKLE1	darkgrey	EC_M8
GO:0003179	4.31E-02	0.98063163	12	50	heart valve morphogenesis	BP	5.09E-01	ROCK2,RB1,TGFB1,ROBO1,HEY1,ACVR1,NOTCH2,TGFB2,BMPR1A,FGFRL1,ROCK1,OLFM1	darkgreen	EC_M4
GO:0060314	4.32E-02	0.987466584	6	20	regulation of ryanodine-sensitive calcium-release channel activity	BP	5.09E-01	FKBP1B,PKD2,CALM1,JPH3,DMD,FKBP1A	darkgreen	EC_M4

GO:0012501	4.01E-02	0.96682037	179	1474	programmed cell death	BP	5.09E-01	TNFAIP8,SEMA6A,TNFAIP3,PLAGL1,TRIM39,AKAP12,FLT4,OXR1,GRAMD4,TPD52L1,PTK2,DAPK1,ATF4,PIM1,TEK,SH3RF3,KIT,TNFRSF21,KDR,SYK,GADD45G,SEMA4D,SHB,IL6,PPT1,PELI1,UTP11,ELMO1,FIGNL1,KLF11,TRAF1,NRP1,PIK3CG,GATA2,DNMT3A,CTSC,NOS3,RALB,ZFAND6,PPARG,GATA3,CXCR4,EDNRB,NET1,TMC8,SFRP1,RPL26,ANKLE2,ARHGEF7,RASSF7,PLK2,RET,MERTK,TNFRSF19,IL1A,IGF1,NR4A1,EIF2AK3,ITCH,SH3RF1,HIP1R,MEF2C,TLR2,IL7,P2RX7,CXCL10,MADD,LMBR1L,PLCG1,NR4A2,HIPK1,ANO6,IFIT2,ADAMTS20,EDN1,STAT1,SNCA,NUAK2,GAS2,MX1,RNF144B,SIK1,HDAC1,ITPRIP,LCK,HYAL2,MEF2A,LAPTM5,NCF1,DYRK3,HCLS1,INPP5D,EAF2,BMX,CFLAR,CASP10,IGFBP3,RPS6KA1,PRKD1,DDX42,ITGA6,DOCK1,PAK2,ELL3,ETV6,MAP3K11,CREB1,TRIM69,USP28,LTF,ADORA2A,ARNT2,ITGA4,DDX3X,TBX1,PYGL,MECOM,ING4,RAMP2,BIRC3,TNFAIP8L3,PPM1F,USP42,PRKCH,HIF1A,EPHA2,ITPKB,CECR2,BID,TNFRSF1B,ARG2,ALDH1A2,MTOR,AR,BNIP2,PSEN1,DAPK2,TAOK2,EFNA1,ATP2A1,PIAS1,S100A14,DNM2,ATG4D,PPP1R13B,CCL2,JAG2,LGALS9,NOS2,IL7R,BFAR,STK26,MYO18A,TNFAIP8L1,ATCAY,TRAF2,TNFRSF25,NOTCH1,PLCG2,STXBP1,DAB2IP,CDH5,MIR23A,VNN1,BCL7B,PMAIP1,TRIM24,DIO3,FCER2,DRAM2,SERPINB9,PERP,E2F3,BMP2,XAF1,IFIT3,RNF152,NMNAT1,EVA1A	turquoise	EC_M6
GO:0032967	4.33E-02	0.987425044	6	20	positive regulation of collagen biosynthetic process	BP	5.10E-01	TGFB1,RUNX1,SUCO,TGFB3,PDGFRB,ITGA2	darkgreen	EC_M4
GO:0014855	4.33E-02	0.979773556	13	56	striated muscle cell proliferation	BP	5.10E-01	ANGPT1,PPARD,TGFBR1,AKIRIN1,FGF2,TENM4,RUNX1,TGFB2,PRKAR1A,KPNA1,FGFR2,BMPR1A,WNT2	darkgreen	EC_M4
GO:0002931	4.34E-02	0.980463179	12	51	response to ischemia	BP	5.10E-01	REST,ROCK2,BVES,PANX1,P2RX4,FAIM2,CX3CL1,HYOU1,ABRAXAS2,PPARGC1A,CPEB4,AKT1	darkgreen	EC_M4
GO:0031115	4.02E-02	0.992165898	4	12	negative regulation of microtubule polymerization	BP	5.10E-01	ARHGEF7,DYRK1A,SNCA,CDH5	turquoise	EC_M6
GO:0031111	3.03E-02	0.989630719	8	36	negative regulation of microtubule polymerization or depolymerization	BP	5.11E-01	CLASP2,CKAP2,TPX2,CAMSAP2,HDGFL3,FKBP4,TAOK1,STMN1	darkgrey	EC_M8
GO:0030308	4.35E-02	0.973019994	29	153	negative regulation of cell growth	BP	5.11E-01	PPARA,PPARD,RTN4,INHBA,RB1,PAK1,TGFB1,SEMA3C,RYK,RNF6,DIP2B,SEMA4F,SPP1,SEMA4G,ADIPOR1,HYAL1,CAPRIN2,TGFB2,RBBP7,TNR,TSPYL2,NPPB,SMARCA4,CRLF3,PLXNA3,ENO1,CDKN1A,DACT3,NME6	darkgreen	EC_M4
GO:0055069	4.36E-02	0.985709419	7	25	zinc ion homeostasis	BP	5.11E-01	SLC39A7,SLC39A4,SLC30A9,AP3B1,SLC39A10,SLC39A6,SLC30A7	darkgreen	EC_M4
GO:0060193	4.36E-02	0.981156845	11	46	positive regulation of lipase activity	BP	5.11E-01	GNAQ,PLAA,FGF2,ABHD5,ARL1,HTR2B,FGFR2,FGFR3,PDGFRB,NMUR1,ARF4	darkgreen	EC_M4
GO:0090162	4.04E-02	0.986829886	7	27	establishment of epithelial cell polarity	BP	5.11E-01	CYTH1,FERMT1,FRMD4B,SYNE4,TCF15,OPHN1,MYO18A	turquoise	EC_M6
GO:0052548	4.05E-02	0.973298984	37	270	regulation of endopeptidase activity	BP	5.11E-01	TNFAIP8,GRAMD4,DAPK1,SYK,C5,CST7,PPARG,TIMP4,ITIH5,NR4A1,EIF2AK3,SH3RF1,HIP1R,SOX7,SNCA,HDAC1,LCK,LAPTM5,CFLAR,CASP10,RPS6KA1,PAK2,CST6,LTF,ADORA2A,DDX3X,BIRC3,PPM1F,BID,PAPLN,EFNA1,LGALS9,PMAIP1,SERPINB9,PSMB9,PERP,BST2	turquoise	EC_M6
GO:0061371	4.05E-02	0.983340033	10	47	determination of heart left/right asymmetry	BP	5.11E-01	MEF2C,NDRG4,VANGL2,MMP21,HIF1A,PSEN1,NOTCH1,ENG,SOX17,SOX18	turquoise	EC_M6
GO:0097284	4.05E-02	0.991910016	4	13	hepatocyte apoptotic process	BP	5.11E-01	PIK3CG,DNMT3A,CFLAR,BID	turquoise	EC_M6
GO:0043117	4.37E-02	0.989247099	5	15	positive regulation of vascular permeability	BP	5.12E-01	ANGPT1,PTP4A3,VEGFA,TGFB1,TJP1	darkgreen	EC_M4
GO:1990266	4.07E-02	0.980783343	13	74	neutrophil migration	BP	5.12E-01	SYK,CXCL8,PIK3CG,CXCL10,THBS4,EDN1,JAM3,PECAM1,MCU,RAC1,DAPK2,CCL2,PDE4B	turquoise	EC_M6
GO:0045648	4.07E-02	0.986566458	7	29	positive regulation of erythrocyte differentiation	BP	5.12E-01	TAL1,GATA2,STAT1,ETS1,INPP5D,HIF1A,ISG15	turquoise	EC_M6

GO:0006355	4.08E-02	0.965053321	292	2411	regulation of transcription, DNA-templated	BP	5.12E-01	ZNF516,TNFRSF11A,BAZ2A,TCF4,TNFAIP3,PLAGL1,CNOT6,FZD6,ZBED4,NOTCH4,TCF20,TCF7,NCOA7,L3MBTL3,ATF4,PDGFB,KDM4C,PIM1,NFYA,MDFI,PRDM4,DDX58,NFX1,KIT,ADGRF1,NFIL3,SYK,SEMA4D,IL6,NFE2L3,REL,CREB5,PPIE,BACH2,PELI1,FOXP2,KLF11,LANCL2,NOBOX,TLR4,TRAF1,CREM,TEAD2,SDCBP,MYCN,KTI12,ZBTB24,BEND5,TAL1,PHF10,EPC1,GATA2,DNMT3A,PRKD2,SAP130,DACH1,PPARG,GATA3,TAF3,ACVR2B,IRAK2,GZF1,EDNRB,SETD5,IKBKB,JAG1,NFKBIB,PDE2A,TMEM173,SFRP1,NR1D2,CD36,CDK17,ARID5A,TCF15,RET,CHD7,IL1A,DNMT3B,IGF1,NR4A1,EIF2AK3,HHEX,KDM3A,ITCH,ERBIN,SMAD1,ZBTB38,POU6F1,STOX2,SOX7,HMBOX1,HEXB,MEF2C,SMARCD1,TLR2,WWTR1,ZNF704,CXCL10,ADGRG3,KMT2D,SFMBT1,SCAF4,NLRC5,ACTR5,ANKRD2,NR4A2,TOX2,DLL4,CDYL,BAZ2B,SOX13,YAF2,EDN1,STAT1,DYRK1A,SNCA,JARID2,ERG,ETS2,CDK18,NFKB2,DCAF1,PRDM15,ETS1,CD200,SIK1,HDAC1,PPP3CA,HYAL2,SLC39A8,TESMIN,MEF2A,BCL9,HLX,NCF1,USP13,FABP4,HCLS1,NFATC2,ZFHX2,EAF2,GMEB1,CD86,KDM2A,CIART,MN1,TGIF1,PARP15,POU2F3,ZSCAN29,CFLAR,TRAK2,ADCY1,ARID1A,RPS6KA1,PROX1,SS18L1,PRKD1,ZNF148,ZSCAN2,HELZ2,MLXIPL,ITGA6,DLG1,ELL3,ETV6,HOXD10,RNF2,CREB1,TET1,LTF,ADORA2A,ARNT2,MYEF2,IL1RAP,BCOR,DDX3X,RFXANK,ETV4,TBX1,MECOM,ING4,CDK16,SPINDOC,LDB2,FGF23,PPM1F,POU2F1,WAS,TEAD4,PRKCH,HIF1A,KDM5A,ZBTB18,APBB2,JUP,BCL6B,KDM5C,TLR6,RSC1A1,RGS14,MAFK,SPIN2A,MTOR,AR,CASZ1,PSEN1,CBX1,HOXB5,HOXB6,HOXB7,HOXB8,HOXB9,ZNF410,FBXW11,BATF,KRBA2,EFNA1,PIAS1,RPS6KA5,SMAD2,TLE3,DNM2,Y	turquoise	EC_M6
GO:0014047	4.08E-02	0.992020538	4	12	glutamate secretion	BP	5.12E-01	APBA1,SNCA,ADORA2A,STXBP1	turquoise	EC_M6
GO:0006937	4.08E-02	0.977298026	20	121	regulation of muscle contraction	BP	5.12E-01	KIT,DOCK4,TRPM4,PIK3CG,ARHGAP42,PDE4D,NR4A1,GUCY1A1,STC1,EDN1,KCNJ2,DLG1,CALCRL,JUP,ATP2A1,DSG2,DSC2,ADRB2,PDE4B,SLC8A3	turquoise	EC_M6
GO:0021879	4.08E-02	0.986497382	7	29	forebrain neuron differentiation	BP	5.12E-01	NRP1,GATA2,DCLK2,PROX1,B2M,LHX6,SHANK3	turquoise	EC_M6
GO:0007519	4.38E-02	0.974270935	24	122	skeletal muscle tissue development	BP	5.12E-01	HIVEP3,MYH14,AKIRIN1,BVES,RB1,TGFB1,CTNNB1,DCN,ANKRD1,FLNB,HOMER1,DNER,ATF3,CASQ1,SIX4,MYLPF,MYOCD,FBXO22,MYOM1,PRKAA1,DMD,IGSF8,SRPK3,CFL2	darkgreen	EC_M4
GO:0009593	4.09E-02	0.986310065	7	32	detection of chemical stimulus	BP	5.12E-01	TLR4,P2RX2,SCARB1,TLR2,RTP4,TLR6,TLR1	turquoise	EC_M6
GO:0110110	4.38E-02	0.984230807	8	30	positive regulation of animal organ morphogenesis	BP	5.12E-01	FGF2,CTNNB1,ROBO1,SIX4,NGFR,GDNF,WNT2,FGF10	darkgreen	EC_M4
GO:1903725	4.10E-02	0.986324021	7	31	regulation of phospholipid metabolic process	BP	5.13E-01	PDGFB,ADGRF5,MTMR2,RAB38,SCARB1,CHRM5,LPCAT1	turquoise	EC_M6
GO:0030001	4.11E-02	0.968938751	80	587	metal ion transport	BP	5.13E-01	TRDN,KLHL3,PDGFB,IREB2,TSPAN13,GJA4,TRPM4,PIK3CG,KCNRG,CXCR4,NDFIP2,SPTBN4,P2RY6,KCNK6,P2RX2,SLC4A11,MICU3,PDE4D,CHD7,CP,P2RX7,GPM6A,CXCL10,TPCN1,PLCG1,STC1,KCNJ3,SLC38A1,ANO6,ZDHHC13,KCNJ15,SNCA,SIK1,LCK,PPP3CA,HECW2,SLC39A8,KCNJ2,SLC12A7,SCN3A,SLC9A8,EPB41,PRKD1,MELTF,DLG1,KCNK7,B2M,LTF,ADORA2A,HOMER3,MCU,CALCRL,SLC9A7,WNK4,RAMP2,CRACR2A,LMTK2,HEPH,PSEN1,KCNN3,CHRNB2,ATP2A1,CD19,DNM2,SCNN1B,ADRB2,CCL2,KCNJ18,PDE4B,PLCG2,SLC25A25,SLC8A3,KCNH2,SCN2B,KCNK5,RGS4,KCNA6,CACNG5,FXYD6,KCNG2	turquoise	EC_M6
GO:0043123	4.11E-02	0.975763324	24	153	positive regulation of I-kappaB kinase/NF-kappaB signaling	BP	5.13E-01	REL,PELI1,TLR4,NDFIP2,IKBKB,CD36,PLK2,TNFRSF19,ZDHHC13,TIFA,CFLAR,CASP10,PRKD1,MAP3K3,LTF,TMEM106A,BIRC3,TLR6,LGALS9,TRAF2,PLEKHG5,TRIM34,TRIM62,BST2	turquoise	EC_M6
GO:0050885	4.39E-02	0.983049518	9	34	neuromuscular process controlling balance	BP	5.13E-01	SHANK1,APP,ADCY5,TNR,NLGN2,CLN8,PAFAH1B1,JPH3,AARS	darkgreen	EC_M4
GO:0001738	4.40E-02	0.97651602	18	84	morphogenesis of a polarized epithelium	BP	5.13E-01	AHI1,RSPO3,PLEKHA4,ACTG1,RYK,PRICKLE2,FAT1,NKD1,BRSK2,FZD2,SMURF1,EXOC5,DAAM1,WDR1,MSN,TTC8,LAMA3,PAFAH1B1	darkgreen	EC_M4
GO:0010469	4.11E-02	0.977508544	19	112	regulation of signaling receptor activity	BP	5.13E-01	DAPK1,DLGAP3,NRP1,PPARG,HBEGF,PDE4D,MEF2C,EDN1,HDAC1,NCF1,HIF1A,PSEN1,ADRB2,CCL2,RELN,LYPD6,SHANK3,CACNG5,GSG1L	turquoise	EC_M6
GO:0045778	4.12E-02	0.983933821	9	42	positive regulation of ossification	BP	5.14E-01	ACVR2B,MEF2C,P2RX7,ANO6,LTF,ADRB2,ISG15,BMP2,BMP6	turquoise	EC_M6
GO:0022029	4.42E-02	0.981813732	10	40	telencephalon cell migration	BP	5.16E-01	SUN2,MDGA1,SRF,RTN4,GLI3,CTNNB1,CXCL12,ROBO1,TNR,PAFAH1B1	darkgreen	EC_M4

GO:0010466	4.44E-02	0.972940847	27	145	negative regulation of peptidase activity	BP	5.16E-01	SERPINB2,LRP1,VEGFA,PICALM,SERPINH1,CRIM1,SPINT2,PRNP,SPINK5,APP,UCHL5,ANOS1,COL6A3,RPS6KA3,SERPINE1,TIMP1,TMED10,ROCK1,USP14,AKT1,PARK7,MIR24-1,SPINK2,SERPINF1,SERPINB5,A2M,TIMP3	darkgreen	EC_M4
GO:0003382	4.44E-02	0.985455549	7	24	epithelial cell morphogenesis	BP	5.16E-01	COL15A1,SPINT2,FAT1,PALLD,FLNB,CLIC4,PLOD3	darkgreen	EC_M4
GO:0032414	4.45E-02	0.976662532	17	80	positive regulation of ion transmembrane transporter activity	BP	5.16E-01	KIF5B,ACTN4,STIM1,ATP1B3,ABCC8,CACNB3,CHP1,PKD2,CASQ1,HTT,KCNA1,HAP1,STIM2,CALM1,VMP1,DMD,TMSB4X	darkgreen	EC_M4
GO:0003300	4.45E-02	0.977164338	16	75	cardiac muscle hypertrophy	BP	5.16E-01	PPARA,LEP,ROCK2,PAK1,P2RX4,ATP2A2,ATP2B4,TWF1,PDLIM5,HTR2B,PRKCA,GLRX3,TCAP,PIN1,ROCK1,MIR24-1	darkgreen	EC_M4
GO:1900076	4.45E-02	0.979858263	12	51	regulation of cellular response to insulin stimulus	BP	5.16E-01	LEP,PAK1,OSBPL8,ATP2B1,SORBS1,USO1,PTPN11,OPA1,ADIPOR1,PRKAA1,PTPN2,MYO1C	darkgreen	EC_M4
GO:0048566	4.45E-02	0.985312059	7	25	embryonic digestive tract development	BP	5.16E-01	GLI3,GLI2,RARB,ADA,TGFB2,FGFR2,FGF10	darkgreen	EC_M4
GO:1902745	4.46E-02	0.98388641	8	30	positive regulation of lamellipodium organization	BP	5.16E-01	AKIRIN1,ACTR2,ACTR3,PIK3CA,AUTS2,CORO1C,NCKAP1,ARPC2	darkgreen	EC_M4
GO:0009891	4.14E-02	0.965533914	190	1533	positive regulation of biosynthetic process	BP	5.16E-01	ZNF516,TCF4,PLAGL1,AKAP12,CNOT6,ZBED4,NOTCH4,TCF20,NCOA7,ATF4,PDGFB,BRPF3,PIM1,NFYA,PRDM4,DDX58,ADGRF5,KDR,NFIL3,IL6,REL,CREB5,CXCL8,TLR4,IL15,TEAD2,MYCN,TAL1,PHF10,EPC1,GATA2,PRKD2,RAB38,NOS3,PPARG,GATA3,IKBKB,JAG1,P2RY6,NFKBIB,TMEM173,SFRP1,NR1D2,RPL26,CD36,SIRT3,TCF15,SCARB1,RET,CHD7,IL1A,IGF1,NR4A1,EIF2AK3,ABHD6,HHEX,KDM3A,SMAD1,ZBTB38,CLCN3,STOX2,SOX7,HMBOX1,HEXB,MEF2C,SMARCD1,TLR2,WWTR1,CXCL10,KMT2D,NLRC5,NR4A2,TOX2,YAF2,EDN1,STAT1,DYRK1A,SNCA,ERG,ETS2,SLC45A3,NFKB2,PRDM15,ABCG1,ETS1,HDAC1,PPP3CA,HYAL2,MEF2A,BCL9,NCF1,HCLS1,NFATC2,EAF2,GMEB1,CD86,POU2F3,ARID1A,RPS6KA1,PROX1,SS18L1,PRKD1,ZNF148,YTHDF1,HELZ2,MLXIPL,ITGA6,ELL3,ETV6,HOXD10,CREB1,TET1,ARNT2,DDX3X,RFXANK,ETV4,TBX1,TMEM106A,MECOM,ING4,LDB2,FGF23,POU2F1,WAS,RAC1,HIF1A,KDM5A,ZBTB18,APBB2,JUP,RAB27A,TLR6,MTOR,AR,CASZ1,PSEN1,HOXB5,HOXB7,HOXB9,FBXW11,BATF,SLC51B,PIAS1,RPS6KA5,SMAD2,DNM2,YY1,ARID3B,LYL1,TNIP1,ZNF521,ABLIM3,ADRB2,CCL2,YES1,RAI1,NOS2,ELF4,MRTFB,DOT1L,AP3D1,HCFC1,NOTCH1,MED16,IRF8,PLCG2,S1PR1,ENG,MTF2,NFAT5,DAB2IP,ARID4A,ZNF24,TRIM24,SOX17,KCNH2,ZNF850,ANKRD49,CDK13,SOX18,MAFF,TRIM62,FCER2,ZNF229,E2F3,BMP2,GLIS3,ZNF71,BMP6,FLI1	turquoise	EC_M6
GO:0031053	4.14E-02	0.991846188	4	12	primary miRNA processing	BP	5.16E-01	IL6,SMAD1,DDX3X,SMAD2	turquoise	EC_M6
GO:0007100	3.07E-02	0.994406621	4	13	mitotic centrosome separation	BP	5.16E-01	KIF11,CHEK1,NEK2,AURKA	darkgrey	EC_M8
GO:0140467	4.49E-02	0.986840678	6	20	integrated stress response signaling	BP	5.17E-01	NCK2,TMED2,EIF2S1,PTPN2,OMA1,HSPA5	darkgreen	EC_M4
GO:0051412	4.49E-02	0.988866004	5	15	response to corticosterone	BP	5.17E-01	ATP2B1,CCND1,NTRK3,CALM1,CDKN1A	darkgreen	EC_M4
GO:0006470	4.49E-02	0.969691272	41	228	protein dephosphorylation	BP	5.17E-01	PTP4A3,NUAK1,PTP4A1,ROCK2,FKBP1B,TGFB1,MTMR14,PPP1R12A,ACPP,SMPD1,PPP2CB,PPP2R2B,MTMR6,HSP90B1,PPP2R2A,PTPN11,PTPN22,CRY2,PPP3CC,SLC39A10,DAPP1,EYA2,PPP6R3,GSK3B,PPP1R11,PPP1R3D,PTPN6,HTT,PPP2R5E,PTPN21,CALM1,PPP4R4,PIN1,DUSP14,PDGFRB,ROCK1,PTPN2,PGP,FKBP1A,PTPN12,DUSP22	darkgreen	EC_M4
GO:0060078	4.50E-02	0.975887027	18	85	regulation of postsynaptic membrane potential	BP	5.17E-01	UNC13B,RIMS1,SHANK1,RAB3GAP1,P2RX4,APP,GLRB,CACNB3,CHRNA7,GSK3B,MPP2,KCNA1,CHRNE,NLGN2,STX1B,GRIA1,AKT1,INSYN1	darkgreen	EC_M4
GO:0006506	4.50E-02	0.983626212	8	31	GPI anchor biosynthetic process	BP	5.17E-01	TMEM246,PIGP,PIGZ,PIGL,DPM2,PIGK,DPM1,PIGB	darkgreen	EC_M4
GO:0150115	4.53E-02	0.974828883	20	95	cell-substrate junction organization	BP	5.18E-01	LRP1,VEGFA,DST,ROCK2,ACTG1,SORBS1,PTPN11,PHLDB2,CTTN,MMP14,CORO1C,IQGAP1,FN1,VCL,ACTN1,ROCK1,ITGA2,DAPK3,THY1,DUSP22	darkgreen	EC_M4
GO:0035336	4.53E-02	0.985005319	7	25	long-chain fatty-acyl-CoA metabolic process	BP	5.18E-01	ACSL6,DGAT2,FAR1,HACD2,ACSL4,ACOT7,HACD1	darkgreen	EC_M4

GO:0070885	4.54E-02	0.988700048	5	15	negative regulation of calcineurin-NFAT signaling cascade	BP	5.18E-01	DYRK2,PRNP,CHP1,ATP2B4,GSK3B	darkgreen	EC_M4
GO:0106057	4.54E-02	0.988700048	5	15	negative regulation of calcineurin-mediated signaling	BP	5.18E-01	DYRK2,PRNP,CHP1,ATP2B4,GSK3B	darkgreen	EC_M4
GO:0035308	4.55E-02	0.983486441	8	30	negative regulation of protein dephosphorylation	BP	5.18E-01	ROCK2,FKBP1B,CRY2,GSK3B,PPP1R11,PPP4R4,ROCK1,FKBP1A	darkgreen	EC_M4
GO:0060438	4.56E-02	0.988646179	5	15	trachea development	BP	5.18E-01	SRF,CTNNB1,LEF1,MAP2K1,EDA	darkgreen	EC_M4
GO:0060412	4.56E-02	0.98228036	9	34	ventricular septum morphogenesis	BP	5.18E-01	TGFBR1,ROBO1,HEY1,ACVR1,TGFB2,FGFR2,FZD2,BMPR1A,FGFRL1	darkgreen	EC_M4
GO:0046849	4.57E-02	0.977084711	15	69	bone remodeling	BP	5.18E-01	LEP,NCDN,TGFB1,CTNNB1,SPP1,NOTCH2,LRP5,PRKCA,TFRC,GPR137,SUCO,TGFB3,ATP6AP1,DEF8,SNX10	darkgreen	EC_M4
GO:0007218	4.57E-02	0.982107609	9	36	neuropeptide signaling pathway	BP	5.18E-01	GPR84,GLRB,RAPGEF2,CPE,NPPB,SSTR5,NMUR1,NXPH3,PROKR2	darkgreen	EC_M4
GO:0040020	3.08E-02	0.992626268	5	21	regulation of meiotic nuclear division	BP	5.18E-01	FBXO43,FBXO5,CDC20,TRIP13,RAD51AP1	darkgrey	EC_M8
GO:0051704	4.17E-02	0.967831266	91	709	multi-organism process	BP	5.18E-01	TSPAN8,SPAG1,QKI,PICK1,KIT,FIGNL1,CAV2,NOBOX,CREM,TUT4,IZUMO1,SPATA6,DNMT3A,NECTIN2,PSG3,ITGB4,EDNRB,SPTBN4,FCHSD2,DND1,GOLGA3,FCHSD1,MERTK,IGF1,KDM3A,SMAD1,STOX2,HEXB,ANGPT2,DHH,KMT2D,SFMBT1,TPCN1,STC1,SLC38A1,CDYL,IFIT1,EDN1,JAM3,GAS2,ETS1,HYAL2,TESMIN,LRMP,PLEKHA1,ZNF148,OAZ3,ZSCAN2,ARHGDIB,ACE,LIMK2,ELL3,ETV6,HOXD10,RNF2,SSTR1,DDX3X,IGFBP5,RAMP2,CDK16,BIRC3,USP42,KDM5A,ADIPOR2,CECR2,SPIN2A,MTOR,AR,FBXW11,ITGA3,PGF,CCDC42,PIAS1,DNM2,YY1,CYP1A1,JAG2,LGALS9,SPEF2,SPIRE1,NOTCH1,STXBP1,STRBP,ARID4A,CD46,SOX17,TDRP,ANKRD49,MAFF,TBC1D20,BST2	turquoise	EC_M6
GO:0001759	4.59E-02	0.986481695	6	20	organ induction	BP	5.18E-01	FGF2,CTNNB1,ROBO1,GDNF,WNT2,FGF10	darkgreen	EC_M4
GO:0071425	4.18E-02	0.987589538	6	24	hematopoietic stem cell proliferation	BP	5.19E-01	PIM1,CD34,ACE,ETV6,MECOM,ATXN1L	turquoise	EC_M6
GO:1901201	4.60E-02	0.988519892	5	15	regulation of extracellular matrix assembly	BP	5.19E-01	TGFB1,PHLDB2,DAG1,PHLDB1,HAS2	darkgreen	EC_M4
GO:0097479	4.60E-02	0.979918119	11	46	synaptic vesicle localization	BP	5.19E-01	AP3S1,KIF5B,PICALM,PCDH17,CTNNB1,PCLO,AP3B1,BRSK2,SLC2A4,NLGN2,BLOC1S2	darkgreen	EC_M4
GO:0050867	4.61E-02	0.968983202	40	226	positive regulation of cell activation	BP	5.19E-01	IL23A,HLA-DRA,FAM49B,LGALS1,LEP,DOCK8,NCK2,SOCS5,GLI3,GLI2,TGFB1,AXL,IL15RA,IRS2,HSPH1,VAMP8,PTPN11,AP3B1,PTPN22,ADA,RUNX1,SLC39A10,MMP14,PIK3CA,LEF1,TP53BP1,TFRC,PTPN6,IGFBP2,CD74,PDGFRB,AKT1,CD40LG,VAMP7,AP1G1,BCL10,THY1,CDKN1A,TSLP,FGF10	darkgreen	EC_M4
GO:0050806	4.19E-02	0.97747639	18	104	positive regulation of synaptic transmission	BP	5.20E-01	RIMS2,GRIK2,PLK2,SNCA,DTNBP1,SHANK2,ADCY1,YTHDF1,CREB1,ADORA2A,RGS14,CCL2,STXBP1,SLC8A3,RELN,SHANK3,STX3,CACNG5	turquoise	EC_M6
GO:0000422	3.10E-03	0.998843605	16	71	autophagy of mitochondrion	BP	5.20E-01	ATG12,SQSTM1,TSPO,CAMKK2,HTRA2,FIS1,ATG2A,PHB2,ATG9A,HDAC6,WDR45,ATP13A2,GABARAP,GBA,SREBF1,TAZ	cyan	EC_M2
GO:0061726	3.10E-03	0.998843605	16	71	mitochondrion disassembly	BP	5.20E-01	ATG12,SQSTM1,TSPO,CAMKK2,HTRA2,FIS1,ATG2A,PHB2,ATG9A,HDAC6,WDR45,ATP13A2,GABARAP,GBA,SREBF1,TAZ	cyan	EC_M2

GO:0051252	3.22E-03	0.997383019	328	2654	regulation of RNA metabolic process	BP	5.20E-01	NFATC1,ZNF236,TSHZ1,SMARCC2,STAT6,MAP3K5,CDK4,CITED2,MAML1,SQSTM1,IRAK3,OSR2,HSPA1B,EHMT2,ZBTB12,HDAC10,ATF6B,RXRB,RING1,ZHX2,ZBTB22,MYC,TOB2,SMAD5,EP300,KDM3B,ZC3H3,ZNF76,DDX17,NFIB,HSF1,CCND3,TRERF1,CDKN2A,HMOX1,WASL,KLF9,JAK2,ZBTB45,ZNF135,ZNF667,ZNF865,EFEMP1,ZNF692,ANXA4,AKNA,MED25,IL4I1,ING3,ARHGEF5,IRF3,LPIN1,FOXO3,KLF15,NOP53,MAML2,BICRA,ZC3HAV1,KMT2E,BBS7,MEIS3,TRIM13,IRF2BP1,SIX5,FOSB,ZNF746,NR2C2,ZNF862,TSC22D1,FASTK,ZNF575,NAA16,MLH1,ZNF574,WBP2,KMT2C,MYD88,CELF2,TADA3,RPS3,BRPF1,DYRK1B,EID2,ZNF219,MED29,SAMD4B,SIRT2,RPTOR,APBB3,NUP98,TRIM21,CYP1B1,HGF,APBB1,DEAF1,HOXC4,NCOA4,PCBP2,LIN37,TARBP2,KMT2B,RBCK1,RARG,SDR16C5,ZNF740,LMO2,POFUT1,URI1,PIK3R1,NCOA2,ATF1,KDM2B,ZFHX4,CAMKK2,SCAND1,SETD6,MCPH1,SSBP2,FNIP2,ARL2BP,SHOX2,NEK4,ZHX3,VDR,RBM12B,MMS19,LPIN3,FOXF2,F2RL1,SORBS3,HNF4A,TP53INP1,PIDD1,HR,TRIM45,FASTKD3,ELK4,LGR4,RBM15B,ICE1,SP140,TCF7L2,DIS3L2,PUM1,TXNIP,AIP,SOX5,ADNP,SF3B4,ZNF217,QARS,DTX3L,PARP14,ECM1,RCOR3,ARNT,PRMT2,RIPK3,GABPB2,PER2,TWIST2,VPS72,HDAC4,USF1,SMARCD2,POGZ,USP21,PATZ1,RNASEL,CCDC85B,MAGEF1,CDKN1B,TCEA3,KAT5,LRP6,YBX3,NFKBIA,NFE2L2,PHC1,HEXIM2,BATF2,EXOSC7,PCBD1,CHCHD10,MEN1,PHB2,TSC22D4,SAV1,TAF6,CTDSP1,EID1,SSBP4,RNF25,FGF7,NELFA,ZSCAN25,ZNF394,TNFRSF1A,STK16,STAT5A,KAT6B,CREG1,NR2F6,HDAC6,PQBP1,CAMTA2,TFE3,PELP1,ZNF12,WIZ,ZBTB25,RARA,FOXK1,AC090517.4,ZBTB17,GPS2,THRA,KLF3,MED	cyan	EC_M2
GO:0006644	3.24E-03	0.998050388	51	314	phospholipid metabolic process	BP	5.20E-01	DGKA,PIP4K2C,CHKB,ABHD16A,AGPAT1,GPAA1,PIGO,TTC7A,SLC44A1,LPIN1,SPHK2,TAMM41,PIP4P1,GPCPD1,PLPP5,PTDSS2,PIK3R1,PLBD2,GPAT3,PIGT,PLA2G4B,OSBPL5,GPAM,CHKA,ABHD4,MTMR11,PIGM,IDH1,PHB2,ABHD12B,SMPD4,PLA2G16,ABHD8,PLSCR3,PIGH,PIP4K2B,DPM3,AC138894.1,MTMR4,MPPE1,MTMR1,PNPLA7,TAZ,PIGQ,INPP5E,PLPPR3,VAC14,LCAT,ABCA2,PLPP3,DNAJC19	cyan	EC_M2
GO:0016575	3.27E-03	0.998775283	16	71	histone deacetylation	BP	5.20E-01	HDAC10,TRERF1,SPHK2,TADA3,SIRT2,SDR16C5,ELK4,RCOR3,PER2,HDAC4,HDAC6,CTBP1,PER1,ZBTB7B,SIN3A,SREBF1	cyan	EC_M2
GO:0046486	3.31E-03	0.998004643	51	317	glycerolipid metabolic process	BP	5.20E-01	DGKA,PIP4K2C,CHKB,ABHD16A,AGPAT1,GPAA1,PIGO,TTC7A,SLC44A1,LPIN1,LIPE,TAMM41,PIP4P1,GPCPD1,DDHD2,PTDSS2,PIK3R1,GPAT3,LPIN3,PIGT,PLA2G4B,OSBPL5,GPAM,CHKA,ABHD4,GPX1,MTMR11,PIGM,PHB2,ABHD12B,SMPD4,PLA2G16,ABHD8,CTDNEP1,PLSCR3,PIGH,PIP4K2B,DPM3,AC138894.1,MTMR4,SREBF1,MPPE1,MTMR1,PNPLA7,TAZ,LMF1,PIGQ,INPP5E,VAC14,LCAT,DNAJC19	cyan	EC_M2
GO:0032933	3.31E-03	0.999506027	6	15	SREBP signaling pathway	BP	5.20E-01	EIF2A,ERLIN1,SCAP,ARHGEF10L,ZBTB7B,SREBF1	cyan	EC_M2
GO:0071501	3.31E-03	0.999506027	6	15	cellular response to sterol depletion	BP	5.20E-01	EIF2A,ERLIN1,SCAP,ARHGEF10L,ZBTB7B,SREBF1	cyan	EC_M2
GO:0009128	3.34E-03	0.999644657	5	10	purine nucleoside monophosphate catabolic process	BP	5.20E-01	DNPH1,PNP,XDH,NT5C2,NT5C	cyan	EC_M2
GO:0090263	4.62E-02	0.974639519	19	92	positive regulation of canonical Wnt signaling pathway	BP	5.20E-01	RSPO3,VCP,PLEKHA4,FGF2,TGFB1,ZEB2,ILK,CAPRIN2,FGFR2,FZD9,ATP6AP2,COL1A1,GSKIP,PIN1,DAPK3,CSNK1G2,WLS,EDA,FGF10	darkgreen	EC_M4
GO:0044060	4.63E-02	0.986320462	6	20	regulation of endocrine process	BP	5.20E-01	LEP,INHBA,C1QTNF1,PTPN11,CRY2,LINC02210-CRHR1	darkgreen	EC_M4
GO:2000479	4.63E-02	0.988410017	5	15	regulation of cAMP-dependent protein kinase activity	BP	5.20E-01	RAPGEF2,ATP2B4,PRKAR1A,SPATC1L,HTT	darkgreen	EC_M4
GO:0006094	4.63E-02	0.978058863	13	58	gluconeogenesis	BP	5.20E-01	PPARA,LEP,DGAT2,KAT2B,SELENOS,ATF3,TPI1,PPARGC1A,PTPN2,PGP,FAM3A,RANBP2,NNMT	darkgreen	EC_M4
GO:0043536	4.64E-02	0.978788034	12	53	positive regulation of blood vessel endothelial cell migration	BP	5.20E-01	ANGPT1,NUS1,VEGFA,FGF2,TGFB1,P2RX4,PRKCA,HSPB1,AKT3,AKT1,TMSB4X,PIK3C2A	darkgreen	EC_M4
GO:0002115	4.65E-02	0.988361079	5	15	store-operated calcium entry	BP	5.20E-01	STIM1,HOMER1,CASQ1,STIM2,SARAF	darkgreen	EC_M4
GO:0061003	4.65E-02	0.986400195	6	19	positive regulation of dendritic spine morphogenesis	BP	5.20E-01	ACTR2,DHX36,OPA1,DNM1L,CAPRIN2,PAFAH1B1	darkgreen	EC_M4
GO:0034763	4.65E-02	0.974894478	18	88	negative regulation of transmembrane transport	BP	5.20E-01	UBQLN1,LEP,FKBP1B,AKT2,IRS2,ARL6IP5,REM1,CACNB3,PKD2,SELENOS,ISCU,PEA15,LINC02210-CRHR1,CALM1,AKT1,MIR24-1,CBARP,GEM	darkgreen	EC_M4

GO:0042180	4.66E-02	0.971197466	28	150	cellular ketone metabolic process	BP	5.20E-01	HSD17B6,PPARA,PPARD,REST,MFSD2A,DKKL1,SLC7A11,DGAT2,AKT2,IRS2,DKK3,RDH10,ATP2B4,CPT1A,COQ10B,WDTC1,COMT,TPI1,HSD17B1,HSD17B10,STARD3,PPARGC1A,CYP46A1,CD74,AKT1,HAGH,PARK7,GLO1	darkgreen	EC_M4
GO:1903975	4.67E-02	0.990678188	4	11	regulation of glial cell migration	BP	5.21E-01	LRP1,P2RX4,CX3CL1,IDH2	darkgreen	EC_M4
GO:0046688	4.68E-02	0.984290998	7	26	response to copper ion	BP	5.21E-01	ATP7B,PRNP,MAP1LC3A,APP,LOXL2,BACE1,PARK7	darkgreen	EC_M4
GO:0010822	4.70E-02	0.977676332	13	59	positive regulation of mitochondrion organization	BP	5.21E-01	VPS35,MGARP,DCN,SLC25A4,OPA1,DNM1L,GSK3B,FAM162A,HTT,PPARGC1A,MIEF2,PARK7,BNIP3	darkgreen	EC_M4
GO:0019722	4.70E-02	0.970896711	28	147	calcium-mediated signaling	BP	5.21E-01	DYRK2,FKBP1B,PRNP,P2RX4,ATP2A2,PPP3CC,ADA,CHP1,ATP2B4,ADGRB2,HTR2B,GSK3B,CMKLR1,CASQ1,MAPT,HTT,CACNA1C,ADGRL1,TMEM100,CALM1,PRKAA1,BCAP31,CDH13,DMD,TMEM38B,NMUR1,TMEM38A,CCR6	darkgreen	EC_M4
GO:0009309	4.71E-02	0.984178877	7	26	amine biosynthetic process	BP	5.21E-01	AZIN1,AZIN2,TGFB2,SMS,AGMAT,PARK7,NNMT	darkgreen	EC_M4
GO:0042401	4.71E-02	0.984178877	7	26	cellular biogenic amine biosynthetic process	BP	5.21E-01	AZIN1,AZIN2,TGFB2,SMS,AGMAT,PARK7,NNMT	darkgreen	EC_M4
GO:0048286	4.72E-02	0.981481141	9	35	lung alveolus development	BP	5.21E-01	SLC7A11,ADA,MAN1A2,FGFR2,TGFB3,MYOCD,TMEM38B,HOPX,FGF10	darkgreen	EC_M4
GO:0002204	3.12E-02	0.989164963	8	40	somatic recombination of immunoglobulin genes involved in immune response	BP	5.22E-01	MSH2,PAXIP1,RIF1,NBN,NSD2,EXO1,THOC1,RNF8	darkgrey	EC_M8
GO:0002208	3.12E-02	0.989164963	8	40	somatic diversification of immunoglobulins involved in immune response	BP	5.22E-01	MSH2,PAXIP1,RIF1,NBN,NSD2,EXO1,THOC1,RNF8	darkgrey	EC_M8
GO:0045190	3.12E-02	0.989164963	8	40	isotype switching	BP	5.22E-01	MSH2,PAXIP1,RIF1,NBN,NSD2,EXO1,THOC1,RNF8	darkgrey	EC_M8
GO:0046822	4.73E-02	0.973945994	19	94	regulation of nucleocytoplasmic transport	BP	5.22E-01	ANGPT1,LEP,RAB23,MDFIC,GLI3,IWS1,TGFB1,PPP1R12A,UFM1,HDAC3,RIOK2,PTPN11,CHP1,GSK3B,SUPT6H,FLNA,PARK7,TXN,ZPR1	darkgreen	EC_M4
GO:1904869	4.73E-02	0.990509675	4	11	regulation of protein localization to Cajal body	BP	5.22E-01	TCP1,CCT8,DKC1,LARP7	darkgreen	EC_M4
GO:1904871	4.73E-02	0.990509675	4	11	positive regulation of protein localization to Cajal body	BP	5.22E-01	TCP1,CCT8,DKC1,LARP7	darkgreen	EC_M4
GO:0060041	4.74E-02	0.972681752	22	108	retina development in camera-type eye	BP	5.22E-01	AHI1,RORB,MFSD2A,SMARCD3,ATP2B1,SLC4A5,OPA1,ATP2B4,TGFB2,LRP5,CLIC4,OBSL1,ROM1,CLN8,MED1,TTC8,SMARCA4,PDGFRB,GNB1,NPHP4,SERPINF1,THY1	darkgreen	EC_M4
GO:0072676	4.21E-02	0.980011208	13	72	lymphocyte migration	BP	5.22E-01	PIK3CG,LRCH1,GATA3,RET,CXCL10,CD200,F11R,APOD,ITGA4,STK10,CCL2,S1PR1,MSMP	turquoise	EC_M6
GO:0014068	4.21E-02	0.980133054	13	66	positive regulation of phosphatidylinositol 3-kinase signaling	BP	5.22E-01	PTK2,PDGFB,TEK,KIT,KDR,SEMA4D,PRR5L,IGF1,NCF1,HCLS1,FGR,SELP,RELN	turquoise	EC_M6
GO:0046931	3.12E-02	0.992637805	5	19	pore complex assembly	BP	5.22E-01	NUP107,NUP205,NUP93,CCT3,NDC1	darkgrey	EC_M8
GO:0090481	4.74E-02	0.990483809	4	11	pyrimidine nucleotide-sugar transmembrane transport	BP	5.22E-01	SLC35A1,SLC35A5,SLC35B1,SLC35A3	darkgreen	EC_M4
GO:0007263	4.22E-02	0.989278666	5	18	nitric oxide mediated signal transduction	BP	5.23E-01	NOS3,CD36,GUCY1A1,GUCY1B1,NOS2	turquoise	EC_M6
GO:0045637	4.23E-02	0.974986822	24	152	regulation of myeloid cell differentiation	BP	5.23E-01	TLR4,TAL1,GATA2,JAG1,SFRP1,TMEM178A,MEF2C,STAT1,ETS1,HCLS1,INPP5D,FES,CREB1,B2M,LTF,HIF1A,ITPKB,MTOR,HOXB8,GPR68,RCOR1,ISG15,CA2,HIST1H4D	turquoise	EC_M6
GO:0045063	4.23E-02	0.991431365	4	13	T-helper 1 cell differentiation	BP	5.23E-01	HLX,CRACR2A,MTOR,SEMA4A	turquoise	EC_M6
GO:0045687	4.23E-02	0.985766803	7	31	positive regulation of glial cell differentiation	BP	5.23E-01	CXCR4,TLR2,HDAC1,TNFRSF1B,MTOR,NOTCH1,BMP2	turquoise	EC_M6

GO:1990874	4.76E-02	0.978118231	12	54	vascular associated smooth muscle cell proliferation	BP	5.23E-01	FAM19A5,FGF2,PAK1,MMP2,BMPR1A,TPM1,TGFB3,CNN1,MYOCD,GNAI3,CDKN1A,PRKG1	darkgreen	EC_M4
GO:0070141	4.77E-02	0.990419905	4	11	response to UV-A	BP	5.23E-01	MMP2,CCND1,TIMP1,AKT1	darkgreen	EC_M4
GO:0009132	4.78E-02	0.973631519	19	94	nucleoside diphosphate metabolic process	BP	5.23E-01	PPARA,CMPK1,ENTPD6,PFKP,NUDT16,APP,PFKM,PGAM4,ENTPD4,NUDT18,ZBTB20,TPI1,PPARGC1A,PKM,AK7,PRKAA1,ENO1,SLC2A6,NME6	darkgreen	EC_M4
GO:2001259	4.78E-02	0.978082913	12	52	positive regulation of cation channel activity	BP	5.23E-01	KIF5B,STIM1,ABCC8,CACNB3,PKD2,CASQ1,HTT,KCNA1,HAP1,STIM2,CALM1,TMSB4X	darkgreen	EC_M4
GO:0033599	4.78E-02	0.987920361	5	15	regulation of mammary gland epithelial cell proliferation	BP	5.23E-01	RTN4,ZNF703,ROBO1,KDM5B,CCND1	darkgreen	EC_M4
GO:0051447	3.14E-02	0.994139663	4	14	negative regulation of meiotic cell cycle	BP	5.24E-01	FBXO43,FBXO5,TTK,TRIP13	darkgrey	EC_M8
GO:1904659	4.80E-02	0.974579357	17	80	glucose transmembrane transport	BP	5.24E-01	PPARD,LEP,MFSD4B,AKT2,OSBPL8,IRS2,SORBS1,PTPN11,SELENOS,SLC2A10,PEA15,CLTCL1,PRKCI,SLC2A4,AKT1,SLC2A6,SLC2A8	darkgreen	EC_M4
GO:0099565	4.80E-02	0.975118062	16	74	chemical synaptic transmission, postsynaptic	BP	5.24E-01	UNC13B,RIMS1,SHANK1,RAB3GAP1,P2RX4,APP,GLRB,CACNB3,CHRNA7,GSK3B,MPP2,CHRNE,NLGN2,STX1B,AKT1,INSYN1	darkgreen	EC_M4
GO:0071248	4.83E-02	0.970357841	27	141	cellular response to metal ion	BP	5.25E-01	SLC12A2,SCN5A,LIG4,PRNP,SMPD1,MAP1LC3A,RYR3,P2RX4,APP,PKD2,NCSTN,IQGAP1,CLIC4,BACE1,TFR2,HSD17B1,KCNA1,EIF2S1,PPARGC1A,SYT11,AKT1,PRKAA1,HSPA5,FSTL3,SERPINF1,BNIP3,CACYBP	darkgreen	EC_M4
GO:0032970	4.83E-02	0.965043312	58	337	regulation of actin filament-based process	BP	5.25E-01	LRP1,ABRACL,FAM49B,MYH9,NCK2,TGFBR1,SHANK1,ACTR2,ROCK2,MYLK3,PAK1,ACTR3,SCN5A,PLEKHG2,ACTG1,CDC42EP3,ILK,CXCL12,PFN2,RASA1,ATP2A2,CX3CL1,TWF1,MAGEL2,PHLDB2,TJP1,NOTCH2,CTTN,PKP2,LMOD1,PIK3CA,NTRK3,SYNPO2,IQGAP1,ARPC5,NCKAP1,ARPC2,SYNPO2L,CAPZB,WDR1,CACNA1C,CGNL1,TPM1,TGFB3,GPR65,TTC8,PDGFRB,ROCK1,FLII,DAPK3,MYO1C,FLNA,SPTAN1,TMSB4X,DSTN,HCN4,COTL1,CFL2	darkgreen	EC_M4
GO:0002532	4.26E-02	0.981253667	11	59	production of molecular mediator involved in inflammatory response	BP	5.25E-01	SYK,IL6,TLR4,CD36,SNAP23,NCF1,APOD,HIF1A,TLR6,NOS2,GBP5	turquoise	EC_M6
GO:0010642	4.84E-02	0.990224514	4	11	negative regulation of platelet-derived growth factor receptor signaling pathway	BP	5.26E-01	LRP1,MYOCD,PTPN2,PTPN12	darkgreen	EC_M4
GO:0055076	4.86E-02	0.972233154	21	107	transition metal ion homeostasis	BP	5.27E-01	SLC39A7,SLC39A4,SLC30A9,SLC31A1,ATP7B,PICALM,PRNP,APP,AP3B1,SLC39A10,ISCU,HJV,TFRC,GLRX3,TFR2,FBXL5,SLC39A6,ATP6AP1,SLC30A7,ATP6V0D1,SLC22A17	darkgreen	EC_M4
GO:0034219	4.86E-02	0.973676443	18	86	carbohydrate transmembrane transport	BP	5.27E-01	PPARD,LEP,SLC17A5,MFSD4B,AKT2,OSBPL8,IRS2,SORBS1,PTPN11,SELENOS,SLC2A10,PEA15,CLTCL1,PRKCI,SLC2A4,AKT1,SLC2A6,SLC2A8	darkgreen	EC_M4
GO:0032528	4.86E-02	0.985456558	6	20	microvillus organization	BP	5.27E-01	ATP8B1,RAPGEF2,PTPN11,TNIK,PLD1,GLDN	darkgreen	EC_M4
GO:0071867	4.87E-02	0.975366172	15	69	response to monoamine	BP	5.27E-01	VPS35,HTR5A,APLP1,APP,ADCY6,ATP2B4,HTR2B,GSK3B,ADCY5,NCSTN,PPARGC1A,GNAL,GNB1,FLNA,GNAI3	darkgreen	EC_M4
GO:0071869	4.87E-02	0.975366172	15	69	response to catecholamine	BP	5.27E-01	VPS35,HTR5A,APLP1,APP,ADCY6,ATP2B4,HTR2B,GSK3B,ADCY5,NCSTN,PPARGC1A,GNAL,GNB1,FLNA,GNAI3	darkgreen	EC_M4
GO:0001890	4.87E-02	0.971370691	23	117	placenta development	BP	5.27E-01	RSPO3,PPARD,LEP,SPINT2,DCN,TMED2,HEY1,ADA,SPP1,PKD2,NOTCH2,LEF1,BMP7,FGFR2,VASH2,HTRA1,MED1,MAP2K1,AKT1,PLAC1,PKD1,BSG,WNT2	darkgreen	EC_M4
GO:0010508	4.89E-02	0.972063995	21	105	positive regulation of autophagy	BP	5.29E-01	MAP3K7,SESN3,RAB3GAP1,DCN,TP53INP2,SLC25A4,MTDH,GSK3B,ATF6,PAFAH1B2,HTT,SPTLC2,ROCK1,PRKAA1,IKBKG,PARK7,GPSM1,GNAI3,BNIP3,PIK3C2A,ORMDL3	darkgreen	EC_M4
GO:0007163	4.89E-02	0.967898985	35	189	establishment or maintenance of cell polarity	BP	5.29E-01	FAM49B,CTNNA1,MYH9,DOCK8,DST,ACTR2,ROCK2,ITGB1,LIN7B,PDCD6IP,PAK1,ACTR3,SPINT2,ILK,FAT1,MAP1B,BRSK2,FEZ1,GSK3B,SNX27,CLIC4,ARPC5,FAM89B,HTT,PRKCI,WDR1,MSN,DOCK2,TTC8,ROCK1,PAFAH1B1,PKD1,PDLIM1,ARF4,FGF10	darkgreen	EC_M4
GO:0006473	3.43E-03	0.998248802	30	168	protein acetylation	BP	5.29E-01	BAG6,JADE2,EP300,HINT2,ING3,KLF15,SPHK2,NAA16,WBP2,TADA3,BRPF1,APBB1,TAF10,SDR16C5,KAT5,POR,EID1,KAT6B,MED24,CTBP1,KAT8,KAT7,PER1,SGF29,NUPR1,CRTC2,SIN3A,CREBBP,WDR5,GTF3C4	cyan	EC_M2

GO:0031000	4.90E-02	0.987526538	5	15	response to caffeine	BP	5.29E-01	RYR3,PPARGC1A,PRKAA1,TMEM38B,TMEM38A	darkgreen	EC_M4
GO:0031641	4.30E-02	0.984232178	8	36	regulation of myelination	BP	5.29E-01	TNFRSF21,MTMR2,CST7,CTSC,EIF2AK3,DLG1,TNFRSF1B,MTOR	turquoise	EC_M6
GO:0034605	4.91E-02	0.977338245	12	53	cellular response to heat	BP	5.30E-01	VCP,PDCL3,CLPB,ATP2A2,DHX36,GSK3B,RBBP7,MAPT,EIF2S1,IRAK1,HSBP1,CDKN1A	darkgreen	EC_M4
GO:0010001	4.31E-02	0.973622498	27	173	glial cell differentiation	BP	5.30E-01	IFNGR1,PICK1,TNFRSF21,IL6,TLR4,MYCN,TAL1,ITGB4,CXCR4,MMP24,TLR2,SOX13,TSPAN2,SLC45A3,HDAC1,ADORA2A,MPP5,TNFRSF1B,MTOR,PSEN1,EIF2B2,B4GALT6,SH3TC2,NOTCH1,SLC8A3,RELN,BMP2	turquoise	EC_M6
GO:1904753	4.92E-02	0.990016749	4	11	negative regulation of vascular associated smooth muscle cell migration	BP	5.30E-01	FAM19A5,TPM1,MYOCD,PRKG1	darkgreen	EC_M4
GO:0014911	4.31E-02	0.984211391	8	35	positive regulation of smooth muscle cell migration	BP	5.30E-01	PDGFB,DOCK4,TLR4,NRP1,P2RY6,IGF1,SEMA6D,IGFBP5	turquoise	EC_M6
GO:0043393	4.93E-02	0.968781141	30	162	regulation of protein binding	BP	5.31E-01	LRP1,ANGPT1,PPARA,TGFBR1,MAPRE3,SMARCD3,DZIP1,PPP2CB,ATP2A2,APP,RAPGEF2,AMFR,AKTIP,CBLB,CAPRIN2,GSK3B,LRPAP1,GREM2,CRMP1,NES,TEX14,PIN1,ROCK1,AKT1,PKD1,PARK7,FKBP1A,LOX,CDKN1A,HOPX	darkgreen	EC_M4
GO:0014897	4.35E-02	0.97865016	14	77	striated muscle hypertrophy	BP	5.33E-01	IGF1,EDN1,JARID2,PDE9A,PPP3CA,MEF2A,INPP5F,IGFBP5,TNFRSF1B,MTOR,YY1,NOTCH1,MIR23A,RGS4	turquoise	EC_M6
GO:0030168	4.35E-02	0.976985271	17	96	platelet activation	BP	5.33E-01	FZD6,PDGFB,TEC,SYK,IL6,TLR4,PIK3CG,NOS3,MERTK,DGKZ,LCK,F11R,SELP,VWF,PEAR1,PLCG2,STXBP1	turquoise	EC_M6
GO:0043550	4.35E-02	0.980174158	12	58	regulation of lipid kinase activity	BP	5.33E-01	PTK2,PDGFB,TEK,KIT,DGKZ,FGR,PPP2R5A,PRKD1,TNFAIP8L3,CD19,PIK3R5,DAB2IP	turquoise	EC_M6
GO:0048706	4.95E-02	0.971647143	21	105	embryonic skeletal system development	BP	5.33E-01	RUNX2,TGFBR1,GLI3,MEGF8,CTNNB1,SULF1,RDH10,HYAL1,SLC2A10,SATB2,MMP14,BMP7,FGFR2,PRRX1,TULP3,SIX4,COL1A1,TGFB3,COL11A1,SLC39A3,CHST11	darkgreen	EC_M4
GO:0030073	4.96E-02	0.969153027	28	149	insulin secretion	BP	5.33E-01	LRP1,PPARD,LEP,CLOCK,REST,FKBP1B,KIF5B,IRS2,PCLO,OSBP,SELENOT,ABCC8,PTPN11,PFKM,BRSK2,DYNLL1,CPT1A,LRP5,HADH,ADCY5,SIDT2,PTPRN,NLGN2,SYTL4,ACSL4,MIDN,SSTR5,PARK7	darkgreen	EC_M4
GO:0001913	4.35E-02	0.988641479	5	20	T cell mediated cytotoxicity	BP	5.33E-01	CTSC,NECTIN2,B2M,RAB27A,IL7R	turquoise	EC_M6
GO:0044872	4.36E-02	0.991092213	4	13	lipoprotein localization	BP	5.33E-01	PPARG,CD36,MTTP,UNC119	turquoise	EC_M6
GO:0048278	4.36E-02	0.981696836	10	50	vesicle docking	BP	5.33E-01	STX11,TSNARE1,CAV2,RAB3B,RALB,NDRG4,RAB3D,STXBP1,STX3,VAMP3	turquoise	EC_M6
GO:0051604	4.96E-02	0.966378894	40	230	protein maturation	BP	5.33E-01	SEC11C,MYH9,ADAMTS3,GLI3,FKBP1B,SERPINH1,KAT2B,PRNP,CPXM1,CIAO1,PCSK1,CPE,CHAC1,CPN1,RUNX1,CASP7,MMP14,HJV,GALNT2,NCSTN,SEC11A,BACE1,PCSK7,GLRX3,PRDX4,SERPINE1,ATP6AP2,TFR2,COMP,ERO1A,ECE1,TSPAN17,TMEM98,OMA1,ENO1,F3,GLG1,CIAO2B,FKBP1A,SPCS3	darkgreen	EC_M4
GO:0070233	4.38E-02	0.991045008	4	13	negative regulation of T cell apoptotic process	BP	5.33E-01	HIF1A,ARG2,EFNA1,IL7R	turquoise	EC_M6
GO:0031629	4.38E-02	0.988640418	5	19	synaptic vesicle fusion to presynaptic active zone membrane	BP	5.33E-01	STX11,SNAP23,SNAP29,STXBP1,STX3	turquoise	EC_M6
GO:0099500	4.38E-02	0.988640418	5	19	vesicle fusion to plasma membrane	BP	5.33E-01	STX11,SNAP23,SNAP29,STXBP1,STX3	turquoise	EC_M6
GO:0086019	4.98E-02	0.983277888	7	24	cell-cell signaling involved in cardiac conduction	BP	5.34E-01	SCN5A,CACNA1D,PKP2,CACNA1C,CACNA1G,FLNA,HCN4	darkgreen	EC_M4
GO:0035988	4.98E-02	0.985002261	6	20	chondrocyte proliferation	BP	5.34E-01	MMP14,LEF1,GOLGB1,COMP,FGFR3,PBXIP1	darkgreen	EC_M4
GO:2001243	4.98E-02	0.973396937	17	83	negative regulation of intrinsic apoptotic signaling pathway	BP	5.34E-01	RRM2B,CREB3,TPT1,CXCL12,OPA1,SELENOS,HYOU1,HSPB1,SYVN1,VDAC2,ARHGEF2,TAF9B,CD74,AKT1,PARK7,ENO1,PTTG1IP	darkgreen	EC_M4
GO:0036336	4.40E-02	0.988687826	5	18	dendritic cell migration	BP	5.35E-01	TRPM4,PIK3CG,CXCR4,ANO6,LGALS9	turquoise	EC_M6

GO:0050804	5.01E-02	0.963977367	55	317	modulation of chemical synaptic transmission	BP	5.35E-01	RNF19A,SLC12A2,ADGRB1,SRF,RIMS1,SHANK1,NCDN,SLC7A11,ITGB1,KIF5B,PCDH17,RAB3GAP1,PRNP,MAP1B,ATP2A2,DLGAP4,APP,RAPGEF2,CX3CL1,CACNB3,HOMER1,VPS18,CHRNA7,EIF4E,DNM1L,GSK3B,KRAS,NCSTN,SYAP1,BACE1,MAPT,EIF4EBP2,TNR,MPP2,ATAD1,ERC1,HAP1,IQSEC2,NLGN2,STX1B,SYT11,RAB11A,CALM1,GRIA1,NPTN,CYP46A1,BEGAIN,ABR,TPRG1L,NTNG2,JPH3,YWHAH,KCNMB4,MAP1A,LRRTM2	darkgreen	EC_M4
GO:0032703	4.42E-02	0.98843878	5	20	negative regulation of interleukin-2 production	BP	5.35E-01	TNFAIP3,GATA3,LAPTM5,CD34,HOMER3	turquoise	EC_M6
GO:0034204	4.42E-02	0.982665529	9	39	lipid translocation	BP	5.35E-01	ATP8A1,ATP11A,P2RX7,ANO6,ANO3,ABCG1,ATP9A,ATP8B4,TMEM30B	turquoise	EC_M6
GO:0097191	4.43E-02	0.973046122	26	173	extrinsic apoptotic signaling pathway	BP	5.35E-01	TNFAIP3,TRAF1,NRP1,NOS3,TMC8,SFRP1,RET,IL1A,IGF1,SH3RF1,IL7,P2RX7,MADD,HIPK1,ITPRIP,HYAL2,CFLAR,ITGA6,PAK2,DDX3X,BID,TNFRSF1B,AR,TRAF2,DAB2IP,PMAIP1	turquoise	EC_M6
GO:0071295	4.44E-02	0.985034545	7	29	cellular response to vitamin	BP	5.35E-01	PIM1,IL15,SFRP1,MN1,FES,FGF23,TRIM24	turquoise	EC_M6
GO:0050999	5.02E-02	0.981348053	8	31	regulation of nitric-oxide synthase activity	BP	5.36E-01	NUS1,LEP,ATP2B4,HTR2B,KRAS,CALM1,AKT1,NPR3	darkgreen	EC_M4
GO:0006865	5.02E-02	0.971642686	20	99	amino acid transport	BP	5.36E-01	SLC12A2,SLC22A4,LEP,SLC17A5,SLC7A11,ITGB1,SLC36A4,RAB3GAP1,SLC38A10,ARL6IP5,SERINC3,SLC3A2,SLC6A13,PRAF2,CLN8,SFXN1,SLC25A44,SLC7A3,SLC36A1,XK	darkgreen	EC_M4
GO:1901998	5.03E-02	0.981265888	8	31	toxin transport	BP	5.36E-01	TCP1,GGA1,COPB2,CCT8,SCFD1,SLC29A4,HSPA5,BNIP3	darkgreen	EC_M4
GO:1901021	5.04E-02	0.979895769	9	36	positive regulation of calcium ion transmembrane transporter activity	BP	5.36E-01	STIM1,CACNB3,PKD2,CASQ1,HTT,HAP1,STIM2,CALM1,VMP1	darkgreen	EC_M4
GO:2000831	5.05E-02	0.989667269	4	11	regulation of steroid hormone secretion	BP	5.36E-01	C1QTNF1,PTPN11,CRY2,LINC02210-CRHR1	darkgreen	EC_M4
GO:0042698	5.05E-02	0.977609646	11	46	ovulation cycle	BP	5.36E-01	LEP,INHBA,AXL,PTX3,TGFB2,SGPL1,PTPRN,TGFB3,HSPA5,SERPINF1,HAS2	darkgreen	EC_M4
GO:0010518	5.05E-02	0.978644579	10	41	positive regulation of phospholipase activity	BP	5.36E-01	GNAQ,PLAA,FGF2,ARL1,HTR2B,FGFR2,FGFR3,PDGFRB,NMUR1,ARF4	darkgreen	EC_M4
GO:1900182	5.07E-02	0.973501888	16	76	positive regulation of protein localization to nucleus	BP	5.36E-01	TCP1,LEP,GLI3,TGFB1,HDAC3,PINX1,CCT8,DKC1,LARP7,TFRC,NGFR,AKT1,FLNA,PARK7,ZPR1,ORMDL3	darkgreen	EC_M4
GO:0048008	4.45E-02	0.981316764	10	48	platelet-derived growth factor receptor signaling pathway	BP	5.36E-01	PDGFB,NRP1,HIP1R,NDRG4,TIPARP,SNCA,PLEKHA1,APOD,BCR,RGS14	turquoise	EC_M6
GO:2000679	5.08E-02	0.986730433	5	16	positive regulation of transcription regulatory region DNA binding	BP	5.36E-01	RB1,TGFB1,CTNNB1,DDRGK1,PARK7	darkgreen	EC_M4
GO:0050775	5.09E-02	0.979715072	9	35	positive regulation of dendrite morphogenesis	BP	5.37E-01	RAB21,ACTR2,ILK,DHX36,OPA1,DNM1L,CAPRIN2,OBSL1,PAFAH1B1	darkgreen	EC_M4
GO:0000045	5.10E-02	0.972134012	18	88	autophagosome assembly	BP	5.37E-01	UBQLN1,RAB23,RAB7A,RAB3GAP1,TMEM41B,MAP1LC3A,TP53INP2,ATP2A2,ATG13,SEC22B,FEZ1,WIPI1,SYNPO2,SCFD1,SMURF1,WIPI2,VMP1,GABARAPL2	darkgreen	EC_M4
GO:0030148	5.11E-02	0.972613677	17	83	sphingolipid biosynthetic process	BP	5.37E-01	B4GALNT1,LARGE1,ACER3,SMPD1,ST8SIA4,CERS5,B3GALT1,HACD2,SGPL1,SGMS1,CLN8,SPTLC2,PRKAA1,CSNK1G2,HACD1,PEMT,ORMDL3	darkgreen	EC_M4
GO:0010563	5.12E-02	0.96251067	60	356	negative regulation of phosphorus metabolic process	BP	5.37E-01	LRP1,ANGPT1,PPARA,SH3GL2,GNAQ,CDKN2B,NCK2,SOCS5,SH3BP5L,ROCK2,INHBA,FKBP1B,RB1,TGFB1,GPD1L,KAT2B,IRS2,PRNP,SMPD1,ILK,TMED2,PTPN22,TSG101,CRY2,CHP1,DBNDD2,CBLB,DYNLL1,TGFB2,LRP5,PRKAR1A,GSK3B,CORO1C,NTRK3,BMP7,PPP1R11,HSPB1,MAPT,DNAJC10,PTPN6,FKBP8,PPP1R36,PPARGC1A,SRCIN1,CALM1,PPP4R4,GSKIP,MYOCD,ROCK1,AKT1,PTPN2,MIDN,PARK7,MIR24-1,DMD,FKBP1A,THY1,CDKN1A,DUSP22,PMEPA1	darkgreen	EC_M4

GO:0045936	5.12E-02	0.96251067	60	356	negative regulation of phosphate metabolic process	BP	5.37E-01	LRP1,ANGPT1,PPARA,SH3GL2,GNAQ,CDKN2B,NCK2,SOCS5,SH3BP5L,ROCK2,INHBA,FKBP1B,RB1,TGFB1,GPD1L,KAT2B,IRS2,PRNP,SMPD1,ILK,TMED2,PTPN22,TSG101,CRY2,CHP1,DBNDD2,CBLB,DYNLL1,TGFB2,LRP5,PRKAR1A,GSK3B,CORO1C,NTRK3,BMP7,PPP1R11,HSPB1,MAPT,DNAJC10,PTPN6,FKBP8,PPP1R36,PPARGC1A,SRCIN1,CALM1,PPP4R4,GSKIP,MYOCD,ROCK1,AKT1,PTPN2,MIDN,PARK7,MIR24-1,DMD,FKBP1A,THY1,CDKN1A,DUSP22,PMEPA1	darkgreen	EC_M4
GO:0070593	5.13E-02	0.986764855	5	15	dendrite self-avoidance	BP	5.38E-01	PALLD,NPTN,BSG,NEXN,VSTM2L	darkgreen	EC_M4
GO:1900017	4.47E-02	0.988477211	5	18	positive regulation of cytokine production involved in inflammatory response	BP	5.38E-01	IL6,TLR4,HIF1A,TLR6,GBP5	turquoise	EC_M6
GO:0051963	5.15E-02	0.972471864	17	79	regulation of synapse assembly	BP	5.39E-01	ADGRB1,MDGA1,TPBG,LRRC4B,VPS35,FARP1,APP,AMIGO2,PDLIM5,NTRK3,SIX4,NLGN2,NTNG2,LRRC24,UBE2V2,LHFPL4,LRRTM2	darkgreen	EC_M4
GO:0008284	5.16E-02	0.959051077	108	681	positive regulation of cell population proliferation	BP	5.39E-01	IL23A,PPARD,LEP,VEGFA,RUNX2,NCK2,PDCL3,TGFBR1,COPS9,RTN4,EMC10,GLI3,ITGB1,FGF2,SRPK2,GLI2,SMARCD3,PAK1,TGFB1,SCN5A,WDR48,ACER3,CTNNB1,LBH,AKT2,FAM98A,LIG4,IRS2,ZNF703,CCKBR,DDRGK1,ILK,CIAO1,CNOT7,CXCL12,FAM98B,CX3CL1,PTPN22,MMP2,ADA,SLC39A10,CHRNA7,TJP1,KDM5B,NOTCH2,MARCKSL1,HYAL1,CCND1,TGFB2,LRP5,HTR2B,LEF1,PRKCA,KRAS,NTRK3,ATF3,FGFR2,VASH2,OSM,HTRA1,TFRC,FZD9,FN1,PTPN6,IGFBP2,PDCD10,ITGAV,COMP,PRKCI,FGFR3,PRRX1,TIMP1,PTPRN,MNAT1,AKT3,BMPR1A,MED1,PPARGC1A,NES,NGFR,TGFB3,SHC1,PBXIP1,MYOCD,CD74,PDGFRB,AKT1,UBE2A,ITGA2,PRKAA1,OSMR,CD40LG,MYDGF,ROGDI,PLXNB3,IRAK1,FLNA,GNAI3,CDH13,F3,GDNF,HAS2,CDKN1A,TSLP,CAPNS1,DERL2,WNT2,FGF10	darkgreen	EC_M4
GO:0051249	5.16E-02	0.96351034	49	287	regulation of lymphocyte activation	BP	5.39E-01	IL23A,HLA-DRA,FAM49B,LGALS1,LEP,DOCK8,NCK2,SOCS5,GLI3,INHBA,GLI2,TGFB1,AXL,CTNNB1,IL15RA,IRS2,PRNP,HSPH1,SPINK5,LMO1,IL20RB,LOXL3,PTPN11,AP3B1,PTPN22,ADA,RUNX1,CBLB,SLC39A10,PRKAR1A,MMP14,PIK3CA,LEF1,SLAMF8,TP53BP1,TFRC,PTPN6,IGFBP2,CD74,AKT1,SUPT6H,PTPN2,CD40LG,AP1G1,BCL10,THY1,CDKN1A,FGL2,FGF10	darkgreen	EC_M4
GO:0010469	5.16E-02	0.969871657	22	112	regulation of signaling receptor activity	BP	5.39E-01	PPARA,LY6E,NCK2,SOCS5,SHANK1,DKKL1,ITGB1,DKK3,DLGAP4,APP,HOMER1,TSG101,CBLB,SERPINE1,ADRA2C,LRPAP1,GREM2,NLGN2,PPARGC1A,SHC1,BEGAIN,PARK7	darkgreen	EC_M4
GO:0010033	4.48E-02	0.96161583	273	2273	response to organic substance	BP	5.39E-01	TNFRSF11A,SEMA6A,IFNGR1,TNFAIP3,AKAP12,FLT4,TCF7,PTK2,DAPK1,ATF4,PDGFB,MAPK13,KDM4C,MICALL1,PIM1,CSF2RB,TEK,DDX58,DNAJB5,KIT,TNFRSF21,KDR,NFIL3,SYK,IL6,REL,CXCL8,KBTBD2,PELI1,FOXP2,CAV2,KLF11,GRB10,LANCL2,TSPAN12,TLR4,TRAF1,IL15,TEAD2,SDCBP,NOCT,TRPM4,NRP1,PIK3CG,GATA2,DNMT3A,PRKD2,CTSC,LRCH1,PRCP,NOS3,RALB,ZFAND6,PPARG,TIMP4,GATA3,ACVR2B,TBC1D4,CXCR4,IRAK2,EDNRB,TIE1,IKBKB,SEL1L2,JAG1,P2RY6,P2RY2,NFKBIB,PDE2A,TMEM173,SFRP1,NR1D2,FERMT1,ASH2L,P2RX2,CD36,ARID5A,SCARB1,RET,TNFRSF19,GRAMD1A,IL1A,DNMT3B,IGF1,NR4A1,EIF2AK3,HHEX,KDM3A,ERBIN,UFSP2,SMAD1,OCLN,CHRM5,GPD1,MEF2C,SMARCD1,TLR2,IL7,P2RX7,VEGFC,CXCL10,ANGPT2,SOGA1,DHH,KMT2D,TIPARP,OAS3,NLRC5,THBS4,PLCG1,STC1,RAPGEF3,ST3GAL6,NR4A2,HIPK1,DLL4,AMPD1,IFIT2,IFIT1,EDN1,STAT1,CD58,EHD4,SNCA,JARID2,DTNBP1,RBBP5,NFKB2,MX2,MX1,ADCY2,ABCG1,ETS1,HDAC1,PPP3CA,HYAL2,ANO1,SHOC2,RFTN2,LAPTM5,USP13,FABP4,PABPN1,HCLS1,CD86,POLG,MN1,TGIF1,SH3BP4,CFLAR,ADCY1,FES,ARID1A,PRKD1,IL10RA,MLXIPL,ITGA6,PAK2,PTPRE,PDK3,CREB1,B2M,EIF4A2,SSTR1,LTF,CCRL2,ADORA2A,ARNT2,CYBB,IL1RAP,ADAMTS7,ITGA4,IGFBP5,TBX1,CALCRL,RAMP2,CDK16,MMP13,BIRC3,SELE,SELP,PLVAP,BAIAP2L1,FGF23,CPEB2,WAS,RAC1,HIF1A,ADSS,JUP,ADIPOR2,EPHA2,MMRN2,BID,TLR6,DDI2,MPP5,TNFRSF1B,ALDH1A2,MTOR,AR,RNF40,PSEN1,FGF18,ITGA3,PGF,EIF2B2,CHRNB2,RPS6KA5,S100A14,GPR68,SMAD2,DNM2,SCNN1B,YY1,CYP1A1,TNIP1,COL4A6,FAM83G,ADRB2,CCL2,IL13RA1,YES1,PDE4B,JAK1,LGALS9	turquoise	EC_M6
GO:1902287	4.48E-02	0.990938917	4	12	semaphorin-plexin signaling pathway involved in axon guidance	BP	5.39E-01	NRP1,PLXND1,SEMA3F,PLXNA2	turquoise	EC_M6
GO:0030198	4.49E-02	0.970044358	38	250	extracellular matrix organization	BP	5.39E-01	SERAC1,IL6,CAV2,TIE1,FERMT1,NTN4,RIC8A,ADAMTS9,TCF15,MMP24,TLL2,TLL1,ADAMTS20,GAS2,NFKB2,ETS1,SLC39A8,CFLAR,MMP21,MELTF,ADAMTS7,RAMP2,MMP13,MMP1,FSCN1,TNFRSF1B,SMOC1,PAPLN,THSD4,COL4A6,COL4A5,MMP28,NOTCH1,ENG,GFOD2,CARMIL2,ATXN1L,BMP2	turquoise	EC_M6

GO:0031641	5.17E-02	0.979254006	9	36	regulation of myelination	BP	5.39E-01	TENM4,CTNNB1,RARB,LGI4,DAG1,DICER1,AKT1,TMEM98,ZPR1	darkgreen	EC_M4
GO:0009134	5.18E-02	0.989293318	4	11	nucleoside diphosphate catabolic process	BP	5.40E-01	ENTPD6,NUDT16,ENTPD4,NUDT18	darkgreen	EC_M4
GO:0060350	5.19E-02	0.97689307	11	46	endochondral bone morphogenesis	BP	5.40E-01	NAB2,RUNX2,SERPINH1,RARB,IMPAD1,MMP14,COMP,FGFR3,THBS3,COL1A1,ANXA6	darkgreen	EC_M4
GO:0051259	5.21E-02	0.966308223	32	174	protein complex oligomerization	BP	5.41E-01	CBY1,COL1A2,PDCD6IP,KCTD21,PRNP,KCTD16,RYR3,HOMER1,OPA1,GLS,CHP1,PKD2,CALHM1,DNM1L,KCTD10,DISP1,POLQ,TP53BP1,OSBPL2,MPP2,KCNA1,ROM1,UGDH,HSD17B10,CD74,TNFAIP1,ABCA3,PKD1,ZNHIT6,BCL10,SIGMAR1,NACC2	darkgreen	EC_M4
GO:0007613	5.22E-02	0.971482342	18	86	memory	BP	5.41E-01	SRF,SHANK1,PRNP,PJA2,ABCC8,CHRNA7,MAPT,EIF4EBP2,COMT,KCNK4,ATAD1,SLC2A4,SYT11,GRIA1,JPH3,SERPINF1,LRRN4,MAP1A	darkgreen	EC_M4
GO:0099177	5.22E-02	0.962329186	55	318	regulation of trans-synaptic signaling	BP	5.41E-01	RNF19A,SLC12A2,ADGRB1,SRF,RIMS1,SHANK1,NCDN,SLC7A11,ITGB1,KIF5B,PCDH17,RAB3GAP1,PRNP,MAP1B,ATP2A2,DLGAP4,APP,RAPGEF2,CX3CL1,CACNB3,HOMER1,VPS18,CHRNA7,EIF4E,DNM1L,GSK3B,KRAS,NCSTN,SYAP1,BACE1,MAPT,EIF4EBP2,TNR,MPP2,ATAD1,ERC1,HAP1,IQSEC2,NLGN2,STX1B,SYT11,RAB11A,CALM1,GRIA1,NPTN,CYP46A1,BEGAIN,ABR,TPRG1L,NTNG2,JPH3,YWHAH,KCNMB4,MAP1A,LRRTM2	darkgreen	EC_M4
GO:0098751	5.24E-02	0.980405732	8	30	bone cell development	BP	5.42E-01	SRF,PTPN11,NOTCH2,PTPN6,FBN1,MED1,PAFAH1B1,ATP6AP1	darkgreen	EC_M4
GO:0007416	5.26E-02	0.967784557	26	132	synapse assembly	BP	5.42E-01	PLXNB2,ADGRB1,MDGA1,TPBG,LRRC4B,VPS35,PCDH17,FARP1,PCLO,RYK,MAP1B,APP,AMIGO2,PDLIM5,DNER,NTRK3,SIX4,OBSL1,NLGN2,NPTN,NTNG2,PCDHB2,LRRC24,UBE2V2,LHFPL4,LRRTM2	darkgreen	EC_M4
GO:0006521	5.26E-02	0.989077661	4	11	regulation of cellular amino acid metabolic process	BP	5.42E-01	SLC7A11,ATP2B4,COMT,PARK7	darkgreen	EC_M4
GO:0030902	5.27E-02	0.968767247	23	116	hindbrain development	BP	5.42E-01	AHI1,WHRN,ITGB1,FGF2,GLI2,PAK1,SCN5A,CTNNB1,FAIM2,PTPN11,PSMG1,LEF1,BMP7,NCSTN,HAP1,PPARGC1A,MAP2K1,SDF4,FLNA,RERE,AARS,HSPA5,WLS	darkgreen	EC_M4
GO:1900026	4.54E-02	0.984580007	7	30	positive regulation of substrate adhesion-dependent cell spreading	BP	5.43E-01	NRP1,UNC13D,ARHGEF7,NEDD9,DOCK1,RAC1,DNM2	turquoise	EC_M6
GO:0043502	4.54E-02	0.978259347	13	71	regulation of muscle adaptation	BP	5.43E-01	NOS3,IGF1,EDN1,JARID2,PDE9A,PPP3CA,MEF2A,IGFBP5,TNFRSF1B,MTOR,YY1,NOTCH1,RGS4	turquoise	EC_M6
GO:0010556	3.57E-03	0.99708212	350	2846	regulation of macromolecule biosynthetic process	BP	5.44E-01	NFATC1,ZNF236,TSHZ1,ORMDL2,PYM1,SMARCC2,STAT6,MAP3K5,CDK4,CITED2,MAML1,SQSTM1,IRAK3,OSR2,HSPA1B,EHMT2,ZBTB12,HDAC10,ATF6B,EIF3H,RXRB,RING1,ZHX2,ZBTB22,MYC,TOB2,SMAD5,EP300,KDM3B,ZNF76,DDX17,NFIB,EIF3D,HSF1,CCND3,TRERF1,CDKN2A,HMOX1,WASL,KLF9,JAK2,ZBTB45,ZNF135,ZNF667,ZNF865,RPS9,MALSU1,EFEMP1,ZNF692,ANXA4,AKNA,MED25,IL4I1,ING3,ARHGEF5,AP2A1,IRF3,LPIN1,FOXO3,KLF15,SPHK2,NOP53,MAML2,BICRA,KMT2E,BBS7,MEIS3,TRIM13,IRF2BP1,SIX5,FOSB,ZNF746,NR2C2,ZNF862,TSC22D1,ZNF575,MIF4GD,NAA16,MLH1,UNK,ZNF574,WBP2,KMT2C,MYD88,TADA3,RPS3,BRPF1,DYRK1B,EID2,ZNF219,MED29,SAMD4B,SIRT2,RPTOR,APBB3,EIF3K,NUP98,TRIM21,CYP1B1,HGF,APBB1,DEAF1,HOXC4,NCOA4,LIN37,TARBP2,KMT2B,RBCK1,RARG,SDR16C5,ZNF740,EIF4B,LMO2,POFUT1,NECAB3,URI1,PIK3R1,NCOA2,ATF1,KDM2B,ZFHX4,CAMKK2,SCAND1,EIF2A,SETD6,MCPH1,SSBP2,FNIP2,ARL2BP,SHOX2,NEK4,ZHX3,VDR,MMS19,LPIN3,FOXF2,F2RL1,SORBS3,ORMDL1,HNF4A,TP53INP1,PIDD1,HR,ACOT8,PCIF1,TRIM45,PARP3,FASTKD3,ELK4,LGR4,ICE1,IRS1,SP140,TCF7L2,DIS3L2,PUM1,TXNIP,AIP,SOX5,ADNP,RPS6KB2,ZNF217,QARS,DTX3L,PARP14,EIF4H,ECM1,RCOR3,ARNT,PRMT2,RIPK3,GABPB2,PER2,TWIST2,VPS72,HDAC4,USF1,SMARCD2,POGZ,USP21,PATZ1,RNASEL,CCDC85B,MAGEF1,CDKN1B,TCEA3,KAT5,LRP6,YBX3,NFKBIA,NFE2L2,PHC1,HEXIM2,SOAT1,BATF2,EXOSC7,PCBD1,CHCHD10,MEN1,PHB2,TSC22D4,SAV1,TAF6,CTDSP1,EID1,SSBP4,RNF25,FGF7,NELFA,ZSCAN25,ZNF394,TNFRSF1A,STK16,STAT5A,KAT6B,CREG1,NR2F6,HDAC6,PQBP1,CAMT	cyan	EC_M2
GO:0070988	4.55E-02	0.979971826	11	52	demethylation	BP	5.44E-01	KDM4C,KDM4D,GATA3,KDM3A,JARID2,KDM2A,TET1,KDM5A,KDM5C,CYP1A1,USP7	turquoise	EC_M6
GO:0006337	3.28E-02	0.993897473	4	13	nucleosome disassembly	BP	5.44E-01	SUPT16H,ARID2,SMARCC1,SMARCE1	darkgrey	EC_M8
GO:0002043	4.55E-02	0.986086584	6	25	blood vessel endothelial cell proliferation involved in sprouting angiogenesis	BP	5.45E-01	GATA2,PPP1R16B,DLL4,EPHA2,NRARP,MIR23A	turquoise	EC_M6

GO:0034764	4.57E-02	0.972488476	25	155	positive regulation of transmembrane transport	BP	5.45E-01	TRDN,P2RY6,IGF1,OCLN,P2RX7,CXCL10,PLCG1,ANO6,SNCA,KCNJ2,MEF2A,WNK4,CRACR2A,ADIPOR2,PSEN1,ATP2A1,CD19,DNM2,ADRB2,CCL2,PLCG2,CA2,RELN,KCNH2,SHANK3	turquoise	EC_M6
GO:0042044	4.57E-02	0.988025407	5	19	fluid transport	BP	5.45E-01	EDNRB,SLC4A11,EDN1,HYAL2,SCNN1B	turquoise	EC_M6
GO:0044409	4.57E-02	0.975103534	18	106	entry into host	BP	5.45E-01	CXCL8,CAV2,NRP1,NECTIN2,CXCR4,EFNB2,SCARB1,ITCH,TPCN1,HYAL2,CD86,F11R,EPHA2,LGALS9,HAVCR1,TRIM34,CD46,TRIM62	turquoise	EC_M6
GO:0021517	5.33E-02	0.979927536	8	31	ventral spinal cord development	BP	5.46E-01	GLI3,GLI2,TCTN1,SCYL1,MDGA2,CLN8,LMO4,LBX1	darkgreen	EC_M4
GO:0071732	4.59E-02	0.990447336	4	13	cellular response to nitric oxide	BP	5.46E-01	GUCY1B1,CFLAR,DNM2,TRAF2	turquoise	EC_M6
GO:0006505	5.35E-02	0.979767841	8	32	GPI anchor metabolic process	BP	5.46E-01	TMEM246,PIGP,PIGZ,PIGL,DPM2,PIGK,DPM1,PIGB	darkgreen	EC_M4
GO:0042102	4.60E-02	0.980360624	10	55	positive regulation of T cell proliferation	BP	5.47E-01	SYK,IL6,IL15,IL1A,IGF1,ICOSLG,CD86,EFNB1,LGALS9,CD46	turquoise	EC_M6
GO:1902043	5.35E-02	0.988813432	4	11	positive regulation of extrinsic apoptotic signaling pathway via death domain receptors	BP	5.47E-01	ATF3,PEA15,SKIL,TIMP3	darkgreen	EC_M4
GO:0045880	5.36E-02	0.979764534	8	31	positive regulation of smoothened signaling pathway	BP	5.47E-01	CTNNA1,UCHL5,CHSY1,ARMC9,PRRX1,GORAB,EVC,RAB34	darkgreen	EC_M4
GO:0060428	5.37E-02	0.981415812	7	26	lung epithelium development	BP	5.47E-01	RCN3,FGFR2,MAP2K1,PKD1,TMEM38B,WNT2,FGF10	darkgreen	EC_M4
GO:0008285	5.37E-02	0.958342251	88	548	negative regulation of cell population proliferation	BP	5.47E-01	B4GALT7,ENPP3,FAM19A5,ADGRB1,PPARD,CTNNA1,CDKN2B,SRF,NCK2,REST,P3H1,TGFBR1,TPBG,AKIRIN1,TES,GLI3,INHBA,FGF2,RB1,CGREF1,PAK1,TGFB1,CTNNB1,RARB,KAT2B,SPINT2,PRNP,ILK,TMEM127,CNOT7,IL20RB,SULF1,ROBO1,PINX1,CELF1,APP,RAPGEF2,ABCC8,TSG101,BAP1,PKD2,CBLB,BRD7,IFT57,NDN,FAP,CDC73,TGFB2,PRKAR1A,BMP7,IDH2,FGFR2,OSM,S100A11,OVOL1,PRDX4,COMT,PTPN6,PDCD10,MAGED1,MED1,PPARGC1A,FGFRL1,TGFB1I1,NGFR,TPM1,TGFB3,MAP2K1,CNN1,DICER1,CLMN,MYOCD,PTPN2,NPR3,LDOC1,TAX1BP3,SSTR5,CDH13,MIR24-1,SERPINF1,PEMT,CD164,LBX1,CDKN1A,NACC2,P3H3,PRKG1,FGF10	darkgreen	EC_M4
GO:0002320	4.61E-02	0.988293871	5	16	lymphoid progenitor cell differentiation	BP	5.47E-01	KIT,GATA3,SOS2,BATF,NOTCH1	turquoise	EC_M6
GO:0014046	4.62E-02	0.987765351	5	20	dopamine secretion	BP	5.47E-01	SNCA,DTNBP1,SNCG,SYT7,CHRNB2	turquoise	EC_M6
GO:0014059	4.62E-02	0.987765351	5	20	regulation of dopamine secretion	BP	5.47E-01	SNCA,DTNBP1,SNCG,SYT7,CHRNB2	turquoise	EC_M6
GO:0048732	5.38E-02	0.960851872	57	335	gland development	BP	5.47E-01	CDO1,LY6E,SRF,VEGFA,ASNS,TGFBR1,RTN4,GLI3,DBP,PLXNA1,FGF2,GLI2,TGFB1,CTNNB1,LBH,AKT2,IRS2,ZNF703,SEMA3C,CCKBR,SULF1,DKK3,ROBO1,ADA,PKD2,KDM5B,NOTCH2,CCND1,CPT1A,TGFB2,LRP5,DAG1,PIK3CA,LEF1,KRAS,BMP7,FGFR2,ARID5B,SRP54,QDPR,SIX4,BMPR1A,MED1,MSN,TGFB3,MAP2K1,AKT1,ITGA2,PKD1,LMO4,WLS,FSTL3,SERPINF1,SERPINB5,EDA,WNT2,FGF10	darkgreen	EC_M4
GO:0071679	5.38E-02	0.988740582	4	11	commissural neuron axon guidance	BP	5.47E-01	VEGFA,RYK,DAG1,GDNF	darkgreen	EC_M4
GO:0009141	5.39E-02	0.970835002	17	86	nucleoside triphosphate metabolic process	BP	5.47E-01	ENPP3,RRM2B,PPARA,VCP,RAB23,CMPK1,TGFB1,NUDT16,OPA1,ENTPD4,ADA,PPARGC1A,AK7,ENO1,GNAI3,TMSB4X,NME6	darkgreen	EC_M4
GO:0019915	5.40E-02	0.972960524	14	65	lipid storage	BP	5.47E-01	B4GALNT1,PPARA,PPARD,PLIN2,LEP,LDAH,ABHD5,DGAT2,OSBPL8,CRY2,CPT1A,ITGAV,TMEM159,PTPN2	darkgreen	EC_M4
GO:0043542	5.40E-02	0.964208566	35	195	endothelial cell migration	BP	5.47E-01	ANGPT1,NUS1,PTP4A3,ADGRB1,MYH9,SRF,VEGFA,TGFBR1,EMC10,ROCK2,RRAS,ITGB1,FGF2,TGFB1,DCN,STARD13,ROBO1,P2RX4,LOXL2,ATP2B4,FAP,FSTL1,PIK3CA,PRKCA,HSPB1,DNAJA4,S100P,PDCD10,AKT3,AKT1,CDH13,MIR24-1,SERPINF1,TMSB4X,PIK3C2A	darkgreen	EC_M4
GO:0010633	5.40E-02	0.973764289	13	59	negative regulation of epithelial cell migration	BP	5.47E-01	ADGRB1,FGF2,TGFB1,DCN,STARD13,PFN2,ATP2B4,ADIPOR1,CORO1C,DNAJA4,PDCD10,MIR24-1,SERPINF1	darkgreen	EC_M4
GO:0006878	5.40E-02	0.98867321	4	11	cellular copper ion homeostasis	BP	5.47E-01	SLC31A1,ATP7B,PRNP,APP	darkgreen	EC_M4

GO:2000106	5.42E-02	0.973693212	13	59	regulation of leukocyte apoptotic process	BP	5.47E-01	TCP1,DOCK8,SLC7A11,AXL,IRS2,CXCL12,RAPGEF2,ADA,SLC39A10,SELENOS,CD74,BCL10,ORMDL3	darkgreen	EC_M4
GO:0010714	5.42E-02	0.983181819	6	21	positive regulation of collagen metabolic process	BP	5.47E-01	TGFB1,RUNX1,SUCO,TGFB3,PDGFRB,ITGA2	darkgreen	EC_M4
GO:0018393	3.31E-02	0.981958083	20	134	internal peptidyl-lysine acetylation	BP	5.48E-01	ZNF451,ZMPSTE24,PAXIP1,KAT14,EPC2,KAT6A,TAF7,BRCA2,TAF9,MCRS1,CHEK1,NAA50,MCM3AP,HAT1,ING5,BRCA1,TAF1,OGT,CTCF,AC118549.1	darkgrey	EC_M8
GO:0001732	3.31E-02	0.993622102	4	15	formation of cytoplasmic translation initiation complex	BP	5.48E-01	EIF3E,EIF3A,EIF3B,EIF5	darkgrey	EC_M8
GO:0010565	5.44E-02	0.969487661	19	95	regulation of cellular ketone metabolic process	BP	5.49E-01	PPARA,PPARD,REST,MFSD2A,DKKL1,SLC7A11,DGAT2,AKT2,IRS2,DKK3,RDH10,ATP2B4,CPT1A,WDTC1,COMT,PPARGC1A,CD74,AKT1,PARK7	darkgreen	EC_M4
GO:0014896	4.63E-02	0.97701763	14	78	muscle hypertrophy	BP	5.49E-01	IGF1,EDN1,JARID2,PDE9A,PPP3CA,MEF2A,INPP5F,IGFBP5,TNFRSF1B,MTOR,YY1,NOTCH1,MIR23A,RGS4	turquoise	EC_M6
GO:0010038	5.46E-02	0.961414938	48	279	response to metal ion	BP	5.50E-01	CYB5A,SLC12A2,ATP7B,ENTPD6,SCN5A,STIM1,LIG4,PRNP,SMPD1,PPP2CB,MAP1LC3A,RYR3,P2RX4,APP,ABCC8,HOMER1,LOXL2,PKD2,CCND1,NCSTN,IQGAP1,UROS,CLIC4,BACE1,MAPT,TFR2,IGFBP2,HSD17B1,QDPR,KCNA1,MNAT1,EIF2S1,PPARGC1A,SYT11,KCNMB1,CACNA1G,CALM1,AKT1,PRKAA1,PARK7,BSG,AHCYL1,HSPA5,FSTL3,SERPINF1,BNIP3,CACYBP,KCNMB4	darkgreen	EC_M4
GO:0045761	5.47E-02	0.983121919	6	20	regulation of adenylate cyclase activity	BP	5.51E-01	STIM1,CACNA1D,LINC02210-CRHR1,CACNA1C,NPR3,GNAI3	darkgreen	EC_M4
GO:0021515	4.67E-02	0.984038007	7	30	cell differentiation in spinal cord	BP	5.52E-01	TAL1,GATA2,DLL4,SOX13,HOXD10,ZC4H2,NOTCH1	turquoise	EC_M6
GO:0019882	4.67E-02	0.979072702	11	61	antigen processing and presentation	BP	5.52E-01	RAB3B,IKBKB,YTHDF1,ACE,B2M,WAS,RAB27A,DNM2,AP3D1,ERAP2,RAB32	turquoise	EC_M6
GO:0060670	5.50E-02	0.988409544	4	11	branching involved in labyrinthine layer morphogenesis	BP	5.52E-01	RSPO3,SPINT2,TMED2,FGFR2	darkgreen	EC_M4
GO:0014897	5.50E-02	0.970878513	16	77	striated muscle hypertrophy	BP	5.53E-01	PPARA,LEP,ROCK2,PAK1,P2RX4,ATP2A2,ATP2B4,TWF1,PDLIM5,HTR2B,PRKCA,GLRX3,TCAP,PIN1,ROCK1,MIR24-1	darkgreen	EC_M4
GO:0009120	3.66E-03	0.999601139	5	10	deoxyribonucleoside metabolic process	BP	5.53E-01	PNP,XDH,DGUOK,DPYD,TK2	cyan	EC_M2
GO:0045892	4.69E-02	0.96238082	121	943	negative regulation of transcription, DNA-templated	BP	5.54E-01	PLAGL1,NOTCH4,TCF7,L3MBTL3,ATF4,PDGFB,MDFI,NFX1,NFIL3,SEMA4D,NFE2L3,BACH2,FOXP2,KLF11,LANCL2,CREM,SDCBP,BEND5,TAL1,PHF10,EPC1,GATA2,DNMT3A,SAP130,DACH1,PPARG,GATA3,TAF3,ACVR2B,GZF1,EDNRB,PDE2A,SFRP1,NR1D2,CD36,ARID5A,DNMT3B,HHEX,ZBTB38,POU6F1,SOX7,HMBOX1,MEF2C,WWTR1,SFMBT1,SCAF4,ANKRD2,NR4A2,DLL4,CDYL,SOX13,YAF2,EDN1,STAT1,SNCA,JARID2,ETS2,DCAF1,HDAC1,MEF2A,FABP4,HCLS1,NFATC2,KDM2A,CIART,TGIF1,PARP15,PROX1,ZNF148,MLXIPL,DLG1,ETV6,RNF2,CREB1,BCOR,MECOM,ING4,SPINDOC,LDB2,PPM1F,POU2F1,ZBTB18,APBB2,BCL6B,KDM5C,MAFK,AR,PSEN1,CBX1,HOXB8,FBXW11,EFNA1,PIAS1,RPS6KA5,SMAD2,TLE3,YY1,HMG20A,RCOR1,HCFC1,NRARP,NOTCH1,IRF8,MTF2,ZBTB34,DAB2IP,ZBTB33,ARID4A,ZNF24,ATXN1L,TRIM24,SOX18,PASD1,COMMD7,TGIF2,BMP2,GLIS3,ZNF10,CBX2,BMP6,ZNF266	turquoise	EC_M6
GO:0060537	4.70E-02	0.96714321	47	323	muscle tissue development	BP	5.54E-01	PIM1,PGM5,CAV2,SVIL,NR1D2,EFNB2,P2RX2,LMOD3,ADAMTS9,EPHB1,CHD7,IGF1,NR4A1,SMAD1,MEF2C,NDRG4,TLL2,TIPARP,DLL4,EDN1,JARID2,SIK1,PPP3CA,MEF2A,BCL9,NRAP,HLX,CFLAR,PROX1,DLG1,HOXD10,CREB1,IGFBP5,TBX1,ZBTB18,ALDH1A2,MTOR,YY1,DSG2,MRTFB,NOTCH1,S1PR1,ENG,MIR23A,MAFF,RGS4,BMP2	turquoise	EC_M6
GO:0045061	5.55E-02	0.985137474	5	16	thymic T cell selection	BP	5.56E-01	SRF,GLI3,DOCK2,CD74,PTPN2	darkgreen	EC_M4
GO:0031341	4.72E-02	0.980945524	9	46	regulation of cell killing	BP	5.56E-01	SYK,NECTIN2,P2RX7,B2M,LGALS9,NOS2,IL7R,FCER2,SERPINB9	turquoise	EC_M6

GO:0098771	4.73E-02	0.964728495	68	502	inorganic ion homeostasis	BP	5.57E-01	DMXL1,TRDN,IREB2,PPT1,CAV2,TRPM4,PIK3CG,GATA2,RAB38,GPR4,CXCR4,EDNRB,TMC8,P2RY6,P2RX2,CD36,TMEM178A,MICU3,PDE4D,CHD7,IL1A,CLCN3,HEXB,CP,P2RX7,CXCL10,TPCN1,PLCG1,STC1,EDN1,SNCA,LCK,HYAL2,SLC39A8,KCNJ2,TESMIN,SLC12A7,HCRTR1,SLC9A8,PRKD1,MELTF,B2M,LTF,CCRL2,ADORA2A,MCU,SLC9A7,FGF23,HIF1A,HEPH,PSEN1,ATP2A1,CD19,NPR1,S100A14,SCNN1B,HMOX2,AP3D1,PLCG2,S1PR1,CDH5,SLC8A3,TRIM24,CA2,KCNH2,LPAR6,RAB39A,BMP6	turquoise	EC_M6
GO:0038061	4.73E-02	0.975087543	16	99	NIK/NF-kappaB signaling	BP	5.57E-01	LRRC19,REL,TLR4,TLR2,ADGRG3,EDN1,NFKB2,LAPTM5,CD86,DDX3X,BIRC3,TLR6,LGALS9,TRAF2,NFAT5,COPS8	turquoise	EC_M6
GO:1903363	4.74E-02	0.978754582	11	60	negative regulation of cellular protein catabolic process	BP	5.57E-01	TRIM39,LAPTM4B,RGP1,SDCBP,FAM192A,OPHN1,PSEN1,EFNA1,USP7,DAB2IP,MGAT3	turquoise	EC_M6
GO:0050866	4.74E-02	0.972684338	21	136	negative regulation of cell activation	BP	5.57E-01	TNFAIP3,PDGFB,ADGRF5,TNFRSF21,PELI1,CST7,NOS3,SFRP1,MERTK,ITCH,CD200,LAPTM5,HLX,INPP5D,CD86,FGR,DLG1,ADORA2A,ARG2,LGALS9,NRARP	turquoise	EC_M6
GO:0051254	4.74E-02	0.960717485	170	1357	positive regulation of RNA metabolic process	BP	5.57E-01	ZNF516,TCF4,PLAGL1,CNOT6,ZBED4,NOTCH4,TCF20,NCOA7,ATF4,PDGFB,PIM1,NFYA,PRDM4,DDX58,NFIL3,IL6,REL,CREB5,TLR4,TEAD2,MYCN,NOCT,TUT4,TAL1,PHF10,EPC1,GATA2,PRKD2,PPARG,GATA3,IKBKB,JAG1,NFKBIB,TMEM173,SFRP1,NR1D2,DND1,PRR5L,TCF15,RET,CHD7,IL1A,IGF1,NR4A1,EIF2AK3,HHEX,KDM3A,SMAD1,ZBTB38,STOX2,SOX7,HMBOX1,HEXB,MEF2C,SMARCD1,TLR2,WWTR1,CXCL10,KMT2D,NLRC5,NR4A2,TOX2,YAF2,EDN1,STAT1,DYRK1A,ERG,ETS2,NFKB2,PRDM15,ETS1,HDAC1,PPP3CA,HYAL2,MEF2A,BCL9,NCF1,HCLS1,NFATC2,EAF2,GMEB1,CD86,POU2F3,ARID1A,RPS6KA1,PROX1,SS18L1,PRKD1,ZNF148,YTHDF1,HELZ2,MLXIPL,ITGA6,ELL3,ETV6,HOXD10,MOV10,CREB1,TET1,ARNT2,DDX3X,RFXANK,ETV4,TBX1,MECOM,ING4,LDB2,FGF23,POU2F1,WAS,HIF1A,KDM5A,ZBTB18,APBB2,JUP,TNFRSF1B,MTOR,AR,CASZ1,PSEN1,HOXB5,HOXB7,HOXB9,FBXW11,BATF,PIAS1,RPS6KA5,SMAD2,CNOT8,DNM2,YY1,ARID3B,LYL1,TNIP1,ZNF521,ABLIM3,ADRB2,YES1,RAI1,ELF4,MRTFB,DOT1L,AP3D1,HCFC1,NOTCH1,MED16,IRF8,S1PR1,ENG,MTF2,NFAT5,DAB2IP,ARID4A,ZNF24,TRIM24,SOX17,KCNH2,ZNF850,ANKRD49,CDK13,SOX18,MAFF,TRIM62,ZNF229,E2F3,BMP2,GLIS3,ZNF71,BMP6,FLI1	turquoise	EC_M6
GO:1904752	5.57E-02	0.980547677	7	26	regulation of vascular associated smooth muscle cell migration	BP	5.57E-01	LRP1,FAM19A5,PAK1,IQGAP1,TPM1,MYOCD,PRKG1	darkgreen	EC_M4
GO:0010927	4.76E-02	0.975110992	16	87	cellular component assembly involved in morphogenesis	BP	5.58E-01	PGM5,TMOD1,MTMR2,NECTIN2,ITGB4,LMOD3,TLR2,EDN1,MEF2A,NRAP,CFLAR,PROX1,TMOD4,TMOD2,MPP5,TBC1D20	turquoise	EC_M6
GO:0001504	5.59E-02	0.982503289	6	21	neurotransmitter uptake	BP	5.59E-01	ITGB1,SLC6A13,SLC29A4,CLN8,PARK7,GDNF	darkgreen	EC_M4
GO:0009889	3.74E-03	0.996925418	369	3010	regulation of biosynthetic process	BP	5.59E-01	NFATC1,ZNF236,TSHZ1,ORMDL2,PYM1,SMARCC2,STAT6,MAP3K5,CDK4,CITED2,MAML1,SQSTM1,IRAK3,OSR2,HSPA1B,EHMT2,ZBTB12,HDAC10,ATF6B,EIF3H,RXRB,TSPO,RING1,ZHX2,ZBTB22,MYC,TOB2,SMAD5,EP300,KDM3B,ZNF76,DDX17,NFIB,EIF3D,HSF1,CCND3,TRERF1,CDKN2A,HMOX1,WASL,KLF9,JAK2,ZBTB45,ZNF135,ZNF667,ZNF865,RPS9,MALSU1,EFEMP1,ZNF692,ANXA4,AKNA,MED25,IL4I1,COQ3,ING3,ARHGEF5,AP2A1,IRF3,LPIN1,FOXO3,KLF15,SPHK2,NOP53,MAML2,BICRA,KMT2E,BBS7,MEIS3,TRIM13,IRF2BP1,SIX5,FOSB,ZNF746,NR2C2,ZNF862,TSC22D1,ZNF575,MIF4GD,NAA16,MLH1,UNK,ZNF574,WBP2,KMT2C,MYD88,CYGB,TADA3,RPS3,BRPF1,DYRK1B,EID2,ZNF219,MED29,SAMD4B,SIRT2,RPTOR,APBB3,EIF3K,NUP98,TRIM21,CYP1B1,HGF,APBB1,DEAF1,HOXC4,NCOA4,LIN37,TARBP2,KMT2B,RBCK1,RARG,SDR16C5,ZNF740,EIF4B,LMO2,POFUT1,NECAB3,URI1,PIK3R1,NCOA2,ATF1,KDM2B,ZFHX4,CAMKK2,SCAND1,EIF2A,SETD6,MCPH1,SSBP2,FNIP2,ARL2BP,SHOX2,NEK4,ZHX3,VDR,MMS19,LPIN3,FOXF2,F2RL1,SORBS3,ORMDL1,HNF4A,TP53INP1,PIDD1,ERLIN1,HR,ACOT8,PCIF1,TRIM45,CD47,PARP3,FASTKD3,ELK4,LGR4,ICE1,IRS1,SP140,TCF7L2,DIS3L2,PUM1,TXNIP,AIP,SOX5,ADNP,RPS6KB2,ZNF217,QARS,DTX3L,PARP14,EIF4H,ECM1,RCOR3,ARNT,PRMT2,RIPK3,GABPB2,PER2,TWIST2,VPS72,HDAC4,USF1,SMARCD2,POGZ,CLCN2,USP21,DNAJC30,PATZ1,RNASEL,CCDC85B,MAGEF1,SCAP,CDKN1B,TCEA3,KAT5,LRP6,YBX3,NFKBIA,POR,NFE2L2,IDH1,PHC1,HEXIM2,ACADL,SOAT1,BATF2,EXOSC7,PCBD1,CHCHD10,MEN1,PHB2,TSC22D4,SAV1,TAF6,CTDSP1,EID1,SSBP4,RNF25,FGF7,NELFA,ZSCAN25	cyan	EC_M2

GO:0042491	4.77E-02	0.983661516	7	29	inner ear auditory receptor cell differentiation	BP	5.59E-01	MYCN,MYO7A,JAG1,USH1C,SEC24B,JAG2,NOTCH1	turquoise	EC_M6
GO:0060419	4.78E-02	0.976216599	14	77	heart growth	BP	5.59E-01	PIM1,IGF1,SMAD1,MEF2C,NDRG4,EDN1,JARID2,PROX1,MTOR,YY1,NOTCH1,S1PR1,MIR23A,RGS4	turquoise	EC_M6
GO:0043062	4.78E-02	0.967929452	38	251	extracellular structure organization	BP	5.59E-01	SERAC1,IL6,CAV2,TIE1,FERMT1,NTN4,RIC8A,ADAMTS9,TCF15,MMP24,TLL2,TLL1,ADAMTS20,GAS2,NFKB2,ETS1,SLC39A8,CFLAR,MMP21,MELTF,ADAMTS7,RAMP2,MMP13,MMP1,FSCN1,TNFRSF1B,SMOC1,PAPLN,THSD4,COL4A6,COL4A5,MMP28,NOTCH1,ENG,GFOD2,CARMIL2,ATXN1L,BMP2	turquoise	EC_M6
GO:0007033	5.61E-02	0.96429775	29	157	vacuole organization	BP	5.59E-01	UBQLN1,RAB23,RAB7A,RAB3GAP1,ACPP,TMEM41B,MAP1LC3A,TP53INP2,ATP2A2,ARSB,ATG13,AP3B1,VPS18,AKTIP,RAB7B,SEC22B,FEZ1,WIPI1,SYNPO2,SCFD1,ATP6AP2,SMURF1,WIPI2,CLN8,CLN6,VMP1,RAB34,MBTPS1,GABARAPL2	darkgreen	EC_M4
GO:0007565	5.62E-02	0.965258149	26	139	female pregnancy	BP	5.59E-01	PPARD,TEAD3,LEP,VEGFA,TMED2,LNPEP,ABCC8,MMP2,SPP1,KPNA6,KRAS,LINC02210-CRHR1,COMT,IGFBP2,ADRA2C,TIMP1,MED1,MAGED2,TGFB3,UBE2Q1,VMP1,ACSL4,AKT1,UBE2A,ITGA2,BSG	darkgreen	EC_M4
GO:0000422	5.63E-02	0.970832388	15	71	autophagy of mitochondrion	BP	5.59E-01	MAP1LC3A,SLC25A4,ATG13,CTTN,DNM1L,CISD2,WIPI1,HTT,WIPI2,PPARGC1A,FUNDC2,PARK7,BNIP3,GABARAPL2,TOMM7	darkgreen	EC_M4
GO:0061726	5.63E-02	0.970832388	15	71	mitochondrion disassembly	BP	5.59E-01	MAP1LC3A,SLC25A4,ATG13,CTTN,DNM1L,CISD2,WIPI1,HTT,WIPI2,PPARGC1A,FUNDC2,PARK7,BNIP3,GABARAPL2,TOMM7	darkgreen	EC_M4
GO:0042407	3.39E-02	0.993407972	4	15	cristae formation	BP	5.59E-01	CHCHD3,LETM1,AFG3L2,DNAJC11	darkgrey	EC_M8
GO:0007034	5.64E-02	0.965117782	26	137	vacuolar transport	BP	5.60E-01	LRP1,AP3S1,VCP,VPS41,VPS35,RAB7A,TRAK1,LEPROTL1,VPS37A,ARSB,AP3B1,TSG101,VPS18,AKTIP,VPS39,RAB7B,HSPA8,SNX27,TMEM50A,SMURF1,CLEC16A,MGRN1,VAMP7,AP1G1,ATP6V0D1,LEPROT	darkgreen	EC_M4
GO:0008340	3.40E-02	0.993600934	4	13	determination of adult lifespan	BP	5.60E-01	MSH2,ZMPSTE24,RAD54L,IDE	darkgrey	EC_M8
GO:0036499	5.65E-02	0.984781338	5	16	PERK-mediated unfolded protein response	BP	5.60E-01	NCK2,TMED2,EIF2S1,PTPN2,HSPA5	darkgreen	EC_M4
GO:0090314	5.67E-02	0.980103126	7	26	positive regulation of protein targeting to membrane	BP	5.60E-01	PAK1,AKT2,PRNP,CACNB3,CHP1,MIEF2,MYO1C	darkgreen	EC_M4
GO:0019219	3.82E-03	0.996871664	350	2852	regulation of nucleobase-containing compound metabolic process	BP	5.61E-01	NFATC1,ZNF236,TSHZ1,SMARCC2,STAT6,MAP3K5,CDK4,CITED2,MAML1,SQSTM1,IRAK3,HELB,OSR2,HSPA1B,EHMT2,ZBTB12,HDAC10,ATF6B,RXRB,RING1,ZHX2,ZBTB22,MYC,TOB2,SMAD5,EP300,KDM3B,ZC3H3,ZNF76,DDX17,NFIB,HSF1,CCND3,TRERF1,CDKN2A,HMOX1,WASL,KLF9,JAK2,ZBTB45,ZNF135,ZNF667,ZNF865,EFEMP1,ZNF692,CYREN,ANXA4,AKNA,MED25,IL4I1,ING3,ARHGEF5,IRF3,LPIN1,FOXO3,KLF15,SPHK2,NOP53,MAML2,BICRA,ZC3HAV1,KMT2E,BBS7,MEIS3,HMCES,TRIM13,IRF2BP1,SIX5,FOSB,ZNF746,NR2C2,ZNF862,TSC22D1,FASTK,XRCC1,ZNF575,NAA16,MLH1,ZNF574,WBP2,KMT2C,MYD88,CELF2,BABAM2,TADA3,RPS3,BRPF1,DYRK1B,EID2,ZNF219,MED29,SAMD4B,SIRT2,RPTOR,APBB3,NUP98,TRIM21,CYP1B1,NDFIP1,HGF,APBB1,DEAF1,HOXC4,NCOA4,PCBP2,LIN37,SLC15A4,TARBP2,KMT2B,RBCK1,RARG,SDR16C5,ZNF740,LMO2,POFUT1,URI1,PIK3R1,NCOA2,ATF1,KDM2B,ZFHX4,CAMKK2,SCAND1,SETD6,MCPH1,SSBP2,FNIP2,PRKAG1,ARL2BP,SHOX2,NEK4,ZHX3,VDR,RBM12B,MMS19,LPIN3,FOXF2,F2RL1,SORBS3,HNF4A,TP53INP1,PIDD1,HR,TRIM45,PARP3,FASTKD3,ELK4,LGR4,RBM15B,ICE1,SP140,TCF7L2,DIS3L2,PUM1,TXNIP,AIP,SOX5,ADNP,SF3B4,ZNF217,QARS,DTX3L,PARP14,ECM1,RCOR3,ARNT,PRMT2,RIPK3,GABPB2,PER2,TWIST2,VPS72,HDAC4,USF1,SMARCD2,POGZ,USP21,DNAJC30,PATZ1,RNASEL,CCDC85B,MAGEF1,CDKN1B,MGMT,TCEA3,KAT5,LRP6,YBX3,NFKBIA,NFE2L2,PHC1,HEXIM2,BATF2,EXOSC7,PCBD1,CHCHD10,MEN1,TMEM161A,PHB2,TSC22D4,SAV1,TAF6,CTDSP1,EID1,SSBP4,RNF25,FGF7,NELFA,ZSCAN25,ZNF394,TNFRSF1A,STK16,STAT5A,KAT6B,CREG1,BABAM1,N	cyan	EC_M2
GO:1901798	3.83E-03	0.999163654	8	24	positive regulation of signal transduction by p53 class mediator	BP	5.61E-01	RPS7,SPRED1,RPL11,CDK5RAP3,RPS15,HEXIM1,RPL23,RPS20	cyan	EC_M2
GO:0006497	3.87E-03	0.998427516	18	81	protein lipidation	BP	5.62E-01	ATG12,GPAA1,PIGO,CLIP3,PIGT,PIGM,ZDHHC24,GLUL,ZDHHC8,WDR45,PIGH,GBA,DPM3,MPPE1,PIGQ,ZDHHC12,ZDHHC1,MAP6D1	cyan	EC_M2
GO:0045861	5.71E-02	0.961511796	37	213	negative regulation of proteolysis	BP	5.62E-01	SERPINB2,LRP1,VEGFA,PDCL3,DNAJC1,PICALM,SERPINH1,CRIM1,SPINT2,PRNP,DDRGK1,SPINK5,APP,CHAC1,TM4SF20,UCHL5,ANOS1,COL6A3,RPS6KA3,SERPINE1,TIMP1,CLN8,TMED10,ROCK1,USP14,AKT1,TMEM98,PARK7,GLG1,MIR24-1,SPINK2,SERPINF1,SERPINB5,A2M,TIMP3,GABARAPL2,MAP1A	darkgreen	EC_M4

GO:0060080	5.71E-02	0.987794196	4	11	inhibitory postsynaptic potential	BP	5.62E-01	UNC13B,RIMS1,NLGN2,INSYN1	darkgreen	EC_M4
GO:1900746	5.72E-02	0.981995962	6	21	regulation of vascular endothelial growth factor signaling pathway	BP	5.62E-01	CD63,PTP4A3,ADAMTS3,DCN,ROBO1,MYO1C	darkgreen	EC_M4
GO:0070098	4.82E-02	0.981687039	8	40	chemokine-mediated signaling pathway	BP	5.62E-01	CXCL8,CXCR4,CXCL10,EDN1,CCRL2,HIF1A,CCL2,ACKR1	turquoise	EC_M6
GO:0071287	5.73E-02	0.987751915	4	11	cellular response to manganese ion	BP	5.62E-01	APP,BACE1,EIF2S1,HSPA5	darkgreen	EC_M4
GO:0018023	3.42E-02	0.987913237	8	39	peptidyl-lysine trimethylation	BP	5.63E-01	PWP1,NSD3,SMYD5,TET2,SETD2,SUV39H1,OGT,ATRX	darkgrey	EC_M8
GO:0060218	3.43E-02	0.990091613	6	28	hematopoietic stem cell differentiation	BP	5.63E-01	HSPA9,UFL1,PUS7,METTL3,PRKDC,XRCC5	darkgrey	EC_M8
GO:0010885	4.85E-02	0.98955942	4	14	regulation of cholesterol storage	BP	5.64E-01	PPARG,CD36,SCARB1,ABCG1	turquoise	EC_M6
GO:0050808	4.86E-02	0.965610633	50	329	synapse organization	BP	5.65E-01	PICK1,LINGO2,ADGRF1,SEMA4D,DLGAP3,SDCBP,NRCAM,NRP1,MTMR2,PLXND1,SETD5,EFNB2,P2RX2,EPHB1,MEF2C,TLR2,GPM6A,SEPT11,TANC1,SPARCL1,SNCA,SHANK2,SEMA3F,IL10RA,CNKSR2,DLG1,IL1RAP,DNM3,APBB2,SNCG,CHRNB1,LGI2,ZC4H2,OPHN1,PSEN1,SEMA4A,ABI3,ITGA3,ARHGEF15,EFNA1,CHRNB2,ZDHHC15,COL4A5,SLITRK2,DAB2IP,ADGRL2,SLC8A3,RELN,ABHD17C,SHANK3	turquoise	EC_M6
GO:0003180	4.86E-02	0.983306259	7	29	aortic valve morphogenesis	BP	5.65E-01	NOS3,GATA3,TIE1,JAG1,DLL4,EFNA1,NOTCH1	turquoise	EC_M6
GO:0032768	5.77E-02	0.974883947	10	43	regulation of monooxygenase activity	BP	5.65E-01	NUS1,LEP,ATP2B4,HTR2B,KRAS,CALM1,AKT1,NPR3,PARK7,GDNF	darkgreen	EC_M4
GO:0031400	5.78E-02	0.956350933	70	426	negative regulation of protein modification process	BP	5.65E-01	LRP1,ANGPT1,ADGRB1,SH3GL2,GNAQ,CDKN2B,PLAA,NCK2,P3H1,SOCS5,COPS9,SH3BP5L,ROCK2,FKBP1B,RB1,CRTAP,TGFB1,SPOPL,CTNNB1,GPD1L,KAT2B,PRNP,SMPD1,ILK,FXYD1,SNTA1,TMED2,PINX1,PTPN22,TSG101,CRY2,CHP1,ATP2B4,DBNDD2,CBLB,TRIP12,LRP5,PRKAR1A,GSK3B,CORO1C,NTRK3,BMP7,PPP1R11,HSPB1,MAPT,DNAJC10,PTPN6,FKBP8,PPARGC1A,SRCIN1,CALM1,PPP4R4,GSKIP,MYOCD,ROCK1,AKT1,PRKAA1,SUPT6H,PTPN2,NXN,PARK7,MIR24-1,PRMT6,DMD,FKBP1A,FRY,THY1,CDKN1A,DUSP22,PMEPA1	darkgreen	EC_M4
GO:0006906	5.79E-02	0.967831864	18	90	vesicle fusion	BP	5.65E-01	STX7,VPS41,KIF5B,RAB7A,AKT2,VAMP8,USO1,CHP1,VPS39,RAB7B,SEC22B,STX1B,SYT11,SOX30,RAB34,VAMP7,GNAI3,STXBP6	darkgreen	EC_M4
GO:0032968	5.79E-02	0.987578142	4	11	positive regulation of transcription elongation from RNA polymerase II promoter	BP	5.65E-01	CTNNB1,CDC73,CDK12,SUPT6H	darkgreen	EC_M4
GO:0010464	5.80E-02	0.979558069	7	26	regulation of mesenchymal cell proliferation	BP	5.65E-01	CTNNB1,IRS2,LRP5,FGFR2,PRRX1,BMPR1A,WNT2	darkgreen	EC_M4
GO:0097186	5.80E-02	0.984263511	5	16	amelogenesis	BP	5.65E-01	PPARA,CNNM4,RELT,STIM1,FAM20C	darkgreen	EC_M4
GO:0002092	5.81E-02	0.981638447	6	21	positive regulation of receptor internalization	BP	5.66E-01	CD63,AHI1,ANGPT1,VEGFA,AP2M1,ATAD1	darkgreen	EC_M4
GO:0044092	4.87E-02	0.961648015	101	806	negative regulation of molecular function	BP	5.66E-01	TNFAIP8,TNFAIP3,PHACTR2,PTPRB,FZD6,TRDN,C6orf106,PDGFB,CSNK1E,PIM1,MDFI,FAM122A,SEMA4D,PELI1,TCAF2,C5,RASIP1,KCNRG,CST7,LRCH1,RALB,PPARG,TIMP4,TAF3,ITIH5,IRAK2,EDNRB,NDFIP2,TMC8,IKBKB,NFKBIB,SFRP1,SH3BP5,NR4A1,HHEX,ITCH,ERBIN,SH3RF1,WWTR1,IL7,ADGRG3,FAM192A,NLRC5,IQGAP2,IFIT2,IFIT1,SNCA,DTNBP1,CD200,SIK1,ITPRIP,PPP3CA,HYAL2,HECW2,FABP4,SH3BP4,PPP2R5A,RPS6KA1,PROX1,F11R,OAZ3,ACE,PAK2,CST6,RNF2,B2M,CTTNBP2NL,EIF4A2,LTF,ADORA2A,ITGA4,DDX3X,BIRC3,CPEB2,LMTK2,PPM1F,RCC2,KDM5A,RGS14,PSEN1,PAPLN,AMOT,ADRB2,ARHGAP28,FAM122C,USP7,NOTCH1,CNN3,TERF2,DAB2IP,DUS2,SLC8A3,GMFG,LYPD6,SERPINB9,COMMD7,BST2,BMP2,INKA1,DUSP7,PPP1R2	turquoise	EC_M6
GO:0035195	4.89E-02	0.973557345	17	107	gene silencing by miRNA	BP	5.67E-01	CNOT6,IL6,MYCN,TUT4,PUM2,PPARG,DND1,SMAD1,PPP3CA,RBM4,MOV10,DDX3X,SMAD2,CNOT8,MIR23A,MIR99A,MIR1307	turquoise	EC_M6

GO:0071407	5.84E-02	0.955499645	74	448	cellular response to organic cyclic compound	BP	5.68E-01	PPARA,PPARD,CTNNA1,LGALS1,LEP,CLOCK,REST,ACTR2,ROCK2,INHBA,VPS35,MGARP,PGRMC2,GLI2,PAK1,TGFB1,HTR5A,CTNNB1,LBH,UFM1,ATP2B1,ZNF703,UBA5,DDRGK1,ARL6IP5,APLP1,RNF6,ANKRD1,HSP90B1,RYR3,P2RX4,HEY1,APP,RAPGEF2,ADCY6,CRY2,VPS18,ATP2B4,SPP1,PKD2,EIF4E,DNM1L,HTR2B,GSK3B,CASP7,DAG1,BMP7,ADCY5,NCSTN,STRN3,MED1,PPARGC1A,MSN,KCNMB1,COL1A1,SMARCA4,AKT1,ITGA2,GNAL,GNB1,FLNA,SSTR5,PARK7,HCN2,GNAI3,HSPA5,SERPINF1,TMEM38B,TMEM38A,FAM210B,YWHAH,PMEPA1,HCN4,MBD2	darkgreen	EC_M4
GO:1902893	4.91E-02	0.979056095	10	47	regulation of pri-miRNA transcription by RNA polymerase II	BP	5.68E-01	PDGFB,GATA2,PPARG,GATA3,SMAD1,ETS1,HIF1A,YY1,MRTFB,BMP2	turquoise	EC_M6
GO:0014074	4.92E-02	0.972558719	19	109	response to purine-containing compound	BP	5.68E-01	TEK,TRPM4,PIK3CG,P2RY6,P2RY2,PDE2A,P2RX2,DNMT3B,GPD1,P2RX7,STC1,RAPGEF3,STAT1,HDAC1,ADORA2A,IGFBP5,ADSS,RAPGEF1,SLC8A3	turquoise	EC_M6
GO:0072001	5.86E-02	0.958832548	45	256	renal system development	BP	5.69E-01	AHI1,RRM2B,ANGPT1,VEGFA,TGFBR1,GLI3,FGF2,SEC61A1,GLI2,SNX17,CTNNB1,RARB,DCN,ILK,ADAMTS6,SULF1,RDH10,GDF6,PKD2,ARL3,NOTCH2,TGFB2,BMP7,AP1B1,BICC1,FGFR2,IQGAP1,ARID5B,OVOL1,SGPL1,FBN1,SIX4,ACTA2,MAGED1,MYO1E,TMED10,TTC8,MYOCD,PDGFRB,PKD1,FSTL3,SERPINF1,GDNF,HAS2,FGF10	darkgreen	EC_M4
GO:0007606	5.87E-02	0.974364517	10	43	sensory perception of chemical stimulus	BP	5.69E-01	REEP2,CALHM1,LEF1,FZD2,TTC8,GNAL,GNB1,SCNN1D,OR5AN1,OR6N2	darkgreen	EC_M4
GO:0035088	5.88E-02	0.974365637	10	42	establishment or maintenance of apical/basal cell polarity	BP	5.69E-01	LIN7B,PDCD6IP,ILK,FAT1,CLIC4,PRKCI,WDR1,MSN,TTC8,ARF4	darkgreen	EC_M4
GO:0061245	5.88E-02	0.974365637	10	42	establishment or maintenance of bipolar cell polarity	BP	5.69E-01	LIN7B,PDCD6IP,ILK,FAT1,CLIC4,PRKCI,WDR1,MSN,TTC8,ARF4	darkgreen	EC_M4
GO:0046326	5.89E-02	0.977325986	8	31	positive regulation of glucose import	BP	5.69E-01	AKT2,OSBPL8,IRS2,SORBS1,PTPN11,CLTCL1,PRKCI,AKT1	darkgreen	EC_M4
GO:0097352	5.89E-02	0.975671963	9	37	autophagosome maturation	BP	5.69E-01	UBQLN1,VCP,MAP1LC3A,VAMP8,ATP2A2,TSG101,CALM1,VMP1,CLEC16A	darkgreen	EC_M4
GO:0015849	5.90E-02	0.960392103	36	203	organic acid transport	BP	5.69E-01	ATP8B1,SLC12A2,PPARA,SLC22A4,PPARD,PLIN2,LEP,SLC17A5,MFSD2A,SLC7A11,ITGB1,SLC36A4,RAB3GAP1,AKT2,SLC38A10,IRS2,ARL6IP5,P2RX4,SLC26A7,SERINC3,PLA2R1,CPT1A,EPRS,SLC3A2,SLC6A13,PRAF2,SLC29A4,CLN8,SFXN1,SLC25A44,SLC7A3,ACSL4,AKT1,PRKAA1,SLC36A1,XK	darkgreen	EC_M4
GO:0015807	5.90E-02	0.973022417	11	48	L-amino acid transport	BP	5.69E-01	SLC7A11,ITGB1,SLC36A4,ARL6IP5,SERINC3,SLC3A2,PRAF2,CLN8,SFXN1,SLC7A3,SLC36A1	darkgreen	EC_M4
GO:0030850	5.91E-02	0.975589091	9	37	prostate gland development	BP	5.69E-01	GLI3,GLI2,CTNNB1,SULF1,BMP7,FGFR2,SERPINF1,SERPINB5,FGF10	darkgreen	EC_M4
GO:0030517	5.91E-02	0.97565087	9	36	negative regulation of axon extension	BP	5.69E-01	RTN4,SEMA3C,RYK,RNF6,DIP2B,SEMA4F,SEMA4G,TNR,PLXNA3	darkgreen	EC_M4
GO:0014808	5.91E-02	0.979050598	7	26	release of sequestered calcium ion into cytosol by sarcoplasmic reticulum	BP	5.69E-01	FKBP1B,CASQ1,CACNA1C,CALM1,DMD,TMEM38B,TMEM38A	darkgreen	EC_M4
GO:1903514	5.91E-02	0.979050598	7	26	release of sequestered calcium ion into cytosol by endoplasmic reticulum	BP	5.69E-01	FKBP1B,CASQ1,CACNA1C,CALM1,DMD,TMEM38B,TMEM38A	darkgreen	EC_M4
GO:0010675	5.92E-02	0.964889687	22	114	regulation of cellular carbohydrate metabolic process	BP	5.69E-01	DYRK2,PPARA,LEP,DGAT2,AKT2,C1QTNF1,KAT2B,IRS2,SORBS1,ADIPOR1,SELENOS,GSK3B,PPP1R3D,PPP1R3F,PPARGC1A,AKT1,PTPN2,PGP,MIDN,FAM3A,RANBP2,NNMT	darkgreen	EC_M4
GO:0045778	5.92E-02	0.974180321	10	42	positive regulation of ossification	BP	5.69E-01	FBN2,TGFB1,ATP2B1,PTPN11,ACVR1,TGFB2,BMP7,FZD9,BMPR1A,TGFB3	darkgreen	EC_M4
GO:1903053	5.94E-02	0.974006697	10	43	regulation of extracellular matrix organization	BP	5.70E-01	LRP1,RB1,TGFB1,RUNX1,FAP,PHLDB2,SLC2A10,DAG1,PHLDB1,HAS2	darkgreen	EC_M4
GO:0003203	5.95E-02	0.977061391	8	31	endocardial cushion morphogenesis	BP	5.70E-01	TGFBR1,ROBO1,HEY1,ACVR1,TGFB2,BMP7,BMPR1A,TMEM100	darkgreen	EC_M4

GO:0060669	5.95E-02	0.981082653	6	21	embryonic placenta morphogenesis	BP	5.70E-01	RSPO3,SPINT2,TMED2,LEF1,BMP7,FGFR2	darkgreen	EC_M4
GO:0050927	4.94E-02	0.986655206	5	20	positive regulation of positive chemotaxis	BP	5.71E-01	KDR,CXCL8,VEGFC,PGF,S1PR1	turquoise	EC_M6
GO:0061339	4.95E-02	0.98476023	6	23	establishment or maintenance of monopolar cell polarity	BP	5.71E-01	SYNE4,TCF15,FSCN1,OPHN1,PATJ,CARMIL2	turquoise	EC_M6
GO:0042445	4.95E-02	0.971331733	21	133	hormone metabolic process	BP	5.71E-01	RDH16,ALDH8A1,HSD17B3,SRD5A3,GATA3,SCARB1,TIPARP,PLEKHA1,ACE,HIF1A,ALDH1A2,SCNN1B,DHRS11,CYP1A1,CHST9,DIO3,BMP2,SRD5A2,BMP6,AKR1D1,RBP1	turquoise	EC_M6
GO:0043367	4.95E-02	0.977736014	11	56	CD4-positive, alpha-beta T cell differentiation	BP	5.71E-01	IL6,RSAD2,GATA3,SH3RF1,HLX,CD86,CRACR2A,MTOR,SEMA4A,BATF,LGALS9	turquoise	EC_M6
GO:2000010	5.96E-02	0.983688327	5	16	positive regulation of protein localization to cell surface	BP	5.71E-01	GPD1L,RIC3,TM9SF4,AKT1,MAP1A	darkgreen	EC_M4
GO:0009247	5.97E-02	0.970453808	13	61	glycolipid biosynthetic process	BP	5.71E-01	B4GALNT1,LARGE1,TMEM246,ST8SIA4,PIGP,B3GALT1,PIGZ,PIGL,PRKAA1,DPM2,PIGK,DPM1,PIGB	darkgreen	EC_M4
GO:0014896	5.98E-02	0.967943694	16	78	muscle hypertrophy	BP	5.71E-01	PPARA,LEP,ROCK2,PAK1,P2RX4,ATP2A2,ATP2B4,TWF1,PDLIM5,HTR2B,PRKCA,GLRX3,TCAP,PIN1,ROCK1,MIR24-1	darkgreen	EC_M4
GO:0060602	6.00E-02	0.98355036	5	16	branch elongation of an epithelium	BP	5.72E-01	RDH10,FGFR2,SIX4,MED1,FGF10	darkgreen	EC_M4
GO:0035050	4.97E-02	0.97769672	11	55	embryonic heart tube development	BP	5.72E-01	TEAD2,MEF2C,NDRG4,VANGL2,HIF1A,PSEN1,NOTCH1,ENG,SOX17,SOX18,PIFO	turquoise	EC_M6
GO:0022612	6.03E-02	0.965747524	19	95	gland morphogenesis	BP	5.73E-01	GLI3,PLXNA1,GLI2,TGFB1,SEMA3C,SULF1,KDM5B,NOTCH2,TGFB2,LRP5,DAG1,BMP7,FGFR2,MED1,MSN,TGFB3,SERPINB5,EDA,FGF10	darkgreen	EC_M4
GO:1902414	6.03E-02	0.967013817	17	82	protein localization to cell junction	BP	5.73E-01	SHANK1,KIF5B,ACTG1,PCLO,TJP1,DAG1,MPP4,HSPB1,MAPT,VCL,IQSEC2,CGNL1,NLGN2,STX1B,RAB11A,FLNA,MAP1A	darkgreen	EC_M4
GO:0042789	6.03E-02	0.986867025	4	11	mRNA transcription by RNA polymerase II	BP	5.73E-01	AP3B1,MED1,SUPT6H,FLNA	darkgreen	EC_M4
GO:0008016	6.03E-02	0.962043531	27	144	regulation of heart contraction	BP	5.73E-01	BVES,FKBP1B,SCN5A,GPD1L,ATP2B1,FXYD1,SNTA1,P2RX4,ATP2A2,CACNA1D,ADA,ATP2B4,PKP2,TGFB2,GLRX3,CACNA1C,HSPB7,TPM1,CACNA1G,ADRA1B,CALM1,FLNA,DMD,TMEM38B,TMEM38A,HOPX,HCN4	darkgreen	EC_M4
GO:0006165	6.04E-02	0.968350373	15	72	nucleoside diphosphate phosphorylation	BP	5.73E-01	PPARA,CMPK1,PFKP,APP,PFKM,PGAM4,ZBTB20,TPI1,PPARGC1A,PKM,AK7,PRKAA1,ENO1,SLC2A6,NME6	darkgreen	EC_M4
GO:0051125	4.98E-02	0.984434964	6	25	regulation of actin nucleation	BP	5.73E-01	PICK1,FCHSD2,HIP1R,AP1AR,WAS,GMFG	turquoise	EC_M6
GO:0051938	6.05E-02	0.986808584	4	11	L-glutamate import	BP	5.74E-01	SLC7A11,ITGB1,ARL6IP5,CLN8	darkgreen	EC_M4
GO:0098712	6.05E-02	0.986808584	4	11	L-glutamate import across plasma membrane	BP	5.74E-01	SLC7A11,ITGB1,ARL6IP5,CLN8	darkgreen	EC_M4
GO:0031345	6.06E-02	0.961526351	28	150	negative regulation of cell projection organization	BP	5.74E-01	LRP1,LGALS1,RTN4,PRNP,SEMA3C,DPYSL3,RYK,RNF6,DIP2B,SEMA4F,PFN2,TLX2,RAPGEF2,ADCY6,SPP1,SEMA4G,TNR,CAPZB,CRMP1,STX1B,EVI5L,PLXNB3,PAFAH1B1,FLNA,PLXNA3,ITM2C,THY1,YWHAH	darkgreen	EC_M4
GO:0051099	6.06E-02	0.962174078	26	141	positive regulation of binding	BP	5.74E-01	LRP1,RB1,MAPRE3,TGFB1,CTNNB1,DDRGK1,PPP2CB,PINX1,APP,RAPGEF2,AMFR,AKTIP,CAPRIN2,GSK3B,EIF2S1,EIF3C,CALM1,MYOCD,PIN1,ITGA2,PKD1,PARK7,GPSM1,FKBP1A,TXN,MBD2	darkgreen	EC_M4
GO:0050688	4.99E-02	0.97853748	10	50	regulation of defense response to virus	BP	5.74E-01	TNFAIP3,C6orf106,DDX58,IL15,SEC14L1,TMEM173,ITCH,IFIT1,STAT1,RNF125	turquoise	EC_M6
GO:0120032	5.00E-02	0.969566512	25	158	regulation of plasma membrane bounded cell projection assembly	BP	5.74E-01	TBC1D30,PODXL,CDC42EP1,KIT,NRP1,OCLN,P2RX7,GPM6A,PPP1R16B,CEP97,SRGAP2C,LIMK2,DNM3,WAS,RAC1,RCC2,FSCN1,EPHA2,MTOR,ABI3,RABEP2,DNM2,FNBP1L,CARMIL2,RALA	turquoise	EC_M6
GO:0001919	5.01E-02	0.986663562	5	18	regulation of receptor recycling	BP	5.74E-01	SNCA,AP1AR,INPP5F,PSEN1,VAMP3	turquoise	EC_M6

GO:0045913	5.01E-02	0.977412726	11	57	positive regulation of carbohydrate metabolic process	BP	5.74E-01	PDGFB,P2RY6,IGF1,GPD1,P2RX7,SNCA,SLC45A3,MLXIPL,HIF1A,PSEN1,PMAIP1	turquoise	EC_M6
GO:0002052	6.09E-02	0.983255667	5	16	positive regulation of neuroblast proliferation	BP	5.75E-01	VEGFA,GLI3,SMARCD3,CTNNB1,CX3CL1	darkgreen	EC_M4
GO:0034754	6.10E-02	0.967173983	16	79	cellular hormone metabolic process	BP	5.75E-01	HSD17B6,REST,DGAT2,DKK3,RDH10,SPP1,SGPL1,COMT,ECE1,HSD17B1,CYP19A1,HSD17B10,STARD3,MED1,PPARGC1A,CYP46A1	darkgreen	EC_M4
GO:0010889	6.11E-02	0.986310089	4	12	regulation of sequestering of triglyceride	BP	5.75E-01	PPARA,PLIN2,ABHD5,OSBPL8	darkgreen	EC_M4
GO:1904589	6.12E-02	0.970654809	12	55	regulation of protein import	BP	5.76E-01	ANGPT1,LEP,RAB23,MDFIC,GLI3,TGFB1,UFM1,HDAC3,CHP1,HSPA8,FLNA,ZPR1	darkgreen	EC_M4
GO:0050854	6.13E-02	0.973017969	10	43	regulation of antigen receptor-mediated signaling pathway	BP	5.77E-01	PRNP,PTPN22,ADA,CBLB,SLC39A10,PTPN6,PTPN2,IKBKG,BCL10,THY1	darkgreen	EC_M4
GO:0071415	6.13E-02	0.986578968	4	11	cellular response to purine-containing compound	BP	5.77E-01	RYR3,PPARGC1A,TMEM38B,TMEM38A	darkgreen	EC_M4
GO:0000154	3.53E-02	0.989611004	6	31	rRNA modification	BP	5.77E-01	DIMT1,GAR1,FTSJ3,METTL16,NAF1,TFB2M	darkgrey	EC_M8
GO:0060512	6.14E-02	0.980310437	6	21	prostate gland morphogenesis	BP	5.77E-01	GLI2,SULF1,BMP7,FGFR2,SERPINB5,FGF10	darkgreen	EC_M4
GO:0071280	6.16E-02	0.986144464	4	12	cellular response to copper ion	BP	5.77E-01	PRNP,MAP1LC3A,APP,BACE1	darkgreen	EC_M4
GO:0050930	6.17E-02	0.986103309	4	12	induction of positive chemotaxis	BP	5.78E-01	VEGFA,CXCL12,ARTN,FGF10	darkgreen	EC_M4
GO:0006082	4.02E-03	0.99715797	101	689	organic acid metabolic process	BP	5.78E-01	ZADH2,GFPT2,HSD17B8,EP300,MPST,ETFB,PTGR1,IL4I1,LPIN1,ACSS1,SEPHS1,ECHDC3,ACAA1,BCKDHA,CYGB,HYI,CSGALNACT1,PNP,HACL1,CYP1B1,GNPDA1,APIP,PEPD,THNSL2,CBR4,ACSS2,PRKAG1,LPIN3,HNF4A,FARS2,ERLIN1,PLA2G4B,ACOT8,WARS2,LONP2,ALDH5A1,IRS1,ACADS,CARMIL1,GPAM,GPX1,QARS,DALRD3,ABCB11,PC,ARNT,CTPS2,PER2,HDAC4,GLUL,SCAP,EHHADH,POR,IDH1,ACADL,MTHFS,PCBD1,PRODH,ATIC,PECR,NAT8L,CYP27A1,TNFRSF1A,STAT5A,FADS3,FOXK1,ILVBL,EPHX1,TECR,MTHFR,MRI1,CRABP2,OSBPL7,HSD3B7,ACOT2,ALDH6A1,ABCD4,GCDH,NUPR1,AC138894.1,SCPEP1,SLC27A3,ACSM3,ACACA,INSR,SREBF1,SLC46A1,ACAA2,CPT2,ASPA,GAMT,JMJD8,GSTM4,DPYD,KARS,PTGES2,LDHD,FPGS,SULT1C4,ZBTB7A,SCP2	cyan	EC_M2
GO:0043154	5.07E-02	0.977996934	10	55	negative regulation of cysteine-type endopeptidase activity involved in apoptotic process	BP	5.78E-01	TNFAIP8,NR4A1,SH3RF1,SNCA,RPS6KA1,PAK2,ADORA2A,DDX3X,BIRC3,SERPINB9	turquoise	EC_M6
GO:0016050	5.07E-02	0.965977313	37	255	vesicle organization	BP	5.78E-01	STX11,LAPTM4B,TSNARE1,CD2AP,SNAP91,CAV2,SDCBP,RAB38,TMCC1,NECTIN2,TBC1D4,MYO7A,CLCN3,DYSF,SNCA,DTNBP1,ABCG1,SNAP23,LYST,SEC24B,LRMP,CD34,CREB1,ALS2CL,SNAP29,RAB27A,SYT7,ZDHHC15,MYO18A,AP3D1,STXBP1,FNBP1L,RAB32,STX3,TBC1D20,VAMP3,RAB39A	turquoise	EC_M6
GO:0006816	5.07E-02	0.965246708	41	282	calcium ion transport	BP	5.78E-01	TRDN,PDGFB,TSPAN13,GJA4,TRPM4,PIK3CG,CXCR4,P2RY6,P2RX2,MICU3,PDE4D,CHD7,P2RX7,GPM6A,CXCL10,TPCN1,PLCG1,STC1,ANO6,SNCA,LCK,PPP3CA,EPB41,PRKD1,ADORA2A,HOMER3,MCU,CALCRL,RAMP2,CRACR2A,PSEN1,CHRNB2,ATP2A1,CD19,CCL2,PDE4B,PLCG2,SLC25A25,SLC8A3,RGS4,CACNG5	turquoise	EC_M6
GO:0031032	5.07E-02	0.968183389	28	172	actomyosin structure organization	BP	5.78E-01	LIMCH1,PGM5,TMOD1,NRP1,SFRP1,LMOD3,FHDC1,RAPGEF3,EDN1,MEF2A,NRAP,EPB41,CFLAR,PROX1,TMOD4,F11R,PAK2,PPM1F,WAS,TMOD2,RAC1,MTOR,ARHGEF15,CDC42BPB,ARHGAP28,MYO18A,S1PR1,CNN3	turquoise	EC_M6
GO:0043409	5.08E-02	0.97052958	21	131	negative regulation of MAPK cascade	BP	5.78E-01	SEMA6A,RNF149,TLR4,PPARG,SFRP1,SIRT3,ITCH,P2RX7,DUSP8,PRDM15,HYAL2,DUSP16,DLG1,MECOM,RGS14,EFNA1,TNIP1,DAB2IP,DUSP5,BMP2,DUSP7	turquoise	EC_M6
GO:0042159	5.08E-02	0.988887818	4	14	lipoprotein catabolic process	BP	5.78E-01	PPT1,NOTUM,ABHD13,ABHD17C	turquoise	EC_M6
GO:0017158	6.19E-02	0.977860935	7	26	regulation of calcium ion-dependent exocytosis	BP	5.79E-01	REST,RAB3GAP1,ATP2A2,SYT11,CACNA1G,SCAMP5,CBARP	darkgreen	EC_M4

GO:0043094	4.07E-03	0.999112211	8	23	cellular metabolic compound salvage	BP	5.79E-01	PNP,APIP,MRI1,APRT,FUK,TK2,PDXK,UPP1	cyan	EC_M2
GO:0061298	5.09E-02	0.986699408	5	16	retina vasculature development in camera-type eye	BP	5.79E-01	NRP1,ACVR2B,HIF1A,RHOJ,ARHGEF15	turquoise	EC_M6
GO:0046718	5.11E-02	0.972306434	17	100	viral entry into host cell	BP	5.80E-01	CAV2,NRP1,NECTIN2,CXCR4,EFNB2,SCARB1,ITCH,TPCN1,HYAL2,CD86,F11R,EPHA2,LGALS9,HAVCR1,TRIM34,CD46,TRIM62	turquoise	EC_M6
GO:0060100	5.11E-02	0.98920318	4	12	positive regulation of phagocytosis, engulfment	BP	5.80E-01	GATA2,CD36,ANO6,PLCG2	turquoise	EC_M6
GO:1905155	5.11E-02	0.98920318	4	12	positive regulation of membrane invagination	BP	5.80E-01	GATA2,CD36,ANO6,PLCG2	turquoise	EC_M6
GO:0042063	5.12E-02	0.966226819	34	230	gliogenesis	BP	5.81E-01	IFNGR1,PICK1,TNFRSF21,IL6,TLR4,MYCN,TAL1,ITGB4,CXCR4,MMP24,HEXB,TLR2,SOX13,TSPAN2,SLC45A3,SRGAP2C,HDAC1,CREB1,ADORA2A,PRKCH,MPP5,TNFRSF1B,MTOR,PSEN1,EIF2B2,B4GALT6,SH3TC2,CCL2,NOTCH1,DAB2IP,SLC8A3,ADGRG1,RELN,BMP2	turquoise	EC_M6
GO:0002686	5.14E-02	0.981846869	7	33	negative regulation of leukocyte migration	BP	5.81E-01	C5,LRCH1,CD200,RIN3,APOD,MMP28,CCL2	turquoise	EC_M6
GO:0045619	5.14E-02	0.971078457	19	117	regulation of lymphocyte differentiation	BP	5.81E-01	TCF7,SYK,IL15,GATA3,SFRP1,SH3RF1,IL7,SOX13,HLX,INPP5D,CD86,SOS2,ITPKB,LGALS9,IL7R,AP3D1,NRARP,VNN1,CD46	turquoise	EC_M6
GO:0035774	5.14E-02	0.983804317	6	25	positive regulation of insulin secretion involved in cellular response to glucose stimulus	BP	5.81E-01	TRPM4,ABCG1,ANO1,RAC1,HIF1A,GPR68	turquoise	EC_M6
GO:0050796	6.22E-02	0.961915042	24	127	regulation of insulin secretion	BP	5.81E-01	LRP1,PPARD,LEP,CLOCK,REST,FKBP1B,KIF5B,IRS2,OSBP,ABCC8,PTPN11,PFKM,BRSK2,DYNLL1,CPT1A,LRP5,HADH,ADCY5,SIDT2,NLGN2,SYTL4,ACSL4,MIDN,SSTR5	darkgreen	EC_M4
GO:0061001	6.23E-02	0.972590346	10	42	regulation of dendritic spine morphogenesis	BP	5.81E-01	ACTR2,DHX36,OPA1,PDLIM5,DNM1L,CAPRIN2,KIF1A,TANC2,SRCIN1,PAFAH1B1	darkgreen	EC_M4
GO:0051606	5.17E-02	0.969920034	21	135	detection of stimulus	BP	5.82E-01	PTK2,KIT,TLR4,GRIK2,DACH1,CXCR4,P2RX2,SCARB1,EPHB1,MMP24,TLR2,ANO3,ANO1,RTP4,JUP,TLR6,GNGT2,UNC119,PIEZO2,PIEZO1,TLR1	turquoise	EC_M6
GO:1902745	5.18E-02	0.981867675	7	30	positive regulation of lamellipodium organization	BP	5.82E-01	ARHGEF7,OCLN,RAC1,FSCN1,MTOR,DNM2,CARMIL2	turquoise	EC_M6
GO:0080164	5.18E-02	0.97874281	9	45	regulation of nitric oxide metabolic process	BP	5.82E-01	TLR4,CD36,EDN1,CD34,TMEM106A,RAC1,TLR6,MTOR,DNM2	turquoise	EC_M6
GO:0009308	5.18E-02	0.974548468	13	77	amine metabolic process	BP	5.82E-01	SNCAIP,ODC1,GATA3,NR4A2,SNCA,SLC7A7,OAZ3,GDPD3,CHRNB2,NPR1,GDPD1,CYP1A1,ATCAY	turquoise	EC_M6
GO:0120163	5.19E-02	0.978717855	9	44	negative regulation of cold-induced thermogenesis	BP	5.82E-01	ATF4,TLR4,IL15,LAMA4,NOVA2,ACVR2B,ABHD6,TLE3,NOTCH1	turquoise	EC_M6
GO:0032615	5.20E-02	0.98006778	8	38	interleukin-12 production	BP	5.82E-01	SYK,TLR4,CD36,TLR2,LAPTM5,LGALS9,IRF8,PLCG2	turquoise	EC_M6
GO:0032655	5.20E-02	0.98006778	8	38	regulation of interleukin-12 production	BP	5.82E-01	SYK,TLR4,CD36,TLR2,LAPTM5,LGALS9,IRF8,PLCG2	turquoise	EC_M6

GO:0019538	6.25E-02	0.943908732	589	4113	protein metabolic process	BP	5.83E-01	SERPINB2,SEC11C,IL23A,NAB2,LRP1,TRIM36,HBS1L,B4GALT7,AGAP2,CANX,TAB2,CAND1,DYRK2,RNF19A,PRRC1,AZIN1,PLXNB2,ANGPT1,PPARA,P4HA2,ROS1,NUS1,PHF20L1,HABP4,PTP4A3,ADGRB1,PPARD,C6orf89,UBQLN1,MTCH1,GGA1,KLHDC10,SH3GL2,GNAQ,MYH9,LEP,CDKN2B,PLAA,LARGE1,NUAK1,CDK14,VEGFA,VCP,C2orf40,NCK2,CLOCK,PDCL3,CHST10,REST,EIF5B,PTP4A1,P3H1,TGFBR1,ERP44,TMEM246,SOCS5,ERLEC1,PSME4,COPS9,UBA6,RTN4,CYCS,ADAMTS3,SLC35A1,GARS,MINDY4,UBE2J1,MAP3K7,SH3BP5L,GFPT1,PCYOX1,GLI3,ROCK2,INHBA,RCN3,PREP,MYLK3,VPS35,CUL2,FKBP1B,FGF2,SRPK2,RPN1,DNAJC1,RAB7A,PSMC2,POMGNT1,GSAP,ABHD12,PRSS23,RB1,ITM2B,EED,IWS1,PICALM,MARK4,MAPRE3,CRTAP,EIF2B3,PDCD6IP,TMUB1,KCTD21,CHPF2,NUB1,PAK1,TGFB1,AXL,RAB3GAP1,LMO7,WDR48,UBXN4,ST6GALNAC2,SPOPL,CTNNB1,TRAK1,DGAT2,SERPINH1,AKT2,GPD1L,STT3B,MTMR14,CANT1,OSBPL8,NPLOC4,PPP1R12A,KAT2B,RNF121,TMTC4,FAM98A,CRIM1,PSMD8,GPS1,SPINT2,SUMF1,KDELC1,UFM1,TMTC3,PRNP,CHFR,CARS2,NUDT16,DCN,HDAC3,DCLK1,ACPP,UBA5,DDRGK1,SMPD1,PPP2CB,ILK,RBPMS,MMP17,ARL6IP5,PPP2R2B,UBA3,CPXM1,SPINK5,CIAO1,TUSC3,PSMD6,CDK8,CNOT7,RNF6,VPS37A,IMPAD1,MTMR6,FXYD1,HECTD2,ADAMTS6,SNTA1,PJA2,HSP90B1,TMED2,EIF2S2,TP53INP2,MAST4,OSBP,ST8SIA4,EIF4G2,SULF1,RNF181,GRK7,RIOK2,LNPEP,KCMF1,PCSK1,ROBO1,TRPC4AP,B3GNT4,DIP2B,PINX1,PLOD2,ELOC,CDKL2,LOXL3,RNF13,PFN2,SELENOT,CELF1,CCNJ,MMP15,APP,ART3,ATXN2,FAM98B,DHX36,RAPGEF2,TET3,RPN2,MAPKAPK5,CX3CL1,KLHL2,PPP2R	darkgreen	EC_M4
GO:0055057	6.26E-02	0.986189542	4	11	neuroblast division	BP	5.83E-01	TEAD3,LEF1,FGFR2,ARHGEF2	darkgreen	EC_M4
GO:0050771	5.21E-02	0.976524219	11	52	negative regulation of axonogenesis	BP	5.84E-01	SEMA6A,CDKL3,SEMA4D,NRP1,SEMA3F,TRAK2,SEMA6D,PSEN1,SEMA4A,SEMA6B,ARHGAP4	turquoise	EC_M6
GO:0001709	5.22E-02	0.981702007	7	30	cell fate determination	BP	5.84E-01	NOTCH4,GATA2,GATA3,JAG1,MEF2C,PROX1,SOX17	turquoise	EC_M6
GO:0033146	6.28E-02	0.975386018	8	32	regulation of intracellular estrogen receptor signaling pathway	BP	5.85E-01	PAK1,LBH,UFM1,UBA5,DDRGK1,VPS18,STRN3,MED1	darkgreen	EC_M4
GO:1903076	6.30E-02	0.964629448	18	90	regulation of protein localization to plasma membrane	BP	5.85E-01	LRP1,STX7,ITGB1,KIF5B,PICALM,TGFB1,ATP2C1,PRNP,VAMP8,SORBS1,ATP2B4,MMP14,AP2M1,LRRC15,PRKCI,RAB11A,CLTC,AKT1	darkgreen	EC_M4
GO:0032008	6.31E-02	0.973541732	9	38	positive regulation of TOR signaling	BP	5.85E-01	LEP,RHEB,SEC13,HDAC3,PIK3CA,GPR137,OTUD5,AKT3,CLEC16A	darkgreen	EC_M4
GO:0001938	6.31E-02	0.96594807	16	78	positive regulation of endothelial cell proliferation	BP	5.85E-01	VEGFA,PDCL3,TGFBR1,EMC10,FGF2,CXCL12,HTR2B,PRKCA,VASH2,AKT3,AKT1,MYDGF,PLXNB3,CDH13,F3,WNT2	darkgreen	EC_M4
GO:0033993	6.32E-02	0.949573824	104	657	response to lipid	BP	5.86E-01	CDO1,TAB2,PPARA,PPARD,MTAP,LEP,PLAA,RORB,CLOCK,REST,TGFBR1,ROCK2,INHBA,MGARP,PGRMC2,GLB1,PAK1,TGFB1,AXL,HTR5A,HNMT,CTNNB1,LBH,DGAT2,UFM1,ATP2B1,DCN,ZNF703,UBA5,DDRGK1,RNF6,ANKRD1,CD180,MAP1B,HEY1,ATP2A2,MMP15,CX3CL1,ABCC8,ADCY6,PTPN22,CRY2,VPS18,MTDH,SPP1,CPN1,PSPH,EIF4E,CDC73,DNM1L,SELENOS,CCND1,CPT1A,TGFB2,DAG1,PRKCA,KRAS,NTRK3,BMP7,ADCY5,FGFR2,RBBP7,STRN3,RPS6KA3,SERPINE1,COMT,KCNK4,IGFBP2,UPF1,CYP19A1,SGMS1,OTUD5,MED1,PPARGC1A,MSN,KCNMB1,COL1A1,TGFB3,SOX30,CALM1,SMARCA4,PDGFRB,RBBP8,AKT1,ITGA2,PRKAA1,LDOC1,ABCA3,IRAK1,SSTR5,PARK7,AGL,BCL10,LOXL1,SERPINF1,LOX,IL36G,CDKN1A,FAM210B,YWHAH,PMEPA1,WNT2,FGF10,MBD2	darkgreen	EC_M4
GO:0090085	6.33E-02	0.985624079	4	12	regulation of protein deubiquitination	BP	5.86E-01	VCP,WDR48,TANK,PARK7	darkgreen	EC_M4
GO:0002064	6.33E-02	0.958325604	32	173	epithelial cell development	BP	5.86E-01	CDSN,ROS1,VEGFA,CLOCK,COL15A1,WHRN,ROCK2,RHEB,SLC4A7,RARB,SPINT2,FAT1,PALLD,FLNB,SLC4A5,RAPGEF2,TJP1,NOTCH2,GSK3B,IQGAP1,CLIC4,SIDT2,PLOD3,VCL,ACTA2,MSN,MYO1E,ROCK1,AKT1,PAFAH1B1,FLNA,CDKN1A	darkgreen	EC_M4
GO:1902692	6.34E-02	0.979506159	6	21	regulation of neuroblast proliferation	BP	5.86E-01	CTNNA1,VEGFA,GLI3,SMARCD3,CTNNB1,CX3CL1	darkgreen	EC_M4
GO:0061512	6.34E-02	0.969452309	12	54	protein localization to cilium	BP	5.86E-01	GGA1,DZIP1,TCTN1,ARL3,TULP3,ROM1,ATP6V1D,NPHP4,TCTN2,SNX10,ARF4,CC2D2B	darkgreen	EC_M4
GO:0031102	6.34E-02	0.970582599	11	49	neuron projection regeneration	BP	5.86E-01	LRP1,CTNNA1,FKBP1B,MAP1B,SPP1,DAG1,TNR,PRRX1,MAP2K1,FLNA,THY1	darkgreen	EC_M4

GO:0007600	6.34E-02	0.953698635	54	320	sensory perception	BP	5.86E-01	ATP8B1,COL11A2,REEP2,RORB,CNNM4,RIMS1,SHANK1,MYH14,WHRN,EML2,TMTC4,LUM,ACPP,CXCL12,GRK7,RDH10,P2RX4,CACNA1D,GLRB,CACNB3,OPA1,NDN,CHRNA7,CALHM1,LEF1,RLBP1,CRYBA4,ATF6,BACE1,FZD2,COMT,KCNK4,ADRA2C,KCNA1,WDR1,ROM1,NLGN2,CLN8,COL1A1,TMEM100,CLN6,TTC8,TRPV2,ITGA2,GNAL,PITPNA,GNB1,SCNN1D,COL11A1,TIMP3,LHFPL4,OTOR,OR5AN1,OR6N2	darkgreen	EC_M4
GO:0035556	6.35E-02	0.944587398	307	2063	intracellular signal transduction	BP	5.86E-01	IL23A,LRP1,ARHGEF25,TAB2,DYRK2,RAB21,RRM2B,ANGPT1,PPARA,ROS1,FAM49B,PTP4A3,PPARD,TEAD3,UBQLN1,LGALS1,KLHDC10,ARHGAP39,LEP,NUAK1,VEGFA,DOCK8,SLC35B2,UNC13B,COL1A2,NCK2,CLOCK,EXOC1,CREB3,TGFBR1,SOCS5,RTN4,TPBG,EMC10,MAP3K7,SH3BP5L,MDFIC,ROCK2,RRAS,RCN3,CUL2,ITGB1,FKBP1B,FGF2,SRPK2,SESN3,ARHGEF4,RB1,MARK4,TPT1,DGKH,RHEB,PAK1,TGFB1,AXL,HTR5A,SEC13,CTNNB1,LBH,AKT2,DYNC1LI1,GPD1L,CANT1,C1QTNF1,ARHGEF17,OSBPL8,CXXC5,CLPB,PSD2,NPLOC4,ACTN4,ANAPC15,SPRED3,GPS1,ATP2C1,PRNP,CHFR,DCN,CDC42EP3,HDAC3,DCLK1,PCLO,STARD13,CAVIN3,DDRGK1,SMPD1,PPP2CB,ILK,ARL6IP5,RYK,TMEM127,TMEM9B,ST5,AKIP1,CXCL12,SLC20A1,ANKRD1,PJA2,MRAS,MAST4,MRVI1,ROBO1,WWC2,TEAD1,DOK1,P2RX4,RNF13,RASA1,ATP2A2,USO1,RTKN,APP,DHX36,RAPGEF2,MAPKAPK5,CX3CL1,HUNK,PTPN11,ADCY6,RALGAPB,OPA1,ARHGAP1,PTPN22,PPP3CC,CHAC1,GDF6,SERINC3,MTDH,ADA,CHP1,ATP2B4,PLA2R1,CBLB,WSB2,TANK,BRSK2,DOK2,CHRNA7,ARL3,DOCK10,ADIPOR1,NOTCH2,DNM1L,SELENOS,CCND1,TGFB2,ADGRB2,KCTD10,ARHGAP31,HTR2B,PRKAR1A,GSK3B,INPP5A,DAG1,PIK3CA,AUTS2,PRKCA,CMKLR1,KRAS,NTRK3,WWC3,SPATC1L,BMP7,ADCY5,HYOU1,GOLT1B,ATF3,FGFR2,ASB1,OSM,CASQ1,PEA15,TP53BP1,SYAP1,IQGAP1,TFRC,FARP2,RPS6KA3,PRDX4,HSPB1,MAPT,SYVN1,EIF4EBP2,ATP6AP2,ZNF385B,DNAJC10,FN1,NCKAP1,MAP4K5,PTPN6,GPR137,GNB1L,PDCD10,ITGAV,ADRA2C,HTT,FKBP8,ERO1A,COL3A1,PRKCI,SKIL,SYNPO2L,FGFR3,ARAF,TNIK,PLD1,VDAC2,MYO9B,FMN2,SGMS1,OTUD5,AKT3,ERC1,CACNA1	darkgreen	EC_M4
GO:0002090	6.35E-02	0.971857614	10	43	regulation of receptor internalization	BP	5.86E-01	CD63,AHI1,ANGPT1,MKLN1,VEGFA,ATXN2,AP2M1,LRPAP1,ATAD1,LRRTM2	darkgreen	EC_M4
GO:0086010	6.37E-02	0.975187393	8	30	membrane depolarization during action potential	BP	5.87E-01	SCN5A,SLMAP,CACNA1D,CACNA1C,CACNA1G,HCN2,YWHAH,HCN4	darkgreen	EC_M4
GO:0042157	6.38E-02	0.962244664	21	111	lipoprotein metabolic process	BP	5.87E-01	LRP1,PPARA,LEP,TMEM246,ABHD12,RAB3GAP1,DGAT2,PIGP,WIPI1,PIGZ,OLR1,ITGAV,ZDHHC4,WIPI2,PIGL,ZDHHC9,DPM2,PEMT,PIGK,DPM1,PIGB	darkgreen	EC_M4
GO:0050863	6.38E-02	0.956786263	36	206	regulation of T cell activation	BP	5.87E-01	IL23A,HLA-DRA,FAM49B,LGALS1,LEP,DOCK8,NCK2,SOCS5,GLI3,GLI2,CTNNB1,PRNP,HSPH1,SPINK5,LMO1,IL20RB,LOXL3,PTPN11,AP3B1,PTPN22,ADA,RUNX1,CBLB,PRKAR1A,PIK3CA,LEF1,TFRC,PTPN6,IGFBP2,CD74,AKT1,PTPN2,CD40LG,BCL10,THY1,FGL2	darkgreen	EC_M4
GO:1903405	6.39E-02	0.985441671	4	12	protein localization to nuclear body	BP	5.87E-01	TCP1,CCT8,DKC1,LARP7	darkgreen	EC_M4
GO:1904867	6.39E-02	0.985441671	4	12	protein localization to Cajal body	BP	5.87E-01	TCP1,CCT8,DKC1,LARP7	darkgreen	EC_M4
GO:0008645	6.39E-02	0.964712194	17	83	hexose transmembrane transport	BP	5.87E-01	PPARD,LEP,MFSD4B,AKT2,OSBPL8,IRS2,SORBS1,PTPN11,SELENOS,SLC2A10,PEA15,CLTCL1,PRKCI,SLC2A4,AKT1,SLC2A6,SLC2A8	darkgreen	EC_M4
GO:0002089	5.26E-02	0.985653422	5	19	lens morphogenesis in camera-type eye	BP	5.88E-01	ATF4,HIPK1,PROX1,EPHA2,TBC1D20	turquoise	EC_M6
GO:0010002	6.41E-02	0.982094831	5	16	cardioblast differentiation	BP	5.88E-01	SRF,REST,DHX36,TGFB2,MYOCD	darkgreen	EC_M4

GO:1901566	6.41E-02	0.94567253	195	1317	organonitrogen compound biosynthetic process	BP	5.88E-01	CDO1,HBS1L,B4GALT7,B4GALNT1,AZIN1,PLXNB2,ANGPT1,PPARA,ACSL6,NUS1,HABP4,PSAT1,MTAP,LARGE1,VCP,SLC35B2,NCK2,ASNS,CHST10,EIF5B,TMEM246,MFSD2A,GARS,UBE2J1,GFPT1,ROCK2,INHBA,SLC7A11,CMPK1,RPN1,DNAJC1,POMGNT1,ITM2B,EIF2B3,CHPF2,TGFB1,RAB3GAP1,ST6GALNAC2,ACER3,CTNNB1,TRAK1,AKT2,STT3B,CANT1,TMTC4,KDELC1,TMTC3,NAXD,CARS2,SMPD1,TUSC3,CNOT7,CHPT1,TMED2,EIF2S2,ST8SIA4,EIF4G2,PCSK1,B3GNT4,CHST2,PLOD2,CERS5,ALDH18A1,SELENOT,CELF1,APP,ATXN2,DHX36,TET3,RPN2,MAPKAPK5,ADCY6,DPY19L4,LPCAT2,CHAC1,SERINC3,ADA,GLS,CHP1,ATP2B4,CSDE1,TMEM14C,PIGP,MAN1A2,TENT4B,MRPS17,PSPH,CARS,EIF4E,AZIN2,CHSY1,HYAL1,SLC2A10,CAPRIN2,TGFB2,EPRS,B4GALT4,TYW5,WIPI1,MOCS3,B3GALT1,IDH2,ADCY5,HACD2,GALNT2,MAN2A2,NCSTN,UROS,PIGZ,RPS6KA3,SMS,EDEM3,PCYT1B,HSPB1,EIF3J,SLC25A16,PLOD3,EIF4EBP2,SGPL1,MRPS14,ZC3H15,UPF1,CHCHD1,SAMD4A,DDOST,QDPR,PLD1,SGMS1,ZDHHC4,B3GAT3,UGDH,WIPI2,AGMAT,CLN8,EIF2S1,CHST12,PPARGC1A,NPPB,GALNT16,CPEB4,XYLT2,EIF3C,SPTLC2,GALNT10,CLTC,PKM,SLC44A2,GALNT1,MPI,PIGL,ACSL4,PDGFRB,ROCK1,AKT1,PGRMC1,MOCS2,ITGA2,PRKAA1,ZDHHC9,TARS,DAPK3,CSNK1G2,HAGH,ACOT7,TMLHE,PARK7,ENO1,SARS,POMT1,DPM2,AARS,BARHL2,NFE2L1,DMD,HACD1,AGO3,TPK1,ITM2C,PEMT,HAS2,TMA7,EIF1,PIGK,DPM1,TMSB4X,NNMT,MRPL51,NME6,PIGB,ADI1,ORMDL3,SLC35C1,CHST11,DENR,HS2ST1	darkgreen	EC_M4
GO:0050892	5.28E-02	0.983370946	6	24	intestinal absorption	BP	5.89E-01	IREB2,TJP2,CD36,SCARB1,EPB41,F11R	turquoise	EC_M6
GO:0043410	6.45E-02	0.952194421	59	356	positive regulation of MAPK cascade	BP	5.90E-01	LRP1,ANGPT1,KLHDC10,LEP,VEGFA,TGFBR1,TPBG,EMC10,MAP3K7,ROCK2,FGF2,PAK1,TGFB1,CTNNB1,C1QTNF1,CAVIN3,ILK,ARL6IP5,RYK,ST5,PJA2,ROBO1,APP,RAPGEF2,CX3CL1,PTPN11,PTPN22,GDF6,CHRNA7,NOTCH2,TGFB2,HTR2B,PRKCA,KRAS,NTRK3,FGFR2,OSM,IQGAP1,PDCD10,ADRA2C,FGFR3,TNIK,MAGED1,SHC1,ADRA1B,MAP2K1,NPTN,CD74,PDGFRB,ROCK1,MYDGF,IRAK1,ATP6AP1,BCAR3,MIR24-1,CCL16,GLIPR2,DUSP22,FGF10	darkgreen	EC_M4
GO:0045197	6.45E-02	0.972893788	9	37	establishment or maintenance of epithelial cell apical/basal polarity	BP	5.90E-01	LIN7B,PDCD6IP,ILK,FAT1,PRKCI,WDR1,MSN,TTC8,ARF4	darkgreen	EC_M4
GO:1990573	5.32E-02	0.979651803	8	35	potassium ion import across plasma membrane	BP	5.91E-01	KCNJ3,KCNJ15,KCNJ2,SLC12A7,DLG1,WNK4,KCNJ18,KCNH2	turquoise	EC_M6
GO:0051253	5.33E-02	0.956706606	131	1032	negative regulation of RNA metabolic process	BP	5.91E-01	PLAGL1,NOTCH4,TCF7,L3MBTL3,ATF4,PDGFB,MDFI,NFX1,NFIL3,SEMA4D,IL6,NFE2L3,PTBP3,BACH2,FOXP2,KLF11,LANCL2,CREM,SDCBP,NOCT,BEND5,TAL1,PHF10,EPC1,GATA2,DNMT3A,SAP130,DACH1,PPARG,GATA3,TAF3,ACVR2B,GZF1,EDNRB,PDE2A,SFRP1,NR1D2,DND1,CD36,ARID5A,DNMT3B,HHEX,ZBTB38,POU6F1,SOX7,HMBOX1,MEF2C,WWTR1,SFMBT1,SCAF4,ANKRD2,NR4A2,DLL4,CDYL,SOX13,YAF2,EDN1,STAT1,DYRK1A,SNCA,JARID2,ETS2,DCAF1,HDAC1,PPP3CA,MEF2A,FABP4,HCLS1,NFATC2,KDM2A,CIART,TGIF1,PARP15,PROX1,ZNF148,MLXIPL,DLG1,ETV6,RNF2,CREB1,EIF4A2,BCOR,MECOM,ING4,SPINDOC,LDB2,PPM1F,POU2F1,ZBTB18,APBB2,BCL6B,KDM5C,MAFK,MTOR,AR,RNF40,PSEN1,CBX1,HOXB8,FBXW11,EFNA1,PIAS1,RPS6KA5,SMAD2,TLE3,YY1,HMG20A,RCOR1,HCFC1,NRARP,TRAF2,NOTCH1,IRF8,MTF2,ZBTB34,DAB2IP,ZBTB33,ARID4A,ZNF24,ATXN1L,TRIM24,SOX18,PASD1,COMMD7,TGIF2,BMP2,GLIS3,ZNF10,CBX2,BMP6,ZNF266	turquoise	EC_M6
GO:0051567	5.33E-02	0.981285588	7	29	histone H3-K9 methylation	BP	5.91E-01	KDM4D,SETD5,DNMT3B,KDM3A,JARID2,MECOM,ARID4A	turquoise	EC_M6
GO:0055001	5.33E-02	0.967692249	24	150	muscle cell development	BP	5.91E-01	PGM5,TMOD1,CAV2,P2RX2,LMOD3,IGF1,DYSF,EDN1,PPP3CA,MEF2A,NRAP,NFATC2,CFLAR,PROX1,TMOD4,RAMP2,TMOD2,CHRNB1,MTOR,YY1,NOTCH1,ENG,MIR23A,RGS4	turquoise	EC_M6
GO:0002292	5.34E-02	0.976785062	10	50	T cell differentiation involved in immune response	BP	5.91E-01	IL6,GATA3,HLX,CD86,CRACR2A,MTOR,SEMA4A,BATF,LGALS9,CD46	turquoise	EC_M6
GO:0120033	5.34E-02	0.983031706	6	25	negative regulation of plasma membrane bounded cell projection assembly	BP	5.91E-01	TBC1D30,CEP97,SRGAP2C,LIMK2,ABI3,DNM2	turquoise	EC_M6

GO:0051146	5.34E-02	0.964905522	33	220	striated muscle cell differentiation	BP	5.91E-01	PGM5,TMOD1,CAV2,EFNB2,ATP11A,P2RX2,LMOD3,IGF1,MEF2C,CXCL10,TANC1,EDN1,SIK1,PPP3CA,MEF2A,BCL9,NRAP,NFATC2,PLEKHO1,CFLAR,PROX1,TMOD4,MYEF2,IGFBP5,TBX1,TMOD2,MTOR,YY1,NOTCH1,PIEZO1,MIR23A,RGS4,BMP2	turquoise	EC_M6
GO:0015718	5.35E-02	0.974567003	12	66	monocarboxylic acid transport	BP	5.91E-01	TNFRSF11A,SYK,SLCO2B1,CD36,SLCO2A1,EDN1,SLC16A14,SLC16A9,ACE,SLC16A13,SLC51B,NOS2	turquoise	EC_M6
GO:0071887	6.47E-02	0.964900194	16	79	leukocyte apoptotic process	BP	5.91E-01	TCP1,DOCK8,GLI3,SLC7A11,AXL,IRS2,CXCL12,RAPGEF2,ADA,SLC39A10,SELENOS,CASP7,CD74,AKT1,BCL10,ORMDL3	darkgreen	EC_M4
GO:0120193	6.48E-02	0.968690857	12	54	tight junction organization	BP	5.91E-01	SRF,TGFBR1,ROCK2,PDCD6IP,ACTG1,ABCC8,MTDH,TJP1,TGFB3,ROCK1,MYO1C,NPHP4	darkgreen	EC_M4
GO:0097530	5.36E-02	0.971991223	15	91	granulocyte migration	BP	5.92E-01	PTK2,SYK,CXCL8,PIK3CG,CXCL10,THBS4,EDN1,JAM3,PECAM1,MCU,RAC1,DAPK2,S100A14,CCL2,PDE4B	turquoise	EC_M6
GO:0061082	5.37E-02	0.983022572	6	24	myeloid leukocyte cytokine production	BP	5.92E-01	DDX58,KIT,TLR4,CD36,LAPTM5,PLCG2	turquoise	EC_M6
GO:0002285	6.49E-02	0.960104659	24	127	lymphocyte activation involved in immune response	BP	5.92E-01	IL23A,HLA-DRA,LGALS1,SOCS5,APBB1IP,TGFB1,LIG4,CD180,LOXL3,CDH17,ADA,DOCK10,NOTCH2,LEF1,TP53BP1,TFRC,CD74,TMEM98,SUPT6H,CD40LG,VAMP7,AP1G1,FGL2,CCR6	darkgreen	EC_M4
GO:0008652	6.50E-02	0.968470231	12	56	cellular amino acid biosynthetic process	BP	5.92E-01	PSAT1,MTAP,ASNS,PLOD2,ALDH18A1,SERINC3,GLS,ATP2B4,PSPH,PLOD3,PARK7,ADI1	darkgreen	EC_M4
GO:1904816	6.50E-02	0.985106753	4	12	positive regulation of protein localization to chromosome, telomeric region	BP	5.92E-01	TCP1,CCT8,GNL3,DKC1	darkgreen	EC_M4
GO:0048841	5.38E-02	0.981060909	7	29	regulation of axon extension involved in axon guidance	BP	5.93E-01	SEMA6A,SEMA4D,NRP1,SEMA3F,SEMA6D,SEMA4A,SEMA6B	turquoise	EC_M6
GO:0050886	6.51E-02	0.969637685	11	49	endocrine process	BP	5.93E-01	LEP,INHBA,C1QTNF1,RAB11FIP1,PTPN11,CRY2,KRAS,LINC02210-CRHR1,ATP6AP2,ECE1,RASL10B	darkgreen	EC_M4
GO:0001570	6.52E-02	0.965521365	15	71	vasculogenesis	BP	5.93E-01	VEGFA,RRAS,TGFB1,CTNNB1,HEY1,RASA1,RAPGEF2,HIF1AN,SGPL1,ITGAV,MYO1E,TMEM100,MYOCD,PDGFRB,HAS2	darkgreen	EC_M4
GO:0019752	4.23E-03	0.997022904	97	659	carboxylic acid metabolic process	BP	5.94E-01	ZADH2,GFPT2,HSD17B8,EP300,MPST,ETFB,PTGR1,IL4I1,LPIN1,ACSS1,SEPHS1,ECHDC3,ACAA1,BCKDHA,CYGB,HYI,CSGALNACT1,HACL1,CYP1B1,GNPDA1,APIP,PEPD,THNSL2,CBR4,ACSS2,PRKAG1,LPIN3,HNF4A,FARS2,ERLIN1,PLA2G4B,ACOT8,WARS2,LONP2,ALDH5A1,IRS1,ACADS,GPAM,GPX1,QARS,DALRD3,ABCB11,PC,ARNT,CTPS2,PER2,HDAC4,GLUL,SCAP,EHHADH,POR,IDH1,ACADL,MTHFS,PCBD1,PRODH,ATIC,PECR,NAT8L,CYP27A1,TNFRSF1A,FADS3,FOXK1,ILVBL,EPHX1,TECR,MTHFR,MRI1,CRABP2,OSBPL7,HSD3B7,ACOT2,ALDH6A1,ABCD4,GCDH,NUPR1,AC138894.1,SCPEP1,SLC27A3,ACSM3,ACACA,INSR,SREBF1,SLC46A1,ACAA2,CPT2,ASPA,GAMT,JMJD8,GSTM4,DPYD,KARS,PTGES2,LDHD,FPGS,ZBTB7A,SCP2	cyan	EC_M2
GO:0006351	4.25E-03	0.996542128	312	2521	transcription, DNA-templated	BP	5.94E-01	NFATC1,ZNF236,TSHZ1,SMARCC2,STAT6,MAP3K5,CDK4,CITED2,MAML1,SQSTM1,IRAK3,OSR2,HSPA1B,EHMT2,ZBTB12,HDAC10,ATF6B,RXRB,RING1,ZHX2,ZBTB22,MYC,TOB2,SMAD5,EP300,KDM3B,ZNF76,DDX17,NFIB,HSF1,CCND3,TRERF1,CDKN2A,HMOX1,WASL,KLF9,POLR1C,JAK2,ZBTB45,ZNF135,ZNF667,ZNF865,EFEMP1,ZNF692,ANXA4,AKNA,MED25,IL4I1,ING3,ARHGEF5,IRF3,LPIN1,FOXO3,KLF15,NOP53,MAML2,BICRA,KMT2E,BBS7,MEIS3,TRIM13,IRF2BP1,SIX5,FOSB,ZNF746,NR2C2,ZNF862,TSC22D1,ZNF575,NAA16,GTF3C2,ZNF574,WBP2,KMT2C,MYD88,TADA3,RPS3,BRPF1,DYRK1B,EID2,ZNF219,MED29,SIRT2,RPTOR,APBB3,NUP98,TRIM21,CYP1B1,HGF,APBB1,TAF10,DEAF1,HOXC4,NCOA4,LIN37,KMT2B,RBCK1,RARG,SDR16C5,ZNF740,LMO2,POFUT1,URI1,PIK3R1,NCOA2,ATF1,KDM2B,ZFHX4,CAMKK2,SCAND1,SETD6,MCPH1,SSBP2,FNIP2,ARL2BP,SHOX2,NEK4,ZHX3,VDR,MMS19,LPIN3,FOXF2,F2RL1,SORBS3,HNF4A,TP53INP1,PIDD1,HR,TRIM45,NPM3,ELK4,LGR4,ICE1,SP140,TCF7L2,TXNIP,AIP,SOX5,ADNP,ZNF217,QARS,DTX3L,PARP14,ECM1,RCOR3,ARNT,PRMT2,RIPK3,GABPB2,PER2,TWIST2,VPS72,HDAC4,USF1,SMARCD2,POGZ,USP21,PATZ1,RNASEL,CCDC85B,MAGEF1,CDKN1B,TCEA3,KAT5,LRP6,YBX3,NFKBIA,NFE2L2,POLR2J2,PHC1,HEXIM2,BATF2,PCBD1,CHCHD10,MEN1,PHB2,TSC22D4,SAV1,TAF6,CTDSP1,EID1,SSBP4,RNF25,FGF7,NELFA,ZSCAN25,ZNF394,TNFRSF1A,STK16,STAT5A,KAT6B,CREG1,NR2F6,HDAC6,PQBP1,CAMTA2,TFE3,PELP1,ZNF12,WIZ,ZBTB25,RARA,FOXK1,AC090517.4,ZBTB17,GPS2,THRA,KLF3,MED24,PRDM2,ARHGEF11,SPIN4,UIMC1,CC2D1A,ZNF771,MLLT6,CTBP1,CRABP2,ZNF6	cyan	EC_M2

GO:0007611	5.40E-02	0.965625937	29	183	learning or memory	BP	5.94E-01	ATP8A1,KIT,ADGRF1,PPT1,RIC8A,PLK2,MEF2C,TLR2,NDRG4,TANC1,SHANK2,ADCY1,YTHDF1,PTCHD1,CREB1,B2M,TMOD2,HIF1A,RGS14,MTOR,PSEN1,ITGA3,CHRNB2,PIAS1,GIT1,SLC8A3,ATXN1L,RELN,SHANK3	turquoise	EC_M6
GO:0007162	5.40E-02	0.964732417	32	215	negative regulation of cell adhesion	BP	5.94E-01	SEMA6A,NOTCH4,PTK2,PODXL,TNFRSF21,SEMA4D,PELI1,PLXND1,JAG1,ITCH,ANGPT2,JAM3,LAPTM5,HLX,AP1AR,CD86,PLXNA2,ARHGDIB,MELTF,DLG1,SIPA1,APOD,ADORA2A,PPM1F,RCC2,ARG2,LGALS9,NRARP,NOTCH1,BMP2,CBLL1,BMP6	turquoise	EC_M6
GO:0060491	5.41E-02	0.966810207	25	159	regulation of cell projection assembly	BP	5.94E-01	TBC1D30,PODXL,CDC42EP1,KIT,NRP1,OCLN,P2RX7,GPM6A,PPP1R16B,CEP97,SRGAP2C,LIMK2,DNM3,WAS,RAC1,RCC2,FSCN1,EPHA2,MTOR,ABI3,RABEP2,DNM2,FNBP1L,CARMIL2,RALA	turquoise	EC_M6
GO:0002711	5.41E-02	0.982572822	6	27	positive regulation of T cell mediated immunity	BP	5.94E-01	IL6,RSAD2,NECTIN2,GATA3,B2M,TRAF2	turquoise	EC_M6
GO:0034134	5.43E-02	0.987857172	4	14	toll-like receptor 2 signaling pathway	BP	5.95E-01	TNFAIP3,TLR2,TLR6,TLR1	turquoise	EC_M6
GO:0002689	5.44E-02	0.987824824	4	14	negative regulation of leukocyte chemotaxis	BP	5.95E-01	C5,RIN3,MMP28,CCL2	turquoise	EC_M6
GO:1902622	5.45E-02	0.98039247	7	34	regulation of neutrophil migration	BP	5.95E-01	CXCL8,THBS4,EDN1,JAM3,MCU,RAC1,DAPK2	turquoise	EC_M6
GO:0061614	5.46E-02	0.976226766	10	48	pri-miRNA transcription by RNA polymerase II	BP	5.95E-01	PDGFB,GATA2,PPARG,GATA3,SMAD1,ETS1,HIF1A,YY1,MRTFB,BMP2	turquoise	EC_M6
GO:0002090	5.46E-02	0.977439438	9	43	regulation of receptor internalization	BP	5.95E-01	PICK1,SYK,SDCBP,MTMR2,RIN3,SELE,OPHN1,PLCG2,GSG1L	turquoise	EC_M6
GO:0001954	6.55E-02	0.96941561	11	49	positive regulation of cell-matrix adhesion	BP	5.96E-01	VEGFA,RRAS,ILK,CX3CL1,PTPN11,GSK3B,DAG1,IQGAP1,ROCK1,CDH13,THY1	darkgreen	EC_M4
GO:1902600	6.56E-02	0.961018809	21	115	proton transmembrane transport	BP	5.96E-01	CYB5A,ATP6V1C1,ATP6V1F,SLC17A5,ATP6V0B,ACTN4,TMCO3,SLC25A4,SLC33A1,CHP1,ATP6V1B2,SLC2A10,ATP6V1D,COX6A2,ATP6AP1,PARK7,ATP6V0D1,SURF1,SLC36A1,TMSB4X,ATP6V0E1	darkgreen	EC_M4
GO:0030879	6.57E-02	0.960536425	22	114	mammary gland development	BP	5.96E-01	CDO1,VEGFA,RTN4,GLI3,FGF2,GLI2,LBH,AKT2,IRS2,ZNF703,ROBO1,KDM5B,CCND1,LRP5,LEF1,FGFR2,MED1,TGFB3,AKT1,ITGA2,WNT2,FGF10	darkgreen	EC_M4
GO:0008207	6.57E-02	0.978388789	6	22	C21-steroid hormone metabolic process	BP	5.96E-01	REST,DKK3,HSD17B10,STARD3,PPARGC1A,CYP46A1	darkgreen	EC_M4
GO:1901836	3.66E-02	0.992706924	4	15	regulation of transcription of nucleolar large rRNA by RNA polymerase I	BP	5.96E-01	PWP1,NCL,NOL11,DDX11	darkgrey	EC_M8
GO:0019319	6.58E-02	0.966965869	13	61	hexose biosynthetic process	BP	5.96E-01	PPARA,LEP,DGAT2,KAT2B,SELENOS,ATF3,TPI1,PPARGC1A,PTPN2,PGP,FAM3A,RANBP2,NNMT	darkgreen	EC_M4
GO:0006760	3.67E-02	0.990751835	5	22	folic acid-containing compound metabolic process	BP	5.96E-01	MTHFD1L,DHFR,TYMS,SLC25A32,GART	darkgrey	EC_M8
GO:0071868	6.59E-02	0.965938186	14	66	cellular response to monoamine stimulus	BP	5.97E-01	VPS35,HTR5A,APLP1,APP,ADCY6,ATP2B4,HTR2B,GSK3B,ADCY5,NCSTN,GNAL,GNB1,FLNA,GNAI3	darkgreen	EC_M4
GO:0071870	6.59E-02	0.965938186	14	66	cellular response to catecholamine stimulus	BP	5.97E-01	VPS35,HTR5A,APLP1,APP,ADCY6,ATP2B4,HTR2B,GSK3B,ADCY5,NCSTN,GNAL,GNB1,FLNA,GNAI3	darkgreen	EC_M4
GO:0090646	6.60E-02	0.984805353	4	12	mitochondrial tRNA processing	BP	5.97E-01	MTO1,TRIT1,TRNT1,HSD17B10	darkgreen	EC_M4
GO:0050918	5.49E-02	0.977109206	9	47	positive chemotaxis	BP	5.97E-01	PDGFB,KDR,CXCL8,NRP1,VEGFC,CXCL10,ANGPT2,PGF,S1PR1	turquoise	EC_M6
GO:0019827	3.68E-02	0.980852633	17	106	stem cell population maintenance	BP	5.98E-01	FANCC,ZC3H13,WDR43,HOOK3,METTL3,RIF1,CUL4A,CNOT1,SMC3,ASPM,DDX6,MED28,LEO1,SMC1A,MED6,NIPBL,SRRT	darkgrey	EC_M8
GO:0038127	6.65E-02	0.961115584	20	102	ERBB signaling pathway	BP	6.00E-01	NCK2,SOCS5,RTN4,RAB7A,TGFB1,PTPN11,TSG101,CBLB,PIK3CA,IQGAP1,HAP1,SHC1,AKT1,PTPN2,RHBDF1,CDH13,BCAR3,PTPN12,ARF4,PIK3C2A	darkgreen	EC_M4

GO:0050794	6.65E-02	0.939912522	1071	7581	regulation of cellular process	BP	6.00E-01	SERPINB2,CD63,LMAN1,IL23A,ATP8B1,NAB2,LRP1,STX7,AP3S1,TRIM36,HBS1L,B4GALT7,AHI1,ARHGEF25,AGAP2,ABRACL,ENPP3,TAB2,CAND1,DYRK2,IER3,RAB21,CDSN,RNF19A,RRM2B,PRRC1,SLC12A2,AZIN1,ATP6V1C1,PLXNB2,FBN2,ANGPT1,FAM19A5,TCP1,PPARA,HLA-DRA,ROS1,NUS1,RSPO3,CXCL14,FAM49B,PHF20L1,MRTFA,TRAPPC9,HABP4,PACSIN1,GNG5,PTP4A3,REEP2,ADGRB1,LY6E,SCUBE3,PPARD,CTNNA1,TEAD3,SUN2,CBY1,MKLN1,C6orf89,UBQLN1,MTCH1,LGALS1,MDGA1,KLHDC10,TXN2,SH3GL2,GNAQ,MYH9,ARHGAP39,LEP,CDKN2B,PLAA,RORB,BTBD11,SLC30A9,KIAA1324L,SRF,BAG1,NUAK1,CDK14,VEGFA,DOCK8,VCP,SLC35B2,RUNX2,RASL11B,UNC13B,COL1A2,C2orf40,NCK2,CLOCK,EXOC1,CREB3,ASNS,PDCL3,REST,EIF5B,ASPN,INPP4A,ZSCAN18,DST,RAB23,SNX13,PTP4A1,NLRP5,COL15A1,P3H1,TGFBR1,ERP44,HIVEP3,SOCS5,RIMS1,TMEM30A,ERLEC1,PSME4,COPS9,RTN4,CYCS,TPBG,SHANK1,ADAMTS3,LRRC4B,EMC10,TAX1BP1,MFSD2A,GGCT,UBE2J1,MAP3K7,SH3BP5L,AKIRIN1,ACTR2,THAP5,KIDINS220,MDFIC,TES,GLI3,NCDN,ROCK2,INHBA,BVES,RRAS,ZYX,RCN3,MYLK3,DKKL1,VPS35,CUL2,MGARP,SLC7A11,ITGB1,PGRMC2,PLEKHA4,DBP,FKBP1B,PLXNA1,FGF2,SRPK2,KIF5B,NAPA,SESN3,ORC5,DNAJC1,RAB7A,PSMC2,ARHGEF4,GSAP,APBB1IP,FBLN2,EML2,ZNF398,RB1,ITM2B,ABHD5,EED,IWS1,PICALM,MARK4,TPT1,NUFIP1,ZNF296,MAPRE3,CRTAP,CGREF1,EIF2B3,PTCH2,PDCD6IP,TENM4,DGKH,KCTD21,GLI2,MEGF8,ATP6V0B,SMARCD3,PCDH17,NUB1,RHEB,SNX17,PAK1,ACTR3,TGFB1,AXL,RAB3GAP1,SCN5A,LMO7,WDR48,HTR5A,SEC13,SFMBT	darkgreen	EC_M4
GO:0017014	6.66E-02	0.984615547	4	12	protein nitrosylation	BP	6.00E-01	SNTA1,ATP2B4,DMD,TXN	darkgreen	EC_M4
GO:0018119	6.66E-02	0.984615547	4	12	peptidyl-cysteine S-nitrosylation	BP	6.00E-01	SNTA1,ATP2B4,DMD,TXN	darkgreen	EC_M4
GO:0051955	5.53E-02	0.982265305	6	25	regulation of amino acid transport	BP	6.00E-01	SNCA,ADORA2A,PSEN1,SLC43A2,STXBP1,RGS4	turquoise	EC_M6
GO:0048167	5.54E-02	0.96667867	23	143	regulation of synaptic plasticity	BP	6.01E-01	RIMS2,ATF4,PICK1,KIT,GRIK2,PLK2,ABHD6,MCTP1,MEF2C,SNCA,SHANK2,ADCY1,YTHDF1,CREB1,ADORA2A,SYT7,RGS14,PSEN1,STXBP1,SLC8A3,RELN,SHANK3,STX3	turquoise	EC_M6
GO:0046889	5.54E-02	0.974431746	11	61	positive regulation of lipid biosynthetic process	BP	6.01E-01	ADGRF5,RAB38,SIRT3,SCARB1,ABHD6,SLC45A3,ABCG1,MLXIPL,CREB1,MTOR,BMP6	turquoise	EC_M6
GO:0055117	5.55E-02	0.974488633	11	58	regulation of cardiac muscle contraction	BP	6.01E-01	TRPM4,PIK3CG,PDE4D,STC1,KCNJ2,DLG1,JUP,ATP2A1,DSG2,DSC2,PDE4B	turquoise	EC_M6
GO:0006661	6.68E-02	0.959763052	22	115	phosphatidylinositol biosynthetic process	BP	6.02E-01	INPP4A,TMEM246,SOCS5,FAM126A,FGF2,MTMR14,IMPAD1,MTMR6,TMEM150A,PIGP,HTR2B,PIK3CA,PITPNM1,PIGZ,PIGL,OCRL,DPM2,PIGK,DPM1,PIGB,PIK3C2A,CDIPT	darkgreen	EC_M4
GO:0051250	5.57E-02	0.970034616	16	99	negative regulation of lymphocyte activation	BP	6.03E-01	TNFAIP3,TNFRSF21,PELI1,SFRP1,MERTK,ITCH,LAPTM5,HLX,INPP5D,CD86,FGR,DLG1,ADORA2A,ARG2,LGALS9,NRARP	turquoise	EC_M6
GO:0090280	5.57E-02	0.987418691	4	14	positive regulation of calcium ion import	BP	6.03E-01	PDGFB,STC1,ATP2A1,CCL2	turquoise	EC_M6
GO:0061647	3.72E-02	0.986661554	8	38	histone H3-K9 modification	BP	6.03E-01	ZNF451,SETDB2,RIF1,CHEK1,BRCA1,SUV39H1,ATRX,EHMT1	darkgrey	EC_M8

GO:0051246	6.70E-02	0.941632294	288	1953	regulation of protein metabolic process	BP	6.03E-01	SERPINB2,IL23A,NAB2,LRP1,HBS1L,AGAP2,TAB2,RNF19A,PRRC1,AZIN1,PLXNB2,ANGPT1,HABP4,ADGRB1,C6orf89,UBQLN1,MTCH1,GGA1,SH3GL2,GNAQ,MYH9,LEP,CDKN2B,PLAA,NUAK1,VEGFA,VCP,NCK2,PDCL3,REST,EIF5B,P3H1,TGFBR1,SOCS5,PSME4,COPS9,RTN4,CYCS,MAP3K7,SH3BP5L,ROCK2,INHBA,RCN3,VPS35,FKBP1B,FGF2,DNAJC1,RAB7A,PSMC2,GSAP,RB1,ITM2B,IWS1,PICALM,MAPRE3,CRTAP,NUB1,PAK1,TGFB1,RAB3GAP1,WDR48,SPOPL,CTNNB1,DGAT2,SERPINH1,AKT2,GPD1L,OSBPL8,PPP1R12A,KAT2B,CRIM1,SPINT2,TMTC3,PRNP,CHFR,HDAC3,DDRGK1,SMPD1,ILK,RBPMS,ARL6IP5,SPINK5,CNOT7,FXYD1,SNTA1,HSP90B1,TMED2,OSBP,EIF4G2,ROBO1,DIP2B,PINX1,PFN2,SELENOT,CELF1,CCNJ,APP,ATXN2,DHX36,RAPGEF2,MAPKAPK5,CX3CL1,PTPN11,PTX3,GNL3,GTF2H1,PTPN22,TSG101,ACVR1,CRY2,CHAC1,GDF6,AKTIP,CHP1,ATP2B4,PKD2,RUNX1,CSDE1,TWF1,DBNDD2,CBLB,NKD1,BRD7,IFT57,TENT4B,TANK,TOLLIP,PSMD14,NDN,SLC39A10,CHRNA7,EIF4E,AZIN2,TM4SF20,UCHL5,TRIP12,SLC2A10,CCND1,CAPRIN2,SEC22B,TGFB2,PPP6R3,PSMD1,LRP5,EPRS,HTR2B,PRKAR1A,GSK3B,MMP14,DAG1,PIK3CA,AUTS2,CORO1C,ANOS1,KRAS,NTRK3,FAM162A,SPATC1L,BMP7,NEDD8,PPP1R11,COL6A3,OSM,NCSTN,SYAP1,PSMD2,ADRM1,IQGAP1,TFRC,RPS6KA3,EGLN3,HSPB1,MAPT,SERPINE1,EIF4EBP2,DNAJC10,TFR2,PCOLCE,FN1,PTPN6,PDCD10,UPF1,ITGAV,ADRA2C,DOK7,HTT,FKBP8,SAMD4A,FGFR3,RTN3,ARAF,SMURF1,TIMP1,TNIK,PLD1,FBXL5,FMN2,MNAT1,HAP1,MAGED1,BMPR1A,TSPYL2,CLN8,EIF2S1,CDK12,PPARGC1A,MSN,RNF111,SRCIN1,CPEB4,ARHGEF2,NGFR,SPOP,TMED10,TGFB3,EIF3C,	darkgreen	EC_M4
GO:0035418	6.70E-02	0.967396744	12	54	protein localization to synapse	BP	6.03E-01	SHANK1,KIF5B,PCLO,DAG1,MPP4,HSPB1,MAPT,IQSEC2,NLGN2,STX1B,RAB11A,MAP1A	darkgreen	EC_M4
GO:1901889	6.71E-02	0.975525109	7	26	negative regulation of cell junction assembly	BP	6.03E-01	LRP1,ROCK2,PHLDB2,MMP14,CORO1C,ROCK1,DUSP22	darkgreen	EC_M4
GO:0045851	6.71E-02	0.971489078	9	38	pH reduction	BP	6.03E-01	RAB7A,CCKBR,SLAMF8,CLIC4,ATP6AP2,CLN6,ATP6AP1,ATP6V0D1,ATP6V0E1	darkgreen	EC_M4
GO:0097659	4.36E-03	0.996449974	312	2522	nucleic acid-templated transcription	BP	6.04E-01	NFATC1,ZNF236,TSHZ1,SMARCC2,STAT6,MAP3K5,CDK4,CITED2,MAML1,SQSTM1,IRAK3,OSR2,HSPA1B,EHMT2,ZBTB12,HDAC10,ATF6B,RXRB,RING1,ZHX2,ZBTB22,MYC,TOB2,SMAD5,EP300,KDM3B,ZNF76,DDX17,NFIB,HSF1,CCND3,TRERF1,CDKN2A,HMOX1,WASL,KLF9,POLR1C,JAK2,ZBTB45,ZNF135,ZNF667,ZNF865,EFEMP1,ZNF692,ANXA4,AKNA,MED25,IL4I1,ING3,ARHGEF5,IRF3,LPIN1,FOXO3,KLF15,NOP53,MAML2,BICRA,KMT2E,BBS7,MEIS3,TRIM13,IRF2BP1,SIX5,FOSB,ZNF746,NR2C2,ZNF862,TSC22D1,ZNF575,NAA16,GTF3C2,ZNF574,WBP2,KMT2C,MYD88,TADA3,RPS3,BRPF1,DYRK1B,EID2,ZNF219,MED29,SIRT2,RPTOR,APBB3,NUP98,TRIM21,CYP1B1,HGF,APBB1,TAF10,DEAF1,HOXC4,NCOA4,LIN37,KMT2B,RBCK1,RARG,SDR16C5,ZNF740,LMO2,POFUT1,URI1,PIK3R1,NCOA2,ATF1,KDM2B,ZFHX4,CAMKK2,SCAND1,SETD6,MCPH1,SSBP2,FNIP2,ARL2BP,SHOX2,NEK4,ZHX3,VDR,MMS19,LPIN3,FOXF2,F2RL1,SORBS3,HNF4A,TP53INP1,PIDD1,HR,TRIM45,NPM3,ELK4,LGR4,ICE1,SP140,TCF7L2,TXNIP,AIP,SOX5,ADNP,ZNF217,QARS,DTX3L,PARP14,ECM1,RCOR3,ARNT,PRMT2,RIPK3,GABPB2,PER2,TWIST2,VPS72,HDAC4,USF1,SMARCD2,POGZ,USP21,PATZ1,RNASEL,CCDC85B,MAGEF1,CDKN1B,TCEA3,KAT5,LRP6,YBX3,NFKBIA,NFE2L2,POLR2J2,PHC1,HEXIM2,BATF2,PCBD1,CHCHD10,MEN1,PHB2,TSC22D4,SAV1,TAF6,CTDSP1,EID1,SSBP4,RNF25,FGF7,NELFA,ZSCAN25,ZNF394,TNFRSF1A,STK16,STAT5A,KAT6B,CREG1,NR2F6,HDAC6,PQBP1,CAMTA2,TFE3,PELP1,ZNF12,WIZ,ZBTB25,RARA,FOXK1,AC090517.4,ZBTB17,GPS2,THRA,KLF3,MED24,PRDM2,ARHGEF11,SPIN4,UIMC1,CC2D1A,ZNF771,MLLT6,CTBP1,CRABP2,ZNF6	cyan	EC_M2
GO:0086002	5.59E-02	0.976922829	9	41	cardiac muscle cell action potential involved in contraction	BP	6.04E-01	TRPM4,KCNJ3,KCNJ2,DLG1,JUP,DSG2,DSC2,KCNH2,SCN2B	turquoise	EC_M6
GO:0090342	3.74E-02	0.986465749	8	42	regulation of cell aging	BP	6.05E-01	FBXO5,PNPT1,ZMPSTE24,PRKDC,RBL1,EEF1E1,FOXM1,RSL1D1	darkgrey	EC_M8
GO:1903900	3.75E-02	0.98080377	16	104	regulation of viral life cycle	BP	6.06E-01	SRPK1,TRIM28,PPIH,TOP2B,IFIT5,PDE12,FAM208A,PPID,DDX5,TOP2A,HACD3,LARP1,ILF3,FMR1,PKN2,TRIM59	darkgrey	EC_M8
GO:0045671	5.62E-02	0.984377094	5	19	negative regulation of osteoclast differentiation	BP	6.06E-01	TLR4,SFRP1,TMEM178A,INPP5D,LTF	turquoise	EC_M6
GO:0099072	5.62E-02	0.976651824	9	43	regulation of postsynaptic membrane neurotransmitter receptor levels	BP	6.06E-01	CPT1C,ERBIN,DLG1,DNM3,OPHN1,AP2B1,STX3,CACNG5,GSG1L	turquoise	EC_M6
GO:0046653	3.76E-02	0.992334254	4	16	tetrahydrofolate metabolic process	BP	6.07E-01	MTHFD1L,DHFR,TYMS,GART	darkgrey	EC_M8

GO:1904738	6.75E-02	0.975186657	7	27	vascular associated smooth muscle cell migration	BP	6.07E-01	LRP1,FAM19A5,PAK1,IQGAP1,TPM1,MYOCD,PRKG1	darkgreen	EC_M4
GO:0006475	3.77E-02	0.979103843	20	136	internal protein amino acid acetylation	BP	6.07E-01	ZNF451,ZMPSTE24,PAXIP1,KAT14,EPC2,KAT6A,TAF7,BRCA2,TAF9,MCRS1,CHEK1,NAA50,MCM3AP,HAT1,ING5,BRCA1,TAF1,OGT,CTCF,AC118549.1	darkgrey	EC_M8
GO:2001239	6.77E-02	0.972929288	8	33	regulation of extrinsic apoptotic signaling pathway in absence of ligand	BP	6.07E-01	CTNNA1,INHBA,CX3CL1,EYA2,AKT1,DAPK3,GDNF,FGF10	darkgreen	EC_M4
GO:0019400	6.77E-02	0.980517072	5	17	alditol metabolic process	BP	6.07E-01	LEP,DGAT2,MOGAT3,TPI1,PGP	darkgreen	EC_M4
GO:0017157	6.78E-02	0.956832478	27	147	regulation of exocytosis	BP	6.07E-01	RAB21,REST,RAB7A,PDCD6IP,RAB3GAP1,RAB2B,PCLO,VAMP8,ATP2A2,TSG101,VPS18,LGI3,DNM1L,GSK3B,BACE1,HAP1,STX1B,SYT11,CACNA1G,CALM1,SYTL4,SCAMP5,ATP6AP1,VAMP7,AP1G1,CBARP,STXBP6	darkgreen	EC_M4
GO:0010638	6.78E-02	0.948309212	70	428	positive regulation of organelle organization	BP	6.07E-01	LMAN1,TCP1,NCK2,TGFBR1,ACTR2,ROCK2,MYLK3,VPS35,MGARP,KIF5B,RB1,MARK4,PDCD6IP,PAK1,ACTR3,RAB3GAP1,CTNNB1,AKT2,DZIP1,NAV3,LIG4,DCN,CDC42EP3,SLC25A4,OSBP,MAP1B,PFN2,DHX36,CCT8,MAPKAPK5,CX3CL1,GNL3,OPA1,DKC1,MAGEL2,ARL3,CTTN,DNM1L,LMOD1,LRP5,GSK3B,NEK7,NTRK3,FAM162A,SYNPO2,ARPC5,MAPT,NCKAP1,ARPC2,HTT,SYNPO2L,WDR1,STX18,HAP1,ANAPC4,PPARGC1A,MSN,NES,TPM1,TGFB3,GPR65,PDGFRB,MIEF2,MYO1C,FLNA,PLXNA3,PARK7,BNIP3,DSTN,CFL2	darkgreen	EC_M4
GO:0030212	6.79E-02	0.977489643	6	22	hyaluronan metabolic process	BP	6.08E-01	FGF2,TGFB1,HYAL1,CLTC,AKT1,HAS2	darkgreen	EC_M4
GO:0042063	6.79E-02	0.952809285	40	230	gliogenesis	BP	6.08E-01	NAB2,LRP1,SUN2,RTN4,GLI3,FGF2,EIF2B3,TENM4,RHEB,TGFB1,CTNNB1,AKT2,ILK,LGI4,P2RX4,APP,CX3CL1,ABCC8,PTPN11,NDN,DNER,TGFB2,MMP14,DAG1,LEF1,KRAS,NTRK3,IDH2,NCSTN,MAPT,PRKCI,WDR1,MAP2K1,DICER1,AKT1,TMEM98,MXRA8,PAFAH1B1,BNIP3,FGF10	darkgreen	EC_M4
GO:0032814	6.81E-02	0.977407505	6	22	regulation of natural killer cell activation	BP	6.08E-01	IL23A,LEP,AXL,IL15RA,PTPN22,AP1G1	darkgreen	EC_M4
GO:0150116	6.81E-02	0.964663764	14	65	regulation of cell-substrate junction organization	BP	6.09E-01	LRP1,VEGFA,ROCK2,ACTG1,PTPN11,PHLDB2,MMP14,CORO1C,IQGAP1,VCL,ROCK1,DAPK3,THY1,DUSP22	darkgreen	EC_M4
GO:0007409	5.66E-02	0.959300772	51	341	axonogenesis	BP	6.09E-01	SEMA6A,KIF5A,CDKL3,PTK2,SEMA4D,NRCAM,NRP1,NOVA2,PLXND1,GATA3,SPTBN4,FEZ2,EFNB2,EPHB1,RET,KIF13B,NR4A2,SEMA3F,ROBO4,PLXNA2,TRAK2,ADCY1,KALRN,LAMA5,YTHDF1,PAK2,LAMC2,CREB1,SEMA6D,EPHB4,ITGA4,LMTK2,RAC1,EPHA2,OPHN1,EFNB1,PSEN1,SEMA4A,TAOK2,EFNA1,CHRNB2,RPS6KA5,DNM2,B4GALT6,SEMA6B,SLITRK2,ARHGAP4,NOTCH1,STXBP1,VASP,RELN	turquoise	EC_M6
GO:0101023	5.68E-02	0.984146065	5	19	vascular endothelial cell proliferation	BP	6.11E-01	PPARG,MEF2C,PLCG1,ITGA4,CCL2	turquoise	EC_M6
GO:1905562	5.68E-02	0.984146065	5	19	regulation of vascular endothelial cell proliferation	BP	6.11E-01	PPARG,MEF2C,PLCG1,ITGA4,CCL2	turquoise	EC_M6
GO:0017145	5.70E-02	0.981389811	6	27	stem cell division	BP	6.12E-01	KIT,WWTR1,PRDM15,NAP1L2,NOTCH1,SOX17	turquoise	EC_M6
GO:0035710	5.70E-02	0.972605496	12	65	CD4-positive, alpha-beta T cell activation	BP	6.12E-01	IL6,RSAD2,GATA3,SH3RF1,HLX,CD86,CRACR2A,ARG2,MTOR,SEMA4A,BATF,LGALS9	turquoise	EC_M6
GO:0055017	5.71E-02	0.97164863	13	72	cardiac muscle tissue growth	BP	6.12E-01	PIM1,IGF1,SMAD1,MEF2C,NDRG4,EDN1,JARID2,MTOR,YY1,NOTCH1,S1PR1,MIR23A,RGS4	turquoise	EC_M6
GO:0034260	5.72E-02	0.979444315	7	31	negative regulation of GTPase activity	BP	6.12E-01	LRCH1,SH3BP4,F11R,CPEB2,RCC2,AMOT,DAB2IP	turquoise	EC_M6
GO:1900273	5.72E-02	0.984035567	5	19	positive regulation of long-term synaptic potentiation	BP	6.12E-01	ADCY1,CREB1,ADORA2A,RELN,SHANK3	turquoise	EC_M6
GO:0002286	5.72E-02	0.972545073	12	65	T cell activation involved in immune response	BP	6.12E-01	IL6,GATA3,HLX,CD86,CRACR2A,RAB27A,MTOR,PSEN1,SEMA4A,BATF,LGALS9,CD46	turquoise	EC_M6
GO:0007416	5.75E-02	0.965432346	23	132	synapse assembly	BP	6.14E-01	LINGO2,ADGRF1,SEMA4D,SDCBP,NRCAM,PLXND1,SETD5,EFNB2,EPHB1,MEF2C,TLR2,GPM6A,SNCA,SHANK2,IL1RAP,DNM3,LGI2,SEMA4A,ABI3,CHRNB2,SLITRK2,ADGRL2,SHANK3	turquoise	EC_M6

GO:0043551	5.75E-02	0.974774652	10	47	regulation of phosphatidylinositol 3-kinase activity	BP	6.14E-01	PTK2,PDGFB,TEK,KIT,FGR,PRKD1,TNFAIP8L3,CD19,PIK3R5,DAB2IP	turquoise	EC_M6
GO:0060997	6.90E-02	0.96628201	12	54	dendritic spine morphogenesis	BP	6.16E-01	SHANK1,ACTR2,DHX36,OPA1,PDLIM5,DOCK10,DNM1L,CAPRIN2,KIF1A,TANC2,SRCIN1,PAFAH1B1	darkgreen	EC_M4
GO:0010811	5.78E-02	0.96821992	17	100	positive regulation of cell-substrate adhesion	BP	6.16E-01	PDGFB,TEK,KDR,NRP1,UNC13D,SFRP1,FERMT1,ARHGEF7,CD36,NEDD9,ITGA6,DOCK1,PPM1F,RAC1,JUP,ITGA3,DNM2	turquoise	EC_M6
GO:0043931	5.78E-02	0.983787605	5	19	ossification involved in bone maturation	BP	6.16E-01	SEMA4D,IGF1,LTF,RFLNB,BMP2	turquoise	EC_M6
GO:0050794	3.84E-02	0.966485935	755	7581	regulation of cellular process	BP	6.17E-01	CDK2,PAN2,ONECUT2,C18orf54,MEX3C,YTHDC2,BCLAF1,CLK4,RPA3,USP15,HNRNPH1,PPIL4,LATS1,NUP107,CCT2,MDC1,DDX39B,FBXO43,FBXO5,CSNK1G3,ZNF608,SCAF8,UBR5,EIF3E,EBAG9,MAP3K4,GTSE1,RAD21,DSCC1,MRPL13,TBC1D32,HSF2,ATAD2,XRCC6,PHF5A,AKAP7,KIF20A,CDC25C,TNRC6B,HSPA9,TUT7,NAA35,SRPK1,UHRF2,SRSF3,PLEC,PSIP1,DENND4C,UBR2,IFT74,HMGXB4,POT1,PWP1,ZNF300,TOPORS,SMU1,DBF4,MTERF1,POLH,AKAP9,GCC2,PUM3,RFX3,ARHGEF39,CENPQ,IL1R1,CKS2,MIOS,ICK,MELK,TRIM28,NCBP1,ZNF451,ANP32B,LRPPRC,FOXJ3,ZNF189,MSH2,FBXO11,SP4,SENP6,ASB3,CDCA7L,PHIP,YTHDC1,MTIF2,TTK,PPP4R3B,IBTK,HNRNPA2B1,PNPT1,RUFY3,ZMPSTE24,RLF,USP34,SYNCRIP,ZNF292,XPO1,PABPC4,CCT4,COMMD1,CASP8AP2,ETAA1,ANLN,TIA1,ZNF350,ITGB1BP1,THRAP3,CAPZA2,EZH2,UFL1,CUL1,FAM3C,CCNC,CLSPN,ASCC3,CNTRL,CASP2,ORC6,DDX1,SHCBP1,NAA15,SMC6,ELF2,ORC1,GEN1,WDR35,NRDC,PLK4,ATAD2B,DHFR,CDCA7,KIAA1109,RINT1,STIL,PUS7,HDAC2,MCM2,CEP57,CCNA2,EXOSC9,MLLT10,SAE1,CEP295,DNAJC2,CHORDC1,RNASEH2B,SETDB2,SYMPK,MASTL,YME1L1,GRIP2,CLASP2,TRDMT1,DDIAS,CNOT10,NUP85,KIF2C,ELF1,RSF1,PPM1G,BORA,DIS3,CCNT2,PAXIP1,FANCD2,ULK4,NCAPG2,GDI2,WDR43,CDC20,BIRC5,FNTA,PARP2,HOOK3,POLR3F,GTPBP4,RAD18,IPO5,EPC2,SUPT16H,CHD8,KAT6A,HNRNPL,METTL3,RIF1,NAP1L1,ZDHHC17,E2F7,PRPF40A,ALYREF,CKAP2,STRN,CEP290,NSD3,CEBPZ,KITLG,SUPT20H,TAF7,EXOSC8,PRKD3,NR2C1,DNAJC13,RFC3,CUL4A,CACNA2D1,BRCA2,PPP4R2,LARS,TOPBP1,ELK3,RRP8,CDC16,TCERG1,MAPK8,PRKDC,APAF1	darkgrey	EC_M8
GO:0030851	5.81E-02	0.981147057	6	25	granulocyte differentiation	BP	6.17E-01	L3MBTL3,TAL1,GATA2,HCLS1,INPP5D,LBR	turquoise	EC_M6
GO:0045332	5.82E-02	0.977349204	8	35	phospholipid translocation	BP	6.18E-01	ATP8A1,ATP11A,P2RX7,ANO6,ANO3,ATP9A,ATP8B4,TMEM30B	turquoise	EC_M6
GO:2000112	4.55E-03	0.9962606	346	2822	regulation of cellular macromolecule biosynthetic process	BP	6.20E-01	NFATC1,ZNF236,TSHZ1,PYM1,SMARCC2,STAT6,MAP3K5,CDK4,CITED2,MAML1,SQSTM1,IRAK3,OSR2,HSPA1B,EHMT2,ZBTB12,HDAC10,ATF6B,EIF3H,RXRB,RING1,ZHX2,ZBTB22,MYC,TOB2,SMAD5,EP300,KDM3B,ZNF76,DDX17,NFIB,EIF3D,HSF1,CCND3,TRERF1,CDKN2A,HMOX1,WASL,KLF9,JAK2,ZBTB45,ZNF135,ZNF667,ZNF865,RPS9,MALSU1,EFEMP1,ZNF692,ANXA4,AKNA,MED25,IL4I1,ING3,ARHGEF5,IRF3,LPIN1,FOXO3,KLF15,NOP53,MAML2,BICRA,KMT2E,BBS7,MEIS3,TRIM13,IRF2BP1,SIX5,FOSB,ZNF746,NR2C2,ZNF862,TSC22D1,ZNF575,MIF4GD,NAA16,MLH1,UNK,ZNF574,WBP2,KMT2C,MYD88,TADA3,RPS3,BRPF1,DYRK1B,EID2,ZNF219,MED29,SAMD4B,SIRT2,RPTOR,APBB3,EIF3K,NUP98,TRIM21,CYP1B1,HGF,APBB1,DEAF1,HOXC4,NCOA4,LIN37,TARBP2,KMT2B,RBCK1,RARG,SDR16C5,ZNF740,EIF4B,LMO2,POFUT1,NECAB3,URI1,PIK3R1,NCOA2,ATF1,KDM2B,ZFHX4,CAMKK2,SCAND1,EIF2A,SETD6,MCPH1,SSBP2,FNIP2,ARL2BP,SHOX2,NEK4,ZHX3,VDR,MMS19,LPIN3,FOXF2,F2RL1,SORBS3,HNF4A,TP53INP1,PIDD1,HR,ACOT8,PCIF1,TRIM45,PARP3,FASTKD3,ELK4,LGR4,ICE1,IRS1,SP140,TCF7L2,DIS3L2,PUM1,TXNIP,AIP,SOX5,ADNP,RPS6KB2,ZNF217,QARS,DTX3L,PARP14,EIF4H,ECM1,RCOR3,ARNT,PRMT2,RIPK3,GABPB2,PER2,TWIST2,VPS72,HDAC4,USF1,SMARCD2,POGZ,USP21,PATZ1,RNASEL,CDC85B,MAGEF1,CDKN1B,TCEA3,KAT5,LRP6,YBX3,NFKBIA,NFE2L2,PHC1,HEXIM2,SOAT1,BATF2,EXOSC7,PCBD1,CHCHD10,MEN1,PHB2,TSC22D4,SAV1,TAF6,CTDSP1,EID1,SSBP4,RNF25,FGF7,NELFA,ZSCAN25,ZNF394,TNFRSF1A,STK16,STAT5A,KAT6B,CREG1,NR2F6,HDAC6,PQBP1,CAMTA2,TFE3,PELP1,ZNF12,WIZ,	cyan	EC_M2
GO:0052548	6.96E-02	0.950429709	45	270	regulation of endopeptidase activity	BP	6.20E-01	SERPINB2,MTCH1,VEGFA,VCP,REST,CYCS,ROCK2,PICALM,SERPINH1,CRIM1,SPINT2,PRNP,ARL6IP5,SPINK5,ROBO1,APP,IFT57,PSMD14,UCHL5,ANOS1,FAM162A,COL6A3,NCSTN,ADRM1,RPS6KA3,EGLN3,MAPT,SERPINE1,TIMP1,NGFR,TMED10,UACA,ROCK1,USP14,AKT1,BCAP31,PARK7,F3,BCL10,MIR24-1,SPINK2,SERPINF1,SERPINB5,A2M,TIMP3	darkgreen	EC_M4
GO:0060831	6.98E-02	0.983617806	4	12	smoothened signaling pathway involved in dorsal/ventral neural tube patterning	BP	6.21E-01	GLI3,GLI2,GORAB,TULP3	darkgreen	EC_M4

GO:0016197	6.98E-02	0.952118242	37	209	endosomal transport	BP	6.21E-01	AGAP2,GGA1,VCP,BVES,VPS35,RAB7A,PICALM,SNX17,DCLK1,LEPROTL1,WASHC2A,ARL1,TMEM87B,ARHGAP1,AP3B1,VPS39,MAGEL2,RAB7B,DNM1L,CORO1C,SNX27,TMEM50A,CLTCL1,AP5M1,ERC1,SNX8,RAB11A,CLTC,SNX18,CLEC16A,MYO5B,SRL,RAB6A,BLOC1S2,VPS26B,ARFRP1,LEPROT	darkgreen	EC_M4
GO:0000041	7.00E-02	0.960781288	17	86	transition metal ion transport	BP	6.23E-01	SLC39A7,SLC39A4,SLC30A9,SLC31A1,PGRMC2,ATP7B,ATP2C1,ARHGAP1,SLC39A10,ISCU,TFRC,TFR2,CLTC,SLC39A6,SLC30A7,SLC22A17,SLC39A3	darkgreen	EC_M4
GO:0022602	7.01E-02	0.97188828	8	32	ovulation cycle process	BP	6.23E-01	LEP,INHBA,PTX3,TGFB2,SGPL1,PTPRN,TGFB3,HSPA5	darkgreen	EC_M4
GO:0060048	7.02E-02	0.958653619	20	103	cardiac muscle contraction	BP	6.23E-01	FKBP1B,SCN5A,GPD1L,SNTA1,P2RX4,ATP2A2,CACNA1D,PKP2,PIK3CA,CACNA1C,TCAP,TPM1,CACNA1G,ADRA1B,CALM1,FLNA,DMD,TMEM38B,TMEM38A,HCN4	darkgreen	EC_M4
GO:0097320	5.89E-02	0.983568552	5	18	plasma membrane tubulation	BP	6.24E-01	PACSIN2,ASAP1,MICALL1,DNM2,FNBP1L	turquoise	EC_M6
GO:0051014	7.03E-02	0.983463205	4	12	actin filament severing	BP	6.24E-01	MYH9,FLII,DSTN,CFL2	darkgreen	EC_M4
GO:0071071	5.90E-02	0.986245125	4	15	regulation of phospholipid biosynthetic process	BP	6.24E-01	PDGFB,ADGRF5,RAB38,LPCAT1	turquoise	EC_M6
GO:0030213	7.03E-02	0.983439164	4	12	hyaluronan biosynthetic process	BP	6.24E-01	TGFB1,HYAL1,CLTC,HAS2	darkgreen	EC_M4
GO:0051937	5.90E-02	0.976468657	8	43	catecholamine transport	BP	6.24E-01	RAB3B,CHRM5,SNCA,DTNBP1,ADORA2A,SNCG,SYT7,CHRNB2	turquoise	EC_M6
GO:0010613	5.91E-02	0.980623086	6	26	positive regulation of cardiac muscle hypertrophy	BP	6.24E-01	IGF1,EDN1,PDE9A,PPP3CA,MEF2A,MTOR	turquoise	EC_M6
GO:0014742	5.91E-02	0.980623086	6	26	positive regulation of muscle hypertrophy	BP	6.24E-01	IGF1,EDN1,PDE9A,PPP3CA,MEF2A,MTOR	turquoise	EC_M6
GO:0071360	5.94E-02	0.986114923	4	15	cellular response to exogenous dsRNA	BP	6.25E-01	DDX58,RALB,TMEM173,IFIT1	turquoise	EC_M6
GO:0010950	5.95E-02	0.965784795	19	127	positive regulation of endopeptidase activity	BP	6.25E-01	GRAMD4,DAPK1,SYK,PPARG,EIF2AK3,HIP1R,SOX7,SNCA,LCK,LAPTM5,CFLAR,CASP10,DDX3X,PPM1F,BID,EFNA1,LGALS9,PMAIP1,PERP	turquoise	EC_M6
GO:0031349	5.95E-02	0.962606413	26	177	positive regulation of defense response	BP	6.25E-01	TNFRSF11A,MAPK13,SYK,IL6,TLR4,IL15,PIK3CG,CTSC,GPR4,NECTIN2,IKBKB,PDE2A,TMEM173,MEF2C,TLR2,NLRC5,SNCA,ETS1,HYAL2,FABP4,PAK2,TNIP1,PLCG2,LRSAM1,GBP5,TRIM62	turquoise	EC_M6
GO:1990868	5.96E-02	0.974729197	9	48	response to chemokine	BP	6.25E-01	CXCL8,LRCH1,CXCR4,CXCL10,EDN1,CCRL2,HIF1A,CCL2,ACKR1	turquoise	EC_M6
GO:1990869	5.96E-02	0.974729197	9	48	cellular response to chemokine	BP	6.25E-01	CXCL8,LRCH1,CXCR4,CXCL10,EDN1,CCRL2,HIF1A,CCL2,ACKR1	turquoise	EC_M6
GO:0050909	5.97E-02	0.982726827	5	22	sensory perception of taste	BP	6.25E-01	P2RX2,CD36,PLCB2,RTP4,SCNN1B	turquoise	EC_M6
GO:0071402	5.98E-02	0.98034642	6	26	cellular response to lipoprotein particle stimulus	BP	6.25E-01	SYK,TLR4,PPARG,CD36,ABCG1,TLR6	turquoise	EC_M6
GO:0001956	5.98E-02	0.986186055	4	14	positive regulation of neurotransmitter secretion	BP	6.25E-01	SNCA,DTNBP1,ADORA2A,STXBP1	turquoise	EC_M6
GO:0003143	5.98E-02	0.973424695	10	50	embryonic heart tube morphogenesis	BP	6.25E-01	TEAD2,MEF2C,NDRG4,VANGL2,HIF1A,PSEN1,NOTCH1,ENG,SOX17,SOX18	turquoise	EC_M6
GO:0060969	5.99E-02	0.98304665	5	19	negative regulation of gene silencing	BP	6.25E-01	IL6,PPARG,DND1,PPP3CA,TET1	turquoise	EC_M6
GO:0000132	3.90E-02	0.988471662	6	26	establishment of mitotic spindle orientation	BP	6.27E-01	CENPA,SPDL1,PLK1,NDC80,SAPCD2,MAD2L1	darkgrey	EC_M8
GO:0015749	7.08E-02	0.960336984	17	84	monosaccharide transmembrane transport	BP	6.28E-01	PPARD,LEP,MFSD4B,AKT2,OSBPL8,IRS2,SORBS1,PTPN11,SELENOS,SLC2A10,PEA15,CLTCL1,PRKCI,SLC2A4,AKT1,SLC2A6,SLC2A8	darkgreen	EC_M4
GO:0007389	6.03E-02	0.957171948	46	329	pattern specification process	BP	6.29E-01	MFNG,MDFI,NRP1,ACVR2B,SFRP1,TCF15,HHEX,SMAD1,MEF2C,NDRG4,STC1,HIPK1,DLL4,EDN1,ETS2,SEMA3F,PLXNA2,VANGL2,MMP21,HOXD10,RNF2,BCOR,TBX1,HIF1A,LFNG,ALDH1A2,AR,EFNB1,PSEN1,HOXB5,HOXB6,HOXB7,HOXB8,HOXB9,SMAD2,YY1,NRARP,NOTCH1,ENG,MTF2,ADGRG1,SOX17,RELN,SOX18,BMP2,PIFO	turquoise	EC_M6
GO:0042573	6.04E-02	0.98600105	4	14	retinoic acid metabolic process	BP	6.29E-01	ALDH8A1,ALDH1A2,CYP1A1,RBP1	turquoise	EC_M6

GO:0001659	6.04E-02	0.963721768	22	141	temperature homeostasis	BP	6.29E-01	ZNF516,TNFRSF11A,ATF4,SYK,GADD45G,GRB10,TLR4,IL15,LAMA4,NOVA2,ACVR2B,CXCR4,EDNRB,CD36,IL1A,ABHD6,KDM3A,FABP4,ADIPOR2,TLE3,ADRB2,NOTCH1	turquoise	EC_M6
GO:0010821	7.10E-02	0.956357812	23	124	regulation of mitochondrion organization	BP	6.29E-01	FAM49B,VPS35,MGARP,DCN,SLC25A4,OPA1,CTTN,DNM1L,GSK3B,FAM162A,TFRC,FZD9,MAPT,C11orf65,HTT,GHITM,PPARGC1A,MIEF2,AKT1,OMA1,PARK7,PRMT6,BNIP3	darkgreen	EC_M4
GO:0007020	3.93E-02	0.9869773	7	32	microtubule nucleation	BP	6.29E-01	AKAP9,CLASP2,TUBGCP3,TPX2,CENPJ,CKAP5,HAUS2	darkgrey	EC_M8
GO:0032774	4.72E-03	0.996150207	313	2534	RNA biosynthetic process	BP	6.30E-01	NFATC1,ZNF236,TSHZ1,SMARCC2,STAT6,MAP3K5,CDK4,CITED2,MAML1,SQSTM1,IRAK3,HELB,OSR2,HSPA1B,EHMT2,ZBTB12,HDAC10,ATF6B,RXRB,RING1,ZHX2,ZBTB22,MYC,TOB2,SMAD5,EP300,KDM3B,ZNF76,DDX17,NFIB,HSF1,CCND3,TRERF1,CDKN2A,HMOX1,WASL,KLF9,POLR1C,JAK2,ZBTB45,ZNF135,ZNF667,ZNF865,EFEMP1,ZNF692,ANXA4,AKNA,MED25,IL4I1,ING3,ARHGEF5,IRF3,LPIN1,FOXO3,KLF15,NOP53,MAML2,BICRA,KMT2E,BBS7,MEIS3,TRIM13,IRF2BP1,SIX5,FOSB,ZNF746,NR2C2,ZNF862,TSC22D1,ZNF575,NAA16,GTF3C2,ZNF574,WBP2,KMT2C,MYD88,TADA3,RPS3,BRPF1,DYRK1B,EID2,ZNF219,MED29,SIRT2,RPTOR,APBB3,NUP98,TRIM21,CYP1B1,HGF,APBB1,TAF10,DEAF1,HOXC4,NCOA4,LIN37,KMT2B,RBCK1,RARG,SDR16C5,ZNF740,LMO2,POFUT1,URI1,PIK3R1,NCOA2,ATF1,KDM2B,ZFHX4,CAMKK2,SCAND1,SETD6,MCPH1,SSBP2,FNIP2,ARL2BP,SHOX2,NEK4,ZHX3,VDR,MMS19,LPIN3,FOXF2,F2RL1,SORBS3,HNF4A,TP53INP1,PIDD1,HR,TRIM45,NPM3,ELK4,LGR4,ICE1,SP140,TCF7L2,TXNIP,AIP,SOX5,ADNP,ZNF217,QARS,DTX3L,PARP14,ECM1,RCOR3,ARNT,PRMT2,RIPK3,GABPB2,PER2,TWIST2,VPS72,HDAC4,USF1,SMARCD2,POGZ,USP21,PATZ1,RNASEL,CCDC85B,MAGEF1,CDKN1B,TCEA3,KAT5,LRP6,YBX3,NFKBIA,NFE2L2,POLR2J2,PHC1,HEXIM2,BATF2,PCBD1,CHCHD10,MEN1,PHB2,TSC22D4,SAV1,TAF6,CTDSP1,EID1,SSBP4,RNF25,FGF7,NELFA,ZSCAN25,ZNF394,TNFRSF1A,STK16,STAT5A,KAT6B,CREG1,NR2F6,HDAC6,PQBP1,CAMTA2,TFE3,PELP1,ZNF12,WIZ,ZBTB25,RARA,FOXK1,AC090517.4,ZBTB17,GPS2,THRA,KLF3,MED24,PRDM2,ARHGEF11,SPIN4,UIMC1,CC2D1A,ZNF771,MLLT6,CTBP1,CRABP2	cyan	EC_M2
GO:0031057	6.06E-02	0.976051265	8	37	negative regulation of histone modification	BP	6.31E-01	KDM4D,DNMT3B,KDM3A,SNCA,JARID2,BCOR,KDM5A,MTF2	turquoise	EC_M6
GO:0007026	3.96E-02	0.988402514	6	24	negative regulation of microtubule depolymerization	BP	6.33E-01	CLASP2,CKAP2,TPX2,CAMSAP2,HDGFL3,TAOK1	darkgrey	EC_M8
GO:1903587	7.21E-02	0.978633012	5	18	regulation of blood vessel endothelial cell proliferation involved in sprouting angiogenesis	BP	6.37E-01	VEGFA,FGF2,PDCD10,NGFR,MIR24-1	darkgreen	EC_M4
GO:0060047	7.21E-02	0.952211475	31	172	heart contraction	BP	6.37E-01	BVES,FKBP1B,SCN5A,GPD1L,ATP2B1,FXYD1,SNTA1,P2RX4,ATP2A2,CACNA1D,ADA,ATP2B4,PKP2,DNM1L,TGFB2,PIK3CA,GLRX3,CACNA1C,HSPB7,TCAP,TPM1,CACNA1G,ADRA1B,CALM1,SGCD,FLNA,DMD,TMEM38B,TMEM38A,HOPX,HCN4	darkgreen	EC_M4
GO:0006941	7.21E-02	0.955099147	24	128	striated muscle contraction	BP	6.37E-01	MYH14,FKBP1B,SCN5A,GPD1L,SNTA1,P2RX4,ATP2A2,CACNA1D,HOMER1,PKP2,PIK3CA,CASQ1,CACNA1C,TCAP,TPM1,CACNA1G,ADRA1B,CALM1,DTNA,FLNA,DMD,TMEM38B,TMEM38A,HCN4	darkgreen	EC_M4
GO:0071364	7.22E-02	0.96886614	9	38	cellular response to epidermal growth factor stimulus	BP	6.37E-01	PTPN11,SYAP1,IQGAP1,MED1,COL1A1,AKT1,PTPN12,DUSP22,ZPR1	darkgreen	EC_M4
GO:0006664	7.24E-02	0.959257884	17	87	glycolipid metabolic process	BP	6.37E-01	B4GALNT1,LARGE1,TMEM246,GLB1,SUMF1,SMPD1,ST8SIA4,PIGP,B3GALT1,PIGZ,CLN6,PIGL,PRKAA1,DPM2,PIGK,DPM1,PIGB	darkgreen	EC_M4
GO:1903509	7.24E-02	0.959257884	17	87	liposaccharide metabolic process	BP	6.37E-01	B4GALNT1,LARGE1,TMEM246,GLB1,SUMF1,SMPD1,ST8SIA4,PIGP,B3GALT1,PIGZ,CLN6,PIGL,PRKAA1,DPM2,PIGK,DPM1,PIGB	darkgreen	EC_M4
GO:0002573	7.25E-02	0.953052782	28	154	myeloid leukocyte differentiation	BP	6.37E-01	IL23A,MYH9,VEGFA,INHBA,OSTM1,RB1,TGFB1,CTNNB1,APP,AP3B1,RUNX1,NOTCH2,LEF1,PRKCA,TCTA,TFRC,FARP2,SRP54,GPR137,FBN1,MED1,CD74,PTPN2,PAFAH1B1,ATP6AP1,FSTL3,SNX10,GLO1	darkgreen	EC_M4
GO:0010453	7.25E-02	0.97872107	5	17	regulation of cell fate commitment	BP	6.37E-01	IL23A,FGF2,LOXL3,BMPR1A,LMO4	darkgreen	EC_M4
GO:0022408	7.25E-02	0.954814037	24	130	negative regulation of cell-cell adhesion	BP	6.37E-01	CDSN,PPARA,VEGFA,SOCS5,GLI3,TGFB1,C1QTNF1,SPINT2,PRNP,ZNF703,CXCL12,IL20RB,LOXL3,PTPN22,RUNX1,CBLB,PRKAR1A,TNR,PTPN6,CD74,AKT1,PTPN2,FGL2,PRKG1	darkgreen	EC_M4
GO:1901222	7.25E-02	0.960866015	15	75	regulation of NIK/NF-kappaB signaling	BP	6.37E-01	IL23A,PTP4A3,ACTN4,ILK,AKIP1,APP,PTPN22,ADIPOR1,BMP7,TSPAN6,UACA,IRAK1,AGO3,EDA,TMSB4X	darkgreen	EC_M4

GO:0007018	7.25E-02	0.947580111	48	283	microtubule-based movement	BP	6.37E-01	AP3S1,RAB21,SUN2,DYNC1I1,DST,ACTR2,MGARP,CFAP100,KIF5B,COPG1,ACTR3,TRAK1,DYNC1LI1,KIF2A,MAP1B,APP,PGAM4,OPA1,AP3B1,ATP2B4,DNAH1,DNAH5,IFT57,ARL3,FEZ1,DNAAF5,HSPA8,DYNC1I2,KIF1A,HSPB1,MAPT,HTT,KTN1,ACTR10,FMN2,HAP1,IFT43,ARMCX3,PAFAH1B1,NPHP4,VAMP7,HSBP1,DYNLRB2,DYNC1LI2,RAB6A,BLOC1S2,CCR6,MAP1A	darkgreen	EC_M4
GO:0006605	7.26E-02	0.948337148	44	262	protein targeting	BP	6.37E-01	LMAN1,HSPA4,SIL1,PEX6,SRP72,VPS41,MGARP,SEC61A1,RAB7A,PAK1,SRP68,TRAK1,AKT2,PRNP,VPS37A,TRAM1,CACNB3,SSR3,ATG13,AP3B1,BAP1,CHP1,SRPRA,HSPA8,TOMM40L,SRP54,C11orf65,PRKCI,SMURF1,ZDHHC4,HAP1,SSR2,LTBP2,MIEF2,PRKAA1,ZDHHC9,MYO1C,BCAP31,HSPA5,SPCS3,TIMM22,LEPROT,GRPEL2,TOMM7	darkgreen	EC_M4
GO:0070166	7.26E-02	0.982721274	4	12	enamel mineralization	BP	6.37E-01	PPARA,CNNM4,STIM1,FAM20C	darkgreen	EC_M4
GO:0140056	7.27E-02	0.961836693	14	67	organelle localization by membrane tethering	BP	6.37E-01	STX7,UNC13B,BVES,ESYT2,SNPH,VAMP8,ATP2A2,USO1,VPS18,VAPB,STX1B,CALM1,VMP1,AHCYL1	darkgreen	EC_M4
GO:0070371	7.27E-02	0.949385929	39	227	ERK1 and ERK2 cascade	BP	6.37E-01	LRP1,ANGPT1,ROS1,TPBG,RRAS,FGF2,TGFB1,SPRED3,CAVIN3,ST5,APP,RAPGEF2,CX3CL1,PTPN11,PTPN22,CHRNA7,NOTCH2,HTR2B,PRKCA,ATF3,FGFR2,FN1,PTPN6,ITGAV,FGFR3,SHC1,MAP2K1,NPTN,PIN1,CD74,PDGFRB,PTPN2,ATP6AP1,MIR24-1,CCL16,TIMP3,DUSP4,GLIPR2,FGF10	darkgreen	EC_M4
GO:0046939	7.27E-02	0.960778577	15	74	nucleotide phosphorylation	BP	6.37E-01	PPARA,CMPK1,PFKP,APP,PFKM,PGAM4,ZBTB20,TPI1,PPARGC1A,PKM,AK7,PRKAA1,ENO1,SLC2A6,NME6	darkgreen	EC_M4
GO:0071786	7.27E-02	0.978636863	5	17	endoplasmic reticulum tubular network organization	BP	6.37E-01	REEP2,RTN4,RAB3GAP1,ZFYVE27,RTN3	darkgreen	EC_M4
GO:0001935	7.27E-02	0.955187413	23	123	endothelial cell proliferation	BP	6.37E-01	LEP,VEGFA,PDCL3,TGFBR1,EMC10,FGF2,CXCL12,SULF1,LOXL2,HTR2B,MMP14,PRKCA,VASH2,PDCD10,AKT3,NGFR,AKT1,MYDGF,PLXNB3,CDH13,F3,MIR24-1,WNT2	darkgreen	EC_M4
GO:1902285	6.15E-02	0.985887687	4	13	semaphorin-plexin signaling pathway involved in neuron projection guidance	BP	6.38E-01	NRP1,PLXND1,SEMA3F,PLXNA2	turquoise	EC_M6
GO:1901385	7.31E-02	0.972489093	7	28	regulation of voltage-gated calcium channel activity	BP	6.40E-01	REM1,CACNB3,LINC02210-CRHR1,CALM1,DMD,CBARP,GEM	darkgreen	EC_M4
GO:0034654	4.83E-03	0.996018613	349	2841	nucleobase-containing compound biosynthetic process	BP	6.40E-01	NFATC1,ZNF236,TSHZ1,SMARCC2,STAT6,MAP3K5,CDK4,CITED2,MAML1,SQSTM1,IRAK3,HELB,GFPT2,OSR2,HSPA1B,EHMT2,ZBTB12,HDAC10,ATF6B,RXRB,RING1,ZHX2,ZBTB22,PMM1,MYC,TOB2,SMAD5,EP300,KDM3B,ZNF76,DDX17,NFIB,HSF1,CCND3,TRERF1,CDKN2A,HMOX1,WASL,KLF9,POLR1C,JAK2,ZBTB45,ZNF135,ZNF667,ZNF865,EFEMP1,ZNF692,ANXA4,AKNA,MED25,IL4I1,ING3,ARHGEF5,IRF3,LPIN1,FOXO3,KLF15,SPHK2,NOP53,MAML2,BICRA,KMT2E,BBS7,MEIS3,TRIM13,ACSS1,IRF2BP1,SIX5,FOSB,ZNF746,NR2C2,ZNF862,TSC22D1,ZNF575,NAA16,GTF3C2,ZNF574,WBP2,KMT2C,MYD88,TADA3,RPS3,TEP1,BRPF1,PNP,DYRK1B,EID2,ZNF219,MED29,SIRT2,RPTOR,APBB3,NUP98,TRIM21,CYP1B1,GNPDA1,HGF,APBB1,TAF10,DEAF1,HOXC4,NCOA4,LIN37,KMT2B,RBCK1,RARG,SDR16C5,ZNF740,LMO2,POFUT1,URI1,PIK3R1,ACSS2,NCOA2,ATF1,TNKS1BP1,POLE4,KDM2B,ZFHX4,CAMKK2,SCAND1,SETD6,MCPH1,SSBP2,FNIP2,DGUOK,ARL2BP,SHOX2,NEK4,ZHX3,VDR,MMS19,LPIN3,FOXF2,F2RL1,SORBS3,HNF4A,TP53INP1,PIDD1,HR,TRIM45,NPM3,PARP3,ELK4,LGR4,ICE1,SP140,TCF7L2,TXNIP,AIP,SOX5,ADNP,ZNF217,QARS,IMPDH2,DTX3L,PARP14,ECM1,RCOR3,ARNT,PRMT2,RIPK3,CTPS2,GABPB2,PER2,TWIST2,VPS72,HDAC4,USF1,SMARCD2,POGZ,USP21,DNAJC30,PATZ1,RNASEL,CCDC85B,MAGEF1,CDKN1B,TCEA3,KAT5,LRP6,YBX3,NFKBIA,NFE2L2,POLR2J2,PHC1,HEXIM2,BATF2,PCBD1,CHCHD10,MEN1,ATIC,PHB2,TSC22D4,SAV1,TAF6,CTDSP1,EID1,SSBP4,RNF25,FGF7,NELFA,ZSCAN25,ZNF394,TNFRSF1A,STK16,STAT5A,KAT6B,CREG1,NR2F6,HDAC6,PQBP1,CAMTA2,UQCC3,TFE3,PELP1,ZNF12,WIZ,ZBTB25,R	cyan	EC_M2
GO:0060216	6.18E-02	0.98251269	5	18	definitive hemopoiesis	BP	6.40E-01	TEK,TAL1,GATA2,HIPK1,LYL1	turquoise	EC_M6
GO:0060986	7.32E-02	0.972564758	7	27	endocrine hormone secretion	BP	6.41E-01	LEP,INHBA,C1QTNF1,RAB11FIP1,PTPN11,CRY2,LINC02210-CRHR1	darkgreen	EC_M4
GO:1903959	6.19E-02	0.982309001	5	19	regulation of anion transmembrane transport	BP	6.41E-01	TCAF2,MTOR,PSEN1,SLC43A2,RGS4	turquoise	EC_M6
GO:0043403	7.34E-02	0.972353995	7	28	skeletal muscle tissue regeneration	BP	6.42E-01	PPARD,LARGE1,AKIRIN1,DAG1,KPNA1,SGCA,HOPX	darkgreen	EC_M4

GO:0001892	7.36E-02	0.961279576	14	67	embryonic placenta development	BP	6.42E-01	RSPO3,SPINT2,TMED2,HEY1,PKD2,LEF1,BMP7,FGFR2,VASH2,MED1,MAP2K1,AKT1,PKD1,WNT2	darkgreen	EC_M4
GO:1905037	7.36E-02	0.957740197	18	92	autophagosome organization	BP	6.42E-01	UBQLN1,RAB23,RAB7A,RAB3GAP1,TMEM41B,MAP1LC3A,TP53INP2,ATP2A2,ATG13,SEC22B,FEZ1,WIPI1,SYNPO2,SCFD1,SMURF1,WIPI2,VMP1,GABARAPL2	darkgreen	EC_M4
GO:0001675	7.36E-02	0.982394019	4	12	acrosome assembly	BP	6.42E-01	SOX30,PAFAH1B1,CCDC136,SPINK2	darkgreen	EC_M4
GO:0042551	7.38E-02	0.968029739	9	38	neuron maturation	BP	6.44E-01	MTCH1,RB1,LGI4,APP,CX3CL1,OPA1,FARP2,C1QL1,GLDN	darkgreen	EC_M4
GO:0043549	6.23E-02	0.951664608	81	612	regulation of kinase activity	BP	6.44E-01	TNFRSF11A,TNFAIP3,PTPRB,FLT4,TPD52L1,PTK2,PDGFB,TEK,KIT,KDR,SYK,TLR4,CCNY,RASIP1,PIK3CG,TAL1,CAB39L,RALB,PPARG,TIE1,SFRP1,HBEGF,SH3BP5,EPHB1,RET,MERTK,IGF1,HHEX,WWTR1,VEGFC,MADD,NLRC5,DGKZ,CCNJL,NEDD9,EDN1,SNCA,DTNBP1,HYAL2,NCF1,FABP4,CD86,FGR,PPP2R5A,PROX1,PRKD1,ACE,PAK2,DLG1,MAP3K11,LTF,ADORA2A,EPHB4,DDX3X,LDB2,TNFAIP8L3,LMTK2,PPM1F,EPHA2,TLR6,RGS14,MTOR,PSEN1,FGF18,TAOK2,EFNA1,CD19,PIK3R5,ADRB2,UNC119,TRAF2,DAB2IP,SLC8A3,RELN,ALKAL1,MMD,DAZAP2,COPS8,BMP2,DUSP7,PIFO	turquoise	EC_M6
GO:0032633	6.24E-02	0.981839298	5	21	interleukin-4 production	BP	6.44E-01	SYK,GATA3,CD86,IL1RAP,LGALS9	turquoise	EC_M6
GO:0032673	6.24E-02	0.981839298	5	21	regulation of interleukin-4 production	BP	6.44E-01	SYK,GATA3,CD86,IL1RAP,LGALS9	turquoise	EC_M6
GO:0072091	6.24E-02	0.976931392	7	33	regulation of stem cell proliferation	BP	6.44E-01	PIM1,FERMT1,VEGFC,ACE,ATXN1L,SOX17,SOX18	turquoise	EC_M6
GO:0055065	6.25E-02	0.953603464	59	438	metal ion homeostasis	BP	6.44E-01	TRDN,IREB2,CAV2,TRPM4,PIK3CG,GATA2,GPR4,CXCR4,EDNRB,TMC8,P2RY6,P2RX2,CD36,TMEM178A,MICU3,PDE4D,CHD7,IL1A,HEXB,CP,P2RX7,CXCL10,TPCN1,PLCG1,STC1,EDN1,SNCA,LCK,HYAL2,SLC39A8,KCNJ2,TESMIN,SLC12A7,HCRTR1,PRKD1,MELTF,B2M,LTF,CCRL2,ADORA2A,MCU,HIF1A,HEPH,PSEN1,ATP2A1,CD19,NPR1,S100A14,SCNN1B,HMOX2,AP3D1,PLCG2,S1PR1,CDH5,SLC8A3,TRIM24,KCNH2,LPAR6,BMP6	turquoise	EC_M6
GO:0015914	6.25E-02	0.968553283	13	70	phospholipid transport	BP	6.44E-01	ATP8A1,ATP11A,SCARB1,PITPNM2,P2RX7,ANO6,ANO3,ABCG1,MTTP,ATP9A,ATP8B4,TNFAIP8L3,TMEM30B	turquoise	EC_M6
GO:0014706	6.25E-02	0.955822696	44	307	striated muscle tissue development	BP	6.44E-01	PIM1,PGM5,CAV2,SVIL,NR1D2,EFNB2,P2RX2,LMOD3,ADAMTS9,EPHB1,CHD7,IGF1,NR4A1,SMAD1,MEF2C,NDRG4,TLL2,DLL4,EDN1,JARID2,SIK1,PPP3CA,MEF2A,BCL9,NRAP,HLX,CFLAR,PROX1,HOXD10,CREB1,TBX1,ZBTB18,ALDH1A2,MTOR,YY1,DSG2,MRTFB,NOTCH1,S1PR1,ENG,MIR23A,MAFF,RGS4,BMP2	turquoise	EC_M6
GO:0060840	6.26E-02	0.966813039	15	81	artery development	BP	6.44E-01	ADGRF5,NRP1,PLXND1,ACVR2B,JAG1,PDE2A,ADAMTS9,CHD7,DLL4,SEC24B,PROX1,TBX1,AP2B1,NOTCH1,ENG	turquoise	EC_M6
GO:0006476	4.92E-03	0.997937348	18	88	protein deacetylation	BP	6.45E-01	HDAC10,EP300,TRERF1,SPHK2,TADA3,SIRT2,SDR16C5,SPRED1,ELK4,RCOR3,PER2,HDAC4,HDAC6,CTBP1,PER1,ZBTB7B,SIN3A,SREBF1	cyan	EC_M2
GO:0033189	6.27E-02	0.985517884	4	13	response to vitamin A	BP	6.45E-01	DNMT3A,DNMT3B,ALDH1A2,CYP1A1	turquoise	EC_M6
GO:2000058	4.04E-02	0.977382167	20	143	regulation of ubiquitin-dependent protein catabolic process	BP	6.46E-01	CDK2,LATS1,XPO1,COMMD1,UFL1,CDC20,TAF9,PBK,UBE3A,SMARCC1,COP1,UBE2K,TAF1,OGT,PLK1,PSMD10,GLMN,AURKA,RNF139,RCHY1	darkgrey	EC_M8
GO:0001921	6.28E-02	0.985494106	4	13	positive regulation of receptor recycling	BP	6.46E-01	SNCA,INPP5F,PSEN1,VAMP3	turquoise	EC_M6
GO:0002294	6.28E-02	0.973230752	9	45	CD4-positive, alpha-beta T cell differentiation involved in immune response	BP	6.46E-01	IL6,GATA3,HLX,CD86,CRACR2A,MTOR,SEMA4A,BATF,LGALS9	turquoise	EC_M6
GO:0060841	6.29E-02	0.985447549	4	13	venous blood vessel development	BP	6.46E-01	ACVR2B,PROX1,NOTCH1,ENG	turquoise	EC_M6
GO:0007616	7.42E-02	0.9721243	7	27	long-term memory	BP	6.47E-01	SRF,SHANK1,PRNP,PJA2,SLC2A4,GRIA1,LRRN4	darkgreen	EC_M4
GO:2000353	6.30E-02	0.98496787	4	15	positive regulation of endothelial cell apoptotic process	BP	6.47E-01	PLCG1,ANO6,ITGA4,CCL2	turquoise	EC_M6
GO:1904861	7.42E-02	0.974783465	6	22	excitatory synapse assembly	BP	6.47E-01	PLXNB2,LRRC4B,NTRK3,NLGN2,NPTN,LRRTM2	darkgreen	EC_M4

GO:0051930	6.32E-02	0.981546887	5	21	regulation of sensory perception of pain	BP	6.47E-01	MGLL,EDNRB,EDN1,ZFHX2,MTOR	turquoise	EC_M6
GO:0006826	6.33E-02	0.972856366	9	48	iron ion transport	BP	6.47E-01	IREB2,CP,SLC39A8,MELTF,B2M,LTF,LMTK2,HEPH,DNM2	turquoise	EC_M6
GO:0033273	6.33E-02	0.967961331	13	75	response to vitamin	BP	6.47E-01	PIM1,IL15,DNMT3A,SFRP1,DNMT3B,CXCL10,STC1,MN1,FES,FGF23,ALDH1A2,CYP1A1,TRIM24	turquoise	EC_M6
GO:0008210	6.33E-02	0.984868418	4	15	estrogen metabolic process	BP	6.47E-01	TIPARP,PLEKHA1,DHRS11,CYP1A1	turquoise	EC_M6
GO:0061097	6.34E-02	0.96795296	13	74	regulation of protein tyrosine kinase activity	BP	6.47E-01	PDGFB,HBEGF,SH3BP5,NEDD9,HYAL2,NCF1,ACE,PAK2,PSEN1,EFNA1,UNC119,RELN,ALKAL1	turquoise	EC_M6
GO:0023057	6.34E-02	0.948078036	131	1048	negative regulation of signaling	BP	6.47E-01	SEMA6A,TNFAIP3,TRIM39,FZD6,CTHRC1,GRAMD4,PICK1,MDFI,CD2AP,RNF149,IL6,CXCL8,RGS3,ARHGAP25,FIGNL1,CAV2,GRB10,TLR4,NRP1,RASIP1,ARHGAP42,MTMR2,GATA2,NOS3,PPARG,GATA3,SEC14L1,PDE2A,SFRP1,NOTUM,FERMT1,SIRT3,PRR5L,PLK2,PDE4D,FAM3D,IL1A,IGF1,ABHD6,HHEX,ITCH,ERBIN,SH3RF1,WWTR1,IL7,P2RX7,NDRG4,LMBR1L,ADGRG3,OAS3,NLRC5,NR4A2,DGKZ,DLL4,SOX13,EDN1,STAT1,DYRK1A,SNCA,DUSP8,PRDM15,SHANK2,HDAC1,ITPRIP,PPP3CA,HYAL2,LAPTM5,DYRK3,AP1AR,INPP5D,SH3BP4,CFLAR,INPP5F,IGFBP3,PLEKHA1,SRMS,ITGA6,PTPRE,DUSP16,DLG1,ELL3,APOD,LTF,TSPAN15,DDX3X,IGFBP5,HOMER3,MECOM,FGF23,HIF1A,EPHA2,MMRN2,BID,TLR6,MCC,LFNG,RGS14,MTOR,AR,PSEN1,ITGA3,EFNA1,CCDC88C,TLE3,DNM2,TNIP1,MARK3,KIF26A,ADRB2,PDE4B,BFAR,TNFAIP8L1,ISG15,NRARP,EGFL7,NOTCH1,STXBP1,DAB2IP,MIR99A,OAS1,VNN1,CD46,SOX17,SHANK3,RGS4,RGS16,DUSP5,BMP2,RNF125,DUSP7,RNF152	turquoise	EC_M6
GO:0045844	6.35E-02	0.985014601	4	14	positive regulation of striated muscle tissue development	BP	6.47E-01	LMOD3,MEF2C,CREB1,MRTFB	turquoise	EC_M6
GO:0048636	6.35E-02	0.985014601	4	14	positive regulation of muscle organ development	BP	6.47E-01	LMOD3,MEF2C,CREB1,MRTFB	turquoise	EC_M6
GO:0051299	4.06E-02	0.991739236	4	14	centrosome separation	BP	6.47E-01	KIF11,CHEK1,NEK2,AURKA	darkgrey	EC_M8
GO:0050789	4.07E-02	0.964496585	784	7907	regulation of biological process	BP	6.47E-01	CDK2,PAN2,ONECUT2,C18orf54,MEX3C,ME2,YTHDC2,BCLAF1,CLK4,RPA3,USP15,HNRNPH1,PPIL4,LATS1,NUP107,CCT2,MDC1,DDX39B,FBXO43,FBXO5,CSNK1G3,ZNF608,SCAF8,UBR5,EIF3E,EBAG9,MAP3K4,GTSE1,RAD21,DSCC1,MRPL13,TBC1D32,HSF2,ATAD2,XRCC6,PHF5A,AKAP7,KIF20A,CDC25C,TNRC6B,HSPA9,TUT7,NAA35,MATR3,SRPK1,UHRF2,SRSF3,PLEC,PSIP1,DENND4C,UBR2,IFT74,HMGXB4,POT1,PWP1,ZNF300,TOPORS,SMU1,DBF4,POLR3B,XPO5,MTERF1,POLH,AKAP9,GCC2,PUM3,RFX3,ARHGEF39,CENPQ,IL1R1,CKS2,MIOS,ICK,MELK,TRIM28,NCBP1,ZNF451,ANP32B,LRPPRC,PPIH,FOXJ3,ZNF189,MSH2,FBXO11,SP4,SENP6,ASB3,CDCA7L,PHIP,YTHDC1,MTIF2,TTK,PPP4R3B,IBTK,HNRNPA2B1,PNPT1,RUFY3,PUS10,ZMPSTE24,RLF,USP34,SYNCRIP,ZNF292,XPO1,PABPC4,CCT4,COMMD1,CASP8AP2,ETAA1,ANLN,TIA1,ZNF350,ITGB1BP1,THRAP3,CAPZA2,EZH2,UFL1,CUL1,FAM3C,CCNC,CLSPN,ASCC3,ZMYM4,CNTRL,CASP2,ORC6,DDX1,SHCBP1,NAA15,SMC6,ELF2,ORC1,GEN1,WDR35,NRDC,PLK4,ATAD2B,DHFR,CDCA7,KIAA1109,RINT1,STIL,PUS7,HDAC2,MCM2,CEP57,CCNA2,EXOSC9,MLLT10,SAE1,CEP295,DNAJC2,CHORDC1,RNASEH2B,SETDB2,SYMPK,MASTL,YME1L1,GRIP2,ZC3H13,CLASP2,TRDMT1,DDIAS,CNOT10,NUP85,KIF2C,ELF1,UPF2,RSF1,PPM1G,BORA,DIS3,CCNT2,PAXIP1,FANCD2,XRN2,ULK4,NCAPG2,GDI2,WDR43,CDC20,BIRC5,FNTA,PARP2,HOOK3,POLR3F,GTPBP4,RAD18,IPO5,EPC2,SUPT16H,CHD8,KAT6A,HNRNPL,METTL3,RIF1,NAP1L1,TOP2B,ZDHHC17,E2F7,PRPF40A,ALYREF,CKAP2,STRN,CEP290,NSD3,CEBPZ,KITLG,SUPT20H,TAF7,EXOSC8,PRKD3,NR2C1,DNAJC13,RFC3,CUL4A,CACNA2D1,BRCA	darkgrey	EC_M8
GO:0033962	4.07E-02	0.989793978	5	18	P-body assembly	BP	6.47E-01	PAN2,PAN3,LSM14A,CNOT1,DDX6	darkgrey	EC_M8
GO:0021532	7.45E-02	0.971845726	7	28	neural tube patterning	BP	6.49E-01	GLI3,GLI2,TCTN1,ATP6AP2,FKBP8,GORAB,TULP3	darkgreen	EC_M4
GO:2001222	7.46E-02	0.969658978	8	32	regulation of neuron migration	BP	6.49E-01	PLXNB2,PLAA,RAPGEF2,CX3CL1,COL3A1,ARHGEF2,FLNA,NTNG2	darkgreen	EC_M4
GO:0008356	7.46E-02	0.982067098	4	12	asymmetric cell division	BP	6.49E-01	TEAD3,ACTR2,ACTR3,ARHGEF2	darkgreen	EC_M4
GO:0035924	7.46E-02	0.961692178	13	62	cellular response to vascular endothelial growth factor stimulus	BP	6.49E-01	CD63,NUS1,PTP4A3,VEGFA,ADAMTS3,DCN,ROBO1,ATP2B4,PIK3CA,HSPB1,PDGFRB,AKT1,MYO1C	darkgreen	EC_M4
GO:0010470	7.47E-02	0.98203346	4	12	regulation of gastrulation	BP	6.49E-01	TENM4,PHLDB2,DAG1,PHLDB1	darkgreen	EC_M4

GO:1901564	7.48E-02	0.932377933	679	4789	organonitrogen compound metabolic process	BP	6.49E-01	SERPINB2,SEC11C,IL23A,NAB2,LRP1,CDO1,TRIM36,HBS1L,B4GALT7,B4GALNT1,AGAP2,CANX,ENPP3,TAB2,CAND1,DYRK2,RNF19A,PRRC1,AZIN1,PLXNB2,ANGPT1,PPARA,ACSL6,P4HA2,SLC22A4,ROS1,NUS1,SERINC1,PHF20L1,HABP4,PTP4A3,ADGRB1,LY6E,PPARD,C6orf89,UBQLN1,MTCH1,GGA1,KLHDC10,SH3GL2,PSAT1,GNAQ,MYH9,MTAP,LEP,CDKN2B,PLAA,LARGE1,NUAK1,CDK14,ALDH1L2,VEGFA,VCP,SLC35B2,C2orf40,NCK2,CLOCK,ASNS,PDCL3,CHST10,REST,EIF5B,RAB23,PTP4A1,P3H1,TGFBR1,ERP44,TMEM246,SOCS5,ERLEC1,PSME4,COPS9,UBA6,RTN4,CYCS,ADAMTS3,MFSD2A,SLC35A1,GARS,MINDY4,UBE2J1,MAP3K7,SH3BP5L,GFPT1,PCYOX1,GLI3,ROCK2,INHBA,RCN3,PREP,MYLK3,VPS35,CUL2,SLC7A11,FKBP1B,FGF2,CMPK1,SRPK2,RPN1,DNAJC1,RAB7A,PSMC2,POMGNT1,GAD2,GSAP,ABHD12,PRSS23,RB1,ITM2B,EED,IWS1,PICALM,MARK4,MMACHC,MAPRE3,CRTAP,GLB1,EIF2B3,PDCD6IP,TMUB1,KCTD21,CHPF2,NUB1,PAK1,TGFB1,AXL,RAB3GAP1,LMO7,WDR48,UBXN4,ST6GALNAC2,HNMT,SPOPL,ACER3,CTNNB1,TRAK1,DGAT2,SERPINH1,AKT2,GPD1L,OSBPL10,STT3B,MTMR14,PFKP,CANT1,OSBPL8,NPLOC4,PPP1R12A,KAT2B,RNF121,TMTC4,FAM98A,CRIM1,PSMD8,GPS1,SPINT2,SUMF1,KDELC1,UFM1,TMTC3,PRNP,CHFR,NAXD,CARS2,NUDT16,DCN,HDAC3,DCLK1,ACPP,UBA5,DDRGK1,SMPD1,PPP2CB,ILK,RBPMS,MMP17,ARL6IP5,PPP2R2B,UBA3,CPXM1,SPINK5,CIAO1,TUSC3,PSMD6,CDK8,CNOT7,RNF6,VPS37A,IMPAD1,MTMR6,FXYD1,CHPT1,HECTD2,ADAMTS6,SNTA1,PJA2,HSP90B1,TMED2,EIF2S2,TP53INP2,MAST4,OSBP,ST8SIA4,EIF4G2,SULF1,RNF181,GRK7,RIOK2,LNPEP,KCMF1,PCSK1,ROBO1	darkgreen	EC_M4
GO:0051701	6.38E-02	0.960932948	23	150	biological process involved in interaction with host	BP	6.50E-01	CXCL8,CAV2,NRP1,NECTIN2,CXCR4,EFNB2,SCARB1,ITCH,TPCN1,ANO6,IFIT1,HYAL2,CD86,F11R,THOC3,EPHA2,TNIP1,LGALS9,HAVCR1,TRIM34,CD46,TRIM62,SERPINB9	turquoise	EC_M6
GO:0032872	6.40E-02	0.960832612	23	148	regulation of stress-activated MAPK cascade	BP	6.50E-01	FLT4,TPD52L1,SH3RF3,GADD45G,TLR4,TRAF1,SDCBP,SFRP1,EPHB1,TNFRSF19,ITCH,SH3RF1,NCF1,MAP3K3,DLG1,MAP3K11,MECOM,CRACR2A,RELL1,TAOK2,TRAF2,DAB2IP,BMP2	turquoise	EC_M6
GO:0006919	6.40E-02	0.970908653	10	57	activation of cysteine-type endopeptidase activity involved in apoptotic process	BP	6.50E-01	PPARG,EIF2AK3,HIP1R,SNCA,LCK,LAPTM5,CFLAR,CASP10,BID,PMAIP1	turquoise	EC_M6
GO:0021783	6.41E-02	0.98484737	4	14	preganglionic parasympathetic fiber development	BP	6.50E-01	NRP1,NAV2,SEMA3F,TBX1	turquoise	EC_M6
GO:0036507	7.50E-02	0.977758962	5	17	protein demannosylation	BP	6.50E-01	AMFR,MAN1A2,MARCH6,EDEM3,SYVN1	darkgreen	EC_M4
GO:0036508	7.50E-02	0.977758962	5	17	protein alpha-1,2-demannosylation	BP	6.50E-01	AMFR,MAN1A2,MARCH6,EDEM3,SYVN1	darkgreen	EC_M4
GO:0048820	7.50E-02	0.981927322	4	12	hair follicle maturation	BP	6.50E-01	CTNNB1,SPINK5,TRPC4AP,TGFB2	darkgreen	EC_M4
GO:0090407	7.52E-02	0.941998502	72	452	organophosphate biosynthetic process	BP	6.51E-01	RRM2B,PPARA,ACSL6,NUS1,SERINC1,PPARD,VCP,SLC35B2,INPP4A,TMEM246,SOCS5,FAM126A,MFSD2A,GARS,FGF2,CMPK1,SELENOI,ABHD5,DGKH,TGFB1,MTMR14,NAXD,IMPAD1,MTMR6,CHPT1,TMEM150A,FAR1,ADCY6,LPCAT2,ADA,CHP1,PIGP,AGPAT3,HTR2B,PIK3CA,PITPNM1,MOCS3,ETNK1,IDH2,ADCY5,HACD2,PIGZ,PCYT1B,SLC25A16,TPI1,PLD1,SGMS1,PPARGC1A,NPPB,SPTLC2,SLC44A2,AK7,PIGL,ACSL4,MOCS2,OCRL,ACOT7,ENO1,DPM2,HACD1,TMEM38B,TPK1,PEMT,PIGK,DPM1,TMSB4X,NNMT,NME6,PIGB,PIK3C2A,ORMDL3,CDIPT	darkgreen	EC_M4
GO:0032355	7.53E-02	0.955192333	20	105	response to estradiol	BP	6.52E-01	LEP,TGFB1,HTR5A,CTNNB1,ZNF703,MAP1B,MMP15,CCND1,BMP7,STRN3,IGFBP2,CYP19A1,PPARGC1A,COL1A1,PDGFRB,RBBP8,ITGA2,FAM210B,FGF10,MBD2	darkgreen	EC_M4
GO:0021544	7.53E-02	0.977631764	5	17	subpallium development	BP	6.52E-01	GLI3,INHBA,SLC7A11,RARB,ZSWIM6	darkgreen	EC_M4
GO:0001936	7.54E-02	0.954483926	21	111	regulation of endothelial cell proliferation	BP	6.52E-01	LEP,VEGFA,PDCL3,TGFBR1,EMC10,FGF2,CXCL12,SULF1,HTR2B,PRKCA,VASH2,PDCD10,AKT3,NGFR,AKT1,MYDGF,PLXNB3,CDH13,F3,MIR24-1,WNT2	darkgreen	EC_M4
GO:1902905	7.55E-02	0.949411267	32	179	positive regulation of supramolecular fiber organization	BP	6.52E-01	NCK2,TGFBR1,ACTR2,ROCK2,MYLK3,RB1,PAK1,ACTR3,NAV3,CDC42EP3,MAP1B,PFN2,APP,CX3CL1,MAGEL2,ARL3,CTTN,LMOD1,SYNPO2,ARPC5,MAPT,NCKAP1,ARPC2,SYNPO2L,WDR1,TPM1,TGFB3,GPR65,MYO1C,FLNA,DSTN,CFL2	darkgreen	EC_M4
GO:1903522	7.57E-02	0.949235932	32	179	regulation of blood circulation	BP	6.53E-01	LEP,BVES,FKBP1B,SCN5A,GPD1L,ATP2B1,FXYD1,SNTA1,P2RX4,ATP2A2,CACNA1D,ADA,ATP2B4,PKP2,TGFB2,GLRX3,ADRA2C,ECE1,CACNA1C,HSPB7,TPM1,CACNA1G,ADRA1B,CALM1,AKT1,FLNA,DMD,TMEM38B,TMEM38A,HOPX,HCN4,KCNMB4	darkgreen	EC_M4

GO:0001822	7.59E-02	0.946014763	43	248	kidney development	BP	6.54E-01	AHI1,RRM2B,ANGPT1,VEGFA,TGFBR1,GLI3,FGF2,SEC61A1,GLI2,SNX17,CTNNB1,RARB,DCN,ILK,ADAMTS6,SULF1,RDH10,GDF6,PKD2,ARL3,NOTCH2,TGFB2,BMP7,AP1B1,BICC1,FGFR2,IQGAP1,ARID5B,OVOL1,SGPL1,FBN1,SIX4,ACTA2,MAGED1,MYO1E,TMED10,PDGFRB,PKD1,FSTL3,SERPINF1,GDNF,HAS2,FGF10	darkgreen	EC_M4
GO:0010718	6.47E-02	0.972261587	9	45	positive regulation of epithelial to mesenchymal transition	BP	6.56E-01	IL6,SDCBP,JAG1,WWTR1,MTOR,SMAD2,NOTCH1,ENG,BMP2	turquoise	EC_M6
GO:0003407	7.63E-02	0.963427834	11	49	neural retina development	BP	6.56E-01	AHI1,RORB,SMARCD3,ATP2B1,ATP2B4,TGFB2,OBSL1,ROM1,TTC8,SMARCA4,THY1	darkgreen	EC_M4
GO:0098840	7.63E-02	0.981505882	4	12	protein transport along microtubule	BP	6.56E-01	KIF5B,KIF1A,HSPB1,MAP1A	darkgreen	EC_M4
GO:0099118	7.63E-02	0.981505882	4	12	microtubule-based protein transport	BP	6.56E-01	KIF5B,KIF1A,HSPB1,MAP1A	darkgreen	EC_M4
GO:0050709	7.63E-02	0.963261807	11	51	negative regulation of protein secretion	BP	6.56E-01	REST,FKBP1B,RAB11FIP1,ABCC8,PTPN11,HADH,IDH2,SRCIN1,SYTL4,MIDN,RHBDF1	darkgreen	EC_M4
GO:1904894	7.64E-02	0.9709519	7	28	positive regulation of receptor signaling pathway via STAT	BP	6.56E-01	IL23A,LEP,TGFB1,ADIPOR1,CRLF3,TSLP,OCIAD2	darkgreen	EC_M4
GO:0046827	4.13E-02	0.99144079	4	15	positive regulation of protein export from nucleus	BP	6.57E-01	ANP32B,XPO4,CTDSPL2,TPR	darkgrey	EC_M8
GO:0060716	7.66E-02	0.97714287	5	17	labyrinthine layer blood vessel development	BP	6.58E-01	TMED2,HEY1,VASH2,AKT1,WNT2	darkgreen	EC_M4
GO:0090316	7.68E-02	0.951341307	25	136	positive regulation of intracellular protein transport	BP	6.58E-01	LEP,TMEM30A,GLI3,KIF5B,PAK1,TGFB1,AKT2,PRNP,HDAC3,TM9SF4,CACNB3,ATG13,BAP1,CHP1,GSK3B,PDCD10,B3GAT3,MIEF2,PRKAA1,MYO1C,BCAP31,FLNA,LEPROT,ZPR1,TOMM7	darkgreen	EC_M4
GO:0010934	6.52E-02	0.984481041	4	14	macrophage cytokine production	BP	6.59E-01	TLR4,CD36,LAPTM5,PLCG2	turquoise	EC_M6
GO:0010935	6.52E-02	0.984481041	4	14	regulation of macrophage cytokine production	BP	6.59E-01	TLR4,CD36,LAPTM5,PLCG2	turquoise	EC_M6
GO:0032418	6.53E-02	0.968978883	11	62	lysosome localization	BP	6.59E-01	KIT,SYK,PIK3CG,GATA2,GAB2,UNC13D,SNAP23,FGR,FES,LGALS9,STXBP1	turquoise	EC_M6
GO:0098719	7.69E-02	0.981312359	4	12	sodium ion import across plasma membrane	BP	6.59E-01	SLC12A2,HCN2,MIR24-1,HCN4	darkgreen	EC_M4
GO:0016577	6.55E-02	0.976008116	7	28	histone demethylation	BP	6.59E-01	KDM4C,KDM4D,KDM3A,JARID2,KDM2A,KDM5A,KDM5C	turquoise	EC_M6
GO:0010638	6.55E-02	0.95131769	58	428	positive regulation of organelle organization	BP	6.59E-01	CNOT6,MTSS1,ASAP1,PDGFB,BAIAP2L2,PICK1,CDC42EP1,TEK,LIMCH1,KDR,SDCBP,NRP1,TAL1,RALB,FCHSD2,SFRP1,FCHSD1,LMOD3,IL1A,IGF1,HIP1R,OCLN,HMBOX1,P2RX7,RAPGEF3,IQGAP2,EDN1,SNCA,ABCG1,KIAA1551,ZDHHC6,HCLS1,AP1AR,FES,PROX1,DLG1,MCU,BAIAP2L1,PPM1F,WAS,RAC1,HIF1A,FSCN1,BID,MTOR,ARHGEF15,ADCK1,ADRB2,SPIRE1,GIT1,LRSAM1,TERF2,CARMIL2,PMAIP1,VASP,GMFG,RALA,ARPC5L	turquoise	EC_M6
GO:0040008	6.55E-02	0.950058036	69	518	regulation of growth	BP	6.59E-01	SEMA6A,RIMS2,CDKL3,PIM1,FAM122A,SEMA4D,PPT1,VPS54,SDCBP,NRCAM,NRP1,ITSN2,EPC1,CXCR4,NET1,TMC8,SPTBN4,GPAT4,SFRP1,HBEGF,CHD7,IGF1,ITCH,MEF2C,IL7,TLL2,NEDD9,EDN1,JARID2,DCAF1,HYAL2,SEMA3F,HLX,EAF2,SH3BP4,RPS6KA1,PROX1,PAK2,SIPA1,CREB1,SEMA6D,DDX3X,IGFBP5,ING4,CDA,PPM1F,HIF1A,ADIPOR2,MTOR,AR,SEMA4A,TAOK2,NPR1,DNM2,YY1,KIF26A,ADRB2,RAI1,SEMA6B,ARHGAP4,NOTCH1,SLC25A33,DAB2IP,SOX17,OSGIN2,DIO3,RGS4,BST2,GNG4	turquoise	EC_M6
GO:0032890	6.56E-02	0.971689147	9	47	regulation of organic acid transport	BP	6.60E-01	TNFRSF11A,SYK,EDN1,SNCA,ADORA2A,PSEN1,SLC43A2,STXBP1,RGS4	turquoise	EC_M6
GO:0060099	6.57E-02	0.984587853	4	13	regulation of phagocytosis, engulfment	BP	6.60E-01	GATA2,CD36,ANO6,PLCG2	turquoise	EC_M6
GO:0061572	6.57E-02	0.959729903	23	141	actin filament bundle organization	BP	6.60E-01	MTSS1,BAIAP2L2,LIMCH1,NRP1,SFRP1,FHDC1,USH1C,RAPGEF3,NEDD9,F11R,PAK2,BAIAP2L1,PPM1F,WAS,RAC1,FSCN1,MTOR,ARHGEF15,ARHGAP28,SPIRE1,RFLNB,S1PR1,SHANK3	turquoise	EC_M6

GO:0044089	7.71E-02	0.940472414	72	441	positive regulation of cellular component biogenesis	BP	6.60E-01	CAND1,ADGRB1,SRF,VEGFA,VCP,UNC13B,NCK2,TGFBR1,TPBG,LRRC4B,AKIRIN1,ACTR2,ROCK2,RAB7A,MARK4,PDCD6IP,PAK1,ACTR3,TGFB1,RAB3GAP1,DZIP1,NAV3,FAM98A,CDC42EP3,DPYSL3,MAP1B,PFN2,CX3CL1,PTPN11,CDH17,AMIGO2,TSG101,MAGEL2,ARL3,PHLDB2,CTTN,HYAL1,LMOD1,GSK3B,DAG1,PIK3CA,AUTS2,PRKCA,BMP7,SYNPO2,PHLDB1,IQGAP1,TFRC,ARPC5,MAPT,NCKAP1,ARPC2,HTT,SYNPO2L,STX18,HAP1,NLGN2,MSN,TPM1,TGFB3,GPR65,ROCK1,MYO1C,ABCA3,NPHP4,FLNA,PARK7,DEF8,LRRC24,UBE2V2,THY1,LRRTM2	darkgreen	EC_M4
GO:0001732	5.21E-03	0.999141944	6	15	formation of cytoplasmic translation initiation complex	BP	6.62E-01	EIF3H,EIF3L,EIF3D,EIF3K,EIF3F,EIF3G	cyan	EC_M2
GO:0120034	7.74E-02	0.95526675	18	92	positive regulation of plasma membrane bounded cell projection assembly	BP	6.62E-01	SRF,TGFBR1,AKIRIN1,ACTR2,MARK4,ACTR3,DZIP1,FAM98A,CDC42EP3,DPYSL3,PIK3CA,AUTS2,NCKAP1,ARPC2,HTT,HAP1,TGFB3,DEF8	darkgreen	EC_M4
GO:0009896	5.30E-03	0.996546038	63	420	positive regulation of catabolic process	BP	6.62E-01	PIP4K2C,BAG6,SNX9,HSPA1B,HSF1,VPS28,HMOX1,FOXO3,SESN1,NOP53,ZC3HAV1,BBS7,TRIM13,MLH1,EGLN2,SAMD4B,SIRT2,TRIM21,NDFIP1,PLEKHF1,SH3D19,CAMKK2,RNFT2,TP53INP1,IRS1,DIS3L2,PUM1,AC013489.1,DTX3L,RHBDD3,ARNT,MAGEF1,CDKN1B,KAT5,NFE2L2,EXOSC7,VSIR,PHB2,GABARAP,ZFP36L1,PIP4K2B,OSBPL7,GBA,NUPR1,TNRC6A,KEAP1,SNX33,INSR,PARN,RILP,FZR1,DVL1,AXIN1,ZER1,ABCA2,RACK1,TRIM65,RNFT1,TRIB2,HSPB8,ZFP36L2,STUB1,EXOSC5	cyan	EC_M2
GO:1902287	7.75E-02	0.981123943	4	12	semaphorin-plexin signaling pathway involved in axon guidance	BP	6.63E-01	PLXNB2,PLXNA1,PLXNB3,PLXNA3	darkgreen	EC_M4
GO:0008033	4.18E-02	0.978196529	16	117	tRNA processing	BP	6.64E-01	POP1,PUS10,DDX1,PUS7,TRDMT1,TSEN2,CDK5RAP1,NSUN2,TRMT10A,ZBTB8OS,QTRT2,TARBP1,SSB,TSEN15,THG1L,THUMPD1	darkgrey	EC_M8
GO:0006805	5.37E-03	0.998340764	11	41	xenobiotic metabolic process	BP	6.65E-01	ACAA1,CYP1B1,HNF4A,FMO5,AIP,ABCB11,POR,EPHX1,GSTM4,SULT1C4,AHRR	cyan	EC_M2
GO:0090042	7.78E-02	0.98102818	4	12	tubulin deacetylation	BP	6.65E-01	MAPT,PRKAA1,FLNA,FRY	darkgreen	EC_M4
GO:0045165	7.78E-02	0.948056584	31	172	cell fate commitment	BP	6.65E-01	IL23A,TEAD3,RUNX2,TGFBR1,IFRD1,GLI3,ITGB1,FGF2,PTCH2,TENM4,GLI2,CTNNB1,LOXL3,ACVR1,NOTCH2,CDC73,EYA2,SATB2,NTRK3,SLAMF8,FGFR2,FKBP8,PRRX1,BMPR1A,ARHGEF2,TGFB1I1,BARHL2,LMO4,LBX1,WNT2,FGF10	darkgreen	EC_M4
GO:0048754	6.64E-02	0.960825154	20	121	branching morphogenesis of an epithelial tube	BP	6.65E-01	CELSR1,NOTCH4,KDR,MYCN,NRP1,RASIP1,PLXND1,GZF1,TIE1,DLL4,EDN1,LAMA5,DLG1,EPHA2,AR,HOXB7,NRARP,NOTCH1,ENG,BMP2	turquoise	EC_M6
GO:0045912	7.80E-02	0.965716781	9	39	negative regulation of carbohydrate metabolic process	BP	6.66E-01	PPARA,TGFB1,SELENOS,GSK3B,PPARGC1A,CLTC,PGP,MIDN,FAM3A	darkgreen	EC_M4
GO:2000279	4.20E-02	0.985868191	7	32	negative regulation of DNA biosynthetic process	BP	6.66E-01	POT1,DNAJC2,XRN1,TERF1,HNRNPU,DCP2,EXOSC10	darkgrey	EC_M8

GO:0009892	6.68E-02	0.942142693	268	2254	negative regulation of metabolic process	BP	6.67E-01	BAZ2A,TNFAIP8,TNFAIP3,PLAGL1,TRIM39,PTPRB,LAPTM4B,CNOT6,OXR1,NOTCH4,TCF7,NCOA7,L3MBTL3,C6orf106,DAPK1,ATF4,PDGFB,MDFI,IREB2,NFX1,TNFRSF21,KDR,CD2AP,NFIL3,SEMA4D,RGP1,IL6,IGF2BP3,NFE2L3,REL,PTBP3,CXCL8,BACH2,FOXP2,KLF11,GRB10,LANCL2,TLR4,C5,IL15,CREM,SDCBP,MYCN,NOCT,TUT4,PUM2,RASIP1,BEND5,PIK3CG,TAL1,LAMA4,PHF10,MTMR2,EPC1,KDM4D,GATA2,DNMT3A,SAP130,CST7,NOVA2,GGA3,DACH1,PPARG,TIMP4,GATA3,TAF3,ACVR2B,ITIH5,GZF1,EDNRB,NDFIP2,IKBKB,PDE2A,SFRP1,HBEGF,NR1D2,DND1,FEZ2,FERMT1,SH3BP5,ANKLE2,CD36,SIRT3,ARID5A,PRR5L,PDE4D,MERTK,DNMT3B,IGF1,NR4A1,EIF2AK3,ABHD6,HHEX,KDM3A,ITCH,ERBIN,SH3RF1,SMAD1,ZBTB38,POU6F1,OCLN,SOX7,HMBOX1,ENC1,MEF2C,WWTR1,SOGA1,SFMBT1,FAM192A,SCAF4,TIPARP,OAS3,PPP1R16B,ANKRD2,NR4A2,DLL4,CDYL,SOX13,YAF2,EDN1,STAT1,DYRK1A,SNCA,JARID2,DTNBP1,ETS2,DCAF1,CD200,LPCAT1,SIK1,HDAC1,KIAA1551,PPP3CA,HYAL2,MEF2A,RIN3,USP30,LAPTM5,FABP4,HCLS1,NFATC2,AP1AR,INPP5D,CD34,KDM2A,CIART,TGIF1,PARP15,WDR6,CFLAR,INPP5F,IGFBP3,PPP2R5A,RBM4,RPS6KA1,PROX1,ZNF148,YTHDF1,IL10RA,HELZ2,MLXIPL,ACE,PAK2,CST6,SENP2,DLG1,SMG7,APOD,ETV6,MOV10,RNF2,CREB1,EIF4A2,TET1,LTF,ADORA2A,MYEF2,BCOR,DDX3X,IGFBP5,HOMER3,MECOM,ING4,CDA,SPINDOC,BIRC3,LDB2,CPEB2,PPM1F,POU2F1,RAC1,HIF1A,KDM5A,ZBTB18,APBB2,ADIPOR2,BCL6B,EPHA2,KDM5C,CASC3,TLR6,RGS14,ARG2,MAFK,MTOR,AR,OPHN1,RNF40,PSEN1,PAPLN,CBX1,HOXB8,FBXW11,EFNA1,PIAS1,RPS6KA5,SMAD2,TLE3,CNOT8,YY1,HMG20A,R	turquoise	EC_M6
GO:0001937	6.68E-02	0.974818957	7	34	negative regulation of endothelial cell proliferation	BP	6.67E-01	CAV2,PPARG,MEF2C,DLL4,STAT1,VASH1,CCL2	turquoise	EC_M6
GO:0001959	6.70E-02	0.961515062	18	119	regulation of cytokine-mediated signaling pathway	BP	6.68E-01	TNFAIP3,SYK,IL6,PPARG,CXCR4,IRAK2,IKBKB,IL7,OAS3,NLRC5,HIPK1,EDN1,LAPTM5,HIF1A,ISG15,TRAF2,MIR99A,OAS1	turquoise	EC_M6
GO:0006953	6.71E-02	0.980041388	5	21	acute-phase response	BP	6.69E-01	TNFRSF11A,IL6,EDNRB,IL1A,F8	turquoise	EC_M6
GO:1904646	6.72E-02	0.974651394	7	34	cellular response to amyloid-beta	BP	6.69E-01	TLR4,CD36,IGF1,ITGA4,TLR6,PSEN1,ADRB2	turquoise	EC_M6
GO:0051895	7.86E-02	0.976367465	5	17	negative regulation of focal adhesion assembly	BP	6.70E-01	LRP1,PHLDB2,MMP14,CORO1C,DUSP22	darkgreen	EC_M4
GO:0150118	7.86E-02	0.976367465	5	17	negative regulation of cell-substrate junction organization	BP	6.70E-01	LRP1,PHLDB2,MMP14,CORO1C,DUSP22	darkgreen	EC_M4
GO:0032925	7.87E-02	0.976343129	5	17	regulation of activin receptor signaling pathway	BP	6.70E-01	ACVR1,NOMO1,FSTL3,FKBP1A,FGF10	darkgreen	EC_M4
GO:0008202	5.46E-03	0.996910418	37	217	steroid metabolic process	BP	6.70E-01	TSPO,HSD17B8,CYB5R3,PDGFRA,PLPP6,HINT2,FDXR,LIPE,ACAA1,PIP4P1,LAMTOR1,CYP1B1,ERLIN1,ACOT8,SRD5A1,OSBPL5,CYB5R1,FMO5,ABCB11,CLCN2,SCAP,POR,ACADL,SOAT1,CYP27A1,OSBPL7,HSD3B7,GBA,ERG28,SREBF1,LEPR,ACAA2,RXRA,LCAT,ABCA2,STUB1,SCP2	cyan	EC_M2
GO:0006790	7.87E-02	0.943337465	45	270	sulfur compound metabolic process	BP	6.70E-01	CDO1,ANGPT1,ACSL6,AHCYL2,MTAP,SLC35B2,SLC7A11,MMACHC,GLB1,CHPF2,DGAT2,DCN,ACPP,ARL6IP5,CIAO1,IMPAD1,B3GNT4,CHST2,FAR1,CHAC1,GLRX2,CHSY1,HYAL1,ISCU,TPST2,HACD2,GLRX3,SMS,COMT,UGDH,CHST12,XYLT2,ACSL4,EGFLAM,HAGH,ACOT7,PARK7,AHCYL1,CIAO2B,HACD1,TPK1,PEMT,GLO1,ADI1,CHST11	darkgreen	EC_M4
GO:0045185	7.89E-02	0.955989235	16	80	maintenance of protein location	BP	6.70E-01	FBN2,SUN2,DZIP1,LTBP1,PKD2,TWF1,FBN1,NBL1,KDELR2,AKT1,PKD1,FLNA,PARK7,HSPA5,TXN,TMSB4X	darkgreen	EC_M4
GO:0032252	7.91E-02	0.980607183	4	12	secretory granule localization	BP	6.72E-01	KIF5B,USO1,KIF1A,TANC2	darkgreen	EC_M4
GO:2000369	6.77E-02	0.980147051	5	19	regulation of clathrin-dependent endocytosis	BP	6.72E-01	SNAP91,HIP1R,BMP2K,DNM2,UNC119	turquoise	EC_M6
GO:0072089	6.77E-02	0.969036423	10	54	stem cell proliferation	BP	6.72E-01	PIM1,FERMT1,VEGFC,CD34,ACE,ETV6,MECOM,ATXN1L,SOX17,SOX18	turquoise	EC_M6
GO:0046685	6.78E-02	0.9766708	6	29	response to arsenic-containing substance	BP	6.72E-01	ATF4,MAPK13,RBM4,DDX3X,CPEB2,CYP1A1	turquoise	EC_M6

GO:2000107	6.79E-02	0.974335159	7	34	negative regulation of leukocyte apoptotic process	BP	6.72E-01	MERTK,HCLS1,HIF1A,ITPKB,ARG2,EFNA1,IL7R	turquoise	EC_M6
GO:0060548	6.80E-02	0.94602208	94	754	negative regulation of cell death	BP	6.72E-01	TNFAIP8,TNFAIP3,FLT4,OXR1,NCOA7,PTK2,ATF4,PIM1,TEK,KIT,KDR,SEMA4D,IL6,REL,PPT1,PELI1,FIGNL1,TEAD2,NRP1,PIK3CG,GATA2,NOS3,ZFAND6,GATA3,EDNRB,SFRP1,ANKLE2,SPEF1,PLK2,MERTK,IL1A,IGF1,NR4A1,ITCH,SH3RF1,HIP1R,MEF2C,IL7,NR4A2,ADAMTS20,EDN1,SNCA,NUAK2,PM20D1,RNF144B,CD200,HDAC1,ITPRIP,LAPTM5,DYRK3,HCLS1,CD34,CFLAR,RPS6KA1,PRKD1,ITGA6,PAK2,ELL3,CREB1,LTF,ADORA2A,ARNT2,DDX3X,TBX1,MECOM,RAMP2,BIRC3,PRKCH,HIF1A,ITPKB,BID,TNFRSF1B,ARG2,MTOR,AR,BNIP2,PSEN1,EFNA1,PIAS1,AP2B1,CCL2,IL7R,BFAR,MYO18A,TRAF2,NOTCH1,PLCG2,STXBP1,DUS2,CDH5,VNN1,SERPINB9,IFIT3,NMNAT1	turquoise	EC_M6
GO:0019932	7.92E-02	0.944770504	38	218	second-messenger-mediated signaling	BP	6.72E-01	DYRK2,VEGFA,FKBP1B,HTR5A,PRNP,PCLO,MRVI1,P2RX4,ATP2A2,RAPGEF2,PPP3CC,ADA,CHP1,ATP2B4,ADGRB2,HTR2B,GSK3B,INPP5A,CMKLR1,CASQ1,MAPT,EIF4EBP2,HTT,CACNA1C,ADGRL1,NPPB,TMEM100,CALM1,PRKAA1,MGRN1,BCAP31,CDH13,DMD,TMEM38B,NMUR1,TMEM38A,CCR6,PRKG1	darkgreen	EC_M4
GO:0099601	6.82E-02	0.970535094	9	44	regulation of neurotransmitter receptor activity	BP	6.73E-01	DAPK1,DLGAP3,MEF2C,ADRB2,CCL2,RELN,SHANK3,CACNG5,GSG1L	turquoise	EC_M6
GO:0072567	6.82E-02	0.983289075	4	15	chemokine (C-X-C motif) ligand 2 production	BP	6.73E-01	TLR4,OAS3,HDAC1,OAS1	turquoise	EC_M6
GO:2000341	6.82E-02	0.983289075	4	15	regulation of chemokine (C-X-C motif) ligand 2 production	BP	6.73E-01	TLR4,OAS3,HDAC1,OAS1	turquoise	EC_M6
GO:0015732	6.82E-02	0.983510569	4	14	prostaglandin transport	BP	6.73E-01	TNFRSF11A,SLCO2A1,EDN1,NOS2	turquoise	EC_M6
GO:0014075	6.83E-02	0.974004942	7	36	response to amine	BP	6.73E-01	NR4A1,NR4A2,HDAC1,PPP3CA,ADORA2A,ADIPOR2,RGS4	turquoise	EC_M6
GO:0021795	6.84E-02	0.97428122	7	32	cerebral cortex cell migration	BP	6.73E-01	EFHC1,SRGAP2C,PSEN1,LHX6,DAB2IP,ADGRG1,RELN	turquoise	EC_M6
GO:1903055	7.93E-02	0.972382728	6	23	positive regulation of extracellular matrix organization	BP	6.73E-01	RB1,TGFB1,RUNX1,PHLDB2,DAG1,PHLDB1	darkgreen	EC_M4
GO:0046434	6.87E-02	0.959739619	19	126	organophosphate catabolic process	BP	6.75E-01	PDE7B,SYNJ2,INPP4B,MTMR2,PDE2A,SCARB1,PDE4D,ABHD6,GPD1,ENTPD1,PLCG1,PDE9A,INPP5F,PGM2,GDPD3,GDPD1,PDE4B,PLCG2,PLA2G4E	turquoise	EC_M6
GO:0051894	6.88E-02	0.976683765	6	25	positive regulation of focal adhesion assembly	BP	6.75E-01	TEK,KDR,NRP1,SFRP1,PPM1F,RAC1	turquoise	EC_M6
GO:0036475	6.88E-02	0.976448383	6	27	neuron death in response to oxidative stress	BP	6.75E-01	OXR1,NCOA7,ATF4,TLR4,HIF1A,TLR6	turquoise	EC_M6
GO:0014015	6.89E-02	0.969931996	9	48	positive regulation of gliogenesis	BP	6.76E-01	IL6,CXCR4,TLR2,HDAC1,PRKCH,TNFRSF1B,MTOR,NOTCH1,BMP2	turquoise	EC_M6
GO:0098698	7.97E-02	0.97592179	5	17	postsynaptic specialization assembly	BP	6.76E-01	LRRC4B,NTRK3,NLGN2,NTNG2,LRRTM2	darkgreen	EC_M4
GO:0006893	7.98E-02	0.959897769	12	56	Golgi to plasma membrane transport	BP	6.76E-01	GGA1,EXOC1,ATP2C1,EXOC6B,ARL3,EXOC5,BLZF1,RAB31,RAB34,C16orf70,STXBP6,ARFRP1	darkgreen	EC_M4
GO:1901841	7.99E-02	0.979943002	4	13	regulation of high voltage-gated calcium channel activity	BP	6.76E-01	REM1,CACNB3,CALM1,GEM	darkgreen	EC_M4
GO:0007266	7.99E-02	0.950834346	22	116	Rho protein signal transduction	BP	6.76E-01	ARHGEF25,COL1A2,ROCK2,ITGB1,CDC42EP3,STARD13,ROBO1,RTKN,ARHGAP1,KCTD10,COL3A1,SYNPO2L,MYO9B,ARHGEF2,NGFR,GPR65,PDGFRB,ROCK1,TNFAIP1,TAX1BP3,CDH13,ARHGAP29	darkgreen	EC_M4
GO:1903533	8.00E-02	0.957280186	14	69	regulation of protein targeting	BP	6.76E-01	LMAN1,PAK1,AKT2,PRNP,CACNB3,ATG13,BAP1,CHP1,C11orf65,MIEF2,PRKAA1,MYO1C,LEPROT,TOMM7	darkgreen	EC_M4
GO:1901550	8.00E-02	0.980291403	4	12	regulation of endothelial cell development	BP	6.76E-01	VEGFA,ROCK2,VCL,ROCK1	darkgreen	EC_M4
GO:1903140	8.00E-02	0.980291403	4	12	regulation of establishment of endothelial barrier	BP	6.76E-01	VEGFA,ROCK2,VCL,ROCK1	darkgreen	EC_M4
GO:0051593	5.55E-03	0.999558221	4	7	response to folic acid	BP	6.76E-01	FOLR2,MGMT,MTHFR,EEF2	cyan	EC_M2

GO:0045995	8.01E-02	0.961202274	11	50	regulation of embryonic development	BP	6.76E-01	NLRP5,TENM4,CTNNB1,DHX36,PHLDB2,TRIP12,DAG1,PHLDB1,LAMA3,PAFAH1B1,WNT2	darkgreen	EC_M4
GO:0010632	8.01E-02	0.944613071	36	208	regulation of epithelial cell migration	BP	6.77E-01	ANGPT1,NUS1,ADGRB1,VEGFA,RTN4,EMC10,ROCK2,RRAS,FGF2,TGFB1,DCN,STARD13,P2RX4,PFN2,ARSB,ATP2B4,ADIPOR1,HYAL1,TGFB2,CORO1C,PRKCA,HSPB1,DNAJA4,PDCD10,AKT3,RAB11A,AKT1,ITGA2,MIR24-1,SERPINF1,HAS2,TMSB4X,GLIPR2,CCR6,PIK3C2A,FGF10	darkgreen	EC_M4
GO:1901616	6.92E-02	0.96816525	10	56	organic hydroxy compound catabolic process	BP	6.78E-01	SYNJ2,SRD5A3,INPP4B,MTMR2,SCARB1,FAH,FGF23,DIO3,SCARF1,AKR1D1	turquoise	EC_M6
GO:0003300	6.92E-02	0.96446059	13	75	cardiac muscle hypertrophy	BP	6.78E-01	IGF1,EDN1,JARID2,PDE9A,PPP3CA,MEF2A,INPP5F,TNFRSF1B,MTOR,YY1,NOTCH1,MIR23A,RGS4	turquoise	EC_M6
GO:0021536	6.93E-02	0.969786563	9	47	diencephalon development	BP	6.78E-01	NRP1,GATA2,NR4A2,ETS1,PTCHD1,CREB1,ALDH1A2,CHRNB2,BMP2	turquoise	EC_M6
GO:0043393	6.96E-02	0.956477396	24	162	regulation of protein binding	BP	6.79E-01	CTHRC1,PDGFB,CSNK1E,MFNG,NRP1,PLXND1,RALB,TMC8,TMEM173,PLK2,HIP1R,FAM192A,IFIT2,IFIT1,DTNBP1,EPB41,ACE,B2M,ITGA4,RAMP2,PSEN1,MARK3,ADRB2,BMP2	turquoise	EC_M6
GO:0030239	6.97E-02	0.968086193	10	51	myofibril assembly	BP	6.81E-01	PGM5,TMOD1,LMOD3,EDN1,MEF2A,NRAP,CFLAR,PROX1,TMOD4,TMOD2	turquoise	EC_M6
GO:0060191	8.09E-02	0.957905325	13	63	regulation of lipase activity	BP	6.81E-01	LRP1,GNAQ,PLAA,FGF2,ABHD5,ARL1,PLA2R1,HTR2B,FGFR2,FGFR3,PDGFRB,NMUR1,ARF4	darkgreen	EC_M4
GO:0048875	8.10E-02	0.979967826	4	12	chemical homeostasis within a tissue	BP	6.81E-01	VEGFA,RCN3,HOMER1,ABCA3	darkgreen	EC_M4
GO:0061025	8.10E-02	0.94938533	23	125	membrane fusion	BP	6.81E-01	STX7,RIMS1,VPS41,KIF5B,NAPA,RAB7A,AKT2,VAMP8,USO1,CHP1,VPS39,RAB7B,DNM1L,SEC22B,STX18,STX1B,SYT11,SOX30,NAPG,RAB34,VAMP7,GNAI3,STXBP6	darkgreen	EC_M4
GO:0060391	8.10E-02	0.975401117	5	17	positive regulation of SMAD protein signal transduction	BP	6.81E-01	TGFBR1,TGFB1,RBPMS,BMPR1A,TGFB3	darkgreen	EC_M4
GO:0009185	8.12E-02	0.954467946	16	81	ribonucleoside diphosphate metabolic process	BP	6.81E-01	PPARA,CMPK1,PFKP,NUDT16,APP,PFKM,PGAM4,ENTPD4,NUDT18,ZBTB20,TPI1,PPARGC1A,PKM,PRKAA1,ENO1,SLC2A6	darkgreen	EC_M4
GO:0071634	8.12E-02	0.966236249	8	33	regulation of transforming growth factor beta production	BP	6.81E-01	LTBP1,LUM,CX3CL1,TGFB2,ATP6AP2,FN1,ITGAV,BMPR1A	darkgreen	EC_M4
GO:0046634	6.99E-02	0.96633914	11	63	regulation of alpha-beta T cell activation	BP	6.82E-01	SYK,GATA3,ITCH,SH3RF1,HLX,CD86,ADORA2A,ITPKB,ARG2,LGALS9,AP3D1	turquoise	EC_M6
GO:0090114	8.14E-02	0.968563737	7	28	COPII-coated vesicle budding	BP	6.83E-01	SEC24A,SEC13,SEC23B,TMED2,VAPB,SEC23A,TMED10	darkgreen	EC_M4
GO:0030316	8.15E-02	0.95636997	14	68	osteoclast differentiation	BP	6.83E-01	IL23A,OSTM1,CTNNB1,NOTCH2,TCTA,TFRC,FARP2,GPR137,FBN1,PAFAH1B1,ATP6AP1,FSTL3,SNX10,GLO1	darkgreen	EC_M4
GO:0072010	7.04E-02	0.979121396	5	19	glomerular epithelium development	BP	6.84E-01	PODXL,JAG1,CD34,PECAM1,PROM1	turquoise	EC_M6
GO:0032409	7.04E-02	0.953871249	29	198	regulation of transporter activity	BP	6.84E-01	TRDN,DAPK1,PIM1,TCAF2,KCNRG,PPARG,NDFIP2,P2RY6,PDE4D,MEF2C,SNCA,PM20D1,HECW2,DLG1,CTTNBP2NL,WNK4,CRACR2A,ATP2A1,DNM2,ADRB2,CCL2,PDE4B,PLCG2,RELN,SCN2B,SHANK3,CACNG5,FXYD6,GSG1L	turquoise	EC_M6
GO:0001947	7.04E-02	0.96928444	9	45	heart looping	BP	6.84E-01	MEF2C,NDRG4,VANGL2,HIF1A,PSEN1,NOTCH1,ENG,SOX17,SOX18	turquoise	EC_M6
GO:1903792	7.05E-02	0.982764395	4	14	negative regulation of anion transport	BP	6.84E-01	TCAF2,MTOR,SLC43A2,RGS4	turquoise	EC_M6
GO:0010830	7.05E-02	0.973186744	7	33	regulation of myotube differentiation	BP	6.84E-01	ATP11A,LMOD3,SIK1,TBX1,MTOR,NOTCH1,PIEZO1	turquoise	EC_M6
GO:0044003	7.06E-02	0.982506435	4	15	modulation by symbiont of host process	BP	6.84E-01	ANO6,HYAL2,TNIP1,SERPINB9	turquoise	EC_M6

GO:0097659	7.06E-02	0.938364931	300	2522	nucleic acid-templated transcription	BP	6.84E-01	ZNF516,TNFRSF11A,BAZ2A,TCF4,TNFAIP3,PLAGL1,CNOT6,FZD6,ZBED4,NOTCH4,TCF20,TCF7,NCOA7,L3MBTL3,ATF4,PDGFB,KDM4C,PIM1,NFYA,MDFI,PRDM4,DDX58,NFX1,KIT,ADGRF1,NFIL3,SYK,SEMA4D,POLR2B,IL6,NFE2L3,REL,CREB5,PPIE,BACH2,PELI1,FOXP2,KLF11,LANCL2,NOBOX,TLR4,TRAF1,CREM,TEAD2,SDCBP,MYCN,NOCT,KTI12,ZBTB24,BEND5,TAL1,PHF10,EPC1,GATA2,DNMT3A,PRKD2,SAP130,DACH1,PPARG,GATA3,TAF3,ACVR2B,IRAK2,GZF1,EDNRB,SETD5,IKBKB,JAG1,NFKBIB,PDE2A,TMEM173,SFRP1,NR1D2,CD36,CDK17,PHRF1,ARID5A,TCF15,RET,CHD7,IL1A,DNMT3B,IGF1,NR4A1,EIF2AK3,HHEX,KDM3A,ITCH,ERBIN,SMAD1,ZBTB38,POU6F1,STOX2,SOX7,HMBOX1,HEXB,MEF2C,SMARCD1,TLR2,WWTR1,ZNF704,CXCL10,ADGRG3,KMT2D,SFMBT1,SCAF4,NLRC5,ACTR5,ANKRD2,NR4A2,TOX2,DLL4,CDYL,BAZ2B,SOX13,YAF2,EDN1,STAT1,DYRK1A,SNCA,JARID2,ERG,ETS2,CDK18,NFKB2,DCAF1,PRDM15,ETS1,CD200,SIK1,HDAC1,PPP3CA,HYAL2,SLC39A8,TESMIN,MEF2A,BCL9,GTF2E1,HLX,NCF1,USP13,FABP4,HCLS1,NFATC2,ZFHX2,EAF2,GMEB1,CD86,KDM2A,CIART,MN1,TGIF1,PARP15,POU2F3,ZSCAN29,CFLAR,TRAK2,ADCY1,ARID1A,RPS6KA1,PROX1,SS18L1,PRKD1,ZNF148,ZSCAN2,HELZ2,MLXIPL,ITGA6,DLG1,ELL3,ETV6,HOXD10,RNF2,CREB1,EIF4A2,TET1,LTF,ADORA2A,ARNT2,MYEF2,IL1RAP,BCOR,DDX3X,RFXANK,ETV4,TBX1,MECOM,ING4,CDK16,SPINDOC,LDB2,FGF23,PPM1F,POU2F1,WAS,TEAD4,PRKCH,HIF1A,KDM5A,ZBTB18,APBB2,JUP,BCL6B,KDM5C,TLR6,RSC1A1,RGS14,MAFK,SPIN2A,MTOR,AR,CASZ1,PSEN1,CBX1,HOXB5,HOXB6,HOXB7,HOXB8,HOXB9,ZNF410,FBXW11,BATF,KRBA2,EFNA1,	turquoise	EC_M6
GO:0002287	7.08E-02	0.969014146	9	46	alpha-beta T cell activation involved in immune response	BP	6.84E-01	IL6,GATA3,HLX,CD86,CRACR2A,MTOR,SEMA4A,BATF,LGALS9	turquoise	EC_M6
GO:0002293	7.08E-02	0.969014146	9	46	alpha-beta T cell differentiation involved in immune response	BP	6.84E-01	IL6,GATA3,HLX,CD86,CRACR2A,MTOR,SEMA4A,BATF,LGALS9	turquoise	EC_M6
GO:0042267	7.11E-02	0.972924924	7	33	natural killer cell mediated cytotoxicity	BP	6.84E-01	NECTIN2,UNC13D,RNF19B,LYST,RAB27A,LGALS9,SERPINB9	turquoise	EC_M6
GO:1904645	7.11E-02	0.970633558	8	41	response to amyloid-beta	BP	6.84E-01	TLR4,CD36,IGF1,ITGA4,MMP13,TLR6,PSEN1,ADRB2	turquoise	EC_M6
GO:0010878	7.12E-02	0.98209828	4	16	cholesterol storage	BP	6.84E-01	PPARG,CD36,SCARB1,ABCG1	turquoise	EC_M6
GO:0006910	7.12E-02	0.982304169	4	15	phagocytosis, recognition	BP	6.84E-01	CD36,SCARB1,PEAR1,MYO18A	turquoise	EC_M6
GO:0035194	7.12E-02	0.959509363	17	112	post-transcriptional gene silencing by RNA	BP	6.84E-01	CNOT6,IL6,MYCN,TUT4,PUM2,PPARG,DND1,SMAD1,PPP3CA,RBM4,MOV10,DDX3X,SMAD2,CNOT8,MIR23A,MIR99A,MIR1307	turquoise	EC_M6
GO:0018230	7.13E-02	0.978297105	5	22	peptidyl-L-cysteine S-palmitoylation	BP	6.84E-01	ZDHHC14,ZDHHC6,ZDHHC18,ZDHHC15,ZDHHC22	turquoise	EC_M6
GO:0018231	7.13E-02	0.978297105	5	22	peptidyl-S-diacylglycerol-L-cysteine biosynthetic process from peptidyl-cysteine	BP	6.84E-01	ZDHHC14,ZDHHC6,ZDHHC18,ZDHHC15,ZDHHC22	turquoise	EC_M6
GO:0048667	7.13E-02	0.946033269	64	446	cell morphogenesis involved in neuron differentiation	BP	6.84E-01	SEMA6A,KIF5A,CDKL3,PTK2,SEMA4D,NRCAM,NRP1,NOVA2,PLXND1,GATA3,MYO7A,SPTBN4,FEZ2,EFNB2,EPHB1,RET,PREX2,KIF13B,MEF2C,NR4A2,DTNBP1,PPP3CA,HECW2,MEF2A,SEMA3F,ROBO4,SEC24B,PLXNA2,TRAK2,ADCY1,KALRN,SS18L1,LAMA5,YTHDF1,PAK2,LAMC2,CREB1,SEMA6D,EPHB4,ITGA4,DNM3,LMTK2,RAC1,EPHA2,OPHN1,EFNB1,PSEN1,SEMA4A,ABI3,TAOK2,EFNA1,CHRNB2,ZDHHC15,RPS6KA5,DNM2,B4GALT6,SEMA6B,SLITRK2,ARHGAP4,NOTCH1,STXBP1,VASP,RELN,SHANK3	turquoise	EC_M6
GO:0061756	7.14E-02	0.97269904	7	34	leukocyte adhesion to vascular endothelial cell	BP	6.84E-01	IL6,ETS1,SLC39A8,ITGA4,SELE,SELP,NFAT5	turquoise	EC_M6
GO:0007613	7.14E-02	0.961275498	15	86	memory	BP	6.84E-01	ADGRF1,PLK2,ADCY1,YTHDF1,PTCHD1,CREB1,RGS14,MTOR,PSEN1,ITGA3,CHRNB2,SLC8A3,ATXN1L,RELN,SHANK3	turquoise	EC_M6
GO:0090559	7.15E-02	0.966790798	10	59	regulation of membrane permeability	BP	6.84E-01	LAPTM4B,TJP2,RASIP1,HIP1R,JAM3,LAPTM5,F11R,BID,MTOR,PMAIP1	turquoise	EC_M6

GO:0010468	7.20E-02	0.936294547	401	3428	regulation of gene expression	BP	6.84E-01	ZNF516,TNFRSF11A,BAZ2A,TCF4,IFNGR1,TNFAIP3,PLAGL1,TRIM39,AKAP12,TSPAN8,LAPTM4B,CNOT6,FLT4,FZD6,RIMS2,ZBED4,QKI,NOTCH4,AFDN,TCF20,TCF7,NCOA7,L3MBTL3,C6orf106,DAPK1,ATF4,PDGFB,MAPK13,CSNK1E,KDM4C,PIM1,NFYA,MDFI,LRRC19,IREB2,PRDM4,DDX58,NFX1,APBA1,KIT,ADGRF1,TNFRSF21,KDR,CD2AP,NFIL3,SYK,SEMA4D,IL6,IGF2BP3,NFE2L3,REL,CREB5,PTBP3,CXCL8,PPIE,BACH2,PELI1,RSAD2,FOXP2,KLF11,LANCL2,NOBOX,TLR4,TRAF1,C5,IL15,CREM,TEAD2,SDCBP,MYCN,NOCT,TUT4,TRPM4,KTI12,ZBTB24,PUM2,BEND5,PIK3CG,TAL1,PHF10,EPC1,GATA2,DNMT3A,PRKD2,SAP130,CST7,NOVA2,NOS3,DACH1,PPARG,GATA3,TAF3,ACVR2B,IRAK2,GZF1,EDNRB,PPP1CB,NDFIP2,SETD5,IKBKB,JAG1,NFKBIB,PDE2A,TMEM173,SFRP1,NR1D2,DND1,FERMT1,RPL26,CD36,CDK17,ARID5A,PRR5L,TCF15,RET,PDE4D,MERTK,CHD7,IL1A,DNMT3B,IGF1,NR4A1,KLKB1,EIF2AK3,HHEX,KDM3A,ITCH,ERBIN,SMAD1,ZBTB38,POU6F1,OCLN,STOX2,SOX7,HMBOX1,ENC1,HEXB,MEF2C,SMARCD1,TLR2,WWTR1,IL7,P2RX7,ZNF704,CXCL10,ADGRG3,DHH,KMT2D,SFMBT1,SCAF4,TIPARP,OAS3,NLRC5,ACTR5,ANKRD2,NR4A2,TOX2,DLL4,CDYL,BAZ2B,SOX13,YAF2,EDN1,STAT1,CD58,DYRK1A,SNCA,JARID2,DTNBP1,ERG,ETS2,CDK18,NFKB2,DCAF1,PRDM15,ETS1,CD200,SIK1,HDAC1,KIAA1551,PPP3CA,HYAL2,SLC39A8,ICOSLG,TESMIN,MEF2A,BCL9,LAPTM5,HLX,NCF1,USP13,FABP4,HCLS1,NFATC2,ZFHX2,INPP5D,EAF2,GMEB1,CD34,CD86,KDM2A,CIART,MN1,TGIF1,PARP15,POU2F3,ZSCAN29,FGR,CFLAR,TRAK2,ADCY1,ARID1A,RBM4,RPS6KA1,PROX1,SS18L1,PRKD1,ZNF148,F11R,YTHDF1,ZSCAN2,HELZ2,MLXIPL,ACE,ITGA	turquoise	EC_M6
GO:0008625	7.20E-02	0.96504267	11	67	extrinsic apoptotic signaling pathway via death domain receptors	BP	6.84E-01	TNFAIP3,NOS3,TMC8,SFRP1,MADD,ITPRIP,CFLAR,DDX3X,BID,DAB2IP,PMAIP1	turquoise	EC_M6
GO:0001649	7.21E-02	0.952995296	28	190	osteoblast differentiation	BP	6.84E-01	CTHRC1,ASF1A,PTK2,ATF4,SEMA4D,IL6,FIGNL1,NOCT,TRPM4,PPARG,ACVR2B,JAG1,SFRP1,IGF1,SMAD1,MEF2C,WWTR1,VEGFC,DHH,PRKD1,LTF,IGFBP5,FGF23,EPHA2,SMOC1,NOTCH1,BMP2,BMP6	turquoise	EC_M6
GO:0002285	7.23E-02	0.95679722	20	127	lymphocyte activation involved in immune response	BP	6.84E-01	MFNG,IL6,TLR4,UNC13D,GATA3,GAPT,HLX,CD86,CRACR2A,RAB27A,LFNG,MTOR,PSEN1,SEMA4A,BATF,CD19,LGALS9,IRF8,PLCG2,CD46	turquoise	EC_M6
GO:1903556	7.24E-02	0.96987856	8	43	negative regulation of tumor necrosis factor superfamily cytokine production	BP	6.84E-01	TNFAIP3,C6orf106,TLR4,IGF1,CD34,LTF,ARG2,LGALS9	turquoise	EC_M6
GO:0048844	7.24E-02	0.965180374	11	57	artery morphogenesis	BP	6.84E-01	ADGRF5,NRP1,JAG1,ADAMTS9,CHD7,DLL4,SEC24B,PROX1,TBX1,NOTCH1,ENG	turquoise	EC_M6
GO:0001574	7.25E-02	0.981868647	4	15	ganglioside biosynthetic process	BP	6.84E-01	ST3GAL5,B4GALT6,ST6GALNAC3,ST8SIA6	turquoise	EC_M6
GO:0002223	7.25E-02	0.982396102	4	13	stimulatory C-type lectin receptor signaling pathway	BP	6.84E-01	SYK,IKBKB,PAK2,PLCG2	turquoise	EC_M6
GO:1990840	7.25E-02	0.982396102	4	13	response to lectin	BP	6.84E-01	SYK,IKBKB,PAK2,PLCG2	turquoise	EC_M6
GO:1990858	7.25E-02	0.982396102	4	13	cellular response to lectin	BP	6.84E-01	SYK,IKBKB,PAK2,PLCG2	turquoise	EC_M6
GO:0032486	7.28E-02	0.982291056	4	13	Rap protein signal transduction	BP	6.84E-01	SGSM3,PLK2,RAPGEF3,RAPGEF1	turquoise	EC_M6

GO:0043067	7.29E-02	0.939752565	135	1119	regulation of programmed cell death	BP	6.84E-01	TNFAIP8,TNFAIP3,TRIM39,AKAP12,FLT4,OXR1,TPD52L1,PTK2,DAPK1,ATF4,PIM1,TEK,KIT,KDR,SYK,GADD45G,SEMA4D,IL6,PPT1,PELI1,UTP11,FIGNL1,KLF11,TRAF1,NRP1,PIK3CG,GATA2,CTSC,NOS3,ZFAND6,PPARG,GATA3,CXCR4,EDNRB,NET1,TMC8,SFRP1,RPL26,ANKLE2,ARHGEF7,PLK2,RET,MERTK,IL1A,IGF1,NR4A1,EIF2AK3,ITCH,SH3RF1,HIP1R,MEF2C,IL7,CXCL10,MADD,PLCG1,NR4A2,ANO6,IFIT2,ADAMTS20,EDN1,STAT1,SNCA,NUAK2,RNF144B,SIK1,HDAC1,ITPRIP,LCK,HYAL2,LAPTM5,DYRK3,HCLS1,INPP5D,CFLAR,CASP10,IGFBP3,RPS6KA1,DDX42,ITGA6,PAK2,ELL3,MAP3K11,CREB1,LTF,ADORA2A,ARNT2,ITGA4,DDX3X,TBX1,MECOM,ING4,RAMP2,BIRC3,TNFAIP8L3,USP42,PRKCH,HIF1A,ITPKB,BID,TNFRSF1B,ARG2,ALDH1A2,MTOR,AR,BNIP2,PSEN1,DAPK2,EFNA1,PIAS1,DNM2,CCL2,LGALS9,NOS2,IL7R,BFAR,STK26,MYO18A,TNFAIP8L1,TRAF2,TNFRSF25,NOTCH1,PLCG2,STXBP1,DAB2IP,CDH5,MIR23A,VNN1,PMAIP1,TRIM24,FCER2,SERPINB9,E2F3,BMP2,IFIT3,NMNAT1	turquoise	EC_M6
GO:0042310	8.20E-02	0.957259952	13	62	vasoconstriction	BP	6.85E-01	LEP,ATP2B1,SNTA1,RAP1GDS1,HTR2B,ADRA2C,COMP,ECE1,ACTA2,ADRA1B,AKT1,PIK3C2A,KCNMB4	darkgreen	EC_M4
GO:0099623	8.21E-02	0.974990316	5	17	regulation of cardiac muscle cell membrane repolarization	BP	6.85E-01	SCN5A,SNTA1,CACNA1D,WDR1,FLNA	darkgreen	EC_M4
GO:0051482	7.33E-02	0.98137582	4	16	positive regulation of cytosolic calcium ion concentration involved in phospholipase C-activating G protein-coupled signaling pathway	BP	6.86E-01	GPR4,EDN1,S1PR1,LPAR6	turquoise	EC_M6
GO:0044106	7.36E-02	0.962855929	12	74	cellular amine metabolic process	BP	6.87E-01	SNCAIP,ODC1,GATA3,NR4A2,SNCA,SLC7A7,OAZ3,GDPD3,CHRNB2,NPR1,GDPD1,ATCAY	turquoise	EC_M6
GO:0006266	4.34E-02	0.990861717	4	15	DNA ligation	BP	6.87E-01	XRCC6,APTX,RAD51,TOP2A	darkgrey	EC_M8
GO:2000810	8.24E-02	0.974876397	5	17	regulation of bicellular tight junction assembly	BP	6.87E-01	ROCK2,TJP1,ROCK1,MYO1C,NPHP4	darkgreen	EC_M4
GO:0001776	7.39E-02	0.962798051	12	70	leukocyte homeostasis	BP	6.87E-01	TNFAIP3,IL6,GAPT,MERTK,MEF2C,JAM3,SOS2,HIF1A,ITPKB,PDE4B,LGALS9,PMAIP1	turquoise	EC_M6
GO:0032774	7.39E-02	0.935386745	301	2534	RNA biosynthetic process	BP	6.87E-01	ZNF516,TNFRSF11A,BAZ2A,TCF4,TNFAIP3,PLAGL1,CNOT6,FZD6,ZBED4,NOTCH4,TCF20,TCF7,NCOA7,L3MBTL3,ATF4,PDGFB,KDM4C,PIM1,NFYA,MDFI,PRDM4,DDX58,NFX1,KIT,ADGRF1,NFIL3,SYK,SEMA4D,POLR2B,IL6,NFE2L3,REL,CREB5,PPIE,BACH2,PELI1,FOXP2,KLF11,LANCL2,NOBOX,TLR4,TRAF1,CREM,TEAD2,SDCBP,MYCN,NOCT,KTI12,ZBTB24,BEND5,TAL1,PHF10,EPC1,GATA2,DNMT3A,PRKD2,SAP130,DACH1,PPARG,GATA3,TAF3,ACVR2B,IRAK2,GZF1,EDNRB,SETD5,IKBKB,JAG1,NFKBIB,PDE2A,TMEM173,SFRP1,NR1D2,CD36,CDK17,PHRF1,ARID5A,TCF15,RET,CHD7,IL1A,DNMT3B,IGF1,NR4A1,EIF2AK3,HHEX,KDM3A,ITCH,ERBIN,SMAD1,ZBTB38,POU6F1,STOX2,SOX7,HMBOX1,HEXB,MEF2C,SMARCD1,TLR2,WWTR1,ZNF704,CXCL10,ADGRG3,KMT2D,SFMBT1,SCAF4,NLRC5,ACTR5,ANKRD2,NR4A2,TOX2,DLL4,CDYL,BAZ2B,SOX13,YAF2,EDN1,STAT1,DYRK1A,SNCA,JARID2,ERG,ETS2,CDK18,NFKB2,DCAF1,PRDM15,ETS1,CD200,SIK1,HDAC1,PPP3CA,HYAL2,SLC39A8,TESMIN,MEF2A,BCL9,GTF2E1,HLX,NCF1,USP13,FABP4,HCLS1,NFATC2,ZFHX2,EAF2,GMEB1,CD86,KDM2A,CIART,MN1,TGIF1,PARP15,POU2F3,ZSCAN29,CFLAR,TRAK2,ADCY1,ARID1A,RPS6KA1,PROX1,SS18L1,PRKD1,ZNF148,ZSCAN2,HELZ2,MLXIPL,ITGA6,DLG1,ELL3,ETV6,HOXD10,RNF2,CREB1,EIF4A2,TET1,LTF,ADORA2A,ARNT2,MYEF2,IL1RAP,BCOR,DDX3X,RFXANK,ETV4,TBX1,MECOM,ING4,CDK16,SPINDOC,LDB2,FGF23,PPM1F,POU2F1,WAS,TEAD4,PRKCH,HIF1A,KDM5A,ZBTB18,APBB2,JUP,BCL6B,KDM5C,TLR6,RSC1A1,TNFRSF1B,RGS14,MAFK,SPIN2A,MTOR,AR,CASZ1,PSEN1,CBX1,HOXB5,HOXB6,HOXB7,HOXB8,HOXB9,ZNF410,FBXW11,BATF,KRB	turquoise	EC_M6
GO:0090596	8.26E-02	0.943140943	35	198	sensory organ morphogenesis	BP	6.88E-01	AHI1,FBN2,RORB,VEGFA,MFSD2A,WHRN,GLI3,FGF2,GLI2,CTNNB1,RARB,FAT1,NKD1,NOTCH2,LRP5,SHROOM2,BMP7,FGFR2,FZD2,FBN1,PRKCI,COL5A2,PRRX1,SIX4,OBSL1,ROM1,TCAP,TTC8,COL5A1,COL11A1,BCAR3,THY1,WNT2,COL8A2,FGF10	darkgreen	EC_M4
GO:0009067	8.27E-02	0.974482456	5	18	aspartate family amino acid biosynthetic process	BP	6.88E-01	MTAP,ASNS,PLOD2,PLOD3,ADI1	darkgreen	EC_M4

GO:0030730	8.27E-02	0.978966417	4	13	sequestering of triglyceride	BP	6.88E-01	PPARA,PLIN2,ABHD5,OSBPL8	darkgreen	EC_M4
GO:0031098	7.41E-02	0.951163746	29	197	stress-activated protein kinase signaling cascade	BP	6.88E-01	FLT4,TPD52L1,MAPK13,SH3RF3,GADD45G,TLR4,TRAF1,SDCBP,IKBKB,SFRP1,EPHB1,TNFRSF19,ITCH,SH3RF1,NCF1,MAP3K3,DLG1,MAP3K11,MECOM,CRACR2A,RELL1,TAOK2,CCDC88C,SH2D3A,LGALS9,TRAF2,SH2D3C,DAB2IP,BMP2	turquoise	EC_M6
GO:0003222	7.41E-02	0.981862594	4	13	ventricular trabecula myocardium morphogenesis	BP	6.88E-01	CHD7,DLL4,NOTCH1,ENG	turquoise	EC_M6
GO:0032270	7.43E-02	0.938653379	131	1066	positive regulation of cellular protein metabolic process	BP	6.88E-01	TNFRSF11A,TNFAIP3,FLT4,GRAMD4,TPD52L1,PTK2,DAPK1,PDGFB,AGTPBP1,CSNK1E,TEK,LIMCH1,FAM122A,TEC,KIT,KDR,SYK,SEMA4D,IL6,PELI1,TLR4,IL15,CCNY,SDCBP,NRP1,PIK3CG,TAL1,PRKD2,CTSC,CAB39L,RALB,PPARG,GATA3,EDNRB,NDFIP2,HBEGF,RPL26,ANKLE2,CD36,PRR5L,PLK2,RET,DNMT3B,IGF1,KLKB1,EIF2AK3,HIP1R,SOX7,VEGFC,MADD,THBS4,PPP1R16B,RAPGEF3,NEDD9,EDN1,DYRK1A,EHD4,SNCA,JARID2,RNF144B,RNF19B,HDAC1,LCK,LAPTM5,NCF1,USP13,HCLS1,PECAM1,CD86,CFLAR,CASP10,PPP2R5A,PROX1,PRKD1,YTHDF1,ACE,LIMK2,PAK2,MELTF,SENP2,DLG1,MAP3K11,TET1,LTF,DDX3X,TBX1,BIRC3,FGF23,PPM1F,RAC1,ADIPOR2,BID,TLR6,TNFRSF1B,MTOR,RNF40,PSEN1,FBXW11,FGF18,PGF,TAOK2,EFNA1,SLC51B,NAP1L2,PIK3R5,PIAS1,RPS6KA5,TNIP1,ADRB2,YES1,SH2D3A,LGALS9,UNC119,TRAF2,PLCG2,ENG,SH2D3C,MTF2,DAB2IP,CDH5,PRSS22,PMAIP1,RELN,ALKAL1,FGF16,MMD,PERP,DAZAP2,COPS8,BMP2,BMP6	turquoise	EC_M6
GO:0016239	8.28E-02	0.958119025	12	56	positive regulation of macroautophagy	BP	6.88E-01	MAP3K7,SESN3,RAB3GAP1,DCN,SLC25A4,PAFAH1B2,HTT,SPTLC2,IKBKG,GPSM1,GNAI3,BNIP3	darkgreen	EC_M4
GO:0090174	8.29E-02	0.95152849	18	94	organelle membrane fusion	BP	6.89E-01	STX7,VPS41,KIF5B,RAB7A,AKT2,VAMP8,USO1,CHP1,VPS39,RAB7B,SEC22B,STX1B,SYT11,SOX30,RAB34,VAMP7,GNAI3,STXBP6	darkgreen	EC_M4
GO:0048486	7.45E-02	0.981175976	4	15	parasympathetic nervous system development	BP	6.89E-01	NRP1,NAV2,SEMA3F,TBX1	turquoise	EC_M6
GO:0045429	7.47E-02	0.973863451	6	27	positive regulation of nitric oxide biosynthetic process	BP	6.89E-01	TLR4,CD36,EDN1,TLR6,MTOR,DNM2	turquoise	EC_M6
GO:2001238	7.47E-02	0.970881023	7	37	positive regulation of extrinsic apoptotic signaling pathway	BP	6.89E-01	SFRP1,RET,HYAL2,PAK2,BID,TRAF2,PMAIP1	turquoise	EC_M6
GO:0052547	7.48E-02	0.947700851	38	294	regulation of peptidase activity	BP	6.89E-01	TNFAIP8,GRAMD4,DAPK1,SYK,C5,CST7,PPARG,TIMP4,ITIH5,NR4A1,EIF2AK3,SH3RF1,HIP1R,SOX7,SNCA,HDAC1,LCK,LAPTM5,CFLAR,CASP10,RPS6KA1,PAK2,CST6,LTF,ADORA2A,DDX3X,BIRC3,PPM1F,BID,PAPLN,EFNA1,LGALS9,PRSS22,PMAIP1,SERPINB9,PSMB9,PERP,BST2	turquoise	EC_M6
GO:0090303	7.49E-02	0.968635576	8	42	positive regulation of wound healing	BP	6.89E-01	PTK2,CXCR4,HBEGF,FERMT1,CD36,OCLN,ANO6,MTOR	turquoise	EC_M6
GO:0035089	7.49E-02	0.977146873	5	20	establishment of apical/basal cell polarity	BP	6.89E-01	SYNE4,TCF15,FSCN1,OPHN1,PATJ	turquoise	EC_M6
GO:0010648	7.50E-02	0.938166095	130	1047	negative regulation of cell communication	BP	6.89E-01	SEMA6A,TNFAIP3,TRIM39,FZD6,CTHRC1,GRAMD4,PICK1,MDFI,CD2AP,RNF149,IL6,CXCL8,RGS3,ARHGAP25,FIGNL1,CAV2,GRB10,TLR4,NRP1,RASIP1,ARHGAP42,MTMR2,GATA2,NOS3,PPARG,GATA3,SEC14L1,PDE2A,SFRP1,NOTUM,FERMT1,SIRT3,PRR5L,PLK2,PDE4D,FAM3D,IL1A,IGF1,ABHD6,HHEX,ITCH,ERBIN,SH3RF1,WWTR1,IL7,P2RX7,NDRG4,LMBR1L,ADGRG3,OAS3,NLRC5,NR4A2,DGKZ,DLL4,SOX13,EDN1,STAT1,DYRK1A,SNCA,DUSP8,PRDM15,SHANK2,HDAC1,ITPRIP,PPP3CA,HYAL2,LAPTM5,DYRK3,INPP5D,SH3BP4,CFLAR,INPP5F,IGFBP3,PLEKHA1,SRMS,ITGA6,PTPRE,DUSP16,DLG1,ELL3,APOD,LTF,TSPAN15,DDX3X,IGFBP5,HOMER3,MECOM,FGF23,HIF1A,EPHA2,MMRN2,BID,TLR6,MCC,LFNG,RGS14,MTOR,AR,PSEN1,ITGA3,EFNA1,CCDC88C,TLE3,DNM2,TNIP1,MARK3,KIF26A,ADRB2,PDE4B,BFAR,TNFAIP8L1,ISG15,NRARP,EGFL7,NOTCH1,STXBP1,DAB2IP,MIR99A,OAS1,VNN1,CD46,SOX17,SHANK3,RGS4,RGS16,DUSP5,BMP2,RNF125,DUSP7,RNF152	turquoise	EC_M6
GO:0042249	7.50E-02	0.981274341	4	14	establishment of planar polarity of embryonic epithelium	BP	6.89E-01	CTHRC1,CELSR1,SFRP1,SEC24B	turquoise	EC_M6

GO:0061337	7.50E-02	0.960076725	14	76	cardiac conduction	BP	6.89E-01	TRDN,TRPM4,SPTBN4,PDE4D,KCNJ3,KCNJ2,MEF2A,JUP,ATP2A1,DSG2,DSC2,SLC8A3,KCNH2,SCN2B	turquoise	EC_M6
GO:0002763	8.31E-02	0.962944312	9	40	positive regulation of myeloid leukocyte differentiation	BP	6.89E-01	IL23A,RB1,TGFB1,RUNX1,NOTCH2,LEF1,PRKCA,CD74,ATP6AP1	darkgreen	EC_M4
GO:0060284	7.52E-02	0.944289286	54	384	regulation of cell development	BP	6.90E-01	SEMA6A,CDKL3,KIT,SEMA4D,IL6,MYCN,NRP1,PLXND1,UNC13D,CXCR4,EDNRB,IKBKB,ARHGEF7,LMOD3,RET,CHD7,IGF1,TLR2,VEGFC,DLL4,NEDD9,HDAC1,PPP3CA,SEMA3F,AP1AR,PLXNA2,TRAK2,SS18L1,F11R,DOCK1,MELTF,ELL3,B2M,LTF,SEMA6D,PRKCH,RAC1,HIF1A,RCC2,TNFRSF1B,RGS14,MTOR,PSEN1,SEMA4A,GPR68,DNM2,SEMA6B,RFLNB,ARHGAP4,NOTCH1,CDH5,RELN,SHANK3,BMP2	turquoise	EC_M6
GO:0006915	7.53E-02	0.936437608	171	1436	apoptotic process	BP	6.90E-01	TNFAIP8,SEMA6A,TNFAIP3,PLAGL1,TRIM39,AKAP12,FLT4,OXR1,GRAMD4,TPD52L1,PTK2,DAPK1,ATF4,PIM1,TEK,SH3RF3,TNFRSF21,KDR,GADD45G,SEMA4D,SHB,IL6,PPT1,UTP11,ELMO1,FIGNL1,KLF11,TRAF1,NRP1,PIK3CG,GATA2,DNMT3A,CTSC,NOS3,RALB,ZFAND6,PPARG,GATA3,CXCR4,EDNRB,NET1,TMC8,SFRP1,RPL26,ANKLE2,ARHGEF7,RASSF7,PLK2,RET,MERTK,TNFRSF19,IL1A,IGF1,NR4A1,EIF2AK3,ITCH,SH3RF1,HIP1R,MEF2C,TLR2,IL7,P2RX7,CXCL10,MADD,LMBR1L,PLCG1,NR4A2,HIPK1,ANO6,IFIT2,ADAMTS20,EDN1,STAT1,SNCA,NUAK2,GAS2,MX1,RNF144B,SIK1,HDAC1,ITPRIP,LCK,HYAL2,MEF2A,NCF1,DYRK3,HCLS1,INPP5D,EAF2,BMX,CFLAR,CASP10,IGFBP3,RPS6KA1,PRKD1,DDX42,ITGA6,DOCK1,PAK2,ELL3,ETV6,MAP3K11,CREB1,TRIM69,USP28,LTF,ADORA2A,ARNT2,ITGA4,DDX3X,TBX1,MECOM,ING4,RAMP2,BIRC3,TNFAIP8L3,PPM1F,USP42,PRKCH,HIF1A,EPHA2,ITPKB,CECR2,BID,TNFRSF1B,ARG2,ALDH1A2,MTOR,AR,BNIP2,PSEN1,DAPK2,TAOK2,EFNA1,ATP2A1,PIAS1,S100A14,DNM2,ATG4D,PPP1R13B,CCL2,JAG2,LGALS9,IL7R,BFAR,STK26,MYO18A,TNFAIP8L1,ATCAY,TRAF2,TNFRSF25,NOTCH1,STXBP1,DAB2IP,CDH5,MIR23A,VNN1,BCL7B,PMAIP1,TRIM24,DIO3,DRAM2,SERPINB9,PERP,E2F3,BMP2,XAF1,IFIT3,RNF152,NMNAT1,EVA1A	turquoise	EC_M6
GO:0097106	7.54E-02	0.973826669	6	25	postsynaptic density organization	BP	6.90E-01	DLG1,IL1RAP,OPHN1,ABI3,RELN,SHANK3	turquoise	EC_M6
GO:0055002	7.54E-02	0.96494256	10	52	striated muscle cell development	BP	6.90E-01	PGM5,TMOD1,LMOD3,EDN1,MEF2A,NRAP,CFLAR,PROX1,TMOD4,TMOD2	turquoise	EC_M6
GO:0010761	7.54E-02	0.966583743	9	46	fibroblast migration	BP	6.90E-01	AKAP12,PTK2,ARHGEF7,PRR5L,HYAL2,RAC1,RCC2,ADIPOR2,ARHGAP4	turquoise	EC_M6
GO:0043065	7.55E-02	0.944088415	53	410	positive regulation of apoptotic process	BP	6.90E-01	TNFAIP8,TRIM39,AKAP12,TPD52L1,DAPK1,ATF4,GADD45G,IL6,UTP11,KLF11,CTSC,PPARG,NET1,SFRP1,RPL26,ARHGEF7,RET,NR4A1,HIP1R,MEF2C,PLCG1,ANO6,IFIT2,SNCA,SIK1,HDAC1,LCK,HYAL2,INPP5D,IGFBP3,ITGA6,PAK2,MAP3K11,CREB1,ADORA2A,ITGA4,DDX3X,ING4,BID,TNFRSF1B,ALDH1A2,PSEN1,DAPK2,DNM2,CCL2,LGALS9,TRAF2,NOTCH1,DAB2IP,MIR23A,PMAIP1,E2F3,BMP2	turquoise	EC_M6
GO:0019233	8.33E-02	0.956402872	13	63	sensory perception of pain	BP	6.91E-01	ACPP,CXCL12,P2RX4,CACNB3,NDN,BACE1,COMT,KCNK4,ADRA2C,KCNA1,NLGN2,TMEM100,ITGA2	darkgreen	EC_M4
GO:0090382	8.34E-02	0.970574968	6	23	phagosome maturation	BP	6.91E-01	RAB7A,RAB7B,SLAMF8,SYT11,RAB31,RAB34	darkgreen	EC_M4
GO:0045926	8.35E-02	0.942763536	34	195	negative regulation of growth	BP	6.91E-01	PPARA,PPARD,RTN4,INHBA,RB1,PAK1,TGFB1,SEMA3C,RYK,RNF6,WWC2,DIP2B,SEMA4F,SPP1,SEMA4G,NKD1,ADIPOR1,NOTCH2,HYAL1,CAPRIN2,TGFB2,WWC3,RBBP7,TNR,FGFR3,TSPYL2,NPPB,SMARCA4,CRLF3,PLXNA3,ENO1,CDKN1A,DACT3,NME6	darkgreen	EC_M4
GO:0050819	7.57E-02	0.970331011	7	38	negative regulation of coagulation	BP	6.91E-01	TSPAN8,PDGFB,NOS3,KLKB1,PROCR,EDN1,CD34	turquoise	EC_M6
GO:1902904	7.59E-02	0.953223946	22	139	negative regulation of supramolecular fiber organization	BP	6.92E-01	PICK1,TMOD1,SVIL,SPTBN4,ARHGEF7,SPEF1,LMOD3,HIP1R,DYRK1A,SNCA,TMOD4,F11R,PAK2,WAS,TMOD2,ARHGAP28,S1PR1,CARMIL2,CDH5,GMFG,KANK3,SHANK3	turquoise	EC_M6
GO:0051931	7.60E-02	0.976407599	5	22	regulation of sensory perception	BP	6.92E-01	MGLL,EDNRB,EDN1,ZFHX2,MTOR	turquoise	EC_M6
GO:0007140	4.41E-02	0.984856035	7	35	male meiotic nuclear division	BP	6.93E-01	UBR2,FOXJ3,BRCA2,KIF18A,TRIP13,SPO11,FANCA	darkgrey	EC_M8
GO:0034244	4.41E-02	0.990664229	4	15	negative regulation of transcription elongation from RNA polymerase II promoter	BP	6.93E-01	EZH2,TCERG1,HNRNPU,RNF8	darkgrey	EC_M8

GO:0010822	7.62E-02	0.964134593	10	59	positive regulation of mitochondrion organization	BP	6.93E-01	KDR,HIP1R,ZDHHC6,MCU,HIF1A,BID,ADCK1,SPIRE1,PMAIP1,RALA	turquoise	EC_M6
GO:0030888	7.63E-02	0.967934866	8	42	regulation of B cell proliferation	BP	6.93E-01	TNFRSF21,PELI1,TLR4,MEF2C,IL7,NFATC2,INPP5D,CHRNB2	turquoise	EC_M6
GO:0043331	7.65E-02	0.970154466	7	35	response to dsRNA	BP	6.94E-01	DDX58,PELI1,RALB,TMEM173,IFIT1,RFTN2,PMAIP1	turquoise	EC_M6
GO:0060965	7.66E-02	0.980462297	4	15	negative regulation of gene silencing by miRNA	BP	6.94E-01	IL6,PPARG,DND1,PPP3CA	turquoise	EC_M6
GO:0021953	8.40E-02	0.94797468	22	116	central nervous system neuron differentiation	BP	6.94E-01	MDGA1,LEP,GLI3,INHBA,GLI2,DCLK1,ZSWIM6,FAIM2,TCTN1,RAPGEF2,SATB2,FGFR2,SCYL1,MAPT,MDGA2,TULP3,CLN8,PAFAH1B1,PLXNA3,LMO4,LHX8,LBX1	darkgreen	EC_M4
GO:0034284	8.40E-02	0.944241015	29	164	response to monosaccharide	BP	6.94E-01	LRP1,PPARD,LGALS1,TXN2,LEP,SRF,UNC13B,FKBP1B,KIF5B,EIF2B3,IRS2,OSBP,SELENOT,OPA1,BRSK2,DYNLL1,SELENOS,ZBTB20,LRP5,PIK3CA,ADCY5,SIDT2,PTPRN,PPARGC1A,SMARCA4,ITGA2,PRKAA1,SERPINF1,COL6A2	darkgreen	EC_M4
GO:0002335	8.41E-02	0.970284306	6	23	mature B cell differentiation	BP	6.94E-01	LGALS1,CDH17,ADA,DOCK10,NOTCH2,SLAMF8	darkgreen	EC_M4
GO:0043500	8.42E-02	0.951580218	17	88	muscle adaptation	BP	6.94E-01	PPARA,ROCK2,PAK1,SCN5A,P2RX4,ATP2A2,ATP2B4,TWF1,DAG1,PRKCA,CASQ1,GLRX3,TCAP,PPARGC1A,SGCA,PIN1,ROCK1	darkgreen	EC_M4
GO:2000177	8.42E-02	0.955950571	13	62	regulation of neural precursor cell proliferation	BP	6.94E-01	CTNNA1,VEGFA,GLI3,FGF2,GLI2,SMARCD3,CTNNB1,SPINT2,ILK,CX3CL1,FZD9,NES,FLNA	darkgreen	EC_M4
GO:0010560	8.43E-02	0.973824361	5	18	positive regulation of glycoprotein biosynthetic process	BP	6.94E-01	CTNNB1,CHP1,SLC2A10,NCSTN,POMT1	darkgreen	EC_M4
GO:0016074	8.44E-02	0.978379643	4	13	sno(s)RNA metabolic process	BP	6.95E-01	NUDT16,DKC1,TENT4B,LARP7	darkgreen	EC_M4
GO:0033687	7.70E-02	0.972854629	6	27	osteoblast proliferation	BP	6.96E-01	CTHRC1,FIGNL1,SFRP1,MN1,LTF,BMP2	turquoise	EC_M6
GO:0070302	7.72E-02	0.95170425	23	151	regulation of stress-activated protein kinase signaling cascade	BP	6.98E-01	FLT4,TPD52L1,SH3RF3,GADD45G,TLR4,TRAF1,SDCBP,SFRP1,EPHB1,TNFRSF19,ITCH,SH3RF1,NCF1,MAP3K3,DLG1,MAP3K11,MECOM,CRACR2A,RELL1,TAOK2,TRAF2,DAB2IP,BMP2	turquoise	EC_M6
GO:0034629	4.45E-02	0.988233204	5	21	cellular protein-containing complex localization	BP	6.98E-01	AKAP9,MIOS,CEP72,NDC1,HASPIN	darkgrey	EC_M8
GO:0044070	8.50E-02	0.956760901	12	57	regulation of anion transport	BP	6.98E-01	ATP8B1,SLC12A2,LEP,ITGB1,RAB3GAP1,PRNP,ARL6IP5,P2RX4,CRY2,PLA2R1,PRKAA1,AHCYL1	darkgreen	EC_M4
GO:1903019	8.50E-02	0.978170736	4	13	negative regulation of glycoprotein metabolic process	BP	6.98E-01	ITM2B,PTX3,SLC2A10,ITM2C	darkgreen	EC_M4
GO:0046485	8.51E-02	0.973515103	5	18	ether lipid metabolic process	BP	6.98E-01	CHPT1,FAR1,LPCAT2,PAFAH1B1,TMEM189	darkgreen	EC_M4
GO:0032956	8.51E-02	0.937087596	51	304	regulation of actin cytoskeleton organization	BP	6.99E-01	LRP1,FAM49B,NCK2,TGFBR1,SHANK1,ACTR2,ROCK2,MYLK3,PAK1,ACTR3,PLEKHG2,ACTG1,CDC42EP3,ILK,CXCL12,PFN2,RASA1,CX3CL1,TWF1,MAGEL2,PHLDB2,TJP1,NOTCH2,CTTN,LMOD1,PIK3CA,NTRK3,SYNPO2,IQGAP1,ARPC5,NCKAP1,ARPC2,SYNPO2L,CAPZB,WDR1,CGNL1,TPM1,TGFB3,GPR65,TTC8,PDGFRB,ROCK1,FLII,DAPK3,MYO1C,FLNA,SPTAN1,TMSB4X,DSTN,COTL1,CFL2	darkgreen	EC_M4
GO:0015800	7.75E-02	0.967565112	8	39	acidic amino acid transport	BP	6.99E-01	APBA1,SLC3A1,SNCA,ADORA2A,PSEN1,STXBP1,LRRC8C,LRRC8D	turquoise	EC_M6
GO:0014070	7.75E-02	0.938332871	90	703	response to organic cyclic compound	BP	6.99E-01	ATF4,PDGFB,KDM4C,PIM1,TEK,DDX58,IL6,PELI1,FOXP2,CAV2,IL15,TRPM4,PIK3CG,DNMT3A,RALB,CXCR4,EDNRB,P2RY6,P2RY2,PDE2A,TMEM173,SFRP1,P2RX2,CD36,GRAMD1A,DNMT3B,KDM3A,UFSP2,SMAD1,CHRM5,GPD1,MEF2C,TLR2,P2RX7,CXCL10,ANGPT2,DHH,KMT2D,TIPARP,STC1,RAPGEF3,IFIT1,EDN1,STAT1,SNCA,DTNBP1,ADCY2,ETS1,HDAC1,RFTN2,MN1,CFLAR,ADCY1,FES,ARID1A,ITGA6,PAK2,SSTR1,ADORA2A,ARNT2,CYBB,IGFBP5,RAMP2,FGF23,ADSS,JUP,BID,ALDH1A2,MTOR,AR,CHRNB2,SMAD2,DNM2,SCNN1B,CYP1A1,CCL2,PDE4B,HCFC1,NOTCH1,RAPGEF1,ENG,STXBP1,PMAIP1,SLC8A3,TRIM24,CA2,RGS4,BMP2,GNG2,BMP6	turquoise	EC_M6

GO:0006464	4.47E-02	0.9615669	298	2859	cellular protein modification process	BP	6.99E-01	CDK2,MEX3C,CLK4,USP15,SHPRH,PPIL4,LATS1,FBXO43,FBXO5,CSNK1G3,UBR5,MAP3K4,DSCC1,XRCC6,CDC25C,NAA35,SRPK1,UHRF2,PPIL1,PLEC,UBR2,PWP1,TOPORS,DBF4,AKAP9,PUM3,CKS2,C1GALT1,ICK,MELK,TRIM28,ZNF451,PPIH,FBXO11,SENP6,ASB3,PHIP,TTK,PPP4R3B,IBTK,ZMPSTE24,RLF,USP34,COMMD1,DPY19L1,FKBP15,ETAA1,ITGB1BP1,EZH2,UFL1,CUL1,USP45,CLSPN,NAA15,PLK4,HDAC2,CCNA2,SAE1,CEP295,CHORDC1,SETDB2,SYMPK,MASTL,COG3,CAD,ALG8,PPM1G,BORA,CCNT2,PAXIP1,ULK4,NCAPG2,CDC20,BIRC5,NKTR,FNTA,PARP2,KAT14,GTPBP4,RAD18,IPO5,EPC2,KAT6A,RIF1,ZDHHC17,TPP2,NSD3,KITLG,TAF7,PRKD3,CUL4A,METAP2,BRCA2,PPP4R2,CDC16,MAPK8,PRKDC,CCNB1,ERCC6,PAN3,SCYL2,LNX2,BUB1,TPX2,ANAPC1,UBA2,IPO7,TNKS2,SGK3,PARP4,CDK5RAP1,PPWD1,FER,NCK1,ARFGEF1,RNF141,OTUD4,ZFP91,GALNT6,TAF9,ATR,USP25,HLTF,PBK,ESCO2,ANAPC7,MCRS1,SH2B3,LTN1,USP16,PPID,MOB1A,NAA25,CPNE3,BUB1B,CCNL1,SMYD5,CDCA2,NBN,SENP1,TOP1,ABRAXAS1,RAD51,RIPK1,PRPF4B,CCNE2,TRIM33,RIOK1,CHUK,STK4,MORC3,UBE2C,GXYLT1,HERC5,TAOK3,OGA,SMARCAD1,BMPR1B,UBE3A,CIT,CUL3,METAP1,ALG10,UBE2T,SLK,CHEK1,ASB7,NAA50,STT3A,PTP4A2,CENPE,TET2,TRIP11,PPP2R2D,TRAIP,COPS7B,KIF14,PRMT5,AJUBA,UBR1,FXR1,FANCI,MCM3AP,NEK2,DTL,HMBS,ALS2,PPIG,BLM,UBR3,G2E3,CDKL5,HAT1,CDK1,CDC25A,PDE8A,ING5,BAZ1B,SETD2,CTDSPL2,IVNS1ABP,PIKFYVE,CDC27,ABL2,FANCM,DBF4B,LRR1,IL12A,COP1,XRCC5,BRCA1,CUL5,CDCA3,CNOT9,CDKN3,NSD2,DCUN1D5,TRPM7,TIPRL,DCAF6,BOD1L1,ECT2,AIPL1,TBL1XR1,SUV39H1,FKBP4,LEO1	darkgrey	EC_M8
GO:0036211	4.47E-02	0.9615669	298	2859	protein modification process	BP	6.99E-01	CDK2,MEX3C,CLK4,USP15,SHPRH,PPIL4,LATS1,FBXO43,FBXO5,CSNK1G3,UBR5,MAP3K4,DSCC1,XRCC6,CDC25C,NAA35,SRPK1,UHRF2,PPIL1,PLEC,UBR2,PWP1,TOPORS,DBF4,AKAP9,PUM3,CKS2,C1GALT1,ICK,MELK,TRIM28,ZNF451,PPIH,FBXO11,SENP6,ASB3,PHIP,TTK,PPP4R3B,IBTK,ZMPSTE24,RLF,USP34,COMMD1,DPY19L1,FKBP15,ETAA1,ITGB1BP1,EZH2,UFL1,CUL1,USP45,CLSPN,NAA15,PLK4,HDAC2,CCNA2,SAE1,CEP295,CHORDC1,SETDB2,SYMPK,MASTL,COG3,CAD,ALG8,PPM1G,BORA,CCNT2,PAXIP1,ULK4,NCAPG2,CDC20,BIRC5,NKTR,FNTA,PARP2,KAT14,GTPBP4,RAD18,IPO5,EPC2,KAT6A,RIF1,ZDHHC17,TPP2,NSD3,KITLG,TAF7,PRKD3,CUL4A,METAP2,BRCA2,PPP4R2,CDC16,MAPK8,PRKDC,CCNB1,ERCC6,PAN3,SCYL2,LNX2,BUB1,TPX2,ANAPC1,UBA2,IPO7,TNKS2,SGK3,PARP4,CDK5RAP1,PPWD1,FER,NCK1,ARFGEF1,RNF141,OTUD4,ZFP91,GALNT6,TAF9,ATR,USP25,HLTF,PBK,ESCO2,ANAPC7,MCRS1,SH2B3,LTN1,USP16,PPID,MOB1A,NAA25,CPNE3,BUB1B,CCNL1,SMYD5,CDCA2,NBN,SENP1,TOP1,ABRAXAS1,RAD51,RIPK1,PRPF4B,CCNE2,TRIM33,RIOK1,CHUK,STK4,MORC3,UBE2C,GXYLT1,HERC5,TAOK3,OGA,SMARCAD1,BMPR1B,UBE3A,CIT,CUL3,METAP1,ALG10,UBE2T,SLK,CHEK1,ASB7,NAA50,STT3A,PTP4A2,CENPE,TET2,TRIP11,PPP2R2D,TRAIP,COPS7B,KIF14,PRMT5,AJUBA,UBR1,FXR1,FANCI,MCM3AP,NEK2,DTL,HMBS,ALS2,PPIG,BLM,UBR3,G2E3,CDKL5,HAT1,CDK1,CDC25A,PDE8A,ING5,BAZ1B,SETD2,CTDSPL2,IVNS1ABP,PIKFYVE,CDC27,ABL2,FANCM,DBF4B,LRR1,IL12A,COP1,XRCC5,BRCA1,CUL5,CDCA3,CNOT9,CDKN3,NSD2,DCUN1D5,TRPM7,TIPRL,DCAF6,BOD1L1,ECT2,AIPL1,TBL1XR1,SUV39H1,FKBP4,LEO1	darkgrey	EC_M8
GO:0006983	8.54E-02	0.978049939	4	13	ER overload response	BP	7.00E-01	SELENOS,GSK3B,TMCO1,HSPA5	darkgreen	EC_M4
GO:0043279	8.56E-02	0.9515958	16	81	response to alkaloid	BP	7.01E-01	HNMT,SMPD1,MAP1LC3A,RYR3,PPP2R2A,HOMER1,ADA,CPT1A,CASP7,PEA15,PPARGC1A,TMED10,PRKA1,HSPA5,TMEM38B,TMEM38A	darkgreen	EC_M4
GO:0007189	8.57E-02	0.951610488	16	80	adenylate cyclase-activating G protein-coupled receptor signaling pathway	BP	7.01E-01	ADGRB1,GNAQ,APLP1,RAPGEF2,ADCY6,ATP2B4,ADGRB2,PRKCA,ADCY5,LINC02210-CRHR1,ADRA2C,ADGRL1,ADRA1B,GPR65,GNAL,GNB1	darkgreen	EC_M4

GO:0044085	8.57E-02	0.923905243	357	2454	cellular component biogenesis	BP	7.01E-01	ATP8B1,LRP1,STX7,AHI1,CAND1,PLXNB2,ANGPT1,TCP1,HLA-DRA,HSPA4,SERINC1,FAM49B,ADGRB1,CTNNA1,CBY1,UBQLN1,MDGA1,SH3GL2,TBCC,SRF,VEGFA,VCP,UNC13B,COL1A2,NCK2,REST,DST,RAB23,TGFBR1,RIMS1,CENPC,RTN4,TPBG,SHANK1,COX18,LRRC4B,MFSD2A,CALD1,AKIRIN1,ACTR2,ROCK2,ZYX,MYLK3,VPS35,CFAP100,ITGB1,SRPK2,NAPA,RAB7A,ARHGEF4,EML2,RB1,PICALM,MARK4,NUFIP1,MAPRE3,PDCD6IP,TENM4,KCTD21,PCDH17,PAK1,ACTR3,TGFB1,RAB3GAP1,CTNNB1,DZIP1,PLEKHG2,DNAJB13,FARP1,ACTG1,NAV3,STAM2,KAT2B,FAM98A,PRNP,NUDT16,CDC42EP3,HDAC3,ZNF703,PCLO,KCTD16,ILK,DPYSL3,ERI1,RYK,TTC17,CIAO1,GTF3A,CNOT7,RIC3,VPS37A,CXCL12,TM9SF4,SACS,KIF2A,TMEM41B,ANKRD1,HSP90B1,TMED2,EIF2S2,MAP1LC3A,TP53INP2,VAMP8,RIOK2,CTNND1,MAP1B,TEAD1,RYR3,ANKRA2,SORBS1,PFN2,RASA1,ATP2A2,CELF1,TCTN1,RTKN,SDAD1,APP,ATXN2,PLEKHA7,CCT8,RAPGEF2,CX3CL1,PTPN11,NEFM,HOMER1,OPA1,CDH17,ATG13,AMIGO2,ZFYVE27,TSG101,DKC1,MTDH,GLS,CHP1,PKD2,CSDE1,TWF1,DNAH1,DNAH5,IFT57,TENT4B,PSMG1,PDLIM5,MAGEL2,RAB7B,ARL3,RAP1GDS1,DYNLL1,PHLDB2,TJP1,CALHM1,CTTN,PKP2,CDC73,DNM1L,DNER,TROVE2,HYAL1,LMOD1,SEC22B,SBDS,FBLN5,EPRS,KCTD10,FEZ1,ARMC9,PRKAR1A,WIPI1,GSK3B,MMP14,DAG1,PIK3CA,AUTS2,DNAAF5,CORO1C,ISCU,PRKCA,NEK7,DISP1,NTRK3,POLQ,HSPA8,PRPF3,BMP7,NDUFB3,ZRSR2,AP2M1,SYNPO2,CASQ1,ITGB5,TP53BP1,PHLDB1,OSBPL2,GORASP2,ADRM1,IQGAP1,SCFD1,STRAP,QSOX1,ABRAXAS2,TFRC,FARP2,GLRX3,SRP54,ARPC5,LSG	darkgreen	EC_M4
GO:0009129	4.50E-02	0.990187051	4	17	pyrimidine nucleoside monophosphate metabolic process	BP	7.02E-01	CAD,UMPS,TYMS,DHODH	darkgrey	EC_M8
GO:0051603	4.50E-02	0.966128999	73	639	proteolysis involved in cellular protein catabolic process	BP	7.02E-01	CDK2,USP15,LATS1,UBR2,TOPORS,FBXO11,ZMPSTE24,USP34,XPO1,COMMD1,FBXO48,UFL1,CUL1,USP45,ASCC3,YME1L1,CDC20,VPS36,CUL4A,PSMD13,CDC16,ANAPC1,IDE,TAF9,USP25,PBK,ANAPC7,LTN1,USP16,UBE2C,UBE3A,CUL3,KIF14,UBR1,PSMD12,DTL,UBR3,PSMD4,CCDC47,SMARCC1,PSMA6,CDC27,COP1,CUL5,PSME3,TBL1XR1,UBE2K,NEDD4,USP3,TAF1,CLPX,OGT,RLIM,RBBP6,PLK1,APPBP2,PSMD10,MIB1,COPS3,CUL4B,UHRF1,FMR1,CCNF,PSMA5,GLMN,USP33,AURKA,RNF139,RNF8,GID4,UBXN2A,PSMD7,RCHY1	darkgrey	EC_M8
GO:0048598	7.82E-02	0.940389191	64	469	embryonic morphogenesis	BP	7.02E-01	FZD6,CTHRC1,CELSR1,MDFI,TEAD2,MYCN,TAL1,GATA2,ITGA8,ITGB4,GATA3,MYO7A,LMBR1,SFRP1,FREM2,RIC8A,RET,CHD7,SMAD1,SOX7,MEF2C,VEGFC,NDRG4,USH1C,HIPK1,DLL4,EDN1,ETS2,HDAC1,HLX,SEC24B,PROX1,VANGL2,LAMA5,DLG1,HOXD10,RNF2,ITGA4,TBX1,HIF1A,EPHA2,CECR2,ALDH1A2,AR,PSEN1,HOXB5,HOXB6,HOXB7,HOXB8,HOXB9,ITGA3,EFNA1,SMAD2,AMOT,JAG2,NOTCH1,ENG,AFF3,VASP,SOX17,SOX18,TGIF2,DUSP5,RALA	turquoise	EC_M6
GO:0009306	7.82E-02	0.94543829	37	266	protein secretion	BP	7.02E-01	RIMS2,PICK1,CD2AP,ICA1,IL6,RSAD2,TLR4,TRPM4,RAB3B,LLGL2,SFRP1,SIRT3,FAM3D,IL1A,IGF1,TLR2,VEGFC,ABCG1,CD200,MTTP,HDAC1,PPP3CA,ANO1,ADORA2A,MCU,CDK16,RAC1,HIF1A,SNCG,SYT7,RAB3D,GPR68,SMAD2,NOS2,MYO18A,RPH3AL,BMP6	turquoise	EC_M6
GO:0035592	7.82E-02	0.94543829	37	266	establishment of protein localization to extracellular region	BP	7.02E-01	RIMS2,PICK1,CD2AP,ICA1,IL6,RSAD2,TLR4,TRPM4,RAB3B,LLGL2,SFRP1,SIRT3,FAM3D,IL1A,IGF1,TLR2,VEGFC,ABCG1,CD200,MTTP,HDAC1,PPP3CA,ANO1,ADORA2A,MCU,CDK16,RAC1,HIF1A,SNCG,SYT7,RAB3D,GPR68,SMAD2,NOS2,MYO18A,RPH3AL,BMP6	turquoise	EC_M6
GO:2001233	7.83E-02	0.945057806	38	292	regulation of apoptotic signaling pathway	BP	7.02E-01	TNFAIP3,TRIM39,TPD52L1,FIGNL1,TRAF1,NRP1,CTSC,NOS3,TMC8,SFRP1,RPL26,RET,IL1A,IGF1,EIF2AK3,SH3RF1,IL7,MADD,NR4A2,HDAC1,ITPRIP,LCK,HYAL2,CFLAR,ITGA6,PAK2,ELL3,ADORA2A,DDX3X,HIF1A,BID,AR,PSEN1,DAPK2,TRAF2,DAB2IP,VNN1,PMAIP1	turquoise	EC_M6
GO:0090158	8.61E-02	0.977787468	4	13	endoplasmic reticulum membrane organization	BP	7.02E-01	RTN4,TMED2,VAPB,STX18	darkgreen	EC_M4
GO:2001026	8.63E-02	0.973012072	5	18	regulation of endothelial cell chemotaxis	BP	7.03E-01	VEGFA,FGF2,P2RX4,HSPB1,TMSB4X	darkgreen	EC_M4
GO:0090278	8.63E-02	0.963473805	8	34	negative regulation of peptide hormone secretion	BP	7.03E-01	LEP,REST,FKBP1B,ABCC8,PTPN11,HADH,SYTL4,MIDN	darkgreen	EC_M4
GO:0033143	8.64E-02	0.95450415	13	63	regulation of intracellular steroid hormone receptor signaling pathway	BP	7.03E-01	CLOCK,PAK1,LBH,UFM1,UBA5,DDRGK1,RNF6,CRY2,VPS18,STRN3,MED1,SMARCA4,PARK7	darkgreen	EC_M4

GO:0034341	8.65E-02	0.949154802	18	95	response to interferon-gamma	BP	7.03E-01	ACTR2,ZYX,KIF5B,SEC61A1,NUB1,ACTR3,ACTG1,FLNB,CX3CL1,RAB7B,EPRS,MED1,CD74,PTPN2,DAPK3,MYO1C,CCL16,VPS26B	darkgreen	EC_M4
GO:0042306	8.65E-02	0.95736023	11	52	regulation of protein import into nucleus	BP	7.03E-01	ANGPT1,LEP,RAB23,MDFIC,GLI3,TGFB1,UFM1,HDAC3,CHP1,FLNA,ZPR1	darkgreen	EC_M4
GO:0051403	7.85E-02	0.948402642	28	190	stress-activated MAPK cascade	BP	7.03E-01	FLT4,TPD52L1,MAPK13,SH3RF3,GADD45G,TLR4,TRAF1,SDCBP,IKBKB,SFRP1,EPHB1,TNFRSF19,ITCH,SH3RF1,NCF1,MAP3K3,DLG1,MAP3K11,MECOM,CRACR2A,RELL1,TAOK2,SH2D3A,LGALS9,TRAF2,SH2D3C,DAB2IP,BMP2	turquoise	EC_M6
GO:0072593	7.85E-02	0.94962095	25	176	reactive oxygen species metabolic process	BP	7.03E-01	PDGFB,SYK,TLR4,MYCN,PRCP,NOS3,CD36,SIRT3,CLCN3,EDN1,SNCA,NCF1,CFLAR,PDK3,CYBB,RAC1,HIF1A,RAB27A,TLR6,ARG2,CYP1A1,NOS2,SLC25A33,PLCG2,PMAIP1	turquoise	EC_M6
GO:0002088	8.66E-02	0.954397833	13	63	lens development in camera-type eye	BP	7.03E-01	TGFBR1,SLC7A11,FGF2,CTNNB1,SPRED3,FAT1,KDM5B,SHROOM2,CRYBA4,SKIL,MED1,BCAR3,WNT2	darkgreen	EC_M4
GO:0031331	5.82E-03	0.99628677	56	366	positive regulation of cellular catabolic process	BP	7.03E-01	PIP4K2C,BAG6,SNX9,HSPA1B,HSF1,HMOX1,FOXO3,SESN1,NOP53,ZC3HAV1,BBS7,TRIM13,MLH1,SAMD4B,SIRT2,TRIM21,PLEKHF1,SH3D19,CAMKK2,RNFT2,TP53INP1,IRS1,DIS3L2,PUM1,AC013489.1,DTX3L,ARNT,MAGEF1,KAT5,NFE2L2,EXOSC7,PHB2,GABARAP,ZFP36L1,PIP4K2B,OSBPL7,GBA,NUPR1,TNRC6A,KEAP1,SNX33,INSR,PARN,FZR1,DVL1,AXIN1,ZER1,ABCA2,RACK1,TRIM65,RNFT1,TRIB2,HSPB8,ZFP36L2,STUB1,EXOSC5	cyan	EC_M2
GO:1903018	8.67E-02	0.963142826	8	35	regulation of glycoprotein metabolic process	BP	7.03E-01	ITM2B,CTNNB1,PTX3,CHP1,SLC2A10,NCSTN,POMT1,ITM2C	darkgreen	EC_M4
GO:0002068	8.68E-02	0.969069	6	23	glandular epithelial cell development	BP	7.03E-01	CLOCK,RHEB,RARB,GSK3B,SIDT2,AKT1	darkgreen	EC_M4
GO:0002474	7.88E-02	0.979473809	4	16	antigen processing and presentation of peptide antigen via MHC class I	BP	7.04E-01	IKBKB,ACE,B2M,ERAP2	turquoise	EC_M6
GO:0043506	7.90E-02	0.964696144	9	46	regulation of JUN kinase activity	BP	7.04E-01	TNFRSF11A,SYK,SFRP1,EDN1,DTNBP1,MAP3K11,TLR6,TRAF2,DAB2IP	turquoise	EC_M6
GO:0060333	7.91E-02	0.974894036	5	24	interferon-gamma-mediated signaling pathway	BP	7.04E-01	IFNGR1,PPARG,NLRC5,STAT1,JAK1	turquoise	EC_M6
GO:0009968	7.91E-02	0.935048842	121	973	negative regulation of signal transduction	BP	7.04E-01	SEMA6A,TNFAIP3,TRIM39,FZD6,CTHRC1,GRAMD4,MDFI,CD2AP,RNF149,IL6,CXCL8,RGS3,ARHGAP25,FIGNL1,CAV2,GRB10,TLR4,NRP1,RASIP1,ARHGAP42,MTMR2,GATA2,NOS3,PPARG,GATA3,SEC14L1,PDE2A,SFRP1,NOTUM,FERMT1,SIRT3,PRR5L,PDE4D,IL1A,IGF1,HHEX,ITCH,ERBIN,SH3RF1,WWTR1,IL7,P2RX7,NDRG4,LMBR1L,ADGRG3,OAS3,NLRC5,NR4A2,DGKZ,DLL4,SOX13,STAT1,DYRK1A,SNCA,DUSP8,PRDM15,SHANK2,HDAC1,ITPRIP,HYAL2,LAPTM5,DYRK3,INPP5D,SH3BP4,CFLAR,INPP5F,IGFBP3,PLEKHA1,SRMS,ITGA6,PTPRE,DUSP16,DLG1,ELL3,APOD,LTF,TSPAN15,DDX3X,IGFBP5,HOMER3,MECOM,HIF1A,EPHA2,MMRN2,BID,TLR6,MCC,LFNG,RGS14,MTOR,AR,PSEN1,ITGA3,EFNA1,CCDC88C,TLE3,DNM2,TNIP1,MARK3,KIF26A,ADRB2,PDE4B,BFAR,TNFAIP8L1,ISG15,NRARP,EGFL7,NOTCH1,DAB2IP,MIR99A,OAS1,VNN1,CD46,SOX17,RGS4,RGS16,DUSP5,BMP2,RNF125,DUSP7,RNF152	turquoise	EC_M6
GO:0048821	7.91E-02	0.97191017	6	27	erythrocyte development	BP	7.04E-01	L3MBTL3,ADGRF5,PTBP3,TAL1,ALAS1,ARID4A	turquoise	EC_M6
GO:1905898	8.70E-02	0.965690182	7	29	positive regulation of response to endoplasmic reticulum stress	BP	7.04E-01	UBQLN1,NCK2,SERINC3,ATF6,PTPN2,BCAP31,TMEM259	darkgreen	EC_M4
GO:0032965	8.71E-02	0.965676378	7	29	regulation of collagen biosynthetic process	BP	7.04E-01	PPARD,TGFB1,RUNX1,SUCO,TGFB3,PDGFRB,ITGA2	darkgreen	EC_M4
GO:0043112	8.71E-02	0.944538487	24	131	receptor metabolic process	BP	7.04E-01	CD63,LRP1,AHI1,ANGPT1,MKLN1,VEGFA,BVES,ITGB1,PICALM,TGFB1,TBC1D16,ATXN2,HTR2B,GRK3,AP2M1,TFRC,LRPAP1,ECE1,SMURF1,ATAD1,CLTC,GRIA1,RAB31,LRRTM2	darkgreen	EC_M4
GO:0003323	8.72E-02	0.972648299	5	18	type B pancreatic cell development	BP	7.04E-01	CLOCK,RHEB,GSK3B,SIDT2,AKT1	darkgreen	EC_M4
GO:0019226	8.73E-02	0.958831842	10	44	transmission of nerve impulse	BP	7.04E-01	MYH14,FKBP1B,SCN5A,P2RX4,TNR,KCNA1,CACNA1G,ITGA2,PAFAH1B1,KCNMB4	darkgreen	EC_M4
GO:0035510	4.52E-02	0.988083907	5	20	DNA dealkylation	BP	7.05E-01	TRIM28,ASCC3,TDG,OTUD4,TET2	darkgrey	EC_M8
GO:0061138	7.92E-02	0.950354903	23	147	morphogenesis of a branching epithelium	BP	7.05E-01	CELSR1,NOTCH4,KDR,MYCN,NRP1,RASIP1,PLXND1,GZF1,TIE1,SFRP1,NTN4,DLL4,EDN1,PROX1,LAMA5,DLG1,EPHA2,AR,HOXB7,NRARP,NOTCH1,ENG,BMP2	turquoise	EC_M6

GO:0106106	7.92E-02	0.952779346	19	123	cold-induced thermogenesis	BP	7.05E-01	ZNF516,ATF4,SYK,GADD45G,GRB10,TLR4,IL15,LAMA4,NOVA2,ACVR2B,CXCR4,CD36,ABHD6,KDM3A,FABP4,ADIPOR2,TLE3,ADRB2,NOTCH1	turquoise	EC_M6
GO:0120161	7.92E-02	0.952779346	19	123	regulation of cold-induced thermogenesis	BP	7.05E-01	ZNF516,ATF4,SYK,GADD45G,GRB10,TLR4,IL15,LAMA4,NOVA2,ACVR2B,CXCR4,CD36,ABHD6,KDM3A,FABP4,ADIPOR2,TLE3,ADRB2,NOTCH1	turquoise	EC_M6
GO:0003151	7.94E-02	0.961075944	11	59	outflow tract morphogenesis	BP	7.06E-01	NRP1,PLXND1,JAG1,MEF2C,SEC24B,TBX1,HIF1A,NOTCH1,ENG,SOX17,SOX18	turquoise	EC_M6
GO:2000377	7.94E-02	0.953317164	18	120	regulation of reactive oxygen species metabolic process	BP	7.06E-01	PDGFB,SYK,TLR4,MYCN,PRCP,CD36,SIRT3,CLCN3,SNCA,CFLAR,PDK3,RAC1,HIF1A,RAB27A,TLR6,ARG2,SLC25A33,PLCG2	turquoise	EC_M6
GO:0006351	7.96E-02	0.930256607	299	2521	transcription, DNA-templated	BP	7.06E-01	ZNF516,TNFRSF11A,BAZ2A,TCF4,TNFAIP3,PLAGL1,CNOT6,FZD6,ZBED4,NOTCH4,TCF20,TCF7,NCOA7,L3MBTL3,ATF4,PDGFB,KDM4C,PIM1,NFYA,MDFI,PRDM4,DDX58,NFX1,KIT,ADGRF1,NFIL3,SYK,SEMA4D,POLR2B,IL6,NFE2L3,REL,CREB5,PPIE,BACH2,PELI1,FOXP2,KLF11,LANCL2,NOBOX,TLR4,TRAF1,CREM,TEAD2,SDCBP,MYCN,NOCT,KTI12,ZBTB24,BEND5,TAL1,PHF10,EPC1,GATA2,DNMT3A,PRKD2,SAP130,DACH1,PPARG,GATA3,TAF3,ACVR2B,IRAK2,GZF1,EDNRB,SETD5,IKBKB,JAG1,NFKBIB,PDE2A,TMEM173,SFRP1,NR1D2,CD36,CDK17,PHRF1,ARID5A,TCF15,RET,CHD7,IL1A,DNMT3B,IGF1,NR4A1,EIF2AK3,HHEX,KDM3A,ITCH,ERBIN,SMAD1,ZBTB38,POU6F1,STOX2,SOX7,HMBOX1,HEXB,MEF2C,SMARCD1,TLR2,WWTR1,ZNF704,CXCL10,ADGRG3,KMT2D,SFMBT1,SCAF4,NLRC5,ACTR5,ANKRD2,NR4A2,TOX2,DLL4,CDYL,BAZ2B,SOX13,YAF2,EDN1,STAT1,DYRK1A,SNCA,JARID2,ERG,ETS2,CDK18,NFKB2,DCAF1,PRDM15,ETS1,CD200,SIK1,HDAC1,PPP3CA,HYAL2,SLC39A8,TESMIN,MEF2A,BCL9,GTF2E1,HLX,NCF1,USP13,FABP4,HCLS1,NFATC2,ZFHX2,EAF2,GMEB1,CD86,KDM2A,CIART,MN1,TGIF1,PARP15,POU2F3,ZSCAN29,CFLAR,TRAK2,ADCY1,ARID1A,RPS6KA1,PROX1,SS18L1,PRKD1,ZNF148,ZSCAN2,HELZ2,MLXIPL,ITGA6,DLG1,ELL3,ETV6,HOXD10,RNF2,CREB1,TET1,LTF,ADORA2A,ARNT2,MYEF2,IL1RAP,BCOR,DDX3X,RFXANK,ETV4,TBX1,MECOM,ING4,CDK16,SPINDOC,LDB2,FGF23,PPM1F,POU2F1,WAS,TEAD4,PRKCH,HIF1A,KDM5A,ZBTB18,APBB2,JUP,BCL6B,KDM5C,TLR6,RSC1A1,RGS14,MAFK,SPIN2A,MTOR,AR,CASZ1,PSEN1,CBX1,HOXB5,HOXB6,HOXB7,HOXB8,HOXB9,ZNF410,FBXW11,BATF,KRBA2,EFNA1,PIAS1,RPS	turquoise	EC_M6
GO:0051924	7.97E-02	0.948869546	25	172	regulation of calcium ion transport	BP	7.06E-01	TRDN,PDGFB,TSPAN13,PIK3CG,CXCR4,P2RY6,P2RX2,PDE4D,CHD7,P2RX7,CXCL10,PLCG1,STC1,SNCA,EPB41,PRKD1,ADORA2A,HOMER3,CRACR2A,ATP2A1,CD19,CCL2,PDE4B,PLCG2,RGS4	turquoise	EC_M6
GO:0016573	5.90E-03	0.997118981	24	131	histone acetylation	BP	7.07E-01	JADE2,EP300,ING3,SPHK2,WBP2,TADA3,BRPF1,APBB1,TAF10,SDR16C5,KAT5,EID1,KAT6B,MED24,CTBP1,KAT8,KAT7,PER1,SGF29,CRTC2,SIN3A,CREBBP,WDR5,GTF3C4	cyan	EC_M2
GO:0002313	8.77E-02	0.972422145	5	18	mature B cell differentiation involved in immune response	BP	7.08E-01	LGALS1,CDH17,ADA,DOCK10,NOTCH2	darkgreen	EC_M4
GO:0035773	8.79E-02	0.954962881	12	58	insulin secretion involved in cellular response to glucose stimulus	BP	7.08E-01	LRP1,PPARD,FKBP1B,KIF5B,OSBP,SELENOT,BRSK2,DYNLL1,LRP5,ADCY5,SIDT2,PTPRN	darkgreen	EC_M4
GO:0001775	8.79E-02	0.928366084	107	692	cell activation	BP	7.08E-01	IL23A,LRP1,ENPP3,HLA-DRA,FAM49B,LGALS1,MYH9,LEP,SRF,DOCK8,RUNX2,NCK2,NLRP5,TGFBR1,SOCS5,GLI3,INHBA,SLC7A11,ITGB1,FKBP1B,APBB1IP,DGKH,GLI2,TGFB1,AXL,CTNNB1,IL15RA,C1QTNF1,ACTG1,LIG4,IRS2,PRNP,ILK,HSPH1,SPINK5,LMO1,PJA2,IL20RB,CD180,VAMP8,LOXL3,P2RX4,MYL9,APP,CX3CL1,PTPN11,CDH17,AP3B1,PTPN22,ADA,RUNX1,CBLB,TOLLIP,SLC39A10,DOCK10,NOTCH2,PRKAR1A,MMP14,PIK3CA,LEF1,PRKCA,SLAMF8,KPNA1,TP53BP1,NCSTN,SNX27,TFRC,FZD9,HSPB1,MAPT,SYVN1,ITGA2B,FN1,PTPN6,IGFBP2,ADRA2C,COMP,COL3A1,DDOST,TIMP1,VCL,MSN,SYT11,DOCK2,CD74,PDGFRB,AKT1,TMEM98,SUPT6H,PTPN2,CD40LG,FLNA,VAMP7,AP1G1,LMO4,BCL10,FKBP1A,CD151,CSRP1,THY1,CDKN1A,DNAJB9,FGL2,CCR6,TSLP,PRKG1,FGF10	darkgreen	EC_M4
GO:0002011	8.83E-02	0.956438358	11	50	morphogenesis of an epithelial sheet	BP	7.08E-01	MRTFA,SRF,MEGF8,PHLDB2,NOTCH2,DAG1,BMP7,PDCD10,FLNA,COL5A1,CD151	darkgreen	EC_M4
GO:0031648	8.02E-02	0.965922176	8	42	protein destabilization	BP	7.09E-01	GGA3,SNCA,MYLIP,SENP2,FBXW11,CCDC88C,SOX17,BMP2	turquoise	EC_M6
GO:0043368	8.84E-02	0.968305957	6	23	positive T cell selection	BP	7.09E-01	IL23A,SRF,LOXL3,DOCK2,CD74,PTPN2	darkgreen	EC_M4
GO:0014741	8.03E-02	0.971223636	6	28	negative regulation of muscle hypertrophy	BP	7.09E-01	JARID2,IGFBP5,TNFRSF1B,YY1,NOTCH1,RGS4	turquoise	EC_M6

GO:1901224	8.04E-02	0.963655374	9	51	positive regulation of NIK/NF-kappaB signaling	BP	7.09E-01	LRRC19,TLR4,TLR2,EDN1,LAPTM5,CD86,DDX3X,LGALS9,NFAT5	turquoise	EC_M6
GO:0046718	8.87E-02	0.946872701	19	100	viral entry into host cell	BP	7.10E-01	LY6E,LGALS1,ITGB1,AXL,SIGLEC1,SMPD1,VAMP8,PTX3,VPS18,DAG1,ITGB5,NECTIN4,TFRC,ITGAV,SLC3A2,CD74,ITGA2,BSG,FCN1	darkgreen	EC_M4
GO:0051928	8.06E-02	0.956349681	14	86	positive regulation of calcium ion transport	BP	7.11E-01	TRDN,PDGFB,P2RY6,P2RX2,P2RX7,CXCL10,PLCG1,STC1,SNCA,CRACR2A,ATP2A1,CD19,CCL2,PLCG2	turquoise	EC_M6
GO:0072498	8.91E-02	0.976748364	4	13	embryonic skeletal joint development	BP	7.11E-01	CTNNB1,HYAL1,SLC2A10,BMP7	darkgreen	EC_M4
GO:0030514	8.91E-02	0.959767174	9	39	negative regulation of BMP signaling pathway	BP	7.11E-01	CRIM1,HTRA1,FBN1,SKIL,SMURF1,NBL1,GREM2,CHRDL1,FSTL3	darkgreen	EC_M4
GO:0009308	8.91E-02	0.950305612	15	77	amine metabolic process	BP	7.11E-01	AZIN1,LY6E,SLC7A11,HNMT,ATP2B4,AZIN2,TGFB2,SMS,COMT,MAOA,AOC3,SLC29A4,AGMAT,PARK7,NNMT	darkgreen	EC_M4
GO:0032231	8.93E-02	0.947374469	18	92	regulation of actin filament bundle assembly	BP	7.12E-01	TGFBR1,SHANK1,ROCK2,PAK1,ACTG1,PFN2,CX3CL1,PHLDB2,TJP1,SYNPO2,SYNPO2L,CGNL1,TPM1,TGFB3,GPR65,TTC8,ROCK1,FLNA	darkgreen	EC_M4
GO:0031664	8.09E-02	0.978518378	4	17	regulation of lipopolysaccharide-mediated signaling pathway	BP	7.13E-01	TNFAIP3,CD36,LTF,BMP6	turquoise	EC_M6
GO:0043117	8.12E-02	0.978879668	4	15	positive regulation of vascular permeability	BP	7.13E-01	TJP2,PDE2A,OCLN,BMP6	turquoise	EC_M6
GO:0000122	8.12E-02	0.935324786	89	694	negative regulation of transcription by RNA polymerase II	BP	7.13E-01	PLAGL1,NOTCH4,ATF4,PDGFB,MDFI,NFX1,NFIL3,SEMA4D,NFE2L3,BACH2,FOXP2,KLF11,CREM,SDCBP,TAL1,EPC1,GATA2,DNMT3A,SAP130,DACH1,PPARG,GATA3,TAF3,ACVR2B,GZF1,EDNRB,PDE2A,NR1D2,CD36,ARID5A,DNMT3B,HHEX,POU6F1,HMBOX1,MEF2C,WWTR1,SCAF4,ANKRD2,NR4A2,DLL4,SOX13,EDN1,STAT1,SNCA,JARID2,ETS2,DCAF1,HDAC1,MEF2A,HCLS1,NFATC2,TGIF1,PARP15,PROX1,ZNF148,MLXIPL,DLG1,ETV6,RNF2,BCOR,LDB2,ZBTB18,APBB2,BCL6B,MAFK,AR,PSEN1,HOXB8,EFNA1,PIAS1,YY1,HMG20A,HCFC1,NRARP,NOTCH1,IRF8,MTF2,ZBTB34,DAB2IP,ZBTB33,ATXN1L,SOX18,TGIF2,BMP2,GLIS3,ZNF10,CBX2,BMP6,ZNF266	turquoise	EC_M6
GO:0035561	4.59E-02	0.987861728	5	20	regulation of chromatin binding	BP	7.13E-01	BTAF1,GMNN,DDX11,WAPL,CDT1	darkgrey	EC_M8
GO:0035036	4.59E-02	0.98752351	5	24	sperm-egg recognition	BP	7.13E-01	CCT2,CCT4,CCT7,CCT5,CCT3	darkgrey	EC_M8
GO:0030212	8.13E-02	0.974253433	5	22	hyaluronan metabolic process	BP	7.14E-01	PDGFB,PIM1,IL15,ITIH5,HYAL2	turquoise	EC_M6
GO:0007044	8.14E-02	0.954096972	16	89	cell-substrate junction assembly	BP	7.14E-01	PTK2,TEK,LIMCH1,KDR,NRP1,ITGB4,SFRP1,THSD1,ARHGEF7,ITGA6,APOD,BCR,PPM1F,RAC1,RCC2,TAOK2	turquoise	EC_M6
GO:0036293	8.14E-02	0.943321368	36	258	response to decreased oxygen levels	BP	7.14E-01	PICK1,TEK,DNMT3A,CXCR4,SFRP1,P2RX2,DNMT3B,TLR2,VEGFC,ANGPT2,STC1,NR4A2,EDN1,ETS1,CD34,CFLAR,APOLD1,PDK3,CREB1,ARNT2,CYBB,RAMP2,CPEB2,HIF1A,MTOR,BNIP2,PGF,CHRNB2,CYP1A1,NOS2,LONP1,HMOX2,NOTCH1,PMAIP1,SLC8A3,BMP2	turquoise	EC_M6
GO:1903204	8.15E-02	0.978527231	4	16	negative regulation of oxidative stress-induced neuron death	BP	7.14E-01	OXR1,NCOA7,ATF4,HIF1A	turquoise	EC_M6
GO:0032480	8.19E-02	0.974189195	5	21	negative regulation of type I interferon production	BP	7.15E-01	C6orf106,REL,ITCH,YY1,RNF125	turquoise	EC_M6
GO:0002673	8.19E-02	0.973832739	5	23	regulation of acute inflammatory response	BP	7.15E-01	TNFRSF11A,IL6,PIK3CG,EDNRB,KLKB1	turquoise	EC_M6
GO:1902170	8.22E-02	0.978513098	4	15	cellular response to reactive nitrogen species	BP	7.15E-01	GUCY1B1,CFLAR,DNM2,TRAF2	turquoise	EC_M6
GO:0006482	8.25E-02	0.96779544	7	30	protein demethylation	BP	7.15E-01	KDM4C,KDM4D,KDM3A,JARID2,KDM2A,KDM5A,KDM5C	turquoise	EC_M6
GO:0008214	8.25E-02	0.96779544	7	30	protein dealkylation	BP	7.15E-01	KDM4C,KDM4D,KDM3A,JARID2,KDM2A,KDM5A,KDM5C	turquoise	EC_M6
GO:0007586	8.25E-02	0.959126495	11	63	digestion	BP	7.15E-01	IREB2,TJP2,TLR4,CD36,SCARB1,HIP1R,CHRM5,EPB41,F11R,WNK4,AKR1D1	turquoise	EC_M6

GO:0046640	8.26E-02	0.973712077	5	22	regulation of alpha-beta T cell proliferation	BP	7.15E-01	SYK,ITCH,SH3RF1,ARG2,LGALS9	turquoise	EC_M6
GO:0006418	4.62E-02	0.983876279	7	37	tRNA aminoacylation for protein translation	BP	7.16E-01	NARS,DARS,LARS,YARS,IARS2,DARS2,RARS	darkgrey	EC_M8
GO:0034629	8.29E-02	0.973762555	5	21	cellular protein-containing complex localization	BP	7.16E-01	EXOC3L2,RALB,DLG1,RCC2,EXOC3L1	turquoise	EC_M6
GO:0045921	8.30E-02	0.958817023	11	63	positive regulation of exocytosis	BP	7.16E-01	SYK,SDCBP,GATA2,GAB2,UNC13D,SNCA,FGR,RAB27A,SYT7,RAB3D,STXBP1	turquoise	EC_M6
GO:0007254	8.30E-02	0.948873591	21	134	JNK cascade	BP	7.16E-01	FLT4,TPD52L1,SH3RF3,GADD45G,TLR4,TRAF1,SDCBP,SFRP1,EPHB1,TNFRSF19,ITCH,SH3RF1,NCF1,MAP3K11,MECOM,CRACR2A,TAOK2,SH2D3A,TRAF2,SH2D3C,DAB2IP	turquoise	EC_M6
GO:0043433	8.31E-02	0.950150996	19	126	negative regulation of DNA-binding transcription factor activity	BP	7.16E-01	TNFAIP3,FZD6,PIM1,MDFI,PELI1,TAF3,IRAK2,NFKBIB,ITCH,ERBIN,ADGRG3,NLRC5,CD200,SIK1,PROX1,RNF2,USP7,DAB2IP,COMMD7	turquoise	EC_M6
GO:1904375	9.01E-02	0.945113547	20	107	regulation of protein localization to cell periphery	BP	7.17E-01	LRP1,STX7,ITGB1,KIF5B,PICALM,TGFB1,ATP2C1,PRNP,VAMP8,SORBS1,ATP2B4,MMP14,DAG1,AP2M1,LRRC15,PRKCI,IQSEC2,RAB11A,CLTC,AKT1	darkgreen	EC_M4
GO:0008544	9.01E-02	0.937410732	35	201	epidermis development	BP	7.17E-01	NAB2,CDSN,PPARA,PPARD,KIAA1324L,WHRN,ROCK2,INHBA,PTCH2,GLI2,CTNNB1,SLC4A7,MAFG,ATP2C1,SPINK5,PALLD,FLNB,TRPC4AP,ATP2A2,AP3B1,TSG101,TGFB2,FGFR2,CLIC4,OVOL1,PLOD3,GORAB,MED1,NGFR,MAP2K1,LAMA3,ROCK1,PAFAH1B1,EDA,FGF10	darkgreen	EC_M4
GO:0098900	9.01E-02	0.959225211	9	39	regulation of action potential	BP	7.17E-01	SCN5A,ATP2A2,CACNB3,PKP2,CACNA1C,CALM1,FLNA,KCNAB2,HCN4	darkgreen	EC_M4
GO:0001764	8.32E-02	0.950299024	19	113	neuron migration	BP	7.17E-01	SEMA6A,CELSR1,NRCAM,NRP1,GATA2,GATA3,CXCR4,SH3RF1,MEF2C,GPM6A,NR4A2,SRGAP2C,RAC1,PSEN1,ITGA3,LHX6,DAB2IP,ADGRG1,RELN	turquoise	EC_M6
GO:0032330	9.02E-02	0.957003981	10	46	regulation of chondrocyte differentiation	BP	7.17E-01	RUNX2,TGFBR1,GLI3,GLI2,CTNNB1,RARB,PTPN11,LOXL2,GDF6,GLG1	darkgreen	EC_M4
GO:1905523	9.03E-02	0.971365343	5	18	positive regulation of macrophage migration	BP	7.18E-01	AKIRIN1,P2RX4,CX3CL1,MMP14,CMKLR1	darkgreen	EC_M4
GO:1901863	8.34E-02	0.978077254	4	15	positive regulation of muscle tissue development	BP	7.18E-01	LMOD3,MEF2C,CREB1,MRTFB	turquoise	EC_M6
GO:0061041	8.37E-02	0.95321581	15	96	regulation of wound healing	BP	7.20E-01	TNFAIP3,TSPAN8,PTK2,PDGFB,NOS3,CXCR4,HBEGF,FERMT1,CD36,KLKB1,OCLN,ANO6,EDN1,CD34,MTOR	turquoise	EC_M6
GO:0002228	8.39E-02	0.96666849	7	34	natural killer cell mediated immunity	BP	7.20E-01	NECTIN2,UNC13D,RNF19B,LYST,RAB27A,LGALS9,SERPINB9	turquoise	EC_M6
GO:0001881	8.40E-02	0.969676067	6	27	receptor recycling	BP	7.20E-01	SNCA,AP1AR,INPP5F,LMTK2,PSEN1,VAMP3	turquoise	EC_M6
GO:0045807	8.41E-02	0.954222991	14	82	positive regulation of endocytosis	BP	7.20E-01	PICK1,SYK,PPT1,GATA2,CD36,ANO6,SNCA,PPP3CA,B2M,SELE,DNM2,AP2B1,PLCG2,CBLL1	turquoise	EC_M6
GO:1901522	8.42E-02	0.973215204	5	21	positive regulation of transcription from RNA polymerase II promoter involved in cellular response to chemical stimulus	BP	7.20E-01	ATF4,SMAD1,HIF1A,NOTCH1,BMP2	turquoise	EC_M6
GO:0003002	8.44E-02	0.941383539	35	251	regionalization	BP	7.20E-01	MDFI,NRP1,ACVR2B,SFRP1,TCF15,HHEX,MEF2C,HIPK1,DLL4,EDN1,ETS2,SEMA3F,PLXNA2,HOXD10,RNF2,TBX1,LFNG,ALDH1A2,AR,PSEN1,HOXB5,HOXB6,HOXB7,HOXB8,HOXB9,SMAD2,YY1,NRARP,NOTCH1,MTF2,ADGRG1,SOX17,RELN,BMP2,PIFO	turquoise	EC_M6
GO:0051281	8.45E-02	0.969223196	6	29	positive regulation of release of sequestered calcium ion into cytosol	BP	7.20E-01	TRDN,P2RY6,CXCL10,PLCG1,SNCA,CD19	turquoise	EC_M6

GO:0002220	8.46E-02	0.977979154	4	14	innate immune response activating cell surface receptor signaling pathway	BP	7.20E-01	SYK,IKBKB,PAK2,PLCG2	turquoise	EC_M6
GO:0007156	8.47E-02	0.951983428	16	88	homophilic cell adhesion via plasma membrane adhesion molecules	BP	7.20E-01	CELSR1,NECTIN2,PCDH1,PCDH12,RET,ROBO4,ESAM,CDH24,CADM3,PECAM1,HMCN1,PCDH11X,DSG2,DSC2,DSC3,CDH5	turquoise	EC_M6
GO:0071320	8.49E-02	0.963882564	8	37	cellular response to cAMP	BP	7.20E-01	PIK3CG,PDE2A,GPD1,STC1,RAPGEF3,IGFBP5,RAPGEF1,SLC8A3	turquoise	EC_M6
GO:0050879	8.50E-02	0.963611029	8	40	multicellular organismal movement	BP	7.20E-01	VPS54,NR4A1,KCNJ2,SYNM,CHRNB1,MTOR,ATP2A1,SLC8A3	turquoise	EC_M6
GO:0050881	8.50E-02	0.963611029	8	40	musculoskeletal movement	BP	7.20E-01	VPS54,NR4A1,KCNJ2,SYNM,CHRNB1,MTOR,ATP2A1,SLC8A3	turquoise	EC_M6
GO:0110020	8.50E-02	0.952688798	15	86	regulation of actomyosin structure organization	BP	7.20E-01	LIMCH1,NRP1,SFRP1,RAPGEF3,EDN1,PROX1,F11R,PAK2,PPM1F,WAS,RAC1,MTOR,ARHGEF15,ARHGAP28,S1PR1	turquoise	EC_M6
GO:0061384	8.52E-02	0.96911272	6	27	heart trabecula morphogenesis	BP	7.20E-01	TEK,CHD7,DLL4,NOTCH1,S1PR1,ENG	turquoise	EC_M6
GO:0016441	8.54E-02	0.950117047	17	115	posttranscriptional gene silencing	BP	7.20E-01	CNOT6,IL6,MYCN,TUT4,PUM2,PPARG,DND1,SMAD1,PPP3CA,RBM4,MOV10,DDX3X,SMAD2,CNOT8,MIR23A,MIR99A,MIR1307	turquoise	EC_M6
GO:0010605	8.54E-02	0.925690568	248	2090	negative regulation of macromolecule metabolic process	BP	7.20E-01	BAZ2A,TNFAIP8,TNFAIP3,PLAGL1,TRIM39,PTPRB,LAPTM4B,CNOT6,OXR1,NOTCH4,TCF7,NCOA7,L3MBTL3,C6orf106,DAPK1,ATF4,PDGFB,MDFI,IREB2,NFX1,TNFRSF21,KDR,CD2AP,NFIL3,SEMA4D,RGP1,IL6,IGF2BP3,NFE2L3,REL,PTBP3,CXCL8,BACH2,FOXP2,KLF11,LANCL2,TLR4,C5,CREM,SDCBP,MYCN,NOCT,TUT4,PUM2,RASIP1,BEND5,TAL1,PHF10,MTMR2,EPC1,KDM4D,GATA2,DNMT3A,SAP130,CST7,GGA3,DACH1,PPARG,TIMP4,GATA3,TAF3,ACVR2B,ITIH5,GZF1,EDNRB,NDFIP2,IKBKB,PDE2A,SFRP1,HBEGF,NR1D2,DND1,FERMT1,SH3BP5,CD36,ARID5A,PRR5L,PDE4D,MERTK,DNMT3B,IGF1,NR4A1,EIF2AK3,HHEX,KDM3A,ITCH,ERBIN,SH3RF1,SMAD1,ZBTB38,POU6F1,OCLN,SOX7,HMBOX1,ENC1,MEF2C,WWTR1,SFMBT1,FAM192A,SCAF4,TIPARP,OAS3,PPP1R16B,ANKRD2,NR4A2,DLL4,CDYL,SOX13,YAF2,EDN1,STAT1,DYRK1A,SNCA,JARID2,DTNBP1,ETS2,DCAF1,CD200,HDAC1,KIAA1551,PPP3CA,HYAL2,MEF2A,RIN3,LAPTM5,FABP4,HCLS1,NFATC2,AP1AR,INPP5D,CD34,KDM2A,CIART,TGIF1,PARP15,INPP5F,IGFBP3,RBM4,RPS6KA1,PROX1,ZNF148,YTHDF1,HELZ2,MLXIPL,ACE,PAK2,CST6,SENP2,DLG1,SMG7,APOD,ETV6,MOV10,RNF2,CREB1,EIF4A2,TET1,LTF,ADORA2A,MYEF2,BCOR,DDX3X,IGFBP5,HOMER3,MECOM,ING4,SPINDOC,BIRC3,LDB2,CPEB2,PPM1F,POU2F1,RAC1,HIF1A,KDM5A,ZBTB18,APBB2,ADIPOR2,BCL6B,EPHA2,KDM5C,CASC3,TLR6,RGS14,ARG2,MAFK,MTOR,AR,OPHN1,RNF40,PSEN1,PAPLN,CBX1,HOXB8,FBXW11,EFNA1,PIAS1,RPS6KA5,SMAD2,TLE3,CNOT8,YY1,HMG20A,RCOR1,LIG3,LGALS9,NOS2,GIT1,USP7,ISG15,HCFC1,NRARP,TRAF2,NOTCH1,IRF8,ENG,MTF2,ZBTB34,TERF2,DAB2IP,MIR23A,MIR9	turquoise	EC_M6
GO:0051452	8.55E-02	0.965693436	7	36	intracellular pH reduction	BP	7.20E-01	DMXL1,PPT1,RAB38,CLCN3,SLC9A7,CA2,RAB39A	turquoise	EC_M6
GO:0022029	8.55E-02	0.963302497	8	40	telencephalon cell migration	BP	7.20E-01	EFHC1,CXCR4,SRGAP2C,PSEN1,LHX6,DAB2IP,ADGRG1,RELN	turquoise	EC_M6
GO:0051494	8.56E-02	0.946493202	22	138	negative regulation of cytoskeleton organization	BP	7.20E-01	PICK1,TMOD1,SVIL,SPTBN4,ARHGEF7,SPEF1,LMOD3,HIP1R,DYRK1A,SNCA,TMOD4,F11R,PAK2,WAS,TMOD2,ARHGAP28,S1PR1,CARMIL2,CDH5,GMFG,KANK3,SHANK3	turquoise	EC_M6
GO:0042391	8.56E-02	0.93840538	42	302	regulation of membrane potential	BP	7.20E-01	RIMS2,TRDN,KDR,GRIK2,TRPM4,MTMR2,KCNK6,P2RX2,SLC4A11,CD36,MEF2C,P2RX7,KCNJ3,EDN1,SNCA,PPP3CA,SLC39A8,KCNJ2,SCN3A,INSYN2,DLG1,KCNK7,ADORA2A,JUP,BID,CHRNB1,PSEN1,CHRNB2,DSG2,DSC2,ADRB2,PIEZO2,SLC25A33,PIEZO1,PMAIP1,SLC8A3,RELN,KCNH2,SCN2B,KCNK5,SHANK3,RGS4	turquoise	EC_M6
GO:0035176	8.57E-02	0.966109833	7	31	social behavior	BP	7.20E-01	SHANK2,PTCHD1,TBX1,MTOR,CHRNB2,ATXN1L,SHANK3	turquoise	EC_M6
GO:0051281	9.08E-02	0.963845159	7	29	positive regulation of release of sequestered calcium ion into cytosol	BP	7.20E-01	CX3CL1,PKD2,CASQ1,HTT,HAP1,CALM1,THY1	darkgreen	EC_M4
GO:0045806	9.13E-02	0.958377238	9	41	negative regulation of endocytosis	BP	7.21E-01	PACSIN1,PICALM,ATXN2,ITGAV,LRPAP1,STX1B,SYT11,SCAMP5,LRRTM2	darkgreen	EC_M4
GO:0045649	9.14E-02	0.970920972	5	18	regulation of macrophage differentiation	BP	7.21E-01	INHBA,RB1,TGFB1,PRKCA,PTPN2	darkgreen	EC_M4

GO:0033622	8.59E-02	0.972502885	5	21	integrin activation	BP	7.21E-01	RASIP1,FERMT1,JAM3,SELP,PIEZO1	turquoise	EC_M6
GO:0048878	8.61E-02	0.929985919	105	832	chemical homeostasis	BP	7.21E-01	DMXL1,TRDN,KLHL3,IREB2,ADGRF5,KDR,IL6,PPT1,CAV2,TRPM4,PIK3CG,GATA2,RAB38,GPR4,PRCP,PPARG,CXCR4,EDNRB,TMC8,P2RY6,P2RY2,NR1D2,P2RX2,SLC4A11,CD36,TMEM178A,MICU3,SCARB1,MALL,PDE4D,CHD7,IL1A,CLCN3,HEXB,CP,P2RX7,CXCL10,TPCN1,PLCG1,STC1,IFIT1,EDN1,STAT1,SNCA,MYLIP,ABCG1,LPCAT1,MTTP,LCK,PPP3CA,HYAL2,ANO1,SLC39A8,KCNJ2,TESMIN,SLC12A7,HCRTR1,SLC9A8,FABP4,POLG,PRKD1,MLXIPL,MELTF,PDK3,B2M,LTF,CCRL2,ADORA2A,DDX3X,IGFBP5,MCU,PYGL,SLC9A7,WNK4,CDK16,FGF23,RAC1,HIF1A,ADIPOR2,HEPH,PSEN1,ADCK1,ATP2A1,CD19,NPR1,S100A14,GPR68,SCNN1B,HMOX2,AP3D1,PLCG2,S1PR1,CDH5,OAS1,SLC8A3,LRRC8D,TRIM24,CA2,KCNH2,SCN2B,LPAR6,TBC1D20,RAB39A,BMP6,RBP1	turquoise	EC_M6
GO:1905153	8.61E-02	0.977438853	4	14	regulation of membrane invagination	BP	7.21E-01	GATA2,CD36,ANO6,PLCG2	turquoise	EC_M6
GO:0010952	8.61E-02	0.947197133	20	142	positive regulation of peptidase activity	BP	7.21E-01	GRAMD4,DAPK1,SYK,PPARG,EIF2AK3,HIP1R,SOX7,SNCA,LCK,LAPTM5,CFLAR,CASP10,DDX3X,PPM1F,BID,EFNA1,LGALS9,PRSS22,PMAIP1,PERP	turquoise	EC_M6
GO:0002312	9.15E-02	0.951298594	13	64	B cell activation involved in immune response	BP	7.21E-01	LGALS1,TGFB1,LIG4,CD180,CDH17,ADA,DOCK10,NOTCH2,TP53BP1,TFRC,SUPT6H,CD40LG,CCR6	darkgreen	EC_M4
GO:0043297	9.16E-02	0.954344022	11	52	apical junction assembly	BP	7.21E-01	CTNNA1,SRF,ROCK2,PDCD6IP,MTDH,TJP1,VCL,WDR1,ROCK1,MYO1C,NPHP4	darkgreen	EC_M4
GO:0010811	9.17E-02	0.944789649	19	100	positive regulation of cell-substrate adhesion	BP	7.21E-01	VEGFA,RRAS,FBLN2,ILK,EDIL3,CX3CL1,PTPN11,GSK3B,DAG1,IQGAP1,COL26A1,FN1,ARPC2,ROCK1,EGFLAM,FLNA,CDH13,THY1,HAS2	darkgreen	EC_M4
GO:0055123	9.19E-02	0.944672622	19	99	digestive system development	BP	7.22E-01	AHI1,GLI3,RB1,GLI2,MEGF8,CTNNB1,RARB,CCKBR,ADA,TGFB2,CLMP,FGFR2,SRP54,TGFB3,MYOCD,PKD1,WLS,CDKN1A,FGF10	darkgreen	EC_M4
GO:0071806	9.22E-02	0.953851926	11	54	protein transmembrane transport	BP	7.22E-01	PEX6,SEC61A1,AKT2,TRAM1,TOMM40L,SRP54,TMED10,HSPA5,TIMM22,GRPEL2,TOMM7	darkgreen	EC_M4
GO:0048025	4.66E-02	0.989821978	4	16	negative regulation of mRNA splicing, via spliceosome	BP	7.22E-01	RBMX,RNPS1,PTBP1,SRSF7	darkgrey	EC_M8
GO:1905521	9.25E-02	0.962998861	7	29	regulation of macrophage migration	BP	7.22E-01	AKIRIN1,P2RX4,CX3CL1,MMP14,CMKLR1,SLAMF8,MIR24-1	darkgreen	EC_M4
GO:0016311	9.26E-02	0.930075041	56	342	dephosphorylation	BP	7.22E-01	PTP4A3,NUAK1,INPP4A,PTP4A1,ROCK2,FKBP1B,ENTPD6,TGFB1,MTMR14,PPP1R12A,NUDT16,ACPP,SMPD1,PPP2CB,PPP2R2B,IMPAD1,MTMR6,HSP90B1,PPP2R2A,PTPN11,PIP4P2,PTPN22,CRY2,PPP3CC,CHP1,PSPH,SLC39A10,DAPP1,EYA2,TGFB2,PPP6R3,GSK3B,INPP5A,PPP1R11,PPP1R3D,PTPN6,HTT,PPP2R5E,PPP1R36,PTPN21,CALM1,PPP4R4,PIN1,DUSP14,PDGFRB,ROCK1,ITGA2,LPIN2,NT5M,OCRL,PTPN2,PGP,FKBP1A,PTPN12,DUSP4,DUSP22	darkgreen	EC_M4
GO:0021801	9.27E-02	0.970381807	5	18	cerebral cortex radial glia-guided migration	BP	7.22E-01	SUN2,RTN4,GLI3,CTNNB1,PAFAH1B1	darkgreen	EC_M4
GO:0022030	9.27E-02	0.970381807	5	18	telencephalon glial cell migration	BP	7.22E-01	SUN2,RTN4,GLI3,CTNNB1,PAFAH1B1	darkgreen	EC_M4
GO:0032986	4.67E-02	0.989911639	4	15	protein-DNA complex disassembly	BP	7.23E-01	SUPT16H,ARID2,SMARCC1,SMARCE1	darkgrey	EC_M8
GO:0043687	9.31E-02	0.962558786	7	30	post-translational protein modification	BP	7.23E-01	P3H1,CRTAP,STT3B,SUMF1,UBA3,FAM20C,WIPI2	darkgreen	EC_M4
GO:0010761	9.31E-02	0.955348197	10	46	fibroblast migration	BP	7.23E-01	ITGB1,FGF2,PAK1,TGFB1,ILK,CORO1C,ARID5B,SGPL1,AKT1,PDLIM1	darkgreen	EC_M4
GO:0030510	9.32E-02	0.947773516	15	74	regulation of BMP signaling pathway	BP	7.23E-01	KIAA1324L,CRIM1,ILK,SULF1,NOTCH2,FSTL1,HTRA1,FBN1,FKBP8,SKIL,SMURF1,NBL1,GREM2,CHRDL1,FSTL3	darkgreen	EC_M4
GO:0046488	9.33E-02	0.939539873	25	138	phosphatidylinositol metabolic process	BP	7.23E-01	INPP4A,TMEM246,SOCS5,FAM126A,FGF2,MTMR14,IMPAD1,MTMR6,TMEM150A,PIP4P2,PIGP,HTR2B,INPP5A,PIK3CA,PITPNM1,PIGZ,PIGL,PDGFRB,OCRL,DPM2,PIGK,DPM1,PIGB,PIK3C2A,CDIPT	darkgreen	EC_M4
GO:0050849	9.33E-02	0.970113052	5	18	negative regulation of calcium-mediated signaling	BP	7.23E-01	DYRK2,PRNP,CHP1,ATP2B4,GSK3B	darkgreen	EC_M4
GO:0048333	9.38E-02	0.965848242	6	23	mesodermal cell differentiation	BP	7.25E-01	INHBA,ITGB1,EYA2,FGFR2,BMPR1A,ITGA2	darkgreen	EC_M4

GO:0050906	9.38E-02	0.953042638	11	52	detection of stimulus involved in sensory perception	BP	7.25E-01	WHRN,CXCL12,CACNB3,BACE1,KCNK4,KCNA1,ROM1,ITGA2,COL11A1,OR5AN1,OR6N2	darkgreen	EC_M4
GO:0021879	9.39E-02	0.962293063	7	29	forebrain neuron differentiation	BP	7.25E-01	INHBA,ZSWIM6,RAPGEF2,SATB2,FGFR2,PLXNA3,LHX8	darkgreen	EC_M4
GO:1900542	6.16E-03	0.997801011	13	56	regulation of purine nucleotide metabolic process	BP	7.26E-01	EP300,SPHK2,PRKAG1,ARNT,HDAC4,DNAJC30,NUPR1,INSR,TAZ,JMJD8,MT-CO2,ZBTB7A,PID1	cyan	EC_M2
GO:0046040	6.28E-03	0.99889997	6	16	IMP metabolic process	BP	7.26E-01	PNP,XDH,NT5C2,ATIC,APRT,NT5C	cyan	EC_M2
GO:1901654	6.29E-03	0.99676792	27	151	response to ketone	BP	7.26E-01	CDK4,TSPO,HSF1,TRERF1,KLF9,FOXO3,SPHK2,FOSB,WBP2,NCOA4,NCOA2,SRD5A1,TXNIP,RPL27,CASP9,GBA,FOS,ADAM15,ACACA,SIN3A,SREBF1,TBXA2R,TFAP4,NDOR1,CYBA,TGFBR3,PTGFR	cyan	EC_M2
GO:0016125	6.30E-03	0.997025969	22	114	sterol metabolic process	BP	7.26E-01	CYB5R3,PLPP6,FDXR,LIPE,PIP4P1,CYP1B1,ERLIN1,OSBPL5,CYB5R1,FMO5,SCAP,POR,ACADL,SOAT1,CYP27A1,GBA,ERG28,SREBF1,LEPR,ACAA2,RXRA,LCAT	cyan	EC_M2
GO:0050435	8.71E-02	0.960038951	9	50	amyloid-beta metabolic process	BP	7.26E-01	IFNGR1,GGA3,IGF1,DYRK1A,ABCG1,ACE,PSEN1,EFNA1,MGAT3	turquoise	EC_M6
GO:1904037	8.71E-02	0.968002354	6	29	positive regulation of epithelial cell apoptotic process	BP	7.26E-01	IL6,PLCG1,ANO6,HDAC1,ITGA4,CCL2	turquoise	EC_M6
GO:0030890	8.76E-02	0.967659835	6	30	positive regulation of B cell proliferation	BP	7.26E-01	PELI1,TLR4,MEF2C,IL7,NFATC2,CHRNB2	turquoise	EC_M6
GO:0071731	8.77E-02	0.976266198	4	16	response to nitric oxide	BP	7.26E-01	GUCY1B1,CFLAR,DNM2,TRAF2	turquoise	EC_M6
GO:0021872	8.79E-02	0.964549101	7	35	forebrain generation of neurons	BP	7.26E-01	NRP1,GATA2,DCLK2,PROX1,B2M,LHX6,SHANK3	turquoise	EC_M6
GO:0001954	8.82E-02	0.959468656	9	49	positive regulation of cell-matrix adhesion	BP	7.26E-01	TEK,KDR,NRP1,SFRP1,FERMT1,CD36,PPM1F,RAC1,JUP	turquoise	EC_M6
GO:0010769	8.82E-02	0.951704323	14	80	regulation of cell morphogenesis involved in differentiation	BP	7.26E-01	CDKL3,PTK2,NRP1,UNC13D,ARHGEF7,NEDD9,AP1AR,SS18L1,DOCK1,MELTF,RAC1,RCC2,DNM2,RELN	turquoise	EC_M6
GO:0022600	8.86E-02	0.957260491	10	56	digestive system process	BP	7.26E-01	IREB2,TJP2,TLR4,CD36,SCARB1,HIP1R,CHRM5,EPB41,F11R,WNK4	turquoise	EC_M6
GO:0031399	8.89E-02	0.925395574	145	1182	regulation of protein modification process	BP	7.26E-01	TNFRSF11A,TNFAIP3,PTPRB,FLT4,OXR1,TPD52L1,NCOA7,PTK2,PDGFB,TEK,LIMCH1,TEC,KIT,KDR,SYK,SEMA4D,PPP6R1,IL6,PELI1,TLR4,IL15,CCNY,SDCBP,NRP1,RASIP1,PIK3CG,TAL1,KDM4D,GATA2,PRKD2,CAB39L,RALB,PPARG,GATA3,EDNRB,NDFIP2,SPTBN4,SETD5,IKBKB,SFRP1,HBEGF,SH3BP5,ANKLE2,CD36,PRR5L,RET,PDE4D,DNMT3B,IGF1,EIF2AK3,HHEX,KDM3A,ITCH,OCLN,WWTR1,IL7,VEGFC,MADD,THBS4,PPP1R16B,RAPGEF3,CCNJL,CDYL,NEDD9,EDN1,DYRK1A,EHD4,SNCA,JARID2,DTNBP1,HDAC1,HYAL2,LAPTM5,NCF1,FABP4,HCLS1,PECAM1,CD86,FGR,INPP5F,IGFBP3,PPP2R5A,PROX1,PRKD1,MLXIPL,ACE,LIMK2,PAK2,SENP2,DLG1,MAP3K11,TET1,LTF,ADORA2A,BCOR,DDX3X,TBX1,BIRC3,FGF23,PPM1F,RAC1,HIF1A,KDM5A,ADIPOR2,ITPKB,TLR6,RGS14,MTOR,RNF40,PSEN1,FGF18,PGF,TAOK2,EFNA1,SLC51B,NAP1L2,PIK3R5,PIAS1,RPS6KA5,CCDC88C,TNIP1,HMG20A,ADRB2,YES1,SH2D3A,UNC119,ISG15,TRAF2,PLCG2,ENG,SH2D3C,MTF2,DAB2IP,CDH5,SLC8A3,RELN,ALKAL1,FGF16,MMD,DAZAP2,COPS8,BMP2,DUSP7,BMP6,PPP1R2	turquoise	EC_M6
GO:0043123	9.41E-02	0.937752062	27	153	positive regulation of I-kappaB kinase/NF-kappaB signaling	BP	7.26E-01	TAB2,LGALS1,SLC35B2,MAP3K7,CTNNB1,CANT1,CXXC5,ATP2C1,TMEM9B,SLC20A1,DHX36,CX3CL1,MTDH,HTR2B,GOLT1B,TFRC,TSPAN6,SLC44A2,CD74,IRAK1,FLNA,IKBKG,BCL10,WLS,FKBP1A,C18orf32,EDA	darkgreen	EC_M4
GO:0001964	9.42E-02	0.974894016	4	13	startle response	BP	7.26E-01	GLRB,BACE1,KCNA1,NPAS1	darkgreen	EC_M4
GO:0060079	9.43E-02	0.948273235	14	69	excitatory postsynaptic potential	BP	7.26E-01	RIMS1,SHANK1,RAB3GAP1,P2RX4,APP,GLRB,CACNB3,CHRNA7,GSK3B,MPP2,CHRNE,NLGN2,STX1B,AKT1	darkgreen	EC_M4
GO:0035909	9.43E-02	0.962239853	7	28	aorta morphogenesis	BP	7.26E-01	LRP1,SRF,HEY1,TGFB2,FKBP10,BMPR1A,PDGFRB	darkgreen	EC_M4
GO:0090313	9.44E-02	0.962040197	7	29	regulation of protein targeting to membrane	BP	7.26E-01	PAK1,AKT2,PRNP,CACNB3,CHP1,MIEF2,MYO1C	darkgreen	EC_M4
GO:0043114	9.44E-02	0.956743154	9	40	regulation of vascular permeability	BP	7.26E-01	ANGPT1,PTP4A3,SH3GL2,VEGFA,MYLK3,TGFB1,ABCC8,TJP1,NPPB	darkgreen	EC_M4

GO:0055117	9.46E-02	0.950902827	12	58	regulation of cardiac muscle contraction	BP	7.27E-01	FKBP1B,SCN5A,P2RX4,ATP2A2,PKP2,CACNA1C,ADRA1B,CALM1,DMD,TMEM38B,TMEM38A,HCN4	darkgreen	EC_M4
GO:0043534	9.46E-02	0.941919634	20	108	blood vessel endothelial cell migration	BP	7.27E-01	ANGPT1,NUS1,MYH9,SRF,VEGFA,ITGB1,FGF2,TGFB1,STARD13,ROBO1,P2RX4,ATP2B4,PRKCA,HSPB1,PDCD10,AKT3,AKT1,MIR24-1,TMSB4X,PIK3C2A	darkgreen	EC_M4
GO:0019637	9.48E-02	0.922010134	113	743	organophosphate metabolic process	BP	7.28E-01	ENPP3,RRM2B,PPARA,ACSL6,NUS1,SERINC1,PPARD,PLAA,VCP,SLC35B2,INPP4A,RAB23,TMEM246,SOCS5,FAM126A,MFSD2A,GARS,GFPT1,FGF2,CMPK1,SELENOI,ENTPD6,ABHD12,ABHD5,DGKH,TGFB1,RBKS,DGAT2,GPD1L,OSBPL10,MTMR14,PFKP,OSBPL8,NAXD,NUDT16,ACPP,SMPD1,IMPAD1,MTMR6,CHPT1,TMEM150A,FAR1,APP,ADCY6,PIP4P2,PFKM,PGAM4,OPA1,ENTPD4,LPCAT2,SERINC3,ADA,CHP1,NUDT18,PIGP,AGPAT3,ZBTB20,HTR2B,ITPK1,INPP5A,PIK3CA,PITPNM1,MOCS3,ETNK1,TPST2,IDH2,ADCY5,HACD2,PIGZ,PCYT1B,REXO2,SLC25A16,TPI1,PLD1,SGMS1,CLN8,PPARGC1A,NPPB,SPTLC2,PKM,SLC44A2,AK7,PIGL,ACSL4,PDGFRB,PNPLA6,MOCS2,NT5M,PRKAA1,OCRL,PAFAH1B1,ABCA3,PGP,ACOT7,PARK7,ENO1,SLC2A6,GNAI3,DPM2,PLA2G15,HACD1,TMEM38B,TPK1,PEMT,PIGK,DPM1,TMSB4X,NNMT,NME6,PIGB,PIK3C2A,ORMDL3,CDIPT	darkgreen	EC_M4
GO:0015908	9.50E-02	0.950499614	12	60	fatty acid transport	BP	7.29E-01	PPARA,PPARD,PLIN2,MFSD2A,AKT2,IRS2,P2RX4,PLA2R1,CPT1A,EPRS,ACSL4,AKT1	darkgreen	EC_M4
GO:1905477	9.52E-02	0.944247042	17	88	positive regulation of protein localization to membrane	BP	7.29E-01	LRP1,ITGB1,KIF5B,PAK1,AKT2,ATP2C1,PRNP,SORBS1,CACNB3,CHP1,ATP2B4,PRKCI,RAB11A,MIEF2,AKT1,MYO1C,MESD	darkgreen	EC_M4
GO:0001958	9.53E-02	0.965130014	6	23	endochondral ossification	BP	7.29E-01	NAB2,RUNX2,IMPAD1,MMP14,FGFR3,COL1A1	darkgreen	EC_M4
GO:0036075	9.53E-02	0.965130014	6	23	replacement ossification	BP	7.29E-01	NAB2,RUNX2,IMPAD1,MMP14,FGFR3,COL1A1	darkgreen	EC_M4
GO:0034162	9.54E-02	0.974486155	4	13	toll-like receptor 9 signaling pathway	BP	7.29E-01	RTN4,PTPN22,RAB7B,IRAK1	darkgreen	EC_M4
GO:0006022	8.95E-02	0.948564504	16	98	aminoglycan metabolic process	BP	7.30E-01	PDGFB,PIM1,IL15,HS6ST1,ITIH5,LYVE1,HEXB,ST3GAL6,CHST1,HYAL2,B3GNT7,GAL3ST3,FUCA1,CHST7,HS3ST1,CHST9	turquoise	EC_M6
GO:0033238	8.95E-02	0.97065302	5	23	regulation of cellular amine metabolic process	BP	7.30E-01	NR4A2,SNCA,SLC7A7,CHRNB2,ATCAY	turquoise	EC_M6
GO:0043491	8.97E-02	0.943506478	22	147	protein kinase B signaling	BP	7.30E-01	PTK2,TEK,KDR,PIK3CG,GATA3,HBEGF,RET,MERTK,IGF1,HYAL2,HCLS1,INPP5F,PLEKHA1,DLG1,IGFBP5,TNFAIP8L3,EPHA2,PEAR1,MTOR,PIK3R5,CCL2,ENG	turquoise	EC_M6
GO:0038095	8.98E-02	0.971077482	5	20	Fc-epsilon receptor signaling pathway	BP	7.30E-01	SYK,IKBKB,MAP3K1,PLCG1,PLCG2	turquoise	EC_M6
GO:0051017	9.00E-02	0.943414965	22	139	actin filament bundle assembly	BP	7.30E-01	MTSS1,BAIAP2L2,LIMCH1,NRP1,SFRP1,FHDC1,USH1C,RAPGEF3,NEDD9,F11R,PAK2,BAIAP2L1,PPM1F,WAS,RAC1,FSCN1,MTOR,ARHGEF15,ARHGAP28,SPIRE1,S1PR1,SHANK3	turquoise	EC_M6
GO:0001666	9.00E-02	0.937455054	34	244	response to hypoxia	BP	7.30E-01	TEK,DNMT3A,CXCR4,SFRP1,P2RX2,DNMT3B,TLR2,VEGFC,ANGPT2,STC1,NR4A2,EDN1,ETS1,CD34,CFLAR,APOLD1,PDK3,CREB1,ARNT2,CYBB,RAMP2,CPEB2,HIF1A,MTOR,PGF,CHRNB2,CYP1A1,NOS2,LONP1,HMOX2,NOTCH1,PMAIP1,SLC8A3,BMP2	turquoise	EC_M6
GO:0048679	9.55E-02	0.964846633	6	24	regulation of axon regeneration	BP	7.30E-01	LRP1,FKBP1B,SPP1,TNR,MAP2K1,FLNA	darkgreen	EC_M4
GO:0007041	9.55E-02	0.942170914	19	100	lysosomal transport	BP	7.30E-01	LRP1,VCP,VPS41,VPS35,RAB7A,TRAK1,ARSB,AP3B1,TSG101,VPS18,AKTIP,VPS39,RAB7B,HSPA8,SNX27,CLEC16A,MGRN1,VAMP7,AP1G1	darkgreen	EC_M4
GO:0007215	9.56E-02	0.9586293	8	34	glutamate receptor signaling pathway	BP	7.30E-01	GNAQ,PRNP,APP,CX3CL1,HOMER1,NCSTN,CPEB4,GRIA1	darkgreen	EC_M4
GO:0009749	9.56E-02	0.936684846	27	153	response to glucose	BP	7.30E-01	LRP1,PPARD,LGALS1,TXN2,SRF,UNC13B,FKBP1B,KIF5B,EIF2B3,IRS2,OSBP,SELENOT,OPA1,BRSK2,DYNLL1,SELENOS,ZBTB20,LRP5,PIK3CA,ADCY5,SIDT2,PTPRN,PPARGC1A,SMARCA4,PRKAA1,SERPINF1,COL6A2	darkgreen	EC_M4
GO:0007517	9.02E-02	0.936623747	36	259	muscle organ development	BP	7.30E-01	CAV2,SVIL,HBEGF,NR1D2,P2RX2,LMOD3,TCF15,EPHB1,CHD7,IGF1,NR4A1,POU6F1,MEF2C,CXCL10,TLL2,SFMBT1,ANKRD2,DLL4,PPP3CA,MEF2A,BCL9,HLX,CFLAR,PROX1,LAMA5,HOXD10,CREB1,TBX1,TEAD4,ZBTB18,MRTFB,NOTCH1,S1PR1,ENG,MAFF,BMP2	turquoise	EC_M6
GO:0031579	9.03E-02	0.975069903	4	17	membrane raft organization	BP	7.30E-01	PACSIN2,PPT1,CAV2,DLG1	turquoise	EC_M6
GO:0070977	9.03E-02	0.970661246	5	21	bone maturation	BP	7.30E-01	SEMA4D,IGF1,LTF,RFLNB,BMP2	turquoise	EC_M6

GO:0030856	9.04E-02	0.946965951	17	106	regulation of epithelial cell differentiation	BP	7.30E-01	NOTCH4,MYCN,GATA3,IKBKB,JAG1,WWTR1,STAT1,PRKX,F11R,APOLD1,ETV4,PROM1,PRKCH,NOTCH1,CDH5,MAFF,BMP6	turquoise	EC_M6
GO:1990845	9.04E-02	0.944371983	20	132	adaptive thermogenesis	BP	7.30E-01	ZNF516,ATF4,SYK,GADD45G,GRB10,TLR4,IL15,LAMA4,NOVA2,ACVR2B,CXCR4,CD36,ABHD6,KDM3A,PM20D1,FABP4,ADIPOR2,TLE3,ADRB2,NOTCH1	turquoise	EC_M6
GO:0033194	9.05E-02	0.975243896	4	16	response to hydroperoxide	BP	7.30E-01	OXR1,DAPK1,CD36,PRKD1	turquoise	EC_M6
GO:0032088	9.05E-02	0.954194947	11	67	negative regulation of NF-kappaB transcription factor activity	BP	7.30E-01	TNFAIP3,PELI1,IRAK2,NFKBIB,ITCH,ERBIN,NLRC5,CD200,USP7,DAB2IP,COMMD7	turquoise	EC_M6
GO:0071599	9.57E-02	0.97436389	4	13	otic vesicle development	BP	7.30E-01	AHI1,FGFR2,TCAP,FGF10	darkgreen	EC_M4
GO:0060828	9.59E-02	0.932547588	36	210	regulation of canonical Wnt signaling pathway	BP	7.30E-01	RSPO3,GNAQ,CDK14,VCP,TPBG,GLI3,DKKL1,PLEKHA4,FGF2,TGFB1,ZEB2,ZNF703,ILK,CTNND1,DKK3,AMFR,NKD1,CAPRIN2,GSK3B,KPNA1,BICC1,FGFR2,FZD9,ATP6AP2,IGFBP2,OTUD5,COL1A1,GSKIP,PIN1,DAPK3,CSNK1G2,NPHP4,WLS,EDA,DACT3,FGF10	darkgreen	EC_M4
GO:0050879	9.59E-02	0.95587688	9	40	multicellular organismal movement	BP	7.30E-01	MYH14,ATP2A2,HOMER1,CASQ1,COMP,CLN8,TCAP,DMD,MAP1A	darkgreen	EC_M4
GO:0050881	9.59E-02	0.95587688	9	40	musculoskeletal movement	BP	7.30E-01	MYH14,ATP2A2,HOMER1,CASQ1,COMP,CLN8,TCAP,DMD,MAP1A	darkgreen	EC_M4
GO:0010883	9.61E-02	0.955713445	9	41	regulation of lipid storage	BP	7.30E-01	PPARA,PPARD,PLIN2,LEP,ABHD5,OSBPL8,CPT1A,ITGAV,PTPN2	darkgreen	EC_M4
GO:0065007	9.61E-02	0.912732963	1174	8375	biological regulation	BP	7.30E-01	SERPINB2,CD63,LMAN1,IL23A,ATP8B1,HSD17B6,NAB2,LRP1,STX7,AP3S1,CDO1,TRIM36,HBS1L,B4GALT7,AHI1,ARHGEF25,AGAP2,ABRACL,ENPP3,TAB2,TBC1D9B,CAND1,DYRK2,IER3,RAB21,CDSN,RNF19A,RRM2B,PRRC1,DENND6B,SLC12A2,AZIN1,ATP6V1C1,PLXNB2,SKIV2L,FBN2,ANGPT1,FAM19A5,TCP1,PPARA,HLA-DRA,SLC22A4,SLC39A7,ROS1,AFF4,NUS1,SMDT1,SERINC1,RSPO3,CXCL14,FAM49B,PHF20L1,MRTFA,KCNK9,TRAPPC9,HABP4,PACSIN1,GNG5,PTP4A3,REEP2,ADGRB1,LY6E,SCUBE3,PPARD,CTNNA1,TEAD3,SIL1,GOLM1,SUN2,CBY1,MKLN1,C6orf89,UBQLN1,MTCH1,LGALS1,MDGA1,GGA1,KLHDC10,TXN2,SH3GL2,GNAQ,PLIN2,SLC39A4,MYH9,ARHGAP39,LEP,CDKN2B,PLAA,LARGE1,PEX6,RORB,BTBD11,SLC30A9,KIAA1324L,SRF,BAG1,NUAK1,CDK14,VEGFA,DOCK8,VCP,SLC35B2,RUNX2,RASL11B,UNC13B,COL1A2,TPM2,C2orf40,NCK2,CLOCK,EXOC1,CREB3,ASNS,PDCL3,REST,EIF5B,ASPN,INPP4A,ZSCAN18,CNNM4,DST,RAB23,SNX13,PTP4A1,NLRP5,COL15A1,P3H1,TGFBR1,ERP44,HIVEP3,ISOC2,SOCS5,RIMS1,TMEM30A,ERLEC1,PSME4,COPS9,RTN4,CYCS,TPBG,SHANK1,ADAMTS3,LRRC4B,EMC10,TAX1BP1,MFSD2A,GGCT,UBE2J1,MAP3K7,MYH14,SH3BP5L,AKIRIN1,ACTR2,SLC31A1,THAP5,GFPT1,KIDINS220,WHRN,MDFIC,TES,GLI3,NCDN,ROCK2,INHBA,BVES,RRAS,ZYX,RCN3,MYLK3,DKKL1,VPS35,CUL2,MGARP,SLC7A11,ITGB1,PGRMC2,LIN7B,PLEKHA4,DBP,FKBP1B,PLXNA1,FGF2,SRPK2,KIF5B,NAPA,SESN3,ORC5,DNAJC1,RAB7A,PANX1,PSMC2,ARHGEF4,ATP7B,GAD2,GSAP,APBB1IP,ABHD12,FBLN2,EML2,ZNF398,RB1,ITM2B,ABHD5,EED,IWS1,PICALM,M	darkgreen	EC_M4
GO:0036010	9.66E-02	0.964547191	6	23	protein localization to endosome	BP	7.30E-01	TMEM30A,ROCK2,VPS35,WASHC2A,TOLLIP,MSN	darkgreen	EC_M4
GO:0001895	9.07E-02	0.960361124	8	43	retina homeostasis	BP	7.30E-01	LYZ,POC1B,CLCN3,USH1C,B2M,LTF,PROM1,DRAM2	turquoise	EC_M6
GO:0060113	9.08E-02	0.958088533	9	48	inner ear receptor cell differentiation	BP	7.31E-01	CTHRC1,MYCN,MYO7A,JAG1,USH1C,SEC24B,CECR2,JAG2,NOTCH1	turquoise	EC_M6
GO:0003094	9.08E-02	0.970447416	5	21	glomerular filtration	BP	7.31E-01	PDGFB,ADGRF5,CD34,MCAM,TBC1D8B	turquoise	EC_M6
GO:0046635	9.70E-02	0.95520401	9	41	positive regulation of alpha-beta T cell activation	BP	7.31E-01	IL23A,HLA-DRA,SOCS5,GLI3,HSPH1,AP3B1,PTPN22,ADA,RUNX1	darkgreen	EC_M4

GO:1901565	9.73E-02	0.917453189	160	1069	organonitrogen compound catabolic process	BP	7.32E-01	LRP1,CDO1,AGAP2,CANX,RNF19A,AZIN1,ADGRB1,UBQLN1,GGA1,KLHDC10,PLAA,ALDH1L2,VCP,C2orf40,CLOCK,PDCL3,SOCS5,ERLEC1,PSME4,UBA6,UBE2J1,PCYOX1,RCN3,VPS35,CUL2,FGF2,RAB7A,PSMC2,GAD2,ABHD12,GLB1,PDCD6IP,TMUB1,KCTD21,NUB1,TGFB1,UBXN4,HNMT,SPOPL,ACER3,CTNNB1,STT3B,NPLOC4,RNF121,PSMD8,TMTC3,CHFR,NUDT16,DDRGK1,SMPD1,PPP2CB,PSMD6,RNF6,VPS37A,HSP90B1,TP53INP2,LNPEP,TRPC4AP,ELOC,RNF13,TET3,ENTPD4,AMFR,TSG101,CHAC1,ADA,GLS,BAP1,ATP2B4,CPN1,NUDT18,TTC3,CBLB,NKD1,TOLLIP,PSMD14,BRSK2,MARCH6,AZIN2,FAP,UCHL5,SELENOS,TRIP12,HYAL1,SEC22B,PSMD1,UBE3B,KCTD10,GSK3B,HSPA8,NEDD8,PPP1R11,SYNPO2,PSMD2,ADRM1,UBE4A,RHBDD2,EDEM3,SYVN1,SGPL1,COMT,DNAJC10,MAOA,DNAJB12,USP48,ECE1,QDPR,ARAF,SMURF1,TIMP1,FBXL5,JKAMP,FMN2,ASRGL1,HSD17B10,CLN8,ANAPC4,MSN,RNF111,SLC25A44,TGFB1I1,SPOP,FBXL22,SEL1L,CLN6,ARIH1,PIN1,FBXO22,ROCK1,PCYOX1L,USP14,AKT1,PSMD11,UBE2A,NT5M,TNFAIP1,OMA1,BCAP31,PKD1,FLNA,ACOT7,TMEM259,PARK7,RHBDF1,USP10,USP20,HSPA5,PLA2G15,FBXO27,BNIP3,UBE2V2,TIMP3,DNAJB9,PTTG1IP,UBE2D2,DDA1,GABARAPL2,UBE2G2,MAP1A,DERL2	darkgreen	EC_M4
GO:1905144	9.75E-02	0.963913761	6	24	response to acetylcholine	BP	7.33E-01	LY6E,GNAQ,ROCK2,ATP2B4,CHRNA7,GNB1	darkgreen	EC_M4
GO:1905145	9.75E-02	0.963913761	6	24	cellular response to acetylcholine	BP	7.33E-01	LY6E,GNAQ,ROCK2,ATP2B4,CHRNA7,GNB1	darkgreen	EC_M4
GO:0048661	9.76E-02	0.944733601	15	78	positive regulation of smooth muscle cell proliferation	BP	7.33E-01	FGF2,PAK1,TGFB1,CX3CL1,MMP2,FGFR2,MNAT1,BMPR1A,PPARGC1A,PDGFRB,AKT1,ITGA2,IRAK1,GNAI3,CDH13	darkgreen	EC_M4
GO:0060996	9.77E-02	0.942626335	17	87	dendritic spine development	BP	7.33E-01	NCK2,UBA6,SHANK1,ACTR2,EIF4G2,DHX36,OPA1,PDLIM5,DOCK10,DNM1L,CAPRIN2,KIF1A,TANC2,SRCIN1,ACSL4,PAFAH1B1,ARF4	darkgreen	EC_M4
GO:0098900	9.12E-02	0.96040166	8	39	regulation of action potential	BP	7.33E-01	TRPM4,CD36,KCNJ2,DLG1,JUP,CHRNB2,DSG2,DSC2	turquoise	EC_M6
GO:0046364	9.79E-02	0.947252059	13	65	monosaccharide biosynthetic process	BP	7.34E-01	PPARA,LEP,DGAT2,KAT2B,SELENOS,ATF3,TPI1,PPARGC1A,PTPN2,PGP,FAM3A,RANBP2,NNMT	darkgreen	EC_M4
GO:0046365	9.80E-02	0.960049505	7	30	monosaccharide catabolic process	BP	7.34E-01	GALT,GLB1,RBKS,PFKP,PFKM,LRP5,ENO1	darkgreen	EC_M4
GO:0007009	9.81E-02	0.941307029	18	94	plasma membrane organization	BP	7.35E-01	ATP8B1,FER1L6,PACSIN1,SH3GL2,MYH9,AKT2,SMPD1,ATP2A2,TGFB2,ARV1,OSBPL2,SYT11,SYTL4,ANXA6,AKT1,SYPL2,COL5A1,MTSS1L	darkgreen	EC_M4
GO:0002762	9.16E-02	0.96270214	7	35	negative regulation of myeloid leukocyte differentiation	BP	7.35E-01	TLR4,GATA2,SFRP1,TMEM178A,INPP5D,LTF,GPR68	turquoise	EC_M6
GO:0044403	9.19E-02	0.93798049	29	206	biological process involved in symbiotic interaction	BP	7.36E-01	LRRC19,CXCL8,CAV2,NRP1,NECTIN2,CXCR4,EFNB2,SCARB1,ITCH,TPCN1,ANO6,IFIT1,HDAC1,HYAL2,CD86,POU2F3,F11R,LTF,THOC3,EPHA2,SCNN1B,TNIP1,LGALS9,HAVCR1,TRIM34,CD46,TRIM62,SERPINB9,TBC1D20	turquoise	EC_M6
GO:0062013	9.20E-02	0.947845358	15	96	positive regulation of small molecule metabolic process	BP	7.36E-01	NOS3,PPARG,P2RY6,IGF1,GPD1,P2RX7,SNCA,SLC45A3,ABCG1,MLXIPL,HIF1A,PSEN1,NOS2,PMAIP1,BMP6	turquoise	EC_M6
GO:0071380	9.85E-02	0.973323458	4	13	cellular response to prostaglandin E stimulus	BP	7.37E-01	ADCY6,AKT1,PRKAA1,GNB1	darkgreen	EC_M4
GO:0050433	9.23E-02	0.965205861	6	32	regulation of catecholamine secretion	BP	7.37E-01	SNCA,DTNBP1,ADORA2A,SNCG,SYT7,CHRNB2	turquoise	EC_M6
GO:0045920	9.24E-02	0.96943117	5	23	negative regulation of exocytosis	BP	7.37E-01	SNCA,ATP9A,RSC1A1,LGALS9,NOTCH1	turquoise	EC_M6
GO:0009220	4.80E-02	0.989320673	4	17	pyrimidine ribonucleotide biosynthetic process	BP	7.38E-01	CTPS1,CAD,UMPS,DHODH	darkgrey	EC_M8
GO:0046474	6.45E-03	0.996469913	32	185	glycerophospholipid biosynthetic process	BP	7.38E-01	DGKA,PIP4K2C,CHKB,AGPAT1,GPAA1,PIGO,TTC7A,SLC44A1,LPIN1,TAMM41,PTDSS2,PIK3R1,GPAT3,PIGT,GPAM,CHKA,ABHD4,PIGM,ABHD8,PLSCR3,PIGH,PIP4K2B,DPM3,AC138894.1,MTMR4,MPPE1,MTMR1,TAZ,PIGQ,INPP5E,VAC14,LCAT	cyan	EC_M2
GO:0071392	4.80E-02	0.984918855	6	28	cellular response to estradiol stimulus	BP	7.38E-01	CCNA2,DDX18,KIF18A,H2AFZ,AIFM1,HNRNPD	darkgrey	EC_M8
GO:0060306	9.88E-02	0.963513786	6	23	regulation of membrane repolarization	BP	7.39E-01	SCN5A,SNTA1,CACNA1D,CACNB3,WDR1,FLNA	darkgreen	EC_M4

GO:0045744	9.28E-02	0.961893618	7	37	negative regulation of G protein-coupled receptor signaling pathway	BP	7.39E-01	CXCL8,SNCA,RGS14,DNM2,ADRB2,PDE4B,RGS4	turquoise	EC_M6
GO:0051446	4.81E-02	0.989382042	4	16	positive regulation of meiotic cell cycle	BP	7.39E-01	YTHDC2,CDC25C,CDC25A,RAD51AP1	darkgrey	EC_M8
GO:0042306	4.82E-02	0.9802684	9	52	regulation of protein import into nucleus	BP	7.39E-01	UBR5,TRIM28,IPO5,NUP58,NUP54,NOLC1,CDK1,TPR,ECT2	darkgrey	EC_M8
GO:0039528	9.32E-02	0.965020845	6	30	cytoplasmic pattern recognition receptor signaling pathway in response to virus	BP	7.40E-01	DDX58,PUM2,SEC14L1,OAS3,BIRC3,RNF125	turquoise	EC_M6
GO:0071243	9.34E-02	0.973678068	4	18	cellular response to arsenic-containing substance	BP	7.40E-01	ATF4,MAPK13,DDX3X,CPEB2	turquoise	EC_M6
GO:0061383	9.35E-02	0.959104467	8	40	trabecula morphogenesis	BP	7.40E-01	TEK,SEMA4D,SFRP1,CHD7,DLL4,NOTCH1,S1PR1,ENG	turquoise	EC_M6
GO:0070423	9.35E-02	0.969104782	5	22	nucleotide-binding oligomerization domain containing signaling pathway	BP	7.40E-01	TNFAIP3,TLR4,ITCH,ERBIN,BIRC3	turquoise	EC_M6
GO:0070232	9.37E-02	0.968707364	5	24	regulation of T cell apoptotic process	BP	7.40E-01	HIF1A,ARG2,EFNA1,LGALS9,IL7R	turquoise	EC_M6
GO:0045428	9.38E-02	0.958678877	8	43	regulation of nitric oxide biosynthetic process	BP	7.40E-01	TLR4,CD36,EDN1,CD34,RAC1,TLR6,MTOR,DNM2	turquoise	EC_M6
GO:0032438	9.38E-02	0.968804573	5	23	melanosome organization	BP	7.40E-01	RAB38,DTNBP1,LYST,AP3D1,RAB32	turquoise	EC_M6
GO:0032922	9.39E-02	0.954229612	10	55	circadian regulation of gene expression	BP	7.40E-01	ATF4,CSNK1E,NOCT,PPP1CB,HDAC1,KDM2A,CIART,KDM5A,RAI1,PASD1	turquoise	EC_M6
GO:0097435	9.40E-02	0.925158514	82	622	supramolecular fiber organization	BP	7.41E-01	TRDN,MTSS1,ARHGAP18,BAIAP2L2,PICK1,CDC42EP1,LIMCH1,PGM5,CD2AP,TMOD1,ARHGAP25,ELMO1,FAM49A,NRP1,SVIL,MSRB2,MYO7A,SPTBN4,FCHSD2,SFRP1,ARHGEF7,SPEF1,FCHSD1,CD36,LMOD3,HIP1R,OCLN,FHDC1,TLL2,TLL1,USH1C,RAPGEF3,IQGAP2,NEDD9,EDN1,DYRK1A,SNCA,GAS2,MEF2A,NRAP,ESAM,HCLS1,AP1AR,CFLAR,FES,PROX1,TMOD4,F11R,LIMK2,PAK2,DLG1,B2M,MCU,BAIAP2L1,PPM1F,WAS,TMOD2,RAC1,FSCN1,SHROOM4,BID,MICAL3,MTOR,PSEN1,ARHGEF15,CCDC88C,THSD4,ARHGAP28,SPIRE1,GIT1,MTM1,RFLNB,S1PR1,CARMIL2,CDH5,VASP,GMFG,KANK3,SHANK3,RGS4,GBA2,ARPC5L	turquoise	EC_M6
GO:0010498	4.83E-02	0.965501083	51	434	proteasomal protein catabolic process	BP	7.41E-01	CDK2,UBR2,TOPORS,XPO1,COMMD1,FBXO48,UFL1,CUL1,ASCC3,CDC20,CUL4A,CDC16,ANAPC1,TAF9,USP25,PBK,ANAPC7,LTN1,UBE2C,CUL3,KIF14,UBR1,PSMD12,UBR3,PSMD4,CCDC47,SMARCC1,PSMA6,CDC27,COP1,CUL5,PSME3,TBL1XR1,UBE2K,NEDD4,TAF1,OGT,PLK1,APPBP2,PSMD10,CUL4B,FMR1,CCNF,PSMA5,GLMN,AURKA,RNF139,GID4,UBXN2A,PSMD7,RCHY1	darkgrey	EC_M8
GO:0060976	9.93E-02	0.954090329	9	39	coronary vasculature development	BP	7.42E-01	SRF,VEGFA,TGFBR1,FGF2,MEGF8,SNX17,ADAMTS6,MYOCD,PDGFRB	darkgreen	EC_M4
GO:0060070	9.94E-02	0.927885943	42	250	canonical Wnt signaling pathway	BP	7.42E-01	RSPO3,GNAQ,CDK14,VCP,TPBG,GLI3,DKKL1,PLEKHA4,FGF2,TGFB1,CTNNB1,ZEB2,ZNF703,ILK,RYK,CTNND1,DKK3,AMFR,NKD1,CAPRIN2,LRP5,GSK3B,LEF1,KPNA1,BICC1,FGFR2,FZD9,ATP6AP2,FZD2,IGFBP2,OTUD5,COL1A1,GSKIP,PIN1,DAPK3,CSNK1G2,NPHP4,WLS,EDA,DACT3,WNT2,FGF10	darkgreen	EC_M4
GO:0006885	9.95E-02	0.944919432	14	70	regulation of pH	BP	7.42E-01	RAB7A,SLC4A7,MAFG,CCKBR,TM9SF4,SLC4A5,CHP1,SLAMF8,CLIC4,ATP6AP2,CLN6,ATP6AP1,ATP6V0D1,ATP6V0E1	darkgreen	EC_M4
GO:0048640	9.42E-02	0.946555864	15	90	negative regulation of developmental growth	BP	7.42E-01	SEMA6A,CDKL3,SEMA4D,NRP1,TLL2,JARID2,SEMA3F,SEMA6D,SEMA4A,YY1,ADRB2,RAI1,SEMA6B,ARHGAP4,RGS4	turquoise	EC_M6
GO:0006110	9.44E-02	0.961401013	7	34	regulation of glycolytic process	BP	7.42E-01	IGF1,GPD1,P2RX7,MLXIPL,HIF1A,PSEN1,GIT1	turquoise	EC_M6

GO:1902041	9.96E-02	0.953606694	9	42	regulation of extrinsic apoptotic signaling pathway via death domain receptors	BP	7.43E-01	FAIM2,GSK3B,ATF3,PEA15,SERPINE1,SKIL,ARHGEF2,PARK7,TIMP3	darkgreen	EC_M4
GO:0009126	4.85E-02	0.982797637	7	39	purine nucleoside monophosphate metabolic process	BP	7.43E-01	PPAT,PAICS,DCK,GMPS,PFAS,HPRT1,GART	darkgrey	EC_M8
GO:0043087	9.98E-02	0.9260522	48	285	regulation of GTPase activity	BP	7.43E-01	TBC1D9B,PLXNB2,DOCK8,SNX13,BVES,ITGB1,PLXNA1,PICALM,RAB3GAP1,AKT2,TBC1D16,TMED2,RASA1,RAPGEF2,CX3CL1,RALGAPB,CBLB,RAP1GDS1,DOCK10,GSK3B,CORO1C,ADAP1,NTRK3,SGSM1,IQGAP1,RASAL2,ZC3H15,EIF2S1,PBXIP1,GPR65,TTC8,PIN1,EVI5L,SNX18,OCRL,ABR,PLXNB3,PAFAH1B1,TAX1BP3,PLXNA3,GPSM1,ARHGAP29,BCAR3,MTSS1L,CCL16,THY1,CHN1,PRKG1	darkgreen	EC_M4
GO:0097062	9.45E-02	0.974048922	4	15	dendritic spine maintenance	BP	7.43E-01	PICK1,MTMR2,TANC1,ITGA3	turquoise	EC_M6
GO:0002067	9.99E-02	0.956174011	8	35	glandular epithelial cell differentiation	BP	7.43E-01	CLOCK,FGF2,RHEB,RARB,GSK3B,FGFR2,SIDT2,AKT1	darkgreen	EC_M4
GO:0001818	9.99E-02	0.930579507	33	195	negative regulation of cytokine production	BP	7.43E-01	IL23A,ANGPT1,PPARA,SOCS5,INHBA,TGFB1,AXL,NAV3,NPLOC4,PRNP,ATP2B1,IL20RB,CX3CL1,PTPN22,CHID1,CHRNA7,SELENOS,SLC2A10,TGFB2,LEF1,CMKLR1,PPP1R11,ASB1,FN1,PTPN6,OTUD5,SYT11,TGFB3,DICER1,NPTN,SARS,MIR24-1,TMSB4X	darkgreen	EC_M4
GO:0030641	9.99E-02	0.946029337	13	64	regulation of cellular pH	BP	7.43E-01	RAB7A,SLC4A7,MAFG,TM9SF4,SLC4A5,CHP1,SLAMF8,CLIC4,ATP6AP2,CLN6,ATP6AP1,ATP6V0D1,ATP6V0E1	darkgreen	EC_M4
GO:1904031	1.00E-01	0.95888053	7	30	positive regulation of cyclin-dependent protein kinase activity	BP	7.44E-01	MAPRE3,PKD2,CCND1,MNAT1,AKT1,PKD1,CDKN1A	darkgreen	EC_M4
GO:0048846	1.01E-01	0.955954704	8	34	axon extension involved in axon guidance	BP	7.44E-01	VEGFA,MEGF8,SEMA3C,RYK,CXCL12,SEMA4F,SEMA4G,PLXNA3	darkgreen	EC_M4
GO:1902284	1.01E-01	0.955954704	8	34	neuron projection extension involved in neuron projection guidance	BP	7.44E-01	VEGFA,MEGF8,SEMA3C,RYK,CXCL12,SEMA4F,SEMA4G,PLXNA3	darkgreen	EC_M4
GO:0032270	1.01E-01	0.914508335	160	1066	positive regulation of cellular protein metabolic process	BP	7.44E-01	IL23A,NAB2,LRP1,AGAP2,TAB2,RNF19A,PRRC1,PLXNB2,ANGPT1,HABP4,C6orf89,UBQLN1,MTCH1,MYH9,LEP,VEGFA,VCP,NCK2,REST,TGFBR1,SOCS5,PSME4,CYCS,MAP3K7,ROCK2,INHBA,RCN3,VPS35,FGF2,PSMC2,PICALM,MAPRE3,NUB1,PAK1,TGFB1,RAB3GAP1,WDR48,CTNNB1,AKT2,OSBPL8,PPP1R12A,TMTC3,PRNP,CHFR,HDAC3,DDRGK1,SMPD1,ILK,RBPMS,ARL6IP5,OSBP,EIF4G2,ROBO1,DIP2B,PFN2,APP,DHX36,RAPGEF2,CX3CL1,PTPN11,GNL3,PTPN22,ACVR1,GDF6,AKTIP,CHP1,ATP2B4,PKD2,CBLB,BRD7,IFT57,TENT4B,TANK,TOLLIP,PSMD14,NDN,SLC39A10,CHRNA7,CCND1,CAPRIN2,TGFB2,HTR2B,GSK3B,MMP14,DAG1,PIK3CA,AUTS2,KRAS,NTRK3,FAM162A,SPATC1L,BMP7,OSM,NCSTN,SYAP1,ADRM1,IQGAP1,TFRC,EGLN3,MAPT,PCOLCE,FN1,PDCD10,ADRA2C,DOK7,SAMD4A,FGFR3,ARAF,SMURF1,TNIK,PLD1,FBXL5,MNAT1,MAGED1,BMPR1A,PPARGC1A,MSN,RNF111,SRCIN1,ARHGEF2,NGFR,TGFB3,EIF3C,MAP2K1,CLN6,CALM1,UACA,PKM,ARIH1,NPTN,PIN1,CD74,FBXO22,PDGFRB,AKT1,ITGA2,PRKAA1,MYDGF,BCAP31,TNFRSF18,PKD1,IRAK1,TMEM259,PARK7,ENO1,POMT1,F3,BCAR3,BARHL2,HSPA5,BCL10,FKBP1A,UBE2V2,CDKN1A,TXN,NNMT,PTTG1IP,TSLP,DDA1,FGF10	darkgreen	EC_M4
GO:1902285	1.01E-01	0.972535132	4	13	semaphorin-plexin signaling pathway involved in neuron projection guidance	BP	7.44E-01	PLXNB2,PLXNA1,PLXNB3,PLXNA3	darkgreen	EC_M4

GO:0030030	9.49E-02	0.91965525	156	1226	cell projection organization	BP	7.45E-01	SEMA6A,KIF5A,TBC1D30,SNX2,SPAG1,CTHRC1,RIMS2,PARVG,PACSIN2,CDKL3,MTSS1,ASAP1,PTK2,PODXL,PICK1,MICALL1,CDC42EP1,KIT,ADGRF1,TNFRSF21,CD2AP,SEMA4D,IL6,SDCBP,NRCAM,NRP1,SPATA6,ITSN2,MTMR2,NOVA2,FGD5,PLXND1,NECTIN2,ITGB4,GATA3,MYO7A,CXCR4,SPTBN4,SFRP1,FEZ2,EFNB2,POC1B,ARHGEF7,SPEF1,FGD6,PRAG1,EPHB1,PLK2,RET,PREX2,OCLN,KIF13B,FHDC1,ENC1,MEF2C,WWTR1,P2RX7,NDRG4,GPM6A,USH1C,PPP1R16B,NR4A2,ANO6,TANC1,CEP97,TSPAN2,JAM3,EHD4,DTNBP1,MYLIP,SRGAP2C,PPP3CA,HECW2,CCDC28B,MEF2A,SEMA3F,ROBO4,SEC24B,ABLIM1,JHY,PLEKHO1,PLXNA2,CFLAR,INPP5F,TRAK2,ADCY1,KALRN,FES,PLEKHA1,SS18L1,PRKD1,VANGL2,LAMA5,YTHDF1,ITGA6,LIMK2,PAK2,LAMC2,APOD,MOV10,CREB1,B2M,ADORA2A,SEMA6D,EPHB4,ITGA4,DNM3,CDK16,SNAP29,LMTK2,WAS,RAC1,RCC2,RHOJ,FSCN1,EPHA2,CECR2,FGD1,MTOR,OPHN1,EFNB1,PSEN1,SEMA4A,ABI3,ITGA3,TAOK2,EFNA1,CHRNB2,ZDHHC15,RABEP2,RPS6KA5,DNM2,B4GALT6,ABLIM3,SPEF2,STK26,SEMA6B,SLITRK2,ATCAY,ARHGAP4,NOTCH1,RAPGEF1,S1PR1,STXBP1,FNBP1L,DAB2IP,CARMIL2,VASP,RELN,ALKAL1,SHANK3,STX3,PDCL,RALA,SCARF1,PIFO	turquoise	EC_M6
GO:0045123	9.50E-02	0.95557642	9	50	cellular extravasation	BP	7.46E-01	PIK3CG,JAM3,PECAM1,F11R,ITGA4,SELE,SELP,PLVAP,CCL2	turquoise	EC_M6
GO:0043470	9.51E-02	0.95815928	8	41	regulation of carbohydrate catabolic process	BP	7.46E-01	PPP1CB,IGF1,GPD1,P2RX7,MLXIPL,HIF1A,PSEN1,GIT1	turquoise	EC_M6
GO:0043408	1.01E-01	0.919725966	79	502	regulation of MAPK cascade	BP	7.46E-01	LRP1,ANGPT1,ROS1,KLHDC10,LEP,VEGFA,TGFBR1,TPBG,EMC10,MAP3K7,MDFIC,ROCK2,RRAS,FGF2,PAK1,TGFB1,CTNNB1,LBH,C1QTNF1,SPRED3,HDAC3,CAVIN3,SMPD1,ILK,ARL6IP5,RYK,ST5,PJA2,ROBO1,APP,RAPGEF2,CX3CL1,PTPN11,PTPN22,GDF6,CHRNA7,NOTCH2,TGFB2,HTR2B,DAG1,PRKCA,KRAS,NTRK3,BMP7,ATF3,FGFR2,OSM,IQGAP1,ATP6AP2,FN1,PTPN6,PDCD10,ADRA2C,FGFR3,TNIK,MAGED1,SHC1,ADRA1B,MAP2K1,NPTN,PIN1,CD74,PDGFRB,ROCK1,AKT1,PTPN2,MYDGF,PAFAH1B1,IRAK1,ATP6AP1,BCAR3,MIR24-1,CCL16,DUSP2,TIMP3,DUSP4,GLIPR2,DUSP22,FGF10	darkgreen	EC_M4
GO:1990173	1.01E-01	0.971930401	4	14	protein localization to nucleoplasm	BP	7.46E-01	TCP1,CCT8,DKC1,LARP7	darkgreen	EC_M4
GO:0010463	1.01E-01	0.955521966	8	35	mesenchymal cell proliferation	BP	7.46E-01	CTNNB1,IRS2,LRP5,BMP7,FGFR2,PRRX1,BMPR1A,WNT2	darkgreen	EC_M4
GO:2000772	4.88E-02	0.982768192	7	37	regulation of cellular senescence	BP	7.46E-01	FBXO5,PNPT1,ZMPSTE24,PRKDC,RBL1,EEF1E1,RSL1D1	darkgrey	EC_M8
GO:0008016	9.52E-02	0.939609345	22	144	regulation of heart contraction	BP	7.46E-01	TRDN,TRPM4,PIK3CG,SPTBN4,HBEGF,PDE4D,STC1,KCNJ3,EDN1,KCNJ2,MEF2A,DLG1,JUP,ATP2A1,DSG2,DSC2,PDE4B,SLC8A3,KCNH2,SCN2B,RGS4,KCNG2	turquoise	EC_M6
GO:0045646	9.54E-02	0.957883579	8	42	regulation of erythrocyte differentiation	BP	7.47E-01	TAL1,GATA2,STAT1,ETS1,INPP5D,B2M,HIF1A,ISG15	turquoise	EC_M6
GO:2000772	9.55E-02	0.960520399	7	37	regulation of cellular senescence	BP	7.47E-01	PLK2,MAP3K3,B2M,ARG2,ABI3,VASH1,TERF2	turquoise	EC_M6
GO:0006650	9.55E-02	0.932823194	35	257	glycerophospholipid metabolic process	BP	7.47E-01	SYNJ2,SERAC1,PDGFB,PIP5K1B,PIGF,INPP4B,PIK3CG,MTMR2,RAB38,PGS1,GPAT4,CDS2,SCARB1,ABHD6,PITPNM2,CHRM5,SERINC5,PLCB2,PLCG1,DGKZ,PIK3C2B,MTMR10,LPCAT1,PLA1A,INPP5D,BMX,INPP5F,PLD2,ITPKB,GDPD3,GDPD1,MTMR12,MTM1,PLCG2,PLA2G4E	turquoise	EC_M6
GO:0007566	1.01E-01	0.955236674	8	36	embryo implantation	BP	7.47E-01	PPARD,VEGFA,MMP2,SPP1,TIMP1,UBE2Q1,VMP1,BSG	darkgreen	EC_M4
GO:0036035	1.01E-01	0.972235674	4	13	osteoclast development	BP	7.47E-01	NOTCH2,FBN1,PAFAH1B1,ATP6AP1	darkgreen	EC_M4
GO:0042326	1.02E-01	0.923741157	51	311	negative regulation of phosphorylation	BP	7.48E-01	LRP1,ANGPT1,PPARA,SH3GL2,GNAQ,CDKN2B,NCK2,SOCS5,SH3BP5L,INHBA,RB1,TGFB1,GPD1L,KAT2B,IRS2,PRNP,SMPD1,ILK,TMED2,PTPN22,TSG101,CHP1,DBNDD2,CBLB,DYNLL1,LRP5,PRKAR1A,CORO1C,NTRK3,BMP7,HSPB1,MAPT,DNAJC10,PTPN6,FKBP8,PPARGC1A,SRCIN1,CALM1,GSKIP,MYOCD,ROCK1,AKT1,PTPN2,MIDN,PARK7,MIR24-1,DMD,THY1,CDKN1A,DUSP22,PMEPA1	darkgreen	EC_M4
GO:0060973	1.02E-01	0.972150063	4	13	cell migration involved in heart development	BP	7.48E-01	BVES,SEMA3C,BMP7,PDGFRB	darkgreen	EC_M4
GO:0042181	1.02E-01	0.954856248	8	36	ketone biosynthetic process	BP	7.50E-01	REST,DKKL1,DKK3,COQ10B,TPI1,HSD17B1,STARD3,PPARGC1A	darkgreen	EC_M4

GO:0019953	4.92E-02	0.963404971	65	565	sexual reproduction	BP	7.52E-01	YTHDC2,CCT2,FBXO5,HSF2,KIFC1,CDC25C,FANCC,TUT7,SRPK1,UBR2,FOXJ3,MSH2,YTHDC1,TTK,CCT4,HDAC2,CEP57,WDR33,ACOX1,XRN2,METTL3,TUBGCP3,BRCA2,CCNB1,RACGAP1,RPS6,CCT7,KNL1,CCT5,BMPR1B,UBE3A,KIF18A,NSUN2,SNRPA1,KHDRBS1,SLC9B1,TRIP13,INTS13,ASPM,SPO11,TIAL1,DDX6,CDK1,SEPT2,FOSL1,ZFX,DDX20,RPL10L,C17orf53,TOP2A,ASF1B,CCNB2,CCT3,ATRX,PTTG1,PLK1,BRIP1,NDC1,DAZAP1,FANCA,ODF2,HIST1H1A,AURKA,RNF8,PAIP2	darkgrey	EC_M8
GO:1904031	4.93E-02	0.984301508	6	30	positive regulation of cyclin-dependent protein kinase activity	BP	7.52E-01	CKS2,CCNT2,CCNB1,CDK5RAP1,CDC6,PSMD10	darkgrey	EC_M8
GO:0007600	9.64E-02	0.929609487	43	320	sensory perception	BP	7.53E-01	KIT,PPT1,GRIK2,MGLL,MYO7A,CXCR4,EDNRB,SPTBN4,P2RX2,CD36,EPHB1,CHD7,MARVELD2,MMP24,HEXB,P2RX7,TMEM150C,USH1C,PLCB2,DLL4,IMPG2,NAV2,EDN1,ANO1,ABLIM1,ZFHX2,HMCN1,ADORA2A,RTP4,CCDC50,TBX1,MTOR,HOXB8,CHRNB2,SCNN1B,CCL2,UNC119,PIEZO2,PKHD1L1,GRAP,DRAM2,RGS16,PDCL	turquoise	EC_M6
GO:0034983	6.66E-03	0.999441264	4	7	peptidyl-lysine deacetylation	BP	7.53E-01	HDAC10,SIRT2,HDAC4,HDAC6	cyan	EC_M2
GO:0043436	6.68E-03	0.995206935	98	677	oxoacid metabolic process	BP	7.53E-01	ZADH2,GFPT2,HSD17B8,EP300,MPST,ETFB,PTGR1,IL4I1,LPIN1,ACSS1,SEPHS1,ECHDC3,ACAA1,BCKDHA,CYGB,HYI,CSGALNACT1,HACL1,CYP1B1,GNPDA1,APIP,PEPD,THNSL2,CBR4,ACSS2,PRKAG1,LPIN3,HNF4A,FARS2,ERLIN1,PLA2G4B,ACOT8,WARS2,LONP2,ALDH5A1,IRS1,ACADS,GPAM,GPX1,QARS,DALRD3,ABCB11,PC,ARNT,CTPS2,PER2,HDAC4,GLUL,SCAP,EHHADH,POR,IDH1,ACADL,MTHFS,PCBD1,PRODH,ATIC,PECR,NAT8L,CYP27A1,TNFRSF1A,FADS3,FOXK1,ILVBL,EPHX1,TECR,MTHFR,MRI1,CRABP2,OSBPL7,HSD3B7,ACOT2,ALDH6A1,ABCD4,GCDH,NUPR1,AC138894.1,SCPEP1,SLC27A3,ACSM3,ACACA,INSR,SREBF1,SLC46A1,ACAA2,CPT2,ASPA,GAMT,JMJD8,GSTM4,DPYD,KARS,PTGES2,LDHD,FPGS,SULT1C4,ZBTB7A,SCP2	cyan	EC_M2
GO:0097193	1.02E-01	0.926037741	40	242	intrinsic apoptotic signaling pathway	BP	7.53E-01	DYRK2,RRM2B,UBQLN1,NCK2,CREB3,CUL2,TPT1,ARL6IP5,CXCL12,OPA1,CHAC1,SERINC3,BRSK2,DNM1L,SELENOS,HYOU1,HSPB1,SYVN1,ZNF385B,DNAJC10,PDCD10,ERO1A,SKIL,VDAC2,SGMS1,ARHGEF2,TAF9B,GSKIP,CD74,AKT1,PTPN2,BCAP31,PARK7,ENO1,BNIP3,CDKN1A,NACC2,PTTG1IP,PLAGL2,TMEM109	darkgreen	EC_M4
GO:0051954	9.67E-02	0.967705455	5	22	positive regulation of amine transport	BP	7.54E-01	RAB3B,ADORA2A,PSEN1,CHRNB2,STXBP1	turquoise	EC_M6
GO:1902932	9.70E-02	0.972546344	4	17	positive regulation of alcohol biosynthetic process	BP	7.54E-01	P2RY6,SNCA,ABCG1,BMP6	turquoise	EC_M6
GO:0035315	9.71E-02	0.96001588	7	34	hair cell differentiation	BP	7.55E-01	MYCN,MYO7A,JAG1,USH1C,SEC24B,JAG2,NOTCH1	turquoise	EC_M6
GO:0032434	4.96E-02	0.972986638	17	121	regulation of proteasomal ubiquitin-dependent protein catabolic process	BP	7.56E-01	CDK2,XPO1,COMMD1,UFL1,CDC20,TAF9,PBK,SMARCC1,COP1,UBE2K,TAF1,OGT,PLK1,PSMD10,GLMN,AURKA,RCHY1	darkgrey	EC_M8
GO:0006906	9.74E-02	0.945531465	14	90	vesicle fusion	BP	7.56E-01	STX11,TSNARE1,CAV2,TBC1D4,DYSF,SNCA,SNAP23,LRMP,SNAP29,SYT7,STXBP1,STX3,VAMP3,RAB39A	turquoise	EC_M6
GO:0051491	9.75E-02	0.963388213	6	27	positive regulation of filopodium assembly	BP	7.56E-01	NRP1,GPM6A,DNM3,FSCN1,FNBP1L,RALA	turquoise	EC_M6
GO:0007200	9.75E-02	0.953987391	9	53	phospholipase C-activating G protein-coupled receptor signaling pathway	BP	7.56E-01	GPR4,EDNRB,P2RY6,P2RY2,EDN1,ANO1,LTB4R,S1PR1,LPAR6	turquoise	EC_M6
GO:0035637	9.76E-02	0.94040131	19	114	multicellular organismal signaling	BP	7.56E-01	TRDN,TRPM4,SPTBN4,PDE4D,CHRM5,KCNJ3,JAM3,KCNJ2,MEF2A,SCN3A,JUP,ATP2A1,DSG2,DSC2,S1PR1,SLC8A3,KCNH2,SCN2B,CACNG5	turquoise	EC_M6
GO:0009309	9.77E-02	0.966627103	5	26	amine biosynthetic process	BP	7.56E-01	ODC1,GATA3,NR4A2,SNCA,OAZ3	turquoise	EC_M6
GO:0042401	9.77E-02	0.966627103	5	26	cellular biogenic amine biosynthetic process	BP	7.56E-01	ODC1,GATA3,NR4A2,SNCA,OAZ3	turquoise	EC_M6

GO:0071902	9.78E-02	0.935799303	25	170	positive regulation of protein serine/threonine kinase activity	BP	7.56E-01	TNFRSF11A,TPD52L1,PDGFB,KIT,SYK,TLR4,CCNY,PIK3CG,CAB39L,RALB,EDN1,SNCA,PROX1,MAP3K11,LTF,DDX3X,TLR6,PSEN1,FGF18,PIK3R5,ADRB2,TRAF2,DAB2IP,DAZAP2,BMP2	turquoise	EC_M6
GO:1902476	9.80E-02	0.959648211	7	33	chloride transmembrane transport	BP	7.57E-01	CLCN3,ANO6,ANO5,ANO3,ANO1,SLC12A7,SLC26A1	turquoise	EC_M6
GO:0071692	9.83E-02	0.930007368	37	271	protein localization to extracellular region	BP	7.57E-01	RIMS2,PICK1,CD2AP,ICA1,IL6,RSAD2,TLR4,TRPM4,RAB3B,LLGL2,SFRP1,SIRT3,FAM3D,IL1A,IGF1,TLR2,VEGFC,ABCG1,CD200,MTTP,HDAC1,PPP3CA,ANO1,ADORA2A,MCU,CDK16,RAC1,HIF1A,SNCG,SYT7,RAB3D,GPR68,SMAD2,NOS2,MYO18A,RPH3AL,BMP6	turquoise	EC_M6
GO:0030522	9.83E-02	0.932353222	31	217	intracellular receptor signaling pathway	BP	7.57E-01	TNFAIP3,KDM4C,PIM1,DDX58,TLR4,PUM2,PRCP,PPARG,SEC14L1,SFRP1,NR1D2,NR4A1,KDM3A,ITCH,ERBIN,UFSP2,KMT2D,OAS3,NR4A2,HDAC1,TIFA,MN1,ARID1A,PDK3,TBX1,BIRC3,ALDH1A2,AR,TRIM24,BMP2,RNF125	turquoise	EC_M6
GO:0060149	9.83E-02	0.972305206	4	16	negative regulation of posttranscriptional gene silencing	BP	7.57E-01	IL6,PPARG,DND1,PPP3CA	turquoise	EC_M6
GO:0060967	9.83E-02	0.972305206	4	16	negative regulation of gene silencing by RNA	BP	7.57E-01	IL6,PPARG,DND1,PPP3CA	turquoise	EC_M6
GO:0070482	9.84E-02	0.929542425	38	280	response to oxygen levels	BP	7.57E-01	PICK1,TEK,DNMT3A,CXCR4,SFRP1,P2RX2,DNMT3B,TLR2,VEGFC,ANGPT2,STC1,NR4A2,EDN1,ETS1,HDAC1,CD34,POLG,CFLAR,APOLD1,PDK3,CREB1,ARNT2,CYBB,RAMP2,CPEB2,HIF1A,MTOR,BNIP2,PGF,CHRNB2,CYP1A1,NOS2,LONP1,HMOX2,NOTCH1,PMAIP1,SLC8A3,BMP2	turquoise	EC_M6
GO:1903531	9.86E-02	0.943419297	15	99	negative regulation of secretion by cell	BP	7.58E-01	RSAD2,SFRP1,FAM3D,EDN1,SNCA,CD200,HDAC1,PPP3CA,ATP9A,FGF23,RSC1A1,TNFRSF1B,LGALS9,NOTCH1,VAMP3	turquoise	EC_M6
GO:0048261	1.03E-01	0.960892369	6	25	negative regulation of receptor-mediated endocytosis	BP	7.58E-01	PICALM,ATXN2,ITGAV,LRPAP1,SYT11,LRRTM2	darkgreen	EC_M4
GO:1903580	9.88E-02	0.962429458	6	29	positive regulation of ATP metabolic process	BP	7.59E-01	IGF1,GPD1,P2RX7,MLXIPL,HIF1A,PSEN1	turquoise	EC_M6
GO:0030855	1.04E-01	0.918936826	69	430	epithelial cell differentiation	BP	7.59E-01	AHI1,CDSN,ROS1,VEGFA,CLOCK,COL15A1,WHRN,ROCK2,SLC7A11,FGF2,PTCH2,RHEB,CTNNB1,LBH,AKT2,SLC4A7,RARB,MAFG,SPRED3,SPINT2,ZNF703,SPINK5,FAT1,PALLD,FLNB,HEY1,SLC4A5,RAPGEF2,TSG101,ACVR1,TOLLIP,TJP1,KDM5B,NOTCH2,CCND1,CPT1A,GSK3B,FSTL1,FAM20C,TAGLN2,BMP7,FGFR2,IQGAP1,CLIC4,TAGLN,SIDT2,OVOL1,PLOD3,SERPINE1,FZD2,SKIL,VCL,ACTA2,MED1,MSN,MYO1E,TGFB1I1,MAP2K1,TMEM100,TTC8,ROCK1,AKT1,ITGA2,PAFAH1B1,FLNA,TMEM38B,GDNF,CDKN1A,FGF10	darkgreen	EC_M4
GO:0001963	9.90E-02	0.971533032	4	18	synaptic transmission, dopaminergic	BP	7.60E-01	RAB3B,SNCA,ADORA2A,CHRNB2	turquoise	EC_M6
GO:0014823	9.90E-02	0.951117204	10	57	response to activity	BP	7.60E-01	IL6,CXCR4,DNMT3B,ANGPT2,EDN1,CREB1,HIF1A,MTOR,SLC25A25,BMP6	turquoise	EC_M6
GO:0000083	9.91E-02	0.971739823	4	17	regulation of transcription involved in G1/S transition of mitotic cell cycle	BP	7.60E-01	KLF11,CDK17,CDK18,CDK16	turquoise	EC_M6
GO:0001654	9.91E-02	0.928438415	40	280	eye development	BP	7.60E-01	ATF4,AGTPBP1,TMOD1,TSPAN12,NRP1,GATA3,ACVR2B,MYO7A,JAG1,FREM2,EPHB1,RET,MERTK,CHD7,GPM6A,USH1C,HIPK1,DLL4,LPCAT1,HDAC1,PROX1,LIMK2,DLG1,PROM1,HIF1A,RHOJ,EPHA2,ALDH1A2,SMOC1,PSEN1,ARHGEF15,TTLL5,YY1,CYP1A1,SLC25A25,RAB18,DIO3,TGIF2,TBC1D20,BMP6	turquoise	EC_M6
GO:0046488	9.93E-02	0.937398753	21	138	phosphatidylinositol metabolic process	BP	7.60E-01	SYNJ2,PDGFB,PIP5K1B,PIGF,INPP4B,PIK3CG,MTMR2,PITPNM2,CHRM5,PLCB2,PLCG1,PIK3C2B,MTMR10,INPP5D,BMX,INPP5F,ITPKB,MTMR12,MTM1,PLCG2,PLA2G4E	turquoise	EC_M6
GO:0099084	9.94E-02	0.96247514	6	27	postsynaptic specialization organization	BP	7.60E-01	DLG1,IL1RAP,OPHN1,ABI3,RELN,SHANK3	turquoise	EC_M6
GO:0051656	5.00E-02	0.965478079	42	334	establishment of organelle localization	BP	7.60E-01	KIF3A,KIFC1,CENPQ,LRPPRC,XPO1,CDCA8,CENPA,CLASP2,KIF2C,HOOK3,CCNB1,ESPL1,RRS1,FER,ABCE1,TERF1,FAM83D,NUSAP1,KIF18A,CUL3,CENPE,TRIP11,KIF14,CFL1,SPO11,CENPF,NUF2,CDCA5,NMD3,DLGAP5,HNRNPU,INCENP,MAD1L1,KPNB1,KIF22,SPDL1,AURKB,PLK1,NDC80,SAPCD2,CDT1,MAD2L1	darkgrey	EC_M8

GO:0002758	9.95E-02	0.972164405	4	15	innate immune response-activating signal transduction	BP	7.60E-01	SYK,IKBKB,PAK2,PLCG2	turquoise	EC_M6
GO:0032990	9.96E-02	0.921435405	74	536	cell part morphogenesis	BP	7.61E-01	SEMA6A,KIF5A,SNX2,RIMS2,PACSIN2,CDKL3,PTK2,SEMA4D,NRCAM,NRP1,ITSN2,NOVA2,PLXND1,NECTIN2,GATA3,CXCR4,SPTBN4,FEZ2,EFNB2,ARHGEF7,EPHB1,RET,PREX2,KIF13B,GPM6A,NR4A2,DTNBP1,PPP3CA,HECW2,MEF2A,SEMA3F,ROBO4,PLEKHO1,PLXNA2,TRAK2,ADCY1,KALRN,SS18L1,LAMA5,YTHDF1,PAK2,LAMC2,MOV10,CREB1,ADORA2A,SEMA6D,EPHB4,ITGA4,DNM3,LMTK2,RAC1,EPHA2,OPHN1,EFNB1,PSEN1,SEMA4A,ABI3,TAOK2,EFNA1,CHRNB2,ZDHHC15,RPS6KA5,DNM2,B4GALT6,SEMA6B,SLITRK2,MTM1,ARHGAP4,NOTCH1,STXBP1,DAB2IP,VASP,RELN,SHANK3	turquoise	EC_M6
GO:0043010	9.97E-02	0.929727896	35	242	camera-type eye development	BP	7.61E-01	ATF4,AGTPBP1,TMOD1,TSPAN12,NRP1,GATA3,ACVR2B,JAG1,EPHB1,RET,MERTK,CHD7,GPM6A,USH1C,HIPK1,DLL4,LPCAT1,HDAC1,PROX1,LIMK2,DLG1,PROM1,HIF1A,RHOJ,EPHA2,ALDH1A2,PSEN1,ARHGEF15,TTLL5,YY1,CYP1A1,SLC25A25,DIO3,TGIF2,TBC1D20	turquoise	EC_M6
GO:0040036	1.04E-01	0.96085338	6	24	regulation of fibroblast growth factor receptor signaling pathway	BP	7.61E-01	RUNX2,FGF2,CTNNB1,SULF1,NGFR,NPTN	darkgreen	EC_M4
GO:0002066	1.04E-01	0.953937745	8	35	columnar/cuboidal epithelial cell development	BP	7.62E-01	ROS1,CLOCK,RHEB,RARB,GSK3B,SIDT2,AKT1,CDKN1A	darkgreen	EC_M4
GO:0033688	1.00E-01	0.96618155	5	22	regulation of osteoblast proliferation	BP	7.62E-01	CTHRC1,SFRP1,MN1,LTF,BMP2	turquoise	EC_M6
GO:0048705	1.00E-01	0.932750455	27	186	skeletal system morphogenesis	BP	7.62E-01	MDFI,TEK,MYCN,SFRP1,TCF15,MEF2C,TIPARP,STC1,HYAL2,FGR,PLEKHA1,DLG1,HOXD10,LTF,TBX1,MMP13,PSEN1,HOXB5,HOXB6,HOXB7,HOXB8,HOXB9,FGF18,SMAD2,SPEF2,RFLNB,BMP6	turquoise	EC_M6
GO:0097345	1.04E-01	0.960487209	6	25	mitochondrial outer membrane permeabilization	BP	7.63E-01	EYA2,GSK3B,FZD9,VDAC2,BNIP3,BLOC1S2	darkgreen	EC_M4
GO:0032102	1.04E-01	0.923406825	44	266	negative regulation of response to external stimulus	BP	7.63E-01	SERPINB2,ENPP3,SLC12A2,FAM19A5,PPARA,PPARD,LEP,SOCS5,VPS35,FGF2,RB1,C1QTNF1,NPLOC4,SEMA3C,SPINK5,RYK,CNOT7,IL20RB,ROBO1,SEMA4F,CX3CL1,ABCC8,CACNB3,ADA,SPP1,SEMA4G,FAP,PHLDB2,SELENOS,SLAMF8,HTRA1,BACE1,SERPINE1,TNR,NBL1,SYT11,TSPAN6,PTPN2,PLXNA3,FAM3A,SERPINF1,A2M,FGL2,PRKG1	darkgreen	EC_M4
GO:0031445	5.04E-02	0.988995007	4	14	regulation of heterochromatin assembly	BP	7.64E-01	TRIM28,SETDB2,FAM208A,TPR	darkgrey	EC_M8
GO:0120261	5.04E-02	0.988995007	4	14	regulation of heterochromatin organization	BP	7.64E-01	TRIM28,SETDB2,FAM208A,TPR	darkgrey	EC_M8
GO:0009056	6.84E-03	0.994468293	254	2000	catabolic process	BP	7.65E-01	PYM1,ATG12,PIP4K2C,OS9,CDK4,ADAMTS2,SQSTM1,IRAK3,BAG6,ALDH7A1,ABHD16A,SNX9,HSPA1B,EHMT2,HDAC10,RNF5,EIF3H,TSPO,ZHX2,EP300,NRBP2,MPST,SHARPIN,FOXRED2,HSF1,FBXL6,VPS28,HMOX1,CUL7,CUL9,DNPH1,HINT2,EEF1A1,SLC44A1,ETFB,RPS7,IL4I1,TBC1D17,LPIN1,FOXO3,SESN1,DHDH,NOP53,ZC3HAV1,BBS7,TRIM13,SMG9,FASTK,AGAP3,MLH1,LIPE,PAFAH1B3,ACAA1,MYD88,DMAC2,BCKDHA,EGLN2,PNP,TIMP2,SAMD4B,GAA,XDH,SIRT2,RPTOR,LRTOMT,HGS,UBAC2,TBC1D5,HACL1,GPCPD1,TRIM21,DDHD2,CYP1B1,GNPDA1,NDFIP1,HGF,SMUG1,PCBP2,PSMF1,RBCK1,KCTD6,PEPD,THNSL2,RMND5A,SBF2,PLEKHF1,FBXO8,NEIL2,SH3D19,CHDH,CAMKK2,HTRA2,PRKAG1,PLBD2,TMEM67,LPIN3,FOXF2,RNFT2,TP53INP1,ERLIN1,PLA2G4B,ACOT8,FBXW4,LONP2,TEX264,FASTKD3,SRD5A1,NT5C2,ALDH5A1,IRS1,ACADS,HYAL3,TPCN2,TCIRG1,ABHD4,UBA7,DIS3L2,PUM1,GPX1,AC013489.1,USP19,DCAF11,DTX3L,ABCB11,ANPEP,MCL1,RNF26,RHBDD3,ARNT,HDAC4,GPC1,USP21,GLUL,RNASEL,MAGEF1,EHHADH,RPL11,CDKN1B,KAT5,LRIG2,YBX3,POR,NFE2L2,IDH1,OVGP1,FIS1,CAPN1,ACADL,VPS51,MTHFS,EXOSC7,CTSH,ATG2A,PRODH,VSIR,PHB2,ABHD12B,UBA52,DDHD1,CYP27A1,SMPD4,NAGLU,ATG9A,PLA2G16,ANKZF1,MVB12A,USE1,HDAC6,UBXN1,WASHC1,WDR45,ATP13A2,RNF216,FBXL18,GABARAP,FOXK1,ILVBL,EPHX1,THRA,ZFP36L1,PIP4K2B,OSBPL7,CDK5RAP3,KAT8,ALDH6A1,ABCD4,GBA,ADAM15,GCDH,NUPR1,AC138894.1,TRIM25,MTMR4,TNRC6A,HEXA,KEAP1,UBL7,ULK3,SNX33,ANKRD9,INSR,SREBF1,FBXO4,OXCT1,LEPR,TMEM199,CLPP,PARN,ACAA2,IDS,CPT2,RILP,FZR1,DVL1,AS	cyan	EC_M2
GO:0045048	1.05E-01	0.96460878	5	20	protein insertion into ER membrane	BP	7.65E-01	EMC10,TRAM1,EMC4,EMC1,EMC7	darkgreen	EC_M4
GO:1900744	1.01E-01	0.957676684	7	37	regulation of p38MAPK cascade	BP	7.66E-01	GADD45G,NCF1,MAP3K3,DLG1,RELL1,DAB2IP,BMP2	turquoise	EC_M6

GO:0010558	1.02E-01	0.914312892	141	1150	negative regulation of macromolecule biosynthetic process	BP	7.69E-01	PLAGL1,CNOT6,NOTCH4,TCF7,L3MBTL3,DAPK1,ATF4,PDGFB,MDFI,IREB2,NFX1,NFIL3,SEMA4D,IGF2BP3,NFE2L3,BACH2,FOXP2,KLF11,LANCL2,CREM,SDCBP,NOCT,TUT4,BEND5,TAL1,PHF10,EPC1,GATA2,DNMT3A,SAP130,DACH1,PPARG,GATA3,TAF3,ACVR2B,GZF1,EDNRB,PDE2A,SFRP1,HBEGF,NR1D2,DND1,CD36,ARID5A,DNMT3B,EIF2AK3,HHEX,ZBTB38,POU6F1,SOX7,HMBOX1,ENC1,MEF2C,WWTR1,SFMBT1,SCAF4,ANKRD2,NR4A2,DLL4,CDYL,SOX13,YAF2,EDN1,STAT1,SNCA,JARID2,ETS2,DCAF1,HDAC1,MEF2A,FABP4,HCLS1,NFATC2,KDM2A,CIART,TGIF1,PARP15,RBM4,PROX1,ZNF148,YTHDF1,MLXIPL,DLG1,ETV6,MOV10,RNF2,CREB1,EIF4A2,BCOR,DDX3X,IGFBP5,MECOM,ING4,SPINDOC,LDB2,CPEB2,PPM1F,POU2F1,ZBTB18,APBB2,BCL6B,KDM5C,MAFK,AR,PSEN1,CBX1,HOXB8,FBXW11,EFNA1,PIAS1,RPS6KA5,SMAD2,TLE3,CNOT8,YY1,HMG20A,RCOR1,LIG3,HCFC1,NRARP,NOTCH1,IRF8,MTF2,ZBTB34,TERF2,DAB2IP,ZBTB33,ARID4A,ZNF24,ATXN1L,TRIM24,SOX18,PASD1,COMMD7,TGIF2,BMP2,GLIS3,ZNF10,CBX2,BMP6,ZNF266	turquoise	EC_M6
GO:0018105	1.05E-01	0.923116196	42	249	peptidyl-serine phosphorylation	BP	7.70E-01	DYRK2,ANGPT1,VEGFA,NCK2,TGFBR1,ROCK2,SRPK2,PAK1,TGFB1,AKT2,GPD1L,DCLK1,ILK,MAST4,PFN2,APP,MAPKAPK5,ATP2B4,BRSK2,CAPRIN2,GSK3B,PIK3CA,PRKCA,NTRK3,OSM,TFRC,RPS6KA3,PDCD10,PRKCI,ARAF,AKT3,ROCK1,AKT1,PRKAA1,NLK,PNCK,PKD1,CSNK1G2,PARK7,BCAR3,DMD,TXN	darkgreen	EC_M4
GO:0099558	1.06E-01	0.970695039	4	13	maintenance of synapse structure	BP	7.70E-01	PCLO,C1QL1,ERC1,SDF4	darkgreen	EC_M4
GO:0032469	1.06E-01	0.960021056	6	24	endoplasmic reticulum calcium ion homeostasis	BP	7.70E-01	TMTC4,ATP2A2,APP,RAP1GDS1,TMCO1,BCAP31	darkgreen	EC_M4
GO:0021700	1.06E-01	0.925359713	35	204	developmental maturation	BP	7.70E-01	CD63,MTCH1,LEP,VEGFA,RUNX2,UNC13B,SHANK1,RB1,PICALM,AXL,CTNNB1,SPINK5,LGI4,TRPC4AP,MAP1B,APP,CX3CL1,OPA1,MMP2,TGFB2,DAG1,FARP2,C1QL1,ATP6AP2,FGFR3,PTPRN,GLDN,ADGRL1,THBS3,RERE,AP1G1,SNX10,CDKN1A,FAM210B,CCR6	darkgreen	EC_M4
GO:0021535	1.06E-01	0.970623974	4	13	cell migration in hindbrain	BP	7.70E-01	ITGB1,LEF1,FLNA,RERE	darkgreen	EC_M4
GO:0051560	1.06E-01	0.95962804	6	25	mitochondrial calcium ion homeostasis	BP	7.71E-01	SMDT1,C19orf12,RAP1GDS1,ANXA6,BCAP31,BNIP3	darkgreen	EC_M4
GO:0001736	1.06E-01	0.942134937	13	64	establishment of planar polarity	BP	7.71E-01	RSPO3,PLEKHA4,RYK,PRICKLE2,NKD1,BRSK2,FZD2,SMURF1,EXOC5,DAAM1,WDR1,TTC8,PAFAH1B1	darkgreen	EC_M4
GO:0007164	1.06E-01	0.942134937	13	64	establishment of tissue polarity	BP	7.71E-01	RSPO3,PLEKHA4,RYK,PRICKLE2,NKD1,BRSK2,FZD2,SMURF1,EXOC5,DAAM1,WDR1,TTC8,PAFAH1B1	darkgreen	EC_M4
GO:0051260	1.06E-01	0.931373202	23	128	protein homooligomerization	BP	7.71E-01	CBY1,PDCD6IP,KCTD21,PRNP,KCTD16,RYR3,GLS,PKD2,CALHM1,KCTD10,DISP1,POLQ,TP53BP1,OSBPL2,MPP2,KCNA1,ROM1,HSD17B10,TNFAIP1,ABCA3,BCL10,SIGMAR1,NACC2	darkgreen	EC_M4
GO:0070585	1.06E-01	0.934511724	19	107	protein localization to mitochondrion	BP	7.71E-01	LMAN1,HSPA4,COX18,MGARP,ATG13,AP3B1,BAP1,DNM1L,TOMM40L,MAPT,C11orf65,CALM1,AKT1,PRKAA1,BCAP31,TIMM22,LEPROT,GRPEL2,TOMM7	darkgreen	EC_M4
GO:0055017	1.07E-01	0.940153559	14	72	cardiac muscle tissue growth	BP	7.71E-01	PPARA,TGFBR1,FGF2,TENM4,PAK1,RUNX1,PDLIM5,TGFB2,PRKAR1A,FGFR2,BMPR1A,PIN1,MIR24-1,WNT2	darkgreen	EC_M4
GO:0070849	1.07E-01	0.94969976	9	41	response to epidermal growth factor	BP	7.71E-01	PTPN11,SYAP1,IQGAP1,MED1,COL1A1,AKT1,PTPN12,DUSP22,ZPR1	darkgreen	EC_M4
GO:0019220	1.07E-01	0.908986696	162	1074	regulation of phosphate metabolic process	BP	7.71E-01	IL23A,NAB2,LRP1,AGAP2,TAB2,PRRC1,PLXNB2,ANGPT1,PPARA,ROS1,SH3GL2,GNAQ,LEP,CDKN2B,NUAK1,VEGFA,VCP,NCK2,PDCL3,TGFBR1,SOCS5,MFSD2A,MAP3K7,SH3BP5L,ROCK2,INHBA,FKBP1B,FGF2,RB1,MAPRE3,PAK1,TGFB1,AXL,AKT2,GPD1L,OSBPL8,PPP1R12A,KAT2B,IRS2,PRNP,HDAC3,CCKBR,DDRGK1,SMPD1,ILK,RBPMS,RYK,CNOT7,HSP90B1,TMED2,OSBP,ROBO1,PFN2,CCNJ,APP,RAPGEF2,CX3CL1,PTPN11,GTF2H1,PTPN22,TSG101,ACVR1,CRY2,GDF6,AKTIP,CHP1,ATP2B4,PKD2,TWF1,DBNDD2,CBLB,SLC39A10,CHRNA7,DYNLL1,CCND1,CAPRIN2,TGFB2,PPP6R3,ZBTB20,LRP5,HTR2B,PRKAR1A,GSK3B,DAG1,PIK3CA,CORO1C,KRAS,NTRK3,SLAMF8,SPATC1L,BMP7,PPP1R11,FGFR2,OSM,SYAP1,IQGAP1,TFRC,HSPB1,MAPT,DNAJC10,FN1,PTPN6,PDCD10,ADRA2C,DOK7,HTT,FKBP8,FGFR3,ARAF,TNIK,MNAT1,PPP1R36,MAGED1,BMPR1A,TSPYL2,CDK12,PPARGC1A,SRCIN1,ARHGEF2,TGFB3,SHC1,CCNG1,MAP2K1,CALM1,PPP4R4,GSKIP,NPTN,MYOCD,PIN1,CD74,PDGFRB,ROCK1,AKT1,ITGA2,PRKAA1,PTPN2,MYDGF,TNFRSF18,ABCA3,PKD1,IRAK1,MIDN,PARK7,ENO1,SLC2A6,BCAR3,LMO4,BCL10,MIR24-1,RANBP2,DMD,FKBP1A,LOX,PPP1R14A,THY1,CDKN1A,TXN,TMSB4X,DUSP22,TSLP,PMEPA1,FGF10	darkgreen	EC_M4

GO:0051174	1.07E-01	0.908986696	162	1074	regulation of phosphorus metabolic process	BP	7.71E-01	IL23A,NAB2,LRP1,AGAP2,TAB2,PRRC1,PLXNB2,ANGPT1,PPARA,ROS1,SH3GL2,GNAQ,LEP,CDKN2B,NUAK1,VEGFA,VCP,NCK2,PDCL3,TGFBR1,SOCS5,MFSD2A,MAP3K7,SH3BP5L,ROCK2,INHBA,FKBP1B,FGF2,RB1,MAPRE3,PAK1,TGFB1,AXL,AKT2,GPD1L,OSBPL8,PPP1R12A,KAT2B,IRS2,PRNP,HDAC3,CCKBR,DDRGK1,SMPD1,ILK,RBPMS,RYK,CNOT7,HSP90B1,TMED2,OSBP,ROBO1,PFN2,CCNJ,APP,RAPGEF2,CX3CL1,PTPN11,GTF2H1,PTPN22,TSG101,ACVR1,CRY2,GDF6,AKTIP,CHP1,ATP2B4,PKD2,TWF1,DBNDD2,CBLB,SLC39A10,CHRNA7,DYNLL1,CCND1,CAPRIN2,TGFB2,PPP6R3,ZBTB20,LRP5,HTR2B,PRKAR1A,GSK3B,DAG1,PIK3CA,CORO1C,KRAS,NTRK3,SLAMF8,SPATC1L,BMP7,PPP1R11,FGFR2,OSM,SYAP1,IQGAP1,TFRC,HSPB1,MAPT,DNAJC10,FN1,PTPN6,PDCD10,ADRA2C,DOK7,HTT,FKBP8,FGFR3,ARAF,TNIK,MNAT1,PPP1R36,MAGED1,BMPR1A,TSPYL2,CDK12,PPARGC1A,SRCIN1,ARHGEF2,TGFB3,SHC1,CCNG1,MAP2K1,CALM1,PPP4R4,GSKIP,NPTN,MYOCD,PIN1,CD74,PDGFRB,ROCK1,AKT1,ITGA2,PRKAA1,PTPN2,MYDGF,TNFRSF18,ABCA3,PKD1,IRAK1,MIDN,PARK7,ENO1,SLC2A6,BCAR3,LMO4,BCL10,MIR24-1,RANBP2,DMD,FKBP1A,LOX,PPP1R14A,THY1,CDKN1A,TXN,TMSB4X,DUSP22,TSLP,PMEPA1,FGF10	darkgreen	EC_M4
GO:0045620	1.07E-01	0.95227838	8	36	negative regulation of lymphocyte differentiation	BP	7.71E-01	SOCS5,GLI3,INHBA,LOXL3,RUNX1,CD74,PTPN2,FGL2	darkgreen	EC_M4
GO:0071711	1.07E-01	0.955500564	7	30	basement membrane organization	BP	7.71E-01	ITGB1,SPINT2,PHLDB2,DAG1,PHLDB1,PLOD3,NTNG2	darkgreen	EC_M4
GO:0007632	1.07E-01	0.952373736	8	35	visual behavior	BP	7.71E-01	SLC7A11,ITGB1,APP,ABCC8,KRAS,NPTN,NPHP4,LRRN4	darkgreen	EC_M4
GO:0009135	1.07E-01	0.938614168	15	78	purine nucleoside diphosphate metabolic process	BP	7.71E-01	PPARA,PFKP,NUDT16,APP,PFKM,PGAM4,ENTPD4,NUDT18,ZBTB20,TPI1,PPARGC1A,PKM,PRKAA1,ENO1,SLC2A6	darkgreen	EC_M4
GO:0009179	1.07E-01	0.938614168	15	78	purine ribonucleoside diphosphate metabolic process	BP	7.71E-01	PPARA,PFKP,NUDT16,APP,PFKM,PGAM4,ENTPD4,NUDT18,ZBTB20,TPI1,PPARGC1A,PKM,PRKAA1,ENO1,SLC2A6	darkgreen	EC_M4
GO:1904814	1.07E-01	0.969691004	4	14	regulation of protein localization to chromosome, telomeric region	BP	7.71E-01	TCP1,CCT8,GNL3,DKC1	darkgreen	EC_M4
GO:1904263	1.07E-01	0.96967914	4	14	positive regulation of TORC1 signaling	BP	7.71E-01	SEC13,GPR137,OTUD5,CLEC16A	darkgreen	EC_M4
GO:0019228	1.07E-01	0.959584595	6	23	neuronal action potential	BP	7.71E-01	MYH14,SCN5A,P2RX4,KCNA1,CACNA1G,KCNMB4	darkgreen	EC_M4
GO:0061082	1.07E-01	0.95916448	6	24	myeloid leukocyte cytokine production	BP	7.71E-01	TGFB1,DHX36,TGFB2,TGFB3,CD74,BCL10	darkgreen	EC_M4
GO:0035335	1.08E-01	0.938236355	15	77	peptidyl-tyrosine dephosphorylation	BP	7.71E-01	PTP4A3,PTP4A1,MTMR14,ACPP,MTMR6,PTPN11,PTPN22,EYA2,PTPN6,PTPN21,DUSP14,PTPN2,PGP,PTPN12,DUSP22	darkgreen	EC_M4
GO:0071604	1.08E-01	0.951977897	8	35	transforming growth factor beta production	BP	7.71E-01	LTBP1,LUM,CX3CL1,TGFB2,ATP6AP2,FN1,ITGAV,BMPR1A	darkgreen	EC_M4
GO:0003222	1.08E-01	0.969832612	4	13	ventricular trabecula myocardium morphogenesis	BP	7.71E-01	TGFBR1,TGFB2,BMPR1A,MED1	darkgreen	EC_M4
GO:0010828	1.08E-01	0.951774315	8	35	positive regulation of glucose transmembrane transport	BP	7.71E-01	AKT2,OSBPL8,IRS2,SORBS1,PTPN11,CLTCL1,PRKCI,AKT1	darkgreen	EC_M4

GO:0008150	1.08E-01	0.909606195	1669	12183	biological_process	BP	7.71E-01	CYB5A,SERPINB2,CD63,LMAN1,SEC11C,IL23A,ATP8B1,HSD17B6,NAB2,LRP1,STX7,AP3S1,CDO1,TRIM36,HBS1L,B4GALT7,AHI1,ARHGEF25,B4GALNT1,AGAP2,ABRACL,CANX,ENPP3,TAB2,TBC1D9B,CAND1,DYRK2,IER3,RAB21,CDSN,RABL2A,RNF19A,MIOX,IPCEF1,RRM2B,PRRC1,DENND6B,SLC12A2,AZIN1,ATP6V1C1,PLXNB2,SKIV2L,FBN2,ANGPT1,ISOC1,FAM19A5,TCP1,PPARA,ACSL6,HLA-DRA,P4HA2,SLC22A4,RNASET2,NUP50,TAF2,CALHM5,COL11A2,SLC39A7,ROS1,AFF4,NUS1,SLC35F1,HSPA4,SMDT1,SERINC1,SEC24A,FER1L6,TATDN1,RSPO3,CXCL14,FAM49B,PHF20L1,MRTFA,KCNK9,TRAPPC9,HABP4,PACSIN1,GNG5,SLC45A4,PTP4A3,REEP2,ADGRB1,LY6E,SCUBE3,PPARD,CTNNA1,TEAD3,SIL1,GOLM1,SUN2,CBY1,MKLN1,TMEM184B,C6orf89,UBQLN1,POLR2F,MTCH1,LGALS1,MDGA1,GGA1,KLHDC10,AHCYL2,TXN2,SH3GL2,PSAT1,GNAQ,FLNC,PLIN2,SLC39A4,ATP6V1F,MYH9,CALU,PRUNE2,ARHGAP39,MTAP,LEP,CDKN2B,PLAA,TBCC,LARGE1,PEX6,RORB,BTBD11,SLC30A9,KIAA1324L,SRF,BAG1,NUAK1,CDK14,FAM189A2,ALDH1L2,DCTN3,VEGFA,GALT,DOCK8,VCP,SLC35B2,RUNX2,RASL11B,UNC13B,RUSC2,COL1A2,TPM2,C2orf40,NCK2,CLOCK,DYNC1I1,EXOC1,CREB3,ASNS,PDCL3,CHST10,SRP72,REST,EIF5B,TXNDC9,ASPN,INPP4A,TMEM131,ZSCAN18,CNNM4,LMAN2L,DST,RAB23,SNX13,NANS,PTP4A1,NLRP5,COL15A1,P3H1,TGFBR1,ERP44,HIVEP3,ISOC2,TMEM246,SOCS5,RIMS1,MTO1,SLC17A5,LAIR1,TMEM30A,ERLEC1,PSME4,FAM126A,COPS9,CENPC,UBA6,RTN4,CFAP36,CYCS,TPBG,SHANK1,ADAMTS3,COX18,LRRC4B,EMC10,TAX1BP1,MFSD2A,TRIT1,SLC35A1,GGCT,GARS,MINDY4	darkgreen	EC_M4
GO:1902004	1.08E-01	0.969148785	4	14	positive regulation of amyloid-beta formation	BP	7.71E-01	ROCK2,GSAP,PICALM,CHRNA7	darkgreen	EC_M4
GO:0038061	1.08E-01	0.934000222	18	99	NIK/NF-kappaB signaling	BP	7.71E-01	IL23A,PTP4A3,MAP3K7,ACTN4,ILK,AKIP1,APP,PTPN22,ADIPOR1,BMP7,TSPAN6,UACA,DICER1,AKT1,IRAK1,AGO3,EDA,TMSB4X	darkgreen	EC_M4
GO:0003401	1.09E-01	0.958587469	6	24	axis elongation	BP	7.71E-01	RDH10,NKD1,FGFR2,SIX4,MED1,FGF10	darkgreen	EC_M4
GO:0032388	1.09E-01	0.925318998	30	173	positive regulation of intracellular transport	BP	7.71E-01	RAB21,LEP,TMEM30A,GLI3,KIF5B,PAK1,TGFB1,AKT2,PRNP,HDAC3,TM9SF4,RIOK2,CACNB3,ATG13,BAP1,CHP1,FEZ1,GSK3B,PDCD10,STX18,B3GAT3,MSN,MIEF2,PRKAA1,MYO1C,BCAP31,FLNA,LEPROT,ZPR1,TOMM7	darkgreen	EC_M4
GO:0008206	1.09E-01	0.951101821	8	36	bile acid metabolic process	BP	7.71E-01	ATP8B1,LEP,OSBP,ARV1,OSBPL2,HSD17B10,CYP46A1,AMACR	darkgreen	EC_M4
GO:0000413	1.09E-01	0.95397949	7	32	protein peptidyl-prolyl isomerization	BP	7.71E-01	FKBP1B,FKBP2,FKBP8,FKBP10,PIN1,RANBP2,FKBP1A	darkgreen	EC_M4
GO:0051098	1.09E-01	0.918272336	48	296	regulation of binding	BP	7.71E-01	LRP1,ANGPT1,PPARA,HABP4,TGFBR1,RB1,MAPRE3,SMARCD3,TGFB1,CTNNB1,DZIP1,DDRGK1,PPP2CB,PINX1,HEY1,ATP2A2,APP,RAPGEF2,AMFR,AKTIP,CBLB,CAPRIN2,GSK3B,LEF1,LRPAP1,GREM2,CRMP1,EIF2S1,NES,EIF3C,PBXIP1,CALM1,TEX14,MYOCD,PIN1,ROCK1,AKT1,ITGA2,PKD1,PARK7,GPSM1,FKBP1A,LOX,CDKN1A,TXN,TMSB4X,HOPX,MBD2	darkgreen	EC_M4
GO:0060562	1.09E-01	0.919084696	45	269	epithelial tube morphogenesis	BP	7.71E-01	AHI1,PLXNB2,SRF,VEGFA,GLI3,FGF2,GLI2,MEGF8,PAK1,TGFB1,CTNNB1,SPINT2,STARD13,ILK,TMED2,RDH10,TCTN1,OPA1,ACVR1,PKD2,IFT57,KDM5B,NOTCH2,TGFB2,LRP5,MMP14,DAG1,LEF1,KRAS,BMP7,FGFR2,CLIC4,FZD2,TULP3,SIX4,MAGED1,TCAP,MED1,PKD1,LMO4,BCL10,LBX1,GDNF,WNT2,FGF10	darkgreen	EC_M4
GO:0003170	1.10E-01	0.941477989	12	58	heart valve development	BP	7.71E-01	ROCK2,RB1,TGFB1,ROBO1,HEY1,ACVR1,NOTCH2,TGFB2,BMPR1A,FGFRL1,ROCK1,OLFM1	darkgreen	EC_M4
GO:0090025	1.10E-01	0.962343087	5	20	regulation of monocyte chemotaxis	BP	7.71E-01	CREB3,CXCL12,SLAMF8,SERPINE1,NBL1	darkgreen	EC_M4
GO:0033598	1.10E-01	0.957940711	6	24	mammary gland epithelial cell proliferation	BP	7.71E-01	RTN4,ZNF703,ROBO1,KDM5B,CCND1,MED1	darkgreen	EC_M4
GO:0007249	1.10E-01	0.921205806	37	222	I-kappaB kinase/NF-kappaB signaling	BP	7.71E-01	TAB2,ANGPT1,LGALS1,SLC35B2,MAP3K7,ROCK2,CTNNB1,CANT1,CXXC5,ATP2C1,DDRGK1,TMEM9B,SLC20A1,DHX36,CX3CL1,MTDH,TANK,HTR2B,GOLT1B,TFRC,PRDX4,HSPB1,ERC1,TSPAN6,SLC44A2,CD74,ROCK1,AKT1,IRAK1,FLNA,IKBKG,USP10,BCL10,WLS,FKBP1A,C18orf32,EDA	darkgreen	EC_M4
GO:0055070	1.10E-01	0.968420124	4	14	copper ion homeostasis	BP	7.71E-01	SLC31A1,ATP7B,PRNP,APP	darkgreen	EC_M4
GO:0031103	1.10E-01	0.947526561	9	42	axon regeneration	BP	7.71E-01	LRP1,CTNNA1,FKBP1B,MAP1B,SPP1,DAG1,TNR,MAP2K1,FLNA	darkgreen	EC_M4
GO:0001505	1.10E-01	0.926258692	26	147	regulation of neurotransmitter levels	BP	7.71E-01	UNC13B,RIMS1,ITGB1,LIN7B,NAPA,GAD2,RAB3GAP1,HNMT,PCLO,SNPH,ATP2A2,VPS18,DNM1L,GSK3B,BACE1,COMT,MAOA,SLC6A13,SLC29A4,CLN8,STX1B,SYT11,CALM1,PARK7,GDNF,KCNMB4	darkgreen	EC_M4
GO:0072028	1.11E-01	0.939222378	13	65	nephron morphogenesis	BP	7.71E-01	AHI1,VEGFA,GLI3,FGF2,CTNNB1,ILK,PKD2,BMP7,SIX4,MAGED1,PDGFRB,PKD1,GDNF	darkgreen	EC_M4
GO:0001755	1.11E-01	0.944967555	10	47	neural crest cell migration	BP	7.71E-01	SEMA3C,SEMA4F,ACVR1,SEMA4G,HTR2B,CORO1C,BMP7,FN1,ANXA6,GDNF	darkgreen	EC_M4

GO:0071816	1.11E-01	0.967737892	4	15	tail-anchored membrane protein insertion into ER membrane	BP	7.71E-01	EMC10,EMC4,EMC1,EMC7	darkgreen	EC_M4
GO:0006606	1.11E-01	0.927966489	23	129	protein import into nucleus	BP	7.71E-01	ANGPT1,NUP50,LEP,RAB23,MDFIC,GLI3,TGFB1,SEC13,UFM1,HDAC3,CRY2,CHP1,CBLB,KPNA6,KPNA1,MED1,AKT1,FLNA,RANBP2,KPNA4,CDKN1A,PTTG1IP,ZPR1	darkgreen	EC_M4
GO:0032941	1.11E-01	0.957202733	6	25	secretion by tissue	BP	7.71E-01	VAMP8,SLC4A5,ADA,COPA,NPR3,FGF10	darkgreen	EC_M4
GO:0050865	1.11E-01	0.913811797	61	385	regulation of cell activation	BP	7.71E-01	IL23A,ENPP3,HLA-DRA,FAM49B,LGALS1,LEP,DOCK8,NCK2,SOCS5,GLI3,INHBA,GLI2,TGFB1,AXL,CTNNB1,IL15RA,C1QTNF1,IRS2,PRNP,HSPH1,SPINK5,LMO1,PJA2,IL20RB,VAMP8,LOXL3,CX3CL1,PTPN11,AP3B1,PTPN22,ADA,RUNX1,CBLB,SLC39A10,PRKAR1A,MMP14,PIK3CA,LEF1,PRKCA,SLAMF8,TP53BP1,TFRC,PTPN6,IGFBP2,SYT11,CD74,PDGFRB,AKT1,SUPT6H,PTPN2,CD40LG,VAMP7,AP1G1,LMO4,BCL10,THY1,CDKN1A,FGL2,TSLP,PRKG1,FGF10	darkgreen	EC_M4
GO:0098659	1.11E-01	0.93713543	14	71	inorganic cation import across plasma membrane	BP	7.71E-01	SLC12A2,KCNK9,SLC39A4,SLC31A1,PRNP,ATP1B3,ATP2B4,SLC39A10,ISCU,SLC39A6,TRPV2,HCN2,MIR24-1,HCN4	darkgreen	EC_M4
GO:0099587	1.11E-01	0.93713543	14	71	inorganic ion import across plasma membrane	BP	7.71E-01	SLC12A2,KCNK9,SLC39A4,SLC31A1,PRNP,ATP1B3,ATP2B4,SLC39A10,ISCU,SLC39A6,TRPV2,HCN2,MIR24-1,HCN4	darkgreen	EC_M4
GO:0009987	1.12E-01	0.903350717	1604	11676	cellular process	BP	7.71E-01	CYB5A,SERPINB2,CD63,LMAN1,SEC11C,IL23A,ATP8B1,HSD17B6,NAB2,LRP1,STX7,AP3S1,CDO1,TRIM36,HBS1L,B4GALT7,AHI1,ARHGEF25,B4GALNT1,AGAP2,ABRACL,CANX,ENPP3,TAB2,TBC1D9B,CAND1,DYRK2,IER3,RAB21,CDSN,RABL2A,RNF19A,MIOX,IPCEF1,RRM2B,PRRC1,SLC12A2,AZIN1,ATP6V1C1,PLXNB2,SKIV2L,FBN2,ANGPT1,FAM19A5,TCP1,PPARA,ACSL6,HLA-DRA,P4HA2,SLC22A4,RNASET2,NUP50,TAF2,CALHM5,COL11A2,SLC39A7,ROS1,AFF4,NUS1,SLC35F1,HSPA4,SMDT1,SERINC1,SEC24A,FER1L6,TATDN1,RSPO3,CXCL14,FAM49B,PHF20L1,MRTFA,KCNK9,TRAPPC9,HABP4,PACSIN1,GNG5,PTP4A3,REEP2,ADGRB1,LY6E,SCUBE3,PPARD,CTNNA1,TEAD3,SIL1,GOLM1,SUN2,CBY1,MKLN1,C6orf89,UBQLN1,POLR2F,MTCH1,LGALS1,MDGA1,GGA1,KLHDC10,AHCYL2,TXN2,SH3GL2,PSAT1,GNAQ,FLNC,SLC39A4,ATP6V1F,MYH9,PRUNE2,ARHGAP39,MTAP,LEP,CDKN2B,PLAA,TBCC,LARGE1,PEX6,RORB,BTBD11,SLC30A9,KIAA1324L,SRF,BAG1,NUAK1,CDK14,ALDH1L2,DCTN3,VEGFA,GALT,DOCK8,VCP,SLC35B2,RUNX2,RASL11B,UNC13B,COL1A2,TPM2,C2orf40,NCK2,CLOCK,DYNC1I1,EXOC1,CREB3,ASNS,PDCL3,CHST10,SRP72,REST,EIF5B,TXNDC9,ASPN,INPP4A,ZSCAN18,CNNM4,LMAN2L,DST,RAB23,SNX13,NANS,PTP4A1,NLRP5,COL15A1,P3H1,TGFBR1,ERP44,HIVEP3,TMEM246,SOCS5,RIMS1,MTO1,SLC17A5,TMEM30A,ERLEC1,PSME4,FAM126A,COPS9,CENPC,UBA6,RTN4,CYCS,TPBG,SHANK1,ADAMTS3,COX18,LRRC4B,EMC10,TAX1BP1,MFSD2A,TRIT1,SLC35A1,GGCT,GARS,MINDY4,UBE2J1,MAP3K7,MYH14,SH3BP5L,CALD1,AKIRIN1,ACTR2,SLC31A1,RNASEH1,THAP5,GFPT1,GMCL1,SNRNP27,IFRD1,KIDINS220,WHRN,	darkgreen	EC_M4
GO:0055013	1.12E-01	0.938400577	13	65	cardiac muscle cell development	BP	7.71E-01	PPARA,SRF,VEGFA,BVES,MYLK3,PAK1,PDLIM5,OBSL1,BMPR1A,TCAP,PIN1,PDGFRB,MIR24-1	darkgreen	EC_M4
GO:0016331	1.12E-01	0.927355134	23	127	morphogenesis of embryonic epithelium	BP	7.71E-01	PLXNB2,GLI2,TGFB1,CTNNB1,SPINT2,TMED2,SULF1,RDH10,TCTN1,OPA1,IFT57,TGFB2,BMP7,FGFR2,FZD2,TULP3,SIX4,TGFB1I1,LMO4,BCL10,GDNF,WNT2,FGF10	darkgreen	EC_M4
GO:2000463	1.12E-01	0.956932736	6	24	positive regulation of excitatory postsynaptic potential	BP	7.71E-01	RIMS1,SHANK1,CACNB3,CHRNA7,NLGN2,STX1B	darkgreen	EC_M4
GO:0050771	1.12E-01	0.941954471	11	52	negative regulation of axonogenesis	BP	7.71E-01	RTN4,SEMA3C,RYK,RNF6,DIP2B,SEMA4F,SPP1,SEMA4G,TNR,PLXNA3,THY1	darkgreen	EC_M4
GO:0006700	1.12E-01	0.967588276	4	14	C21-steroid hormone biosynthetic process	BP	7.71E-01	REST,DKK3,STARD3,PPARGC1A	darkgreen	EC_M4
GO:1901605	1.13E-01	0.92484502	25	146	alpha-amino acid metabolic process	BP	7.71E-01	CDO1,AZIN1,PSAT1,MTAP,ASNS,GFPT1,SLC7A11,GAD2,PLOD2,ALDH18A1,SERINC3,GLS,ATP2B4,PSPH,AZIN2,SMS,PLOD3,COMT,QDPR,ASRGL1,AGMAT,HSD17B10,PARK7,PEMT,ADI1	darkgreen	EC_M4
GO:0060021	1.13E-01	0.935967295	14	71	roof of mouth development	BP	7.71E-01	COL11A2,TGFBR1,GLI3,INHBA,LOXL3,TGFB2,SATB2,LEF1,ARID5B,SGPL1,FZD2,PRRX1,BMPR1A,TGFB3	darkgreen	EC_M4
GO:0048640	1.13E-01	0.931733909	17	90	negative regulation of developmental growth	BP	7.71E-01	PPARA,RTN4,PAK1,SEMA3C,RYK,RNF6,WWC2,DIP2B,SEMA4F,SPP1,SEMA4G,NKD1,WWC3,TNR,FGFR3,PLXNA3,CDKN1A	darkgreen	EC_M4

GO:0051291	1.14E-01	0.960934176	5	19	protein heterooligomerization	BP	7.71E-01	COL1A2,PKD2,CALHM1,ROM1,PKD1	darkgreen	EC_M4
GO:0010559	1.14E-01	0.951728013	7	31	regulation of glycoprotein biosynthetic process	BP	7.71E-01	ITM2B,CTNNB1,CHP1,SLC2A10,NCSTN,POMT1,ITM2C	darkgreen	EC_M4
GO:1902042	1.14E-01	0.955441521	6	26	negative regulation of extrinsic apoptotic signaling pathway via death domain receptors	BP	7.71E-01	FAIM2,GSK3B,PEA15,SERPINE1,ARHGEF2,PARK7	darkgreen	EC_M4
GO:0009144	1.14E-01	0.938380483	12	63	purine nucleoside triphosphate metabolic process	BP	7.71E-01	PPARA,VCP,RAB23,TGFB1,NUDT16,OPA1,ADA,PPARGC1A,ENO1,GNAI3,TMSB4X,NME6	darkgreen	EC_M4
GO:0002694	1.14E-01	0.912266165	56	352	regulation of leukocyte activation	BP	7.71E-01	IL23A,ENPP3,HLA-DRA,FAM49B,LGALS1,LEP,DOCK8,NCK2,SOCS5,GLI3,INHBA,GLI2,TGFB1,AXL,CTNNB1,IL15RA,IRS2,PRNP,HSPH1,SPINK5,LMO1,PJA2,IL20RB,VAMP8,LOXL3,CX3CL1,PTPN11,AP3B1,PTPN22,ADA,RUNX1,CBLB,SLC39A10,PRKAR1A,MMP14,PIK3CA,LEF1,SLAMF8,TP53BP1,TFRC,PTPN6,IGFBP2,SYT11,CD74,AKT1,SUPT6H,PTPN2,CD40LG,VAMP7,AP1G1,BCL10,THY1,CDKN1A,FGL2,TSLP,FGF10	darkgreen	EC_M4
GO:0010812	1.14E-01	0.940449496	11	53	negative regulation of cell-substrate adhesion	BP	7.71E-01	LRP1,LGALS1,ACTN4,RASA1,CX3CL1,PHLDB2,MMP14,CORO1C,SERPINE1,COL1A1,DUSP22	darkgreen	EC_M4
GO:0010596	1.14E-01	0.942567188	10	48	negative regulation of endothelial cell migration	BP	7.71E-01	ADGRB1,FGF2,TGFB1,DCN,STARD13,ATP2B4,DNAJA4,PDCD10,MIR24-1,SERPINF1	darkgreen	EC_M4
GO:0030534	1.14E-01	0.931056849	17	90	adult behavior	BP	7.71E-01	PPARA,LEP,SHANK1,SLC7A11,ABHD12,PCDH17,MAFG,CXCL12,LGI4,APP,GLRB,HOMER1,NCSTN,CLN8,PAFAH1B1,PARK7,GDNF	darkgreen	EC_M4
GO:0046889	1.14E-01	0.938272653	12	61	positive regulation of lipid biosynthetic process	BP	7.71E-01	PPARA,MFSD2A,DGAT2,RDH10,SORBS1,CHP1,ZBTB20,HTR2B,PPARGC1A,CD74,AKT1,PRKAA1	darkgreen	EC_M4
GO:0051493	1.15E-01	0.909079827	73	457	regulation of cytoskeleton organization	BP	7.71E-01	LRP1,TRIM36,FAM49B,NCK2,TGFBR1,SHANK1,ACTR2,ROCK2,MYLK3,EML2,MAPRE3,PAK1,ACTR3,PLEKHG2,ACTG1,NAV3,KAT2B,CDC42EP3,ILK,CXCL12,MAP1B,PFN2,RASA1,CX3CL1,TWF1,MAGEL2,ARL3,PHLDB2,TJP1,NOTCH2,CTTN,LMOD1,GSK3B,PIK3CA,NTRK3,SYNPO2,PHLDB1,IQGAP1,CLIC4,ARPC5,MAPT,NCKAP1,ARPC2,SYNPO2L,CAPZB,WDR1,CGNL1,NES,ARHGEF2,TPM1,TGFB3,GPR65,TTC8,CLTC,PDGFRB,ROCK1,FLII,PRKAA1,TUBB4A,DAPK3,MYO1C,PAFAH1B1,PKD1,FLNA,PLXNA3,SPTAN1,NEXN,SKA2,TMSB4X,DSTN,MAP1A,COTL1,CFL2	darkgreen	EC_M4
GO:0001709	1.15E-01	0.951436234	7	30	cell fate determination	BP	7.71E-01	IFRD1,PTCH2,CTNNB1,NOTCH2,PRRX1,BARHL2,LBX1	darkgreen	EC_M4
GO:0019320	1.15E-01	0.955360856	6	25	hexose catabolic process	BP	7.71E-01	GALT,GLB1,PFKP,PFKM,LRP5,ENO1	darkgreen	EC_M4
GO:0071396	1.15E-01	0.909709683	67	422	cellular response to lipid	BP	7.71E-01	PPARA,PPARD,LEP,PLAA,RORB,CLOCK,REST,ROCK2,INHBA,MGARP,PGRMC2,PAK1,TGFB1,AXL,LBH,DGAT2,UFM1,ATP2B1,ZNF703,UBA5,DDRGK1,RNF6,ANKRD1,CD180,HEY1,CX3CL1,ADCY6,PTPN22,CRY2,VPS18,MTDH,SPP1,EIF4E,CDC73,DNM1L,SELENOS,CPT1A,DAG1,PRKCA,NTRK3,BMP7,ADCY5,FGFR2,STRN3,SERPINE1,KCNK4,UPF1,SGMS1,MED1,PPARGC1A,MSN,KCNMB1,COL1A1,SMARCA4,AKT1,ITGA2,LDOC1,IRAK1,SSTR5,PARK7,BCL10,SERPINF1,IL36G,FAM210B,YWHAH,PMEPA1,WNT2	darkgreen	EC_M4
GO:0038034	1.16E-01	0.941824406	10	49	signal transduction in absence of ligand	BP	7.71E-01	CTNNA1,INHBA,CX3CL1,EYA2,GSK3B,ITGAV,AKT1,DAPK3,GDNF,FGF10	darkgreen	EC_M4
GO:0097192	1.16E-01	0.941824406	10	49	extrinsic apoptotic signaling pathway in absence of ligand	BP	7.71E-01	CTNNA1,INHBA,CX3CL1,EYA2,GSK3B,ITGAV,AKT1,DAPK3,GDNF,FGF10	darkgreen	EC_M4
GO:0003015	1.16E-01	0.919353363	31	180	heart process	BP	7.71E-01	BVES,FKBP1B,SCN5A,GPD1L,ATP2B1,FXYD1,SNTA1,P2RX4,ATP2A2,CACNA1D,ADA,ATP2B4,PKP2,DNM1L,TGFB2,PIK3CA,GLRX3,CACNA1C,HSPB7,TCAP,TPM1,CACNA1G,ADRA1B,CALM1,SGCD,FLNA,DMD,TMEM38B,TMEM38A,HOPX,HCN4	darkgreen	EC_M4
GO:0048593	1.16E-01	0.928710526	18	96	camera-type eye morphogenesis	BP	7.71E-01	AHI1,RORB,VEGFA,MFSD2A,GLI3,CTNNB1,FAT1,NOTCH2,LRP5,SHROOM2,BMP7,OBSL1,ROM1,TTC8,BCAR3,THY1,WNT2,COL8A2	darkgreen	EC_M4

GO:0045671	1.16E-01	0.959768678	5	19	negative regulation of osteoclast differentiation	BP	7.71E-01	CTNNB1,TCTA,GPR137,FBN1,FSTL3	darkgreen	EC_M4
GO:0001754	1.16E-01	0.950491657	7	30	eye photoreceptor cell differentiation	BP	7.71E-01	RORB,VEGFA,NKD1,PRKCI,ROM1,TTC8,THY1	darkgreen	EC_M4
GO:0007338	1.16E-01	0.930880774	16	86	single fertilization	BP	7.71E-01	TRIM36,TCP1,MYH9,UNC13B,NLRP5,RIMS1,DKKL1,WDR48,GLRB,CCT8,AP3B1,KDM5B,VDAC2,PARK7,CCDC136,IGSF8	darkgreen	EC_M4
GO:0048259	1.16E-01	0.930896458	16	85	regulation of receptor-mediated endocytosis	BP	7.71E-01	CD63,AHI1,RAB21,ANGPT1,MKLN1,SH3GL2,VEGFA,PICALM,ATXN2,AP2M1,SERPINE1,ITGAV,LRPAP1,ATAD1,SYT11,LRRTM2	darkgreen	EC_M4
GO:1904380	1.17E-01	0.96590991	4	14	endoplasmic reticulum mannose trimming	BP	7.71E-01	AMFR,MARCH6,EDEM3,SYVN1	darkgreen	EC_M4
GO:0072075	1.17E-01	0.965822133	4	14	metanephric mesenchyme development	BP	7.71E-01	PKD2,BMP7,SIX4,PDGFRB	darkgreen	EC_M4
GO:0044106	1.17E-01	0.933358782	14	74	cellular amine metabolic process	BP	7.71E-01	AZIN1,LY6E,SLC7A11,HNMT,ATP2B4,AZIN2,TGFB2,SMS,COMT,MAOA,SLC29A4,AGMAT,PARK7,NNMT	darkgreen	EC_M4
GO:0006096	1.17E-01	0.936767318	12	60	glycolytic process	BP	7.71E-01	PPARA,PFKP,APP,PFKM,PGAM4,ZBTB20,TPI1,PPARGC1A,PKM,PRKAA1,ENO1,SLC2A6	darkgreen	EC_M4
GO:0006757	1.17E-01	0.936767318	12	60	ATP generation from ADP	BP	7.71E-01	PPARA,PFKP,APP,PFKM,PGAM4,ZBTB20,TPI1,PPARGC1A,PKM,PRKAA1,ENO1,SLC2A6	darkgreen	EC_M4
GO:0010763	1.17E-01	0.965723702	4	14	positive regulation of fibroblast migration	BP	7.71E-01	ITGB1,PAK1,TGFB1,AKT1	darkgreen	EC_M4
GO:0097696	1.17E-01	0.926871639	19	105	receptor signaling pathway via STAT	BP	7.71E-01	IL23A,LEP,SOCS5,TGFB1,CNOT7,OSBP,PKD2,ADIPOR1,OSM,FGFR3,ELP2,CRLF3,NLK,PTPN2,TNFRSF18,PKD1,TSLP,OCIAD2,LEPROT	darkgreen	EC_M4
GO:0015813	1.17E-01	0.959312675	5	19	L-glutamate transmembrane transport	BP	7.71E-01	SLC7A11,ITGB1,ARL6IP5,PRAF2,CLN8	darkgreen	EC_M4
GO:0016482	1.17E-01	0.920520987	27	153	cytosolic transport	BP	7.71E-01	RAB21,GGA1,ACTR2,VPS35,KIF5B,RAB7A,WASHC2A,ARL1,TMEM87B,AKTIP,MAGEL2,RAB7B,WIPI1,KIF1A,TANC2,CLTCL1,ERC1,SNX8,MSN,MAP2K1,CLTC,MYO1D,FLNA,AP1G1,RAB6A,VPS26B,ARFRP1	darkgreen	EC_M4
GO:1903038	1.17E-01	0.930161639	16	87	negative regulation of leukocyte cell-cell adhesion	BP	7.71E-01	PPARA,SOCS5,GLI3,PRNP,CXCL12,IL20RB,LOXL3,PTPN22,RUNX1,CBLB,PRKAR1A,PTPN6,CD74,AKT1,PTPN2,FGL2	darkgreen	EC_M4
GO:0042110	1.18E-01	0.910981354	50	310	T cell activation	BP	7.71E-01	IL23A,HLA-DRA,FAM49B,LGALS1,MYH9,LEP,SRF,DOCK8,RUNX2,NCK2,SOCS5,GLI3,FKBP1B,APBB1IP,GLI2,CTNNB1,LIG4,PRNP,HSPH1,SPINK5,LMO1,IL20RB,LOXL3,PTPN11,AP3B1,PTPN22,ADA,RUNX1,CBLB,PRKAR1A,PIK3CA,LEF1,NCSTN,TFRC,PTPN6,IGFBP2,DDOST,MSN,DOCK2,CD74,AKT1,TMEM98,PTPN2,CD40LG,BCL10,FKBP1A,CD151,THY1,FGL2,CCR6	darkgreen	EC_M4
GO:0007259	1.18E-01	0.927590699	18	98	receptor signaling pathway via JAK-STAT	BP	7.71E-01	IL23A,LEP,SOCS5,CNOT7,OSBP,PKD2,ADIPOR1,OSM,FGFR3,ELP2,CRLF3,NLK,PTPN2,TNFRSF18,PKD1,TSLP,OCIAD2,LEPROT	darkgreen	EC_M4
GO:0030282	1.18E-01	0.927542483	18	97	bone mineralization	BP	7.71E-01	FBN2,LEP,COL1A2,ASPN,TGFB1,ATP2B1,ACVR1,SBDS,BMP7,FGFR2,FZD9,COMP,FGFR3,BMPR1A,TGFB3,TMEM38B,LOX,SNX10	darkgreen	EC_M4
GO:0003333	1.18E-01	0.934403794	13	66	amino acid transmembrane transport	BP	7.71E-01	SLC22A4,SLC7A11,ITGB1,SLC36A4,SLC38A10,ARL6IP5,SLC3A2,SLC6A13,PRAF2,CLN8,SFXN1,SLC7A3,SLC36A1	darkgreen	EC_M4
GO:0010934	1.18E-01	0.965166805	4	14	macrophage cytokine production	BP	7.71E-01	TGFB1,TGFB2,TGFB3,CD74	darkgreen	EC_M4
GO:0010935	1.18E-01	0.965166805	4	14	regulation of macrophage cytokine production	BP	7.71E-01	TGFB1,TGFB2,TGFB3,CD74	darkgreen	EC_M4

GO:0032269	1.19E-01	0.900553755	115	760	negative regulation of cellular protein metabolic process	BP	7.71E-01	SERPINB2,LRP1,ANGPT1,ADGRB1,SH3GL2,GNAQ,CDKN2B,PLAA,VEGFA,NCK2,PDCL3,P3H1,SOCS5,COPS9,SH3BP5L,ROCK2,FKBP1B,DNAJC1,RB1,PICALM,CRTAP,TGFB1,SPOPL,CTNNB1,SERPINH1,GPD1L,KAT2B,CRIM1,SPINT2,PRNP,DDRGK1,SMPD1,ILK,SPINK5,CNOT7,FXYD1,SNTA1,TMED2,PINX1,CELF1,APP,DHX36,PTPN22,TSG101,CRY2,CHAC1,CHP1,ATP2B4,DBNDD2,CBLB,TENT4B,EIF4E,TM4SF20,UCHL5,TRIP12,CAPRIN2,LRP5,EPRS,PRKAR1A,GSK3B,CORO1C,ANOS1,NTRK3,BMP7,PPP1R11,COL6A3,RPS6KA3,HSPB1,MAPT,SERPINE1,EIF4EBP2,DNAJC10,PTPN6,UPF1,FKBP8,SAMD4A,TIMP1,CLN8,EIF2S1,PPARGC1A,SRCIN1,CPEB4,TMED10,CALM1,PPP4R4,GSKIP,MYOCD,ROCK1,USP14,AKT1,TMEM98,PRKAA1,SUPT6H,PTPN2,NXN,DAPK3,PARK7,GLG1,MIR24-1,PRMT6,DMD,FKBP1A,SPINK2,SERPINF1,SERPINB5,AGO3,A2M,FRY,TIMP3,THY1,CDKN1A,DUSP22,PMEPA1,GABARAPL2,MAP1A	darkgreen	EC_M4
GO:0051495	1.20E-01	0.915347879	33	194	positive regulation of cytoskeleton organization	BP	7.71E-01	NCK2,TGFBR1,ACTR2,ROCK2,MYLK3,PAK1,ACTR3,NAV3,CDC42EP3,MAP1B,PFN2,CX3CL1,MAGEL2,ARL3,CTTN,LMOD1,NTRK3,SYNPO2,ARPC5,MAPT,NCKAP1,ARPC2,SYNPO2L,WDR1,NES,TPM1,TGFB3,GPR65,MYO1C,FLNA,PLXNA3,DSTN,CFL2	darkgreen	EC_M4
GO:0071346	1.20E-01	0.929759157	15	80	cellular response to interferon-gamma	BP	7.71E-01	ACTR2,ZYX,KIF5B,ACTR3,ACTG1,FLNB,CX3CL1,RAB7B,EPRS,MED1,PTPN2,DAPK3,MYO1C,CCL16,VPS26B	darkgreen	EC_M4
GO:0061437	1.20E-01	0.952667357	6	25	renal system vasculature development	BP	7.71E-01	ANGPT1,PKD2,NOTCH2,BMP7,ACTA2,PDGFRB	darkgreen	EC_M4
GO:0061440	1.20E-01	0.952667357	6	25	kidney vasculature development	BP	7.71E-01	ANGPT1,PKD2,NOTCH2,BMP7,ACTA2,PDGFRB	darkgreen	EC_M4
GO:0061311	1.20E-01	0.952646032	6	25	cell surface receptor signaling pathway involved in heart development	BP	7.71E-01	TGFB1,CTNNB1,HEY1,ACVR1,NOTCH2,BMPR1A	darkgreen	EC_M4
GO:0045116	1.20E-01	0.96403801	4	15	protein neddylation	BP	7.71E-01	COPS9,UBA3,NEDD8,NAE1	darkgreen	EC_M4
GO:1903020	1.20E-01	0.957615836	5	20	positive regulation of glycoprotein metabolic process	BP	7.71E-01	CTNNB1,CHP1,SLC2A10,NCSTN,POMT1	darkgreen	EC_M4
GO:0045765	1.21E-01	0.912043734	38	229	regulation of angiogenesis	BP	7.71E-01	SLC12A2,FAM19A5,ADGRB1,LEP,VEGFA,PDCL3,EMC10,ROCK2,RRAS,ITGB1,FGF2,CTNNB1,STIM1,DCN,SPINK5,SULF1,ABCC8,MTDH,ATP2B4,RUNX1,CHRNA7,TJP1,HYAL1,TGFB2,ADGRB2,PRKCA,VASH2,HSPB1,SERPINE1,AKT3,NPPB,PKM,ROCK1,MYDGF,SARS,F3,MIR24-1,SERPINF1	darkgreen	EC_M4
GO:0044092	1.21E-01	0.898189071	121	806	negative regulation of molecular function	BP	7.71E-01	SERPINB2,LRP1,CAND1,PPARA,HABP4,LY6E,UBQLN1,GNAQ,CDKN2B,VEGFA,SOCS5,TAX1BP1,SH3BP5L,ROCK2,DKKL1,FKBP1B,RB1,PICALM,SERPINH1,PPP1R12A,KAT2B,CRIM1,GPS1,SPINT2,IRS2,PRNP,SMPD1,ILK,SPINK5,REM1,TMED2,DKK3,PINX1,HEY1,ATP2A2,RTKN,APP,CACNB3,PTX3,PTPN22,TSG101,CRY2,CHP1,ATP2B4,PKD2,PLA2R1,DBNDD2,CBLB,NDN,DYNLL1,PPP1R12B,UCHL5,TGFB2,LRP5,PRKAR1A,GSK3B,LEF1,CORO1C,ANOS1,CMKLR1,BMP7,PPP1R11,WDTC1,COL6A3,IQGAP1,RPS6KA3,HSPB1,LINC02210-CRHR1,MAPT,SERPINE1,PTPN6,ITGAV,LRPAP1,AOC3,TIMP1,MNAT1,CRMP1,PPP1R36,EIF2S1,PPARGC1A,SRCIN1,NES,TMED10,TTC8,CALM1,TEX14,PPP4R4,GSKIP,MYOCD,PIN1,ROCK1,USP14,AKT1,PTPN2,NPR3,FAM122B,PLXNB3,PKD1,IRAK1,FLNA,MIDN,PARK7,GPSM1,GNAI3,MIR24-1,FKBP1A,CBARP,SPINK2,SERPINF1,SERPINB5,A2M,FRY,TIMP3,PPP1R14A,THY1,PPP1R1A,CDKN1A,TMSB4X,DUSP22,ORMDL3,GEM	darkgreen	EC_M4
GO:0010524	1.22E-01	0.943855968	8	37	positive regulation of calcium ion transport into cytosol	BP	7.71E-01	P2RX4,CX3CL1,PKD2,CASQ1,HTT,HAP1,CALM1,THY1	darkgreen	EC_M4
GO:0090110	1.22E-01	0.96362263	4	14	COPII-coated vesicle cargo loading	BP	7.71E-01	SEC24A,SEC13,SEC23B,SEC23A	darkgreen	EC_M4
GO:0032026	1.23E-01	0.963225552	4	14	response to magnesium ion	BP	7.71E-01	ENTPD6,RYR3,CCND1,KCNA1	darkgreen	EC_M4
GO:0019395	1.23E-01	0.926025737	16	87	fatty acid oxidation	BP	7.71E-01	PPARA,PPARD,LEP,MFSD2A,DGAT2,AKT2,IRS2,ADIPOR1,CPT1A,HADH,ACOX3,HSD17B10,PPARGC1A,AKT1,PRKAA1,AMACR	darkgreen	EC_M4

GO:0003198	1.23E-01	0.963159517	4	14	epithelial to mesenchymal transition involved in endocardial cushion formation	BP	7.71E-01	TGFBR1,ACVR1,TGFB2,TMEM100	darkgreen	EC_M4
GO:0009755	1.24E-01	0.917097443	25	142	hormone-mediated signaling pathway	BP	7.71E-01	PPARA,PPARD,CLOCK,PGRMC2,PAK1,LBH,RARB,UFM1,UBA5,DDRGK1,RNF6,PTPN11,GTF2H1,CRY2,VPS18,ADIPOR1,BMP7,STRN3,LINC02210-CRHR1,MED1,SMARCA4,SSTR5,PARK7,YWHAH,PMEPA1	darkgreen	EC_M4
GO:0009268	1.24E-01	0.946302188	7	31	response to pH	BP	7.71E-01	ABCC8,ARSB,CHP1,HYAL1,KCNK4,GPR65,SERPINF1	darkgreen	EC_M4
GO:0002761	1.24E-01	0.925526531	16	86	regulation of myeloid leukocyte differentiation	BP	7.71E-01	IL23A,INHBA,RB1,TGFB1,CTNNB1,RUNX1,NOTCH2,LEF1,PRKCA,TCTA,GPR137,FBN1,CD74,PTPN2,ATP6AP1,FSTL3	darkgreen	EC_M4
GO:0045176	1.24E-01	0.962838853	4	14	apical protein localization	BP	7.71E-01	ATP8B1,NAPA,SHROOM2,ARF4	darkgreen	EC_M4
GO:0021756	1.24E-01	0.962816974	4	14	striatum development	BP	7.71E-01	INHBA,SLC7A11,RARB,ZSWIM6	darkgreen	EC_M4
GO:0045217	1.25E-01	0.962670359	4	14	cell-cell junction maintenance	BP	7.71E-01	WHRN,PLEKHA7,TJP1,NLGN2	darkgreen	EC_M4
GO:0060411	1.25E-01	0.931863557	12	59	cardiac septum morphogenesis	BP	7.71E-01	TGFBR1,SEMA3C,ROBO1,HEY1,ACVR1,NOTCH2,TGFB2,BMP7,FGFR2,FZD2,BMPR1A,FGFRL1	darkgreen	EC_M4
GO:1904427	1.25E-01	0.933752065	11	55	positive regulation of calcium ion transmembrane transport	BP	7.71E-01	STIM1,CX3CL1,CACNB3,PKD2,CASQ1,HTT,HAP1,STIM2,CALM1,VMP1,THY1	darkgreen	EC_M4
GO:1903729	1.25E-01	0.962519006	4	14	regulation of plasma membrane organization	BP	7.71E-01	ATP8B1,SH3GL2,MYH9,SYTL4	darkgreen	EC_M4
GO:0043535	1.25E-01	0.924777039	16	87	regulation of blood vessel endothelial cell migration	BP	7.71E-01	ANGPT1,NUS1,VEGFA,FGF2,TGFB1,STARD13,P2RX4,ATP2B4,PRKCA,HSPB1,PDCD10,AKT3,AKT1,MIR24-1,TMSB4X,PIK3C2A	darkgreen	EC_M4
GO:0010035	1.25E-01	0.900652689	68	435	response to inorganic substance	BP	7.71E-01	CYB5A,SLC12A2,VCP,FKBP1B,SELENOW,ATP7B,ENTPD6,AXL,SCN5A,STIM1,LIG4,PRNP,SMPD1,PPP2CB,MAP1LC3A,MAP1B,RYR3,P2RX4,APP,DHX36,ABCC8,PTPN11,HOMER1,LOXL2,ADA,BAP1,ATP2B4,PKD2,GLRX2,CCND1,FBLN5,PIK3CA,BMP7,NCSTN,IQGAP1,UROS,CLIC4,BACE1,MAPT,TFR2,IGFBP2,PDCD10,HSD17B1,QDPR,KCNA1,MNAT1,EIF2S1,PPARGC1A,SYT11,KCNMB1,COL1A1,CACNA1G,CALM1,GRIA1,PDGFRB,AKT1,PRKAA1,PARK7,BSG,AHCYL1,HSPA5,FSTL3,SERPINF1,BNIP3,CACYBP,SIGMAR1,TXN,KCNMB4	darkgreen	EC_M4
GO:0021924	1.25E-01	0.962313758	4	14	cell proliferation in external granule layer	BP	7.71E-01	FGF2,GLI2,PSMG1,RERE	darkgreen	EC_M4
GO:0021930	1.25E-01	0.962313758	4	14	cerebellar granule cell precursor proliferation	BP	7.71E-01	FGF2,GLI2,PSMG1,RERE	darkgreen	EC_M4
GO:0046676	1.25E-01	0.945393851	7	31	negative regulation of insulin secretion	BP	7.71E-01	REST,FKBP1B,ABCC8,PTPN11,HADH,SYTL4,MIDN	darkgreen	EC_M4
GO:0061035	1.26E-01	0.931041813	12	61	regulation of cartilage development	BP	7.71E-01	LEP,RUNX2,TGFBR1,GLI3,GLI2,CTNNB1,RARB,PTPN11,LOXL2,GDF6,BMP1,GLG1	darkgreen	EC_M4
GO:0002065	1.26E-01	0.931008322	12	61	columnar/cuboidal epithelial cell differentiation	BP	7.71E-01	ROS1,CLOCK,FGF2,RHEB,RARB,GSK3B,FAM20C,FGFR2,SIDT2,SERPINE1,AKT1,CDKN1A	darkgreen	EC_M4
GO:0150146	1.26E-01	0.962130216	4	14	cell junction disassembly	BP	7.71E-01	TGFBR1,CX3CL1,ABCC8,TGFB3	darkgreen	EC_M4
GO:0048524	1.26E-01	0.933065438	11	55	positive regulation of viral process	BP	7.71E-01	PPARA,LGALS1,MDFIC,SRPK2,RAB7A,AXL,CNOT7,TSG101,KPNA6,VAPB,CD74	darkgreen	EC_M4
GO:0097107	1.26E-01	0.962083886	4	14	postsynaptic density assembly	BP	7.71E-01	LRRC4B,NTRK3,NLGN2,LRRTM2	darkgreen	EC_M4
GO:0032413	1.26E-01	0.935415733	10	49	negative regulation of ion transmembrane transporter activity	BP	7.71E-01	UBQLN1,FKBP1B,REM1,CACNB3,PKD2,LINC02210-CRHR1,CALM1,MIR24-1,CBARP,GEM	darkgreen	EC_M4
GO:0072655	1.26E-01	0.921501094	18	103	establishment of protein localization to mitochondrion	BP	7.71E-01	LMAN1,HSPA4,COX18,MGARP,ATG13,AP3B1,BAP1,TOMM40L,MAPT,C11orf65,CALM1,AKT1,PRKAA1,BCAP31,TIMM22,LEPROT,GRPEL2,TOMM7	darkgreen	EC_M4

GO:0002687	1.27E-01	0.921122245	18	101	positive regulation of leukocyte migration	BP	7.71E-01	VEGFA,DOCK8,CREB3,AKIRIN1,CXCL12,P2RX4,APP,CX3CL1,DNM1L,MMP14,CMKLR1,SERPINE1,ITGA2B,CD74,ITGA2,TNFRSF18,THY1,CCR6	darkgreen	EC_M4
GO:0007040	1.27E-01	0.930419108	12	61	lysosome organization	BP	7.71E-01	RAB7A,ACPP,ARSB,AP3B1,VPS18,AKTIP,RAB7B,ATP6AP2,CLN8,CLN6,RAB34,MBTPS1	darkgreen	EC_M4
GO:0080171	1.27E-01	0.930419108	12	61	lytic vacuole organization	BP	7.71E-01	RAB7A,ACPP,ARSB,AP3B1,VPS18,AKTIP,RAB7B,ATP6AP2,CLN8,CLN6,RAB34,MBTPS1	darkgreen	EC_M4
GO:2000651	1.27E-01	0.961781999	4	14	positive regulation of sodium ion transmembrane transporter activity	BP	7.71E-01	KIF5B,ACTN4,CHP1,DMD	darkgreen	EC_M4
GO:0048568	1.27E-01	0.901999638	55	341	embryonic organ development	BP	7.71E-01	AHI1,FBN2,RSPO3,TEAD3,SRF,VEGFA,RUNX2,TGFBR1,WHRN,GLI3,GLI2,MEGF8,TGFB1,WDR48,CTNNB1,RARB,SPINT2,TMED2,TEAD1,RDH10,HEY1,ACVR1,ADA,PKD2,IFT57,NOTCH2,HYAL1,TGFB2,SATB2,MMP14,LEF1,BMP7,FGFR2,VASH2,FZD2,FBN1,PRRX1,TULP3,SIX4,BMPR1A,TCAP,MED1,NES,TGFB3,MAP2K1,AKT1,PKD1,COL11A1,LBX1,GDNF,SLC39A3,CHST11,WNT2,FGF10,MBD2	darkgreen	EC_M4
GO:0051899	1.27E-01	0.928045572	13	66	membrane depolarization	BP	7.71E-01	SCN5A,GPD1L,DCN,SLMAP,P2RX4,CACNA1D,FZD9,CACNA1C,CACNA1G,PARK7,HCN2,YWHAH,HCN4	darkgreen	EC_M4
GO:0006909	1.27E-01	0.912021098	28	163	phagocytosis	BP	7.71E-01	LRP1,ADGRB1,MYH9,LEP,ITGB1,RAB7A,PAK1,AXL,IL15RA,TM9SF4,PTX3,PIP4P2,TXNDC5,RAB7B,ELMO2,PIK3CA,CORO1C,ITGAV,LMAN2,SYT11,DOCK2,ICAM5,ITGA2,RAB31,RAB34,VAMP7,FCN1,MESD	darkgreen	EC_M4
GO:0051385	1.28E-01	0.948563419	6	26	response to mineralocorticoid	BP	7.71E-01	ATP2B1,CCND1,KRAS,NTRK3,CALM1,CDKN1A	darkgreen	EC_M4
GO:0060351	1.28E-01	0.948696633	6	25	cartilage development involved in endochondral bone morphogenesis	BP	7.71E-01	SERPINH1,RARB,COMP,THBS3,COL1A1,ANXA6	darkgreen	EC_M4
GO:0031663	1.28E-01	0.936820781	9	44	lipopolysaccharide-mediated signaling pathway	BP	7.71E-01	TGFB1,CD180,CX3CL1,PTPN22,MTDH,PRKCA,AKT1,IRAK1,BCL10	darkgreen	EC_M4
GO:1901099	1.28E-01	0.948252376	6	26	negative regulation of signal transduction in absence of ligand	BP	7.71E-01	CTNNA1,CX3CL1,EYA2,AKT1,GDNF,FGF10	darkgreen	EC_M4
GO:2001240	1.28E-01	0.948252376	6	26	negative regulation of extrinsic apoptotic signaling pathway in absence of ligand	BP	7.71E-01	CTNNA1,CX3CL1,EYA2,AKT1,GDNF,FGF10	darkgreen	EC_M4
GO:0045737	1.29E-01	0.947822796	6	26	positive regulation of cyclin-dependent protein serine/threonine kinase activity	BP	7.71E-01	MAPRE3,PKD2,CCND1,MNAT1,AKT1,PKD1	darkgreen	EC_M4
GO:0035809	1.29E-01	0.96027367	4	15	regulation of urine volume	BP	7.71E-01	ADCY6,NPPB,NPR3,HAS2	darkgreen	EC_M4
GO:0061951	1.29E-01	0.930980248	11	55	establishment of protein localization to plasma membrane	BP	7.71E-01	GGA1,VPS35,RAB7A,ATP2C1,GORASP2,BLZF1,RAB11A,RAB31,RAB34,C16orf70,ARFRP1	darkgreen	EC_M4
GO:2000649	1.29E-01	0.936068732	9	43	regulation of sodium ion transmembrane transporter activity	BP	7.71E-01	KIF5B,GPD1L,ACTN4,FXYD1,SLMAP,CHP1,MIR24-1,DMD,YWHAH	darkgreen	EC_M4
GO:0046649	1.30E-01	0.896195178	73	470	lymphocyte activation	BP	7.71E-01	IL23A,HLA-DRA,FAM49B,LGALS1,MYH9,LEP,SRF,DOCK8,RUNX2,NCK2,SOCS5,GLI3,INHBA,ITGB1,FKBP1B,APBB1IP,GLI2,TGFB1,AXL,CTNNB1,IL15RA,LIG4,IRS2,PRNP,HSPH1,SPINK5,LMO1,IL20RB,CD180,LOXL3,PTPN11,CDH17,AP3B1,PTPN22,ADA,RUNX1,CBLB,SLC39A10,DOCK10,NOTCH2,PRKAR1A,MMP14,PIK3CA,LEF1,SLAMF8,TP53BP1,NCSTN,SNX27,TFRC,FZD9,SYVN1,PTPN6,IGFBP2,DDOST,MSN,DOCK2,CD74,AKT1,TMEM98,SUPT6H,PTPN2,CD40LG,VAMP7,AP1G1,BCL10,FKBP1A,CD151,THY1,CDKN1A,DNAJB9,FGL2,CCR6,FGF10	darkgreen	EC_M4

GO:0045987	1.30E-01	0.953095546	5	20	positive regulation of smooth muscle contraction	BP	7.71E-01	SRF,ADA,CTTN,MYOCD,ITGA2	darkgreen	EC_M4
GO:0009593	1.30E-01	0.942646752	7	32	detection of chemical stimulus	BP	7.71E-01	STIM1,KCNMB1,TGFB3,CALM1,OR5AN1,OR6N2,KCNMB4	darkgreen	EC_M4
GO:0044539	1.30E-01	0.959811194	4	15	long-chain fatty acid import into cell	BP	7.71E-01	AKT2,IRS2,EPRS,AKT1	darkgreen	EC_M4
GO:0140354	1.30E-01	0.959811194	4	15	lipid import into cell	BP	7.71E-01	AKT2,IRS2,EPRS,AKT1	darkgreen	EC_M4
GO:0090141	1.30E-01	0.952901502	5	20	positive regulation of mitochondrial fission	BP	7.71E-01	VPS35,DCN,DNM1L,MIEF2,BNIP3	darkgreen	EC_M4
GO:2001244	1.30E-01	0.935222898	9	46	positive regulation of intrinsic apoptotic signaling pathway	BP	7.71E-01	NCK2,SERINC3,DNM1L,SKIL,PTPN2,BCAP31,PARK7,NACC2,PLAGL2	darkgreen	EC_M4
GO:0034766	1.30E-01	0.925921832	13	68	negative regulation of ion transmembrane transport	BP	7.71E-01	UBQLN1,FKBP1B,ARL6IP5,REM1,CACNB3,PKD2,ISCU,LINC02210-CRHR1,CALM1,AKT1,MIR24-1,CBARP,GEM	darkgreen	EC_M4
GO:0090398	1.31E-01	0.92568383	13	68	cellular senescence	BP	7.71E-01	CDKN2B,SRF,NUAK1,C2orf40,MAPKAPK5,OPA1,PLA2R1,KRAS,AKT3,BMPR1A,MAP2K1,PRMT6,CDKN1A	darkgreen	EC_M4
GO:1903351	1.31E-01	0.929805654	11	55	cellular response to dopamine	BP	7.71E-01	VPS35,HTR5A,ADCY6,HTR2B,GSK3B,ADCY5,NCSTN,GNAL,GNB1,FLNA,GNAI3	darkgreen	EC_M4
GO:0030501	1.31E-01	0.938165915	8	37	positive regulation of bone mineralization	BP	7.71E-01	FBN2,TGFB1,ATP2B1,ACVR1,BMP7,FZD9,BMPR1A,TGFB3	darkgreen	EC_M4
GO:1904063	1.32E-01	0.927102063	12	62	negative regulation of cation transmembrane transport	BP	7.71E-01	UBQLN1,FKBP1B,REM1,CACNB3,PKD2,ISCU,LINC02210-CRHR1,CALM1,AKT1,MIR24-1,CBARP,GEM	darkgreen	EC_M4
GO:0022011	1.32E-01	0.946675722	6	25	myelination in peripheral nervous system	BP	7.71E-01	AKT2,ILK,LGI4,DAG1,DICER1,AKT1	darkgreen	EC_M4
GO:0032292	1.32E-01	0.946675722	6	25	peripheral nervous system axon ensheathment	BP	7.71E-01	AKT2,ILK,LGI4,DAG1,DICER1,AKT1	darkgreen	EC_M4
GO:0060972	1.32E-01	0.952140123	5	20	left/right pattern formation	BP	7.71E-01	AHI1,MEGF8,IFT57,NOTCH2,PKD1L1	darkgreen	EC_M4
GO:0001914	1.32E-01	0.959119448	4	15	regulation of T cell mediated cytotoxicity	BP	7.71E-01	IL23A,STX7,HLA-DRA,FAM49B	darkgreen	EC_M4
GO:0030901	1.32E-01	0.924918149	13	69	midbrain development	BP	7.71E-01	FGF2,CTNNB1,RYK,DYNLL1,INA,UCHL5,FGFR2,CALM1,SYPL2,HSPA5,WLS,YWHAH,WNT2	darkgreen	EC_M4
GO:0050901	1.32E-01	0.952047355	5	20	leukocyte tethering or rolling	BP	7.71E-01	LEP,ITGB1,CXCL12,CHST2,ROCK1	darkgreen	EC_M4
GO:0035994	1.32E-01	0.951965091	5	20	response to muscle stretch	BP	7.71E-01	CTNNB1,ANKRD1,PIK3CA,TCAP,DMD	darkgreen	EC_M4
GO:0044706	1.33E-01	0.908753488	27	159	multi-multicellular organism process	BP	7.71E-01	PPARD,TEAD3,LEP,VEGFA,DKKL1,TMED2,LNPEP,ABCC8,MMP2,SPP1,KPNA6,KRAS,LINC02210-CRHR1,COMT,IGFBP2,ADRA2C,TIMP1,MED1,MAGED2,TGFB3,UBE2Q1,VMP1,ACSL4,AKT1,UBE2A,ITGA2,BSG	darkgreen	EC_M4
GO:0009746	1.33E-01	0.908721992	27	159	response to hexose	BP	7.71E-01	LRP1,PPARD,LGALS1,TXN2,SRF,UNC13B,FKBP1B,KIF5B,EIF2B3,IRS2,OSBP,SELENOT,OPA1,BRSK2,DYNLL1,SELENOS,ZBTB20,LRP5,PIK3CA,ADCY5,SIDT2,PTPRN,PPARGC1A,SMARCA4,PRKAA1,SERPINF1,COL6A2	darkgreen	EC_M4
GO:0097191	1.33E-01	0.907281866	29	173	extrinsic apoptotic signaling pathway	BP	7.71E-01	CTNNA1,TGFBR1,INHBA,TGFB1,FAIM2,CX3CL1,ACVR1,CTTN,EYA2,TGFB2,GSK3B,ATF3,PEA15,PDIA3,SERPINE1,ITGAV,HTT,SKIL,FGFR3,ARHGEF2,AKT1,DAPK3,PARK7,BCL10,ITM2C,GDNF,TIMP3,BLOC1S2,FGF10	darkgreen	EC_M4
GO:0051170	1.33E-01	0.911693441	23	132	import into nucleus	BP	7.71E-01	ANGPT1,NUP50,LEP,RAB23,MDFIC,GLI3,TGFB1,SEC13,UFM1,HDAC3,CRY2,CHP1,CBLB,KPNA6,KPNA1,MED1,AKT1,FLNA,RANBP2,KPNA4,CDKN1A,PTTG1IP,ZPR1	darkgreen	EC_M4
GO:0021915	1.33E-01	0.911337238	23	130	neural tube development	BP	7.71E-01	PLXNB2,GLI3,GLI2,TGFB1,SPINT2,SEMA3C,TMED2,TCTN1,OPA1,PKD2,IFT57,TGFB2,ITPK1,BMP7,PLOD3,ATP6AP2,FZD2,FKBP8,GORAB,TULP3,PKD1,LMO4,BCL10	darkgreen	EC_M4

GO:1901615	1.33E-01	0.895459228	60	382	organic hydroxy compound metabolic process	BP	7.71E-01	ATP8B1,HSD17B6,MIOX,NUS1,LY6E,PPARD,PSAT1,LEP,REST,INPP4A,DKKL1,SLC7A11,FGF2,EBPL,SNX17,ACER3,DGAT2,ZEB2,ACPP,SMPD1,IMPAD1,OSBP,DKK3,RDH10,APP,RAPGEF2,MRS2,TGFB2,LRP5,ITPK1,INPP5A,ARV1,IDH2,OSBPL2,HDLBP,MOGAT3,COMT,MAOA,TPI1,HSD17B1,QDPR,CYP19A1,HSD17B10,CLN8,STARD3,MED1,SPTLC2,CLN6,CYP46A1,PRKAA1,OCRL,PGP,HAGH,PARK7,MBTPS1,DPM2,NFE2L1,TPK1,AMACR,DPM1	darkgreen	EC_M4
GO:1905330	1.34E-01	0.928018086	11	56	regulation of morphogenesis of an epithelium	BP	7.71E-01	VEGFA,FGF2,CTNNB1,SULF1,NKD1,BMP7,SIX4,MAGED1,GDNF,WNT2,FGF10	darkgreen	EC_M4
GO:0033043	1.34E-01	0.885750929	143	950	regulation of organelle organization	BP	7.71E-01	LMAN1,LRP1,TRIM36,TCP1,FAM49B,NCK2,TGFBR1,SHANK1,LRRC4B,ACTR2,ROCK2,MYLK3,VPS35,MGARP,KIF5B,RAB7A,EML2,RB1,MARK4,MAPRE3,PDCD6IP,PAK1,ACTR3,RAB3GAP1,CTNNB1,AKT2,DYNC1LI1,DZIP1,ZEB2,PLEKHG2,ACTG1,NAV3,ANAPC15,KAT2B,LIG4,CHFR,DCN,CDC42EP3,ILK,CXCL12,SLC25A4,OSBP,RIOK2,MAP1B,PINX1,PFN2,RASA1,DHX36,CCT8,MAPKAPK5,CX3CL1,GNL3,OPA1,TSG101,DKC1,TWF1,TENT4B,MAGEL2,ARL3,PHLDB2,TJP1,NOTCH2,CTTN,DNM1L,LMOD1,SEC22B,LRP5,FEZ1,PRKAR1A,GSK3B,PIK3CA,NEK7,NTRK3,FAM162A,BMP7,SYNPO2,PHLDB1,IQGAP1,SCFD1,CLIC4,TFRC,FZD9,ARPC5,MAPT,NCKAP1,C11orf65,PDCD10,UPF1,ARPC2,HTT,SYNPO2L,CAPZB,WDR1,MNAT1,OBSL1,STX18,GHITM,HAP1,SLC2A4,CGNL1,ANAPC4,PPARGC1A,MSN,NES,ARHGEF2,SYT11,TPM1,TGFB3,MAP2K1,GPR65,TTC8,TEX14,CLTC,PIN1,PDGFRB,MIEF2,ROCK1,FLII,EVI5L,AKT1,PRKAA1,TUBB4A,OMA1,CLEC16A,DAPK3,MYO1C,PAFAH1B1,PKD1,FLNA,PLXNA3,PARK7,SPTAN1,NEXN,PRMT6,BNIP3,SKA2,TMSB4X,DSTN,NME6,MAP1A,COTL1,CFL2,LRRTM2	darkgreen	EC_M4
GO:1900102	1.34E-01	0.958060811	4	15	negative regulation of endoplasmic reticulum unfolded protein response	BP	7.71E-01	NCK2,DDRGK1,HSPA5,DNAJB9	darkgreen	EC_M4
GO:0061178	1.34E-01	0.930110397	10	50	regulation of insulin secretion involved in cellular response to glucose stimulus	BP	7.71E-01	LRP1,PPARD,FKBP1B,KIF5B,OSBP,BRSK2,DYNLL1,LRP5,ADCY5,SIDT2	darkgreen	EC_M4
GO:1902683	1.35E-01	0.95785014	4	15	regulation of receptor localization to synapse	BP	7.71E-01	STX7,DAG1,IQSEC2,NPTN	darkgreen	EC_M4
GO:0045581	1.35E-01	0.939955939	7	32	negative regulation of T cell differentiation	BP	7.71E-01	SOCS5,GLI3,LOXL3,RUNX1,CD74,PTPN2,FGL2	darkgreen	EC_M4
GO:0031016	1.36E-01	0.929348745	10	50	pancreas development	BP	7.71E-01	CLOCK,RHEB,CTNNB1,SELENOT,GSK3B,SIDT2,SRP54,AKT1,WLS,FGF10	darkgreen	EC_M4
GO:0048168	1.36E-01	0.93547141	8	38	regulation of neuronal synaptic plasticity	BP	7.71E-01	NCDN,RAB3GAP1,APP,KRAS,SYAP1,RAB11A,NPTN,JPH3	darkgreen	EC_M4
GO:0007043	1.36E-01	0.914619939	18	99	cell-cell junction assembly	BP	7.71E-01	CTNNA1,SRF,ROCK2,PDCD6IP,CTNNB1,ACTG1,ZNF703,CTNND1,MTDH,TJP1,PKP2,PRKCA,VCL,WDR1,TLN2,ROCK1,MYO1C,NPHP4	darkgreen	EC_M4
GO:0035270	1.36E-01	0.917119004	16	87	endocrine system development	BP	7.71E-01	LY6E,SRF,CLOCK,TGFBR1,FGF2,GLI2,RHEB,DKK3,GSK3B,ARID5B,SIDT2,BMPR1A,MAP2K1,AKT1,FSTL3,FGF10	darkgreen	EC_M4
GO:0060740	1.36E-01	0.950052243	5	20	prostate gland epithelium morphogenesis	BP	7.71E-01	GLI2,SULF1,BMP7,FGFR2,FGF10	darkgreen	EC_M4
GO:0090322	1.36E-01	0.943718074	6	27	regulation of superoxide metabolic process	BP	7.71E-01	TGFB1,FBLN5,BMP7,MAPT,AKT1,GNAI3	darkgreen	EC_M4
GO:0036120	1.37E-01	0.94975826	5	20	cellular response to platelet-derived growth factor stimulus	BP	7.71E-01	HYAL1,SYAP1,PDGFRB,MTSS1L,HAS2	darkgreen	EC_M4
GO:1901223	1.37E-01	0.949357578	5	21	negative regulation of NIK/NF-kappaB signaling	BP	7.71E-01	ADIPOR1,BMP7,TSPAN6,UACA,TMSB4X	darkgreen	EC_M4
GO:0045103	1.37E-01	0.939047789	7	31	intermediate filament-based process	BP	7.71E-01	DST,NEFM,INA,PKP2,SYNC,CLN8,NES	darkgreen	EC_M4

GO:1901342	1.37E-01	0.899096195	38	232	regulation of vasculature development	BP	7.71E-01	SLC12A2,FAM19A5,ADGRB1,LEP,VEGFA,PDCL3,EMC10,ROCK2,RRAS,ITGB1,FGF2,CTNNB1,STIM1,DCN,SPINK5,SULF1,ABCC8,MTDH,ATP2B4,RUNX1,CHRNA7,TJP1,HYAL1,TGFB2,ADGRB2,PRKCA,VASH2,HSPB1,SERPINE1,AKT3,NPPB,PKM,ROCK1,MYDGF,SARS,F3,MIR24-1,SERPINF1	darkgreen	EC_M4
GO:0044409	1.37E-01	0.912232467	19	106	entry into host	BP	7.71E-01	LY6E,LGALS1,ITGB1,AXL,SIGLEC1,SMPD1,VAMP8,PTX3,VPS18,DAG1,ITGB5,NECTIN4,TFRC,ITGAV,SLC3A2,CD74,ITGA2,BSG,FCN1	darkgreen	EC_M4
GO:0008542	1.37E-01	0.938712028	7	31	visual learning	BP	7.71E-01	SLC7A11,ITGB1,APP,ABCC8,KRAS,NPTN,LRRN4	darkgreen	EC_M4
GO:0006942	1.38E-01	0.921182342	13	68	regulation of striated muscle contraction	BP	7.71E-01	FKBP1B,SCN5A,P2RX4,ATP2A2,PKP2,CASQ1,CACNA1C,ADRA1B,CALM1,DMD,TMEM38B,TMEM38A,HCN4	darkgreen	EC_M4
GO:0042147	1.38E-01	0.917455293	15	80	retrograde transport, endosome to Golgi	BP	7.71E-01	GGA1,VPS35,RAB7A,WASHC2A,ARL1,TMEM87B,MAGEL2,RAB7B,CLTCL1,ERC1,SNX8,CLTC,RAB6A,VPS26B,ARFRP1	darkgreen	EC_M4
GO:0030225	1.38E-01	0.938218909	7	32	macrophage differentiation	BP	7.71E-01	VEGFA,INHBA,RB1,TGFB1,APP,PRKCA,PTPN2	darkgreen	EC_M4
GO:0007176	1.38E-01	0.943060677	6	26	regulation of epidermal growth factor-activated receptor activity	BP	7.71E-01	NCK2,SOCS5,APP,TSG101,CBLB,SHC1	darkgreen	EC_M4
GO:0006968	1.38E-01	0.942602323	6	27	cellular defense response	BP	7.71E-01	RAB23,ITGB1,LGALS3BP,ADORA2B,BCL10,CCR6	darkgreen	EC_M4
GO:0019098	1.39E-01	0.956158523	4	15	reproductive behavior	BP	7.71E-01	APP,UBE2Q1,NPAS1,MBD2	darkgreen	EC_M4
GO:0033673	1.39E-01	0.900842328	32	191	negative regulation of kinase activity	BP	7.71E-01	GNAQ,CDKN2B,SOCS5,SH3BP5L,RB1,KAT2B,IRS2,SMPD1,ILK,PTPN22,TSG101,CHP1,DBNDD2,CBLB,LRP5,PRKAR1A,CORO1C,BMP7,HSPB1,MAPT,PTPN6,SRCIN1,GSKIP,MYOCD,AKT1,PTPN2,MIDN,PARK7,MIR24-1,THY1,CDKN1A,DUSP22	darkgreen	EC_M4
GO:0032816	1.39E-01	0.95596882	4	15	positive regulation of natural killer cell activation	BP	7.71E-01	IL23A,AXL,IL15RA,AP1G1	darkgreen	EC_M4
GO:0120032	1.39E-01	0.903547314	27	158	regulation of plasma membrane bounded cell projection assembly	BP	7.71E-01	ATP8B1,SRF,TGFBR1,AKIRIN1,ACTR2,MARK4,ACTR3,DZIP1,FAM98A,CDC42EP3,DPYSL3,PFN2,TWF1,PIK3CA,AUTS2,CORO1C,NCKAP1,ARPC2,HTT,CAPZB,PLD1,HAP1,TGFB3,EVI5L,MYO10,PLXNB3,DEF8	darkgreen	EC_M4
GO:0045862	1.40E-01	0.893739165	47	297	positive regulation of proteolysis	BP	7.71E-01	RNF19A,UBQLN1,MTCH1,MYH9,VCP,REST,SOCS5,PSME4,CYCS,ROCK2,RCN3,PSMC2,PICALM,NUB1,WDR48,TMTC3,CHFR,DDRGK1,ARL6IP5,ROBO1,APP,IFT57,TANK,PSMD14,GSK3B,MMP14,FAM162A,NCSTN,ADRM1,EGLN3,MAPT,PCOLCE,FN1,SMURF1,NGFR,CLN6,UACA,ARIH1,FBXO22,AKT1,BCAP31,TMEM259,ENO1,F3,BCL10,UBE2V2,DDA1	darkgreen	EC_M4
GO:0017038	1.40E-01	0.901839274	29	174	protein import	BP	7.71E-01	ANGPT1,NUP50,HSPA4,LEP,PEX6,RAB23,MDFIC,GLI3,TGFB1,SEC13,UFM1,HDAC3,CRY2,CHP1,CBLB,KPNA6,HSPA8,KPNA1,TOMM40L,MED1,AKT1,FLNA,RANBP2,KPNA4,CDKN1A,PTTG1IP,ZPR1,GRPEL2,TOMM7	darkgreen	EC_M4
GO:0062149	1.40E-01	0.948084464	5	20	detection of stimulus involved in sensory perception of pain	BP	7.71E-01	CXCL12,CACNB3,BACE1,KCNA1,ITGA2	darkgreen	EC_M4
GO:0002719	1.41E-01	0.955352251	4	15	negative regulation of cytokine production involved in immune response	BP	7.71E-01	ANGPT1,TGFB1,TGFB2,TGFB3	darkgreen	EC_M4
GO:0048565	1.41E-01	0.912518564	17	92	digestive tract development	BP	7.71E-01	AHI1,GLI3,RB1,GLI2,MEGF8,CTNNB1,RARB,CCKBR,ADA,TGFB2,CLMP,FGFR2,TGFB3,MYOCD,PKD1,CDKN1A,FGF10	darkgreen	EC_M4
GO:0010566	1.41E-01	0.955283958	4	15	regulation of ketone biosynthetic process	BP	7.71E-01	REST,DKKL1,DKK3,PPARGC1A	darkgreen	EC_M4
GO:0031295	1.41E-01	0.941254159	6	27	T cell costimulation	BP	7.71E-01	LGALS1,PTPN11,PIK3CA,PTPN6,AKT1,CD40LG	darkgreen	EC_M4
GO:0006956	1.41E-01	0.941230027	6	27	complement activation	BP	7.71E-01	CFI,C6,A2M,CD59,MFAP4,FCN1	darkgreen	EC_M4
GO:0006576	1.41E-01	0.920459958	12	64	cellular biogenic amine metabolic process	BP	7.71E-01	AZIN1,LY6E,HNMT,AZIN2,TGFB2,SMS,COMT,MAOA,SLC29A4,AGMAT,PARK7,NNMT	darkgreen	EC_M4
GO:0009791	1.41E-01	0.918479509	13	68	post-embryonic development	BP	7.71E-01	VEGFA,TGFBR1,SEMA3C,IMPAD1,NDN,KDM5B,FGFR2,ARID5B,SGPL1,FBN1,MYO1E,PLAGL2,CHST11	darkgreen	EC_M4

GO:0035050	1.42E-01	0.922572892	11	55	embryonic heart tube development	BP	7.71E-01	AHI1,SRF,MEGF8,CTNNB1,TMED2,ACVR1,PKD2,IFT57,NOTCH2,MED1,LBX1	darkgreen	EC_M4
GO:0043457	1.42E-01	0.931512517	8	39	regulation of cellular respiration	BP	7.71E-01	VCP,PIK3CA,ISCU,PPARGC1A,AKT1,PARK7,BNIP3,COX7A2	darkgreen	EC_M4
GO:0090504	1.42E-01	0.936024976	7	31	epiboly	BP	7.71E-01	MRTFA,MEGF8,PHLDB2,PDCD10,FLNA,COL5A1,CD151	darkgreen	EC_M4
GO:0006892	1.42E-01	0.909823065	18	99	post-Golgi vesicle-mediated transport	BP	7.71E-01	AP3S1,GGA1,EXOC1,ATP2C1,EXOC6B,ARL3,WIPI1,SCFD1,SCAMP3,EXOC5,BLZF1,MYO5A,RAB31,RAB34,AP1G1,C16orf70,STXBP6,ARFRP1	darkgreen	EC_M4
GO:0030033	1.43E-01	0.954378601	4	15	microvillus assembly	BP	7.71E-01	ATP8B1,RAPGEF2,TNIK,PLD1	darkgreen	EC_M4
GO:1905314	1.43E-01	0.93145906	8	37	semi-lunar valve development	BP	7.71E-01	ROCK2,RB1,TGFB1,ROBO1,HEY1,NOTCH2,TGFB2,ROCK1	darkgreen	EC_M4
GO:0009894	1.43E-01	0.879043715	121	806	regulation of catabolic process	BP	7.71E-01	LRP1,AGAP2,TAB2,RNF19A,AZIN1,ATP6V1C1,PPARA,ADGRB1,UBQLN1,GGA1,LEP,VCP,EXOC1,PDCL3,SOCS5,MFSD2A,MAP3K7,ROCK2,VPS35,ITGB1,SESN3,RAB7A,PSMC2,ABHD5,ATP6V0B,NUB1,RHEB,RAB3GAP1,AKT2,IRS2,TMTC3,CHFR,DCN,DDRGK1,CNOT7,TP53INP2,SLC25A4,EIF4G2,CELF1,APP,DHX36,PTPN22,DKC1,MTDH,ATP2B4,CBLB,NKD1,TENT5C,TENT4B,PSMD14,ATP6V1B2,AZIN2,FAP,CTTN,DNM1L,UCHL5,SEC22B,CPT1A,CISD2,ZBTB20,PSMD1,FEZ1,HTR2B,GSK3B,PIK3CA,PRKCA,PPP1R3D,PSMD2,SCFD1,QSOX1,ATF6,PAFAH1B2,HSPB1,MAPT,GPR137,UPF1,HTT,SAMD4A,ARAF,SMURF1,TIMP1,FBXL5,FMN2,ATP6V1D,PPARGC1A,MSN,SPTLC2,ARIH1,PIN1,FBXO22,ROCK1,USP14,AKT1,PRKAA1,CLEC16A,DAPK3,BCAP31,PKD1,FLNA,IKBKG,TMEM259,PARK7,GPSM1,RHBDF1,SLC2A6,GNAI3,USP10,ATP6V0D1,BNIP3,UBE2V2,GDNF,TIMP3,VPS26B,PTTG1IP,DDA1,CAPNS1,GABARAPL2,PIK3C2A,ORMDL3,ATP6V0E1,MAP1A	darkgreen	EC_M4
GO:1902106	1.43E-01	0.91734942	13	69	negative regulation of leukocyte differentiation	BP	7.71E-01	SOCS5,GLI3,INHBA,CTNNB1,LOXL3,RUNX1,TCTA,GPR137,FBN1,CD74,PTPN2,FSTL3,FGL2	darkgreen	EC_M4
GO:0032516	1.43E-01	0.954111731	4	15	positive regulation of phosphoprotein phosphatase activity	BP	7.71E-01	PPP1R12A,SLC39A10,CALM1,PDGFRB	darkgreen	EC_M4
GO:0032984	1.43E-01	0.896575638	33	195	protein-containing complex disassembly	BP	7.71E-01	UBQLN1,VCP,KIF5B,NAPA,SMARCD3,NCKAP5,NAV3,KIF2A,MAP1LC3A,VAMP8,MAP1B,ATP2A2,TSG101,TWF1,LMOD1,GSK3B,PIK3CA,HSPA8,UPF1,CAPZB,WDR1,NES,ARHGEF2,CALM1,SMARCA4,VMP1,FLII,CLEC16A,SPTAN1,BNIP3,DSTN,MAP1A,CFL2	darkgreen	EC_M4
GO:0006469	1.43E-01	0.898933681	29	172	negative regulation of protein kinase activity	BP	7.71E-01	GNAQ,CDKN2B,SOCS5,SH3BP5L,RB1,KAT2B,SMPD1,ILK,PTPN22,TSG101,CHP1,DBNDD2,CBLB,LRP5,PRKAR1A,CORO1C,BMP7,HSPB1,PTPN6,SRCIN1,GSKIP,MYOCD,AKT1,PTPN2,PARK7,MIR24-1,THY1,CDKN1A,DUSP22	darkgreen	EC_M4
GO:0021799	1.44E-01	0.940077188	6	26	cerebral cortex radially oriented cell migration	BP	7.71E-01	SUN2,MDGA1,RTN4,GLI3,CTNNB1,PAFAH1B1	darkgreen	EC_M4
GO:0002076	1.44E-01	0.953950148	4	15	osteoblast development	BP	7.71E-01	RUNX2,GLI2,LRP5,SATB2	darkgreen	EC_M4
GO:0003417	1.44E-01	0.953795216	4	15	growth plate cartilage development	BP	7.71E-01	RARB,COMP,THBS3,ANXA6	darkgreen	EC_M4
GO:1903350	1.44E-01	0.921197724	11	56	response to dopamine	BP	7.71E-01	VPS35,HTR5A,ADCY6,HTR2B,GSK3B,ADCY5,NCSTN,GNAL,GNB1,FLNA,GNAI3	darkgreen	EC_M4
GO:0150105	1.44E-01	0.946044357	5	20	protein localization to cell-cell junction	BP	7.71E-01	ACTG1,TJP1,VCL,CGNL1,FLNA	darkgreen	EC_M4
GO:0045921	1.46E-01	0.917756355	12	63	positive regulation of exocytosis	BP	7.71E-01	RAB7A,PDCD6IP,RAB2B,VAMP8,TSG101,DNM1L,CACNA1G,SYTL4,SCAMP5,ATP6AP1,VAMP7,AP1G1	darkgreen	EC_M4
GO:0030219	1.46E-01	0.925930156	9	44	megakaryocyte differentiation	BP	7.71E-01	CDKN2B,SRF,UBA5,PTPN11,RAB7B,PTPN6,MED1,PRMT6,LOX	darkgreen	EC_M4
GO:0050730	1.46E-01	0.894959307	32	193	regulation of peptidyl-tyrosine phosphorylation	BP	7.71E-01	IL23A,ANGPT1,LEP,VEGFA,NCK2,PDCL3,SOCS5,SH3BP5L,TGFB1,PRNP,CNOT7,OSBP,APP,PTPN11,TSG101,ACVR1,CBLB,OSM,IQGAP1,PTPN6,DOK7,FGFR3,SRCIN1,ARHGEF2,SHC1,CD74,PTPN2,TNFRSF18,THY1,DUSP22,TSLP,FGF10	darkgreen	EC_M4
GO:0001708	1.46E-01	0.917477321	12	62	cell fate specification	BP	7.71E-01	GLI3,ITGB1,FGF2,PTCH2,TENM4,GLI2,CTNNB1,EYA2,NTRK3,FKBP8,BMPR1A,LMO4	darkgreen	EC_M4
GO:0030010	1.46E-01	0.902389328	22	123	establishment of cell polarity	BP	7.71E-01	FAM49B,MYH9,DOCK8,ROCK2,ITGB1,PAK1,FAT1,MAP1B,BRSK2,FEZ1,GSK3B,SNX27,FAM89B,HTT,PRKCI,MSN,DOCK2,TTC8,ROCK1,PAFAH1B1,PKD1,FGF10	darkgreen	EC_M4
GO:0031279	1.46E-01	0.933367199	7	32	regulation of cyclase activity	BP	7.71E-01	STIM1,CACNA1D,LINC02210-CRHR1,CACNA1C,CALM1,NPR3,GNAI3	darkgreen	EC_M4

GO:0051247	1.47E-01	0.872109921	168	1140	positive regulation of protein metabolic process	BP	7.71E-01	IL23A,NAB2,LRP1,AGAP2,TAB2,RNF19A,PRRC1,PLXNB2,ANGPT1,HABP4,C6orf89,UBQLN1,MTCH1,GGA1,MYH9,LEP,VEGFA,VCP,NCK2,REST,TGFBR1,SOCS5,PSME4,CYCS,MAP3K7,ROCK2,INHBA,RCN3,VPS35,FGF2,RAB7A,PSMC2,GSAP,PICALM,MAPRE3,NUB1,PAK1,TGFB1,RAB3GAP1,WDR48,CTNNB1,AKT2,OSBPL8,PPP1R12A,TMTC3,PRNP,CHFR,HDAC3,DDRGK1,SMPD1,ILK,RBPMS,ARL6IP5,OSBP,EIF4G2,ROBO1,DIP2B,PFN2,APP,DHX36,RAPGEF2,CX3CL1,PTPN11,GNL3,PTPN22,ACVR1,GDF6,AKTIP,CHP1,ATP2B4,PKD2,CBLB,NKD1,BRD7,IFT57,TENT4B,TANK,TOLLIP,PSMD14,NDN,SLC39A10,CHRNA7,SLC2A10,CCND1,CAPRIN2,SEC22B,TGFB2,HTR2B,GSK3B,MMP14,DAG1,PIK3CA,AUTS2,KRAS,NTRK3,FAM162A,SPATC1L,BMP7,OSM,NCSTN,SYAP1,ADRM1,IQGAP1,TFRC,EGLN3,MAPT,TFR2,PCOLCE,FN1,PDCD10,ADRA2C,DOK7,SAMD4A,FGFR3,ARAF,SMURF1,TNIK,PLD1,FBXL5,MNAT1,MAGED1,BMPR1A,PPARGC1A,MSN,RNF111,SRCIN1,ARHGEF2,NGFR,TGFB3,EIF3C,MAP2K1,CLN6,CALM1,UACA,PKM,ARIH1,NPTN,PIN1,CD74,FBXO22,PDGFRB,AKT1,ITGA2,PRKAA1,MYDGF,BCAP31,TNFRSF18,PKD1,IRAK1,TMEM259,PARK7,ENO1,POMT1,F3,BCAR3,BARHL2,HSPA5,BCL10,FKBP1A,PEMT,UBE2V2,CDKN1A,TXN,NNMT,PTTG1IP,TSLP,DDA1,FGF10	darkgreen	EC_M4
GO:0060491	1.47E-01	0.897265933	27	159	regulation of cell projection assembly	BP	7.71E-01	ATP8B1,SRF,TGFBR1,AKIRIN1,ACTR2,MARK4,ACTR3,DZIP1,FAM98A,CDC42EP3,DPYSL3,PFN2,TWF1,PIK3CA,AUTS2,CORO1C,NCKAP1,ARPC2,HTT,CAPZB,PLD1,HAP1,TGFB3,EVI5L,MYO10,PLXNB3,DEF8	darkgreen	EC_M4
GO:0021795	1.47E-01	0.932844841	7	32	cerebral cortex cell migration	BP	7.71E-01	SUN2,MDGA1,RTN4,GLI3,CTNNB1,ROBO1,PAFAH1B1	darkgreen	EC_M4
GO:0046850	1.48E-01	0.928486541	8	38	regulation of bone remodeling	BP	7.71E-01	LEP,SPP1,PRKCA,TFRC,GPR137,SUCO,ATP6AP1,DEF8	darkgreen	EC_M4
GO:0036296	1.48E-01	0.937669491	6	27	response to increased oxygen levels	BP	7.71E-01	COL1A1,PDGFRB,ATP6AP1,ATP6V0D1,BNIP3,CDKN1A	darkgreen	EC_M4
GO:0046513	1.48E-01	0.921541802	10	51	ceramide biosynthetic process	BP	7.71E-01	B4GALNT1,SMPD1,ST8SIA4,CERS5,B3GALT1,SGMS1,CLN8,SPTLC2,PRKAA1,ORMDL3	darkgreen	EC_M4
GO:0071577	1.48E-01	0.943987251	5	21	zinc ion transmembrane transport	BP	7.71E-01	SLC39A7,SLC39A4,SLC39A10,SLC39A6,SLC39A3	darkgreen	EC_M4
GO:1905209	1.48E-01	0.952053515	4	15	positive regulation of cardiocyte differentiation	BP	7.71E-01	TGFB1,DHX36,TGFB2,MYOCD	darkgreen	EC_M4
GO:0032268	1.48E-01	0.867989694	263	1819	regulation of cellular protein metabolic process	BP	7.71E-01	SERPINB2,IL23A,NAB2,LRP1,HBS1L,AGAP2,TAB2,RNF19A,PRRC1,PLXNB2,ANGPT1,HABP4,ADGRB1,C6orf89,UBQLN1,MTCH1,SH3GL2,GNAQ,MYH9,LEP,CDKN2B,PLAA,NUAK1,VEGFA,VCP,NCK2,PDCL3,REST,EIF5B,P3H1,TGFBR1,SOCS5,PSME4,COPS9,CYCS,MAP3K7,SH3BP5L,ROCK2,INHBA,RCN3,VPS35,FKBP1B,FGF2,DNAJC1,PSMC2,RB1,IWS1,PICALM,MAPRE3,CRTAP,NUB1,PAK1,TGFB1,RAB3GAP1,WDR48,SPOPL,CTNNB1,SERPINH1,AKT2,GPD1L,OSBPL8,PPP1R12A,KAT2B,CRIM1,SPINT2,TMTC3,PRNP,CHFR,HDAC3,DDRGK1,SMPD1,ILK,RBPMS,ARL6IP5,SPINK5,CNOT7,FXYD1,SNTA1,HSP90B1,TMED2,OSBP,EIF4G2,ROBO1,DIP2B,PINX1,PFN2,SELENOT,CELF1,CCNJ,APP,ATXN2,DHX36,RAPGEF2,MAPKAPK5,CX3CL1,PTPN11,GNL3,GTF2H1,PTPN22,TSG101,ACVR1,CRY2,CHAC1,GDF6,AKTIP,CHP1,ATP2B4,PKD2,CSDE1,TWF1,DBNDD2,CBLB,BRD7,IFT57,TENT4B,TANK,TOLLIP,PSMD14,NDN,SLC39A10,CHRNA7,EIF4E,TM4SF20,UCHL5,TRIP12,CCND1,CAPRIN2,TGFB2,PPP6R3,LRP5,EPRS,HTR2B,PRKAR1A,GSK3B,MMP14,DAG1,PIK3CA,AUTS2,CORO1C,ANOS1,KRAS,NTRK3,FAM162A,SPATC1L,BMP7,PPP1R11,COL6A3,OSM,NCSTN,SYAP1,ADRM1,IQGAP1,TFRC,RPS6KA3,EGLN3,HSPB1,MAPT,SERPINE1,EIF4EBP2,DNAJC10,PCOLCE,FN1,PTPN6,PDCD10,UPF1,ADRA2C,DOK7,HTT,FKBP8,SAMD4A,FGFR3,ARAF,SMURF1,TIMP1,TNIK,PLD1,FBXL5,MNAT1,MAGED1,BMPR1A,TSPYL2,CLN8,EIF2S1,CDK12,PPARGC1A,MSN,RNF111,SRCIN1,CPEB4,ARHGEF2,NGFR,TMED10,TGFB3,EIF3C,SHC1,CCNG1,MAP2K1,CLN6,CALM1,UACA,PPP4R4,PKM,ARIH1,GSKIP,NPTN,MYOCD,PIN1,CD74,FBXO22,PDGFRB,ROCK1,USP14,AKT1,TMEM98,ITGA2,PR	darkgreen	EC_M4
GO:0001655	1.48E-01	0.886982424	46	283	urogenital system development	BP	7.71E-01	AHI1,RRM2B,ANGPT1,VEGFA,TGFBR1,GLI3,FGF2,SEC61A1,GLI2,SNX17,CTNNB1,RARB,DCN,ILK,ADAMTS6,SULF1,RDH10,GDF6,PKD2,ARL3,NOTCH2,TGFB2,BMP7,AP1B1,BICC1,FGFR2,IQGAP1,ARID5B,OVOL1,SGPL1,FBN1,SIX4,ACTA2,MAGED1,MYO1E,TMED10,TTC8,MYOCD,PDGFRB,PKD1,FSTL3,SERPINF1,SERPINB5,GDNF,HAS2,FGF10	darkgreen	EC_M4
GO:0006635	1.48E-01	0.915837258	12	64	fatty acid beta-oxidation	BP	7.71E-01	PPARA,PPARD,LEP,MFSD2A,AKT2,IRS2,CPT1A,HADH,ACOX3,HSD17B10,AKT1,AMACR	darkgreen	EC_M4
GO:0097062	1.48E-01	0.951884005	4	15	dendritic spine maintenance	BP	7.71E-01	VPS35,PRNP,HOMER1,CTTN	darkgreen	EC_M4

GO:0009225	1.48E-01	0.932072065	7	33	nucleotide-sugar metabolic process	BP	7.71E-01	GALT,NANS,GFPT1,UGDH,MPI,SLC35A3,DPM1	darkgreen	EC_M4
GO:0070925	1.48E-01	0.875443364	107	705	organelle assembly	BP	7.71E-01	STX7,AHI1,UBQLN1,SRF,RAB23,CENPC,LRRC4B,ACTR2,MYLK3,CFAP100,ITGB1,RAB7A,MARK4,MAPRE3,PDCD6IP,ACTR3,RAB3GAP1,DZIP1,DNAJB13,ACTG1,STAM2,KAT2B,HDAC3,CNOT7,VPS37A,KIF2A,TMEM41B,ANKRD1,MAP1LC3A,TP53INP2,ATP2A2,TCTN1,ATXN2,ATG13,TSG101,CSDE1,DNAH1,DNAH5,IFT57,RAB7B,ARL3,DYNLL1,TROVE2,LMOD1,SEC22B,SBDS,FEZ1,ARMC9,PRKAR1A,WIPI1,DNAAF5,NEK7,NTRK3,SYNPO2,CASQ1,GORASP2,SCFD1,ABRAXAS2,FARP2,MAPT,HTT,SYNPO2L,GORAB,SMURF1,EXOC5,WDR1,SIX4,OBSL1,STX18,HAP1,WIPI2,NLGN2,ATP6V1D,TCAP,EIF2S1,MSN,TPM1,IFT43,RAB11A,SOX30,TTC8,LRRC49,VMP1,TMEM159,PDGFRB,FLII,EVI5L,PRKAA1,OCRL,RAB34,PAFAH1B1,ABCA3,FLNA,NTNG2,ATP6V0D1,TCTN2,CCDC136,SPINK2,SNX10,ARF4,CSRP1,ABT1,CC2D2B,MZT1,GABARAPL2,CFL2,LRRTM2	darkgreen	EC_M4
GO:1900006	1.49E-01	0.951758979	4	15	positive regulation of dendrite development	BP	7.71E-01	PACSIN1,OPA1,BMP7,IQGAP1	darkgreen	EC_M4
GO:0046626	1.49E-01	0.920972448	10	50	regulation of insulin receptor signaling pathway	BP	7.71E-01	LEP,SESN3,PAK1,OSBPL8,SORBS1,PTPN11,OPA1,ADIPOR1,PRKAA1,PTPN2	darkgreen	EC_M4
GO:1903902	1.49E-01	0.943463343	5	21	positive regulation of viral life cycle	BP	7.71E-01	LGALS1,AXL,TSG101,KPNA6,CD74	darkgreen	EC_M4
GO:0003309	1.49E-01	0.9434368	5	21	type B pancreatic cell differentiation	BP	7.71E-01	CLOCK,RHEB,GSK3B,SIDT2,AKT1	darkgreen	EC_M4
GO:0035883	1.49E-01	0.9434368	5	21	enteroendocrine cell differentiation	BP	7.71E-01	CLOCK,RHEB,GSK3B,SIDT2,AKT1	darkgreen	EC_M4
GO:0060603	1.49E-01	0.937255729	6	26	mammary gland duct morphogenesis	BP	7.71E-01	GLI2,KDM5B,LRP5,FGFR2,MED1,FGF10	darkgreen	EC_M4
GO:0001953	1.49E-01	0.932002391	7	32	negative regulation of cell-matrix adhesion	BP	7.71E-01	LRP1,RASA1,PHLDB2,MMP14,CORO1C,SERPINE1,DUSP22	darkgreen	EC_M4
GO:0051145	1.49E-01	0.917954702	11	57	smooth muscle cell differentiation	BP	7.71E-01	MRTFA,SRF,VEGFA,SMARCD3,CTNNB1,HEY1,ACVR1,FGFR2,COMP,MYOCD,FGF10	darkgreen	EC_M4
GO:0045859	1.49E-01	0.878568224	79	513	regulation of protein kinase activity	BP	7.71E-01	IL23A,NAB2,AGAP2,TAB2,PRRC1,ANGPT1,GNAQ,LEP,CDKN2B,VEGFA,NCK2,SOCS5,MAP3K7,SH3BP5L,FGF2,RB1,MAPRE3,PAK1,TGFB1,OSBPL8,KAT2B,PRNP,SMPD1,ILK,ROBO1,CCNJ,APP,RAPGEF2,GTF2H1,PTPN22,TSG101,CHP1,ATP2B4,PKD2,DBNDD2,CBLB,CCND1,TGFB2,LRP5,HTR2B,PRKAR1A,DAG1,PIK3CA,CORO1C,KRAS,NTRK3,SPATC1L,BMP7,SYAP1,IQGAP1,HSPB1,PTPN6,PDCD10,ADRA2C,DOK7,HTT,MNAT1,MAGED1,BMPR1A,TSPYL2,CDK12,SRCIN1,SHC1,CCNG1,MAP2K1,CALM1,GSKIP,MYOCD,PDGFRB,AKT1,PTPN2,PKD1,IRAK1,PARK7,MIR24-1,THY1,CDKN1A,TXN,DUSP22	darkgreen	EC_M4
GO:1900101	1.49E-01	0.936738213	6	27	regulation of endoplasmic reticulum unfolded protein response	BP	7.71E-01	NCK2,DDRGK1,ATF6,PTPN2,HSPA5,DNAJB9	darkgreen	EC_M4
GO:0010611	1.50E-01	0.920272422	10	51	regulation of cardiac muscle hypertrophy	BP	7.71E-01	PPARA,ROCK2,PAK1,P2RX4,ATP2B4,TWF1,PRKCA,GLRX3,PIN1,ROCK1	darkgreen	EC_M4
GO:1903715	1.50E-01	0.936081356	6	28	regulation of aerobic respiration	BP	7.72E-01	VCP,ISCU,AKT1,PARK7,BNIP3,COX7A2	darkgreen	EC_M4
GO:0016485	1.50E-01	0.894076083	28	170	protein processing	BP	7.73E-01	SEC11C,MYH9,ADAMTS3,GLI3,PRNP,CPXM1,PCSK1,CPE,CHAC1,CPN1,RUNX1,CASP7,MMP14,HJV,NCSTN,SEC11A,BACE1,PCSK7,SERPINE1,ATP6AP2,COMP,ECE1,TMEM98,OMA1,ENO1,F3,GLG1,SPCS3	darkgreen	EC_M4
GO:0032233	1.51E-01	0.916753891	11	56	positive regulation of actin filament bundle assembly	BP	7.73E-01	TGFBR1,ROCK2,PAK1,PFN2,CX3CL1,SYNPO2,SYNPO2L,TPM1,TGFB3,GPR65,FLNA	darkgreen	EC_M4
GO:0010001	1.51E-01	0.892952407	29	173	glial cell differentiation	BP	7.74E-01	NAB2,LRP1,GLI3,FGF2,EIF2B3,TENM4,RHEB,TGFB1,CTNNB1,AKT2,ILK,LGI4,APP,PTPN11,DNER,DAG1,LEF1,KRAS,NTRK3,NCSTN,MAPT,WDR1,MAP2K1,DICER1,AKT1,TMEM98,MXRA8,BNIP3,FGF10	darkgreen	EC_M4
GO:1904892	1.03E-01	0.948924031	10	59	regulation of receptor signaling pathway via STAT	BP	7.74E-01	KIT,IL6,PPARG,RET,INPP5F,IL10RA,IL7R,DOT1L,NOTCH1,MIR99A	turquoise	EC_M6

GO:0002526	1.51E-01	0.916305554	11	58	acute inflammatory response	BP	7.74E-01	MYLK3,IL20RB,ACVR1,SELENOS,OSM,CD163,TFR2,FN1,OSMR,PARK7,F3	darkgreen	EC_M4
GO:0018149	1.51E-01	0.950540786	4	15	peptide cross-linking	BP	7.74E-01	DCN,FN1,COL3A1,EGFLAM	darkgreen	EC_M4
GO:0021534	1.51E-01	0.95047069	4	15	cell proliferation in hindbrain	BP	7.74E-01	FGF2,GLI2,PSMG1,RERE	darkgreen	EC_M4
GO:0071496	1.52E-01	0.885242136	43	267	cellular response to external stimulus	BP	7.74E-01	PPARA,HABP4,TXN2,SLC39A4,LEP,CDKN2B,SRF,NUAK1,ASNS,MFSD2A,VPS41,SESN3,AXL,ATP2B1,ANKRD1,MAP1LC3A,CACNB3,PTPN11,BRSK2,DNM1L,GLRX2,WIPI1,DAG1,TLR5,HSPA8,ATF3,COMT,KCNK4,WIPI2,EIF2S1,MED1,CPEB4,COL1A1,MYOCD,AKT1,ITGA2,PRKAA1,CLEC16A,HSPA5,BCL10,BNIP3,CDKN1A,GABARAPL2	darkgreen	EC_M4
GO:0034440	1.52E-01	0.905808873	16	90	lipid oxidation	BP	7.74E-01	PPARA,PPARD,LEP,MFSD2A,DGAT2,AKT2,IRS2,ADIPOR1,CPT1A,HADH,ACOX3,HSD17B10,PPARGC1A,AKT1,PRKAA1,AMACR	darkgreen	EC_M4
GO:0031532	1.52E-01	0.904382197	17	94	actin cytoskeleton reorganization	BP	7.74E-01	MKLN1,MYH9,PAK1,ATP2C1,BRSK2,TJP1,NOTCH2,CTTN,AUTS2,NTRK3,FARP2,TNIK,SHC1,GPR65,DAPK3,FLNA,FGF10	darkgreen	EC_M4
GO:0031329	1.52E-01	0.872617178	104	689	regulation of cellular catabolic process	BP	7.74E-01	LRP1,TAB2,RNF19A,ATP6V1C1,PPARA,UBQLN1,LEP,VCP,EXOC1,PDCL3,SOCS5,MFSD2A,MAP3K7,ROCK2,VPS35,SESN3,PSMC2,ABHD5,ATP6V0B,NUB1,RHEB,RAB3GAP1,AKT2,IRS2,TMTC3,CHFR,DCN,DDRGK1,CNOT7,TP53INP2,SLC25A4,EIF4G2,CELF1,APP,DHX36,PTPN22,DKC1,MTDH,ATP2B4,TENT5C,TENT4B,PSMD14,ATP6V1B2,CTTN,DNM1L,UCHL5,SEC22B,CPT1A,CISD2,ZBTB20,FEZ1,HTR2B,GSK3B,PIK3CA,PRKCA,PPP1R3D,SCFD1,QSOX1,ATF6,PAFAH1B2,HSPB1,MAPT,GPR137,UPF1,HTT,SAMD4A,ARAF,SMURF1,TIMP1,FBXL5,ATP6V1D,MSN,SPTLC2,ARIH1,FBXO22,ROCK1,USP14,AKT1,PRKAA1,CLEC16A,DAPK3,BCAP31,PKD1,IKBKG,TMEM259,PARK7,GPSM1,RHBDF1,GNAI3,USP10,ATP6V0D1,BNIP3,UBE2V2,GDNF,TIMP3,VPS26B,PTTG1IP,DDA1,CAPNS1,GABARAPL2,PIK3C2A,ORMDL3,ATP6V0E1,MAP1A	darkgreen	EC_M4
GO:0051220	1.52E-01	0.941783355	5	21	cytoplasmic sequestering of protein	BP	7.74E-01	DZIP1,PKD2,PKD1,FLNA,TMSB4X	darkgreen	EC_M4
GO:0007186	1.52E-01	0.877078268	71	458	G protein-coupled receptor signaling pathway	BP	7.75E-01	LRP1,FAM19A5,GNG5,ADGRB1,GNAQ,RORB,ROCK2,DGKH,HTR5A,ACPP,KCTD16,CCKBR,GPR84,APLP1,CXCL12,GRK7,P2RY14,CACNA1D,GLRB,RAPGEF2,CX3CL1,CPE,ADCY6,HOMER1,ADA,ATP2B4,ADGRB2,HTR2B,PRKCA,CMKLR1,GRK3,ADCY5,FZD9,LINC02210-CRHR1,FZD2,GPR82,PTPN6,GNB1L,ADRA2C,ECE1,TULP3,ADGRL1,NPPB,ADRA1B,GPR65,CALM1,ADORA2B,PDGFRB,ROCK1,AKT1,GNAL,TM2D1,NPR3,MGRN1,GNB1,FLNA,GPR153,SSTR5,AHCYL1,GNAI3,USP20,BCAR3,CCL16,NMUR1,SIGMAR1,OR5AN1,OR6N2,NXPH3,FCN1,CCR6,PROKR2	darkgreen	EC_M4
GO:0045851	1.03E-01	0.956551371	7	38	pH reduction	BP	7.75E-01	DMXL1,PPT1,RAB38,CLCN3,SLC9A7,CA2,RAB39A	turquoise	EC_M6
GO:0038066	1.03E-01	0.953479434	8	44	p38MAPK cascade	BP	7.75E-01	GADD45G,NCF1,MAP3K3,DLG1,RELL1,LGALS9,DAB2IP,BMP2	turquoise	EC_M6
GO:0055067	1.03E-01	0.938279052	17	108	monovalent inorganic cation homeostasis	BP	7.75E-01	DMXL1,PPT1,RAB38,EDNRB,IL1A,CLCN3,EDN1,KCNJ2,SLC12A7,SLC9A8,ADORA2A,SLC9A7,NPR1,SCNN1B,CA2,KCNH2,RAB39A	turquoise	EC_M6
GO:0070076	1.03E-01	0.960992163	6	25	histone lysine demethylation	BP	7.75E-01	KDM4C,KDM4D,KDM3A,KDM2A,KDM5A,KDM5C	turquoise	EC_M6
GO:0030004	1.53E-01	0.906785802	15	81	cellular monovalent inorganic cation homeostasis	BP	7.76E-01	SLC12A2,RAB7A,SLC4A7,MAFG,TM9SF4,ATP1B3,SLC4A5,CHP1,SLAMF8,CLIC4,ATP6AP2,CLN6,ATP6AP1,ATP6V0D1,ATP6V0E1	darkgreen	EC_M4
GO:0051443	5.15E-02	0.985623128	5	23	positive regulation of ubiquitin-protein transferase activity	BP	7.76E-01	TOPORS,CDC20,UBE2C,DCUN1D5,PLK1	darkgrey	EC_M8
GO:0050869	1.04E-01	0.964346135	5	24	negative regulation of B cell activation	BP	7.76E-01	TNFAIP3,TNFRSF21,SFRP1,LAPTM5,INPP5D	turquoise	EC_M6
GO:0016242	1.54E-01	0.929154666	7	32	negative regulation of macroautophagy	BP	7.78E-01	SEC22B,FEZ1,PIK3CA,SCFD1,QSOX1,AKT1,CLEC16A	darkgreen	EC_M4
GO:0050994	1.54E-01	0.924384598	8	39	regulation of lipid catabolic process	BP	7.78E-01	PPARA,MFSD2A,ABHD5,AKT2,IRS2,CPT1A,AKT1,PRKAA1	darkgreen	EC_M4
GO:0042303	1.54E-01	0.907788184	14	75	molting cycle	BP	7.78E-01	CLOCK,INHBA,PTCH2,GLI2,CTNNB1,SPINK5,TRPC4AP,TGFB2,FGFR2,FARP2,GORAB,NGFR,EDA,FGF10	darkgreen	EC_M4
GO:0042633	1.54E-01	0.907788184	14	75	hair cycle	BP	7.78E-01	CLOCK,INHBA,PTCH2,GLI2,CTNNB1,SPINK5,TRPC4AP,TGFB2,FGFR2,FARP2,GORAB,NGFR,EDA,FGF10	darkgreen	EC_M4

GO:0008202	1.54E-01	0.886614022	35	217	steroid metabolic process	BP	7.78E-01	ATP8B1,HSD17B6,PPARD,LEP,REST,DKKL1,EBPL,SNX17,DGAT2,SMPD1,OSBP,DKK3,APP,SPP1,LRP5,ARV1,OSBPL2,HDLBP,SGPL1,COMT,HSD17B1,CYP19A1,ACBD3,HSD17B10,CLN8,STARD3,MED1,PPARGC1A,CLN6,CYP46A1,PRKAA1,MBTPS1,NFE2L1,AMACR,YWHAH	darkgreen	EC_M4
GO:1900271	1.54E-01	0.928627447	7	33	regulation of long-term synaptic potentiation	BP	7.78E-01	PRNP,APP,CHRNA7,GSK3B,NCSTN,NPTN,CYP46A1	darkgreen	EC_M4
GO:0030203	1.04E-01	0.939705932	15	93	glycosaminoglycan metabolic process	BP	7.79E-01	PDGFB,PIM1,IL15,HS6ST1,ITIH5,LYVE1,HEXB,ST3GAL6,CHST1,HYAL2,B3GNT7,FUCA1,CHST7,HS3ST1,CHST9	turquoise	EC_M6
GO:0046637	1.05E-01	0.952680483	8	44	regulation of alpha-beta T cell differentiation	BP	7.79E-01	SYK,GATA3,SH3RF1,HLX,CD86,ITPKB,LGALS9,AP3D1	turquoise	EC_M6
GO:1903035	1.55E-01	0.911369757	12	64	negative regulation of response to wounding	BP	7.80E-01	SERPINB2,SLC12A2,FAM19A5,FGF2,C1QTNF1,ABCC8,SPP1,FAP,PHLDB2,SERPINE1,TNR,PRKG1	darkgreen	EC_M4
GO:0098657	1.05E-01	0.931900321	23	155	import into cell	BP	7.80E-01	TRPM4,RAB3B,CD36,KCNJ3,SLC38A1,KCNJ15,SNCA,SLC39A8,KCNJ2,SLC12A7,IL10RA,DLG1,ITGA4,SLC9A7,WNK4,PSEN1,KCNJ18,SLC43A2,LRSAM1,LRRC8C,SLC8A3,KCNH2,RGS4	turquoise	EC_M6
GO:1903036	1.05E-01	0.949758437	9	53	positive regulation of response to wounding	BP	7.80E-01	PTK2,CXCR4,HBEGF,FERMT1,CD36,OCLN,ANO6,MTOR,SCARF1	turquoise	EC_M6
GO:0043405	1.05E-01	0.932506199	22	146	regulation of MAP kinase activity	BP	7.81E-01	TNFRSF11A,TPD52L1,PDGFB,KIT,SYK,TLR4,PIK3CG,PPARG,SFRP1,EDN1,DTNBP1,HYAL2,MAP3K11,TLR6,RGS14,PSEN1,FGF18,PIK3R5,TRAF2,DAB2IP,BMP2,DUSP7	turquoise	EC_M6
GO:0030539	1.05E-01	0.969328115	4	17	male genitalia development	BP	7.81E-01	HSD17B3,AR,SRD5A2,BMP6	turquoise	EC_M6
GO:0009628	1.05E-01	0.913015354	109	891	response to abiotic stimulus	BP	7.81E-01	ZNF516,TNFRSF11A,AKAP12,PTK2,ATF4,MAPK13,PICK1,TEK,KIT,FIGNL1,TLR4,GRIK2,KDM4D,DNMT3A,GPR4,NOS3,GATA3,CXCR4,PPP1CB,NET1,PDE2A,SFRP1,RPL26,P2RX2,SLC4A11,RIC8A,MAP3K1,EPHB1,IL1A,DNMT3B,IGF1,NR4A1,EIF2AK3,MMP24,TLR2,VEGFC,NDRG4,CXCL10,ANGPT2,TMEM150C,ACTR5,STC1,KCNJ3,NR4A2,TANC1,EDN1,STAT1,ANO3,ETS1,SIK1,HDAC1,HYAL2,ANO1,ICOSLG,KCNJ2,CD34,POLG,CFLAR,RBM4,F11R,APOLD1,SIPA1,PDK3,CREB1,USP28,ARNT2,CYBB,DDX3X,RAMP2,MMP1,CPEB2,RAC1,HIF1A,ADSS,JUP,RGS14,MTOR,BNIP2,GNGT2,PGF,EIF2B2,CHRNB2,PIAS1,GPR68,DNM2,YY1,CYP1A1,ADRB2,NOS2,UNC119,PIEZO2,LONP1,HMOX2,NOTCH1,PIEZO1,ENG,RFWD3,LRRC8C,STRBP,NFAT5,PMAIP1,SLC8A3,LRRC8D,CA2,FGF16,SHANK3,BMP2,BMP6,NABP1	turquoise	EC_M6
GO:0006357	1.05E-01	0.908322033	215	1784	regulation of transcription by RNA polymerase II	BP	7.81E-01	ZNF516,TCF4,PLAGL1,ZBED4,NOTCH4,TCF20,TCF7,NCOA7,ATF4,PDGFB,KDM4C,NFYA,MDFI,PRDM4,DDX58,NFX1,NFIL3,SEMA4D,IL6,NFE2L3,REL,CREB5,BACH2,FOXP2,KLF11,NOBOX,TLR4,CREM,TEAD2,SDCBP,MYCN,KTI12,ZBTB24,TAL1,PHF10,EPC1,GATA2,DNMT3A,PRKD2,SAP130,DACH1,PPARG,GATA3,TAF3,ACVR2B,GZF1,EDNRB,IKBKB,JAG1,PDE2A,TMEM173,NR1D2,CD36,ARID5A,TCF15,CHD7,IL1A,DNMT3B,IGF1,NR4A1,HHEX,KDM3A,SMAD1,ZBTB38,POU6F1,STOX2,SOX7,HMBOX1,HEXB,MEF2C,SMARCD1,TLR2,WWTR1,ZNF704,CXCL10,KMT2D,SCAF4,NLRC5,ANKRD2,NR4A2,TOX2,DLL4,BAZ2B,SOX13,EDN1,STAT1,SNCA,JARID2,ERG,ETS2,NFKB2,DCAF1,PRDM15,ETS1,HDAC1,PPP3CA,HYAL2,MEF2A,BCL9,HLX,HCLS1,NFATC2,ZFHX2,EAF2,GMEB1,KDM2A,TGIF1,PARP15,POU2F3,ZSCAN29,TRAK2,ARID1A,RPS6KA1,PROX1,SS18L1,PRKD1,ZNF148,ZSCAN2,HELZ2,MLXIPL,ITGA6,DLG1,ELL3,ETV6,HOXD10,RNF2,CREB1,TET1,ARNT2,MYEF2,BCOR,DDX3X,RFXANK,ETV4,TBX1,MECOM,LDB2,POU2F1,WAS,TEAD4,HIF1A,ZBTB18,APBB2,JUP,BCL6B,MAFK,AR,CASZ1,PSEN1,HOXB5,HOXB6,HOXB7,HOXB8,HOXB9,ZNF410,BATF,EFNA1,PIAS1,RPS6KA5,SMAD2,YY1,ARID3B,LYL1,TNIP1,HMG20A,RCOR1,ZNF521,ABLIM3,ADRB2,YES1,RAI1,ELF4,MRTFB,MAMLD1,DOT1L,AP3D1,HCFC1,NRARP,NOTCH1,MED16,IRF8,S1PR1,ENG,MTF2,ZBTB34,ZNF821,NFAT5,LHX6,DAB2IP,ZBTB33,ARID4A,ZNF24,ATXN1L,SOX17,ZNF850,CDK13,SOX18,ZNF260,MAFF,ZNF229,PASD1,TGIF2,ZNF570,ZNF789,E2F3,ZKSCAN1,BMP2,GLIS3,ZNF10,CBX2,ZNF792,ZNF71,BMP6,FLI1,ZNF266	turquoise	EC_M6
GO:0002702	1.06E-01	0.943263687	12	75	positive regulation of production of molecular mediator of immune response	BP	7.81E-01	DDX58,KIT,IL6,RSAD2,TLR4,GATA3,CD36,LAPTM5,CD86,B2M,TRAF2,PLCG2	turquoise	EC_M6
GO:0072189	1.06E-01	0.969486527	4	16	ureter development	BP	7.81E-01	GATA3,RET,ALDH1A2,SOX17	turquoise	EC_M6

GO:0022408	1.06E-01	0.93447812	19	130	negative regulation of cell-cell adhesion	BP	7.81E-01	NOTCH4,PTK2,PODXL,TNFRSF21,PELI1,JAG1,ITCH,LAPTM5,HLX,CD86,DLG1,ADORA2A,PPM1F,ARG2,LGALS9,NRARP,NOTCH1,BMP2,BMP6	turquoise	EC_M6
GO:1903034	1.06E-01	0.935358757	18	123	regulation of response to wounding	BP	7.81E-01	TNFAIP3,TSPAN8,PTK2,PDGFB,NOS3,CXCR4,HBEGF,FERMT1,CD36,KLKB1,OCLN,ANO6,EDN1,CD34,INPP5F,MTOR,GIT1,SCARF1	turquoise	EC_M6
GO:0120036	1.06E-01	0.91029061	152	1193	plasma membrane bounded cell projection organization	BP	7.81E-01	SEMA6A,KIF5A,TBC1D30,SNX2,SPAG1,CTHRC1,RIMS2,CDKL3,MTSS1,ASAP1,PTK2,PODXL,PICK1,MICALL1,CDC42EP1,KIT,ADGRF1,TNFRSF21,CD2AP,SEMA4D,IL6,SDCBP,NRCAM,NRP1,SPATA6,ITSN2,MTMR2,NOVA2,FGD5,PLXND1,NECTIN2,ITGB4,GATA3,MYO7A,CXCR4,SPTBN4,SFRP1,FEZ2,EFNB2,POC1B,ARHGEF7,SPEF1,FGD6,PRAG1,EPHB1,PLK2,RET,PREX2,OCLN,KIF13B,FHDC1,ENC1,MEF2C,WWTR1,P2RX7,NDRG4,GPM6A,USH1C,PPP1R16B,NR4A2,ANO6,TANC1,CEP97,TSPAN2,JAM3,EHD4,DTNBP1,MYLIP,SRGAP2C,PPP3CA,HECW2,CCDC28B,MEF2A,SEMA3F,ROBO4,SEC24B,ABLIM1,JHY,PLEKHO1,PLXNA2,CFLAR,INPP5F,TRAK2,ADCY1,KALRN,FES,PLEKHA1,SS18L1,PRKD1,VANGL2,LAMA5,YTHDF1,ITGA6,LIMK2,PAK2,LAMC2,APOD,MOV10,CREB1,B2M,ADORA2A,SEMA6D,EPHB4,ITGA4,DNM3,CDK16,SNAP29,LMTK2,WAS,RAC1,RCC2,FSCN1,EPHA2,CECR2,FGD1,MTOR,OPHN1,EFNB1,PSEN1,SEMA4A,ABI3,ITGA3,TAOK2,EFNA1,CHRNB2,ZDHHC15,RABEP2,RPS6KA5,DNM2,B4GALT6,ABLIM3,SPEF2,STK26,SEMA6B,SLITRK2,ATCAY,ARHGAP4,NOTCH1,RAPGEF1,S1PR1,STXBP1,FNBP1L,DAB2IP,CARMIL2,VASP,RELN,ALKAL1,SHANK3,STX3,RALA,SCARF1,PIFO	turquoise	EC_M6
GO:0048858	1.06E-01	0.916475538	72	520	cell projection morphogenesis	BP	7.81E-01	SEMA6A,KIF5A,SNX2,RIMS2,PACSIN2,CDKL3,PTK2,SEMA4D,NRCAM,NRP1,ITSN2,NOVA2,PLXND1,GATA3,CXCR4,SPTBN4,FEZ2,EFNB2,ARHGEF7,EPHB1,RET,PREX2,KIF13B,GPM6A,NR4A2,DTNBP1,PPP3CA,HECW2,MEF2A,SEMA3F,ROBO4,PLEKHO1,PLXNA2,TRAK2,ADCY1,KALRN,SS18L1,LAMA5,YTHDF1,PAK2,LAMC2,MOV10,CREB1,ADORA2A,SEMA6D,EPHB4,ITGA4,DNM3,LMTK2,RAC1,EPHA2,OPHN1,EFNB1,PSEN1,SEMA4A,ABI3,TAOK2,EFNA1,CHRNB2,ZDHHC15,RPS6KA5,DNM2,B4GALT6,SEMA6B,SLITRK2,ARHGAP4,NOTCH1,STXBP1,DAB2IP,VASP,RELN,SHANK3	turquoise	EC_M6
GO:1901881	1.55E-01	0.948727008	4	15	positive regulation of protein depolymerization	BP	7.81E-01	WDR1,NES,DSTN,CFL2	darkgreen	EC_M4
GO:1902170	5.19E-02	0.988406732	4	15	cellular response to reactive nitrogen species	BP	7.81E-01	CDK2,CCNA2,AIFM1,HNRNPD	darkgrey	EC_M8
GO:2001235	1.06E-01	0.939610395	14	98	positive regulation of apoptotic signaling pathway	BP	7.82E-01	TRIM39,TPD52L1,CTSC,SFRP1,RPL26,RET,LCK,HYAL2,PAK2,ADORA2A,BID,TRAF2,DAB2IP,PMAIP1	turquoise	EC_M6
GO:0006464	1.06E-01	0.905646907	335	2859	cellular protein modification process	BP	7.82E-01	TNFRSF11A,BAZ2A,RMND5B,TNFAIP3,FBXO30,TRIM39,PTPRB,FLT4,ZDHHC14,DCAF13,OXR1,CDKL3,TPD52L1,NCOA7,RNF146,KLHL3,PTK2,DAPK1,PDGFB,AGTPBP1,MAPK13,BRPF3,CSNK1E,PICK1,KDM4C,PIM1,TEK,PRDM4,LIMCH1,NFX1,DCAF12,TEC,SH3RF3,LNX1,KIT,KDR,SRD5A3,SYK,SEMA4D,RNF149,MGAT4A,RIMKLA,PPP6R1,PIGF,IL6,PPT1,PPIE,PELI1,TLR4,TRAF1,IL15,CCNY,SDCBP,TRPM4,NRP1,RASIP1,PIK3CG,TAL1,MTMR2,EPC1,KDM4D,GATA2,PRKD2,CAB39L,SNRK,RALB,PTPN4,PPARG,GATA3,ACVR2B,IRAK2,EDNRB,PPP1CB,NDFIP2,SPTBN4,TIE1,SETD5,IKBKB,SEL1L2,SFRP1,HBEGF,NOTUM,SH3BP5,ABHD13,ANKLE2,ASH2L,CD36,SIRT3,CDK17,NEURL3,EOGT,GALNT7,PCMTD1,PRAG1,PRR5L,MAP3K1,EPHB1,PLK2,RET,PDE4D,MERTK,DNMT3B,IGF1,EIF2AK3,HHEX,KDM3A,ITCH,SH3RF1,ST3GAL5,SMAD1,GALNT18,OCLN,DCLK2,ENC1,TLR2,WWTR1,IL7,VEGFC,MADD,KMT2D,RSPRY1,BMP2K,TIPARP,KANSL2,THBS4,PPP1R16B,RAPGEF3,ST3GAL6,HIPK1,CCNJL,ZDHHC13,CDYL,NEDD9,EDN1,DYRK1A,EHD4,SNCA,JARID2,DTNBP1,MYLIP,ERG,RBBP5,NUAK2,CDK18,DCAF1,RNF144B,RNF19B,MTTP,SIK1,HDAC1,LCK,PPP3CA,HYAL2,HECW2,SLC39A8,ZDHHC6,ADPRH,B3GNT7,USP30,PRKX,LAPTM5,RNF123,NCF1,DYRK3,USP13,FABP4,HCLS1,PECAM1,CD86,KDM2A,B3GNT5,ASB9,PARP15,HHAT,BMX,FGR,MAP3K6,TGM1,INPP5F,TRAK2,KALRN,IGFBP3,ZDHHC18,FES,PPP2R5A,RPS6KA1,PROX1,PRKD1,PDIK1L,RNF215,MAP3K3,MGAT1,SRMS,DCAF7,MLXIPL,ACE,LIMK2,PAK2,PTPRE,SENP2,DLG1,MAP3K11,STYK1,PDK3,RNF2,CREB1,CTTNBP2NL,TRIM69,USP28,ST6GAL1,KANSL1,TET1,LTF,BCR,ADORA2A,P3H2,PALD1,BCOR,ALG9	turquoise	EC_M6

GO:0036211	1.06E-01	0.905646907	335	2859	protein modification process	BP	7.82E-01	TNFRSF11A,BAZ2A,RMND5B,TNFAIP3,FBXO30,TRIM39,PTPRB,FLT4,ZDHHC14,DCAF13,OXR1,CDKL3,TPD52L1,NCOA7,RNF146,KLHL3,PTK2,DAPK1,PDGFB,AGTPBP1,MAPK13,BRPF3,CSNK1E,PICK1,KDM4C,PIM1,TEK,PRDM4,LIMCH1,NFX1,DCAF12,TEC,SH3RF3,LNX1,KIT,KDR,SRD5A3,SYK,SEMA4D,RNF149,MGAT4A,RIMKLA,PPP6R1,PIGF,IL6,PPT1,PPIE,PELI1,TLR4,TRAF1,IL15,CCNY,SDCBP,TRPM4,NRP1,RASIP1,PIK3CG,TAL1,MTMR2,EPC1,KDM4D,GATA2,PRKD2,CAB39L,SNRK,RALB,PTPN4,PPARG,GATA3,ACVR2B,IRAK2,EDNRB,PPP1CB,NDFIP2,SPTBN4,TIE1,SETD5,IKBKB,SEL1L2,SFRP1,HBEGF,NOTUM,SH3BP5,ABHD13,ANKLE2,ASH2L,CD36,SIRT3,CDK17,NEURL3,EOGT,GALNT7,PCMTD1,PRAG1,PRR5L,MAP3K1,EPHB1,PLK2,RET,PDE4D,MERTK,DNMT3B,IGF1,EIF2AK3,HHEX,KDM3A,ITCH,SH3RF1,ST3GAL5,SMAD1,GALNT18,OCLN,DCLK2,ENC1,TLR2,WWTR1,IL7,VEGFC,MADD,KMT2D,RSPRY1,BMP2K,TIPARP,KANSL2,THBS4,PPP1R16B,RAPGEF3,ST3GAL6,HIPK1,CCNJL,ZDHHC13,CDYL,NEDD9,EDN1,DYRK1A,EHD4,SNCA,JARID2,DTNBP1,MYLIP,ERG,RBBP5,NUAK2,CDK18,DCAF1,RNF144B,RNF19B,MTTP,SIK1,HDAC1,LCK,PPP3CA,HYAL2,HECW2,SLC39A8,ZDHHC6,ADPRH,B3GNT7,USP30,PRKX,LAPTM5,RNF123,NCF1,DYRK3,USP13,FABP4,HCLS1,PECAM1,CD86,KDM2A,B3GNT5,ASB9,PARP15,HHAT,BMX,FGR,MAP3K6,TGM1,INPP5F,TRAK2,KALRN,IGFBP3,ZDHHC18,FES,PPP2R5A,RPS6KA1,PROX1,PRKD1,PDIK1L,RNF215,MAP3K3,MGAT1,SRMS,DCAF7,MLXIPL,ACE,LIMK2,PAK2,PTPRE,SENP2,DLG1,MAP3K11,STYK1,PDK3,RNF2,CREB1,CTTNBP2NL,TRIM69,USP28,ST6GAL1,KANSL1,TET1,LTF,BCR,ADORA2A,P3H2,PALD1,BCOR,ALG9	turquoise	EC_M6
GO:1902042	1.06E-01	0.962794413	5	26	negative regulation of extrinsic apoptotic signaling pathway via death domain receptors	BP	7.82E-01	TNFAIP3,NOS3,ITPRIP,CFLAR,DDX3X	turquoise	EC_M6
GO:0035872	1.06E-01	0.963282937	5	23	nucleotide-binding domain, leucine rich repeat containing receptor signaling pathway	BP	7.82E-01	TNFAIP3,TLR4,ITCH,ERBIN,BIRC3	turquoise	EC_M6
GO:0060421	1.07E-01	0.954984923	7	35	positive regulation of heart growth	BP	7.83E-01	PIM1,IGF1,MEF2C,EDN1,PROX1,MTOR,NOTCH1	turquoise	EC_M6
GO:0098815	1.07E-01	0.954986067	7	34	modulation of excitatory postsynaptic potential	BP	7.84E-01	RIMS2,MTMR2,ADRB2,SLC8A3,RELN,SHANK3,RGS4	turquoise	EC_M6
GO:0042220	1.07E-01	0.954801688	7	35	response to cocaine	BP	7.84E-01	DNMT3A,DNMT3B,SNCA,MTOR,CHRNB2,DNM2,RGS4	turquoise	EC_M6
GO:0051099	1.07E-01	0.932333223	20	141	positive regulation of binding	BP	7.85E-01	CTHRC1,MFNG,NRP1,KDM4D,PLXND1,RALB,PPARG,GATA3,TMEM173,PLK2,IGF1,HIP1R,HMBOX1,EPB41,ACE,B2M,RAMP2,PSEN1,MARK3,BMP2	turquoise	EC_M6
GO:2000113	1.07E-01	0.909395692	140	1144	negative regulation of cellular macromolecule biosynthetic process	BP	7.85E-01	PLAGL1,CNOT6,NOTCH4,TCF7,L3MBTL3,DAPK1,ATF4,PDGFB,MDFI,IREB2,NFX1,NFIL3,SEMA4D,IGF2BP3,NFE2L3,BACH2,FOXP2,KLF11,LANCL2,CREM,SDCBP,NOCT,TUT4,BEND5,TAL1,PHF10,EPC1,GATA2,DNMT3A,SAP130,DACH1,PPARG,GATA3,TAF3,ACVR2B,GZF1,EDNRB,PDE2A,SFRP1,HBEGF,NR1D2,DND1,CD36,ARID5A,DNMT3B,EIF2AK3,HHEX,ZBTB38,POU6F1,SOX7,HMBOX1,ENC1,MEF2C,WWTR1,SFMBT1,SCAF4,ANKRD2,NR4A2,DLL4,CDYL,SOX13,YAF2,EDN1,STAT1,SNCA,JARID2,ETS2,DCAF1,HDAC1,MEF2A,FABP4,HCLS1,NFATC2,KDM2A,CIART,TGIF1,PARP15,RBM4,PROX1,ZNF148,YTHDF1,MLXIPL,DLG1,ETV6,MOV10,RNF2,CREB1,BCOR,DDX3X,IGFBP5,MECOM,ING4,SPINDOC,LDB2,CPEB2,PPM1F,POU2F1,ZBTB18,APBB2,BCL6B,KDM5C,MAFK,AR,PSEN1,CBX1,HOXB8,FBXW11,EFNA1,PIAS1,RPS6KA5,SMAD2,TLE3,CNOT8,YY1,HMG20A,RCOR1,LIG3,HCFC1,NRARP,NOTCH1,IRF8,MTF2,ZBTB34,TERF2,DAB2IP,ZBTB33,ARID4A,ZNF24,ATXN1L,TRIM24,SOX18,PASD1,COMMD7,TGIF2,BMP2,GLIS3,ZNF10,CBX2,BMP6,ZNF266	turquoise	EC_M6
GO:0044344	1.07E-01	0.939085998	14	86	cellular response to fibroblast growth factor stimulus	BP	7.85E-01	CXCL8,PRKD2,GATA3,SFRP1,NR4A1,DLL4,SNCA,HYAL2,SHOC2,TBX1,FGF23,FGF18,CCL2,FGF16	turquoise	EC_M6
GO:0045746	1.56E-01	0.927349576	7	33	negative regulation of Notch signaling pathway	BP	7.86E-01	HEY1,CHAC1,HIF1AN,MMP14,BMP7,AKT1,SLC35C1	darkgreen	EC_M4
GO:0045058	1.57E-01	0.927288563	7	33	T cell selection	BP	7.86E-01	IL23A,SRF,GLI3,LOXL3,DOCK2,CD74,PTPN2	darkgreen	EC_M4

GO:0065002	1.57E-01	0.918716547	9	47	intracellular protein transmembrane transport	BP	7.86E-01	PEX6,SEC61A1,AKT2,TRAM1,TOMM40L,SRP54,HSPA5,GRPEL2,TOMM7	darkgreen	EC_M4
GO:0032091	1.08E-01	0.941881426	12	76	negative regulation of protein binding	BP	7.86E-01	PDGFB,CSNK1E,RALB,TMC8,FAM192A,IFIT2,IFIT1,DTNBP1,ACE,B2M,ITGA4,ADRB2	turquoise	EC_M6
GO:0051693	1.08E-01	0.954455158	7	34	actin filament capping	BP	7.86E-01	TMOD1,SVIL,SPTBN4,LMOD3,TMOD4,TMOD2,CARMIL2	turquoise	EC_M6
GO:0032495	1.08E-01	0.968199773	4	17	response to muramyl dipeptide	BP	7.86E-01	TNFAIP3,JAG1,ERBIN,NOTCH1	turquoise	EC_M6
GO:0050803	1.08E-01	0.927403498	26	170	regulation of synapse structure or activity	BP	7.87E-01	LINGO2,SEMA4D,PPT1,SETD5,EPHB1,MEF2C,TLR2,GPM6A,SEPT11,SNCA,SEMA3F,IL10RA,IL1RAP,DNM3,SEMA4A,ABI3,ARHGEF15,EFNA1,CHRNB2,ZDHHC15,SLITRK2,DAB2IP,ADGRL2,RELN,ABHD17C,SHANK3	turquoise	EC_M6
GO:0045824	1.09E-01	0.950235432	8	47	negative regulation of innate immune response	BP	7.87E-01	TNFAIP3,PPARG,OAS3,NLRC5,LGALS9,ISG15,OAS1,SERPINB9	turquoise	EC_M6
GO:1905709	7.09E-03	0.999326306	4	8	negative regulation of membrane permeability	BP	7.87E-01	HEG1,ACAA2,NOL3,BCL2L2	cyan	EC_M2
GO:0031113	1.09E-01	0.947753233	9	49	regulation of microtubule polymerization	BP	7.87E-01	ARHGEF7,OCLN,DYRK1A,SNCA,FES,RAC1,GIT1,CDH5,GBA2	turquoise	EC_M6
GO:0048568	1.09E-01	0.918931411	46	341	embryonic organ development	BP	7.87E-01	FZD6,CTHRC1,ATF4,PDGFB,MDFI,KIT,KDR,CXCL8,TEAD2,MYCN,TAL1,GATA2,GATA3,MYO7A,PCDH12,CHD7,MEF2C,NDRG4,USH1C,HIPK1,EDN1,HLX,SEC24B,PROX1,VANGL2,DLG1,HOXD10,TBX1,TEAD4,HIF1A,EPHA2,ALDH1A2,PSEN1,HOXB5,HOXB6,HOXB7,HOXB8,HOXB9,EFNA1,VASH1,SMAD2,NOTCH1,ENG,SOX17,SOX18,PIFO	turquoise	EC_M6
GO:0006576	1.09E-01	0.944820212	10	64	cellular biogenic amine metabolic process	BP	7.87E-01	SNCAIP,ODC1,GATA3,NR4A2,SNCA,OAZ3,GDPD3,CHRNB2,NPR1,GDPD1	turquoise	EC_M6
GO:0050789	1.57E-01	0.854818807	1105	7907	regulation of biological process	BP	7.88E-01	SERPINB2,CD63,LMAN1,IL23A,ATP8B1,NAB2,LRP1,STX7,AP3S1,TRIM36,HBS1L,B4GALT7,AHI1,ARHGEF25,AGAP2,ABRACL,ENPP3,TAB2,CAND1,DYRK2,IER3,RAB21,CDSN,RNF19A,RRM2B,PRRC1,SLC12A2,AZIN1,ATP6V1C1,PLXNB2,SKIV2L,FBN2,ANGPT1,FAM19A5,TCP1,PPARA,HLA-DRA,ROS1,AFF4,NUS1,RSPO3,CXCL14,FAM49B,PHF20L1,MRTFA,TRAPPC9,HABP4,PACSIN1,GNG5,PTP4A3,REEP2,ADGRB1,LY6E,SCUBE3,PPARD,CTNNA1,TEAD3,GOLM1,SUN2,CBY1,MKLN1,C6orf89,UBQLN1,MTCH1,LGALS1,MDGA1,GGA1,KLHDC10,TXN2,SH3GL2,GNAQ,PLIN2,MYH9,ARHGAP39,LEP,CDKN2B,PLAA,RORB,BTBD11,SLC30A9,KIAA1324L,SRF,BAG1,NUAK1,CDK14,VEGFA,DOCK8,VCP,SLC35B2,RUNX2,RASL11B,UNC13B,COL1A2,C2orf40,NCK2,CLOCK,EXOC1,CREB3,ASNS,PDCL3,REST,EIF5B,ASPN,INPP4A,ZSCAN18,DST,RAB23,SNX13,PTP4A1,NLRP5,COL15A1,P3H1,TGFBR1,ERP44,HIVEP3,SOCS5,RIMS1,TMEM30A,ERLEC1,PSME4,COPS9,RTN4,CYCS,TPBG,SHANK1,ADAMTS3,LRRC4B,EMC10,TAX1BP1,MFSD2A,GGCT,UBE2J1,MAP3K7,MYH14,SH3BP5L,AKIRIN1,ACTR2,THAP5,GFPT1,KIDINS220,WHRN,MDFIC,TES,GLI3,NCDN,ROCK2,INHBA,BVES,RRAS,ZYX,RCN3,MYLK3,DKKL1,VPS35,CUL2,MGARP,SLC7A11,ITGB1,PGRMC2,PLEKHA4,DBP,FKBP1B,PLXNA1,FGF2,SRPK2,KIF5B,NAPA,SESN3,ORC5,DNAJC1,RAB7A,PANX1,PSMC2,ARHGEF4,GSAP,APBB1IP,ABHD12,FBLN2,EML2,ZNF398,RB1,ITM2B,ABHD5,EED,IWS1,PICALM,MARK4,TPT1,NUFIP1,ZNF296,MAPRE3,CRTAP,CGREF1,EIF2B3,PTCH2,PDCD6IP,TENM4,DGKH,KCTD21,GLI2,MEGF8,ATP6V0B,SMARCD3,PCDH17,NUB1,RHEB,SNX17,PAK1,ACTR3,T	darkgreen	EC_M4
GO:0150063	1.09E-01	0.920494661	40	282	visual system development	BP	7.88E-01	ATF4,AGTPBP1,TMOD1,TSPAN12,NRP1,GATA3,ACVR2B,MYO7A,JAG1,FREM2,EPHB1,RET,MERTK,CHD7,GPM6A,USH1C,HIPK1,DLL4,LPCAT1,HDAC1,PROX1,LIMK2,DLG1,PROM1,HIF1A,RHOJ,EPHA2,ALDH1A2,SMOC1,PSEN1,ARHGEF15,TTLL5,YY1,CYP1A1,SLC25A25,RAB18,DIO3,TGIF2,TBC1D20,BMP6	turquoise	EC_M6
GO:0006346	1.09E-01	0.968015477	4	16	DNA methylation-dependent heterochromatin assembly	BP	7.88E-01	DNMT3A,HDAC1,KIAA1551,TET1	turquoise	EC_M6
GO:0001502	1.58E-01	0.947618347	4	15	cartilage condensation	BP	7.89E-01	BMP1,PKD1,COL11A1,OTOR	darkgreen	EC_M4
GO:0033622	1.58E-01	0.938691609	5	21	integrin activation	BP	7.90E-01	CXCL12,CX3CL1,CDH17,FARP2,FN1	darkgreen	EC_M4
GO:0039535	1.10E-01	0.967474652	4	17	regulation of RIG-I signaling pathway	BP	7.90E-01	PUM2,SEC14L1,BIRC3,RNF125	turquoise	EC_M6
GO:0060420	1.10E-01	0.944548535	10	58	regulation of heart growth	BP	7.90E-01	PIM1,IGF1,MEF2C,EDN1,JARID2,PROX1,MTOR,YY1,NOTCH1,RGS4	turquoise	EC_M6

GO:0051592	1.59E-01	0.895259226	20	114	response to calcium ion	BP	7.92E-01	ENTPD6,SCN5A,STIM1,SMPD1,RYR3,HOMER1,PKD2,CCND1,NCSTN,IQGAP1,CLIC4,MNAT1,SYT11,KCNMB1,CALM1,PRKAA1,AHCYL1,HSPA5,CACYBP,KCNMB4	darkgreen	EC_M4
GO:0051252	1.10E-01	0.902216079	312	2654	regulation of RNA metabolic process	BP	7.92E-01	ZNF516,TNFRSF11A,BAZ2A,TCF4,TNFAIP3,PLAGL1,CNOT6,FZD6,ZBED4,QKI,NOTCH4,TCF20,TCF7,NCOA7,L3MBTL3,ATF4,PDGFB,KDM4C,PIM1,NFYA,MDFI,PRDM4,DDX58,NFX1,KIT,ADGRF1,NFIL3,SYK,SEMA4D,IL6,IGF2BP3,NFE2L3,REL,CREB5,PTBP3,PPIE,BACH2,PELI1,FOXP2,KLF11,LANCL2,NOBOX,TLR4,TRAF1,CREM,TEAD2,SDCBP,MYCN,NOCT,TUT4,KTI12,ZBTB24,PUM2,BEND5,TAL1,PHF10,EPC1,GATA2,DNMT3A,PRKD2,SAP130,NOVA2,DACH1,PPARG,GATA3,TAF3,ACVR2B,IRAK2,GZF1,EDNRB,SETD5,IKBKB,JAG1,NFKBIB,PDE2A,TMEM173,SFRP1,NR1D2,DND1,CD36,CDK17,ARID5A,PRR5L,TCF15,RET,CHD7,IL1A,DNMT3B,IGF1,NR4A1,EIF2AK3,HHEX,KDM3A,ITCH,ERBIN,SMAD1,ZBTB38,POU6F1,STOX2,SOX7,HMBOX1,HEXB,MEF2C,SMARCD1,TLR2,WWTR1,ZNF704,CXCL10,ADGRG3,KMT2D,SFMBT1,SCAF4,OAS3,NLRC5,ACTR5,ANKRD2,NR4A2,TOX2,DLL4,CDYL,BAZ2B,SOX13,YAF2,EDN1,STAT1,DYRK1A,SNCA,JARID2,ERG,ETS2,CDK18,NFKB2,DCAF1,PRDM15,ETS1,CD200,SIK1,HDAC1,PPP3CA,HYAL2,SLC39A8,TESMIN,MEF2A,BCL9,HLX,NCF1,USP13,FABP4,HCLS1,NFATC2,ZFHX2,EAF2,GMEB1,CD86,KDM2A,CIART,MN1,TGIF1,PARP15,POU2F3,ZSCAN29,CFLAR,TRAK2,ADCY1,ARID1A,RBM4,RPS6KA1,PROX1,SS18L1,PRKD1,ZNF148,YTHDF1,ZSCAN2,HELZ2,MLXIPL,ITGA6,DLG1,ELL3,ETV6,HOXD10,MOV10,RNF2,CREB1,RBM7,EIF4A2,TET1,LTF,ADORA2A,ARNT2,MYEF2,IL1RAP,BCOR,DDX3X,RFXANK,ETV4,TBX1,MECOM,ING4,CDK16,SPINDOC,LDB2,FGF23,PPM1F,POU2F1,WAS,TEAD4,PRKCH,HIF1A,KDM5A,ZBTB18,APBB2,JUP,BCL6B,KDM5C,TLR6,RSC1A1,TNFRSF1B,RGS14,MAFK,SPIN2A,MTOR,AR,RNF40,CASZ1,PSEN1,	turquoise	EC_M6
GO:1902532	1.10E-01	0.916019911	53	407	negative regulation of intracellular signal transduction	BP	7.92E-01	SEMA6A,TNFAIP3,TRIM39,CD2AP,RNF149,ARHGAP25,FIGNL1,TLR4,RASIP1,ARHGAP42,PPARG,SEC14L1,PDE2A,SFRP1,SIRT3,PDE4D,ITCH,ERBIN,P2RX7,ADGRG3,STAT1,DYRK1A,DUSP8,PRDM15,SHANK2,HDAC1,HYAL2,DYRK3,SH3BP4,PLEKHA1,DUSP16,DLG1,ELL3,DDX3X,HOMER3,MECOM,HIF1A,EPHA2,BID,RGS14,MTOR,ITGA3,EFNA1,TNIP1,MARK3,TNFAIP8L1,DAB2IP,VNN1,DUSP5,BMP2,RNF125,DUSP7,RNF152	turquoise	EC_M6
GO:0044766	1.11E-01	0.967021145	4	17	multi-organism transport	BP	7.92E-01	CAV2,TPCN1,IFIT1,BST2	turquoise	EC_M6
GO:1902579	1.11E-01	0.967021145	4	17	multi-organism localization	BP	7.92E-01	CAV2,TPCN1,IFIT1,BST2	turquoise	EC_M6
GO:0031294	1.59E-01	0.931257893	6	28	lymphocyte costimulation	BP	7.92E-01	LGALS1,PTPN11,PIK3CA,PTPN6,AKT1,CD40LG	darkgreen	EC_M4
GO:0003184	1.59E-01	0.947123451	4	15	pulmonary valve morphogenesis	BP	7.92E-01	ROBO1,HEY1,NOTCH2,TGFB2	darkgreen	EC_M4
GO:0010557	1.11E-01	0.904536483	173	1433	positive regulation of macromolecule biosynthetic process	BP	7.92E-01	ZNF516,TCF4,PLAGL1,AKAP12,CNOT6,ZBED4,NOTCH4,TCF20,NCOA7,ATF4,PDGFB,BRPF3,PIM1,NFYA,PRDM4,DDX58,KDR,NFIL3,IL6,REL,CREB5,TLR4,IL15,TEAD2,MYCN,TAL1,PHF10,EPC1,GATA2,PRKD2,PPARG,GATA3,IKBKB,JAG1,NFKBIB,TMEM173,SFRP1,NR1D2,RPL26,TCF15,RET,CHD7,IL1A,IGF1,NR4A1,EIF2AK3,HHEX,KDM3A,SMAD1,ZBTB38,STOX2,SOX7,HMBOX1,HEXB,MEF2C,SMARCD1,TLR2,WWTR1,CXCL10,KMT2D,NLRC5,NR4A2,TOX2,YAF2,EDN1,STAT1,DYRK1A,ERG,ETS2,NFKB2,PRDM15,ETS1,HDAC1,PPP3CA,HYAL2,MEF2A,BCL9,NCF1,HCLS1,NFATC2,EAF2,GMEB1,CD86,POU2F3,ARID1A,RPS6KA1,PROX1,SS18L1,PRKD1,ZNF148,YTHDF1,HELZ2,MLXIPL,ITGA6,ELL3,ETV6,HOXD10,CREB1,TET1,ARNT2,DDX3X,RFXANK,ETV4,TBX1,TMEM106A,MECOM,ING4,LDB2,FGF23,POU2F1,WAS,RAC1,HIF1A,KDM5A,ZBTB18,APBB2,JUP,MTOR,AR,CASZ1,PSEN1,HOXB5,HOXB7,HOXB9,FBXW11,BATF,SLC51B,PIAS1,RPS6KA5,SMAD2,DNM2,YY1,ARID3B,LYL1,TNIP1,ZNF521,ABLIM3,ADRB2,CCL2,YES1,RAI1,ELF4,MRTFB,DOT1L,AP3D1,HCFC1,NOTCH1,MED16,IRF8,S1PR1,ENG,MTF2,NFAT5,DAB2IP,ARID4A,ZNF24,TRIM24,SOX17,KCNH2,ZNF850,ANKRD49,CDK13,SOX18,MAFF,TRIM62,FCER2,ZNF229,E2F3,BMP2,GLIS3,ZNF71,BMP6,FLI1	turquoise	EC_M6
GO:0010460	1.12E-01	0.966778062	4	17	positive regulation of heart rate	BP	7.93E-01	TRPM4,PDE4D,EDN1,RGS4	turquoise	EC_M6
GO:1905063	1.12E-01	0.966755998	4	17	regulation of vascular associated smooth muscle cell differentiation	BP	7.93E-01	PDGFB,KIT,NFATC2,ENG	turquoise	EC_M6
GO:0015748	1.12E-01	0.933516604	16	102	organophosphate ester transport	BP	7.93E-01	ATP8A1,ATP11A,SCARB1,PITPNM2,P2RX7,ANO6,ANO3,ABCG1,MTTP,ATP9A,ATP8B4,TNFAIP8L3,TMEM30B,SLC25A33,SLC25A25,LRRC8C	turquoise	EC_M6
GO:0022404	1.59E-01	0.908396164	12	63	molting cycle process	BP	7.93E-01	INHBA,GLI2,CTNNB1,SPINK5,TRPC4AP,TGFB2,FGFR2,FARP2,GORAB,NGFR,EDA,FGF10	darkgreen	EC_M4

GO:0022405	1.59E-01	0.908396164	12	63	hair cycle process	BP	7.93E-01	INHBA,GLI2,CTNNB1,SPINK5,TRPC4AP,TGFB2,FGFR2,FARP2,GORAB,NGFR,EDA,FGF10	darkgreen	EC_M4
GO:0036092	1.59E-01	0.946871197	4	15	phosphatidylinositol-3-phosphate biosynthetic process	BP	7.93E-01	INPP4A,PIK3CA,OCRL,PIK3C2A	darkgreen	EC_M4
GO:0003338	1.59E-01	0.931093728	6	27	metanephros morphogenesis	BP	7.93E-01	CTNNB1,PKD2,PDGFRB,PKD1,GDNF,FGF10	darkgreen	EC_M4
GO:0036119	1.60E-01	0.937846207	5	21	response to platelet-derived growth factor	BP	7.94E-01	HYAL1,SYAP1,PDGFRB,MTSS1L,HAS2	darkgreen	EC_M4
GO:0006081	1.60E-01	0.916874734	9	46	cellular aldehyde metabolic process	BP	7.94E-01	REST,ESD,DKK3,RDH10,IDH2,TPI1,HAGH,PARK7,GLO1	darkgreen	EC_M4
GO:0043507	1.12E-01	0.952117976	7	35	positive regulation of JUN kinase activity	BP	7.94E-01	TNFRSF11A,SYK,EDN1,MAP3K11,TLR6,TRAF2,DAB2IP	turquoise	EC_M6
GO:0061162	1.12E-01	0.960865922	5	22	establishment of monopolar cell polarity	BP	7.94E-01	SYNE4,TCF15,FSCN1,OPHN1,PATJ	turquoise	EC_M6
GO:2001224	1.60E-01	0.946584754	4	15	positive regulation of neuron migration	BP	7.94E-01	PLAA,RAPGEF2,ARHGEF2,FLNA	darkgreen	EC_M4
GO:0061042	1.60E-01	0.946014907	4	16	vascular wound healing	BP	7.94E-01	SLC12A2,FAM19A5,VEGFA,SERPINE1	darkgreen	EC_M4
GO:0000165	1.60E-01	0.867338386	88	579	MAPK cascade	BP	7.95E-01	LRP1,ANGPT1,ROS1,KLHDC10,LEP,VEGFA,TGFBR1,TPBG,EMC10,MAP3K7,MDFIC,ROCK2,RRAS,FGF2,PAK1,TGFB1,CTNNB1,LBH,C1QTNF1,SPRED3,GPS1,HDAC3,CAVIN3,SMPD1,ILK,ARL6IP5,RYK,ST5,PJA2,ROBO1,APP,RAPGEF2,MAPKAPK5,CX3CL1,PTPN11,PTPN22,GDF6,CHRNA7,NOTCH2,TGFB2,HTR2B,DAG1,PRKCA,KRAS,NTRK3,BMP7,ATF3,FGFR2,OSM,PEA15,IQGAP1,ATP6AP2,FN1,MAP4K5,PTPN6,PDCD10,ITGAV,ADRA2C,FGFR3,ARAF,TNIK,MAGED1,SHC1,ADRA1B,MAP2K1,NPTN,PIN1,ADORA2B,CD74,PDGFRB,ROCK1,AKT1,NLK,ZDHHC9,PTPN2,MYDGF,PAFAH1B1,IRAK1,ATP6AP1,BCAR3,MIR24-1,CCL16,DUSP2,TIMP3,DUSP4,GLIPR2,DUSP22,FGF10	darkgreen	EC_M4
GO:0048713	1.60E-01	0.930643113	6	27	regulation of oligodendrocyte differentiation	BP	7.95E-01	TENM4,RHEB,CTNNB1,DAG1,WDR1,TMEM98	darkgreen	EC_M4
GO:0055094	1.12E-01	0.960274728	5	24	response to lipoprotein particle	BP	7.95E-01	SYK,TLR4,PPARG,CD36,TLR6	turquoise	EC_M6
GO:0097734	1.12E-01	0.965895937	4	19	extracellular exosome biogenesis	BP	7.95E-01	SDCBP,ATP9A,CD34,RAB27A	turquoise	EC_M6
GO:0031399	1.60E-01	0.859844464	174	1182	regulation of protein modification process	BP	7.95E-01	IL23A,NAB2,LRP1,AGAP2,TAB2,PRRC1,PLXNB2,ANGPT1,ADGRB1,C6orf89,UBQLN1,SH3GL2,GNAQ,LEP,CDKN2B,PLAA,NUAK1,VEGFA,VCP,NCK2,PDCL3,P3H1,TGFBR1,SOCS5,COPS9,MAP3K7,SH3BP5L,ROCK2,INHBA,FKBP1B,FGF2,RB1,IWS1,MAPRE3,CRTAP,PAK1,TGFB1,RAB3GAP1,WDR48,SPOPL,CTNNB1,AKT2,GPD1L,OSBPL8,PPP1R12A,KAT2B,PRNP,CHFR,HDAC3,DDRGK1,SMPD1,ILK,RBPMS,CNOT7,FXYD1,SNTA1,HSP90B1,TMED2,OSBP,ROBO1,DIP2B,PINX1,PFN2,CCNJ,APP,RAPGEF2,CX3CL1,PTPN11,GNL3,GTF2H1,PTPN22,TSG101,ACVR1,CRY2,GDF6,AKTIP,CHP1,ATP2B4,PKD2,TWF1,DBNDD2,CBLB,BRD7,TANK,TOLLIP,NDN,SLC39A10,CHRNA7,TRIP12,CCND1,CAPRIN2,TGFB2,PPP6R3,LRP5,HTR2B,PRKAR1A,GSK3B,DAG1,PIK3CA,AUTS2,CORO1C,KRAS,NTRK3,SPATC1L,BMP7,PPP1R11,OSM,SYAP1,IQGAP1,TFRC,HSPB1,MAPT,DNAJC10,FN1,PTPN6,PDCD10,ADRA2C,DOK7,HTT,FKBP8,FGFR3,ARAF,TNIK,MNAT1,MAGED1,BMPR1A,TSPYL2,CDK12,PPARGC1A,RNF111,SRCIN1,ARHGEF2,TGFB3,SHC1,CCNG1,MAP2K1,CALM1,PPP4R4,GSKIP,NPTN,MYOCD,PIN1,CD74,PDGFRB,ROCK1,AKT1,PRKAA1,SUPT6H,PTPN2,MYDGF,NXN,TNFRSF18,PKD1,IRAK1,PARK7,POMT1,BCAR3,HSPA5,BCL10,MIR24-1,PRMT6,DMD,FKBP1A,FRY,LOX,THY1,CDKN1A,TXN,NNMT,DUSP22,PTTG1IP,TSLP,PMEPA1,FGF10	darkgreen	EC_M4
GO:0001573	1.13E-01	0.96009777	5	24	ganglioside metabolic process	BP	7.96E-01	ST3GAL5,HEXB,B4GALT6,ST6GALNAC3,ST8SIA6	turquoise	EC_M6
GO:0060419	1.61E-01	0.902873242	14	77	heart growth	BP	7.96E-01	PPARA,TGFBR1,FGF2,TENM4,PAK1,RUNX1,PDLIM5,TGFB2,PRKAR1A,FGFR2,BMPR1A,PIN1,MIR24-1,WNT2	darkgreen	EC_M4
GO:0061045	1.61E-01	0.91276227	10	52	negative regulation of wound healing	BP	7.97E-01	SERPINB2,SLC12A2,FAM19A5,FGF2,C1QTNF1,ABCC8,FAP,PHLDB2,SERPINE1,PRKG1	darkgreen	EC_M4

GO:1903204	1.61E-01	0.945571226	4	16	negative regulation of oxidative stress-induced neuron death	BP	7.97E-01	REST,SLC7A11,CTNNB1,PARK7	darkgreen	EC_M4
GO:2000757	5.31E-02	0.987894076	4	16	negative regulation of peptidyl-lysine acetylation	BP	7.97E-01	ZNF451,HDAC2,TAF7,BRCA1	darkgrey	EC_M8
GO:0006644	1.13E-01	0.916913155	41	314	phospholipid metabolic process	BP	7.97E-01	SYNJ2,SERAC1,PDGFB,PIP5K1B,ADGRF5,SRD5A3,PIGF,INPP4B,PIK3CG,MTMR2,RAB38,PGS1,GPAT4,CDS2,SCARB1,ABHD6,PITPNM2,CHRM5,HEXB,SERINC5,PLCB2,PLCG1,DGKZ,PIK3C2B,SNCA,MTMR10,LPCAT1,PLA1A,INPP5D,BMX,INPP5F,TNFAIP8L3,SAMD8,PLD2,ITPKB,GDPD3,GDPD1,MTMR12,MTM1,PLCG2,PLA2G4E	turquoise	EC_M6
GO:0048753	1.13E-01	0.959850871	5	24	pigment granule organization	BP	7.98E-01	RAB38,DTNBP1,LYST,AP3D1,RAB32	turquoise	EC_M6
GO:1903364	1.61E-01	0.889806241	23	137	positive regulation of cellular protein catabolic process	BP	7.98E-01	LRP1,RNF19A,UBQLN1,VCP,SOCS5,VPS35,PSMC2,NUB1,TMTC3,CHFR,DDRGK1,GSK3B,SMURF1,FBXL5,MSN,ARIH1,FBXO22,AKT1,BCAP31,TMEM259,UBE2V2,PTTG1IP,DDA1	darkgreen	EC_M4
GO:0006474	5.32E-02	0.988004706	4	15	N-terminal protein amino acid acetylation	BP	7.98E-01	NAA35,NAA15,NAA25,NAA50	darkgrey	EC_M8
GO:0010634	1.61E-01	0.888686678	24	143	positive regulation of epithelial cell migration	BP	7.99E-01	ANGPT1,NUS1,VEGFA,RTN4,EMC10,ROCK2,RRAS,FGF2,TGFB1,P2RX4,HYAL1,TGFB2,PRKCA,HSPB1,AKT3,RAB11A,AKT1,ITGA2,HAS2,TMSB4X,GLIPR2,CCR6,PIK3C2A,FGF10	darkgreen	EC_M4
GO:0010720	1.14E-01	0.919713352	33	231	positive regulation of cell development	BP	7.99E-01	CDKL3,KIT,SEMA4D,IL6,NRP1,PLXND1,UNC13D,CXCR4,ARHGEF7,LMOD3,RET,TLR2,VEGFC,NEDD9,HDAC1,PLXNA2,SS18L1,F11R,DOCK1,ELL3,PRKCH,RAC1,HIF1A,TNFRSF1B,RGS14,MTOR,GPR68,DNM2,NOTCH1,CDH5,RELN,SHANK3,BMP2	turquoise	EC_M6
GO:0062009	1.14E-01	0.960324174	5	21	secondary palate development	BP	7.99E-01	CHD7,DLG1,TBX1,SMAD2,JAG2	turquoise	EC_M6
GO:1901185	1.62E-01	0.929836196	6	27	negative regulation of ERBB signaling pathway	BP	8.00E-01	SOCS5,RAB7A,TSG101,CBLB,PTPN2,PTPN12	darkgreen	EC_M4
GO:0007005	1.62E-01	0.869801912	65	436	mitochondrion organization	BP	8.00E-01	LMAN1,HSPA4,FAM49B,COX18,GGCT,MYH14,VPS35,MGARP,TRAK1,DCN,PPP2CB,SLC25A46,MAP1LC3A,SLC25A4,SLC4A5,OPA1,ATG13,AP3B1,BAP1,CTTN,DNM1L,EYA2,CISD2,FEZ1,WIPI1,GSK3B,FAM162A,NDUFB3,TOMM40L,TFRC,FZD9,MAPT,C11orf65,HTT,GABPB1,VDAC2,AKT3,GHITM,HAP1,WIPI2,HSD17B10,CLN8,PPARGC1A,CALM1,ARMCX3,MIEF2,AKT1,ATPAF2,PRKAA1,OMA1,BCAP31,FUNDC2,PARK7,PRMT6,SURF1,BNIP3,COX7A2,BLOC1S2,COA1,GABARAPL2,TIMM22,LEPROT,GRPEL2,NDUFAF4,TOMM7	darkgreen	EC_M4
GO:0036092	1.14E-01	0.966491425	4	15	phosphatidylinositol-3-phosphate biosynthetic process	BP	8.00E-01	SYNJ2,INPP4B,PIK3CG,PIK3C2B	turquoise	EC_M6
GO:2000027	1.14E-01	0.930752208	17	108	regulation of animal organ morphogenesis	BP	8.00E-01	FZD6,CTHRC1,CELSR1,GATA3,SFRP1,SPEF1,NTN4,EDN1,SEC24B,JHY,CD34,VANGL2,TBX1,RAC1,AR,HOXB7,BMP2	turquoise	EC_M6
GO:0060999	1.62E-01	0.91949142	8	38	positive regulation of dendritic spine development	BP	8.01E-01	SHANK1,ACTR2,EIF4G2,DHX36,OPA1,DNM1L,CAPRIN2,PAFAH1B1	darkgreen	EC_M4
GO:0090398	5.35E-02	0.975295516	11	68	cellular senescence	BP	8.01E-01	CDK2,FBXO5,PNPT1,ZMPSTE24,SMC6,KAT6A,MAPK8,PRKDC,RBL1,EEF1E1,RSL1D1	darkgrey	EC_M8
GO:0031331	1.62E-01	0.871512351	57	366	positive regulation of cellular catabolic process	BP	8.01E-01	LRP1,RNF19A,PPARA,UBQLN1,VCP,SOCS5,MAP3K7,ROCK2,VPS35,SESN3,PSMC2,ABHD5,NUB1,RAB3GAP1,AKT2,IRS2,TMTC3,CHFR,DCN,DDRGK1,CNOT7,TP53INP2,SLC25A4,CELF1,APP,DHX36,MTDH,TENT4B,CPT1A,ZBTB20,GSK3B,ATF6,PAFAH1B2,UPF1,HTT,SAMD4A,SMURF1,FBXL5,MSN,SPTLC2,ARIH1,FBXO22,ROCK1,AKT1,PRKAA1,BCAP31,IKBKG,TMEM259,PARK7,GPSM1,GNAI3,BNIP3,UBE2V2,PTTG1IP,DDA1,PIK3C2A,ORMDL3	darkgreen	EC_M4
GO:0070664	1.14E-01	0.944239126	9	53	negative regulation of leukocyte proliferation	BP	8.02E-01	TNFAIP3,TNFRSF21,PELI1,ITCH,INPP5D,CD86,DLG1,ARG2,LGALS9	turquoise	EC_M6
GO:0007259	1.14E-01	0.932840067	15	98	receptor signaling pathway via JAK-STAT	BP	8.02E-01	KIT,IL6,IL15,RET,IGF1,STAT1,HDAC1,HCLS1,INPP5F,IL10RA,PIAS1,CCL2,JAK1,DOT1L,NOTCH1	turquoise	EC_M6

GO:1903587	1.15E-01	0.965310936	4	18	regulation of blood vessel endothelial cell proliferation involved in sprouting angiogenesis	BP	8.02E-01	GATA2,PPP1R16B,DLL4,MIR23A	turquoise	EC_M6
GO:0014912	1.15E-01	0.959277841	5	24	negative regulation of smooth muscle cell migration	BP	8.02E-01	MEF2C,NDRG4,IGFBP3,SEMA6D,IGFBP5	turquoise	EC_M6
GO:0006835	1.15E-01	0.941826557	10	56	dicarboxylic acid transport	BP	8.02E-01	APBA1,SLC3A1,SNCA,ADORA2A,SLC26A1,PSEN1,STXBP1,LRRC8C,LRRC8D,SLC25A21	turquoise	EC_M6
GO:0070936	1.15E-01	0.94163407	10	58	protein K48-linked ubiquitination	BP	8.03E-01	TNFAIP3,RNF146,KLHL3,PELI1,ITCH,BFAR,ZNRF1,UBE2G1,UBE2R2,RNF152	turquoise	EC_M6
GO:0034332	1.15E-01	0.947381466	8	39	adherens junction organization	BP	8.03E-01	MTSS1,HIPK1,JAM3,CDH24,PAK2,RAMP2,CDH5,BMP6	turquoise	EC_M6
GO:0031175	1.15E-01	0.905162056	103	784	neuron projection development	BP	8.03E-01	SEMA6A,KIF5A,CTHRC1,RIMS2,CDKL3,ASAP1,PTK2,MICALL1,ADGRF1,TNFRSF21,SEMA4D,IL6,NRCAM,NRP1,ITSN2,NOVA2,PLXND1,GATA3,MYO7A,CXCR4,SPTBN4,SFRP1,FEZ2,EFNB2,PRAG1,EPHB1,PLK2,RET,PREX2,KIF13B,ENC1,MEF2C,NDRG4,GPM6A,USH1C,NR4A2,TSPAN2,JAM3,DTNBP1,MYLIP,SRGAP2C,PPP3CA,HECW2,MEF2A,SEMA3F,ROBO4,SEC24B,PLXNA2,CFLAR,INPP5F,TRAK2,ADCY1,KALRN,FES,SS18L1,PRKD1,LAMA5,YTHDF1,ITGA6,PAK2,LAMC2,APOD,MOV10,CREB1,B2M,ADORA2A,SEMA6D,EPHB4,ITGA4,DNM3,CDK16,LMTK2,RAC1,EPHA2,CECR2,MTOR,OPHN1,EFNB1,PSEN1,SEMA4A,ABI3,ITGA3,TAOK2,EFNA1,CHRNB2,ZDHHC15,RPS6KA5,DNM2,B4GALT6,SEMA6B,SLITRK2,ATCAY,ARHGAP4,NOTCH1,RAPGEF1,STXBP1,DAB2IP,VASP,RELN,ALKAL1,SHANK3,STX3,SCARF1	turquoise	EC_M6
GO:0008037	1.15E-01	0.932458817	15	97	cell recognition	BP	8.03E-01	TNFRSF21,CNTNAP3,NRCAM,NRP1,IZUMO1,CXCR4,PCDH12,CD36,SCARB1,EPHB1,ROBO4,PECAM1,DLG1,PEAR1,MYO18A	turquoise	EC_M6
GO:0042088	1.15E-01	0.958731052	5	25	T-helper 1 type immune response	BP	8.03E-01	TLR4,HLX,CRACR2A,MTOR,SEMA4A	turquoise	EC_M6
GO:0043473	1.15E-01	0.936983029	12	75	pigmentation	BP	8.03E-01	KIT,RAB38,MYO7A,EDNRB,MEF2C,ADAMTS20,DTNBP1,LYST,USP13,RAB27A,AP3D1,RAB32	turquoise	EC_M6
GO:0060048	1.15E-01	0.93097711	16	103	cardiac muscle contraction	BP	8.03E-01	TRPM4,PIK3CG,CXCR4,PDE4D,STC1,KCNJ3,KCNJ2,DLG1,JUP,MTOR,ATP2A1,DSG2,DSC2,PDE4B,KCNH2,SCN2B	turquoise	EC_M6
GO:0014904	1.15E-01	0.953896682	6	31	myotube cell development	BP	8.03E-01	CAV2,P2RX2,LMOD3,IGF1,PPP3CA,NFATC2	turquoise	EC_M6
GO:0003018	1.16E-01	0.920152218	29	200	vascular process in circulatory system	BP	8.03E-01	AKAP12,TEK,ATP8A1,TJP2,DOCK4,TRPM4,ARHGAP42,GPR4,NOS3,EDNRB,SLCO2B1,P2RY2,PDE2A,CD36,OCLN,GUCY1A1,SLC38A1,EDN1,ACE,ADORA2A,RAMP2,PLVAP,NPR1,SCNN1B,ADRB2,YES1,CDH5,MIR23A,BMP6	turquoise	EC_M6
GO:2001224	1.16E-01	0.965826156	4	15	positive regulation of neuron migration	BP	8.03E-01	SEMA6A,SRGAP2C,DAB2IP,RELN	turquoise	EC_M6
GO:0048143	1.16E-01	0.96482003	4	18	astrocyte activation	BP	8.03E-01	IFNGR1,IL6,ADORA2A,PSEN1	turquoise	EC_M6
GO:0034340	1.16E-01	0.946136016	8	47	response to type I interferon	BP	8.03E-01	OAS3,NLRC5,IFIT1,STAT1,MX1,JAK1,ISG15,OAS1	turquoise	EC_M6
GO:0051961	1.16E-01	0.929570181	17	106	negative regulation of nervous system development	BP	8.03E-01	SEMA6A,CDKL3,SEMA4D,IL6,MYCN,NRP1,TLR2,PPP3CA,SEMA3F,TRAK2,B2M,SEMA6D,PSEN1,SEMA4A,SEMA6B,ARHGAP4,NOTCH1	turquoise	EC_M6
GO:0034694	1.16E-01	0.959090015	5	22	response to prostaglandin	BP	8.03E-01	P2RY6,SFRP1,EDN1,YY1,GNG2	turquoise	EC_M6
GO:0032309	1.16E-01	0.95864818	5	24	icosanoid secretion	BP	8.03E-01	TNFRSF11A,SYK,EDN1,ACE,NOS2	turquoise	EC_M6
GO:0007276	1.16E-01	0.909902629	59	469	gamete generation	BP	8.03E-01	TSPAN8,QKI,PICK1,KIT,FIGNL1,NOBOX,CREM,TUT4,SPATA6,DNMT3A,NECTIN2,DND1,GOLGA3,MERTK,IGF1,KDM3A,SMAD1,HEXB,ANGPT2,DHH,KMT2D,SFMBT1,CDYL,JAM3,GAS2,TESMIN,PLEKHA1,ZNF148,OAZ3,ZSCAN2,ACE,LIMK2,ELL3,ETV6,RNF2,SSTR1,DDX3X,CDK16,BIRC3,USP42,KDM5A,SPIN2A,MTOR,AR,FBXW11,CCDC42,PIAS1,DNM2,YY1,JAG2,SPEF2,SPIRE1,NOTCH1,STRBP,ARID4A,SOX17,TDRP,ANKRD49,TBC1D20	turquoise	EC_M6
GO:0048041	1.16E-01	0.933508271	14	81	focal adhesion assembly	BP	8.03E-01	PTK2,TEK,LIMCH1,KDR,NRP1,SFRP1,THSD1,ARHGEF7,APOD,BCR,PPM1F,RAC1,RCC2,TAOK2	turquoise	EC_M6
GO:0050864	1.16E-01	0.932992204	14	93	regulation of B cell activation	BP	8.03E-01	TNFAIP3,TNFRSF21,SYK,IL6,PELI1,TLR4,SFRP1,MEF2C,IL7,LAPTM5,NFATC2,INPP5D,CHRNB2,CD19	turquoise	EC_M6
GO:0051953	1.63E-01	0.94457145	4	16	negative regulation of amine transport	BP	8.04E-01	LEP,ARL6IP5,ADRA2C,SYT11	darkgreen	EC_M4

GO:0071711	1.17E-01	0.953461958	6	30	basement membrane organization	BP	8.06E-01	CAV2,FERMT1,NTN4,RIC8A,GAS2,RAMP2	turquoise	EC_M6
GO:0060047	1.17E-01	0.921792026	25	172	heart contraction	BP	8.06E-01	TRDN,TRPM4,PIK3CG,CXCR4,SPTBN4,HBEGF,PDE4D,STC1,KCNJ3,EDN1,KCNJ2,MEF2A,ACE,DLG1,JUP,MTOR,ATP2A1,DSG2,DSC2,PDE4B,SLC8A3,KCNH2,SCN2B,RGS4,KCNG2	turquoise	EC_M6
GO:0051246	1.17E-01	0.897845615	227	1953	regulation of protein metabolic process	BP	8.06E-01	TNFRSF11A,TNFAIP8,IFNGR1,TNFAIP3,TRIM39,PTPRB,LAPTM4B,CNOT6,FLT4,OXR1,GRAMD4,QKI,TPD52L1,NCOA7,SGSM3,PTK2,DAPK1,ATF4,PDGFB,AGTPBP1,CSNK1E,TEK,IREB2,LIMCH1,DDX58,FAM122A,TEC,KIT,KDR,SYK,SEMA4D,RGP1,PPP6R1,IL6,IGF2BP3,PELI1,ODC1,TLR4,C5,IL15,CCNY,SDCBP,NOCT,TUT4,NRP1,PUM2,RASIP1,PIK3CG,TAL1,KDM4D,GATA2,PRKD2,CST7,CTSC,CAB39L,GGA3,RALB,PPARG,TIMP4,GATA3,ITIH5,EDNRB,NDFIP2,SPTBN4,SETD5,IKBKB,SFRP1,HBEGF,DND1,RPL26,SH3BP5,ANKLE2,CD36,PRR5L,PLK2,RET,PDE4D,DNMT3B,IGF1,NR4A1,KLKB1,EIF2AK3,HHEX,KDM3A,ITCH,SH3RF1,HIP1R,OCLN,SOX7,ENC1,WWTR1,IL7,VEGFC,MADD,FAM192A,TIPARP,THBS4,PPP1R16B,RAPGEF3,CCNJL,CDYL,NEDD9,EDN1,DYRK1A,EHD4,SNCA,JARID2,DTNBP1,MYLIP,RNF144B,ABCG1,RNF19B,LPCAT1,HDAC1,LCK,HYAL2,HECW2,LAPTM5,NCF1,USP13,FABP4,HCLS1,PECAM1,CD86,ASB9,FGR,CFLAR,CASP10,INPP5F,IGFBP3,PPP2R5A,RBM4,RPS6KA1,PROX1,PRKD1,YTHDF1,OAZ3,MLXIPL,ACE,LIMK2,PAK2,CST6,MELTF,SENP2,DLG1,APOD,MAP3K11,MOV10,EIF4A2,TET1,LTF,ADORA2A,BCOR,DDX3X,IGFBP5,TBX1,SECISBP2L,BIRC3,FGF23,CPEB2,PPM1F,RAC1,HIF1A,KDM5A,ADIPOR2,ITPKB,CASC3,BID,TLR6,TNFRSF1B,RGS14,FBXL20,MTOR,OPHN1,RNF40,PSEN1,PAPLN,FBXW11,FGF18,PGF,EIF2B2,TAOK2,EFNA1,SLC51B,NAP1L2,PIK3R5,PIAS1,RPS6KA5,CCDC88C,CNOT8,TNIP1,HMG20A,ADRB2,YES1,SH2D3A,LGALS9,NOS2,UNC119,USP7,ISG15,TRAF2,PLCG2,DPH5,ENG,SH2D3C,MTF2,DAB2IP,CDH5,PRSS22,PMAIP1,SLC8A3,SOX17,RELN,ALKAL1,FGF16,MMD,SERPINB9,MGAT3,PSMB9,PERP,DAZAP2,COPS8,BST	turquoise	EC_M6
GO:0050909	1.64E-01	0.935434848	5	22	sensory perception of taste	BP	8.06E-01	REEP2,CALHM1,LEF1,GNB1,SCNN1D	darkgreen	EC_M4
GO:0042325	1.64E-01	0.858684101	144	967	regulation of phosphorylation	BP	8.06E-01	IL23A,NAB2,LRP1,AGAP2,TAB2,PRRC1,PLXNB2,ANGPT1,PPARA,ROS1,SH3GL2,GNAQ,LEP,CDKN2B,VEGFA,NCK2,PDCL3,TGFBR1,SOCS5,MAP3K7,SH3BP5L,ROCK2,INHBA,FGF2,RB1,MAPRE3,PAK1,TGFB1,AXL,AKT2,GPD1L,OSBPL8,KAT2B,IRS2,PRNP,HDAC3,CCKBR,DDRGK1,SMPD1,ILK,RBPMS,RYK,CNOT7,TMED2,OSBP,ROBO1,PFN2,CCNJ,APP,RAPGEF2,CX3CL1,PTPN11,GTF2H1,PTPN22,TSG101,ACVR1,GDF6,AKTIP,CHP1,ATP2B4,PKD2,TWF1,DBNDD2,CBLB,CHRNA7,DYNLL1,CCND1,CAPRIN2,TGFB2,ZBTB20,LRP5,HTR2B,PRKAR1A,DAG1,PIK3CA,CORO1C,KRAS,NTRK3,SLAMF8,SPATC1L,BMP7,FGFR2,OSM,SYAP1,IQGAP1,TFRC,HSPB1,MAPT,DNAJC10,FN1,PTPN6,PDCD10,ADRA2C,DOK7,HTT,FKBP8,FGFR3,ARAF,TNIK,MNAT1,MAGED1,BMPR1A,TSPYL2,CDK12,PPARGC1A,SRCIN1,ARHGEF2,TGFB3,SHC1,CCNG1,MAP2K1,CALM1,GSKIP,NPTN,MYOCD,PIN1,CD74,PDGFRB,ROCK1,AKT1,PRKAA1,PTPN2,MYDGF,TNFRSF18,PKD1,IRAK1,MIDN,PARK7,SLC2A6,BCAR3,LMO4,BCL10,MIR24-1,RANBP2,DMD,LOX,PPP1R14A,THY1,CDKN1A,TXN,DUSP22,TSLP,PMEPA1,FGF10	darkgreen	EC_M4
GO:0035265	1.17E-01	0.925525766	20	134	organ growth	BP	8.06E-01	PIM1,IGF1,SMAD1,MEF2C,IL7,NDRG4,STC1,EDN1,JARID2,HLX,PROX1,MMP13,MTOR,AR,SMAD2,YY1,NOTCH1,S1PR1,MIR23A,RGS4	turquoise	EC_M6
GO:0050432	1.17E-01	0.952639429	6	34	catecholamine secretion	BP	8.06E-01	SNCA,DTNBP1,ADORA2A,SNCG,SYT7,CHRNB2	turquoise	EC_M6
GO:0034143	1.17E-01	0.964133374	4	18	regulation of toll-like receptor 4 signaling pathway	BP	8.06E-01	TNFAIP3,PELI1,LTF,DAB2IP	turquoise	EC_M6
GO:0002861	1.17E-01	0.957928621	5	24	regulation of inflammatory response to antigenic stimulus	BP	8.06E-01	SYK,PLK2,PLCG1,FGR,YES1	turquoise	EC_M6
GO:0003007	1.17E-01	0.918777028	29	197	heart morphogenesis	BP	8.06E-01	PTK2,PIM1,TEK,TEAD2,NRP1,PLXND1,NOS3,GATA3,JAG1,CHD7,MEF2C,NDRG4,DLL4,SEC24B,PROX1,VANGL2,EPHB4,TBX1,HIF1A,ALDH1A2,MTOR,PSEN1,EFNA1,NOTCH1,S1PR1,ENG,SOX17,SOX18,BMP2	turquoise	EC_M6
GO:0071715	1.17E-01	0.953011478	6	30	icosanoid transport	BP	8.06E-01	TNFRSF11A,SYK,SLCO2A1,EDN1,ACE,NOS2	turquoise	EC_M6

GO:0044703	1.18E-01	0.904683562	84	688	multi-organism reproductive process	BP	8.06E-01	TSPAN8,SPAG1,QKI,PICK1,KIT,FIGNL1,NOBOX,CREM,TUT4,IZUMO1,SPATA6,DNMT3A,NECTIN2,PSG3,ITGB4,EDNRB,SPTBN4,DND1,GOLGA3,MERTK,IGF1,KDM3A,SMAD1,STOX2,HEXB,ANGPT2,DHH,KMT2D,SFMBT1,STC1,SLC38A1,CDYL,EDN1,JAM3,GAS2,ETS1,TESMIN,LRMP,PLEKHA1,ZNF148,OAZ3,ZSCAN2,ARHGDIB,ACE,LIMK2,ELL3,ETV6,HOXD10,RNF2,SSTR1,DDX3X,IGFBP5,RAMP2,CDK16,BIRC3,USP42,KDM5A,ADIPOR2,CECR2,SPIN2A,MTOR,AR,FBXW11,ITGA3,PGF,CCDC42,PIAS1,DNM2,YY1,CYP1A1,JAG2,LGALS9,SPEF2,SPIRE1,NOTCH1,STXBP1,STRBP,ARID4A,CD46,SOX17,TDRP,ANKRD49,MAFF,TBC1D20	turquoise	EC_M6
GO:0120255	1.18E-01	0.96403214	4	17	olefinic compound biosynthetic process	BP	8.09E-01	HSD17B3,BMP2,SRD5A2,BMP6	turquoise	EC_M6
GO:0001933	1.64E-01	0.874135302	44	276	negative regulation of protein phosphorylation	BP	8.09E-01	LRP1,ANGPT1,SH3GL2,GNAQ,CDKN2B,NCK2,SOCS5,SH3BP5L,RB1,TGFB1,GPD1L,KAT2B,PRNP,SMPD1,ILK,TMED2,PTPN22,TSG101,CHP1,DBNDD2,CBLB,LRP5,PRKAR1A,CORO1C,NTRK3,BMP7,HSPB1,DNAJC10,PTPN6,FKBP8,PPARGC1A,SRCIN1,CALM1,GSKIP,MYOCD,AKT1,PTPN2,PARK7,MIR24-1,DMD,THY1,CDKN1A,DUSP22,PMEPA1	darkgreen	EC_M4
GO:0042093	1.18E-01	0.944897684	8	44	T-helper cell differentiation	BP	8.09E-01	IL6,GATA3,HLX,CD86,CRACR2A,MTOR,SEMA4A,BATF	turquoise	EC_M6
GO:0032101	1.64E-01	0.862335412	97	646	regulation of response to external stimulus	BP	8.09E-01	SERPINB2,IL23A,LRP1,ENPP3,SLC12A2,FAM19A5,PPARA,FAM49B,PPARD,LEP,VEGFA,CLOCK,CREB3,SOCS5,TPBG,MAP3K7,AKIRIN1,ZYX,VPS35,FKBP1B,FGF2,ABHD12,RB1,MEGF8,PAK1,TGFB1,C1QTNF1,NPLOC4,SEMA3C,SPINK5,RYK,CNOT7,CXCL12,IL20RB,CD180,VAMP8,GRK7,ROBO1,SEMA4F,P2RX4,CELF1,APP,CX3CL1,ABCC8,CACNB3,PTPN11,PTPN22,POLR3D,ADA,BAP1,SPP1,SEMA4G,FAP,PHLDB2,DNM1L,SELENOS,PRKCA,CMKLR1,NTRK3,SLAMF8,OSM,HTRA1,BACE1,HSPB1,SERPINE1,TNR,PTPN6,NBL1,MED1,SYT11,MAP2K1,TSPAN6,CD74,PDGFRB,USP14,ITGA2,OSMR,PTPN2,FLNA,PLXNA3,FAM3A,VAMP7,PARK7,CDH13,F3,AP1G1,SERPINF1,A2M,FGL2,ARTN,TMSB4X,FCN1,CCR6,TSLP,HOPX,PRKG1,FGF10	darkgreen	EC_M4
GO:0014743	1.65E-01	0.910280908	10	52	regulation of muscle hypertrophy	BP	8.10E-01	PPARA,ROCK2,PAK1,P2RX4,ATP2B4,TWF1,PRKCA,GLRX3,PIN1,ROCK1	darkgreen	EC_M4
GO:0010810	1.19E-01	0.919628275	26	175	regulation of cell-substrate adhesion	BP	8.11E-01	PTK2,PDGFB,TEK,LIMCH1,KDR,NRP1,UNC13D,JAG1,SFRP1,FERMT1,ARHGEF7,CD36,ANGPT2,NEDD9,AP1AR,ITGA6,DOCK1,MELTF,APOD,PPM1F,RAC1,RCC2,JUP,ITGA3,DNM2,NOTCH1	turquoise	EC_M6
GO:0042417	1.19E-01	0.957303977	5	24	dopamine metabolic process	BP	8.11E-01	SNCAIP,NR4A2,SNCA,CHRNB2,NPR1	turquoise	EC_M6
GO:0060998	1.19E-01	0.93941943	10	54	regulation of dendritic spine development	BP	8.11E-01	ASAP1,PLK2,MEF2C,SRGAP2C,DNM3,MTOR,PSEN1,EFNA1,RELN,SHANK3	turquoise	EC_M6
GO:0031110	5.43E-02	0.973083969	13	79	regulation of microtubule polymerization or depolymerization	BP	8.11E-01	AKAP9,CLASP2,CKAP2,TPX2,SKA3,CKAP5,STMND1,CAMSAP2,HDGFL3,FKBP4,TAOK1,STMN1,DRG1	darkgrey	EC_M8
GO:0006706	1.19E-01	0.962986075	4	20	steroid catabolic process	BP	8.11E-01	SCARB1,FGF23,SCARF1,AKR1D1	turquoise	EC_M6
GO:0031328	1.19E-01	0.897022098	180	1503	positive regulation of cellular biosynthetic process	BP	8.12E-01	ZNF516,TCF4,PLAGL1,CNOT6,ZBED4,NOTCH4,TCF20,NCOA7,ATF4,PDGFB,BRPF3,PIM1,NFYA,PRDM4,DDX58,ADGRF5,NFIL3,IL6,REL,CREB5,CXCL8,TLR4,IL15,TEAD2,MYCN,TAL1,PHF10,EPC1,GATA2,PRKD2,RAB38,NOS3,PPARG,GATA3,IKBKB,JAG1,P2RY6,NFKBIB,TMEM173,SFRP1,NR1D2,RPL26,CD36,SIRT3,TCF15,SCARB1,RET,CHD7,IL1A,IGF1,NR4A1,EIF2AK3,HHEX,KDM3A,SMAD1,ZBTB38,STOX2,SOX7,HMBOX1,HEXB,MEF2C,SMARCD1,TLR2,WWTR1,CXCL10,KMT2D,NLRC5,NR4A2,TOX2,YAF2,EDN1,STAT1,DYRK1A,SNCA,ERG,ETS2,SLC45A3,NFKB2,PRDM15,ETS1,HDAC1,PPP3CA,HYAL2,MEF2A,BCL9,NCF1,HCLS1,NFATC2,EAF2,GMEB1,CD86,POU2F3,ARID1A,RPS6KA1,PROX1,SS18L1,PRKD1,ZNF148,YTHDF1,HELZ2,MLXIPL,ITGA6,ELL3,ETV6,HOXD10,CREB1,TET1,ARNT2,DDX3X,RFXANK,ETV4,TBX1,MECOM,ING4,LDB2,FGF23,POU2F1,WAS,RAC1,HIF1A,KDM5A,ZBTB18,APBB2,JUP,TLR6,MTOR,AR,CASZ1,PSEN1,HOXB5,HOXB7,HOXB9,FBXW11,BATF,SLC51B,PIAS1,RPS6KA5,SMAD2,DNM2,YY1,ARID3B,LYL1,TNIP1,ZNF521,ABLIM3,ADRB2,YES1,RAI1,NOS2,ELF4,MRTFB,DOT1L,AP3D1,HCFC1,NOTCH1,MED16,IRF8,S1PR1,ENG,MTF2,NFAT5,DAB2IP,ARID4A,ZNF24,TRIM24,SOX17,KCNH2,ZNF850,ANKRD49,CDK13,SOX18,MAFF,TRIM62,ZNF229,E2F3,BMP2,GLIS3,ZNF71,BMP6,FLI1	turquoise	EC_M6

GO:0008285	1.19E-01	0.905587002	68	548	negative regulation of cell population proliferation	BP	8.12E-01	TNFAIP3,MTSS1,AGTPBP1,TNFRSF21,IL6,CXCL8,PELI1,CAV2,KLF11,IL15,GATA2,NOS3,DACH1,PPARG,GATA3,SFRP1,FERMT1,EPHB1,IL1A,ITCH,SMAD1,SOX7,MEF2C,TLR2,NDRG4,TP53I11,DLL4,STAT1,JARID2,ETS1,CD200,HDAC1,INPP5D,EAF2,CD86,MN1,WDR6,SH3BP4,IGFBP3,PROX1,DLG1,APOD,B2M,SSTR1,ADORA2A,P3H2,IGFBP5,ING4,MCC,ARG2,ALDH1A2,AR,VASH1,NPR1,SMAD2,PPP2R5C,CCL2,LGALS9,NOTCH1,DAB2IP,CDH5,MIR23A,ADGRG1,TRIM24,E2F3,COPS8,BMP2,IFIT3	turquoise	EC_M6
GO:0098901	1.19E-01	0.957484299	5	22	regulation of cardiac muscle cell action potential	BP	8.12E-01	TRPM4,DLG1,JUP,DSG2,DSC2	turquoise	EC_M6
GO:0002027	1.19E-01	0.934504241	12	73	regulation of heart rate	BP	8.12E-01	TRPM4,SPTBN4,PDE4D,KCNJ3,EDN1,KCNJ2,JUP,DSG2,DSC2,KCNH2,SCN2B,RGS4	turquoise	EC_M6
GO:0051171	7.40E-03	0.993731674	487	4082	regulation of nitrogen compound metabolic process	BP	8.13E-01	NFATC1,ZNF236,TSHZ1,ORMDL2,PYM1,SMARCC2,STAT6,MAP3K5,CDK4,CITED2,CTDSP2,MAML1,SQSTM1,SASH1,IRAK3,HELB,OSR2,BAG6,PPP6R2,SNX9,HSPA1B,EHMT2,ZBTB12,HDAC10,ATF6B,EIF3H,RPS6KA2,DEPTOR,RXRB,TSPO,RING1,ZHX2,ZBTB22,MYC,TOB2,SMAD5,EP300,KDM3B,ZC3H3,ZNF76,TAB1,DDX17,STK38,NFIB,EIF3D,HSF1,VPS28,CCND3,PPP1R16A,TRERF1,CDKN2A,HMOX1,WASL,KLF9,JAK2,HINT2,RECK,ZBTB45,ZNF135,ZNF667,ZNF865,RPS9,EEF1A1,CD109,GPNMB,MALSU1,STAP1,EFEMP1,ZNF692,CYREN,RPS7,ANTXR1,ANXA4,AKNA,MED25,IL4I1,AKT1S1,ING3,ARHGEF5,AP2A1,IRF3,LPIN1,GSN,PARD3,FOXO3,KLF15,SPHK2,SPRY1,NOP53,MAML2,BICRA,ZC3HAV1,KMT2E,BBS7,MEIS3,HMCES,TRIM13,IRF2BP1,SIX5,FOSB,ZNF746,NR2C2,ZNF862,TSC22D1,FASTK,XRCC1,ZNF575,MIF4GD,NAA16,MLH1,UNK,ZNF574,WBP2,KMT2C,MYD88,CELF2,EGLN2,LTBP4,BABAM2,TADA3,RPS3,BRPF1,DYRK1B,EID2,TGFBR2,ZNF219,TIMP2,MED29,SAMD4B,XDH,SIRT2,RPTOR,HGS,APBB3,ASPSCR1,EIF3K,NUP98,FGFR1,TRIM21,CYP1B1,NDFIP1,SPRY4,HGF,APBB1,DEAF1,HOXC4,CLIP3,NCOA4,PCBP2,LIN37,SLC15A4,PSMF1,TARBP2,KMT2B,RBCK1,RARG,SDR16C5,ZNF740,EIF4B,LMO2,POFUT1,LATS2,NECAB3,URI1,PIK3R1,NCOA2,CAST,PCOLCE2,ATF1,TNKS1BP1,SH3D19,ARRDC3,KDM2B,ZFHX4,CAMKK2,SCAND1,HTRA2,EIF2A,SETD6,MCPH1,PDGFC,SPRED1,SSBP2,CCNG2,FNIP2,PRKAG1,ARL2BP,TIAM1,SHOX2,NEK4,PROS1,ZHX3,VDR,RBM12B,MMS19,LPIN3,FOXF2,F2RL1,RNFT2,SORBS3,ORMDL1,HNF4A,TP53INP1,PIDD1,HR,NGF,ACOT8,PCIF1,TRIM45,CD47,PARP3,FASTKD3,ELK4,LGR4,RBM15B,ICE1,CSTB,SP140,	cyan	EC_M2
GO:0006140	7.43E-03	0.997277758	13	57	regulation of nucleotide metabolic process	BP	8.13E-01	EP300,SPHK2,PRKAG1,ARNT,HDAC4,DNAJC30,NUPR1,INSR,TAZ,JMJD8,MT-CO2,ZBTB7A,PID1	cyan	EC_M2
GO:0098742	1.20E-01	0.920242045	24	152	cell-cell adhesion via plasma-membrane adhesion molecules	BP	8.15E-01	CELSR1,NECTIN2,PCDH1,PCDH12,RIC8A,RET,MMP24,SPARCL1,ROBO4,ESAM,CDH24,CADM3,PECAM1,HMCN1,IL1RAP,SELE,SELP,PCDH11X,DSG2,DSC2,DSC3,SLITRK2,CDH5,BMP2	turquoise	EC_M6
GO:0048311	1.20E-01	0.96366862	4	16	mitochondrion distribution	BP	8.15E-01	MEF2A,TRAK2,MTM1,ATCAY	turquoise	EC_M6
GO:0045055	1.66E-01	0.88180215	28	167	regulated exocytosis	BP	8.15E-01	MYH9,UNC13B,REST,NLRP5,RIMS1,VPS41,NAPA,RAB3GAP1,RAB11FIP1,PCLO,SNPH,VAMP8,ATP2A2,VPS18,DNM1L,GSK3B,BACE1,STX1B,SYT11,TMED10,CACNA1G,CALM1,SCAMP5,RAB31,SDF4,VAMP7,AP1G1,CBARP	darkgreen	EC_M4
GO:0034308	1.20E-01	0.938120886	10	61	primary alcohol metabolic process	BP	8.15E-01	RDH16,IGF1,GPD1,ALDH1A2,GDPD3,GDPD1,SCNN1B,CYP1A1,BMP2,BMP6	turquoise	EC_M6
GO:0051048	1.20E-01	0.92725013	16	110	negative regulation of secretion	BP	8.15E-01	RSAD2,SFRP1,FAM3D,EDN1,SNCA,CD200,HDAC1,PPP3CA,ATP9A,WNK4,FGF23,RSC1A1,TNFRSF1B,LGALS9,NOTCH1,VAMP3	turquoise	EC_M6
GO:0003015	1.21E-01	0.917847863	26	180	heart process	BP	8.15E-01	TRDN,TRPM4,PIK3CG,CXCR4,SPTBN4,HBEGF,PDE4D,WWTR1,STC1,KCNJ3,EDN1,KCNJ2,MEF2A,ACE,DLG1,JUP,MTOR,ATP2A1,DSG2,DSC2,PDE4B,SLC8A3,KCNH2,SCN2B,RGS4,KCNG2	turquoise	EC_M6
GO:0016082	1.21E-01	0.962902184	4	17	synaptic vesicle priming	BP	8.15E-01	SNCA,SNAP23,SNAP29,STXBP1	turquoise	EC_M6
GO:0048846	1.21E-01	0.947319724	7	34	axon extension involved in axon guidance	BP	8.15E-01	SEMA6A,SEMA4D,NRP1,SEMA3F,SEMA6D,SEMA4A,SEMA6B	turquoise	EC_M6
GO:1902284	1.21E-01	0.947319724	7	34	neuron projection extension involved in neuron projection guidance	BP	8.15E-01	SEMA6A,SEMA4D,NRP1,SEMA3F,SEMA6D,SEMA4A,SEMA6B	turquoise	EC_M6
GO:0003009	1.21E-01	0.951137343	6	30	skeletal muscle contraction	BP	8.15E-01	NR4A1,KCNJ2,SYNM,CHRNB1,ATP2A1,SLC8A3	turquoise	EC_M6

GO:0018105	1.21E-01	0.912893257	35	249	peptidyl-serine phosphorylation	BP	8.15E-01	PDGFB,MAPK13,CSNK1E,SYK,IL6,PRKD2,SPTBN4,IKBKB,PLK2,RET,PDE4D,EIF2AK3,DCLK2,RAPGEF3,HIPK1,DYRK1A,SNCA,PRKX,DYRK3,HCLS1,INPP5F,RPS6KA1,PRKD1,MLXIPL,PAK2,PDK3,LMTK2,PPM1F,PRKCH,MTOR,SBK1,RPS6KA5,MARK3,SH2D3A,SH2D3C	turquoise	EC_M6
GO:0098659	1.21E-01	0.933208292	12	71	inorganic cation import across plasma membrane	BP	8.16E-01	TRPM4,KCNJ3,KCNJ15,SLC39A8,KCNJ2,SLC12A7,DLG1,SLC9A7,WNK4,KCNJ18,SLC8A3,KCNH2	turquoise	EC_M6
GO:0099587	1.21E-01	0.933208292	12	71	inorganic ion import across plasma membrane	BP	8.16E-01	TRPM4,KCNJ3,KCNJ15,SLC39A8,KCNJ2,SLC12A7,DLG1,SLC9A7,WNK4,KCNJ18,SLC8A3,KCNH2	turquoise	EC_M6
GO:1901890	1.22E-01	0.929804664	14	82	positive regulation of cell junction assembly	BP	8.16E-01	TEK,LINGO2,KDR,SEMA4D,NRP1,SFRP1,EPHB1,IL1RAP,PPM1F,RAC1,EPHA2,SEMA4A,SLITRK2,ADGRL2	turquoise	EC_M6
GO:0030193	1.22E-01	0.942445329	8	50	regulation of blood coagulation	BP	8.16E-01	TSPAN8,PDGFB,NOS3,CD36,KLKB1,ANO6,EDN1,CD34	turquoise	EC_M6
GO:1904892	1.66E-01	0.906157256	11	59	regulation of receptor signaling pathway via STAT	BP	8.16E-01	IL23A,LEP,TGFB1,ADIPOR1,ELP2,CRLF3,NLK,PTPN2,TSLP,OCIAD2,LEPROT	darkgreen	EC_M4
GO:0002706	1.22E-01	0.929056839	14	92	regulation of lymphocyte mediated immunity	BP	8.16E-01	IL6,RSAD2,TRPM4,NECTIN2,GATA3,B2M,WAS,TNFRSF1B,LGALS9,IL7R,TRAF2,CD46,FCER2,SERPINB9	turquoise	EC_M6
GO:0048489	1.22E-01	0.946046007	7	38	synaptic vesicle transport	BP	8.17E-01	KIF5A,SNAP91,SNCA,DTNBP1,RAB27A,DNM2,AP3D1	turquoise	EC_M6
GO:0002822	1.23E-01	0.927085137	15	102	regulation of adaptive immune response based on somatic recombination of immune receptors built from immunoglobulin superfamily domains	BP	8.18E-01	TNFAIP3,IL6,RSAD2,TRPM4,NECTIN2,GATA3,MEF2C,HLX,B2M,WAS,TNFRSF1B,IL7R,TRAF2,CD46,FCER2	turquoise	EC_M6
GO:0007519	1.23E-01	0.923358562	18	122	skeletal muscle tissue development	BP	8.18E-01	CAV2,SVIL,NR1D2,P2RX2,LMOD3,EPHB1,NR4A1,MEF2C,TLL2,PPP3CA,BCL9,HLX,CFLAR,HOXD10,TBX1,ZBTB18,NOTCH1,MAFF	turquoise	EC_M6
GO:0010977	1.23E-01	0.923476982	18	116	negative regulation of neuron projection development	BP	8.18E-01	SEMA6A,CDKL3,SEMA4D,NRP1,EFNB2,PRAG1,MYLIP,SEMA3F,INPP5F,TRAK2,B2M,SEMA6D,DNM3,PSEN1,SEMA4A,EFNA1,SEMA6B,ARHGAP4	turquoise	EC_M6
GO:0071260	1.23E-01	0.934098584	11	69	cellular response to mechanical stimulus	BP	8.18E-01	TLR4,PDE2A,MAP3K1,TMEM150C,KCNJ2,F11R,RAC1,PIEZO2,PIEZO1,ENG,BMP6	turquoise	EC_M6
GO:0120039	1.23E-01	0.902156631	71	517	plasma membrane bounded cell projection morphogenesis	BP	8.18E-01	SEMA6A,KIF5A,SNX2,RIMS2,CDKL3,PTK2,SEMA4D,NRCAM,NRP1,ITSN2,NOVA2,PLXND1,GATA3,CXCR4,SPTBN4,FEZ2,EFNB2,ARHGEF7,EPHB1,RET,PREX2,KIF13B,GPM6A,NR4A2,DTNBP1,PPP3CA,HECW2,MEF2A,SEMA3F,ROBO4,PLEKHO1,PLXNA2,TRAK2,ADCY1,KALRN,SS18L1,LAMA5,YTHDF1,PAK2,LAMC2,MOV10,CREB1,ADORA2A,SEMA6D,EPHB4,ITGA4,DNM3,LMTK2,RAC1,EPHA2,OPHN1,EFNB1,PSEN1,SEMA4A,ABI3,TAOK2,EFNA1,CHRNB2,ZDHHC15,RPS6KA5,DNM2,B4GALT6,SEMA6B,SLITRK2,ARHGAP4,NOTCH1,STXBP1,DAB2IP,VASP,RELN,SHANK3	turquoise	EC_M6
GO:0031327	1.23E-01	0.89516177	145	1198	negative regulation of cellular biosynthetic process	BP	8.18E-01	PLAGL1,CNOT6,NOTCH4,TCF7,L3MBTL3,DAPK1,ATF4,PDGFB,MDFI,IREB2,NFX1,NFIL3,SEMA4D,IGF2BP3,NFE2L3,BACH2,FOXP2,KLF11,LANCL2,CREM,SDCBP,NOCT,TUT4,BEND5,TAL1,PHF10,EPC1,GATA2,DNMT3A,SAP130,DACH1,PPARG,GATA3,TAF3,ACVR2B,GZF1,EDNRB,PDE2A,SFRP1,HBEGF,NR1D2,DND1,CD36,ARID5A,DNMT3B,EIF2AK3,HHEX,ZBTB38,POU6F1,SOX7,HMBOX1,ENC1,MEF2C,WWTR1,SOGA1,SFMBT1,SCAF4,ANKRD2,NR4A2,DLL4,CDYL,SOX13,YAF2,EDN1,STAT1,SNCA,JARID2,ETS2,DCAF1,LPCAT1,SIK1,HDAC1,MEF2A,FABP4,HCLS1,NFATC2,CD34,KDM2A,CIART,TGIF1,PARP15,RBM4,PROX1,ZNF148,YTHDF1,MLXIPL,DLG1,ETV6,MOV10,RNF2,CREB1,EIF4A2,BCOR,DDX3X,IGFBP5,MECOM,ING4,SPINDOC,LDB2,CPEB2,PPM1F,POU2F1,ZBTB18,APBB2,BCL6B,KDM5C,MAFK,AR,PSEN1,CBX1,HOXB8,FBXW11,EFNA1,PIAS1,RPS6KA5,SMAD2,TLE3,CNOT8,YY1,HMG20A,RCOR1,LIG3,HCFC1,NRARP,NOTCH1,IRF8,MTF2,ZBTB34,TERF2,DAB2IP,ZBTB33,ARID4A,ZNF24,ATXN1L,TRIM24,SOX18,PASD1,COMMD7,TGIF2,BMP2,GLIS3,ZNF10,CBX2,BMP6,ZNF266	turquoise	EC_M6

GO:0043406	1.24E-01	0.926708184	15	93	positive regulation of MAP kinase activity	BP	8.18E-01	TNFRSF11A,TPD52L1,PDGFB,KIT,SYK,TLR4,PIK3CG,EDN1,MAP3K11,TLR6,PSEN1,FGF18,PIK3R5,TRAF2,DAB2IP	turquoise	EC_M6
GO:0071407	1.24E-01	0.903549046	59	448	cellular response to organic cyclic compound	BP	8.18E-01	ATF4,PDGFB,KDM4C,PIM1,DDX58,CAV2,IL15,TRPM4,PIK3CG,RALB,P2RY6,P2RY2,PDE2A,TMEM173,SFRP1,GRAMD1A,DNMT3B,KDM3A,UFSP2,SMAD1,CHRM5,GPD1,MEF2C,P2RX7,KMT2D,TIPARP,STC1,RAPGEF3,IFIT1,EDN1,STAT1,SNCA,DTNBP1,ADCY2,HDAC1,MN1,CFLAR,ADCY1,FES,ARID1A,ITGA6,PAK2,SSTR1,IGFBP5,FGF23,JUP,AR,DNM2,SCNN1B,CYP1A1,CCL2,PDE4B,HCFC1,RAPGEF1,SLC8A3,TRIM24,RGS4,BMP2,GNG2	turquoise	EC_M6
GO:0071359	1.24E-01	0.961439849	4	18	cellular response to dsRNA	BP	8.18E-01	DDX58,RALB,TMEM173,IFIT1	turquoise	EC_M6
GO:0050807	1.24E-01	0.91647612	25	165	regulation of synapse organization	BP	8.18E-01	LINGO2,SEMA4D,SETD5,EPHB1,MEF2C,TLR2,GPM6A,SEPT11,SNCA,SEMA3F,IL10RA,IL1RAP,DNM3,SEMA4A,ABI3,ARHGEF15,EFNA1,CHRNB2,ZDHHC15,SLITRK2,DAB2IP,ADGRL2,RELN,ABHD17C,SHANK3	turquoise	EC_M6
GO:0009582	1.24E-01	0.929564546	13	83	detection of abiotic stimulus	BP	8.18E-01	PTK2,KIT,GRIK2,CXCR4,EPHB1,MMP24,ANO3,ANO1,JUP,GNGT2,UNC119,PIEZO2,PIEZO1	turquoise	EC_M6
GO:0061311	1.25E-01	0.954357362	5	25	cell surface receptor signaling pathway involved in heart development	BP	8.18E-01	JAG1,DLL4,NOTCH1,SOX17,BMP2	turquoise	EC_M6
GO:0021885	1.25E-01	0.941398433	8	43	forebrain cell migration	BP	8.18E-01	EFHC1,CXCR4,SRGAP2C,PSEN1,LHX6,DAB2IP,ADGRG1,RELN	turquoise	EC_M6
GO:0048333	1.25E-01	0.954632449	5	23	mesodermal cell differentiation	BP	8.18E-01	TAL1,ITGA8,ITGB4,SMAD1,ITGA3	turquoise	EC_M6
GO:0046475	1.25E-01	0.954104861	5	25	glycerophospholipid catabolic process	BP	8.18E-01	SCARB1,INPP5F,GDPD3,GDPD1,PLA2G4E	turquoise	EC_M6
GO:1901215	1.25E-01	0.916843269	23	161	negative regulation of neuron death	BP	8.18E-01	OXR1,NCOA7,ATF4,REL,PPT1,NRP1,MEF2C,NR4A2,SNCA,PM20D1,CD200,HDAC1,CD34,CREB1,ADORA2A,HIF1A,TNFRSF1B,PSEN1,AP2B1,CCL2,TRAF2,STXBP1,NMNAT1	turquoise	EC_M6
GO:0031960	1.67E-01	0.888801503	20	117	response to corticosteroid	BP	8.19E-01	CDO1,REST,GLB1,HNMT,ATP2B1,HEY1,CPN1,EIF4E,CCND1,KRAS,NTRK3,IGFBP2,COL1A1,SOX30,CALM1,ABCA3,SSTR5,AGL,SERPINF1,CDKN1A	darkgreen	EC_M4
GO:0060038	1.67E-01	0.912336664	9	45	cardiac muscle cell proliferation	BP	8.19E-01	TGFBR1,FGF2,TENM4,RUNX1,TGFB2,PRKAR1A,FGFR2,BMPR1A,WNT2	darkgreen	EC_M4
GO:0060339	1.25E-01	0.960447472	4	19	negative regulation of type I interferon-mediated signaling pathway	BP	8.19E-01	OAS3,NLRC5,ISG15,OAS1	turquoise	EC_M6
GO:0019395	7.54E-03	0.996695445	18	87	fatty acid oxidation	BP	8.19E-01	ETFB,ACAA1,CYGB,HACL1,ACOT8,LONP2,IRS1,ACADS,ABCB11,EHHADH,POR,ACADL,ILVBL,ABCD4,GCDH,ACAA2,CPT2,SCP2	cyan	EC_M2
GO:0035024	5.51E-02	0.98466035	5	20	negative regulation of Rho protein signal transduction	BP	8.20E-01	CCDC125,CUL3,ABL2,SCAI,STMN1	darkgrey	EC_M8
GO:0031498	5.51E-02	0.987440916	4	15	chromatin disassembly	BP	8.20E-01	SUPT16H,ARID2,SMARCC1,SMARCE1	darkgrey	EC_M8
GO:0043967	5.52E-02	0.97555498	10	58	histone H4 acetylation	BP	8.20E-01	ZMPSTE24,TAF7,BRCA2,MCRS1,NAA50,HAT1,ING5,BRCA1,OGT,AC118549.1	darkgrey	EC_M8
GO:0031398	5.52E-02	0.970866821	15	102	positive regulation of protein ubiquitination	BP	8.20E-01	TOPORS,COMMD1,PAXIP1,CDC20,UBE2C,UBE3A,CUL3,FANCI,FANCM,BRCA1,DCUN1D5,PLK1,PSMD10,XIAP,RCHY1	darkgrey	EC_M8
GO:0002181	5.53E-02	0.971389032	14	111	cytoplasmic translation	BP	8.20E-01	EIF3E,METTL3,DHX29,NCK1,RPS6,EIF3A,DHX9,RPL22L1,EIF3B,EIF4A1,EIF5,GSPT1,FMR1,DRG1	darkgrey	EC_M8
GO:0032232	1.26E-01	0.948851057	6	29	negative regulation of actin filament bundle assembly	BP	8.20E-01	F11R,PAK2,WAS,ARHGAP28,S1PR1,SHANK3	turquoise	EC_M6
GO:0150115	1.26E-01	0.923821201	16	95	cell-substrate junction organization	BP	8.20E-01	PTK2,TEK,LIMCH1,KDR,NRP1,ITGB4,SFRP1,THSD1,ARHGEF7,ITGA6,APOD,BCR,PPM1F,RAC1,RCC2,TAOK2	turquoise	EC_M6
GO:0061025	1.26E-01	0.920610577	18	125	membrane fusion	BP	8.21E-01	STX11,TSNARE1,CAV2,IZUMO1,NECTIN2,TBC1D4,TIE1,DYSF,SNCA,HYAL2,SNAP23,LRMP,SNAP29,SYT7,STXBP1,STX3,VAMP3,RAB39A	turquoise	EC_M6
GO:0010742	1.27E-01	0.953365554	5	25	macrophage derived foam cell differentiation	BP	8.21E-01	PPARG,CD36,STAT1,ABCG1,PRKCH	turquoise	EC_M6
GO:0090077	1.27E-01	0.953365554	5	25	foam cell differentiation	BP	8.21E-01	PPARG,CD36,STAT1,ABCG1,PRKCH	turquoise	EC_M6
GO:0042219	1.68E-01	0.932894707	5	23	cellular modified amino acid catabolic process	BP	8.22E-01	ALDH1L2,PCYOX1,ABHD12,CHAC1,PCYOX1L	darkgreen	EC_M4

GO:0045862	1.27E-01	0.906773019	38	297	positive regulation of proteolysis	BP	8.23E-01	GRAMD4,PTK2,DAPK1,AGTPBP1,CSNK1E,FAM122A,SYK,CTSC,PPARG,PLK2,KLKB1,EIF2AK3,HIP1R,SOX7,SNCA,RNF144B,RNF19B,LCK,LAPTM5,USP13,CFLAR,CASP10,MELTF,DDX3X,PPM1F,BID,TNFRSF1B,RNF40,PSEN1,FBXW11,EFNA1,PIAS1,TNIP1,LGALS9,DAB2IP,PRSS22,PMAIP1,PERP	turquoise	EC_M6
GO:0048880	1.27E-01	0.906029449	40	286	sensory system development	BP	8.23E-01	ATF4,AGTPBP1,TMOD1,TSPAN12,NRP1,GATA3,ACVR2B,MYO7A,JAG1,FREM2,EPHB1,RET,MERTK,CHD7,GPM6A,USH1C,HIPK1,DLL4,LPCAT1,HDAC1,PROX1,LIMK2,DLG1,PROM1,HIF1A,RHOJ,EPHA2,ALDH1A2,SMOC1,PSEN1,ARHGEF15,TTLL5,YY1,CYP1A1,SLC25A25,RAB18,DIO3,TGIF2,TBC1D20,BMP6	turquoise	EC_M6
GO:0000096	1.68E-01	0.926017168	6	28	sulfur amino acid metabolic process	BP	8.23E-01	CDO1,MTAP,SLC7A11,SMS,COMT,ADI1	darkgreen	EC_M4
GO:0071887	1.27E-01	0.928933765	12	79	leukocyte apoptotic process	BP	8.23E-01	TNFRSF21,IL6,MERTK,MEF2C,LMBR1L,HCLS1,HIF1A,ITPKB,ARG2,EFNA1,LGALS9,IL7R	turquoise	EC_M6
GO:0002433	1.28E-01	0.953299491	5	23	immune response-regulating cell surface receptor signaling pathway involved in phagocytosis	BP	8.24E-01	PTK2,SYK,FGR,PLD2,YES1	turquoise	EC_M6
GO:0038096	1.28E-01	0.953299491	5	23	Fc-gamma receptor signaling pathway involved in phagocytosis	BP	8.24E-01	PTK2,SYK,FGR,PLD2,YES1	turquoise	EC_M6
GO:0050732	1.68E-01	0.911377384	9	46	negative regulation of peptidyl-tyrosine phosphorylation	BP	8.24E-01	SOCS5,SH3BP5L,TSG101,CBLB,PTPN6,SRCIN1,PTPN2,THY1,DUSP22	darkgreen	EC_M4
GO:0009062	7.67E-03	0.996638013	18	86	fatty acid catabolic process	BP	8.24E-01	ETFB,LPIN1,ACAA1,HACL1,LPIN3,ACOT8,LONP2,IRS1,ACADS,ABCB11,EHHADH,ACADL,ILVBL,ABCD4,GCDH,ACAA2,CPT2,SCP2	cyan	EC_M2
GO:0071230	7.70E-03	0.997159206	13	59	cellular response to amino acid stimulus	BP	8.24E-01	HSF1,PDGFRA,SESN1,RPTOR,LAMTOR1,PDGFC,NSMF,CYBA,LAMTOR2,SIX1,BCL2L2,CEBPB,PDGFD	cyan	EC_M2
GO:0060445	1.28E-01	0.959688405	4	18	branching involved in salivary gland morphogenesis	BP	8.25E-01	NRP1,PLXND1,NTN4,LAMA5	turquoise	EC_M6
GO:1903670	1.28E-01	0.947744657	6	29	regulation of sprouting angiogenesis	BP	8.25E-01	SEMA6A,ADAMTS9,SMAD1,PPP1R16B,RHOJ,JAK1	turquoise	EC_M6
GO:0002685	1.69E-01	0.88197203	25	152	regulation of leukocyte migration	BP	8.26E-01	VEGFA,DOCK8,CREB3,AKIRIN1,CXCL12,CHST2,P2RX4,APP,CX3CL1,ADA,DNM1L,MMP14,CMKLR1,SLAMF8,SERPINE1,ITGA2B,NBL1,MSN,CD74,AKT1,ITGA2,TNFRSF18,MIR24-1,THY1,CCR6	darkgreen	EC_M4
GO:0051261	5.58E-02	0.969979142	16	99	protein depolymerization	BP	8.26E-01	KIF24,CAPZA2,CLASP2,KIF2C,CKAP2,TPX2,CKAP5,STMND1,KIF18A,CAMSAP2,CFL1,HDGFL3,KIF18B,TAOK1,TMOD3,STMN1	darkgrey	EC_M8
GO:0002360	1.28E-01	0.959408176	4	18	T cell lineage commitment	BP	8.27E-01	IL6,IL7,MTOR,BATF	turquoise	EC_M6
GO:0006584	1.29E-01	0.946965014	6	32	catecholamine metabolic process	BP	8.27E-01	SNCAIP,GATA3,NR4A2,SNCA,CHRNB2,NPR1	turquoise	EC_M6
GO:0009712	1.29E-01	0.946965014	6	32	catechol-containing compound metabolic process	BP	8.27E-01	SNCAIP,GATA3,NR4A2,SNCA,CHRNB2,NPR1	turquoise	EC_M6
GO:0140056	1.29E-01	0.930502527	11	67	organelle localization by membrane tethering	BP	8.27E-01	STX11,TSNARE1,CAV2,RAB3B,RALB,NDRG4,SNAP29,RAB3D,STXBP1,STX3,VAMP3	turquoise	EC_M6
GO:0071805	1.29E-01	0.91691212	20	131	potassium ion transmembrane transport	BP	8.28E-01	KCNRG,KCNK6,KCNJ3,ANO6,KCNJ15,KCNJ2,SLC12A7,SLC9A8,DLG1,KCNK7,SLC9A7,WNK4,KCNN3,DNM2,KCNJ18,KCNH2,KCNK5,RGS4,KCNA6,KCNG2	turquoise	EC_M6
GO:1903672	1.69E-01	0.941640268	4	16	positive regulation of sprouting angiogenesis	BP	8.28E-01	VEGFA,FGF2,TJP1,PKM	darkgreen	EC_M4
GO:1903322	5.60E-02	0.969069113	17	121	positive regulation of protein modification by small protein conjugation or removal	BP	8.28E-01	TOPORS,COMMD1,SAE1,PAXIP1,CDC20,UBA2,UBE2C,UBE3A,CUL3,FANCI,FANCM,BRCA1,DCUN1D5,PLK1,PSMD10,XIAP,RCHY1	darkgrey	EC_M8

GO:0090174	1.29E-01	0.924230926	14	94	organelle membrane fusion	BP	8.28E-01	STX11,TSNARE1,CAV2,TBC1D4,DYSF,SNCA,SNAP23,LRMP,SNAP29,SYT7,STXBP1,STX3,VAMP3,RAB39A	turquoise	EC_M6
GO:0001958	1.30E-01	0.952369869	5	23	endochondral ossification	BP	8.28E-01	TEK,MEF2C,MMP13,FGF18,BMP6	turquoise	EC_M6
GO:0036075	1.30E-01	0.952369869	5	23	replacement ossification	BP	8.28E-01	TEK,MEF2C,MMP13,FGF18,BMP6	turquoise	EC_M6
GO:0055072	1.30E-01	0.929586011	11	72	iron ion homeostasis	BP	8.28E-01	IREB2,CP,HYAL2,SLC39A8,MELTF,B2M,LTF,HIF1A,HEPH,HMOX2,BMP6	turquoise	EC_M6
GO:0030195	1.30E-01	0.945945897	6	35	negative regulation of blood coagulation	BP	8.28E-01	TSPAN8,PDGFB,NOS3,KLKB1,EDN1,CD34	turquoise	EC_M6
GO:0003159	1.30E-01	0.958499921	4	19	morphogenesis of an endothelium	BP	8.28E-01	PRKD2,CXCR4,CXCL10,DLL4	turquoise	EC_M6
GO:0061154	1.30E-01	0.958499921	4	19	endothelial tube morphogenesis	BP	8.28E-01	PRKD2,CXCR4,CXCL10,DLL4	turquoise	EC_M6
GO:0022898	1.30E-01	0.910958471	26	186	regulation of transmembrane transporter activity	BP	8.28E-01	TRDN,DAPK1,PIM1,TCAF2,KCNRG,P2RY6,PDE4D,MEF2C,SNCA,PM20D1,HECW2,DLG1,WNK4,CRACR2A,ATP2A1,DNM2,ADRB2,CCL2,PDE4B,PLCG2,RELN,SCN2B,SHANK3,CACNG5,FXYD6,GSG1L	turquoise	EC_M6
GO:0030225	1.30E-01	0.946168123	6	32	macrophage differentiation	BP	8.28E-01	L3MBTL3,IL15,GATA2,TLR2,TSPAN2,HCLS1	turquoise	EC_M6
GO:0044282	1.69E-01	0.869824028	44	284	small molecule catabolic process	BP	8.28E-01	HSD17B6,CDO1,MIOX,PPARA,PPARD,LEP,ALDH1L2,GALT,MFSD2A,GAD2,ESD,GLB1,ABHD1,SNX17,HNMT,RBKS,AKT2,PFKP,IRS2,IMPAD1,PFKM,ENTPD4,ADA,GLS,ATP2B4,CPT1A,LRP5,INPP5A,HADH,TPI1,ACOX3,QDPR,ASRGL1,HSD17B10,SLC25A44,CYP46A1,AKT1,LPIN2,OCRL,HAGH,PARK7,ENO1,PLA2G15,AMACR	darkgreen	EC_M4
GO:0001932	1.70E-01	0.854748963	126	845	regulation of protein phosphorylation	BP	8.28E-01	IL23A,NAB2,LRP1,AGAP2,TAB2,PRRC1,PLXNB2,ANGPT1,SH3GL2,GNAQ,LEP,CDKN2B,VEGFA,NCK2,PDCL3,TGFBR1,SOCS5,MAP3K7,SH3BP5L,ROCK2,INHBA,FGF2,RB1,MAPRE3,PAK1,TGFB1,AKT2,GPD1L,OSBPL8,KAT2B,PRNP,HDAC3,DDRGK1,SMPD1,ILK,RBPMS,CNOT7,TMED2,OSBP,ROBO1,PFN2,CCNJ,APP,RAPGEF2,CX3CL1,PTPN11,GTF2H1,PTPN22,TSG101,ACVR1,GDF6,AKTIP,CHP1,ATP2B4,PKD2,TWF1,DBNDD2,CBLB,CHRNA7,CCND1,CAPRIN2,TGFB2,LRP5,HTR2B,PRKAR1A,DAG1,PIK3CA,CORO1C,KRAS,NTRK3,SPATC1L,BMP7,OSM,SYAP1,IQGAP1,TFRC,HSPB1,DNAJC10,FN1,PTPN6,PDCD10,ADRA2C,DOK7,HTT,FKBP8,FGFR3,ARAF,TNIK,MNAT1,MAGED1,BMPR1A,TSPYL2,CDK12,PPARGC1A,SRCIN1,ARHGEF2,TGFB3,SHC1,CCNG1,MAP2K1,CALM1,GSKIP,NPTN,MYOCD,PIN1,CD74,PDGFRB,AKT1,PRKAA1,PTPN2,MYDGF,TNFRSF18,PKD1,IRAK1,PARK7,BCAR3,MIR24-1,DMD,LOX,THY1,CDKN1A,TXN,DUSP22,TSLP,PMEPA1,FGF10	darkgreen	EC_M4
GO:0006793	1.70E-01	0.846961426	312	2169	phosphorus metabolic process	BP	8.28E-01	IL23A,NAB2,LRP1,AGAP2,ENPP3,TAB2,DYRK2,RRM2B,PRRC1,PLXNB2,ANGPT1,PPARA,ACSL6,ROS1,NUS1,SERINC1,PTP4A3,PPARD,SH3GL2,GNAQ,LEP,CDKN2B,PLAA,NUAK1,CDK14,VEGFA,GALT,VCP,SLC35B2,NCK2,PDCL3,INPP4A,RAB23,NANS,PTP4A1,TGFBR1,TMEM246,SOCS5,FAM126A,MFSD2A,GARS,MAP3K7,SH3BP5L,GFPT1,ROCK2,INHBA,MYLK3,FKBP1B,FGF2,CMPK1,SRPK2,SELENOI,ENTPD6,ABHD12,RB1,ABHD5,MARK4,MAPRE3,DGKH,PAK1,TGFB1,AXL,RBKS,DGAT2,AKT2,GPD1L,OSBPL10,MTMR14,PFKP,OSBPL8,PPP1R12A,KAT2B,IRS2,PRNP,NAXD,NUDT16,HDAC3,DCLK1,ACPP,CCKBR,DDRGK1,SMPD1,PPP2CB,ILK,RBPMS,PPP2R2B,RYK,CDK8,CNOT7,IMPAD1,MTMR6,SLC20A1,CHPT1,HSP90B1,TMED2,MAST4,OSBP,TMEM150A,GRK7,RIOK2,ROBO1,FAR1,ALDH18A1,CDKL2,PFN2,CCNJ,APP,RAPGEF2,MAPKAPK5,CX3CL1,PPP2R2A,HUNK,PTPN11,ADCY6,PIP4P2,PFKM,PGAM4,OPA1,ENTPD4,GTF2H1,PTPN22,TSG101,ACVR1,CRY2,PPP3CC,LPCAT2,GDF6,SERINC3,ADA,AKTIP,CHP1,ATP2B4,PKD2,TWF1,NUDT18,DBNDD2,PIGP,CBLB,TOLLIP,BRSK2,PSPH,SLC39A10,CHRNA7,DYNLL1,DAPP1,AGPAT3,EYA2,CCND1,CAPRIN2,TGFB2,PPP6R3,ZBTB20,PRKAB2,LRP5,HTR2B,PRKAR1A,ITPK1,GSK3B,INPP5A,FAM20C,DAG1,PIK3CA,CORO1C,PRKCA,NEK7,KRAS,PITPNM1,NTRK3,MOCS3,GRK3,ETNK1,TPST2,SLAMF8,SPATC1L,BMP7,IDH2,ADCY5,HACD2,PPP1R11,PPP1R3D,FGFR2,OSM,SYAP1,IQGAP1,TFRC,PIGZ,RPS6KA3,PRDX4,PCYT1B,REXO2,HSPB1,SCYL1,MAPT,SLC25A16,DNAJC10,FN1,MAP4K5,PTPN6,TPI1,PDCD10,ADRA2C,DOK7,HTT,FKBP8,PRKCI,FGFR3,ARAF,TNIK,PLD1,SGMS1,MNAT1,PPP2R5E,AKT3,ERC1,UGDH,PPP1R3	darkgreen	EC_M4
GO:0071731	5.61E-02	0.986983807	4	16	response to nitric oxide	BP	8.29E-01	CDK2,CCNA2,AIFM1,HNRNPD	darkgrey	EC_M8
GO:0090140	1.30E-01	0.946219224	6	31	regulation of mitochondrial fission	BP	8.29E-01	KDR,PPARG,MCU,INF2,SPIRE1,RALA	turquoise	EC_M6
GO:0051650	1.30E-01	0.914715413	21	143	establishment of vesicle localization	BP	8.29E-01	KIF5A,FAM91A1,SNAP91,MYO7A,EXOC6,SNCA,DTNBP1,SNAP23,LRMP,AP1AR,TRAK2,LIMK2,ITGA4,SNAP29,RAB27A,PSEN1,FBXW11,DNM2,BICDL2,AP3D1,FNBP1L	turquoise	EC_M6

GO:0050873	1.31E-01	0.941291366	7	40	brown fat cell differentiation	BP	8.29E-01	ZNF516,PIM1,SOX13,FABP4,MTOR,ADRB2,LRG1	turquoise	EC_M6
GO:0048261	1.31E-01	0.951431832	5	25	negative regulation of receptor-mediated endocytosis	BP	8.29E-01	SDCBP,MTMR2,RIN3,RAC1,UNC119	turquoise	EC_M6
GO:0006040	1.31E-01	0.945787739	6	32	amino sugar metabolic process	BP	8.29E-01	CHST1,MGAT1,NPL,ST6GAL1,CHST7,MGAT3	turquoise	EC_M6
GO:0006884	1.31E-01	0.951365855	5	24	cell volume homeostasis	BP	8.29E-01	CLCN3,P2RX7,ANO6,SLC12A7,SHANK3	turquoise	EC_M6
GO:0014829	1.31E-01	0.951277255	5	24	vascular associated smooth muscle contraction	BP	8.29E-01	DOCK4,ARHGAP42,EDNRB,EDN1,SCNN1B	turquoise	EC_M6
GO:0060251	1.31E-01	0.950996177	5	25	regulation of glial cell proliferation	BP	8.29E-01	IL6,CREB1,PRKCH,MTOR,NOTCH1	turquoise	EC_M6
GO:0036465	1.32E-01	0.928601852	11	64	synaptic vesicle recycling	BP	8.29E-01	SYNJ2,SNAP91,ITSN2,SNCA,DNM3,PLD2,SNCG,SYT7,OPHN1,DNM2,AP3D1	turquoise	EC_M6
GO:0009620	1.32E-01	0.950221482	5	27	response to fungus	BP	8.29E-01	SYK,TLR4,LTF,PLCG2,TRIM62	turquoise	EC_M6
GO:0032720	1.32E-01	0.940218609	7	41	negative regulation of tumor necrosis factor production	BP	8.29E-01	TNFAIP3,C6orf106,TLR4,IGF1,CD34,ARG2,LGALS9	turquoise	EC_M6
GO:0009914	1.32E-01	0.907124881	29	212	hormone transport	BP	8.29E-01	RIMS2,PICK1,ICA1,IL6,TRPM4,GATA3,SFRP1,SIRT3,CHD7,FAM3D,EDN1,ABCG1,HDAC1,PPP3CA,ANO1,SNAP23,SLCO4A1,CREB1,MCU,CDK16,FGF23,RAC1,HIF1A,SYT7,GPR68,SMAD2,NOS2,EXOC3L1,BMP6	turquoise	EC_M6
GO:0046683	1.32E-01	0.920653026	15	94	response to organophosphorus	BP	8.29E-01	TEK,TRPM4,PIK3CG,P2RY6,P2RY2,PDE2A,P2RX2,GPD1,P2RX7,STC1,RAPGEF3,STAT1,IGFBP5,RAPGEF1,SLC8A3	turquoise	EC_M6
GO:0032231	1.32E-01	0.920718495	15	92	regulation of actin filament bundle assembly	BP	8.29E-01	MTSS1,LIMCH1,NRP1,SFRP1,RAPGEF3,F11R,PAK2,PPM1F,WAS,RAC1,MTOR,ARHGEF15,ARHGAP28,S1PR1,SHANK3	turquoise	EC_M6
GO:0055021	1.32E-01	0.93328827	9	53	regulation of cardiac muscle tissue growth	BP	8.29E-01	PIM1,IGF1,MEF2C,EDN1,JARID2,MTOR,YY1,NOTCH1,RGS4	turquoise	EC_M6
GO:0150117	1.33E-01	0.945330278	6	29	positive regulation of cell-substrate junction organization	BP	8.29E-01	TEK,KDR,NRP1,SFRP1,PPM1F,RAC1	turquoise	EC_M6
GO:0009581	1.33E-01	0.923668152	13	84	detection of external stimulus	BP	8.29E-01	PTK2,KIT,GRIK2,CXCR4,EPHB1,MMP24,ANO3,ANO1,JUP,GNGT2,UNC119,PIEZO2,PIEZO1	turquoise	EC_M6
GO:0007520	1.33E-01	0.950358312	5	25	myoblast fusion	BP	8.29E-01	CXCL10,TANC1,NFATC2,PLEKHO1,CFLAR	turquoise	EC_M6
GO:1900271	1.33E-01	0.944579788	6	33	regulation of long-term synaptic potentiation	BP	8.29E-01	ADCY1,YTHDF1,CREB1,ADORA2A,RELN,SHANK3	turquoise	EC_M6
GO:0050680	1.33E-01	0.918695765	16	108	negative regulation of epithelial cell proliferation	BP	8.29E-01	MTSS1,CAV2,PPARG,GATA3,SFRP1,MEF2C,DLL4,STAT1,EAF2,DLG1,B2M,MCC,AR,VASH1,CCL2,DAB2IP	turquoise	EC_M6
GO:1900046	1.33E-01	0.935845715	8	51	regulation of hemostasis	BP	8.29E-01	TSPAN8,PDGFB,NOS3,CD36,KLKB1,ANO6,EDN1,CD34	turquoise	EC_M6
GO:0009890	1.34E-01	0.885633282	147	1222	negative regulation of biosynthetic process	BP	8.29E-01	PLAGL1,CNOT6,NOTCH4,TCF7,L3MBTL3,DAPK1,ATF4,PDGFB,MDFI,IREB2,NFX1,NFIL3,SEMA4D,IL6,IGF2BP3,NFE2L3,BACH2,FOXP2,KLF11,LANCL2,CREM,SDCBP,NOCT,TUT4,BEND5,TAL1,PHF10,EPC1,GATA2,DNMT3A,SAP130,DACH1,PPARG,GATA3,TAF3,ACVR2B,GZF1,EDNRB,PDE2A,SFRP1,HBEGF,NR1D2,DND1,CD36,ARID5A,DNMT3B,EIF2AK3,HHEX,ZBTB38,POU6F1,SOX7,HMBOX1,ENC1,MEF2C,WWTR1,SOGA1,SFMBT1,SCAF4,ANKRD2,NR4A2,DLL4,CDYL,SOX13,YAF2,EDN1,STAT1,SNCA,JARID2,ETS2,DCAF1,LPCAT1,SIK1,HDAC1,MEF2A,FABP4,HCLS1,NFATC2,CD34,KDM2A,CIART,TGIF1,PARP15,CFLAR,RBM4,PROX1,ZNF148,YTHDF1,MLXIPL,DLG1,ETV6,MOV10,RNF2,CREB1,EIF4A2,BCOR,DDX3X,IGFBP5,MECOM,ING4,SPINDOC,LDB2,CPEB2,PPM1F,POU2F1,ZBTB18,APBB2,BCL6B,KDM5C,MAFK,AR,PSEN1,CBX1,HOXB8,FBXW11,EFNA1,PIAS1,RPS6KA5,SMAD2,TLE3,CNOT8,YY1,HMG20A,RCOR1,LIG3,HCFC1,NRARP,NOTCH1,IRF8,MTF2,ZBTB34,TERF2,DAB2IP,ZBTB33,ARID4A,ZNF24,ATXN1L,TRIM24,SOX18,PASD1,COMMD7,TGIF2,BMP2,GLIS3,ZNF10,CBX2,BMP6,ZNF266	turquoise	EC_M6
GO:0002437	1.34E-01	0.939284685	7	40	inflammatory response to antigenic stimulus	BP	8.29E-01	SYK,GATA3,PLK2,PLCG1,FGR,YES1,NOTCH1	turquoise	EC_M6

GO:0045935	1.34E-01	0.883574425	178	1489	positive regulation of nucleobase-containing compound metabolic process	BP	8.29E-01	ZNF516,TCF4,PLAGL1,CNOT6,ZBED4,NOTCH4,TCF20,NCOA7,ATF4,PDGFB,PIM1,NFYA,PRDM4,DDX58,NFIL3,IL6,REL,CREB5,TLR4,TEAD2,MYCN,NOCT,TUT4,TAL1,PHF10,EPC1,KDM4D,GATA2,PRKD2,NOS3,PPARG,GATA3,IKBKB,JAG1,NFKBIB,TMEM173,SFRP1,NR1D2,DND1,PRR5L,TCF15,RET,CHD7,IL1A,IGF1,NR4A1,EIF2AK3,HHEX,KDM3A,SMAD1,ZBTB38,STOX2,SOX7,HMBOX1,GPD1,HEXB,MEF2C,SMARCD1,TLR2,WWTR1,P2RX7,CXCL10,KMT2D,NLRC5,NR4A2,TOX2,YAF2,EDN1,STAT1,DYRK1A,ERG,ETS2,NFKB2,PRDM15,ETS1,HDAC1,PPP3CA,HYAL2,MEF2A,BCL9,NCF1,HCLS1,NFATC2,EAF2,GMEB1,CD86,POU2F3,ARID1A,RPS6KA1,PROX1,SS18L1,PRKD1,ZNF148,YTHDF1,HELZ2,MLXIPL,ITGA6,ELL3,ETV6,HOXD10,MOV10,CREB1,TET1,ARNT2,DDX3X,RFXANK,ETV4,TBX1,MECOM,ING4,LDB2,FGF23,POU2F1,WAS,HIF1A,KDM5A,ZBTB18,APBB2,JUP,TNFRSF1B,MTOR,AR,CASZ1,PSEN1,HOXB5,HOXB7,HOXB9,FBXW11,BATF,PIAS1,RPS6KA5,SMAD2,CNOT8,DNM2,YY1,ARID3B,LYL1,TNIP1,ZNF521,ABLIM3,ADRB2,YES1,RAI1,NOS2,ELF4,MRTFB,SPIRE1,USP7,DOT1L,AP3D1,HCFC1,NOTCH1,MED16,IRF8,S1PR1,ENG,MTF2,NFAT5,TERF2,DAB2IP,ARID4A,ZNF24,TRIM24,SOX17,KCNH2,ZNF850,ANKRD49,CDK13,SOX18,MAFF,TRIM62,ZNF229,E2F3,BMP2,GLIS3,ZNF71,BMP6,FLI1	turquoise	EC_M6
GO:0042490	1.35E-01	0.932014395	9	52	mechanoreceptor differentiation	BP	8.29E-01	CTHRC1,MYCN,MYO7A,JAG1,USH1C,SEC24B,CECR2,JAG2,NOTCH1	turquoise	EC_M6
GO:0055093	1.35E-01	0.956307015	4	19	response to hyperoxia	BP	8.29E-01	DNMT3B,HDAC1,POLG,CYP1A1	turquoise	EC_M6
GO:0098661	1.35E-01	0.931636574	9	51	inorganic anion transmembrane transport	BP	8.29E-01	SLC4A11,CLCN3,ANO6,ANO5,ANO3,ANO1,SLC12A7,MTOR,SLC26A1	turquoise	EC_M6
GO:0046890	1.36E-01	0.913987316	18	127	regulation of lipid biosynthetic process	BP	8.29E-01	PDGFB,ADGRF5,RAB38,SIRT3,SCARB1,ABHD6,DHH,SLC45A3,ABCG1,LPCAT1,SIK1,PROX1,MLXIPL,CREB1,SAMD8,MTOR,BMP2,BMP6	turquoise	EC_M6
GO:0021533	1.36E-01	0.956549416	4	17	cell differentiation in hindbrain	BP	8.29E-01	AGTPBP1,GATA2,PROX1,OPHN1	turquoise	EC_M6
GO:0036010	1.36E-01	0.949229191	5	23	protein localization to endosome	BP	8.29E-01	PACSIN2,MICALL1,NRP1,ABHD17C,MGAT3	turquoise	EC_M6
GO:0045746	1.36E-01	0.942717431	6	33	negative regulation of Notch signaling pathway	BP	8.29E-01	GATA2,DLL4,TSPAN15,LFNG,NRARP,EGFL7	turquoise	EC_M6
GO:0051489	1.36E-01	0.934478804	8	44	regulation of filopodium assembly	BP	8.29E-01	NRP1,GPM6A,PPP1R16B,SRGAP2C,DNM3,FSCN1,FNBP1L,RALA	turquoise	EC_M6
GO:0021602	1.37E-01	0.948624564	5	24	cranial nerve morphogenesis	BP	8.29E-01	NRP1,EPHB1,SEMA3F,TBX1,CHRNB2	turquoise	EC_M6
GO:0045596	1.37E-01	0.891569763	63	489	negative regulation of cell differentiation	BP	8.29E-01	SEMA6A,NOTCH4,CDKL3,PDGFB,SEMA4D,IL6,TLR4,MYCN,NOCT,TRPM4,NRP1,GATA2,PPARG,GATA3,EDNRB,JAG1,SFRP1,TMEM178A,TCF15,EPHB1,IGF1,SMAD1,WWTR1,VEGFC,CXCL10,ANKRD2,STAT1,ABCG1,PPP3CA,SEMA3F,HLX,NFATC2,AP1AR,INPP5D,TRAK2,MELTF,B2M,LTF,SEMA6D,ADAMTS7,IGFBP5,TBX1,FGF23,RCC2,ITPKB,PSEN1,SEMA4A,HOXB8,EFNA1,GPR68,YY1,HMG20A,SEMA6B,RFLNB,ARHGAP4,NRARP,NOTCH1,DAB2IP,CDK13,RGS4,TRIM62,HIST1H4D,BMP2	turquoise	EC_M6
GO:0048708	1.37E-01	0.927510628	10	60	astrocyte differentiation	BP	8.29E-01	IFNGR1,IL6,TLR4,MYCN,TAL1,TSPAN2,ADORA2A,PSEN1,NOTCH1,BMP2	turquoise	EC_M6

GO:0009889	1.37E-01	0.876639618	348	3010	regulation of biosynthetic process	BP	8.29E-01	ZNF516,TNFRSF11A,BAZ2A,TCF4,TNFAIP3,PLAGL1,AKAP12,CNOT6,FZD6,ZBED4,QKI,NOTCH4,TCF20,TCF7,NCOA7,L3MBTL3,DAPK1,ATF4,PDGFB,BRPF3,KDM4C,PIM1,NFYA,MDFI,IREB2,PRDM4,DDX58,NFX1,ADGRF5,KIT,ADGRF1,KDR,NFIL3,SYK,SEMA4D,IL6,IGF2BP3,NFE2L3,REL,CREB5,CXCL8,PPIE,BACH2,PELI1,FOXP2,KLF11,LANCL2,NOBOX,TLR4,TRAF1,IL15,CREM,TEAD2,SDCBP,MYCN,NOCT,TUT4,KTI12,ZBTB24,PUM2,BEND5,TAL1,PHF10,EPC1,GATA2,DNMT3A,PRKD2,SAP130,RAB38,NOS3,DACH1,PPARG,GATA3,TAF3,ACVR2B,IRAK2,GZF1,EDNRB,PPP1CB,SETD5,IKBKB,JAG1,P2RY6,NFKBIB,PDE2A,TMEM173,SFRP1,HBEGF,NR1D2,DND1,RPL26,CD36,SIRT3,CDK17,ARID5A,TCF15,SCARB1,RET,CHD7,IL1A,DNMT3B,IGF1,NR4A1,EIF2AK3,ABHD6,HHEX,KDM3A,ITCH,ERBIN,SMAD1,ZBTB38,CLCN3,POU6F1,STOX2,SOX7,HMBOX1,ENC1,HEXB,MEF2C,SMARCD1,TLR2,WWTR1,ZNF704,CXCL10,SOGA1,ADGRG3,DHH,KMT2D,SFMBT1,SCAF4,NLRC5,ACTR5,ANKRD2,NR4A2,TOX2,DLL4,CDYL,BAZ2B,SOX13,YAF2,EDN1,STAT1,DYRK1A,SNCA,JARID2,ERG,ETS2,CDK18,SLC45A3,NFKB2,DCAF1,PRDM15,ABCG1,ETS1,CD200,LPCAT1,SIK1,HDAC1,PPP3CA,HYAL2,SLC39A8,TESMIN,MEF2A,BCL9,HLX,NCF1,USP13,FABP4,HCLS1,NFATC2,ZFHX2,EAF2,GMEB1,CD34,CD86,KDM2A,CIART,MN1,TGIF1,PARP15,POU2F3,ZSCAN29,CFLAR,TRAK2,ADCY1,ARID1A,RBM4,RPS6KA1,PROX1,SS18L1,PRKD1,ZNF148,YTHDF1,ZSCAN2,HELZ2,MLXIPL,ITGA6,DLG1,ELL3,ETV6,HOXD10,MOV10,PDK3,RNF2,CREB1,EIF4A2,TET1,LTF,ADORA2A,ARNT2,MYEF2,IL1RAP,BCOR,DDX3X,RFXANK,IGFBP5,ETV4,TBX1,TMEM106A,MECOM,ING4,SECISBP2L,CDK16,SPINDOC,L	turquoise	EC_M6
GO:0071702	1.37E-01	0.879148877	221	1891	organic substance transport	BP	8.29E-01	TNFRSF11A,KIF5A,STX11,TBC1D30,SNX2,SNX24,ZDHHC14,SERAC1,RIMS2,QKI,FAM91A1,SGSM3,FAM53C,TSNARE1,PICK1,MICALL1,TBC1D22B,ATP8A1,APBA1,UBAP1,CD2AP,SYK,TRAM2,ICA1,SLC3A1,IL6,IGF2BP3,SNAP91,PPT1,VPS54,RSAD2,GRB10,TLR4,TCAF2,TBC1D9,SDCBP,TRPM4,RAB3B,OSBPL9,ANKRD50,RAB38,GGA3,LLGL2,USP6NL,ZFAND6,PPARG,TBC1D4,MYO7A,SEC14L1,NDFIP2,SLCO2B1,FCHSD2,SFRP1,FERMT1,ATP11A,SLC4A11,CD36,SIRT3,SLCO2A1,ADAMTS9,PRR5L,SCARB1,GRAMD1A,CHD7,FAM3D,IL1A,IGF1,HHEX,EXOC6,ERBIN,PITPNM2,SLCO5A1,OCLN,CHRM5,KIF13B,TLR2,P2RX7,VEGFC,TMEM144,SERINC5,MTX3,RAPGEF3,AP4B1,SLC38A1,ANO6,ZC3H11A,IFIT1,DOP1B,EDN1,SNCA,DTNBP1,MFSD4A,SLC45A3,ANO3,MX2,ABCG1,CD200,MTTP,HDAC1,PPP3CA,HYAL2,ANO1,SLC16A14,SLC39A8,MEF2A,RFTN2,ZDHHC6,ADPRH,SNAP23,SLC7A7,LYST,LAPTM5,NCF1,SEC24B,FABP4,PABPN1,HCLS1,AP1AR,ATP9A,TRAK2,ZDHHC18,SLC16A9,PRKD1,RNF215,SLCO4A1,OAZ3,IL10RA,ACE,APOLD1,SENP2,DUSP16,SMG7,APOD,ZW10,OSBPL6,ADORA2A,RTP4,NECAP1,THOC3,HOMER3,MCU,RUNDC1,CALCRL,RAMP2,ATP8B4,CDK16,SNAP29,SPNS3,TNFAIP8L3,LMTK2,PPM1F,NUP88,SLC2A9,TMEM30B,RAC1,HIF1A,JUP,RAB27A,ADIPOR2,SLC16A13,SNCG,SYT7,CASC3,BID,AP1S3,TBC1D1,RSC1A1,SLC26A1,PSEN1,TAOK2,SLC51B,CHRNB2,ZDHHC15,RABEP2,RAB3D,GPR68,SMAD2,DNM2,ATG4D,CA4,TBC1D8B,AP2B1,ABLIM3,SH3TC2,YES1,NOS2,UNC119,SPIRE1,MYO18A,MTM1,RPH3AL,SLC43A2,SGSM2,AP3D1,NOTCH1,SLC25A33,NUP214,TBC1D13,SLC25A25,STXBP1,LRSAM1,LRRC8C,RIPOR1,EXOC3L1,RASSF9,LR	turquoise	EC_M6
GO:0033173	1.38E-01	0.937041339	7	38	calcineurin-NFAT signaling cascade	BP	8.29E-01	IGF1,PPP3CA,NFATC2,HOMER3,MTOR,PLCG2,NFAT5	turquoise	EC_M6
GO:0050768	1.39E-01	0.914746	16	102	negative regulation of neurogenesis	BP	8.29E-01	SEMA6A,CDKL3,SEMA4D,IL6,MYCN,NRP1,PPP3CA,SEMA3F,TRAK2,B2M,SEMA6D,PSEN1,SEMA4A,SEMA6B,ARHGAP4,NOTCH1	turquoise	EC_M6
GO:0050691	1.39E-01	0.947414791	5	25	regulation of defense response to virus by host	BP	8.29E-01	TNFAIP3,DDX58,IL15,TMEM173,STAT1	turquoise	EC_M6
GO:0051640	1.39E-01	0.891007011	58	447	organelle localization	BP	8.29E-01	KIF5A,STX11,FAM91A1,TSNARE1,AGTPBP1,KIT,SYK,SNAP91,CAV2,RAB3B,PIK3CG,GATA2,KIF3C,ARHGAP21,NECTIN2,GAB2,LLGL2,RALB,UNC13D,MYO7A,EML4,EXOC6,NDRG4,SNCA,DTNBP1,MEF2A,SNAP23,LRMP,AP1AR,FGR,TRAK2,FES,LIMK2,DLG1,ZW10,ITGA4,SNAP29,NUP88,HIF1A,SYNE2,RAB27A,MTOR,PSEN1,FBXW11,ATP2A1,RAB3D,DNM2,LGALS9,SPIRE1,MTM1,ATCAY,BICDL2,AP3D1,STXBP1,FNBP1L,RIPOR1,STX3,VAMP3	turquoise	EC_M6
GO:0046503	1.39E-01	0.932347307	8	47	glycerolipid catabolic process	BP	8.29E-01	PIK3CG,MGLL,SCARB1,ABHD6,INPP5F,GDPD3,GDPD1,PLA2G4E	turquoise	EC_M6
GO:0038094	1.39E-01	0.947306236	5	24	Fc-gamma receptor signaling pathway	BP	8.29E-01	PTK2,SYK,FGR,PLD2,YES1	turquoise	EC_M6
GO:0051570	1.40E-01	0.954743091	4	17	regulation of histone H3-K9 methylation	BP	8.29E-01	KDM4D,DNMT3B,KDM3A,JARID2	turquoise	EC_M6
GO:0001763	1.40E-01	0.905654464	23	157	morphogenesis of a branching structure	BP	8.29E-01	CELSR1,NOTCH4,KDR,MYCN,NRP1,RASIP1,PLXND1,GZF1,TIE1,SFRP1,NTN4,DLL4,EDN1,PROX1,LAMA5,DLG1,EPHA2,AR,HOXB7,NRARP,NOTCH1,ENG,BMP2	turquoise	EC_M6

GO:1904427	1.40E-01	0.928359685	9	55	positive regulation of calcium ion transmembrane transport	BP	8.29E-01	TRDN,P2RY6,CXCL10,PLCG1,SNCA,CRACR2A,ATP2A1,CD19,PLCG2	turquoise	EC_M6
GO:0046879	1.41E-01	0.901287009	28	206	hormone secretion	BP	8.29E-01	RIMS2,PICK1,ICA1,IL6,TRPM4,GATA3,SFRP1,SIRT3,CHD7,FAM3D,EDN1,ABCG1,HDAC1,PPP3CA,ANO1,SNAP23,CREB1,MCU,CDK16,FGF23,RAC1,HIF1A,SYT7,GPR68,SMAD2,NOS2,EXOC3L1,BMP6	turquoise	EC_M6
GO:0032268	1.41E-01	0.876229542	211	1819	regulation of cellular protein metabolic process	BP	8.29E-01	TNFRSF11A,TNFAIP8,TNFAIP3,TRIM39,PTPRB,LAPTM4B,CNOT6,FLT4,OXR1,GRAMD4,QKI,TPD52L1,NCOA7,PTK2,DAPK1,ATF4,PDGFB,AGTPBP1,CSNK1E,TEK,IREB2,LIMCH1,FAM122A,TEC,KIT,KDR,SYK,SEMA4D,RGP1,PPP6R1,IL6,IGF2BP3,PELI1,TLR4,C5,IL15,CCNY,SDCBP,NOCT,TUT4,NRP1,PUM2,RASIP1,PIK3CG,TAL1,KDM4D,GATA2,PRKD2,CST7,CTSC,CAB39L,RALB,PPARG,TIMP4,GATA3,ITIH5,EDNRB,NDFIP2,SPTBN4,SETD5,IKBKB,SFRP1,HBEGF,DND1,RPL26,SH3BP5,ANKLE2,CD36,PRR5L,PLK2,RET,PDE4D,DNMT3B,IGF1,NR4A1,KLKB1,EIF2AK3,HHEX,KDM3A,ITCH,SH3RF1,HIP1R,OCLN,SOX7,ENC1,WWTR1,IL7,VEGFC,MADD,FAM192A,THBS4,PPP1R16B,RAPGEF3,CCNJL,CDYL,NEDD9,EDN1,DYRK1A,EHD4,SNCA,JARID2,DTNBP1,MYLIP,RNF144B,RNF19B,HDAC1,LCK,HYAL2,LAPTM5,NCF1,USP13,FABP4,HCLS1,PECAM1,CD86,FGR,CFLAR,CASP10,INPP5F,IGFBP3,PPP2R5A,RBM4,RPS6KA1,PROX1,PRKD1,YTHDF1,MLXIPL,ACE,LIMK2,PAK2,CST6,MELTF,SENP2,DLG1,MAP3K11,MOV10,EIF4A2,TET1,LTF,ADORA2A,BCOR,DDX3X,IGFBP5,TBX1,SECISBP2L,BIRC3,FGF23,CPEB2,PPM1F,RAC1,HIF1A,KDM5A,ADIPOR2,ITPKB,CASC3,BID,TLR6,TNFRSF1B,RGS14,MTOR,OPHN1,RNF40,PSEN1,PAPLN,FBXW11,FGF18,PGF,EIF2B2,TAOK2,EFNA1,SLC51B,NAP1L2,PIK3R5,PIAS1,RPS6KA5,CCDC88C,CNOT8,TNIP1,HMG20A,ADRB2,YES1,SH2D3A,LGALS9,UNC119,USP7,ISG15,TRAF2,PLCG2,DPH5,ENG,SH2D3C,MTF2,DAB2IP,CDH5,PRSS22,PMAIP1,SLC8A3,RELN,ALKAL1,FGF16,MMD,SERPINB9,MGAT3,PERP,DAZAP2,COPS8,BST2,BMP2,DUSP7,BMP6,PPP1R2	turquoise	EC_M6
GO:0003215	1.41E-01	0.953898571	4	18	cardiac right ventricle morphogenesis	BP	8.29E-01	GATA3,JAG1,CHD7,NOTCH1	turquoise	EC_M6
GO:0048661	1.41E-01	0.919800073	12	78	positive regulation of smooth muscle cell proliferation	BP	8.29E-01	PDGFB,IL6,TLR4,P2RY6,HBEGF,IGF1,EDN1,STAT1,IGFBP5,CALCRL,MTOR,S1PR1	turquoise	EC_M6
GO:0006873	1.42E-01	0.888775916	57	444	cellular ion homeostasis	BP	8.29E-01	DMXL1,TRDN,IREB2,PPT1,CAV2,TRPM4,PIK3CG,GATA2,RAB38,GPR4,CXCR4,EDNRB,P2RY6,P2RY2,P2RX2,SLC4A11,CD36,TMEM178A,MICU3,PDE4D,CHD7,IL1A,CLCN3,HEXB,CP,P2RX7,CXCL10,TPCN1,PLCG1,STC1,EDN1,SNCA,LCK,SLC39A8,KCNJ2,TESMIN,HCRTR1,SLC9A8,PRKD1,CCRL2,MCU,SLC9A7,FGF23,HIF1A,HEPH,PSEN1,ATP2A1,CD19,AP3D1,PLCG2,S1PR1,CDH5,SLC8A3,CA2,LPAR6,RAB39A,BMP6	turquoise	EC_M6
GO:0002691	1.42E-01	0.945562793	5	26	regulation of cellular extravasation	BP	8.29E-01	JAM3,ITGA4,SELE,SELP,PLVAP	turquoise	EC_M6
GO:1900745	1.42E-01	0.945347886	5	26	positive regulation of p38MAPK cascade	BP	8.30E-01	GADD45G,NCF1,MAP3K3,RELL1,BMP2	turquoise	EC_M6
GO:0070555	1.70E-01	0.890775839	17	98	response to interleukin-1	BP	8.30E-01	MAP3K7,MYLK3,HNMT,SMPD1,ANKRD1,APP,CX3CL1,TANK,TOLLIP,HYAL1,PRKCA,UPF1,PRKCI,IRAK1,USP10,CCL16,HAS2	darkgreen	EC_M4
GO:0014002	1.43E-01	0.939431207	6	32	astrocyte development	BP	8.30E-01	IFNGR1,IL6,TLR4,TSPAN2,ADORA2A,PSEN1	turquoise	EC_M6
GO:0090175	1.43E-01	0.927178977	9	51	regulation of establishment of planar polarity	BP	8.30E-01	FZD6,CTHRC1,CELSR1,SFRP1,SPEF1,SEC24B,JHY,VANGL2,RAC1	turquoise	EC_M6
GO:0051384	1.70E-01	0.889103863	18	104	response to glucocorticoid	BP	8.31E-01	CDO1,REST,GLB1,HNMT,ATP2B1,HEY1,CPN1,EIF4E,CCND1,KRAS,NTRK3,IGFBP2,CALM1,ABCA3,SSTR5,AGL,SERPINF1,CDKN1A	darkgreen	EC_M4
GO:0048284	1.43E-01	0.90989792	17	120	organelle fusion	BP	8.31E-01	STX11,TSNARE1,CAV2,TBC1D4,DYSF,SNCA,ZDHHC6,SNAP23,USP30,LRMP,SNAP29,SYT7,ADCK1,STXBP1,STX3,VAMP3,RAB39A	turquoise	EC_M6
GO:0032504	1.43E-01	0.884907664	70	571	multicellular organism reproduction	BP	8.31E-01	TSPAN8,QKI,PICK1,KIT,FIGNL1,NOBOX,CREM,TUT4,IZUMO1,SPATA6,DNMT3A,NECTIN2,TIMP4,EDNRB,SFRP1,DND1,GOLGA3,MERTK,IGF1,KDM3A,SMAD1,STOX2,HEXB,ANGPT2,DHH,KMT2D,SFMBT1,STC1,CDYL,EDN1,JAM3,GAS2,ETS1,TESMIN,PLEKHA1,ZNF148,OAZ3,ZSCAN2,ACE,LIMK2,ELL3,ETV6,RNF2,SSTR1,DDX3X,CDK16,BIRC3,USP42,KDM5A,SPIN2A,MTOR,AR,FBXW11,ITGA3,CCDC42,PIAS1,DNM2,YY1,CYP1A1,JAG2,LGALS9,SPEF2,SPIRE1,NOTCH1,STRBP,ARID4A,SOX17,TDRP,ANKRD49,TBC1D20	turquoise	EC_M6

GO:0002761	1.43E-01	0.916350198	13	86	regulation of myeloid leukocyte differentiation	BP	8.32E-01	TLR4,TAL1,GATA2,SFRP1,TMEM178A,HCLS1,INPP5D,FES,CREB1,LTF,MTOR,GPR68,CA2	turquoise	EC_M6
GO:0031669	1.71E-01	0.876523042	30	182	cellular response to nutrient levels	BP	8.32E-01	PPARA,TXN2,SLC39A4,LEP,CDKN2B,SRF,NUAK1,ASNS,MFSD2A,VPS41,SESN3,ATP2B1,MAP1LC3A,CACNB3,BRSK2,DNM1L,WIPI1,HSPA8,ATF3,COMT,WIPI2,EIF2S1,MED1,CPEB4,COL1A1,PRKAA1,CLEC16A,HSPA5,CDKN1A,GABARAPL2	darkgreen	EC_M4
GO:2000810	1.44E-01	0.953099671	4	17	regulation of bicellular tight junction assembly	BP	8.33E-01	IKBKB,F11R,PRKCH,EPHA2	turquoise	EC_M6
GO:0002534	1.44E-01	0.933735255	7	40	cytokine production involved in inflammatory response	BP	8.33E-01	IL6,TLR4,APOD,HIF1A,TLR6,NOS2,GBP5	turquoise	EC_M6
GO:1900015	1.44E-01	0.933735255	7	40	regulation of cytokine production involved in inflammatory response	BP	8.33E-01	IL6,TLR4,APOD,HIF1A,TLR6,NOS2,GBP5	turquoise	EC_M6
GO:1900047	1.44E-01	0.93817231	6	36	negative regulation of hemostasis	BP	8.33E-01	TSPAN8,PDGFB,NOS3,KLKB1,EDN1,CD34	turquoise	EC_M6
GO:0051656	1.44E-01	0.890869795	44	334	establishment of organelle localization	BP	8.33E-01	KIF5A,FAM91A1,AGTPBP1,KIT,SYK,SNAP91,PIK3CG,GATA2,KIF3C,ARHGAP21,NECTIN2,GAB2,LLGL2,UNC13D,MYO7A,EML4,EXOC6,SNCA,DTNBP1,SNAP23,LRMP,AP1AR,FGR,TRAK2,FES,LIMK2,DLG1,ZW10,ITGA4,SNAP29,NUP88,HIF1A,SYNE2,RAB27A,PSEN1,FBXW11,DNM2,LGALS9,SPIRE1,BICDL2,AP3D1,STXBP1,FNBP1L,RIPOR1	turquoise	EC_M6
GO:0021801	1.44E-01	0.95256314	4	18	cerebral cortex radial glia-guided migration	BP	8.33E-01	SRGAP2C,DAB2IP,ADGRG1,RELN	turquoise	EC_M6
GO:0022030	1.44E-01	0.95256314	4	18	telencephalon glial cell migration	BP	8.33E-01	SRGAP2C,DAB2IP,ADGRG1,RELN	turquoise	EC_M6
GO:0015800	1.71E-01	0.913844384	8	39	acidic amino acid transport	BP	8.34E-01	SLC12A2,SLC7A11,ITGB1,RAB3GAP1,ARL6IP5,SLC6A13,PRAF2,CLN8	darkgreen	EC_M4
GO:2001135	1.44E-01	0.952482267	4	18	regulation of endocytic recycling	BP	8.34E-01	NDRG4,ATP9A,INPP5F,PLA2G4E	turquoise	EC_M6
GO:2000463	1.44E-01	0.944901306	5	24	positive regulation of excitatory postsynaptic potential	BP	8.34E-01	RIMS2,ADRB2,RELN,SHANK3,RGS4	turquoise	EC_M6
GO:0042130	1.44E-01	0.933489189	7	40	negative regulation of T cell proliferation	BP	8.34E-01	TNFRSF21,PELI1,ITCH,CD86,DLG1,ARG2,LGALS9	turquoise	EC_M6
GO:0090277	1.71E-01	0.900007929	12	65	positive regulation of peptide hormone secretion	BP	8.34E-01	LRP1,PPARD,KIF5B,IRS2,OSBP,SELENOT,PFKM,DYNLL1,TFR2,NLGN2,ACSL4,RASL10B	darkgreen	EC_M4
GO:0043549	1.71E-01	0.85678336	93	612	regulation of kinase activity	BP	8.35E-01	IL23A,NAB2,AGAP2,TAB2,PRRC1,ANGPT1,ROS1,GNAQ,LEP,CDKN2B,VEGFA,NCK2,SOCS5,MAP3K7,SH3BP5L,FGF2,RB1,MAPRE3,PAK1,TGFB1,AXL,OSBPL8,KAT2B,IRS2,PRNP,CCKBR,SMPD1,ILK,RYK,ROBO1,CCNJ,APP,RAPGEF2,GTF2H1,PTPN22,TSG101,CHP1,ATP2B4,PKD2,DBNDD2,CBLB,CCND1,TGFB2,LRP5,HTR2B,PRKAR1A,DAG1,PIK3CA,CORO1C,KRAS,NTRK3,SLAMF8,SPATC1L,BMP7,FGFR2,SYAP1,IQGAP1,HSPB1,MAPT,PTPN6,PDCD10,ADRA2C,DOK7,HTT,FGFR3,MNAT1,MAGED1,BMPR1A,TSPYL2,CDK12,SRCIN1,SHC1,CCNG1,MAP2K1,CALM1,GSKIP,MYOCD,CD74,PDGFRB,AKT1,PTPN2,PKD1,IRAK1,MIDN,PARK7,LMO4,BCL10,MIR24-1,RANBP2,THY1,CDKN1A,TXN,DUSP22	darkgreen	EC_M4
GO:0051338	1.71E-01	0.855107173	106	706	regulation of transferase activity	BP	8.35E-01	IL23A,NAB2,AGAP2,TAB2,PRRC1,ANGPT1,TCP1,ROS1,SERINC1,GNAQ,LEP,CDKN2B,VEGFA,NCK2,SOCS5,MAP3K7,SH3BP5L,FGF2,RB1,MAPRE3,PAK1,TGFB1,AXL,CTNNB1,OSBPL8,KAT2B,IRS2,PRNP,CCKBR,SMPD1,ILK,RYK,ROBO1,PINX1,CCNJ,APP,RAPGEF2,MAPKAPK5,GTF2H1,PTPN22,TSG101,DKC1,CHP1,ATP2B4,PKD2,DBNDD2,CBLB,CHRNA7,DNM1L,CCND1,TGFB2,LRP5,HTR2B,PRKAR1A,GSK3B,DAG1,PIK3CA,CORO1C,NEK7,KRAS,NTRK3,SLAMF8,SPATC1L,BMP7,FGFR2,SYAP1,IQGAP1,HSPB1,MAPT,PTPN6,PDCD10,ADRA2C,DOK7,HTT,FGFR3,MNAT1,PPP1R3F,MAGED1,BMPR1A,TSPYL2,CDK12,SRCIN1,SHC1,CCNG1,MAP2K1,CALM1,GSKIP,MYOCD,PIN1,CD74,PDGFRB,AKT1,PTPN2,PKD1,IRAK1,MIDN,PARK7,LMO4,BCL10,MIR24-1,RANBP2,THY1,CDKN1A,TXN,DUSP22,ORMDL3	darkgreen	EC_M4

GO:0043116	1.71E-01	0.940590589	4	16	negative regulation of vascular permeability	BP	8.35E-01	ANGPT1,SH3GL2,VEGFA,ABCC8	darkgreen	EC_M4
GO:2001237	1.45E-01	0.917487391	12	79	negative regulation of extrinsic apoptotic signaling pathway	BP	8.36E-01	TNFAIP3,NRP1,NOS3,IL1A,IGF1,SH3RF1,IL7,ITPRIP,CFLAR,ITGA6,DDX3X,AR	turquoise	EC_M6
GO:0031324	1.45E-01	0.872447886	222	1896	negative regulation of cellular metabolic process	BP	8.36E-01	TNFAIP8,TNFAIP3,PLAGL1,TRIM39,PTPRB,LAPTM4B,CNOT6,OXR1,NOTCH4,TCF7,NCOA7,L3MBTL3,DAPK1,ATF4,PDGFB,MDFI,IREB2,NFX1,NFIL3,SEMA4D,RGP1,IL6,IGF2BP3,NFE2L3,PTBP3,BACH2,FOXP2,KLF11,GRB10,LANCL2,C5,CREM,SDCBP,MYCN,NOCT,TUT4,RASIP1,BEND5,PIK3CG,TAL1,PHF10,MTMR2,EPC1,KDM4D,GATA2,DNMT3A,SAP130,CST7,GGA3,DACH1,PPARG,TIMP4,GATA3,TAF3,ACVR2B,ITIH5,GZF1,EDNRB,IKBKB,PDE2A,SFRP1,HBEGF,NR1D2,DND1,FEZ2,SH3BP5,ANKLE2,CD36,SIRT3,ARID5A,PRR5L,PDE4D,DNMT3B,IGF1,NR4A1,EIF2AK3,HHEX,KDM3A,SH3RF1,ZBTB38,POU6F1,OCLN,SOX7,HMBOX1,ENC1,MEF2C,WWTR1,SOGA1,SFMBT1,FAM192A,SCAF4,PPP1R16B,ANKRD2,NR4A2,DLL4,CDYL,SOX13,YAF2,EDN1,STAT1,DYRK1A,SNCA,JARID2,DTNBP1,ETS2,DCAF1,LPCAT1,SIK1,HDAC1,PPP3CA,HYAL2,MEF2A,RIN3,USP30,FABP4,HCLS1,NFATC2,AP1AR,CD34,KDM2A,CIART,TGIF1,PARP15,WDR6,CFLAR,INPP5F,IGFBP3,PPP2R5A,RBM4,RPS6KA1,PROX1,ZNF148,YTHDF1,IL10RA,MLXIPL,PAK2,CST6,SENP2,DLG1,APOD,ETV6,MOV10,RNF2,CREB1,EIF4A2,LTF,ADORA2A,BCOR,DDX3X,IGFBP5,MECOM,ING4,CDA,SPINDOC,BIRC3,LDB2,CPEB2,PPM1F,POU2F1,HIF1A,KDM5A,ZBTB18,APBB2,BCL6B,KDM5C,RGS14,MAFK,MTOR,AR,OPHN1,RNF40,PSEN1,PAPLN,CBX1,HOXB8,FBXW11,EFNA1,PIAS1,RPS6KA5,SMAD2,TLE3,CNOT8,YY1,HMG20A,RCOR1,LIG3,GIT1,USP7,MTM1,ATCAY,ISG15,HCFC1,NRARP,TRAF2,NOTCH1,IRF8,ENG,MTF2,ZBTB34,TERF2,DAB2IP,ZBTB33,SLC8A3,ARID4A,ZNF24,ATXN1L,TRIM24,SOX18,PASD1,SERPINB9,COMMD7,MGAT3,TGIF2,BST2,BMP2,GLIS3,ZNF10,DUSP7,CBX2,NMNAT1,BMP6,Z	turquoise	EC_M6
GO:0071774	1.45E-01	0.913516998	14	91	response to fibroblast growth factor	BP	8.36E-01	CXCL8,PRKD2,GATA3,SFRP1,NR4A1,DLL4,SNCA,HYAL2,SHOC2,TBX1,FGF23,FGF18,CCL2,FGF16	turquoise	EC_M6
GO:0021545	1.45E-01	0.933044396	7	38	cranial nerve development	BP	8.38E-01	NRP1,EPHB1,CHD7,NAV2,SEMA3F,TBX1,CHRNB2	turquoise	EC_M6
GO:0060977	1.46E-01	0.952653288	4	16	coronary vasculature morphogenesis	BP	8.38E-01	NRP1,SEC24B,TBX1,NOTCH1	turquoise	EC_M6
GO:0032482	1.46E-01	0.951818501	4	18	Rab protein signal transduction	BP	8.38E-01	SGSM3,DENND3,MADD,RAB39A	turquoise	EC_M6
GO:0010039	1.46E-01	0.943688866	5	26	response to iron ion	BP	8.38E-01	SNCA,B2M,HIF1A,CYP1A1,BMP6	turquoise	EC_M6
GO:0006672	1.72E-01	0.894498582	14	78	ceramide metabolic process	BP	8.38E-01	B4GALNT1,ACER3,SMPD1,ST8SIA4,CERS5,B3GALT1,SGPL1,SGMS1,CLN8,SPTLC2,CLN6,PRKAA1,PLA2G15,ORMDL3	darkgreen	EC_M4
GO:0009062	1.72E-01	0.892342024	15	86	fatty acid catabolic process	BP	8.39E-01	PPARA,PPARD,LEP,MFSD2A,ABHD1,AKT2,IRS2,CPT1A,HADH,ACOX3,HSD17B10,AKT1,LPIN2,PLA2G15,AMACR	darkgreen	EC_M4
GO:0055023	1.46E-01	0.937676719	6	31	positive regulation of cardiac muscle tissue growth	BP	8.40E-01	PIM1,IGF1,MEF2C,EDN1,MTOR,NOTCH1	turquoise	EC_M6
GO:0051954	1.73E-01	0.930531464	5	22	positive regulation of amine transport	BP	8.40E-01	SLC12A2,ITGB1,RAB3GAP1,CXCL12,GDNF	darkgreen	EC_M4
GO:0010256	1.46E-01	0.885623364	54	422	endomembrane system organization	BP	8.41E-01	STX11,LAPTM4B,PACSIN2,TRDN,MTSS1,ASAP1,BAIAP2L2,MICALL1,TRAM2,CAV2,IZUMO1,ARHGAP21,RAB38,TMCC1,NECTIN2,USP6NL,CXCR4,TIE1,ANKLE2,ARHGEF7,EIF2AK3,CLCN3,FHDC1,P2RX7,DYSF,ANO6,DOP1B,DTNBP1,ANO3,ABCG1,SNAP23,LYST,PRKD1,TOR1AIP1,CREB1,ALS2CL,ZW10,ATP8B4,SNAP29,BAIAP2L1,RAB27A,SYT7,MPP5,AR,DNM2,SH3TC2,MYO18A,AP3D1,STXBP1,FNBP1L,RAB43,RAB18,STX3,TBC1D20	turquoise	EC_M6
GO:0043555	1.47E-01	0.951369647	4	18	regulation of translation in response to stress	BP	8.42E-01	EIF2AK3,RBM4,RPS6KA1,DDX3X	turquoise	EC_M6
GO:0021983	1.47E-01	0.943378278	5	25	pituitary gland development	BP	8.42E-01	GATA2,ETS1,CREB1,ALDH1A2,BMP2	turquoise	EC_M6
GO:0016458	1.47E-01	0.900263966	23	169	gene silencing	BP	8.42E-01	BAZ2A,CNOT6,IL6,MYCN,TUT4,PUM2,DNMT3A,PPARG,DND1,SMAD1,HDAC1,KIAA1551,PPP3CA,RBM4,MOV10,TET1,DDX3X,SMAD2,CNOT8,MIR23A,MIR99A,MIR1307,HIST1H3F	turquoise	EC_M6

GO:0045934	1.47E-01	0.874224789	135	1115	negative regulation of nucleobase-containing compound metabolic process	BP	8.42E-01	PLAGL1,NOTCH4,TCF7,L3MBTL3,ATF4,PDGFB,MDFI,NFX1,NFIL3,SEMA4D,IL6,NFE2L3,PTBP3,BACH2,FOXP2,KLF11,LANCL2,CREM,SDCBP,NOCT,BEND5,TAL1,PHF10,EPC1,GATA2,DNMT3A,SAP130,DACH1,PPARG,GATA3,TAF3,ACVR2B,GZF1,EDNRB,PDE2A,SFRP1,NR1D2,DND1,CD36,ARID5A,DNMT3B,HHEX,ZBTB38,POU6F1,SOX7,HMBOX1,MEF2C,WWTR1,SFMBT1,SCAF4,ANKRD2,NR4A2,DLL4,CDYL,SOX13,YAF2,EDN1,STAT1,DYRK1A,SNCA,JARID2,ETS2,DCAF1,HDAC1,PPP3CA,MEF2A,FABP4,HCLS1,NFATC2,KDM2A,CIART,TGIF1,PARP15,PROX1,ZNF148,MLXIPL,DLG1,ETV6,RNF2,CREB1,EIF4A2,BCOR,MECOM,ING4,CDA,SPINDOC,LDB2,PPM1F,POU2F1,ZBTB18,APBB2,BCL6B,KDM5C,MAFK,MTOR,AR,RNF40,PSEN1,CBX1,HOXB8,FBXW11,EFNA1,PIAS1,RPS6KA5,SMAD2,TLE3,YY1,HMG20A,RCOR1,GIT1,HCFC1,NRARP,TRAF2,NOTCH1,IRF8,MTF2,ZBTB34,TERF2,DAB2IP,ZBTB33,ARID4A,ZNF24,ATXN1L,TRIM24,SOX18,PASD1,COMMD7,TGIF2,BMP2,GLIS3,ZNF10,CBX2,NMNAT1,BMP6,ZNF266	turquoise	EC_M6
GO:0050982	1.47E-01	0.931754484	7	40	detection of mechanical stimulus	BP	8.42E-01	PTK2,KIT,CXCR4,ANO3,JUP,PIEZO2,PIEZO1	turquoise	EC_M6
GO:0051931	1.73E-01	0.930277821	5	22	regulation of sensory perception	BP	8.42E-01	ACPP,CACNB3,COMT,ADRA2C,TMEM100	darkgreen	EC_M4
GO:0046638	1.73E-01	0.917023396	7	34	positive regulation of alpha-beta T cell differentiation	BP	8.42E-01	IL23A,HLA-DRA,SOCS5,GLI3,AP3B1,ADA,RUNX1	darkgreen	EC_M4
GO:0034390	1.48E-01	0.950262774	4	20	smooth muscle cell apoptotic process	BP	8.43E-01	PPARG,IGF1,EDN1,E2F3	turquoise	EC_M6
GO:0034391	1.48E-01	0.950262774	4	20	regulation of smooth muscle cell apoptotic process	BP	8.43E-01	PPARG,IGF1,EDN1,E2F3	turquoise	EC_M6
GO:0032331	1.74E-01	0.930079501	5	22	negative regulation of chondrocyte differentiation	BP	8.43E-01	TGFBR1,GLI2,CTNNB1,RARB,PTPN11	darkgreen	EC_M4
GO:0032006	1.74E-01	0.89154897	15	84	regulation of TOR signaling	BP	8.43E-01	ROS1,LEP,SESN3,RHEB,SEC13,HDAC3,TMEM127,MAPKAPK5,PIK3CA,GPR137,ARAF,OTUD5,AKT3,PRKAA1,CLEC16A	darkgreen	EC_M4
GO:0010719	1.48E-01	0.942586297	5	26	negative regulation of epithelial to mesenchymal transition	BP	8.43E-01	GATA3,SFRP1,EFNA1,DAB2IP,TRIM62	turquoise	EC_M6
GO:0015914	1.74E-01	0.895843487	13	70	phospholipid transport	BP	8.43E-01	ATP8B1,TMEM30A,MFSD2A,OSBPL10,OSBPL8,PITPNM1,PITPNB,PRELID3B,OSBPL2,ATP11C,PITPNA,ABCA3,PLEKHA3	darkgreen	EC_M4
GO:0007498	1.74E-01	0.88963085	16	89	mesoderm development	BP	8.44E-01	SRF,VEGFA,INHBA,ITGB1,TLX2,ACVR1,EYA2,PRKAR1A,LEF1,BMP7,FGFR2,OVOL1,BMPR1A,ITGA2,WLS,MESD	darkgreen	EC_M4
GO:0002701	1.74E-01	0.929915936	5	22	negative regulation of production of molecular mediator of immune response	BP	8.44E-01	ANGPT1,TGFB1,SPINK5,TGFB2,TGFB3	darkgreen	EC_M4
GO:0010677	1.74E-01	0.916546788	7	34	negative regulation of cellular carbohydrate metabolic process	BP	8.44E-01	PPARA,SELENOS,GSK3B,PPARGC1A,PGP,MIDN,FAM3A	darkgreen	EC_M4
GO:1905476	1.74E-01	0.922378899	6	28	negative regulation of protein localization to membrane	BP	8.44E-01	PICALM,TGFB1,AP2M1,FZD9,LRRC15,CLTC	darkgreen	EC_M4
GO:0008637	1.74E-01	0.890861043	15	87	apoptotic mitochondrial changes	BP	8.44E-01	GGCT,VPS35,PPP2CB,SLC25A4,OPA1,DNM1L,EYA2,GSK3B,FAM162A,FZD9,VDAC2,GHITM,AKT1,BNIP3,BLOC1S2	darkgreen	EC_M4
GO:0021762	1.74E-01	0.916178476	7	35	substantia nigra development	BP	8.44E-01	FGF2,DYNLL1,INA,CALM1,SYPL2,HSPA5,YWHAH	darkgreen	EC_M4
GO:0051348	7.95E-03	0.995412854	36	219	negative regulation of transferase activity	BP	8.45E-01	PIP4K2C,IRAK3,DEPTOR,STK38,CDKN2A,RPS7,AKT1S1,SPRY1,SPRY4,TARBP2,LATS2,SPRED1,PARP3,QARS,DTX3L,HEG1,CEP85,RPL11,CDKN1B,LRP6,HEXIM2,MEN1,WASHC1,PIP4K2B,CDK5RAP3,MVP,GBA,TFAP4,DVL1,PHPT1,RPS15,HEXIM1,RACK1,RPL23,TRIB2,RPS20	cyan	EC_M2
GO:0046164	1.48E-01	0.931007675	7	41	alcohol catabolic process	BP	8.45E-01	SYNJ2,SRD5A3,INPP4B,MTMR2,SCARB1,SCARF1,AKR1D1	turquoise	EC_M6

GO:0051453	1.75E-01	0.90035289	11	58	regulation of intracellular pH	BP	8.45E-01	RAB7A,SLC4A7,TM9SF4,SLC4A5,CHP1,SLAMF8,CLIC4,ATP6AP2,CLN6,ATP6V0D1,ATP6V0E1	darkgreen	EC_M4
GO:0071676	1.48E-01	0.950208914	4	19	negative regulation of mononuclear cell migration	BP	8.46E-01	LRCH1,CD200,APOD,CCL2	turquoise	EC_M6
GO:0021695	1.75E-01	0.911336224	8	39	cerebellar cortex development	BP	8.46E-01	WHRN,GLI2,FAIM2,PTPN11,MAP2K1,RERE,AARS,HSPA5	darkgreen	EC_M4
GO:0051480	1.48E-01	0.89438555	29	212	regulation of cytosolic calcium ion concentration	BP	8.46E-01	TRDN,CAV2,TRPM4,PIK3CG,GATA2,GPR4,CXCR4,EDNRB,P2RY6,P2RX2,CD36,TMEM178A,PDE4D,CHD7,P2RX7,CXCL10,TPCN1,PLCG1,EDN1,SNCA,LCK,HCRTR1,PRKD1,CCRL2,CD19,PLCG2,S1PR1,SLC8A3,LPAR6	turquoise	EC_M6
GO:0006007	1.75E-01	0.938845695	4	16	glucose catabolic process	BP	8.46E-01	PFKP,PFKM,LRP5,ENO1	darkgreen	EC_M4
GO:0035584	1.75E-01	0.938800833	4	16	calcium-mediated signaling using intracellular calcium source	BP	8.46E-01	FKBP1B,PRNP,ADGRL1,BCAP31	darkgreen	EC_M4
GO:0046620	1.49E-01	0.914427713	12	78	regulation of organ growth	BP	8.48E-01	PIM1,IGF1,MEF2C,IL7,EDN1,JARID2,HLX,PROX1,MTOR,YY1,NOTCH1,RGS4	turquoise	EC_M6
GO:0048745	1.49E-01	0.950520805	4	17	smooth muscle tissue development	BP	8.48E-01	TIPARP,PROX1,DLG1,ENG	turquoise	EC_M6
GO:0032945	1.49E-01	0.926152389	8	48	negative regulation of mononuclear cell proliferation	BP	8.48E-01	TNFRSF21,PELI1,ITCH,INPP5D,CD86,DLG1,ARG2,LGALS9	turquoise	EC_M6
GO:0050672	1.49E-01	0.926152389	8	48	negative regulation of lymphocyte proliferation	BP	8.48E-01	TNFRSF21,PELI1,ITCH,INPP5D,CD86,DLG1,ARG2,LGALS9	turquoise	EC_M6
GO:2001014	1.49E-01	0.95004637	4	18	regulation of skeletal muscle cell differentiation	BP	8.48E-01	NR1D2,EPHB1,MEF2C,TBX1	turquoise	EC_M6
GO:2000310	1.49E-01	0.950038487	4	18	regulation of NMDA receptor activity	BP	8.48E-01	DAPK1,MEF2C,CCL2,RELN	turquoise	EC_M6
GO:0090075	1.49E-01	0.941926393	5	25	relaxation of muscle	BP	8.48E-01	PDE4D,GUCY1A1,KCNJ2,ATP2A1,PDE4B	turquoise	EC_M6
GO:0051646	1.49E-01	0.926273099	8	46	mitochondrion localization	BP	8.48E-01	AGTPBP1,NECTIN2,MEF2A,TRAK2,HIF1A,ATP2A1,MTM1,ATCAY	turquoise	EC_M6
GO:0043268	1.50E-01	0.936150773	6	29	positive regulation of potassium ion transport	BP	8.48E-01	ANO6,KCNJ2,DLG1,WNK4,DNM2,KCNH2	turquoise	EC_M6
GO:0031343	1.50E-01	0.941388182	5	27	positive regulation of cell killing	BP	8.48E-01	SYK,NECTIN2,B2M,NOS2,FCER2	turquoise	EC_M6
GO:0006939	1.50E-01	0.911907417	13	84	smooth muscle contraction	BP	8.48E-01	KIT,DOCK4,ARHGAP42,EDNRB,P2RX2,GUCY1A1,EDN1,DLG1,CALCRL,APBB2,CHRNB2,SCNN1B,ADRB2	turquoise	EC_M6
GO:0045619	1.76E-01	0.882067165	20	117	regulation of lymphocyte differentiation	BP	8.48E-01	IL23A,HLA-DRA,SOCS5,GLI3,INHBA,GLI2,AXL,IL15RA,SPINK5,LOXL3,AP3B1,ADA,RUNX1,MMP14,LEF1,SLAMF8,PTPN6,CD74,PTPN2,FGL2	darkgreen	EC_M4
GO:1903317	1.76E-01	0.902632512	10	54	regulation of protein maturation	BP	8.48E-01	MYH9,PRNP,CHAC1,RUNX1,MMP14,SERPINE1,TFR2,TMEM98,ENO1,GLG1	darkgreen	EC_M4
GO:1903707	1.76E-01	0.894202064	13	72	negative regulation of hemopoiesis	BP	8.48E-01	SOCS5,GLI3,INHBA,CTNNB1,LOXL3,RUNX1,TCTA,GPR137,FBN1,CD74,PTPN2,FSTL3,FGL2	darkgreen	EC_M4
GO:0086009	1.76E-01	0.915608993	7	33	membrane repolarization	BP	8.49E-01	SCN5A,SNTA1,ATP1B3,CACNA1D,CACNB3,WDR1,FLNA	darkgreen	EC_M4
GO:0014044	1.76E-01	0.921462726	6	27	Schwann cell development	BP	8.50E-01	AKT2,ILK,LGI4,DAG1,DICER1,AKT1	darkgreen	EC_M4
GO:0071357	1.50E-01	0.929664308	7	42	cellular response to type I interferon	BP	8.50E-01	OAS3,NLRC5,IFIT1,STAT1,JAK1,ISG15,OAS1	turquoise	EC_M6
GO:0050857	1.51E-01	0.948794253	4	20	positive regulation of antigen receptor-mediated signaling pathway	BP	8.51E-01	PRKD2,NECTIN2,LCK,PRKCH	turquoise	EC_M6

GO:0009896	1.51E-01	0.882092418	53	420	positive regulation of catabolic process	BP	8.52E-01	TNFAIP3,CNOT6,SGSM3,PTK2,DAPK1,AGTPBP1,CSNK1E,FAM122A,KDR,IL6,NOCT,TUT4,CTSC,GGA3,RALB,TMEM173,DND1,PRR5L,PLK2,IGF1,ITCH,GPD1,P2RX7,TIPARP,TPCN1,SNCA,MYLIP,RNF144B,RNF19B,LPCAT1,HECW2,LAPTM5,USP13,ASB9,SH3BP4,PRKD1,YTHDF1,OAZ3,MLXIPL,MOV10,HIF1A,BID,TNFRSF1B,RNF40,PSEN1,PIAS1,CNOT8,ADRB2,LRSAM1,DAB2IP,TRIM34,SOX17,RNF152	turquoise	EC_M6
GO:1903078	1.77E-01	0.902118029	10	52	positive regulation of protein localization to plasma membrane	BP	8.52E-01	LRP1,ITGB1,KIF5B,ATP2C1,PRNP,SORBS1,ATP2B4,PRKCI,RAB11A,AKT1	darkgreen	EC_M4
GO:1902110	1.77E-01	0.920592814	6	29	positive regulation of mitochondrial membrane permeability involved in apoptotic process	BP	8.52E-01	EYA2,GSK3B,FZD9,VDAC2,BNIP3,BLOC1S2	darkgreen	EC_M4
GO:0010827	1.77E-01	0.898885829	11	58	regulation of glucose transmembrane transport	BP	8.52E-01	LEP,AKT2,OSBPL8,IRS2,SORBS1,PTPN11,SELENOS,PEA15,CLTCL1,PRKCI,AKT1	darkgreen	EC_M4
GO:1901879	1.77E-01	0.891321788	14	75	regulation of protein depolymerization	BP	8.52E-01	NAV3,MAP1B,TWF1,LMOD1,PIK3CA,CAPZB,WDR1,NES,ARHGEF2,FLII,SPTAN1,DSTN,MAP1A,CFL2	darkgreen	EC_M4
GO:0045665	1.77E-01	0.901924706	10	52	negative regulation of neuron differentiation	BP	8.52E-01	REST,GLI3,ZNF536,HEY1,APP,EIF4E,GSK3B,BMP7,MED1,LBX1	darkgreen	EC_M4
GO:0010955	1.77E-01	0.927749732	5	23	negative regulation of protein processing	BP	8.52E-01	PRNP,CHAC1,SERPINE1,TMEM98,GLG1	darkgreen	EC_M4
GO:1903318	1.77E-01	0.927749732	5	23	negative regulation of protein maturation	BP	8.52E-01	PRNP,CHAC1,SERPINE1,TMEM98,GLG1	darkgreen	EC_M4
GO:0031056	5.78E-02	0.967358345	18	124	regulation of histone modification	BP	8.53E-01	UBR5,ZNF451,ZMPSTE24,PAXIP1,NCAPG2,RIF1,NSD3,TAF7,MAPK8,CCNB1,CHEK1,BRCA1,NSD1,OGT,ATRX,NIPBL,FMR1,CTCF	darkgrey	EC_M8
GO:1904667	8.08E-03	0.998869505	5	11	negative regulation of ubiquitin protein ligase activity	BP	8.53E-01	RPS7,RPL11,RPS15,RPL23,RPS20	cyan	EC_M2
GO:0032479	5.80E-02	0.971855396	12	75	regulation of type I interferon production	BP	8.53E-01	POLR3B,POLR3F,TOMM70,CHUK,TRAIP,DHX9,SETD2,POLA1,DHX33,POLR3A,G3BP1,RIOK3	darkgrey	EC_M8
GO:0032606	5.80E-02	0.971855396	12	75	type I interferon production	BP	8.53E-01	POLR3B,POLR3F,TOMM70,CHUK,TRAIP,DHX9,SETD2,POLA1,DHX33,POLR3A,G3BP1,RIOK3	darkgrey	EC_M8
GO:0006749	1.78E-01	0.909135113	8	42	glutathione metabolic process	BP	8.53E-01	SLC7A11,MMACHC,ARL6IP5,CHAC1,GLRX2,HAGH,PARK7,GLO1	darkgreen	EC_M4
GO:0007398	1.78E-01	0.937551101	4	16	ectoderm development	BP	8.53E-01	TPT1,CTNNB1,SMURF1,BMPR1A	darkgreen	EC_M4
GO:0032720	1.78E-01	0.909129015	8	41	negative regulation of tumor necrosis factor production	BP	8.53E-01	AXL,CX3CL1,PTPN22,CHRNA7,SELENOS,PTPN6,SYT11,DICER1	darkgreen	EC_M4
GO:0042908	1.78E-01	0.920181827	6	28	xenobiotic transport	BP	8.54E-01	ATP8B1,SLC22A4,TMEM30A,ATP7B,SLC29A4,ABCA3	darkgreen	EC_M4
GO:0046638	1.51E-01	0.934152278	6	34	positive regulation of alpha-beta T cell differentiation	BP	8.54E-01	SYK,HLX,CD86,ITPKB,LGALS9,AP3D1	turquoise	EC_M6
GO:0031050	1.52E-01	0.924971804	8	46	dsRNA processing	BP	8.54E-01	IL6,MYCN,TUT4,PUM2,SMAD1,PPP3CA,DDX3X,SMAD2	turquoise	EC_M6
GO:0035196	1.52E-01	0.924971804	8	46	production of miRNAs involved in gene silencing by miRNA	BP	8.54E-01	IL6,MYCN,TUT4,PUM2,SMAD1,PPP3CA,DDX3X,SMAD2	turquoise	EC_M6
GO:0070918	1.52E-01	0.924971804	8	46	production of small RNA involved in gene silencing by RNA	BP	8.54E-01	IL6,MYCN,TUT4,PUM2,SMAD1,PPP3CA,DDX3X,SMAD2	turquoise	EC_M6
GO:0010522	1.52E-01	0.915140346	11	71	regulation of calcium ion transport into cytosol	BP	8.54E-01	TRDN,P2RY6,P2RX2,PDE4D,CHD7,P2RX7,CXCL10,PLCG1,SNCA,PRKD1,CD19	turquoise	EC_M6

GO:0007160	1.52E-01	0.892608042	28	192	cell-matrix adhesion	BP	8.54E-01	PARVG,PTK2,TEK,LIMCH1,KDR,NRP1,BCAM,ITGA8,ITGB4,JAG1,SFRP1,FERMT1,THSD1,ARHGEF7,CD36,ADAMTS9,LYVE1,JAM3,CD34,APOD,BCR,ITGA4,PPM1F,RAC1,RCC2,JUP,ITGA3,TAOK2	turquoise	EC_M6
GO:2000401	1.52E-01	0.928867899	7	41	regulation of lymphocyte migration	BP	8.54E-01	LRCH1,CXCL10,CD200,APOD,ITGA4,STK10,CCL2	turquoise	EC_M6
GO:0010803	1.52E-01	0.933785134	6	35	regulation of tumor necrosis factor-mediated signaling pathway	BP	8.54E-01	TNFAIP3,SYK,IKBKB,HIPK1,LAPTM5,TRAF2	turquoise	EC_M6
GO:1901575	8.16E-03	0.993486629	210	1628	organic substance catabolic process	BP	8.55E-01	PYM1,OS9,CDK4,SQSTM1,IRAK3,BAG6,ALDH7A1,ABHD16A,SNX9,HSPA1B,RNF5,EIF3H,ZHX2,EP300,MPST,SHARPIN,FOXRED2,HSF1,FBXL6,VPS28,HMOX1,CUL7,CUL9,DNPH1,HINT2,EEF1A1,SLC44A1,ETFB,RPS7,IL4I1,LPIN1,DHDH,NOP53,ZC3HAV1,BBS7,TRIM13,SMG9,FASTK,AGAP3,MLH1,LIPE,PAFAH1B3,ACAA1,MYD88,DMAC2,BCKDHA,EGLN2,PNP,TIMP2,SAMD4B,GAA,XDH,SIRT2,LRTOMT,HGS,UBAC2,HACL1,GPCPD1,DDHD2,CYP1B1,GNPDA1,NDFIP1,SMUG1,PCBP2,PSMF1,RBCK1,KCTD6,THNSL2,RMND5A,FBXO8,NEIL2,SH3D19,CHDH,HTRA2,PRKAG1,PLBD2,TMEM67,LPIN3,FOXF2,RNFT2,ERLIN1,PLA2G4B,ACOT8,FBXW4,LONP2,FASTKD3,SRD5A1,NT5C2,ALDH5A1,IRS1,ACADS,HYAL3,TCIRG1,ABHD4,UBA7,DIS3L2,PUM1,GPX1,AC013489.1,USP19,DCAF11,DTX3L,ABCB11,ANPEP,RNF26,RHBDD3,ARNT,HDAC4,GPC1,USP21,GLUL,RNASEL,MAGEF1,EHHADH,RPL11,CDKN1B,KAT5,LRIG2,YBX3,NFE2L2,IDH1,OVGP1,CAPN1,ACADL,MTHFS,EXOSC7,CTSH,PRODH,ABHD12B,UBA52,DDHD1,CYP27A1,SMPD4,NAGLU,PLA2G16,ANKZF1,MVB12A,USE1,HDAC6,UBXN1,ATP13A2,RNF216,FBXL18,GABARAP,FOXK1,ILVBL,THRA,ZFP36L1,OSBPL7,CDK5RAP3,ALDH6A1,ABCD4,GBA,GCDH,NUPR1,AC138894.1,TRIM25,TNRC6A,HEXA,KEAP1,UBL7,SNX33,ANKRD9,INSR,FBXO4,OXCT1,TMEM199,CLPP,PARN,ACAA2,IDS,CPT2,RILP,FZR1,DVL1,ASPA,PNPLA7,ABHD17A,ANAPC2,JMJD8,INPP5E,AXIN1,UBE4B,RNF166,ZER1,DPYD,LDHD,EDC4,ABCA2,BSCL2,SULT1C4,AGO4,ZBTB7A,RNH1,RACK1,RNF130,FUT4,NT5C,TENT5B,CST3,RNFT1,RPL23,TRIB2,FBXO6,TRIR,ZFP36L2,STUB1,UPP1,SCP2,EXOSC5	cyan	EC_M2
GO:0086003	1.79E-01	0.897865996	11	57	cardiac muscle cell contraction	BP	8.55E-01	SCN5A,GPD1L,SNTA1,ATP2A2,CACNA1D,PKP2,PIK3CA,CACNA1C,CACNA1G,FLNA,HCN4	darkgreen	EC_M4
GO:0062009	1.79E-01	0.927678509	5	21	secondary palate development	BP	8.56E-01	COL11A2,TGFB2,LEF1,FZD2,TGFB3	darkgreen	EC_M4
GO:0071219	1.79E-01	0.877080245	22	133	cellular response to molecule of bacterial origin	BP	8.56E-01	FAM49B,PPARD,PLAA,TGFB1,AXL,ANKRD1,CD180,CX3CL1,PTPN22,MTDH,CDC73,SELENOS,PRKCA,SERPINE1,UPF1,SGMS1,PPARGC1A,AKT1,LDOC1,IRAK1,BCL10,IL36G	darkgreen	EC_M4
GO:0071222	1.79E-01	0.878231446	21	126	cellular response to lipopolysaccharide	BP	8.56E-01	PPARD,PLAA,TGFB1,AXL,ANKRD1,CD180,CX3CL1,PTPN22,MTDH,CDC73,SELENOS,PRKCA,SERPINE1,UPF1,SGMS1,PPARGC1A,AKT1,LDOC1,IRAK1,BCL10,IL36G	darkgreen	EC_M4
GO:0050850	1.53E-01	0.947909752	4	20	positive regulation of calcium-mediated signaling	BP	8.57E-01	SYK,P2RX2,IGF1,PLCG2	turquoise	EC_M6
GO:0140112	1.53E-01	0.947417288	4	21	extracellular vesicle biogenesis	BP	8.59E-01	SDCBP,ATP9A,CD34,RAB27A	turquoise	EC_M6
GO:0060993	1.79E-01	0.889416231	14	77	kidney morphogenesis	BP	8.59E-01	AHI1,VEGFA,GLI3,FGF2,CTNNB1,ILK,PKD2,BMP7,SIX4,MAGED1,PDGFRB,PKD1,GDNF,FGF10	darkgreen	EC_M4
GO:0010977	1.80E-01	0.878865589	20	116	negative regulation of neuron projection development	BP	8.60E-01	LRP1,LGALS1,RTN4,SEMA3C,DPYSL3,RYK,RNF6,DIP2B,SEMA4F,ADCY6,SPP1,SEMA4G,TNR,CRMP1,STX1B,PAFAH1B1,FLNA,PLXNA3,ITM2C,THY1	darkgreen	EC_M4
GO:0051353	1.54E-01	0.923360618	8	50	positive regulation of oxidoreductase activity	BP	8.61E-01	SIRT3,SCARB1,EDN1,SNCA,FGF23,HIF1A,TERF2,FCER2	turquoise	EC_M6
GO:0050905	1.54E-01	0.903917413	16	103	neuromuscular process	BP	8.61E-01	AGTPBP1,VPS54,MYO7A,TCF15,NR4A1,HEXB,TMEM150C,USH1C,KCNJ2,SYNM,HOXD10,ADORA2A,CHRNB1,MTOR,ATP2A1,SLC8A3	turquoise	EC_M6
GO:0035148	1.80E-01	0.876099402	22	129	tube formation	BP	8.61E-01	PLXNB2,VEGFA,FGF2,TGFB1,CTNNB1,SPINT2,TMED2,TCTN1,OPA1,IFT57,TGFB2,BMP7,FGFR2,FZD2,TULP3,SIX4,TCAP,LMO4,BCL10,GDNF,EDA,FGF10	darkgreen	EC_M4
GO:0008543	1.80E-01	0.896335629	11	59	fibroblast growth factor receptor signaling pathway	BP	8.62E-01	RUNX2,FGF2,CTNNB1,SULF1,PTPN11,FGFR2,FGFR3,FGFRL1,NGFR,NPTN,FGF10	darkgreen	EC_M4

GO:0016570	8.28E-03	0.994571102	58	399	histone modification	BP	8.62E-01	EHMT2,HDAC10,RING1,JADE2,EP300,KDM3B,TRERF1,JAK2,ING3,SPHK2,KMT2E,WBP2,KMT2C,TADA3,BRPF1,SIRT2,APBB1,TAF10,KMT2B,SDR16C5,KDM2B,SETD6,HR,WDR82,ELK4,DTX3L,RCOR3,PRMT2,PER2,HDAC4,USP21,KAT5,PHC1,MEN1,KDM6A,EID1,NELFA,KAT6B,HDAC6,MED24,PRDM2,UIMC1,MTHFR,MLLT6,CTBP1,KAT8,KAT7,PER1,ZBTB7B,SGF29,CRTC2,SIN3A,SREBF1,CXXC1,CREBBP,WDR5,GTF3C4,SETDB1	cyan	EC_M2
GO:0032757	1.81E-01	0.907344689	8	41	positive regulation of interleukin-8 production	BP	8.62E-01	LEP,TLR5,SERPINE1,CD74,PARK7,F3,BCL10,FCN1	darkgreen	EC_M4
GO:0060996	1.54E-01	0.907231011	14	87	dendritic spine development	BP	8.63E-01	ASAP1,EPHB1,PLK2,MEF2C,SRGAP2C,PAK2,DNM3,MTOR,PSEN1,ABI3,EFNA1,ZDHHC15,RELN,SHANK3	turquoise	EC_M6
GO:0060039	1.54E-01	0.948287711	4	17	pericardium development	BP	8.63E-01	DLL4,SMAD2,NOTCH1,BMP2	turquoise	EC_M6
GO:0006383	5.89E-02	0.978178372	7	39	transcription by RNA polymerase III	BP	8.63E-01	POLR3F,CHD8,ZC3H8,ZNF143,BDP1,IVNS1ABP,ICE2	darkgrey	EC_M8
GO:0030837	1.54E-01	0.91960235	9	53	negative regulation of actin filament polymerization	BP	8.63E-01	TMOD1,SVIL,SPTBN4,LMOD3,HIP1R,TMOD4,TMOD2,CARMIL2,KANK3	turquoise	EC_M6
GO:0021549	1.81E-01	0.888010059	14	77	cerebellum development	BP	8.65E-01	WHRN,GLI2,PAK1,SCN5A,FAIM2,PTPN11,NCSTN,HAP1,PPARGC1A,MAP2K1,SDF4,RERE,AARS,HSPA5	darkgreen	EC_M4
GO:0034097	1.82E-01	0.8469528	98	659	response to cytokine	BP	8.65E-01	SLC12A2,ANGPT1,PTP4A3,LEP,SRF,DOCK8,SOCS5,MAP3K7,ACTR2,ZYX,MYLK3,KIF5B,SEC61A1,NUB1,ACTR3,AXL,HNMT,IL15RA,PFKP,ACTG1,ACTN4,SMPD1,ILK,DPYSL3,CNOT7,CXCL12,ANKRD1,IL20RB,MRAS,FLNB,ROBO1,DOK1,APP,CX3CL1,PTPN11,TANK,TOLLIP,RAB7B,INA,ADIPOR1,KDM5B,TROVE2,HYAL1,EPRS,GSK3B,LEF1,PRKCA,CMKLR1,KRAS,OSM,TFRC,ARID5B,PDIA3,PTPN6,ZC3H15,PDCD10,UPF1,STIP1,COL3A1,PRKCI,SKIL,DDOST,TIMP1,PTPRN,SGMS1,GREM2,SLC2A4,MED1,PPARGC1A,ARHGEF2,COL1A1,TEX14,ACSL4,CD74,AKT1,OSMR,PTPN2,DAPK3,MYO1C,PAFAH1B1,TNFRSF18,IRAK1,USP10,F3,HSPA5,MIR24-1,CCL16,LOX,SNX10,CACYBP,IL36G,TIMP3,HAS2,VPS26B,TMSB4X,CCR6,TSLP,UBE2G2	darkgreen	EC_M4
GO:0007528	1.82E-01	0.907022455	8	39	neuromuscular junction development	BP	8.65E-01	UNC13B,SNTA1,APP,CACNB3,FZD9,DOK7,SIX4,MESD	darkgreen	EC_M4
GO:0002793	1.82E-01	0.892525571	12	66	positive regulation of peptide secretion	BP	8.65E-01	LRP1,PPARD,KIF5B,IRS2,OSBP,SELENOT,PFKM,DYNLL1,TFR2,NLGN2,ACSL4,RASL10B	darkgreen	EC_M4
GO:0045621	1.82E-01	0.889846379	13	72	positive regulation of lymphocyte differentiation	BP	8.65E-01	IL23A,HLA-DRA,SOCS5,GLI3,GLI2,AXL,IL15RA,AP3B1,ADA,RUNX1,MMP14,LEF1,CD74	darkgreen	EC_M4
GO:0051492	1.55E-01	0.90853445	13	80	regulation of stress fiber assembly	BP	8.66E-01	LIMCH1,NRP1,SFRP1,RAPGEF3,F11R,PAK2,PPM1F,WAS,RAC1,MTOR,ARHGEF15,ARHGAP28,S1PR1	turquoise	EC_M6
GO:0048146	1.82E-01	0.906188677	8	41	positive regulation of fibroblast proliferation	BP	8.67E-01	LIG4,FN1,NGFR,CD74,PDGFRB,CDKN1A,WNT2,FGF10	darkgreen	EC_M4
GO:0009988	1.83E-01	0.911134995	7	35	cell-cell recognition	BP	8.67E-01	TCP1,DOCK8,NCK2,CCT8,VDAC2,MSN,DOCK2	darkgreen	EC_M4
GO:0006687	1.83E-01	0.901769156	9	47	glycosphingolipid metabolic process	BP	8.67E-01	B4GALNT1,LARGE1,GLB1,SUMF1,SMPD1,ST8SIA4,B3GALT1,CLN6,PRKAA1	darkgreen	EC_M4
GO:0003254	1.83E-01	0.911294162	7	34	regulation of membrane depolarization	BP	8.67E-01	SCN5A,GPD1L,DCN,SLMAP,FZD9,CACNA1G,HCN4	darkgreen	EC_M4
GO:0071902	1.83E-01	0.868103012	28	170	positive regulation of protein serine/threonine kinase activity	BP	8.67E-01	VEGFA,MAP3K7,FGF2,MAPRE3,PAK1,TGFB1,ILK,ROBO1,RAPGEF2,ATP2B4,PKD2,CCND1,HTR2B,KRAS,NTRK3,SPATC1L,SYAP1,IQGAP1,PDCD10,MNAT1,MAGED1,BMPR1A,MAP2K1,CALM1,PDGFRB,AKT1,PKD1,IRAK1	darkgreen	EC_M4
GO:0070231	1.55E-01	0.931798146	6	36	T cell apoptotic process	BP	8.67E-01	LMBR1L,HIF1A,ARG2,EFNA1,LGALS9,IL7R	turquoise	EC_M6
GO:0032892	1.55E-01	0.938608086	5	26	positive regulation of organic acid transport	BP	8.68E-01	TNFRSF11A,EDN1,ADORA2A,PSEN1,STXBP1	turquoise	EC_M6
GO:0048268	1.55E-01	0.947240534	4	18	clathrin coat assembly	BP	8.68E-01	SNAP91,HIP1R,FCHO1,AP2B1	turquoise	EC_M6
GO:0050922	1.83E-01	0.901449503	9	46	negative regulation of chemotaxis	BP	8.69E-01	SEMA3C,RYK,ROBO1,SEMA4F,SEMA4G,SLAMF8,NBL1,PTPN2,PLXNA3	darkgreen	EC_M4

GO:0072175	1.83E-01	0.876182069	20	116	epithelial tube formation	BP	8.69E-01	PLXNB2,FGF2,TGFB1,CTNNB1,SPINT2,TMED2,TCTN1,OPA1,IFT57,TGFB2,BMP7,FGFR2,FZD2,TULP3,SIX4,TCAP,LMO4,BCL10,GDNF,FGF10	darkgreen	EC_M4
GO:0040014	1.56E-01	0.921922434	8	47	regulation of multicellular organism growth	BP	8.71E-01	SPTBN4,GPAT4,CHD7,IGF1,CREB1,ADRB2,RAI1,DIO3	turquoise	EC_M6
GO:0003008	1.79E-03	0.999649665	8	1240	system process	BP	8.71E-01	CHRNA5,ABCG5,ABCG8,TRPC3,FGFBP3,CAMK4,INAVA,CACNG7	violet	EC_M7
GO:1990090	1.84E-01	0.90094981	9	46	cellular response to nerve growth factor stimulus	BP	8.72E-01	SH3GL2,KIDINS220,APP,RAPGEF2,NTRK3,MAPT,AKT1,HSPA5,DYNC1LI2	darkgreen	EC_M4
GO:0046685	1.84E-01	0.916341825	6	29	response to arsenic-containing substance	BP	8.72E-01	VCP,DHX36,ATF3,UROS,SERPINF1,CDKN1A	darkgreen	EC_M4
GO:0009299	1.84E-01	0.93432001	4	16	mRNA transcription	BP	8.72E-01	AP3B1,MED1,SUPT6H,FLNA	darkgreen	EC_M4
GO:0051000	1.57E-01	0.946251319	4	19	positive regulation of nitric-oxide synthase activity	BP	8.72E-01	SCARB1,HIF1A,TERF2,FCER2	turquoise	EC_M6
GO:0060122	1.57E-01	0.937788398	5	26	inner ear receptor cell stereocilium organization	BP	8.72E-01	CTHRC1,MYO7A,USH1C,SEC24B,CECR2	turquoise	EC_M6
GO:0031128	1.84E-01	0.916414204	6	28	developmental induction	BP	8.72E-01	FGF2,CTNNB1,ROBO1,GDNF,WNT2,FGF10	darkgreen	EC_M4
GO:0071880	1.85E-01	0.934170027	4	16	adenylate cyclase-activating adrenergic receptor signaling pathway	BP	8.72E-01	RAPGEF2,ATP2B4,ADRA2C,ADRA1B	darkgreen	EC_M4
GO:0051348	1.85E-01	0.861689966	35	219	negative regulation of transferase activity	BP	8.72E-01	GNAQ,CDKN2B,SOCS5,SH3BP5L,RB1,KAT2B,IRS2,SMPD1,ILK,PINX1,PTPN22,TSG101,CHP1,DBNDD2,CBLB,LRP5,PRKAR1A,GSK3B,CORO1C,BMP7,HSPB1,MAPT,PTPN6,SRCIN1,GSKIP,MYOCD,AKT1,PTPN2,MIDN,PARK7,MIR24-1,THY1,CDKN1A,DUSP22,ORMDL3	darkgreen	EC_M4
GO:0048812	1.57E-01	0.873097821	68	503	neuron projection morphogenesis	BP	8.73E-01	SEMA6A,KIF5A,RIMS2,CDKL3,PTK2,SEMA4D,NRCAM,NRP1,ITSN2,NOVA2,PLXND1,GATA3,CXCR4,SPTBN4,FEZ2,EFNB2,EPHB1,RET,PREX2,KIF13B,GPM6A,NR4A2,DTNBP1,PPP3CA,HECW2,MEF2A,SEMA3F,ROBO4,PLXNA2,TRAK2,ADCY1,KALRN,SS18L1,LAMA5,YTHDF1,PAK2,LAMC2,MOV10,CREB1,ADORA2A,SEMA6D,EPHB4,ITGA4,DNM3,LMTK2,RAC1,EPHA2,OPHN1,EFNB1,PSEN1,SEMA4A,ABI3,TAOK2,EFNA1,CHRNB2,ZDHHC15,RPS6KA5,DNM2,B4GALT6,SEMA6B,SLITRK2,ARHGAP4,NOTCH1,STXBP1,DAB2IP,VASP,RELN,SHANK3	turquoise	EC_M6
GO:1904645	1.85E-01	0.904418165	8	41	response to amyloid-beta	BP	8.75E-01	LRP1,PRNP,APP,MMP2,CHRNA7,GSK3B,BACE1,NGFR	darkgreen	EC_M4
GO:0051648	1.59E-01	0.892296706	22	154	vesicle localization	BP	8.76E-01	KIF5A,FAM91A1,SNAP91,PIK3CG,MYO7A,EXOC6,SNCA,DTNBP1,SNAP23,LRMP,AP1AR,TRAK2,LIMK2,ITGA4,SNAP29,RAB27A,PSEN1,FBXW11,DNM2,BICDL2,AP3D1,FNBP1L	turquoise	EC_M6
GO:0120034	1.59E-01	0.903616147	14	92	positive regulation of plasma membrane bounded cell projection assembly	BP	8.76E-01	CDC42EP1,KIT,NRP1,OCLN,P2RX7,GPM6A,DNM3,RAC1,FSCN1,MTOR,DNM2,FNBP1L,CARMIL2,RALA	turquoise	EC_M6
GO:0010951	1.59E-01	0.89657037	18	137	negative regulation of endopeptidase activity	BP	8.76E-01	TNFAIP8,C5,CST7,TIMP4,ITIH5,NR4A1,SH3RF1,SNCA,RPS6KA1,PAK2,CST6,LTF,ADORA2A,DDX3X,BIRC3,PAPLN,SERPINB9,BST2	turquoise	EC_M6
GO:0071695	1.86E-01	0.866698408	27	163	anatomical structure maturation	BP	8.76E-01	MTCH1,LEP,VEGFA,RUNX2,RB1,AXL,CTNNB1,SPINK5,LGI4,TRPC4AP,APP,CX3CL1,OPA1,MMP2,TGFB2,DAG1,FARP2,C1QL1,ATP6AP2,FGFR3,GLDN,THBS3,RERE,SNX10,CDKN1A,FAM210B,CCR6	darkgreen	EC_M4
GO:0098743	1.86E-01	0.93350496	4	16	cell aggregation	BP	8.76E-01	BMP1,PKD1,COL11A1,OTOR	darkgreen	EC_M4
GO:0055006	1.86E-01	0.886961005	13	71	cardiac cell development	BP	8.77E-01	PPARA,SRF,VEGFA,BVES,MYLK3,PAK1,PDLIM5,OBSL1,BMPR1A,TCAP,PIN1,PDGFRB,MIR24-1	darkgreen	EC_M4
GO:1903793	1.59E-01	0.936484299	5	26	positive regulation of anion transport	BP	8.78E-01	TNFRSF11A,EDN1,ADORA2A,PSEN1,STXBP1	turquoise	EC_M6

GO:0019222	1.59E-01	0.854260907	549	4858	regulation of metabolic process	BP	8.78E-01	ZNF516,TNFRSF11A,BAZ2A,TCF4,TNFAIP8,IFNGR1,TNFAIP3,PLAGL1,TRIM39,AKAP12,PTPRB,TSPAN8,LAPTM4B,CNOT6,FLT4,FZD6,RIMS2,OXR1,ZBED4,GRAMD4,QKI,NOTCH4,AFDN,TCF20,TCF7,TPD52L1,NCOA7,L3MBTL3,SGSM3,PTK2,C6orf106,DAPK1,ATF4,PDGFB,AGTPBP1,MAPK13,BRPF3,CSNK1E,PICK1,KDM4C,PIM1,NFYA,MDFI,LRRC19,TEK,IREB2,PRDM4,LIMCH1,DDX58,NFX1,APBA1,DCAF12,FAM122A,TEC,ADGRF5,KIT,ADGRF1,TNFRSF21,KDR,CD2AP,NFIL3,SYK,GADD45G,SEMA4D,RGP1,PPP6R1,IL6,IGF2BP3,NFE2L3,REL,CREB5,PTBP3,CXCL8,PPIE,BACH2,PELI1,RSAD2,FIGNL1,FOXP2,KLF11,ODC1,GRB10,LANCL2,NOBOX,TLR4,TRAF1,C5,IL15,CCNY,CREM,TEAD2,SDCBP,MYCN,NOCT,TUT4,TRPM4,NRP1,KTI12,ZBTB24,PUM2,RASIP1,BEND5,PIK3CG,TAL1,LAMA4,PHF10,MTMR2,EPC1,KDM4D,GATA2,DNMT3A,PRKD2,SAP130,CST7,NOVA2,CTSC,RAB38,CAB39L,PRCP,NOS3,GGA3,RALB,DACH1,PPARG,TIMP4,GATA3,TAF3,ACVR2B,ITIH5,CXCR4,IRAK2,GZF1,EDNRB,PPP1CB,NDFIP2,SPTBN4,TIE1,SETD5,IKBKB,JAG1,P2RY6,NFKBIB,PDE2A,TMEM173,SFRP1,HBEGF,NR1D2,DND1,FEZ2,FERMT1,RPL26,SH3BP5,ANKLE2,CD36,SIRT3,CDK17,ARID5A,PRR5L,TCF15,SCARB1,EPHB1,PLK2,DEPP1,RET,PDE4D,MERTK,CHD7,IL1A,DNMT3B,IGF1,NR4A1,KLKB1,EIF2AK3,ABHD6,HHEX,KDM3A,ITCH,ERBIN,SH3RF1,SMAD1,ZBTB38,CLCN3,HIP1R,POU6F1,OCLN,STOX2,CHRM5,SOX7,HMBOX1,GPD1,ENC1,HEXB,MEF2C,SMARCD1,TLR2,WWTR1,IL7,P2RX7,VEGFC,ZNF704,CXCL10,SOGA1,MADD,ADGRG3,DHH,KMT2D,SFMBT1,FAM192A,SCAF4,TIPARP,OAS3,NLRC5,THBS4,ACTR5,PPP1R16B,TPCN1,RAPGEF3,ANKRD2,NR4A2,DGKZ,TOX2,DLL4,	turquoise	EC_M6
GO:0048663	1.86E-01	0.909080134	7	34	neuron fate commitment	BP	8.78E-01	TGFBR1,GLI3,GLI2,SATB2,NTRK3,PRRX1,LBX1	darkgreen	EC_M4
GO:0090151	1.86E-01	0.922328274	5	24	establishment of protein localization to mitochondrial membrane	BP	8.78E-01	HSPA4,COX18,AP3B1,CALM1,TIMM22	darkgreen	EC_M4
GO:0035337	1.87E-01	0.908569913	7	35	fatty-acyl-CoA metabolic process	BP	8.79E-01	ACSL6,DGAT2,FAR1,HACD2,ACSL4,ACOT7,HACD1	darkgreen	EC_M4
GO:0008585	1.87E-01	0.886357088	13	72	female gonad development	BP	8.79E-01	CTNNA1,LEP,VEGFA,INHBA,PTX3,MMP14,ARID5B,PCYT1B,SGPL1,PTPRN,CYP19A1,HSPA5,LHX8	darkgreen	EC_M4
GO:0071379	1.87E-01	0.93305268	4	16	cellular response to prostaglandin stimulus	BP	8.79E-01	ADCY6,AKT1,PRKAA1,GNB1	darkgreen	EC_M4
GO:0031344	1.60E-01	0.870250309	70	538	regulation of cell projection organization	BP	8.79E-01	SEMA6A,TBC1D30,CDKL3,PODXL,CDC42EP1,KIT,SEMA4D,NRP1,PLXND1,GATA3,SFRP1,EFNB2,ARHGEF7,PRAG1,RET,OCLN,KIF13B,ENC1,P2RX7,NDRG4,GPM6A,USH1C,PPP1R16B,CEP97,MYLIP,SRGAP2C,PPP3CA,HECW2,SEMA3F,PLXNA2,CFLAR,INPP5F,TRAK2,FES,SS18L1,PRKD1,ITGA6,LIMK2,PAK2,MOV10,B2M,SEMA6D,DNM3,WAS,RAC1,RCC2,FSCN1,EPHA2,MTOR,PSEN1,SEMA4A,ABI3,ITGA3,EFNA1,CHRNB2,ZDHHC15,RABEP2,DNM2,SEMA6B,ARHGAP4,RAPGEF1,FNBP1L,DAB2IP,CARMIL2,RELN,ALKAL1,SHANK3,RALA,SCARF1,PIFO	turquoise	EC_M6
GO:0006027	1.87E-01	0.932304905	4	17	glycosaminoglycan catabolic process	BP	8.80E-01	FGF2,GLB1,TGFB1,HYAL1	darkgreen	EC_M4
GO:0030511	1.87E-01	0.914765094	6	28	positive regulation of transforming growth factor beta receptor signaling pathway	BP	8.80E-01	CDKN2B,ZEB2,SLC2A10,RNF111,TGFB1I1,MYOCD	darkgreen	EC_M4
GO:1903846	1.87E-01	0.914765094	6	28	positive regulation of cellular response to transforming growth factor beta stimulus	BP	8.80E-01	CDKN2B,ZEB2,SLC2A10,RNF111,TGFB1I1,MYOCD	darkgreen	EC_M4
GO:0048512	1.60E-01	0.935720677	5	27	circadian behavior	BP	8.80E-01	NR1D2,CIART,ADORA2A,MTOR,CHRNB2	turquoise	EC_M6
GO:0046031	1.88E-01	0.888322995	12	66	ADP metabolic process	BP	8.81E-01	PPARA,PFKP,APP,PFKM,PGAM4,ZBTB20,TPI1,PPARGC1A,PKM,PRKAA1,ENO1,SLC2A6	darkgreen	EC_M4
GO:0038179	1.88E-01	0.90820972	7	34	neurotrophin signaling pathway	BP	8.81E-01	KIDINS220,RAPGEF2,PTPN11,ZFYVE27,NDN,NTRK3,HAP1	darkgreen	EC_M4
GO:0035601	8.57E-03	0.996080723	19	99	protein deacylation	BP	8.81E-01	HDAC10,EP300,TRERF1,SPHK2,TADA3,SIRT2,SDR16C5,SPRED1,ELK4,RCOR3,PER2,HDAC4,HDAC6,CTBP1,PER1,ZBTB7B,SIN3A,SREBF1,ABHD17A	cyan	EC_M2
GO:0009264	8.58E-03	0.997800102	8	26	deoxyribonucleotide catabolic process	BP	8.81E-01	DNPH1,PNP,XDH,SMUG1,NEIL2,DPYD,NT5C,UPP1	cyan	EC_M2

GO:0070897	6.04E-02	0.979678535	6	31	transcription preinitiation complex assembly	BP	8.81E-01	TAF1B,TAF7,TAF9,BDP1,UBTF,TAF1	darkgrey	EC_M8
GO:0071900	1.88E-01	0.852949119	47	300	regulation of protein serine/threonine kinase activity	BP	8.81E-01	CDKN2B,VEGFA,MAP3K7,FGF2,RB1,MAPRE3,PAK1,TGFB1,KAT2B,SMPD1,ILK,ROBO1,CCNJ,RAPGEF2,GTF2H1,PTPN22,TSG101,ATP2B4,PKD2,CCND1,LRP5,HTR2B,PRKAR1A,KRAS,NTRK3,SPATC1L,BMP7,SYAP1,IQGAP1,PTPN6,PDCD10,HTT,MNAT1,MAGED1,BMPR1A,CDK12,CCNG1,MAP2K1,CALM1,MYOCD,PDGFRB,AKT1,PKD1,IRAK1,MIR24-1,THY1,CDKN1A	darkgreen	EC_M4
GO:0032727	1.61E-01	0.94477772	4	18	positive regulation of interferon-alpha production	BP	8.82E-01	DDX58,TLR4,STAT1,DDX3X	turquoise	EC_M6
GO:0045638	1.61E-01	0.911776133	10	64	negative regulation of myeloid cell differentiation	BP	8.82E-01	TLR4,GATA2,SFRP1,TMEM178A,INPP5D,LTF,ITPKB,HOXB8,GPR68,HIST1H4D	turquoise	EC_M6
GO:0007202	1.61E-01	0.94392991	4	20	activation of phospholipase C activity	BP	8.82E-01	P2RY6,PLCB2,CD86,SELE	turquoise	EC_M6
GO:0098773	1.88E-01	0.887996858	12	65	skin epidermis development	BP	8.82E-01	INHBA,GLI2,CTNNB1,SPINK5,TRPC4AP,AP3B1,TGFB2,FGFR2,GORAB,NGFR,EDA,FGF10	darkgreen	EC_M4
GO:0001941	1.88E-01	0.907859433	7	34	postsynaptic membrane organization	BP	8.82E-01	SLC7A11,GLRB,FZD9,NLGN2,GDNF,LHFPL4,MESD	darkgreen	EC_M4
GO:0000003	1.88E-01	0.835715554	151	1031	reproduction	BP	8.82E-01	TRIM36,B4GALNT1,TCP1,ROS1,AFF4,RSPO3,PPARD,CTNNA1,TEAD3,SUN2,MYH9,LEP,VEGFA,UNC13B,CLOCK,NLRP5,TGFBR1,RIMS1,PSME4,CENPC,UBE2J1,ACTR2,GMCL1,GLI3,INHBA,DKKL1,ITGB1,PANX1,ZNF296,CRTAP,GLI2,ACTR3,AXL,WDR48,CTNNB1,DZIP1,SPINT2,CHFR,DCN,KATNAL1,TMED2,EIF2S2,SULF1,LNPEP,RDH10,CDKL2,HEY1,CELF1,APP,GLRB,DHX36,CCT8,ABCC8,PTPN11,PTX3,PGAM4,RAD54B,AP3B1,ACVR1,MMP2,ADA,ATP2B4,SPP1,PKD2,CSDE1,DNAH1,SYCP2L,DNAH5,DYNLL1,AZIN2,KDM5B,NOTCH2,CCND1,KPNA6,TGFB2,PRKAR1A,MMP14,LEF1,KRAS,LARP7,SPATC1L,BMP7,FGFR2,VASH2,ASB1,HTRA1,GORASP2,OSBP2,CLIC4,ARID5B,PAFAH1B2,OVOL1,PRDX4,PCYT1B,LINC02210-CRHR1,SGPL1,COMT,PTPN6,IGFBP2,ADRA2C,SKIL,TIMP1,PTPRN,CYP19A1,VDAC2,FMN2,SIX4,BMPR1A,MED1,MAGED2,MYCBPAP,TGFB3,UBE2Q1,MAP2K1,SOX30,DIAPH2,TEX14,VMP1,MYOCD,ACSL4,PDGFRB,RBBP8,AKT1,UBE2A,ITGA2,RAI14,PLAC1,MMP23B,BCAP31,PAFAH1B1,PKD1,NPHP4,FLNA,PARK7,BSG,SLC2A8,HSPA5,LHX8,FSTL3,CCDC136,GGN,SPINK2,SERPINF1,SERPINB5,IGSF8,HAS2,CCR6,WNT2,NPAS1,FGF10,MBD2	darkgreen	EC_M4
GO:0010556	1.61E-01	0.854770569	329	2846	regulation of macromolecule biosynthetic process	BP	8.82E-01	ZNF516,TNFRSF11A,BAZ2A,TCF4,TNFAIP3,PLAGL1,AKAP12,CNOT6,FZD6,ZBED4,QKI,NOTCH4,TCF20,TCF7,NCOA7,L3MBTL3,DAPK1,ATF4,PDGFB,BRPF3,KDM4C,PIM1,NFYA,MDFI,IREB2,PRDM4,DDX58,NFX1,KIT,ADGRF1,KDR,NFIL3,SYK,SEMA4D,IL6,IGF2BP3,NFE2L3,REL,CREB5,PPIE,BACH2,PELI1,FOXP2,KLF11,LANCL2,NOBOX,TLR4,TRAF1,IL15,CREM,TEAD2,SDCBP,MYCN,NOCT,TUT4,KTI12,ZBTB24,PUM2,BEND5,TAL1,PHF10,EPC1,GATA2,DNMT3A,PRKD2,SAP130,DACH1,PPARG,GATA3,TAF3,ACVR2B,IRAK2,GZF1,EDNRB,PPP1CB,SETD5,IKBKB,JAG1,NFKBIB,PDE2A,TMEM173,SFRP1,HBEGF,NR1D2,DND1,RPL26,CD36,SIRT3,CDK17,ARID5A,TCF15,RET,CHD7,IL1A,DNMT3B,IGF1,NR4A1,EIF2AK3,HHEX,KDM3A,ITCH,ERBIN,SMAD1,ZBTB38,POU6F1,STOX2,SOX7,HMBOX1,ENC1,HEXB,MEF2C,SMARCD1,TLR2,WWTR1,ZNF704,CXCL10,ADGRG3,KMT2D,SFMBT1,SCAF4,NLRC5,ACTR5,ANKRD2,NR4A2,TOX2,DLL4,CDYL,BAZ2B,SOX13,YAF2,EDN1,STAT1,DYRK1A,SNCA,JARID2,ERG,ETS2,CDK18,NFKB2,DCAF1,PRDM15,ETS1,CD200,SIK1,HDAC1,PPP3CA,HYAL2,SLC39A8,TESMIN,MEF2A,BCL9,HLX,NCF1,USP13,FABP4,HCLS1,NFATC2,ZFHX2,EAF2,GMEB1,CD86,KDM2A,CIART,MN1,TGIF1,PARP15,POU2F3,ZSCAN29,CFLAR,TRAK2,ADCY1,ARID1A,RBM4,RPS6KA1,PROX1,SS18L1,PRKD1,ZNF148,YTHDF1,ZSCAN2,HELZ2,MLXIPL,ITGA6,DLG1,ELL3,ETV6,HOXD10,MOV10,RNF2,CREB1,EIF4A2,TET1,LTF,ADORA2A,ARNT2,MYEF2,IL1RAP,BCOR,DDX3X,RFXANK,IGFBP5,ETV4,TBX1,TMEM106A,MECOM,ING4,SECISBP2L,CDK16,SPINDOC,LDB2,FGF23,CPEB2,SAMD8,PPM1F,POU2F1,WAS,TEAD4,PRKCH,RAC1,HIF1A,KDM5A,ZBTB18,APBB2,JUP,	turquoise	EC_M6
GO:0003181	1.89E-01	0.921792469	5	22	atrioventricular valve morphogenesis	BP	8.83E-01	HEY1,ACVR1,TGFB2,BMPR1A,OLFM1	darkgreen	EC_M4
GO:1900181	1.61E-01	0.935183066	5	27	negative regulation of protein localization to nucleus	BP	8.83E-01	C6orf106,FERMT1,CD36,DCLK2,APOD	turquoise	EC_M6

GO:0002673	1.89E-01	0.921344206	5	23	regulation of acute inflammatory response	BP	8.84E-01	IL20RB,SELENOS,OSM,OSMR,PARK7	darkgreen	EC_M4
GO:0035025	1.89E-01	0.921602735	5	22	positive regulation of Rho protein signal transduction	BP	8.84E-01	ROBO1,COL3A1,SYNPO2L,GPR65,PDGFRB	darkgreen	EC_M4
GO:0003197	1.89E-01	0.90189259	8	40	endocardial cushion development	BP	8.85E-01	TGFBR1,ROBO1,HEY1,ACVR1,TGFB2,BMP7,BMPR1A,TMEM100	darkgreen	EC_M4
GO:0009743	1.90E-01	0.860865668	30	186	response to carbohydrate	BP	8.86E-01	LRP1,PPARD,LGALS1,TXN2,LEP,SRF,UNC13B,FKBP1B,KIF5B,EIF2B3,IRS2,OSBP,MAP1B,SELENOT,OPA1,BRSK2,DYNLL1,SELENOS,ZBTB20,LRP5,PIK3CA,ADCY5,SIDT2,PTPRN,PPARGC1A,SMARCA4,ITGA2,PRKAA1,SERPINF1,COL6A2	darkgreen	EC_M4
GO:0042157	1.62E-01	0.898619997	15	111	lipoprotein metabolic process	BP	8.87E-01	ZDHHC14,PIGF,PPT1,NOTUM,ABHD13,ZDHHC13,MTTP,ZDHHC6,HHAT,ZDHHC18,APOLD1,APOD,ZDHHC15,ABHD17C,ZDHHC22	turquoise	EC_M6
GO:0006366	1.63E-01	0.85597364	220	1862	transcription by RNA polymerase II	BP	8.87E-01	ZNF516,TCF4,PLAGL1,ZBED4,NOTCH4,TCF20,TCF7,NCOA7,ATF4,PDGFB,KDM4C,NFYA,MDFI,PRDM4,DDX58,NFX1,NFIL3,SEMA4D,POLR2B,IL6,NFE2L3,REL,CREB5,BACH2,FOXP2,KLF11,NOBOX,TLR4,CREM,TEAD2,SDCBP,MYCN,NOCT,KTI12,ZBTB24,TAL1,PHF10,EPC1,GATA2,DNMT3A,PRKD2,SAP130,DACH1,PPARG,GATA3,TAF3,ACVR2B,GZF1,EDNRB,IKBKB,JAG1,PDE2A,TMEM173,NR1D2,CD36,PHRF1,ARID5A,TCF15,CHD7,IL1A,DNMT3B,IGF1,NR4A1,HHEX,KDM3A,SMAD1,ZBTB38,POU6F1,STOX2,SOX7,HMBOX1,HEXB,MEF2C,SMARCD1,TLR2,WWTR1,ZNF704,CXCL10,KMT2D,SCAF4,NLRC5,ANKRD2,NR4A2,TOX2,DLL4,BAZ2B,SOX13,EDN1,STAT1,SNCA,JARID2,ERG,ETS2,NFKB2,DCAF1,PRDM15,ETS1,HDAC1,PPP3CA,HYAL2,MEF2A,BCL9,GTF2E1,HLX,HCLS1,NFATC2,ZFHX2,EAF2,GMEB1,KDM2A,TGIF1,PARP15,POU2F3,ZSCAN29,TRAK2,ARID1A,RPS6KA1,PROX1,SS18L1,PRKD1,ZNF148,ZSCAN2,HELZ2,MLXIPL,ITGA6,DLG1,ELL3,ETV6,HOXD10,RNF2,CREB1,TET1,ARNT2,MYEF2,BCOR,DDX3X,RFXANK,ETV4,TBX1,MECOM,LDB2,POU2F1,WAS,TEAD4,HIF1A,ZBTB18,APBB2,JUP,BCL6B,MAFK,AR,CASZ1,PSEN1,HOXB5,HOXB6,HOXB7,HOXB8,HOXB9,ZNF410,BATF,EFNA1,PIAS1,RPS6KA5,SMAD2,YY1,ARID3B,LYL1,TNIP1,HMG20A,RCOR1,ZNF521,ABLIM3,ADRB2,YES1,RAI1,ELF4,MRTFB,MAMLD1,DOT1L,AP3D1,HCFC1,NRARP,NOTCH1,MED16,IRF8,S1PR1,ENG,MTF2,ZBTB34,ZNF821,NFAT5,LHX6,DAB2IP,ZBTB33,ARID4A,ZNF24,ATXN1L,TRIM24,SOX17,ZNF850,CDK13,SOX18,ZNF260,MAFF,ZNF229,PASD1,TGIF2,ZNF570,ZNF789,E2F3,ZKSCAN1,BMP2,GLIS3,ZNF10,CBX2,ZNF792,ZNF71,BMP6,FLI1,ZNF266	turquoise	EC_M6
GO:0048813	1.63E-01	0.89269743	19	126	dendrite morphogenesis	BP	8.87E-01	CDKL3,SEMA4D,NRP1,EPHB1,PREX2,DTNBP1,PPP3CA,HECW2,MEF2A,TRAK2,SS18L1,DNM3,ABI3,TAOK2,EFNA1,CHRNB2,ZDHHC15,RELN,SHANK3	turquoise	EC_M6
GO:0048246	1.63E-01	0.934373652	5	27	macrophage chemotaxis	BP	8.87E-01	PTK2,C5,EDNRB,MMP28,CCL2	turquoise	EC_M6
GO:0060255	1.63E-01	0.85101818	507	4468	regulation of macromolecule metabolic process	BP	8.87E-01	ZNF516,TNFRSF11A,BAZ2A,TCF4,TNFAIP8,IFNGR1,TNFAIP3,PLAGL1,TRIM39,AKAP12,PTPRB,TSPAN8,LAPTM4B,CNOT6,FLT4,FZD6,RIMS2,OXR1,ZBED4,GRAMD4,QKI,NOTCH4,AFDN,TCF20,TCF7,TPD52L1,NCOA7,L3MBTL3,SGSM3,PTK2,C6orf106,DAPK1,ATF4,PDGFB,AGTPBP1,MAPK13,BRPF3,CSNK1E,PICK1,KDM4C,PIM1,NFYA,MDFI,LRRC19,TEK,IREB2,PRDM4,LIMCH1,DDX58,NFX1,APBA1,FAM122A,TEC,KIT,ADGRF1,TNFRSF21,KDR,CD2AP,NFIL3,SYK,SEMA4D,RGP1,PPP6R1,IL6,IGF2BP3,NFE2L3,REL,CREB5,PTBP3,CXCL8,PPIE,BACH2,PELI1,RSAD2,FIGNL1,FOXP2,KLF11,ODC1,LANCL2,NOBOX,TLR4,TRAF1,C5,IL15,CCNY,CREM,TEAD2,SDCBP,MYCN,NOCT,TUT4,TRPM4,NRP1,KTI12,ZBTB24,PUM2,RASIP1,BEND5,PIK3CG,TAL1,PHF10,MTMR2,EPC1,KDM4D,GATA2,DNMT3A,PRKD2,SAP130,CST7,NOVA2,CTSC,CAB39L,NOS3,GGA3,RALB,DACH1,PPARG,TIMP4,GATA3,TAF3,ACVR2B,ITIH5,IRAK2,GZF1,EDNRB,PPP1CB,NDFIP2,SPTBN4,SETD5,IKBKB,JAG1,NFKBIB,PDE2A,TMEM173,SFRP1,HBEGF,NR1D2,DND1,FERMT1,RPL26,SH3BP5,ANKLE2,CD36,SIRT3,CDK17,ARID5A,PRR5L,TCF15,PLK2,RET,PDE4D,MERTK,CHD7,IL1A,DNMT3B,IGF1,NR4A1,KLKB1,EIF2AK3,HHEX,KDM3A,ITCH,ERBIN,SH3RF1,SMAD1,ZBTB38,HIP1R,POU6F1,OCLN,STOX2,CHRM5,SOX7,HMBOX1,ENC1,HEXB,MEF2C,SMARCD1,TLR2,WWTR1,IL7,P2RX7,VEGFC,ZNF704,CXCL10,MADD,ADGRG3,DHH,KMT2D,SFMBT1,FAM192A,SCAF4,TIPARP,OAS3,NLRC5,THBS4,ACTR5,PPP1R16B,RAPGEF3,ANKRD2,NR4A2,TOX2,DLL4,CCNJL,CDYL,BAZ2B,SOX13,NEDD9,YAF2,EDN1,STAT1,CD58,DYRK1A,EHD4,SNCA,JARID2,DTNBP1,MYLIP,ERG,ETS2,CDK18,NFKB2,DCAF1,RNF144B,P	turquoise	EC_M6
GO:0002218	1.63E-01	0.927737681	6	33	activation of innate immune response	BP	8.87E-01	SYK,TLR4,IKBKB,TMEM173,PAK2,PLCG2	turquoise	EC_M6
GO:2000737	1.63E-01	0.943591981	4	18	negative regulation of stem cell differentiation	BP	8.87E-01	JAG1,TCF15,NOTCH1,CDK13	turquoise	EC_M6

GO:0090087	1.63E-01	0.890906775	20	144	regulation of peptide transport	BP	8.87E-01	PICK1,ICA1,IL6,TRPM4,SFRP1,SIRT3,CHD7,FAM3D,ABCG1,HDAC1,PPP3CA,ANO1,MCU,CDK16,RAC1,HIF1A,SYT7,GPR68,NOS2,CA2	turquoise	EC_M6
GO:2000379	1.63E-01	0.913380268	9	57	positive regulation of reactive oxygen species metabolic process	BP	8.87E-01	PDGFB,SYK,TLR4,CD36,CLCN3,SNCA,RAB27A,TLR6,PLCG2	turquoise	EC_M6
GO:0001558	1.63E-01	0.874487426	44	339	regulation of cell growth	BP	8.87E-01	SEMA6A,RIMS2,CDKL3,FAM122A,SEMA4D,PPT1,SDCBP,NRCAM,NRP1,ITSN2,CXCR4,NET1,TMC8,SFRP1,HBEGF,IGF1,ITCH,EDN1,HYAL2,SEMA3F,EAF2,SH3BP4,RPS6KA1,SIPA1,SEMA6D,DDX3X,IGFBP5,CDA,ADIPOR2,MTOR,SEMA4A,TAOK2,NPR1,DNM2,YY1,KIF26A,SEMA6B,ARHGAP4,SLC25A33,SOX17,OSGIN2,RGS4,BST2,GNG4	turquoise	EC_M6
GO:0006821	1.64E-01	0.910279456	10	60	chloride transport	BP	8.87E-01	P2RY6,CLCN3,ANO6,CLIC6,ANO5,ANO3,ANO1,SLC12A7,WNK4,SLC26A1	turquoise	EC_M6
GO:0072583	1.90E-01	0.897002666	9	46	clathrin-dependent endocytosis	BP	8.87E-01	CANX,SH3GL2,PICALM,SIGLEC1,AP2A2,AP2M1,SYT11,CLTC,GPR107	darkgreen	EC_M4
GO:1904358	1.90E-01	0.906293947	7	35	positive regulation of telomere maintenance via telomere lengthening	BP	8.87E-01	TCP1,CTNNB1,DHX36,CCT8,MAPKAPK5,DKC1,NEK7	darkgreen	EC_M4
GO:0007017	1.91E-01	0.837605017	107	715	microtubule-based process	BP	8.88E-01	AP3S1,TRIM36,RAB21,RNF19A,SUN2,MYH9,DCTN3,VCP,DYNC1I1,TXNDC9,DST,NLRP5,ACTR2,ROCK2,MGARP,CFAP100,ITGB1,KIF5B,COPG1,EML2,MARK4,MAPRE3,PDCD6IP,PAK1,ACTR3,NCKAP5,CTNNB1,TRAK1,CLIP4,DYNC1LI1,DNAJB13,NAV3,PPP1R12A,KAT2B,TACC1,HDAC3,DCTN6,KATNAL1,KIF2A,MAP1B,APP,PGAM4,OPA1,AP3B1,TUBB2A,CHP1,ATP2B4,PKD2,DNAH1,DNAH5,IFT57,BRSK2,ARL3,DYNLL1,PHLDB2,SBDS,FEZ1,GSK3B,DAG1,DNAAF5,NEK7,HSPA8,PHLDB1,DYNC1I2,KIF1A,ABRAXAS2,HSPB1,MAPT,HTT,KTN1,TUBA4A,ACTR10,FMN2,OBSL1,CRMP1,HAP1,CCSER2,ARHGEF2,IFT43,RAB11A,LRRC49,CLTC,ARMCX3,EML1,SS18,ROCK1,PRKAA1,TUBB4A,PAFAH1B1,PKD1,NPHP4,FLNA,VAMP7,HSBP1,DYNLRB2,DYNC1LI2,MAP7D1,RAB6A,SKA2,BLOC1S2,MZT1,CCR6,TUBA1C,ZPR1,RMDN1,MAP1A,FGF10	darkgreen	EC_M4
GO:0048169	1.91E-01	0.930449512	4	17	regulation of long-term neuronal synaptic plasticity	BP	8.88E-01	APP,KRAS,RAB11A,NPTN	darkgreen	EC_M4
GO:0070534	1.64E-01	0.916835531	8	50	protein K63-linked ubiquitination	BP	8.88E-01	PELI1,TRAF1,ITCH,DDX3X,BFAR,TRAF2,UBE2G1,RNF152	turquoise	EC_M6
GO:0010640	1.64E-01	0.942372176	4	20	regulation of platelet-derived growth factor receptor signaling pathway	BP	8.89E-01	HIP1R,NDRG4,SNCA,APOD	turquoise	EC_M6
GO:0007010	1.64E-01	0.858046347	145	1177	cytoskeleton organization	BP	8.89E-01	PARD6G,SEMA6A,TNFAIP3,PHACTR2,SPAG1,CELSR1,PARVG,PACSIN2,TRDN,MTSS1,ARHGAP18,PTK2,BAIAP2L2,PICK1,CDC42EP1,STRIP2,TEK,LIMCH1,PGM5,KIT,CD2AP,EFHC1,THSD7A,TMOD1,VPS54,CEP68,ARHGAP25,ELMO1,FIGNL1,SDCBP,FAM49A,NRP1,SVIL,MSRB2,FGD5,NECTIN2,LLGL2,MYO7A,SPTBN4,IKBKB,FCHSD2,SFRP1,POC1B,ARHGEF7,SPEF1,FCHSD1,ARAP3,ARHGAP26,FGD6,EML4,RASSF7,LMOD3,PLK2,ERBIN,HIP1R,OCLN,DCLK2,FHDC1,P2RX7,USH1C,RAPGEF3,IQGAP2,CEP97,NEDD9,EDN1,DYRK1A,JAM3,SNCA,DTNBP1,NUAK2,GAS2,SRGAP2C,RASSF1,MEF2A,SYNM,NRAP,ESAM,ABLIM1,HCLS1,AP1AR,EPB41,JHY,CFLAR,FES,PROX1,TMOD4,F11R,ARHGDIB,LIMK2,PAK2,DLG1,SIPA1,ZW10,MCU,BAIAP2L1,PPM1F,WAS,TMOD2,RAC1,RHOJ,PLD2,FSCN1,SHROOM4,CECR2,MICAL3,RGS14,FGD1,MTOR,OPHN1,STARD8,FBXW11,ABI3,TAOK2,ARHGEF15,TTLL5,CCDC88C,CCDC69,CDC42BPB,AMOT,MARK3,ABLIM3,INF2,CCL2,ARHGAP28,SPEF2,PATJ,SPIRE1,MYO18A,GIT1,MTM1,RFLNB,ARHGAP4,S1PR1,CNN3,CARMIL2,CDH5,VASP,GMFG,KANK3,SHANK3,RGS4,GBA2,RALA,BST2,ARPC5L	turquoise	EC_M6
GO:0070071	8.72E-03	0.998705016	5	12	proton-transporting two-sector ATPase complex assembly	BP	8.89E-01	ATP23,PIP4P1,OXA1L,TMEM199,ATP5F1D	cyan	EC_M2
GO:0036498	1.91E-01	0.930236996	4	17	IRE1-mediated unfolded protein response	BP	8.89E-01	DDRGK1,VAPB,HSPA5,DNAJB9	darkgreen	EC_M4

GO:0070168	1.91E-01	0.920329491	5	22	negative regulation of biomineral tissue development	BP	8.89E-01	ASPN,ROCK2,TGFB1,HEY1,ROCK1	darkgreen	EC_M4
GO:0110150	1.91E-01	0.920329491	5	22	negative regulation of biomineralization	BP	8.89E-01	ASPN,ROCK2,TGFB1,HEY1,ROCK1	darkgreen	EC_M4
GO:0031929	1.91E-01	0.872855572	18	105	TOR signaling	BP	8.89E-01	ROS1,LEP,SESN3,RHEB,SEC13,HDAC3,TMEM127,MAPKAPK5,PIK3CA,SYAP1,EIF4EBP2,GPR137,ARAF,OTUD5,AKT3,AKT1,PRKAA1,CLEC16A	darkgreen	EC_M4
GO:0098901	1.92E-01	0.920175963	5	22	regulation of cardiac muscle cell action potential	BP	8.90E-01	ATP2A2,PKP2,CACNA1C,CALM1,HCN4	darkgreen	EC_M4
GO:0048638	1.65E-01	0.877705468	35	257	regulation of developmental growth	BP	8.90E-01	SEMA6A,RIMS2,CDKL3,PIM1,SEMA4D,NRCAM,NRP1,ITSN2,CXCR4,SPTBN4,GPAT4,CHD7,IGF1,MEF2C,IL7,TLL2,EDN1,JARID2,SEMA3F,HLX,PROX1,CREB1,SEMA6D,MTOR,AR,SEMA4A,DNM2,YY1,ADRB2,RAI1,SEMA6B,ARHGAP4,NOTCH1,DIO3,RGS4	turquoise	EC_M6
GO:1901264	1.92E-01	0.888127215	11	61	carbohydrate derivative transport	BP	8.90E-01	SLC35B2,MFSD2A,SLC35A1,SLC25A4,SLC33A1,CALHM1,SLC35A5,SLC29A4,SLC35B1,SLC35A3,SLC35C1	darkgreen	EC_M4
GO:0046677	1.92E-01	0.899746551	8	41	response to antibiotic	BP	8.91E-01	ACTR2,PPP2CB,KDM5B,HYAL1,CPT1A,UROS,AOC3,HSPA5	darkgreen	EC_M4
GO:0002920	1.92E-01	0.929568146	4	17	regulation of humoral immune response	BP	8.91E-01	SPINK5,PTPN6,A2M,CD59	darkgreen	EC_M4
GO:0086065	1.92E-01	0.895330204	9	46	cell communication involved in cardiac conduction	BP	8.91E-01	FKBP1B,SCN5A,CACNA1D,PKP2,CACNA1C,CACNA1G,CALM1,FLNA,HCN4	darkgreen	EC_M4
GO:1903522	1.65E-01	0.884249168	25	179	regulation of blood circulation	BP	8.92E-01	TRDN,DOCK4,TRPM4,PIK3CG,ARHGAP42,SPTBN4,HBEGF,PDE4D,STC1,KCNJ3,EDN1,KCNJ2,MEF2A,ACE,DLG1,JUP,ATP2A1,DSG2,DSC2,PDE4B,SLC8A3,KCNH2,SCN2B,RGS4,KCNG2	turquoise	EC_M6
GO:1990868	1.93E-01	0.894836973	9	48	response to chemokine	BP	8.92E-01	SLC12A2,DOCK8,CXCL12,ROBO1,CX3CL1,CMKLR1,CCL16,LOX,CCR6	darkgreen	EC_M4
GO:1990869	1.93E-01	0.894836973	9	48	cellular response to chemokine	BP	8.92E-01	SLC12A2,DOCK8,CXCL12,ROBO1,CX3CL1,CMKLR1,CCL16,LOX,CCR6	darkgreen	EC_M4
GO:0046039	1.93E-01	0.929277196	4	17	GTP metabolic process	BP	8.93E-01	RAB23,OPA1,GNAI3,NME6	darkgreen	EC_M4
GO:0030072	1.66E-01	0.885743606	23	168	peptide hormone secretion	BP	8.93E-01	RIMS2,PICK1,ICA1,IL6,TRPM4,SFRP1,SIRT3,CHD7,FAM3D,EDN1,ABCG1,HDAC1,PPP3CA,ANO1,MCU,CDK16,RAC1,HIF1A,SYT7,GPR68,SMAD2,NOS2,EXOC3L1	turquoise	EC_M6
GO:0010614	1.66E-01	0.932911138	5	27	negative regulation of cardiac muscle hypertrophy	BP	8.93E-01	JARID2,TNFRSF1B,YY1,NOTCH1,RGS4	turquoise	EC_M6
GO:2000404	1.66E-01	0.932483948	5	28	regulation of T cell migration	BP	8.94E-01	LRCH1,CXCL10,CD200,APOD,ITGA4	turquoise	EC_M6
GO:0055074	1.66E-01	0.874808205	38	291	calcium ion homeostasis	BP	8.94E-01	TRDN,CAV2,TRPM4,PIK3CG,GATA2,GPR4,CXCR4,EDNRB,P2RY6,P2RX2,CD36,TMEM178A,MICU3,PDE4D,CHD7,HEXB,P2RX7,CXCL10,TPCN1,PLCG1,STC1,EDN1,SNCA,LCK,HCRTR1,PRKD1,CCRL2,MCU,PSEN1,ATP2A1,CD19,S100A14,PLCG2,S1PR1,CDH5,SLC8A3,TRIM24,LPAR6	turquoise	EC_M6
GO:0010770	1.93E-01	0.884331421	12	65	positive regulation of cell morphogenesis involved in differentiation	BP	8.94E-01	RAB21,ACTR2,ILK,DHX36,OPA1,DNM1L,CAPRIN2,ARPC2,OBSL1,PAFAH1B1,FLNA,HAS2	darkgreen	EC_M4
GO:0051205	1.94E-01	0.898485814	8	44	protein insertion into membrane	BP	8.95E-01	HSPA4,COX18,EMC10,TRAM1,EMC4,EMC1,EMC7,TIMM22	darkgreen	EC_M4
GO:0050854	1.66E-01	0.919878745	7	43	regulation of antigen receptor-mediated signaling pathway	BP	8.95E-01	PRKD2,NECTIN2,DGKZ,LCK,LAPTM5,PRKCH,CD19	turquoise	EC_M6
GO:1904037	1.94E-01	0.910636679	6	29	positive regulation of epithelial cell apoptotic process	BP	8.95E-01	PLA2R1,PRKCI,EIF2S1,PPARGC1A,CD40LG,MIR24-1	darkgreen	EC_M4
GO:0090288	1.94E-01	0.878748818	14	78	negative regulation of cellular response to growth factor stimulus	BP	8.95E-01	FGF2,CRIM1,DCN,SULF1,ATP2B4,HTRA1,FBN1,SKIL,SMURF1,NBL1,GREM2,NGFR,CHRDL1,FSTL3	darkgreen	EC_M4
GO:0061162	1.94E-01	0.918894764	5	22	establishment of monopolar cell polarity	BP	8.95E-01	FAT1,MAP1B,PRKCI,MSN,TTC8	darkgreen	EC_M4

GO:0031668	1.94E-01	0.855152541	33	205	cellular response to extracellular stimulus	BP	8.95E-01	PPARA,TXN2,SLC39A4,LEP,CDKN2B,SRF,NUAK1,ASNS,MFSD2A,VPS41,SESN3,AXL,ATP2B1,MAP1LC3A,CACNB3,BRSK2,DNM1L,GLRX2,WIPI1,HSPA8,ATF3,COMT,WIPI2,EIF2S1,MED1,CPEB4,COL1A1,MYOCD,PRKAA1,CLEC16A,HSPA5,CDKN1A,GABARAPL2	darkgreen	EC_M4
GO:0042733	1.94E-01	0.893997876	9	47	embryonic digit morphogenesis	BP	8.95E-01	GLI3,GLI2,CTNNB1,IMPAD1,LRP5,ECE1,TULP3,BMPR1A,CHST11	darkgreen	EC_M4
GO:0006796	1.94E-01	0.823878212	308	2150	phosphate-containing compound metabolic process	BP	8.95E-01	IL23A,NAB2,LRP1,AGAP2,ENPP3,TAB2,DYRK2,RRM2B,PRRC1,PLXNB2,ANGPT1,PPARA,ACSL6,ROS1,NUS1,SERINC1,PTP4A3,PPARD,SH3GL2,GNAQ,LEP,CDKN2B,PLAA,NUAK1,CDK14,VEGFA,VCP,SLC35B2,NCK2,PDCL3,INPP4A,RAB23,PTP4A1,TGFBR1,TMEM246,SOCS5,FAM126A,MFSD2A,GARS,MAP3K7,SH3BP5L,GFPT1,ROCK2,INHBA,MYLK3,FKBP1B,FGF2,CMPK1,SRPK2,SELENOI,ENTPD6,ABHD12,RB1,ABHD5,MARK4,MAPRE3,DGKH,PAK1,TGFB1,AXL,RBKS,DGAT2,AKT2,GPD1L,OSBPL10,MTMR14,PFKP,OSBPL8,PPP1R12A,KAT2B,IRS2,PRNP,NAXD,NUDT16,HDAC3,DCLK1,ACPP,CCKBR,DDRGK1,SMPD1,PPP2CB,ILK,RBPMS,PPP2R2B,RYK,CDK8,CNOT7,IMPAD1,MTMR6,SLC20A1,CHPT1,HSP90B1,TMED2,MAST4,OSBP,TMEM150A,GRK7,RIOK2,ROBO1,FAR1,ALDH18A1,CDKL2,PFN2,CCNJ,APP,RAPGEF2,MAPKAPK5,CX3CL1,PPP2R2A,HUNK,PTPN11,ADCY6,PIP4P2,PFKM,PGAM4,OPA1,ENTPD4,GTF2H1,PTPN22,TSG101,ACVR1,CRY2,PPP3CC,LPCAT2,GDF6,SERINC3,ADA,AKTIP,CHP1,ATP2B4,PKD2,TWF1,NUDT18,DBNDD2,PIGP,CBLB,TOLLIP,BRSK2,PSPH,SLC39A10,CHRNA7,DYNLL1,DAPP1,AGPAT3,EYA2,CCND1,CAPRIN2,TGFB2,PPP6R3,ZBTB20,PRKAB2,LRP5,HTR2B,PRKAR1A,ITPK1,GSK3B,INPP5A,FAM20C,DAG1,PIK3CA,CORO1C,PRKCA,NEK7,KRAS,PITPNM1,NTRK3,MOCS3,GRK3,ETNK1,TPST2,SLAMF8,SPATC1L,BMP7,IDH2,ADCY5,HACD2,PPP1R11,PPP1R3D,FGFR2,OSM,SYAP1,IQGAP1,TFRC,PIGZ,RPS6KA3,PRDX4,PCYT1B,REXO2,HSPB1,SCYL1,MAPT,SLC25A16,DNAJC10,FN1,MAP4K5,PTPN6,TPI1,PDCD10,ADRA2C,DOK7,HTT,FKBP8,PRKCI,FGFR3,ARAF,TNIK,PLD1,SGMS1,MNAT1,PPP2R5E,AKT3,ERC1,PPP1R36,MAGED1,BMPR	darkgreen	EC_M4
GO:1902001	1.94E-01	0.928631645	4	17	fatty acid transmembrane transport	BP	8.95E-01	AKT2,IRS2,CPT1A,AKT1	darkgreen	EC_M4
GO:0046890	1.94E-01	0.866045797	21	127	regulation of lipid biosynthetic process	BP	8.95E-01	PPARA,LEP,REST,MFSD2A,DKKL1,DGAT2,DKK3,RDH10,SORBS1,CHP1,ZBTB20,HTR2B,WDTC1,PPARGC1A,CD74,AKT1,PRKAA1,ABCA3,FAM3A,MBTPS1,ORMDL3	darkgreen	EC_M4
GO:0016052	1.95E-01	0.868732802	19	113	carbohydrate catabolic process	BP	8.96E-01	MIOX,PPARA,GALT,GLB1,RBKS,PFKP,APP,PFKM,PGAM4,ZBTB20,LRP5,PPP1R3D,TPI1,PPARGC1A,PKM,PRKAA1,ENO1,SLC2A6,AGL	darkgreen	EC_M4
GO:0010743	1.67E-01	0.941006796	4	20	regulation of macrophage derived foam cell differentiation	BP	8.97E-01	PPARG,CD36,ABCG1,PRKCH	turquoise	EC_M6
GO:0007129	6.17E-02	0.976878144	7	39	homologous chromosome pairing at meiosis	BP	8.98E-01	RAD21,FANCD2,CCNE2,TRIP13,SPO11,BRIP1,NDC1	darkgrey	EC_M8
GO:0006903	1.67E-01	0.91946953	7	42	vesicle targeting	BP	8.98E-01	FAM91A1,EXOC6,SNAP23,LRMP,AP1AR,SNAP29,PSEN1	turquoise	EC_M6
GO:0045577	1.67E-01	0.940820845	4	20	regulation of B cell differentiation	BP	8.98E-01	SYK,SFRP1,IL7,INPP5D	turquoise	EC_M6
GO:0006896	1.68E-01	0.94070777	4	20	Golgi to vacuole transport	BP	8.98E-01	VPS54,LAPTM5,RNF215,AP3D1	turquoise	EC_M6
GO:0120035	1.68E-01	0.864089583	68	524	regulation of plasma membrane bounded cell projection organization	BP	8.98E-01	SEMA6A,TBC1D30,CDKL3,PODXL,CDC42EP1,KIT,SEMA4D,NRP1,PLXND1,GATA3,SFRP1,EFNB2,ARHGEF7,PRAG1,RET,OCLN,KIF13B,ENC1,P2RX7,NDRG4,GPM6A,USH1C,PPP1R16B,CEP97,MYLIP,SRGAP2C,PPP3CA,HECW2,SEMA3F,PLXNA2,CFLAR,INPP5F,TRAK2,FES,SS18L1,PRKD1,ITGA6,LIMK2,PAK2,B2M,SEMA6D,DNM3,WAS,RAC1,RCC2,FSCN1,EPHA2,MTOR,PSEN1,SEMA4A,ABI3,ITGA3,EFNA1,CHRNB2,ZDHHC15,RABEP2,DNM2,SEMA6B,ARHGAP4,RAPGEF1,FNBP1L,DAB2IP,CARMIL2,RELN,ALKAL1,SHANK3,RALA,SCARF1	turquoise	EC_M6
GO:0009064	1.68E-01	0.910456441	9	59	glutamine family amino acid metabolic process	BP	8.98E-01	RIMKLA,NOS3,AADAT,SLC39A8,SLC7A7,FAH,ARG2,NOS2,ATCAY	turquoise	EC_M6
GO:0034383	1.68E-01	0.940001987	4	22	low-density lipoprotein particle clearance	BP	8.98E-01	CD36,SCARB1,MYLIP,LIPA	turquoise	EC_M6
GO:0048806	1.68E-01	0.924790528	6	34	genitalia development	BP	8.98E-01	HSD17B3,MERTK,CHD7,AR,SRD5A2,BMP6	turquoise	EC_M6
GO:0090342	1.68E-01	0.9189477	7	42	regulation of cell aging	BP	8.98E-01	PLK2,MAP3K3,B2M,ARG2,ABI3,VASH1,TERF2	turquoise	EC_M6
GO:0031670	1.68E-01	0.919129196	7	40	cellular response to nutrient	BP	8.98E-01	PIM1,IL15,SFRP1,MN1,FES,FGF23,TRIM24	turquoise	EC_M6
GO:0000002	1.68E-01	0.940362516	4	20	mitochondrial genome maintenance	BP	8.98E-01	MEF2A,LIG3,LONP1,SLC25A33	turquoise	EC_M6

GO:0043124	1.68E-01	0.924565789	6	34	negative regulation of I-kappaB kinase/NF-kappaB signaling	BP	8.98E-01	TNFAIP3,TRIM39,STAT1,HDAC1,TNIP1,DAB2IP	turquoise	EC_M6
GO:0021782	1.69E-01	0.898619653	13	86	glial cell development	BP	8.98E-01	IFNGR1,PICK1,IL6,TLR4,ITGB4,TLR2,TSPAN2,ADORA2A,MPP5,PSEN1,EIF2B2,B4GALT6,SH3TC2	turquoise	EC_M6
GO:0034389	1.69E-01	0.940235338	4	20	lipid droplet organization	BP	8.98E-01	CDS2,ZFYVE1,RAB18,TBC1D20	turquoise	EC_M6
GO:0097720	1.69E-01	0.918930587	7	40	calcineurin-mediated signaling	BP	8.98E-01	IGF1,PPP3CA,NFATC2,HOMER3,MTOR,PLCG2,NFAT5	turquoise	EC_M6
GO:0045088	1.69E-01	0.886589636	20	148	regulation of innate immune response	BP	8.98E-01	TNFAIP3,SYK,TLR4,NECTIN2,PPARG,IKBKB,TMEM173,OAS3,NLRC5,NCF1,FGR,PAK2,BIRC3,LGALS9,ISG15,PLCG2,OAS1,GBP5,TRIM62,SERPINB9	turquoise	EC_M6
GO:0007608	1.95E-01	0.928086026	4	17	sensory perception of smell	BP	8.98E-01	FZD2,TTC8,OR5AN1,OR6N2	darkgreen	EC_M4
GO:0001658	1.96E-01	0.892800549	9	47	branching involved in ureteric bud morphogenesis	BP	8.98E-01	VEGFA,GLI3,FGF2,CTNNB1,ILK,PKD2,SIX4,MAGED1,GDNF	darkgreen	EC_M4
GO:0051590	1.96E-01	0.927746374	4	17	positive regulation of neurotransmitter transport	BP	8.98E-01	ITGB1,RAB3GAP1,DNM1L,STX1B	darkgreen	EC_M4
GO:0003298	1.96E-01	0.916767255	5	24	physiological muscle hypertrophy	BP	8.98E-01	PPARA,PAK1,PDLIM5,PIN1,MIR24-1	darkgreen	EC_M4
GO:0003301	1.96E-01	0.916767255	5	24	physiological cardiac muscle hypertrophy	BP	8.98E-01	PPARA,PAK1,PDLIM5,PIN1,MIR24-1	darkgreen	EC_M4
GO:0061049	1.96E-01	0.916767255	5	24	cell growth involved in cardiac muscle cell development	BP	8.98E-01	PPARA,PAK1,PDLIM5,PIN1,MIR24-1	darkgreen	EC_M4
GO:0061037	1.96E-01	0.909018236	6	29	negative regulation of cartilage development	BP	8.98E-01	LEP,TGFBR1,GLI2,CTNNB1,RARB,PTPN11	darkgreen	EC_M4
GO:0031323	1.69E-01	0.845503032	498	4391	regulation of cellular metabolic process	BP	8.99E-01	ZNF516,TNFRSF11A,BAZ2A,TCF4,TNFAIP8,IFNGR1,TNFAIP3,PLAGL1,TRIM39,PTPRB,LAPTM4B,CNOT6,FLT4,FZD6,OXR1,ZBED4,GRAMD4,QKI,NOTCH4,TCF20,TCF7,TPD52L1,NCOA7,L3MBTL3,PTK2,DAPK1,ATF4,PDGFB,AGTPBP1,BRPF3,CSNK1E,PICK1,KDM4C,PIM1,NFYA,MDFI,TEK,IREB2,PRDM4,LIMCH1,DDX58,NFX1,DCAF12,FAM122A,TEC,ADGRF5,KIT,ADGRF1,KDR,NFIL3,SYK,SEMA4D,RGP1,PPP6R1,IL6,IGF2BP3,NFE2L3,REL,CREB5,PTBP3,CXCL8,PPIE,BACH2,PELI1,FIGNL1,FOXP2,KLF11,GRB10,LANCL2,NOBOX,TLR4,TRAF1,C5,IL15,CCNY,CREM,TEAD2,SDCBP,MYCN,NOCT,TUT4,NRP1,KTI12,ZBTB24,PUM2,RASIP1,BEND5,PIK3CG,TAL1,PHF10,MTMR2,EPC1,KDM4D,GATA2,DNMT3A,PRKD2,SAP130,CST7,NOVA2,CTSC,RAB38,CAB39L,PRCP,NOS3,GGA3,RALB,DACH1,PPARG,TIMP4,GATA3,TAF3,ACVR2B,ITIH5,IRAK2,GZF1,EDNRB,PPP1CB,NDFIP2,SPTBN4,TIE1,SETD5,IKBKB,JAG1,P2RY6,NFKBIB,PDE2A,TMEM173,SFRP1,HBEGF,NR1D2,DND1,FEZ2,RPL26,SH3BP5,ANKLE2,CD36,SIRT3,CDK17,ARID5A,PRR5L,TCF15,SCARB1,EPHB1,PLK2,DEPP1,RET,PDE4D,MERTK,CHD7,IL1A,DNMT3B,IGF1,NR4A1,KLKB1,EIF2AK3,HHEX,KDM3A,ITCH,ERBIN,SH3RF1,SMAD1,ZBTB38,CLCN3,HIP1R,POU6F1,OCLN,STOX2,CHRM5,SOX7,HMBOX1,GPD1,ENC1,HEXB,MEF2C,SMARCD1,TLR2,WWTR1,IL7,P2RX7,VEGFC,ZNF704,CXCL10,SOGA1,MADD,ADGRG3,KMT2D,SFMBT1,FAM192A,SCAF4,OAS3,NLRC5,THBS4,ACTR5,PPP1R16B,TPCN1,RAPGEF3,ANKRD2,NR4A2,DGKZ,TOX2,DLL4,CCNJL,CDYL,BAZ2B,SOX13,NEDD9,YAF2,EDN1,STAT1,DYRK1A,EHD4,SNCA,JARID2,DTNBP1,MYLIP,ERG,ETS2,CDK18,SLC45A3,NFKB2,DCAF1,RNF144B,PRDM15,ABCG1,ETS1,	turquoise	EC_M6
GO:0034243	1.97E-01	0.908471182	6	29	regulation of transcription elongation from RNA polymerase II promoter	BP	9.02E-01	CTNNB1,EAF1,CDC73,LARP7,CDK12,SUPT6H	darkgreen	EC_M4
GO:2000179	1.70E-01	0.918338314	7	39	positive regulation of neural precursor cell proliferation	BP	9.02E-01	VEGFC,DLL4,PROX1,ELL3,HIF1A,NOTCH1,ADGRG1	turquoise	EC_M6
GO:0043628	1.97E-01	0.901614132	7	36	ncRNA 3'-end processing	BP	9.02E-01	TRNT1,ERI1,DKC1,TENT4B,LARP7,HSD17B10,INTS6L	darkgreen	EC_M4
GO:0050796	1.70E-01	0.888513407	18	127	regulation of insulin secretion	BP	9.02E-01	PICK1,ICA1,IL6,TRPM4,SFRP1,SIRT3,FAM3D,ABCG1,HDAC1,PPP3CA,ANO1,MCU,CDK16,RAC1,HIF1A,SYT7,GPR68,NOS2	turquoise	EC_M6

GO:0043401	1.98E-01	0.866573928	19	111	steroid hormone mediated signaling pathway	BP	9.02E-01	PPARA,PPARD,CLOCK,PGRMC2,PAK1,LBH,UFM1,UBA5,DDRGK1,RNF6,CRY2,VPS18,BMP7,STRN3,MED1,SMARCA4,PARK7,YWHAH,PMEPA1	darkgreen	EC_M4
GO:0070169	1.98E-01	0.896304797	8	41	positive regulation of biomineral tissue development	BP	9.02E-01	FBN2,TGFB1,ATP2B1,ACVR1,BMP7,FZD9,BMPR1A,TGFB3	darkgreen	EC_M4
GO:0032438	1.98E-01	0.916274723	5	23	melanosome organization	BP	9.03E-01	ZEB2,AP3B1,SHROOM2,AP1G1,BLOC1S2	darkgreen	EC_M4
GO:0001961	1.70E-01	0.917604395	7	43	positive regulation of cytokine-mediated signaling pathway	BP	9.03E-01	CXCR4,IL7,NLRC5,EDN1,LAPTM5,HIF1A,TRAF2	turquoise	EC_M6
GO:0098815	1.98E-01	0.90159662	7	34	modulation of excitatory postsynaptic potential	BP	9.03E-01	RIMS1,SHANK1,APP,CACNB3,CHRNA7,NLGN2,STX1B	darkgreen	EC_M4
GO:0030003	1.71E-01	0.864728308	55	434	cellular cation homeostasis	BP	9.05E-01	DMXL1,TRDN,IREB2,PPT1,CAV2,TRPM4,PIK3CG,GATA2,RAB38,GPR4,CXCR4,EDNRB,P2RY6,P2RX2,SLC4A11,CD36,TMEM178A,MICU3,PDE4D,CHD7,IL1A,CLCN3,HEXB,CP,P2RX7,CXCL10,TPCN1,PLCG1,STC1,EDN1,SNCA,LCK,SLC39A8,KCNJ2,TESMIN,HCRTR1,SLC9A8,PRKD1,CCRL2,MCU,SLC9A7,HIF1A,HEPH,PSEN1,ATP2A1,CD19,AP3D1,PLCG2,S1PR1,CDH5,SLC8A3,CA2,LPAR6,RAB39A,BMP6	turquoise	EC_M6
GO:0031333	1.71E-01	0.889622178	17	115	negative regulation of protein-containing complex assembly	BP	9.05E-01	TMOD1,SVIL,TMC8,SPTBN4,ARHGEF7,LMOD3,HIP1R,DYRK1A,JAM3,SNCA,TMOD4,DDX3X,TMOD2,STXBP1,CARMIL2,CDH5,KANK3	turquoise	EC_M6
GO:1903077	1.99E-01	0.915587225	5	23	negative regulation of protein localization to plasma membrane	BP	9.05E-01	PICALM,TGFB1,AP2M1,LRRC15,CLTC	darkgreen	EC_M4
GO:0048864	1.99E-01	0.877327648	13	72	stem cell development	BP	9.05E-01	FGF2,SEMA3C,RDH10,SEMA4F,ACVR1,SEMA4G,HTR2B,CORO1C,BMP7,FN1,BMPR1A,ANXA6,GDNF	darkgreen	EC_M4
GO:0035914	1.71E-01	0.912707129	8	48	skeletal muscle cell differentiation	BP	9.05E-01	NR1D2,EPHB1,NR4A1,MEF2C,BCL9,TBX1,NOTCH1,MAFF	turquoise	EC_M6
GO:0071496	1.71E-01	0.872528854	35	267	cellular response to external stimulus	BP	9.05E-01	ATF4,PICK1,PIM1,TLR4,IL15,RALB,PDE2A,SFRP1,MAP3K1,EIF2AK3,TMEM150C,NR4A2,NUAK2,SIK1,KCNJ2,MN1,FES,PRKD1,F11R,SIPA1,ITGA4,FGF23,RAC1,MTOR,ZFYVE1,KIF26A,PIEZO2,STK26,PIEZO1,ENG,RIPOR1,PMAIP1,TRIM24,RNF152,BMP6	turquoise	EC_M6
GO:0030101	1.71E-01	0.917107041	7	43	natural killer cell activation	BP	9.05E-01	NFIL3,IL15,UNC13D,MERTK,FGR,RAB27A,ELF4	turquoise	EC_M6
GO:0039531	1.71E-01	0.939065206	4	20	regulation of viral-induced cytoplasmic pattern recognition receptor signaling pathway	BP	9.05E-01	PUM2,SEC14L1,BIRC3,RNF125	turquoise	EC_M6
GO:1902116	1.71E-01	0.923175338	6	33	negative regulation of organelle assembly	BP	9.05E-01	TBC1D30,FEZ2,CEP97,LIMK2,DNM2,MTM1	turquoise	EC_M6
GO:0043266	1.99E-01	0.883034206	11	59	regulation of potassium ion transport	BP	9.06E-01	CD63,ITGB1,KIF5B,PRNP,ATP1B3,CACNA1D,ABCC8,KCNA1,KCNMB1,FLNA,KCNAB2	darkgreen	EC_M4
GO:0043616	2.00E-01	0.900437213	7	35	keratinocyte proliferation	BP	9.06E-01	PPARD,NOTCH2,FGFR2,MED1,CDH13,HAS2,FGF10	darkgreen	EC_M4
GO:0030325	2.00E-01	0.925776549	4	17	adrenal gland development	BP	9.06E-01	LY6E,DKK3,ARID5B,FSTL3	darkgreen	EC_M4
GO:0072074	2.00E-01	0.925775787	4	17	kidney mesenchyme development	BP	9.06E-01	PKD2,BMP7,SIX4,PDGFRB	darkgreen	EC_M4
GO:0060977	2.00E-01	0.926413572	4	16	coronary vasculature morphogenesis	BP	9.06E-01	VEGFA,TGFBR1,FGF2,PDGFRB	darkgreen	EC_M4
GO:0051588	2.00E-01	0.876637419	13	73	regulation of neurotransmitter transport	BP	9.07E-01	RIMS1,ITGB1,RAB3GAP1,ATP2A2,VPS18,DNM1L,GSK3B,BACE1,STX1B,SYT11,CALM1,GDNF,KCNMB4	darkgreen	EC_M4
GO:0007035	2.00E-01	0.914858658	5	23	vacuolar acidification	BP	9.08E-01	CLIC4,ATP6AP2,CLN6,ATP6V0D1,ATP6V0E1	darkgreen	EC_M4

GO:0090190	2.01E-01	0.925384546	4	17	positive regulation of branching involved in ureteric bud morphogenesis	BP	9.08E-01	VEGFA,SIX4,MAGED1,GDNF	darkgreen	EC_M4
GO:1990089	2.01E-01	0.889680604	9	47	response to nerve growth factor	BP	9.08E-01	SH3GL2,KIDINS220,APP,RAPGEF2,NTRK3,MAPT,AKT1,HSPA5,DYNC1LI2	darkgreen	EC_M4
GO:0071241	2.01E-01	0.854457562	27	165	cellular response to inorganic substance	BP	9.08E-01	SLC12A2,SCN5A,LIG4,PRNP,SMPD1,MAP1LC3A,RYR3,P2RX4,APP,PKD2,NCSTN,IQGAP1,CLIC4,BACE1,TFR2,HSD17B1,KCNA1,EIF2S1,PPARGC1A,SYT11,AKT1,PRKAA1,HSPA5,FSTL3,SERPINF1,BNIP3,CACYBP	darkgreen	EC_M4
GO:0008064	2.01E-01	0.855346571	26	159	regulation of actin polymerization or depolymerization	BP	9.08E-01	FAM49B,NCK2,ACTR2,ACTR3,PLEKHG2,CDC42EP3,CXCL12,PFN2,RASA1,TWF1,MAGEL2,CTTN,LMOD1,PIK3CA,ARPC5,NCKAP1,ARPC2,CAPZB,WDR1,FLII,MYO1C,SPTAN1,TMSB4X,DSTN,COTL1,CFL2	darkgreen	EC_M4
GO:0032332	2.01E-01	0.925174742	4	17	positive regulation of chondrocyte differentiation	BP	9.08E-01	RUNX2,GLI3,LOXL2,GDF6	darkgreen	EC_M4
GO:0048260	2.01E-01	0.894023367	8	41	positive regulation of receptor-mediated endocytosis	BP	9.08E-01	CD63,AHI1,RAB21,ANGPT1,VEGFA,AP2M1,SERPINE1,ATAD1	darkgreen	EC_M4
GO:0072012	2.01E-01	0.914404646	5	23	glomerulus vasculature development	BP	9.08E-01	ANGPT1,NOTCH2,BMP7,ACTA2,PDGFRB	darkgreen	EC_M4
GO:0032103	2.01E-01	0.842865068	44	286	positive regulation of response to external stimulus	BP	9.08E-01	IL23A,LRP1,VEGFA,CLOCK,CREB3,TPBG,MAP3K7,AKIRIN1,FKBP1B,FGF2,MEGF8,PAK1,TGFB1,CXCL12,CD180,VAMP8,P2RX4,APP,CX3CL1,POLR3D,DNM1L,PRKCA,CMKLR1,NTRK3,OSM,HSPB1,SERPINE1,MED1,CD74,PDGFRB,ITGA2,OSMR,FLNA,VAMP7,PARK7,CDH13,F3,AP1G1,ARTN,TMSB4X,FCN1,CCR6,TSLP,FGF10	darkgreen	EC_M4
GO:0030834	2.02E-01	0.888987927	9	46	regulation of actin filament depolymerization	BP	9.11E-01	TWF1,LMOD1,PIK3CA,CAPZB,WDR1,FLII,SPTAN1,DSTN,CFL2	darkgreen	EC_M4
GO:0061298	2.02E-01	0.925270996	4	16	retina vasculature development in camera-type eye	BP	9.11E-01	LRP5,CLIC4,ROM1,PDGFRB	darkgreen	EC_M4
GO:0007548	2.02E-01	0.848268967	33	206	sex differentiation	BP	9.12E-01	CTNNA1,LEP,VEGFA,TGFBR1,INHBA,AXL,WDR48,CTNNB1,EIF2S2,RDH10,CDKL2,PTPN11,PTX3,CSDE1,CCND1,TGFB2,MMP14,ASB1,ARID5B,PRDX4,PCYT1B,SGPL1,PTPRN,CYP19A1,SIX4,BMPR1A,PDGFRB,PKD1,FLNA,HSPA5,LHX8,FSTL3,FGF10	darkgreen	EC_M4
GO:0033238	2.02E-01	0.913712643	5	23	regulation of cellular amine metabolic process	BP	9.12E-01	SLC7A11,ATP2B4,COMT,PARK7,NNMT	darkgreen	EC_M4
GO:0014911	2.02E-01	0.898585073	7	35	positive regulation of smooth muscle cell migration	BP	9.12E-01	LRP1,PAK1,IQGAP1,S100A11,PDGFRB,ITGA2,HAS2	darkgreen	EC_M4
GO:0030808	2.03E-01	0.913096861	5	24	regulation of nucleotide biosynthetic process	BP	9.12E-01	PPARA,VCP,PPARGC1A,ENO1,TMSB4X	darkgreen	EC_M4
GO:1900371	2.03E-01	0.913096861	5	24	regulation of purine nucleotide biosynthetic process	BP	9.12E-01	PPARA,VCP,PPARGC1A,ENO1,TMSB4X	darkgreen	EC_M4
GO:0002762	2.03E-01	0.898161369	7	35	negative regulation of myeloid leukocyte differentiation	BP	9.13E-01	INHBA,CTNNB1,TCTA,GPR137,FBN1,PTPN2,FSTL3	darkgreen	EC_M4
GO:0010460	2.03E-01	0.924012061	4	17	positive regulation of heart rate	BP	9.13E-01	ATP2A2,ADA,TPM1,ADRA1B	darkgreen	EC_M4
GO:0016358	1.73E-01	0.874615634	29	204	dendrite development	BP	9.13E-01	CDKL3,ASAP1,SEMA4D,NRP1,EPHB1,PLK2,PREX2,MEF2C,DTNBP1,SRGAP2C,PPP3CA,HECW2,MEF2A,TRAK2,SS18L1,PAK2,LAMC2,DNM3,MTOR,PSEN1,ABI3,TAOK2,EFNA1,CHRNB2,ZDHHC15,DAB2IP,RELN,SHANK3,SCARF1	turquoise	EC_M6
GO:1903825	2.03E-01	0.863608576	18	106	organic acid transmembrane transport	BP	9.13E-01	SLC22A4,SLC7A11,ITGB1,SLC36A4,AKT2,SLC38A10,IRS2,ARL6IP5,CPT1A,SLC3A2,SLC6A13,PRAF2,SLC29A4,CLN8,SFXN1,SLC7A3,AKT1,SLC36A1	darkgreen	EC_M4
GO:0034109	1.73E-01	0.900254887	11	71	homotypic cell-cell adhesion	BP	9.14E-01	TJP2,SYK,IL6,PIK3CG,F11R,JUP,PEAR1,DSG2,DSC2,JAK1,STXBP1	turquoise	EC_M6

GO:0021799	1.74E-01	0.928823145	5	26	cerebral cortex radially oriented cell migration	BP	9.14E-01	SRGAP2C,LHX6,DAB2IP,ADGRG1,RELN	turquoise	EC_M6
GO:0034367	1.74E-01	0.937375664	4	21	protein-containing complex remodeling	BP	9.14E-01	SCARB1,ABCG1,MTTP,GTF2E1	turquoise	EC_M6
GO:0009205	2.04E-01	0.882915672	10	57	purine ribonucleoside triphosphate metabolic process	BP	9.15E-01	PPARA,VCP,RAB23,TGFB1,OPA1,PPARGC1A,ENO1,GNAI3,TMSB4X,NME6	darkgreen	EC_M4
GO:0002042	2.04E-01	0.883074814	10	55	cell migration involved in sprouting angiogenesis	BP	9.15E-01	SRF,VEGFA,ITGB1,FGF2,STARD13,ROBO1,PDCD10,AKT3,AKT1,PIK3C2A	darkgreen	EC_M4
GO:0035307	2.04E-01	0.897588533	7	35	positive regulation of protein dephosphorylation	BP	9.15E-01	TGFB1,PPP1R12A,SMPD1,SLC39A10,CALM1,PIN1,PDGFRB	darkgreen	EC_M4
GO:0014014	2.04E-01	0.904265559	6	29	negative regulation of gliogenesis	BP	9.15E-01	CTNNB1,ABCC8,NTRK3,IDH2,DICER1,TMEM98	darkgreen	EC_M4
GO:0033619	2.04E-01	0.886879855	9	48	membrane protein proteolysis	BP	9.16E-01	MYH9,TGFB1,DAG1,NCSTN,BACE1,TIMP1,ROCK1,MBTPS1,TIMP3	darkgreen	EC_M4
GO:0042177	2.04E-01	0.864372106	17	101	negative regulation of protein catabolic process	BP	9.16E-01	AGAP2,AZIN1,ADGRB1,PDCL3,DDRGK1,AZIN2,UCHL5,TIMP1,FMN2,PIN1,ROCK1,USP14,FLNA,PARK7,TIMP3,GABARAPL2,MAP1A	darkgreen	EC_M4
GO:0033059	1.74E-01	0.915031894	7	43	cellular pigmentation	BP	9.17E-01	RAB38,MYO7A,DTNBP1,LYST,RAB27A,AP3D1,RAB32	turquoise	EC_M6
GO:0052126	1.74E-01	0.885158402	18	127	movement in host environment	BP	9.17E-01	CXCL8,CAV2,NRP1,NECTIN2,CXCR4,EFNB2,SCARB1,ITCH,TPCN1,HYAL2,CD86,F11R,EPHA2,LGALS9,HAVCR1,TRIM34,CD46,TRIM62	turquoise	EC_M6
GO:0008610	1.75E-01	0.85816371	65	544	lipid biosynthetic process	BP	9.20E-01	SYNJ2,SERAC1,QKI,HSD17B3,PDGFB,PIP5K1B,ADGRF5,SRD5A3,AGMO,PIGF,INPP4B,OSBPL9,PIK3CG,MTMR2,MGLL,PRKD2,RAB38,PGS1,GPAT4,CDS2,SIRT3,SCARB1,ELOVL7,ABHD6,PITPNM2,ST3GAL5,HEXB,DHH,SERINC5,DGKZ,PIK3C2B,EDN1,SLC45A3,ABCG1,LPCAT1,SIK1,INPP5D,B3GNT5,BMX,INPP5F,PROX1,PRKD1,MLXIPL,GAL3ST3,CREB1,OSBPL6,SAMD8,PLD2,ITPKB,LBR,ALDH1A2,MTOR,DHRS11,CYP1A1,B4GALT6,MTM1,PLCG2,PTGS1,ST6GALNAC3,BMP2,SRD5A2,BMP6,AKR1D1,RBP1,ST8SIA6	turquoise	EC_M6
GO:0072078	2.06E-01	0.878281477	11	60	nephron tubule morphogenesis	BP	9.21E-01	AHI1,VEGFA,GLI3,FGF2,CTNNB1,ILK,PKD2,SIX4,MAGED1,PKD1,GDNF	darkgreen	EC_M4
GO:0014068	2.06E-01	0.875069294	12	66	positive regulation of phosphatidylinositol 3-kinase signaling	BP	9.21E-01	ANGPT1,PPARD,LEP,FGF2,DCN,TGFB2,NTRK3,OSM,FN1,PTPN6,PDGFRB,MYDGF	darkgreen	EC_M4
GO:0046128	2.06E-01	0.921879758	4	18	purine ribonucleoside metabolic process	BP	9.22E-01	MTAP,ACPP,ADA,PEMT	darkgreen	EC_M4
GO:0043069	1.76E-01	0.854415831	81	681	negative regulation of programmed cell death	BP	9.22E-01	TNFAIP8,TNFAIP3,FLT4,OXR1,PTK2,PIM1,TEK,KIT,KDR,SEMA4D,IL6,PPT1,PELI1,FIGNL1,NRP1,PIK3CG,GATA2,NOS3,ZFAND6,GATA3,EDNRB,SFRP1,ANKLE2,PLK2,MERTK,IL1A,IGF1,NR4A1,ITCH,SH3RF1,HIP1R,MEF2C,IL7,NR4A2,ADAMTS20,EDN1,SNCA,NUAK2,RNF144B,HDAC1,ITPRIP,LAPTM5,DYRK3,HCLS1,CFLAR,RPS6KA1,ITGA6,PAK2,ELL3,LTF,ADORA2A,ARNT2,DDX3X,TBX1,MECOM,RAMP2,BIRC3,PRKCH,HIF1A,ITPKB,BID,ARG2,MTOR,AR,BNIP2,PSEN1,EFNA1,PIAS1,CCL2,IL7R,BFAR,MYO18A,TRAF2,NOTCH1,PLCG2,STXBP1,CDH5,VNN1,SERPINB9,IFIT3,NMNAT1	turquoise	EC_M6
GO:0009065	1.76E-01	0.936273119	4	21	glutamine family amino acid catabolic process	BP	9.23E-01	NOS3,FAH,ARG2,NOS2	turquoise	EC_M6
GO:0032715	2.07E-01	0.902465149	6	30	negative regulation of interleukin-6 production	BP	9.23E-01	SOCS5,CX3CL1,PTPN22,SELENOS,PTPN6,SYT11	darkgreen	EC_M4
GO:0043271	2.07E-01	0.864612559	16	93	negative regulation of ion transport	BP	9.23E-01	UBQLN1,LEP,FKBP1B,ARL6IP5,REM1,CACNB3,PKD2,PLA2R1,ISCU,LINC02210-CRHR1,SYT11,CALM1,AKT1,MIR24-1,CBARP,GEM	darkgreen	EC_M4
GO:0032770	1.76E-01	0.927233423	5	27	positive regulation of monooxygenase activity	BP	9.24E-01	SCARB1,FGF23,HIF1A,TERF2,FCER2	turquoise	EC_M6

GO:0022414	2.07E-01	0.818599354	150	1029	reproductive process	BP	9.24E-01	TRIM36,B4GALNT1,TCP1,ROS1,AFF4,RSPO3,PPARD,CTNNA1,TEAD3,SUN2,MYH9,LEP,VEGFA,UNC13B,CLOCK,NLRP5,TGFBR1,RIMS1,PSME4,CENPC,UBE2J1,ACTR2,GMCL1,GLI3,INHBA,DKKL1,ITGB1,PANX1,ZNF296,CRTAP,GLI2,ACTR3,AXL,WDR48,CTNNB1,DZIP1,SPINT2,CHFR,DCN,KATNAL1,TMED2,EIF2S2,SULF1,LNPEP,RDH10,CDKL2,HEY1,CELF1,APP,GLRB,DHX36,CCT8,ABCC8,PTPN11,PTX3,PGAM4,RAD54B,AP3B1,ACVR1,MMP2,ADA,ATP2B4,SPP1,PKD2,CSDE1,DNAH1,SYCP2L,DNAH5,DYNLL1,AZIN2,KDM5B,NOTCH2,CCND1,KPNA6,TGFB2,PRKAR1A,MMP14,LEF1,KRAS,LARP7,SPATC1L,BMP7,FGFR2,VASH2,ASB1,HTRA1,GORASP2,OSBP2,CLIC4,ARID5B,PAFAH1B2,OVOL1,PRDX4,PCYT1B,LINC02210-CRHR1,SGPL1,COMT,PTPN6,IGFBP2,ADRA2C,SKIL,TIMP1,PTPRN,CYP19A1,VDAC2,FMN2,SIX4,BMPR1A,MED1,MAGED2,MYCBPAP,TGFB3,UBE2Q1,MAP2K1,SOX30,DIAPH2,TEX14,VMP1,MYOCD,ACSL4,PDGFRB,RBBP8,AKT1,UBE2A,ITGA2,RAI14,PLAC1,BCAP31,PAFAH1B1,PKD1,NPHP4,FLNA,PARK7,BSG,SLC2A8,HSPA5,LHX8,FSTL3,CCDC136,GGN,SPINK2,SERPINF1,SERPINB5,IGSF8,HAS2,CCR6,WNT2,NPAS1,FGF10,MBD2	darkgreen	EC_M4
GO:0021510	1.76E-01	0.898225397	11	70	spinal cord development	BP	9.25E-01	TAL1,GATA2,DLL4,SOX13,PROX1,HOXD10,ZC4H2,MTOR,HOXB8,NOTCH1,RELN	turquoise	EC_M6
GO:0042398	2.07E-01	0.895034766	7	37	cellular modified amino acid biosynthetic process	BP	9.25E-01	SLC7A11,PLOD2,CHAC1,PLOD3,HAGH,TMLHE,PARK7	darkgreen	EC_M4
GO:0072273	2.08E-01	0.910641191	5	23	metanephric nephron morphogenesis	BP	9.26E-01	CTNNB1,PKD2,PDGFRB,PKD1,GDNF	darkgreen	EC_M4
GO:1904589	6.41E-02	0.972138181	9	55	regulation of protein import	BP	9.27E-01	UBR5,TRIM28,IPO5,NUP58,NUP54,NOLC1,CDK1,TPR,ECT2	darkgrey	EC_M8
GO:2000106	1.77E-01	0.904206035	9	59	regulation of leukocyte apoptotic process	BP	9.27E-01	MERTK,MEF2C,HCLS1,HIF1A,ITPKB,ARG2,EFNA1,LGALS9,IL7R	turquoise	EC_M6
GO:0031954	1.77E-01	0.927155736	5	25	positive regulation of protein autophosphorylation	BP	9.27E-01	PDGFB,VEGFC,ACE,DDX3X,TAOK2	turquoise	EC_M6
GO:0019953	1.78E-01	0.855143995	68	565	sexual reproduction	BP	9.28E-01	TSPAN8,SPAG1,QKI,PICK1,KIT,FIGNL1,NOBOX,CREM,TUT4,IZUMO1,SPATA6,DNMT3A,NECTIN2,EDNRB,SPTBN4,DND1,GOLGA3,MERTK,IGF1,KDM3A,SMAD1,HEXB,ANGPT2,DHH,KMT2D,SFMBT1,CDYL,JAM3,GAS2,TESMIN,LRMP,PLEKHA1,ZNF148,OAZ3,ZSCAN2,ACE,LIMK2,ELL3,ETV6,HOXD10,RNF2,SSTR1,DDX3X,CDK16,BIRC3,USP42,KDM5A,CECR2,SPIN2A,MTOR,AR,FBXW11,CCDC42,PIAS1,DNM2,YY1,JAG2,SPEF2,SPIRE1,NOTCH1,STXBP1,STRBP,ARID4A,CD46,SOX17,TDRP,ANKRD49,TBC1D20	turquoise	EC_M6
GO:0030835	1.78E-01	0.913599187	7	38	negative regulation of actin filament depolymerization	BP	9.28E-01	TMOD1,SVIL,SPTBN4,LMOD3,TMOD4,TMOD2,CARMIL2	turquoise	EC_M6
GO:0006821	2.08E-01	0.876577816	11	60	chloride transport	BP	9.28E-01	ATP8B1,SLC12A2,PCYOX1,ANO10,PRNP,FXYD1,GLRB,SLC26A7,CLIC4,CLCC1,NMUR1	darkgreen	EC_M4
GO:0002700	1.78E-01	0.887343829	15	106	regulation of production of molecular mediator of immune response	BP	9.28E-01	DDX58,KIT,IL6,RSAD2,TLR4,TRPM4,GATA3,CD36,LAPTM5,CD86,B2M,TNFRSF1B,TRAF2,PLCG2,BST2	turquoise	EC_M6
GO:0009790	1.78E-01	0.849194745	104	841	embryo development	BP	9.28E-01	FZD6,CTHRC1,CELSR1,ATF4,PDGFB,PICK1,MFNG,MDFI,APBA1,ADGRF5,KIT,KDR,CXCL8,C5,TEAD2,MYCN,NOCT,NRP1,TAL1,LAMA4,GATA2,DNMT3A,ITGA8,ITGB4,GATA3,MYO7A,LMBR1,TIE1,SFRP1,FREM2,ATP11A,PCDH12,RIC8A,TCF15,RET,CHD7,IGF1,SMAD1,STOX2,SOX7,MEF2C,VEGFC,NDRG4,USH1C,PLCG1,HIPK1,DLL4,EDN1,ETS2,HDAC1,SEMA3F,HLX,SEC24B,PLXNA2,PROX1,VANGL2,LAMA5,MGAT1,DLG1,HOXD10,RNF2,TET1,ARNT2,BCOR,ITGA4,TBX1,RUNDC1,TEAD4,HIF1A,EPHA2,CECR2,LFNG,ALDH1A2,AR,EFNB1,PSEN1,HOXB5,HOXB6,HOXB7,HOXB8,HOXB9,ITGA3,EFNA1,VASH1,SMAD2,AMOT,JAG2,NRARP,NOTCH1,CMIP,ENG,TERF2,AFF3,VASP,SOX17,SOX18,LPAR6,MAFF,TGIF2,DUSP5,RALA,BMP2,PIFO,ST8SIA6	turquoise	EC_M6
GO:0071243	2.08E-01	0.920680984	4	18	cellular response to arsenic-containing substance	BP	9.29E-01	VCP,DHX36,ATF3,UROS	darkgreen	EC_M4
GO:0032288	1.78E-01	0.935776922	4	19	myelin assembly	BP	9.29E-01	MTMR2,ITGB4,TLR2,MPP5	turquoise	EC_M6

GO:0019884	1.78E-01	0.934692661	4	22	antigen processing and presentation of exogenous antigen	BP	9.29E-01	IKBKB,B2M,DNM2,AP3D1	turquoise	EC_M6
GO:0007589	2.09E-01	0.872797272	12	68	body fluid secretion	BP	9.30E-01	CDO1,SLC22A4,VEGFA,VAMP8,SLC4A5,ADA,CCND1,COPA,MED1,NPPB,NPR3,FGF10	darkgreen	EC_M4
GO:0061097	2.09E-01	0.869865449	13	74	regulation of protein tyrosine kinase activity	BP	9.30E-01	NCK2,SOCS5,SH3BP5L,PRNP,APP,TSG101,CBLB,DOK7,SRCIN1,SHC1,PTPN2,THY1,DUSP22	darkgreen	EC_M4
GO:0120192	2.09E-01	0.883642414	9	48	tight junction assembly	BP	9.31E-01	SRF,ROCK2,PDCD6IP,ACTG1,MTDH,TJP1,ROCK1,MYO1C,NPHP4	darkgreen	EC_M4
GO:0050868	1.79E-01	0.895740977	11	76	negative regulation of T cell activation	BP	9.33E-01	TNFRSF21,PELI1,ITCH,LAPTM5,HLX,CD86,DLG1,ADORA2A,ARG2,LGALS9,NRARP	turquoise	EC_M6
GO:0007205	1.80E-01	0.934388447	4	21	protein kinase C-activating G protein-coupled receptor signaling pathway	BP	9.33E-01	PICK1,DGKZ,EDN1,ADORA2A	turquoise	EC_M6
GO:0042177	1.80E-01	0.888025529	14	101	negative regulation of protein catabolic process	BP	9.33E-01	TRIM39,LAPTM4B,RGP1,SDCBP,TIMP4,FAM192A,SNCA,OPHN1,PSEN1,EFNA1,NOS2,USP7,DAB2IP,MGAT3	turquoise	EC_M6
GO:0051338	1.80E-01	0.850080601	86	706	regulation of transferase activity	BP	9.33E-01	TNFRSF11A,TNFAIP3,PTPRB,FLT4,TPD52L1,PTK2,PDGFB,TEK,KIT,KDR,SYK,TLR4,CCNY,RASIP1,PIK3CG,TAL1,CAB39L,RALB,PPARG,TIE1,SFRP1,HBEGF,SH3BP5,EPHB1,RET,MERTK,IGF1,HHEX,HMBOX1,WWTR1,VEGFC,MADD,SERINC5,NLRC5,DGKZ,CCNJL,NEDD9,EDN1,SNCA,DTNBP1,CD200,HYAL2,NCF1,FABP4,PABPN1,CD86,FGR,PPP2R5A,PROX1,PRKD1,ACE,PAK2,DLG1,MAP3K11,EIF4A2,LTF,ADORA2A,EPHB4,DDX3X,LDB2,TNFAIP8L3,LMTK2,PPM1F,EPHA2,TLR6,RGS14,MTOR,PSEN1,FGF18,TAOK2,EFNA1,CD19,PIK3R5,ADRB2,UNC119,TRAF2,DAB2IP,SLC8A3,RELN,ALKAL1,MMD,DAZAP2,COPS8,BMP2,DUSP7,PIFO	turquoise	EC_M6
GO:0006656	2.10E-01	0.908791934	5	24	phosphatidylcholine biosynthetic process	BP	9.34E-01	MFSD2A,CHPT1,PCYT1B,SLC44A2,PEMT	darkgreen	EC_M4
GO:0009199	2.10E-01	0.874776185	11	64	ribonucleoside triphosphate metabolic process	BP	9.34E-01	PPARA,VCP,RAB23,TGFB1,OPA1,ENTPD4,PPARGC1A,ENO1,GNAI3,TMSB4X,NME6	darkgreen	EC_M4
GO:0044272	2.10E-01	0.853521544	21	127	sulfur compound biosynthetic process	BP	9.34E-01	CDO1,ANGPT1,ACSL6,MTAP,SLC35B2,SLC7A11,CHPF2,B3GNT4,CHST2,CHAC1,CHSY1,HACD2,UGDH,CHST12,XYLT2,ACSL4,HAGH,HACD1,TPK1,ADI1,CHST11	darkgreen	EC_M4
GO:0034695	2.10E-01	0.920312129	4	17	response to prostaglandin E	BP	9.34E-01	ADCY6,AKT1,PRKAA1,GNB1	darkgreen	EC_M4
GO:1904738	1.81E-01	0.924714951	5	27	vascular associated smooth muscle cell migration	BP	9.34E-01	PDGFB,DOCK4,GRB10,MEF2C,IGFBP5	turquoise	EC_M6
GO:0060070	1.81E-01	0.86558995	33	250	canonical Wnt signaling pathway	BP	9.34E-01	FZD6,CTHRC1,TCF7,RNF146,CSNK1E,FZD8,CCNY,TRPM4,GATA3,SFRP1,NOTUM,FERMT1,HHEX,SOX7,WWTR1,LMBR1L,NR4A2,SOX13,PRDM15,HDAC1,BCL9,DDX3X,JUP,MCC,PSEN1,CCDC88C,TLE3,NRARP,NOTCH1,DAB2IP,SOX17,LYPD6,BMP2	turquoise	EC_M6
GO:0007622	1.81E-01	0.924279918	5	28	rhythmic behavior	BP	9.34E-01	NR1D2,CIART,ADORA2A,MTOR,CHRNB2	turquoise	EC_M6
GO:1990182	2.10E-01	0.919651507	4	18	exosomal secretion	BP	9.34E-01	RAB7A,PDCD6IP,TSG101,RAB11A	darkgreen	EC_M4
GO:0035855	2.10E-01	0.920209162	4	17	megakaryocyte development	BP	9.34E-01	SRF,PTPN11,PTPN6,MED1	darkgreen	EC_M4
GO:0030832	2.11E-01	0.846951878	26	160	regulation of actin filament length	BP	9.36E-01	FAM49B,NCK2,ACTR2,ACTR3,PLEKHG2,CDC42EP3,CXCL12,PFN2,RASA1,TWF1,MAGEL2,CTTN,LMOD1,PIK3CA,ARPC5,NCKAP1,ARPC2,CAPZB,WDR1,FLII,MYO1C,SPTAN1,TMSB4X,DSTN,COTL1,CFL2	darkgreen	EC_M4
GO:0051963	1.82E-01	0.889499038	13	79	regulation of synapse assembly	BP	9.37E-01	LINGO2,SEMA4D,SETD5,EPHB1,MEF2C,TLR2,SNCA,IL1RAP,SEMA4A,ABI3,CHRNB2,SLITRK2,ADGRL2	turquoise	EC_M6
GO:0010507	2.11E-01	0.871097574	12	67	negative regulation of autophagy	BP	9.37E-01	TAB2,LEP,EIF4G2,PTPN22,SEC22B,FEZ1,HTR2B,PIK3CA,SCFD1,QSOX1,AKT1,CLEC16A	darkgreen	EC_M4
GO:0051817	1.82E-01	0.897110518	10	67	modulation of process of other organism involved in symbiotic interaction	BP	9.37E-01	LRRC19,CAV2,ANO6,HDAC1,HYAL2,POU2F3,LTF,TNIP1,SERPINB9,TBC1D20	turquoise	EC_M6

GO:0090141	1.82E-01	0.933359838	4	20	positive regulation of mitochondrial fission	BP	9.37E-01	KDR,MCU,SPIRE1,RALA	turquoise	EC_M6
GO:0048609	1.82E-01	0.851001076	68	563	multicellular organismal reproductive process	BP	9.37E-01	TSPAN8,QKI,PICK1,KIT,FIGNL1,NOBOX,CREM,TUT4,SPATA6,DNMT3A,NECTIN2,TIMP4,EDNRB,DND1,GOLGA3,MERTK,IGF1,KDM3A,SMAD1,STOX2,HEXB,ANGPT2,DHH,KMT2D,SFMBT1,STC1,CDYL,EDN1,JAM3,GAS2,ETS1,TESMIN,PLEKHA1,ZNF148,OAZ3,ZSCAN2,ACE,LIMK2,ELL3,ETV6,RNF2,SSTR1,DDX3X,CDK16,BIRC3,USP42,KDM5A,SPIN2A,MTOR,AR,FBXW11,ITGA3,CCDC42,PIAS1,DNM2,YY1,CYP1A1,JAG2,LGALS9,SPEF2,SPIRE1,NOTCH1,STRBP,ARID4A,SOX17,TDRP,ANKRD49,TBC1D20	turquoise	EC_M6
GO:0007204	1.83E-01	0.869335003	26	194	positive regulation of cytosolic calcium ion concentration	BP	9.37E-01	TRDN,TRPM4,PIK3CG,GATA2,GPR4,CXCR4,EDNRB,P2RY6,P2RX2,CD36,PDE4D,CHD7,P2RX7,CXCL10,TPCN1,PLCG1,EDN1,SNCA,LCK,PRKD1,CCRL2,CD19,PLCG2,S1PR1,SLC8A3,LPAR6	turquoise	EC_M6
GO:0097305	2.12E-01	0.844526265	28	174	response to alcohol	BP	9.38E-01	CDO1,PPARA,CTNNA1,LEP,TGFBR1,ACTR2,INHBA,GLB1,TGFB1,CTNNB1,ATP2B1,ADCY6,PSMD14,CCND1,CPT1A,DAG1,NTRK3,ADCY5,FGFR2,SLC2A4,KCNMB1,CALM1,AKT1,PRKAA1,GNB1,SDF4,PEMT,CDKN1A	darkgreen	EC_M4
GO:0002040	2.12E-01	0.855190721	19	114	sprouting angiogenesis	BP	9.39E-01	ANGPT1,RSPO3,SRF,VEGFA,ITGB1,FGF2,STARD13,ROBO1,LOXL2,TJP1,LEF1,PDCD10,AKT3,NGFR,PKM,AKT1,CDH13,MIR24-1,PIK3C2A	darkgreen	EC_M4
GO:0019637	9.27E-03	0.993197776	104	743	organophosphate metabolic process	BP	9.39E-01	DGKA,PIP4K2C,GFPT2,CHKB,ABHD16A,AGPAT1,EP300,GPAA1,DNPH1,PIGO,TTC7A,SLC44A1,UGP2,LPIN1,SPHK2,ACSS1,TAMM41,PIP4P1,PNP,XDH,GPCPD1,PLPP5,PTDSS2,SMUG1,PIK3R1,ACSS2,NEIL2,DGUOK,PRKAG1,PLBD2,GPAT3,PIGT,PLA2G4B,ACOT8,OSBPL5,NT5C2,GPAM,CHKA,ABHD4,MTMR11,PIGM,IMPDH2,ARNT,CTPS2,HDAC4,DNAJC30,IDH1,ATIC,G6PC3,PHB2,ABHD12B,SMPD4,PLA2G16,PGLS,ABHD8,UQCC3,FOXK1,TECR,PLSCR3,PIGH,PIP4K2B,NAXE,PNPO,ACOT2,DPM3,GCDH,FLAD1,NUPR1,AC138894.1,PARP16,MTMR4,MOCOS,ACSM3,ACACA,INSR,MPPE1,MTMR1,PNPLA7,ATP5F1D,TAZ,JMJD8,PIGQ,INPP5E,PLPPR3,APRT,DPYD,KARS,FUK,VAC14,LCAT,TK2,MT-CO2,MT-ATP6,ABCA2,SULT1C4,ZBTB7A,NT5C,PLPP3,PID1,PDXK,NUDT2,UPP1,DNAJC19,RPIA	cyan	EC_M2
GO:0051346	1.83E-01	0.865240934	30	242	negative regulation of hydrolase activity	BP	9.40E-01	TNFAIP8,SEMA4D,C5,CST7,LRCH1,TIMP4,ITIH5,IKBKB,NR4A1,SH3RF1,SNCA,SH3BP4,RPS6KA1,F11R,PAK2,CST6,LTF,ADORA2A,DDX3X,BIRC3,CPEB2,RCC2,KDM5A,PAPLN,AMOT,CNN3,TERF2,DAB2IP,SERPINB9,BST2	turquoise	EC_M6
GO:1905517	1.84E-01	0.915246248	6	36	macrophage migration	BP	9.40E-01	PTK2,C5,EDNRB,CD200,MMP28,CCL2	turquoise	EC_M6
GO:0098727	1.84E-01	0.881543206	16	108	maintenance of cell number	BP	9.40E-01	KIT,TUT4,TAL1,GATA2,JAG1,SFRP1,TCF15,IGF1,KDM3A,MMP24,BCL9,PROX1,TET1,LDB2,NOTCH1,MTF2	turquoise	EC_M6
GO:0006664	1.84E-01	0.889862888	12	87	glycolipid metabolic process	BP	9.40E-01	KIT,PIGF,ST3GAL5,HEXB,ST3GAL6,B3GNT5,GAL3ST3,FUCA1,B4GALT6,ST6GALNAC3,GBA2,ST8SIA6	turquoise	EC_M6
GO:1903509	1.84E-01	0.889862888	12	87	liposaccharide metabolic process	BP	9.40E-01	KIT,PIGF,ST3GAL5,HEXB,ST3GAL6,B3GNT5,GAL3ST3,FUCA1,B4GALT6,ST6GALNAC3,GBA2,ST8SIA6	turquoise	EC_M6
GO:0051960	1.84E-01	0.856388518	46	344	regulation of nervous system development	BP	9.40E-01	SEMA6A,CDKL3,LINGO2,KIT,TNFRSF21,SEMA4D,IL6,MYCN,NRP1,MTMR2,CST7,CTSC,PLXND1,CXCR4,EPHB1,CHD7,EIF2AK3,TLR2,VEGFC,DLL4,HDAC1,PPP3CA,SEMA3F,PLXNA2,TRAK2,SS18L1,DLG1,ELL3,B2M,SEMA6D,IL1RAP,PRKCH,HIF1A,TNFRSF1B,RGS14,MTOR,PSEN1,SEMA4A,SEMA6B,SLITRK2,ARHGAP4,NOTCH1,ADGRL2,RELN,SHANK3,BMP2	turquoise	EC_M6
GO:1905517	2.12E-01	0.89202696	7	36	macrophage migration	BP	9.40E-01	AKIRIN1,P2RX4,CX3CL1,MMP14,CMKLR1,SLAMF8,MIR24-1	darkgreen	EC_M4
GO:0048016	1.84E-01	0.904013921	8	48	inositol phosphate-mediated signaling	BP	9.41E-01	IGF1,EDN1,PPP3CA,NFATC2,HOMER3,MTOR,PLCG2,NFAT5	turquoise	EC_M6

GO:0015031	1.84E-01	0.839165759	152	1301	protein transport	BP	9.41E-01	KIF5A,STX11,TBC1D30,SNX2,SNX24,ZDHHC14,RIMS2,FAM91A1,SGSM3,FAM53C,TSNARE1,PICK1,MICALL1,TBC1D22B,APBA1,UBAP1,CD2AP,SYK,TRAM2,ICA1,IL6,SNAP91,PPT1,VPS54,RSAD2,TLR4,TCAF2,TBC1D9,SDCBP,TRPM4,RAB3B,ANKRD50,RAB38,GGA3,LLGL2,USP6NL,ZFAND6,PPARG,TBC1D4,MYO7A,NDFIP2,FCHSD2,SFRP1,FERMT1,CD36,SIRT3,ADAMTS9,PRR5L,FAM3D,IL1A,IGF1,EXOC6,ERBIN,KIF13B,TLR2,VEGFC,MTX3,RAPGEF3,AP4B1,IFIT1,DOP1B,MX2,ABCG1,CD200,MTTP,HDAC1,PPP3CA,HYAL2,ANO1,ZDHHC6,ADPRH,SNAP23,LYST,LAPTM5,NCF1,SEC24B,HCLS1,AP1AR,TRAK2,ZDHHC18,PRKD1,RNF215,OAZ3,IL10RA,SENP2,DUSP16,APOD,ZW10,ADORA2A,RTP4,NECAP1,HOMER3,MCU,RUNDC1,CALCRL,RAMP2,CDK16,SNAP29,LMTK2,PPM1F,NUP88,TMEM30B,RAC1,HIF1A,JUP,RAB27A,SNCG,SYT7,BID,AP1S3,TBC1D1,RSC1A1,PSEN1,TAOK2,SLC51B,ZDHHC15,RABEP2,RAB3D,GPR68,SMAD2,DNM2,ATG4D,TBC1D8B,AP2B1,ABLIM3,SH3TC2,NOS2,UNC119,SPIRE1,MYO18A,MTM1,RPH3AL,SGSM2,AP3D1,NOTCH1,NUP214,TBC1D13,STXBP1,LRSAM1,RIPOR1,RASSF9,RAB43,RAB18,NUPL2,RAB32,STX3,E2F3,VAMP3,CACNG5,RAB39A,BMP6,ZDHHC22	turquoise	EC_M6
GO:0051496	1.85E-01	0.903990017	8	47	positive regulation of stress fiber assembly	BP	9.41E-01	LIMCH1,NRP1,SFRP1,RAPGEF3,PPM1F,RAC1,MTOR,ARHGEF15	turquoise	EC_M6
GO:0055078	1.85E-01	0.91476012	6	34	sodium ion homeostasis	BP	9.41E-01	EDNRB,IL1A,EDN1,ADORA2A,NPR1,SCNN1B	turquoise	EC_M6
GO:0019751	2.13E-01	0.862078938	15	88	polyol metabolic process	BP	9.42E-01	MIOX,LEP,INPP4A,FGF2,ACER3,DGAT2,IMPAD1,ITPK1,INPP5A,MOGAT3,TPI1,QDPR,SPTLC2,OCRL,PGP	darkgreen	EC_M4
GO:0030201	2.13E-01	0.898803891	6	29	heparan sulfate proteoglycan metabolic process	BP	9.43E-01	CTNNB1,SULF1,B3GAT3,UGDH,XYLT2,HS2ST1	darkgreen	EC_M4
GO:0003171	2.13E-01	0.907399974	5	23	atrioventricular valve development	BP	9.43E-01	HEY1,ACVR1,TGFB2,BMPR1A,OLFM1	darkgreen	EC_M4
GO:0048738	1.85E-01	0.867232969	26	186	cardiac muscle tissue development	BP	9.43E-01	PIM1,EFNB2,ADAMTS9,CHD7,IGF1,SMAD1,MEF2C,NDRG4,DLL4,EDN1,JARID2,SIK1,MEF2A,NRAP,PROX1,CREB1,ALDH1A2,MTOR,YY1,DSG2,NOTCH1,S1PR1,ENG,MIR23A,RGS4,BMP2	turquoise	EC_M6
GO:0001885	2.13E-01	0.87665941	10	53	endothelial cell development	BP	9.43E-01	VEGFA,COL15A1,ROCK2,RAPGEF2,TJP1,CLIC4,PLOD3,VCL,MSN,ROCK1	darkgreen	EC_M4
GO:1903556	2.13E-01	0.885377054	8	43	negative regulation of tumor necrosis factor superfamily cytokine production	BP	9.43E-01	AXL,CX3CL1,PTPN22,CHRNA7,SELENOS,PTPN6,SYT11,DICER1	darkgreen	EC_M4
GO:0014037	2.13E-01	0.891475726	7	35	Schwann cell differentiation	BP	9.44E-01	NAB2,AKT2,ILK,LGI4,DAG1,DICER1,AKT1	darkgreen	EC_M4
GO:0042306	1.86E-01	0.902723393	8	52	regulation of protein import into nucleus	BP	9.44E-01	FERMT1,CD36,HYAL2,HCLS1,PRKD1,APOD,JUP,PSEN1	turquoise	EC_M6
GO:0090090	1.86E-01	0.879691912	16	113	negative regulation of canonical Wnt signaling pathway	BP	9.44E-01	FZD6,CTHRC1,SFRP1,NOTUM,FERMT1,WWTR1,LMBR1L,SOX13,HDAC1,MCC,CCDC88C,TLE3,NOTCH1,DAB2IP,SOX17,BMP2	turquoise	EC_M6
GO:0000768	1.86E-01	0.913910908	6	34	syncytium formation by plasma membrane fusion	BP	9.45E-01	CXCL10,RAPGEF3,TANC1,NFATC2,PLEKHO1,CFLAR	turquoise	EC_M6
GO:0140253	1.86E-01	0.913910908	6	34	cell-cell fusion	BP	9.45E-01	CXCL10,RAPGEF3,TANC1,NFATC2,PLEKHO1,CFLAR	turquoise	EC_M6
GO:0052372	2.14E-01	0.897947434	6	30	modulation by symbiont of entry into host	BP	9.45E-01	LY6E,LGALS1,PTX3,ITGAV,CD74,FCN1	darkgreen	EC_M4
GO:0006406	1.87E-01	0.901928641	8	53	mRNA export from nucleus	BP	9.45E-01	HHEX,ZC3H11A,PABPN1,SMG7,THOC3,NUP88,NUP214,FYTTD1	turquoise	EC_M6
GO:0071427	1.87E-01	0.901928641	8	53	mRNA-containing ribonucleoprotein complex export from nucleus	BP	9.45E-01	HHEX,ZC3H11A,PABPN1,SMG7,THOC3,NUP88,NUP214,FYTTD1	turquoise	EC_M6
GO:0051893	1.87E-01	0.894425796	10	60	regulation of focal adhesion assembly	BP	9.45E-01	PTK2,TEK,LIMCH1,KDR,NRP1,SFRP1,APOD,PPM1F,RAC1,RCC2	turquoise	EC_M6
GO:0090109	1.87E-01	0.894425796	10	60	regulation of cell-substrate junction assembly	BP	9.45E-01	PTK2,TEK,LIMCH1,KDR,NRP1,SFRP1,APOD,PPM1F,RAC1,RCC2	turquoise	EC_M6

GO:0008207	1.87E-01	0.93032576	4	22	C21-steroid hormone metabolic process	BP	9.45E-01	SCNN1B,BMP2,BMP6,AKR1D1	turquoise	EC_M6
GO:0072006	2.14E-01	0.851993304	20	118	nephron development	BP	9.45E-01	AHI1,ANGPT1,VEGFA,GLI3,FGF2,SEC61A1,CTNNB1,ILK,SULF1,PKD2,NOTCH2,BMP7,IQGAP1,SIX4,ACTA2,MAGED1,MYO1E,PDGFRB,PKD1,GDNF	darkgreen	EC_M4
GO:0022612	1.87E-01	0.882734904	14	95	gland morphogenesis	BP	9.46E-01	TNFAIP3,IL6,NRP1,PLXND1,SFRP1,NTN4,CFLAR,RPS6KA1,PROX1,LAMA5,IGFBP5,EPHA2,AR,NOTCH1	turquoise	EC_M6
GO:0050900	2.14E-01	0.834716547	38	245	leukocyte migration	BP	9.46E-01	SLC12A2,MYH9,LEP,VEGFA,DOCK8,CREB3,AKIRIN1,ITGB1,CXCL12,CHST2,P2RX4,APP,CX3CL1,ADA,DNM1L,SBDS,TGFB2,MMP14,CMKLR1,SLAMF8,SRP54,SERPINE1,ITGA2B,NBL1,WDR1,MSN,GP2,CD74,ROCK1,AKT1,ITGA2,TNFRSF18,BSG,MIR24-1,CCL16,THY1,ARTN,CCR6	darkgreen	EC_M4
GO:0051279	1.87E-01	0.897318751	9	58	regulation of release of sequestered calcium ion into cytosol	BP	9.47E-01	TRDN,P2RY6,PDE4D,CHD7,CXCL10,PLCG1,SNCA,PRKD1,CD19	turquoise	EC_M6
GO:0042981	1.88E-01	0.837846384	126	1092	regulation of apoptotic process	BP	9.47E-01	TNFAIP8,TNFAIP3,TRIM39,AKAP12,FLT4,OXR1,TPD52L1,PTK2,DAPK1,ATF4,PIM1,TEK,KDR,GADD45G,SEMA4D,IL6,PPT1,UTP11,FIGNL1,KLF11,TRAF1,NRP1,PIK3CG,GATA2,CTSC,NOS3,ZFAND6,PPARG,GATA3,EDNRB,NET1,TMC8,SFRP1,RPL26,ANKLE2,ARHGEF7,PLK2,RET,MERTK,IL1A,IGF1,NR4A1,EIF2AK3,ITCH,SH3RF1,HIP1R,MEF2C,IL7,CXCL10,MADD,PLCG1,NR4A2,ANO6,IFIT2,ADAMTS20,EDN1,STAT1,SNCA,NUAK2,RNF144B,SIK1,HDAC1,ITPRIP,LCK,HYAL2,DYRK3,HCLS1,INPP5D,CFLAR,CASP10,IGFBP3,RPS6KA1,DDX42,ITGA6,PAK2,ELL3,MAP3K11,CREB1,LTF,ADORA2A,ARNT2,ITGA4,DDX3X,TBX1,ING4,RAMP2,BIRC3,TNFAIP8L3,USP42,PRKCH,HIF1A,ITPKB,BID,TNFRSF1B,ARG2,ALDH1A2,MTOR,AR,BNIP2,PSEN1,DAPK2,EFNA1,PIAS1,DNM2,CCL2,LGALS9,IL7R,BFAR,STK26,MYO18A,TNFAIP8L1,TRAF2,TNFRSF25,NOTCH1,STXBP1,DAB2IP,CDH5,MIR23A,VNN1,PMAIP1,TRIM24,SERPINB9,E2F3,BMP2,IFIT3,NMNAT1	turquoise	EC_M6
GO:0032928	2.15E-01	0.917325516	4	18	regulation of superoxide anion generation	BP	9.47E-01	TGFB1,MAPT,AKT1,GNAI3	darkgreen	EC_M4
GO:0046486	1.88E-01	0.855390494	40	317	glycerolipid metabolic process	BP	9.48E-01	SYNJ2,SERAC1,PDGFB,PIP5K1B,AGMO,PIGF,INPP4B,PIK3CG,MTMR2,MGLL,RAB38,PGS1,GPAT4,CDS2,SCARB1,ABHD6,PITPNM2,CHRM5,GPD1,SERINC5,PLCB2,PLCG1,DGKZ,PIK3C2B,MTMR10,LPCAT1,MTTP,SIK1,PLA1A,INPP5D,BMX,INPP5F,PLD2,ITPKB,GDPD3,GDPD1,MTMR12,MTM1,PLCG2,PLA2G4E	turquoise	EC_M6
GO:0030890	2.15E-01	0.89734268	6	30	positive regulation of B cell proliferation	BP	9.48E-01	IRS2,ADA,SLC39A10,TFRC,CD74,CDKN1A	darkgreen	EC_M4
GO:0032768	1.88E-01	0.90623018	7	43	regulation of monooxygenase activity	BP	9.48E-01	SCARB1,IL1A,SNCA,FGF23,HIF1A,TERF2,FCER2	turquoise	EC_M6
GO:0052372	1.88E-01	0.919641741	5	30	modulation by symbiont of entry into host	BP	9.48E-01	CXCL8,NECTIN2,LGALS9,TRIM34,TRIM62	turquoise	EC_M6
GO:0045670	2.15E-01	0.884486036	8	42	regulation of osteoclast differentiation	BP	9.48E-01	IL23A,CTNNB1,NOTCH2,TCTA,GPR137,FBN1,ATP6AP1,FSTL3	darkgreen	EC_M4
GO:0032677	2.15E-01	0.874960869	10	56	regulation of interleukin-8 production	BP	9.49E-01	LEP,PTPN22,TLR5,SERPINE1,CD74,PARK7,F3,BCL10,TMSB4X,FCN1	darkgreen	EC_M4
GO:0018198	2.15E-01	0.884088771	8	43	peptidyl-cysteine modification	BP	9.49E-01	SNTA1,ATP2B4,ZDHHC4,ZDHHC9,PARK7,DMD,RAB6A,TXN	darkgreen	EC_M4
GO:0010821	9.43E-03	0.995283624	23	124	regulation of mitochondrion organization	BP	9.49E-01	SQSTM1,TSPO,EP300,DDHD2,HGF,CAMKK2,HTRA2,GPX1,FIS1,CHCHD10,VAT1,DDHD1,HDAC6,ATP13A2,PLSCR3,SREBF1,ACAA2,RHOT2,ZNF205,NOL3,PID1,SLC35F6,BCL2L2	cyan	EC_M2
GO:1904894	1.89E-01	0.919644971	5	28	positive regulation of receptor signaling pathway via STAT	BP	9.50E-01	KIT,IL6,IL10RA,IL7R,NOTCH1	turquoise	EC_M6
GO:0032689	2.16E-01	0.905430356	5	24	negative regulation of interferon-gamma production	BP	9.50E-01	INHBA,AXL,PRNP,IL20RB,MIR24-1	darkgreen	EC_M4
GO:0046651	2.16E-01	0.840678811	28	177	lymphocyte proliferation	BP	9.51E-01	IL23A,LEP,DOCK8,NCK2,FKBP1B,CTNNB1,IRS2,PRNP,LMO1,IL20RB,CD180,PTPN22,ADA,CBLB,SLC39A10,PRKAR1A,LEF1,NCSTN,TFRC,PTPN6,IGFBP2,MSN,DOCK2,CD74,CD40LG,CD151,CDKN1A,FGF10	darkgreen	EC_M4

GO:0110151	2.16E-01	0.883708309	8	42	positive regulation of biomineralization	BP	9.51E-01	FBN2,TGFB1,ATP2B1,ACVR1,BMP7,FZD9,BMPR1A,TGFB3	darkgreen	EC_M4
GO:1902993	2.16E-01	0.916478718	4	18	positive regulation of amyloid precursor protein catabolic process	BP	9.51E-01	ROCK2,GSAP,PICALM,CHRNA7	darkgreen	EC_M4
GO:0032418	2.16E-01	0.870355759	11	62	lysosome localization	BP	9.52E-01	MYH9,KIF5B,FAM98A,VAMP8,HPS6,KXD1,SYTL4,RAB34,VAMP7,DEF8,BLOC1S2	darkgreen	EC_M4
GO:0003180	2.17E-01	0.896524438	6	29	aortic valve morphogenesis	BP	9.53E-01	ROCK2,RB1,TGFB1,ROBO1,HEY1,ROCK1	darkgreen	EC_M4
GO:0009226	2.17E-01	0.916142014	4	18	nucleotide-sugar biosynthetic process	BP	9.53E-01	NANS,GFPT1,UGDH,MPI	darkgreen	EC_M4
GO:0046636	1.90E-01	0.918676864	5	29	negative regulation of alpha-beta T cell activation	BP	9.53E-01	ITCH,HLX,ADORA2A,ARG2,LGALS9	turquoise	EC_M6
GO:0002790	1.91E-01	0.865811685	23	172	peptide secretion	BP	9.53E-01	RIMS2,PICK1,ICA1,IL6,TRPM4,SFRP1,SIRT3,CHD7,FAM3D,EDN1,ABCG1,HDAC1,PPP3CA,ANO1,MCU,CDK16,RAC1,HIF1A,SYT7,GPR68,SMAD2,NOS2,EXOC3L1	turquoise	EC_M6
GO:0097150	1.91E-01	0.929906919	4	18	neuronal stem cell population maintenance	BP	9.53E-01	JAG1,MMP24,PROX1,NOTCH1	turquoise	EC_M6
GO:0007187	1.91E-01	0.928312654	4	22	G protein-coupled receptor signaling pathway, coupled to cyclic nucleotide second messenger	BP	9.53E-01	CHRM5,SSTR1,CALCRL,CCL2	turquoise	EC_M6
GO:0002347	1.91E-01	0.928187857	4	22	response to tumor cell	BP	9.54E-01	DAPK1,NECTIN2,LAPTM5,ABI3	turquoise	EC_M6
GO:0043586	2.17E-01	0.915861261	4	18	tongue development	BP	9.55E-01	GLI3,CTNNB1,LEF1,SIX4	darkgreen	EC_M4
GO:0046596	2.17E-01	0.904473436	5	24	regulation of viral entry into host cell	BP	9.55E-01	LY6E,LGALS1,PTX3,CD74,FCN1	darkgreen	EC_M4
GO:0061333	1.91E-01	0.891023988	10	63	renal tubule morphogenesis	BP	9.55E-01	MTSS1,KLHL3,GATA3,GZF1,MEF2C,LAMA5,DLG1,WNK4,HOXB7,BMP2	turquoise	EC_M6
GO:0052126	2.18E-01	0.847489096	21	127	movement in host environment	BP	9.56E-01	LY6E,LGALS1,ITGB1,RAB7A,AXL,SIGLEC1,SMPD1,VAMP8,PTX3,TSG101,VPS18,DAG1,ITGB5,NECTIN4,TFRC,ITGAV,SLC3A2,CD74,ITGA2,BSG,FCN1	darkgreen	EC_M4
GO:0006611	2.18E-01	0.877450493	9	49	protein export from nucleus	BP	9.56E-01	TGFB1,PTPN11,CHP1,GSK3B,SMURF1,PKD1,PARK7,AHCYL1,TXN	darkgreen	EC_M4
GO:0032729	1.92E-01	0.910485711	6	35	positive regulation of interferon-gamma production	BP	9.56E-01	TLR4,PDE4D,PDE4B,LGALS9,ISG15,IRF8	turquoise	EC_M6
GO:0003044	1.92E-01	0.917610674	5	30	regulation of systemic arterial blood pressure mediated by a chemical signal	BP	9.56E-01	NOS3,PDE4D,EDN1,ACE,ADRB2	turquoise	EC_M6
GO:0060914	1.92E-01	0.918303805	5	27	heart formation	BP	9.56E-01	PIM1,MEF2C,NOTCH1,SOX17,BMP2	turquoise	EC_M6
GO:0060828	1.92E-01	0.859922647	28	210	regulation of canonical Wnt signaling pathway	BP	9.56E-01	FZD6,CTHRC1,RNF146,CSNK1E,CCNY,TRPM4,SFRP1,NOTUM,FERMT1,HHEX,SOX7,WWTR1,LMBR1L,SOX13,PRDM15,HDAC1,DDX3X,JUP,MCC,PSEN1,CCDC88C,TLE3,NRARP,NOTCH1,DAB2IP,SOX17,LYPD6,BMP2	turquoise	EC_M6
GO:0030866	2.18E-01	0.88830678	7	35	cortical actin cytoskeleton organization	BP	9.57E-01	ROCK2,PDCD6IP,CAVIN3,EPB41L1,RTKN,NCKAP1,ROCK1	darkgreen	EC_M4
GO:0002431	1.93E-01	0.917871761	5	27	Fc receptor mediated stimulatory signaling pathway	BP	9.58E-01	PTK2,SYK,FGR,PLD2,YES1	turquoise	EC_M6
GO:0050906	1.93E-01	0.897928254	8	52	detection of stimulus involved in sensory perception	BP	9.58E-01	KIT,GRIK2,CXCR4,EPHB1,MMP24,ANO1,RTP4,PIEZO2	turquoise	EC_M6
GO:0050805	1.93E-01	0.90346744	7	42	negative regulation of synaptic transmission	BP	9.58E-01	PICK1,MTMR2,PLK2,ABHD6,SHANK2,STXBP1,SHANK3	turquoise	EC_M6

GO:0006357	9.59E-03	0.992266211	222	1784	regulation of transcription by RNA polymerase II	BP	9.58E-01	NFATC1,ZNF236,TSHZ1,SMARCC2,STAT6,CDK4,CITED2,MAML1,SQSTM1,OSR2,EHMT2,ZBTB12,HDAC10,ATF6B,RXRB,ZHX2,ZBTB22,MYC,SMAD5,EP300,KDM3B,ZNF76,DDX17,NFIB,HSF1,CCND3,TRERF1,HMOX1,WASL,KLF9,ZBTB45,ZNF135,ZNF667,ZNF865,ZNF692,ANXA4,AKNA,MED25,IL4I1,IRF3,LPIN1,FOXO3,KLF15,NOP53,MAML2,BBS7,MEIS3,IRF2BP1,SIX5,FOSB,ZNF746,NR2C2,TSC22D1,ZNF575,ZNF574,WBP2,KMT2C,TADA3,EID2,ZNF219,MED29,SIRT2,HGF,APBB1,DEAF1,HOXC4,LIN37,RARG,SDR16C5,ZNF740,LMO2,URI1,PIK3R1,NCOA2,ATF1,KDM2B,ZFHX4,MCPH1,SSBP2,FNIP2,SHOX2,ZHX3,VDR,LPIN3,FOXF2,F2RL1,SORBS3,HNF4A,HR,ELK4,SP140,TCF7L2,TXNIP,SOX5,ADNP,ZNF217,ECM1,RCOR3,ARNT,GABPB2,PER2,TWIST2,VPS72,HDAC4,USF1,SMARCD2,POGZ,PATZ1,RNASEL,MAGEF1,KAT5,LRP6,YBX3,NFKBIA,NFE2L2,HEXIM2,BATF2,MEN1,TSC22D4,CTDSP1,EID1,SSBP4,NELFA,ZSCAN25,ZNF394,TNFRSF1A,STK16,STAT5A,KAT6B,CREG1,NR2F6,CAMTA2,TFE3,PELP1,ZNF12,WIZ,ZBTB25,RARA,FOXK1,AC090517.4,ZBTB17,GPS2,THRA,KLF3,MED24,PRDM2,CC2D1A,ZNF771,MLLT6,CTBP1,ZNF646,CREBRF,CDK5RAP3,GLMP,KAT8,CHD3,KAT7,PER1,FOS,ZBTB7B,IRF2BPL,EBF1,KANK2,CRTC2,VEZF1,PPM1D,SIN3A,ZNF358,PPARGC1B,SREBF1,ZNF414,EFCAB7,RFX2,ZNF449,GLIS2,TFAP4,CREBBP,DVL1,TRPV1,TCF3,PER3,ZNF276,RXRA,ZFP90,THAP11,E2F4,ABCA2,JUND,ZNF205,HEXIM1,ZNF444,ZBTB7A,AHRR,FOXJ2,ZNF580,RPL23,GLI4,REPIN1,ZNF688,ZNF432,SIX1,ZNF48,ZNF768,TSC22D3,ZNF45,ZNF333,ZNF514,ZNF74,ZNF879,PID1,ZNF250,ZBTB47,ZNF524,ZNF84,CEBPB,NSMCE3,MZF1,ZNF446,ZNF239,GTF2H4,	cyan	EC_M2
GO:0046427	2.19E-01	0.903704705	5	24	positive regulation of receptor signaling pathway via JAK-STAT	BP	9.59E-01	IL23A,LEP,ADIPOR1,CRLF3,OCIAD2	darkgreen	EC_M4
GO:0043039	6.69E-02	0.974409243	7	40	tRNA aminoacylation	BP	9.59E-01	NARS,DARS,LARS,YARS,IARS2,DARS2,RARS	darkgrey	EC_M8
GO:0019217	2.19E-01	0.868607096	11	62	regulation of fatty acid metabolic process	BP	9.59E-01	PPARA,PPARD,MFSD2A,DGAT2,AKT2,IRS2,CPT1A,WDTC1,PPARGC1A,CD74,AKT1	darkgreen	EC_M4
GO:0051452	2.19E-01	0.887666324	7	36	intracellular pH reduction	BP	9.59E-01	RAB7A,SLAMF8,CLIC4,ATP6AP2,CLN6,ATP6V0D1,ATP6V0E1	darkgreen	EC_M4
GO:0050871	1.93E-01	0.893098636	9	61	positive regulation of B cell activation	BP	9.59E-01	SYK,IL6,PELI1,TLR4,MEF2C,IL7,NFATC2,INPP5D,CHRNB2	turquoise	EC_M6
GO:0045933	2.19E-01	0.894505748	6	30	positive regulation of muscle contraction	BP	9.60E-01	SRF,ADA,CTTN,MYOCD,ITGA2,ENO1	darkgreen	EC_M4
GO:1901616	2.19E-01	0.871898744	10	56	organic hydroxy compound catabolic process	BP	9.60E-01	HSD17B6,MIOX,SNX17,IMPAD1,INPP5A,COMT,MAOA,TPI1,CYP46A1,OCRL	darkgreen	EC_M4
GO:0071216	2.19E-01	0.840887958	25	157	cellular response to biotic stimulus	BP	9.60E-01	FAM49B,PPARD,PLAA,TGFB1,AXL,ANKRD1,CD180,CX3CL1,PTPN22,MTDH,CDC73,SELENOS,GSK3B,PRKCA,TMCO1,SERPINE1,UPF1,SGMS1,PPARGC1A,AKT1,LDOC1,IRAK1,HSPA5,BCL10,IL36G	darkgreen	EC_M4
GO:0001756	1.94E-01	0.897714864	8	48	somitogenesis	BP	9.62E-01	SFRP1,TCF15,PLXNA2,LFNG,ALDH1A2,PSEN1,NRARP,NOTCH1	turquoise	EC_M6
GO:1902686	2.20E-01	0.893779853	6	31	mitochondrial outer membrane permeabilization involved in programmed cell death	BP	9.62E-01	EYA2,GSK3B,FZD9,VDAC2,BNIP3,BLOC1S2	darkgreen	EC_M4
GO:0034205	1.94E-01	0.902305798	7	44	amyloid-beta formation	BP	9.63E-01	IFNGR1,GGA3,IGF1,DYRK1A,ABCG1,PSEN1,EFNA1	turquoise	EC_M6
GO:0032148	2.20E-01	0.902702061	5	24	activation of protein kinase B activity	BP	9.63E-01	OSBPL8,NTRK3,ADRA2C,PARK7,TXN	darkgreen	EC_M4
GO:0045601	2.21E-01	0.893760947	6	29	regulation of endothelial cell differentiation	BP	9.64E-01	VEGFA,ROCK2,CTNNB1,VCL,TMEM100,ROCK1	darkgreen	EC_M4
GO:0033273	2.21E-01	0.860725091	13	75	response to vitamin	BP	9.64E-01	PPARD,LEP,FKBP1B,ATP2B1,MAP1B,ADA,SPP1,CCND1,BMP7,MED1,COL1A1,ITGA2,PEMT	darkgreen	EC_M4
GO:0035088	1.95E-01	0.902147318	7	42	establishment or maintenance of apical/basal cell polarity	BP	9.65E-01	SYNE4,TCF15,ERBIN,DLG1,FSCN1,OPHN1,PATJ	turquoise	EC_M6
GO:0061245	1.95E-01	0.902147318	7	42	establishment or maintenance of bipolar cell polarity	BP	9.65E-01	SYNE4,TCF15,ERBIN,DLG1,FSCN1,OPHN1,PATJ	turquoise	EC_M6
GO:0035767	2.21E-01	0.902235391	5	24	endothelial cell chemotaxis	BP	9.65E-01	VEGFA,FGF2,P2RX4,HSPB1,TMSB4X	darkgreen	EC_M4

GO:0060538	1.95E-01	0.868829506	18	132	skeletal muscle organ development	BP	9.65E-01	CAV2,SVIL,NR1D2,P2RX2,LMOD3,EPHB1,NR4A1,MEF2C,TLL2,PPP3CA,BCL9,HLX,CFLAR,HOXD10,TBX1,ZBTB18,NOTCH1,MAFF	turquoise	EC_M6
GO:0048002	1.95E-01	0.915607963	5	30	antigen processing and presentation of peptide antigen	BP	9.65E-01	IKBKB,ACE,B2M,DNM2,ERAP2	turquoise	EC_M6
GO:0006509	2.21E-01	0.886002268	7	36	membrane protein ectodomain proteolysis	BP	9.65E-01	MYH9,DAG1,NCSTN,BACE1,TIMP1,ROCK1,TIMP3	darkgreen	EC_M4
GO:0001912	2.21E-01	0.902059983	5	24	positive regulation of leukocyte mediated cytotoxicity	BP	9.65E-01	IL23A,STX7,HLA-DRA,FAM49B,AP1G1	darkgreen	EC_M4
GO:0006688	2.22E-01	0.901943231	5	24	glycosphingolipid biosynthetic process	BP	9.65E-01	B4GALNT1,LARGE1,ST8SIA4,B3GALT1,PRKAA1	darkgreen	EC_M4
GO:0086002	2.22E-01	0.880048127	8	41	cardiac muscle cell action potential involved in contraction	BP	9.65E-01	SCN5A,GPD1L,SNTA1,CACNA1D,PKP2,CACNA1C,CACNA1G,FLNA	darkgreen	EC_M4
GO:0048663	1.96E-01	0.908225061	6	34	neuron fate commitment	BP	9.66E-01	GATA2,DLL4,HOXD10,ZNF521,JAG2,NOTCH1	turquoise	EC_M6
GO:0061339	2.22E-01	0.902242692	5	23	establishment or maintenance of monopolar cell polarity	BP	9.66E-01	FAT1,MAP1B,PRKCI,MSN,TTC8	darkgreen	EC_M4
GO:0042311	2.22E-01	0.892364008	6	31	vasodilation	BP	9.66E-01	PPARD,PLOD3,NPPB,KCNMB1,NPR3,PRKG1	darkgreen	EC_M4
GO:1902254	2.22E-01	0.913119708	4	18	negative regulation of intrinsic apoptotic signaling pathway by p53 class mediator	BP	9.66E-01	RRM2B,TAF9B,CD74,PTTG1IP	darkgreen	EC_M4
GO:0030101	2.22E-01	0.879254757	8	43	natural killer cell activation	BP	9.66E-01	IL23A,LEP,AXL,IL15RA,PTPN22,SNX27,VAMP7,AP1G1	darkgreen	EC_M4
GO:0008053	2.22E-01	0.901511341	5	24	mitochondrial fusion	BP	9.66E-01	OPA1,DNM1L,TFRC,OMA1,BNIP3	darkgreen	EC_M4
GO:0043330	1.96E-01	0.915054852	5	30	response to exogenous dsRNA	BP	9.67E-01	DDX58,RALB,TMEM173,IFIT1,RFTN2	turquoise	EC_M6
GO:0051208	1.96E-01	0.878264676	13	87	sequestering of calcium ion	BP	9.67E-01	TRDN,P2RY6,PDE4D,CHD7,CXCL10,TPCN1,PLCG1,SNCA,LCK,PRKD1,PSEN1,CD19,PLCG2	turquoise	EC_M6
GO:0001782	1.97E-01	0.915165146	5	29	B cell homeostasis	BP	9.67E-01	TNFAIP3,GAPT,MEF2C,SOS2,HIF1A	turquoise	EC_M6
GO:0001738	1.97E-01	0.878204004	13	84	morphogenesis of a polarized epithelium	BP	9.67E-01	FZD6,CTHRC1,CELSR1,SFRP1,SPEF1,SYNE4,TCF15,SEC24B,JHY,VANGL2,LAMA5,RAC1,OPHN1	turquoise	EC_M6
GO:0051353	2.23E-01	0.873744206	9	50	positive regulation of oxidoreductase activity	BP	9.67E-01	NUS1,HTR2B,KRAS,CALM1,AKT1,NPR3,PARK7,GNAI3,GDNF	darkgreen	EC_M4
GO:0003148	2.23E-01	0.901600659	5	23	outflow tract septum morphogenesis	BP	9.67E-01	SEMA3C,ROBO1,TGFB2,FGFR2,BMPR1A	darkgreen	EC_M4
GO:0021987	2.23E-01	0.854215917	15	87	cerebral cortex development	BP	9.67E-01	LRP1,TRAPPC9,SUN2,MDGA1,RTN4,GLI3,CTNNB1,TACC1,ROBO1,CELF1,GSK3B,COL3A1,PAFAH1B1,FLNA,BNIP3	darkgreen	EC_M4
GO:0045859	1.97E-01	0.839131397	63	513	regulation of protein kinase activity	BP	9.67E-01	TNFRSF11A,TNFAIP3,PTPRB,TPD52L1,PTK2,PDGFB,KIT,SYK,TLR4,CCNY,RASIP1,PIK3CG,TAL1,CAB39L,RALB,PPARG,SFRP1,HBEGF,SH3BP5,IGF1,HHEX,WWTR1,VEGFC,MADD,CCNJL,NEDD9,EDN1,SNCA,DTNBP1,HYAL2,NCF1,FABP4,CD86,FGR,PROX1,ACE,PAK2,DLG1,MAP3K11,LTF,ADORA2A,DDX3X,PPM1F,TLR6,RGS14,MTOR,PSEN1,FGF18,TAOK2,EFNA1,PIK3R5,ADRB2,UNC119,TRAF2,DAB2IP,SLC8A3,RELN,ALKAL1,MMD,DAZAP2,COPS8,BMP2,DUSP7	turquoise	EC_M6
GO:0008206	1.97E-01	0.906845324	6	36	bile acid metabolic process	BP	9.67E-01	KIT,OSBPL9,PROX1,OSBPL6,GBA2,AKR1D1	turquoise	EC_M6
GO:0043122	2.23E-01	0.832021087	31	197	regulation of I-kappaB kinase/NF-kappaB signaling	BP	9.68E-01	TAB2,ANGPT1,LGALS1,SLC35B2,MAP3K7,CTNNB1,CANT1,CXXC5,ATP2C1,TMEM9B,SLC20A1,DHX36,CX3CL1,MTDH,TANK,HTR2B,GOLT1B,TFRC,HSPB1,TSPAN6,SLC44A2,CD74,IRAK1,FLNA,IKBKG,USP10,BCL10,WLS,FKBP1A,C18orf32,EDA	darkgreen	EC_M4
GO:1903747	2.23E-01	0.878555954	8	43	regulation of establishment of protein localization to mitochondrion	BP	9.68E-01	LMAN1,ATG13,BAP1,MAPT,C11orf65,PRKAA1,LEPROT,TOMM7	darkgreen	EC_M4

GO:0016486	2.24E-01	0.912242103	4	18	peptide hormone processing	BP	9.69E-01	PCSK1,PCSK7,ATP6AP2,ECE1	darkgreen	EC_M4
GO:0140448	2.24E-01	0.912242103	4	18	signaling receptor ligand precursor processing	BP	9.69E-01	PCSK1,PCSK7,ATP6AP2,ECE1	darkgreen	EC_M4
GO:1904292	2.24E-01	0.912198186	4	18	regulation of ERAD pathway	BP	9.69E-01	UBQLN1,USP14,BCAP31,TMEM259	darkgreen	EC_M4
GO:0050868	2.24E-01	0.858324298	13	76	negative regulation of T cell activation	BP	9.69E-01	SOCS5,GLI3,PRNP,IL20RB,LOXL3,PTPN22,RUNX1,CBLB,PRKAR1A,PTPN6,CD74,PTPN2,FGL2	darkgreen	EC_M4
GO:0110020	2.24E-01	0.853393132	15	86	regulation of actomyosin structure organization	BP	9.69E-01	TGFBR1,ROCK2,MYLK3,PAK1,ACTG1,PFN2,PHLDB2,TJP1,SYNPO2L,CGNL1,TPM1,TGFB3,GPR65,TTC8,ROCK1	darkgreen	EC_M4
GO:0007628	1.98E-01	0.925281558	4	20	adult walking behavior	BP	9.69E-01	OXR1,AGTPBP1,SPTBN4,CHD7	turquoise	EC_M6
GO:0007276	6.80E-02	0.949796638	54	469	gamete generation	BP	9.70E-01	YTHDC2,FBXO5,HSF2,KIFC1,CDC25C,FANCC,TUT7,SRPK1,UBR2,FOXJ3,MSH2,YTHDC1,TTK,CEP57,WDR33,ACOX1,XRN2,METTL3,BRCA2,CCNB1,RACGAP1,RPS6,KNL1,BMPR1B,KIF18A,NSUN2,SNRPA1,KHDRBS1,TRIP13,ASPM,SPO11,TIAL1,DDX6,SEPT2,FOSL1,ZFX,DDX20,RPL10L,C17orf53,TOP2A,ASF1B,CCNB2,ATRX,PTTG1,PLK1,BRIP1,NDC1,DAZAP1,FANCA,ODF2,HIST1H1A,AURKA,RNF8,PAIP2	darkgrey	EC_M8
GO:0043624	6.81E-02	0.96080802	18	119	cellular protein complex disassembly	BP	9.71E-01	KIF24,CAPZA2,CLASP2,KIF2C,CKAP2,TPX2,ABCE1,CKAP5,STMND1,KIF18A,CAMSAP2,CFL1,HDGFL3,KIF18B,GSPT1,TAOK1,TMOD3,STMN1	darkgrey	EC_M8
GO:0006949	1.99E-01	0.906220352	6	35	syncytium formation	BP	9.71E-01	CXCL10,RAPGEF3,TANC1,NFATC2,PLEKHO1,CFLAR	turquoise	EC_M6
GO:0003177	1.99E-01	0.925224779	4	19	pulmonary valve development	BP	9.71E-01	NOS3,JAG1,TNFRSF1B,NOTCH1	turquoise	EC_M6
GO:0048260	1.99E-01	0.899778084	7	41	positive regulation of receptor-mediated endocytosis	BP	9.71E-01	PICK1,SYK,PPT1,B2M,SELE,DNM2,PLCG2	turquoise	EC_M6
GO:0001942	2.25E-01	0.864274843	11	61	hair follicle development	BP	9.72E-01	INHBA,GLI2,CTNNB1,SPINK5,TRPC4AP,TGFB2,FGFR2,GORAB,NGFR,EDA,FGF10	darkgreen	EC_M4
GO:0120254	1.99E-01	0.878559208	12	86	olefinic compound metabolic process	BP	9.72E-01	RDH16,ALDH8A1,HSD17B3,MGLL,ABHD6,ALDH1A2,SCNN1B,CYP1A1,PTGS1,BMP2,SRD5A2,BMP6	turquoise	EC_M6
GO:0044273	2.25E-01	0.899400593	5	25	sulfur compound catabolic process	BP	9.72E-01	CDO1,GLB1,CHAC1,HYAL1,ACOT7	darkgreen	EC_M4
GO:2000677	2.25E-01	0.883182264	7	37	regulation of transcription regulatory region DNA binding	BP	9.72E-01	RB1,TGFB1,CTNNB1,DDRGK1,HEY1,PARK7,TMSB4X	darkgreen	EC_M4
GO:0003009	2.25E-01	0.890589812	6	30	skeletal muscle contraction	BP	9.72E-01	MYH14,ATP2A2,HOMER1,CASQ1,TCAP,DMD	darkgreen	EC_M4
GO:0070167	2.26E-01	0.857041141	13	74	regulation of biomineral tissue development	BP	9.72E-01	FBN2,ASPN,ROCK2,TGFB1,ATP2B1,HEY1,ACVR1,BMP7,FZD9,COMP,BMPR1A,TGFB3,ROCK1	darkgreen	EC_M4
GO:0010155	2.26E-01	0.910411956	4	19	regulation of proton transport	BP	9.72E-01	ACTN4,CHP1,PARK7,TMSB4X	darkgreen	EC_M4
GO:0048016	2.26E-01	0.871634355	9	48	inositol phosphate-mediated signaling	BP	9.72E-01	DYRK2,PRNP,PPP3CC,CHP1,ATP2B4,ADGRB2,GSK3B,INPP5A,NMUR1	darkgreen	EC_M4
GO:0032436	2.26E-01	0.853894236	14	83	positive regulation of proteasomal ubiquitin-dependent protein catabolic process	BP	9.72E-01	RNF19A,UBQLN1,VCP,SOCS5,NUB1,CHFR,DDRGK1,GSK3B,ARIH1,FBXO22,AKT1,BCAP31,UBE2V2,DDA1	darkgreen	EC_M4
GO:0006730	2.26E-01	0.889760069	6	31	one-carbon metabolic process	BP	9.72E-01	AHCYL2,ALDH1L2,MTHFD2,SFXN1,AHCYL1,CA13	darkgreen	EC_M4
GO:0001823	2.26E-01	0.854018589	14	81	mesonephros development	BP	9.72E-01	VEGFA,GLI3,FGF2,CTNNB1,RARB,ILK,PKD2,BMP7,FGFR2,SIX4,MAGED1,PKD1,GDNF,FGF10	darkgreen	EC_M4
GO:0048753	2.27E-01	0.898869808	5	24	pigment granule organization	BP	9.72E-01	ZEB2,AP3B1,SHROOM2,AP1G1,BLOC1S2	darkgreen	EC_M4
GO:0010769	2.27E-01	0.853358017	14	80	regulation of cell morphogenesis involved in differentiation	BP	9.72E-01	RAB21,ACTR2,ACTN4,ILK,DHX36,OPA1,DNM1L,CAPRIN2,CORO1C,ARPC2,OBSL1,PAFAH1B1,FLNA,HAS2	darkgreen	EC_M4
GO:0033059	2.27E-01	0.875750955	8	43	cellular pigmentation	BP	9.72E-01	CD63,ZEB2,AP3B1,SHROOM2,MYO5A,RAB11A,AP1G1,BLOC1S2	darkgreen	EC_M4

GO:0002043	2.28E-01	0.897967851	5	25	blood vessel endothelial cell proliferation involved in sprouting angiogenesis	BP	9.72E-01	VEGFA,FGF2,PDCD10,NGFR,MIR24-1	darkgreen	EC_M4
GO:1901861	2.28E-01	0.888975307	6	30	regulation of muscle tissue development	BP	9.72E-01	TGFB1,CTNNB1,LEF1,PPARGC1A,PRKAA1,LOX	darkgreen	EC_M4
GO:0006418	2.28E-01	0.881336886	7	37	tRNA aminoacylation for protein translation	BP	9.72E-01	GARS,CARS2,CARS,EPRS,TARS,SARS,AARS	darkgreen	EC_M4
GO:0007006	2.28E-01	0.847542525	16	99	mitochondrial membrane organization	BP	9.72E-01	HSPA4,COX18,SLC25A4,OPA1,AP3B1,EYA2,GSK3B,FZD9,VDAC2,GHITM,CLN8,CALM1,OMA1,BNIP3,BLOC1S2,TIMM22	darkgreen	EC_M4
GO:0051492	2.29E-01	0.852316113	14	80	regulation of stress fiber assembly	BP	9.72E-01	TGFBR1,ROCK2,PAK1,ACTG1,PFN2,PHLDB2,TJP1,SYNPO2L,CGNL1,TPM1,TGFB3,GPR65,TTC8,ROCK1	darkgreen	EC_M4
GO:0046660	2.29E-01	0.849393651	15	88	female sex differentiation	BP	9.72E-01	CTNNA1,LEP,VEGFA,INHBA,AXL,PTX3,MMP14,ARID5B,PCYT1B,SGPL1,PTPRN,CYP19A1,HSPA5,LHX8,FGF10	darkgreen	EC_M4
GO:0046545	2.29E-01	0.854379941	13	75	development of primary female sexual characteristics	BP	9.72E-01	CTNNA1,LEP,VEGFA,INHBA,PTX3,MMP14,ARID5B,PCYT1B,SGPL1,PTPRN,CYP19A1,HSPA5,LHX8	darkgreen	EC_M4
GO:0060998	2.31E-01	0.864082144	10	54	regulation of dendritic spine development	BP	9.72E-01	SHANK1,ACTR2,EIF4G2,DHX36,OPA1,DNM1L,CAPRIN2,KIF1A,TANC2,PAFAH1B1	darkgreen	EC_M4
GO:1990776	2.31E-01	0.896482767	5	24	response to angiotensin	BP	9.72E-01	ROCK2,PTPN11,IQGAP1,ROCK1,AHCYL1	darkgreen	EC_M4
GO:0060042	2.31E-01	0.873578932	8	42	retina morphogenesis in camera-type eye	BP	9.72E-01	AHI1,RORB,MFSD2A,LRP5,OBSL1,ROM1,TTC8,THY1	darkgreen	EC_M4
GO:0007265	2.31E-01	0.815765362	46	296	Ras protein signal transduction	BP	9.72E-01	ARHGEF25,RAB21,COL1A2,ROCK2,RRAS,ITGB1,FGF2,RB1,PSD2,CDC42EP3,STARD13,PPP2CB,MRAS,ROBO1,DOK1,RASA1,RTKN,RAPGEF2,MAPKAPK5,ARHGAP1,DOK2,NOTCH2,TGFB2,KCTD10,AUTS2,KRAS,FARP2,NCKAP1,COL3A1,SYNPO2L,PLD1,MYO9B,IQSEC2,ARHGEF2,NGFR,GPR65,PDGFRB,ROCK1,TNFAIP1,GNB1,TAX1BP3,PARK7,CDH13,ARHGAP29,CDKN1A,FGF10	darkgreen	EC_M4
GO:0043268	2.32E-01	0.887040946	6	29	positive regulation of potassium ion transport	BP	9.72E-01	KIF5B,ATP1B3,ABCC8,KCNA1,KCNMB1,FLNA	darkgreen	EC_M4
GO:0071542	2.32E-01	0.895942154	5	24	dopaminergic neuron differentiation	BP	9.72E-01	VEGFA,CTNNB1,RYK,GSK3B,WNT2	darkgreen	EC_M4
GO:0002521	2.32E-01	0.810644951	58	383	leukocyte differentiation	BP	9.72E-01	IL23A,HLA-DRA,LGALS1,MYH9,LEP,SRF,VEGFA,RUNX2,SOCS5,GLI3,INHBA,RRAS,OSTM1,ITGB1,RB1,GLI2,TGFB1,AXL,CTNNB1,IL15RA,LIG4,SPINK5,LOXL3,APP,CDH17,AP3B1,PTPN22,ADA,RUNX1,DOCK10,NOTCH2,MMP14,LEF1,PRKCA,TCTA,SLAMF8,TFRC,FARP2,FZD9,SRP54,SYVN1,PTPN6,GPR137,FBN1,MED1,DOCK2,CD74,TMEM98,PTPN2,CD40LG,PAFAH1B1,ATP6AP1,FSTL3,SNX10,GLO1,DNAJB9,FGL2,CCR6	darkgreen	EC_M4
GO:1902229	2.32E-01	0.886275196	6	31	regulation of intrinsic apoptotic signaling pathway in response to DNA damage	BP	9.72E-01	TPT1,CXCL12,SKIL,TAF9B,CD74,NACC2	darkgreen	EC_M4
GO:0006919	2.32E-01	0.862860953	10	57	activation of cysteine-type endopeptidase activity involved in apoptotic process	BP	9.72E-01	MTCH1,VCP,CYCS,ROBO1,IFT57,FAM162A,EGLN3,MAPT,NGFR,F3	darkgreen	EC_M4
GO:0001649	2.32E-01	0.825545166	30	190	osteoblast differentiation	BP	9.72E-01	FBN2,RORB,RUNX2,REST,GLI3,FGF2,PSMC2,GLI2,CTNNB1,CRIM1,ILK,TP53INP2,ACVR1,SPP1,LRP5,SATB2,LEF1,COL6A1,BMP7,FGFR2,MRC2,SUCO,TPM4,BMPR1A,PDLIM7,COL1A1,CLTC,AKT1,ATP6AP1,LOX	darkgreen	EC_M4
GO:0014829	2.32E-01	0.895390279	5	24	vascular associated smooth muscle contraction	BP	9.72E-01	ATP2B1,RAP1GDS1,COMP,ACTA2,PIK3C2A	darkgreen	EC_M4
GO:0032637	2.33E-01	0.861990095	10	57	interleukin-8 production	BP	9.72E-01	LEP,PTPN22,TLR5,SERPINE1,CD74,PARK7,F3,BCL10,TMSB4X,FCN1	darkgreen	EC_M4

GO:0090311	2.33E-01	0.872012115	8	42	regulation of protein deacetylation	BP	9.72E-01	C6orf89,VEGFA,NDN,MAPT,PRKAA1,PRMT6,FRY,NNMT	darkgreen	EC_M4
GO:1901800	2.34E-01	0.841252024	17	104	positive regulation of proteasomal protein catabolic process	BP	9.72E-01	RNF19A,UBQLN1,VCP,SOCS5,PSMC2,NUB1,TMTC3,CHFR,DDRGK1,GSK3B,ARIH1,FBXO22,AKT1,BCAP31,TMEM259,UBE2V2,DDA1	darkgreen	EC_M4
GO:0043555	2.35E-01	0.906163271	4	18	regulation of translation in response to stress	BP	9.72E-01	NCK2,TMED2,RPS6KA3,EIF2S1	darkgreen	EC_M4
GO:0010628	2.35E-01	0.795958887	117	803	positive regulation of gene expression	BP	9.72E-01	IL23A,LRP1,PLXNB2,ANGPT1,FAM49B,HABP4,PPARD,MYH9,LEP,VEGFA,RUNX2,NCK2,PDCL3,TGFBR1,RIMS1,MAP3K7,WHRN,ROCK2,INHBA,VPS35,FGF2,SRPK2,PANX1,TGFB1,CTNNB1,C1QTNF1,ACTG1,KAT2B,FAM98A,MAFG,LUM,DDRGK1,TMED2,IL20RB,EIF4G2,SULF1,ROBO1,BCDIN3D,HEY1,CELF1,APP,FAM98B,DHX36,ACTG2,CX3CL1,ABCC8,PTPN11,PTPN22,POLR3D,PKD2,RUNX1,PLA2R1,TENT5C,TENT4B,RAB7B,KDM5B,SLC2A10,KPNA6,HTR2B,GSK3B,MMP14,LEF1,TLR5,KRAS,NTRK3,HSPA8,BMP7,ATF3,OSM,TP53BP1,TFRC,HSPB1,SERPINE1,ATP6AP2,DNAJA4,TFR2,FN1,PDCD10,SAMD4A,PLD1,SGMS1,ACTA2,BMPR1A,MED1,PPARGC1A,MSN,ARHGEF2,TMED10,EIF3C,MAP2K1,PKM,MYOCD,SCAMP5,CD74,ROCK1,AKT1,ITGA2,MYOM1,PRKAA1,CD40LG,IRAK1,PARK7,ENO1,FUBP3,F3,BARHL2,BCL10,AGO3,IL36G,GDNF,EDA,DNAJB9,FCN1,TSLP,ZPR1,OLFM1,FGF10	darkgreen	EC_M4
GO:0051057	2.35E-01	0.860760659	10	55	positive regulation of small GTPase mediated signal transduction	BP	9.72E-01	ROBO1,NOTCH2,AUTS2,KRAS,ITGAV,COL3A1,SYNPO2L,GPR65,PDGFRB,FGF10	darkgreen	EC_M4
GO:0032943	2.35E-01	0.824718572	28	179	mononuclear cell proliferation	BP	9.72E-01	IL23A,LEP,DOCK8,NCK2,FKBP1B,CTNNB1,IRS2,PRNP,LMO1,IL20RB,CD180,PTPN22,ADA,CBLB,SLC39A10,PRKAR1A,LEF1,NCSTN,TFRC,PTPN6,IGFBP2,MSN,DOCK2,CD74,CD40LG,CD151,CDKN1A,FGF10	darkgreen	EC_M4
GO:0046634	2.35E-01	0.8562686	11	63	regulation of alpha-beta T cell activation	BP	9.72E-01	IL23A,HLA-DRA,SOCS5,GLI3,HSPH1,LOXL3,AP3B1,PTPN22,ADA,RUNX1,CBLB	darkgreen	EC_M4
GO:0032922	2.36E-01	0.860377684	10	55	circadian regulation of gene expression	BP	9.72E-01	PPARA,CLOCK,GFPT1,HDAC3,CAVIN3,CRY2,MAGED1,PPARGC1A,NGFR,BHLHE41	darkgreen	EC_M4
GO:0036258	2.36E-01	0.892845085	5	25	multivesicular body assembly	BP	9.72E-01	PDCD6IP,STAM2,VPS37A,TSG101,RAB11A	darkgreen	EC_M4
GO:0045453	2.36E-01	0.864395791	9	50	bone resorption	BP	9.72E-01	NCDN,CTNNB1,SPP1,PRKCA,TFRC,GPR137,ATP6AP1,DEF8,SNX10	darkgreen	EC_M4
GO:0046579	2.36E-01	0.864215033	9	49	positive regulation of Ras protein signal transduction	BP	9.72E-01	ROBO1,NOTCH2,AUTS2,KRAS,COL3A1,SYNPO2L,GPR65,PDGFRB,FGF10	darkgreen	EC_M4
GO:0008154	2.37E-01	0.822476202	29	183	actin polymerization or depolymerization	BP	9.72E-01	FAM49B,NCK2,ACTR2,ACTR3,PLEKHG2,CDC42EP3,TTC17,CXCL12,PFN2,RASA1,TWF1,MAGEL2,CTTN,LMOD1,PIK3CA,SPATC1L,ARPC5,NCKAP1,ARPC2,CAPZB,WDR1,ENAH,FLII,MYO1C,SPTAN1,TMSB4X,DSTN,COTL1,CFL2	darkgreen	EC_M4
GO:0072088	2.37E-01	0.855199187	11	62	nephron epithelium morphogenesis	BP	9.72E-01	AHI1,VEGFA,GLI3,FGF2,CTNNB1,ILK,PKD2,SIX4,MAGED1,PKD1,GDNF	darkgreen	EC_M4
GO:0061136	2.37E-01	0.825114103	26	166	regulation of proteasomal protein catabolic process	BP	9.72E-01	RNF19A,UBQLN1,VCP,SOCS5,PSMC2,NUB1,TMTC3,CHFR,DDRGK1,PSMD14,UCHL5,GSK3B,ARAF,ARIH1,FBXO22,USP14,AKT1,BCAP31,PKD1,TMEM259,PARK7,RHBDF1,UBE2V2,DDA1,GABARAPL2,MAP1A	darkgreen	EC_M4
GO:0051261	2.37E-01	0.838858598	17	99	protein depolymerization	BP	9.72E-01	NCKAP5,NAV3,KIF2A,MAP1B,TWF1,LMOD1,PIK3CA,HSPA8,CAPZB,WDR1,NES,ARHGEF2,FLII,SPTAN1,DSTN,MAP1A,CFL2	darkgreen	EC_M4
GO:0006090	2.38E-01	0.848035585	13	76	pyruvate metabolic process	BP	9.72E-01	PPARA,ME3,PFKP,APP,PFKM,PGAM4,ZBTB20,TPI1,PPARGC1A,PKM,PRKAA1,ENO1,SLC2A6	darkgreen	EC_M4
GO:0055067	2.38E-01	0.836034731	18	108	monovalent inorganic cation homeostasis	BP	9.72E-01	SLC12A2,RAB7A,SLC4A7,MAFG,CCKBR,TM9SF4,ATP1B3,SLC4A5,CHP1,SLAMF8,CLIC4,ATP6AP2,COMT,NPPB,CLN6,ATP6AP1,ATP6V0D1,ATP6V0E1	darkgreen	EC_M4
GO:0032410	2.38E-01	0.858256494	10	57	negative regulation of transporter activity	BP	9.72E-01	UBQLN1,FKBP1B,REM1,CACNB3,PKD2,LINC02210-CRHR1,CALM1,MIR24-1,CBARP,GEM	darkgreen	EC_M4
GO:0043001	2.38E-01	0.874581832	7	37	Golgi to plasma membrane protein transport	BP	9.72E-01	GGA1,ATP2C1,BLZF1,RAB31,RAB34,C16orf70,ARFRP1	darkgreen	EC_M4

GO:0051155	2.38E-01	0.874480537	7	37	positive regulation of striated muscle cell differentiation	BP	9.72E-01	MYLK3,TGFB1,MMP14,MORF4L2,MYOCD,PIN1,HOPX	darkgreen	EC_M4
GO:0051653	2.39E-01	0.862420259	9	49	spindle localization	BP	9.72E-01	MYH9,NLRP5,ACTR2,ITGB1,ACTR3,HTT,FMN2,PAFAH1B1,FGF10	darkgreen	EC_M4
GO:0048268	2.39E-01	0.903710155	4	18	clathrin coat assembly	BP	9.72E-01	PICALM,CLINT1,CLTC,PIK3C2A	darkgreen	EC_M4
GO:1903146	2.40E-01	0.881418615	6	30	regulation of autophagy of mitochondrion	BP	9.72E-01	SLC25A4,CTTN,DNM1L,HTT,PARK7,BNIP3	darkgreen	EC_M4
GO:0048843	2.40E-01	0.890903897	5	24	negative regulation of axon extension involved in axon guidance	BP	9.72E-01	SEMA3C,RYK,SEMA4F,SEMA4G,PLXNA3	darkgreen	EC_M4
GO:0003215	2.40E-01	0.903314081	4	18	cardiac right ventricle morphogenesis	BP	9.72E-01	SMARCD3,SEMA3C,TGFB2,BMPR1A	darkgreen	EC_M4
GO:0002683	2.40E-01	0.809037302	44	290	negative regulation of immune system process	BP	9.72E-01	ENPP3,ANGPT1,SOCS5,GLI3,INHBA,TGFB1,AXL,CTNNB1,NPLOC4,PRNP,SPINK5,CNOT7,CXCL12,IL20RB,LOXL3,CX3CL1,PTPN22,ADA,RUNX1,CBLB,SELENOS,TGFB2,PRKAR1A,TCTA,SLAMF8,HTRA1,PTPN6,GPR137,FBN1,COL3A1,NBL1,SYT11,TGFB3,TSPAN6,CD74,AKT1,PTPN2,FAM3A,MIR24-1,FSTL3,A2M,CD59,THY1,FGL2	darkgreen	EC_M4
GO:0110149	2.40E-01	0.845901476	13	75	regulation of biomineralization	BP	9.72E-01	FBN2,ASPN,ROCK2,TGFB1,ATP2B1,HEY1,ACVR1,BMP7,FZD9,COMP,BMPR1A,TGFB3,ROCK1	darkgreen	EC_M4
GO:1903202	2.40E-01	0.87271594	7	38	negative regulation of oxidative stress-induced cell death	BP	9.72E-01	REST,SLC7A11,CTNNB1,HSPB1,AKT1,PARK7,TXN	darkgreen	EC_M4
GO:0097734	2.41E-01	0.902023297	4	19	extracellular exosome biogenesis	BP	9.72E-01	RAB7A,PDCD6IP,TSG101,RAB11A	darkgreen	EC_M4
GO:0042059	2.41E-01	0.890151296	5	24	negative regulation of epidermal growth factor receptor signaling pathway	BP	9.72E-01	SOCS5,RAB7A,TSG101,CBLB,PTPN2	darkgreen	EC_M4
GO:0045104	2.41E-01	0.880476075	6	30	intermediate filament cytoskeleton organization	BP	9.72E-01	DST,NEFM,INA,PKP2,CLN8,NES	darkgreen	EC_M4
GO:0071498	2.41E-01	0.902380368	4	18	cellular response to fluid shear stress	BP	9.72E-01	SOCS5,HDAC3,PKD2,HAS2	darkgreen	EC_M4
GO:1902107	2.42E-01	0.832586461	18	110	positive regulation of leukocyte differentiation	BP	9.72E-01	IL23A,HLA-DRA,SOCS5,GLI3,RB1,GLI2,TGFB1,AXL,IL15RA,AP3B1,ADA,RUNX1,NOTCH2,MMP14,LEF1,PRKCA,CD74,ATP6AP1	darkgreen	EC_M4
GO:1903708	2.42E-01	0.832586461	18	110	positive regulation of hemopoiesis	BP	9.72E-01	IL23A,HLA-DRA,SOCS5,GLI3,RB1,GLI2,TGFB1,AXL,IL15RA,AP3B1,ADA,RUNX1,NOTCH2,MMP14,LEF1,PRKCA,CD74,ATP6AP1	darkgreen	EC_M4
GO:0015012	2.42E-01	0.889362106	5	24	heparan sulfate proteoglycan biosynthetic process	BP	9.72E-01	CTNNB1,B3GAT3,UGDH,XYLT2,HS2ST1	darkgreen	EC_M4
GO:0006629	2.43E-01	0.7857606	141	982	lipid metabolic process	BP	9.72E-01	ATP8B1,HSD17B6,LRP1,B4GALNT1,AGAP2,PPARA,ACSL6,SLC22A4,NUS1,SERINC1,PPARD,GOLM1,LEP,PLAA,LARGE1,REST,INPP4A,TMEM246,SOCS5,FAM126A,MFSD2A,DKKL1,LDAH,FGF2,RAB7A,SELENOI,EBPL,ABHD12,ABHD5,GLB1,ABHD1,DGKH,SNX17,TGFB1,ACER3,DGAT2,AKT2,OSBPL10,MTMR14,OSBPL8,SUMF1,IRS2,SMPD1,IMPAD1,MTMR6,CHPT1,OSBP,ST8SIA4,TMEM150A,DKK3,RDH10,FAR1,CERS5,SORBS1,APP,PTPN11,PIP4P2,LPCAT2,SERINC3,CHP1,SPP1,PIGP,ADIPOR1,AGPAT3,CPT1A,ZBTB20,PRKAB2,LRP5,B4GALT4,HTR2B,INPP5A,PIK3CA,HADH,PITPNM1,RLBP1,ETNK1,B3GALT1,PITPNB,ARV1,HACD2,WDTC1,OSBPL2,HDLBP,PIGZ,PAFAH1B2,PCYT1B,MOGAT3,SGPL1,COMT,ACOX3,HSD17B1,FGFR3,PLD1,CYP19A1,SGMS1,ACBD3,HSD17B10,CLN8,STARD3,MED1,PPARGC1A,SPTLC2,SEL1L,CLN6,SLC44A2,CYP46A1,PIGL,ACSL4,CD74,PDGFRB,PNPLA6,AKT1,LPIN2,PRKAA1,OCRL,OMA1,PITPNA,PAFAH1B1,ABCA3,PGP,CSNK1G2,ACOT7,FAM3A,MBTPS1,DPM2,PLA2G15,NFE2L1,HACD1,TMEM38B,PEMT,AMACR,PIGK,DPM1,YWHAH,PIGB,PLAGL2,TMEM189,PIK3C2A,ORMDL3,SLC35C1,CDIPT	darkgreen	EC_M4

GO:0006520	2.43E-01	0.812444275	34	224	cellular amino acid metabolic process	BP	9.72E-01	CDO1,AZIN1,PSAT1,MTAP,ASNS,GARS,GFPT1,SLC7A11,GAD2,HNMT,CARS2,PLOD2,ALDH18A1,SERINC3,GLS,ATP2B4,PSPH,CARS,AZIN2,EPRS,SMS,PLOD3,COMT,QDPR,ASRGL1,AGMAT,HSD17B10,SLC25A44,TARS,PARK7,SARS,AARS,PEMT,ADI1	darkgreen	EC_M4
GO:0045639	2.43E-01	0.843543635	13	77	positive regulation of myeloid cell differentiation	BP	9.72E-01	IL23A,INHBA,RB1,TGFB1,RUNX1,RAB7B,NOTCH2,LEF1,PRKCA,MED1,CD74,ATP6AP1,FAM210B	darkgreen	EC_M4
GO:0001825	2.44E-01	0.87855554	6	31	blastocyst formation	BP	9.72E-01	SRF,RTN4,ADA,SKIL,SUPT6H,HOPX	darkgreen	EC_M4
GO:0043627	2.44E-01	0.854181736	10	57	response to estrogen	BP	9.72E-01	CTNNA1,GLI3,ARSB,CCND1,MMP14,COMT,IGFBP2,PTPRN,TGFB3,PDGFRB	darkgreen	EC_M4
GO:0010562	2.45E-01	0.789494055	99	669	positive regulation of phosphorus metabolic process	BP	9.72E-01	IL23A,NAB2,AGAP2,TAB2,PRRC1,ANGPT1,PPARA,ROS1,LEP,VEGFA,VCP,TGFBR1,MAP3K7,ROCK2,INHBA,FGF2,MAPRE3,PAK1,TGFB1,AXL,AKT2,OSBPL8,PPP1R12A,PRNP,HDAC3,DDRGK1,SMPD1,ILK,RBPMS,RYK,OSBP,ROBO1,PFN2,APP,RAPGEF2,CX3CL1,PTPN11,ACVR1,GDF6,AKTIP,CHP1,ATP2B4,PKD2,SLC39A10,CHRNA7,CCND1,CAPRIN2,TGFB2,ZBTB20,HTR2B,DAG1,PIK3CA,KRAS,NTRK3,SPATC1L,BMP7,FGFR2,OSM,SYAP1,IQGAP1,TFRC,MAPT,PDCD10,ADRA2C,DOK7,FGFR3,ARAF,TNIK,MNAT1,MAGED1,BMPR1A,PPARGC1A,SRCIN1,ARHGEF2,TGFB3,MAP2K1,CALM1,NPTN,PIN1,CD74,PDGFRB,AKT1,ITGA2,PRKAA1,MYDGF,TNFRSF18,PKD1,IRAK1,PARK7,ENO1,BCAR3,LMO4,BCL10,RANBP2,CDKN1A,TXN,TMSB4X,TSLP,FGF10	darkgreen	EC_M4
GO:0045937	2.45E-01	0.789494055	99	669	positive regulation of phosphate metabolic process	BP	9.72E-01	IL23A,NAB2,AGAP2,TAB2,PRRC1,ANGPT1,PPARA,ROS1,LEP,VEGFA,VCP,TGFBR1,MAP3K7,ROCK2,INHBA,FGF2,MAPRE3,PAK1,TGFB1,AXL,AKT2,OSBPL8,PPP1R12A,PRNP,HDAC3,DDRGK1,SMPD1,ILK,RBPMS,RYK,OSBP,ROBO1,PFN2,APP,RAPGEF2,CX3CL1,PTPN11,ACVR1,GDF6,AKTIP,CHP1,ATP2B4,PKD2,SLC39A10,CHRNA7,CCND1,CAPRIN2,TGFB2,ZBTB20,HTR2B,DAG1,PIK3CA,KRAS,NTRK3,SPATC1L,BMP7,FGFR2,OSM,SYAP1,IQGAP1,TFRC,MAPT,PDCD10,ADRA2C,DOK7,FGFR3,ARAF,TNIK,MNAT1,MAGED1,BMPR1A,PPARGC1A,SRCIN1,ARHGEF2,TGFB3,MAP2K1,CALM1,NPTN,PIN1,CD74,PDGFRB,AKT1,ITGA2,PRKAA1,MYDGF,TNFRSF18,PKD1,IRAK1,PARK7,ENO1,BCAR3,LMO4,BCL10,RANBP2,CDKN1A,TXN,TMSB4X,TSLP,FGF10	darkgreen	EC_M4
GO:0015748	2.45E-01	0.832615275	17	102	organophosphate ester transport	BP	9.72E-01	ATP8B1,SLC35B2,TMEM30A,MFSD2A,OSBPL10,OSBPL8,SLC25A4,SLC33A1,CALHM1,PITPNM1,PITPNB,PRELID3B,OSBPL2,ATP11C,PITPNA,ABCA3,PLEKHA3	darkgreen	EC_M4
GO:0009310	2.45E-01	0.899837665	4	19	amine catabolic process	BP	9.72E-01	HNMT,ATP2B4,COMT,MAOA	darkgreen	EC_M4
GO:0009895	2.45E-01	0.807095923	39	254	negative regulation of catabolic process	BP	9.72E-01	AGAP2,TAB2,AZIN1,PPARA,ADGRB1,LEP,PDCL3,MFSD2A,DDRGK1,EIF4G2,DHX36,PTPN22,DKC1,ATP2B4,TENT5C,TENT4B,AZIN2,UCHL5,SEC22B,FEZ1,HTR2B,PIK3CA,SCFD1,QSOX1,TIMP1,FMN2,PPARGC1A,PIN1,ROCK1,USP14,AKT1,PRKAA1,CLEC16A,FLNA,PARK7,GDNF,TIMP3,GABARAPL2,MAP1A	darkgreen	EC_M4
GO:0044319	2.46E-01	0.877427503	6	30	wound healing, spreading of cells	BP	9.72E-01	MRTFA,PHLDB2,PDCD10,FLNA,COL5A1,CD151	darkgreen	EC_M4
GO:0090505	2.46E-01	0.877427503	6	30	epiboly involved in wound healing	BP	9.72E-01	MRTFA,PHLDB2,PDCD10,FLNA,COL5A1,CD151	darkgreen	EC_M4
GO:0042752	2.46E-01	0.836036052	15	89	regulation of circadian rhythm	BP	9.72E-01	PPARA,GNAQ,RORB,CLOCK,ROCK2,HDAC3,CRY2,ADA,MAGEL2,ARNTL2,GSK3B,MAGED1,PPARGC1A,FBXL22,PRKAA1	darkgreen	EC_M4
GO:0070661	2.46E-01	0.812360704	31	201	leukocyte proliferation	BP	9.72E-01	IL23A,ENPP3,LEP,DOCK8,NCK2,FKBP1B,CTNNB1,IRS2,PRNP,LMO1,IL20RB,CD180,CX3CL1,PTPN22,ADA,CBLB,SLC39A10,PRKAR1A,LEF1,NCSTN,TFRC,PTPN6,IGFBP2,MSN,DOCK2,CD74,NPR3,CD40LG,CD151,CDKN1A,FGF10	darkgreen	EC_M4
GO:0006979	2.47E-01	0.798996946	53	357	response to oxidative stress	BP	9.72E-01	IPCEF1,RRM2B,UBQLN1,TXN2,REST,SLC7A11,FKBP1B,SESN3,AXL,CTNNB1,PRNP,PPP2CB,RBPMS,ARL6IP5,MAP1LC3A,C19orf12,ATP2A2,APP,PTPN11,MMP2,ADA,PKD2,PLA2R1,GLRX2,SELENOS,HYAL1,FBLN5,MMP14,BMP7,PYROXD1,PRDX4,HSPB1,MAPT,PDCD10,ERO1A,PTPRN,CLN8,EIF2S1,PPARGC1A,TPM1,COL1A1,GSKIP,PDGFRB,AKT1,PRKAA1,PARK7,NFE2L1,BNIP3,PDLIM1,MSRB3,SIGMAR1,TXN,RBM11	darkgreen	EC_M4
GO:0034063	2.47E-01	0.886347969	5	24	stress granule assembly	BP	9.72E-01	ATXN2,CSDE1,MAPT,EIF2S1,PRKAA1	darkgreen	EC_M4
GO:0035774	2.47E-01	0.885819765	5	25	positive regulation of insulin secretion involved in cellular response to glucose stimulus	BP	9.72E-01	LRP1,PPARD,KIF5B,OSBP,DYNLL1	darkgreen	EC_M4

GO:0007188	2.47E-01	0.823147718	21	129	adenylate cyclase-modulating G protein-coupled receptor signaling pathway	BP	9.72E-01	ADGRB1,GNAQ,HTR5A,APLP1,CACNA1D,RAPGEF2,ADCY6,ATP2B4,ADGRB2,PRKCA,ADCY5,LINC02210-CRHR1,ADRA2C,ADGRL1,ADRA1B,GPR65,GNAL,NPR3,GNB1,FLNA,GNAI3	darkgreen	EC_M4
GO:0006026	2.47E-01	0.898277965	4	19	aminoglycan catabolic process	BP	9.72E-01	FGF2,GLB1,TGFB1,HYAL1	darkgreen	EC_M4
GO:0060443	2.48E-01	0.868013445	7	37	mammary gland morphogenesis	BP	9.72E-01	GLI3,GLI2,KDM5B,LRP5,FGFR2,MED1,FGF10	darkgreen	EC_M4
GO:0070584	2.48E-01	0.898097627	4	19	mitochondrion morphogenesis	BP	9.72E-01	MYH14,OPA1,DNM1L,FEZ1	darkgreen	EC_M4
GO:0071383	2.48E-01	0.815713101	26	163	cellular response to steroid hormone stimulus	BP	9.72E-01	PPARA,PPARD,CLOCK,REST,MGARP,PGRMC2,PAK1,LBH,UFM1,ATP2B1,UBA5,DDRGK1,RNF6,HEY1,CRY2,VPS18,EIF4E,BMP7,STRN3,MED1,SMARCA4,SSTR5,PARK7,SERPINF1,YWHAH,PMEPA1	darkgreen	EC_M4
GO:0043547	2.48E-01	0.809812294	32	202	positive regulation of GTPase activity	BP	9.72E-01	TBC1D9B,DOCK8,SNX13,ITGB1,PICALM,RAB3GAP1,AKT2,TBC1D16,RASA1,RAPGEF2,CX3CL1,RALGAPB,RAP1GDS1,DOCK10,GSK3B,CORO1C,ADAP1,NTRK3,SGSM1,ZC3H15,GPR65,PIN1,EVI5L,SNX18,ABR,TAX1BP3,ARHGAP29,BCAR3,MTSS1L,CCL16,THY1,CHN1	darkgreen	EC_M4
GO:0009954	2.49E-01	0.88473912	5	25	proximal/distal pattern formation	BP	9.72E-01	GLI3,GLI2,CTNNB1,CHSY1,FGF10	darkgreen	EC_M4
GO:0070613	2.49E-01	0.85465065	9	52	regulation of protein processing	BP	9.72E-01	MYH9,PRNP,CHAC1,RUNX1,MMP14,SERPINE1,TMEM98,ENO1,GLG1	darkgreen	EC_M4
GO:0014032	2.49E-01	0.842413117	12	69	neural crest cell development	BP	9.72E-01	SEMA3C,RDH10,SEMA4F,ACVR1,SEMA4G,HTR2B,CORO1C,BMP7,FN1,BMPR1A,ANXA6,GDNF	darkgreen	EC_M4
GO:0002263	2.49E-01	0.811797344	29	185	cell activation involved in immune response	BP	9.72E-01	IL23A,LRP1,ENPP3,HLA-DRA,LGALS1,SOCS5,APBB1IP,TGFB1,LIG4,CD180,VAMP8,LOXL3,APP,CDH17,ADA,DOCK10,NOTCH2,LEF1,TP53BP1,TFRC,DOCK2,CD74,TMEM98,SUPT6H,CD40LG,VAMP7,AP1G1,FGL2,CCR6	darkgreen	EC_M4
GO:0031647	2.49E-01	0.80445456	38	249	regulation of protein stability	BP	9.72E-01	RAB21,TCP1,GNAQ,PEX6,BAG1,PDCL3,P3H1,ISOC2,RTN4,VPS35,CRTAP,PRNP,CHFR,HDAC3,DDRGK1,ATP1B3,CTNND1,PINX1,PFN2,CCT8,CHP1,CDC73,KRAS,HSPA8,SYVN1,PDCD10,HTT,DDOST,PPARGC1A,TAF9B,SEL1L,PIN1,NLK,NAPG,FLNA,PARK7,DPM2,TOMM7	darkgreen	EC_M4
GO:0021680	2.49E-01	0.884836789	5	24	cerebellar Purkinje cell layer development	BP	9.72E-01	WHRN,FAIM2,RERE,AARS,HSPA5	darkgreen	EC_M4
GO:0071636	2.50E-01	0.89700166	4	19	positive regulation of transforming growth factor beta production	BP	9.72E-01	LUM,CX3CL1,ATP6AP2,BMPR1A	darkgreen	EC_M4
GO:0009267	2.50E-01	0.821077939	21	130	cellular response to starvation	BP	9.72E-01	PPARA,SLC39A4,NUAK1,ASNS,MFSD2A,VPS41,SESN3,MAP1LC3A,BRSK2,WIPI1,HSPA8,ATF3,COMT,WIPI2,EIF2S1,CPEB4,PRKAA1,CLEC16A,HSPA5,CDKN1A,GABARAPL2	darkgreen	EC_M4
GO:0002697	2.50E-01	0.80812785	32	206	regulation of immune effector process	BP	9.72E-01	IL23A,STX7,ENPP3,ANGPT1,HLA-DRA,FAM49B,LEP,SOCS5,MAP3K7,TGFB1,SPINK5,IL20RB,VAMP8,LOXL3,DHX36,PTPN22,TGFB2,SLAMF8,TP53BP1,TFRC,PTPN6,TGFB3,CD74,SUPT6H,CD40LG,VAMP7,AP1G1,BCL10,A2M,CD59,DNAJB9,FGL2	darkgreen	EC_M4
GO:0098876	2.50E-01	0.820569125	21	129	vesicle-mediated transport to the plasma membrane	BP	9.72E-01	GGA1,EXOC1,BVES,VPS35,RAB7A,SNX17,ATP2C1,EXOC6B,ARHGAP1,VPS39,ARL3,SNX27,EXOC5,BLZF1,STX1B,RAB11A,RAB31,RAB34,C16orf70,STXBP6,ARFRP1	darkgreen	EC_M4
GO:0071345	2.51E-01	0.78650485	84	574	cellular response to cytokine stimulus	BP	9.72E-01	SLC12A2,ANGPT1,PTP4A3,LEP,DOCK8,SOCS5,MAP3K7,ACTR2,ZYX,MYLK3,KIF5B,ACTR3,AXL,IL15RA,PFKP,ACTG1,ACTN4,ILK,DPYSL3,CNOT7,CXCL12,ANKRD1,IL20RB,MRAS,FLNB,ROBO1,DOK1,CX3CL1,PTPN11,TANK,TOLLIP,RAB7B,INA,ADIPOR1,KDM5B,TROVE2,HYAL1,EPRS,GSK3B,LEF1,CMKLR1,KRAS,OSM,TFRC,ARID5B,PDIA3,PTPN6,ZC3H15,PDCD10,UPF1,STIP1,PTPRN,SGMS1,GREM2,SLC2A4,MED1,PPARGC1A,ARHGEF2,COL1A1,TEX14,CD74,AKT1,OSMR,PTPN2,DAPK3,MYO1C,PAFAH1B1,TNFRSF18,IRAK1,USP10,F3,HSPA5,MIR24-1,CCL16,LOX,SNX10,CACYBP,IL36G,HAS2,VPS26B,TMSB4X,CCR6,TSLP,UBE2G2	darkgreen	EC_M4
GO:0070830	2.51E-01	0.858662422	8	44	bicellular tight junction assembly	BP	9.72E-01	SRF,ROCK2,PDCD6IP,MTDH,TJP1,ROCK1,MYO1C,NPHP4	darkgreen	EC_M4
GO:0002548	2.51E-01	0.864692169	7	40	monocyte chemotaxis	BP	9.72E-01	CREB3,CXCL12,CX3CL1,SLAMF8,SERPINE1,NBL1,CCL16	darkgreen	EC_M4
GO:0086091	2.52E-01	0.873575535	6	30	regulation of heart rate by cardiac conduction	BP	9.72E-01	SCN5A,CACNA1D,PKP2,CACNA1C,CACNA1G,HCN4	darkgreen	EC_M4
GO:0006413	2.52E-01	0.828929135	16	99	translational initiation	BP	9.72E-01	HABP4,NCK2,EIF5B,EIF2B3,TMED2,EIF2S2,EIF4G2,CSDE1,EIF4E,HSPB1,EIF3J,EIF4EBP2,EIF2S1,EIF3C,EIF1,DENR	darkgreen	EC_M4

GO:1902532	2.52E-01	0.791676421	61	407	negative regulation of intracellular signal transduction	BP	9.72E-01	DYRK2,RRM2B,PPARA,FAM49B,CREB3,ITGB1,SESN3,TPT1,GPD1L,NPLOC4,SPRED3,PRNP,HDAC3,CAVIN3,SMPD1,PPP2CB,TMEM127,CXCL12,WWC2,RASA1,MAPKAPK5,OPA1,PTPN22,CHP1,ATP2B4,TANK,ADIPOR1,SELENOS,TGFB2,KCTD10,GSK3B,DAG1,WWC3,BMP7,HYOU1,ATF3,HSPB1,SYVN1,PTPN6,VDAC2,CGNL1,ARHGEF2,TAF9B,TSPAN6,UACA,PIN1,CD74,AKT1,PRKAA1,TNFAIP1,PTPN2,MGRN1,PAFAH1B1,PARK7,ENO1,USP10,DUSP2,TIMP3,DUSP4,TMSB4X,PTTG1IP	darkgreen	EC_M4
GO:0055093	2.52E-01	0.895778193	4	19	response to hyperoxia	BP	9.72E-01	COL1A1,PDGFRB,BNIP3,CDKN1A	darkgreen	EC_M4
GO:1904376	2.52E-01	0.882980691	5	25	negative regulation of protein localization to cell periphery	BP	9.72E-01	PICALM,TGFB1,AP2M1,LRRC15,CLTC	darkgreen	EC_M4
GO:0048511	2.52E-01	0.804292118	35	223	rhythmic process	BP	9.72E-01	PPARA,GNAQ,LEP,RORB,CLOCK,GFPT1,ROCK2,INHBA,DBP,AXL,KAT2B,HDAC3,CAVIN3,PTX3,CRY2,ADA,MAGEL2,KDM5B,DNM1L,TGFB2,ARNTL2,GSK3B,SGPL1,PTPRN,MAGED1,PPARGC1A,NGFR,FBXL22,TGFB3,PRKAA1,HSPA5,SERPINF1,HAS2,BHLHE41,PROKR2	darkgreen	EC_M4
GO:0000963	2.52E-01	0.895557369	4	19	mitochondrial RNA processing	BP	9.72E-01	MTO1,TRIT1,TRNT1,HSD17B10	darkgreen	EC_M4
GO:0048066	2.52E-01	0.872490702	6	32	developmental pigmentation	BP	9.72E-01	CD63,GLI3,ZEB2,HPS6,ATP6AP2,AP1G1	darkgreen	EC_M4
GO:0051955	2.52E-01	0.882550075	5	25	regulation of amino acid transport	BP	9.72E-01	SLC12A2,LEP,ITGB1,RAB3GAP1,ARL6IP5	darkgreen	EC_M4
GO:0033144	2.53E-01	0.872575875	6	31	negative regulation of intracellular steroid hormone receptor signaling pathway	BP	9.72E-01	CLOCK,LBH,CRY2,VPS18,STRN3,SMARCA4	darkgreen	EC_M4
GO:0046637	2.53E-01	0.857817891	8	44	regulation of alpha-beta T cell differentiation	BP	9.72E-01	IL23A,HLA-DRA,SOCS5,GLI3,LOXL3,AP3B1,ADA,RUNX1	darkgreen	EC_M4
GO:0007422	2.53E-01	0.839644096	12	69	peripheral nervous system development	BP	9.72E-01	NAB2,ADGRB1,AKT2,ILK,LGI4,RUNX1,ADGRB2,DAG1,DICER1,AKT1,GDNF,ARTN	darkgreen	EC_M4
GO:0070265	2.53E-01	0.85748894	8	45	necrotic cell death	BP	9.72E-01	SLC25A4,DNM1L,ITPK1,FZD9,SLC6A13,ARHGEF2,BNIP3,TMEM123	darkgreen	EC_M4
GO:0048871	2.53E-01	0.791442646	57	378	multicellular organismal homeostasis	BP	9.72E-01	SLC12A2,ANGPT1,SH3GL2,LEP,SRF,VEGFA,MFSD2A,WHRN,NCDN,RCN3,ITGB1,RB1,RHEB,CTNNB1,ACTG1,HDAC3,IL20RB,TP53INP2,LNPEP,P2RX4,ABCC8,PTPN11,ADCY6,HOMER1,SPP1,NDN,ADIPOR1,TJP1,PRKAB2,PIK3CA,HADH,PRKCA,CMKLR1,KRAS,STRAP,TFRC,HSPB1,GPR137,VCL,AKT3,CLN8,PPARGC1A,SLC25A44,TRPV2,PRKAA1,NPR3,OMA1,MYO5B,ABCA3,NPHP4,ATP6AP1,DEF8,DMD,ADAMTS5,PEMT,SNX10,HAS2	darkgreen	EC_M4
GO:1901379	2.54E-01	0.846377574	10	56	regulation of potassium ion transmembrane transport	BP	9.72E-01	CD63,ITGB1,PRNP,ATP1B3,CACNA1D,ABCC8,KCNA1,KCNMB1,FLNA,KCNAB2	darkgreen	EC_M4
GO:0010950	2.54E-01	0.818805615	20	127	positive regulation of endopeptidase activity	BP	9.72E-01	MTCH1,VCP,REST,CYCS,ROCK2,PICALM,ARL6IP5,ROBO1,IFT57,PSMD14,FAM162A,NCSTN,ADRM1,EGLN3,MAPT,NGFR,UACA,BCAP31,F3,BCL10	darkgreen	EC_M4
GO:0046639	2.55E-01	0.89395207	4	19	negative regulation of alpha-beta T cell differentiation	BP	9.72E-01	SOCS5,GLI3,LOXL3,RUNX1	darkgreen	EC_M4
GO:0009069	2.55E-01	0.880552412	5	26	serine family amino acid metabolic process	BP	9.72E-01	CDO1,PSAT1,SLC7A11,SERINC3,PSPH	darkgreen	EC_M4
GO:0002252	2.55E-01	0.791180285	54	360	immune effector process	BP	9.72E-01	IL23A,LRP1,STX7,ENPP3,ANGPT1,HLA-DRA,FAM49B,LGALS1,LEP,SOCS5,MAP3K7,KIF5B,APBB1IP,PAK1,TGFB1,LIG4,SPINK5,IL20RB,CD180,VAMP8,LOXL3,APP,DHX36,PTX3,CDH17,PTPN22,ADA,DOCK10,NOTCH2,TGFB2,LEF1,CFI,SLAMF8,TP53BP1,TFRC,PTPN6,WDR1,DOCK2,TGFB3,CD74,TMEM98,C6,SUPT6H,CD40LG,VAMP7,AP1G1,BCL10,A2M,CD59,DNAJB9,FGL2,MFAP4,FCN1,CCR6	darkgreen	EC_M4
GO:0002532	2.56E-01	0.844565379	10	59	production of molecular mediator involved in inflammatory response	BP	9.72E-01	PPARA,LEP,VAMP8,BAP1,CHID1,KPNA6,SLAMF8,SERPINE1,PBXIP1,VAMP7	darkgreen	EC_M4

GO:0070228	2.57E-01	0.861341509	7	38	regulation of lymphocyte apoptotic process	BP	9.72E-01	DOCK8,IRS2,ADA,SLC39A10,CD74,BCL10,ORMDL3	darkgreen	EC_M4
GO:0061842	2.57E-01	0.869560457	6	31	microtubule organizing center localization	BP	9.72E-01	SUN2,KIF5B,ARPC5,PAFAH1B1,DYNC1LI2,RANBP2	darkgreen	EC_M4
GO:0030858	2.57E-01	0.854429387	8	45	positive regulation of epithelial cell differentiation	BP	9.72E-01	AHI1,FGF2,PTCH2,CTNNB1,SERPINE1,MED1,TMEM100,GDNF	darkgreen	EC_M4
GO:0016042	2.57E-01	0.800382984	34	225	lipid catabolic process	BP	9.72E-01	HSD17B6,LRP1,PPARA,PPARD,LEP,MFSD2A,LDAH,RAB7A,ABHD12,ABHD5,ABHD1,SNX17,ACER3,AKT2,IRS2,SMPD1,SPP1,CPT1A,HADH,PAFAH1B2,SGPL1,ACOX3,PLD1,CYP19A1,HSD17B10,CYP46A1,PNPLA6,AKT1,LPIN2,PRKAA1,PAFAH1B1,PLA2G15,AMACR,YWHAH	darkgreen	EC_M4
GO:0046174	2.57E-01	0.879437692	5	25	polyol catabolic process	BP	9.72E-01	MIOX,IMPAD1,INPP5A,TPI1,OCRL	darkgreen	EC_M4
GO:1990000	2.57E-01	0.878992779	5	26	amyloid fibril formation	BP	9.72E-01	APP,CHRNA7,BACE1,MAPT,FKBP1A	darkgreen	EC_M4
GO:1902105	2.58E-01	0.803426938	30	193	regulation of leukocyte differentiation	BP	9.72E-01	IL23A,HLA-DRA,SOCS5,GLI3,INHBA,RB1,GLI2,TGFB1,AXL,CTNNB1,IL15RA,SPINK5,LOXL3,AP3B1,ADA,RUNX1,NOTCH2,MMP14,LEF1,PRKCA,TCTA,SLAMF8,PTPN6,GPR137,FBN1,CD74,PTPN2,ATP6AP1,FSTL3,FGL2	darkgreen	EC_M4
GO:0021884	2.58E-01	0.892199462	4	19	forebrain neuron development	BP	9.72E-01	RAPGEF2,FGFR2,PLXNA3,LHX8	darkgreen	EC_M4
GO:0003006	2.58E-01	0.776988573	100	685	developmental process involved in reproduction	BP	9.72E-01	B4GALNT1,ROS1,AFF4,RSPO3,PPARD,CTNNA1,LEP,VEGFA,CLOCK,TGFBR1,PSME4,UBE2J1,GMCL1,GLI3,INHBA,PANX1,ZNF296,CRTAP,GLI2,AXL,WDR48,CTNNB1,DZIP1,SPINT2,DCN,KATNAL1,TMED2,EIF2S2,SULF1,RDH10,CDKL2,HEY1,CELF1,DHX36,PTPN11,PTX3,AP3B1,ACVR1,ADA,ATP2B4,SPP1,PKD2,CSDE1,DNAH1,DYNLL1,AZIN2,KDM5B,NOTCH2,CCND1,TGFB2,MMP14,LEF1,LARP7,SPATC1L,BMP7,FGFR2,VASH2,ASB1,HTRA1,GORASP2,OSBP2,ARID5B,PAFAH1B2,OVOL1,PRDX4,PCYT1B,SGPL1,PTPN6,SKIL,PTPRN,CYP19A1,FMN2,SIX4,BMPR1A,MED1,MYCBPAP,MAP2K1,SOX30,DIAPH2,MYOCD,PDGFRB,AKT1,RAI14,PLAC1,BCAP31,PAFAH1B1,PKD1,FLNA,BSG,HSPA5,LHX8,FSTL3,CCDC136,GGN,SPINK2,SERPINF1,SERPINB5,CCR6,WNT2,FGF10	darkgreen	EC_M4
GO:0035338	2.58E-01	0.891969572	4	19	long-chain fatty-acyl-CoA biosynthetic process	BP	9.72E-01	ACSL6,HACD2,ACSL4,HACD1	darkgreen	EC_M4
GO:0061912	2.58E-01	0.83898007	11	63	selective autophagy	BP	9.72E-01	UBQLN1,RAB7A,UFM1,UBA5,DDRGK1,SLC25A4,ATG13,HTT,WIPI2,SPTLC2,TOMM7	darkgreen	EC_M4
GO:0034110	2.59E-01	0.87826855	5	25	regulation of homotypic cell-cell adhesion	BP	9.72E-01	LGALS1,C1QTNF1,ZNF703,PRKCA,PRKG1	darkgreen	EC_M4
GO:0061333	2.59E-01	0.838294974	11	63	renal tubule morphogenesis	BP	9.72E-01	AHI1,VEGFA,GLI3,FGF2,CTNNB1,ILK,PKD2,SIX4,MAGED1,PKD1,GDNF	darkgreen	EC_M4
GO:1902473	2.60E-01	0.891081053	4	19	regulation of protein localization to synapse	BP	9.72E-01	DAG1,MAPT,IQSEC2,NLGN2	darkgreen	EC_M4
GO:0035023	2.60E-01	0.834136225	12	69	regulation of Rho protein signal transduction	BP	9.72E-01	ARHGEF25,ITGB1,STARD13,ROBO1,KCTD10,COL3A1,SYNPO2L,MYO9B,ARHGEF2,GPR65,PDGFRB,TNFAIP1	darkgreen	EC_M4
GO:0019934	2.60E-01	0.890953656	4	19	cGMP-mediated signaling	BP	9.72E-01	MRVI1,HTR2B,NPPB,PRKG1	darkgreen	EC_M4
GO:0015711	2.60E-01	0.797085461	35	228	organic anion transport	BP	9.72E-01	ATP8B1,SLC12A2,LEP,SLC35B2,SLC17A5,MFSD2A,SLC35A1,SLC7A11,ITGB1,SLC36A4,RAB3GAP1,SLC4A7,ARL6IP5,SLC25A4,P2RX4,SLC4A5,SLC33A1,SLC26A7,SERINC3,PLA2R1,CALHM1,SLC2A10,SLC3A2,SLC6A13,PRAF2,CLN8,SFXN1,SLC7A3,SLC35B1,PRKAA1,SLC2A6,SLC35A3,SLC2A8,SLC36A1,PLEKHA3	darkgreen	EC_M4
GO:0080164	2.61E-01	0.851831469	8	45	regulation of nitric oxide metabolic process	BP	9.72E-01	ROCK2,P2RX4,PTX3,ATP2B4,PKD2,DYNLL1,SELENOS,AKT1	darkgreen	EC_M4
GO:0048538	2.61E-01	0.858677873	7	38	thymus development	BP	9.72E-01	SRF,TGFBR1,CTNNB1,SIX4,MAP2K1,LMO4,FGF10	darkgreen	EC_M4
GO:0050766	2.61E-01	0.858613077	7	38	positive regulation of phagocytosis	BP	9.72E-01	LRP1,IL15RA,PTX3,LMAN2,DOCK2,ITGA2,RAB31	darkgreen	EC_M4
GO:0045823	2.61E-01	0.877185178	5	25	positive regulation of heart contraction	BP	9.72E-01	ATP2A2,ADA,TGFB2,TPM1,ADRA1B	darkgreen	EC_M4
GO:0010042	2.61E-01	0.890174322	4	19	response to manganese ion	BP	9.72E-01	APP,BACE1,EIF2S1,HSPA5	darkgreen	EC_M4

GO:0051341	2.61E-01	0.82938017	13	79	regulation of oxidoreductase activity	BP	9.72E-01	NUS1,LEP,ATP2B4,HTR2B,KRAS,SLAMF8,AOC3,CALM1,AKT1,NPR3,PARK7,GNAI3,GDNF	darkgreen	EC_M4
GO:0008631	2.61E-01	0.857913503	7	39	intrinsic apoptotic signaling pathway in response to oxidative stress	BP	9.72E-01	UBQLN1,ARL6IP5,HSPB1,PDCD10,GSKIP,AKT1,PARK7	darkgreen	EC_M4
GO:0070286	2.61E-01	0.876757189	5	25	axonemal dynein complex assembly	BP	9.72E-01	CFAP100,DNAH1,DNAH5,DNAAF5,LRRC49	darkgreen	EC_M4
GO:0090189	2.62E-01	0.889822829	4	19	regulation of branching involved in ureteric bud morphogenesis	BP	9.72E-01	VEGFA,SIX4,MAGED1,GDNF	darkgreen	EC_M4
GO:0032212	2.62E-01	0.865799331	6	32	positive regulation of telomere maintenance via telomerase	BP	9.72E-01	TCP1,CTNNB1,CCT8,MAPKAPK5,DKC1,NEK7	darkgreen	EC_M4
GO:0060219	2.63E-01	0.889308981	4	19	camera-type eye photoreceptor cell differentiation	BP	9.72E-01	RORB,ROM1,TTC8,THY1	darkgreen	EC_M4
GO:0014074	2.63E-01	0.815847732	18	109	response to purine-containing compound	BP	9.72E-01	CDO1,PANX1,HSP90B1,RYR3,P2RX4,APP,RAPGEF2,PKD2,PTPRN,PPARGC1A,COL1A1,PRKAA1,HCN2,BSG,HSPA5,TMEM38B,TMEM38A,HCN4	darkgreen	EC_M4
GO:0035637	2.63E-01	0.813881534	19	114	multicellular organismal signaling	BP	9.72E-01	MYH14,FKBP1B,SCN5A,ATP2B1,P2RX4,ATP2A2,CACNA1D,ATP2B4,PKP2,TNR,KCNA1,CACNA1C,CACNA1G,CALM1,ITGA2,PAFAH1B1,FLNA,HCN4,KCNMB4	darkgreen	EC_M4
GO:0090075	2.63E-01	0.875828036	5	25	relaxation of muscle	BP	9.72E-01	P2RX4,ATP2A2,ABCC8,PIK3CA,PRKG1	darkgreen	EC_M4
GO:0015909	2.63E-01	0.849723444	8	46	long-chain fatty acid transport	BP	9.72E-01	PLIN2,MFSD2A,AKT2,IRS2,PLA2R1,CPT1A,EPRS,AKT1	darkgreen	EC_M4
GO:0036257	2.63E-01	0.875107804	5	26	multivesicular body organization	BP	9.72E-01	PDCD6IP,STAM2,VPS37A,TSG101,RAB11A	darkgreen	EC_M4
GO:0001569	2.64E-01	0.875355675	5	25	branching involved in blood vessel morphogenesis	BP	9.72E-01	SRF,VEGFA,CTNNB1,ACVR1,LEF1	darkgreen	EC_M4
GO:0006066	2.64E-01	0.790982662	39	258	alcohol metabolic process	BP	9.72E-01	MIOX,NUS1,PPARD,LEP,REST,INPP4A,FGF2,SNX17,ACER3,DGAT2,ACPP,SMPD1,IMPAD1,DKK3,RDH10,APP,LRP5,ITPK1,INPP5A,ARV1,IDH2,HDLBP,MOGAT3,TPI1,QDPR,CLN8,STARD3,SPTLC2,CLN6,CYP46A1,PRKAA1,OCRL,PGP,PARK7,MBTPS1,DPM2,NFE2L1,TPK1,DPM1	darkgreen	EC_M4
GO:0042445	2.64E-01	0.809034939	21	133	hormone metabolic process	BP	9.72E-01	HSD17B6,LEP,REST,DGAT2,DKK3,PCSK1,RDH10,PTPN11,SPP1,PCSK7,SGPL1,ATP6AP2,COMT,ECE1,HSD17B1,CYP19A1,HSD17B10,STARD3,MED1,PPARGC1A,CYP46A1	darkgreen	EC_M4
GO:0061036	2.64E-01	0.874897066	5	25	positive regulation of cartilage development	BP	9.72E-01	RUNX2,GLI3,LOXL2,GDF6,BMP1	darkgreen	EC_M4
GO:0080135	2.64E-01	0.774574237	81	553	regulation of cellular response to stress	BP	9.72E-01	LRP1,IER3,UBQLN1,KLHDC10,LEP,NUAK1,VEGFA,NCK2,CREB3,REST,EMC10,ACTR2,MDFIC,ROCK2,SLC7A11,FKBP1B,TPT1,WDR48,CTNNB1,HDAC3,DDRGK1,ARL6IP5,CXCL12,ANKRD1,PJA2,TMED2,RNF13,APP,OPA1,AMFR,PTPN22,GDF6,SERINC3,SPP1,PLA2R1,SELENOS,TRIP12,EYA2,TGFB2,FBLN5,GSK3B,KRAS,POLQ,BMP7,HYOU1,TP53BP1,ATF6,HSPB1,MAPT,SYVN1,TNR,PDCD10,SKIL,PRRX1,TNIK,FMN2,AKT3,BMPR1A,ARHGEF2,TAF9B,MAP2K1,CD74,USP14,AKT1,PTPN2,BCAP31,PAFAH1B1,FLNA,TMEM259,PARK7,ENO1,HSPA5,UBE2V2,THY1,EFHD1,DNAJB9,TXN,NACC2,DUSP22,PTTG1IP,FGF10	darkgreen	EC_M4
GO:0050821	2.65E-01	0.803788389	24	154	protein stabilization	BP	9.72E-01	RAB21,TCP1,GNAQ,PEX6,BAG1,PDCL3,P3H1,RTN4,CRTAP,ATP1B3,CTNND1,PFN2,CCT8,CHP1,SYVN1,PDCD10,PPARGC1A,TAF9B,SEL1L,PIN1,NLK,NAPG,FLNA,PARK7	darkgreen	EC_M4
GO:1901890	2.65E-01	0.823311853	14	82	positive regulation of cell junction assembly	BP	9.72E-01	ADGRB1,VEGFA,TPBG,LRRC4B,PTPN11,AMIGO2,IQGAP1,NLGN2,ROCK1,NPHP4,LRRC24,UBE2V2,THY1,LRRTM2	darkgreen	EC_M4
GO:0003143	2.66E-01	0.842737725	9	50	embryonic heart tube morphogenesis	BP	9.72E-01	AHI1,SRF,MEGF8,TMED2,ACVR1,PKD2,IFT57,NOTCH2,LBX1	darkgreen	EC_M4
GO:0042098	2.66E-01	0.809411015	20	127	T cell proliferation	BP	9.72E-01	IL23A,LEP,DOCK8,NCK2,FKBP1B,CTNNB1,PRNP,LMO1,IL20RB,PTPN22,CBLB,PRKAR1A,NCSTN,TFRC,PTPN6,IGFBP2,MSN,DOCK2,CD40LG,CD151	darkgreen	EC_M4

GO:0048863	2.66E-01	0.799448189	27	171	stem cell differentiation	BP	9.72E-01	SRF,RUNX2,REST,FGF2,CTNNB1,LBH,SEMA3C,RDH10,SEMA4F,DHX36,ACVR1,SEMA4G,TGFB2,HTR2B,CORO1C,BMP7,FGFR2,OSM,FN1,MEOX1,BMPR1A,CDK12,MYOCD,ANXA6,PSMD11,A2M,GDNF	darkgreen	EC_M4
GO:0048489	2.66E-01	0.854921078	7	38	synaptic vesicle transport	BP	9.72E-01	AP3S1,KIF5B,PICALM,CTNNB1,AP3B1,SLC2A4,BLOC1S2	darkgreen	EC_M4
GO:0002684	2.66E-01	0.773274478	79	542	positive regulation of immune system process	BP	9.72E-01	IL23A,STX7,ENPP3,HLA-DRA,FAM49B,LGALS1,LEP,VEGFA,DOCK8,NCK2,CREB3,SOCS5,MAP3K7,AKIRIN1,GLI3,RB1,EIF2B3,GLI2,PAK1,TGFB1,AXL,IL15RA,IRS2,PRNP,HSPH1,CXCL12,VAMP8,P2RX4,APP,DHX36,CX3CL1,CACNB3,PTPN11,AP3B1,PTPN22,POLR3D,ADA,RUNX1,CBLB,SLC39A10,NOTCH2,DNM1L,TGFB2,MMP14,PIK3CA,LEF1,PRKCA,CMKLR1,CFI,TP53BP1,TFRC,SERPINE1,ITGA2B,PTPN6,IGFBP2,MED1,CD74,AKT1,ITGA2,C6,PTPN2,CD40LG,TNFRSF18,ATP6AP1,IKBKG,VAMP7,PARK7,AP1G1,BCL10,A2M,CD59,THY1,CDKN1A,DNAJB9,MFAP4,FCN1,CCR6,TSLP,FGF10	darkgreen	EC_M4
GO:0009855	2.67E-01	0.816975712	16	97	determination of bilateral symmetry	BP	9.72E-01	AHI1,SRF,MEGF8,CTNNB1,TMED2,ACVR1,PKD2,DNAH5,IFT57,NOTCH2,AP1B1,BICC1,PKD1L1,NBL1,LBX1,FGF10	darkgreen	EC_M4
GO:0046883	2.00E-01	0.858661559	23	174	regulation of hormone secretion	BP	9.73E-01	PICK1,ICA1,IL6,TRPM4,SFRP1,SIRT3,CHD7,FAM3D,EDN1,ABCG1,HDAC1,PPP3CA,ANO1,CREB1,MCU,CDK16,FGF23,RAC1,HIF1A,SYT7,GPR68,NOS2,BMP6	turquoise	EC_M6
GO:0051497	2.00E-01	0.914120203	5	26	negative regulation of stress fiber assembly	BP	9.73E-01	F11R,PAK2,WAS,ARHGAP28,S1PR1	turquoise	EC_M6
GO:0062149	2.00E-01	0.924250219	4	20	detection of stimulus involved in sensory perception of pain	BP	9.73E-01	CXCR4,EPHB1,MMP24,ANO1	turquoise	EC_M6
GO:0015804	2.00E-01	0.905243579	6	36	neutral amino acid transport	BP	9.73E-01	SLC3A1,LLGL2,SERINC5,SLC38A1,SLC43A2,RGS4	turquoise	EC_M6
GO:0031047	2.00E-01	0.866668934	17	129	gene silencing by RNA	BP	9.73E-01	CNOT6,IL6,MYCN,TUT4,PUM2,PPARG,DND1,SMAD1,PPP3CA,RBM4,MOV10,DDX3X,SMAD2,CNOT8,MIR23A,MIR99A,MIR1307	turquoise	EC_M6
GO:0003279	2.68E-01	0.818825627	15	88	cardiac septum development	BP	9.74E-01	TGFBR1,SNX17,SEMA3C,ADAMTS6,ROBO1,HEY1,ACVR1,NOTCH2,TGFB2,BMP7,FGFR2,FZD2,BMPR1A,FGFRL1,LMO4	darkgreen	EC_M4
GO:0044281	2.68E-01	0.758968158	183	1301	small molecule metabolic process	BP	9.74E-01	ATP8B1,HSD17B6,CDO1,ENPP3,DYRK2,MIOX,RRM2B,AZIN1,PPARA,ACSL6,NUS1,PPARD,AHCYL2,TXN2,PSAT1,MTAP,LEP,PLAA,ALDH1L2,GALT,VCP,SLC35B2,ASNS,REST,INPP4A,RAB23,NANS,MFSD2A,GARS,GFPT1,DKKL1,SLC7A11,FGF2,CMPK1,GAD2,ENTPD6,ABHD12,ME3,ABHD5,ESD,MMACHC,GLB1,ABHD1,SNX17,TGFB1,HNMT,RBKS,ACER3,DGAT2,AKT2,PFKP,C1QTNF1,KAT2B,IRS2,NAXD,CARS2,NUDT16,ACPP,SMPD1,IMPAD1,CHPT1,OSBP,DKK3,RDH10,PLOD2,FAR1,SORBS1,ALDH18A1,APP,MTHFD2,HECTD4,ADCY6,PFKM,PGAM4,OPA1,ENTPD4,LPCAT2,SERINC3,ADA,GLS,ATP2B4,NUDT18,PSPH,MRS2,CARS,ADIPOR1,AZIN2,SELENOS,CPT1A,ZBTB20,PRKAB2,LRP5,EPRS,COQ10B,ITPK1,GSK3B,INPP5A,PIK3CA,HADH,RLBP1,TPST2,CMAS,ARV1,IDH2,ADCY5,HACD2,WDTC1,MAN2A2,PPP1R3D,ATF3,OSBPL2,UROS,HDLBP,SMS,REXO2,SLC25A16,PLOD3,MOGAT3,SGPL1,COMT,TPI1,ACOX3,HSD17B1,QDPR,ASRGL1,PPP1R3F,UGDH,AGMAT,HSD17B10,CLN8,STARD3,PPARGC1A,NPPB,SFXN1,SLC25A44,SPTLC2,CLN6,PKM,AK7,CYP46A1,MPI,ACSL4,CD74,AKT1,LPIN2,NT5M,PRKAA1,OCRL,TARS,PTPN2,OMA1,PAFAH1B1,PGP,HAGH,MIDN,ACOT7,FAM3A,PARK7,ENO1,SLC2A6,AHCYL1,GNAI3,SARS,MBTPS1,SLC35A3,DPM2,AARS,PLA2G15,RANBP2,NFE2L1,HACD1,TPK1,PEMT,GLO1,AMACR,DPM1,TXN,TMSB4X,NNMT,CA13,NME6,TMEM189,ADI1	darkgreen	EC_M4
GO:0070227	2.68E-01	0.840360902	9	52	lymphocyte apoptotic process	BP	9.74E-01	DOCK8,GLI3,IRS2,ADA,SLC39A10,CD74,AKT1,BCL10,ORMDL3	darkgreen	EC_M4
GO:0050806	2.68E-01	0.813087185	17	104	positive regulation of synaptic transmission	BP	9.74E-01	RIMS1,KIF5B,RAB3GAP1,PRNP,APP,CHRNA7,DNM1L,GSK3B,NCSTN,TNR,MPP2,HAP1,NLGN2,STX1B,NPTN,CYP46A1,LRRTM2	darkgreen	EC_M4
GO:1903578	2.69E-01	0.830575733	11	66	regulation of ATP metabolic process	BP	9.74E-01	PPARA,VCP,APP,DNM1L,ZBTB20,PPARGC1A,PRKAA1,ENO1,SLC2A6,COX7A2,TMSB4X	darkgreen	EC_M4
GO:0010613	2.69E-01	0.871196815	5	26	positive regulation of cardiac muscle hypertrophy	BP	9.75E-01	ROCK2,TWF1,PRKCA,PIN1,ROCK1	darkgreen	EC_M4

GO:0014742	2.69E-01	0.871196815	5	26	positive regulation of muscle hypertrophy	BP	9.75E-01	ROCK2,TWF1,PRKCA,PIN1,ROCK1	darkgreen	EC_M4
GO:0002705	2.01E-01	0.88060137	11	76	positive regulation of leukocyte mediated immunity	BP	9.75E-01	DDX58,KIT,IL6,RSAD2,NECTIN2,GATA3,B2M,NOS2,TRAF2,PLCG2,FCER2	turquoise	EC_M6
GO:0032528	2.01E-01	0.923689358	4	20	microvillus organization	BP	9.75E-01	PODXL,USH1C,FSCN1,STK26	turquoise	EC_M6
GO:0003209	2.01E-01	0.913383054	5	26	cardiac atrium morphogenesis	BP	9.75E-01	DLL4,PROX1,NOTCH1,ENG,BMP2	turquoise	EC_M6
GO:0032147	2.69E-01	0.812218364	17	105	activation of protein kinase activity	BP	9.75E-01	IL23A,AGAP2,PRRC1,ANGPT1,LEP,VEGFA,MAP3K7,OSBPL8,PRNP,TGFB2,PIK3CA,NTRK3,ADRA2C,MAP2K1,IRAK1,PARK7,TXN	darkgreen	EC_M4
GO:0042509	2.70E-01	0.839201938	9	52	regulation of tyrosine phosphorylation of STAT protein	BP	9.75E-01	IL23A,LEP,CNOT7,OSBP,OSM,FGFR3,PTPN2,TNFRSF18,TSLP	darkgreen	EC_M4
GO:0071277	2.70E-01	0.829923432	11	63	cellular response to calcium ion	BP	9.75E-01	SCN5A,SMPD1,RYR3,PKD2,NCSTN,IQGAP1,CLIC4,SYT11,PRKAA1,HSPA5,CACYBP	darkgreen	EC_M4
GO:0032228	2.70E-01	0.884670417	4	19	regulation of synaptic transmission, GABAergic	BP	9.75E-01	KIF5B,KRAS,HAP1,NLGN2	darkgreen	EC_M4
GO:0045860	2.71E-01	0.780659979	46	305	positive regulation of protein kinase activity	BP	9.75E-01	IL23A,NAB2,AGAP2,TAB2,PRRC1,ANGPT1,LEP,VEGFA,MAP3K7,FGF2,MAPRE3,PAK1,TGFB1,OSBPL8,PRNP,ILK,ROBO1,RAPGEF2,ATP2B4,PKD2,CCND1,TGFB2,HTR2B,DAG1,PIK3CA,KRAS,NTRK3,SPATC1L,SYAP1,IQGAP1,PDCD10,ADRA2C,DOK7,MNAT1,MAGED1,BMPR1A,SRCIN1,MAP2K1,CALM1,PDGFRB,AKT1,PKD1,IRAK1,PARK7,CDKN1A,TXN	darkgreen	EC_M4
GO:0043647	2.01E-01	0.898038243	7	42	inositol phosphate metabolic process	BP	9.75E-01	SYNJ2,INPP4B,MTMR2,P2RY6,SNCA,ITPKB,PLCG2	turquoise	EC_M6
GO:1901381	2.01E-01	0.913472634	5	25	positive regulation of potassium ion transmembrane transport	BP	9.76E-01	ANO6,KCNJ2,WNK4,DNM2,KCNH2	turquoise	EC_M6
GO:0006308	2.02E-01	0.921720475	4	25	DNA catabolic process	BP	9.76E-01	IL6,ISG20,CECR2,NMNAT1	turquoise	EC_M6
GO:0071108	2.71E-01	0.859916926	6	31	protein K48-linked deubiquitination	BP	9.76E-01	MINDY4,BAP1,OTUD5,PARK7,USP20,MINDY3	darkgreen	EC_M4
GO:0099068	2.71E-01	0.870362824	5	25	postsynapse assembly	BP	9.76E-01	LRRC4B,NTRK3,NLGN2,NTNG2,LRRTM2	darkgreen	EC_M4
GO:0099622	2.71E-01	0.870302284	5	25	cardiac muscle cell membrane repolarization	BP	9.76E-01	SCN5A,SNTA1,CACNA1D,WDR1,FLNA	darkgreen	EC_M4
GO:0009566	2.72E-01	0.808161276	18	113	fertilization	BP	9.76E-01	TRIM36,TCP1,MYH9,UNC13B,NLRP5,RIMS1,DKKL1,WDR48,GLRB,CCT8,AP3B1,KDM5B,CLIC4,VDAC2,UBE2Q1,PARK7,CCDC136,IGSF8	darkgreen	EC_M4
GO:0045124	2.72E-01	0.859216916	6	32	regulation of bone resorption	BP	9.76E-01	SPP1,PRKCA,TFRC,GPR137,ATP6AP1,DEF8	darkgreen	EC_M4
GO:0051894	2.72E-01	0.869895717	5	25	positive regulation of focal adhesion assembly	BP	9.76E-01	VEGFA,PTPN11,IQGAP1,ROCK1,THY1	darkgreen	EC_M4
GO:1903214	2.72E-01	0.850075043	7	39	regulation of protein targeting to mitochondrion	BP	9.76E-01	LMAN1,ATG13,BAP1,C11orf65,PRKAA1,LEPROT,TOMM7	darkgreen	EC_M4
GO:0002819	2.02E-01	0.869098538	15	111	regulation of adaptive immune response	BP	9.77E-01	TNFAIP3,IL6,RSAD2,TRPM4,NECTIN2,GATA3,MEF2C,HLX,B2M,WAS,TNFRSF1B,IL7R,TRAF2,CD46,FCER2	turquoise	EC_M6
GO:1901136	2.02E-01	0.866735205	16	126	carbohydrate derivative catabolic process	BP	9.77E-01	PDE7B,PDE2A,ADAMTS9,PDE4D,LYVE1,GPD1,HEXB,PDE9A,HYAL2,MGAT1,FUCA1,NPL,CDA,PGM2,PDE4B,GBA2	turquoise	EC_M6
GO:0032892	2.74E-01	0.867870051	5	26	positive regulation of organic acid transport	BP	9.78E-01	SLC12A2,ITGB1,RAB3GAP1,P2RX4,PLA2R1	darkgreen	EC_M4
GO:1905039	2.74E-01	0.808130315	17	105	carboxylic acid transmembrane transport	BP	9.78E-01	SLC22A4,SLC7A11,ITGB1,SLC36A4,AKT2,SLC38A10,IRS2,ARL6IP5,CPT1A,SLC3A2,SLC6A13,PRAF2,CLN8,SFXN1,SLC7A3,AKT1,SLC36A1	darkgreen	EC_M4
GO:1900745	2.74E-01	0.867842217	5	26	positive regulation of p38MAPK cascade	BP	9.78E-01	LEP,VEGFA,EMC10,PJA2,GDF6	darkgreen	EC_M4
GO:0019933	2.03E-01	0.897367004	7	40	cAMP-mediated signaling	BP	9.79E-01	PDE7B,RIMS2,PDE2A,PDE4D,RAPGEF3,ADCY2,ADCY1	turquoise	EC_M6

GO:0040018	2.03E-01	0.922644711	4	20	positive regulation of multicellular organism growth	BP	9.79E-01	SPTBN4,CHD7,CREB1,DIO3	turquoise	EC_M6
GO:0046425	2.75E-01	0.835191442	9	52	regulation of receptor signaling pathway via JAK-STAT	BP	9.79E-01	IL23A,LEP,ADIPOR1,ELP2,CRLF3,NLK,PTPN2,OCIAD2,LEPROT	darkgreen	EC_M4
GO:0030031	2.03E-01	0.836109846	56	443	cell projection assembly	BP	9.79E-01	TBC1D30,SPAG1,PARVG,MTSS1,ASAP1,PODXL,CDC42EP1,KIT,CD2AP,SDCBP,NRP1,SPATA6,FGD5,ITGB4,POC1B,ARHGEF7,SPEF1,FGD6,OCLN,FHDC1,WWTR1,P2RX7,GPM6A,PPP1R16B,ANO6,CEP97,EHD4,SRGAP2C,CCDC28B,ABLIM1,JHY,VANGL2,LAMA5,LIMK2,DNM3,SNAP29,WAS,RAC1,RCC2,RHOJ,FSCN1,EPHA2,FGD1,MTOR,OPHN1,ABI3,RABEP2,DNM2,ABLIM3,SPEF2,STK26,NOTCH1,S1PR1,FNBP1L,CARMIL2,RALA	turquoise	EC_M6
GO:0060976	2.03E-01	0.897213958	7	39	coronary vasculature development	BP	9.79E-01	NRP1,PLXND1,SEC24B,MMP21,TBX1,AP2B1,NOTCH1	turquoise	EC_M6
GO:0050687	2.05E-01	0.92122278	4	21	negative regulation of defense response to virus	BP	9.79E-01	C6orf106,SEC14L1,ITCH,RNF125	turquoise	EC_M6
GO:0002028	2.05E-01	0.881288063	10	66	regulation of sodium ion transport	BP	9.79E-01	SPTBN4,P2RX7,SIK1,HECW2,DLG1,WNK4,DNM2,ADRB2,SCN2B,FXYD6	turquoise	EC_M6
GO:0090276	2.05E-01	0.859551841	19	140	regulation of peptide hormone secretion	BP	9.79E-01	PICK1,ICA1,IL6,TRPM4,SFRP1,SIRT3,CHD7,FAM3D,ABCG1,HDAC1,PPP3CA,ANO1,MCU,CDK16,RAC1,HIF1A,SYT7,GPR68,NOS2	turquoise	EC_M6
GO:0051223	2.06E-01	0.835606765	51	412	regulation of protein transport	BP	9.79E-01	PICK1,CD2AP,ICA1,IL6,RSAD2,TLR4,TCAF2,TRPM4,LLGL2,NDFIP2,SFRP1,FERMT1,CD36,SIRT3,PRR5L,FAM3D,IL1A,IGF1,TLR2,VEGFC,RAPGEF3,ABCG1,CD200,HDAC1,PPP3CA,HYAL2,ANO1,HCLS1,PRKD1,OAZ3,APOD,ADORA2A,MCU,CDK16,PPM1F,TMEM30B,RAC1,HIF1A,JUP,SYT7,RSC1A1,PSEN1,SLC51B,GPR68,ABLIM3,SH3TC2,NOS2,MYO18A,RPH3AL,RIPOR1,BMP6	turquoise	EC_M6
GO:0060317	2.06E-01	0.910105541	5	28	cardiac epithelial to mesenchymal transition	BP	9.79E-01	JAG1,EFNA1,NOTCH1,ENG,BMP2	turquoise	EC_M6
GO:0045730	2.06E-01	0.919931179	4	23	respiratory burst	BP	9.79E-01	PIK3CG,NCF1,CYBB,RAC1	turquoise	EC_M6
GO:0048710	2.06E-01	0.920629873	4	21	regulation of astrocyte differentiation	BP	9.79E-01	IL6,MYCN,NOTCH1,BMP2	turquoise	EC_M6
GO:0042311	2.07E-01	0.908880048	5	31	vasodilation	BP	9.79E-01	NOS3,EDNRB,GUCY1A1,ADORA2A,ADRB2	turquoise	EC_M6
GO:0055006	2.08E-01	0.876091085	11	71	cardiac cell development	BP	9.79E-01	JAG1,IGF1,EDN1,MEF2A,NRAP,PROX1,MTOR,YY1,NOTCH1,MIR23A,RGS4	turquoise	EC_M6
GO:0007163	2.08E-01	0.849047891	26	189	establishment or maintenance of cell polarity	BP	9.79E-01	PARD6G,PARVG,PTK2,TEK,LLGL2,GATA3,CYTH1,FERMT1,FRMD4B,SYNE4,TCF15,EPHB1,ERBIN,JAM3,DLG1,ZW10,RAC1,FSCN1,OPHN1,FBXW11,CDC42BPB,AMOT,PATJ,MYO18A,CARMIL2,CDH5	turquoise	EC_M6
GO:0071214	6.89E-02	0.953455787	33	267	cellular response to abiotic stimulus	BP	9.79E-01	XRCC6,PLEC,POLH,ZMPSTE24,CASP8AP2,CASP2,METTL3,MAPK8,ATR,PBK,PPID,TENT2,RAD51,SLC38A2,INO80,NUCKS1,CHEK1,NFKB1,RGS9,INTS7,BLM,CDC25A,XRCC5,LETM1,RAD51AP1,ECT2,AIPL1,NEDD4,AURKB,TAF1,CUL4B,NIPBL,FMR1	darkgrey	EC_M8
GO:0104004	6.89E-02	0.953455787	33	267	cellular response to environmental stimulus	BP	9.79E-01	XRCC6,PLEC,POLH,ZMPSTE24,CASP8AP2,CASP2,METTL3,MAPK8,ATR,PBK,PPID,TENT2,RAD51,SLC38A2,INO80,NUCKS1,CHEK1,NFKB1,RGS9,INTS7,BLM,CDC25A,XRCC5,LETM1,RAD51AP1,ECT2,AIPL1,NEDD4,AURKB,TAF1,CUL4B,NIPBL,FMR1	darkgrey	EC_M8
GO:0009147	6.90E-02	0.982351215	4	19	pyrimidine nucleoside triphosphate metabolic process	BP	9.80E-01	CTPS1,CAD,DCTPP1,TYMS	darkgrey	EC_M8
GO:0045736	6.91E-02	0.978902812	5	24	negative regulation of cyclin-dependent protein serine/threonine kinase activity	BP	9.80E-01	LATS1,IPO5,IPO7,CDK5RAP1,PLK1	darkgrey	EC_M8
GO:1904030	6.91E-02	0.978902812	5	24	negative regulation of cyclin-dependent protein kinase activity	BP	9.80E-01	LATS1,IPO5,IPO7,CDK5RAP1,PLK1	darkgrey	EC_M8
GO:0006813	2.08E-01	0.856034178	20	140	potassium ion transport	BP	9.80E-01	KCNRG,KCNK6,KCNJ3,ANO6,KCNJ15,KCNJ2,SLC12A7,SLC9A8,DLG1,KCNK7,SLC9A7,WNK4,KCNN3,DNM2,KCNJ18,KCNH2,KCNK5,RGS4,KCNA6,KCNG2	turquoise	EC_M6
GO:0097164	1.01E-02	0.998400844	5	13	ammonium ion metabolic process	BP	9.80E-01	ALDH7A1,SLC44A1,CHDH,SRD5A1,CHKA	cyan	EC_M2

GO:0048147	1.01E-02	0.997614143	7	23	negative regulation of fibroblast proliferation	BP	9.80E-01	MYC,MED25,TP53INP1,IFI30,NUPR1,FBXO4,EMD	cyan	EC_M2
GO:0070932	1.03E-02	0.997946281	6	17	histone H3 deacetylation	BP	9.80E-01	SIRT2,ELK4,PER2,HDAC4,HDAC6,PER1	cyan	EC_M2
GO:0072329	1.05E-02	0.995087859	19	95	monocarboxylic acid catabolic process	BP	9.80E-01	ETFB,LPIN1,ACAA1,HACL1,LPIN3,ACOT8,LONP2,IRS1,ACADS,ABCB11,EHHADH,ACADL,ILVBL,ABCD4,GCDH,ACAA2,CPT2,LDHD,SCP2	cyan	EC_M2
GO:0031100	1.06E-02	0.99594892	13	58	animal organ regeneration	BP	9.80E-01	CDK4,HMOX1,LPIN1,TGFBR2,RPS16,HGF,SULF2,ATIC,FPGS,TGFBR3,MT-CYB,RPS15,CEBPB	cyan	EC_M2
GO:0046386	1.06E-02	0.997142763	8	27	deoxyribose phosphate catabolic process	BP	9.80E-01	DNPH1,PNP,XDH,SMUG1,NEIL2,DPYD,NT5C,UPP1	cyan	EC_M2
GO:0006577	1.08E-02	0.998253938	5	13	amino-acid betaine metabolic process	BP	9.80E-01	ALDH7A1,CHDH,POR,ACADL,CPT2	cyan	EC_M2
GO:0045017	1.08E-02	0.99368643	35	214	glycerolipid biosynthetic process	BP	9.80E-01	DGKA,PIP4K2C,CHKB,AGPAT1,GPAA1,PIGO,TTC7A,SLC44A1,LPIN1,TAMM41,PTDSS2,PIK3R1,GPAT3,LPIN3,PIGT,GPAM,CHKA,ABHD4,PIGM,ABHD8,CTDNEP1,PLSCR3,PIGH,PIP4K2B,DPM3,AC138894.1,MTMR4,SREBF1,MPPE1,MTMR1,TAZ,PIGQ,INPP5E,VAC14,LCAT	cyan	EC_M2
GO:0006635	1.08E-02	0.995663111	14	64	fatty acid beta-oxidation	BP	9.80E-01	ETFB,ACAA1,ACOT8,LONP2,IRS1,ACADS,ABCB11,EHHADH,ACADL,ABCD4,GCDH,ACAA2,CPT2,SCP2	cyan	EC_M2
GO:0061512	1.09E-02	0.995966825	12	54	protein localization to cilium	BP	9.80E-01	TTC26,IFT122,TTC21A,FAM149B1,DYNC2H1,CC2D2A,CROCC,WDR19,TMEM107,SPATA7,EFCAB7,INPP5E	cyan	EC_M2
GO:0034440	1.10E-02	0.994994208	18	90	lipid oxidation	BP	9.80E-01	ETFB,ACAA1,CYGB,HACL1,ACOT8,LONP2,IRS1,ACADS,ABCB11,EHHADH,POR,ACADL,ILVBL,ABCD4,GCDH,ACAA2,CPT2,SCP2	cyan	EC_M2
GO:1904292	1.10E-02	0.997743712	6	18	regulation of ERAD pathway	BP	9.80E-01	BAG6,RNFT2,USP19,NFE2L2,UBXN1,RNFT1	cyan	EC_M2
GO:0032570	1.12E-02	0.996918248	8	29	response to progesterone	BP	9.80E-01	TSPO,TRERF1,FOSB,WBP2,NCOA2,TXNIP,FOS,SREBF1	cyan	EC_M2
GO:0008299	2.08E-01	0.918594037	4	23	isoprenoid biosynthetic process	BP	9.80E-01	SRD5A3,ALDH1A2,CYP1A1,RBP1	turquoise	EC_M6
GO:0097061	2.09E-01	0.875445767	11	70	dendritic spine organization	BP	9.80E-01	PICK1,MTMR2,EPHB1,TANC1,DNM3,ABI3,ITGA3,EFNA1,ZDHHC15,RELN,SHANK3	turquoise	EC_M6
GO:0032069	6.92E-02	0.982573533	4	17	regulation of nuclease activity	BP	9.81E-01	TCEA1,ABCE1,TERF1,DDX11	darkgrey	EC_M8
GO:0061326	2.76E-01	0.817844885	13	77	renal tubule development	BP	9.81E-01	AHI1,VEGFA,GLI3,FGF2,CTNNB1,ILK,PKD2,NOTCH2,SIX4,MAGED1,TTC8,PKD1,GDNF	darkgreen	EC_M4
GO:0033173	2.76E-01	0.847645866	7	38	calcineurin-NFAT signaling cascade	BP	9.81E-01	DYRK2,PRNP,PPP3CC,CHP1,ATP2B4,ADGRB2,GSK3B	darkgreen	EC_M4
GO:1903052	1.15E-02	0.994260457	22	118	positive regulation of proteolysis involved in cellular protein catabolic process	BP	9.81E-01	BAG6,HSPA1B,NOP53,BBS7,SIRT2,RNFT2,AC013489.1,MAGEF1,NFE2L2,GABARAP,OSBPL7,GBA,NUPR1,KEAP1,FZR1,DVL1,AXIN1,ZER1,RACK1,RNFT1,TRIB2,STUB1	cyan	EC_M2
GO:0003093	1.15E-02	0.998647209	4	9	regulation of glomerular filtration	BP	9.81E-01	F2RL1,CORO2B,EMP2,CYBA	cyan	EC_M2
GO:0000027	1.16E-02	0.997596654	6	18	ribosomal large subunit assembly	BP	9.82E-01	RPL3,NOP53,RPL11,RPL23A,TRAF7,DDX28	cyan	EC_M2
GO:0032434	2.77E-01	0.801566425	19	121	regulation of proteasomal ubiquitin-dependent protein catabolic process	BP	9.82E-01	RNF19A,UBQLN1,VCP,SOCS5,NUB1,CHFR,DDRGK1,UCHL5,GSK3B,ARAF,ARIH1,FBXO22,USP14,AKT1,BCAP31,PARK7,UBE2V2,DDA1,MAP1A	darkgreen	EC_M4
GO:1903793	2.77E-01	0.866024199	5	26	positive regulation of anion transport	BP	9.82E-01	SLC12A2,ITGB1,RAB3GAP1,P2RX4,PLA2R1	darkgreen	EC_M4
GO:0007616	2.09E-01	0.908174253	5	27	long-term memory	BP	9.83E-01	ADCY1,PTCHD1,RGS14,MTOR,RELN	turquoise	EC_M6
GO:1902475	2.78E-01	0.83946535	8	45	L-alpha-amino acid transmembrane transport	BP	9.83E-01	SLC7A11,ITGB1,SLC36A4,ARL6IP5,SLC3A2,PRAF2,CLN8,SLC7A3	darkgreen	EC_M4
GO:1901800	1.17E-02	0.99437601	20	104	positive regulation of proteasomal protein catabolic process	BP	9.84E-01	BAG6,HSPA1B,NOP53,BBS7,SIRT2,RNFT2,AC013489.1,NFE2L2,GABARAP,OSBPL7,GBA,NUPR1,KEAP1,FZR1,DVL1,ZER1,RACK1,RNFT1,TRIB2,STUB1	cyan	EC_M2
GO:1904377	2.78E-01	0.828178537	10	58	positive regulation of protein localization to cell periphery	BP	9.84E-01	LRP1,ITGB1,KIF5B,ATP2C1,PRNP,SORBS1,ATP2B4,PRKCI,RAB11A,AKT1	darkgreen	EC_M4

GO:2000736	2.10E-01	0.886782354	8	50	regulation of stem cell differentiation	BP	9.84E-01	TEAD2,JAG1,SLC4A11,TCF15,KDM3A,ITCH,NOTCH1,CDK13	turquoise	EC_M6
GO:0033674	2.78E-01	0.769312691	56	373	positive regulation of kinase activity	BP	9.85E-01	IL23A,NAB2,AGAP2,TAB2,PRRC1,ANGPT1,ROS1,LEP,VEGFA,MAP3K7,FGF2,MAPRE3,PAK1,TGFB1,AXL,OSBPL8,PRNP,ILK,RYK,ROBO1,RAPGEF2,ATP2B4,PKD2,CCND1,TGFB2,HTR2B,DAG1,PIK3CA,KRAS,NTRK3,SPATC1L,FGFR2,SYAP1,IQGAP1,MAPT,PDCD10,ADRA2C,DOK7,FGFR3,MNAT1,MAGED1,BMPR1A,SRCIN1,MAP2K1,CALM1,CD74,PDGFRB,AKT1,PKD1,IRAK1,PARK7,LMO4,BCL10,RANBP2,CDKN1A,TXN	darkgreen	EC_M4
GO:0071875	2.79E-01	0.879554057	4	19	adrenergic receptor signaling pathway	BP	9.85E-01	RAPGEF2,ATP2B4,ADRA2C,ADRA1B	darkgreen	EC_M4
GO:0006839	2.79E-01	0.784093917	31	210	mitochondrial transport	BP	9.85E-01	LMAN1,HSPA4,SMDT1,COX18,VPS35,MGARP,SLC25A4,OPA1,ATG13,BAP1,TMEM14C,MRS2,EYA2,CPT1A,GSK3B,TOMM40L,FZD9,SLC25A16,C11orf65,VDAC2,STARD3,SFXN1,AKT1,PRKAA1,BCAP31,BNIP3,BLOC1S2,TIMM22,LEPROT,GRPEL2,TOMM7	darkgreen	EC_M4
GO:0048144	2.79E-01	0.82262654	11	66	fibroblast proliferation	BP	9.85E-01	B4GALT7,CTNNB1,LIG4,CDC73,FN1,NGFR,CD74,PDGFRB,CDKN1A,WNT2,FGF10	darkgreen	EC_M4
GO:0048145	2.79E-01	0.82262654	11	66	regulation of fibroblast proliferation	BP	9.85E-01	B4GALT7,CTNNB1,LIG4,CDC73,FN1,NGFR,CD74,PDGFRB,CDKN1A,WNT2,FGF10	darkgreen	EC_M4
GO:0002686	2.79E-01	0.85380471	6	33	negative regulation of leukocyte migration	BP	9.85E-01	CXCL12,ADA,SLAMF8,NBL1,AKT1,MIR24-1	darkgreen	EC_M4
GO:0062014	2.79E-01	0.818389715	12	73	negative regulation of small molecule metabolic process	BP	9.85E-01	PPARA,REST,MFSD2A,DKKL1,DGAT2,DKK3,ATP2B4,WDTC1,PPARGC1A,AKT1,PGP,FAM3A	darkgreen	EC_M4
GO:0009126	1.17E-02	0.996147248	10	39	purine nucleoside monophosphate metabolic process	BP	9.85E-01	DNPH1,PNP,XDH,DGUOK,NT5C2,IMPDH2,ATIC,APRT,NT5C,NUDT2	cyan	EC_M2
GO:0032958	2.10E-01	0.91828112	4	21	inositol phosphate biosynthetic process	BP	9.85E-01	P2RY6,SNCA,ITPKB,PLCG2	turquoise	EC_M6
GO:0071398	2.10E-01	0.907116937	5	29	cellular response to fatty acid	BP	9.85E-01	SMARCD1,EDN1,PDK3,CREB1,ADIPOR2	turquoise	EC_M6
GO:0000079	2.80E-01	0.814559015	13	80	regulation of cyclin-dependent protein serine/threonine kinase activity	BP	9.86E-01	CDKN2B,MAPRE3,KAT2B,CCNJ,GTF2H1,PKD2,CCND1,MNAT1,CCNG1,MYOCD,AKT1,PKD1,CDKN1A	darkgreen	EC_M4
GO:0034314	2.80E-01	0.853320562	6	33	Arp2/3 complex-mediated actin nucleation	BP	9.86E-01	ACTR2,ACTR3,MAGEL2,ARPC5,NCKAP1,ARPC2	darkgreen	EC_M4
GO:0001817	2.80E-01	0.762541137	72	492	regulation of cytokine production	BP	9.86E-01	IL23A,LRP1,ANGPT1,PPARA,FAM49B,LEP,SOCS5,UBE2J1,MAP3K7,ROCK2,INHBA,PANX1,TGFB1,AXL,NAV3,NPLOC4,LTBP1,PRNP,ATP2B1,LUM,IL20RB,SULF1,APP,DHX36,CX3CL1,ABCC8,PTPN11,PTPN22,POLR3D,BAP1,CHID1,RUNX1,PLA2R1,RAB7B,CHRNA7,SELENOS,SLC2A10,KPNA6,TGFB2,HTR2B,LEF1,TLR5,CMKLR1,PPP1R11,ASB1,OSM,HSPB1,SERPINE1,ATP6AP2,FN1,PTPN6,ITGAV,OTUD5,BMPR1A,ARHGEF2,SYT11,TMED10,TGFB3,DICER1,NPTN,SCAMP5,CD74,CD40LG,IRAK1,PARK7,SARS,F3,BCL10,MIR24-1,TMSB4X,FCN1,TSLP	darkgreen	EC_M4
GO:0009799	2.80E-01	0.805809108	16	98	specification of symmetry	BP	9.87E-01	AHI1,SRF,MEGF8,CTNNB1,TMED2,ACVR1,PKD2,DNAH5,IFT57,NOTCH2,AP1B1,BICC1,PKD1L1,NBL1,LBX1,FGF10	darkgreen	EC_M4
GO:0001913	2.80E-01	0.877811893	4	20	T cell mediated cytotoxicity	BP	9.87E-01	IL23A,STX7,HLA-DRA,FAM49B	darkgreen	EC_M4
GO:0002704	2.12E-01	0.905922226	5	30	negative regulation of leukocyte mediated immunity	BP	9.88E-01	LGALS9,IL7R,CD46,SERPINB9,BST2	turquoise	EC_M6
GO:0008361	2.12E-01	0.850115907	22	157	regulation of cell size	BP	9.88E-01	SEMA6A,CDKL3,SEMA4D,NRCAM,NRP1,RET,CLCN3,P2RX7,ANO6,EDN1,SLC12A7,SEMA3F,CREB1,SEMA6D,RAC1,MTOR,SEMA4A,DNM2,IL7R,SEMA6B,ARHGAP4,SHANK3	turquoise	EC_M6
GO:0070229	2.13E-01	0.916604232	4	22	negative regulation of lymphocyte apoptotic process	BP	9.88E-01	HIF1A,ARG2,EFNA1,IL7R	turquoise	EC_M6

GO:0032233	2.13E-01	0.879968042	9	56	positive regulation of actin filament bundle assembly	BP	9.88E-01	MTSS1,LIMCH1,NRP1,SFRP1,RAPGEF3,PPM1F,RAC1,MTOR,ARHGEF15	turquoise	EC_M6
GO:0001910	2.13E-01	0.896635201	6	38	regulation of leukocyte mediated cytotoxicity	BP	9.88E-01	NECTIN2,B2M,LGALS9,NOS2,IL7R,SERPINB9	turquoise	EC_M6
GO:0006775	2.13E-01	0.916326441	4	22	fat-soluble vitamin metabolic process	BP	9.88E-01	FGF23,ALDH1A2,CYP1A1,RBP1	turquoise	EC_M6
GO:0030073	2.13E-01	0.851548543	20	149	insulin secretion	BP	9.88E-01	RIMS2,PICK1,ICA1,IL6,TRPM4,SFRP1,SIRT3,FAM3D,ABCG1,HDAC1,PPP3CA,ANO1,MCU,CDK16,RAC1,HIF1A,SYT7,GPR68,SMAD2,NOS2	turquoise	EC_M6
GO:0070252	2.81E-01	0.817057912	12	70	actin-mediated cell contraction	BP	9.89E-01	SCN5A,GPD1L,SNTA1,ATP2A2,CACNA1D,PKP2,PIK3CA,CACNA1C,TPM1,CACNA1G,FLNA,HCN4	darkgreen	EC_M4
GO:0009896	2.81E-01	0.764249294	62	420	positive regulation of catabolic process	BP	9.89E-01	LRP1,RNF19A,PPARA,UBQLN1,GGA1,VCP,SOCS5,MAP3K7,ROCK2,VPS35,SESN3,RAB7A,PSMC2,ABHD5,NUB1,RAB3GAP1,AKT2,IRS2,TMTC3,CHFR,DCN,DDRGK1,CNOT7,TP53INP2,SLC25A4,CELF1,APP,DHX36,MTDH,CBLB,NKD1,TENT4B,SEC22B,CPT1A,ZBTB20,GSK3B,ATF6,PAFAH1B2,UPF1,HTT,SAMD4A,SMURF1,FBXL5,MSN,SPTLC2,ARIH1,FBXO22,ROCK1,AKT1,PRKAA1,BCAP31,IKBKG,TMEM259,PARK7,GPSM1,GNAI3,BNIP3,UBE2V2,PTTG1IP,DDA1,PIK3C2A,ORMDL3	darkgreen	EC_M4
GO:0001836	2.81E-01	0.835983812	8	48	release of cytochrome c from mitochondria	BP	9.89E-01	GGCT,OPA1,DNM1L,FAM162A,FZD9,GHITM,AKT1,BNIP3	darkgreen	EC_M4
GO:0022037	2.81E-01	0.81018212	14	84	metencephalon development	BP	9.89E-01	WHRN,GLI2,PAK1,SCN5A,FAIM2,PTPN11,NCSTN,HAP1,PPARGC1A,MAP2K1,SDF4,RERE,AARS,HSPA5	darkgreen	EC_M4
GO:0043277	2.14E-01	0.896345685	6	37	apoptotic cell clearance	BP	9.89E-01	CD36,SCARB1,MERTK,RAC1,PEAR1,CCL2	turquoise	EC_M6
GO:0035051	2.14E-01	0.856075963	17	119	cardiocyte differentiation	BP	9.90E-01	JAG1,EFNB2,IGF1,MEF2C,EDN1,SIK1,MEF2A,NRAP,PROX1,MTOR,YY1,NOTCH1,MIR23A,SOX17,SOX18,RGS4,BMP2	turquoise	EC_M6
GO:0060968	2.14E-01	0.878699226	9	59	regulation of gene silencing	BP	9.90E-01	IL6,MYCN,PUM2,PPARG,DND1,KIAA1551,PPP3CA,TET1,HIST1H3F	turquoise	EC_M6
GO:0007040	2.15E-01	0.878140076	9	61	lysosome organization	BP	9.90E-01	LAPTM4B,PPT1,MYO7A,HEXB,LYST,LAPTM5,SYT7,MTOR,RAB39A	turquoise	EC_M6
GO:0080171	2.15E-01	0.878140076	9	61	lytic vacuole organization	BP	9.90E-01	LAPTM4B,PPT1,MYO7A,HEXB,LYST,LAPTM5,SYT7,MTOR,RAB39A	turquoise	EC_M6
GO:0030808	2.15E-01	0.914534723	4	24	regulation of nucleotide biosynthetic process	BP	9.90E-01	NOS3,SNCA,PDK3,NOS2	turquoise	EC_M6
GO:1900371	2.15E-01	0.914534723	4	24	regulation of purine nucleotide biosynthetic process	BP	9.90E-01	NOS3,SNCA,PDK3,NOS2	turquoise	EC_M6
GO:0032620	2.15E-01	0.914428981	4	24	interleukin-17 production	BP	9.90E-01	IL6,TLR4,IL15,ARG2	turquoise	EC_M6
GO:0032660	2.15E-01	0.914428981	4	24	regulation of interleukin-17 production	BP	9.90E-01	IL6,TLR4,IL15,ARG2	turquoise	EC_M6
GO:0006629	2.16E-01	0.814070868	112	982	lipid metabolic process	BP	9.91E-01	RDH16,ALDH8A1,SYNJ2,SERAC1,QKI,PTK2,HSD17B3,PDGFB,TEK,PIP5K1B,ADGRF5,KIT,SRD5A3,AGMO,PIGF,PPT1,KBTBD2,CPT1C,INPP4B,OSBPL9,PIK3CG,MTMR2,MGLL,PRKD2,RAB38,PPARG,PGS1,GDPD5,GPAT4,NR1D2,PLCB4,CDS2,CD36,SIRT3,SCARB1,ELOVL7,ABHD6,PITPNM2,ST3GAL5,CHRM5,GPD1,HEXB,DHH,ACOT12,TIPARP,SERINC5,PLCB2,PLCG1,ST3GAL6,DGKZ,PIK3C2B,EDN1,SNCA,SLC45A3,PM20D1,ABCG1,MTMR10,LPCAT1,MTTP,SIK1,PLA1A,INPP5D,B3GNT5,BMX,FGR,INPP5F,PPP2R5A,PROX1,PLEKHA1,PRKD1,MLXIPL,GAL3ST3,FUCA1,APOD,PDK3,CREB1,OSBPL6,ALG9,HSPG2,TNFAIP8L3,FGF23,SAMD8,LIPA,PLD2,CYP4F8,ADIPOR2,ITPKB,LBR,ALDH1A2,MTOR,GDPD3,CD19,GDPD1,SCNN1B,DHRS11,CYP1A1,B4GALT6,MTMR12,MTM1,PLCG2,PTGS1,DAB2IP,ST6GALNAC3,PLA2G4E,GBA2,BMP2,SCARF1,SRD5A2,BMP6,AKR1D1,RBP1,ST8SIA6	turquoise	EC_M6
GO:1903169	2.16E-01	0.85872988	15	110	regulation of calcium ion transmembrane transport	BP	9.91E-01	TRDN,TSPAN13,PIK3CG,P2RY6,PDE4D,CHD7,CXCL10,PLCG1,SNCA,PRKD1,CRACR2A,ATP2A1,CD19,PDE4B,PLCG2	turquoise	EC_M6
GO:0031571	7.01E-02	0.978420866	5	25	mitotic G1 DNA damage checkpoint signaling	BP	9.91E-01	CDK2,GTSE1,CASP2,PRKDC,RPA2	darkgrey	EC_M8

GO:0032785	7.01E-02	0.982284531	4	17	negative regulation of DNA-templated transcription, elongation	BP	9.91E-01	EZH2,TCERG1,HNRNPU,RNF8	darkgrey	EC_M8
GO:0050857	2.82E-01	0.876556169	4	20	positive regulation of antigen receptor-mediated signaling pathway	BP	9.91E-01	ADA,SLC39A10,IKBKG,BCL10	darkgreen	EC_M4
GO:0030162	2.16E-01	0.822950855	62	538	regulation of proteolysis	BP	9.91E-01	TNFAIP8,TRIM39,GRAMD4,PTK2,DAPK1,AGTPBP1,CSNK1E,FAM122A,SYK,C5,SDCBP,CST7,CTSC,PPARG,TIMP4,ITIH5,PLK2,NR4A1,KLKB1,EIF2AK3,ITCH,SH3RF1,HIP1R,SOX7,FAM192A,SNCA,RNF144B,RNF19B,HDAC1,LCK,LAPTM5,USP13,CFLAR,CASP10,RPS6KA1,PAK2,CST6,MELTF,LTF,ADORA2A,DDX3X,BIRC3,PPM1F,BID,TNFRSF1B,OPHN1,RNF40,PSEN1,PAPLN,FBXW11,EFNA1,PIAS1,TNIP1,LGALS9,USP7,DAB2IP,PRSS22,PMAIP1,SERPINB9,PSMB9,PERP,BST2	turquoise	EC_M6
GO:0016331	2.16E-01	0.852669678	18	127	morphogenesis of embryonic epithelium	BP	9.91E-01	FZD6,CTHRC1,CELSR1,TEAD2,GATA3,SFRP1,RET,VEGFC,SEC24B,VANGL2,LAMA5,HIF1A,CECR2,ALDH1A2,AR,JAG2,VASP,RALA	turquoise	EC_M6
GO:0035019	2.83E-01	0.851678799	6	32	somatic stem cell population maintenance	BP	9.91E-01	REST,LBH,LIG4,LRP5,BMP7,FGF10	darkgreen	EC_M4
GO:0008654	1.19E-02	0.992939088	36	222	phospholipid biosynthetic process	BP	9.92E-01	DGKA,PIP4K2C,CHKB,AGPAT1,GPAA1,PIGO,TTC7A,SLC44A1,LPIN1,SPHK2,TAMM41,PTDSS2,PIK3R1,GPAT3,PIGT,GPAM,CHKA,ABHD4,PIGM,IDH1,PLA2G16,ABHD8,PLSCR3,PIGH,PIP4K2B,DPM3,AC138894.1,MTMR4,MPPE1,MTMR1,TAZ,PIGQ,INPP5E,VAC14,LCAT,ABCA2	cyan	EC_M2
GO:0019082	2.83E-01	0.862120783	5	26	viral protein processing	BP	9.92E-01	CANX,ST6GALNAC2,GSK3B,GALNT1,SPCS3	darkgreen	EC_M4
GO:0035282	2.17E-01	0.868980392	11	74	segmentation	BP	9.92E-01	NRP1,SFRP1,TCF15,SEMA3F,PLXNA2,LFNG,ALDH1A2,PSEN1,NRARP,NOTCH1,MTF2	turquoise	EC_M6
GO:0018209	2.17E-01	0.833763769	35	267	peptidyl-serine modification	BP	9.92E-01	PDGFB,MAPK13,CSNK1E,SYK,IL6,PRKD2,SPTBN4,IKBKB,PLK2,RET,PDE4D,EIF2AK3,DCLK2,RAPGEF3,HIPK1,DYRK1A,SNCA,PRKX,DYRK3,HCLS1,INPP5F,RPS6KA1,PRKD1,MLXIPL,PAK2,PDK3,LMTK2,PPM1F,PRKCH,MTOR,SBK1,RPS6KA5,MARK3,SH2D3A,SH2D3C	turquoise	EC_M6
GO:0002092	2.17E-01	0.914494013	4	21	positive regulation of receptor internalization	BP	9.92E-01	PICK1,SYK,SELE,PLCG2	turquoise	EC_M6
GO:2001257	2.17E-01	0.855441908	16	116	regulation of cation channel activity	BP	9.92E-01	TRDN,DAPK1,KCNRG,P2RY6,PDE4D,MEF2C,DLG1,CRACR2A,ADRB2,CCL2,PDE4B,PLCG2,RELN,SHANK3,CACNG5,GSG1L	turquoise	EC_M6
GO:0072202	2.17E-01	0.914065326	4	22	cell differentiation involved in metanephros development	BP	9.92E-01	PDGFB,WWTR1,STAT1,CD34	turquoise	EC_M6
GO:0034614	1.20E-02	0.99387772	23	124	cellular response to reactive oxygen species	BP	9.92E-01	STAT6,MAP3K5,ERCC6L2,HSF1,PDGFRA,FOXO3,AGAP3,RPS3,CYP1B1,HGF,CAMKK2,RIPK3,CCS,NFE2L2,ANKZF1,HDAC6,FOS,PPARGC1B,RACK1,ZNF580,ADPRHL2,PDGFD,MPV17	cyan	EC_M2
GO:0002791	2.18E-01	0.849629816	19	142	regulation of peptide secretion	BP	9.93E-01	PICK1,ICA1,IL6,TRPM4,SFRP1,SIRT3,CHD7,FAM3D,ABCG1,HDAC1,PPP3CA,ANO1,MCU,CDK16,RAC1,HIF1A,SYT7,GPR68,NOS2	turquoise	EC_M6
GO:0010719	2.84E-01	0.861618567	5	26	negative regulation of epithelial to mesenchymal transition	BP	9.94E-01	SPRED3,ADIPOR1,TGFB2,VASN,DACT3	darkgreen	EC_M4
GO:0009612	2.19E-01	0.843213614	23	176	response to mechanical stimulus	BP	9.95E-01	PTK2,KIT,TLR4,CXCR4,PDE2A,MAP3K1,CXCL10,ANGPT2,TMEM150C,EDN1,STAT1,ANO3,ETS1,KCNJ2,F11R,RAC1,JUP,PIEZO2,PIEZO1,ENG,STRBP,SHANK3,BMP6	turquoise	EC_M6
GO:0008654	2.19E-01	0.8378098	28	222	phospholipid biosynthetic process	BP	9.95E-01	SYNJ2,SERAC1,PDGFB,PIP5K1B,ADGRF5,SRD5A3,PIGF,INPP4B,PIK3CG,MTMR2,RAB38,PGS1,GPAT4,CDS2,PITPNM2,HEXB,SERINC5,DGKZ,PIK3C2B,LPCAT1,INPP5D,BMX,INPP5F,SAMD8,PLD2,ITPKB,MTM1,PLCG2	turquoise	EC_M6
GO:0010466	2.19E-01	0.849469613	18	145	negative regulation of peptidase activity	BP	9.95E-01	TNFAIP8,C5,CST7,TIMP4,ITIH5,NR4A1,SH3RF1,SNCA,RPS6KA1,PAK2,CST6,LTF,ADORA2A,DDX3X,BIRC3,PAPLN,SERPINB9,BST2	turquoise	EC_M6
GO:1901214	2.20E-01	0.83367604	32	251	regulation of neuron death	BP	9.95E-01	OXR1,NCOA7,ATF4,REL,PPT1,TLR4,NRP1,GATA3,EFNB2,EPHB1,MEF2C,NR4A2,SNCA,PM20D1,CD200,HDAC1,CD34,MAP3K11,CREB1,ADORA2A,HIF1A,SNCG,TLR6,TNFRSF1B,MTOR,PSEN1,AP2B1,PPP1R13B,CCL2,TRAF2,STXBP1,NMNAT1	turquoise	EC_M6
GO:0009066	2.20E-01	0.89171577	6	43	aspartate family amino acid metabolic process	BP	9.95E-01	ATF4,AADAT,ADSS,PIPOX,MTHFD2L,SLC25A21	turquoise	EC_M6

GO:0006874	2.20E-01	0.830802151	36	283	cellular calcium ion homeostasis	BP	9.95E-01	TRDN,CAV2,TRPM4,PIK3CG,GATA2,GPR4,CXCR4,EDNRB,P2RY6,P2RX2,CD36,TMEM178A,MICU3,PDE4D,CHD7,HEXB,P2RX7,CXCL10,TPCN1,PLCG1,STC1,EDN1,SNCA,LCK,HCRTR1,PRKD1,CCRL2,MCU,PSEN1,ATP2A1,CD19,PLCG2,S1PR1,CDH5,SLC8A3,LPAR6	turquoise	EC_M6
GO:0072507	2.20E-01	0.827779302	41	323	divalent inorganic cation homeostasis	BP	9.95E-01	TRDN,CAV2,TRPM4,PIK3CG,GATA2,GPR4,CXCR4,EDNRB,TMC8,P2RY6,P2RX2,CD36,TMEM178A,MICU3,PDE4D,CHD7,HEXB,P2RX7,CXCL10,TPCN1,PLCG1,STC1,EDN1,SNCA,LCK,SLC39A8,HCRTR1,PRKD1,CCRL2,MCU,PSEN1,ATP2A1,CD19,S100A14,AP3D1,PLCG2,S1PR1,CDH5,SLC8A3,TRIM24,LPAR6	turquoise	EC_M6
GO:0045736	1.22E-02	0.996972813	7	24	negative regulation of cyclin-dependent protein serine/threonine kinase activity	BP	9.95E-01	CDKN2A,LATS2,CDKN1B,HEXIM2,MEN1,TFAP4,HEXIM1	cyan	EC_M2
GO:1904030	1.22E-02	0.996972813	7	24	negative regulation of cyclin-dependent protein kinase activity	BP	9.95E-01	CDKN2A,LATS2,CDKN1B,HEXIM2,MEN1,TFAP4,HEXIM1	cyan	EC_M2
GO:0002446	2.20E-01	0.912182866	4	23	neutrophil mediated immunity	BP	9.95E-01	SYK,IL6,ACE,SCNN1B	turquoise	EC_M6
GO:0006865	2.20E-01	0.857835783	14	99	amino acid transport	BP	9.95E-01	APBA1,SLC3A1,LLGL2,SERINC5,SLC38A1,SNCA,SLC7A7,ADORA2A,PSEN1,SLC43A2,STXBP1,LRRC8C,LRRC8D,RGS4	turquoise	EC_M6
GO:0106027	2.20E-01	0.863588382	12	78	neuron projection organization	BP	9.95E-01	PICK1,MTMR2,EPHB1,TANC1,DNM3,PSEN1,ABI3,ITGA3,EFNA1,ZDHHC15,RELN,SHANK3	turquoise	EC_M6
GO:0002381	7.05E-02	0.970594731	8	48	immunoglobulin production involved in immunoglobulin-mediated immune response	BP	9.95E-01	MSH2,PAXIP1,RIF1,NBN,NSD2,EXO1,THOC1,RNF8	darkgrey	EC_M8
GO:0046825	7.06E-02	0.978108927	5	26	regulation of protein export from nucleus	BP	9.97E-01	ANP32B,XPO1,XPO4,CTDSPL2,TPR	darkgrey	EC_M8
GO:0003407	2.21E-01	0.879300098	8	49	neural retina development	BP	9.97E-01	TSPAN12,GPM6A,USH1C,HIPK1,DLL4,PROM1,PSEN1,DIO3	turquoise	EC_M6
GO:1901889	2.21E-01	0.901438689	5	26	negative regulation of cell junction assembly	BP	9.97E-01	IKBKB,TLR2,ACE,APOD,RCC2	turquoise	EC_M6
GO:0007498	2.21E-01	0.859476488	13	89	mesoderm development	BP	9.97E-01	TEAD2,TAL1,ITGA8,ITGB4,TIE1,TCF15,SMAD1,ETS2,BMX,TBX1,EPHA2,ITGA3,SMAD2	turquoise	EC_M6
GO:0050856	2.22E-01	0.899649819	5	31	regulation of T cell receptor signaling pathway	BP	9.98E-01	PRKD2,NECTIN2,DGKZ,LCK,LAPTM5	turquoise	EC_M6
GO:0051149	2.85E-01	0.827229605	9	53	positive regulation of muscle cell differentiation	BP	9.98E-01	MYLK3,SMARCD3,TGFB1,MMP14,MORF4L2,SETD3,MYOCD,PIN1,HOPX	darkgreen	EC_M4
GO:0006836	2.86E-01	0.789296072	22	139	neurotransmitter transport	BP	9.99E-01	UNC13B,RIMS1,ITGB1,LIN7B,NAPA,RAB3GAP1,PCLO,SNPH,ATP2A2,VPS18,DNM1L,GSK3B,BACE1,SLC6A13,SLC29A4,CLN8,STX1B,SYT11,CALM1,PARK7,GDNF,KCNMB4	darkgreen	EC_M4
GO:0039694	7.10E-02	0.978173293	5	24	viral RNA genome replication	BP	9.99E-01	TRIM28,TOP2B,FAM208A,TOP2A,FMR1	darkgrey	EC_M8
GO:0000002	2.86E-01	0.874359585	4	20	mitochondrial genome maintenance	BP	9.99E-01	SLC25A4,OPA1,AKT3,PPARGC1A	darkgreen	EC_M4

GO:0051171	2.23E-01	0.794830461	460	4082	regulation of nitrogen compound metabolic process	BP	1.00E+00	ZNF516,TNFRSF11A,BAZ2A,TCF4,TNFAIP8,IFNGR1,TNFAIP3,PLAGL1,TRIM39,PTPRB,LAPTM4B,CNOT6,FLT4,FZD6,OXR1,ZBED4,GRAMD4,QKI,NOTCH4,TCF20,TCF7,TPD52L1,NCOA7,L3MBTL3,SGSM3,PTK2,DAPK1,ATF4,PDGFB,AGTPBP1,CSNK1E,KDM4C,PIM1,NFYA,MDFI,TEK,IREB2,PRDM4,LIMCH1,DDX58,NFX1,FAM122A,TEC,KIT,ADGRF1,KDR,NFIL3,SYK,SEMA4D,RGP1,PPP6R1,IL6,IGF2BP3,NFE2L3,REL,CREB5,PTBP3,PPIE,BACH2,PELI1,FIGNL1,FOXP2,KLF11,ODC1,LANCL2,NOBOX,TLR4,TRAF1,C5,IL15,CCNY,CREM,TEAD2,SDCBP,MYCN,NOCT,TUT4,NRP1,KTI12,ZBTB24,PUM2,RASIP1,BEND5,PIK3CG,TAL1,PHF10,EPC1,KDM4D,GATA2,DNMT3A,PRKD2,SAP130,CST7,NOVA2,CTSC,RAB38,CAB39L,NOS3,GGA3,RALB,DACH1,PPARG,TIMP4,GATA3,TAF3,ACVR2B,ITIH5,IRAK2,GZF1,EDNRB,NDFIP2,SPTBN4,SETD5,IKBKB,JAG1,NFKBIB,PDE2A,TMEM173,SFRP1,HBEGF,NR1D2,DND1,RPL26,SH3BP5,ANKLE2,CD36,SIRT3,CDK17,ARID5A,PRR5L,TCF15,SCARB1,PLK2,RET,PDE4D,CHD7,IL1A,DNMT3B,IGF1,NR4A1,KLKB1,EIF2AK3,HHEX,KDM3A,ITCH,ERBIN,SH3RF1,SMAD1,ZBTB38,HIP1R,POU6F1,OCLN,STOX2,SOX7,HMBOX1,GPD1,ENC1,HEXB,MEF2C,SMARCD1,TLR2,WWTR1,IL7,P2RX7,VEGFC,ZNF704,CXCL10,MADD,ADGRG3,KMT2D,SFMBT1,FAM192A,SCAF4,TIPARP,OAS3,NLRC5,THBS4,ACTR5,PPP1R16B,RAPGEF3,ANKRD2,NR4A2,TOX2,DLL4,CCNJL,CDYL,BAZ2B,SOX13,NEDD9,YAF2,EDN1,STAT1,DYRK1A,EHD4,SNCA,JARID2,DTNBP1,MYLIP,ERG,ETS2,CDK18,NFKB2,DCAF1,RNF144B,PRDM15,ABCG1,ETS1,RNF19B,CD200,LPCAT1,SIK1,HDAC1,LCK,PPP3CA,HYAL2,HECW2,SLC39A8,TESMIN,MEF2A,BCL9,SLC7A7,LAPTM5,HLX,NCF1,US	turquoise	EC_M6
GO:0051172	2.23E-01	0.800395128	204	1770	negative regulation of nitrogen compound metabolic process	BP	1.00E+00	TNFAIP8,TNFAIP3,PLAGL1,TRIM39,PTPRB,LAPTM4B,CNOT6,OXR1,NOTCH4,TCF7,NCOA7,L3MBTL3,DAPK1,ATF4,PDGFB,MDFI,IREB2,NFX1,NFIL3,SEMA4D,RGP1,IL6,IGF2BP3,NFE2L3,PTBP3,BACH2,FOXP2,KLF11,LANCL2,C5,CREM,SDCBP,NOCT,TUT4,RASIP1,BEND5,TAL1,PHF10,EPC1,KDM4D,GATA2,DNMT3A,SAP130,CST7,GGA3,DACH1,PPARG,TIMP4,GATA3,TAF3,ACVR2B,ITIH5,GZF1,EDNRB,IKBKB,PDE2A,SFRP1,HBEGF,NR1D2,DND1,SH3BP5,CD36,ARID5A,PRR5L,PDE4D,DNMT3B,IGF1,NR4A1,EIF2AK3,HHEX,KDM3A,SH3RF1,ZBTB38,POU6F1,OCLN,SOX7,HMBOX1,ENC1,MEF2C,WWTR1,SFMBT1,FAM192A,SCAF4,PPP1R16B,ANKRD2,NR4A2,DLL4,CDYL,SOX13,YAF2,EDN1,STAT1,DYRK1A,SNCA,JARID2,DTNBP1,ETS2,DCAF1,LPCAT1,HDAC1,PPP3CA,HYAL2,MEF2A,FABP4,HCLS1,NFATC2,CD34,KDM2A,CIART,TGIF1,PARP15,INPP5F,IGFBP3,RBM4,RPS6KA1,PROX1,ZNF148,YTHDF1,MLXIPL,PAK2,CST6,SENP2,DLG1,APOD,ETV6,MOV10,RNF2,CREB1,EIF4A2,LTF,ADORA2A,BCOR,DDX3X,IGFBP5,MECOM,ING4,CDA,SPINDOC,BIRC3,LDB2,CPEB2,PPM1F,POU2F1,KDM5A,ZBTB18,APBB2,BCL6B,KDM5C,RGS14,MAFK,MTOR,AR,OPHN1,RNF40,PSEN1,PAPLN,CBX1,HOXB8,FBXW11,EFNA1,PIAS1,RPS6KA5,SMAD2,TLE3,CNOT8,YY1,HMG20A,RCOR1,NOS2,GIT1,USP7,ATCAY,ISG15,HCFC1,NRARP,TRAF2,NOTCH1,IRF8,ENG,MTF2,ZBTB34,TERF2,DAB2IP,ZBTB33,SLC8A3,ARID4A,ZNF24,ATXN1L,TRIM24,SOX18,PASD1,SERPINB9,COMMD7,MGAT3,TGIF2,BST2,BMP2,GLIS3,ZNF10,DUSP7,CBX2,NMNAT1,BMP6,ZNF266	turquoise	EC_M6
GO:0043651	2.51E-04	1	13	13	linoleic acid metabolic process	BP	1.00E+00	ELOVL5,PNPLA8,ELOVL1,ELOVL2,ALOX15,FADS2,ALOX15B,ALOXE3,CYP2J2,ABCD1,ALOX12,FADS1,ELOVL3	grey	EC_M10
GO:0006636	5.83E-04	0.999854505	29	36	unsaturated fatty acid biosynthetic process	BP	1.00E+00	AVPR1A,ANXA1,ELOVL5,EDN2,PNPLA8,PIBF1,ELOVL1,SCD,ELOVL2,PLA2G4F,PTGIS,ELOVL6,PLA2G3,PTGS2,PLA2G4A,DAGLB,ALOX15,FADS2,DEGS1,ALOX15B,ABCD1,DECR2,ALOX12,FADS1,FABP5,ELOVL3,SCD5,ELOVL4,PTGES	grey	EC_M10
GO:0140131	9.96E-04	1	11	11	positive regulation of lymphocyte chemotaxis	BP	1.00E+00	CCL27,ADAM17,OXSR1,PTK2B,STK39,WNK1,TMEM102,ADAM10,CCL5,CCL7,WNT5A	grey	EC_M10
GO:0033865	1.11E-03	0.999452528	70	102	nucleoside bisphosphate metabolic process	BP	1.00E+00	ENPP1,HSD17B4,PPT2,CROT,PDK4,ELOVL5,PPCS,AASS,DLD,SUCLA2,ELOVL1,OXSM,FASN,PANK2,HSD17B12,PDHX,PANK1,PDHB,ACSL1,MCCC2,FITM2,HMGCR,SLC35B3,ELOVL2,ABHD14B,MCEE,TPST1,MVK,FAR2,BPNT1,ACACB,ELOVL6,THEM5,PDHA1,PDK1,HMGCL,ACOT9,DLAT,ACSBG1,ACAT1,COASY,MPC2,PAPSS2,ACLY,PDK2,DLST,PANK3,ACSF2,PMVK,PPCDC,SLC26A2,HMGCS1,ACSBG2,ACOT11,ABCD1,PANK4,ACSF3,MVD,MLYCD,NUDT7,SULT1C3,ACOT6,ACOT4,SLC25A1,DGAT1,ELOVL3,ACSL3,NUDT19,ELOVL4,DCAKD	grey	EC_M10

GO:0033875	1.11E-03	0.999452528	70	102	ribonucleoside bisphosphate metabolic process	BP	1.00E+00	ENPP1,HSD17B4,PPT2,CROT,PDK4,ELOVL5,PPCS,AASS,DLD,SUCLA2,ELOVL1,OXSM,FASN,PANK2,HSD17B12,PDHX,PANK1,PDHB,ACSL1,MCCC2,FITM2,HMGCR,SLC35B3,ELOVL2,ABHD14B,MCEE,TPST1,MVK,FAR2,BPNT1,ACACB,ELOVL6,THEM5,PDHA1,PDK1,HMGCL,ACOT9,DLAT,ACSBG1,ACAT1,COASY,MPC2,PAPSS2,ACLY,PDK2,DLST,PANK3,ACSF2,PMVK,PPCDC,SLC26A2,HMGCS1,ACSBG2,ACOT11,ABCD1,PANK4,ACSF3,MVD,MLYCD,NUDT7,SULT1C3,ACOT6,ACOT4,SLC25A1,DGAT1,ELOVL3,ACSL3,NUDT19,ELOVL4,DCAKD	grey	EC_M10
GO:0034032	1.11E-03	0.999452528	70	102	purine nucleoside bisphosphate metabolic process	BP	1.00E+00	ENPP1,HSD17B4,PPT2,CROT,PDK4,ELOVL5,PPCS,AASS,DLD,SUCLA2,ELOVL1,OXSM,FASN,PANK2,HSD17B12,PDHX,PANK1,PDHB,ACSL1,MCCC2,FITM2,HMGCR,SLC35B3,ELOVL2,ABHD14B,MCEE,TPST1,MVK,FAR2,BPNT1,ACACB,ELOVL6,THEM5,PDHA1,PDK1,HMGCL,ACOT9,DLAT,ACSBG1,ACAT1,COASY,MPC2,PAPSS2,ACLY,PDK2,DLST,PANK3,ACSF2,PMVK,PPCDC,SLC26A2,HMGCS1,ACSBG2,ACOT11,ABCD1,PANK4,ACSF3,MVD,MLYCD,NUDT7,SULT1C3,ACOT6,ACOT4,SLC25A1,DGAT1,ELOVL3,ACSL3,NUDT19,ELOVL4,DCAKD	grey	EC_M10
GO:0001539	2.03E-03	0.999063458	55	79	cilium or flagellum-dependent cell motility	BP	1.00E+00	TTLL1,UBE2B,CATSPER3,DNAH8,VPS13A,CFAP69,RSPH9,DNAJA1,DNAH11,INPP5B,ARMC2,APOB,ADCY3,ENKUR,DRC1,RSPH6A,TTLL3,TPPP2,MKKS,CEP131,YIF1B,CFAP54,TMF1,ING2,CFAP20,CCDC65,PLTP,DPCD,ROPN1L,DNAH7,CFAP43,SLC9B2,CFAP46,CFAP45,TSSK4,IQCG,CELF3,PLA2G3,CATSPER2,SORD,TTC12,DNAAF2,SPAG16,EFHC2,RGN,MNS1,ASH1L,BBS4,DNAH3,TEKT3,CATSPERD,WDR63,ATP1A4,CCSAP,MEIG1	grey	EC_M10
GO:0060285	2.03E-03	0.999063458	55	79	cilium-dependent cell motility	BP	1.00E+00	TTLL1,UBE2B,CATSPER3,DNAH8,VPS13A,CFAP69,RSPH9,DNAJA1,DNAH11,INPP5B,ARMC2,APOB,ADCY3,ENKUR,DRC1,RSPH6A,TTLL3,TPPP2,MKKS,CEP131,YIF1B,CFAP54,TMF1,ING2,CFAP20,CCDC65,PLTP,DPCD,ROPN1L,DNAH7,CFAP43,SLC9B2,CFAP46,CFAP45,TSSK4,IQCG,CELF3,PLA2G3,CATSPER2,SORD,TTC12,DNAAF2,SPAG16,EFHC2,RGN,MNS1,ASH1L,BBS4,DNAH3,TEKT3,CATSPERD,WDR63,ATP1A4,CCSAP,MEIG1	grey	EC_M10
GO:0015936	2.52E-03	0.999670526	15	17	coenzyme A metabolic process	BP	1.00E+00	CROT,PPCS,PANK2,PANK1,MCCC2,HMGCR,ACAT1,COASY,ACLY,PANK3,PPCDC,PANK4,NUDT7,NUDT19,DCAKD	grey	EC_M10
GO:0060294	2.61E-03	0.998848722	48	68	cilium movement involved in cell motility	BP	1.00E+00	TTLL1,UBE2B,CATSPER3,VPS13A,CFAP69,RSPH9,DNAJA1,DNAH11,INPP5B,ARMC2,APOB,ADCY3,ENKUR,RSPH6A,TTLL3,TPPP2,MKKS,CEP131,YIF1B,CFAP54,TMF1,ING2,CFAP20,PLTP,DPCD,ROPN1L,CFAP43,SLC9B2,CFAP46,CFAP45,TSSK4,IQCG,CELF3,PLA2G3,CATSPER2,SORD,TTC12,SPAG16,RGN,MNS1,ASH1L,BBS4,TEKT3,CATSPERD,WDR63,ATP1A4,CCSAP,MEIG1	grey	EC_M10
GO:0034755	2.76E-03	0.999635435	15	17	iron ion transmembrane transport	BP	1.00E+00	STEAP2,SLC6A9,SLC11A2,SLC25A37,SLC40A1,SLC39A14,HFE,ABCC5,SLC11A1,ABCB6,ABCB7,ATP7A,MCOLN1,LCN2,SLC25A28	grey	EC_M10
GO:0010820	3.47E-03	1	9	9	positive regulation of T cell chemotaxis	BP	1.00E+00	CCL27,ADAM17,OXSR1,STK39,WNK1,TMEM102,ADAM10,CCL5,WNT5A	grey	EC_M10
GO:0007214	4.18E-03	0.999661893	11	12	gamma-aminobutyric acid signaling pathway	BP	1.00E+00	GABRA1,PHF24,GABBR2,PLCL2,GNAI2,PLCL1,GPR156,GABBR1,GABRE,GABRA3,SHISA7	grey	EC_M10
GO:0072087	4.73E-03	0.999132411	16	19	renal vesicle development	BP	1.00E+00	SMO,SIX2,OSR1,SOX9,PAX8,FMN1,GREM1,PAX2,SALL1,WNT4,BMP4,KIF26B,CITED1,LHX1,SOX8,LIF	grey	EC_M10
GO:0070778	5.12E-03	1	8	8	L-aspartate transmembrane transport	BP	1.00E+00	SLC1A1,SLC25A13,SLC1A4,SLC1A5,SLC38A7,SLC25A12,SLC25A18,SLC1A3	grey	EC_M10
Other	5.74E-03	0.995286162	533	928			1.00E+00		grey	EC_M10
GO:0048240	6.28E-03	0.999451003	11	12	sperm capacitation	BP	1.00E+00	CATSPER3,TCP11,DLD,ROPN1L,ABHD2,SLC26A6,CATSPER2,CABYR,PRKACA,CATSPERD,PCSK4	grey	EC_M10
GO:0001578	7.07E-03	0.99627996	57	86	microtubule bundle formation	BP	1.00E+00	ZNF207,TTLL1,UBE2B,LRGUK,DNAH8,CFAP69,RSPH9,WDR92,CDK5RAP2,ARMC2,MAPRE1,ARMC4,CCDC114,DRC1,RSPH6A,CLASP1,FOXJ1,TTLL3,CEP131,SPAST,NUMA1,YIF1B,AAAS,CLIP1,CCDC65,DNAH7,CFAP43,ZMYND10,NAV1,TPPP,CFAP46,GAS2L1,IQCG,PLA2G3,MAP2,TTC12,TOGARAM1,DNAAF2,SPAG16,MAP1S,TTC25,DNAAF4,MNS1,DNAL1,TTLL6,TRIM46,CCDC151,CAPN6,WDR78,MTCL1,C16orf71,PSRC1,DNAAF1,HYDIN,WDR63,TPPP3,MEIG1	grey	EC_M10
GO:0033683	7.08E-03	1	8	8	nucleotide-excision repair, DNA incision	BP	1.00E+00	XPA,ERCC1,ERCC2,ERCC3,OGG1,FAN1,ERCC4,NTHL1	grey	EC_M10
GO:0006107	7.31E-03	1	8	8	oxaloacetate metabolic process	BP	1.00E+00	MDH1,MDH2,GOT2,NIT2,GOT1,PCK2,MDH1B,ACLY	grey	EC_M10

GO:0016126	7.44E-03	0.996905274	35	49	sterol biosynthetic process	BP	1.00E+00	SQLE,CYP51A1,APOB,EBP,APOE,PRKAG2,INSIG1,ERLIN2,CYP7A1,FDFT1,SOD1,HMGCR,DHCR7,MVK,MSMO1,SC5D,LSS,HSD17B7,MBTPS2,TM7SF2,LPCAT3,CH25H,ACLY,PMVK,FAXDC2,HMGCS1,PRKAA2,DHCR24,NSDHL,G6PD,MVD,FDPS,IDI1,INSIG2,FGF1	grey	EC_M10
GO:0072077	7.52E-03	0.998546287	15	18	renal vesicle morphogenesis	BP	1.00E+00	SMO,SIX2,SOX9,PAX8,FMN1,GREM1,PAX2,SALL1,WNT4,BMP4,KIF26B,CITED1,LHX1,SOX8,LIF	grey	EC_M10
GO:0010232	9.52E-03	0.995193727	47	71	vascular transport	BP	1.00E+00	SLC16A7,AVPR1A,AGER,GJA1,ABCB1,SLC1A1,SLC2A1,SLC4A4,SLC1A4,SLC8A2,SLC1A5,SLC6A6,SLC9A3R1,APOE,SLC5A6,SLC6A9,SLC24A3,ABCC4,SLC7A2,SLC16A12,LRP3,SLC4A8,ABCC2,SLC5A3,ABCG2,SLC2A13,SLC38A3,SLCO3A1,SLC19A1,ABCC9,ABCC5,LRP2,ATP1A2,SLCO1C1,SLC29A2,SLC27A1,SLC38A5,ABCC3,ABCC1,SLC1A3,SLC6A17,SLC7A5,SLC27A4,SLC22A5,ATP1A4,FABP5,KCNJ8	grey	EC_M10
GO:0006695	9.55E-03	0.996175763	31	43	cholesterol biosynthetic process	BP	1.00E+00	CYP51A1,APOB,EBP,APOE,INSIG1,ERLIN2,CYP7A1,FDFT1,SOD1,HMGCR,DHCR7,MVK,MSMO1,SC5D,LSS,HSD17B7,MBTPS2,TM7SF2,LPCAT3,ACLY,PMVK,HMGCS1,PRKAA2,DHCR24,NSDHL,G6PD,MVD,FDPS,IDI1,INSIG2,FGF1	grey	EC_M10
GO:1902653	9.55E-03	0.996175763	31	43	secondary alcohol biosynthetic process	BP	1.00E+00	CYP51A1,APOB,EBP,APOE,INSIG1,ERLIN2,CYP7A1,FDFT1,SOD1,HMGCR,DHCR7,MVK,MSMO1,SC5D,LSS,HSD17B7,MBTPS2,TM7SF2,LPCAT3,ACLY,PMVK,HMGCS1,PRKAA2,DHCR24,NSDHL,G6PD,MVD,FDPS,IDI1,INSIG2,FGF1	grey	EC_M10
GO:0006858	1.02E-02	0.996752469	22	29	extracellular transport	BP	1.00E+00	TTLL1,KIF27,DNAH11,SLC9A3R1,NEK10,DPCD,ROPN1L,CFAP43,CFAP45,CABYR,DNAAF2,SPAG16,STK36,ADCY10,TTC25,DNAAF4,KIAA0556,CFAP53,ARRDC1,DNAAF1,WWP2,SPA17	grey	EC_M10
GO:0006084	1.08E-02	0.996543624	22	29	acetyl-CoA metabolic process	BP	1.00E+00	PDK4,PPCS,AASS,DLD,FASN,PDHX,PDHB,MVK,ACACB,PDHA1,PDK1,DLAT,ACAT1,MPC2,ACLY,PDK2,DLST,PMVK,HMGCS1,MVD,MLYCD,NUDT7	grey	EC_M10
GO:0006633	1.10E-02	0.993592038	69	107	fatty acid biosynthetic process	BP	1.00E+00	AVPR1A,LTC4S,MCAT,HACD4,ANXA1,PDK4,ELOVL5,EDN2,NR1H2,PNPLA8,APOC1,PRKAG2,PIBF1,INSIG1,ELOVL1,OXSM,FASN,ERLIN2,HSD17B12,TRIB3,CYP7A1,NR1H3,SCD,ELOVL2,PLA2G4F,PRKAB1,LPL,ACACB,PTGIS,ELOVL6,ABHD2,MECR,XBP1,LPGAT1,PLA2G3,PTGS2,PLA2G4A,MID1IP1,ACSBG1,RGN,DAGLB,ACLY,ALOX15,LIAS,ACADVL,FADS2,DEGS1,LIPC,ALOX15B,ALOXE3,NDUFAB1,ABHD3,ACSBG2,PRKAA2,ABCD1,DECR2,ACSF3,MLYCD,ALOX12,ACOT4,FADS1,FADS6,FABP5,ELOVL3,SCD5,ACSL3,ELOVL4,PTGES,INSIG2	grey	EC_M10
GO:0003351	1.11E-02	0.996697768	20	26	epithelial cilium movement involved in extracellular fluid movement	BP	1.00E+00	TTLL1,KIF27,DNAH11,SLC9A3R1,NEK10,DPCD,ROPN1L,CFAP43,CFAP45,CABYR,DNAAF2,SPAG16,STK36,ADCY10,TTC25,DNAAF4,KIAA0556,CFAP53,DNAAF1,SPA17	grey	EC_M10
GO:0061004	1.11E-02	1	7	7	pattern specification involved in kidney development	BP	1.00E+00	OSR1,PAX8,PAX2,IRX1,IRX2,BMP4,IRX3	grey	EC_M10
GO:0072048	1.11E-02	1	7	7	renal system pattern specification	BP	1.00E+00	OSR1,PAX8,PAX2,IRX1,IRX2,BMP4,IRX3	grey	EC_M10
GO:0006637	1.15E-02	0.993923938	52	78	acyl-CoA metabolic process	BP	1.00E+00	HSD17B4,PPT2,PDK4,ELOVL5,PPCS,AASS,DLD,SUCLA2,ELOVL1,OXSM,FASN,HSD17B12,PDHX,PDHB,ACSL1,FITM2,ELOVL2,MCEE,MVK,FAR2,ACACB,ELOVL6,THEM5,PDHA1,PDK1,HMGCL,ACOT9,DLAT,ACSBG1,ACAT1,MPC2,ACLY,PDK2,DLST,ACSF2,PMVK,HMGCS1,ACSBG2,ACOT11,ABCD1,ACSF3,MVD,MLYCD,NUDT7,ACOT6,ACOT4,SLC25A1,DGAT1,ELOVL3,ACSL3,NUDT19,ELOVL4	grey	EC_M10
GO:0035383	1.15E-02	0.993923938	52	78	thioester metabolic process	BP	1.00E+00	HSD17B4,PPT2,PDK4,ELOVL5,PPCS,AASS,DLD,SUCLA2,ELOVL1,OXSM,FASN,HSD17B12,PDHX,PDHB,ACSL1,FITM2,ELOVL2,MCEE,MVK,FAR2,ACACB,ELOVL6,THEM5,PDHA1,PDK1,HMGCL,ACOT9,DLAT,ACSBG1,ACAT1,MPC2,ACLY,PDK2,DLST,ACSF2,PMVK,HMGCS1,ACSBG2,ACOT11,ABCD1,ACSF3,MVD,MLYCD,NUDT7,ACOT6,ACOT4,SLC25A1,DGAT1,ELOVL3,ACSL3,NUDT19,ELOVL4	grey	EC_M10
GO:0150104	1.23E-02	0.993699481	46	70	transport across blood-brain barrier	BP	1.00E+00	SLC16A7,AVPR1A,AGER,ABCB1,SLC1A1,SLC2A1,SLC4A4,SLC1A4,SLC8A2,SLC1A5,SLC6A6,SLC9A3R1,APOE,SLC5A6,SLC6A9,SLC24A3,ABCC4,SLC7A2,SLC16A12,LRP3,SLC4A8,ABCC2,SLC5A3,ABCG2,SLC2A13,SLC38A3,SLCO3A1,SLC19A1,ABCC9,ABCC5,LRP2,ATP1A2,SLCO1C1,SLC29A2,SLC27A1,SLC38A5,ABCC3,ABCC1,SLC1A3,SLC6A17,SLC7A5,SLC27A4,SLC22A5,ATP1A4,FABP5,KCNJ8	grey	EC_M10
GO:0035082	1.25E-02	0.994172847	38	55	axoneme assembly	BP	1.00E+00	TTLL1,UBE2B,LRGUK,DNAH8,CFAP69,RSPH9,WDR92,ARMC2,ARMC4,CCDC114,DRC1,RSPH6A,FOXJ1,TTLL3,CEP131,YIF1B,CCDC65,DNAH7,CFAP43,ZMYND10,CFAP46,IQCG,PLA2G3,TTC12,TOGARAM1,DNAAF2,SPAG16,TTC25,DNAAF4,MNS1,DNAL1,CCDC151,WDR78,C16orf71,DNAAF1,HYDIN,WDR63,MEIG1	grey	EC_M10

GO:0032543	1.29E-02	0.993339553	49	72	mitochondrial translation	BP	1.00E+00	SHMT2,TSFM,MRPS18B,RMND1,MTRF1L,MRPL2,MRPS18A,AARS2,NOA1,RARS2,QRSL1,MRPL58,MTRF1,HARS,MTIF3,MRPS27,GATB,GFM2,MRPS28,GFM1,NSUN3,MRPL43,GATC,RPUSD4,YARS2,MRPS35,MRPL52,RCC1L,MRPL47,MRPS11,MRPS21,TARS2,NGRN,MTG2,TACO1,LARS2,COA3,MPV17L2,C1QBP,TUFM,ALKBH1,EARS2,TRUB2,MRPS6,MRPS15,UQCC2,MRPS16,TRMT10C,MRPL16	grey	EC_M10
GO:0015740	1.32E-02	0.996809541	15	19	C4-dicarboxylate transport	BP	1.00E+00	SLC1A1,SLC25A13,SLC1A4,SLC1A5,SLC25A30,SLC38A7,NTSR1,SLC25A12,SLC25A11,SLC25A18,LRRC8E,SLC1A3,SLC25A14,ABAT,LRRC8A	grey	EC_M10
GO:0044828	1.32E-02	1	7	7	negative regulation by host of viral genome replication	BP	1.00E+00	PRKN,ZC3H12A,EIF2AK4,CCNK,VAPA,MIR222,CCL8	grey	EC_M10
GO:0019933	1.34E-02	0.994528228	28	40	cAMP-mediated signaling	BP	1.00E+00	ADGRG6,UBE2B,TCP11,AHR,EPHA5,SOX9,GIPR,SLC9A3R1,RNF157,PDE7A,ADM,FFAR4,PDE3B,CAP2,RGS2,PDE3A,CRTC3,RAPGEF4,AKAP6,GNAI1,DGKQ,PDE4A,MAPK7,KSR1,CAP1,CDC34,CRH,RASD2	grey	EC_M10
GO:0072348	1.49E-02	0.993833807	29	41	sulfur compound transport	BP	1.00E+00	SLC26A10,GJA1,SLC1A1,SLC1A4,SLC13A4,SLC26A5,SLC6A6,SLC25A30,SLC9A3R1,SLC25A19,SLC5A6,ABCC4,SLC26A11,SLC25A26,SLC38A7,SLC35B3,ABCG2,ABCC5,SLC26A6,MGST1,SLC19A2,SLC27A1,SLC25A11,ABCC1,SLC26A2,SLC25A14,ABCD1,CTNS,LRRC8A	grey	EC_M10
GO:0050686	1.51E-02	0.995351124	19	25	negative regulation of mRNA processing	BP	1.00E+00	NELFE,U2AF2,RNF20,SRSF10,SUPT5H,SFSWAP,CTR9,CCNT1,SRSF6,PCBP4,SRSF9,SRSF4,BARD1,RBM10,C1QBP,ZC3H14,CDK9,SRSF12,SAP18	grey	EC_M10
GO:0007185	1.55E-02	1	6	6	transmembrane receptor protein tyrosine phosphatase signaling pathway	BP	1.00E+00	FRS2,PTPRD,PTN,PTPRF,TRIO,PTPRU	grey	EC_M10
GO:0009584	1.62E-02	0.994964042	19	25	detection of visible light	BP	1.00E+00	OPN1SW,PRPH2,CAMKMT,GJA10,ATP8A2,RGS9BP,RRH,SAG,SEMA5B,NMT1,GRK4,OPN3,GUCY2D,GNAT2,ABCA4,REEP6,NMT2,ELOVL4,GPR88	grey	EC_M10
GO:0008033	1.65E-02	0.989923525	75	117	tRNA processing	BP	1.00E+00	ELAC1,METTL1,ADAT2,RPP21,TRMU,TRMT12,TRMT11,C9orf64,RTCB,RPP25L,AARS2,THADA,TSEN34,ELP1,GRSF1,DUS4L,NSUN6,TSEN54,THUMPD3,DPH3,DUS1L,METTL6,PUS1,THUMPD2,NAT10,RPP14,ELP4,POP4,LSM6,CLP1,ELP3,TPRKB,NSUN3,OSGEPL1,RPP40,CDKAL1,POP5,RPUSD4,WDR4,PUS3,TYW1B,TRUB1,RTRAF,LCMT2,METTL8,ELP6,METTL2B,KIAA0391,TRMT1L,POP7,TRMT112,TRMT44,TRPT1,ALKBH8,DTWD1,FTSJ1,GTPBP3,TRMT5,MTFMT,ALKBH1,ELAC2,PUSL1,POLR3K,TRMT13,ADAT1,URM1,CTU1,RPP38,TRMT10B,TP53RK,TRMT10C,RPP30,TRMT61B,OSGEP,TYW3	grey	EC_M10
GO:0000244	1.71E-02	0.996273458	14	17	spliceosomal tri-snRNP complex assembly	BP	1.00E+00	PRPF31,SRSF10,AAR2,SART3,PRPF6,PRPF19,PRPF8,RNU5E-1,RNU5B-1,USP4,RNU4ATAC,RNU4-2,SRSF12,RNU4-1	grey	EC_M10
GO:1901017	1.74E-02	0.997115722	11	13	negative regulation of potassium ion transmembrane transporter activity	BP	1.00E+00	NEDD4L,CAV1,OXSR1,KCNE3,KCNE2,CASQ2,CAB39,ACTN2,STK39,KCNE4,CRBN	grey	EC_M10
GO:0048857	1.77E-02	0.992064516	32	46	neural nucleus development	BP	1.00E+00	MBP,BCL2,SCRIB,CKB,TTBK1,GLUD1,NDRG2,NDUFS3,SUDS3,COX6B1,ALDH1A3,GNB4,CCDC14,ATF2,MAOB,YWHAQ,CNP,ACTB,HOXB1,HOXB2,CALM2,CDK5R1,RAD1,G6PD,SEC16A,ATP5PB,MAPKAP1,RHOA,ATP5PF,CALM3,BASP1,FGF9	grey	EC_M10
GO:0048733	1.83E-02	1	6	6	sebaceous gland development	BP	1.00E+00	SMAD4,EXT1,ZDHHC21,RBPJ,ASH1L,GATA6	grey	EC_M10
GO:1900019	1.86E-02	1	6	6	regulation of protein kinase C activity	BP	1.00E+00	ROR2,EGFR,TCIM,CEMIP,WNT5A,C3orf58	grey	EC_M10
GO:1900020	1.86E-02	1	6	6	positive regulation of protein kinase C activity	BP	1.00E+00	ROR2,EGFR,TCIM,CEMIP,WNT5A,C3orf58	grey	EC_M10
GO:0061436	1.89E-02	0.995882507	13	16	establishment of skin barrier	BP	1.00E+00	UGCG,GRHL1,MET,ELOVL1,CYP26B1,KDF1,SFN,GRHL3,ABCA12,TMEM79,ALOXE3,ALOX12,CLDN1	grey	EC_M10
GO:0010819	1.92E-02	0.99800506	9	10	regulation of T cell chemotaxis	BP	1.00E+00	CCL27,ADAM17,OXSR1,STK39,WNK1,TMEM102,ADAM10,CCL5,WNT5A	grey	EC_M10
GO:0021957	1.93E-02	1	6	6	corticospinal tract morphogenesis	BP	1.00E+00	SCN1B,FBXO45,NIN,EPHA4,SLIT2,CDH11	grey	EC_M10
GO:0050482	1.99E-02	0.995630034	13	16	arachidonic acid secretion	BP	1.00E+00	PLA2G6,ANXA1,PNPLA8,PLA2G4F,NTSR1,PLA2G3,PLA2G4A,NMUR2,PROCA1,ATP5PF,PLA2G2C,BDKRB2,NMB	grey	EC_M10
GO:1903963	1.99E-02	0.995630034	13	16	arachidonate transport	BP	1.00E+00	PLA2G6,ANXA1,PNPLA8,PLA2G4F,NTSR1,PLA2G3,PLA2G4A,NMUR2,PROCA1,ATP5PF,PLA2G2C,BDKRB2,NMB	grey	EC_M10

GO:0006086	2.05E-02	0.99783065	9	10	acetyl-CoA biosynthetic process from pyruvate	BP	1.00E+00	PDK4,DLD,PDHX,PDHB,PDHA1,PDK1,DLAT,MPC2,PDK2	grey	EC_M10
GO:0030317	2.14E-02	0.989223876	39	58	flagellated sperm motility	BP	1.00E+00	TTLL1,UBE2B,CATSPER3,VPS13A,CFAP69,DNAJA1,DNAH11,INPP5B,ARMC2,APOB,ADCY3,ENKUR,TTLL3,TPPP2,CEP131,YIF1B,TMF1,ING2,PLTP,DPCD,ROPN1L,CFAP43,SLC9B2,CFAP45,TSSK4,IQCG,CELF3,PLA2G3,CATSPER2,SORD,TTC12,SPAG16,RGN,MNS1,ASH1L,TEKT3,CATSPERD,ATP1A4,MEIG1	grey	EC_M10
GO:0097722	2.14E-02	0.989223876	39	58	sperm motility	BP	1.00E+00	TTLL1,UBE2B,CATSPER3,VPS13A,CFAP69,DNAJA1,DNAH11,INPP5B,ARMC2,APOB,ADCY3,ENKUR,TTLL3,TPPP2,CEP131,YIF1B,TMF1,ING2,PLTP,DPCD,ROPN1L,CFAP43,SLC9B2,CFAP45,TSSK4,IQCG,CELF3,PLA2G3,CATSPER2,SORD,TTC12,SPAG16,RGN,MNS1,ASH1L,TEKT3,CATSPERD,ATP1A4,MEIG1	grey	EC_M10
GO:0043271	2.15E-02	0.987322059	59	93	negative regulation of ion transport	BP	1.00E+00	BCL2,NEDD4L,GOPC,SRI,WNK2,THADA,PRKCE,CAV1,HECW1,SFRP4,KEL,OSR1,SLC26A5,SLC9A3R1,BIN1,SLC6A9,OXSR1,KCNE3,SLC43A1,TMBIM6,PTK2B,PACSIN3,KCNE2,CASQ2,GSTO1,RGS2,CAB39,ACTN2,ADRA2A,STK39,TRIM27,CAMK2D,ATP1A2,NTSR1,PTGS2,SESTD1,EPHB2,MAOB,YWHAQ,GNB5,WNK1,SERPINE2,WNK3,ATP7A,CALM2,ABAT,GABRE,INPP5K,RRAD,KCNE4,GPR35,ATP5PF,CALM3,PLN,CLIC2,CRBN,ICAM1,SLC30A1,PPIF	grey	EC_M10
GO:0006085	2.17E-02	0.995155551	13	16	acetyl-CoA biosynthetic process	BP	1.00E+00	PDK4,PPCS,DLD,PDHX,PDHB,PDHA1,PDK1,DLAT,ACAT1,MPC2,ACLY,PDK2,MLYCD	grey	EC_M10
GO:0055092	2.18E-02	0.988661946	42	63	sterol homeostasis	BP	1.00E+00	APOM,SREBF2,TTC39B,CYP39A1,HDAC9,ABCA1,NR1H2,CAV1,APOB,CNBP,APOE,INSIG1,TSKU,COMMD9,SOAT2,CYP7A1,CYP7B1,RALY,LIMA1,EPHX2,NR1H3,HMGCR,LPL,ABCA5,FABP3,NR5A2,GRAMD1B,XBP1,ABCG4,EHD1,NR1D1,LIPC,DISP3,RORA,NPC2,LDLR,NPC1,TMEM97,ABCD1,MIR27B,MIR33B,LDLRAP1	grey	EC_M10
GO:0046985	2.27E-02	1	6	6	positive regulation of hemoglobin biosynthetic process	BP	1.00E+00	KLF4,PRMT1,SLC6A9,SLC25A37,LDB1,ABCB10	grey	EC_M10
GO:0019852	2.30E-02	1	6	6	L-ascorbic acid metabolic process	BP	1.00E+00	GCLC,SLC2A1,AKR1A1,SLC23A2,GSTO1,RGN	grey	EC_M10
GO:1901334	2.30E-02	1	6	6	lactone metabolic process	BP	1.00E+00	GCLC,SLC2A1,AKR1A1,SLC23A2,GSTO1,RGN	grey	EC_M10
GO:0097104	2.31E-02	0.997586662	8	9	postsynaptic membrane assembly	BP	1.00E+00	LRP4,NLGN4Y,EPHB2,NRXN2,NLGN1,NLGN3,CDH2,CEL	grey	EC_M10
GO:0043949	2.35E-02	0.993835367	14	18	regulation of cAMP-mediated signaling	BP	1.00E+00	UBE2B,TCP11,GIPR,RNF157,FFAR4,PDE3B,RGS2,PDE3A,CRTC3,GNAI1,PDE4A,CDC34,CRH,RASD2	grey	EC_M10
GO:0048710	2.40E-02	0.99285989	16	21	regulation of astrocyte differentiation	BP	1.00E+00	TTBK1,BIN1,KDM4A,IL6ST,CLCF1,EPHA4,NR1D1,SERPINE2,PRPF19,LDLR,MBD1,DAB1,CNTN2,GPR37L1,NOG,LIF	grey	EC_M10
GO:0002026	2.41E-02	0.993656125	14	18	regulation of the force of heart contraction	BP	1.00E+00	EDN2,CAV1,ADM,ATP1A1,RYR2,MYH7,GRK2,CAMK2D,ATP1A2,SLC9A1,MYL4,SLC8A1,CHGA,PLN	grey	EC_M10
GO:0071709	2.43E-02	0.989445913	27	39	membrane assembly	BP	1.00E+00	FLOT1,PTPRD,CHMP2A,SPTBN1,UGCG,CAV1,SPAST,RFTN1,STX2,NSFL1C,UBXN2B,LRP4,CHMP7,REEP4,NLGN4Y,EPHB2,NRXN2,NLGN1,ANXA2,GAK,STX4,NLGN3,CDH2,CEL,VPS4A,TLCD1,REEP3	grey	EC_M10
GO:0043267	2.44E-02	0.99355949	14	18	negative regulation of potassium ion transport	BP	1.00E+00	NEDD4L,CAV1,KEL,BIN1,OXSR1,KCNE3,PTK2B,KCNE2,CASQ2,CAB39,ACTN2,STK39,KCNE4,CRBN	grey	EC_M10
GO:0021762	2.44E-02	0.99001224	25	35	substantia nigra development	BP	1.00E+00	MBP,CKB,TTBK1,GLUD1,NDRG2,NDUFS3,SUDS3,COX6B1,GNB4,CCDC14,MAOB,YWHAQ,CNP,ACTB,CALM2,RAD1,G6PD,SEC16A,ATP5PB,MAPKAP1,RHOA,ATP5PF,CALM3,BASP1,FGF9	grey	EC_M10
GO:1905907	2.50E-02	0.997224128	9	10	negative regulation of amyloid fibril formation	BP	1.00E+00	PFDN6,APOE,PFDN1,PFDN5,PFDN2,CRYAB,PFDN4,LDLR,VBP1	grey	EC_M10
GO:0031441	2.53E-02	0.99730429	8	9	negative regulation of mRNA 3'-end processing	BP	1.00E+00	NELFE,RNF20,SUPT5H,CTR9,CCNT1,BARD1,ZC3H14,CDK9	grey	EC_M10
GO:0033866	2.53E-02	0.987071234	38	57	nucleoside bisphosphate biosynthetic process	BP	1.00E+00	PPT2,PDK4,ELOVL5,PPCS,DLD,ELOVL1,PANK2,HSD17B12,PDHX,PANK1,PDHB,ACSL1,SLC35B3,ELOVL2,ACACB,ELOVL6,PDHA1,PDK1,DLAT,ACSBG1,ACAT1,COASY,MPC2,PAPSS2,ACLY,PDK2,PANK3,PPCDC,SLC26A2,ACSBG2,PANK4,ACSF3,MLYCD,SLC25A1,ELOVL3,ACSL3,ELOVL4,DCAKD	grey	EC_M10

GO:0034030	2.53E-02	0.987071234	38	57	ribonucleoside bisphosphate biosynthetic process	BP	1.00E+00	PPT2,PDK4,ELOVL5,PPCS,DLD,ELOVL1,PANK2,HSD17B12,PDHX,PANK1,PDHB,ACSL1,SLC35B3,ELOVL2,ACACB,ELOVL6,PDHA1,PDK1,DLAT,ACSBG1,ACAT1,COASY,MPC2,PAPSS2,ACLY,PDK2,PANK3,PPCDC,SLC26A2,ACSBG2,PANK4,ACSF3,MLYCD,SLC25A1,ELOVL3,ACSL3,ELOVL4,DCAKD	grey	EC_M10
GO:0034033	2.53E-02	0.987071234	38	57	purine nucleoside bisphosphate biosynthetic process	BP	1.00E+00	PPT2,PDK4,ELOVL5,PPCS,DLD,ELOVL1,PANK2,HSD17B12,PDHX,PANK1,PDHB,ACSL1,SLC35B3,ELOVL2,ACACB,ELOVL6,PDHA1,PDK1,DLAT,ACSBG1,ACAT1,COASY,MPC2,PAPSS2,ACLY,PDK2,PANK3,PPCDC,SLC26A2,ACSBG2,PANK4,ACSF3,MLYCD,SLC25A1,ELOVL3,ACSL3,ELOVL4,DCAKD	grey	EC_M10
GO:0140115	2.54E-02	0.987757816	33	49	export across plasma membrane	BP	1.00E+00	GJA1,ABCB1,ABCA1,SLC4A4,SLC8A2,ATP1A3,ABCC4,KCNE3,ATP4A,SLC40A1,ABCC2,KCNE2,ABCG2,ATP1A1,KCNIP2,PDZK1,ABCC5,ATP1A2,SLC9A1,KCND3,SLC27A1,CALM2,ABCC1,RALBP1,ATP2B3,MIR21,MIR186,KCNE4,SLC8A1,SLC22A5,ATP1A4,CALM3,KCNT2	grey	EC_M10
GO:0071827	2.57E-02	0.989897782	23	32	plasma lipoprotein particle organization	BP	1.00E+00	APOM,PLA2G7,ABCA1,NR1H2,APOB,APOC1,APOE,NR1H4,SOAT2,AGTR1,PLTP,LPL,ABCA5,AGT,PLA2G3,LPCAT3,PRKACA,LIPC,MPO,ABCA7,PRKACB,DGAT1,ACSL3	grey	EC_M10
GO:0030104	2.63E-02	0.988453337	27	39	water homeostasis	BP	1.00E+00	NEDD4L,EXT1,AKR1B1,WFS1,TFAP2B,UGCG,GRHL1,MET,AKAP11,AQP11,ELOVL1,CYP26B1,EXT2,TRPV4,KDF1,SFN,GRHL3,ABCA12,SCNN1A,PRKACA,TMEM79,ALOXE3,INPP5K,PRKACB,ALOX12,AQP3,CLDN1	grey	EC_M10
GO:0046942	2.68E-02	0.981435074	104	173	carboxylic acid transport	BP	1.00E+00	SLC26A10,SLC35D3,SLC16A7,AVPR1A,ARG1,GJA1,NTRK2,PLA2G6,SLC16A8,ANXA1,ABCB4,ABCC10,SLC1A1,SLC25A13,SLC1A4,PNPLA8,SLC17A7,SLC26A5,SLC1A5,SLC6A6,SLC25A30,SLC9A3R1,SLC16A5,SLC5A6,SLC6A9,SLC22A13,SLC25A38,ABCC4,SLC6A15,SLC25A15,SLC23A2,TRPC4,SLC16A3,SLC38A9,SLC5A8,SLC7A2,CYP7A1,SLC16A12,SLC10A7,SLC43A1,SLC38A7,SFXN5,ABCC2,ABCG2,SFXN3,PLA2G4F,GRM2,RGS2,SLC38A3,MAP2K6,SLC16A6,SLCO3A1,SV2A,SLC19A1,AGT,ABCC5,ACE2,LRP2,ATP1A2,SLC26A6,SLCO1A2,SLCO1C1,NTSR1,SLC25A12,PLA2G3,PLA2G4A,PSAP,SLC11A1,PQLC2,SLC19A2,SLC27A1,SLC38A5,MPC2,ARL6IP1,SLC38A6,SLC25A11,SLC25A18,GIPC1,ABCC3,NMUR2,SLC6A7,ABCC1,SLC26A2,LRRC8E,EMB,SLC1A3,SLC25A14,PROCA1,ABAT,CTNS,SLC16A4,SLC6A17,SLC7A5,LRRC8A,SLC27A4,SLC7A6,SLC25A1,ATP5PF,PLA2G2C,SLC35D1,BDKRB2,SLC27A5,PTGES,NMB	grey	EC_M10
GO:0042632	2.78E-02	0.985258177	41	62	cholesterol homeostasis	BP	1.00E+00	APOM,SREBF2,TTC39B,CYP39A1,HDAC9,ABCA1,NR1H2,CAV1,APOB,CNBP,APOE,INSIG1,TSKU,COMMD9,SOAT2,CYP7A1,CYP7B1,RALY,LIMA1,EPHX2,NR1H3,HMGCR,LPL,ABCA5,FABP3,NR5A2,GRAMD1B,XBP1,ABCG4,EHD1,NR1D1,LIPC,DISP3,RORA,NPC2,LDLR,NPC1,TMEM97,MIR27B,MIR33B,LDLRAP1	grey	EC_M10
GO:0042304	2.82E-02	0.9893332	21	29	regulation of fatty acid biosynthetic process	BP	1.00E+00	AVPR1A,ANXA1,PDK4,ELOVL5,NR1H2,APOC1,PIBF1,INSIG1,ERLIN2,TRIB3,CYP7A1,NR1H3,LPGAT1,PLA2G3,PTGS2,MID1IP1,RGN,ACADVL,ABCD1,FABP5,INSIG2	grey	EC_M10
GO:0072283	2.85E-02	0.993417388	12	15	metanephric renal vesicle morphogenesis	BP	1.00E+00	SMO,SIX2,PAX8,FMN1,GREM1,PAX2,SALL1,BMP4,KIF26B,CITED1,LHX1,LIF	grey	EC_M10
GO:0097006	2.87E-02	0.985585665	36	53	regulation of plasma lipoprotein particle levels	BP	1.00E+00	APOM,VLDLR,PLA2G7,ABCA1,NR1H2,APOB,FGF21,APOC1,APOE,NR1H4,SOAT2,AGTR1,PLTP,LPL,GPLD1,ABCA5,AGT,PLA2G3,EHD1,LPCAT3,PRKACA,LIPC,ANXA2,MPO,LDLR,KHSRP,ABCA7,PRKACB,MIR199A1,MIR27B,MIR27A,DGAT1,MIR33B,ACSL3,NCEH1,LDLRAP1	grey	EC_M10
GO:2000403	2.92E-02	0.991012364	16	21	positive regulation of lymphocyte migration	BP	1.00E+00	CCL27,ADAM17,OXSR1,JAM2,PTK2B,STK39,ADAM8,WNK1,TMEM102,ADAM10,CCL5,CCL7,TNFRSF14,RHOA,FADD,WNT5A	grey	EC_M10
GO:0009249	2.94E-02	1	6	6	protein lipoylation	BP	1.00E+00	LIPT1,LIPT2,NNAT,LIAS,NDUFAB1,GCSH	grey	EC_M10
GO:0045989	3.02E-02	0.996618687	8	9	positive regulation of striated muscle contraction	BP	1.00E+00	ATP1A1,GSTO1,RGS2,TRPV4,ACE2,ACTN3,CHGA,CTGF	grey	EC_M10
GO:0009311	3.03E-02	0.985726116	30	44	oligosaccharide metabolic process	BP	1.00E+00	NEU1,NAGA,FBP1,B4GALT1,ST6GAL2,MGAM,BRAF,ST3GAL3,NEU3,MOGS,ST3GAL4,MANBA,ST8SIA2,ST8SIA1,MGAT2,B3GALNT1,GAL3ST4,FUT8,MPDU1,IDUA,ST8SIA5,GLA,MAN2C1,MAN1B1,ST6GALNAC4,ST3GAL2,CTBS,ST6GALNAC5,FBP2,AL157935.2	grey	EC_M10
GO:0050891	3.06E-02	0.986819721	25	36	multicellular organismal water homeostasis	BP	1.00E+00	EXT1,AKR1B1,WFS1,TFAP2B,UGCG,GRHL1,MET,AKAP11,ELOVL1,CYP26B1,EXT2,TRPV4,KDF1,SFN,GRHL3,ABCA12,SCNN1A,PRKACA,TMEM79,ALOXE3,INPP5K,PRKACB,ALOX12,AQP3,CLDN1	grey	EC_M10
GO:0070886	3.08E-02	0.994385471	10	12	positive regulation of calcineurin-NFAT signaling cascade	BP	1.00E+00	LMCD1,NRG1,STIMATE,SPPL3,CIB1,SLC9A1,AKAP6,CHERP,CAMTA1,C10orf71	grey	EC_M10

GO:0106058	3.08E-02	0.994385471	10	12	positive regulation of calcineurin-mediated signaling	BP	1.00E+00	LMCD1,NRG1,STIMATE,SPPL3,CIB1,SLC9A1,AKAP6,CHERP,CAMTA1,C10orf71	grey	EC_M10
GO:0042908	3.18E-02	0.987872832	20	28	xenobiotic transport	BP	1.00E+00	GJA1,ABCB4,ABCB1,ABCC10,SLC2A1,ABCB5,EBP,ABCC4,ABCC2,ABCG2,PDZK1,ABCC5,ABCC3,ABCC1,RALBP1,SLC7A5,MIR186,SLC22A5,ABCA8,CLDN1	grey	EC_M10
GO:1901568	3.19E-02	0.983622285	36	54	fatty acid derivative metabolic process	BP	1.00E+00	HSD17B4,PPT2,ELOVL5,HMGCLL1,ELOVL1,ACSS3,HSD17B12,AACS,PAM,GGT7,ACSL1,HPGD,FITM2,ELOVL2,FAR2,ELOVL6,THEM5,HMGCL,ACSBG1,ACAT1,GGT6,ALOX15,ALOX15B,ACSBG2,ABCD1,ACSF3,NUDT7,ALOX12,SLC25A1,DGAT1,ELOVL3,ACSL3,NUDT19,ELOVL4,LYPLA2,SLC27A5	grey	EC_M10
GO:0002072	3.19E-02	1	5	5	optic cup morphogenesis involved in camera-type eye development	BP	1.00E+00	TFAP2A,PAX2,ALDH1A3,PHACTR4,WNT5A	grey	EC_M10
GO:1902017	3.27E-02	0.984235646	30	45	regulation of cilium assembly	BP	1.00E+00	CEP120,MAPK15,KCTD17,CEP135,DYNC2LI1,CCDC88A,SEPT7,ATG5,ARHGAP35,SEPT9,VDAC3,IFT88,CYLD,TBC1D7,ZMYND10,MAP4,TAPT1,SDCCAG8,MNS1,CNTROB,CCDC151,BBS4,ATG3,CCP110,IFT20,WRAP73,ENTR1,ATMIN,ODF2L,YAP1	grey	EC_M10
GO:0036151	3.30E-02	0.993872931	10	12	phosphatidylcholine acyl-chain remodeling	BP	1.00E+00	TMEM86B,MBOAT7,NR1H2,MBOAT2,DBI,PLB1,LPCAT4,NR1H3,PLA2G4A,LPCAT3	grey	EC_M10
GO:1901623	3.34E-02	0.992023568	12	15	regulation of lymphocyte chemotaxis	BP	1.00E+00	CCL27,ADAM17,OXSR1,PTK2B,SLC8B1,STK39,WNK1,TMEM102,ADAM10,CCL5,CCL7,WNT5A	grey	EC_M10
GO:0018200	3.35E-02	0.989450611	16	21	peptidyl-glutamic acid modification	BP	1.00E+00	TTLL1,CEP41,AGBL3,AGBL5,TTLL3,AGBL2,CFAP20,VKORC1L1,TTLL13P,PARP1,TTLL6,TPGS2,NAA10,TTLL11,TTLL7,FOLH1	grey	EC_M10
GO:0042596	3.39E-02	0.98945219	15	20	fear response	BP	1.00E+00	BCL2,MAPK8IP2,EXT1,GJA1,USP46,SLC1A1,NPAS2,APOE,ASIC1,ADRA2A,ATP1A2,EIF4G1,MECP2,DPP4,CRH	grey	EC_M10
GO:0048708	3.41E-02	0.981667699	39	60	astrocyte differentiation	BP	1.00E+00	AGER,SMO,TTBK1,CDK6,ROR2,GCM1,FPR2,EGFR,DLL1,C5AR1,SOX9,VIM,BIN1,KDM4A,IL6ST,BACE2,TTC21B,CLCF1,GRN,STAT3,MAPK1,EPHA4,NR1D1,SERPINE2,PRPF19,MAPK3,LDLR,S100A8,MBD1,DAB1,SOX8,LAMC3,ABL1,CNTN2,CNTF,GPR37L1,NOG,POU3F2,LIF	grey	EC_M10
GO:1903312	3.62E-02	0.979026106	47	74	negative regulation of mRNA metabolic process	BP	1.00E+00	PABPC1,NELFE,HNRNPA0,MAPK14,SECISBP2,U2AF2,RNF20,SRSF10,NBAS,TRAF3IP2,DHX34,SUPT5H,ZFP36,BAG4,PKP3,SFSWAP,E2F1,CTR9,CCNT1,SRSF6,RBM24,PCBP4,IKBKE,SRSF9,TENT4A,TIRAP,AXIN2,MAPKAPK2,SRSF4,TRAF5,ANGEL2,EIF4ENIF1,BARD1,SLC11A1,RBM10,C1QBP,TARDBP,IGF2BP1,TOB1,ZC3H14,PAIP1,CIRBP,CDK9,SRSF12,YBX1,TENT5A,SAP18	grey	EC_M10
GO:0071716	3.64E-02	1	5	5	leukotriene transport	BP	1.00E+00	ABCC10,ABCC4,ABCC2,ABCC3,ABCC1	grey	EC_M10
GO:0046165	3.65E-02	0.976694287	67	108	alcohol biosynthetic process	BP	1.00E+00	MOXD1,CYP27B1,IP6K3,AKR1B1,CYP51A1,SPTLC1,IPPK,PLEK,APOB,EBP,APOE,HRH1,SPHK1,INSIG1,ITPKC,SPTLC3,ERLIN2,CYP7A1,ASAH1,PPIP5K2,FDFT1,PTH,CYP2R1,P2RY1,PTK2B,SOD1,GOT1,HMGCR,ITPKA,DHCR7,MVK,IP6K1,MSMO1,GPER1,SC5D,PCK2,LSS,IP6K2,NTSR1,PPIP5K1,HSD17B7,MBTPS2,PTS,TM7SF2,LPCAT3,WNT4,ISYNA1,GCH1,ACLY,DGKQ,PMVK,HMGCS1,IMPA2,PRKAA2,DHCR24,NSDHL,G6PD,H6PD,MVD,GFI1,DHDDS,ACER2,FDPS,IDI1,INSIG2,FGF1,PCBD2	grey	EC_M10
GO:0033564	3.67E-02	1	5	5	anterior/posterior axon guidance	BP	1.00E+00	LHX9,UNC5B,UNC5A,NTN1,LHX1	grey	EC_M10
GO:0097090	3.67E-02	0.99586778	7	8	presynaptic membrane organization	BP	1.00E+00	PTPRD,LRP4,NLGN4Y,NLGN1,NLGN3,CEL,CNTN2	grey	EC_M10
GO:1905749	3.71E-02	1	5	5	regulation of endosome to plasma membrane protein transport	BP	1.00E+00	ZDHHC2,GRIPAP1,AKAP5,ARHGAP44,GRASP	grey	EC_M10
GO:0042769	3.73E-02	0.992884663	10	12	DNA damage response, detection of DNA damage	BP	1.00E+00	MRPS9,DNAJA1,PNKP,MRPS26,NEK1,MRPS35,MRPS11,PARP1,USP1,SOX4	grey	EC_M10
GO:0060900	3.76E-02	0.995749751	7	8	embryonic camera-type eye formation	BP	1.00E+00	FRS2,TFAP2A,PAX2,ALDH1A3,PHACTR4,STRA6,WNT5A	grey	EC_M10
GO:0051388	3.81E-02	1	5	5	positive regulation of neurotrophin TRK receptor signaling pathway	BP	1.00E+00	WASF1,TMEM108,CYFIP1,PPP2R5B,CYFIP2	grey	EC_M10

GO:1901380	3.83E-02	0.989179348	13	17	negative regulation of potassium ion transmembrane transport	BP	1.00E+00	NEDD4L,CAV1,KEL,BIN1,OXSR1,KCNE3,KCNE2,CASQ2,CAB39,ACTN2,STK39,KCNE4,CRBN	grey	EC_M10
GO:0001682	3.87E-02	0.992557298	10	12	tRNA 5'-leader removal	BP	1.00E+00	RPP21,RPP25L,RPP14,POP4,RPP40,POP5,KIAA0391,POP7,RPP38,RPP30	grey	EC_M10
GO:0072174	3.91E-02	1	5	5	metanephric tubule formation	BP	1.00E+00	SOX9,PAX8,PAX2,WNT4,SOX8	grey	EC_M10
GO:0002820	3.91E-02	0.983217103	23	33	negative regulation of adaptive immune response	BP	1.00E+00	ARG1,IL1RL1,AHR,ZC3H12A,FOXJ1,NCKAP1L,NOD2,HFE,TRIM27,BCL6,RC3H1,TNFSF18,PPP3CB,FOXP3,ALOX15,IL27RA,TBX21,IL4R,SMAD7,TNFRSF14,CD55,HAVCR2,RC3H2	grey	EC_M10
GO:0071715	4.00E-02	0.98351863	21	30	icosanoid transport	BP	1.00E+00	PLA2G6,ANXA1,ABCC10,PNPLA8,ABCC4,ABCC2,PLA2G4F,MAP2K6,SLCO3A1,NTSR1,PLA2G3,PLA2G4A,ABCC3,NMUR2,ABCC1,PROCA1,ATP5PF,PLA2G2C,BDKRB2,PTGES,NMB	grey	EC_M10
GO:0034115	4.08E-02	1	5	5	negative regulation of heterotypic cell-cell adhesion	BP	1.00E+00	MBP,KLF4,WNK1,MAP2K5,MAPK7	grey	EC_M10
GO:0070587	4.08E-02	1	5	5	regulation of cell-cell adhesion involved in gastrulation	BP	1.00E+00	MBP,KLF4,WNK1,MAP2K5,MAPK7	grey	EC_M10
GO:0043652	4.13E-02	0.989945853	11	14	engulfment of apoptotic cell	BP	1.00E+00	MEGF10,RAC2,RHOG,XKR6,THBS1,RHOBTB2,RHOBTB1,ALOX15,ABCA7,XKR9,XKR5	grey	EC_M10
GO:0006352	4.13E-02	0.973529849	65	105	DNA-templated transcription, initiation	BP	1.00E+00	TBPL1,MED23,ESR1,TFB1M,MED30,POLR3H,TAF11,TAF8,POLR1E,NAT14,PIH1D1,TBP,POLR2D,ERCC1,MED4,ERCC3,GTF2F2,BRF2,GTF2E2,MITF,POLR2I,NCOA6,CDK7,CCNH,WNT10B,TAF5,CRCP,PSMC6,TFAM,TAF4,ATF7IP,E2F2,SMARCB1,MED14,POLR2G,TAF6L,GTF2A2,TP53,MAZ,MED12,SNAPC5,GTF3C1,MED7,TAF4B,BRF1,GTF2F1,RRN3,JUN,CTNNBIP1,TAF13,GTF3C5,SETX,MED27,TAF1C,DR1,GTF2B,TAF12,CAVIN1,HIST1H4B,HIST2H4A,HIST1H4H,CDK9,HIST1H4A,NKX2-5,HIST1H4F	grey	EC_M10
GO:0042471	4.15E-02	0.97438791	55	89	ear morphogenesis	BP	1.00E+00	LRIG3,SCRIB,TRIOBP,PTK7,ROR2,DLX5,SIX2,MYO6,KCNQ4,HOXA1,HOXA2,WDPCP,SOBP,OSR1,MYO3A,SOX9,SLC9A3R1,USH1G,INSIG1,SPRY2,SLITRK6,ATP8A2,HPN,PAX8,EYA1,PLS1,FZD3,SOD1,COL2A1,TFAP2A,PAX2,PDZD7,ALDH1A3,GBX2,MYO3B,GRHL3,STRC,PRKRA,EPHB2,NKX3-2,MAPK1,MSX1,DVL2,MAPK3,POU3F4,NTN1,GSC,TPRN,CHRNA10,FOXG1,FRZB,NOG,WNT5A,INSIG2,ZIC1	grey	EC_M10
GO:0060426	4.19E-02	1	5	5	lung vasculature development	BP	1.00E+00	TCF21,STRA6,ERRFI1,FOXF1,LIF	grey	EC_M10
GO:1903598	4.21E-02	1	5	5	positive regulation of gap junction assembly	BP	1.00E+00	CAV1,TBX5,AGT,ACE2,IRX3	grey	EC_M10
GO:0070286	4.22E-02	0.984172097	18	25	axonemal dynein complex assembly	BP	1.00E+00	DNAH8,WDR92,ARMC4,CCDC114,DRC1,CCDC65,DNAH7,ZMYND10,TTC12,DNAAF2,TTC25,DNAAF4,DNAL1,CCDC151,WDR78,C16orf71,DNAAF1,WDR63	grey	EC_M10
GO:0046951	4.23E-02	1	5	5	ketone body biosynthetic process	BP	1.00E+00	HMGCLL1,ACSS3,AACS,HMGCL,SLC27A5	grey	EC_M10
GO:1900364	4.26E-02	0.995003449	7	8	negative regulation of mRNA polyadenylation	BP	1.00E+00	NELFE,RNF20,SUPT5H,CTR9,CCNT1,ZC3H14,CDK9	grey	EC_M10
GO:0003352	4.26E-02	0.986177341	15	20	regulation of cilium movement	BP	1.00E+00	CFAP69,DNAH11,ARMC4,DRC1,TPPP2,MKKS,CFAP20,CCDC65,CFAP43,CFAP45,RGN,TTLL6,BBS4,DNAAF1,CCSAP	grey	EC_M10
GO:0048247	4.29E-02	0.981349909	23	33	lymphocyte chemotaxis	BP	1.00E+00	CCL27,ADAM17,OXSR1,GAS6,CYP7B1,PTK2B,CCL17,SLC8B1,STK39,ADAM8,CH25H,WNK1,CXCL16,TMEM102,ADAM10,CCL14,CCL5,CCL7,CCL25,PIK3CD,CCL8,WNT5A,CCL22	grey	EC_M10
GO:0150012	4.31E-02	0.994919204	7	8	positive regulation of neuron projection arborization	BP	1.00E+00	GRIP1,FZD4,DVL3,MAP3K13,DLG4,DVL2,WNT5A	grey	EC_M10
GO:0043576	4.32E-02	0.989352703	11	14	regulation of respiratory gaseous exchange	BP	1.00E+00	TSHZ3,FTO,SLC5A3,ATP1A2,MTG2,NTSR1,PASK,NLGN1,NLGN3,MECP2,PBX3	grey	EC_M10
GO:1900117	4.37E-02	0.989224626	11	14	regulation of execution phase of apoptosis	BP	1.00E+00	ZC3H12A,DLC1,BCL2L1,FZD3,RNF34,PTGIS,TP53BP2,TP53,DFFA,RFFL,CIDEA	grey	EC_M10

GO:0034220	4.37E-02	0.962824756	427	765	ion transmembrane transport	BP	1.00E+00	NETO1,SLC39A5,NEDD4L,ATP5F1B,STAC3,KCNN2,SLC26A10,SLC35D3,SLC16A7,UTRN,ATP5MGL,NIPAL2,MAPK8IP2,KCNV1,PKDREJ,CALHM4,GOPC,GJA1,BAK1,CATSPER3,ATP5PD,ITPR3,SLC25A17,PKD2L2,SLC35B4,KCNJ4,PLA2G6,SLC16A8,CYC1,CHRNA3,TRPM6,ABCB1,SRI,STEAP2,GABRA1,ATP10D,CNGA1,SLC41A2,TMEM63B,CLIC5,SLC1A1,TMEM165,SLC25A13,WNK2,COX5B,THADA,PRKCE,ATP6V1E2,KCNQ5,TMC4,KCNQ4,PTPN3,SLC4A4,NDUFA4,SLC1A4,SLC13A4,CAV1,ATP6V1C2,KCNF1,HECW1,TCAF1,CLCN1,KEL,SLC17A7,UCP1,STOM,ATG5,SLC41A3,OSR1,BAX,FYN,KCNJ14,GRIN2D,SLC8A2,SLC26A5,SLC1A5,CACNB2,SLC6A6,SLC39A12,SLC25A30,ATP6V0E2,SLC9A3R1,BIN1,GRIN2C,SLC4A2,ABCB8,ASIC3,SLC25A19,STAC,SLC5A6,SLC6A9,ATP1A3,CCT8L2,OXSR1,TMEM163,ATP5F1C,SCN1A,SLC25A38,TMC6,EHD3,SLC24A3,ABCC4,CHRNA6,SLC20A2,KCNE3,VDAC3,UCP2,SLC26A11,SLC30A6,COX7B,CACNB4,RYR1,NALCN,FGF14,SLC6A15,SLC25A15,SLC23A2,TRPC4,SLC16A3,DIAPH1,CLIC1,GAS6,SLC25A3,SLC25A26,SLC38A9,KCNIP3,ANO4,SLC5A8,SLC7A2,ATP6V1H,ATP4A,LYN,COX4I2,HPN,SCN1B,SLC16A12,MICU2,ATP6V0A2,SCN8A,SLC4A8,SLC43A1,SLC12A6,SLC11A2,GLRX,P2RX3,TRPC1,SLC9A9,JPH1,ORAI1,ASIC1,SLC38A7,HVCN1,PTK2B,SLC39A13,KCNJ11,WWP1,STIMATE,SFXN5,SLC8B1,IFNGR2,SLC25A37,F2R,TESC,SLC40A1,SLC6A2,SLC39A14,COX15,KCNK15,ABCC2,KCNE2,SLC35B3,CASQ2,ATP1A1,SFXN3,MCUR1,SLC12A5,KCNIP2,ADAMTS8,RYR2,ATP10A,FAM173B,NIPSNAP2,SLC41A1,COX6B1,SLC4A10,SLC35C2,TRPM1,CAPN3,CACNA2D2,SLC37A1,CALHM2,SLC6A3,GSTO1,RGS2,ATP6V1A,CAB39,SLC38A3,TR	grey	EC_M10
GO:0002725	4.38E-02	1	5	5	negative regulation of T cell cytokine production	BP	1.00E+00	ARG1,HFE,FOXP3,TBX21,SMAD7	grey	EC_M10
GO:0070940	4.39E-02	1	5	5	dephosphorylation of RNA polymerase II C-terminal domain	BP	1.00E+00	CTDP1,RPAP2,RPRD1B,RPRD1A,SSU72	grey	EC_M10
GO:0099541	4.44E-02	1	5	5	trans-synaptic signaling by lipid	BP	1.00E+00	PLCB1,F2R,DAGLB,DAGLA,FABP5	grey	EC_M10
GO:0099542	4.44E-02	1	5	5	trans-synaptic signaling by endocannabinoid	BP	1.00E+00	PLCB1,F2R,DAGLB,DAGLA,FABP5	grey	EC_M10
GO:0015985	4.51E-02	0.986598949	14	18	energy coupled proton transport, down electrochemical gradient	BP	1.00E+00	ATP5F1B,ATP5MGL,ATP5PD,ATP5F1C,FAM173B,ATP6V1A,ATP5MC3,ATP5F1A,ATP6V0C,ATP5PB,VPS9D1,ATP5ME,ATP5MF,ATP5PF	grey	EC_M10
GO:0015986	4.51E-02	0.986598949	14	18	ATP synthesis coupled proton transport	BP	1.00E+00	ATP5F1B,ATP5MGL,ATP5PD,ATP5F1C,FAM173B,ATP6V1A,ATP5MC3,ATP5F1A,ATP6V0C,ATP5PB,VPS9D1,ATP5ME,ATP5MF,ATP5PF	grey	EC_M10
GO:1905208	4.52E-02	0.99124515	9	11	negative regulation of cardiocyte differentiation	BP	1.00E+00	SMAD4,FRS2,EGFR,DLL1,SOX6,PRICKLE1,FZD7,DKK1,MIR222	grey	EC_M10
GO:0120197	4.54E-02	1	5	5	mucociliary clearance	BP	1.00E+00	TTLL1,NEK10,CFAP43,SPAG16,TTC25	grey	EC_M10
GO:0003338	4.59E-02	0.981663881	19	27	metanephros morphogenesis	BP	1.00E+00	SMO,SIX2,SOX9,FOXJ1,PAX8,WT1,FMN1,GREM1,FRAS1,PAX2,SALL1,WNT4,BMP4,KIF26B,CITED1,LHX1,SOX8,BASP1,LIF	grey	EC_M10
GO:0072033	4.61E-02	0.994464314	7	8	renal vesicle formation	BP	1.00E+00	SMO,SOX9,FMN1,SALL1,WNT4,KIF26B,SOX8	grey	EC_M10
GO:0070129	4.63E-02	0.983358356	17	23	regulation of mitochondrial translation	BP	1.00E+00	SHMT2,TSFM,RMND1,MRPS27,NSUN3,RPUSD4,RCC1L,NGRN,MTG2,TACO1,COA3,MPV17L2,C1QBP,ALKBH1,TRUB2,UQCC2,TRMT10C	grey	EC_M10
GO:0045723	4.63E-02	0.988408289	11	14	positive regulation of fatty acid biosynthetic process	BP	1.00E+00	AVPR1A,ANXA1,ELOVL5,NR1H2,NR1H3,LPGAT1,PLA2G3,PTGS2,MID1IP1,RGN,ABCD1	grey	EC_M10
GO:2000726	4.68E-02	0.994355589	7	8	negative regulation of cardiac muscle cell differentiation	BP	1.00E+00	SMAD4,FRS2,DLL1,SOX6,FZD7,DKK1,MIR222	grey	EC_M10
GO:0098792	4.71E-02	0.990761532	9	11	xenophagy	BP	1.00E+00	TBK1,NOD1,OPTN,RIPK2,NOD2,LGALS8,ATG16L1,CALCOCO2,MAPK3	grey	EC_M10
GO:0030889	4.73E-02	0.990728254	9	11	negative regulation of B cell proliferation	BP	1.00E+00	PAWR,LYN,CASP3,MNDA,RC3H1,ATM,PKN1,BTK,TNFRSF13B	grey	EC_M10
GO:0035513	4.77E-02	1	5	5	oxidative RNA demethylation	BP	1.00E+00	JMJD6,ALKBH3,FTO,ALKBH1,ALKBH5	grey	EC_M10
GO:0003356	4.79E-02	0.990573214	9	11	regulation of cilium beat frequency	BP	1.00E+00	DNAH11,ARMC4,MKKS,CFAP20,CFAP43,CFAP45,BBS4,DNAAF1,CCSAP	grey	EC_M10

GO:1902358	4.84E-02	0.990440111	9	11	sulfate transmembrane transport	BP	1.00E+00	SLC26A10,SLC13A4,SLC26A5,SLC25A30,SLC26A11,SLC26A6,SLC25A11,SLC26A2,SLC25A14	grey	EC_M10
GO:0034114	4.85E-02	0.987754762	11	14	regulation of heterotypic cell-cell adhesion	BP	1.00E+00	MBP,FLOT1,AGER,KLF4,CD44,GCNT2,WNK1,ALOX15,MAP2K5,MAPK7,FGA	grey	EC_M10
GO:0036265	4.88E-02	1	5	5	RNA (guanine-N7)-methylation	BP	1.00E+00	RNMT,METTL1,BUD23,WDR4,TRMT112	grey	EC_M10
GO:0015718	4.90E-02	0.971743562	42	66	monocarboxylic acid transport	BP	1.00E+00	SLC16A7,PLA2G6,SLC16A8,ANXA1,ABCB4,PNPLA8,SLC9A3R1,SLC16A5,SLC5A6,SLC22A13,ABCC4,SLC16A3,SLC5A8,CYP7A1,SLC16A12,SLC10A7,ABCC2,ABCG2,PLA2G4F,MAP2K6,SLC16A6,SLCO3A1,SLC26A6,SLCO1A2,SLCO1C1,NTSR1,PLA2G3,PLA2G4A,SLC27A1,MPC2,ABCC3,NMUR2,EMB,PROCA1,SLC16A4,SLC27A4,ATP5PF,PLA2G2C,BDKRB2,SLC27A5,PTGES,NMB	grey	EC_M10
GO:0071825	4.91E-02	0.977535095	24	35	protein-lipid complex subunit organization	BP	1.00E+00	APOM,PLA2G7,ABCA1,NR1H2,APOB,BIN1,APOC1,APOE,NR1H4,SOAT2,AGTR1,PLTP,LPL,ABCA5,AGT,PLA2G3,LPCAT3,PRKACA,LIPC,MPO,ABCA7,PRKACB,DGAT1,ACSL3	grey	EC_M10
GO:0021988	4.92E-02	0.982605809	15	21	olfactory lobe development	BP	1.00E+00	EXT1,DLX5,SEMA3A,ROBO2,RPGRIP1L,SALL1,LRRK2,ATP1A2,DLX2,SLIT2,SEMA7A,SKI,LHX2,ERBB4,WNT5A	grey	EC_M10
GO:0044091	4.93E-02	0.975119931	29	44	membrane biogenesis	BP	1.00E+00	PEX3,FLOT1,PTPRD,CHMP2A,SPTBN1,UGCG,CAV1,SPAST,RFTN1,STX2,NSFL1C,UBXN2B,LRP4,CHMP7,PEX16,REEP4,NLGN4Y,EPHB2,NRXN2,NLGN1,ANXA2,GAK,STX4,NLGN3,CDH2,CEL,VPS4A,TLCD1,REEP3	grey	EC_M10
GO:0045724	4.93E-02	0.982551241	15	21	positive regulation of cilium assembly	BP	1.00E+00	CEP120,KCTD17,CEP135,CCDC88A,SEPT7,ARHGAP35,SEPT9,ZMYND10,TAPT1,MNS1,BBS4,CCP110,WRAP73,ENTR1,ATMIN	grey	EC_M10
GO:0098754	4.94E-02	0.969065855	57	90	detoxification	BP	1.00E+00	LTC4S,APOM,SOD2,MGST3,PRXL2C,ABCB1,TXNRD1,PRDX1,CAT,ALB,PXDN,MGST2,GPX7,AKR1A1,APOE,GSTK1,PRDX3,PXDNL,GLRX,SOD1,GPX8,ABCC2,ABCG2,GSTO1,SLC30A10,PDZK1,MARC2,MARC1,SESN2,MGST1,ADH5,PTGS2,TXNRD2,PRDX6,GCH1,ABCB6,PRXL2A,RDH11,SOD3,PRDX2,GSTZ1,ATP7A,MPO,NNT,RALBP1,TXNDC2,GSTM3,NQO1,MIR21,MIR186,GSR,SLC22A5,CLIC2,PTGES,ADH4,GPX3,SLC30A1	grey	EC_M10
GO:0072521	4.97E-02	0.960987568	185	319	purine-containing compound metabolic process	BP	1.00E+00	ENPP1,NME1,ATP5F1B,HSD17B4,SHMT2,ATP5MGL,PPT2,PDE10A,ATP5PD,GUK1,PRXL2C,NUDT3,FBP1,LRGUK,GTPBP1,OARD1,GUCA1B,IMPDH1,GNMT,GDA,CROT,ENPP4,PDK4,SLC25A13,GAPDH,NPR2,ELOVL5,PPCS,RHOQ,SLC4A4,NT5E,BPGM,AASS,DLD,ADCY3,NUDT15,SUCLA2,DLG2,LACC1,DHTKD1,PRKAG2,GALK1,ATP5F1C,ELOVL1,OXSM,FASN,PANK2,HSD17B12,OGDHL,PDHX,PANK1,PDE7A,GPI,PDHB,AHCY,AMPD3,ACSL1,MCCC2,PTH,SAMHD1,FITM2,ENTPD7,HMGCR,NUDT9,SLC35B3,ELOVL2,ADCY7,GMPR,LRRK2,ABHD14B,FAM173B,MCEE,NME4,AK2,ATP6V1A,TPST1,MVK,PFKL,FAR2,BPNT1,PTHLH,ACACB,MFN1,ELOVL6,PFKFB2,PRPS2,ADAL,GMPR2,PDE5A,ACTN3,SLC25A12,THEM5,PDHA1,PDK1,HMGCL,MAGI3,KDM1A,ATP5MC3,ACOT9,EFL1,HK1,DLAT,ACSBG1,PDE1A,ATP5S,CASK,BAD,ENO2,ACAT1,MACROD1,GUCY1A2,COASY,PINK1,STAT3,NME7,ADK,MPC2,ADCY10,PAPSS2,ENO3,ACLY,MTHFD1,PARP1,CARD11,NUDT1,HK3,RORA,GUCY2D,PKLR,PDK2,DLST,PANK3,ACSF2,PMVK,ATP5F1A,ADPGK,PDE4A,PPCDC,NCOR1,SLC26A2,PRPSAP2,SHMT1,HMGCS1,PGM1,ACSBG2,PRKAA2,ACOT11,ADCY9,ABCD1,ATP6V0C,PANK4,NME3,MPP1,ATP5PB,AMPD2,VPS9D1,ACSF3,CBFA2T3,MVD,MLYCD,NUDT7,AK1,AK5,RHOA,SULT1C3,ACOT6,ATP5ME,ACOT4,SLC25A1,ATP5MF,ATP5PF,DGAT1,ENTPD5,PDZD3,ELOVL3,ACSL3,NUDT19,ELOVL4,MACROD2,PRPSAP1,NUDT4B,ITPA,HINT1,DCAKD,DDIT4	grey	EC_M10
GO:0042073	4.99E-02	0.977315435	23	34	intraciliary transport	BP	1.00E+00	IFT27,DYNC2LI1,LCA5,BBS12,IFT172,WDR60,CEP131,TUB,PCM1,KIF3B,IFT88,IFT81,IFT52,TTC21B,TRAF3IP1,IFT46,RPGR,IFT20,HSPB11,SSNA1,WDR34,SSX2IP,TTC30A	grey	EC_M10
GO:1904896	5.07E-02	0.993726389	7	8	ESCRT complex disassembly	BP	1.00E+00	VPS4B,VTA1,CHMP2A,CHMP2B,CHMP7,CHMP1A,VPS4A	grey	EC_M10
GO:1904903	5.07E-02	0.993726389	7	8	ESCRT III complex disassembly	BP	1.00E+00	VPS4B,VTA1,CHMP2A,CHMP2B,CHMP7,CHMP1A,VPS4A	grey	EC_M10
GO:0007379	5.13E-02	0.986864442	11	14	segment specification	BP	1.00E+00	MLLT3,MEOX2,HOXA2,COBL,OSR1,DLL1,BMI1,IRX1,IRX2,DVL2,IRX3	grey	EC_M10
GO:0061732	5.15E-02	1	5	5	mitochondrial acetyl-CoA biosynthetic process from pyruvate	BP	1.00E+00	DLD,PDHX,PDHB,PDHA1,MPC2	grey	EC_M10
GO:1903427	5.22E-02	0.986597297	11	14	negative regulation of reactive oxygen species biosynthetic process	BP	1.00E+00	FYN,SLC30A10,STAT3,ABCB7,ABCD1,TRAP1,CTNS,MIR21,RHOA,APOPT1,AC140504.1	grey	EC_M10

GO:0035384	5.22E-02	0.973976407	28	42	thioester biosynthetic process	BP	1.00E+00	PPT2,PDK4,ELOVL5,PPCS,DLD,ELOVL1,HSD17B12,PDHX,PDHB,ACSL1,ELOVL2,ACACB,ELOVL6,PDHA1,PDK1,DLAT,ACSBG1,ACAT1,MPC2,ACLY,PDK2,ACSBG2,ACSF3,MLYCD,SLC25A1,ELOVL3,ACSL3,ELOVL4	grey	EC_M10
GO:0071616	5.22E-02	0.973976407	28	42	acyl-CoA biosynthetic process	BP	1.00E+00	PPT2,PDK4,ELOVL5,PPCS,DLD,ELOVL1,HSD17B12,PDHX,PDHB,ACSL1,ELOVL2,ACACB,ELOVL6,PDHA1,PDK1,DLAT,ACSBG1,ACAT1,MPC2,ACLY,PDK2,ACSBG2,ACSF3,MLYCD,SLC25A1,ELOVL3,ACSL3,ELOVL4	grey	EC_M10
GO:0010884	5.31E-02	0.982343811	14	19	positive regulation of lipid storage	BP	1.00E+00	SREBF2,ZC3H12A,NR1H2,APOB,ASXL2,FITM2,LPL,IKBKE,ACACB,FITM1,EHD1,C3,CIDEA,PLIN3	grey	EC_M10
GO:0072161	5.31E-02	0.99333148	7	8	mesenchymal cell differentiation involved in kidney development	BP	1.00E+00	TCF21,SIX2,OSR1,PAX2,WNT4,BMP4,AMER1	grey	EC_M10
GO:2001012	5.31E-02	0.99333148	7	8	mesenchymal cell differentiation involved in renal system development	BP	1.00E+00	TCF21,SIX2,OSR1,PAX2,WNT4,BMP4,AMER1	grey	EC_M10
GO:1901387	5.35E-02	0.989165757	9	11	positive regulation of voltage-gated calcium channel activity	BP	1.00E+00	STAC3,CACNB2,STAC,EHD3,KCNE3,FGF14,KCNE2,NIPSNAP2,STAC2	grey	EC_M10
GO:0007605	5.37E-02	0.964937057	62	102	sensory perception of sound	BP	1.00E+00	MBP,MYO1A,EYA4,NDUFB9,TRIOBP,WFS1,CLIC5,MYO6,KCNQ4,GSDME,HOXA1,SOBP,SLC26A5,MYO3A,FZD4,MYO7B,SLC9A3R1,USH1G,SPRY2,SLITRK6,MKKS,THRB,AP000812.5,EPYC,DIAPH1,SNAI2,TUB,HPN,PPIP5K2,CASP3,TIMM10,EYA1,ADGRV1,SCARB2,SOD1,COL2A1,TFAP2A,PDZD7,PGAP1,LRP2,USP53,MYO3B,COCH,STRC,HOMER2,CEMIP,CDH23,POU3F4,CRYM,ASIC2,SLC1A3,TPRN,CDC14A,SRRM4,PJVK,CHRNA10,SERPINB6,DDIT3,TIMM9,TIMM8B,ICAM1,OTOS	grey	EC_M10
GO:0044281	5.40E-02	0.952111216	722	1301	small molecule metabolic process	BP	1.00E+00	ENPP1,NME1,RDH5,KDSR,CS,APOF,GLS2,ATP5F1B,HSD17B4,MOXD1,SHMT2,TBPL1,MARS,CYP27B1,AIG1,FUCA2,AVPR1A,LTC4S,ATP5MGL,ARG1,VARS2,TPH2,RIDA,CPT1B,DDAH2,VARS,DPYS,SLC27A6,ACAT2,TNXB,PPT2,PRKN,PDE10A,MGST3,PNPLA3,MCAT,SMPDL3A,SREBF2,SQLE,ECHDC1,ACO2,ATP5PD,SLC25A17,ST3GAL1,GUK1,IP6K3,PRXL2C,NUDT3,EGR1,AKR1B1,SLC35B4,FBP1,LRGUK,PYCR3,TSTA3,APOBEC3H,GTPBP1,MAPK14,PLA2G6,GCAT,TST,TTC39B,OARD1,GUCA1B,GPT,IMPDH1,HACD4,NMRK1,GNMT,ANXA1,ACO1,CRY1,GDA,CROT,B4GALT1,ABCC10,GNPDA2,APPL2,CYP51A1,AARS2,ENPP4,CYP39A1,VLDLR,PLA2G7,CA9,UXS1,MUT,PDK4,TFAP2B,SPTLC1,SLC25A13,GAPDH,SDHAF3,AASDH,NPR2,ELOVL5,GCLC,IPPK,LIPT1,GNE,IARS,HMGCLL1,GRHPR,ALDH1B1,SLC2A1,CAT,LMBRD1,PPCS,EDN2,TMEM86B,PRKCE,RHOQ,RDH13,ABCA1,BCKDHB,OSBPL3,PGM3,SLC4A4,ME1,PEX13,NT5E,RARS2,MDH1,NT5C3A,BPGM,INPP5B,PLEK,NR1H2,DDC,CAV1,AASS,PNPLA8,GPT2,MGST2,QRSL1,DLD,APOB,BCAT2,PRKAR2B,ADCY3,GLUD1,NAPEPLD,UGGT1,FAAH,HADHA,EBP,ACAD9,NOX4,SOX9,CDADC1,NUDT15,SUCLA2,RUBCNL,CYP27C1,DLG2,AKR1A1,CUBN,LACC1,APOC1,APOE,HPDL,SLC25A19,EPM2AIP1,NUDT5,DHTKD1,PRKAG2,GALK1,SLC22A13,HRH1,ATP5F1C,XYLB,PIBF1,SPHK1,INSIG1,ITPKC,COQ8B,ENTPD3,GCKR,PFKFB3,TSKU,PLB1,ELOVL1,TGDS,OGG1,LIPT2,UGGT2,PGM2L1,SPTLC3,ECH1,OXSM,INPPL1,IDO2,IDO1,ACSS3,PCCA,MDH2,SIRT7,HARS,PYCR1,DCXR,BTD,SLC25A15,FASN,SLC23A2,PANK2,ERLIN2,GALM,FUT10,AMDHD1,B3GLCT,SNAI2,PPP1R3B,HSD17B12,OGDHL,PARG,NR1H4,PLPP1,PDHX,	grey	EC_M10
GO:0003128	5.50E-02	0.983594392	12	16	heart field specification	BP	1.00E+00	EXT1,FOXH1,WNT11,ROBO2,AXIN2,LRP2,GATA5,BMP4,DKK1,RBPJ,ISL1,WNT5A	grey	EC_M10
GO:0033079	5.51E-02	0.993000362	7	8	immature T cell proliferation	BP	1.00E+00	BMI1,TMEM131L,RIPK2,WNT4,BMP4,FOXP3,ERBB2	grey	EC_M10
GO:0015937	5.54E-02	0.988691273	9	11	coenzyme A biosynthetic process	BP	1.00E+00	PPCS,PANK2,PANK1,ACAT1,COASY,PANK3,PPCDC,PANK4,DCAKD	grey	EC_M10
GO:2000480	5.56E-02	0.992925858	7	8	negative regulation of cAMP-dependent protein kinase activity	BP	1.00E+00	PRKAR2B,PRKAG2,PKIA,PKIG,PRKAR2A,PPP1R1B,PKIB	grey	EC_M10
GO:0010996	5.59E-02	0.983267522	12	16	response to auditory stimulus	BP	1.00E+00	XPA,PTN,SLC26A5,XPC,SLITRK6,ATP8A2,KIAA0319,ATP1A2,USP53,NRXN2,STRA6,SLC1A3	grey	EC_M10
GO:0010873	5.61E-02	1	5	5	positive regulation of cholesterol esterification	BP	1.00E+00	APOC1,APOE,AGTR1,AGT,STARD4	grey	EC_M10

GO:2001279	5.61E-02	0.992843907	7	8	regulation of unsaturated fatty acid biosynthetic process	BP	1.00E+00	AVPR1A,ANXA1,PIBF1,PLA2G3,PTGS2,ABCD1,FABP5	grey	EC_M10
GO:0060192	5.66E-02	0.988363215	9	11	negative regulation of lipase activity	BP	1.00E+00	ANXA1,HDAC9,APOC1,RGS2,BICD1,ANXA8,ANGPTL4,SORT1,ABL1	grey	EC_M10
GO:0036363	5.69E-02	0.988716032	8	10	transforming growth factor beta activation	BP	1.00E+00	HSP90AB1,ITGB8,LRRC32,ITGB6,FURIN,LTBP3,GATA6,NRROS	grey	EC_M10
GO:0048243	5.69E-02	0.988283177	9	11	norepinephrine secretion	BP	1.00E+00	ADRA2B,FFAR3,P2RY1,NISCH,ADRA2A,KCNB1,AGT,STX1A,CRH	grey	EC_M10
GO:0006415	5.70E-02	0.982876705	12	16	translational termination	BP	1.00E+00	MTRF1L,JMJD4,MRPL58,MTRF1,C12orf65,GFM2,OGFOD1,ETF1,EIF5A,C19orf66,GLE1,EIF5A2	grey	EC_M10
GO:0000387	5.70E-02	0.972463283	26	38	spliceosomal snRNP assembly	BP	1.00E+00	PRPF31,SRSF10,SNRPD2,GEMIN7,SNRPB,AAR2,SART3,SART1,PRPF6,GEMIN2,LSM4,CLNS1A,PRPF19,SNRPD3,SNRPD1,PRPF8,PRMT7,RNU5E-1,RNU5B-1,USP4,RNU4ATAC,GEMIN8,SNRPC,RNU4-2,SRSF12,RNU4-1	grey	EC_M10
GO:0006839	5.73E-02	0.957125453	125	210	mitochondrial transport	BP	1.00E+00	TIMM21,BCL2,ATP5F1B,HEBP2,CPT1B,HSPA1L,DYNLT1,PRKN,SAMM50,SREBF2,BAK1,ATP5PD,RAC2,TST,FBXO7,SLC25A13,TMEM14A,GCLC,SLC25A51,UCP1,BAX,AGK,CHCHD4,SLC25A30,TOMM40,DNAJC15,ATP5F1C,SLC25A38,TIMM50,USP36,UCP2,SLC25A15,BAG4,SLC25A3,BCL2L1,MIPEP,MICU2,IMMP1L,PDCD5,TIMM10,FBXW7,GDAP1,CSNK2A2,SFXN5,SLC8B1,SLC25A37,TOMM34,SFXN3,MCUR1,LRRK2,FAM173B,SIRT4,MAIP1,MCUB,TOMM20,HPS4,PARL,ARIH2,SLC25A20,SFXN4,ABCB10,BAG3,SLC9A1,VPS11,SLC25A12,THEM4,STPG1,ATF2,MTX2,KIF1BP,NMT1,NDUFA13,BAD,MICU1,BCS1L,PINK1,MUL1,STAT3,MPC2,CNP,PSEN2,SLC25A18,TMEM102,HUWE1,MFN2,NPEPPS,TP53,HAX1,ABCB7,TIMM29,ATP5F1A,TOMM22,CAMK2A,TIMM44,SLC25A5,RHOT1,SLC25A23,SLC25A41,ALKBH7,SLC25A14,PAM16,DNLZ,ATP5PB,SPG7,NAIF1,SH3GLB1,MIR29A,ATP5ME,SLC25A1,ATP5MF,UBE2D3,ATP5PF,BNIP3L,SLC25A28,TOMM20L,TIMM10B,TIMM23B,BCL2L11,TIMM9,TIMM8B,MRPL18,PPIF,UBE2J2,ATP5IF1,AC140504.1	grey	EC_M10
GO:0003016	5.77E-02	0.975072579	20	29	respiratory system process	BP	1.00E+00	CCBE1,TTLL1,ZFAND5,HOXA5,YWHAZ,NEK10,TSHZ3,TNNC1,FTO,CFAP43,ATP1A2,MTG2,ADH5,SPAG16,RAB3A,NLGN1,TTC25,NLGN3,MECP2,PBX3	grey	EC_M10
GO:0032958	5.90E-02	0.978427364	15	21	inositol phosphate biosynthetic process	BP	1.00E+00	IP6K3,IPPK,PLEK,HRH1,ITPKC,PPIP5K2,PTH,P2RY1,PTK2B,ITPKA,IP6K1,GPER1,IP6K2,NTSR1,PPIP5K1	grey	EC_M10
GO:1902410	5.91E-02	0.979924954	14	19	mitotic cytokinetic process	BP	1.00E+00	VPS4B,MITD1,CHMP2A,NUP62,CHMP6,CHMP3,CHMP2B,CHMP4C,CHMP7,KLHDC8B,CNTROB,CHMP1A,VPS4A,RHOA	grey	EC_M10
GO:1905508	5.96E-02	0.972388979	22	33	protein localization to microtubule organizing center	BP	1.00E+00	DCTN2,NUP62,MAPRE1,PIBF1,CEP131,NUMA1,CSNK1D,CEP83,NSFL1C,UBXN2B,PCM1,APC,BICD1,DISC1,CCDC14,CEP350,KIAA0753,BBS4,CEP192,WRAP73,C2CD3,PARD6A	grey	EC_M10
GO:1901570	5.98E-02	0.970449108	26	39	fatty acid derivative biosynthetic process	BP	1.00E+00	PPT2,ELOVL5,HMGCLL1,ELOVL1,ACSS3,HSD17B12,AACS,PAM,GGT7,ACSL1,ELOVL2,FAR2,ELOVL6,HMGCL,ACSBG1,GGT6,ALOX15,ALOX15B,ACSBG2,ACSF3,ALOX12,SLC25A1,ELOVL3,ACSL3,ELOVL4,SLC27A5	grey	EC_M10
GO:0034368	6.00E-02	0.979532987	14	19	protein-lipid complex remodeling	BP	1.00E+00	APOM,PLA2G7,APOB,APOC1,APOE,NR1H4,AGTR1,PLTP,LPL,ABCA5,AGT,PLA2G3,LIPC,MPO	grey	EC_M10
GO:0034369	6.00E-02	0.979532987	14	19	plasma lipoprotein particle remodeling	BP	1.00E+00	APOM,PLA2G7,APOB,APOC1,APOE,NR1H4,AGTR1,PLTP,LPL,ABCA5,AGT,PLA2G3,LIPC,MPO	grey	EC_M10
GO:0050433	6.04E-02	0.972576758	22	32	regulation of catecholamine secretion	BP	1.00E+00	PRKN,SYT17,SYT5,SYT1,SYT9,ADRA2B,FFAR3,P2RY1,SYT8,GRM2,ADRA2A,KCNB1,GRK2,SYT12,AGT,STX1A,RAB3A,PINK1,SYT4,ABAT,CHGA,CRH	grey	EC_M10
GO:0042472	6.05E-02	0.963158916	45	73	inner ear morphogenesis	BP	1.00E+00	LRIG3,SCRIB,TRIOBP,PTK7,ROR2,DLX5,MYO6,KCNQ4,HOXA1,WDPCP,SOBP,MYO3A,SOX9,SLC9A3R1,USH1G,INSIG1,SPRY2,SLITRK6,ATP8A2,HPN,PAX8,EYA1,PLS1,FZD3,SOD1,COL2A1,TFAP2A,PAX2,PDZD7,ALDH1A3,GBX2,MYO3B,GRHL3,STRC,EPHB2,DVL2,POU3F4,NTN1,TPRN,CHRNA10,FOXG1,FRZB,WNT5A,INSIG2,ZIC1	grey	EC_M10
GO:0006099	6.12E-02	0.974324288	19	27	tricarboxylic acid cycle	BP	1.00E+00	CS,ACO2,ACO1,MDH1,SUCLA2,DHTKD1,MDH2,OGDHL,PDHB,SDHA,PDHA1,MDH1B,DLAT,IDH3A,SDHB,DLST,NNT,IDH3G,SDHAF2	grey	EC_M10
GO:1901096	6.19E-02	0.983859601	10	13	regulation of autophagosome maturation	BP	1.00E+00	UVRAG,TMEM39A,RUBCN,FYCO1,TBC1D25,PHF23,UBQLN4,CALCOCO2,SMCR8,IRGM	grey	EC_M10
GO:0009312	6.26E-02	0.980798905	12	16	oligosaccharide biosynthetic process	BP	1.00E+00	FBP1,B4GALT1,ST3GAL3,ST3GAL4,MGAT2,B3GALNT1,FUT8,MPDU1,ST3GAL2,ST6GALNAC5,FBP2,AL157935.2	grey	EC_M10

GO:0046686	6.28E-02	0.966587456	31	47	response to cadmium ion	BP	1.00E+00	ARG1,MAPK9,SOD2,DAXX,GCLC,CAT,ALAD,MTF1,EGFR,PPP5C,ERCC1,OGG1,PCNA,GPI,GSS,PTH,SOD1,GOT1,TERT,DTYMK,SORD,ABCB6,MAPK1,NUDT1,MAPK3,ATP7A,SPARC,NPC1,JUN,UBE2D3,SLC30A1	grey	EC_M10
GO:1904386	6.32E-02	1	4	4	response to L-phenylalanine derivative	BP	1.00E+00	GCLC,CRLS1,PPP1R9B,GCLM	grey	EC_M10
GO:0010801	6.37E-02	0.980359561	12	16	negative regulation of peptidyl-threonine phosphorylation	BP	1.00E+00	PPP2R5D,SPRED2,SPRY2,DMTN,EIF4G1,DGKQ,CALM2,SMAD7,INPP5K,CALM3,CADM4,DDIT4	grey	EC_M10
GO:0045599	6.37E-02	0.966910526	28	43	negative regulation of fat cell differentiation	BP	1.00E+00	ENPP1,ZFPM2,WISP1,MKX,BBS12,INSIG1,FOXO1,TRIB3,ASXL1,LRP3,E2F1,ARNTL,WNT10B,TRIO,TRPV4,GPER1,FERMT2,RORA,JDP2,SMAD3,SORT1,MIR27B,MIR27A,WNT5A,DDIT3,YAP1,C1QL4,ZFPM1	grey	EC_M10
GO:0021889	6.38E-02	0.991977873	6	7	olfactory bulb interneuron differentiation	BP	1.00E+00	DLX5,ROBO2,SALL1,SLIT2,ERBB4,WNT5A	grey	EC_M10
GO:0016191	6.44E-02	1	4	4	synaptic vesicle uncoating	BP	1.00E+00	SYNJ1,GAK,DNAJC6,SH3GL1	grey	EC_M10
GO:0072093	6.47E-02	1	4	4	metanephric renal vesicle formation	BP	1.00E+00	SMO,FMN1,SALL1,KIF26B	grey	EC_M10
GO:0071300	6.51E-02	0.96382002	33	52	cellular response to retinoic acid	BP	1.00E+00	ADNP2,SNW1,PTK7,ABCA1,KLF4,HOXA2,TNC,GJB3,OSR1,SOX9,FZD4,WNT11,FZD10,LYN,PTK2B,EPHA3,WNT10B,CYP26B1,TESC,PAX2,FZD7,WNT3,BRINP2,NDUFA13,ATM,WNT5B,SLC6A4,SETX,TWF2,WNT5A,ZNF35,WNT9A,YAP1	grey	EC_M10
GO:0062125	6.51E-02	0.972426011	19	27	regulation of mitochondrial gene expression	BP	1.00E+00	SHMT2,TSFM,RMND1,C6orf203,MRPS27,NSUN3,RPUSD4,RCC1L,NGRN,MTG2,TACO1,COA3,MPV17L2,C1QBP,ALKBH1,METTL4,TRUB2,UQCC2,TRMT10C	grey	EC_M10
GO:0098660	6.53E-02	0.944931431	321	574	inorganic ion transmembrane transport	BP	1.00E+00	NETO1,SLC39A5,NEDD4L,ATP5F1B,STAC3,KCNN2,SLC26A10,UTRN,ATP5MGL,NIPAL2,KCNV1,PKDREJ,CATSPER3,ATP5PD,ITPR3,PKD2L2,KCNJ4,PLA2G6,CYC1,TRPM6,SRI,STEAP2,GABRA1,SLC41A2,SLC1A1,TMEM165,WNK2,COX5B,THADA,PRKCE,ATP6V1E2,KCNQ5,KCNQ4,PTPN3,SLC4A4,NDUFA4,SLC13A4,CAV1,ATP6V1C2,KCNF1,HECW1,CLCN1,KEL,SLC17A7,UCP1,STOM,ATG5,OSR1,BAX,FYN,KCNJ14,GRIN2D,SLC8A2,SLC26A5,CACNB2,SLC6A6,SLC39A12,SLC25A30,ATP6V0E2,SLC9A3R1,BIN1,GRIN2C,ABCB8,ASIC3,STAC,SLC5A6,SLC6A9,ATP1A3,CCT8L2,OXSR1,TMEM163,ATP5F1C,SCN1A,EHD3,SLC24A3,SLC20A2,KCNE3,UCP2,SLC26A11,SLC30A6,COX7B,CACNB4,RYR1,NALCN,FGF14,SLC6A15,TRPC4,DIAPH1,GAS6,SLC25A3,KCNIP3,ANO4,ATP6V1H,ATP4A,LYN,COX4I2,HPN,SCN1B,MICU2,ATP6V0A2,SCN8A,SLC4A8,SLC12A6,SLC11A2,GLRX,P2RX3,TRPC1,SLC9A9,JPH1,ORAI1,ASIC1,HVCN1,PTK2B,SLC39A13,KCNJ11,STIMATE,SLC8B1,SLC25A37,F2R,TESC,SLC40A1,SLC6A2,SLC39A14,COX15,KCNK15,ABCC2,KCNE2,CASQ2,ATP1A1,MCUR1,SLC12A5,KCNIP2,ADAMTS8,RYR2,FAM173B,NIPSNAP2,SLC41A1,COX6B1,SLC4A10,TRPM1,CAPN3,CACNA2D2,SLC37A1,SLC6A3,GSTO1,ATP6V1A,CAB39,TRPV4,SLC9B2,ACTN2,ADRA2A,SLC30A10,HFE,SLC37A2,MAIP1,COX17,SCN2A,ITPR2,KCNB1,MCUB,SCN9A,CLCN4,KCNK1,KCNG1,SCN7A,UBASH3B,STK39,GPER1,KLHL24,RHCG,TRPM8,CAMK2D,ABCC9,ABCC5,SCN4A,ATP1A2,SLC26A6,SLC9A1,UQCRC1,CCDC51,KCNK2,NTSR1,SCN4B,TCN2,RHCE,KCNQ2,TACO1,NIPAL3,AKAP6,CATSPER2,ATP5MC3,COX6A1,KCND3,ORAI2,SLC30A4,CEMIP,CLDN16,SLC12A9,SESTD1,ATP13A1,ATP5S,MICU1	grey	EC_M10
GO:0060122	6.56E-02	0.972435312	18	26	inner ear receptor cell stereocilium organization	BP	1.00E+00	SCRIB,TRIOBP,IFT27,SLC9A3R1,USH1G,TSKU,IFT88,PLS1,ADGRV1,SOD1,PDZD7,STRC,CDH23,MKS1,IFT20,TPRN,PJVK,SDC4	grey	EC_M10
GO:0006901	6.58E-02	0.979589369	12	16	vesicle coating	BP	1.00E+00	TMED9,ARFGAP3,MAPK15,CSNK1D,ARFGAP2,TFG,GBF1,KLHL12,PEF1,PDCD6,PPP6C,SAR1B	grey	EC_M10
GO:0030242	6.58E-02	0.991639783	6	7	autophagy of peroxisome	BP	1.00E+00	ACBD5,PIK3R4,RB1CC1,PIK3C3,TRAPPC8,PJVK	grey	EC_M10
[truncated: 617,577 more chars]
