# Supplementary material for: Locational memory of macrovessel vascular cells is transcriptionally imprinted
Source: Sci Rep. 2023 Aug 10;13:13028. doi: 10.1038/s41598-023-38880-6 (PMC10415317; doi:10.1038/s41598-023-38880-6)
Supplement: Supplementary file 9 — Supplementary Table 12. [file 41598_2023_38880_MOESM9_ESM.pdf]

Supplemental Table 12. Gene ontology terms for modules in the vascular smooth muscle cell dataset.

category	over_represented_pvalue	under_represented_pvalue	numDEInCat	numInCat	term	ontology	p.adjust	GeneID	module	module_num
GO:0006695	9.172E-07	1.00	14	43	cholesterol biosynthetic process	BP	0.00462	INSIG1,FDFT1,SOD1,DHCR7,MVK,LSS,ACLY,PMVK,HMGCS1,DHCR24,NSDHL,MVD,FDPS,IDI1	yellow	VSMC_M3
GO:1902653	9.172E-07	1.00	14	43	secondary alcohol biosynthetic process	BP	0.00462	INSIG1,FDFT1,SOD1,DHCR7,MVK,LSS,ACLY,PMVK,HMGCS1,DHCR24,NSDHL,MVD,FDPS,IDI1	yellow	VSMC_M3
GO:1902652	2.158E-06	1.00	23	111	secondary alcohol metabolic process	BP	0.00815	SREBF2,SQLE,PPARD,FDXR,SNX17,INSIG1,IDH3B,SCARB1,FDFT1,SOD1,DHCR7,MVK,LSS,IDH1,ACLY,PMVK,HMGCS1,DHCR24,NSDHL,MVD,GBA2,FDPS,IDI1	yellow	VSMC_M3
GO:0008203	6.583E-06	1.00	21	103	cholesterol metabolic process	BP	0.01988	SREBF2,SQLE,PPARD,FDXR,SNX17,INSIG1,SCARB1,FDFT1,SOD1,DHCR7,MVK,LSS,ACLY,PMVK,HMGCS1,DHCR24,NSDHL,MVD,GBA2,FDPS,IDI1	yellow	VSMC_M3
GO:0016197	3.140E-05	1.00	30	209	endosomal transport	BP	0.07074	PQLC1,SQSTM1,SNX9,SCRIB,VPS28,TOM1,EIPR1,SNX3,EHD2,SNX17,LAMTOR1,DCTN1,RAB35,TRIM27,PLA2G3,PTPN23,VPS51,MVB12A,WASHC1,CCDC22,SNX8,SNF8,STX10,SNX18,DENND1C,AP3D1,PLA2G4E,AP5B1,ARFRP1,PHETA2	yellow	VSMC_M3
GO:0016125	3.279E-05	1.00	21	114	sterol metabolic process	BP	0.07074	SREBF2,SQLE,PPARD,FDXR,SNX17,INSIG1,SCARB1,FDFT1,SOD1,DHCR7,MVK,LSS,ACLY,PMVK,HMGCS1,DHCR24,NSDHL,MVD,GBA2,FDPS,IDI1	yellow	VSMC_M3
GO:0009240	4.774E-05	1.00	4	4	isopentenyl diphosphate biosynthetic process	BP	0.08009	MVK,PMVK,MVD,IDI1	yellow	VSMC_M3
GO:0046490	4.774E-05	1.00	4	4	isopentenyl diphosphate metabolic process	BP	0.08009	MVK,PMVK,MVD,IDI1	yellow	VSMC_M3
GO:0032456	1.161E-04	1.00	14	70	endocytic recycling	BP	0.17528	SCRIB,EIPR1,SNX3,EHD2,SNX17,RAB35,PLA2G3,PTPN23,VPS51,WASHC1,CCDC22,SNF8,DENND1C,PLA2G4E	yellow	VSMC_M3
GO:1903725	1.861E-04	1.00	9	31	regulation of phospholipid metabolic process	BP	0.25544	SCARB1,CHP1,FABP3,PDGFA,IDH1,PHB2,CAPN2,MTMR4,TAZ	yellow	VSMC_M3
GO:0034660	2.410E-04	1.00	50	412	ncRNA metabolic process	BP	0.30327	NHP2,TRMT12,BOP1,SND1,RTCB,AARS2,ZC3H12A,KTI12,METTL3,PUS1,TARBP2,NAT10,RRS1,LSM6,GATB,NPM3,CARS,WDR4,RRP1B,MOCS3,NGDN,QARS,PSTK,RPS21,FTSJ3,BUD23,SART1,RELA,DDX52,TRMT112,NOP14,MRTO4,GTPBP3,ELP5,SEPSECS,INTS1,RRNAD1,TRMT1,FARSA,RPL7A,KHSRP,DUS3L,TBL3,TSR3,SARS,CTU2,RBFA,PIN4,TRIR,OSGEP	yellow	VSMC_M3
GO:0009451	2.882E-04	1.00	23	141	RNA modification	BP	0.33477	NHP2,TRMT12,AARS2,KTI12,METTL3,PUS1,NAT10,PCIF1,RBM15B,WDR4,MOCS3,FTSJ3,BUD23,TRMT2A,TRMT112,GTPBP3,ELP5,RRNAD1,TRMT1,DUS3L,TSR3,CTU2,OSGEP	yellow	VSMC_M3
GO:0006412	3.888E-04	1.00	62	548	translation	BP	0.38755	CDK4,EEF1D,RPL3,RPL8,MRPS18A,AARS2,SECISBP2,RPS9,CNOT3,ZC3H12A,UNK,METTL3,DND1,RPL29,EIF4EBP1,TARBP2,NAT10,EIF4B,GATB,RPLP2,MRPL43,PCIF1,CARS,DCPS,APEH,NGDN,QARS,EIF3A,LSM14B,PSTK,RPS21,TACO1,MRPL49,IGFBP5,MPV17L2,C1QBP,AIMP2,SEPSECS,NANOS3,PER1,FARSA,TUFM,RPL37,RPL7A,EEF2K,MRPL4,EIF3G,C19orf66,AKT1,PAIP1,KHSRP,MRPL20,TRAP1,MKNK2,MRPL28,SARS,RBM8A,EEF1G,MRPL34,ZNF385A,MRPS16,ZAR1L	yellow	VSMC_M3
GO:0034641	4.107E-04	1.00	390	4878	cellular nitrogen compound metabolic process	BP	0.38755	ENPP1,NAB2,NHP2,CDK4,SQSTM1,DHX16,ZNF608,HDAC10,NELFE,CERK,FNIP1,ATF6B,PBX2,RING1,SREBF2,TRMT12,H2AFY,HNRNPA0,TRIM11,GUK1,TRAPPC9,NUDT3,PPARD,FANCE,EEF1D,TSTA3,RPL3,MPST,BOP1,SLC52A2,RPL8,SND1,RTCB,DNPH1,MRPS18A,AARS2,SECISBP2,PHF2,RPS9,CNOT3,KLF4,HOXA1,FHL3,ZC3H12A,FIGNL1,BLVRA,PNKP,CPT1C,SNRNP70,CC2D1B,KTI12,KMT2E,SIX5,ZNF282,GEMIN7,ZNF296,RELB,MUTYH,DMAP1,CIC,GTF3C2,GALK1,UNK,BLVRB,OGG1,SERTAD1,TTC5,METTL3,CXXC5,FAAP100,DND1,FASN,SMOX,RPL29,EIF4EBP1,GNPDA1,PUS1,GSR,DEAF1,SMUG1,HOXC4,OGDHL,POLR1D,TARBP2,KMT2B,C12orf10,NAT10,HM13,ZNF740,PDE4D,EIF4B,RRS1,SWAP70,RALY,ACSL1,ZBTB38,ARL6IP4,LSM6,SLC11A2,TNKS1BP1,PINX1,GATB,GREM1,KDM2B,PRPF40B,MCRS1,POLR2C,CX3CL1,WNT10B,SOD1,ACTR5,ANKRD2,EGR3,CCAR2,DNAJC17,DLL4,RPLP2,CHP1,MRPL43,YAF2,HTATIP2,PCIF1,NPM3,PCBP4,KDM1B,CHCHD2,RBM15B,CARS,SRSF9,AK2,DCPS,IRX1,WDR4,GSTO1,RRP1B,TMTC1,MVK,BRD9,RBM6,ZCCHC17,PAPSS1,APEH,PABPN1,GPX1,AIP,MOCS3,CLCF1,NGDN,SRRD,TSHZ2,QARS,SLC19A1,IMPDH2,PIR,ANPEP,TRIM27,EIF3A,ZNRD1,PRMT2,THOC5,RIPK3,KHNYN,TWIST2,LSM14B,SF3A1,ADRM1,PSTK,RPS21,SMARCD2,FTSJ3,BRMS1,PPOX,SEC11A,BUD23,TACO1,GMEB2,CEP164,SART1,CCDC85B,PRPF6,PBX1,RNASEH2C,KAT5,RELA,ATP5MC3,ZNF511,DDX52,IDH1,LRWD1,ALKBH4,ARNT2,MRPL49,ZFPL1,LIMD1,SGPL1,SMARCB1,CHCHD10,SLC25A39,TRMT2A,PBX4,UBTF,GATAD2A,ATIC,THOC3,MEF2B,PHB2,TRMT112,NDP,IGFBP5,BAD,CTDSP1,MLF2,OTUB1,NOP14,EID1,SSBP4,METTL13,MPV17L2,ZNF394,LGALS3,[illegible]	yellow	VSMC_M3
GO:0006694	5.508E-04	1.00	20	126	steroid biosynthetic process	BP	0.46207	SQLE,FDXR,INSIG1,SCARB1,FDFT1,SOD1,DHCR7,MVK,LSS,PBX1,HSD17B1,ACLY,ARMC5,PMVK,HMGCS1,DHCR24,NSDHL,MVD,FDPS,IDI1	yellow	VSMC_M3

GO:1901360	7.447E-04	1.00	364	4575	organic cyclic compound metabolic process	BP	0.59179	ENPP1,NAB2,NHP2,CDK4,SQSTM1,DHX16,ZNF608,HDAC10,NELFE,FNIP1,ATF6B,PBX2,RING1,SREBF2,TRMT12,SQLE,H2AFY,HNRNPA0,TRIM11,GUK1,TRAPPC9,NUDT3,PPARD,FANCE,TSTA3,BOP1,SLC52A2,SND1,RTCB,DNPH1,AARS2,PDGFRA,SECISBP2,PHF2,CNOT3,KLF4,HOXA1,FHL3,ZC3H12A,FIGNL1,BLVRA,PNKP,SNRNP70,CC2D1B,KTI12,KMT2E,SIX5,ZNF282,GEMIN7,ZNF296,RELB,MUTYH,FDXR,DMAP1,CIC,GTF3C2,SNX17,GALK1,INSIG1,BLVRB,OGG1,SERTAD1,TTC5,METTL3,CXXC5,FAAP100,LAMTOR1,DND1,PYCR1,FASN,GNPDA1,PUS1,DEAF1,SMUG1,HOXC4,OGDHL,POLR1D,TARBP2,KMT2B,SCARB1,C12orf10,NAT10,ZNF740,PDE4D,RRS1,SWAP70,RALY,ACSL1,ZBTB38,ARL6IP4,LSM6,SLC11A2,FDFT1,TNKS1BP1,PINX1,GATB,GREM1,KDM2B,PRPF40B,MCRS1,POLR2C,CX3CL1,WNT10B,SOD1,ACTR5,ANKRD2,EGR3,CCAR2,DNAJC17,DLL4,CHP1,YAF2,HTATIP2,PCIF1,NPM3,PCBP4,KDM1B,CHCHD2,RBM15B,CARS,SRSF9,AK2,DCPS,IRX1,DHCR7,WDR4,GSTO1,RRP1B,TMTC1,MVK,BRD9,RBM6,ZCCHC17,PAPSS1,PABPN1,AIP,MOCS3,CLCF1,NGDN,SRRD,TSHZ2,QARS,SLC19A1,IMPDH2,LSS,PIR,TRIM27,ZNRD1,PRMT2,THOC5,RIPK3,KHNYN,TWIST2,SF3A1,ADRM1,PSTK,RPS21,SMARCD2,FTSJ3,BRMS1,PPOX,BUD23,GMEB2,THAP4,CEP164,SART1,CCDC85B,PRPF6,PBX1,RNASEH2C,KAT5,RELA,ATP5MC3,ZNF511,DDX52,LRWD1,ALKBH4,ARNT2,ZFPL1,LIMD1,SGPL1,SMARCB1,CHCHD10,SLC25A39,TRMT2A,PBX4,UBTF,GATAD2A,ATIC,THOC3,MEF2B,PHB2,TRMT112,NDP,BAD,CTDSP1,MLF2,OTUB1,NOP14,EID1,SSBP4,METTL13,HSD17B1,ZNF394,LGALS3,C11orf95,BUD31,MRTO4,TNFRSF1A,ASMTL,GTPBP3,BABAM1,TEAD4,C1QBP,CSTF2T,TAF6L,CHERP,ZBTB[illegible]	yellow	VSMC_M3
GO:0043043	8.316E-04	1.00	62	566	peptide biosynthetic process	BP	0.60782	CDK4,EEF1D,RPL3,RPL8,MRPS18A,AARS2,SECISBP2,RPS9,CNOT3,ZC3H12A,UNK,METTL3,DND1,RPL29,EIF4EBP1,TARBP2,NAT10,EIF4B,GATB,RPLP2,MRPL43,PCIF1,CARS,DCPS,APEH,NGDN,QARS,EIF3A,LSM14B,PSTK,RPS21,TACO1,MRPL49,IGFBP5,MPV17L2,C1QBP,AIMP2,SEPSECS,NANOS3,PER1,FARSA,TUFM,RPL37,RPL7A,EEF2K,MRPL4,EIF3G,C19orf66,AKT1,PAIP1,KHSRP,MRPL20,TRAP1,MKNK2,MRPL28,SARS,RBM8A,EEF1G,MRPL34,ZNF385A,MRPS16,ZAR1L	yellow	VSMC_M3
GO:0006396	8.454E-04	1.00	96	950	RNA processing	BP	0.60782	NHP2,DHX16,NELFE,TRMT12,HNRNPA0,BOP1,RTCB,AARS2,ZC3H12A,SNRNP70,KTI12,GEMIN7,METTL3,PUS1,TARBP2,NAT10,RRS1,RALY,ARL6IP4,LSM6,PRPF40B,CCAR2,NPM3,PCBP4,RBM15B,SRSF9,DCPS,WDR4,RRP1B,TMTC1,RBM6,PABPN1,MOCS3,NGDN,THOC5,SF3A1,RPS21,FTSJ3,BUD23,SART1,PRPF6,DDX52,TRMT2A,THOC3,TRMT112,NOP14,LGALS3,BUD31,MRTO4,GTPBP3,C1QBP,CSTF2T,CHERP,GPKOW,ELP5,INTS1,POLR2A,CWC25,SLTM,RRNAD1,CD2BP2,TRMT1,RPL7A,RAVER1,RBM22,ZNF830,KHSRP,DUS3L,SAFB,SAFB2,MAP2K2,CACTIN,CDK11B,SSU72,AURKAIP1,TBL3,TSR3,FAM50A,SARS,CTU2,SNORD24,SNORD3J,RBM8A,RBFA,PIN4,SNORD83B,SNORD56,SNORA61,SNORD17,SNORD32A,SNORD12,SNORA71D,SNORA50C,SNORD94,AKAP17A,OSGEP	yellow	VSMC_M3
GO:0008299	9.429E-04	1.00	7	23	isoprenoid biosynthetic process	BP	0.64713	MVK,LSS,PMVK,HMGCS1,MVD,FDPS,IDI1	yellow	VSMC_M3
GO:0006518	1.032E-03	1.00	72	688	peptide metabolic process	BP	0.67764	CDK4,EEF1D,RPL3,RPL8,MRPS18A,AARS2,SECISBP2,RPS9,CNOT3,ZC3H12A,UNK,METTL3,DND1,RPL29,EIF4EBP1,GSR,TARBP2,NAT10,HM13,EIF4B,GATB,SOD1,RPLP2,MRPL43,PCIF1,CARS,DCPS,GSTO1,APEH,GPX1,NGDN,QARS,ANPEP,EIF3A,LSM14B,PSTK,RPS21,SEC11A,TACO1,RELA,IDH1,MRPL49,IGFBP5,MPV17L2,C1QBP,AIMP2,SEPSECS,NANOS3,PER1,FARSA,TUFM,RPL37,RPL7A,EEF2K,MRPL4,EIF3G,C19orf66,AKT1,PAIP1,KHSRP,MRPL20,TRAP1,CTNS,MKNK2,MRPL28,SARS,RBM8A,EEF1G,MRPL34,ZNF385A,MRPS16,ZAR1L	yellow	VSMC_M3
GO:0046596	1.235E-03	1.00	7	24	regulation of viral entry into host cell	BP	0.73793	TRIM11,LGALS1,SNX3,P4HB,TRIM27,TRIM25,MID2	yellow	VSMC_M3
GO:0046165	1.261E-03	1.00	17	108	alcohol biosynthetic process	BP	0.73793	INSIG1,FDFT1,SOD1,DHCR7,MVK,PCK2,LSS,ISYNA1,ACLY,PMVK,HMGCS1,DHCR24,NSDHL,PGP,MVD,FDPS,IDI1	yellow	VSMC_M3
GO:0043414	1.271E-03	1.00	31	254	macromolecule methylation	BP	0.73793	NELFE,TRMT12,H2AFY,KMT2E,DMAP1,METTL3,KMT2B,SETD1B,PCIF1,KDM1B,RBM15B,WDR4,ETFBKMT,PRMT2,FTSJ3,BUD23,SMARCB1,TRMT2A,GATAD2A,TRMT112,GTPBP3,MTA2,MTHFR,RRNAD1,TRMT1,CARM1,COPRS,METTL22,EHMT1,MBD3,NTMT1	yellow	VSMC_M3
GO:0008202	1.349E-03	1.00	28	217	steroid metabolic process	BP	0.75439	SREBF2,SQLE,PPARD,PDGFRA,FDXR,SNX17,INSIG1,LAMTOR1,SCARB1,FDFT1,SOD1,DHCR7,MVK,LSS,PBX1,SGPL1,HSD17B1,ACLY,ARMC5,PMVK,HMGCS1,DHCR24,NSDHL,MVD,GBA2,FDPS,IDI1,STUB1	yellow	VSMC_M3
GO:0008654	1.422E-03	1.00	28	222	phospholipid biosynthetic process	BP	0.75981	PPARD,PIGO,SOCS5,TTC7A,INPPL1,PCYT2,PTDSS2,DGKZ,CHP1,CISH,MVK,FABP3,PDGFA,IDH1,ISYNA1,CAPN2,PIP4K2B,DPM3,PMVK,MTMR4,HMGCS1,TAZ,MVD,SLC44A3,VAC14,FADS1,FDPS,IDI1	yellow	VSMC_M3
GO:0019637	1.698E-03	1.00	74	743	organophosphate metabolic process	BP	0.85155	ENPP1,GUK1,NUDT3,PPARD,DNPH1,PIGO,SOCS5,TTC7A,GALK1,PLCD1,OGG1,INPPL1,PCYT2,FASN,PTDSS2,SMUG1,OGDHL,SCARB1,PDE4D,ACSL1,FDFT1,TKT,DGKZ,CHP1,CISH,AK2,MVK,FABP3,PAPSS1,PDGFA,MOCS3,IMPDH2,PLA2G3,ATP5MC3,IDH1,ATIC,PHB2,BAD,PLCB3,ISYNA1,ASMTL,UQCC3,CNP,ACLY,FOXK1,CAPN2,PIP4K2B,PGD,ACSF2,DPM3,FLAD1,PMVK,ATP5PD,MTMR4,HMGCS1,PGM1,GIT1,PGP,ATP5F1D,TAZ,JMJD8,AMPD2,APRT,MVD,AK1,SLC44A3,VAC14,PLA2G4E,FADS1,NT5C,TALDO1,FDPS,IDI1,ITPA	yellow	VSMC_M3
GO:0032259	1.766E-03	1.00	34	290	methylation	BP	0.85155	NELFE,TRMT12,H2AFY,KMT2E,DMAP1,METTL3,KMT2B,SETD1B,PCIF1,KDM1B,RBM15B,WDR4,ETFBKMT,PRMT2,FTSJ3,BUD23,SMARCB1,TRMT2A,GATAD2A,TRMT112,METTL13,ASMTL,GTPBP3,COMTD1,MTA2,MTHFR,RRNAD1,TRMT1,CARM1,COPRS,METTL22,EHMT1,MBD3,NTMT1	yellow	VSMC_M3

GO:0009126	1.934E-03	1.00	9	39	purine nucleoside monophosphate metabolic process	BP	0.85155	GUK1,DNPH1,AK2,IMPDH2,ATIC,AMPD2,APRT,AK1,NT5C	yellow	VSMC_M3
GO:0006644	1.974E-03	1.00	36	314	phospholipid metabolic process	BP	0.85155	PPARD,PIGO,SOCS5,TTC7A,PLCD1,INPPL1,PCYT2,PTDSS2,SCARB1,FDFT1,DGKZ,CHP1,CISH,MVK,FABP3,PDGFA,PLA2G3,IDH1,PHB2,PLCB3,ISYNA1,CAPN2,PIP4K2B,DPM3,PMVK,MTMR4,HMGCS1,PGP,TAZ,MVD,SLC44A3,VAC14,PLA2G4E,FADS1,FDPS,IDI1	yellow	VSMC_M3
GO:0043603	2.054E-03	1.00	88	897	cellular amide metabolic process	BP	0.85381	CDK4,CERK,EEF1D,RPL3,RPL8,DNPH1,MRPS18A,AARS2,SECISBP2,RPS9,CNOT3,ZC3H12A,UNK,METTL3,DND1,FASN,RPL29,EIF4EBP1,GNPDA1,GSR,TARBP2,NAT10,HM13,EIF4B,ACSL1,GATB,SOD1,RPLP2,MRPL43,PCIF1,CARS,DCPS,GSTO1,MVK,APEH,GPX1,NGDN,QARS,SLC19A1,ANPEP,EIF3A,LSM14B,PSTK,RPS21,SEC11A,TACO1,RELA,IDH1,MRPL49,SGPL1,IGFBP5,MPV17L2,C1QBP,AIMP2,ACLY,SEPSECS,NANOS3,PER1,ACSF2,FARSA,PMVK,TUFM,RPL37,RPL7A,EEF2K,MRPL4,EIF3G,C19orf66,AKT1,PAIP1,HMGCS1,KHSRP,MRPL20,TRAP1,CTNS,MKNK2,MRPL28,SARS,MVD,RBM8A,EEF1G,PLA2G4E,NT5C,MRPL34,ZNF385A,GBA2,MRPS16,ZAR1L	yellow	VSMC_M3
GO:0000478	2.149E-03	1.00	5	15	endonucleolytic cleavage involved in rRNA processing	BP	0.85381	BOP1,RRS1,RPS21,NOP14,TBL3	yellow	VSMC_M3
GO:0000479	2.149E-03	1.00	5	15	endonucleolytic cleavage of tricistronic rRNA transcript (SSU-rRNA, 5.8S rRNA, LSU-rRNA)	BP	0.85381	BOP1,RRS1,RPS21,NOP14,TBL3	yellow	VSMC_M3
GO:0009303	2.233E-03	1.00	8	33	rRNA transcription	BP	0.86457	H2AFY,GTF3C2,NPM3,SMARCB1,SMARCA4,BRF1,GTF3C5,CAVIN1	yellow	VSMC_M3
GO:0006399	2.380E-03	1.00	23	162	tRNA metabolic process	BP	0.88731	TRMT12,RTCB,AARS2,KTI12,PUS1,NAT10,LSM6,GATB,CARS,WDR4,MOCS3,QARS,PSTK,TRMT112,GTPBP3,ELP5,SEPSECS,TRMT1,FARSA,DUS3L,SARS,CTU2,OSGEP	yellow	VSMC_M3
GO:0016071	2.409E-03	1.00	59	586	mRNA metabolic process	BP	0.88731	DHX16,NELFE,HNRNPA0,SND1,SECISBP2,CNOT3,ZC3H12A,SNRNP70,GEMIN7,METTL3,DND1,PUS1,RALY,ARL6IP4,LSM6,PRPF40B,CCAR2,PCIF1,PCBP4,RBM15B,SRSF9,DCPS,RRP1B,RBM6,PABPN1,ZNRD1,THOC5,SF3A1,SART1,PRPF6,THOC3,LGALS3,BUD31,MRTO4,C1QBP,CSTF2T,GPKOW,CWC25,SLTM,NANOS3,CD2BP2,RAVER1,RBM22,ZNF830,AKT1,PAIP1,KHSRP,SAFB,SAFB2,CARHSP1,CACTIN,CDK11B,SSU72,AURKAIP1,FAM50A,RBM8A,RNH1,TENT5B,AKAP17A	yellow	VSMC_M3
GO:0009058	2.699E-03	1.00	343	4393	biosynthetic process	BP	0.9358	ENPP1,NAB2,NHP2,CDK4,SQSTM1,ZNF608,HDAC10,NELFE,FNIP1,ATF6B,PBX2,RING1,SREBF2,SQLE,H2AFY,TRIM11,GUK1,TRAPPC9,PPARD,EEF1D,TSTA3,RPL3,MPST,RPL8,SND1,B4GALT1,MRPS18A,AARS2,PIGO,SECISBP2,PHF2,SOCS5,TTC7A,RPS9,CNOT3,KLF4,HOXA1,FHL3,ZC3H12A,PNKP,RCN3,CC2D1B,KTI12,MGLL,KMT2E,SIX5,ZNF282,ZNF296,RELB,SERP2,FDXR,EMILIN1,SEPHS1,DMAP1,CIC,GTF3C2,UNK,INSIG1,GALNT14,OGG1,SERTAD1,TTC5,METTL3,PHLDA1,CXXC5,INPPL1,PCYT2,DND1,PYCR1,FASN,SMOX,RPL29,EIF4EBP1,GNPDA1,PTDSS2,DEAF1,HOXC4,POLR1D,TARBP2,KMT2B,AACS,SCARB1,NAT10,ZNF740,EIF4B,RALY,ACSL1,ZBTB38,SLC11A2,FDFT1,TNKS1BP1,PINX1,GATB,GREM1,KDM2B,MCRS1,TKT,POLR2C,CX3CL1,WNT10B,SOD1,ACTR5,ANKRD2,DGKZ,EGR3,CCAR2,DNAJC17,DLL4,ACCS,RPLP2,CHP1,SCD,MRPL43,YAF2,HTATIP2,PCIF1,NPM3,KDM1B,CHCHD2,SLC35C2,CARS,CISH,ST3GAL4,AK2,DCPS,IRX1,DHCR7,SELENOS,RRP1B,TMTC1,MVK,BRD9,FABP3,PAPSS1,PDGFA,APEH,AIP,MOCS3,NGDN,SRRD,TSHZ2,QARS,PCK2,IMPDH2,LSS,PIR,TRIM27,EIF3A,ZNRD1,PRMT2,THOC5,RIPK3,TWIST2,LSM14B,ADRM1,PSTK,RPS21,SMARCD2,EXTL1,BRMS1,PPOX,TACO1,PLA2G3,GMEB2,CCDC85B,PRPF6,PBX1,KAT5,RELA,MGST3,ATP5MC3,ZNF511,IDH1,LRWD1,ARNT2,MRPL49,ZFPL1,LIMD1,MID1IP1,ZDHHC3,SGPL1,SMARCB1,CHCHD10,SLC25A39,PBX4,UBTF,GATAD2A,ATIC,MEF2B,PHB2,NDP,IGFBP5,CTDSP1,MLF2,EID1,ISYNA1,SSBP4,METTL13,MPV17L2,HSD17B1,ZNF394,C11orf95,BUD31,TNFRSF1A,TEAD4,C1QBP,AIMP2,TAF6L,ZBTB49,UQCC3,MTA2,ZNF316,ACLY,MED11,PELP1,CCDC22,FADS2,GLUD1,FOXK1,SDE2,ILVBL,KLF3,PKN1,CAPN2,S[illegible]	yellow	VSMC_M3
GO:0046718	2.716E-03	1.00	15	100	viral entry into host cell	BP	0.9358	TRIM11,LGALS1,SLC52A2,SNX3,P4HB,SIGLEC1,SCARB1,ITGB7,TYRO3,NECTIN1,ANPEP,TRIM27,TRIM25,MID2,CLDN6	yellow	VSMC_M3
GO:1901576	2.727E-03	1.00	338	4324	organic substance biosynthetic process	BP	0.9358	ENPP1,NAB2,NHP2,CDK4,SQSTM1,ZNF608,HDAC10,NELFE,FNIP1,ATF6B,PBX2,RING1,SREBF2,SQLE,H2AFY,TRIM11,GUK1,TRAPPC9,PPARD,EEF1D,TSTA3,RPL3,RPL8,SND1,B4GALT1,MRPS18A,AARS2,PIGO,SECISBP2,PHF2,SOCS5,TTC7A,RPS9,CNOT3,KLF4,HOXA1,FHL3,ZC3H12A,PNKP,CC2D1B,KTI12,MGLL,KMT2E,SIX5,ZNF282,ZNF296,RELB,SERP2,FDXR,SEPHS1,DMAP1,CIC,GTF3C2,UNK,INSIG1,GALNT14,OGG1,SERTAD1,TTC5,METTL3,PHLDA1,CXXC5,INPPL1,PCYT2,DND1,PYCR1,FASN,SMOX,RPL29,EIF4EBP1,GNPDA1,PTDSS2,DEAF1,HOXC4,POLR1D,TARBP2,KMT2B,AACS,SCARB1,NAT10,ZNF740,EIF4B,RALY,ACSL1,ZBTB38,SLC11A2,FDFT1,TNKS1BP1,PINX1,GATB,GREM1,KDM2B,MCRS1,TKT,POLR2C,CX3CL1,WNT10B,SOD1,ACTR5,ANKRD2,DGKZ,EGR3,CCAR2,DNAJC17,DLL4,RPLP2,CHP1,SCD,MRPL43,YAF2,HTATIP2,PCIF1,NPM3,KDM1B,CHCHD2,SLC35C2,CARS,CISH,ST3GAL4,AK2,DCPS,IRX1,DHCR7,SELENOS,RRP1B,TMTC1,MVK,BRD9,FABP3,PAPSS1,PDGFA,APEH,AIP,MOCS3,NGDN,SRRD,TSHZ2,QARS,PCK2,IMPDH2,LSS,PIR,TRIM27,EIF3A,ZNRD1,PRMT2,THOC5,RIPK3,TWIST2,LSM14B,ADRM1,PSTK,RPS21,SMARCD2,EXTL1,BRMS1,PPOX,TACO1,PLA2G3,GMEB2,CCDC85B,PRPF6,PBX1,KAT5,RELA,MGST3,ATP5MC3,ZNF511,IDH1,LRWD1,ARNT2,MRPL49,ZFPL1,LIMD1,MID1IP1,ZDHHC3,SGPL1,SMARCB1,CHCHD10,SLC25A39,PBX4,UBTF,GATAD2A,ATIC,MEF2B,PHB2,NDP,IGFBP5,CTDSP1,MLF2,EID1,ISYNA1,SSBP4,METTL13,MPV17L2,HSD17B1,ZNF394,C11orf95,BUD31,TNFRSF1A,TEAD4,C1QBP,AIMP2,TAF6L,ZBTB49,UQCC3,MTA2,ZNF316,ACLY,MED11,PELP1,CCDC22,FADS2,GLUD1,FOXK1,SDE2,ILVBL,KLF3,PKN1,CAPN2,SEPSECS,POLR2A,MTHFR,PIP[illegible]	yellow	VSMC_M3

GO:0006361	3.020E-03	1.00	4	11	transcription initiation from RNA polymerase I promoter	BP	0.95194	SMARCB1,UBTF,SMARCA4,CAVIN1	yellow	VSMC_M3
GO:0042303	3.086E-03	1.00	12	75	molting cycle	BP	0.95194	NUMA1,LRIG1,WNT10B,PDGFA,MPZL3,RELA,IGFBP5,TMEM79,PER1,NSDHL,TRADD,NOM1	yellow	VSMC_M3
GO:0042633	3.086E-03	1.00	12	75	hair cycle	BP	0.95194	NUMA1,LRIG1,WNT10B,PDGFA,MPZL3,RELA,IGFBP5,TMEM79,PER1,NSDHL,TRADD,NOM1	yellow	VSMC_M3
GO:0044539	3.089E-03	1.00	5	15	long-chain fatty acid import into cell	BP	0.95194	ACSL1,FABP3,SPX,AKT1,SLC27A4	yellow	VSMC_M3
GO:0140354	3.089E-03	1.00	5	15	lipid import into cell	BP	0.95194	ACSL1,FABP3,SPX,AKT1,SLC27A4	yellow	VSMC_M3
GO:0006397	3.266E-03	1.00	44	407	mRNA processing	BP	0.96947	DHX16,NELFE,HNRNPA0,SNRNP70,GEMIN7,METTL3,RALY,ARL6IP4,LSM6,PRPF40B,CCAR2,PCBP4,RBM15B,SRSF9,DCPS,RRP1B,RBM6,PABPN1,THOC5,SF3A1,SART1,PRPF6,THOC3,LGALS3,BUD31,C1QBP,CSTF2T,GPKOW,CWC25,SLTM,CD2BP2,RAVER1,RBM22,ZNF830,KHSRP,SAFB,SAFB2,CACTIN,CDK11B,SSU72,AURKAIP1,FAM50A,RBM8A,AKAP17A	yellow	VSMC_M3
GO:0046483	3.275E-03	1.00	343	4376	heterocycle metabolic process	BP	0.96947	ENPP1,NAB2,NHP2,CDK4,SQSTM1,DHX16,ZNF608,HDAC10,NELFE,FNIP1,ATF6B,PBX2,RING1,SREBF2,TRMT12,H2AFY,HNRNPA0,TRIM11,GUK1,TRAPPC9,NUDT3,PPARD,FANCE,TSTA3,BOP1,SLC52A2,SND1,RTCB,DNPH1,AARS2,SECISBP2,PHF2,CNOT3,KLF4,HOXA1,FHL3,ZC3H12A,FIGNL1,BLVRA,PNKP,SNRNP70,CC2D1B,KTI12,KMT2E,SIX5,ZNF282,GEMIN7,ZNF296,RELB,MUTYH,DMAP1,CIC,GTF3C2,GALK1,BLVRB,OGG1,SERTAD1,TTC5,METTL3,CXXC5,FAAP100,DND1,PYCR1,FASN,GNPDA1,PUS1,DEAF1,SMUG1,HOXC4,OGDHL,POLR1D,TARBP2,KMT2B,C12orf10,NAT10,ZNF740,PDE4D,RRS1,SWAP70,RALY,ACSL1,ZBTB38,ARL6IP4,LSM6,SLC11A2,TNKS1BP1,PINX1,GATB,GREM1,KDM2B,PRPF40B,MCRS1,POLR2C,CX3CL1,WNT10B,ACTR5,ANKRD2,EGR3,CCAR2,DNAJC17,DLL4,CHP1,YAF2,HTATIP2,PCIF1,NPM3,PCBP4,KDM1B,CHCHD2,RBM15B,CARS,SRSF9,AK2,DCPS,IRX1,WDR4,GSTO1,RRP1B,TMTC1,MVK,BRD9,RBM6,ZCCHC17,PAPSS1,PABPN1,AIP,MOCS3,CLCF1,NGDN,SRRD,TSHZ2,QARS,SLC19A1,IMPDH2,PIR,TRIM27,ZNRD1,PRMT2,THOC5,RIPK3,KHNYN,TWIST2,SF3A1,ADRM1,PSTK,RPS21,SMARCD2,FTSJ3,BRMS1,PPOX,BUD23,GMEB2,CEP164,SART1,CCDC85B,PRPF6,PBX1,RNASEH2C,KAT5,RELA,ATP5MC3,ZNF511,DDX52,LRWD1,ALKBH4,ARNT2,ZFPL1,LIMD1,SMARCB1,CHCHD10,SLC25A39,TRMT2A,PBX4,UBTF,GATAD2A,ATIC,THOC3,MEF2B,PHB2,TRMT112,NDP,BAD,CTDSP1,MLF2,OTUB1,NOP14,EID1,SSBP4,METTL13,ZNF394,LGALS3,C11orf95,BUD31,MRTO4,TNFRSF1A,ASMTL,GTPBP3,BABAM1,TEAD4,C1QBP,CSTF2T,TAF6L,CHERP,ZBTB49,UQCC3,CNP,GPKOW,MTA2,ZNF316,ACLY,MED11,PELP1,CCDC22,FOXK1,ELP5,KLF3,PKN1,SEPSECS,[illegible]	yellow	VSMC_M3
GO:0071071	3.384E-03	1.00	5	15	regulation of phospholipid biosynthetic process	BP	0.98255	CHP1,FABP3,PDGFA,IDH1,CAPN2	yellow	VSMC_M3
GO:0045880	3.486E-03	1.00	7	31	positive regulation of smoothened signaling pathway	BP	0.98981	TXNDC15,EVC,TEDC1,RAB34,TEDC2,GAS8,NDST1	yellow	VSMC_M3
GO:0009150	3.591E-03	1.00	33	286	purine ribonucleotide metabolic process	BP	0.98981	ENPP1,GUK1,NUDT3,GALK1,FASN,OGDHL,PDE4D,ACSL1,AK2,MVK,PAPSS1,IMPDH2,ATP5MC3,ATIC,BAD,UQCC3,ACLY,FOXK1,ACSF2,PMVK,ATP5PD,HMGCS1,PGM1,GIT1,ATP5F1D,TAZ,JMJD8,AMPD2,APRT,MVD,AK1,NT5C,ITPA	yellow	VSMC_M3
GO:0098876	3.671E-03	1.00	17	129	vesicle-mediated transport to the plasma membrane	BP	0.98981	SCRIB,EIPR1,SNX3,EHD2,SNX17,RAB35,PLA2G3,PTPN23,VPS51,WASHC1,CCDC22,SNF8,CSK,DENND1C,RAB34,PLA2G4E,ARFRP1	yellow	VSMC_M3
GO:0044403	3.671E-03	1.00	25	206	biological process involved in symbiotic interaction	BP	0.98981	TRIM11,LGALS1,SLC52A2,ZC3H12A,SNX3,P4HB,SIGLEC1,SCARB1,BCL2L1,ITGB7,TYRO3,RRP1B,GPX1,NECTIN1,ANPEP,TRIM27,THOC5,SMARCB1,THOC3,PHB2,TRIM25,SMARCA4,MID2,CLDN6,CARD9	yellow	VSMC_M3
GO:0008610	3.963E-03	1.00	55	544	lipid biosynthetic process	BP	1	CDK4,SQLE,PPARD,PIGO,SOCS5,TTC7A,MGLL,FDXR,INSIG1,INPPL1,PCYT2,FASN,PTDSS2,SCARB1,ACSL1,FDFT1,SOD1,DGKZ,CHP1,SCD,CISH,ST3GAL4,DHCR7,MVK,FABP3,PDGFA,PCK2,LSS,PLA2G3,PBX1,IDH1,MID1IP1,SGPL1,ISYNA1,HSD17B1,ACLY,FADS2,CAPN2,PIP4K2B,ARMC5,DPM3,PMVK,MTMR4,AKT1,HMGCS1,DHCR24,NSDHL,CSNK1G2,TAZ,MVD,SLC44A3,VAC14,FADS1,FDPS,IDI1	yellow	VSMC_M3
GO:0042407	4.038E-03	1.00	5	15	cristae formation	BP	1	SAMM50,CHCHD10,LETM1,UQCC3,TAZ	yellow	VSMC_M3
GO:0009167	4.171E-03	1.00	8	36	purine ribonucleoside monophosphate metabolic process	BP	1	GUK1,AK2,IMPDH2,ATIC,AMPD2,APRT,AK1,NT5C	yellow	VSMC_M3
GO:1903727	4.243E-03	1.00	4	10	positive regulation of phospholipid metabolic process	BP	1	CHP1,FABP3,CAPN2,TAZ	yellow	VSMC_M3
GO:0044249	4.387E-03	1.00	332	4271	cellular biosynthetic process	BP	1	ENPP1,NAB2,NHP2,CDK4,SQSTM1,ZNF608,HDAC10,NELFE,FNIP1,ATF6B,PBX2,RING1,SREBF2,H2AFY,TRIM11,GUK1,TRAPPC9,PPARD,EEF1D,TSTA3,RPL3,MPST,RPL8,SND1,B4GALT1,MRPS18A,AARS2,PIGO,SECISBP2,PHF2,SOCS5,TTC7A,RPS9,CNOT3,KLF4,HOXA1,FHL3,ZC3H12A,PNKP,CC2D1B,KTI12,MGLL,KMT2E,SIX5,ZNF282,ZNF296,RELB,SERP2,FDXR,SEPHS1,DMAP1,CIC,GTF3C2,UNK,INSIG1,GALNT14,OGG1,SERTAD1,TTC5,METTL3,PHLDA1,CXXC5,INPPL1,PCYT2,DND1,PYCR1,FASN,SMOX,RPL29,EIF4EBP1,GNPDA1,PTDSS2,DEAF1,HOXC4,POLR1D,TARBP2,KMT2B,AACS,SCARB1,NAT10,ZNF740,EIF4B,RALY,ACSL1,ZBTB38,SLC11A2,TNKS1BP1,PINX1,GATB,GREM1,KDM2B,MCRS1,TKT,POLR2C,CX3CL1,WNT10B,SOD1,ACTR5,ANKRD2,DGKZ,EGR3,CCAR2,DNAJC17,DLL4,RPLP2,CHP1,SCD,MRPL43,YAF2,HTATIP2,PCIF1,NPM3,KDM1B,CHCHD2,SLC35C2,CARS,CISH,ST3GAL4,AK2,DCPS,IRX1,SELENOS,RRP1B,TMTC1,MVK,BRD9,FABP3,PAPSS1,PDGFA,APEH,AIP,MOCS3,NGDN,SRRD,TSHZ2,QARS,PCK2,IMPDH2,LSS,PIR,TRIM27,EIF3A,ZNRD1,PRMT2,THOC5,RIPK3,TWIST2,LSM14B,ADRM1,PSTK,RPS21,SMARCD2,EXTL1,BRMS1,PPOX,TACO1,PLA2G3,GMEB2,CCDC85B,PRPF6,PBX1,KAT5,RELA,MGST3,ATP5MC3,ZNF511,IDH1,LRWD1,ARNT2,MRPL49,ZFPL1,LIMD1,MID1IP1,ZDHHC3,SGPL1,SMARCB1,CHCHD10,SLC25A39,PBX4,UBTF,GATAD2A,ATIC,MEF2B,PHB2,NDP,IGFBP5,CTDSP1,MLF2,EID1,ISYNA1,SSBP4,METTL13,MPV17L2,HSD17B1,ZNF394,C11orf95,BUD31,TNFRSF1A,TEAD4,C1QBP,AIMP2,TAF6L,ZBTB49,UQCC3,MTA2,ZNF316,ACLY,MED11,PELP1,CCDC22,FADS2,GLUD1,FOXK1,SDE2,ILVBL,KLF3,PKN1,CAPN2,SEPSECS,POLR2A,MTHFR,PIP4K2B,MAD2L2,[illegible]	yellow	VSMC_M3

GO:0001101	4.449E-03	1.00	15	107	response to acid chemical	BP	1	PLEC,PDGFRA,KRT8,BAIAP2,LAMTOR1,BCL2L1,COL6A1,RELA,SIPA1,BAD,LAMTOR4,CD9,CAPN2,MTHFR,NSMF	yellow	VSMC_M3
GO:0006400	4.524E-03	1.00	14	85	tRNA modification	BP	1	TRMT12,AARS2,KTI12,PUS1,NAT10,WDR4,MOCS3,TRMT112,GTPBP3,ELP5,TRMT1,DUS3L,CTU2,OSGEP	yellow	VSMC_M3
GO:0044409	4.681E-03	1.00	15	106	entry into host	BP	1	TRIM11,LGALS1,SLC52A2,SNX3,P4HB,SIGLEC1,SCARB1,ITGB7,TYRO3,NECTIN1,ANPEP,TRIM27,TRIM25,MID2,CLDN6	yellow	VSMC_M3
GO:0034470	4.725E-03	1.00	39	345	ncRNA processing	BP	1	NHP2,TRMT12,BOP1,RTCB,AARS2,KTI12,METTL3,PUS1,TARBP2,NAT10,RRS1,LSM6,NPM3,WDR4,RRP1B,MOCS3,NGDN,RPS21,FTSJ3,BUD23,SART1,DDX52,TRMT112,NOP14,MRTO4,GTPBP3,ELP5,INTS1,RRNAD1,TRMT1,RPL7A,DUS3L,TBL3,TSR3,SARS,CTU2,RBFA,PIN4,OSGEP	yellow	VSMC_M3
GO:0006163	4.825E-03	1.00	34	302	purine nucleotide metabolic process	BP	1	ENPP1,GUK1,NUDT3,DNPH1,GALK1,FASN,OGDHL,PDE4D,ACSL1,AK2,MVK,PAPSS1,IMPDH2,ATP5MC3,ATIC,BAD,UQCC3,ACLY,FOXK1,ACSF2,PMVK,ATP5PD,HMGCS1,PGM1,GIT1,ATP5F1D,TAZ,JMJD8,AMPD2,APRT,MVD,AK1,NT5C,ITPA	yellow	VSMC_M3
GO:0052372	4.916E-03	1.00	7	30	modulation by symbiont of entry into host	BP	1	TRIM11,LGALS1,SNX3,P4HB,TRIM27,TRIM25,MID2	yellow	VSMC_M3
GO:0030490	5.202E-03	1.00	9	46	maturation of SSU-rRNA	BP	1	NAT10,RRS1,LSM6,NGDN,RPS21,DDX52,NOP14,TBL3,TSR3	yellow	VSMC_M3
GO:0071704	5.233E-03	1.00	601	8083	organic substance metabolic process	BP	1	ENPP1,CD63,NAB2,NHP2,CDK4,CTDSP2,SQSTM1,DHX16,ZNF608,LMF2,BAG6,SBF1,SNX9,HDAC10,NELFE,ACAT2,CERK,FNIP1,ATF6B,PBX2,PRR5,TTLL12,RING1,SREBF2,JADE2,TRMT12,SQLE,H2AFY,HNRNPA0,TRIM11,SGSM3,GUK1,TRAPPC9,NUDT3,PPARD,FANCE,EEF1D,TSTA3,RPL3,SCRIB,PLEC,MPST,SHARPIN,BOP1,SLC52A2,VPS28,RPL8,SND1,CUL7,RTCB,DNPH1,B4GALT1,MRPS18A,AARS2,PIGO,PDGFRA,IL1R1,SECISBP2,PHF2,GRHPR,SSC5D,HSPBP1,SOCS5,TTC7A,RPS9,CNOT3,KLF4,HOXA1,JOSD2,PTGR1,PSMB3,FHL3,SPRED2,EIPR1,MEAF6,ZC3H12A,FIGNL1,BLVRA,PNKP,CPT1C,RCN3,SNRNP70,CC2D1B,SNX3,KTI12,MICAL1,MGLL,KMT2E,WDFY2,ACAD9,SIX5,ZNF282,GEMIN7,ZNF296,RELB,SLC9A3R1,SERP2,NAT9,MUTYH,FDXR,EMILIN1,SEPHS1,TMUB1,RNF220,DMAP1,PAFAH1B3,CIC,GTF3C2,SNX17,GALK1,PPM1G,UNK,PLCD1,BCKDHA,INSIG1,GALNT14,BLVRB,OGG1,SERTAD1,TTC5,PSMC4,KAT14,P4HA3,SEL1L2,METTL3,PHLDA1,CXXC5,INPPL1,FAAP100,LAMTOR1,P4HB,PCYT2,DND1,PYCR1,FASN,SMOX,RPL29,EIF4EBP1,DELE1,GNPDA1,CAVIN3,PUS1,GSR,PTDSS2,DEAF1,SMUG1,HOXC4,IDH3B,OGDHL,POLR1D,LDLRAD3,TARBP2,KMT2B,AACS,SCARB1,C12orf10,NAT10,HM13,ZNF740,PDE4D,TNS2,EIF4B,PCCB,RRS1,PEPD,SWAP70,LRP3,RALY,ACSL1,ZBTB38,ARL6IP4,LSM6,SLC11A2,FDFT1,TNKS1BP1,MMP24,PINX1,GATB,SETD1B,GREM1,KDM2B,PDE3B,PRPF40B,MCRS1,TKT,POLR2C,CX3CL1,WNT10B,FKBP11,SOD1,PACSIN3,ACTR5,ANKRD2,DGKZ,EGR3,CCAR2,DNAJC17,DLL4,ACCS,RPLP2,CHP1,TYRO3,SCD,MRPL43,YAF2,HTATIP2,PCIF1,PPM1M,NPM3,PCBP4,KDM1B,CHCHD2,SLC35C2,RBM15B,CARS,TSPAN5,CISH,SRSF9,ST3GAL4,AK2,RNF19B,DC[illegible]	yellow	VSMC_M3
GO:0006725	5.331E-03	1.00	343	4410	cellular aromatic compound metabolic process	BP	1	ENPP1,NAB2,NHP2,CDK4,SQSTM1,DHX16,ZNF608,HDAC10,NELFE,FNIP1,ATF6B,PBX2,RING1,SREBF2,TRMT12,SQLE,H2AFY,HNRNPA0,TRIM11,GUK1,TRAPPC9,NUDT3,PPARD,FANCE,TSTA3,BOP1,SND1,RTCB,DNPH1,AARS2,SECISBP2,PHF2,CNOT3,KLF4,HOXA1,FHL3,ZC3H12A,FIGNL1,BLVRA,PNKP,SNRNP70,CC2D1B,KTI12,KMT2E,SIX5,ZNF282,GEMIN7,ZNF296,RELB,MUTYH,DMAP1,CIC,GTF3C2,GALK1,BLVRB,OGG1,SERTAD1,TTC5,METTL3,CXXC5,FAAP100,DND1,FASN,GNPDA1,PUS1,DEAF1,SMUG1,HOXC4,OGDHL,POLR1D,TARBP2,KMT2B,C12orf10,NAT10,ZNF740,PDE4D,RRS1,SWAP70,RALY,ACSL1,ZBTB38,ARL6IP4,LSM6,SLC11A2,TNKS1BP1,PINX1,GATB,GREM1,KDM2B,PRPF40B,MCRS1,POLR2C,CX3CL1,WNT10B,ACTR5,ANKRD2,EGR3,CCAR2,DNAJC17,DLL4,CHP1,YAF2,HTATIP2,PCIF1,NPM3,PCBP4,KDM1B,CHCHD2,RBM15B,CARS,SRSF9,AK2,DCPS,IRX1,WDR4,RRP1B,TMTC1,MVK,BRD9,RBM6,ZCCHC17,PAPSS1,PABPN1,AIP,MOCS3,CLCF1,NGDN,SRRD,TSHZ2,QARS,SLC19A1,IMPDH2,PIR,TRIM27,ZNRD1,PRMT2,THOC5,RIPK3,KHNYN,TWIST2,SF3A1,ADRM1,PSTK,RPS21,SMARCD2,FTSJ3,BRMS1,PPOX,BUD23,GMEB2,THAP4,CEP164,SART1,CCDC85B,PRPF6,PBX1,RNASEH2C,KAT5,RELA,ATP5MC3,ZNF511,DDX52,LRWD1,ALKBH4,ARNT2,ZFPL1,LIMD1,SMARCB1,CHCHD10,SLC25A39,TRMT2A,PBX4,UBTF,GATAD2A,ATIC,THOC3,MEF2B,PHB2,TRMT112,NDP,BAD,CTDSP1,MLF2,OTUB1,NOP14,EID1,SSBP4,METTL13,ZNF394,LGALS3,C11orf95,BUD31,MRTO4,TNFRSF1A,ASMTL,GTPBP3,BABAM1,TEAD4,C1QBP,CSTF2T,TAF6L,CHERP,ZBTB49,UQCC3,CNP,GPKOW,MTA2,ZNF316,ACLY,MED11,PELP1,CCDC22,FOXK1,ELP5,KLF3,PKN1,SEPSECS,INTS1,POLR[illegible]	yellow	VSMC_M3
GO:0006629	5.629E-03	1.00	90	982	lipid metabolic process	BP	1	CDK4,ACAT2,CERK,SREBF2,SQLE,H2AFY,PPARD,PIGO,PDGFRA,SOCS5,TTC7A,PTGR1,CPT1C,MGLL,ACAD9,FDXR,PAFAH1B3,SNX17,PLCD1,INSIG1,INPPL1,LAMTOR1,PCYT2,FASN,PTDSS2,AACS,SCARB1,PCCB,ACSL1,FDFT1,PDE3B,WNT10B,SOD1,DGKZ,CHP1,SCD,CISH,ST3GAL4,DHCR7,ETFBKMT,MVK,FABP3,PDGFA,GPX1,PCK2,LSS,PLA2G3,PBX1,MGST3,IDH1,MID1IP1,SGPL1,PHB2,PLCB3,ISYNA1,HSD17B1,TNFRSF1A,WASHC1,ACLY,CYP4F8,FADS2,ILVBL,CAPN2,PIP4K2B,MPDU1,CRABP2,ARMC5,ACSF2,DPM3,PMVK,SLC27A3,MTMR4,AKT1,HMGCS1,ALKBH7,DHCR24,NSDHL,PGP,CSNK1G2,TAZ,MVD,SLC27A4,SLC44A3,VAC14,PLA2G4E,FADS1,GBA2,FDPS,IDI1,STUB1	yellow	VSMC_M3
GO:0007249	5.641E-03	1.00	26	222	I-kappaB kinase/NF-kappaB signaling	BP	1	SQSTM1,EEF1D,LGALS1,SHARPIN,ZC3H12A,RELB,CXXC5,CX3CL1,TRIM27,RIPK3,RELA,RHOC,MAP3K14,TNFRSF1A,CCDC22,TBKBP1,PER1,TRIM25,S100A4,MID2,AKT1,CACTIN,IRAK1,JMJD8,CARD9,TRADD	yellow	VSMC_M3
GO:0045292	5.660E-03	1.00	6	24	mRNA cis splicing, via spliceosome	BP	1	PRPF40B,DCPS,SF3A1,SART1,RBM22,CACTIN	yellow	VSMC_M3

GO:0044238	5.666E-03	1.00	570	7641	primary metabolic process	BP	1	ENPP1,NAB2,NHP2,CDK4,CTDSP2,SQSTM1,DHX16,ZNF608,LMF2,BAG6,SBF1,SNX9,HDAC10,NELFE,ACAT2,CERK,FNIP1,ATF6B,PBX2,PRR5,TTLL12,RING1,SREBF2,JADE2,TRMT12,SQLE,H2AFY,HNRNPA0,TRIM11,SGSM3,GUK1,TRAPPC9,NUDT3,PPARD,FANCE,EEF1D,TSTA3,RPL3,PLEC,MPST,SHARPIN,BOP1,VPS28,RPL8,SND1,CUL7,RTCB,DNPH1,B4GALT1,MRPS18A,AARS2,PIGO,PDGFRA,SECISBP2,PHF2,HSPBP1,SOCS5,TTC7A,RPS9,CNOT3,KLF4,HOXA1,JOSD2,PTGR1,PSMB3,FHL3,SPRED2,EIPR1,MEAF6,ZC3H12A,FIGNL1,PNKP,CPT1C,RCN3,SNRNP70,CC2D1B,SNX3,KTI12,MICAL1,MGLL,KMT2E,WDFY2,ACAD9,SIX5,ZNF282,GEMIN7,ZNF296,RELB,SLC9A3R1,SERP2,NAT9,MUTYH,FDXR,EMILIN1,SEPHS1,TMUB1,RNF220,DMAP1,PAFAH1B3,CIC,GTF3C2,SNX17,GALK1,PPM1G,UNK,PLCD1,BCKDHA,INSIG1,GALNT14,OGG1,SERTAD1,TTC5,PSMC4,KAT14,P4HA3,SEL1L2,METTL3,PHLDA1,CXXC5,INPPL1,FAAP100,LAMTOR1,P4HB,PCYT2,DND1,PYCR1,FASN,RPL29,EIF4EBP1,DELE1,GNPDA1,PUS1,PTDSS2,DEAF1,SMUG1,HOXC4,IDH3B,OGDHL,POLR1D,LDLRAD3,TARBP2,KMT2B,AACS,SCARB1,C12orf10,NAT10,HM13,ZNF740,PDE4D,TNS2,EIF4B,PCCB,RRS1,PEPD,SWAP70,RALY,ACSL1,ZBTB38,ARL6IP4,LSM6,FDFT1,TNKS1BP1,MMP24,PINX1,GATB,SETD1B,GREM1,KDM2B,PDE3B,PRPF40B,MCRS1,POLR2C,CX3CL1,WNT10B,FKBP11,SOD1,PACSIN3,ACTR5,ANKRD2,DGKZ,EGR3,CCAR2,DNAJC17,DLL4,ACCS,RPLP2,CHP1,TYRO3,SCD,MRPL43,YAF2,HTATIP2,PCIF1,PPM1M,NPM3,PCBP4,KDM1B,CHCHD2,SLC35C2,RBM15B,CARS,TSPAN5,CISH,SRSF9,ST3GAL4,AK2,RNF19B,DCPS,IRX1,DHCR7,WDR4,SELENOS,GSTO1,RRP1B,ETFBKMT,MANBA,TMTC1,MVK,BRD9,RBM6,SD [illegible]	yellow	VSMC_M3
GO:0031952	5.740E-03	1.00	7	37	regulation of protein autophosphorylation	BP	1	ENPP1,TNKS1BP1,GREM1,CHP1,PDGFA,MVP,EEF2K	yellow	VSMC_M3
GO:0009415	6.010E-03	1.00	4	13	response to water	BP	1	PLEC,KRT8,SIPA1,CD9	yellow	VSMC_M3
GO:0043436	6.089E-03	1.00	67	677	oxoacid metabolic process	BP	1	ENPP1,ACAT2,PPARD,MPST,AARS2,GRHPR,PTGR1,CPT1C,MGLL,ACAD9,SEPHS1,GALK1,BCKDHA,INSIG1,PYCR1,FASN,GNPDA1,IDH3B,OGDHL,AACS,PCCB,PEPD,ACSL1,GATB,ACCS,SCD,CARS,GSTO1,ETFBKMT,SDHA,FABP3,PAPSS1,GPX1,QARS,PCK2,SLC19A1,PLA2G3,THAP4,MGST3,IDH1,MID1IP1,SGPL1,ATIC,TNFRSF1A,ACLY,CYP4F8,FADS2,GLUD1,FOXK1,ILVBL,MTHFR,CRABP2,PGD,ACSF2,FARSA,SLC27A3,AKT1,PGM1,ALKBH7,GIT1,BLMH,CTNS,JMJD8,SARS,SLC27A4,FADS1,GBA2	yellow	VSMC_M3
GO:0008544	6.287E-03	1.00	23	201	epidermis development	BP	1	NAB2,H2AFY,PPARD,SCRIB,PLEC,SHARPIN,KLF4,WDPCP,SLC9A3R1,NUMA1,WNT10B,SOD1,PDGFA,RELA,IGFBP5,KRT10,CASP14,CRABP2,TMEM79,PPL,NSDHL,TRADD,NOM1	yellow	VSMC_M3
GO:0051701	6.300E-03	1.00	19	150	biological process involved in interaction with host	BP	1	TRIM11,LGALS1,SLC52A2,SNX3,P4HB,SIGLEC1,SCARB1,BCL2L1,ITGB7,TYRO3,NECTIN1,ANPEP,TRIM27,THOC5,THOC3,PHB2,TRIM25,MID2,CLDN6	yellow	VSMC_M3
GO:0071229	6.465E-03	1.00	10	65	cellular response to acid chemical	BP	1	PLEC,PDGFRA,BAIAP2,LAMTOR1,BCL2L1,COL6A1,SIPA1,LAMTOR4,CAPN2,NSMF	yellow	VSMC_M3
GO:0006139	6.488E-03	0.99	332	4274	nucleobase-containing compound metabolic process	BP	1	ENPP1,NAB2,NHP2,CDK4,SQSTM1,DHX16,ZNF608,HDAC10,NELFE,FNIP1,ATF6B,PBX2,RING1,SREBF2,TRMT12,H2AFY,HNRNPA0,TRIM11,GUK1,TRAPPC9,NUDT3,PPARD,FANCE,TSTA3,BOP1,SND1,RTCB,DNPH1,AARS2,SECISBP2,PHF2,CNOT3,KLF4,HOXA1,FHL3,ZC3H12A,FIGNL1,PNKP,SNRNP70,CC2D1B,KTI12,KMT2E,SIX5,ZNF282,GEMIN7,ZNF296,RELB,MUTYH,DMAP1,CIC,GTF3C2,GALK1,OGG1,SERTAD1,TTC5,METTL3,CXXC5,FAAP100,DND1,FASN,GNPDA1,PUS1,DEAF1,SMUG1,HOXC4,OGDHL,POLR1D,TARBP2,KMT2B,C12orf10,NAT10,ZNF740,PDE4D,RRS1,SWAP70,RALY,ACSL1,ZBTB38,ARL6IP4,LSM6,TNKS1BP1,PINX1,GATB,GREM1,KDM2B,PRPF40B,MCRS1,POLR2C,CX3CL1,WNT10B,ACTR5,ANKRD2,EGR3,CCAR2,DNAJC17,DLL4,CHP1,YAF2,HTATIP2,PCIF1,NPM3,PCBP4,KDM1B,CHCHD2,RBM15B,CARS,SRSF9,AK2,DCPS,IRX1,WDR4,RRP1B,TMTC1,MVK,BRD9,RBM6,ZCCHC17,PAPSS1,PABPN1,AIP,MOCS3,CLCF1,NGDN,TSHZ2,QARS,IMPDH2,PIR,TRIM27,ZNRD1,PRMT2,THOC5,RIPK3,KHNYN,TWIST2,SF3A1,ADRM1,PSTK,RPS21,SMARCD2,FTSJ3,BRMS1,BUD23,GMEB2,CEP164,SART1,CCDC85B,PRPF6,PBX1,RNASEH2C,KAT5,RELA,ATP5MC3,ZNF511,DDX52,LRWD1,ALKBH4,ARNT2,ZFPL1,LIMD1,SMARCB1,CHCHD10,TRMT2A,PBX4,UBTF,GATAD2A,ATIC,THOC3,MEF2B,PHB2,TRMT112,NDP,BAD,CTDSP1,MLF2,OTUB1,NOP14,EID1,SSBP4,METTL13,ZNF394,LGALS3,C11orf95,BUD31,MRTO4,TNFRSF1A,ASMTL,GTPBP3,BABAM1,TEAD4,C1QBP,CSTF2T,TAF6L,CHERP,ZBTB49,UQCC3,CNP,GPKOW,MTA2,ZNF316,ACLY,MED11,PELP1,CCDC22,FOXK1,ELP5,KLF3,PKN1,SEPSECS,INTS1,POLR2A,CWC25,MAD2L2,SLTM,NANOS3,CTBP1,FBXL19,RRNAD1,CRABP2 [illegible]	yellow	VSMC_M3
GO:0001510	6.990E-03	1.00	12	73	RNA methylation	BP	1	TRMT12,METTL3,PCIF1,RBM15B,WDR4,FTSJ3,BUD23,TRMT2A,TRMT112,GTPBP3,RRNAD1,TRMT1	yellow	VSMC_M3
GO:0022404	7.156E-03	1.00	10	63	molting cycle process	BP	1	NUMA1,LRIG1,WNT10B,PDGFA,RELA,IGFBP5,TMEM79,NSDHL,TRADD,NOM1	yellow	VSMC_M3
GO:0022405	7.156E-03	1.00	10	63	hair cycle process	BP	1	NUMA1,LRIG1,WNT10B,PDGFA,RELA,IGFBP5,TMEM79,NSDHL,TRADD,NOM1	yellow	VSMC_M3
GO:0031424	7.215E-03	1.00	4	13	keratinization	BP	1	SHARPIN,CASP14,TMEM79,PPL	yellow	VSMC_M3
GO:0002098	7.266E-03	1.00	5	17	tRNA wobble uridine modification	BP	1	KTI12,MOCS3,GTPBP3,ELP5,CTU2	yellow	VSMC_M3
GO:0046597	7.285E-03	1.00	4	12	negative regulation of viral entry into host cell	BP	1	TRIM11,SNX3,TRIM25,MID2	yellow	VSMC_M3
GO:0019752	7.290E-03	1.00	65	659	carboxylic acid metabolic process	BP	1	ACAT2,PPARD,MPST,AARS2,GRHPR,PTGR1,CPT1C,MGLL,ACAD9,SEPHS1,GALK1,BCKDHA,INSIG1,PYCR1,FASN,GNPDA1,IDH3B,OGDHL,AACS,PCCB,PEPD,ACSL1,GATB,ACCS,SCD,CARS,GSTO1,ETFBKMT,SDHA,FABP3,GPX1,QARS,PCK2,SLC19A1,PLA2G3,THAP4,MGST3,IDH1,MID1IP1,SGPL1,ATIC,TNFRSF1A,ACLY,CYP4F8,FADS2,GLUD1,FOXK1,ILVBL,MTHFR,CRABP2,PGD,ACSF2,FARSA,SLC27A3,AKT1,PGM1,ALKBH7,GIT1,BLMH,CTNS,JMJD8,SARS,SLC27A4,FADS1,GBA2	yellow	VSMC_M3
GO:0008033	7.581E-03	1.00	17	117	tRNA processing	BP	1	TRMT12,RTCB,AARS2,KTI12,PUS1,NAT10,LSM6,WDR4,MOCS3,TRMT112,GTPBP3,ELP5,TRMT1,DUS3L,SARS,CTU2,OSGEP	yellow	VSMC_M3
GO:0007288	7.748E-03	1.00	5	18	sperm axoneme assembly	BP	1	CEP131,YIF1B,IQCG,PLA2G3,MEIG1	yellow	VSMC_M3

GO:0120316	7.748E-03	1.00	5	18	sperm flagellum assembly	BP	1	CEP131,YIF1B,IQCG,PLA2G3,MEIG1	yellow	VSMC_M3
GO:0000027	7.795E-03	1.00	5	18	ribosomal large subunit assembly	BP	1	RPL3,BOP1,RRS1,MRTO4,MRPL20	yellow	VSMC_M3
GO:0010467	7.983E-03	0.99	344	4455	gene expression	BP	1	ENPP1,NAB2,NHP2,CDK4,SQSTM1,DHX16,ZNF608,LMF2,HDAC10,NELFE,FNIP1,ATF6B,PBX2,RING1,SREBF2,TRMT12,H2AFY,HNRNPA0,TRIM11,TRAPPC9,PPARD,EEF1D,RPL3,SCRIB,PLEC,BOP1,RPL8,SND1,RTCB,MRPS18A,AARS2,PDGFRA,IL1R1,SECISBP2,PHF2,SSC5D,SOCS5,RPS9,CNOT3,KLF4,HOXA1,FHL3,ZC3H12A,SNRNP70,CC2D1B,KTI12,KMT2E,SIX5,ZNF282,GEMIN7,ZNF296,RELB,EMILIN1,DMAP1,CIC,GTF3C2,UNK,OGG1,SERTAD1,TTC5,METTL3,CXXC5,INPPL1,DND1,RPL29,EIF4EBP1,CAVIN3,PUS1,DEAF1,HOXC4,POLR1D,LDLRAD3,TARBP2,KMT2B,NAT10,HM13,ZNF740,PDE4D,EIF4B,RRS1,SWAP70,LRP3,RALY,ZBTB38,ARL6IP4,LSM6,GATB,GREM1,KDM2B,PRPF40B,MCRS1,POLR2C,CX3CL1,WNT10B,SOD1,ACTR5,ANKRD2,EGR3,CCAR2,DNAJC17,DLL4,RPLP2,CHP1,MRPL43,YAF2,HTATIP2,PCIF1,NPM3,PCBP4,KDM1B,CHCHD2,SLC35C2,RBM15B,CARS,TSPAN5,SRSF9,DCPS,IRX1,WDR4,SELENOS,RRP1B,TMTC1,BRD9,RBM6,ZCCHC17,MMP14,C14orf93,APEH,PABPN1,GPX1,AIP,MOCS3,CLCF1,NGDN,TSHZ2,QARS,PIR,TRIM27,EIF3A,ZNRD1,PRMT2,THOC5,RIPK3,TWIST2,LSM14B,SF3A1,ADRM1,PSTK,RPS21,SMARCD2,FTSJ3,BRMS1,SEC11A,BUD23,TACO1,PLA2G3,GMEB2,SART1,CCDC85B,PRPF6,PBX1,KAT5,RELA,ZNF511,DDX52,ARNT2,MRPL49,ZFPL1,LIMD1,SMARCB1,CHCHD10,TRMT2A,PBX4,UBTF,GATAD2A,THOC3,MEF2B,PHB2,TRMT112,NDP,IGFBP5,BAD,CTDSP1,MLF2,NOP14,EID1,SSBP4,METTL13,MPV17L2,ZNF394,LGALS3,C11orf95,BUD31,MRTO4,PLAU,TNFRSF1A,GTPBP3,TEAD4,C1QBP,NUP88,CSTF2T,AIMP2,TAF6L,CHERP,ZBTB49,GPKOW,MTA2,ZNF316,MED11,PELP1,CCDC22,FOXK1,ELP5,SDE2,KLF3,PKN1,CAPN2,SEPSECS,INTS1,PO[illegible]	yellow	VSMC_M3
GO:0044255	8.503E-03	0.99	70	743	cellular lipid metabolic process	BP	1	ACAT2,CERK,PPARD,PIGO,SOCS5,TTC7A,PTGR1,CPT1C,MGLL,ACAD9,PLCD1,INSIG1,INPPL1,LAMTOR1,PCYT2,FASN,PTDSS2,AACS,SCARB1,PCCB,ACSL1,FDFT1,DGKZ,CHP1,SCD,CISH,ST3GAL4,ETFBKMT,MVK,FABP3,PDGFA,GPX1,PCK2,LSS,PLA2G3,IDH1,MID1IP1,SGPL1,PHB2,PLCB3,ISYNA1,TNFRSF1A,ACLY,CYP4F8,FADS2,ILVBL,CAPN2,PIP4K2B,MPDU1,CRABP2,ACSF2,DPM3,PMVK,SLC27A3,MTMR4,AKT1,HMGCS1,ALKBH7,PGP,CSNK1G2,TAZ,MVD,SLC27A4,SLC44A3,VAC14,PLA2G4E,FADS1,GBA2,FDPS,IDI1	yellow	VSMC_M3
GO:0009987	8.538E-03	0.99	829	11676	cellular process	BP	1	ENPP1,PQLC1,CD63,ESYT1,NAB2,DCTN2,NHP2,CDK4,CTDSP2,RUFY1,SQSTM1,GNL1,DHX16,CCHCR1,SPAG1,ZNF608,BAG6,NCAPH2,SBF1,SNX9,CTHRC1,HDAC10,NELFE,ACAT2,CERK,FNIP1,ATF6B,PBX2,PDLIM4,PRR5,SAMM50,TTLL12,RING1,SREBF2,JADE2,TRMT12,NDUFB9,SQLE,TXNDC15,H2AFY,ITPR3,HNRNPA0,TRIM11,SGSM3,GUK1,TRAPPC9,NUDT3,PACSIN1,DEF6,PPARD,FANCE,EEF1D,TSTA3,RPL3,SCRIB,PLEC,LGALS1,MPST,IFT27,SHARPIN,BOP1,SLC52A2,VPS28,RPL8,SND1,TOM1,TBCC,CUL7,RTCB,PPP1R14B,DNPH1,B4GALT1,MRPS18A,AARS2,PIGO,PDGFRA,IL1R1,SECISBP2,PHF2,GRHPR,COL15A1,EPN1,COL19A1,SSC5D,HSPBP1,SOCS5,TTC7A,RPS9,CNOT3,ASNA1,KLF4,DBNL,TPBG,HOXA1,JOSD2,PTGR1,PSMB3,PLEKHA8,WDPCP,CCM2,FHL3,SPRED2,EIPR1,RGS3,MEAF6,ZC3H12A,FIGNL1,PDIA4,BLVRA,PDIA6,PNKP,CPT1C,ZYX,RCN3,SNRNP70,CC2D1B,SNX3,KTI12,MICAL1,PLEKHA4,EMP3,MGLL,EHD2,FAM120B,KMT2E,ANXA5,KRT8,WDFY2,ACAD9,SIX5,ZNF282,TMEM43,GEMIN7,ZNF296,RELB,SLC9A3R1,SERP2,NAT9,MUTYH,PLXND1,FDXR,EMILIN1,SLC16A5,SEPHS1,TMUB1,RNF220,DMAP1,SLC30A3,CIC,GTF3C2,SNX17,GALK1,H3F3B,PPM1G,UNK,PLCD1,DLEC1,CD79A,BCKDHA,INSIG1,MYRIP,SEC13,SEPT9,GALNT14,BLVRB,OGG1,SERTAD1,TTC5,PSMC4,KAT14,ARHGEF40,P4HA3,UCP2,SEL1L2,METTL3,PHLDA1,BAIAP2,CEP131,CXXC5,INPPL1,FAAP100,TSPAN10,LAMTOR1,NUMA1,P4HB,PCYT2,DND1,PYCR1,YIF1B,FASN,CCDC57,RAB20,SMOX,RPL29,EIF4EBP1,ARAP3,SIGLEC1,DELE1,GNPDA1,MORN2,CAVIN3,PUS1,RIC8A,GSR,PTDSS2,ARHGAP22,DEAF1,TBCB,SMUG1,HOXC4,IDH3B,SDHAF1,OGDHL,POLR1D,ARHGAP33,LDLRAD3,TARBP2,KMT2B,A[illegible]	yellow	VSMC_M3
GO:0044237	8.597E-03	0.99	588	7918	cellular metabolic process	BP	1	ENPP1,CD63,NAB2,NHP2,CDK4,CTDSP2,SQSTM1,DHX16,ZNF608,BAG6,SBF1,SNX9,HDAC10,NELFE,ACAT2,CERK,FNIP1,ATF6B,PBX2,PRR5,TTLL12,RING1,SREBF2,JADE2,TRMT12,NDUFB9,SQLE,H2AFY,HNRNPA0,TRIM11,GUK1,TRAPPC9,NUDT3,PPARD,FANCE,EEF1D,TSTA3,RPL3,SCRIB,PLEC,MPST,SHARPIN,BOP1,SLC52A2,VPS28,RPL8,SND1,CUL7,RTCB,PPP1R14B,DNPH1,B4GALT1,MRPS18A,AARS2,PIGO,PDGFRA,SECISBP2,PHF2,GRHPR,HSPBP1,SOCS5,TTC7A,RPS9,CNOT3,KLF4,HOXA1,JOSD2,PTGR1,PSMB3,FHL3,SPRED2,EIPR1,MEAF6,ZC3H12A,FIGNL1,BLVRA,PNKP,CPT1C,RCN3,SNRNP70,CC2D1B,KTI12,MICAL1,MGLL,KMT2E,WDFY2,ACAD9,SIX5,ZNF282,GEMIN7,ZNF296,RELB,SLC9A3R1,SERP2,NAT9,MUTYH,FDXR,EMILIN1,SEPHS1,TMUB1,RNF220,DMAP1,CIC,GTF3C2,GALK1,PPM1G,UNK,PLCD1,BCKDHA,INSIG1,GALNT14,BLVRB,OGG1,SERTAD1,TTC5,PSMC4,KAT14,P4HA3,SEL1L2,METTL3,PHLDA1,CXXC5,INPPL1,FAAP100,LAMTOR1,P4HB,PCYT2,DND1,PYCR1,FASN,SMOX,RPL29,EIF4EBP1,GNPDA1,CAVIN3,PUS1,GSR,PTDSS2,DEAF1,SMUG1,HOXC4,IDH3B,OGDHL,POLR1D,TARBP2,KMT2B,AACS,SCARB1,C12orf10,NAT10,HM13,ZNF740,PDE4D,TNS2,EIF4B,PCCB,RRS1,PEPD,SWAP70,RALY,PLEKHF1,UQCRFS1,ACSL1,ZBTB38,ARL6IP4,LSM6,SLC11A2,FDFT1,TNKS1BP1,PINX1,GATB,SETD1B,GREM1,KDM2B,PRPF40B,MCRS1,TKT,POLR2C,CX3CL1,WNT10B,FKBP11,SOD1,PACSIN3,ACTR5,ANKRD2,DGKZ,EGR3,CCAR2,DNAJC17,DLL4,ACCS,RPLP2,CHP1,TYRO3,SCD,MRPL43,YAF2,HTATIP2,PCIF1,PPM1M,NPM3,PCBP4,KDM1B,CHCHD2,SLC35C2,RBM15B,CARS,CISH,SRSF9,ST3GAL4,AK2,RNF19B,DCPS,IRX1,WDR4,SELENOS,GSTO1,RRP1B,ETFBKMT,MANBA,TMTC[illegible]	yellow	VSMC_M3

GO:0006082	8.637E-03	0.99	67	689	organic acid metabolic process	BP	1	ENPP1,ACAT2,PPARD,MPST,AARS2,GRHPR,PTGR1,CPT1C,MGLL,ACAD9,SEPHS1,GALK1,BCKDHA,INSIG1,PYCR1,FASN,GNPDA1,IDH3B,OGDHL,AACS,PCCB,PEPD,ACSL1,GATB,ACCS,SCD,CARS,GSTO1,ETFBKMT,SDHA,FABP3,PAPSS1,GPX1,QARS,PCK2,SLC19A1,PLA2G3,THAP4,MGST3,IDH1,MID1IP1,SGPL1,ATIC,TNFRSF1A,ACLY,CYP4F8,FADS2,GLUD1,FOXK1,ILVBL,MTHFR,CRABP2,PGD,ACSF2,FARSA,SLC27A3,AKT1,PGM1,ALKBH7,GIT1,BLMH,CTNS,JMJD8,SARS,SLC27A4,FADS1,GBA2	yellow	VSMC_M3
GO:0002093	8.767E-03	1.00	4	13	auditory receptor cell morphogenesis	BP	1	SCRIB,WDPCP,SLC9A3R1,SOD1	yellow	VSMC_M3
GO:0009259	8.789E-03	0.99	33	302	ribonucleotide metabolic process	BP	1	ENPP1,GUK1,NUDT3,GALK1,FASN,OGDHL,PDE4D,ACSL1,AK2,MVK,PAPSS1,IMPDH2,ATP5MC3,ATIC,BAD,UQCC3,ACLY,FOXK1,ACSF2,PMVK,ATP5PD,HMGCS1,PGM1,GIT1,ATP5F1D,TAZ,JMJD8,AMPD2,APRT,MVD,AK1,NT5C,ITPA	yellow	VSMC_M3
GO:0019693	8.807E-03	0.99	34	312	ribose phosphate metabolic process	BP	1	ENPP1,GUK1,NUDT3,GALK1,FASN,OGDHL,PDE4D,ACSL1,TKT,AK2,MVK,PAPSS1,IMPDH2,ATP5MC3,ATIC,BAD,UQCC3,ACLY,FOXK1,ACSF2,PMVK,ATP5PD,HMGCS1,PGM1,GIT1,ATP5F1D,TAZ,JMJD8,AMPD2,APRT,MVD,AK1,NT5C,ITPA	yellow	VSMC_M3
GO:0000469	9.012E-03	1.00	6	26	cleavage involved in rRNA processing	BP	1	NHP2,BOP1,RRS1,RPS21,NOP14,TBL3	yellow	VSMC_M3
GO:0044281	9.298E-03	0.99	116	1301	small molecule metabolic process	BP	1	ENPP1,ACAT2,SREBF2,SQLE,GUK1,NUDT3,PPARD,TSTA3,MPST,SLC52A2,DNPH1,B4GALT1,AARS2,GRHPR,PTGR1,CPT1C,MGLL,ACAD9,FDXR,SEPHS1,SNX17,GALK1,BCKDHA,INSIG1,OGG1,INPPL1,PYCR1,FASN,GNPDA1,SMUG1,IDH3B,OGDHL,AACS,SCARB1,PDE4D,PCCB,PEPD,ACSL1,FDFT1,GATB,SOD1,ACCS,SCD,CARS,AK2,DHCR7,SELENOS,GSTO1,ETFBKMT,MVK,SDHA,FABP3,PAPSS1,GPX1,QARS,PCK2,SLC19A1,IMPDH2,LSS,PLA2G3,THAP4,MGST3,ATP5MC3,IDH1,MID1IP1,SGPL1,ATIC,BAD,ISYNA1,HSD17B1,TNFRSF1A,ASMTL,UQCC3,CNP,ACLY,CYP4F8,FADS2,GLUD1,FOXK1,ILVBL,MTHFR,CRABP2,PGD,COQ6,ACSF2,FARSA,FLAD1,PMVK,ATP5PD,SLC27A3,AKT1,HMGCS1,PGM1,ALKBH7,GIT1,DHCR24,BLMH,NSDHL,CTNS,PGP,MIDN,ATP5F1D,TAZ,JMJD8,AMPD2,SARS,APRT,MVD,SLC27A4,AK1,FADS1,NT5C,GBA2,FDPS,IDI1,ITPA	yellow	VSMC_M3
GO:0000154	9.652E-03	1.00	7	31	rRNA modification	BP	1	NHP2,NAT10,FTSJ3,BUD23,TRMT112,RRNAD1,TSR3	yellow	VSMC_M3
GO:0050728	0.01	1.00	13	91	negative regulation of inflammatory response	BP	1	PPARD,SHARPIN,SOCS5,KLF4,CX3CL1,SOD1,TYRO3,SELENOS,MVK,GPX1,PSMB4,TNFRSF1A,GIT1	yellow	VSMC_M3
GO:1903901	0.01	1.00	4	13	negative regulation of viral life cycle	BP	1	TRIM11,SNX3,TRIM25,MID2	yellow	VSMC_M3
GO:0072350	0.01	1.00	4	13	tricarboxylic acid metabolic process	BP	1	IDH3B,IDH1,ACLY,GLUD1	yellow	VSMC_M3
GO:0098781	0.01	1.00	9	53	ncRNA transcription	BP	1	H2AFY,GTF3C2,NPM3,SMARCB1,SMARCA4,BRF1,GTF3C5,CAVIN1,SNAPC2	yellow	VSMC_M3
GO:0072521	0.01	0.99	34	319	purine-containing compound metabolic process	BP	1	ENPP1,GUK1,NUDT3,DNPH1,GALK1,FASN,OGDHL,PDE4D,ACSL1,AK2,MVK,PAPSS1,IMPDH2,ATP5MC3,ATIC,BAD,UQCC3,ACLY,FOXK1,ACSF2,PMVK,ATP5PD,HMGCS1,PGM1,GIT1,ATP5F1D,TAZ,JMJD8,AMPD2,APRT,MVD,AK1,NT5C,ITPA	yellow	VSMC_M3
GO:0019080	0.01	1.00	12	86	viral gene expression	BP	1	TRIM11,TARBP2,ST3GAL4,RRP1B,TRIM27,EIF3A,SMARCB1,SMARCA4,EIF3G,C19orf66,MID2,MAN1B1	yellow	VSMC_M3
GO:0042147	0.01	1.00	11	80	retrograde transport, endosome to Golgi	BP	1	PQLC1,EIPR1,SNX3,DCTN1,TRIM27,VPS51,WASHC1,SNX8,STX10,ARFRP1,PHETA2	yellow	VSMC_M3
GO:0090407	0.01	0.99	45	452	organophosphate biosynthetic process	BP	1	GUK1,PPARD,PIGO,SOCS5,TTC7A,INPPL1,PCYT2,PTDSS2,ACSL1,TKT,DGKZ,CHP1,CISH,AK2,MVK,FABP3,PAPSS1,PDGFA,MOCS3,IMPDH2,ATP5MC3,IDH1,ATIC,ISYNA1,UQCC3,ACLY,CAPN2,PIP4K2B,DPM3,FLAD1,PMVK,ATP5PD,MTMR4,HMGCS1,ATP5F1D,TAZ,AMPD2,APRT,MVD,AK1,SLC44A3,VAC14,FADS1,FDPS,IDI1	yellow	VSMC_M3
GO:0032008	0.01	1.00	7	38	positive regulation of TOR signaling	BP	1	FNIP1,SEC13,LAMTOR1,LAMTOR4,CTNS,TELO2,WDR24	yellow	VSMC_M3
GO:0007097	0.01	1.00	4	19	nuclear migration	BP	1	SLC9A3R1,DCTN1,SYNE3,TMEM201	yellow	VSMC_M3
GO:0019216	0.01	0.99	27	251	regulation of lipid metabolic process	BP	1	CDK4,H2AFY,PPARD,PDGFRA,INSIG1,LAMTOR1,SCARB1,PDE3B,SOD1,DGKZ,CHP1,DHCR7,ETFBKMT,FABP3,PDGFA,PLA2G3,IDH1,MID1IP1,PHB2,WASHC1,CAPN2,PIP4K2B,ARMC5,MTMR4,AKT1,TAZ,STUB1	yellow	VSMC_M3
GO:0006631	0.01	0.99	30	271	fatty acid metabolic process	BP	1	ACAT2,PPARD,PTGR1,CPT1C,MGLL,ACAD9,INSIG1,FASN,AACS,PCCB,ACSL1,SCD,ETFBKMT,FABP3,GPX1,PCK2,PLA2G3,MID1IP1,SGPL1,TNFRSF1A,ACLY,CYP4F8,FADS2,ILVBL,ACSF2,SLC27A3,AKT1,ALKBH7,SLC27A4,FADS1	yellow	VSMC_M3
GO:0006066	0.01	0.99	28	258	alcohol metabolic process	BP	1	SREBF2,SQLE,NUDT3,PPARD,FDXR,SNX17,GALK1,INSIG1,IDH3B,SCARB1,FDFT1,SOD1,DHCR7,MVK,PCK2,LSS,IDH1,ISYNA1,ACLY,PMVK,HMGCS1,DHCR24,NSDHL,PGP,MVD,GBA2,FDPS,IDI1	yellow	VSMC_M3
GO:0042254	0.01	0.99	29	256	ribosome biogenesis	BP	1	NHP2,RPL3,BOP1,NAT10,RRS1,LSM6,NPM3,RRP1B,NGDN,RPS21,FTSJ3,BUD23,SART1,DDX52,TRMT112,NOP14,MPV17L2,MRTO4,C1QBP,NUP88,RRNAD1,RPL7A,AATF,MRPL20,TBL3,TSR3,RBFA,PIN4,NOM1	yellow	VSMC_M3
GO:0031348	0.01	0.99	18	149	negative regulation of defense response	BP	1	TTLL12,PPARD,SHARPIN,SOCS5,KLF4,METTL3,TARBP2,CX3CL1,SOD1,TYRO3,SELENOS,MVK,GPX1,PSMB4,TNFRSF1A,C1QBP,GIT1,CACTIN	yellow	VSMC_M3
GO:1903321	0.01	0.99	12	82	negative regulation of protein modification by small protein conjugation or removal	BP	1	SQSTM1,VPS28,PINX1,CHP1,LIMK1,RELA,OTUB1,UBXN1,MAD2L2,RPL37,AKT1,HMG20B	yellow	VSMC_M3
GO:0002097	0.02	1.00	5	20	tRNA wobble base modification	BP	1	KTI12,MOCS3,GTPBP3,ELP5,CTU2	yellow	VSMC_M3
GO:0006084	0.02	1.00	6	29	acetyl-CoA metabolic process	BP	1	FASN,MVK,ACLY,PMVK,HMGCS1,MVD	yellow	VSMC_M3
GO:0050775	0.02	1.00	6	35	positive regulation of dendrite morphogenesis	BP	1	CUL7,DBNL,BAIAP2,DBN1,EEF2K,ANAPC2	yellow	VSMC_M3
GO:0032787	0.02	0.99	41	404	monocarboxylic acid metabolic process	BP	1	ACAT2,PPARD,GRHPR,PTGR1,CPT1C,MGLL,ACAD9,GALK1,INSIG1,FASN,OGDHL,AACS,PCCB,ACSL1,SCD,ETFBKMT,FABP3,GPX1,PCK2,PLA2G3,IDH1,MID1IP1,SGPL1,TNFRSF1A,ACLY,CYP4F8,FADS2,FOXK1,ILVBL,CRABP2,PGD,ACSF2,SLC27A3,AKT1,PGM1,ALKBH7,GIT1,JMJD8,SLC27A4,FADS1,GBA2	yellow	VSMC_M3

GO:0043604	0.02	0.99	64	672	amide biosynthetic process	BP	1	CDK4,EEF1D,RPL3,RPL8,MRPS18A,AARS2,SECISBP2,RPS9,CNOT3,ZC3H12A,UNK,METTL3,DND1,RPL29,EIF4EBP1,TARBP2,NAT10,EIF4B,ACSL1,GATB,RPLP2,MRPL43,PCIF1,CARS,DCPS,APEH,NGDN,QARS,EIF3A,LSM14B,PSTK,RPS21,TACO1,MRPL49,IGFBP5,MPV17L2,C1QBP,AIMP2,ACLY,SEPSECS,NANOS3,PER1,FARSA,TUFM,RPL37,RPL7A,EEF2K,MRPL4,EIF3G,C19orf66,AKT1,PAIP1,KHSRP,MRPL20,TRAP1,MKNK2,MRPL28,SARS,RBM8A,EEF1G,MRPL34,ZNF385A,MRPS16,ZAR1L	yellow	VSMC_M3
GO:0016070	0.02	0.99	269	3480	RNA metabolic process	BP	1	ENPP1,NAB2,NHP2,CDK4,SQSTM1,DHX16,ZNF608,HDAC10,NELFE,FNIP1,ATF6B,PBX2,RING1,SREBF2,TRMT12,H2AFY,HNRNPA0,TRIM11,TRAPPC9,NUDT3,PPARD,BOP1,SND1,RTCB,AARS2,SECISBP2,PHF2,CNOT3,KLF4,HOXA1,FHL3,ZC3H12A,SNRNP70,CC2D1B,KTI12,KMT2E,SIX5,ZNF282,GEMIN7,ZNF296,RELB,DMAP1,CIC,GTF3C2,OGG1,SERTAD1,TTC5,METTL3,CXXC5,DND1,PUS1,DEAF1,HOXC4,POLR1D,TARBP2,KMT2B,NAT10,ZNF740,RRS1,RALY,ZBTB38,ARL6IP4,LSM6,GATB,GREM1,KDM2B,PRPF40B,MCRS1,POLR2C,CX3CL1,WNT10B,ACTR5,ANKRD2,EGR3,CCAR2,DNAJC17,DLL4,CHP1,YAF2,HTATIP2,PCIF1,NPM3,PCBP4,KDM1B,CHCHD2,RBM15B,CARS,SRSF9,DCPS,IRX1,WDR4,RRP1B,TMTC1,BRD9,RBM6,ZCCHC17,PABPN1,AIP,MOCS3,NGDN,TSHZ2,QARS,PIR,TRIM27,ZNRD1,PRMT2,THOC5,RIPK3,KHNYN,TWIST2,SF3A1,ADRM1,PSTK,RPS21,SMARCD2,FTSJ3,BRMS1,BUD23,GMEB2,SART1,CCDC85B,PRPF6,PBX1,RNASEH2C,KAT5,RELA,ZNF511,DDX52,ARNT2,ZFPL1,LIMD1,SMARCB1,CHCHD10,TRMT2A,PBX4,UBTF,GATAD2A,THOC3,MEF2B,PHB2,TRMT112,NDP,CTDSP1,MLF2,NOP14,EID1,SSBP4,METTL13,ZNF394,LGALS3,C11orf95,BUD31,MRTO4,TNFRSF1A,GTPBP3,TEAD4,C1QBP,CSTF2T,TAF6L,CHERP,ZBTB49,GPKOW,MTA2,ZNF316,MED11,PELP1,CCDC22,FOXK1,ELP5,KLF3,PKN1,SEPSECS,INTS1,POLR2A,CWC25,MAD2L2,SLTM,NANOS3,CTBP1,FBXL19,RRNAD1,CRABP2,SP2,ARHGEF2,SNF8,CD2BP2,PER1,NFIX,TRMT1,FARSA,IRF2BPL,TRIM25,SMARCA4,CARM1,RPL7A,RAVER1,AATF,MID2,RBM22,ZNF521,ZNF358,ZNF830,AKT1,BRF1,PAIP1,KHSRP,GTF2F1,RFX2,DUS3L,SAFB,SAFB2,CARHSP1,MYDGF,HDGFL2,SIRT6,MAP2K2,CACTIN,CDK11B,SSU72[illegible]	yellow	VSMC_M3
GO:0043122	0.02	0.99	22	197	regulation of I-kappaB kinase/NF-kappaB signaling	BP	1	SQSTM1,EEF1D,LGALS1,SHARPIN,ZC3H12A,CXXC5,CX3CL1,TRIM27,RELA,RHOC,MAP3K14,TNFRSF1A,CCDC22,PER1,TRIM25,S100A4,MID2,CACTIN,IRAK1,JMJD8,CARD9,TRADD	yellow	VSMC_M3
GO:0001942	0.02	0.99	9	61	hair follicle development	BP	1	NUMA1,WNT10B,PDGFA,RELA,IGFBP5,TMEM79,NSDHL,TRADD,NOM1	yellow	VSMC_M3
GO:0009117	0.02	0.99	39	381	nucleotide metabolic process	BP	1	ENPP1,GUK1,NUDT3,DNPH1,GALK1,OGG1,FASN,SMUG1,OGDHL,PDE4D,ACSL1,AK2,MVK,PAPSS1,IMPDH2,ATP5MC3,ATIC,BAD,ASMTL,UQCC3,CNP,ACLY,FOXK1,ACSF2,FLAD1,PMVK,ATP5PD,HMGCS1,PGM1,GIT1,ATP5F1D,TAZ,JMJD8,AMPD2,APRT,MVD,AK1,NT5C,ITPA	yellow	VSMC_M3
GO:0030833	0.02	0.99	17	145	regulation of actin filament polymerization	BP	1	SNX9,DBNL,BAIAP2,CAPG,CORO1B,TRIM27,ARPIN,ARPC1B,WASHC1,DBN1,CORO1A,MIR214,TWF2,ELN,CDC42EP5,GBA2,ARPC5L	yellow	VSMC_M3
GO:0061003	0.02	1.00	4	19	positive regulation of dendritic spine morphogenesis	BP	1	DBNL,BAIAP2,DBN1,EEF2K	yellow	VSMC_M3
GO:0048814	0.02	0.99	8	57	regulation of dendrite morphogenesis	BP	1	CUL7,DBNL,BAIAP2,DBN1,EEF2K,ANAPC2,STK11,NSMF	yellow	VSMC_M3
GO:0007007	0.02	1.00	7	35	inner mitochondrial membrane organization	BP	1	SAMM50,CHCHD10,LETM1,UQCC3,MTX1,TAZ,TIMM22	yellow	VSMC_M3
GO:0008064	0.02	0.99	18	159	regulation of actin polymerization or depolymerization	BP	1	SNX9,DBNL,BAIAP2,SWAP70,CAPG,CORO1B,TRIM27,ARPIN,ARPC1B,WASHC1,DBN1,CORO1A,MIR214,TWF2,ELN,CDC42EP5,GBA2,ARPC5L	yellow	VSMC_M3
GO:0046033	0.02	1.00	4	14	AMP metabolic process	BP	1	AK2,AMPD2,APRT,AK1	yellow	VSMC_M3
GO:0030832	0.02	0.99	18	160	regulation of actin filament length	BP	1	SNX9,DBNL,BAIAP2,SWAP70,CAPG,CORO1B,TRIM27,ARPIN,ARPC1B,WASHC1,DBN1,CORO1A,MIR214,TWF2,ELN,CDC42EP5,GBA2,ARPC5L	yellow	VSMC_M3
GO:0070542	0.02	0.99	8	49	response to fatty acid	BP	1	CDK4,ZC3H12A,UCP2,AACS,ACSL1,SCD,FABP3,BAD	yellow	VSMC_M3
GO:0043123	0.02	0.99	18	153	positive regulation of I-kappaB kinase/NF-kappaB signaling	BP	1	EEF1D,LGALS1,SHARPIN,CXXC5,CX3CL1,TRIM27,RELA,RHOC,MAP3K14,TNFRSF1A,CCDC22,TRIM25,S100A4,MID2,IRAK1,JMJD8,CARD9,TRADD	yellow	VSMC_M3
GO:0006897	0.02	0.99	40	444	endocytosis	BP	1	ENPP1,CD63,RUFY1,SNX9,PACSIN1,SCRIB,VPS28,TOM1,EPN1,SSC5D,DBNL,EHD2,SNX17,INPPL1,SIGLEC1,LDLRAD3,SCARB1,BCL2L1,LRP3,GREM1,PACSIN3,AP2A2,RIN3,AP1G2,TNK2,MRC2,SCARF2,LGALS3,CD9,FCHO1,CLTB,RABEP2,EEF2K,SNX18,DENND1C,RAB34,ANKRD13B,CSNK1G2,RABEPK,GSG1L	yellow	VSMC_M3
GO:0016570	0.02	0.99	37	399	histone modification	BP	1	HDAC10,NELFE,RING1,JADE2,H2AFY,PHF2,MEAF6,KMT2E,DMAP1,KAT14,KMT2B,SETD1B,KDM2B,MCRS1,KDM1B,PRMT2,BRMS1,KAT5,SMARCB1,TRMT112,OTUB1,EID1,TAF6L,MTA2,PKN1,MTHFR,CTBP1,FBXL19,PER1,SGF29,CARM1,COPRS,SIRT6,EHMT1,MBD3,NTMT1,THAP7	yellow	VSMC_M3
GO:0071404	0.02	1.00	4	16	cellular response to low-density lipoprotein particle stimulus	BP	1	SREBF2,SOCS5,CD9,HMGCS1	yellow	VSMC_M3
GO:0046627	0.02	1.00	5	25	negative regulation of insulin receptor signaling pathway	BP	1	ENPP1,TNS2,RELA,PIP4K2B,SLC27A4	yellow	VSMC_M3
GO:0070897	0.02	0.99	6	31	transcription preinitiation complex assembly	BP	1	WNT10B,SMARCB1,UBTF,TAF6L,SMARCA4,BRF1	yellow	VSMC_M3

GO:0048585	0.02	0.98	101	1229	negative regulation of response to stimulus	BP	1	ENPP1,CTHRC1,FNIP1,ATF6B,TTLL12,PPARD,SHARPIN,PDGFRA,SOCS5,KLF4,TPBG,SPRED2,RGS3,ZC3H12A,FIGNL1,SLC9A3R1,EMILIN1,INSIG1,OGG1,UCP2,METTL3,PYCR1,CAVIN3,TARBP2,BCL2L1,PDE4D,TNS2,GREM1,PDE3B,CX3CL1,SOD1,DGKZ,CCAR2,DLL4,CHP1,TYRO3,CISH,SELENOS,ITPRIP,MVK,RIN3,PDGFA,MMP14,GPX1,CORO1B,QARS,TRIM27,EIF3A,SPX,THOC5,PSMB4,STMN3,RELA,LIMD1,PHB2,IGFBP5,IFI35,OTUB1,LGALS3,CD9,PLAU,TNFRSF1A,C1QBP,UBXN1,CCDC22,PALM3,AMER1,PIP4K2B,MAD2L2,NANOS3,ARHGEF2,MVP,PER1,MTMR4,SMARCA4,AATF,CXorf57,CSK,AKT1,GIT1,CACTIN,VASN,HIC1,TRAP1,MNT,STK11,SLC27A4,TGFBR3,MIR214,RF00024,PTPRR,IGFBP6,JAGN1,ZNF385A,TXNDC12,CDC34,APCDD1L,RGS16,DUSP5,STUB1,RGS18	yellow	VSMC_M3
GO:0051647	0.02	1.00	4	21	nucleus localization	BP	1	SLC9A3R1,DCTN1,SYNE3,TMEM201	yellow	VSMC_M3
GO:0042274	0.02	0.99	10	64	ribosomal small subunit biogenesis	BP	1	NAT10,RRS1,LSM6,NGDN,RPS21,DDX52,NOP14,TBL3,TSR3,NOM1	yellow	VSMC_M3
GO:0006753	0.02	0.99	39	387	nucleoside phosphate metabolic process	BP	1	ENPP1,GUK1,NUDT3,DNPH1,GALK1,OGG1,FASN,SMUG1,OGDHL,PDE4D,ACSL1,AK2,MVK,PAPSS1,IMPDH2,ATP5MC3,ATIC,BAD,ASMTL,UQCC3,CNP,ACLY,FOXK1,ACSF2,FLAD1,PMVK,ATP5PD,HMGCS1,PGM1,GIT1,ATP5F1D,TAZ,JMJD8,AMPD2,APRT,MVD,AK1,NT5C,ITPA	yellow	VSMC_M3
GO:0016032	0.02	0.99	32	325	viral process	BP	1	TRIM11,LGALS1,SLC52A2,VPS28,ZC3H12A,SNX3,P4HB,SIGLEC1,TARBP2,SCARB1,BCL2L1,ITGB7,TYRO3,ST3GAL4,RRP1B,NECTIN1,ANPEP,TRIM27,EIF3A,BANF1,SMARCB1,PHB2,MVB12A,CTBP1,SNF8,TRIM25,SMARCA4,EIF3G,C19orf66,MID2,CLDN6,MAN1B1	yellow	VSMC_M3
GO:0055094	0.02	1.00	5	24	response to lipoprotein particle	BP	1	SREBF2,SOCS5,CD9,AKT1,HMGCS1	yellow	VSMC_M3
GO:1901566	0.02	0.98	114	1317	organonitrogen compound biosynthetic process	BP	1	CDK4,GUK1,EEF1D,RPL3,RPL8,B4GALT1,MRPS18A,AARS2,PIGO,SECISBP2,RPS9,CNOT3,KLF4,ZC3H12A,SERP2,SEPHS1,UNK,GALNT14,METTL3,PHLDA1,DND1,PYCR1,SMOX,RPL29,EIF4EBP1,PTDSS2,TARBP2,NAT10,EIF4B,ACSL1,SLC11A2,GATB,RPLP2,CHP1,MRPL43,PCIF1,SLC35C2,CARS,ST3GAL4,AK2,DCPS,TMTC1,FABP3,PAPSS1,APEH,MOCS3,NGDN,SRRD,QARS,IMPDH2,EIF3A,LSM14B,PSTK,RPS21,EXTL1,PPOX,TACO1,ATP5MC3,MRPL49,ZDHHC3,SGPL1,SLC25A39,ATIC,IGFBP5,MPV17L2,C1QBP,AIMP2,UQCC3,ACLY,GLUD1,ILVBL,CAPN2,SEPSECS,MTHFR,NANOS3,MPDU1,PER1,DPM3,FARSA,FLAD1,TUFM,ATP5PD,RPL37,RPL7A,EEF2K,AATF,MRPL4,EIF3G,C19orf66,AKT1,PAIP1,KHSRP,MRPL20,TRAP1,MKNK2,CSNK1G2,MAN1B1,ATP5F1D,TAZ,MRPL28,AMPD2,SARS,APRT,POMT1,AK1,SLC44A3,B3GNT9,NDST1,RBM8A,EEF1G,MRPL34,ZNF385A,MRPS16,ZAR1L	yellow	VSMC_M3
GO:0006482	0.02	1.00	5	30	protein demethylation	BP	1	PHF2,KDM2B,KDM1B,ALKBH4,FBXL19	yellow	VSMC_M3
GO:0008214	0.02	1.00	5	30	protein dealkylation	BP	1	PHF2,KDM2B,KDM1B,ALKBH4,FBXL19	yellow	VSMC_M3
GO:0060117	0.02	1.00	4	17	auditory receptor cell development	BP	1	SCRIB,WDPCP,SLC9A3R1,SOD1	yellow	VSMC_M3
GO:1902175	0.02	1.00	5	23	regulation of oxidative stress-induced intrinsic apoptotic signaling pathway	BP	1	P4HB,SOD1,GPX1,AKT1,TRAP1	yellow	VSMC_M3
GO:0008152	0.02	0.98	621	8467	metabolic process	BP	1	ENPP1,CD63,NAB2,NHP2,CDK4,CTDSP2,SQSTM1,DHX16,ZNF608,LMF2,BAG6,SBF1,SNX9,HDAC10,NELFE,ACAT2,CERK,FNIP1,ATF6B,PBX2,PRR5,TTLL12,RING1,SREBF2,JADE2,TRMT12,NDUFB9,SQLE,H2AFY,HNRNPA0,TRIM11,SGSM3,GUK1,TRAPPC9,NUDT3,PPARD,FANCE,EEF1D,TSTA3,RPL3,SCRIB,PLEC,MPST,SHARPIN,BOP1,SLC52A2,VPS28,RPL8,SND1,CUL7,RTCB,PPP1R14B,DNPH1,B4GALT1,MRPS18A,AARS2,PIGO,PDGFRA,IL1R1,SECISBP2,PHF2,GRHPR,SSC5D,HSPBP1,SOCS5,TTC7A,RPS9,CNOT3,KLF4,HOXA1,JOSD2,PTGR1,PSMB3,FHL3,SPRED2,EIPR1,MEAF6,ZC3H12A,FIGNL1,BLVRA,PNKP,CPT1C,RCN3,SNRNP70,CC2D1B,SNX3,KTI12,MICAL1,MGLL,KMT2E,WDFY2,ACAD9,SIX5,ZNF282,GEMIN7,ZNF296,RELB,SLC9A3R1,SERP2,NAT9,MUTYH,FDXR,EMILIN1,SEPHS1,TMUB1,RNF220,DMAP1,PAFAH1B3,CIC,GTF3C2,SNX17,GALK1,PPM1G,UNK,PLCD1,BCKDHA,INSIG1,GALNT14,BLVRB,OGG1,SERTAD1,TTC5,PSMC4,KAT14,P4HA3,UCP2,SEL1L2,METTL3,PHLDA1,CXXC5,INPPL1,FAAP100,LAMTOR1,P4HB,PCYT2,DND1,PYCR1,FASN,SMOX,RPL29,EIF4EBP1,DELE1,GNPDA1,CAVIN3,PUS1,GSR,PTDSS2,DEAF1,SMUG1,HOXC4,IDH3B,OGDHL,POLR1D,LDLRAD3,TARBP2,KMT2B,AACS,SCARB1,C12orf10,NAT10,HM13,ZNF740,PDE4D,TNS2,EIF4B,PCCB,RRS1,PEPD,SWAP70,LRP3,RALY,PLEKHF1,UQCRFS1,ACSL1,ZBTB38,ARL6IP4,LSM6,SLC11A2,FDFT1,TNKS1BP1,MMP24,PINX1,GATB,SETD1B,GREM1,KDM2B,PDE3B,PRPF40B,MCRS1,TKT,POLR2C,CX3CL1,WNT10B,FKBP11,SOD1,PACSIN3,ACTR5,ANKRD2,DGKZ,EGR3,CCAR2,DNAJC17,DLL4,ACCS,RPLP2,CHP1,TYRO3,SCD,MRPL43,YAF2,HTATIP2,PCIF1,PPM1M,NPM3,PCBP4,KDM1B,CHCHD2,SLC35C2,RBM15B,CARS,TS[illegible]	yellow	VSMC_M3
GO:0032271	0.02	0.99	21	199	regulation of protein polymerization	BP	1	SNX9,DBNL,BAIAP2,NUMA1,CAPG,DCTN1,CORO1B,TRIM27,ARPIN,ARPC1B,WASHC1,DBN1,RANGRF,CORO1A,GIT1,MIR214,TWF2,ELN,CDC42EP5,GBA2,ARPC5L	yellow	VSMC_M3
GO:0008625	0.02	0.99	10	67	extrinsic apoptotic signaling pathway via death domain receptors	BP	1	DELE1,BCL2L1,ITPRIP,GPX1,ZDHHC3,BAD,LGALS3,TNFRSF1A,ARHGEF2,TRADD	yellow	VSMC_M3
GO:0046040	0.02	1.00	4	16	IMP metabolic process	BP	1	ATIC,AMPD2,APRT,NT5C	yellow	VSMC_M3
GO:0052126	0.02	0.99	15	127	movement in host environment	BP	1	TRIM11,LGALS1,SLC52A2,SNX3,P4HB,SIGLEC1,SCARB1,ITGB7,TYRO3,NECTIN1,ANPEP,TRIM27,TRIM25,MID2,CLDN6	yellow	VSMC_M3
GO:0097320	0.02	1.00	4	18	plasma membrane tubulation	BP	1	SNX9,PACSIN1,EHD2,PACSIN3	yellow	VSMC_M3
GO:0032495	0.02	1.00	4	17	response to muramyl dipeptide	BP	1	RELA,ARHGEF2,LDOC1,CARD9	yellow	VSMC_M3
GO:0045684	0.02	0.99	5	25	positive regulation of epidermis development	BP	1	H2AFY,PPARD,NUMA1,KRT10,TMEM79	yellow	VSMC_M3
GO:0071498	0.02	1.00	4	18	cellular response to fluid shear stress	BP	1	SREBF2,PLEC,SOCS5,KLF4	yellow	VSMC_M3
GO:0006304	0.02	0.99	11	84	DNA modification	BP	1	KMT2E,MUTYH,DMAP1,OGG1,SMUG1,KDM1B,ALKBH4,GATAD2A,MTA2,EHMT1,MBD3	yellow	VSMC_M3
GO:2001135	0.02	1.00	4	18	regulation of endocytic recycling	BP	1	EIPR1,EHD2,PLA2G3,PLA2G4E	yellow	VSMC_M3

GO:1901362	0.02	0.98	230	3012	organic cyclic compound biosynthetic process	BP	1	ENPP1,NAB2,NHP2,CDK4,SQSTM1,ZNF608,HDAC10,NELFE,FNIP1,ATF6B,PBX2,RING1,SREBF2,SQLE,H2AFY,TRIM11,GUK1,TRAPPC9,PPARD,TSTA3,SND1,PHF2,CNOT3,KLF4,HOXA1,FHL3,ZC3H12A,PNKP,CC2D1B,KTI12,KMT2E,SIX5,ZNF282,ZNF296,RELB,FDXR,DMAP1,CIC,GTF3C2,INSIG1,OGG1,SERTAD1,TTC5,CXXC5,PYCR1,GNPDA1,DEAF1,HOXC4,POLR1D,KMT2B,SCARB1,NAT10,ZNF740,RALY,ACSL1,ZBTB38,SLC11A2,FDFT1,TNKS1BP1,PINX1,GREM1,KDM2B,MCRS1,POLR2C,CX3CL1,WNT10B,SOD1,ACTR5,ANKRD2,EGR3,CCAR2,DNAJC17,DLL4,CHP1,YAF2,HTATIP2,NPM3,KDM1B,CHCHD2,AK2,IRX1,DHCR7,RRP1B,MVK,BRD9,PAPSS1,AIP,MOCS3,SRRD,TSHZ2,QARS,IMPDH2,LSS,PIR,TRIM27,ZNRD1,PRMT2,THOC5,RIPK3,TWIST2,ADRM1,PSTK,SMARCD2,BRMS1,PPOX,GMEB2,CCDC85B,PRPF6,PBX1,KAT5,RELA,ATP5MC3,ZNF511,ARNT2,ZFPL1,LIMD1,SMARCB1,CHCHD10,SLC25A39,PBX4,UBTF,GATAD2A,ATIC,MEF2B,PHB2,NDP,CTDSP1,MLF2,EID1,SSBP4,METTL13,HSD17B1,ZNF394,C11orf95,BUD31,TNFRSF1A,TEAD4,C1QBP,TAF6L,ZBTB49,UQCC3,MTA2,ZNF316,ACLY,MED11,PELP1,CCDC22,FOXK1,KLF3,PKN1,SEPSECS,POLR2A,MAD2L2,SLTM,CTBP1,FBXL19,CRABP2,SP2,ARHGEF2,ARMC5,SNF8,PER1,NFIX,FLAD1,IRF2BPL,PMVK,TRIM25,ATP5PD,USP43,SMARCA4,CARM1,AATF,MID2,ZNF521,ZNF358,AKT1,BRF1,HMGCS1,KHSRP,GTF2F1,RFX2,SAFB,SAFB2,DHCR24,MYDGF,HDGFL2,SIRT6,MAP2K2,NSDHL,CACTIN,CDK11B,SSU72,HIC1,MNT,CTNS,EHMT1,AP3D1,IRAK1,MBD3,ATP5F1D,TELO2,TAZ,MED16,CARD9,AMPD2,SARS,APRT,GTF3C5,MVD,AK1,TRADD,RF00024,ERBB4,JUND,PTMA,KLF13,FADS1,ZNF783,HOXC5,CAVIN1,IER5,AKAP17A,FDPS,ZNF[illegible]	yellow	VSMC_M3
GO:0098773	0.02	0.99	9	65	skin epidermis development	BP	1	NUMA1,WNT10B,PDGFA,RELA,IGFBP5,TMEM79,NSDHL,TRADD,NOM1	yellow	VSMC_M3
GO:1901617	0.02	0.99	20	178	organic hydroxy compound biosynthetic process	BP	1	SQLE,INSIG1,FDFT1,SOD1,DHCR7,MVK,PCK2,LSS,ISYNA1,HSD17B1,ACLY,PMVK,HMGCS1,DHCR24,NSDHL,CTNS,PGP,MVD,FDPS,IDI1	yellow	VSMC_M3
GO:0060119	0.02	0.99	6	35	inner ear receptor cell development	BP	1	CTHRC1,SCRIB,IFT27,WDPCP,SLC9A3R1,SOD1	yellow	VSMC_M3
GO:0000466	0.02	0.99	5	24	maturation of 5.8S rRNA from tricistronic rRNA transcript (SSU-rRNA, 5.8S rRNA, LSU-rRNA)	BP	1	BOP1,RRS1,RPS21,FTSJ3,NOP14	yellow	VSMC_M3
GO:0046782	0.02	1.00	4	17	regulation of viral transcription	BP	1	TRIM11,TARBP2,TRIM27,MID2	yellow	VSMC_M3
GO:0006091	0.02	0.98	40	392	generation of precursor metabolites and energy	BP	1	ENPP1,NDUFB9,PPARD,TSTA3,PLEC,FDXR,GALK1,GNPDA1,CAVIN3,GSR,IDH3B,OGDHL,AACS,UQCRFS1,TKT,CHCHD2,SELENOS,ETFBKMT,SDHA,NDUFV1,UQCRC1,TACO1,IDH1,CHCHD10,PHB2,UQCC3,FOXK1,PGD,ATP5PD,FDX2,AKT1,PGM1,GIT1,TRAP1,NDUFS7,ATP5F1D,TAZ,JMJD8,SURF1,TALDO1	yellow	VSMC_M3
GO:0034637	0.03	0.99	9	63	cellular carbohydrate biosynthetic process	BP	1	ENPP1,B4GALT1,SELENOS,PCK2,ISYNA1,AKT1,PGP,B3GNT9,NDST1	yellow	VSMC_M3
GO:0060122	0.03	0.99	5	26	inner ear receptor cell stereocilium organization	BP	1	CTHRC1,SCRIB,IFT27,SLC9A3R1,SOD1	yellow	VSMC_M3
GO:0006414	0.03	0.99	8	50	translational elongation	BP	1	EEF1D,SECISBP2,RPLP2,SEPSECS,TUFM,EEF2K,C19orf66,EEF1G	yellow	VSMC_M3
GO:0008380	0.03	0.98	36	356	RNA splicing	BP	1	DHX16,RTCB,SNRNP70,GEMIN7,METTL3,RALY,ARL6IP4,LSM6,PRPF40B,CCAR2,PCBP4,RBM15B,SRSF9,DCPS,RRP1B,RBM6,THOC5,SF3A1,SART1,PRPF6,THOC3,LGALS3,BUD31,C1QBP,GPKOW,POLR2A,CWC25,CD2BP2,RAVER1,RBM22,ZNF830,KHSRP,CACTIN,FAM50A,RBM8A,AKAP17A	yellow	VSMC_M3
GO:1900077	0.03	0.99	5	27	negative regulation of cellular response to insulin stimulus	BP	1	ENPP1,TNS2,RELA,PIP4K2B,SLC27A4	yellow	VSMC_M3
GO:0045444	0.03	0.99	20	188	fat cell differentiation	BP	1	ENPP1,PPARD,PDGFRA,KLF4,ZC3H12A,FAM120B,WDFY2,INSIG1,AACS,LRP3,WNT10B,FABP3,GPX1,CCDC85B,TMEM120A,CTBP1,CARM1,AKT1,TRIB2,ZNF385A	yellow	VSMC_M3
GO:0022613	0.03	0.98	38	376	ribonucleoprotein complex biogenesis	BP	1	NHP2,RPL3,BOP1,GEMIN7,TARBP2,NAT10,EIF4B,RRS1,LSM6,NPM3,SRSF9,RRP1B,NGDN,EIF3A,SF3A1,RPS21,FTSJ3,BUD23,SART1,PRPF6,DDX52,TRMT112,NOP14,MPV17L2,MRTO4,C1QBP,NUP88,RRNAD1,CD2BP2,RPL7A,AATF,EIF3G,MRPL20,TBL3,TSR3,RBFA,PIN4,NOM1	yellow	VSMC_M3
GO:0046777	0.03	0.98	20	204	protein autophosphorylation	BP	1	ENPP1,PDGFRA,TNKS1BP1,GREM1,CHP1,TYRO3,HTATIP2,PDGFA,RIPK3,MEX3B,MVP,EEF2K,CSK,AKT1,MAP2K2,MKNK2,CSNK1G2,IRAK1,STK11,ERBB4	yellow	VSMC_M3
GO:1903311	0.03	0.98	25	247	regulation of mRNA metabolic process	BP	1	NELFE,HNRNPA0,SECISBP2,CNOT3,ZC3H12A,SNRNP70,METTL3,DND1,PCBP4,RBM15B,SRSF9,DCPS,C1QBP,SLTM,NANOS3,RAVER1,AKT1,PAIP1,KHSRP,SAFB,SAFB2,CARHSP1,CDK11B,RBM8A,TENT5B	yellow	VSMC_M3
GO:0071402	0.03	0.99	5	26	cellular response to lipoprotein particle stimulus	BP	1	SREBF2,SOCS5,CD9,AKT1,HMGCS1	yellow	VSMC_M3
GO:0010761	0.03	0.99	7	46	fibroblast migration	BP	1	PLEC,WDPCP,SGPL1,RCC2,MTA2,AKT1,TMEM201	yellow	VSMC_M3
GO:0016192	0.03	0.98	95	1192	vesicle-mediated transport	BP	1	ENPP1,PQLC1,CD63,RUFY1,SQSTM1,SNX9,SGSM3,PACSIN1,SCRIB,VPS28,TOM1,EPN1,SSC5D,DBNL,EIPR1,SNX3,MICAL1,EHD2,SNX17,INSIG1,SEC13,INPPL1,LAMTOR1,YIF1B,RAB20,ARAP3,SIGLEC1,LDLRAD3,SCARB1,BCL2L1,LRP3,GREM1,DCTN1,SOD1,PACSIN3,HPS1,CHP1,TYRO3,AP2A2,RAB35,EXOC3,RIN3,AP1G2,SYT12,TRIM27,LIMK1,TNK2,PLA2G3,PTPN23,MRC2,SCYL1,VPS51,ZFPL1,COPE,SCARF2,LGALS3,CD9,FCHO1,MVB12A,ANXA11,WASHC1,AP1M1,RAB3IL1,CCDC22,SNX8,LMAN2,PIP4K2B,CLTB,TMEM79,SNF8,RANGRF,STX10,CORO1A,RABEP2,TMED1,EEF2K,CSK,ATG3,SNX18,DENND1C,RAB34,GIT1,ANKRD13B,MAP2K2,AP3D1,CSNK1G2,RABEPK,TRAPPC5,PLA2G4E,AP5B1,JAGN1,UNC13A,ARFRP1,PHETA2,GSG1L	yellow	VSMC_M3
GO:0044270	0.03	0.98	35	361	cellular nitrogen compound catabolic process	BP	1	ENPP1,HNRNPA0,NUDT3,MPST,SND1,DNPH1,SECISBP2,CNOT3,ZC3H12A,BLVRA,BLVRB,OGG1,METTL3,DND1,SMUG1,PDE4D,LSM6,PCBP4,DCPS,ZCCHC17,RNASEH2C,MRTO4,CNP,NANOS3,DFFA,AKT1,PAIP1,KHSRP,CARHSP1,RBM8A,RNH1,NT5C,TENT5B,TRIR,ITPA	yellow	VSMC_M3
GO:1902903	0.03	0.98	30	327	regulation of supramolecular fiber organization	BP	1	SNX9,DBNL,EMILIN1,BAIAP2,NUMA1,SWAP70,CAPG,DCTN1,CX3CL1,GPX1,CORO1B,TRIM27,ARPIN,LIMK1,PFDN2,RHOC,MID1IP1,ARPC1B,WASHC1,DBN1,ARHGEF2,RANGRF,CORO1A,GIT1,MIR214,TWF2,ELN,CDC42EP5,GBA2,ARPC5L	yellow	VSMC_M3

GO:0046034	0.03	0.98	25	219	ATP metabolic process	BP	1	ENPP1,NDUFB9,GUK1,FIGNL1,GALK1,OGDHL,CHCHD2,AK2,SDHA,NDUFV1,UQCRC1,ATP5MC3,CHCHD10,BAD,UQCC3,FOXK1,ATP5PD,PGM1,GIT1,CTNS,NDUFS7,ATP5F1D,TAZ,JMJD8,AK1	yellow	VSMC_M3
GO:0007080	0.03	0.99	7	43	mitotic metaphase plate congression	BP	1	DCTN2,RRS1,PINX1,NUDC,EML3,MAD1L1,MIS12	yellow	VSMC_M3
GO:0042634	0.03	0.99	4	20	regulation of hair cycle	BP	1	NUMA1,WNT10B,PER1,TRADD	yellow	VSMC_M3
GO:0019083	0.03	0.99	7	45	viral transcription	BP	1	TRIM11,TARBP2,RRP1B,TRIM27,SMARCB1,SMARCA4,MID2	yellow	VSMC_M3
GO:0045010	0.03	0.99	7	44	actin nucleation	BP	1	CORO1B,TRIM27,ARPIN,ARPC1B,WASHC1,CORO1A,ARPC5L	yellow	VSMC_M3
GO:0016072	0.03	0.98	23	206	rRNA metabolic process	BP	1	NHP2,BOP1,NAT10,RRS1,LSM6,NPM3,RRP1B,NGDN,RPS21,FTSJ3,BUD23,SART1,DDX52,TRMT112,NOP14,MRTO4,RRNAD1,RPL7A,TBL3,TSR3,RBFA,PIN4,TRIR	yellow	VSMC_M3
GO:0071230	0.03	0.99	8	59	cellular response to amino acid stimulus	BP	1	PDGFRA,BAIAP2,LAMTOR1,BCL2L1,COL6A1,LAMTOR4,CAPN2,NSMF	yellow	VSMC_M3
GO:0006807	0.03	0.97	535	7284	nitrogen compound metabolic process	BP	1	ENPP1,NAB2,NHP2,CDK4,CTDSP2,SQSTM1,DHX16,ZNF608,LMF2,BAG6,SBF1,SNX9,HDAC10,NELFE,CERK,FNIP1,ATF6B,PBX2,PRR5,TTLL12,RING1,SREBF2,JADE2,TRMT12,H2AFY,HNRNPA0,TRIM11,SGSM3,GUK1,TRAPPC9,NUDT3,PPARD,FANCE,EEF1D,TSTA3,RPL3,PLEC,MPST,SHARPIN,BOP1,SLC52A2,VPS28,RPL8,SND1,CUL7,RTCB,DNPH1,B4GALT1,MRPS18A,AARS2,PIGO,PDGFRA,SECISBP2,PHF2,HSPBP1,SOCS5,RPS9,CNOT3,KLF4,HOXA1,JOSD2,PSMB3,FHL3,SPRED2,EIPR1,MEAF6,ZC3H12A,FIGNL1,BLVRA,PNKP,CPT1C,RCN3,SNRNP70,CC2D1B,SNX3,KTI12,MICAL1,KMT2E,WDFY2,SIX5,ZNF282,GEMIN7,ZNF296,RELB,SLC9A3R1,SERP2,NAT9,MUTYH,EMILIN1,SEPHS1,TMUB1,RNF220,DMAP1,CIC,GTF3C2,GALK1,PPM1G,UNK,BCKDHA,GALNT14,BLVRB,OGG1,SERTAD1,TTC5,PSMC4,KAT14,P4HA3,SEL1L2,METTL3,PHLDA1,CXXC5,FAAP100,P4HB,DND1,PYCR1,FASN,SMOX,RPL29,EIF4EBP1,DELE1,GNPDA1,PUS1,GSR,PTDSS2,DEAF1,SMUG1,HOXC4,OGDHL,POLR1D,LDLRAD3,TARBP2,KMT2B,SCARB1,C12orf10,NAT10,HM13,ZNF740,PDE4D,TNS2,EIF4B,RRS1,PEPD,SWAP70,RALY,ACSL1,ZBTB38,ARL6IP4,LSM6,SLC11A2,TNKS1BP1,MMP24,PINX1,GATB,SETD1B,GREM1,KDM2B,PRPF40B,MCRS1,POLR2C,CX3CL1,WNT10B,FKBP11,SOD1,PACSIN3,ACTR5,ANKRD2,EGR3,CCAR2,DNAJC17,DLL4,ACCS,RPLP2,CHP1,TYRO3,MRPL43,YAF2,HTATIP2,PCIF1,PPM1M,NPM3,PCBP4,KDM1B,CHCHD2,SLC35C2,RBM15B,CARS,TSPAN5,CISH,SRSF9,ST3GAL4,AK2,RNF19B,DCPS,IRX1,WDR4,SELENOS,GSTO1,RRP1B,ETFBKMT,MANBA,TMTC1,MVK,BRD9,RBM6,STK32C,FABP3,ZCCHC17,PAPSS1,PDGFA,MMP14,APEH,PABPN1,GPX1,AIP,HCCS,MOCS3,CLCF1,NGDN,SRRD,TSHZ2,QA[illegible]	yellow	VSMC_M3
GO:0008154	0.03	0.98	19	183	actin polymerization or depolymerization	BP	1	SNX9,DBNL,MICAL1,BAIAP2,SWAP70,CAPG,CORO1B,TRIM27,ARPIN,ARPC1B,WASHC1,DBN1,CORO1A,MIR214,TWF2,ELN,CDC42EP5,GBA2,ARPC5L	yellow	VSMC_M3
GO:0071353	0.03	0.99	5	26	cellular response to interleukin-4	BP	1	CDK4,RPL3,FASN,IMPDH2,CORO1A	yellow	VSMC_M3
GO:0006353	0.03	0.99	4	19	DNA-templated transcription, termination	BP	1	ZNRD1,POLR2A,SSU72,CAVIN1	yellow	VSMC_M3
GO:0090304	0.03	0.97	296	3916	nucleic acid metabolic process	BP	1	ENPP1,NAB2,NHP2,CDK4,SQSTM1,DHX16,ZNF608,HDAC10,NELFE,FNIP1,ATF6B,PBX2,RING1,SREBF2,TRMT12,H2AFY,HNRNPA0,TRIM11,TRAPPC9,NUDT3,PPARD,FANCE,BOP1,SND1,RTCB,AARS2,SECISBP2,PHF2,CNOT3,KLF4,HOXA1,FHL3,ZC3H12A,FIGNL1,PNKP,SNRNP70,CC2D1B,KTI12,KMT2E,SIX5,ZNF282,GEMIN7,ZNF296,RELB,MUTYH,DMAP1,CIC,GTF3C2,OGG1,SERTAD1,TTC5,METTL3,CXXC5,FAAP100,DND1,PUS1,DEAF1,SMUG1,HOXC4,POLR1D,TARBP2,KMT2B,C12orf10,NAT10,ZNF740,RRS1,SWAP70,RALY,ZBTB38,ARL6IP4,LSM6,TNKS1BP1,PINX1,GATB,GREM1,KDM2B,PRPF40B,MCRS1,POLR2C,CX3CL1,WNT10B,ACTR5,ANKRD2,EGR3,CCAR2,DNAJC17,DLL4,CHP1,YAF2,HTATIP2,PCIF1,NPM3,PCBP4,KDM1B,CHCHD2,RBM15B,CARS,SRSF9,DCPS,IRX1,WDR4,RRP1B,TMTC1,BRD9,RBM6,ZCCHC17,PABPN1,AIP,MOCS3,CLCF1,NGDN,TSHZ2,QARS,PIR,TRIM27,ZNRD1,PRMT2,THOC5,RIPK3,KHNYN,TWIST2,SF3A1,ADRM1,PSTK,RPS21,SMARCD2,FTSJ3,BRMS1,BUD23,GMEB2,CEP164,SART1,CCDC85B,PRPF6,PBX1,RNASEH2C,KAT5,RELA,ZNF511,DDX52,LRWD1,ALKBH4,ARNT2,ZFPL1,LIMD1,SMARCB1,CHCHD10,TRMT2A,PBX4,UBTF,GATAD2A,THOC3,MEF2B,PHB2,TRMT112,NDP,CTDSP1,MLF2,OTUB1,NOP14,EID1,SSBP4,METTL13,ZNF394,LGALS3,C11orf95,BUD31,MRTO4,TNFRSF1A,GTPBP3,BABAM1,TEAD4,C1QBP,CSTF2T,TAF6L,CHERP,ZBTB49,GPKOW,MTA2,ZNF316,MED11,PELP1,CCDC22,FOXK1,ELP5,KLF3,PKN1,SEPSECS,INTS1,POLR2A,CWC25,MAD2L2,SLTM,NANOS3,CTBP1,FBXL19,RRNAD1,CRABP2,DFFA,SP2,ARHGEF2,SNF8,CD2BP2,PER1,NFIX,TRMT1,PPP4C,FARSA,SLX1A,IRF2BPL,TRIM25,USP43,SMARCA4,CARM1,RPL7A,RAVER1,AATF,CXorf[illegible]	yellow	VSMC_M3
GO:0031397	0.03	0.99	10	72	negative regulation of protein ubiquitination	BP	1	SQSTM1,VPS28,PINX1,CHP1,LIMK1,OTUB1,UBXN1,MAD2L2,RPL37,AKT1	yellow	VSMC_M3
GO:0046031	0.04	0.99	9	66	ADP metabolic process	BP	1	GALK1,OGDHL,AK2,BAD,FOXK1,PGM1,GIT1,JMJD8,AK1	yellow	VSMC_M3
GO:0051310	0.04	0.99	8	55	metaphase plate congression	BP	1	DCTN2,NUMA1,RRS1,PINX1,NUDC,EML3,MAD1L1,MIS12	yellow	VSMC_M3
GO:0018393	0.04	0.98	15	134	internal peptidyl-lysine acetylation	BP	1	BAG6,JADE2,MEAF6,DMAP1,KAT14,MCRS1,KAT5,SMARCB1,EID1,TAF6L,CTBP1,PER1,SGF29,MBD3,THAP7	yellow	VSMC_M3
GO:0001885	0.04	0.99	7	53	endothelial cell development	BP	1	COL15A1,CCM2,FASN,PDE4D,GPX1,TNFRSF1A,TJP3	yellow	VSMC_M3
GO:0009161	0.04	0.99	8	51	ribonucleoside monophosphate metabolic process	BP	1	GUK1,AK2,IMPDH2,ATIC,AMPD2,APRT,AK1,NT5C	yellow	VSMC_M3
GO:0006325	0.04	0.98	28	301	chromatin organization	BP	1	BAG6,HDAC10,H2AFY,PHF2,KMT2E,DMAP1,H3F3B,ACTR5,NPM3,BRD9,SMARCD2,BUD23,RELA,LRWD1,ALKBH4,ZNHIT1,SMARCB1,BABAM1,TAF6L,MTA2,H3F3A,SMARCA4,SAFB,UBN1,HMG20B,EHMT1,MBD3,FAM50A	yellow	VSMC_M3
GO:0019058	0.04	0.98	24	239	viral life cycle	BP	1	TRIM11,LGALS1,SLC52A2,VPS28,ZC3H12A,SNX3,P4HB,SIGLEC1,TARBP2,SCARB1,ITGB7,TYRO3,NECTIN1,ANPEP,TRIM27,BANF1,SMARCB1,MVB12A,CTBP1,SNF8,TRIM25,C19orf66,MID2,CLDN6	yellow	VSMC_M3

GO:0032102	0.04	0.98	26	266	negative regulation of response to external stimulus	BP	1	TTLL12,PPARD,SHARPIN,PDGFRA,SOCS5,KLF4,METTL3,TARBP2,GREM1,CX3CL1,SOD1,TYRO3,SELENOS,MVK,RIN3,PDGFA,GPX1,CORO1B,SPX,PSMB4,CD9,PLAU,TNFRSF1A,C1QBP,GIT1,CACTIN	yellow	VSMC_M3
GO:0006720	0.04	0.98	10	73	isoprenoid metabolic process	BP	1	PPARD,FDFT1,MVK,LSS,CRABP2,PMVK,HMGCS1,MVD,FDPS,IDI1	yellow	VSMC_M3
GO:0030041	0.04	0.98	17	160	actin filament polymerization	BP	1	SNX9,DBNL,BAIAP2,CAPG,CORO1B,TRIM27,ARPIN,ARPC1B,WASHC1,DBN1,CORO1A,MIR214,TWF2,ELN,CDC42EP5,GBA2,ARPC5L	yellow	VSMC_M3
GO:0050773	0.04	0.98	10	87	regulation of dendrite development	BP	1	PACSIN1,CUL7,DBNL,BAIAP2,DBN1,CARM1,EEF2K,ANAPC2,STK11,NSMF	yellow	VSMC_M3
GO:0046700	0.04	0.97	34	357	heterocycle catabolic process	BP	1	ENPP1,HNRNPA0,NUDT3,SND1,DNPH1,SECISBP2,CNOT3,ZC3H12A,BLVRA,BLVRB,OGG1,METTL3,DND1,SMUG1,PDE4D,LSM6,PCBP4,DCPS,ZCCHC17,RNASEH2C,MRTO4,CNP,NANOS3,DFFA,AKT1,PAIP1,KHSRP,CARHSP1,RBM8A,RNH1,NT5C,TENT5B,TRIR,ITPA	yellow	VSMC_M3
GO:2001242	0.04	0.98	16	135	regulation of intrinsic apoptotic signaling pathway	BP	1	FIGNL1,SLC9A3R1,P4HB,BCL2L1,PLEKHF1,SOD1,CCAR2,SELENOS,GPX1,RIPK3,BAD,ARHGEF2,AKT1,TRAP1,ZNF385A,TXNDC12	yellow	VSMC_M3
GO:0016571	0.04	0.98	13	118	histone methylation	BP	1	NELFE,H2AFY,KMT2E,KMT2B,SETD1B,PRMT2,SMARCB1,TRMT112,MTHFR,CARM1,COPRS,EHMT1,NTMT1	yellow	VSMC_M3
GO:0070050	0.04	0.99	4	22	neuron cellular homeostasis	BP	1	DCTN1,CX3CL1,TYRO3,TSPOAP1	yellow	VSMC_M3
GO:0006364	0.04	0.98	22	198	rRNA processing	BP	1	NHP2,BOP1,NAT10,RRS1,LSM6,NPM3,RRP1B,NGDN,RPS21,FTSJ3,BUD23,SART1,DDX52,TRMT112,NOP14,MRTO4,RRNAD1,RPL7A,TBL3,TSR3,RBFA,PIN4	yellow	VSMC_M3
GO:0042472	0.04	0.98	9	73	inner ear morphogenesis	BP	1	CTHRC1,SCRIB,HOXA1,WDPCP,SLC9A3R1,INSIG1,LRIG1,SOD1,TTC39C	yellow	VSMC_M3
GO:0006749	0.04	0.99	7	42	glutathione metabolic process	BP	1	GSR,SOD1,GSTO1,GPX1,IDH1,CTNS,EEF1G	yellow	VSMC_M3
GO:0009168	0.04	0.99	4	18	purine ribonucleoside monophosphate biosynthetic process	BP	1	IMPDH2,ATIC,AMPD2,APRT	yellow	VSMC_M3
GO:0044271	0.04	0.97	260	3436	cellular nitrogen compound biosynthetic process	BP	1	ENPP1,NAB2,NHP2,CDK4,SQSTM1,ZNF608,HDAC10,NELFE,FNIP1,ATF6B,PBX2,RING1,SREBF2,H2AFY,TRIM11,GUK1,TRAPPC9,PPARD,EEF1D,TSTA3,RPL3,RPL8,SND1,MRPS18A,AARS2,SECISBP2,PHF2,RPS9,CNOT3,KLF4,HOXA1,FHL3,ZC3H12A,PNKP,CC2D1B,KTI12,KMT2E,SIX5,ZNF282,ZNF296,RELB,DMAP1,CIC,GTF3C2,UNK,OGG1,SERTAD1,TTC5,METTL3,CXXC5,DND1,SMOX,RPL29,EIF4EBP1,GNPDA1,DEAF1,HOXC4,POLR1D,TARBP2,KMT2B,NAT10,ZNF740,EIF4B,RALY,ACSL1,ZBTB38,SLC11A2,TNKS1BP1,PINX1,GATB,GREM1,KDM2B,MCRS1,POLR2C,CX3CL1,WNT10B,ACTR5,ANKRD2,EGR3,CCAR2,DNAJC17,DLL4,RPLP2,CHP1,MRPL43,YAF2,HTATIP2,PCIF1,NPM3,KDM1B,CHCHD2,CARS,AK2,DCPS,IRX1,RRP1B,BRD9,PAPSS1,APEH,AIP,NGDN,SRRD,TSHZ2,QARS,IMPDH2,PIR,TRIM27,EIF3A,ZNRD1,PRMT2,THOC5,RIPK3,TWIST2,LSM14B,ADRM1,PSTK,RPS21,SMARCD2,BRMS1,PPOX,TACO1,GMEB2,CCDC85B,PRPF6,PBX1,KAT5,RELA,ATP5MC3,ZNF511,ARNT2,MRPL49,ZFPL1,LIMD1,SMARCB1,CHCHD10,SLC25A39,PBX4,UBTF,GATAD2A,ATIC,MEF2B,PHB2,NDP,IGFBP5,CTDSP1,MLF2,EID1,SSBP4,METTL13,MPV17L2,ZNF394,C11orf95,BUD31,TNFRSF1A,TEAD4,C1QBP,AIMP2,TAF6L,ZBTB49,UQCC3,MTA2,ZNF316,ACLY,MED11,PELP1,CCDC22,FOXK1,KLF3,PKN1,SEPSECS,POLR2A,MAD2L2,SLTM,NANOS3,CTBP1,FBXL19,CRABP2,SP2,ARHGEF2,SNF8,PER1,NFIX,FARSA,FLAD1,IRF2BPL,TUFM,TRIM25,ATP5PD,USP43,RPL37,SMARCA4,CARM1,RPL7A,EEF2K,AATF,MRPL4,EIF3G,C19orf66,MID2,ZNF521,ZNF358,AKT1,BRF1,PAIP1,KHSRP,GTF2F1,RFX2,SAFB,SAFB2,MYDGF,HDGFL2,SIRT6,MAP2K2,CACTIN,CDK11B,SSU72,HIC1,MRPL20,TRAP1,MNT,EHMT1,AP3D1,M[illegible]	yellow	VSMC_M3
GO:0061001	0.04	0.99	6	42	regulation of dendritic spine morphogenesis	BP	1	DBNL,BAIAP2,ARHGAP33,NGEF,DBN1,EEF2K	yellow	VSMC_M3
GO:0006475	0.04	0.98	15	136	internal protein amino acid acetylation	BP	1	BAG6,JADE2,MEAF6,DMAP1,KAT14,MCRS1,KAT5,SMARCB1,EID1,TAF6L,CTBP1,PER1,SGF29,MBD3,THAP7	yellow	VSMC_M3
GO:0033559	0.04	0.98	9	64	unsaturated fatty acid metabolic process	BP	1	PTGR1,MGLL,SCD,GPX1,PLA2G3,TNFRSF1A,CYP4F8,FADS2,FADS1	yellow	VSMC_M3
GO:0009135	0.04	0.98	10	78	purine nucleoside diphosphate metabolic process	BP	1	GUK1,GALK1,OGDHL,AK2,BAD,FOXK1,PGM1,GIT1,JMJD8,AK1	yellow	VSMC_M3
GO:0009179	0.04	0.98	10	78	purine ribonucleoside diphosphate metabolic process	BP	1	GUK1,GALK1,OGDHL,AK2,BAD,FOXK1,PGM1,GIT1,JMJD8,AK1	yellow	VSMC_M3
GO:0007224	0.04	0.98	13	119	smoothened signaling pathway	BP	1	ENPP1,TXNDC15,IFT27,WDPCP,WNT10B,EVC,TEDC1,RAB34,NSDHL,TEDC2,SSNA1,GAS8,NDST1	yellow	VSMC_M3
GO:0034655	0.04	0.97	32	335	nucleobase-containing compound catabolic process	BP	1	ENPP1,HNRNPA0,NUDT3,SND1,DNPH1,SECISBP2,CNOT3,ZC3H12A,OGG1,METTL3,DND1,SMUG1,PDE4D,LSM6,PCBP4,DCPS,ZCCHC17,RNASEH2C,MRTO4,CNP,NANOS3,DFFA,AKT1,PAIP1,KHSRP,CARHSP1,RBM8A,RNH1,NT5C,TENT5B,TRIR,ITPA	yellow	VSMC_M3
GO:0034405	0.04	0.99	5	31	response to fluid shear stress	BP	1	SREBF2,PLEC,SOCS5,KLF4,AKT1	yellow	VSMC_M3
GO:0034622	0.04	0.97	72	830	cellular protein-containing complex assembly	BP	1	SPAG1,SNX9,SAMM50,NDUFB9,H2AFY,RPL3,BOP1,TBCC,DBNL,ACAD9,GEMIN7,H3F3B,BAIAP2,NUMA1,CCDC57,SDHAF1,TARBP2,EIF4B,RRS1,UQCRFS1,CAPG,GREM1,DCTN1,WNT10B,SRSF9,CORO1B,HCCS,TRIM27,ARPIN,EIF3A,SF3A1,ADRM1,TACO1,SART1,PRPF6,MID1IP1,SMARCB1,UBTF,ARPC1B,MRTO4,FCHO1,TAF6L,UQCC3,WASHC1,H3F3A,DBN1,CLTB,CD2BP2,RANGRF,CORO1A,SMARCA4,EIF3G,BRF1,GIT1,UBN1,MRPL20,NDUFS7,ATP5F1D,TAZ,TGFBR3,MIR214,TWF2,SURF1,MIS12,ELN,UNC13A,CDC42EP5,GBA2,TUBGCP2,STUB1,CCDC115,ARPC5L	yellow	VSMC_M3
GO:0042168	0.04	0.99	6	37	heme metabolic process	BP	1	BLVRA,BLVRB,SLC11A2,SRRD,PPOX,SLC25A39	yellow	VSMC_M3
GO:1902905	0.04	0.98	18	179	positive regulation of supramolecular fiber organization	BP	1	SNX9,BAIAP2,NUMA1,SWAP70,DCTN1,CX3CL1,GPX1,CORO1B,TRIM27,ARPIN,LIMK1,RHOC,ARPC1B,WASHC1,CORO1A,GIT1,CDC42EP5,ARPC5L	yellow	VSMC_M3
GO:0006898	0.04	0.98	19	192	receptor-mediated endocytosis	BP	1	CD63,SNX9,SCRIB,DBNL,SNX17,SIGLEC1,LDLRAD3,LRP3,GREM1,AP2A2,RIN3,AP1G2,TNK2,CD9,FCHO1,CLTB,ANKRD13B,RABEPK,GSG1L	yellow	VSMC_M3
GO:0043648	0.04	0.98	10	73	dicarboxylic acid metabolic process	BP	1	GRHPR,IDH3B,OGDHL,SDHA,PCK2,SLC19A1,IDH1,ATIC,ACLY,GLUD1	yellow	VSMC_M3

GO:0044782	0.04	0.97	27	288	cilium organization	BP	1	SPAG1,TXNDC15,IFT27,WDPCP,EHD2,SLC9A3R1,SEPT9,CEP131,YIF1B,CCDC57,DCTN1,CCDC32,CCDC28B,PCNT,IQCG,PLA2G3,CEP164,PTPN23,FAM161B,RABEP2,ATG3,TTC39C,RFX2,SSNA1,GAS8,WDR34,MEIG1	yellow	VSMC_M3
GO:0009913	0.04	0.98	13	120	epidermal cell differentiation	BP	1	H2AFY,SCRIB,PLEC,SHARPIN,KLF4,WDPCP,SLC9A3R1,NUMA1,SOD1,KRT10,CASP14,TMEM79,PPL	yellow	VSMC_M3
GO:0043588	0.04	0.98	17	171	skin development	BP	1	H2AFY,PLEC,SHARPIN,NUMA1,WNT10B,PDGFA,RELA,IGFBP5,KRT10,CASP14,TMEM79,DHCR24,PPL,NSDHL,SLC27A4,TRADD,NOM1	yellow	VSMC_M3
GO:0007020	0.04	0.99	5	32	microtubule nucleation	BP	1	CCDC57,DCTN1,RANGRF,GIT1,TUBGCP2	yellow	VSMC_M3
GO:0010586	0.04	0.99	4	23	miRNA metabolic process	BP	1	SND1,ZC3H12A,RELA,KHSRP	yellow	VSMC_M3
GO:0051258	0.04	0.97	24	250	protein polymerization	BP	1	SNX9,DBNL,BAIAP2,NUMA1,CCDC57,CAPG,DCTN1,CORO1B,TRIM27,ARPIN,MID1IP1,ARPC1B,WASHC1,DBN1,RANGRF,CORO1A,GIT1,MIR214,TWF2,ELN,CDC42EP5,GBA2,TUBGCP2,ARPC5L	yellow	VSMC_M3
GO:0015909	0.05	0.98	7	46	long-chain fatty acid transport	BP	1	ACSL1,FABP3,SPX,PLA2G3,SLC27A3,AKT1,SLC27A4	yellow	VSMC_M3
GO:0050994	0.05	0.99	6	39	regulation of lipid catabolic process	BP	1	CDK4,SCARB1,PDE3B,ETFBKMT,IDH1,AKT1	yellow	VSMC_M3
GO:0032273	0.05	0.98	13	116	positive regulation of protein polymerization	BP	1	SNX9,BAIAP2,NUMA1,DCTN1,CORO1B,TRIM27,ARPIN,ARPC1B,WASHC1,CORO1A,GIT1,CDC42EP5,ARPC5L	yellow	VSMC_M3
GO:0070670	0.05	0.99	5	28	response to interleukin-4	BP	1	CDK4,RPL3,FASN,IMPDH2,CORO1A	yellow	VSMC_M3
GO:0043200	0.05	0.98	11	93	response to amino acid	BP	1	PDGFRA,BAIAP2,LAMTOR1,BCL2L1,COL6A1,RELA,BAD,LAMTOR4,CAPN2,MTHFR,NSMF	yellow	VSMC_M3
GO:0050772	0.05	0.98	8	68	positive regulation of axonogenesis	BP	1	DBNL,PLXND1,LIMK1,DBN1,CRABP2,ANAPC2,STK11,TWF2	yellow	VSMC_M3
GO:0010770	0.05	0.98	8	65	positive regulation of cell morphogenesis involved in differentiation	BP	1	CUL7,DBNL,BAIAP2,P4HB,C1QBP,DBN1,EEF2K,ANAPC2	yellow	VSMC_M3
GO:0018027	0.05	0.99	4	21	peptidyl-lysine dimethylation	BP	1	KMT2B,SETD1B,SMARCB1,EHMT1	yellow	VSMC_M3
GO:0001676	0.05	0.98	9	66	long-chain fatty acid metabolic process	BP	1	MGLL,ACAD9,ACSL1,GPX1,FADS2,ACSF2,SLC27A3,SLC27A4,FADS1	yellow	VSMC_M3
GO:0038061	0.05	0.98	12	99	NIK/NF-kappaB signaling	BP	1	ZC3H12A,RELB,GREM1,RIPK3,RELA,MAP3K14,PHB2,IFI35,PPP4C,AKT1,IRAK1,TRADD	yellow	VSMC_M3
GO:0009185	0.05	0.98	10	81	ribonucleoside diphosphate metabolic process	BP	1	GUK1,GALK1,OGDHL,AK2,BAD,FOXK1,PGM1,GIT1,JMJD8,AK1	yellow	VSMC_M3
GO:0002181	0.05	0.97	14	111	cytoplasmic translation	BP	1	RPL3,RPL8,RPS9,UNK,METTL3,RPL29,EIF4B,RPLP2,EIF3A,RPS21,RPL37,RPL7A,EIF3G,ZNF385A	yellow	VSMC_M3
GO:0032609	0.05	0.98	8	59	interferon-gamma production	BP	1	SCRIB,IL1R1,ZC3H12A,PDE4D,TRIM27,RIPK3,C1QBP,CD276	yellow	VSMC_M3
GO:0032649	0.05	0.98	8	59	regulation of interferon-gamma production	BP	1	SCRIB,IL1R1,ZC3H12A,PDE4D,TRIM27,RIPK3,C1QBP,CD276	yellow	VSMC_M3
GO:0010608	0.05	0.96	43	480	posttranscriptional regulation of gene expression	BP	1	CDK4,HNRNPA0,SECISBP2,RPS9,CNOT3,ZC3H12A,UNK,METTL3,DND1,EIF4EBP1,TARBP2,NAT10,EIF4B,PCIF1,PCBP4,DCPS,ZCCHC17,NGDN,LSM14B,TACO1,LIMD1,IGFBP5,MPV17L2,C1QBP,SEPSECS,NANOS3,PER1,CDC37,C19orf66,AKT1,PAIP1,KHSRP,CARHSP1,MAP2K2,TRAP1,MKNK2,MIR214,MIR155,RBM8A,MIR374B,TENT5B,ZNF385A,ZAR1L	yellow	VSMC_M3
GO:0042255	0.05	0.98	7	45	ribosome assembly	BP	1	RPL3,BOP1,RRS1,MPV17L2,MRTO4,C1QBP,MRPL20	yellow	VSMC_M3
GO:0006305	0.05	0.98	7	53	DNA alkylation	BP	1	KMT2E,DMAP1,KDM1B,GATAD2A,MTA2,EHMT1,MBD3	yellow	VSMC_M3
GO:0006306	0.05	0.98	7	53	DNA methylation	BP	1	KMT2E,DMAP1,KDM1B,GATAD2A,MTA2,EHMT1,MBD3	yellow	VSMC_M3
GO:1903902	0.05	0.99	4	21	positive regulation of viral life cycle	BP	1	TRIM11,LGALS1,P4HB,TYRO3	yellow	VSMC_M3
GO:0051668	0.05	0.96	46	537	localization within membrane	BP	1	SQSTM1,BAG6,SAMM50,PACSIN1,SCRIB,TTC7A,ASNA1,TPBG,EIPR1,SNX3,EHD2,SLC9A3R1,SNX17,YIF1B,BCL2L1,ITGB7,PLEKHF1,RAPSN,CHP1,RAB35,TSPAN5,MMP14,PLA2G3,PTPN23,VPS51,PEX5,ZDHHC3,YJEFN3,LGALS3,TNFRSF1A,WASHC1,CCDC22,SNF8,RANGRF,SYNE3,CSK,ATG3,AKT1,PACS2,DENND1C,RAB34,AP3D1,SSNA1,PLA2G4E,ARFRP1,TIMM22	yellow	VSMC_M3
GO:0018394	0.05	0.97	15	143	peptidyl-lysine acetylation	BP	1	BAG6,JADE2,MEAF6,DMAP1,KAT14,MCRS1,KAT5,SMARCB1,EID1,TAF6L,CTBP1,PER1,SGF29,MBD3,THAP7	yellow	VSMC_M3
GO:1902600	0.05	0.97	14	115	proton transmembrane transport	BP	1	SLC9A3R1,UCP2,UQCRFS1,SLC11A2,CHP1,UQCRC1,TACO1,ATP5MC3,PHB2,ATP5PD,CTNS,NDUFS7,ATP5F1D,SURF1	yellow	VSMC_M3
GO:0002090	0.05	0.98	6	43	regulation of receptor internalization	BP	1	CD63,SCRIB,GREM1,RIN3,ANKRD13B,GSG1L	yellow	VSMC_M3
GO:1904994	0.05	0.99	4	21	regulation of leukocyte adhesion to vascular endothelial cell	BP	1	KLF4,ST3GAL4,RELA,IRAK1	yellow	VSMC_M3
GO:0043903	0.05	0.98	7	49	regulation of biological process involved in symbiotic interaction	BP	1	TRIM11,LGALS1,SNX3,P4HB,TRIM27,TRIM25,MID2	yellow	VSMC_M3
GO:0030838	0.06	0.98	10	82	positive regulation of actin filament polymerization	BP	1	SNX9,BAIAP2,CORO1B,TRIM27,ARPIN,ARPC1B,WASHC1,CORO1A,CDC42EP5,ARPC5L	yellow	VSMC_M3
GO:0006473	0.06	0.97	17	168	protein acetylation	BP	1	BAG6,JADE2,MEAF6,NAT9,DMAP1,KAT14,NAT10,MCRS1,KAT5,SMARCB1,EID1,TAF6L,CTBP1,PER1,SGF29,MBD3,THAP7	yellow	VSMC_M3
GO:0032715	0.06	0.98	5	30	negative regulation of interleukin-6 production	BP	1	SOCS5,ZC3H12A,CX3CL1,SELENOS,CSK	yellow	VSMC_M3
GO:0016573	0.06	0.97	14	131	histone acetylation	BP	1	JADE2,MEAF6,DMAP1,KAT14,MCRS1,KAT5,SMARCB1,EID1,TAF6L,CTBP1,PER1,SGF29,MBD3,THAP7	yellow	VSMC_M3
GO:0014037	0.06	0.98	5	35	Schwann cell differentiation	BP	1	NAB2,PLEC,SOD1,RELA,AKT1	yellow	VSMC_M3
GO:0045599	0.06	0.98	6	43	negative regulation of fat cell differentiation	BP	1	ENPP1,INSIG1,LRP3,WNT10B,CCDC85B,TRIB2	yellow	VSMC_M3
GO:0030307	0.06	0.97	14	134	positive regulation of cell growth	BP	1	DNPH1,DBNL,H3F3B,KDM2B,MMP14,LIMK1,H3F3A,DBN1,CRABP2,AKT1,HDGFL2,ANAPC2,TWF2,UNC13A	yellow	VSMC_M3
GO:0055086	0.06	0.96	41	439	nucleobase-containing small molecule metabolic process	BP	1	ENPP1,GUK1,NUDT3,TSTA3,DNPH1,GALK1,OGG1,FASN,GNPDA1,SMUG1,OGDHL,PDE4D,ACSL1,AK2,MVK,PAPSS1,IMPDH2,ATP5MC3,ATIC,BAD,ASMTL,UQCC3,CNP,ACLY,FOXK1,ACSF2,FLAD1,PMVK,ATP5PD,HMGCS1,PGM1,GIT1,ATP5F1D,TAZ,JMJD8,AMPD2,APRT,MVD,AK1,NT5C,ITPA	yellow	VSMC_M3
GO:0010155	0.06	0.99	4	19	regulation of proton transport	BP	1	SLC9A3R1,CHP1,TACO1,PHB2	yellow	VSMC_M3
GO:0042273	0.06	0.98	8	56	ribosomal large subunit biogenesis	BP	1	NHP2,RPL3,BOP1,RRS1,FTSJ3,MRTO4,RPL7A,MRPL20	yellow	VSMC_M3
GO:1902532	0.06	0.96	36	407	negative regulation of intracellular signal transduction	BP	1	FNIP1,KLF4,SPRED2,ZC3H12A,FIGNL1,SLC9A3R1,EMILIN1,CAVIN3,BCL2L1,PDE4D,PDE3B,CCAR2,CHP1,SELENOS,GPX1,QARS,EIF3A,THOC5,STMN3,RELA,LIMD1,IFI35,C1QBP,CCDC22,ARHGEF2,PER1,CSK,AKT1,CACTIN,TRAP1,STK11,PTPRR,ZNF385A,TXNDC12,CDC34,DUSP5	yellow	VSMC_M3

GO:0045598	0.06	0.97	12	109	regulation of fat cell differentiation	BP	1	ENPP1,PPARD,ZC3H12A,WDFY2,INSIG1,LRP3,WNT10B,CCDC85B,CARM1,AKT1,TRIB2,ZNF385A	yellow	VSMC_M3
GO:0009123	0.06	0.98	9	65	nucleoside monophosphate metabolic process	BP	1	GUK1,DNPH1,AK2,IMPDH2,ATIC,AMPD2,APRT,AK1,NT5C	yellow	VSMC_M3
GO:0009127	0.06	0.99	4	20	purine nucleoside monophosphate biosynthetic process	BP	1	IMPDH2,ATIC,AMPD2,APRT	yellow	VSMC_M3
GO:0018205	0.06	0.96	29	313	peptidyl-lysine modification	BP	1	BAG6,HDAC10,NELFE,JADE2,H2AFY,MEAF6,KMT2E,DMAP1,KAT14,KMT2B,SETD1B,MCRS1,ETFBKMT,TRIM27,KAT5,RELA,SMARCB1,TRMT112,EID1,TAF6L,CTBP1,PER1,SGF29,METTL22,HMG20B,EHMT1,MBD3,LOXL1,THAP7	yellow	VSMC_M3
GO:0016577	0.06	0.99	4	28	histone demethylation	BP	1	PHF2,KDM2B,KDM1B,FBXL19	yellow	VSMC_M3
GO:0032869	0.06	0.97	16	161	cellular response to insulin stimulus	BP	1	ENPP1,CDK4,INSIG1,UCP2,BAIAP2,TNS2,PDE3B,SELENOS,PCK2,CAPN10,RELA,PIP4K2B,EEF2K,AKT1,APRT,SLC27A4	yellow	VSMC_M3
GO:0016482	0.06	0.97	15	153	cytosolic transport	BP	1	PQLC1,EIPR1,SNX3,DCTN1,TRIM27,PTPN23,VPS51,WASHC1,SNX8,SNF8,STX10,CORO1A,MAP2K2,ARFRP1,PHETA2	yellow	VSMC_M3
GO:0043162	0.06	0.99	4	21	ubiquitin-dependent protein catabolic process via the multivesicular body sorting pathway	BP	1	VPS28,PTPN23,MVB12A,SNF8	yellow	VSMC_M3
GO:0010769	0.06	0.97	9	80	regulation of cell morphogenesis involved in differentiation	BP	1	CUL7,DBNL,BAIAP2,P4HB,C1QBP,RCC2,DBN1,EEF2K,ANAPC2	yellow	VSMC_M3
GO:1901564	0.06	0.95	356	4789	organonitrogen compound metabolic process	BP	1	ENPP1,NAB2,CDK4,CTDSP2,SQSTM1,LMF2,BAG6,SBF1,SNX9,HDAC10,NELFE,CERK,FNIP1,PRR5,TTLL12,RING1,JADE2,H2AFY,TRIM11,SGSM3,GUK1,NUDT3,PPARD,EEF1D,RPL3,PLEC,MPST,SHARPIN,SLC52A2,VPS28,RPL8,CUL7,DNPH1,B4GALT1,MRPS18A,AARS2,PIGO,PDGFRA,SECISBP2,PHF2,HSPBP1,SOCS5,RPS9,CNOT3,KLF4,JOSD2,PSMB3,SPRED2,EIPR1,MEAF6,ZC3H12A,BLVRA,PNKP,CPT1C,RCN3,SNRNP70,SNX3,MICAL1,KMT2E,WDFY2,SLC9A3R1,SERP2,NAT9,EMILIN1,SEPHS1,TMUB1,RNF220,DMAP1,GALK1,PPM1G,UNK,BCKDHA,GALNT14,BLVRB,OGG1,SERTAD1,PSMC4,KAT14,P4HA3,SEL1L2,METTL3,PHLDA1,P4HB,DND1,PYCR1,FASN,SMOX,RPL29,EIF4EBP1,DELE1,GNPDA1,GSR,PTDSS2,SMUG1,OGDHL,LDLRAD3,TARBP2,KMT2B,SCARB1,NAT10,HM13,PDE4D,TNS2,EIF4B,PEPD,SWAP70,ACSL1,SLC11A2,TNKS1BP1,MMP24,PINX1,GATB,SETD1B,GREM1,KDM2B,MCRS1,CX3CL1,WNT10B,FKBP11,SOD1,PACSIN3,CCAR2,ACCS,RPLP2,CHP1,TYRO3,MRPL43,HTATIP2,PCIF1,PPM1M,KDM1B,SLC35C2,CARS,TSPAN5,CISH,ST3GAL4,AK2,RNF19B,DCPS,SELENOS,GSTO1,ETFBKMT,MANBA,TMTC1,MVK,STK32C,FABP3,PAPSS1,PDGFA,MMP14,APEH,GPX1,AIP,HCCS,MOCS3,CLCF1,NGDN,SRRD,QARS,SLC19A1,ASB9,IMPDH2,PSME2,ANPEP,TRIM27,EIF3A,LIMK1,RABGGTA,PRMT2,RIPK3,LSM14B,ADRM1,DPP3,PSTK,RPS21,CAPN10,PSMB4,EXTL1,PFDN2,BRMS1,PPOX,SEC11A,TNK2,TACO1,PLA2G3,THAP4,PTPN23,KAT5,RELA,ATP5MC3,SCYL1,IDH1,ALKBH4,MEX3B,MAP3K14,MRPL49,ZDHHC3,SGPL1,SMARCB1,SLC25A39,ATIC,PHB2,TRMT112,IGFBP5,BAD,FKBP2,CTDSP1,OTUB1,EID1,COPS6,MPV17L2,LGALS3,PLAU,TNFRSF1A,MVB12A,BABAM1,C1QBP,AIMP2,[illegible]	yellow	VSMC_M3
GO:0046890	0.06	0.97	14	127	regulation of lipid biosynthetic process	BP	1	CDK4,INSIG1,SCARB1,SOD1,CHP1,DHCR7,FABP3,PDGFA,PLA2G3,IDH1,MID1IP1,CAPN2,ARMC5,AKT1	yellow	VSMC_M3
GO:1903312	0.06	0.97	9	74	negative regulation of mRNA metabolic process	BP	1	NELFE,HNRNPA0,SECISBP2,DND1,PCBP4,SRSF9,C1QBP,PAIP1,TENT5B	yellow	VSMC_M3
GO:0006778	0.06	0.98	6	40	porphyrin-containing compound metabolic process	BP	1	BLVRA,BLVRB,SLC11A2,SRRD,PPOX,SLC25A39	yellow	VSMC_M3
GO:0044728	0.06	0.97	8	67	DNA methylation or demethylation	BP	1	KMT2E,DMAP1,KDM1B,ALKBH4,GATAD2A,MTA2,EHMT1,MBD3	yellow	VSMC_M3
GO:0043550	0.06	0.98	7	58	regulation of lipid kinase activity	BP	1	PDGFRA,SOCS5,KLF4,DGKZ,CISH,WASHC1,PIP4K2B	yellow	VSMC_M3
GO:0090502	0.06	0.97	8	62	RNA phosphodiester bond hydrolysis, endonucleolytic	BP	1	BOP1,SND1,ZC3H12A,RRS1,KHNYN,RPS21,NOP14,TBL3	yellow	VSMC_M3
GO:0000423	0.06	0.98	4	24	mitophagy	BP	1	SQSTM1,PHB2,CDC37,TAZ	yellow	VSMC_M3
GO:0036314	0.06	0.98	4	25	response to sterol	BP	1	INSIG1,AACS,PMVK,HMGCS1	yellow	VSMC_M3
GO:0048015	0.06	0.97	13	133	phosphatidylinositol-mediated signaling	BP	1	PRR5,PPARD,PDGFRA,KLF4,SLC9A3R1,PLCD1,TYRO3,PDGFA,PLCB3,C1QBP,AKT1,MYDGF,ERBB4	yellow	VSMC_M3
GO:1901292	0.06	0.97	9	69	nucleoside phosphate catabolic process	BP	1	ENPP1,NUDT3,DNPH1,OGG1,SMUG1,PDE4D,CNP,NT5C,ITPA	yellow	VSMC_M3
GO:0071222	0.06	0.97	14	126	cellular response to lipopolysaccharide	BP	1	CDK4,PPARD,ZC3H12A,SCARB1,CX3CL1,SELENOS,PABPN1,RELA,CAPN2,AKT1,GIT1,LDOC1,CACTIN,IRAK1	yellow	VSMC_M3
GO:0060765	0.06	0.98	4	24	regulation of androgen receptor signaling pathway	BP	1	PRMT2,PKN1,SMARCA4,SAFB2	yellow	VSMC_M3
GO:1901361	0.07	0.95	36	395	organic cyclic compound catabolic process	BP	1	ENPP1,HNRNPA0,NUDT3,SND1,DNPH1,SECISBP2,CNOT3,ZC3H12A,BLVRA,SNX17,BLVRB,OGG1,METTL3,DND1,SMUG1,SCARB1,PDE4D,LSM6,PCBP4,DCPS,ZCCHC17,RNASEH2C,MRTO4,CNP,NANOS3,DFFA,AKT1,PAIP1,KHSRP,CARHSP1,RBM8A,RNH1,NT5C,TENT5B,TRIR,ITPA	yellow	VSMC_M3
GO:0006479	0.07	0.96	15	148	protein methylation	BP	1	NELFE,H2AFY,KMT2E,KMT2B,SETD1B,ETFBKMT,PRMT2,SMARCB1,TRMT112,MTHFR,CARM1,COPRS,METTL22,EHMT1,NTMT1	yellow	VSMC_M3
GO:0008213	0.07	0.96	15	148	protein alkylation	BP	1	NELFE,H2AFY,KMT2E,KMT2B,SETD1B,ETFBKMT,PRMT2,SMARCB1,TRMT112,MTHFR,CARM1,COPRS,METTL22,EHMT1,NTMT1	yellow	VSMC_M3
GO:0043039	0.07	0.98	6	40	tRNA aminoacylation	BP	1	AARS2,GATB,CARS,QARS,FARSA,SARS	yellow	VSMC_M3
GO:0046486	0.07	0.96	29	317	glycerolipid metabolic process	BP	1	PIGO,SOCS5,TTC7A,MGLL,PLCD1,INSIG1,INPPL1,PCYT2,PTDSS2,SCARB1,ACSL1,DGKZ,CISH,FABP3,PDGFA,GPX1,PCK2,PLA2G3,PHB2,PLCB3,CAPN2,PIP4K2B,DPM3,MTMR4,PGP,TAZ,SLC44A3,VAC14,PLA2G4E	yellow	VSMC_M3
GO:0046488	0.07	0.96	14	138	phosphatidylinositol metabolic process	BP	1	PIGO,SOCS5,TTC7A,PLCD1,INPPL1,CISH,PDGFA,PLA2G3,PLCB3,PIP4K2B,DPM3,MTMR4,VAC14,PLA2G4E	yellow	VSMC_M3
GO:0043966	0.07	0.97	7	54	histone H3 acetylation	BP	1	JADE2,MEAF6,KAT14,SMARCB1,TAF6L,PER1,SGF29	yellow	VSMC_M3
GO:0002064	0.07	0.96	16	173	epithelial cell development	BP	1	SCRIB,PLEC,B4GALT1,COL15A1,WDPCP,CCM2,SLC9A3R1,FASN,PDE4D,SOD1,GPX1,BAD,TNFRSF1A,TMEM79,AKT1,TJP3	yellow	VSMC_M3
GO:0031167	0.07	0.98	4	20	rRNA methylation	BP	1	FTSJ3,BUD23,TRMT112,RRNAD1	yellow	VSMC_M3

GO:0032438	0.07	0.98	4	23	melanosome organization	BP	1	HPS1,BLOC1S4,AP1M1,AP3D1	yellow	VSMC_M3
GO:0015908	0.07	0.97	8	60	fatty acid transport	BP	1	PPARD,ACSL1,FABP3,SPX,PLA2G3,SLC27A3,AKT1,SLC27A4	yellow	VSMC_M3
GO:0009152	0.07	0.96	15	137	purine ribonucleotide biosynthetic process	BP	1	GUK1,ACSL1,AK2,PAPSS1,IMPDH2,ATP5MC3,ATIC,UQCC3,ACLY,ATP5PD,ATP5F1D,TAZ,AMPD2,APRT,AK1	yellow	VSMC_M3
GO:0033059	0.07	0.97	6	43	cellular pigmentation	BP	1	CD63,DCTN2,HPS1,BLOC1S4,AP1M1,AP3D1	yellow	VSMC_M3
GO:0046939	0.07	0.97	9	74	nucleotide phosphorylation	BP	1	GUK1,PNKP,GALK1,OGDHL,FOXK1,PGM1,GIT1,JMJD8,AK1	yellow	VSMC_M3
GO:0043038	0.07	0.97	6	41	amino acid activation	BP	1	AARS2,GATB,CARS,QARS,FARSA,SARS	yellow	VSMC_M3
GO:0007006	0.07	0.96	12	99	mitochondrial membrane organization	BP	1	SAMM50,BCL2L1,CHCHD10,BAD,LETM1,UQCC3,CNP,MTX1,ALKBH7,TAZ,SPG7,TIMM22	yellow	VSMC_M3
GO:0045773	0.07	0.98	5	37	positive regulation of axon extension	BP	1	DBNL,LIMK1,DBN1,ANAPC2,TWF2	yellow	VSMC_M3
GO:0019439	0.07	0.95	34	375	aromatic compound catabolic process	BP	1	ENPP1,HNRNPA0,NUDT3,SND1,DNPH1,SECISBP2,CNOT3,ZC3H12A,BLVRA,BLVRB,OGG1,METTL3,DND1,SMUG1,PDE4D,LSM6,PCBP4,DCPS,ZCCHC17,RNASEH2C,MRTO4,CNP,NANOS3,DFFA,AKT1,PAIP1,KHSRP,CARHSP1,RBM8A,RNH1,NT5C,TENT5B,TRIR,ITPA	yellow	VSMC_M3
GO:2000191	0.07	0.98	4	23	regulation of fatty acid transport	BP	1	ACSL1,FABP3,PLA2G3,AKT1	yellow	VSMC_M3
GO:0048753	0.07	0.98	4	24	pigment granule organization	BP	1	HPS1,BLOC1S4,AP1M1,AP3D1	yellow	VSMC_M3
GO:0006417	0.07	0.95	31	341	regulation of translation	BP	1	CDK4,SECISBP2,RPS9,CNOT3,ZC3H12A,UNK,METTL3,DND1,EIF4EBP1,TARBP2,NAT10,EIF4B,PCIF1,DCPS,NGDN,LSM14B,TACO1,IGFBP5,MPV17L2,C1QBP,SEPSECS,NANOS3,PER1,C19orf66,AKT1,PAIP1,KHSRP,TRAP1,MKNK2,RBM8A,ZNF385A	yellow	VSMC_M3
GO:0031400	0.07	0.95	37	426	negative regulation of protein modification process	BP	1	ENPP1,CTDSP2,SQSTM1,H2AFY,PLEC,VPS28,SOCS5,SPRED2,ZC3H12A,PNKP,MICAL1,EMILIN1,TARBP2,PDE4D,SWAP70,PINX1,GREM1,CHP1,QARS,TRIM27,LIMK1,RELA,SMARCB1,CTDSP1,OTUB1,LGALS3,UBXN1,PKN1,MAD2L2,CTBP1,MVP,RPL37,AKT1,CACTIN,HMG20B,TRIB2,THAP7	yellow	VSMC_M3
GO:2001243	0.07	0.96	10	83	negative regulation of intrinsic apoptotic signaling pathway	BP	1	FIGNL1,BCL2L1,CCAR2,SELENOS,GPX1,ARHGEF2,AKT1,TRAP1,ZNF385A,TXNDC12	yellow	VSMC_M3
GO:1902749	0.08	0.96	10	87	regulation of cell cycle G2/M phase transition	BP	1	CDK4,H2AFY,PINX1,WNT10B,PBX1,BABAM1,RCC2,MIIP,ZNF830,NABP2	yellow	VSMC_M3
GO:0070373	0.08	0.97	7	53	negative regulation of ERK1 and ERK2 cascade	BP	1	KLF4,SPRED2,SLC9A3R1,EMILIN1,EIF3A,CSK,PTPRR	yellow	VSMC_M3
GO:0014912	0.08	0.98	4	24	negative regulation of smooth muscle cell migration	BP	1	PPARD,CORO1B,IGFBP5,MIR214	yellow	VSMC_M3
GO:0006401	0.08	0.95	22	237	RNA catabolic process	BP	1	HNRNPA0,SND1,SECISBP2,CNOT3,ZC3H12A,METTL3,DND1,LSM6,PCBP4,DCPS,ZCCHC17,RNASEH2C,MRTO4,NANOS3,AKT1,PAIP1,KHSRP,CARHSP1,RBM8A,RNH1,TENT5B,TRIR	yellow	VSMC_M3
GO:0006402	0.08	0.95	19	201	mRNA catabolic process	BP	1	HNRNPA0,SND1,SECISBP2,CNOT3,ZC3H12A,METTL3,DND1,LSM6,PCBP4,DCPS,MRTO4,NANOS3,AKT1,PAIP1,KHSRP,CARHSP1,RBM8A,RNH1,TENT5B	yellow	VSMC_M3
GO:0044283	0.08	0.95	38	414	small molecule biosynthetic process	BP	1	MGLL,FDXR,SEPHS1,INSIG1,PYCR1,FASN,AACS,FDFT1,SOD1,SCD,DHCR7,SELENOS,MVK,PCK2,LSS,PLA2G3,MGST3,MID1IP1,ISYNA1,HSD17B1,ACLY,FADS2,GLUD1,ILVBL,MTHFR,PGD,COQ6,PMVK,HMGCS1,PGM1,DHCR24,NSDHL,PGP,APRT,MVD,FADS1,FDPS,IDI1	yellow	VSMC_M3
GO:0010677	0.08	0.98	5	34	negative regulation of cellular carbohydrate metabolic process	BP	1	ENPP1,SELENOS,GIT1,PGP,MIDN	yellow	VSMC_M3
GO:0009166	0.08	0.97	8	61	nucleotide catabolic process	BP	1	NUDT3,DNPH1,OGG1,SMUG1,PDE4D,CNP,NT5C,ITPA	yellow	VSMC_M3
GO:0006650	0.08	0.95	24	257	glycerophospholipid metabolic process	BP	1	PIGO,SOCS5,TTC7A,PLCD1,INPPL1,PCYT2,PTDSS2,SCARB1,DGKZ,CISH,FABP3,PDGFA,PLA2G3,PHB2,PLCB3,CAPN2,PIP4K2B,DPM3,MTMR4,PGP,TAZ,SLC44A3,VAC14,PLA2G4E	yellow	VSMC_M3
GO:0048017	0.08	0.96	13	137	inositol lipid-mediated signaling	BP	1	PRR5,PPARD,PDGFRA,KLF4,SLC9A3R1,PLCD1,TYRO3,PDGFA,PLCB3,C1QBP,AKT1,MYDGF,ERBB4	yellow	VSMC_M3
GO:0014074	0.08	0.96	11	109	response to purine-containing compound	BP	1	AACS,WNT10B,SOD1,SLC26A6,RELA,IGFBP5,PER1,CARM1,EEF2K,HMGCS1,SLC6A4	yellow	VSMC_M3
GO:0034314	0.08	0.97	5	33	Arp2/3 complex-mediated actin nucleation	BP	1	CORO1B,TRIM27,ARPC1B,WASHC1,ARPC5L	yellow	VSMC_M3
GO:0006690	0.08	0.97	8	62	icosanoid metabolic process	BP	1	PTGR1,MGLL,GPX1,PLA2G3,MGST3,TNFRSF1A,CYP4F8,FADS1	yellow	VSMC_M3
GO:0061756	0.08	0.97	5	34	leukocyte adhesion to vascular endothelial cell	BP	1	KLF4,ITGB7,ST3GAL4,RELA,IRAK1	yellow	VSMC_M3
GO:0018208	0.08	0.97	7	48	peptidyl-proline modification	BP	1	P4HA3,P4HB,FKBP11,AIP,FKBP2,NTMT1,PIN4	yellow	VSMC_M3
GO:0008589	0.08	0.97	8	71	regulation of smoothened signaling pathway	BP	1	ENPP1,TXNDC15,EVC,TEDC1,RAB34,TEDC2,GAS8,NDST1	yellow	VSMC_M3
GO:0007520	0.08	0.98	4	25	myoblast fusion	BP	1	EHD2,PLEKHO1,CD9,CAPN2	yellow	VSMC_M3
GO:0042440	0.08	0.96	8	65	pigment metabolic process	BP	1	BLVRA,BLVRB,SLC11A2,SRRD,PPOX,SLC25A39,CTNS,APRT	yellow	VSMC_M3
GO:0046320	0.08	0.98	4	26	regulation of fatty acid oxidation	BP	1	PPARD,ETFBKMT,FABP3,AKT1	yellow	VSMC_M3
GO:0031057	0.08	0.97	5	37	negative regulation of histone modification	BP	1	H2AFY,SMARCB1,OTUB1,CTBP1,THAP7	yellow	VSMC_M3
GO:0051495	0.08	0.95	18	194	positive regulation of cytoskeleton organization	BP	1	SNX9,BAIAP2,NUMA1,SWAP70,DCTN1,CX3CL1,CORO1B,TRIM27,ARPIN,LIMK1,PFDN2,RHOC,ARPC1B,WASHC1,CORO1A,GIT1,CDC42EP5,ARPC5L	yellow	VSMC_M3
GO:0014065	0.08	0.96	11	111	phosphatidylinositol 3-kinase signaling	BP	1	PRR5,PPARD,PDGFRA,KLF4,SLC9A3R1,TYRO3,PDGFA,C1QBP,AKT1,MYDGF,ERBB4	yellow	VSMC_M3
GO:0007009	0.08	0.96	10	94	plasma membrane organization	BP	1	SNX9,PACSIN1,PLEC,EHD2,SLC9A3R1,BAIAP2,SOD1,PACSIN3,CD9,AKT1	yellow	VSMC_M3
GO:0032024	0.08	0.97	7	56	positive regulation of insulin secretion	BP	1	PPARD,MYRIP,AACS,PCK2,CAPN10,BAD,GLUD1	yellow	VSMC_M3
GO:0045454	0.08	0.97	5	32	cell redox homeostasis	BP	1	GSR,SELENOS,GPX1,TXNRD2,GIT1	yellow	VSMC_M3
GO:0032729	0.08	0.97	5	35	positive regulation of interferon-gamma production	BP	1	SCRIB,IL1R1,PDE4D,TRIM27,CD276	yellow	VSMC_M3
GO:0000470	0.08	0.98	4	23	maturation of LSU-rRNA	BP	1	NHP2,BOP1,FTSJ3,RPL7A	yellow	VSMC_M3
GO:0051235	0.08	0.95	22	248	maintenance of location	BP	1	ENPP1,SREBF2,SQLE,ITPR3,PPARD,ZC3H12A,SLC30A3,INSIG1,SCARB1,PDE4D,CX3CL1,GSTO1,CHCHD10,LETM1,CHERP,CCDC22,DBN1,CORO1A,AKT1,ALKBH7,AP3D1,TWF2	yellow	VSMC_M3
GO:0110053	0.09	0.95	21	237	regulation of actin filament organization	BP	1	SNX9,DBNL,BAIAP2,SWAP70,CAPG,CX3CL1,CORO1B,TRIM27,ARPIN,LIMK1,RHOC,ARPC1B,WASHC1,DBN1,CORO1A,MIR214,TWF2,ELN,CDC42EP5,GBA2,ARPC5L	yellow	VSMC_M3
GO:0048813	0.09	0.95	12	126	dendrite morphogenesis	BP	1	CUL7,DBNL,TPBG,BAIAP2,ARHGAP33,SLC11A2,NGEF,DBN1,EEF2K,ANAPC2,STK11,NSMF	yellow	VSMC_M3
GO:0090501	0.09	0.95	13	124	RNA phosphodiester bond hydrolysis	BP	1	NHP2,BOP1,SND1,ZC3H12A,RRS1,DCPS,ZNRD1,KHNYN,RPS21,NOP14,CSTF2T,TBL3,TRIR	yellow	VSMC_M3

GO:0043967	0.09	0.96	7	58	histone H4 acetylation	BP	1	JADE2,MEAF6,DMAP1,MCRS1,SMARCB1,CTBP1,PER1	yellow	VSMC_M3
GO:0060113	0.09	0.97	6	48	inner ear receptor cell differentiation	BP	1	CTHRC1,SCRIB,IFT27,WDPCP,SLC9A3R1,SOD1	yellow	VSMC_M3
GO:0031122	0.09	0.97	6	52	cytoplasmic microtubule organization	BP	1	NUMA1,DCTN1,CHP1,CLIP2,STMN3,TUBGCP2	yellow	VSMC_M3
GO:0036503	0.09	0.96	11	99	ERAD pathway	BP	1	BAG6,RCN3,TMUB1,SEL1L2,HM13,SELENOS,UBXN1,TRIM25,MAN1B1,FBXO6,STUB1	yellow	VSMC_M3
GO:0099175	0.09	0.96	8	73	regulation of postsynapse organization	BP	1	DBNL,BAIAP2,ARHGAP22,ARHGAP33,NGEF,LRFN4,DBN1,EEF2K	yellow	VSMC_M3
GO:1902806	0.09	0.95	12	115	regulation of cell cycle G1/S phase transition	BP	1	CTDSP2,KLF4,KMT2E,CDK2AP2,PSME2,PRMT2,PHB2,CTDSP1,METTL13,SDE2,AKT1,MIR214	yellow	VSMC_M3
GO:0044458	0.09	0.97	5	33	motile cilium assembly	BP	1	CEP131,YIF1B,IQCG,PLA2G3,MEIG1	yellow	VSMC_M3
GO:1901798	0.09	0.97	4	24	positive regulation of signal transduction by p53 class mediator	BP	1	SPRED2,RPL37,HIC1,ZNF385A	yellow	VSMC_M3
GO:0031929	0.09	0.95	11	105	TOR signaling	BP	1	FNIP1,PRR5,SEC13,LAMTOR1,EIF4EBP1,LAMTOR4,AKT1,CTNS,STK11,TELO2,WDR24	yellow	VSMC_M3
GO:0070482	0.09	0.94	25	280	response to oxygen levels	BP	1	CDK4,PPARD,UCP2,P4HB,EIF4EBP1,SLC11A2,CHCHD2,MMP14,ARNT2,LIMD1,PHB2,BAD,PLAU,CAPN2,MTHFR,EEF2K,AKT1,SLC6A4,VASN,IRAK1,TGFBR3,MIR214,RF00024,STUB1,CCDC115	yellow	VSMC_M3
GO:0071219	0.09	0.95	14	133	cellular response to molecule of bacterial origin	BP	1	CDK4,PPARD,ZC3H12A,SCARB1,CX3CL1,SELENOS,PABPN1,RELA,CAPN2,AKT1,GIT1,LDOC1,CACTIN,IRAK1	yellow	VSMC_M3
GO:0000375	0.09	0.94	25	259	RNA splicing, via transesterification reactions	BP	1	DHX16,SNRNP70,GEMIN7,METTL3,RALY,LSM6,PRPF40B,PCBP4,RBM15B,SRSF9,DCPS,RBM6,SF3A1,SART1,PRPF6,BUD31,C1QBP,GPKOW,CWC25,CD2BP2,RAVER1,RBM22,KHSRP,CACTIN,RBM8A	yellow	VSMC_M3
GO:0035082	0.09	0.96	7	55	axoneme assembly	BP	1	SPAG1,CEP131,YIF1B,IQCG,PLA2G3,GAS8,MEIG1	yellow	VSMC_M3
GO:0032506	0.09	0.97	5	35	cytokinetic process	BP	1	SNX9,PLEC,ALKBH4,ANXA11,SNX18	yellow	VSMC_M3
GO:0022604	0.09	0.94	22	261	regulation of cell morphogenesis	BP	1	CUL7,DBNL,WDPCP,SLC9A3R1,PLXND1,BAIAP2,P4HB,CORO1B,PLEKHO1,ARPIN,RHOC,FMNL1,LIMD1,C1QBP,RCC2,DBN1,CORO1A,SYNE3,EEF2K,ANAPC2,UNC13A,CDC42EP5	yellow	VSMC_M3
GO:0009954	0.09	0.97	4	25	proximal/distal pattern formation	BP	1	PBX2,GREM1,IRX1,PBX1	yellow	VSMC_M3
GO:0036294	0.09	0.95	13	129	cellular response to decreased oxygen levels	BP	1	PPARD,P4HB,EIF4EBP1,CHCHD2,PHB2,BAD,EEF2K,AKT1,VASN,IRAK1,MIR214,RF00024,STUB1	yellow	VSMC_M3
GO:0030216	0.09	0.96	8	74	keratinocyte differentiation	BP	1	H2AFY,PLEC,SHARPIN,NUMA1,KRT10,CASP14,TMEM79,PPL	yellow	VSMC_M3
GO:0016050	0.09	0.94	23	255	vesicle organization	BP	1	SQSTM1,VPS28,SNX3,INSIG1,SEC13,LAMTOR1,RAB20,PLEKHF1,HPS1,CHP1,PLA2G3,BLOC1S4,MVB12A,AP1M1,PIP4K2B,SNF8,STX10,CORO1A,RFX2,RAB34,AP3D1,UNC13A,PHETA2	yellow	VSMC_M3
GO:0000462	0.09	0.97	5	34	maturation of SSU-rRNA from tricistronic rRNA transcript (SSU-rRNA, 5.8S rRNA, LSU-rRNA)	BP	1	RRS1,NGDN,RPS21,NOP14,TBL3	yellow	VSMC_M3
GO:1903900	0.10	0.95	11	104	regulation of viral life cycle	BP	1	TRIM11,LGALS1,SNX3,P4HB,TARBP2,TYRO3,TRIM27,BANF1,TRIM25,C19orf66,MID2	yellow	VSMC_M3
GO:0032088	0.10	0.96	8	67	negative regulation of NF-kappaB transcription factor activity	BP	1	KLF4,ZC3H12A,CHP1,PRMT2,BRMS1,CCDC22,CACTIN,IRAK1	yellow	VSMC_M3
GO:0000460	0.10	0.97	5	34	maturation of 5.8S rRNA	BP	1	BOP1,RRS1,RPS21,FTSJ3,NOP14	yellow	VSMC_M3
GO:0009968	0.10	0.92	76	973	negative regulation of signal transduction	BP	1	ENPP1,CTHRC1,FNIP1,ATF6B,TTLL12,SOCS5,KLF4,TPBG,SPRED2,RGS3,ZC3H12A,FIGNL1,SLC9A3R1,EMILIN1,INSIG1,METTL3,CAVIN3,BCL2L1,PDE4D,TNS2,GREM1,PDE3B,CX3CL1,DGKZ,CCAR2,DLL4,CHP1,TYRO3,CISH,SELENOS,ITPRIP,MMP14,GPX1,QARS,EIF3A,THOC5,STMN3,RELA,LIMD1,PHB2,IGFBP5,IFI35,LGALS3,C1QBP,CCDC22,PALM3,AMER1,PIP4K2B,MAD2L2,NANOS3,ARHGEF2,MVP,PER1,MTMR4,SMARCA4,AATF,CSK,AKT1,CACTIN,VASN,HIC1,TRAP1,MNT,STK11,SLC27A4,TGFBR3,PTPRR,IGFBP6,ZNF385A,TXNDC12,CDC34,APCDD1L,RGS16,DUSP5,STUB1,RGS18	yellow	VSMC_M3
GO:0050686	0.10	0.97	4	25	negative regulation of mRNA processing	BP	1	NELFE,PCBP4,SRSF9,C1QBP	yellow	VSMC_M3
GO:0070199	0.10	0.97	4	24	establishment of protein localization to chromosome	BP	1	H2AFY,LRWD1,SGF29,NABP2	yellow	VSMC_M3
GO:0001666	0.10	0.94	22	244	response to hypoxia	BP	1	PPARD,UCP2,P4HB,EIF4EBP1,SLC11A2,CHCHD2,MMP14,ARNT2,LIMD1,PHB2,BAD,PLAU,CAPN2,MTHFR,AKT1,SLC6A4,VASN,IRAK1,TGFBR3,MIR214,RF00024,STUB1	yellow	VSMC_M3
GO:0048701	0.10	0.97	5	40	embryonic cranial skeleton morphogenesis	BP	1	PDGFRA,HOXA1,MMP14,SLC39A1,NDST1	yellow	VSMC_M3
GO:0045017	0.10	0.94	20	214	glycerolipid biosynthetic process	BP	1	PIGO,SOCS5,TTC7A,INPPL1,PCYT2,PTDSS2,SCARB1,ACSL1,DGKZ,CISH,FABP3,PDGFA,PCK2,CAPN2,PIP4K2B,DPM3,MTMR4,TAZ,SLC44A3,VAC14	yellow	VSMC_M3
GO:1903649	0.10	0.97	4	28	regulation of cytoplasmic transport	BP	1	EIPR1,SNX3,PTPN23,MAP2K2	yellow	VSMC_M3
GO:0036293	0.10	0.94	23	258	response to decreased oxygen levels	BP	1	PPARD,UCP2,P4HB,EIF4EBP1,SLC11A2,CHCHD2,MMP14,ARNT2,LIMD1,PHB2,BAD,PLAU,CAPN2,MTHFR,EEF2K,AKT1,SLC6A4,VASN,IRAK1,TGFBR3,MIR214,RF00024,STUB1	yellow	VSMC_M3
GO:0071453	0.10	0.94	14	142	cellular response to oxygen levels	BP	1	PPARD,P4HB,EIF4EBP1,CHCHD2,PHB2,BAD,EEF2K,AKT1,VASN,IRAK1,MIR214,RF00024,STUB1,CCDC115	yellow	VSMC_M3
GO:0043467	0.10	0.95	11	104	regulation of generation of precursor metabolites and energy	BP	1	ENPP1,CAVIN3,CHCHD2,SELENOS,ETFBKMT,TACO1,PHB2,AKT1,GIT1,TRAP1,JMJD8	yellow	VSMC_M3
GO:0009144	0.10	0.95	8	63	purine nucleoside triphosphate metabolic process	BP	1	GUK1,IMPDH2,ATP5MC3,UQCC3,ATP5PD,ATP5F1D,TAZ,ITPA	yellow	VSMC_M3

GO:0023051	0.10	0.91	182	2524	regulation of signaling	BP	1	ENPP1,CD63,CDK4,SQSTM1,CTHRC1,NELFE,FNIP1,ATF6B,PRR5,TTLL12,SREBF2,TXNDC15,ITPR3,SGSM3,DEF6,PPARD,EEF1D,SCRIB,LGALS1,SHARPIN,BOP1,PDGFRA,IL1R1,SOCS5,KLF4,TPBG,SPRED2,EIPR1,RGS3,ZC3H12A,FIGNL1,RCN3,SNX3,PLEKHA4,MGLL,SLC9A3R1,EMILIN1,RNF220,INSIG1,MYRIP,SEC13,ARHGEF40,UCP2,METTL3,BAIAP2,CXXC5,LAMTOR1,NUMA1,P4HB,CAVIN3,ARHGAP22,AACS,BCL2L1,PDE4D,TNS2,RRS1,SWAP70,PLEKHF1,GREM1,PDE3B,RAPSN,CX3CL1,WNT10B,SOD1,DGKZ,CCAR2,DLL4,CHP1,TYRO3,SLC35C2,TSPAN5,CISH,SELENOS,ITPRIP,PDGFA,MMP14,GPX1,AIP,NGEF,SYT12,QARS,PCK2,TRIM27,EIF3A,PRMT2,THOC5,RIPK3,CAPN10,PLA2G3,STMN3,PTPN23,RELA,SIPA1,RHOC,MAP3K14,LIMD1,ZDHHC3,SMARCB1,YJEFN3,PHB2,IGFBP5,BAD,LAMTOR4,IFI35,LGALS3,BUD31,PLAU,TNFRSF1A,MVB12A,C1QBP,CHERP,EVC,CCDC22,GLUD1,PKN1,DBN1,PALM3,AMER1,PIP4K2B,MAD2L2,NANOS3,ARHGEF2,MVP,PER1,TRIM25,RPL37,MTMR4,S100A4,SMARCA4,CARM1,CDC37,AATF,CSK,MID2,AKT1,TEDC1,RAB34,SAFB2,GIT1,SLC6A4,MYDGF,MAP2K2,CACTIN,VASN,HIC1,TRAP1,NCLN,MNT,CTNS,TEDC2,CSNK1G2,IRAK1,ANAPC2,MIDN,STK11,TELO2,WDR24,JMJD8,CARD9,GAS8,SLC27A4,TGFBR3,TRADD,NDST1,TNFRSF12A,ERBB4,PTPRR,IGFBP6,JAGN1,UNC13A,ZNF385A,TXNDC12,NSMF,CDC34,APCDD1L,ARHGEF1,RGS16,DUSP5,GLIPR2,STUB1,RGS18,GSG1L	yellow	VSMC_M3
GO:0010971	0.10	0.97	4	25	positive regulation of G2/M transition of mitotic cell cycle	BP	1	CDK4,WNT10B,PBX1,RCC2	yellow	VSMC_M3
GO:0061013	0.10	0.94	14	144	regulation of mRNA catabolic process	BP	1	HNRNPA0,SECISBP2,CNOT3,ZC3H12A,METTL3,DND1,PCBP4,DCPS,NANOS3,AKT1,PAIP1,KHSRP,CARHSP1,TENT5B	yellow	VSMC_M3
GO:0002237	0.10	0.93	22	236	response to molecule of bacterial origin	BP	1	CDK4,HNRNPA0,PPARD,SSC5D,ZC3H12A,SCARB1,CX3CL1,SELENOS,PABPN1,PCK2,RELA,CNP,CAPN2,PALM3,AKT1,GIT1,LDOC1,CACTIN,IRAK1,CARD9,JUND,LOXL1	yellow	VSMC_M3
GO:0008150	0.10	0.92	854	12183	biological_process	BP	1	ENPP1,PQLC1,CD63,ESYT1,NAB2,DCTN2,NHP2,CDK4,CTDSP2,RUFY1,SQSTM1,GNL1,DHX16,CCHCR1,SPAG1,ZNF608,LMF2,BAG6,NCAPH2,SBF1,SNX9,CTHRC1,HDAC10,NELFE,ACAT2,CERK,FNIP1,ATF6B,PBX2,PDLIM4,PRR5,SAMM50,TTLL12,RING1,SREBF2,JADE2,TRMT12,NDUFB9,SQLE,TXNDC15,H2AFY,ITPR3,HNRNPA0,TRIM11,SGSM3,GUK1,TRAPPC9,NUDT3,PACSIN1,DEF6,PPARD,FANCE,EEF1D,TIGD5,TSTA3,RPL3,SCRIB,PLEC,LGALS1,MPST,IFT27,SHARPIN,HGH1,BOP1,SLC52A2,VPS28,RPL8,SND1,TOM1,TBCC,CUL7,RTCB,PPP1R14B,DNPH1,B4GALT1,MRPS18A,AARS2,PIGO,PDGFRA,IL1R1,SECISBP2,PHF2,GRHPR,COL15A1,EPN1,COL19A1,SSC5D,HSPBP1,SOCS5,TTC7A,RPS9,CNOT3,ASNA1,KLF4,DBNL,TPBG,HOXA1,JOSD2,PTGR1,PSMB3,PLEKHA8,WDPCP,CCM2,FHL3,SPRED2,EIPR1,RGS3,MEAF6,ZC3H12A,FIGNL1,PDIA4,BLVRA,PDIA6,PNKP,CPT1C,ZYX,RCN3,SNRNP70,CC2D1B,SNX3,KTI12,MICAL1,PLEKHA4,EMP3,MGLL,EHD2,FAM120B,KMT2E,ANXA5,KRT8,WDFY2,ACAD9,SIX5,ZNF282,TMEM43,C17orf80,TTYH2,GEMIN7,ZNF296,RELB,SLC9A3R1,SERP2,NAT9,MUTYH,PLXND1,FDXR,EMILIN1,SLC16A5,SEPHS1,TMUB1,RNF220,DMAP1,SLC30A3,PAFAH1B3,CIC,GTF3C2,SNX17,GALK1,H3F3B,PPM1G,UNK,PLCD1,DLEC1,CD79A,BCKDHA,INSIG1,MYRIP,SEC13,MFSD11,SEPT9,GALNT14,BLVRB,OGG1,SERTAD1,TTC5,PSMC4,KAT14,ARHGEF40,P4HA3,UCP2,SEL1L2,METTL3,PHLDA1,BAIAP2,CEP131,CXXC5,INPPL1,FAAP100,TSPAN10,LAMTOR1,NUMA1,P4HB,PCYT2,DND1,PYCR1,YIF1B,FASN,CCDC57,RAB20,SMOX,RPL29,PXMP2,EIF4EBP1,ARAP3,SIGLEC1,DELE1,GNPDA1,MORN2,CAVIN3,PUS1,RIC8A,GSR,PTDSS2,ARHGAP22,DEAF1,TBCB,SMUG1,HOXC4,IDH3B,L [illegible]	yellow	VSMC_M3
GO:0040029	0.10	0.95	9	86	regulation of gene expression, epigenetic	BP	1	H2AFY,PHF2,SERTAD1,METTL3,KDM1B,RBM15B,GPX1,TRIM27,MBD3	yellow	VSMC_M3
GO:0017004	0.10	0.96	5	31	cytochrome complex assembly	BP	1	UQCRFS1,HCCS,TACO1,UQCC3,SURF1	yellow	VSMC_M3
GO:0046390	0.10	0.94	16	156	ribose phosphate biosynthetic process	BP	1	GUK1,ACSL1,TKT,AK2,PAPSS1,IMPDH2,ATP5MC3,ATIC,UQCC3,ACLY,ATP5PD,ATP5F1D,TAZ,AMPD2,APRT,AK1	yellow	VSMC_M3
GO:0006783	0.10	0.97	4	26	heme biosynthetic process	BP	1	SLC11A2,SRRD,PPOX,SLC25A39	yellow	VSMC_M3
GO:1901990	0.10	0.93	22	237	regulation of mitotic cell cycle phase transition	BP	1	CDK4,CTDSP2,KLF4,KMT2E,PINX1,WNT10B,CDK2AP2,PSME2,PRMT2,PBX1,PHB2,CTDSP1,BABAM1,RCC2,SDE2,MIIP,MAD1L1,MAD2L2,ZNF830,AKT1,MIR214,NABP2	yellow	VSMC_M3
GO:0007032	0.11	0.95	8	71	endosome organization	BP	1	SQSTM1,VPS28,SNX3,LAMTOR1,PLEKHF1,MVB12A,SNF8,PHETA2	yellow	VSMC_M3
GO:0031503	0.11	0.94	17	186	protein-containing complex localization	BP	1	SCRIB,IFT27,CEP131,RRS1,RBM15B,EXOC3,PABPN1,THOC5,ZDHHC3,THOC3,NUP88,RCC2,DBN1,AP3D1,SSNA1,WDR34,GSG1L	yellow	VSMC_M3
GO:0050684	0.11	0.94	12	118	regulation of mRNA processing	BP	1	NELFE,SNRNP70,PCBP4,RBM15B,SRSF9,C1QBP,SLTM,RAVER1,SAFB,SAFB2,CDK11B,RBM8A	yellow	VSMC_M3
GO:0050777	0.11	0.94	13	128	negative regulation of immune response	BP	1	TTLL12,SOCS5,ZC3H12A,METTL3,TARBP2,TYRO3,SELENOS,GPX1,TRIM27,PSMB4,LGALS3,C1QBP,CACTIN	yellow	VSMC_M3
GO:0051303	0.11	0.95	8	68	establishment of chromosome localization	BP	1	DCTN2,NUMA1,RRS1,PINX1,NUDC,EML3,MAD1L1,MIS12	yellow	VSMC_M3
GO:0042491	0.11	0.97	4	29	inner ear auditory receptor cell differentiation	BP	1	SCRIB,WDPCP,SLC9A3R1,SOD1	yellow	VSMC_M3
GO:1905517	0.11	0.96	5	36	macrophage migration	BP	1	EMILIN1,CX3CL1,MMP14,LGALS3,CD9	yellow	VSMC_M3
GO:1902807	0.11	0.95	7	60	negative regulation of cell cycle G1/S phase transition	BP	1	CTDSP2,KLF4,CDK2AP2,PRMT2,CTDSP1,METTL13,SDE2	yellow	VSMC_M3
GO:0060997	0.11	0.96	6	54	dendritic spine morphogenesis	BP	1	DBNL,BAIAP2,ARHGAP33,NGEF,DBN1,EEF2K	yellow	VSMC_M3

GO:0010646	0.11	0.91	181	2513	regulation of cell communication	BP	1	ENPP1,CD63,CDK4,SQSTM1,CTHRC1,NELFE,FNIP1,ATF6B,PRR5,TTLL12,SREBF2,TXNDC15,ITPR3,SGSM3,DEF6,PPARD,EEF1D,LGALS1,SHARPIN,BOP1,PDGFRA,IL1R1,SOCS5,KLF4,TPBG,SPRED2,EIPR1,RGS3,ZC3H12A,FIGNL1,RCN3,SNX3,PLEKHA4,MGLL,SLC9A3R1,EMILIN1,RNF220,INSIG1,MYRIP,SEC13,ARHGEF40,UCP2,METTL3,BAIAP2,CXXC5,LAMTOR1,NUMA1,P4HB,CAVIN3,ARHGAP22,AACS,BCL2L1,PDE4D,TNS2,RRS1,SWAP70,PLEKHF1,GREM1,PDE3B,RAPSN,CX3CL1,WNT10B,SOD1,DGKZ,CCAR2,DLL4,CHP1,TYRO3,SLC35C2,TSPAN5,CISH,SELENOS,ITPRIP,PDGFA,MMP14,GPX1,AIP,NGEF,SYT12,QARS,PCK2,TRIM27,EIF3A,PRMT2,THOC5,RIPK3,CAPN10,PLA2G3,STMN3,PTPN23,RELA,SIPA1,RHOC,MAP3K14,LIMD1,ZDHHC3,SMARCB1,YJEFN3,PHB2,IGFBP5,BAD,LAMTOR4,IFI35,LGALS3,BUD31,PLAU,TNFRSF1A,MVB12A,C1QBP,CHERP,EVC,CCDC22,GLUD1,PKN1,DBN1,PALM3,AMER1,PIP4K2B,MAD2L2,NANOS3,ARHGEF2,MVP,PER1,TRIM25,RPL37,MTMR4,S100A4,SMARCA4,CARM1,CDC37,AATF,CSK,MID2,AKT1,TEDC1,RAB34,SAFB2,GIT1,SLC6A4,MYDGF,MAP2K2,CACTIN,VASN,HIC1,TRAP1,NCLN,MNT,CTNS,TEDC2,CSNK1G2,IRAK1,ANAPC2,MIDN,STK11,TELO2,WDR24,JMJD8,CARD9,GAS8,SLC27A4,TGFBR3,TRADD,NDST1,TNFRSF12A,ERBB4,PTPRR,IGFBP6,JAGN1,UNC13A,ZNF385A,TXNDC12,NSMF,CDC34,APCDD1L,ARHGEF1,RGS16,DUSP5,GLIPR2,STUB1,RGS18,GSG1L	yellow	VSMC_M3
GO:0046626	0.11	0.96	6	50	regulation of insulin receptor signaling pathway	BP	1	ENPP1,CDK4,TNS2,RELA,PIP4K2B,SLC27A4	yellow	VSMC_M3
GO:0032543	0.11	0.95	9	72	mitochondrial translation	BP	1	MRPS18A,AARS2,GATB,MRPL43,TACO1,MPV17L2,C1QBP,TUFM,MRPS16	yellow	VSMC_M3
GO:0071456	0.11	0.94	12	119	cellular response to hypoxia	BP	1	PPARD,P4HB,EIF4EBP1,CHCHD2,PHB2,BAD,AKT1,VASN,IRAK1,MIR214,RF00024,STUB1	yellow	VSMC_M3
GO:0051092	0.11	0.94	12	119	positive regulation of NF-kappaB transcription factor activity	BP	1	TRAPPC9,GREM1,CX3CL1,TRIM27,RIPK3,RELA,ARHGEF2,TRIM25,MID2,IRAK1,CARD9,TRADD	yellow	VSMC_M3
GO:0045682	0.11	0.96	6	50	regulation of epidermis development	BP	1	NAB2,H2AFY,PPARD,NUMA1,KRT10,TMEM79	yellow	VSMC_M3
GO:0010389	0.11	0.95	9	82	regulation of G2/M transition of mitotic cell cycle	BP	1	CDK4,PINX1,WNT10B,PBX1,BABAM1,RCC2,MIIP,ZNF830,NABP2	yellow	VSMC_M3
GO:0098927	0.11	0.96	5	40	vesicle-mediated transport between endosomal compartments	BP	1	SNX3,PTPN23,SNF8,CORO1A,MAP2K2	yellow	VSMC_M3
GO:0009141	0.11	0.94	10	86	nucleoside triphosphate metabolic process	BP	1	ENPP1,GUK1,IMPDH2,ATP5MC3,UQCC3,ATP5PD,ATP5F1D,TAZ,AK1,ITPA	yellow	VSMC_M3
GO:0050931	0.11	0.96	4	28	pigment cell differentiation	BP	1	ENPP1,CD63,AP1M1,AP3D1	yellow	VSMC_M3
GO:0043491	0.11	0.93	14	147	protein kinase B signaling	BP	1	KLF4,TPBG,RCN3,SLC9A3R1,CAVIN3,CX3CL1,TYRO3,PDGFA,GPX1,IGFBP5,C1QBP,AKT1,MYDGF,STK11	yellow	VSMC_M3
GO:0007346	0.11	0.92	31	360	regulation of mitotic cell cycle	BP	1	CDK4,CTDSP2,TTLL12,SCRIB,CUL7,KLF4,KMT2E,SLC9A3R1,CCDC57,EIF4EBP1,PINX1,WNT10B,CDK2AP2,PSME2,PRMT2,PBX1,PHB2,CTDSP1,BABAM1,RCC2,SDE2,MIIP,MAD1L1,MAD2L2,AATF,ZNF830,AKT1,CDK11B,AURKAIP1,MIR214,NABP2	yellow	VSMC_M3
GO:0010833	0.11	0.95	8	73	telomere maintenance via telomere lengthening	BP	1	NHP2,PNKP,NAT10,TNKS1BP1,PINX1,SLX1A,TELO2,RF00024	yellow	VSMC_M3
GO:0010243	0.12	0.91	62	793	response to organonitrogen compound	BP	1	ENPP1,CDK4,BAG6,SREBF2,PDGFRA,SOCS5,KLF4,RCN3,SLC9A3R1,TMUB1,INSIG1,OGG1,UCP2,SEL1L2,BAIAP2,INPPL1,LAMTOR1,AACS,HM13,BCL2L1,TNS2,PDE3B,WNT10B,SOD1,SRSF9,SELENOS,FABP3,PCK2,COL6A1,SLC26A6,UQCRC1,CAPN10,RELA,MGST3,IGFBP5,BAD,LAMTOR4,CD9,UBXN1,KLF3,CAPN2,MTHFR,PIP4K2B,ARHGEF2,PER1,TRIM25,CARM1,EEF2K,CSK,AKT1,HMGCS1,SLC6A4,LDOC1,MAN1B1,CARD9,APRT,SLC27A4,TGFBR3,JUND,NSMF,FBXO6,STUB1	yellow	VSMC_M3
GO:0045912	0.12	0.96	5	39	negative regulation of carbohydrate metabolic process	BP	1	ENPP1,SELENOS,GIT1,PGP,MIDN	yellow	VSMC_M3
GO:0006360	0.12	0.95	6	50	transcription by RNA polymerase I	BP	1	H2AFY,ZNRD1,SMARCB1,UBTF,SMARCA4,CAVIN1	yellow	VSMC_M3
GO:0042490	0.12	0.95	6	52	mechanoreceptor differentiation	BP	1	CTHRC1,SCRIB,IFT27,WDPCP,SLC9A3R1,SOD1	yellow	VSMC_M3
GO:0001578	0.12	0.94	9	86	microtubule bundle formation	BP	1	SPAG1,CEP131,NUMA1,YIF1B,CHP1,IQCG,PLA2G3,GAS8,MEIG1	yellow	VSMC_M3
GO:1904888	0.12	0.95	6	54	cranial skeletal system development	BP	1	PDGFRA,HOXA1,INSIG1,MMP14,SLC39A1,NDST1	yellow	VSMC_M3
GO:0006352	0.12	0.94	11	105	DNA-templated transcription, initiation	BP	1	CDK4,WNT10B,SMARCB1,UBTF,TAF6L,SMARCA4,BRF1,GTF2F1,MED16,GTF3C5,CAVIN1	yellow	VSMC_M3
GO:0050792	0.12	0.94	12	119	regulation of viral process	BP	1	TRIM11,LGALS1,SNX3,P4HB,TARBP2,TYRO3,TRIM27,BANF1,SNF8,TRIM25,C19orf66,MID2	yellow	VSMC_M3
GO:0034340	0.12	0.95	6	47	response to type I interferon	BP	1	TTLL12,METTL3,CDC37,C19orf66,CACTIN,IRAK1	yellow	VSMC_M3
GO:0033143	0.12	0.95	7	63	regulation of intracellular steroid hormone receptor signaling pathway	BP	1	PRMT2,PHB2,PKN1,PER1,SMARCA4,CARM1,SAFB2	yellow	VSMC_M3

GO:0034645	0.12	0.90	258	3533	cellular macromolecule biosynthetic process	BP	1	ENPP1,NAB2,NHP2,CDK4,SQSTM1,ZNF608,HDAC10,NELFE,FNIP1,ATF6B,PBX2,RING1,SREBF2,H2AFY,TRIM11,TRAPPC9,PPARD,EEF1D,RPL3,RPL8,SND1,B4GALT1,MRPS18A,AARS2,PIGO,SECISBP2,PHF2,RPS9,CNOT3,KLF4,HOXA1,FHL3,ZC3H12A,PNKP,CC2D1B,KTI12,KMT2E,SIX5,ZNF282,ZNF296,RELB,SERP2,DMAP1,CIC,GTF3C2,UNK,GALNT14,OGG1,SERTAD1,TTC5,METTL3,PHLDA1,CXXC5,DND1,RPL29,EIF4EBP1,DEAF1,HOXC4,POLR1D,TARBP2,KMT2B,NAT10,ZNF740,EIF4B,RALY,ZBTB38,TNKS1BP1,PINX1,GATB,GREM1,KDM2B,MCRS1,POLR2C,CX3CL1,WNT10B,ACTR5,ANKRD2,EGR3,CCAR2,DNAJC17,DLL4,RPLP2,CHP1,MRPL43,YAF2,HTATIP2,PCIF1,NPM3,KDM1B,CHCHD2,SLC35C2,CARS,ST3GAL4,DCPS,IRX1,SELENOS,RRP1B,TMTC1,BRD9,APEH,AIP,NGDN,TSHZ2,QARS,PIR,TRIM27,EIF3A,ZNRD1,PRMT2,THOC5,RIPK3,TWIST2,LSM14B,ADRM1,PSTK,RPS21,SMARCD2,EXTL1,BRMS1,TACO1,GMEB2,CCDC85B,PRPF6,PBX1,KAT5,RELA,ZNF511,LRWD1,ARNT2,MRPL49,ZFPL1,LIMD1,ZDHHC3,SMARCB1,CHCHD10,PBX4,UBTF,GATAD2A,MEF2B,PHB2,NDP,IGFBP5,CTDSP1,MLF2,EID1,SSBP4,METTL13,MPV17L2,ZNF394,C11orf95,BUD31,TNFRSF1A,TEAD4,C1QBP,AIMP2,TAF6L,ZBTB49,MTA2,ZNF316,MED11,PELP1,CCDC22,FOXK1,SDE2,KLF3,PKN1,SEPSECS,POLR2A,MAD2L2,SLTM,NANOS3,MPDU1,CTBP1,FBXL19,CRABP2,SP2,ARHGEF2,SNF8,PER1,NFIX,DPM3,FARSA,IRF2BPL,TUFM,TRIM25,USP43,RPL37,SMARCA4,CARM1,RPL7A,EEF2K,AATF,MRPL4,EIF3G,C19orf66,MID2,ZNF521,ZNF358,ZNF830,AKT1,BRF1,PAIP1,KHSRP,GTF2F1,RFX2,SAFB,SAFB2,MYDGF,HDGFL2,SIRT6,MAP2K2,CACTIN,CDK11B,SSU72,HIC1,MRPL20,TRAP1,MNT,EHMT1,AP3D1,MKNK[illegible]	yellow	VSMC_M3
GO:0031110	0.12	0.94	8	79	regulation of microtubule polymerization or depolymerization	BP	1	NUMA1,DCTN1,STMN3,MID1IP1,ARHGEF2,RANGRF,GIT1,GBA2	yellow	VSMC_M3
GO:0000377	0.12	0.92	24	255	RNA splicing, via transesterification reactions with bulged adenosine as nucleophile	BP	1	DHX16,SNRNP70,GEMIN7,METTL3,RALY,LSM6,PRPF40B,PCBP4,RBM15B,SRSF9,DCPS,RBM6,SF3A1,SART1,PRPF6,BUD31,C1QBP,GPKOW,CWC25,CD2BP2,RAVER1,RBM22,CACTIN,RBM8A	yellow	VSMC_M3
GO:0000398	0.12	0.92	24	255	mRNA splicing, via spliceosome	BP	1	DHX16,SNRNP70,GEMIN7,METTL3,RALY,LSM6,PRPF40B,PCBP4,RBM15B,SRSF9,DCPS,RBM6,SF3A1,SART1,PRPF6,BUD31,C1QBP,GPKOW,CWC25,CD2BP2,RAVER1,RBM22,CACTIN,RBM8A	yellow	VSMC_M3
GO:0048732	0.12	0.92	28	335	gland development	BP	1	SCRIB,MPST,PDGFRA,PHF2,SLC9A3R1,PLXND1,UCP2,FASN,DEAF1,AACS,SOD1,PDGFA,GPX1,PCK2,RIPK3,PBX1,RELA,PHB2,IGFBP5,MAD1L1,ARMC5,MTX1,AKT1,HMGCS1,STK11,APRT,TGFBR3,ERBB4	yellow	VSMC_M3
GO:0042471	0.12	0.94	9	89	ear morphogenesis	BP	1	CTHRC1,SCRIB,HOXA1,WDPCP,SLC9A3R1,INSIG1,LRIG1,SOD1,TTC39C	yellow	VSMC_M3
GO:2000045	0.12	0.94	10	97	regulation of G1/S transition of mitotic cell cycle	BP	1	CTDSP2,KLF4,KMT2E,CDK2AP2,PSME2,PRMT2,CTDSP1,SDE2,AKT1,MIR214	yellow	VSMC_M3
GO:0031334	0.12	0.92	18	199	positive regulation of protein-containing complex assembly	BP	1	SNX9,FNIP1,BAIAP2,NUMA1,DCTN1,WNT10B,CORO1B,TRIM27,ARPIN,RHOC,LGALS3,ARPC1B,WASHC1,CORO1A,GIT1,CDC42EP5,STUB1,ARPC5L	yellow	VSMC_M3
GO:0045834	0.12	0.93	11	109	positive regulation of lipid metabolic process	BP	1	PPARD,PDGFRA,SCARB1,DGKZ,CHP1,FABP3,PLA2G3,MID1IP1,CAPN2,AKT1,TAZ	yellow	VSMC_M3
GO:0050000	0.12	0.94	8	70	chromosome localization	BP	1	DCTN2,NUMA1,RRS1,PINX1,NUDC,EML3,MAD1L1,MIS12	yellow	VSMC_M3
GO:2000780	0.12	0.96	4	29	negative regulation of double-strand break repair	BP	1	OGG1,OTUB1,MAD2L2,CXorf57	yellow	VSMC_M3
GO:0009132	0.12	0.94	10	94	nucleoside diphosphate metabolic process	BP	1	GUK1,GALK1,OGDHL,AK2,BAD,FOXK1,PGM1,GIT1,JMJD8,AK1	yellow	VSMC_M3
GO:0006164	0.12	0.93	15	149	purine nucleotide biosynthetic process	BP	1	GUK1,ACSL1,AK2,PAPSS1,IMPDH2,ATP5MC3,ATIC,UQCC3,ACLY,ATP5PD,ATP5F1D,TAZ,AMPD2,APRT,AK1	yellow	VSMC_M3
GO:1902751	0.12	0.96	4	27	positive regulation of cell cycle G2/M phase transition	BP	1	CDK4,WNT10B,PBX1,RCC2	yellow	VSMC_M3
GO:0002931	0.12	0.95	6	51	response to ischemia	BP	1	SQSTM1,EIF4EBP1,CX3CL1,EEF2K,AKT1,STUB1	yellow	VSMC_M3
GO:2001234	0.12	0.92	18	187	negative regulation of apoptotic signaling pathway	BP	1	FIGNL1,BCL2L1,CX3CL1,CCAR2,SELENOS,ITPRIP,GPX1,QARS,RELA,LGALS3,NANOS3,ARHGEF2,AATF,AKT1,TRAP1,MNT,ZNF385A,TXNDC12	yellow	VSMC_M3
GO:0009260	0.12	0.93	15	149	ribonucleotide biosynthetic process	BP	1	GUK1,ACSL1,AK2,PAPSS1,IMPDH2,ATP5MC3,ATIC,UQCC3,ACLY,ATP5PD,ATP5F1D,TAZ,AMPD2,APRT,AK1	yellow	VSMC_M3
GO:0071470	0.12	0.95	5	40	cellular response to osmotic stress	BP	1	RELB,AQP5,BAD,LETM1,ARHGEF2	yellow	VSMC_M3
GO:1902042	0.12	0.96	4	26	negative regulation of extrinsic apoptotic signaling pathway via death domain receptors	BP	1	BCL2L1,ITPRIP,GPX1,ARHGEF2	yellow	VSMC_M3
GO:0009966	0.12	0.89	162	2237	regulation of signal transduction	BP	1	ENPP1,CD63,CDK4,SQSTM1,CTHRC1,NELFE,FNIP1,ATF6B,PRR5,TTLL12,SREBF2,TXNDC15,SGSM3,DEF6,PPARD,EEF1D,LGALS1,SHARPIN,BOP1,PDGFRA,IL1R1,SOCS5,KLF4,TPBG,SPRED2,RGS3,ZC3H12A,FIGNL1,RCN3,SNX3,PLEKHA4,MGLL,SLC9A3R1,EMILIN1,RNF220,INSIG1,SEC13,ARHGEF40,METTL3,BAIAP2,CXXC5,LAMTOR1,NUMA1,P4HB,CAVIN3,ARHGAP22,BCL2L1,PDE4D,TNS2,RRS1,SWAP70,PLEKHF1,GREM1,PDE3B,CX3CL1,WNT10B,SOD1,DGKZ,CCAR2,DLL4,CHP1,TYRO3,SLC35C2,TSPAN5,CISH,SELENOS,ITPRIP,PDGFA,MMP14,GPX1,AIP,NGEF,QARS,TRIM27,EIF3A,PRMT2,THOC5,RIPK3,STMN3,RELA,SIPA1,RHOC,MAP3K14,LIMD1,ZDHHC3,SMARCB1,YJEFN3,PHB2,IGFBP5,BAD,LAMTOR4,IFI35,LGALS3,BUD31,PLAU,TNFRSF1A,MVB12A,C1QBP,CHERP,EVC,CCDC22,PKN1,PALM3,AMER1,PIP4K2B,MAD2L2,NANOS3,ARHGEF2,MVP,PER1,TRIM25,RPL37,MTMR4,S100A4,SMARCA4,CARM1,CDC37,AATF,CSK,MID2,AKT1,TEDC1,RAB34,SAFB2,GIT1,MYDGF,MAP2K2,CACTIN,VASN,HIC1,TRAP1,NCLN,MNT,CTNS,TEDC2,CSNK1G2,IRAK1,STK11,TELO2,WDR24,JMJD8,CARD9,GAS8,SLC27A4,TGFBR3,TRADD,NDST1,TNFRSF12A,ERBB4,PTPRR,IGFBP6,ZNF385A,TXNDC12,CDC34,APCDD1L,ARHGEF1,RGS16,DUSP5,GLIPR2,STUB1,RGS18,GSG1L	yellow	VSMC_M3
GO:0007004	0.12	0.94	7	63	telomere maintenance via telomerase	BP	1	NHP2,PNKP,NAT10,TNKS1BP1,PINX1,TELO2,RF00024	yellow	VSMC_M3

GO:0010648	0.13	0.90	80	1047	negative regulation of cell communication	BP	1	ENPP1,CTHRC1,FNIP1,ATF6B,TTLL12,SOCS5,KLF4,TPBG,SPRED2,RGS3,ZC3H12A,FIGNL1,SLC9A3R1,EMILIN1,INSIG1,UCP2,METTL3,CAVIN3,BCL2L1,PDE4D,TNS2,GREM1,PDE3B,CX3CL1,DGKZ,CCAR2,DLL4,CHP1,TYRO3,CISH,SELENOS,ITPRIP,MMP14,GPX1,QARS,EIF3A,THOC5,STMN3,RELA,LIMD1,PHB2,IGFBP5,IFI35,LGALS3,C1QBP,CCDC22,PALM3,AMER1,PIP4K2B,MAD2L2,NANOS3,ARHGEF2,MVP,PER1,MTMR4,SMARCA4,AATF,CSK,AKT1,SLC6A4,CACTIN,VASN,HIC1,TRAP1,MNT,MIDN,STK11,SLC27A4,TGFBR3,PTPRR,IGFBP6,JAGN1,ZNF385A,TXNDC12,CDC34,APCDD1L,RGS16,DUSP5,STUB1,RGS18	yellow	VSMC_M3
GO:0045833	0.13	0.94	8	72	negative regulation of lipid metabolic process	BP	1	INSIG1,PDE3B,SOD1,ETFBKMT,PDGFA,WASHC1,PIP4K2B,AKT1	yellow	VSMC_M3
GO:0006418	0.13	0.95	5	37	tRNA aminoacylation for protein translation	BP	1	AARS2,CARS,QARS,FARSA,SARS	yellow	VSMC_M3
GO:0023057	0.13	0.90	80	1048	negative regulation of signaling	BP	1	ENPP1,CTHRC1,FNIP1,ATF6B,TTLL12,SOCS5,KLF4,TPBG,SPRED2,RGS3,ZC3H12A,FIGNL1,SLC9A3R1,EMILIN1,INSIG1,UCP2,METTL3,CAVIN3,BCL2L1,PDE4D,TNS2,GREM1,PDE3B,CX3CL1,DGKZ,CCAR2,DLL4,CHP1,TYRO3,CISH,SELENOS,ITPRIP,MMP14,GPX1,QARS,EIF3A,THOC5,STMN3,RELA,LIMD1,PHB2,IGFBP5,IFI35,LGALS3,C1QBP,CCDC22,PALM3,AMER1,PIP4K2B,MAD2L2,NANOS3,ARHGEF2,MVP,PER1,MTMR4,SMARCA4,AATF,CSK,AKT1,SLC6A4,CACTIN,VASN,HIC1,TRAP1,MNT,MIDN,STK11,SLC27A4,TGFBR3,PTPRR,IGFBP6,JAGN1,ZNF385A,TXNDC12,CDC34,APCDD1L,RGS16,DUSP5,STUB1,RGS18	yellow	VSMC_M3
GO:0051896	0.13	0.93	12	125	regulation of protein kinase B signaling	BP	1	KLF4,TPBG,RCN3,SLC9A3R1,CAVIN3,CX3CL1,PDGFA,GPX1,IGFBP5,C1QBP,MYDGF,STK11	yellow	VSMC_M3
GO:0048525	0.13	0.94	7	61	negative regulation of viral process	BP	1	TRIM11,SNX3,TRIM27,BANF1,TRIM25,C19orf66,MID2	yellow	VSMC_M3
GO:0010563	0.13	0.91	30	356	negative regulation of phosphorus metabolic process	BP	1	ENPP1,CTDSP2,H2AFY,PLEC,SOCS5,SPRED2,ZC3H12A,MICAL1,EMILIN1,TARBP2,PDE4D,SWAP70,GREM1,CHP1,PDGFA,QARS,TRIM27,PPP1R35,CTDSP1,LGALS3,WASHC1,PKN1,PIP4K2B,MVP,CSK,AKT1,GIT1,CACTIN,MIDN,TRIB2	yellow	VSMC_M3
GO:0045936	0.13	0.91	30	356	negative regulation of phosphate metabolic process	BP	1	ENPP1,CTDSP2,H2AFY,PLEC,SOCS5,SPRED2,ZC3H12A,MICAL1,EMILIN1,TARBP2,PDE4D,SWAP70,GREM1,CHP1,PDGFA,QARS,TRIM27,PPP1R35,CTDSP1,LGALS3,WASHC1,PKN1,PIP4K2B,MVP,CSK,AKT1,GIT1,CACTIN,MIDN,TRIB2	yellow	VSMC_M3
GO:0006278	0.13	0.94	7	64	RNA-dependent DNA biosynthetic process	BP	1	NHP2,PNKP,NAT10,TNKS1BP1,PINX1,TELO2,RF00024	yellow	VSMC_M3
GO:0043487	0.13	0.92	14	148	regulation of RNA stability	BP	1	HNRNPA0,CNOT3,ZC3H12A,METTL3,DND1,PCBP4,DCPS,ZCCHC17,NANOS3,AKT1,PAIP1,KHSRP,CARHSP1,TENT5B	yellow	VSMC_M3
GO:0033013	0.13	0.95	6	49	tetrapyrrole metabolic process	BP	1	BLVRA,BLVRB,SLC11A2,SRRD,PPOX,SLC25A39	yellow	VSMC_M3
GO:0006195	0.13	0.95	5	38	purine nucleotide catabolic process	BP	1	NUDT3,DNPH1,PDE4D,NT5C,ITPA	yellow	VSMC_M3
GO:0098869	0.13	0.94	8	67	cellular oxidant detoxification	BP	1	GSR,SOD1,SELENOS,GSTO1,GPX1,MGST3,TXNRD2,PRXL2A	yellow	VSMC_M3
GO:0043488	0.13	0.92	13	137	regulation of mRNA stability	BP	1	HNRNPA0,CNOT3,ZC3H12A,METTL3,DND1,PCBP4,DCPS,NANOS3,AKT1,PAIP1,KHSRP,CARHSP1,TENT5B	yellow	VSMC_M3
GO:1901987	0.13	0.91	27	311	regulation of cell cycle phase transition	BP	1	CDK4,CTDSP2,H2AFY,KLF4,KMT2E,PINX1,WNT10B,CCAR2,CDK2AP2,PSME2,PRMT2,THOC5,PBX1,PHB2,CTDSP1,METTL13,BABAM1,RCC2,SDE2,MIIP,MAD1L1,MAD2L2,ZNF830,AKT1,MIR214,NABP2,FBXO6	yellow	VSMC_M3
GO:0044267	0.13	0.88	272	3725	cellular protein metabolic process	BP	1	ENPP1,NAB2,CDK4,CTDSP2,SQSTM1,BAG6,SBF1,SNX9,HDAC10,NELFE,FNIP1,PRR5,TTLL12,RING1,JADE2,H2AFY,TRIM11,EEF1D,RPL3,PLEC,SHARPIN,VPS28,RPL8,CUL7,B4GALT1,MRPS18A,AARS2,PIGO,PDGFRA,SECISBP2,PHF2,HSPBP1,SOCS5,RPS9,CNOT3,KLF4,JOSD2,PSMB3,SPRED2,EIPR1,MEAF6,ZC3H12A,PNKP,RCN3,MICAL1,KMT2E,WDFY2,SLC9A3R1,SERP2,NAT9,EMILIN1,SEPHS1,TMUB1,RNF220,DMAP1,PPM1G,UNK,GALNT14,SERTAD1,PSMC4,KAT14,P4HA3,SEL1L2,METTL3,P4HB,DND1,RPL29,EIF4EBP1,TARBP2,KMT2B,NAT10,HM13,PDE4D,TNS2,EIF4B,SWAP70,ACSL1,TNKS1BP1,PINX1,GATB,SETD1B,GREM1,KDM2B,MCRS1,CX3CL1,WNT10B,FKBP11,SOD1,PACSIN3,CCAR2,RPLP2,CHP1,TYRO3,MRPL43,HTATIP2,PCIF1,PPM1M,KDM1B,SLC35C2,CARS,CISH,ST3GAL4,RNF19B,DCPS,SELENOS,ETFBKMT,MANBA,TMTC1,STK32C,PDGFA,MMP14,APEH,GPX1,AIP,HCCS,MOCS3,CLCF1,NGDN,QARS,ASB9,PSME2,TRIM27,EIF3A,LIMK1,RABGGTA,PRMT2,RIPK3,LSM14B,ADRM1,PSTK,RPS21,PSMB4,EXTL1,BRMS1,SEC11A,TNK2,TACO1,PTPN23,KAT5,RELA,SCYL1,ALKBH4,MEX3B,MAP3K14,MRPL49,ZDHHC3,SMARCB1,PHB2,TRMT112,IGFBP5,BAD,FKBP2,CTDSP1,OTUB1,EID1,COPS6,MPV17L2,LGALS3,TNFRSF1A,MVB12A,BABAM1,C1QBP,AIMP2,TAF6L,UBXN1,WASHC1,MTA2,MED11,CCDC22,USP27X,KRT10,SDE2,PKN1,CAPN2,SEPSECS,AMER1,MTHFR,MAD2L2,NANOS3,MPDU1,CTBP1,FBXL19,ARHGEF2,SNF8,RUSC1,MVP,PER1,DPM3,PPP4C,FARSA,SGF29,IRF2BPL,TUFM,TRIM25,USP43,RPL37,MTMR4,CARM1,SENP8,RPL7A,EEF2K,CDC37,MRPL4,EIF3G,C19orf66,CSK,MID2,ATG3,ANKRD9,AKT1,COPRS,PAIP1,KHSRP,SGK494,DHCR24,BLMH,MYDGF,ME[illegible]	yellow	VSMC_M3
GO:0008593	0.13	0.94	8	76	regulation of Notch signaling pathway	BP	1	SREBF2,METTL3,DLL4,SLC35C2,TSPAN5,MMP14,YJEFN3,AKT1	yellow	VSMC_M3
GO:0051591	0.13	0.94	7	68	response to cAMP	BP	1	WNT10B,SLC26A6,RELA,IGFBP5,PER1,CARM1,EEF2K	yellow	VSMC_M3
GO:1905508	0.13	0.96	4	33	protein localization to microtubule organizing center	BP	1	DCTN2,CEP131,NUMA1,KIAA0753	yellow	VSMC_M3
GO:0006779	0.13	0.96	4	28	porphyrin-containing compound biosynthetic process	BP	1	SLC11A2,SRRD,PPOX,SLC25A39	yellow	VSMC_M3
GO:0033014	0.13	0.96	4	28	tetrapyrrole biosynthetic process	BP	1	SLC11A2,SRRD,PPOX,SLC25A39	yellow	VSMC_M3
GO:0009205	0.13	0.94	7	57	purine ribonucleoside triphosphate metabolic process	BP	1	IMPDH2,ATP5MC3,UQCC3,ATP5PD,ATP5F1D,TAZ,ITPA	yellow	VSMC_M3
GO:0032868	0.14	0.91	18	206	response to insulin	BP	1	ENPP1,CDK4,INSIG1,UCP2,BAIAP2,INPPL1,TNS2,PDE3B,SELENOS,FABP3,PCK2,CAPN10,RELA,PIP4K2B,EEF2K,AKT1,APRT,SLC27A4	yellow	VSMC_M3

GO:0046474	0.14	0.91	17	185	glycerophospholipid biosynthetic process	BP	1	PIGO,SOCS5,TTC7A,INPPL1,PCYT2,PTDSS2,DGKZ,CISH,FABP3,PDGFA,CAPN2,PIP4K2B,DPM3,MTMR4,TAZ,SLC44A3,VAC14	yellow	VSMC_M3
GO:0018130	0.14	0.88	211	2900	heterocycle biosynthetic process	BP	1	ENPP1,NAB2,NHP2,CDK4,SQSTM1,ZNF608,HDAC10,NELFE,FNIP1,ATF6B,PBX2,RING1,SREBF2,H2AFY,TRIM11,GUK1,TRAPPC9,PPARD,TSTA3,SND1,PHF2,CNOT3,KLF4,HOXA1,FHL3,ZC3H12A,PNKP,CC2D1B,KTI12,KMT2E,SIX5,ZNF282,ZNF296,RELB,DMAP1,CIC,GTF3C2,OGG1,SERTAD1,TTC5,CXXC5,PYCR1,GNPDA1,DEAF1,HOXC4,POLR1D,KMT2B,NAT10,ZNF740,RALY,ACSL1,ZBTB38,SLC11A2,TNKS1BP1,PINX1,GREM1,KDM2B,MCRS1,POLR2C,CX3CL1,WNT10B,ACTR5,ANKRD2,EGR3,CCAR2,DNAJC17,DLL4,CHP1,YAF2,HTATIP2,NPM3,KDM1B,CHCHD2,AK2,IRX1,RRP1B,BRD9,PAPSS1,AIP,MOCS3,SRRD,TSHZ2,QARS,IMPDH2,PIR,TRIM27,ZNRD1,PRMT2,THOC5,RIPK3,TWIST2,ADRM1,PSTK,SMARCD2,BRMS1,PPOX,GMEB2,CCDC85B,PRPF6,PBX1,KAT5,RELA,ATP5MC3,ZNF511,ARNT2,ZFPL1,LIMD1,SMARCB1,CHCHD10,SLC25A39,PBX4,UBTF,GATAD2A,ATIC,MEF2B,PHB2,NDP,CTDSP1,MLF2,EID1,SSBP4,METTL13,ZNF394,C11orf95,BUD31,TNFRSF1A,TEAD4,C1QBP,TAF6L,ZBTB49,UQCC3,MTA2,ZNF316,ACLY,MED11,PELP1,CCDC22,FOXK1,KLF3,PKN1,SEPSECS,POLR2A,MAD2L2,SLTM,CTBP1,FBXL19,CRABP2,SP2,ARHGEF2,SNF8,PER1,NFIX,FLAD1,IRF2BPL,TRIM25,ATP5PD,USP43,SMARCA4,CARM1,AATF,MID2,ZNF521,ZNF358,AKT1,BRF1,KHSRP,GTF2F1,RFX2,SAFB,SAFB2,MYDGF,HDGFL2,SIRT6,MAP2K2,CACTIN,CDK11B,SSU72,HIC1,MNT,EHMT1,AP3D1,IRAK1,MBD3,ATP5F1D,TELO2,TAZ,MED16,CARD9,AMPD2,SARS,APRT,GTF3C5,AK1,TRADD,RF00024,ERBB4,JUND,PTMA,KLF13,FADS1,ZNF783,HOXC5,CAVIN1,IER5,AKAP17A,ZNF250,THAP7,NSMCE3,ERF,SNAPC2,ZSCAN1	yellow	VSMC_M3
GO:0014812	0.14	0.93	8	79	muscle cell migration	BP	1	PPARD,PDGFA,CORO1B,PLEKHO1,IGFBP5,AAMP,PLAU,MIR214	yellow	VSMC_M3
GO:0043933	0.14	0.89	105	1370	protein-containing complex subunit organization	BP	1	SPAG1,SNX9,FNIP1,SAMM50,NDUFB9,H2AFY,RPL3,PLEC,BOP1,TBCC,DBNL,ZC3H12A,MICAL1,ACAD9,GEMIN7,SLC9A3R1,DMAP1,H3F3B,BAIAP2,NUMA1,CCDC57,EIF4EBP1,SDHAF1,TARBP2,SCARB1,EIF4B,RRS1,SWAP70,UQCRFS1,CAPG,GREM1,AQP5,DCTN1,CX3CL1,WNT10B,CHP1,SRSF9,LIX1L,APEH,CORO1B,HCCS,TRIM27,ARPIN,EIF3A,SF3A1,ADRM1,SMARCD2,TACO1,PLA2G3,STMN3,SART1,PRPF6,RHOC,TMEM120A,ZNHIT1,MID1IP1,PEX5,SMARCB1,CHCHD10,UBTF,BAD,LETM1,LGALS3,ARPC1B,MRTO4,FCHO1,AIMP2,TAF6L,UQCC3,WASHC1,KRT10,H3F3A,DBN1,CLTB,ARHGEF2,CD2BP2,RANGRF,FARSA,CORO1A,SMARCA4,EIF3G,C19orf66,BRF1,GIT1,UBN1,MRPL20,NCLN,NDUFS7,ATP5F1D,TAZ,CARD9,SPG7,TGFBR3,MIR214,TWF2,SURF1,MIS12,ELN,UNC13A,CDC42EP5,GBA2,TUBGCP2,STUB1,CCDC115,ARPC5L	yellow	VSMC_M3
GO:0019217	0.14	0.94	7	62	regulation of fatty acid metabolic process	BP	1	PPARD,INSIG1,ETFBKMT,FABP3,PLA2G3,MID1IP1,AKT1	yellow	VSMC_M3
GO:2001021	0.14	0.93	8	72	negative regulation of response to DNA damage stimulus	BP	1	OGG1,BCL2L1,CCAR2,THOC5,OTUB1,MAD2L2,CXorf57,ZNF385A	yellow	VSMC_M3
GO:0043543	0.14	0.91	18	202	protein acylation	BP	1	BAG6,JADE2,MEAF6,NAT9,DMAP1,KAT14,NAT10,MCRS1,KAT5,ZDHHC3,SMARCB1,EID1,TAF6L,CTBP1,PER1,SGF29,MBD3,THAP7	yellow	VSMC_M3
GO:0043484	0.14	0.92	12	126	regulation of RNA splicing	BP	1	SNRNP70,PCBP4,RBM15B,SRSF9,RRP1B,C1QBP,POLR2A,RAVER1,RBM22,FAM50A,RBM8A,AKAP17A	yellow	VSMC_M3
GO:0006739	0.14	0.95	4	27	NADP metabolic process	BP	1	TKT,IDH1,PGD,TALDO1	yellow	VSMC_M3
GO:1905521	0.14	0.95	4	29	regulation of macrophage migration	BP	1	EMILIN1,CX3CL1,MMP14,CD9	yellow	VSMC_M3
GO:0097435	0.14	0.89	47	622	supramolecular fiber organization	BP	1	SNX9,PACSIN1,PLEC,PDGFRA,DBNL,ZYX,MICAL1,KRT8,EMILIN1,BAIAP2,INPPL1,NUMA1,CCDC57,SWAP70,CAPG,GREM1,DCTN1,CX3CL1,CHP1,GPX1,CORO1B,CLIP2,TRIM27,ARPIN,LIMK1,RIPK3,PFDN2,STMN3,RHOC,MID1IP1,ARPC1B,WASHC1,DBN1,MAD2L2,ARHGEF2,RANGRF,CORO1A,MYO19,GIT1,MIR214,TWF2,LOXL1,ELN,CDC42EP5,GBA2,TUBGCP2,ARPC5L	yellow	VSMC_M3
GO:0060612	0.14	0.95	4	32	adipose tissue development	BP	1	CDK4,PPARD,AACS,AMER1	yellow	VSMC_M3
GO:0014909	0.14	0.93	7	67	smooth muscle cell migration	BP	1	PPARD,PDGFA,CORO1B,IGFBP5,AAMP,PLAU,MIR214	yellow	VSMC_M3
GO:0051091	0.14	0.91	18	204	positive regulation of DNA-binding transcription factor activity	BP	1	TRAPPC9,GREM1,CX3CL1,WNT10B,TRIM27,RIPK3,RELA,SMARCB1,PHB2,BUD31,ARHGEF2,TRIM25,SMARCA4,MID2,AKT1,IRAK1,CARD9,TRADD	yellow	VSMC_M3
GO:0016042	0.14	0.91	21	225	lipid catabolic process	BP	1	CDK4,ACAT2,PPARD,CPT1C,MGLL,PAFAH1B3,SNX17,PLCD1,SCARB1,PCCB,PDE3B,ETFBKMT,PCK2,IDH1,SGPL1,PLCB3,ILVBL,AKT1,SLC27A4,PLA2G4E,GBA2	yellow	VSMC_M3
GO:0009394	0.14	0.95	5	35	2'-deoxyribonucleotide metabolic process	BP	1	GUK1,DNPH1,OGG1,SMUG1,NT5C	yellow	VSMC_M3
GO:0045600	0.14	0.94	6	53	positive regulation of fat cell differentiation	BP	1	PPARD,ZC3H12A,WDFY2,CARM1,AKT1,ZNF385A	yellow	VSMC_M3

GO:0043412	0.14	0.88	219	3029	macromolecule modification	BP	1	ENPP1,NAB2,NHP2,CDK4,CTDSP2,SQSTM1,BAG6,SBF1,SNX9,HDAC10,NELFE,FNIP1,PRR5,TTLL12,RING1,JADE2,TRMT12,H2AFY,TRIM11,PLEC,SHARPIN,VPS28,CUL7,B4GALT1,AARS2,PIGO,PDGFRA,PHF2,HSPBP1,SOCS5,JOSD2,SPRED2,EIPR1,MEAF6,ZC3H12A,PNKP,KTI12,MICAL1,KMT2E,WDFY2,SLC9A3R1,SERP2,NAT9,MUTYH,EMILIN1,SEPHS1,RNF220,DMAP1,PPM1G,GALNT14,OGG1,SERTAD1,KAT14,P4HA3,SEL1L2,METTL3,P4HB,PUS1,SMUG1,TARBP2,KMT2B,NAT10,PDE4D,TNS2,SWAP70,ACSL1,TNKS1BP1,PINX1,SETD1B,GREM1,KDM2B,MCRS1,CX3CL1,FKBP11,SOD1,CCAR2,CHP1,TYRO3,HTATIP2,PCIF1,PPM1M,KDM1B,SLC35C2,RBM15B,CISH,ST3GAL4,RNF19B,WDR4,ETFBKMT,MANBA,TMTC1,STK32C,PDGFA,GPX1,AIP,HCCS,MOCS3,CLCF1,QARS,ASB9,TRIM27,LIMK1,RABGGTA,PRMT2,RIPK3,EXTL1,FTSJ3,BRMS1,BUD23,TNK2,PTPN23,KAT5,RELA,SCYL1,ALKBH4,MEX3B,MAP3K14,ZDHHC3,SMARCB1,TRMT2A,GATAD2A,PHB2,TRMT112,FKBP2,CTDSP1,OTUB1,EID1,COPS6,LGALS3,TNFRSF1A,GTPBP3,BABAM1,AIMP2,TAF6L,UBXN1,WASHC1,MTA2,MED11,USP27X,KRT10,ELP5,SDE2,PKN1,AMER1,MTHFR,MAD2L2,MPDU1,CTBP1,FBXL19,RRNAD1,ARHGEF2,RUSC1,MVP,PER1,DPM3,TRMT1,PPP4C,SGF29,IRF2BPL,TRIM25,USP43,RPL37,MTMR4,CARM1,SENP8,EEF2K,CDC37,CSK,MID2,ATG3,ANKRD9,AKT1,COPRS,SGK494,DUS3L,BLMH,MYDGF,METTL22,SIRT6,MAP2K2,CACTIN,HMG20B,CDK11B,SSU72,EHMT1,PGP,MKNK2,CSNK1G2,IRAK1,ANAPC2,SPSB3,MBD3,MAN1B1,STK11,TELO2,TSR3,UBAC1,CTU2,POMT1,NTMT1,ZNRF1,TGFBR3,B3GNT9,NDST1,ERBB4,PTPRR,LOXL1,PIN4,TRIM7,TRIB2,NSMF,CDC34,FBXO6,DUSP5,THAP7,STUB1,NSMC[illegible]	yellow	VSMC_M3
GO:0045931	0.14	0.92	10	97	positive regulation of mitotic cell cycle	BP	1	CDK4,KMT2E,CCDC57,EIF4EBP1,WNT10B,PBX1,PHB2,RCC2,AKT1,MIR214	yellow	VSMC_M3
GO:0097194	0.14	0.93	7	60	execution phase of apoptosis	BP	1	SHARPIN,ZC3H12A,BCL2L1,CAPN10,CASP14,DFFA,AKT1	yellow	VSMC_M3
GO:0046907	0.14	0.88	95	1261	intracellular transport	BP	1	PQLC1,CD63,SQSTM1,CCHCR1,SNX9,SAMM50,SREBF2,SGSM3,SCRIB,IFT27,VPS28,TOM1,ASNA1,PLEKHA8,EIPR1,ZC3H12A,SNX3,EHD2,SLC9A3R1,DMAP1,SNX17,INSIG1,MYRIP,SEC13,CEP131,LAMTOR1,NUMA1,YIF1B,RAB20,HM13,RRS1,DCTN1,SOD1,CHP1,AP2A2,HTATIP2,RAB35,RBM15B,SELENOS,FABP3,PABPN1,AIP,AP1G2,TRIM27,PCNT,THOC5,CAPN10,PLA2G3,PTPN23,VPS51,PEX5,ZDHHC3,THOC3,BLOC1S4,PHB2,COPE,MVB12A,NUP88,WASHC1,AP1M1,CCDC22,SNX8,LMAN2,RAB24,CLTB,ARHGEF2,SNF8,RANGRF,STX10,CORO1A,ATP5PD,TMED1,SYNE3,CDC37,MYO19,ATG3,RBM22,AKT1,SNX18,DENND1C,RANBP3,MAP2K2,AP3D1,SSNA1,ATP5F1D,STK11,TMEM201,SPG7,WDR34,TRAPPC5,PLA2G4E,AP5B1,ARFRP1,TIMM22,PHETA2	yellow	VSMC_M3
GO:0046889	0.14	0.93	7	61	positive regulation of lipid biosynthetic process	BP	1	SCARB1,CHP1,FABP3,PLA2G3,MID1IP1,CAPN2,AKT1	yellow	VSMC_M3
GO:1902750	0.14	0.94	6	52	negative regulation of cell cycle G2/M phase transition	BP	1	H2AFY,PINX1,BABAM1,MIIP,ZNF830,NABP2	yellow	VSMC_M3
GO:0046683	0.14	0.93	9	94	response to organophosphorus	BP	1	WNT10B,SOD1,SLC26A6,RELA,IGFBP5,PER1,CARM1,EEF2K,SLC6A4	yellow	VSMC_M3
GO:0006099	0.14	0.95	4	27	tricarboxylic acid cycle	BP	1	IDH3B,OGDHL,SDHA,IDH1	yellow	VSMC_M3
GO:0031324	0.14	0.88	140	1896	negative regulation of cellular metabolic process	BP	1	ENPP1,NAB2,CTDSP2,SQSTM1,ZNF608,BAG6,HDAC10,NELFE,FNIP1,RING1,SREBF2,H2AFY,HNRNPA0,TRIM11,PPARD,PLEC,VPS28,SECISBP2,SOCS5,CNOT3,KLF4,SPRED2,ZC3H12A,PNKP,SNRNP70,CC2D1B,MICAL1,SIX5,ZNF282,ZNF296,RELB,EMILIN1,DMAP1,CIC,UNK,INSIG1,OGG1,METTL3,CXXC5,DND1,EIF4EBP1,CAVIN3,DEAF1,TARBP2,NAT10,PDE4D,SWAP70,ZBTB38,PINX1,GREM1,KDM2B,WNT10B,ANKRD2,CCAR2,DNAJC17,DLL4,CHP1,YAF2,PCIF1,PCBP4,KDM1B,SRSF9,DCPS,IRX1,SELENOS,ETFBKMT,RIN3,ZCCHC17,PDGFA,GPX1,QARS,TRIM27,LIMK1,PRMT2,TWIST2,BRMS1,CCDC85B,KAT5,RELA,LIMD1,SMARCB1,GATAD2A,PHB2,PPP1R35,IGFBP5,CTDSP1,OTUB1,EID1,METTL13,LGALS3,C11orf95,C1QBP,ZBTB49,UBXN1,WASHC1,MTA2,FOXK1,KLF3,PKN1,PIP4K2B,MAD2L2,NANOS3,CTBP1,DFFA,SP2,MVP,PER1,NFIX,SLX1A,IRF2BPL,RPL37,SMARCA4,AATF,CXorf57,C19orf66,CSK,ZNF830,AKT1,PAIP1,KHSRP,GIT1,ANKRD13B,DHCR24,SIRT6,CACTIN,HMG20B,HIC1,TRAP1,MNT,CTNS,EHMT1,PGP,MBD3,MIDN,SARS,TENT5B,TRIB2,THAP7,NSMCE3,ERF	yellow	VSMC_M3
GO:0043403	0.14	0.95	4	28	skeletal muscle tissue regeneration	BP	1	PPARD,WNT10B,GPX1,CD9	yellow	VSMC_M3
GO:1901135	0.14	0.89	64	791	carbohydrate derivative metabolic process	BP	1	ENPP1,CERK,GUK1,NUDT3,PPARD,TSTA3,DNPH1,B4GALT1,PIGO,SERP2,GALK1,GALNT14,OGG1,PHLDA1,FASN,GNPDA1,SMUG1,OGDHL,PDE4D,ACSL1,TKT,CHP1,SLC35C2,ST3GAL4,AK2,MANBA,TMTC1,MVK,PAPSS1,IMPDH2,EXTL1,ATP5MC3,ATIC,BAD,UQCC3,ACLY,FOXK1,MPDU1,PGD,ACSF2,DPM3,PMVK,ATP5PD,AATF,AKT1,HMGCS1,PGM1,GIT1,MAN1B1,ATP5F1D,TAZ,JMJD8,AMPD2,APRT,MVD,POMT1,AK1,B3GNT9,NDST1,NT5C,TALDO1,FBXO6,GBA2,ITPA	yellow	VSMC_M3
GO:1903573	0.14	0.94	5	39	negative regulation of response to endoplasmic reticulum stress	BP	1	ATF6B,BCL2L1,SELENOS,UBXN1,TXNDC12	yellow	VSMC_M3
GO:0008631	0.14	0.94	5	39	intrinsic apoptotic signaling pathway in response to oxidative stress	BP	1	P4HB,SOD1,GPX1,AKT1,TRAP1	yellow	VSMC_M3
GO:2000779	0.14	0.93	8	76	regulation of double-strand break repair	BP	1	FIGNL1,PNKP,OGG1,OTUB1,MAD2L2,PPP4C,CXorf57,SIRT6	yellow	VSMC_M3

GO:0045934	0.14	0.88	84	1115	negative regulation of nucleobase-containing compound metabolic process	BP	1	NAB2,SQSTM1,ZNF608,HDAC10,NELFE,FNIP1,RING1,SREBF2,H2AFY,HNRNPA0,TRIM11,PPARD,SECISBP2,KLF4,ZC3H12A,CC2D1B,SIX5,ZNF282,ZNF296,RELB,DMAP1,CIC,OGG1,CXXC5,DND1,DEAF1,NAT10,ZBTB38,PINX1,GREM1,KDM2B,WNT10B,ANKRD2,CCAR2,DNAJC17,DLL4,YAF2,PCBP4,KDM1B,SRSF9,IRX1,ZCCHC17,QARS,TRIM27,PRMT2,TWIST2,BRMS1,CCDC85B,KAT5,RELA,LIMD1,GATAD2A,PHB2,OTUB1,EID1,METTL13,C11orf95,C1QBP,ZBTB49,MTA2,FOXK1,KLF3,MAD2L2,CTBP1,DFFA,SP2,PER1,NFIX,SLX1A,IRF2BPL,SMARCA4,CXorf57,PAIP1,GIT1,SIRT6,HIC1,MNT,EHMT1,MBD3,SARS,TENT5B,THAP7,NSMCE3,ERF	yellow	VSMC_M3
GO:0009890	0.15	0.88	92	1222	negative regulation of biosynthetic process	BP	1	ENPP1,NAB2,SQSTM1,ZNF608,HDAC10,NELFE,FNIP1,RING1,SREBF2,H2AFY,TRIM11,PPARD,CNOT3,KLF4,ZC3H12A,CC2D1B,SIX5,ZNF282,ZNF296,RELB,EMILIN1,DMAP1,CIC,UNK,INSIG1,METTL3,CXXC5,DND1,EIF4EBP1,DEAF1,NAT10,ZBTB38,PINX1,GREM1,KDM2B,WNT10B,SOD1,ANKRD2,CCAR2,DNAJC17,DLL4,YAF2,PCIF1,KDM1B,DCPS,IRX1,SELENOS,PDGFA,QARS,TRIM27,PRMT2,TWIST2,BRMS1,CCDC85B,KAT5,RELA,LIMD1,GATAD2A,PHB2,IGFBP5,EID1,METTL13,C11orf95,C1QBP,ZBTB49,MTA2,FOXK1,KLF3,MAD2L2,NANOS3,CTBP1,SP2,PER1,NFIX,IRF2BPL,SMARCA4,AATF,C19orf66,ZNF830,KHSRP,SIRT6,HIC1,TRAP1,MNT,CTNS,EHMT1,PGP,MBD3,SARS,THAP7,NSMCE3,ERF	yellow	VSMC_M3
GO:0009264	0.15	0.95	4	26	deoxyribonucleotide catabolic process	BP	1	DNPH1,OGG1,SMUG1,NT5C	yellow	VSMC_M3
GO:0043551	0.15	0.94	5	47	regulation of phosphatidylinositol 3-kinase activity	BP	1	PDGFRA,SOCS5,KLF4,CISH,WASHC1	yellow	VSMC_M3
GO:0009892	0.15	0.87	165	2254	negative regulation of metabolic process	BP	1	ENPP1,NAB2,CTDSP2,SQSTM1,ZNF608,BAG6,HDAC10,NELFE,FNIP1,RING1,SREBF2,H2AFY,HNRNPA0,TRIM11,PPARD,PLEC,VPS28,SND1,SECISBP2,PHF2,SSC5D,SOCS5,CNOT3,KLF4,SPRED2,ZC3H12A,PNKP,SNRNP70,CC2D1B,SNX3,MICAL1,SIX5,ZNF282,ZNF296,RELB,EMILIN1,DMAP1,CIC,UNK,INSIG1,OGG1,METTL3,CXXC5,INPPL1,DND1,EIF4EBP1,CAVIN3,DEAF1,TARBP2,NAT10,PDE4D,SWAP70,LRP3,ZBTB38,LSM6,PINX1,GREM1,KDM2B,PDE3B,CX3CL1,WNT10B,SOD1,ANKRD2,CCAR2,DNAJC17,DLL4,CHP1,YAF2,PCIF1,PCBP4,KDM1B,SLC35C2,SRSF9,DCPS,IRX1,SELENOS,ETFBKMT,RIN3,ZCCHC17,PDGFA,GPX1,QARS,TRIM27,LIMK1,PRMT2,TWIST2,PFDN2,BRMS1,PLA2G3,CCDC85B,KAT5,RELA,LIMD1,SMARCB1,GATAD2A,PHB2,PPP1R35,IGFBP5,CTDSP1,OTUB1,EID1,METTL13,LGALS3,C11orf95,MRTO4,C1QBP,ZBTB49,UBXN1,WASHC1,MTA2,FOXK1,KLF3,PKN1,PIP4K2B,MAD2L2,NANOS3,CTBP1,DFFA,SP2,MVP,PER1,NFIX,SLX1A,IRF2BPL,RPL37,SMARCA4,AATF,CXorf57,C19orf66,CSK,ZNF830,AKT1,PAIP1,KHSRP,GIT1,ANKRD13B,DHCR24,CARHSP1,SIRT6,MAP2K2,CACTIN,HMG20B,HIC1,TRAP1,MNT,CTNS,EHMT1,PGP,ANAPC2,MBD3,MIDN,STK11,SARS,SLC27A4,MIR214,MIR155,RBM8A,RNH1,MIR374B,TENT5B,TRIB2,THAP7,NSMCE3,ERF,ZAR1L	yellow	VSMC_M3
GO:0006692	0.15	0.95	4	29	prostanoid metabolic process	BP	1	PTGR1,PLA2G3,TNFRSF1A,CYP4F8	yellow	VSMC_M3
GO:0006693	0.15	0.95	4	29	prostaglandin metabolic process	BP	1	PTGR1,PLA2G3,TNFRSF1A,CYP4F8	yellow	VSMC_M3
GO:1903131	0.15	0.90	25	297	mononuclear cell differentiation	BP	1	NCAPH2,FNIP1,LGALS1,SOCS5,ZC3H12A,RELB,CD79A,METTL3,FASN,SOD1,EGR3,DLL4,TYRO3,MMP14,CLCF1,PIR,THOC5,RIPK3,SART1,PBX1,BAD,ARMC5,DOCK11,AP3D1,STK11	yellow	VSMC_M3
GO:0006383	0.15	0.94	5	39	transcription by RNA polymerase III	BP	1	NAB2,GTF3C2,BRF1,GTF3C5,SNAPC2	yellow	VSMC_M3
GO:0030866	0.15	0.95	4	35	cortical actin cytoskeleton organization	BP	1	PLEC,EHD2,CAVIN3,FMNL1	yellow	VSMC_M3
GO:0045806	0.15	0.94	5	41	negative regulation of endocytosis	BP	1	PACSIN1,PACSIN3,RIN3,LGALS3,ANKRD13B	yellow	VSMC_M3
GO:1901224	0.15	0.93	6	51	positive regulation of NIK/NF-kappaB signaling	BP	1	GREM1,RELA,PHB2,IFI35,IRAK1,TRADD	yellow	VSMC_M3
GO:0009206	0.15	0.93	6	46	purine ribonucleoside triphosphate biosynthetic process	BP	1	IMPDH2,ATP5MC3,UQCC3,ATP5PD,ATP5F1D,TAZ	yellow	VSMC_M3
GO:0045927	0.15	0.90	17	199	positive regulation of growth	BP	1	PPARD,DNPH1,DBNL,H3F3B,KDM2B,MMP14,LIMK1,H3F3A,DBN1,CRABP2,AKT1,HDGFL2,ANAPC2,TGFBR3,ERBB4,TWF2,UNC13A	yellow	VSMC_M3
GO:0010883	0.15	0.94	5	41	regulation of lipid storage	BP	1	SREBF2,PPARD,ZC3H12A,SCARB1,ALKBH7	yellow	VSMC_M3
GO:0045738	0.15	0.95	4	31	negative regulation of DNA repair	BP	1	OGG1,OTUB1,MAD2L2,CXorf57	yellow	VSMC_M3
GO:1901575	0.15	0.87	124	1628	organic substance catabolic process	BP	1	ENPP1,CDK4,SQSTM1,BAG6,SNX9,ACAT2,HNRNPA0,SGSM3,NUDT3,PPARD,MPST,SHARPIN,VPS28,SND1,CUL7,DNPH1,SECISBP2,HSPBP1,SOCS5,CNOT3,PSMB3,ZC3H12A,BLVRA,CPT1C,RCN3,SNRNP70,SNX3,MGLL,TMUB1,PAFAH1B3,SNX17,GALK1,PLCD1,BCKDHA,BLVRB,OGG1,PSMC4,SEL1L2,METTL3,DND1,SMOX,GNPDA1,SMUG1,OGDHL,SCARB1,HM13,PDE4D,PCCB,LSM6,PDE3B,WNT10B,PACSIN3,CCAR2,PCBP4,RNF19B,DCPS,SELENOS,ETFBKMT,MANBA,ZCCHC17,GPX1,PCK2,ASB9,PSME2,ANPEP,ADRM1,PSMB4,PTPN23,RNASEH2C,KAT5,RELA,IDH1,SGPL1,BAD,PLCB3,MRTO4,MVB12A,UBXN1,CNP,CCDC22,USP27X,GLUD1,FOXK1,ILVBL,CAPN2,AMER1,MAD2L2,NANOS3,FBXL19,DFFA,PGD,SNF8,TRIM25,USP43,ANKRD9,AKT1,PAIP1,KHSRP,PGM1,GIT1,CARHSP1,BLMH,SIRT6,ANAPC2,SPSB3,MAN1B1,JMJD8,SLC27A4,ZNRF1,PSMB10,CTRL,RBM8A,RNH1,PLA2G4E,NT5C,TENT5B,TRIB2,CDC34,FBXO6,GBA2,TRIR,STUB1,ITPA,CCDC115	yellow	VSMC_M3
GO:1901992	0.15	0.92	8	73	positive regulation of mitotic cell cycle phase transition	BP	1	CDK4,KMT2E,WNT10B,PBX1,PHB2,RCC2,AKT1,MIR214	yellow	VSMC_M3
GO:0140053	0.15	0.91	11	101	mitochondrial gene expression	BP	1	MRPS18A,AARS2,PUS1,GATB,MRPL43,TACO1,CHCHD10,MPV17L2,C1QBP,TUFM,MRPS16	yellow	VSMC_M3
GO:0097191	0.15	0.90	16	173	extrinsic apoptotic signaling pathway	BP	1	KRT8,DELE1,BCL2L1,CX3CL1,ITPRIP,GPX1,RELA,ZDHHC3,BAD,LGALS3,TNFRSF1A,ARHGEF2,AKT1,MKNK2,TRADD,TNFRSF12A	yellow	VSMC_M3
GO:0050819	0.15	0.94	5	38	negative regulation of coagulation	BP	1	PDGFRA,ANXA5,PDGFA,CD9,PLAU	yellow	VSMC_M3
GO:0061180	0.15	0.93	6	58	mammary gland epithelium development	BP	1	SCRIB,DEAF1,GPX1,PHB2,AKT1,ERBB4	yellow	VSMC_M3
GO:0062125	0.15	0.95	4	27	regulation of mitochondrial gene expression	BP	1	TACO1,CHCHD10,MPV17L2,C1QBP	yellow	VSMC_M3

GO:0019915	0.15	0.93	7	65	lipid storage	BP	1	ENPP1,SREBF2,SQLE,PPARD,ZC3H12A,SCARB1,ALKBH7	yellow	VSMC_M3
GO:0030574	0.15	0.95	4	31	collagen catabolic process	BP	1	PEPD,MMP24,MMP14,MRC2	yellow	VSMC_M3
GO:0042326	0.15	0.89	26	311	negative regulation of phosphorylation	BP	1	ENPP1,CTDSP2,H2AFY,PLEC,SOCS5,SPRED2,ZC3H12A,MICAL1,EMILIN1,TARBP2,PDE4D,GREM1,CHP1,QARS,TRIM27,CTDSP1,WASHC1,PKN1,PIP4K2B,MVP,CSK,AKT1,GIT1,CACTIN,MIDN,TRIB2	yellow	VSMC_M3
GO:1901988	0.15	0.90	18	201	negative regulation of cell cycle phase transition	BP	1	CTDSP2,H2AFY,KLF4,PINX1,CCAR2,CDK2AP2,PRMT2,THOC5,CTDSP1,METTL13,BABAM1,SDE2,MIIP,MAD1L1,MAD2L2,ZNF830,NABP2,FBXO6	yellow	VSMC_M3
GO:0031109	0.15	0.92	10	109	microtubule polymerization or depolymerization	BP	1	NUMA1,CCDC57,DCTN1,STMN3,MID1IP1,ARHGEF2,RANGRF,GIT1,GBA2,TUBGCP2	yellow	VSMC_M3
GO:0030098	0.15	0.90	22	259	lymphocyte differentiation	BP	1	NCAPH2,FNIP1,LGALS1,SOCS5,ZC3H12A,RELB,CD79A,METTL3,SOD1,EGR3,DLL4,TYRO3,MMP14,CLCF1,RIPK3,SART1,PBX1,BAD,ARMC5,DOCK11,AP3D1,STK11	yellow	VSMC_M3
GO:0072583	0.15	0.94	5	46	clathrin-dependent endocytosis	BP	1	SIGLEC1,AP2A2,TNK2,FCHO1,CLTB	yellow	VSMC_M3
GO:0072659	0.15	0.90	20	239	protein localization to plasma membrane	BP	1	SQSTM1,PACSIN1,SCRIB,TTC7A,TPBG,EHD2,SLC9A3R1,BCL2L1,PLEKHF1,TSPAN5,MMP14,ZDHHC3,LGALS3,TNFRSF1A,RANGRF,CSK,AKT1,PACS2,RAB34,ARFRP1	yellow	VSMC_M3
GO:0019438	0.15	0.86	210	2902	aromatic compound biosynthetic process	BP	1	ENPP1,NAB2,NHP2,CDK4,SQSTM1,ZNF608,HDAC10,NELFE,FNIP1,ATF6B,PBX2,RING1,SREBF2,H2AFY,TRIM11,GUK1,TRAPPC9,PPARD,TSTA3,SND1,PHF2,CNOT3,KLF4,HOXA1,FHL3,ZC3H12A,PNKP,CC2D1B,KTI12,KMT2E,SIX5,ZNF282,ZNF296,RELB,DMAP1,CIC,GTF3C2,OGG1,SERTAD1,TTC5,CXXC5,GNPDA1,DEAF1,HOXC4,POLR1D,KMT2B,NAT10,ZNF740,RALY,ACSL1,ZBTB38,SLC11A2,TNKS1BP1,PINX1,GREM1,KDM2B,MCRS1,POLR2C,CX3CL1,WNT10B,ACTR5,ANKRD2,EGR3,CCAR2,DNAJC17,DLL4,CHP1,YAF2,HTATIP2,NPM3,KDM1B,CHCHD2,AK2,IRX1,RRP1B,BRD9,PAPSS1,AIP,SRRD,TSHZ2,QARS,IMPDH2,PIR,TRIM27,ZNRD1,PRMT2,THOC5,RIPK3,TWIST2,ADRM1,PSTK,SMARCD2,BRMS1,PPOX,GMEB2,CCDC85B,PRPF6,PBX1,KAT5,RELA,ATP5MC3,ZNF511,ARNT2,ZFPL1,LIMD1,SMARCB1,CHCHD10,SLC25A39,PBX4,UBTF,GATAD2A,ATIC,MEF2B,PHB2,NDP,CTDSP1,MLF2,EID1,SSBP4,METTL13,ZNF394,C11orf95,BUD31,TNFRSF1A,TEAD4,C1QBP,TAF6L,ZBTB49,UQCC3,MTA2,ZNF316,ACLY,MED11,PELP1,CCDC22,FOXK1,KLF3,PKN1,SEPSECS,POLR2A,MAD2L2,SLTM,CTBP1,FBXL19,CRABP2,SP2,ARHGEF2,SNF8,PER1,NFIX,FLAD1,IRF2BPL,TRIM25,ATP5PD,USP43,SMARCA4,CARM1,AATF,MID2,ZNF521,ZNF358,AKT1,BRF1,KHSRP,GTF2F1,RFX2,SAFB,SAFB2,MYDGF,HDGFL2,SIRT6,MAP2K2,CACTIN,CDK11B,SSU72,HIC1,MNT,CTNS,EHMT1,AP3D1,IRAK1,MBD3,ATP5F1D,TELO2,TAZ,MED16,CARD9,AMPD2,SARS,APRT,GTF3C5,AK1,TRADD,RF00024,ERBB4,JUND,PTMA,KLF13,FADS1,ZNF783,HOXC5,CAVIN1,IER5,AKAP17A,ZNF250,THAP7,NSMCE3,ERF,SNAPC2,ZSCAN1	yellow	VSMC_M3
GO:1902533	0.16	0.88	57	731	positive regulation of intracellular signal transduction	BP	1	NELFE,FNIP1,PRR5,PPARD,EEF1D,LGALS1,SHARPIN,PDGFRA,TPBG,SPRED2,ZC3H12A,SLC9A3R1,SEC13,CXXC5,LAMTOR1,CAVIN3,PLEKHF1,GREM1,CX3CL1,SOD1,CCAR2,PDGFA,GPX1,TRIM27,RIPK3,RELA,RHOC,MAP3K14,PHB2,IGFBP5,BAD,LAMTOR4,IFI35,TNFRSF1A,C1QBP,CHERP,CCDC22,TRIM25,RPL37,S100A4,CSK,MID2,MYDGF,MAP2K2,HIC1,CTNS,IRAK1,TELO2,WDR24,JMJD8,CARD9,TRADD,NDST1,ERBB4,IGFBP6,ZNF385A,GLIPR2	yellow	VSMC_M3
GO:0046148	0.16	0.93	6	52	pigment biosynthetic process	BP	1	SLC11A2,SRRD,PPOX,SLC25A39,CTNS,APRT	yellow	VSMC_M3
GO:0035904	0.16	0.94	5	50	aorta development	BP	1	PLXND1,SNX17,DLL4,NDST1,LOXL1	yellow	VSMC_M3
GO:0032535	0.16	0.89	26	325	regulation of cellular component size	BP	1	CDK4,SNX9,DBNL,SLC9A3R1,BAIAP2,SWAP70,CAPG,RIN3,CORO1B,TRIM27,ARPIN,LIMK1,LAMTOR4,ARPC1B,WASHC1,DBN1,CRABP2,CORO1A,AKT1,ANAPC2,MIR214,TWF2,ELN,CDC42EP5,GBA2,ARPC5L	yellow	VSMC_M3
GO:0046386	0.16	0.94	4	27	deoxyribose phosphate catabolic process	BP	1	DNPH1,OGG1,SMUG1,NT5C	yellow	VSMC_M3
GO:0060966	0.16	0.94	5	42	regulation of gene silencing by RNA	BP	1	ZC3H12A,DND1,TARBP2,LIMD1,MAP2K2	yellow	VSMC_M3
GO:0090277	0.16	0.92	7	65	positive regulation of peptide hormone secretion	BP	1	PPARD,MYRIP,AACS,PCK2,CAPN10,BAD,GLUD1	yellow	VSMC_M3
GO:0006356	0.16	0.94	4	31	regulation of transcription by RNA polymerase I	BP	1	H2AFY,SMARCB1,UBTF,SMARCA4	yellow	VSMC_M3
GO:0009059	0.16	0.86	258	3569	macromolecule biosynthetic process	BP	1	ENPP1,NAB2,NHP2,CDK4,SQSTM1,ZNF608,HDAC10,NELFE,FNIP1,ATF6B,PBX2,RING1,SREBF2,H2AFY,TRIM11,TRAPPC9,PPARD,EEF1D,RPL3,RPL8,SND1,B4GALT1,MRPS18A,AARS2,PIGO,SECISBP2,PHF2,RPS9,CNOT3,KLF4,HOXA1,FHL3,ZC3H12A,PNKP,CC2D1B,KTI12,KMT2E,SIX5,ZNF282,ZNF296,RELB,SERP2,DMAP1,CIC,GTF3C2,UNK,GALNT14,OGG1,SERTAD1,TTC5,METTL3,PHLDA1,CXXC5,DND1,RPL29,EIF4EBP1,DEAF1,HOXC4,POLR1D,TARBP2,KMT2B,NAT10,ZNF740,EIF4B,RALY,ZBTB38,TNKS1BP1,PINX1,GATB,GREM1,KDM2B,MCRS1,POLR2C,CX3CL1,WNT10B,ACTR5,ANKRD2,EGR3,CCAR2,DNAJC17,DLL4,RPLP2,CHP1,MRPL43,YAF2,HTATIP2,PCIF1,NPM3,KDM1B,CHCHD2,SLC35C2,CARS,ST3GAL4,DCPS,IRX1,SELENOS,RRP1B,TMTC1,BRD9,APEH,AIP,NGDN,TSHZ2,QARS,PIR,TRIM27,EIF3A,ZNRD1,PRMT2,THOC5,RIPK3,TWIST2,LSM14B,ADRM1,PSTK,RPS21,SMARCD2,EXTL1,BRMS1,TACO1,GMEB2,CCDC85B,PRPF6,PBX1,KAT5,RELA,ZNF511,LRWD1,ARNT2,MRPL49,ZFPL1,LIMD1,ZDHHC3,SMARCB1,CHCHD10,PBX4,UBTF,GATAD2A,MEF2B,PHB2,NDP,IGFBP5,CTDSP1,MLF2,EID1,SSBP4,METTL13,MPV17L2,ZNF394,C11orf95,BUD31,TNFRSF1A,TEAD4,C1QBP,AIMP2,TAF6L,ZBTB49,MTA2,ZNF316,MED11,PELP1,CCDC22,FOXK1,SDE2,KLF3,PKN1,SEPSECS,POLR2A,MAD2L2,SLTM,NANOS3,MPDU1,CTBP1,FBXL19,CRABP2,SP2,ARHGEF2,SNF8,PER1,NFIX,DPM3,FARSA,IRF2BPL,TUFM,TRIM25,USP43,RPL37,SMARCA4,CARM1,RPL7A,EEF2K,AATF,MRPL4,EIF3G,C19orf66,MID2,ZNF521,ZNF358,ZNF830,AKT1,BRF1,PAIP1,KHSRP,GTF2F1,RFX2,SAFB,SAFB2,MYDGF,HDGFL2,SIRT6,MAP2K2,CACTIN,CDK11B,SSU72,HIC1,MRPL20,TRAP1,MNT,EHMT1,AP3D1,MKNK[illegible]	yellow	VSMC_M3
GO:0007281	0.16	0.90	16	177	germ cell development	BP	1	SBF1,SIX5,METTL3,CEP131,DND1,YIF1B,DEAF1,BCL2L1,SLC26A6,IQCG,PLA2G3,NANOS3,ZNF830,AKT1,RFX2,MEIG1	yellow	VSMC_M3

GO:0009145	0.16	0.93	6	47	purine nucleoside triphosphate biosynthetic process	BP	1	IMPDH2,ATP5MC3,UQCC3,ATP5PD,ATP5F1D,TAZ	yellow	VSMC_M3
GO:0003176	0.16	0.94	4	34	aortic valve development	BP	1	EMILIN1,DLL4,TNFRSF1A,ELN	yellow	VSMC_M3
GO:0006790	0.16	0.89	24	270	sulfur compound metabolic process	BP	1	ENPP1,MPST,MICAL1,FASN,GSR,ACSL1,SOD1,ST3GAL4,GSTO1,MVK,PAPSS1,GPX1,IDH1,ACLY,MTHFR,ACSF2,PMVK,HMGCS1,BLMH,CTNS,NUBP2,MVD,NDST1,EEF1G	yellow	VSMC_M3
GO:0032496	0.16	0.89	20	225	response to lipopolysaccharide	BP	1	CDK4,HNRNPA0,PPARD,ZC3H12A,SCARB1,CX3CL1,SELENOS,PABPN1,PCK2,RELA,CNP,CAPN2,PALM3,AKT1,GIT1,LDOC1,CACTIN,IRAK1,JUND,LOXL1	yellow	VSMC_M3
GO:0033108	0.16	0.91	10	83	mitochondrial respiratory chain complex assembly	BP	1	SAMM50,NDUFB9,ACAD9,SDHAF1,UQCRFS1,TACO1,UQCC3,NDUFS7,TAZ,SURF1	yellow	VSMC_M3
GO:0071320	0.16	0.94	4	37	cellular response to cAMP	BP	1	WNT10B,SLC26A6,IGFBP5,EEF2K	yellow	VSMC_M3
GO:1903320	0.16	0.89	19	211	regulation of protein modification by small protein conjugation or removal	BP	1	SQSTM1,VPS28,HSPBP1,ZC3H12A,PINX1,CHP1,LIMK1,RELA,OTUB1,AIMP2,UBXN1,WASHC1,AMER1,MAD2L2,RPL37,AKT1,HMG20B,STUB1,NSMCE3	yellow	VSMC_M3
GO:0051090	0.16	0.88	27	328	regulation of DNA-binding transcription factor activity	BP	1	ENPP1,TRAPPC9,KLF4,ZC3H12A,GREM1,CX3CL1,WNT10B,CHP1,TRIM27,PRMT2,RIPK3,BRMS1,RELA,SMARCB1,PHB2,BUD31,CCDC22,MAD2L2,ARHGEF2,TRIM25,SMARCA4,MID2,AKT1,CACTIN,IRAK1,CARD9,TRADD	yellow	VSMC_M3
GO:0019692	0.16	0.93	5	37	deoxyribose phosphate metabolic process	BP	1	GUK1,DNPH1,OGG1,SMUG1,NT5C	yellow	VSMC_M3
GO:0018022	0.16	0.91	10	108	peptidyl-lysine methylation	BP	1	NELFE,H2AFY,KMT2E,KMT2B,SETD1B,ETFBKMT,SMARCB1,TRMT112,METTL22,EHMT1	yellow	VSMC_M3
GO:0090305	0.16	0.89	19	213	nucleic acid phosphodiester bond hydrolysis	BP	1	ENPP1,NHP2,BOP1,SND1,ZC3H12A,PNKP,OGG1,C12orf10,RRS1,DCPS,ZNRD1,KHNYN,RPS21,NOP14,CSTF2T,DFFA,SLX1A,TBL3,TRIR	yellow	VSMC_M3
GO:0060337	0.16	0.93	5	41	type I interferon signaling pathway	BP	1	TTLL12,METTL3,CDC37,CACTIN,IRAK1	yellow	VSMC_M3
GO:0032720	0.16	0.93	5	41	negative regulation of tumor necrosis factor production	BP	1	ZC3H12A,CX3CL1,SELENOS,TRIM27,CACTIN	yellow	VSMC_M3
GO:0051651	0.16	0.90	14	163	maintenance of location in cell	BP	1	ITPR3,SLC30A3,INSIG1,PDE4D,CX3CL1,GSTO1,CHCHD10,LETM1,CHERP,DBN1,CORO1A,AKT1,AP3D1,TWF2	yellow	VSMC_M3
GO:0034654	0.16	0.86	205	2841	nucleobase-containing compound biosynthetic process	BP	1	ENPP1,NAB2,NHP2,CDK4,SQSTM1,ZNF608,HDAC10,NELFE,FNIP1,ATF6B,PBX2,RING1,SREBF2,H2AFY,TRIM11,GUK1,TRAPPC9,PPARD,TSTA3,SND1,PHF2,CNOT3,KLF4,HOXA1,FHL3,ZC3H12A,PNKP,CC2D1B,KTI12,KMT2E,SIX5,ZNF282,ZNF296,RELB,DMAP1,CIC,GTF3C2,OGG1,SERTAD1,TTC5,CXXC5,GNPDA1,DEAF1,HOXC4,POLR1D,KMT2B,NAT10,ZNF740,RALY,ACSL1,ZBTB38,TNKS1BP1,PINX1,GREM1,KDM2B,MCRS1,POLR2C,CX3CL1,WNT10B,ACTR5,ANKRD2,EGR3,CCAR2,DNAJC17,DLL4,CHP1,YAF2,HTATIP2,NPM3,KDM1B,CHCHD2,AK2,IRX1,RRP1B,BRD9,PAPSS1,AIP,TSHZ2,QARS,IMPDH2,PIR,TRIM27,ZNRD1,PRMT2,THOC5,RIPK3,TWIST2,ADRM1,PSTK,SMARCD2,BRMS1,GMEB2,CCDC85B,PRPF6,PBX1,KAT5,RELA,ATP5MC3,ZNF511,ARNT2,ZFPL1,LIMD1,SMARCB1,CHCHD10,PBX4,UBTF,GATAD2A,ATIC,MEF2B,PHB2,NDP,CTDSP1,MLF2,EID1,SSBP4,METTL13,ZNF394,C11orf95,BUD31,TNFRSF1A,TEAD4,C1QBP,TAF6L,ZBTB49,UQCC3,MTA2,ZNF316,ACLY,MED11,PELP1,CCDC22,FOXK1,KLF3,PKN1,SEPSECS,POLR2A,MAD2L2,SLTM,CTBP1,FBXL19,CRABP2,SP2,ARHGEF2,SNF8,PER1,NFIX,FLAD1,IRF2BPL,TRIM25,ATP5PD,USP43,SMARCA4,CARM1,AATF,MID2,ZNF521,ZNF358,AKT1,BRF1,KHSRP,GTF2F1,RFX2,SAFB,SAFB2,MYDGF,HDGFL2,SIRT6,MAP2K2,CACTIN,CDK11B,SSU72,HIC1,MNT,EHMT1,AP3D1,IRAK1,MBD3,ATP5F1D,TELO2,TAZ,MED16,CARD9,AMPD2,SARS,APRT,GTF3C5,AK1,TRADD,RF00024,ERBB4,JUND,PTMA,KLF13,FADS1,ZNF783,HOXC5,CAVIN1,IER5,AKAP17A,ZNF250,THAP7,NSMCE3,ERF,SNAPC2,ZSCAN1	yellow	VSMC_M3
GO:1902893	0.17	0.93	5	47	regulation of pri-miRNA transcription by RNA polymerase II	BP	1	SREBF2,PPARD,KLF4,RELA,SMARCA4	yellow	VSMC_M3
GO:0060271	0.17	0.89	22	266	cilium assembly	BP	1	SPAG1,TXNDC15,WDPCP,EHD2,SEPT9,CEP131,YIF1B,CCDC57,DCTN1,CCDC28B,PCNT,IQCG,PLA2G3,CEP164,PTPN23,RABEP2,ATG3,TTC39C,RFX2,GAS8,WDR34,MEIG1	yellow	VSMC_M3
GO:0072329	0.17	0.91	10	95	monocarboxylic acid catabolic process	BP	1	ACAT2,PPARD,CPT1C,PCCB,ETFBKMT,PCK2,ILVBL,PGD,AKT1,SLC27A4	yellow	VSMC_M3
GO:0046902	0.17	0.92	6	50	regulation of mitochondrial membrane permeability	BP	1	BCL2L1,CHCHD10,BAD,CNP,ALKBH7,SPG7	yellow	VSMC_M3
GO:0032963	0.17	0.91	8	78	collagen metabolic process	BP	1	PPARD,RCN3,EMILIN1,TNS2,PEPD,MMP24,MMP14,MRC2	yellow	VSMC_M3
GO:0070227	0.17	0.92	6	52	lymphocyte apoptotic process	BP	1	FNIP1,RIPK3,LGALS3,PKN1,DFFA,AKT1	yellow	VSMC_M3
GO:0007015	0.17	0.88	29	373	actin filament organization	BP	1	SNX9,PACSIN1,PLEC,DBNL,ZYX,MICAL1,BAIAP2,INPPL1,SWAP70,CAPG,CX3CL1,CORO1B,TRIM27,ARPIN,LIMK1,RHOC,ARPC1B,WASHC1,DBN1,MAD2L2,ARHGEF2,CORO1A,MYO19,MIR214,TWF2,ELN,CDC42EP5,GBA2,ARPC5L	yellow	VSMC_M3
GO:0035315	0.17	0.94	4	34	hair cell differentiation	BP	1	SCRIB,WDPCP,SLC9A3R1,SOD1	yellow	VSMC_M3
GO:0010569	0.17	0.93	5	43	regulation of double-strand break repair via homologous recombination	BP	1	FIGNL1,MAD2L2,PPP4C,CXorf57,SIRT6	yellow	VSMC_M3
GO:0000768	0.17	0.94	4	34	syncytium formation by plasma membrane fusion	BP	1	EHD2,PLEKHO1,CD9,CAPN2	yellow	VSMC_M3
GO:0140253	0.17	0.94	4	34	cell-cell fusion	BP	1	EHD2,PLEKHO1,CD9,CAPN2	yellow	VSMC_M3
GO:0002183	0.17	0.94	4	30	cytoplasmic translational initiation	BP	1	METTL3,EIF4B,EIF3A,EIF3G	yellow	VSMC_M3
GO:0042073	0.17	0.94	4	34	intraciliary transport	BP	1	IFT27,CEP131,SSNA1,WDR34	yellow	VSMC_M3
GO:2001237	0.17	0.91	8	79	negative regulation of extrinsic apoptotic signaling pathway	BP	1	BCL2L1,CX3CL1,ITPRIP,GPX1,RELA,LGALS3,ARHGEF2,AKT1	yellow	VSMC_M3

GO:0023056	0.17	0.86	93	1258	positive regulation of signaling	BP	1	CD63,SQSTM1,NELFE,FNIP1,PRR5,TXNDC15,ITPR3,PPARD,EEF1D,SCRIB,LGALS1,SHARPIN,PDGFRA,IL1R1,TPBG,SPRED2,ZC3H12A,PLEKHA4,SLC9A3R1,RNF220,MYRIP,SEC13,BAIAP2,CXXC5,LAMTOR1,NUMA1,CAVIN3,AACS,PLEKHF1,GREM1,RAPSN,CX3CL1,WNT10B,SOD1,CCAR2,DLL4,SLC35C2,TSPAN5,PDGFA,GPX1,SYT12,PCK2,TRIM27,RIPK3,CAPN10,PLA2G3,PTPN23,RELA,RHOC,MAP3K14,ZDHHC3,SMARCB1,PHB2,IGFBP5,BAD,LAMTOR4,IFI35,TNFRSF1A,C1QBP,CHERP,EVC,CCDC22,GLUD1,AMER1,TRIM25,RPL37,S100A4,SMARCA4,CSK,MID2,TEDC1,RAB34,MYDGF,MAP2K2,HIC1,CTNS,TEDC2,CSNK1G2,IRAK1,STK11,TELO2,WDR24,JMJD8,CARD9,GAS8,TGFBR3,TRADD,NDST1,TNFRSF12A,ERBB4,IGFBP6,ZNF385A,GLIPR2	yellow	VSMC_M3
GO:0072522	0.17	0.89	15	157	purine-containing compound biosynthetic process	BP	1	GUK1,ACSL1,AK2,PAPSS1,IMPDH2,ATP5MC3,ATIC,UQCC3,ACLY,ATP5PD,ATP5F1D,TAZ,AMPD2,APRT,AK1	yellow	VSMC_M3
GO:0002793	0.17	0.92	7	66	positive regulation of peptide secretion	BP	1	PPARD,MYRIP,AACS,PCK2,CAPN10,BAD,GLUD1	yellow	VSMC_M3
GO:0045333	0.17	0.89	19	191	cellular respiration	BP	1	NDUFB9,PLEC,IDH3B,OGDHL,UQCRFS1,CHCHD2,SDHA,NDUFV1,UQCRC1,IDH1,CHCHD10,UQCC3,ATP5PD,AKT1,TRAP1,NDUFS7,ATP5F1D,TAZ,SURF1	yellow	VSMC_M3
GO:0014066	0.17	0.91	8	86	regulation of phosphatidylinositol 3-kinase signaling	BP	1	PRR5,PPARD,PDGFRA,KLF4,SLC9A3R1,PDGFA,MYDGF,ERBB4	yellow	VSMC_M3
GO:0030488	0.17	0.93	5	39	tRNA methylation	BP	1	TRMT12,WDR4,TRMT112,GTPBP3,TRMT1	yellow	VSMC_M3
GO:2000134	0.17	0.92	6	56	negative regulation of G1/S transition of mitotic cell cycle	BP	1	CTDSP2,KLF4,CDK2AP2,PRMT2,CTDSP1,SDE2	yellow	VSMC_M3
GO:0009262	0.17	0.93	5	38	deoxyribonucleotide metabolic process	BP	1	GUK1,DNPH1,OGG1,SMUG1,NT5C	yellow	VSMC_M3
GO:0022412	0.17	0.88	21	247	cellular process involved in reproduction in multicellular organism	BP	1	NCAPH2,SBF1,FIGNL1,SIX5,METTL3,CEP131,DND1,YIF1B,DEAF1,BCL2L1,KDM1B,SLC26A6,IQCG,PLA2G3,CD9,NANOS3,ZNF830,AKT1,RFX2,TAZ,MEIG1	yellow	VSMC_M3
GO:0051817	0.17	0.91	7	67	modulation of process of other organism involved in symbiotic interaction	BP	1	ZC3H12A,BCL2L1,RRP1B,SMARCB1,PHB2,SMARCA4,CARD9	yellow	VSMC_M3
GO:0009636	0.17	0.89	16	174	response to toxic substance	BP	1	CDK4,MPST,GSR,ARHGAP33,SOD1,SRSF9,SELENOS,GSTO1,GPX1,MGST3,TXNRD2,CNP,PRXL2A,SLC6A4,BLMH,EHMT1	yellow	VSMC_M3
GO:0030433	0.17	0.91	8	78	ubiquitin-dependent ERAD pathway	BP	1	BAG6,TMUB1,SEL1L2,SELENOS,TRIM25,MAN1B1,FBXO6,STUB1	yellow	VSMC_M3
GO:0051348	0.17	0.88	19	219	negative regulation of transferase activity	BP	1	H2AFY,PLEC,SOCS5,TARBP2,PINX1,CHP1,QARS,TRIM27,LIMK1,WASHC1,PKN1,PIP4K2B,MAD2L2,MVP,RPL37,CSK,AKT1,MIDN,TRIB2	yellow	VSMC_M3
GO:0032200	0.17	0.90	12	130	telomere organization	BP	1	NHP2,PNKP,H3F3B,NAT10,TNKS1BP1,PINX1,H3F3A,SLX1A,SIRT6,TELO2,RF00024,NABP2	yellow	VSMC_M3
GO:0060976	0.18	0.93	4	39	coronary vasculature development	BP	1	PLXND1,SNX17,TGFBR3,NDST1	yellow	VSMC_M3
GO:2000113	0.18	0.85	85	1144	negative regulation of cellular macromolecule biosynthetic process	BP	1	ENPP1,NAB2,SQSTM1,ZNF608,HDAC10,NELFE,FNIP1,RING1,SREBF2,H2AFY,TRIM11,PPARD,CNOT3,KLF4,ZC3H12A,CC2D1B,SIX5,ZNF282,ZNF296,RELB,DMAP1,CIC,UNK,METTL3,CXXC5,DND1,EIF4EBP1,DEAF1,NAT10,ZBTB38,PINX1,GREM1,KDM2B,WNT10B,ANKRD2,CCAR2,DNAJC17,DLL4,YAF2,PCIF1,KDM1B,DCPS,IRX1,SELENOS,QARS,TRIM27,PRMT2,TWIST2,BRMS1,CCDC85B,KAT5,RELA,LIMD1,GATAD2A,PHB2,IGFBP5,EID1,METTL13,C11orf95,C1QBP,ZBTB49,MTA2,FOXK1,KLF3,MAD2L2,NANOS3,CTBP1,SP2,PER1,NFIX,IRF2BPL,SMARCA4,AATF,C19orf66,ZNF830,KHSRP,SIRT6,HIC1,MNT,EHMT1,MBD3,SARS,THAP7,NSMCE3,ERF	yellow	VSMC_M3
GO:0061028	0.18	0.93	4	38	establishment of endothelial barrier	BP	1	FASN,PDE4D,TNFRSF1A,TJP3	yellow	VSMC_M3
GO:0071357	0.18	0.92	5	42	cellular response to type I interferon	BP	1	TTLL12,METTL3,CDC37,CACTIN,IRAK1	yellow	VSMC_M3
GO:0048066	0.18	0.93	4	32	developmental pigmentation	BP	1	ENPP1,CD63,AP1M1,AP3D1	yellow	VSMC_M3
GO:0061614	0.18	0.92	5	48	pri-miRNA transcription by RNA polymerase II	BP	1	SREBF2,PPARD,KLF4,RELA,SMARCA4	yellow	VSMC_M3
GO:0043254	0.18	0.87	29	361	regulation of protein-containing complex assembly	BP	1	SNX9,FNIP1,DBNL,BAIAP2,NUMA1,EIF4EBP1,CAPG,DCTN1,WNT10B,CORO1B,TRIM27,ARPIN,RHOC,PEX5,LGALS3,ARPC1B,WASHC1,DBN1,RANGRF,CORO1A,GIT1,NCLN,MIR214,TWF2,ELN,CDC42EP5,GBA2,STUB1,ARPC5L	yellow	VSMC_M3
GO:0031327	0.18	0.85	89	1198	negative regulation of cellular biosynthetic process	BP	1	ENPP1,NAB2,SQSTM1,ZNF608,HDAC10,NELFE,FNIP1,RING1,SREBF2,H2AFY,TRIM11,PPARD,CNOT3,KLF4,ZC3H12A,CC2D1B,SIX5,ZNF282,ZNF296,RELB,DMAP1,CIC,UNK,INSIG1,METTL3,CXXC5,DND1,EIF4EBP1,DEAF1,NAT10,ZBTB38,PINX1,GREM1,KDM2B,WNT10B,ANKRD2,CCAR2,DNAJC17,DLL4,YAF2,PCIF1,KDM1B,DCPS,IRX1,SELENOS,PDGFA,QARS,TRIM27,PRMT2,TWIST2,BRMS1,CCDC85B,KAT5,RELA,LIMD1,GATAD2A,PHB2,IGFBP5,EID1,METTL13,C11orf95,C1QBP,ZBTB49,MTA2,FOXK1,KLF3,MAD2L2,NANOS3,CTBP1,SP2,PER1,NFIX,IRF2BPL,SMARCA4,AATF,C19orf66,ZNF830,KHSRP,SIRT6,HIC1,MNT,CTNS,EHMT1,PGP,MBD3,SARS,THAP7,NSMCE3,ERF	yellow	VSMC_M3
GO:0032527	0.18	0.92	5	43	protein exit from endoplasmic reticulum	BP	1	INSIG1,SEC13,HM13,SELENOS,RANGRF	yellow	VSMC_M3
GO:0006805	0.18	0.92	5	41	xenobiotic metabolic process	BP	1	GUK1,SMOX,ACSL1,GSTO1,AIP	yellow	VSMC_M3
GO:1901991	0.18	0.89	13	142	negative regulation of mitotic cell cycle phase transition	BP	1	CTDSP2,KLF4,PINX1,CDK2AP2,PRMT2,CTDSP1,BABAM1,SDE2,MIIP,MAD1L1,MAD2L2,ZNF830,NABP2	yellow	VSMC_M3
GO:0046434	0.18	0.89	12	126	organophosphate catabolic process	BP	1	ENPP1,NUDT3,DNPH1,OGG1,SMUG1,SCARB1,PDE4D,IDH1,CNP,PLA2G4E,NT5C,ITPA	yellow	VSMC_M3
GO:0006754	0.18	0.92	5	39	ATP biosynthetic process	BP	1	ATP5MC3,UQCC3,ATP5PD,ATP5F1D,TAZ	yellow	VSMC_M3
GO:1902895	0.18	0.93	4	37	positive regulation of pri-miRNA transcription by RNA polymerase II	BP	1	SREBF2,KLF4,RELA,SMARCA4	yellow	VSMC_M3
GO:1902041	0.18	0.92	5	42	regulation of extrinsic apoptotic signaling pathway via death domain receptors	BP	1	BCL2L1,ITPRIP,GPX1,LGALS3,ARHGEF2	yellow	VSMC_M3
GO:0009062	0.18	0.90	9	86	fatty acid catabolic process	BP	1	ACAT2,PPARD,CPT1C,PCCB,ETFBKMT,PCK2,ILVBL,AKT1,SLC27A4	yellow	VSMC_M3
GO:1903432	0.18	0.93	4	32	regulation of TORC1 signaling	BP	1	SEC13,CTNS,STK11,TELO2	yellow	VSMC_M3
GO:0060999	0.19	0.93	4	38	positive regulation of dendritic spine development	BP	1	DBNL,BAIAP2,DBN1,EEF2K	yellow	VSMC_M3
GO:0006949	0.19	0.93	4	35	syncytium formation	BP	1	EHD2,PLEKHO1,CD9,CAPN2	yellow	VSMC_M3
GO:0014910	0.19	0.91	6	60	regulation of smooth muscle cell migration	BP	1	PPARD,PDGFA,CORO1B,IGFBP5,PLAU,MIR214	yellow	VSMC_M3

GO:0010647	0.19	0.84	92	1251	positive regulation of cell communication	BP	1	CD63,SQSTM1,NELFE,FNIP1,PRR5,TXNDC15,ITPR3,PPARD,EEF1D,LGALS1,SHARPIN,PDGFRA,IL1R1,TPBG,SPRED2,ZC3H12A,PLEKHA4,SLC9A3R1,RNF220,MYRIP,SEC13,BAIAP2,CXXC5,LAMTOR1,NUMA1,CAVIN3,AACS,PLEKHF1,GREM1,RAPSN,CX3CL1,WNT10B,SOD1,CCAR2,DLL4,SLC35C2,TSPAN5,PDGFA,GPX1,SYT12,PCK2,TRIM27,RIPK3,CAPN10,PLA2G3,PTPN23,RELA,RHOC,MAP3K14,ZDHHC3,SMARCB1,PHB2,IGFBP5,BAD,LAMTOR4,IFI35,TNFRSF1A,C1QBP,CHERP,EVC,CCDC22,GLUD1,AMER1,TRIM25,RPL37,S100A4,SMARCA4,CSK,MID2,TEDC1,RAB34,MYDGF,MAP2K2,HIC1,CTNS,TEDC2,CSNK1G2,IRAK1,STK11,TELO2,WDR24,JMJD8,CARD9,GAS8,TGFBR3,TRADD,NDST1,TNFRSF12A,ERBB4,IGFBP6,ZNF385A,GLIPR2	yellow	VSMC_M3
GO:0097193	0.19	0.87	21	242	intrinsic apoptotic signaling pathway	BP	1	BAG6,FIGNL1,SLC9A3R1,P4HB,BCL2L1,PLEKHF1,SOD1,CCAR2,SELENOS,GPX1,RIPK3,ZNF385B,BAD,TNFRSF1A,ARHGEF2,AKT1,HIC1,TRAP1,STK11,ZNF385A,TXNDC12	yellow	VSMC_M3
GO:0044419	0.19	0.84	73	948	biological process involved in interspecies interaction between organisms	BP	1	CDK4,TTLL12,HNRNPA0,TRIM11,PPARD,LGALS1,SLC52A2,PPP1R14B,SSC5D,ZC3H12A,ZYX,SNX3,KRT8,RELB,METTL3,BAIAP2,P4HB,RAB20,SIGLEC1,TARBP2,SCARB1,BCL2L1,ITGB7,CX3CL1,TYRO3,RNF19B,SELENOS,RRP1B,PABPN1,GPX1,PCK2,NECTIN1,ANPEP,TRIM27,SLC26A6,THOC5,RIPK3,TNK2,BANF1,RELA,MAP3K14,SMARCB1,THOC3,PHB2,IFI35,LGALS3,TNFRSF1A,C1QBP,CNP,CAPN2,PALM3,TBKBP1,ARHGEF2,ARMC5,CORO1A,TRIM25,SMARCA4,CDC37,C19orf66,MID2,AKT1,GTF2F1,GIT1,LDOC1,CACTIN,CLDN6,IRAK1,C8G,CARD9,JUND,LOXL1,EEF1G,JAGN1	yellow	VSMC_M3
GO:0080164	0.19	0.92	5	45	regulation of nitric oxide metabolic process	BP	1	KLF4,ZC3H12A,SELENOS,AKT1,KHSRP	yellow	VSMC_M3
GO:0001558	0.19	0.86	27	339	regulation of cell growth	BP	1	ENPP1,PPARD,DNPH1,DBNL,H3F3B,LAMTOR1,GREM1,KDM2B,CCAR2,CISH,MMP14,LIMK1,CCDC85B,SIPA1,IGFBP5,H3F3A,DBN1,MAD2L2,CRABP2,SMARCA4,AKT1,HDGFL2,CDK11B,ANAPC2,STK11,TWF2,UNC13A	yellow	VSMC_M3
GO:0070988	0.19	0.92	5	52	demethylation	BP	1	PHF2,KDM2B,KDM1B,ALKBH4,FBXL19	yellow	VSMC_M3
GO:0007162	0.19	0.87	18	215	negative regulation of cell adhesion	BP	1	SCRIB,LGALS1,SOCS5,KLF4,ZC3H12A,PLXND1,METTL3,SWAP70,PDE3B,CX3CL1,MMP14,SIPA1,LGALS3,CD9,RCC2,MAD1L1,MAD2L2,AKT1	yellow	VSMC_M3
GO:0031113	0.19	0.92	5	49	regulation of microtubule polymerization	BP	1	NUMA1,DCTN1,RANGRF,GIT1,GBA2	yellow	VSMC_M3
GO:0010558	0.19	0.84	85	1150	negative regulation of macromolecule biosynthetic process	BP	1	ENPP1,NAB2,SQSTM1,ZNF608,HDAC10,NELFE,FNIP1,RING1,SREBF2,H2AFY,TRIM11,PPARD,CNOT3,KLF4,ZC3H12A,CC2D1B,SIX5,ZNF282,ZNF296,RELB,DMAP1,CIC,UNK,METTL3,CXXC5,DND1,EIF4EBP1,DEAF1,NAT10,ZBTB38,PINX1,GREM1,KDM2B,WNT10B,ANKRD2,CCAR2,DNAJC17,DLL4,YAF2,PCIF1,KDM1B,DCPS,IRX1,SELENOS,QARS,TRIM27,PRMT2,TWIST2,BRMS1,CCDC85B,KAT5,RELA,LIMD1,GATAD2A,PHB2,IGFBP5,EID1,METTL13,C11orf95,C1QBP,ZBTB49,MTA2,FOXK1,KLF3,MAD2L2,NANOS3,CTBP1,SP2,PER1,NFIX,IRF2BPL,SMARCA4,AATF,C19orf66,ZNF830,KHSRP,SIRT6,HIC1,MNT,EHMT1,MBD3,SARS,THAP7,NSMCE3,ERF	yellow	VSMC_M3
GO:0030031	0.19	0.86	34	443	cell projection assembly	BP	1	SPAG1,TXNDC15,DBNL,WDPCP,EMP3,EHD2,SLC9A3R1,SEPT9,CEP131,INPPL1,YIF1B,CCDC57,CAPG,DCTN1,CCDC28B,PDGFA,PCNT,IQCG,PLA2G3,CEP164,PTPN23,WASHC1,RCC2,RABEP2,ATG3,TTC39C,DOCK11,RFX2,GAS8,WDR34,MIR214,TWF2,CDC42EP5,MEIG1	yellow	VSMC_M3
GO:0006996	0.19	0.83	209	2943	organelle organization	BP	1	ESYT1,DCTN2,NHP2,SQSTM1,SPAG1,BAG6,NCAPH2,SNX9,HDAC10,PDLIM4,SAMM50,SREBF2,NDUFB9,SQLE,TXNDC15,H2AFY,PACSIN1,RPL3,PLEC,IFT27,SHARPIN,BOP1,VPS28,CUL7,PDGFRA,PHF2,ASNA1,DBNL,WDPCP,FHL3,FIGNL1,PNKP,ZYX,SNX3,MICAL1,EHD2,KMT2E,KRT8,ACAD9,TMEM43,SLC9A3R1,DMAP1,H3F3B,INSIG1,SEC13,SEPT9,BAIAP2,CEP131,INPPL1,LAMTOR1,NUMA1,YIF1B,CCDC57,RAB20,ARAP3,CAVIN3,SDHAF1,NAT10,BCL2L1,SPRYD3,RRS1,SWAP70,PLEKHF1,UQCRFS1,CAPG,TNKS1BP1,PINX1,DCTN1,CX3CL1,SOD1,PACSIN3,ACTR5,CCDC32,CCAR2,HPS1,CHP1,NPM3,CHCHD2,CCDC28B,BRD9,PDGFA,CDK2AP2,GPX1,AIP,CORO1B,LRFN4,CLIP2,TRIM27,ARPIN,PCNT,LIMK1,NUDC,CAPN10,SMARCD2,IQCG,PFDN2,BUD23,TACO1,PLA2G3,STMN3,CEP164,BANF1,PTPN23,RELA,SIPA1,RHOC,LRWD1,ALKBH4,ZNHIT1,FMNL1,VPS51,SAC3D1,LIMD1,MID1IP1,PEX5,SMARCB1,CHCHD10,BLOC1S4,PHB2,NDP,BAD,MPV17L2,LETM1,ARPC1B,MRTO4,MVB12A,KIAA0753,BABAM1,C1QBP,TAF6L,UQCC3,WASHC1,CNP,AP1M1,EML3,MTA2,H3F3A,DBN1,CAPN2,LMAN2,MAD1L1,PIP4K2B,MAD2L2,FAM161B,ARHGEF2,SNF8,MTX1,RANGRF,STX10,CORO1A,SLX1A,RABEP2,SMARCA4,TMED1,SYNE3,CDC37,MYO19,EML1,ATG3,TTC39C,INF2,ZNF830,AKT1,PACS2,ALKBH7,RFX2,RAB34,SAFB,GIT1,SIRT6,PPL,MAP2K2,UBN1,HMG20B,MRPL20,AURKAIP1,EHMT1,AP3D1,SSNA1,MBD3,NDUFS7,ATP5F1D,TELO2,TAZ,FAM50A,GAS8,SPG7,NTMT1,WDR34,MIR214,RF00024,NABP2,ERBB4,TWF2,SURF1,MAP7D1,JAGN1,MIS12,ELN,UNC13A,CDC42EP5,GBA2,TUBGCP2,MEIG1,TIMM22,ITPA,PHETA2,CCDC115,ARPC5L	yellow	VSMC_M3
GO:0043124	0.19	0.93	4	34	negative regulation of I-kappaB kinase/NF-kappaB signaling	BP	1	ZC3H12A,CCDC22,PER1,CACTIN	yellow	VSMC_M3
GO:0051493	0.19	0.86	34	457	regulation of cytoskeleton organization	BP	1	SNX9,PDGFRA,DBNL,BAIAP2,NUMA1,SWAP70,CAPG,DCTN1,CX3CL1,PDGFA,CDK2AP2,CORO1B,TRIM27,ARPIN,LIMK1,PFDN2,STMN3,RHOC,MID1IP1,ARPC1B,WASHC1,EML3,DBN1,CAPN2,ARHGEF2,RANGRF,CORO1A,GIT1,MIR214,TWF2,ELN,CDC42EP5,GBA2,ARPC5L	yellow	VSMC_M3
GO:1905314	0.19	0.93	4	37	semi-lunar valve development	BP	1	EMILIN1,DLL4,TNFRSF1A,ELN	yellow	VSMC_M3

GO:0006793	0.19	0.83	157	2169	phosphorus metabolic process	BP	1	ENPP1,NAB2,CDK4,CTDSP2,SQSTM1,SBF1,SNX9,CERK,FNIP1,PRR5,H2AFY,GUK1,NUDT3,PPARD,TSTA3,PLEC,PPP1R14B,DNPH1,PIGO,PDGFRA,SOCS5,TTC7A,KLF4,SPRED2,ZC3H12A,PNKP,MICAL1,WDFY2,SLC9A3R1,EMILIN1,SEPHS1,GALK1,PPM1G,PLCD1,OGG1,SERTAD1,INPPL1,PCYT2,FASN,GNPDA1,PTDSS2,SMUG1,OGDHL,TARBP2,SCARB1,PDE4D,TNS2,SWAP70,ACSL1,FDFT1,TNKS1BP1,GREM1,TKT,CX3CL1,SOD1,DGKZ,CHP1,TYRO3,HTATIP2,PPM1M,CISH,AK2,MVK,STK32C,FABP3,PAPSS1,PDGFA,MOCS3,CLCF1,QARS,IMPDH2,TRIM27,LIMK1,RIPK3,PSTK,TNK2,PLA2G3,PTPN23,ATP5MC3,SCYL1,IDH1,MEX3B,MAP3K14,LIMD1,ATIC,PHB2,PPP1R35,BAD,PLCB3,CTDSP1,ISYNA1,LGALS3,TNFRSF1A,ASMTL,UQCC3,WASHC1,CNP,ACLY,FOXK1,PKN1,CAPN2,PIP4K2B,MAD2L2,CTBP1,PGD,ARHGEF2,MVP,ACSF2,DPM3,PPP4C,FLAD1,PMVK,ATP5PD,MTMR4,HDHD2,EEF2K,CDC37,CSK,AKT1,HMGCS1,PGM1,SGK494,GIT1,MYDGF,MAP2K2,CACTIN,CDK11B,SSU72,PGP,MKNK2,CSNK1G2,IRAK1,MIDN,ATP5F1D,STK11,TELO2,TAZ,JMJD8,AMPD2,APRT,MVD,AK1,SLC44A3,VAC14,TGFBR3,ERBB4,PTPRR,PLA2G4E,FADS1,NT5C,TRIB2,NSMF,TALDO1,DUSP5,FDPS,IDI1,ITPA	yellow	VSMC_M3
GO:0071887	0.19	0.90	8	79	leukocyte apoptotic process	BP	1	FNIP1,SELENOS,RIPK3,LGALS3,PKN1,DFFA,PLEKHO2,AKT1	yellow	VSMC_M3
GO:0009156	0.19	0.93	4	30	ribonucleoside monophosphate biosynthetic process	BP	1	IMPDH2,ATIC,AMPD2,APRT	yellow	VSMC_M3
GO:0046785	0.19	0.90	7	74	microtubule polymerization	BP	1	NUMA1,CCDC57,DCTN1,RANGRF,GIT1,GBA2,TUBGCP2	yellow	VSMC_M3
GO:1903556	0.19	0.92	5	43	negative regulation of tumor necrosis factor superfamily cytokine production	BP	1	ZC3H12A,CX3CL1,SELENOS,TRIM27,CACTIN	yellow	VSMC_M3
GO:0022407	0.19	0.86	24	293	regulation of cell-cell adhesion	BP	1	SCRIB,LGALS1,SOCS5,KLF4,ZC3H12A,METTL3,SWAP70,CX3CL1,EGR3,ST3GAL4,SART1,PTPN23,RELA,BAD,LGALS3,CD9,MAD1L1,MAD2L2,CORO1A,CD276,CSK,AKT1,AP3D1,IRAK1	yellow	VSMC_M3
GO:0106027	0.19	0.90	7	78	neuron projection organization	BP	1	DBNL,BAIAP2,ARHGAP33,DCTN1,NGEF,DBN1,EEF2K	yellow	VSMC_M3
GO:0014902	0.19	0.90	8	86	myotube differentiation	BP	1	PLEC,EHD2,WNT10B,MMP14,GPX1,PLEKHO1,CD9,CAPN2	yellow	VSMC_M3
GO:0008585	0.19	0.90	7	72	female gonad development	BP	1	PDGFRA,BCL2L1,SOD1,MMP14,IDH1,SGPL1,ZNF830	yellow	VSMC_M3
GO:0072523	0.19	0.91	5	43	purine-containing compound catabolic process	BP	1	NUDT3,DNPH1,PDE4D,NT5C,ITPA	yellow	VSMC_M3
GO:0030855	0.20	0.85	33	430	epithelial cell differentiation	BP	1	H2AFY,SCRIB,PLEC,SHARPIN,DNPH1,B4GALT1,COL15A1,KLF4,WDPCP,CCM2,SPRED2,SLC9A3R1,NUMA1,FASN,PDE4D,GREM1,WNT10B,SOD1,GPX1,TAGLN2,PCK2,BAD,LGALS3,TNFRSF1A,AIMP2,KRT10,CASP14,TMEM79,AKT1,SAFB2,PPL,TJP3,ERBB4	yellow	VSMC_M3
GO:0030879	0.20	0.89	10	114	mammary gland development	BP	1	SCRIB,FASN,DEAF1,GPX1,PHB2,IGFBP5,MTX1,AKT1,APRT,ERBB4	yellow	VSMC_M3
GO:0050764	0.20	0.90	6	59	regulation of phagocytosis	BP	1	SNX3,SCARB1,SOD1,LMAN2,CSK,ATG3	yellow	VSMC_M3
GO:0007517	0.20	0.86	21	259	muscle organ development	BP	1	PLEC,COL19A1,FHL3,WNT10B,ANKRD2,EGR3,DLL4,GPX1,MEF2B,TEAD4,EVC,FOXK1,PRR14,COPRS,SIRT6,HMG20B,TAZ,GTF3C5,TGFBR3,IGSF8,ELN	yellow	VSMC_M3
GO:0010762	0.20	0.92	4	35	regulation of fibroblast migration	BP	1	WDPCP,RCC2,MTA2,AKT1	yellow	VSMC_M3
GO:1903201	0.20	0.90	6	56	regulation of oxidative stress-induced cell death	BP	1	P4HB,PYCR1,SOD1,GPX1,AKT1,TRAP1	yellow	VSMC_M3
GO:0009894	0.20	0.84	61	806	regulation of catabolic process	BP	1	CDK4,BAG6,SNX9,SREBF2,HNRNPA0,SGSM3,VPS28,SECISBP2,HSPBP1,SOCS5,CNOT3,ZC3H12A,SNRNP70,SNX3,PSMC4,METTL3,DND1,SCARB1,PLEKHF1,PDE3B,WNT10B,PACSIN3,CCAR2,PCBP4,RNF19B,DCPS,ETFBKMT,ZCCHC17,GPX1,ASB9,PSME2,TRIM27,KAT5,RELA,IDH1,PHB2,BAD,UBXN1,WASHC1,CCDC22,FOXK1,AMER1,PIP4K2B,MAD2L2,NANOS3,DFFA,SNF8,MTMR4,MID2,AKT1,PAIP1,KHSRP,GIT1,CARHSP1,SIRT6,STK11,WDR24,JMJD8,TENT5B,TRIB2,STUB1	yellow	VSMC_M3
GO:0006721	0.20	0.90	6	56	terpenoid metabolic process	BP	1	PPARD,FDFT1,LSS,CRABP2,HMGCS1,FDPS	yellow	VSMC_M3
GO:0033865	0.20	0.88	10	102	nucleoside bisphosphate metabolic process	BP	1	ENPP1,FASN,ACSL1,MVK,PAPSS1,ACLY,ACSF2,PMVK,HMGCS1,MVD	yellow	VSMC_M3
GO:0033875	0.20	0.88	10	102	ribonucleoside bisphosphate metabolic process	BP	1	ENPP1,FASN,ACSL1,MVK,PAPSS1,ACLY,ACSF2,PMVK,HMGCS1,MVD	yellow	VSMC_M3
GO:0034032	0.20	0.88	10	102	purine nucleoside bisphosphate metabolic process	BP	1	ENPP1,FASN,ACSL1,MVK,PAPSS1,ACLY,ACSF2,PMVK,HMGCS1,MVD	yellow	VSMC_M3
GO:0032006	0.20	0.89	8	84	regulation of TOR signaling	BP	1	FNIP1,SEC13,LAMTOR1,LAMTOR4,CTNS,STK11,TELO2,WDR24	yellow	VSMC_M3
GO:0072698	0.20	0.91	5	52	protein localization to microtubule cytoskeleton	BP	1	DCTN2,CEP131,NUMA1,KIAA0753,MID2	yellow	VSMC_M3
GO:0060968	0.20	0.90	6	59	regulation of gene silencing	BP	1	PHF2,ZC3H12A,DND1,TARBP2,LIMD1,MAP2K2	yellow	VSMC_M3
GO:0031396	0.20	0.87	16	180	regulation of protein ubiquitination	BP	1	SQSTM1,VPS28,HSPBP1,PINX1,CHP1,LIMK1,OTUB1,AIMP2,UBXN1,WASHC1,AMER1,MAD2L2,RPL37,AKT1,STUB1,NSMCE3	yellow	VSMC_M3
GO:1901989	0.20	0.89	9	91	positive regulation of cell cycle phase transition	BP	1	CDK4,KMT2E,WNT10B,THOC5,PBX1,PHB2,RCC2,AKT1,MIR214	yellow	VSMC_M3

GO:0009056	0.20	0.82	148	2000	catabolic process	BP	1	ENPP1,CDK4,SQSTM1,BAG6,SNX9,HDAC10,ACAT2,SREBF2,HNRNPA0,SGSM3,NUDT3,PPARD,MPST,SHARPIN,VPS28,SND1,CUL7,DNPH1,SECISBP2,HSPBP1,SOCS5,CNOT3,PSMB3,ZC3H12A,BLVRA,CPT1C,RCN3,SNRNP70,SNX3,MGLL,TMUB1,PAFAH1B3,SNX17,GALK1,PLCD1,BCKDHA,BLVRB,OGG1,PSMC4,SEL1L2,METTL3,DND1,SMOX,GNPDA1,SMUG1,OGDHL,SCARB1,HM13,PDE4D,PCCB,PEPD,PLEKHF1,ACSL1,LSM6,MMP24,PDE3B,WNT10B,PACSIN3,CCAR2,PCBP4,RNF19B,DCPS,SELENOS,GSTO1,ETFBKMT,MANBA,ZCCHC17,MMP14,LIX1L,GPX1,PCK2,ASB9,PSME2,ANPEP,TRIM27,ADRM1,PSMB4,PTPN23,MRC2,RNASEH2C,KAT5,RELA,IDH1,VPS51,SGPL1,PHB2,BAD,PLCB3,MRTO4,MVB12A,UBXN1,WASHC1,CNP,CCDC22,USP27X,GLUD1,FOXK1,ILVBL,CAPN2,RAB24,AMER1,PIP4K2B,MAD2L2,NANOS3,FBXL19,DFFA,PGD,SNF8,TRIM25,USP43,MTMR4,CDC37,MID2,ATG3,ANKRD9,AKT1,PACS2,PAIP1,KHSRP,PGM1,GIT1,CARHSP1,BLMH,SIRT6,ANAPC2,SPSB3,MAN1B1,STK11,TAZ,WDR24,JMJD8,SLC27A4,ZNRF1,PSMB10,CTRL,RBM8A,RNH1,PLA2G4E,NT5C,TENT5B,TRIB2,CDC34,FBXO6,GBA2,TRIR,STUB1,ITPA,CCDC115	yellow	VSMC_M3
GO:0019219	0.20	0.82	203	2852	regulation of nucleobase-containing compound metabolic process	BP	1	ENPP1,NAB2,CDK4,SQSTM1,ZNF608,HDAC10,NELFE,FNIP1,ATF6B,PBX2,RING1,SREBF2,H2AFY,HNRNPA0,TRIM11,TRAPPC9,PPARD,SND1,SECISBP2,PHF2,CNOT3,KLF4,HOXA1,FHL3,ZC3H12A,FIGNL1,PNKP,SNRNP70,CC2D1B,KTI12,KMT2E,SIX5,ZNF282,ZNF296,RELB,DMAP1,CIC,OGG1,SERTAD1,TTC5,METTL3,CXXC5,DND1,DEAF1,HOXC4,TARBP2,KMT2B,NAT10,ZNF740,RALY,ZBTB38,PINX1,GREM1,KDM2B,MCRS1,CX3CL1,WNT10B,ACTR5,ANKRD2,EGR3,CCAR2,DNAJC17,DLL4,CHP1,YAF2,HTATIP2,PCBP4,KDM1B,CHCHD2,RBM15B,SRSF9,DCPS,IRX1,RRP1B,BRD9,ZCCHC17,AIP,CLCF1,TSHZ2,QARS,PIR,TRIM27,PRMT2,THOC5,RIPK3,TWIST2,SMARCD2,BRMS1,BUD23,GMEB2,CCDC85B,PRPF6,PBX1,KAT5,RELA,ZNF511,ARNT2,ZFPL1,LIMD1,SMARCB1,CHCHD10,PBX4,UBTF,GATAD2A,MEF2B,PHB2,TRMT112,NDP,CTDSP1,MLF2,OTUB1,EID1,SSBP4,METTL13,ZNF394,C11orf95,BUD31,TNFRSF1A,BABAM1,TEAD4,C1QBP,TAF6L,ZBTB49,MTA2,ZNF316,MED11,PELP1,CCDC22,FOXK1,KLF3,PKN1,POLR2A,MAD2L2,SLTM,NANOS3,CTBP1,FBXL19,CRABP2,DFFA,SP2,ARHGEF2,SNF8,PER1,NFIX,PPP4C,SLX1A,IRF2BPL,TRIM25,SMARCA4,CARM1,RAVER1,AATF,CXorf57,MID2,RBM22,ZNF521,ZNF358,AKT1,BRF1,PAIP1,KHSRP,GTF2F1,RFX2,SAFB,SAFB2,GIT1,CARHSP1,MYDGF,HDGFL2,SIRT6,MAP2K2,CACTIN,CDK11B,SSU72,HIC1,MNT,EHMT1,AP3D1,IRAK1,MBD3,TAZ,FAM50A,JMJD8,MED16,CARD9,SARS,TRADD,NABP2,RBM8A,ERBB4,JUND,KLF13,FADS1,ZNF783,HOXC5,TENT5B,IER5,AKAP17A,ZNF250,THAP7,NSMCE3,ERF,ZSCAN1	yellow	VSMC_M3
GO:0044242	0.20	0.87	15	164	cellular lipid catabolic process	BP	1	ACAT2,PPARD,CPT1C,MGLL,SCARB1,PCCB,ETFBKMT,PCK2,IDH1,SGPL1,ILVBL,AKT1,SLC27A4,PLA2G4E,GBA2	yellow	VSMC_M3
GO:0006637	0.20	0.89	8	78	acyl-CoA metabolic process	BP	1	FASN,ACSL1,MVK,ACLY,ACSF2,PMVK,HMGCS1,MVD	yellow	VSMC_M3
GO:0035383	0.20	0.89	8	78	thioester metabolic process	BP	1	FASN,ACSL1,MVK,ACLY,ACSF2,PMVK,HMGCS1,MVD	yellow	VSMC_M3
GO:0031663	0.20	0.91	5	44	lipopolysaccharide-mediated signaling pathway	BP	1	SCARB1,CX3CL1,AKT1,CACTIN,IRAK1	yellow	VSMC_M3
GO:0009199	0.20	0.89	7	64	ribonucleoside triphosphate metabolic process	BP	1	IMPDH2,ATP5MC3,UQCC3,ATP5PD,ATP5F1D,TAZ,ITPA	yellow	VSMC_M3
GO:0071331	0.20	0.88	10	112	cellular response to hexose stimulus	BP	1	PPARD,LGALS1,UCP2,AACS,PCK2,SLC26A6,SMARCB1,BAD,SMARCA4,JAGN1	yellow	VSMC_M3
GO:0045746	0.20	0.92	4	33	negative regulation of Notch signaling pathway	BP	1	METTL3,DLL4,MMP14,AKT1	yellow	VSMC_M3
GO:0001667	0.20	0.85	28	360	ameboidal-type cell migration	BP	1	PPARD,TSTA3,PLEC,KLF4,TPBG,WDPCP,ZC3H12A,PLXND1,RIC8A,SCARB1,ITGB7,GREM1,EGR3,DLL4,GPX1,CORO1B,PTPN23,SGPL1,AAMP,C1QBP,RCC2,MTA2,PKN1,AKT1,TMEM201,ERBB4,PTPRR,GLIPR2	yellow	VSMC_M3
GO:0007010	0.21	0.83	83	1177	cytoskeleton organization	BP	1	DCTN2,SPAG1,SNX9,PDLIM4,PACSIN1,PLEC,CUL7,PDGFRA,DBNL,WDPCP,FHL3,FIGNL1,ZYX,MICAL1,EHD2,KRT8,SLC9A3R1,BAIAP2,CEP131,INPPL1,NUMA1,YIF1B,CCDC57,ARAP3,CAVIN3,NAT10,SPRYD3,SWAP70,CAPG,DCTN1,CX3CL1,SOD1,PACSIN3,CHP1,PDGFA,CDK2AP2,CORO1B,CLIP2,TRIM27,ARPIN,PCNT,LIMK1,NUDC,CAPN10,IQCG,PFDN2,PLA2G3,STMN3,SIPA1,RHOC,ALKBH4,FMNL1,SAC3D1,LIMD1,MID1IP1,ARPC1B,KIAA0753,WASHC1,CNP,EML3,DBN1,CAPN2,MAD2L2,ARHGEF2,RANGRF,CORO1A,SYNE3,MYO19,EML1,INF2,GIT1,PPL,GAS8,NTMT1,MIR214,TWF2,MAP7D1,ELN,CDC42EP5,GBA2,TUBGCP2,MEIG1,ARPC5L	yellow	VSMC_M3
GO:0035924	0.21	0.90	6	62	cellular response to vascular endothelial growth factor stimulus	BP	1	CD63,PDGFRA,EGR3,DLL4,RELA,AKT1	yellow	VSMC_M3
GO:0060998	0.21	0.91	5	54	regulation of dendritic spine development	BP	1	DBNL,BAIAP2,NGEF,DBN1,EEF2K	yellow	VSMC_M3
GO:0065003	0.21	0.83	92	1210	protein-containing complex assembly	BP	1	SPAG1,SNX9,FNIP1,SAMM50,NDUFB9,H2AFY,RPL3,BOP1,TBCC,DBNL,ZC3H12A,ACAD9,GEMIN7,SLC9A3R1,H3F3B,BAIAP2,NUMA1,CCDC57,EIF4EBP1,SDHAF1,TARBP2,EIF4B,RRS1,UQCRFS1,CAPG,GREM1,AQP5,DCTN1,CX3CL1,WNT10B,CHP1,SRSF9,CORO1B,HCCS,TRIM27,ARPIN,EIF3A,SF3A1,ADRM1,TACO1,SART1,PRPF6,RHOC,TMEM120A,MID1IP1,PEX5,SMARCB1,CHCHD10,UBTF,BAD,LETM1,LGALS3,ARPC1B,MRTO4,FCHO1,AIMP2,TAF6L,UQCC3,WASHC1,KRT10,H3F3A,DBN1,CLTB,CD2BP2,RANGRF,FARSA,CORO1A,SMARCA4,EIF3G,BRF1,GIT1,UBN1,MRPL20,NCLN,NDUFS7,ATP5F1D,TAZ,CARD9,SPG7,TGFBR3,MIR214,TWF2,SURF1,MIS12,ELN,UNC13A,CDC42EP5,GBA2,TUBGCP2,STUB1,CCDC115,ARPC5L	yellow	VSMC_M3
GO:1903037	0.21	0.86	18	206	regulation of leukocyte cell-cell adhesion	BP	1	SCRIB,LGALS1,SOCS5,KLF4,ZC3H12A,EGR3,ST3GAL4,SART1,RELA,BAD,LGALS3,MAD1L1,CORO1A,CD276,CSK,AKT1,AP3D1,IRAK1	yellow	VSMC_M3

GO:0034248	0.21	0.84	32	395	regulation of cellular amide metabolic process	BP	1	CDK4,SECISBP2,RPS9,CNOT3,ZC3H12A,UNK,METTL3,DND1,EIF4EBP1,TARBP2,NAT10,EIF4B,PCIF1,DCPS,NGDN,LSM14B,TACO1,RELA,IGFBP5,MPV17L2,C1QBP,SEPSECS,NANOS3,PER1,C19orf66,AKT1,PAIP1,KHSRP,TRAP1,MKNK2,RBM8A,ZNF385A	yellow	VSMC_M3
GO:0009311	0.21	0.91	5	44	oligosaccharide metabolic process	BP	1	B4GALT1,ST3GAL4,MANBA,MPDU1,MAN1B1	yellow	VSMC_M3
GO:0006796	0.21	0.82	155	2150	phosphate-containing compound metabolic process	BP	1	ENPP1,NAB2,CDK4,CTDSP2,SQSTM1,SBF1,SNX9,CERK,FNIP1,PRR5,H2AFY,GUK1,NUDT3,PPARD,PLEC,PPP1R14B,DNPH1,PIGO,PDGFRA,SOCS5,TTC7A,KLF4,SPRED2,ZC3H12A,PNKP,MICAL1,WDFY2,SLC9A3R1,EMILIN1,SEPHS1,GALK1,PPM1G,PLCD1,OGG1,SERTAD1,INPPL1,PCYT2,FASN,PTDSS2,SMUG1,OGDHL,TARBP2,SCARB1,PDE4D,TNS2,SWAP70,ACSL1,FDFT1,TNKS1BP1,GREM1,TKT,CX3CL1,SOD1,DGKZ,CHP1,TYRO3,HTATIP2,PPM1M,CISH,AK2,MVK,STK32C,FABP3,PAPSS1,PDGFA,MOCS3,CLCF1,QARS,IMPDH2,TRIM27,LIMK1,RIPK3,PSTK,TNK2,PLA2G3,PTPN23,ATP5MC3,SCYL1,IDH1,MEX3B,MAP3K14,LIMD1,ATIC,PHB2,PPP1R35,BAD,PLCB3,CTDSP1,ISYNA1,LGALS3,TNFRSF1A,ASMTL,UQCC3,WASHC1,CNP,ACLY,FOXK1,PKN1,CAPN2,PIP4K2B,MAD2L2,CTBP1,PGD,ARHGEF2,MVP,ACSF2,DPM3,PPP4C,FLAD1,PMVK,ATP5PD,MTMR4,HDHD2,EEF2K,CDC37,CSK,AKT1,HMGCS1,PGM1,SGK494,GIT1,MYDGF,MAP2K2,CACTIN,CDK11B,SSU72,PGP,MKNK2,CSNK1G2,IRAK1,MIDN,ATP5F1D,STK11,TELO2,TAZ,JMJD8,AMPD2,APRT,MVD,AK1,SLC44A3,VAC14,TGFBR3,ERBB4,PTPRR,PLA2G4E,FADS1,NT5C,TRIB2,NSMF,TALDO1,DUSP5,FDPS,IDI1,ITPA	yellow	VSMC_M3
GO:0009142	0.21	0.89	7	62	nucleoside triphosphate biosynthetic process	BP	1	IMPDH2,ATP5MC3,UQCC3,ATP5PD,ATP5F1D,TAZ,AK1	yellow	VSMC_M3
GO:0051568	0.21	0.91	5	51	histone H3-K4 methylation	BP	1	NELFE,H2AFY,KMT2E,KMT2B,SETD1B	yellow	VSMC_M3
GO:0045930	0.21	0.86	16	186	negative regulation of mitotic cell cycle	BP	1	CTDSP2,SCRIB,KLF4,SLC9A3R1,PINX1,CDK2AP2,PRMT2,CTDSP1,BABAM1,SDE2,MIIP,MAD1L1,MAD2L2,ZNF830,AURKAIP1,NABP2	yellow	VSMC_M3
GO:1903039	0.21	0.87	13	143	positive regulation of leukocyte cell-cell adhesion	BP	1	LGALS1,SOCS5,EGR3,ST3GAL4,SART1,RELA,BAD,CORO1A,CD276,CSK,AKT1,AP3D1,IRAK1	yellow	VSMC_M3
GO:0030512	0.21	0.90	6	64	negative regulation of transforming growth factor beta receptor signaling pathway	BP	1	SPRED2,EMILIN1,MTMR4,VASN,TGFBR3,STUB1	yellow	VSMC_M3
GO:0050818	0.21	0.90	6	54	regulation of coagulation	BP	1	PDGFRA,ANXA5,ST3GAL4,PDGFA,CD9,PLAU	yellow	VSMC_M3
GO:0071326	0.21	0.87	10	113	cellular response to monosaccharide stimulus	BP	1	PPARD,LGALS1,UCP2,AACS,PCK2,SLC26A6,SMARCB1,BAD,SMARCA4,JAGN1	yellow	VSMC_M3
GO:1901615	0.21	0.84	31	382	organic hydroxy compound metabolic process	BP	1	SREBF2,SQLE,NUDT3,PPARD,PTGR1,FDXR,SNX17,GALK1,INSIG1,IDH3B,SCARB1,FDFT1,SOD1,DHCR7,MVK,PCK2,LSS,IDH1,ISYNA1,HSD17B1,ACLY,PMVK,HMGCS1,DHCR24,NSDHL,CTNS,PGP,MVD,GBA2,FDPS,IDI1	yellow	VSMC_M3
GO:0043154	0.21	0.90	6	55	negative regulation of cysteine-type endopeptidase activity involved in apoptotic process	BP	1	FNIP1,KLF4,MICAL1,GPX1,AKT1,DHCR24	yellow	VSMC_M3
GO:1901698	0.21	0.83	63	845	response to nitrogen compound	BP	1	ENPP1,CDK4,BAG6,SREBF2,PDGFRA,SOCS5,KLF4,RCN3,SLC9A3R1,TMUB1,INSIG1,OGG1,UCP2,SEL1L2,BAIAP2,INPPL1,LAMTOR1,AACS,HM13,BCL2L1,TNS2,PDE3B,WNT10B,SOD1,SRSF9,SELENOS,FABP3,PCK2,COL6A1,SLC26A6,UQCRC1,CAPN10,RELA,MGST3,IGFBP5,BAD,LAMTOR4,CD9,UBXN1,KLF3,CAPN2,MTHFR,PIP4K2B,ARHGEF2,PER1,TRIM25,CARM1,EEF2K,CSK,AKT1,HMGCS1,SLC6A4,LDOC1,MAN1B1,CARD9,APRT,SLC27A4,TGFBR3,JUND,NSMF,FBXO6,STUB1,NSMCE3	yellow	VSMC_M3
GO:0002686	0.21	0.91	4	33	negative regulation of leukocyte migration	BP	1	EMILIN1,GREM1,RIN3,AKT1	yellow	VSMC_M3
GO:0071216	0.21	0.86	14	157	cellular response to biotic stimulus	BP	1	CDK4,PPARD,ZC3H12A,SCARB1,CX3CL1,SELENOS,PABPN1,RELA,CAPN2,AKT1,GIT1,LDOC1,CACTIN,IRAK1	yellow	VSMC_M3
GO:0030521	0.21	0.91	4	37	androgen receptor signaling pathway	BP	1	PRMT2,PKN1,SMARCA4,SAFB2	yellow	VSMC_M3
GO:0046649	0.22	0.83	37	470	lymphocyte activation	BP	1	BAG6,NCAPH2,FNIP1,SCRIB,LGALS1,SOCS5,ZC3H12A,RELB,CD79A,METTL3,SWAP70,SOD1,EGR3,DLL4,TYRO3,MMP14,CLCF1,IMPDH2,RIPK3,SART1,PBX1,PHB2,BAD,LGALS3,PKN1,MAD1L1,MAD2L2,ARMC5,CORO1A,CD276,CSK,DOCK11,AKT1,AP3D1,STK11,PSMB10,AKAP17A	yellow	VSMC_M3
GO:0006096	0.22	0.89	6	60	glycolytic process	BP	1	GALK1,OGDHL,FOXK1,PGM1,GIT1,JMJD8	yellow	VSMC_M3
GO:0006757	0.22	0.89	6	60	ATP generation from ADP	BP	1	GALK1,OGDHL,FOXK1,PGM1,GIT1,JMJD8	yellow	VSMC_M3
GO:0030317	0.22	0.89	6	58	flagellated sperm motility	BP	1	CEP131,YIF1B,IQCG,PLA2G3,GAS8,MEIG1	yellow	VSMC_M3
GO:0097722	0.22	0.89	6	58	sperm motility	BP	1	CEP131,YIF1B,IQCG,PLA2G3,GAS8,MEIG1	yellow	VSMC_M3
GO:0000910	0.22	0.86	12	141	cytokinesis	BP	1	SNX9,PLEC,CUL7,SEPT9,BCL2L1,RAB35,RHOC,ALKBH4,ANXA11,MYO19,SNX18,GIT1	yellow	VSMC_M3
GO:0051276	0.22	0.83	51	682	chromosome organization	BP	1	DCTN2,NHP2,BAG6,NCAPH2,HDAC10,H2AFY,PHF2,PNKP,KMT2E,DMAP1,H3F3B,NUMA1,NAT10,RRS1,TNKS1BP1,PINX1,ACTR5,NPM3,BRD9,NUDC,SMARCD2,BUD23,BANF1,RELA,LRWD1,ALKBH4,ZNHIT1,SMARCB1,PHB2,BABAM1,TAF6L,EML3,MTA2,H3F3A,MAD1L1,MAD2L2,SLX1A,SMARCA4,ZNF830,SAFB,SIRT6,UBN1,HMG20B,EHMT1,MBD3,TELO2,FAM50A,RF00024,NABP2,MIS12,ITPA	yellow	VSMC_M3
GO:0022900	0.22	0.86	15	149	electron transport chain	BP	1	NDUFB9,TSTA3,PLEC,GSR,IDH3B,UQCRFS1,CHCHD2,SDHA,NDUFV1,UQCRC1,UQCC3,FDX2,NDUFS7,TAZ,SURF1	yellow	VSMC_M3
GO:0022618	0.22	0.86	15	163	ribonucleoprotein complex assembly	BP	1	RPL3,BOP1,GEMIN7,TARBP2,EIF4B,RRS1,SRSF9,EIF3A,SF3A1,SART1,PRPF6,MRTO4,CD2BP2,EIF3G,MRPL20	yellow	VSMC_M3
GO:1901136	0.22	0.86	12	126	carbohydrate derivative catabolic process	BP	1	NUDT3,DNPH1,OGG1,GNPDA1,SMUG1,PDE4D,MANBA,MAN1B1,NT5C,FBXO6,GBA2,ITPA	yellow	VSMC_M3
GO:0006476	0.22	0.88	8	88	protein deacetylation	BP	1	HDAC10,SPRED2,CCAR2,BRMS1,MTA2,CTBP1,PER1,SIRT6	yellow	VSMC_M3
GO:0019933	0.22	0.91	4	40	cAMP-mediated signaling	BP	1	SLC9A3R1,PDE4D,PDE3B,CDC34	yellow	VSMC_M3
GO:0048639	0.22	0.87	11	134	positive regulation of developmental growth	BP	1	PPARD,DBNL,LIMK1,DBN1,CRABP2,AKT1,ANAPC2,TGFBR3,ERBB4,TWF2,UNC13A	yellow	VSMC_M3
GO:0006575	0.22	0.86	13	136	cellular modified amino acid metabolic process	BP	1	CPT1C,GSR,PTDSS2,SOD1,GSTO1,GPX1,SLC19A1,PLA2G3,IDH1,ATIC,MTHFR,CTNS,EEF1G	yellow	VSMC_M3
GO:0045022	0.22	0.91	4	37	early endosome to late endosome transport	BP	1	SNX3,PTPN23,SNF8,MAP2K2	yellow	VSMC_M3

GO:1902369	0.22	0.89	6	60	negative regulation of RNA catabolic process	BP	1	HNRNPA0,SECISBP2,DND1,ZCCHC17,PAIP1,TENT5B	yellow	VSMC_M3
GO:2001233	0.22	0.84	25	292	regulation of apoptotic signaling pathway	BP	1	FIGNL1,SLC9A3R1,P4HB,BCL2L1,PLEKHF1,CX3CL1,SOD1,CCAR2,SELENOS,ITPRIP,GPX1,QARS,RIPK3,RELA,BAD,LGALS3,NANOS3,ARHGEF2,AATF,AKT1,TRAP1,MNT,TNFRSF12A,ZNF385A,TXNDC12	yellow	VSMC_M3
GO:0046660	0.22	0.88	8	88	female sex differentiation	BP	1	PDGFRA,BCL2L1,SOD1,TYRO3,MMP14,IDH1,SGPL1,ZNF830	yellow	VSMC_M3
GO:0009201	0.22	0.89	6	52	ribonucleoside triphosphate biosynthetic process	BP	1	IMPDH2,ATP5MC3,UQCC3,ATP5PD,ATP5F1D,TAZ	yellow	VSMC_M3
GO:0097190	0.22	0.83	38	471	apoptotic signaling pathway	BP	1	BAG6,PPARD,FIGNL1,KRT8,SLC9A3R1,P4HB,DELE1,BCL2L1,PLEKHF1,CX3CL1,SOD1,CCAR2,SELENOS,ITPRIP,GPX1,QARS,RIPK3,RELA,ZDHHC3,SGPL1,CHCHD10,ZNF385B,BAD,LGALS3,TNFRSF1A,NANOS3,ARHGEF2,AATF,AKT1,HIC1,TRAP1,MNT,MKNK2,STK11,TRADD,TNFRSF12A,ZNF385A,TXNDC12	yellow	VSMC_M3
GO:0044260	0.22	0.80	418	5916	cellular macromolecule metabolic process	BP	1	ENPP1,CD63,NAB2,NHP2,CDK4,CTDSP2,SQSTM1,ZNF608,BAG6,SBF1,SNX9,HDAC10,NELFE,FNIP1,ATF6B,PBX2,PRR5,TTLL12,RING1,SREBF2,JADE2,TRMT12,H2AFY,HNRNPA0,TRIM11,TRAPPC9,PPARD,FANCE,EEF1D,RPL3,SCRIB,PLEC,SHARPIN,VPS28,RPL8,SND1,CUL7,B4GALT1,MRPS18A,AARS2,PIGO,PDGFRA,SECISBP2,PHF2,HSPBP1,SOCS5,RPS9,CNOT3,KLF4,HOXA1,JOSD2,PSMB3,FHL3,SPRED2,EIPR1,MEAF6,ZC3H12A,FIGNL1,PNKP,RCN3,CC2D1B,KTI12,MICAL1,KMT2E,WDFY2,SIX5,ZNF282,ZNF296,RELB,SLC9A3R1,SERP2,NAT9,MUTYH,EMILIN1,SEPHS1,TMUB1,RNF220,DMAP1,CIC,GTF3C2,PPM1G,UNK,GALNT14,OGG1,SERTAD1,TTC5,PSMC4,KAT14,P4HA3,SEL1L2,METTL3,PHLDA1,CXXC5,FAAP100,LAMTOR1,P4HB,DND1,RPL29,EIF4EBP1,DEAF1,SMUG1,HOXC4,POLR1D,TARBP2,KMT2B,NAT10,HM13,ZNF740,PDE4D,TNS2,EIF4B,SWAP70,RALY,ACSL1,ZBTB38,LSM6,TNKS1BP1,PINX1,GATB,SETD1B,GREM1,KDM2B,MCRS1,POLR2C,CX3CL1,WNT10B,FKBP11,SOD1,PACSIN3,ACTR5,ANKRD2,EGR3,CCAR2,DNAJC17,DLL4,RPLP2,CHP1,TYRO3,MRPL43,YAF2,HTATIP2,PCIF1,PPM1M,NPM3,PCBP4,KDM1B,CHCHD2,SLC35C2,RBM15B,CARS,CISH,ST3GAL4,RNF19B,DCPS,IRX1,WDR4,SELENOS,RRP1B,ETFBKMT,MANBA,TMTC1,BRD9,RIN3,STK32C,ZCCHC17,PDGFA,MMP14,APEH,GPX1,AIP,HCCS,MOCS3,CLCF1,NGDN,TSHZ2,QARS,ASB9,PSME2,PIR,TRIM27,EIF3A,LIMK1,ZNRD1,RABGGTA,PRMT2,THOC5,RIPK3,TWIST2,LSM14B,ADRM1,PSTK,RPS21,PSMB4,SMARCD2,EXTL1,FTSJ3,BRMS1,SEC11A,BUD23,TNK2,TACO1,GMEB2,CEP164,CCDC85B,PRPF6,PTPN23,PBX1,RNASEH2C,KAT5,RELA,ZNF511,SCYL1,LRWD1,ALKBH4,MEX3B,MAP3K14,ARNT2,MRPL49,ZFPL[illegible]	yellow	VSMC_M3
GO:0051253	0.22	0.82	76	1032	negative regulation of RNA metabolic process	BP	1	NAB2,SQSTM1,ZNF608,HDAC10,NELFE,FNIP1,RING1,SREBF2,H2AFY,HNRNPA0,TRIM11,PPARD,SECISBP2,KLF4,ZC3H12A,CC2D1B,SIX5,ZNF282,ZNF296,RELB,DMAP1,CIC,CXXC5,DND1,DEAF1,ZBTB38,GREM1,KDM2B,WNT10B,ANKRD2,CCAR2,DNAJC17,DLL4,YAF2,PCBP4,KDM1B,SRSF9,IRX1,ZCCHC17,QARS,TRIM27,PRMT2,TWIST2,BRMS1,CCDC85B,KAT5,RELA,LIMD1,GATAD2A,PHB2,EID1,METTL13,C11orf95,C1QBP,ZBTB49,MTA2,FOXK1,KLF3,MAD2L2,CTBP1,SP2,PER1,NFIX,IRF2BPL,SMARCA4,PAIP1,SIRT6,HIC1,MNT,EHMT1,MBD3,SARS,TENT5B,THAP7,NSMCE3,ERF	yellow	VSMC_M3
GO:0007159	0.22	0.85	20	234	leukocyte cell-cell adhesion	BP	1	TSTA3,SCRIB,LGALS1,SOCS5,KLF4,ZC3H12A,ITGB7,EGR3,ST3GAL4,SART1,RELA,BAD,LGALS3,MAD1L1,CORO1A,CD276,CSK,AKT1,AP3D1,IRAK1	yellow	VSMC_M3
GO:1990778	0.22	0.84	22	279	protein localization to cell periphery	BP	1	SQSTM1,PACSIN1,SCRIB,TTC7A,TPBG,EHD2,SLC9A3R1,NUMA1,BCL2L1,PLEKHF1,RAPSN,TSPAN5,MMP14,ZDHHC3,LGALS3,TNFRSF1A,RANGRF,CSK,AKT1,PACS2,RAB34,ARFRP1	yellow	VSMC_M3
GO:0045732	0.22	0.85	17	203	positive regulation of protein catabolic process	BP	1	BAG6,SNX9,SGSM3,VPS28,HSPBP1,SOCS5,PSMC4,PACSIN3,RNF19B,ASB9,CCDC22,AMER1,SNF8,AKT1,SIRT6,TRIB2,STUB1	yellow	VSMC_M3
GO:0050807	0.22	0.86	13	165	regulation of synapse organization	BP	1	DBNL,TPBG,BAIAP2,ARHGAP22,ARHGAP33,DCTN1,NGEF,IGSF9,LRFN4,NECTIN1,DBN1,EEF2K,ANAPC2	yellow	VSMC_M3
GO:0014068	0.22	0.89	6	66	positive regulation of phosphatidylinositol 3-kinase signaling	BP	1	PRR5,PPARD,PDGFRA,PDGFA,MYDGF,ERBB4	yellow	VSMC_M3
GO:0051252	0.22	0.80	188	2654	regulation of RNA metabolic process	BP	1	ENPP1,NAB2,CDK4,SQSTM1,ZNF608,HDAC10,NELFE,FNIP1,ATF6B,PBX2,RING1,SREBF2,H2AFY,HNRNPA0,TRIM11,TRAPPC9,PPARD,SND1,SECISBP2,PHF2,CNOT3,KLF4,HOXA1,FHL3,ZC3H12A,SNRNP70,CC2D1B,KTI12,KMT2E,SIX5,ZNF282,ZNF296,RELB,DMAP1,CIC,OGG1,SERTAD1,TTC5,METTL3,CXXC5,DND1,DEAF1,HOXC4,TARBP2,KMT2B,ZNF740,RALY,ZBTB38,GREM1,KDM2B,MCRS1,CX3CL1,WNT10B,ACTR5,ANKRD2,EGR3,CCAR2,DNAJC17,DLL4,CHP1,YAF2,HTATIP2,PCBP4,KDM1B,CHCHD2,RBM15B,SRSF9,DCPS,IRX1,RRP1B,BRD9,ZCCHC17,AIP,TSHZ2,QARS,PIR,TRIM27,PRMT2,THOC5,RIPK3,TWIST2,SMARCD2,BRMS1,BUD23,GMEB2,CCDC85B,PRPF6,PBX1,KAT5,RELA,ZNF511,ARNT2,ZFPL1,LIMD1,SMARCB1,CHCHD10,PBX4,UBTF,GATAD2A,MEF2B,PHB2,TRMT112,NDP,CTDSP1,MLF2,EID1,SSBP4,METTL13,ZNF394,C11orf95,BUD31,TNFRSF1A,TEAD4,C1QBP,TAF6L,ZBTB49,MTA2,ZNF316,MED11,PELP1,CCDC22,FOXK1,KLF3,PKN1,POLR2A,MAD2L2,SLTM,NANOS3,CTBP1,FBXL19,CRABP2,SP2,ARHGEF2,SNF8,PER1,NFIX,IRF2BPL,TRIM25,SMARCA4,CARM1,RAVER1,AATF,MID2,RBM22,ZNF521,ZNF358,AKT1,BRF1,PAIP1,KHSRP,GTF2F1,RFX2,SAFB,SAFB2,CARHSP1,MYDGF,HDGFL2,SIRT6,MAP2K2,CACTIN,CDK11B,SSU72,HIC1,MNT,EHMT1,AP3D1,IRAK1,MBD3,FAM50A,MED16,CARD9,SARS,TRADD,RBM8A,ERBB4,JUND,KLF13,FADS1,ZNF783,HOXC5,TENT5B,IER5,AKAP17A,ZNF250,THAP7,NSMCE3,ERF,ZSCAN1	yellow	VSMC_M3
GO:0034728	0.22	0.87	9	97	nucleosome organization	BP	1	H2AFY,DMAP1,H3F3B,SMARCD2,ZNHIT1,SMARCB1,H3F3A,SMARCA4,UBN1	yellow	VSMC_M3
GO:1902373	0.22	0.90	5	49	negative regulation of mRNA catabolic process	BP	1	HNRNPA0,SECISBP2,DND1,PAIP1,TENT5B	yellow	VSMC_M3

GO:0046545	0.22	0.88	7	75	development of primary female sexual characteristics	BP	1	PDGFRA,BCL2L1,SOD1,MMP14,IDH1,SGPL1,ZNF830	yellow	VSMC_M3
GO:0031060	0.23	0.89	5	52	regulation of histone methylation	BP	1	NELFE,H2AFY,KMT2E,SMARCB1,MTHFR	yellow	VSMC_M3
GO:0010605	0.23	0.80	150	2090	negative regulation of macromolecule metabolic process	BP	1	ENPP1,NAB2,CTDSP2,SQSTM1,ZNF608,BAG6,HDAC10,NELFE,FNIP1,RING1,SREBF2,H2AFY,HNRNPA0,TRIM11,PPARD,PLEC,VPS28,SND1,SECISBP2,PHF2,SSC5D,SOCS5,CNOT3,KLF4,SPRED2,ZC3H12A,PNKP,SNRNP70,CC2D1B,SNX3,MICAL1,SIX5,ZNF282,ZNF296,RELB,EMILIN1,DMAP1,CIC,UNK,OGG1,METTL3,CXXC5,INPPL1,DND1,EIF4EBP1,DEAF1,TARBP2,NAT10,PDE4D,SWAP70,LRP3,ZBTB38,LSM6,PINX1,GREM1,KDM2B,CX3CL1,WNT10B,ANKRD2,CCAR2,DNAJC17,DLL4,CHP1,YAF2,PCIF1,PCBP4,KDM1B,SLC35C2,SRSF9,DCPS,IRX1,SELENOS,RIN3,ZCCHC17,GPX1,QARS,TRIM27,LIMK1,PRMT2,TWIST2,PFDN2,BRMS1,PLA2G3,CCDC85B,KAT5,RELA,LIMD1,SMARCB1,GATAD2A,PHB2,IGFBP5,CTDSP1,OTUB1,EID1,METTL13,LGALS3,C11orf95,MRTO4,C1QBP,ZBTB49,UBXN1,MTA2,FOXK1,KLF3,PKN1,MAD2L2,NANOS3,CTBP1,DFFA,SP2,MVP,PER1,NFIX,SLX1A,IRF2BPL,RPL37,SMARCA4,AATF,CXorf57,C19orf66,CSK,ZNF830,AKT1,PAIP1,KHSRP,GIT1,ANKRD13B,DHCR24,CARHSP1,SIRT6,MAP2K2,CACTIN,HMG20B,HIC1,MNT,EHMT1,ANAPC2,MBD3,SARS,MIR214,MIR155,RBM8A,RNH1,MIR374B,TENT5B,TRIB2,THAP7,NSMCE3,ERF,ZAR1L	yellow	VSMC_M3
GO:0061383	0.23	0.90	4	40	trabecula morphogenesis	BP	1	GREM1,WNT10B,DLL4,TGFBR3	yellow	VSMC_M3
GO:0006661	0.23	0.86	10	115	phosphatidylinositol biosynthetic process	BP	1	PIGO,SOCS5,TTC7A,INPPL1,CISH,PDGFA,PIP4K2B,DPM3,MTMR4,VAC14	yellow	VSMC_M3
GO:0022409	0.23	0.85	15	176	positive regulation of cell-cell adhesion	BP	1	LGALS1,SOCS5,CX3CL1,EGR3,ST3GAL4,SART1,PTPN23,RELA,BAD,CORO1A,CD276,CSK,AKT1,AP3D1,IRAK1	yellow	VSMC_M3
GO:0060338	0.23	0.90	4	34	regulation of type I interferon-mediated signaling pathway	BP	1	TTLL12,METTL3,CDC37,CACTIN	yellow	VSMC_M3
GO:0061640	0.23	0.87	8	87	cytoskeleton-dependent cytokinesis	BP	1	SNX9,PLEC,CUL7,SEPT9,RAB35,RHOC,ALKBH4,SNX18	yellow	VSMC_M3
GO:0044380	0.23	0.89	5	55	protein localization to cytoskeleton	BP	1	DCTN2,CEP131,NUMA1,KIAA0753,MID2	yellow	VSMC_M3
GO:1902531	0.23	0.80	94	1309	regulation of intracellular signal transduction	BP	1	SQSTM1,NELFE,FNIP1,PRR5,SGSM3,DEF6,PPARD,EEF1D,LGALS1,SHARPIN,BOP1,PDGFRA,KLF4,TPBG,SPRED2,ZC3H12A,FIGNL1,RCN3,SLC9A3R1,EMILIN1,SEC13,ARHGEF40,CXXC5,LAMTOR1,P4HB,CAVIN3,ARHGAP22,BCL2L1,PDE4D,RRS1,SWAP70,PLEKHF1,GREM1,PDE3B,CX3CL1,SOD1,CCAR2,CHP1,SELENOS,PDGFA,GPX1,AIP,NGEF,QARS,TRIM27,EIF3A,THOC5,RIPK3,STMN3,RELA,SIPA1,RHOC,MAP3K14,LIMD1,PHB2,IGFBP5,BAD,LAMTOR4,IFI35,TNFRSF1A,C1QBP,CHERP,CCDC22,ARHGEF2,PER1,TRIM25,RPL37,S100A4,CSK,MID2,AKT1,MYDGF,MAP2K2,CACTIN,HIC1,TRAP1,CTNS,IRAK1,STK11,TELO2,WDR24,JMJD8,CARD9,TRADD,NDST1,ERBB4,PTPRR,IGFBP6,ZNF385A,TXNDC12,CDC34,ARHGEF1,DUSP5,GLIPR2	yellow	VSMC_M3
GO:0009154	0.23	0.90	4	35	purine ribonucleotide catabolic process	BP	1	NUDT3,PDE4D,NT5C,ITPA	yellow	VSMC_M3
GO:0097300	0.23	0.90	4	35	programmed necrotic cell death	BP	1	RIPK3,ARHGEF2,ALKBH7,MIR214	yellow	VSMC_M3
GO:0006165	0.23	0.88	7	72	nucleoside diphosphate phosphorylation	BP	1	GALK1,OGDHL,FOXK1,PGM1,GIT1,JMJD8,AK1	yellow	VSMC_M3
GO:1901796	0.23	0.87	8	83	regulation of signal transduction by p53 class mediator	BP	1	BOP1,SPRED2,RRS1,RPL37,AKT1,HIC1,STK11,ZNF385A	yellow	VSMC_M3
GO:0060627	0.23	0.82	31	403	regulation of vesicle-mediated transport	BP	1	CD63,RUFY1,PACSIN1,SCRIB,VPS28,EIPR1,SNX3,MICAL1,EHD2,SNX17,SCARB1,GREM1,SOD1,PACSIN3,RIN3,SYT12,TNK2,PLA2G3,PTPN23,LGALS3,LMAN2,SNF8,CORO1A,EEF2K,CSK,ATG3,GIT1,ANKRD13B,MAP2K2,PLA2G4E,GSG1L	yellow	VSMC_M3
GO:0050727	0.23	0.84	20	239	regulation of inflammatory response	BP	1	PPARD,SHARPIN,IL1R1,SOCS5,KLF4,ZYX,MGLL,CX3CL1,SOD1,TYRO3,SELENOS,MVK,GPX1,PSMB4,PLA2G3,RELA,IFI35,TNFRSF1A,GIT1,TRADD	yellow	VSMC_M3
GO:0043473	0.23	0.87	7	75	pigmentation	BP	1	ENPP1,CD63,DCTN2,HPS1,BLOC1S4,AP1M1,AP3D1	yellow	VSMC_M3
GO:0008286	0.23	0.87	8	93	insulin receptor signaling pathway	BP	1	ENPP1,CDK4,BAIAP2,TNS2,RELA,PIP4K2B,AKT1,SLC27A4	yellow	VSMC_M3
GO:0031529	0.23	0.89	5	49	ruffle organization	BP	1	DBNL,WDPCP,INPPL1,CORO1B,RCC2	yellow	VSMC_M3
GO:0071300	0.23	0.89	5	52	cellular response to retinoic acid	BP	1	KLF4,WNT10B,PHB2,SLC6A4,TWF2	yellow	VSMC_M3
GO:0009060	0.23	0.84	16	161	aerobic respiration	BP	1	NDUFB9,IDH3B,OGDHL,CHCHD2,SDHA,NDUFV1,UQCRC1,IDH1,CHCHD10,UQCC3,ATP5PD,AKT1,NDUFS7,ATP5F1D,TAZ,SURF1	yellow	VSMC_M3
GO:0032434	0.23	0.85	11	121	regulation of proteasomal ubiquitin-dependent protein catabolic process	BP	1	BAG6,HSPBP1,SOCS5,WNT10B,CCAR2,RNF19B,UBXN1,AKT1,SIRT6,TRIB2,STUB1	yellow	VSMC_M3
GO:0051881	0.23	0.87	7	66	regulation of mitochondrial membrane potential	BP	1	UCP2,PYCR1,BCL2L1,SOD1,BAD,AKT1,CTNS	yellow	VSMC_M3
GO:0050796	0.24	0.85	11	127	regulation of insulin secretion	BP	1	PPARD,EIPR1,MYRIP,UCP2,AACS,PCK2,CAPN10,BAD,GLUD1,MIDN,JAGN1	yellow	VSMC_M3
GO:1900076	0.24	0.89	5	51	regulation of cellular response to insulin stimulus	BP	1	ENPP1,TNS2,RELA,PIP4K2B,SLC27A4	yellow	VSMC_M3
GO:0040008	0.24	0.81	39	518	regulation of growth	BP	1	ENPP1,CDK4,PPARD,DNPH1,SOCS5,DBNL,DMAP1,H3F3B,LAMTOR1,BCL2L1,GREM1,KDM2B,TKT,SOD1,CCAR2,CISH,MMP14,LIMK1,CCDC85B,KAT5,SIPA1,SGPL1,IGFBP5,H3F3A,DBN1,MAD2L2,CRABP2,SMARCA4,AKT1,SLC6A4,HDGFL2,CDK11B,ANAPC2,STK11,TGFBR3,ERBB4,TWF2,UNC13A,NSMCE3	yellow	VSMC_M3
GO:0006081	0.24	0.89	5	46	cellular aldehyde metabolic process	BP	1	GRHPR,TKT,RELA,IDH1,TALDO1	yellow	VSMC_M3
GO:0007040	0.24	0.88	6	61	lysosome organization	BP	1	LAMTOR1,RAB20,HPS1,CORO1A,RAB34,CCDC115	yellow	VSMC_M3
GO:0080171	0.24	0.88	6	61	lytic vacuole organization	BP	1	LAMTOR1,RAB20,HPS1,CORO1A,RAB34,CCDC115	yellow	VSMC_M3
GO:0022406	0.24	0.87	7	76	membrane docking	BP	1	ESYT1,SNX3,CHP1,STX10,PACS2,RABEPK,UNC13A	yellow	VSMC_M3
GO:0051209	0.24	0.87	7	82	release of sequestered calcium ion into cytosol	BP	1	ITPR3,PDE4D,CX3CL1,GSTO1,LETM1,CHERP,CORO1A	yellow	VSMC_M3
GO:2001057	0.24	0.88	6	61	reactive nitrogen species metabolic process	BP	1	KLF4,ZC3H12A,SELENOS,THAP4,AKT1,KHSRP	yellow	VSMC_M3
GO:0032956	0.24	0.82	23	304	regulation of actin cytoskeleton organization	BP	1	SNX9,PDGFRA,DBNL,BAIAP2,SWAP70,CAPG,CX3CL1,PDGFA,CORO1B,TRIM27,ARPIN,LIMK1,RHOC,ARPC1B,WASHC1,DBN1,CORO1A,MIR214,TWF2,ELN,CDC42EP5,GBA2,ARPC5L	yellow	VSMC_M3
GO:0008360	0.24	0.85	11	134	regulation of cell shape	BP	1	WDPCP,SLC9A3R1,PLXND1,BAIAP2,PLEKHO1,RHOC,FMNL1,LIMD1,CORO1A,SYNE3,CDC42EP5	yellow	VSMC_M3
GO:0044843	0.24	0.84	15	180	cell cycle G1/S phase transition	BP	1	CDK4,CTDSP2,KLF4,KMT2E,EIF4EBP1,CDK2AP2,PSME2,PRMT2,PHB2,CTDSP1,METTL13,SDE2,AKT1,MIR214,CDC34	yellow	VSMC_M3

GO:0051702	0.24	0.87	6	61	biological process involved in interaction with symbiont	BP	1	ZC3H12A,RRP1B,GPX1,SMARCB1,SMARCA4,CARD9	yellow	VSMC_M3
GO:0071824	0.24	0.84	14	163	protein-DNA complex subunit organization	BP	1	H2AFY,DMAP1,H3F3B,WNT10B,SMARCD2,ZNHIT1,SMARCB1,UBTF,TAF6L,H3F3A,SMARCA4,BRF1,UBN1,MIS12	yellow	VSMC_M3
GO:0071396	0.24	0.81	32	422	cellular response to lipid	BP	1	CDK4,PPARD,KLF4,ZC3H12A,INSIG1,AACS,SCARB1,CX3CL1,WNT10B,SELENOS,PABPN1,PCK2,PRMT2,KAT5,RELA,PHB2,BAD,PKN1,CAPN2,PER1,SMARCA4,CARM1,AKT1,HMGCS1,SAFB,SAFB2,GIT1,SLC6A4,LDOC1,CACTIN,IRAK1,TWF2	yellow	VSMC_M3
GO:0046887	0.25	0.86	8	86	positive regulation of hormone secretion	BP	1	PPARD,MYRIP,AACS,PCK2,CAPN10,PLA2G3,BAD,GLUD1	yellow	VSMC_M3
GO:0050852	0.25	0.86	8	85	T cell receptor signaling pathway	BP	1	ZC3H12A,PDE4D,DGKZ,RELA,LGALS3,CD276,CSK,STK11	yellow	VSMC_M3
GO:0007566	0.25	0.89	4	36	embryo implantation	BP	1	PPARD,RPL29,SOD1,C1QBP	yellow	VSMC_M3
GO:0032388	0.25	0.84	14	173	positive regulation of intracellular transport	BP	1	SREBF2,EIPR1,ZC3H12A,EHD2,DMAP1,CEP131,NUMA1,CHP1,PCNT,CAPN10,PTPN23,RBM22,STK11,PLA2G4E	yellow	VSMC_M3
GO:0033273	0.25	0.86	7	75	response to vitamin	BP	1	PPARD,OGG1,DCPS,RELA,MTHFR,TRIM25,HMGCS1	yellow	VSMC_M3
GO:0018023	0.25	0.89	4	39	peptidyl-lysine trimethylation	BP	1	KMT2E,SETD1B,ETFBKMT,SMARCB1	yellow	VSMC_M3
GO:0046323	0.25	0.88	5	54	glucose import	BP	1	ENPP1,SELENOS,CAPN10,AKT1,SLC27A4	yellow	VSMC_M3
GO:0046916	0.25	0.86	8	86	cellular transition metal ion homeostasis	BP	1	TTC7A,SLC30A3,SLC11A2,SOD1,CCDC22,ANKRD9,AP3D1,CCDC115	yellow	VSMC_M3
GO:0048538	0.25	0.89	4	38	thymus development	BP	1	SOD1,RIPK3,PBX1,MAD1L1	yellow	VSMC_M3
GO:0032970	0.25	0.81	25	337	regulation of actin filament-based process	BP	1	SNX9,PDGFRA,DBNL,BAIAP2,PDE4D,SWAP70,CAPG,CX3CL1,PDGFA,CORO1B,TRIM27,ARPIN,LIMK1,RHOC,ARPC1B,WASHC1,DBN1,RANGRF,CORO1A,MIR214,TWF2,ELN,CDC42EP5,GBA2,ARPC5L	yellow	VSMC_M3
GO:1990748	0.25	0.86	8	79	cellular detoxification	BP	1	GSR,SOD1,SELENOS,GSTO1,GPX1,MGST3,TXNRD2,PRXL2A	yellow	VSMC_M3
GO:0048568	0.25	0.81	26	341	embryonic organ development	BP	1	CTHRC1,PBX2,SCRIB,PDGFRA,EPN1,HOXA1,WDPCP,KRT8,SLC9A3R1,INSIG1,HOXC4,LRIG1,KDM2B,SOD1,PDGFA,MMP14,THOC5,PBX1,PBX4,TEAD4,SLC39A1,TTC39C,AKT1,NSDHL,MBD3,NDST1	yellow	VSMC_M3
GO:0062014	0.25	0.86	7	73	negative regulation of small molecule metabolic process	BP	1	INSIG1,SOD1,ETFBKMT,AKT1,GIT1,PGP,SLC27A4	yellow	VSMC_M3
GO:0050769	0.25	0.83	14	181	positive regulation of neurogenesis	BP	1	CUL7,DBNL,PLXND1,BAIAP2,CX3CL1,CLCF1,LIMK1,RELA,DBN1,CRABP2,EEF2K,ANAPC2,STK11,TWF2	yellow	VSMC_M3
GO:0000413	0.25	0.89	4	32	protein peptidyl-prolyl isomerization	BP	1	FKBP11,AIP,FKBP2,PIN4	yellow	VSMC_M3
GO:0051283	0.25	0.86	7	83	negative regulation of sequestering of calcium ion	BP	1	ITPR3,PDE4D,CX3CL1,GSTO1,LETM1,CHERP,CORO1A	yellow	VSMC_M3
GO:0001706	0.25	0.89	4	43	endoderm formation	BP	1	H2AFY,MMP14,COL6A1,DUSP5	yellow	VSMC_M3
GO:0097061	0.25	0.87	6	70	dendritic spine organization	BP	1	DBNL,BAIAP2,ARHGAP33,NGEF,DBN1,EEF2K	yellow	VSMC_M3
GO:0097178	0.25	0.89	4	38	ruffle assembly	BP	1	DBNL,WDPCP,INPPL1,RCC2	yellow	VSMC_M3
GO:0032774	0.25	0.77	179	2534	RNA biosynthetic process	BP	1	ENPP1,NAB2,CDK4,SQSTM1,ZNF608,HDAC10,NELFE,FNIP1,ATF6B,PBX2,RING1,SREBF2,H2AFY,TRIM11,TRAPPC9,PPARD,SND1,PHF2,CNOT3,KLF4,HOXA1,FHL3,ZC3H12A,CC2D1B,KTI12,KMT2E,SIX5,ZNF282,ZNF296,RELB,DMAP1,CIC,GTF3C2,OGG1,SERTAD1,TTC5,CXXC5,DEAF1,HOXC4,POLR1D,KMT2B,ZNF740,RALY,ZBTB38,GREM1,KDM2B,MCRS1,POLR2C,CX3CL1,WNT10B,ACTR5,ANKRD2,EGR3,CCAR2,DNAJC17,DLL4,CHP1,YAF2,HTATIP2,NPM3,KDM1B,CHCHD2,IRX1,RRP1B,BRD9,AIP,TSHZ2,QARS,PIR,TRIM27,ZNRD1,PRMT2,THOC5,RIPK3,TWIST2,ADRM1,PSTK,SMARCD2,BRMS1,GMEB2,CCDC85B,PRPF6,PBX1,KAT5,RELA,ZNF511,ARNT2,ZFPL1,LIMD1,SMARCB1,CHCHD10,PBX4,UBTF,GATAD2A,MEF2B,PHB2,NDP,CTDSP1,MLF2,EID1,SSBP4,METTL13,ZNF394,C11orf95,BUD31,TNFRSF1A,TEAD4,C1QBP,TAF6L,ZBTB49,MTA2,ZNF316,MED11,PELP1,CCDC22,FOXK1,KLF3,PKN1,SEPSECS,POLR2A,MAD2L2,SLTM,CTBP1,FBXL19,CRABP2,SP2,ARHGEF2,SNF8,PER1,NFIX,IRF2BPL,TRIM25,SMARCA4,CARM1,AATF,MID2,ZNF521,ZNF358,AKT1,BRF1,KHSRP,GTF2F1,RFX2,SAFB,SAFB2,MYDGF,HDGFL2,SIRT6,MAP2K2,CACTIN,CDK11B,SSU72,HIC1,MNT,EHMT1,AP3D1,IRAK1,MBD3,MED16,CARD9,SARS,GTF3C5,TRADD,ERBB4,JUND,PTMA,KLF13,FADS1,ZNF783,HOXC5,CAVIN1,IER5,AKAP17A,ZNF250,THAP7,NSMCE3,ERF,SNAPC2,ZSCAN1	yellow	VSMC_M3
GO:0050803	0.25	0.83	13	170	regulation of synapse structure or activity	BP	1	DBNL,TPBG,BAIAP2,ARHGAP22,ARHGAP33,DCTN1,NGEF,IGSF9,LRFN4,NECTIN1,DBN1,EEF2K,ANAPC2	yellow	VSMC_M3
GO:0010256	0.26	0.80	32	422	endomembrane system organization	BP	1	ESYT1,SQSTM1,BAG6,SNX9,PACSIN1,PLEC,VPS28,CUL7,ASNA1,SNX3,EHD2,TMEM43,SLC9A3R1,BAIAP2,LAMTOR1,PLEKHF1,DCTN1,SOD1,PACSIN3,PLA2G3,VPS51,CD9,MVB12A,LMAN2,SNF8,TMED1,AKT1,RFX2,MAP2K2,AP3D1,JAGN1,PHETA2	yellow	VSMC_M3
GO:0002250	0.26	0.81	20	244	adaptive immune response	BP	1	GNL1,IL1R1,SOCS5,DBNL,ZC3H12A,RELB,CD79A,SWAP70,RNF19B,CLCF1,TRIM27,RIPK3,SIPA1,OTUB1,C1QBP,PKN1,MAD2L2,CSK,C8G,CARD9	yellow	VSMC_M3
GO:1900182	0.26	0.86	7	76	positive regulation of protein localization to nucleus	BP	1	ZC3H12A,DMAP1,PINX1,MCRS1,RBM22,AKT1,STK11	yellow	VSMC_M3
GO:0009895	0.26	0.81	20	254	negative regulation of catabolic process	BP	1	BAG6,HNRNPA0,SECISBP2,SNRNP70,SNX3,DND1,PDE3B,CCAR2,ETFBKMT,ZCCHC17,RELA,UBXN1,WASHC1,FOXK1,MAD2L2,DFFA,AKT1,PAIP1,GIT1,TENT5B	yellow	VSMC_M3
GO:0035821	0.26	0.86	7	75	modulation of process of other organism	BP	1	ZC3H12A,BCL2L1,RRP1B,SMARCB1,PHB2,SMARCA4,CARD9	yellow	VSMC_M3
GO:0001701	0.26	0.81	24	314	in utero embryonic development	BP	1	PDGFRA,EPN1,CNOT3,KLF4,CCM2,KRT8,UNK,PSMC4,RIC8A,HM13,BCL2L1,STMN3,ARNT2,SMARCB1,DBN1,CAPN2,ARMC5,SLC39A1,ZNF830,AKT1,NSDHL,SLC35E2B,MBD3,PTPRR	yellow	VSMC_M3

GO:0009628	0.26	0.78	66	891	response to abiotic stimulus	BP	1	CDK4,PPARD,EEF1D,PLEC,HOXA1,FIGNL1,PNKP,KRT8,RELB,OGG1,UCP2,METTL3,P4HB,EIF4EBP1,RIC8A,BCL2L1,SLC11A2,TNKS1BP1,MMP24,AQP5,SOD1,ACTR5,CCAR2,CHP1,CHCHD2,MMP14,GPX1,KAT5,RELA,SIPA1,TMEM120A,MAP3K14,ARNT2,LIMD1,PHB2,BAD,LETM1,CD9,PLAU,TNFRSF1A,BABAM1,SDE2,PKN1,CAPN2,MTHFR,ARHGEF2,PER1,EEF2K,AKT1,HMGCS1,SLC6A4,PPL,VASN,CTNS,IRAK1,STK11,TGFBR3,MIR214,RF00024,NABP2,JUND,NSMF,IER5,STUB1,NSMCE3,CCDC115	yellow	VSMC_M3
GO:0050866	0.26	0.83	12	136	negative regulation of cell activation	BP	1	SCRIB,PDGFRA,SOCS5,ZC3H12A,EMILIN1,CX3CL1,TYRO3,PDGFA,LGALS3,CD9,PKN1,MAD1L1	yellow	VSMC_M3
GO:0045123	0.26	0.87	5	50	cellular extravasation	BP	1	IL1R1,ITGB7,CX3CL1,ST3GAL4,RIPK3	yellow	VSMC_M3
GO:0030195	0.26	0.89	4	35	negative regulation of blood coagulation	BP	1	PDGFRA,PDGFA,CD9,PLAU	yellow	VSMC_M3
GO:0090311	0.26	0.88	4	42	regulation of protein deacetylation	BP	1	SPRED2,CCAR2,BRMS1,CTBP1	yellow	VSMC_M3
GO:1901653	0.26	0.81	21	270	cellular response to peptide	BP	1	ENPP1,CDK4,KLF4,INSIG1,UCP2,BAIAP2,TNS2,PDE3B,SELENOS,PCK2,CAPN10,RELA,KLF3,PIP4K2B,ARHGEF2,EEF2K,CSK,AKT1,LDOC1,APRT,SLC27A4	yellow	VSMC_M3
GO:0030100	0.26	0.82	14	174	regulation of endocytosis	BP	1	CD63,RUFY1,PACSIN1,SCRIB,VPS28,SNX17,GREM1,PACSIN3,RIN3,TNK2,LGALS3,EEF2K,ANKRD13B,GSG1L	yellow	VSMC_M3
GO:0071347	0.26	0.85	7	75	cellular response to interleukin-1	BP	1	IL1R1,ZC3H12A,CX3CL1,RELA,OTUB1,CACTIN,IRAK1	yellow	VSMC_M3
GO:0007422	0.26	0.86	6	69	peripheral nervous system development	BP	1	NAB2,PLEC,SOD1,EGR3,RELA,AKT1	yellow	VSMC_M3
GO:1902904	0.26	0.83	11	139	negative regulation of supramolecular fiber organization	BP	1	EMILIN1,SWAP70,CAPG,CORO1B,ARPIN,PFDN2,MID1IP1,ARHGEF2,CORO1A,MIR214,TWF2	yellow	VSMC_M3
GO:0033692	0.26	0.87	5	52	cellular polysaccharide biosynthetic process	BP	1	ENPP1,SELENOS,AKT1,B3GNT9,NDST1	yellow	VSMC_M3
GO:0097305	0.26	0.82	14	174	response to alcohol	BP	1	CDK4,KLF4,DMAP1,OGG1,EIF4EBP1,AACS,BCL2L1,SOD1,BAD,PMVK,TUFM,AKT1,HMGCS1,TGFBR3	yellow	VSMC_M3
GO:0006636	0.26	0.88	4	36	unsaturated fatty acid biosynthetic process	BP	1	SCD,PLA2G3,FADS2,FADS1	yellow	VSMC_M3
GO:0002028	0.26	0.86	6	66	regulation of sodium ion transport	BP	1	SLC9A3R1,CHP1,NKAIN1,PER1,RANGRF,AKT1	yellow	VSMC_M3
GO:0010972	0.26	0.87	5	51	negative regulation of G2/M transition of mitotic cell cycle	BP	1	PINX1,BABAM1,MIIP,ZNF830,NABP2	yellow	VSMC_M3
GO:0071826	0.26	0.82	15	170	ribonucleoprotein complex subunit organization	BP	1	RPL3,BOP1,GEMIN7,TARBP2,EIF4B,RRS1,SRSF9,EIF3A,SF3A1,SART1,PRPF6,MRTO4,CD2BP2,EIF3G,MRPL20	yellow	VSMC_M3
GO:0006338	0.26	0.82	14	175	chromatin remodeling	BP	1	H2AFY,DMAP1,H3F3B,ACTR5,NPM3,SMARCD2,ZNHIT1,SMARCB1,TAF6L,MTA2,H3F3A,SMARCA4,UBN1,MBD3	yellow	VSMC_M3
GO:0051282	0.26	0.85	7	84	regulation of sequestering of calcium ion	BP	1	ITPR3,PDE4D,CX3CL1,GSTO1,LETM1,CHERP,CORO1A	yellow	VSMC_M3
GO:0090559	0.26	0.86	6	59	regulation of membrane permeability	BP	1	BCL2L1,CHCHD10,BAD,CNP,ALKBH7,SPG7	yellow	VSMC_M3
GO:0006090	0.27	0.85	7	76	pyruvate metabolic process	BP	1	GALK1,OGDHL,PCK2,FOXK1,PGM1,GIT1,JMJD8	yellow	VSMC_M3
GO:0071417	0.27	0.79	34	466	cellular response to organonitrogen compound	BP	1	ENPP1,CDK4,PDGFRA,KLF4,SLC9A3R1,INSIG1,UCP2,BAIAP2,LAMTOR1,BCL2L1,TNS2,PDE3B,WNT10B,SOD1,SELENOS,PCK2,COL6A1,SLC26A6,CAPN10,RELA,IGFBP5,LAMTOR4,KLF3,CAPN2,PIP4K2B,ARHGEF2,EEF2K,CSK,AKT1,SLC6A4,LDOC1,APRT,SLC27A4,NSMF	yellow	VSMC_M3
GO:0007286	0.27	0.84	9	100	spermatid development	BP	1	SBF1,SIX5,CEP131,YIF1B,SLC26A6,IQCG,PLA2G3,RFX2,MEIG1	yellow	VSMC_M3
GO:0016458	0.27	0.82	14	169	gene silencing	BP	1	SND1,PHF2,CNOT3,ZC3H12A,METTL3,DND1,TARBP2,LIMD1,MAP2K2,MBD3,MIR214,MIR155,MIR374B,ZAR1L	yellow	VSMC_M3
GO:0010508	0.27	0.84	9	105	positive regulation of autophagy	BP	1	ZC3H12A,PLEKHF1,TRIM27,KAT5,PHB2,BAD,PIP4K2B,MID2,STK11	yellow	VSMC_M3
GO:0009889	0.27	0.76	212	3010	regulation of biosynthetic process	BP	1	ENPP1,NAB2,CDK4,SQSTM1,ZNF608,HDAC10,NELFE,FNIP1,ATF6B,PBX2,RING1,SREBF2,H2AFY,TRIM11,TRAPPC9,PPARD,SND1,SECISBP2,PHF2,RPS9,CNOT3,KLF4,HOXA1,FHL3,ZC3H12A,PNKP,CC2D1B,KTI12,KMT2E,SIX5,ZNF282,ZNF296,RELB,EMILIN1,DMAP1,CIC,UNK,INSIG1,OGG1,SERTAD1,TTC5,METTL3,CXXC5,DND1,EIF4EBP1,DEAF1,HOXC4,TARBP2,KMT2B,SCARB1,NAT10,ZNF740,EIF4B,RALY,ZBTB38,PINX1,GREM1,KDM2B,MCRS1,CX3CL1,WNT10B,SOD1,ACTR5,ANKRD2,EGR3,CCAR2,DNAJC17,DLL4,CHP1,YAF2,HTATIP2,PCIF1,KDM1B,CHCHD2,DCPS,IRX1,DHCR7,SELENOS,RRP1B,BRD9,FABP3,PDGFA,AIP,NGDN,SRRD,TSHZ2,QARS,PIR,TRIM27,PRMT2,THOC5,RIPK3,TWIST2,LSM14B,SMARCD2,BRMS1,TACO1,PLA2G3,GMEB2,CCDC85B,PRPF6,PBX1,KAT5,RELA,ZNF511,IDH1,ARNT2,ZFPL1,LIMD1,MID1IP1,SMARCB1,CHCHD10,PBX4,UBTF,GATAD2A,MEF2B,PHB2,NDP,IGFBP5,CTDSP1,MLF2,EID1,SSBP4,METTL13,MPV17L2,ZNF394,C11orf95,BUD31,TNFRSF1A,TEAD4,C1QBP,TAF6L,ZBTB49,MTA2,ZNF316,MED11,PELP1,CCDC22,FOXK1,KLF3,PKN1,CAPN2,SEPSECS,POLR2A,MAD2L2,SLTM,NANOS3,CTBP1,FBXL19,CRABP2,SP2,ARHGEF2,ARMC5,SNF8,PER1,NFIX,IRF2BPL,TRIM25,SMARCA4,CARM1,AATF,C19orf66,MID2,ZNF521,ZNF358,ZNF830,AKT1,BRF1,PAIP1,KHSRP,GTF2F1,RFX2,SAFB,SAFB2,MYDGF,HDGFL2,SIRT6,MAP2K2,CACTIN,CDK11B,SSU72,HIC1,TRAP1,MNT,CTNS,EHMT1,PGP,AP3D1,MKNK2,IRAK1,MBD3,TAZ,MED16,CARD9,SARS,POMT1,TRADD,RBM8A,ERBB4,JUND,KLF13,FADS1,ZNF783,HOXC5,ZNF385A,IER5,AKAP17A,ZNF250,THAP7,NSMCE3,ERF,ZSCAN1	yellow	VSMC_M3
GO:0009896	0.27	0.79	32	420	positive regulation of catabolic process	BP	1	BAG6,SNX9,SGSM3,VPS28,HSPBP1,SOCS5,CNOT3,ZC3H12A,PSMC4,METTL3,DND1,PLEKHF1,PACSIN3,RNF19B,DCPS,ASB9,TRIM27,KAT5,PHB2,BAD,CCDC22,AMER1,PIP4K2B,NANOS3,SNF8,MID2,AKT1,KHSRP,SIRT6,STK11,TRIB2,STUB1	yellow	VSMC_M3
GO:0038034	0.27	0.87	5	49	signal transduction in absence of ligand	BP	1	BCL2L1,CX3CL1,BAD,AKT1,MKNK2	yellow	VSMC_M3
GO:0097192	0.27	0.87	5	49	extrinsic apoptotic signaling pathway in absence of ligand	BP	1	BCL2L1,CX3CL1,BAD,AKT1,MKNK2	yellow	VSMC_M3
GO:0042632	0.27	0.86	6	62	cholesterol homeostasis	BP	1	SREBF2,INSIG1,LAMTOR1,SCARB1,RALY,FABP3	yellow	VSMC_M3
GO:0051851	0.27	0.87	5	51	modulation by host of symbiont process	BP	1	ZC3H12A,RRP1B,SMARCB1,SMARCA4,CARD9	yellow	VSMC_M3
GO:0043409	0.27	0.83	11	131	negative regulation of MAPK cascade	BP	1	KLF4,SPRED2,SLC9A3R1,EMILIN1,QARS,EIF3A,PER1,CSK,AKT1,PTPRR,DUSP5	yellow	VSMC_M3

GO:0120031	0.27	0.79	32	435	plasma membrane bounded cell projection assembly	BP	1	SPAG1,TXNDC15,DBNL,WDPCP,EMP3,EHD2,SLC9A3R1,SEPT9,CEP131,INPPL1,YIF1B,CCDC57,DCTN1,CCDC28B,PCNT,IQCG,PLA2G3,CEP164,PTPN23,WASHC1,RCC2,RABEP2,ATG3,TTC39C,DOCK11,RFX2,GAS8,WDR34,MIR214,TWF2,CDC42EP5,MEIG1	yellow	VSMC_M3
GO:0030518	0.27	0.84	8	96	intracellular steroid hormone receptor signaling pathway	BP	1	PRMT2,PHB2,PKN1,PER1,SMARCA4,CARM1,SAFB,SAFB2	yellow	VSMC_M3
GO:0001824	0.27	0.84	8	86	blastocyst development	BP	1	CNOT3,KLF4,PSMC4,STMN3,SMARCB1,CAPN2,ZNF830,SLC35E2B	yellow	VSMC_M3
GO:0000723	0.27	0.83	10	118	telomere maintenance	BP	1	NHP2,PNKP,NAT10,TNKS1BP1,PINX1,SLX1A,SIRT6,TELO2,RF00024,NABP2	yellow	VSMC_M3
GO:2000378	0.27	0.88	4	37	negative regulation of reactive oxygen species metabolic process	BP	1	AATF,AKT1,TRAP1,CTNS	yellow	VSMC_M3
GO:0043489	0.27	0.87	5	52	RNA stabilization	BP	1	HNRNPA0,DND1,ZCCHC17,PAIP1,TENT5B	yellow	VSMC_M3
GO:0070231	0.27	0.88	4	36	T cell apoptotic process	BP	1	RIPK3,LGALS3,DFFA,AKT1	yellow	VSMC_M3
GO:1902882	0.27	0.85	7	74	regulation of response to oxidative stress	BP	1	H2AFY,P4HB,PYCR1,SOD1,GPX1,AKT1,TRAP1	yellow	VSMC_M3
GO:0031326	0.27	0.75	208	2955	regulation of cellular biosynthetic process	BP	1	ENPP1,NAB2,CDK4,SQSTM1,ZNF608,HDAC10,NELFE,FNIP1,ATF6B,PBX2,RING1,SREBF2,H2AFY,TRIM11,TRAPPC9,PPARD,SND1,SECISBP2,PHF2,RPS9,CNOT3,KLF4,HOXA1,FHL3,ZC3H12A,PNKP,CC2D1B,KTI12,KMT2E,SIX5,ZNF282,ZNF296,RELB,DMAP1,CIC,UNK,INSIG1,OGG1,SERTAD1,TTC5,METTL3,CXXC5,DND1,EIF4EBP1,DEAF1,HOXC4,TARBP2,KMT2B,SCARB1,NAT10,ZNF740,EIF4B,RALY,ZBTB38,PINX1,GREM1,KDM2B,MCRS1,CX3CL1,WNT10B,ACTR5,ANKRD2,EGR3,CCAR2,DNAJC17,DLL4,CHP1,YAF2,HTATIP2,PCIF1,KDM1B,CHCHD2,DCPS,IRX1,SELENOS,RRP1B,BRD9,FABP3,PDGFA,AIP,NGDN,SRRD,TSHZ2,QARS,PIR,TRIM27,PRMT2,THOC5,RIPK3,TWIST2,LSM14B,SMARCD2,BRMS1,TACO1,PLA2G3,GMEB2,CCDC85B,PRPF6,PBX1,KAT5,RELA,ZNF511,IDH1,ARNT2,ZFPL1,LIMD1,MID1IP1,SMARCB1,CHCHD10,PBX4,UBTF,GATAD2A,MEF2B,PHB2,NDP,IGFBP5,CTDSP1,MLF2,EID1,SSBP4,METTL13,MPV17L2,ZNF394,C11orf95,BUD31,TNFRSF1A,TEAD4,C1QBP,TAF6L,ZBTB49,MTA2,ZNF316,MED11,PELP1,CCDC22,FOXK1,KLF3,PKN1,CAPN2,SEPSECS,POLR2A,MAD2L2,SLTM,NANOS3,CTBP1,FBXL19,CRABP2,SP2,ARHGEF2,SNF8,PER1,NFIX,IRF2BPL,TRIM25,SMARCA4,CARM1,AATF,C19orf66,MID2,ZNF521,ZNF358,ZNF830,AKT1,BRF1,PAIP1,KHSRP,GTF2F1,RFX2,SAFB,SAFB2,MYDGF,HDGFL2,SIRT6,MAP2K2,CACTIN,CDK11B,SSU72,HIC1,TRAP1,MNT,CTNS,EHMT1,PGP,AP3D1,MKNK2,IRAK1,MBD3,TAZ,MED16,CARD9,SARS,POMT1,TRADD,RBM8A,ERBB4,JUND,KLF13,FADS1,ZNF783,HOXC5,ZNF385A,IER5,AKAP17A,ZNF250,THAP7,NSMCE3,ERF,ZSCAN1	yellow	VSMC_M3
GO:0001541	0.27	0.88	4	39	ovarian follicle development	BP	1	BCL2L1,SOD1,MMP14,ZNF830	yellow	VSMC_M3
GO:0071375	0.27	0.81	17	219	cellular response to peptide hormone stimulus	BP	1	ENPP1,CDK4,INSIG1,UCP2,BAIAP2,TNS2,PDE3B,SELENOS,PCK2,CAPN10,RELA,PIP4K2B,EEF2K,CSK,AKT1,APRT,SLC27A4	yellow	VSMC_M3
GO:0030865	0.27	0.86	5	55	cortical cytoskeleton organization	BP	1	PLEC,EHD2,CAVIN3,RHOC,FMNL1	yellow	VSMC_M3
GO:0016358	0.27	0.81	15	204	dendrite development	BP	1	PACSIN1,CUL7,DBNL,TPBG,BAIAP2,ARHGAP33,SLC11A2,NGEF,IGSF9,DBN1,CARM1,EEF2K,ANAPC2,STK11,NSMF	yellow	VSMC_M3
GO:2000117	0.27	0.85	6	60	negative regulation of cysteine-type endopeptidase activity	BP	1	FNIP1,KLF4,MICAL1,GPX1,AKT1,DHCR24	yellow	VSMC_M3
GO:0071840	0.27	0.75	333	4827	cellular component organization or biogenesis	BP	1	ENPP1,PQLC1,CD63,ESYT1,DCTN2,NHP2,CDK4,RUFY1,SQSTM1,SPAG1,BAG6,NCAPH2,SNX9,CTHRC1,HDAC10,FNIP1,PDLIM4,SAMM50,SREBF2,NDUFB9,SQLE,TXNDC15,H2AFY,PACSIN1,PPARD,RPL3,SCRIB,PLEC,LGALS1,IFT27,SHARPIN,BOP1,VPS28,TBCC,CUL7,DNPH1,B4GALT1,PDGFRA,PHF2,COL15A1,COL19A1,ASNA1,KLF4,DBNL,TPBG,PLEKHA8,WDPCP,CCM2,FHL3,ZC3H12A,FIGNL1,PNKP,ZYX,SNX3,MICAL1,EMP3,EHD2,KMT2E,KRT8,ACAD9,TMEM43,GEMIN7,SLC9A3R1,PLXND1,EMILIN1,DMAP1,SNX17,H3F3B,INSIG1,SEC13,SEPT9,BAIAP2,CEP131,INPPL1,LAMTOR1,NUMA1,YIF1B,CCDC57,RAB20,EIF4EBP1,ARAP3,CAVIN3,RIC8A,ARHGAP22,SDHAF1,ARHGAP33,TARBP2,SCARB1,NAT10,BCL2L1,SPRYD3,EIF4B,RRS1,SWAP70,PLEKHF1,UQCRFS1,LSM6,CAPG,SLC11A2,TNKS1BP1,MMP24,PINX1,GREM1,KDM2B,AQP5,DCTN1,RAPSN,CX3CL1,WNT10B,SOD1,PACSIN3,ACTR5,CCDC32,CCAR2,HPS1,CHP1,NPM3,RAB35,CHCHD2,CISH,SRSF9,RRP1B,CCDC28B,BRD9,RIN3,PDGFA,MMP14,CDK2AP2,LIX1L,APEH,GPX1,AIP,CORO1B,NGEF,HCCS,NGDN,PLEKHO1,IGSF9,LRFN4,CLIP2,NECTIN1,TRIM27,ARPIN,PCNT,EIF3A,LIMK1,NUDC,RIPK3,SF3A1,ADRM1,RPS21,CAPN10,SMARCD2,IQCG,PFDN2,FTSJ3,MPZL3,BUD23,TNK2,TACO1,PLA2G3,STMN3,CEP164,BANF1,SART1,CCDC85B,PRPF6,PTPN23,RELA,SIPA1,RHOC,TMEM120A,DDX52,LRWD1,ALKBH4,ZNHIT1,FMNL1,VPS51,SAC3D1,LIMD1,MID1IP1,PEX5,SMARCB1,CHCHD10,YJEFN3,UBTF,BLOC1S4,PHB2,TRMT112,NDP,IGFBP5,C12orf57,BAD,LAMTOR4,NOP14,MPV17L2,LETM1,LGALS3,ARPC1B,CD9,MRTO4,TNFRSF1A,FCHO1,MVB12A,KIAA0753,BABAM1,C1QBP,NUP88,AIMP2,TAF6L,UQCC3,WASHC1,RCC2,CNP,A[illegible]	yellow	VSMC_M3
GO:0006942	0.28	0.85	6	68	regulation of striated muscle contraction	BP	1	ZC3H12A,PDE4D,GSTO1,SCN4A,C12orf57,RANGRF	yellow	VSMC_M3
GO:1900047	0.28	0.88	4	36	negative regulation of hemostasis	BP	1	PDGFRA,PDGFA,CD9,PLAU	yellow	VSMC_M3
GO:0090087	0.28	0.82	12	144	regulation of peptide transport	BP	1	PPARD,EIPR1,MYRIP,UCP2,AACS,PCK2,SLC26A6,CAPN10,BAD,GLUD1,MIDN,JAGN1	yellow	VSMC_M3
GO:0034968	0.28	0.84	8	98	histone lysine methylation	BP	1	NELFE,H2AFY,KMT2E,KMT2B,SETD1B,SMARCB1,TRMT112,EHMT1	yellow	VSMC_M3
GO:0010948	0.28	0.80	19	237	negative regulation of cell cycle process	BP	1	CTDSP2,H2AFY,KLF4,PINX1,CCAR2,CDK2AP2,PRMT2,THOC5,CTDSP1,METTL13,BABAM1,SDE2,MIIP,MAD1L1,MAD2L2,ZNF830,AURKAIP1,NABP2,FBXO6	yellow	VSMC_M3
GO:0035305	0.28	0.87	4	40	negative regulation of dephosphorylation	BP	1	SWAP70,CHP1,PPP1R35,LGALS3	yellow	VSMC_M3
GO:0048839	0.28	0.82	11	139	inner ear development	BP	1	CTHRC1,SCRIB,IFT27,HOXA1,WDPCP,CCM2,SLC9A3R1,INSIG1,LRIG1,SOD1,TTC39C	yellow	VSMC_M3

GO:0071214	0.28	0.80	21	267	cellular response to abiotic stimulus	BP	1	EEF1D,PLEC,FIGNL1,RELB,METTL3,BCL2L1,TNKS1BP1,AQP5,ACTR5,CHP1,SIPA1,MAP3K14,BAD,LETM1,TNFRSF1A,SDE2,ARHGEF2,AKT1,STK11,NSMF,NSMCE3	yellow	VSMC_M3
GO:0104004	0.28	0.80	21	267	cellular response to environmental stimulus	BP	1	EEF1D,PLEC,FIGNL1,RELB,METTL3,BCL2L1,TNKS1BP1,AQP5,ACTR5,CHP1,SIPA1,MAP3K14,BAD,LETM1,TNFRSF1A,SDE2,ARHGEF2,AKT1,STK11,NSMF,NSMCE3	yellow	VSMC_M3
GO:1905952	0.28	0.82	10	118	regulation of lipid localization	BP	1	SREBF2,PPARD,ZC3H12A,LAMTOR1,SCARB1,ACSL1,FABP3,PLA2G3,YJEFN3,AKT1	yellow	VSMC_M3
GO:0071322	0.28	0.82	10	121	cellular response to carbohydrate stimulus	BP	1	PPARD,LGALS1,UCP2,AACS,PCK2,SLC26A6,SMARCB1,BAD,SMARCA4,JAGN1	yellow	VSMC_M3
GO:0060964	0.28	0.87	4	39	regulation of gene silencing by miRNA	BP	1	ZC3H12A,DND1,LIMD1,MAP2K2	yellow	VSMC_M3
GO:0038202	0.28	0.87	4	40	TORC1 signaling	BP	1	SEC13,CTNS,STK11,TELO2	yellow	VSMC_M3
GO:0051053	0.28	0.83	8	94	negative regulation of DNA metabolic process	BP	1	OGG1,NAT10,PINX1,OTUB1,MAD2L2,DFFA,SLX1A,CXorf57	yellow	VSMC_M3
GO:2001244	0.28	0.86	5	46	positive regulation of intrinsic apoptotic signaling pathway	BP	1	SLC9A3R1,PLEKHF1,SOD1,RIPK3,BAD	yellow	VSMC_M3
GO:0016051	0.28	0.81	13	155	carbohydrate biosynthetic process	BP	1	ENPP1,B4GALT1,ST3GAL4,SELENOS,PCK2,ISYNA1,MPDU1,PGD,AKT1,PGM1,PGP,B3GNT9,NDST1	yellow	VSMC_M3
GO:0002260	0.28	0.86	5	50	lymphocyte homeostasis	BP	1	RIPK3,PKN1,CORO1A,DOCK11,AKT1	yellow	VSMC_M3
GO:0006457	0.28	0.81	15	166	protein folding	BP	1	TBCC,HSPBP1,PDIA4,PDIA6,SNRNP70,P4HB,FKBP11,AIP,NUDC,PFDN2,FKBP2,MPDU1,DFFA,CDC37,TRAP1	yellow	VSMC_M3
GO:0044085	0.28	0.75	174	2454	cellular component biogenesis	BP	1	NHP2,SPAG1,BAG6,SNX9,FNIP1,SAMM50,NDUFB9,SQLE,TXNDC15,H2AFY,RPL3,PLEC,BOP1,VPS28,TBCC,PDGFRA,PHF2,DBNL,TPBG,WDPCP,ZC3H12A,ZYX,MICAL1,EMP3,EHD2,KRT8,ACAD9,GEMIN7,SLC9A3R1,PLXND1,EMILIN1,H3F3B,SEPT9,BAIAP2,CEP131,INPPL1,NUMA1,YIF1B,CCDC57,RAB20,EIF4EBP1,SDHAF1,TARBP2,NAT10,EIF4B,RRS1,SWAP70,UQCRFS1,LSM6,CAPG,GREM1,AQP5,DCTN1,CX3CL1,WNT10B,HPS1,CHP1,NPM3,SRSF9,RRP1B,CCDC28B,PDGFA,MMP14,CORO1B,HCCS,NGDN,LRFN4,NECTIN1,TRIM27,ARPIN,PCNT,EIF3A,LIMK1,SF3A1,ADRM1,RPS21,IQCG,FTSJ3,BUD23,TACO1,PLA2G3,CEP164,SART1,PRPF6,PTPN23,RHOC,TMEM120A,DDX52,SAC3D1,LIMD1,MID1IP1,PEX5,SMARCB1,CHCHD10,UBTF,TRMT112,BAD,NOP14,MPV17L2,LETM1,LGALS3,ARPC1B,CD9,MRTO4,FCHO1,MVB12A,KIAA0753,C1QBP,NUP88,AIMP2,TAF6L,UQCC3,WASHC1,RCC2,EML3,KRT10,H3F3A,DBN1,PIP4K2B,CLTB,RRNAD1,SNF8,CD2BP2,RANGRF,FARSA,CORO1A,SLX1A,RABEP2,SMARCA4,RPL7A,EEF2K,AATF,EIF3G,ATG3,TTC39C,DOCK11,BRF1,PACS2,RFX2,RAB34,GIT1,UBN1,MRPL20,NCLN,CLDN6,TBL3,NUBP2,NDUFS7,ATP5F1D,TAZ,TSR3,CARD9,GAS8,SPG7,WDR34,TGFBR3,MIR214,ERBB4,TWF2,SURF1,RBFA,PIN4,MIS12,ELN,UNC13A,CDC42EP5,CDC34,NOM1,GBA2,TUBGCP2,MEIG1,STUB1,CCDC115,ARPC5L	yellow	VSMC_M3
GO:1901880	0.28	0.86	5	62	negative regulation of protein depolymerization	BP	1	SWAP70,CAPG,MID1IP1,ARHGEF2,TWF2	yellow	VSMC_M3
GO:0055092	0.28	0.85	6	63	sterol homeostasis	BP	1	SREBF2,INSIG1,LAMTOR1,SCARB1,RALY,FABP3	yellow	VSMC_M3
GO:0006351	0.28	0.74	177	2521	transcription, DNA-templated	BP	1	ENPP1,NAB2,CDK4,SQSTM1,ZNF608,HDAC10,NELFE,FNIP1,ATF6B,PBX2,RING1,SREBF2,H2AFY,TRIM11,TRAPPC9,PPARD,SND1,PHF2,CNOT3,KLF4,HOXA1,FHL3,ZC3H12A,CC2D1B,KTI12,KMT2E,SIX5,ZNF282,ZNF296,RELB,DMAP1,CIC,GTF3C2,OGG1,SERTAD1,TTC5,CXXC5,DEAF1,HOXC4,POLR1D,KMT2B,ZNF740,RALY,ZBTB38,GREM1,KDM2B,MCRS1,POLR2C,CX3CL1,WNT10B,ACTR5,ANKRD2,EGR3,CCAR2,DNAJC17,DLL4,CHP1,YAF2,HTATIP2,NPM3,KDM1B,CHCHD2,IRX1,RRP1B,BRD9,AIP,TSHZ2,QARS,PIR,TRIM27,ZNRD1,PRMT2,THOC5,RIPK3,TWIST2,ADRM1,SMARCD2,BRMS1,GMEB2,CCDC85B,PRPF6,PBX1,KAT5,RELA,ZNF511,ARNT2,ZFPL1,LIMD1,SMARCB1,CHCHD10,PBX4,UBTF,GATAD2A,MEF2B,PHB2,NDP,CTDSP1,MLF2,EID1,SSBP4,METTL13,ZNF394,C11orf95,BUD31,TNFRSF1A,TEAD4,C1QBP,TAF6L,ZBTB49,MTA2,ZNF316,MED11,PELP1,CCDC22,FOXK1,KLF3,PKN1,POLR2A,MAD2L2,SLTM,CTBP1,FBXL19,CRABP2,SP2,ARHGEF2,SNF8,PER1,NFIX,IRF2BPL,TRIM25,SMARCA4,CARM1,AATF,MID2,ZNF521,ZNF358,AKT1,BRF1,KHSRP,GTF2F1,RFX2,SAFB,SAFB2,MYDGF,HDGFL2,SIRT6,MAP2K2,CACTIN,CDK11B,SSU72,HIC1,MNT,EHMT1,AP3D1,IRAK1,MBD3,MED16,CARD9,SARS,GTF3C5,TRADD,ERBB4,JUND,PTMA,KLF13,FADS1,ZNF783,HOXC5,CAVIN1,IER5,AKAP17A,ZNF250,THAP7,NSMCE3,ERF,SNAPC2,ZSCAN1	yellow	VSMC_M3
GO:0045668	0.28	0.87	4	41	negative regulation of osteoblast differentiation	BP	1	GREM1,TWIST2,LIMD1,IGFBP5	yellow	VSMC_M3
GO:0051726	0.28	0.76	61	827	regulation of cell cycle	BP	1	CDK4,CTDSP2,TTLL12,H2AFY,SCRIB,BOP1,SND1,CUL7,KLF4,FIGNL1,KMT2E,SLC9A3R1,PPM1G,SERTAD1,METTL3,CEP131,NUMA1,CCDC57,EIF4EBP1,NAT10,BCL2L1,PINX1,DCTN1,WNT10B,CCAR2,CDK2AP2,PSME2,PRMT2,THOC5,PBX1,SIPA1,PHB2,CTDSP1,METTL13,BABAM1,ZBTB49,RCC2,EML3,SDE2,MIIP,MAD1L1,MAD2L2,NANOS3,CTBP1,CDC37,MYO19,AATF,ZNF830,AKT1,GIT1,SLC6A4,DHCR24,CDK11B,AURKAIP1,MNT,STK11,MIR214,NABP2,JUND,FBXO6,AC093323.1	yellow	VSMC_M3
GO:0043401	0.29	0.82	9	111	steroid hormone mediated signaling pathway	BP	1	PPARD,PRMT2,PHB2,PKN1,PER1,SMARCA4,CARM1,SAFB,SAFB2	yellow	VSMC_M3
GO:1901222	0.29	0.84	7	75	regulation of NIK/NF-kappaB signaling	BP	1	ZC3H12A,GREM1,RELA,PHB2,IFI35,IRAK1,TRADD	yellow	VSMC_M3
GO:0031331	0.29	0.78	28	366	positive regulation of cellular catabolic process	BP	1	BAG6,SNX9,HSPBP1,SOCS5,CNOT3,ZC3H12A,PSMC4,METTL3,DND1,PLEKHF1,PACSIN3,RNF19B,DCPS,TRIM27,KAT5,PHB2,BAD,CCDC22,AMER1,PIP4K2B,NANOS3,MID2,AKT1,KHSRP,SIRT6,STK11,TRIB2,STUB1	yellow	VSMC_M3

GO:0097659	0.29	0.74	177	2522	nucleic acid-templated transcription	BP	1	ENPP1,NAB2,CDK4,SQSTM1,ZNF608,HDAC10,NELFE,FNIP1,ATF6B,PBX2,RING1,SREBF2,H2AFY,TRIM11,TRAPPC9,PPARD,SND1,PHF2,CNOT3,KLF4,HOXA1,FHL3,ZC3H12A,CC2D1B,KTI12,KMT2E,SIX5,ZNF282,ZNF296,RELB,DMAP1,CIC,GTF3C2,OGG1,SERTAD1,TTC5,CXXC5,DEAF1,HOXC4,POLR1D,KMT2B,ZNF740,RALY,ZBTB38,GREM1,KDM2B,MCRS1,POLR2C,CX3CL1,WNT10B,ACTR5,ANKRD2,EGR3,CCAR2,DNAJC17,DLL4,CHP1,YAF2,HTATIP2,NPM3,KDM1B,CHCHD2,IRX1,RRP1B,BRD9,AIP,TSHZ2,QARS,PIR,TRIM27,ZNRD1,PRMT2,THOC5,RIPK3,TWIST2,ADRM1,SMARCD2,BRMS1,GMEB2,CCDC85B,PRPF6,PBX1,KAT5,RELA,ZNF511,ARNT2,ZFPL1,LIMD1,SMARCB1,CHCHD10,PBX4,UBTF,GATAD2A,MEF2B,PHB2,NDP,CTDSP1,MLF2,EID1,SSBP4,METTL13,ZNF394,C11orf95,BUD31,TNFRSF1A,TEAD4,C1QBP,TAF6L,ZBTB49,MTA2,ZNF316,MED11,PELP1,CCDC22,FOXK1,KLF3,PKN1,POLR2A,MAD2L2,SLTM,CTBP1,FBXL19,CRABP2,SP2,ARHGEF2,SNF8,PER1,NFIX,IRF2BPL,TRIM25,SMARCA4,CARM1,AATF,MID2,ZNF521,ZNF358,AKT1,BRF1,KHSRP,GTF2F1,RFX2,SAFB,SAFB2,MYDGF,HDGFL2,SIRT6,MAP2K2,CACTIN,CDK11B,SSU72,HIC1,MNT,EHMT1,AP3D1,IRAK1,MBD3,MED16,CARD9,SARS,GTF3C5,TRADD,ERBB4,JUND,PTMA,KLF13,FADS1,ZNF783,HOXC5,CAVIN1,IER5,AKAP17A,ZNF250,THAP7,NSMCE3,ERF,SNAPC2,ZSCAN1	yellow	VSMC_M3
GO:0019538	0.29	0.74	292	4113	protein metabolic process	BP	1	ENPP1,NAB2,CDK4,CTDSP2,SQSTM1,LMF2,BAG6,SBF1,SNX9,HDAC10,NELFE,FNIP1,PRR5,TTLL12,RING1,JADE2,H2AFY,TRIM11,SGSM3,PPARD,EEF1D,RPL3,PLEC,SHARPIN,VPS28,RPL8,CUL7,B4GALT1,MRPS18A,AARS2,PIGO,PDGFRA,SECISBP2,PHF2,HSPBP1,SOCS5,RPS9,CNOT3,KLF4,JOSD2,PSMB3,SPRED2,EIPR1,MEAF6,ZC3H12A,PNKP,RCN3,SNRNP70,SNX3,MICAL1,KMT2E,WDFY2,SLC9A3R1,SERP2,NAT9,EMILIN1,SEPHS1,TMUB1,RNF220,DMAP1,PPM1G,UNK,GALNT14,SERTAD1,PSMC4,KAT14,P4HA3,SEL1L2,METTL3,PHLDA1,P4HB,DND1,RPL29,EIF4EBP1,DELE1,LDLRAD3,TARBP2,KMT2B,NAT10,HM13,PDE4D,TNS2,EIF4B,PEPD,SWAP70,ACSL1,TNKS1BP1,MMP24,PINX1,GATB,SETD1B,GREM1,KDM2B,MCRS1,CX3CL1,WNT10B,FKBP11,SOD1,PACSIN3,CCAR2,RPLP2,CHP1,TYRO3,MRPL43,HTATIP2,PCIF1,PPM1M,KDM1B,SLC35C2,CARS,TSPAN5,CISH,ST3GAL4,RNF19B,DCPS,SELENOS,ETFBKMT,MANBA,TMTC1,STK32C,PDGFA,MMP14,APEH,GPX1,AIP,HCCS,MOCS3,CLCF1,NGDN,QARS,ASB9,PSME2,ANPEP,TRIM27,EIF3A,LIMK1,RABGGTA,PRMT2,RIPK3,LSM14B,ADRM1,DPP3,PSTK,RPS21,CAPN10,PSMB4,EXTL1,PFDN2,BRMS1,SEC11A,TNK2,TACO1,PTPN23,KAT5,RELA,SCYL1,ALKBH4,MEX3B,MAP3K14,MRPL49,ZDHHC3,SMARCB1,PHB2,TRMT112,IGFBP5,BAD,FKBP2,CTDSP1,OTUB1,EID1,COPS6,MPV17L2,LGALS3,PLAU,TNFRSF1A,MVB12A,BABAM1,C1QBP,AIMP2,TAF6L,UBXN1,WASHC1,MTA2,MED11,CCDC22,USP27X,KRT10,SDE2,CASP14,PKN1,CAPN2,SEPSECS,AMER1,MTHFR,MAD2L2,NANOS3,MPDU1,CTBP1,FBXL19,ARHGEF2,SNF8,RUSC1,MVP,PER1,DPM3,PPP4C,FARSA,SGF29,IRF2BPL,TUFM,TRIM25,USP43,RPL37,MTMR4,CARM1,SENP8,R[illegible]	yellow	VSMC_M3
GO:0070925	0.29	0.76	51	705	organelle assembly	BP	1	SPAG1,SQLE,TXNDC15,RPL3,PLEC,BOP1,VPS28,PDGFRA,DBNL,WDPCP,EHD2,KRT8,SEPT9,CEP131,NUMA1,YIF1B,CCDC57,RAB20,RRS1,DCTN1,HPS1,CCDC28B,LRFN4,PCNT,IQCG,PLA2G3,CEP164,PTPN23,SAC3D1,LIMD1,MPV17L2,MRTO4,MVB12A,KIAA0753,C1QBP,EML3,PIP4K2B,SNF8,CORO1A,RABEP2,ATG3,TTC39C,PACS2,RFX2,RAB34,MRPL20,GAS8,WDR34,MIS12,TUBGCP2,MEIG1	yellow	VSMC_M3
GO:0061136	0.29	0.80	14	166	regulation of proteasomal protein catabolic process	BP	1	BAG6,HSPBP1,SOCS5,PSMC4,WNT10B,CCAR2,RNF19B,GPX1,PSME2,UBXN1,AKT1,SIRT6,TRIB2,STUB1	yellow	VSMC_M3
GO:0022408	0.29	0.81	11	130	negative regulation of cell-cell adhesion	BP	1	SCRIB,SOCS5,KLF4,ZC3H12A,METTL3,SWAP70,LGALS3,CD9,MAD1L1,MAD2L2,AKT1	yellow	VSMC_M3
GO:0022904	0.29	0.82	10	99	respiratory electron transport chain	BP	1	NDUFB9,PLEC,UQCRFS1,CHCHD2,SDHA,NDUFV1,UQCRC1,UQCC3,NDUFS7,TAZ	yellow	VSMC_M3
GO:0050863	0.29	0.79	17	206	regulation of T cell activation	BP	1	SCRIB,LGALS1,SOCS5,ZC3H12A,METTL3,SOD1,EGR3,RIPK3,SART1,BAD,LGALS3,MAD1L1,CORO1A,CD276,CSK,AKT1,AP3D1	yellow	VSMC_M3
GO:0001678	0.29	0.81	10	124	cellular glucose homeostasis	BP	1	PPARD,LGALS1,UCP2,AACS,PCK2,SMARCB1,BAD,FOXK1,SMARCA4,JAGN1	yellow	VSMC_M3
GO:1903202	0.29	0.87	4	38	negative regulation of oxidative stress-induced cell death	BP	1	PYCR1,GPX1,AKT1,TRAP1	yellow	VSMC_M3
GO:0051172	0.29	0.74	126	1770	negative regulation of nitrogen compound metabolic process	BP	1	ENPP1,NAB2,CTDSP2,SQSTM1,ZNF608,BAG6,HDAC10,NELFE,FNIP1,RING1,SREBF2,H2AFY,HNRNPA0,TRIM11,PPARD,PLEC,VPS28,SECISBP2,SOCS5,CNOT3,KLF4,SPRED2,ZC3H12A,PNKP,SNRNP70,CC2D1B,SNX3,MICAL1,SIX5,ZNF282,ZNF296,RELB,EMILIN1,DMAP1,CIC,UNK,OGG1,METTL3,CXXC5,DND1,EIF4EBP1,DEAF1,TARBP2,NAT10,PDE4D,SWAP70,ZBTB38,PINX1,GREM1,KDM2B,WNT10B,ANKRD2,CCAR2,DNAJC17,DLL4,CHP1,YAF2,PCIF1,PCBP4,KDM1B,SRSF9,DCPS,IRX1,ZCCHC17,GPX1,QARS,TRIM27,LIMK1,PRMT2,TWIST2,PFDN2,BRMS1,CCDC85B,KAT5,RELA,LIMD1,SMARCB1,GATAD2A,PHB2,IGFBP5,CTDSP1,OTUB1,EID1,METTL13,LGALS3,C11orf95,C1QBP,ZBTB49,UBXN1,MTA2,FOXK1,KLF3,PKN1,MAD2L2,NANOS3,CTBP1,DFFA,SP2,MVP,PER1,NFIX,SLX1A,IRF2BPL,RPL37,SMARCA4,AATF,CXorf57,C19orf66,AKT1,PAIP1,KHSRP,GIT1,DHCR24,SIRT6,CACTIN,HMG20B,HIC1,MNT,EHMT1,MBD3,SARS,TENT5B,TRIB2,THAP7,NSMCE3,ERF	yellow	VSMC_M3
GO:0048232	0.29	0.77	29	371	male gamete generation	BP	1	BAG6,SBF1,IFT27,FIGNL1,SIX5,ZNF296,PAFAH1B3,DLEC1,METTL3,CEP131,DND1,YIF1B,MORN2,BCL2L1,KDM2B,SOD1,TYRO3,TRIM27,SLC26A6,IQCG,PLA2G3,SGPL1,BAD,NANOS3,RFX2,STK11,TAZ,FAM50A,MEIG1	yellow	VSMC_M3

GO:0043624	0.29	0.82	9	119	cellular protein complex disassembly	BP	1	MICAL1,SWAP70,CAPG,APEH,STMN3,MID1IP1,ARHGEF2,C19orf66,TWF2	yellow	VSMC_M3
GO:0061157	0.29	0.83	7	79	mRNA destabilization	BP	1	CNOT3,ZC3H12A,METTL3,DND1,DCPS,NANOS3,KHSRP	yellow	VSMC_M3
GO:0051208	0.29	0.83	7	87	sequestering of calcium ion	BP	1	ITPR3,PDE4D,CX3CL1,GSTO1,LETM1,CHERP,CORO1A	yellow	VSMC_M3
GO:0045445	0.29	0.84	6	67	myoblast differentiation	BP	1	PPARD,PLEC,LGALS1,GREM1,WNT10B,ANKRD2	yellow	VSMC_M3
GO:0031047	0.29	0.81	11	129	gene silencing by RNA	BP	1	SND1,CNOT3,ZC3H12A,METTL3,DND1,TARBP2,LIMD1,MAP2K2,MIR214,MIR155,MIR374B	yellow	VSMC_M3
GO:0010629	0.29	0.75	56	766	negative regulation of gene expression	BP	1	H2AFY,HNRNPA0,SND1,SECISBP2,PHF2,SSC5D,SOCS5,CNOT3,KLF4,ZC3H12A,RELB,EMILIN1,UNK,METTL3,INPPL1,DND1,EIF4EBP1,TARBP2,LRP3,LSM6,CX3CL1,DLL4,PCIF1,PCBP4,SLC35C2,SRSF9,DCPS,SELENOS,TRIM27,PLA2G3,KAT5,LIMD1,IGFBP5,MRTO4,C1QBP,NANOS3,C19orf66,CSK,AKT1,PAIP1,KHSRP,GIT1,CARHSP1,MAP2K2,CACTIN,ANAPC2,MBD3,SARS,MIR214,MIR155,RBM8A,RNH1,MIR374B,TENT5B,TRIB2,ZAR1L	yellow	VSMC_M3
GO:0051926	0.29	0.86	4	39	negative regulation of calcium ion transport	BP	1	PACSIN3,GSTO1,TRIM27,AKT1	yellow	VSMC_M3
GO:0010035	0.30	0.77	33	435	response to inorganic substance	BP	1	CDK4,ITPR3,PLEC,KLF4,ZC3H12A,KRT8,SLC30A3,OGG1,UCP2,PYCR1,SLC11A2,SOD1,GPX1,SYT12,RIPK3,RELA,SIPA1,ATIC,TXNRD2,BAD,HSD17B1,CD9,ANXA11,CAPN2,S100A16,EEF2K,AKT1,HMGCS1,TRAP1,ATP5F1D,JUND,OSER1,NSMCE3	yellow	VSMC_M3
GO:0007283	0.30	0.77	28	357	spermatogenesis	BP	1	BAG6,SBF1,IFT27,SIX5,ZNF296,PAFAH1B3,DLEC1,METTL3,CEP131,DND1,YIF1B,MORN2,BCL2L1,KDM2B,SOD1,TYRO3,TRIM27,SLC26A6,IQCG,PLA2G3,SGPL1,BAD,NANOS3,RFX2,STK11,TAZ,FAM50A,MEIG1	yellow	VSMC_M3
GO:2000058	0.30	0.80	12	143	regulation of ubiquitin-dependent protein catabolic process	BP	1	BAG6,HSPBP1,SOCS5,WNT10B,CCAR2,RNF19B,UBXN1,CCDC22,AKT1,SIRT6,TRIB2,STUB1	yellow	VSMC_M3
GO:0002685	0.30	0.80	13	152	regulation of leukocyte migration	BP	1	IL1R1,EMILIN1,SWAP70,GREM1,CX3CL1,ST3GAL4,RIN3,MMP14,RIPK3,LGALS3,CD9,C1QBP,AKT1	yellow	VSMC_M3
GO:0000082	0.30	0.80	13	160	G1/S transition of mitotic cell cycle	BP	1	CDK4,CTDSP2,KLF4,KMT2E,EIF4EBP1,CDK2AP2,PSME2,PRMT2,CTDSP1,SDE2,AKT1,MIR214,CDC34	yellow	VSMC_M3
GO:0008347	0.30	0.86	4	40	glial cell migration	BP	1	SCRIB,CX3CL1,MMP14,CD9	yellow	VSMC_M3
GO:0070372	0.30	0.79	17	211	regulation of ERK1 and ERK2 cascade	BP	1	NELFE,PDGFRA,KLF4,TPBG,SPRED2,SLC9A3R1,EMILIN1,CAVIN3,CX3CL1,PDGFA,EIF3A,PHB2,CSK,CARD9,ERBB4,PTPRR,GLIPR2	yellow	VSMC_M3
GO:0016441	0.30	0.81	10	115	posttranscriptional gene silencing	BP	1	ZC3H12A,METTL3,DND1,TARBP2,LIMD1,MAP2K2,MIR214,MIR155,MIR374B,ZAR1L	yellow	VSMC_M3
GO:0009790	0.30	0.75	59	841	embryo development	BP	1	BAG6,CTHRC1,PBX2,H2AFY,SCRIB,PDGFRA,EPN1,CNOT3,KLF4,HOXA1,WDPCP,CCM2,KRT8,SLC9A3R1,RNF220,UNK,INSIG1,PSMC4,RIC8A,DEAF1,HOXC4,LRIG1,HM13,BCL2L1,GREM1,KDM2B,SOD1,DLL4,IRX1,PDGFA,MMP14,COL6A1,THOC5,STMN3,PBX1,ARNT2,SMARCB1,PBX4,TEAD4,DBN1,CAPN2,MTHFR,CRABP2,ARMC5,SLC39A1,AATF,TTC39C,ZNF358,ZNF830,AKT1,NSDHL,SLC35E2B,EHMT1,MBD3,NDST1,ERBB4,PTPRR,ARFRP1,DUSP5	yellow	VSMC_M3
GO:0033993	0.30	0.75	48	657	response to lipid	BP	1	CDK4,HNRNPA0,PPARD,KLF4,ZC3H12A,INSIG1,OGG1,UCP2,AACS,SCARB1,ACSL1,CX3CL1,WNT10B,SCD,SELENOS,FABP3,PABPN1,PCK2,PRMT2,KAT5,RELA,IDH1,ARNT2,PHB2,BAD,ARPC1B,CNP,PKN1,CAPN2,PALM3,PER1,PMVK,TRIM25,SMARCA4,CARM1,AKT1,HMGCS1,SAFB,SAFB2,GIT1,SLC6A4,LDOC1,CACTIN,IRAK1,MBD3,TWF2,JUND,LOXL1	yellow	VSMC_M3
GO:0046324	0.30	0.86	4	44	regulation of glucose import	BP	1	ENPP1,SELENOS,CAPN10,AKT1	yellow	VSMC_M3
GO:0001818	0.30	0.79	16	195	negative regulation of cytokine production	BP	1	SSC5D,SOCS5,KLF4,ZC3H12A,RELB,CX3CL1,SELENOS,TRIM27,KAT5,C1QBP,CSK,GIT1,CACTIN,SARS,MIR155,TRIB2	yellow	VSMC_M3
GO:0002534	0.30	0.86	4	40	cytokine production involved in inflammatory response	BP	1	ZC3H12A,PLA2G3,PER1,CARD9	yellow	VSMC_M3
GO:1900015	0.30	0.86	4	40	regulation of cytokine production involved in inflammatory response	BP	1	ZC3H12A,PLA2G3,PER1,CARD9	yellow	VSMC_M3
GO:0071333	0.30	0.81	9	110	cellular response to glucose stimulus	BP	1	PPARD,LGALS1,UCP2,AACS,PCK2,SMARCB1,BAD,SMARCA4,JAGN1	yellow	VSMC_M3
GO:0006282	0.30	0.81	10	118	regulation of DNA repair	BP	1	HDAC10,FIGNL1,PNKP,OGG1,OTUB1,BABAM1,MAD2L2,PPP4C,CXorf57,SIRT6	yellow	VSMC_M3
GO:0045814	0.30	0.86	4	43	negative regulation of gene expression, epigenetic	BP	1	H2AFY,PHF2,TRIM27,MBD3	yellow	VSMC_M3
GO:0006333	0.30	0.81	9	106	chromatin assembly or disassembly	BP	1	H2AFY,PHF2,H3F3B,SMARCD2,SMARCB1,MTA2,H3F3A,SMARCA4,UBN1	yellow	VSMC_M3
GO:0006284	0.30	0.86	4	39	base-excision repair	BP	1	PNKP,MUTYH,OGG1,SMUG1	yellow	VSMC_M3
GO:0015980	0.30	0.77	22	259	energy derivation by oxidation of organic compounds	BP	1	ENPP1,NDUFB9,PLEC,CAVIN3,IDH3B,OGDHL,UQCRFS1,CHCHD2,SELENOS,SDHA,NDUFV1,UQCRC1,IDH1,CHCHD10,UQCC3,ATP5PD,AKT1,TRAP1,NDUFS7,ATP5F1D,TAZ,SURF1	yellow	VSMC_M3
GO:0080134	0.30	0.74	71	970	regulation of response to stress	BP	1	BAG6,HDAC10,ATF6B,TTLL12,H2AFY,PPARD,SHARPIN,PDGFRA,IL1R1,SOCS5,KLF4,SPRED2,ZC3H12A,FIGNL1,PNKP,ZYX,MGLL,INSIG1,OGG1,METTL3,P4HB,PYCR1,TARBP2,BCL2L1,CX3CL1,SOD1,CCAR2,TYRO3,CHCHD2,ST3GAL4,SELENOS,MVK,PDGFA,GPX1,QARS,THOC5,PSMB4,PLA2G3,RELA,BAD,IFI35,OTUB1,LETM1,CD9,PLAU,TNFRSF1A,BABAM1,C1QBP,UBXN1,MAD2L2,ARHGEF2,PER1,PPP4C,CDC37,CXorf57,AKT1,GIT1,SIRT6,MAP2K2,CACTIN,HIC1,TRAP1,CARD9,TRADD,MIR214,RF00024,TNFRSF12A,IGFBP6,ZNF385A,TXNDC12,IER5	yellow	VSMC_M3
GO:0032507	0.30	0.84	5	55	maintenance of protein location in cell	BP	1	INSIG1,CHCHD10,DBN1,AKT1,TWF2	yellow	VSMC_M3

GO:0010468	0.30	0.72	239	3428	regulation of gene expression	BP	1	ENPP1,NAB2,CDK4,SQSTM1,ZNF608,HDAC10,NELFE,FNIP1,ATF6B,PBX2,RING1,SREBF2,H2AFY,HNRNPA0,TRIM11,TRAPPC9,PPARD,SCRIB,SND1,PDGFRA,IL1R1,SECISBP2,PHF2,SSC5D,SOCS5,RPS9,CNOT3,KLF4,HOXA1,FHL3,ZC3H12A,SNRNP70,CC2D1B,KTI12,KMT2E,SIX5,ZNF282,ZNF296,RELB,EMILIN1,DMAP1,CIC,UNK,OGG1,SERTAD1,TTC5,METTL3,CXXC5,INPPL1,DND1,EIF4EBP1,CAVIN3,DEAF1,HOXC4,LDLRAD3,TARBP2,KMT2B,NAT10,ZNF740,PDE4D,EIF4B,LRP3,RALY,ZBTB38,LSM6,GREM1,KDM2B,MCRS1,CX3CL1,WNT10B,SOD1,ACTR5,ANKRD2,EGR3,CCAR2,DNAJC17,DLL4,CHP1,YAF2,HTATIP2,PCIF1,PCBP4,KDM1B,CHCHD2,SLC35C2,RBM15B,SRSF9,DCPS,IRX1,SELENOS,RRP1B,BRD9,ZCCHC17,MMP14,C14orf93,GPX1,AIP,CLCF1,NGDN,TSHZ2,QARS,PIR,TRIM27,PRMT2,THOC5,RIPK3,TWIST2,LSM14B,SMARCD2,BRMS1,BUD23,TACO1,PLA2G3,GMEB2,CCDC85B,PRPF6,PBX1,KAT5,RELA,ZNF511,ARNT2,ZFPL1,LIMD1,SMARCB1,CHCHD10,PBX4,UBTF,GATAD2A,MEF2B,PHB2,TRMT112,NDP,IGFBP5,CTDSP1,MLF2,EID1,SSBP4,METTL13,MPV17L2,ZNF394,C11orf95,BUD31,MRTO4,TNFRSF1A,TEAD4,C1QBP,TAF6L,ZBTB49,MTA2,ZNF316,MED11,PELP1,CCDC22,FOXK1,KLF3,PKN1,CAPN2,SEPSECS,POLR2A,MAD2L2,SLTM,NANOS3,CTBP1,FBXL19,CRABP2,SP2,ARHGEF2,SNF8,PER1,NFIX,IRF2BPL,TRIM25,SMARCA4,CARM1,CDC37,CD276,RAVER1,AATF,C19orf66,CSK,MID2,RBM22,ZNF521,ZNF358,AKT1,BRF1,PAIP1,KHSRP,GTF2F1,RFX2,SAFB,SAFB2,GIT1,SLC6A4,CARHSP1,MYDGF,HDGFL2,SIRT6,MAP2K2,CACTIN,HMG20B,CDK11B,SSU72,HIC1,TRAP1,MNT,EHMT1,AP3D1,MKNK2,IRAK1,ANAPC2,MBD3,FAM50A,MED16,CARD9,SARS,TRADD,MIR214,MI[illegible]	yellow	VSMC_M3
GO:0048259	0.30	0.82	7	85	regulation of receptor-mediated endocytosis	BP	1	CD63,SCRIB,GREM1,RIN3,TNK2,ANKRD13B,GSG1L	yellow	VSMC_M3
GO:0015718	0.30	0.83	6	66	monocarboxylic acid transport	BP	1	SLC9A3R1,SLC16A5,SLC16A14,SLC26A6,PLA2G3,SLC27A4	yellow	VSMC_M3
GO:0060147	0.30	0.86	4	41	regulation of posttranscriptional gene silencing	BP	1	ZC3H12A,DND1,LIMD1,MAP2K2	yellow	VSMC_M3
GO:0051649	0.30	0.73	118	1679	establishment of localization in cell	BP	1	PQLC1,CD63,DCTN2,SQSTM1,CCHCR1,SNX9,SAMM50,SREBF2,ITPR3,SGSM3,PACSIN1,PPARD,SCRIB,IFT27,VPS28,TOM1,ASNA1,PLEKHA8,EIPR1,ZC3H12A,SNX3,EHD2,SLC9A3R1,DMAP1,SNX17,INSIG1,MYRIP,SEC13,UCP2,CEP131,LAMTOR1,NUMA1,YIF1B,RAB20,HM13,PDE4D,RRS1,SLC11A2,PINX1,DCTN1,CX3CL1,SOD1,CHP1,AP2A2,HTATIP2,RAB35,RBM15B,SELENOS,GSTO1,FABP3,PABPN1,AIP,AP1G2,SYT12,TRIM27,PCNT,NUDC,THOC5,CAPN10,PLA2G3,PTPN23,VPS51,PEX5,ZDHHC3,THOC3,BLOC1S4,PHB2,BAD,COPE,LETM1,MVB12A,NUP88,CHERP,WASHC1,AP1M1,EML3,CCDC22,SNX8,LMAN2,RAB24,MAD1L1,CLTB,ARHGEF2,SNF8,RANGRF,STX10,CORO1A,ATP5PD,TMED1,SYNE3,CDC37,MYO19,ATG3,RBM22,AKT1,SNX18,DENND1C,RANBP3,GIT1,SLC6A4,MAP2K2,AP3D1,SSNA1,ATP5F1D,STK11,TMEM201,SPG7,WDR34,TRAPPC5,PLA2G4E,AP5B1,JAGN1,MIS12,UNC13A,ARFRP1,TIMM22,PHETA2,GSG1L	yellow	VSMC_M3
GO:0062197	0.31	0.77	22	282	cellular response to chemical stress	BP	1	PLEC,PDGFRA,KLF4,ZC3H12A,RELB,P4HB,PYCR1,GSR,AQP5,SOD1,ANKRD2,CHCHD2,SELENOS,GPX1,RIPK3,RELA,BAD,LETM1,ARHGEF2,AKT1,TRAP1,OSER1	yellow	VSMC_M3
GO:0070371	0.31	0.78	18	227	ERK1 and ERK2 cascade	BP	1	NELFE,PDGFRA,KLF4,TPBG,SPRED2,SLC9A3R1,EMILIN1,CAVIN3,CX3CL1,PDGFA,EIF3A,PHB2,CSK,MAP2K2,CARD9,ERBB4,PTPRR,GLIPR2	yellow	VSMC_M3
GO:0010720	0.31	0.78	17	231	positive regulation of cell development	BP	1	CUL7,DBNL,PLXND1,BAIAP2,P4HB,CX3CL1,CLCF1,LIMK1,RELA,BAD,C1QBP,DBN1,CRABP2,EEF2K,ANAPC2,STK11,TWF2	yellow	VSMC_M3
GO:0010564	0.31	0.75	38	515	regulation of cell cycle process	BP	1	CDK4,CTDSP2,H2AFY,SND1,CUL7,KLF4,KMT2E,CEP131,NUMA1,NAT10,BCL2L1,PINX1,DCTN1,WNT10B,CCAR2,CDK2AP2,PSME2,PRMT2,THOC5,PBX1,PHB2,CTDSP1,METTL13,BABAM1,RCC2,EML3,SDE2,MIIP,MAD1L1,MAD2L2,MYO19,ZNF830,AKT1,GIT1,AURKAIP1,MIR214,NABP2,FBXO6	yellow	VSMC_M3
GO:0016525	0.31	0.82	7	82	negative regulation of angiogenesis	BP	1	EPN1,KLF4,EMILIN1,PDE3B,YJEFN3,SARS,MIR214	yellow	VSMC_M3
GO:0051128	0.31	0.72	129	1877	regulation of cellular component organization	BP	1	ENPP1,CD63,RUFY1,SQSTM1,SNX9,FNIP1,SREBF2,H2AFY,PACSIN1,PPARD,SCRIB,LGALS1,VPS28,CUL7,DNPH1,PDGFRA,PHF2,KLF4,DBNL,TPBG,WDPCP,PNKP,SNX3,EHD2,PLXND1,EMILIN1,SNX17,H3F3B,SEPT9,BAIAP2,CEP131,LAMTOR1,NUMA1,EIF4EBP1,ARHGAP22,ARHGAP33,NAT10,BCL2L1,SWAP70,CAPG,PINX1,GREM1,KDM2B,DCTN1,RAPSN,CX3CL1,WNT10B,PACSIN3,CCAR2,CISH,RIN3,PDGFA,MMP14,CDK2AP2,GPX1,CORO1B,NGEF,PLEKHO1,IGSF9,LRFN4,NECTIN1,TRIM27,ARPIN,LIMK1,PFDN2,TNK2,PLA2G3,STMN3,CCDC85B,PTPN23,SIPA1,RHOC,ALKBH4,MID1IP1,PEX5,CHCHD10,IGFBP5,BAD,LGALS3,ARPC1B,TNFRSF1A,WASHC1,RCC2,EML3,H3F3A,DBN1,CAPN2,MAD1L1,PIP4K2B,MAD2L2,CRABP2,ARHGEF2,RANGRF,CORO1A,SLX1A,RABEP2,SMARCA4,CARM1,EEF2K,MYO19,C19orf66,ATG3,DOCK11,INF2,AKT1,GIT1,ANKRD13B,HDGFL2,SIRT6,MAP2K2,CDK11B,AURKAIP1,NCLN,ACAP3,ANAPC2,STK11,NEGR1,MIR214,NABP2,TWF2,ELN,UNC13A,CDC42EP5,NSMF,CDC34,GBA2,STUB1,ARPC5L,GSG1L	yellow	VSMC_M3
GO:1901607	0.31	0.84	5	49	alpha-amino acid biosynthetic process	BP	1	SEPHS1,PYCR1,GLUD1,ILVBL,MTHFR	yellow	VSMC_M3

GO:1901700	0.31	0.73	88	1234	response to oxygen-containing compound	BP	1	ENPP1,CDK4,HNRNPA0,PPARD,PLEC,LGALS1,PDGFRA,KLF4,ZC3H12A,KRT8,SLC9A3R1,DMAP1,INSIG1,OGG1,UCP2,BAIAP2,INPPL1,LAMTOR1,PYCR1,EIF4EBP1,AACS,SCARB1,BCL2L1,TNS2,ACSL1,PDE3B,CX3CL1,WNT10B,SOD1,SCD,DCPS,SELENOS,FABP3,PABPN1,GPX1,PCK2,COL6A1,SLC26A6,RIPK3,CAPN10,KAT5,RELA,SIPA1,ARNT2,SMARCB1,TXNRD2,PHB2,IGFBP5,BAD,LAMTOR4,ARPC1B,CD9,CNP,KLF3,CAPN2,PALM3,MTHFR,PIP4K2B,ARHGEF2,PER1,PMVK,TUFM,TRIM25,SMARCA4,CARM1,EEF2K,CSK,AKT1,HMGCS1,GIT1,SLC6A4,LDOC1,CACTIN,TRAP1,IRAK1,MBD3,MAN1B1,CARD9,APRT,SLC27A4,TGFBR3,TWF2,JUND,LOXL1,JAGN1,COL6A2,NSMF,OSER1	yellow	VSMC_M3
GO:0048524	0.31	0.84	5	55	positive regulation of viral process	BP	1	TRIM11,LGALS1,P4HB,TARBP2,TYRO3	yellow	VSMC_M3
GO:0009261	0.31	0.85	4	40	ribonucleotide catabolic process	BP	1	NUDT3,PDE4D,NT5C,ITPA	yellow	VSMC_M3
GO:0033673	0.31	0.78	15	191	negative regulation of kinase activity	BP	1	H2AFY,PLEC,SOCS5,TARBP2,CHP1,QARS,TRIM27,WASHC1,PKN1,PIP4K2B,MVP,CSK,AKT1,MIDN,TRIB2	yellow	VSMC_M3
GO:0030193	0.31	0.84	5	50	regulation of blood coagulation	BP	1	PDGFRA,ST3GAL4,PDGFA,CD9,PLAU	yellow	VSMC_M3
GO:0002042	0.31	0.84	5	55	cell migration involved in sprouting angiogenesis	BP	1	KLF4,GREM1,EGR3,DLL4,AKT1	yellow	VSMC_M3
GO:0048146	0.31	0.85	4	41	positive regulation of fibroblast proliferation	BP	1	CDK4,PDGFRA,PDGFA,S100A6	yellow	VSMC_M3
GO:0044265	0.31	0.73	69	947	cellular macromolecule catabolic process	BP	1	SQSTM1,BAG6,HNRNPA0,SHARPIN,VPS28,SND1,CUL7,SECISBP2,HSPBP1,SOCS5,CNOT3,PSMB3,ZC3H12A,RCN3,TMUB1,PSMC4,SEL1L2,METTL3,DND1,HM13,LSM6,WNT10B,CCAR2,PCBP4,RNF19B,DCPS,SELENOS,MANBA,ZCCHC17,GPX1,PSME2,ADRM1,PSMB4,PTPN23,RNASEH2C,KAT5,MRTO4,MVB12A,UBXN1,CCDC22,USP27X,CAPN2,AMER1,NANOS3,FBXL19,DFFA,SNF8,TRIM25,USP43,ANKRD9,AKT1,PAIP1,KHSRP,CARHSP1,SIRT6,ANAPC2,SPSB3,MAN1B1,ZNRF1,PSMB10,RBM8A,RNH1,TENT5B,TRIB2,CDC34,FBXO6,TRIR,STUB1,CCDC115	yellow	VSMC_M3
GO:0016049	0.31	0.75	29	399	cell growth	BP	1	ENPP1,PPARD,DNPH1,DBNL,H3F3B,LAMTOR1,GREM1,KDM2B,CCAR2,CISH,MMP14,LIMK1,PRMT2,CCDC85B,SIPA1,IGFBP5,H3F3A,DBN1,MAD2L2,CRABP2,SMARCA4,AKT1,HDGFL2,SIRT6,CDK11B,ANAPC2,STK11,TWF2,UNC13A	yellow	VSMC_M3
GO:0009124	0.31	0.85	4	37	nucleoside monophosphate biosynthetic process	BP	1	IMPDH2,ATIC,AMPD2,APRT	yellow	VSMC_M3
GO:0048255	0.31	0.85	4	43	mRNA stabilization	BP	1	HNRNPA0,DND1,PAIP1,TENT5B	yellow	VSMC_M3
GO:0097237	0.31	0.81	8	85	cellular response to toxic substance	BP	1	GSR,SOD1,SELENOS,GSTO1,GPX1,MGST3,TXNRD2,PRXL2A	yellow	VSMC_M3
GO:0030217	0.31	0.78	14	179	T cell differentiation	BP	1	NCAPH2,SOCS5,ZC3H12A,RELB,METTL3,SOD1,EGR3,DLL4,RIPK3,SART1,BAD,ARMC5,AP3D1,STK11	yellow	VSMC_M3
GO:2001020	0.31	0.77	16	200	regulation of response to DNA damage stimulus	BP	1	HDAC10,SPRED2,FIGNL1,PNKP,OGG1,BCL2L1,CCAR2,THOC5,OTUB1,BABAM1,MAD2L2,PPP4C,CXorf57,SIRT6,HIC1,ZNF385A	yellow	VSMC_M3
GO:0080090	0.32	0.71	293	4206	regulation of primary metabolic process	BP	1	ENPP1,NAB2,CDK4,CTDSP2,SQSTM1,ZNF608,BAG6,SNX9,HDAC10,NELFE,FNIP1,ATF6B,PBX2,PRR5,RING1,SREBF2,H2AFY,HNRNPA0,TRIM11,SGSM3,TRAPPC9,PPARD,PLEC,VPS28,SND1,PDGFRA,SECISBP2,PHF2,HSPBP1,SOCS5,RPS9,CNOT3,KLF4,HOXA1,FHL3,SPRED2,ZC3H12A,FIGNL1,PNKP,RCN3,SNRNP70,CC2D1B,SNX3,KTI12,MICAL1,KMT2E,WDFY2,SIX5,ZNF282,ZNF296,RELB,SLC9A3R1,EMILIN1,DMAP1,CIC,UNK,INSIG1,OGG1,SERTAD1,TTC5,PSMC4,METTL3,CXXC5,LAMTOR1,DND1,EIF4EBP1,DELE1,DEAF1,HOXC4,LDLRAD3,TARBP2,KMT2B,SCARB1,NAT10,ZNF740,PDE4D,EIF4B,SWAP70,RALY,ACSL1,ZBTB38,TNKS1BP1,PINX1,GREM1,KDM2B,PDE3B,MCRS1,CX3CL1,WNT10B,SOD1,PACSIN3,ACTR5,ANKRD2,DGKZ,EGR3,CCAR2,DNAJC17,DLL4,CHP1,YAF2,HTATIP2,PCIF1,PCBP4,KDM1B,CHCHD2,RBM15B,SRSF9,RNF19B,DCPS,IRX1,DHCR7,SELENOS,RRP1B,ETFBKMT,BRD9,FABP3,ZCCHC17,PDGFA,MMP14,GPX1,AIP,CLCF1,NGDN,TSHZ2,QARS,ASB9,PSME2,PIR,TRIM27,LIMK1,PRMT2,THOC5,RIPK3,TWIST2,LSM14B,ADRM1,SMARCD2,PFDN2,BRMS1,BUD23,TNK2,TACO1,PLA2G3,GMEB2,CCDC85B,PRPF6,PBX1,KAT5,RELA,ZNF511,IDH1,ARNT2,ZFPL1,LIMD1,MID1IP1,SMARCB1,CHCHD10,PBX4,UBTF,GATAD2A,MEF2B,PHB2,TRMT112,NDP,IGFBP5,BAD,CTDSP1,MLF2,OTUB1,EID1,SSBP4,METTL13,MPV17L2,ZNF394,LGALS3,C11orf95,BUD31,TNFRSF1A,BABAM1,TEAD4,C1QBP,AIMP2,TAF6L,ZBTB49,UBXN1,WASHC1,MTA2,ZNF316,MED11,PELP1,CCDC22,FOXK1,KLF3,PKN1,CAPN2,SEPSECS,POLR2A,AMER1,MTHFR,PIP4K2B,MAD2L2,SLTM,NANOS3,CTBP1,FBXL19,CRABP2,DFFA,SP2,ARHGEF2,ARMC5,SNF8,MVP,PER1,NFIX,PPP4C,SLX1A,IRF2BPL,TRIM25,[illegible]	yellow	VSMC_M3
GO:0045446	0.32	0.81	7	91	endothelial cell differentiation	BP	1	COL15A1,CCM2,FASN,PDE4D,GPX1,TNFRSF1A,TJP3	yellow	VSMC_M3
GO:0071426	0.32	0.82	6	66	ribonucleoprotein complex export from nucleus	BP	1	RRS1,RBM15B,PABPN1,THOC5,THOC3,NUP88	yellow	VSMC_M3
GO:0000387	0.32	0.85	4	38	spliceosomal snRNP assembly	BP	1	GEMIN7,SART1,PRPF6,CD2BP2	yellow	VSMC_M3
GO:1901800	0.32	0.80	9	104	positive regulation of proteasomal protein catabolic process	BP	1	BAG6,HSPBP1,SOCS5,PSMC4,RNF19B,AKT1,SIRT6,TRIB2,STUB1	yellow	VSMC_M3
GO:0062012	0.32	0.76	19	243	regulation of small molecule metabolic process	BP	1	ENPP1,PPARD,INSIG1,SOD1,DHCR7,SELENOS,ETFBKMT,FABP3,PLA2G3,MID1IP1,BAD,FOXK1,AKT1,GIT1,PGP,MIDN,TAZ,JMJD8,SLC27A4	yellow	VSMC_M3
GO:0006406	0.32	0.83	5	53	mRNA export from nucleus	BP	1	RBM15B,PABPN1,THOC5,THOC3,NUP88	yellow	VSMC_M3
GO:0071427	0.32	0.83	5	53	mRNA-containing ribonucleoprotein complex export from nucleus	BP	1	RBM15B,PABPN1,THOC5,THOC3,NUP88	yellow	VSMC_M3
GO:1903052	0.32	0.79	10	118	positive regulation of proteolysis involved in cellular protein catabolic process	BP	1	BAG6,HSPBP1,SOCS5,PSMC4,RNF19B,CCDC22,AKT1,SIRT6,TRIB2,STUB1	yellow	VSMC_M3
GO:0042698	0.32	0.84	4	46	ovulation cycle	BP	1	PDGFRA,TYRO3,SGPL1,ZNF830	yellow	VSMC_M3
GO:0061014	0.32	0.81	7	82	positive regulation of mRNA catabolic process	BP	1	CNOT3,ZC3H12A,METTL3,DND1,DCPS,NANOS3,KHSRP	yellow	VSMC_M3

GO:0043207	0.32	0.73	61	820	response to external biotic stimulus	BP	1	CDK4,TTLL12,HNRNPA0,TRIM11,PPARD,PPP1R14B,SSC5D,ZC3H12A,ZYX,SNX3,KRT8,RELB,METTL3,BAIAP2,RAB20,TARBP2,SCARB1,BCL2L1,CX3CL1,TYRO3,RNF19B,SELENOS,RRP1B,PABPN1,GPX1,PCK2,TRIM27,SLC26A6,RIPK3,TNK2,BANF1,RELA,MAP3K14,PHB2,IFI35,LGALS3,TNFRSF1A,C1QBP,CNP,CAPN2,PALM3,TBKBP1,ARHGEF2,ARMC5,CORO1A,TRIM25,CDC37,C19orf66,MID2,AKT1,GTF2F1,GIT1,LDOC1,CACTIN,IRAK1,C8G,CARD9,JUND,LOXL1,EEF1G,JAGN1	yellow	VSMC_M3
GO:0051707	0.32	0.73	61	820	response to other organism	BP	1	CDK4,TTLL12,HNRNPA0,TRIM11,PPARD,PPP1R14B,SSC5D,ZC3H12A,ZYX,SNX3,KRT8,RELB,METTL3,BAIAP2,RAB20,TARBP2,SCARB1,BCL2L1,CX3CL1,TYRO3,RNF19B,SELENOS,RRP1B,PABPN1,GPX1,PCK2,TRIM27,SLC26A6,RIPK3,TNK2,BANF1,RELA,MAP3K14,PHB2,IFI35,LGALS3,TNFRSF1A,C1QBP,CNP,CAPN2,PALM3,TBKBP1,ARHGEF2,ARMC5,CORO1A,TRIM25,CDC37,C19orf66,MID2,AKT1,GTF2F1,GIT1,LDOC1,CACTIN,IRAK1,C8G,CARD9,JUND,LOXL1,EEF1G,JAGN1	yellow	VSMC_M3
GO:0008637	0.32	0.80	8	87	apoptotic mitochondrial changes	BP	1	BCL2L1,CCAR2,GPX1,CHCHD10,BAD,ATG3,AKT1,ERBB4	yellow	VSMC_M3
GO:0001933	0.32	0.76	21	276	negative regulation of protein phosphorylation	BP	1	ENPP1,CTDSP2,H2AFY,PLEC,SOCS5,SPRED2,ZC3H12A,MICAL1,EMILIN1,TARBP2,PDE4D,GREM1,CHP1,QARS,TRIM27,CTDSP1,PKN1,MVP,AKT1,CACTIN,TRIB2	yellow	VSMC_M3
GO:1900407	0.32	0.82	6	66	regulation of cellular response to oxidative stress	BP	1	P4HB,PYCR1,SOD1,GPX1,AKT1,TRAP1	yellow	VSMC_M3
GO:0009967	0.32	0.72	80	1127	positive regulation of signal transduction	BP	1	CD63,NELFE,FNIP1,PRR5,TXNDC15,PPARD,EEF1D,LGALS1,SHARPIN,PDGFRA,IL1R1,TPBG,SPRED2,ZC3H12A,PLEKHA4,SLC9A3R1,RNF220,SEC13,BAIAP2,CXXC5,LAMTOR1,NUMA1,CAVIN3,PLEKHF1,GREM1,CX3CL1,WNT10B,SOD1,CCAR2,DLL4,SLC35C2,TSPAN5,PDGFA,GPX1,TRIM27,RIPK3,RELA,RHOC,MAP3K14,SMARCB1,PHB2,IGFBP5,BAD,LAMTOR4,IFI35,TNFRSF1A,C1QBP,CHERP,EVC,CCDC22,AMER1,TRIM25,RPL37,S100A4,SMARCA4,CSK,MID2,TEDC1,RAB34,MYDGF,MAP2K2,HIC1,CTNS,TEDC2,CSNK1G2,IRAK1,STK11,TELO2,WDR24,JMJD8,CARD9,GAS8,TGFBR3,TRADD,NDST1,TNFRSF12A,ERBB4,IGFBP6,ZNF385A,GLIPR2	yellow	VSMC_M3
GO:1990090	0.32	0.84	4	46	cellular response to nerve growth factor stimulus	BP	1	RAB35,CORO1A,EEF2K,AKT1	yellow	VSMC_M3
GO:0060688	0.32	0.84	4	42	regulation of morphogenesis of a branching structure	BP	1	GREM1,PDGFA,PHB2,SIRT6	yellow	VSMC_M3
GO:0044839	0.32	0.78	11	132	cell cycle G2/M phase transition	BP	1	CDK4,H2AFY,CCDC57,PINX1,WNT10B,PBX1,BABAM1,RCC2,MIIP,ZNF830,NABP2	yellow	VSMC_M3
GO:0031329	0.32	0.73	50	689	regulation of cellular catabolic process	BP	1	BAG6,SNX9,SREBF2,HNRNPA0,SECISBP2,HSPBP1,SOCS5,CNOT3,ZC3H12A,SNRNP70,PSMC4,METTL3,DND1,SCARB1,PLEKHF1,WNT10B,PACSIN3,CCAR2,PCBP4,RNF19B,DCPS,ETFBKMT,ZCCHC17,GPX1,PSME2,TRIM27,KAT5,IDH1,PHB2,BAD,UBXN1,WASHC1,CCDC22,FOXK1,AMER1,PIP4K2B,NANOS3,DFFA,MTMR4,MID2,AKT1,PAIP1,KHSRP,CARHSP1,SIRT6,STK11,WDR24,TENT5B,TRIB2,STUB1	yellow	VSMC_M3
GO:2000181	0.32	0.81	7	83	negative regulation of blood vessel morphogenesis	BP	1	EPN1,KLF4,EMILIN1,PDE3B,YJEFN3,SARS,MIR214	yellow	VSMC_M3
GO:0098609	0.32	0.73	41	592	cell-cell adhesion	BP	1	TSTA3,SCRIB,LGALS1,PDGFRA,COL19A1,SOCS5,KLF4,ZC3H12A,METTL3,BAIAP2,SIGLEC1,RIC8A,ITGB7,SWAP70,MMP24,CX3CL1,EGR3,TYRO3,ST3GAL4,IGSF9,LRFN4,NECTIN1,SART1,PTPN23,RELA,BLOC1S4,BAD,SCARF2,LGALS3,CD9,MAD1L1,MAD2L2,CORO1A,CD276,CSK,AKT1,TJP3,CLDN6,AP3D1,IRAK1,NEGR1	yellow	VSMC_M3
GO:0050779	0.32	0.81	7	82	RNA destabilization	BP	1	CNOT3,ZC3H12A,METTL3,DND1,DCPS,NANOS3,KHSRP	yellow	VSMC_M3
GO:1904377	0.32	0.83	5	58	positive regulation of protein localization to cell periphery	BP	1	SQSTM1,NUMA1,LGALS3,RANGRF,AKT1	yellow	VSMC_M3
GO:0006970	0.33	0.81	6	71	response to osmotic stress	BP	1	RELB,AQP5,BAD,LETM1,PKN1,ARHGEF2	yellow	VSMC_M3
GO:0051341	0.33	0.81	7	79	regulation of oxidoreductase activity	BP	1	SCARB1,HM13,ETFBKMT,RIPK3,TACO1,PHB2,AKT1	yellow	VSMC_M3
GO:0080135	0.33	0.73	41	553	regulation of cellular response to stress	BP	1	BAG6,HDAC10,ATF6B,KLF4,SPRED2,ZC3H12A,FIGNL1,PNKP,INSIG1,OGG1,P4HB,PYCR1,BCL2L1,SOD1,CCAR2,CHCHD2,SELENOS,GPX1,QARS,THOC5,BAD,OTUB1,LETM1,BABAM1,UBXN1,MAD2L2,ARHGEF2,PER1,PPP4C,CXorf57,AKT1,SIRT6,MAP2K2,HIC1,TRAP1,CARD9,RF00024,IGFBP6,ZNF385A,TXNDC12,IER5	yellow	VSMC_M3
GO:0048515	0.33	0.79	9	106	spermatid differentiation	BP	1	SBF1,SIX5,CEP131,YIF1B,SLC26A6,IQCG,PLA2G3,RFX2,MEIG1	yellow	VSMC_M3
GO:0090066	0.33	0.74	30	418	regulation of anatomical structure size	BP	1	CDK4,SNX9,PPARD,DBNL,SLC9A3R1,BAIAP2,SWAP70,CAPG,SOD1,RIN3,GPX1,CORO1B,TRIM27,ARPIN,LIMK1,LAMTOR4,ARPC1B,WASHC1,DBN1,CRABP2,CORO1A,AKT1,SLC6A4,ANAPC2,MIR214,TWF2,ELN,CDC42EP5,GBA2,ARPC5L	yellow	VSMC_M3
GO:1900046	0.33	0.83	5	51	regulation of hemostasis	BP	1	PDGFRA,ST3GAL4,PDGFA,CD9,PLAU	yellow	VSMC_M3
GO:0042176	0.33	0.74	26	342	regulation of protein catabolic process	BP	1	BAG6,SNX9,SGSM3,VPS28,HSPBP1,SOCS5,SNRNP70,SNX3,PSMC4,WNT10B,PACSIN3,CCAR2,RNF19B,GPX1,ASB9,PSME2,RELA,UBXN1,CCDC22,AMER1,MAD2L2,SNF8,AKT1,SIRT6,TRIB2,STUB1	yellow	VSMC_M3
GO:2001022	0.33	0.80	8	95	positive regulation of response to DNA damage stimulus	BP	1	HDAC10,SPRED2,PNKP,CCAR2,BABAM1,MAD2L2,HIC1,ZNF385A	yellow	VSMC_M3
GO:0070507	0.33	0.78	10	135	regulation of microtubule cytoskeleton organization	BP	1	NUMA1,DCTN1,CDK2AP2,STMN3,MID1IP1,EML3,ARHGEF2,RANGRF,GIT1,GBA2	yellow	VSMC_M3
GO:0000422	0.33	0.81	6	71	autophagy of mitochondrion	BP	1	SQSTM1,SREBF2,PHB2,CDC37,ATG3,TAZ	yellow	VSMC_M3
GO:0061726	0.33	0.81	6	71	mitochondrion disassembly	BP	1	SQSTM1,SREBF2,PHB2,CDC37,ATG3,TAZ	yellow	VSMC_M3
GO:0051271	0.33	0.76	18	241	negative regulation of cellular component movement	BP	1	PPARD,KLF4,SLC9A3R1,EMILIN1,GREM1,CX3CL1,DLL4,RIN3,CORO1B,ARPIN,PTPN23,IGFBP5,CD9,MIIP,AKT1,TGFBR3,MIR214,PTPRR	yellow	VSMC_M3
GO:1902808	0.33	0.84	4	41	positive regulation of cell cycle G1/S phase transition	BP	1	KMT2E,PHB2,AKT1,MIR214	yellow	VSMC_M3
GO:0030258	0.33	0.77	14	171	lipid modification	BP	1	ACAT2,CERK,PPARD,CPT1C,INPPL1,LAMTOR1,DGKZ,ST3GAL4,ETFBKMT,FABP3,ILVBL,MTMR4,AKT1,GBA2	yellow	VSMC_M3

GO:0000122	0.33	0.72	50	694	negative regulation of transcription by RNA polymerase II	BP	1	SQSTM1,ZNF608,HDAC10,NELFE,FNIP1,SREBF2,H2AFY,PPARD,KLF4,CC2D1B,ZNF282,ZNF296,DMAP1,CXXC5,DEAF1,KDM2B,WNT10B,ANKRD2,DNAJC17,DLL4,KDM1B,IRX1,TRIM27,BRMS1,KAT5,RELA,GATAD2A,EID1,METTL13,C1QBP,ZBTB49,MTA2,FOXK1,KLF3,MAD2L2,CTBP1,SP2,PER1,NFIX,IRF2BPL,SMARCA4,SIRT6,HIC1,MNT,EHMT1,MBD3,SARS,THAP7,NSMCE3,ERF	yellow	VSMC_M3
GO:0008089	0.33	0.84	4	44	anterograde axonal transport	BP	1	SOD1,BLOC1S4,AP3D1,SPG7	yellow	VSMC_M3
GO:0044089	0.33	0.74	31	441	positive regulation of cellular component biogenesis	BP	1	SNX9,FNIP1,TPBG,EMILIN1,SEPT9,BAIAP2,NUMA1,SWAP70,DCTN1,CX3CL1,WNT10B,CORO1B,TRIM27,ARPIN,LIMK1,RHOC,LGALS3,ARPC1B,WASHC1,PIP4K2B,SNF8,CORO1A,SLX1A,EEF2K,DOCK11,GIT1,TWF2,CDC42EP5,CDC34,STUB1,ARPC5L	yellow	VSMC_M3
GO:0000271	0.33	0.82	5	58	polysaccharide biosynthetic process	BP	1	ENPP1,SELENOS,AKT1,B3GNT9,NDST1	yellow	VSMC_M3
GO:0006470	0.33	0.76	17	228	protein dephosphorylation	BP	1	CTDSP2,SBF1,PPM1G,TNS2,SWAP70,PPM1M,PTPN23,CTDSP1,LGALS3,PPP4C,MTMR4,SSU72,PGP,STK11,PTPRR,NSMF,DUSP5	yellow	VSMC_M3
GO:0045428	0.33	0.84	4	43	regulation of nitric oxide biosynthetic process	BP	1	KLF4,ZC3H12A,AKT1,KHSRP	yellow	VSMC_M3
GO:0030036	0.33	0.73	41	600	actin cytoskeleton organization	BP	1	SNX9,PDLIM4,PACSIN1,PLEC,PDGFRA,DBNL,FHL3,ZYX,MICAL1,EHD2,KRT8,SLC9A3R1,BAIAP2,INPPL1,CAVIN3,SWAP70,CAPG,CX3CL1,PDGFA,CORO1B,TRIM27,ARPIN,LIMK1,CAPN10,RHOC,ALKBH4,FMNL1,ARPC1B,WASHC1,DBN1,MAD2L2,ARHGEF2,CORO1A,MYO19,INF2,MIR214,TWF2,ELN,CDC42EP5,GBA2,ARPC5L	yellow	VSMC_M3
GO:0050714	0.33	0.79	8	100	positive regulation of protein secretion	BP	1	PPARD,MYRIP,AACS,PCK2,CAPN10,PTPN23,BAD,GLUD1	yellow	VSMC_M3
GO:0061024	0.33	0.72	44	599	membrane organization	BP	1	PQLC1,BAG6,SNX9,SAMM50,PACSIN1,PLEC,ASNA1,PLEKHA8,SNX3,EHD2,TMEM43,SLC9A3R1,INSIG1,SEC13,BAIAP2,RAB20,BCL2L1,DCTN1,RAPSN,SOD1,PACSIN3,CHP1,ALKBH4,CHCHD10,YJEFN3,BAD,LETM1,CD9,UQCC3,CNP,PIP4K2B,MTX1,STX10,CORO1A,AKT1,SNX18,ALKBH7,RAB34,DHCR24,AP3D1,TAZ,SPG7,GBA2,TIMM22	yellow	VSMC_M3
GO:0065004	0.33	0.78	11	132	protein-DNA complex assembly	BP	1	H2AFY,H3F3B,WNT10B,SMARCB1,UBTF,TAF6L,H3F3A,SMARCA4,BRF1,UBN1,MIS12	yellow	VSMC_M3
GO:0071166	0.33	0.81	6	67	ribonucleoprotein complex localization	BP	1	RRS1,RBM15B,PABPN1,THOC5,THOC3,NUP88	yellow	VSMC_M3
GO:0090630	0.33	0.80	7	93	activation of GTPase activity	BP	1	SGSM3,SCRIB,ARHGAP22,NGEF,SIPA1,RCC2,SLC27A4	yellow	VSMC_M3
GO:0007423	0.33	0.73	29	415	sensory organ development	BP	1	CDK4,CTHRC1,PBX2,RING1,SCRIB,IFT27,PDGFRA,KLF4,HOXA1,WDPCP,CCM2,SPRED2,SIX5,SLC9A3R1,INSIG1,LRIG1,KDM2B,AQP5,WNT10B,SOD1,DLL4,IMPDH2,NECTIN1,PBX1,PBX4,C12orf57,SMARCA4,TTC39C,CTNS	yellow	VSMC_M3
GO:1901343	0.34	0.80	7	84	negative regulation of vasculature development	BP	1	EPN1,KLF4,EMILIN1,PDE3B,YJEFN3,SARS,MIR214	yellow	VSMC_M3
GO:0035601	0.34	0.79	8	99	protein deacylation	BP	1	HDAC10,SPRED2,CCAR2,BRMS1,MTA2,CTBP1,PER1,SIRT6	yellow	VSMC_M3
GO:0007005	0.34	0.73	35	436	mitochondrion organization	BP	1	SQSTM1,SAMM50,SREBF2,NDUFB9,PLEC,SHARPIN,ACAD9,SDHAF1,BCL2L1,UQCRFS1,CCAR2,CHCHD2,GPX1,AIP,TACO1,CHCHD10,PHB2,BAD,LETM1,UQCC3,CNP,MTX1,CDC37,MYO19,ATG3,INF2,AKT1,ALKBH7,NDUFS7,ATP5F1D,TAZ,SPG7,ERBB4,SURF1,TIMM22	yellow	VSMC_M3
GO:1902743	0.34	0.83	4	45	regulation of lamellipodium organization	BP	1	CORO1B,ARPIN,MIR214,TWF2	yellow	VSMC_M3
GO:0045892	0.34	0.71	67	943	negative regulation of transcription, DNA-templated	BP	1	NAB2,SQSTM1,ZNF608,HDAC10,NELFE,FNIP1,RING1,SREBF2,H2AFY,TRIM11,PPARD,KLF4,CC2D1B,SIX5,ZNF282,ZNF296,RELB,DMAP1,CIC,CXXC5,DEAF1,ZBTB38,GREM1,KDM2B,WNT10B,ANKRD2,CCAR2,DNAJC17,DLL4,YAF2,KDM1B,IRX1,QARS,TRIM27,PRMT2,TWIST2,BRMS1,CCDC85B,KAT5,RELA,LIMD1,GATAD2A,PHB2,EID1,METTL13,C11orf95,C1QBP,ZBTB49,MTA2,FOXK1,KLF3,MAD2L2,CTBP1,SP2,PER1,NFIX,IRF2BPL,SMARCA4,SIRT6,HIC1,MNT,EHMT1,MBD3,SARS,THAP7,NSMCE3,ERF	yellow	VSMC_M3
GO:0031346	0.34	0.74	21	302	positive regulation of cell projection organization	BP	1	PACSIN1,CUL7,DBNL,SNX3,PLXND1,SEPT9,BAIAP2,CX3CL1,CORO1B,LIMK1,PLA2G3,WASHC1,DBN1,CRABP2,EEF2K,DOCK11,ANAPC2,STK11,NEGR1,TWF2,CDC42EP5	yellow	VSMC_M3
GO:0016575	0.34	0.80	6	71	histone deacetylation	BP	1	HDAC10,BRMS1,MTA2,CTBP1,PER1,SIRT6	yellow	VSMC_M3
GO:2000060	0.34	0.79	8	95	positive regulation of ubiquitin-dependent protein catabolic process	BP	1	HSPBP1,SOCS5,RNF19B,CCDC22,AKT1,SIRT6,TRIB2,STUB1	yellow	VSMC_M3
GO:2000112	0.34	0.69	196	2822	regulation of cellular macromolecule biosynthetic process	BP	1	ENPP1,NAB2,CDK4,SQSTM1,ZNF608,HDAC10,NELFE,FNIP1,ATF6B,PBX2,RING1,SREBF2,H2AFY,TRIM11,TRAPPC9,PPARD,SND1,SECISBP2,PHF2,RPS9,CNOT3,KLF4,HOXA1,FHL3,ZC3H12A,PNKP,CC2D1B,KTI12,KMT2E,SIX5,ZNF282,ZNF296,RELB,DMAP1,CIC,UNK,OGG1,SERTAD1,TTC5,METTL3,CXXC5,DND1,EIF4EBP1,DEAF1,HOXC4,TARBP2,KMT2B,NAT10,ZNF740,EIF4B,RALY,ZBTB38,PINX1,GREM1,KDM2B,MCRS1,CX3CL1,WNT10B,ACTR5,ANKRD2,EGR3,CCAR2,DNAJC17,DLL4,CHP1,YAF2,HTATIP2,PCIF1,KDM1B,CHCHD2,DCPS,IRX1,SELENOS,RRP1B,BRD9,AIP,NGDN,TSHZ2,QARS,PIR,TRIM27,PRMT2,THOC5,RIPK3,TWIST2,LSM14B,SMARCD2,BRMS1,TACO1,GMEB2,CCDC85B,PRPF6,PBX1,KAT5,RELA,ZNF511,ARNT2,ZFPL1,LIMD1,SMARCB1,CHCHD10,PBX4,UBTF,GATAD2A,MEF2B,PHB2,NDP,IGFBP5,CTDSP1,MLF2,EID1,SSBP4,METTL13,MPV17L2,ZNF394,C11orf95,BUD31,TNFRSF1A,TEAD4,C1QBP,TAF6L,ZBTB49,MTA2,ZNF316,MED11,PELP1,CCDC22,FOXK1,KLF3,PKN1,SEPSECS,POLR2A,MAD2L2,SLTM,NANOS3,CTBP1,FBXL19,CRABP2,SP2,ARHGEF2,SNF8,PER1,NFIX,IRF2BPL,TRIM25,SMARCA4,CARM1,AATF,C19orf66,MID2,ZNF521,ZNF358,ZNF830,AKT1,BRF1,PAIP1,KHSRP,GTF2F1,RFX2,SAFB,SAFB2,MYDGF,HDGFL2,SIRT6,MAP2K2,CACTIN,CDK11B,SSU72,HIC1,TRAP1,MNT,EHMT1,AP3D1,MKNK2,IRAK1,MBD3,MED16,CARD9,SARS,POMT1,TRADD,RBM8A,ERBB4,JUND,KLF13,FADS1,ZNF783,HOXC5,ZNF385A,IER5,AKAP17A,ZNF250,THAP7,NSMCE3,ERF,ZSCAN1	yellow	VSMC_M3

GO:0044248	0.34	0.70	126	1755	cellular catabolic process	BP	1	ENPP1,SQSTM1,BAG6,SNX9,HDAC10,ACAT2,SREBF2,HNRNPA0,NUDT3,PPARD,MPST,SHARPIN,VPS28,SND1,CUL7,DNPH1,SECISBP2,HSPBP1,SOCS5,CNOT3,PSMB3,ZC3H12A,BLVRA,CPT1C,RCN3,SNRNP70,MGLL,TMUB1,BCKDHA,BLVRB,OGG1,PSMC4,SEL1L2,METTL3,DND1,GNPDA1,SMUG1,SCARB1,HM13,PDE4D,PCCB,PLEKHF1,ACSL1,LSM6,WNT10B,PACSIN3,CCAR2,PCBP4,RNF19B,DCPS,SELENOS,GSTO1,ETFBKMT,MANBA,ZCCHC17,LIX1L,GPX1,PCK2,PSME2,ANPEP,TRIM27,ADRM1,PSMB4,PTPN23,RNASEH2C,KAT5,IDH1,VPS51,SGPL1,PHB2,BAD,MRTO4,MVB12A,UBXN1,WASHC1,CNP,CCDC22,USP27X,GLUD1,FOXK1,ILVBL,CAPN2,RAB24,AMER1,PIP4K2B,NANOS3,FBXL19,DFFA,PGD,SNF8,TRIM25,USP43,MTMR4,CDC37,MID2,ATG3,ANKRD9,AKT1,PACS2,PAIP1,KHSRP,CARHSP1,BLMH,SIRT6,ANAPC2,SPSB3,MAN1B1,STK11,TAZ,WDR24,SLC27A4,ZNRF1,PSMB10,RBM8A,RNH1,PLA2G4E,NT5C,TENT5B,TRIB2,CDC34,FBXO6,GBA2,TRIR,STUB1,ITPA,CCDC115	yellow	VSMC_M3
GO:0048583	0.34	0.69	196	2834	regulation of response to stimulus	BP	1	ENPP1,CD63,CDK4,SQSTM1,BAG6,CTHRC1,HDAC10,NELFE,FNIP1,ATF6B,PRR5,TTLL12,SREBF2,TXNDC15,H2AFY,SGSM3,DEF6,PPARD,EEF1D,LGALS1,SHARPIN,BOP1,PDGFRA,IL1R1,SOCS5,KLF4,TPBG,SPRED2,RGS3,ZC3H12A,FIGNL1,PNKP,ZYX,RCN3,SNX3,PLEKHA4,MGLL,SLC9A3R1,EMILIN1,RNF220,CD79A,INSIG1,SEC13,OGG1,ARHGEF40,UCP2,METTL3,BAIAP2,CXXC5,LAMTOR1,NUMA1,P4HB,PYCR1,CAVIN3,ARHGAP22,TARBP2,BCL2L1,PDE4D,TNS2,RRS1,SWAP70,PLEKHF1,GREM1,PDE3B,CX3CL1,WNT10B,SOD1,DGKZ,CCAR2,DLL4,CHP1,TYRO3,CHCHD2,SLC35C2,TSPAN5,CISH,ST3GAL4,SELENOS,ITPRIP,MVK,RIN3,PDGFA,MMP14,GPX1,AIP,CORO1B,NGEF,CLCF1,QARS,TRIM27,EIF3A,LIMK1,PRMT2,SPX,THOC5,RIPK3,PSMB4,PLA2G3,STMN3,RELA,SIPA1,RHOC,MAP3K14,LIMD1,ZDHHC3,SMARCB1,YJEFN3,PHB2,IGFBP5,BAD,LAMTOR4,IFI35,OTUB1,LETM1,LGALS3,BUD31,CD9,PLAU,TNFRSF1A,MVB12A,BABAM1,C1QBP,CHERP,UBXN1,EVC,CCDC22,PKN1,PALM3,AMER1,PIP4K2B,MAD2L2,NANOS3,ARHGEF2,MVP,PER1,PPP4C,TRIM25,RPL37,MTMR4,S100A4,SMARCA4,CARM1,CDC37,CD276,AATF,CXorf57,CSK,MID2,AKT1,TEDC1,RAB34,SAFB2,GIT1,MYDGF,SIRT6,MAP2K2,CACTIN,VASN,HIC1,TRAP1,NCLN,MNT,CTNS,TEDC2,CSNK1G2,IRAK1,C8G,STK11,TELO2,WDR24,JMJD8,CARD9,GAS8,SLC27A4,TGFBR3,TRADD,MIR214,RF00024,NDST1,TNFRSF12A,ERBB4,PTPRR,IGFBP6,JAGN1,ZNF385A,TXNDC12,CDC34,IER5,APCDD1L,ARHGEF1,RGS16,DUSP5,GLIPR2,STUB1,RGS18,GSG1L	yellow	VSMC_M3
GO:0031623	0.34	0.79	7	85	receptor internalization	BP	1	CD63,SCRIB,GREM1,RIN3,CD9,ANKRD13B,GSG1L	yellow	VSMC_M3
GO:0033344	0.34	0.83	4	42	cholesterol efflux	BP	1	SREBF2,LAMTOR1,SCARB1,YJEFN3	yellow	VSMC_M3
GO:0060294	0.34	0.80	6	68	cilium movement involved in cell motility	BP	1	CEP131,YIF1B,IQCG,PLA2G3,GAS8,MEIG1	yellow	VSMC_M3
GO:0032635	0.34	0.79	8	96	interleukin-6 production	BP	1	SOCS5,ZC3H12A,CX3CL1,SELENOS,CAPN2,ARHGEF2,CSK,CARD9	yellow	VSMC_M3
GO:0032675	0.34	0.79	8	96	regulation of interleukin-6 production	BP	1	SOCS5,ZC3H12A,CX3CL1,SELENOS,CAPN2,ARHGEF2,CSK,CARD9	yellow	VSMC_M3
GO:0031638	0.34	0.83	4	38	zymogen activation	BP	1	MMP14,BAD,PLAU,DHCR24	yellow	VSMC_M3
GO:0090090	0.34	0.78	9	113	negative regulation of canonical Wnt signaling pathway	BP	1	CTHRC1,TPBG,SLC9A3R1,GREM1,LIMD1,AMER1,MAD2L2,STK11,IGFBP6	yellow	VSMC_M3
GO:1990089	0.34	0.83	4	47	response to nerve growth factor	BP	1	RAB35,CORO1A,EEF2K,AKT1	yellow	VSMC_M3
GO:0009607	0.34	0.71	63	856	response to biotic stimulus	BP	1	CDK4,TTLL12,HNRNPA0,TRIM11,PPARD,PPP1R14B,SSC5D,KLF4,ZC3H12A,ZYX,SNX3,KRT8,RELB,DLEC1,METTL3,BAIAP2,RAB20,TARBP2,SCARB1,BCL2L1,CX3CL1,TYRO3,RNF19B,SELENOS,RRP1B,PABPN1,GPX1,PCK2,TRIM27,SLC26A6,RIPK3,TNK2,BANF1,RELA,MAP3K14,PHB2,IFI35,LGALS3,TNFRSF1A,C1QBP,CNP,CAPN2,PALM3,TBKBP1,ARHGEF2,ARMC5,CORO1A,TRIM25,CDC37,C19orf66,MID2,AKT1,GTF2F1,GIT1,LDOC1,CACTIN,IRAK1,C8G,CARD9,JUND,LOXL1,EEF1G,JAGN1	yellow	VSMC_M3
GO:0006641	0.34	0.80	6	70	triglyceride metabolic process	BP	1	MGLL,INSIG1,SCARB1,ACSL1,GPX1,PCK2	yellow	VSMC_M3
GO:0007584	0.34	0.77	11	137	response to nutrient	BP	1	PPARD,OGG1,AACS,ACSL1,DCPS,RELA,MTHFR,TRIM25,HMGCS1,SLC6A4,SLC27A4	yellow	VSMC_M3
GO:0051028	0.34	0.78	9	109	mRNA transport	BP	1	SEC13,RBM15B,PABPN1,THOC5,THOC3,NUP88,MVP,KHSRP,RBM8A	yellow	VSMC_M3
GO:0031050	0.35	0.83	4	46	dsRNA processing	BP	1	ZC3H12A,METTL3,TARBP2,MAP2K2	yellow	VSMC_M3
GO:0035196	0.35	0.83	4	46	production of miRNAs involved in gene silencing by miRNA	BP	1	ZC3H12A,METTL3,TARBP2,MAP2K2	yellow	VSMC_M3
GO:0070918	0.35	0.83	4	46	production of small RNA involved in gene silencing by RNA	BP	1	ZC3H12A,METTL3,TARBP2,MAP2K2	yellow	VSMC_M3
GO:1903507	0.35	0.70	67	945	negative regulation of nucleic acid-templated transcription	BP	1	NAB2,SQSTM1,ZNF608,HDAC10,NELFE,FNIP1,RING1,SREBF2,H2AFY,TRIM11,PPARD,KLF4,CC2D1B,SIX5,ZNF282,ZNF296,RELB,DMAP1,CIC,CXXC5,DEAF1,ZBTB38,GREM1,KDM2B,WNT10B,ANKRD2,CCAR2,DNAJC17,DLL4,YAF2,KDM1B,IRX1,QARS,TRIM27,PRMT2,TWIST2,BRMS1,CCDC85B,KAT5,RELA,LIMD1,GATAD2A,PHB2,EID1,METTL13,C11orf95,C1QBP,ZBTB49,MTA2,FOXK1,KLF3,MAD2L2,CTBP1,SP2,PER1,NFIX,IRF2BPL,SMARCA4,SIRT6,HIC1,MNT,EHMT1,MBD3,SARS,THAP7,NSMCE3,ERF	yellow	VSMC_M3
GO:0042246	0.35	0.81	5	53	tissue regeneration	BP	1	PPARD,WNT10B,GPX1,CD9,ERBB4	yellow	VSMC_M3
GO:0002285	0.35	0.77	10	127	lymphocyte activation involved in immune response	BP	1	LGALS1,SOCS5,ZC3H12A,RELB,SWAP70,CLCF1,LGALS3,MAD2L2,CORO1A,DOCK11	yellow	VSMC_M3
GO:0006633	0.35	0.77	9	107	fatty acid biosynthetic process	BP	1	MGLL,INSIG1,FASN,SCD,PLA2G3,MID1IP1,ACLY,FADS2,FADS1	yellow	VSMC_M3

GO:0006355	0.35	0.69	167	2411	regulation of transcription, DNA-templated	BP	1	ENPP1,NAB2,CDK4,SQSTM1,ZNF608,HDAC10,NELFE,FNIP1,ATF6B,PBX2,RING1,SREBF2,H2AFY,TRIM11,TRAPPC9,PPARD,SND1,PHF2,CNOT3,KLF4,HOXA1,FHL3,ZC3H12A,CC2D1B,KTI12,KMT2E,SIX5,ZNF282,ZNF296,RELB,DMAP1,CIC,OGG1,SERTAD1,TTC5,CXXC5,DEAF1,HOXC4,KMT2B,ZNF740,RALY,ZBTB38,GREM1,KDM2B,MCRS1,CX3CL1,WNT10B,ACTR5,ANKRD2,EGR3,CCAR2,DNAJC17,DLL4,CHP1,YAF2,HTATIP2,KDM1B,CHCHD2,IRX1,RRP1B,BRD9,AIP,TSHZ2,QARS,PIR,TRIM27,PRMT2,THOC5,RIPK3,TWIST2,SMARCD2,BRMS1,GMEB2,CCDC85B,PRPF6,PBX1,KAT5,RELA,ZNF511,ARNT2,ZFPL1,LIMD1,SMARCB1,CHCHD10,PBX4,UBTF,GATAD2A,MEF2B,PHB2,NDP,CTDSP1,MLF2,EID1,SSBP4,METTL13,ZNF394,C11orf95,BUD31,TNFRSF1A,TEAD4,C1QBP,TAF6L,ZBTB49,MTA2,ZNF316,MED11,PELP1,CCDC22,FOXK1,KLF3,PKN1,POLR2A,MAD2L2,SLTM,CTBP1,FBXL19,CRABP2,SP2,ARHGEF2,SNF8,PER1,NFIX,IRF2BPL,TRIM25,SMARCA4,CARM1,AATF,MID2,ZNF521,ZNF358,AKT1,BRF1,KHSRP,GTF2F1,RFX2,SAFB,SAFB2,MYDGF,HDGFL2,SIRT6,MAP2K2,CACTIN,CDK11B,SSU72,HIC1,MNT,EHMT1,AP3D1,IRAK1,MBD3,MED16,CARD9,SARS,TRADD,ERBB4,JUND,KLF13,FADS1,ZNF783,HOXC5,IER5,AKAP17A,ZNF250,THAP7,NSMCE3,ERF,ZSCAN1	yellow	VSMC_M3
GO:0043170	0.35	0.68	494	7034	macromolecule metabolic process	BP	1	ENPP1,CD63,NAB2,NHP2,CDK4,CTDSP2,SQSTM1,DHX16,ZNF608,LMF2,BAG6,SBF1,SNX9,HDAC10,NELFE,FNIP1,ATF6B,PBX2,PRR5,TTLL12,RING1,SREBF2,JADE2,TRMT12,H2AFY,HNRNPA0,TRIM11,SGSM3,TRAPPC9,NUDT3,PPARD,FANCE,EEF1D,RPL3,SCRIB,PLEC,SHARPIN,BOP1,VPS28,RPL8,SND1,CUL7,RTCB,B4GALT1,MRPS18A,AARS2,PIGO,PDGFRA,IL1R1,SECISBP2,PHF2,SSC5D,HSPBP1,SOCS5,RPS9,CNOT3,KLF4,HOXA1,JOSD2,PSMB3,FHL3,SPRED2,EIPR1,MEAF6,ZC3H12A,FIGNL1,PNKP,RCN3,SNRNP70,CC2D1B,SNX3,KTI12,MICAL1,KMT2E,WDFY2,SIX5,ZNF282,GEMIN7,ZNF296,RELB,SLC9A3R1,SERP2,NAT9,MUTYH,EMILIN1,SEPHS1,TMUB1,RNF220,DMAP1,CIC,GTF3C2,PPM1G,UNK,GALNT14,OGG1,SERTAD1,TTC5,PSMC4,KAT14,P4HA3,SEL1L2,METTL3,PHLDA1,CXXC5,INPPL1,FAAP100,LAMTOR1,P4HB,DND1,RPL29,EIF4EBP1,DELE1,CAVIN3,PUS1,DEAF1,SMUG1,HOXC4,POLR1D,LDLRAD3,TARBP2,KMT2B,C12orf10,NAT10,HM13,ZNF740,PDE4D,TNS2,EIF4B,RRS1,PEPD,SWAP70,LRP3,RALY,ACSL1,ZBTB38,ARL6IP4,LSM6,TNKS1BP1,MMP24,PINX1,GATB,SETD1B,GREM1,KDM2B,PRPF40B,MCRS1,POLR2C,CX3CL1,WNT10B,FKBP11,SOD1,PACSIN3,ACTR5,ANKRD2,EGR3,CCAR2,DNAJC17,DLL4,RPLP2,CHP1,TYRO3,MRPL43,YAF2,HTATIP2,PCIF1,PPM1M,NPM3,PCBP4,KDM1B,CHCHD2,SLC35C2,RBM15B,CARS,TSPAN5,CISH,SRSF9,ST3GAL4,RNF19B,DCPS,IRX1,WDR4,SELENOS,RRP1B,ETFBKMT,MANBA,TMTC1,BRD9,RBM6,RIN3,STK32C,ZCCHC17,PDGFA,MMP14,C14orf93,APEH,PABPN1,GPX1,AIP,HCCS,MOCS3,CLCF1,NGDN,TSHZ2,QARS,ASB9,PSME2,PIR,ANPEP,TRIM27,EIF3A,LIMK1,ZNRD1,RABGGTA,PRMT2,THOC5,RIPK3,KHNYN,TWIST2,LSM14B,SF3A1,[illegible]	yellow	VSMC_M3
GO:0051897	0.35	0.79	7	88	positive regulation of protein kinase B signaling	BP	1	TPBG,CX3CL1,PDGFA,GPX1,IGFBP5,C1QBP,MYDGF	yellow	VSMC_M3
GO:0006984	0.35	0.82	4	44	ER-nucleus signaling pathway	BP	1	ATF6B,SREBF2,INSIG1,SELENOS	yellow	VSMC_M3
GO:0042542	0.35	0.77	10	118	response to hydrogen peroxide	BP	1	KLF4,PYCR1,SOD1,GPX1,RIPK3,RELA,BAD,CAPN2,TRAP1,OSER1	yellow	VSMC_M3
GO:0090276	0.35	0.76	11	140	regulation of peptide hormone secretion	BP	1	PPARD,EIPR1,MYRIP,UCP2,AACS,PCK2,CAPN10,BAD,GLUD1,MIDN,JAGN1	yellow	VSMC_M3
GO:1903506	0.35	0.68	167	2412	regulation of nucleic acid-templated transcription	BP	1	ENPP1,NAB2,CDK4,SQSTM1,ZNF608,HDAC10,NELFE,FNIP1,ATF6B,PBX2,RING1,SREBF2,H2AFY,TRIM11,TRAPPC9,PPARD,SND1,PHF2,CNOT3,KLF4,HOXA1,FHL3,ZC3H12A,CC2D1B,KTI12,KMT2E,SIX5,ZNF282,ZNF296,RELB,DMAP1,CIC,OGG1,SERTAD1,TTC5,CXXC5,DEAF1,HOXC4,KMT2B,ZNF740,RALY,ZBTB38,GREM1,KDM2B,MCRS1,CX3CL1,WNT10B,ACTR5,ANKRD2,EGR3,CCAR2,DNAJC17,DLL4,CHP1,YAF2,HTATIP2,KDM1B,CHCHD2,IRX1,RRP1B,BRD9,AIP,TSHZ2,QARS,PIR,TRIM27,PRMT2,THOC5,RIPK3,TWIST2,SMARCD2,BRMS1,GMEB2,CCDC85B,PRPF6,PBX1,KAT5,RELA,ZNF511,ARNT2,ZFPL1,LIMD1,SMARCB1,CHCHD10,PBX4,UBTF,GATAD2A,MEF2B,PHB2,NDP,CTDSP1,MLF2,EID1,SSBP4,METTL13,ZNF394,C11orf95,BUD31,TNFRSF1A,TEAD4,C1QBP,TAF6L,ZBTB49,MTA2,ZNF316,MED11,PELP1,CCDC22,FOXK1,KLF3,PKN1,POLR2A,MAD2L2,SLTM,CTBP1,FBXL19,CRABP2,SP2,ARHGEF2,SNF8,PER1,NFIX,IRF2BPL,TRIM25,SMARCA4,CARM1,AATF,MID2,ZNF521,ZNF358,AKT1,BRF1,KHSRP,GTF2F1,RFX2,SAFB,SAFB2,MYDGF,HDGFL2,SIRT6,MAP2K2,CACTIN,CDK11B,SSU72,HIC1,MNT,EHMT1,AP3D1,IRAK1,MBD3,MED16,CARD9,SARS,TRADD,ERBB4,JUND,KLF13,FADS1,ZNF783,HOXC5,IER5,AKAP17A,ZNF250,THAP7,NSMCE3,ERF,ZSCAN1	yellow	VSMC_M3
GO:0060840	0.35	0.79	6	81	artery development	BP	1	HOXA1,PLXND1,SNX17,DLL4,NDST1,LOXL1	yellow	VSMC_M3
GO:1902679	0.35	0.70	67	946	negative regulation of RNA biosynthetic process	BP	1	NAB2,SQSTM1,ZNF608,HDAC10,NELFE,FNIP1,RING1,SREBF2,H2AFY,TRIM11,PPARD,KLF4,CC2D1B,SIX5,ZNF282,ZNF296,RELB,DMAP1,CIC,CXXC5,DEAF1,ZBTB38,GREM1,KDM2B,WNT10B,ANKRD2,CCAR2,DNAJC17,DLL4,YAF2,KDM1B,IRX1,QARS,TRIM27,PRMT2,TWIST2,BRMS1,CCDC85B,KAT5,RELA,LIMD1,GATAD2A,PHB2,EID1,METTL13,C11orf95,C1QBP,ZBTB49,MTA2,FOXK1,KLF3,MAD2L2,CTBP1,SP2,PER1,NFIX,IRF2BPL,SMARCA4,SIRT6,HIC1,MNT,EHMT1,MBD3,SARS,THAP7,NSMCE3,ERF	yellow	VSMC_M3
GO:0035195	0.35	0.77	9	107	gene silencing by miRNA	BP	1	ZC3H12A,METTL3,DND1,TARBP2,LIMD1,MAP2K2,MIR214,MIR155,MIR374B	yellow	VSMC_M3
GO:0051972	0.35	0.82	4	42	regulation of telomerase activity	BP	1	KLF4,PNKP,PINX1,GREM1	yellow	VSMC_M3
GO:0003158	0.35	0.77	8	107	endothelium development	BP	1	COL15A1,CCM2,FASN,PDE4D,DLL4,GPX1,TNFRSF1A,TJP3	yellow	VSMC_M3
GO:0030178	0.35	0.76	11	142	negative regulation of Wnt signaling pathway	BP	1	CTHRC1,TPBG,SLC9A3R1,GREM1,LIMD1,AMER1,MAD2L2,HIC1,STK11,IGFBP6,APCDD1L	yellow	VSMC_M3

GO:0072331	0.36	0.76	11	139	signal transduction by p53 class mediator	BP	1	BAG6,BOP1,SPRED2,RRS1,KAT5,ZNF385B,RPL37,AKT1,HIC1,STK11,ZNF385A	yellow	VSMC_M3
GO:0032204	0.36	0.79	6	74	regulation of telomere maintenance	BP	1	PNKP,NAT10,PINX1,SLX1A,SIRT6,NABP2	yellow	VSMC_M3
GO:1905954	0.36	0.79	6	73	positive regulation of lipid localization	BP	1	SREBF2,ZC3H12A,SCARB1,ACSL1,FABP3,PLA2G3	yellow	VSMC_M3
GO:0043112	0.36	0.76	10	131	receptor metabolic process	BP	1	CD63,SCRIB,LAMTOR1,GREM1,RIN3,CD9,MVB12A,ANKRD13B,PHETA2,GSG1L	yellow	VSMC_M3
GO:0002521	0.36	0.71	28	383	leukocyte differentiation	BP	1	NCAPH2,FNIP1,LGALS1,SOCS5,ZC3H12A,RELB,CD79A,METTL3,FASN,SOD1,EGR3,DLL4,TYRO3,MMP14,CLCF1,PIR,THOC5,RIPK3,PLA2G3,SART1,PBX1,BAD,PRXL2A,ARMC5,DOCK11,AP3D1,STK11,JAGN1	yellow	VSMC_M3
GO:2001141	0.36	0.67	167	2416	regulation of RNA biosynthetic process	BP	1	ENPP1,NAB2,CDK4,SQSTM1,ZNF608,HDAC10,NELFE,FNIP1,ATF6B,PBX2,RING1,SREBF2,H2AFY,TRIM11,TRAPPC9,PPARD,SND1,PHF2,CNOT3,KLF4,HOXA1,FHL3,ZC3H12A,CC2D1B,KTI12,KMT2E,SIX5,ZNF282,ZNF296,RELB,DMAP1,CIC,OGG1,SERTAD1,TTC5,CXXC5,DEAF1,HOXC4,KMT2B,ZNF740,RALY,ZBTB38,GREM1,KDM2B,MCRS1,CX3CL1,WNT10B,ACTR5,ANKRD2,EGR3,CCAR2,DNAJC17,DLL4,CHP1,YAF2,HTATIP2,KDM1B,CHCHD2,IRX1,RRP1B,BRD9,AIP,TSHZ2,QARS,PIR,TRIM27,PRMT2,THOC5,RIPK3,TWIST2,SMARCD2,BRMS1,GMEB2,CCDC85B,PRPF6,PBX1,KAT5,RELA,ZNF511,ARNT2,ZFPL1,LIMD1,SMARCB1,CHCHD10,PBX4,UBTF,GATAD2A,MEF2B,PHB2,NDP,CTDSP1,MLF2,EID1,SSBP4,METTL13,ZNF394,C11orf95,BUD31,TNFRSF1A,TEAD4,C1QBP,TAF6L,ZBTB49,MTA2,ZNF316,MED11,PELP1,CCDC22,FOXK1,KLF3,PKN1,POLR2A,MAD2L2,SLTM,CTBP1,FBXL19,CRABP2,SP2,ARHGEF2,SNF8,PER1,NFIX,IRF2BPL,TRIM25,SMARCA4,CARM1,AATF,MID2,ZNF521,ZNF358,AKT1,BRF1,KHSRP,GTF2F1,RFX2,SAFB,SAFB2,MYDGF,HDGFL2,SIRT6,MAP2K2,CACTIN,CDK11B,SSU72,HIC1,MNT,EHMT1,AP3D1,IRAK1,MBD3,MED16,CARD9,SARS,TRADD,ERBB4,JUND,KLF13,FADS1,ZNF783,HOXC5,IER5,AKAP17A,ZNF250,THAP7,NSMCE3,ERF,ZSCAN1	yellow	VSMC_M3
GO:0051298	0.36	0.80	5	64	centrosome duplication	BP	1	CEP131,CCDC57,NAT10,SAC3D1,KIAA0753	yellow	VSMC_M3
GO:2001252	0.36	0.79	6	72	positive regulation of chromosome organization	BP	1	H2AFY,PNKP,NUMA1,SLX1A,SIRT6,NABP2	yellow	VSMC_M3
GO:0045580	0.36	0.77	8	98	regulation of T cell differentiation	BP	1	SOCS5,ZC3H12A,METTL3,SOD1,EGR3,SART1,BAD,AP3D1	yellow	VSMC_M3
GO:0051641	0.36	0.67	158	2295	cellular localization	BP	1	PQLC1,CD63,ESYT1,DCTN2,SQSTM1,CCHCR1,BAG6,SNX9,SAMM50,SREBF2,H2AFY,ITPR3,SGSM3,PACSIN1,PPARD,SCRIB,PLEC,IFT27,VPS28,TOM1,TTC7A,ASNA1,TPBG,PLEKHA8,EIPR1,ZC3H12A,SNX3,EHD2,SLC9A3R1,DMAP1,SLC30A3,SNX17,INSIG1,MYRIP,SEC13,SEPT9,UCP2,CEP131,TSPAN10,LAMTOR1,NUMA1,YIF1B,RAB20,HM13,BCL2L1,ITGB7,PDE4D,RRS1,PLEKHF1,SLC11A2,PINX1,MCRS1,DCTN1,RAPSN,CX3CL1,SOD1,CHP1,AP2A2,HTATIP2,RAB35,RBM15B,TSPAN5,SELENOS,GSTO1,EXOC3,FABP3,MMP14,PABPN1,AIP,AP1G2,CORO1B,SYT12,TRIM27,PCNT,NUDC,THOC5,CAPN10,PLA2G3,PTPN23,SCYL1,LRWD1,VPS51,PEX5,ZDHHC3,CHCHD10,YJEFN3,THOC3,BLOC1S4,PHB2,BAD,COPE,LAMTOR4,LETM1,LGALS3,TNFRSF1A,MVB12A,KIAA0753,NUP88,CHERP,WASHC1,RCC2,AP1M1,EML3,CCDC22,DBN1,SNX8,LMAN2,RAB24,MAD1L1,CLTB,ARHGEF2,SNF8,RANGRF,STX10,CORO1A,SGF29,ATP5PD,TMED1,SYNE3,CDC37,MYO19,CSK,MID2,ATG3,RBM22,AKT1,SNX18,PACS2,DENND1C,RANBP3,RAB34,GIT1,SLC6A4,MAP2K2,AP3D1,SSNA1,ATP5F1D,STK11,TMEM201,GAS8,SPG7,WDR34,RABEPK,NABP2,ERBB4,TWF2,TRAPPC5,PLA2G4E,AP5B1,JAGN1,MIS12,UNC13A,ZNF385A,ARFRP1,MEIG1,TIMM22,PHETA2,GSG1L	yellow	VSMC_M3
GO:0030101	0.36	0.81	4	43	natural killer cell activation	BP	1	BAG6,TYRO3,PBX1,CORO1A	yellow	VSMC_M3
GO:0008630	0.36	0.77	7	84	intrinsic apoptotic signaling pathway in response to DNA damage	BP	1	BAG6,BCL2L1,CCAR2,BAD,TNFRSF1A,HIC1,ZNF385A	yellow	VSMC_M3
GO:1903364	0.37	0.75	11	137	positive regulation of cellular protein catabolic process	BP	1	BAG6,HSPBP1,SOCS5,PSMC4,RNF19B,CCDC22,AMER1,AKT1,SIRT6,TRIB2,STUB1	yellow	VSMC_M3
GO:0070555	0.37	0.76	8	98	response to interleukin-1	BP	1	IL1R1,ZC3H12A,CX3CL1,RELA,OTUB1,MTHFR,CACTIN,IRAK1	yellow	VSMC_M3
GO:0097553	0.37	0.77	7	95	calcium ion transmembrane import into cytosol	BP	1	ITPR3,PDE4D,CX3CL1,GSTO1,LETM1,CHERP,CORO1A	yellow	VSMC_M3
GO:0090199	0.37	0.81	4	39	regulation of release of cytochrome c from mitochondria	BP	1	BCL2L1,GPX1,BAD,AKT1	yellow	VSMC_M3
GO:0030042	0.37	0.81	4	51	actin filament depolymerization	BP	1	MICAL1,SWAP70,CAPG,TWF2	yellow	VSMC_M3
GO:0002683	0.37	0.71	22	290	negative regulation of immune system process	BP	1	TTLL12,SCRIB,SOCS5,ZC3H12A,EMILIN1,METTL3,TARBP2,GREM1,CX3CL1,DGKZ,TYRO3,SELENOS,RIN3,GPX1,TRIM27,PSMB4,LGALS3,C1QBP,PKN1,MAD1L1,AKT1,CACTIN	yellow	VSMC_M3
GO:0098754	0.37	0.76	8	90	detoxification	BP	1	GSR,SOD1,SELENOS,GSTO1,GPX1,MGST3,TXNRD2,PRXL2A	yellow	VSMC_M3
GO:0050865	0.37	0.70	29	385	regulation of cell activation	BP	1	SCRIB,LGALS1,PDGFRA,SOCS5,ZC3H12A,EMILIN1,METTL3,CX3CL1,SOD1,EGR3,TYRO3,PDGFA,MMP14,CLCF1,RIPK3,PLA2G3,SART1,BAD,LGALS3,CD9,PKN1,MAD1L1,MAD2L2,CORO1A,CD276,CSK,AKT1,AP3D1,JUND	yellow	VSMC_M3
GO:0043433	0.37	0.75	10	126	negative regulation of DNA-binding transcription factor activity	BP	1	KLF4,ZC3H12A,CHP1,PRMT2,BRMS1,PHB2,CCDC22,MAD2L2,CACTIN,IRAK1	yellow	VSMC_M3
GO:0048008	0.37	0.81	4	48	platelet-derived growth factor receptor signaling pathway	BP	1	PDGFRA,SLC9A3R1,PDGFA,SGPL1	yellow	VSMC_M3
GO:0017148	0.37	0.74	12	158	negative regulation of translation	BP	1	CNOT3,ZC3H12A,UNK,METTL3,DND1,EIF4EBP1,PCIF1,DCPS,IGFBP5,NANOS3,C19orf66,KHSRP	yellow	VSMC_M3
GO:0046470	0.37	0.79	5	55	phosphatidylcholine metabolic process	BP	1	SCARB1,FABP3,PLA2G3,CAPN2,SLC44A3	yellow	VSMC_M3
GO:0032436	0.37	0.77	7	83	positive regulation of proteasomal ubiquitin-dependent protein catabolic process	BP	1	HSPBP1,SOCS5,RNF19B,AKT1,SIRT6,TRIB2,STUB1	yellow	VSMC_M3
GO:0034502	0.37	0.77	7	86	protein localization to chromosome	BP	1	H2AFY,PINX1,LRWD1,RCC2,SGF29,NABP2,MIS12	yellow	VSMC_M3
GO:0048024	0.37	0.77	7	85	regulation of mRNA splicing, via spliceosome	BP	1	SNRNP70,PCBP4,RBM15B,SRSF9,C1QBP,RAVER1,RBM8A	yellow	VSMC_M3
GO:0043242	0.37	0.79	5	70	negative regulation of protein-containing complex disassembly	BP	1	SWAP70,CAPG,MID1IP1,ARHGEF2,TWF2	yellow	VSMC_M3

GO:0051261	0.37	0.77	7	99	protein depolymerization	BP	1	MICAL1,SWAP70,CAPG,STMN3,MID1IP1,ARHGEF2,TWF2	yellow	VSMC_M3
GO:0051249	0.37	0.71	22	287	regulation of lymphocyte activation	BP	1	SCRIB,LGALS1,SOCS5,ZC3H12A,METTL3,SOD1,EGR3,TYRO3,MMP14,CLCF1,RIPK3,SART1,BAD,LGALS3,PKN1,MAD1L1,MAD2L2,CORO1A,CD276,CSK,AKT1,AP3D1	yellow	VSMC_M3
GO:0030858	0.37	0.81	4	45	positive regulation of epithelial cell differentiation	BP	1	H2AFY,NUMA1,WNT10B,BAD	yellow	VSMC_M3
GO:0002791	0.37	0.74	11	142	regulation of peptide secretion	BP	1	PPARD,EIPR1,MYRIP,UCP2,AACS,PCK2,CAPN10,BAD,GLUD1,MIDN,JAGN1	yellow	VSMC_M3
GO:0009165	0.37	0.72	16	198	nucleotide biosynthetic process	BP	1	GUK1,ACSL1,AK2,PAPSS1,IMPDH2,ATP5MC3,ATIC,UQCC3,ACLY,FLAD1,ATP5PD,ATP5F1D,TAZ,AMPD2,APRT,AK1	yellow	VSMC_M3
GO:1901655	0.37	0.78	6	77	cellular response to ketone	BP	1	CDK4,KLF4,AACS,DCPS,PCK2,AKT1	yellow	VSMC_M3
GO:0000086	0.37	0.75	10	124	G2/M transition of mitotic cell cycle	BP	1	CDK4,CCDC57,PINX1,WNT10B,PBX1,BABAM1,RCC2,MIIP,ZNF830,NABP2	yellow	VSMC_M3
GO:0008104	0.37	0.66	140	2028	protein localization	BP	1	CD63,DCTN2,RUFY1,SQSTM1,CCHCR1,BAG6,SNX9,SAMM50,SREBF2,H2AFY,SGSM3,GUK1,PACSIN1,PPARD,SCRIB,PLEC,IFT27,VPS28,TOM1,TTC7A,ASNA1,TPBG,PLEKHA8,WDPCP,EIPR1,ZC3H12A,PDIA4,RCN3,SNX3,EHD2,SLC9A3R1,SERP2,DMAP1,SNX17,INSIG1,MYRIP,SEC13,SEPT9,UCP2,BAIAP2,CEP131,TSPAN10,LAMTOR1,NUMA1,YIF1B,RAB20,ARHGAP33,AACS,HM13,BCL2L1,ITGB7,RRS1,SWAP70,PLEKHF1,PINX1,MCRS1,RAPSN,CHP1,AP2A2,SNX21,RAB35,TSPAN5,APBA2,SELENOS,EXOC3,MMP14,AIP,AP1G2,CORO1B,PCK2,NECTIN1,TRIM27,PCNT,CAPN10,PTPN23,SCYL1,LRWD1,VPS51,PEX5,ZDHHC3,CHCHD10,PHB2,BAD,COPE,LAMTOR4,LGALS3,TNFRSF1A,MVB12A,KIAA0753,NUP88,WASHC1,RCC2,AP1M1,RAB3IL1,CCDC22,GLUD1,DBN1,SNX8,LMAN2,RAB24,CLTB,ARHGEF2,SNF8,MVP,MTX1,RANGRF,STX10,SGF29,RABEP2,TRIM25,TMED1,SYNE3,CDC37,CSK,MID2,ATG3,RBM22,AKT1,SNX18,PACS2,RANBP3,RAB34,DHCR24,TJP3,CTNS,AP3D1,SSNA1,MIDN,STK11,GAS8,NABP2,ERBB4,TWF2,AP5B1,JAGN1,CAVIN1,MIS12,ARFRP1,MEIG1,TIMM22	yellow	VSMC_M3
GO:0035335	0.37	0.78	6	77	peptidyl-tyrosine dephosphorylation	BP	1	TNS2,PTPN23,MTMR4,PGP,PTPRR,DUSP5	yellow	VSMC_M3
GO:0016043	0.37	0.65	316	4652	cellular component organization	BP	1	ENPP1,PQLC1,CD63,ESYT1,DCTN2,NHP2,CDK4,RUFY1,SQSTM1,SPAG1,BAG6,NCAPH2,SNX9,CTHRC1,HDAC10,FNIP1,PDLIM4,SAMM50,SREBF2,NDUFB9,SQLE,TXNDC15,H2AFY,PACSIN1,PPARD,RPL3,SCRIB,PLEC,LGALS1,IFT27,SHARPIN,BOP1,VPS28,TBCC,CUL7,DNPH1,B4GALT1,PDGFRA,PHF2,COL15A1,COL19A1,ASNA1,KLF4,DBNL,TPBG,PLEKHA8,WDPCP,CCM2,FHL3,ZC3H12A,FIGNL1,PNKP,ZYX,SNX3,MICAL1,EMP3,EHD2,KMT2E,KRT8,ACAD9,TMEM43,GEMIN7,SLC9A3R1,PLXND1,EMILIN1,DMAP1,SNX17,H3F3B,INSIG1,SEC13,SEPT9,BAIAP2,CEP131,INPPL1,LAMTOR1,NUMA1,YIF1B,CCDC57,RAB20,EIF4EBP1,ARAP3,CAVIN3,RIC8A,ARHGAP22,SDHAF1,ARHGAP33,TARBP2,SCARB1,NAT10,BCL2L1,SPRYD3,EIF4B,RRS1,SWAP70,PLEKHF1,UQCRFS1,CAPG,SLC11A2,TNKS1BP1,MMP24,PINX1,GREM1,KDM2B,AQP5,DCTN1,RAPSN,CX3CL1,WNT10B,SOD1,PACSIN3,ACTR5,CCDC32,CCAR2,HPS1,CHP1,NPM3,RAB35,CHCHD2,CISH,SRSF9,CCDC28B,BRD9,RIN3,PDGFA,MMP14,CDK2AP2,LIX1L,APEH,GPX1,AIP,CORO1B,NGEF,HCCS,PLEKHO1,IGSF9,LRFN4,CLIP2,NECTIN1,TRIM27,ARPIN,PCNT,EIF3A,LIMK1,NUDC,RIPK3,SF3A1,ADRM1,CAPN10,SMARCD2,IQCG,PFDN2,MPZL3,BUD23,TNK2,TACO1,PLA2G3,STMN3,CEP164,BANF1,SART1,CCDC85B,PRPF6,PTPN23,RELA,SIPA1,RHOC,TMEM120A,LRWD1,ALKBH4,ZNHIT1,FMNL1,VPS51,SAC3D1,LIMD1,MID1IP1,PEX5,SMARCB1,CHCHD10,YJEFN3,UBTF,BLOC1S4,PHB2,NDP,IGFBP5,C12orf57,BAD,LAMTOR4,MPV17L2,LETM1,LGALS3,ARPC1B,CD9,MRTO4,TNFRSF1A,FCHO1,MVB12A,KIAA0753,BABAM1,C1QBP,AIMP2,TAF6L,UQCC3,WASHC1,RCC2,CNP,AP1M1,EML3,MTA2,KRT10,H3F3A,DBN1,CAPN2,LMAN2,MAD1L1,[illegible]	yellow	VSMC_M3
GO:0098732	0.37	0.76	8	102	macromolecule deacylation	BP	1	HDAC10,SPRED2,CCAR2,BRMS1,MTA2,CTBP1,PER1,SIRT6	yellow	VSMC_M3
GO:0051962	0.38	0.72	15	216	positive regulation of nervous system development	BP	1	CUL7,DBNL,TPBG,PLXND1,BAIAP2,CX3CL1,CLCF1,LIMK1,RELA,DBN1,CRABP2,EEF2K,ANAPC2,STK11,TWF2	yellow	VSMC_M3
GO:0098742	0.38	0.74	10	152	cell-cell adhesion via plasma-membrane adhesion molecules	BP	1	KLF4,RIC8A,MMP24,CX3CL1,IGSF9,LRFN4,NECTIN1,PTPN23,SCARF2,CLDN6	yellow	VSMC_M3
GO:0090596	0.38	0.72	14	198	sensory organ morphogenesis	BP	1	CTHRC1,RING1,SCRIB,HOXA1,WDPCP,SLC9A3R1,INSIG1,LRIG1,KDM2B,AQP5,SOD1,NECTIN1,C12orf57,TTC39C	yellow	VSMC_M3
GO:0032623	0.38	0.80	4	43	interleukin-2 production	BP	1	PDE4D,TRIM27,KAT5,CARD9	yellow	VSMC_M3
GO:0032663	0.38	0.80	4	43	regulation of interleukin-2 production	BP	1	PDE4D,TRIM27,KAT5,CARD9	yellow	VSMC_M3
GO:1901652	0.38	0.70	27	375	response to peptide	BP	1	ENPP1,CDK4,KLF4,INSIG1,UCP2,BAIAP2,INPPL1,TNS2,PDE3B,SELENOS,FABP3,PCK2,CAPN10,RELA,IGFBP5,KLF3,PIP4K2B,ARHGEF2,EEF2K,CSK,AKT1,LDOC1,CARD9,APRT,SLC27A4,TGFBR3,JUND	yellow	VSMC_M3
GO:0045471	0.38	0.76	7	87	response to ethanol	BP	1	DMAP1,OGG1,EIF4EBP1,AACS,SOD1,BAD,TUFM	yellow	VSMC_M3
GO:1901293	0.38	0.72	16	199	nucleoside phosphate biosynthetic process	BP	1	GUK1,ACSL1,AK2,PAPSS1,IMPDH2,ATP5MC3,ATIC,UQCC3,ACLY,FLAD1,ATP5PD,ATP5F1D,TAZ,AMPD2,APRT,AK1	yellow	VSMC_M3
GO:0002819	0.38	0.74	9	111	regulation of adaptive immune response	BP	1	IL1R1,SOCS5,ZC3H12A,CLCF1,TRIM27,RIPK3,PKN1,MAD2L2,CARD9	yellow	VSMC_M3
GO:0051438	0.38	0.80	4	43	regulation of ubiquitin-protein transferase activity	BP	1	LIMK1,MAD2L2,RPL37,STUB1	yellow	VSMC_M3
GO:0043086	0.38	0.68	41	555	negative regulation of catalytic activity	BP	1	FNIP1,H2AFY,PLEC,PPP1R14B,HSPBP1,SOCS5,KLF4,SPRED2,MICAL1,ANXA5,TARBP2,PINX1,CCAR2,CHP1,ITPRIP,RRP1B,ETFBKMT,GPX1,AIP,QARS,TRIM27,LIMK1,TNK2,PPP1R35,LGALS3,UBXN1,WASHC1,RCC2,PKN1,PIP4K2B,MAD2L2,DFFA,MVP,RPL37,CSK,AKT1,DHCR24,MIDN,SLC27A4,RNH1,TRIB2	yellow	VSMC_M3
GO:0042110	0.38	0.70	23	310	T cell activation	BP	1	NCAPH2,SCRIB,LGALS1,SOCS5,ZC3H12A,RELB,METTL3,SOD1,EGR3,DLL4,RIPK3,SART1,BAD,LGALS3,MAD1L1,ARMC5,CORO1A,CD276,CSK,AKT1,AP3D1,STK11,PSMB10	yellow	VSMC_M3

GO:1903050	0.38	0.72	15	192	regulation of proteolysis involved in cellular protein catabolic process	BP	1	BAG6,HSPBP1,SOCS5,PSMC4,WNT10B,CCAR2,RNF19B,GPX1,PSME2,UBXN1,CCDC22,AKT1,SIRT6,TRIB2,STUB1	yellow	VSMC_M3
GO:0000070	0.38	0.73	11	143	mitotic sister chromatid segregation	BP	1	DCTN2,NCAPH2,H2AFY,NUMA1,RRS1,PINX1,NUDC,EML3,MAD1L1,MAD2L2,MIS12	yellow	VSMC_M3
GO:1905897	0.38	0.77	6	70	regulation of response to endoplasmic reticulum stress	BP	1	BAG6,ATF6B,BCL2L1,SELENOS,UBXN1,TXNDC12	yellow	VSMC_M3
GO:0035065	0.38	0.80	4	45	regulation of histone acetylation	BP	1	SMARCB1,EID1,CTBP1,THAP7	yellow	VSMC_M3
GO:0060429	0.38	0.67	55	810	epithelium development	BP	1	CTHRC1,H2AFY,SCRIB,PLEC,SHARPIN,DNPH1,B4GALT1,COL15A1,KLF4,WDPCP,CCM2,SPRED2,RCN3,PLEKHA4,SLC9A3R1,PLXND1,RNF220,NUMA1,FASN,DEAF1,PDE4D,GREM1,KDM2B,WNT10B,SOD1,DLL4,IRX1,PDGFA,MMP14,GPX1,TAGLN2,PCK2,PBX1,RELA,RHOC,PHB2,IGFBP5,BAD,LGALS3,TNFRSF1A,AIMP2,KRT10,CASP14,MTHFR,TMEM79,ZNF358,AKT1,SAFB2,SIRT6,PPL,NSDHL,TJP3,TRADD,ERBB4,NOM1	yellow	VSMC_M3
GO:0045621	0.38	0.77	6	72	positive regulation of lymphocyte differentiation	BP	1	SOCS5,EGR3,MMP14,SART1,BAD,AP3D1	yellow	VSMC_M3
GO:0016052	0.39	0.74	9	113	carbohydrate catabolic process	BP	1	GALK1,OGDHL,MANBA,BAD,FOXK1,PGD,PGM1,GIT1,JMJD8	yellow	VSMC_M3
GO:0016579	0.39	0.75	8	108	protein deubiquitination	BP	1	JOSD2,ZC3H12A,OTUB1,BABAM1,UBXN1,USP27X,USP43,SENP8	yellow	VSMC_M3
GO:0070647	0.39	0.66	57	806	protein modification by small protein conjugation or removal	BP	1	SQSTM1,RING1,JADE2,TRIM11,SHARPIN,VPS28,CUL7,HSPBP1,SOCS5,JOSD2,EIPR1,ZC3H12A,RNF220,SEL1L2,PINX1,KDM2B,CHP1,CISH,RNF19B,MOCS3,ASB9,TRIM27,LIMK1,RELA,OTUB1,COPS6,BABAM1,AIMP2,UBXN1,WASHC1,MED11,USP27X,SDE2,AMER1,MAD2L2,RUSC1,IRF2BPL,TRIM25,USP43,RPL37,SENP8,MID2,ATG3,ANKRD9,AKT1,BLMH,HMG20B,ANAPC2,SPSB3,UBAC1,CTU2,ZNRF1,TRIM7,CDC34,FBXO6,STUB1,NSMCE3	yellow	VSMC_M3
GO:0030336	0.39	0.71	16	223	negative regulation of cell migration	BP	1	PPARD,KLF4,SLC9A3R1,EMILIN1,GREM1,CX3CL1,DLL4,RIN3,CORO1B,ARPIN,PTPN23,IGFBP5,MIIP,AKT1,MIR214,PTPRR	yellow	VSMC_M3
GO:0009057	0.39	0.66	78	1102	macromolecule catabolic process	BP	1	SQSTM1,BAG6,SNX9,HNRNPA0,SGSM3,SHARPIN,VPS28,SND1,CUL7,SECISBP2,HSPBP1,SOCS5,CNOT3,PSMB3,ZC3H12A,RCN3,SNRNP70,SNX3,TMUB1,PSMC4,SEL1L2,METTL3,DND1,HM13,LSM6,WNT10B,PACSIN3,CCAR2,PCBP4,RNF19B,DCPS,SELENOS,MANBA,ZCCHC17,GPX1,ASB9,PSME2,ADRM1,PSMB4,PTPN23,RNASEH2C,KAT5,RELA,MRTO4,MVB12A,UBXN1,CCDC22,USP27X,CAPN2,AMER1,MAD2L2,NANOS3,FBXL19,DFFA,SNF8,TRIM25,USP43,ANKRD9,AKT1,PAIP1,KHSRP,CARHSP1,SIRT6,ANAPC2,SPSB3,MAN1B1,ZNRF1,PSMB10,CTRL,RBM8A,RNH1,TENT5B,TRIB2,CDC34,FBXO6,TRIR,STUB1,CCDC115	yellow	VSMC_M3
GO:0010556	0.39	0.64	196	2846	regulation of macromolecule biosynthetic process	BP	1	ENPP1,NAB2,CDK4,SQSTM1,ZNF608,HDAC10,NELFE,FNIP1,ATF6B,PBX2,RING1,SREBF2,H2AFY,TRIM11,TRAPPC9,PPARD,SND1,SECISBP2,PHF2,RPS9,CNOT3,KLF4,HOXA1,FHL3,ZC3H12A,PNKP,CC2D1B,KTI12,KMT2E,SIX5,ZNF282,ZNF296,RELB,DMAP1,CIC,UNK,OGG1,SERTAD1,TTC5,METTL3,CXXC5,DND1,EIF4EBP1,DEAF1,HOXC4,TARBP2,KMT2B,NAT10,ZNF740,EIF4B,RALY,ZBTB38,PINX1,GREM1,KDM2B,MCRS1,CX3CL1,WNT10B,ACTR5,ANKRD2,EGR3,CCAR2,DNAJC17,DLL4,CHP1,YAF2,HTATIP2,PCIF1,KDM1B,CHCHD2,DCPS,IRX1,SELENOS,RRP1B,BRD9,AIP,NGDN,TSHZ2,QARS,PIR,TRIM27,PRMT2,THOC5,RIPK3,TWIST2,LSM14B,SMARCD2,BRMS1,TACO1,GMEB2,CCDC85B,PRPF6,PBX1,KAT5,RELA,ZNF511,ARNT2,ZFPL1,LIMD1,SMARCB1,CHCHD10,PBX4,UBTF,GATAD2A,MEF2B,PHB2,NDP,IGFBP5,CTDSP1,MLF2,EID1,SSBP4,METTL13,MPV17L2,ZNF394,C11orf95,BUD31,TNFRSF1A,TEAD4,C1QBP,TAF6L,ZBTB49,MTA2,ZNF316,MED11,PELP1,CCDC22,FOXK1,KLF3,PKN1,SEPSECS,POLR2A,MAD2L2,SLTM,NANOS3,CTBP1,FBXL19,CRABP2,SP2,ARHGEF2,SNF8,PER1,NFIX,IRF2BPL,TRIM25,SMARCA4,CARM1,AATF,C19orf66,MID2,ZNF521,ZNF358,ZNF830,AKT1,BRF1,PAIP1,KHSRP,GTF2F1,RFX2,SAFB,SAFB2,MYDGF,HDGFL2,SIRT6,MAP2K2,CACTIN,CDK11B,SSU72,HIC1,TRAP1,MNT,EHMT1,AP3D1,MKNK2,IRAK1,MBD3,MED16,CARD9,SARS,POMT1,TRADD,RBM8A,ERBB4,JUND,KLF13,FADS1,ZNF783,HOXC5,ZNF385A,IER5,AKAP17A,ZNF250,THAP7,NSMCE3,ERF,ZSCAN1	yellow	VSMC_M3
GO:0010498	0.39	0.68	32	434	proteasomal protein catabolic process	BP	1	BAG6,SHARPIN,HSPBP1,SOCS5,PSMB3,RCN3,TMUB1,PSMC4,SEL1L2,HM13,WNT10B,CCAR2,RNF19B,SELENOS,GPX1,PSME2,PSMB4,KAT5,UBXN1,FBXL19,TRIM25,ANKRD9,AKT1,SIRT6,SPSB3,MAN1B1,ZNRF1,PSMB10,TRIB2,CDC34,FBXO6,STUB1	yellow	VSMC_M3
GO:0001649	0.39	0.71	14	190	osteoblast differentiation	BP	1	CTHRC1,SND1,FIGNL1,FASN,LRP3,GREM1,WNT10B,COL6A1,TWIST2,MRC2,LIMD1,IGFBP5,AKT1,JUND	yellow	VSMC_M3

GO:0033036	0.39	0.64	162	2356	macromolecule localization	BP	1	ENPP1,PQLC1,CD63,ESYT1,DCTN2,NHP2,RUFY1,SQSTM1,CCHCR1,BAG6,SNX9,SAMM50,SREBF2,SQLE,H2AFY,SGSM3,GUK1,PACSIN1,PPARD,SCRIB,PLEC,IFT27,VPS28,TOM1,TTC7A,ASNA1,TPBG,PLEKHA8,WDPCP,EIPR1,ZC3H12A,PDIA4,RCN3,SNX3,EHD2,SLC9A3R1,SERP2,DMAP1,SNX17,INSIG1,MYRIP,SEC13,SEPT9,UCP2,BAIAP2,CEP131,TSPAN10,LAMTOR1,NUMA1,YIF1B,RAB20,ARHGAP33,AACS,SCARB1,HM13,BCL2L1,ITGB7,RRS1,SWAP70,PLEKHF1,ACSL1,PINX1,MCRS1,RAPSN,CHP1,AP2A2,SNX21,RAB35,RBM15B,TSPAN5,APBA2,SELENOS,EXOC3,FABP3,MMP14,PABPN1,AIP,AP1G2,CORO1B,PCK2,NECTIN1,TRIM27,PCNT,SPX,THOC5,CAPN10,PLA2G3,PRPF6,PTPN23,SCYL1,LRWD1,VPS51,PEX5,ZDHHC3,CHCHD10,YJEFN3,THOC3,PHB2,BAD,COPE,LAMTOR4,LGALS3,TNFRSF1A,MVB12A,KIAA0753,NUP88,WASHC1,RCC2,AP1M1,RAB3IL1,CCDC22,GLUD1,DBN1,SNX8,LMAN2,RAB24,CLTB,ARHGEF2,SNF8,MVP,MTX1,RANGRF,STX10,SGF29,RABEP2,TRIM25,SLC27A3,TMED1,SYNE3,CDC37,CSK,MID2,ATG3,RBM22,AKT1,SNX18,PACS2,KHSRP,ALKBH7,RANBP3,RAB34,DHCR24,TJP3,CTNS,AP3D1,SSNA1,MIDN,STK11,GAS8,SLC27A4,NABP2,RBM8A,ERBB4,TWF2,AP5B1,JAGN1,CAVIN1,MIS12,ZNF385A,ARFRP1,MEIG1,TIMM22	yellow	VSMC_M3
GO:0009746	0.39	0.72	12	159	response to hexose	BP	1	PPARD,LGALS1,UCP2,AACS,SELENOS,PCK2,SLC26A6,SMARCB1,BAD,SMARCA4,JAGN1,COL6A2	yellow	VSMC_M3
GO:1903076	0.39	0.75	7	90	regulation of protein localization to plasma membrane	BP	1	SQSTM1,BCL2L1,MMP14,LGALS3,RANGRF,CSK,AKT1	yellow	VSMC_M3
GO:0032502	0.39	0.63	309	4589	developmental process	BP	1	ENPP1,CD63,NAB2,CDK4,SQSTM1,CCHCR1,BAG6,NCAPH2,SBF1,CTHRC1,HDAC10,FNIP1,PBX2,PDLIM4,RING1,H2AFY,TRIM11,TRAPPC9,PACSIN1,PPARD,SCRIB,PLEC,LGALS1,MPST,IFT27,SHARPIN,SND1,TBCC,CUL7,DNPH1,B4GALT1,PDGFRA,SECISBP2,PHF2,COL15A1,EPN1,COL19A1,SOCS5,TTC7A,CNOT3,KLF4,DBNL,TPBG,HOXA1,WDPCP,CCM2,FHL3,SPRED2,ZC3H12A,FIGNL1,RCN3,SNX3,PLEKHA4,EHD2,FAM120B,KMT2E,KRT8,WDFY2,SIX5,ZNF296,RELB,SLC9A3R1,PLXND1,EMILIN1,RNF220,PAFAH1B3,CIC,SNX17,UNK,DLEC1,CD79A,INSIG1,OGG1,PSMC4,UCP2,METTL3,BAIAP2,CEP131,INPPL1,NUMA1,P4HB,DND1,YIF1B,FASN,RPL29,EIF4EBP1,MORN2,RIC8A,ARHGAP22,DEAF1,TBCB,HOXC4,LRIG1,ARHGAP33,AACS,HM13,BCL2L1,ITGB7,PDE4D,TNS2,SWAP70,LRP3,CAPG,SLC11A2,MMP24,GREM1,KDM2B,AQP5,PDE3B,DCTN1,CX3CL1,WNT10B,SOD1,CCDC32,ANKRD2,EGR3,DLL4,TYRO3,HTATIP2,RAB35,APBA2,IRX1,SDHA,FABP3,PAPSS1,PDGFA,MMP14,C14orf93,GPX1,CORO1B,NGEF,HCCS,CLCF1,PLEKHO1,TAGLN2,IGSF9,QARS,LRFN4,PCK2,COL6A1,IMPDH2,PIR,NECTIN1,ANPEP,TRIM27,ARPIN,LIMK1,SLC26A6,PRMT2,THOC5,RIPK3,TWIST2,EXTL1,IQCG,TNK2,PLA2G3,STMN3,SART1,CCDC85B,MRC2,PBX1,KAT5,RELA,RHOC,TMEM120A,SCYL1,IDH1,FMNL1,ARNT2,VPS51,LIMD1,SGPL1,SMARCB1,PBX4,YJEFN3,ATIC,BLOC1S4,MEF2B,PHB2,NDP,IGFBP5,C12orf57,BAD,AAMP,CTDSP1,EID1,HSD17B1,LGALS3,CD9,TNFRSF1A,TEAD4,C1QBP,AIMP2,TAF6L,CHERP,EVC,RCC2,CNP,AP1M1,PRXL2A,KRT10,GLUD1,FOXK1,CASP14,KLF3,PKN1,DBN1,CAPN2,MAD1L1,AMER1,MTHFR,MAD2L2,NANOS3,PRR14,CTBP1,CRABP2,SP2,TMEM79,ARHGEF2,ARMC5,MTX1,[illegible]	yellow	VSMC_M3
GO:0046209	0.40	0.77	5	60	nitric oxide metabolic process	BP	1	KLF4,ZC3H12A,SELENOS,AKT1,KHSRP	yellow	VSMC_M3
GO:1904659	0.40	0.76	6	80	glucose transmembrane transport	BP	1	ENPP1,PPARD,SELENOS,CAPN10,AKT1,SLC27A4	yellow	VSMC_M3
GO:0034976	0.40	0.70	17	222	response to endoplasmic reticulum stress	BP	1	BAG6,ATF6B,PDIA4,RCN3,SERP2,TMUB1,SEL1L2,P4HB,HM13,BCL2L1,SELENOS,UBXN1,TRIM25,MAN1B1,TXNDC12,FBXO6,STUB1	yellow	VSMC_M3
GO:0032446	0.40	0.66	50	703	protein modification by small protein conjugation	BP	1	SQSTM1,RING1,JADE2,TRIM11,SHARPIN,VPS28,CUL7,HSPBP1,SOCS5,EIPR1,RNF220,SEL1L2,PINX1,KDM2B,CHP1,CISH,RNF19B,MOCS3,ASB9,TRIM27,LIMK1,RELA,OTUB1,AIMP2,UBXN1,WASHC1,MED11,SDE2,AMER1,MAD2L2,RUSC1,IRF2BPL,TRIM25,RPL37,MID2,ATG3,ANKRD9,AKT1,BLMH,HMG20B,ANAPC2,SPSB3,UBAC1,CTU2,ZNRF1,TRIM7,CDC34,FBXO6,STUB1,NSMCE3	yellow	VSMC_M3
GO:0006493	0.40	0.76	6	74	protein O-linked glycosylation	BP	1	GALNT14,SLC35C2,ST3GAL4,TMTC1,DPM3,POMT1	yellow	VSMC_M3
GO:0035194	0.40	0.73	9	112	post-transcriptional gene silencing by RNA	BP	1	ZC3H12A,METTL3,DND1,TARBP2,LIMD1,MAP2K2,MIR214,MIR155,MIR374B	yellow	VSMC_M3
GO:0010976	0.40	0.72	10	140	positive regulation of neuron projection development	BP	1	PACSIN1,DBNL,SNX3,BAIAP2,CX3CL1,PLA2G3,DBN1,EEF2K,NEGR1,TWF2	yellow	VSMC_M3
GO:0022411	0.40	0.68	26	375	cellular component disassembly	BP	1	SQSTM1,SREBF2,SHARPIN,MICAL1,SWAP70,CAPG,DCTN1,CX3CL1,MMP14,LIX1L,APEH,CAPN10,SMARCD2,STMN3,MID1IP1,SMARCB1,PHB2,WASHC1,DFFA,ARHGEF2,SMARCA4,CDC37,C19orf66,ATG3,TAZ,TWF2	yellow	VSMC_M3
GO:0045582	0.40	0.77	5	60	positive regulation of T cell differentiation	BP	1	SOCS5,EGR3,SART1,BAD,AP3D1	yellow	VSMC_M3
GO:1901699	0.40	0.67	35	508	cellular response to nitrogen compound	BP	1	ENPP1,CDK4,PDGFRA,KLF4,SLC9A3R1,INSIG1,UCP2,BAIAP2,LAMTOR1,BCL2L1,TNS2,PDE3B,WNT10B,SOD1,SELENOS,PCK2,COL6A1,SLC26A6,CAPN10,RELA,IGFBP5,LAMTOR4,KLF3,CAPN2,PIP4K2B,ARHGEF2,EEF2K,CSK,AKT1,SLC6A4,LDOC1,APRT,SLC27A4,NSMF,NSMCE3	yellow	VSMC_M3
GO:0019218	0.40	0.75	6	73	regulation of steroid metabolic process	BP	1	INSIG1,LAMTOR1,SOD1,DHCR7,ARMC5,STUB1	yellow	VSMC_M3
GO:0002088	0.40	0.77	5	63	lens development in camera-type eye	BP	1	CDK4,SPRED2,SIX5,NECTIN1,CTNS	yellow	VSMC_M3
GO:0000302	0.40	0.70	14	177	response to reactive oxygen species	BP	1	PDGFRA,KLF4,UCP2,PYCR1,SOD1,GPX1,RIPK3,RELA,TXNRD2,BAD,CAPN2,AKT1,TRAP1,OSER1	yellow	VSMC_M3
GO:0051170	0.40	0.72	10	132	import into nucleus	BP	1	ZC3H12A,DMAP1,SEC13,CHP1,HTATIP2,PHB2,NUP88,RANGRF,RBM22,AKT1	yellow	VSMC_M3
GO:0061077	0.40	0.77	5	55	chaperone-mediated protein folding	BP	1	PDIA4,FKBP11,FKBP2,DFFA,TRAP1	yellow	VSMC_M3

GO:0051248	0.40	0.65	58	813	negative regulation of protein metabolic process	BP	1	ENPP1,CTDSP2,SQSTM1,BAG6,FNIP1,H2AFY,PLEC,VPS28,SOCS5,CNOT3,KLF4,SPRED2,ZC3H12A,PNKP,SNRNP70,SNX3,MICAL1,EMILIN1,UNK,METTL3,DND1,EIF4EBP1,TARBP2,PDE4D,SWAP70,PINX1,GREM1,CCAR2,CHP1,PCIF1,DCPS,GPX1,QARS,TRIM27,LIMK1,PFDN2,RELA,SMARCB1,IGFBP5,CTDSP1,OTUB1,LGALS3,UBXN1,PKN1,MAD2L2,NANOS3,CTBP1,MVP,RPL37,AATF,C19orf66,AKT1,KHSRP,DHCR24,CACTIN,HMG20B,TRIB2,THAP7	yellow	VSMC_M3
GO:0000281	0.40	0.76	5	63	mitotic cytokinesis	BP	1	SNX9,CUL7,RAB35,RHOC,SNX18	yellow	VSMC_M3
GO:0010970	0.40	0.72	10	139	transport along microtubule	BP	1	IFT27,CEP131,SOD1,BLOC1S4,AP3D1,SSNA1,STK11,TMEM201,SPG7,WDR34	yellow	VSMC_M3
GO:0006909	0.40	0.71	12	163	phagocytosis	BP	1	SNX3,RAB20,SCARB1,SOD1,TYRO3,LIMK1,ANXA11,LMAN2,CORO1A,CSK,ATG3,RAB34	yellow	VSMC_M3
GO:0042773	0.40	0.73	8	83	ATP synthesis coupled electron transport	BP	1	NDUFB9,CHCHD2,SDHA,NDUFV1,UQCRC1,UQCC3,NDUFS7,TAZ	yellow	VSMC_M3
GO:0042775	0.40	0.73	8	83	mitochondrial ATP synthesis coupled electron transport	BP	1	NDUFB9,CHCHD2,SDHA,NDUFV1,UQCRC1,UQCC3,NDUFS7,TAZ	yellow	VSMC_M3
GO:0006464	0.40	0.63	196	2859	cellular protein modification process	BP	1	ENPP1,NAB2,CDK4,CTDSP2,SQSTM1,BAG6,SBF1,SNX9,HDAC10,NELFE,FNIP1,PRR5,TTLL12,RING1,JADE2,H2AFY,TRIM11,PLEC,SHARPIN,VPS28,CUL7,B4GALT1,PIGO,PDGFRA,PHF2,HSPBP1,SOCS5,JOSD2,SPRED2,EIPR1,MEAF6,ZC3H12A,PNKP,MICAL1,KMT2E,WDFY2,SLC9A3R1,SERP2,NAT9,EMILIN1,SEPHS1,RNF220,DMAP1,PPM1G,GALNT14,SERTAD1,KAT14,P4HA3,SEL1L2,P4HB,TARBP2,KMT2B,NAT10,PDE4D,TNS2,SWAP70,ACSL1,TNKS1BP1,PINX1,SETD1B,GREM1,KDM2B,MCRS1,CX3CL1,FKBP11,SOD1,CCAR2,CHP1,TYRO3,HTATIP2,PPM1M,KDM1B,SLC35C2,CISH,ST3GAL4,RNF19B,ETFBKMT,MANBA,TMTC1,STK32C,PDGFA,GPX1,AIP,HCCS,MOCS3,CLCF1,QARS,ASB9,TRIM27,LIMK1,RABGGTA,PRMT2,RIPK3,EXTL1,BRMS1,TNK2,PTPN23,KAT5,RELA,SCYL1,ALKBH4,MEX3B,MAP3K14,ZDHHC3,SMARCB1,PHB2,TRMT112,FKBP2,CTDSP1,OTUB1,EID1,COPS6,LGALS3,TNFRSF1A,BABAM1,AIMP2,TAF6L,UBXN1,WASHC1,MTA2,MED11,USP27X,KRT10,SDE2,PKN1,AMER1,MTHFR,MAD2L2,MPDU1,CTBP1,FBXL19,ARHGEF2,RUSC1,MVP,PER1,DPM3,PPP4C,SGF29,IRF2BPL,TRIM25,USP43,RPL37,MTMR4,CARM1,SENP8,EEF2K,CDC37,CSK,MID2,ATG3,ANKRD9,AKT1,COPRS,SGK494,BLMH,MYDGF,METTL22,SIRT6,MAP2K2,CACTIN,HMG20B,CDK11B,SSU72,EHMT1,PGP,MKNK2,CSNK1G2,IRAK1,ANAPC2,SPSB3,MBD3,MAN1B1,STK11,TELO2,UBAC1,CTU2,POMT1,NTMT1,ZNRF1,TGFBR3,B3GNT9,NDST1,ERBB4,PTPRR,LOXL1,PIN4,TRIM7,TRIB2,NSMF,CDC34,FBXO6,DUSP5,THAP7,STUB1,NSMCE3,RCE1	yellow	VSMC_M3
GO:0036211	0.40	0.63	196	2859	protein modification process	BP	1	ENPP1,NAB2,CDK4,CTDSP2,SQSTM1,BAG6,SBF1,SNX9,HDAC10,NELFE,FNIP1,PRR5,TTLL12,RING1,JADE2,H2AFY,TRIM11,PLEC,SHARPIN,VPS28,CUL7,B4GALT1,PIGO,PDGFRA,PHF2,HSPBP1,SOCS5,JOSD2,SPRED2,EIPR1,MEAF6,ZC3H12A,PNKP,MICAL1,KMT2E,WDFY2,SLC9A3R1,SERP2,NAT9,EMILIN1,SEPHS1,RNF220,DMAP1,PPM1G,GALNT14,SERTAD1,KAT14,P4HA3,SEL1L2,P4HB,TARBP2,KMT2B,NAT10,PDE4D,TNS2,SWAP70,ACSL1,TNKS1BP1,PINX1,SETD1B,GREM1,KDM2B,MCRS1,CX3CL1,FKBP11,SOD1,CCAR2,CHP1,TYRO3,HTATIP2,PPM1M,KDM1B,SLC35C2,CISH,ST3GAL4,RNF19B,ETFBKMT,MANBA,TMTC1,STK32C,PDGFA,GPX1,AIP,HCCS,MOCS3,CLCF1,QARS,ASB9,TRIM27,LIMK1,RABGGTA,PRMT2,RIPK3,EXTL1,BRMS1,TNK2,PTPN23,KAT5,RELA,SCYL1,ALKBH4,MEX3B,MAP3K14,ZDHHC3,SMARCB1,PHB2,TRMT112,FKBP2,CTDSP1,OTUB1,EID1,COPS6,LGALS3,TNFRSF1A,BABAM1,AIMP2,TAF6L,UBXN1,WASHC1,MTA2,MED11,USP27X,KRT10,SDE2,PKN1,AMER1,MTHFR,MAD2L2,MPDU1,CTBP1,FBXL19,ARHGEF2,RUSC1,MVP,PER1,DPM3,PPP4C,SGF29,IRF2BPL,TRIM25,USP43,RPL37,MTMR4,CARM1,SENP8,EEF2K,CDC37,CSK,MID2,ATG3,ANKRD9,AKT1,COPRS,SGK494,BLMH,MYDGF,METTL22,SIRT6,MAP2K2,CACTIN,HMG20B,CDK11B,SSU72,EHMT1,PGP,MKNK2,CSNK1G2,IRAK1,ANAPC2,SPSB3,MBD3,MAN1B1,STK11,TELO2,UBAC1,CTU2,POMT1,NTMT1,ZNRF1,TGFBR3,B3GNT9,NDST1,ERBB4,PTPRR,LOXL1,PIN4,TRIM7,TRIB2,NSMF,CDC34,FBXO6,DUSP5,THAP7,STUB1,NSMCE3,RCE1	yellow	VSMC_M3
GO:0043434	0.40	0.68	22	308	response to peptide hormone	BP	1	ENPP1,CDK4,INSIG1,UCP2,BAIAP2,INPPL1,TNS2,PDE3B,SELENOS,FABP3,PCK2,CAPN10,RELA,IGFBP5,PIP4K2B,EEF2K,CSK,AKT1,APRT,SLC27A4,TGFBR3,JUND	yellow	VSMC_M3
GO:0006119	0.40	0.71	11	119	oxidative phosphorylation	BP	1	NDUFB9,CHCHD2,SDHA,NDUFV1,UQCRC1,CHCHD10,UQCC3,ATP5PD,NDUFS7,ATP5F1D,TAZ	yellow	VSMC_M3
GO:0033077	0.41	0.76	5	61	T cell differentiation in thymus	BP	1	NCAPH2,SOD1,EGR3,RIPK3,STK11	yellow	VSMC_M3
GO:0008652	0.41	0.76	5	56	cellular amino acid biosynthetic process	BP	1	SEPHS1,PYCR1,GLUD1,ILVBL,MTHFR	yellow	VSMC_M3
GO:0007276	0.41	0.66	34	469	gamete generation	BP	1	BAG6,NCAPH2,SBF1,IFT27,FIGNL1,SIX5,ZNF296,PAFAH1B3,DLEC1,METTL3,CEP131,DND1,YIF1B,MORN2,DEAF1,BCL2L1,KDM2B,SOD1,TYRO3,KDM1B,TRIM27,SLC26A6,IQCG,PLA2G3,SGPL1,BAD,NANOS3,ZNF830,AKT1,RFX2,STK11,TAZ,FAM50A,MEIG1	yellow	VSMC_M3
GO:0032890	0.41	0.78	4	47	regulation of organic acid transport	BP	1	ACSL1,FABP3,PLA2G3,AKT1	yellow	VSMC_M3
GO:0005996	0.41	0.69	15	199	monosaccharide metabolic process	BP	1	ENPP1,PPARD,B4GALT1,GALK1,INPPL1,SELENOS,GSTO1,PCK2,BAD,FOXK1,PGD,AKT1,PGM1,PGP,MIDN	yellow	VSMC_M3
GO:0048662	0.41	0.78	4	44	negative regulation of smooth muscle cell proliferation	BP	1	PPARD,KLF4,IGFBP5,MIR214	yellow	VSMC_M3
GO:0048706	0.41	0.73	8	105	embryonic skeletal system development	BP	1	PDGFRA,HOXA1,DEAF1,HOXC4,MMP14,PBX1,SLC39A1,NDST1	yellow	VSMC_M3
GO:0032870	0.41	0.66	31	450	cellular response to hormone stimulus	BP	1	ENPP1,CDK4,PPARD,INSIG1,UCP2,BAIAP2,TNS2,ACSL1,PDE3B,WNT10B,SELENOS,PCK2,PRMT2,CAPN10,RELA,PHB2,PELP1,PKN1,PIP4K2B,PER1,SMARCA4,CARM1,EEF2K,CSK,AKT1,HMGCS1,SAFB,SAFB2,APRT,SLC27A4,NSMF	yellow	VSMC_M3

GO:0031330	0.41	0.69	15	207	negative regulation of cellular catabolic process	BP	1	BAG6,HNRNPA0,SECISBP2,SNRNP70,DND1,CCAR2,ETFBKMT,ZCCHC17,UBXN1,WASHC1,FOXK1,DFFA,AKT1,PAIP1,TENT5B	yellow	VSMC_M3
GO:0051168	0.41	0.71	10	131	nuclear export	BP	1	CCHCR1,RRS1,CHP1,RBM15B,PABPN1,THOC5,THOC3,NUP88,RBM22,RANBP3	yellow	VSMC_M3
GO:0006520	0.41	0.68	18	224	cellular amino acid metabolic process	BP	1	MPST,AARS2,SEPHS1,BCKDHA,PYCR1,PEPD,GATB,ACCS,CARS,QARS,THAP4,GLUD1,ILVBL,MTHFR,FARSA,BLMH,CTNS,SARS	yellow	VSMC_M3
GO:0120035	0.41	0.66	35	524	regulation of plasma membrane bounded cell projection organization	BP	1	PACSIN1,LGALS1,CUL7,KLF4,DBNL,WDPCP,SNX3,PLXND1,SEPT9,BAIAP2,ARHGAP33,CX3CL1,CORO1B,NGEF,ARPIN,LIMK1,PLA2G3,WASHC1,RCC2,DBN1,CRABP2,RABEP2,CARM1,EEF2K,ATG3,DOCK11,AKT1,ACAP3,ANAPC2,STK11,NEGR1,MIR214,TWF2,CDC42EP5,NSMF	yellow	VSMC_M3
GO:0032269	0.41	0.64	54	760	negative regulation of cellular protein metabolic process	BP	1	ENPP1,CTDSP2,SQSTM1,BAG6,FNIP1,H2AFY,PLEC,VPS28,SOCS5,CNOT3,KLF4,SPRED2,ZC3H12A,PNKP,MICAL1,EMILIN1,UNK,METTL3,DND1,EIF4EBP1,TARBP2,PDE4D,SWAP70,PINX1,GREM1,CCAR2,CHP1,PCIF1,DCPS,GPX1,QARS,TRIM27,LIMK1,RELA,SMARCB1,IGFBP5,CTDSP1,OTUB1,LGALS3,UBXN1,PKN1,MAD2L2,NANOS3,CTBP1,MVP,RPL37,C19orf66,AKT1,KHSRP,DHCR24,CACTIN,HMG20B,TRIB2,THAP7	yellow	VSMC_M3
GO:1901983	0.41	0.76	5	63	regulation of protein acetylation	BP	1	KAT5,SMARCB1,EID1,CTBP1,THAP7	yellow	VSMC_M3
GO:0043009	0.42	0.65	36	522	chordate embryonic development	BP	1	CTHRC1,SCRIB,PDGFRA,EPN1,CNOT3,KLF4,HOXA1,CCM2,KRT8,RNF220,UNK,PSMC4,RIC8A,DEAF1,HOXC4,HM13,BCL2L1,KDM2B,MMP14,STMN3,PBX1,ARNT2,SMARCB1,DBN1,CAPN2,MTHFR,ARMC5,SLC39A1,ZNF358,ZNF830,AKT1,NSDHL,SLC35E2B,MBD3,NDST1,PTPRR	yellow	VSMC_M3
GO:0050918	0.42	0.77	4	47	positive chemotaxis	BP	1	SCRIB,CX3CL1,LGALS3,CORO1A	yellow	VSMC_M3
GO:0072657	0.42	0.65	33	466	protein localization to membrane	BP	1	SQSTM1,BAG6,SAMM50,PACSIN1,SCRIB,TTC7A,ASNA1,TPBG,EHD2,SLC9A3R1,YIF1B,BCL2L1,ITGB7,PLEKHF1,RAPSN,CHP1,TSPAN5,MMP14,PEX5,ZDHHC3,LGALS3,TNFRSF1A,RANGRF,SYNE3,CSK,ATG3,AKT1,PACS2,RAB34,AP3D1,SSNA1,ARFRP1,TIMM22	yellow	VSMC_M3
GO:0048522	0.42	0.61	287	4212	positive regulation of cellular process	BP	1	CD63,NAB2,CDK4,SQSTM1,BAG6,SNX9,CTHRC1,HDAC10,NELFE,FNIP1,ATF6B,PBX2,PRR5,SREBF2,TXNDC15,H2AFY,ITPR3,PACSIN1,PPARD,EEF1D,TSTA3,SCRIB,LGALS1,SHARPIN,VPS28,CUL7,DNPH1,B4GALT1,PDGFRA,IL1R1,PHF2,HSPBP1,SOCS5,RPS9,CNOT3,KLF4,DBNL,TPBG,HOXA1,SPRED2,EIPR1,ZC3H12A,PNKP,RCN3,SNRNP70,SNX3,PLEKHA4,EHD2,KMT2E,WDFY2,SIX5,ZNF296,SLC9A3R1,PLXND1,EMILIN1,RNF220,DMAP1,H3F3B,MYRIP,SEC13,SEPT9,SERTAD1,TTC5,PSMC4,UCP2,METTL3,PHLDA1,BAIAP2,CEP131,CXXC5,LAMTOR1,NUMA1,P4HB,DND1,CCDC57,EIF4EBP1,CAVIN3,DEAF1,HOXC4,KMT2B,AACS,SCARB1,NAT10,BCL2L1,SWAP70,LRP3,PLEKHF1,ACSL1,ZBTB38,TNKS1BP1,PINX1,GREM1,KDM2B,MCRS1,DCTN1,RAPSN,CX3CL1,WNT10B,SOD1,PACSIN3,DGKZ,EGR3,CCAR2,DLL4,CHP1,TYRO3,YAF2,HTATIP2,PCIF1,KDM1B,CHCHD2,SLC35C2,TSPAN5,ST3GAL4,RNF19B,DCPS,GSTO1,RRP1B,FABP3,PDGFA,MMP14,GPX1,AIP,CORO1B,CLCF1,SYT12,PCK2,PSME2,TRIM27,ARPIN,PCNT,LIMK1,SLC26A6,PRMT2,THOC5,RIPK3,TWIST2,ADRM1,CAPN10,SMARCD2,PFDN2,BRMS1,BUD23,TNK2,PLA2G3,GMEB2,SART1,PRPF6,PTPN23,PBX1,KAT5,RELA,RHOC,MAP3K14,ARNT2,MID1IP1,ZDHHC3,SMARCB1,CHCHD10,PBX4,UBTF,MEF2B,PHB2,TRMT112,NDP,IGFBP5,BAD,LAMTOR4,IFI35,AAMP,SSBP4,MPV17L2,LGALS3,BUD31,ARPC1B,PLAU,TNFRSF1A,BABAM1,C1QBP,AIMP2,TAF6L,CHERP,ZBTB49,WASHC1,EVC,RCC2,MTA2,PELP1,CCDC22,USP27X,GLUD1,FOXK1,ELP5,H3F3A,PKN1,DBN1,CAPN2,LMAN2,POLR2A,AMER1,PIP4K2B,MAD2L2,NANOS3,CTBP1,CRABP2,DFFA,ARHGEF2,SNF8,PER1,NFIX,RANGRF,CORO1A,SLX1A,IRF2BPL,TRIM25,RPL37,S1[illegible]	yellow	VSMC_M3
GO:0070265	0.42	0.77	4	45	necrotic cell death	BP	1	RIPK3,ARHGEF2,ALKBH7,MIR214	yellow	VSMC_M3
GO:0045619	0.42	0.71	9	117	regulation of lymphocyte differentiation	BP	1	SOCS5,ZC3H12A,METTL3,SOD1,EGR3,MMP14,SART1,BAD,AP3D1	yellow	VSMC_M3
GO:0016567	0.42	0.64	46	656	protein ubiquitination	BP	1	SQSTM1,RING1,JADE2,TRIM11,SHARPIN,VPS28,CUL7,HSPBP1,SOCS5,EIPR1,RNF220,SEL1L2,PINX1,KDM2B,CHP1,CISH,RNF19B,ASB9,TRIM27,LIMK1,OTUB1,AIMP2,UBXN1,WASHC1,MED11,SDE2,AMER1,MAD2L2,RUSC1,IRF2BPL,TRIM25,RPL37,MID2,ATG3,ANKRD9,AKT1,BLMH,ANAPC2,SPSB3,UBAC1,ZNRF1,TRIM7,CDC34,FBXO6,STUB1,NSMCE3	yellow	VSMC_M3
GO:0010812	0.42	0.77	4	53	negative regulation of cell-substrate adhesion	BP	1	LGALS1,CX3CL1,MMP14,RCC2	yellow	VSMC_M3
GO:0006639	0.42	0.73	7	92	acylglycerol metabolic process	BP	1	MGLL,INSIG1,SCARB1,ACSL1,DGKZ,GPX1,PCK2	yellow	VSMC_M3
GO:0031667	0.42	0.66	26	362	response to nutrient levels	BP	1	FNIP1,SREBF2,PPARD,PLEC,ZC3H12A,OGG1,UCP2,EIF4EBP1,AACS,ACSL1,SOD1,DCPS,PCK2,SPX,RELA,FOXK1,MTHFR,TRIM25,AKT1,HMGCS1,SLC6A4,SIRT6,MBD3,WDR24,SLC27A4,FADS1	yellow	VSMC_M3
GO:0051051	0.42	0.66	23	314	negative regulation of transport	BP	1	ENPP1,SREBF2,PACSIN1,SNX3,SLC9A3R1,INSIG1,UCP2,PACSIN3,CHP1,SELENOS,GSTO1,RIN3,TRIM27,SPX,LGALS3,TNFRSF1A,CORO1A,CSK,ATG3,AKT1,ANKRD13B,MIDN,JAGN1	yellow	VSMC_M3

GO:0022607	0.42	0.61	153	2230	cellular component assembly	BP	1	SPAG1,BAG6,SNX9,FNIP1,SAMM50,NDUFB9,SQLE,TXNDC15,H2AFY,RPL3,PLEC,BOP1,VPS28,TBCC,PDGFRA,PHF2,DBNL,TPBG,WDPCP,ZC3H12A,ZYX,MICAL1,EMP3,EHD2,KRT8,ACAD9,GEMIN7,SLC9A3R1,PLXND1,EMILIN1,H3F3B,SEPT9,BAIAP2,CEP131,INPPL1,NUMA1,YIF1B,CCDC57,RAB20,EIF4EBP1,SDHAF1,TARBP2,EIF4B,RRS1,SWAP70,UQCRFS1,CAPG,GREM1,AQP5,DCTN1,CX3CL1,WNT10B,HPS1,CHP1,SRSF9,CCDC28B,PDGFA,MMP14,CORO1B,HCCS,LRFN4,NECTIN1,TRIM27,ARPIN,PCNT,EIF3A,LIMK1,SF3A1,ADRM1,IQCG,TACO1,PLA2G3,CEP164,SART1,PRPF6,PTPN23,RHOC,TMEM120A,SAC3D1,LIMD1,MID1IP1,PEX5,SMARCB1,CHCHD10,UBTF,BAD,MPV17L2,LETM1,LGALS3,ARPC1B,CD9,MRTO4,FCHO1,MVB12A,KIAA0753,C1QBP,AIMP2,TAF6L,UQCC3,WASHC1,RCC2,EML3,KRT10,H3F3A,DBN1,PIP4K2B,CLTB,SNF8,CD2BP2,RANGRF,FARSA,CORO1A,SLX1A,RABEP2,SMARCA4,EEF2K,EIF3G,ATG3,TTC39C,DOCK11,BRF1,PACS2,RFX2,RAB34,GIT1,UBN1,MRPL20,NCLN,CLDN6,NUBP2,NDUFS7,ATP5F1D,TAZ,CARD9,GAS8,SPG7,WDR34,TGFBR3,MIR214,ERBB4,TWF2,SURF1,MIS12,ELN,UNC13A,CDC42EP5,CDC34,GBA2,TUBGCP2,MEIG1,STUB1,CCDC115,ARPC5L	yellow	VSMC_M3
GO:0010927	0.43	0.73	6	87	cellular component assembly involved in morphogenesis	BP	1	PLEC,PDGFRA,KRT8,PLA2G3,CD9,RFX2	yellow	VSMC_M3
GO:0030073	0.43	0.69	11	149	insulin secretion	BP	1	PPARD,EIPR1,MYRIP,UCP2,AACS,PCK2,CAPN10,BAD,GLUD1,MIDN,JAGN1	yellow	VSMC_M3
GO:0045185	0.43	0.73	6	80	maintenance of protein location	BP	1	INSIG1,CHCHD10,CCDC22,DBN1,AKT1,TWF2	yellow	VSMC_M3
GO:0097306	0.43	0.74	5	71	cellular response to alcohol	BP	1	CDK4,KLF4,AACS,AKT1,HMGCS1	yellow	VSMC_M3
GO:0031056	0.43	0.70	9	124	regulation of histone modification	BP	1	NELFE,H2AFY,KMT2E,SMARCB1,OTUB1,EID1,MTHFR,CTBP1,THAP7	yellow	VSMC_M3
GO:0044706	0.43	0.69	12	159	multi-multicellular organism process	BP	1	PPARD,B4GALT1,EPN1,UCP2,RPL29,SOD1,SLC19A1,IGFBP5,C1QBP,CAPN2,AKT1,SLC6A4	yellow	VSMC_M3
GO:0016054	0.43	0.67	15	186	organic acid catabolic process	BP	1	ACAT2,PPARD,MPST,CPT1C,BCKDHA,GNPDA1,PCCB,ETFBKMT,PCK2,GLUD1,ILVBL,PGD,AKT1,BLMH,SLC27A4	yellow	VSMC_M3
GO:0007586	0.43	0.74	5	63	digestion	BP	1	SCARB1,AQP5,PIR,SLC26A6,ZNF830	yellow	VSMC_M3
GO:0007519	0.43	0.70	9	122	skeletal muscle tissue development	BP	1	PLEC,COL19A1,WNT10B,GPX1,HMG20B,TAZ,GTF3C5,IGSF8,ELN	yellow	VSMC_M3
GO:0070527	0.43	0.76	4	51	platelet aggregation	BP	1	PDGFRA,TYRO3,BLOC1S4,CD9	yellow	VSMC_M3
GO:0045321	0.43	0.63	42	586	leukocyte activation	BP	1	BAG6,NCAPH2,FNIP1,SCRIB,LGALS1,SOCS5,ZC3H12A,KMT2E,RELB,CD79A,METTL3,SWAP70,CX3CL1,SOD1,EGR3,DLL4,TYRO3,MMP14,CLCF1,IMPDH2,RIPK3,PLA2G3,SART1,PBX1,PHB2,BAD,IFI35,LGALS3,PKN1,MAD1L1,MAD2L2,ARMC5,CORO1A,CD276,CSK,DOCK11,AKT1,AP3D1,STK11,PSMB10,JUND,AKAP17A	yellow	VSMC_M3
GO:0097529	0.43	0.69	11	142	myeloid leukocyte migration	BP	1	IL1R1,EMILIN1,SWAP70,GREM1,CX3CL1,RIN3,MMP14,LGALS3,CD9,C1QBP,JAGN1	yellow	VSMC_M3
GO:1904375	0.43	0.71	8	107	regulation of protein localization to cell periphery	BP	1	SQSTM1,NUMA1,BCL2L1,MMP14,LGALS3,RANGRF,CSK,AKT1	yellow	VSMC_M3
GO:0034284	0.43	0.68	12	164	response to monosaccharide	BP	1	PPARD,LGALS1,UCP2,AACS,SELENOS,PCK2,SLC26A6,SMARCB1,BAD,SMARCA4,JAGN1,COL6A2	yellow	VSMC_M3
GO:0050851	0.43	0.70	9	117	antigen receptor-mediated signaling pathway	BP	1	ZC3H12A,CD79A,PDE4D,DGKZ,RELA,LGALS3,CD276,CSK,STK11	yellow	VSMC_M3
GO:0070646	0.43	0.70	9	125	protein modification by small protein removal	BP	1	JOSD2,ZC3H12A,OTUB1,COPS6,BABAM1,UBXN1,USP27X,USP43,SENP8	yellow	VSMC_M3
GO:1903038	0.43	0.72	7	87	negative regulation of leukocyte cell-cell adhesion	BP	1	SCRIB,SOCS5,KLF4,ZC3H12A,LGALS3,MAD1L1,AKT1	yellow	VSMC_M3
GO:0046854	0.43	0.76	4	54	phosphatidylinositol phosphate biosynthetic process	BP	1	SOCS5,TTC7A,CISH,PIP4K2B	yellow	VSMC_M3
GO:0022603	0.43	0.63	50	761	regulation of anatomical structure morphogenesis	BP	1	CTHRC1,CUL7,EPN1,KLF4,DBNL,WDPCP,ZC3H12A,PLEKHA4,SLC9A3R1,PLXND1,EMILIN1,BAIAP2,P4HB,ARHGAP33,GREM1,PDE3B,HTATIP2,PDGFA,CORO1B,NGEF,PLEKHO1,ARPIN,LIMK1,RHOC,FMNL1,LIMD1,YJEFN3,PHB2,C1QBP,RCC2,PKN1,DBN1,CRABP2,CORO1A,SYNE3,EEF2K,MYO19,INF2,MYDGF,SIRT6,ANAPC2,STK11,JMJD8,SARS,MIR214,TWF2,RNH1,UNC13A,CDC42EP5,NSMF	yellow	VSMC_M3
GO:0035556	0.43	0.60	139	2063	intracellular signal transduction	BP	1	RUFY1,SQSTM1,BAG6,NELFE,FNIP1,PRR5,ITPR3,SGSM3,DEF6,PPARD,EEF1D,LGALS1,SHARPIN,BOP1,PDGFRA,SOCS5,KLF4,DBNL,TPBG,CCM2,SPRED2,ZC3H12A,FIGNL1,RCN3,RELB,SLC9A3R1,EMILIN1,PLCD1,SEC13,ARHGEF40,CXXC5,LAMTOR1,P4HB,EIF4EBP1,CAVIN3,ARHGAP22,ARHGAP33,BCL2L1,PDE4D,TNS2,RRS1,SWAP70,PLEKHF1,GREM1,DOK1,PDE3B,CX3CL1,SOD1,DGKZ,CCAR2,CHP1,TYRO3,RAB35,CISH,SELENOS,GSTO1,STK32C,PDGFA,PABPN1,GPX1,AIP,NGEF,QARS,ASB9,TRIM27,EIF3A,LIMK1,THOC5,RIPK3,TNK2,STMN3,KAT5,RELA,SIPA1,RHOC,MAP3K14,LIMD1,ZNF385B,PHB2,IGFBP5,BAD,PLCB3,LAMTOR4,IFI35,TNFRSF1A,BABAM1,TEAD4,C1QBP,CHERP,CCDC22,SDE2,PKN1,MAD1L1,MAD2L2,TBKBP1,ARHGEF2,PER1,RANGRF,PPP4C,TRIM25,RPL37,S100A4,CSK,MID2,DOCK11,ZNF830,AKT1,DHCR24,CARHSP1,MYDGF,MAP2K2,CACTIN,HIC1,TRAP1,CTNS,MKNK2,IRAK1,STK11,TELO2,WDR24,JMJD8,CARD9,TGFBR3,TRADD,NDST1,NABP2,ERBB4,PTPRR,IGFBP6,UNC13A,ZNF385A,TXNDC12,CDC42EP5,CDC34,FBXO6,ARHGEF1,DUSP5,GLIPR2,RCE1	yellow	VSMC_M3
GO:0002822	0.43	0.71	8	102	regulation of adaptive immune response based on somatic recombination of immune receptors built from immunoglobulin superfamily domains	BP	1	IL1R1,SOCS5,ZC3H12A,CLCF1,RIPK3,PKN1,MAD2L2,CARD9	yellow	VSMC_M3
GO:0006767	0.43	0.76	4	47	water-soluble vitamin metabolic process	BP	1	SLC52A2,GSTO1,SLC19A1,FLAD1	yellow	VSMC_M3
GO:0008645	0.43	0.72	6	83	hexose transmembrane transport	BP	1	ENPP1,PPARD,SELENOS,CAPN10,AKT1,SLC27A4	yellow	VSMC_M3
GO:0032206	0.44	0.76	4	48	positive regulation of telomere maintenance	BP	1	PNKP,SLX1A,SIRT6,NABP2	yellow	VSMC_M3
GO:0006638	0.44	0.71	7	93	neutral lipid metabolic process	BP	1	MGLL,INSIG1,SCARB1,ACSL1,DGKZ,GPX1,PCK2	yellow	VSMC_M3

GO:0048514	0.44	0.63	34	508	blood vessel morphogenesis	BP	1	CUL7,B4GALT1,PDGFRA,COL15A1,EPN1,KLF4,HOXA1,CCM2,ZC3H12A,PLXND1,EMILIN1,ARHGAP22,GREM1,PDE3B,CX3CL1,EGR3,DLL4,HTATIP2,PDGFA,MMP14,GPX1,ANPEP,SGPL1,YJEFN3,AAMP,AKT1,MYDGF,SIRT6,JMJD8,SARS,TGFBR3,MIR214,TNFRSF12A,RNH1	yellow	VSMC_M3
GO:0050900	0.44	0.66	18	245	leukocyte migration	BP	1	PLEC,B4GALT1,IL1R1,EMILIN1,ITGB7,SWAP70,GREM1,CX3CL1,ST3GAL4,RIN3,MMP14,RIPK3,LGALS3,CD9,C1QBP,CORO1A,AKT1,JAGN1	yellow	VSMC_M3
GO:0060996	0.44	0.72	6	87	dendritic spine development	BP	1	DBNL,BAIAP2,ARHGAP33,NGEF,DBN1,EEF2K	yellow	VSMC_M3
GO:0030029	0.44	0.62	43	662	actin filament-based process	BP	1	SNX9,PDLIM4,PACSIN1,PLEC,PDGFRA,DBNL,FHL3,ZYX,MICAL1,EHD2,KRT8,SLC9A3R1,BAIAP2,INPPL1,CAVIN3,PDE4D,SWAP70,CAPG,CX3CL1,PDGFA,CORO1B,TRIM27,ARPIN,LIMK1,CAPN10,RHOC,ALKBH4,FMNL1,ARPC1B,WASHC1,DBN1,MAD2L2,ARHGEF2,RANGRF,CORO1A,MYO19,INF2,MIR214,TWF2,ELN,CDC42EP5,GBA2,ARPC5L	yellow	VSMC_M3
GO:1901879	0.44	0.73	5	75	regulation of protein depolymerization	BP	1	SWAP70,CAPG,MID1IP1,ARHGEF2,TWF2	yellow	VSMC_M3
GO:0019395	0.44	0.71	7	87	fatty acid oxidation	BP	1	ACAT2,PPARD,CPT1C,ETFBKMT,FABP3,ILVBL,AKT1	yellow	VSMC_M3
GO:0120162	0.44	0.72	6	78	positive regulation of cold-induced thermogenesis	BP	1	CNOT3,UCP2,ACSL1,SCD,SIRT6,EHMT1	yellow	VSMC_M3
GO:0045824	0.44	0.75	4	47	negative regulation of innate immune response	BP	1	TTLL12,METTL3,TYRO3,CACTIN	yellow	VSMC_M3
GO:0001525	0.44	0.64	29	428	angiogenesis	BP	1	B4GALT1,PDGFRA,COL15A1,EPN1,KLF4,ZC3H12A,PLXND1,EMILIN1,ARHGAP22,GREM1,PDE3B,CX3CL1,EGR3,DLL4,HTATIP2,PDGFA,MMP14,GPX1,ANPEP,YJEFN3,AAMP,AKT1,MYDGF,SIRT6,JMJD8,SARS,MIR214,TNFRSF12A,RNH1	yellow	VSMC_M3
GO:1903078	0.44	0.75	4	52	positive regulation of protein localization to plasma membrane	BP	1	SQSTM1,LGALS3,RANGRF,AKT1	yellow	VSMC_M3
GO:0061178	0.44	0.75	4	50	regulation of insulin secretion involved in cellular response to glucose stimulus	BP	1	PPARD,UCP2,BAD,JAGN1	yellow	VSMC_M3
GO:0015749	0.44	0.72	6	84	monosaccharide transmembrane transport	BP	1	ENPP1,PPARD,SELENOS,CAPN10,AKT1,SLC27A4	yellow	VSMC_M3
GO:0045184	0.44	0.60	97	1395	establishment of protein localization	BP	1	CD63,RUFY1,CCHCR1,BAG6,SNX9,SAMM50,SREBF2,H2AFY,SGSM3,GUK1,PPARD,SCRIB,IFT27,VPS28,TOM1,ASNA1,PLEKHA8,WDPCP,EIPR1,ZC3H12A,PDIA4,RCN3,SNX3,SERP2,DMAP1,SNX17,INSIG1,MYRIP,SEC13,UCP2,CEP131,TSPAN10,YIF1B,RAB20,ARHGAP33,AACS,HM13,RAPSN,CHP1,AP2A2,SNX21,RAB35,APBA2,SELENOS,EXOC3,AIP,AP1G2,PCK2,PCNT,CAPN10,PTPN23,LRWD1,VPS51,PEX5,ZDHHC3,PHB2,BAD,COPE,MVB12A,NUP88,WASHC1,RCC2,AP1M1,RAB3IL1,CCDC22,GLUD1,SNX8,LMAN2,RAB24,CLTB,ARHGEF2,SNF8,MVP,MTX1,RANGRF,STX10,SGF29,RABEP2,TMED1,SYNE3,CDC37,CSK,ATG3,RBM22,AKT1,SNX18,RANBP3,RAB34,CTNS,AP3D1,MIDN,NABP2,AP5B1,JAGN1,CAVIN1,ARFRP1,TIMM22	yellow	VSMC_M3
GO:1903035	0.44	0.73	5	64	negative regulation of response to wounding	BP	1	PDGFRA,PDGFA,CD9,PLAU,GIT1	yellow	VSMC_M3
GO:0009755	0.44	0.68	10	142	hormone-mediated signaling pathway	BP	1	PPARD,ACSL1,PRMT2,PHB2,PKN1,PER1,SMARCA4,CARM1,SAFB,SAFB2	yellow	VSMC_M3
GO:0002460	0.44	0.67	13	173	adaptive immune response based on somatic recombination of immune receptors built from immunoglobulin superfamily domains	BP	1	GNL1,IL1R1,SOCS5,ZC3H12A,RELB,SWAP70,CLCF1,RIPK3,C1QBP,PKN1,MAD2L2,C8G,CARD9	yellow	VSMC_M3
GO:0032784	0.44	0.75	4	49	regulation of DNA-templated transcription, elongation	BP	1	NELFE,CCAR2,THOC5,GTF2F1	yellow	VSMC_M3
GO:0042593	0.44	0.67	13	183	glucose homeostasis	BP	1	PPARD,LGALS1,UCP2,AACS,PCK2,SMARCB1,IGFBP5,BAD,FOXK1,SMARCA4,AKT1,STK11,JAGN1	yellow	VSMC_M3
GO:0045667	0.44	0.70	8	109	regulation of osteoblast differentiation	BP	1	CTHRC1,LRP3,GREM1,WNT10B,TWIST2,LIMD1,IGFBP5,JUND	yellow	VSMC_M3
GO:0014855	0.44	0.75	4	56	striated muscle cell proliferation	BP	1	PPARD,SIX5,TGFBR3,ERBB4	yellow	VSMC_M3
GO:0032984	0.44	0.67	13	195	protein-containing complex disassembly	BP	1	MICAL1,SWAP70,CAPG,LIX1L,APEH,SMARCD2,STMN3,MID1IP1,SMARCB1,ARHGEF2,SMARCA4,C19orf66,TWF2	yellow	VSMC_M3
GO:0045786	0.45	0.64	22	306	negative regulation of cell cycle	BP	1	CTDSP2,H2AFY,SCRIB,KLF4,SLC9A3R1,PINX1,CCAR2,CDK2AP2,PRMT2,THOC5,SIPA1,CTDSP1,METTL13,BABAM1,SDE2,MIIP,MAD1L1,MAD2L2,ZNF830,AURKAIP1,NABP2,FBXO6	yellow	VSMC_M3
GO:0036473	0.45	0.72	6	76	cell death in response to oxidative stress	BP	1	P4HB,PYCR1,SOD1,GPX1,AKT1,TRAP1	yellow	VSMC_M3
GO:0006888	0.45	0.69	8	106	endoplasmic reticulum to Golgi vesicle-mediated transport	BP	1	INSIG1,SEC13,YIF1B,COPE,LMAN2,RANGRF,TMED1,TRAPPC5	yellow	VSMC_M3
GO:0016311	0.45	0.64	24	342	dephosphorylation	BP	1	CTDSP2,SBF1,PNKP,PPM1G,INPPL1,TNS2,SWAP70,CHP1,PPM1M,RIPK3,PTPN23,PPP1R35,CTDSP1,LGALS3,PPP4C,MTMR4,HDHD2,SSU72,PGP,STK11,PTPRR,NT5C,NSMF,DUSP5	yellow	VSMC_M3
GO:0002312	0.45	0.73	5	64	B cell activation involved in immune response	BP	1	LGALS1,SWAP70,CLCF1,MAD2L2,DOCK11	yellow	VSMC_M3
GO:0050810	0.45	0.75	4	50	regulation of steroid biosynthetic process	BP	1	INSIG1,SOD1,DHCR7,ARMC5	yellow	VSMC_M3
GO:0006611	0.45	0.75	4	49	protein export from nucleus	BP	1	CCHCR1,CHP1,RBM22,RANBP3	yellow	VSMC_M3
GO:0033500	0.45	0.66	13	184	carbohydrate homeostasis	BP	1	PPARD,LGALS1,UCP2,AACS,PCK2,SMARCB1,IGFBP5,BAD,FOXK1,SMARCA4,AKT1,STK11,JAGN1	yellow	VSMC_M3
GO:1903313	0.45	0.69	8	105	positive regulation of mRNA metabolic process	BP	1	CNOT3,ZC3H12A,SNRNP70,METTL3,DND1,DCPS,NANOS3,KHSRP	yellow	VSMC_M3
GO:0002429	0.45	0.67	11	148	immune response-activating cell surface receptor signaling pathway	BP	1	BAG6,ZC3H12A,CD79A,PDE4D,DGKZ,LIMK1,RELA,LGALS3,CD276,CSK,STK11	yellow	VSMC_M3
GO:0002757	0.45	0.67	11	148	immune response-activating signal transduction	BP	1	BAG6,ZC3H12A,CD79A,PDE4D,DGKZ,LIMK1,RELA,LGALS3,CD276,CSK,STK11	yellow	VSMC_M3
GO:0008285	0.45	0.62	39	548	negative regulation of cell population proliferation	BP	1	PPARD,SCRIB,B4GALT1,KLF4,TPBG,SIX5,SLC9A3R1,DLEC1,INPPL1,TNS2,PINX1,GREM1,KDM2B,WNT10B,DLL4,FABP3,SMARCB1,PHB2,IGFBP5,IFI35,CD9,AIMP2,CHERP,ZBTB49,PKN1,MAD1L1,CTBP1,CSK,SLC6A4,DHCR24,LDOC1,MNT,STK11,GAS8,TGFBR3,MIR214,ERBB4,KLF13,IGFBP6	yellow	VSMC_M3

GO:0019220	0.45	0.60	73	1074	regulation of phosphate metabolic process	BP	1	ENPP1,NAB2,CTDSP2,SQSTM1,SNX9,FNIP1,PRR5,H2AFY,PLEC,PPP1R14B,PDGFRA,SOCS5,KLF4,SPRED2,ZC3H12A,MICAL1,WDFY2,SLC9A3R1,EMILIN1,SERTAD1,TARBP2,SCARB1,PDE4D,SWAP70,ACSL1,TNKS1BP1,GREM1,CX3CL1,SOD1,DGKZ,CHP1,TYRO3,CISH,FABP3,PDGFA,CLCF1,QARS,TRIM27,RIPK3,TNK2,IDH1,PHB2,PPP1R35,BAD,CTDSP1,LGALS3,TNFRSF1A,WASHC1,PKN1,CAPN2,PIP4K2B,MAD2L2,ARHGEF2,MVP,MTMR4,EEF2K,CDC37,CSK,AKT1,GIT1,MYDGF,MAP2K2,CACTIN,IRAK1,MIDN,STK11,TELO2,TAZ,JMJD8,VAC14,ERBB4,TRIB2,NSMF	yellow	VSMC_M3
GO:0051174	0.45	0.60	73	1074	regulation of phosphorus metabolic process	BP	1	ENPP1,NAB2,CTDSP2,SQSTM1,SNX9,FNIP1,PRR5,H2AFY,PLEC,PPP1R14B,PDGFRA,SOCS5,KLF4,SPRED2,ZC3H12A,MICAL1,WDFY2,SLC9A3R1,EMILIN1,SERTAD1,TARBP2,SCARB1,PDE4D,SWAP70,ACSL1,TNKS1BP1,GREM1,CX3CL1,SOD1,DGKZ,CHP1,TYRO3,CISH,FABP3,PDGFA,CLCF1,QARS,TRIM27,RIPK3,TNK2,IDH1,PHB2,PPP1R35,BAD,CTDSP1,LGALS3,TNFRSF1A,WASHC1,PKN1,CAPN2,PIP4K2B,MAD2L2,ARHGEF2,MVP,MTMR4,EEF2K,CDC37,CSK,AKT1,GIT1,MYDGF,MAP2K2,CACTIN,IRAK1,MIDN,STK11,TELO2,TAZ,JMJD8,VAC14,ERBB4,TRIB2,NSMF	yellow	VSMC_M3
GO:0090101	0.45	0.70	7	101	negative regulation of transmembrane receptor protein serine/threonine kinase signaling pathway	BP	1	SPRED2,EMILIN1,GREM1,MTMR4,VASN,TGFBR3,STUB1	yellow	VSMC_M3
GO:0017015	0.45	0.70	7	102	regulation of transforming growth factor beta receptor signaling pathway	BP	1	SPRED2,EMILIN1,MTMR4,VASN,STK11,TGFBR3,STUB1	yellow	VSMC_M3
GO:0021510	0.45	0.72	5	70	spinal cord development	BP	1	MPST,DCTN1,DLL4,SCYL1,AKT1	yellow	VSMC_M3
GO:0040007	0.45	0.60	50	742	growth	BP	1	ENPP1,CDK4,PPARD,PLEC,DNPH1,SOCS5,DBNL,CCM2,DMAP1,H3F3B,LAMTOR1,BCL2L1,TNS2,GREM1,KDM2B,TKT,WNT10B,SOD1,CCAR2,CISH,MMP14,GPX1,LIMK1,PRMT2,CCDC85B,KAT5,SIPA1,SGPL1,IGFBP5,CD9,EVC,H3F3A,DBN1,MAD2L2,CRABP2,SP2,SMARCA4,ZNF830,AKT1,SLC6A4,HDGFL2,SIRT6,CDK11B,ANAPC2,STK11,TGFBR3,ERBB4,TWF2,UNC13A,NSMCE3	yellow	VSMC_M3
GO:0001539	0.46	0.71	6	79	cilium or flagellum-dependent cell motility	BP	1	CEP131,YIF1B,IQCG,PLA2G3,GAS8,MEIG1	yellow	VSMC_M3
GO:0060285	0.46	0.71	6	79	cilium-dependent cell motility	BP	1	CEP131,YIF1B,IQCG,PLA2G3,GAS8,MEIG1	yellow	VSMC_M3
GO:0002832	0.46	0.71	6	77	negative regulation of response to biotic stimulus	BP	1	TTLL12,METTL3,TARBP2,TYRO3,C1QBP,CACTIN	yellow	VSMC_M3
GO:0050870	0.46	0.67	10	130	positive regulation of T cell activation	BP	1	LGALS1,SOCS5,EGR3,SART1,BAD,CORO1A,CD276,CSK,AKT1,AP3D1	yellow	VSMC_M3
GO:0001568	0.46	0.61	38	576	blood vessel development	BP	1	CUL7,B4GALT1,PDGFRA,COL15A1,EPN1,KLF4,HOXA1,CCM2,ZC3H12A,PLXND1,EMILIN1,SNX17,ARHGAP22,GREM1,PDE3B,CX3CL1,EGR3,DLL4,HTATIP2,PDGFA,MMP14,GPX1,ANPEP,SGPL1,YJEFN3,AAMP,AKT1,MYDGF,SIRT6,NSDHL,JMJD8,SARS,TGFBR3,MIR214,NDST1,TNFRSF12A,LOXL1,RNH1	yellow	VSMC_M3
GO:0001944	0.46	0.61	40	607	vasculature development	BP	1	CUL7,B4GALT1,PDGFRA,COL15A1,EPN1,KLF4,HOXA1,CCM2,ZC3H12A,PLXND1,EMILIN1,SNX17,RIC8A,ARHGAP22,GREM1,PDE3B,CX3CL1,EGR3,DLL4,HTATIP2,PDGFA,MMP14,GPX1,ANPEP,SGPL1,YJEFN3,AAMP,AKT1,MYDGF,SIRT6,NSDHL,STK11,JMJD8,SARS,TGFBR3,MIR214,NDST1,TNFRSF12A,LOXL1,RNH1	yellow	VSMC_M3
GO:0019953	0.46	0.61	40	565	sexual reproduction	BP	1	SPAG1,BAG6,NCAPH2,SBF1,IFT27,B4GALT1,FIGNL1,SIX5,ZNF296,PAFAH1B3,DLEC1,METTL3,CEP131,DND1,YIF1B,GNPDA1,MORN2,DEAF1,BCL2L1,KDM2B,SOD1,TYRO3,KDM1B,TRIM27,SLC26A6,IQCG,PLA2G3,SGPL1,BAD,CD9,NANOS3,ZNF830,AKT1,RFX2,SLC6A4,STK11,TAZ,FAM50A,IGSF8,MEIG1	yellow	VSMC_M3
GO:0009749	0.46	0.66	11	153	response to glucose	BP	1	PPARD,LGALS1,UCP2,AACS,SELENOS,PCK2,SMARCB1,BAD,SMARCA4,JAGN1,COL6A2	yellow	VSMC_M3
GO:0048511	0.46	0.64	15	223	rhythmic process	BP	1	CDK4,PDGFRA,RELB,METTL3,CAVIN3,EGR3,CCAR2,TYRO3,SRRD,IMPDH2,SGPL1,PER1,ZNF830,SLC6A4,JUND	yellow	VSMC_M3
GO:0048193	0.46	0.64	18	259	Golgi vesicle transport	BP	1	SNX3,INSIG1,SEC13,YIF1B,AP1G2,SCYL1,VPS51,COPE,CCDC22,SNX8,LMAN2,RANGRF,TMED1,CSK,RAB34,AP3D1,TRAPPC5,ARFRP1	yellow	VSMC_M3
GO:0060761	0.46	0.72	5	61	negative regulation of response to cytokine stimulus	BP	1	TTLL12,KLF4,METTL3,PALM3,CACTIN	yellow	VSMC_M3
GO:0010324	0.46	0.73	4	54	membrane invagination	BP	1	SNX9,SNX3,ALKBH4,SNX18	yellow	VSMC_M3
GO:0010631	0.46	0.63	18	261	epithelial cell migration	BP	1	PPARD,TSTA3,KLF4,WDPCP,ZC3H12A,PLXND1,SCARB1,GREM1,EGR3,DLL4,GPX1,CORO1B,PTPN23,AAMP,PKN1,AKT1,PTPRR,GLIPR2	yellow	VSMC_M3
GO:0007492	0.46	0.73	4	59	endoderm development	BP	1	H2AFY,MMP14,COL6A1,DUSP5	yellow	VSMC_M3
GO:0140056	0.46	0.71	5	67	organelle localization by membrane tethering	BP	1	ESYT1,STX10,PACS2,RABEPK,UNC13A	yellow	VSMC_M3
GO:0042886	0.46	0.64	15	210	amide transport	BP	1	PPARD,PLEKHA8,EIPR1,SLC9A3R1,MYRIP,UCP2,AACS,PCK2,SLC19A1,SLC26A6,CAPN10,BAD,GLUD1,MIDN,JAGN1	yellow	VSMC_M3
GO:0048598	0.46	0.61	31	469	embryonic morphogenesis	BP	1	CTHRC1,PBX2,H2AFY,SCRIB,PDGFRA,KLF4,HOXA1,WDPCP,SLC9A3R1,INSIG1,RIC8A,DEAF1,HOXC4,LRIG1,GREM1,KDM2B,SOD1,DLL4,IRX1,MMP14,COL6A1,PBX1,MTHFR,CRABP2,ARMC5,SLC39A1,TTC39C,ZNF358,NDST1,ARFRP1,DUSP5	yellow	VSMC_M3
GO:0000819	0.47	0.65	12	168	sister chromatid segregation	BP	1	DCTN2,NCAPH2,H2AFY,NUMA1,RRS1,PINX1,NUDC,PHB2,EML3,MAD1L1,MAD2L2,MIS12	yellow	VSMC_M3

GO:0031323	0.47	0.56	300	4391	regulation of cellular metabolic process	BP	1	ENPP1,CD63,NAB2,CDK4,CTDSP2,SQSTM1,ZNF608,BAG6,SNX9,HDAC10,NELFE,FNIP1,ATF6B,PBX2,PRR5,RING1,SREBF2,H2AFY,HNRNPA0,TRIM11,TRAPPC9,PPARD,SCRIB,PLEC,VPS28,SND1,PPP1R14B,PDGFRA,SECISBP2,PHF2,HSPBP1,SOCS5,RPS9,CNOT3,KLF4,HOXA1,FHL3,SPRED2,ZC3H12A,FIGNL1,PNKP,RCN3,SNRNP70,CC2D1B,KTI12,MICAL1,KMT2E,WDFY2,SIX5,ZNF282,ZNF296,RELB,SLC9A3R1,EMILIN1,DMAP1,CIC,UNK,INSIG1,OGG1,SERTAD1,TTC5,PSMC4,METTL3,CXXC5,LAMTOR1,DND1,EIF4EBP1,CAVIN3,DEAF1,HOXC4,TARBP2,KMT2B,SCARB1,NAT10,ZNF740,PDE4D,EIF4B,SWAP70,RALY,PLEKHF1,ACSL1,ZBTB38,TNKS1BP1,PINX1,GREM1,KDM2B,MCRS1,CX3CL1,WNT10B,SOD1,PACSIN3,ACTR5,ANKRD2,DGKZ,EGR3,CCAR2,DNAJC17,DLL4,CHP1,TYRO3,YAF2,HTATIP2,PCIF1,PCBP4,KDM1B,CHCHD2,RBM15B,CISH,SRSF9,RNF19B,DCPS,IRX1,SELENOS,RRP1B,ETFBKMT,BRD9,RIN3,FABP3,ZCCHC17,PDGFA,MMP14,GPX1,AIP,CLCF1,NGDN,SRRD,TSHZ2,QARS,PSME2,PIR,TRIM27,LIMK1,PRMT2,THOC5,RIPK3,TWIST2,LSM14B,ADRM1,SMARCD2,BRMS1,BUD23,TNK2,TACO1,PLA2G3,GMEB2,CCDC85B,PRPF6,PBX1,KAT5,RELA,ZNF511,IDH1,ARNT2,ZFPL1,LIMD1,MID1IP1,SMARCB1,CHCHD10,PBX4,UBTF,GATAD2A,MEF2B,PHB2,TRMT112,PPP1R35,NDP,IGFBP5,BAD,CTDSP1,MLF2,OTUB1,EID1,SSBP4,METTL13,MPV17L2,ZNF394,LGALS3,C11orf95,BUD31,TNFRSF1A,BABAM1,TEAD4,C1QBP,AIMP2,TAF6L,ZBTB49,UBXN1,WASHC1,MTA2,ZNF316,MED11,PELP1,CCDC22,FOXK1,KLF3,PKN1,CAPN2,SEPSECS,POLR2A,AMER1,MTHFR,PIP4K2B,MAD2L2,SLTM,NANOS3,CTBP1,FBXL19,CRABP2,DFFA,SP2,ARHGEF2,SNF8,MVP,PER1,NFIX,PPP4C,SLX1A,IRF2BPL,[illegible]	yellow	VSMC_M3
GO:0034219	0.47	0.70	6	86	carbohydrate transmembrane transport	BP	1	ENPP1,PPARD,SELENOS,CAPN10,AKT1,SLC27A4	yellow	VSMC_M3
GO:0032368	0.47	0.68	7	95	regulation of lipid transport	BP	1	SREBF2,LAMTOR1,ACSL1,FABP3,PLA2G3,YJEFN3,AKT1	yellow	VSMC_M3
GO:0090132	0.47	0.63	18	262	epithelium migration	BP	1	PPARD,TSTA3,KLF4,WDPCP,ZC3H12A,PLXND1,SCARB1,GREM1,EGR3,DLL4,GPX1,CORO1B,PTPN23,AAMP,PKN1,AKT1,PTPRR,GLIPR2	yellow	VSMC_M3
GO:0002637	0.47	0.73	4	48	regulation of immunoglobulin production	BP	1	CLCF1,PHB2,PKN1,MAD2L2	yellow	VSMC_M3
GO:2001236	0.47	0.67	9	121	regulation of extrinsic apoptotic signaling pathway	BP	1	BCL2L1,CX3CL1,ITPRIP,GPX1,RELA,LGALS3,ARHGEF2,AKT1,TNFRSF12A	yellow	VSMC_M3
GO:0051052	0.47	0.62	21	297	regulation of DNA metabolic process	BP	1	HDAC10,KLF4,FIGNL1,PNKP,OGG1,NAT10,PINX1,GREM1,KDM1B,CLCF1,OTUB1,BABAM1,MAD2L2,DFFA,PPP4C,SLX1A,CXorf57,AKT1,SIRT6,MBD3,NABP2	yellow	VSMC_M3
GO:0030183	0.47	0.68	7	96	B cell differentiation	BP	1	FNIP1,LGALS1,CD79A,MMP14,CLCF1,BAD,DOCK11	yellow	VSMC_M3
GO:0009991	0.47	0.61	27	385	response to extracellular stimulus	BP	1	FNIP1,SREBF2,PPARD,PLEC,ZC3H12A,OGG1,UCP2,EIF4EBP1,AACS,ACSL1,SOD1,DCPS,PCK2,SPX,RELA,SIPA1,FOXK1,MTHFR,TRIM25,AKT1,HMGCS1,SLC6A4,SIRT6,MBD3,WDR24,SLC27A4,FADS1	yellow	VSMC_M3
GO:0032233	0.47	0.72	4	56	positive regulation of actin filament bundle assembly	BP	1	SWAP70,CX3CL1,LIMK1,RHOC	yellow	VSMC_M3
GO:0048856	0.47	0.56	278	4193	anatomical structure development	BP	1	ENPP1,NAB2,CDK4,BAG6,NCAPH2,SBF1,CTHRC1,HDAC10,FNIP1,PBX2,PDLIM4,RING1,H2AFY,TRIM11,TRAPPC9,PACSIN1,PPARD,SCRIB,PLEC,LGALS1,MPST,IFT27,SHARPIN,TBCC,CUL7,DNPH1,B4GALT1,PDGFRA,SECISBP2,PHF2,COL15A1,EPN1,COL19A1,SOCS5,TTC7A,CNOT3,KLF4,DBNL,TPBG,HOXA1,WDPCP,CCM2,FHL3,SPRED2,ZC3H12A,RCN3,SNX3,PLEKHA4,EHD2,KMT2E,KRT8,SIX5,RELB,SLC9A3R1,PLXND1,EMILIN1,RNF220,PAFAH1B3,CIC,SNX17,UNK,CD79A,INSIG1,PSMC4,UCP2,METTL3,BAIAP2,CEP131,INPPL1,NUMA1,P4HB,DND1,YIF1B,FASN,RPL29,EIF4EBP1,RIC8A,ARHGAP22,DEAF1,TBCB,HOXC4,LRIG1,ARHGAP33,AACS,HM13,BCL2L1,ITGB7,PDE4D,TNS2,SWAP70,CAPG,SLC11A2,MMP24,GREM1,KDM2B,AQP5,PDE3B,DCTN1,CX3CL1,WNT10B,SOD1,CCDC32,ANKRD2,EGR3,DLL4,TYRO3,HTATIP2,RAB35,APBA2,IRX1,SDHA,PAPSS1,PDGFA,MMP14,C14orf93,GPX1,CORO1B,NGEF,HCCS,CLCF1,PLEKHO1,TAGLN2,IGSF9,QARS,LRFN4,PCK2,COL6A1,IMPDH2,PIR,NECTIN1,ANPEP,ARPIN,LIMK1,SLC26A6,PRMT2,THOC5,RIPK3,EXTL1,IQCG,PLA2G3,STMN3,SART1,PBX1,RELA,RHOC,SCYL1,IDH1,FMNL1,ARNT2,VPS51,LIMD1,SGPL1,SMARCB1,PBX4,YJEFN3,ATIC,BLOC1S4,MEF2B,PHB2,NDP,IGFBP5,C12orf57,BAD,AAMP,CTDSP1,HSD17B1,LGALS3,CD9,TNFRSF1A,TEAD4,C1QBP,AIMP2,CHERP,EVC,RCC2,CNP,PRXL2A,KRT10,GLUD1,FOXK1,CASP14,KLF3,PKN1,DBN1,CAPN2,MAD1L1,AMER1,MTHFR,MAD2L2,NANOS3,PRR14,CRABP2,TMEM79,ARHGEF2,ARMC5,MTX1,CORO1A,IRF2BPL,SLC39A1,S100A4,S100A6,SMARCA4,CARM1,SYNE3,EEF2K,MYO19,EML1,AATF,CSK,ZNF521,TTC39C,ZNF358,DOCK11,INF2,ZNF830,AKT1,COPRS,HMGCS1,RFX2,SAFB2,GIT1[illegible]	yellow	VSMC_M3

GO:0051171	0.47	0.56	279	4082	regulation of nitrogen compound metabolic process	BP	1	ENPP1,NAB2,CDK4,CTDSP2,SQSTM1,ZNF608,BAG6,SNX9,HDAC10,NELFE,FNIP1,ATF6B,PBX2,PRR5,RING1,SREBF2,H2AFY,HNRNPA0,TRIM11,SGSM3,TRAPPC9,PPARD,PLEC,VPS28,SND1,SECISBP2,PHF2,HSPBP1,SOCS5,RPS9,CNOT3,KLF4,HOXA1,FHL3,SPRED2,ZC3H12A,FIGNL1,PNKP,RCN3,SNRNP70,CC2D1B,SNX3,KTI12,MICAL1,KMT2E,WDFY2,SIX5,ZNF282,ZNF296,RELB,SLC9A3R1,EMILIN1,DMAP1,CIC,UNK,OGG1,SERTAD1,TTC5,PSMC4,METTL3,CXXC5,DND1,EIF4EBP1,DELE1,DEAF1,HOXC4,LDLRAD3,TARBP2,KMT2B,SCARB1,NAT10,ZNF740,PDE4D,EIF4B,SWAP70,RALY,ACSL1,ZBTB38,TNKS1BP1,PINX1,GREM1,KDM2B,MCRS1,CX3CL1,WNT10B,SOD1,PACSIN3,ACTR5,ANKRD2,EGR3,CCAR2,DNAJC17,DLL4,CHP1,YAF2,HTATIP2,PCIF1,PCBP4,KDM1B,CHCHD2,RBM15B,SRSF9,RNF19B,DCPS,IRX1,SELENOS,RRP1B,BRD9,FABP3,ZCCHC17,PDGFA,MMP14,GPX1,AIP,CLCF1,NGDN,SRRD,TSHZ2,QARS,ASB9,PSME2,PIR,TRIM27,LIMK1,PRMT2,THOC5,RIPK3,TWIST2,LSM14B,ADRM1,SMARCD2,PFDN2,BRMS1,BUD23,TNK2,TACO1,GMEB2,CCDC85B,PRPF6,PBX1,KAT5,RELA,ZNF511,ARNT2,ZFPL1,LIMD1,SMARCB1,CHCHD10,PBX4,UBTF,GATAD2A,MEF2B,PHB2,TRMT112,NDP,IGFBP5,BAD,CTDSP1,MLF2,OTUB1,EID1,SSBP4,METTL13,MPV17L2,ZNF394,LGALS3,C11orf95,BUD31,TNFRSF1A,BABAM1,TEAD4,C1QBP,AIMP2,TAF6L,ZBTB49,UBXN1,WASHC1,MTA2,ZNF316,MED11,PELP1,CCDC22,FOXK1,KLF3,PKN1,CAPN2,SEPSECS,POLR2A,AMER1,MTHFR,MAD2L2,SLTM,NANOS3,CTBP1,FBXL19,CRABP2,DFFA,SP2,ARHGEF2,SNF8,MVP,PER1,NFIX,PPP4C,SLX1A,IRF2BPL,TRIM25,RPL37,SMARCA4,CARM1,EEF2K,CDC37,RAVER1,AATF,CXorf57,C19orf66,CSK,MID2,RB[illegible]	yellow	VSMC_M3
GO:0031344	0.47	0.60	35	538	regulation of cell projection organization	BP	1	PACSIN1,LGALS1,CUL7,KLF4,DBNL,WDPCP,SNX3,PLXND1,SEPT9,BAIAP2,ARHGAP33,CX3CL1,CORO1B,NGEF,ARPIN,LIMK1,PLA2G3,WASHC1,RCC2,DBN1,CRABP2,RABEP2,CARM1,EEF2K,ATG3,DOCK11,AKT1,ACAP3,ANAPC2,STK11,NEGR1,MIR214,TWF2,CDC42EP5,NSMF	yellow	VSMC_M3
GO:0043583	0.47	0.65	11	162	ear development	BP	1	CTHRC1,SCRIB,IFT27,HOXA1,WDPCP,CCM2,SLC9A3R1,INSIG1,LRIG1,SOD1,TTC39C	yellow	VSMC_M3
GO:0010906	0.47	0.68	7	96	regulation of glucose metabolic process	BP	1	ENPP1,SELENOS,BAD,FOXK1,AKT1,PGP,MIDN	yellow	VSMC_M3
GO:0003170	0.47	0.72	4	58	heart valve development	BP	1	EMILIN1,DLL4,TNFRSF1A,ELN	yellow	VSMC_M3
GO:0044772	0.48	0.61	25	350	mitotic cell cycle phase transition	BP	1	CDK4,CTDSP2,KLF4,KMT2E,CCDC57,EIF4EBP1,PINX1,WNT10B,CDK2AP2,PSME2,PRMT2,PBX1,PHB2,CTDSP1,BABAM1,RCC2,SDE2,MIIP,MAD1L1,MAD2L2,ZNF830,AKT1,MIR214,NABP2,CDC34	yellow	VSMC_M3
GO:0002040	0.48	0.67	8	114	sprouting angiogenesis	BP	1	EPN1,KLF4,GREM1,EGR3,DLL4,YJEFN3,AKT1,JMJD8	yellow	VSMC_M3
GO:0043161	0.48	0.61	26	368	proteasome-mediated ubiquitin-dependent protein catabolic process	BP	1	BAG6,SHARPIN,HSPBP1,SOCS5,PSMB3,TMUB1,PSMC4,SEL1L2,WNT10B,CCAR2,RNF19B,SELENOS,KAT5,UBXN1,FBXL19,TRIM25,ANKRD9,AKT1,SIRT6,SPSB3,MAN1B1,ZNRF1,TRIB2,CDC34,FBXO6,STUB1	yellow	VSMC_M3
GO:0060021	0.48	0.70	5	71	roof of mouth development	BP	1	PDGFRA,WDPCP,INSIG1,SGPL1,TGFBR3	yellow	VSMC_M3
GO:0051205	0.48	0.72	4	44	protein insertion into membrane	BP	1	BAG6,SAMM50,ASNA1,TIMM22	yellow	VSMC_M3
GO:0034440	0.48	0.67	7	90	lipid oxidation	BP	1	ACAT2,PPARD,CPT1C,ETFBKMT,FABP3,ILVBL,AKT1	yellow	VSMC_M3
GO:0019935	0.48	0.72	4	57	cyclic-nucleotide-mediated signaling	BP	1	SLC9A3R1,PDE4D,PDE3B,CDC34	yellow	VSMC_M3
GO:0009617	0.48	0.60	26	362	response to bacterium	BP	1	CDK4,HNRNPA0,PPARD,SSC5D,ZC3H12A,SNX3,BAIAP2,SCARB1,CX3CL1,SELENOS,PABPN1,GPX1,PCK2,RELA,TNFRSF1A,CNP,CAPN2,PALM3,AKT1,GIT1,LDOC1,CACTIN,IRAK1,CARD9,JUND,LOXL1	yellow	VSMC_M3
GO:0070059	0.48	0.72	4	50	intrinsic apoptotic signaling pathway in response to endoplasmic reticulum stress	BP	1	BAG6,BCL2L1,SELENOS,TXNDC12	yellow	VSMC_M3
GO:1903844	0.48	0.67	7	104	regulation of cellular response to transforming growth factor beta stimulus	BP	1	SPRED2,EMILIN1,MTMR4,VASN,STK11,TGFBR3,STUB1	yellow	VSMC_M3
GO:0030155	0.48	0.59	36	541	regulation of cell adhesion	BP	1	TSTA3,SCRIB,LGALS1,SOCS5,KLF4,WDPCP,ZC3H12A,PLXND1,EMILIN1,METTL3,P4HB,SWAP70,GREM1,PDE3B,CX3CL1,EGR3,ST3GAL4,MMP14,SART1,PTPN23,RELA,SIPA1,BAD,LGALS3,CD9,PLAU,C1QBP,RCC2,MAD1L1,MAD2L2,CORO1A,CD276,CSK,AKT1,AP3D1,IRAK1	yellow	VSMC_M3
GO:2000146	0.48	0.62	16	234	negative regulation of cell motility	BP	1	PPARD,KLF4,SLC9A3R1,EMILIN1,GREM1,CX3CL1,DLL4,RIN3,CORO1B,ARPIN,PTPN23,IGFBP5,MIIP,AKT1,MIR214,PTPRR	yellow	VSMC_M3
GO:0030030	0.48	0.57	79	1226	cell projection organization	BP	1	SPAG1,CTHRC1,TXNDC15,PACSIN1,SCRIB,LGALS1,IFT27,CUL7,KLF4,DBNL,TPBG,WDPCP,SNX3,EMP3,EHD2,SLC9A3R1,PLXND1,SEPT9,BAIAP2,CEP131,INPPL1,YIF1B,CCDC57,ARHGAP33,CAPG,SLC11A2,DCTN1,CX3CL1,SOD1,CCDC32,RAB35,CCDC28B,PDGFA,CORO1B,NGEF,PLEKHO1,IGSF9,NECTIN1,ARPIN,PCNT,LIMK1,IQCG,PLA2G3,STMN3,CEP164,PTPN23,BLOC1S4,C12orf57,WASHC1,RCC2,CNP,DBN1,CRABP2,FAM161B,CORO1A,RABEP2,S100A6,CARM1,EEF2K,ATG3,TTC39C,DOCK11,AKT1,RFX2,RAB34,ACAP3,SSNA1,ANAPC2,NUBP2,STK11,GAS8,WDR34,NEGR1,MIR214,TWF2,UNC13A,CDC42EP5,NSMF,MEIG1	yellow	VSMC_M3
GO:0051494	0.48	0.66	9	138	negative regulation of cytoskeleton organization	BP	1	SWAP70,CAPG,CORO1B,ARPIN,MID1IP1,ARHGEF2,CORO1A,MIR214,TWF2	yellow	VSMC_M3
GO:0099173	0.48	0.66	9	137	postsynapse organization	BP	1	DBNL,BAIAP2,ARHGAP22,ARHGAP33,RAPSN,NGEF,LRFN4,DBN1,EEF2K	yellow	VSMC_M3
GO:0006979	0.48	0.60	26	357	response to oxidative stress	BP	1	H2AFY,PDGFRA,KLF4,ZC3H12A,PNKP,OGG1,UCP2,P4HB,PYCR1,GSR,SOD1,ANKRD2,CHCHD2,SELENOS,MMP14,GPX1,RIPK3,RELA,IDH1,TXNRD2,BAD,CAPN2,AKT1,DHCR24,TRAP1,OSER1	yellow	VSMC_M3

GO:0048518	0.48	0.55	307	4533	positive regulation of biological process	BP	1	CD63,NAB2,NHP2,CDK4,SQSTM1,BAG6,SNX9,CTHRC1,HDAC10,NELFE,FNIP1,ATF6B,PBX2,PRR5,SREBF2,TXNDC15,H2AFY,ITPR3,HNRNPA0,TRIM11,SGSM3,PACSIN1,PPARD,EEF1D,TSTA3,SCRIB,LGALS1,SHARPIN,VPS28,CUL7,DNPH1,B4GALT1,PDGFRA,IL1R1,SECISBP2,PHF2,HSPBP1,SOCS5,RPS9,CNOT3,KLF4,DBNL,TPBG,HOXA1,SPRED2,EIPR1,ZC3H12A,PNKP,RCN3,SNRNP70,SNX3,PLEKHA4,EHD2,KMT2E,WDFY2,SIX5,ZNF296,RELB,SLC9A3R1,PLXND1,EMILIN1,RNF220,DMAP1,SLC30A3,H3F3B,CD79A,MYRIP,SEC13,SEPT9,SERTAD1,TTC5,PSMC4,UCP2,METTL3,PHLDA1,BAIAP2,CEP131,CXXC5,LAMTOR1,NUMA1,P4HB,DND1,CCDC57,EIF4EBP1,CAVIN3,DEAF1,HOXC4,TARBP2,KMT2B,AACS,SCARB1,NAT10,BCL2L1,PDE4D,SWAP70,LRP3,PLEKHF1,ACSL1,ZBTB38,TNKS1BP1,PINX1,GREM1,KDM2B,MCRS1,DCTN1,RAPSN,CX3CL1,WNT10B,SOD1,PACSIN3,DGKZ,EGR3,CCAR2,DLL4,CHP1,TYRO3,SCD,YAF2,HTATIP2,PCIF1,KDM1B,CHCHD2,SLC35C2,TSPAN5,ST3GAL4,RNF19B,DCPS,GSTO1,RRP1B,FABP3,PDGFA,MMP14,C14orf93,GPX1,AIP,CORO1B,CLCF1,SYT12,PCK2,ASB9,PSME2,TRIM27,ARPIN,PCNT,LIMK1,SLC26A6,PRMT2,SPX,THOC5,RIPK3,TWIST2,ADRM1,CAPN10,SMARCD2,PFDN2,BRMS1,BUD23,TNK2,PLA2G3,GMEB2,SART1,PRPF6,PTPN23,PBX1,KAT5,RELA,RHOC,MAP3K14,ARNT2,LIMD1,MID1IP1,ZDHHC3,SMARCB1,CHCHD10,PBX4,UBTF,MEF2B,PHB2,TRMT112,NDP,IGFBP5,BAD,LAMTOR4,IFI35,AAMP,SSBP4,MPV17L2,LGALS3,BUD31,ARPC1B,PLAU,TNFRSF1A,BABAM1,C1QBP,AIMP2,TAF6L,CHERP,ZBTB49,WASHC1,EVC,RCC2,MTA2,PELP1,CCDC22,USP27X,KRT10,GLUD1,FOXK1,ELP5,H3F3A,PKN1,DBN1,CAPN2,LMAN2,POLR2A,AMER1,PIP4K2B,MA[illegible]	yellow	VSMC_M3
GO:0044770	0.48	0.59	30	426	cell cycle phase transition	BP	1	CDK4,CTDSP2,H2AFY,KLF4,KMT2E,CCDC57,EIF4EBP1,PINX1,WNT10B,CCAR2,CDK2AP2,PSME2,PRMT2,THOC5,PBX1,PHB2,CTDSP1,METTL13,BABAM1,RCC2,SDE2,MIIP,MAD1L1,MAD2L2,ZNF830,AKT1,MIR214,NABP2,CDC34,FBXO6	yellow	VSMC_M3
GO:0009792	0.48	0.59	36	537	embryo development ending in birth or egg hatching	BP	1	CTHRC1,SCRIB,PDGFRA,EPN1,CNOT3,KLF4,HOXA1,CCM2,KRT8,RNF220,UNK,PSMC4,RIC8A,DEAF1,HOXC4,HM13,BCL2L1,KDM2B,MMP14,STMN3,PBX1,ARNT2,SMARCB1,DBN1,CAPN2,MTHFR,ARMC5,SLC39A1,ZNF358,ZNF830,AKT1,NSDHL,SLC35E2B,MBD3,NDST1,PTPRR	yellow	VSMC_M3
GO:0007623	0.48	0.64	11	160	circadian rhythm	BP	1	CDK4,RELB,METTL3,CAVIN3,EGR3,CCAR2,SRRD,IMPDH2,PER1,SLC6A4,JUND	yellow	VSMC_M3
GO:0043087	0.48	0.61	18	285	regulation of GTPase activity	BP	1	SBF1,SNX9,SGSM3,SCRIB,PLXND1,ARAP3,ARHGAP22,CX3CL1,SOD1,RRP1B,NGEF,STMN3,SIPA1,RCC2,DOCK11,SNX18,SLC27A4,RGS16	yellow	VSMC_M3
GO:0061061	0.49	0.59	34	504	muscle structure development	BP	1	PDLIM4,PPARD,PLEC,LGALS1,PDGFRA,COL19A1,FHL3,EHD2,KRT8,GREM1,WNT10B,ANKRD2,EGR3,DLL4,MMP14,GPX1,PLEKHO1,MEF2B,IGFBP5,CD9,TEAD4,EVC,FOXK1,CAPN2,PRR14,AKT1,COPRS,SIRT6,HMG20B,TAZ,GTF3C5,TGFBR3,IGSF8,ELN	yellow	VSMC_M3
GO:0061045	0.49	0.71	4	52	negative regulation of wound healing	BP	1	PDGFRA,PDGFA,CD9,PLAU	yellow	VSMC_M3
GO:0006893	0.49	0.71	4	56	Golgi to plasma membrane transport	BP	1	CCDC22,CSK,RAB34,ARFRP1	yellow	VSMC_M3
GO:1901264	0.49	0.69	5	61	carbohydrate derivative transport	BP	1	GUK1,PLEKHA8,SCARB1,SLC35C2,SLC19A1	yellow	VSMC_M3
GO:0010565	0.49	0.67	7	95	regulation of cellular ketone metabolic process	BP	1	PPARD,INSIG1,ETFBKMT,FABP3,PLA2G3,MID1IP1,AKT1	yellow	VSMC_M3
GO:0010001	0.49	0.63	12	173	glial cell differentiation	BP	1	NAB2,HDAC10,PLEC,METTL3,MMP24,SOD1,CLCF1,RELA,CD9,CNP,CSK,AKT1	yellow	VSMC_M3
GO:0006006	0.49	0.64	11	155	glucose metabolic process	BP	1	ENPP1,PPARD,INPPL1,SELENOS,PCK2,BAD,FOXK1,AKT1,PGM1,PGP,MIDN	yellow	VSMC_M3
GO:2000756	0.49	0.71	4	53	regulation of peptidyl-lysine acetylation	BP	1	SMARCB1,EID1,CTBP1,THAP7	yellow	VSMC_M3
GO:1903362	0.49	0.61	16	222	regulation of cellular protein catabolic process	BP	1	BAG6,HSPBP1,SOCS5,PSMC4,WNT10B,CCAR2,RNF19B,GPX1,PSME2,UBXN1,CCDC22,AMER1,AKT1,SIRT6,TRIB2,STUB1	yellow	VSMC_M3
GO:0031347	0.49	0.59	28	397	regulation of defense response	BP	1	TTLL12,PPARD,SHARPIN,IL1R1,SOCS5,KLF4,ZC3H12A,ZYX,MGLL,METTL3,TARBP2,CX3CL1,SOD1,TYRO3,SELENOS,MVK,GPX1,PSMB4,PLA2G3,RELA,IFI35,TNFRSF1A,C1QBP,CDC37,GIT1,CACTIN,CARD9,TRADD	yellow	VSMC_M3
GO:0001952	0.49	0.66	7	105	regulation of cell-matrix adhesion	BP	1	TSTA3,WDPCP,GREM1,CX3CL1,MMP14,PLAU,RCC2	yellow	VSMC_M3
GO:0006354	0.49	0.67	6	80	DNA-templated transcription, elongation	BP	1	NELFE,SERTAD1,CCAR2,THOC5,ADRM1,GTF2F1	yellow	VSMC_M3
GO:0043547	0.49	0.62	13	202	positive regulation of GTPase activity	BP	1	SNX9,SGSM3,SCRIB,ARAP3,ARHGAP22,CX3CL1,NGEF,SIPA1,RCC2,DOCK11,SNX18,SLC27A4,RGS16	yellow	VSMC_M3
GO:1903047	0.49	0.57	42	608	mitotic cell cycle process	BP	1	DCTN2,CDK4,CTDSP2,NCAPH2,SNX9,H2AFY,CUL7,KLF4,KMT2E,NUMA1,CCDC57,EIF4EBP1,RRS1,PINX1,DCTN1,WNT10B,RAB35,CDK2AP2,PSME2,PCNT,NUDC,PRMT2,PBX1,RHOC,PHB2,CTDSP1,BABAM1,RCC2,EML3,SDE2,MIIP,MAD1L1,MAD2L2,EML1,ZNF830,AKT1,SNX18,AURKAIP1,MIR214,NABP2,MIS12,CDC34	yellow	VSMC_M3
GO:0055076	0.49	0.65	8	107	transition metal ion homeostasis	BP	1	TTC7A,SLC30A3,SLC11A2,SOD1,CCDC22,ANKRD9,AP3D1,CCDC115	yellow	VSMC_M3
GO:0051893	0.49	0.71	4	60	regulation of focal adhesion assembly	BP	1	WDPCP,GREM1,MMP14,RCC2	yellow	VSMC_M3
GO:0090109	0.49	0.71	4	60	regulation of cell-substrate junction assembly	BP	1	WDPCP,GREM1,MMP14,RCC2	yellow	VSMC_M3
GO:0001775	0.49	0.57	48	692	cell activation	BP	1	BAG6,NCAPH2,FNIP1,SCRIB,LGALS1,PDGFRA,SOCS5,ZC3H12A,KMT2E,RELB,EMILIN1,CD79A,METTL3,SWAP70,CX3CL1,SOD1,DGKZ,EGR3,DLL4,TYRO3,PDGFA,MMP14,CLCF1,IMPDH2,RIPK3,PLA2G3,SART1,PBX1,BLOC1S4,PHB2,BAD,IFI35,LGALS3,CD9,PKN1,MAD1L1,MAD2L2,ARMC5,CORO1A,CD276,CSK,DOCK11,AKT1,AP3D1,STK11,PSMB10,JUND,AKAP17A	yellow	VSMC_M3
GO:0043068	0.49	0.58	30	422	positive regulation of programmed cell death	BP	1	CDK4,SQSTM1,FNIP1,SCRIB,B4GALT1,ZC3H12A,SLC9A3R1,PHLDA1,PLEKHF1,RAPSN,WNT10B,SOD1,CCAR2,HTATIP2,RRP1B,PRMT2,RIPK3,CAPN10,BRMS1,BAD,TNFRSF1A,C1QBP,USP27X,CAPN2,DFFA,AKT1,SLC27A4,TRADD,TNFRSF12A,CDC34	yellow	VSMC_M3
GO:0034109	0.49	0.69	5	71	homotypic cell-cell adhesion	BP	1	LGALS1,PDGFRA,TYRO3,BLOC1S4,CD9	yellow	VSMC_M3
GO:0070085	0.49	0.62	14	194	glycosylation	BP	1	B4GALT1,SERP2,GALNT14,CHP1,SLC35C2,ST3GAL4,TMTC1,EXTL1,MPDU1,DPM3,MAN1B1,POMT1,B3GNT9,GBA2	yellow	VSMC_M3

GO:0030097	0.49	0.57	42	618	hemopoiesis	BP	1	NCAPH2,FNIP1,LGALS1,PDGFRA,SOCS5,TTC7A,KLF4,ZC3H12A,KMT2E,RELB,CD79A,METTL3,FASN,SLC11A2,WNT10B,SOD1,EGR3,DLL4,TYRO3,MMP14,CLCF1,PIR,THOC5,RIPK3,PLA2G3,SART1,PBX1,SGPL1,YJEFN3,BAD,PRXL2A,ARMC5,EML1,DOCK11,AP3D1,MKNK2,STK11,TAZ,TGFBR3,KLF13,JAGN1,ZNF385A	yellow	VSMC_M3
GO:0001654	0.50	0.60	18	280	eye development	BP	1	CDK4,PBX2,RING1,PDGFRA,KLF4,WDPCP,SPRED2,SIX5,KDM2B,AQP5,DLL4,IMPDH2,NECTIN1,PBX1,PBX4,C12orf57,SMARCA4,CTNS	yellow	VSMC_M3
GO:0090130	0.50	0.60	18	266	tissue migration	BP	1	PPARD,TSTA3,KLF4,WDPCP,ZC3H12A,PLXND1,SCARB1,GREM1,EGR3,DLL4,GPX1,CORO1B,PTPN23,AAMP,PKN1,AKT1,PTPRR,GLIPR2	yellow	VSMC_M3
GO:0019827	0.50	0.66	7	106	stem cell population maintenance	BP	1	CNOT3,KLF4,METTL3,MMP24,KDM2B,TAF6L,ZNF358	yellow	VSMC_M3
GO:0044703	0.50	0.56	48	688	multi-organism reproductive process	BP	1	SPAG1,BAG6,NCAPH2,SBF1,PPARD,IFT27,B4GALT1,EPN1,FIGNL1,SIX5,ZNF296,PAFAH1B3,DLEC1,UCP2,METTL3,CEP131,DND1,YIF1B,RPL29,GNPDA1,MORN2,DEAF1,BCL2L1,KDM2B,SOD1,TYRO3,KDM1B,SLC19A1,TRIM27,SLC26A6,IQCG,PLA2G3,SGPL1,IGFBP5,BAD,CD9,C1QBP,CAPN2,NANOS3,ZNF830,AKT1,RFX2,SLC6A4,STK11,TAZ,FAM50A,IGSF8,MEIG1	yellow	VSMC_M3
GO:1901565	0.50	0.55	75	1069	organonitrogen compound catabolic process	BP	1	SQSTM1,BAG6,SNX9,SGSM3,NUDT3,MPST,SHARPIN,VPS28,CUL7,DNPH1,HSPBP1,SOCS5,PSMB3,BLVRA,RCN3,SNRNP70,SNX3,TMUB1,BCKDHA,BLVRB,OGG1,PSMC4,SEL1L2,SMOX,GNPDA1,SMUG1,SCARB1,HM13,PDE4D,WNT10B,PACSIN3,CCAR2,RNF19B,SELENOS,MANBA,GPX1,ASB9,PSME2,ANPEP,ADRM1,PSMB4,PTPN23,KAT5,RELA,SGPL1,MVB12A,UBXN1,CCDC22,USP27X,GLUD1,CAPN2,AMER1,MAD2L2,FBXL19,SNF8,TRIM25,USP43,ANKRD9,AKT1,BLMH,SIRT6,ANAPC2,SPSB3,MAN1B1,ZNRF1,PSMB10,CTRL,NT5C,TRIB2,CDC34,FBXO6,GBA2,STUB1,ITPA,CCDC115	yellow	VSMC_M3
GO:0042306	0.50	0.70	4	52	regulation of protein import into nucleus	BP	1	ZC3H12A,DMAP1,CHP1,RBM22	yellow	VSMC_M3
GO:0007565	0.50	0.63	10	139	female pregnancy	BP	1	PPARD,EPN1,UCP2,RPL29,SOD1,SLC19A1,IGFBP5,C1QBP,CAPN2,AKT1	yellow	VSMC_M3
GO:0048704	0.50	0.67	6	83	embryonic skeletal system morphogenesis	BP	1	PDGFRA,HOXA1,HOXC4,MMP14,SLC39A1,NDST1	yellow	VSMC_M3
GO:0046364	0.50	0.68	5	65	monosaccharide biosynthetic process	BP	1	SELENOS,PCK2,PGD,PGM1,PGP	yellow	VSMC_M3
GO:0048534	0.50	0.56	44	649	hematopoietic or lymphoid organ development	BP	1	NCAPH2,FNIP1,LGALS1,PDGFRA,SOCS5,TTC7A,KLF4,ZC3H12A,KMT2E,RELB,CD79A,METTL3,FASN,SLC11A2,WNT10B,SOD1,EGR3,DLL4,TYRO3,MMP14,CLCF1,PIR,THOC5,RIPK3,PLA2G3,SART1,PBX1,SGPL1,YJEFN3,BAD,PRXL2A,PKN1,MAD1L1,ARMC5,EML1,DOCK11,AP3D1,MKNK2,STK11,TAZ,TGFBR3,KLF13,JAGN1,ZNF385A	yellow	VSMC_M3
GO:0042770	0.50	0.62	11	155	signal transduction in response to DNA damage	BP	1	SPRED2,CCAR2,THOC5,KAT5,BABAM1,SDE2,MAD2L2,ZNF830,HIC1,ZNF385A,FBXO6	yellow	VSMC_M3
GO:0002252	0.50	0.58	25	360	immune effector process	BP	1	GNL1,PLEC,LGALS1,IL1R1,SOCS5,ZC3H12A,KMT2E,RELB,SWAP70,RNF19B,CLCF1,LIMK1,RIPK3,PLA2G3,PHB2,IFI35,LGALS3,C1QBP,PKN1,MAD2L2,CORO1A,DOCK11,C8G,CARD9,JAGN1	yellow	VSMC_M3
GO:0015833	0.50	0.61	13	186	peptide transport	BP	1	PPARD,EIPR1,SLC9A3R1,MYRIP,UCP2,AACS,PCK2,SLC26A6,CAPN10,BAD,GLUD1,MIDN,JAGN1	yellow	VSMC_M3
GO:0000956	0.50	0.65	7	102	nuclear-transcribed mRNA catabolic process	BP	1	SECISBP2,CNOT3,ZC3H12A,DCPS,MRTO4,NANOS3,RBM8A	yellow	VSMC_M3
GO:0010975	0.50	0.58	24	376	regulation of neuron projection development	BP	1	PACSIN1,LGALS1,CUL7,KLF4,DBNL,SNX3,PLXND1,BAIAP2,ARHGAP33,CX3CL1,NGEF,LIMK1,PLA2G3,DBN1,CRABP2,CARM1,EEF2K,AKT1,ACAP3,ANAPC2,STK11,NEGR1,TWF2,NSMF	yellow	VSMC_M3
GO:0006635	0.50	0.68	5	64	fatty acid beta-oxidation	BP	1	ACAT2,PPARD,CPT1C,ETFBKMT,AKT1	yellow	VSMC_M3
GO:0046395	0.50	0.61	14	182	carboxylic acid catabolic process	BP	1	ACAT2,PPARD,CPT1C,BCKDHA,GNPDA1,PCCB,ETFBKMT,PCK2,GLUD1,ILVBL,PGD,AKT1,BLMH,SLC27A4	yellow	VSMC_M3
GO:0061951	0.50	0.70	4	55	establishment of protein localization to plasma membrane	BP	1	SCRIB,CSK,RAB34,ARFRP1	yellow	VSMC_M3
GO:0044262	0.50	0.60	16	229	cellular carbohydrate metabolic process	BP	1	ENPP1,B4GALT1,GALK1,SELENOS,PCK2,IDH1,BAD,ISYNA1,FOXK1,PGD,AKT1,GIT1,PGP,MIDN,B3GNT9,NDST1	yellow	VSMC_M3
GO:0060402	0.50	0.65	7	107	calcium ion transport into cytosol	BP	1	ITPR3,PDE4D,CX3CL1,GSTO1,LETM1,CHERP,CORO1A	yellow	VSMC_M3
GO:0042116	0.50	0.68	5	69	macrophage activation	BP	1	ZC3H12A,CX3CL1,PLA2G3,IFI35,JUND	yellow	VSMC_M3
GO:0043367	0.50	0.70	4	56	CD4-positive, alpha-beta T cell differentiation	BP	1	SOCS5,ZC3H12A,RELB,ARMC5	yellow	VSMC_M3
GO:0150063	0.51	0.59	18	282	visual system development	BP	1	CDK4,PBX2,RING1,PDGFRA,KLF4,WDPCP,SPRED2,SIX5,KDM2B,AQP5,DLL4,IMPDH2,NECTIN1,PBX1,PBX4,C12orf57,SMARCA4,CTNS	yellow	VSMC_M3
GO:0006367	0.51	0.68	5	65	transcription initiation from RNA polymerase II promoter	BP	1	CDK4,WNT10B,TAF6L,GTF2F1,MED16	yellow	VSMC_M3
GO:0006606	0.51	0.63	9	129	protein import into nucleus	BP	1	ZC3H12A,DMAP1,SEC13,CHP1,PHB2,NUP88,RANGRF,RBM22,AKT1	yellow	VSMC_M3
GO:0070301	0.51	0.66	6	79	cellular response to hydrogen peroxide	BP	1	KLF4,PYCR1,RIPK3,RELA,TRAP1,OSER1	yellow	VSMC_M3
GO:0043065	0.51	0.57	29	410	positive regulation of apoptotic process	BP	1	CDK4,SQSTM1,FNIP1,SCRIB,B4GALT1,ZC3H12A,SLC9A3R1,PHLDA1,PLEKHF1,RAPSN,WNT10B,SOD1,CCAR2,RRP1B,PRMT2,RIPK3,CAPN10,BRMS1,BAD,TNFRSF1A,C1QBP,USP27X,CAPN2,DFFA,AKT1,SLC27A4,TRADD,TNFRSF12A,CDC34	yellow	VSMC_M3
GO:0010827	0.51	0.69	4	58	regulation of glucose transmembrane transport	BP	1	ENPP1,SELENOS,CAPN10,AKT1	yellow	VSMC_M3
GO:1903829	0.51	0.60	16	241	positive regulation of cellular protein localization	BP	1	SQSTM1,SREBF2,VPS28,ZC3H12A,DMAP1,NUMA1,PINX1,MCRS1,CHP1,LGALS3,RANGRF,RBM22,AKT1,STK11,GAS8,ERBB4	yellow	VSMC_M3
GO:0043524	0.51	0.64	8	115	negative regulation of neuron apoptotic process	BP	1	BCL2L1,KDM2B,CX3CL1,SOD1,TYRO3,CLCF1,PLA2G3,CORO1A	yellow	VSMC_M3
GO:0070664	0.51	0.69	4	53	negative regulation of leukocyte proliferation	BP	1	SCRIB,GREM1,PKN1,MAD1L1	yellow	VSMC_M3
GO:0000018	0.51	0.66	6	83	regulation of DNA recombination	BP	1	FIGNL1,CLCF1,MAD2L2,PPP4C,CXorf57,SIRT6	yellow	VSMC_M3
GO:0098930	0.51	0.69	4	58	axonal transport	BP	1	SOD1,BLOC1S4,AP3D1,SPG7	yellow	VSMC_M3
GO:0071345	0.51	0.56	40	574	cellular response to cytokine stimulus	BP	1	CDK4,TTLL12,RPL3,SHARPIN,IL1R1,SOCS5,KLF4,ZC3H12A,ZYX,KRT8,METTL3,P4HB,FASN,RAB20,ACSL1,DOK1,CX3CL1,LSP1,CLCF1,PCK2,IMPDH2,SLC26A6,RELA,ATIC,BAD,OTUB1,TNFRSF1A,CAPN2,PALM3,ARHGEF2,CORO1A,TRIM25,CDC37,AKT1,KHSRP,CACTIN,IRAK1,TRADD,JAGN1,CDC34	yellow	VSMC_M3

GO:0006486	0.51	0.60	13	182	protein glycosylation	BP	1	B4GALT1,SERP2,GALNT14,CHP1,SLC35C2,ST3GAL4,TMTC1,EXTL1,MPDU1,DPM3,MAN1B1,POMT1,B3GNT9	yellow	VSMC_M3
GO:0043413	0.51	0.60	13	182	macromolecule glycosylation	BP	1	B4GALT1,SERP2,GALNT14,CHP1,SLC35C2,ST3GAL4,TMTC1,EXTL1,MPDU1,DPM3,MAN1B1,POMT1,B3GNT9	yellow	VSMC_M3
GO:0010821	0.51	0.63	9	124	regulation of mitochondrion organization	BP	1	SQSTM1,SREBF2,BCL2L1,GPX1,CHCHD10,BAD,MYO19,INF2,AKT1	yellow	VSMC_M3
GO:0048562	0.51	0.60	15	225	embryonic organ morphogenesis	BP	1	CTHRC1,SCRIB,PDGFRA,HOXA1,WDPCP,SLC9A3R1,INSIG1,HOXC4,LRIG1,KDM2B,SOD1,MMP14,SLC39A1,TTC39C,NDST1	yellow	VSMC_M3
GO:0006511	0.51	0.56	37	542	ubiquitin-dependent protein catabolic process	BP	1	SQSTM1,BAG6,SHARPIN,VPS28,CUL7,HSPBP1,SOCS5,PSMB3,TMUB1,PSMC4,SEL1L2,WNT10B,CCAR2,RNF19B,SELENOS,ADRM1,PTPN23,KAT5,MVB12A,UBXN1,CCDC22,USP27X,FBXL19,SNF8,TRIM25,USP43,ANKRD9,AKT1,SIRT6,ANAPC2,SPSB3,MAN1B1,ZNRF1,TRIB2,CDC34,FBXO6,STUB1	yellow	VSMC_M3
GO:0002694	0.51	0.57	25	352	regulation of leukocyte activation	BP	1	SCRIB,LGALS1,SOCS5,ZC3H12A,METTL3,CX3CL1,SOD1,EGR3,TYRO3,MMP14,CLCF1,RIPK3,PLA2G3,SART1,BAD,LGALS3,PKN1,MAD1L1,MAD2L2,CORO1A,CD276,CSK,AKT1,AP3D1,JUND	yellow	VSMC_M3
GO:0019318	0.51	0.60	13	185	hexose metabolic process	BP	1	ENPP1,PPARD,B4GALT1,GALK1,INPPL1,SELENOS,PCK2,BAD,FOXK1,AKT1,PGM1,PGP,MIDN	yellow	VSMC_M3
GO:0006900	0.51	0.69	4	55	vesicle budding from membrane	BP	1	SNX3,INSIG1,SEC13,AP3D1	yellow	VSMC_M3
GO:1901137	0.51	0.56	35	494	carbohydrate derivative biosynthetic process	BP	1	GUK1,TSTA3,B4GALT1,PIGO,SERP2,GALNT14,PHLDA1,GNPDA1,ACSL1,TKT,CHP1,SLC35C2,ST3GAL4,AK2,TMTC1,PAPSS1,IMPDH2,EXTL1,ATP5MC3,ATIC,UQCC3,ACLY,MPDU1,DPM3,ATP5PD,AATF,MAN1B1,ATP5F1D,TAZ,AMPD2,APRT,POMT1,AK1,B3GNT9,NDST1	yellow	VSMC_M3
GO:0099111	0.51	0.61	11	165	microtubule-based transport	BP	1	IFT27,SLC9A3R1,CEP131,SOD1,BLOC1S4,AP3D1,SSNA1,STK11,TMEM201,SPG7,WDR34	yellow	VSMC_M3
GO:0006839	0.51	0.59	16	210	mitochondrial transport	BP	1	SAMM50,SREBF2,UCP2,BCL2L1,SFXN5,AIP,CHCHD10,BAD,LETM1,CNP,ATP5PD,AKT1,ALKBH7,ATP5F1D,SPG7,TIMM22	yellow	VSMC_M3
GO:0030163	0.52	0.54	56	815	protein catabolic process	BP	1	SQSTM1,BAG6,SNX9,SGSM3,SHARPIN,VPS28,CUL7,HSPBP1,SOCS5,PSMB3,RCN3,SNRNP70,SNX3,TMUB1,PSMC4,SEL1L2,HM13,WNT10B,PACSIN3,CCAR2,RNF19B,SELENOS,MANBA,GPX1,ASB9,PSME2,ADRM1,PSMB4,PTPN23,KAT5,RELA,MVB12A,UBXN1,CCDC22,USP27X,CAPN2,AMER1,MAD2L2,FBXL19,SNF8,TRIM25,USP43,ANKRD9,AKT1,SIRT6,ANAPC2,SPSB3,MAN1B1,ZNRF1,PSMB10,CTRL,TRIB2,CDC34,FBXO6,STUB1,CCDC115	yellow	VSMC_M3
GO:0060828	0.52	0.59	14	210	regulation of canonical Wnt signaling pathway	BP	1	CTHRC1,TPBG,PLEKHA4,SLC9A3R1,RNF220,GREM1,WNT10B,CCAR2,LIMD1,AMER1,MAD2L2,CSNK1G2,STK11,IGFBP6	yellow	VSMC_M3
GO:0009411	0.52	0.62	9	127	response to UV	BP	1	METTL3,ACTR5,CCAR2,GPX1,RELA,SDE2,AKT1,STK11,NSMCE3	yellow	VSMC_M3
GO:0010675	0.52	0.63	8	114	regulation of cellular carbohydrate metabolic process	BP	1	ENPP1,SELENOS,BAD,FOXK1,AKT1,GIT1,PGP,MIDN	yellow	VSMC_M3
GO:0006405	0.52	0.66	5	69	RNA export from nucleus	BP	1	RBM15B,PABPN1,THOC5,THOC3,NUP88	yellow	VSMC_M3
GO:0051224	0.52	0.64	7	96	negative regulation of protein transport	BP	1	SNX3,INSIG1,UCP2,CHP1,CSK,MIDN,JAGN1	yellow	VSMC_M3
GO:0001704	0.52	0.65	6	93	formation of primary germ layer	BP	1	H2AFY,KLF4,MMP14,COL6A1,ARMC5,DUSP5	yellow	VSMC_M3
GO:0032886	0.52	0.59	13	202	regulation of microtubule-based process	BP	1	CEP131,NUMA1,NAT10,DCTN1,CDK2AP2,STMN3,MID1IP1,EML3,ARHGEF2,RANGRF,GIT1,STK11,GBA2	yellow	VSMC_M3
GO:0001836	0.52	0.68	4	48	release of cytochrome c from mitochondria	BP	1	BCL2L1,GPX1,BAD,AKT1	yellow	VSMC_M3
GO:0000902	0.52	0.54	49	799	cell morphogenesis	BP	1	PACSIN1,SCRIB,PLEC,TBCC,CUL7,COL15A1,DBNL,TPBG,WDPCP,SLC9A3R1,PLXND1,UNK,METTL3,BAIAP2,P4HB,ARHGAP33,ITGB7,SLC11A2,GREM1,SOD1,TYRO3,CORO1B,NGEF,PLEKHO1,IGSF9,NECTIN1,ARPIN,LIMK1,RHOC,FMNL1,LIMD1,C12orf57,C1QBP,RCC2,CNP,DBN1,CRABP2,ARHGEF2,CORO1A,S100A6,SYNE3,EEF2K,ANAPC2,STK11,PSMB10,TWF2,UNC13A,CDC42EP5,NSMF	yellow	VSMC_M3
GO:0006809	0.52	0.68	4	55	nitric oxide biosynthetic process	BP	1	KLF4,ZC3H12A,AKT1,KHSRP	yellow	VSMC_M3
GO:0035303	0.52	0.63	7	106	regulation of dephosphorylation	BP	1	SWAP70,CHP1,RIPK3,PPP1R35,LGALS3,MTMR4,NSMF	yellow	VSMC_M3
GO:0051603	0.52	0.54	44	639	proteolysis involved in cellular protein catabolic process	BP	1	SQSTM1,BAG6,SHARPIN,VPS28,CUL7,HSPBP1,SOCS5,PSMB3,RCN3,TMUB1,PSMC4,SEL1L2,HM13,WNT10B,CCAR2,RNF19B,SELENOS,GPX1,PSME2,ADRM1,PSMB4,PTPN23,KAT5,MVB12A,UBXN1,CCDC22,USP27X,CAPN2,FBXL19,SNF8,TRIM25,USP43,ANKRD9,AKT1,SIRT6,ANAPC2,SPSB3,MAN1B1,ZNRF1,PSMB10,TRIB2,CDC34,FBXO6,STUB1	yellow	VSMC_M3
GO:0098727	0.52	0.63	7	108	maintenance of cell number	BP	1	CNOT3,KLF4,METTL3,MMP24,KDM2B,TAF6L,ZNF358	yellow	VSMC_M3
GO:0007160	0.52	0.60	12	192	cell-matrix adhesion	BP	1	CD63,TSTA3,WDPCP,ZYX,EMILIN1,SIGLEC1,ITGB7,GREM1,CX3CL1,MMP14,PLAU,RCC2	yellow	VSMC_M3
GO:0051704	0.52	0.54	49	709	multi-organism process	BP	1	SPAG1,BAG6,NCAPH2,SBF1,PPARD,IFT27,B4GALT1,EPN1,FIGNL1,SIX5,ZNF296,PAFAH1B3,DLEC1,UCP2,METTL3,CEP131,DND1,YIF1B,RPL29,SIGLEC1,GNPDA1,MORN2,DEAF1,BCL2L1,KDM2B,SOD1,TYRO3,KDM1B,SLC19A1,TRIM27,SLC26A6,IQCG,PLA2G3,SGPL1,IGFBP5,BAD,CD9,C1QBP,CAPN2,NANOS3,ZNF830,AKT1,RFX2,SLC6A4,STK11,TAZ,FAM50A,IGSF8,MEIG1	yellow	VSMC_M3
GO:0051017	0.53	0.61	9	139	actin filament bundle assembly	BP	1	ZYX,MICAL1,BAIAP2,SWAP70,CX3CL1,CORO1B,LIMK1,RHOC,ELN	yellow	VSMC_M3
GO:0071260	0.53	0.66	5	69	cellular response to mechanical stimulus	BP	1	PLEC,MAP3K14,BAD,TNFRSF1A,AKT1	yellow	VSMC_M3
GO:0045727	0.53	0.62	8	109	positive regulation of translation	BP	1	CDK4,RPS9,METTL3,NAT10,PCIF1,MPV17L2,C1QBP,PAIP1	yellow	VSMC_M3
GO:0051279	0.53	0.68	4	58	regulation of release of sequestered calcium ion into cytosol	BP	1	PDE4D,CX3CL1,GSTO1,CORO1A	yellow	VSMC_M3
GO:0002768	0.53	0.60	11	158	immune response-regulating cell surface receptor signaling pathway	BP	1	BAG6,ZC3H12A,CD79A,PDE4D,DGKZ,LIMK1,RELA,LGALS3,CD276,CSK,STK11	yellow	VSMC_M3
GO:0045669	0.53	0.67	4	56	positive regulation of osteoblast differentiation	BP	1	CTHRC1,LRP3,WNT10B,JUND	yellow	VSMC_M3
GO:1904356	0.53	0.67	4	57	regulation of telomere maintenance via telomere lengthening	BP	1	PNKP,NAT10,PINX1,SLX1A	yellow	VSMC_M3

GO:0006357	0.53	0.51	119	1784	regulation of transcription by RNA polymerase II	BP	1	CDK4,SQSTM1,ZNF608,HDAC10,NELFE,FNIP1,ATF6B,PBX2,SREBF2,H2AFY,PPARD,PHF2,KLF4,HOXA1,ZC3H12A,CC2D1B,KTI12,SIX5,ZNF282,ZNF296,RELB,DMAP1,CIC,SERTAD1,TTC5,CXXC5,DEAF1,HOXC4,ZNF740,ZBTB38,GREM1,KDM2B,MCRS1,CX3CL1,WNT10B,ANKRD2,EGR3,DNAJC17,DLL4,HTATIP2,KDM1B,CHCHD2,IRX1,RRP1B,BRD9,TSHZ2,TRIM27,TWIST2,SMARCD2,BRMS1,GMEB2,PRPF6,PBX1,KAT5,RELA,ZNF511,ARNT2,SMARCB1,PBX4,GATAD2A,MEF2B,CTDSP1,EID1,SSBP4,METTL13,ZNF394,BUD31,TNFRSF1A,TEAD4,C1QBP,TAF6L,ZBTB49,MTA2,ZNF316,MED11,PELP1,FOXK1,KLF3,PKN1,MAD2L2,SLTM,CTBP1,FBXL19,SP2,ARHGEF2,SNF8,PER1,NFIX,IRF2BPL,SMARCA4,AATF,ZNF521,ZNF358,AKT1,GTF2F1,RFX2,SAFB,SAFB2,MYDGF,HDGFL2,SIRT6,SSU72,HIC1,MNT,EHMT1,AP3D1,MBD3,MED16,SARS,JUND,KLF13,ZNF783,HOXC5,IER5,ZNF250,THAP7,NSMCE3,ERF,ZSCAN1	yellow	VSMC_M3
GO:1901701	0.53	0.53	59	887	cellular response to oxygen-containing compound	BP	1	ENPP1,CDK4,PPARD,PLEC,LGALS1,PDGFRA,KLF4,ZC3H12A,SLC9A3R1,INSIG1,UCP2,BAIAP2,LAMTOR1,PYCR1,AACS,SCARB1,BCL2L1,TNS2,PDE3B,CX3CL1,WNT10B,SOD1,DCPS,SELENOS,PABPN1,PCK2,COL6A1,SLC26A6,RIPK3,CAPN10,KAT5,RELA,SIPA1,SMARCB1,PHB2,IGFBP5,BAD,LAMTOR4,KLF3,CAPN2,PIP4K2B,ARHGEF2,SMARCA4,EEF2K,CSK,AKT1,HMGCS1,GIT1,SLC6A4,LDOC1,CACTIN,TRAP1,IRAK1,APRT,SLC27A4,TWF2,JAGN1,NSMF,OSER1	yellow	VSMC_M3
GO:0022600	0.53	0.67	4	56	digestive system process	BP	1	SCARB1,AQP5,SLC26A6,ZNF830	yellow	VSMC_M3
GO:0055117	0.53	0.67	4	58	regulation of cardiac muscle contraction	BP	1	ZC3H12A,PDE4D,GSTO1,RANGRF	yellow	VSMC_M3
GO:0046883	0.53	0.59	12	174	regulation of hormone secretion	BP	1	PPARD,EIPR1,MYRIP,UCP2,AACS,PCK2,CAPN10,PLA2G3,BAD,GLUD1,MIDN,JAGN1	yellow	VSMC_M3
GO:0048880	0.53	0.57	18	286	sensory system development	BP	1	CDK4,PBX2,RING1,PDGFRA,KLF4,WDPCP,SPRED2,SIX5,KDM2B,AQP5,DLL4,IMPDH2,NECTIN1,PBX1,PBX4,C12orf57,SMARCA4,CTNS	yellow	VSMC_M3
GO:0001776	0.53	0.65	5	70	leukocyte homeostasis	BP	1	RIPK3,PKN1,CORO1A,DOCK11,AKT1	yellow	VSMC_M3
GO:1901214	0.53	0.57	17	251	regulation of neuron death	BP	1	BCL2L1,KDM2B,RAPSN,CX3CL1,SOD1,CHP1,TYRO3,CLCF1,PLA2G3,BAD,AIMP2,CAPN2,CORO1A,AKT1,DHCR24,NSMF,CDC34	yellow	VSMC_M3
GO:0005975	0.53	0.54	32	458	carbohydrate metabolic process	BP	1	ENPP1,PPARD,B4GALT1,GALK1,INPPL1,GNPDA1,OGDHL,ST3GAL4,SELENOS,GSTO1,MANBA,PCK2,TPD52L2,IDH1,BAD,ISYNA1,FOXK1,MPDU1,PGD,DPM3,AKT1,PGM1,GIT1,PGP,MAN1B1,MIDN,JMJD8,POMT1,B3GNT9,NDST1,TALDO1,GBA2	yellow	VSMC_M3
GO:0030705	0.53	0.59	11	173	cytoskeleton-dependent intracellular transport	BP	1	IFT27,CEP131,SOD1,BLOC1S4,MYO19,AP3D1,SSNA1,STK11,TMEM201,SPG7,WDR34	yellow	VSMC_M3
GO:0090150	0.53	0.57	15	210	establishment of protein localization to membrane	BP	1	BAG6,SAMM50,SCRIB,ASNA1,YIF1B,RAPSN,CHP1,PEX5,ZDHHC3,SYNE3,CSK,ATG3,RAB34,ARFRP1,TIMM22	yellow	VSMC_M3
GO:0034614	0.53	0.60	9	124	cellular response to reactive oxygen species	BP	1	PDGFRA,KLF4,PYCR1,SOD1,RIPK3,RELA,AKT1,TRAP1,OSER1	yellow	VSMC_M3
GO:0051222	0.53	0.57	16	241	positive regulation of protein transport	BP	1	SREBF2,PPARD,VPS28,ZC3H12A,DMAP1,MYRIP,CEP131,AACS,CHP1,PCK2,PCNT,CAPN10,PTPN23,BAD,GLUD1,RBM22	yellow	VSMC_M3
GO:0050770	0.54	0.61	8	132	regulation of axonogenesis	BP	1	DBNL,PLXND1,LIMK1,DBN1,CRABP2,ANAPC2,STK11,TWF2	yellow	VSMC_M3
GO:0008361	0.54	0.59	10	157	regulation of cell size	BP	1	CDK4,DBNL,SLC9A3R1,LIMK1,LAMTOR4,DBN1,CRABP2,AKT1,ANAPC2,TWF2	yellow	VSMC_M3
GO:0060538	0.54	0.60	9	132	skeletal muscle organ development	BP	1	PLEC,COL19A1,WNT10B,GPX1,HMG20B,TAZ,GTF3C5,IGSF8,ELN	yellow	VSMC_M3
GO:0044282	0.54	0.55	21	284	small molecule catabolic process	BP	1	ACAT2,NUDT3,PPARD,MPST,CPT1C,SNX17,GALK1,BCKDHA,GNPDA1,SCARB1,PCCB,ETFBKMT,PCK2,BAD,GLUD1,FOXK1,ILVBL,PGD,AKT1,BLMH,SLC27A4	yellow	VSMC_M3
GO:0061387	0.54	0.63	6	95	regulation of extent of cell growth	BP	1	DBNL,LIMK1,DBN1,CRABP2,ANAPC2,TWF2	yellow	VSMC_M3
GO:0090257	0.54	0.58	12	179	regulation of muscle system process	BP	1	KLF4,ZC3H12A,PDE4D,SOD1,GSTO1,SCN4A,SPX,IGFBP5,C12orf57,TNFRSF1A,RANGRF,MIR214	yellow	VSMC_M3
GO:0048519	0.54	0.49	266	3961	negative regulation of biological process	BP	1	ENPP1,NAB2,CTDSP2,SQSTM1,ZNF608,BAG6,CTHRC1,HDAC10,NELFE,FNIP1,ATF6B,TTLL12,RING1,SREBF2,H2AFY,ITPR3,HNRNPA0,TRIM11,PACSIN1,PPARD,SCRIB,PLEC,LGALS1,SHARPIN,VPS28,SND1,B4GALT1,PDGFRA,SECISBP2,PHF2,EPN1,SSC5D,SOCS5,CNOT3,KLF4,TPBG,SPRED2,RGS3,ZC3H12A,FIGNL1,PNKP,SNRNP70,CC2D1B,SNX3,MICAL1,ANXA5,SIX5,ZNF282,ZNF296,RELB,SLC9A3R1,PLXND1,EMILIN1,DMAP1,CIC,UNK,DLEC1,INSIG1,OGG1,UCP2,METTL3,CXXC5,INPPL1,DND1,PYCR1,EIF4EBP1,CAVIN3,DEAF1,TARBP2,NAT10,BCL2L1,PDE4D,TNS2,SWAP70,LRP3,ZBTB38,LSM6,CAPG,PINX1,GREM1,KDM2B,PDE3B,CX3CL1,WNT10B,SOD1,PACSIN3,ANKRD2,DGKZ,EGR3,CCAR2,DNAJC17,DLL4,CHP1,TYRO3,YAF2,HTATIP2,PCIF1,PCBP4,KDM1B,SLC35C2,CISH,SRSF9,DCPS,IRX1,SELENOS,GSTO1,ITPRIP,RRP1B,ETFBKMT,MVK,RIN3,FABP3,ZCCHC17,PDGFA,MMP14,CDK2AP2,GPX1,CORO1B,NGEF,CLCF1,QARS,TRIM27,ARPIN,EIF3A,LIMK1,PRMT2,SPX,THOC5,TWIST2,PSMB4,PFDN2,BRMS1,PLA2G3,STMN3,BANF1,CCDC85B,PTPN23,PBX1,KAT5,RELA,SIPA1,ARNT2,LIMD1,MID1IP1,PEX5,SMARCB1,YJEFN3,GATAD2A,PHB2,PPP1R35,IGFBP5,IFI35,CTDSP1,OTUB1,EID1,METTL13,LETM1,LGALS3,C11orf95,CD9,MRTO4,PLAU,TNFRSF1A,BABAM1,C1QBP,AIMP2,CHERP,ZBTB49,UBXN1,WASHC1,RCC2,MTA2,CCDC22,FOXK1,SDE2,KLF3,PKN1,MIIP,MAD1L1,PALM3,AMER1,PIP4K2B,MAD2L2,NANOS3,CTBP1,DFFA,SP2,ARHGEF2,MVP,PER1,NFIX,CORO1A,SLX1A,IRF2BPL,TRIM25,RPL37,MTMR4,SMARCA4,CARM1,EEF2K,AATF,CXorf57,C19orf66,CSK,MID2,ATG3,ZNF830,AKT1,PAIP1,KHSRP,GIT1,ANKRD13B,SLC6A4,DHCR24,CARHSP1,MYDGF,LDOC1,SIRT6,MAP2K2,[illegible]	yellow	VSMC_M3
GO:0061572	0.54	0.60	9	141	actin filament bundle organization	BP	1	ZYX,MICAL1,BAIAP2,SWAP70,CX3CL1,CORO1B,LIMK1,RHOC,ELN	yellow	VSMC_M3
GO:0033627	0.54	0.66	4	60	cell adhesion mediated by integrin	BP	1	ITGB7,SWAP70,PDE3B,PLAU	yellow	VSMC_M3
GO:0001838	0.54	0.61	7	107	embryonic epithelial tube formation	BP	1	CTHRC1,SCRIB,DEAF1,GREM1,KDM2B,IRX1,MTHFR	yellow	VSMC_M3
GO:0006403	0.54	0.58	12	171	RNA localization	BP	1	NHP2,SEC13,RBM15B,PABPN1,THOC5,PRPF6,THOC3,NUP88,MVP,KHSRP,RBM8A,ZNF385A	yellow	VSMC_M3

GO:0043069	0.54	0.52	47	681	negative regulation of programmed cell death	BP	1	BAG6,FNIP1,PPARD,KLF4,ZC3H12A,FIGNL1,MICAL1,ANXA5,OGG1,UCP2,BCL2L1,GREM1,KDM2B,CX3CL1,SOD1,EGR3,CCAR2,TYRO3,HTATIP2,DCPS,SELENOS,ITPRIP,GPX1,CLCF1,QARS,TWIST2,PLA2G3,RELA,ARNT2,PHB2,LGALS3,NANOS3,DFFA,ARHGEF2,CORO1A,EEF2K,AATF,ZNF830,AKT1,DHCR24,MYDGF,TRAP1,MNT,MIR214,PTMA,ZNF385A,TXNDC12	yellow	VSMC_M3
GO:0051453	0.54	0.66	4	58	regulation of intracellular pH	BP	1	RAB20,CHP1,SLC26A6,CCDC115	yellow	VSMC_M3
GO:0032504	0.54	0.52	39	571	multicellular organism reproduction	BP	1	BAG6,NCAPH2,SBF1,PPARD,IFT27,B4GALT1,PDGFRA,FIGNL1,SIX5,ZNF296,PAFAH1B3,DLEC1,METTL3,CEP131,DND1,YIF1B,MORN2,DEAF1,BCL2L1,KDM2B,SOD1,TYRO3,KDM1B,TRIM27,SLC26A6,IQCG,PLA2G3,SGPL1,BAD,CD9,NANOS3,ZNF830,AKT1,RFX2,SLC6A4,STK11,TAZ,FAM50A,MEIG1	yellow	VSMC_M3
GO:1900180	0.54	0.60	8	116	regulation of protein localization to nucleus	BP	1	ZC3H12A,DMAP1,PINX1,MCRS1,CHP1,RBM22,AKT1,STK11	yellow	VSMC_M3
GO:0001889	0.54	0.60	8	116	liver development	BP	1	MPST,PHF2,UCP2,AACS,PCK2,RELA,HMGCS1,TGFBR3	yellow	VSMC_M3
GO:1904589	0.55	0.66	4	55	regulation of protein import	BP	1	ZC3H12A,DMAP1,CHP1,RBM22	yellow	VSMC_M3
GO:0010810	0.55	0.58	11	175	regulation of cell-substrate adhesion	BP	1	TSTA3,LGALS1,WDPCP,EMILIN1,P4HB,GREM1,CX3CL1,MMP14,PLAU,C1QBP,RCC2	yellow	VSMC_M3
GO:0090263	0.55	0.62	6	92	positive regulation of canonical Wnt signaling pathway	BP	1	PLEKHA4,RNF220,WNT10B,CCAR2,AMER1,CSNK1G2	yellow	VSMC_M3
GO:1905330	0.55	0.66	4	56	regulation of morphogenesis of an epithelium	BP	1	GREM1,PDGFA,PHB2,SIRT6	yellow	VSMC_M3
GO:0046632	0.55	0.64	5	74	alpha-beta T cell differentiation	BP	1	SOCS5,ZC3H12A,RELB,ARMC5,AP3D1	yellow	VSMC_M3
GO:0051254	0.55	0.50	90	1357	positive regulation of RNA metabolic process	BP	1	SQSTM1,NELFE,ATF6B,PBX2,SREBF2,PPARD,PHF2,CNOT3,KLF4,HOXA1,ZC3H12A,SNRNP70,KMT2E,SIX5,ZNF296,SERTAD1,TTC5,METTL3,DND1,DEAF1,HOXC4,KMT2B,ZBTB38,GREM1,MCRS1,CX3CL1,WNT10B,EGR3,CCAR2,YAF2,HTATIP2,KDM1B,CHCHD2,DCPS,RRP1B,AIP,TRIM27,PRMT2,THOC5,RIPK3,SMARCD2,BUD23,GMEB2,PRPF6,PBX1,KAT5,RELA,ARNT2,SMARCB1,CHCHD10,PBX4,UBTF,MEF2B,TRMT112,NDP,SSBP4,BUD31,TNFRSF1A,TAF6L,ZBTB49,MTA2,PELP1,FOXK1,PKN1,POLR2A,MAD2L2,NANOS3,ARHGEF2,PER1,NFIX,IRF2BPL,TRIM25,SMARCA4,CARM1,AATF,MID2,RBM22,ZNF521,AKT1,BRF1,KHSRP,GTF2F1,MYDGF,MAP2K2,AP3D1,MED16,ERBB4,JUND,KLF13,IER5	yellow	VSMC_M3
GO:0061351	0.55	0.61	7	111	neural precursor cell proliferation	BP	1	KDM2B,CX3CL1,DLL4,DBN1,ARHGEF2,EML1,SLC6A4	yellow	VSMC_M3
GO:0006366	0.55	0.49	124	1862	transcription by RNA polymerase II	BP	1	CDK4,SQSTM1,ZNF608,HDAC10,NELFE,FNIP1,ATF6B,PBX2,SREBF2,H2AFY,PPARD,PHF2,KLF4,HOXA1,ZC3H12A,CC2D1B,KTI12,SIX5,ZNF282,ZNF296,RELB,DMAP1,CIC,SERTAD1,TTC5,CXXC5,DEAF1,HOXC4,ZNF740,ZBTB38,GREM1,KDM2B,MCRS1,POLR2C,CX3CL1,WNT10B,ANKRD2,EGR3,DNAJC17,DLL4,HTATIP2,KDM1B,CHCHD2,IRX1,RRP1B,BRD9,TSHZ2,PIR,TRIM27,TWIST2,ADRM1,SMARCD2,BRMS1,GMEB2,PRPF6,PBX1,KAT5,RELA,ZNF511,ARNT2,SMARCB1,PBX4,GATAD2A,MEF2B,CTDSP1,EID1,SSBP4,METTL13,ZNF394,BUD31,TNFRSF1A,TEAD4,C1QBP,TAF6L,ZBTB49,MTA2,ZNF316,MED11,PELP1,FOXK1,KLF3,PKN1,POLR2A,MAD2L2,SLTM,CTBP1,FBXL19,SP2,ARHGEF2,SNF8,PER1,NFIX,IRF2BPL,SMARCA4,AATF,ZNF521,ZNF358,AKT1,GTF2F1,RFX2,SAFB,SAFB2,MYDGF,HDGFL2,SIRT6,SSU72,HIC1,MNT,EHMT1,AP3D1,MBD3,MED16,SARS,JUND,KLF13,ZNF783,HOXC5,IER5,ZNF250,THAP7,NSMCE3,ERF,SNAPC2,ZSCAN1	yellow	VSMC_M3
GO:0060401	0.55	0.60	8	126	cytosolic calcium ion transport	BP	1	ITPR3,PDE4D,CX3CL1,GSTO1,LETM1,CHERP,CORO1A,AKT1	yellow	VSMC_M3
GO:0044264	0.55	0.63	5	75	cellular polysaccharide metabolic process	BP	1	ENPP1,SELENOS,AKT1,B3GNT9,NDST1	yellow	VSMC_M3
GO:0022610	0.55	0.50	66	1050	biological adhesion	BP	1	CD63,PPARD,TSTA3,SCRIB,LGALS1,B4GALT1,PDGFRA,COL15A1,COL19A1,SOCS5,KLF4,TPBG,WDPCP,ZC3H12A,ZYX,PLXND1,EMILIN1,METTL3,BAIAP2,INPPL1,P4HB,SIGLEC1,RIC8A,SCARB1,ITGB7,SWAP70,MMP24,GREM1,PDE3B,CX3CL1,EGR3,TYRO3,ST3GAL4,MMP14,IGSF9,LRFN4,COL6A1,NECTIN1,MPZL3,SART1,PTPN23,RELA,SIPA1,BLOC1S4,BAD,SCARF2,LGALS3,CD9,PLAU,C1QBP,RCC2,MAD1L1,MAD2L2,CORO1A,CD276,AATF,CSK,AKT1,CD99L2,TJP3,CLDN6,AP3D1,IRAK1,NEGR1,TNFRSF12A,COL6A2	yellow	VSMC_M3
GO:0097006	0.55	0.65	4	53	regulation of plasma lipoprotein particle levels	BP	1	SCARB1,PLA2G3,CSK,KHSRP	yellow	VSMC_M3
GO:0032268	0.55	0.49	124	1819	regulation of cellular protein metabolic process	BP	1	ENPP1,NAB2,CDK4,CTDSP2,SQSTM1,BAG6,SNX9,NELFE,FNIP1,PRR5,H2AFY,PLEC,VPS28,SECISBP2,HSPBP1,SOCS5,RPS9,CNOT3,KLF4,SPRED2,ZC3H12A,PNKP,RCN3,MICAL1,KMT2E,WDFY2,SLC9A3R1,EMILIN1,UNK,SERTAD1,PSMC4,METTL3,DND1,EIF4EBP1,TARBP2,NAT10,PDE4D,EIF4B,SWAP70,ACSL1,TNKS1BP1,PINX1,GREM1,CX3CL1,WNT10B,SOD1,PACSIN3,CCAR2,CHP1,PCIF1,RNF19B,DCPS,PDGFA,MMP14,GPX1,CLCF1,NGDN,QARS,PSME2,TRIM27,LIMK1,RIPK3,LSM14B,ADRM1,BRMS1,TNK2,TACO1,KAT5,RELA,SMARCB1,PHB2,IGFBP5,BAD,CTDSP1,OTUB1,EID1,MPV17L2,LGALS3,TNFRSF1A,C1QBP,AIMP2,UBXN1,WASHC1,CCDC22,PKN1,SEPSECS,AMER1,MTHFR,MAD2L2,NANOS3,CTBP1,ARHGEF2,MVP,PER1,RPL37,EEF2K,CDC37,C19orf66,CSK,AKT1,PAIP1,KHSRP,DHCR24,MYDGF,SIRT6,MAP2K2,CACTIN,HMG20B,TRAP1,AURKAIP1,MKNK2,IRAK1,STK11,TELO2,CARD9,POMT1,RBM8A,ERBB4,TRIB2,ZNF385A,NSMF,THAP7,STUB1,NSMCE3	yellow	VSMC_M3
GO:0045765	0.55	0.56	15	229	regulation of angiogenesis	BP	1	EPN1,KLF4,ZC3H12A,PLXND1,EMILIN1,GREM1,PDE3B,HTATIP2,YJEFN3,MYDGF,SIRT6,JMJD8,SARS,MIR214,RNH1	yellow	VSMC_M3
GO:0035264	0.55	0.60	7	108	multicellular organism growth	BP	1	CDK4,PLEC,CCM2,TNS2,SOD1,SGPL1,SP2	yellow	VSMC_M3

GO:0031399	0.55	0.50	79	1182	regulation of protein modification process	BP	1	ENPP1,NAB2,CTDSP2,SQSTM1,SNX9,NELFE,FNIP1,PRR5,H2AFY,PLEC,VPS28,HSPBP1,SOCS5,SPRED2,ZC3H12A,PNKP,MICAL1,KMT2E,WDFY2,SLC9A3R1,EMILIN1,SERTAD1,TARBP2,PDE4D,SWAP70,ACSL1,TNKS1BP1,PINX1,GREM1,CX3CL1,SOD1,CCAR2,CHP1,PDGFA,CLCF1,QARS,TRIM27,LIMK1,RIPK3,BRMS1,TNK2,KAT5,RELA,SMARCB1,PHB2,CTDSP1,OTUB1,EID1,LGALS3,TNFRSF1A,AIMP2,UBXN1,WASHC1,PKN1,AMER1,MTHFR,MAD2L2,CTBP1,ARHGEF2,MVP,RPL37,EEF2K,CDC37,CSK,AKT1,MYDGF,MAP2K2,CACTIN,HMG20B,IRAK1,STK11,TELO2,POMT1,ERBB4,TRIB2,NSMF,THAP7,STUB1,NSMCE3	yellow	VSMC_M3
GO:0051607	0.55	0.57	12	174	defense response to virus	BP	1	TRIM11,ZC3H12A,TARBP2,BCL2L1,RIPK3,RELA,MAP3K14,PHB2,C1QBP,ARMC5,C19orf66,CARD9	yellow	VSMC_M3
GO:0140546	0.55	0.57	12	174	defense response to symbiont	BP	1	TRIM11,ZC3H12A,TARBP2,BCL2L1,RIPK3,RELA,MAP3K14,PHB2,C1QBP,ARMC5,C19orf66,CARD9	yellow	VSMC_M3
GO:0019725	0.55	0.51	42	638	cellular homeostasis	BP	1	ENPP1,ITPR3,PPARD,LGALS1,PDGFRA,TTC7A,SLC9A3R1,SLC30A3,UCP2,RAB20,GSR,AACS,PDE4D,TNS2,SWAP70,SLC11A2,DCTN1,CX3CL1,SOD1,CHP1,TYRO3,SELENOS,GSTO1,GPX1,PCK2,SLC26A6,SMARCB1,TXNRD2,BAD,LETM1,CHERP,CCDC22,FOXK1,CORO1A,TSPOAP1,SMARCA4,ANKRD9,PACS2,GIT1,AP3D1,JAGN1,CCDC115	yellow	VSMC_M3
GO:0019941	0.55	0.52	37	550	modification-dependent protein catabolic process	BP	1	SQSTM1,BAG6,SHARPIN,VPS28,CUL7,HSPBP1,SOCS5,PSMB3,TMUB1,PSMC4,SEL1L2,WNT10B,CCAR2,RNF19B,SELENOS,ADRM1,PTPN23,KAT5,MVB12A,UBXN1,CCDC22,USP27X,FBXL19,SNF8,TRIM25,USP43,ANKRD9,AKT1,SIRT6,ANAPC2,SPSB3,MAN1B1,ZNRF1,TRIB2,CDC34,FBXO6,STUB1	yellow	VSMC_M3
GO:0071897	0.55	0.58	10	150	DNA biosynthetic process	BP	1	NHP2,KLF4,PNKP,NAT10,TNKS1BP1,PINX1,GREM1,USP43,TELO2,RF00024	yellow	VSMC_M3
GO:0120036	0.55	0.50	75	1193	plasma membrane bounded cell projection organization	BP	1	SPAG1,CTHRC1,TXNDC15,PACSIN1,SCRIB,LGALS1,IFT27,CUL7,KLF4,DBNL,TPBG,WDPCP,SNX3,EMP3,EHD2,SLC9A3R1,PLXND1,SEPT9,BAIAP2,CEP131,INPPL1,YIF1B,CCDC57,ARHGAP33,SLC11A2,DCTN1,CX3CL1,SOD1,CCDC32,RAB35,CCDC28B,CORO1B,NGEF,PLEKHO1,IGSF9,NECTIN1,ARPIN,PCNT,LIMK1,IQCG,PLA2G3,STMN3,CEP164,PTPN23,BLOC1S4,C12orf57,WASHC1,RCC2,CNP,DBN1,CRABP2,FAM161B,CORO1A,RABEP2,S100A6,CARM1,EEF2K,ATG3,TTC39C,DOCK11,AKT1,RFX2,ACAP3,SSNA1,ANAPC2,STK11,GAS8,WDR34,NEGR1,MIR214,TWF2,UNC13A,CDC42EP5,NSMF,MEIG1	yellow	VSMC_M3
GO:0042177	0.55	0.60	7	101	negative regulation of protein catabolic process	BP	1	BAG6,SNRNP70,SNX3,CCAR2,RELA,UBXN1,MAD2L2	yellow	VSMC_M3
GO:0140694	0.55	0.54	20	305	non-membrane-bounded organelle assembly	BP	1	SQLE,RPL3,PLEC,BOP1,PDGFRA,DBNL,KRT8,NUMA1,CCDC57,RRS1,SAC3D1,LIMD1,MPV17L2,MRTO4,KIAA0753,C1QBP,EML3,MRPL20,MIS12,TUBGCP2	yellow	VSMC_M3
GO:0150116	0.55	0.65	4	65	regulation of cell-substrate junction organization	BP	1	WDPCP,GREM1,MMP14,RCC2	yellow	VSMC_M3
GO:0006879	0.56	0.65	4	56	cellular iron ion homeostasis	BP	1	TTC7A,SLC11A2,SOD1,CCDC115	yellow	VSMC_M3
GO:0045216	0.56	0.58	9	146	cell-cell junction organization	BP	1	PLEC,CCM2,NECTIN1,PTPN23,RHOC,CD9,CSK,TJP3,CLDN6	yellow	VSMC_M3
GO:1904950	0.56	0.60	7	100	negative regulation of establishment of protein localization	BP	1	SNX3,INSIG1,UCP2,CHP1,CSK,MIDN,JAGN1	yellow	VSMC_M3
GO:0051251	0.56	0.56	13	186	positive regulation of lymphocyte activation	BP	1	LGALS1,SOCS5,EGR3,MMP14,CLCF1,SART1,BAD,MAD2L2,CORO1A,CD276,CSK,AKT1,AP3D1	yellow	VSMC_M3
GO:0031589	0.56	0.54	18	294	cell-substrate adhesion	BP	1	CD63,PPARD,TSTA3,LGALS1,WDPCP,ZYX,EMILIN1,P4HB,SIGLEC1,ITGB7,GREM1,CX3CL1,TYRO3,MMP14,PLAU,C1QBP,RCC2,CORO1A	yellow	VSMC_M3
GO:0000278	0.56	0.50	49	729	mitotic cell cycle	BP	1	DCTN2,CDK4,CTDSP2,NCAPH2,SNX9,TTLL12,H2AFY,SCRIB,CUL7,KLF4,KMT2E,SLC9A3R1,NUMA1,CCDC57,EIF4EBP1,RRS1,PINX1,DCTN1,WNT10B,RAB35,CDK2AP2,PSME2,PCNT,NUDC,PRMT2,PBX1,RHOC,PHB2,CTDSP1,BABAM1,NUP88,RCC2,EML3,SDE2,MIIP,MAD1L1,MAD2L2,EML1,AATF,ZNF830,AKT1,SNX18,CDK11B,AURKAIP1,MIR214,NABP2,MIS12,CDC34,TUBGCP2	yellow	VSMC_M3
GO:0060759	0.56	0.58	9	128	regulation of response to cytokine stimulus	BP	1	TTLL12,SHARPIN,IL1R1,KLF4,METTL3,PALM3,CDC37,CACTIN,IRAK1	yellow	VSMC_M3
GO:0010633	0.56	0.64	4	59	negative regulation of epithelial cell migration	BP	1	KLF4,DLL4,PTPN23,PTPRR	yellow	VSMC_M3
GO:0070661	0.56	0.55	14	201	leukocyte proliferation	BP	1	SCRIB,CD79A,GREM1,CX3CL1,CLCF1,IMPDH2,RIPK3,LGALS3,PKN1,MAD1L1,ARMC5,CORO1A,CD276,PSMB10	yellow	VSMC_M3
GO:0051250	0.56	0.59	7	99	negative regulation of lymphocyte activation	BP	1	SCRIB,SOCS5,ZC3H12A,TYRO3,LGALS3,PKN1,MAD1L1	yellow	VSMC_M3
GO:0030326	0.56	0.60	6	96	embryonic limb morphogenesis	BP	1	PBX2,WDPCP,GREM1,PBX1,CRABP2,ZNF358	yellow	VSMC_M3
GO:0035113	0.56	0.60	6	96	embryonic appendage morphogenesis	BP	1	PBX2,WDPCP,GREM1,PBX1,CRABP2,ZNF358	yellow	VSMC_M3
GO:0099518	0.56	0.64	4	65	vesicle cytoskeletal trafficking	BP	1	BLOC1S4,MYO19,AP3D1,STK11	yellow	VSMC_M3
GO:0035773	0.56	0.64	4	58	insulin secretion involved in cellular response to glucose stimulus	BP	1	PPARD,UCP2,BAD,JAGN1	yellow	VSMC_M3
GO:0006368	0.56	0.64	4	58	transcription elongation from RNA polymerase II promoter	BP	1	NELFE,SERTAD1,ADRM1,GTF2F1	yellow	VSMC_M3
GO:0072665	0.56	0.64	4	60	protein localization to vacuole	BP	1	VPS28,LAMTOR4,SNF8,AP3D1	yellow	VSMC_M3

GO:0051246	0.56	0.47	133	1953	regulation of protein metabolic process	BP	1	ENPP1,NAB2,CDK4,CTDSP2,SQSTM1,BAG6,SNX9,NELFE,FNIP1,PRR5,H2AFY,SGSM3,PLEC,VPS28,SECISBP2,HSPBP1,SOCS5,RPS9,CNOT3,KLF4,SPRED2,ZC3H12A,PNKP,RCN3,SNRNP70,SNX3,MICAL1,KMT2E,WDFY2,SLC9A3R1,EMILIN1,UNK,SERTAD1,PSMC4,METTL3,DND1,EIF4EBP1,DELE1,LDLRAD3,TARBP2,NAT10,PDE4D,EIF4B,SWAP70,ACSL1,TNKS1BP1,PINX1,GREM1,CX3CL1,WNT10B,SOD1,PACSIN3,CCAR2,CHP1,PCIF1,RNF19B,DCPS,PDGFA,MMP14,GPX1,CLCF1,NGDN,QARS,ASB9,PSME2,TRIM27,LIMK1,RIPK3,LSM14B,ADRM1,PFDN2,BRMS1,TNK2,TACO1,KAT5,RELA,SMARCB1,PHB2,IGFBP5,BAD,CTDSP1,OTUB1,EID1,MPV17L2,LGALS3,TNFRSF1A,C1QBP,AIMP2,UBXN1,WASHC1,CCDC22,PKN1,SEPSECS,AMER1,MTHFR,MAD2L2,NANOS3,CTBP1,ARHGEF2,SNF8,MVP,PER1,RPL37,EEF2K,CDC37,AATF,C19orf66,CSK,AKT1,PAIP1,KHSRP,DHCR24,MYDGF,SIRT6,MAP2K2,CACTIN,HMG20B,TRAP1,AURKAIP1,MKNK2,IRAK1,STK11,TELO2,CARD9,POMT1,RBM8A,ERBB4,TRIB2,ZNF385A,NSMF,THAP7,STUB1,NSMCE3	yellow	VSMC_M3
GO:0006766	0.57	0.62	5	71	vitamin metabolic process	BP	1	PPARD,SLC52A2,GSTO1,SLC19A1,FLAD1	yellow	VSMC_M3
GO:1901654	0.57	0.56	10	151	response to ketone	BP	1	CDK4,KLF4,AACS,BCL2L1,DCPS,PCK2,RELA,BAD,AKT1,TGFBR3	yellow	VSMC_M3
GO:0002520	0.57	0.50	46	694	immune system development	BP	1	NCAPH2,FNIP1,LGALS1,PDGFRA,SOCS5,TTC7A,KLF4,ZC3H12A,KMT2E,RELB,CD79A,METTL3,FASN,SWAP70,SLC11A2,WNT10B,SOD1,EGR3,DLL4,TYRO3,MMP14,CLCF1,PIR,THOC5,RIPK3,PLA2G3,SART1,PBX1,SGPL1,YJEFN3,BAD,PRXL2A,PKN1,MAD1L1,MAD2L2,ARMC5,EML1,DOCK11,AP3D1,MKNK2,STK11,TAZ,TGFBR3,KLF13,JAGN1,ZNF385A	yellow	VSMC_M3
GO:2000177	0.57	0.64	4	62	regulation of neural precursor cell proliferation	BP	1	KDM2B,CX3CL1,DLL4,SLC6A4	yellow	VSMC_M3
GO:0097581	0.57	0.61	5	78	lamellipodium organization	BP	1	CORO1B,PLEKHO1,ARPIN,MIR214,TWF2	yellow	VSMC_M3
GO:0048869	0.57	0.47	198	3033	cellular developmental process	BP	1	ENPP1,CD63,NAB2,SQSTM1,CCHCR1,BAG6,NCAPH2,SBF1,CTHRC1,HDAC10,FNIP1,PBX2,H2AFY,TRIM11,TRAPPC9,PACSIN1,PPARD,SCRIB,PLEC,LGALS1,IFT27,SHARPIN,SND1,CUL7,DNPH1,B4GALT1,PDGFRA,SECISBP2,COL15A1,COL19A1,SOCS5,CNOT3,KLF4,DBNL,TPBG,WDPCP,CCM2,SPRED2,ZC3H12A,FIGNL1,SNX3,EHD2,FAM120B,KMT2E,KRT8,WDFY2,SIX5,RELB,SLC9A3R1,PLXND1,UNK,DLEC1,CD79A,INSIG1,METTL3,BAIAP2,CEP131,NUMA1,P4HB,DND1,YIF1B,FASN,MORN2,ARHGAP22,DEAF1,TBCB,ARHGAP33,AACS,BCL2L1,ITGB7,PDE4D,LRP3,SLC11A2,MMP24,GREM1,CX3CL1,WNT10B,SOD1,ANKRD2,EGR3,DLL4,TYRO3,HTATIP2,RAB35,IRX1,FABP3,MMP14,C14orf93,GPX1,CORO1B,NGEF,CLCF1,PLEKHO1,TAGLN2,IGSF9,PCK2,COL6A1,PIR,NECTIN1,ANPEP,ARPIN,LIMK1,SLC26A6,PRMT2,THOC5,RIPK3,TWIST2,IQCG,TNK2,PLA2G3,STMN3,SART1,CCDC85B,MRC2,PBX1,KAT5,RELA,TMEM120A,SCYL1,LIMD1,SGPL1,SMARCB1,PBX4,BLOC1S4,MEF2B,IGFBP5,C12orf57,BAD,AAMP,CTDSP1,EID1,LGALS3,CD9,TNFRSF1A,C1QBP,AIMP2,RCC2,CNP,AP1M1,PRXL2A,KRT10,FOXK1,CASP14,DBN1,CAPN2,AMER1,MAD2L2,NANOS3,CTBP1,CRABP2,TMEM79,ARHGEF2,ARMC5,S100A4,S100A6,CARM1,EEF2K,EML1,CSK,ZNF521,DOCK11,ZNF830,AKT1,RFX2,SAFB2,GIT1,SLC6A4,SIRT6,PPL,TJP3,HMG20B,VASN,ACAP3,MNT,AP3D1,ANAPC2,STK11,GTF3C5,TGFBR3,NEGR1,MIR214,RF00024,TNFRSF12A,ERBB4,TWF2,JUND,KLF13,FADS1,JAGN1,UNC13A,TRIB2,ZNF385A,NSMF,GBA2,GLIPR2,TUBGCP2,MEIG1,ERF	yellow	VSMC_M3
GO:0043010	0.57	0.54	15	242	camera-type eye development	BP	1	CDK4,RING1,PDGFRA,KLF4,WDPCP,SPRED2,SIX5,KDM2B,AQP5,DLL4,IMPDH2,NECTIN1,C12orf57,SMARCA4,CTNS	yellow	VSMC_M3
GO:0043627	0.57	0.63	4	57	response to estrogen	BP	1	MMP14,ARPC1B,PELP1,TRIM25	yellow	VSMC_M3
GO:0030111	0.57	0.53	18	277	regulation of Wnt signaling pathway	BP	1	CTHRC1,TPBG,SNX3,PLEKHA4,SLC9A3R1,RNF220,GREM1,WNT10B,CCAR2,LIMD1,AMER1,MAD2L2,SMARCA4,HIC1,CSNK1G2,STK11,IGFBP6,APCDD1L	yellow	VSMC_M3
GO:0048609	0.57	0.50	38	563	multicellular organismal reproductive process	BP	1	BAG6,NCAPH2,SBF1,PPARD,IFT27,B4GALT1,PDGFRA,FIGNL1,SIX5,ZNF296,PAFAH1B3,DLEC1,METTL3,CEP131,DND1,YIF1B,MORN2,DEAF1,BCL2L1,KDM2B,SOD1,TYRO3,KDM1B,TRIM27,SLC26A6,IQCG,PLA2G3,SGPL1,BAD,NANOS3,ZNF830,AKT1,RFX2,SLC6A4,STK11,TAZ,FAM50A,MEIG1	yellow	VSMC_M3
GO:0034599	0.57	0.53	16	234	cellular response to oxidative stress	BP	1	PDGFRA,KLF4,ZC3H12A,P4HB,PYCR1,GSR,SOD1,ANKRD2,CHCHD2,SELENOS,GPX1,RIPK3,RELA,AKT1,TRAP1,OSER1	yellow	VSMC_M3
GO:0061008	0.57	0.57	8	119	hepaticobiliary system development	BP	1	MPST,PHF2,UCP2,AACS,PCK2,RELA,HMGCS1,TGFBR3	yellow	VSMC_M3
GO:0072676	0.57	0.61	5	72	lymphocyte migration	BP	1	PLEC,ITGB7,CX3CL1,RIPK3,AKT1	yellow	VSMC_M3
GO:0007272	0.57	0.58	7	110	ensheathment of neurons	BP	1	NAB2,PPARD,PLEC,SOD1,CD9,AKT1,MARVELD1	yellow	VSMC_M3
GO:0008366	0.57	0.58	7	110	axon ensheathment	BP	1	NAB2,PPARD,PLEC,SOD1,CD9,AKT1,MARVELD1	yellow	VSMC_M3
GO:0048705	0.57	0.55	12	186	skeletal system morphogenesis	BP	1	NAB2,PDGFRA,HOXA1,INSIG1,INPPL1,HOXC4,GREM1,WNT10B,MMP14,SGPL1,SLC39A1,NDST1	yellow	VSMC_M3
GO:0009416	0.57	0.53	16	239	response to light stimulus	BP	1	HOXA1,OGG1,METTL3,RIC8A,ACTR5,CCAR2,GPX1,RELA,SDE2,PER1,AKT1,HMGCS1,CTNS,STK11,JUND,NSMCE3	yellow	VSMC_M3
GO:0006937	0.57	0.57	8	121	regulation of muscle contraction	BP	1	ZC3H12A,PDE4D,SOD1,GSTO1,SCN4A,SPX,C12orf57,RANGRF	yellow	VSMC_M3
GO:1901342	0.58	0.53	15	232	regulation of vasculature development	BP	1	EPN1,KLF4,ZC3H12A,PLXND1,EMILIN1,GREM1,PDE3B,HTATIP2,YJEFN3,MYDGF,SIRT6,JMJD8,SARS,MIR214,RNH1	yellow	VSMC_M3
GO:0042063	0.58	0.53	15	230	gliogenesis	BP	1	NAB2,HDAC10,SCRIB,PLEC,METTL3,MMP24,CX3CL1,SOD1,MMP14,CLCF1,RELA,CD9,CNP,CSK,AKT1	yellow	VSMC_M3
GO:0002366	0.58	0.54	12	181	leukocyte activation involved in immune response	BP	1	LGALS1,SOCS5,ZC3H12A,RELB,SWAP70,CLCF1,PLA2G3,IFI35,LGALS3,MAD2L2,CORO1A,DOCK11	yellow	VSMC_M3
GO:0032677	0.58	0.63	4	56	regulation of interleukin-8 production	BP	1	SSC5D,KLF4,RELA,CACTIN	yellow	VSMC_M3
GO:0002449	0.58	0.55	11	159	lymphocyte mediated immunity	BP	1	GNL1,IL1R1,SWAP70,RNF19B,CLCF1,RIPK3,C1QBP,MAD2L2,CORO1A,C8G,CARD9	yellow	VSMC_M3
GO:0071356	0.58	0.55	11	163	cellular response to tumor necrosis factor	BP	1	SHARPIN,ZC3H12A,KRT8,CX3CL1,PCK2,RELA,TNFRSF1A,ARHGEF2,AKT1,CACTIN,TRADD	yellow	VSMC_M3

GO:2001251	0.58	0.60	5	77	negative regulation of chromosome organization	BP	1	NAT10,PINX1,MAD1L1,MAD2L2,SLX1A	yellow	VSMC_M3
GO:0007155	0.58	0.47	65	1044	cell adhesion	BP	1	CD63,PPARD,TSTA3,SCRIB,LGALS1,B4GALT1,PDGFRA,COL15A1,COL19A1,SOCS5,KLF4,TPBG,WDPCP,ZC3H12A,ZYX,PLXND1,EMILIN1,METTL3,BAIAP2,INPPL1,P4HB,SIGLEC1,RIC8A,ITGB7,SWAP70,MMP24,GREM1,PDE3B,CX3CL1,EGR3,TYRO3,ST3GAL4,MMP14,IGSF9,LRFN4,COL6A1,NECTIN1,MPZL3,SART1,PTPN23,RELA,SIPA1,BLOC1S4,BAD,SCARF2,LGALS3,CD9,PLAU,C1QBP,RCC2,MAD1L1,MAD2L2,CORO1A,CD276,AATF,CSK,AKT1,CD99L2,TJP3,CLDN6,AP3D1,IRAK1,NEGR1,TNFRSF12A,COL6A2	yellow	VSMC_M3
GO:0061912	0.58	0.62	4	63	selective autophagy	BP	1	SQSTM1,PHB2,CDC37,TAZ	yellow	VSMC_M3
GO:0009314	0.58	0.50	24	359	response to radiation	BP	1	EEF1D,HOXA1,FIGNL1,PNKP,OGG1,METTL3,RIC8A,BCL2L1,TNKS1BP1,ACTR5,CCAR2,GPX1,KAT5,RELA,BABAM1,SDE2,PER1,AKT1,HMGCS1,CTNS,STK11,NABP2,JUND,NSMCE3	yellow	VSMC_M3
GO:0046394	0.58	0.53	15	213	carboxylic acid biosynthetic process	BP	1	MGLL,SEPHS1,INSIG1,PYCR1,FASN,SCD,PLA2G3,MGST3,MID1IP1,ACLY,FADS2,GLUD1,ILVBL,MTHFR,FADS1	yellow	VSMC_M3
GO:0007049	0.58	0.46	94	1405	cell cycle	BP	1	DCTN2,CDK4,CTDSP2,BAG6,NCAPH2,SNX9,PRR5,TTLL12,H2AFY,SGSM3,SCRIB,PLEC,BOP1,SND1,CUL7,KLF4,FIGNL1,KMT2E,SLC9A3R1,PPM1G,SEPT9,SERTAD1,METTL3,CEP131,NUMA1,CCDC57,EIF4EBP1,NAT10,BCL2L1,PDCD2L,RRS1,PINX1,DCTN1,WNT10B,CCAR2,RAB35,CDK2AP2,PSME2,PCNT,NUDC,PRMT2,THOC5,CEP164,PBX1,SIPA1,RHOC,ALKBH4,SAC3D1,SMARCB1,PHB2,CTDSP1,EID1,METTL13,KIAA0753,BABAM1,NUP88,ZBTB49,ANXA11,RCC2,EML3,SDE2,MIIP,MAD1L1,MAD2L2,NANOS3,CTBP1,ARHGEF2,GADD45GIP1,CDC37,MYO19,EML1,AATF,ZNF830,AKT1,SNX18,GIT1,SLC6A4,DHCR24,HMG20B,CDK11B,AURKAIP1,MNT,ANAPC2,STK11,NTMT1,MIR214,NABP2,JUND,MIS12,CDC34,FBXO6,TUBGCP2,MARVELD1,AC093323.1	yellow	VSMC_M3
GO:0044087	0.58	0.48	51	801	regulation of cellular component biogenesis	BP	1	SNX9,FNIP1,PHF2,DBNL,TPBG,WDPCP,EMILIN1,SEPT9,BAIAP2,NUMA1,EIF4EBP1,SWAP70,CAPG,GREM1,DCTN1,CX3CL1,WNT10B,MMP14,CORO1B,LRFN4,NECTIN1,TRIM27,ARPIN,LIMK1,RHOC,PEX5,LGALS3,ARPC1B,WASHC1,RCC2,EML3,DBN1,PIP4K2B,SNF8,RANGRF,CORO1A,SLX1A,RABEP2,EEF2K,ATG3,DOCK11,GIT1,NCLN,MIR214,TWF2,ELN,CDC42EP5,CDC34,GBA2,STUB1,ARPC5L	yellow	VSMC_M3
GO:0048870	0.58	0.47	79	1234	cell motility	BP	1	CD63,CTHRC1,PPARD,TSTA3,SCRIB,PLEC,B4GALT1,PDGFRA,IL1R1,KLF4,TPBG,WDPCP,ZC3H12A,SLC9A3R1,PLXND1,EMILIN1,UNK,CEP131,YIF1B,RIC8A,SCARB1,ITGB7,SWAP70,GREM1,CX3CL1,DGKZ,EGR3,DLL4,TYRO3,ST3GAL4,RIN3,PDGFA,MMP14,GPX1,CORO1B,PLEKHO1,ARPIN,RIPK3,TWIST2,IQCG,PLA2G3,PTPN23,RHOC,FMNL1,LIMD1,SGPL1,PHB2,IGFBP5,AAMP,LGALS3,CD9,PLAU,C1QBP,WASHC1,RCC2,MTA2,ELP5,PKN1,MIIP,ARHGEF2,CORO1A,AKT1,ACAP3,TMEM201,GAS8,TGFBR3,TRADD,MIR214,ERBB4,PTPRR,IGFBP6,JAGN1,CAVIN1,IGSF8,NSMF,GLIPR2,TUBGCP2,MEIG1,ARPC5L	yellow	VSMC_M3
GO:0051674	0.58	0.47	79	1234	localization of cell	BP	1	CD63,CTHRC1,PPARD,TSTA3,SCRIB,PLEC,B4GALT1,PDGFRA,IL1R1,KLF4,TPBG,WDPCP,ZC3H12A,SLC9A3R1,PLXND1,EMILIN1,UNK,CEP131,YIF1B,RIC8A,SCARB1,ITGB7,SWAP70,GREM1,CX3CL1,DGKZ,EGR3,DLL4,TYRO3,ST3GAL4,RIN3,PDGFA,MMP14,GPX1,CORO1B,PLEKHO1,ARPIN,RIPK3,TWIST2,IQCG,PLA2G3,PTPN23,RHOC,FMNL1,LIMD1,SGPL1,PHB2,IGFBP5,AAMP,LGALS3,CD9,PLAU,C1QBP,WASHC1,RCC2,MTA2,ELP5,PKN1,MIIP,ARHGEF2,CORO1A,AKT1,ACAP3,TMEM201,GAS8,TGFBR3,TRADD,MIR214,ERBB4,PTPRR,IGFBP6,JAGN1,CAVIN1,IGSF8,NSMF,GLIPR2,TUBGCP2,MEIG1,ARPC5L	yellow	VSMC_M3
GO:0070374	0.58	0.55	10	145	positive regulation of ERK1 and ERK2 cascade	BP	1	NELFE,PDGFRA,TPBG,CAVIN3,CX3CL1,PDGFA,PHB2,CARD9,ERBB4,GLIPR2	yellow	VSMC_M3
GO:0002253	0.58	0.53	13	191	activation of immune response	BP	1	BAG6,ZC3H12A,CD79A,PDE4D,DGKZ,LIMK1,RELA,LGALS3,C1QBP,CD276,CSK,C8G,STK11	yellow	VSMC_M3
GO:0070727	0.58	0.46	97	1462	cellular macromolecule localization	BP	1	CD63,DCTN2,SQSTM1,CCHCR1,BAG6,SNX9,SAMM50,SREBF2,H2AFY,SGSM3,PACSIN1,SCRIB,PLEC,IFT27,VPS28,TTC7A,ASNA1,TPBG,ZC3H12A,EHD2,SLC9A3R1,DMAP1,SNX17,INSIG1,MYRIP,SEC13,SEPT9,CEP131,TSPAN10,LAMTOR1,NUMA1,YIF1B,RAB20,HM13,BCL2L1,ITGB7,RRS1,PLEKHF1,PINX1,MCRS1,RAPSN,CHP1,AP2A2,RAB35,TSPAN5,SELENOS,EXOC3,MMP14,AIP,AP1G2,CORO1B,PCNT,SCYL1,LRWD1,PEX5,ZDHHC3,CHCHD10,PHB2,LAMTOR4,LGALS3,TNFRSF1A,KIAA0753,NUP88,RCC2,AP1M1,DBN1,SNX8,RAB24,CLTB,ARHGEF2,SNF8,RANGRF,STX10,SGF29,TMED1,SYNE3,CDC37,CSK,MID2,ATG3,RBM22,AKT1,PACS2,RANBP3,RAB34,AP3D1,SSNA1,STK11,GAS8,NABP2,ERBB4,TWF2,MIS12,ZNF385A,ARFRP1,MEIG1,TIMM22	yellow	VSMC_M3
GO:0001738	0.58	0.60	5	84	morphogenesis of a polarized epithelium	BP	1	CTHRC1,SCRIB,PLEKHA4,SLC9A3R1,ERBB4	yellow	VSMC_M3
GO:0034249	0.58	0.54	12	183	negative regulation of cellular amide metabolic process	BP	1	CNOT3,ZC3H12A,UNK,METTL3,DND1,EIF4EBP1,PCIF1,DCPS,IGFBP5,NANOS3,C19orf66,KHSRP	yellow	VSMC_M3
GO:0007044	0.58	0.60	5	89	cell-substrate junction assembly	BP	1	PLEC,WDPCP,GREM1,MMP14,RCC2	yellow	VSMC_M3

GO:0060255	0.58	0.44	301	4468	regulation of macromolecule metabolic process	BP	1	ENPP1,CD63,NAB2,CDK4,CTDSP2,SQSTM1,ZNF608,BAG6,SNX9,HDAC10,NELFE,FNIP1,ATF6B,PBX2,PRR5,RING1,SREBF2,H2AFY,HNRNPA0,TRIM11,SGSM3,TRAPPC9,PPARD,SCRIB,PLEC,VPS28,SND1,PDGFRA,IL1R1,SECISBP2,PHF2,SSC5D,HSPBP1,SOCS5,RPS9,CNOT3,KLF4,HOXA1,FHL3,SPRED2,ZC3H12A,FIGNL1,PNKP,RCN3,SNRNP70,CC2D1B,SNX3,KTI12,MICAL1,KMT2E,WDFY2,SIX5,ZNF282,ZNF296,RELB,SLC9A3R1,EMILIN1,DMAP1,CIC,UNK,OGG1,SERTAD1,TTC5,PSMC4,METTL3,CXXC5,INPPL1,LAMTOR1,DND1,EIF4EBP1,DELE1,CAVIN3,DEAF1,HOXC4,LDLRAD3,TARBP2,KMT2B,NAT10,ZNF740,PDE4D,EIF4B,SWAP70,LRP3,RALY,ACSL1,ZBTB38,LSM6,TNKS1BP1,PINX1,GREM1,KDM2B,MCRS1,CX3CL1,WNT10B,SOD1,PACSIN3,ACTR5,ANKRD2,EGR3,CCAR2,DNAJC17,DLL4,CHP1,YAF2,HTATIP2,PCIF1,PCBP4,KDM1B,CHCHD2,SLC35C2,RBM15B,SRSF9,RNF19B,DCPS,IRX1,SELENOS,RRP1B,BRD9,RIN3,ZCCHC17,PDGFA,MMP14,C14orf93,GPX1,AIP,CLCF1,NGDN,TSHZ2,QARS,ASB9,PSME2,PIR,TRIM27,LIMK1,PRMT2,THOC5,RIPK3,TWIST2,LSM14B,ADRM1,SMARCD2,PFDN2,BRMS1,BUD23,TNK2,TACO1,PLA2G3,GMEB2,CCDC85B,PRPF6,PBX1,KAT5,RELA,ZNF511,ARNT2,ZFPL1,LIMD1,SMARCB1,CHCHD10,PBX4,UBTF,GATAD2A,MEF2B,PHB2,TRMT112,NDP,IGFBP5,BAD,CTDSP1,MLF2,OTUB1,EID1,SSBP4,METTL13,MPV17L2,ZNF394,LGALS3,C11orf95,BUD31,MRTO4,TNFRSF1A,BABAM1,TEAD4,C1QBP,AIMP2,TAF6L,ZBTB49,UBXN1,WASHC1,MTA2,ZNF316,MED11,PELP1,CCDC22,FOXK1,KLF3,PKN1,CAPN2,SEPSECS,POLR2A,AMER1,MTHFR,MAD2L2,SLTM,NANOS3,CTBP1,FBXL19,CRABP2,DFFA,SP2,ARHGEF2,SNF8,MVP,PER1,NFIX,PPP4C,SLX1A,IRF2BPL,TRIM25,[illegible]	yellow	VSMC_M3
GO:0006974	0.58	0.48	47	699	cellular response to DNA damage stimulus	BP	1	GNL1,BAG6,HDAC10,FANCE,SPRED2,ZC3H12A,FIGNL1,PNKP,MUTYH,DMAP1,OGG1,TTC5,METTL3,FAAP100,SMUG1,BCL2L1,ZBTB38,TNKS1BP1,MCRS1,ACTR5,CCAR2,WDR4,THOC5,CEP164,KAT5,BAD,OTUB1,TNFRSF1A,BABAM1,SDE2,MAD2L2,PPP4C,SLX1A,USP43,AATF,CXorf57,ZNF830,AKT1,ALKBH7,SIRT6,HIC1,STK11,NABP2,ZNF385A,FBXO6,STUB1,NSMCE3	yellow	VSMC_M3
GO:0002696	0.58	0.52	15	218	positive regulation of leukocyte activation	BP	1	LGALS1,SOCS5,EGR3,MMP14,CLCF1,PLA2G3,SART1,BAD,MAD2L2,CORO1A,CD276,CSK,AKT1,AP3D1,JUND	yellow	VSMC_M3
GO:0007219	0.59	0.55	9	138	Notch signaling pathway	BP	1	SREBF2,EPN1,METTL3,DLL4,SLC35C2,TSPAN5,MMP14,YJEFN3,AKT1	yellow	VSMC_M3
GO:0044257	0.59	0.48	46	685	cellular protein catabolic process	BP	1	SQSTM1,BAG6,SHARPIN,VPS28,CUL7,HSPBP1,SOCS5,PSMB3,RCN3,TMUB1,PSMC4,SEL1L2,HM13,WNT10B,CCAR2,RNF19B,SELENOS,GPX1,PSME2,ADRM1,PSMB4,PTPN23,KAT5,MVB12A,UBXN1,CCDC22,USP27X,CAPN2,AMER1,FBXL19,SNF8,TRIM25,USP43,ANKRD9,AKT1,SIRT6,ANAPC2,SPSB3,MAN1B1,ZNRF1,PSMB10,TRIB2,CDC34,FBXO6,STUB1,CCDC115	yellow	VSMC_M3
GO:0032637	0.59	0.62	4	57	interleukin-8 production	BP	1	SSC5D,KLF4,RELA,CACTIN	yellow	VSMC_M3
GO:0106106	0.59	0.56	8	123	cold-induced thermogenesis	BP	1	CNOT3,UCP2,ACSL1,WNT10B,SCD,SIRT6,EHMT1,STK11	yellow	VSMC_M3
GO:0120161	0.59	0.56	8	123	regulation of cold-induced thermogenesis	BP	1	CNOT3,UCP2,ACSL1,WNT10B,SCD,SIRT6,EHMT1,STK11	yellow	VSMC_M3
GO:0032355	0.59	0.57	7	105	response to estradiol	BP	1	OGG1,KAT5,ARNT2,BAD,ARPC1B,SLC6A4,MBD3	yellow	VSMC_M3
GO:0043066	0.59	0.47	45	662	negative regulation of apoptotic process	BP	1	BAG6,FNIP1,PPARD,KLF4,ZC3H12A,FIGNL1,MICAL1,ANXA5,OGG1,UCP2,BCL2L1,GREM1,KDM2B,CX3CL1,SOD1,EGR3,CCAR2,TYRO3,HTATIP2,SELENOS,ITPRIP,GPX1,CLCF1,QARS,TWIST2,PLA2G3,RELA,ARNT2,PHB2,LGALS3,NANOS3,DFFA,ARHGEF2,CORO1A,EEF2K,AATF,ZNF830,AKT1,DHCR24,MYDGF,TRAP1,MNT,PTMA,ZNF385A,TXNDC12	yellow	VSMC_M3
GO:0043534	0.59	0.57	7	108	blood vessel endothelial cell migration	BP	1	KLF4,SCARB1,GREM1,EGR3,DLL4,GPX1,AKT1	yellow	VSMC_M3
GO:0022612	0.59	0.58	6	95	gland morphogenesis	BP	1	SCRIB,SLC9A3R1,PLXND1,PDGFA,PHB2,IGFBP5	yellow	VSMC_M3
GO:2000106	0.59	0.61	4	59	regulation of leukocyte apoptotic process	BP	1	FNIP1,SELENOS,RIPK3,LGALS3	yellow	VSMC_M3
GO:0016064	0.59	0.58	6	85	immunoglobulin mediated immune response	BP	1	SWAP70,CLCF1,C1QBP,MAD2L2,C8G,CARD9	yellow	VSMC_M3
GO:0006109	0.59	0.55	9	139	regulation of carbohydrate metabolic process	BP	1	ENPP1,SELENOS,BAD,FOXK1,AKT1,GIT1,PGP,MIDN,JMJD8	yellow	VSMC_M3
GO:0031100	0.59	0.61	4	58	animal organ regeneration	BP	1	CDK4,UCP2,ATIC,TGFBR3	yellow	VSMC_M3
GO:0019221	0.59	0.50	21	306	cytokine-mediated signaling pathway	BP	1	TTLL12,SHARPIN,IL1R1,SOCS5,KRT8,METTL3,P4HB,ACSL1,DOK1,CX3CL1,CLCF1,RELA,BAD,TNFRSF1A,PALM3,CDC37,AKT1,CACTIN,IRAK1,TRADD,JAGN1	yellow	VSMC_M3
GO:0010212	0.59	0.55	9	137	response to ionizing radiation	BP	1	EEF1D,FIGNL1,BCL2L1,TNKS1BP1,GPX1,KAT5,BABAM1,STK11,NABP2	yellow	VSMC_M3
GO:0042325	0.59	0.46	63	967	regulation of phosphorylation	BP	1	ENPP1,NAB2,CTDSP2,SQSTM1,SNX9,FNIP1,PRR5,H2AFY,PLEC,PPP1R14B,PDGFRA,SOCS5,KLF4,SPRED2,ZC3H12A,MICAL1,WDFY2,SLC9A3R1,EMILIN1,SERTAD1,TARBP2,PDE4D,ACSL1,TNKS1BP1,GREM1,CX3CL1,SOD1,DGKZ,CHP1,TYRO3,CISH,PDGFA,CLCF1,QARS,TRIM27,RIPK3,TNK2,PHB2,BAD,CTDSP1,TNFRSF1A,WASHC1,PKN1,PIP4K2B,MAD2L2,ARHGEF2,MVP,EEF2K,CDC37,CSK,AKT1,GIT1,MYDGF,MAP2K2,CACTIN,IRAK1,MIDN,STK11,TELO2,JMJD8,VAC14,ERBB4,TRIB2	yellow	VSMC_M3
GO:0030072	0.59	0.53	11	168	peptide hormone secretion	BP	1	PPARD,EIPR1,MYRIP,UCP2,AACS,PCK2,CAPN10,BAD,GLUD1,MIDN,JAGN1	yellow	VSMC_M3
GO:0030516	0.59	0.59	5	83	regulation of axon extension	BP	1	DBNL,LIMK1,DBN1,ANAPC2,TWF2	yellow	VSMC_M3

GO:0019222	0.59	0.43	328	4858	regulation of metabolic process	BP	1	ENPP1,CD63,NAB2,CDK4,CTDSP2,SQSTM1,ZNF608,BAG6,SNX9,HDAC10,NELFE,FNIP1,ATF6B,PBX2,PRR5,RING1,SREBF2,H2AFY,HNRNPA0,TRIM11,SGSM3,TRAPPC9,PPARD,SCRIB,PLEC,VPS28,SND1,PPP1R14B,PDGFRA,IL1R1,SECISBP2,PHF2,SSC5D,HSPBP1,SOCS5,RPS9,CNOT3,KLF4,HOXA1,FHL3,SPRED2,ZC3H12A,FIGNL1,PNKP,RCN3,SNRNP70,CC2D1B,SNX3,KTI12,MICAL1,KMT2E,WDFY2,SIX5,ZNF282,ZNF296,RELB,SLC9A3R1,EMILIN1,DMAP1,CIC,UNK,INSIG1,OGG1,SERTAD1,TTC5,PSMC4,UCP2,METTL3,CXXC5,INPPL1,LAMTOR1,DND1,EIF4EBP1,DELE1,CAVIN3,DEAF1,HOXC4,LDLRAD3,TARBP2,KMT2B,SCARB1,NAT10,ZNF740,PDE4D,EIF4B,SWAP70,LRP3,RALY,PLEKHF1,ACSL1,ZBTB38,LSM6,TNKS1BP1,PINX1,GREM1,KDM2B,PDE3B,MCRS1,CX3CL1,WNT10B,SOD1,PACSIN3,ACTR5,ANKRD2,DGKZ,EGR3,CCAR2,DNAJC17,DLL4,CHP1,TYRO3,SCD,YAF2,HTATIP2,PCIF1,PCBP4,KDM1B,CHCHD2,SLC35C2,RBM15B,CISH,SRSF9,RNF19B,DCPS,IRX1,DHCR7,SELENOS,RRP1B,ETFBKMT,BRD9,RIN3,FABP3,ZCCHC17,PDGFA,MMP14,C14orf93,GPX1,AIP,CLCF1,NGDN,SRRD,TSHZ2,QARS,ASB9,PSME2,PIR,TRIM27,LIMK1,PRMT2,THOC5,RIPK3,TWIST2,LSM14B,ADRM1,SMARCD2,PFDN2,BRMS1,BUD23,TNK2,TACO1,PLA2G3,GMEB2,CCDC85B,PRPF6,PBX1,KAT5,RELA,ZNF511,IDH1,ARNT2,ZFPL1,LIMD1,MID1IP1,SMARCB1,CHCHD10,PBX4,UBTF,GATAD2A,MEF2B,PHB2,TRMT112,PPP1R35,NDP,IGFBP5,BAD,CTDSP1,MLF2,OTUB1,EID1,SSBP4,METTL13,MPV17L2,ZNF394,LGALS3,C11orf95,BUD31,MRTO4,TNFRSF1A,BABAM1,TEAD4,C1QBP,AIMP2,TAF6L,ZBTB49,UBXN1,WASHC1,MTA2,ZNF316,MED11,PELP1,CCDC22,FOXK1,KLF3,PKN1,CAPN2,SEPSECS,POLR2A,AMER1,[illegible]	yellow	VSMC_M3
GO:0016477	0.59	0.46	71	1120	cell migration	BP	1	CD63,CTHRC1,PPARD,TSTA3,SCRIB,PLEC,B4GALT1,PDGFRA,IL1R1,KLF4,TPBG,WDPCP,ZC3H12A,SLC9A3R1,PLXND1,EMILIN1,UNK,RIC8A,SCARB1,ITGB7,SWAP70,GREM1,CX3CL1,DGKZ,EGR3,DLL4,TYRO3,ST3GAL4,RIN3,PDGFA,MMP14,GPX1,CORO1B,PLEKHO1,ARPIN,RIPK3,TWIST2,PTPN23,RHOC,FMNL1,LIMD1,SGPL1,PHB2,IGFBP5,AAMP,LGALS3,CD9,PLAU,C1QBP,WASHC1,RCC2,MTA2,ELP5,PKN1,MIIP,ARHGEF2,CORO1A,AKT1,ACAP3,TMEM201,TGFBR3,TRADD,MIR214,ERBB4,PTPRR,IGFBP6,JAGN1,NSMF,GLIPR2,TUBGCP2,ARPC5L	yellow	VSMC_M3
GO:1902414	0.59	0.59	5	82	protein localization to cell junction	BP	1	SCRIB,BAIAP2,RAPSN,NECTIN1,TJP3	yellow	VSMC_M3
GO:0034613	0.59	0.45	96	1451	cellular protein localization	BP	1	CD63,DCTN2,SQSTM1,CCHCR1,BAG6,SNX9,SAMM50,SREBF2,H2AFY,SGSM3,PACSIN1,SCRIB,PLEC,IFT27,VPS28,TTC7A,ASNA1,TPBG,ZC3H12A,EHD2,SLC9A3R1,DMAP1,SNX17,INSIG1,MYRIP,SEC13,SEPT9,CEP131,TSPAN10,LAMTOR1,NUMA1,YIF1B,RAB20,HM13,BCL2L1,ITGB7,RRS1,PLEKHF1,PINX1,MCRS1,RAPSN,CHP1,AP2A2,RAB35,TSPAN5,SELENOS,EXOC3,MMP14,AIP,AP1G2,CORO1B,PCNT,SCYL1,LRWD1,PEX5,ZDHHC3,CHCHD10,PHB2,LAMTOR4,LGALS3,TNFRSF1A,KIAA0753,NUP88,RCC2,AP1M1,DBN1,SNX8,RAB24,CLTB,ARHGEF2,SNF8,RANGRF,STX10,SGF29,TMED1,SYNE3,CDC37,CSK,MID2,ATG3,RBM22,AKT1,PACS2,RANBP3,RAB34,AP3D1,SSNA1,STK11,GAS8,NABP2,ERBB4,TWF2,MIS12,ARFRP1,MEIG1,TIMM22	yellow	VSMC_M3
GO:0003341	0.60	0.56	7	103	cilium movement	BP	1	SLC9A3R1,CEP131,YIF1B,IQCG,PLA2G3,GAS8,MEIG1	yellow	VSMC_M3
GO:0043523	0.60	0.53	11	169	regulation of neuron apoptotic process	BP	1	BCL2L1,KDM2B,RAPSN,CX3CL1,SOD1,TYRO3,CLCF1,PLA2G3,CORO1A,NSMF,CDC34	yellow	VSMC_M3
GO:0006094	0.60	0.61	4	58	gluconeogenesis	BP	1	SELENOS,PCK2,PGM1,PGP	yellow	VSMC_M3
GO:0034250	0.60	0.54	9	130	positive regulation of cellular amide metabolic process	BP	1	CDK4,RPS9,METTL3,NAT10,PCIF1,RELA,MPV17L2,C1QBP,PAIP1	yellow	VSMC_M3
GO:0001822	0.60	0.51	15	248	kidney development	BP	1	BAG6,MPST,IFT27,PDGFRA,WDPCP,PLXND1,SNX17,TNS2,GREM1,IRX1,PDGFA,PBX1,SGPL1,AMER1,ERBB4	yellow	VSMC_M3
GO:0043244	0.60	0.57	6	107	regulation of protein-containing complex disassembly	BP	1	SWAP70,CAPG,MID1IP1,ARHGEF2,C19orf66,TWF2	yellow	VSMC_M3
GO:0120032	0.60	0.53	10	158	regulation of plasma membrane bounded cell projection assembly	BP	1	WDPCP,SEPT9,WASHC1,RCC2,RABEP2,ATG3,DOCK11,MIR214,TWF2,CDC42EP5	yellow	VSMC_M3
GO:0035148	0.60	0.55	8	129	tube formation	BP	1	CTHRC1,SCRIB,HOXA1,DEAF1,GREM1,KDM2B,IRX1,MTHFR	yellow	VSMC_M3
GO:0016053	0.60	0.51	15	215	organic acid biosynthetic process	BP	1	MGLL,SEPHS1,INSIG1,PYCR1,FASN,SCD,PLA2G3,MGST3,MID1IP1,ACLY,FADS2,GLUD1,ILVBL,MTHFR,FADS1	yellow	VSMC_M3
GO:0072594	0.60	0.49	24	355	establishment of protein localization to organelle	BP	1	SAMM50,SREBF2,H2AFY,SCRIB,VPS28,ASNA1,ZC3H12A,DMAP1,SEC13,TSPAN10,CHP1,AIP,LRWD1,PEX5,PHB2,NUP88,SNF8,RANGRF,SGF29,RBM22,AKT1,AP3D1,NABP2,TIMM22	yellow	VSMC_M3
GO:0030168	0.60	0.57	6	96	platelet activation	BP	1	PDGFRA,DGKZ,TYRO3,PDGFA,BLOC1S4,CD9	yellow	VSMC_M3
GO:0010942	0.60	0.47	32	470	positive regulation of cell death	BP	1	CDK4,SQSTM1,FNIP1,SCRIB,B4GALT1,ZC3H12A,SLC9A3R1,UCP2,PHLDA1,PLEKHF1,RAPSN,WNT10B,SOD1,CCAR2,HTATIP2,RRP1B,PRMT2,RIPK3,CAPN10,BRMS1,BAD,TNFRSF1A,C1QBP,AIMP2,USP27X,CAPN2,DFFA,AKT1,SLC27A4,TRADD,TNFRSF12A,CDC34	yellow	VSMC_M3
GO:0045935	0.60	0.44	98	1489	positive regulation of nucleobase-containing compound metabolic process	BP	1	SQSTM1,HDAC10,NELFE,ATF6B,PBX2,SREBF2,PPARD,PHF2,CNOT3,KLF4,HOXA1,ZC3H12A,PNKP,SNRNP70,KMT2E,SIX5,ZNF296,SERTAD1,TTC5,METTL3,DND1,DEAF1,HOXC4,KMT2B,ZBTB38,GREM1,MCRS1,CX3CL1,WNT10B,EGR3,CCAR2,YAF2,HTATIP2,KDM1B,CHCHD2,DCPS,RRP1B,AIP,CLCF1,TRIM27,PRMT2,THOC5,RIPK3,SMARCD2,BUD23,GMEB2,PRPF6,PBX1,KAT5,RELA,ARNT2,SMARCB1,CHCHD10,PBX4,UBTF,MEF2B,TRMT112,NDP,SSBP4,BUD31,TNFRSF1A,BABAM1,TAF6L,ZBTB49,MTA2,PELP1,FOXK1,PKN1,POLR2A,MAD2L2,NANOS3,ARHGEF2,PER1,NFIX,SLX1A,IRF2BPL,TRIM25,SMARCA4,CARM1,AATF,MID2,RBM22,ZNF521,AKT1,BRF1,KHSRP,GTF2F1,MYDGF,SIRT6,MAP2K2,AP3D1,TAZ,MED16,NABP2,ERBB4,JUND,KLF13,IER5	yellow	VSMC_M3

GO:0002376	0.60	0.44	112	1656	immune system process	BP	1	ENPP1,SQSTM1,GNL1,BAG6,NCAPH2,FNIP1,TTLL12,TRIM11,SCRIB,PLEC,LGALS1,PPP1R14B,B4GALT1,PDGFRA,IL1R1,SSC5D,SOCS5,TTC7A,KLF4,DBNL,ZC3H12A,ZYX,KMT2E,RELB,EMILIN1,CD79A,METTL3,INPPL1,FASN,RAB20,TARBP2,BCL2L1,ITGB7,PDE4D,SWAP70,SLC11A2,GREM1,CX3CL1,WNT10B,SOD1,DGKZ,EGR3,DLL4,TYRO3,RAB35,ST3GAL4,RNF19B,SELENOS,RIN3,MMP14,GPX1,CLCF1,IMPDH2,PIR,NECTIN1,TRIM27,LIMK1,SLC26A6,THOC5,RIPK3,PSMB4,TNK2,PLA2G3,SART1,PBX1,RELA,SIPA1,MAP3K14,SGPL1,YJEFN3,PHB2,BAD,IFI35,OTUB1,LGALS3,CD9,TNFRSF1A,C1QBP,PRXL2A,PKN1,MAD1L1,MAD2L2,TBKBP1,SP2,ARHGEF2,ARMC5,CORO1A,TRIM25,CDC37,CD276,EML1,C19orf66,CSK,MID2,DOCK11,AKT1,RAB34,CACTIN,AP3D1,MKNK2,IRAK1,C8G,STK11,TAZ,CARD9,TGFBR3,PSMB10,JUND,KLF13,JAGN1,ZNF385A,AKAP17A	yellow	VSMC_M3
GO:0050657	0.60	0.53	9	135	nucleic acid transport	BP	1	SEC13,RBM15B,PABPN1,THOC5,THOC3,NUP88,MVP,KHSRP,RBM8A	yellow	VSMC_M3
GO:0050658	0.60	0.53	9	135	RNA transport	BP	1	SEC13,RBM15B,PABPN1,THOC5,THOC3,NUP88,MVP,KHSRP,RBM8A	yellow	VSMC_M3
GO:0060491	0.60	0.53	10	159	regulation of cell projection assembly	BP	1	WDPCP,SEPT9,WASHC1,RCC2,RABEP2,ATG3,DOCK11,MIR214,TWF2,CDC42EP5	yellow	VSMC_M3
GO:0006892	0.60	0.56	6	99	post-Golgi vesicle-mediated transport	BP	1	AP1G2,CCDC22,CSK,RAB34,AP3D1,ARFRP1	yellow	VSMC_M3
GO:0006913	0.60	0.49	17	257	nucleocytoplasmic transport	BP	1	CCHCR1,ZC3H12A,DMAP1,SEC13,RRS1,CHP1,HTATIP2,RBM15B,PABPN1,THOC5,THOC3,PHB2,NUP88,RANGRF,RBM22,AKT1,RANBP3	yellow	VSMC_M3
GO:0051169	0.60	0.49	17	257	nuclear transport	BP	1	CCHCR1,ZC3H12A,DMAP1,SEC13,RRS1,CHP1,HTATIP2,RBM15B,PABPN1,THOC5,THOC3,PHB2,NUP88,RANGRF,RBM22,AKT1,RANBP3	yellow	VSMC_M3
GO:0046651	0.60	0.51	12	177	lymphocyte proliferation	BP	1	SCRIB,CD79A,CLCF1,IMPDH2,RIPK3,LGALS3,PKN1,MAD1L1,ARMC5,CORO1A,CD276,PSMB10	yellow	VSMC_M3
GO:0010717	0.60	0.58	5	80	regulation of epithelial to mesenchymal transition	BP	1	SPRED2,GREM1,MAD2L2,VASN,GLIPR2	yellow	VSMC_M3
GO:0043632	0.60	0.46	37	561	modification-dependent macromolecule catabolic process	BP	1	SQSTM1,BAG6,SHARPIN,VPS28,CUL7,HSPBP1,SOCS5,PSMB3,TMUB1,PSMC4,SEL1L2,WNT10B,CCAR2,RNF19B,SELENOS,ADRM1,PTPN23,KAT5,MVB12A,UBXN1,CCDC22,USP27X,FBXL19,SNF8,TRIM25,USP43,ANKRD9,AKT1,SIRT6,ANAPC2,SPSB3,MAN1B1,ZNRF1,TRIB2,CDC34,FBXO6,STUB1	yellow	VSMC_M3
GO:0001659	0.61	0.53	9	141	temperature homeostasis	BP	1	CNOT3,UCP2,ACSL1,WNT10B,SCD,GPX1,SIRT6,EHMT1,STK11	yellow	VSMC_M3
GO:0002263	0.61	0.51	12	185	cell activation involved in immune response	BP	1	LGALS1,SOCS5,ZC3H12A,RELB,SWAP70,CLCF1,PLA2G3,IFI35,LGALS3,MAD2L2,CORO1A,DOCK11	yellow	VSMC_M3
GO:0031099	0.61	0.52	10	150	regeneration	BP	1	CDK4,PPARD,KLF4,UCP2,WNT10B,GPX1,ATIC,CD9,TGFBR3,ERBB4	yellow	VSMC_M3
GO:0051130	0.61	0.45	55	870	positive regulation of cellular component organization	BP	1	CD63,SNX9,FNIP1,H2AFY,PACSIN1,VPS28,CUL7,DBNL,TPBG,PNKP,SNX3,EHD2,PLXND1,EMILIN1,SEPT9,BAIAP2,NUMA1,SWAP70,GREM1,DCTN1,CX3CL1,WNT10B,GPX1,CORO1B,TRIM27,ARPIN,LIMK1,PFDN2,PLA2G3,PTPN23,RHOC,CHCHD10,BAD,LGALS3,ARPC1B,WASHC1,DBN1,CAPN2,PIP4K2B,CRABP2,CORO1A,SLX1A,EEF2K,DOCK11,GIT1,SIRT6,ANAPC2,STK11,NEGR1,NABP2,TWF2,CDC42EP5,CDC34,STUB1,ARPC5L	yellow	VSMC_M3
GO:0010876	0.61	0.48	20	311	lipid localization	BP	1	ENPP1,PQLC1,ESYT1,SREBF2,SQLE,PPARD,PLEKHA8,ZC3H12A,LAMTOR1,SCARB1,ACSL1,FABP3,SPX,PLA2G3,VPS51,YJEFN3,SLC27A3,AKT1,ALKBH7,SLC27A4	yellow	VSMC_M3
GO:0015031	0.61	0.44	87	1301	protein transport	BP	1	CD63,RUFY1,CCHCR1,SNX9,SAMM50,SREBF2,SGSM3,GUK1,PPARD,SCRIB,IFT27,VPS28,TOM1,ASNA1,PLEKHA8,EIPR1,ZC3H12A,PDIA4,RCN3,SNX3,SERP2,DMAP1,SNX17,INSIG1,MYRIP,SEC13,UCP2,CEP131,YIF1B,RAB20,ARHGAP33,AACS,HM13,CHP1,AP2A2,SNX21,RAB35,APBA2,SELENOS,EXOC3,AIP,AP1G2,PCK2,PCNT,CAPN10,PTPN23,VPS51,PEX5,ZDHHC3,PHB2,BAD,COPE,MVB12A,NUP88,WASHC1,AP1M1,RAB3IL1,CCDC22,GLUD1,SNX8,LMAN2,RAB24,CLTB,ARHGEF2,SNF8,MVP,MTX1,RANGRF,STX10,RABEP2,TMED1,CDC37,CSK,ATG3,RBM22,AKT1,SNX18,RANBP3,RAB34,CTNS,AP3D1,MIDN,AP5B1,JAGN1,CAVIN1,ARFRP1,TIMM22	yellow	VSMC_M3
GO:0048667	0.61	0.47	26	446	cell morphogenesis involved in neuron differentiation	BP	1	SCRIB,CUL7,DBNL,TPBG,WDPCP,SLC9A3R1,PLXND1,UNK,BAIAP2,ARHGAP33,SLC11A2,SOD1,NGEF,IGSF9,NECTIN1,LIMK1,C12orf57,CNP,DBN1,CRABP2,S100A6,EEF2K,ANAPC2,STK11,TWF2,NSMF	yellow	VSMC_M3
GO:0001843	0.61	0.57	5	82	neural tube closure	BP	1	CTHRC1,SCRIB,DEAF1,KDM2B,MTHFR	yellow	VSMC_M3
GO:0009743	0.61	0.51	12	186	response to carbohydrate	BP	1	PPARD,LGALS1,UCP2,AACS,SELENOS,PCK2,SLC26A6,SMARCB1,BAD,SMARCA4,JAGN1,COL6A2	yellow	VSMC_M3
GO:0046620	0.61	0.57	5	78	regulation of organ growth	BP	1	SOD1,AKT1,SLC6A4,TGFBR3,ERBB4	yellow	VSMC_M3
GO:0006906	0.61	0.55	6	90	vesicle fusion	BP	1	RAB20,CHP1,PIP4K2B,STX10,CORO1A,RAB34	yellow	VSMC_M3
GO:0072028	0.61	0.59	4	65	nephron morphogenesis	BP	1	GREM1,IRX1,PBX1,ERBB4	yellow	VSMC_M3

GO:0030154	0.61	0.42	192	2966	cell differentiation	BP	1	ENPP1,CD63,NAB2,SQSTM1,CCHCR1,BAG6,NCAPH2,SBF1,CTHRC1,HDAC10,FNIP1,PBX2,H2AFY,TRIM11,TRAPPC9,PACSIN1,PPARD,SCRIB,PLEC,LGALS1,IFT27,SHARPIN,SND1,CUL7,DNPH1,B4GALT1,PDGFRA,SECISBP2,COL15A1,COL19A1,SOCS5,CNOT3,KLF4,DBNL,TPBG,WDPCP,CCM2,SPRED2,ZC3H12A,FIGNL1,SNX3,EHD2,FAM120B,KMT2E,KRT8,WDFY2,SIX5,RELB,SLC9A3R1,PLXND1,UNK,DLEC1,CD79A,INSIG1,METTL3,BAIAP2,CEP131,NUMA1,P4HB,DND1,YIF1B,FASN,MORN2,ARHGAP22,DEAF1,TBCB,ARHGAP33,AACS,BCL2L1,ITGB7,PDE4D,LRP3,SLC11A2,MMP24,GREM1,CX3CL1,WNT10B,SOD1,ANKRD2,EGR3,DLL4,TYRO3,HTATIP2,RAB35,IRX1,FABP3,MMP14,C14orf93,GPX1,NGEF,CLCF1,PLEKHO1,TAGLN2,IGSF9,PCK2,COL6A1,PIR,NECTIN1,ANPEP,LIMK1,SLC26A6,PRMT2,THOC5,RIPK3,TWIST2,IQCG,TNK2,PLA2G3,STMN3,SART1,CCDC85B,MRC2,PBX1,RELA,TMEM120A,SCYL1,LIMD1,SGPL1,SMARCB1,PBX4,BLOC1S4,MEF2B,IGFBP5,C12orf57,BAD,AAMP,CTDSP1,EID1,LGALS3,CD9,TNFRSF1A,C1QBP,AIMP2,RCC2,CNP,AP1M1,PRXL2A,KRT10,FOXK1,CASP14,DBN1,CAPN2,AMER1,MAD2L2,NANOS3,CTBP1,CRABP2,TMEM79,ARHGEF2,ARMC5,S100A4,S100A6,CARM1,EEF2K,EML1,CSK,ZNF521,DOCK11,ZNF830,AKT1,RFX2,SAFB2,GIT1,SLC6A4,SIRT6,PPL,TJP3,HMG20B,VASN,ACAP3,AP3D1,ANAPC2,STK11,GTF3C5,TGFBR3,NEGR1,TNFRSF12A,ERBB4,TWF2,JUND,KLF13,FADS1,JAGN1,UNC13A,TRIB2,ZNF385A,NSMF,GBA2,GLIPR2,TUBGCP2,MEIG1,ERF	yellow	VSMC_M3
GO:0009101	0.61	0.49	16	245	glycoprotein biosynthetic process	BP	1	B4GALT1,SERP2,GALNT14,PHLDA1,CHP1,SLC35C2,ST3GAL4,TMTC1,EXTL1,MPDU1,DPM3,AATF,MAN1B1,POMT1,B3GNT9,NDST1	yellow	VSMC_M3
GO:0050905	0.61	0.55	6	103	neuromuscular process	BP	1	HOXA1,DCTN1,GSTO1,SCN4A,BLOC1S4,C12orf57	yellow	VSMC_M3
GO:0019724	0.62	0.55	6	87	B cell mediated immunity	BP	1	SWAP70,CLCF1,C1QBP,MAD2L2,C8G,CARD9	yellow	VSMC_M3
GO:0051338	0.62	0.44	46	706	regulation of transferase activity	BP	1	NAB2,SNX9,H2AFY,PLEC,PDGFRA,SOCS5,KLF4,PNKP,SLC9A3R1,SERTAD1,TARBP2,ACSL1,PINX1,GREM1,SOD1,DGKZ,CHP1,TYRO3,CISH,PDGFA,PABPN1,QARS,TRIM27,LIMK1,RIPK3,PHB2,BAD,WASHC1,PKN1,PIP4K2B,MAD2L2,MVP,RPL37,CDC37,CSK,AKT1,MAP2K2,IRAK1,MIDN,STK11,TELO2,JMJD8,VAC14,ERBB4,TRIB2,STUB1	yellow	VSMC_M3
GO:0001960	0.62	0.59	4	58	negative regulation of cytokine-mediated signaling pathway	BP	1	TTLL12,METTL3,PALM3,CACTIN	yellow	VSMC_M3
GO:0000904	0.62	0.45	33	562	cell morphogenesis involved in differentiation	BP	1	SCRIB,CUL7,COL15A1,DBNL,TPBG,WDPCP,SLC9A3R1,PLXND1,UNK,METTL3,BAIAP2,P4HB,ARHGAP33,ITGB7,SLC11A2,SOD1,TYRO3,NGEF,IGSF9,NECTIN1,LIMK1,C12orf57,C1QBP,RCC2,CNP,DBN1,CRABP2,S100A6,EEF2K,ANAPC2,STK11,TWF2,NSMF	yellow	VSMC_M3
GO:0019646	0.62	0.55	6	75	aerobic electron transport chain	BP	1	NDUFB9,SDHA,NDUFV1,UQCRC1,UQCC3,NDUFS7	yellow	VSMC_M3
GO:0002695	0.62	0.53	8	118	negative regulation of leukocyte activation	BP	1	SCRIB,SOCS5,ZC3H12A,CX3CL1,TYRO3,LGALS3,PKN1,MAD1L1	yellow	VSMC_M3
GO:0007052	0.62	0.54	7	109	mitotic spindle organization	BP	1	DCTN2,NUMA1,DCTN1,PCNT,NUDC,EML3,EML1	yellow	VSMC_M3
GO:0032640	0.62	0.53	7	111	tumor necrosis factor production	BP	1	ZC3H12A,CX3CL1,SELENOS,TRIM27,ARHGEF2,CACTIN,CARD9	yellow	VSMC_M3
GO:0032680	0.62	0.53	7	111	regulation of tumor necrosis factor production	BP	1	ZC3H12A,CX3CL1,SELENOS,TRIM27,ARHGEF2,CACTIN,CARD9	yellow	VSMC_M3
GO:0072175	0.62	0.53	7	116	epithelial tube formation	BP	1	CTHRC1,SCRIB,DEAF1,GREM1,KDM2B,IRX1,MTHFR	yellow	VSMC_M3
GO:0030641	0.62	0.58	4	64	regulation of cellular pH	BP	1	RAB20,CHP1,SLC26A6,CCDC115	yellow	VSMC_M3
GO:0009725	0.62	0.44	42	652	response to hormone	BP	1	ENPP1,CDK4,PPARD,INSIG1,UCP2,BAIAP2,INPPL1,TNS2,ACSL1,PDE3B,WNT10B,SELENOS,FABP3,MMP14,PCK2,PRMT2,CAPN10,RELA,IDH1,PHB2,IGFBP5,BAD,ARPC1B,PELP1,PKN1,PIP4K2B,PER1,TRIM25,SMARCA4,CARM1,EEF2K,CSK,AKT1,HMGCS1,SAFB,SAFB2,DHCR24,APRT,SLC27A4,TGFBR3,JUND,NSMF	yellow	VSMC_M3
GO:0002532	0.62	0.58	4	59	production of molecular mediator involved in inflammatory response	BP	1	ZC3H12A,PLA2G3,PER1,CARD9	yellow	VSMC_M3
GO:0007612	0.62	0.55	6	104	learning	BP	1	TPBG,CIC,RIC8A,TACO1,GIT1,CTNS	yellow	VSMC_M3
GO:0032943	0.62	0.50	12	179	mononuclear cell proliferation	BP	1	SCRIB,CD79A,CLCF1,IMPDH2,RIPK3,LGALS3,PKN1,MAD1L1,ARMC5,CORO1A,CD276,PSMB10	yellow	VSMC_M3
GO:0051236	0.62	0.52	9	138	establishment of RNA localization	BP	1	SEC13,RBM15B,PABPN1,THOC5,THOC3,NUP88,MVP,KHSRP,RBM8A	yellow	VSMC_M3
GO:0006914	0.62	0.46	29	450	autophagy	BP	1	SQSTM1,HDAC10,SREBF2,VPS28,ZC3H12A,SNRNP70,PLEKHF1,LIX1L,TRIM27,KAT5,VPS51,PHB2,BAD,MVB12A,WASHC1,FOXK1,RAB24,PIP4K2B,SNF8,MTMR4,CDC37,MID2,ATG3,AKT1,PACS2,STK11,TAZ,WDR24,STUB1	yellow	VSMC_M3
GO:0061919	0.62	0.46	29	450	process utilizing autophagic mechanism	BP	1	SQSTM1,HDAC10,SREBF2,VPS28,ZC3H12A,SNRNP70,PLEKHF1,LIX1L,TRIM27,KAT5,VPS51,PHB2,BAD,MVB12A,WASHC1,FOXK1,RAB24,PIP4K2B,SNF8,MTMR4,CDC37,MID2,ATG3,AKT1,PACS2,STK11,TAZ,WDR24,STUB1	yellow	VSMC_M3
GO:0045862	0.62	0.47	20	297	positive regulation of proteolysis	BP	1	BAG6,SNX9,HSPBP1,SOCS5,ZC3H12A,RCN3,PSMC4,PACSIN3,RNF19B,MMP14,PSME2,ADRM1,BAD,CCDC22,AKT1,SIRT6,AURKAIP1,CARD9,TRIB2,STUB1	yellow	VSMC_M3
GO:0009791	0.62	0.58	4	68	post-embryonic development	BP	1	KLF4,INPPL1,SGPL1,C12orf57	yellow	VSMC_M3
GO:0008643	0.62	0.53	7	112	carbohydrate transport	BP	1	ENPP1,PPARD,SELENOS,SLC26A6,CAPN10,AKT1,SLC27A4	yellow	VSMC_M3
GO:0001837	0.62	0.52	8	131	epithelial to mesenchymal transition	BP	1	CUL7,SPRED2,GREM1,MAD2L2,S100A4,VASN,TGFBR3,GLIPR2	yellow	VSMC_M3
GO:0050808	0.62	0.47	19	329	synapse organization	BP	1	DBNL,TPBG,PLXND1,BAIAP2,ARHGAP22,ARHGAP33,DCTN1,RAPSN,CX3CL1,NGEF,IGSF9,LRFN4,NECTIN1,CHCHD10,DBN1,EEF2K,ANAPC2,ERBB4,UNC13A	yellow	VSMC_M3

GO:0048584	0.62	0.42	99	1488	positive regulation of response to stimulus	BP	1	CD63,BAG6,HDAC10,NELFE,FNIP1,PRR5,TXNDC15,PPARD,EEF1D,LGALS1,SHARPIN,PDGFRA,IL1R1,SOCS5,TPBG,SPRED2,ZC3H12A,PNKP,PLEKHA4,SLC9A3R1,RNF220,CD79A,SEC13,BAIAP2,CXXC5,LAMTOR1,NUMA1,CAVIN3,PDE4D,SWAP70,PLEKHF1,GREM1,CX3CL1,WNT10B,SOD1,DGKZ,CCAR2,DLL4,SLC35C2,TSPAN5,ST3GAL4,PDGFA,GPX1,CLCF1,TRIM27,LIMK1,RIPK3,PLA2G3,RELA,RHOC,MAP3K14,SMARCB1,PHB2,IGFBP5,BAD,LAMTOR4,IFI35,LGALS3,TNFRSF1A,BABAM1,C1QBP,CHERP,EVC,CCDC22,AMER1,MAD2L2,TRIM25,RPL37,S100A4,SMARCA4,CD276,CSK,MID2,TEDC1,RAB34,MYDGF,MAP2K2,HIC1,CTNS,TEDC2,CSNK1G2,IRAK1,C8G,STK11,TELO2,WDR24,JMJD8,CARD9,GAS8,TGFBR3,TRADD,MIR214,NDST1,TNFRSF12A,ERBB4,IGFBP6,ZNF385A,IER5,GLIPR2	yellow	VSMC_M3
GO:0001959	0.62	0.52	8	119	regulation of cytokine-mediated signaling pathway	BP	1	TTLL12,SHARPIN,IL1R1,METTL3,PALM3,CDC37,CACTIN,IRAK1	yellow	VSMC_M3
GO:1903034	0.62	0.52	8	123	regulation of response to wounding	BP	1	PDGFRA,KLF4,ST3GAL4,PDGFA,CD9,PLAU,GIT1,TNFRSF12A	yellow	VSMC_M3
GO:0048661	0.62	0.56	5	78	positive regulation of smooth muscle cell proliferation	BP	1	CX3CL1,IGFBP5,AKT1,IRAK1,MIR214	yellow	VSMC_M3
GO:1990266	0.62	0.56	5	74	neutrophil migration	BP	1	IL1R1,CX3CL1,LGALS3,C1QBP,JAGN1	yellow	VSMC_M3
GO:0060606	0.62	0.56	5	83	tube closure	BP	1	CTHRC1,SCRIB,DEAF1,KDM2B,MTHFR	yellow	VSMC_M3
GO:0050767	0.62	0.47	17	285	regulation of neurogenesis	BP	1	TRIM11,CUL7,DBNL,PLXND1,BAIAP2,CX3CL1,DLL4,CLCF1,LIMK1,RELA,CTDSP1,DBN1,CRABP2,EEF2K,ANAPC2,STK11,TWF2	yellow	VSMC_M3
GO:0051592	0.62	0.53	7	114	response to calcium ion	BP	1	ITPR3,SYT12,BAD,ANXA11,S100A16,EEF2K,JUND	yellow	VSMC_M3
GO:0003006	0.63	0.44	45	685	developmental process involved in reproduction	BP	1	BAG6,SBF1,PPARD,IFT27,CUL7,B4GALT1,PDGFRA,KRT8,SIX5,ZNF296,PAFAH1B3,DLEC1,METTL3,CEP131,DND1,YIF1B,MORN2,DEAF1,BCL2L1,KDM2B,SOD1,TYRO3,MMP14,TRIM27,SLC26A6,IQCG,PLA2G3,PBX1,IDH1,SGPL1,PHB2,BAD,NANOS3,IRF2BPL,ZNF830,AKT1,HMGCS1,RFX2,SAFB2,DHCR24,NSDHL,STK11,TAZ,FAM50A,MEIG1	yellow	VSMC_M3
GO:0006469	0.63	0.50	11	172	negative regulation of protein kinase activity	BP	1	H2AFY,PLEC,SOCS5,TARBP2,CHP1,QARS,TRIM27,PKN1,MVP,AKT1,TRIB2	yellow	VSMC_M3
GO:0030330	0.63	0.57	4	63	DNA damage response, signal transduction by p53 class mediator	BP	1	SPRED2,KAT5,HIC1,ZNF385A	yellow	VSMC_M3
GO:0048523	0.63	0.40	242	3647	negative regulation of cellular process	BP	1	ENPP1,NAB2,CTDSP2,SQSTM1,ZNF608,BAG6,CTHRC1,HDAC10,NELFE,FNIP1,ATF6B,TTLL12,RING1,SREBF2,H2AFY,ITPR3,HNRNPA0,TRIM11,PACSIN1,PPARD,SCRIB,PLEC,LGALS1,VPS28,B4GALT1,PDGFRA,SECISBP2,PHF2,SOCS5,CNOT3,KLF4,TPBG,SPRED2,RGS3,ZC3H12A,FIGNL1,PNKP,SNRNP70,CC2D1B,SNX3,MICAL1,ANXA5,SIX5,ZNF282,ZNF296,RELB,SLC9A3R1,PLXND1,EMILIN1,DMAP1,CIC,UNK,DLEC1,INSIG1,OGG1,UCP2,METTL3,CXXC5,INPPL1,DND1,PYCR1,EIF4EBP1,CAVIN3,DEAF1,TARBP2,NAT10,BCL2L1,PDE4D,TNS2,SWAP70,LRP3,ZBTB38,CAPG,PINX1,GREM1,KDM2B,PDE3B,CX3CL1,WNT10B,SOD1,PACSIN3,ANKRD2,DGKZ,EGR3,CCAR2,DNAJC17,DLL4,CHP1,TYRO3,YAF2,HTATIP2,PCIF1,PCBP4,KDM1B,CISH,SRSF9,DCPS,IRX1,SELENOS,GSTO1,ITPRIP,ETFBKMT,RIN3,FABP3,ZCCHC17,PDGFA,MMP14,CDK2AP2,GPX1,CORO1B,NGEF,CLCF1,QARS,TRIM27,ARPIN,EIF3A,LIMK1,PRMT2,THOC5,TWIST2,PFDN2,BRMS1,PLA2G3,STMN3,CCDC85B,PTPN23,PBX1,KAT5,RELA,SIPA1,ARNT2,LIMD1,MID1IP1,PEX5,SMARCB1,GATAD2A,PHB2,PPP1R35,IGFBP5,IFI35,CTDSP1,OTUB1,EID1,METTL13,LETM1,LGALS3,C11orf95,CD9,TNFRSF1A,BABAM1,C1QBP,AIMP2,CHERP,ZBTB49,UBXN1,WASHC1,RCC2,MTA2,CCDC22,FOXK1,SDE2,KLF3,PKN1,MIIP,MAD1L1,PALM3,AMER1,PIP4K2B,MAD2L2,NANOS3,CTBP1,DFFA,SP2,ARHGEF2,MVP,PER1,NFIX,CORO1A,SLX1A,IRF2BPL,RPL37,MTMR4,SMARCA4,CARM1,EEF2K,AATF,CXorf57,C19orf66,CSK,ATG3,ZNF830,AKT1,PAIP1,KHSRP,GIT1,ANKRD13B,SLC6A4,DHCR24,MYDGF,LDOC1,SIRT6,CACTIN,HMG20B,VASN,HIC1,TRAP1,AURKAIP1,MNT,CTNS,EHMT1,PGP,MBD3,MIDN,STK11,GAS8,SARS,SLC27A4,TGFBR3,MIR214,R[illegible]	yellow	VSMC_M3
GO:0034644	0.63	0.55	5	77	cellular response to UV	BP	1	METTL3,ACTR5,SDE2,STK11,NSMCE3	yellow	VSMC_M3
GO:0050871	0.63	0.57	4	61	positive regulation of B cell activation	BP	1	MMP14,CLCF1,BAD,MAD2L2	yellow	VSMC_M3
GO:0150104	0.63	0.57	4	70	transport across blood-brain barrier	BP	1	SLC9A3R1,LRP3,SLC19A1,SLC27A4	yellow	VSMC_M3
GO:0002790	0.63	0.49	11	172	peptide secretion	BP	1	PPARD,EIPR1,MYRIP,UCP2,AACS,PCK2,CAPN10,BAD,GLUD1,MIDN,JAGN1	yellow	VSMC_M3
GO:0033209	0.63	0.55	5	75	tumor necrosis factor-mediated signaling pathway	BP	1	SHARPIN,KRT8,RELA,TNFRSF1A,TRADD	yellow	VSMC_M3
GO:0032526	0.63	0.55	5	84	response to retinoic acid	BP	1	KLF4,WNT10B,PHB2,SLC6A4,TWF2	yellow	VSMC_M3
GO:0010507	0.63	0.57	4	67	negative regulation of autophagy	BP	1	SNRNP70,WASHC1,FOXK1,AKT1	yellow	VSMC_M3
GO:1901216	0.63	0.55	5	78	positive regulation of neuron death	BP	1	RAPSN,BAD,AIMP2,CAPN2,CDC34	yellow	VSMC_M3
GO:0042594	0.63	0.50	10	158	response to starvation	BP	1	FNIP1,SREBF2,ZC3H12A,UCP2,EIF4EBP1,AACS,PCK2,FOXK1,WDR24,FADS1	yellow	VSMC_M3
GO:0009653	0.63	0.41	128	2050	anatomical structure morphogenesis	BP	1	NAB2,CTHRC1,PBX2,RING1,H2AFY,PACSIN1,SCRIB,PLEC,TBCC,CUL7,B4GALT1,PDGFRA,COL15A1,EPN1,CNOT3,KLF4,DBNL,TPBG,HOXA1,WDPCP,CCM2,ZC3H12A,PLEKHA4,EHD2,KRT8,SLC9A3R1,PLXND1,EMILIN1,UNK,INSIG1,METTL3,BAIAP2,INPPL1,P4HB,RIC8A,ARHGAP22,DEAF1,HOXC4,LRIG1,ARHGAP33,BCL2L1,ITGB7,SLC11A2,GREM1,KDM2B,AQP5,PDE3B,CX3CL1,WNT10B,SOD1,EGR3,DLL4,TYRO3,HTATIP2,IRX1,PDGFA,MMP14,GPX1,CORO1B,NGEF,HCCS,PLEKHO1,IGSF9,COL6A1,NECTIN1,ANPEP,ARPIN,LIMK1,PLA2G3,PBX1,RELA,RHOC,FMNL1,VPS51,LIMD1,SGPL1,PBX4,YJEFN3,PHB2,IGFBP5,C12orf57,AAMP,CD9,TNFRSF1A,C1QBP,RCC2,CNP,PKN1,DBN1,CAPN2,MTHFR,CRABP2,TMEM79,ARHGEF2,ARMC5,CORO1A,SLC39A1,S100A6,SYNE3,EEF2K,MYO19,TTC39C,ZNF358,INF2,AKT1,RFX2,SLC6A4,MYDGF,SIRT6,ANAPC2,STK11,TAZ,JMJD8,SARS,TGFBR3,PSMB10,MIR214,NDST1,TNFRSF12A,ERBB4,TWF2,RNH1,ELN,UNC13A,CDC42EP5,NSMF,ARFRP1,DUSP5	yellow	VSMC_M3
GO:0007605	0.63	0.53	6	102	sensory perception of sound	BP	1	NDUFB9,HOXA1,SLC9A3R1,LRIG1,SOD1,GPX1	yellow	VSMC_M3
GO:0071466	0.63	0.53	6	92	cellular response to xenobiotic stimulus	BP	1	GUK1,SMOX,ACSL1,GSTO1,AIP,RBM22	yellow	VSMC_M3

GO:0034097	0.63	0.43	44	659	response to cytokine	BP	1	CDK4,TTLL12,RPL3,SHARPIN,IL1R1,SOCS5,KLF4,ZC3H12A,ZYX,KRT8,RELB,METTL3,P4HB,FASN,RAB20,BCL2L1,ACSL1,DOK1,CX3CL1,LSP1,CLCF1,PCK2,IMPDH2,SLC26A6,RELA,ATIC,BAD,OTUB1,TNFRSF1A,CAPN2,PALM3,MTHFR,ARHGEF2,CORO1A,TRIM25,CDC37,C19orf66,AKT1,KHSRP,CACTIN,IRAK1,TRADD,JAGN1,CDC34	yellow	VSMC_M3
GO:0071383	0.63	0.50	10	163	cellular response to steroid hormone stimulus	BP	1	PPARD,PCK2,PRMT2,PHB2,PKN1,PER1,SMARCA4,CARM1,SAFB,SAFB2	yellow	VSMC_M3
GO:0008088	0.63	0.57	4	68	axo-dendritic transport	BP	1	SOD1,BLOC1S4,AP3D1,SPG7	yellow	VSMC_M3
GO:0098586	0.63	0.57	4	63	cellular response to virus	BP	1	ZC3H12A,RRP1B,PHB2,C1QBP	yellow	VSMC_M3
GO:0008277	0.63	0.53	6	96	regulation of G protein-coupled receptor signaling pathway	BP	1	RGS3,MGLL,ZDHHC3,GIT1,RGS16,RGS18	yellow	VSMC_M3
GO:0050868	0.63	0.55	5	76	negative regulation of T cell activation	BP	1	SCRIB,SOCS5,ZC3H12A,LGALS3,MAD1L1	yellow	VSMC_M3
GO:0007033	0.63	0.49	10	157	vacuole organization	BP	1	LAMTOR1,RAB20,HPS1,NDP,PIP4K2B,CORO1A,ATG3,PACS2,RAB34,CCDC115	yellow	VSMC_M3
GO:0005976	0.63	0.54	5	83	polysaccharide metabolic process	BP	1	ENPP1,SELENOS,AKT1,B3GNT9,NDST1	yellow	VSMC_M3
GO:0072332	0.63	0.57	4	63	intrinsic apoptotic signaling pathway by p53 class mediator	BP	1	BAG6,ZNF385B,STK11,ZNF385A	yellow	VSMC_M3
GO:0019319	0.63	0.57	4	61	hexose biosynthetic process	BP	1	SELENOS,PCK2,PGM1,PGP	yellow	VSMC_M3
GO:0006955	0.64	0.41	68	1012	immune response	BP	1	ENPP1,GNL1,BAG6,TTLL12,TRIM11,PLEC,LGALS1,PPP1R14B,IL1R1,SSC5D,SOCS5,DBNL,ZC3H12A,ZYX,RELB,CD79A,METTL3,RAB20,TARBP2,BCL2L1,PDE4D,SWAP70,CX3CL1,DGKZ,TYRO3,RNF19B,SELENOS,GPX1,CLCF1,NECTIN1,TRIM27,LIMK1,SLC26A6,RIPK3,PSMB4,TNK2,PLA2G3,RELA,SIPA1,MAP3K14,PHB2,IFI35,OTUB1,LGALS3,TNFRSF1A,C1QBP,PKN1,MAD2L2,TBKBP1,SP2,ARHGEF2,ARMC5,CORO1A,TRIM25,CDC37,CD276,C19orf66,CSK,MID2,DOCK11,CACTIN,IRAK1,C8G,STK11,CARD9,TGFBR3,PSMB10,JAGN1	yellow	VSMC_M3
GO:0002286	0.64	0.56	4	65	T cell activation involved in immune response	BP	1	SOCS5,ZC3H12A,RELB,LGALS3	yellow	VSMC_M3
GO:0050867	0.64	0.47	15	226	positive regulation of cell activation	BP	1	LGALS1,SOCS5,EGR3,MMP14,CLCF1,PLA2G3,SART1,BAD,MAD2L2,CORO1A,CD276,CSK,AKT1,AP3D1,JUND	yellow	VSMC_M3
GO:0035710	0.64	0.56	4	65	CD4-positive, alpha-beta T cell activation	BP	1	SOCS5,ZC3H12A,RELB,ARMC5	yellow	VSMC_M3
GO:0000226	0.64	0.43	33	522	microtubule cytoskeleton organization	BP	1	DCTN2,SPAG1,CUL7,FIGNL1,CEP131,NUMA1,YIF1B,CCDC57,NAT10,DCTN1,CHP1,CDK2AP2,CLIP2,PCNT,NUDC,IQCG,PLA2G3,STMN3,SAC3D1,MID1IP1,KIAA0753,CNP,EML3,ARHGEF2,RANGRF,EML1,GIT1,GAS8,NTMT1,MAP7D1,GBA2,TUBGCP2,MEIG1	yellow	VSMC_M3
GO:0022402	0.64	0.41	62	951	cell cycle process	BP	1	DCTN2,CDK4,CTDSP2,BAG6,NCAPH2,SNX9,H2AFY,PLEC,SND1,CUL7,KLF4,FIGNL1,KMT2E,SEPT9,CEP131,NUMA1,CCDC57,EIF4EBP1,NAT10,BCL2L1,RRS1,PINX1,DCTN1,WNT10B,CCAR2,RAB35,CDK2AP2,PSME2,PCNT,NUDC,PRMT2,THOC5,PBX1,RHOC,ALKBH4,SAC3D1,PHB2,CTDSP1,METTL13,KIAA0753,BABAM1,ANXA11,RCC2,EML3,SDE2,MIIP,MAD1L1,MAD2L2,MYO19,EML1,ZNF830,AKT1,SNX18,GIT1,AURKAIP1,NTMT1,MIR214,NABP2,MIS12,CDC34,FBXO6,TUBGCP2	yellow	VSMC_M3
GO:1990542	0.64	0.52	6	84	mitochondrial transmembrane transport	BP	1	UCP2,SFXN5,LETM1,ATP5PD,AKT1,ATP5F1D	yellow	VSMC_M3
GO:0010638	0.64	0.44	27	428	positive regulation of organelle organization	BP	1	SNX9,H2AFY,PNKP,SEPT9,BAIAP2,NUMA1,SWAP70,DCTN1,CX3CL1,CORO1B,TRIM27,ARPIN,LIMK1,PFDN2,RHOC,CHCHD10,BAD,ARPC1B,WASHC1,PIP4K2B,CORO1A,SLX1A,GIT1,SIRT6,NABP2,CDC42EP5,ARPC5L	yellow	VSMC_M3
GO:0010232	0.64	0.56	4	71	vascular transport	BP	1	SLC9A3R1,LRP3,SLC19A1,SLC27A4	yellow	VSMC_M3
GO:0000380	0.64	0.56	4	66	alternative mRNA splicing, via spliceosome	BP	1	RBM15B,SRSF9,RAVER1,RBM8A	yellow	VSMC_M3
GO:0060284	0.64	0.44	23	384	regulation of cell development	BP	1	TRIM11,CUL7,DBNL,PLXND1,BAIAP2,P4HB,CX3CL1,DLL4,CLCF1,LIMK1,RELA,BAD,CTDSP1,TNFRSF1A,C1QBP,RCC2,DBN1,CRABP2,EEF2K,AKT1,ANAPC2,STK11,TWF2	yellow	VSMC_M3
GO:0042180	0.64	0.48	10	150	cellular ketone metabolic process	BP	1	PPARD,FDXR,INSIG1,ETFBKMT,FABP3,PLA2G3,MID1IP1,HSD17B1,COQ6,AKT1	yellow	VSMC_M3
GO:0007369	0.65	0.50	8	138	gastrulation	BP	1	H2AFY,KLF4,RIC8A,MMP14,COL6A1,ARMC5,ARFRP1,DUSP5	yellow	VSMC_M3
GO:0150115	0.65	0.53	5	95	cell-substrate junction organization	BP	1	PLEC,WDPCP,GREM1,MMP14,RCC2	yellow	VSMC_M3
GO:0018193	0.65	0.41	65	997	peptidyl-amino acid modification	BP	1	BAG6,HDAC10,NELFE,FNIP1,JADE2,H2AFY,PDGFRA,SOCS5,SPRED2,MEAF6,KMT2E,DMAP1,KAT14,P4HA3,P4HB,KMT2B,PDE4D,TNKS1BP1,SETD1B,GREM1,MCRS1,FKBP11,TYRO3,ETFBKMT,STK32C,PDGFA,AIP,CLCF1,TRIM27,PRMT2,TNK2,KAT5,RELA,SCYL1,ZDHHC3,SMARCB1,TRMT112,FKBP2,EID1,TNFRSF1A,TAF6L,PKN1,MAD2L2,CTBP1,ARHGEF2,MVP,PER1,DPM3,SGF29,CARM1,CSK,AKT1,COPRS,METTL22,MAP2K2,HMG20B,EHMT1,MKNK2,CSNK1G2,MBD3,NTMT1,ERBB4,LOXL1,PIN4,THAP7	yellow	VSMC_M3
GO:0010921	0.65	0.55	4	68	regulation of phosphatase activity	BP	1	CHP1,RIPK3,PPP1R35,LGALS3	yellow	VSMC_M3
GO:0015849	0.65	0.46	13	203	organic acid transport	BP	1	PPARD,SLC9A3R1,ACSL1,SFXN5,FABP3,SLC19A1,SLC26A6,SPX,PLA2G3,SLC27A3,AKT1,CTNS,SLC27A4	yellow	VSMC_M3
GO:0040013	0.65	0.45	16	261	negative regulation of locomotion	BP	1	PPARD,KLF4,SLC9A3R1,EMILIN1,GREM1,CX3CL1,DLL4,RIN3,CORO1B,ARPIN,PTPN23,IGFBP5,MIIP,AKT1,MIR214,PTPRR	yellow	VSMC_M3
GO:0019233	0.65	0.55	4	63	sensory perception of pain	BP	1	MGLL,MMP24,SPX,TMEM120A	yellow	VSMC_M3
GO:1904951	0.65	0.45	16	257	positive regulation of establishment of protein localization	BP	1	SREBF2,PPARD,VPS28,ZC3H12A,DMAP1,MYRIP,CEP131,AACS,CHP1,PCK2,PCNT,CAPN10,PTPN23,BAD,GLUD1,RBM22	yellow	VSMC_M3
GO:0090174	0.65	0.51	6	94	organelle membrane fusion	BP	1	RAB20,CHP1,PIP4K2B,STX10,CORO1A,RAB34	yellow	VSMC_M3
GO:0006259	0.65	0.41	49	741	DNA metabolic process	BP	1	NHP2,HDAC10,FANCE,KLF4,FIGNL1,PNKP,KMT2E,MUTYH,DMAP1,OGG1,TTC5,FAAP100,SMUG1,NAT10,SWAP70,TNKS1BP1,PINX1,GREM1,MCRS1,ACTR5,KDM1B,CLCF1,CEP164,KAT5,LRWD1,ALKBH4,SMARCB1,GATAD2A,OTUB1,BABAM1,MTA2,MAD2L2,DFFA,PPP4C,SLX1A,USP43,CXorf57,ZNF830,AKT1,SIRT6,EHMT1,MBD3,TELO2,RF00024,NABP2,CDC34,FBXO6,STUB1,NSMCE3	yellow	VSMC_M3
GO:0072001	0.65	0.45	15	256	renal system development	BP	1	BAG6,MPST,IFT27,PDGFRA,WDPCP,PLXND1,SNX17,TNS2,GREM1,IRX1,PDGFA,PBX1,SGPL1,AMER1,ERBB4	yellow	VSMC_M3

GO:0050954	0.65	0.50	7	120	sensory perception of mechanical stimulus	BP	1	NDUFB9,HOXA1,SLC9A3R1,LRIG1,SOD1,GPX1,TMEM120A	yellow	VSMC_M3
GO:0001570	0.65	0.55	4	71	vasculogenesis	BP	1	CUL7,CCM2,SGPL1,TGFBR3	yellow	VSMC_M3
GO:0007416	0.65	0.50	7	132	synapse assembly	BP	1	DBNL,TPBG,PLXND1,LRFN4,NECTIN1,EEF2K,ERBB4	yellow	VSMC_M3
GO:0071479	0.65	0.55	4	64	cellular response to ionizing radiation	BP	1	EEF1D,FIGNL1,BCL2L1,TNKS1BP1	yellow	VSMC_M3
GO:0034330	0.65	0.42	31	541	cell junction organization	BP	1	PLEC,DBNL,TPBG,WDPCP,CCM2,PLXND1,BAIAP2,ARHGAP22,ARHGAP33,GREM1,DCTN1,RAPSN,CX3CL1,MMP14,NGEF,IGSF9,LRFN4,NECTIN1,PTPN23,RHOC,CHCHD10,CD9,RCC2,DBN1,EEF2K,CSK,TJP3,CLDN6,ANAPC2,ERBB4,UNC13A	yellow	VSMC_M3
GO:1902107	0.65	0.50	7	110	positive regulation of leukocyte differentiation	BP	1	SOCS5,EGR3,MMP14,PLA2G3,SART1,BAD,AP3D1	yellow	VSMC_M3
GO:1903708	0.65	0.50	7	110	positive regulation of hemopoiesis	BP	1	SOCS5,EGR3,MMP14,PLA2G3,SART1,BAD,AP3D1	yellow	VSMC_M3
GO:0014020	0.65	0.52	5	86	primary neural tube formation	BP	1	CTHRC1,SCRIB,DEAF1,KDM2B,MTHFR	yellow	VSMC_M3
GO:0048754	0.65	0.50	7	121	branching morphogenesis of an epithelial tube	BP	1	PLXND1,GREM1,DLL4,MMP14,PBX1,PHB2,SIRT6	yellow	VSMC_M3
GO:0033002	0.65	0.47	11	174	muscle cell proliferation	BP	1	PPARD,KLF4,SIX5,CX3CL1,IGFBP5,AKT1,IRAK1,TGFBR3,MIR214,ERBB4,ELN	yellow	VSMC_M3
GO:0030198	0.65	0.45	15	250	extracellular matrix organization	BP	1	B4GALT1,PDGFRA,COL15A1,COL19A1,EMILIN1,RIC8A,MMP24,GREM1,MMP14,MPZL3,TNFRSF1A,WASHC1,POMT1,LOXL1,ELN	yellow	VSMC_M3
GO:0048513	0.66	0.38	163	2529	animal organ development	BP	1	ENPP1,NAB2,CDK4,BAG6,NCAPH2,CTHRC1,FNIP1,PBX2,PDLIM4,RING1,H2AFY,TRAPPC9,PPARD,SCRIB,PLEC,LGALS1,MPST,IFT27,SHARPIN,CUL7,PDGFRA,SECISBP2,PHF2,EPN1,COL19A1,SOCS5,TTC7A,KLF4,HOXA1,WDPCP,CCM2,FHL3,SPRED2,ZC3H12A,RCN3,PLEKHA4,KMT2E,KRT8,SIX5,RELB,SLC9A3R1,PLXND1,EMILIN1,PAFAH1B3,CIC,SNX17,CD79A,INSIG1,UCP2,METTL3,BAIAP2,INPPL1,NUMA1,FASN,EIF4EBP1,DEAF1,HOXC4,LRIG1,AACS,BCL2L1,TNS2,SLC11A2,GREM1,KDM2B,AQP5,DCTN1,WNT10B,SOD1,ANKRD2,EGR3,DLL4,TYRO3,IRX1,PDGFA,MMP14,GPX1,HCCS,CLCF1,QARS,PCK2,IMPDH2,PIR,NECTIN1,THOC5,RIPK3,PLA2G3,SART1,PBX1,RELA,SCYL1,IDH1,ARNT2,VPS51,SGPL1,PBX4,YJEFN3,ATIC,MEF2B,PHB2,IGFBP5,C12orf57,BAD,HSD17B1,CD9,TNFRSF1A,TEAD4,AIMP2,EVC,CNP,PRXL2A,KRT10,GLUD1,FOXK1,CASP14,PKN1,MAD1L1,AMER1,MAD2L2,PRR14,TMEM79,ARMC5,MTX1,SLC39A1,S100A4,SMARCA4,EML1,CSK,TTC39C,DOCK11,ZNF830,AKT1,COPRS,HMGCS1,SAFB2,GIT1,SLC6A4,DHCR24,SIRT6,PPL,NSDHL,HMG20B,VASN,CTNS,AP3D1,MKNK2,MBD3,STK11,TAZ,APRT,GTF3C5,SLC27A4,TGFBR3,TRADD,NDST1,ERBB4,KLF13,JAGN1,IGSF8,ELN,ZNF385A,NOM1,GLIPR2,TUBGCP2	yellow	VSMC_M3
GO:0019882	0.66	0.54	4	61	antigen processing and presentation	BP	1	RELB,RAB35,RAB34,AP3D1	yellow	VSMC_M3
GO:0098542	0.66	0.41	38	574	defense response to other organism	BP	1	TTLL12,TRIM11,PPP1R14B,SSC5D,ZC3H12A,ZYX,RELB,METTL3,RAB20,TARBP2,BCL2L1,CX3CL1,TYRO3,RNF19B,TRIM27,SLC26A6,RIPK3,TNK2,RELA,MAP3K14,PHB2,IFI35,LGALS3,TNFRSF1A,C1QBP,TBKBP1,ARHGEF2,ARMC5,CORO1A,TRIM25,CDC37,C19orf66,MID2,CACTIN,IRAK1,C8G,CARD9,JAGN1	yellow	VSMC_M3
GO:0071706	0.66	0.49	7	115	tumor necrosis factor superfamily cytokine production	BP	1	ZC3H12A,CX3CL1,SELENOS,TRIM27,ARHGEF2,CACTIN,CARD9	yellow	VSMC_M3
GO:1903555	0.66	0.49	7	115	regulation of tumor necrosis factor superfamily cytokine production	BP	1	ZC3H12A,CX3CL1,SELENOS,TRIM27,ARHGEF2,CACTIN,CARD9	yellow	VSMC_M3
GO:1990845	0.66	0.48	8	132	adaptive thermogenesis	BP	1	CNOT3,UCP2,ACSL1,WNT10B,SCD,SIRT6,EHMT1,STK11	yellow	VSMC_M3
GO:0038127	0.66	0.50	6	102	ERBB signaling pathway	BP	1	SOCS5,MVB12A,MVP,AKT1,PTPRR,STUB1	yellow	VSMC_M3
GO:0034446	0.66	0.52	5	89	substrate adhesion-dependent cell spreading	BP	1	P4HB,ITGB7,TYRO3,C1QBP,RCC2	yellow	VSMC_M3
GO:0043062	0.66	0.44	15	251	extracellular structure organization	BP	1	B4GALT1,PDGFRA,COL15A1,COL19A1,EMILIN1,RIC8A,MMP24,GREM1,MMP14,MPZL3,TNFRSF1A,WASHC1,POMT1,LOXL1,ELN	yellow	VSMC_M3
GO:0048660	0.66	0.48	8	124	regulation of smooth muscle cell proliferation	BP	1	PPARD,KLF4,CX3CL1,IGFBP5,AKT1,IRAK1,MIR214,ELN	yellow	VSMC_M3
GO:0009100	0.66	0.43	19	299	glycoprotein metabolic process	BP	1	PPARD,B4GALT1,SERP2,GALNT14,PHLDA1,CHP1,SLC35C2,ST3GAL4,MANBA,TMTC1,EXTL1,MPDU1,DPM3,AATF,MAN1B1,POMT1,B3GNT9,NDST1,FBXO6	yellow	VSMC_M3
GO:0045229	0.66	0.44	15	251	external encapsulating structure organization	BP	1	B4GALT1,PDGFRA,COL15A1,COL19A1,EMILIN1,RIC8A,MMP24,GREM1,MMP14,MPZL3,TNFRSF1A,WASHC1,POMT1,LOXL1,ELN	yellow	VSMC_M3
GO:0021782	0.66	0.52	5	86	glial cell development	BP	1	HDAC10,PLEC,SOD1,CD9,AKT1	yellow	VSMC_M3
GO:0007169	0.66	0.41	30	499	transmembrane receptor protein tyrosine kinase signaling pathway	BP	1	ENPP1,CD63,CDK4,PDGFRA,SOCS5,SLC9A3R1,EMILIN1,BAIAP2,TNS2,GREM1,DOK1,TYRO3,PDGFA,NGEF,TNK2,RELA,SGPL1,IGFBP5,MVB12A,PIP4K2B,MVP,AKT1,GIT1,SLC27A4,TRADD,NDST1,ERBB4,PTPRR,IGFBP6,STUB1	yellow	VSMC_M3
GO:0050778	0.66	0.42	20	306	positive regulation of immune response	BP	1	BAG6,IL1R1,SOCS5,ZC3H12A,CD79A,PDE4D,DGKZ,CLCF1,LIMK1,PLA2G3,RELA,IFI35,LGALS3,C1QBP,MAD2L2,CD276,CSK,C8G,STK11,CARD9	yellow	VSMC_M3
GO:0033043	0.66	0.39	60	950	regulation of organelle organization	BP	1	SQSTM1,SNX9,SREBF2,H2AFY,CUL7,PDGFRA,DBNL,PNKP,SEPT9,BAIAP2,NUMA1,NAT10,BCL2L1,SWAP70,CAPG,PINX1,DCTN1,CX3CL1,PDGFA,CDK2AP2,GPX1,CORO1B,LRFN4,TRIM27,ARPIN,LIMK1,PFDN2,STMN3,RHOC,MID1IP1,CHCHD10,BAD,ARPC1B,WASHC1,EML3,DBN1,CAPN2,MAD1L1,PIP4K2B,MAD2L2,ARHGEF2,RANGRF,CORO1A,SLX1A,RABEP2,MYO19,ATG3,INF2,AKT1,GIT1,SIRT6,MAP2K2,AURKAIP1,MIR214,NABP2,TWF2,ELN,CDC42EP5,GBA2,ARPC5L	yellow	VSMC_M3
GO:0061041	0.66	0.50	6	96	regulation of wound healing	BP	1	PDGFRA,ST3GAL4,PDGFA,CD9,PLAU,TNFRSF12A	yellow	VSMC_M3
GO:0048709	0.66	0.53	4	67	oligodendrocyte differentiation	BP	1	HDAC10,CD9,CNP,CSK	yellow	VSMC_M3
GO:0007229	0.67	0.51	5	92	integrin-mediated signaling pathway	BP	1	CD63,CCM2,ZYX,ITGB7,RCC2	yellow	VSMC_M3
GO:0045785	0.67	0.42	19	306	positive regulation of cell adhesion	BP	1	TSTA3,LGALS1,SOCS5,EMILIN1,P4HB,CX3CL1,EGR3,ST3GAL4,SART1,PTPN23,RELA,BAD,C1QBP,CORO1A,CD276,CSK,AKT1,AP3D1,IRAK1	yellow	VSMC_M3
GO:0043542	0.67	0.44	12	195	endothelial cell migration	BP	1	TSTA3,KLF4,ZC3H12A,PLXND1,SCARB1,GREM1,EGR3,DLL4,GPX1,CORO1B,AAMP,AKT1	yellow	VSMC_M3

GO:0045087	0.67	0.40	30	459	innate immune response	BP	1	TTLL12,TRIM11,PPP1R14B,SSC5D,ZYX,RELB,METTL3,RAB20,CX3CL1,TYRO3,RNF19B,TRIM27,SLC26A6,TNK2,RELA,PHB2,IFI35,LGALS3,C1QBP,TBKBP1,ARHGEF2,CORO1A,TRIM25,CDC37,C19orf66,MID2,CACTIN,IRAK1,C8G,CARD9	yellow	VSMC_M3
GO:0010639	0.67	0.42	18	297	negative regulation of organelle organization	BP	1	NAT10,BCL2L1,SWAP70,CAPG,PINX1,GPX1,CORO1B,ARPIN,MID1IP1,MAD1L1,MAD2L2,ARHGEF2,CORO1A,SLX1A,AKT1,AURKAIP1,MIR214,TWF2	yellow	VSMC_M3
GO:0001503	0.67	0.42	20	328	ossification	BP	1	ENPP1,NAB2,CTHRC1,SND1,FIGNL1,INPPL1,FASN,LRP3,GREM1,WNT10B,MMP14,COL6A1,TWIST2,MRC2,PBX1,LIMD1,IGFBP5,AKT1,GIT1,JUND	yellow	VSMC_M3
GO:0071495	0.67	0.38	63	1018	cellular response to endogenous stimulus	BP	1	ENPP1,CDK4,PPARD,PDGFRA,KLF4,SPRED2,ZYX,SLC9A3R1,EMILIN1,INSIG1,UCP2,BAIAP2,LAMTOR1,NUMA1,BCL2L1,TNS2,ACSL1,GREM1,PDE3B,WNT10B,SOD1,EGR3,DLL4,RAB35,SELENOS,CORO1B,PCK2,COL6A1,SLC26A6,PRMT2,CAPN10,RELA,PHB2,IGFBP5,LAMTOR4,PELP1,KLF3,PKN1,CAPN2,PIP4K2B,ARHGEF2,PER1,CORO1A,MTMR4,SMARCA4,CARM1,EEF2K,CSK,AKT1,HMGCS1,SAFB,SAFB2,SLC6A4,LDOC1,VASN,STK11,APRT,SLC27A4,TGFBR3,NDST1,ERBB4,NSMF,STUB1	yellow	VSMC_M3
GO:0071674	0.67	0.47	8	126	mononuclear cell migration	BP	1	PLEC,ITGB7,GREM1,CX3CL1,RIPK3,LGALS3,C1QBP,AKT1	yellow	VSMC_M3
GO:0017038	0.67	0.45	11	174	protein import	BP	1	SAMM50,ZC3H12A,DMAP1,SEC13,CHP1,PEX5,PHB2,NUP88,RANGRF,RBM22,AKT1	yellow	VSMC_M3
GO:0071346	0.67	0.50	5	80	cellular response to interferon-gamma	BP	1	ZYX,RAB20,CX3CL1,SLC26A6,CDC37	yellow	VSMC_M3
GO:0140029	0.67	0.52	4	66	exocytic process	BP	1	SCRIB,PLA2G3,RABEPK,UNC13A	yellow	VSMC_M3
GO:0007029	0.67	0.50	5	80	endoplasmic reticulum organization	BP	1	ESYT1,BAG6,ASNA1,LMAN2,JAGN1	yellow	VSMC_M3
GO:0042060	0.67	0.41	20	329	wound healing	BP	1	PPARD,SCRIB,PLEC,B4GALT1,PDGFRA,ANXA5,SCARB1,CX3CL1,DGKZ,TYRO3,ST3GAL4,PDGFA,GPX1,CORO1B,RHOC,BLOC1S4,CD9,PLAU,PPL,TNFRSF12A	yellow	VSMC_M3
GO:0034341	0.67	0.48	6	95	response to interferon-gamma	BP	1	ZYX,RAB20,CX3CL1,SLC26A6,CDC37,C19orf66	yellow	VSMC_M3
GO:0006302	0.67	0.43	14	225	double-strand break repair	BP	1	FIGNL1,PNKP,OGG1,TNKS1BP1,ACTR5,KAT5,OTUB1,BABAM1,MAD2L2,PPP4C,SLX1A,CXorf57,SIRT6,NABP2	yellow	VSMC_M3
GO:0010257	0.68	0.52	4	53	NADH dehydrogenase complex assembly	BP	1	NDUFB9,ACAD9,NDUFS7,TAZ	yellow	VSMC_M3
GO:0032981	0.68	0.52	4	53	mitochondrial respiratory chain complex I assembly	BP	1	NDUFB9,ACAD9,NDUFS7,TAZ	yellow	VSMC_M3
GO:0050790	0.68	0.36	119	1823	regulation of catalytic activity	BP	1	NAB2,CDK4,SBF1,SNX9,FNIP1,RING1,H2AFY,SGSM3,DEF6,EEF1D,SCRIB,PLEC,PPP1R14B,PDGFRA,HSPBP1,SOCS5,KLF4,DBNL,SPRED2,RGS3,PNKP,RCN3,SNRNP70,MICAL1,ANXA5,SLC9A3R1,PLXND1,SERTAD1,ARHGEF40,LAMTOR1,ARAP3,DELE1,RIC8A,ARHGAP22,ARHGAP33,TARBP2,SCARB1,HM13,SWAP70,ACSL1,MMP24,PINX1,GREM1,CX3CL1,SOD1,DGKZ,CCAR2,HPS1,CHP1,TYRO3,CISH,ITPRIP,RRP1B,ETFBKMT,RIN3,PDGFA,PABPN1,GPX1,AIP,NGEF,QARS,PSME2,TRIM27,LIMK1,RIPK3,ADRM1,TNK2,TACO1,STMN3,SIPA1,RHOC,MID1IP1,PHB2,PPP1R35,BAD,LAMTOR4,LGALS3,AIMP2,UBXN1,WASHC1,RCC2,RAB3IL1,PKN1,PIP4K2B,MAD2L2,DFFA,ARHGEF2,MVP,RANGRF,DPM3,RABEP2,RPL37,CDC37,CSK,DOCK11,AKT1,SNX18,DENND1C,GTF2F1,RANBP3,GIT1,DHCR24,MAP2K2,ACAP3,IRAK1,MIDN,STK11,TELO2,JMJD8,CARD9,SLC27A4,VAC14,ERBB4,RNH1,TRIB2,ARHGEF1,RGS16,STUB1,RGS18	yellow	VSMC_M3
GO:0048144	0.68	0.52	4	66	fibroblast proliferation	BP	1	CDK4,PDGFRA,PDGFA,S100A6	yellow	VSMC_M3
GO:0048145	0.68	0.52	4	66	regulation of fibroblast proliferation	BP	1	CDK4,PDGFRA,PDGFA,S100A6	yellow	VSMC_M3
GO:0021700	0.68	0.43	12	204	developmental maturation	BP	1	CD63,GREM1,CX3CL1,WNT10B,SLC26A6,PLA2G3,AP1M1,TMEM79,AP3D1,ANAPC2,UNC13A,NOM1	yellow	VSMC_M3
GO:0002824	0.68	0.52	4	64	positive regulation of adaptive immune response based on somatic recombination of immune receptors built from immunoglobulin superfamily domains	BP	1	IL1R1,CLCF1,MAD2L2,CARD9	yellow	VSMC_M3
GO:0001764	0.68	0.48	6	113	neuron migration	BP	1	UNK,CX3CL1,ARHGEF2,ACAP3,NSMF,TUBGCP2	yellow	VSMC_M3
GO:0070997	0.68	0.41	18	288	neuron death	BP	1	BCL2L1,KDM2B,RAPSN,CX3CL1,SOD1,CHP1,TYRO3,GPX1,CLCF1,PLA2G3,BAD,AIMP2,CAPN2,CORO1A,AKT1,DHCR24,NSMF,CDC34	yellow	VSMC_M3
GO:0032386	0.68	0.41	18	290	regulation of intracellular transport	BP	1	SREBF2,EIPR1,ZC3H12A,SNX3,EHD2,DMAP1,INSIG1,CEP131,NUMA1,CHP1,PCNT,CAPN10,PLA2G3,PTPN23,RBM22,MAP2K2,STK11,PLA2G4E	yellow	VSMC_M3
GO:0016485	0.68	0.44	11	170	protein processing	BP	1	LDLRAD3,HM13,MMP14,SEC11A,BAD,PLAU,SDE2,CAPN2,DHCR24,SPG7,RCE1	yellow	VSMC_M3
GO:1905477	0.68	0.49	5	88	positive regulation of protein localization to membrane	BP	1	SQSTM1,CHP1,LGALS3,RANGRF,AKT1	yellow	VSMC_M3
GO:0034754	0.68	0.49	5	79	cellular hormone metabolic process	BP	1	PDGFRA,SCARB1,SGPL1,HSD17B1,CRABP2	yellow	VSMC_M3
GO:0042113	0.68	0.43	12	193	B cell activation	BP	1	FNIP1,LGALS1,CD79A,SWAP70,MMP14,CLCF1,PHB2,BAD,PKN1,MAD2L2,DOCK11,AKAP17A	yellow	VSMC_M3
GO:0006612	0.68	0.48	6	97	protein targeting to membrane	BP	1	ASNA1,YIF1B,CHP1,PEX5,ZDHHC3,ATG3	yellow	VSMC_M3
GO:0048638	0.68	0.42	15	257	regulation of developmental growth	BP	1	CDK4,PPARD,DBNL,SOD1,LIMK1,SGPL1,DBN1,CRABP2,AKT1,SLC6A4,ANAPC2,TGFBR3,ERBB4,TWF2,UNC13A	yellow	VSMC_M3
GO:0051963	0.68	0.51	4	79	regulation of synapse assembly	BP	1	TPBG,LRFN4,NECTIN1,EEF2K	yellow	VSMC_M3
GO:0051146	0.69	0.42	13	220	striated muscle cell differentiation	BP	1	PLEC,PDGFRA,EHD2,KRT8,GREM1,WNT10B,MMP14,GPX1,PLEKHO1,IGFBP5,CD9,CAPN2,AKT1	yellow	VSMC_M3
GO:0071675	0.69	0.49	5	81	regulation of mononuclear cell migration	BP	1	GREM1,RIPK3,LGALS3,C1QBP,AKT1	yellow	VSMC_M3
GO:0002443	0.69	0.42	14	221	leukocyte mediated immunity	BP	1	GNL1,IL1R1,KMT2E,SWAP70,RNF19B,CLCF1,RIPK3,PLA2G3,C1QBP,MAD2L2,CORO1A,C8G,CARD9,JAGN1	yellow	VSMC_M3
GO:0019079	0.69	0.47	6	99	viral genome replication	BP	1	ZC3H12A,TARBP2,BANF1,SMARCB1,CTBP1,C19orf66	yellow	VSMC_M3
GO:0060070	0.69	0.41	15	250	canonical Wnt signaling pathway	BP	1	CTHRC1,KLF4,TPBG,PLEKHA4,SLC9A3R1,RNF220,GREM1,WNT10B,CCAR2,LIMD1,AMER1,MAD2L2,CSNK1G2,STK11,IGFBP6	yellow	VSMC_M3

GO:0009605	0.69	0.35	116	1770	response to external stimulus	BP	1	CD63,CDK4,FNIP1,TTLL12,SREBF2,HNRNPA0,TRIM11,PPARD,SCRIB,PLEC,SHARPIN,PPP1R14B,PDGFRA,IL1R1,SSC5D,SOCS5,KLF4,TPBG,ZC3H12A,ZYX,SNX3,MGLL,KRT8,RELB,PLXND1,OGG1,UCP2,METTL3,BAIAP2,RAB20,EIF4EBP1,TARBP2,AACS,SCARB1,BCL2L1,SWAP70,ACSL1,MMP24,GREM1,CX3CL1,SOD1,EGR3,TYRO3,LSP1,ST3GAL4,RNF19B,DCPS,SELENOS,RRP1B,MVK,RIN3,PDGFA,MMP14,PABPN1,GPX1,CORO1B,IGSF9,PCK2,NECTIN1,TRIM27,SLC26A6,SPX,RIPK3,PSMB4,TNK2,PLA2G3,BANF1,RELA,SIPA1,TMEM120A,MAP3K14,PHB2,BAD,IFI35,LGALS3,CD9,PLAU,TNFRSF1A,C1QBP,CNP,AP1M1,FOXK1,CAPN2,PALM3,MTHFR,TBKBP1,ARHGEF2,ARMC5,PER1,CORO1A,TRIM25,CDC37,C19orf66,MID2,AKT1,HMGCS1,GTF2F1,GIT1,SLC6A4,LDOC1,SIRT6,PPL,CACTIN,AP3D1,IRAK1,MBD3,C8G,WDR24,CARD9,SLC27A4,TRADD,JUND,LOXL1,EEF1G,FADS1,JAGN1	yellow	VSMC_M3
GO:0009410	0.69	0.40	20	314	response to xenobiotic stimulus	BP	1	GUK1,LGALS1,OGG1,SMOX,AACS,ACSL1,SOD1,GSTO1,FABP3,GPX1,AIP,PPOX,RELA,BAD,MTHFR,RBM22,HMGCS1,SLC6A4,BLMH,CARD9	yellow	VSMC_M3
GO:0055088	0.69	0.46	7	116	lipid homeostasis	BP	1	SREBF2,RCN3,INSIG1,LAMTOR1,SCARB1,RALY,FABP3	yellow	VSMC_M3
GO:0043549	0.69	0.37	38	612	regulation of kinase activity	BP	1	NAB2,SNX9,H2AFY,PLEC,PDGFRA,SOCS5,KLF4,SLC9A3R1,SERTAD1,TARBP2,ACSL1,GREM1,SOD1,DGKZ,CHP1,TYRO3,CISH,PDGFA,QARS,TRIM27,RIPK3,PHB2,BAD,WASHC1,PKN1,PIP4K2B,MVP,CDC37,CSK,AKT1,MAP2K2,IRAK1,MIDN,STK11,TELO2,VAC14,ERBB4,TRIB2	yellow	VSMC_M3
GO:0006446	0.69	0.50	4	66	regulation of translational initiation	BP	1	METTL3,EIF4EBP1,EIF4B,PAIP1	yellow	VSMC_M3
GO:0033044	0.69	0.43	10	168	regulation of chromosome organization	BP	1	H2AFY,PNKP,NUMA1,NAT10,PINX1,MAD1L1,MAD2L2,SLX1A,SIRT6,NABP2	yellow	VSMC_M3
GO:0045739	0.69	0.50	4	66	positive regulation of DNA repair	BP	1	HDAC10,PNKP,BABAM1,MAD2L2	yellow	VSMC_M3
GO:0042098	0.69	0.45	8	127	T cell proliferation	BP	1	SCRIB,RIPK3,LGALS3,MAD1L1,ARMC5,CORO1A,CD276,PSMB10	yellow	VSMC_M3
GO:0045893	0.69	0.35	79	1244	positive regulation of transcription, DNA-templated	BP	1	SQSTM1,NELFE,ATF6B,PBX2,SREBF2,PPARD,PHF2,KLF4,HOXA1,ZC3H12A,KMT2E,SIX5,ZNF296,SERTAD1,TTC5,DEAF1,HOXC4,KMT2B,ZBTB38,GREM1,MCRS1,CX3CL1,WNT10B,EGR3,CCAR2,YAF2,HTATIP2,KDM1B,CHCHD2,RRP1B,AIP,TRIM27,PRMT2,THOC5,RIPK3,SMARCD2,GMEB2,PRPF6,PBX1,KAT5,RELA,ARNT2,SMARCB1,CHCHD10,PBX4,UBTF,MEF2B,NDP,SSBP4,BUD31,TNFRSF1A,TAF6L,ZBTB49,MTA2,PELP1,FOXK1,PKN1,MAD2L2,ARHGEF2,PER1,NFIX,IRF2BPL,TRIM25,SMARCA4,CARM1,AATF,MID2,ZNF521,AKT1,BRF1,GTF2F1,MYDGF,MAP2K2,AP3D1,MED16,ERBB4,JUND,KLF13,IER5	yellow	VSMC_M3
GO:1903508	0.69	0.35	79	1244	positive regulation of nucleic acid-templated transcription	BP	1	SQSTM1,NELFE,ATF6B,PBX2,SREBF2,PPARD,PHF2,KLF4,HOXA1,ZC3H12A,KMT2E,SIX5,ZNF296,SERTAD1,TTC5,DEAF1,HOXC4,KMT2B,ZBTB38,GREM1,MCRS1,CX3CL1,WNT10B,EGR3,CCAR2,YAF2,HTATIP2,KDM1B,CHCHD2,RRP1B,AIP,TRIM27,PRMT2,THOC5,RIPK3,SMARCD2,GMEB2,PRPF6,PBX1,KAT5,RELA,ARNT2,SMARCB1,CHCHD10,PBX4,UBTF,MEF2B,NDP,SSBP4,BUD31,TNFRSF1A,TAF6L,ZBTB49,MTA2,PELP1,FOXK1,PKN1,MAD2L2,ARHGEF2,PER1,NFIX,IRF2BPL,TRIM25,SMARCA4,CARM1,AATF,MID2,ZNF521,AKT1,BRF1,GTF2F1,MYDGF,MAP2K2,AP3D1,MED16,ERBB4,JUND,KLF13,IER5	yellow	VSMC_M3
GO:0033554	0.69	0.35	103	1561	cellular response to stress	BP	1	SQSTM1,GNL1,BAG6,HDAC10,FNIP1,ATF6B,SREBF2,PPARD,FANCE,PLEC,PDGFRA,SOCS5,KLF4,CCM2,SPRED2,ZC3H12A,FIGNL1,PDIA4,PNKP,RCN3,RELB,SERP2,MUTYH,TMUB1,DMAP1,INSIG1,OGG1,TTC5,UCP2,SEL1L2,METTL3,FAAP100,P4HB,PYCR1,EIF4EBP1,GSR,SMUG1,HM13,BCL2L1,ZBTB38,TNKS1BP1,AQP5,MCRS1,SOD1,ACTR5,ANKRD2,CCAR2,CHCHD2,WDR4,SELENOS,GPX1,QARS,THOC5,RIPK3,CEP164,KAT5,RELA,SIPA1,PHB2,BAD,OTUB1,LETM1,TNFRSF1A,BABAM1,UBXN1,SDE2,MAD2L2,ARHGEF2,PER1,PPP4C,SLX1A,TRIM25,USP43,EEF2K,CDC37,AATF,CXorf57,ZNF830,AKT1,ALKBH7,SIRT6,MAP2K2,VASN,HIC1,TRAP1,IRAK1,MAN1B1,STK11,WDR24,CARD9,MIR214,RF00024,NABP2,FADS1,IGFBP6,ZNF385A,TXNDC12,CDC42EP5,FBXO6,IER5,OSER1,STUB1,NSMCE3	yellow	VSMC_M3
GO:0006885	0.69	0.50	4	70	regulation of pH	BP	1	RAB20,CHP1,SLC26A6,CCDC115	yellow	VSMC_M3
GO:0051270	0.69	0.36	51	830	regulation of cellular component movement	BP	1	PPARD,TSTA3,PDGFRA,IL1R1,KLF4,WDPCP,ZC3H12A,SLC9A3R1,PLXND1,EMILIN1,SCARB1,PDE4D,SWAP70,GREM1,CX3CL1,DLL4,ST3GAL4,RIN3,PDGFA,MMP14,CORO1B,ARPIN,RIPK3,TWIST2,PTPN23,RHOC,IGFBP5,AAMP,LGALS3,CD9,PLAU,C1QBP,WASHC1,RCC2,MTA2,ELP5,PKN1,MIIP,ARHGEF2,RANGRF,CORO1A,AKT1,STK11,TGFBR3,TRADD,MIR214,ERBB4,PTPRR,CAVIN1,NSMF,GLIPR2	yellow	VSMC_M3
GO:0030334	0.69	0.36	45	735	regulation of cell migration	BP	1	PPARD,TSTA3,PDGFRA,IL1R1,KLF4,WDPCP,ZC3H12A,SLC9A3R1,PLXND1,EMILIN1,SCARB1,SWAP70,GREM1,CX3CL1,DLL4,ST3GAL4,RIN3,PDGFA,MMP14,CORO1B,ARPIN,RIPK3,TWIST2,PTPN23,RHOC,IGFBP5,AAMP,LGALS3,CD9,PLAU,C1QBP,WASHC1,RCC2,MTA2,ELP5,MIIP,ARHGEF2,CORO1A,AKT1,TRADD,MIR214,ERBB4,PTPRR,NSMF,GLIPR2	yellow	VSMC_M3
GO:0051054	0.69	0.42	11	176	positive regulation of DNA metabolic process	BP	1	HDAC10,KLF4,PNKP,GREM1,CLCF1,BABAM1,MAD2L2,SLX1A,AKT1,SIRT6,NABP2	yellow	VSMC_M3
GO:0007051	0.69	0.43	10	165	spindle organization	BP	1	DCTN2,NUMA1,DCTN1,PCNT,NUDC,SAC3D1,EML3,EML1,NTMT1,TUBGCP2	yellow	VSMC_M3
GO:0002821	0.69	0.50	4	66	positive regulation of adaptive immune response	BP	1	IL1R1,CLCF1,MAD2L2,CARD9	yellow	VSMC_M3
GO:0035239	0.69	0.36	42	699	tube morphogenesis	BP	1	CTHRC1,SCRIB,CUL7,B4GALT1,PDGFRA,COL15A1,EPN1,KLF4,HOXA1,CCM2,ZC3H12A,PLXND1,EMILIN1,ARHGAP22,DEAF1,GREM1,KDM2B,PDE3B,CX3CL1,EGR3,DLL4,HTATIP2,IRX1,PDGFA,MMP14,GPX1,ANPEP,PBX1,SGPL1,YJEFN3,PHB2,AAMP,MTHFR,AKT1,MYDGF,SIRT6,JMJD8,SARS,TGFBR3,MIR214,TNFRSF12A,RNH1	yellow	VSMC_M3
GO:0048659	0.70	0.44	8	128	smooth muscle cell proliferation	BP	1	PPARD,KLF4,CX3CL1,IGFBP5,AKT1,IRAK1,MIR214,ELN	yellow	VSMC_M3

GO:0030177	0.70	0.45	7	124	positive regulation of Wnt signaling pathway	BP	1	PLEKHA4,RNF220,WNT10B,CCAR2,AMER1,SMARCA4,CSNK1G2	yellow	VSMC_M3
GO:1902680	0.70	0.35	79	1246	positive regulation of RNA biosynthetic process	BP	1	SQSTM1,NELFE,ATF6B,PBX2,SREBF2,PPARD,PHF2,KLF4,HOXA1,ZC3H12A,KMT2E,SIX5,ZNF296,SERTAD1,TTC5,DEAF1,HOXC4,KMT2B,ZBTB38,GREM1,MCRS1,CX3CL1,WNT10B,EGR3,CCAR2,YAF2,HTATIP2,KDM1B,CHCHD2,RRP1B,AIP,TRIM27,PRMT2,THOC5,RIPK3,SMARCD2,GMEB2,PRPF6,PBX1,KAT5,RELA,ARNT2,SMARCB1,CHCHD10,PBX4,UBTF,MEF2B,NDP,SSBP4,BUD31,TNFRSF1A,TAF6L,ZBTB49,MTA2,PELP1,FOXK1,PKN1,MAD2L2,ARHGEF2,PER1,NFIX,IRF2BPL,TRIM25,SMARCA4,CARM1,AATF,MID2,ZNF521,AKT1,BRF1,GTF2F1,MYDGF,MAP2K2,AP3D1,MED16,ERBB4,JUND,KLF13,IER5	yellow	VSMC_M3
GO:1903008	0.70	0.46	6	101	organelle disassembly	BP	1	SQSTM1,SREBF2,PHB2,CDC37,ATG3,TAZ	yellow	VSMC_M3
GO:0009615	0.70	0.40	16	253	response to virus	BP	1	TRIM11,ZC3H12A,TARBP2,BCL2L1,RRP1B,RIPK3,BANF1,RELA,MAP3K14,PHB2,C1QBP,ARMC5,C19orf66,GTF2F1,CARD9,EEF1G	yellow	VSMC_M3
GO:0090068	0.70	0.42	11	180	positive regulation of cell cycle process	BP	1	CDK4,H2AFY,KMT2E,NUMA1,WNT10B,THOC5,PBX1,PHB2,RCC2,AKT1,MIR214	yellow	VSMC_M3
GO:0030324	0.70	0.44	8	141	lung development	BP	1	BAG6,PDGFRA,RCN3,EIF4EBP1,PDGFA,MMP14,IGFBP5,AIMP2	yellow	VSMC_M3
GO:0007589	0.70	0.49	4	68	body fluid secretion	BP	1	AQP5,MTX1,APRT,ERBB4	yellow	VSMC_M3
GO:0060322	0.70	0.36	37	624	head development	BP	1	BAG6,PBX2,TRAPPC9,SCRIB,SHARPIN,PDGFRA,SECISBP2,PAFAH1B3,CIC,METTL3,BAIAP2,KDM2B,CCDC32,TYRO3,MMP14,QARS,PBX1,ARNT2,VPS51,SGPL1,PBX4,ATIC,C12orf57,BAD,CD9,CNP,GLUD1,EML1,CSK,HMGCS1,GIT1,SLC6A4,CTNS,MBD3,NDST1,ERBB4,TUBGCP2	yellow	VSMC_M3
GO:0050708	0.70	0.41	12	200	regulation of protein secretion	BP	1	PPARD,EIPR1,MYRIP,UCP2,AACS,PCK2,CAPN10,PTPN23,BAD,GLUD1,MIDN,JAGN1	yellow	VSMC_M3
GO:0043067	0.70	0.35	74	1119	regulation of programmed cell death	BP	1	CDK4,SQSTM1,BAG6,FNIP1,PPARD,SCRIB,LGALS1,B4GALT1,KLF4,ZC3H12A,FIGNL1,MICAL1,ANXA5,SLC9A3R1,OGG1,UCP2,PHLDA1,P4HB,BCL2L1,PLEKHF1,GREM1,KDM2B,RAPSN,CX3CL1,WNT10B,SOD1,EGR3,CCAR2,TYRO3,HTATIP2,DCPS,SELENOS,ITPRIP,RRP1B,GPX1,CLCF1,QARS,PRMT2,RIPK3,TWIST2,CAPN10,BRMS1,PLA2G3,RELA,ARNT2,PHB2,BAD,LGALS3,TNFRSF1A,C1QBP,USP27X,CAPN2,NANOS3,DFFA,ARHGEF2,CORO1A,EEF2K,AATF,ZNF830,AKT1,DHCR24,MYDGF,TRAP1,MNT,CARD9,SLC27A4,TRADD,MIR214,TNFRSF12A,PTMA,ZNF385A,TXNDC12,NSMF,CDC34	yellow	VSMC_M3
GO:0035270	0.70	0.47	5	87	endocrine system development	BP	1	PDGFRA,PBX1,BAD,ARMC5,AKT1	yellow	VSMC_M3
GO:0010506	0.70	0.39	16	268	regulation of autophagy	BP	1	SREBF2,ZC3H12A,SNRNP70,PLEKHF1,TRIM27,KAT5,PHB2,BAD,WASHC1,FOXK1,PIP4K2B,MTMR4,MID2,AKT1,STK11,WDR24	yellow	VSMC_M3
GO:0050789	0.70	0.32	528	7907	regulation of biological process	BP	1	ENPP1,CD63,NAB2,NHP2,CDK4,CTDSP2,RUFY1,SQSTM1,GNL1,ZNF608,BAG6,SNX9,CTHRC1,HDAC10,NELFE,FNIP1,ATF6B,PBX2,PRR5,TTLL12,RING1,SREBF2,SQLE,TXNDC15,H2AFY,ITPR3,HNRNPA0,TRIM11,SGSM3,TRAPPC9,PACSIN1,DEF6,PPARD,EEF1D,TSTA3,SCRIB,PLEC,LGALS1,IFT27,SHARPIN,BOP1,VPS28,SND1,TOM1,CUL7,PPP1R14B,DNPH1,B4GALT1,PDGFRA,IL1R1,SECISBP2,PHF2,COL15A1,EPN1,SSC5D,HSPBP1,SOCS5,RPS9,CNOT3,KLF4,DBNL,TPBG,HOXA1,WDPCP,CCM2,FHL3,SPRED2,EIPR1,RGS3,ZC3H12A,FIGNL1,PNKP,ZYX,RCN3,SNRNP70,CC2D1B,SNX3,KTI12,MICAL1,PLEKHA4,MGLL,EHD2,FAM120B,KMT2E,ANXA5,KRT8,WDFY2,SIX5,ZNF282,ZNF296,RELB,SLC9A3R1,SERP2,PLXND1,EMILIN1,RNF220,DMAP1,SLC30A3,CIC,SNX17,H3F3B,PPM1G,UNK,PLCD1,DLEC1,CD79A,INSIG1,MYRIP,SEC13,SEPT9,OGG1,SERTAD1,TTC5,PSMC4,ARHGEF40,UCP2,METTL3,PHLDA1,BAIAP2,CEP131,CXXC5,INPPL1,LAMTOR1,NUMA1,P4HB,DND1,PYCR1,CCDC57,EIF4EBP1,ARAP3,DELE1,CAVIN3,RIC8A,GSR,ARHGAP22,DEAF1,HOXC4,ARHGAP33,LDLRAD3,TARBP2,KMT2B,AACS,SCARB1,NAT10,BCL2L1,ITGB7,ZNF740,PDE4D,SPRYD3,TNS2,EIF4B,RRS1,SWAP70,LRP3,RALY,PLEKHF1,ACSL1,ZBTB38,LSM6,CAPG,TNKS1BP1,PINX1,GREM1,KDM2B,DOK1,PDE3B,MCRS1,DCTN1,TKT,RAPSN,CX3CL1,WNT10B,SOD1,PACSIN3,ACTR5,ANKRD2,DGKZ,EGR3,CCAR2,DNAJC17,DLL4,CHP1,TYRO3,SCD,YAF2,HTATIP2,PCIF1,PCBP4,LSP1,RAB35,KDM1B,CHCHD2,SLC35C2,RBM15B,TSPAN5,CISH,SRSF9,ST3GAL4,RNF19B,DCPS,IRX1,DHCR7,SELENOS,GSTO1,ITPRIP,RRP1B,ETFBKMT,MVK,BRD9,RIN3,STK32C,FABP3,ZCCHC17,NKAIN1,PDGFA,MMP14,C14orf93,CDK2AP2,PABPN1,GPX [illegible]	yellow	VSMC_M3
GO:0048646	0.70	0.35	53	877	anatomical structure formation involved in morphogenesis	BP	1	CTHRC1,H2AFY,SCRIB,PLEC,B4GALT1,PDGFRA,COL15A1,EPN1,CNOT3,KLF4,HOXA1,ZC3H12A,EHD2,KRT8,PLXND1,EMILIN1,ARHGAP22,DEAF1,GREM1,KDM2B,PDE3B,CX3CL1,WNT10B,EGR3,DLL4,HTATIP2,IRX1,PDGFA,MMP14,GPX1,PLEKHO1,COL6A1,NECTIN1,ANPEP,PLA2G3,YJEFN3,AAMP,CD9,PKN1,CAPN2,MTHFR,ARMC5,AKT1,RFX2,MYDGF,SIRT6,JMJD8,SARS,TGFBR3,MIR214,TNFRSF12A,RNH1,DUSP5	yellow	VSMC_M3
GO:0010522	0.70	0.49	4	71	regulation of calcium ion transport into cytosol	BP	1	PDE4D,CX3CL1,GSTO1,CORO1A	yellow	VSMC_M3
GO:0030856	0.70	0.45	6	106	regulation of epithelial cell differentiation	BP	1	H2AFY,SPRED2,NUMA1,WNT10B,BAD,TNFRSF1A	yellow	VSMC_M3
GO:0043502	0.70	0.49	4	71	regulation of muscle adaptation	BP	1	KLF4,IGFBP5,TNFRSF1A,MIR214	yellow	VSMC_M3
GO:0016310	0.70	0.34	91	1440	phosphorylation	BP	1	ENPP1,NAB2,CDK4,CTDSP2,SQSTM1,SNX9,CERK,FNIP1,PRR5,H2AFY,GUK1,PLEC,PPP1R14B,PDGFRA,SOCS5,KLF4,SPRED2,ZC3H12A,PNKP,MICAL1,WDFY2,SLC9A3R1,EMILIN1,SEPHS1,GALK1,SERTAD1,OGDHL,TARBP2,PDE4D,ACSL1,TNKS1BP1,GREM1,CX3CL1,SOD1,DGKZ,CHP1,TYRO3,HTATIP2,CISH,AK2,MVK,STK32C,PAPSS1,PDGFA,CLCF1,QARS,TRIM27,LIMK1,RIPK3,PSTK,TNK2,SCYL1,MEX3B,MAP3K14,LIMD1,PHB2,BAD,CTDSP1,TNFRSF1A,WASHC1,FOXK1,PKN1,PIP4K2B,MAD2L2,CTBP1,ARHGEF2,MVP,PMVK,EEF2K,CDC37,CSK,AKT1,PGM1,SGK494,GIT1,MYDGF,MAP2K2,CACTIN,CDK11B,MKNK2,CSNK1G2,IRAK1,MIDN,STK11,TELO2,JMJD8,AK1,VAC14,TGFBR3,ERBB4,TRIB2	yellow	VSMC_M3
GO:0051402	0.70	0.41	12	198	neuron apoptotic process	BP	1	BCL2L1,KDM2B,RAPSN,CX3CL1,SOD1,TYRO3,GPX1,CLCF1,PLA2G3,CORO1A,NSMF,CDC34	yellow	VSMC_M3

GO:0009719	0.70	0.34	75	1202	response to endogenous stimulus	BP	1	ENPP1,CDK4,SREBF2,PPARD,PDGFRA,SOCS5,KLF4,SPRED2,ZYX,SLC9A3R1,EMILIN1,INSIG1,UCP2,BAIAP2,INPPL1,LAMTOR1,NUMA1,BCL2L1,TNS2,ACSL1,GREM1,PDE3B,WNT10B,SOD1,EGR3,DLL4,RAB35,SELENOS,FABP3,MMP14,CORO1B,PCK2,COL6A1,SLC26A6,PRMT2,CAPN10,RELA,IDH1,PHB2,IGFBP5,BAD,LAMTOR4,ARPC1B,CD9,PELP1,KLF3,PKN1,CAPN2,PIP4K2B,ARHGEF2,PER1,CORO1A,TRIM25,MTMR4,SMARCA4,CARM1,EEF2K,CSK,AKT1,HMGCS1,SAFB,SAFB2,SLC6A4,DHCR24,LDOC1,VASN,STK11,APRT,SLC27A4,TGFBR3,NDST1,ERBB4,JUND,NSMF,STUB1	yellow	VSMC_M3
GO:0098657	0.70	0.42	9	155	import into cell	BP	1	VPS28,ACSL1,SLC11A2,FABP3,SLC19A1,SPX,AKT1,SLC6A4,SLC27A4	yellow	VSMC_M3
GO:0072330	0.70	0.42	9	144	monocarboxylic acid biosynthetic process	BP	1	MGLL,INSIG1,FASN,SCD,PLA2G3,MID1IP1,ACLY,FADS2,FADS1	yellow	VSMC_M3
GO:0048468	0.71	0.33	97	1587	cell development	BP	1	SBF1,CTHRC1,HDAC10,PBX2,TRIM11,PACSIN1,SCRIB,PLEC,LGALS1,IFT27,CUL7,B4GALT1,PDGFRA,SECISBP2,COL15A1,KLF4,DBNL,TPBG,WDPCP,CCM2,SNX3,KRT8,SIX5,SLC9A3R1,PLXND1,UNK,METTL3,BAIAP2,CEP131,P4HB,DND1,YIF1B,FASN,DEAF1,ARHGAP33,BCL2L1,ITGB7,PDE4D,SLC11A2,CX3CL1,WNT10B,SOD1,DLL4,TYRO3,RAB35,IRX1,GPX1,NGEF,CLCF1,IGSF9,NECTIN1,LIMK1,SLC26A6,PRMT2,IQCG,PLA2G3,STMN3,PBX1,RELA,SCYL1,LIMD1,PBX4,BLOC1S4,C12orf57,BAD,CTDSP1,CD9,TNFRSF1A,C1QBP,RCC2,CNP,DBN1,NANOS3,CRABP2,TMEM79,S100A6,CARM1,EEF2K,ZNF830,AKT1,RFX2,GIT1,SIRT6,TJP3,ACAP3,ANAPC2,STK11,TGFBR3,NEGR1,ERBB4,TWF2,JUND,UNC13A,ZNF385A,NSMF,GBA2,MEIG1	yellow	VSMC_M3
GO:0051604	0.71	0.39	15	230	protein maturation	BP	1	LMF2,LDLRAD3,HM13,TSPAN5,MMP14,AIP,SEC11A,BAD,PLAU,SDE2,CAPN2,DHCR24,SPG7,STUB1,RCE1	yellow	VSMC_M3
GO:0051241	0.71	0.35	47	743	negative regulation of multicellular organismal process	BP	1	ENPP1,TRIM11,PDGFRA,EPN1,SSC5D,SOCS5,KLF4,SPRED2,ZC3H12A,ANXA5,RELB,EMILIN1,PDE4D,GREM1,PDE3B,CX3CL1,WNT10B,SOD1,DLL4,SELENOS,PDGFA,TRIM27,SPX,PLA2G3,PTPN23,KAT5,YJEFN3,PHB2,IGFBP5,CTDSP1,CD9,PLAU,TNFRSF1A,C1QBP,MAD2L2,CSK,KHSRP,GIT1,SLC6A4,CACTIN,VASN,STK11,SARS,MIR214,MIR155,PTPRR,TRIB2	yellow	VSMC_M3
GO:0050890	0.71	0.40	12	214	cognition	BP	1	ITPR3,TPBG,HOXA1,CIC,RIC8A,SLC11A2,ST3GAL4,TACO1,C12orf57,GIT1,SLC6A4,CTNS	yellow	VSMC_M3
GO:0042221	0.71	0.32	192	2921	response to chemical	BP	1	ENPP1,CD63,CDK4,BAG6,ATF6B,TTLL12,SREBF2,SQLE,ITPR3,HNRNPA0,GUK1,PPARD,RPL3,SCRIB,PLEC,LGALS1,MPST,SHARPIN,PDGFRA,IL1R1,SSC5D,SOCS5,KLF4,TPBG,PTGR1,SPRED2,ZC3H12A,ZYX,RCN3,KRT8,RELB,SLC9A3R1,SERP2,PLXND1,EMILIN1,TMUB1,DMAP1,SLC30A3,INSIG1,OGG1,UCP2,SEL1L2,METTL3,BAIAP2,INPPL1,LAMTOR1,NUMA1,P4HB,PYCR1,FASN,RAB20,SMOX,EIF4EBP1,GSR,ARHGAP33,AACS,SCARB1,HM13,BCL2L1,TNS2,SWAP70,ACSL1,SLC11A2,GREM1,DOK1,AQP5,PDE3B,CX3CL1,WNT10B,SOD1,ANKRD2,EGR3,DLL4,SCD,LSP1,RAB35,CHCHD2,SRSF9,DCPS,SELENOS,GSTO1,RIN3,FABP3,PDGFA,MMP14,PABPN1,GPX1,AIP,CORO1B,CLCF1,IGSF9,SYT12,PCK2,COL6A1,IMPDH2,NECTIN1,SLC26A6,PRMT2,SPX,UQCRC1,RIPK3,CAPN10,PPOX,KAT5,RELA,SIPA1,MGST3,IDH1,ARNT2,SMARCB1,ATIC,TXNRD2,PHB2,IGFBP5,BAD,LAMTOR4,OTUB1,HSD17B1,LETM1,LGALS3,ARPC1B,CD9,PLAU,TNFRSF1A,C1QBP,UBXN1,ANXA11,CNP,PRXL2A,PELP1,KLF3,PKN1,CAPN2,PALM3,MTHFR,PIP4K2B,ARHGEF2,PER1,CORO1A,PMVK,TUFM,TRIM25,MTMR4,S100A16,SMARCA4,CARM1,EEF2K,CDC37,C19orf66,CSK,RBM22,AKT1,HMGCS1,KHSRP,SAFB,SAFB2,GIT1,SLC6A4,DHCR24,BLMH,LDOC1,CACTIN,VASN,TRAP1,EHMT1,MKNK2,IRAK1,MBD3,MAN1B1,ATP5F1D,STK11,CARD9,APRT,SLC27A4,TGFBR3,TRADD,MIR214,RF00024,NDST1,ERBB4,TWF2,JUND,LOXL1,JAGN1,COL6A2,NSMF,CDC34,FBXO6,OSER1,STUB1,NSMCE3,CCDC115	yellow	VSMC_M3
GO:0060349	0.71	0.48	4	76	bone morphogenesis	BP	1	NAB2,INSIG1,INPPL1,MMP14	yellow	VSMC_M3
GO:0030901	0.71	0.48	4	69	midbrain development	BP	1	KDM2B,CNP,GLUD1,NDST1	yellow	VSMC_M3
GO:0002377	0.71	0.46	5	81	immunoglobulin production	BP	1	SWAP70,CLCF1,PHB2,PKN1,MAD2L2	yellow	VSMC_M3
GO:0098813	0.71	0.39	14	230	nuclear chromosome segregation	BP	1	DCTN2,BAG6,NCAPH2,H2AFY,NUMA1,RRS1,PINX1,NUDC,PHB2,RCC2,EML3,MAD1L1,MAD2L2,MIS12	yellow	VSMC_M3
GO:0001841	0.71	0.46	5	92	neural tube formation	BP	1	CTHRC1,SCRIB,DEAF1,KDM2B,MTHFR	yellow	VSMC_M3
GO:0016331	0.71	0.43	7	127	morphogenesis of embryonic epithelium	BP	1	CTHRC1,SCRIB,DEAF1,GREM1,KDM2B,IRX1,MTHFR	yellow	VSMC_M3
GO:0051656	0.71	0.37	20	334	establishment of organelle localization	BP	1	DCTN2,SCRIB,SLC9A3R1,NUMA1,RRS1,PINX1,DCTN1,CHP1,NUDC,PLA2G3,BLOC1S4,NUP88,EML3,MAD1L1,SYNE3,MYO19,AP3D1,STK11,TMEM201,MIS12	yellow	VSMC_M3
GO:0042981	0.71	0.33	72	1092	regulation of apoptotic process	BP	1	CDK4,SQSTM1,BAG6,FNIP1,PPARD,SCRIB,LGALS1,B4GALT1,KLF4,ZC3H12A,FIGNL1,MICAL1,ANXA5,SLC9A3R1,OGG1,UCP2,PHLDA1,P4HB,BCL2L1,PLEKHF1,GREM1,KDM2B,RAPSN,CX3CL1,WNT10B,SOD1,EGR3,CCAR2,TYRO3,HTATIP2,SELENOS,ITPRIP,RRP1B,GPX1,CLCF1,QARS,PRMT2,RIPK3,TWIST2,CAPN10,BRMS1,PLA2G3,RELA,ARNT2,PHB2,BAD,LGALS3,TNFRSF1A,C1QBP,USP27X,CAPN2,NANOS3,DFFA,ARHGEF2,CORO1A,EEF2K,AATF,ZNF830,AKT1,DHCR24,MYDGF,TRAP1,MNT,CARD9,SLC27A4,TRADD,TNFRSF12A,PTMA,ZNF385A,TXNDC12,NSMF,CDC34	yellow	VSMC_M3
GO:0001935	0.71	0.43	7	123	endothelial cell proliferation	BP	1	SCARB1,EGR3,DLL4,MMP14,AKT1,MYDGF,SIRT6	yellow	VSMC_M3
GO:0006323	0.71	0.43	7	119	DNA packaging	BP	1	NCAPH2,H2AFY,PHF2,H3F3B,BANF1,H3F3A,UBN1	yellow	VSMC_M3
GO:0007548	0.71	0.39	12	206	sex differentiation	BP	1	PDGFRA,BCL2L1,SOD1,TYRO3,MMP14,PBX1,IDH1,SGPL1,ZNF830,HMGCS1,SAFB2,DHCR24	yellow	VSMC_M3
GO:0002444	0.71	0.47	4	70	myeloid leukocyte mediated immunity	BP	1	KMT2E,PLA2G3,CARD9,JAGN1	yellow	VSMC_M3
GO:0042552	0.72	0.44	6	109	myelination	BP	1	NAB2,PLEC,SOD1,CD9,AKT1,MARVELD1	yellow	VSMC_M3

GO:0002764	0.72	0.37	17	278	immune response-regulating signaling pathway	BP	1	BAG6,ZC3H12A,CD79A,PDE4D,DGKZ,TYRO3,LIMK1,RELA,PHB2,IFI35,LGALS3,C1QBP,CD276,CSK,CACTIN,IRAK1,STK11	yellow	VSMC_M3
GO:0051640	0.72	0.36	27	447	organelle localization	BP	1	ESYT1,DCTN2,SCRIB,SLC9A3R1,LAMTOR1,NUMA1,RRS1,PINX1,DCTN1,CHP1,NUDC,PLA2G3,BLOC1S4,NUP88,EML3,MAD1L1,STX10,SYNE3,MYO19,PACS2,RAB34,AP3D1,STK11,TMEM201,RABEPK,MIS12,UNC13A	yellow	VSMC_M3
GO:0001655	0.72	0.38	16	283	urogenital system development	BP	1	BAG6,MPST,IFT27,PDGFRA,WDPCP,PLXND1,SNX17,TNS2,GREM1,IRX1,PDGFA,PBX1,SGPL1,AMER1,STK11,ERBB4	yellow	VSMC_M3
GO:0031497	0.72	0.45	5	84	chromatin assembly	BP	1	H2AFY,PHF2,H3F3B,H3F3A,UBN1	yellow	VSMC_M3
GO:0001656	0.72	0.47	4	74	metanephros development	BP	1	PDGFRA,GREM1,IRX1,PDGFA	yellow	VSMC_M3
GO:0048167	0.72	0.41	8	143	regulation of synaptic plasticity	BP	1	SQSTM1,ITPR3,BAIAP2,CX3CL1,SYT12,DBN1,ANAPC2,NSMF	yellow	VSMC_M3
GO:1901657	0.72	0.47	4	60	glycosyl compound metabolic process	BP	1	DNPH1,ATIC,APRT,GBA2	yellow	VSMC_M3
GO:0120034	0.72	0.45	5	92	positive regulation of plasma membrane bounded cell projection assembly	BP	1	SEPT9,WASHC1,DOCK11,TWF2,CDC42EP5	yellow	VSMC_M3
GO:2000145	0.72	0.34	47	772	regulation of cell motility	BP	1	PPARD,TSTA3,PDGFRA,IL1R1,KLF4,WDPCP,ZC3H12A,SLC9A3R1,PLXND1,EMILIN1,SCARB1,SWAP70,GREM1,CX3CL1,DLL4,ST3GAL4,RIN3,PDGFA,MMP14,CORO1B,ARPIN,RIPK3,TWIST2,PTPN23,RHOC,IGFBP5,AAMP,LGALS3,CD9,PLAU,C1QBP,WASHC1,RCC2,MTA2,ELP5,PKN1,MIIP,ARHGEF2,CORO1A,AKT1,TRADD,MIR214,ERBB4,PTPRR,CAVIN1,NSMF,GLIPR2	yellow	VSMC_M3
GO:0071478	0.72	0.40	9	150	cellular response to radiation	BP	1	EEF1D,FIGNL1,METTL3,BCL2L1,TNKS1BP1,ACTR5,SDE2,STK11,NSMCE3	yellow	VSMC_M3
GO:0048041	0.72	0.47	4	81	focal adhesion assembly	BP	1	WDPCP,GREM1,MMP14,RCC2	yellow	VSMC_M3
GO:0006952	0.72	0.33	62	949	defense response	BP	1	TTLL12,HNRNPA0,TRIM11,PPARD,SHARPIN,PPP1R14B,B4GALT1,IL1R1,SSC5D,SOCS5,KLF4,ZC3H12A,ZYX,MGLL,RELB,DLEC1,OGG1,METTL3,RAB20,SIGLEC1,TARBP2,BCL2L1,CX3CL1,SOD1,TYRO3,LSP1,RNF19B,SELENOS,MVK,GPX1,TRIM27,SLC26A6,RIPK3,PSMB4,TNK2,PLA2G3,RELA,SCYL1,MAP3K14,PHB2,IFI35,LGALS3,TNFRSF1A,C1QBP,TBKBP1,ARHGEF2,ARMC5,PER1,CORO1A,TRIM25,CDC37,C19orf66,MID2,AKT1,GIT1,CACTIN,IRAK1,C8G,CARD9,TRADD,NDST1,JAGN1	yellow	VSMC_M3
GO:0048731	0.72	0.31	226	3556	system development	BP	1	ENPP1,NAB2,CDK4,BAG6,NCAPH2,CTHRC1,HDAC10,FNIP1,PBX2,PDLIM4,RING1,H2AFY,TRIM11,TRAPPC9,PACSIN1,PPARD,SCRIB,PLEC,LGALS1,MPST,IFT27,SHARPIN,CUL7,B4GALT1,PDGFRA,SECISBP2,PHF2,COL15A1,EPN1,COL19A1,SOCS5,TTC7A,KLF4,DBNL,TPBG,HOXA1,WDPCP,CCM2,FHL3,SPRED2,ZC3H12A,RCN3,SNX3,PLEKHA4,KMT2E,KRT8,SIX5,RELB,SLC9A3R1,PLXND1,EMILIN1,RNF220,PAFAH1B3,CIC,SNX17,UNK,CD79A,INSIG1,UCP2,METTL3,BAIAP2,INPPL1,NUMA1,FASN,EIF4EBP1,RIC8A,ARHGAP22,DEAF1,TBCB,HOXC4,LRIG1,ARHGAP33,AACS,BCL2L1,TNS2,SWAP70,CAPG,SLC11A2,MMP24,GREM1,KDM2B,AQP5,PDE3B,DCTN1,CX3CL1,WNT10B,SOD1,ANKRD2,EGR3,DLL4,TYRO3,HTATIP2,RAB35,APBA2,IRX1,SDHA,PAPSS1,PDGFA,MMP14,GPX1,NGEF,HCCS,CLCF1,IGSF9,QARS,LRFN4,PCK2,IMPDH2,PIR,NECTIN1,ANPEP,LIMK1,THOC5,RIPK3,EXTL1,PLA2G3,STMN3,SART1,PBX1,RELA,SCYL1,IDH1,ARNT2,VPS51,SGPL1,SMARCB1,PBX4,YJEFN3,ATIC,BLOC1S4,MEF2B,PHB2,NDP,IGFBP5,C12orf57,BAD,AAMP,CTDSP1,HSD17B1,CD9,TNFRSF1A,TEAD4,AIMP2,CHERP,EVC,CNP,PRXL2A,KRT10,GLUD1,FOXK1,CASP14,PKN1,DBN1,MAD1L1,AMER1,MTHFR,MAD2L2,PRR14,CRABP2,TMEM79,ARHGEF2,ARMC5,MTX1,IRF2BPL,SLC39A1,S100A4,S100A6,SMARCA4,CARM1,EEF2K,EML1,CSK,ZNF521,TTC39C,ZNF358,DOCK11,ZNF830,AKT1,COPRS,HMGCS1,SAFB2,GIT1,SLC6A4,DHCR24,MYDGF,SIRT6,PPL,NSDHL,HMG20B,VASN,ACAP3,CTNS,AP3D1,MKNK2,ANAPC2,MBD3,STK11,TAZ,JMJD8,SPG7,SARS,APRT,GTF3C5,SLC27A4,TGFBR3,TRADD,NEGR1,MIR214,NDST1,TNFRSF12A,ERBB4,TWF2,LOXL1,KLF13,RNH1,JAGN1,IGSF8,ELN,UNC13A,ZNF385A,NSMF,N[illegible]	yellow	VSMC_M3
GO:0034612	0.72	0.39	11	181	response to tumor necrosis factor	BP	1	SHARPIN,ZC3H12A,KRT8,CX3CL1,PCK2,RELA,TNFRSF1A,ARHGEF2,AKT1,CACTIN,TRADD	yellow	VSMC_M3
GO:0006413	0.72	0.43	6	99	translational initiation	BP	1	METTL3,EIF4EBP1,EIF4B,EIF3A,EIF3G,PAIP1	yellow	VSMC_M3
GO:0031532	0.72	0.44	5	94	actin cytoskeleton reorganization	BP	1	PDLIM4,PLEC,PDGFRA,BAIAP2,CAPN10	yellow	VSMC_M3
GO:0060541	0.72	0.40	9	163	respiratory system development	BP	1	BAG6,PDGFRA,WDPCP,RCN3,EIF4EBP1,PDGFA,MMP14,IGFBP5,AIMP2	yellow	VSMC_M3
GO:0007017	0.73	0.33	44	715	microtubule-based process	BP	1	DCTN2,SPAG1,IFT27,CUL7,FIGNL1,SLC9A3R1,CEP131,NUMA1,YIF1B,CCDC57,NAT10,DCTN1,SOD1,CHP1,CDK2AP2,SRRD,CLIP2,PCNT,NUDC,IQCG,PLA2G3,STMN3,SAC3D1,MID1IP1,BLOC1S4,KIAA0753,CNP,EML3,ARHGEF2,RANGRF,EML1,GIT1,AP3D1,SSNA1,STK11,TMEM201,GAS8,SPG7,NTMT1,WDR34,MAP7D1,GBA2,TUBGCP2,MEIG1	yellow	VSMC_M3
GO:0006334	0.73	0.46	4	68	nucleosome assembly	BP	1	H2AFY,H3F3B,H3F3A,UBN1	yellow	VSMC_M3
GO:0030323	0.73	0.40	8	145	respiratory tube development	BP	1	BAG6,PDGFRA,RCN3,EIF4EBP1,PDGFA,MMP14,IGFBP5,AIMP2	yellow	VSMC_M3
GO:0012501	0.73	0.31	97	1474	programmed cell death	BP	1	CDK4,SQSTM1,BAG6,FNIP1,PPARD,SCRIB,LGALS1,SHARPIN,B4GALT1,KLF4,ZC3H12A,FIGNL1,MICAL1,ANXA5,KRT8,SLC9A3R1,OGG1,UCP2,PHLDA1,P4HB,DELE1,BCL2L1,PLEKHF1,GREM1,KDM2B,RAPSN,CX3CL1,WNT10B,SOD1,EGR3,CCAR2,TYRO3,HTATIP2,DCPS,SELENOS,ITPRIP,RRP1B,GPX1,CLCF1,QARS,PRMT2,RIPK3,TWIST2,CAPN10,BRMS1,PLA2G3,RELA,ARNT2,ZDHHC3,SGPL1,CHCHD10,ZNF385B,PHB2,BAD,LGALS3,TNFRSF1A,C1QBP,AIMP2,USP27X,CASP14,PKN1,CAPN2,SLTM,NANOS3,BCL7C,DFFA,TMEM79,ARHGEF2,PLEKHO2,CORO1A,EEF2K,AATF,ATG3,ZNF830,AKT1,PACS2,ALKBH7,DHCR24,MYDGF,CDK11B,HIC1,TRAP1,MNT,MKNK2,STK11,CARD9,SPG7,SLC27A4,TRADD,MIR214,TNFRSF12A,ERBB4,PTMA,ZNF385A,TXNDC12,NSMF,CDC34	yellow	VSMC_M3
GO:0060993	0.73	0.45	4	77	kidney morphogenesis	BP	1	GREM1,IRX1,PBX1,ERBB4	yellow	VSMC_M3
GO:0009408	0.73	0.43	5	84	response to heat	BP	1	SOD1,AKT1,IRAK1,IER5,STUB1	yellow	VSMC_M3

GO:0050776	0.73	0.33	34	538	regulation of immune response	BP	1	BAG6,TTLL12,IL1R1,SOCS5,ZC3H12A,CD79A,METTL3,TARBP2,PDE4D,DGKZ,TYRO3,SELENOS,GPX1,CLCF1,TRIM27,LIMK1,RIPK3,PSMB4,PLA2G3,RELA,PHB2,IFI35,LGALS3,C1QBP,PKN1,MAD2L2,CDC37,CD276,CSK,CACTIN,IRAK1,C8G,STK11,CARD9	yellow	VSMC_M3
GO:0031058	0.73	0.45	4	75	positive regulation of histone modification	BP	1	NELFE,KMT2E,SMARCB1,CTBP1	yellow	VSMC_M3
GO:0061138	0.73	0.40	8	147	morphogenesis of a branching epithelium	BP	1	PLXND1,GREM1,DLL4,PDGFA,MMP14,PBX1,PHB2,SIRT6	yellow	VSMC_M3
GO:0007275	0.73	0.29	243	3815	multicellular organism development	BP	1	ENPP1,NAB2,CDK4,BAG6,NCAPH2,CTHRC1,HDAC10,FNIP1,PBX2,PDLIM4,RING1,H2AFY,TRIM11,TRAPPC9,PACSIN1,PPARD,SCRIB,PLEC,LGALS1,MPST,IFT27,SHARPIN,CUL7,B4GALT1,PDGFRA,SECISBP2,PHF2,COL15A1,EPN1,COL19A1,SOCS5,TTC7A,CNOT3,KLF4,DBNL,TPBG,HOXA1,WDPCP,CCM2,FHL3,SPRED2,ZC3H12A,RCN3,SNX3,PLEKHA4,KMT2E,KRT8,SIX5,RELB,SLC9A3R1,PLXND1,EMILIN1,RNF220,PAFAH1B3,CIC,SNX17,UNK,CD79A,INSIG1,PSMC4,UCP2,METTL3,BAIAP2,INPPL1,NUMA1,FASN,RPL29,EIF4EBP1,RIC8A,ARHGAP22,DEAF1,TBCB,HOXC4,LRIG1,ARHGAP33,AACS,HM13,BCL2L1,TNS2,SWAP70,CAPG,SLC11A2,MMP24,GREM1,KDM2B,AQP5,PDE3B,DCTN1,CX3CL1,WNT10B,SOD1,ANKRD2,EGR3,DLL4,TYRO3,HTATIP2,RAB35,APBA2,IRX1,SDHA,PAPSS1,PDGFA,MMP14,GPX1,NGEF,HCCS,CLCF1,IGSF9,QARS,LRFN4,PCK2,COL6A1,IMPDH2,PIR,NECTIN1,ANPEP,LIMK1,THOC5,RIPK3,EXTL1,PLA2G3,STMN3,SART1,PBX1,RELA,SCYL1,IDH1,ARNT2,VPS51,SGPL1,SMARCB1,PBX4,YJEFN3,ATIC,BLOC1S4,MEF2B,PHB2,NDP,IGFBP5,C12orf57,BAD,AAMP,CTDSP1,HSD17B1,CD9,TNFRSF1A,TEAD4,C1QBP,AIMP2,CHERP,EVC,CNP,PRXL2A,KRT10,GLUD1,FOXK1,CASP14,KLF3,PKN1,DBN1,CAPN2,MAD1L1,AMER1,MTHFR,MAD2L2,PRR14,CRABP2,TMEM79,ARHGEF2,ARMC5,MTX1,IRF2BPL,SLC39A1,S100A4,S100A6,SMARCA4,CARM1,EEF2K,EML1,AATF,CSK,ZNF521,TTC39C,ZNF358,DOCK11,ZNF830,AKT1,COPRS,HMGCS1,SAFB2,GIT1,SLC6A4,DHCR24,MYDGF,SIRT6,PPL,NSDHL,HMG20B,SLC35E2B,VASN,ACAP3,MNT,CTNS,EHMT1,AP3D1,MKNK2,ANAPC2,MBD3,STK11,TAZ,JMJD8,SPG7,SARS,APRT,GTF3C5,POMT1,SLC27A4,TGFBR3,TRADD,NEGR1,MIR214,ND[illegible]	yellow	VSMC_M3
GO:0000724	0.73	0.40	7	124	double-strand break repair via homologous recombination	BP	1	FIGNL1,MAD2L2,PPP4C,SLX1A,CXorf57,SIRT6,NABP2	yellow	VSMC_M3
GO:0031328	0.73	0.30	96	1503	positive regulation of cellular biosynthetic process	BP	1	CDK4,SQSTM1,NELFE,ATF6B,PBX2,SREBF2,PPARD,PHF2,RPS9,KLF4,HOXA1,ZC3H12A,PNKP,KMT2E,SIX5,ZNF296,SERTAD1,TTC5,METTL3,DEAF1,HOXC4,KMT2B,SCARB1,NAT10,ZBTB38,GREM1,MCRS1,CX3CL1,WNT10B,EGR3,CCAR2,CHP1,YAF2,HTATIP2,PCIF1,KDM1B,CHCHD2,RRP1B,FABP3,AIP,TRIM27,PRMT2,THOC5,RIPK3,SMARCD2,PLA2G3,GMEB2,PRPF6,PBX1,KAT5,RELA,ARNT2,MID1IP1,SMARCB1,CHCHD10,PBX4,UBTF,MEF2B,NDP,SSBP4,MPV17L2,BUD31,TNFRSF1A,C1QBP,TAF6L,ZBTB49,MTA2,PELP1,FOXK1,PKN1,CAPN2,MAD2L2,ARHGEF2,PER1,NFIX,IRF2BPL,TRIM25,SMARCA4,CARM1,AATF,MID2,ZNF521,AKT1,BRF1,PAIP1,GTF2F1,MYDGF,MAP2K2,AP3D1,TAZ,MED16,POMT1,ERBB4,JUND,KLF13,IER5	yellow	VSMC_M3
GO:0006941	0.73	0.40	7	128	striated muscle contraction	BP	1	ZC3H12A,PDE4D,GSTO1,SCN4A,C12orf57,RANGRF,TAZ	yellow	VSMC_M3
GO:0044092	0.73	0.31	52	806	negative regulation of molecular function	BP	1	FNIP1,H2AFY,PLEC,PPP1R14B,HSPBP1,SOCS5,KLF4,SPRED2,ZC3H12A,MICAL1,ANXA5,SLC9A3R1,TARBP2,PINX1,CCAR2,CHP1,GSTO1,ITPRIP,RRP1B,ETFBKMT,GPX1,AIP,QARS,TRIM27,LIMK1,PRMT2,BRMS1,TNK2,PHB2,PPP1R35,LGALS3,UBXN1,WASHC1,RCC2,CCDC22,PKN1,PIP4K2B,MAD2L2,DFFA,MVP,RPL37,CSK,AKT1,GTF2F1,DHCR24,CACTIN,IRAK1,MIDN,SLC27A4,RNH1,TRIB2,STUB1	yellow	VSMC_M3
GO:0048593	0.74	0.43	5	96	camera-type eye morphogenesis	BP	1	RING1,KDM2B,AQP5,NECTIN1,C12orf57	yellow	VSMC_M3
GO:0045597	0.74	0.32	39	639	positive regulation of cell differentiation	BP	1	CTHRC1,H2AFY,PPARD,CUL7,SOCS5,DBNL,ZC3H12A,WDFY2,PLXND1,BAIAP2,NUMA1,P4HB,LRP3,GREM1,CX3CL1,WNT10B,EGR3,MMP14,CLCF1,LIMK1,PLA2G3,SART1,RELA,BAD,C1QBP,DBN1,CRABP2,ARHGEF2,CARM1,EEF2K,AKT1,HMG20B,AP3D1,ANAPC2,STK11,TWF2,JUND,ZNF385A,GLIPR2	yellow	VSMC_M3
GO:0035107	0.74	0.41	6	115	appendage morphogenesis	BP	1	PBX2,WDPCP,GREM1,PBX1,CRABP2,ZNF358	yellow	VSMC_M3
GO:0035108	0.74	0.41	6	115	limb morphogenesis	BP	1	PBX2,WDPCP,GREM1,PBX1,CRABP2,ZNF358	yellow	VSMC_M3
GO:0000077	0.74	0.41	6	105	DNA damage checkpoint signaling	BP	1	CCAR2,THOC5,BABAM1,SDE2,ZNF830,FBXO6	yellow	VSMC_M3
GO:0021915	0.74	0.40	7	130	neural tube development	BP	1	CTHRC1,SCRIB,RNF220,DEAF1,KDM2B,MTHFR,ZNF358	yellow	VSMC_M3
GO:0015931	0.74	0.37	11	181	nucleobase-containing compound transport	BP	1	SEC13,SLC35C2,RBM15B,PABPN1,SLC19A1,THOC5,THOC3,NUP88,MVP,KHSRP,RBM8A	yellow	VSMC_M3
GO:0007338	0.74	0.42	5	86	single fertilization	BP	1	SPAG1,B4GALT1,GNPDA1,CD9,IGSF8	yellow	VSMC_M3
GO:0120254	0.74	0.42	5	86	olefinic compound metabolic process	BP	1	MGLL,GPX1,HSD17B1,FADS2,FADS1	yellow	VSMC_M3
GO:0055082	0.74	0.32	34	564	cellular chemical homeostasis	BP	1	ENPP1,ITPR3,PPARD,LGALS1,PDGFRA,TTC7A,SLC9A3R1,SLC30A3,UCP2,RAB20,AACS,PDE4D,SWAP70,SLC11A2,CX3CL1,SOD1,CHP1,GSTO1,PCK2,SLC26A6,SMARCB1,BAD,LETM1,CHERP,CCDC22,FOXK1,CORO1A,TSPOAP1,SMARCA4,ANKRD9,PACS2,AP3D1,JAGN1,CCDC115	yellow	VSMC_M3
GO:0050680	0.74	0.41	6	108	negative regulation of epithelial cell proliferation	BP	1	PPARD,WNT10B,DLL4,PHB2,STK11,TGFBR3	yellow	VSMC_M3
GO:0008584	0.74	0.41	6	111	male gonad development	BP	1	PDGFRA,BCL2L1,MMP14,SGPL1,HMGCS1,SAFB2	yellow	VSMC_M3
GO:0040012	0.74	0.31	48	796	regulation of locomotion	BP	1	PPARD,TSTA3,PDGFRA,IL1R1,KLF4,TPBG,WDPCP,ZC3H12A,SLC9A3R1,PLXND1,EMILIN1,SCARB1,SWAP70,GREM1,CX3CL1,DLL4,ST3GAL4,RIN3,PDGFA,MMP14,CORO1B,ARPIN,RIPK3,TWIST2,PTPN23,RHOC,IGFBP5,AAMP,LGALS3,CD9,PLAU,C1QBP,WASHC1,RCC2,MTA2,ELP5,PKN1,MIIP,ARHGEF2,CORO1A,AKT1,TRADD,MIR214,ERBB4,PTPRR,CAVIN1,NSMF,GLIPR2	yellow	VSMC_M3
GO:0034763	0.74	0.42	5	88	negative regulation of transmembrane transport	BP	1	ENPP1,SLC9A3R1,SELENOS,GSTO1,AKT1	yellow	VSMC_M3
GO:0046661	0.74	0.39	7	128	male sex differentiation	BP	1	PDGFRA,BCL2L1,MMP14,SGPL1,HMGCS1,SAFB2,DHCR24	yellow	VSMC_M3
GO:0006869	0.74	0.35	16	273	lipid transport	BP	1	PQLC1,ESYT1,SREBF2,PPARD,PLEKHA8,LAMTOR1,SCARB1,ACSL1,FABP3,SPX,PLA2G3,VPS51,YJEFN3,SLC27A3,AKT1,SLC27A4	yellow	VSMC_M3

GO:0051129	0.74	0.32	34	569	negative regulation of cellular component organization	BP	1	PACSIN1,LGALS1,PHF2,EMILIN1,EIF4EBP1,NAT10,BCL2L1,SWAP70,CAPG,PINX1,PACSIN3,RIN3,MMP14,GPX1,CORO1B,NGEF,ARPIN,PFDN2,MID1IP1,PEX5,LGALS3,TNFRSF1A,RCC2,MAD1L1,MAD2L2,ARHGEF2,CORO1A,SLX1A,CARM1,AKT1,ANKRD13B,AURKAIP1,MIR214,TWF2	yellow	VSMC_M3
GO:0030968	0.74	0.44	4	69	endoplasmic reticulum unfolded protein response	BP	1	ATF6B,SERP2,SELENOS,STUB1	yellow	VSMC_M3
GO:0065007	0.74	0.28	559	8375	biological regulation	BP	1	ENPP1,PQLC1,CD63,NAB2,NHP2,CDK4,CTDSP2,RUFY1,SQSTM1,GNL1,ZNF608,BAG6,SBF1,SNX9,CTHRC1,HDAC10,NELFE,FNIP1,ATF6B,PBX2,PRR5,TTLL12,RING1,SREBF2,SQLE,TXNDC15,H2AFY,ITPR3,HNRNPA0,TRIM11,SGSM3,TRAPPC9,PACSIN1,DEF6,PPARD,EEF1D,TSTA3,SCRIB,PLEC,LGALS1,IFT27,SHARPIN,BOP1,VPS28,SND1,TOM1,CUL7,PPP1R14B,DNPH1,B4GALT1,AARS2,PDGFRA,IL1R1,SECISBP2,PHF2,COL15A1,EPN1,SSC5D,HSPBP1,SOCS5,TTC7A,RPS9,CNOT3,KLF4,DBNL,TPBG,HOXA1,WDPCP,CCM2,FHL3,SPRED2,EIPR1,RGS3,ZC3H12A,FIGNL1,PNKP,CPT1C,ZYX,RCN3,SNRNP70,CC2D1B,SNX3,KTI12,MICAL1,PLEKHA4,MGLL,EHD2,FAM120B,KMT2E,ANXA5,KRT8,WDFY2,SIX5,ZNF282,ZNF296,RELB,SLC9A3R1,SERP2,PLXND1,EMILIN1,RNF220,DMAP1,SLC30A3,CIC,SNX17,H3F3B,PPM1G,UNK,PLCD1,DLEC1,CD79A,INSIG1,MYRIP,SEC13,SEPT9,OGG1,SERTAD1,TTC5,PSMC4,ARHGEF40,UCP2,METTL3,PHLDA1,BAIAP2,CEP131,CXXC5,INPPL1,LAMTOR1,NUMA1,P4HB,DND1,PYCR1,CCDC57,RAB20,EIF4EBP1,ARAP3,DELE1,CAVIN3,RIC8A,GSR,ARHGAP22,DEAF1,HOXC4,ARHGAP33,LDLRAD3,TARBP2,KMT2B,AACS,SCARB1,NAT10,HM13,BCL2L1,ITGB7,ZNF740,PDE4D,SPRYD3,TNS2,EIF4B,RRS1,SWAP70,LRP3,RALY,PLEKHF1,ACSL1,ZBTB38,LSM6,CAPG,SLC11A2,TNKS1BP1,MMP24,PINX1,GREM1,KDM2B,DOK1,AQP5,PDE3B,MCRS1,DCTN1,TKT,RAPSN,CX3CL1,WNT10B,SOD1,PACSIN3,ACTR5,ANKRD2,DGKZ,EGR3,CCAR2,DNAJC17,HPS1,DLL4,CHP1,TYRO3,SCD,YAF2,HTATIP2,PCIF1,PCBP4,LSP1,RAB35,KDM1B,CHCHD2,SLC35C2,RBM15B,TSPAN5,CISH,SRSF9,TMEM183A,ST3GAL4,RNF19B,DCPS,IRX1,DHCR7,SELENOS,GSTO1,ITPRIP,RRP1B,ETFBKMT,MVK,BR[illegible]	yellow	VSMC_M3
GO:0019751	0.74	0.42	5	88	polyol metabolic process	BP	1	NUDT3,GALK1,PCK2,ISYNA1,PGP	yellow	VSMC_M3
GO:0003018	0.75	0.36	11	200	vascular process in circulatory system	BP	1	PPARD,PLEC,SLC9A3R1,LRP3,SOD1,GPX1,SLC19A1,AKT1,SLC6A4,TJP3,SLC27A4	yellow	VSMC_M3
GO:0051346	0.75	0.35	15	242	negative regulation of hydrolase activity	BP	1	FNIP1,KLF4,MICAL1,CHP1,RRP1B,GPX1,AIP,PPP1R35,LGALS3,UBXN1,RCC2,DFFA,AKT1,DHCR24,SLC27A4	yellow	VSMC_M3
GO:0046546	0.75	0.40	6	112	development of primary male sexual characteristics	BP	1	PDGFRA,BCL2L1,MMP14,SGPL1,HMGCS1,SAFB2	yellow	VSMC_M3
GO:0051301	0.75	0.32	32	525	cell division	BP	1	CDK4,SNX9,PLEC,CUL7,SEPT9,NUMA1,BCL2L1,DCTN1,RAB35,PDGFA,CDK2AP2,NUDC,CEP164,RHOC,ALKBH4,SAC3D1,BABAM1,ANXA11,RCC2,EML3,SDE2,MAD1L1,MAD2L2,ARHGEF2,MYO19,AATF,ZNF830,SNX18,GIT1,ANAPC2,TAZ,MIS12	yellow	VSMC_M3
GO:2001235	0.75	0.40	6	98	positive regulation of apoptotic signaling pathway	BP	1	SLC9A3R1,PLEKHF1,SOD1,RIPK3,BAD,TNFRSF12A	yellow	VSMC_M3
GO:0007098	0.75	0.40	6	115	centrosome cycle	BP	1	CEP131,CCDC57,NAT10,DCTN1,SAC3D1,KIAA0753	yellow	VSMC_M3
GO:0042129	0.75	0.40	6	102	regulation of T cell proliferation	BP	1	SCRIB,RIPK3,LGALS3,MAD1L1,CORO1A,CD276	yellow	VSMC_M3
GO:0002687	0.75	0.40	6	101	positive regulation of leukocyte migration	BP	1	IL1R1,SWAP70,CX3CL1,MMP14,LGALS3,C1QBP	yellow	VSMC_M3
GO:0030010	0.75	0.40	6	123	establishment of cell polarity	BP	1	SCRIB,WDPCP,SLC9A3R1,NUMA1,DCTN1,STK11	yellow	VSMC_M3
GO:0000725	0.75	0.38	7	126	recombinational repair	BP	1	FIGNL1,MAD2L2,PPP4C,SLX1A,CXorf57,SIRT6,NABP2	yellow	VSMC_M3
GO:2000377	0.75	0.38	7	120	regulation of reactive oxygen species metabolic process	BP	1	ZC3H12A,SOD1,RIPK3,AATF,AKT1,TRAP1,CTNS	yellow	VSMC_M3
GO:0051960	0.75	0.33	19	344	regulation of nervous system development	BP	1	TRIM11,CUL7,DBNL,TPBG,PLXND1,BAIAP2,CX3CL1,DLL4,CLCF1,LIMK1,RELA,CTDSP1,DBN1,CRABP2,EEF2K,AKT1,ANAPC2,STK11,TWF2	yellow	VSMC_M3
GO:0016079	0.75	0.42	4	79	synaptic vesicle exocytosis	BP	1	SCRIB,SYT12,GIT1,UNC13A	yellow	VSMC_M3
GO:0007179	0.75	0.37	8	156	transforming growth factor beta receptor signaling pathway	BP	1	SPRED2,ZYX,EMILIN1,MTMR4,VASN,STK11,TGFBR3,STUB1	yellow	VSMC_M3
GO:0046879	0.75	0.35	12	206	hormone secretion	BP	1	PPARD,EIPR1,MYRIP,UCP2,AACS,PCK2,CAPN10,PLA2G3,BAD,GLUD1,MIDN,JAGN1	yellow	VSMC_M3
GO:0043271	0.75	0.40	5	93	negative regulation of ion transport	BP	1	SLC9A3R1,PACSIN3,GSTO1,TRIM27,AKT1	yellow	VSMC_M3
GO:0009611	0.75	0.32	25	422	response to wounding	BP	1	PPARD,SCRIB,PLEC,LGALS1,B4GALT1,PDGFRA,KLF4,ANXA5,SCARB1,CX3CL1,SOD1,DGKZ,TYRO3,ST3GAL4,PDGFA,GPX1,CORO1B,RHOC,BLOC1S4,PHB2,CD9,PLAU,GIT1,PPL,TNFRSF12A	yellow	VSMC_M3
GO:0009612	0.75	0.36	10	176	response to mechanical stimulus	BP	1	PLEC,MMP14,RELA,TMEM120A,MAP3K14,BAD,TNFRSF1A,AKT1,PPL,JUND	yellow	VSMC_M3
GO:0007568	0.75	0.33	15	252	aging	BP	1	OGG1,UCP2,CX3CL1,SOD1,KAT5,RELA,IGFBP5,CNP,CARM1,AKT1,MNT,IRAK1,MBD3,RF00024,JUND	yellow	VSMC_M3
GO:0050793	0.75	0.28	115	1852	regulation of developmental process	BP	1	ENPP1,NAB2,CDK4,BAG6,CTHRC1,FNIP1,H2AFY,TRIM11,PACSIN1,PPARD,CUL7,PDGFRA,EPN1,SOCS5,CNOT3,KLF4,DBNL,TPBG,WDPCP,SPRED2,ZC3H12A,PLEKHA4,EHD2,WDFY2,SLC9A3R1,PLXND1,EMILIN1,INSIG1,METTL3,BAIAP2,NUMA1,P4HB,ARHGAP33,LRP3,GREM1,KDM2B,PDE3B,CX3CL1,WNT10B,SOD1,ANKRD2,EGR3,DLL4,HTATIP2,PDGFA,MMP14,CORO1B,NGEF,CLCF1,PLEKHO1,ARPIN,LIMK1,TWIST2,PLA2G3,SART1,CCDC85B,PBX1,RELA,RHOC,FMNL1,LIMD1,SGPL1,YJEFN3,PHB2,IGFBP5,BAD,CTDSP1,EID1,TNFRSF1A,C1QBP,TAF6L,RCC2,PRXL2A,KRT10,PKN1,DBN1,CAPN2,MAD2L2,CRABP2,TMEM79,ARHGEF2,CORO1A,CARM1,SYNE3,EEF2K,MYO19,INF2,AKT1,SLC6A4,MYDGF,SIRT6,HMG20B,VASN,EHMT1,AP3D1,ANAPC2,STK11,JMJD8,SARS,TGFBR3,TRADD,MIR214,RF00024,ERBB4,TWF2,JUND,KLF13,RNH1,FADS1,UNC13A,TRIB2,ZNF385A,CDC42EP5,NSMF,GLIPR2	yellow	VSMC_M3
GO:0001938	0.76	0.42	4	78	positive regulation of endothelial cell proliferation	BP	1	EGR3,AKT1,MYDGF,SIRT6	yellow	VSMC_M3
GO:0033138	0.76	0.42	4	74	positive regulation of peptidyl-serine phosphorylation	BP	1	FNIP1,TNKS1BP1,MAD2L2,AKT1	yellow	VSMC_M3
GO:0072384	0.76	0.42	4	78	organelle transport along microtubule	BP	1	BLOC1S4,AP3D1,STK11,TMEM201	yellow	VSMC_M3
GO:1901215	0.76	0.36	9	161	negative regulation of neuron death	BP	1	BCL2L1,KDM2B,CX3CL1,SOD1,TYRO3,CLCF1,PLA2G3,CORO1A,AKT1	yellow	VSMC_M3

GO:0000075	0.76	0.35	9	155	cell cycle checkpoint signaling	BP	1	CCAR2,THOC5,BABAM1,SDE2,MAD1L1,MAD2L2,ZNF830,NABP2,FBXO6	yellow	VSMC_M3
GO:0060548	0.76	0.29	48	754	negative regulation of cell death	BP	1	BAG6,FNIP1,PPARD,KLF4,ZC3H12A,FIGNL1,MICAL1,ANXA5,OGG1,UCP2,PYCR1,BCL2L1,GREM1,KDM2B,CX3CL1,SOD1,EGR3,CCAR2,TYRO3,HTATIP2,DCPS,SELENOS,ITPRIP,GPX1,CLCF1,QARS,TWIST2,PLA2G3,RELA,ARNT2,PHB2,LGALS3,NANOS3,DFFA,ARHGEF2,CORO1A,EEF2K,AATF,ZNF830,AKT1,DHCR24,MYDGF,TRAP1,MNT,MIR214,PTMA,ZNF385A,TXNDC12	yellow	VSMC_M3
GO:0050670	0.76	0.36	8	137	regulation of lymphocyte proliferation	BP	1	SCRIB,CLCF1,RIPK3,LGALS3,PKN1,MAD1L1,CORO1A,CD276	yellow	VSMC_M3
GO:0055072	0.76	0.41	4	72	iron ion homeostasis	BP	1	TTC7A,SLC11A2,SOD1,CCDC115	yellow	VSMC_M3
GO:0040011	0.76	0.27	82	1349	locomotion	BP	1	CD63,CTHRC1,PPARD,TSTA3,SCRIB,PLEC,B4GALT1,PDGFRA,IL1R1,KLF4,TPBG,WDPCP,ZC3H12A,SLC9A3R1,PLXND1,EMILIN1,UNK,CEP131,YIF1B,RIC8A,SCARB1,ITGB7,SWAP70,GREM1,CX3CL1,DGKZ,EGR3,DLL4,TYRO3,LSP1,ST3GAL4,RIN3,PDGFA,MMP14,GPX1,CORO1B,PLEKHO1,IGSF9,NECTIN1,ARPIN,RIPK3,TWIST2,IQCG,PLA2G3,PTPN23,RHOC,FMNL1,LIMD1,SGPL1,PHB2,IGFBP5,AAMP,LGALS3,CD9,PLAU,C1QBP,WASHC1,RCC2,MTA2,ELP5,PKN1,MIIP,ARHGEF2,CORO1A,AKT1,ACAP3,TMEM201,GAS8,TGFBR3,TRADD,MIR214,ERBB4,PTPRR,IGFBP6,JAGN1,CAVIN1,IGSF8,NSMF,GLIPR2,TUBGCP2,MEIG1,ARPC5L	yellow	VSMC_M3
GO:0071241	0.76	0.35	9	165	cellular response to inorganic substance	BP	1	OGG1,SOD1,SYT12,BAD,HSD17B1,EEF2K,AKT1,JUND,NSMCE3	yellow	VSMC_M3
GO:0006954	0.77	0.30	31	497	inflammatory response	BP	1	HNRNPA0,PPARD,SHARPIN,B4GALT1,IL1R1,SOCS5,KLF4,ZC3H12A,ZYX,MGLL,RELB,OGG1,SIGLEC1,CX3CL1,SOD1,TYRO3,SELENOS,MVK,GPX1,PSMB4,PLA2G3,RELA,SCYL1,IFI35,TNFRSF1A,PER1,AKT1,GIT1,CARD9,TRADD,NDST1	yellow	VSMC_M3
GO:0071407	0.77	0.30	26	448	cellular response to organic cyclic compound	BP	1	CDK4,PPARD,LGALS1,KLF4,ZC3H12A,SLC9A3R1,INSIG1,AACS,WNT10B,SOD1,PCK2,SLC26A6,PRMT2,KAT5,PHB2,IGFBP5,PKN1,PER1,SMARCA4,CARM1,EEF2K,AKT1,HMGCS1,SAFB,SAFB2,SLC6A4	yellow	VSMC_M3
GO:0050794	0.77	0.26	502	7581	regulation of cellular process	BP	1	ENPP1,CD63,NAB2,CDK4,CTDSP2,RUFY1,SQSTM1,GNL1,ZNF608,BAG6,SNX9,CTHRC1,HDAC10,NELFE,FNIP1,ATF6B,PBX2,PRR5,TTLL12,RING1,SREBF2,SQLE,TXNDC15,H2AFY,ITPR3,HNRNPA0,TRIM11,SGSM3,TRAPPC9,PACSIN1,DEF6,PPARD,EEF1D,TSTA3,SCRIB,PLEC,LGALS1,IFT27,SHARPIN,BOP1,VPS28,SND1,TOM1,CUL7,PPP1R14B,DNPH1,B4GALT1,PDGFRA,IL1R1,SECISBP2,PHF2,COL15A1,EPN1,HSPBP1,SOCS5,RPS9,CNOT3,KLF4,DBNL,TPBG,HOXA1,WDPCP,CCM2,FHL3,SPRED2,EIPR1,RGS3,ZC3H12A,FIGNL1,PNKP,ZYX,RCN3,SNRNP70,CC2D1B,SNX3,KTI12,MICAL1,PLEKHA4,MGLL,EHD2,FAM120B,KMT2E,ANXA5,KRT8,WDFY2,SIX5,ZNF282,ZNF296,RELB,SLC9A3R1,SERP2,PLXND1,EMILIN1,RNF220,DMAP1,SLC30A3,CIC,SNX17,H3F3B,PPM1G,UNK,PLCD1,DLEC1,CD79A,INSIG1,MYRIP,SEC13,SEPT9,OGG1,SERTAD1,TTC5,PSMC4,ARHGEF40,UCP2,METTL3,PHLDA1,BAIAP2,CEP131,CXXC5,INPPL1,LAMTOR1,NUMA1,P4HB,DND1,PYCR1,CCDC57,EIF4EBP1,ARAP3,DELE1,CAVIN3,RIC8A,GSR,ARHGAP22,DEAF1,HOXC4,ARHGAP33,TARBP2,KMT2B,AACS,SCARB1,NAT10,BCL2L1,ITGB7,ZNF740,PDE4D,SPRYD3,TNS2,EIF4B,RRS1,SWAP70,LRP3,RALY,PLEKHF1,ACSL1,ZBTB38,CAPG,TNKS1BP1,PINX1,GREM1,KDM2B,DOK1,PDE3B,MCRS1,DCTN1,RAPSN,CX3CL1,WNT10B,SOD1,PACSIN3,ACTR5,ANKRD2,DGKZ,EGR3,CCAR2,DNAJC17,DLL4,CHP1,TYRO3,YAF2,HTATIP2,PCIF1,PCBP4,LSP1,RAB35,KDM1B,CHCHD2,SLC35C2,RBM15B,TSPAN5,CISH,SRSF9,ST3GAL4,RNF19B,DCPS,IRX1,SELENOS,GSTO1,ITPRIP,RRP1B,ETFBKMT,BRD9,RIN3,STK32C,FABP3,ZCCHC17,PDGFA,MMP14,CDK2AP2,PABPN1,GPX1,AIP,CORO1B,NGEF,CLCF1,NGDN,SRRD,PLEKHO1,TSHZ2,IGSF9,SYT[illegible]	yellow	VSMC_M3
GO:0070663	0.77	0.35	9	153	regulation of leukocyte proliferation	BP	1	SCRIB,GREM1,CLCF1,RIPK3,LGALS3,PKN1,MAD1L1,CORO1A,CD276	yellow	VSMC_M3
GO:0006664	0.77	0.39	5	87	glycolipid metabolic process	BP	1	CERK,PIGO,ST3GAL4,DPM3,GBA2	yellow	VSMC_M3
GO:1903509	0.77	0.39	5	87	liposaccharide metabolic process	BP	1	CERK,PIGO,ST3GAL4,DPM3,GBA2	yellow	VSMC_M3
GO:0010469	0.77	0.37	6	112	regulation of signaling receptor activity	BP	1	SOCS5,PDE4D,GREM1,BUD31,PLAU,GSG1L	yellow	VSMC_M3
GO:0071705	0.77	0.27	104	1609	nitrogen compound transport	BP	1	CD63,RUFY1,CCHCR1,SNX9,SAMM50,SREBF2,SGSM3,GUK1,PPARD,SCRIB,IFT27,SLC52A2,VPS28,TOM1,ASNA1,PLEKHA8,EIPR1,ZC3H12A,PDIA4,RCN3,SNX3,SLC9A3R1,SERP2,DMAP1,SNX17,INSIG1,MYRIP,SEC13,UCP2,CEP131,YIF1B,RAB20,ARHGAP33,AACS,HM13,SFXN5,CHP1,AP2A2,SNX21,RAB35,SLC35C2,RBM15B,APBA2,SELENOS,EXOC3,PABPN1,AIP,AP1G2,SYT12,PCK2,SLC19A1,PCNT,SLC26A6,THOC5,CAPN10,RHCE,PLA2G3,PTPN23,VPS51,PEX5,ZDHHC3,THOC3,PHB2,BAD,COPE,MVB12A,NUP88,WASHC1,AP1M1,RAB3IL1,CCDC22,GLUD1,SNX8,LMAN2,RAB24,CLTB,ARHGEF2,SNF8,MVP,MTX1,RANGRF,STX10,RABEP2,TMED1,CDC37,CSK,ATG3,RBM22,AKT1,SNX18,KHSRP,RANBP3,RAB34,SLC6A4,CTNS,AP3D1,MIDN,SLC44A3,RBM8A,AP5B1,JAGN1,CAVIN1,ARFRP1,TIMM22	yellow	VSMC_M3
GO:1905475	0.77	0.35	8	147	regulation of protein localization to membrane	BP	1	SQSTM1,BCL2L1,CHP1,MMP14,LGALS3,RANGRF,CSK,AKT1	yellow	VSMC_M3
GO:0008406	0.77	0.34	9	166	gonad development	BP	1	PDGFRA,BCL2L1,SOD1,MMP14,IDH1,SGPL1,ZNF830,HMGCS1,SAFB2	yellow	VSMC_M3

GO:0010557	0.77	0.27	90	1433	positive regulation of macromolecule biosynthetic process	BP	1	CDK4,SQSTM1,NELFE,ATF6B,PBX2,SREBF2,PPARD,PHF2,RPS9,KLF4,HOXA1,ZC3H12A,PNKP,KMT2E,SIX5,ZNF296,SERTAD1,TTC5,METTL3,DEAF1,HOXC4,KMT2B,NAT10,ZBTB38,GREM1,MCRS1,CX3CL1,WNT10B,EGR3,CCAR2,CHP1,YAF2,HTATIP2,PCIF1,KDM1B,CHCHD2,RRP1B,AIP,TRIM27,PRMT2,THOC5,RIPK3,SMARCD2,GMEB2,PRPF6,PBX1,KAT5,RELA,ARNT2,SMARCB1,CHCHD10,PBX4,UBTF,MEF2B,NDP,SSBP4,MPV17L2,BUD31,TNFRSF1A,C1QBP,TAF6L,ZBTB49,MTA2,PELP1,FOXK1,PKN1,MAD2L2,ARHGEF2,PER1,NFIX,IRF2BPL,TRIM25,SMARCA4,CARM1,AATF,MID2,ZNF521,AKT1,BRF1,PAIP1,GTF2F1,MYDGF,MAP2K2,AP3D1,MED16,POMT1,ERBB4,JUND,KLF13,IER5	yellow	VSMC_M3
GO:0048771	0.77	0.36	7	129	tissue remodeling	BP	1	CTHRC1,GREM1,DLL4,MMP14,IGFBP5,CSK,MIR214	yellow	VSMC_M3
GO:0065008	0.77	0.26	177	2770	regulation of biological quality	BP	1	ENPP1,PQLC1,CDK4,SQSTM1,BAG6,SNX9,SREBF2,ITPR3,HNRNPA0,PPARD,SCRIB,PLEC,LGALS1,AARS2,PDGFRA,TTC7A,RPS9,CNOT3,DBNL,TPBG,WDPCP,EIPR1,ZC3H12A,CPT1C,RCN3,KMT2E,ANXA5,SLC9A3R1,PLXND1,SLC30A3,INSIG1,MYRIP,UCP2,METTL3,BAIAP2,LAMTOR1,DND1,PYCR1,RAB20,GSR,ARHGAP22,ARHGAP33,AACS,SCARB1,BCL2L1,PDE4D,TNS2,SWAP70,RALY,ACSL1,CAPG,SLC11A2,PINX1,AQP5,DCTN1,CX3CL1,WNT10B,SOD1,DGKZ,CCAR2,CHP1,TYRO3,SCD,PCBP4,TMEM183A,ST3GAL4,DCPS,DHCR7,SELENOS,GSTO1,RIN3,FABP3,ZCCHC17,PDGFA,GPX1,CORO1B,NGEF,PLEKHO1,IGSF9,SYT12,LRFN4,PCK2,LSS,NECTIN1,ANPEP,TRIM27,ARPIN,LIMK1,SCN4A,SLC26A6,SPX,RIPK3,CAPN10,PLA2G3,RHOC,FMNL1,LIMD1,SGPL1,SMARCB1,CHCHD10,BLOC1S4,TXNRD2,PHB2,IGFBP5,BAD,PLCB3,LAMTOR4,HSD17B1,LETM1,ARPC1B,CD9,PLAU,CHERP,WASHC1,CNP,CCDC22,USP27X,GLUD1,FOXK1,PKN1,DBN1,NANOS3,CRABP2,TMEM79,SNF8,MTX1,RANGRF,DPM3,CORO1A,TSPOAP1,SMARCA4,SYNE3,EEF2K,CDC37,CSK,ANKRD9,DOCK11,ZNF830,AKT1,PACS2,PAIP1,KHSRP,ALKBH7,GIT1,SLC6A4,CARHSP1,SIRT6,TJP3,NCLN,CTNS,EHMT1,AP3D1,ANAPC2,MIDN,STK11,TELO2,CARD9,AMPD2,SPG7,APRT,TGFBR3,MIR214,ERBB4,TWF2,ARMCX1,KLF13,TENT5B,JAGN1,ELN,UNC13A,CDC42EP5,NSMF,GBA2,STUB1,CCDC115,ARPC5L,GSG1L	yellow	VSMC_M3
GO:0007264	0.77	0.30	24	437	small GTPase mediated signal transduction	BP	1	RUFY1,SQSTM1,SGSM3,DEF6,DBNL,ARHGEF40,ARHGAP22,ARHGAP33,SWAP70,DOK1,CHP1,RAB35,NGEF,LIMK1,TNK2,STMN3,SIPA1,RHOC,ARHGEF2,RANGRF,DOCK11,DHCR24,CDC42EP5,ARHGEF1	yellow	VSMC_M3
GO:0061025	0.77	0.36	7	125	membrane fusion	BP	1	RAB20,CHP1,CD9,PIP4K2B,STX10,CORO1A,RAB34	yellow	VSMC_M3
GO:0032944	0.77	0.35	8	138	regulation of mononuclear cell proliferation	BP	1	SCRIB,CLCF1,RIPK3,LGALS3,PKN1,MAD1L1,CORO1A,CD276	yellow	VSMC_M3
GO:0060326	0.77	0.33	12	206	cell chemotaxis	BP	1	PLEC,PDGFRA,TPBG,SWAP70,GREM1,CX3CL1,EGR3,RIN3,CORO1B,LGALS3,C1QBP,CORO1A	yellow	VSMC_M3
GO:0003279	0.77	0.40	4	88	cardiac septum development	BP	1	PLXND1,SNX17,TGFBR3,NDST1	yellow	VSMC_M3
GO:0043255	0.77	0.40	4	78	regulation of carbohydrate biosynthetic process	BP	1	ENPP1,SELENOS,AKT1,PGP	yellow	VSMC_M3
GO:0030522	0.77	0.32	12	217	intracellular receptor signaling pathway	BP	1	PPARD,FAM120B,PRMT2,RELA,PHB2,C1QBP,PKN1,PER1,SMARCA4,CARM1,SAFB,SAFB2	yellow	VSMC_M3
GO:0045787	0.78	0.31	14	243	positive regulation of cell cycle	BP	1	CDK4,H2AFY,KMT2E,NUMA1,CCDC57,EIF4EBP1,WNT10B,THOC5,PBX1,PHB2,RCC2,AKT1,SLC6A4,MIR214	yellow	VSMC_M3
GO:1903532	0.78	0.33	11	196	positive regulation of secretion by cell	BP	1	PPARD,MYRIP,AACS,PCK2,CAPN10,PLA2G3,PTPN23,BAD,GLUD1,SNF8,SLC6A4	yellow	VSMC_M3
GO:0030004	0.78	0.39	4	81	cellular monovalent inorganic cation homeostasis	BP	1	RAB20,CHP1,SLC26A6,CCDC115	yellow	VSMC_M3
GO:0062207	0.78	0.39	4	77	regulation of pattern recognition receptor signaling pathway	BP	1	TYRO3,IFI35,C1QBP,CACTIN	yellow	VSMC_M3
GO:0030301	0.78	0.39	4	77	cholesterol transport	BP	1	SREBF2,LAMTOR1,SCARB1,YJEFN3	yellow	VSMC_M3
GO:0002702	0.78	0.39	4	75	positive regulation of production of molecular mediator of immune response	BP	1	IL1R1,CLCF1,PHB2,MAD2L2	yellow	VSMC_M3
GO:0043281	0.78	0.33	9	153	regulation of cysteine-type endopeptidase activity involved in apoptotic process	BP	1	FNIP1,KLF4,MICAL1,DELE1,GPX1,BAD,AKT1,DHCR24,CARD9	yellow	VSMC_M3
GO:0007018	0.78	0.30	16	283	microtubule-based movement	BP	1	IFT27,SLC9A3R1,CEP131,YIF1B,SOD1,IQCG,PLA2G3,BLOC1S4,AP3D1,SSNA1,STK11,TMEM201,GAS8,SPG7,WDR34,MEIG1	yellow	VSMC_M3
GO:1901888	0.78	0.34	8	165	regulation of cell junction assembly	BP	1	TPBG,WDPCP,GREM1,MMP14,LRFN4,NECTIN1,RCC2,EEF2K	yellow	VSMC_M3
GO:0006886	0.78	0.27	46	739	intracellular protein transport	BP	1	CCHCR1,SNX9,SAMM50,SREBF2,SGSM3,SCRIB,IFT27,VPS28,ASNA1,ZC3H12A,DMAP1,SNX17,INSIG1,MYRIP,SEC13,CEP131,YIF1B,RAB20,HM13,CHP1,AP2A2,SELENOS,AIP,AP1G2,PCNT,PEX5,ZDHHC3,PHB2,NUP88,AP1M1,SNX8,RAB24,CLTB,ARHGEF2,SNF8,RANGRF,STX10,TMED1,CDC37,ATG3,RBM22,AKT1,RANBP3,AP3D1,ARFRP1,TIMM22	yellow	VSMC_M3
GO:0045807	0.78	0.39	4	82	positive regulation of endocytosis	BP	1	CD63,VPS28,GREM1,EEF2K	yellow	VSMC_M3
GO:0048675	0.78	0.37	5	106	axon extension	BP	1	DBNL,LIMK1,DBN1,ANAPC2,TWF2	yellow	VSMC_M3
GO:0044272	0.78	0.35	7	127	sulfur compound biosynthetic process	BP	1	MPST,ACSL1,ST3GAL4,PAPSS1,ACLY,MTHFR,NDST1	yellow	VSMC_M3
GO:0048588	0.78	0.32	10	195	developmental cell growth	BP	1	DBNL,LIMK1,PRMT2,DBN1,CRABP2,SIRT6,ANAPC2,STK11,TWF2,UNC13A	yellow	VSMC_M3
GO:0034504	0.78	0.31	14	244	protein localization to nucleus	BP	1	ZC3H12A,DMAP1,SEC13,RRS1,PINX1,MCRS1,CHP1,CHCHD10,PHB2,NUP88,RANGRF,RBM22,AKT1,STK11	yellow	VSMC_M3
GO:0032989	0.78	0.28	33	609	cellular component morphogenesis	BP	1	PACSIN1,PLEC,CUL7,PDGFRA,DBNL,TPBG,KRT8,PLXND1,BAIAP2,ARHGAP33,BCL2L1,SLC11A2,CORO1B,NGEF,PLEKHO1,IGSF9,NECTIN1,ARPIN,LIMK1,PLA2G3,C12orf57,CD9,CNP,DBN1,CRABP2,S100A6,EEF2K,RFX2,ANAPC2,STK11,TWF2,UNC13A,NSMF	yellow	VSMC_M3
GO:0031123	0.78	0.37	5	94	RNA 3'-end processing	BP	1	NELFE,RPS21,CSTF2T,INTS1,SSU72	yellow	VSMC_M3
GO:0097530	0.78	0.37	5	91	granulocyte migration	BP	1	IL1R1,CX3CL1,LGALS3,C1QBP,JAGN1	yellow	VSMC_M3
GO:0009914	0.78	0.31	12	212	hormone transport	BP	1	PPARD,EIPR1,MYRIP,UCP2,AACS,PCK2,CAPN10,PLA2G3,BAD,GLUD1,MIDN,JAGN1	yellow	VSMC_M3
GO:0031570	0.78	0.35	6	111	DNA integrity checkpoint signaling	BP	1	CCAR2,THOC5,BABAM1,SDE2,ZNF830,FBXO6	yellow	VSMC_M3

GO:0140014	0.78	0.30	14	244	mitotic nuclear division	BP	1	DCTN2,NCAPH2,H2AFY,CUL7,NUMA1,RRS1,PINX1,NUDC,PHB2,EML3,MAD1L1,MAD2L2,AURKAIP1,MIS12	yellow	VSMC_M3
GO:1902105	0.78	0.31	11	193	regulation of leukocyte differentiation	BP	1	SOCS5,ZC3H12A,METTL3,SOD1,EGR3,MMP14,PLA2G3,SART1,BAD,PRXL2A,AP3D1	yellow	VSMC_M3
GO:0035265	0.79	0.34	7	134	organ growth	BP	1	SOD1,EVC,AKT1,SLC6A4,SIRT6,TGFBR3,ERBB4	yellow	VSMC_M3
GO:0007613	0.79	0.38	4	86	memory	BP	1	ITPR3,CIC,SLC6A4,CTNS	yellow	VSMC_M3
GO:0048736	0.79	0.34	7	140	appendage development	BP	1	PBX2,WDPCP,GREM1,PBX1,CRABP2,SLC39A1,ZNF358	yellow	VSMC_M3
GO:0060173	0.79	0.34	7	140	limb development	BP	1	PBX2,WDPCP,GREM1,PBX1,CRABP2,SLC39A1,ZNF358	yellow	VSMC_M3
GO:0007399	0.79	0.24	112	1867	nervous system development	BP	1	NAB2,BAG6,CTHRC1,HDAC10,PBX2,TRIM11,TRAPPC9,PACSIN1,PPARD,SCRIB,PLEC,LGALS1,MPST,IFT27,SHARPIN,CUL7,SECISBP2,KLF4,DBNL,TPBG,HOXA1,WDPCP,ZC3H12A,SNX3,SLC9A3R1,PLXND1,RNF220,PAFAH1B3,CIC,UNK,METTL3,BAIAP2,DEAF1,TBCB,LRIG1,ARHGAP33,CAPG,SLC11A2,MMP24,KDM2B,DCTN1,CX3CL1,WNT10B,SOD1,EGR3,DLL4,TYRO3,RAB35,APBA2,IRX1,SDHA,MMP14,NGEF,CLCF1,IGSF9,QARS,LRFN4,NECTIN1,LIMK1,PLA2G3,STMN3,PBX1,RELA,SCYL1,ARNT2,VPS51,SMARCB1,PBX4,ATIC,BLOC1S4,NDP,C12orf57,BAD,CTDSP1,CD9,CHERP,CNP,GLUD1,DBN1,MTHFR,CRABP2,ARHGEF2,IRF2BPL,S100A6,SMARCA4,CARM1,EEF2K,EML1,CSK,ZNF521,ZNF358,AKT1,HMGCS1,GIT1,SLC6A4,HMG20B,ACAP3,CTNS,ANAPC2,MBD3,STK11,SPG7,NEGR1,NDST1,ERBB4,TWF2,IGSF8,UNC13A,NSMF,GBA2,TUBGCP2,MARVELD1	yellow	VSMC_M3
GO:0008037	0.79	0.36	5	97	cell recognition	BP	1	B4GALT1,SCARB1,IGSF9,LGALS3,CD9	yellow	VSMC_M3
GO:0050864	0.79	0.36	5	93	regulation of B cell activation	BP	1	MMP14,CLCF1,BAD,PKN1,MAD2L2	yellow	VSMC_M3
GO:0071482	0.79	0.36	5	94	cellular response to light stimulus	BP	1	METTL3,ACTR5,SDE2,STK11,NSMCE3	yellow	VSMC_M3
GO:0001763	0.79	0.32	8	157	morphogenesis of a branching structure	BP	1	PLXND1,GREM1,DLL4,PDGFA,MMP14,PBX1,PHB2,SIRT6	yellow	VSMC_M3
GO:0046631	0.79	0.36	5	97	alpha-beta T cell activation	BP	1	SOCS5,ZC3H12A,RELB,ARMC5,AP3D1	yellow	VSMC_M3
GO:0010811	0.79	0.36	5	100	positive regulation of cell-substrate adhesion	BP	1	TSTA3,EMILIN1,P4HB,CX3CL1,C1QBP	yellow	VSMC_M3
GO:1990138	0.79	0.33	7	147	neuron projection extension	BP	1	DBNL,LIMK1,DBN1,ANAPC2,STK11,TWF2,UNC13A	yellow	VSMC_M3
GO:0060041	0.79	0.35	5	108	retina development in camera-type eye	BP	1	PDGFRA,DLL4,IMPDH2,NECTIN1,SMARCA4	yellow	VSMC_M3
GO:2000278	0.79	0.35	5	95	regulation of DNA biosynthetic process	BP	1	KLF4,PNKP,NAT10,PINX1,GREM1	yellow	VSMC_M3
GO:0045137	0.79	0.31	9	170	development of primary sexual characteristics	BP	1	PDGFRA,BCL2L1,SOD1,MMP14,IDH1,SGPL1,ZNF830,HMGCS1,SAFB2	yellow	VSMC_M3
GO:0006814	0.79	0.32	8	161	sodium ion transport	BP	1	SLC9A3R1,CHP1,NKAIN1,SCN4A,PER1,RANGRF,AKT1,SLC6A4	yellow	VSMC_M3
GO:0051983	0.79	0.37	4	78	regulation of chromosome segregation	BP	1	NUMA1,RCC2,MAD1L1,MAD2L2	yellow	VSMC_M3
GO:0035637	0.79	0.35	5	114	multicellular organismal signaling	BP	1	PLEC,PDE4D,SOD1,SCN4A,RANGRF	yellow	VSMC_M3
GO:0050729	0.79	0.35	5	93	positive regulation of inflammatory response	BP	1	CX3CL1,PLA2G3,IFI35,TNFRSF1A,TRADD	yellow	VSMC_M3
GO:1902850	0.79	0.33	7	133	microtubule cytoskeleton organization involved in mitosis	BP	1	DCTN2,NUMA1,DCTN1,PCNT,NUDC,EML3,EML1	yellow	VSMC_M3
GO:0007596	0.80	0.31	9	164	blood coagulation	BP	1	PDGFRA,ANXA5,DGKZ,TYRO3,ST3GAL4,PDGFA,BLOC1S4,CD9,PLAU	yellow	VSMC_M3
GO:0007420	0.80	0.26	33	587	brain development	BP	1	BAG6,PBX2,TRAPPC9,SCRIB,SHARPIN,SECISBP2,PAFAH1B3,CIC,METTL3,BAIAP2,KDM2B,TYRO3,QARS,PBX1,ARNT2,VPS51,PBX4,ATIC,C12orf57,BAD,CD9,CNP,GLUD1,EML1,CSK,HMGCS1,GIT1,SLC6A4,CTNS,MBD3,NDST1,ERBB4,TUBGCP2	yellow	VSMC_M3
GO:0034764	0.80	0.32	8	155	positive regulation of transmembrane transport	BP	1	SLC9A3R1,ACSL1,CX3CL1,CHP1,GSTO1,SLC26A6,CAPN10,AKT1	yellow	VSMC_M3
GO:0043279	0.80	0.36	4	81	response to alkaloid	BP	1	BCL2L1,SRSF9,UQCRC1,RELA	yellow	VSMC_M3
GO:0009891	0.80	0.23	96	1533	positive regulation of biosynthetic process	BP	1	CDK4,SQSTM1,NELFE,ATF6B,PBX2,SREBF2,PPARD,PHF2,RPS9,KLF4,HOXA1,ZC3H12A,PNKP,KMT2E,SIX5,ZNF296,SERTAD1,TTC5,METTL3,DEAF1,HOXC4,KMT2B,SCARB1,NAT10,ZBTB38,GREM1,MCRS1,CX3CL1,WNT10B,EGR3,CCAR2,CHP1,YAF2,HTATIP2,PCIF1,KDM1B,CHCHD2,RRP1B,FABP3,AIP,TRIM27,PRMT2,THOC5,RIPK3,SMARCD2,PLA2G3,GMEB2,PRPF6,PBX1,KAT5,RELA,ARNT2,MID1IP1,SMARCB1,CHCHD10,PBX4,UBTF,MEF2B,NDP,SSBP4,MPV17L2,BUD31,TNFRSF1A,C1QBP,TAF6L,ZBTB49,MTA2,PELP1,FOXK1,PKN1,CAPN2,MAD2L2,ARHGEF2,PER1,NFIX,IRF2BPL,TRIM25,SMARCA4,CARM1,AATF,MID2,ZNF521,AKT1,BRF1,PAIP1,GTF2F1,MYDGF,MAP2K2,AP3D1,TAZ,MED16,POMT1,ERBB4,JUND,KLF13,IER5	yellow	VSMC_M3
GO:0062013	0.80	0.34	5	96	positive regulation of small molecule metabolic process	BP	1	PPARD,PLA2G3,MID1IP1,AKT1,TAZ	yellow	VSMC_M3
GO:0198738	0.80	0.27	21	374	cell-cell signaling by wnt	BP	1	CTHRC1,KLF4,TPBG,SNX3,PLEKHA4,SLC9A3R1,RNF220,GREM1,WNT10B,CCAR2,PTPN23,LIMD1,NDP,AMER1,MAD2L2,SMARCA4,HIC1,CSNK1G2,STK11,IGFBP6,APCDD1L	yellow	VSMC_M3
GO:0051050	0.80	0.25	39	660	positive regulation of transport	BP	1	CD63,SREBF2,PPARD,VPS28,EIPR1,ZC3H12A,EHD2,SLC9A3R1,DMAP1,SLC30A3,MYRIP,CEP131,NUMA1,AACS,ACSL1,GREM1,CX3CL1,SOD1,CHP1,GSTO1,FABP3,PCK2,PCNT,SLC26A6,CAPN10,PLA2G3,PTPN23,CHCHD10,BAD,LGALS3,GLUD1,LMAN2,SNF8,EEF2K,RBM22,AKT1,SLC6A4,STK11,PLA2G4E	yellow	VSMC_M3
GO:0001890	0.80	0.33	6	117	placenta development	BP	1	PPARD,CUL7,KRT8,SOD1,AKT1,NSDHL	yellow	VSMC_M3
GO:0030308	0.81	0.30	8	153	negative regulation of cell growth	BP	1	ENPP1,PPARD,GREM1,CCAR2,CCDC85B,SIPA1,SMARCA4,STK11	yellow	VSMC_M3
GO:0007599	0.81	0.30	9	166	hemostasis	BP	1	PDGFRA,ANXA5,DGKZ,TYRO3,ST3GAL4,PDGFA,BLOC1S4,CD9,PLAU	yellow	VSMC_M3
GO:0050817	0.81	0.30	9	166	coagulation	BP	1	PDGFRA,ANXA5,DGKZ,TYRO3,ST3GAL4,PDGFA,BLOC1S4,CD9,PLAU	yellow	VSMC_M3
GO:0090316	0.81	0.31	7	136	positive regulation of intracellular protein transport	BP	1	SREBF2,ZC3H12A,DMAP1,CEP131,CHP1,PCNT,RBM22	yellow	VSMC_M3
GO:1903828	0.81	0.33	5	99	negative regulation of cellular protein localization	BP	1	H2AFY,INSIG1,BCL2L1,CHP1,CSK	yellow	VSMC_M3
GO:0060048	0.81	0.33	5	103	cardiac muscle contraction	BP	1	ZC3H12A,PDE4D,GSTO1,RANGRF,TAZ	yellow	VSMC_M3
GO:0007611	0.81	0.29	9	183	learning or memory	BP	1	ITPR3,TPBG,CIC,RIC8A,SLC11A2,TACO1,GIT1,SLC6A4,CTNS	yellow	VSMC_M3

GO:0060341	0.81	0.24	40	675	regulation of cellular localization	BP	1	SQSTM1,SREBF2,H2AFY,PPARD,VPS28,EIPR1,ZC3H12A,SNX3,EHD2,DMAP1,INSIG1,UCP2,CEP131,NUMA1,BCL2L1,PDE4D,PINX1,MCRS1,CX3CL1,CHP1,GSTO1,MMP14,PCNT,CAPN10,PLA2G3,PTPN23,BAD,LGALS3,MAD1L1,RANGRF,CORO1A,CSK,RBM22,AKT1,MAP2K2,STK11,GAS8,ERBB4,PLA2G4E,JAGN1	yellow	VSMC_M3
GO:0032880	0.81	0.23	41	693	regulation of protein localization	BP	1	SQSTM1,SREBF2,H2AFY,PPARD,VPS28,WDPCP,EIPR1,ZC3H12A,SNX3,DMAP1,INSIG1,MYRIP,UCP2,CEP131,NUMA1,AACS,BCL2L1,SWAP70,PINX1,MCRS1,CHP1,MMP14,PCK2,TRIM27,PCNT,CAPN10,PTPN23,BAD,LGALS3,GLUD1,RANGRF,STX10,TRIM25,CSK,RBM22,AKT1,MIDN,STK11,GAS8,ERBB4,JAGN1	yellow	VSMC_M3
GO:0006915	0.81	0.22	92	1436	apoptotic process	BP	1	CDK4,SQSTM1,BAG6,FNIP1,PPARD,SCRIB,LGALS1,SHARPIN,B4GALT1,KLF4,ZC3H12A,FIGNL1,MICAL1,ANXA5,KRT8,SLC9A3R1,OGG1,UCP2,PHLDA1,P4HB,DELE1,BCL2L1,PLEKHF1,GREM1,KDM2B,RAPSN,CX3CL1,WNT10B,SOD1,EGR3,CCAR2,TYRO3,HTATIP2,SELENOS,ITPRIP,RRP1B,GPX1,CLCF1,QARS,PRMT2,RIPK3,TWIST2,CAPN10,BRMS1,PLA2G3,RELA,ARNT2,ZDHHC3,SGPL1,CHCHD10,ZNF385B,PHB2,BAD,LGALS3,TNFRSF1A,C1QBP,AIMP2,USP27X,CASP14,PKN1,CAPN2,SLTM,NANOS3,BCL7C,DFFA,ARHGEF2,PLEKHO2,CORO1A,EEF2K,AATF,ATG3,ZNF830,AKT1,PACS2,DHCR24,MYDGF,CDK11B,HIC1,TRAP1,MNT,MKNK2,STK11,CARD9,SLC27A4,TRADD,TNFRSF12A,ERBB4,PTMA,ZNF385A,TXNDC12,NSMF,CDC34	yellow	VSMC_M3
GO:0009306	0.81	0.26	15	266	protein secretion	BP	1	PPARD,EIPR1,PDIA4,RCN3,MYRIP,UCP2,AACS,PCK2,CAPN10,PTPN23,BAD,GLUD1,MIDN,JAGN1,CAVIN1	yellow	VSMC_M3
GO:0035592	0.81	0.26	15	266	establishment of protein localization to extracellular region	BP	1	PPARD,EIPR1,PDIA4,RCN3,MYRIP,UCP2,AACS,PCK2,CAPN10,PTPN23,BAD,GLUD1,MIDN,JAGN1,CAVIN1	yellow	VSMC_M3
GO:0006281	0.82	0.24	27	455	DNA repair	BP	1	HDAC10,FANCE,FIGNL1,PNKP,MUTYH,DMAP1,OGG1,TTC5,FAAP100,SMUG1,TNKS1BP1,MCRS1,ACTR5,CEP164,KAT5,OTUB1,BABAM1,MAD2L2,PPP4C,SLX1A,USP43,CXorf57,SIRT6,NABP2,FBXO6,STUB1,NSMCE3	yellow	VSMC_M3
GO:0032879	0.82	0.21	129	2074	regulation of localization	BP	1	ENPP1,CD63,NHP2,RUFY1,SQSTM1,SREBF2,H2AFY,ITPR3,PACSIN1,PPARD,TSTA3,SCRIB,VPS28,PDGFRA,IL1R1,KLF4,WDPCP,EIPR1,ZC3H12A,SNX3,MICAL1,EHD2,SLC9A3R1,PLXND1,EMILIN1,DMAP1,SLC30A3,SNX17,INSIG1,MYRIP,UCP2,CEP131,LAMTOR1,NUMA1,AACS,SCARB1,BCL2L1,PDE4D,SWAP70,ACSL1,PINX1,GREM1,MCRS1,CX3CL1,SOD1,PACSIN3,DLL4,CHP1,ST3GAL4,SELENOS,GSTO1,RIN3,FABP3,NKAIN1,PDGFA,MMP14,CORO1B,SYT12,PCK2,TRIM27,ARPIN,PCNT,SCN4A,SLC26A6,SPX,RIPK3,TWIST2,CAPN10,TNK2,TACO1,PLA2G3,PTPN23,RHOC,ZDHHC3,CHCHD10,YJEFN3,PHB2,IGFBP5,BAD,AAMP,LETM1,LGALS3,CD9,PLAU,TNFRSF1A,C1QBP,CHERP,WASHC1,RCC2,MTA2,GLUD1,ELP5,PKN1,DBN1,LMAN2,MIIP,MAD1L1,ARHGEF2,SNF8,PER1,RANGRF,STX10,CORO1A,TRIM25,EEF2K,CSK,ATG3,RBM22,AKT1,ALKBH7,GIT1,ANKRD13B,SLC6A4,MAP2K2,AP3D1,MIDN,STK11,GAS8,TGFBR3,TRADD,MIR214,ERBB4,PTPRR,PLA2G4E,JAGN1,CAVIN1,NSMF,GLIPR2,GSG1L	yellow	VSMC_M3
GO:0010817	0.82	0.25	19	331	regulation of hormone levels	BP	1	PPARD,PDGFRA,EIPR1,MYRIP,UCP2,AACS,SCARB1,DHCR7,PCK2,CAPN10,PLA2G3,SGPL1,BAD,HSD17B1,GLUD1,CRABP2,MIDN,JAGN1,STUB1	yellow	VSMC_M3
GO:0002221	0.82	0.30	7	134	pattern recognition receptor signaling pathway	BP	1	TYRO3,RELA,PHB2,IFI35,C1QBP,CACTIN,IRAK1	yellow	VSMC_M3
GO:0048666	0.82	0.23	49	877	neuron development	BP	1	CTHRC1,PBX2,PACSIN1,SCRIB,LGALS1,IFT27,CUL7,SECISBP2,KLF4,DBNL,TPBG,WDPCP,SNX3,SLC9A3R1,PLXND1,UNK,BAIAP2,ARHGAP33,SLC11A2,CX3CL1,SOD1,RAB35,NGEF,IGSF9,NECTIN1,LIMK1,PLA2G3,STMN3,PBX1,SCYL1,PBX4,BLOC1S4,C12orf57,CNP,DBN1,CRABP2,S100A6,CARM1,EEF2K,AKT1,GIT1,ACAP3,ANAPC2,STK11,NEGR1,TWF2,UNC13A,NSMF,GBA2	yellow	VSMC_M3
GO:0051234	0.82	0.20	222	3429	establishment of localization	BP	1	ENPP1,PQLC1,CD63,ESYT1,DCTN2,RUFY1,SQSTM1,CCHCR1,BAG6,SNX9,SAMM50,SREBF2,H2AFY,ITPR3,SGSM3,GUK1,PACSIN1,PPARD,SCRIB,IFT27,SLC52A2,VPS28,TOM1,EPN1,SSC5D,ASNA1,DBNL,PLEKHA8,WDPCP,EIPR1,ZC3H12A,PDIA4,RCN3,SNX3,MICAL1,EHD2,TTYH2,SLC9A3R1,SERP2,SLC16A5,DMAP1,SLC30A3,SNX17,INSIG1,MYRIP,SEC13,UCP2,CEP131,INPPL1,TSPAN10,LAMTOR1,NUMA1,YIF1B,RAB20,ARAP3,SIGLEC1,ARHGAP33,LDLRAD3,AACS,SCARB1,HM13,BCL2L1,PDE4D,RRS1,LRP3,UQCRFS1,ACSL1,SLC11A2,PINX1,GREM1,AQP5,DCTN1,RAPSN,CX3CL1,SOD1,PACSIN3,SFXN5,DNAJC17,HPS1,CHP1,TYRO3,AP2A2,SNX21,HTATIP2,RAB35,SLC35C2,RBM15B,APBA2,SELENOS,GSTO1,SLC16A14,EXOC3,RIN3,FABP3,NKAIN1,PABPN1,AIP,AP1G2,SYT12,PCK2,SLC19A1,NECTIN1,TRIM27,PCNT,LIMK1,SCN4A,SLC26A6,NUDC,SPX,THOC5,UQCRC1,CAPN10,RHCE,TNK2,TACO1,PLA2G3,PTPN23,MRC2,ATP5MC3,TMEM120A,SCYL1,LRWD1,VPS51,ZFPL1,PEX5,ZDHHC3,CHCHD10,YJEFN3,THOC3,BLOC1S4,PHB2,BAD,COPE,SCARF2,LETM1,LGALS3,CD9,TNFRSF1A,FCHO1,MVB12A,NUP88,CHERP,ANXA11,WASHC1,RCC2,CNP,AP1M1,EML3,RAB3IL1,CCDC22,GLUD1,SNX8,LMAN2,RAB24,MAD1L1,PIP4K2B,CLTB,TMEM79,ARHGEF2,SNF8,MVP,PER1,MTX1,RANGRF,STX10,CORO1A,SGF29,RABEP2,SLC39A1,ATP5PD,SLC27A3,S100A6,TMED1,SYNE3,EEF2K,CDC37,MYO19,CSK,ATG3,RBM22,AKT1,SNX18,DENND1C,KHSRP,ALKBH7,RANBP3,RAB34,GIT1,ANKRD13B,SLC6A4,MAP2K2,SLC35E2B,CTNS,AP3D1,CSNK1G2,SSNA1,NDUFS7,MIDN,ATP5F1D,STK11,TMEM201,SPG7,APRT,WDR34,SLC27A4,SLC44A3,RABEPK,NABP2,RBM8A,ERBB4,SURF1,TRAPPC5,PLA2G4E,[illegible]	yellow	VSMC_M3

GO:0000165	0.82	0.23	34	579	MAPK cascade	BP	1	NELFE,PDGFRA,KLF4,TPBG,CCM2,SPRED2,ZC3H12A,SLC9A3R1,EMILIN1,LAMTOR1,CAVIN3,CX3CL1,SOD1,PDGFA,PABPN1,QARS,EIF3A,MAP3K14,PHB2,PER1,CSK,AKT1,MYDGF,MAP2K2,IRAK1,CARD9,NDST1,ERBB4,PTPRR,IGFBP6,CDC42EP5,DUSP5,GLIPR2,RCE1	yellow	VSMC_M3
GO:0070887	0.82	0.21	141	2230	cellular response to chemical stimulus	BP	1	ENPP1,CD63,CDK4,BAG6,ATF6B,TTLL12,GUK1,PPARD,RPL3,PLEC,LGALS1,SHARPIN,PDGFRA,IL1R1,SOCS5,KLF4,TPBG,SPRED2,ZC3H12A,ZYX,KRT8,RELB,SLC9A3R1,SERP2,EMILIN1,INSIG1,OGG1,UCP2,METTL3,BAIAP2,LAMTOR1,NUMA1,P4HB,PYCR1,FASN,RAB20,SMOX,EIF4EBP1,GSR,AACS,SCARB1,BCL2L1,TNS2,SWAP70,ACSL1,GREM1,DOK1,AQP5,PDE3B,CX3CL1,WNT10B,SOD1,ANKRD2,EGR3,DLL4,LSP1,RAB35,CHCHD2,DCPS,SELENOS,GSTO1,RIN3,PABPN1,GPX1,AIP,CORO1B,CLCF1,SYT12,PCK2,COL6A1,IMPDH2,SLC26A6,PRMT2,RIPK3,CAPN10,KAT5,RELA,SIPA1,MGST3,SMARCB1,ATIC,TXNRD2,PHB2,IGFBP5,BAD,LAMTOR4,OTUB1,HSD17B1,LETM1,LGALS3,TNFRSF1A,C1QBP,PRXL2A,PELP1,KLF3,PKN1,CAPN2,PALM3,PIP4K2B,ARHGEF2,PER1,CORO1A,TRIM25,MTMR4,SMARCA4,CARM1,EEF2K,CDC37,CSK,RBM22,AKT1,HMGCS1,KHSRP,SAFB,SAFB2,GIT1,SLC6A4,LDOC1,CACTIN,VASN,TRAP1,MKNK2,IRAK1,STK11,APRT,SLC27A4,TGFBR3,TRADD,MIR214,RF00024,NDST1,ERBB4,TWF2,JUND,JAGN1,NSMF,CDC34,OSER1,STUB1,NSMCE3,CCDC115	yellow	VSMC_M3
GO:0009566	0.82	0.30	6	113	fertilization	BP	1	SPAG1,B4GALT1,GNPDA1,BCL2L1,CD9,IGSF8	yellow	VSMC_M3
GO:0071248	0.82	0.29	7	141	cellular response to metal ion	BP	1	OGG1,SOD1,SYT12,HSD17B1,EEF2K,AKT1,JUND	yellow	VSMC_M3
GO:0060562	0.82	0.26	14	269	epithelial tube morphogenesis	BP	1	CTHRC1,SCRIB,CCM2,PLXND1,DEAF1,GREM1,KDM2B,DLL4,IRX1,MMP14,PBX1,PHB2,MTHFR,SIRT6	yellow	VSMC_M3
GO:0031023	0.82	0.30	6	127	microtubule organizing center organization	BP	1	CEP131,CCDC57,NAT10,DCTN1,SAC3D1,KIAA0753	yellow	VSMC_M3
GO:0035150	0.82	0.31	5	100	regulation of tube size	BP	1	PPARD,SOD1,GPX1,AKT1,SLC6A4	yellow	VSMC_M3
GO:0035296	0.82	0.31	5	100	regulation of tube diameter	BP	1	PPARD,SOD1,GPX1,AKT1,SLC6A4	yellow	VSMC_M3
GO:0097746	0.82	0.31	5	100	blood vessel diameter maintenance	BP	1	PPARD,SOD1,GPX1,AKT1,SLC6A4	yellow	VSMC_M3
GO:0051173	0.82	0.20	148	2331	positive regulation of nitrogen compound metabolic process	BP	1	NAB2,CDK4,SQSTM1,BAG6,SNX9,HDAC10,NELFE,FNIP1,ATF6B,PBX2,PRR5,SREBF2,SGSM3,PPARD,VPS28,PHF2,HSPBP1,SOCS5,RPS9,CNOT3,KLF4,HOXA1,ZC3H12A,PNKP,RCN3,SNRNP70,KMT2E,WDFY2,SIX5,ZNF296,SERTAD1,TTC5,PSMC4,METTL3,DND1,DEAF1,HOXC4,KMT2B,NAT10,ACSL1,ZBTB38,TNKS1BP1,GREM1,MCRS1,CX3CL1,WNT10B,PACSIN3,EGR3,CCAR2,CHP1,YAF2,HTATIP2,PCIF1,KDM1B,CHCHD2,RNF19B,DCPS,RRP1B,PDGFA,MMP14,AIP,CLCF1,ASB9,PSME2,TRIM27,PRMT2,THOC5,RIPK3,ADRM1,SMARCD2,BRMS1,BUD23,TNK2,GMEB2,PRPF6,PBX1,KAT5,RELA,ARNT2,SMARCB1,CHCHD10,PBX4,UBTF,MEF2B,PHB2,TRMT112,NDP,BAD,SSBP4,MPV17L2,BUD31,TNFRSF1A,BABAM1,C1QBP,AIMP2,TAF6L,ZBTB49,MTA2,PELP1,CCDC22,FOXK1,PKN1,CAPN2,POLR2A,AMER1,MAD2L2,NANOS3,CTBP1,ARHGEF2,SNF8,PER1,NFIX,SLX1A,IRF2BPL,TRIM25,SMARCA4,CARM1,AATF,CSK,MID2,RBM22,ZNF521,AKT1,BRF1,PAIP1,KHSRP,GTF2F1,MYDGF,SIRT6,MAP2K2,AURKAIP1,AP3D1,IRAK1,STK11,TELO2,TAZ,MED16,CARD9,POMT1,NABP2,ERBB4,JUND,KLF13,TRIB2,NSMF,IER5,STUB1,NSMCE3	yellow	VSMC_M3
GO:0032990	0.82	0.23	28	536	cell part morphogenesis	BP	1	PACSIN1,PLEC,CUL7,DBNL,TPBG,PLXND1,BAIAP2,ARHGAP33,BCL2L1,SLC11A2,CORO1B,NGEF,PLEKHO1,IGSF9,NECTIN1,ARPIN,LIMK1,C12orf57,CNP,DBN1,CRABP2,S100A6,EEF2K,ANAPC2,STK11,TWF2,UNC13A,NSMF	yellow	VSMC_M3
GO:0032101	0.82	0.22	39	646	regulation of response to external stimulus	BP	1	TTLL12,PPARD,SHARPIN,PDGFRA,IL1R1,SOCS5,KLF4,TPBG,ZC3H12A,ZYX,MGLL,METTL3,TARBP2,SWAP70,GREM1,CX3CL1,SOD1,TYRO3,ST3GAL4,SELENOS,MVK,RIN3,PDGFA,GPX1,CORO1B,SPX,PSMB4,PLA2G3,RELA,IFI35,CD9,PLAU,TNFRSF1A,C1QBP,CDC37,GIT1,CACTIN,CARD9,TRADD	yellow	VSMC_M3
GO:0031647	0.82	0.25	14	249	regulation of protein stability	BP	1	BAG6,PINX1,WNT10B,CCAR2,CHP1,TMEM183A,LSS,PHB2,USP27X,DPM3,CDC37,NCLN,TELO2,STUB1	yellow	VSMC_M3
GO:0001906	0.82	0.33	4	78	cell killing	BP	1	BCL2L1,RNF19B,RIPK3,CORO1A	yellow	VSMC_M3
GO:0001817	0.82	0.23	29	492	regulation of cytokine production	BP	1	SCRIB,IL1R1,SSC5D,SOCS5,KLF4,ZC3H12A,RELB,PDE4D,CX3CL1,SOD1,SELENOS,TRIM27,RIPK3,PLA2G3,KAT5,RELA,C1QBP,CAPN2,ARHGEF2,PER1,CD276,CSK,GIT1,CACTIN,IRAK1,CARD9,SARS,MIR155,TRIB2	yellow	VSMC_M3
GO:1904019	0.82	0.33	4	86	epithelial cell apoptotic process	BP	1	KRT8,BCL2L1,CAPN10,BAD	yellow	VSMC_M3
GO:0051403	0.83	0.27	10	190	stress-activated MAPK cascade	BP	1	CCM2,ZC3H12A,QARS,PER1,AKT1,MAP2K2,IRAK1,CARD9,IGFBP6,CDC42EP5	yellow	VSMC_M3
GO:0050806	0.83	0.31	5	104	positive regulation of synaptic transmission	BP	1	SQSTM1,ITPR3,RAPSN,SYT12,ZDHHC3	yellow	VSMC_M3
GO:0032231	0.83	0.32	4	92	regulation of actin filament bundle assembly	BP	1	SWAP70,CX3CL1,LIMK1,RHOC	yellow	VSMC_M3
GO:0042445	0.83	0.28	7	133	hormone metabolic process	BP	1	PDGFRA,SCARB1,DHCR7,SGPL1,HSD17B1,CRABP2,STUB1	yellow	VSMC_M3
GO:0035295	0.83	0.21	49	849	tube development	BP	1	BAG6,CTHRC1,SCRIB,CUL7,B4GALT1,PDGFRA,COL15A1,EPN1,KLF4,HOXA1,CCM2,ZC3H12A,RCN3,PLXND1,EMILIN1,RNF220,EIF4EBP1,ARHGAP22,DEAF1,GREM1,KDM2B,PDE3B,CX3CL1,EGR3,DLL4,HTATIP2,IRX1,PDGFA,MMP14,GPX1,ANPEP,PBX1,SGPL1,YJEFN3,PHB2,IGFBP5,AAMP,AIMP2,MTHFR,ZNF358,AKT1,MYDGF,SIRT6,JMJD8,SARS,TGFBR3,MIR214,TNFRSF12A,RNH1	yellow	VSMC_M3
GO:0048762	0.83	0.26	10	198	mesenchymal cell differentiation	BP	1	CUL7,SPRED2,GREM1,AMER1,MAD2L2,S100A4,VASN,TGFBR3,ERBB4,GLIPR2	yellow	VSMC_M3
GO:0071692	0.83	0.24	15	271	protein localization to extracellular region	BP	1	PPARD,EIPR1,PDIA4,RCN3,MYRIP,UCP2,AACS,PCK2,CAPN10,PTPN23,BAD,GLUD1,MIDN,JAGN1,CAVIN1	yellow	VSMC_M3

GO:0002009	0.83	0.23	22	412	morphogenesis of an epithelium	BP	1	CTHRC1,SCRIB,KLF4,CCM2,PLEKHA4,SLC9A3R1,PLXND1,DEAF1,GREM1,KDM2B,DLL4,IRX1,PDGFA,MMP14,PBX1,RHOC,PHB2,IGFBP5,MTHFR,TMEM79,SIRT6,ERBB4	yellow	VSMC_M3
GO:0040017	0.83	0.23	26	462	positive regulation of locomotion	BP	1	TSTA3,PDGFRA,IL1R1,TPBG,ZC3H12A,SCARB1,SWAP70,CX3CL1,PDGFA,MMP14,TWIST2,RHOC,IGFBP5,AAMP,LGALS3,PLAU,C1QBP,WASHC1,ELP5,ARHGEF2,CORO1A,AKT1,TRADD,CAVIN1,NSMF,GLIPR2	yellow	VSMC_M3
GO:0051650	0.83	0.28	7	143	establishment of vesicle localization	BP	1	DCTN2,SCRIB,CHP1,BLOC1S4,MYO19,AP3D1,STK11	yellow	VSMC_M3
GO:0023052	0.83	0.19	271	4258	signaling	BP	1	ENPP1,CD63,CDK4,RUFY1,SQSTM1,GNL1,BAG6,CTHRC1,NELFE,FNIP1,ATF6B,PDLIM4,PRR5,TTLL12,SREBF2,TXNDC15,ITPR3,SGSM3,NUDT3,DEF6,PPARD,EEF1D,SCRIB,PLEC,LGALS1,IFT27,SHARPIN,BOP1,TOM1,PDGFRA,IL1R1,COL15A1,EPN1,SOCS5,KLF4,DBNL,TPBG,WDPCP,CCM2,SPRED2,EIPR1,RGS3,ZC3H12A,FIGNL1,ZYX,RCN3,SNX3,MICAL1,PLEKHA4,MGLL,FAM120B,ANXA5,KRT8,RELB,SLC9A3R1,SERP2,PLXND1,EMILIN1,RNF220,SNX17,PLCD1,CD79A,INSIG1,MYRIP,SEC13,ARHGEF40,UCP2,METTL3,BAIAP2,CXXC5,LAMTOR1,NUMA1,P4HB,EIF4EBP1,ARAP3,DELE1,CAVIN3,RIC8A,ARHGAP22,ARHGAP33,AACS,SCARB1,BCL2L1,ITGB7,PDE4D,SPRYD3,TNS2,RRS1,SWAP70,PLEKHF1,ACSL1,GREM1,DOK1,PDE3B,RAPSN,CX3CL1,WNT10B,SOD1,DGKZ,EGR3,CCAR2,DLL4,CHP1,TYRO3,LSP1,RAB35,SLC35C2,TSPAN5,CISH,APBA2,SELENOS,GSTO1,ITPRIP,RIN3,STK32C,PDGFA,MMP14,PABPN1,GPX1,AIP,NGEF,CLCF1,SYT12,QARS,PCK2,ASB9,ANPEP,TRIM27,PCNT,EIF3A,LIMK1,SCN4A,PRMT2,SPX,THOC5,RIPK3,CAPN10,TNK2,PLA2G3,STMN3,PTPN23,KAT5,RELA,SIPA1,RHOC,MAP3K14,LIMD1,ZDHHC3,SGPL1,SMARCB1,CHCHD10,ZNF385B,YJEFN3,GNB2,PHB2,NDP,IGFBP5,BAD,PLCB3,LAMTOR4,IFI35,SH3BP2,LGALS3,BUD31,PLAU,TNFRSF1A,MVB12A,BABAM1,TEAD4,C1QBP,CHERP,EVC,RCC2,CNP,CCDC22,GLUD1,SDE2,PKN1,DBN1,MAD1L1,PALM3,AMER1,PIP4K2B,MAD2L2,NANOS3,TBKBP1,CRABP2,ARHGEF2,MVP,PER1,RANGRF,PPP4C,CORO1A,RABEP2,TRIM25,RPL37,MTMR4,S100A4,S100A6,SMARCA4,CARM1,TMED1,CDC37,CD276,AATF,CSK,MID2,DOCK11,ZNF830,AKT1,TEDC1,RAB34,SAFB,SAFB2,GIT1,SLC6A4,DHCR24,CARHSP1,MYDGF,MAP2K2,NSDHL [illegible]	yellow	VSMC_M3
GO:0043408	0.83	0.22	29	502	regulation of MAPK cascade	BP	1	NELFE,PDGFRA,KLF4,TPBG,SPRED2,ZC3H12A,SLC9A3R1,EMILIN1,LAMTOR1,CAVIN3,CX3CL1,SOD1,PDGFA,QARS,EIF3A,PHB2,PER1,CSK,AKT1,MYDGF,MAP2K2,IRAK1,CARD9,NDST1,ERBB4,PTPRR,IGFBP6,DUSP5,GLIPR2	yellow	VSMC_M3
GO:0030595	0.83	0.27	8	150	leukocyte chemotaxis	BP	1	PLEC,SWAP70,GREM1,CX3CL1,RIN3,LGALS3,C1QBP,CORO1A	yellow	VSMC_M3
GO:0023061	0.83	0.23	18	325	signal release	BP	1	PPARD,SCRIB,EIPR1,MYRIP,UCP2,AACS,SYT12,PCK2,CAPN10,PLA2G3,PTPN23,BAD,GLUD1,GIT1,SLC6A4,MIDN,JAGN1,UNC13A	yellow	VSMC_M3
GO:0050878	0.83	0.24	15	273	regulation of body fluid levels	BP	1	PLEC,PDGFRA,ANXA5,AQP5,DGKZ,TYRO3,ST3GAL4,PDGFA,BLOC1S4,CD9,PLAU,TMEM79,MTX1,APRT,ERBB4	yellow	VSMC_M3
GO:0031398	0.83	0.29	5	102	positive regulation of protein ubiquitination	BP	1	HSPBP1,AIMP2,AMER1,STUB1,NSMCE3	yellow	VSMC_M3
GO:0051094	0.84	0.20	58	982	positive regulation of developmental process	BP	1	CTHRC1,H2AFY,PACSIN1,PPARD,CUL7,SOCS5,KLF4,DBNL,TPBG,ZC3H12A,EHD2,WDFY2,PLXND1,BAIAP2,NUMA1,P4HB,LRP3,GREM1,KDM2B,CX3CL1,WNT10B,EGR3,MMP14,CORO1B,CLCF1,LIMK1,PLA2G3,SART1,RELA,BAD,TNFRSF1A,C1QBP,KRT10,DBN1,CAPN2,MAD2L2,CRABP2,TMEM79,ARHGEF2,CARM1,EEF2K,AKT1,MYDGF,SIRT6,HMG20B,AP3D1,ANAPC2,STK11,JMJD8,TGFBR3,TRADD,MIR214,ERBB4,TWF2,JUND,UNC13A,ZNF385A,GLIPR2	yellow	VSMC_M3
GO:0031032	0.84	0.26	8	172	actomyosin structure organization	BP	1	PLEC,PDGFRA,ZYX,KRT8,LIMK1,RHOC,ALKBH4,ELN	yellow	VSMC_M3
GO:0099003	0.84	0.27	7	151	vesicle-mediated transport in synapse	BP	1	PACSIN1,SCRIB,SYT12,GIT1,AP3D1,UNC13A,GSG1L	yellow	VSMC_M3
GO:0031325	0.84	0.19	157	2483	positive regulation of cellular metabolic process	BP	1	CD63,NAB2,CDK4,SQSTM1,BAG6,SNX9,HDAC10,NELFE,FNIP1,ATF6B,PBX2,PRR5,SREBF2,PPARD,SCRIB,PDGFRA,PHF2,HSPBP1,SOCS5,RPS9,CNOT3,KLF4,HOXA1,ZC3H12A,PNKP,RCN3,SNRNP70,KMT2E,WDFY2,SIX5,ZNF296,SERTAD1,TTC5,PSMC4,METTL3,DND1,DEAF1,HOXC4,KMT2B,SCARB1,NAT10,PLEKHF1,ACSL1,ZBTB38,TNKS1BP1,GREM1,MCRS1,CX3CL1,WNT10B,SOD1,PACSIN3,DGKZ,EGR3,CCAR2,CHP1,TYRO3,YAF2,HTATIP2,PCIF1,KDM1B,CHCHD2,RNF19B,DCPS,RRP1B,FABP3,PDGFA,MMP14,AIP,CLCF1,PSME2,TRIM27,PRMT2,THOC5,RIPK3,ADRM1,SMARCD2,BRMS1,BUD23,TNK2,PLA2G3,GMEB2,PRPF6,PBX1,KAT5,RELA,ARNT2,MID1IP1,SMARCB1,CHCHD10,PBX4,UBTF,MEF2B,PHB2,TRMT112,NDP,BAD,SSBP4,MPV17L2,BUD31,TNFRSF1A,BABAM1,C1QBP,AIMP2,TAF6L,ZBTB49,MTA2,PELP1,CCDC22,FOXK1,PKN1,CAPN2,POLR2A,AMER1,PIP4K2B,MAD2L2,NANOS3,CTBP1,ARHGEF2,PER1,NFIX,SLX1A,IRF2BPL,TRIM25,SMARCA4,CARM1,AATF,CSK,MID2,RBM22,ZNF521,AKT1,BRF1,PAIP1,KHSRP,GTF2F1,MYDGF,SIRT6,MAP2K2,AURKAIP1,AP3D1,IRAK1,STK11,TELO2,TAZ,MED16,CARD9,POMT1,VAC14,NABP2,ERBB4,JUND,KLF13,TRIB2,NSMF,IER5,STUB1,NSMCE3	yellow	VSMC_M3
GO:2000027	0.84	0.29	5	108	regulation of animal organ morphogenesis	BP	1	CTHRC1,PLEKHA4,GREM1,PDGFA,PHB2	yellow	VSMC_M3
GO:0046942	0.84	0.26	9	173	carboxylic acid transport	BP	1	SLC9A3R1,SLC16A5,SFXN5,SLC16A14,SLC19A1,SLC26A6,PLA2G3,CTNS,SLC27A4	yellow	VSMC_M3
GO:0030278	0.84	0.31	4	91	regulation of ossification	BP	1	ENPP1,GREM1,WNT10B,PBX1	yellow	VSMC_M3
GO:0007059	0.84	0.23	16	288	chromosome segregation	BP	1	DCTN2,BAG6,NCAPH2,H2AFY,NUMA1,RRS1,PINX1,NUDC,BANF1,PHB2,RCC2,EML3,MAD1L1,MAD2L2,NTMT1,MIS12	yellow	VSMC_M3
GO:0032147	0.84	0.29	5	105	activation of protein kinase activity	BP	1	GREM1,RIPK3,PHB2,IRAK1,STK11	yellow	VSMC_M3
GO:0033135	0.84	0.28	5	104	regulation of peptidyl-serine phosphorylation	BP	1	FNIP1,PDE4D,TNKS1BP1,MAD2L2,AKT1	yellow	VSMC_M3

GO:0009888	0.84	0.19	82	1389	tissue development	BP	1	ENPP1,NAB2,CDK4,CTHRC1,H2AFY,PPARD,SCRIB,PLEC,SHARPIN,CUL7,DNPH1,B4GALT1,PDGFRA,COL15A1,COL19A1,KLF4,WDPCP,CCM2,SPRED2,RCN3,PLEKHA4,SLC9A3R1,PLXND1,RNF220,NUMA1,FASN,DEAF1,AACS,PDE4D,GREM1,KDM2B,WNT10B,SOD1,DLL4,IRX1,PDGFA,MMP14,GPX1,TAGLN2,PCK2,COL6A1,NECTIN1,PBX1,RELA,RHOC,PHB2,IGFBP5,BAD,LGALS3,TNFRSF1A,AIMP2,EVC,KRT10,CASP14,AMER1,MTHFR,MAD2L2,CRABP2,TMEM79,ARMC5,S100A4,ZNF358,AKT1,SAFB2,DHCR24,SIRT6,PPL,NSDHL,TJP3,HMG20B,VASN,MBD3,TAZ,GTF3C5,TGFBR3,TRADD,ERBB4,IGSF8,ELN,NOM1,DUSP5,GLIPR2	yellow	VSMC_M3
GO:0050896	0.84	0.17	384	5876	response to stimulus	BP	1	ENPP1,CD63,CDK4,RUFY1,SQSTM1,GNL1,BAG6,CTHRC1,HDAC10,NELFE,FNIP1,ATF6B,PRR5,TTLL12,SREBF2,SQLE,TXNDC15,H2AFY,ITPR3,HNRNPA0,TRIM11,SGSM3,GUK1,DEF6,PPARD,FANCE,EEF1D,RPL3,SCRIB,PLEC,LGALS1,MPST,IFT27,SHARPIN,BOP1,TOM1,PPP1R14B,B4GALT1,PDGFRA,IL1R1,COL15A1,EPN1,SSC5D,SOCS5,KLF4,DBNL,TPBG,HOXA1,PTGR1,WDPCP,CCM2,SPRED2,RGS3,ZC3H12A,FIGNL1,PDIA4,PNKP,ZYX,RCN3,SNX3,MICAL1,PLEKHA4,MGLL,FAM120B,ANXA5,KRT8,RELB,SLC9A3R1,SERP2,MUTYH,PLXND1,EMILIN1,TMUB1,RNF220,DMAP1,SLC30A3,SNX17,PLCD1,DLEC1,CD79A,INSIG1,SEC13,OGG1,TTC5,ARHGEF40,UCP2,SEL1L2,METTL3,BAIAP2,CXXC5,INPPL1,FAAP100,LAMTOR1,NUMA1,P4HB,PYCR1,FASN,RAB20,SMOX,EIF4EBP1,ARAP3,SIGLEC1,DELE1,CAVIN3,RIC8A,GSR,ARHGAP22,SMUG1,ARHGAP33,TARBP2,AACS,SCARB1,HM13,BCL2L1,ITGB7,PDE4D,SPRYD3,TNS2,RRS1,SWAP70,PLEKHF1,ACSL1,ZBTB38,SLC11A2,TNKS1BP1,MMP24,GREM1,DOK1,AQP5,PDE3B,MCRS1,CX3CL1,WNT10B,SOD1,ACTR5,ANKRD2,DGKZ,EGR3,CCAR2,HPS1,DLL4,CHP1,TYRO3,SCD,LSP1,RAB35,CHCHD2,SLC35C2,TSPAN5,CISH,SRSF9,ST3GAL4,RNF19B,DCPS,WDR4,SELENOS,GSTO1,ITPRIP,RRP1B,MVK,RIN3,STK32C,FABP3,PDGFA,MMP14,PABPN1,GPX1,AIP,CORO1B,NGEF,CLCF1,IGSF9,SYT12,QARS,PCK2,ASB9,COL6A1,IMPDH2,NECTIN1,ANPEP,TRIM27,PCNT,EIF3A,LIMK1,SLC26A6,PRMT2,SPX,THOC5,UQCRC1,RIPK3,CAPN10,PSMB4,PPOX,TNK2,PLA2G3,STMN3,CEP164,BANF1,KAT5,RELA,SIPA1,MGST3,RHOC,TMEM120A,SCYL1,IDH1,MAP3K14,ARNT2,LIMD1,ZDHHC3,SGPL1,SMARCB1,CHCHD10,ZNF385B,YJEFN3,GNB2,ATIC,BLOC1S4,TXNRD2,PHB[illegible]	yellow	VSMC_M3
GO:0010941	0.84	0.19	77	1223	regulation of cell death	BP	1	CDK4,SQSTM1,BAG6,FNIP1,PPARD,SCRIB,LGALS1,B4GALT1,KLF4,ZC3H12A,FIGNL1,MICAL1,ANXA5,SLC9A3R1,OGG1,UCP2,PHLDA1,P4HB,PYCR1,BCL2L1,PLEKHF1,GREM1,KDM2B,RAPSN,CX3CL1,WNT10B,SOD1,EGR3,CCAR2,CHP1,TYRO3,HTATIP2,DCPS,SELENOS,ITPRIP,RRP1B,GPX1,CLCF1,QARS,PRMT2,RIPK3,TWIST2,CAPN10,BRMS1,PLA2G3,RELA,ARNT2,PHB2,BAD,LGALS3,TNFRSF1A,C1QBP,AIMP2,USP27X,CAPN2,NANOS3,DFFA,ARHGEF2,CORO1A,EEF2K,AATF,ZNF830,AKT1,DHCR24,MYDGF,TRAP1,MNT,CARD9,SLC27A4,TRADD,MIR214,TNFRSF12A,PTMA,ZNF385A,TXNDC12,NSMF,CDC34	yellow	VSMC_M3
GO:0045926	0.84	0.24	10	195	negative regulation of growth	BP	1	ENPP1,PPARD,GREM1,CCAR2,CCDC85B,SIPA1,IGFBP5,SMARCA4,SLC6A4,STK11	yellow	VSMC_M3
GO:0007173	0.84	0.30	4	90	epidermal growth factor receptor signaling pathway	BP	1	SOCS5,MVB12A,MVP,AKT1	yellow	VSMC_M3
GO:0007093	0.84	0.27	6	117	mitotic cell cycle checkpoint signaling	BP	1	BABAM1,SDE2,MAD1L1,MAD2L2,ZNF830,NABP2	yellow	VSMC_M3
GO:0002697	0.84	0.24	11	206	regulation of immune effector process	BP	1	IL1R1,SOCS5,ZC3H12A,CLCF1,RIPK3,PLA2G3,PHB2,LGALS3,C1QBP,PKN1,MAD2L2	yellow	VSMC_M3
GO:0050821	0.84	0.25	8	154	protein stabilization	BP	1	BAG6,WNT10B,CHP1,PHB2,USP27X,CDC37,NCLN,TELO2	yellow	VSMC_M3
GO:0051304	0.85	0.29	4	85	chromosome separation	BP	1	NCAPH2,NUMA1,MAD1L1,MAD2L2	yellow	VSMC_M3
GO:0055067	0.85	0.28	5	108	monovalent inorganic cation homeostasis	BP	1	RAB20,CHP1,SLC26A6,SPX,CCDC115	yellow	VSMC_M3
GO:0014070	0.85	0.19	41	703	response to organic cyclic compound	BP	1	CDK4,PPARD,LGALS1,KLF4,ZC3H12A,SLC9A3R1,INSIG1,OGG1,AACS,BCL2L1,ACSL1,WNT10B,SOD1,MMP14,PCK2,SLC26A6,PRMT2,KAT5,RELA,IDH1,ARNT2,PHB2,IGFBP5,BAD,ARPC1B,PKN1,MTHFR,PER1,PMVK,TRIM25,SMARCA4,CARM1,EEF2K,AKT1,HMGCS1,SAFB,SAFB2,SLC6A4,MBD3,CARD9,JUND	yellow	VSMC_M3
GO:0001932	0.85	0.19	50	845	regulation of protein phosphorylation	BP	1	ENPP1,NAB2,CTDSP2,SQSTM1,SNX9,FNIP1,PRR5,H2AFY,PLEC,SOCS5,SPRED2,ZC3H12A,MICAL1,WDFY2,SLC9A3R1,EMILIN1,SERTAD1,TARBP2,PDE4D,ACSL1,TNKS1BP1,GREM1,CX3CL1,SOD1,CHP1,PDGFA,CLCF1,QARS,TRIM27,RIPK3,TNK2,PHB2,CTDSP1,TNFRSF1A,PKN1,MAD2L2,ARHGEF2,MVP,EEF2K,CDC37,CSK,AKT1,MYDGF,MAP2K2,CACTIN,IRAK1,STK11,TELO2,ERBB4,TRIB2	yellow	VSMC_M3
GO:0051272	0.85	0.20	26	466	positive regulation of cellular component movement	BP	1	TSTA3,PDGFRA,IL1R1,ZC3H12A,SCARB1,SWAP70,CX3CL1,PDGFA,MMP14,TWIST2,RHOC,IGFBP5,AAMP,LGALS3,PLAU,C1QBP,WASHC1,ELP5,ARHGEF2,CORO1A,AKT1,STK11,TRADD,CAVIN1,NSMF,GLIPR2	yellow	VSMC_M3
GO:0022414	0.85	0.18	63	1029	reproductive process	BP	1	SPAG1,BAG6,NCAPH2,SBF1,PPARD,IFT27,CUL7,B4GALT1,PDGFRA,EPN1,FIGNL1,KRT8,SIX5,ZNF296,PAFAH1B3,DLEC1,UCP2,METTL3,CEP131,NUMA1,DND1,YIF1B,RPL29,GNPDA1,MORN2,DEAF1,BCL2L1,KDM2B,SOD1,TYRO3,KDM1B,MMP14,SLC19A1,TRIM27,SLC26A6,IQCG,PLA2G3,PBX1,IDH1,SGPL1,PHB2,IGFBP5,BAD,CD9,C1QBP,CAPN2,NANOS3,IRF2BPL,ZNF830,AKT1,HMGCS1,RFX2,SAFB2,SLC6A4,DHCR24,NSDHL,STK11,TAZ,FAM50A,GAS8,IGSF8,TUBGCP2,MEIG1	yellow	VSMC_M3

GO:0051247	0.85	0.18	70	1140	positive regulation of protein metabolic process	BP	1	NAB2,CDK4,SQSTM1,BAG6,SNX9,NELFE,FNIP1,PRR5,SGSM3,VPS28,HSPBP1,SOCS5,RPS9,KLF4,ZC3H12A,RCN3,KMT2E,WDFY2,PSMC4,METTL3,NAT10,ACSL1,TNKS1BP1,GREM1,CX3CL1,PACSIN3,CHP1,PCIF1,RNF19B,PDGFA,MMP14,CLCF1,ASB9,PSME2,RIPK3,ADRM1,BRMS1,TNK2,KAT5,RELA,SMARCB1,PHB2,BAD,MPV17L2,TNFRSF1A,C1QBP,AIMP2,CCDC22,AMER1,MAD2L2,CTBP1,ARHGEF2,SNF8,CSK,AKT1,PAIP1,MYDGF,SIRT6,MAP2K2,AURKAIP1,IRAK1,STK11,TELO2,CARD9,POMT1,ERBB4,TRIB2,NSMF,STUB1,NSMCE3	yellow	VSMC_M3
GO:0006887	0.85	0.22	14	266	exocytosis	BP	1	SCRIB,MICAL1,EXOC3,SYT12,PLA2G3,WASHC1,RAB3IL1,TMEM79,SNF8,CORO1A,GIT1,RABEPK,JAGN1,UNC13A	yellow	VSMC_M3
GO:0006950	0.85	0.17	175	2715	response to stress	BP	1	CDK4,SQSTM1,GNL1,BAG6,HDAC10,FNIP1,ATF6B,TTLL12,SREBF2,H2AFY,HNRNPA0,TRIM11,PPARD,FANCE,SCRIB,PLEC,LGALS1,SHARPIN,PPP1R14B,B4GALT1,PDGFRA,IL1R1,SSC5D,SOCS5,KLF4,CCM2,SPRED2,ZC3H12A,FIGNL1,PDIA4,PNKP,ZYX,RCN3,MGLL,ANXA5,KRT8,RELB,SERP2,MUTYH,TMUB1,DMAP1,DLEC1,INSIG1,OGG1,TTC5,UCP2,SEL1L2,METTL3,FAAP100,P4HB,PYCR1,RAB20,EIF4EBP1,SIGLEC1,GSR,SMUG1,TARBP2,AACS,SCARB1,HM13,BCL2L1,ZBTB38,SLC11A2,TNKS1BP1,AQP5,MCRS1,CX3CL1,SOD1,ACTR5,ANKRD2,DGKZ,CCAR2,TYRO3,LSP1,CHCHD2,ST3GAL4,RNF19B,WDR4,SELENOS,MVK,PDGFA,MMP14,GPX1,CORO1B,QARS,PCK2,TRIM27,SLC26A6,THOC5,RIPK3,PSMB4,TNK2,PLA2G3,CEP164,KAT5,RELA,SIPA1,RHOC,SCYL1,IDH1,MAP3K14,ARNT2,LIMD1,BLOC1S4,TXNRD2,PHB2,BAD,IFI35,OTUB1,LETM1,LGALS3,CD9,PLAU,TNFRSF1A,BABAM1,C1QBP,UBXN1,FOXK1,SDE2,PKN1,CAPN2,MTHFR,MAD2L2,TBKBP1,ARHGEF2,ARMC5,PER1,PPP4C,CORO1A,SLX1A,TRIM25,USP43,EEF2K,CDC37,AATF,CXorf57,C19orf66,MID2,ZNF830,AKT1,ALKBH7,GIT1,SLC6A4,DHCR24,SIRT6,PPL,MAP2K2,CACTIN,VASN,HIC1,TRAP1,IRAK1,MAN1B1,C8G,STK11,WDR24,CARD9,TGFBR3,TRADD,MIR214,RF00024,NDST1,NABP2,TNFRSF12A,FADS1,IGFBP6,JAGN1,ZNF385A,TXNDC12,CDC42EP5,FBXO6,IER5,OSER1,STUB1,NSMCE3	yellow	VSMC_M3
GO:0051047	0.85	0.23	11	211	positive regulation of secretion	BP	1	PPARD,MYRIP,AACS,PCK2,CAPN10,PLA2G3,PTPN23,BAD,GLUD1,SNF8,SLC6A4	yellow	VSMC_M3
GO:0030038	0.85	0.28	4	95	contractile actin filament bundle assembly	BP	1	ZYX,LIMK1,RHOC,ELN	yellow	VSMC_M3
GO:0043149	0.85	0.28	4	95	stress fiber assembly	BP	1	ZYX,LIMK1,RHOC,ELN	yellow	VSMC_M3
GO:0035725	0.85	0.27	5	120	sodium ion transmembrane transport	BP	1	SLC9A3R1,CHP1,SCN4A,RANGRF,SLC6A4	yellow	VSMC_M3
GO:0006928	0.85	0.17	92	1565	movement of cell or subcellular component	BP	1	CD63,CTHRC1,PPARD,TSTA3,SCRIB,PLEC,IFT27,B4GALT1,PDGFRA,IL1R1,KLF4,TPBG,WDPCP,ZC3H12A,SLC9A3R1,PLXND1,EMILIN1,UNK,CEP131,YIF1B,RIC8A,SCARB1,ITGB7,PDE4D,SWAP70,GREM1,CX3CL1,SOD1,DGKZ,EGR3,DLL4,TYRO3,ST3GAL4,RIN3,PDGFA,MMP14,GPX1,CORO1B,PLEKHO1,IGSF9,NECTIN1,ARPIN,RIPK3,TWIST2,IQCG,PLA2G3,PTPN23,RHOC,FMNL1,LIMD1,SGPL1,BLOC1S4,PHB2,IGFBP5,AAMP,LGALS3,CD9,PLAU,C1QBP,WASHC1,RCC2,MTA2,ELP5,PKN1,MIIP,ARHGEF2,RANGRF,CORO1A,MYO19,AKT1,ACAP3,AP3D1,SSNA1,STK11,TMEM201,GAS8,SPG7,WDR34,TGFBR3,TRADD,MIR214,ERBB4,PTPRR,IGFBP6,JAGN1,CAVIN1,IGSF8,NSMF,GLIPR2,TUBGCP2,MEIG1,ARPC5L	yellow	VSMC_M3
GO:0048589	0.85	0.20	27	501	developmental growth	BP	1	CDK4,PPARD,PLEC,DBNL,CCM2,TNS2,WNT10B,SOD1,GPX1,LIMK1,PRMT2,SGPL1,CD9,EVC,DBN1,CRABP2,SP2,ZNF830,AKT1,SLC6A4,SIRT6,ANAPC2,STK11,TGFBR3,ERBB4,TWF2,UNC13A	yellow	VSMC_M3
GO:0048284	0.85	0.26	6	120	organelle fusion	BP	1	RAB20,CHP1,PIP4K2B,STX10,CORO1A,RAB34	yellow	VSMC_M3
GO:0000041	0.85	0.28	4	86	transition metal ion transport	BP	1	SLC30A3,SLC11A2,NECTIN1,SLC39A1	yellow	VSMC_M3
GO:0015918	0.85	0.28	4	89	sterol transport	BP	1	SREBF2,LAMTOR1,SCARB1,YJEFN3	yellow	VSMC_M3
GO:0000003	0.85	0.18	63	1031	reproduction	BP	1	SPAG1,BAG6,NCAPH2,SBF1,PPARD,IFT27,CUL7,B4GALT1,PDGFRA,EPN1,FIGNL1,KRT8,SIX5,ZNF296,PAFAH1B3,DLEC1,UCP2,METTL3,CEP131,NUMA1,DND1,YIF1B,RPL29,GNPDA1,MORN2,DEAF1,BCL2L1,KDM2B,SOD1,TYRO3,KDM1B,MMP14,SLC19A1,TRIM27,SLC26A6,IQCG,PLA2G3,PBX1,IDH1,SGPL1,PHB2,IGFBP5,BAD,CD9,C1QBP,CAPN2,NANOS3,IRF2BPL,ZNF830,AKT1,HMGCS1,RFX2,SAFB2,SLC6A4,DHCR24,NSDHL,STK11,TAZ,FAM50A,GAS8,IGSF8,TUBGCP2,MEIG1	yellow	VSMC_M3
GO:0001816	0.85	0.19	29	504	cytokine production	BP	1	SCRIB,IL1R1,SSC5D,SOCS5,KLF4,ZC3H12A,RELB,PDE4D,CX3CL1,SOD1,SELENOS,TRIM27,RIPK3,PLA2G3,KAT5,RELA,C1QBP,CAPN2,ARHGEF2,PER1,CD276,CSK,GIT1,CACTIN,IRAK1,CARD9,SARS,MIR155,TRIB2	yellow	VSMC_M3
GO:1903322	0.85	0.26	6	121	positive regulation of protein modification by small protein conjugation or removal	BP	1	HSPBP1,ZC3H12A,AIMP2,AMER1,STUB1,NSMCE3	yellow	VSMC_M3

GO:0010033	0.85	0.17	143	2273	response to organic substance	BP	1	ENPP1,CD63,CDK4,BAG6,ATF6B,TTLL12,SREBF2,SQLE,HNRNPA0,PPARD,RPL3,LGALS1,SHARPIN,PDGFRA,IL1R1,SSC5D,SOCS5,KLF4,SPRED2,ZC3H12A,ZYX,RCN3,KRT8,RELB,SLC9A3R1,SERP2,EMILIN1,TMUB1,DMAP1,INSIG1,OGG1,UCP2,SEL1L2,METTL3,BAIAP2,INPPL1,LAMTOR1,NUMA1,P4HB,FASN,RAB20,EIF4EBP1,AACS,SCARB1,HM13,BCL2L1,TNS2,ACSL1,GREM1,DOK1,PDE3B,CX3CL1,WNT10B,SOD1,EGR3,DLL4,SCD,LSP1,RAB35,SRSF9,DCPS,SELENOS,FABP3,MMP14,PABPN1,CORO1B,CLCF1,PCK2,COL6A1,IMPDH2,SLC26A6,PRMT2,UQCRC1,CAPN10,KAT5,RELA,MGST3,IDH1,ARNT2,SMARCB1,ATIC,PHB2,IGFBP5,BAD,LAMTOR4,OTUB1,ARPC1B,CD9,TNFRSF1A,UBXN1,CNP,PELP1,KLF3,PKN1,CAPN2,PALM3,MTHFR,PIP4K2B,ARHGEF2,PER1,CORO1A,PMVK,TUFM,TRIM25,MTMR4,SMARCA4,CARM1,EEF2K,CDC37,C19orf66,CSK,AKT1,HMGCS1,KHSRP,SAFB,SAFB2,GIT1,SLC6A4,DHCR24,LDOC1,CACTIN,VASN,IRAK1,MBD3,MAN1B1,STK11,CARD9,APRT,SLC27A4,TGFBR3,TRADD,NDST1,ERBB4,TWF2,JUND,LOXL1,JAGN1,COL6A2,NSMF,CDC34,FBXO6,STUB1,NSMCE3	yellow	VSMC_M3
GO:0010604	0.85	0.17	163	2586	positive regulation of macromolecule metabolic process	BP	1	CD63,NAB2,CDK4,SQSTM1,BAG6,SNX9,HDAC10,NELFE,FNIP1,ATF6B,PBX2,PRR5,SREBF2,H2AFY,HNRNPA0,SGSM3,PPARD,SCRIB,VPS28,IL1R1,SECISBP2,PHF2,HSPBP1,SOCS5,RPS9,CNOT3,KLF4,HOXA1,ZC3H12A,PNKP,RCN3,SNRNP70,KMT2E,WDFY2,SIX5,ZNF296,RELB,EMILIN1,SERTAD1,TTC5,PSMC4,METTL3,DND1,DEAF1,HOXC4,KMT2B,NAT10,PDE4D,LRP3,ACSL1,ZBTB38,TNKS1BP1,GREM1,MCRS1,CX3CL1,WNT10B,SOD1,PACSIN3,EGR3,CCAR2,DLL4,CHP1,YAF2,HTATIP2,PCIF1,KDM1B,CHCHD2,RNF19B,DCPS,RRP1B,PDGFA,MMP14,C14orf93,AIP,CLCF1,ASB9,PSME2,TRIM27,PRMT2,THOC5,RIPK3,ADRM1,SMARCD2,BRMS1,BUD23,TNK2,PLA2G3,GMEB2,PRPF6,PBX1,KAT5,RELA,ARNT2,SMARCB1,CHCHD10,PBX4,UBTF,MEF2B,PHB2,TRMT112,NDP,BAD,SSBP4,MPV17L2,BUD31,TNFRSF1A,BABAM1,C1QBP,AIMP2,TAF6L,ZBTB49,MTA2,PELP1,CCDC22,FOXK1,PKN1,POLR2A,AMER1,MAD2L2,NANOS3,CTBP1,ARHGEF2,SNF8,PER1,NFIX,SLX1A,IRF2BPL,TRIM25,SMARCA4,CARM1,CD276,AATF,CSK,MID2,RBM22,ZNF521,AKT1,BRF1,PAIP1,KHSRP,GTF2F1,SLC6A4,MYDGF,SIRT6,MAP2K2,AURKAIP1,AP3D1,IRAK1,STK11,TELO2,MED16,CARD9,POMT1,NABP2,ERBB4,JUND,KLF13,TENT5B,TRIB2,NSMF,IER5,STUB1,NSMCE3	yellow	VSMC_M3
GO:0060348	0.86	0.24	8	172	bone development	BP	1	NAB2,INSIG1,INPPL1,GREM1,MMP14,HSD17B1,AMER1,ZNF385A	yellow	VSMC_M3
GO:0022008	0.86	0.17	72	1272	neurogenesis	BP	1	NAB2,CTHRC1,HDAC10,PBX2,TRIM11,TRAPPC9,PACSIN1,SCRIB,PLEC,LGALS1,IFT27,CUL7,SECISBP2,KLF4,DBNL,TPBG,WDPCP,SNX3,SLC9A3R1,PLXND1,UNK,METTL3,BAIAP2,ARHGAP33,SLC11A2,MMP24,CX3CL1,WNT10B,SOD1,DLL4,RAB35,IRX1,MMP14,NGEF,CLCF1,IGSF9,NECTIN1,LIMK1,PLA2G3,STMN3,PBX1,RELA,SCYL1,PBX4,BLOC1S4,C12orf57,CTDSP1,CD9,CNP,DBN1,CRABP2,ARHGEF2,S100A6,CARM1,EEF2K,EML1,CSK,ZNF521,AKT1,GIT1,SLC6A4,HMG20B,ACAP3,ANAPC2,STK11,NEGR1,ERBB4,TWF2,UNC13A,NSMF,GBA2,TUBGCP2	yellow	VSMC_M3
GO:0050673	0.86	0.21	17	316	epithelial cell proliferation	BP	1	PPARD,B4GALT1,DEAF1,SCARB1,WNT10B,EGR3,DLL4,MMP14,GPX1,PHB2,IGFBP5,BAD,AKT1,MYDGF,SIRT6,STK11,TGFBR3	yellow	VSMC_M3
GO:0016055	0.86	0.20	20	373	Wnt signaling pathway	BP	1	CTHRC1,KLF4,TPBG,SNX3,PLEKHA4,SLC9A3R1,RNF220,GREM1,WNT10B,CCAR2,LIMD1,NDP,AMER1,MAD2L2,SMARCA4,HIC1,CSNK1G2,STK11,IGFBP6,APCDD1L	yellow	VSMC_M3
GO:0043500	0.86	0.28	4	88	muscle adaptation	BP	1	KLF4,IGFBP5,TNFRSF1A,MIR214	yellow	VSMC_M3
GO:0034329	0.86	0.21	15	318	cell junction assembly	BP	1	PLEC,DBNL,TPBG,WDPCP,PLXND1,GREM1,MMP14,LRFN4,NECTIN1,RHOC,CD9,RCC2,EEF2K,CLDN6,ERBB4	yellow	VSMC_M3
GO:0010595	0.86	0.26	5	105	positive regulation of endothelial cell migration	BP	1	TSTA3,ZC3H12A,SCARB1,AAMP,AKT1	yellow	VSMC_M3
GO:2000116	0.86	0.23	9	167	regulation of cysteine-type endopeptidase activity	BP	1	FNIP1,KLF4,MICAL1,DELE1,GPX1,BAD,AKT1,DHCR24,CARD9	yellow	VSMC_M3
GO:0018107	0.86	0.28	4	98	peptidyl-threonine phosphorylation	BP	1	SPRED2,TNKS1BP1,PKN1,AKT1	yellow	VSMC_M3
GO:0120039	0.86	0.19	26	517	plasma membrane bounded cell projection morphogenesis	BP	1	PACSIN1,CUL7,DBNL,TPBG,PLXND1,BAIAP2,ARHGAP33,SLC11A2,CORO1B,NGEF,PLEKHO1,IGSF9,NECTIN1,ARPIN,LIMK1,C12orf57,CNP,DBN1,CRABP2,S100A6,EEF2K,ANAPC2,STK11,TWF2,UNC13A,NSMF	yellow	VSMC_M3
GO:0010959	0.86	0.21	14	263	regulation of metal ion transport	BP	1	CD63,SLC9A3R1,PDE4D,CX3CL1,PACSIN3,CHP1,GSTO1,NKAIN1,TRIM27,LGALS3,PER1,RANGRF,CORO1A,AKT1	yellow	VSMC_M3
GO:0045055	0.86	0.23	8	167	regulated exocytosis	BP	1	SCRIB,MICAL1,SYT12,PLA2G3,TMEM79,CORO1A,GIT1,UNC13A	yellow	VSMC_M3
GO:0032270	0.86	0.17	65	1066	positive regulation of cellular protein metabolic process	BP	1	NAB2,CDK4,SQSTM1,BAG6,SNX9,NELFE,FNIP1,PRR5,HSPBP1,SOCS5,RPS9,KLF4,ZC3H12A,RCN3,KMT2E,WDFY2,PSMC4,METTL3,NAT10,ACSL1,TNKS1BP1,GREM1,CX3CL1,PACSIN3,CHP1,PCIF1,RNF19B,PDGFA,MMP14,CLCF1,PSME2,RIPK3,ADRM1,BRMS1,TNK2,KAT5,SMARCB1,PHB2,BAD,MPV17L2,TNFRSF1A,C1QBP,AIMP2,CCDC22,AMER1,MAD2L2,CTBP1,ARHGEF2,CSK,AKT1,PAIP1,MYDGF,SIRT6,MAP2K2,AURKAIP1,IRAK1,STK11,TELO2,CARD9,POMT1,ERBB4,TRIB2,NSMF,STUB1,NSMCE3	yellow	VSMC_M3
GO:0031098	0.86	0.22	10	197	stress-activated protein kinase signaling cascade	BP	1	CCM2,ZC3H12A,QARS,PER1,AKT1,MAP2K2,IRAK1,CARD9,IGFBP6,CDC42EP5	yellow	VSMC_M3
GO:0010038	0.86	0.21	15	279	response to metal ion	BP	1	CDK4,ITPR3,SLC30A3,OGG1,SLC11A2,SOD1,SYT12,BAD,HSD17B1,ANXA11,S100A16,EEF2K,AKT1,ATP5F1D,JUND	yellow	VSMC_M3
GO:0072006	0.86	0.26	5	118	nephron development	BP	1	PDGFRA,GREM1,IRX1,PBX1,ERBB4	yellow	VSMC_M3
GO:0007626	0.86	0.25	6	132	locomotory behavior	BP	1	C12orf10,SOD1,CNP,GIT1,CTNS,NEGR1	yellow	VSMC_M3
GO:0048592	0.86	0.26	5	118	eye morphogenesis	BP	1	RING1,KDM2B,AQP5,NECTIN1,C12orf57	yellow	VSMC_M3
GO:0006997	0.86	0.26	5	112	nucleus organization	BP	1	PLEC,PHF2,TMEM43,NUMA1,DCTN1	yellow	VSMC_M3

GO:0008219	0.86	0.16	100	1587	cell death	BP	1	CDK4,SQSTM1,BAG6,FNIP1,PPARD,SCRIB,LGALS1,SHARPIN,B4GALT1,KLF4,ZC3H12A,FIGNL1,MICAL1,EMP3,ANXA5,KRT8,SLC9A3R1,OGG1,UCP2,PHLDA1,P4HB,PYCR1,DELE1,BCL2L1,PLEKHF1,GREM1,KDM2B,RAPSN,CX3CL1,WNT10B,SOD1,EGR3,CCAR2,CHP1,TYRO3,HTATIP2,DCPS,SELENOS,ITPRIP,RRP1B,GPX1,CLCF1,QARS,PRMT2,RIPK3,TWIST2,CAPN10,BRMS1,PLA2G3,RELA,ARNT2,ZDHHC3,SGPL1,CHCHD10,ZNF385B,PHB2,BAD,LGALS3,TNFRSF1A,C1QBP,AIMP2,USP27X,CASP14,PKN1,CAPN2,SLTM,NANOS3,BCL7C,DFFA,TMEM79,ARHGEF2,PLEKHO2,CORO1A,EEF2K,AATF,ATG3,ZNF830,AKT1,PACS2,ALKBH7,DHCR24,MYDGF,CDK11B,HIC1,TRAP1,MNT,MKNK2,STK11,CARD9,SPG7,SLC27A4,TRADD,MIR214,TNFRSF12A,ERBB4,PTMA,ZNF385A,TXNDC12,NSMF,CDC34	yellow	VSMC_M3
GO:2000147	0.86	0.19	25	455	positive regulation of cell motility	BP	1	TSTA3,PDGFRA,IL1R1,ZC3H12A,SCARB1,SWAP70,CX3CL1,PDGFA,MMP14,TWIST2,RHOC,IGFBP5,AAMP,LGALS3,PLAU,C1QBP,WASHC1,ELP5,ARHGEF2,CORO1A,AKT1,TRADD,CAVIN1,NSMF,GLIPR2	yellow	VSMC_M3
GO:0060249	0.86	0.21	11	218	anatomical structure homeostasis	BP	1	RCN3,DCTN1,CX3CL1,SOD1,TYRO3,CORO1A,TSPOAP1,CSK,ZNF830,TJP3,STK11	yellow	VSMC_M3
GO:0051223	0.86	0.19	23	412	regulation of protein transport	BP	1	SREBF2,PPARD,VPS28,EIPR1,ZC3H12A,SNX3,DMAP1,INSIG1,MYRIP,UCP2,CEP131,AACS,CHP1,PCK2,PCNT,CAPN10,PTPN23,BAD,GLUD1,CSK,RBM22,MIDN,JAGN1	yellow	VSMC_M3
GO:0048872	0.86	0.21	12	229	homeostasis of number of cells	BP	1	KMT2E,SLC11A2,SOD1,RIPK3,PKN1,CORO1A,DOCK11,AKT1,CARD9,TGFBR3,ARMCX1,KLF13	yellow	VSMC_M3
GO:0001936	0.86	0.25	5	111	regulation of endothelial cell proliferation	BP	1	EGR3,DLL4,AKT1,MYDGF,SIRT6	yellow	VSMC_M3
GO:0009266	0.87	0.23	7	137	response to temperature stimulus	BP	1	UCP2,MMP24,SOD1,AKT1,IRAK1,IER5,STUB1	yellow	VSMC_M3
GO:0000209	0.87	0.21	10	203	protein polyubiquitination	BP	1	SHARPIN,RNF19B,TRIM27,OTUB1,RUSC1,BLMH,ANAPC2,ZNRF1,CDC34,STUB1	yellow	VSMC_M3
GO:0002700	0.87	0.25	5	106	regulation of production of molecular mediator of immune response	BP	1	IL1R1,CLCF1,PHB2,PKN1,MAD2L2	yellow	VSMC_M3
GO:0015711	0.87	0.20	12	228	organic anion transport	BP	1	SLC52A2,PLEKHA8,SLC9A3R1,SLC16A5,SFXN5,SLC35C2,SLC16A14,SLC19A1,SLC26A6,PLA2G3,CTNS,SLC27A4	yellow	VSMC_M3
GO:0031333	0.87	0.25	5	115	negative regulation of protein-containing complex assembly	BP	1	EIF4EBP1,CAPG,PEX5,MIR214,TWF2	yellow	VSMC_M3
GO:0048858	0.87	0.18	26	520	cell projection morphogenesis	BP	1	PACSIN1,CUL7,DBNL,TPBG,PLXND1,BAIAP2,ARHGAP33,SLC11A2,CORO1B,NGEF,PLEKHO1,IGSF9,NECTIN1,ARPIN,LIMK1,C12orf57,CNP,DBN1,CRABP2,S100A6,EEF2K,ANAPC2,STK11,TWF2,UNC13A,NSMF	yellow	VSMC_M3
GO:0060560	0.87	0.22	9	200	developmental growth involved in morphogenesis	BP	1	DBNL,LIMK1,DBN1,CRABP2,SIRT6,ANAPC2,STK11,TWF2,UNC13A	yellow	VSMC_M3
GO:0090092	0.87	0.21	9	193	regulation of transmembrane receptor protein serine/threonine kinase signaling pathway	BP	1	SPRED2,EMILIN1,NUMA1,GREM1,MTMR4,VASN,STK11,TGFBR3,STUB1	yellow	VSMC_M3
GO:0001501	0.87	0.18	22	413	skeletal system development	BP	1	NAB2,PDGFRA,COL19A1,HOXA1,INSIG1,INPPL1,DEAF1,HOXC4,GREM1,WNT10B,PAPSS1,MMP14,EXTL1,PBX1,SGPL1,HSD17B1,TEAD4,EVC,AMER1,SLC39A1,NDST1,ZNF385A	yellow	VSMC_M3
GO:0099504	0.87	0.23	6	138	synaptic vesicle cycle	BP	1	PACSIN1,SCRIB,SYT12,GIT1,AP3D1,UNC13A	yellow	VSMC_M3
GO:0007154	0.87	0.15	272	4300	cell communication	BP	1	ENPP1,CD63,CDK4,RUFY1,SQSTM1,GNL1,BAG6,CTHRC1,NELFE,FNIP1,ATF6B,PDLIM4,PRR5,TTLL12,SREBF2,TXNDC15,ITPR3,SGSM3,NUDT3,DEF6,PPARD,EEF1D,SCRIB,PLEC,LGALS1,IFT27,SHARPIN,BOP1,TOM1,PDGFRA,IL1R1,COL15A1,EPN1,SOCS5,KLF4,DBNL,TPBG,WDPCP,CCM2,SPRED2,EIPR1,RGS3,ZC3H12A,FIGNL1,ZYX,RCN3,SNX3,MICAL1,PLEKHA4,MGLL,FAM120B,ANXA5,KRT8,RELB,SLC9A3R1,SERP2,PLXND1,EMILIN1,RNF220,SNX17,PLCD1,CD79A,INSIG1,MYRIP,SEC13,ARHGEF40,UCP2,METTL3,BAIAP2,CXXC5,LAMTOR1,NUMA1,P4HB,EIF4EBP1,ARAP3,DELE1,CAVIN3,RIC8A,ARHGAP22,ARHGAP33,AACS,SCARB1,BCL2L1,ITGB7,PDE4D,SPRYD3,TNS2,RRS1,SWAP70,PLEKHF1,ACSL1,GREM1,DOK1,PDE3B,RAPSN,CX3CL1,WNT10B,SOD1,DGKZ,EGR3,CCAR2,DLL4,CHP1,TYRO3,LSP1,RAB35,SLC35C2,TSPAN5,CISH,APBA2,DCPS,SELENOS,GSTO1,ITPRIP,RIN3,STK32C,PDGFA,MMP14,PABPN1,GPX1,AIP,NGEF,CLCF1,SYT12,QARS,PCK2,ASB9,ANPEP,TRIM27,PCNT,EIF3A,LIMK1,SCN4A,PRMT2,SPX,THOC5,RIPK3,CAPN10,TNK2,PLA2G3,STMN3,PTPN23,KAT5,RELA,SIPA1,RHOC,MAP3K14,LIMD1,ZDHHC3,SGPL1,SMARCB1,CHCHD10,ZNF385B,YJEFN3,GNB2,PHB2,NDP,IGFBP5,BAD,PLCB3,LAMTOR4,IFI35,SH3BP2,LGALS3,BUD31,PLAU,TNFRSF1A,MVB12A,BABAM1,TEAD4,C1QBP,CHERP,EVC,RCC2,CNP,CCDC22,GLUD1,SDE2,PKN1,DBN1,MAD1L1,PALM3,AMER1,PIP4K2B,MAD2L2,NANOS3,TBKBP1,CRABP2,ARHGEF2,MVP,PER1,RANGRF,PPP4C,CORO1A,RABEP2,TRIM25,RPL37,MTMR4,S100A4,S100A6,SMARCA4,CARM1,TMED1,CDC37,CD276,AATF,CSK,MID2,DOCK11,ZNF830,AKT1,TEDC1,RAB34,SAFB,SAFB2,GIT1,SLC6A4,DHCR24,CARHSP1,MYDGF,MAP2K2,[illegible]	yellow	VSMC_M3
GO:0006310	0.87	0.20	13	245	DNA recombination	BP	1	HDAC10,FIGNL1,SWAP70,MCRS1,ACTR5,CLCF1,MAD2L2,PPP4C,SLX1A,CXorf57,SIRT6,NABP2,NSMCE3	yellow	VSMC_M3
GO:1901605	0.87	0.22	8	146	alpha-amino acid metabolic process	BP	1	MPST,SEPHS1,PYCR1,THAP4,GLUD1,ILVBL,MTHFR,BLMH	yellow	VSMC_M3
GO:0030182	0.87	0.16	59	1067	neuron differentiation	BP	1	CTHRC1,PBX2,TRAPPC9,PACSIN1,SCRIB,LGALS1,IFT27,CUL7,SECISBP2,KLF4,DBNL,TPBG,WDPCP,SNX3,SLC9A3R1,PLXND1,UNK,BAIAP2,ARHGAP33,SLC11A2,CX3CL1,WNT10B,SOD1,DLL4,RAB35,IRX1,NGEF,IGSF9,NECTIN1,LIMK1,PLA2G3,STMN3,PBX1,SCYL1,PBX4,BLOC1S4,C12orf57,CTDSP1,CNP,DBN1,CRABP2,ARHGEF2,S100A6,CARM1,EEF2K,ZNF521,AKT1,GIT1,SLC6A4,HMG20B,ACAP3,ANAPC2,STK11,NEGR1,ERBB4,TWF2,UNC13A,NSMF,GBA2	yellow	VSMC_M3
GO:0032872	0.87	0.22	7	148	regulation of stress-activated MAPK cascade	BP	1	ZC3H12A,QARS,PER1,AKT1,MAP2K2,CARD9,IGFBP6	yellow	VSMC_M3
GO:0009267	0.87	0.23	6	130	cellular response to starvation	BP	1	FNIP1,SREBF2,ZC3H12A,UCP2,WDR24,FADS1	yellow	VSMC_M3
GO:0030335	0.87	0.18	24	443	positive regulation of cell migration	BP	1	TSTA3,PDGFRA,IL1R1,ZC3H12A,SCARB1,SWAP70,CX3CL1,PDGFA,MMP14,TWIST2,RHOC,IGFBP5,AAMP,LGALS3,PLAU,C1QBP,WASHC1,ELP5,ARHGEF2,CORO1A,AKT1,TRADD,NSMF,GLIPR2	yellow	VSMC_M3

GO:0033365	0.87	0.16	43	737	protein localization to organelle	BP	1	DCTN2,BAG6,SAMM50,SREBF2,H2AFY,SCRIB,VPS28,ASNA1,ZC3H12A,DMAP1,INSIG1,SEC13,CEP131,TSPAN10,NUMA1,RRS1,PINX1,MCRS1,CHP1,RAB35,AIP,LRWD1,PEX5,ZDHHC3,CHCHD10,PHB2,LAMTOR4,KIAA0753,NUP88,RCC2,SNF8,RANGRF,SGF29,MID2,RBM22,AKT1,AP3D1,STK11,GAS8,NABP2,MIS12,ARFRP1,TIMM22	yellow	VSMC_M3
GO:0002684	0.87	0.17	32	542	positive regulation of immune system process	BP	1	BAG6,LGALS1,IL1R1,SOCS5,ZC3H12A,CD79A,PDE4D,SWAP70,CX3CL1,DGKZ,EGR3,MMP14,CLCF1,LIMK1,PLA2G3,SART1,RELA,PHB2,BAD,IFI35,LGALS3,C1QBP,MAD2L2,CORO1A,CD276,CSK,AKT1,AP3D1,C8G,STK11,CARD9,JUND	yellow	VSMC_M3
GO:0007600	0.87	0.18	17	320	sensory perception	BP	1	NDUFB9,ITPR3,HOXA1,MGLL,SLC9A3R1,LRIG1,MMP24,WNT10B,SOD1,HPS1,DLL4,GPX1,RABGGTA,SPX,TMEM120A,NDP,RGS16	yellow	VSMC_M3
GO:0051716	0.87	0.14	324	5042	cellular response to stimulus	BP	1	ENPP1,CD63,CDK4,RUFY1,SQSTM1,GNL1,BAG6,CTHRC1,HDAC10,NELFE,FNIP1,ATF6B,PRR5,TTLL12,SREBF2,TXNDC15,ITPR3,SGSM3,GUK1,DEF6,PPARD,FANCE,EEF1D,RPL3,PLEC,LGALS1,IFT27,SHARPIN,BOP1,TOM1,PDGFRA,IL1R1,COL15A1,EPN1,SOCS5,KLF4,DBNL,TPBG,WDPCP,CCM2,SPRED2,RGS3,ZC3H12A,FIGNL1,PDIA4,PNKP,ZYX,RCN3,SNX3,MICAL1,PLEKHA4,MGLL,FAM120B,ANXA5,KRT8,RELB,SLC9A3R1,SERP2,MUTYH,PLXND1,EMILIN1,TMUB1,RNF220,DMAP1,SNX17,PLCD1,CD79A,INSIG1,SEC13,OGG1,TTC5,ARHGEF40,UCP2,SEL1L2,METTL3,BAIAP2,CXXC5,FAAP100,LAMTOR1,NUMA1,P4HB,PYCR1,FASN,RAB20,SMOX,EIF4EBP1,ARAP3,DELE1,CAVIN3,RIC8A,GSR,ARHGAP22,SMUG1,ARHGAP33,AACS,SCARB1,HM13,BCL2L1,ITGB7,PDE4D,SPRYD3,TNS2,RRS1,SWAP70,PLEKHF1,ACSL1,ZBTB38,TNKS1BP1,GREM1,DOK1,AQP5,PDE3B,MCRS1,CX3CL1,WNT10B,SOD1,ACTR5,ANKRD2,DGKZ,EGR3,CCAR2,DLL4,CHP1,TYRO3,LSP1,RAB35,CHCHD2,SLC35C2,TSPAN5,CISH,DCPS,WDR4,SELENOS,GSTO1,ITPRIP,RIN3,STK32C,PDGFA,MMP14,PABPN1,GPX1,AIP,CORO1B,NGEF,CLCF1,SYT12,QARS,PCK2,ASB9,COL6A1,IMPDH2,ANPEP,TRIM27,PCNT,EIF3A,LIMK1,SLC26A6,PRMT2,SPX,THOC5,RIPK3,CAPN10,TNK2,STMN3,CEP164,KAT5,RELA,SIPA1,MGST3,RHOC,MAP3K14,LIMD1,ZDHHC3,SGPL1,SMARCB1,CHCHD10,ZNF385B,YJEFN3,GNB2,ATIC,TXNRD2,PHB2,NDP,IGFBP5,BAD,PLCB3,LAMTOR4,IFI35,OTUB1,SH3BP2,HSD17B1,LETM1,LGALS3,BUD31,CD9,PLAU,TNFRSF1A,MVB12A,BABAM1,TEAD4,C1QBP,CHERP,UBXN1,EVC,RCC2,AP1M1,PRXL2A,PELP1,CCDC22,SDE2,KLF3,PKN1,CAPN2,MAD1L1,PALM3,AMER1,PIP4K2B,MAD2L2,NANOS3,TBKBP1,CRAB[illegible]	yellow	VSMC_M3
GO:0002244	0.87	0.25	4	96	hematopoietic progenitor cell differentiation	BP	1	FNIP1,PDGFRA,METTL3,EML1	yellow	VSMC_M3
GO:1903169	0.88	0.23	5	110	regulation of calcium ion transmembrane transport	BP	1	PDE4D,CX3CL1,GSTO1,CORO1A,AKT1	yellow	VSMC_M3
GO:0048878	0.88	0.16	48	832	chemical homeostasis	BP	1	ENPP1,SREBF2,ITPR3,PPARD,PLEC,LGALS1,PDGFRA,TTC7A,RCN3,SLC9A3R1,SLC30A3,INSIG1,UCP2,LAMTOR1,RAB20,AACS,SCARB1,PDE4D,SWAP70,RALY,SLC11A2,CX3CL1,SOD1,CHP1,GSTO1,FABP3,PCK2,SLC26A6,SPX,SMARCB1,IGFBP5,BAD,LETM1,CHERP,CCDC22,FOXK1,TMEM79,RANGRF,CORO1A,TSPOAP1,SMARCA4,ANKRD9,AKT1,PACS2,AP3D1,STK11,JAGN1,CCDC115	yellow	VSMC_M3
GO:0060047	0.88	0.21	8	172	heart contraction	BP	1	ZC3H12A,PDE4D,SOD1,GSTO1,GPX1,SPX,RANGRF,TAZ	yellow	VSMC_M3
GO:0009893	0.88	0.14	177	2815	positive regulation of metabolic process	BP	1	CD63,NAB2,CDK4,SQSTM1,BAG6,SNX9,HDAC10,NELFE,FNIP1,ATF6B,PBX2,PRR5,SREBF2,H2AFY,HNRNPA0,SGSM3,PPARD,SCRIB,VPS28,PDGFRA,IL1R1,SECISBP2,PHF2,HSPBP1,SOCS5,RPS9,CNOT3,KLF4,HOXA1,ZC3H12A,PNKP,RCN3,SNRNP70,KMT2E,WDFY2,SIX5,ZNF296,RELB,EMILIN1,SERTAD1,TTC5,PSMC4,UCP2,METTL3,DND1,DEAF1,HOXC4,KMT2B,SCARB1,NAT10,PDE4D,LRP3,PLEKHF1,ACSL1,ZBTB38,TNKS1BP1,GREM1,MCRS1,CX3CL1,WNT10B,SOD1,PACSIN3,DGKZ,EGR3,CCAR2,DLL4,CHP1,TYRO3,SCD,YAF2,HTATIP2,PCIF1,KDM1B,CHCHD2,RNF19B,DCPS,RRP1B,FABP3,PDGFA,MMP14,C14orf93,AIP,CLCF1,ASB9,PSME2,TRIM27,PRMT2,THOC5,RIPK3,ADRM1,SMARCD2,BRMS1,BUD23,TNK2,PLA2G3,GMEB2,PRPF6,PBX1,KAT5,RELA,ARNT2,MID1IP1,SMARCB1,CHCHD10,PBX4,UBTF,MEF2B,PHB2,TRMT112,NDP,BAD,SSBP4,MPV17L2,BUD31,TNFRSF1A,BABAM1,C1QBP,AIMP2,TAF6L,ZBTB49,MTA2,PELP1,CCDC22,FOXK1,PKN1,CAPN2,POLR2A,AMER1,PIP4K2B,MAD2L2,NANOS3,CTBP1,ARHGEF2,SNF8,PER1,NFIX,SLX1A,IRF2BPL,TRIM25,SMARCA4,CARM1,CD276,AATF,CSK,MID2,RBM22,ZNF521,AKT1,BRF1,PAIP1,KHSRP,GTF2F1,SLC6A4,MYDGF,SIRT6,MAP2K2,AURKAIP1,EHMT1,AP3D1,IRAK1,STK11,TELO2,TAZ,MED16,CARD9,POMT1,VAC14,NABP2,ERBB4,JUND,KLF13,TENT5B,TRIB2,NSMF,IER5,STUB1,NSMCE3	yellow	VSMC_M3
GO:1903305	0.88	0.24	4	95	regulation of regulated secretory pathway	BP	1	MICAL1,SYT12,PLA2G3,GIT1	yellow	VSMC_M3
GO:0045944	0.88	0.15	52	904	positive regulation of transcription by RNA polymerase II	BP	1	SQSTM1,NELFE,ATF6B,PBX2,SREBF2,PPARD,KLF4,HOXA1,ZC3H12A,SIX5,ZNF296,SERTAD1,TTC5,HOXC4,ZBTB38,GREM1,MCRS1,CX3CL1,WNT10B,EGR3,HTATIP2,KDM1B,CHCHD2,RRP1B,GMEB2,PRPF6,PBX1,KAT5,RELA,ARNT2,SMARCB1,MEF2B,SSBP4,TNFRSF1A,ZBTB49,MTA2,PELP1,ARHGEF2,PER1,NFIX,IRF2BPL,SMARCA4,AATF,ZNF521,AKT1,GTF2F1,MYDGF,AP3D1,MED16,JUND,KLF13,IER5	yellow	VSMC_M3
GO:0034620	0.88	0.24	4	86	cellular response to unfolded protein	BP	1	ATF6B,SERP2,SELENOS,STUB1	yellow	VSMC_M3
GO:0051648	0.88	0.21	7	154	vesicle localization	BP	1	DCTN2,SCRIB,CHP1,BLOC1S4,MYO19,AP3D1,STK11	yellow	VSMC_M3
GO:0006873	0.88	0.17	24	444	cellular ion homeostasis	BP	1	ENPP1,ITPR3,PDGFRA,TTC7A,SLC9A3R1,SLC30A3,RAB20,PDE4D,SWAP70,SLC11A2,CX3CL1,SOD1,CHP1,GSTO1,SLC26A6,LETM1,CHERP,CCDC22,CORO1A,TSPOAP1,ANKRD9,PACS2,AP3D1,CCDC115	yellow	VSMC_M3
GO:0045766	0.88	0.21	6	133	positive regulation of angiogenesis	BP	1	KLF4,ZC3H12A,GREM1,MYDGF,SIRT6,JMJD8	yellow	VSMC_M3
GO:1904018	0.88	0.21	6	133	positive regulation of vasculature development	BP	1	KLF4,ZC3H12A,GREM1,MYDGF,SIRT6,JMJD8	yellow	VSMC_M3

GO:0048699	0.88	0.15	65	1177	generation of neurons	BP	1	CTHRC1,PBX2,TRIM11,TRAPPC9,PACSIN1,SCRIB,LGALS1,IFT27,CUL7,SECISBP2,KLF4,DBNL,TPBG,WDPCP,SNX3,SLC9A3R1,PLXND1,UNK,METTL3,BAIAP2,ARHGAP33,SLC11A2,CX3CL1,WNT10B,SOD1,DLL4,RAB35,IRX1,NGEF,CLCF1,IGSF9,NECTIN1,LIMK1,PLA2G3,STMN3,PBX1,RELA,SCYL1,PBX4,BLOC1S4,C12orf57,CTDSP1,CNP,DBN1,CRABP2,ARHGEF2,S100A6,CARM1,EEF2K,EML1,ZNF521,AKT1,GIT1,SLC6A4,HMG20B,ACAP3,ANAPC2,STK11,NEGR1,ERBB4,TWF2,UNC13A,NSMF,GBA2,TUBGCP2	yellow	VSMC_M3
GO:0035967	0.88	0.22	5	103	cellular response to topologically incorrect protein	BP	1	BAG6,ATF6B,SERP2,SELENOS,STUB1	yellow	VSMC_M3
GO:0050678	0.88	0.18	14	275	regulation of epithelial cell proliferation	BP	1	PPARD,B4GALT1,DEAF1,WNT10B,EGR3,DLL4,GPX1,PHB2,BAD,AKT1,MYDGF,SIRT6,STK11,TGFBR3	yellow	VSMC_M3
GO:0070302	0.88	0.20	7	151	regulation of stress-activated protein kinase signaling cascade	BP	1	ZC3H12A,QARS,PER1,AKT1,MAP2K2,CARD9,IGFBP6	yellow	VSMC_M3
GO:0048871	0.88	0.17	20	378	multicellular organismal homeostasis	BP	1	PLEC,CNOT3,RCN3,UCP2,TNS2,ACSL1,SLC11A2,WNT10B,SOD1,SCD,GPX1,TMEM79,CORO1A,CSK,ZNF830,SIRT6,TJP3,EHMT1,STK11,AMPD2	yellow	VSMC_M3
GO:0002682	0.88	0.14	55	915	regulation of immune system process	BP	1	BAG6,TTLL12,SCRIB,LGALS1,IL1R1,SOCS5,ZC3H12A,EMILIN1,CD79A,METTL3,TARBP2,PDE4D,SWAP70,GREM1,CX3CL1,SOD1,DGKZ,EGR3,TYRO3,ST3GAL4,SELENOS,RIN3,MMP14,GPX1,CLCF1,TRIM27,LIMK1,RIPK3,PSMB4,PLA2G3,SART1,RELA,PHB2,BAD,IFI35,LGALS3,CD9,C1QBP,PRXL2A,PKN1,MAD1L1,MAD2L2,CORO1A,CDC37,CD276,CSK,AKT1,CACTIN,AP3D1,IRAK1,C8G,STK11,CARD9,JUND,KLF13	yellow	VSMC_M3
GO:0002224	0.89	0.23	4	92	toll-like receptor signaling pathway	BP	1	TYRO3,IFI35,CACTIN,IRAK1	yellow	VSMC_M3
GO:0051179	0.89	0.13	293	4586	localization	BP	1	ENPP1,PQLC1,CD63,ESYT1,DCTN2,NHP2,RUFY1,SQSTM1,CCHCR1,BAG6,SNX9,CTHRC1,SAMM50,SREBF2,SQLE,H2AFY,ITPR3,SGSM3,GUK1,PACSIN1,PPARD,TSTA3,SCRIB,PLEC,IFT27,SLC52A2,VPS28,TOM1,B4GALT1,PDGFRA,IL1R1,EPN1,SSC5D,TTC7A,ASNA1,KLF4,DBNL,TPBG,PLEKHA8,WDPCP,EIPR1,ZC3H12A,PDIA4,RCN3,SNX3,MICAL1,EHD2,TTYH2,SLC9A3R1,SERP2,PLXND1,EMILIN1,SLC16A5,DMAP1,SLC30A3,SNX17,UNK,INSIG1,MYRIP,SEC13,SEPT9,UCP2,BAIAP2,CEP131,INPPL1,TSPAN10,LAMTOR1,NUMA1,YIF1B,RAB20,ARAP3,SIGLEC1,RIC8A,ARHGAP33,LDLRAD3,AACS,SCARB1,HM13,BCL2L1,ITGB7,PDE4D,RRS1,SWAP70,LRP3,PLEKHF1,UQCRFS1,ACSL1,SLC11A2,PINX1,GREM1,AQP5,MCRS1,DCTN1,RAPSN,CX3CL1,SOD1,PACSIN3,SFXN5,DGKZ,EGR3,DNAJC17,HPS1,DLL4,CHP1,TYRO3,AP2A2,SNX21,HTATIP2,RAB35,SLC35C2,RBM15B,TSPAN5,APBA2,ST3GAL4,SELENOS,GSTO1,SLC16A14,EXOC3,RIN3,FABP3,NKAIN1,PDGFA,MMP14,PABPN1,GPX1,AIP,AP1G2,CORO1B,PLEKHO1,SYT12,PCK2,SLC19A1,NECTIN1,TRIM27,ARPIN,PCNT,LIMK1,SCN4A,SLC26A6,NUDC,SPX,THOC5,UQCRC1,RIPK3,TWIST2,CAPN10,IQCG,RHCE,TNK2,TACO1,PLA2G3,PRPF6,PTPN23,MRC2,ATP5MC3,RHOC,TMEM120A,SCYL1,LRWD1,FMNL1,VPS51,ZFPL1,LIMD1,PEX5,ZDHHC3,SGPL1,CHCHD10,YJEFN3,THOC3,BLOC1S4,PHB2,IGFBP5,BAD,COPE,LAMTOR4,AAMP,SCARF2,LETM1,LGALS3,CD9,PLAU,TNFRSF1A,FCHO1,MVB12A,KIAA0753,C1QBP,NUP88,CHERP,ANXA11,WASHC1,RCC2,CNP,AP1M1,EML3,MTA2,RAB3IL1,CCDC22,GLUD1,ELP5,PKN1,DBN1,SNX8,LMAN2,MIIP,RAB24,MAD1L1,PIP4K2B,CLTB,TMEM79,ARHGEF2,SNF8,MVP,PER1,MTX1,RANGRF,STX10,CORO1A,SGF29,[illegible]	yellow	VSMC_M3
GO:0043410	0.89	0.16	19	356	positive regulation of MAPK cascade	BP	1	NELFE,PDGFRA,TPBG,ZC3H12A,LAMTOR1,CAVIN3,CX3CL1,SOD1,PDGFA,PHB2,CSK,MYDGF,MAP2K2,IRAK1,CARD9,NDST1,ERBB4,IGFBP6,GLIPR2	yellow	VSMC_M3
GO:0009887	0.89	0.15	43	784	animal organ morphogenesis	BP	1	NAB2,CTHRC1,PBX2,RING1,SCRIB,PDGFRA,HOXA1,WDPCP,PLEKHA4,SLC9A3R1,PLXND1,INSIG1,INPPL1,HOXC4,LRIG1,GREM1,KDM2B,AQP5,WNT10B,SOD1,DLL4,IRX1,PDGFA,MMP14,HCCS,NECTIN1,PBX1,RELA,VPS51,SGPL1,PBX4,PHB2,IGFBP5,C12orf57,SLC39A1,TTC39C,SLC6A4,SIRT6,TAZ,TGFBR3,NDST1,ERBB4,ELN	yellow	VSMC_M3
GO:0048608	0.89	0.17	17	330	reproductive structure development	BP	1	PPARD,CUL7,PDGFRA,KRT8,BCL2L1,SOD1,TYRO3,MMP14,IDH1,SGPL1,ZNF830,AKT1,HMGCS1,SAFB2,DHCR24,NSDHL,STK11	yellow	VSMC_M3
GO:0060537	0.89	0.16	16	323	muscle tissue development	BP	1	PLEC,PDGFRA,COL19A1,GREM1,WNT10B,DLL4,GPX1,IGFBP5,SIRT6,HMG20B,TAZ,GTF3C5,TGFBR3,ERBB4,IGSF8,ELN	yellow	VSMC_M3
GO:0072655	0.89	0.21	5	103	establishment of protein localization to mitochondrion	BP	1	SAMM50,SREBF2,AIP,AKT1,TIMM22	yellow	VSMC_M3
GO:0010632	0.89	0.18	10	208	regulation of epithelial cell migration	BP	1	TSTA3,KLF4,ZC3H12A,SCARB1,DLL4,PTPN23,AAMP,AKT1,PTPRR,GLIPR2	yellow	VSMC_M3
GO:0051480	0.89	0.18	10	212	regulation of cytosolic calcium ion concentration	BP	1	ITPR3,PDGFRA,PDE4D,SWAP70,CX3CL1,GSTO1,LETM1,CHERP,CORO1A,TSPOAP1	yellow	VSMC_M3
GO:0006022	0.89	0.22	4	98	aminoglycan metabolic process	BP	1	ST3GAL4,AKT1,B3GNT9,NDST1	yellow	VSMC_M3
GO:0033674	0.89	0.16	19	373	positive regulation of kinase activity	BP	1	NAB2,SNX9,PDGFRA,ACSL1,GREM1,DGKZ,TYRO3,PDGFA,RIPK3,PHB2,BAD,CSK,AKT1,MAP2K2,IRAK1,STK11,TELO2,VAC14,ERBB4	yellow	VSMC_M3
GO:0018210	0.89	0.22	4	104	peptidyl-threonine modification	BP	1	SPRED2,TNKS1BP1,PKN1,AKT1	yellow	VSMC_M3
GO:0002706	0.89	0.22	4	92	regulation of lymphocyte mediated immunity	BP	1	IL1R1,CLCF1,RIPK3,MAD2L2	yellow	VSMC_M3
GO:0061458	0.89	0.16	17	333	reproductive system development	BP	1	PPARD,CUL7,PDGFRA,KRT8,BCL2L1,SOD1,TYRO3,MMP14,IDH1,SGPL1,ZNF830,AKT1,HMGCS1,SAFB2,DHCR24,NSDHL,STK11	yellow	VSMC_M3
GO:0007030	0.89	0.21	5	122	Golgi organization	BP	1	CUL7,VPS51,LMAN2,TMED1,MAP2K2	yellow	VSMC_M3
GO:0051048	0.89	0.21	5	110	negative regulation of secretion	BP	1	UCP2,SPX,TNFRSF1A,MIDN,JAGN1	yellow	VSMC_M3
GO:0006626	0.89	0.22	4	86	protein targeting to mitochondrion	BP	1	SAMM50,SREBF2,AIP,TIMM22	yellow	VSMC_M3

GO:0007165	0.89	0.12	246	3919	signal transduction	BP	1	ENPP1,CD63,CDK4,RUFY1,SQSTM1,GNL1,BAG6,CTHRC1,NELFE,FNIP1,ATF6B,PRR5,TTLL12,SREBF2,TXNDC15,ITPR3,SGSM3,DEF6,PPARD,EEF1D,LGALS1,IFT27,SHARPIN,BOP1,TOM1,PDGFRA,IL1R1,COL15A1,EPN1,SOCS5,KLF4,DBNL,TPBG,WDPCP,CCM2,SPRED2,RGS3,ZC3H12A,FIGNL1,ZYX,RCN3,SNX3,MICAL1,PLEKHA4,MGLL,FAM120B,ANXA5,KRT8,RELB,SLC9A3R1,SERP2,PLXND1,EMILIN1,RNF220,SNX17,PLCD1,CD79A,INSIG1,SEC13,ARHGEF40,METTL3,BAIAP2,CXXC5,LAMTOR1,NUMA1,P4HB,EIF4EBP1,ARAP3,DELE1,CAVIN3,RIC8A,ARHGAP22,ARHGAP33,SCARB1,BCL2L1,ITGB7,PDE4D,SPRYD3,TNS2,RRS1,SWAP70,PLEKHF1,ACSL1,GREM1,DOK1,PDE3B,CX3CL1,WNT10B,SOD1,DGKZ,CCAR2,DLL4,CHP1,TYRO3,LSP1,RAB35,SLC35C2,TSPAN5,CISH,SELENOS,GSTO1,ITPRIP,RIN3,STK32C,PDGFA,MMP14,PABPN1,GPX1,AIP,NGEF,CLCF1,QARS,ASB9,ANPEP,TRIM27,PCNT,EIF3A,LIMK1,PRMT2,SPX,THOC5,RIPK3,TNK2,STMN3,KAT5,RELA,SIPA1,RHOC,MAP3K14,LIMD1,ZDHHC3,SGPL1,SMARCB1,CHCHD10,ZNF385B,YJEFN3,GNB2,PHB2,NDP,IGFBP5,BAD,PLCB3,LAMTOR4,IFI35,SH3BP2,LGALS3,BUD31,PLAU,TNFRSF1A,MVB12A,BABAM1,TEAD4,C1QBP,CHERP,EVC,RCC2,CCDC22,SDE2,PKN1,MAD1L1,PALM3,AMER1,PIP4K2B,MAD2L2,NANOS3,TBKBP1,CRABP2,ARHGEF2,MVP,PER1,RANGRF,PPP4C,CORO1A,RABEP2,TRIM25,RPL37,MTMR4,S100A4,S100A6,SMARCA4,CARM1,TMED1,CDC37,CD276,AATF,CSK,MID2,DOCK11,ZNF830,AKT1,TEDC1,RAB34,SAFB,SAFB2,GIT1,DHCR24,CARHSP1,MYDGF,MAP2K2,NSDHL,CACTIN,VASN,HIC1,TRAP1,NCLN,MNT,CTNS,TEDC2,MKNK2,CSNK1G2,IRAK1,SSNA1,STK11,TELO2,WDR24,JMJD8,CARD9,GAS8,SLC27A4,VAC14,TG[illegible]	yellow	VSMC_M3
GO:0048545	0.89	0.16	13	258	response to steroid hormone	BP	1	PPARD,PCK2,PRMT2,RELA,IDH1,PHB2,BAD,PKN1,PER1,SMARCA4,CARM1,SAFB,SAFB2	yellow	VSMC_M3
GO:0051056	0.89	0.17	11	259	regulation of small GTPase mediated signal transduction	BP	1	SQSTM1,SGSM3,DEF6,ARHGEF40,ARHGAP22,SWAP70,NGEF,STMN3,SIPA1,ARHGEF2,ARHGEF1	yellow	VSMC_M3
GO:0007204	0.90	0.18	9	194	positive regulation of cytosolic calcium ion concentration	BP	1	ITPR3,PDGFRA,PDE4D,SWAP70,CX3CL1,GSTO1,LETM1,CHERP,CORO1A	yellow	VSMC_M3
GO:0071702	0.90	0.12	117	1891	organic substance transport	BP	1	ENPP1,PQLC1,CD63,ESYT1,RUFY1,CCHCR1,SNX9,SAMM50,SREBF2,SGSM3,GUK1,PPARD,SCRIB,IFT27,SLC52A2,VPS28,TOM1,ASNA1,PLEKHA8,EIPR1,ZC3H12A,PDIA4,RCN3,SNX3,SLC9A3R1,SERP2,SLC16A5,DMAP1,SNX17,INSIG1,MYRIP,SEC13,UCP2,CEP131,LAMTOR1,YIF1B,RAB20,ARHGAP33,AACS,SCARB1,HM13,ACSL1,AQP5,SFXN5,CHP1,AP2A2,SNX21,RAB35,SLC35C2,RBM15B,APBA2,SELENOS,SLC16A14,EXOC3,FABP3,PABPN1,AIP,AP1G2,SYT12,PCK2,SLC19A1,PCNT,SLC26A6,SPX,THOC5,CAPN10,PLA2G3,PTPN23,VPS51,PEX5,ZDHHC3,YJEFN3,THOC3,PHB2,BAD,COPE,MVB12A,NUP88,WASHC1,AP1M1,RAB3IL1,CCDC22,GLUD1,SNX8,LMAN2,RAB24,CLTB,ARHGEF2,SNF8,MVP,MTX1,RANGRF,STX10,RABEP2,SLC27A3,TMED1,CDC37,CSK,ATG3,RBM22,AKT1,SNX18,KHSRP,RANBP3,RAB34,SLC6A4,CTNS,AP3D1,MIDN,SLC27A4,SLC44A3,RBM8A,AP5B1,JAGN1,CAVIN1,ARFRP1,TIMM22	yellow	VSMC_M3
GO:1904062	0.90	0.16	12	241	regulation of cation transmembrane transport	BP	1	CD63,SLC9A3R1,PDE4D,CX3CL1,CHP1,GSTO1,TACO1,PHB2,RANGRF,CORO1A,AKT1,GSG1L	yellow	VSMC_M3
GO:0007034	0.90	0.19	6	137	vacuolar transport	BP	1	VPS28,AP1G2,VPS51,MVB12A,SNF8,AP3D1	yellow	VSMC_M3
GO:0050731	0.90	0.19	6	136	positive regulation of peptidyl-tyrosine phosphorylation	BP	1	GREM1,CLCF1,TNK2,TNFRSF1A,ARHGEF2,ERBB4	yellow	VSMC_M3
GO:0014706	0.90	0.16	15	307	striated muscle tissue development	BP	1	PLEC,PDGFRA,COL19A1,GREM1,WNT10B,DLL4,GPX1,SIRT6,HMG20B,TAZ,GTF3C5,TGFBR3,ERBB4,IGSF8,ELN	yellow	VSMC_M3
GO:0065009	0.90	0.12	142	2307	regulation of molecular function	BP	1	ENPP1,NAB2,CDK4,SBF1,SNX9,CTHRC1,FNIP1,RING1,H2AFY,SGSM3,TRAPPC9,DEF6,EEF1D,SCRIB,PLEC,PPP1R14B,PDGFRA,HSPBP1,SOCS5,KLF4,DBNL,SPRED2,RGS3,ZC3H12A,PNKP,RCN3,SNRNP70,MICAL1,ANXA5,SLC9A3R1,PLXND1,SERTAD1,ARHGEF40,LAMTOR1,ARAP3,DELE1,RIC8A,ARHGAP22,ARHGAP33,TARBP2,SCARB1,HM13,PDE4D,SWAP70,ACSL1,MMP24,PINX1,GREM1,CX3CL1,WNT10B,SOD1,DGKZ,CCAR2,HPS1,CHP1,TYRO3,CISH,GSTO1,ITPRIP,RRP1B,ETFBKMT,RIN3,PDGFA,PABPN1,GPX1,AIP,NGEF,QARS,PSME2,TRIM27,LIMK1,PRMT2,RIPK3,ADRM1,BRMS1,TNK2,TACO1,STMN3,RELA,SIPA1,RHOC,MID1IP1,SMARCB1,PHB2,PPP1R35,BAD,LAMTOR4,LGALS3,BUD31,PLAU,AIMP2,UBXN1,WASHC1,RCC2,RAB3IL1,CCDC22,PKN1,PIP4K2B,MAD2L2,DFFA,ARHGEF2,MVP,RANGRF,DPM3,RABEP2,TRIM25,RPL37,SMARCA4,CARM1,CDC37,CSK,MID2,DOCK11,AKT1,SNX18,DENND1C,GTF2F1,RANBP3,GIT1,DHCR24,MAP2K2,CACTIN,ACAP3,IRAK1,MIDN,STK11,TELO2,JMJD8,CARD9,GAS8,SLC27A4,VAC14,TGFBR3,TRADD,ERBB4,RNH1,TRIB2,ARHGEF1,RGS16,STUB1,RGS18,GSG1L	yellow	VSMC_M3
GO:0046903	0.90	0.13	38	673	secretion	BP	1	PPARD,SCRIB,EIPR1,PDIA4,RCN3,MICAL1,SLC9A3R1,MYRIP,UCP2,AACS,AQP5,TYRO3,EXOC3,SYT12,PCK2,SLC26A6,SPX,CAPN10,PLA2G3,PTPN23,BAD,TNFRSF1A,WASHC1,RAB3IL1,GLUD1,TMEM79,SNF8,MTX1,CORO1A,GIT1,SLC6A4,MIDN,APRT,RABEPK,ERBB4,JAGN1,CAVIN1,UNC13A	yellow	VSMC_M3
GO:0045088	0.90	0.18	7	148	regulation of innate immune response	BP	1	TTLL12,METTL3,TYRO3,IFI35,CDC37,CACTIN,CARD9	yellow	VSMC_M3
GO:0007569	0.90	0.21	4	98	cell aging	BP	1	SOD1,KAT5,MNT,RF00024	yellow	VSMC_M3
GO:0048738	0.90	0.17	8	186	cardiac muscle tissue development	BP	1	PLEC,PDGFRA,GREM1,DLL4,SIRT6,TAZ,TGFBR3,ERBB4	yellow	VSMC_M3
GO:0003015	0.90	0.17	8	180	heart process	BP	1	ZC3H12A,PDE4D,SOD1,GSTO1,GPX1,SPX,RANGRF,TAZ	yellow	VSMC_M3
GO:1902115	0.90	0.18	7	161	regulation of organelle assembly	BP	1	SEPT9,NUMA1,LRFN4,EML3,PIP4K2B,RABEP2,ATG3	yellow	VSMC_M3

GO:0072359	0.90	0.12	49	902	circulatory system development	BP	1	PDLIM4,PPARD,PLEC,CUL7,B4GALT1,PDGFRA,COL15A1,EPN1,KLF4,HOXA1,WDPCP,CCM2,ZC3H12A,PLXND1,EMILIN1,SNX17,RIC8A,ARHGAP22,GREM1,PDE3B,CX3CL1,EGR3,DLL4,HTATIP2,PDGFA,MMP14,GPX1,ANPEP,SGPL1,YJEFN3,AAMP,TNFRSF1A,AKT1,MYDGF,SIRT6,NSDHL,MBD3,STK11,TAZ,JMJD8,SARS,TGFBR3,MIR214,NDST1,TNFRSF12A,ERBB4,LOXL1,RNH1,ELN	yellow	VSMC_M3
GO:0018108	0.90	0.15	14	287	peptidyl-tyrosine phosphorylation	BP	1	PDGFRA,SOCS5,GREM1,TYRO3,PDGFA,CLCF1,TNK2,SCYL1,TNFRSF1A,ARHGEF2,MVP,CSK,MAP2K2,ERBB4	yellow	VSMC_M3
GO:0002573	0.90	0.17	7	154	myeloid leukocyte differentiation	BP	1	RELB,FASN,PIR,THOC5,PLA2G3,PRXL2A,JAGN1	yellow	VSMC_M3
GO:0046822	0.90	0.20	4	94	regulation of nucleocytoplasmic transport	BP	1	ZC3H12A,DMAP1,CHP1,RBM22	yellow	VSMC_M3
GO:0070585	0.91	0.19	5	107	protein localization to mitochondrion	BP	1	SAMM50,SREBF2,AIP,AKT1,TIMM22	yellow	VSMC_M3
GO:0051259	0.91	0.16	8	174	protein complex oligomerization	BP	1	ZC3H12A,AQP5,CHP1,TMEM120A,LETM1,KRT10,FARSA,CARD9	yellow	VSMC_M3
GO:0042127	0.91	0.12	74	1230	regulation of cell population proliferation	BP	1	CDK4,BAG6,CTHRC1,SQLE,PPARD,SCRIB,B4GALT1,PDGFRA,RPS9,KLF4,TPBG,SIX5,SLC9A3R1,DLEC1,SERTAD1,CEP131,INPPL1,DEAF1,BCL2L1,TNS2,PINX1,GREM1,KDM2B,CX3CL1,WNT10B,EGR3,DLL4,FABP3,PDGFA,GPX1,CLCF1,RIPK3,PBX1,RELA,ARNT2,SMARCB1,PHB2,IGFBP5,BAD,IFI35,LGALS3,CD9,PLAU,AIMP2,CHERP,ZBTB49,PKN1,MAD1L1,CTBP1,ARHGEF2,CORO1A,S100A6,CARM1,CD276,CSK,AKT1,SLC6A4,DHCR24,MYDGF,LDOC1,SIRT6,MNT,IRAK1,STK11,GAS8,MVD,TGFBR3,MIR214,ERBB4,JUND,KLF13,IGFBP6,ELN,IER5	yellow	VSMC_M3
GO:2000026	0.91	0.12	57	1014	regulation of multicellular organismal development	BP	1	ENPP1,BAG6,H2AFY,TRIM11,PPARD,CUL7,EPN1,SOCS5,KLF4,DBNL,TPBG,WDPCP,SPRED2,ZC3H12A,PLXND1,EMILIN1,METTL3,BAIAP2,NUMA1,GREM1,PDE3B,CX3CL1,WNT10B,SOD1,EGR3,DLL4,HTATIP2,MMP14,CLCF1,LIMK1,PLA2G3,SART1,RELA,YJEFN3,BAD,CTDSP1,TNFRSF1A,PRXL2A,DBN1,CRABP2,EEF2K,AKT1,MYDGF,SIRT6,EHMT1,AP3D1,ANAPC2,STK11,JMJD8,SARS,TGFBR3,TRADD,MIR214,ERBB4,TWF2,KLF13,RNH1	yellow	VSMC_M3
GO:0045595	0.91	0.12	66	1153	regulation of cell differentiation	BP	1	ENPP1,CTHRC1,FNIP1,H2AFY,TRIM11,PPARD,CUL7,PDGFRA,SOCS5,KLF4,DBNL,SPRED2,ZC3H12A,WDFY2,PLXND1,INSIG1,METTL3,BAIAP2,NUMA1,P4HB,LRP3,GREM1,CX3CL1,WNT10B,SOD1,ANKRD2,EGR3,DLL4,MMP14,CLCF1,LIMK1,TWIST2,PLA2G3,SART1,CCDC85B,PBX1,RELA,LIMD1,IGFBP5,BAD,CTDSP1,EID1,TNFRSF1A,C1QBP,RCC2,PRXL2A,DBN1,MAD2L2,CRABP2,ARHGEF2,CARM1,EEF2K,AKT1,SLC6A4,HMG20B,VASN,AP3D1,ANAPC2,STK11,TWF2,JUND,KLF13,FADS1,TRIB2,ZNF385A,GLIPR2	yellow	VSMC_M3
GO:0007088	0.91	0.20	4	96	regulation of mitotic nuclear division	BP	1	CUL7,MAD1L1,MAD2L2,AURKAIP1	yellow	VSMC_M3
GO:0006810	0.91	0.11	208	3300	transport	BP	1	ENPP1,PQLC1,CD63,ESYT1,DCTN2,RUFY1,SQSTM1,CCHCR1,SNX9,SAMM50,SREBF2,ITPR3,SGSM3,GUK1,PACSIN1,PPARD,SCRIB,IFT27,SLC52A2,VPS28,TOM1,EPN1,SSC5D,ASNA1,DBNL,PLEKHA8,EIPR1,ZC3H12A,PDIA4,RCN3,SNX3,MICAL1,EHD2,TTYH2,SLC9A3R1,SERP2,SLC16A5,DMAP1,SLC30A3,SNX17,INSIG1,MYRIP,SEC13,UCP2,CEP131,INPPL1,LAMTOR1,NUMA1,YIF1B,RAB20,ARAP3,SIGLEC1,ARHGAP33,LDLRAD3,AACS,SCARB1,HM13,BCL2L1,PDE4D,RRS1,LRP3,UQCRFS1,ACSL1,SLC11A2,GREM1,AQP5,DCTN1,CX3CL1,SOD1,PACSIN3,SFXN5,DNAJC17,HPS1,CHP1,TYRO3,AP2A2,SNX21,HTATIP2,RAB35,SLC35C2,RBM15B,APBA2,SELENOS,GSTO1,SLC16A14,EXOC3,RIN3,FABP3,NKAIN1,PABPN1,AIP,AP1G2,SYT12,PCK2,SLC19A1,NECTIN1,TRIM27,PCNT,LIMK1,SCN4A,SLC26A6,SPX,THOC5,UQCRC1,CAPN10,RHCE,TNK2,TACO1,PLA2G3,PTPN23,MRC2,ATP5MC3,TMEM120A,SCYL1,VPS51,ZFPL1,PEX5,ZDHHC3,CHCHD10,YJEFN3,THOC3,BLOC1S4,PHB2,BAD,COPE,SCARF2,LETM1,LGALS3,CD9,TNFRSF1A,FCHO1,MVB12A,NUP88,CHERP,ANXA11,WASHC1,CNP,AP1M1,RAB3IL1,CCDC22,GLUD1,SNX8,LMAN2,RAB24,PIP4K2B,CLTB,TMEM79,ARHGEF2,SNF8,MVP,PER1,MTX1,RANGRF,STX10,CORO1A,RABEP2,SLC39A1,ATP5PD,SLC27A3,S100A6,TMED1,SYNE3,EEF2K,CDC37,MYO19,CSK,ATG3,RBM22,AKT1,SNX18,DENND1C,KHSRP,ALKBH7,RANBP3,RAB34,GIT1,ANKRD13B,SLC6A4,MAP2K2,SLC35E2B,CTNS,AP3D1,CSNK1G2,SSNA1,NDUFS7,MIDN,ATP5F1D,STK11,TMEM201,SPG7,APRT,WDR34,SLC27A4,SLC44A3,RABEPK,RBM8A,ERBB4,SURF1,TRAPPC5,PLA2G4E,AP5B1,JAGN1,CAVIN1,UNC13A,ARFRP1,TIMM22,PHETA2,GSG1L	yellow	VSMC_M3
GO:0051924	0.91	0.16	8	172	regulation of calcium ion transport	BP	1	PDE4D,CX3CL1,PACSIN3,GSTO1,TRIM27,LGALS3,CORO1A,AKT1	yellow	VSMC_M3
GO:0051347	0.91	0.13	23	441	positive regulation of transferase activity	BP	1	NAB2,SNX9,PDGFRA,KLF4,PNKP,ACSL1,GREM1,DGKZ,TYRO3,PDGFA,PABPN1,RIPK3,PHB2,BAD,CSK,AKT1,MAP2K2,IRAK1,STK11,TELO2,VAC14,ERBB4,STUB1	yellow	VSMC_M3
GO:0007259	0.91	0.19	4	98	receptor signaling pathway via JAK-STAT	BP	1	SOCS5,CLCF1,TNFRSF1A,ERBB4	yellow	VSMC_M3
GO:0010466	0.91	0.17	7	145	negative regulation of peptidase activity	BP	1	FNIP1,KLF4,MICAL1,GPX1,UBXN1,AKT1,DHCR24	yellow	VSMC_M3
GO:0002688	0.91	0.19	4	92	regulation of leukocyte chemotaxis	BP	1	SWAP70,GREM1,RIN3,C1QBP	yellow	VSMC_M3
GO:0015748	0.91	0.19	4	102	organophosphate ester transport	BP	1	PQLC1,PLEKHA8,SCARB1,SLC19A1	yellow	VSMC_M3
GO:0016236	0.91	0.14	13	265	macroautophagy	BP	1	SQSTM1,HDAC10,VPS28,LIX1L,PHB2,MVB12A,PIP4K2B,SNF8,CDC37,ATG3,AKT1,PACS2,TAZ	yellow	VSMC_M3
GO:0051049	0.91	0.11	75	1276	regulation of transport	BP	1	ENPP1,CD63,RUFY1,SREBF2,PACSIN1,PPARD,SCRIB,VPS28,EIPR1,ZC3H12A,SNX3,MICAL1,EHD2,SLC9A3R1,DMAP1,SLC30A3,SNX17,INSIG1,MYRIP,UCP2,CEP131,LAMTOR1,NUMA1,AACS,SCARB1,PDE4D,ACSL1,GREM1,CX3CL1,SOD1,PACSIN3,CHP1,SELENOS,GSTO1,RIN3,FABP3,NKAIN1,SYT12,PCK2,TRIM27,PCNT,SCN4A,SLC26A6,SPX,CAPN10,TNK2,TACO1,PLA2G3,PTPN23,CHCHD10,YJEFN3,PHB2,BAD,LGALS3,TNFRSF1A,GLUD1,LMAN2,SNF8,PER1,RANGRF,CORO1A,EEF2K,CSK,ATG3,RBM22,AKT1,GIT1,ANKRD13B,SLC6A4,MAP2K2,MIDN,STK11,PLA2G4E,JAGN1,GSG1L	yellow	VSMC_M3
GO:0007601	0.91	0.18	5	123	visual perception	BP	1	HPS1,DLL4,RABGGTA,NDP,RGS16	yellow	VSMC_M3

GO:0032412	0.91	0.16	8	178	regulation of ion transmembrane transporter activity	BP	1	SLC9A3R1,PDE4D,CHP1,GSTO1,TACO1,PHB2,RANGRF,GSG1L	yellow	VSMC_M3
GO:0030218	0.91	0.19	4	102	erythrocyte differentiation	BP	1	KMT2E,SLC11A2,TGFBR3,KLF13	yellow	VSMC_M3
GO:0010594	0.91	0.16	7	157	regulation of endothelial cell migration	BP	1	TSTA3,KLF4,ZC3H12A,SCARB1,DLL4,AAMP,AKT1	yellow	VSMC_M3
GO:0018212	0.91	0.14	14	290	peptidyl-tyrosine modification	BP	1	PDGFRA,SOCS5,GREM1,TYRO3,PDGFA,CLCF1,TNK2,SCYL1,TNFRSF1A,ARHGEF2,MVP,CSK,MAP2K2,ERBB4	yellow	VSMC_M3
GO:0050730	0.91	0.15	9	193	regulation of peptidyl-tyrosine phosphorylation	BP	1	SOCS5,GREM1,PDGFA,CLCF1,TNK2,TNFRSF1A,ARHGEF2,MVP,ERBB4	yellow	VSMC_M3
GO:0002831	0.91	0.14	11	224	regulation of response to biotic stimulus	BP	1	TTLL12,ZC3H12A,METTL3,TARBP2,CX3CL1,TYRO3,IFI35,C1QBP,CDC37,CACTIN,CARD9	yellow	VSMC_M3
GO:0071695	0.91	0.16	7	163	anatomical structure maturation	BP	1	GREM1,CX3CL1,WNT10B,SLC26A6,PLA2G3,TMEM79,NOM1	yellow	VSMC_M3
GO:0051345	0.91	0.12	23	440	positive regulation of hydrolase activity	BP	1	SNX9,SGSM3,SCRIB,PDGFRA,RCN3,ARAP3,ARHGAP22,CX3CL1,NGEF,PSME2,RIPK3,ADRM1,SIPA1,RHOC,PHB2,BAD,RCC2,DOCK11,AKT1,SNX18,CARD9,SLC27A4,RGS16	yellow	VSMC_M3
GO:0071496	0.91	0.14	13	267	cellular response to external stimulus	BP	1	FNIP1,SREBF2,PLEC,ZC3H12A,UCP2,DCPS,SIPA1,MAP3K14,BAD,TNFRSF1A,AKT1,WDR24,FADS1	yellow	VSMC_M3
GO:0010562	0.92	0.11	36	669	positive regulation of phosphorus metabolic process	BP	1	NAB2,SQSTM1,SNX9,FNIP1,PRR5,PDGFRA,WDFY2,ACSL1,TNKS1BP1,GREM1,CX3CL1,DGKZ,CHP1,TYRO3,FABP3,PDGFA,CLCF1,RIPK3,TNK2,PHB2,BAD,TNFRSF1A,CAPN2,MAD2L2,ARHGEF2,CSK,AKT1,MYDGF,MAP2K2,IRAK1,STK11,TELO2,TAZ,VAC14,ERBB4,NSMF	yellow	VSMC_M3
GO:0045937	0.92	0.11	36	669	positive regulation of phosphate metabolic process	BP	1	NAB2,SQSTM1,SNX9,FNIP1,PRR5,PDGFRA,WDFY2,ACSL1,TNKS1BP1,GREM1,CX3CL1,DGKZ,CHP1,TYRO3,FABP3,PDGFA,CLCF1,RIPK3,TNK2,PHB2,BAD,TNFRSF1A,CAPN2,MAD2L2,ARHGEF2,CSK,AKT1,MYDGF,MAP2K2,IRAK1,STK11,TELO2,TAZ,VAC14,ERBB4,NSMF	yellow	VSMC_M3
GO:0051225	0.92	0.18	4	101	spindle assembly	BP	1	NUMA1,SAC3D1,EML3,TUBGCP2	yellow	VSMC_M3
GO:0070201	0.92	0.12	23	436	regulation of establishment of protein localization	BP	1	SREBF2,PPARD,VPS28,EIPR1,ZC3H12A,SNX3,DMAP1,INSIG1,MYRIP,UCP2,CEP131,AACS,CHP1,PCK2,PCNT,CAPN10,PTPN23,BAD,GLUD1,CSK,RBM22,MIDN,JAGN1	yellow	VSMC_M3
GO:0007292	0.92	0.18	4	101	female gamete generation	BP	1	NCAPH2,METTL3,NANOS3,ZNF830	yellow	VSMC_M3
GO:0010628	0.92	0.11	46	803	positive regulation of gene expression	BP	1	CDK4,H2AFY,HNRNPA0,PPARD,SCRIB,IL1R1,SECISBP2,PHF2,RPS9,KLF4,ZC3H12A,SNRNP70,RELB,EMILIN1,SERTAD1,METTL3,DND1,NAT10,PDE4D,LRP3,CX3CL1,SOD1,DLL4,PCIF1,MMP14,C14orf93,CLCF1,TRIM27,PLA2G3,RELA,PHB2,MPV17L2,C1QBP,POLR2A,MAD2L2,ARHGEF2,SNF8,CD276,RBM22,AKT1,PAIP1,SLC6A4,MAP2K2,IRAK1,CARD9,TENT5B	yellow	VSMC_M3
GO:0090287	0.92	0.14	10	230	regulation of cellular response to growth factor stimulus	BP	1	CD63,SPRED2,EMILIN1,NUMA1,GREM1,MTMR4,VASN,STK11,TGFBR3,STUB1	yellow	VSMC_M3
GO:0098662	0.92	0.11	29	541	inorganic cation transmembrane transport	BP	1	CD63,ITPR3,SLC9A3R1,SLC30A3,UCP2,PDE4D,UQCRFS1,SLC11A2,CX3CL1,CHP1,GSTO1,SCN4A,UQCRC1,RHCE,TACO1,ATP5MC3,PHB2,LETM1,CHERP,RANGRF,CORO1A,SLC39A1,ATP5PD,AKT1,SLC6A4,CTNS,NDUFS7,ATP5F1D,SURF1	yellow	VSMC_M3
GO:0050953	0.92	0.16	5	126	sensory perception of light stimulus	BP	1	HPS1,DLL4,RABGGTA,NDP,RGS16	yellow	VSMC_M3
GO:0003205	0.92	0.16	5	135	cardiac chamber development	BP	1	PLXND1,SNX17,DLL4,TGFBR3,NDST1	yellow	VSMC_M3
GO:0010634	0.92	0.15	6	143	positive regulation of epithelial cell migration	BP	1	TSTA3,ZC3H12A,SCARB1,AAMP,AKT1,GLIPR2	yellow	VSMC_M3
GO:0002274	0.92	0.14	7	156	myeloid leukocyte activation	BP	1	ZC3H12A,KMT2E,RELB,CX3CL1,PLA2G3,IFI35,JUND	yellow	VSMC_M3
GO:0032940	0.92	0.10	31	574	secretion by cell	BP	1	PPARD,SCRIB,EIPR1,PDIA4,RCN3,MICAL1,MYRIP,UCP2,AACS,TYRO3,EXOC3,SYT12,PCK2,CAPN10,PLA2G3,PTPN23,BAD,TNFRSF1A,WASHC1,RAB3IL1,GLUD1,TMEM79,SNF8,CORO1A,GIT1,SLC6A4,MIDN,RABEPK,JAGN1,CAVIN1,UNC13A	yellow	VSMC_M3
GO:0043270	0.92	0.14	8	182	positive regulation of ion transport	BP	1	SLC9A3R1,CX3CL1,CHP1,GSTO1,PLA2G3,LGALS3,AKT1,SLC6A4	yellow	VSMC_M3
GO:0031401	0.93	0.10	41	747	positive regulation of protein modification process	BP	1	NAB2,SQSTM1,SNX9,NELFE,FNIP1,PRR5,HSPBP1,ZC3H12A,KMT2E,WDFY2,ACSL1,TNKS1BP1,GREM1,CX3CL1,CHP1,PDGFA,CLCF1,RIPK3,BRMS1,TNK2,KAT5,SMARCB1,PHB2,TNFRSF1A,AIMP2,AMER1,MAD2L2,CTBP1,ARHGEF2,CSK,AKT1,MYDGF,MAP2K2,IRAK1,STK11,TELO2,POMT1,ERBB4,NSMF,STUB1,NSMCE3	yellow	VSMC_M3
GO:1903531	0.93	0.16	4	99	negative regulation of secretion by cell	BP	1	UCP2,TNFRSF1A,MIDN,JAGN1	yellow	VSMC_M3
GO:0048729	0.93	0.11	24	490	tissue morphogenesis	BP	1	CTHRC1,SCRIB,KLF4,CCM2,PLEKHA4,SLC9A3R1,PLXND1,DEAF1,GREM1,KDM2B,DLL4,IRX1,PDGFA,MMP14,PBX1,RHOC,PHB2,IGFBP5,MTHFR,TMEM79,ARMC5,SIRT6,TGFBR3,ERBB4	yellow	VSMC_M3
GO:0031175	0.93	0.10	40	784	neuron projection development	BP	1	CTHRC1,PACSIN1,SCRIB,LGALS1,IFT27,CUL7,KLF4,DBNL,TPBG,SNX3,SLC9A3R1,PLXND1,BAIAP2,ARHGAP33,SLC11A2,CX3CL1,SOD1,RAB35,NGEF,IGSF9,NECTIN1,LIMK1,PLA2G3,STMN3,BLOC1S4,C12orf57,CNP,DBN1,CRABP2,S100A6,CARM1,EEF2K,AKT1,ACAP3,ANAPC2,STK11,NEGR1,TWF2,UNC13A,NSMF	yellow	VSMC_M3
GO:0002440	0.93	0.14	6	138	production of molecular mediator of immune response	BP	1	IL1R1,SWAP70,CLCF1,PHB2,PKN1,MAD2L2	yellow	VSMC_M3
GO:0007417	0.93	0.10	42	795	central nervous system development	BP	1	BAG6,HDAC10,PBX2,TRAPPC9,SCRIB,MPST,SHARPIN,SECISBP2,PAFAH1B3,CIC,METTL3,BAIAP2,CAPG,KDM2B,DCTN1,DLL4,TYRO3,CLCF1,QARS,PBX1,SCYL1,ARNT2,VPS51,PBX4,ATIC,C12orf57,BAD,CD9,CNP,GLUD1,EML1,CSK,AKT1,HMGCS1,GIT1,SLC6A4,CTNS,MBD3,NDST1,ERBB4,GBA2,TUBGCP2	yellow	VSMC_M3

GO:0008283	0.93	0.09	86	1442	cell population proliferation	BP	1	CDK4,BAG6,CTHRC1,SQLE,PPARD,SCRIB,BOP1,B4GALT1,PDGFRA,RPS9,KLF4,TPBG,FIGNL1,SIX5,SLC9A3R1,DLEC1,CD79A,SERTAD1,CEP131,INPPL1,DEAF1,SCARB1,BCL2L1,TNS2,PINX1,GREM1,KDM2B,CX3CL1,WNT10B,EGR3,DLL4,FABP3,PDGFA,MMP14,GPX1,CLCF1,IMPDH2,RIPK3,PBX1,RELA,ARNT2,SMARCB1,YJEFN3,PHB2,IGFBP5,BAD,IFI35,LGALS3,CD9,PLAU,AIMP2,CHERP,ZBTB49,PKN1,DBN1,MAD1L1,CTBP1,ARHGEF2,ARMC5,CORO1A,S100A6,CARM1,CD276,EML1,CSK,AKT1,SLC6A4,DHCR24,MYDGF,LDOC1,SIRT6,MNT,IRAK1,STK11,GAS8,MVD,TGFBR3,PSMB10,MIR214,NDST1,ERBB4,JUND,KLF13,IGFBP6,ELN,IER5	yellow	VSMC_M3
GO:0030003	0.93	0.10	22	434	cellular cation homeostasis	BP	1	ITPR3,PDGFRA,TTC7A,SLC30A3,RAB20,PDE4D,SWAP70,SLC11A2,CX3CL1,SOD1,CHP1,GSTO1,SLC26A6,LETM1,CHERP,CCDC22,CORO1A,TSPOAP1,ANKRD9,PACS2,AP3D1,CCDC115	yellow	VSMC_M3
GO:0006875	0.93	0.11	19	381	cellular metal ion homeostasis	BP	1	ITPR3,PDGFRA,TTC7A,SLC30A3,PDE4D,SWAP70,SLC11A2,CX3CL1,SOD1,GSTO1,LETM1,CHERP,CCDC22,CORO1A,TSPOAP1,ANKRD9,PACS2,AP3D1,CCDC115	yellow	VSMC_M3
GO:0008217	0.93	0.14	5	122	regulation of blood pressure	BP	1	PDE4D,SOD1,ANPEP,SPX,PLCB3	yellow	VSMC_M3
GO:0071560	0.93	0.13	8	199	cellular response to transforming growth factor beta stimulus	BP	1	SPRED2,ZYX,EMILIN1,MTMR4,VASN,STK11,TGFBR3,STUB1	yellow	VSMC_M3
GO:0045859	0.93	0.10	27	513	regulation of protein kinase activity	BP	1	NAB2,SNX9,H2AFY,PLEC,SOCS5,SLC9A3R1,SERTAD1,TARBP2,ACSL1,GREM1,SOD1,CHP1,PDGFA,QARS,TRIM27,RIPK3,PHB2,PKN1,MVP,CDC37,CSK,AKT1,MAP2K2,IRAK1,STK11,TELO2,TRIB2	yellow	VSMC_M3
GO:0031668	0.93	0.12	9	205	cellular response to extracellular stimulus	BP	1	FNIP1,SREBF2,PLEC,ZC3H12A,UCP2,DCPS,SIPA1,WDR24,FADS1	yellow	VSMC_M3
GO:0021953	0.93	0.15	4	116	central nervous system neuron differentiation	BP	1	DLL4,SCYL1,C12orf57,GBA2	yellow	VSMC_M3
GO:0002699	0.93	0.13	6	142	positive regulation of immune effector process	BP	1	IL1R1,SOCS5,CLCF1,PLA2G3,PHB2,MAD2L2	yellow	VSMC_M3
GO:0071310	0.93	0.08	105	1784	cellular response to organic substance	BP	1	ENPP1,CD63,CDK4,BAG6,ATF6B,TTLL12,PPARD,RPL3,LGALS1,SHARPIN,PDGFRA,IL1R1,SOCS5,KLF4,SPRED2,ZC3H12A,ZYX,KRT8,SLC9A3R1,SERP2,EMILIN1,INSIG1,UCP2,METTL3,BAIAP2,LAMTOR1,NUMA1,P4HB,FASN,RAB20,AACS,SCARB1,BCL2L1,TNS2,ACSL1,GREM1,DOK1,PDE3B,CX3CL1,WNT10B,SOD1,EGR3,DLL4,LSP1,RAB35,DCPS,SELENOS,PABPN1,CORO1B,CLCF1,PCK2,COL6A1,IMPDH2,SLC26A6,PRMT2,CAPN10,KAT5,RELA,SMARCB1,ATIC,PHB2,IGFBP5,BAD,LAMTOR4,OTUB1,TNFRSF1A,PELP1,KLF3,PKN1,CAPN2,PALM3,PIP4K2B,ARHGEF2,PER1,CORO1A,TRIM25,MTMR4,SMARCA4,CARM1,EEF2K,CDC37,CSK,AKT1,HMGCS1,KHSRP,SAFB,SAFB2,GIT1,SLC6A4,LDOC1,CACTIN,VASN,IRAK1,STK11,APRT,SLC27A4,TGFBR3,TRADD,NDST1,ERBB4,TWF2,JAGN1,NSMF,CDC34,STUB1	yellow	VSMC_M3
GO:0043393	0.93	0.13	7	162	regulation of protein binding	BP	1	CTHRC1,PLXND1,AKT1,GTF2F1,GAS8,TGFBR3,STUB1	yellow	VSMC_M3
GO:0072593	0.93	0.12	8	176	reactive oxygen species metabolic process	BP	1	ZC3H12A,SOD1,GPX1,RIPK3,AATF,AKT1,TRAP1,CTNS	yellow	VSMC_M3
GO:0022898	0.93	0.12	8	186	regulation of transmembrane transporter activity	BP	1	SLC9A3R1,PDE4D,CHP1,GSTO1,TACO1,PHB2,RANGRF,GSG1L	yellow	VSMC_M3
GO:0007269	0.93	0.15	4	110	neurotransmitter secretion	BP	1	SCRIB,SYT12,GIT1,UNC13A	yellow	VSMC_M3
GO:0099643	0.93	0.15	4	110	signal release from synapse	BP	1	SCRIB,SYT12,GIT1,UNC13A	yellow	VSMC_M3
GO:0015698	0.94	0.15	4	113	inorganic anion transport	BP	1	ENPP1,ASNA1,TTYH2,SLC26A6	yellow	VSMC_M3
GO:0097696	0.94	0.14	4	105	receptor signaling pathway via STAT	BP	1	SOCS5,CLCF1,TNFRSF1A,ERBB4	yellow	VSMC_M3
GO:1903827	0.94	0.09	23	454	regulation of cellular protein localization	BP	1	SQSTM1,SREBF2,H2AFY,VPS28,ZC3H12A,DMAP1,INSIG1,CEP131,NUMA1,BCL2L1,PINX1,MCRS1,CHP1,MMP14,PCNT,LGALS3,RANGRF,CSK,RBM22,AKT1,STK11,GAS8,ERBB4	yellow	VSMC_M3
GO:0010951	0.94	0.13	6	137	negative regulation of endopeptidase activity	BP	1	FNIP1,KLF4,MICAL1,GPX1,AKT1,DHCR24	yellow	VSMC_M3
GO:0071902	0.94	0.12	7	170	positive regulation of protein serine/threonine kinase activity	BP	1	ACSL1,PDGFA,CSK,AKT1,MAP2K2,IRAK1,TELO2	yellow	VSMC_M3
GO:0030162	0.94	0.09	30	538	regulation of proteolysis	BP	1	BAG6,SNX9,FNIP1,HSPBP1,SOCS5,KLF4,ZC3H12A,RCN3,MICAL1,PSMC4,DELE1,LDLRAD3,WNT10B,PACSIN3,CCAR2,RNF19B,MMP14,GPX1,PSME2,ADRM1,BAD,UBXN1,CCDC22,AKT1,DHCR24,SIRT6,AURKAIP1,CARD9,TRIB2,STUB1	yellow	VSMC_M3
GO:0030902	0.94	0.14	4	116	hindbrain development	BP	1	SCRIB,KDM2B,ATIC,SLC6A4	yellow	VSMC_M3
GO:0048812	0.94	0.09	23	503	neuron projection morphogenesis	BP	1	PACSIN1,CUL7,DBNL,TPBG,PLXND1,BAIAP2,ARHGAP33,SLC11A2,NGEF,IGSF9,NECTIN1,LIMK1,C12orf57,CNP,DBN1,CRABP2,S100A6,EEF2K,ANAPC2,STK11,TWF2,UNC13A,NSMF	yellow	VSMC_M3
GO:1905114	0.94	0.09	22	448	cell surface receptor signaling pathway involved in cell-cell signaling	BP	1	CTHRC1,KLF4,TPBG,SNX3,PLEKHA4,SLC9A3R1,RNF220,BAIAP2,GREM1,WNT10B,CCAR2,LIMD1,NDP,AMER1,MAD2L2,SMARCA4,AKT1,HIC1,CSNK1G2,STK11,IGFBP6,APCDD1L	yellow	VSMC_M3
GO:0071559	0.94	0.11	8	205	response to transforming growth factor beta	BP	1	SPRED2,ZYX,EMILIN1,MTMR4,VASN,STK11,TGFBR3,STUB1	yellow	VSMC_M3
GO:0051336	0.94	0.08	41	754	regulation of hydrolase activity	BP	1	SBF1,SNX9,FNIP1,SGSM3,SCRIB,PDGFRA,KLF4,RCN3,SNRNP70,MICAL1,PLXND1,ARAP3,DELE1,ARHGAP22,CX3CL1,SOD1,CHP1,RRP1B,GPX1,AIP,NGEF,PSME2,RIPK3,ADRM1,STMN3,SIPA1,RHOC,PHB2,PPP1R35,BAD,LGALS3,UBXN1,RCC2,DFFA,DOCK11,AKT1,SNX18,DHCR24,CARD9,SLC27A4,RGS16	yellow	VSMC_M3
GO:0046467	0.94	0.12	5	120	membrane lipid biosynthetic process	BP	1	PIGO,ST3GAL4,SGPL1,DPM3,CSNK1G2	yellow	VSMC_M3
GO:0060485	0.94	0.10	10	235	mesenchyme development	BP	1	CUL7,SPRED2,GREM1,AMER1,MAD2L2,S100A4,VASN,TGFBR3,ERBB4,GLIPR2	yellow	VSMC_M3
GO:0098660	0.94	0.08	30	574	inorganic ion transmembrane transport	BP	1	CD63,ITPR3,SLC9A3R1,SLC30A3,UCP2,PDE4D,UQCRFS1,SLC11A2,CX3CL1,CHP1,GSTO1,SCN4A,SLC26A6,UQCRC1,RHCE,TACO1,ATP5MC3,PHB2,LETM1,CHERP,RANGRF,CORO1A,SLC39A1,ATP5PD,AKT1,SLC6A4,CTNS,NDUFS7,ATP5F1D,SURF1	yellow	VSMC_M3
GO:0007254	0.94	0.12	5	134	JNK cascade	BP	1	PER1,AKT1,IRAK1,CARD9,CDC42EP5	yellow	VSMC_M3
GO:0034101	0.94	0.13	4	111	erythrocyte homeostasis	BP	1	KMT2E,SLC11A2,TGFBR3,KLF13	yellow	VSMC_M3
GO:0050920	0.94	0.11	7	167	regulation of chemotaxis	BP	1	PDGFRA,TPBG,SWAP70,GREM1,RIN3,CORO1B,C1QBP	yellow	VSMC_M3

GO:0042592	0.94	0.07	74	1295	homeostatic process	BP	1	ENPP1,SREBF2,ITPR3,PPARD,PLEC,LGALS1,PDGFRA,TTC7A,CNOT3,RCN3,KMT2E,SLC9A3R1,SLC30A3,INSIG1,UCP2,LAMTOR1,RAB20,GSR,AACS,SCARB1,PDE4D,TNS2,SWAP70,RALY,ACSL1,SLC11A2,DCTN1,CX3CL1,WNT10B,SOD1,CHP1,TYRO3,SCD,SELENOS,GSTO1,FABP3,GPX1,PCK2,SLC26A6,SPX,RIPK3,SMARCB1,TXNRD2,IGFBP5,BAD,LETM1,CHERP,CCDC22,FOXK1,PKN1,TMEM79,RANGRF,CORO1A,TSPOAP1,SMARCA4,CSK,ANKRD9,DOCK11,ZNF830,AKT1,PACS2,GIT1,SIRT6,TJP3,EHMT1,AP3D1,STK11,CARD9,AMPD2,TGFBR3,ARMCX1,KLF13,JAGN1,CCDC115	yellow	VSMC_M3
GO:0032501	0.94	0.06	311	5003	multicellular organismal process	BP	1	ENPP1,NAB2,CDK4,BAG6,NCAPH2,SBF1,CTHRC1,HDAC10,FNIP1,PBX2,PDLIM4,RING1,NDUFB9,H2AFY,ITPR3,TRIM11,TRAPPC9,PACSIN1,PPARD,TSTA3,SCRIB,PLEC,LGALS1,MPST,IFT27,SHARPIN,SND1,CUL7,B4GALT1,PDGFRA,IL1R1,SECISBP2,PHF2,GRHPR,COL15A1,EPN1,COL19A1,SSC5D,SOCS5,TTC7A,CNOT3,KLF4,DBNL,TPBG,HOXA1,WDPCP,CCM2,FHL3,SPRED2,ZC3H12A,FIGNL1,RCN3,SNX3,PLEKHA4,MGLL,KMT2E,ANXA5,KRT8,SIX5,ZNF296,RELB,SLC9A3R1,PLXND1,EMILIN1,RNF220,PAFAH1B3,CIC,SNX17,UNK,DLEC1,CD79A,INSIG1,PSMC4,UCP2,METTL3,BAIAP2,CEP131,INPPL1,NUMA1,DND1,YIF1B,FASN,RPL29,EIF4EBP1,MORN2,CAVIN3,RIC8A,ARHGAP22,DEAF1,TBCB,HOXC4,LRIG1,ARHGAP33,AACS,SCARB1,HM13,BCL2L1,PDE4D,TNS2,SWAP70,LRP3,ACSL1,CAPG,SLC11A2,MMP24,GREM1,KDM2B,AQP5,PDE3B,DCTN1,CX3CL1,WNT10B,SOD1,ANKRD2,DGKZ,EGR3,HPS1,DLL4,TYRO3,SCD,HTATIP2,RAB35,KDM1B,APBA2,ST3GAL4,IRX1,SELENOS,GSTO1,SDHA,PAPSS1,PDGFA,MMP14,PABPN1,GPX1,CORO1B,NGEF,HCCS,CLCF1,IGSF9,QARS,LRFN4,PCK2,SLC19A1,COL6A1,IMPDH2,PIR,NECTIN1,ANPEP,TRIM27,LIMK1,SCN4A,SLC26A6,RABGGTA,SPX,THOC5,RIPK3,TWIST2,EXTL1,IQCG,MPZL3,TACO1,PLA2G3,STMN3,SART1,PTPN23,MRC2,PBX1,KAT5,RELA,TMEM120A,SCYL1,IDH1,ARNT2,VPS51,LIMD1,SGPL1,SMARCB1,PBX4,YJEFN3,ATIC,BLOC1S4,MEF2B,PHB2,NDP,IGFBP5,C12orf57,BAD,PLCB3,AAMP,CTDSP1,HSD17B1,CD9,PLAU,TNFRSF1A,TEAD4,C1QBP,AIMP2,TAF6L,CHERP,EVC,CNP,PRXL2A,KRT10,GLUD1,FOXK1,CASP14,KLF3,PKN1,DBN1,CAPN2,MAD1L1,AMER1,MTHFR,MAD2L2,NANOS3,PRR14,CRABP2,SP2,TMEM79,ARHGEF2,ARMC5,PER1,[illegible]	yellow	VSMC_M3
GO:0050804	0.94	0.09	14	317	modulation of chemical synaptic transmission	BP	1	SQSTM1,ITPR3,BAIAP2,RAPSN,CX3CL1,DGKZ,SYT12,ZDHHC3,DBN1,GIT1,SLC6A4,ANAPC2,UNC13A,NSMF	yellow	VSMC_M3
GO:0003013	0.95	0.08	21	437	circulatory system process	BP	1	PPARD,PLEC,ZC3H12A,SLC9A3R1,PDE4D,LRP3,SOD1,DLL4,GSTO1,GPX1,SLC19A1,ANPEP,SPX,PLCB3,RANGRF,AKT1,SLC6A4,TJP3,TAZ,SLC27A4,ELN	yellow	VSMC_M3
GO:0007266	0.95	0.12	4	116	Rho protein signal transduction	BP	1	LIMK1,ARHGEF2,CDC42EP5,ARHGEF1	yellow	VSMC_M3
GO:0006508	0.95	0.07	77	1295	proteolysis	BP	1	SQSTM1,BAG6,SNX9,FNIP1,SHARPIN,VPS28,CUL7,HSPBP1,SOCS5,KLF4,JOSD2,PSMB3,ZC3H12A,RCN3,MICAL1,TMUB1,PSMC4,SEL1L2,DELE1,LDLRAD3,HM13,PEPD,MMP24,WNT10B,PACSIN3,CCAR2,RNF19B,SELENOS,MMP14,APEH,GPX1,PSME2,ANPEP,ADRM1,DPP3,CAPN10,PSMB4,SEC11A,PTPN23,KAT5,BAD,OTUB1,COPS6,PLAU,MVB12A,BABAM1,UBXN1,CCDC22,USP27X,SDE2,CASP14,CAPN2,FBXL19,SNF8,TRIM25,USP43,SENP8,ANKRD9,AKT1,DHCR24,BLMH,SIRT6,AURKAIP1,NCLN,ANAPC2,SPSB3,MAN1B1,CARD9,SPG7,ZNRF1,PSMB10,CTRL,TRIB2,CDC34,FBXO6,STUB1,RCE1	yellow	VSMC_M3
GO:0099177	0.95	0.09	14	318	regulation of trans-synaptic signaling	BP	1	SQSTM1,ITPR3,BAIAP2,RAPSN,CX3CL1,DGKZ,SYT12,ZDHHC3,DBN1,GIT1,SLC6A4,ANAPC2,UNC13A,NSMF	yellow	VSMC_M3
GO:0035051	0.95	0.12	4	119	cardiocyte differentiation	BP	1	PLEC,PDGFRA,GREM1,TGFBR3	yellow	VSMC_M3
GO:1903706	0.95	0.09	12	258	regulation of hemopoiesis	BP	1	SOCS5,ZC3H12A,METTL3,SOD1,EGR3,MMP14,PLA2G3,SART1,BAD,PRXL2A,AP3D1,KLF13	yellow	VSMC_M3
GO:0050679	0.95	0.11	6	154	positive regulation of epithelial cell proliferation	BP	1	B4GALT1,EGR3,BAD,AKT1,MYDGF,SIRT6	yellow	VSMC_M3
GO:2000241	0.95	0.12	4	105	regulation of reproductive process	BP	1	B4GALT1,METTL3,C1QBP,STK11	yellow	VSMC_M3
GO:0034767	0.95	0.12	4	114	positive regulation of ion transmembrane transport	BP	1	SLC9A3R1,CX3CL1,CHP1,GSTO1	yellow	VSMC_M3
GO:0021537	0.95	0.10	7	184	telencephalon development	BP	1	TRAPPC9,SECISBP2,KDM2B,ATIC,C12orf57,BAD,ERBB4	yellow	VSMC_M3
GO:0002262	0.95	0.11	5	134	myeloid cell homeostasis	BP	1	KMT2E,SLC11A2,SOD1,TGFBR3,KLF13	yellow	VSMC_M3
GO:0051093	0.95	0.07	35	676	negative regulation of developmental process	BP	1	ENPP1,TRIM11,PPARD,EPN1,SOCS5,KLF4,SPRED2,ZC3H12A,EMILIN1,INSIG1,LRP3,GREM1,PDE3B,WNT10B,ANKRD2,NGEF,ARPIN,TWIST2,CCDC85B,PBX1,LIMD1,YJEFN3,IGFBP5,CTDSP1,RCC2,MAD2L2,CARM1,SLC6A4,VASN,STK11,SARS,MIR214,RF00024,KLF13,TRIB2	yellow	VSMC_M3
GO:0006820	0.95	0.08	15	319	anion transport	BP	1	ENPP1,SLC52A2,ASNA1,PLEKHA8,TTYH2,SLC9A3R1,SLC16A5,SFXN5,SLC35C2,SLC16A14,SLC19A1,SLC26A6,PLA2G3,CTNS,SLC27A4	yellow	VSMC_M3
GO:0042327	0.95	0.07	31	612	positive regulation of phosphorylation	BP	1	NAB2,SQSTM1,SNX9,FNIP1,PRR5,PDGFRA,WDFY2,ACSL1,TNKS1BP1,GREM1,CX3CL1,DGKZ,TYRO3,PDGFA,CLCF1,RIPK3,TNK2,PHB2,BAD,TNFRSF1A,MAD2L2,ARHGEF2,CSK,AKT1,MYDGF,MAP2K2,IRAK1,STK11,TELO2,VAC14,ERBB4	yellow	VSMC_M3
GO:0006986	0.95	0.10	5	124	response to unfolded protein	BP	1	ATF6B,SERP2,SELENOS,FBXO6,STUB1	yellow	VSMC_M3
GO:0098771	0.95	0.07	25	502	inorganic ion homeostasis	BP	1	ENPP1,ITPR3,PDGFRA,TTC7A,SLC9A3R1,SLC30A3,RAB20,PDE4D,SWAP70,SLC11A2,CX3CL1,SOD1,CHP1,GSTO1,SLC26A6,SPX,LETM1,CHERP,CCDC22,CORO1A,TSPOAP1,ANKRD9,PACS2,AP3D1,CCDC115	yellow	VSMC_M3
GO:0048469	0.95	0.11	4	116	cell maturation	BP	1	CX3CL1,SLC26A6,PLA2G3,TMEM79	yellow	VSMC_M3
GO:0006836	0.96	0.10	5	139	neurotransmitter transport	BP	1	SCRIB,SYT12,GIT1,SLC6A4,UNC13A	yellow	VSMC_M3
GO:0007163	0.96	0.09	7	189	establishment or maintenance of cell polarity	BP	1	SCRIB,WDPCP,SLC9A3R1,NUMA1,DCTN1,RHOC,STK11	yellow	VSMC_M3
GO:0006936	0.96	0.08	11	256	muscle contraction	BP	1	ZC3H12A,PDE4D,SOD1,ANKRD2,GSTO1,PABPN1,SCN4A,SPX,C12orf57,RANGRF,TAZ	yellow	VSMC_M3
GO:0007409	0.96	0.07	14	341	axonogenesis	BP	1	DBNL,PLXND1,BAIAP2,IGSF9,NECTIN1,LIMK1,C12orf57,CNP,DBN1,CRABP2,S100A6,ANAPC2,STK11,TWF2	yellow	VSMC_M3

GO:0006643	0.96	0.09	7	164	membrane lipid metabolic process	BP	1	CERK,PIGO,ST3GAL4,SGPL1,DPM3,CSNK1G2,GBA2	yellow	VSMC_M3
GO:0008015	0.96	0.07	17	369	blood circulation	BP	1	PPARD,PLEC,ZC3H12A,PDE4D,SOD1,DLL4,GSTO1,GPX1,ANPEP,SPX,PLCB3,RANGRF,AKT1,SLC6A4,TJP3,TAZ,ELN	yellow	VSMC_M3
GO:0035966	0.96	0.09	6	143	response to topologically incorrect protein	BP	1	BAG6,ATF6B,SERP2,SELENOS,FBXO6,STUB1	yellow	VSMC_M3
GO:0051239	0.96	0.05	112	1937	regulation of multicellular organismal process	BP	1	ENPP1,CDK4,BAG6,H2AFY,TRIM11,PPARD,TSTA3,SCRIB,CUL7,PDGFRA,IL1R1,EPN1,SSC5D,SOCS5,CNOT3,KLF4,DBNL,TPBG,WDPCP,SPRED2,ZC3H12A,MGLL,ANXA5,RELB,SLC9A3R1,PLXND1,EMILIN1,UCP2,METTL3,BAIAP2,NUMA1,SCARB1,PDE4D,ACSL1,GREM1,KDM2B,PDE3B,CX3CL1,WNT10B,SOD1,EGR3,DLL4,SCD,HTATIP2,ST3GAL4,SELENOS,GSTO1,PDGFA,MMP14,CLCF1,TRIM27,LIMK1,SCN4A,SPX,RIPK3,PLA2G3,SART1,PTPN23,PBX1,KAT5,RELA,SGPL1,YJEFN3,PHB2,IGFBP5,C12orf57,BAD,AAMP,CTDSP1,CD9,PLAU,TNFRSF1A,C1QBP,TAF6L,PRXL2A,DBN1,CAPN2,MAD2L2,CRABP2,ARHGEF2,PER1,RANGRF,EEF2K,CD276,CSK,AKT1,KHSRP,GIT1,SLC6A4,MYDGF,SIRT6,CACTIN,VASN,EHMT1,AP3D1,IRAK1,ANAPC2,STK11,JMJD8,CARD9,SARS,TGFBR3,TRADD,MIR214,MIR155,ERBB4,TWF2,PTPRR,KLF13,RNH1,TRIB2,GLIPR2	yellow	VSMC_M3
GO:0032409	0.96	0.08	8	198	regulation of transporter activity	BP	1	SLC9A3R1,PDE4D,CHP1,GSTO1,TACO1,PHB2,RANGRF,GSG1L	yellow	VSMC_M3
GO:0051302	0.96	0.09	5	138	regulation of cell division	BP	1	BCL2L1,PDGFA,CDK2AP2,MYO19,GIT1	yellow	VSMC_M3
GO:0050877	0.96	0.06	34	678	nervous system process	BP	1	NDUFB9,ITPR3,PLEC,TPBG,HOXA1,MGLL,SLC9A3R1,CIC,BAIAP2,RIC8A,LRIG1,SLC11A2,MMP24,DCTN1,WNT10B,SOD1,HPS1,DLL4,ST3GAL4,GSTO1,GPX1,SCN4A,RABGGTA,SPX,TACO1,TMEM120A,BLOC1S4,NDP,C12orf57,AKT1,GIT1,SLC6A4,CTNS,RGS16	yellow	VSMC_M3
GO:0008016	0.96	0.09	5	144	regulation of heart contraction	BP	1	ZC3H12A,PDE4D,GSTO1,SPX,RANGRF	yellow	VSMC_M3
GO:0051240	0.96	0.05	57	1050	positive regulation of multicellular organismal process	BP	1	H2AFY,SCRIB,CUL7,IL1R1,SOCS5,CNOT3,KLF4,DBNL,TPBG,ZC3H12A,PLXND1,UCP2,BAIAP2,NUMA1,PDE4D,ACSL1,GREM1,KDM2B,CX3CL1,WNT10B,SOD1,EGR3,SCD,ST3GAL4,GSTO1,MMP14,CLCF1,TRIM27,LIMK1,SPX,PLA2G3,SART1,RELA,BAD,C1QBP,DBN1,MAD2L2,CRABP2,ARHGEF2,EEF2K,CD276,AKT1,MYDGF,SIRT6,EHMT1,AP3D1,IRAK1,ANAPC2,STK11,JMJD8,CARD9,TGFBR3,TRADD,MIR214,ERBB4,TWF2,GLIPR2	yellow	VSMC_M3
GO:0003012	0.96	0.07	15	332	muscle system process	BP	1	KLF4,ZC3H12A,PDE4D,SOD1,ANKRD2,GSTO1,PABPN1,SCN4A,SPX,IGFBP5,C12orf57,TNFRSF1A,RANGRF,TAZ,MIR214	yellow	VSMC_M3
GO:0098739	0.96	0.10	4	123	import across plasma membrane	BP	1	SLC9A3R1,ACSL1,SLC19A1,AKT1	yellow	VSMC_M3
GO:0033157	0.96	0.08	8	196	regulation of intracellular protein transport	BP	1	SREBF2,ZC3H12A,DMAP1,INSIG1,CEP131,CHP1,PCNT,RBM22	yellow	VSMC_M3
GO:0051606	0.96	0.09	5	135	detection of stimulus	BP	1	SSC5D,PNKP,SCARB1,MMP24,TMEM120A	yellow	VSMC_M3
GO:0031669	0.96	0.08	7	182	cellular response to nutrient levels	BP	1	FNIP1,SREBF2,ZC3H12A,UCP2,DCPS,WDR24,FADS1	yellow	VSMC_M3
GO:0002703	0.96	0.09	5	136	regulation of leukocyte mediated immunity	BP	1	IL1R1,CLCF1,RIPK3,PLA2G3,MAD2L2	yellow	VSMC_M3
GO:0045664	0.96	0.09	5	145	regulation of neuron differentiation	BP	1	PBX1,CTDSP1,ARHGEF2,SLC6A4,HMG20B	yellow	VSMC_M3
GO:0006605	0.96	0.07	12	262	protein targeting	BP	1	SAMM50,SREBF2,ASNA1,YIF1B,CHP1,AIP,PEX5,ZDHHC3,CDC37,ATG3,AP3D1,TIMM22	yellow	VSMC_M3
GO:0018105	0.96	0.07	10	249	peptidyl-serine phosphorylation	BP	1	FNIP1,PDE4D,TNKS1BP1,STK32C,PKN1,MAD2L2,AKT1,MAP2K2,MKNK2,CSNK1G2	yellow	VSMC_M3
GO:0007167	0.96	0.05	37	752	enzyme linked receptor protein signaling pathway	BP	1	ENPP1,CD63,CDK4,PDGFRA,SOCS5,SPRED2,ZYX,SLC9A3R1,EMILIN1,BAIAP2,NUMA1,TNS2,GREM1,DOK1,TYRO3,PDGFA,NGEF,TNK2,RELA,SGPL1,IGFBP5,MVB12A,PIP4K2B,MVP,MTMR4,AKT1,GIT1,VASN,STK11,SLC27A4,TGFBR3,TRADD,NDST1,ERBB4,PTPRR,IGFBP6,STUB1	yellow	VSMC_M3
GO:0018209	0.96	0.07	11	267	peptidyl-serine modification	BP	1	FNIP1,PDE4D,TNKS1BP1,STK32C,PKN1,MAD2L2,AKT1,MAP2K2,MKNK2,CSNK1G2,NTMT1	yellow	VSMC_M3
GO:1903530	0.96	0.06	19	399	regulation of secretion by cell	BP	1	PPARD,EIPR1,MICAL1,MYRIP,UCP2,AACS,SYT12,PCK2,CAPN10,PLA2G3,PTPN23,BAD,TNFRSF1A,GLUD1,SNF8,GIT1,SLC6A4,MIDN,JAGN1	yellow	VSMC_M3
GO:0051783	0.96	0.09	4	114	regulation of nuclear division	BP	1	CUL7,MAD1L1,MAD2L2,AURKAIP1	yellow	VSMC_M3
GO:0051046	0.96	0.06	21	434	regulation of secretion	BP	1	PPARD,EIPR1,MICAL1,SLC9A3R1,MYRIP,UCP2,AACS,SYT12,PCK2,SPX,CAPN10,PLA2G3,PTPN23,BAD,TNFRSF1A,GLUD1,SNF8,GIT1,SLC6A4,MIDN,JAGN1	yellow	VSMC_M3
GO:0070588	0.96	0.07	8	211	calcium ion transmembrane transport	BP	1	ITPR3,PDE4D,CX3CL1,GSTO1,LETM1,CHERP,CORO1A,AKT1	yellow	VSMC_M3
GO:0007166	0.96	0.04	111	1935	cell surface receptor signaling pathway	BP	1	ENPP1,CD63,CDK4,BAG6,CTHRC1,TTLL12,SREBF2,TXNDC15,IFT27,SHARPIN,PDGFRA,IL1R1,EPN1,SOCS5,KLF4,TPBG,WDPCP,CCM2,SPRED2,ZC3H12A,ZYX,SNX3,PLEKHA4,KRT8,SLC9A3R1,PLXND1,EMILIN1,RNF220,CD79A,METTL3,BAIAP2,NUMA1,P4HB,DELE1,SCARB1,BCL2L1,ITGB7,PDE4D,SPRYD3,TNS2,ACSL1,GREM1,DOK1,CX3CL1,WNT10B,DGKZ,CCAR2,DLL4,TYRO3,SLC35C2,TSPAN5,ITPRIP,PDGFA,MMP14,GPX1,NGEF,CLCF1,LIMK1,TNK2,RELA,LIMD1,ZDHHC3,SGPL1,YJEFN3,PHB2,NDP,IGFBP5,BAD,LGALS3,TNFRSF1A,MVB12A,EVC,RCC2,PALM3,AMER1,PIP4K2B,MAD2L2,ARHGEF2,MVP,CORO1A,MTMR4,SMARCA4,CDC37,CD276,CSK,AKT1,TEDC1,RAB34,GIT1,NSDHL,CACTIN,VASN,HIC1,TEDC2,MKNK2,CSNK1G2,IRAK1,SSNA1,STK11,GAS8,SLC27A4,TGFBR3,TRADD,NDST1,TNFRSF12A,ERBB4,PTPRR,IGFBP6,JAGN1,APCDD1L,STUB1	yellow	VSMC_M3
GO:0015850	0.97	0.07	7	179	organic hydroxy compound transport	BP	1	SREBF2,LAMTOR1,SCARB1,SYT12,SLC26A6,YJEFN3,SLC6A4	yellow	VSMC_M3
GO:0030900	0.97	0.06	11	282	forebrain development	BP	1	TRAPPC9,SECISBP2,METTL3,KDM2B,TYRO3,ATIC,C12orf57,BAD,CNP,NDST1,ERBB4	yellow	VSMC_M3
GO:0007610	0.97	0.05	18	406	behavior	BP	1	ITPR3,TPBG,HOXA1,CIC,RIC8A,C12orf10,SLC11A2,DCTN1,SOD1,TACO1,C12orf57,CNP,CAPN2,GIT1,SLC6A4,CTNS,APRT,NEGR1	yellow	VSMC_M3
GO:0042692	0.97	0.06	13	303	muscle cell differentiation	BP	1	PLEC,PDGFRA,EHD2,KRT8,GREM1,WNT10B,MMP14,GPX1,PLEKHO1,IGFBP5,CD9,CAPN2,AKT1	yellow	VSMC_M3

GO:0043269	0.97	0.05	21	444	regulation of ion transport	BP	1	CD63,SLC9A3R1,PDE4D,CX3CL1,PACSIN3,CHP1,GSTO1,NKAIN1,SYT12,TRIM27,SCN4A,TACO1,PLA2G3,PHB2,LGALS3,PER1,RANGRF,CORO1A,AKT1,SLC6A4,GSG1L	yellow	VSMC_M3
GO:0034762	0.97	0.05	18	396	regulation of transmembrane transport	BP	1	ENPP1,CD63,SLC9A3R1,PDE4D,ACSL1,CX3CL1,CHP1,SELENOS,GSTO1,SCN4A,SLC26A6,CAPN10,TACO1,PHB2,RANGRF,CORO1A,AKT1,GSG1L	yellow	VSMC_M3
GO:0055001	0.97	0.07	5	150	muscle cell development	BP	1	PLEC,PDGFRA,KRT8,WNT10B,GPX1	yellow	VSMC_M3
GO:0031345	0.97	0.07	5	150	negative regulation of cell projection organization	BP	1	LGALS1,NGEF,ARPIN,CARM1,MIR214	yellow	VSMC_M3
GO:0045596	0.97	0.05	23	489	negative regulation of cell differentiation	BP	1	ENPP1,TRIM11,PPARD,SOCS5,SPRED2,ZC3H12A,INSIG1,LRP3,GREM1,WNT10B,ANKRD2,TWIST2,CCDC85B,PBX1,LIMD1,IGFBP5,CTDSP1,RCC2,MAD2L2,SLC6A4,VASN,KLF13,TRIB2	yellow	VSMC_M3
GO:0001505	0.97	0.07	5	147	regulation of neurotransmitter levels	BP	1	SCRIB,SYT12,GIT1,SLC6A4,UNC13A	yellow	VSMC_M3
GO:0007265	0.97	0.06	12	296	Ras protein signal transduction	BP	1	SQSTM1,SGSM3,DBNL,DOK1,RAB35,LIMK1,STMN3,ARHGEF2,RANGRF,DHCR24,CDC42EP5,ARHGEF1	yellow	VSMC_M3
GO:0031214	0.97	0.08	4	126	biomineral tissue development	BP	1	ENPP1,GREM1,WNT10B,NECTIN1	yellow	VSMC_M3
GO:0045165	0.97	0.07	6	172	cell fate commitment	BP	1	KLF4,WNT10B,DLL4,ARHGEF2,ZNF521,ERBB4	yellow	VSMC_M3
GO:0072503	0.97	0.05	13	306	cellular divalent inorganic cation homeostasis	BP	1	ITPR3,PDGFRA,SLC30A3,PDE4D,SWAP70,CX3CL1,GSTO1,LETM1,CHERP,CORO1A,TSPOAP1,PACS2,AP3D1	yellow	VSMC_M3
GO:0140352	0.97	0.04	31	618	export from cell	BP	1	PPARD,SCRIB,EIPR1,PDIA4,RCN3,MICAL1,MYRIP,UCP2,AACS,TYRO3,EXOC3,SYT12,PCK2,CAPN10,PLA2G3,PTPN23,BAD,TNFRSF1A,WASHC1,RAB3IL1,GLUD1,TMEM79,SNF8,CORO1A,GIT1,SLC6A4,MIDN,RABEPK,JAGN1,CAVIN1,UNC13A	yellow	VSMC_M3
GO:0110148	0.97	0.07	4	127	biomineralization	BP	1	ENPP1,GREM1,WNT10B,NECTIN1	yellow	VSMC_M3
GO:0045860	0.97	0.05	13	305	positive regulation of protein kinase activity	BP	1	NAB2,SNX9,ACSL1,GREM1,PDGFA,RIPK3,PHB2,CSK,AKT1,MAP2K2,IRAK1,STK11,TELO2	yellow	VSMC_M3
GO:0001894	0.97	0.06	7	189	tissue homeostasis	BP	1	RCN3,SOD1,CORO1A,CSK,ZNF830,TJP3,STK11	yellow	VSMC_M3
GO:0045861	0.97	0.05	9	213	negative regulation of proteolysis	BP	1	BAG6,FNIP1,KLF4,MICAL1,CCAR2,GPX1,UBXN1,AKT1,DHCR24	yellow	VSMC_M3
GO:0071363	0.97	0.04	25	545	cellular response to growth factor stimulus	BP	1	CD63,PDGFRA,KLF4,SPRED2,ZYX,EMILIN1,BAIAP2,NUMA1,GREM1,EGR3,DLL4,RAB35,CORO1B,RELA,CORO1A,MTMR4,EEF2K,AKT1,VASN,STK11,TGFBR3,NDST1,ERBB4,TWF2,STUB1	yellow	VSMC_M3
GO:0050801	0.97	0.04	26	542	ion homeostasis	BP	1	ENPP1,ITPR3,PDGFRA,TTC7A,SLC9A3R1,SLC30A3,RAB20,PDE4D,SWAP70,SLC11A2,CX3CL1,SOD1,CHP1,GSTO1,SLC26A6,SPX,LETM1,CHERP,CCDC22,RANGRF,CORO1A,TSPOAP1,ANKRD9,PACS2,AP3D1,CCDC115	yellow	VSMC_M3
GO:0007268	0.97	0.04	21	471	chemical synaptic transmission	BP	1	SQSTM1,PDLIM4,ITPR3,SCRIB,TPBG,BAIAP2,RAPSN,CX3CL1,DGKZ,EGR3,APBA2,SYT12,ZDHHC3,CNP,DBN1,AKT1,GIT1,SLC6A4,ANAPC2,UNC13A,NSMF	yellow	VSMC_M3
GO:0098916	0.97	0.04	21	471	anterograde trans-synaptic signaling	BP	1	SQSTM1,PDLIM4,ITPR3,SCRIB,TPBG,BAIAP2,RAPSN,CX3CL1,DGKZ,EGR3,APBA2,SYT12,ZDHHC3,CNP,DBN1,AKT1,GIT1,SLC6A4,ANAPC2,UNC13A,NSMF	yellow	VSMC_M3
GO:0017157	0.97	0.06	5	147	regulation of exocytosis	BP	1	MICAL1,SYT12,PLA2G3,SNF8,GIT1	yellow	VSMC_M3
GO:0006935	0.97	0.04	19	426	chemotaxis	BP	1	SCRIB,PLEC,PDGFRA,TPBG,PLXND1,SWAP70,GREM1,CX3CL1,EGR3,LSP1,RIN3,PDGFA,CORO1B,IGSF9,NECTIN1,LGALS3,PLAU,C1QBP,CORO1A	yellow	VSMC_M3
GO:0001934	0.97	0.04	26	543	positive regulation of protein phosphorylation	BP	1	NAB2,SQSTM1,SNX9,FNIP1,PRR5,WDFY2,ACSL1,TNKS1BP1,GREM1,CX3CL1,PDGFA,CLCF1,RIPK3,TNK2,PHB2,TNFRSF1A,MAD2L2,ARHGEF2,CSK,AKT1,MYDGF,MAP2K2,IRAK1,STK11,TELO2,ERBB4	yellow	VSMC_M3
GO:0042330	0.97	0.04	19	427	taxis	BP	1	SCRIB,PLEC,PDGFRA,TPBG,PLXND1,SWAP70,GREM1,CX3CL1,EGR3,LSP1,RIN3,PDGFA,CORO1B,IGSF9,NECTIN1,LGALS3,PLAU,C1QBP,CORO1A	yellow	VSMC_M3
GO:0006665	0.97	0.07	4	120	sphingolipid metabolic process	BP	1	CERK,SGPL1,CSNK1G2,GBA2	yellow	VSMC_M3
GO:0006468	0.97	0.03	65	1223	protein phosphorylation	BP	1	ENPP1,NAB2,CDK4,CTDSP2,SQSTM1,SNX9,FNIP1,PRR5,H2AFY,PLEC,PDGFRA,SOCS5,SPRED2,ZC3H12A,MICAL1,WDFY2,SLC9A3R1,EMILIN1,SERTAD1,TARBP2,PDE4D,ACSL1,TNKS1BP1,GREM1,CX3CL1,SOD1,CHP1,TYRO3,HTATIP2,STK32C,PDGFA,CLCF1,QARS,TRIM27,LIMK1,RIPK3,TNK2,SCYL1,MEX3B,MAP3K14,PHB2,CTDSP1,TNFRSF1A,PKN1,MAD2L2,CTBP1,ARHGEF2,MVP,EEF2K,CDC37,CSK,AKT1,SGK494,MYDGF,MAP2K2,CACTIN,CDK11B,MKNK2,CSNK1G2,IRAK1,STK11,TELO2,TGFBR3,ERBB4,TRIB2	yellow	VSMC_M3
GO:0019932	0.98	0.05	8	218	second-messenger-mediated signaling	BP	1	ITPR3,SLC9A3R1,PDE4D,PDE3B,CHP1,GSTO1,CHERP,CDC34	yellow	VSMC_M3
GO:0009952	0.98	0.06	5	150	anterior/posterior pattern specification	BP	1	RING1,HOXC4,KDM2B,PBX1,HOXC5	yellow	VSMC_M3
GO:0000280	0.98	0.04	16	355	nuclear division	BP	1	DCTN2,BAG6,NCAPH2,H2AFY,CUL7,FIGNL1,NUMA1,RRS1,PINX1,NUDC,PHB2,EML3,MAD1L1,MAD2L2,AURKAIP1,MIS12	yellow	VSMC_M3
GO:0003002	0.98	0.05	10	251	regionalization	BP	1	PBX2,RING1,RNF220,HOXC4,GREM1,KDM2B,DLL4,IRX1,PBX1,HOXC5	yellow	VSMC_M3
GO:0051260	0.98	0.06	4	128	protein homooligomerization	BP	1	AQP5,TMEM120A,LETM1,CARD9	yellow	VSMC_M3
GO:0061564	0.98	0.04	15	380	axon development	BP	1	KLF4,DBNL,PLXND1,BAIAP2,IGSF9,NECTIN1,LIMK1,C12orf57,CNP,DBN1,CRABP2,S100A6,ANAPC2,STK11,TWF2	yellow	VSMC_M3
GO:0055065	0.98	0.04	20	438	metal ion homeostasis	BP	1	ITPR3,PDGFRA,TTC7A,SLC30A3,PDE4D,SWAP70,SLC11A2,CX3CL1,SOD1,GSTO1,SPX,LETM1,CHERP,CCDC22,CORO1A,TSPOAP1,ANKRD9,PACS2,AP3D1,CCDC115	yellow	VSMC_M3
GO:0070848	0.98	0.03	26	570	response to growth factor	BP	1	CD63,PDGFRA,KLF4,SPRED2,ZYX,EMILIN1,BAIAP2,NUMA1,GREM1,EGR3,DLL4,RAB35,CORO1B,RELA,CORO1A,MTMR4,EEF2K,AKT1,VASN,STK11,TGFBR3,NDST1,ERBB4,TWF2,CDC34,STUB1	yellow	VSMC_M3
GO:0055080	0.98	0.03	23	494	cation homeostasis	BP	1	ITPR3,PDGFRA,TTC7A,SLC30A3,RAB20,PDE4D,SWAP70,SLC11A2,CX3CL1,SOD1,CHP1,GSTO1,SLC26A6,SPX,LETM1,CHERP,CCDC22,CORO1A,TSPOAP1,ANKRD9,PACS2,AP3D1,CCDC115	yellow	VSMC_M3
GO:0099537	0.98	0.04	21	477	trans-synaptic signaling	BP	1	SQSTM1,PDLIM4,ITPR3,SCRIB,TPBG,BAIAP2,RAPSN,CX3CL1,DGKZ,EGR3,APBA2,SYT12,ZDHHC3,CNP,DBN1,AKT1,GIT1,SLC6A4,ANAPC2,UNC13A,NSMF	yellow	VSMC_M3

GO:0099536	0.98	0.03	22	494	synaptic signaling	BP	1	SQSTM1,PDLIM4,ITPR3,SCRIB,TPBG,BAIAP2,RAPSN,CX3CL1,DGKZ,EGR3,APBA2,SYT12,RELA,ZDHHC3,CNP,DBN1,AKT1,GIT1,SLC6A4,ANAPC2,UNC13A,NSMF	yellow	VSMC_M3
GO:1903522	0.98	0.05	6	179	regulation of blood circulation	BP	1	ZC3H12A,PDE4D,GSTO1,SPX,RANGRF,AKT1	yellow	VSMC_M3
GO:0006816	0.98	0.04	11	282	calcium ion transport	BP	1	ITPR3,PDE4D,CX3CL1,PACSIN3,GSTO1,TRIM27,LETM1,LGALS3,CHERP,CORO1A,AKT1	yellow	VSMC_M3
GO:0030099	0.98	0.04	12	293	myeloid cell differentiation	BP	1	KMT2E,RELB,FASN,SLC11A2,PIR,THOC5,PLA2G3,PRXL2A,TGFBR3,KLF13,JAGN1,ZNF385A	yellow	VSMC_M3
GO:0010952	0.98	0.05	5	142	positive regulation of peptidase activity	BP	1	RCN3,PSME2,ADRM1,BAD,CARD9	yellow	VSMC_M3
GO:0007267	0.98	0.03	60	1146	cell-cell signaling	BP	1	SQSTM1,CTHRC1,PDLIM4,ITPR3,NUDT3,PPARD,SCRIB,KLF4,TPBG,EIPR1,ZYX,SNX3,PLEKHA4,SLC9A3R1,RNF220,MYRIP,UCP2,BAIAP2,AACS,GREM1,RAPSN,CX3CL1,WNT10B,DGKZ,EGR3,CCAR2,APBA2,PDGFA,SYT12,PCK2,CAPN10,PLA2G3,PTPN23,RELA,LIMD1,ZDHHC3,NDP,BAD,CNP,GLUD1,DBN1,AMER1,MAD2L2,RANGRF,SMARCA4,TMED1,AKT1,GIT1,SLC6A4,HIC1,CSNK1G2,ANAPC2,MIDN,STK11,FADS1,IGFBP6,JAGN1,UNC13A,NSMF,APCDD1L	yellow	VSMC_M3
GO:0007178	0.98	0.04	10	272	transmembrane receptor protein serine/threonine kinase signaling pathway	BP	1	SPRED2,ZYX,EMILIN1,NUMA1,GREM1,MTMR4,VASN,STK11,TGFBR3,STUB1	yellow	VSMC_M3
GO:0051100	0.98	0.05	4	129	negative regulation of binding	BP	1	MAD2L2,AKT1,GTF2F1,STUB1	yellow	VSMC_M3
GO:0061448	0.98	0.04	7	203	connective tissue development	BP	1	CDK4,PPARD,AACS,GREM1,WNT10B,EVC,AMER1	yellow	VSMC_M3
GO:0071103	0.98	0.04	7	200	DNA conformation change	BP	1	NCAPH2,H2AFY,PHF2,H3F3B,BANF1,H3F3A,UBN1	yellow	VSMC_M3
GO:0072507	0.98	0.03	13	323	divalent inorganic cation homeostasis	BP	1	ITPR3,PDGFRA,SLC30A3,PDE4D,SWAP70,CX3CL1,GSTO1,LETM1,CHERP,CORO1A,TSPOAP1,PACS2,AP3D1	yellow	VSMC_M3
GO:0048285	0.98	0.03	18	401	organelle fission	BP	1	DCTN2,BAG6,NCAPH2,H2AFY,CUL7,FIGNL1,NUMA1,RRS1,PINX1,NUDC,PHB2,EML3,MAD1L1,MAD2L2,MYO19,INF2,AURKAIP1,MIS12	yellow	VSMC_M3
GO:0006874	0.98	0.03	11	283	cellular calcium ion homeostasis	BP	1	ITPR3,PDGFRA,PDE4D,SWAP70,CX3CL1,GSTO1,LETM1,CHERP,CORO1A,TSPOAP1,PACS2	yellow	VSMC_M3
GO:0052547	0.98	0.03	13	294	regulation of peptidase activity	BP	1	FNIP1,KLF4,RCN3,MICAL1,DELE1,GPX1,PSME2,ADRM1,BAD,UBXN1,AKT1,DHCR24,CARD9	yellow	VSMC_M3
GO:0098655	0.98	0.03	30	623	cation transmembrane transport	BP	1	CD63,ITPR3,SLC9A3R1,SLC30A3,UCP2,PDE4D,UQCRFS1,SLC11A2,CX3CL1,CHP1,GSTO1,SCN4A,UQCRC1,RHCE,TACO1,ATP5MC3,PHB2,LETM1,CHERP,RANGRF,CORO1A,SLC39A1,ATP5PD,AKT1,SLC6A4,CTNS,NDUFS7,ATP5F1D,SURF1,GSG1L	yellow	VSMC_M3
GO:0034765	0.98	0.03	13	323	regulation of ion transmembrane transport	BP	1	CD63,SLC9A3R1,PDE4D,CX3CL1,CHP1,GSTO1,SCN4A,TACO1,PHB2,RANGRF,CORO1A,AKT1,GSG1L	yellow	VSMC_M3
GO:0006261	0.98	0.05	4	137	DNA-dependent DNA replication	BP	1	PNKP,LRWD1,ZNF830,CDC34	yellow	VSMC_M3
GO:0010950	0.98	0.04	4	127	positive regulation of endopeptidase activity	BP	1	PSME2,ADRM1,BAD,CARD9	yellow	VSMC_M3
GO:0043085	0.98	0.02	44	875	positive regulation of catalytic activity	BP	1	NAB2,SNX9,SGSM3,SCRIB,PDGFRA,KLF4,PNKP,RCN3,ARAP3,ARHGAP22,SCARB1,ACSL1,GREM1,CX3CL1,SOD1,DGKZ,TYRO3,PDGFA,PABPN1,NGEF,PSME2,RIPK3,ADRM1,SIPA1,RHOC,MID1IP1,PHB2,BAD,AIMP2,RCC2,CSK,DOCK11,AKT1,SNX18,MAP2K2,IRAK1,STK11,TELO2,CARD9,SLC27A4,VAC14,ERBB4,RGS16,STUB1	yellow	VSMC_M3
GO:0003007	0.99	0.03	6	197	heart morphogenesis	BP	1	PLXND1,DLL4,SIRT6,TAZ,TGFBR3,ELN	yellow	VSMC_M3
GO:0044093	0.99	0.02	60	1151	positive regulation of molecular function	BP	1	NAB2,SNX9,CTHRC1,SGSM3,TRAPPC9,SCRIB,PDGFRA,KLF4,PNKP,RCN3,PLXND1,ARAP3,ARHGAP22,SCARB1,ACSL1,PINX1,GREM1,CX3CL1,WNT10B,SOD1,DGKZ,CHP1,TYRO3,GSTO1,PDGFA,PABPN1,NGEF,PSME2,TRIM27,RIPK3,ADRM1,RELA,SIPA1,RHOC,MID1IP1,SMARCB1,PHB2,BAD,BUD31,AIMP2,RCC2,ARHGEF2,TRIM25,SMARCA4,CSK,MID2,DOCK11,AKT1,SNX18,MAP2K2,IRAK1,STK11,TELO2,CARD9,SLC27A4,VAC14,TRADD,ERBB4,RGS16,STUB1	yellow	VSMC_M3
GO:0031349	0.99	0.03	6	177	positive regulation of defense response	BP	1	CX3CL1,PLA2G3,IFI35,TNFRSF1A,CARD9,TRADD	yellow	VSMC_M3
GO:0055074	0.99	0.03	11	291	calcium ion homeostasis	BP	1	ITPR3,PDGFRA,PDE4D,SWAP70,CX3CL1,GSTO1,LETM1,CHERP,CORO1A,TSPOAP1,PACS2	yellow	VSMC_M3
GO:0043405	0.99	0.03	4	146	regulation of MAP kinase activity	BP	1	PDGFA,CSK,IRAK1,TRIB2	yellow	VSMC_M3
GO:0008284	0.99	0.02	33	681	positive regulation of cell population proliferation	BP	1	CDK4,CTHRC1,PPARD,B4GALT1,PDGFRA,RPS9,SERTAD1,CEP131,BCL2L1,GREM1,CX3CL1,WNT10B,EGR3,DLL4,PDGFA,CLCF1,PBX1,RELA,ARNT2,IGFBP5,BAD,CORO1A,S100A6,CARM1,CD276,AKT1,MYDGF,SIRT6,IRAK1,MVD,TGFBR3,MIR214,ERBB4	yellow	VSMC_M3
GO:0006811	0.99	0.02	53	1026	ion transport	BP	1	ENPP1,CD63,ITPR3,SLC52A2,ASNA1,PLEKHA8,TTYH2,SLC9A3R1,SLC16A5,SLC30A3,UCP2,PDE4D,UQCRFS1,SLC11A2,CX3CL1,PACSIN3,SFXN5,CHP1,SLC35C2,GSTO1,SLC16A14,NKAIN1,SYT12,SLC19A1,NECTIN1,TRIM27,SCN4A,SLC26A6,UQCRC1,RHCE,TACO1,PLA2G3,ATP5MC3,TMEM120A,PHB2,LETM1,LGALS3,CHERP,PER1,RANGRF,CORO1A,SLC39A1,ATP5PD,S100A6,AKT1,SLC6A4,CTNS,NDUFS7,ATP5F1D,SLC27A4,SLC44A3,SURF1,GSG1L	yellow	VSMC_M3
GO:0052548	0.99	0.02	11	270	regulation of endopeptidase activity	BP	1	FNIP1,KLF4,MICAL1,DELE1,GPX1,PSME2,ADRM1,BAD,AKT1,DHCR24,CARD9	yellow	VSMC_M3
GO:0001819	0.99	0.02	12	304	positive regulation of cytokine production	BP	1	SCRIB,IL1R1,PDE4D,CX3CL1,SOD1,TRIM27,PLA2G3,RELA,ARHGEF2,CD276,IRAK1,CARD9	yellow	VSMC_M3
GO:0046578	0.99	0.03	4	161	regulation of Ras protein signal transduction	BP	1	SQSTM1,SGSM3,STMN3,ARHGEF2	yellow	VSMC_M3
GO:0006812	0.99	0.02	39	796	cation transport	BP	1	CD63,ITPR3,SLC9A3R1,SLC30A3,UCP2,PDE4D,UQCRFS1,SLC11A2,CX3CL1,PACSIN3,CHP1,GSTO1,NKAIN1,SYT12,NECTIN1,TRIM27,SCN4A,UQCRC1,RHCE,TACO1,PLA2G3,ATP5MC3,PHB2,LETM1,LGALS3,CHERP,PER1,RANGRF,CORO1A,SLC39A1,ATP5PD,AKT1,SLC6A4,CTNS,NDUFS7,ATP5F1D,SLC44A3,SURF1,GSG1L	yellow	VSMC_M3
GO:0044057	0.99	0.02	16	398	regulation of system process	BP	1	KLF4,ZC3H12A,MGLL,SLC9A3R1,BAIAP2,PDE4D,SOD1,GSTO1,SCN4A,SPX,IGFBP5,C12orf57,TNFRSF1A,RANGRF,AKT1,MIR214	yellow	VSMC_M3
GO:0042391	0.99	0.02	11	302	regulation of membrane potential	BP	1	UCP2,BAIAP2,PYCR1,BCL2L1,SOD1,SCN4A,CHCHD10,BAD,RANGRF,AKT1,CTNS	yellow	VSMC_M3
GO:0051099	0.99	0.03	4	141	positive regulation of binding	BP	1	CTHRC1,KLF4,PLXND1,PINX1	yellow	VSMC_M3
GO:0051321	0.99	0.02	6	192	meiotic cell cycle	BP	1	BAG6,NCAPH2,FIGNL1,METTL3,NUMA1,TUBGCP2	yellow	VSMC_M3
GO:0071900	0.99	0.01	11	300	regulation of protein serine/threonine kinase activity	BP	1	H2AFY,SERTAD1,ACSL1,PDGFA,CDC37,CSK,AKT1,MAP2K2,IRAK1,TELO2,TRIB2	yellow	VSMC_M3

GO:0007186	0.99	0.01	19	458	G protein-coupled receptor signaling pathway	BP	1	ITPR3,RGS3,MGLL,SLC9A3R1,RIC8A,PDE4D,PDE3B,CX3CL1,DGKZ,TYRO3,CISH,ZDHHC3,GNB2,PLCB3,AKT1,GIT1,ARHGEF1,RGS16,RGS18	yellow	VSMC_M3
GO:0051098	0.99	0.01	11	296	regulation of binding	BP	1	CTHRC1,KLF4,PLXND1,PINX1,MAD2L2,CARM1,AKT1,GTF2F1,GAS8,TGFBR3,STUB1	yellow	VSMC_M3
GO:0034220	0.99	0.01	36	765	ion transmembrane transport	BP	1	CD63,ITPR3,SLC9A3R1,SLC30A3,UCP2,PDE4D,UQCRFS1,SLC11A2,CX3CL1,SFXN5,CHP1,SLC35C2,GSTO1,SLC19A1,SCN4A,SLC26A6,UQCRC1,RHCE,TACO1,ATP5MC3,TMEM120A,PHB2,LETM1,CHERP,RANGRF,CORO1A,SLC39A1,ATP5PD,S100A6,AKT1,SLC6A4,CTNS,NDUFS7,ATP5F1D,SURF1,GSG1L	yellow	VSMC_M3
GO:0007507	0.99	0.01	18	474	heart development	BP	1	PDLIM4,PPARD,PLEC,PDGFRA,CCM2,PLXND1,EMILIN1,SNX17,GREM1,DLL4,TNFRSF1A,SIRT6,MBD3,TAZ,TGFBR3,NDST1,ERBB4,ELN	yellow	VSMC_M3
GO:0006260	0.99	0.01	7	230	DNA replication	BP	1	PNKP,ZBTB38,LRWD1,SDE2,NFIX,ZNF830,CDC34	yellow	VSMC_M3
GO:0048863	1.00	0.01	4	171	stem cell differentiation	BP	1	PDGFRA,METTL3,GREM1,ERBB4	yellow	VSMC_M3
GO:0007389	1.00	0.01	11	329	pattern specification process	BP	1	PBX2,RING1,RNF220,HOXC4,GREM1,KDM2B,DLL4,IRX1,PBX1,ERBB4,HOXC5	yellow	VSMC_M3
GO:0055085	1.00	0.00	50	1047	transmembrane transport	BP	1	ENPP1,CD63,ITPR3,PPARD,SLC9A3R1,SLC16A5,SLC30A3,UCP2,SCARB1,PDE4D,UQCRFS1,ACSL1,SLC11A2,AQP5,CX3CL1,SFXN5,CHP1,SLC35C2,SELENOS,GSTO1,SLC16A14,SLC19A1,SCN4A,SLC26A6,UQCRC1,CAPN10,RHCE,TACO1,ATP5MC3,TMEM120A,PEX5,PHB2,LETM1,CHERP,RANGRF,CORO1A,SLC39A1,ATP5PD,S100A6,AKT1,SLC6A4,SLC35E2B,CTNS,NDUFS7,ATP5F1D,SLC27A4,SLC44A3,SURF1,TIMM22,GSG1L	yellow	VSMC_M3
GO:0030001	1.00	0.00	23	587	metal ion transport	BP	1	CD63,ITPR3,SLC9A3R1,SLC30A3,PDE4D,SLC11A2,CX3CL1,PACSIN3,CHP1,GSTO1,NKAIN1,NECTIN1,TRIM27,SCN4A,LETM1,LGALS3,CHERP,PER1,RANGRF,CORO1A,SLC39A1,AKT1,SLC6A4	yellow	VSMC_M3
GO:0003008	1.00	0.00	58	1240	system process	BP	1	NDUFB9,ITPR3,PPARD,PLEC,GRHPR,KLF4,TPBG,HOXA1,ZC3H12A,MGLL,SLC9A3R1,CIC,BAIAP2,RIC8A,LRIG1,SCARB1,PDE4D,LRP3,SLC11A2,MMP24,AQP5,DCTN1,WNT10B,SOD1,ANKRD2,HPS1,DLL4,ST3GAL4,GSTO1,PABPN1,GPX1,SLC19A1,ANPEP,SCN4A,SLC26A6,RABGGTA,SPX,TACO1,TMEM120A,BLOC1S4,NDP,IGFBP5,C12orf57,PLCB3,TNFRSF1A,PKN1,RANGRF,ZNF830,AKT1,GIT1,SLC6A4,TJP3,CTNS,TAZ,SLC27A4,MIR214,ELN,RGS16	yellow	VSMC_M3
GO:0032103	1.00	0.00	9	286	positive regulation of response to external stimulus	BP	1	TPBG,SWAP70,CX3CL1,PLA2G3,IFI35,TNFRSF1A,C1QBP,CARD9,TRADD	yellow	VSMC_M3
GO:0099537	0.00	1.00	288	477	trans-synaptic signaling	BP	1	STAC3,KIF5A,PDE7B,SNCAIP,MAPK8IP2,SLC12A2,RIMS2,PRRT1,PRKN,AGER,SYNGAP1,AKAP7,GRM4,EXOC4,ATF4,SYNGR1,NTRK2,PLA2G6,PICK1,BTBD9,CHRNA3,LRRC4,GRM8,CHRNA5,SRF,GABRA1,APBA1,PHF24,USP46,KIT,SLC1A1,ROR2,GABBR2,BRSK1,PRKCE,RIMS1,RIMS3,ACP4,SHANK1,PPT1,CRHR2,SLC1A4,PTN,DGKI,EGFR,NCDN,GRIK2,DLGAP3,AMPH,SDCBP,SLC7A11,PPFIA3,LIN7B,NTF4,PRKAR2B,GRIN2D,MTMR2,NAPA,SLC8A2,GAD2,CACNB2,DLG2,PCDH8,GRIK5,NAPB,PLAT,CHRNA6,FARP1,FCHSD2,SYT1,CACNB4,RAB5A,LIN7A,PLCB1,PLCL2,RAC3,PRNP,FCHSD1,P2RX2,PTPRA,STX2,SYT9,SNPH,EPHB1,PLK2,HTR7,FAM107A,NR4A1,PXK,ABHD6,CLSTN2,P2RX3,CHRM5,ERC2,MEF2C,ASIC1,P2RX7,DLGAP4,GUCY1A1,GUCY1B1,CUX2,P2RY1,PTK2B,MME,RAPGEF2,EIF2AK4,SYNJ1,HOMER1,CHRM4,F2R,VPS18,SLC6A2,GLS,ITPKA,CYFIP1,DTNBP1,SLC12A5,KCNIP2,CHRM3,LZTS1,SYT8,GRM2,BDNF,SLC4A10,NEURL1,DNM1L,SLC6A3,SORCS3,PPP3CA,SLC12A7,ADRA2A,IGSF11,ADARB1,SNAP23,DAG1,NLGN4Y,SSH1,STAU1,KCNB1,KRAS,BSN,ADNP,DISC1,GPER1,GRIK4,AGT,ATP1A2,NFATC4,RPS6KA1,SYAP1,STX1A,TMEM25,NPAS4,NTSR1,YTHDF1,CNIH2,BACE1,DNAJC5,GLUL,CSPG5,DLG1,CREB1,YWHAG,HTR3B,SPG11,MAPT,RAP1B,BLOC1S6,SRGN,VGF,EIF4EBP2,TNR,NRXN2,CASK,MPP2,BCHE,PNKD,CRTC1,PPP3CB,RAB3A,PINK1,KCNN1,SYN1,SNAP29,STAT3,KCNA1,CD38,NPTX2,MAPK1,CHRM1,TMOD2,NSG1,DAGLB,MINK1,RAB8A,HAP1,EPHA4,RNF216,DLG4,SYT7,IQSEC2,NLGN2,CHRNB1,CNIH3,RGS14,CACNB1,SYNJ2BP,HRH2,CPLX1,STX1B,STX4,PSEN1,CACNA1A,SYT11,NLGN3,KCTD13,MYCBPAP,CACNA1G,AC138894.1,CHRNB2,RAB11A,DGKE,CALM[illegible]	grey	VSMC_M10
GO:0007268	0.00	1.00	284	471	chemical synaptic transmission	BP	1	STAC3,KIF5A,PDE7B,SNCAIP,MAPK8IP2,SLC12A2,RIMS2,PRRT1,PRKN,AGER,SYNGAP1,AKAP7,GRM4,EXOC4,ATF4,SYNGR1,NTRK2,PLA2G6,PICK1,BTBD9,CHRNA3,LRRC4,GRM8,CHRNA5,SRF,GABRA1,APBA1,PHF24,USP46,KIT,SLC1A1,ROR2,GABBR2,BRSK1,PRKCE,RIMS1,RIMS3,ACP4,SHANK1,PPT1,CRHR2,SLC1A4,PTN,DGKI,EGFR,NCDN,GRIK2,DLGAP3,AMPH,SDCBP,SLC7A11,PPFIA3,LIN7B,NTF4,PRKAR2B,GRIN2D,MTMR2,NAPA,SLC8A2,GAD2,CACNB2,DLG2,PCDH8,GRIK5,NAPB,PLAT,CHRNA6,FCHSD2,SYT1,CACNB4,RAB5A,LIN7A,PLCB1,PLCL2,RAC3,PRNP,FCHSD1,P2RX2,PTPRA,STX2,SYT9,SNPH,EPHB1,PLK2,HTR7,FAM107A,NR4A1,PXK,ABHD6,CLSTN2,P2RX3,CHRM5,ERC2,MEF2C,ASIC1,P2RX7,DLGAP4,GUCY1A1,GUCY1B1,CUX2,P2RY1,PTK2B,MME,RAPGEF2,EIF2AK4,SYNJ1,HOMER1,CHRM4,F2R,VPS18,SLC6A2,GLS,ITPKA,CYFIP1,DTNBP1,SLC12A5,KCNIP2,CHRM3,LZTS1,SYT8,GRM2,BDNF,SLC4A10,NEURL1,DNM1L,SLC6A3,SORCS3,PPP3CA,SLC12A7,ADRA2A,IGSF11,ADARB1,SNAP23,NLGN4Y,SSH1,STAU1,KCNB1,KRAS,BSN,ADNP,DISC1,GPER1,GRIK4,AGT,ATP1A2,NFATC4,RPS6KA1,SYAP1,STX1A,TMEM25,NPAS4,NTSR1,YTHDF1,CNIH2,BACE1,DNAJC5,GLUL,CSPG5,DLG1,CREB1,YWHAG,HTR3B,SPG11,MAPT,RAP1B,BLOC1S6,SRGN,VGF,EIF4EBP2,TNR,NRXN2,CASK,MPP2,BCHE,PNKD,CRTC1,PPP3CB,RAB3A,PINK1,KCNN1,SYN1,SNAP29,STAT3,KCNA1,CD38,NPTX2,MAPK1,CHRM1,TMOD2,NSG1,MINK1,RAB8A,HAP1,EPHA4,RNF216,DLG4,SYT7,IQSEC2,NLGN2,CHRNB1,CNIH3,RGS14,CACNB1,SYNJ2BP,HRH2,CPLX1,STX1B,STX4,PSEN1,CACNA1A,SYT11,NLGN3,KCTD13,MYCBPAP,CACNA1G,AC138894.1,CHRNB2,RAB11A,DGKE,CALM1,SNAPIN,PRKCB,RIT2[illegible]	grey	VSMC_M10

GO:0098916	0.00	1.00	284	471	anterograde trans-synaptic signaling	BP	1	STAC3,KIF5A,PDE7B,SNCAIP,MAPK8IP2,SLC12A2,RIMS2,PRRT1,PRKN,AGER,SYNGAP1,AKAP7,GRM4,EXOC4,ATF4,SYNGR1,NTRK2,PLA2G6,PICK1,BTBD9,CHRNA3,LRRC4,GRM8,CHRNA5,SRF,GABRA1,APBA1,PHF24,USP46,KIT,SLC1A1,ROR2,GABBR2,BRSK1,PRKCE,RIMS1,RIMS3,ACP4,SHANK1,PPT1,CRHR2,SLC1A4,PTN,DGKI,EGFR,NCDN,GRIK2,DLGAP3,AMPH,SDCBP,SLC7A11,PPFIA3,LIN7B,NTF4,PRKAR2B,GRIN2D,MTMR2,NAPA,SLC8A2,GAD2,CACNB2,DLG2,PCDH8,GRIK5,NAPB,PLAT,CHRNA6,FCHSD2,SYT1,CACNB4,RAB5A,LIN7A,PLCB1,PLCL2,RAC3,PRNP,FCHSD1,P2RX2,PTPRA,STX2,SYT9,SNPH,EPHB1,PLK2,HTR7,FAM107A,NR4A1,PXK,ABHD6,CLSTN2,P2RX3,CHRM5,ERC2,MEF2C,ASIC1,P2RX7,DLGAP4,GUCY1A1,GUCY1B1,CUX2,P2RY1,PTK2B,MME,RAPGEF2,EIF2AK4,SYNJ1,HOMER1,CHRM4,F2R,VPS18,SLC6A2,GLS,ITPKA,CYFIP1,DTNBP1,SLC12A5,KCNIP2,CHRM3,LZTS1,SYT8,GRM2,BDNF,SLC4A10,NEURL1,DNM1L,SLC6A3,SORCS3,PPP3CA,SLC12A7,ADRA2A,IGSF11,ADARB1,SNAP23,NLGN4Y,SSH1,STAU1,KCNB1,KRAS,BSN,ADNP,DISC1,GPER1,GRIK4,AGT,ATP1A2,NFATC4,RPS6KA1,SYAP1,STX1A,TMEM25,NPAS4,NTSR1,YTHDF1,CNIH2,BACE1,DNAJC5,GLUL,CSPG5,DLG1,CREB1,YWHAG,HTR3B,SPG11,MAPT,RAP1B,BLOC1S6,SRGN,VGF,EIF4EBP2,TNR,NRXN2,CASK,MPP2,BCHE,PNKD,CRTC1,PPP3CB,RAB3A,PINK1,KCNN1,SYN1,SNAP29,STAT3,KCNA1,CD38,NPTX2,MAPK1,CHRM1,TMOD2,NSG1,MINK1,RAB8A,HAP1,EPHA4,RNF216,DLG4,SYT7,IQSEC2,NLGN2,CHRNB1,CNIH3,RGS14,CACNB1,SYNJ2BP,HRH2,CPLX1,STX1B,STX4,PSEN1,CACNA1A,SYT11,NLGN3,KCTD13,MYCBPAP,CACNA1G,AC138894.1,CHRNB2,RAB11A,DGKE,CALM1,SNAPIN,PRKCB,RIT2[illegible]	grey	VSMC_M10
GO:0099536	0.00	1.00	297	494	synaptic signaling	BP	1	STAC3,KIF5A,PDE7B,SNCAIP,MAPK8IP2,SLC12A2,RIMS2,PRRT1,PRKN,AGER,SYNGAP1,AKAP7,GRM4,LY6E,EXOC4,ATF4,SYNGR1,NTRK2,PLA2G6,PICK1,BTBD9,CHRNA3,LRRC4,GRM8,CHRNA5,SRF,GABRA1,APBA1,PHF24,USP46,KIT,SLC1A1,ROR2,GABBR2,BRSK1,PRKCE,RIMS1,RIMS3,ACP4,SHANK1,PPT1,CRHR2,SLC1A4,PTN,DGKI,EGFR,NCDN,GRIK2,DLGAP3,AMPH,SDCBP,SLC7A11,PPFIA3,LIN7B,NTF4,PRKAR2B,GRIN2D,MTMR2,NAPA,SLC8A2,GAD2,CACNB2,DLG2,PCDH8,GRIK5,NAPB,PLAT,KPNA2,CHRNA6,FARP1,FCHSD2,SYT1,CACNB4,RAB5A,LIN7A,PLCB1,PLCL2,RAC3,PRNP,FCHSD1,P2RX2,NRG1,PTPRA,STX2,SYT9,SNPH,EPHB1,PLK2,HTR7,FAM107A,NR4A1,PXK,ABHD6,CLSTN2,P2RX3,CHRM5,ERC2,MEF2C,ASIC1,P2RX7,DLGAP4,GUCY1A1,GUCY1B1,CUX2,P2RY1,PTK2B,MME,RAPGEF2,EIF2AK4,SYNJ1,HOMER1,CHRM4,F2R,VPS18,SLC6A2,GLS,ITPKA,CYFIP1,DTNBP1,SLC12A5,KCNIP2,CHRM3,LZTS1,SYT8,GRM2,BDNF,SLC4A10,NEURL1,DNM1L,SLC6A3,SORCS3,PPP3CA,SLC12A7,ADRA2A,IGSF11,ADARB1,SNAP23,DAG1,NLGN4Y,SSH1,STAU1,KCNB1,KRAS,BSN,ADNP,GRK2,DISC1,GPER1,GRIK4,AGT,ATP1A2,NFATC4,RPS6KA1,SYAP1,STX1A,TMEM25,NPAS4,NTSR1,YTHDF1,CNIH2,BACE1,DNAJC5,GLUL,CSPG5,DLG1,CREB1,YWHAG,HTR3B,SPG11,MAPT,RAP1B,BLOC1S6,SRGN,VGF,EIF4EBP2,TNR,NRXN2,CASK,MPP2,BCHE,PNKD,CRTC1,PPP3CB,RAB3A,PINK1,KCNN1,SYN1,SNAP29,STAT3,KCNA1,CD38,NPTX2,MAPK1,CHRM1,TMOD2,NSG1,DAGLB,MINK1,RAB8A,HAP1,EPHA4,RNF216,DLG4,SYT7,IQSEC2,NLGN2,CHRNB1,CNIH3,RGS14,CACNB1,SYNJ2BP,HRH2,CPLX1,STX1B,STX4,PSEN1,CACNA1A,SYT11,NLGN3,KCTD13,MYCBPAP,CACNA1G,AC138894.1,CH[illegible]	grey	VSMC_M10
GO:0009063	0.00	1.00	60	84	cellular amino acid catabolic process	BP	1	GLS2,SHMT2,ALDH8A1,RIDA,GPT,CARNMT1,PPAT,HMGCLL1,IL4I1,DDO,BCAT2,GAD2,HNMT,IDO2,IDO1,AMDHD1,CSAD,AHCY,MCCC2,SDSL,IVD,HOGA1,HIBCH,GLS,ACAD8,MTRR,ALDH5A1,CBSL,HGD,AMT,MCCC1,ACADSB,OAT,HMGCL,GLUL,FAH,PRODH,HDC,ALDH4A1,MAT1A,ASRGL1,HSD17B10,SLC25A44,ALDH6A1,GSTZ1,CRYM,SHMT1,ENOSF1,NOS2,PIPOX,ABAT,SARDH,GCSH,KYAT1,KYAT3,MIR21,HIBADH,SLC25A21,AUH,HYKK	grey	VSMC_M10
GO:0007031	0.00	1.00	26	32	peroxisome organization	BP	1	PEX7,PEX3,PEX6,PEX1,ZFAND6,HACL1,MAVS,PEX2,PEX16,LONP2,DNM1L,PEX11B,PEX19,FIS1,SEC16B,PLA2G16,PEX26,PEX14,ABCD4,RAB8B,PEX11G,PEX10,ABCD3,PJVK,PEX11A,PEX12	grey	VSMC_M10
GO:0055085	0.00	1.00	604	1047	transmembrane transport	BP	1	TIMM21,SLC39A5,NEDD4L,ATP8B1,MIP,SLC18B1,STAC3,SLC2A12,SLC26A10,PEX7,SLC16A7,ATP5MGL,NIPAL2,MAPK8IP2,CPT1B,SLC12A2,KCNV1,PRRT1,RNF5,TAP1,SLC22A4,CALHM4,GOPC,SLC35F1,SMDT1,BAK1,CATSPER3,PKD2L2,KCNK9,MAPK14,TOMM22,KCNJ4,PLA2G6,PIM1,SLC39A4,ATP6V1F,MFSD3,CHRNA3,CHRNA5,PEX6,TRPM6,ABCB1,SLC25A40,ABCC10,STEAP2,GABRA1,CNGA1,SLC41A2,TMEM63B,PEX1,SLC35B2,CLIC5,SLC25A27,SLC1A1,SLC25A13,TRAM2,WNK2,MFSD14C,CNNM4,TSPAN13,ABCG5,ABCG8,SEC61B,SLC3A1,PRKCE,ATP6V1E2,ABCB5,TMC4,TMEM30A,KCNQ4,IBTK,PNPT1,SHANK1,SLC4A4,SLC46A2,SLC35A1,AQP1,GJA10,SLC1A4,SLC31A2,SLC13A4,GRB10,SEC61G,ATP6V1C2,HECW1,TCAF2,GRIK2,GJB5,CLCN1,KEL,UCP1,ATG5,TRPM4,SLC7A11,OSR1,MFSD8,FGF21,BRAF,MFSD4B,MFSD2B,SPHK2,KCNJ14,GRIN2D,TRPC3,SLC8A2,PANX1,SLC26A5,ATP7B,KCNRG,CACNB2,ANO10,SLC39A12,SLC25A30,ATP6V0E2,SLC4A2,DNAJC15,ASIC3,STAC,ATP6V0B,AQP11,PRKAG2,GRIK5,ATP1A3,CCT8L2,OXSR1,TMEM163,SLC22A13,ATP5F1C,SCN5A,SCN1A,SLC25A38,SEC62,SLC24A3,AKT2,GPD1L,ABCC4,CHRNA6,TIMM50,SLC20A2,SLC4A7,SLC38A10,CACNB4,ASPSCR1,RYR1,NALCN,SLC6A15,SLC25A15,SLC23A2,PRNP,DIAPH1,P2RX2,CLIC1,TMCO3,GAS6,RASA3,ARL6IP5,COX7A1,SLC7A1,SLC25A26,SLC46A3,SLC38A9,MICU3,KCNIP3,ANO4,SLC5A8,ATP6V1H,TRIB3,COX4I2,SLC35A4,CYB5A,SLCO6A1,PIK3R1,ABCB9,SLMAP,SLC43A1,ATP5F1A,SLC43A3,GLRX,P2RX3,TRPC4AP,SLC9A9,RYR3,ORAI1,SORBS1,MEF2C,ASIC1,PEX2,P2RX7,HVCN1,KCNH3,TMEM144,PTK2B,SLC39A13,KCNJ11,SLC33A1,TMEM150C,TPCN1,SLC8B1,HOMER1,IFNGR2,SL[illegible]	grey	VSMC_M10
GO:0006625	0.00	1.00	15	17	protein targeting to peroxisome	BP	1	PEX7,PEX3,PEX6,PEX1,ZFAND6,HACL1,PEX2,PEX16,LONP2,PEX19,PEX26,PEX14,RAB8B,PEX10,PEX12	grey	VSMC_M10

GO:0072662	0.00	1.00	15	17	protein localization to peroxisome	BP	1	PEX7,PEX3,PEX6,PEX1,ZFAND6,HACL1,PEX2,PEX16,LONP2,PEX19,PEX26,PEX14,RAB8B,PEX10,PEX12	grey	VSMC_M10
GO:0072663	0.00	1.00	15	17	establishment of protein localization to peroxisome	BP	1	PEX7,PEX3,PEX6,PEX1,ZFAND6,HACL1,PEX2,PEX16,LONP2,PEX19,PEX26,PEX14,RAB8B,PEX10,PEX12	grey	VSMC_M10
GO:0080154	0.00	1.00	9	9	regulation of fertilization	BP	1	MYH9,CFAP69,NLRP5,PLAT,PLCB1,INTS13,CCDC87,ZP2,LHFPL2	grey	VSMC_M10
GO:0043574	0.00	1.00	17	20	peroxisomal transport	BP	1	PEX7,PEX3,PEX6,PEX1,ZFAND6,HACL1,PEX2,PEX16,LONP2,PEX19,PEX26,PEX14,ABCD4,RAB8B,PEX10,ABCD3,PEX12	grey	VSMC_M10
GO:0006813	0.00	1.00	90	140	potassium ion transport	BP	1	VPS4B,NEDD4L,SLC12A2,KCNV1,KCNK9,KCNJ4,WNK2,KCNQ4,AQP1,KEL,KCNJ14,KCNRG,ATP1A3,CCT8L2,OXSR1,SLC24A3,NALCN,PRNP,KCNIP3,SLC9A9,KCNH3,PTK2B,KCNJ11,KCNJ3,ANO6,KCNK15,KCNE2,PKD2,CASQ2,KCNJ15,SLC12A5,KCNIP2,KCNJ2,SLC12A7,ADRA2A,KCNB1,SLC9A8,KCNK1,KCNG1,ANK2,KLHL24,ATP1A2,SLC9A1,CCDC51,SLC12A8,KCNK2,NOS1AP,CDKN1B,DLG1,KCNK7,NSF,SLC12A9,KCNK4,SLC9A7,KCNN1,ATP1B1,KCNA1,KCNMA1,KCNH4,KCND1,WNK3,ATP1B2,KCNAB3,KCNIP1,HCN3,KCNN3,DNM2,KCNJ18,SLC9A6,RNF207,KCNC4,AMIGO1,TSC1,WWP2,SLC12A4,SLC9A5,MIR21,KCNK3,KCNH2,KCNK5,NETO2,CRBN,RGS4,KCNA6,KCNJ8,KCNT2,HCN4,KCNMB4,KCNG2,KCNJ9	grey	VSMC_M10
GO:0046395	0.00	1.00	116	182	carboxylic acid catabolic process	BP	1	GLS2,HSD17B4,SHMT2,ALDH8A1,AIG1,RIDA,CPT1B,PPARA,MCAT,ECHDC1,GPT,CARNMT1,CROT,GNPDA2,PPAT,HMGCLL1,ETFB,IL4I1,LPIN1,DDO,BCAT2,FAAH,HADHA,GAD2,AKR1A1,ACOX1,XYLB,HNMT,AKT2,IDO2,IDO1,HACL1,AMDHD1,ACOXL,CSAD,ACOX2,AHCY,MCCC2,PEX2,ETFDH,SDSL,IVD,LPIN3,HOGA1,ECI2,HIBCH,GLS,ACAD8,ADTRP,LONP2,MTRR,MCEE,ALDH5A1,IRS1,CBSL,CPT1A,BDH2,HGD,AMT,ABHD2,MCCC1,ABCB11,ACADSB,OAT,HMGCL,GLUL,EHHADH,ACADL,FAH,MTHFS,TYSND1,PRODH,HDC,ALDH4A1,MAT1A,ASRGL1,ACADVL,HSD17B10,SLC25A44,ALDH6A1,ABCD4,GCDH,GSTZ1,CRYM,ABHD3,SHMT1,ENOSF1,LPIN2,NOS2,PIPOX,ACAA2,ABAT,DECR2,SARDH,ACSF3,CEL,MLYCD,GCSH,KYAT1,ABCD3,KYAT3,PLA2G15,ACADM,MIR21,PHYH,ACOT4,HIBADH,SLC25A21,HADHB,AMACR,NUDT19,IDNK,ALDH1L1,ECI1,AUH,HYKK	grey	VSMC_M10
GO:0098659	0.00	1.00	49	71	inorganic cation import across plasma membrane	BP	1	SLC39A5,SLC12A2,KCNK9,KCNJ4,SLC39A4,STEAP2,WNK2,TRPM4,KCNJ14,SLC8A2,SLC39A12,ATP1A3,PRNP,SLC9A9,KCNJ11,KCNJ3,KCNE2,KCNJ15,SLC12A5,KCNJ2,SLC12A7,TRPV4,ISCU,ATP1A2,SLC9A1,SLC12A8,DLG1,SLC12A9,SLC9A7,ATP1B1,WNK3,ATP1B2,FAM155B,SLC5A2,SLC39A6,TRPV2,KCNJ18,SLC9A6,TRPV3,TRPV1,P2RX1,LCN2,SLC12A4,SLC9A5,MIR24-1,KCNH2,KCNJ8,HCN4,KCNJ9	grey	VSMC_M10
GO:0099587	0.00	1.00	49	71	inorganic ion import across plasma membrane	BP	1	SLC39A5,SLC12A2,KCNK9,KCNJ4,SLC39A4,STEAP2,WNK2,TRPM4,KCNJ14,SLC8A2,SLC39A12,ATP1A3,PRNP,SLC9A9,KCNJ11,KCNJ3,KCNE2,KCNJ15,SLC12A5,KCNJ2,SLC12A7,TRPV4,ISCU,ATP1A2,SLC9A1,SLC12A8,DLG1,SLC12A9,SLC9A7,ATP1B1,WNK3,ATP1B2,FAM155B,SLC5A2,SLC39A6,TRPV2,KCNJ18,SLC9A6,TRPV3,TRPV1,P2RX1,LCN2,SLC12A4,SLC9A5,MIR24-1,KCNH2,KCNJ8,HCN4,KCNJ9	grey	VSMC_M10
GO:0003333	0.00	1.00	46	66	amino acid transmembrane transport	BP	1	SLC22A4,SLC1A1,SLC25A13,SLC3A1,SLC1A4,SLC7A11,SLC25A38,SLC38A10,SLC6A15,SLC25A15,ARL6IP5,SLC7A1,SLC38A9,SLC43A1,SLC38A4,SLC38A2,SLC38A1,SFXN2,SLC38A11,SLC38A3,SLC7A7,AGT,ACE2,ATP1A2,PER2,NTSR1,PQLC2,SLC38A5,ARL6IP1,SLC3A2,SLC38A6,PRAF2,CLN8,PSEN1,SLC7A3,SLC6A7,LRRC8E,SLC1A7,SLC7A5,LRRC8A,SLC7A6,SLC36A1,LRRC8D,SLC6A19,RGS4,SLC25A29	grey	VSMC_M10
GO:0045956	0.01	1.00	11	12	positive regulation of calcium ion-dependent exocytosis	BP	1	SYT1,SYT9,HYAL3,KCNB1,STX1A,SYT7,CACNA1G,SCAMP5,CACNA1H,STXBP1,DOC2B	grey	VSMC_M10
GO:0071805	0.01	1.00	84	131	potassium ion transmembrane transport	BP	1	NEDD4L,SLC12A2,KCNV1,KCNK9,KCNJ4,WNK2,KCNQ4,AQP1,KEL,KCNJ14,KCNRG,ATP1A3,CCT8L2,OXSR1,SLC24A3,NALCN,PRNP,KCNIP3,SLC9A9,KCNH3,KCNJ11,KCNJ3,ANO6,KCNK15,KCNE2,PKD2,CASQ2,KCNJ15,SLC12A5,KCNIP2,KCNJ2,SLC12A7,KCNB1,SLC9A8,KCNK1,KCNG1,ANK2,KLHL24,ATP1A2,SLC9A1,CCDC51,SLC12A8,KCNK2,NOS1AP,DLG1,KCNK7,SLC12A9,KCNK4,SLC9A7,KCNN1,ATP1B1,KCNA1,KCNMA1,KCNH4,KCND1,WNK3,ATP1B2,KCNAB3,KCNIP1,HCN3,KCNN3,DNM2,KCNJ18,SLC9A6,RNF207,KCNC4,AMIGO1,WWP2,SLC12A4,SLC9A5,MIR21,KCNK3,KCNH2,KCNK5,NETO2,CRBN,RGS4,KCNA6,KCNJ8,KCNT2,HCN4,KCNMB4,KCNG2,KCNJ9	grey	VSMC_M10
GO:0035435	0.01	1.00	11	12	phosphate ion transmembrane transport	BP	1	SLC25A30,SLC20A2,ADAMTS8,SLC37A1,SLC37A2,SLC37A4,XPR1,ANKH,SLC25A11,SLC25A14,SLC25A3	grey	VSMC_M10
GO:1990573	0.01	1.00	26	35	potassium ion import across plasma membrane	BP	1	SLC12A2,KCNK9,KCNJ4,WNK2,KCNJ14,ATP1A3,KCNJ11,KCNJ3,KCNE2,KCNJ15,SLC12A5,KCNJ2,SLC12A7,ATP1A2,SLC12A8,DLG1,SLC12A9,ATP1B1,WNK3,ATP1B2,KCNJ18,SLC12A4,KCNH2,KCNJ8,HCN4,KCNJ9	grey	VSMC_M10

GO:0016054	0.01	1.00	117	186	organic acid catabolic process	BP	1	GLS2,HSD17B4,SHMT2,ALDH8A1,AIG1,RIDA,CPT1B,PPARA,MCAT,ECHDC1,GPT,CARNMT1,CROT,GNPDA2,PPAT,HMGCLL1,ETFB,IL4I1,LPIN1,DDO,BCAT2,FAAH,HADHA,GAD2,AKR1A1,ACOX1,XYLB,HNMT,AKT2,IDO2,IDO1,HACL1,AMDHD1,ACOXL,CSAD,ACOX2,THNSL2,AHCY,MCCC2,PEX2,ETFDH,SDSL,IVD,LPIN3,HOGA1,ECI2,HIBCH,GLS,ACAD8,ADTRP,LONP2,MTRR,MCEE,ALDH5A1,IRS1,CBSL,CPT1A,BDH2,HGD,AMT,ABHD2,MCCC1,ABCB11,ACADSB,OAT,HMGCL,GLUL,EHHADH,ACADL,FAH,MTHFS,TYSND1,PRODH,HDC,ALDH4A1,MAT1A,ASRGL1,ACADVL,HSD17B10,SLC25A44,ALDH6A1,ABCD4,GCDH,GSTZ1,CRYM,ABHD3,SHMT1,ENOSF1,LPIN2,NOS2,PIPOX,ACAA2,ABAT,DECR2,SARDH,ACSF3,CEL,MLYCD,GCSH,KYAT1,ABCD3,KYAT3,PLA2G15,ACADM,MIR21,PHYH,ACOT4,HIBADH,SLC25A21,HADHB,AMACR,NUDT19,IDNK,ALDH1L1,ECI1,AUH,HYKK	grey	VSMC_M10
GO:0006817	0.01	1.00	15	18	phosphate ion transport	BP	1	SFRP4,SLC25A30,SLC20A2,CRY2,ADAMTS8,SLC37A1,SLC37A2,IP6K2,SLC37A4,XPR1,ANKH,SLC25A11,SLC25A14,SLC25A3,CEBPB	grey	VSMC_M10
GO:1903825	0.01	1.00	69	106	organic acid transmembrane transport	BP	1	SLC16A7,CPT1B,SLC22A4,ABCB1,SLC1A1,SLC25A13,SLC3A1,SLC1A4,SLC7A11,SLC25A30,SLC25A38,AKT2,SLC38A10,SLC6A15,SLC25A15,SLC23A2,ARL6IP5,SLC7A1,SLC38A9,SLC5A8,SLC43A1,SLC38A4,SLC38A2,SLC38A1,SFXN2,SLC38A11,SLC38A3,CPT1A,ACSL5,SLC7A7,AGT,SLC25A20,ABCC5,ACE2,ATP1A2,PER2,NTSR1,SLC16A1,PQLC2,SLC27A1,SLC38A5,MPC2,ARL6IP1,SLC3A2,SLC38A6,PRAF2,SLC25A11,SLC29A4,CLN8,PSEN1,SLC7A3,ABCD4,SLC6A7,LRRC8E,EMB,SLC46A1,SLC25A14,SLC1A7,SLC7A5,LRRC8A,ABCD3,SLC7A6,SLC36A1,LRRC8D,SLC6A19,SLC25A21,SLC25A32,RGS4,SLC25A29	grey	VSMC_M10
GO:0015919	0.01	1.00	15	18	peroxisomal membrane transport	BP	1	PEX7,PEX3,PEX6,PEX1,PEX2,PEX16,LONP2,PEX19,PEX26,PEX14,ABCD4,RAB8B,PEX10,ABCD3,PEX12	grey	VSMC_M10
GO:0042789	0.01	1.00	10	11	mRNA transcription by RNA polymerase II	BP	1	ATF4,FOXE3,C5AR1,TFCP2,AP3B1,STAT3,ZBTB1,MED1,SREBF1,SUPT6H	grey	VSMC_M10
GO:0007494	0.01	1.00	10	11	midgut development	BP	1	SMO,EGFR,RET,CPS1,ALDH1A2,DAB1,FOXL1,FOXF1,ASS1,WNT5A	grey	VSMC_M10
GO:0015807	0.01	1.00	34	48	L-amino acid transport	BP	1	SLC1A1,SLC25A13,SLC3A1,SLC1A4,SLC7A11,SLC25A15,ARL6IP5,SLC7A1,SLC38A9,SLC43A1,SERINC5,SLC38A2,SLC38A1,SLC38A3,SLC7A7,AGT,ACE2,ATP1A2,PER2,NTSR1,SLC11A1,PQLC2,SLC38A5,ARL6IP1,SLC3A2,PRAF2,CLN8,PSEN1,SLC7A3,SLC1A7,SLC7A5,SLC7A6,SLC36A1,SLC25A29	grey	VSMC_M10
GO:0061025	0.01	0.99	80	125	membrane fusion	BP	1	STX7,SEPT8,TSNARE1,TGFBRAP1,STX17,RIMS1,SPACA6,VPS41,HACE1,IZUMO1,NAPA,RAB7A,PIP4K2A,RUBCNL,NECTIN2,GRIK5,TBC1D4,AKT2,YIPF4,SYT1,GAS6,BET1L,STX2,SYT9,NSFL1C,ANKRD27,MYOF,CHMP2B,DYSF,VPS39,RAB7B,DNM1L,SEC22B,VTI1A,SNAP23,MFN1,RCC1L,C2CD5,STX1A,STX6,VPS8,LYZL6,NSF,SPG11,PIKFYVE,BLOC1S6,ANXA7,RAB3A,VAMP1,SNAP29,ANKFY1,STX5,STX18,RAB8A,ATP13A2,CPLANE2,SYT7,ANXA2,CPLX1,STX1B,STX4,BNIP1,SYT11,RAB8B,AC138894.1,SOX30,STX8,SPESP1,NOX5,SNAPIN,NAPG,GNAI3,VAV3,STXBP1,VPS4A,SNAP47,DOC2B,RABIF,VAMP3,RAB39A	grey	VSMC_M10
GO:1905039	0.01	0.99	68	105	carboxylic acid transmembrane transport	BP	1	SLC16A7,CPT1B,SLC22A4,ABCB1,SLC1A1,SLC25A13,SLC3A1,SLC1A4,SLC7A11,SLC25A30,SLC25A38,AKT2,SLC38A10,SLC6A15,SLC25A15,SLC23A2,ARL6IP5,SLC7A1,SLC38A9,SLC5A8,SLC43A1,SLC38A4,SLC38A2,SLC38A1,SFXN2,SLC38A11,SLC38A3,CPT1A,ACSL5,SLC7A7,AGT,SLC25A20,ABCC5,ACE2,ATP1A2,PER2,NTSR1,SLC16A1,PQLC2,SLC27A1,SLC38A5,MPC2,ARL6IP1,SLC3A2,SLC38A6,PRAF2,SLC25A11,CLN8,PSEN1,SLC7A3,ABCD4,SLC6A7,LRRC8E,EMB,SLC46A1,SLC25A14,SLC1A7,SLC7A5,LRRC8A,ABCD3,SLC7A6,SLC36A1,LRRC8D,SLC6A19,SLC25A21,SLC25A32,RGS4,SLC25A29	grey	VSMC_M10
GO:0034220	0.01	0.99	439	765	ion transmembrane transport	BP	1	SLC39A5,NEDD4L,ATP8B1,STAC3,SLC26A10,SLC16A7,ATP5MGL,NIPAL2,MAPK8IP2,SLC12A2,KCNV1,PRRT1,SLC22A4,CALHM4,GOPC,SMDT1,BAK1,CATSPER3,PKD2L2,KCNK9,KCNJ4,PLA2G6,SLC39A4,ATP6V1F,MFSD3,CHRNA3,CHRNA5,TRPM6,ABCB1,STEAP2,GABRA1,CNGA1,SLC41A2,TMEM63B,SLC35B2,CLIC5,SLC1A1,SLC25A13,WNK2,CNNM4,TSPAN13,SLC3A1,PRKCE,ATP6V1E2,TMC4,KCNQ4,IBTK,SHANK1,SLC4A4,SLC35A1,AQP1,SLC1A4,SLC31A2,SLC13A4,ATP6V1C2,HECW1,TCAF2,GRIK2,CLCN1,KEL,UCP1,ATG5,TRPM4,SLC7A11,OSR1,SPHK2,KCNJ14,GRIN2D,TRPC3,SLC8A2,PANX1,SLC26A5,ATP7B,KCNRG,CACNB2,ANO10,SLC39A12,SLC25A30,ATP6V0E2,SLC4A2,ASIC3,STAC,ATP6V0B,GRIK5,ATP1A3,CCT8L2,OXSR1,TMEM163,ATP5F1C,SCN5A,SCN1A,SLC25A38,SLC24A3,GPD1L,ABCC4,CHRNA6,SLC20A2,SLC4A7,CACNB4,RYR1,NALCN,SLC6A15,SLC25A15,SLC23A2,PRNP,DIAPH1,P2RX2,CLIC1,TMCO3,GAS6,RASA3,ARL6IP5,COX7A1,SLC7A1,SLC25A26,SLC38A9,MICU3,KCNIP3,ANO4,SLC5A8,ATP6V1H,COX4I2,CYB5A,SLMAP,SLC43A1,ATP5F1A,GLRX,P2RX3,TRPC4AP,SLC9A9,RYR3,ORAI1,MEF2C,ASIC1,P2RX7,HVCN1,KCNH3,PTK2B,SLC39A13,KCNJ11,SLC33A1,TMEM150C,TPCN1,SLC8B1,HOMER1,IFNGR2,SLC25A37,OPA1,KCNJ3,F2R,TESC,RIPK1,SLC40A1,ANO6,SLC6A2,ZDHHC13,COX15,KCNK15,ABCC2,KCNE2,PKD2,NIPA1,CASQ2,KCNJ15,MCUR1,SLC12A5,KCNIP2,CHRM3,ADAMTS8,RYR2,FAM173B,ATP6V1B2,PM20D1,SLC41A1,COX6B1,SLC4A10,SFXN2,TRPM1,SLC37A1,CALHM2,CALHM1,SLC6A3,LCK,SLC2A10,HECW2,KCNJ2,SLC38A3,SLC9B1,SLC12A7,TRPV4,SLC9B2,ADRA2A,SLC30A10,SLC37A2,TCIRG1,COX17,SCN2A,ISCU,KCNB1,SHROOM2,MC [illegible]	grey	VSMC_M10

GO:1902475	0.01	1.00	32	45	L-alpha-amino acid transmembrane transport	BP	1	SLC1A1,SLC25A13,SLC3A1,SLC1A4,SLC7A11,SLC25A38,SLC25A15,ARL6IP5,SLC7A1,SLC38A9,SLC43A1,SLC38A1,SLC38A3,SLC7A7,AGT,ACE2,ATP1A2,PER2,NTSR1,PQLC2,SLC38A5,ARL6IP1,SLC3A2,PRAF2,CLN8,PSEN1,SLC7A3,SLC1A7,SLC7A5,SLC7A6,RGS4,SLC25A29	grey	VSMC_M10
GO:0006858	0.01	1.00	22	29	extracellular transport	BP	1	LRRC6,RFX3,DNAH11,NEK10,DNAH5,DPCD,ROPN1L,CFAP43,JHY,OFD1,CFAP45,DNAAF2,SPAG16,STK36,ADCY10,TTC25,DNAAF4,SPEF2,CFAP53,ARRDC1,WWP2,SPA17	grey	VSMC_M10
GO:0045741	0.01	1.00	10	11	positive regulation of epidermal growth factor-activated receptor activity	BP	1	EPGN,AREG,TGFA,HBEGF,FBXW7,NEURL1,ADRA2A,NCF1,EGF,EREG	grey	VSMC_M10
GO:0018410	0.01	1.00	10	11	C-terminal protein amino acid modification	BP	1	ATG12,AGTPBP1,ATG5,TTL,GPLD1,ATG16L1,WIPI2,LCMT1,FOLH1,IRGM	grey	VSMC_M10
GO:0001505	0.01	0.99	92	147	regulation of neurotransmitter levels	BP	1	SNCAIP,RIMS2,PRKN,GRM4,AGTPBP1,CHRNA3,CHRNA5,APBA1,BRSK1,RIMS1,RIMS3,PPT1,PPFIA3,LIN7B,NAPA,GAD2,CACNB2,GRIK5,NAPB,HNMT,ZNF219,SYT1,LRTOMT,AP000812.5,RAB5A,LIN7A,STX2,SYT9,SNPH,NR4A1,ERC2,MEF2C,ASIC1,P2RY1,SYNJ1,SLC38A1,VPS18,SLC6A2,DTNBP1,SYT8,ALDH5A1,DNM1L,SLC6A3,ADRA2A,SNAP23,GPER1,ATP1A2,PER2,STX1A,SLC29A2,BACE1,DNAJC5,CSPG5,RAP1B,BLOC1S6,COMT,NRXN2,CASK,BCHE,PNKD,RAB3A,SYN1,SNAP29,DAGLB,SLC29A4,SYT7,CLN8,CPLX1,STX1B,STX4,PSEN1,SYT11,AC138894.1,CALM1,SNAPIN,PRKCB,CAMK2A,STXBP2,ABAT,FMR1,SLC1A7,PIP5K1C,P2RX1,BAIAP3,PARK7,STXBP1,CHRNB4,SNAP47,PDZD11,DOC2B,KCNMB4,PRIMA1	grey	VSMC_M10
GO:0044282	0.01	0.99	172	284	small molecule catabolic process	BP	1	GLS2,HSD17B4,SHMT2,ALDH8A1,CYP27B1,AIG1,RIDA,MIOX,CPT1B,SYNJ2,PPARA,MCAT,ECHDC1,APOBEC3H,GPT,CARNMT1,GDA,CROT,GNPDA2,GALT,ENPP4,SRD5A3,PPAT,HMGCLL1,ADH5,ETFB,IL4I1,LPIN1,INPP4B,DHDH,DDO,BCAT2,MTMR2,FAAH,HADHA,GAD2,CDADC1,ESD,AKR1A1,GLB1,NUDT5,ACOX1,XYLB,HNMT,RBKS,AKT2,PFKP,IDO2,IDO1,HACL1,FUT10,AMDHD1,MTMR7,ACOXL,CSAD,CYP7A1,ACOX2,THNSL2,AHCY,GK5,MCCC2,PEX2,ETFDH,PFKM,SYNJ1,SDSL,IVD,ENTPD4,LPIN3,HOGA1,NQO2,ECI2,HIBCH,GLS,CBR3,ACAD8,GLYCTK,ADTRP,LONP2,MTRR,MCEE,ALDH5A1,IRS1,CBSL,CPT1A,BDH2,INPP5A,ALDH3B2,HGD,AMT,PFKFB2,ABHD2,MCCC1,ABCB11,ACTN3,ACADSB,NTSR1,OAT,MGAT1,INPP5J,HMGCL,GLUL,EHHADH,SORD,RPE,UPB1,ACADL,HK1,FAH,MTHFS,TYSND1,PRODH,PNKD,CYP27A1,HDC,ALDH4A1,MAT1A,ASRGL1,ACADVL,TKFC,HSD17B10,SLC25A44,ALDH6A1,ABCD4,GCDH,GSTZ1,CRYM,CYP46A1,ABHD3,SHMT1,ENOSF1,LPIN2,OXCT1,NOS2,IMPA2,PIPOX,ACAA2,ABAT,INPP5K,TKTL1,PARK7,DECR2,ALDH2,SARDH,ACSF3,CEL,MLYCD,GCSH,KYAT1,ABCD3,KYAT3,PLA2G15,ACADM,MIR21,PHYH,ACOT4,FUT4,HIBADH,SLC25A21,TIGAR,HADHB,AMACR,NUDT19,IDNK,ALDH1L1,ECI1,AUH,HYKK,INPP1,ADH4,PDXP,UPP1	grey	VSMC_M10
GO:0003351	0.01	1.00	20	26	epithelial cilium movement involved in extracellular fluid movement	BP	1	LRRC6,RFX3,DNAH11,NEK10,DNAH5,DPCD,ROPN1L,CFAP43,JHY,OFD1,CFAP45,DNAAF2,SPAG16,STK36,ADCY10,TTC25,DNAAF4,SPEF2,CFAP53,SPA17	grey	VSMC_M10
GO:0007631	0.01	0.99	32	45	feeding behavior	BP	1	ADM2,MCHR1,NTRK2,CNTFR,USP46,NAPEPLD,DACH1,POU4F1,TCF15,HAND2,CFAP20,P2RY1,EIF2AK4,GLS,CPT1A,GIGYF2,TTC21B,OPRD1,AGT,ACE2,PYY,GDF15,STAT3,FOS,UBE2Q1,NMUR2,STRA6,SGIP1,LEPR,NPB,MRAP2,DERL2	grey	VSMC_M10
GO:0099177	0.01	0.99	188	318	regulation of trans-synaptic signaling	BP	1	SNCAIP,MAPK8IP2,SLC12A2,RIMS2,PRRT1,PRKN,AGER,SYNGAP1,AKAP7,GRM4,ATF4,SYNGR1,NTRK2,PLA2G6,PICK1,BTBD9,CHRNA3,LRRC4,GRM8,CHRNA5,SRF,APBA1,PHF24,USP46,KIT,SLC1A1,ROR2,BRSK1,PRKCE,RIMS1,RIMS3,ACP4,SHANK1,CRHR2,PTN,DGKI,EGFR,NCDN,GRIK2,DLGAP3,SLC7A11,PPFIA3,NTF4,PRKAR2B,GRIN2D,MTMR2,SLC8A2,CACNB2,GRIK5,SYT1,RAB5A,PLCB1,PLCL2,PRNP,PTPRA,EPHB1,PLK2,FAM107A,PXK,ABHD6,CLSTN2,P2RX3,ERC2,MEF2C,ASIC1,DLGAP4,CUX2,P2RY1,PTK2B,MME,RAPGEF2,EIF2AK4,HOMER1,VPS18,ITPKA,CYFIP1,DTNBP1,LZTS1,GRM2,BDNF,SLC4A10,NEURL1,DNM1L,SORCS3,PPP3CA,ADRA2A,IGSF11,NLGN4Y,SSH1,STAU1,KCNB1,KRAS,ADNP,DISC1,GPER1,GRIK4,AGT,ATP1A2,NFATC4,SYAP1,STX1A,TMEM25,NPAS4,YTHDF1,CNIH2,BACE1,GLUL,CSPG5,CREB1,YWHAG,MAPT,RAP1B,SRGN,VGF,EIF4EBP2,TNR,CASK,MPP2,BCHE,PNKD,CRTC1,PPP3CB,RAB3A,PINK1,SYN1,STAT3,CD38,NPTX2,MAPK1,NSG1,RAB8A,HAP1,EPHA4,DLG4,SYT7,IQSEC2,NLGN2,RGS14,CPLX1,STX1B,STX4,PSEN1,CACNA1A,SYT11,NLGN3,KCTD13,AC138894.1,CHRNB2,RAB11A,DGKE,CALM1,SNAPIN,PRKCB,CYP46A1,BEGAIN,CAMK2A,CCL2,DLGAP1,ABR,FMR1,LRP8,P2RX1,CACNA1B,PRKCZ,RAB26,MECP2,TPRG1L,BAIAP3,CEL,NTNG2,JPH3,ZDHHC12,CNTNAP4,STXBP1,FABP5,CHRNB4,CNTN2,GRID2,CACNG7,RGS4,CRH,YWHAH,HRAS,SHISA8,CACNG5,KCNMB4,MAP1A,LRRTM2	grey	VSMC_M10
GO:0030878	0.01	1.00	14	17	thyroid gland development	BP	1	SRF,RAF1,PAX8,HOXD3,RAP1GAP,MAPK1,THRA,MAPK3,MAP2K1,SMAD3,HOXB3,SIX1,NKX2-5,HOXA3	grey	VSMC_M10
GO:0000289	0.01	1.00	21	28	nuclear-transcribed mRNA poly(A) tail shortening	BP	1	PAN2,CNOT2,CNOT6,PABPC1,PNLDC1,AGO2,TNRC6B,TUT7,TUT4,MLH1,TNRC6C,PAN3,CNOT1,CNOT6L,TENT4B,EIF4ENIF1,SAMD4A,POLR2G,TOB1,TNRC6A,PARN	grey	VSMC_M10
GO:0006307	0.01	1.00	7	7	DNA dealkylation involved in DNA repair	BP	1	ASCC3,ALKBH3,FTO,ALKBH2,MGMT,ALKBH1,MPG	grey	VSMC_M10
GO:0110095	0.01	1.00	7	7	cellular detoxification of aldehyde	BP	1	ALDH1A1,ADH5,ESD,AKR1A1,RDH11,PARK7,ADH4	grey	VSMC_M10

GO:0050804	0.01	0.99	187	317	modulation of chemical synaptic transmission	BP	1	SNCAIP,MAPK8IP2,SLC12A2,RIMS2,PRRT1,PRKN,AGER,SYNGAP1,AKAP7,GRM4,ATF4,SYNGR1,NTRK2,PLA2G6,PICK1,BTBD9,CHRNA3,LRRC4,GRM8,CHRNA5,SRF,APBA1,PHF24,USP46,KIT,SLC1A1,ROR2,BRSK1,PRKCE,RIMS1,RIMS3,ACP4,SHANK1,CRHR2,PTN,DGKI,EGFR,NCDN,GRIK2,DLGAP3,SLC7A11,PPFIA3,NTF4,PRKAR2B,GRIN2D,MTMR2,SLC8A2,CACNB2,GRIK5,SYT1,RAB5A,PLCB1,PLCL2,PRNP,PTPRA,EPHB1,PLK2,FAM107A,PXK,ABHD6,CLSTN2,P2RX3,ERC2,MEF2C,ASIC1,DLGAP4,CUX2,P2RY1,PTK2B,MME,RAPGEF2,EIF2AK4,HOMER1,VPS18,ITPKA,CYFIP1,DTNBP1,LZTS1,GRM2,BDNF,SLC4A10,NEURL1,DNM1L,SORCS3,PPP3CA,ADRA2A,IGSF11,NLGN4Y,SSH1,STAU1,KCNB1,KRAS,ADNP,DISC1,GPER1,GRIK4,AGT,ATP1A2,NFATC4,SYAP1,STX1A,TMEM25,NPAS4,YTHDF1,CNIH2,BACE1,GLUL,CSPG5,CREB1,YWHAG,MAPT,RAP1B,SRGN,VGF,EIF4EBP2,TNR,CASK,MPP2,BCHE,PNKD,CRTC1,PPP3CB,RAB3A,PINK1,SYN1,STAT3,CD38,NPTX2,MAPK1,NSG1,RAB8A,HAP1,EPHA4,DLG4,SYT7,IQSEC2,NLGN2,RGS14,CPLX1,STX1B,STX4,PSEN1,CACNA1A,SYT11,NLGN3,KCTD13,AC138894.1,CHRNB2,RAB11A,DGKE,CALM1,SNAPIN,PRKCB,CYP46A1,BEGAIN,CAMK2A,CCL2,DLGAP1,ABR,FMR1,LRP8,P2RX1,CACNA1B,PRKCZ,RAB26,MECP2,TPRG1L,BAIAP3,CEL,NTNG2,JPH3,ZDHHC12,CNTNAP4,STXBP1,CHRNB4,CNTN2,GRID2,CACNG7,RGS4,CRH,YWHAH,HRAS,SHISA8,CACNG5,KCNMB4,MAP1A,LRRTM2	grey	VSMC_M10
GO:0042908	0.02	0.99	21	28	xenobiotic transport	BP	1	ATP8B1,SLC22A4,ABCB1,ABCC10,ABCB5,TMEM30A,ATP7B,EBP,ABCC4,ABCC2,ABCG2,ABCB11,ABCC5,SLC29A4,ABCC3,RALBP1,ABCA3,SLC7A5,SLC22A5,ABCA8,CLDN1	grey	VSMC_M10
GO:0035278	0.02	1.00	16	20	miRNA mediated inhibition of translation	BP	1	AGO2,TNRC6B,AGO1,TNRC6C,EIF4E2,DDX6,EIF4ENIF1,STAT3,TNRC6A,MIR29A,MIR21,MIR222,MIR125B1,MIR27B,MIR27A,AGO4	grey	VSMC_M10
GO:0040033	0.02	1.00	16	20	negative regulation of translation, ncRNA-mediated	BP	1	AGO2,TNRC6B,AGO1,TNRC6C,EIF4E2,DDX6,EIF4ENIF1,STAT3,TNRC6A,MIR29A,MIR21,MIR222,MIR125B1,MIR27B,MIR27A,AGO4	grey	VSMC_M10
GO:0045974	0.02	1.00	16	20	regulation of translation, ncRNA-mediated	BP	1	AGO2,TNRC6B,AGO1,TNRC6C,EIF4E2,DDX6,EIF4ENIF1,STAT3,TNRC6A,MIR29A,MIR21,MIR222,MIR125B1,MIR27B,MIR27A,AGO4	grey	VSMC_M10
GO:0001539	0.02	0.99	52	79	cilium or flagellum-dependent cell motility	BP	1	UBE2B,CATSPER3,LRRC6,CFAP69,RFX3,EFHC1,DNAJA1,DNAH11,CABS1,ARMC2,APOB,ADCY3,DRC1,RSPH6A,TTC21A,TTLL3,MKKS,ING2,DNAH6,WDR66,CFAP20,CCDC65,DNAH1,PLTP,DNAH5,DPCD,ROPN1L,NEURL1,CFAP43,SLC9B1,SLC9B2,CFAP46,INTS13,CFAP45,ENO4,TSSK4,CELF3,CATSPER2,SORD,TTC12,DNAAF2,SPAG16,EFHC2,MNS1,DNAH2,DNAH3,TEKT3,SPEF2,CATSPERD,TPGS1,WDR63,CCSAP	grey	VSMC_M10
GO:0060285	0.02	0.99	52	79	cilium-dependent cell motility	BP	1	UBE2B,CATSPER3,LRRC6,CFAP69,RFX3,EFHC1,DNAJA1,DNAH11,CABS1,ARMC2,APOB,ADCY3,DRC1,RSPH6A,TTC21A,TTLL3,MKKS,ING2,DNAH6,WDR66,CFAP20,CCDC65,DNAH1,PLTP,DNAH5,DPCD,ROPN1L,NEURL1,CFAP43,SLC9B1,SLC9B2,CFAP46,INTS13,CFAP45,ENO4,TSSK4,CELF3,CATSPER2,SORD,TTC12,DNAAF2,SPAG16,EFHC2,MNS1,DNAH2,DNAH3,TEKT3,SPEF2,CATSPERD,TPGS1,WDR63,CCSAP	grey	VSMC_M10
GO:0007368	0.02	0.99	58	90	determination of left/right symmetry	BP	1	TBC1D32,LRRC6,SMO,DAAM2,FOXH1,IFT74,SRF,RFX3,ANKS6,DYNC2LI1,DNAH11,ARMC4,BBS7,SETDB2,DRC1,MEGF8,GALNT11,IFT172,FOXJ1,TGFBR2,OVOL2,ACVR2A,MKKS,KIF3B,HAND2,MEF2C,IFT52,RPGRIP1L,PKD2,DNAH5,DPCD,SUFU,OFD1,CFAP45,AP1B1,BICC1,MMP21,DNAAF2,PKD1L1,NKX3-2,TTC25,DNAAF4,PSEN1,TMEM107,SMAD3,CCDC151,MKS1,TBX2,MIB1,CFAP53,RNF207,FOXF1,ENG,PSKH1,LBX1,WNT5A,C2CD3,NKX2-5	grey	VSMC_M10
GO:0045591	0.02	1.00	9	10	positive regulation of regulatory T cell differentiation	BP	1	HLA-DRA,IL4I1,SOX12,DUSP10,BCL6,VSIR,FOXP3,SOCS1,CD46	grey	VSMC_M10
GO:0031547	0.02	1.00	6	6	brain-derived neurotrophic factor receptor signaling pathway	BP	1	FSTL4,NTRK2,RAPGEF2,BDNF,NFATC4,SLC2A4	grey	VSMC_M10
GO:0006855	0.02	1.00	6	6	xenobiotic transmembrane transport	BP	1	ATP8B1,TMEM30A,ABCC2,ABCB11,ABCC3,ABCA3	grey	VSMC_M10
GO:0098656	0.02	0.99	99	161	anion transmembrane transport	BP	1	SLC26A10,SLC16A7,SLC12A2,GOPC,ABCB1,GABRA1,SLC35B2,SLC1A1,SLC25A13,SLC3A1,SLC4A4,SLC35A1,SLC1A4,SLC13A4,TCAF2,CLCN1,SLC7A11,SLC26A5,SLC25A30,SLC4A2,SLC25A38,SLC24A3,ABCC4,SLC20A2,SLC4A7,SLC25A15,SLC23A2,PRNP,ARL6IP5,SLC7A1,SLC38A9,ANO4,SLC5A8,SLC43A1,SLC9A9,RIPK1,ANO6,SLC12A5,ADAMTS8,SLC4A10,SFXN2,SLC37A1,SLC38A3,SLC12A7,SLC37A2,SLC9A8,AGT,ABCC5,ACE2,ATP1A2,SLC9A1,SLC37A4,PER2,SLC12A8,NTSR1,XPR1,SLC16A1,SLC12A9,ANKH,SLC25A42,MFSD10,SLC9A7,PQLC2,SLC27A1,SLC38A5,MPC2,ARL6IP1,SLC3A2,SLC35A2,SLC4A3,PRAF2,SLC25A11,CLN8,SLC26A1,PSEN1,SLC7A3,SLC35B1,SLC26A2,LRRC8E,EMB,SLC25A41,SLC46A1,SLC25A14,SLC9A6,GABRE,GABRA3,SLC1A7,CLCN7,SLC7A5,LRRC8A,SLC12A4,SLC9A5,SLC25A3,LRRC8D,LRRC8B,SLC25A21,SLC25A32,RGS4,SLC25A29	grey	VSMC_M10
GO:0010603	0.02	1.00	6	6	regulation of cytoplasmic mRNA processing body assembly	BP	1	PAN2,CNOT2,CNOT6,PAN3,CNOT1,CNOT6L	grey	VSMC_M10
GO:0010606	0.02	1.00	6	6	positive regulation of cytoplasmic mRNA processing body assembly	BP	1	PAN2,CNOT2,CNOT6,PAN3,CNOT1,CNOT6L	grey	VSMC_M10

GO:0044106	0.02	0.99	49	74	cellular amine metabolic process	BP	1	SNCAIP,ALDH7A1,PRKN,LY6E,AKR1B1,GPR37,SLC1A1,EPAS1,DDC,IL4I1,SLC7A11,NAPEPLD,AANAT,HNMT,IDO2,IDO1,LRTOMT,AP000812.5,NR1H4,HAND2,CHDH,NR4A2,ABCC2,SIRT4,AZIN2,SLC6A3,TGFB2,CHKA,SLC7A7,SMS,COMT,PNKD,GCH1,HDC,SLC29A4,ITGAM,GDPD3,GCDH,AC138894.1,CHRNB2,GDPD1,GDE1,ABAT,OAZ1,MECP2,PARK7,MIR21,NNMT,AOC2	grey	VSMC_M10
GO:0009855	0.02	0.99	62	97	determination of bilateral symmetry	BP	1	TBC1D32,LRRC6,SMO,DAAM2,FOXH1,IFT74,SRF,RFX3,SOSTDC1,ANKS6,DYNC2LI1,DNAH11,ARMC4,BBS7,SETDB2,DRC1,MEGF8,GALNT11,IFT172,FOXJ1,TGFBR2,OVOL2,ACVR2A,MKKS,KIF3B,HAND2,MEF2C,IFT52,RPGRIP1L,PKD2,DNAH5,DPCD,SUFU,OFD1,CFAP45,AP1B1,BICC1,MMP21,AIDA,DNAAF2,PKD1L1,NKX3-2,TTC25,DNAAF4,ALDH1A2,PSEN1,TMEM107,SMAD3,CCDC151,MKS1,TBX2,MIB1,CFAP53,RNF207,FOXF1,ENG,PSKH1,LBX1,WNT5A,DDIT3,C2CD3,NKX2-5	grey	VSMC_M10
GO:0071467	0.02	1.00	13	16	cellular response to pH	BP	1	ASIC1,HVCN1,RAB11FIP5,GPLD1,HYAL1,SLC38A3,SLC9A1,KCNK4,GPR68,TRPV1,PKD1L3,INSRR,IMPACT	grey	VSMC_M10
GO:1900019	0.02	1.00	6	6	regulation of protein kinase C activity	BP	1	ROR2,EGFR,TCIM,CEMIP,WNT5A,C3orf58	grey	VSMC_M10
GO:1900020	0.02	1.00	6	6	positive regulation of protein kinase C activity	BP	1	ROR2,EGFR,TCIM,CEMIP,WNT5A,C3orf58	grey	VSMC_M10
GO:0097164	0.02	1.00	11	13	ammonium ion metabolic process	BP	1	TPH2,ALDH7A1,BTBD9,DDC,HNMT,CHDH,SRD5A1,CHKA,BCHE,HDC,SLC29A4	grey	VSMC_M10
GO:0033278	0.02	1.00	6	6	cell proliferation in midbrain	BP	1	FZD6,RYK,FZD3,WNT5A,PITX3,WNT2	grey	VSMC_M10
GO:0110096	0.02	1.00	9	10	cellular response to aldehyde	BP	1	AKR1B1,ALDH1A1,ADH5,ESD,AKR1A1,RDH11,SCNN1B,PARK7,ADH4	grey	VSMC_M10
GO:0098734	0.02	1.00	11	13	macromolecule depalmitoylation	BP	1	ABHD16A,PPT2,ABHD17B,PPT1,LYPLA1,LYPLAL1,ABHD12B,ABHD17A,ABHD10,ABHD17C,LYPLA2	grey	VSMC_M10
GO:0006884	0.02	0.99	18	24	cell volume homeostasis	BP	1	SLC12A2,AQP1,CLNS1A,AQP11,P2RX7,ANO6,SLC12A5,SLC12A7,TRPV4,SLC12A8,SLC12A9,GNB3,KCNMA1,WNK3,AC138894.1,LRRC8E,LRRC8A,SLC12A4	grey	VSMC_M10
GO:0035095	0.02	1.00	6	6	behavioral response to nicotine	BP	1	PPARA,CHRNA3,CHRNA5,GRM2,CHRNB1,CHRNB2	grey	VSMC_M10
GO:1901678	0.02	1.00	11	13	iron coordination entity transport	BP	1	PGRMC2,ASIC3,HPX,SLC48A1,ABCC5,ABCB6,FLVCR2,SLC46A1,C1orf194,LCN2,SLC22A17	grey	VSMC_M10
GO:0060456	0.02	1.00	6	6	positive regulation of digestive system process	BP	1	AQP1,NR1H2,LPCAT3,TRPV1,SLC22A5,CRH	grey	VSMC_M10
GO:0007269	0.02	0.99	69	110	neurotransmitter secretion	BP	1	SNCAIP,RIMS2,PRKN,GRM4,CHRNA3,CHRNA5,APBA1,BRSK1,RIMS1,RIMS3,PPT1,PPFIA3,LIN7B,NAPA,CACNB2,GRIK5,NAPB,SYT1,RAB5A,LIN7A,STX2,SYT9,SNPH,NR4A1,ERC2,MEF2C,ASIC1,P2RY1,SYNJ1,VPS18,DTNBP1,SYT8,DNM1L,ADRA2A,SNAP23,GPER1,STX1A,BACE1,DNAJC5,CSPG5,RAP1B,BLOC1S6,NRXN2,CASK,PNKD,RAB3A,SYN1,SNAP29,SYT7,CPLX1,STX1B,STX4,PSEN1,SYT11,CALM1,SNAPIN,PRKCB,CAMK2A,STXBP2,FMR1,PIP5K1C,P2RX1,BAIAP3,STXBP1,CHRNB4,SNAP47,PDZD11,DOC2B,KCNMB4	grey	VSMC_M10
GO:0099643	0.02	0.99	69	110	signal release from synapse	BP	1	SNCAIP,RIMS2,PRKN,GRM4,CHRNA3,CHRNA5,APBA1,BRSK1,RIMS1,RIMS3,PPT1,PPFIA3,LIN7B,NAPA,CACNB2,GRIK5,NAPB,SYT1,RAB5A,LIN7A,STX2,SYT9,SNPH,NR4A1,ERC2,MEF2C,ASIC1,P2RY1,SYNJ1,VPS18,DTNBP1,SYT8,DNM1L,ADRA2A,SNAP23,GPER1,STX1A,BACE1,DNAJC5,CSPG5,RAP1B,BLOC1S6,NRXN2,CASK,PNKD,RAB3A,SYN1,SNAP29,SYT7,CPLX1,STX1B,STX4,PSEN1,SYT11,CALM1,SNAPIN,PRKCB,CAMK2A,STXBP2,FMR1,PIP5K1C,P2RX1,BAIAP3,STXBP1,CHRNB4,SNAP47,PDZD11,DOC2B,KCNMB4	grey	VSMC_M10
GO:0048241	0.02	1.00	6	6	epinephrine transport	BP	1	LY6E,ADRA2B,ADRA2A,ADRA2C,SLC29A4,CRH	grey	VSMC_M10
GO:0048304	0.02	1.00	6	6	positive regulation of isotype switching to IgG isotypes	BP	1	MSH2,MLH1,PAXIP1,CD40,CD28,TBX21	grey	VSMC_M10
GO:1903307	0.02	0.99	26	37	positive regulation of regulated secretory pathway	BP	1	SPHK2,CACNB2,GAB2,UNC13D,SYT1,SYT9,F2RL1,DNM1L,HYAL3,KCNB1,FGR,STX1A,SNX4,RAB3A,SYT7,RAB15,STX4,ITGAM,CACNA1G,RAB3D,IL4R,SCAMP5,CACNA1H,STXBP1,AP1G1,DOC2B	grey	VSMC_M10
GO:0006584	0.02	0.99	23	32	catecholamine metabolic process	BP	1	SNCAIP,PRKN,LY6E,AKR1B1,GPR37,SLC1A1,EPAS1,DDC,LRTOMT,AP000812.5,HAND2,NR4A2,SLC6A3,TGFB2,COMT,PNKD,GCH1,HDC,ITGAM,CHRNB2,ABAT,PARK7,AOC2	grey	VSMC_M10
GO:0009712	0.02	0.99	23	32	catechol-containing compound metabolic process	BP	1	SNCAIP,PRKN,LY6E,AKR1B1,GPR37,SLC1A1,EPAS1,DDC,LRTOMT,AP000812.5,HAND2,NR4A2,SLC6A3,TGFB2,COMT,PNKD,GCH1,HDC,ITGAM,CHRNB2,ABAT,PARK7,AOC2	grey	VSMC_M10
GO:0070471	0.02	1.00	6	6	uterine smooth muscle contraction	BP	1	ADRA2B,GPER1,AGT,ADRA2C,SETD3,ABAT	grey	VSMC_M10
GO:0051937	0.03	0.99	30	43	catecholamine transport	BP	1	PRKN,LY6E,MAPK15,SYT1,SYT9,ADRA2B,CHRM5,P2RY1,SLC6A2,DTNBP1,SYT8,GRM2,SLC6A3,ADRA2A,KCNB1,GRK2,AGT,STX1A,ADRA2C,RAB3A,PINK1,ACTB,SLC29A4,SYT7,SYT11,CHRNB2,ABAT,PARK7,CHGA,CRH	grey	VSMC_M10
GO:0009799	0.03	0.98	62	98	specification of symmetry	BP	1	TBC1D32,LRRC6,SMO,DAAM2,FOXH1,IFT74,SRF,RFX3,SOSTDC1,ANKS6,DYNC2LI1,DNAH11,ARMC4,BBS7,SETDB2,DRC1,MEGF8,GALNT11,IFT172,FOXJ1,TGFBR2,OVOL2,ACVR2A,MKKS,KIF3B,HAND2,MEF2C,IFT52,RPGRIP1L,PKD2,DNAH5,DPCD,SUFU,OFD1,CFAP45,AP1B1,BICC1,MMP21,AIDA,DNAAF2,PKD1L1,NKX3-2,TTC25,DNAAF4,ALDH1A2,PSEN1,TMEM107,SMAD3,CCDC151,MKS1,TBX2,MIB1,CFAP53,RNF207,FOXF1,ENG,PSKH1,LBX1,WNT5A,DDIT3,C2CD3,NKX2-5	grey	VSMC_M10
GO:0090174	0.03	0.98	60	94	organelle membrane fusion	BP	1	STX7,SEPT8,TSNARE1,TGFBRAP1,STX17,VPS41,RAB7A,PIP4K2A,RUBCNL,GRIK5,TBC1D4,AKT2,YIPF4,SYT1,STX2,SYT9,ANKRD27,CHMP2B,DYSF,VPS39,RAB7B,SEC22B,VTI1A,SNAP23,MFN1,RCC1L,C2CD5,STX1A,STX6,VPS8,SPG11,PIKFYVE,RAB3A,VAMP1,SNAP29,ANKFY1,STX5,RAB8A,ATP13A2,CPLANE2,SYT7,ANXA2,CPLX1,STX1B,STX4,BNIP1,SYT11,RAB8B,AC138894.1,SOX30,STX8,SNAPIN,GNAI3,VAV3,STXBP1,VPS4A,SNAP47,DOC2B,VAMP3,RAB39A	grey	VSMC_M10

GO:0098660	0.03	0.98	329	574	inorganic ion transmembrane transport	BP	1	SLC39A5,NEDD4L,STAC3,SLC26A10,ATP5MGL,NIPAL2,SLC12A2,KCNV1,SMDT1,CATSPER3,PKD2L2,KCNK9,KCNJ4,PLA2G6,SLC39A4,ATP6V1F,MFSD3,TRPM6,STEAP2,GABRA1,SLC41A2,SLC1A1,WNK2,CNNM4,TSPAN13,PRKCE,ATP6V1E2,KCNQ4,IBTK,SLC4A4,AQP1,SLC31A2,SLC13A4,ATP6V1C2,HECW1,CLCN1,KEL,UCP1,ATG5,TRPM4,OSR1,SPHK2,KCNJ14,GRIN2D,TRPC3,SLC8A2,PANX1,SLC26A5,ATP7B,KCNRG,CACNB2,SLC39A12,SLC25A30,ATP6V0E2,ASIC3,STAC,ATP6V0B,ATP1A3,CCT8L2,OXSR1,TMEM163,ATP5F1C,SCN5A,SCN1A,SLC24A3,GPD1L,SLC20A2,SLC4A7,CACNB4,RYR1,NALCN,SLC6A15,PRNP,DIAPH1,TMCO3,GAS6,RASA3,COX7A1,MICU3,KCNIP3,ANO4,ATP6V1H,COX4I2,CYB5A,SLMAP,ATP5F1A,GLRX,P2RX3,TRPC4AP,SLC9A9,RYR3,ORAI1,ASIC1,P2RX7,HVCN1,KCNH3,PTK2B,SLC39A13,KCNJ11,SLC33A1,TPCN1,SLC8B1,SLC25A37,OPA1,KCNJ3,F2R,TESC,SLC40A1,ANO6,SLC6A2,ZDHHC13,COX15,KCNK15,ABCC2,KCNE2,PKD2,NIPA1,CASQ2,KCNJ15,MCUR1,SLC12A5,KCNIP2,ADAMTS8,RYR2,FAM173B,ATP6V1B2,PM20D1,SLC41A1,COX6B1,SLC4A10,TRPM1,SLC37A1,CALHM1,SLC6A3,LCK,SLC2A10,HECW2,KCNJ2,SLC9B1,SLC12A7,TRPV4,SLC9B2,ADRA2A,SLC30A10,SLC37A2,TCIRG1,COX17,SCN2A,ISCU,KCNB1,SHROOM2,MCUB,SLC9A8,CLCN4,KCNK1,KCNG1,SCN7A,UBASH3B,ANK2,GPER1,KLHL24,RHCG,TRPM8,ABCC5,ATP1A2,SLC9A1,SLC37A4,C4orf3,CASQ1,CCDC51,SLC12A8,KCNK2,NTSR1,XPR1,SCN4B,DNAJC30,NIPAL3,NOS1AP,CATSPER2,DLG1,KCNK7,TMCO1,COX6A1,SLC30A4,CEMIP,SLC12A9,SESTD1,ATP5S,ANKH,PTPN6,KCNK4,MICU1,MCU,SLC11A1,HTT,PKD1L1,COX8A,SLC9A7,ATP6V0A1,KCNN1,ABCB6,TRPM7,ATP1B1,KCNA1,BH[illegible]	grey	VSMC_M10
GO:0098655	0.03	0.98	356	623	cation transmembrane transport	BP	1	SLC39A5,NEDD4L,STAC3,ATP5MGL,NIPAL2,MAPK8IP2,SLC12A2,KCNV1,PRRT1,SLC22A4,CALHM4,SMDT1,CATSPER3,PKD2L2,KCNK9,KCNJ4,PLA2G6,SLC39A4,ATP6V1F,MFSD3,TRPM6,STEAP2,CNGA1,SLC41A2,TMEM63B,SLC1A1,WNK2,CNNM4,TSPAN13,PRKCE,ATP6V1E2,KCNQ4,IBTK,SHANK1,SLC4A4,AQP1,SLC1A4,SLC31A2,ATP6V1C2,HECW1,KEL,UCP1,ATG5,TRPM4,OSR1,SPHK2,KCNJ14,GRIN2D,TRPC3,SLC8A2,PANX1,ATP7B,KCNRG,CACNB2,ANO10,SLC39A12,ATP6V0E2,ASIC3,STAC,ATP6V0B,ATP1A3,CCT8L2,OXSR1,TMEM163,ATP5F1C,SCN5A,SCN1A,SLC25A38,SLC24A3,GPD1L,SLC20A2,SLC4A7,CACNB4,RYR1,NALCN,SLC6A15,SLC25A15,PRNP,DIAPH1,P2RX2,TMCO3,GAS6,RASA3,COX7A1,SLC7A1,SLC25A26,SLC38A9,MICU3,KCNIP3,ATP6V1H,COX4I2,CYB5A,SLMAP,SLC43A1,ATP5F1A,GLRX,P2RX3,TRPC4AP,SLC9A9,RYR3,ORAI1,MEF2C,ASIC1,P2RX7,HVCN1,KCNH3,PTK2B,SLC39A13,KCNJ11,SLC33A1,TPCN1,SLC8B1,HOMER1,IFNGR2,SLC25A37,OPA1,KCNJ3,F2R,TESC,SLC40A1,ANO6,SLC6A2,ZDHHC13,COX15,KCNK15,ABCC2,KCNE2,PKD2,NIPA1,CASQ2,KCNJ15,MCUR1,SLC12A5,KCNIP2,RYR2,FAM173B,ATP6V1B2,PM20D1,SLC41A1,COX6B1,SLC4A10,SFXN2,TRPM1,CALHM2,CALHM1,SLC6A3,LCK,SLC2A10,HECW2,KCNJ2,SLC38A3,SLC9B1,SLC12A7,TRPV4,SLC9B2,ADRA2A,SLC30A10,TCIRG1,COX17,SCN2A,ISCU,KCNB1,SHROOM2,MCUB,SLC9A8,CLCN4,KCNK1,KCNG1,SCN7A,UBASH3B,ANK2,GPER1,KLHL24,RHCG,TRPM8,AGT,SLC25A20,ABCC5,ACE2,ATP1A2,SLC9A1,C4orf3,CASQ1,CCDC51,SLC12A8,KCNK2,NTSR1,SCN4B,CNIH2,DNAJC30,NIPAL3,NOS1AP,CATSPER2,DLG1,KCNK7,TMCO1,COX6A1,SLC30A4,UNC80,CEMIP,SLC12A9,SESTD1,ATP5S,[illegible]	grey	VSMC_M10
GO:0010257	0.03	0.99	37	53	NADH dehydrogenase complex assembly	BP	1	TIMM21,NDUFA6,NDUFB2,NDUFB6,TMEM126B,NDUFC2,DMAC2,NDUFS8,NDUFAF7,NDUFA12,NDUFAF2,NDUFS3,NDUFAF6,FOXRED1,OXA1L,NDUFB4,NDUFB3,NDUFS2,NUBPL,NDUFB11,BCS1L,NDUFA9,NDUFAB1,NDUFB10,MT-ND1,MT-ND2,MT-ND6,NDUFA5,NDUFC1,NDUFAF8,DMAC1,NDUFA3,NDUFB5,NDUFA8,NDUFS5,TMEM126A,COA1	grey	VSMC_M10
GO:0032981	0.03	0.99	37	53	mitochondrial respiratory chain complex I assembly	BP	1	TIMM21,NDUFA6,NDUFB2,NDUFB6,TMEM126B,NDUFC2,DMAC2,NDUFS8,NDUFAF7,NDUFA12,NDUFAF2,NDUFS3,NDUFAF6,FOXRED1,OXA1L,NDUFB4,NDUFB3,NDUFS2,NUBPL,NDUFB11,BCS1L,NDUFA9,NDUFAB1,NDUFB10,MT-ND1,MT-ND2,MT-ND6,NDUFA5,NDUFC1,NDUFAF8,DMAC1,NDUFA3,NDUFB5,NDUFA8,NDUFS5,TMEM126A,COA1	grey	VSMC_M10
GO:0035871	0.03	1.00	8	9	protein K11-linked deubiquitination	BP	1	TNFAIP3,OTUD7A,USP30,YOD1,OTUD7B,USP37,OTUD3,OTUB2	grey	VSMC_M10
GO:2000647	0.03	1.00	6	6	negative regulation of stem cell proliferation	BP	1	FBLN1,OVOL2,FERMT1,KDF1,OVOL1,MIR222	grey	VSMC_M10

GO:0050877	0.03	0.98	384	678	nervous system process	BP	1	RDH5,ATP8B1,MIP,MYO1A,EYA4,STAC3,MAPK8IP2,ALDH7A1,RIMS2,PRRT1,PRKN,AGER,HLA-DRA,COL11A2,SYNGAP1,LRRC6,AGTPBP1,NTRK2,PLA2G6,SOX10,BTBD9,OPN1SW,GUCA1B,RBFOX2,CHRNA3,PRPH2,GRM8,CHRNA5,RORB,SRF,TTBK1,CFAP69,GABRA1,CNGA1,PHF24,CLIC5,KIT,SLC1A1,ADGRF1,TNFRSF21,CNNM4,CLDN19,BRSK1,TNNI3,EPAS1,RIMS1,DNAH11,KCNQ4,GSDME,SHANK1,PPT1,GJA10,MYH14,SLC1A4,WHRN,PTN,EGFR,GRIK2,DLGAP3,CLCN1,LPIN1,SOBP,SLC7A11,NTF4,FIG4,TULP2,PRKAR2B,GRIN2D,ADCY3,MTMR2,BBS7,SLC8A2,C5AR1,SLC26A5,MYO3A,CST7,MYO7B,EML2,OPA3,CACNB2,USH1G,ASIC3,XRCC1,STAC,B4GALT2,CRYGN,ZNF513,GRIK5,SCN5A,SCN1A,MYO7A,MYCBP2,METTL23,POU4F1,SPRY2,CHRNA6,CHD8,MKKS,FSCN2,PDE6G,LRTOMT,AP000812.5,PLCB1,TMTC4,RAC3,PRNP,DDHD2,KERA,LUM,DIAPH1,SPART,P2RX2,ARL6IP5,FAM19A4,ATXN7,TUB,TCF15,EPHB1,PLK2,FAM107A,GPI,NR4A1,CAMK4,CASP3,CHMP2B,MARVELD2,EYA1,P2RX3,CHRM5,CEP250,CCDC66,ADGRV1,MEF2C,ASIC1,P2RX7,NCAM2,JAM2,DLGAP4,CUX2,P2RY1,KIFC3,PTK2B,MME,KCNJ11,EIF2AK4,TMEM150C,NBN,USH1C,SYNJ1,HOMER1,OPA1,AMFR,F2R,NQO2,TNNC1,GLS,IMPG2,CBR3,DOP1B,TFAP2A,PAX2,CYFIP1,SLC12A5,ATXN1,FAM173B,BDNF,SLC4A10,TRPM1,RNF10,CABP1,CALHM1,CFAP43,SLC6A3,ALDH1A3,SORCS3,PPP3CA,LINS1,KCNJ2,PGAP1,TNNI1,SYNM,IGSF11,VTI1A,ADARB1,PDE6D,SCN2A,DAG1,NLGN4Y,SSH1,KRAS,GIGYF2,RRH,LRIT3,ADNP,MYH7,ZFHX2,SCN7A,OPRD1,JHY,GPER1,CFAP45,CRYBA4,NRL,EYA3,TRPM8,AGT,ACE2,ATP1A2,SLC9A1,NIPSNAP1,NFATC4,CASQ1,MYO3B,ACTN3,BBS1,KCNK2,STX1A,T[illegible]	grey	VSMC_M10
GO:0045066	0.03	0.99	14	18	regulatory T cell differentiation	BP	1	HLA-DRA,IL4I1,SOX12,TOX,CD28,DUSP10,BCL6,VSIR,LAG3,FOXP3,SOCS1,DROSHA,MIR21,CD46	grey	VSMC_M10
GO:0098719	0.03	0.99	10	12	sodium ion import across plasma membrane	BP	1	SLC12A2,TRPM4,SLC9A9,SLC9A1,SLC9A7,SLC5A2,SLC9A6,SLC9A5,MIR24-1,HCN4	grey	VSMC_M10
GO:0045055	0.03	0.98	101	167	regulated exocytosis	BP	1	RIMS2,SYNGR1,RAC2,MYH9,CHRNA5,STEAP2,APBA1,KIT,NLRP5,RIMS1,RIMS3,PLEK,VPS41,PPFIA3,SPHK2,NAPA,RAB10,CACNB2,GAB2,GRIK5,UNC13D,NAPB,ABCC4,SYT1,RAB5A,NCKAP1L,STX2,SYT9,SNPH,HCK,FER,ERC2,P2RY1,RASGRP1,RAB11FIP5,SYNJ1,F2RL1,VPS18,DMTN,DTNBP1,SYT8,DNM1L,HYAL3,ADRA2A,SNAP23,KCNB1,RAB11FIP2,LAT2,FGR,CBL,STX1A,SNX4,BACE1,DNAJC5,CSPG5,RAP1B,BLOC1S6,ABCA12,CASK,PTGDR,PPP3CB,RAB3A,SYN1,SNAP29,C12orf4,SYT7,RAB15,CPLX1,STX1B,STX4,PSEN1,ITGAM,SYT11,TMED10,CACNA1G,LAT,RAB3D,IL4R,CALM1,SNAPIN,S100A13,PRKCB,BTK,SCAMP5,STXBP2,PRAM1,FMR1,PIP5K1C,SDF4,PDPK1,P2RX1,RAB26,BAIAP3,CACNA1H,FOXF1,STXBP1,AP1G1,CHGA,SNAP47,DOC2B,VAMP3	grey	VSMC_M10
GO:0003341	0.03	0.98	65	103	cilium movement	BP	1	UBE2B,CATSPER3,LRRC6,CFAP69,RFX3,DNAJA1,DNAH11,CABS1,ARMC2,ARMC4,APOB,ADCY3,DRC1,RSPH6A,TTC21A,CFAP61,TTLL3,MKKS,NEK10,SPEF1,ING2,WDR66,CFAP20,CCDC65,DNAH1,PLTP,DNAH5,DPCD,ROPN1L,NEURL1,CFAP43,ZMYND10,SLC9B1,SLC9B2,CFAP46,INTS13,JHY,OFD1,CFAP45,ENO4,TSSK4,CELF3,CATSPER2,SORD,TTC12,DNAAF2,SPAG16,CFAP70,STK36,ADCY10,TTC25,DNAAF4,MNS1,CCDC151,TEKT3,CAMSAP3,SPEF2,CATSPERD,CFAP53,TPGS1,CELSR2,HYDIN,WDR63,CCSAP,SPA17	grey	VSMC_M10
GO:1901606	0.03	0.98	43	65	alpha-amino acid catabolic process	BP	1	GLS2,SHMT2,ALDH8A1,RIDA,GPT,PPAT,IL4I1,DDO,BCAT2,GAD2,IDO2,IDO1,CSAD,THNSL2,AHCY,SDSL,HOGA1,GLS,MTRR,CBSL,HGD,AMT,ACADSB,OAT,GLUL,FAH,PRODH,ALDH4A1,MAT1A,ASRGL1,HSD17B10,GSTZ1,CRYM,SHMT1,NOS2,PIPOX,SARDH,GCSH,KYAT1,KYAT3,MIR21,SLC25A21,HYKK	grey	VSMC_M10
GO:0046461	0.03	0.99	19	26	neutral lipid catabolic process	BP	1	ABHD16A,PNPLA3,PNPLA5,APOB,FGF21,FAAH,ABHD5,LIPE,DDHD2,ABHD6,GPLD1,ABHD2,SORL1,CPS1,ABHD12B,DAGLB,LIPC,PNPLA4,LYPLA2	grey	VSMC_M10
GO:0046464	0.03	0.99	19	26	acylglycerol catabolic process	BP	1	ABHD16A,PNPLA3,PNPLA5,APOB,FGF21,FAAH,ABHD5,LIPE,DDHD2,ABHD6,GPLD1,ABHD2,SORL1,CPS1,ABHD12B,DAGLB,LIPC,PNPLA4,LYPLA2	grey	VSMC_M10
GO:0002717	0.03	0.99	10	12	positive regulation of natural killer cell mediated immunity	BP	1	CD226,NECTIN2,PVR,RASGRP1,CADM1,IL12A,LAG3,STAT5B,VAV1,AP1G1	grey	VSMC_M10
GO:0045954	0.03	0.99	10	12	positive regulation of natural killer cell mediated cytotoxicity	BP	1	CD226,NECTIN2,PVR,RASGRP1,CADM1,IL12A,LAG3,STAT5B,VAV1,AP1G1	grey	VSMC_M10
GO:0032816	0.03	0.99	12	15	positive regulation of natural killer cell activation	BP	1	IL15,IL15RA,GAS6,TOX,RASGRP1,BLOC1S6,IL12A,STAT5B,ZBTB1,TICAM1,AP1G1,BLOC1S3	grey	VSMC_M10
GO:0033604	0.03	1.00	8	9	negative regulation of catecholamine secretion	BP	1	ADRA2B,P2RY1,ADRA2A,ADRA2C,SYT11,ABAT,CHGA,CRH	grey	VSMC_M10
GO:0140042	0.03	1.00	8	9	lipid droplet formation	BP	1	CDS2,AUP1,CDS1,FITM2,FITM1,ZFYVE1,TMEM159,BSCL2	grey	VSMC_M10
GO:0015802	0.03	0.99	12	15	basic amino acid transport	BP	1	SLC3A1,SLC7A1,SLC38A9,SLC38A3,SLC7A7,AGT,SLC11A1,PQLC2,SLC7A3,AC138894.1,SLC7A6,SLC25A29	grey	VSMC_M10
GO:0055075	0.03	0.99	15	20	potassium ion homeostasis	BP	1	SLC12A2,TFAP2B,ATP1A3,SLC12A5,KCNJ2,SLC12A7,ATP1A2,SLC12A8,SLC12A9,ATP1B1,KCNMA1,ATP1B2,SCNN1B,SLC12A4,KCNH2	grey	VSMC_M10
GO:0016558	0.03	0.99	10	12	protein import into peroxisome matrix	BP	1	PEX7,PEX6,PEX1,PEX2,PEX16,LONP2,PEX26,PEX14,PEX10,PEX12	grey	VSMC_M10
GO:0033344	0.03	0.98	29	42	cholesterol efflux	BP	1	LRP1,APOM,PTCH1,TTC39B,ABCG5,ABCG8,NR1H2,APOB,APOC1,TSKU,SOAT2,NR1H3,PLTP,ABCA5,EGF,ABCG4,SOAT1,ZDHHC8,ABCA12,GPS2,NAXE,NPC1,ABCA3,ABCA7,RXRA,MIR27B,MIR27A,ABCA8,MIR33B	grey	VSMC_M10

GO:0006865	0.03	0.98	62	99	amino acid transport	BP	1	SLC12A2,SLC22A4,NTRK2,APBA1,SLC1A1,SLC25A13,SLC3A1,SLC1A4,SLC7A11,LLGL2,SLC25A38,SLC38A10,SLC6A15,SLC25A15,ARL6IP5,SLC7A1,SLC38A9,SLC43A1,SERINC5,SLC38A4,SLC38A2,SLC38A1,OCA2,GRM2,SFXN2,SLC38A11,SLC38A3,SLC7A7,AGT,ACE2,ATP1A2,PER2,NTSR1,SLC11A1,PQLC2,SLC38A5,ARL6IP1,SLC3A2,SLC38A6,PRAF2,CLN8,PSEN1,SLC25A44,SLC7A3,AC138894.1,SLC25A47,SLC6A7,LRRC8E,ABAT,SLC1A7,TRPV1,SLC7A5,LRRC8A,STXBP1,SLC7A6,SLC36A1,LRRC8D,SLC6A19,SLC25A45,XK,RGS4,SLC25A29	grey	VSMC_M10
GO:0046330	0.03	0.98	44	68	positive regulation of JNK cascade	BP	1	MAP3K5,STK3,AGER,GADD45G,MTURN,NOD1,TLR4,TRAF1,SDCBP,MYD88,PLCB1,SEMA3A,SH3RF2,MFHAS1,TRAF6,PTK2B,RASGRP1,RIPK2,F2RL1,RIPK1,NOD2,TAOK3,TRPV4,NCF1,TRAF5,FZD7,MAP3K11,MBIP,MAP4K2,DUSP19,LTBR,MINK1,TAOK2,TRAF3,TAOK1,SERPINF2,MAPK8IP3,TRAF2,DAB2IP,ZNF622,WNT5A,GADD45A,DUSP22,HRAS	grey	VSMC_M10
GO:0033238	0.04	0.99	17	23	regulation of cellular amine metabolic process	BP	1	PRKN,GPR37,SLC7A11,NR1H4,NR4A2,SIRT4,SLC6A3,SLC7A7,COMT,PNKD,ITGAM,AC138894.1,CHRNB2,ABAT,PARK7,MIR21,NNMT	grey	VSMC_M10
GO:0046185	0.04	1.00	8	9	aldehyde catabolic process	BP	1	ADH5,ESD,AKR1A1,HOGA1,PNKD,PARK7,ADH4,PDXP	grey	VSMC_M10
GO:0006639	0.04	0.98	58	92	acylglycerol metabolic process	BP	1	AVIL,ABHD16A,TNXB,SLC22A4,PNPLA3,PNPLA5,CAT,MBOAT7,NR1H2,DGKI,LPIN1,APOB,FGF21,FAAH,ABHD5,APOC1,LIPE,DDHD2,PANK2,ABHD6,GK5,PLCE1,FBXW7,NR1H3,LPIN3,FITM2,GPLD1,SIK1,CPT1A,GPAM,ABHD2,SORL1,DGKD,LPGAT1,DGKG,PLA2G4A,MOGAT3,CPS1,ABHD12B,GNB3,PLA2G16,SLC27A1,DAGLB,LIPC,DGKQ,APOBR,SEL1L,DGKE,LDLR,SREBF1,LPIN2,C3,LMF1,PLA2G15,PNPLA4,CNEP1R1,LYPLA2,INSIG2	grey	VSMC_M10
GO:0072235	0.04	1.00	5	5	metanephric distal tubule development	BP	1	POU3F3,PAX8,PKD2,PAX2,PKD1	grey	VSMC_M10
GO:0000414	0.04	1.00	5	5	regulation of histone H3-K36 methylation	BP	1	IWS1,PAXIP1,NSD3,BCOR,NSD1	grey	VSMC_M10
GO:0006906	0.04	0.98	57	90	vesicle fusion	BP	1	STX7,SEPT8,TSNARE1,TGFBRAP1,STX17,VPS41,RAB7A,PIP4K2A,RUBCNL,GRIK5,TBC1D4,AKT2,YIPF4,SYT1,STX2,SYT9,ANKRD27,CHMP2B,DYSF,VPS39,RAB7B,SEC22B,VTI1A,SNAP23,C2CD5,STX1A,STX6,VPS8,SPG11,PIKFYVE,RAB3A,VAMP1,SNAP29,ANKFY1,STX5,RAB8A,ATP13A2,CPLANE2,SYT7,ANXA2,CPLX1,STX1B,STX4,SYT11,RAB8B,AC138894.1,SOX30,STX8,SNAPIN,GNAI3,VAV3,STXBP1,VPS4A,SNAP47,DOC2B,VAMP3,RAB39A	grey	VSMC_M10
GO:0071294	0.04	1.00	8	9	cellular response to zinc ion	BP	1	TSPO,MTF1,HVCN1,CREB1,ATP13A2,PARP1,KCNK3,MT1X	grey	VSMC_M10
GO:0006576	0.04	0.98	42	64	cellular biogenic amine metabolic process	BP	1	SNCAIP,ALDH7A1,PRKN,LY6E,AKR1B1,GPR37,SLC1A1,EPAS1,DDC,IL4I1,NAPEPLD,AANAT,HNMT,IDO2,IDO1,LRTOMT,AP000812.5,HAND2,CHDH,NR4A2,AZIN2,SLC6A3,TGFB2,CHKA,SMS,COMT,PNKD,GCH1,HDC,SLC29A4,ITGAM,GDPD3,GCDH,CHRNB2,GDPD1,GDE1,ABAT,OAZ1,MECP2,PARK7,NNMT,AOC2	grey	VSMC_M10
GO:0050957	0.04	1.00	5	5	equilibrioception	BP	1	USH1G,MYO7A,POU4F1,USH1C,CDH23	grey	VSMC_M10
GO:0031064	0.04	1.00	5	5	negative regulation of histone deacetylation	BP	1	SPHK2,KDM5A,FOXP3,SKI,PRMT6	grey	VSMC_M10
GO:0060073	0.04	1.00	5	5	micturition	BP	1	CHRNA3,KCNMA1,CHRNB2,TRPV1,CHRNB4	grey	VSMC_M10
GO:0050774	0.04	1.00	5	5	negative regulation of dendrite morphogenesis	BP	1	TLX2,RAPGEF2,PPP3CA,NFATC4,YWHAH	grey	VSMC_M10
GO:1902816	0.04	1.00	5	5	regulation of protein localization to microtubule	BP	1	ABHD17B,TTBK2,ABHD17A,ABHD17C,MAP1A	grey	VSMC_M10
GO:1902817	0.04	1.00	5	5	negative regulation of protein localization to microtubule	BP	1	ABHD17B,TTBK2,ABHD17A,ABHD17C,MAP1A	grey	VSMC_M10
GO:0050953	0.04	0.97	77	126	sensory perception of light stimulus	BP	1	RDH5,MIP,EYA4,OPN1SW,GUCA1B,PRPH2,GRM8,RORB,CNGA1,CLIC5,CNNM4,CLDN19,EPAS1,RIMS1,PPT1,GJA10,WHRN,TULP2,BBS7,MYO3A,EML2,OPA3,CACNB2,USH1G,CRYGN,ZNF513,MYO7A,MKKS,FSCN2,PDE6G,KERA,LUM,ATXN7,CEP250,CCDC66,ADGRV1,KIFC3,USH1C,OPA1,IMPG2,PAX2,SLC4A10,TRPM1,CABP1,PDE6D,RRH,LRIT3,CRYBA4,NRL,EYA3,MYO3B,BBS1,OAT,PDE6H,HMCN1,CDH23,ROM1,CLN8,DHRS3,GUCY2D,CHRNB2,LCTL,CLN6,MYO9A,UNC119,SLC45A2,PITPNA,GNAT2,LAMC3,HSF4,CRYZ,REEP6,DRAM2,LRAT,AOC2,BBS10,DNAJC19	grey	VSMC_M10
GO:0010804	0.04	0.99	12	15	negative regulation of tumor necrosis factor-mediated signaling pathway	BP	1	GAS6,F2RL1,PELI3,GPS2,PYDC1,PTPN2,PIAS4,MIR27B,MIR24-1,NOL3,CCDC3,HIST1H2BJ	grey	VSMC_M10
GO:0007158	0.04	0.99	11	14	neuron cell-cell adhesion	BP	1	RET,NCAM2,NLGN4Y,TNR,NRXN2,NINJ2,NLGN2,NLGN3,CDK5R1,CEL,NRXN3	grey	VSMC_M10
GO:0021937	0.04	1.00	5	5	cerebellar Purkinje cell-granule cell precursor cell signaling involved in regulation of granule cell precursor cell proliferation	BP	1	GLI1,SMO,GLI2,ZNF423,LHX1	grey	VSMC_M10

GO:0006811	0.04	0.96	574	1026	ion transport	BP	1	VPS4B,SLC39A5,NEDD4L,ATP8B1,STAC3,SLC26A10,CYP27B1,SLC16A7,ATP5MGL,NIPAL2,MAPK8IP2,SLC12A2,SLC27A6,KCNV1,PRRT1,PRKN,SLC22A4,CALHM4,TSPO,GOPC,SMDT1,BAK1,CATSPER3,KLHL3,MCHR1,PKD2L2,KCNK9,MAPK15,NTRK2,KCNJ4,PLA2G6,SLC39A4,ATP6V1F,MFSD3,CHRNA3,CHRNA5,TRPM6,ABCB1,ABCC10,STEAP2,GABRA1,APBA1,CNGA1,SLC41A2,TMEM63B,SLC35B2,CLIC5,SLC1A1,SLC25A13,WNK2,CNNM4,TSPAN13,SLC3A1,PRKCE,ATP6V1E2,TMC4,KCNQ4,IBTK,SHANK1,SLC4A4,CYB5R4,SLC35A1,AQP1,SLC1A4,SLC31A2,BSPRY,SLC13A4,ATP6V1C2,HECW1,TCAF2,GRIK2,CLCN1,SFRP4,KEL,UCP1,ATG5,TRPM4,SLC7A11,OSR1,PGRMC2,MFSD4B,SPHK2,KCNJ14,GRIN2D,TRPC3,SLC8A2,COMMD3,PANX1,SLC26A5,HEPHL1,ATP7B,KCNRG,CACNB2,ANO10,SLC39A12,SLC25A30,ATP6V0E2,SLC4A2,ASIC3,STAC,LLGL2,ATP6V0B,CLNS1A,STEAP3,GRIK5,ATP1A3,CCT8L2,OXSR1,TMEM163,SLC22A13,ATP5F1C,SCN5A,SCN1A,SLC25A38,SEC14L1,SLC24A3,GPD1L,ABCC4,CHRNA6,SLC20A2,SLC4A7,SLC38A10,SYT1,CACNB4,EFHB,RYR1,NALCN,SLC6A15,SLC25A15,SLC23A2,PRNP,TAF7,DIAPH1,P2RX2,CLIC1,TMCO3,GAS6,HPX,RASA3,ARL6IP5,COX7A1,SLC7A1,SLC25A26,SYT9,COMMD9,SLC38A9,MICU3,KCNIP3,ANO4,SLC5A8,ATP6V1H,MFSD5,COX4I2,CYP7A1,CYB5A,PXK,SLCO6A1,SLMAP,SLC43A1,ATP5F1A,GLRX,P2RX3,TRPC4AP,CHRM5,SLC9A9,RYR3,ORAI1,MEF2C,ASIC1,TMBIM6,P2RX7,HVCN1,KCNH3,P2RY1,PTK2B,SLC39A13,KCNJ11,SLC33A1,PLLP,SERINC5,TMEM150C,TPCN1,SLC8B1,HOMER1,IFNGR2,VDR,STC1,SLC25A37,OPA1,SLC48A1,KCNJ3,SLC38A4,SLC38A2,F2R,SLC38A1,TESC,RIPK1,SLC40A1,SLC22A23,ANO6,CRY2,SLC6A2,ZDHHC1[illegible]	grey	VSMC_M10
GO:0048284	0.04	0.97	74	120	organelle fusion	BP	1	STX7,PRKN,SEPT8,BAK1,TSNARE1,TGFBRAP1,STX17,VPS41,RAB7A,PIP4K2A,RUBCNL,GRIK5,TBC1D4,AKT2,YIPF4,SYT1,VPS16,STX2,SYT9,ANKRD27,CHMP2B,DYSF,OPA1,VPS18,VPS39,RAB7B,DNM1L,SEC22B,ZDHHC6,VTI1A,SNAP23,USP30,MFN1,RCC1L,C2CD5,STX1A,STX6,VPS8,SPG11,PIKFYVE,FIS1,RAB3A,MUL1,VAMP1,SNAP29,ANKFY1,STX5,RAB8A,ATP13A2,CPLANE2,SYT7,MFN2,ANXA2,CPLX1,STX1B,STX4,BNIP1,SYT11,RAB8B,AC138894.1,ADCK1,THG1L,SOX30,STX8,SNAPIN,GNAI3,VAV3,MIGA2,STXBP1,VPS4A,SNAP47,DOC2B,VAMP3,RAB39A	grey	VSMC_M10
GO:0007343	0.04	1.00	5	5	egg activation	BP	1	MYH9,NLRP5,PLAT,PLCB1,ZP2	grey	VSMC_M10
GO:0002420	0.04	1.00	5	5	natural killer cell mediated cytotoxicity directed against tumor cell target	BP	1	CD226,NECTIN2,PVR,IL12A,HAVCR2	grey	VSMC_M10
GO:0002423	0.04	1.00	5	5	natural killer cell mediated immune response to tumor cell	BP	1	CD226,NECTIN2,PVR,IL12A,HAVCR2	grey	VSMC_M10
GO:0002855	0.04	1.00	5	5	regulation of natural killer cell mediated immune response to tumor cell	BP	1	CD226,NECTIN2,PVR,IL12A,HAVCR2	grey	VSMC_M10
GO:0002858	0.04	1.00	5	5	regulation of natural killer cell mediated cytotoxicity directed against tumor cell target	BP	1	CD226,NECTIN2,PVR,IL12A,HAVCR2	grey	VSMC_M10
GO:0080144	0.04	1.00	5	5	amino acid homeostasis	BP	1	SLC1A1,SLC7A11,GLS,GRM2,PQLC2	grey	VSMC_M10
GO:0098739	0.04	0.97	75	123	import across plasma membrane	BP	1	SLC39A5,SLC12A2,SLC22A4,KCNK9,KCNJ4,SLC39A4,STEAP2,SLC1A1,WNK2,SLC1A4,TRPM4,SLC7A11,KCNJ14,SLC8A2,SLC39A12,ATP1A3,AKT2,PRNP,ARL6IP5,SLC7A1,SLC43A1,SLC9A9,KCNJ11,KCNJ3,KCNE2,KCNJ15,SLC12A5,SLC2A10,KCNJ2,SLC12A7,TRPV4,ACSL5,ISCU,AGT,ACE2,ATP1A2,SLC9A1,PER2,SLC12A8,NTSR1,DLG1,SLC12A9,SLC9A7,ATP1B1,SLC27A1,ARL6IP1,SLC3A2,CLN8,WNK3,ATP1B2,FAM155B,PSEN1,SLC7A3,SLC5A2,SLC39A6,TRPV2,LRRC8E,KCNJ18,SLC46A1,SLC9A6,TRPV3,TRPV1,P2RX1,SLC7A5,LRRC8A,LCN2,SLC12A4,SLC9A5,MIR24-1,SLC36A1,KCNH2,RGS4,KCNJ8,HCN4,KCNJ9	grey	VSMC_M10
GO:0045217	0.04	0.99	11	14	cell-cell junction maintenance	BP	1	MTSS1,WHRN,KIFC3,PLEKHA7,F2RL1,F2R,NLGN2,CSF1R,CAMSAP3,INAVA,PARD6A	grey	VSMC_M10
GO:0060332	0.04	1.00	5	5	positive regulation of response to interferon-gamma	BP	1	HPX,NLRC5,PARP9,MED1,IRGM	grey	VSMC_M10
GO:0060335	0.04	1.00	5	5	positive regulation of interferon-gamma-mediated signaling pathway	BP	1	HPX,NLRC5,PARP9,MED1,IRGM	grey	VSMC_M10
GO:0072174	0.04	1.00	5	5	metanephric tubule formation	BP	1	SOX9,PAX8,PAX2,WNT4,SOX8	grey	VSMC_M10
GO:0001910	0.04	0.98	26	38	regulation of leukocyte mediated cytotoxicity	BP	1	CD226,STX7,AGER,HLA-DRA,NECTIN2,PVR,SLC22A13,NCKAP1L,RASGRP1,F2RL1,CADM1,IL12A,LAG3,PPP3CB,STAT5B,ITGAM,VAV1,NOS2,IL7R,CRK,DNASE1,AP1G1,FADD,CXCL6,SERPINB9,HAVCR2	grey	VSMC_M10
GO:0070472	0.04	1.00	5	5	regulation of uterine smooth muscle contraction	BP	1	ADRA2B,GPER1,ADRA2C,SETD3,ABAT	grey	VSMC_M10
GO:0045663	0.04	0.99	13	17	positive regulation of myoblast differentiation	BP	1	MAPK14,AKIRIN1,PLCB1,BTG1,SMYD1,MEF2C,HIF1AN,RBM24,CDON,RANBP3L,LRRC8A,MIR199A1,XKR8	grey	VSMC_M10
GO:0045589	0.04	0.99	13	17	regulation of regulatory T cell differentiation	BP	1	HLA-DRA,IL4I1,SOX12,CD28,DUSP10,BCL6,VSIR,LAG3,FOXP3,SOCS1,DROSHA,MIR21,CD46	grey	VSMC_M10
GO:0006376	0.04	0.98	20	28	mRNA splice site selection	BP	1	SF3A3,PUF60,YTHDC1,SRSF10,CELF2,CELF1,SRSF6,CELF3,PRPF39,KHDC4,LUC7L3,SLU7,CELF5,LUC7L,RNVU1-6,NOL3,RNU4ATAC,RF00003,SNRPC,SRSF12	grey	VSMC_M10
GO:0010815	0.04	1.00	5	5	bradykinin catabolic process	BP	1	IDE,CPN1,XPNPEP1,CTSH,ECE1	grey	VSMC_M10
GO:0051938	0.04	0.99	9	11	L-glutamate import	BP	1	SLC1A1,SLC7A11,ARL6IP5,ATP1A2,PER2,NTSR1,ARL6IP1,CLN8,PSEN1	grey	VSMC_M10
GO:0098712	0.04	0.99	9	11	L-glutamate import across plasma membrane	BP	1	SLC1A1,SLC7A11,ARL6IP5,ATP1A2,PER2,NTSR1,ARL6IP1,CLN8,PSEN1	grey	VSMC_M10
GO:0048278	0.05	0.98	33	50	vesicle docking	BP	1	STX7,TSNARE1,EXOC4,SCFD2,STX17,PLEK,BVES,PPFIA3,RAB10,SYTL2,RALB,SYT1,STX2,SNPH,VPS18,KCNB1,STX1A,STX6,NSF,BLOC1S6,RAB3A,STX5,RAB8A,RAB15,STX1B,STX4,RAB8B,AC138894.1,STX8,RAB3D,STXBP2,STXBP1,VAMP3	grey	VSMC_M10
GO:0048242	0.05	1.00	5	5	epinephrine secretion	BP	1	LY6E,ADRA2B,ADRA2A,ADRA2C,CRH	grey	VSMC_M10
GO:0046039	0.05	0.99	13	17	GTP metabolic process	BP	1	NME1,GTPBP1,IMPDH1,RAB23,OPA1,NME4,MFN1,EFL1,NME7,NME3,GNAI3,RHOA,NME6	grey	VSMC_M10

GO:0006836	0.05	0.97	84	139	neurotransmitter transport	BP	1	SNCAIP,RIMS2,PRKN,GRM4,CHRNA3,CHRNA5,APBA1,SLC1A1,BRSK1,RIMS1,RIMS3,PPT1,DDC,PPFIA3,LIN7B,NAPA,CACNB2,GRIK5,NAPB,SYT1,RAB5A,LIN7A,SLC6A15,STX2,SYT9,SNPH,NR4A1,ERC2,MEF2C,ASIC1,P2RY1,SYNJ1,SLC38A2,SLC38A1,VPS18,SLC6A2,DTNBP1,SYT8,DNM1L,SLC6A3,ADRA2A,SNAP23,GPER1,ATP1A2,PER2,STX1A,SLC29A2,BACE1,DNAJC5,CSPG5,RAP1B,BLOC1S6,NRXN2,CASK,PNKD,RAB3A,SYN1,SNAP29,SLC29A4,SYT7,CLN8,CPLX1,STX1B,STX4,PSEN1,SYT11,CALM1,SNAPIN,PRKCB,CAMK2A,SLC6A7,STXBP2,FMR1,SLC1A7,PIP5K1C,P2RX1,BAIAP3,PARK7,STXBP1,CHRNB4,SNAP47,PDZD11,DOC2B,KCNMB4	grey	VSMC_M10
GO:0010700	0.05	1.00	5	5	negative regulation of norepinephrine secretion	BP	1	ADRA2B,P2RY1,ADRA2A,ADRA2C,CRH	grey	VSMC_M10
GO:0060259	0.05	0.99	7	8	regulation of feeding behavior	BP	1	NAPEPLD,CFAP20,EIF2AK4,TTC21B,STAT3,SGIP1,LEPR	grey	VSMC_M10
GO:0098662	0.05	0.96	308	541	inorganic cation transmembrane transport	BP	1	SLC39A5,NEDD4L,STAC3,ATP5MGL,NIPAL2,SLC12A2,KCNV1,SMDT1,CATSPER3,PKD2L2,KCNK9,KCNJ4,PLA2G6,SLC39A4,ATP6V1F,MFSD3,TRPM6,STEAP2,SLC41A2,SLC1A1,WNK2,CNNM4,TSPAN13,PRKCE,ATP6V1E2,KCNQ4,IBTK,SLC4A4,AQP1,SLC31A2,ATP6V1C2,HECW1,KEL,UCP1,ATG5,TRPM4,OSR1,SPHK2,KCNJ14,GRIN2D,TRPC3,SLC8A2,PANX1,ATP7B,KCNRG,CACNB2,SLC39A12,ATP6V0E2,ASIC3,STAC,ATP6V0B,ATP1A3,CCT8L2,OXSR1,TMEM163,ATP5F1C,SCN5A,SCN1A,SLC24A3,GPD1L,SLC20A2,SLC4A7,CACNB4,RYR1,NALCN,SLC6A15,PRNP,DIAPH1,TMCO3,GAS6,RASA3,COX7A1,MICU3,KCNIP3,ATP6V1H,COX4I2,CYB5A,SLMAP,ATP5F1A,GLRX,P2RX3,TRPC4AP,SLC9A9,RYR3,ORAI1,ASIC1,P2RX7,HVCN1,KCNH3,PTK2B,SLC39A13,KCNJ11,SLC33A1,TPCN1,SLC8B1,SLC25A37,OPA1,KCNJ3,F2R,TESC,SLC40A1,ANO6,SLC6A2,ZDHHC13,COX15,KCNK15,ABCC2,KCNE2,PKD2,NIPA1,CASQ2,KCNJ15,MCUR1,SLC12A5,KCNIP2,RYR2,FAM173B,ATP6V1B2,PM20D1,SLC41A1,COX6B1,SLC4A10,TRPM1,CALHM1,SLC6A3,LCK,SLC2A10,HECW2,KCNJ2,SLC9B1,SLC12A7,TRPV4,SLC9B2,ADRA2A,SLC30A10,TCIRG1,COX17,SCN2A,ISCU,KCNB1,SHROOM2,MCUB,SLC9A8,CLCN4,KCNK1,KCNG1,SCN7A,UBASH3B,ANK2,GPER1,KLHL24,RHCG,TRPM8,ABCC5,ATP1A2,SLC9A1,C4orf3,CASQ1,CCDC51,SLC12A8,KCNK2,NTSR1,SCN4B,DNAJC30,NIPAL3,NOS1AP,CATSPER2,DLG1,KCNK7,TMCO1,COX6A1,SLC30A4,CEMIP,SLC12A9,SESTD1,ATP5S,PTPN6,KCNK4,MICU1,MCU,SLC11A1,HTT,PKD1L1,COX8A,SLC9A7,ATP6V0A1,KCNN1,ABCB6,TRPM7,ATP1B1,KCNA1,BHLHA15,KCNMA1,SLC3A2,ASIC4,KCNH4,SLC2A9,GNB5,KCND1,HAP1,PSEN2,ATP6V1D,WNK3,STAC2,CACNB1,ANXA2,A[illegible]	grey	VSMC_M10
GO:0006638	0.05	0.97	58	93	neutral lipid metabolic process	BP	1	AVIL,ABHD16A,TNXB,SLC22A4,PNPLA3,PNPLA5,CAT,MBOAT7,NR1H2,DGKI,LPIN1,APOB,FGF21,FAAH,ABHD5,APOC1,LIPE,DDHD2,PANK2,ABHD6,GK5,PLCE1,FBXW7,NR1H3,LPIN3,FITM2,GPLD1,SIK1,CPT1A,GPAM,ABHD2,SORL1,DGKD,LPGAT1,DGKG,PLA2G4A,MOGAT3,CPS1,ABHD12B,GNB3,PLA2G16,SLC27A1,DAGLB,LIPC,DGKQ,APOBR,SEL1L,DGKE,LDLR,SREBF1,LPIN2,C3,LMF1,PLA2G15,PNPLA4,CNEP1R1,LYPLA2,INSIG2	grey	VSMC_M10
GO:0021940	0.05	0.99	7	8	positive regulation of cerebellar granule cell precursor proliferation	BP	1	GLI1,SMO,GLI2,ZNF423,EGF,LHX1,GPR37L1	grey	VSMC_M10
GO:0000965	0.05	1.00	5	5	mitochondrial RNA 3'-end processing	BP	1	PNPT1,TRNT1,SUPV3L1,HSD17B10,TRMT10C	grey	VSMC_M10
GO:0042219	0.05	0.98	17	23	cellular modified amino acid catabolic process	BP	1	ABHD16A,CHAC2,GGACT,AHCY,HOGA1,ACADL,GGT2,MTHFS,PRODH,ABHD12B,ALDH4A1,PCYOX1L,SARDH,DIO3,ALDH1L1,GGT5,DIO2	grey	VSMC_M10
GO:0006643	0.05	0.97	99	164	membrane lipid metabolic process	BP	1	KDSR,ORMDL2,B4GALNT1,A4GALT,PLA2G6,GPAA1,HACD4,LARGE1,CWH43,KIT,SPTLC1,TMEM246,PIGF,PPP2R1A,PPT1,MGST2,SMPD2,AGK,SPHK2,PRKD2,GLB1,ACER3,ELOVL1,NEU3,SPTLC3,PGAP2,SIRT3,NSMAF,ELOVL7,ASAH1,PIGU,ST8SIA4,HTRA2,SERINC5,ST3GAL6,ORMDL1,COL4A3BP,PIGT,GPLD1,A3GALT2,PGAP1,ST8SIA2,SGMS2,ALDH3B2,B3GALT1,TEX2,B3GNT5,CERS6,PIGM,HACD2,CERS2,PIGV,PIGX,GAL3ST3,PIGZ,ABCA12,PPM1L,B3GALNT1,GAL3ST4,PIGC,SMPD4,SGMS1,SCCPDH,FADS3,TECR,CLN8,PGAP3,DEGS1,PIGH,ALOXE3,HACD3,GALC,CLN6,ST8SIA5,GLA,HEXA,GM2A,PIGL,MPPE1,P2RX1,GBGT1,CEL,ST6GALNAC4,ST3GAL2,PLA2G15,ST6GALNAC5,CYP4F22,ABCA8,ELOVL3,PEMT,ACER2,PYURF,CPTP,NEU2,PIGB,B4GALT3,PIGA,SGPP1,ST8SIA6	grey	VSMC_M10
GO:0051289	0.05	0.98	30	45	protein homotetramerization	BP	1	MIP,SHMT2,CBY1,GNMT,TRPM4,SSBP1,RYR1,APIP,CBR4,RYR3,USP16,GLS,PKD2,SYCP1,ALDH1A3,KCNJ2,OSBPL2,UPB1,CRTC1,MAT1A,HSD17B10,ALDH1A2,THG1L,CRTC2,EVL,ACACA,SHMT1,TRPV1,CRYZ,VASP	grey	VSMC_M10
GO:0043011	0.05	0.99	11	14	myeloid dendritic cell differentiation	BP	1	TGFBR2,TRAF6,CAMK4,ITGB6,BATF2,LTBR,RBPJ,PSEN1,BATF,DHRS2,BATF3	grey	VSMC_M10
GO:0009308	0.05	0.97	49	77	amine metabolic process	BP	1	SNCAIP,ALDH7A1,PRKN,LY6E,AKR1B1,GPR37,SLC1A1,EPAS1,DDC,IL4I1,SLC7A11,NAPEPLD,AANAT,HNMT,IDO2,IDO1,LRTOMT,AP000812.5,NR1H4,HAND2,CHDH,NR4A2,ABCC2,SIRT4,AZIN2,SLC6A3,TGFB2,CHKA,SLC7A7,SMS,COMT,PNKD,GCH1,HDC,SLC29A4,ITGAM,GDPD3,GCDH,AC138894.1,CHRNB2,GDPD1,GDE1,ABAT,OAZ1,MECP2,PARK7,MIR21,NNMT,AOC2	grey	VSMC_M10
GO:0032823	0.05	0.99	7	8	regulation of natural killer cell differentiation	BP	1	IL15,IL15RA,GAS6,TOX,RASGRP1,STAT5B,ZBTB1	grey	VSMC_M10
GO:0032825	0.05	0.99	7	8	positive regulation of natural killer cell differentiation	BP	1	IL15,IL15RA,GAS6,TOX,RASGRP1,STAT5B,ZBTB1	grey	VSMC_M10
GO:0021591	0.05	0.98	19	27	ventricular system development	BP	1	CCDC134,CDK6,MBOAT7,AQP1,SLC7A11,ARMC4,NUMBL,TSKU,RAPGEF2,RPGRIP1L,DNAH5,DPCD,TTC21B,SEMA6D,ATP1B2,MECP2,CELSR2,AK8,HYDIN	grey	VSMC_M10

GO:0048167	0.05	0.97	86	143	regulation of synaptic plasticity	BP	1	RIMS2,PRRT1,AGER,SYNGAP1,ATF4,SYNGR1,NTRK2,PICK1,SRF,KIT,SLC1A1,BRSK1,RIMS1,RIMS3,ACP4,CRHR2,PTN,NCDN,GRIK2,PPFIA3,GRIN2D,SLC8A2,RAB5A,PRNP,PLK2,FAM107A,ABHD6,P2RX3,ERC2,MEF2C,PTK2B,MME,RAPGEF2,EIF2AK4,ITPKA,LZTS1,SLC4A10,NEURL1,SORCS3,IGSF11,SSH1,STAU1,KCNB1,KRAS,AGT,NFATC4,SYAP1,NPAS4,YTHDF1,CREB1,YWHAG,MAPT,VGF,EIF4EBP2,TNR,MPP2,CRTC1,PPP3CB,RAB3A,CD38,MAPK1,NSG1,RAB8A,EPHA4,DLG4,SYT7,RGS14,STX4,PSEN1,AC138894.1,RAB11A,CYP46A1,CAMK2A,FMR1,PRKCZ,MECP2,JPH3,STXBP1,CNTN2,GRID2,CRH,YWHAH,HRAS,SHISA8,MAP1A,LRRTM2	grey	VSMC_M10
GO:0015844	0.05	0.97	32	48	monoamine transport	BP	1	PRKN,LY6E,MAPK15,PICK1,SYT1,SYT9,ADRA2B,CHRM5,P2RY1,SLC6A2,DTNBP1,SYT8,GRM2,SLC6A3,ADRA2A,KCNB1,GRK2,AGT,STX1A,ADRA2C,RAB3A,PINK1,ACTB,SLC29A4,SYT7,SYT11,CHRNB2,ABAT,P2RX1,PARK7,CHGA,CRH	grey	VSMC_M10
GO:0042754	0.05	0.99	9	11	negative regulation of circadian rhythm	BP	1	CRY1,SUV39H2,CRY2,PER2,CIPC,SFPQ,SIN3A,CRH,PASD1	grey	VSMC_M10
GO:0072173	0.05	0.99	7	8	metanephric tubule morphogenesis	BP	1	SOX9,PAX8,PAX2,CXCR2,WNT4,PKD1,SOX8	grey	VSMC_M10
GO:0051560	0.05	0.98	18	25	mitochondrial calcium ion homeostasis	BP	1	SMDT1,SLC25A27,MICU3,IMMT,C19orf12,SLC8B1,MCUR1,RAP1GDS1,MCUB,DISC1,PDZD8,FIS1,MICU1,MCU,ATP2A1,SLC25A23,MAIP1,BCAP31	grey	VSMC_M10
GO:0021559	0.05	0.99	7	8	trigeminal nerve development	BP	1	PLXNA4,POU4F1,SEMA3A,TFAP2A,SEMA3F,ISL1,PLXNA3	grey	VSMC_M10
GO:1990822	0.05	0.99	9	11	basic amino acid transmembrane transport	BP	1	SLC3A1,SLC7A1,SLC38A9,SLC7A7,AGT,PQLC2,SLC7A3,SLC7A6,SLC25A29	grey	VSMC_M10
GO:0019323	0.05	1.00	5	5	pentose catabolic process	BP	1	DHDH,NUDT5,XYLB,RBKS,RPE	grey	VSMC_M10
GO:0050905	0.05	0.97	63	103	neuromuscular process	BP	1	STAC3,PRKN,AGTPBP1,RBFOX2,TNNI3,SHANK1,MYH14,GRIN2D,USH1G,XRCC1,STAC,MYO7A,MYCBP2,POU4F1,CHD8,RAC3,SPART,TCF15,NR4A1,TMEM150C,NBN,USH1C,HOMER1,TNNC1,SLC6A3,ALDH1A3,KCNJ2,TNNI1,SYNM,VTI1A,ADARB1,GIGYF2,MYH7,CASQ1,ACTN3,BACE1,CDH23,TNR,PNKD,CNTNAP1,GCH1,KCNA1,DLG4,NLGN2,CHRNB1,CLN8,TCAP,STAC2,AC138894.1,IGDCC3,ATP2A1,STRA6,GM2A,MECP2,JPH3,AARS,DMD,GRID2,TNNC2,FXN,POMK,GPR88,MAP1A	grey	VSMC_M10
GO:0046655	0.05	0.99	9	11	folic acid metabolic process	BP	1	SHMT2,DHFR,MTHFD2,MTRR,MTHFS,SHMT1,SLC46A1,MTHFD2L,SLC25A32	grey	VSMC_M10
GO:0060287	0.05	0.99	7	8	epithelial cilium movement involved in determination of left/right asymmetry	BP	1	LRRC6,RFX3,DNAH11,OFD1,CFAP45,TTC25,CFAP53	grey	VSMC_M10
GO:0030001	0.05	0.96	331	587	metal ion transport	BP	1	VPS4B,SLC39A5,NEDD4L,STAC3,CYP27B1,NIPAL2,SLC12A2,KCNV1,SLC22A4,TSPO,SMDT1,BAK1,CATSPER3,KLHL3,MCHR1,PKD2L2,KCNK9,KCNJ4,PLA2G6,SLC39A4,TRPM6,STEAP2,SLC41A2,SLC1A1,WNK2,CNNM4,TSPAN13,PRKCE,KCNQ4,IBTK,SLC4A4,AQP1,SLC31A2,BSPRY,SLC13A4,HECW1,KEL,ATG5,TRPM4,OSR1,PGRMC2,MFSD4B,SPHK2,KCNJ14,GRIN2D,TRPC3,SLC8A2,COMMD3,PANX1,HEPHL1,ATP7B,KCNRG,CACNB2,SLC39A12,ASIC3,STAC,STEAP3,ATP1A3,CCT8L2,OXSR1,TMEM163,SCN5A,SCN1A,SLC24A3,GPD1L,SLC20A2,SLC4A7,SLC38A10,CACNB4,EFHB,RYR1,NALCN,SLC6A15,SLC23A2,PRNP,DIAPH1,P2RX2,GAS6,HPX,RASA3,COMMD9,MICU3,KCNIP3,SLC5A8,SLMAP,GLRX,P2RX3,TRPC4AP,SLC9A9,RYR3,ORAI1,ASIC1,TMBIM6,P2RX7,KCNH3,PTK2B,SLC39A13,KCNJ11,TPCN1,SLC8B1,HOMER1,VDR,STC1,SLC25A37,OPA1,SLC48A1,KCNJ3,SLC38A4,SLC38A2,F2R,SLC38A1,TESC,SLC40A1,ANO6,SLC6A2,ZDHHC13,SLC10A6,KCNK15,CUTC,ABCC2,KCNE2,PKD2,NIPA1,CASQ2,KCNJ15,MCUR1,CNTN1,SLC12A5,KCNIP2,RYR2,SLC41A1,SLC4A10,TRPM1,CALHM1,PKP2,DNM1L,SLC6A3,SIK1,LCK,PPP3CA,SLC38A11,HECW2,KCNJ2,SLC38A3,SLC9B1,SLC12A7,TRPV4,SLC9B2,ADRA2A,SLC30A10,COX17,SCN2A,ISCU,KCNB1,SHROOM2,MCUB,SLC9A8,EGF,KCNK1,KCNG1,SCN7A,EPB41,OPRD1,UBASH3B,ANK2,GPER1,KLHL24,TRPM8,AGT,ABCC5,ATP1A2,SLC9A1,PLPP4,C4orf3,CASQ1,CCDC51,SLC12A8,KCNK2,NTSR1,SCN4B,NIPAL3,NOS1AP,MELTF,HOMER2,CATSPER2,CDKN1B,DLG1,KCNK7,TMCO1,NSF,SLC30A4,IL16,CEMIP,SLC12A9,SESTD1,CDH23,CASK,PTPN6,KCNK4,MICU1,MCU,SLC11A1,HTT,PKD1L1,CALCRL,SLC9A7,RAMP2,KCNN1,ABCB6,CAMK2G,TRPM7 [illegible]	grey	VSMC_M10
GO:0090184	0.05	0.99	7	8	positive regulation of kidney development	BP	1	MYC,SOX9,RET,PAX2,WNT4,SOX8,NOG	grey	VSMC_M10
GO:0045006	0.05	1.00	5	5	DNA deamination	BP	1	APOBEC3H,EXOSC4,EXOSC3,CDADC1,EXOSC5	grey	VSMC_M10
GO:0042416	0.05	0.99	7	8	dopamine biosynthetic process	BP	1	GPR37,DDC,NR4A2,SLC6A3,TGFB2,GCH1,PARK7	grey	VSMC_M10
GO:0006835	0.05	0.97	36	56	dicarboxylic acid transport	BP	1	SLC26A10,SLC12A2,NTRK2,APBA1,SLC1A1,SLC25A13,SLC3A1,SLC1A4,SLC7A11,SLC26A5,SLC25A30,ARL6IP5,ABCC2,GRM2,ABCC5,ATP1A2,PER2,NTSR1,ARL6IP1,PRAF2,SLC25A11,CLN8,SLC26A1,PSEN1,SLC26A2,LRRC8E,SLC46A1,SLC25A14,ABAT,SLC1A7,TRPV1,LRRC8A,STXBP1,LRRC8D,SLC25A21,SLC25A32	grey	VSMC_M10
GO:0002715	0.05	0.98	14	19	regulation of natural killer cell mediated immunity	BP	1	CD226,NECTIN2,PVR,RASGRP1,CD96,CADM1,IL12A,LAG3,STAT5B,VAV1,CRK,AP1G1,SERPINB9,HAVCR2	grey	VSMC_M10
GO:1903959	0.05	0.98	14	19	regulation of anion transmembrane transport	BP	1	GOPC,ABCB1,TCAF2,PRNP,ARL6IP5,SLC43A1,RIPK1,AGT,ACE2,ATP1A2,PER2,ARL6IP1,PSEN1,RGS4	grey	VSMC_M10
GO:0051262	0.06	0.97	42	66	protein tetramerization	BP	1	MIP,SHMT2,CPSF6,STK3,HSD17B8,CBY1,GNMT,TRPM6,TRPM4,SSBP1,RYR1,APIP,CBR4,RYR3,USP16,HOMER1,GLS,CUTC,PKD2,STK4,SYCP1,TRPM1,ALDH1A3,KCNJ2,OXA1L,OSBPL2,UPB1,CRTC1,TRPM7,MAT1A,FARSB,HSD17B10,ALDH1A2,THG1L,CRTC2,EVL,ACACA,SHMT1,TRPV1,PKD1,CRYZ,VASP	grey	VSMC_M10
GO:0048172	0.06	0.99	9	11	regulation of short-term neuronal synaptic plasticity	BP	1	SYNGR1,GRIK2,PPFIA3,SLC8A2,SLC4A10,SYAP1,RAB3A,AC138894.1,SHISA8	grey	VSMC_M10
GO:1901186	0.06	0.97	22	32	positive regulation of ERBB signaling pathway	BP	1	RTN4,EPGN,AREG,TGFA,NEU3,HBEGF,PDE6G,HIP1R,FBXW7,NEURL1,ADRA2A,NCF1,EGF,GPER1,DGKD,AGT,CBL,PDE6H,HAP1,RBPJ,MIR29A,EREG	grey	VSMC_M10
GO:0009299	0.06	0.98	12	16	mRNA transcription	BP	1	ATF4,HSF1,FOXE3,C5AR1,TFCP2,AP3B1,STAT3,ZBTB1,MED1,SREBF1,SUPT6H,EREG	grey	VSMC_M10
GO:0042430	0.06	0.99	9	11	indole-containing compound metabolic process	BP	1	TPH2,BTBD9,DDC,IL4I1,AANAT,IDO2,IDO1,SRD5A1,GCDH	grey	VSMC_M10

GO:0021536	0.06	0.97	31	47	diencephalon development	BP	1	GLI1,UQCRQ,PITX1,SMO,PTN,NDNF,GLI2,POU4F1,SEMA3A,NR4A2,SALL1,SRD5A1,SLC6A3,SOX2,GBX2,CREB1,WNT4,TBX19,HAP1,BMPR1A,RBPJ,ALDH1A2,CHRNB2,ISL1,UBB,NOG,WNT5A,POU3F2,OTX1,CRH,TAL2	grey	VSMC_M10
GO:0036480	0.06	0.99	7	8	neuron intrinsic apoptotic signaling pathway in response to oxidative stress	BP	1	PRKN,FBXW7,MCL1,NONO,PINK1,PARP1,PARK7	grey	VSMC_M10
GO:1903376	0.06	0.99	7	8	regulation of oxidative stress-induced neuron intrinsic apoptotic signaling pathway	BP	1	PRKN,FBXW7,MCL1,NONO,PINK1,PARP1,PARK7	grey	VSMC_M10
GO:0046471	0.06	0.98	19	27	phosphatidylglycerol metabolic process	BP	1	SERAC1,PLA2G6,HADHA,TAMM41,CRLS1,CDS2,PLA2G4B,LPCAT1,GPAM,LPGAT1,THEM5,PLA2G4A,SLC27A1,ABCA3,MECP2,PLA2G15,LCLAT1,PTPMT1,DNAJC19	grey	VSMC_M10
GO:1905244	0.06	0.99	9	11	regulation of modification of synaptic structure	BP	1	CTTNBP2,CHMP2B,TIAM1,CYFIP1,CAMKV,GRIPAP1,EPHA4,AC138894.1,RHOA	grey	VSMC_M10
GO:0035270	0.06	0.96	54	87	endocrine system development	BP	1	ONECUT2,GLI1,TSPO,BAK1,PITX1,LY6E,SMO,SRF,CDK6,RFX3,SOX9,GIPR,GLI2,RAF1,MNX1,WNT11,PAX8,DKK3,ARNTL,NKX6-1,ZBED6,SALL1,SRD5A1,SLC6A3,SOX2,SIDT2,HOXD3,CREB1,MEN1,WNT4,RAP1GAP,TBX19,MAPK1,BMPR1A,THRA,RBPJ,ALDH1A2,MAPK3,IL6R,MAP2K1,SMAD3,STRA6,GATA6,ISL1,PDPK1,FSTL3,SOX4,HOXB3,NOG,WNT5A,POU3F2,SIX1,NKX2-5,HOXA3	grey	VSMC_M10
GO:0031629	0.06	0.98	14	19	synaptic vesicle fusion to presynaptic active zone membrane	BP	1	GRIK5,SYT1,STX2,SYT9,SNAP23,STX1A,RAB3A,SNAP29,CPLX1,STX1B,STX4,SNAPIN,STXBP1,SNAP47	grey	VSMC_M10
GO:0099500	0.06	0.98	14	19	vesicle fusion to plasma membrane	BP	1	GRIK5,SYT1,STX2,SYT9,SNAP23,STX1A,RAB3A,SNAP29,CPLX1,STX1B,STX4,SNAPIN,STXBP1,SNAP47	grey	VSMC_M10
GO:0042417	0.06	0.98	17	24	dopamine metabolic process	BP	1	SNCAIP,PRKN,GPR37,SLC1A1,DDC,LRTOMT,AP000812.5,NR4A2,SLC6A3,TGFB2,COMT,PNKD,GCH1,ITGAM,CHRNB2,ABAT,PARK7	grey	VSMC_M10
GO:0045637	0.06	0.96	91	152	regulation of myeloid cell differentiation	BP	1	NME1,HSPA1B,BRD1,IL5,TRIB1,MYC,TOB2,MAPK14,HSF1,ZNF16,RBFOX2,CDK6,ROR2,SMAP1,HOXA7,MTURN,CNOT4,TLR4,FOXO3,POU4F1,ACVR2A,JAG1,NCKAP1L,FOXP1,MITF,PRKDC,IRF7,TRAF6,ACVR1B,CAMK4,PIK3R1,FBXW7,MEF2C,PTK2B,TESC,RIPK1,LDB1,RAB7B,SLC9B2,GPR137B,PIAS3,ACIN1,TCTA,HCLS1,UBASH3B,CIB1,ARNT,THPO,ZBTB46,CREB1,WDR61,ESRRA,GPR137,FBN1,STAT3,STAT5B,LEO1,INHA,TFE3,MED1,HOXB8,KAT7,FOS,HAX1,GPR68,RCOR1,CD74,PPARGC1B,EVI2B,PTPN2,JUN,ATP6AP1,MIR222,MIR125B1,PRMT6,FSTL3,ANKRD54,MAFB,FADD,CASP8,CA2,BGLAP,HIST1H4B,HIST2H4A,HIST1H4H,HIST1H4D,FAM210B,LIF,CEBPB,HIST1H4A,HIST1H4F	grey	VSMC_M10
GO:0016559	0.06	0.99	7	8	peroxisome fission	BP	1	DNM1L,PEX11B,PEX19,FIS1,SEC16B,PEX11G,PEX11A	grey	VSMC_M10
GO:0006812	0.06	0.95	446	796	cation transport	BP	1	VPS4B,SLC39A5,NEDD4L,STAC3,CYP27B1,ATP5MGL,NIPAL2,MAPK8IP2,SLC12A2,KCNV1,PRRT1,PRKN,SLC22A4,CALHM4,TSPO,SMDT1,BAK1,CATSPER3,KLHL3,MCHR1,PKD2L2,KCNK9,MAPK15,KCNJ4,PLA2G6,SLC39A4,ATP6V1F,MFSD3,CHRNA3,TRPM6,STEAP2,CNGA1,SLC41A2,TMEM63B,SLC1A1,WNK2,CNNM4,TSPAN13,PRKCE,ATP6V1E2,KCNQ4,IBTK,SHANK1,SLC4A4,AQP1,SLC1A4,SLC31A2,BSPRY,SLC13A4,ATP6V1C2,HECW1,KEL,UCP1,ATG5,TRPM4,OSR1,PGRMC2,MFSD4B,SPHK2,KCNJ14,GRIN2D,TRPC3,SLC8A2,COMMD3,PANX1,HEPHL1,ATP7B,KCNRG,CACNB2,ANO10,SLC39A12,ATP6V0E2,ASIC3,STAC,ATP6V0B,STEAP3,ATP1A3,CCT8L2,OXSR1,TMEM163,ATP5F1C,SCN5A,SCN1A,SLC25A38,SEC14L1,SLC24A3,GPD1L,SLC20A2,SLC4A7,SLC38A10,SYT1,CACNB4,EFHB,RYR1,NALCN,SLC6A15,SLC25A15,SLC23A2,PRNP,TAF7,DIAPH1,P2RX2,TMCO3,GAS6,HPX,RASA3,COX7A1,SLC7A1,SLC25A26,SYT9,COMMD9,SLC38A9,MICU3,KCNIP3,SLC5A8,ATP6V1H,COX4I2,CYB5A,SLMAP,SLC43A1,ATP5F1A,GLRX,P2RX3,TRPC4AP,CHRM5,SLC9A9,RYR3,ORAI1,MEF2C,ASIC1,TMBIM6,P2RX7,HVCN1,KCNH3,PTK2B,SLC39A13,KCNJ11,SLC33A1,SERINC5,TPCN1,SLC8B1,HOMER1,IFNGR2,VDR,STC1,SLC25A37,OPA1,SLC48A1,KCNJ3,SLC38A4,SLC38A2,F2R,SLC38A1,TESC,SLC40A1,ANO6,SLC6A2,ZDHHC13,SLC10A6,COX15,KCNK15,CUTC,ABCC2,KCNE2,PKD2,NIPA1,CASQ2,KCNJ15,MCUR1,CNTN1,DTNBP1,SLC12A5,KCNIP2,RYR2,SYT8,FAM173B,GRM2,ATP6V1B2,PM20D1,SLC41A1,COX6B1,SLC4A10,SFXN2,TRPM1,CALHM2,CALHM1,PKP2,DNM1L,SLC6A3,SIK1,LCK,SLC2A10,PPP3CA,SLC38A11,HECW2,KCNJ2,SLC38A3,SLC9B1,SLC12A7,TRPV4,SLC9B2,ADRA2A,SLC[illegible]	grey	VSMC_M10
GO:0008045	0.06	0.98	17	24	motor neuron axon guidance	BP	1	PLXNA4,RAC2,HOXA2,MYCBP2,KIF5C,SEMA3A,SLIT1,ALCAM,SEMA3F,EPHA4,NTN1,RNF165,LHX1,NTN3,PLXNA3,NOG,CHN1	grey	VSMC_M10
GO:0007274	0.06	0.98	15	21	neuromuscular synaptic transmission	BP	1	STAC3,CHRNA3,FCHSD2,FCHSD1,P2RX2,P2RX3,ERC2,ADARB1,RAB3A,CHRM1,CHRNB1,CHRNB2,DTNA,STXBP1,CHRNB4	grey	VSMC_M10
GO:0051259	0.06	0.95	103	174	protein complex oligomerization	BP	1	MIP,SHMT2,CPSF6,STK3,KCNV1,HSD17B8,CBY1,PRPH2,GNMT,TRPM6,NINJ1,CHMP2A,PNPT1,TRPM4,SSBP1,VSTM5,KCNRG,KCTD2,KCTD21,AQP11,HGSNAT,TMEM173,SYT1,RYR1,PRNP,ATL2,APIP,KCTD6,KCTD15,CBR4,P2RX3,RYR3,TMEM120B,ASIC1,USP16,HOMER1,OPA1,GLS,CUTC,PKD2,STK4,SYCP1,TWNK,TRPM1,CALHM1,DNM1L,ALDH1A3,KCNJ2,KCTD18,OXA1L,KCNB1,KCNG1,TIFA,HSCB,SLC9A1,OSBPL2,UPB1,EHD1,MPP2,ATL1,MICU1,MCU,CRTC1,TRPM7,KCNA1,KCND1,ROM1,MAT1A,ZBTB1,FARSB,HSD17B10,CARD11,ALDH1A2,KCTD13,THG1L,CRTC2,EVL,ACACA,CD74,SHMT1,RNF135,TNFAIP1,TRPV1,KCTD5,ABCA3,PKD1,KCNC4,LRRC8A,ZNHIT6,BCL10,KCTD19,CRYZ,LRRC8D,NOL3,VASP,BEND3,CLDN1,KCTD1,KCNA6,IKZF4,CLYBL,TRMT61B,KCNG2	grey	VSMC_M10
GO:0000959	0.06	0.97	29	44	mitochondrial RNA metabolic process	BP	1	TFB1M,MTERF1,LRPPRC,MTO1,PNPT1,TRIT1,C6orf203,FOXO3,FASTK,TRNT1,TWNK,WARS2,RPUSD4,YARS2,TARS2,TFAM,FASTKD1,KIAA0391,SUPV3L1,DARS2,TRMT5,HSD17B10,EARS2,PPARGC1B,METTL4,TEFM,SLC25A33,TRMT10C,TRMT61B	grey	VSMC_M10

GO:0033630	0.06	0.98	12	16	positive regulation of cell adhesion mediated by integrin	BP	1	PODXL,IFT74,FERMT1,RAC3,NCKAP1L,RET,TGFB2,CIB1,PTPN6,CCL5,PIEZO1,LIF	grey	VSMC_M10
GO:0072017	0.06	0.99	8	10	distal tubule development	BP	1	KLHL3,TFAP2B,POU3F3,JAG1,PAX8,PKD2,PAX2,PKD1	grey	VSMC_M10
GO:0001912	0.06	0.97	17	24	positive regulation of leukocyte mediated cytotoxicity	BP	1	CD226,STX7,HLA-DRA,NECTIN2,PVR,SLC22A13,RASGRP1,F2RL1,CADM1,IL12A,LAG3,STAT5B,ITGAM,VAV1,NOS2,AP1G1,FADD	grey	VSMC_M10
GO:0050433	0.06	0.97	22	32	regulation of catecholamine secretion	BP	1	PRKN,SYT1,SYT9,ADRA2B,P2RY1,DTNBP1,SYT8,GRM2,ADRA2A,KCNB1,GRK2,AGT,STX1A,ADRA2C,RAB3A,PINK1,SYT7,SYT11,CHRNB2,ABAT,CHGA,CRH	grey	VSMC_M10
GO:0006814	0.06	0.95	95	161	sodium ion transport	BP	1	NEDD4L,SLC12A2,SLC22A4,CATSPER3,KLHL3,WNK2,CNNM4,PRKCE,SLC4A4,SLC13A4,HECW1,TRPM4,OSR1,MFSD4B,SLC8A2,COMMD3,ASIC3,ATP1A3,SCN5A,SCN1A,SLC24A3,GPD1L,SLC20A2,SLC4A7,SLC38A10,NALCN,SLC6A15,SLC23A2,COMMD9,SLC5A8,SLMAP,GLRX,SLC9A9,ASIC1,P2RX7,TPCN1,SLC8B1,SLC38A4,SLC38A2,SLC38A1,TESC,ANO6,SLC6A2,SLC10A6,PKD2,CNTN1,SLC41A1,SLC4A10,PKP2,SLC6A3,SIK1,SLC38A11,HECW2,SLC38A3,SLC9B1,SLC9B2,SCN2A,SHROOM2,SLC9A8,KCNK1,SCN7A,KLHL24,ATP1A2,SLC9A1,SCN4B,DLG1,SLC9A7,ATP1B1,NDUFA9,SLC3A2,ASIC4,SLC38A6,WNK3,ATP1B2,SLC5A2,HCN3,CACNA1G,SLC5A11,DNM2,SCNN1B,SLC6A7,ASIC2,SLC9A6,CACNA1H,SLC9A5,MIR24-1,DMD,SLC22A5,SLC6A19,FGF11,SCN2B,NETO2,YWHAH,HCN4,NKX2-5	grey	VSMC_M10
GO:0007601	0.07	0.96	74	123	visual perception	BP	1	RDH5,MIP,EYA4,OPN1SW,GUCA1B,PRPH2,GRM8,RORB,CNGA1,CLIC5,CNNM4,CLDN19,EPAS1,RIMS1,PPT1,GJA10,TULP2,BBS7,MYO3A,EML2,OPA3,CACNB2,CRYGN,ZNF513,MYO7A,MKKS,FSCN2,PDE6G,KERA,LUM,ATXN7,CEP250,CCDC66,ADGRV1,KIFC3,OPA1,IMPG2,PAX2,SLC4A10,TRPM1,CABP1,PDE6D,RRH,LRIT3,CRYBA4,NRL,EYA3,MYO3B,BBS1,OAT,PDE6H,HMCN1,CDH23,ROM1,CLN8,DHRS3,GUCY2D,CHRNB2,LCTL,CLN6,MYO9A,UNC119,SLC45A2,PITPNA,GNAT2,LAMC3,HSF4,CRYZ,REEP6,DRAM2,LRAT,AOC2,BBS10,DNAJC19	grey	VSMC_M10
GO:0007611	0.07	0.95	107	183	learning or memory	BP	1	MAPK8IP2,PRRT1,PRKN,AGER,SYNGAP1,NTRK2,PLA2G6,BTBD9,SRF,TTBK1,KIT,SLC1A1,ADGRF1,BRSK1,DNAH11,SHANK1,PPT1,PTN,EGFR,SLC7A11,NTF4,PRKAR2B,ADCY3,SLC8A2,B4GALT2,PLCB1,PRNP,DDHD2,ARL6IP5,PLK2,GPI,CAMK4,CASP3,MEF2C,ASIC1,CUX2,MME,EIF2AK4,SYNJ1,AMFR,NQO2,SLC12A5,ATXN1,BDNF,SORCS3,SCN2A,NLGN4Y,KRAS,ADNP,AGT,ATP1A2,NFATC4,KCNK2,NPAS4,NTSR1,YTHDF1,FZD9,CREB1,MAPT,C1QL1,EIF4EBP2,COMT,TNR,NRXN2,KCNK4,BCHE,CRTC1,HTT,PPP3CB,NPTX2,MAPK1,TMOD2,DLG4,SLC2A4,THRA,CLN8,RGS14,PSEN1,SYT11,ITGA3,NLGN3,FOS,AC138894.1,CHRNB2,LDLR,GPRASP2,BHLHB9,STRA6,GM2A,INSR,IFT20,JUN,AFF2,PRKCZ,MECP2,ABCA7,JPH3,LHX8,ATXN1L,CNTN2,POMK,CRH,GPR88,NRXN3,CEBPB,MAP1A,FOXO6	grey	VSMC_M10
GO:0030004	0.07	0.96	50	81	cellular monovalent inorganic cation homeostasis	BP	1	NEDD4L,SLC12A2,TFAP2B,SLC4A4,PPT1,RAB7A,ATP6V0E2,LACC1,SLC4A2,AQP11,ATP1A3,SLC4A7,TMEM106B,MAFG,ATP6V1H,TM9SF4,IL1A,SLC9A9,HVCN1,SLC4A10,KCNJ2,SLC9B1,TMEM9,TCIRG1,SLC9A8,SLAMF8,AGT,ATP1A2,SLC9A1,CLIC4,SLC11A1,SLC9A7,ATP6V0A1,ATP1B1,MAPK1,DMXL2,KCNMA1,SLC4A3,ATP1B2,MAPK3,AC138894.1,CLN6,SNAPIN,C7,TMEM199,SLC9A6,ATP6AP1,SLC9A5,CA2,RAB39A	grey	VSMC_M10
GO:0042157	0.07	0.95	68	111	lipoprotein metabolic process	BP	1	LRP1,ATG12,APOM,ZDHHC14,PPARA,GPAA1,ZDHHC21,APOL4,APOL6,ABHD17B,CWH43,TMEM246,PIGF,PPT1,ATG5,APOB,APOC1,PGAP2,LYPLA1,ZDHHC20,PIGU,ATG10,GOLGA7B,ZDHHC13,PIGT,GPLD1,PGAP1,ZDHHC23,LYPLAL1,ZDHHC6,RABL3,ATG16L1,PIGM,HHAT,ZDHHC18,PIGV,ZDHHC24,PIGX,GLUL,PIGZ,APOLD1,APOD,OLR1,NMT1,ZDHHC8,PIGC,PPM1A,ZDHHC4,WIPI2,PGAP3,PIGH,ZDHHC15,LDLR,PIGL,MPPE1,ABHD17A,ZDHHC12,ZDHHC1,ZDHHC11,ABHD10,PEMT,IRGM,ABHD17C,PYURF,LYPLA2,PIGB,PIGA,MAP6D1	grey	VSMC_M10
GO:0060294	0.07	0.96	43	68	cilium movement involved in cell motility	BP	1	UBE2B,CATSPER3,LRRC6,CFAP69,DNAJA1,DNAH11,CABS1,ARMC2,APOB,ADCY3,RSPH6A,TTC21A,TTLL3,MKKS,ING2,WDR66,CFAP20,DNAH1,PLTP,DNAH5,DPCD,ROPN1L,NEURL1,CFAP43,SLC9B1,SLC9B2,CFAP46,INTS13,CFAP45,ENO4,TSSK4,CELF3,CATSPER2,SORD,TTC12,SPAG16,MNS1,TEKT3,SPEF2,CATSPERD,TPGS1,WDR63,CCSAP	grey	VSMC_M10
GO:0046467	0.07	0.95	73	120	membrane lipid biosynthetic process	BP	1	KDSR,ORMDL2,B4GALNT1,A4GALT,PLA2G6,GPAA1,HACD4,LARGE1,CWH43,SPTLC1,TMEM246,PIGF,SMPD2,AGK,SPHK2,PRKD2,ACER3,ELOVL1,SPTLC3,PGAP2,SIRT3,ELOVL7,ASAH1,PIGU,ST8SIA4,ORMDL1,PIGT,A3GALT2,PGAP1,ST8SIA2,SGMS2,ALDH3B2,B3GALT1,B3GNT5,CERS6,PIGM,HACD2,CERS2,PIGV,PIGX,GAL3ST3,PIGZ,PPM1L,GAL3ST4,PIGC,SMPD4,SGMS1,SCCPDH,CLN8,PGAP3,DEGS1,PIGH,ALOXE3,HACD3,ST8SIA5,PIGL,MPPE1,P2RX1,GBGT1,ST6GALNAC4,ST3GAL2,ST6GALNAC5,CYP4F22,ABCA8,ELOVL3,PEMT,ACER2,PYURF,PIGB,B4GALT3,PIGA,SGPP1,ST8SIA6	grey	VSMC_M10
GO:0086016	0.07	0.99	6	7	AV node cell action potential	BP	1	TRPM4,CACNB2,SCN5A,RYR2,SCN4B,CACNA1G	grey	VSMC_M10
GO:0086027	0.07	0.99	6	7	AV node cell to bundle of His cell signaling	BP	1	TRPM4,CACNB2,SCN5A,RYR2,SCN4B,CACNA1G	grey	VSMC_M10

GO:0006520	0.07	0.95	132	224	cellular amino acid metabolic process	BP	1	GLS2,SHMT2,ALDH8A1,MARS,GFPT2,TPH2,RIDA,DPYS,ATF4,PYCR3,PSAT1,GPT,MTAP,CARNMT1,GNMT,ALDH1A1,SLC25A13,AASDH,PPAT,HMGCLL1,RIMKLA,DDC,IL4I1,QRSL1,SLC7A11,DDO,BCAT2,DHFR,GAD2,HPDL,HNMT,IDO2,IDO1,HARS,HARS2,CARS2,AMDHD1,LARS,NR1H4,APIP,CSAD,THNSL2,AHCY,GSS,MCCC2,MSRA,GMPS,SERINC5,ENOPH1,SDSL,IVD,HOGA1,NIT2,BPHL,ASNSD1,HIBCH,GLS,ACAD8,WARS2,MTRR,PM20D1,SIRT4,GATC,ASL,ALDH5A1,AZIN2,MTR,YARS2,CBSL,SLC7A7,HGD,AMT,MCCC1,TARS2,DALRD3,CTPS2,ACADSB,OAT,HMGCL,GLUL,SMS,UPB1,PLOD3,FAH,CPS1,MTHFS,LARS2,PRODH,COMT,DARS2,AASDHPPT,NAT8L,HDC,ALDH4A1,MAT1A,ASRGL1,FARSB,HSD17B10,SLC25A44,ALDH6A1,GCDH,AC138894.1,GSTZ1,EARS2,CRYM,SHMT1,ADSSL1,ENOSF1,NOS2,PIPOX,ABAT,MECP2,LRRC47,PARK7,SARDH,ASS1,GCSH,KYAT1,KARS,AARS,KYAT3,MIR21,MARS2,MTHFD2L,PEMT,HIBADH,SLC25A21,GGT5,AUH,HYKK,PHGDH,PCBD2	grey	VSMC_M10
GO:0050884	0.07	0.98	10	13	neuromuscular process controlling posture	BP	1	POU4F1,TCF15,TMEM150C,ADARB1,PNKD,CNTNAP1,GCH1,CLN8,MECP2,FXN	grey	VSMC_M10
GO:1902953	0.07	1.00	4	4	positive regulation of ER to Golgi vesicle-mediated transport	BP	1	PGAP1,SORL1,STX18,TBC1D20	grey	VSMC_M10
GO:0050771	0.07	0.96	33	52	negative regulation of axonogenesis	BP	1	SEMA6A,FSTL4,SYNGAP1,SEMA4D,SEMA4C,RTN4,RUFY3,EPHA7,SEMA3D,SPART,SEMA3A,ULK1,RYK,SEMA4F,SEMA3G,SPP1,SEMA4G,KIAA0319,SEMA3F,WNT3,SEMA6D,TNR,PSEN1,SEMA4A,TRIM46,NTN1,CDK5R1,DAB1,ARHGAP4,PLXNA3,THY1,WNT5A,EFNB3	grey	VSMC_M10
GO:0034331	0.07	0.97	19	28	cell junction maintenance	BP	1	SYNGAP1,MTSS1,WHRN,ERC2,KIFC3,PLEKHA7,F2RL1,F2R,BSN,DLG1,C1QL1,RAB3A,NLGN2,CSF1R,CAMSAP3,SDF4,CLDN1,INAVA,PARD6A	grey	VSMC_M10
GO:0006400	0.07	0.95	53	85	tRNA modification	BP	1	ADAT2,TRMU,TRMT11,C9orf64,MTO1,ELP1,PUS10,TRIT1,DUS4L,NSUN6,THUMPD3,DPH3,TRMT6,THUMPD2,TRMT9B,ELP4,ELP3,BCDIN3D,TPRKB,NSUN3,OSGEPL1,CDKAL1,RPUSD4,TRMT10A,QTRT2,TYW1B,TYW5,TRUB1,DALRD3,LCMT2,METTL8,ELP6,METTL2B,TRMT1L,ALKBH8,TRMT5,HSD17B10,MTFMT,ALKBH1,THG1L,QTRT1,ELP2,THUMPD1,METTL6,PUSL1,AARS,DUS2,CTU1,TRMT10B,TRMT61A,TRMT10C,TRMT61B,TYW3	grey	VSMC_M10
GO:0003010	0.07	1.00	4	4	voluntary skeletal muscle contraction	BP	1	SYNM,MYH7,ACTN3,ATP2A1	grey	VSMC_M10
GO:0014721	0.07	1.00	4	4	twitch skeletal muscle contraction	BP	1	SYNM,MYH7,ACTN3,ATP2A1	grey	VSMC_M10
GO:0035562	0.07	0.99	6	7	negative regulation of chromatin binding	BP	1	FBH1,BTAF1,PPP3CA,NFATC4,MEPCE,ZNF304	grey	VSMC_M10
GO:0021936	0.07	0.98	8	10	regulation of cerebellar granule cell precursor proliferation	BP	1	GLI1,SMO,GLI2,ZNF423,EGF,LHX1,GPR37L1,CEND1	grey	VSMC_M10
GO:0030317	0.07	0.96	37	58	flagellated sperm motility	BP	1	UBE2B,CATSPER3,LRRC6,CFAP69,DNAJA1,DNAH11,CABS1,ARMC2,APOB,ADCY3,TTC21A,TTLL3,ING2,WDR66,DNAH1,PLTP,DNAH5,DPCD,ROPN1L,NEURL1,CFAP43,SLC9B1,SLC9B2,INTS13,CFAP45,ENO4,TSSK4,CELF3,CATSPER2,SORD,TTC12,SPAG16,MNS1,TEKT3,SPEF2,CATSPERD,TPGS1	grey	VSMC_M10
GO:0097722	0.07	0.96	37	58	sperm motility	BP	1	UBE2B,CATSPER3,LRRC6,CFAP69,DNAJA1,DNAH11,CABS1,ARMC2,APOB,ADCY3,TTC21A,TTLL3,ING2,WDR66,DNAH1,PLTP,DNAH5,DPCD,ROPN1L,NEURL1,CFAP43,SLC9B1,SLC9B2,INTS13,CFAP45,ENO4,TSSK4,CELF3,CATSPER2,SORD,TTC12,SPAG16,MNS1,TEKT3,SPEF2,CATSPERD,TPGS1	grey	VSMC_M10
GO:0071806	0.07	0.96	35	54	protein transmembrane transport	BP	1	TIMM21,PEX7,TOMM22,PEX6,PEX1,TRAM2,SEC61B,SEC61G,DNAJC15,SEC62,AKT2,TIMM50,PEX2,PEX16,LONP2,C2CD5,MCL1,TOMM40L,GRPEL1,AP4M1,ZFAND2B,TIMM17B,PEX26,PEX14,TMED10,PAM16,PEX10,HSPA5,BLOC1S3,TIMM23B,TRAM1L1,GRPEL2,PEX12,DNAJC19,SEC61A2	grey	VSMC_M10
GO:0048934	0.07	0.98	8	10	peripheral nervous system neuron differentiation	BP	1	ONECUT2,NTRK2,ETV1,POU4F1,HAND2,RUNX3,ISL1,ISL2	grey	VSMC_M10
GO:0048935	0.07	0.98	8	10	peripheral nervous system neuron development	BP	1	ONECUT2,NTRK2,ETV1,POU4F1,HAND2,RUNX3,ISL1,ISL2	grey	VSMC_M10
GO:0060033	0.07	0.98	8	10	anatomical structure regression	BP	1	GLI1,SMAD5,GLI2,FLT1,AMHR2,SLC6A3,BMPR1A,CD248	grey	VSMC_M10
GO:0050432	0.07	0.96	23	34	catecholamine secretion	BP	1	PRKN,LY6E,SYT1,SYT9,ADRA2B,P2RY1,DTNBP1,SYT8,GRM2,ADRA2A,KCNB1,GRK2,AGT,STX1A,ADRA2C,RAB3A,PINK1,SYT7,SYT11,CHRNB2,ABAT,CHGA,CRH	grey	VSMC_M10
GO:0010586	0.07	0.97	16	23	miRNA metabolic process	BP	1	AGO2,TUT7,XPO5,PNPT1,TUT4,BCDIN3D,TENT2,TENT4B,DIS3L2,RC3H1,ZSWIM8,PARN,DROSHA,AGO4,HRAS,RC3H2	grey	VSMC_M10
GO:0042053	0.07	0.98	10	13	regulation of dopamine metabolic process	BP	1	PRKN,GPR37,NR4A2,SLC6A3,COMT,PNKD,ITGAM,CHRNB2,ABAT,PARK7	grey	VSMC_M10
GO:0042069	0.07	0.98	10	13	regulation of catecholamine metabolic process	BP	1	PRKN,GPR37,NR4A2,SLC6A3,COMT,PNKD,ITGAM,CHRNB2,ABAT,PARK7	grey	VSMC_M10
GO:0072289	0.07	1.00	4	4	metanephric nephron tubule formation	BP	1	SOX9,PAX8,PAX2,SOX8	grey	VSMC_M10
GO:0060896	0.07	0.99	6	7	neural plate pattern specification	BP	1	PTCH1,FUZ,BMPR1A,CELSR2,NOG,C2CD3	grey	VSMC_M10
GO:0015874	0.08	0.98	12	16	norepinephrine transport	BP	1	ADRA2B,P2RY1,SLC6A2,SLC6A3,ADRA2A,KCNB1,AGT,STX1A,ADRA2C,ACTB,SLC29A4,CRH	grey	VSMC_M10
GO:0015837	0.08	0.96	37	58	amine transport	BP	1	SLC12A2,PRKN,CHRNA3,DDC,SYT1,ARL6IP5,SYT9,ADRA2B,SLC43A1,P2RY1,DTNBP1,SYT8,GRM2,SLC38A3,ADRA2A,KCNB1,GRK2,RHCG,AGT,ACE2,ATP1A2,PER2,STX1A,NTSR1,ADRA2C,RAB3A,PINK1,ARL6IP1,SYT7,PSEN1,SYT11,CHRNB2,ABAT,STXBP1,CHGA,RGS4,CRH	grey	VSMC_M10
GO:0021938	0.08	1.00	4	4	smoothened signaling pathway involved in regulation of cerebellar granule cell precursor cell proliferation	BP	1	GLI1,SMO,GLI2,ZNF423	grey	VSMC_M10
GO:0046726	0.08	1.00	4	4	positive regulation by virus of viral protein levels in host cell	BP	1	STAU1,DDB1,HACD3,TBC1D20	grey	VSMC_M10
GO:0006904	0.08	0.96	21	31	vesicle docking involved in exocytosis	BP	1	EXOC4,SCFD2,PLEK,PPFIA3,RAB10,SYTL2,RALB,SNPH,VPS18,KCNB1,STX1A,BLOC1S6,RAB3A,RAB8A,RAB15,STX1B,RAB8B,RAB3D,STXBP2,STXBP1,VAMP3	grey	VSMC_M10

GO:0042391	0.08	0.94	173	302	regulation of membrane potential	BP	1	NEDD4L,HEBP2,MAPK8IP2,RIMS2,PRKN,TSPO,BAK1,KCNK9,NTRK2,CHRNA3,CHRNA5,GABRA1,SLC25A27,CLDN19,RIMS1,ABCB5,RIMS3,SHANK1,SLC4A4,MYH14,PTN,GRIK2,BVES,CLCN1,TRPM4,DLD,WISP3,GRIN2D,MTMR2,SLC8A2,SLC26A5,CACNB2,GRIK5,ATP1A3,PRDX3,SCN5A,SCN1A,AKT2,GPD1L,CHRNA6,NALCN,DCN,PANK2,SPART,P2RX2,RNF122,CLIC1,ARL6IP5,FAM19A4,RGS7BP,PXK,SLMAP,GLRX,P2RX3,MEF2C,ASIC1,P2RX7,KCNH3,CUX2,PTK2B,KCNJ11,SLC8B1,KCNJ3,TBX5,KCNK15,KCNE2,DSP,CASQ2,KCNIP2,RYR2,PKP2,PPP3CA,KCNJ2,IGSF11,PPA2,SCN2A,SSH1,MFN1,PIAS3,KCNB1,KCNK1,SCN7A,OPRD1,ANK2,GPER1,GRIK4,IFI6,ATP1A2,SLC9A1,KCNK2,STX1A,TMEM25,NPAS4,NTSR1,SCN4B,CNIH2,NOS1AP,FZD9,DLG1,PPP2R3C,KCNK7,HTR3B,MAPT,STOX1,BCO2,MPP2,KCNK4,CRTC1,CNTNAP1,PINK1,MUL1,ATP1B1,KCNA1,KCNMA1,CHRM1,KCNH4,SLC4A3,DLG4,PARP1,NLGN2,CHRNB1,CNIH3,ATP1B2,STX1B,PSEN1,HCN3,NLGN3,CACNA1G,AC138894.1,CHRNB2,CALM1,DSG2,ASIC2,SEZ6,JUN,ABAT,FMR1,GABRE,GABRA3,GNA15,TRPV1,P2RX1,PRKCZ,MECP2,RNF207,CACNA1H,PARK7,SLC25A33,PIEZO1,PIP5KL1,WWP2,KCNK3,UBB,DMD,GPR35,CHRNB4,MLLT11,CHRNA10,CHRNG,GRID2,BNIP3L,KCNH2,SCN2B,KCNK5,RGS4,GPR88,POPDC2,INSYN1,YWHAH,KCNJ8,GNA14,HCN4,KCNMB4	grey	VSMC_M10
GO:0015747	0.08	1.00	4	4	urate transport	BP	1	SLC22A13,ABCC4,ABCG2,SLC2A9	grey	VSMC_M10
GO:0035907	0.08	0.98	8	10	dorsal aorta development	BP	1	SRF,NKX3-1,BMPR1A,RBPJ,NGFR,ENG,ACVRL1,HEY2	grey	VSMC_M10
GO:0035249	0.08	0.96	36	57	synaptic transmission, glutamatergic	BP	1	MAPK8IP2,PRKN,GRM4,PLA2G6,GRM8,ROR2,SLC1A4,DGKI,EGFR,GRIK2,NAPA,GRIK5,NAPB,SYT1,MEF2C,PTK2B,HOMER1,GRM2,DISC1,GRIK4,ATP1A2,CNIH2,GLUL,TNR,NLGN2,CNIH3,PSEN1,NLGN3,AC138894.1,CCL2,P2RX1,TPRG1L,STXBP1,GRID2,CACNG7,CACNG5	grey	VSMC_M10
GO:0060467	0.08	1.00	4	4	negative regulation of fertilization	BP	1	MYH9,NLRP5,PLAT,ZP2	grey	VSMC_M10
GO:0060468	0.08	1.00	4	4	prevention of polyspermy	BP	1	MYH9,NLRP5,PLAT,ZP2	grey	VSMC_M10
GO:0009452	0.08	0.99	6	7	7-methylguanosine RNA capping	BP	1	RNMT,CMTR1,RNGTT,TGS1,NCBP3,CMTR2	grey	VSMC_M10
GO:0036260	0.08	0.99	6	7	RNA capping	BP	1	RNMT,CMTR1,RNGTT,TGS1,NCBP3,CMTR2	grey	VSMC_M10
GO:0035864	0.08	0.98	8	10	response to potassium ion	BP	1	SLC12A2,HSF1,SLC26A5,DLG2,CARF,DLG4,CACNA1H,ADAMTS13	grey	VSMC_M10
GO:0031034	0.08	1.00	4	4	myosin filament assembly	BP	1	RAP1GDS1,TTN,TCAP,MYH11	grey	VSMC_M10
GO:0006577	0.08	0.98	10	13	amino-acid betaine metabolic process	BP	1	CPT1B,ALDH7A1,SLC22A4,CROT,CHDH,CPT1A,POR,ACADL,TMLHE,ACADM	grey	VSMC_M10
GO:0002857	0.08	1.00	4	4	positive regulation of natural killer cell mediated immune response to tumor cell	BP	1	CD226,NECTIN2,PVR,IL12A	grey	VSMC_M10
GO:0002860	0.08	1.00	4	4	positive regulation of natural killer cell mediated cytotoxicity directed against tumor cell target	BP	1	CD226,NECTIN2,PVR,IL12A	grey	VSMC_M10
GO:1901656	0.08	1.00	4	4	glycoside transport	BP	1	SLC5A2,SLC50A1,ABCC3,RALBP1	grey	VSMC_M10
GO:0032324	0.08	1.00	4	4	molybdopterin cofactor biosynthetic process	BP	1	MOCS1,GPHN,MOCOS,MOCS2	grey	VSMC_M10
GO:0042269	0.08	0.97	13	18	regulation of natural killer cell mediated cytotoxicity	BP	1	CD226,NECTIN2,PVR,RASGRP1,CADM1,IL12A,LAG3,STAT5B,VAV1,CRK,AP1G1,SERPINB9,HAVCR2	grey	VSMC_M10
GO:0090461	0.08	1.00	4	4	glutamate homeostasis	BP	1	SLC1A1,SLC7A11,GLS,GRM2	grey	VSMC_M10
GO:0002834	0.08	0.98	8	10	regulation of response to tumor cell	BP	1	CD226,IL4I1,NECTIN2,PVR,SLC22A13,IL12A,FBXO38,HAVCR2	grey	VSMC_M10
GO:0002837	0.08	0.98	8	10	regulation of immune response to tumor cell	BP	1	CD226,IL4I1,NECTIN2,PVR,SLC22A13,IL12A,FBXO38,HAVCR2	grey	VSMC_M10
GO:0045576	0.08	0.96	31	48	mast cell activation	BP	1	CD226,ENPP3,RAC2,KIT,SPHK2,CD300LF,NECTIN2,GAB2,UNC13D,FER,RASGRP1,SNAP23,LAT2,FGR,CBL,SNX4,PTGDR,C12orf4,LCP2,LAT,IL4R,S100A13,BTK,STXBP2,PDPK1,FOXF1,STXBP1,S100A12,HAVCR1,CHGA,TSLP	grey	VSMC_M10
GO:0009235	0.08	0.98	8	10	cobalamin metabolic process	BP	1	MMACHC,MMAA,MTR,MMAB,ABCD4,CD320,MMADHC,CLYBL	grey	VSMC_M10
GO:2001225	0.08	1.00	4	4	regulation of chloride transport	BP	1	ATP8B1,ABCB1,PRNP,GABRE	grey	VSMC_M10
GO:0060750	0.08	1.00	4	4	epithelial cell proliferation involved in mammary gland duct elongation	BP	1	ESR1,AREG,MED1,WNT5A	grey	VSMC_M10
GO:0060751	0.08	1.00	4	4	branch elongation involved in mammary gland duct branching	BP	1	ESR1,AREG,MED1,WNT5A	grey	VSMC_M10
GO:0033198	0.08	0.97	16	23	response to ATP	BP	1	TRPM4,TRPC3,PANX1,P2RX2,P2RX3,RYR3,P2RX7,P2RY1,KCNJ11,SSH1,DGKQ,PKLR,TAF1,TRPV1,P2RX1,PDXP	grey	VSMC_M10
GO:0050915	0.08	1.00	4	4	sensory perception of sour taste	BP	1	ASIC3,ASIC1,ASIC2,PKD1L3	grey	VSMC_M10
GO:1990809	0.08	1.00	4	4	endoplasmic reticulum tubular network membrane organization	BP	1	RTN4,ATL2,ATL1,ARL6IP1	grey	VSMC_M10
GO:0019433	0.08	0.97	13	18	triglyceride catabolic process	BP	1	PNPLA3,PNPLA5,APOB,FGF21,ABHD5,LIPE,DDHD2,GPLD1,SORL1,CPS1,DAGLB,LIPC,PNPLA4	grey	VSMC_M10
GO:0060075	0.08	0.99	6	7	regulation of resting membrane potential	BP	1	ATP1A3,NALCN,KCNJ2,KCNK1,PSEN1,KCNK5	grey	VSMC_M10
GO:0015809	0.08	0.98	8	10	arginine transport	BP	1	SLC7A1,SLC38A9,AGT,SLC11A1,PQLC2,SLC7A3,AC138894.1,SLC25A29	grey	VSMC_M10
GO:0098953	0.08	1.00	4	4	receptor diffusion trapping	BP	1	DLG4,GPHN,CACNG7,CACNG5	grey	VSMC_M10
GO:0098970	0.08	1.00	4	4	postsynaptic neurotransmitter receptor diffusion trapping	BP	1	DLG4,GPHN,CACNG7,CACNG5	grey	VSMC_M10
GO:0099628	0.08	1.00	4	4	neurotransmitter receptor diffusion trapping	BP	1	DLG4,GPHN,CACNG7,CACNG5	grey	VSMC_M10
GO:0015858	0.08	1.00	4	4	nucleoside transport	BP	1	SLC29A2,SLC29A3,SLC29A4,RSC1A1	grey	VSMC_M10
GO:1904177	0.08	1.00	4	4	regulation of adipose tissue development	BP	1	TRPM4,SORL1,KLF7,PLA2G16	grey	VSMC_M10
GO:0036444	0.08	0.98	10	13	calcium import into the mitochondrion	BP	1	SMDT1,MICU3,OPA1,MCUR1,MCUB,MICU1,MCU,PSEN2,SLC25A23,MAIP1	grey	VSMC_M10
GO:0070973	0.08	0.99	6	7	protein localization to endoplasmic reticulum exit site	BP	1	BCAP29,GBF1,MIA3,SEC16B,BCAP31,SEC16A	grey	VSMC_M10
GO:0014060	0.08	1.00	4	4	regulation of epinephrine secretion	BP	1	ADRA2B,ADRA2A,ADRA2C,CRH	grey	VSMC_M10
GO:0032811	0.08	1.00	4	4	negative regulation of epinephrine secretion	BP	1	ADRA2B,ADRA2A,ADRA2C,CRH	grey	VSMC_M10

GO:0035194	0.08	0.94	69	112	post-transcriptional gene silencing by RNA	BP	1	ZC3H10,ESR1,CNOT6,MOV10L1,AGO2,TNRC6B,TUT7,XPO5,PUS10,SNIP1,AGO1,EGFR,MYCN,TUT4,PUM2,TNRC6C,CLP1,BCDIN3D,CNOT1,CELF1,EIF4E2,RIPK1,PPP3CA,TERT,AJUBA,DDX6,EIF4ENIF1,PRKRA,DDX3X,STAT3,MAP2K1,SMAD3,NRDE2,TNRC6A,CNOT8,NCOR1,DROSHA,FMR1,SPOUT1,MIRLET7A2,MIR29A,MIR199A1,MIR21,MIR222,MIR23A,MIR425,MIR125B1,MIR32,MIR22,MIR27B,MIR24-1,MIR7-1,MIR199A2,MIRLET7D,MIR27A,MIR23B,MIR365B,MIR25,MIR491,MIR24-2,MIRLET7A3,AGO4,MIR33B,MIR339,MIR125A,MIR99B,MIR940,MIR1249,MIR1180	grey	VSMC_M10
GO:1903706	0.08	0.93	149	258	regulation of hemopoiesis	BP	1	NME1,HSPA1B,BRD1,AGER,HLA-DRA,IL5,TCF7,XRCC6,TRIB1,MYC,TOB2,MAPK14,HSF1,ZNF16,RBFOX2,FBXO7,CDK6,ROR2,SMAP1,HOXA7,SLC46A2,MTURN,CNOT4,PTN,IL4I1,TLR4,IL15,FOXO3,CLPTM1,GLI2,FOXJ1,POU4F1,IL15RA,TGFBR2,ACVR2A,JAG1,NCKAP1L,GAS6,FOXP1,MITF,PRKDC,IRF7,TRAF6,SOX12,TOX,ZC3H8,ACVR1B,CAMK4,PIK3R1,FBXW7,MEF2C,TMEM131L,IL7,PTK2B,RASGRP1,RIPK2,AP3B1,TESC,RIPK1,SOX13,LDB1,RAB7B,SLC9B2,GPR137B,PIAS3,HLX,ACIN1,TCTA,HCLS1,UBASH3B,SLAMF8,CIB1,ARNT,THPO,CD28,RUNX3,ZBTB46,PPP2R3C,ADAM8,CREB1,DUSP10,BCL6,WDR61,VSIR,RC3H1,ESRRA,PTPN6,GPR137,FBN1,TNFSF18,LAG3,STAT3,STAT5B,LEO1,INHA,TFE3,FOXP3,ZBTB1,CARD11,MED1,TBX21,HOXB8,KAT7,FOS,ZBTB7B,HAX1,IL4R,GPR68,BTK,RCOR1,CD74,PPARGC1B,NKAP,EVI2B,IL7R,PTPN2,JUN,SOCS1,DROSHA,PRKCZ,ATP6AP1,MIR21,MIR222,MIR125B1,RHOA,PRMT6,FSTL3,ANKRD54,SOX4,MAFB,FADD,CD46,CASP8,CA2,BGLAP,HIST1H4B,HIST2H4A,HIST1H4H,FGL2,TNFSF9,HIST1H4D,FAM210B,LIF,ZFP36L2,CEBPB,HIST1H4A,IL17D,HIST1H4F,RC3H2	grey	VSMC_M10
GO:0006578	0.08	1.00	4	4	amino-acid betaine biosynthetic process	BP	1	ALDH7A1,CHDH,TMLHE,ACADM	grey	VSMC_M10
GO:0072177	0.08	0.99	6	7	mesonephric duct development	BP	1	OSR1,WNT11,PKD2,LHX1,GPC3,PKD1	grey	VSMC_M10
GO:1905616	0.08	0.99	6	7	regulation of miRNA mediated inhibition of translation	BP	1	AGO2,TNRC6B,EIF4E2,DDX6,EIF4ENIF1,STAT3	grey	VSMC_M10
GO:1905618	0.08	0.99	6	7	positive regulation of miRNA mediated inhibition of translation	BP	1	AGO2,TNRC6B,EIF4E2,DDX6,EIF4ENIF1,STAT3	grey	VSMC_M10
GO:0097201	0.08	0.98	8	10	negative regulation of transcription from RNA polymerase II promoter in response to stress	BP	1	NCK2,SIRT2,TMBIM6,HIF1AN,BAG3,DNAJB1,JUN,IMPACT	grey	VSMC_M10
GO:1900747	0.08	0.98	8	10	negative regulation of vascular endothelial growth factor signaling pathway	BP	1	SEMA6A,SPRY2,DCN,PIK3CB,IL12A,DAB2IP,MIR199A1,CADM4	grey	VSMC_M10
GO:0001773	0.08	0.97	13	18	myeloid dendritic cell activation	BP	1	TGFBR2,TRAF6,CAMK4,ITGB6,BATF2,LTBR,RBPJ,PSEN1,DOCK2,BATF,DHRS2,HAVCR2,BATF3	grey	VSMC_M10
GO:0061739	0.08	1.00	4	4	protein lipidation involved in autophagosome assembly	BP	1	ATG5,ATG16L1,WIPI2,IRGM	grey	VSMC_M10
GO:0032730	0.08	1.00	4	4	positive regulation of interleukin-1 alpha production	BP	1	PANX1,IL16,S100A13,ISL1	grey	VSMC_M10
GO:0070291	0.08	1.00	4	4	N-acylethanolamine metabolic process	BP	1	NAPEPLD,GDPD3,GDPD1,GDE1	grey	VSMC_M10
GO:0019230	0.08	1.00	4	4	proprioception	BP	1	POU4F1,TMEM150C,MECP2,FXN	grey	VSMC_M10
GO:1904505	0.08	1.00	4	4	regulation of telomere maintenance in response to DNA damage	BP	1	ERCC1,XRCC1,RTEL1,ERCC4	grey	VSMC_M10
GO:1904506	0.08	1.00	4	4	negative regulation of telomere maintenance in response to DNA damage	BP	1	ERCC1,XRCC1,RTEL1,ERCC4	grey	VSMC_M10
GO:0051952	0.08	0.95	35	55	regulation of amine transport	BP	1	SLC12A2,PRKN,CHRNA3,SYT1,ARL6IP5,SYT9,ADRA2B,SLC43A1,P2RY1,DTNBP1,SYT8,GRM2,SLC38A3,ADRA2A,KCNB1,GRK2,AGT,ACE2,ATP1A2,PER2,STX1A,NTSR1,ADRA2C,RAB3A,PINK1,ARL6IP1,SYT7,PSEN1,SYT11,CHRNB2,ABAT,STXBP1,CHGA,RGS4,CRH	grey	VSMC_M10
GO:0006063	0.08	1.00	4	4	uronic acid metabolic process	BP	1	PRKCE,AKR1A1,XYLB,ABHD10	grey	VSMC_M10
GO:0019585	0.08	1.00	4	4	glucuronate metabolic process	BP	1	PRKCE,AKR1A1,XYLB,ABHD10	grey	VSMC_M10
GO:0002084	0.09	0.98	8	10	protein depalmitoylation	BP	1	ABHD17B,PPT1,LYPLA1,LYPLAL1,ABHD17A,ABHD10,ABHD17C,LYPLA2	grey	VSMC_M10
GO:0006572	0.09	1.00	4	4	tyrosine catabolic process	BP	1	IL4I1,HGD,FAH,GSTZ1	grey	VSMC_M10
GO:0000964	0.09	1.00	4	4	mitochondrial RNA 5'-end processing	BP	1	PNPT1,KIAA0391,HSD17B10,TRMT10C	grey	VSMC_M10
GO:0002836	0.09	0.99	6	7	positive regulation of response to tumor cell	BP	1	CD226,NECTIN2,PVR,SLC22A13,IL12A,FBXO38	grey	VSMC_M10
GO:0002839	0.09	0.99	6	7	positive regulation of immune response to tumor cell	BP	1	CD226,NECTIN2,PVR,SLC22A13,IL12A,FBXO38	grey	VSMC_M10
GO:0002275	0.09	0.95	39	62	myeloid cell activation involved in immune response	BP	1	ENPP3,RAC2,KIT,PRKCE,SPHK2,GAB2,UNC13D,MYD88,NMI,FER,RASGRP1,DYSF,F2RL1,SNAP23,LAT2,FGR,CBL,SNX4,FCER1G,PTGDR,C12orf4,STX4,ITGAM,DOCK2,LAT,IL4R,S100A13,BTK,SCNN1B,STXBP2,PRAM1,TICAM1,DNASE1,PDPK1,FOXF1,KARS,STXBP1,CHGA,HAVCR2	grey	VSMC_M10
GO:0048570	0.09	0.99	6	7	notochord morphogenesis	BP	1	GLI1,GLI2,WNT11,EFNA1,NOG,WNT5A	grey	VSMC_M10
GO:0006844	0.09	1.00	4	4	acyl carnitine transport	BP	1	SLC25A20,SLC25A47,SLC25A45,SLC25A29	grey	VSMC_M10
GO:0051694	0.09	0.99	6	7	pointed-end actin filament capping	BP	1	LMOD2,TMOD1,LMOD3,LMOD1,TMOD4,TMOD2	grey	VSMC_M10
GO:0006548	0.09	1.00	4	4	histidine catabolic process	BP	1	CARNMT1,HNMT,AMDHD1,HDC	grey	VSMC_M10
GO:0018200	0.09	0.97	15	21	peptidyl-glutamic acid modification	BP	1	AGTPBP1,AGBL3,TTLL3,AGBL2,CFAP20,TTLL13P,TTLL4,PARP1,TTLL5,TPGS2,NAA10,TPGS1,TTLL11,TTLL7,FOLH1	grey	VSMC_M10
GO:0071651	0.09	1.00	4	4	positive regulation of chemokine (C-C motif) ligand 5 production	BP	1	MAVS,TRPV4,DDX3X,DEFB124	grey	VSMC_M10
GO:1903826	0.09	0.99	6	7	arginine transmembrane transport	BP	1	SLC7A1,SLC38A9,AGT,PQLC2,SLC7A3,SLC25A29	grey	VSMC_M10
GO:0006000	0.09	0.98	8	10	fructose metabolic process	BP	1	AKR1B1,KHK,GLYCTK,PFKFB2,PFKFB4,SORD,ALDOC,FBP2	grey	VSMC_M10
GO:0009744	0.09	1.00	4	4	response to sucrose	BP	1	PNPLA3,ERCC1,KHK,CALCRL	grey	VSMC_M10
GO:0034285	0.09	1.00	4	4	response to disaccharide	BP	1	PNPLA3,ERCC1,KHK,CALCRL	grey	VSMC_M10

GO:0008033	0.09	0.94	71	117	tRNA processing	BP	1	ADAT2,RPP21,TRMU,TRMT11,C9orf64,MTO1,TSEN34,ELP1,PUS10,TRIT1,DUS4L,NSUN6,THUMPD3,DPH3,TRMT6,TRNT1,THUMPD2,TRMT9B,ELP4,POP4,CLP1,ELP3,BCDIN3D,TPRKB,NSUN3,OSGEPL1,RPP40,CDKAL1,POP5,RPUSD4,TRMT10A,ZBTB8OS,QTRT2,TYW1B,TYW5,TRUB1,RTRAF,DALRD3,LCMT2,METTL8,ELP6,METTL2B,KIAA0391,TRMT1L,POP7,TRPT1,ALKBH8,TRMT5,HSD17B10,MTFMT,ALKBH1,THG1L,QTRT1,ELP2,THUMPD1,METTL6,PUSL1,POLR3K,KARS,ADAT1,AARS,DUS2,CTU1,RPP38,TRMT10B,TP53RK,TRMT61A,TRMT10C,RPP30,TRMT61B,TYW3	grey	VSMC_M10
GO:0036017	0.09	1.00	4	4	response to erythropoietin	BP	1	KIT,CD40,EPOR,MT1X	grey	VSMC_M10
GO:0036018	0.09	1.00	4	4	cellular response to erythropoietin	BP	1	KIT,CD40,EPOR,MT1X	grey	VSMC_M10
GO:1903146	0.09	0.96	20	30	regulation of autophagy of mitochondrion	BP	1	PRKN,TSPO,FBXO7,USP36,FBXW7,HTRA2,CSNK2A2,DNM1L,USP30,FZD5,HTT,PINK1,ATP13A2,VPS13D,VPS13C,HAX1,SREBF1,PARK7,BNIP3L,TIGAR	grey	VSMC_M10
GO:0042538	0.09	0.98	8	10	hyperosmotic salinity response	BP	1	XRCC6,AKR1B1,TRPV4,XRCC5,MICU1,SLC25A23,MT-CYB,CLDN1	grey	VSMC_M10
GO:0090207	0.09	0.96	19	28	regulation of triglyceride metabolic process	BP	1	MBOAT7,NR1H2,LPIN1,FGF21,ABHD5,PANK2,FBXW7,NR1H3,FITM2,GPLD1,SIK1,SORL1,GNB3,SLC27A1,DAGLB,LDLR,SREBF1,C3,CNEP1R1	grey	VSMC_M10
GO:0001754	0.09	0.96	20	30	eye photoreceptor cell differentiation	BP	1	AGTPBP1,NTRK2,RORB,RDH13,PTN,SOX9,MYO7A,DZANK1,FSCN2,CEP290,USH1C,RPGRIP1L,NKD1,NRL,STAT3,ROM1,SOX8,OLFM3,THY1,DIO3	grey	VSMC_M10
GO:0015886	0.09	0.98	8	10	heme transport	BP	1	PGRMC2,HPX,SLC48A1,ABCC5,ABCB6,FLVCR2,SLC46A1,C1orf194	grey	VSMC_M10
GO:0033091	0.09	1.00	4	4	positive regulation of immature T cell proliferation	BP	1	BMI1,IL1A,RIPK2,FOXP3	grey	VSMC_M10
GO:0006552	0.09	0.99	6	7	leucine catabolic process	BP	1	HMGCLL1,MCCC2,IVD,MCCC1,HMGCL,AUH	grey	VSMC_M10
GO:1905395	0.09	1.00	4	4	response to flavonoid	BP	1	DNM1L,IP6K2,FIS1,NQO1	grey	VSMC_M10
GO:0002923	0.09	0.99	6	7	regulation of humoral immune response mediated by circulating immunoglobulin	BP	1	FOXJ1,HPX,NOD2,PTPN6,CD46,FCER2	grey	VSMC_M10
GO:0071609	0.09	0.99	6	7	chemokine (C-C motif) ligand 5 production	BP	1	MAVS,OAS3,TRPV4,DDX3X,MUL1,DEFB124	grey	VSMC_M10
GO:0071649	0.09	0.99	6	7	regulation of chemokine (C-C motif) ligand 5 production	BP	1	MAVS,OAS3,TRPV4,DDX3X,MUL1,DEFB124	grey	VSMC_M10
GO:0045656	0.09	0.99	6	7	negative regulation of monocyte differentiation	BP	1	MYC,CDK6,HOXA7,ZBTB46,GPR68,MIR125B1	grey	VSMC_M10
GO:0140029	0.09	0.94	41	66	exocytic process	BP	1	EXOC4,SCFD2,PLEK,PPFIA3,NAPA,RAB10,SYTL2,RALB,GRIK5,NAPB,SYT1,STX2,SYT9,SNPH,ERC2,EXOC6B,SYNJ1,VPS18,DMTN,SNAP23,KCNB1,STX1A,SNX4,BLOC1S6,RAB3A,SNAP29,RAB8A,RAB15,CPLX1,STX1B,STX4,PSEN1,RAB8B,RAB3D,SNAPIN,BTK,STXBP2,P2RX1,STXBP1,SNAP47,VAMP3	grey	VSMC_M10
GO:0015850	0.09	0.93	105	179	organic hydroxy compound transport	BP	1	VPS4B,ATP8B1,LRP1,SLC16A7,APOM,SERAC1,PRKN,TSPO,PTCH1,LY6E,MAPK15,TTC39B,ABCG5,ABCG8,AQP1,NR1H2,APOB,PIP4K2A,APOC1,ASIC3,AQP11,TSKU,ABCC4,C1QTNF1,SYT1,SYT9,ADRA2B,SLC5A8,SOAT2,CYP7A1,OSBP,CHRM5,LIMA1,P2RY1,NR1H3,SLC6A2,SLC10A6,ABCC2,SPP1,PLTP,DTNBP1,SLC2A13,SYT8,GRM2,SLC6A3,ABCA5,ADRA2A,KCNB1,EGF,GRAMD1B,GRK2,ABCB11,AGT,ABCG4,SLCO1A2,SLCO1C1,OSBPL2,STX1A,OSBP2,SLC16A1,STARD5,OSBPL6,SOAT1,OSBPL1A,ZDHHC8,ABCA12,LPCAT3,ADRA2C,MFSD10,RAB3A,PINK1,ACTB,SLC29A4,SYT7,GPS2,LIPC,ANXA2,OSBPL7,NAXE,SYT11,CHRNB2,ABCC3,SLC5A11,LDLR,STRA6,NPC1,EMB,RALBP1,ABAT,P2RX1,ABCA3,ABCA7,PARK7,RXRA,CEL,ABCD3,VPS4A,MIR27B,MIR27A,CHGA,ABCA8,MIR33B,LDLRAP1,CRH,STARD4	grey	VSMC_M10
GO:0042531	0.09	0.95	26	40	positive regulation of tyrosine phosphorylation of STAT protein	BP	1	HSF1,KIT,IL15,HDAC2,PIBF1,HPX,OSBP,ARL2BP,CD40,HDAC1,HCLS1,PARP9,PARP14,OSM,IL12A,TNFSF18,FGFR3,CTF1,IL6R,CCL5,CSF1R,ISL1,GHR,CNTF,LIF,TSLP	grey	VSMC_M10
GO:0014061	0.09	0.98	8	10	regulation of norepinephrine secretion	BP	1	ADRA2B,P2RY1,ADRA2A,KCNB1,AGT,STX1A,ADRA2C,CRH	grey	VSMC_M10
GO:0042182	0.09	0.98	8	10	ketone catabolic process	BP	1	ALDH8A1,NQO2,CBR3,PNKD,OXCT1,PARK7,KYAT1,KYAT3	grey	VSMC_M10
GO:0060292	0.09	0.96	14	20	long-term synaptic depression	BP	1	PRRT1,AGER,PICK1,SRF,PLK2,ABHD6,PTK2B,SORCS3,KCNB1,MAPT,CD38,FMR1,STXBP1,GRID2	grey	VSMC_M10
GO:0010984	0.09	0.97	12	16	regulation of lipoprotein particle clearance	BP	1	FGF21,APOC1,NR1H4,MYLIP,GPLD1,LRPAP1,ANXA2,MIR199A1,MIR27B,MIR27A,MIR33B,LDLRAP1	grey	VSMC_M10
GO:0099159	0.09	0.99	6	7	regulation of modification of postsynaptic structure	BP	1	CTTNBP2,CHMP2B,TIAM1,CYFIP1,CAMKV,RHOA	grey	VSMC_M10
GO:0050890	0.09	0.93	123	214	cognition	BP	1	MAPK8IP2,PRRT1,PRKN,AGER,HLA-DRA,SYNGAP1,NTRK2,PLA2G6,BTBD9,SRF,TTBK1,KIT,SLC1A1,ADGRF1,BRSK1,DNAH11,SHANK1,PPT1,SLC1A4,PTN,EGFR,SOBP,SLC7A11,NTF4,PRKAR2B,ADCY3,SLC8A2,C5AR1,B4GALT2,METTL23,PLCB1,PRNP,DDHD2,ARL6IP5,PLK2,FAM107A,GPI,CAMK4,CASP3,CHMP2B,MEF2C,ASIC1,CUX2,MME,EIF2AK4,SYNJ1,AMFR,NQO2,CBR3,DOP1B,CYFIP1,SLC12A5,ATXN1,BDNF,SORCS3,LINS1,SCN2A,NLGN4Y,KRAS,ADNP,AGT,ATP1A2,NFATC4,KCNK2,NPAS4,NTSR1,YTHDF1,FZD9,NSUN5,GPR155,CREB1,MAPT,C1QL1,EIF4EBP2,COMT,TNR,NRXN2,KCNK4,BCHE,CRTC1,HTT,PPP3CB,NPTX2,MAPK1,CHRM1,TMOD2,DLG4,SHROOM4,SLC2A4,THRA,CLN8,RGS14,PSEN1,SYT11,ITGA3,NLGN3,FOS,AC138894.1,CHRNB2,LDLR,GPRASP2,BHLHB9,STRA6,GM2A,INSR,IFT20,JUN,AFF2,PRKCZ,MECP2,ABCA7,JPH3,LHX8,ATXN1L,CNTN2,RP9,POMK,CRH,GPR88,NRXN3,CEBPB,MAP1A,FOXO6	grey	VSMC_M10
GO:0046503	0.09	0.95	30	47	glycerolipid catabolic process	BP	1	ABHD16A,PNPLA3,PNPLA5,PLA2G6,APOB,FGF21,FAAH,ABHD5,APOC1,LIPE,DDHD2,ABHD6,PLA2G4B,GPLD1,ABHD2,SORL1,PLA2G4A,CPS1,ABHD12B,SMPD4,DAGLB,LIPC,GDPD3,LDLR,GDPD1,GDE1,PNPLA6,PLA2G15,PNPLA4,LYPLA2	grey	VSMC_M10
GO:0036135	0.09	1.00	4	4	Schwann cell migration	BP	1	LRP1,TIAM1,CERS2,MIR222	grey	VSMC_M10
GO:1900147	0.09	1.00	4	4	regulation of Schwann cell migration	BP	1	LRP1,TIAM1,CERS2,MIR222	grey	VSMC_M10
GO:0008535	0.09	0.96	15	21	respiratory chain complex IV assembly	BP	1	TIMM21,COA5,COX15,COX17,OXA1L,COA6,COA3,BCS1L,COX10,MT-CO3,COX16,APOPT1,COX14,COA1,SCO1	grey	VSMC_M10

GO:0016079	0.10	0.94	48	79	synaptic vesicle exocytosis	BP	1	RIMS2,CHRNA5,APBA1,RIMS1,RIMS3,PPFIA3,NAPA,CACNB2,GRIK5,NAPB,SYT1,RAB5A,STX2,SYT9,SNPH,ERC2,P2RY1,SYNJ1,VPS18,DTNBP1,SYT8,DNM1L,ADRA2A,SNAP23,STX1A,BACE1,DNAJC5,CSPG5,RAP1B,BLOC1S6,CASK,RAB3A,SYN1,SNAP29,SYT7,CPLX1,STX1B,STX4,PSEN1,CALM1,SNAPIN,PRKCB,FMR1,PIP5K1C,P2RX1,STXBP1,SNAP47,DOC2B	grey	VSMC_M10
GO:0048875	0.10	0.97	9	12	chemical homeostasis within a tissue	BP	1	EPAS1,HOMER1,ITGB6,LPCAT1,HOMER2,CTSH,ABCA12,ABCA3,OAS1	grey	VSMC_M10
GO:0006501	0.10	0.99	6	7	C-terminal protein lipidation	BP	1	ATG12,ATG5,GPLD1,ATG16L1,WIPI2,IRGM	grey	VSMC_M10
GO:0009066	0.10	0.95	28	43	aspartate family amino acid metabolic process	BP	1	RIDA,ATF4,MTAP,GNMT,SLC25A13,DDO,APIP,MSRA,ENOPH1,SDSL,NIT2,ASNSD1,MTRR,MTR,SMS,PLOD3,AASDHPPT,NAT8L,MAT1A,ASRGL1,CRYM,ADSSL1,PIPOX,ASS1,MTHFD2L,SLC25A21,HYKK,PHGDH	grey	VSMC_M10
GO:0045670	0.10	0.95	27	42	regulation of osteoclast differentiation	BP	1	TOB2,TLR4,POU4F1,MITF,TRAF6,CAMK4,PIK3R1,FBXW7,SLC9B2,GPR137B,PIAS3,TCTA,UBASH3B,CREB1,ESRRA,GPR137,FBN1,TFE3,FOS,GPR68,PPARGC1B,ATP6AP1,FSTL3,MAFB,CA2,BGLAP,CEBPB	grey	VSMC_M10
GO:0090141	0.10	0.96	14	20	positive regulation of mitochondrial fission	BP	1	PRKN,VPS35,DDHD2,DCN,DNM1L,FIS1,MCU,DDHD1,PINK1,MUL1,MIEF2,RALBP1,RALA,MIEF1	grey	VSMC_M10
GO:0006183	0.10	0.99	6	7	GTP biosynthetic process	BP	1	NME1,IMPDH1,NME4,NME7,NME3,NME6	grey	VSMC_M10
GO:0045742	0.10	0.95	20	30	positive regulation of epidermal growth factor receptor signaling pathway	BP	1	EPGN,AREG,TGFA,NEU3,HBEGF,PDE6G,HIP1R,FBXW7,NEURL1,ADRA2A,NCF1,EGF,GPER1,DGKD,AGT,CBL,PDE6H,HAP1,MIR29A,EREG	grey	VSMC_M10
GO:0006767	0.10	0.94	30	47	water-soluble vitamin metabolic process	BP	1	SHMT2,PSAT1,DHFR,AKR1A1,MMACHC,BTD,SLC23A2,PANK2,MMAA,MTHFD2,MTRR,MTR,MMAB,THTPA,MCCC1,MTHFS,AASDHPPT,PNPO,ABCD4,SHMT1,CD320,SLC46A1,TKTL1,TPK1,MTHFD2L,SLC25A32,MMADHC,NNMT,CLYBL,PDXP	grey	VSMC_M10
GO:0021983	0.10	0.96	17	25	pituitary gland development	BP	1	GLI1,PITX1,GLI2,SALL1,SRD5A1,SLC6A3,SOX2,CREB1,WNT4,TBX19,BMPR1A,RBPJ,ALDH1A2,ISL1,NOG,WNT5A,POU3F2	grey	VSMC_M10
GO:0007259	0.10	0.93	59	98	receptor signaling pathway via JAK-STAT	BP	1	IL5,AKR1B1,HSF1,OCIAD1,KIT,PPP2R1A,IL15,HDAC2,PIBF1,NMI,HPX,RET,FER,PIGU,OSBP,PTK2B,ARL2BP,F2R,PKD2,CD40,HDAC1,MST1,EGF,HCLS1,PARP9,PARP14,AGT,OSM,IL10RA,IL12A,TNFSF18,FGFR3,STAT3,STAT5B,CTF1,IL6R,STAT2,ELP2,CCL5,CSF1R,CCL2,CDK5R1,ISL1,SUZ12,GHR,JAK1,NLK,PTPN2,SOCS1,DAB1,DOT1L,PKD1,CNTF,MIR125A,STAMBP,GADD45A,LIF,TSLP,LEPROT	grey	VSMC_M10
GO:0070509	0.10	0.94	36	58	calcium ion import	BP	1	SMDT1,SPHK2,SLC8A2,CACNB2,PRNP,MICU3,ORAI1,HOMER1,STC1,OPA1,PKD2,MCUR1,TRPV4,MCUB,EGF,PLPP4,CASK,MICU1,MCU,PSEN2,WNK3,FAM155B,CACNA1A,CACNA1G,ATP2A1,TRPV2,CCL2,SLC25A23,MAIP1,TRPV3,TRPV1,P2RX1,CACNA1B,CACNA1H,ATP2C2,CRH	grey	VSMC_M10
GO:0050885	0.10	0.95	22	34	neuromuscular process controlling balance	BP	1	RBFOX2,SHANK1,USH1G,MYO7A,POU4F1,RAC3,NBN,USH1C,ALDH1A3,GIGYF2,CDH23,TNR,CNTNAP1,DLG4,NLGN2,CLN8,AC138894.1,IGDCC3,GM2A,JPH3,AARS,GPR88	grey	VSMC_M10
GO:0006629	0.10	0.91	545	982	lipid metabolic process	BP	1	RDH5,KDSR,ORMDL2,APOF,ATP8B1,RDH16,HSD17B4,LRP1,ALDH8A1,PIP4K2C,PEX7,B4GALNT1,AGAP2,CYP27B1,AVIL,AIG1,LTC4S,ESR1,CPT1B,ABHD16A,SYNJ2,SERAC1,SLC27A6,ALG12,CYP21A2,TNXB,AGPAT4,AGPAT1,QKI,PPARA,ACSL6,SLC22A4,PNPLA3,PNPLA5,SULT4A1,TSPO,MCAT,HSD17B8,A4GALT,ECHDC1,PTK2,HSD17B3,AKR1B1,GOLM1,MAPK14,PLA2G6,GPAA1,TTC39B,HACD4,APOL4,TEK,LARGE1,ALDH1A1,CROT,CWH43,KIT,PLPP6,SRD5A3,SPTLC1,AASDH,HINT2,LIPT1,HMGCLL1,INPP4A,ADH5,CAT,ALG2,PLPPR1,TMEM246,TMEM86B,PRKCE,PIGF,RDH13,MBOAT7,PPP2R1A,FPR2,ETFB,JAZF1,PPT1,PLEK,NR1H2,MBOAT2,DGKI,EGFR,LPIN1,INPP4B,DKKL1,MGST2,SMPD2,DLD,FIG4,LDAH,APOB,AGK,HSD17B14,FGF21,PRKAR2B,SPHK2,TPRA1,MTMR2,RAB7A,PIP4K2A,NAPEPLD,ALG11,FAAH,PRKD2,HADHA,EBP,SELENOI,EBPL,SOX9,OPA3,ABHD5,RUBCNL,CUBN,APOC1,GLB1,DNAJC15,LIPE,ECHDC3,PRKAG2,GDPD4,ACOX1,TAMM41,SLC22A13,PIBF1,ITPKC,CYGB,ACER3,TSKU,ELOVL1,ALK,AKT2,GDPD5,PIP4P1,NEU3,MTMR14,SPTLC3,SIRT2,NR1D2,PLCB1,PLCL2,PGAP2,CRLS1,HACL1,CDS2,PLPP5,DDHD2,PANK2,PDK1,SIRT3,FLT1,NR1H4,MTMR7,TRIB3,LYPLA1,SDR16C5,ACOXL,SOAT2,CYP7A1,NSMAF,ELOVL7,ASAH1,CYP7B1,ACOX2,ABHD6,CEBPA,THNSL2,CBR4,PIGU,PIK3CB,OSBP,PITPNM2,ST8SIA4,PIK3R1,TMEM150A,GK5,RETSAT,DKK3,ATP5F1A,HPGD,PLCE1,CHRM5,MTMR9,FBXW7,LIMA1,SORBS1,GPD1,PEX2,HTRA2,EPHX2,CYP2R1,SLA2,PTK2B,ETFDH,ACOT12,PLCH1,PRKAG1,NR1H3,SERINC5,SYNJ1,COQ2,IVD,LPIN3,CDS1,ST3GAL6,FITM2,ORMDL1,ECI2,COL4A3BP,HSD17B13,ITPKA,SP P1,ERLIN1,PIGT,PLA2G4B,ACAD8,ADTRP,NOD2,PRMT [illegible]	grey	VSMC_M10
GO:0099563	0.10	0.96	14	20	modification of synaptic structure	BP	1	CTTNBP2,DLGAP3,WASF1,CHMP2B,TIAM1,CYFIP1,CAMKV,STAU1,GRIPAP1,EPHA4,AC138894.1,MYH10,ARHGAP44,RHOA	grey	VSMC_M10
GO:0002708	0.10	0.94	38	61	positive regulation of lymphocyte mediated immunity	BP	1	CD226,STX7,HLA-DRA,EXOSC3,MSH2,RSAD2,HMCES,NECTIN2,PVR,MLH1,SLC22A13,PAXIP1,SHLD1,HPX,TRAF6,RASGRP1,NOD2,CD40,DENND1B,CD28,CADM1,FZD5,IL12A,LAG3,STAT5B,FOXP3,ZBTB1,SHLD2,TBX21,BTK,FBXO38,VAV1,C3,PRKCZ,TRAF2,AP1G1,FADD,FCER2	grey	VSMC_M10
GO:0048745	0.10	0.96	12	17	smooth muscle tissue development	BP	1	PTCH1,SMO,ZFAND5,SRF,OSR1,SOX9,PKD2,EFEMP2,DLG1,STRA6,ENG,SIX1	grey	VSMC_M10
GO:0034308	0.10	0.94	38	61	primary alcohol metabolic process	BP	1	RDH5,RDH16,AKR1B1,ALDH1A1,ADH5,RDH13,NAPEPLD,AKR1A1,LIPE,SDR16C5,CBR4,RETSAT,DKK3,GPD1,ALDH1A3,CHKA,ALDH3B2,THTPA,DHRS9,PECR,WNT4,DHRS3,RDH11,ALDH1A2,DGKQ,GDPD3,GDPD1,SCNN1B,GDE1,TKTL1,CACNA1H,PARK7,ALDH2,TPK1,PNPLA4,LRAT,RDH14,ADH4	grey	VSMC_M10
GO:0001914	0.10	0.97	11	15	regulation of T cell mediated cytotoxicity	BP	1	STX7,AGER,HLA-DRA,NECTIN2,PVR,SLC22A13,NCKAP1L,IL12A,PPP3CB,IL7R,FADD	grey	VSMC_M10
GO:0038183	0.10	0.97	9	12	bile acid signaling pathway	BP	1	ABCG5,ABCG8,ABCC4,NR1H4,CYP7A1,VDR,ABCC2,CIDEB,GPBAR1	grey	VSMC_M10

GO:0098661	0.10	0.94	32	51	inorganic anion transmembrane transport	BP	1	SLC26A10,SLC12A2,GABRA1,SLC1A1,SLC13A4,CLCN1,SLC26A5,SLC25A30,SLC20A2,PRNP,ANO4,ANO6,SLC12A5,ADAMTS8,SLC37A1,SLC12A7,SLC37A2,SLC37A4,SLC12A8,XPR1,SLC12A9,ANKH,SLC25A11,SLC26A1,SLC26A2,SLC25A14,GABRE,GABRA3,CLCN7,LRRC8A,SLC12A4,SLC25A3	grey	VSMC_M10
GO:0015698	0.10	0.93	67	113	inorganic anion transport	BP	1	ATP8B1,SLC26A10,SLC12A2,TSPO,ABCB1,ABCC10,GABRA1,CLIC5,SLC1A1,SLC4A4,SLC1A4,SLC13A4,CLCN1,SFRP4,SLC26A5,ANO10,SLC25A30,SLC4A2,CLNS1A,CCT8L2,ABCC4,SLC20A2,SLC4A7,PRNP,CLIC1,ANO4,MFSD5,ANO6,CRY2,ABCC2,SLC12A5,ADAMTS8,SLC4A10,SLC37A1,SLC12A7,SLC37A2,CLCN4,ABCC5,IP6K2,TMEM206,SLC37A4,SLC12A8,XPR1,CLIC4,SLC12A9,ANKH,SLC11A1,ANO8,SLC4A3,SLC25A11,TTYH3,SLC26A1,ABCC3,NMUR2,SLC26A2,LRRC8E,SLC25A14,GABRE,GABRA3,CLCN7,LRRC8A,CLCA2,SLC12A4,SLC25A3,LRRC8D,LRRC8B,CEBPB	grey	VSMC_M10
GO:0046328	0.10	0.93	62	104	regulation of JNK cascade	BP	1	PHLPP1,MAP3K5,STK3,MAPK8IP2,PRKN,AGER,GADD45G,KLHL31,DNAJA1,FKTN,MTURN,NOD1,EGFR,TLR4,TRAF1,SDCBP,MYD88,ZMYND11,PLCB1,SEMA3A,SH3RF2,MFHAS1,TRAF6,EPHB1,PTK2B,RASGRP1,RIPK2,F2RL1,RIPK1,NOD2,TAOK3,TRPV4,PDCD4,NCF1,TRAF5,FZD7,MAGI3,MAP3K11,AIDA,DUSP10,MBIP,MEN1,MAP4K2,DUSP19,PINK1,LTBR,MINK1,GPS2,TAOK2,NCOR1,TRAF3,TAOK1,SERPINF2,MAPK8IP3,TRAF2,MARVELD3,DAB2IP,ZNF622,WNT5A,GADD45A,DUSP22,HRAS	grey	VSMC_M10
GO:0031063	0.10	0.95	18	27	regulation of histone deacetylation	BP	1	C6orf89,SPHK2,PRKD2,TADA3,SDR16C5,ING2,BCL6,PINK1,KDM5A,FOXP3,AKAP8L,AKAP8,JDP2,ZBTB7B,SREBF1,SKI,PRMT6,SAP30BP	grey	VSMC_M10
GO:2000353	0.10	0.96	11	15	positive regulation of endothelial cell apoptotic process	BP	1	FOXO3,ANO6,CD40,PDCD4,GPER1,ITGA4,CCL2,MIR24-1,MIR125A,CD248,RGCC	grey	VSMC_M10
GO:0034381	0.10	0.95	21	31	plasma lipoprotein particle clearance	BP	1	APOM,APOB,FGF21,APOC1,NR1H4,SOAT2,MYLIP,GPLD1,SOAT1,EHD1,LRPAP1,LIPA,LIPC,ANXA2,LDLR,MIR199A1,MIR27B,MIR27A,MIR33B,NCEH1,LDLRAP1	grey	VSMC_M10
GO:0017004	0.10	0.95	21	31	cytochrome complex assembly	BP	1	TIMM21,LYRM7,COA5,UQCC1,COX15,COX17,OXA1L,COA6,COA3,BCS1L,COX10,SLC25A33,CYBA,MT-CO3,COX16,C12orf73,APOPT1,COX14,COA1,UQCC2,SCO1	grey	VSMC_M10
GO:0010803	0.11	0.95	23	35	regulation of tumor necrosis factor-mediated signaling pathway	BP	1	TNFAIP3,HSPA1B,PRKN,TRIM32,IKBKB,GAS6,OTULIN,F2RL1,SPATA2,PELI3,SPPL2A,GPS2,PYDC1,PTPN2,PIAS4,SPPL2B,TRAF2,MIR27B,MIR24-1,NOL3,CASP8,CCDC3,HIST1H2BJ	grey	VSMC_M10
GO:0006641	0.11	0.93	43	70	triglyceride metabolic process	BP	1	TNXB,SLC22A4,PNPLA3,PNPLA5,CAT,MBOAT7,NR1H2,LPIN1,APOB,FGF21,ABHD5,APOC1,LIPE,DDHD2,PANK2,GK5,FBXW7,NR1H3,LPIN3,FITM2,GPLD1,SIK1,CPT1A,GPAM,SORL1,LPGAT1,MOGAT3,CPS1,GNB3,PLA2G16,SLC27A1,DAGLB,LIPC,APOBR,SEL1L,LDLR,SREBF1,LPIN2,C3,LMF1,PNPLA4,CNEP1R1,INSIG2	grey	VSMC_M10
GO:0006541	0.11	0.96	14	20	glutamine metabolic process	BP	1	GLS2,GFPT2,PPAT,GMPS,NIT2,ASNSD1,GLS,SIRT4,CTPS2,GLUL,CPS1,ADSSL1,MECP2,PHGDH	grey	VSMC_M10
GO:0016441	0.11	0.92	70	115	posttranscriptional gene silencing	BP	1	ZC3H10,ESR1,CNOT6,MOV10L1,AGO2,TNRC6B,TUT7,XPO5,PUS10,SNIP1,AGO1,EGFR,MYCN,TUT4,PUM2,TNRC6C,CLP1,BCDIN3D,CNOT1,CELF1,EIF4E2,RIPK1,PPP3CA,TERT,AJUBA,GIGYF2,DDX6,EIF4ENIF1,PRKRA,DDX3X,STAT3,MAP2K1,SMAD3,NRDE2,TNRC6A,CNOT8,NCOR1,DROSHA,FMR1,SPOUT1,MIRLET7A2,MIR29A,MIR199A1,MIR21,MIR222,MIR23A,MIR425,MIR125B1,MIR32,MIR22,MIR27B,MIR24-1,MIR7-1,MIR199A2,MIRLET7D,MIR27A,MIR23B,MIR365B,MIR25,MIR491,MIR24-2,MIRLET7A3,AGO4,MIR33B,MIR339,MIR125A,MIR99B,MIR940,MIR1249,MIR1180	grey	VSMC_M10
GO:1902548	0.11	0.97	9	12	negative regulation of cellular response to vascular endothelial growth factor stimulus	BP	1	SEMA6A,SPRY2,DCN,PIK3CB,IL12A,ADAMTS12,DAB2IP,MIR199A1,CADM4	grey	VSMC_M10
GO:0048002	0.11	0.95	20	30	antigen processing and presentation of peptide antigen	BP	1	HLA-DRA,HLA-DOB,TAP1,HLA-DMA,HLA-DPB1,IKBKB,TRAF6,MARCH8,IDE,ABCB9,ERAP1,MFSD6,FCER1G,PIKFYVE,SLC11A1,CTSV,TAPBPL,DNM2,CD74,ERAP2	grey	VSMC_M10
GO:0030534	0.11	0.93	54	90	adult behavior	BP	1	PRKN,PPARA,AGTPBP1,BTBD9,CHRNA3,CHRNA5,ADAM22,USP46,SLC1A1,TMOD1,SHANK1,PPT1,SLC7A11,NTF4,GRIN2D,HDAC2,ALK,MAFG,LGI4,PREX2,HTRA2,HOMER1,NR4A2,GRM2,GIGYF2,ZFHX2,OPRD1,ATP1A2,SPTBN2,NTSR1,EPS8,HOMER2,NRXN2,EPHA4,SDK1,CHRNB1,CLN8,HOXB8,NLGN3,CHRNB2,NPC1,SEZ6,DAB1,ABAT,MECP2,PARK7,TPGS1,TSC1,CNTN2,FADD,CEND1,FXN,NRXN3,EFNB3	grey	VSMC_M10
GO:1904862	0.11	0.98	7	9	inhibitory synapse assembly	BP	1	GABRA1,SEMA4D,NPAS4,NLGN2,LGI2,SEMA4A,WNT5A	grey	VSMC_M10
GO:0002097	0.11	0.96	14	20	tRNA wobble base modification	BP	1	ADAT2,TRMU,MTO1,ELP1,DPH3,TRMT9B,ELP4,ELP3,NSUN3,ELP6,ALKBH8,ALKBH1,ELP2,CTU1	grey	VSMC_M10
GO:0002761	0.11	0.93	52	86	regulation of myeloid leukocyte differentiation	BP	1	NME1,IL5,TRIB1,MYC,TOB2,HSF1,CDK6,ROR2,HOXA7,TLR4,POU4F1,FOXP1,MITF,IRF7,TRAF6,CAMK4,PIK3R1,FBXW7,TESC,RIPK1,SLC9B2,GPR137B,PIAS3,ACIN1,TCTA,HCLS1,UBASH3B,ZBTB46,CREB1,ESRRA,GPR137,FBN1,INHA,TFE3,FOS,HAX1,GPR68,CD74,PPARGC1B,EVI2B,PTPN2,JUN,ATP6AP1,MIR125B1,FSTL3,MAFB,FADD,CASP8,CA2,BGLAP,LIF,CEBPB	grey	VSMC_M10
GO:0060078	0.11	0.93	51	85	regulation of postsynaptic membrane potential	BP	1	MAPK8IP2,RIMS2,CHRNA3,CHRNA5,GABRA1,RIMS1,SHANK1,GRIK2,GRIN2D,MTMR2,SLC8A2,GRIK5,CHRNA6,P2RX2,RGS7BP,P2RX3,MEF2C,P2RX7,CUX2,PTK2B,PPP3CA,IGSF11,SSH1,GRIK4,STX1A,TMEM25,NPAS4,NTSR1,MPP2,KCNA1,CHRM1,DLG4,NLGN2,CHRNB1,STX1B,NLGN3,CHRNB2,SEZ6,ABAT,GABRE,GABRA3,TRPV1,P2RX1,PRKCZ,MECP2,CHRNB4,CHRNA10,CHRNG,GRID2,RGS4,INSYN1	grey	VSMC_M10

GO:0045639	0.11	0.93	47	77	positive regulation of myeloid cell differentiation	BP	1	HSPA1B,BRD1,IL5,TRIB1,MAPK14,HSF1,ZNF16,ROR2,SMAP1,MTURN,FOXO3,POU4F1,ACVR2A,JAG1,NCKAP1L,PRKDC,TRAF6,ACVR1B,TESC,RIPK1,RAB7B,SLC9B2,ACIN1,HCLS1,ARNT,THPO,CREB1,STAT3,STAT5B,MED1,KAT7,FOS,HAX1,GPR68,RCOR1,CD74,PPARGC1B,EVI2B,JUN,ATP6AP1,MIR222,ANKRD54,FADD,CASP8,CA2,FAM210B,LIF	grey	VSMC_M10
GO:0035640	0.11	0.96	12	17	exploration behavior	BP	1	PRKCE,SLC4A10,ATP1A2,ABL2,TNR,DLG4,NLGN2,ITGA3,ABAT,JPH3,DPP4,CRBN	grey	VSMC_M10
GO:0016578	0.11	0.95	15	22	histone deubiquitination	BP	1	USP15,USP49,SUPT3H,USP36,TAF10,ATXN7,ASXL1,USP16,SART3,USP21,ENY2,ATXN7L3,UIMC1,EPOP,BRCC3	grey	VSMC_M10
GO:0010958	0.11	0.97	9	12	regulation of amino acid import across plasma membrane	BP	1	ARL6IP5,SLC43A1,AGT,ACE2,ATP1A2,PER2,ARL6IP1,PSEN1,RGS4	grey	VSMC_M10
GO:1903789	0.11	0.97	9	12	regulation of amino acid transmembrane transport	BP	1	ARL6IP5,SLC43A1,AGT,ACE2,ATP1A2,PER2,ARL6IP1,PSEN1,RGS4	grey	VSMC_M10
GO:0009247	0.11	0.93	38	61	glycolipid biosynthetic process	BP	1	B4GALNT1,A4GALT,GPAA1,LARGE1,CWH43,TMEM246,PIGF,PGAP2,PIGU,ST8SIA4,PIGT,A3GALT2,PGAP1,ST8SIA2,B3GALT1,B3GNT5,PIGM,PIGV,PIGX,GAL3ST3,PIGZ,GAL3ST4,PIGC,SCCPDH,PGAP3,PIGH,ST8SIA5,PIGL,MPPE1,GBGT1,ST6GALNAC4,ST3GAL2,ST6GALNAC5,PYURF,PIGB,B4GALT3,PIGA,ST8SIA6	grey	VSMC_M10
GO:0060831	0.11	0.97	9	12	smoothened signaling pathway involved in dorsal/ventral neural tube patterning	BP	1	TBC1D32,PTCH1,GLI2,SUFU,GORAB,GPR161,TULP3,WDR19,MKS1	grey	VSMC_M10
GO:0030301	0.11	0.93	47	77	cholesterol transport	BP	1	VPS4B,LRP1,APOM,SERAC1,TSPO,PTCH1,TTC39B,ABCG5,ABCG8,NR1H2,APOB,PIP4K2A,APOC1,TSKU,SOAT2,OSBP,LIMA1,NR1H3,PLTP,ABCA5,EGF,ABCG4,OSBPL2,STARD5,OSBPL6,SOAT1,ZDHHC8,ABCA12,LPCAT3,SYT7,GPS2,LIPC,ANXA2,NAXE,LDLR,NPC1,ABCA3,ABCA7,RXRA,CEL,VPS4A,MIR27B,MIR27A,ABCA8,MIR33B,LDLRAP1,STARD4	grey	VSMC_M10
GO:0018958	0.11	0.93	40	65	phenol-containing compound metabolic process	BP	1	TPH2,SNCAIP,PRKN,LY6E,AKR1B1,BTBD9,GPR37,SLC1A1,EPAS1,DDC,SLC7A11,LRTOMT,AP000812.5,PAX8,HAND2,RAPGEF2,NR4A2,OCA2,SRD5A1,SLC6A3,TGFB2,SLCO1C1,DUOX1,FAH,COMT,PNKD,GCH1,HDC,MED1,ITGAM,CITED1,CHRNB2,CRYM,SLC45A2,ABAT,PARK7,WNT5A,DIO3,AOC2,DIO2	grey	VSMC_M10
GO:0006264	0.11	0.97	9	12	mitochondrial DNA replication	BP	1	SSBP1,MGME1,PRIMPOL,TWNK,POLG2,POLG,LIG3,METTL4,DNAJA3	grey	VSMC_M10
GO:0009583	0.11	0.94	21	32	detection of light stimulus	BP	1	OPN1SW,PRPH2,CAMKMT,GJA10,TRPC3,PLEKHB1,CEP250,CCDC66,CDS1,PITPNM1,RRH,SAG,NMT1,GRK4,ROM1,GUCY2D,ASIC2,UNC119,GNAT2,REEP6,GPR88	grey	VSMC_M10
GO:0009713	0.11	0.97	9	12	catechol-containing compound biosynthetic process	BP	1	GPR37,DDC,HAND2,NR4A2,SLC6A3,TGFB2,GCH1,HDC,PARK7	grey	VSMC_M10
GO:0042423	0.11	0.97	9	12	catecholamine biosynthetic process	BP	1	GPR37,DDC,HAND2,NR4A2,SLC6A3,TGFB2,GCH1,HDC,PARK7	grey	VSMC_M10
GO:0099116	0.11	0.96	11	15	tRNA 5'-end processing	BP	1	RPP21,POP4,RPP40,POP5,KIAA0391,POP7,HSD17B10,THG1L,RPP38,TRMT10C,RPP30	grey	VSMC_M10
GO:0099550	0.11	0.97	7	9	trans-synaptic signaling, modulating synaptic transmission	BP	1	NTRK2,PLAT,GUCY1A1,GUCY1B1,F2R,CSPG5,EFNB3	grey	VSMC_M10
GO:0009205	0.11	0.93	36	57	purine ribonucleoside triphosphate metabolic process	BP	1	NME1,ATP5MGL,PPARA,GTPBP1,IMPDH1,SLC25A13,RAB23,SPHK2,PRKAG2,ATP5F1C,ATP5F1A,DGUOK,OPA1,FAM173B,NME4,MFN1,DNAJC30,EFL1,ATP5S,PINK1,STAT3,NME7,PARP1,PKLR,ATP6V0C,NME3,ATP5PB,VPS9D1,GNAI3,MT-ATP8,MT-ATP6,RHOA,ATP5ME,ATP5MF,NME6,NUDT2	grey	VSMC_M10
GO:0033108	0.11	0.92	52	83	mitochondrial respiratory chain complex assembly	BP	1	TIMM21,LYRM7,NDUFA6,COA5,SDHAF4,NDUFB2,NDUFB6,TMEM126B,NDUFC2,DMAC2,NDUFS8,NDUFAF7,NDUFA12,NDUFAF2,UQCC1,NDUFS3,NDUFAF6,FOXRED1,COX17,OXA1L,NDUFB4,NDUFB3,TFAM,NDUFS2,NUBPL,COA3,NDUFB11,BCS1L,NDUFA9,NDUFAB1,NDUFB10,SLC25A33,MT-ND1,MT-ND2,MT-ND6,NDUFA5,NDUFC1,COX16,NDUFAF8,DMAC1,NDUFA3,NDUFB5,NDUFA8,SDHAF2,C12orf73,NDUFS5,APOPT1,TMEM126A,COX14,COA1,UQCC2,SCO1	grey	VSMC_M10
GO:0010587	0.12	0.97	7	9	miRNA catabolic process	BP	1	PNPT1,TUT4,TENT2,TENT4B,DIS3L2,ZSWIM8,PARN	grey	VSMC_M10
GO:0002577	0.12	0.97	9	12	regulation of antigen processing and presentation	BP	1	HLA-DOB,NOD1,NOD2,YTHDF1,SLC11A1,TAPBPL,WAS,CD74,FGL2	grey	VSMC_M10
GO:1903025	0.12	0.97	9	12	regulation of RNA polymerase II regulatory region sequence-specific DNA binding	BP	1	DDRGK1,FBXW7,NSD1,TAF1,ZBTB7C,LAMTOR5,DDIT3,ZNF593,LIF	grey	VSMC_M10
GO:0010866	0.12	0.96	12	17	regulation of triglyceride biosynthetic process	BP	1	NR1H2,LPIN1,FBXW7,NR1H3,FITM2,GPLD1,SIK1,SLC27A1,LDLR,SREBF1,C3,CNEP1R1	grey	VSMC_M10
GO:0031343	0.12	0.94	18	27	positive regulation of cell killing	BP	1	CD226,STX7,HLA-DRA,NECTIN2,PVR,SLC22A13,RASGRP1,F2RL1,CADM1,IL12A,LAG3,STAT5B,ITGAM,VAV1,NOS2,AP1G1,FADD,FCER2	grey	VSMC_M10
GO:0090646	0.12	0.96	9	12	mitochondrial tRNA processing	BP	1	MTO1,TRIT1,TRNT1,RPUSD4,KIAA0391,TRMT5,HSD17B10,TRMT10C,TRMT61B	grey	VSMC_M10
GO:0044242	0.12	0.91	96	164	cellular lipid catabolic process	BP	1	HSD17B4,LRP1,AIG1,CPT1B,ABHD16A,PPARA,PNPLA3,PNPLA5,MCAT,ECHDC1,PLA2G6,CROT,SRD5A3,ETFB,PPT1,LPIN1,MGST2,SMPD2,APOB,FGF21,NAPEPLD,FAAH,HADHA,ABHD5,APOC1,LIPE,ACOX1,ACER3,AKT2,NEU3,SIRT2,HACL1,DDHD2,ACOXL,ASAH1,ACOX2,ABHD6,PEX2,ETFDH,IVD,LPIN3,ECI2,PLA2G4B,ADTRP,LONP2,GPLD1,MCEE,IRS1,CPT1A,BDH2,ABHD2,SORL1,ABCB11,PLBD1,EHHADH,PLA2G4A,ACADL,BCO2,CPS1,TYSND1,ABHD12B,SMPD4,DAGLB,ACADVL,HSD17B10,LIPC,ABCD4,GDPD3,GCDH,GALC,LDLR,GDPD1,GLA,HEXA,GM2A,GDE1,ABHD3,PNPLA6,LPIN2,ACAA2,DECR2,CEL,MLYCD,ABCD3,PLA2G15,ACADM,PHYH,PNPLA4,HADHB,AMACR,ACER2,NUDT19,ECI1,AUH,LYPLA2,NEU2	grey	VSMC_M10
GO:0051954	0.12	0.95	15	22	positive regulation of amine transport	BP	1	SLC12A2,SYT1,SLC38A3,KCNB1,GRK2,AGT,ACE2,STX1A,NTSR1,PINK1,ARL6IP1,PSEN1,CHRNB2,ABAT,STXBP1	grey	VSMC_M10

GO:0070103	0.12	0.98	6	7	regulation of interleukin-6-mediated signaling pathway	BP	1	RIPK1,PTPN2,GFI1,MIR125B1,MIR125A,C1QTNF4	grey	VSMC_M10
GO:0035725	0.12	0.91	70	120	sodium ion transmembrane transport	BP	1	NEDD4L,SLC12A2,WNK2,CNNM4,PRKCE,SLC4A4,HECW1,TRPM4,OSR1,SLC8A2,ASIC3,ATP1A3,SCN5A,SCN1A,SLC24A3,GPD1L,SLC20A2,SLC4A7,NALCN,SLC6A15,SLMAP,GLRX,SLC9A9,ASIC1,TPCN1,SLC8B1,TESC,ANO6,SLC6A2,PKD2,SLC41A1,SLC4A10,SLC6A3,HECW2,SLC9B1,SLC9B2,SCN2A,SHROOM2,SLC9A8,KCNK1,SCN7A,KLHL24,ATP1A2,SLC9A1,SCN4B,DLG1,SLC9A7,ATP1B1,SLC3A2,ASIC4,WNK3,ATP1B2,SLC5A2,HCN3,CACNA1G,DNM2,SCNN1B,SLC6A7,ASIC2,SLC9A6,CACNA1H,SLC9A5,MIR24-1,DMD,SLC6A19,FGF11,SCN2B,NETO2,YWHAH,HCN4	grey	VSMC_M10
GO:0048490	0.12	0.95	12	17	anterograde synaptic vesicle transport	BP	1	AP3S1,AP3M2,AP3B1,DTNBP1,CNIH2,BLOC1S6,AP3M1,TRIM46,SNAPIN,BLOC1S3,BORCS5,AP3S2	grey	VSMC_M10
GO:0099514	0.12	0.95	12	17	synaptic vesicle cytoskeletal transport	BP	1	AP3S1,AP3M2,AP3B1,DTNBP1,CNIH2,BLOC1S6,AP3M1,TRIM46,SNAPIN,BLOC1S3,BORCS5,AP3S2	grey	VSMC_M10
GO:0099517	0.12	0.95	12	17	synaptic vesicle transport along microtubule	BP	1	AP3S1,AP3M2,AP3B1,DTNBP1,CNIH2,BLOC1S6,AP3M1,TRIM46,SNAPIN,BLOC1S3,BORCS5,AP3S2	grey	VSMC_M10
GO:0070525	0.12	0.96	9	12	tRNA threonylcarbamoyladenosine metabolic process	BP	1	MTO1,TRIT1,TPRKB,OSGEPL1,RPUSD4,TRMT5,HSD17B10,TP53RK,TRMT61B	grey	VSMC_M10
GO:0070071	0.12	0.96	9	12	proton-transporting two-sector ATPase complex assembly	BP	1	ATP23,ATPAF1,PIP4P1,TM9SF4,TMEM9,OXA1L,ATPAF2,TMEM199,VMA21	grey	VSMC_M10
GO:0065002	0.12	0.93	30	47	intracellular protein transmembrane transport	BP	1	TIMM21,PEX7,PEX6,PEX1,TRAM2,SEC61B,SEC61G,DNAJC15,SEC62,AKT2,TIMM50,PEX2,PEX16,LONP2,C2CD5,TOMM40L,GRPEL1,ZFAND2B,TIMM17B,PEX26,PEX14,PAM16,PEX10,HSPA5,TIMM23B,TRAM1L1,GRPEL2,PEX12,DNAJC19,SEC61A2	grey	VSMC_M10
GO:0032814	0.12	0.95	15	22	regulation of natural killer cell activation	BP	1	IL15,PIBF1,IL15RA,GAS6,TOX,RASGRP1,FGR,BLOC1S6,IL12A,STAT5B,ZBTB1,TICAM1,AP1G1,BLOC1S3,HAVCR2	grey	VSMC_M10
GO:0007216	0.12	0.97	7	9	G protein-coupled glutamate receptor signaling pathway	BP	1	GRM4,GRM8,HOMER1,GRM2,TRPM1,HOMER2,DAGLB	grey	VSMC_M10
GO:0003263	0.12	0.97	7	9	cardioblast proliferation	BP	1	PIM1,HAND2,EYA1,TBX5,MKS1,ISL1,SIX1	grey	VSMC_M10
GO:0003264	0.12	0.97	7	9	regulation of cardioblast proliferation	BP	1	PIM1,HAND2,EYA1,TBX5,MKS1,ISL1,SIX1	grey	VSMC_M10
GO:0048712	0.12	0.97	7	9	negative regulation of astrocyte differentiation	BP	1	MYCN,KDM4A,LDLR,MBD1,DAB1,GPR37L1,NOG	grey	VSMC_M10
GO:0007602	0.12	0.95	15	22	phototransduction	BP	1	OPN1SW,CAMKMT,TRPC3,PLEKHB1,CDS1,PITPNM1,RRH,SAG,NMT1,GRK4,GUCY2D,ASIC2,UNC119,GNAT2,GPR88	grey	VSMC_M10
GO:0007600	0.12	0.90	181	320	sensory perception	BP	1	RDH5,ATP8B1,MIP,MYO1A,EYA4,ALDH7A1,COL11A2,BTBD9,OPN1SW,GUCA1B,PRPH2,GRM8,RORB,CFAP69,CNGA1,PHF24,CLIC5,KIT,CNNM4,CLDN19,EPAS1,RIMS1,KCNQ4,GSDME,SHANK1,PPT1,GJA10,MYH14,WHRN,GRIK2,SOBP,TULP2,GRIN2D,ADCY3,BBS7,C5AR1,SLC26A5,MYO3A,MYO7B,EML2,OPA3,CACNB2,USH1G,ASIC3,CRYGN,ZNF513,SCN1A,MYO7A,POU4F1,SPRY2,MKKS,FSCN2,PDE6G,LRTOMT,AP000812.5,TMTC4,KERA,LUM,DIAPH1,P2RX2,FAM19A4,ATXN7,TUB,EPHB1,CASP3,MARVELD2,EYA1,P2RX3,CEP250,CCDC66,ADGRV1,ASIC1,P2RX7,NCAM2,P2RY1,KIFC3,MME,TMEM150C,USH1C,OPA1,F2R,IMPG2,TFAP2A,PAX2,FAM173B,SLC4A10,TRPM1,CABP1,CALHM1,SLC6A3,PGAP1,PDE6D,RRH,LRIT3,ZFHX2,OPRD1,CRYBA4,NRL,EYA3,TRPM8,SLC9A1,NIPSNAP1,MYO3B,BBS1,NTSR1,OAT,COCH,PDE6H,BACE1,STRC,HOMER2,CDKN1B,LRIG2,HMCN1,CEMIP,RTP4,CDH23,COMT,KCNK4,ADRA2C,RAB3A,KCNA1,MAPK1,ROM1,NLGN2,CLN8,DHRS3,HOXB8,GUCY2D,ALOXE3,MAPK3,CHRNB2,LCTL,TMEM100,POU3F4,CLN6,MYO9A,SCNN1B,OTOA,BDKRB1,PDE4A,CRYM,NMUR2,TRPV2,CCL2,ASIC2,UNC119,GNAL,SLC45A2,PITPNA,GNB1,TRPV1,MECP2,GNG13,GNAT2,LAMC3,CDC14A,PKD1L3,HSF4,CRYZ,SRRM4,REEP6,FABP5,PJVK,CHRNA10,TAS2R3,EPS8L2,FXN,SIX1,POMK,DDIT3,DRAM2,OTOR,OR5AN1,LRAT,AOC2,PTGES,TIMM8B,BBS10,OTOS,DNAJC19	grey	VSMC_M10
GO:0006665	0.12	0.91	71	120	sphingolipid metabolic process	BP	1	KDSR,ORMDL2,B4GALNT1,A4GALT,PLA2G6,HACD4,LARGE1,KIT,SPTLC1,PPP2R1A,PPT1,SMPD2,AGK,SPHK2,PRKD2,GLB1,ACER3,ELOVL1,NEU3,SPTLC3,SIRT3,NSMAF,ELOVL7,ASAH1,ST8SIA4,HTRA2,SERINC5,ORMDL1,COL4A3BP,A3GALT2,ST8SIA2,SGMS2,ALDH3B2,B3GALT1,TEX2,CERS6,HACD2,CERS2,ABCA12,PPM1L,B3GALNT1,SMPD4,SGMS1,FADS3,TECR,CLN8,DEGS1,ALOXE3,HACD3,GALC,CLN6,ST8SIA5,GLA,HEXA,GM2A,P2RX1,CEL,ST6GALNAC4,ST3GAL2,PLA2G15,ST6GALNAC5,CYP4F22,ABCA8,ELOVL3,PEMT,ACER2,CPTP,NEU2,B4GALT3,SGPP1,ST8SIA6	grey	VSMC_M10
GO:0015740	0.12	0.95	13	19	C4-dicarboxylate transport	BP	1	SLC12A2,SLC1A1,SLC25A13,SLC3A1,SLC1A4,SLC25A30,NTSR1,SLC25A11,LRRC8E,SLC25A14,ABAT,LRRC8A,LRRC8D	grey	VSMC_M10
GO:0140053	0.12	0.91	61	101	mitochondrial gene expression	BP	1	SHMT2,TSFM,RMND1,TFB1M,MTERF1,LRPPRC,MTO1,MTIF2,PNPT1,TRIT1,QRSL1,C6orf203,FOXO3,FASTK,MTRF1,MRPS12,HARS,TRNT1,MRPS27,MRPS28,NSUN3,TWNK,WARS2,MRPS17,GATC,RPUSD4,YARS2,MRPS35,MRPL52,RCC1L,MRPS11,MRPS21,TARS2,TFAM,FASTKD1,MTG2,KIAA0391,SUPV3L1,LARS2,DARS2,COA3,TRMT5,MRPL44,HSD17B10,ALKBH1,EARS2,PPARGC1B,METTL4,TEFM,NDUFA7,MRPS34,SLC25A33,MRPS2,MRPS6,RPUSD3,MRPL57,MRPL51,UQCC2,TRMT10C,MRPL16,TRMT61B	grey	VSMC_M10
GO:0097151	0.12	0.98	5	6	positive regulation of inhibitory postsynaptic potential	BP	1	RIMS2,RIMS1,NTSR1,NLGN2,ABAT	grey	VSMC_M10
GO:0098828	0.12	0.98	5	6	modulation of inhibitory postsynaptic potential	BP	1	RIMS2,RIMS1,NTSR1,NLGN2,ABAT	grey	VSMC_M10
GO:0061029	0.12	0.97	7	9	eyelid development in camera-type eye	BP	1	OSR2,SRF,HDAC2,TFAP2A,HDAC1,GRHL3,STRA6	grey	VSMC_M10
GO:0014014	0.12	0.94	19	29	negative regulation of gliogenesis	BP	1	TSPO,SOX10,DAAM2,PTN,MYCN,KDM4A,SIRT2,NKX6-1,RNF10,CERS2,DLX1,DUSP10,LDLR,MBD1,DAB1,SKI,GPR37L1,NOG,PITX3	grey	VSMC_M10

GO:0006654	0.12	0.94	19	29	phosphatidic acid biosynthetic process	BP	1	AGPAT4,AGPAT1,PNPLA3,LPIN1,ABHD5,NR1H4,LPCAT1,GPAM,ACP6,ABHD4,GNPAT,DGKD,DGKG,SLC27A1,ABHD8,DGKQ,DGKE,AGPAT2,LCLAT1	grey	VSMC_M10
GO:0002706	0.12	0.91	55	92	regulation of lymphocyte mediated immunity	BP	1	CD226,STX7,AGER,HLA-DRA,EXOSC3,MSH2,RSAD2,IL4I1,TRPM4,HMCES,NECTIN2,PVR,MLH1,SLC22A13,FOXJ1,PAXIP1,SHLD1,NCKAP1L,HPX,TRAF6,RASGRP1,NOD2,CD40,CD96,DENND1B,CD28,CADM1,FZD5,BCL6,IL12A,PTPN6,LAG3,PPP3CB,STAT5B,WAS,FOXP3,ZBTB1,SHLD2,TNFRSF1B,TBX21,BTK,FBXO38,VAV1,C3,IL7R,SUPT6H,CRK,PRKCZ,TRAF2,AP1G1,FADD,CD46,FCER2,SERPINB9,HAVCR2	grey	VSMC_M10
GO:0072282	0.12	0.98	5	6	metanephric nephron tubule morphogenesis	BP	1	SOX9,PAX8,PAX2,PKD1,SOX8	grey	VSMC_M10
GO:0007187	0.12	0.94	15	22	G protein-coupled receptor signaling pathway, coupled to cyclic nucleotide second messenger	BP	1	PTGIR,HTR7,CHRM5,NPY1R,CHRM4,CHRM3,OPRD1,AGT,PTH1R,CALCRL,CHRM1,HRH2,HRH4,CCL2,HTR1D	grey	VSMC_M10
GO:0035195	0.13	0.91	65	107	gene silencing by miRNA	BP	1	ZC3H10,ESR1,CNOT6,AGO2,TNRC6B,TUT7,XPO5,PUS10,SNIP1,AGO1,EGFR,MYCN,TUT4,PUM2,TNRC6C,BCDIN3D,CNOT1,EIF4E2,RIPK1,PPP3CA,TERT,AJUBA,DDX6,EIF4ENIF1,PRKRA,DDX3X,STAT3,MAP2K1,SMAD3,TNRC6A,CNOT8,NCOR1,DROSHA,FMR1,SPOUT1,MIRLET7A2,MIR29A,MIR199A1,MIR21,MIR222,MIR23A,MIR425,MIR125B1,MIR32,MIR22,MIR27B,MIR24-1,MIR7-1,MIR199A2,MIRLET7D,MIR27A,MIR23B,MIR365B,MIR25,MIR491,MIR24-2,MIRLET7A3,AGO4,MIR33B,MIR339,MIR125A,MIR99B,MIR940,MIR1249,MIR1180	grey	VSMC_M10
GO:0015813	0.13	0.95	13	19	L-glutamate transmembrane transport	BP	1	SLC1A1,SLC25A13,SLC3A1,SLC7A11,ARL6IP5,ATP1A2,PER2,NTSR1,ARL6IP1,PRAF2,CLN8,PSEN1,SLC1A7	grey	VSMC_M10
GO:0071285	0.13	0.97	7	9	cellular response to lithium ion	BP	1	TPH2,CEBPA,XRCC4,ADCY7,FABP4,NFATC4,CDKN1B	grey	VSMC_M10
GO:0006551	0.13	0.97	7	9	leucine metabolic process	BP	1	HMGCLL1,BCAT2,MCCC2,IVD,MCCC1,HMGCL,AUH	grey	VSMC_M10
GO:0030299	0.13	0.97	7	9	intestinal cholesterol absorption	BP	1	ABCG5,ABCG8,SOAT2,LIMA1,LPCAT3,LDLR,CEL	grey	VSMC_M10
GO:0044241	0.13	0.97	7	9	lipid digestion	BP	1	ABCG5,ABCG8,SOAT2,LIMA1,LPCAT3,LDLR,CEL	grey	VSMC_M10
GO:0006505	0.13	0.93	21	32	GPI anchor metabolic process	BP	1	GPAA1,CWH43,TMEM246,PIGF,PGAP2,PIGU,PIGT,GPLD1,PGAP1,PIGM,PIGV,PIGX,PIGZ,PIGC,PGAP3,PIGH,PIGL,MPPE1,PYURF,PIGB,PIGA	grey	VSMC_M10
GO:0006612	0.13	0.91	58	97	protein targeting to membrane	BP	1	PEX3,ZDHHC14,SIL1,MICALL1,ZDHHC21,SLC1A1,TRAM2,SEC61B,ITGB1BP1,SEC61G,TCAF2,SDCBP,AQP11,SEC62,AKT2,PRNP,SRP14,ZDHHC20,SSR3,GOLGA7B,PEX16,DMTN,SRPRA,ZDHHC23,ZDHHC6,KCNB1,NCF1,C2CD5,CIB1,ZDHHC18,PEX19,ZDHHC24,VPS37D,PIKFYVE,FIS1,CEMIP,RTP4,ZFAND2B,ARL6IP1,ZDHHC4,PEX26,ITGAM,SSR2,RAB8B,TAOK2,ZDHHC15,MIEF2,CDK5R1,INPP5K,SGTA,ZDHHC12,HSPA5,ZDHHC1,ZDHHC11,TRAM1L1,HRAS,MIEF1,SEC61A2	grey	VSMC_M10
GO:1901605	0.13	0.91	86	146	alpha-amino acid metabolic process	BP	1	GLS2,SHMT2,ALDH8A1,GFPT2,RIDA,ATF4,PYCR3,PSAT1,GPT,MTAP,GNMT,SLC25A13,PPAT,RIMKLA,IL4I1,SLC7A11,DDO,BCAT2,DHFR,GAD2,IDO2,IDO1,AMDHD1,NR1H4,APIP,CSAD,THNSL2,AHCY,MSRA,GMPS,SERINC5,ENOPH1,SDSL,HOGA1,NIT2,ASNSD1,GLS,MTRR,SIRT4,ASL,ALDH5A1,AZIN2,MTR,CBSL,SLC7A7,HGD,AMT,CTPS2,ACADSB,OAT,GLUL,SMS,PLOD3,FAH,CPS1,MTHFS,PRODH,COMT,AASDHPPT,NAT8L,ALDH4A1,MAT1A,ASRGL1,HSD17B10,GCDH,AC138894.1,GSTZ1,CRYM,SHMT1,ADSSL1,NOS2,PIPOX,MECP2,PARK7,SARDH,ASS1,GCSH,KYAT1,KYAT3,MIR21,MTHFD2L,PEMT,SLC25A21,HYKK,PHGDH,PCBD2	grey	VSMC_M10
GO:0032228	0.13	0.95	13	19	regulation of synaptic transmission, GABAergic	BP	1	PHF24,USP46,PRKCE,PLCL2,KRAS,NPAS4,HAP1,NLGN2,AC138894.1,BAIAP3,ZDHHC12,CNTNAP4,STXBP1	grey	VSMC_M10
GO:0051260	0.13	0.91	75	128	protein homooligomerization	BP	1	MIP,SHMT2,KCNV1,CBY1,PRPH2,GNMT,NINJ1,CHMP2A,PNPT1,TRPM4,SSBP1,VSTM5,KCNRG,KCTD2,KCTD21,AQP11,RYR1,PRNP,ATL2,APIP,KCTD6,KCTD15,CBR4,P2RX3,RYR3,ASIC1,USP16,GLS,PKD2,SYCP1,CALHM1,ALDH1A3,KCNJ2,KCTD18,KCNB1,KCNG1,TIFA,OSBPL2,UPB1,EHD1,MPP2,ATL1,MICU1,CRTC1,KCNA1,KCND1,ROM1,MAT1A,ZBTB1,HSD17B10,CARD11,ALDH1A2,KCTD13,THG1L,CRTC2,EVL,ACACA,SHMT1,RNF135,TNFAIP1,TRPV1,KCTD5,ABCA3,KCNC4,BCL10,KCTD19,CRYZ,VASP,BEND3,KCTD1,KCNA6,IKZF4,CLYBL,TRMT61B,KCNG2	grey	VSMC_M10
GO:0070935	0.13	0.96	10	14	3'-UTR-mediated mRNA stabilization	BP	1	MAPK14,MYD88,TENT4B,RBM24,MAPKAPK2,ANGEL2,YBX3,RBM10,TARDBP,METTL16	grey	VSMC_M10
GO:1901160	0.13	0.97	7	9	primary amino compound metabolic process	BP	1	TPH2,BTBD9,DDC,SRD5A1,AZIN2,CHKA,GDE1	grey	VSMC_M10
GO:0042976	0.13	0.97	7	9	activation of Janus kinase activity	BP	1	PIBF1,PTK2B,AGT,IL6R,CCL5,GHR,SOCS1	grey	VSMC_M10
GO:0089718	0.13	0.93	20	31	amino acid import across plasma membrane	BP	1	SLC22A4,SLC1A1,SLC1A4,SLC7A11,ARL6IP5,SLC7A1,SLC43A1,AGT,ACE2,ATP1A2,PER2,NTSR1,ARL6IP1,SLC3A2,CLN8,PSEN1,SLC7A3,SLC7A5,SLC36A1,RGS4	grey	VSMC_M10
GO:0060304	0.13	0.98	5	6	regulation of phosphatidylinositol dephosphorylation	BP	1	MTMR2,CHRM5,MTMR9,MTMR3,MTMR1	grey	VSMC_M10
GO:1902107	0.13	0.91	65	110	positive regulation of leukocyte differentiation	BP	1	AGER,HLA-DRA,IL5,XRCC6,TRIB1,HSF1,ROR2,IL4I1,IL15,GLI2,POU4F1,IL15RA,TGFBR2,NCKAP1L,GAS6,PRKDC,TRAF6,SOX12,TOX,IL7,RASGRP1,RIPK2,AP3B1,TESC,RIPK1,SOX13,SLC9B2,HLX,ACIN1,HCLS1,RUNX3,ZBTB46,PPP2R3C,ADAM8,CREB1,DUSP10,BCL6,VSIR,STAT5B,FOXP3,ZBTB1,FOS,ZBTB7B,HAX1,IL4R,GPR68,BTK,CD74,PPARGC1B,NKAP,EVI2B,IL7R,JUN,SOCS1,PRKCZ,ATP6AP1,MIR21,RHOA,SOX4,FADD,CD46,CASP8,CA2,TNFSF9,LIF	grey	VSMC_M10

GO:1903708	0.13	0.91	65	110	positive regulation of hemopoiesis	BP	1	AGER,HLA-DRA,IL5,XRCC6,TRIB1,HSF1,ROR2,IL4I1,IL15,GLI2,POU4F1,IL15RA,TGFBR2,NCKAP1L,GAS6,PRKDC,TRAF6,SOX12,TOX,IL7,RASGRP1,RIPK2,AP3B1,TESC,RIPK1,SOX13,SLC9B2,HLX,ACIN1,HCLS1,RUNX3,ZBTB46,PPP2R3C,ADAM8,CREB1,DUSP10,BCL6,VSIR,STAT5B,FOXP3,ZBTB1,FOS,ZBTB7B,HAX1,IL4R,GPR68,BTK,CD74,PPARGC1B,NKAP,EVI2B,IL7R,JUN,SOCS1,PRKCZ,ATP6AP1,MIR21,RHOA,SOX4,FADD,CD46,CASP8,CA2,TNFSF9,LIF	grey	VSMC_M10
GO:0006370	0.13	0.98	5	6	7-methylguanosine mRNA capping	BP	1	RNMT,CMTR1,RNGTT,NCBP3,CMTR2	grey	VSMC_M10
GO:0003016	0.13	0.93	19	29	respiratory system process	BP	1	ZFAND5,ADH5,YWHAZ,NEK10,TCF15,AP3B1,TNNC1,FTO,GLS,CFAP43,ATP1A2,MTG2,SPAG16,RAB3A,TTC25,NLGN2,NLGN3,JAG2,MECP2	grey	VSMC_M10
GO:0051606	0.13	0.90	79	135	detection of stimulus	BP	1	TSPO,PKD2L2,PTK2,SMO,OPN1SW,PRPH2,PHF24,KIT,MRPS9,CAMKMT,DNAJA1,CYB5R4,NOD1,GJA10,WHRN,TLR4,GRIK2,TRPC3,ASIC3,DACH1,SCN1A,PLEKHB1,MKKS,SYT1,P2RX2,MRPS26,EPHB1,CEP250,CCDC66,ADGRV1,PTPRJ,NKX6-1,CDS1,PKD2,CASQ2,NOD2,KCNIP2,RYR2,FAP,MRPS35,PITPNM1,MRPS11,RRH,SAG,TRPM8,NTSR1,BACE1,STRC,CADM1,NMT1,RTP4,TTN,KCNK4,GRK4,PKD1L1,KCNA1,ROM1,PARP1,TCAP,GUCY2D,NGFR,TMEM107,CALM1,ASIC2,UNC119,TRPV1,PKD1,GNAT2,PIEZO1,FOXF1,PKD1L3,REEP6,PJVK,CHRNA10,TAS2R3,SOX4,OR5AN1,GPR88,KCNMB4	grey	VSMC_M10
GO:1902105	0.13	0.90	111	193	regulation of leukocyte differentiation	BP	1	NME1,AGER,HLA-DRA,IL5,TCF7,XRCC6,TRIB1,MYC,TOB2,HSF1,FBXO7,CDK6,ROR2,HOXA7,SLC46A2,IL4I1,TLR4,IL15,CLPTM1,GLI2,FOXJ1,POU4F1,IL15RA,TGFBR2,NCKAP1L,GAS6,FOXP1,MITF,PRKDC,IRF7,TRAF6,SOX12,TOX,ZC3H8,CAMK4,PIK3R1,FBXW7,TMEM131L,IL7,RASGRP1,RIPK2,AP3B1,TESC,RIPK1,SOX13,SLC9B2,GPR137B,PIAS3,HLX,ACIN1,TCTA,HCLS1,UBASH3B,SLAMF8,CD28,RUNX3,ZBTB46,PPP2R3C,ADAM8,CREB1,DUSP10,BCL6,VSIR,RC3H1,ESRRA,PTPN6,GPR137,FBN1,TNFSF18,LAG3,STAT5B,INHA,TFE3,FOXP3,ZBTB1,CARD11,TBX21,FOS,ZBTB7B,HAX1,IL4R,GPR68,BTK,CD74,PPARGC1B,NKAP,EVI2B,IL7R,PTPN2,JUN,SOCS1,DROSHA,PRKCZ,ATP6AP1,MIR21,MIR125B1,RHOA,FSTL3,SOX4,MAFB,FADD,CD46,CASP8,CA2,BGLAP,FGL2,TNFSF9,LIF,ZFP36L2,CEBPB,RC3H2	grey	VSMC_M10
GO:0019884	0.13	0.94	15	22	antigen processing and presentation of exogenous antigen	BP	1	HLA-DRA,HLA-DOB,TAP1,HLA-DMA,HLA-DPB1,IKBKB,TRAF6,AP3B1,MFSD6,PSME1,FCER1G,PIKFYVE,CTSV,DNM2,CD74	grey	VSMC_M10
GO:0000353	0.13	1.00	4	4	formation of quadruple SL/U4/U5/U6 snRNP	BP	1	RNU5E-1,RNU5B-1,RNU4-2,RNU4-1	grey	VSMC_M10
GO:0000365	0.13	1.00	4	4	mRNA trans splicing, via spliceosome	BP	1	RNU5E-1,RNU5B-1,RNU4-2,RNU4-1	grey	VSMC_M10
GO:0045291	0.13	1.00	4	4	mRNA trans splicing, SL addition	BP	1	RNU5E-1,RNU5B-1,RNU4-2,RNU4-1	grey	VSMC_M10
GO:0050919	0.13	0.92	25	40	negative chemotaxis	BP	1	SEMA6A,PLXNA4,SEMA4D,SEMA4C,EPHA7,SEMA3D,SEMA3A,NRG1,RYK,ROBO1,ROBO2,SEMA4F,SLIT1,SEMA3G,SEMA4G,LGR6,SEMA3F,SEMA6D,LRTM2,SEMA4A,NTN1,PLXNA3,RHOA,DPP4,WNT5A	grey	VSMC_M10
GO:0031047	0.13	0.90	77	129	gene silencing by RNA	BP	1	ZC3H10,ESR1,CNOT2,CNOT6,PABPC1,MOV10L1,AGO2,TNRC6B,TUT7,XPO5,CNOT11,PUS10,SNIP1,AGO1,EGFR,MYCN,TUT4,PUM2,TNRC6C,TEX15,CLP1,BCDIN3D,CNOT1,CELF1,CNOT6L,EIF4E2,RIPK1,EXD1,PPP3CA,TERT,AJUBA,DDX6,TDRKH,NCBP2,EIF4ENIF1,PRKRA,DDX3X,STAT3,MAP2K1,SMAD3,NRDE2,TNRC6A,CNOT8,NCOR1,DROSHA,FMR1,SPOUT1,MIRLET7A2,MIR29A,MIR199A1,MIR21,MIR222,MIR23A,MIR425,MIR125B1,MIR32,MIR22,MIR27B,MIR24-1,MIR7-1,MIR199A2,MIRLET7D,MIR27A,MIR23B,MIR365B,MIR25,MIR491,MIR24-2,MIRLET7A3,AGO4,MIR33B,MIR339,MIR125A,MIR99B,MIR940,MIR1249,MIR1180	grey	VSMC_M10
GO:0009145	0.13	0.92	30	47	purine nucleoside triphosphate biosynthetic process	BP	1	NME1,ATP5MGL,PPARA,IMPDH1,SLC25A13,SPHK2,PRKAG2,ATP5F1C,ATP5F1A,DGUOK,FAM173B,NME4,DNAJC30,ATP5S,PINK1,STAT3,NME7,ADK,PARP1,PKLR,ATP6V0C,NME3,ATP5PB,VPS9D1,MT-ATP8,MT-ATP6,ATP5ME,ATP5MF,NME6,NUDT2	grey	VSMC_M10
GO:0015822	0.13	0.98	5	6	ornithine transport	BP	1	SLC25A15,SLC7A1,SLC7A3,SLC7A6,SLC25A29	grey	VSMC_M10
GO:0001547	0.13	0.98	5	6	antral ovarian follicle growth	BP	1	ESR1,FOXO3,BMPR1B,GAS2,EREG	grey	VSMC_M10
GO:0031290	0.13	0.95	11	16	retinal ganglion cell axon guidance	BP	1	EPHA7,EPHB1,ROBO2,SEMA4F,SLIT1,ALCAM,BMPR1B,PTPRO,EPHB3,ISL1,ISL2	grey	VSMC_M10
GO:0014883	0.13	0.98	5	6	transition between fast and slow fiber	BP	1	TNNC1,PPP3CA,TNNI1,MYH7,ACTN3	grey	VSMC_M10
GO:0043576	0.13	0.95	10	14	regulation of respiratory gaseous exchange	BP	1	NR4A2,FTO,GLS,ATP1A2,MTG2,NTSR1,PASK,NLGN2,NLGN3,MECP2	grey	VSMC_M10
GO:0031058	0.14	0.91	45	75	positive regulation of histone modification	BP	1	SNW1,PHF1,C6orf89,RNF20,SPHK2,PRKD2,PAXIP1,ARRB1,CHTOP,NSD3,SDR16C5,DNMT3B,ING2,RTF1,JARID2,AUTS2,SART3,TET1,BCL6,WDR61,TADA2B,NELFA,PINK1,FOXP3,AKAP8L,AKAP8,KAT7,MUC1,JDP2,MAPK3,ZBTB7B,OGT,NAP1L2,RPS6KA5,SREBF1,ISL1,FMR1,GLYR1,MECP2,MTF2,SAP30BP,ZNF304,UBE2N,LIF,RPS6KA4	grey	VSMC_M10
GO:0043954	0.14	0.92	29	47	cellular component maintenance	BP	1	SYNGAP1,MTSS1,PICK1,ABHD17B,WHRN,VPS35,MTMR2,PRNP,ERC2,KIFC3,PLEKHA7,HOMER1,F2RL1,F2R,ITPKA,BSN,DLG1,C1QL1,RAB3A,NLGN2,ITGA3,INSR,CSF1R,CAMSAP3,SDF4,PJVK,CLDN1,INAVA,PARD6A	grey	VSMC_M10
GO:0120040	0.14	0.98	5	6	regulation of macrophage proliferation	BP	1	PTK2,MAPK1,MAPK3,CSF1R,IL33	grey	VSMC_M10
GO:0099010	0.14	0.95	10	14	modification of postsynaptic structure	BP	1	CTTNBP2,WASF1,CHMP2B,TIAM1,CYFIP1,CAMKV,STAU1,MYH10,ARHGAP44,RHOA	grey	VSMC_M10
GO:0006120	0.14	0.92	30	46	mitochondrial electron transport, NADH to ubiquinone	BP	1	NDUFA6,DLD,NDUFB2,NDUFB6,DNAJC15,NDUFC2,NDUFS8,NDUFS3,NDUFV3,NDUFB4,ISCU,NDUFB3,NDUFS2,PINK1,NDUFA9,NDUFAB1,NDUFA7,NDUFV2,NDUFB10,PARK7,MT-ND1,MT-ND2,MT-ND6,NDUFA5,NDUFC1,NDUFA3,NDUFB5,NDUFS6,NDUFA8,NDUFS5	grey	VSMC_M10

GO:0032148	0.14	0.94	16	24	activation of protein kinase B activity	BP	1	SLC1A1,ITGB1BP1,NRG1,GAS6,ADRA2B,ADRA2A,ADRA2C,PINK1,INSR,PDPK1,PRKCZ,PARK7,MIR21,WNT5A,TXN,FGF1	grey	VSMC_M10
GO:0045653	0.14	0.97	7	9	negative regulation of megakaryocyte differentiation	BP	1	CIB1,HIST1H4B,HIST2H4A,HIST1H4H,HIST1H4D,HIST1H4A,HIST1H4F	grey	VSMC_M10
GO:0030316	0.14	0.91	41	68	osteoclast differentiation	BP	1	TOB2,MAPK14,CD109,TLR4,OSTM1,GAB2,POU4F1,FOXP1,MITF,TRAF6,CAMK4,PIK3R1,FBXW7,SH3PXD2A,LRRK1,SLC9B2,TCIRG1,GPR137B,PIAS3,TCTA,UBASH3B,FARP2,CREB1,ESRRA,GPR137,FBN1,TFE3,ANXA2,FOS,GPR68,CSF1R,PPARGC1B,GPC3,ATP6AP1,FSTL3,MAFB,CA2,BGLAP,SNX10,GLO1,CEBPB	grey	VSMC_M10
GO:0042159	0.14	0.95	10	14	lipoprotein catabolic process	BP	1	ABHD17B,PPT1,APOB,LYPLA1,LYPLAL1,LDLR,ABHD17A,ABHD10,ABHD17C,LYPLA2	grey	VSMC_M10
GO:0009071	0.14	0.97	7	9	serine family amino acid catabolic process	BP	1	SHMT2,CSAD,THNSL2,CBSL,AMT,SHMT1,GCSH	grey	VSMC_M10
GO:0009144	0.14	0.91	39	63	purine nucleoside triphosphate metabolic process	BP	1	NME1,ATP5MGL,PPARA,GTPBP1,IMPDH1,SLC25A13,RAB23,SPHK2,NUDT15,PRKAG2,ATP5F1C,NUDT16,ATP5F1A,DGUOK,OPA1,FAM173B,NME4,MFN1,DNAJC30,EFL1,ATP5S,PINK1,STAT3,NME7,ADK,PARP1,PKLR,ATP6V0C,NME3,ATP5PB,VPS9D1,GNAI3,MT-ATP8,MT-ATP6,RHOA,ATP5ME,ATP5MF,NME6,NUDT2	grey	VSMC_M10
GO:0045652	0.14	0.93	16	24	regulation of megakaryocyte differentiation	BP	1	ZNF16,MTURN,CNOT4,MEF2C,TESC,RAB7B,CIB1,THPO,RCOR1,PRMT6,HIST1H4B,HIST2H4A,HIST1H4H,HIST1H4D,HIST1H4A,HIST1H4F	grey	VSMC_M10
GO:0009581	0.14	0.90	50	84	detection of external stimulus	BP	1	PKD2L2,PTK2,OPN1SW,PRPH2,PHF24,KIT,CAMKMT,GJA10,WHRN,GRIK2,TRPC3,ASIC3,SCN1A,PLEKHB1,MKKS,EPHB1,CEP250,CCDC66,ADGRV1,CDS1,PKD2,PITPNM1,RRH,SAG,TRPM8,NTSR1,BACE1,STRC,NMT1,TTN,KCNK4,GRK4,PKD1L1,KCNA1,ROM1,TCAP,GUCY2D,NGFR,ASIC2,UNC119,TRPV1,PKD1,GNAT2,PIEZO1,FOXF1,PKD1L3,REEP6,PJVK,CHRNA10,GPR88	grey	VSMC_M10
GO:1903764	0.14	0.98	5	6	regulation of potassium ion export across plasma membrane	BP	1	ANO6,KCNIP2,DLG1,MIR21,KCNH2	grey	VSMC_M10
GO:0021904	0.14	0.94	13	19	dorsal/ventral neural tube patterning	BP	1	TBC1D32,PTCH1,SMO,GLI2,SUFU,FKBP8,GORAB,GPR161,TULP3,WDR19,PSEN1,MKS1,GSC	grey	VSMC_M10
GO:0002036	0.14	0.98	5	6	regulation of L-glutamate import across plasma membrane	BP	1	ARL6IP5,ATP1A2,PER2,ARL6IP1,PSEN1	grey	VSMC_M10
GO:0021794	0.14	0.96	7	9	thalamus development	BP	1	UQCRQ,SMO,PTN,SRD5A1,GBX2,CHRNB2,TAL2	grey	VSMC_M10
GO:0046473	0.14	0.93	20	31	phosphatidic acid metabolic process	BP	1	AGPAT4,AGPAT1,PNPLA3,PLA2G6,LPIN1,ABHD5,NR1H4,LPCAT1,GPAM,ACP6,ABHD4,GNPAT,DGKD,DGKG,SLC27A1,ABHD8,DGKQ,DGKE,AGPAT2,LCLAT1	grey	VSMC_M10
GO:0043379	0.14	0.98	5	6	memory T cell differentiation	BP	1	HLA-DRA,BCL6,TSC1,CD46,FGL2	grey	VSMC_M10
GO:0021785	0.14	0.98	5	6	branchiomotor neuron axon guidance	BP	1	PLXNA4,MYCBP2,SEMA3A,SEMA3F,PLXNA3	grey	VSMC_M10
GO:0070534	0.14	0.91	31	50	protein K63-linked ubiquitination	BP	1	PRKN,RNF5,UBE2B,PELI1,TRAF1,TRAF3IP2,UBE2E2,UBE2O,TRAF6,ZFP91,NOD2,MAGEL2,RNF115,ARIH2,TRAF5,PELI3,RNF168,DDX3X,RNF4,GPS2,PRPF19,OTUB2,TRAF3,RNF135,TRAF2,RNF126,WWP2,UBE2G1,RNF8,UBE2N,RNF152	grey	VSMC_M10
GO:0072087	0.14	0.94	13	19	renal vesicle development	BP	1	SMO,SIX2,OSR1,SOX9,PAX8,FMN1,PAX2,SALL1,WNT4,CITED1,LHX1,SOX8,LIF	grey	VSMC_M10
GO:0050907	0.14	0.96	7	9	detection of chemical stimulus involved in sensory perception	BP	1	ASIC3,RTP4,TRPV1,GNAT2,PKD1L3,TAS2R3,OR5AN1	grey	VSMC_M10
GO:0019228	0.14	0.93	15	23	neuronal action potential	BP	1	MYH14,SCN5A,SCN1A,P2RX3,SCN2A,SCN7A,GPER1,KCNA1,CACNA1G,FMR1,P2RX1,CACNA1H,GPR35,GPR88,KCNMB4	grey	VSMC_M10
GO:0000963	0.14	0.94	13	19	mitochondrial RNA processing	BP	1	MTO1,PNPT1,TRIT1,FASTK,TRNT1,RPUSD4,FASTKD1,KIAA0391,SUPV3L1,TRMT5,HSD17B10,TRMT10C,TRMT61B	grey	VSMC_M10
GO:0014832	0.14	0.98	5	6	urinary bladder smooth muscle contraction	BP	1	PLA2G6,P2RX2,P2RX3,KCNMA1,TRPV1	grey	VSMC_M10
GO:0006362	0.14	0.98	5	6	transcription elongation from RNA polymerase I promoter	BP	1	ERCC2,ERCC3,POU4F1,ERCC6,GTF2H5	grey	VSMC_M10
GO:0070070	0.14	0.98	5	6	proton-transporting V-type ATPase complex assembly	BP	1	PIP4P1,TM9SF4,TMEM9,TMEM199,VMA21	grey	VSMC_M10
GO:0006544	0.14	0.96	7	9	glycine metabolic process	BP	1	SHMT2,RIDA,DHFR,AMT,SHMT1,GCSH,PHGDH	grey	VSMC_M10
GO:0034067	0.14	0.93	18	28	protein localization to Golgi apparatus	BP	1	BICD2,ARL5B,OPTN,ARL5A,PAQR3,GBF1,SORL1,PACS1,OBSL1,VPS13D,ARL5C,GAK,VPS13C,TMED10,ZDHHC15,COG7,IFT20,RIPOR1	grey	VSMC_M10
GO:1903400	0.14	0.98	5	6	L-arginine transmembrane transport	BP	1	SLC7A1,SLC38A9,AGT,SLC7A3,SLC25A29	grey	VSMC_M10
GO:0090280	0.14	0.95	10	14	positive regulation of calcium ion import	BP	1	SPHK2,STC1,CASK,ATP2A1,TRPV2,CCL2,TRPV3,P2RX1,ATP2C2,CRH	grey	VSMC_M10
GO:1902022	0.14	0.98	5	6	L-lysine transport	BP	1	SLC7A1,AGT,PQLC2,SLC7A3,SLC25A29	grey	VSMC_M10
GO:1903401	0.14	0.98	5	6	L-lysine transmembrane transport	BP	1	SLC7A1,AGT,PQLC2,SLC7A3,SLC25A29	grey	VSMC_M10
GO:0043583	0.14	0.89	93	162	ear development	BP	1	ATP8B1,EYA4,OSR2,FZD6,PTK7,SLC25A27,ROR2,DLX5,SIX2,KCNQ4,GSDME,HOXA2,HOXA13,MYCL,BMPER,WHRN,ATG5,MYCN,SOBP,OSR1,SLC26A5,MYO3A,SOX9,USH1G,GLI2,MYO7A,TSKU,SPRY2,SLC4A7,JAG1,LRTOMT,AP000812.5,LIN7A,FREM2,CEP290,TCF15,PAX8,CEBPA,EYA1,PLS1,ADGRV1,FZD3,USH1C,OPA1,RPGRIP1L,TFAP2A,PAX2,ALDH1A3,TGFB2,TRIP11,SHROOM2,MAPKAPK2,SOX2,GBX2,MYO3B,KCNK2,GRHL3,STRC,CDKN1B,STOX1,PRKRA,CDH23,ECE1,NKX3-2,MAPK1,EPHA4,DVL2,WDR19,TCAP,RBPJ,MAPK3,ESRRB,POU3F4,NTN1,MKS1,GSC,STRA6,JAG2,IFT20,MCOLN3,KCNK3,PJVK,CHRNA10,FOXG1,MAFB,NOG,WNT5A,OTX1,SIX1,BCL2L11,INSIG2,HEY2,ZIC1	grey	VSMC_M10
GO:0090036	0.14	0.95	10	14	regulation of protein kinase C signaling	BP	1	PLA2G6,SPHK2,WNT11,GPD1L,ADGRV1,CD40,DGKD,DGKG,SEZ6,WNT5A	grey	VSMC_M10
GO:0060330	0.14	0.95	10	14	regulation of response to interferon-gamma	BP	1	NR1H2,HPX,NR1H3,NLRC5,PARP9,PARP14,MED1,PTPN2,SOCS1,IRGM	grey	VSMC_M10
GO:0060334	0.14	0.95	10	14	regulation of interferon-gamma-mediated signaling pathway	BP	1	NR1H2,HPX,NR1H3,NLRC5,PARP9,PARP14,MED1,PTPN2,SOCS1,IRGM	grey	VSMC_M10

GO:0097696	0.14	0.90	62	105	receptor signaling pathway via STAT	BP	1	IL5,AKR1B1,HSF1,OCIAD1,KIT,PPP2R1A,IL15,HDAC2,PIBF1,NMI,HPX,RET,FER,PIGU,OSBP,PTK2B,ARL2BP,F2R,PKD2,CD40,HDAC1,MST1,EGF,HCLS1,PARP9,PARP14,AGT,OSM,THPO,IL10RA,IL12A,TNFSF18,FGFR3,STAT3,STAT5B,CTF1,IL6R,STAT2,ELP2,CCL5,CSF1R,CCL2,CDK5R1,ISL1,SUZ12,GHR,JAK1,NLK,IL7R,PTPN2,SOCS1,DAB1,DOT1L,PKD1,MIR125B1,CNTF,MIR125A,STAMBP,GADD45A,LIF,TSLP,LEPROT	grey	VSMC_M10
GO:1904263	0.14	0.95	10	14	positive regulation of TORC1 signaling	BP	1	RRAGD,PIP4P1,LARS,GPR137B,SIK3,GPR137,OTUD5,RRAGB,CLEC16A,LAMTOR5	grey	VSMC_M10
GO:0044803	0.14	0.98	5	6	multi-organism membrane organization	BP	1	NECTIN2,FCHSD2,FCHSD1,GAS6,PI4KA	grey	VSMC_M10
GO:0034383	0.14	0.93	15	22	low-density lipoprotein particle clearance	BP	1	APOB,FGF21,NR1H4,SOAT2,MYLIP,SOAT1,EHD1,LIPA,ANXA2,LDLR,MIR199A1,MIR27B,MIR27A,NCEH1,LDLRAP1	grey	VSMC_M10
GO:0051966	0.14	0.92	25	40	regulation of synaptic transmission, glutamatergic	BP	1	MAPK8IP2,GRM4,PLA2G6,GRM8,ROR2,DGKI,EGFR,SYT1,MEF2C,PTK2B,HOMER1,GRM2,DISC1,ATP1A2,GLUL,TNR,NLGN2,PSEN1,NLGN3,AC138894.1,CCL2,TPRG1L,STXBP1,CACNG7,CACNG5	grey	VSMC_M10
GO:0045836	0.15	0.96	7	9	positive regulation of meiotic nuclear division	BP	1	UBE2B,SIRT2,PLCB1,NPM2,WNT4,OOEP,WNT5A	grey	VSMC_M10
GO:0002430	0.15	0.98	5	6	complement receptor mediated signaling pathway	BP	1	FPR2,C5AR2,C5AR1,GPLD1,CMKLR1	grey	VSMC_M10
GO:0050806	0.15	0.90	61	104	positive regulation of synaptic transmission	BP	1	RIMS2,PRRT1,AGER,NTRK2,SLC1A1,ROR2,PRKCE,RIMS1,RIMS3,CRHR2,PTN,EGFR,GRIK2,GRIN2D,SLC8A2,CACNB2,SYT1,PRNP,PLK2,FAM107A,CLSTN2,PTK2B,MME,EIF2AK4,DTNBP1,DNM1L,IGSF11,STAU1,GPER1,NFATC4,STX1A,YTHDF1,GLUL,CREB1,TNR,MPP2,CRTC1,PINK1,MAPK1,NSG1,HAP1,EPHA4,DLG4,NLGN2,RGS14,STX1B,STX4,NLGN3,KCTD13,CYP46A1,CCL2,FMR1,PRKCZ,MECP2,BAIAP3,ZDHHC12,STXBP1,CACNG7,CRH,CACNG5,LRRTM2	grey	VSMC_M10
GO:0036446	0.15	0.98	5	6	myofibroblast differentiation	BP	1	TSKU,TP53INP1,PDCD4,PARP1,MIR21	grey	VSMC_M10
GO:1904760	0.15	0.98	5	6	regulation of myofibroblast differentiation	BP	1	TSKU,TP53INP1,PDCD4,PARP1,MIR21	grey	VSMC_M10
GO:0070584	0.15	0.94	13	19	mitochondrion morphogenesis	BP	1	PNPT1,MYH14,WASF1,SSBP1,PANK2,OPA1,COL4A3BP,DNM1L,POLG2,NUBPL,FIS1,SUPV3L1,MTM1	grey	VSMC_M10
GO:0045046	0.15	0.98	5	6	protein import into peroxisome membrane	BP	1	PEX3,PEX16,PEX19,PEX26,RAB8B	grey	VSMC_M10
GO:0002703	0.15	0.89	79	136	regulation of leukocyte mediated immunity	BP	1	CD226,STX7,AGER,HLA-DRA,RAC2,KIT,EXOSC3,MSH2,RSAD2,IL4I1,TLR4,TRPM4,SPHK2,HMCES,NECTIN2,PVR,GAB2,MLH1,UNC13D,SLC22A13,FOXJ1,PAXIP1,SHLD1,MAVS,NCKAP1L,HPX,TRAF6,FER,RASGRP1,F2RL1,NOD2,CD40,CD96,DENND1B,FGR,SNX4,CD28,CADM1,FZD5,BCL6,IL12A,PTPN6,LAG3,PPP3CB,C12orf4,STAT5B,WAS,FOXP3,ZBTB1,SHLD2,TNFRSF1B,TBX21,STX4,ITGAM,IL4R,BTK,STXBP2,FBXO38,PRAM1,VAV1,C3,NOS2,IL7R,SUPT6H,TICAM1,CRK,DNASE1,PDPK1,PRKCZ,TRAF2,FOXF1,STXBP1,AP1G1,FADD,CXCL6,CD46,FCER2,SERPINB9,HAVCR2	grey	VSMC_M10
GO:0007197	0.15	0.98	5	6	adenylate cyclase-inhibiting G protein-coupled acetylcholine receptor signaling pathway	BP	1	CHRM5,CHRM4,CHRM3,CHRM1,HRH4	grey	VSMC_M10
GO:0046034	0.15	0.88	128	219	ATP metabolic process	BP	1	SHMT2,ENPP3,ATP5MGL,HSPA1B,PPARA,TSPO,UQCRQ,NDUFA6,PRXL2C,SLC25A13,GAPDH,MSH2,SLC4A4,BPGM,DLD,NDUFB2,SPHK2,NDUFB6,DNAJC15,NUDT5,DHTKD1,NDUFC2,PRKAG2,ATP5F1C,PFKP,NDUFS8,NDUFA12,COX7A1,COX4I2,GPI,ATP5F1A,GPD1,P2RX7,NDUFS3,DGUOK,PRKAG1,PFKM,UQCRB,COX15,FAM173B,ATP6V1B2,COX6B1,NDUFV3,DNM1L,ZBTB20,FAM20C,NDUFB4,ISCU,COA6,PFKFB2,MYH7,HSPA8,ENO4,NDUFB3,ARNT,ATP1A2,ACTN3,HDAC4,NDUFS2,DNAJC30,MLXIPL,SDHC,OLA1,COX6A1,FIS1,HK1,ATP5S,COX8A,PRKAG3,PINK1,STAT3,ATP1B1,NDUFA9,ENO3,PARP1,PSEN1,COX6A2,PKLR,OGT,NDUFAB1,ADPGK,UQCRC2,COX5A,NCOR1,INSR,TEFM,NDUFA7,SLC25A23,NDUFV2,ALDOC,NADK,ATP6V0C,NDUFB10,UQCR11,PARK7,SLC25A33,ATP5PB,SLC2A6,VPS9D1,SLC25A25,MT-ND1,MT-ND2,MT-ATP8,MT-ATP6,MT-CO3,MT-ND6,MT-CYB,RHOA,ATP5ME,ATP5MF,NDUFA5,ENTPD5,NDUFC1,BEND3,COX7A2,TIGAR,FXN,NDUFA3,NDUFB5,NDUFS6,NDUFA8,SDHAF2,NDUFS5,UQCC2,NUDT2,COX7A2L,PPIF,NMNAT1	grey	VSMC_M10
GO:0021670	0.15	0.95	8	11	lateral ventricle development	BP	1	CDK6,AQP1,NUMBL,TSKU,RPGRIP1L,DNAH5,DPCD,ATP1B2	grey	VSMC_M10
GO:0018198	0.15	0.91	27	43	peptidyl-cysteine modification	BP	1	ZDHHC14,NCOA7,ZDHHC21,GAPDH,ADH5,PRDX3,ZDHHC20,GOLGA7B,ZDHHC23,ZDHHC6,HHAT,ZDHHC18,ZDHHC24,NOS1AP,ZDHHC8,ZDHHC4,ZDHHC15,RAB3D,S100A8,NOS2,PARK7,ZDHHC12,ZDHHC1,DMD,ZDHHC11,TXN,MAP6D1	grey	VSMC_M10
GO:0042439	0.15	0.98	5	6	ethanolamine-containing compound metabolic process	BP	1	NAPEPLD,CHKA,GDPD3,GDPD1,GDE1	grey	VSMC_M10
GO:0055088	0.15	0.89	68	116	lipid homeostasis	BP	1	ORMDL2,APOM,PNPLA3,PNPLA5,TTC39B,SLC25A27,ABCG5,ABCG8,RTN4,NR1H2,APOB,CNBP,ABHD5,ACOX1,GCKR,TSKU,NR1D2,COMMD9,NR1H4,USF2,ACOXL,SOAT2,CYP7A1,CYP7B1,ACOX2,CEBPA,FBXW7,LIMA1,EPHX2,NR1H3,FITM2,ORMDL1,COL4A3BP,ITGB6,MYLIP,ABCA5,ZBTB20,GPAM,ABHD4,NR5A2,FABP4,GRAMD1B,ABCB11,ABCG4,USF1,MLXIPL,SOAT1,EHD1,DDX3X,ABCA12,ABHD8,ATP13A2,CLN8,FGFR4,LIPC,ADCK1,LDLR,ACSM3,ACACA,NPC1,TMEM97,FAM57A,ABCA3,MIR27B,MIR33B,PNPLA4,LDLRAP1,TLCD1	grey	VSMC_M10
GO:0055064	0.15	0.95	8	11	chloride ion homeostasis	BP	1	SLC12A2,CKB,ABCC2,SLC12A5,SLC12A7,SLC12A8,SLC12A9,SLC12A4	grey	VSMC_M10
GO:0055083	0.15	0.95	8	11	monovalent inorganic anion homeostasis	BP	1	SLC12A2,CKB,ABCC2,SLC12A5,SLC12A7,SLC12A8,SLC12A9,SLC12A4	grey	VSMC_M10
GO:0006555	0.15	0.95	10	14	methionine metabolic process	BP	1	MTAP,GNMT,APIP,MSRA,ENOPH1,MTRR,MTR,SMS,MAT1A,MTHFD2L	grey	VSMC_M10
GO:0072378	0.15	0.98	5	6	blood coagulation, fibrin clot formation	BP	1	FBLN1,FN1,SERPINC1,F12,FGA	grey	VSMC_M10
GO:0032610	0.15	0.98	5	6	interleukin-1 alpha production	BP	1	IL1R2,PANX1,IL16,S100A13,ISL1	grey	VSMC_M10
GO:0032650	0.15	0.98	5	6	regulation of interleukin-1 alpha production	BP	1	IL1R2,PANX1,IL16,S100A13,ISL1	grey	VSMC_M10

GO:0071500	0.15	0.98	5	6	cellular response to nitrosative stress	BP	1	DNAJA1,ATG5,STOX1,PARK7,DDIT3	grey	VSMC_M10
GO:0044255	0.15	0.87	412	743	cellular lipid metabolic process	BP	1	RDH5,KDSR,ORMDL2,RDH16,HSD17B4,LRP1,ALDH8A1,PIP4K2C,PEX7,B4GALNT1,AVIL,AIG1,LTC4S,CPT1B,ABHD16A,SYNJ2,SERAC1,SLC27A6,ALG12,TNXB,AGPAT4,AGPAT1,QKI,PPARA,ACSL6,SLC22A4,PNPLA3,PNPLA5,MCAT,HSD17B8,A4GALT,ECHDC1,AKR1B1,MAPK14,PLA2G6,GPAA1,HACD4,LARGE1,ALDH1A1,CROT,CWH43,KIT,PLPP6,SRD5A3,SPTLC1,AASDH,INPP4A,ADH5,CAT,ALG2,PLPPR1,TMEM246,TMEM86B,PIGF,RDH13,MBOAT7,PPP2R1A,ETFB,PPT1,PLEK,NR1H2,MBOAT2,DGKI,EGFR,LPIN1,INPP4B,MGST2,SMPD2,DLD,FIG4,APOB,AGK,FGF21,PRKAR2B,SPHK2,MTMR2,PIP4K2A,NAPEPLD,ALG11,FAAH,PRKD2,HADHA,SELENOI,SOX9,ABHD5,APOC1,GLB1,LIPE,ECHDC3,PRKAG2,ACOX1,TAMM41,SLC22A13,PIBF1,ITPKC,CYGB,ACER3,ELOVL1,AKT2,PIP4P1,NEU3,MTMR14,SPTLC3,SIRT2,PLCB1,PGAP2,CRLS1,HACL1,CDS2,PLPP5,DDHD2,PANK2,PDK1,SIRT3,NR1H4,MTMR7,TRIB3,LYPLA1,SDR16C5,ACOXL,SOAT2,CYP7A1,NSMAF,ELOVL7,ASAH1,ACOX2,ABHD6,THNSL2,CBR4,PIGU,PIK3CB,PITPNM2,ST8SIA4,PIK3R1,TMEM150A,GK5,RETSAT,HPGD,PLCE1,CHRM5,MTMR9,FBXW7,GPD1,PEX2,HTRA2,EPHX2,SLA2,ETFDH,ACOT12,PRKAG1,NR1H3,SERINC5,SYNJ1,COQ2,IVD,LPIN3,CDS1,ST3GAL6,FITM2,ORMDL1,ECI2,COL4A3BP,ITPKA,ERLIN1,PIGT,PLA2G4B,ADTRP,PRMT3,PRKAB1,LONP2,PM20D1,SIRT4,GPLD1,SRD5A1,MCEE,A3GALT2,IRS1,ALG10,LPCAT1,SIK1,ALDH1A3,PGAP1,CPT1A,BDH2,GPAM,ACSL5,CHKA,PLA1A,ACP6,INPP5A,ABHD4,ST8SIA2,SGMS2,CYP2U1,ALDH3B2,AJUBA,GGPS1,PITPNM1,ABHD2,MSMO1,MTMR11,MECR,B3GALT1,SORL1,TEX2,GNPAT,DGKD,B3GNT5,CERS6,PIGM,FITM1,ABCB11,DHRS9,BMX,AGT,HACD2,FAM126B,IP6K2,LPG[illegible]	grey	VSMC_M10
GO:0099565	0.15	0.90	44	74	chemical synaptic transmission, postsynaptic	BP	1	MAPK8IP2,RIMS2,CHRNA3,CHRNA5,RIMS1,SHANK1,GRIK2,GRIN2D,MTMR2,SLC8A2,GRIK5,CHRNA6,P2RX2,P2RX3,MEF2C,P2RX7,CUX2,PTK2B,PPP3CA,IGSF11,SSH1,STX1A,TMEM25,NPAS4,NTSR1,MPP2,DLG4,NLGN2,CHRNB1,STX1B,NLGN3,CHRNB2,SEZ6,ABAT,TRPV1,P2RX1,PRKCZ,MECP2,CHRNB4,CHRNA10,CHRNG,GRID2,RGS4,INSYN1	grey	VSMC_M10
GO:0002922	0.15	0.98	5	6	positive regulation of humoral immune response	BP	1	PHB,HPX,NOD2,C3,FCER2	grey	VSMC_M10
GO:0048168	0.15	0.91	24	38	regulation of neuronal synaptic plasticity	BP	1	SYNGAP1,SYNGR1,KIT,ACP4,NCDN,GRIK2,PPFIA3,SLC8A2,RAB5A,SLC4A10,NEURL1,KRAS,AGT,SYAP1,RAB3A,RAB8A,DLG4,AC138894.1,RAB11A,CAMK2A,JPH3,CNTN2,HRAS,SHISA8	grey	VSMC_M10
GO:0060080	0.15	0.95	8	11	inhibitory postsynaptic potential	BP	1	RIMS2,RIMS1,NPAS4,NTSR1,NLGN2,NLGN3,ABAT,INSYN1	grey	VSMC_M10
GO:1903793	0.15	0.92	17	26	positive regulation of anion transport	BP	1	SLC12A2,PLA2G6,ABCB1,PLA2R1,SLC38A3,MAP2K6,ABCB11,AGT,ACE2,NTSR1,PLA2G4A,ARL6IP1,PSEN1,ABAT,STXBP1,PTGES,CEBPB	grey	VSMC_M10
GO:0006547	0.15	0.98	5	6	histidine metabolic process	BP	1	CARNMT1,HNMT,AMDHD1,HDC,MTHFD2L	grey	VSMC_M10
GO:0010624	0.15	0.98	5	6	regulation of Schwann cell proliferation	BP	1	SOX10,RNF10,CERS2,SKI,MIR222	grey	VSMC_M10
GO:0050687	0.15	0.93	14	21	negative regulation of defense response to virus	BP	1	C6orf106,SEC14L1,NPLOC4,PCBP2,RNF26,NLRX1,TRAF3IP1,HTRA1,MUL1,FOXP3,TKFC,TSPAN6,RIOK3,FGL2	grey	VSMC_M10
GO:0046839	0.15	0.91	25	40	phospholipid dephosphorylation	BP	1	SYNJ2,PLPP6,PLPPR1,INPP4B,FIG4,MTMR2,PIP4P1,PLPP5,MTMR7,CHRM5,MTMR9,EPHX2,SYNJ1,INPP5A,MTMR11,PLPP4,MTMR3,INPP5J,SACM1L,PLPPR2,MTMR12,MTM1,MTMR1,INPP5K,SGPP1	grey	VSMC_M10
GO:0018243	0.15	0.98	5	6	protein O-linked glycosylation via threonine	BP	1	GALNT11,EOGT,GALNT2,GALNT16,GALNT1	grey	VSMC_M10
GO:0048640	0.15	0.89	53	90	negative regulation of developmental growth	BP	1	SEMA6A,STK3,PPARA,FSTL4,PTCH1,PI16,SEMA4D,SEMA4C,RTN4,EPHA7,TGFBR2,SEMA3D,SPART,SEMA3A,ULK1,RYK,SEMA4F,TLL2,TBX5,SEMA3G,SPP1,STK4,SEMA4G,NKD1,JARID2,KIAA0319,SEMA3F,KCNK2,CDKN1B,WNT3,DUSP10,SEMA6D,TNR,SAV1,MYOZ1,GDF15,FGFR3,SEMA4A,TRIM46,WWC1,NTN1,YY1,CDK5R1,RAI1,ARHGAP4,PLXNA3,MIR199A1,MIR25,NOG,WNT5A,FXN,RGS4,PLAC8	grey	VSMC_M10
GO:0006887	0.15	0.88	150	266	exocytosis	BP	1	VPS4B,RAB21,RIMS2,PRKN,EXOC4,SYNGR1,PLA2G6,RAC2,MYH9,CHRNA5,STEAP2,APBA1,SCFD2,KIT,CHMP2A,NLRP5,STX17,RIMS1,RIMS3,SCRN1,PLEK,VPS41,SDCBP,PPFIA3,LIN7B,SPHK2,NAPA,RAB7A,RAB10,CACNB2,SYTL2,CLASP2,GAB2,LLGL2,RALB,GRIK5,UNC13D,NAPB,ABCC4,ANK1,SYT1,RAB5A,LIN7A,NCKAP1L,STX2,SYT9,SNPH,HCK,FER,TRAPPC11,ERC2,P2RY1,RASGRP1,RAB11FIP5,EXOC6B,SYNJ1,F2RL1,VPS18,LGI3,DMTN,DTNBP1,SYT8,DNM1L,HYAL3,ADRA2A,SNAP23,KCNB1,RAB11FIP2,LAT2,FGR,CBL,SYTL1,PRSS12,STX1A,SNX4,SEPT2,BACE1,DNAJC5,CSPG5,NSF,MIA3,RAP1B,BLOC1S6,ABCA12,CASK,PTGDR,PPP3CB,RAB3A,SYN1,SNAP29,EXOC5,C12orf4,RAB8A,ATP13A2,HAP1,CPLANE2,SYT7,RAB15,RSC1A1,SEPT1,WASHC3,CPLX1,STX1B,STX4,PSEN1,RAB25,ITGAM,SYT11,RAB8B,TMED10,CACNA1G,RAB11A,LAT,RAB3D,IL4R,CALM1,SYTL4,SNAPIN,S100A13,PRKCB,BTK,ARHGAP44,SCAMP5,CCL5,TNFAIP2,STXBP2,PRAM1,FMR1,RPH3AL,PIP5K1C,SDF4,PDPK1,P2RX1,RAB26,ATP6AP1,BAIAP3,CACNA1H,FOXF1,STXBP1,AP1G1,VPS4A,FGA,CHGA,SNAP47,TMEM167A,TMEM167B,DOC2B,RALA,VAMP3,EXOC3L4	grey	VSMC_M10
GO:1903350	0.15	0.90	34	56	response to dopamine	BP	1	PRKN,ATF4,SLC1A1,VPS35,HDAC2,ALK,HTR7,CHRM5,CHRM4,DTNBP1,CHRM3,CALY,HTR3B,MAPK1,CHRM1,GNB5,NSG1,HRH2,HCN3,MAPK3,DNM2,SIN3A,HRH4,GNAL,GNB1,GNA15,PALM,GNAI3,FUK,HTR1D,RGS4,GNA14,GNG2,ARPP19	grey	VSMC_M10
GO:0086067	0.15	0.96	6	8	AV node cell to bundle of His cell communication	BP	1	TRPM4,CACNB2,SCN5A,RYR2,SCN4B,CACNA1G	grey	VSMC_M10
GO:0002227	0.15	0.97	5	6	innate immune response in mucosa	BP	1	NOD2,NOS2,HIST1H2BK,HIST1H2BF,HIST1H2BJ	grey	VSMC_M10
GO:0042220	0.15	0.91	22	35	response to cocaine	BP	1	SLC1A1,HDAC2,DNMT3A,HNMT,DNMT3B,PTK2B,TIAM1,HOMER1,GRM2,SLC6A3,HOMER2,SDK1,CHRNB2,DNM2,MBD1,ABAT,HSPA5,MT-ND6,FADD,RGS4,CRH,PITX3	grey	VSMC_M10
GO:0046856	0.16	0.92	18	28	phosphatidylinositol dephosphorylation	BP	1	SYNJ2,INPP4B,FIG4,MTMR2,PIP4P1,MTMR7,CHRM5,MTMR9,SYNJ1,INPP5A,MTMR11,MTMR3,INPP5J,SACM1L,MTMR12,MTM1,MTMR1,INPP5K	grey	VSMC_M10

GO:0002448	0.16	0.91	25	40	mast cell mediated immunity	BP	1	RAC2,KIT,SPHK2,GAB2,UNC13D,FER,RASGRP1,SNAP23,LAT2,FGR,CBL,SNX4,PTGDR,C12orf4,SPON2,LAT,IL4R,S100A13,BTK,STXBP2,PDPK1,FOXF1,STXBP1,CHGA,SERPINB9	grey	VSMC_M10
GO:0002478	0.16	0.93	13	19	antigen processing and presentation of exogenous peptide antigen	BP	1	HLA-DRA,HLA-DOB,TAP1,HLA-DMA,HLA-DPB1,IKBKB,TRAF6,MFSD6,FCER1G,PIKFYVE,CTSV,DNM2,CD74	grey	VSMC_M10
GO:1904429	0.16	0.97	5	6	regulation of t-circle formation	BP	1	ERCC1,RTEL1,XRCC5,SLX4,TERF2	grey	VSMC_M10
GO:0032874	0.16	0.89	57	97	positive regulation of stress-activated MAPK cascade	BP	1	MAP3K5,STK3,MAPK8IP2,MAP3K4,AGER,GADD45G,SEMA4C,EMC10,MTURN,NOD1,TLR4,TRAF1,SDCBP,MYD88,PLCB1,EIF2AK2,RELL2,SEMA3A,SH3RF2,ARL6IP5,MFHAS1,TRAF6,HAND2,PTK2B,RASGRP1,RIPK2,F2RL1,RIPK1,NOD2,TAOK3,TRPV4,TGFB2,NCF1,TRAF5,FZD7,MAP3K3,MAP3K11,MBIP,MAP4K2,DUSP19,PDCD10,LTBR,MINK1,RELL1,TAOK2,TRAF3,TAOK1,SERPINF2,MAPK8IP3,TRAF2,DAB2IP,ZNF622,WNT5A,GADD45A,INAVA,DUSP22,HRAS	grey	VSMC_M10
GO:0097623	0.16	0.95	8	11	potassium ion export across plasma membrane	BP	1	ANO6,KCNE2,KCNIP2,DLG1,MIR21,KCNH2,KCNK5,KCNT2	grey	VSMC_M10
GO:0051930	0.16	0.93	14	21	regulation of sensory perception of pain	BP	1	GRIN2D,FAM19A4,P2RX3,F2R,FAM173B,ZFHX2,OPRD1,SLC9A1,NTSR1,COMT,ADRA2C,TMEM100,NMUR2,FABP5	grey	VSMC_M10
GO:0098657	0.16	0.88	89	155	import into cell	BP	1	SLC39A5,SLC12A2,PRKN,SLC22A4,KCNK9,MAPK15,KCNJ4,SLC39A4,STEAP2,SLC1A1,WNK2,SLC1A4,TRPM4,SLC7A11,KCNJ14,SLC8A2,SLC39A12,ATP1A3,AKT2,PRNP,ARL6IP5,SLC7A1,SLC43A1,SLC9A9,KCNJ11,KCNJ3,SLC38A1,SLC6A2,KCNE2,KCNJ15,SLC12A5,SLC6A3,SLC2A10,KCNJ2,SLC12A7,TRPV4,ACSL5,ISCU,AGT,ACE2,ATP1A2,SLC9A1,PER2,SLC12A8,NTSR1,SLC29A2,IL10RA,DLG1,SLC12A9,ITGA4,SLC9A7,ATP1B1,SLC27A1,ARL6IP1,SLC3A2,ACTB,SLC29A4,CLN8,WNK3,ATP1B2,FAM155B,PSEN1,NEURL1B,SLC7A3,SLC5A2,NOX5,SLC39A6,TRPV2,LRRC8E,KCNJ18,SLC46A1,SLC9A6,SLC1A7,TRPV3,TRPV1,P2RX1,PARK7,SLC7A5,LRRC8A,LCN2,SLC12A4,SLC9A5,MIR24-1,SLC36A1,KCNH2,RGS4,KCNJ8,HCN4,KCNJ9	grey	VSMC_M10
GO:0072170	0.16	0.94	11	16	metanephric tubule development	BP	1	POU3F3,OSR1,SOX9,PAX8,PKD2,PAX2,CXCR2,WNT4,PKD1,SOX8,LIF	grey	VSMC_M10
GO:0002418	0.16	0.95	8	11	immune response to tumor cell	BP	1	CD226,IL4I1,NECTIN2,PVR,SLC22A13,IL12A,FBXO38,HAVCR2	grey	VSMC_M10
GO:0042135	0.16	0.95	8	11	neurotransmitter catabolic process	BP	1	HNMT,LRTOMT,AP000812.5,ALDH5A1,COMT,BCHE,ABAT,PRIMA1	grey	VSMC_M10
GO:0097186	0.16	0.94	11	16	amelogenesis	BP	1	PPARA,CNNM4,KLK4,SLC4A2,RELT,ITGB6,TCIRG1,FAM20A,FAM20C,AMELX,PERP	grey	VSMC_M10
GO:1903361	0.16	0.97	5	6	protein localization to basolateral plasma membrane	BP	1	LIN7B,RAB10,LIN7A,AP4M1,PDZD11	grey	VSMC_M10
GO:0048681	0.16	0.95	8	11	negative regulation of axon regeneration	BP	1	SPP1,KIAA0319,KREMEN1,CERS2,LRIG2,TNR,EPHA4,RTN4RL1	grey	VSMC_M10
GO:1900424	0.16	0.95	8	11	regulation of defense response to bacterium	BP	1	SIRT2,FOXP1,F2RL1,MAPKBP1,NOD2,SYT11,CYBA,HAVCR2	grey	VSMC_M10
GO:0002762	0.16	0.91	22	35	negative regulation of myeloid leukocyte differentiation	BP	1	NME1,TRIB1,MYC,TOB2,CDK6,HOXA7,TLR4,PIK3R1,FBXW7,GPR137B,PIAS3,TCTA,UBASH3B,ZBTB46,GPR137,FBN1,INHA,GPR68,PTPN2,MIR125B1,FSTL3,MAFB	grey	VSMC_M10
GO:0043922	0.16	0.95	8	11	negative regulation by host of viral transcription	BP	1	HDAC1,ZNF639,POU2F3,PSMC3,TARDBP,CCL5,JUN,INPP5K	grey	VSMC_M10
GO:0006506	0.16	0.91	20	31	GPI anchor biosynthetic process	BP	1	GPAA1,CWH43,TMEM246,PIGF,PGAP2,PIGU,PIGT,PGAP1,PIGM,PIGV,PIGX,PIGZ,PIGC,PGAP3,PIGH,PIGL,MPPE1,PYURF,PIGB,PIGA	grey	VSMC_M10
GO:0045921	0.16	0.89	38	63	positive regulation of exocytosis	BP	1	VPS4B,PLA2G6,CHMP2A,SDCBP,SPHK2,RAB7A,CACNB2,CLASP2,GAB2,UNC13D,SYT1,RAB5A,SYT9,F2RL1,DNM1L,HYAL3,KCNB1,FGR,STX1A,SNX4,RAB3A,ATP13A2,SYT7,RAB15,STX4,ITGAM,CACNA1G,RAB3D,IL4R,SYTL4,SCAMP5,ATP6AP1,CACNA1H,STXBP1,AP1G1,VPS4A,FGA,DOC2B	grey	VSMC_M10
GO:0051151	0.16	0.93	11	16	negative regulation of smooth muscle cell differentiation	BP	1	NFATC1,ANKRD17,PDCD4,FOXO4,RBPMS2,MECP2,NFATC3,MIR21,PRDM6,EREG,HEY2	grey	VSMC_M10
GO:0000038	0.16	0.92	18	28	very long-chain fatty acid metabolic process	BP	1	HSD17B4,SLC27A6,ACSL6,HACD4,ACOX1,ELOVL1,ELOVL7,ACOX2,PEX2,HACD2,ACSBG1,TECR,ACOT2,ABCD4,HACD3,ABCD3,ACOT4,ELOVL3	grey	VSMC_M10
GO:0006521	0.16	0.95	8	11	regulation of cellular amino acid metabolic process	BP	1	SLC7A11,NR1H4,SIRT4,SLC7A7,COMT,AC138894.1,PARK7,MIR21	grey	VSMC_M10
GO:0009582	0.16	0.89	49	83	detection of abiotic stimulus	BP	1	PKD2L2,PTK2,OPN1SW,PRPH2,PHF24,KIT,CAMKMT,GJA10,WHRN,GRIK2,TRPC3,ASIC3,SCN1A,PLEKHB1,MKKS,EPHB1,CEP250,CCDC66,ADGRV1,CDS1,PKD2,PITPNM1,RRH,SAG,TRPM8,NTSR1,BACE1,STRC,NMT1,TTN,KCNK4,GRK4,PKD1L1,KCNA1,ROM1,TCAP,GUCY2D,NGFR,ASIC2,UNC119,TRPV1,PKD1,GNAT2,PIEZO1,PKD1L3,REEP6,PJVK,CHRNA10,GPR88	grey	VSMC_M10
GO:0060079	0.16	0.89	41	69	excitatory postsynaptic potential	BP	1	MAPK8IP2,RIMS2,CHRNA3,CHRNA5,RIMS1,SHANK1,GRIK2,GRIN2D,MTMR2,SLC8A2,GRIK5,CHRNA6,P2RX2,P2RX3,MEF2C,P2RX7,CUX2,PTK2B,PPP3CA,IGSF11,SSH1,STX1A,TMEM25,NPAS4,MPP2,DLG4,NLGN2,CHRNB1,STX1B,NLGN3,CHRNB2,SEZ6,TRPV1,P2RX1,PRKCZ,MECP2,CHRNB4,CHRNA10,CHRNG,GRID2,RGS4	grey	VSMC_M10
GO:0009206	0.16	0.90	29	46	purine ribonucleoside triphosphate biosynthetic process	BP	1	NME1,ATP5MGL,PPARA,IMPDH1,SLC25A13,SPHK2,PRKAG2,ATP5F1C,ATP5F1A,DGUOK,FAM173B,NME4,DNAJC30,ATP5S,PINK1,STAT3,NME7,PARP1,PKLR,ATP6V0C,NME3,ATP5PB,VPS9D1,MT-ATP8,MT-ATP6,ATP5ME,ATP5MF,NME6,NUDT2	grey	VSMC_M10
GO:1902358	0.16	0.95	8	11	sulfate transmembrane transport	BP	1	SLC26A10,SLC13A4,SLC26A5,SLC25A30,SLC25A11,SLC26A1,SLC26A2,SLC25A14	grey	VSMC_M10
GO:0014012	0.16	0.97	5	6	peripheral nervous system axon regeneration	BP	1	TSPO,TNC,CERS2,APOD,MIR222	grey	VSMC_M10
GO:0019883	0.16	0.95	8	11	antigen processing and presentation of endogenous antigen	BP	1	HLA-DRA,TAP1,ATG5,IDE,ABCB9,ERAP1,CD74,ERAP2	grey	VSMC_M10
GO:0002082	0.16	0.93	13	19	regulation of oxidative phosphorylation	BP	1	SHMT2,DNAJC15,COX7A1,ACTN3,MLXIPL,PINK1,SLC25A23,SLC25A33,RHOA,COX7A2,UQCC2,COX7A2L,PPIF	grey	VSMC_M10

GO:0090181	0.16	0.91	18	28	regulation of cholesterol metabolic process	BP	1	TTC39B,APOB,NR1H4,CYP7A1,EPHX2,ERLIN1,FMO5,SCAP,POR,ACADL,LPCAT3,GNB3,ACADVL,DGKQ,LDLR,SREBF1,LDLRAP1,FGF1	grey	VSMC_M10
GO:0051953	0.16	0.93	11	16	negative regulation of amine transport	BP	1	ARL6IP5,ADRA2B,SLC43A1,P2RY1,ADRA2A,ADRA2C,SYT11,ABAT,CHGA,RGS4,CRH	grey	VSMC_M10
GO:0140056	0.16	0.89	40	67	organelle localization by membrane tethering	BP	1	STX7,TSNARE1,EXOC4,SCFD2,STX17,PLEK,BVES,PPFIA3,RAB10,SYTL2,RALB,SYT1,CEP290,STX2,SNPH,VPS18,KCNB1,PDZD8,STX1A,STX6,NSF,BLOC1S6,MOSPD3,RAB3A,SNAP29,STX5,RAB8A,PSEN2,RAB15,STX1B,STX4,RAB8B,AC138894.1,STX8,RAB3D,CALM1,VMP1,STXBP2,STXBP1,VAMP3	grey	VSMC_M10
GO:0007610	0.17	0.86	225	406	behavior	BP	1	MAPK8IP2,ADM2,PRRT1,PRKN,AGER,PPARA,TSPO,SYNGAP1,MCHR1,AGTPBP1,NTRK2,PLA2G6,BTBD9,CHRNA3,CHRNA5,GPR37,SRF,TTBK1,ADAM22,CFAP69,APBA1,CNTFR,USP46,KIT,SLC1A1,ADGRF1,ETV1,TMOD1,BRSK1,PRKCE,DNAH11,SHANK1,PPT1,MYH14,PTN,FOXP2,EGFR,SOBP,SLC7A11,NTF4,FIG4,DDO,PRKAR2B,GRIN2D,HDAC2,ADCY3,SLC8A2,NAPEPLD,B4GALT2,DACH1,POU4F1,ALK,CHD8,MKKS,RPTOR,NR1D2,PLCB1,MAFG,PRNP,DDHD2,P2RX2,NRG1,ARL6IP5,TCF15,PLK2,NPHP1,LGI4,GPI,CAMK4,AHCY,PREX2,CASP3,NCOA2,HAND2,P2RX3,MEF2C,ASIC1,HTRA2,CFAP20,CUX2,P2RY1,MME,EIF2AK4,SYNJ1,HOMER1,AMFR,NR4A2,NQO2,GLS,SLC12A5,ATXN1,GRM2,BDNF,SLC4A10,SRD5A1,SLC6A3,ALDH1A3,SORCS3,CPT1A,ADARB1,SCN2A,NLGN4Y,KRAS,GIGYF2,ADNP,TTC21B,ZFHX2,OPRD1,CIART,USP2,AGT,ACE2,ATP1A2,SPTBN2,NFATC4,HDAC4,KCNK2,NPAS4,NTSR1,EPS8,YTHDF1,FZD9,HOMER2,C3orf70,SLC16A1,CREB1,TRMT1L,SPG11,MAPT,ABL2,C1QL1,EIF4EBP2,ZDHHC8,CDH23,COMT,ANKH,TNR,NRXN2,PYY,KCNK4,BCHE,CRTC1,HTT,PPP3CB,GDF15,STAT3,NPTX2,MAPK1,TMOD2,ASIC4,EPHA4,DLG4,SLC2A4,THRA,SDK1,NLGN2,CHRNB1,CLN8,RGS14,ATP1B2,PSEN1,HOXB8,SYT11,ITGA3,NLGN3,FOS,AC138894.1,CHRNB2,UBE2Q1,CLN6,LDLR,GPRASP2,BHLHB9,NMUR2,STRA6,GM2A,NCOR1,INSR,NPC1,SGIP1,LEPR,IFT20,SEZ6,JUN,DAB1,ABAT,AFF2,VWA1,TRPV1,PRKCZ,MECP2,ABCA7,PER3,PARK7,TPGS1,GPR157,TSC1,JPH3,CNTNAP4,STRBP,ZFHX3,MCOLN3,LHX8,HTR1D,ATXN1L,CNTN2,FADD,DPP4,NPB,CEND1,FXN,CRBN,POMK,CRH,GPR88,NRXN3,APH1B,EFNB3,CEBPB,PITX3,MRAP2,MAP1A,FOXO6,DERL2,RASD2	grey	VSMC_M10
GO:0002501	0.17	0.97	5	6	peptide antigen assembly with MHC protein complex	BP	1	HLA-DRA,HLA-DOB,HLA-DMA,HLA-DPB1,TAPBPL	grey	VSMC_M10
GO:0006228	0.17	0.97	5	6	UTP biosynthetic process	BP	1	NME1,NME4,NME7,NME3,NME6	grey	VSMC_M10
GO:0080009	0.17	0.93	11	16	mRNA methylation	BP	1	CMTR1,TRMT6,WTAP,METTL8,METTL16,CMTR2,METTL14,TRMT61A,TRMT10C,TRMT61B,CBLL1	grey	VSMC_M10
GO:0006165	0.17	0.89	43	72	nucleoside diphosphate phosphorylation	BP	1	NME1,PPARA,PRXL2C,GAPDH,SLC4A4,BPGM,CMPK1,DHTKD1,PRKAG2,PFKP,GPI,GPD1,P2RX7,PRKAG1,PFKM,NME4,ZBTB20,PFKFB2,ENO4,ARNT,ACTN3,HDAC4,MLXIPL,DTYMK,HK1,PRKAG3,STAT3,NME7,ENO3,PSEN1,PKLR,OGT,ADPGK,NCOR1,INSR,ALDOC,NME3,SLC2A6,AK8,ENTPD5,CMPK2,TIGAR,NME6	grey	VSMC_M10
GO:0009249	0.17	0.97	5	6	protein lipoylation	BP	1	LIPT1,NNAT,LIAS,NDUFAB1,GCSH	grey	VSMC_M10
GO:0010692	0.17	0.96	6	8	regulation of alkaline phosphatase activity	BP	1	SEMA4D,MEF2C,GPLD1,TGFB2,SMAD3,PPARGC1B	grey	VSMC_M10
GO:0048247	0.17	0.91	21	33	lymphocyte chemotaxis	BP	1	SLC12A2,CCL27,OXSR1,GAS6,CYP7B1,PTK2B,CCL17,SLC8B1,ADAM8,CH25H,PADI2,CXCL16,TMEM102,CCL14,CCL5,CCL7,CCL2,CCL25,CCL16,WNT5A,CCL22	grey	VSMC_M10
GO:0099633	0.17	0.95	8	11	protein localization to postsynaptic specialization membrane	BP	1	ADAM22,DAG1,DLG1,DLG4,IQSEC2,PRKCZ,CACNG7,HRAS	grey	VSMC_M10
GO:0099645	0.17	0.95	8	11	neurotransmitter receptor localization to postsynaptic specialization membrane	BP	1	ADAM22,DAG1,DLG1,DLG4,IQSEC2,PRKCZ,CACNG7,HRAS	grey	VSMC_M10
GO:0046710	0.17	0.95	8	11	GDP metabolic process	BP	1	DLG2,ENTPD4,NUDT18,MAGI3,DLG1,CASK,CARD11,MPP1	grey	VSMC_M10
GO:0015801	0.17	0.95	8	11	aromatic amino acid transport	BP	1	SLC7A1,OCA2,SLC38A3,ACE2,SLC38A5,SLC3A2,SLC7A5,SLC25A29	grey	VSMC_M10
GO:0009437	0.17	0.95	8	11	carnitine metabolic process	BP	1	CPT1B,SLC22A4,CROT,CPT1A,POR,ACADL,TMLHE,ACADM	grey	VSMC_M10
GO:1905809	0.17	0.96	6	8	negative regulation of synapse organization	BP	1	EPHA7,PTN,ROBO2,SLIT1,NFATC4,WNT5A	grey	VSMC_M10
GO:1903707	0.17	0.88	43	72	negative regulation of hemopoiesis	BP	1	NME1,TRIB1,MYC,TOB2,FBXO7,CDK6,HOXA7,TLR4,FOXJ1,ZC3H8,PIK3R1,FBXW7,TMEM131L,GPR137B,PIAS3,HLX,TCTA,UBASH3B,RUNX3,ZBTB46,BCL6,RC3H1,GPR137,FBN1,TNFSF18,LAG3,INHA,FOXP3,TBX21,ZBTB7B,IL4R,GPR68,CD74,PTPN2,SOCS1,MIR21,MIR222,MIR125B1,FSTL3,MAFB,FGL2,IL17D,RC3H2	grey	VSMC_M10
GO:0048708	0.17	0.89	36	60	astrocyte differentiation	BP	1	LRP1,AGER,SMO,TTBK1,CDK6,ROR2,FPR2,EGFR,TLR4,MYCN,C5AR1,SOX9,KDM4A,TSPAN2,KRAS,TTC21B,MAPT,STAT3,MAPK1,EPHA4,PRPF19,PSEN1,MAPK3,MAP2K1,LDLR,S100A8,MBD1,DAB1,SOX8,LAMC3,CNTN2,CNTF,GPR37L1,NOG,POU3F2,LIF	grey	VSMC_M10
GO:0007612	0.17	0.87	60	104	learning	BP	1	MAPK8IP2,PRKN,SYNGAP1,NTRK2,SRF,KIT,SLC1A1,BRSK1,SHANK1,PPT1,PTN,SLC7A11,PRKAR2B,ADCY3,SLC8A2,B4GALT2,DDHD2,ASIC1,EIF2AK4,SYNJ1,SLC12A5,ATXN1,SORCS3,NLGN4Y,KRAS,AGT,ATP1A2,NPAS4,NTSR1,YTHDF1,CREB1,C1QL1,COMT,TNR,NRXN2,BCHE,HTT,PPP3CB,NPTX2,DLG4,CLN8,RGS14,SYT11,NLGN3,FOS,AC138894.1,CHRNB2,STRA6,INSR,IFT20,JUN,MECP2,ABCA7,JPH3,ATXN1L,CNTN2,CRH,GPR88,NRXN3,MAP1A	grey	VSMC_M10

GO:0016458	0.17	0.87	98	169	gene silencing	BP	1	ZC3H10,BAZ2A,ESR1,CNOT2,CNOT6,PABPC1,MOV10L1,AGO2,H3F3C,TNRC6B,TUT7,XPO5,CNOT11,SCMH1,PUS10,SNIP1,AGO1,EGFR,MYCN,TUT4,PUM2,DNMT3A,SETDB2,TNRC6C,SIRT2,TEX15,CLP1,FAM208A,BCDIN3D,CNOT1,CELF1,CNOT6L,EIF4E2,BAHD1,RIPK1,EXD1,PPHLN1,HDAC1,PPP3CA,TERT,AJUBA,ZNFX1,GIGYF2,DDX6,TDRKH,NCBP2,EIF4ENIF1,TET1,PRKRA,DDX3X,STAT3,PHF8,MAP2K1,SMAD3,NRDE2,TNRC6A,CNOT8,NCOR1,SMCHD1,DROSHA,FMR1,CHMP1A,SPOUT1,MIRLET7A2,MIR29A,MIR199A1,MIR21,MIR222,MIR23A,MIR425,MIR125B1,MIR32,MIR22,MIR27B,MIR24-1,MIR7-1,MIR199A2,MIRLET7D,MIR27A,MIR23B,MIR365B,MIR25,MIR491,MIR24-2,MIRLET7A3,SETDB1,AGO4,MIR33B,MIR339,MIR125A,MIR99B,MIR940,MIR1249,MIR1180,BEND3,HIST1H3F,HIST1H3H,ZNF304	grey	VSMC_M10
GO:0060900	0.17	0.96	6	8	embryonic camera-type eye formation	BP	1	FRS2,TFAP2A,PAX2,ALDH1A3,STRA6,WNT5A	grey	VSMC_M10
GO:0002385	0.17	0.94	8	11	mucosal immune response	BP	1	NOD2,OTUD7B,RAB17,NOS2,HIST1H2BK,IFNLR1,HIST1H2BF,HIST1H2BJ	grey	VSMC_M10
GO:0051446	0.17	0.93	11	16	positive regulation of meiotic cell cycle	BP	1	YTHDC2,UBE2B,MAPK15,TOPAZ1,SIRT2,PLCB1,NPM2,OVOL1,WNT4,OOEP,WNT5A	grey	VSMC_M10
GO:0009451	0.17	0.87	82	141	RNA modification	BP	1	RNMT,ADAT2,TFB1M,TRMU,TRMT11,APOBEC3H,C9orf64,CMTR1,MTO1,ELP1,PUS10,TRIT1,DUS4L,NSUN6,THUMPD3,DPH3,TRMT6,BAG4,NUDT16,WTAP,THUMPD2,ALKBH3,TRMT9B,TGS1,DIMT1,ELP4,ELP3,BCDIN3D,TPRKB,NSUN3,RPUSD2,OSGEPL1,FTO,PUS7L,CDKAL1,RPUSD4,TRMT10A,QTRT2,TYW1B,TYW5,ADARB1,GAR1,TRUB1,DALRD3,LCMT2,METTL8,ELP6,NSUN5,METTL2B,TRMT1L,MEPCE,ALKBH8,TRMT5,HSD17B10,MRM2,ZCCHC4,MTFMT,ALKBH1,THG1L,QTRT1,ELP2,THUMPD1,ALKBH5,METTL4,METTL6,PARN,MRM3,PUSL1,METTL16,RPUSD1,AARS,CMTR2,DUS2,CTU1,METTL14,TRMT10B,TRMT61A,RPUSD3,TRMT10C,TRMT61B,CBLL1,TYW3	grey	VSMC_M10
GO:0006490	0.17	0.93	11	16	oligosaccharide-lipid intermediate biosynthetic process	BP	1	ALG12,SRD5A3,ALG2,ALG11,ALG10,DPAGT1,ALG3,ALG9,ALG6,ALG1,DHDDS	grey	VSMC_M10
GO:0018345	0.17	0.91	18	28	protein palmitoylation	BP	1	ZDHHC14,ZDHHC21,ZDHHC20,GOLGA7B,ZDHHC13,ZDHHC23,ZDHHC6,HHAT,ZDHHC18,ZDHHC24,GLUL,ZDHHC8,ZDHHC4,ZDHHC15,ZDHHC12,ZDHHC1,ZDHHC11,MAP6D1	grey	VSMC_M10
GO:0050768	0.17	0.87	59	102	negative regulation of neurogenesis	BP	1	SEMA6A,TSPO,FSTL4,SYNGAP1,SOX10,DAAM2,SEMA4D,SEMA4C,RTN4,RUFY3,EPHA7,PTN,MYCN,KDM4A,HOOK3,SIRT2,SEMA3D,SPART,SEMA3A,ULK1,RYK,SEMA4F,TLX2,RAPGEF2,NKX6-1,SEMA3G,SPP1,SEMA4G,RNF10,KIAA0319,PPP3CA,SEMA3F,SORL1,CERS2,NFATC4,DLX1,WNT3,DUSP10,SEMA6D,TNR,BMPR1A,PSEN1,SEMA4A,TRIM46,NTN1,LDLR,CDK5R1,MBD1,DAB1,SKI,ARHGAP4,PLXNA3,GPR37L1,NOG,THY1,WNT5A,YWHAH,EFNB3,PITX3	grey	VSMC_M10
GO:0009199	0.17	0.88	39	64	ribonucleoside triphosphate metabolic process	BP	1	NME1,ATP5MGL,PPARA,GTPBP1,IMPDH1,SLC25A13,RAB23,SPHK2,PRKAG2,ATP5F1C,ATP5F1A,DGUOK,OPA1,ENTPD4,FAM173B,NME4,MFN1,CTPS2,DNAJC30,EFL1,ATP5S,PINK1,STAT3,NME7,PARP1,PKLR,ATP6V0C,NME3,ATP5PB,VPS9D1,GNAI3,UCK1,MT-ATP8,MT-ATP6,RHOA,ATP5ME,ATP5MF,NME6,NUDT2	grey	VSMC_M10
GO:0031065	0.17	0.92	12	18	positive regulation of histone deacetylation	BP	1	C6orf89,PRKD2,SDR16C5,ING2,BCL6,PINK1,AKAP8L,AKAP8,JDP2,ZBTB7B,SREBF1,SAP30BP	grey	VSMC_M10
GO:0003009	0.17	0.90	19	30	skeletal muscle contraction	BP	1	STAC3,TNNI3,MYH14,STAC,NR4A1,HOMER1,TNNC1,KCNJ2,TNNI1,SYNM,MYH7,CASQ1,ACTN3,CHRNB1,TCAP,STAC2,ATP2A1,DMD,TNNC2	grey	VSMC_M10
GO:0018095	0.18	0.94	8	11	protein polyglutamylation	BP	1	CFAP20,TTLL13P,TTLL4,TTLL5,TPGS2,TPGS1,TTLL11,TTLL7	grey	VSMC_M10
GO:0048243	0.18	0.94	8	11	norepinephrine secretion	BP	1	ADRA2B,P2RY1,ADRA2A,KCNB1,AGT,STX1A,ADRA2C,CRH	grey	VSMC_M10
GO:0051561	0.18	0.94	8	11	positive regulation of mitochondrial calcium ion concentration	BP	1	MCUR1,RAP1GDS1,FIS1,MICU1,MCU,ATP2A1,SLC25A23,BCAP31	grey	VSMC_M10
GO:0015872	0.18	0.91	18	28	dopamine transport	BP	1	PRKN,MAPK15,SYT1,SYT9,CHRM5,SLC6A2,DTNBP1,SYT8,GRM2,SLC6A3,RAB3A,PINK1,SLC29A4,SYT7,SYT11,CHRNB2,ABAT,PARK7	grey	VSMC_M10
GO:0021801	0.18	0.92	12	18	cerebral cortex radial glia-guided migration	BP	1	SUN2,MBOAT7,RTN4,SRGAP2C,DISC1,CDK5R1,DAB1,LRP8,DAB2IP,ADGRG1,FOXG1,LAMB1	grey	VSMC_M10
GO:0022030	0.18	0.92	12	18	telencephalon glial cell migration	BP	1	SUN2,MBOAT7,RTN4,SRGAP2C,DISC1,CDK5R1,DAB1,LRP8,DAB2IP,ADGRG1,FOXG1,LAMB1	grey	VSMC_M10
GO:0002705	0.18	0.88	45	76	positive regulation of leukocyte mediated immunity	BP	1	CD226,STX7,HLA-DRA,KIT,EXOSC3,MSH2,RSAD2,HMCES,NECTIN2,PVR,MLH1,SLC22A13,PAXIP1,SHLD1,MAVS,HPX,TRAF6,RASGRP1,F2RL1,NOD2,CD40,DENND1B,CD28,CADM1,FZD5,IL12A,LAG3,STAT5B,FOXP3,ZBTB1,SHLD2,TBX21,STX4,ITGAM,BTK,FBXO38,VAV1,C3,NOS2,TICAM1,PRKCZ,TRAF2,AP1G1,FADD,FCER2	grey	VSMC_M10
GO:0006907	0.18	0.92	12	18	pinocytosis	BP	1	PPT1,NR1H2,SNX5,NR1H3,MAPKAPK3,CARMIL1,MAPKAPK2,ANKFY1,STX1B,DOCK2,AC138894.1,DNM2	grey	VSMC_M10
GO:0061098	0.18	0.89	27	44	positive regulation of protein tyrosine kinase activity	BP	1	ERBB3,EPGN,AREG,TGFA,ABI1,HBEGF,PRNP,NRG1,GAS6,ERCC6,FBXW7,NEURL1,ADRA2A,NCF1,EGF,CASS4,AGT,DOK7,EPHA4,DLG4,DVL2,EFNA1,CCL5,CSF1R,UNC119,LRP8,EREG	grey	VSMC_M10
GO:0009062	0.18	0.87	51	86	fatty acid catabolic process	BP	1	HSD17B4,AIG1,CPT1B,PPARA,MCAT,ECHDC1,CROT,ETFB,LPIN1,FAAH,HADHA,ACOX1,AKT2,HACL1,ACOXL,ACOX2,PEX2,ETFDH,IVD,LPIN3,ECI2,ADTRP,LONP2,MCEE,IRS1,CPT1A,BDH2,ABHD2,ABCB11,EHHADH,ACADL,TYSND1,ACADVL,HSD17B10,ABCD4,GCDH,ABHD3,LPIN2,ACAA2,DECR2,CEL,MLYCD,ABCD3,PLA2G15,ACADM,PHYH,HADHB,AMACR,NUDT19,ECI1,AUH	grey	VSMC_M10

GO:1901184	0.18	0.88	40	67	regulation of ERBB signaling pathway	BP	1	RTN4,ACP4,EPGN,AREG,TGFA,EGFR,RAB7A,NEU3,SNX5,HBEGF,PDE6G,FER,HIP1R,FBXW7,PTPRJ,WDR54,NEURL1,ADRA2A,RNF115,NCF1,EGF,GPER1,DGKD,IFI6,AGT,CBL,PDE6H,SNX6,ZFYVE28,SOCS4,HAP1,RBPJ,PSEN1,PTPN2,RNF126,MVB12B,DAB2IP,MIR29A,MIR21,EREG	grey	VSMC_M10
GO:0048499	0.18	0.91	15	23	synaptic vesicle membrane organization	BP	1	SYNGR1,GRIK5,SYT1,STX2,SYT9,SNAP23,STX1A,RAB3A,SNAP29,CPLX1,STX1B,STX4,SNAPIN,STXBP1,SNAP47	grey	VSMC_M10
GO:0048312	0.18	0.95	6	8	intracellular distribution of mitochondria	BP	1	ARMC1,OPA1,DNM1L,MAPT,BHLHA15,CLUH	grey	VSMC_M10
GO:2000311	0.18	0.92	12	18	regulation of AMPA receptor activity	BP	1	MAPK8IP2,PRRT1,SHANK1,MEF2C,CNIH2,MINK1,NLGN2,CNIH3,NLGN3,CACNG7,SHISA8,CACNG5	grey	VSMC_M10
GO:0044275	0.18	0.90	19	30	cellular carbohydrate catabolic process	BP	1	MIOX,MGAM,PYGB,PPP1CB,PPP1R3B,GK5,PFKM,PPP1R3D,TREH,SORD,PYGM,PYGL,TKFC,ENOSF1,AGL,ABHD10,TIGAR,IDNK,NEU2	grey	VSMC_M10
GO:0009072	0.18	0.92	11	16	aromatic amino acid family metabolic process	BP	1	TPH2,IL4I1,HPDL,IDO2,IDO1,HGD,FAH,GCDH,GSTZ1,PARK7,PCBD2	grey	VSMC_M10
GO:0015696	0.18	0.95	6	8	ammonium transport	BP	1	SLC12A2,AQP1,SLC12A7,RHCG,RHBG,SLC12A4	grey	VSMC_M10
GO:0072488	0.18	0.95	6	8	ammonium transmembrane transport	BP	1	SLC12A2,AQP1,SLC12A7,RHCG,RHBG,SLC12A4	grey	VSMC_M10
GO:0051961	0.18	0.87	61	106	negative regulation of nervous system development	BP	1	SEMA6A,TSPO,FSTL4,SYNGAP1,SOX10,DAAM2,SEMA4D,SEMA4C,RTN4,RUFY3,EPHA7,PTN,MYCN,KDM4A,HOOK3,SIRT2,SEMA3D,SPART,SEMA3A,ULK1,RYK,ROBO2,SEMA4F,TLX2,RAPGEF2,SLIT1,NKX6-1,SEMA3G,SPP1,SEMA4G,RNF10,KIAA0319,PPP3CA,SEMA3F,SORL1,CERS2,NFATC4,DLX1,WNT3,DUSP10,SEMA6D,TNR,BMPR1A,PSEN1,SEMA4A,TRIM46,NTN1,LDLR,CDK5R1,MBD1,DAB1,SKI,ARHGAP4,PLXNA3,GPR37L1,NOG,THY1,WNT5A,YWHAH,EFNB3,PITX3	grey	VSMC_M10
GO:0072329	0.18	0.87	56	95	monocarboxylic acid catabolic process	BP	1	HSD17B4,AIG1,CPT1B,PPARA,MCAT,ECHDC1,CROT,ETFB,LPIN1,FAAH,HADHA,AKR1A1,ACOX1,XYLB,AKT2,HACL1,ACOXL,ACOX2,PEX2,ETFDH,IVD,LPIN3,HOGA1,ECI2,ADTRP,LONP2,MCEE,IRS1,CPT1A,BDH2,ABHD2,ABCB11,EHHADH,ACADL,FAH,TYSND1,ACADVL,HSD17B10,ABCD4,GCDH,ABHD3,LPIN2,ACAA2,DECR2,CEL,MLYCD,ABCD3,PLA2G15,ACADM,PHYH,HADHB,AMACR,NUDT19,IDNK,ECI1,AUH	grey	VSMC_M10
GO:0007613	0.18	0.87	50	86	memory	BP	1	PLA2G6,BTBD9,SRF,SLC1A1,ADGRF1,SHANK1,PTN,NTF4,SLC8A2,B4GALT2,PLCB1,PRNP,PLK2,CAMK4,ASIC1,CUX2,EIF2AK4,NQO2,ATXN1,BDNF,SORCS3,SCN2A,ADNP,NFATC4,KCNK2,NPAS4,YTHDF1,CREB1,MAPT,EIF4EBP2,COMT,KCNK4,CRTC1,PPP3CB,SLC2A4,RGS14,PSEN1,SYT11,ITGA3,CHRNB2,LDLR,INSR,PRKCZ,MECP2,ABCA7,JPH3,ATXN1L,CEBPB,MAP1A,FOXO6	grey	VSMC_M10
GO:1900452	0.18	0.95	6	8	regulation of long-term synaptic depression	BP	1	AGER,SORCS3,KCNB1,MAPT,FMR1,GRID2	grey	VSMC_M10
GO:0021795	0.18	0.90	20	32	cerebral cortex cell migration	BP	1	SUN2,EFHC1,MBOAT7,RTN4,POU3F3,ROBO1,SRGAP2C,DISC1,DIXDC1,PSEN1,NDEL1,CDK5R1,DAB1,LRP8,DAB2IP,RHOA,ADGRG1,FOXG1,LAMB1,POU3F2	grey	VSMC_M10
GO:0030641	0.18	0.88	38	64	regulation of cellular pH	BP	1	SLC4A4,PPT1,RAB7A,ATP6V0E2,LACC1,SLC4A2,AQP11,SLC4A7,TMEM106B,MAFG,ATP6V1H,TM9SF4,SLC9A9,HVCN1,SLC4A10,SLC9B1,TMEM9,TCIRG1,SLC9A8,SLAMF8,SLC9A1,CLIC4,SLC11A1,SLC9A7,ATP6V0A1,MAPK1,DMXL2,SLC4A3,MAPK3,AC138894.1,CLN6,SNAPIN,TMEM199,SLC9A6,ATP6AP1,SLC9A5,CA2,RAB39A	grey	VSMC_M10
GO:0051127	0.18	0.93	9	13	positive regulation of actin nucleation	BP	1	WASF1,FCHSD2,FMN1,CYFIP1,MAGEL2,WASF2,WHAMM,NCKAP1,WAS	grey	VSMC_M10
GO:0002495	0.18	0.92	11	16	antigen processing and presentation of peptide antigen via MHC class II	BP	1	HLA-DRA,HLA-DOB,HLA-DMA,HLA-DPB1,TRAF6,MARCH8,FCER1G,PIKFYVE,CTSV,DNM2,CD74	grey	VSMC_M10
GO:0003266	0.18	0.95	6	8	regulation of secondary heart field cardioblast proliferation	BP	1	HAND2,EYA1,TBX5,MKS1,ISL1,SIX1	grey	VSMC_M10
GO:0035912	0.18	0.95	6	8	dorsal aorta morphogenesis	BP	1	SRF,BMPR1A,RBPJ,ENG,ACVRL1,HEY2	grey	VSMC_M10
GO:1903351	0.18	0.88	33	55	cellular response to dopamine	BP	1	PRKN,ATF4,SLC1A1,VPS35,HDAC2,ALK,HTR7,CHRM5,CHRM4,DTNBP1,CHRM3,CALY,HTR3B,MAPK1,CHRM1,GNB5,NSG1,HRH2,HCN3,MAPK3,DNM2,SIN3A,HRH4,GNAL,GNB1,GNA15,PALM,GNAI3,HTR1D,RGS4,GNA14,GNG2,ARPP19	grey	VSMC_M10
GO:0060213	0.18	0.93	9	13	positive regulation of nuclear-transcribed mRNA poly(A) tail shortening	BP	1	PABPC1,AGO2,TNRC6B,TNRC6C,CNOT1,EIF4ENIF1,POLR2G,TOB1,TNRC6A	grey	VSMC_M10
GO:0010807	0.18	0.95	6	8	regulation of synaptic vesicle priming	BP	1	NAPA,NAPB,STX1A,RAB3A,STX1B,STXBP1	grey	VSMC_M10
GO:0009311	0.18	0.89	27	44	oligosaccharide metabolic process	BP	1	ST6GAL2,MGAM,BRAF,NEU3,ST8SIA4,MOGS,ST3GAL6,GANC,ST8SIA2,B3GALT1,TREH,GAL3ST3,MGAT2,B3GALNT1,GAL3ST4,IDUA,ST8SIA5,GLA,GM2A,ST6GALNAC4,ST3GAL2,CTBS,ST6GALNAC5,FBP2,FUT4,NEU2,ST8SIA6	grey	VSMC_M10
GO:1902106	0.18	0.87	41	69	negative regulation of leukocyte differentiation	BP	1	NME1,TRIB1,MYC,TOB2,FBXO7,CDK6,HOXA7,TLR4,FOXJ1,ZC3H8,PIK3R1,FBXW7,TMEM131L,GPR137B,PIAS3,HLX,TCTA,UBASH3B,RUNX3,ZBTB46,BCL6,RC3H1,GPR137,FBN1,TNFSF18,LAG3,INHA,FOXP3,TBX21,ZBTB7B,IL4R,GPR68,CD74,PTPN2,SOCS1,MIR21,MIR125B1,FSTL3,MAFB,FGL2,RC3H2	grey	VSMC_M10
GO:0072176	0.18	0.93	9	13	nephric duct development	BP	1	EPHA7,OSR1,WNT11,PKD2,PAX2,EPHA4,LHX1,GPC3,PKD1	grey	VSMC_M10
GO:0007215	0.18	0.89	21	34	glutamate receptor signaling pathway	BP	1	GRM4,GRM8,SLC1A1,GRIK2,GRIN2D,GRIK5,PLCB1,PRNP,PTK2B,TIAM1,HOMER1,GRM2,TRPM1,KCNB1,GRIK4,HOMER2,DAGLB,AC138894.1,CDK5R1,FMR1,GRID2	grey	VSMC_M10
GO:0000820	0.19	0.95	6	8	regulation of glutamine family amino acid metabolic process	BP	1	SLC7A11,NR1H4,SIRT4,SLC7A7,AC138894.1,MIR21	grey	VSMC_M10
GO:0030890	0.19	0.90	19	30	positive regulation of B cell proliferation	BP	1	IL5,TNFRSF13C,PELI1,TLR4,BMI1,NCKAP1L,MEF2C,IL7,CD40,BCL6,CD38,BST1,CARD11,CHRNB2,CD74,CD320,TICAM1,VAV3,TNFSF13B	grey	VSMC_M10
GO:0014848	0.19	0.95	6	8	urinary tract smooth muscle contraction	BP	1	PLA2G6,P2RX2,P2RX3,KCNMA1,TBX2,TRPV1	grey	VSMC_M10
GO:0046341	0.19	0.93	9	13	CDP-diacylglycerol metabolic process	BP	1	AGPAT4,AGPAT1,TAMM41,CDS2,CDS1,GPAM,AGPAT2,LCLAT1,CDIPT	grey	VSMC_M10
GO:0000272	0.19	0.93	9	13	polysaccharide catabolic process	BP	1	MGAM,PYGB,PPP1CB,PPP1R3B,PFKM,PPP1R3D,PYGM,PYGL,AGL	grey	VSMC_M10
GO:0009251	0.19	0.93	9	13	glucan catabolic process	BP	1	MGAM,PYGB,PPP1CB,PPP1R3B,PFKM,PPP1R3D,PYGM,PYGL,AGL	grey	VSMC_M10

GO:0044247	0.19	0.93	9	13	cellular polysaccharide catabolic process	BP	1	MGAM,PYGB,PPP1CB,PPP1R3B,PFKM,PPP1R3D,PYGM,PYGL,AGL	grey	VSMC_M10
GO:0046530	0.19	0.89	25	41	photoreceptor cell differentiation	BP	1	AGTPBP1,NTRK2,PRPH2,RORB,RDH13,PTN,SOX9,MYO7A,DZANK1,FSCN2,CEP290,USH1C,C8orf37,RPGRIP1L,NKD1,NRL,DLX1,STAT3,ROM1,IFT20,SOX8,OLFM3,CNTF,THY1,DIO3	grey	VSMC_M10
GO:0002753	0.19	0.88	32	53	cytoplasmic pattern recognition receptor signaling pathway	BP	1	TNFAIP3,USP15,HSPA1B,ANKRD17,SLC46A2,TLR4,PUM2,PHB,LACC1,SEC14L1,CLPB,NPLOC4,IRF7,TRAF6,OAS3,RIPK2,OTULIN,NOD2,IFIH1,OASL,MAP2K6,TIFA,ALPK1,NLRX1,BIRC3,TKFC,TSPAN6,RIOK3,RNF135,ZCCHC3,IRGM,INAVA	grey	VSMC_M10
GO:0042796	0.19	0.95	6	8	snRNA transcription by RNA polymerase III	BP	1	SNAPC3,ZC3H8,ELL,ICE2,SNAPC5,SNAPC4	grey	VSMC_M10
GO:0015701	0.19	0.91	13	20	bicarbonate transport	BP	1	SLC26A10,SLC4A4,CYB5R4,SLC26A5,SLC4A2,SLC4A7,SLC4A10,CYB5R1,SLC4A3,SLC26A1,CA4,SLC26A2,CYB5RL	grey	VSMC_M10
GO:0001911	0.19	0.95	6	8	negative regulation of leukocyte mediated cytotoxicity	BP	1	NCKAP1L,PPP3CB,IL7R,CRK,SERPINB9,HAVCR2	grey	VSMC_M10
GO:0090037	0.19	0.95	6	8	positive regulation of protein kinase C signaling	BP	1	PLA2G6,SPHK2,WNT11,ADGRV1,CD40,WNT5A	grey	VSMC_M10
GO:0035561	0.19	0.91	13	20	regulation of chromatin binding	BP	1	ZNF618,MED25,FBH1,BTAF1,HMBOX1,EBF2,PPP3CA,PARP9,DTX3L,NFATC4,MEPCE,DDX11,ZNF304	grey	VSMC_M10
GO:0036230	0.19	0.90	18	28	granulocyte activation	BP	1	ENPP3,CXCL8,IL15,TRAF3IP2,MYD88,F2RL1,FCER1G,CXCR2,STX4,ITGAM,SCNN1B,CCL5,STXBP2,PRAM1,DNASE1,KARS,CXCL6,MIR125A	grey	VSMC_M10
GO:0035865	0.19	0.95	6	8	cellular response to potassium ion	BP	1	SLC12A2,HSF1,DLG2,CARF,DLG4,CACNA1H	grey	VSMC_M10
GO:0072033	0.19	0.95	6	8	renal vesicle formation	BP	1	SMO,SOX9,FMN1,SALL1,WNT4,SOX8	grey	VSMC_M10
GO:0034315	0.19	0.91	12	18	regulation of Arp2/3 complex-mediated actin nucleation	BP	1	PICK1,WASF1,FCHSD2,ARFIP2,HIP1R,CYFIP1,AP1AR,WASF2,NCKAP1,WAS,WDR63,GMFG	grey	VSMC_M10
GO:0072077	0.19	0.91	12	18	renal vesicle morphogenesis	BP	1	SMO,SIX2,SOX9,PAX8,FMN1,PAX2,SALL1,WNT4,CITED1,LHX1,SOX8,LIF	grey	VSMC_M10
GO:0015849	0.19	0.85	115	203	organic acid transport	BP	1	ATP8B1,SLC26A10,SLC16A7,CPT1B,SLC12A2,SLC27A6,PPARA,SLC22A4,NTRK2,PLA2G6,PLIN2,CROT,ABCB1,APBA1,SLC1A1,SLC25A13,SLC3A1,SLC1A4,SLC7A11,SLC26A5,SLC25A30,LLGL2,SLC22A13,SLC25A38,AKT2,ABCC4,SLC38A10,SLC6A15,SLC25A15,SLC23A2,ARL6IP5,SLC7A1,SLC38A9,SLC5A8,CYP7A1,SLC43A1,SERINC5,SLC38A4,SLC38A2,SLC38A1,SLC10A6,ABCC2,PLA2R1,ABCG2,OCA2,GRM2,SFXN2,SLC38A11,SLC38A3,CPT1A,MAP2K6,ACSL5,SLC7A7,FABP4,ABCB11,AGT,SLC25A20,ABCC5,ACE2,ATP1A2,SLCO1A2,SLCO1C1,PER2,NTSR1,SLC16A1,PLA2G4A,FIS1,SLC11A1,PQLC2,SLC27A1,SLC38A5,MPC2,ARL6IP1,SLC3A2,SLC38A6,PRAF2,SLC25A11,SLC29A4,CLN8,SLC26A1,PSEN1,SLC25A44,SLC7A3,ABCD4,AC138894.1,ABCC3,NMUR2,SLC25A47,SLC6A7,SLC26A2,LRRC8E,EMB,SLC46A1,SLC25A14,PROCA1,ABAT,SLC1A7,TRPV1,SLC7A5,LRRC8A,ABCD3,STXBP1,SLC7A6,FABP5,SLC36A1,LRRC8D,SLC6A19,SLC25A45,XK,PLA2G2C,SLC25A21,SLC25A32,RGS4,SLC25A29,PTGES	grey	VSMC_M10
GO:0006497	0.19	0.87	48	81	protein lipidation	BP	1	ATG12,ZDHHC14,GPAA1,ZDHHC21,CWH43,TMEM246,PIGF,ATG5,PGAP2,ZDHHC20,PIGU,ATG10,GOLGA7B,ZDHHC13,PIGT,GPLD1,PGAP1,ZDHHC23,ZDHHC6,RABL3,ATG16L1,PIGM,HHAT,ZDHHC18,PIGV,ZDHHC24,PIGX,GLUL,PIGZ,NMT1,ZDHHC8,PIGC,PPM1A,ZDHHC4,WIPI2,PGAP3,PIGH,ZDHHC15,PIGL,MPPE1,ZDHHC12,ZDHHC1,ZDHHC11,IRGM,PYURF,PIGB,PIGA,MAP6D1	grey	VSMC_M10
GO:0070571	0.19	0.93	9	13	negative regulation of neuron projection regeneration	BP	1	SPP1,KIAA0319,KREMEN1,CERS2,LRIG2,TNR,EPHA4,RTN4RL1,THY1	grey	VSMC_M10
GO:0006672	0.19	0.87	46	78	ceramide metabolic process	BP	1	ORMDL2,B4GALNT1,PLA2G6,SPTLC1,PPP2R1A,SMPD2,AGK,SPHK2,ACER3,ELOVL1,NEU3,SPTLC3,SIRT3,NSMAF,ASAH1,ST8SIA4,HTRA2,ORMDL1,COL4A3BP,ST8SIA2,SGMS2,B3GALT1,CERS6,CERS2,ABCA12,SMPD4,SGMS1,CLN8,DEGS1,ALOXE3,GALC,CLN6,GLA,HEXA,GM2A,P2RX1,CEL,ST6GALNAC4,ST3GAL2,PLA2G15,ST6GALNAC5,CYP4F22,ACER2,NEU2,B4GALT3,ST8SIA6	grey	VSMC_M10
GO:0006820	0.19	0.84	178	319	anion transport	BP	1	ATP8B1,SLC26A10,SLC16A7,SLC12A2,SLC27A6,TSPO,GOPC,NTRK2,PLA2G6,ABCB1,ABCC10,GABRA1,APBA1,SLC35B2,CLIC5,SLC1A1,SLC25A13,SLC3A1,SLC4A4,CYB5R4,SLC35A1,SLC1A4,SLC13A4,TCAF2,CLCN1,SFRP4,SLC7A11,SLC26A5,ANO10,SLC25A30,SLC4A2,ASIC3,LLGL2,CLNS1A,CCT8L2,SLC22A13,SLC25A38,SLC24A3,ABCC4,SLC20A2,SLC4A7,SLC6A15,SLC25A15,SLC23A2,PRNP,CLIC1,ARL6IP5,SLC7A1,SLC38A9,ANO4,SLC5A8,MFSD5,CYP7A1,SLCO6A1,SLC43A1,SLC9A9,SLC33A1,SERINC5,STC1,SLC38A2,SLC38A1,RIPK1,ANO6,CRY2,SLC10A6,COL4A3BP,ABCC2,PLA2R1,ABCG2,SLC12A5,ADAMTS8,GRM2,SLC4A10,SFXN2,CYB5R1,SLC37A1,CALHM1,SLC2A10,SLC38A3,MAP2K6,SLC12A7,SLC37A2,SLC16A6,SLC7A7,SLCO3A1,SLC9A8,CLCN4,ABCB11,AGT,ABCC5,ACE2,IP6K2,ATP1A2,SLC9A1,TMEM206,SLCO1A2,SLCO1C1,SLC37A4,PER2,SLC12A8,NTSR1,XPR1,CLIC4,SLC16A1,PLA2G4A,SLC12A9,ANKH,SLC25A42,SLC11A1,MFSD10,SLC9A7,SLC2A2,PQLC2,SLC27A1,SLC38A5,MPC2,ANO8,ARL6IP1,SLC3A2,SLC2A9,SLC38A6,SLC35A2,SLC4A3,PRAF2,SLC25A11,SLC16A13,CLN8,TTYH3,SLC26A1,PSEN1,SLC7A3,SLC35B1,AC138894.1,ABCC3,NMUR2,CA4,SLC6A7,SLC26A2,LRRC8E,EMB,NOS2,SLC25A23,SLC25A41,SLC46A1,SLC25A14,PROCA1,SLC9A6,ABAT,CYB5RL,GABRE,GABRA3,SLC1A7,TRPV1,CLCN7,SLC2A6,SLC16A4,SLC7A5,LRRC8A,SLC25A25,ABCD3,STXBP1,CLCA2,SLC7A6,SLC12A4,SLC9A5,SLC25A3,SLC36A1,LRRC8D,LRRC8B,CA2,PLA2G2C,SLC25A21,SLC25A32,RGS4,CPTP,SLC25A29,PTGES,CEBPB	grey	VSMC_M10

GO:0009132	0.19	0.86	55	94	nucleoside diphosphate metabolic process	BP	1	NME1,PPARA,PRXL2C,GAPDH,SLC4A4,NT5E,BPGM,CMPK1,ENTPD6,DLG2,NUDT5,DHTKD1,PRKAG2,PFKP,NUDT16,GPI,GPD1,P2RX7,PRKAG1,PFKM,ENTPD4,NUDT18,NME4,ZBTB20,PFKFB2,ENO4,ARNT,ACTN3,HDAC4,MLXIPL,DTYMK,MAGI3,DLG1,HK1,CASK,PRKAG3,STAT3,NME7,ENO3,CARD11,PSEN1,PKLR,OGT,ADPGK,NCOR1,INSR,ALDOC,NME3,MPP1,SLC2A6,AK8,ENTPD5,CMPK2,TIGAR,NME6	grey	VSMC_M10
GO:1902023	0.19	0.95	6	8	L-arginine transport	BP	1	SLC7A1,SLC38A9,AGT,SLC11A1,SLC7A3,SLC25A29	grey	VSMC_M10
GO:0090205	0.19	0.95	6	8	positive regulation of cholesterol metabolic process	BP	1	CYP7A1,SCAP,POR,SREBF1,LDLRAP1,FGF1	grey	VSMC_M10
GO:0018242	0.19	0.95	6	8	protein O-linked glycosylation via serine	BP	1	MGAT5B,POGLUT1,GALNT2,KDELC2,GALNT16,GALNT1	grey	VSMC_M10
GO:0007413	0.19	0.91	13	20	axonal fasciculation	BP	1	TNFRSF21,RTN4,NRCAM,MEGF8,SEMA3A,CASP3,NCAM2,CRTAC1,EPHB3,EPHA4,CDK5R1,AMIGO1,CNTN2	grey	VSMC_M10
GO:0106030	0.19	0.91	13	20	neuron projection fasciculation	BP	1	TNFRSF21,RTN4,NRCAM,MEGF8,SEMA3A,CASP3,NCAM2,CRTAC1,EPHB3,EPHA4,CDK5R1,AMIGO1,CNTN2	grey	VSMC_M10
GO:0033605	0.19	0.95	6	8	positive regulation of catecholamine secretion	BP	1	SYT1,KCNB1,GRK2,STX1A,PINK1,CHRNB2	grey	VSMC_M10
GO:0090219	0.19	0.95	6	8	negative regulation of lipid kinase activity	BP	1	PIP4K2C,PIP4K2A,PPP2R5A,RUBCN,PIK3IP1,DAB2IP	grey	VSMC_M10
GO:0007260	0.19	0.88	32	53	tyrosine phosphorylation of STAT protein	BP	1	HSF1,KIT,PPP2R1A,IL15,HDAC2,PIBF1,HPX,FER,OSBP,ARL2BP,CD40,HDAC1,HCLS1,PARP9,PARP14,OSM,IL12A,TNFSF18,FGFR3,CTF1,IL6R,CCL5,CSF1R,ISL1,SUZ12,GHR,PTPN2,SOCS1,CNTF,MIR125A,LIF,TSLP	grey	VSMC_M10
GO:0007210	0.19	0.93	9	13	serotonin receptor signaling pathway	BP	1	HTR7,CHRM5,CHRM4,CHRM3,HTR3B,CHRM1,HRH2,HRH4,HTR1D	grey	VSMC_M10
GO:0086046	0.19	0.97	4	5	membrane depolarization during SA node cell action potential	BP	1	SCN5A,ANK2,CACNA1G,HCN4	grey	VSMC_M10
GO:0051957	0.19	0.93	9	13	positive regulation of amino acid transport	BP	1	SLC12A2,SLC38A3,AGT,ACE2,NTSR1,ARL6IP1,PSEN1,ABAT,STXBP1	grey	VSMC_M10
GO:0070536	0.19	0.89	20	32	protein K63-linked deubiquitination	BP	1	SHMT2,TNFAIP3,OTUD1,OTUD4,USP25,USP16,ABRAXAS1,OTUD7A,USP13,SPATA2,YOD1,OTUD7B,ABRAXAS2,OTUD5,STAMBPL1,UIMC1,OTUB2,USP20,STAMBP,BRCC3	grey	VSMC_M10
GO:0019321	0.19	0.93	8	11	pentose metabolic process	BP	1	UXS1,DHDH,NUDT5,XYLB,RBKS,RPE,FGGY,RPIA	grey	VSMC_M10
GO:0002634	0.19	0.95	6	8	regulation of germinal center formation	BP	1	TNFAIP3,FOXJ1,MEF2C,BCL6,RC3H1,TNFSF13B	grey	VSMC_M10
GO:0002279	0.19	0.88	24	39	mast cell activation involved in immune response	BP	1	ENPP3,RAC2,KIT,SPHK2,GAB2,UNC13D,FER,RASGRP1,SNAP23,LAT2,FGR,CBL,SNX4,PTGDR,C12orf4,LAT,IL4R,S100A13,BTK,STXBP2,PDPK1,FOXF1,STXBP1,CHGA	grey	VSMC_M10
GO:0030517	0.19	0.89	22	36	negative regulation of axon extension	BP	1	SEMA6A,SEMA4D,SEMA4C,RTN4,SEMA3D,SEMA3A,RYK,SEMA4F,SEMA3G,SEMA4G,KIAA0319,SEMA3F,WNT3,SEMA6D,TNR,SEMA4A,TRIM46,NTN1,CDK5R1,ARHGAP4,PLXNA3,WNT5A	grey	VSMC_M10
GO:0060914	0.19	0.90	17	27	heart formation	BP	1	LEMD2,PIM1,FOXH1,WNT11,HAND2,EYA1,ROBO1,ROBO2,MEF2C,TBX5,AXIN2,BMPR1A,RBPJ,MKS1,ISL1,WNT5A,SIX1	grey	VSMC_M10
GO:0055081	0.19	0.90	17	27	anion homeostasis	BP	1	SLC12A2,CKB,SLC1A1,TFAP2B,SFRP4,SLC7A11,GLS,ABCC2,SLC12A5,GRM2,SLC12A7,SLC12A8,XPR1,CPS1,SLC12A9,FGFR4,SLC12A4	grey	VSMC_M10
GO:0031341	0.19	0.88	28	46	regulation of cell killing	BP	1	CD226,STX7,AGER,HLA-DRA,NECTIN2,PVR,SLC22A13,NCKAP1L,P2RX7,RASGRP1,F2RL1,CADM1,IL12A,LAG3,PPP3CB,STAT5B,ITGAM,VAV1,NOS2,IL7R,CRK,DNASE1,AP1G1,FADD,CXCL6,FCER2,SERPINB9,HAVCR2	grey	VSMC_M10
GO:0042058	0.19	0.87	37	62	regulation of epidermal growth factor receptor signaling pathway	BP	1	EPGN,AREG,TGFA,EGFR,RAB7A,NEU3,SNX5,HBEGF,PDE6G,FER,HIP1R,FBXW7,PTPRJ,WDR54,NEURL1,ADRA2A,RNF115,NCF1,EGF,GPER1,DGKD,IFI6,AGT,CBL,PDE6H,SNX6,ZFYVE28,SOCS4,HAP1,PSEN1,PTPN2,RNF126,MVB12B,DAB2IP,MIR29A,MIR21,EREG	grey	VSMC_M10
GO:0001696	0.19	0.95	6	8	gastric acid secretion	BP	1	CCKBR,HIP1R,CHRM5,HRH2,TRPV1,SNX10	grey	VSMC_M10
GO:0042462	0.19	0.91	13	20	eye photoreceptor cell development	BP	1	NTRK2,RORB,RDH13,MYO7A,DZANK1,FSCN2,CEP290,USH1C,RPGRIP1L,NRL,OLFM3,THY1,DIO3	grey	VSMC_M10
GO:0046928	0.19	0.87	39	66	regulation of neurotransmitter secretion	BP	1	SNCAIP,PRKN,CHRNA3,CHRNA5,APBA1,RIMS1,RIMS3,CACNB2,SYT1,RAB5A,MEF2C,ASIC1,P2RY1,VPS18,DTNBP1,DNM1L,ADRA2A,GPER1,STX1A,BACE1,CSPG5,RAP1B,CASK,PNKD,RAB3A,SYN1,CPLX1,STX1B,SYT11,CALM1,SNAPIN,PRKCB,CAMK2A,FMR1,P2RX1,BAIAP3,STXBP1,CHRNB4,KCNMB4	grey	VSMC_M10
GO:0010667	0.19	0.90	15	23	negative regulation of cardiac muscle cell apoptotic process	BP	1	PPP1R10,RGL2,HSF1,PAX8,HAND2,SIRT5,SIRT4,NFE2L2,PDPK1,MIR199A1,MIR21,MIR24-1,NOL3,NKX2-5,HEY2	grey	VSMC_M10
GO:0021513	0.19	0.95	6	8	spinal cord dorsal/ventral patterning	BP	1	SMO,INTU,GLI2,SUFU,CHRD,TULP3	grey	VSMC_M10
GO:0031630	0.19	0.95	6	8	regulation of synaptic vesicle fusion to presynaptic active zone membrane	BP	1	GRIK5,SYT1,SYT9,RAB3A,CPLX1,STXBP1	grey	VSMC_M10
GO:1901632	0.19	0.95	6	8	regulation of synaptic vesicle membrane organization	BP	1	GRIK5,SYT1,SYT9,RAB3A,CPLX1,STXBP1	grey	VSMC_M10
GO:1904896	0.19	0.95	6	8	ESCRT complex disassembly	BP	1	VPS4B,CHMP2A,CHMP2B,CHMP7,CHMP1A,VPS4A	grey	VSMC_M10
GO:1904903	0.19	0.95	6	8	ESCRT III complex disassembly	BP	1	VPS4B,CHMP2A,CHMP2B,CHMP7,CHMP1A,VPS4A	grey	VSMC_M10
GO:0051125	0.20	0.90	16	25	regulation of actin nucleation	BP	1	WASHC5,PICK1,WASF1,FCHSD2,ARFIP2,HIP1R,FMN1,CYFIP1,MAGEL2,AP1AR,WASF2,WHAMM,NCKAP1,WAS,WDR63,GMFG	grey	VSMC_M10
GO:0071468	0.20	0.95	6	8	cellular response to acidic pH	BP	1	RAB11FIP5,SLC38A3,SLC9A1,TRPV1,PKD1L3,IMPACT	grey	VSMC_M10
GO:1900029	0.20	0.92	9	13	positive regulation of ruffle assembly	BP	1	EPS8L1,CYFIP1,EPS8,NDEL1,EPS8L3,DEF8,CARMIL2,EPS8L2,HRAS	grey	VSMC_M10
GO:0002468	0.20	0.95	6	8	dendritic cell antigen processing and presentation	BP	1	HLA-DRA,NOD1,NOD2,SLC11A1,CD74,FGL2	grey	VSMC_M10
GO:0090660	0.20	0.95	6	8	cerebrospinal fluid circulation	BP	1	LRRC6,CFAP43,JHY,CFAP45,SPAG16,TTC25	grey	VSMC_M10
GO:0002091	0.20	0.92	9	13	negative regulation of receptor internalization	BP	1	SDCBP,MTMR2,WDR54,ANKRD13A,ANKRD13D,LRPAP1,DLG4,MIR199A1,LRRTM2	grey	VSMC_M10
GO:0044070	0.20	0.87	34	57	regulation of anion transport	BP	1	ATP8B1,SLC12A2,GOPC,PLA2G6,ABCB1,TCAF2,SFRP4,PRNP,ARL6IP5,SLC43A1,STC1,RIPK1,CRY2,ABCC2,PLA2R1,GRM2,SLC38A3,MAP2K6,ABCB11,AGT,ACE2,ATP1A2,PER2,NTSR1,PLA2G4A,ARL6IP1,PSEN1,ABAT,GABRE,STXBP1,CA2,RGS4,PTGES,CEBPB	grey	VSMC_M10

GO:0010874	0.20	0.89	19	30	regulation of cholesterol efflux	BP	1	LRP1,PTCH1,TTC39B,NR1H2,NR1H3,PLTP,ABCA5,EGF,ZDHHC8,ABCA12,GPS2,NAXE,ABCA3,ABCA7,RXRA,MIR27B,MIR27A,ABCA8,MIR33B	grey	VSMC_M10
GO:0006851	0.20	0.91	12	18	mitochondrial calcium ion transmembrane transport	BP	1	SMDT1,MICU3,SLC8B1,OPA1,MCUR1,MCUB,MICU1,MCU,BHLHA15,PSEN2,SLC25A23,MAIP1	grey	VSMC_M10
GO:0051932	0.20	0.89	17	27	synaptic transmission, GABAergic	BP	1	GABRA1,PHF24,USP46,PRKCE,PLCL2,RAC3,KRAS,NPAS4,HAP1,NLGN2,AC138894.1,GABRE,GABRA3,BAIAP3,ZDHHC12,CNTNAP4,STXBP1	grey	VSMC_M10
GO:0045821	0.20	0.92	10	15	positive regulation of glycolytic process	BP	1	PRXL2C,SLC4A4,GPD1,P2RX7,ZBTB20,ARNT,MLXIPL,PSEN1,INSR,ENTPD5	grey	VSMC_M10
GO:0070900	0.20	0.95	6	8	mitochondrial tRNA modification	BP	1	MTO1,TRIT1,RPUSD4,TRMT5,HSD17B10,TRMT61B	grey	VSMC_M10
GO:1900864	0.20	0.95	6	8	mitochondrial RNA modification	BP	1	MTO1,TRIT1,RPUSD4,TRMT5,HSD17B10,TRMT61B	grey	VSMC_M10
GO:0086045	0.20	0.97	4	5	membrane depolarization during AV node cell action potential	BP	1	TRPM4,CACNB2,SCN5A,CACNA1G	grey	VSMC_M10
GO:0034058	0.20	0.95	6	8	endosomal vesicle fusion	BP	1	TGFBRAP1,VPS41,VPS39,VPS8,ANKFY1,VPS4A	grey	VSMC_M10
GO:0042133	0.20	0.90	13	20	neurotransmitter metabolic process	BP	1	AGTPBP1,GAD2,HNMT,LRTOMT,AP000812.5,ALDH5A1,SLC6A3,COMT,BCHE,DAGLB,AC138894.1,ABAT,PRIMA1	grey	VSMC_M10
GO:0046689	0.20	0.94	6	8	response to mercury ion	BP	1	SLC1A1,AQP1,ABCC2,PARK7,BSG,MT-CYB	grey	VSMC_M10
GO:0010988	0.20	0.93	8	11	regulation of low-density lipoprotein particle clearance	BP	1	FGF21,NR1H4,MYLIP,ANXA2,MIR199A1,MIR27B,MIR27A,LDLRAP1	grey	VSMC_M10
GO:0019627	0.20	0.94	6	8	urea metabolic process	BP	1	SLC25A15,NR1H4,CEBPA,ASL,CPS1,ASS1	grey	VSMC_M10
GO:0071941	0.20	0.94	6	8	nitrogen cycle metabolic process	BP	1	SLC25A15,NR1H4,CEBPA,ASL,CPS1,ASS1	grey	VSMC_M10
GO:0014010	0.20	0.94	6	8	Schwann cell proliferation	BP	1	SNW1,SOX10,RNF10,CERS2,SKI,MIR222	grey	VSMC_M10
GO:0090158	0.20	0.92	9	13	endoplasmic reticulum membrane organization	BP	1	RTN4,ATL2,MOSPD3,LPCAT3,ATL1,ARL6IP1,STX18,BNIP1,CXorf56	grey	VSMC_M10
GO:0050688	0.20	0.87	30	50	regulation of defense response to virus	BP	1	TNFAIP3,C6orf106,IL15,TRAF3IP2,SEC14L1,TMEM173,NPLOC4,MAVS,PCBP2,TRIM44,EIF2AK4,HERC5,PARP9,DTX3L,RNF26,NLRX1,TRAF3IP1,HTRA1,MUL1,FOXP3,RNF216,TKFC,TSPAN6,SIN3A,TRAF3,RIOK3,ZDHHC1,ZDHHC11,IFNLR1,FGL2	grey	VSMC_M10
GO:0055067	0.20	0.85	62	108	monovalent inorganic cation homeostasis	BP	1	NEDD4L,SLC12A2,TFAP2B,SLC4A4,PPT1,RAB7A,ATP6V0E2,LACC1,SLC4A2,AQP11,ATP1A3,SNX5,SLC4A7,TMEM106B,MAFG,CCKBR,ATP6V1H,TM9SF4,IL1A,SLC9A9,HVCN1,SLC12A5,SLC4A10,KCNJ2,SLC9B1,SLC12A7,TMEM9,TCIRG1,SLC9A8,SCN7A,SLAMF8,RHCG,AGT,ATP1A2,SLC9A1,SLC12A8,CLIC4,SLC12A9,COMT,SLC11A1,SLC9A7,ATP6V0A1,ATP1B1,MAPK1,DMXL2,KCNMA1,SLC4A3,ATP1B2,MAPK3,AC138894.1,CLN6,SNAPIN,SCNN1B,C7,TMEM199,SLC9A6,ATP6AP1,SLC12A4,SLC9A5,CA2,KCNH2,RAB39A	grey	VSMC_M10
GO:0021534	0.20	0.91	10	15	cell proliferation in hindbrain	BP	1	GLI1,SMO,C5AR1,GLI2,ZNF423,EGF,GBX2,LHX1,GPR37L1,CEND1	grey	VSMC_M10
GO:0003338	0.20	0.89	17	27	metanephros morphogenesis	BP	1	SMO,SIX2,SOX9,FOXJ1,PAX8,FMN1,FRAS1,PKD2,PAX2,SALL1,WNT4,CITED1,LHX1,PKD1,SOX8,BASP1,LIF	grey	VSMC_M10
GO:0033683	0.20	0.94	6	8	nucleotide-excision repair, DNA incision	BP	1	ERCC1,ERCC2,ERCC3,FAN1,ERCC4,NTHL1	grey	VSMC_M10
GO:0034389	0.20	0.90	13	20	lipid droplet organization	BP	1	PNPLA3,CIDEC,CDS2,DDHD2,SPART,AUP1,CDS1,FITM2,FITM1,ZFYVE1,TMEM159,BSCL2,TBC1D20	grey	VSMC_M10
GO:0035082	0.20	0.86	33	55	axoneme assembly	BP	1	UBE2B,LRRC6,CFAP69,WDR92,ARMC2,ARMC4,TTC26,DRC1,RSPH6A,FOXJ1,TTLL3,SPEF1,CCDC65,DNAH1,DNAH5,NEURL1,CFAP43,ZMYND10,CFAP46,JHY,TTC12,TOGARAM1,DNAAF2,SPAG16,TTC25,DNAAF4,MNS1,CCDC151,SPEF2,C16orf71,TPGS1,HYDIN,WDR63	grey	VSMC_M10
GO:0006664	0.20	0.85	51	87	glycolipid metabolic process	BP	1	B4GALNT1,A4GALT,GPAA1,LARGE1,CWH43,KIT,TMEM246,PIGF,GLB1,NEU3,PGAP2,PIGU,ST8SIA4,ST3GAL6,PIGT,GPLD1,A3GALT2,PGAP1,ST8SIA2,B3GALT1,B3GNT5,PIGM,PIGV,PIGX,GAL3ST3,PIGZ,B3GALNT1,GAL3ST4,PIGC,SCCPDH,PGAP3,PIGH,GALC,CLN6,ST8SIA5,GLA,HEXA,GM2A,PIGL,MPPE1,GBGT1,ST6GALNAC4,ST3GAL2,ST6GALNAC5,PYURF,CPTP,NEU2,PIGB,B4GALT3,PIGA,ST8SIA6	grey	VSMC_M10
GO:1903509	0.20	0.85	51	87	liposaccharide metabolic process	BP	1	B4GALNT1,A4GALT,GPAA1,LARGE1,CWH43,KIT,TMEM246,PIGF,GLB1,NEU3,PGAP2,PIGU,ST8SIA4,ST3GAL6,PIGT,GPLD1,A3GALT2,PGAP1,ST8SIA2,B3GALT1,B3GNT5,PIGM,PIGV,PIGX,GAL3ST3,PIGZ,B3GALNT1,GAL3ST4,PIGC,SCCPDH,PGAP3,PIGH,GALC,CLN6,ST8SIA5,GLA,HEXA,GM2A,PIGL,MPPE1,GBGT1,ST6GALNAC4,ST3GAL2,ST6GALNAC5,PYURF,CPTP,NEU2,PIGB,B4GALT3,PIGA,ST8SIA6	grey	VSMC_M10
GO:0060211	0.20	0.91	10	15	regulation of nuclear-transcribed mRNA poly(A) tail shortening	BP	1	PABPC1,AGO2,TNRC6B,TNRC6C,CNOT1,TENT4B,EIF4ENIF1,POLR2G,TOB1,TNRC6A	grey	VSMC_M10
GO:0033079	0.20	0.94	6	8	immature T cell proliferation	BP	1	BMI1,IL1A,TMEM131L,RIPK2,WNT4,FOXP3	grey	VSMC_M10
GO:0002251	0.20	0.92	9	13	organ or tissue specific immune response	BP	1	NOD2,OTUD7B,RAB17,IL6R,NOS2,HIST1H2BK,IFNLR1,HIST1H2BF,HIST1H2BJ	grey	VSMC_M10
GO:0098917	0.20	0.94	6	8	retrograde trans-synaptic signaling	BP	1	FARP1,PLCB1,GUCY1A1,DAG1,DAGLB,FABP5	grey	VSMC_M10
GO:0030516	0.20	0.85	48	83	regulation of axon extension	BP	1	LRP1,SEMA6A,RAB21,PLXNA4,SRF,SEMA4D,SEMA4C,RTN4,RUFY3,NRCAM,GOLGA4,MEGF8,SEMA3D,SEMA3A,RYK,TTL,SEMA4F,DPYSL2,NKX6-1,ZFYVE27,SEMA3G,CYFIP1,SEMA4G,KIAA0319,SEMA3F,ADNP,DISC1,CDKL5,WNT3,MAPT,SEMA6D,TNR,FN1,SEMA4A,TRIM46,NDEL1,RAB11A,NTN1,DNM2,SIN3A,TRPV2,CDK5R1,L1CAM,ARHGAP4,PLXNA3,BARHL2,WNT5A,OLFM1	grey	VSMC_M10
GO:0015669	0.20	0.94	6	8	gas transport	BP	1	MYC,AQP1,CYGB,NGB,CA2,HBA2	grey	VSMC_M10
GO:0098971	0.21	0.96	4	5	anterograde dendritic transport of neurotransmitter receptor complex	BP	1	KIF5A,KIF5C,KIF3B,KIF17	grey	VSMC_M10
GO:0043090	0.21	0.88	22	36	amino acid import	BP	1	SLC22A4,SLC1A1,SLC1A4,SLC7A11,SLC25A38,ARL6IP5,SLC7A1,SLC43A1,SFXN2,AGT,ACE2,ATP1A2,PER2,NTSR1,ARL6IP1,SLC3A2,CLN8,PSEN1,SLC7A3,SLC7A5,SLC36A1,RGS4	grey	VSMC_M10
GO:0045837	0.21	0.92	9	13	negative regulation of membrane potential	BP	1	HEBP2,SLC25A27,PTN,RNF122,ARL6IP5,MAPT,TRPV1,PIP5KL1,BNIP3L	grey	VSMC_M10
GO:0051964	0.21	0.96	4	5	negative regulation of synapse assembly	BP	1	EPHA7,ROBO2,SLIT1,WNT5A	grey	VSMC_M10
GO:0006563	0.21	0.94	6	8	L-serine metabolic process	BP	1	SHMT2,PSAT1,SERINC5,CBSL,SHMT1,PHGDH	grey	VSMC_M10
GO:0043129	0.21	0.93	7	10	surfactant homeostasis	BP	1	EPAS1,ITGB6,LPCAT1,CTSH,ABCA12,ABCA3,OAS1	grey	VSMC_M10
GO:0045198	0.21	0.91	10	15	establishment of epithelial cell apical/basal polarity	BP	1	PTK7,SYNE4,TCF15,MYO9A,CAMSAP3,LAMA1,IFT20,FOXF1,RHOA,WNT5A	grey	VSMC_M10

GO:0006513	0.21	0.86	36	61	protein monoubiquitination	BP	1	NEDD4L,PRKN,SKP1,UBE2B,RNF20,UBE3D,FANCL,BMI1,FBXL2,WDR48,UBE2O,UBE2E1,RYBP,KBTBD8,DDB2,MARCH7,PCGF6,KLHL12,NEURL1,PEF1,PDCD6,DTX3L,CBL,RNF168,BCOR,ATXN7L3,LEO1,DDB1,PCGF3,TRIM37,CUL4B,MGRN1,RNF126,UBB,UBE2R2,PEX12	grey	VSMC_M10
GO:0060371	0.21	0.96	4	5	regulation of atrial cardiac muscle cell membrane depolarization	BP	1	SCN5A,TBX5,CACNA1G,SCN2B	grey	VSMC_M10
GO:0090279	0.21	0.89	17	27	regulation of calcium ion import	BP	1	SPHK2,PRNP,HOMER1,STC1,PKD2,EGF,PLPP4,CASK,PSEN2,WNK3,ATP2A1,TRPV2,CCL2,TRPV3,P2RX1,ATP2C2,CRH	grey	VSMC_M10
GO:0032787	0.21	0.82	225	404	monocarboxylic acid metabolic process	BP	1	ATP8B1,HSD17B4,ALDH8A1,AIG1,LTC4S,CPT1B,SLC27A6,TNXB,QKI,PPARA,ACSL6,PNPLA3,MCAT,HSD17B8,ECHDC1,PRXL2C,AKR1B1,MAPK14,HACD4,ALDH1A1,CROT,KIT,GAPDH,AASDH,ADH5,PRKCE,ETFB,SLC4A4,ME1,BPGM,NR1H2,IL4I1,LPIN1,DLD,PRKAR2B,FAAH,HADHA,SOX9,ABHD5,AKR1A1,APOC1,DHTKD1,ECHDC3,PRKAG2,ACOX1,SLC22A13,XYLB,PIBF1,CYGB,HYI,ELOVL1,AKT2,PFKP,BTD,HACL1,PANK2,PDK1,AMDHD1,NR1H4,PDHX,TRIB3,LYPLA1,ACOXL,CYP7A1,ELOVL7,ASAH1,CYP7B1,ACOX2,GPI,ABHD6,THNSL2,CBR4,OSBP,HPGD,GPD1,PEX2,P2RX7,ETFDH,ACOT12,PRKAG1,NR1H3,PFKM,IVD,LPIN3,HOGA1,ECI2,ABCC2,ERLIN1,PLA2G4B,ADTRP,PRMT3,PRKAB1,LONP2,PM20D1,SIRT4,MCEE,ALDH5A1,IRS1,ALDH1A3,CPT1A,BDH2,ZBTB20,GPAM,ACSL5,NR5A2,CYP2U1,PFKFB2,ABHD2,MSMO1,MECR,MCCC1,GNPAT,ENO4,ABCB11,DHRS9,PC,HACD2,ARNT,LPGAT1,PER2,ACTN3,HDAC4,OSBPL2,ACADSB,THEM5,THEM4,MLXIPL,SCAP,EHHADH,POR,PDK3,PLA2G4A,OSBPL6,ACADL,HK1,FAH,BCO2,MTHFS,TYSND1,OSBPL1A,ACSBG1,PECR,PNKD,AASDHPPT,CYP27A1,PRKAG3,STAT3,SLC27A1,MPC2,DAGLB,ENO3,FADS3,LIAS,ACADVL,EPHX1,TECR,HSD17B10,DEGS1,ALDH1A2,FGFR4,LIPC,OSBPL7,PSEN1,ACOT2,AC005520.1,ABCD4,ALOXE3,PKLR,GCDH,MAPK3,OGT,HACD3,SCPEP1,NDUFAB1,ADPGK,CYP46A1,ACSM3,ACACA,NCOR1,CD74,INSR,NPC1,ABHD3,SREBF1,LPIN2,C3,ACSBG2,ALDOC,CYP2J2,ACAA2,ACOT11,ABAT,GAMT,PARK7,DECR2,SLC2A6,ACSF3,CEL,MLYCD,PTGES2,ABCD3,PLA2G15,CES2,ACADM,PHYH,ACOT4,FABP5,FADS6,ENTPD5,ELOVL3,ABHD10,TIGAR,HADHB,AMACR,NUDT19,IDNK,GGT5,ECI1,AUH,LYPLA2,STARD4,PTGES,INSIG2,PH[illegible]	grey	VSMC_M10
GO:0002822	0.21	0.84	59	102	regulation of adaptive immune response based on somatic recombination of immune receptors built from immunoglobulin superfamily domains	BP	1	CD226,STX7,TNFAIP3,AGER,HLA-DRA,EXOSC3,MSH2,RSAD2,IL4I1,TRPM4,HMCES,NECTIN2,PVR,MLH1,SLC22A13,FOXJ1,PAXIP1,SHLD1,NCKAP1L,HPX,TRAF6,MEF2C,RIPK2,NOD2,CD40,HLX,DENND1B,CD28,FZD5,PLA2G4A,BCL6,IL12A,RC3H1,PTPN6,TNFSF18,SLC11A1,PPP3CB,WAS,FOXP3,ZBTB1,SHLD2,TNFRSF1B,TBX21,ZBTB7B,IL4R,BTK,FBXO38,C3,IL7R,SUPT6H,PRKCZ,TRAF2,MIR21,FADD,CD46,FCER2,TNFSF13B,HAVCR2,RC3H2	grey	VSMC_M10
GO:0002714	0.21	0.88	17	27	positive regulation of B cell mediated immunity	BP	1	CD226,EXOSC3,MSH2,HMCES,NECTIN2,MLH1,PAXIP1,SHLD1,HPX,NOD2,CD40,CD28,SHLD2,TBX21,BTK,C3,FCER2	grey	VSMC_M10
GO:0002891	0.21	0.88	17	27	positive regulation of immunoglobulin mediated immune response	BP	1	CD226,EXOSC3,MSH2,HMCES,NECTIN2,MLH1,PAXIP1,SHLD1,HPX,NOD2,CD40,CD28,SHLD2,TBX21,BTK,C3,FCER2	grey	VSMC_M10
GO:0030854	0.21	0.94	6	8	positive regulation of granulocyte differentiation	BP	1	IL5,TRIB1,TESC,HCLS1,HAX1,EVI2B	grey	VSMC_M10
GO:0019646	0.21	0.85	46	75	aerobic electron transport chain	BP	1	UQCRQ,NDUFA6,DLD,NDUFB2,NDUFB6,DNAJC15,NDUFC2,NDUFS8,COX7A1,COX4I2,NDUFS3,UQCRB,COX15,NDUFV3,NDUFB4,ISCU,NDUFB3,NDUFS2,SDHC,COX6A1,COX8A,PINK1,NDUFA9,COX6A2,NDUFAB1,COX5A,NDUFA7,NDUFV2,NDUFB10,UQCR11,PARK7,MT-ND1,MT-ND2,MT-CO3,MT-ND6,MT-CYB,NDUFA5,NDUFC1,COX7A2,NDUFA3,NDUFB5,NDUFS6,NDUFA8,SDHAF2,NDUFS5,COX7A2L	grey	VSMC_M10
GO:0086029	0.21	0.96	4	5	Purkinje myocyte to ventricular cardiac muscle cell signaling	BP	1	TRPM4,SCN5A,CASQ2,RYR2	grey	VSMC_M10
GO:0086068	0.21	0.96	4	5	Purkinje myocyte to ventricular cardiac muscle cell communication	BP	1	TRPM4,SCN5A,CASQ2,RYR2	grey	VSMC_M10
GO:0009074	0.21	0.94	6	8	aromatic amino acid family catabolic process	BP	1	IL4I1,IDO2,IDO1,HGD,FAH,GSTZ1	grey	VSMC_M10
GO:0098935	0.21	0.93	7	10	dendritic transport	BP	1	KIF5A,WASF1,KIF5C,PURA,KIF3B,KIF17,SFPQ	grey	VSMC_M10
GO:0014733	0.21	0.93	7	10	regulation of skeletal muscle adaptation	BP	1	TNNC1,PPP3CA,TNNI1,MYH7,GTF2IRD1,ACTN3,CAMK2G	grey	VSMC_M10
GO:0042572	0.21	0.88	17	27	retinol metabolic process	BP	1	RDH5,RDH16,AKR1B1,ALDH1A1,RDH13,LIPE,SDR16C5,RETSAT,ALDH1A3,DHRS9,DHRS3,RDH11,ALDH1A2,PNPLA4,LRAT,RDH14,ADH4	grey	VSMC_M10
GO:0002072	0.21	0.96	4	5	optic cup morphogenesis involved in camera-type eye development	BP	1	TFAP2A,PAX2,ALDH1A3,WNT5A	grey	VSMC_M10
GO:0070633	0.21	0.89	14	22	transepithelial transport	BP	1	SLC12A2,ABCB1,SLC1A1,CLDN19,AQP1,ACTG1,SLC23A2,ABCC2,ABCG2,GPLD1,RHCG,ACTB,RHBG,SCNN1B	grey	VSMC_M10
GO:0000395	0.21	0.94	6	8	mRNA 5'-splice site recognition	BP	1	PRPF39,RNVU1-6,RNU4ATAC,RF00003,SNRPC,SRSF12	grey	VSMC_M10
GO:0014854	0.21	0.94	6	8	response to inactivity	BP	1	CAT,SCN5A,DAG1,CASQ1,ACTN3,HDAC4	grey	VSMC_M10
GO:0048261	0.21	0.89	16	25	negative regulation of receptor-mediated endocytosis	BP	1	SDCBP,MTMR2,APOC1,NEU3,WDR54,ANKRD13A,ANKRD13D,SH3GL3,LRPAP1,DLG4,SYT11,UNC119,MIR199A1,MIR27B,MIR27A,LRRTM2	grey	VSMC_M10
GO:0001778	0.21	0.93	7	10	plasma membrane repair	BP	1	MYH9,MYOF,DYSF,RAB3A,SYT7,SYT11,SYTL4	grey	VSMC_M10
GO:0090399	0.21	0.91	10	15	replicative senescence	BP	1	CDKN2A,ERCC1,WRN,ATR,MME,PLA2R1,TERT,CHEK2,CTC1,MIR21	grey	VSMC_M10
GO:0001961	0.21	0.86	26	43	positive regulation of cytokine-mediated signaling pathway	BP	1	HSPA1B,PRKN,AGPAT1,TRIM32,CD300LF,MAVS,GAS6,HPX,IRF7,TRIM44,IL7,NLRC5,RIPK2,RIPK1,PARP9,PARP14,MED1,CD74,TRAF2,AGPAT2,GFI1,FADD,IRGM,WNT5A,C1QTNF4,TSLP	grey	VSMC_M10
GO:0070120	0.21	0.96	4	5	ciliary neurotrophic factor-mediated signaling pathway	BP	1	CNTFR,IL6R,LIFR,CNTF	grey	VSMC_M10
GO:0043171	0.21	0.89	14	22	peptide catabolic process	BP	1	LVRN,CHAC2,IDE,ERAP1,CPN1,XPNPEP1,ENPEP,GGT2,CTSH,ECE1,NPEPPS,ADAMTS13,ERAP2,GGT5	grey	VSMC_M10
GO:0071622	0.21	0.87	25	41	regulation of granulocyte chemotaxis	BP	1	PTK2,RAC2,CXCL8,AKIRIN1,C5AR2,C5AR1,NCKAP1L,NOD2,DNM1L,TRPV4,CMKLR1,MOSPD2,MCU,TNFSF18,CXCR2,BST1,MAPK1,DAPK2,MAPK3,S100A14,CD74,CCL5,CSF1R,MPP1,DPP4	grey	VSMC_M10

GO:0072567	0.22	0.91	10	15	chemokine (C-X-C motif) ligand 2 production	BP	1	LRP1,TLR4,MYD88,FOXP1,OAS3,F2RL1,HDAC1,MAP2K5,CD74,OAS1	grey	VSMC_M10
GO:2000341	0.22	0.91	10	15	regulation of chemokine (C-X-C motif) ligand 2 production	BP	1	LRP1,TLR4,MYD88,FOXP1,OAS3,F2RL1,HDAC1,MAP2K5,CD74,OAS1	grey	VSMC_M10
GO:0042795	0.22	0.91	10	15	snRNA transcription by RNA polymerase II	BP	1	SNAPC3,ZC3H8,ZNF143,CDK7,MEPCE,ELL,CC2D1A,ICE2,SNAPC5,SNAPC4	grey	VSMC_M10
GO:2000628	0.22	0.93	7	10	regulation of miRNA metabolic process	BP	1	PNPT1,TENT2,RC3H1,ZSWIM8,DROSHA,HRAS,RC3H2	grey	VSMC_M10
GO:0016052	0.22	0.83	65	113	carbohydrate catabolic process	BP	1	MIOX,PPARA,PRXL2C,GALT,GAPDH,SLC4A4,BPGM,MGAM,DHDH,PYGB,GLB1,NUDT5,DHTKD1,PRKAG2,XYLB,RBKS,PPP1CB,NEU3,PFKP,FUT10,PPP1R3B,GPI,GK5,GPD1,P2RX7,PRKAG1,PFKM,GLYCTK,ZBTB20,PFKFB2,ENO4,ARNT,PPP1R3D,TREH,ACTN3,HDAC4,MLXIPL,SORD,RPE,HK1,PYGM,PYGL,PRKAG3,STAT3,ENO3,TKFC,PSEN1,PKLR,OGT,ADPGK,GM2A,NCOR1,INSR,ENOSF1,ALDOC,TKTL1,SLC2A6,AGL,CTBS,FUT4,ENTPD5,ABHD10,TIGAR,IDNK,NEU2	grey	VSMC_M10
GO:0007351	0.22	0.93	7	10	tripartite regional subdivision	BP	1	FRS2,FAM208A,HOXD8,FZD5,WDR77,WNT5A,BASP1	grey	VSMC_M10
GO:0008595	0.22	0.93	7	10	anterior/posterior axis specification, embryo	BP	1	FRS2,FAM208A,HOXD8,FZD5,WDR77,WNT5A,BASP1	grey	VSMC_M10
GO:0060897	0.22	0.96	4	5	neural plate regionalization	BP	1	FUZ,BMPR1A,CELSR2,NOG	grey	VSMC_M10
GO:0070304	0.22	0.84	57	99	positive regulation of stress-activated protein kinase signaling cascade	BP	1	MAP3K5,STK3,MAPK8IP2,MAP3K4,AGER,GADD45G,SEMA4C,EMC10,MTURN,NOD1,TLR4,TRAF1,SDCBP,MYD88,PLCB1,EIF2AK2,RELL2,SEMA3A,SH3RF2,ARL6IP5,MFHAS1,TRAF6,HAND2,PTK2B,RASGRP1,RIPK2,F2RL1,RIPK1,NOD2,TAOK3,TRPV4,TGFB2,NCF1,TRAF5,FZD7,MAP3K3,MAP3K11,MBIP,MAP4K2,DUSP19,PDCD10,LTBR,MINK1,RELL1,TAOK2,TRAF3,TAOK1,SERPINF2,MAPK8IP3,TRAF2,DAB2IP,ZNF622,WNT5A,GADD45A,INAVA,DUSP22,HRAS	grey	VSMC_M10
GO:0021603	0.22	0.96	4	5	cranial nerve formation	BP	1	ATP8B1,TFAP2A,PAX2,MAFB	grey	VSMC_M10
GO:0015804	0.22	0.87	22	36	neutral amino acid transport	BP	1	SLC1A1,SLC3A1,SLC1A4,LLGL2,SLC25A38,SLC6A15,SLC38A9,SLC43A1,SERINC5,SLC38A2,SLC38A1,SFXN2,SLC38A3,ACE2,SLC38A5,SLC3A2,SLC38A6,SLC6A7,SLC7A5,SLC36A1,SLC6A19,RGS4	grey	VSMC_M10
GO:0006119	0.22	0.83	71	119	oxidative phosphorylation	BP	1	SHMT2,UQCRQ,NDUFA6,MSH2,DLD,NDUFB2,NDUFB6,DNAJC15,NDUFC2,ATP5F1C,NDUFS8,NDUFA12,COX7A1,COX4I2,ATP5F1A,NDUFS3,DGUOK,UQCRB,COX15,FAM173B,COX6B1,NDUFV3,NDUFB4,ISCU,COA6,NDUFB3,ACTN3,NDUFS2,DNAJC30,MLXIPL,SDHC,COX6A1,COX8A,PINK1,NDUFA9,COX6A2,NDUFAB1,UQCRC2,COX5A,TEFM,NDUFA7,SLC25A23,NDUFV2,NDUFB10,UQCR11,PARK7,SLC25A33,ATP5PB,MT-ND1,MT-ND2,MT-ATP8,MT-ATP6,MT-CO3,MT-ND6,MT-CYB,RHOA,ATP5ME,ATP5MF,NDUFA5,NDUFC1,COX7A2,FXN,NDUFA3,NDUFB5,NDUFS6,NDUFA8,SDHAF2,NDUFS5,UQCC2,COX7A2L,PPIF	grey	VSMC_M10
GO:0002709	0.22	0.86	26	43	regulation of T cell mediated immunity	BP	1	STX7,AGER,HLA-DRA,RSAD2,IL4I1,TRPM4,NECTIN2,PVR,SLC22A13,NCKAP1L,TRAF6,NOD2,DENND1B,FZD5,IL12A,PPP3CB,WAS,FOXP3,ZBTB1,TNFRSF1B,TBX21,FBXO38,IL7R,PRKCZ,TRAF2,FADD	grey	VSMC_M10
GO:0010759	0.22	0.90	10	15	positive regulation of macrophage chemotaxis	BP	1	PTK2,AKIRIN1,C5AR1,TRPV4,CMKLR1,TNFSF18,MAPK1,MAPK3,CCL5,CSF1R	grey	VSMC_M10
GO:0033005	0.22	0.90	10	15	positive regulation of mast cell activation	BP	1	CD226,SPHK2,NECTIN2,GAB2,FGR,SNX4,IL4R,STXBP1,HAVCR1,TSLP	grey	VSMC_M10
GO:0098814	0.22	0.92	7	10	spontaneous synaptic transmission	BP	1	SLC12A2,RIMS2,PRKN,AGER,SYT1,NSG1,STX1B	grey	VSMC_M10
GO:0060221	0.22	0.92	7	10	retinal rod cell differentiation	BP	1	NTRK2,RORB,PTN,SOX9,RPGRIP1L,NRL,SOX8	grey	VSMC_M10
GO:0010793	0.22	0.96	4	5	regulation of mRNA export from nucleus	BP	1	IWS1,SETD2,AKAP8L,SUPT6H	grey	VSMC_M10
GO:0120041	0.22	0.96	4	5	positive regulation of macrophage proliferation	BP	1	PTK2,MAPK1,MAPK3,CSF1R	grey	VSMC_M10
GO:0003431	0.22	0.96	4	5	growth plate cartilage chondrocyte development	BP	1	COL27A1,SOX9,TSKU,TGFBR2	grey	VSMC_M10
GO:0030488	0.22	0.86	24	39	tRNA methylation	BP	1	TRMT11,MTO1,NSUN6,THUMPD3,TRMT6,THUMPD2,TRMT9B,BCDIN3D,NSUN3,TRMT10A,DALRD3,LCMT2,METTL8,METTL2B,TRMT1L,ALKBH8,TRMT5,HSD17B10,METTL6,TRMT10B,TRMT61A,TRMT10C,TRMT61B,TYW3	grey	VSMC_M10
GO:0030913	0.22	0.96	4	5	paranodal junction assembly	BP	1	ANK2,GNPAT,CNTNAP1,EPB41L3	grey	VSMC_M10
GO:1901097	0.22	0.96	4	5	negative regulation of autophagosome maturation	BP	1	TMEM39A,RUBCN,PHF23,CLEC16A	grey	VSMC_M10
GO:0006390	0.22	0.90	10	15	mitochondrial transcription	BP	1	TFB1M,MTERF1,C6orf203,FOXO3,TWNK,TFAM,PPARGC1B,METTL4,TEFM,SLC25A33	grey	VSMC_M10
GO:0003056	0.22	0.92	7	10	regulation of vascular associated smooth muscle contraction	BP	1	ZDHHC21,ADRA2B,DOCK5,CHRM3,P2RX1,MIR21,RHOA	grey	VSMC_M10
GO:0003139	0.22	0.92	7	10	secondary heart field specification	BP	1	FOXH1,WNT11,MEF2C,AXIN2,RBPJ,ISL1,WNT5A	grey	VSMC_M10
GO:0050910	0.22	0.92	7	10	detection of mechanical stimulus involved in sensory perception of sound	BP	1	KIT,WHRN,MKKS,ADGRV1,STRC,PJVK,CHRNA10	grey	VSMC_M10
GO:0010359	0.22	0.96	4	5	regulation of anion channel activity	BP	1	GOPC,ABCB1,TCAF2,PRNP	grey	VSMC_M10
GO:0007340	0.22	0.89	14	22	acrosome reaction	BP	1	TRIM36,RIMS1,ADCY3,PLCB1,STX2,HYAL3,ABHD2,CCDC87,PLCD4,RAB3A,AKAP3,SPESP1,CACNA1H,STXBP1	grey	VSMC_M10
GO:1904478	0.22	0.96	4	5	regulation of intestinal absorption	BP	1	ABCG5,ABCG8,EPB41,LPCAT3	grey	VSMC_M10
GO:1902455	0.22	0.96	4	5	negative regulation of stem cell population maintenance	BP	1	ZNF706,PAX8,LOXL2,PAX2	grey	VSMC_M10
GO:0033564	0.22	0.96	4	5	anterior/posterior axon guidance	BP	1	LHX9,UNC5A,NTN1,LHX1	grey	VSMC_M10
GO:0010626	0.22	0.96	4	5	negative regulation of Schwann cell proliferation	BP	1	SOX10,RNF10,CERS2,SKI	grey	VSMC_M10
GO:0035524	0.22	0.96	4	5	proline transmembrane transport	BP	1	SLC1A4,ACE2,SLC6A7,SLC36A1	grey	VSMC_M10
GO:0046835	0.22	0.88	14	22	carbohydrate phosphorylation	BP	1	IPPK,KHK,XYLB,RBKS,PFKM,ITPK1,PFKFB2,PFKFB4,HK1,TKFC,FGGY,GNPTG,FUK,POMK	grey	VSMC_M10
GO:0097070	0.22	0.96	4	5	ductus arteriosus closure	BP	1	TFAP2B,HPGD,STRA6,FOXF1	grey	VSMC_M10
GO:0045745	0.22	0.90	10	15	positive regulation of G protein-coupled receptor signaling pathway	BP	1	KLK14,PHB,PDE6G,GPER1,PDE6H,TMOD2,C3,CNTN2,CHGA,MRAP2	grey	VSMC_M10
GO:0060729	0.22	0.96	4	5	intestinal epithelial structure maintenance	BP	1	TLR4,SOX9,SLC22A5,INAVA	grey	VSMC_M10
GO:0045648	0.22	0.87	18	29	positive regulation of erythrocyte differentiation	BP	1	HSPA1B,BRD1,MAPK14,ZNF16,SMAP1,FOXO3,ACVR2A,NCKAP1L,PRKDC,ACVR1B,ARNT,STAT3,STAT5B,MED1,KAT7,MIR222,ANKRD54,FAM210B	grey	VSMC_M10
GO:1903352	0.22	0.96	4	5	L-ornithine transmembrane transport	BP	1	SLC25A15,SLC7A1,SLC7A3,SLC25A29	grey	VSMC_M10

GO:1900186	0.22	0.96	4	5	negative regulation of clathrin-dependent endocytosis	BP	1	NEU3,SH3GL3,SYT11,UNC119	grey	VSMC_M10
GO:0071716	0.22	0.96	4	5	leukotriene transport	BP	1	ABCC10,ABCC4,ABCC2,ABCC3	grey	VSMC_M10
GO:0061669	0.22	0.96	4	5	spontaneous neurotransmitter secretion	BP	1	RIMS2,PRKN,SYT1,STX1B	grey	VSMC_M10
GO:0060478	0.22	0.96	4	5	acrosomal vesicle exocytosis	BP	1	RIMS1,HYAL3,RAB3A,STXBP1	grey	VSMC_M10
GO:0016042	0.22	0.81	127	225	lipid catabolic process	BP	1	HSD17B4,LRP1,CYP27B1,AIG1,CPT1B,ABHD16A,PPARA,PNPLA3,PNPLA5,MCAT,ECHDC1,PLA2G6,CROT,SRD5A3,HINT2,PRKCE,ETFB,PPT1,LPIN1,MGST2,SMPD2,LDAH,APOB,HSD17B14,FGF21,RAB7A,NAPEPLD,FAAH,HADHA,ABHD5,APOC1,LIPE,ACOX1,ACER3,ALK,AKT2,NEU3,SIRT2,PLCB1,HACL1,DDHD2,ACOXL,CYP7A1,ASAH1,ACOX2,ABHD6,PLCE1,PEX2,ETFDH,PLCH1,IVD,LPIN3,ECI2,SPP1,PLA2G4B,ADTRP,LONP2,GPLD1,SRD5A1,MCEE,PLCXD2,IRS1,CPT1A,BDH2,ADRA2A,PLA1A,ABHD4,ABHD2,SORL1,HSD11B1,ABCB11,PAFAH2,PLBD1,EHHADH,PLA2G4A,ACADL,BCO2,CPS1,TYSND1,ABHD12B,PLCD4,DDHD1,CYP27A1,SMPD4,PLA2G16,LIPA,DAGLB,ACADVL,THRA,HSD17B10,LIPC,ABCD4,GDPD3,GCDH,GALC,LDLR,GDPD1,GLA,HEXA,CYP46A1,GM2A,GDE1,ABHD3,PNPLA6,LPIN2,ACAA2,DECR2,CEL,MLYCD,ABCD3,PLA2G15,ACADM,PHYH,BSCL2,LIPJ,PNPLA4,PLA2G2C,NCEH1,HADHB,AMACR,ACER2,NUDT19,ECI1,AUH,LYPLA2,NEU2,YWHAH	grey	VSMC_M10
GO:0030320	0.22	0.96	4	5	cellular monovalent inorganic anion homeostasis	BP	1	SLC12A2,CKB,ABCC2,SLC12A5	grey	VSMC_M10
GO:0030644	0.22	0.96	4	5	cellular chloride ion homeostasis	BP	1	SLC12A2,CKB,ABCC2,SLC12A5	grey	VSMC_M10
GO:0048489	0.22	0.86	23	38	synaptic vesicle transport	BP	1	AP3S1,KIF5A,PRKN,AP3M2,KIF5C,LIN7A,TMEM230,SYNJ1,AP3B1,DTNBP1,CNIH2,SPG11,BLOC1S6,RAB3A,PINK1,AP3M1,SLC2A4,TRIM46,SNAPIN,DNM2,BLOC1S3,BORCS5,AP3S2	grey	VSMC_M10
GO:1903205	0.22	0.89	13	20	regulation of hydrogen peroxide-induced cell death	BP	1	GPR37,MET,FOXO3,PAWR,RIPK1,PDE8A,NFE2L2,PINK1,STK26,PARK7,MIR21,GPR37L1,TXN	grey	VSMC_M10
GO:0009448	0.23	0.96	4	5	gamma-aminobutyric acid metabolic process	BP	1	ALDH1A1,ALDH5A1,ABAT,PHGDH	grey	VSMC_M10
GO:0044743	0.23	0.87	20	32	protein transmembrane import into intracellular organelle	BP	1	TIMM21,PEX7,PEX6,PEX1,DNAJC15,TIMM50,PEX2,PEX16,LONP2,TOMM40L,GRPEL1,TIMM17B,PEX26,PEX14,PAM16,PEX10,TIMM23B,GRPEL2,PEX12,DNAJC19	grey	VSMC_M10
GO:0061643	0.23	0.96	4	5	chemorepulsion of axon	BP	1	PLXNA4,RYK,NTN1,WNT5A	grey	VSMC_M10
GO:0048843	0.23	0.88	15	24	negative regulation of axon extension involved in axon guidance	BP	1	SEMA6A,SEMA4D,SEMA4C,SEMA3D,SEMA3A,RYK,SEMA4F,SEMA3G,SEMA4G,SEMA3F,WNT3,SEMA6D,SEMA4A,PLXNA3,WNT5A	grey	VSMC_M10
GO:0061087	0.23	0.96	4	5	positive regulation of histone H3-K27 methylation	BP	1	PHF1,OGT,MTF2,ZNF304	grey	VSMC_M10
GO:0006366	0.23	0.79	1005	1862	transcription by RNA polymerase II	BP	1	ADNP2,NFATC1,ZNF236,ZNF407,SMARCC2,ONECUT2,TCF4,TBPL1,GLI1,HIVEP2,PLAGL1,MAML1,ZBTB2,ESR1,CNOT2,OSR2,SNW1,ARID1B,ZBTB12,ZBED4,PRKN,PPARA,MED30,BRD2,TAF2,RXRB,VGLL2,HSF2,TCF7,XRCC6,NCOA7,PITX1,MYC,SMAD5,AGO2,KDM3B,PTCH1,ATF4,RPAP2,CBX7,MAPK14,KDM4C,SOX10,NRF1,TMPRSS6,SMO,SNAPC3,HSF1,NFYA,FOXP4,MDFI,TFEB,IRF5,MED20,CCND3,TAF8,FOXH1,ZNF16,IFT74,RORB,ZNF300,PRDM4,CRY1,DMTF1,SRF,NFX1,NFXL1,SUPT3H,SMARCA2,RFX3,NCK2,FHL2,TFAP2B,NFIL3,SEMA4D,RFX8,DLX5,PHF14,ETV1,ZNF135,RPS14,ZNF154,MEOX2,ZNF550,KANSL3,ZNF268,ZNF667,ZNF787,ZNF691,MACC1,SIX2,FOXJ3,ZNF865,HIVEP3,IL11,ZNF189,EPAS1,EPCAM,SP4,ZNF462,PHIP,HMGN3,NFYC,NFE2L3,HOXA2,HOXA4,HOXA6,HOXA7,HOXA13,REL,ZNF483,JAZF1,CREB5,MYCL,XPO1,AKIRIN2,BACH2,ZNF692,AKIRIN1,MTF1,SNIP1,ANXA4,PCBP1,NR1H2,AKNA,ZNF350,ITGB1BP1,FOXP2,GRHL1,MED25,KLF11,MET,IL4I1,AGO1,POU3F3,EGFR,NOBOX,TLR4,CCNC,BCL2L12,SCAF1,LPIN1,UCP1,CREM,TEAD2,SDCBP,MYCN,ELF2,OSR1,FOXO3,ZXDC,ZBTB24,MKX,MAMSTR,ATAD2B,KDM7A,ZNF438,FOXE3,HDAC2,PHF10,MAML2,BBS7,MLLT10,DNMT3A,BMI1,C5AR1,ZC3H4,PRKD2,CNBP,PHB,FOXA3,SOX9,ZNF786,ZNF746,ERCC1,ZNF467,EED,ERCC2,ERCC3,GTF2F2,PCF11,SUV39H2,UTF1,ZNF404,PLK3,SUMO2,ZNF575,GLI2,ZNF526,RSF1,ZNF513,ZNF574,RAF1,DACH1,TAF3,ACVR2B,KLF5,KLF12,FOXJ1,PAXIP1,CSRNP1,GZF1,METTL23,MNX1,POU4F1,MED8,SS18L2,TADA3,KLF6,ZNF662,ARRB1,THAP1,EID2,OVOL2,ZNF219,ACVR2A,EOMES,IKBKB,MED29,CBX4,BHLHE40,CHD8,JAG1,SIRT2,TOX4,NMI,ZMYND11,TMEM173,R[illegible]	grey	VSMC_M10
GO:0051931	0.23	0.88	14	22	regulation of sensory perception	BP	1	GRIN2D,FAM19A4,P2RX3,F2R,FAM173B,ZFHX2,OPRD1,SLC9A1,NTSR1,COMT,ADRA2C,TMEM100,NMUR2,FABP5	grey	VSMC_M10
GO:0030277	0.23	0.92	7	10	maintenance of gastrointestinal epithelium	BP	1	KIAA1211,TLR4,SOX9,NOD2,PBLD,SLC22A5,INAVA	grey	VSMC_M10
GO:0016082	0.23	0.89	11	17	synaptic vesicle priming	BP	1	NAPA,NAPB,ERC2,SYNJ1,SNAP23,STX1A,RAB3A,SNAP29,STX1B,STXBP1,SNAP47	grey	VSMC_M10
GO:1904251	0.23	0.92	7	10	regulation of bile acid metabolic process	BP	1	KIT,PANK2,NR1H4,CYP7A1,ABCB11,FGFR4,STARD4	grey	VSMC_M10
GO:0036301	0.23	0.96	4	5	macrophage colony-stimulating factor production	BP	1	FOXP1,ISL1,TSLP,HAVCR2	grey	VSMC_M10
GO:1901256	0.23	0.96	4	5	regulation of macrophage colony-stimulating factor production	BP	1	FOXP1,ISL1,TSLP,HAVCR2	grey	VSMC_M10
GO:0042509	0.23	0.85	31	52	regulation of tyrosine phosphorylation of STAT protein	BP	1	HSF1,KIT,PPP2R1A,IL15,HDAC2,PIBF1,HPX,OSBP,ARL2BP,CD40,HDAC1,HCLS1,PARP9,PARP14,OSM,IL12A,TNFSF18,FGFR3,CTF1,IL6R,CCL5,CSF1R,ISL1,SUZ12,GHR,PTPN2,SOCS1,CNTF,MIR125A,LIF,TSLP	grey	VSMC_M10
GO:1903578	0.23	0.84	39	66	regulation of ATP metabolic process	BP	1	SHMT2,PPARA,TSPO,PRXL2C,SLC4A4,SPHK2,DNAJC15,PRKAG2,COX7A1,GPD1,P2RX7,PRKAG1,FAM173B,DNM1L,ZBTB20,ARNT,ACTN3,HDAC4,DNAJC30,MLXIPL,FIS1,PINK1,STAT3,PARP1,PSEN1,OGT,NCOR1,INSR,SLC25A23,SLC25A33,SLC2A6,RHOA,ENTPD5,BEND3,COX7A2,TIGAR,UQCC2,COX7A2L,PPIF	grey	VSMC_M10

GO:0030258	0.23	0.82	97	171	lipid modification	BP	1	HSD17B4,B4GALNT1,CPT1B,SYNJ2,PPARA,MCAT,ECHDC1,MAPK14,CROT,PLPP6,ADH5,PLPPR1,MBOAT7,ETFB,MBOAT2,DGKI,INPP4B,FIG4,AGK,SPHK2,MTMR2,HADHA,SOX9,APOC1,PRKAG2,ACOX1,CYGB,AKT2,PIP4P1,HACL1,PLPP5,MTMR7,ACOXL,SOAT2,ACOX2,CHRM5,MTMR9,PEX2,EPHX2,ETFDH,SYNJ1,IVD,ECI2,LONP2,SIRT4,A3GALT2,IRS1,CPT1A,BDH2,INPP5A,MTMR11,B3GALT1,DGKD,ABCB11,AGT,PLPP4,MTMR3,INPP5J,EHHADH,APOD,POR,DGKG,ACADL,SOAT1,SACM1L,TYSND1,LPCAT3,ACADVL,HSD17B10,DGKQ,ABCD4,GCDH,DGKE,PLPPR2,MTMR12,ACAA2,MTM1,MTMR1,INPP5K,DECR2,ABO,GBGT1,MLYCD,ABCD3,ST3GAL2,ACADM,MIR27B,MIR27A,PHYH,HADHB,AMACR,ECI1,AUH,STARD4,ADH4,SLC35C1,SGPP1	grey	VSMC_M10
GO:0001923	0.23	0.96	4	5	B-1 B cell differentiation	BP	1	CMTM7,PLCL2,SLAMF8,CD19	grey	VSMC_M10
GO:1990166	0.23	0.96	4	5	protein localization to site of double-strand break	BP	1	SLF1,XRCC4,SLF2,IFFO1	grey	VSMC_M10
GO:0001782	0.23	0.87	18	29	B cell homeostasis	BP	1	TNFAIP3,BAK1,TRAF3IP2,NCKAP1L,GAPT,CASP3,MEF2C,DOCK10,PPP2R3C,RC3H1,FOXP3,CD74,BCL10,CAMLG,BCL2L11,SH2B2,TNFSF13B,RC3H2	grey	VSMC_M10
GO:0006498	0.23	0.96	4	5	N-terminal protein lipidation	BP	1	HHAT,NMT1,PPM1A,MAP6D1	grey	VSMC_M10
GO:0022029	0.23	0.86	24	40	telencephalon cell migration	BP	1	SUN2,SRF,EFHC1,MBOAT7,RTN4,POU3F3,NRG1,ROBO1,SRGAP2C,DISC1,DIXDC1,TNR,PSEN1,NDEL1,CDK5R1,DAB1,LRP8,DAB2IP,RHOA,ADGRG1,CNTN2,FOXG1,LAMB1,POU3F2	grey	VSMC_M10
GO:0010469	0.23	0.83	64	112	regulation of signaling receptor activity	BP	1	MAPK8IP2,PRRT1,PPARA,LY6E,NCK2,SHANK1,EPGN,AREG,TGFA,DLGAP3,DKKL1,HDAC2,HBEGF,FAM19A4,ADRA2B,DKK3,FBXW7,MEF2C,DLGAP4,P2RY1,PTK2B,HOMER1,IFNGR2,NEURL1,HDAC1,ADRA2A,DKK2,NCF1,EGF,KLHL24,CBL,ACE2,CNIH2,SNX6,PPP2R5B,ADRA2C,LRPAP1,ZFYVE28,SOCS4,PINK1,NPTX2,GREM2,MINK1,DLG4,NLGN2,CNIH3,PSEN1,NLGN3,TAF1,WFIKKN2,BEGAIN,CCL2,DLGAP1,WFIKKN1,PARK7,MIR125A,NOG,NETO2,CACNG7,CRH,SHISA8,EREG,CNRIP1,CACNG5	grey	VSMC_M10
GO:0048645	0.23	0.84	33	56	animal organ formation	BP	1	FRS2,LEMD2,PIM1,FOXH1,NTF4,SPRY1,GLI2,WNT11,TGFBR2,PAX8,HAND2,EYA1,ROBO1,ROBO2,MEF2C,TBX5,PAX2,AXIN2,NKX3-2,MAPK1,BMPR1A,RBPJ,MAPK3,MAP2K1,MKS1,GATA6,ISL1,NOG,WNT5A,SIX1,FGF1,WNT2,HOXA3	grey	VSMC_M10
GO:0019184	0.23	0.90	10	15	nonribosomal peptide biosynthetic process	BP	1	SLC1A1,AASDH,CHAC2,MGST2,SLC7A11,GSS,BDH2,NFE2L2,GGT6,GGT5	grey	VSMC_M10
GO:0035771	0.23	0.96	4	5	interleukin-4-mediated signaling pathway	BP	1	CD300LF,PARP14,IL4R,PTPN2	grey	VSMC_M10
GO:0031033	0.23	0.96	4	5	myosin filament organization	BP	1	RAP1GDS1,TTN,TCAP,MYH11	grey	VSMC_M10
GO:0032792	0.23	0.96	4	5	negative regulation of CREB transcription factor activity	BP	1	EIF2AK4,SIK1,FOXP3,DDIT3	grey	VSMC_M10
GO:0019233	0.23	0.84	37	63	sensory perception of pain	BP	1	PHF24,GRIN2D,ASIC3,SCN1A,FAM19A4,EPHB1,P2RX3,P2RX7,P2RY1,MME,F2R,FAM173B,ZFHX2,OPRD1,SLC9A1,NIPSNAP1,NTSR1,BACE1,COMT,KCNK4,ADRA2C,KCNA1,MAPK1,NLGN2,HOXB8,ALOXE3,MAPK3,CHRNB2,TMEM100,BDKRB1,NMUR2,CCL2,TRPV1,MECP2,FABP5,POMK,PTGES	grey	VSMC_M10
GO:1903297	0.23	0.96	4	5	regulation of hypoxia-induced intrinsic apoptotic signaling pathway	BP	1	TMBIM6,HYOU1,PINK1,NOL3	grey	VSMC_M10
GO:1903298	0.23	0.96	4	5	negative regulation of hypoxia-induced intrinsic apoptotic signaling pathway	BP	1	TMBIM6,HYOU1,PINK1,NOL3	grey	VSMC_M10
GO:0008361	0.23	0.82	88	157	regulation of cell size	BP	1	LRP1,SEMA6A,RAB21,PRR16,SLC12A2,FSTL4,PLXNA4,SRF,SEMA4D,SEMA4C,RTN4,RUFY3,AQP1,PLEK,EPHA7,KEL,NRCAM,SLC26A5,GOLGA4,MEGF8,CLNS1A,AQP11,RPTOR,SEMA3D,SPART,SEMA3A,LARS,ULK1,RYK,RET,TTL,SEMA4F,P2RX7,DPYSL2,NKX6-1,ZFYVE27,ANO6,SEMA3G,SPP1,CYFIP1,SEMA4G,SLC12A5,BDNF,KIAA0319,SLC12A7,TRPV4,SEMA3F,ADNP,DISC1,SLC12A8,CDKL5,WNT3,CREB1,MAPT,SEMA6D,SLC12A9,TNR,FN1,GNB3,KCNMA1,CLN8,WNK3,SEMA4A,TRIM46,NDEL1,AC138894.1,RAB11A,NTN1,DNM2,SIN3A,TRPV2,LRRC8E,CDK5R1,VAV1,IL7R,RAP1GAP2,L1CAM,ARHGAP4,PLXNA3,TSC1,LAMTOR5,VAV3,LRRC8A,BARHL2,SLC12A4,RHOA,WNT5A,OLFM1	grey	VSMC_M10
GO:0051386	0.23	0.92	7	10	regulation of neurotrophin TRK receptor signaling pathway	BP	1	WASF1,SPRY1,SPRY2,CYFIP1,DOK5,AGT,PPP2R5B	grey	VSMC_M10
GO:0070170	0.23	0.96	4	5	regulation of tooth mineralization	BP	1	SLC4A2,TFAP2A,AMELX,BCOR	grey	VSMC_M10
GO:0050879	0.23	0.86	24	40	multicellular organismal movement	BP	1	STAC3,TNNI3,MYH14,XRCC1,STAC,NR4A1,HOMER1,TNNC1,KCNJ2,TNNI1,SYNM,VTI1A,GIGYF2,MYH7,CASQ1,ACTN3,CHRNB1,CLN8,TCAP,STAC2,ATP2A1,DMD,TNNC2,MAP1A	grey	VSMC_M10
GO:0050881	0.23	0.86	24	40	musculoskeletal movement	BP	1	STAC3,TNNI3,MYH14,XRCC1,STAC,NR4A1,HOMER1,TNNC1,KCNJ2,TNNI1,SYNM,VTI1A,GIGYF2,MYH7,CASQ1,ACTN3,CHRNB1,CLN8,TCAP,STAC2,ATP2A1,DMD,TNNC2,MAP1A	grey	VSMC_M10
GO:0061734	0.23	0.96	4	5	parkin-mediated stimulation of mitophagy in response to mitochondrial depolarization	BP	1	PRKN,OPTN,MFN2,VPS13C	grey	VSMC_M10
GO:0009138	0.23	0.96	4	5	pyrimidine nucleoside diphosphate metabolic process	BP	1	CMPK1,ENTPD4,DTYMK,CMPK2	grey	VSMC_M10
GO:0019695	0.23	0.96	4	5	choline metabolic process	BP	1	ALDH7A1,CHDH,CHKA,BCHE	grey	VSMC_M10
GO:0042428	0.23	0.96	4	5	serotonin metabolic process	BP	1	TPH2,BTBD9,DDC,SRD5A1	grey	VSMC_M10
GO:2000465	0.23	0.96	4	5	regulation of glycogen (starch) synthase activity	BP	1	EPM2AIP1,IGF2,PPP1R3F,INPP5K	grey	VSMC_M10
GO:0035502	0.23	0.96	4	5	metanephric part of ureteric bud development	BP	1	FOXJ1,PKD2,LHX1,BASP1	grey	VSMC_M10
GO:1905563	0.23	0.96	4	5	negative regulation of vascular endothelial cell proliferation	BP	1	FLT1,MEF2C,CCL2,MIR24-1	grey	VSMC_M10
GO:0070669	0.23	0.96	4	5	response to interleukin-2	BP	1	STAT5B,CITED1,JAK1,PTPN2	grey	VSMC_M10
GO:0006349	0.23	0.92	7	10	regulation of gene expression by genetic imprinting	BP	1	DNMT3A,IGF2,PCGF3,MECP2,PRMT7,CTCF,ARID4A	grey	VSMC_M10
GO:0018230	0.23	0.88	14	22	peptidyl-L-cysteine S-palmitoylation	BP	1	ZDHHC14,ZDHHC21,ZDHHC20,GOLGA7B,ZDHHC23,ZDHHC6,ZDHHC18,ZDHHC24,ZDHHC8,ZDHHC4,ZDHHC15,ZDHHC12,ZDHHC1,ZDHHC11	grey	VSMC_M10
GO:0018231	0.23	0.88	14	22	peptidyl-S-diacylglycerol-L-cysteine biosynthetic process from peptidyl-cysteine	BP	1	ZDHHC14,ZDHHC21,ZDHHC20,GOLGA7B,ZDHHC23,ZDHHC6,ZDHHC18,ZDHHC24,ZDHHC8,ZDHHC4,ZDHHC15,ZDHHC12,ZDHHC1,ZDHHC11	grey	VSMC_M10

GO:0016540	0.23	0.90	10	15	protein autoprocessing	BP	1	MYRFL,HTRA2,PIDD1,HJV,SPRTN,PISD,PARP1,F12,FXN,FAM111A	grey	VSMC_M10
GO:0034505	0.23	0.89	11	17	tooth mineralization	BP	1	PPARA,CNNM4,SLC4A2,TFAP2A,ITGB6,TCIRG1,FAM20A,FAM20C,AMELX,BCOR,ALPL	grey	VSMC_M10
GO:0042158	0.23	0.83	49	84	lipoprotein biosynthetic process	BP	1	ATG12,ZDHHC14,GPAA1,ZDHHC21,CWH43,TMEM246,PIGF,ATG5,APOB,PGAP2,ZDHHC20,PIGU,ATG10,GOLGA7B,ZDHHC13,PIGT,GPLD1,PGAP1,ZDHHC23,ZDHHC6,RABL3,ATG16L1,PIGM,HHAT,ZDHHC18,PIGV,ZDHHC24,PIGX,GLUL,PIGZ,NMT1,ZDHHC8,PIGC,PPM1A,ZDHHC4,WIPI2,PGAP3,PIGH,ZDHHC15,PIGL,MPPE1,ZDHHC12,ZDHHC1,ZDHHC11,IRGM,PYURF,PIGB,PIGA,MAP6D1	grey	VSMC_M10
GO:0086015	0.23	0.94	5	7	SA node cell action potential	BP	1	SCN5A,RYR2,ANK2,CACNA1G,HCN4	grey	VSMC_M10
GO:0086018	0.23	0.94	5	7	SA node cell to atrial cardiac muscle cell signaling	BP	1	SCN5A,RYR2,ANK2,CACNA1G,HCN4	grey	VSMC_M10
GO:0050908	0.23	0.92	7	10	detection of light stimulus involved in visual perception	BP	1	PRPH2,GJA10,CEP250,CCDC66,ROM1,GNAT2,REEP6	grey	VSMC_M10
GO:0050962	0.23	0.92	7	10	detection of light stimulus involved in sensory perception	BP	1	PRPH2,GJA10,CEP250,CCDC66,ROM1,GNAT2,REEP6	grey	VSMC_M10
GO:2000427	0.23	0.96	4	5	positive regulation of apoptotic cell clearance	BP	1	CD300LF,CCL2,C3,ABCA7	grey	VSMC_M10
GO:0006488	0.23	0.90	10	15	dolichol-linked oligosaccharide biosynthetic process	BP	1	ALG12,SRD5A3,ALG2,ALG10,DPAGT1,ALG3,ALG9,ALG6,ALG1,DHDDS	grey	VSMC_M10
GO:0009137	0.23	0.96	4	5	purine nucleoside diphosphate catabolic process	BP	1	NT5E,NUDT16,ENTPD4,NUDT18	grey	VSMC_M10
GO:0009181	0.23	0.96	4	5	purine ribonucleoside diphosphate catabolic process	BP	1	NT5E,NUDT16,ENTPD4,NUDT18	grey	VSMC_M10
GO:0033127	0.23	0.92	7	10	regulation of histone phosphorylation	BP	1	UBE2B,AKAP8L,AKAP8,MAPK3,RPS6KA5,FMR1,RPS6KA4	grey	VSMC_M10
GO:0043303	0.23	0.86	23	38	mast cell degranulation	BP	1	RAC2,KIT,SPHK2,GAB2,UNC13D,FER,RASGRP1,SNAP23,LAT2,FGR,CBL,SNX4,PTGDR,C12orf4,LAT,IL4R,S100A13,BTK,STXBP2,PDPK1,FOXF1,STXBP1,CHGA	grey	VSMC_M10
GO:0015985	0.23	0.89	12	18	energy coupled proton transport, down electrochemical gradient	BP	1	ATP5MGL,ATP5F1C,ATP5F1A,FAM173B,DNAJC30,ATP6V0C,ATP5PB,VPS9D1,MT-ATP8,MT-ATP6,ATP5ME,ATP5MF	grey	VSMC_M10
GO:0015986	0.23	0.89	12	18	ATP synthesis coupled proton transport	BP	1	ATP5MGL,ATP5F1C,ATP5F1A,FAM173B,DNAJC30,ATP6V0C,ATP5PB,VPS9D1,MT-ATP8,MT-ATP6,ATP5ME,ATP5MF	grey	VSMC_M10
GO:0043380	0.23	0.96	4	5	regulation of memory T cell differentiation	BP	1	HLA-DRA,BCL6,CD46,FGL2	grey	VSMC_M10
GO:0051409	0.23	0.92	7	10	response to nitrosative stress	BP	1	ADH5,DNAJA1,ATG5,DUSP6,STOX1,PARK7,DDIT3	grey	VSMC_M10
GO:0070493	0.23	0.92	7	10	thrombin-activated receptor signaling pathway	BP	1	PLEK,MET,HPGD,F2RL1,F2R,IQGAP2,DGKQ	grey	VSMC_M10
GO:0097237	0.23	0.83	50	85	cellular response to toxic substance	BP	1	LTC4S,APOM,PRKN,PRXL2C,KDM3B,FANCC,PIM1,ALDH1A1,TXNRD1,PRDX1,ADH5,CAT,ALB,PXDN,MGST2,TXNRD3,DHFR,ESD,AKR1A1,GSTK1,PRDX3,CYGB,PXDNL,ABTB2,GLRX,GPX8,TP53INP1,ABCG2,FBLN5,MARC2,MARC1,OPRD1,NFE2L2,DUOX1,FIS1,GCH1,PINK1,ABCB6,RDH11,SOD3,GSTZ1,MPO,TXNDC2,PARK7,NQO1,MIR21,HBA2,TXN,PTGES,ADH4	grey	VSMC_M10
GO:0030240	0.23	0.96	4	5	skeletal muscle thin filament assembly	BP	1	LMOD3,ACTC1,TTN,TCAP	grey	VSMC_M10
GO:0006004	0.23	0.92	7	10	fucose metabolic process	BP	1	FUCA2,FUT10,POFUT1,FUOM,FUT4,FPGT,POFUT2	grey	VSMC_M10
GO:0031640	0.23	0.88	13	20	killing of cells of other organism	BP	1	GAPDH,MYD88,P2RX7,F2RL1,SCNN1B,NOS2,S100A12,HIST1H2BK,CHGA,CXCL6,FCER2,SERPINB9,HIST1H2BJ	grey	VSMC_M10
GO:1901623	0.23	0.90	10	15	regulation of lymphocyte chemotaxis	BP	1	CCL27,OXSR1,PTK2B,SLC8B1,PADI2,TMEM102,CCL5,CCL7,CCL2,WNT5A	grey	VSMC_M10
GO:0003071	0.24	0.89	10	15	renal system process involved in regulation of systemic arterial blood pressure	BP	1	GAS6,F2RL1,F2R,AGT,PTPRO,CORO2B,SERPINF2,CYBA,HSD11B2,RHOA	grey	VSMC_M10
GO:0046618	0.24	0.92	7	10	xenobiotic export	BP	1	ABCB1,ATP7B,ABCC2,ABCB11,RALBP1,ABCA3,SLC22A5	grey	VSMC_M10
GO:0035518	0.24	0.89	11	17	histone H2A monoubiquitination	BP	1	SKP1,BMI1,RYBP,DDB2,PCGF6,RNF168,BCOR,DDB1,PCGF3,TRIM37,CUL4B	grey	VSMC_M10
GO:0003228	0.24	0.96	4	5	atrial cardiac muscle tissue development	BP	1	ENG,NOG,NKX2-5,WNT2	grey	VSMC_M10
GO:1901668	0.24	0.96	4	5	regulation of superoxide dismutase activity	BP	1	SZT2,SIRT3,PARK7,MIR21	grey	VSMC_M10
GO:0044065	0.24	0.92	7	10	regulation of respiratory system process	BP	1	FTO,GLS,ATP1A2,MTG2,NLGN2,NLGN3,MECP2	grey	VSMC_M10
GO:0002579	0.24	0.96	4	5	positive regulation of antigen processing and presentation	BP	1	NOD1,NOD2,SLC11A1,CD74	grey	VSMC_M10
GO:0002606	0.24	0.96	4	5	positive regulation of dendritic cell antigen processing and presentation	BP	1	NOD1,NOD2,SLC11A1,CD74	grey	VSMC_M10
GO:0018298	0.24	0.96	4	5	protein-chromophore linkage	BP	1	OPN1SW,CRY1,CRY2,RRH	grey	VSMC_M10
GO:0031054	0.24	0.90	8	12	pre-miRNA processing	BP	1	AGO2,TUT7,AGO1,TUT4,BCDIN3D,PRKRA,DROSHA,AGO4	grey	VSMC_M10
GO:0035608	0.24	0.96	4	5	protein deglutamylation	BP	1	AGTPBP1,AGBL3,AGBL2,FOLH1	grey	VSMC_M10
GO:0030259	0.24	0.92	7	10	lipid glycosylation	BP	1	B4GALNT1,A3GALT2,B3GALT1,ABO,GBGT1,ST3GAL2,SLC35C1	grey	VSMC_M10
GO:0021636	0.24	0.96	4	5	trigeminal nerve morphogenesis	BP	1	PLXNA4,SEMA3A,SEMA3F,PLXNA3	grey	VSMC_M10
GO:0021637	0.24	0.96	4	5	trigeminal nerve structural organization	BP	1	PLXNA4,SEMA3A,SEMA3F,PLXNA3	grey	VSMC_M10
GO:0009637	0.24	0.96	4	5	response to blue light	BP	1	CPT1B,CRY1,CRY2,LCN2	grey	VSMC_M10
GO:0001967	0.24	0.91	7	10	suckling behavior	BP	1	CNTFR,DACH1,POU4F1,HAND2,GLS,UBE2Q1,DERL2	grey	VSMC_M10
GO:0032700	0.24	0.91	7	10	negative regulation of interleukin-17 production	BP	1	TLR4,PRNP,NCKAP1L,IL12A,VSIR,FOXP3,DDIT3	grey	VSMC_M10
GO:1905515	0.24	0.84	31	53	non-motile cilium assembly	BP	1	TBC1D32,IFT74,TMEM17,FUZ,MAPRE1,INTU,BBS7,PIBF1,IFT172,CCDC13,MKKS,CEP290,TMEM80,CEP250,IFT52,RPGRIP1L,CLCN4,DISC1,BBS1,TOGARAM1,GORAB,CEP126,EXOC5,HAP1,TMEM107,MKS1,DNM2,WRAP73,C2CD3,CC2D2B,BBS10	grey	VSMC_M10
GO:0001977	0.24	0.91	7	10	renal system process involved in regulation of blood volume	BP	1	GAS6,F2RL1,F2R,PTPRO,CORO2B,CYBA,HSD11B2	grey	VSMC_M10
GO:0070672	0.24	0.95	4	5	response to interleukin-15	BP	1	IL15,IL15RA,PLCB1,STAT5B	grey	VSMC_M10
GO:0034754	0.24	0.83	46	79	cellular hormone metabolic process	BP	1	RDH5,RDH16,HSD17B4,ALDH8A1,ESR1,CYP21A2,TSPO,HSD17B8,HSD17B3,AKR1B1,ALDH1A1,SRD5A3,RDH13,IL4I1,LIPE,AANAT,SDR16C5,RETSAT,DKK3,SPP1,PRMT3,SRD5A1,ALDH1A3,DHRS9,HSD17B7,BCO2,COMT,WNT4,ECE1,STAT5B,HSD17B10,DHRS3,MED1,RDH11,ALDH1A2,DGKQ,SCPEP1,SCNN1B,CYP46A1,DHRS11,CACNA1H,DHRS2,PNPLA4,LRAT,RDH14,ADH4	grey	VSMC_M10
GO:0099541	0.24	0.95	4	5	trans-synaptic signaling by lipid	BP	1	PLCB1,F2R,DAGLB,FABP5	grey	VSMC_M10
GO:0099542	0.24	0.95	4	5	trans-synaptic signaling by endocannabinoid	BP	1	PLCB1,F2R,DAGLB,FABP5	grey	VSMC_M10
GO:0048291	0.24	0.91	7	10	isotype switching to IgG isotypes	BP	1	MSH2,MLH1,PAXIP1,CD40,CD28,FOXP3,TBX21	grey	VSMC_M10
GO:0048302	0.24	0.91	7	10	regulation of isotype switching to IgG isotypes	BP	1	MSH2,MLH1,PAXIP1,CD40,CD28,FOXP3,TBX21	grey	VSMC_M10

GO:1904528	0.24	0.95	4	5	positive regulation of microtubule binding	BP	1	HSF1,MAPRE1,MAPRE3,PPP2CB	grey	VSMC_M10
GO:0017156	0.24	0.84	26	44	calcium-ion regulated exocytosis	BP	1	RIMS2,MYH9,NLRP5,RIMS1,RIMS3,SYT1,SYT9,SYT8,HYAL3,ADRA2A,KCNB1,STX1A,RAP1B,PPP3CB,RAB3A,SYT7,STX1B,SYT11,CACNA1G,SCAMP5,SDF4,BAIAP3,CACNA1H,STXBP1,DOC2B,VAMP3	grey	VSMC_M10
GO:1902177	0.24	0.95	4	5	positive regulation of oxidative stress-induced intrinsic apoptotic signaling pathway	BP	1	FBXW7,MCL1,SFPQ,PARK7	grey	VSMC_M10
GO:0033003	0.24	0.86	19	31	regulation of mast cell activation	BP	1	CD226,ENPP3,RAC2,SPHK2,CD300LF,NECTIN2,GAB2,UNC13D,FER,FGR,SNX4,C12orf4,IL4R,STXBP2,PDPK1,FOXF1,STXBP1,HAVCR1,TSLP	grey	VSMC_M10
GO:0061387	0.24	0.82	54	95	regulation of extent of cell growth	BP	1	LRP1,SEMA6A,RAB21,FSTL4,PLXNA4,SRF,SEMA4D,SEMA4C,RTN4,RUFY3,EPHA7,NRCAM,GOLGA4,MEGF8,SEMA3D,SPART,SEMA3A,ULK1,RYK,TTL,SEMA4F,DPYSL2,NKX6-1,ZFYVE27,SEMA3G,SPP1,CYFIP1,SEMA4G,BDNF,KIAA0319,SEMA3F,ADNP,DISC1,CDKL5,WNT3,MAPT,SEMA6D,TNR,FN1,SEMA4A,TRIM46,NDEL1,RAB11A,NTN1,DNM2,SIN3A,TRPV2,CDK5R1,L1CAM,ARHGAP4,PLXNA3,BARHL2,WNT5A,OLFM1	grey	VSMC_M10
GO:0021910	0.24	0.95	4	5	smoothened signaling pathway involved in ventral spinal cord patterning	BP	1	SMO,GLI2,SUFU,TULP3	grey	VSMC_M10
GO:0120197	0.24	0.95	4	5	mucociliary clearance	BP	1	NEK10,CFAP43,SPAG16,TTC25	grey	VSMC_M10
GO:0002221	0.24	0.81	76	134	pattern recognition receptor signaling pathway	BP	1	TNFAIP3,USP15,ESR1,HSPA1B,GRAMD4,LRRC14,NINJ1,PRKCE,RTN4,ANKRD17,SLC46A2,NOD1,PELI1,RSAD2,TLR4,PUM2,PHB,CD300LF,LACC1,MYD88,IRAK2,SEC14L1,NMI,CLPB,NPLOC4,IRF7,MFHAS1,TRAF6,NR1H4,OTUD4,NR1H3,OAS3,RIPK2,OTULIN,AP3B1,F2RL1,NOD2,CD40,RAB7B,MAPKAPK3,IFIH1,OASL,MAP2K6,UNC93B1,TLR5,MAPKAPK2,TIFA,ALPK1,RAB11FIP2,NLRX1,DDX3X,TNIP2,BIRC3,GPS2,TKFC,WDFY1,TSPAN6,S100A14,BTK,TNIP1,TRAF3,RIOK3,COLEC12,RNF135,GPR108,TICAM1,PDPK1,CYBA,GFI1,BCL10,DAB2IP,OAS1,ZCCHC3,IRGM,INAVA,HAVCR2	grey	VSMC_M10
GO:0009083	0.24	0.89	10	15	branched-chain amino acid catabolic process	BP	1	BCAT2,IVD,HIBCH,ACAD8,ACADSB,HSD17B10,SLC25A44,ALDH6A1,HIBADH,AUH	grey	VSMC_M10
GO:0060760	0.24	0.84	29	49	positive regulation of response to cytokine stimulus	BP	1	HSPA1B,PRKN,AGPAT1,TRIM32,TLR4,CD300LF,MAVS,GAS6,HPX,IRF7,TRIM44,TAF9,IL7,NLRC5,RIPK2,RIPK1,IFIH1,PARP9,PARP14,MED1,CD74,TRAF2,AGPAT2,GFI1,FADD,IRGM,WNT5A,C1QTNF4,TSLP	grey	VSMC_M10
GO:0022038	0.24	0.90	8	12	corpus callosum development	BP	1	TSKU,SZT2,RYK,RPGRIP1L,EPHB3,NSUN5,NIN,RTN4RL1	grey	VSMC_M10
GO:0006528	0.24	0.95	4	5	asparagine metabolic process	BP	1	ATF4,NIT2,ASNSD1,ASRGL1	grey	VSMC_M10
GO:0017157	0.24	0.81	83	147	regulation of exocytosis	BP	1	VPS4B,RAB21,RIMS2,PRKN,PLA2G6,RAC2,CHRNA5,APBA1,CHMP2A,RIMS3,SDCBP,SPHK2,RAB7A,RAB10,CACNB2,CLASP2,GAB2,UNC13D,SYT1,RAB5A,NCKAP1L,SYT9,FER,P2RY1,F2RL1,VPS18,LGI3,DTNBP1,SYT8,DNM1L,HYAL3,ADRA2A,KCNB1,FGR,STX1A,SNX4,SEPT2,BACE1,CSPG5,NSF,RAP1B,CASK,RAB3A,SYN1,C12orf4,RAB8A,ATP13A2,HAP1,CPLANE2,SYT7,RAB15,RSC1A1,SEPT1,CPLX1,STX1B,STX4,ITGAM,SYT11,RAB8B,CACNA1G,RAB3D,IL4R,CALM1,SYTL4,SNAPIN,PRKCB,SCAMP5,STXBP2,PRAM1,FMR1,RPH3AL,PDPK1,P2RX1,RAB26,ATP6AP1,CACNA1H,FOXF1,STXBP1,AP1G1,VPS4A,FGA,DOC2B,RALA	grey	VSMC_M10
GO:0097202	0.24	0.91	7	10	activation of cysteine-type endopeptidase activity	BP	1	BAK1,ASPH,RIPK2,CIDEB,FADD,CASP8,PERP	grey	VSMC_M10
GO:0086005	0.24	0.86	16	26	ventricular cardiac muscle cell action potential	BP	1	NEDD4L,TRPM4,SCN5A,GPD1L,KCNJ3,KCNE2,DSP,RYR2,PKP2,ANK2,NOS1AP,DLG1,DSG2,RNF207,KCNH2,KCNJ8	grey	VSMC_M10
GO:1900426	0.24	0.95	4	5	positive regulation of defense response to bacterium	BP	1	F2RL1,NOD2,CYBA,HAVCR2	grey	VSMC_M10
GO:0039694	0.24	0.87	15	24	viral RNA genome replication	BP	1	APOBEC3H,CXCL8,PCBP1,ATG5,PHB,FBXL2,ATG16L2,RAB5A,PCBP2,FAM208A,ATG16L1,PI4KA,FMR1,INPP5K,SETDB1	grey	VSMC_M10
GO:0006559	0.24	0.95	4	5	L-phenylalanine catabolic process	BP	1	IL4I1,HGD,FAH,GSTZ1	grey	VSMC_M10
GO:1902222	0.24	0.95	4	5	erythrose 4-phosphate/phosphoenolpyruvate family amino acid catabolic process	BP	1	IL4I1,HGD,FAH,GSTZ1	grey	VSMC_M10
GO:0045048	0.24	0.88	13	20	protein insertion into ER membrane	BP	1	EMC2,TRAM2,EMC10,EMC3,EMC4,GET4,EMC6,SGTA,UBL4A,WDR83OS,CAMLG,EMC7,TRAM1L1	grey	VSMC_M10
GO:0070944	0.24	0.95	4	5	neutrophil-mediated killing of bacterium	BP	1	MYD88,F2RL1,SCNN1B,CXCL6	grey	VSMC_M10
GO:0046939	0.24	0.82	43	74	nucleotide phosphorylation	BP	1	NME1,PPARA,PRXL2C,GAPDH,SLC4A4,BPGM,CMPK1,DHTKD1,PRKAG2,PFKP,GPI,GPD1,P2RX7,PRKAG1,PFKM,NME4,ZBTB20,PFKFB2,ENO4,ARNT,ACTN3,HDAC4,MLXIPL,DTYMK,HK1,PRKAG3,STAT3,NME7,ENO3,PSEN1,PKLR,OGT,ADPGK,NCOR1,INSR,ALDOC,NME3,SLC2A6,AK8,ENTPD5,CMPK2,TIGAR,NME6	grey	VSMC_M10
GO:0051620	0.24	0.95	4	5	norepinephrine uptake	BP	1	SLC6A2,SLC6A3,ACTB,SLC29A4	grey	VSMC_M10
GO:0032048	0.24	0.89	10	15	cardiolipin metabolic process	BP	1	PLA2G6,HADHA,TAMM41,CRLS1,THEM5,SLC27A1,MECP2,LCLAT1,PTPMT1,DNAJC19	grey	VSMC_M10
GO:0050796	0.24	0.81	72	127	regulation of insulin secretion	BP	1	LRP1,PRKN,PLA2G6,PICK1,RFX3,TFAP2B,PRKCE,HMGN3,BMP8A,TRPM4,GIPR,SIRT3,SYT9,NR1H4,NDUFAF2,FAM3D,OSBP,ARNTL,KCNJ11,NNAT,TIAM1,PFKM,SLC8B1,NKX6-1,ACVR1C,ZBED6,SIRT4,GPLD1,IRS1,HDAC1,PPP3CA,CPT1A,SLC9B2,ADRA2A,TCIRG1,TCF7L2,KCNB1,PFKFB2,GPER1,PER2,STX1A,GLUL,SIDT2,SLC16A1,KLF7,ENY2,ABCA12,MCU,PPP3CB,CD38,MPC2,SYT7,NLGN2,TARDBP,STX4,SYTL4,GPR68,CCL5,SREBF1,ISL1,OXCT1,NOS2,ABAT,NADK,BAIAP3,LRRC8A,CHGA,SOX4,DPP4,CRH,DOC2B,UQCC2	grey	VSMC_M10
GO:0007214	0.24	0.90	8	12	gamma-aminobutyric acid signaling pathway	BP	1	SLC12A2,GABRA1,PHF24,GABBR2,PLCL2,GPR156,GABRE,GABRA3	grey	VSMC_M10
GO:0001916	0.24	0.91	7	10	positive regulation of T cell mediated cytotoxicity	BP	1	STX7,HLA-DRA,NECTIN2,PVR,SLC22A13,IL12A,FADD	grey	VSMC_M10
GO:0015671	0.24	0.95	4	5	oxygen transport	BP	1	MYC,CYGB,NGB,HBA2	grey	VSMC_M10
GO:0006655	0.24	0.91	7	10	phosphatidylglycerol biosynthetic process	BP	1	PLA2G6,TAMM41,CRLS1,CDS2,GPAM,SLC27A1,PTPMT1	grey	VSMC_M10
GO:0019886	0.24	0.89	10	15	antigen processing and presentation of exogenous peptide antigen via MHC class II	BP	1	HLA-DRA,HLA-DOB,HLA-DMA,HLA-DPB1,TRAF6,FCER1G,PIKFYVE,CTSV,DNM2,CD74	grey	VSMC_M10
GO:0015697	0.25	0.91	7	10	quaternary ammonium group transport	BP	1	SLC22A4,SLC38A2,SLC25A20,SLC25A47,SLC22A5,SLC25A45,SLC25A29	grey	VSMC_M10

GO:0071377	0.25	0.95	4	5	cellular response to glucagon stimulus	BP	1	FGF21,CYSLTR2,CPS1,ASS1	grey	VSMC_M10
GO:0044058	0.25	0.88	11	17	regulation of digestive system process	BP	1	ABCG5,ABCG8,AQP1,NR1H2,HIP1R,NR1H3,EPB41,LPCAT3,TRPV1,SLC22A5,CRH	grey	VSMC_M10
GO:0099601	0.25	0.84	26	44	regulation of neurotransmitter receptor activity	BP	1	MAPK8IP2,PRRT1,SHANK1,DLGAP3,MEF2C,DLGAP4,PTK2B,HOMER1,IFNGR2,KLHL24,CNIH2,PINK1,NPTX2,MINK1,DLG4,NLGN2,CNIH3,NLGN3,BEGAIN,CCL2,DLGAP1,NETO2,CACNG7,CRH,SHISA8,CACNG5	grey	VSMC_M10
GO:0045655	0.25	0.88	11	17	regulation of monocyte differentiation	BP	1	MYC,CDK6,HOXA7,FOXP1,IRF7,ACIN1,ZBTB46,GPR68,CD74,JUN,MIR125B1	grey	VSMC_M10
GO:0035965	0.25	0.95	4	5	cardiolipin acyl-chain remodeling	BP	1	PLA2G6,HADHA,THEM5,LCLAT1	grey	VSMC_M10
GO:1905274	0.25	0.95	4	5	regulation of modification of postsynaptic actin cytoskeleton	BP	1	CTTNBP2,TIAM1,CYFIP1,RHOA	grey	VSMC_M10
GO:1902956	0.25	0.95	4	5	regulation of mitochondrial electron transport, NADH to ubiquinone	BP	1	DNAJC15,ISCU,PINK1,PARK7	grey	VSMC_M10
GO:1903377	0.25	0.95	4	5	negative regulation of oxidative stress-induced neuron intrinsic apoptotic signaling pathway	BP	1	PRKN,NONO,PINK1,PARK7	grey	VSMC_M10
GO:0032732	0.25	0.84	24	40	positive regulation of interleukin-1 production	BP	1	AGER,NOD1,TLR4,HDAC2,PANX1,MYD88,P2RX7,RIPK2,F2RL1,NOD2,HDAC1,FZD5,IL16,HK1,STAT3,PYDC1,TMED10,SMAD3,S100A13,ISL1,CASP8,WNT5A,INAVA,HAVCR2	grey	VSMC_M10
GO:0035513	0.25	0.95	4	5	oxidative RNA demethylation	BP	1	ALKBH3,FTO,ALKBH1,ALKBH5	grey	VSMC_M10
GO:0006801	0.25	0.83	32	54	superoxide metabolic process	BP	1	FANCC,SLC1A1,PRDX1,FPR2,CYB5R4,EGFR,DHFR,FAM19A4,HVCN1,F2RL1,SH3PXD2A,FBLN5,NCF1,AGT,NCF2,NFE2L2,MAPT,DUOX1,GCH1,BST1,SOD3,ITGAM,MPO,NOX5,NOS2,NOXA1,GNAI3,CYBA,NQO1,MIR21,MIR27B,NRROS	grey	VSMC_M10
GO:0010390	0.25	0.86	16	26	histone monoubiquitination	BP	1	SKP1,RNF20,BMI1,UBE2E1,RYBP,DDB2,PCGF6,DTX3L,RNF168,BCOR,ATXN7L3,LEO1,DDB1,PCGF3,TRIM37,CUL4B	grey	VSMC_M10
GO:0002920	0.25	0.88	11	17	regulation of humoral immune response	BP	1	PHB,FOXJ1,HPX,NOD2,PPP2R3C,PTPN6,GATA6,C3,CD46,A2M,FCER2	grey	VSMC_M10
GO:0010533	0.25	0.95	4	5	regulation of activation of Janus kinase activity	BP	1	AGT,IL6R,CCL5,SOCS1	grey	VSMC_M10
GO:0021799	0.25	0.86	16	26	cerebral cortex radially oriented cell migration	BP	1	SUN2,MBOAT7,RTN4,POU3F3,SRGAP2C,DISC1,DIXDC1,NDEL1,CDK5R1,DAB1,LRP8,DAB2IP,ADGRG1,FOXG1,LAMB1,POU3F2	grey	VSMC_M10
GO:0050805	0.25	0.84	25	42	negative regulation of synaptic transmission	BP	1	PRRT1,PRKN,AGER,PLA2G6,PICK1,SRF,MTMR2,PLK2,ABHD6,ASIC1,PTK2B,SORCS3,KCNB1,ADNP,TMEM25,MAPT,RAP1B,TNR,BCHE,PNKD,CD38,SYT11,FMR1,STXBP1,GRID2	grey	VSMC_M10
GO:0070257	0.25	0.95	4	5	positive regulation of mucus secretion	BP	1	PRKCE,EGFR,ATG5,CYBA	grey	VSMC_M10
GO:0006574	0.25	0.95	4	5	valine catabolic process	BP	1	HIBCH,ACAD8,ALDH6A1,HIBADH	grey	VSMC_M10
GO:0042776	0.25	0.90	9	13	mitochondrial ATP synthesis coupled proton transport	BP	1	ATP5F1C,ATP5F1A,FAM173B,DNAJC30,ATP5PB,MT-ATP8,MT-ATP6,ATP5ME,ATP5MF	grey	VSMC_M10
GO:0048169	0.25	0.88	11	17	regulation of long-term neuronal synaptic plasticity	BP	1	SYNGAP1,SYNGR1,KIT,RAB5A,NEURL1,KRAS,AGT,RAB8A,DLG4,RAB11A,HRAS	grey	VSMC_M10
GO:0019336	0.25	0.91	7	10	phenol-containing compound catabolic process	BP	1	LRTOMT,AP000812.5,SLC6A3,FAH,COMT,DIO3,DIO2	grey	VSMC_M10
GO:0050912	0.25	0.95	4	5	detection of chemical stimulus involved in sensory perception of taste	BP	1	RTP4,GNAT2,PKD1L3,TAS2R3	grey	VSMC_M10
GO:0045671	0.25	0.87	12	19	negative regulation of osteoclast differentiation	BP	1	TOB2,TLR4,PIK3R1,FBXW7,GPR137B,PIAS3,TCTA,UBASH3B,GPR137,FBN1,FSTL3,MAFB	grey	VSMC_M10
GO:0009142	0.25	0.82	37	62	nucleoside triphosphate biosynthetic process	BP	1	NME1,TBPL1,ATP5MGL,PPARA,IMPDH1,SLC25A13,SPHK2,CMPK1,PRKAG2,ATP5F1C,ATP5F1A,DGUOK,FAM173B,NME4,CTPS2,DNAJC30,DTYMK,ATP5S,PINK1,STAT3,NME7,ADK,PARP1,PKLR,ATP6V0C,NME3,ATP5PB,VPS9D1,AK8,UCK1,MT-ATP8,MT-ATP6,ATP5ME,ATP5MF,CMPK2,NME6,NUDT2	grey	VSMC_M10
GO:0030148	0.25	0.81	48	83	sphingolipid biosynthetic process	BP	1	KDSR,ORMDL2,B4GALNT1,A4GALT,PLA2G6,HACD4,LARGE1,SPTLC1,SMPD2,AGK,SPHK2,PRKD2,ACER3,ELOVL1,SPTLC3,SIRT3,ELOVL7,ASAH1,ST8SIA4,ORMDL1,A3GALT2,ST8SIA2,SGMS2,ALDH3B2,B3GALT1,CERS6,HACD2,CERS2,PPM1L,SMPD4,SGMS1,CLN8,DEGS1,ALOXE3,HACD3,ST8SIA5,P2RX1,ST6GALNAC4,ST3GAL2,ST6GALNAC5,CYP4F22,ABCA8,ELOVL3,PEMT,ACER2,B4GALT3,SGPP1,ST8SIA6	grey	VSMC_M10
GO:0034334	0.25	0.93	5	7	adherens junction maintenance	BP	1	MTSS1,KIFC3,PLEKHA7,CAMSAP3,INAVA	grey	VSMC_M10
GO:0032373	0.25	0.86	16	26	positive regulation of sterol transport	BP	1	LRP1,PTCH1,NR1H2,NR1H3,PLTP,ABCA5,ZDHHC8,ABCA12,LPCAT3,GPS2,ANXA2,ABCA3,ABCA7,RXRA,ABCA8,LDLRAP1	grey	VSMC_M10
GO:0032376	0.25	0.86	16	26	positive regulation of cholesterol transport	BP	1	LRP1,PTCH1,NR1H2,NR1H3,PLTP,ABCA5,ZDHHC8,ABCA12,LPCAT3,GPS2,ANXA2,ABCA3,ABCA7,RXRA,ABCA8,LDLRAP1	grey	VSMC_M10
GO:0009148	0.25	0.89	10	15	pyrimidine nucleoside triphosphate biosynthetic process	BP	1	NME1,TBPL1,NME4,CTPS2,DTYMK,NME7,NME3,UCK1,CMPK2,NME6	grey	VSMC_M10
GO:0006241	0.25	0.91	7	10	CTP biosynthetic process	BP	1	NME1,NME4,CTPS2,NME7,NME3,UCK1,NME6	grey	VSMC_M10
GO:0050821	0.25	0.80	87	154	protein stabilization	BP	1	TRIM39,RAB21,STK3,ZNF207,HSPA1B,PRKN,UBE2B,PIM1,SMO,PEX6,BAG1,CDC37L1,GAPDH,PDCL3,RTN4,AAK1,PHB,CRTAP,USP36,MCM8,BAG4,TRIM44,PIK3R1,CDK7,HIP1R,TAF9,CTNND1,PARVA,MTMR9,FBXW7,CCNH,TESC,MARCH7,STK4,MORC3,RPL23,TBRG1,RABL3,USP13,USP19,HPS4,ANK2,CHEK2,USP2,DVL3,BAG3,PEX19,RPL11,CREB1,PRKRA,SYVN1,MFSD1,SAV1,PDCD10,TNIP2,PINK1,MUL1,OTUD3,ATP1B1,PIM2,EPHA4,ATP1B2,TMEM88,AHSP,EFNA1,TAF1,TAF9B,SEL1L,SMAD3,SWSAP1,COG7,LAMP2,NLK,NAPG,HCFC1,MAPK8IP3,SEC16A,PER3,PARK7,TSC1,STXBP1,SOX4,BAG2,IRGM,SUGT1,CAMLG,APOPT1	grey	VSMC_M10
GO:0048679	0.25	0.86	15	24	regulation of axon regeneration	BP	1	LRP1,PTN,PTPRF,SPP1,KIAA0319,KREMEN1,CERS2,LRIG2,TNR,EPHA4,NDEL1,MAP2K1,MIR222,CNTF,RTN4RL1	grey	VSMC_M10
GO:0050873	0.25	0.84	24	40	brown fat cell differentiation	BP	1	MAPK14,PIM1,UCP1,NAPEPLD,CEBPA,EBF2,FTO,RREB1,SOX13,TRPV4,FABP4,LAMB3,DUSP10,TFE3,SLC2A4,ALDH6A1,ZBTB7B,PRDM16,SIX1,PLAC8,PEX11A,SH2B2,CEBPB,DIO2	grey	VSMC_M10

GO:0034765	0.25	0.78	178	323	regulation of ion transmembrane transport	BP	1	NEDD4L,STAC3,MAPK8IP2,KCNV1,PRRT1,GOPC,CATSPER3,KCNJ4,PLA2G6,ABCB1,CLIC5,WNK2,TSPAN13,PRKCE,KCNQ4,SHANK1,SLC31A2,HECW1,TCAF2,CLCN1,KEL,ATG5,OSR1,SPHK2,KCNJ14,SLC26A5,KCNRG,CACNB2,STAC,OXSR1,SCN5A,SCN1A,GPD1L,CACNB4,NALCN,PRNP,DIAPH1,CLIC1,ARL6IP5,KCNIP3,SLMAP,SLC43A1,GLRX,MEF2C,P2RX7,HVCN1,KCNH3,PTK2B,KCNJ11,TPCN1,HOMER1,IFNGR2,KCNJ3,F2R,TESC,RIPK1,ANO6,KCNK15,KCNE2,PKD2,CASQ2,KCNJ15,KCNIP2,CHRM3,RYR2,FAM173B,PM20D1,CALHM1,HECW2,KCNJ2,ADRA2A,TCIRG1,COX17,SCN2A,ISCU,KCNB1,KCNG1,SCN7A,UBASH3B,ANK2,GPER1,KLHL24,AGT,ACE2,ATP1A2,SLC9A1,PER2,C4orf3,CASQ1,NTSR1,SCN4B,CNIH2,CLIC4,DNAJC30,NOS1AP,CATSPER2,DLG1,KCNK7,CEMIP,SESTD1,PTPN6,KCNK4,HTT,PINK1,ATP1B1,KCNA1,ARL6IP1,KCNMA1,KCNH4,GNB5,KCND1,MINK1,HAP1,DLG4,PSEN2,NLGN2,CNIH3,WNK3,STAC2,CACNB1,ATP1B2,PSEN1,KCNAB3,CACNA1A,KCNIP1,HCN3,NLGN3,CACNA1G,ATP2A1,CD19,CALM1,DNM2,VMP1,BDKRB1,CCL2,ASIC2,KCNJ18,FMR1,PDPK1,P2RX1,CACNA1B,RNF207,CACNA1H,PARK7,KCNC4,AMIGO1,CYBA,JPH3,LCN2,WWP2,RRAD,MIR21,MIR24-1,DMD,GPR35,JPH2,KCNH2,FGF11,THY1,SCN2B,KCNK5,NETO2,CACNG7,CRBN,RGS4,CRH,KCNA6,APOPT1,YWHAH,TMEM109,SHISA8,KCNJ8,CACNG5,HCN4,GEM,PPIF,KCNG2,KCNJ9	grey	VSMC_M10
GO:0014029	0.25	0.90	8	12	neural crest formation	BP	1	FUZ,SOX9,KBTBD8,KLHL12,PEF1,PDCD6,GSC,TCOF1	grey	VSMC_M10
GO:0009202	0.25	0.95	4	5	deoxyribonucleoside triphosphate biosynthetic process	BP	1	TBPL1,DTYMK,ADK,CMPK2	grey	VSMC_M10
GO:1903026	0.25	0.95	4	5	negative regulation of RNA polymerase II regulatory region sequence-specific DNA binding	BP	1	FBXW7,TAF1,DDIT3,ZNF593	grey	VSMC_M10
GO:0016024	0.25	0.89	8	12	CDP-diacylglycerol biosynthetic process	BP	1	AGPAT4,AGPAT1,TAMM41,CDS2,CDS1,GPAM,AGPAT2,LCLAT1	grey	VSMC_M10
GO:0005980	0.25	0.89	8	12	glycogen catabolic process	BP	1	PYGB,PPP1CB,PPP1R3B,PFKM,PPP1R3D,PYGM,PYGL,AGL	grey	VSMC_M10
GO:0006357	0.26	0.76	961	1784	regulation of transcription by RNA polymerase II	BP	1	ADNP2,NFATC1,ZNF236,ZNF407,SMARCC2,ONECUT2,TCF4,GLI1,HIVEP2,PLAGL1,MAML1,ZBTB2,ESR1,CNOT2,OSR2,SNW1,ARID1B,ZBTB12,ZBED4,PRKN,PPARA,MED30,BRD2,RXRB,VGLL2,HSF2,TCF7,XRCC6,NCOA7,PITX1,MYC,SMAD5,AGO2,KDM3B,PTCH1,ATF4,RPAP2,CBX7,MAPK14,KDM4C,SOX10,NRF1,TMPRSS6,SMO,HSF1,NFYA,FOXP4,MDFI,TFEB,IRF5,MED20,CCND3,FOXH1,ZNF16,IFT74,RORB,ZNF300,PRDM4,CRY1,DMTF1,SRF,NFX1,NFXL1,SUPT3H,SMARCA2,RFX3,NCK2,FHL2,TFAP2B,NFIL3,SEMA4D,RFX8,DLX5,PHF14,ETV1,ZNF135,RPS14,ZNF154,MEOX2,ZNF550,KANSL3,ZNF268,ZNF667,ZNF787,ZNF691,MACC1,SIX2,FOXJ3,ZNF865,HIVEP3,IL11,ZNF189,EPAS1,EPCAM,SP4,ZNF462,PHIP,HMGN3,NFYC,NFE2L3,HOXA2,HOXA4,HOXA6,HOXA7,HOXA13,REL,ZNF483,JAZF1,CREB5,MYCL,XPO1,AKIRIN2,BACH2,ZNF692,AKIRIN1,MTF1,SNIP1,ANXA4,PCBP1,NR1H2,AKNA,ZNF350,ITGB1BP1,FOXP2,GRHL1,MED25,KLF11,MET,IL4I1,AGO1,POU3F3,EGFR,NOBOX,TLR4,CCNC,BCL2L12,LPIN1,UCP1,CREM,TEAD2,SDCBP,MYCN,ELF2,OSR1,FOXO3,ZXDC,ZBTB24,MKX,MAMSTR,ATAD2B,KDM7A,ZNF438,FOXE3,HDAC2,PHF10,MAML2,BBS7,MLLT10,DNMT3A,BMI1,ZC3H4,PRKD2,CNBP,PHB,FOXA3,SOX9,ZNF786,ZNF746,ERCC1,ZNF467,EED,SUV39H2,UTF1,ZNF404,PLK3,SUMO2,ZNF575,GLI2,ZNF526,RSF1,ZNF513,ZNF574,RAF1,DACH1,TAF3,ACVR2B,KLF5,KLF12,FOXJ1,PAXIP1,CSRNP1,GZF1,METTL23,MNX1,POU4F1,MED8,SS18L2,TADA3,KLF6,ZNF662,ARRB1,THAP1,EID2,OVOL2,ZNF219,ACVR2A,EOMES,IKBKB,MED29,CBX4,BHLHE40,CHD8,JAG1,SIRT2,TOX4,TMEM173,RARB,PURA,NR1D2,PAWR,ZIC5,SIRT7,MAFG,DPF1,ALX1,TAF7,LUM,DCN,MAVS,SOHLH[illegible]	grey	VSMC_M10
GO:0010986	0.26	0.95	4	5	positive regulation of lipoprotein particle clearance	BP	1	GPLD1,ANXA2,MIR33B,LDLRAP1	grey	VSMC_M10
GO:0002763	0.26	0.84	24	40	positive regulation of myeloid leukocyte differentiation	BP	1	IL5,TRIB1,HSF1,ROR2,POU4F1,TRAF6,TESC,RIPK1,SLC9B2,ACIN1,HCLS1,CREB1,FOS,HAX1,GPR68,CD74,PPARGC1B,EVI2B,JUN,ATP6AP1,FADD,CASP8,CA2,LIF	grey	VSMC_M10
GO:0031062	0.26	0.85	17	28	positive regulation of histone methylation	BP	1	SNW1,PHF1,RNF20,PAXIP1,CHTOP,NSD3,DNMT3B,RTF1,JARID2,AUTS2,TET1,WDR61,NELFA,OGT,MECP2,MTF2,ZNF304	grey	VSMC_M10
GO:0007176	0.26	0.85	16	26	regulation of epidermal growth factor-activated receptor activity	BP	1	NCK2,EPGN,AREG,TGFA,HBEGF,FBXW7,NEURL1,ADRA2A,NCF1,EGF,CBL,SNX6,ZFYVE28,SOCS4,PSEN1,EREG	grey	VSMC_M10
GO:0035641	0.26	0.89	8	12	locomotory exploration behavior	BP	1	PRKCE,SLC4A10,ATP1A2,TNR,DLG4,NLGN2,DPP4,CRBN	grey	VSMC_M10
GO:0021889	0.26	0.93	5	7	olfactory bulb interneuron differentiation	BP	1	DLX5,ROBO1,ROBO2,SALL1,WNT5A	grey	VSMC_M10
GO:0070166	0.26	0.89	8	12	enamel mineralization	BP	1	PPARA,CNNM4,SLC4A2,ITGB6,TCIRG1,FAM20A,FAM20C,AMELX	grey	VSMC_M10
GO:0002098	0.26	0.87	11	17	tRNA wobble uridine modification	BP	1	TRMU,MTO1,ELP1,DPH3,TRMT9B,ELP4,ELP3,ELP6,ALKBH8,ELP2,CTU1	grey	VSMC_M10
GO:0072205	0.26	0.92	5	7	metanephric collecting duct development	BP	1	PTCH1,AKR1B1,PAX8,PAX2,PKD1	grey	VSMC_M10
GO:0060333	0.26	0.86	15	24	interferon-gamma-mediated signaling pathway	BP	1	NR1H2,NMI,HPX,HCK,NR1H3,NLRC5,IFNGR2,SP100,PARP9,PARP14,MED1,JAK1,PTPN2,SOCS1,IRGM	grey	VSMC_M10
GO:0030219	0.26	0.83	26	44	megakaryocyte differentiation	BP	1	MPIG6B,ZNF16,SRF,KIT,IL11,MTURN,CNOT4,PIP4K2A,ABI1,MEF2C,TESC,RAB7B,CIB1,WASF2,THPO,SP3,PTPN6,MED1,RCOR1,PRMT6,HIST1H4B,HIST2H4A,HIST1H4H,HIST1H4D,HIST1H4A,HIST1H4F	grey	VSMC_M10
GO:0002467	0.26	0.89	8	12	germinal center formation	BP	1	TNFAIP3,UNC13D,FOXJ1,MEF2C,NFKB2,BCL6,RC3H1,TNFSF13B	grey	VSMC_M10
GO:0010875	0.26	0.87	12	19	positive regulation of cholesterol efflux	BP	1	LRP1,PTCH1,NR1H2,NR1H3,PLTP,ZDHHC8,ABCA12,GPS2,ABCA3,ABCA7,RXRA,ABCA8	grey	VSMC_M10
GO:0043299	0.26	0.82	30	51	leukocyte degranulation	BP	1	RAC2,KIT,SPHK2,GAB2,UNC13D,NCKAP1L,HCK,FER,RASGRP1,F2RL1,SNAP23,LAT2,FGR,CBL,SNX4,PTGDR,C12orf4,STX4,ITGAM,LAT,IL4R,S100A13,BTK,STXBP2,PRAM1,PDPK1,FOXF1,STXBP1,AP1G1,CHGA	grey	VSMC_M10

GO:0002791	0.26	0.79	80	142	regulation of peptide secretion	BP	1	LRP1,PRKN,PLA2G6,PICK1,RFX3,TFAP2B,PRKCE,HMGN3,BMP8A,EGFR,TRPM4,GIPR,AIMP1,SIRT3,SYT9,NR1H4,NDUFAF2,FAM3D,PAX8,OSBP,ARNTL,KCNJ11,NNAT,TIAM1,PFKM,SLC8B1,NKX6-1,ACVR1C,ZBED6,SIRT4,GPLD1,IRS1,HDAC1,PPP3CA,CPT1A,SLC9B2,ADRA2A,TCIRG1,TCF7L2,KCNB1,PFKFB2,GPER1,PER2,STX1A,PASK,GLUL,SIDT2,SLC16A1,KLF7,ENY2,ABCA12,MCU,PPP3CB,CD38,MPC2,SYT7,NLGN2,TARDBP,STX4,RAB8B,SYTL4,GPR68,S100A8,CD74,CCL5,SREBF1,ISL1,OXCT1,NOS2,ABAT,NADK,BAIAP3,LRRC8A,FGA,CHGA,SOX4,DPP4,CRH,DOC2B,UQCC2	grey	VSMC_M10
GO:0015918	0.26	0.80	51	89	sterol transport	BP	1	VPS4B,LRP1,APOM,SERAC1,TSPO,PTCH1,TTC39B,ABCG5,ABCG8,NR1H2,APOB,PIP4K2A,APOC1,TSKU,SOAT2,OSBP,LIMA1,NR1H3,PLTP,ABCA5,EGF,GRAMD1B,ABCG4,OSBPL2,OSBP2,STARD5,OSBPL6,SOAT1,OSBPL1A,ZDHHC8,ABCA12,LPCAT3,SYT7,GPS2,LIPC,ANXA2,OSBPL7,NAXE,LDLR,NPC1,ABCA3,ABCA7,RXRA,CEL,VPS4A,MIR27B,MIR27A,ABCA8,MIR33B,LDLRAP1,STARD4	grey	VSMC_M10
GO:0002399	0.26	0.95	4	5	MHC class II protein complex assembly	BP	1	HLA-DRA,HLA-DOB,HLA-DMA,HLA-DPB1	grey	VSMC_M10
GO:0002503	0.26	0.95	4	5	peptide antigen assembly with MHC class II protein complex	BP	1	HLA-DRA,HLA-DOB,HLA-DMA,HLA-DPB1	grey	VSMC_M10
GO:2000660	0.26	0.95	4	5	negative regulation of interleukin-1-mediated signaling pathway	BP	1	IL1R2,OTUD4,MIR21,MIR27A	grey	VSMC_M10
GO:0035235	0.26	0.88	9	14	ionotropic glutamate receptor signaling pathway	BP	1	GRIK2,GRIN2D,GRIK5,PTK2B,TIAM1,GRIK4,AC138894.1,CDK5R1,GRID2	grey	VSMC_M10
GO:1903305	0.26	0.80	54	95	regulation of regulated secretory pathway	BP	1	RAC2,CHRNA5,APBA1,RIMS3,SPHK2,CACNB2,GAB2,UNC13D,SYT1,RAB5A,NCKAP1L,SYT9,FER,P2RY1,F2RL1,VPS18,DTNBP1,SYT8,DNM1L,HYAL3,ADRA2A,KCNB1,FGR,STX1A,SNX4,BACE1,CSPG5,RAP1B,CASK,RAB3A,SYN1,C12orf4,SYT7,RAB15,STX4,ITGAM,SYT11,CACNA1G,RAB3D,IL4R,CALM1,SNAPIN,PRKCB,SCAMP5,STXBP2,PRAM1,FMR1,PDPK1,P2RX1,CACNA1H,FOXF1,STXBP1,AP1G1,DOC2B	grey	VSMC_M10
GO:0042461	0.26	0.85	17	28	photoreceptor cell development	BP	1	NTRK2,PRPH2,RORB,RDH13,MYO7A,DZANK1,FSCN2,CEP290,USH1C,C8orf37,RPGRIP1L,NRL,ROM1,IFT20,OLFM3,THY1,DIO3	grey	VSMC_M10
GO:0031103	0.26	0.83	25	42	axon regeneration	BP	1	LRP1,TSPO,PTN,TNC,DHFR,PTPRF,CHL1,SPP1,KIAA0319,MTR,GAP43,DAG1,KREMEN1,CERS2,LRIG2,APOD,TNR,EPHA4,NDEL1,MAP2K1,ISL1,MAPK8IP3,MIR222,CNTF,RTN4RL1	grey	VSMC_M10
GO:0000288	0.26	0.82	31	53	nuclear-transcribed mRNA catabolic process, deadenylation-dependent decay	BP	1	PAN2,CNOT2,CNOT6,PABPC1,PNLDC1,AGO2,TNRC6B,TUT7,EXOSC3,TUT4,MLH1,TNRC6C,LSM1,PAN3,CNOT1,CNOT6L,TENT4B,EIF4ENIF1,RC3H1,SAMD4A,POLR2G,NT5C3B,DCP1B,TOB1,TNRC6A,CNOT8,PARN,NANOS1,ZFP36L2,RC3H2,EXOSC5	grey	VSMC_M10
GO:0046645	0.26	0.92	5	7	positive regulation of gamma-delta T cell activation	BP	1	NCKAP1L,SOX13,NOD2,STAT5B,SOX4	grey	VSMC_M10
GO:1901018	0.26	0.88	9	14	positive regulation of potassium ion transmembrane transporter activity	BP	1	KCNIP2,ANK2,NOS1AP,ATP1B1,KCNA1,ATP1B2,DNM2,RNF207,AMIGO1	grey	VSMC_M10
GO:0016198	0.26	0.92	5	7	axon choice point recognition	BP	1	ROBO1,ROBO2,GAP43,FOXG1,EFNB3	grey	VSMC_M10
GO:0008089	0.26	0.83	26	44	anterograde axonal transport	BP	1	AP3S1,KIF5A,RAB21,KIF3A,AGTPBP1,MGARP,DLG2,AP3M2,KIF5C,KIF3B,AP3B1,DTNBP1,CNIH2,CALY,BLOC1S6,AP3M1,KIF1C,HAP1,TRIM46,SNAPIN,MAPK8IP3,TERF2,BLOC1S3,BORCS5,AP3S2,MAP1A	grey	VSMC_M10
GO:0030322	0.26	0.89	8	12	stabilization of membrane potential	BP	1	KCNK9,KCNK15,KCNK1,KCNK2,KCNK7,KCNK4,KCNK3,KCNK5	grey	VSMC_M10
GO:0098664	0.26	0.89	8	12	G protein-coupled serotonin receptor signaling pathway	BP	1	HTR7,CHRM5,CHRM4,CHRM3,CHRM1,HRH2,HRH4,HTR1D	grey	VSMC_M10
GO:0097120	0.26	0.82	27	46	receptor localization to synapse	BP	1	STX7,KIF5A,ADAM22,DLG2,KIF5C,ANKS1B,KIF3B,DAG1,CNIH2,DLG1,KIF17,NSG1,GRIPAP1,RAB8A,DLG4,IQSEC2,GPHN,SYNJ2BP,CPLX1,STX1B,RAB11A,ARHGAP44,PRKCZ,CEP112,CACNG7,HRAS,CACNG5	grey	VSMC_M10
GO:0048793	0.26	0.92	5	7	pronephros development	BP	1	OSR1,CEP290,PAX8,PAX2,LHX1	grey	VSMC_M10
GO:0071816	0.26	0.88	10	15	tail-anchored membrane protein insertion into ER membrane	BP	1	EMC2,EMC10,EMC3,EMC4,GET4,EMC6,SGTA,UBL4A,CAMLG,EMC7	grey	VSMC_M10
GO:0008272	0.26	0.89	8	12	sulfate transport	BP	1	SLC26A10,SLC13A4,SLC26A5,SLC25A30,SLC25A11,SLC26A1,SLC26A2,SLC25A14	grey	VSMC_M10
GO:1903818	0.26	0.92	5	7	positive regulation of voltage-gated potassium channel activity	BP	1	KCNIP2,NOS1AP,KCNA1,RNF207,AMIGO1	grey	VSMC_M10
GO:0021884	0.26	0.86	12	19	forebrain neuron development	BP	1	UQCRQ,NDNF,SEMA3A,DCLK2,RAPGEF2,SLC4A10,DISC1,GBX2,PLXNA3,LHX8,UBB,FOXG1	grey	VSMC_M10
GO:0099640	0.26	0.92	5	7	axo-dendritic protein transport	BP	1	KIF5A,DLG2,KIF5C,MAPK8IP3,MAP1A	grey	VSMC_M10
GO:0099641	0.26	0.92	5	7	anterograde axonal protein transport	BP	1	KIF5A,DLG2,KIF5C,MAPK8IP3,MAP1A	grey	VSMC_M10
GO:0048791	0.27	0.88	9	14	calcium ion-regulated exocytosis of neurotransmitter	BP	1	RIMS2,RIMS1,RIMS3,SYT1,SYT8,SYT7,STX1B,STXBP1,DOC2B	grey	VSMC_M10
GO:0043266	0.27	0.81	34	59	regulation of potassium ion transport	BP	1	NEDD4L,WNK2,KEL,KCNRG,OXSR1,PRNP,KCNIP3,PTK2B,ANO6,KCNE2,CASQ2,KCNIP2,KCNJ2,ADRA2A,KCNG1,ANK2,KLHL24,NOS1AP,DLG1,ATP1B1,KCNA1,WNK3,ATP1B2,KCNAB3,KCNIP1,DNM2,RNF207,AMIGO1,WWP2,MIR21,KCNH2,NETO2,CRBN,RGS4	grey	VSMC_M10
GO:0061014	0.27	0.80	47	82	positive regulation of mRNA catabolic process	BP	1	PAN2,CNOT2,CNOT6,RIDA,PABPC1,PNLDC1,AGO2,TNRC6B,TUT7,GTPBP1,EXOSC3,PNPT1,TUT4,ZC3HAV1,MLH1,TNRC6C,LSM1,PAN3,CNOT1,CELF1,CNOT6L,FTO,TENT4B,RBM24,DIS3L2,GIGYF2,YTHDF2,YTHDF1,EIF4ENIF1,RC3H1,UPF1,SAMD4A,POLR2G,NT5C3B,DCP1B,TARDBP,TOB1,TNRC6A,CNOT8,PARN,METTL16,PLEKHN1,METTL14,NANOS1,ZFP36L2,RC3H2,EXOSC5	grey	VSMC_M10
GO:0002756	0.27	0.89	8	12	MyD88-independent toll-like receptor signaling pathway	BP	1	PRKCE,TLR4,CD300LF,IRF7,CD40,RAB11FIP2,TRAF3,TICAM1	grey	VSMC_M10

GO:0090087	0.27	0.79	81	144	regulation of peptide transport	BP	1	LRP1,PRKN,PLA2G6,PICK1,RFX3,TFAP2B,PRKCE,HMGN3,BMP8A,EGFR,TRPM4,GIPR,AIMP1,SIRT3,SYT9,NR1H4,NDUFAF2,FAM3D,PAX8,OSBP,ARNTL,KCNJ11,NNAT,TIAM1,PFKM,SLC8B1,NKX6-1,ACVR1C,ZBED6,SIRT4,GPLD1,IRS1,HDAC1,PPP3CA,CPT1A,SLC9B2,ADRA2A,TCIRG1,TCF7L2,KCNB1,PFKFB2,GPER1,PER2,STX1A,PASK,GLUL,SIDT2,SLC16A1,KLF7,ENY2,ABCA12,MCU,PPP3CB,CD38,MPC2,SYT7,NLGN2,TARDBP,STX4,RAB8B,SYTL4,GPR68,S100A8,CD74,CCL5,SREBF1,ISL1,OXCT1,NOS2,ABAT,NADK,BAIAP3,LRRC8A,FGA,CHGA,SOX4,DPP4,CA2,CRH,DOC2B,UQCC2	grey	VSMC_M10
GO:0046513	0.27	0.82	30	51	ceramide biosynthetic process	BP	1	ORMDL2,B4GALNT1,PLA2G6,SPTLC1,SMPD2,AGK,SPHK2,ELOVL1,SPTLC3,SIRT3,ASAH1,ST8SIA4,ORMDL1,ST8SIA2,SGMS2,B3GALT1,CERS6,CERS2,SMPD4,SGMS1,CLN8,DEGS1,ALOXE3,P2RX1,ST6GALNAC4,ST3GAL2,ST6GALNAC5,CYP4F22,B4GALT3,ST8SIA6	grey	VSMC_M10
GO:0015824	0.27	0.92	5	7	proline transport	BP	1	SLC1A4,SLC6A15,ACE2,SLC6A7,SLC36A1	grey	VSMC_M10
GO:0015820	0.27	0.92	5	7	leucine transport	BP	1	LLGL2,SLC6A15,SLC43A1,SLC3A2,SLC7A5	grey	VSMC_M10
GO:0002819	0.27	0.79	63	111	regulation of adaptive immune response	BP	1	CD226,STX7,TNFAIP3,AGER,HLA-DRA,EXOSC3,MSH2,RSAD2,IL4I1,TRPM4,HMCES,NECTIN2,PVR,MLH1,SLC22A13,FOXJ1,PAXIP1,SHLD1,NCKAP1L,HPX,IRF7,TRAF6,MEF2C,EIF2AK4,RIPK2,NOD2,ADCY7,CD40,HLX,DENND1B,CD28,FZD5,DUSP10,PLA2G4A,BCL6,IL12A,RC3H1,PTPN6,TNFSF18,SLC11A1,PPP3CB,WAS,FOXP3,ZBTB1,SHLD2,TNFRSF1B,TBX21,ZBTB7B,IL4R,BTK,FBXO38,C3,IL7R,SUPT6H,PRKCZ,TRAF2,MIR21,FADD,CD46,FCER2,TNFSF13B,HAVCR2,RC3H2	grey	VSMC_M10
GO:0099111	0.27	0.78	92	165	microtubule-based transport	BP	1	AP3S1,KIF5A,RAB21,KIF3A,LRRC6,AGTPBP1,SUN2,COPG2,IFT74,APBA1,RFX3,BICD2,DYNC2LI1,LRPPRC,DNAH11,MGARP,WASF1,TTC26,KIF3C,ARHGAP21,COPG1,KLC3,DLG2,IFT172,TTC21A,WDR60,AP3M2,KIF5C,NEK10,PURA,TUB,KIF3B,IFT81,RASGRP1,OPA1,AP3B1,IFT52,DNAH5,DTNBP1,DPCD,ROPN1L,KIF13A,BICDL1,CFAP43,TTC21B,HSPA8,JHY,OFD1,CFAP45,BAG3,TRAF3IP1,CNIH2,CALY,TTC30A,SPG11,MAPT,BLOC1S6,DNAAF2,SPAG16,MREG,HTT,STK36,KIF17,UXT,AP3M1,ADCY10,KIF1C,TTC25,HAP1,DNAAF4,WDR19,PEX14,TRIM46,NDEL1,AC138894.1,SNAPIN,SFPQ,CAMSAP3,IFT20,SPEF2,CFAP53,HSPB11,BICDL2,PRKCZ,MAPK8IP3,TERF2,UBB,BLOC1S3,BORCS5,AP3S2,MAP1A,SPA17	grey	VSMC_M10
GO:0050779	0.27	0.80	47	82	RNA destabilization	BP	1	PAN2,CNOT2,CNOT6,RIDA,PABPC1,DXO,PNLDC1,AGO2,TNRC6B,TUT7,EXOSC3,PNPT1,TUT4,MLH1,TNRC6C,LSM1,PAN3,CNOT1,CELF1,CNOT6L,FTO,TENT4B,RBM24,DIS3L2,GIGYF2,YTHDF2,YTHDF1,EIF4ENIF1,RC3H1,UPF1,SAMD4A,POLR2G,NT5C3B,DCP1B,TNFRSF1B,TARDBP,TOB1,TNRC6A,CNOT8,PARN,METTL16,PLEKHN1,METTL14,NANOS1,ZFP36L2,RC3H2,EXOSC5	grey	VSMC_M10
GO:0010839	0.27	0.89	8	12	negative regulation of keratinocyte proliferation	BP	1	CD109,INTU,VDR,KDF1,SFN,CASK,MED1,MIR125B1	grey	VSMC_M10
GO:0032620	0.27	0.85	15	24	interleukin-17 production	BP	1	TLR4,IL15,PHB,MYD88,PRNP,NCKAP1L,NR1H4,NOD2,OSM,IL12A,VSIR,FOXP3,ZBTB7B,SLC7A5,DDIT3	grey	VSMC_M10
GO:0032660	0.27	0.85	15	24	regulation of interleukin-17 production	BP	1	TLR4,IL15,PHB,MYD88,PRNP,NCKAP1L,NR1H4,NOD2,OSM,IL12A,VSIR,FOXP3,ZBTB7B,SLC7A5,DDIT3	grey	VSMC_M10
GO:0046460	0.27	0.83	22	37	neutral lipid biosynthetic process	BP	1	AVIL,PNPLA3,NR1H2,LPIN1,PLCE1,FBXW7,NR1H3,LPIN3,FITM2,GPLD1,SIK1,GPAM,LPGAT1,PLA2G4A,MOGAT3,SLC27A1,LDLR,SREBF1,LPIN2,C3,PLA2G15,CNEP1R1	grey	VSMC_M10
GO:0046463	0.27	0.83	22	37	acylglycerol biosynthetic process	BP	1	AVIL,PNPLA3,NR1H2,LPIN1,PLCE1,FBXW7,NR1H3,LPIN3,FITM2,GPLD1,SIK1,GPAM,LPGAT1,PLA2G4A,MOGAT3,SLC27A1,LDLR,SREBF1,LPIN2,C3,PLA2G15,CNEP1R1	grey	VSMC_M10
GO:0071868	0.27	0.81	38	66	cellular response to monoamine stimulus	BP	1	PRKN,ATF4,SLC1A1,VPS35,HDAC2,ALK,SIRT2,HTR7,CHRM5,CHRM4,DTNBP1,CHRM3,RYR2,SRD5A1,SLC9A1,CALY,HTR3B,MAPK1,CHRM1,GNB5,NSG1,HRH2,PKLR,HCN3,MAPK3,DNM2,SIN3A,HRH4,GNAL,GNB1,GNA15,PALM,GNAI3,HTR1D,RGS4,GNA14,GNG2,ARPP19	grey	VSMC_M10
GO:0071870	0.27	0.81	38	66	cellular response to catecholamine stimulus	BP	1	PRKN,ATF4,SLC1A1,VPS35,HDAC2,ALK,SIRT2,HTR7,CHRM5,CHRM4,DTNBP1,CHRM3,RYR2,SRD5A1,SLC9A1,CALY,HTR3B,MAPK1,CHRM1,GNB5,NSG1,HRH2,PKLR,HCN3,MAPK3,DNM2,SIN3A,HRH4,GNAL,GNB1,GNA15,PALM,GNAI3,HTR1D,RGS4,GNA14,GNG2,ARPP19	grey	VSMC_M10
GO:0009220	0.27	0.87	11	17	pyrimidine ribonucleotide biosynthetic process	BP	1	NME1,CMPK1,NME4,CTPS2,NME7,UPRT,NME3,UCK1,DHODH,NME6,UPP1	grey	VSMC_M10
GO:0015800	0.27	0.83	23	39	acidic amino acid transport	BP	1	SLC12A2,NTRK2,APBA1,SLC1A1,SLC25A13,SLC3A1,SLC7A11,ARL6IP5,GRM2,ATP1A2,PER2,NTSR1,ARL6IP1,PRAF2,CLN8,PSEN1,LRRC8E,ABAT,SLC1A7,TRPV1,LRRC8A,STXBP1,LRRC8D	grey	VSMC_M10
GO:0072234	0.27	0.88	9	14	metanephric nephron tubule development	BP	1	POU3F3,OSR1,SOX9,PAX8,PKD2,PAX2,PKD1,SOX8,LIF	grey	VSMC_M10
GO:0010665	0.27	0.83	20	33	regulation of cardiac muscle cell apoptotic process	BP	1	MAP3K5,PPP1R10,RGL2,HSF1,PAX8,HAND2,SIRT5,SIRT4,AGT,NFE2L2,CXCR2,CAMK2A,PDPK1,MIR199A1,MIR21,MIR24-1,NOL3,TIGAR,NKX2-5,HEY2	grey	VSMC_M10
GO:0007172	0.27	0.92	5	7	signal complex assembly	BP	1	MAPK8IP2,PTK2,NCK2,PTK2B,PXN	grey	VSMC_M10
GO:0036148	0.27	0.92	5	7	phosphatidylglycerol acyl-chain remodeling	BP	1	SERAC1,CRLS1,PLA2G4B,LPCAT1,LPGAT1	grey	VSMC_M10
GO:0006766	0.27	0.80	41	71	vitamin metabolic process	BP	1	SHMT2,CYP27B1,PSAT1,DHFR,AKR1A1,MMACHC,BTD,SLC23A2,PANK2,MMAA,CYP2R1,MTHFD2,CBR3,PRMT3,PLTP,MTRR,MTR,MMAB,SNAI1,THTPA,MCCC1,MTHFS,AASDHPPT,CYP27A1,ALDH1A2,PNPO,ABCD4,SHMT1,CD320,SLC46A1,PIAS4,TKTL1,GFI1,TPK1,MTHFD2L,SLC25A32,MMADHC,LRAT,NNMT,CLYBL,PDXP	grey	VSMC_M10
GO:0051890	0.27	0.92	5	7	regulation of cardioblast differentiation	BP	1	TBX5,PRICKLE1,TGFB2,GATA6,NKX2-5	grey	VSMC_M10

GO:0044458	0.27	0.83	20	33	motile cilium assembly	BP	1	UBE2B,LRRC6,CFAP69,ARMC2,INTU,SPATA6,RSPH6A,FOXJ1,DNAH1,NEURL1,CFAP43,ZMYND10,JHY,TTC12,SPAG16,MNS1,BBOF1,MKS1,SPEF2,TPGS1	grey	VSMC_M10
GO:0002576	0.27	0.92	5	7	platelet degranulation	BP	1	PLEK,ABCC4,DMTN,P2RX1,STXBP1	grey	VSMC_M10
GO:0070498	0.27	0.84	16	26	interleukin-1-mediated signaling pathway	BP	1	IL1R2,VRK2,MYD88,IRAK2,IKBKB,PLCB1,ST18,TRAF6,OTUD4,TOLLIP,TNIP2,MAPK3,RPS6KA5,MIR21,MIR27A,RPS6KA4	grey	VSMC_M10
GO:1904354	0.27	0.92	5	7	negative regulation of telomere capping	BP	1	ERCC1,XRCC1,NBN,ERCC4,TERF2	grey	VSMC_M10
GO:0021924	0.27	0.88	9	14	cell proliferation in external granule layer	BP	1	GLI1,SMO,GLI2,ZNF423,EGF,GBX2,LHX1,GPR37L1,CEND1	grey	VSMC_M10
GO:0021930	0.27	0.88	9	14	cerebellar granule cell precursor proliferation	BP	1	GLI1,SMO,GLI2,ZNF423,EGF,GBX2,LHX1,GPR37L1,CEND1	grey	VSMC_M10
GO:0150094	0.27	0.92	5	7	amyloid-beta clearance by cellular catabolic process	BP	1	LRP1,IDE,MME,LDLR,ABCA7	grey	VSMC_M10
GO:0071639	0.27	0.92	5	7	positive regulation of monocyte chemotactic protein-1 production	BP	1	AGER,OAS3,NOD2,TRPV4,OAS1	grey	VSMC_M10
GO:0007254	0.27	0.78	75	134	JNK cascade	BP	1	PHLPP1,MAP3K5,STK3,MAPK8IP2,PRKN,AGER,TRIB1,ROR2,GADD45G,KLHL31,DNAJA1,FKTN,MTURN,NOD1,EGFR,TLR4,TRAF1,SDCBP,MYD88,ZMYND11,PLCB1,MAP4K1,GPS1,SEMA3A,SH3RF2,ERCC6,MFHAS1,TRAF6,EPHB1,PTK2B,RASGRP1,RIPK2,F2RL1,RIPK1,NOD2,TAOK3,TRPV4,PDCD4,NCF1,TRAF5,STRADB,FZD7,MAGI3,MAP3K11,AIDA,DUSP10,MBIP,MEN1,MAP4K2,DUSP19,PINK1,LTBR,MINK1,GPS2,TAOK2,HACD3,SMAD3,NCOR1,TRAF3,ADORA2B,SH2D3A,PTGER4,TAOK1,ARHGEF6,SERPINF2,MAPK8IP3,TRAF2,MARVELD3,DAB2IP,ZNF622,WNT5A,GADD45A,DUSP22,HRAS,MAP2K7	grey	VSMC_M10
GO:0017014	0.27	0.88	8	12	protein nitrosylation	BP	1	NCOA7,GAPDH,ADH5,NOS1AP,S100A8,NOS2,DMD,TXN	grey	VSMC_M10
GO:0018119	0.27	0.88	8	12	peptidyl-cysteine S-nitrosylation	BP	1	NCOA7,GAPDH,ADH5,NOS1AP,S100A8,NOS2,DMD,TXN	grey	VSMC_M10
GO:1903748	0.27	0.92	5	7	negative regulation of establishment of protein localization to mitochondrion	BP	1	DNAJA1,BAG4,BAG3,MAPT,C11orf65	grey	VSMC_M10
GO:0006082	0.27	0.75	378	689	organic acid metabolic process	BP	1	ATP8B1,GLS2,HSD17B4,SHMT2,ALDH8A1,MARS,AIG1,LTC4S,GFPT2,TPH2,RIDA,CPT1B,DPYS,SLC27A6,TNXB,QKI,PPARA,ACSL6,PNPLA3,MCAT,HSD17B8,ECHDC1,ACO2,PRXL2C,AKR1B1,ATF4,PYCR3,MAPK14,PSAT1,GPT,HACD4,MTAP,CARNMT1,GNMT,ALDH1A1,CROT,ABCC10,GNPDA2,SLC35B2,KIT,SLC25A13,GAPDH,AASDH,PPAT,LIPT1,HMGCLL1,ADH5,RIMKLA,PRKCE,ETFB,SLC4A4,ME1,BPGM,NR1H2,DDC,IL4I1,LPIN1,MGST2,QRSL1,SLC7A11,DLD,DDO,BCAT2,NANP,PRKAR2B,DHFR,FAAH,HADHA,GAD2,SOX9,ABHD5,AKR1A1,APOC1,HPDL,DHTKD1,ECHDC3,PRKAG2,ACOX1,SLC22A13,XYLB,PIBF1,CYGB,GCKR,HYI,HNMT,ELOVL1,CSGALNACT1,AKT2,PFKP,IDO2,IDO1,MDH2,HARS,HARS2,BTD,HACL1,SLC23A2,CARS2,PANK2,PDK1,AMDHD1,LARS,SUCLG2,NR1H4,PDHX,APIP,TRIB3,LYPLA1,ACOXL,CSAD,CYP7A1,ELOVL7,ASAH1,CYP7B1,ACOX2,GPI,ABHD6,THNSL2,AHCY,CBR4,OSBP,GSS,MCCC2,HPGD,MSRA,GPD1,PEX2,P2RX7,CYP2R1,ETFDH,ACOT12,MTHFD2,PRKAG1,GMPS,NR1H3,SERINC5,ENOPH1,PFKM,SDSL,IVD,LPIN3,HOGA1,NIT2,BPHL,ASNSD1,ECI2,HIBCH,GLS,ABCC2,ERLIN1,ABCG2,PLA2G4B,ACAD8,ADTRP,PRMT3,PRKAB1,WARS2,LONP2,MTRR,PM20D1,SIRT4,GATC,ASL,MCEE,ALDH5A1,AZIN2,IRS1,MTR,YARS2,CARMIL1,CBSL,ALDH1A3,CPT1A,TPST1,BDH2,ZBTB20,GPAM,PPA2,ACSL5,NR5A2,SLC7A7,CYP2U1,HGD,MARC2,MARC1,MAPKAPK2,AMT,PFKFB2,ABHD2,MSMO1,MECR,MCCC1,GNPAT,CMAS,ENO4,TARS2,DALRD3,ABCB11,DHRS9,PC,HACD2,ARNT,LPGAT1,CTPS2,PER2,ACTN3,HDAC4,OSBPL2,ACADSB,OAT,THEM5,THEM4,MLXIPL,HMGCL,GLUL,D2HGDH,SCAP,EHHADH,SMS,MDH1B,POR,PDK3,PLA2G4A,OSBPL6,UPB1,ACADL,PLOD3,G[illegible]	grey	VSMC_M10
GO:0090715	0.27	0.92	5	7	immunological memory formation process	BP	1	HLA-DRA,BCL6,TSC1,CD46,FGL2	grey	VSMC_M10
GO:0035701	0.27	0.92	5	7	hematopoietic stem cell migration	BP	1	KIT,GAS6,JAM2,GPLD1,BCL11B	grey	VSMC_M10
GO:0036151	0.27	0.88	8	12	phosphatidylcholine acyl-chain remodeling	BP	1	TMEM86B,MBOAT7,NR1H2,MBOAT2,NR1H3,LPCAT1,PLA2G4A,LPCAT3	grey	VSMC_M10
GO:0033622	0.27	0.85	13	21	integrin activation	BP	1	TLN1,PLEK,ITGB1BP1,FERMT1,CDH17,COL16A1,FARP2,RAP1B,FN1,FERMT3,FBLIM1,PTGER4,PIEZO1	grey	VSMC_M10
GO:0032239	0.27	0.87	9	14	regulation of nucleobase-containing compound transport	BP	1	CPSF6,IWS1,RIPK1,NCBP2,SETD2,AKAP8L,RSC1A1,NRDE2,SUPT6H	grey	VSMC_M10
GO:0032418	0.27	0.80	36	62	lysosome localization	BP	1	RAC2,TFEB,MYH9,KIT,SPHK2,GAB2,UNC13D,PIP4P1,TMEM106B,FER,RASGRP1,SNAP23,LAT2,FGR,CBL,SNX4,KLC2,PTGDR,KXD1,RAB3A,C12orf4,BORCS6,NDEL1,LAT,IL4R,SYTL4,SNAPIN,S100A13,BTK,STXBP2,PDPK1,DEF8,FOXF1,STXBP1,CHGA,BORCS5	grey	VSMC_M10
GO:0034393	0.27	0.88	8	12	positive regulation of smooth muscle cell apoptotic process	BP	1	CDKN2A,ARRB1,STK4,PDCD4,IL12A,RBM10,MFN2,MIR24-1	grey	VSMC_M10
GO:0009084	0.28	0.88	8	12	glutamine family amino acid biosynthetic process	BP	1	GLS2,PYCR3,GLS,ASL,OAT,GLUL,AC138894.1,ASS1	grey	VSMC_M10
GO:1901381	0.28	0.84	15	25	positive regulation of potassium ion transmembrane transport	BP	1	WNK2,ANO6,KCNIP2,KCNJ2,ANK2,NOS1AP,ATP1B1,KCNA1,WNK3,ATP1B2,DNM2,RNF207,AMIGO1,MIR21,KCNH2	grey	VSMC_M10
GO:0060242	0.28	0.92	5	7	contact inhibition	BP	1	TSPO,SMO,DACH1,PTPRJ,FAP	grey	VSMC_M10
GO:0060245	0.28	0.92	5	7	detection of cell density	BP	1	TSPO,SMO,DACH1,PTPRJ,FAP	grey	VSMC_M10
GO:0048537	0.28	0.92	5	7	mucosa-associated lymphoid tissue development	BP	1	RET,TOX,STAT5B,FOXL1,ARTN	grey	VSMC_M10
GO:0048541	0.28	0.92	5	7	Peyer's patch development	BP	1	RET,TOX,STAT5B,FOXL1,ARTN	grey	VSMC_M10
GO:0046036	0.28	0.88	8	12	CTP metabolic process	BP	1	NME1,ENTPD4,NME4,CTPS2,NME7,NME3,UCK1,NME6	grey	VSMC_M10
GO:0006221	0.28	0.84	15	24	pyrimidine nucleotide biosynthetic process	BP	1	NME1,TBPL1,CMPK1,NME4,CTPS2,DTYMK,NME7,UPRT,NME3,UCK1,DHODH,CMPK2,NME6,DCTD,UPP1	grey	VSMC_M10
GO:0042501	0.28	0.92	5	7	serine phosphorylation of STAT protein	BP	1	RET,CDK5R1,NLK,GADD45A,LIF	grey	VSMC_M10
GO:0032372	0.28	0.89	7	10	negative regulation of sterol transport	BP	1	ABCG5,ABCG8,APOC1,EGF,MIR27B,MIR27A,MIR33B	grey	VSMC_M10
GO:0032375	0.28	0.89	7	10	negative regulation of cholesterol transport	BP	1	ABCG5,ABCG8,APOC1,EGF,MIR27B,MIR27A,MIR33B	grey	VSMC_M10
GO:0015803	0.28	0.92	5	7	branched-chain amino acid transport	BP	1	SLC38A9,SLC43A1,SLC3A2,SLC25A44,SLC7A5	grey	VSMC_M10
GO:1900246	0.28	0.92	5	7	positive regulation of RIG-I signaling pathway	BP	1	USP15,ANKRD17,PUM2,OASL,ZCCHC3	grey	VSMC_M10

GO:0010758	0.28	0.85	13	21	regulation of macrophage chemotaxis	BP	1	PTK2,AKIRIN1,C5AR1,MTUS1,PTK2B,TRPV4,CMKLR1,SLAMF8,TNFSF18,MAPK1,MAPK3,CCL5,CSF1R	grey	VSMC_M10
GO:0045717	0.28	0.88	8	12	negative regulation of fatty acid biosynthetic process	BP	1	APOC1,PIBF1,TRIB3,CYP7A1,ERLIN1,ACADL,ACADVL,INSIG2	grey	VSMC_M10
GO:0050996	0.28	0.88	8	12	positive regulation of lipid catabolic process	BP	1	PPARA,PRKCE,FGF21,ABHD5,AKT2,IRS1,CPT1A,DAGLB	grey	VSMC_M10
GO:0010917	0.28	0.88	8	12	negative regulation of mitochondrial membrane potential	BP	1	HEBP2,SLC25A27,RNF122,ARL6IP5,MAPT,TRPV1,PIP5KL1,BNIP3L	grey	VSMC_M10
GO:0033235	0.28	0.88	8	12	positive regulation of protein sumoylation	BP	1	TOLLIP,PIAS3,ARNT,HDAC4,MUL1,PIAS4,RWDD3,RASD2	grey	VSMC_M10
GO:0006885	0.28	0.79	40	70	regulation of pH	BP	1	SLC4A4,PPT1,RAB7A,ATP6V0E2,LACC1,SLC4A2,AQP11,SLC4A7,TMEM106B,MAFG,CCKBR,ATP6V1H,TM9SF4,SLC9A9,HVCN1,SLC4A10,SLC9B1,TMEM9,TCIRG1,SLC9A8,SLAMF8,RHCG,SLC9A1,CLIC4,SLC11A1,SLC9A7,ATP6V0A1,MAPK1,DMXL2,SLC4A3,MAPK3,AC138894.1,CLN6,SNAPIN,TMEM199,SLC9A6,ATP6AP1,SLC9A5,CA2,RAB39A	grey	VSMC_M10
GO:0097250	0.28	0.94	4	5	mitochondrial respirasome assembly	BP	1	HIGD1A,COX7A1,COX7A2,COX7A2L	grey	VSMC_M10
GO:2000785	0.28	0.82	22	37	regulation of autophagosome assembly	BP	1	PIP4K2C,RNF5,PIP4K2A,RALB,RAB5A,FEZ2,ULK1,TBC1D12,SEC22B,TMEM39A,ADPRH,MTMR3,RAB1B,PIKFYVE,TBC1D14,PINK1,PHF23,SMCR8,IFT20,MTM1,KIAA1324,SH3GLB1	grey	VSMC_M10
GO:0002573	0.28	0.77	86	154	myeloid leukocyte differentiation	BP	1	NME1,IL5,TRIB1,MYC,TOB2,L3MBTL3,MAPK14,HSF1,MYH9,CDK6,KIT,ROR2,CD109,HOXA7,TLR4,IL15,OSTM1,GAB2,POU4F1,TGFBR2,FOXP1,MITF,IRF7,TRAF6,CAMK4,CEBPA,PIK3R1,FBXW7,AP3B1,F2RL1,TESC,RIPK1,TSPAN2,ITGB6,SH3PXD2A,LRRK1,SLC9B2,TCIRG1,GPR137B,PIAS3,ACIN1,TCTA,HCLS1,UBASH3B,FARP2,ZBTB46,SP3,CREB1,BATF2,ESRRA,GPR137,FBN1,LTBR,INHA,TFE3,PARP1,RBPJ,MED1,ANXA2,PSEN1,FOS,BATF,HAX1,GPR68,CD74,CSF1R,PPARGC1B,EVI2B,PTPN2,GPC3,JUN,ATP6AP1,MIR125B1,FSTL3,MAFB,FADD,CASP8,CA2,DHRS2,BGLAP,SNX10,GLO1,NRROS,LIF,CEBPB,BATF3	grey	VSMC_M10
GO:0010664	0.28	0.84	16	26	negative regulation of striated muscle cell apoptotic process	BP	1	PPP1R10,RGL2,HSF1,PAX8,HAND2,SIRT5,SIRT4,BAG3,NFE2L2,PDPK1,MIR199A1,MIR21,MIR24-1,NOL3,NKX2-5,HEY2	grey	VSMC_M10
GO:0061909	0.28	0.91	5	7	autophagosome-lysosome fusion	BP	1	PIP4K2A,RUBCNL,VPS39,ATP13A2,AC138894.1	grey	VSMC_M10
GO:0009201	0.28	0.80	31	52	ribonucleoside triphosphate biosynthetic process	BP	1	NME1,ATP5MGL,PPARA,IMPDH1,SLC25A13,SPHK2,PRKAG2,ATP5F1C,ATP5F1A,DGUOK,FAM173B,NME4,CTPS2,DNAJC30,ATP5S,PINK1,STAT3,NME7,PARP1,PKLR,ATP6V0C,NME3,ATP5PB,VPS9D1,UCK1,MT-ATP8,MT-ATP6,ATP5ME,ATP5MF,NME6,NUDT2	grey	VSMC_M10
GO:0070127	0.28	0.91	5	7	tRNA aminoacylation for mitochondrial protein translation	BP	1	WARS2,YARS2,TARS2,DARS2,EARS2	grey	VSMC_M10
GO:0045542	0.28	0.91	5	7	positive regulation of cholesterol biosynthetic process	BP	1	CYP7A1,SCAP,POR,SREBF1,FGF1	grey	VSMC_M10
GO:0106120	0.28	0.91	5	7	positive regulation of sterol biosynthetic process	BP	1	CYP7A1,SCAP,POR,SREBF1,FGF1	grey	VSMC_M10
GO:0002824	0.28	0.80	37	64	positive regulation of adaptive immune response based on somatic recombination of immune receptors built from immunoglobulin superfamily domains	BP	1	CD226,STX7,HLA-DRA,EXOSC3,MSH2,RSAD2,HMCES,NECTIN2,PVR,MLH1,SLC22A13,PAXIP1,SHLD1,HPX,TRAF6,RIPK2,NOD2,CD40,DENND1B,CD28,FZD5,PLA2G4A,IL12A,SLC11A1,FOXP3,ZBTB1,SHLD2,TBX21,BTK,FBXO38,C3,PRKCZ,TRAF2,MIR21,FADD,FCER2,TNFSF13B	grey	VSMC_M10
GO:1903961	0.28	0.91	5	7	positive regulation of anion transmembrane transport	BP	1	ABCB1,AGT,ACE2,ARL6IP1,PSEN1	grey	VSMC_M10
GO:0007350	0.28	0.87	9	14	blastoderm segmentation	BP	1	FRS2,SEMA3A,FAM208A,SEMA3F,HOXD8,FZD5,WDR77,WNT5A,BASP1	grey	VSMC_M10
GO:0010662	0.28	0.82	21	35	regulation of striated muscle cell apoptotic process	BP	1	MAP3K5,PPP1R10,RGL2,HSF1,PAX8,HAND2,SIRT5,SIRT4,AGT,BAG3,NFE2L2,CXCR2,CAMK2A,PDPK1,MIR199A1,MIR21,MIR24-1,NOL3,TIGAR,NKX2-5,HEY2	grey	VSMC_M10
GO:0000244	0.28	0.86	11	17	spliceosomal tri-snRNP complex assembly	BP	1	PRPF31,SRSF10,AAR2,SART3,PRPF19,RNU5E-1,RNU5B-1,RNU4ATAC,RNU4-2,SRSF12,RNU4-1	grey	VSMC_M10
GO:0030388	0.28	0.91	5	7	fructose 1,6-bisphosphate metabolic process	BP	1	PFKP,PFKM,ALDOC,FBP2,TIGAR	grey	VSMC_M10
GO:0072044	0.28	0.89	6	9	collecting duct development	BP	1	PTCH1,AKR1B1,TFAP2B,PAX8,PAX2,PKD1	grey	VSMC_M10
GO:1903792	0.28	0.87	9	14	negative regulation of anion transport	BP	1	GOPC,TCAF2,SFRP4,ARL6IP5,SLC43A1,PLA2R1,ABAT,GABRE,RGS4	grey	VSMC_M10
GO:0002604	0.28	0.91	5	7	regulation of dendritic cell antigen processing and presentation	BP	1	NOD1,NOD2,SLC11A1,CD74,FGL2	grey	VSMC_M10
GO:0035666	0.29	0.91	5	7	TRIF-dependent toll-like receptor signaling pathway	BP	1	TLR4,CD300LF,CD40,TRAF3,TICAM1	grey	VSMC_M10
GO:0071379	0.29	0.86	10	16	cellular response to prostaglandin stimulus	BP	1	PRKCE,TNC,APOB,PTGER2,AKAP8,ACACA,PTGER4,GNB1,PTGFR,GNG2	grey	VSMC_M10
GO:1900746	0.29	0.84	13	21	regulation of vascular endothelial growth factor signaling pathway	BP	1	SEMA6A,PTP4A3,ADAMTS3,TSPAN12,SPRY2,DCN,PIK3CB,ROBO1,IL12A,DAB2IP,MIR199A1,MIR21,CADM4	grey	VSMC_M10
GO:0071318	0.29	0.87	9	14	cellular response to ATP	BP	1	TRPM4,P2RX3,RYR3,P2RX7,P2RY1,SSH1,TAF1,TRPV1,PDXP	grey	VSMC_M10
GO:0021532	0.29	0.83	17	28	neural tube patterning	BP	1	TBC1D32,PTCH1,SMO,BMI1,GLI2,RPGRIP1L,SUFU,GBX2,FKBP8,GORAB,GPR161,TULP3,WDR19,PSEN1,TMEM107,MKS1,GSC	grey	VSMC_M10
GO:1904415	0.29	0.91	5	7	regulation of xenophagy	BP	1	NOD1,OPTN,RIPK2,NOD2,MAPK3	grey	VSMC_M10
GO:1904417	0.29	0.91	5	7	positive regulation of xenophagy	BP	1	NOD1,OPTN,RIPK2,NOD2,MAPK3	grey	VSMC_M10
GO:0009191	0.29	0.91	5	7	ribonucleoside diphosphate catabolic process	BP	1	NT5E,NUDT5,NUDT16,ENTPD4,NUDT18	grey	VSMC_M10
GO:0001956	0.29	0.87	9	14	positive regulation of neurotransmitter secretion	BP	1	CACNB2,SYT1,DTNBP1,DNM1L,GPER1,STX1A,STX1B,BAIAP3,STXBP1	grey	VSMC_M10
GO:0043029	0.29	0.83	17	28	T cell homeostasis	BP	1	SLC46A2,NCKAP1L,ZC3H8,CASP3,GPAM,TCIRG1,PPP2R3C,RC3H1,PPP3CB,STAT5B,FOXP3,DNAJA3,FADD,TSC22D3,BCL2L11,SLC39A3,RC3H2	grey	VSMC_M10
GO:0002821	0.29	0.79	38	66	positive regulation of adaptive immune response	BP	1	CD226,STX7,HLA-DRA,EXOSC3,MSH2,RSAD2,HMCES,NECTIN2,PVR,MLH1,SLC22A13,PAXIP1,SHLD1,HPX,TRAF6,EIF2AK4,RIPK2,NOD2,CD40,DENND1B,CD28,FZD5,PLA2G4A,IL12A,SLC11A1,FOXP3,ZBTB1,SHLD2,TBX21,BTK,FBXO38,C3,PRKCZ,TRAF2,MIR21,FADD,FCER2,TNFSF13B	grey	VSMC_M10
GO:1990314	0.29	0.91	5	7	cellular response to insulin-like growth factor stimulus	BP	1	SYAP1,CREB1,FBN1,CRK,SLC25A33	grey	VSMC_M10

GO:2000638	0.29	0.91	5	7	regulation of SREBP signaling pathway	BP	1	C12orf49,FBXW7,AMFR,ZBTB7B,INSIG2	grey	VSMC_M10
GO:0045623	0.29	0.86	9	14	negative regulation of T-helper cell differentiation	BP	1	HLX,BCL6,RC3H1,TNFSF18,FOXP3,TBX21,ZBTB7B,IL4R,RC3H2	grey	VSMC_M10
GO:0014874	0.29	0.88	8	12	response to stimulus involved in regulation of muscle adaptation	BP	1	SCN5A,DAG1,AGT,CASQ1,ACTN3,HDAC4,PRKAG3,NOL3	grey	VSMC_M10
GO:0038030	0.29	0.91	5	7	non-canonical Wnt signaling pathway via MAPK cascade	BP	1	NKD1,DVL3,FZD7,WNT4,WNT5A	grey	VSMC_M10
GO:0090324	0.29	0.91	5	7	negative regulation of oxidative phosphorylation	BP	1	DNAJC15,ACTN3,MLXIPL,RHOA,PPIF	grey	VSMC_M10
GO:0010896	0.29	0.91	5	7	regulation of triglyceride catabolic process	BP	1	FGF21,ABHD5,GPLD1,SORL1,DAGLB	grey	VSMC_M10
GO:0033083	0.29	0.91	5	7	regulation of immature T cell proliferation	BP	1	BMI1,IL1A,TMEM131L,RIPK2,FOXP3	grey	VSMC_M10
GO:0033617	0.29	0.85	11	17	mitochondrial cytochrome c oxidase assembly	BP	1	TIMM21,COA5,COX17,OXA1L,COA3,BCS1L,COX16,APOPT1,COX14,COA1,SCO1	grey	VSMC_M10
GO:1901029	0.29	0.91	5	7	negative regulation of mitochondrial outer membrane permeabilization involved in apoptotic signaling pathway	BP	1	TMEM14A,FZD9,ACAA2,SLC35F6,AC140504.1	grey	VSMC_M10
GO:0070831	0.29	0.86	9	14	basement membrane assembly	BP	1	PXDN,CLASP2,DAG1,LAMB3,PHLDB1,PLOD3,RAMP2,NTNG2,LAMB1	grey	VSMC_M10
GO:0033240	0.29	0.91	5	7	positive regulation of cellular amine metabolic process	BP	1	GPR37,NR1H4,COMT,ABAT,PARK7	grey	VSMC_M10
GO:0022900	0.29	0.76	86	149	electron transport chain	BP	1	RDH16,P4HA2,UQCRQ,NDUFA6,AKR1B1,SLC25A13,ADH5,ETFB,ME1,DLD,NDUFB2,NDUFB6,AKR1A1,DNAJC15,NDUFC2,IDO1,NDUFS8,NDUFA12,COX7A1,COX4I2,ASPH,CYB5A,GPD1,NDUFS3,ETFDH,CIAPIN1,DGUOK,LOXL2,NQO2,UQCRB,COX15,COX6B1,SRD5A1,NDUFV3,GLRX2,NDUFB4,ISCU,NCF1,COA6,POLG2,PDIA5,NDUFB3,NDUFS2,SDHC,NCF2,POR,COX6A1,FDX1,COX8A,PINK1,NDUFA9,ALDH4A1,SDHB,DHRS3,DEGS1,COX6A2,COX11,NDUFAB1,COX5A,NDUFA7,NDUFV2,ENOX2,NDUFB10,UQCR11,PARK7,ALDH2,CYBA,MT-ND1,MT-ND2,MT-CO3,MT-ND6,MT-CYB,NDUFA5,DHRS2,NDUFC1,COX7A2,ETFRF1,NDUFA3,NDUFB5,NDUFS6,NDUFA8,SDHAF2,NDUFS5,AOC2,PHGDH,COX7A2L	grey	VSMC_M10
GO:0072273	0.29	0.83	14	23	metanephric nephron morphogenesis	BP	1	SMO,SIX2,SOX9,PAX8,FMN1,PKD2,PAX2,SALL1,WNT4,CITED1,LHX1,PKD1,SOX8,LIF	grey	VSMC_M10
GO:0098754	0.29	0.78	52	90	detoxification	BP	1	LTC4S,APOM,PRXL2C,KDM3B,FANCC,PIM1,ALDH1A1,ABCB1,TXNRD1,PRDX1,ADH5,CAT,ALB,PXDN,MGST2,TXNRD3,DHFR,ATP7B,ESD,AKR1A1,GSTK1,PRDX3,CYGB,PXDNL,GLRX,GPX8,TP53INP1,ABCC2,ABCG2,SLC30A10,FBLN5,MARC2,MARC1,NFE2L2,DUOX1,GCH1,ABCB6,RDH11,SOD3,GSTZ1,MPO,RALBP1,TXNDC2,PARK7,NQO1,MIR21,MT1X,SLC22A5,HBA2,TXN,PTGES,ADH4	grey	VSMC_M10
GO:0051683	0.29	0.91	5	7	establishment of Golgi localization	BP	1	YWHAZ,ARHGAP21,COPG1,PDCD10,RIPOR1	grey	VSMC_M10
GO:0042368	0.29	0.91	5	7	vitamin D biosynthetic process	BP	1	CYP27B1,CYP2R1,SNAI1,CYP27A1,GFI1	grey	VSMC_M10
GO:0010002	0.29	0.85	10	16	cardioblast differentiation	BP	1	SRF,NRG1,TBX5,PRICKLE1,TGFB2,RBPJ,TBX2,GATA6,ISL1,NKX2-5	grey	VSMC_M10
GO:0021979	0.29	0.91	5	7	hypothalamus cell differentiation	BP	1	NDNF,SEMA3A,HAP1,UBB,POU3F2	grey	VSMC_M10
GO:0006635	0.29	0.79	37	64	fatty acid beta-oxidation	BP	1	HSD17B4,CPT1B,PPARA,MCAT,ECHDC1,CROT,ETFB,HADHA,ACOX1,AKT2,ACOXL,ACOX2,PEX2,ETFDH,IVD,ECI2,LONP2,IRS1,CPT1A,BDH2,ABCB11,EHHADH,ACADL,TYSND1,ACADVL,HSD17B10,ABCD4,GCDH,ACAA2,DECR2,MLYCD,ABCD3,ACADM,HADHB,AMACR,ECI1,AUH	grey	VSMC_M10
GO:0007389	0.29	0.74	180	329	pattern specification process	BP	1	GLI1,FRS2,TBC1D32,SYNGAP1,SMAD5,LRRC6,PTCH1,MFNG,SMO,DAAM2,MDFI,FOXH1,IFT74,SRF,RFX3,ROR2,MEOX2,SOSTDC1,ANKS6,DYNC2LI1,SIX2,SCMH1,DNAH11,HOXA2,HOXA4,HOXA6,HOXA7,FUZ,OSR1,NTF4,ARMC4,INTU,SPRY1,BBS7,BMI1,SETDB2,DRC1,ABI1,GLI2,MEGF8,PCDH8,GALNT11,XRCC2,ACVR2B,IFT172,FOXJ1,WNT11,TGFBR2,OVOL2,ACVR2A,BHLHE40,MKKS,SEMA3A,PRKDC,HOXC6,TCF15,POFUT1,KIF3B,PAX8,HAND2,EYA1,ROBO1,ROBO2,FAM208A,MEF2C,STC1,NKX3-1,TBX5,IFT52,RPGRIP1L,PKD2,RIPPLY3,PAX2,NKD1,DNAH5,DPCD,LDB1,BMPR1B,FBXL15,SUFU,IRX2,PGAP1,SEMA3F,POGLUT1,SATB2,AXIN2,SNAI1,TTC21B,OFD1,CFAP45,AP1B1,GBX2,BICC1,HES6,MMP21,CHRD,DLX1,GRHL3,WNT3,HOXD8,HOXD4,HOXD3,AIDA,FZD5,BCOR,DNAAF2,CRAMP1,MEOX1,PKD1L1,FKBP8,GORAB,GPR161,NKX3-2,TULP3,TTC25,DNAAF4,MNS1,DVL2,BMPR1A,WDR19,TCAP,RBPJ,ALDH1A2,RNF111,PSEN1,HOXB5,HOXB6,HOXB8,HOXB9,ITGAM,TMEM107,CITED1,SMAD3,CCDC151,MKS1,GSC,TBX2,LHX1,YY1,CDX1,MIB1,NLE1,ISL1,C3,GPC3,CFAP53,HSPB11,HES4,SKI,APC2,RNF207,WDR77,ALX3,CELSR2,FOXF1,ENG,MTF2,PSKH1,ADGRG1,OOEP,FOXG1,MAFB,ACVRL1,HOXB3,NOG,LBX1,WNT5A,BASP1,OTX1,SIX1,DDIT3,C2CD3,APC,NKX2-5,FGF1,PIFO,HEY2,WNT2,HOXA3,ZIC1	grey	VSMC_M10
GO:0098926	0.29	0.82	18	30	postsynaptic signal transduction	BP	1	LY6E,CHRNA3,KPNA2,PLCB1,NRG1,CHRM5,CHRM4,CHRM3,GRK2,CRTC1,STAT3,CHRM1,CHRNB2,HRH4,CDK5R1,GNB1,GNA15,PRR7	grey	VSMC_M10
GO:0070431	0.29	0.86	9	14	nucleotide-binding oligomerization domain containing 2 signaling pathway	BP	1	TNFAIP3,HSPA1B,TLR4,LACC1,RIPK2,OTULIN,NOD2,IRGM,INAVA	grey	VSMC_M10
GO:0001682	0.29	0.87	8	12	tRNA 5'-leader removal	BP	1	RPP21,POP4,RPP40,POP5,KIAA0391,POP7,RPP38,RPP30	grey	VSMC_M10
GO:0002712	0.29	0.81	23	39	regulation of B cell mediated immunity	BP	1	CD226,EXOSC3,MSH2,HMCES,NECTIN2,MLH1,FOXJ1,PAXIP1,SHLD1,HPX,NOD2,CD40,CD28,BCL6,PTPN6,FOXP3,SHLD2,TBX21,BTK,C3,SUPT6H,CD46,FCER2	grey	VSMC_M10
GO:0002889	0.29	0.81	23	39	regulation of immunoglobulin mediated immune response	BP	1	CD226,EXOSC3,MSH2,HMCES,NECTIN2,MLH1,FOXJ1,PAXIP1,SHLD1,HPX,NOD2,CD40,CD28,BCL6,PTPN6,FOXP3,SHLD2,TBX21,BTK,C3,SUPT6H,CD46,FCER2	grey	VSMC_M10
GO:1901509	0.29	0.91	5	7	regulation of endothelial tube morphogenesis	BP	1	FOXP1,ADAMTS12,LCN2,MIR21,FGF1	grey	VSMC_M10
GO:0099072	0.29	0.80	25	43	regulation of postsynaptic membrane neurotransmitter receptor levels	BP	1	ADAM22,USP46,EPS15,CTNND1,DAG1,AP2M1,EPS8,DLG1,CALY,DNM3,NSG1,GRIPAP1,RAB8A,DLG4,IQSEC2,GPHN,CPLX1,STX1B,RAB11A,ARHGAP44,AP2B1,PRKCZ,CACNG7,HRAS,CACNG5	grey	VSMC_M10

GO:0002863	0.29	0.91	5	7	positive regulation of inflammatory response to antigenic stimulus	BP	1	CD28,BTK,C3,PARK7,KARS	grey	VSMC_M10
GO:0035418	0.29	0.79	31	54	protein localization to synapse	BP	1	KIF5A,ABHD17B,ADAM22,SHANK1,DLG2,KIF5C,DAG1,BSN,MPP4,DLG1,MAPT,NRXN2,NSG1,GRIPAP1,RAB8A,DLG4,IQSEC2,NLGN2,CPLX1,STX1B,ZDHHC15,RAB11A,ARHGAP44,ASIC2,PRKCZ,MAPK8IP3,ZDHHC12,WNT5A,CACNG7,HRAS,MAP1A	grey	VSMC_M10
GO:0000050	0.29	0.91	5	7	urea cycle	BP	1	SLC25A15,CEBPA,ASL,CPS1,ASS1	grey	VSMC_M10
GO:0061157	0.29	0.78	45	79	mRNA destabilization	BP	1	PAN2,CNOT2,CNOT6,RIDA,PABPC1,PNLDC1,AGO2,TNRC6B,TUT7,EXOSC3,PNPT1,TUT4,MLH1,TNRC6C,LSM1,PAN3,CNOT1,CELF1,CNOT6L,FTO,TENT4B,RBM24,DIS3L2,GIGYF2,YTHDF2,YTHDF1,EIF4ENIF1,RC3H1,UPF1,SAMD4A,POLR2G,NT5C3B,DCP1B,TARDBP,TOB1,TNRC6A,CNOT8,PARN,METTL16,PLEKHN1,METTL14,NANOS1,ZFP36L2,RC3H2,EXOSC5	grey	VSMC_M10
GO:0048308	0.30	0.86	9	14	organelle inheritance	BP	1	YWHAZ,PLK3,GBF1,PDCD10,MAPK1,STX5,MAPK3,MAP2K1,PLK5	grey	VSMC_M10
GO:0048313	0.30	0.86	9	14	Golgi inheritance	BP	1	YWHAZ,PLK3,GBF1,PDCD10,MAPK1,STX5,MAPK3,MAP2K1,PLK5	grey	VSMC_M10
GO:0097501	0.30	0.91	5	7	stress response to metal ion	BP	1	SLC30A10,ABCB6,PARK7,HSPA5,MT1X	grey	VSMC_M10
GO:0090136	0.30	0.85	10	16	epithelial cell-cell adhesion	BP	1	SERPINB8,SRF,KIT,BVES,KIFC3,PLEKHA7,DSP,ITGB5,VCL,CAMSAP3	grey	VSMC_M10
GO:0045668	0.30	0.80	24	41	negative regulation of osteoblast differentiation	BP	1	PTCH1,RORB,CDK6,SEMA4D,HOXA2,AREG,TRPM4,SOX9,HAND2,VEGFC,HDAC7,SUFU,AXIN2,HDAC4,CHRD,MEN1,RIOX1,TOB1,CITED1,SMAD3,TWSG1,RANBP3L,SKI,NOG	grey	VSMC_M10
GO:1902547	0.30	0.83	14	23	regulation of cellular response to vascular endothelial growth factor stimulus	BP	1	SEMA6A,PTP4A3,ADAMTS3,TSPAN12,SPRY2,DCN,PIK3CB,ROBO1,IL12A,ADAMTS12,DAB2IP,MIR199A1,MIR21,CADM4	grey	VSMC_M10
GO:0006110	0.30	0.81	20	34	regulation of glycolytic process	BP	1	PPARA,PRXL2C,SLC4A4,PRKAG2,GPD1,P2RX7,PRKAG1,ZBTB20,ARNT,ACTN3,HDAC4,MLXIPL,STAT3,PSEN1,OGT,NCOR1,INSR,SLC2A6,ENTPD5,TIGAR	grey	VSMC_M10
GO:1902600	0.30	0.76	66	115	proton transmembrane transport	BP	1	ATP5MGL,ATP6V1F,MFSD3,ATP6V1E2,ATP6V1C2,UCP1,SPHK2,ATP6V0E2,ATP6V0B,ATP1A3,ATP5F1C,TMCO3,COX7A1,ATP6V1H,COX4I2,CYB5A,ATP5F1A,SLC9A9,HVCN1,SLC33A1,TESC,COX15,FAM173B,ATP6V1B2,PM20D1,COX6B1,SLC4A10,SLC2A10,SLC9B1,SLC9B2,TCIRG1,COX17,SLC9A8,CLCN4,ATP1A2,SLC9A1,DNAJC30,COX6A1,ATP5S,COX8A,SLC9A7,ATP6V0A1,SLC2A9,ATP6V1D,COX6A2,NOX5,COX5A,SLC46A1,SLC9A6,ATP6V0C,ATP6AP1,PARK7,ATP5PB,VPS9D1,SLC9A5,MT-ATP8,MT-ATP6,MT-CO3,MT-CYB,SLC25A3,ATP5ME,SLC36A1,ATP5MF,APOPT1,COX7A2L,PPIF	grey	VSMC_M10
GO:0072655	0.30	0.77	59	103	establishment of protein localization to mitochondrion	BP	1	TIMM21,PRKN,TSPO,HSPA4,TOMM22,RAC2,FBXO7,DNAJA1,MGARP,AGK,DNAJC15,PITRM1,TIMM50,USP36,BAG4,MIPEP,IMMP1L,FBXW7,HTRA2,CSNK2A2,AP3B1,TOMM34,SIRT4,OXA1L,HPS4,ARIH2,BAG3,TOMM40L,FZD5,MAPT,FIS1,NMT1,HK1,GRPEL1,C11orf65,BCS1L,PINK1,TIMM17B,MFN2,NPEPPS,HAX1,CALM1,TIMM29,SREBF1,MAIP1,PAM16,BCAP31,SH3GLB1,MICALL2,BNIP3L,TIMM10B,TIMM23B,DDIT3,RALA,TIMM8B,RHOU,LEPROT,GRPEL2,DNAJC19	grey	VSMC_M10
GO:0006586	0.30	0.91	5	7	indolalkylamine metabolic process	BP	1	IL4I1,AANAT,IDO2,IDO1,GCDH	grey	VSMC_M10
GO:0033080	0.30	0.91	5	7	immature T cell proliferation in thymus	BP	1	BMI1,IL1A,TMEM131L,WNT4,FOXP3	grey	VSMC_M10
GO:0046942	0.30	0.75	96	173	carboxylic acid transport	BP	1	ATP8B1,SLC26A10,SLC16A7,SLC12A2,NTRK2,PLA2G6,ABCC10,APBA1,SLC1A1,SLC25A13,SLC3A1,SLC1A4,SLC7A11,SLC26A5,SLC25A30,LLGL2,SLC22A13,SLC25A38,ABCC4,SLC6A15,SLC25A15,SLC23A2,ARL6IP5,SLC7A1,SLC38A9,SLC5A8,CYP7A1,SLC43A1,SERINC5,SLC38A2,SLC38A1,SLC10A6,ABCC2,PLA2R1,ABCG2,GRM2,SFXN2,SLC38A3,MAP2K6,SLC16A6,SLC7A7,SLCO3A1,ABCB11,AGT,ABCC5,ACE2,ATP1A2,SLCO1A2,SLCO1C1,PER2,NTSR1,SLC16A1,PLA2G4A,SLC11A1,PQLC2,SLC27A1,SLC38A5,MPC2,ARL6IP1,SLC3A2,SLC38A6,PRAF2,SLC25A11,SLC16A13,CLN8,SLC26A1,PSEN1,SLC7A3,AC138894.1,ABCC3,NMUR2,SLC6A7,SLC26A2,LRRC8E,EMB,NOS2,SLC46A1,SLC25A14,PROCA1,ABAT,SLC1A7,TRPV1,SLC16A4,SLC7A5,LRRC8A,ABCD3,STXBP1,SLC7A6,SLC36A1,LRRC8D,PLA2G2C,SLC25A21,SLC25A32,RGS4,SLC25A29,PTGES	grey	VSMC_M10
GO:0031338	0.30	0.83	13	21	regulation of vesicle fusion	BP	1	GRIK5,TBC1D4,AKT2,SYT1,SYT9,ANKRD27,C2CD5,RAB3A,CPLANE2,ANXA2,CPLX1,STXBP1,DOC2B	grey	VSMC_M10
GO:2000121	0.30	0.91	5	7	regulation of removal of superoxide radicals	BP	1	DHFR,FBLN5,NFE2L2,GCH1,MIR21	grey	VSMC_M10
GO:0006883	0.30	0.85	10	16	cellular sodium ion homeostasis	BP	1	NEDD4L,SLC12A2,ATP1A3,IL1A,AGT,ATP1A2,SLC9A1,ATP1B1,ATP1B2,C7	grey	VSMC_M10
GO:0039528	0.30	0.81	18	30	cytoplasmic pattern recognition receptor signaling pathway in response to virus	BP	1	USP15,ANKRD17,PUM2,PHB,SEC14L1,CLPB,NPLOC4,IRF7,OAS3,IFIH1,OASL,NLRX1,BIRC3,TKFC,TSPAN6,RIOK3,RNF135,ZCCHC3	grey	VSMC_M10
GO:1901379	0.30	0.79	32	56	regulation of potassium ion transmembrane transport	BP	1	NEDD4L,WNK2,KEL,KCNRG,OXSR1,PRNP,KCNIP3,ANO6,KCNE2,CASQ2,KCNIP2,KCNJ2,KCNG1,ANK2,KLHL24,NOS1AP,DLG1,ATP1B1,KCNA1,WNK3,ATP1B2,KCNAB3,KCNIP1,DNM2,RNF207,AMIGO1,WWP2,MIR21,KCNH2,NETO2,CRBN,RGS4	grey	VSMC_M10
GO:1901031	0.30	0.82	17	28	regulation of response to reactive oxygen species	BP	1	GPR37,MET,FOXO3,DHFR,SESN3,PAWR,RIPK1,FBLN5,PDE8A,NFE2L2,GCH1,PINK1,STK26,PARK7,MIR21,GPR37L1,TXN	grey	VSMC_M10
GO:0042492	0.30	0.89	6	9	gamma-delta T cell differentiation	BP	1	TCF7,NCKAP1L,SOX13,STAT5B,JAG2,SOX4	grey	VSMC_M10
GO:0032371	0.30	0.79	27	46	regulation of sterol transport	BP	1	LRP1,TSPO,PTCH1,TTC39B,ABCG5,ABCG8,NR1H2,APOC1,NR1H3,PLTP,ABCA5,EGF,OSBPL6,ZDHHC8,ABCA12,LPCAT3,GPS2,ANXA2,NAXE,ABCA3,ABCA7,RXRA,MIR27B,MIR27A,ABCA8,MIR33B,LDLRAP1	grey	VSMC_M10
GO:0032374	0.30	0.79	27	46	regulation of cholesterol transport	BP	1	LRP1,TSPO,PTCH1,TTC39B,ABCG5,ABCG8,NR1H2,APOC1,NR1H3,PLTP,ABCA5,EGF,OSBPL6,ZDHHC8,ABCA12,LPCAT3,GPS2,ANXA2,NAXE,ABCA3,ABCA7,RXRA,MIR27B,MIR27A,ABCA8,MIR33B,LDLRAP1	grey	VSMC_M10

GO:0042471	0.30	0.77	50	89	ear morphogenesis	BP	1	OSR2,FZD6,PTK7,ROR2,DLX5,SIX2,KCNQ4,HOXA2,WHRN,SOBP,OSR1,MYO3A,SOX9,USH1G,GLI2,MYO7A,SPRY2,CEP290,PAX8,EYA1,PLS1,FZD3,USH1C,TFAP2A,PAX2,ALDH1A3,GBX2,MYO3B,GRHL3,STRC,STOX1,PRKRA,NKX3-2,MAPK1,DVL2,WDR19,TCAP,MAPK3,POU3F4,NTN1,GSC,CHRNA10,FOXG1,MAFB,NOG,WNT5A,OTX1,SIX1,INSIG2,ZIC1	grey	VSMC_M10
GO:0014002	0.30	0.81	19	32	astrocyte development	BP	1	LRP1,AGER,SMO,TTBK1,CDK6,ROR2,FPR2,EGFR,TLR4,C5AR1,TSPAN2,KRAS,MAPT,PSEN1,LDLR,S100A8,LAMC3,CNTF,POU3F2	grey	VSMC_M10
GO:0045672	0.30	0.86	9	14	positive regulation of osteoclast differentiation	BP	1	POU4F1,TRAF6,SLC9B2,CREB1,FOS,GPR68,PPARGC1B,ATP6AP1,CA2	grey	VSMC_M10
GO:0051873	0.30	0.90	5	7	killing by host of symbiont cells	BP	1	GAPDH,MYD88,F2RL1,SCNN1B,CXCL6	grey	VSMC_M10
GO:0051883	0.30	0.90	5	7	killing of cells in other organism involved in symbiotic interaction	BP	1	GAPDH,MYD88,F2RL1,SCNN1B,CXCL6	grey	VSMC_M10
GO:0045342	0.30	0.89	6	9	MHC class II biosynthetic process	BP	1	NFX1,TLR4,HDAC2,TAF7,SLC11A1,CIITA	grey	VSMC_M10
GO:0071475	0.30	0.90	5	7	cellular hyperosmotic salinity response	BP	1	XRCC6,AKR1B1,XRCC5,MICU1,SLC25A23	grey	VSMC_M10
GO:0043300	0.30	0.81	19	32	regulation of leukocyte degranulation	BP	1	RAC2,SPHK2,GAB2,UNC13D,NCKAP1L,FER,F2RL1,FGR,SNX4,C12orf4,STX4,ITGAM,IL4R,STXBP2,PRAM1,PDPK1,FOXF1,STXBP1,AP1G1	grey	VSMC_M10
GO:0006570	0.30	0.90	5	7	tyrosine metabolic process	BP	1	IL4I1,HGD,FAH,GSTZ1,PCBD2	grey	VSMC_M10
GO:0030073	0.30	0.75	83	149	insulin secretion	BP	1	LRP1,RIMS2,PRKN,PLA2G6,PICK1,RFX3,TFAP2B,PRKCE,HMGN3,CYB5R4,BMP8A,TRPM4,GIPR,RAF1,SIRT3,SYT9,NR1H4,NDUFAF2,FAM3D,OSBP,ARNTL,KCNJ11,NNAT,TIAM1,RAB11FIP5,PFKM,SLC8B1,NKX6-1,ACVR1C,ZBED6,SIRT4,GPLD1,IRS1,HDAC1,PPP3CA,CPT1A,SLC9B2,ADRA2A,TCIRG1,TCF7L2,KCNB1,PFKFB2,GPER1,RAB11FIP2,PER2,STX1A,GLUL,SIDT2,SLC16A1,KLF7,VGF,ENY2,ABCA12,MCU,PPP3CB,CAMK2G,PTPRN,CD38,MPC2,ILDR2,SYT7,NLGN2,CPLX1,TARDBP,STX4,SYTL4,GPR68,CCL5,SREBF1,ISL1,OXCT1,NOS2,ABAT,NADK,BAIAP3,PARK7,LRRC8A,CHGA,SOX4,DPP4,CRH,DOC2B,UQCC2	grey	VSMC_M10
GO:0070942	0.30	0.90	5	7	neutrophil mediated cytotoxicity	BP	1	MYD88,F2RL1,SCNN1B,DNASE1,CXCL6	grey	VSMC_M10
GO:0007035	0.30	0.83	14	23	vacuolar acidification	BP	1	PPT1,ATP6V0E2,TMEM106B,ATP6V1H,TMEM9,TCIRG1,CLIC4,SLC11A1,ATP6V0A1,DMXL2,AC138894.1,CLN6,SNAPIN,TMEM199	grey	VSMC_M10
GO:0042994	0.30	0.86	9	14	cytoplasmic sequestering of transcription factor	BP	1	MDFI,NFKBIE,FAF1,NFKBIB,PKD2,SUFU,KEAP1,PSMD10,PKD1	grey	VSMC_M10
GO:0015808	0.30	0.90	5	7	L-alanine transport	BP	1	SLC1A4,SLC38A3,SLC38A5,SLC3A2,SLC36A1	grey	VSMC_M10
GO:0043268	0.30	0.81	17	29	positive regulation of potassium ion transport	BP	1	WNK2,ANO6,KCNIP2,KCNJ2,ADRA2A,ANK2,NOS1AP,DLG1,ATP1B1,KCNA1,WNK3,ATP1B2,DNM2,RNF207,AMIGO1,MIR21,KCNH2	grey	VSMC_M10
GO:0009185	0.30	0.77	46	81	ribonucleoside diphosphate metabolic process	BP	1	PPARA,PRXL2C,GAPDH,SLC4A4,NT5E,BPGM,CMPK1,DLG2,NUDT5,DHTKD1,PRKAG2,PFKP,NUDT16,GPI,GPD1,P2RX7,PRKAG1,PFKM,ENTPD4,NUDT18,ZBTB20,PFKFB2,ENO4,ARNT,ACTN3,HDAC4,MLXIPL,MAGI3,DLG1,HK1,CASK,PRKAG3,STAT3,ENO3,CARD11,PSEN1,PKLR,OGT,ADPGK,NCOR1,INSR,ALDOC,MPP1,SLC2A6,ENTPD5,TIGAR	grey	VSMC_M10
GO:0046643	0.30	0.88	6	9	regulation of gamma-delta T cell activation	BP	1	TCF7,NCKAP1L,SOX13,NOD2,STAT5B,SOX4	grey	VSMC_M10
GO:0046697	0.30	0.83	14	23	decidualization	BP	1	CYP27B1,PTN,VDR,STC1,SPP1,DEDD,MEN1,CTSV,MAPK1,MAPK3,EPOR,BSG,TCF23,LIF	grey	VSMC_M10
GO:0016101	0.30	0.79	29	50	diterpenoid metabolic process	BP	1	RDH5,RDH16,LRP1,ALDH8A1,AKR1B1,ALDH1A1,ADH5,RDH13,EGFR,NAPEPLD,LIPE,SDR16C5,RETSAT,PRMT3,SRD5A1,ALDH1A3,DHRS9,BCO2,PECR,DHRS3,RDH11,ALDH1A2,SCPEP1,LRP8,PNPLA4,CRH,LRAT,RDH14,ADH4	grey	VSMC_M10
GO:0034397	0.30	0.90	5	7	telomere localization	BP	1	UBE2B,CCDC155,MLH1,SPO11,MAJIN	grey	VSMC_M10
GO:0090220	0.30	0.90	5	7	chromosome localization to nuclear envelope involved in homologous chromosome segregation	BP	1	UBE2B,CCDC155,MLH1,SPO11,MAJIN	grey	VSMC_M10
GO:0040020	0.30	0.83	13	21	regulation of meiotic nuclear division	BP	1	FBXO43,UBE2B,SIRT2,PLCB1,MOS,NPM2,PRKAR1A,GPR3,WNT4,FZR1,OOEP,WNT5A,LIF	grey	VSMC_M10
GO:0007379	0.30	0.85	9	14	segment specification	BP	1	MEOX2,HOXA2,OSR1,BMI1,IRX2,MEOX1,DVL2,MTF2,MAFB	grey	VSMC_M10
GO:0010172	0.31	0.88	6	9	embryonic body morphogenesis	BP	1	FUZ,CLASP2,MAB21L2,DAG1,PHLDB1,GREM2	grey	VSMC_M10
GO:0072528	0.31	0.81	17	28	pyrimidine-containing compound biosynthetic process	BP	1	NME1,TBPL1,CMPK1,NME4,CTPS2,DTYMK,CPS1,NME7,UPRT,NME3,UCK1,DHODH,TPK1,CMPK2,NME6,DCTD,UPP1	grey	VSMC_M10
GO:0060602	0.31	0.85	10	16	branch elongation of an epithelium	BP	1	ESR1,AREG,TNC,SPRY1,SOX9,SPRY2,MED1,WNT5A,SIX1,FGF1	grey	VSMC_M10
GO:0000966	0.31	0.83	13	21	RNA 5'-end processing	BP	1	RPP21,PNPT1,POP4,RPP40,POP5,NOP9,KIAA0391,POP7,HSD17B10,THG1L,RPP38,TRMT10C,RPP30	grey	VSMC_M10
GO:0046838	0.31	0.84	10	16	phosphorylated carbohydrate dephosphorylation	BP	1	SYNJ2,INPP4B,MTMR2,MTMR7,SYNJ1,INPP5A,INPP5J,IMPA2,INPP5K,INPP1	grey	VSMC_M10
GO:0046855	0.31	0.84	10	16	inositol phosphate dephosphorylation	BP	1	SYNJ2,INPP4B,MTMR2,MTMR7,SYNJ1,INPP5A,INPP5J,IMPA2,INPP5K,INPP1	grey	VSMC_M10
GO:0007342	0.31	0.90	5	7	fusion of sperm to egg plasma membrane involved in single fertilization	BP	1	SPACA6,IZUMO1,LYZL6,SPESP1,NOX5	grey	VSMC_M10
GO:0010669	0.31	0.84	10	16	epithelial structure maintenance	BP	1	SRF,KIAA1211,TLR4,SOX9,NOD2,LDB1,PBLD,MKS1,SLC22A5,INAVA	grey	VSMC_M10
GO:1903588	0.31	0.90	5	7	negative regulation of blood vessel endothelial cell proliferation involved in sprouting angiogenesis	BP	1	IL12A,PDCD10,NGFR,MIR222,MIR24-1	grey	VSMC_M10
GO:0031204	0.31	0.90	5	7	posttranslational protein targeting to membrane, translocation	BP	1	SEC61B,SEC61G,SEC62,HSPA5,SEC61A2	grey	VSMC_M10
GO:0070561	0.31	0.88	6	9	vitamin D receptor signaling pathway	BP	1	CYP27B1,SNW1,PIM1,VDR,MED1,TRIM24	grey	VSMC_M10
GO:0035860	0.31	0.88	6	9	glial cell-derived neurotrophic factor receptor signaling pathway	BP	1	GFRA3,GFRA4,RET,SULF2,GFRA1,GDF15	grey	VSMC_M10
GO:0015721	0.31	0.84	10	16	bile acid and bile salt transport	BP	1	ATP8B1,ABCC4,CYP7A1,SLC10A6,ABCC2,ABCB11,SLCO1A2,SLCO1C1,ABCC3,ABCD3	grey	VSMC_M10
GO:0046051	0.31	0.90	5	7	UTP metabolic process	BP	1	NME1,NME4,NME7,NME3,NME6	grey	VSMC_M10
GO:0044351	0.31	0.88	6	9	macropinocytosis	BP	1	MAPKAPK3,CARMIL1,MAPKAPK2,STX1B,DOCK2,DNM2	grey	VSMC_M10

GO:0090276	0.31	0.75	78	140	regulation of peptide hormone secretion	BP	1	LRP1,PRKN,PLA2G6,PICK1,RFX3,TFAP2B,PRKCE,HMGN3,BMP8A,EGFR,TRPM4,GIPR,AIMP1,SIRT3,SYT9,NR1H4,NDUFAF2,FAM3D,PAX8,OSBP,ARNTL,KCNJ11,NNAT,TIAM1,PFKM,SLC8B1,NKX6-1,ACVR1C,ZBED6,SIRT4,GPLD1,IRS1,HDAC1,PPP3CA,CPT1A,SLC9B2,ADRA2A,TCIRG1,TCF7L2,KCNB1,PFKFB2,GPER1,PER2,STX1A,PASK,GLUL,SIDT2,SLC16A1,KLF7,ENY2,ABCA12,MCU,PPP3CB,CD38,MPC2,SYT7,NLGN2,TARDBP,STX4,RAB8B,SYTL4,GPR68,CCL5,SREBF1,ISL1,OXCT1,NOS2,ABAT,NADK,BAIAP3,LRRC8A,FGA,CHGA,SOX4,DPP4,CRH,DOC2B,UQCC2	grey	VSMC_M10
GO:1903203	0.31	0.82	14	23	regulation of oxidative stress-induced neuron death	BP	1	PRKN,NCOA7,ATF4,FBXO7,TLR4,SLC7A11,FBXW7,MCL1,NONO,PINK1,PARP1,PARK7,TSC1,TLDC1	grey	VSMC_M10
GO:2000601	0.31	0.88	6	9	positive regulation of Arp2/3 complex-mediated actin nucleation	BP	1	WASF1,FCHSD2,CYFIP1,WASF2,NCKAP1,WAS	grey	VSMC_M10
GO:0060759	0.31	0.75	72	128	regulation of response to cytokine stimulus	BP	1	TNFAIP3,HSPA1B,PRKN,AGPAT1,IL1R2,VRK2,PXDN,NR1H2,TRIM32,TLR4,CD300LF,IRAK2,IKBKB,MAVS,GAS6,HPX,IRF7,TRIM44,OTUD4,TAF9,ROBO1,IL7,NR1H3,OAS3,NLRC5,RIPK2,OTULIN,F2RL1,RIPK1,IFIH1,SPATA2,YTHDF2,PARP9,PARP14,PELI3,PTPN6,MUL1,SPPL2A,PADI2,GPS2,USP18,MED1,PYDC1,DCST1,MAP2K5,STAT2,CD74,CCL5,MAPK7,PTPN2,SOCS1,PIAS4,SPPL2B,TRAF2,AGPAT2,GFI1,MIR21,MIR125B1,MIR27B,MIR24-1,MIR27A,OAS1,NOL3,FADD,CASP8,MIR125A,IRGM,WNT5A,C1QTNF4,CCDC3,TSLP,HIST1H2BJ	grey	VSMC_M10
GO:0006972	0.31	0.82	14	23	hyperosmotic response	BP	1	SLC12A2,XRCC6,AKR1B1,AQP1,HNMT,ICOSLG,TRPV4,YBX3,XRCC5,MICU1,SLC25A23,PDPK1,MT-CYB,CLDN1	grey	VSMC_M10
GO:0001508	0.31	0.76	55	99	action potential	BP	1	NEDD4L,NTRK2,CLDN19,MYH14,CLCN1,TRPM4,SLC8A2,CACNB2,SCN5A,SCN1A,GPD1L,SLMAP,P2RX3,KCNJ3,KCNE2,DSP,KCNIP2,RYR2,PKP2,KCNJ2,SCN2A,KCNB1,SCN7A,ANK2,GPER1,ATP1A2,SLC9A1,NTSR1,SCN4B,NOS1AP,DLG1,CNTNAP1,ATP1B1,KCNA1,SLC4A3,CACNA1G,AC138894.1,CHRNB2,CALM1,DSG2,FMR1,GNA15,P2RX1,RNF207,CACNA1H,DMD,GPR35,KCNH2,SCN2B,GPR88,YWHAH,KCNJ8,GNA14,HCN4,KCNMB4	grey	VSMC_M10
GO:0014870	0.31	0.90	5	7	response to muscle inactivity	BP	1	SCN5A,DAG1,CASQ1,ACTN3,HDAC4	grey	VSMC_M10
GO:0014877	0.31	0.90	5	7	response to muscle inactivity involved in regulation of muscle adaptation	BP	1	SCN5A,DAG1,CASQ1,ACTN3,HDAC4	grey	VSMC_M10
GO:0014894	0.31	0.90	5	7	response to denervation involved in regulation of muscle adaptation	BP	1	SCN5A,DAG1,CASQ1,ACTN3,HDAC4	grey	VSMC_M10
GO:0048839	0.31	0.75	77	139	inner ear development	BP	1	ATP8B1,EYA4,FZD6,PTK7,SLC25A27,ROR2,DLX5,KCNQ4,GSDME,HOXA13,MYCL,BMPER,WHRN,ATG5,MYCN,SOBP,SLC26A5,MYO3A,SOX9,USH1G,GLI2,MYO7A,TSKU,SPRY2,SLC4A7,JAG1,LRTOMT,AP000812.5,LIN7A,FREM2,CEP290,PAX8,CEBPA,EYA1,PLS1,ADGRV1,FZD3,USH1C,OPA1,RPGRIP1L,TFAP2A,PAX2,ALDH1A3,TGFB2,TRIP11,MAPKAPK2,SOX2,GBX2,MYO3B,KCNK2,GRHL3,STRC,CDKN1B,STOX1,CDH23,EPHA4,DVL2,TCAP,RBPJ,ESRRB,POU3F4,NTN1,MKS1,JAG2,IFT20,MCOLN3,KCNK3,PJVK,CHRNA10,FOXG1,MAFB,WNT5A,OTX1,SIX1,INSIG2,HEY2,ZIC1	grey	VSMC_M10
GO:0070585	0.31	0.75	61	107	protein localization to mitochondrion	BP	1	TIMM21,MTCH2,PRKN,TSPO,HSPA4,TOMM22,RAC2,FBXO7,DNAJA1,MGARP,AGK,DNAJC15,PITRM1,TIMM50,USP36,BAG4,MIPEP,IMMP1L,FBXW7,HTRA2,CSNK2A2,AP3B1,TOMM34,SIRT4,DNM1L,OXA1L,HPS4,ARIH2,BAG3,TOMM40L,FZD5,MAPT,FIS1,NMT1,HK1,GRPEL1,C11orf65,BCS1L,PINK1,TIMM17B,MFN2,NPEPPS,HAX1,CALM1,TIMM29,SREBF1,MAIP1,PAM16,BCAP31,SH3GLB1,MICALL2,BNIP3L,TIMM10B,TIMM23B,DDIT3,RALA,TIMM8B,RHOU,LEPROT,GRPEL2,DNAJC19	grey	VSMC_M10
GO:2000351	0.31	0.79	23	39	regulation of endothelial cell apoptotic process	BP	1	TNFAIP3,TEK,FOXO3,FGF21,NDNF,GAS6,ANO6,CD40,TERT,PDCD4,GPER1,NFE2L2,ITGA4,TNIP2,RAMP2,MAPK7,CCL2,PDPK1,MIR24-1,FGA,MIR125A,CD248,RGCC	grey	VSMC_M10
GO:0021520	0.31	0.90	5	7	spinal cord motor neuron cell fate specification	BP	1	GLI2,MNX1,SUFU,ISL1,ISL2	grey	VSMC_M10
GO:0002260	0.31	0.78	29	50	lymphocyte homeostasis	BP	1	TNFAIP3,BAK1,SLC46A2,TRAF3IP2,NCKAP1L,GAPT,ZC3H8,CASP3,MEF2C,DOCK10,GPAM,TCIRG1,PPP2R3C,RC3H1,PPP3CB,STAT5B,FOXP3,LAT,CD74,DNAJA3,BCL10,FADD,CAMLG,TSC22D3,BCL2L11,SH2B2,SLC39A3,TNFSF13B,RC3H2	grey	VSMC_M10
GO:0033762	0.31	0.85	9	14	response to glucagon	BP	1	CRY1,FGF21,CYSLTR2,ABCC2,CREB1,CPS1,SREBF1,ASS1,MT-CYB	grey	VSMC_M10
GO:0042475	0.31	0.77	36	63	odontogenesis of dentin-containing tooth	BP	1	PPARA,TNFRSF11B,SMO,CNNM4,SOSTDC1,KLK4,TNC,HDAC2,SLC4A2,GLI2,SCN5A,ACVR2A,RELT,TRAF6,HAND2,ITGB6,HDAC1,TCIRG1,FAM20A,FAM20C,AMELX,HTRA1,LAMA5,DLX1,BMPR1A,NGFR,DLX3,BCL11B,JAG2,ROGDI,BSG,ANKRD11,LHX8,CA2,BCL2L11,PERP	grey	VSMC_M10
GO:0002274	0.31	0.74	87	156	myeloid leukocyte activation	BP	1	CD226,ENPP3,AGER,RAC2,TTBK1,KIT,PRKCE,FPR2,CXCL8,TLR4,IL15,TRAF3IP2,SPHK2,C5AR1,CST7,CD300LF,NECTIN2,GAB2,UNC13D,MYD88,TGFBR2,NMI,FOXP1,MFHAS1,TRAF6,CAMK4,CEBPA,FER,RASGRP1,NR1H3,DYSF,IFNGR2,F2RL1,ITGB6,CD200,GPR137B,SNAP23,LAT2,FGR,CBL,SNX4,FCER1G,MAPT,PLA2G4A,BATF2,CXCR2,SLC11A1,PTGDR,TNIP2,LTBR,C12orf4,RBPJ,STX4,PSEN1,ITGAM,SYT11,LCP2,DOCK2,BATF,LAT,IL4R,LDLR,S100A13,BTK,SCNN1B,CD74,CCL5,STXBP2,PRAM1,TICAM1,DNASE1,TRPV1,PDPK1,FOXF1,KARS,STXBP1,S100A12,HAVCR1,CHGA,CXCL6,DHRS2,MIR125A,WNT5A,IL33,TSLP,HAVCR2,BATF3	grey	VSMC_M10
GO:0006882	0.31	0.82	14	23	cellular zinc ion homeostasis	BP	1	SLC39A5,SLC39A4,SLC1A1,SLC39A12,SLC39A13,AP3B1,LCK,SLC30A10,SLC30A4,ATP13A2,S100A8,SLC39A6,SLC30A7,MT1X	grey	VSMC_M10
GO:0045581	0.31	0.80	19	32	negative regulation of T cell differentiation	BP	1	FOXJ1,ZC3H8,TMEM131L,HLX,RUNX3,BCL6,RC3H1,TNFSF18,LAG3,FOXP3,TBX21,ZBTB7B,IL4R,CD74,PTPN2,SOCS1,MIR21,FGL2,RC3H2	grey	VSMC_M10

GO:0070286	0.31	0.81	15	25	axonemal dynein complex assembly	BP	1	LRRC6,WDR92,ARMC4,DRC1,CCDC65,DNAH1,DNAH5,ZMYND10,TTC12,DNAAF2,TTC25,DNAAF4,CCDC151,C16orf71,WDR63	grey	VSMC_M10
GO:0046174	0.31	0.81	15	25	polyol catabolic process	BP	1	MIOX,SYNJ2,INPP4B,MTMR2,MTMR7,GK5,SYNJ1,INPP5A,NTSR1,INPP5J,SORD,TKFC,IMPA2,INPP5K,INPP1	grey	VSMC_M10
GO:0046425	0.31	0.78	30	52	regulation of receptor signaling pathway via JAK-STAT	BP	1	IL5,AKR1B1,OCIAD1,KIT,PPP2R1A,PIBF1,RET,PIGU,PTK2B,F2R,MST1,EGF,PARP14,AGT,IL10RA,IL6R,ELP2,CCL5,CDK5R1,SUZ12,GHR,NLK,PTPN2,SOCS1,DAB1,DOT1L,MIR125A,GADD45A,LIF,LEPROT	grey	VSMC_M10
GO:0046514	0.31	0.84	10	16	ceramide catabolic process	BP	1	ACER3,NEU3,ASAH1,GALC,GLA,HEXA,GM2A,CEL,ACER2,NEU2	grey	VSMC_M10
GO:0061436	0.31	0.84	10	16	establishment of skin barrier	BP	1	GRHL1,MET,ELOVL1,LSR,KDF1,SFN,GRHL3,ABCA12,ALOXE3,CLDN1	grey	VSMC_M10
GO:1904953	0.31	0.88	6	9	Wnt signaling pathway involved in midbrain dopaminergic neuron differentiation	BP	1	CSNK1E,FZD1,RYK,WNT3,WNT5A,WNT2	grey	VSMC_M10
GO:2000320	0.31	0.88	6	9	negative regulation of T-helper 17 cell differentiation	BP	1	RC3H1,TNFSF18,FOXP3,TBX21,ZBTB7B,RC3H2	grey	VSMC_M10
GO:0010452	0.31	0.86	7	11	histone H3-K36 methylation	BP	1	IWS1,PAXIP1,NSD3,SETD2,BCOR,NSD1,SETD3	grey	VSMC_M10
GO:1903715	0.31	0.81	17	28	regulation of aerobic respiration	BP	1	SHMT2,DNAJC15,COX7A1,IDE,TRPV4,ISCU,ACTN3,MLXIPL,PINK1,SLC25A23,PARK7,SLC25A33,RHOA,COX7A2,UQCC2,COX7A2L,PPIF	grey	VSMC_M10
GO:0043436	0.32	0.71	370	677	oxoacid metabolic process	BP	1	ATP8B1,GLS2,HSD17B4,SHMT2,ALDH8A1,MARS,AIG1,LTC4S,GFPT2,TPH2,RIDA,CPT1B,DPYS,SLC27A6,TNXB,QKI,PPARA,ACSL6,PNPLA3,MCAT,HSD17B8,ECHDC1,ACO2,PRXL2C,AKR1B1,ATF4,PYCR3,MAPK14,PSAT1,GPT,HACD4,MTAP,CARNMT1,GNMT,ALDH1A1,CROT,ABCC10,GNPDA2,SLC35B2,KIT,SLC25A13,GAPDH,AASDH,PPAT,LIPT1,HMGCLL1,ADH5,RIMKLA,PRKCE,ETFB,SLC4A4,ME1,BPGM,NR1H2,DDC,IL4I1,LPIN1,MGST2,QRSL1,SLC7A11,DLD,DDO,BCAT2,NANP,PRKAR2B,DHFR,FAAH,HADHA,GAD2,SOX9,ABHD5,AKR1A1,APOC1,HPDL,DHTKD1,ECHDC3,PRKAG2,ACOX1,SLC22A13,XYLB,PIBF1,CYGB,HYI,HNMT,ELOVL1,CSGALNACT1,AKT2,PFKP,IDO2,IDO1,MDH2,HARS,HARS2,BTD,HACL1,SLC23A2,CARS2,PANK2,PDK1,AMDHD1,LARS,SUCLG2,NR1H4,PDHX,APIP,TRIB3,LYPLA1,ACOXL,CSAD,CYP7A1,ELOVL7,ASAH1,CYP7B1,ACOX2,GPI,ABHD6,THNSL2,AHCY,CBR4,OSBP,GSS,MCCC2,HPGD,MSRA,GPD1,PEX2,P2RX7,ETFDH,ACOT12,MTHFD2,PRKAG1,GMPS,NR1H3,SERINC5,ENOPH1,PFKM,SDSL,IVD,LPIN3,HOGA1,NIT2,BPHL,ASNSD1,ECI2,HIBCH,GLS,ABCC2,ERLIN1,PLA2G4B,ACAD8,ADTRP,PRMT3,PRKAB1,WARS2,LONP2,MTRR,PM20D1,SIRT4,GATC,ASL,MCEE,ALDH5A1,AZIN2,IRS1,MTR,YARS2,CBSL,ALDH1A3,CPT1A,TPST1,BDH2,ZBTB20,GPAM,PPA2,ACSL5,NR5A2,SLC7A7,CYP2U1,HGD,MARC2,MARC1,MAPKAPK2,AMT,PFKFB2,ABHD2,MSMO1,MECR,MCCC1,GNPAT,CMAS,ENO4,TARS2,DALRD3,ABCB11,DHRS9,PC,HACD2,ARNT,LPGAT1,CTPS2,PER2,ACTN3,HDAC4,OSBPL2,ACADSB,OAT,THEM5,THEM4,MLXIPL,HMGCL,GLUL,D2HGDH,SCAP,EHHADH,SMS,MDH1B,POR,PDK3,PLA2G4A,OSBPL6,UPB1,ACADL,PLOD3,GGT2,HK1,FAH,BCO2,CPS1,MTHFS,T[illegible]	grey	VSMC_M10
GO:0006816	0.32	0.73	154	282	calcium ion transport	BP	1	STAC3,CYP27B1,TSPO,SMDT1,BAK1,CATSPER3,MCHR1,PKD2L2,PLA2G6,TRPM6,TSPAN13,PRKCE,IBTK,BSPRY,ATG5,TRPM4,SPHK2,GRIN2D,TRPC3,SLC8A2,PANX1,CACNB2,STAC,SLC24A3,CACNB4,EFHB,RYR1,NALCN,PRNP,DIAPH1,P2RX2,GAS6,RASA3,MICU3,P2RX3,TRPC4AP,RYR3,ORAI1,ASIC1,TMBIM6,P2RX7,PTK2B,TPCN1,SLC8B1,HOMER1,VDR,STC1,OPA1,F2R,ANO6,KCNE2,PKD2,CASQ2,MCUR1,RYR2,TRPM1,CALHM1,DNM1L,LCK,PPP3CA,TRPV4,ADRA2A,MCUB,EGF,EPB41,OPRD1,UBASH3B,ANK2,GPER1,TRPM8,AGT,ATP1A2,SLC9A1,PLPP4,C4orf3,CASQ1,NTSR1,NOS1AP,HOMER2,CATSPER2,TMCO1,IL16,CEMIP,SESTD1,CDH23,CASK,PTPN6,MICU1,MCU,HTT,PKD1L1,CALCRL,RAMP2,CAMK2G,TRPM7,ATP1B1,BHLHA15,SLC3A2,GNB5,HAP1,PSEN2,WNK3,STAC2,CACNB1,ANXA2,FAM155B,PSEN1,CACNA1A,CACNA1G,CHRNB2,ATP2A1,CD19,CALM1,PRKCB,VMP1,BDKRB1,NMUR2,TRPV2,CAMK2A,CCL5,CCL2,SLC25A23,MAIP1,FMR1,INPP5K,ATP2B3,TRPV3,TRPV1,PDPK1,P2RX1,CACNA1B,PKD1,CACNA1H,CYBA,JPH3,ATP2C2,SLC25A25,PKD1L3,MCOLN3,RRAD,MIR21,RHOA,DMD,GPR35,NOL3,CHRNA10,JPH2,THY1,CACNG7,RGS4,DDIT3,CRH,CACNG5,GEM	grey	VSMC_M10
GO:0050691	0.32	0.81	15	25	regulation of defense response to virus by host	BP	1	TNFAIP3,IL15,TRAF3IP2,TMEM173,MAVS,TRIM44,EIF2AK4,PARP9,DTX3L,MUL1,RNF216,SIN3A,ZDHHC1,ZDHHC11,IFNLR1	grey	VSMC_M10
GO:0002396	0.32	0.90	5	7	MHC protein complex assembly	BP	1	HLA-DRA,HLA-DOB,HLA-DMA,HLA-DPB1,TAPBPL	grey	VSMC_M10
GO:1904526	0.32	0.88	6	9	regulation of microtubule binding	BP	1	HSF1,MAPRE1,MAPRE3,PPP2CB,TTBK2,MARK2	grey	VSMC_M10
GO:0099003	0.32	0.74	83	151	vesicle-mediated transport in synapse	BP	1	CANX,SYNJ2,RIMS2,BTBD9,CHRNA5,APBA1,USP46,BRSK1,RIMS1,RIMS3,DDC,AMPH,PPFIA3,EPS15,NAPA,CACNB2,GRIK5,NAPB,ACTG1,SYT1,RAB5A,STX2,SYT9,SNPH,ERC2,FCHO2,P2RY1,SYNJ1,AP3B1,VPS18,CYFIP1,DTNBP1,SYT8,DNM1L,ADRA2A,SNAP23,NLGN4Y,BSN,AP2M1,STX1A,BACE1,DNAJC5,CSPG5,CALY,RAP1B,BLOC1S6,CASK,DNM3,PPP3CB,RAB3A,SYN1,SNAP29,NSG1,GRIPAP1,RAB8A,ACTB,SYT7,SLC2A4,NLGN2,DGKQ,GAK,CPLX1,STX1B,STX4,PSEN1,SYT11,NLGN3,RAB11A,CALM1,SNAPIN,PRKCB,DNM2,AP2B1,CDK5R1,FMR1,PIP5K1C,P2RX1,CEL,STXBP1,BTBD8,DENND1A,SNAP47,DOC2B	grey	VSMC_M10
GO:0039530	0.32	0.88	6	9	MDA-5 signaling pathway	BP	1	ANKRD17,IRF7,OAS3,IFIH1,TKFC,RIOK3	grey	VSMC_M10
GO:0021511	0.32	0.88	6	9	spinal cord patterning	BP	1	SMO,INTU,GLI2,SUFU,CHRD,TULP3	grey	VSMC_M10
GO:0042398	0.32	0.79	22	37	cellular modified amino acid biosynthetic process	BP	1	ALDH7A1,CKB,SLC1A1,CHAC2,MGST2,SLC7A11,DHFR,GSS,CHDH,CKMT2,CKMT1B,NFE2L2,PLOD3,MTHFS,GCH1,GGT6,SLC27A1,GAMT,TMLHE,PARK7,ACADM,GGT5	grey	VSMC_M10
GO:0031342	0.32	0.88	6	9	negative regulation of cell killing	BP	1	NCKAP1L,PPP3CB,IL7R,CRK,SERPINB9,HAVCR2	grey	VSMC_M10
GO:0051955	0.32	0.81	15	25	regulation of amino acid transport	BP	1	SLC12A2,ARL6IP5,SLC43A1,GRM2,SLC38A3,AGT,ACE2,ATP1A2,PER2,NTSR1,ARL6IP1,PSEN1,ABAT,STXBP1,RGS4	grey	VSMC_M10

GO:0070316	0.32	0.88	6	9	regulation of G0 to G1 transition	BP	1	PPP2R5B,RHNO1,MED1,SOX15,FOXO4,DAB2IP	grey	VSMC_M10
GO:1901032	0.32	0.85	9	14	negative regulation of response to reactive oxygen species	BP	1	GPR37,MET,PDE8A,NFE2L2,PINK1,PARK7,MIR21,GPR37L1,TXN	grey	VSMC_M10
GO:1903206	0.32	0.85	9	14	negative regulation of hydrogen peroxide-induced cell death	BP	1	GPR37,MET,PDE8A,NFE2L2,PINK1,PARK7,MIR21,GPR37L1,TXN	grey	VSMC_M10
GO:0045833	0.32	0.76	41	72	negative regulation of lipid metabolic process	BP	1	ORMDL2,PIP4K2C,CYP27B1,DKKL1,PIP4K2A,SOX9,APOC1,SLC22A13,PIBF1,ALK,TRIB3,CYP7A1,DKK3,FBXW7,ORMDL1,ERLIN1,PRMT3,SIRT4,GPLD1,LPCAT1,SIK1,ADRA2A,SNAI1,SORL1,GPER1,PPP2R5A,RUBCN,PIK3IP1,SCAP,APOD,ACADL,WNT4,SLC27A1,ACADVL,ASXL3,NCOR1,GFI1,DAB2IP,BSCL2,INSIG2,CCDC3	grey	VSMC_M10
GO:0070528	0.32	0.81	14	23	protein kinase C signaling	BP	1	PLA2G6,PLEK,SPHK2,WNT11,GPD1L,ADGRV1,CD40,DGKD,DGKG,DGKQ,SEZ6,PRKCZ,ADGRG1,WNT5A	grey	VSMC_M10
GO:0051453	0.32	0.77	33	58	regulation of intracellular pH	BP	1	SLC4A4,PPT1,RAB7A,ATP6V0E2,SLC4A2,AQP11,SLC4A7,TMEM106B,ATP6V1H,TM9SF4,SLC9A9,HVCN1,SLC4A10,SLC9B1,TMEM9,TCIRG1,SLC9A8,SLAMF8,SLC9A1,CLIC4,SLC11A1,SLC9A7,ATP6V0A1,DMXL2,SLC4A3,AC138894.1,CLN6,SNAPIN,TMEM199,SLC9A6,SLC9A5,CA2,RAB39A	grey	VSMC_M10
GO:0051709	0.32	0.88	6	9	regulation of killing of cells of other organism	BP	1	P2RX7,F2RL1,NOS2,CXCL6,FCER2,SERPINB9	grey	VSMC_M10
GO:0001959	0.32	0.74	67	119	regulation of cytokine-mediated signaling pathway	BP	1	TNFAIP3,HSPA1B,PRKN,AGPAT1,IL1R2,VRK2,PXDN,NR1H2,TRIM32,CD300LF,IRAK2,IKBKB,MAVS,GAS6,HPX,IRF7,TRIM44,OTUD4,ROBO1,IL7,NR1H3,OAS3,NLRC5,RIPK2,OTULIN,F2RL1,RIPK1,SPATA2,YTHDF2,PARP9,PARP14,PELI3,PTPN6,MUL1,SPPL2A,PADI2,GPS2,USP18,MED1,PYDC1,DCST1,STAT2,CD74,CCL5,PTPN2,SOCS1,PIAS4,SPPL2B,TRAF2,AGPAT2,GFI1,MIR21,MIR125B1,MIR27B,MIR24-1,MIR27A,OAS1,NOL3,FADD,CASP8,MIR125A,IRGM,WNT5A,C1QTNF4,CCDC3,TSLP,HIST1H2BJ	grey	VSMC_M10
GO:0001919	0.32	0.83	11	18	regulation of receptor recycling	BP	1	BVES,EPS15,OPTN,TBC1D16,AP1AR,NSF,ECE1,NSG1,ANXA2,PSEN1,VAMP3	grey	VSMC_M10
GO:0099504	0.32	0.74	76	138	synaptic vesicle cycle	BP	1	CANX,SYNJ2,RIMS2,BTBD9,CHRNA5,APBA1,BRSK1,RIMS1,RIMS3,DDC,AMPH,PPFIA3,NAPA,CACNB2,GRIK5,NAPB,ACTG1,SYT1,RAB5A,STX2,SYT9,SNPH,ERC2,FCHO2,P2RY1,SYNJ1,AP3B1,VPS18,CYFIP1,DTNBP1,SYT8,DNM1L,ADRA2A,SNAP23,NLGN4Y,BSN,AP2M1,STX1A,BACE1,DNAJC5,CSPG5,RAP1B,BLOC1S6,CASK,DNM3,PPP3CB,RAB3A,SYN1,SNAP29,GRIPAP1,ACTB,SYT7,SLC2A4,NLGN2,DGKQ,GAK,CPLX1,STX1B,STX4,PSEN1,SYT11,NLGN3,CALM1,SNAPIN,PRKCB,DNM2,CDK5R1,FMR1,PIP5K1C,P2RX1,CEL,STXBP1,BTBD8,DENND1A,SNAP47,DOC2B	grey	VSMC_M10
GO:0022600	0.32	0.77	32	56	digestive system process	BP	1	KIAA1211,ABCG5,ABCG8,AQP1,NR1H2,TLR4,SOX9,CCKBR,SOAT2,HIP1R,PLS1,CHRM5,LIMA1,NR1H3,VDR,NOD2,CHRM3,ADRA2A,EPB41,COPA,PBLD,LPCAT3,CHRM1,HRH2,LDLR,SLC46A1,TRPV1,CEL,SLC22A5,SNX10,INAVA,CRH	grey	VSMC_M10
GO:0045686	0.32	0.83	11	18	negative regulation of glial cell differentiation	BP	1	DAAM2,MYCN,KDM4A,NKX6-1,DLX1,DUSP10,LDLR,MBD1,DAB1,GPR37L1,NOG	grey	VSMC_M10
GO:0006821	0.32	0.77	34	60	chloride transport	BP	1	ATP8B1,SLC26A10,SLC12A2,TSPO,ABCB1,GABRA1,CLIC5,SLC1A1,CLCN1,SLC26A5,ANO10,CLNS1A,PRNP,CLIC1,ANO4,ANO6,SLC12A5,SLC4A10,SLC12A7,CLCN4,TMEM206,SLC12A8,CLIC4,SLC12A9,ANO8,TTYH3,SLC26A1,SLC26A2,GABRE,GABRA3,CLCN7,LRRC8A,CLCA2,SLC12A4	grey	VSMC_M10
GO:0046929	0.32	0.87	6	9	negative regulation of neurotransmitter secretion	BP	1	PRKN,ASIC1,RAP1B,PNKD,SYT11,FMR1	grey	VSMC_M10
GO:0090335	0.32	0.83	11	18	regulation of brown fat cell differentiation	BP	1	MAPK14,PIM1,NAPEPLD,FTO,RREB1,SOX13,TRPV4,DUSP10,TFE3,ZBTB7B,SIX1	grey	VSMC_M10
GO:0048841	0.32	0.80	17	29	regulation of axon extension involved in axon guidance	BP	1	SEMA6A,PLXNA4,SEMA4D,SEMA4C,MEGF8,SEMA3D,SEMA3A,RYK,SEMA4F,SEMA3G,SEMA4G,SEMA3F,WNT3,SEMA6D,SEMA4A,PLXNA3,WNT5A	grey	VSMC_M10
GO:0071867	0.32	0.76	39	69	response to monoamine	BP	1	PRKN,ATF4,SLC1A1,VPS35,HDAC2,ALK,SIRT2,HTR7,CHRM5,CHRM4,DTNBP1,CHRM3,RYR2,SRD5A1,SLC9A1,CALY,HTR3B,MAPK1,CHRM1,GNB5,NSG1,HRH2,PKLR,HCN3,MAPK3,DNM2,SIN3A,HRH4,GNAL,GNB1,GNA15,PALM,GNAI3,FUK,HTR1D,RGS4,GNA14,GNG2,ARPP19	grey	VSMC_M10
GO:0071869	0.32	0.76	39	69	response to catecholamine	BP	1	PRKN,ATF4,SLC1A1,VPS35,HDAC2,ALK,SIRT2,HTR7,CHRM5,CHRM4,DTNBP1,CHRM3,RYR2,SRD5A1,SLC9A1,CALY,HTR3B,MAPK1,CHRM1,GNB5,NSG1,HRH2,PKLR,HCN3,MAPK3,DNM2,SIN3A,HRH4,GNAL,GNB1,GNA15,PALM,GNAI3,FUK,HTR1D,RGS4,GNA14,GNG2,ARPP19	grey	VSMC_M10
GO:0032429	0.32	0.87	6	9	regulation of phospholipase A2 activity	BP	1	LRP1,PPT1,EGFR,PLA2R1,ANXA8,NMUR2	grey	VSMC_M10

GO:0019752	0.32	0.71	360	659	carboxylic acid metabolic process	BP	1	ATP8B1,GLS2,HSD17B4,SHMT2,ALDH8A1,MARS,AIG1,LTC4S,GFPT2,TPH2,RIDA,CPT1B,DPYS,SLC27A6,TNXB,QKI,PPARA,ACSL6,PNPLA3,MCAT,HSD17B8,ECHDC1,ACO2,PRXL2C,AKR1B1,ATF4,PYCR3,MAPK14,PSAT1,GPT,HACD4,MTAP,CARNMT1,GNMT,ALDH1A1,CROT,ABCC10,GNPDA2,KIT,SLC25A13,GAPDH,AASDH,PPAT,LIPT1,HMGCLL1,ADH5,RIMKLA,PRKCE,ETFB,SLC4A4,ME1,BPGM,NR1H2,DDC,IL4I1,LPIN1,MGST2,QRSL1,SLC7A11,DLD,DDO,BCAT2,NANP,PRKAR2B,DHFR,FAAH,HADHA,GAD2,SOX9,ABHD5,AKR1A1,APOC1,HPDL,DHTKD1,ECHDC3,PRKAG2,ACOX1,SLC22A13,XYLB,PIBF1,CYGB,HYI,HNMT,ELOVL1,CSGALNACT1,AKT2,PFKP,IDO2,IDO1,MDH2,HARS,HARS2,BTD,HACL1,SLC23A2,CARS2,PANK2,PDK1,AMDHD1,LARS,SUCLG2,NR1H4,PDHX,APIP,TRIB3,LYPLA1,ACOXL,CSAD,CYP7A1,ELOVL7,ASAH1,CYP7B1,ACOX2,GPI,ABHD6,THNSL2,AHCY,CBR4,OSBP,GSS,MCCC2,HPGD,MSRA,GPD1,PEX2,P2RX7,ETFDH,ACOT12,MTHFD2,PRKAG1,GMPS,NR1H3,SERINC5,ENOPH1,PFKM,SDSL,IVD,LPIN3,HOGA1,NIT2,BPHL,ASNSD1,ECI2,HIBCH,GLS,ABCC2,ERLIN1,PLA2G4B,ACAD8,ADTRP,PRMT3,PRKAB1,WARS2,LONP2,MTRR,PM20D1,SIRT4,GATC,ASL,MCEE,ALDH5A1,AZIN2,IRS1,MTR,YARS2,CBSL,ALDH1A3,CPT1A,BDH2,ZBTB20,GPAM,ACSL5,NR5A2,SLC7A7,CYP2U1,HGD,MAPKAPK2,AMT,PFKFB2,ABHD2,MSMO1,MECR,MCCC1,GNPAT,CMAS,ENO4,TARS2,DALRD3,ABCB11,DHRS9,PC,HACD2,ARNT,LPGAT1,CTPS2,PER2,ACTN3,HDAC4,OSBPL2,ACADSB,OAT,THEM5,THEM4,MLXIPL,HMGCL,GLUL,D2HGDH,SCAP,EHHADH,SMS,MDH1B,POR,PDK3,PLA2G4A,OSBPL6,UPB1,ACADL,PLOD3,GGT2,HK1,FAH,BCO2,CPS1,MTHFS,TYSND1,LARS2,OSBPL1A,PRODH,ID[illegible]	grey	VSMC_M10
GO:0005984	0.32	0.87	6	9	disaccharide metabolic process	BP	1	MGAM,BRAF,GANC,TREH,IDUA,FBP2	grey	VSMC_M10
GO:0000012	0.32	0.87	6	9	single strand break repair	BP	1	XRCC1,ERCC6,ERCC8,PARP1,TDP1,TERF2	grey	VSMC_M10
GO:0001779	0.32	0.83	11	18	natural killer cell differentiation	BP	1	NFIL3,IL15,IL15RA,GAS6,TOX,RASGRP1,RABL3,SP3,STAT5B,ZBTB1,KAT7	grey	VSMC_M10
GO:0072178	0.32	0.87	6	9	nephric duct morphogenesis	BP	1	EPHA7,OSR1,PAX2,EPHA4,LHX1,GPC3	grey	VSMC_M10
GO:0098915	0.32	0.87	6	9	membrane repolarization during ventricular cardiac muscle cell action potential	BP	1	KCNJ3,KCNE2,NOS1AP,RNF207,KCNH2,KCNJ8	grey	VSMC_M10
GO:0072577	0.32	0.78	25	43	endothelial cell apoptotic process	BP	1	MAP3K5,TNFAIP3,TEK,FOXO3,FGF21,NDNF,GAS6,ANO6,CD40,TERT,PDCD4,GPER1,NFE2L2,ITGA4,TNIP2,RAMP2,MAPK7,CCL2,PDPK1,DAB2IP,MIR24-1,FGA,MIR125A,CD248,RGCC	grey	VSMC_M10
GO:1903204	0.32	0.83	10	16	negative regulation of oxidative stress-induced neuron death	BP	1	PRKN,NCOA7,ATF4,FBXO7,SLC7A11,NONO,PINK1,PARK7,TSC1,TLDC1	grey	VSMC_M10
GO:0032024	0.32	0.77	32	56	positive regulation of insulin secretion	BP	1	LRP1,PLA2G6,PRKCE,TRPM4,GIPR,SIRT3,OSBP,NNAT,PFKM,NKX6-1,GPLD1,TCF7L2,PFKFB2,GPER1,GLUL,MCU,PPP3CB,CD38,MPC2,NLGN2,TARDBP,STX4,GPR68,ISL1,OXCT1,ABAT,NADK,BAIAP3,LRRC8A,SOX4,CRH,DOC2B	grey	VSMC_M10
GO:0043242	0.32	0.76	39	70	negative regulation of protein-containing complex disassembly	BP	1	AVIL,LMOD2,KIAA1211,TMOD1,PLEKHH2,SPTBN1,ADD2,CLASP2,TRIM54,NAV3,SPEF1,LMOD3,ATXN7,LIMA1,KATNB1,SCAF4,DMTN,CARMIL1,LMOD1,ADD3,TMEM39A,TTBK2,CIB1,SPTBN2,TMOD4,EPS8,RUBCN,CAPZA1,TMOD2,PHF23,SPTB,CAMSAP3,TAOK1,CLEC16A,APC2,CAMSAP1,CARMIL2,APC,MAP6D1	grey	VSMC_M10
GO:0052646	0.33	0.87	6	9	alditol phosphate metabolic process	BP	1	GPD1L,ABHD6,GK5,GPD1,GPAM,ACP6	grey	VSMC_M10
GO:0006335	0.33	0.83	10	16	DNA replication-dependent nucleosome assembly	BP	1	ASF1A,RBBP4,HIST1H4B,HIST2H4A,HIST1H4H,HIST1H3F,HIST1H3H,HIST1H4D,HIST1H4A,HIST1H4F	grey	VSMC_M10
GO:0034723	0.33	0.83	10	16	DNA replication-dependent nucleosome organization	BP	1	ASF1A,RBBP4,HIST1H4B,HIST2H4A,HIST1H4H,HIST1H3F,HIST1H3H,HIST1H4D,HIST1H4A,HIST1H4F	grey	VSMC_M10
GO:0071545	0.33	0.82	11	18	inositol phosphate catabolic process	BP	1	SYNJ2,INPP4B,MTMR2,MTMR7,SYNJ1,INPP5A,NTSR1,INPP5J,IMPA2,INPP5K,INPP1	grey	VSMC_M10
GO:0072207	0.33	0.82	11	18	metanephric epithelium development	BP	1	POU3F3,OSR1,SOX9,PAX8,PKD2,PAX2,CXCR2,WNT4,PKD1,SOX8,LIF	grey	VSMC_M10
GO:0002483	0.33	0.87	6	9	antigen processing and presentation of endogenous peptide antigen	BP	1	HLA-DRA,TAP1,IDE,ABCB9,ERAP1,ERAP2	grey	VSMC_M10
GO:0030007	0.33	0.86	7	11	cellular potassium ion homeostasis	BP	1	SLC12A2,ATP1A3,KCNJ2,ATP1A2,ATP1B1,KCNMA1,ATP1B2	grey	VSMC_M10
GO:0009584	0.33	0.80	15	25	detection of visible light	BP	1	OPN1SW,PRPH2,CAMKMT,GJA10,CEP250,CCDC66,RRH,SAG,NMT1,GRK4,ROM1,GUCY2D,GNAT2,REEP6,GPR88	grey	VSMC_M10
GO:1901215	0.33	0.73	89	161	negative regulation of neuron death	BP	1	ERBB3,LRP1,AGAP2,PRKN,PPARA,SYNGAP1,NCOA7,ATF4,NTRK2,NRBP2,HSF1,FBXO7,CNTFR,SLC1A1,TFAP2B,MSH2,REL,PPT1,VPS35,SLC7A11,NTF4,FGF21,NDNF,C5AR1,XRCC2,POU4F1,ARRB1,SLC23A2,GPI,GBE1,MEF2C,FAIM2,HTRA2,PTK2B,NR4A2,F2R,PM20D1,BDNF,CD200,HDAC1,TERT,SLC30A10,INPP5A,KRAS,ADNP,SORL1,CBL,HYOU1,NFATC4,DLX1,DNAJC5,FZD9,SNX6,ADAM8,CREB1,NONO,PINK1,STAT3,ATP13A2,TNFRSF1B,PSEN1,AC138894.1,CITED1,ALKBH1,GPRASP2,BHLHB9,AP2B1,CCL2,ISL1,GHR,PDPK1,MECP2,TRAF2,IKBKG,TMEM259,PARK7,TSC1,TLDC1,STXBP1,AARS,RHOA,CHGA,CNTF,PSMC1,TIGAR,SIX1,HRAS,CEBPB,NMNAT1	grey	VSMC_M10
GO:1900016	0.33	0.84	9	14	negative regulation of cytokine production involved in inflammatory response	BP	1	PPARA,IL1R2,CHID1,ADCY7,PDCD4,APOD,MIR222,C5orf30,MIR125A	grey	VSMC_M10
GO:0098974	0.33	0.87	6	9	postsynaptic actin cytoskeleton organization	BP	1	FARP1,ACTG1,INA,WASF2,NOS1AP,ACTB	grey	VSMC_M10
GO:0099188	0.33	0.87	6	9	postsynaptic cytoskeleton organization	BP	1	FARP1,ACTG1,INA,WASF2,NOS1AP,ACTB	grey	VSMC_M10
GO:0042733	0.33	0.77	27	47	embryonic digit morphogenesis	BP	1	OSR2,FZD6,TBC1D32,ROR2,MYCN,OSR1,INTU,HDAC2,GLI2,LMBR1,FREM2,HAND2,IFT52,SALL1,HDAC1,ECE1,TULP3,BMPR1A,TMEM107,MKS1,TBX2,CREBBP,TMEM231,NOG,WNT5A,C2CD3,BCL2L11	grey	VSMC_M10
GO:0055069	0.33	0.80	15	25	zinc ion homeostasis	BP	1	SLC39A5,PRKN,SLC39A4,SLC1A1,SLC39A12,SLC39A13,AP3B1,LCK,SLC30A10,SLC30A4,ATP13A2,S100A8,SLC39A6,SLC30A7,MT1X	grey	VSMC_M10
GO:0002021	0.33	0.82	11	18	response to dietary excess	BP	1	BMP8A,UCP1,ACVR1C,TRPV4,SORL1,VGF,GDF15,NMUR2,SGIP1,TRPV1,SLC25A25	grey	VSMC_M10

GO:0045596	0.33	0.70	265	489	negative regulation of cell differentiation	BP	1	NFATC1,NME1,SEMA6A,FRS2,PPARA,FBLN1,TSPO,FSTL4,SYNGAP1,TRIB1,MYC,TOB2,PTCH1,SOX10,PI16,SMO,DAAM2,FBXO7,RORB,CDK6,SEMA4D,S1PR3,SEMA4C,SOSTDC1,SIX2,RTN4,RUFY3,HOXA2,HOXA7,ANKRD17,AREG,AKIRIN1,EPHA7,NR1H2,PTN,ITGB1BP1,EGFR,FUZ,TLR4,CDK5RAP2,MYCN,TRPM4,OSR1,FOXO3,MKX,SPRY1,FOXE3,SOX9,GLI2,KDM4A,FOXJ1,TSKU,SPRY2,HOOK3,OVOL2,JAG1,SIRT2,RARB,SRA1,SPRED3,LSM1,SEMA3D,SPART,SEMA3A,ULK1,RYK,TCF15,TRIB3,EPHB1,ZC3H8,ASXL1,ZNF536,PIK3R1,HAND2,IL17RD,ARNTL,FBXW7,ADGRV1,TMEM131L,SEMA4F,VEGFC,TLX2,SOX6,PTK2B,RAPGEF2,CCL17,EIF2AK4,NR1H3,RUNX1T1,HDAC7,NKX6-1,TRIO,TBX5,SRSF6,TP53INP1,SEMA3G,SPP1,ZBED6,DMTN,SEMA4G,PRICKLE1,LDB1,IGF2,BDNF,SUFU,RNF10,KIAA0319,PKP2,NEPRO,PPP3CA,SINHCAF,USF3,CCND1,TRPV4,SEMA3F,PDCD4,ABCA5,TGFB2,PTHLH,GPR137B,AXIN2,PIAS3,HLX,KRAS,TCTA,SNAI1,SOX2,AP1AR,YTHDF2,UBASH3B,SORL1,GPER1,CIB1,CERS2,NFATC4,FZD7,DDX6,HDAC4,CHRD,RUNX3,DLX1,ZBTB46,MELTF,EIF4ENIF1,WNT3,NFE2L2,DUSP10,BCL6,SEMA6D,DIXDC1,MMP11,MEN1,WDR61,TNR,RC3H1,GPR137,FBN1,TNFSF18,WNT4,LAG3,COL5A2,RAP1GAP,STAT3,NKX3-2,MAPK1,BHLHA15,STAT5B,LEO1,INHA,FOXP3,BMPR1A,GPS2,RBPJ,CDK12,MED1,TBX21,PSEN1,RIOX1,SEMA4A,HOXB8,TGFB1I1,FOXO4,JDP2,TRIM46,EFNA1,RBPMS2,ZBTB7B,TOB1,ESRRB,CITED1,SMAD3,NTN1,IL4R,LDLR,GPR68,YY1,CD74,MIB1,CDK5R1,ISL1,SUZ12,TWSG1,RANBP3L,MBNL3,ADAMTS12,PTPN2,SOCS1,MBD1,DAB1,RFLNB,TRPV1,SKI,ARHGAP4,MECP2,PLX[illegible]	grey	VSMC_M10
GO:0009218	0.33	0.81	14	23	pyrimidine ribonucleotide metabolic process	BP	1	NME1,DPYS,CMPK1,ENTPD4,NME4,CTPS2,UPB1,NME7,UPRT,NME3,UCK1,DHODH,NME6,UPP1	grey	VSMC_M10
GO:0042773	0.33	0.75	49	83	ATP synthesis coupled electron transport	BP	1	UQCRQ,NDUFA6,DLD,NDUFB2,NDUFB6,DNAJC15,NDUFC2,NDUFS8,NDUFA12,COX7A1,COX4I2,NDUFS3,DGUOK,UQCRB,COX15,NDUFV3,NDUFB4,ISCU,COA6,NDUFB3,NDUFS2,SDHC,COX6A1,COX8A,PINK1,NDUFA9,COX6A2,NDUFAB1,COX5A,NDUFA7,NDUFV2,NDUFB10,UQCR11,PARK7,MT-ND1,MT-ND2,MT-CO3,MT-ND6,MT-CYB,NDUFA5,NDUFC1,COX7A2,NDUFA3,NDUFB5,NDUFS6,NDUFA8,SDHAF2,NDUFS5,COX7A2L	grey	VSMC_M10
GO:0042775	0.33	0.75	49	83	mitochondrial ATP synthesis coupled electron transport	BP	1	UQCRQ,NDUFA6,DLD,NDUFB2,NDUFB6,DNAJC15,NDUFC2,NDUFS8,NDUFA12,COX7A1,COX4I2,NDUFS3,DGUOK,UQCRB,COX15,NDUFV3,NDUFB4,ISCU,COA6,NDUFB3,NDUFS2,SDHC,COX6A1,COX8A,PINK1,NDUFA9,COX6A2,NDUFAB1,COX5A,NDUFA7,NDUFV2,NDUFB10,UQCR11,PARK7,MT-ND1,MT-ND2,MT-CO3,MT-ND6,MT-CYB,NDUFA5,NDUFC1,COX7A2,NDUFA3,NDUFB5,NDUFS6,NDUFA8,SDHAF2,NDUFS5,COX7A2L	grey	VSMC_M10
GO:0019882	0.33	0.76	35	61	antigen processing and presentation	BP	1	HLA-DRA,HLA-DOB,PSMB8,TAP1,HLA-DMA,HLA-DPB1,NOD1,ATG5,RAB10,IKBKB,TRAF6,MARCH8,IDE,ABCB9,ERAP1,AP3B1,MFSD6,NOD2,PSME1,RAB4A,YTHDF1,FCER1G,PIKFYVE,CTSH,SLC11A1,CTSV,TAPBPL,WAS,RAB8B,DNM2,CD74,RAB33A,ERAP2,FGL2,RAB32	grey	VSMC_M10
GO:0006073	0.33	0.76	33	58	cellular glucan metabolic process	BP	1	GNMT,MGAM,PYGB,RUBCNL,KHK,EPM2AIP1,PRKAG2,PPP1CB,AKT2,PPP1R3B,GBE1,SORBS1,PFKM,IGF2,NHLRC1,PHKB,IRS1,PPP1R3E,PPP1R3D,PER2,PASK,PHKA2,PYGM,PYGL,PRKAG3,PPP1R3F,PHKA1,INSR,LEPR,INPP5K,AGL,ACADM,PPP1R1A	grey	VSMC_M10
GO:0044042	0.33	0.76	33	58	glucan metabolic process	BP	1	GNMT,MGAM,PYGB,RUBCNL,KHK,EPM2AIP1,PRKAG2,PPP1CB,AKT2,PPP1R3B,GBE1,SORBS1,PFKM,IGF2,NHLRC1,PHKB,IRS1,PPP1R3E,PPP1R3D,PER2,PASK,PHKA2,PYGM,PYGL,PRKAG3,PPP1R3F,PHKA1,INSR,LEPR,INPP5K,AGL,ACADM,PPP1R1A	grey	VSMC_M10
GO:0006283	0.33	0.87	6	9	transcription-coupled nucleotide-excision repair	BP	1	ERCC2,ERCC3,POLR2I,ERCC6,ERCC8,XAB2	grey	VSMC_M10
GO:0070936	0.33	0.76	33	58	protein K48-linked ubiquitination	BP	1	NEDD4L,TNFAIP3,PRKN,RNF5,SKP1,UBE2B,RNF146,KLHL3,PELI1,TRIM32,HACE1,KBTBD6,UBE2E2,UBE2E1,NMI,TRIM44,AMFR,TTC3,RNF115,DTX3L,ARIH2,SYVN1,RNF4,RNF216,UBE2Q2,FBXO38,RNF126,UBE2G1,RNF8,UBE2G2,UBE2R2,RNF152,UBE2D1	grey	VSMC_M10
GO:0045649	0.33	0.82	11	18	regulation of macrophage differentiation	BP	1	TRIB1,HSF1,ROR2,RIPK1,HCLS1,ZBTB46,INHA,PTPN2,FADD,CASP8,LIF	grey	VSMC_M10
GO:0006868	0.33	0.87	6	9	glutamine transport	BP	1	SLC1A4,SLC38A2,SLC38A1,SLC38A3,SLC38A5,SLC38A6	grey	VSMC_M10
GO:1903299	0.33	0.87	6	9	regulation of hexokinase activity	BP	1	PRKN,GCKR,PFKFB2,DUSP12,COX11,TIGAR	grey	VSMC_M10
GO:0003093	0.33	0.87	6	9	regulation of glomerular filtration	BP	1	GAS6,F2RL1,F2R,PTPRO,CORO2B,CYBA	grey	VSMC_M10
GO:2000650	0.33	0.85	7	11	negative regulation of sodium ion transmembrane transporter activity	BP	1	NEDD4L,PRKCE,HECW1,OSR1,HECW2,ATP1A2,MIR24-1	grey	VSMC_M10
GO:0007213	0.33	0.83	10	16	G protein-coupled acetylcholine receptor signaling pathway	BP	1	PLCB1,CHRM5,CHRM4,CHRM3,GRK2,CHRM1,HRH4,CDK5R1,GNB1,GNA15	grey	VSMC_M10
GO:0016246	0.33	0.85	7	11	RNA interference	BP	1	AGO2,XPO5,CLP1,CELF1,TERT,PRKRA,NRDE2	grey	VSMC_M10
GO:0046629	0.33	0.85	7	11	gamma-delta T cell activation	BP	1	TCF7,NCKAP1L,SOX13,NOD2,STAT5B,JAG2,SOX4	grey	VSMC_M10
GO:0098885	0.33	0.87	6	9	modification of postsynaptic actin cytoskeleton	BP	1	CTTNBP2,WASF1,TIAM1,CYFIP1,MYH10,RHOA	grey	VSMC_M10
GO:0009135	0.33	0.75	44	78	purine nucleoside diphosphate metabolic process	BP	1	PPARA,PRXL2C,GAPDH,SLC4A4,NT5E,BPGM,DLG2,DHTKD1,PRKAG2,PFKP,NUDT16,GPI,GPD1,P2RX7,PRKAG1,PFKM,ENTPD4,NUDT18,ZBTB20,PFKFB2,ENO4,ARNT,ACTN3,HDAC4,MLXIPL,MAGI3,DLG1,HK1,CASK,PRKAG3,STAT3,ENO3,CARD11,PSEN1,PKLR,OGT,ADPGK,NCOR1,INSR,ALDOC,MPP1,SLC2A6,ENTPD5,TIGAR	grey	VSMC_M10
GO:0009179	0.33	0.75	44	78	purine ribonucleoside diphosphate metabolic process	BP	1	PPARA,PRXL2C,GAPDH,SLC4A4,NT5E,BPGM,DLG2,DHTKD1,PRKAG2,PFKP,NUDT16,GPI,GPD1,P2RX7,PRKAG1,PFKM,ENTPD4,NUDT18,ZBTB20,PFKFB2,ENO4,ARNT,ACTN3,HDAC4,MLXIPL,MAGI3,DLG1,HK1,CASK,PRKAG3,STAT3,ENO3,CARD11,PSEN1,PKLR,OGT,ADPGK,NCOR1,INSR,ALDOC,MPP1,SLC2A6,ENTPD5,TIGAR	grey	VSMC_M10
GO:0019226	0.33	0.77	25	44	transmission of nerve impulse	BP	1	NTRK2,CLDN19,MYH14,CLCN1,SCN5A,SCN1A,P2RX3,CHRM5,SCN2A,SCN7A,GPER1,AGT,TNR,CNTNAP1,KCNA1,CACNA1G,OTOA,FMR1,P2RX1,CACNA1H,GPR35,CACNG7,GPR88,CACNG5,KCNMB4	grey	VSMC_M10

GO:0008206	0.33	0.78	21	36	bile acid metabolic process	BP	1	ATP8B1,KIT,PANK2,NR1H4,CYP7A1,CYP7B1,OSBP,NR5A2,ABCB11,OSBPL2,OSBPL6,OSBPL1A,CYP27A1,HSD17B10,FGFR4,OSBPL7,CYP46A1,NPC1,ABCD3,AMACR,STARD4	grey	VSMC_M10
GO:0045945	0.33	0.85	7	11	positive regulation of transcription by RNA polymerase III	BP	1	CHD8,RPTOR,ZC3H8,ZNF143,CEBPA,ELL,ICE2	grey	VSMC_M10
GO:0090208	0.33	0.83	10	16	positive regulation of triglyceride metabolic process	BP	1	NR1H2,FGF21,ABHD5,NR1H3,GPLD1,SLC27A1,DAGLB,LDLR,SREBF1,CNEP1R1	grey	VSMC_M10
GO:0071287	0.33	0.85	7	11	cellular response to manganese ion	BP	1	PRKN,A3GALT2,SLC30A10,BACE1,ATP13A2,SLFN14,HSPA5	grey	VSMC_M10
GO:0036474	0.33	0.80	14	23	cell death in response to hydrogen peroxide	BP	1	GPR37,MET,FOXO3,PAWR,RIPK1,PDE8A,NFE2L2,PDCD10,PINK1,STK26,PARK7,MIR21,GPR37L1,TXN	grey	VSMC_M10
GO:0009110	0.33	0.82	10	16	vitamin biosynthetic process	BP	1	CYP27B1,PSAT1,AKR1A1,CYP2R1,PLTP,SNAI1,CYP27A1,PNPO,GFI1,TPK1	grey	VSMC_M10
GO:0010043	0.34	0.78	19	32	response to zinc ion	BP	1	TSPO,MTF1,KHK,AANAT,HVCN1,SLC39A13,SLC30A10,D2HGDH,CREB1,SLC30A4,CPS1,ATP13A2,PARP1,S100A8,ASS1,KCNK3,MT1X,CA2,BGLAP	grey	VSMC_M10
GO:0033561	0.34	0.82	11	18	regulation of water loss via skin	BP	1	SRF,GRHL1,MET,ELOVL1,LSR,KDF1,SFN,GRHL3,ABCA12,ALOXE3,CLDN1	grey	VSMC_M10
GO:0009301	0.34	0.82	11	18	snRNA transcription	BP	1	RPAP2,SNAPC3,ZC3H8,ZNF143,CDK7,MEPCE,ELL,CC2D1A,ICE2,SNAPC5,SNAPC4	grey	VSMC_M10
GO:0006096	0.34	0.75	34	60	glycolytic process	BP	1	PPARA,PRXL2C,GAPDH,SLC4A4,BPGM,DHTKD1,PRKAG2,PFKP,GPI,GPD1,P2RX7,PRKAG1,PFKM,ZBTB20,PFKFB2,ENO4,ARNT,ACTN3,HDAC4,MLXIPL,HK1,PRKAG3,STAT3,ENO3,PSEN1,PKLR,OGT,ADPGK,NCOR1,INSR,ALDOC,SLC2A6,ENTPD5,TIGAR	grey	VSMC_M10
GO:0006757	0.34	0.75	34	60	ATP generation from ADP	BP	1	PPARA,PRXL2C,GAPDH,SLC4A4,BPGM,DHTKD1,PRKAG2,PFKP,GPI,GPD1,P2RX7,PRKAG1,PFKM,ZBTB20,PFKFB2,ENO4,ARNT,ACTN3,HDAC4,MLXIPL,HK1,PRKAG3,STAT3,ENO3,PSEN1,PKLR,OGT,ADPGK,NCOR1,INSR,ALDOC,SLC2A6,ENTPD5,TIGAR	grey	VSMC_M10
GO:0050982	0.34	0.77	23	40	detection of mechanical stimulus	BP	1	PKD2L2,PTK2,PHF24,KIT,WHRN,ASIC3,SCN1A,MKKS,ADGRV1,PKD2,BACE1,STRC,TTN,KCNK4,PKD1L1,KCNA1,TCAP,ASIC2,PKD1,PIEZO1,PKD1L3,PJVK,CHRNA10	grey	VSMC_M10
GO:0090183	0.34	0.79	16	27	regulation of kidney development	BP	1	MYC,SMO,SIX2,OSR1,SOX9,RET,PAX8,PAX2,AGT,WNT4,MAGED1,LHX1,SOX8,NOG,BASP1,SIX1	grey	VSMC_M10
GO:0001886	0.34	0.85	7	11	endothelial cell morphogenesis	BP	1	HOXA13,MET,ARHGEF26,STC1,CLIC4,PLOD3,TNMD	grey	VSMC_M10
GO:0045026	0.34	0.86	6	9	plasma membrane fusion	BP	1	SPACA6,IZUMO1,LYZL6,NSF,SPESP1,NOX5	grey	VSMC_M10
GO:1904292	0.34	0.81	11	18	regulation of ERAD pathway	BP	1	AQP11,USP25,RNFT2,USP13,USP19,RNF185,NFE2L2,BCAP31,SGTA,TMEM259,RNFT1	grey	VSMC_M10
GO:1901620	0.34	0.86	6	9	regulation of smoothened signaling pathway involved in dorsal/ventral neural tube patterning	BP	1	GLI2,SUFU,GORAB,GPR161,TULP3,MKS1	grey	VSMC_M10
GO:0045620	0.34	0.77	21	36	negative regulation of lymphocyte differentiation	BP	1	FBXO7,FOXJ1,ZC3H8,TMEM131L,HLX,RUNX3,BCL6,RC3H1,TNFSF18,LAG3,INHA,FOXP3,TBX21,ZBTB7B,IL4R,CD74,PTPN2,SOCS1,MIR21,FGL2,RC3H2	grey	VSMC_M10
GO:0040018	0.34	0.81	12	20	positive regulation of multicellular organism growth	BP	1	SMO,MKKS,PLS1,CELF1,IGF2,SLC6A3,GPAM,CREB1,STAT5B,GHR,DIO3,POU3F2	grey	VSMC_M10
GO:0061844	0.34	0.83	9	14	antimicrobial humoral immune response mediated by antimicrobial peptide	BP	1	CXCL14,GAPDH,CXCL8,NOD2,S100A12,HIST1H2BK,CXCL6,HIST1H2BF,HIST1H2BJ	grey	VSMC_M10
GO:0003222	0.34	0.83	8	13	ventricular trabecula myocardium morphogenesis	BP	1	FOXH1,NRG1,TGFB2,BMPR1A,RBPJ,MED1,ENG,HEY2	grey	VSMC_M10
GO:0090160	0.34	0.85	7	11	Golgi to lysosome transport	BP	1	RBSN,CCDC91,AP4M1,ANKFY1,GAK,AC138894.1,AP1G1	grey	VSMC_M10
GO:0036475	0.34	0.79	16	27	neuron death in response to oxidative stress	BP	1	PRKN,NCOA7,ATF4,FBXO7,SLC1A1,TLR4,SLC7A11,ARL6IP5,FBXW7,MCL1,NONO,PINK1,PARP1,PARK7,TSC1,TLDC1	grey	VSMC_M10
GO:0042362	0.34	0.86	6	9	fat-soluble vitamin biosynthetic process	BP	1	CYP27B1,CYP2R1,PLTP,SNAI1,CYP27A1,GFI1	grey	VSMC_M10
GO:0061000	0.34	0.85	7	11	negative regulation of dendritic spine development	BP	1	FSTL4,ASAP1,HDAC2,PLK2,SRGAP2C,DNM3,EFNA1	grey	VSMC_M10
GO:0018202	0.34	0.86	6	9	peptidyl-histidine modification	BP	1	DPH2,DPH3,HIF1AN,SETD3,DPH1,METTL18	grey	VSMC_M10
GO:0140115	0.34	0.76	28	49	export across plasma membrane	BP	1	ABCB1,SLC4A4,SLC8A2,ATP7B,ATP1A3,ABCC4,SLC40A1,ANO6,ABCC2,KCNE2,ABCG2,KCNIP2,ABCC5,ATP1A2,SLC9A1,DLG1,ATP1B1,SLC27A1,SLC29A4,ATP1B2,CALM1,RALBP1,ATP2B3,MIR21,SLC22A5,KCNH2,KCNK5,KCNT2	grey	VSMC_M10
GO:0015711	0.34	0.71	125	228	organic anion transport	BP	1	ATP8B1,SLC26A10,SLC16A7,SLC12A2,SLC27A6,NTRK2,PLA2G6,ABCC10,APBA1,SLC35B2,SLC1A1,SLC25A13,SLC3A1,SLC4A4,CYB5R4,SLC35A1,SLC1A4,SLC7A11,SLC26A5,SLC25A30,SLC4A2,ASIC3,LLGL2,SLC22A13,SLC25A38,ABCC4,SLC4A7,SLC6A15,SLC25A15,SLC23A2,ARL6IP5,SLC7A1,SLC38A9,SLC5A8,CYP7A1,SLCO6A1,SLC43A1,SLC33A1,SERINC5,SLC38A2,SLC38A1,SLC10A6,COL4A3BP,ABCC2,PLA2R1,ABCG2,GRM2,SLC4A10,SFXN2,CYB5R1,CALHM1,SLC2A10,SLC38A3,MAP2K6,SLC16A6,SLC7A7,SLCO3A1,ABCB11,AGT,ABCC5,ACE2,ATP1A2,SLCO1A2,SLCO1C1,PER2,NTSR1,SLC16A1,PLA2G4A,SLC25A42,SLC11A1,MFSD10,SLC2A2,PQLC2,SLC27A1,SLC38A5,MPC2,ARL6IP1,SLC3A2,SLC2A9,SLC38A6,SLC35A2,SLC4A3,PRAF2,SLC25A11,SLC16A13,CLN8,SLC26A1,PSEN1,SLC7A3,SLC35B1,AC138894.1,ABCC3,NMUR2,CA4,SLC6A7,SLC26A2,LRRC8E,EMB,NOS2,SLC25A23,SLC25A41,SLC46A1,SLC25A14,PROCA1,ABAT,CYB5RL,SLC1A7,TRPV1,SLC2A6,SLC16A4,SLC7A5,LRRC8A,SLC25A25,ABCD3,STXBP1,SLC7A6,SLC36A1,LRRC8D,PLA2G2C,SLC25A21,SLC25A32,RGS4,CPTP,SLC25A29,PTGES	grey	VSMC_M10
GO:0090317	0.34	0.77	20	34	negative regulation of intracellular protein transport	BP	1	RAB23,ITGB1BP1,UBAC2,FERMT1,BAG4,PKIA,DMTN,SUFU,CABP1,SP100,YOD1,BAG3,APOD,DERL3,C11orf65,INPP5K,PARK7,TXN,UBE2G2,DERL2	grey	VSMC_M10
GO:0051657	0.34	0.85	7	11	maintenance of organelle location	BP	1	ALB,ARHGAP21,TBCCD1,ATP2A1,GPSM2,SPOUT1,POLR2M	grey	VSMC_M10
GO:0070129	0.34	0.80	14	23	regulation of mitochondrial translation	BP	1	SHMT2,TSFM,RMND1,LRPPRC,MRPS27,NSUN3,RPUSD4,RCC1L,MTG2,COA3,ALKBH1,RPUSD3,UQCC2,TRMT10C	grey	VSMC_M10

GO:0030858	0.34	0.76	26	45	positive regulation of epithelial cell differentiation	BP	1	CYP27B1,PTCH1,RFX3,SFRP4,SOX9,PTCH2,FOXJ1,OVOL2,BTG1,PAX8,VDR,NKX6-1,PAX2,KDF1,SFN,H2AFY2,MED1,TMEM100,VEZF1,LHX1,MIR21,MIR125B1,ACVRL1,MIR99B,ZBED2,LIF	grey	VSMC_M10
GO:0006699	0.34	0.79	16	27	bile acid biosynthetic process	BP	1	NR1H4,CYP7A1,CYP7B1,OSBP,ABCB11,OSBPL2,OSBPL6,OSBPL1A,CYP27A1,HSD17B10,FGFR4,OSBPL7,CYP46A1,ABCD3,AMACR,STARD4	grey	VSMC_M10
GO:0090313	0.34	0.78	17	29	regulation of protein targeting to membrane	BP	1	SLC1A1,ITGB1BP1,TCAF2,AKT2,PRNP,DMTN,KCNB1,C2CD5,CIB1,FIS1,CEMIP,ITGAM,MIEF2,CDK5R1,INPP5K,HRAS,MIEF1	grey	VSMC_M10
GO:0009141	0.34	0.73	49	86	nucleoside triphosphate metabolic process	BP	1	NME1,TBPL1,ENPP3,ATP5MGL,PPARA,SMPDL3A,GTPBP1,IMPDH1,SLC25A13,RAB23,SPHK2,CMPK1,NUDT15,PRKAG2,ATP5F1C,NUDT16,ATP5F1A,DGUOK,OPA1,ENTPD4,FAM173B,NME4,MFN1,CTPS2,DNAJC30,DTYMK,EFL1,ATP5S,PINK1,STAT3,NME7,ADK,PARP1,PKLR,ATP6V0C,NME3,ATP5PB,VPS9D1,GNAI3,AK8,UCK1,MT-ATP8,MT-ATP6,RHOA,ATP5ME,ATP5MF,CMPK2,NME6,NUDT2	grey	VSMC_M10
GO:0042447	0.34	0.86	6	9	hormone catabolic process	BP	1	IDE,SPP1,SRD5A1,ECE1,DIO3,DIO2	grey	VSMC_M10
GO:0051588	0.34	0.74	41	73	regulation of neurotransmitter transport	BP	1	SNCAIP,PRKN,CHRNA3,CHRNA5,APBA1,RIMS1,RIMS3,CACNB2,SYT1,RAB5A,MEF2C,ASIC1,P2RY1,VPS18,DTNBP1,DNM1L,ADRA2A,GPER1,ATP1A2,PER2,STX1A,BACE1,CSPG5,RAP1B,CASK,PNKD,RAB3A,SYN1,CPLX1,STX1B,SYT11,CALM1,SNAPIN,PRKCB,CAMK2A,FMR1,P2RX1,BAIAP3,STXBP1,CHRNB4,KCNMB4	grey	VSMC_M10
GO:1905208	0.34	0.84	7	11	negative regulation of cardiocyte differentiation	BP	1	FRS2,EGFR,SOX6,PRICKLE1,FZD7,MIR222,HEY2	grey	VSMC_M10
GO:1905065	0.34	0.86	6	9	positive regulation of vascular associated smooth muscle cell differentiation	BP	1	KIT,GPER1,EFEMP2,ENG,MIR21,MIR125B1	grey	VSMC_M10
GO:0045851	0.34	0.77	22	38	pH reduction	BP	1	PPT1,RAB7A,ATP6V0E2,AQP11,TMEM106B,CCKBR,ATP6V1H,TMEM9,TCIRG1,SLAMF8,CLIC4,SLC11A1,SLC9A7,ATP6V0A1,DMXL2,AC138894.1,CLN6,SNAPIN,TMEM199,ATP6AP1,CA2,RAB39A	grey	VSMC_M10
GO:0002711	0.34	0.78	16	27	positive regulation of T cell mediated immunity	BP	1	STX7,HLA-DRA,RSAD2,NECTIN2,PVR,SLC22A13,TRAF6,DENND1B,FZD5,IL12A,FOXP3,ZBTB1,FBXO38,PRKCZ,TRAF2,FADD	grey	VSMC_M10
GO:0006367	0.35	0.74	37	65	transcription initiation from RNA polymerase II promoter	BP	1	ESR1,MED30,TAF2,TAF8,SRF,ERCC1,ERCC3,GTF2F2,PAXIP1,TAF7,GTF2E2,TAF10,POLR2I,ERCC6,CDK7,TAF9,CCNH,TAF5,GTF2E1,TAF4,E2F2,CREB1,MED14,TAF6,POLR2G,THRA,MED24,MED1,TAF1,MED7,TAF4B,TAF13,MED27,TAF12,NKX2-5,HEY2,GTF2H5	grey	VSMC_M10
GO:0014819	0.35	0.84	7	11	regulation of skeletal muscle contraction	BP	1	NR4A1,KCNJ2,MYH7,CASQ1,ACTN3,ATP2A1,DMD	grey	VSMC_M10
GO:1901616	0.35	0.75	32	56	organic hydroxy compound catabolic process	BP	1	CYP27B1,MIOX,SYNJ2,SRD5A3,INPP4B,MTMR2,LRTOMT,AP000812.5,MTMR7,CYP7A1,GK5,SYNJ1,SLC6A3,INPP5A,ALDH3B2,NTSR1,INPP5J,SORD,FAH,COMT,CYP27A1,TKFC,CYP46A1,IMPA2,INPP5K,ALDH2,CEL,DIO3,INPP1,ADH4,PDXP,DIO2	grey	VSMC_M10
GO:0035510	0.35	0.80	12	20	DNA dealkylation	BP	1	APOBEC3H,STPG4,ASCC3,ALKBH3,TOX,OTUD4,FTO,ALKBH2,MGMT,TET1,ALKBH1,MPG	grey	VSMC_M10
GO:0001976	0.35	0.86	6	9	nervous system process involved in regulation of systemic arterial blood pressure	BP	1	TNNI3,P2RX2,AGT,ACE2,ASIC2,MECP2	grey	VSMC_M10
GO:0003356	0.35	0.84	7	11	regulation of cilium beat frequency	BP	1	DNAH11,ARMC4,MKKS,CFAP20,CFAP43,CFAP45,CCSAP	grey	VSMC_M10
GO:0070050	0.35	0.80	13	22	neuron cellular homeostasis	BP	1	PRKN,SLC8A2,CACNB2,CHMP2B,P2RY1,SLC6A2,NELL2,DNAJB2,FGGY,TAOK1,ATP2B3,P2RX1,MAP1A	grey	VSMC_M10
GO:1902476	0.35	0.77	19	33	chloride transmembrane transport	BP	1	SLC26A10,SLC12A2,GABRA1,SLC1A1,CLCN1,SLC26A5,ANO4,ANO6,SLC12A5,SLC12A7,SLC12A8,SLC12A9,SLC26A1,SLC26A2,GABRE,GABRA3,CLCN7,LRRC8A,SLC12A4	grey	VSMC_M10
GO:0070588	0.35	0.70	115	211	calcium ion transmembrane transport	BP	1	STAC3,SMDT1,CATSPER3,PKD2L2,PLA2G6,TRPM6,TSPAN13,PRKCE,IBTK,ATG5,TRPM4,GRIN2D,TRPC3,SLC8A2,PANX1,CACNB2,STAC,SLC24A3,CACNB4,RYR1,NALCN,PRNP,DIAPH1,GAS6,RASA3,MICU3,TRPC4AP,RYR3,ORAI1,ASIC1,P2RX7,PTK2B,TPCN1,SLC8B1,OPA1,F2R,ANO6,KCNE2,PKD2,CASQ2,MCUR1,RYR2,TRPM1,CALHM1,LCK,TRPV4,ADRA2A,MCUB,UBASH3B,ANK2,GPER1,TRPM8,ATP1A2,SLC9A1,C4orf3,CASQ1,NTSR1,NOS1AP,CATSPER2,TMCO1,CEMIP,SESTD1,PTPN6,MICU1,MCU,HTT,PKD1L1,TRPM7,ATP1B1,BHLHA15,GNB5,HAP1,PSEN2,STAC2,CACNB1,ANXA2,FAM155B,PSEN1,CACNA1A,CACNA1G,ATP2A1,CD19,CALM1,VMP1,BDKRB1,TRPV2,SLC25A23,MAIP1,FMR1,ATP2B3,TRPV3,TRPV1,PDPK1,P2RX1,CACNA1B,PKD1,CACNA1H,CYBA,JPH3,ATP2C2,SLC25A25,PKD1L3,MCOLN3,RRAD,MIR21,DMD,GPR35,NOL3,CHRNA10,JPH2,THY1,CACNG7,DDIT3,CACNG5,GEM	grey	VSMC_M10
GO:0060231	0.35	0.81	11	18	mesenchymal to epithelial transition	BP	1	SMO,SIX2,TCF15,PAX8,PAX2,SALL1,FZD7,WNT4,CITED1,BASP1,LIF	grey	VSMC_M10

GO:0006839	0.35	0.70	117	210	mitochondrial transport	BP	1	TIMM21,HEBP2,CPT1B,PRKN,TSPO,HSPA4,SMDT1,BAK1,TOMM22,RAC2,FBXO7,SLC25A13,TMEM14A,PNPT1,UCP1,VPS35,MGARP,AGK,SLC25A30,DNAJC15,ATP5F1C,SLC25A38,PITRM1,TIMM50,USP36,SLC25A15,BAG4,MICU3,MIPEP,IMMP1L,HIP1R,ATP5F1A,FBXW7,HTRA2,CSNK2A2,SLC8B1,SLC25A37,OPA1,TOMM34,TMEM14C,MCUR1,FAM173B,SFXN2,SIRT4,CPT1A,OXA1L,MCUB,HPS4,ARIH2,SLC25A20,BAG3,SLC9A1,TOMM40L,THEM4,DNAJC30,STPG1,FZD9,FZD5,SLC25A16,FIS1,KIF1BP,NMT1,GRPEL1,C11orf65,MICU1,MCU,BCS1L,PINK1,MUL1,STAT3,MPC2,TIMM17B,PSEN2,TMEM102,MFN2,NPEPPS,PSEN1,HAX1,TIMM29,CAMK2A,SREBF1,SLC25A23,MAIP1,SLC25A41,SLC25A14,ACAA2,PAM16,BCAP31,SLC25A33,ATP5PB,NAIF1,SH3GLB1,MIR29A,MT-ATP8,MT-ATP6,MICALL2,SLC25A3,ATP5ME,NOL3,ATP5MF,BNIP3L,SLC25A21,SLC25A32,TIMM10B,TIMM23B,BCL2L11,SLC25A29,SLC35F6,BCL2L2,TIMM8B,RHOU,MRPL18,LEPROT,GRPEL2,PPIF,AC140504.1,DNAJC19	grey	VSMC_M10
GO:0150104	0.35	0.74	39	70	transport across blood-brain barrier	BP	1	LRP1,SLC16A7,SLC12A2,AGER,ABCB1,SLC1A1,SLC4A4,SLC1A4,SLC8A2,SLC24A3,ABCC4,SLC7A1,SLC38A2,SLC38A1,ABCC2,ABCG2,SLC2A13,SLC2A10,SLC38A3,SLCO3A1,ABCC5,ATP1A2,SLCO1C1,SLC29A2,SLC16A1,SLC27A1,SLC38A5,SLC4A3,SLC29A4,SLC2A4,ATP1B2,SLC7A3,ABCC3,INSR,LEPR,SLC7A5,FABP5,SLC22A5,KCNJ8	grey	VSMC_M10
GO:0048143	0.35	0.81	11	18	astrocyte activation	BP	1	LRP1,AGER,SMO,TTBK1,FPR2,EGFR,C5AR1,MAPT,PSEN1,LDLR,CNTF	grey	VSMC_M10
GO:0042474	0.35	0.81	11	18	middle ear morphogenesis	BP	1	OSR2,SIX2,HOXA2,OSR1,EYA1,PRKRA,NKX3-2,GSC,NOG,SIX1,INSIG2	grey	VSMC_M10
GO:0010977	0.35	0.72	64	116	negative regulation of neuron projection development	BP	1	LRP1,SEMA6A,FSTL4,SYNGAP1,SEMA4D,SEMA4C,RTN4,RUFY3,ACP4,LPAR1,EPHA7,HDAC2,TSKU,SEMA3D,SPART,SEMA3A,ULK1,DPYSL3,RYK,CBFA2T2,SEMA4F,DGUOK,PAQR3,SEMA3G,SPP1,SEMA4G,MYLIP,KIAA0319,TRPV4,SEMA3F,KREMEN1,CIB1,CERS2,PTPRO,INPP5J,LRIG2,WNT3,SEMA6D,TNR,DNM3,CD38,CRMP1,EPHA4,GAK,STX1B,PSEN1,SEMA4A,TRIM46,EFNA1,NTN1,SNAPIN,RIT2,CDK5R1,THOC2,DAB1,ARHGAP4,PLXNA3,TSC1,GFI1,RHOA,RTN4RL1,THY1,WNT5A,EFNB3	grey	VSMC_M10
GO:1990542	0.35	0.73	48	84	mitochondrial transmembrane transport	BP	1	TIMM21,CPT1B,SMDT1,SLC25A13,PNPT1,UCP1,DNAJC15,ATP5F1C,SLC25A38,TIMM50,SLC25A15,MICU3,ATP5F1A,SLC8B1,SLC25A37,OPA1,MCUR1,FAM173B,SFXN2,CPT1A,MCUB,SLC25A20,TOMM40L,DNAJC30,GRPEL1,MICU1,MCU,MPC2,TIMM17B,PSEN2,SLC25A23,MAIP1,SLC25A41,PAM16,SLC25A33,ATP5PB,MT-ATP8,MT-ATP6,SLC25A3,ATP5ME,ATP5MF,SLC25A21,SLC25A32,TIMM23B,SLC25A29,MRPL18,GRPEL2,DNAJC19	grey	VSMC_M10
GO:0010659	0.35	0.77	21	36	cardiac muscle cell apoptotic process	BP	1	MAP3K5,PPP1R10,RGL2,HSF1,PAX8,HAND2,SIRT5,SIRT4,AGT,NFE2L2,CXCR2,CAMK2A,GNB1,PDPK1,MIR199A1,MIR21,MIR24-1,NOL3,TIGAR,NKX2-5,HEY2	grey	VSMC_M10
GO:0032369	0.35	0.79	14	23	negative regulation of lipid transport	BP	1	CRY1,ABCG5,ABCG8,NR1H2,APOC1,AKT2,NR1H3,CRY2,PLA2R1,EGF,FIS1,MIR27B,MIR27A,MIR33B	grey	VSMC_M10
GO:0090103	0.35	0.80	12	20	cochlea morphogenesis	BP	1	PTK7,MYO3A,SOX9,GLI2,EYA1,PAX2,MYO3B,GRHL3,DVL2,POU3F4,WNT5A,SIX1	grey	VSMC_M10
GO:0046488	0.35	0.71	76	138	phosphatidylinositol metabolic process	BP	1	PIP4K2C,SYNJ2,GPAA1,CWH43,INPP4A,TMEM246,PIGF,MBOAT7,PLEK,INPP4B,FIG4,MTMR2,PIP4K2A,ITPKC,PIP4P1,MTMR14,PLCB1,PGAP2,MTMR7,PIGU,PIK3CB,PITPNM2,PIK3R1,TMEM150A,CHRM5,MTMR9,SLA2,SYNJ1,CDS1,ITPKA,PIGT,GPLD1,PGAP1,INPP5A,PITPNM1,MTMR11,PIGM,BMX,FAM126B,IP6K2,LPGAT1,PIGV,MTMR3,INPP5J,PIGX,PIGZ,PIKFYVE,SACM1L,PIGC,PLCD4,SOCS4,PI4KA,SLC27A1,PGAP3,PIGH,PI4K2B,SOCS7,DGKE,TTC7B,PIGL,CSF1R,MPPE1,IMPA2,MTMR12,SOCS1,MTM1,MTMR1,INPP5K,PIP5K1C,PIP5KL1,PYURF,LCLAT1,PIGB,PIGA,INPP1,CDIPT	grey	VSMC_M10
GO:0098789	0.35	0.84	7	11	pre-mRNA cleavage required for polyadenylation	BP	1	CPSF6,CPSF1,CPSF2,NCBP2,CPSF4,AP002990.1,CSTF2	grey	VSMC_M10
GO:0031102	0.35	0.75	28	49	neuron projection regeneration	BP	1	LRP1,TSPO,PTN,TNC,DHFR,PTPRF,CHL1,ULK1,SPP1,KIAA0319,MTR,GAP43,DAG1,KREMEN1,CERS2,LRIG2,APOD,TNR,EPHA4,NDEL1,MAP2K1,ISL1,OMG,MAPK8IP3,MIR222,CNTF,RTN4RL1,THY1	grey	VSMC_M10
GO:0006631	0.35	0.69	149	271	fatty acid metabolic process	BP	1	HSD17B4,AIG1,LTC4S,CPT1B,SLC27A6,TNXB,QKI,PPARA,ACSL6,PNPLA3,MCAT,HSD17B8,ECHDC1,AKR1B1,MAPK14,HACD4,CROT,AASDH,ADH5,ETFB,NR1H2,LPIN1,DLD,PRKAR2B,FAAH,HADHA,SOX9,ABHD5,APOC1,ECHDC3,PRKAG2,ACOX1,SLC22A13,PIBF1,CYGB,ELOVL1,AKT2,HACL1,PANK2,PDK1,TRIB3,LYPLA1,ACOXL,CYP7A1,ELOVL7,ASAH1,ACOX2,ABHD6,THNSL2,CBR4,HPGD,PEX2,ETFDH,ACOT12,PRKAG1,NR1H3,IVD,LPIN3,ECI2,ERLIN1,PLA2G4B,ADTRP,PRKAB1,LONP2,PM20D1,SIRT4,MCEE,IRS1,CPT1A,BDH2,GPAM,ACSL5,CYP2U1,ABHD2,MSMO1,MECR,GNPAT,ABCB11,HACD2,LPGAT1,PER2,ACADSB,THEM5,THEM4,MLXIPL,SCAP,EHHADH,POR,PDK3,PLA2G4A,ACADL,TYSND1,ACSBG1,PECR,PRKAG3,SLC27A1,DAGLB,FADS3,LIAS,ACADVL,EPHX1,TECR,HSD17B10,DEGS1,LIPC,ACOT2,AC005520.1,ABCD4,ALOXE3,GCDH,MAPK3,HACD3,NDUFAB1,ACSM3,ACACA,NCOR1,CD74,ABHD3,SREBF1,LPIN2,C3,ACSBG2,CYP2J2,ACAA2,ACOT11,DECR2,ACSF3,CEL,MLYCD,PTGES2,ABCD3,PLA2G15,CES2,ACADM,PHYH,ACOT4,FABP5,FADS6,ELOVL3,HADHB,AMACR,NUDT19,GGT5,ECI1,AUH,LYPLA2,PTGES,INSIG2,ADH4	grey	VSMC_M10
GO:0061548	0.35	0.84	7	11	ganglion development	BP	1	SEMA3A,FZD3,SEMA3F,TULP3,TUBB3,SIX1,RGS4	grey	VSMC_M10
GO:0070254	0.35	0.86	6	9	mucus secretion	BP	1	PRKCE,EGFR,ATG5,TRAF3IP2,SCNN1B,CYBA	grey	VSMC_M10

GO:0071621	0.35	0.73	43	76	granulocyte chemotaxis	BP	1	PTK2,RAC2,CXCL8,AKIRIN1,C5AR2,C5AR1,NCKAP1L,CCL17,NOD2,GBF1,DNM1L,TRPV4,TGFB2,CMKLR1,MOSPD2,FCER1G,PIKFYVE,MCU,TNFSF18,CXCR2,BST1,MAPK1,DAPK2,MAPK3,S100A14,S100A8,CD74,CCL14,CCL5,CSF1R,CCL7,CCL2,CCL25,VAV1,PIP5K1C,MPP1,BSG,VAV3,CCL16,S100A12,DPP4,CXCL6,CCL22	grey	VSMC_M10
GO:1902644	0.35	0.84	7	11	tertiary alcohol metabolic process	BP	1	AKR1B1,AKR1A1,CBR4,DKK3,WNT4,DGKQ,CACNA1H	grey	VSMC_M10
GO:0090385	0.35	0.84	7	11	phagosome-lysosome fusion	BP	1	RAB7A,RAB7B,SPG11,PIKFYVE,SYT7,AC138894.1,RAB39A	grey	VSMC_M10
GO:0051238	0.35	0.86	6	9	sequestering of metal ion	BP	1	SLC30A4,FTH1,S100A8,SLC30A7,LCN2,FTL	grey	VSMC_M10
GO:0033555	0.35	0.76	24	42	multicellular organismal response to stress	BP	1	MAPK8IP2,TSPO,USP46,SLC1A1,P2RX2,RET,P2RX3,MEF2C,ASIC1,NR4A2,SLC6A2,KIAA0319,PPP3CA,ADRA2A,ATP1A2,COMT,GCH1,ASIC4,NMUR2,VWA1,TRPV1,MECP2,DPP4,CRH	grey	VSMC_M10
GO:0042119	0.35	0.78	15	25	neutrophil activation	BP	1	CXCL8,IL15,TRAF3IP2,MYD88,F2RL1,FCER1G,CXCR2,ITGAM,SCNN1B,CCL5,STXBP2,PRAM1,DNASE1,CXCL6,MIR125A	grey	VSMC_M10
GO:0006896	0.35	0.80	12	20	Golgi to vacuole transport	BP	1	AP3S1,VPS52,RBSN,CCDC91,VTI1A,RNF215,AP4M1,ANKFY1,GAK,AC138894.1,AP1G1,AP3S2	grey	VSMC_M10
GO:0007026	0.35	0.79	14	24	negative regulation of microtubule depolymerization	BP	1	CLASP2,TRIM54,NAV3,SPEF1,ATXN7,KATNB1,TTBK2,CIB1,CAMSAP3,TAOK1,APC2,CAMSAP1,APC,MAP6D1	grey	VSMC_M10
GO:0098792	0.35	0.84	7	11	xenophagy	BP	1	NOD1,OPTN,RIPK2,NOD2,ATG16L1,WIPI2,MAPK3	grey	VSMC_M10
GO:0007288	0.35	0.80	11	18	sperm axoneme assembly	BP	1	UBE2B,CFAP69,ARMC2,DNAH1,NEURL1,CFAP43,TTC12,SPAG16,MNS1,SPEF2,TPGS1	grey	VSMC_M10
GO:0120316	0.35	0.80	11	18	sperm flagellum assembly	BP	1	UBE2B,CFAP69,ARMC2,DNAH1,NEURL1,CFAP43,TTC12,SPAG16,MNS1,SPEF2,TPGS1	grey	VSMC_M10
GO:0036035	0.35	0.82	8	13	osteoclast development	BP	1	FOXP1,FBXW7,LRRK1,SLC9B2,FBN1,ANXA2,GPR68,ATP6AP1	grey	VSMC_M10
GO:0001921	0.35	0.82	8	13	positive regulation of receptor recycling	BP	1	BVES,EPS15,NSF,ECE1,NSG1,ANXA2,PSEN1,VAMP3	grey	VSMC_M10
GO:0098856	0.35	0.84	7	11	intestinal lipid absorption	BP	1	ABCG5,ABCG8,SOAT2,LIMA1,LPCAT3,LDLR,CEL	grey	VSMC_M10
GO:0090140	0.36	0.77	18	31	regulation of mitochondrial fission	BP	1	PRKN,VPS35,DDHD2,DCN,DNM1L,MAPT,FIS1,C11orf65,MCU,DDHD1,PINK1,MUL1,STAT2,MIEF2,RALBP1,DHODH,RALA,MIEF1	grey	VSMC_M10
GO:0086010	0.36	0.77	17	30	membrane depolarization during action potential	BP	1	TRPM4,CACNB2,SCN5A,SCN1A,SLMAP,KCNJ2,SCN2A,SCN7A,ANK2,ATP1A2,SCN4B,CACNA1G,CACNA1H,KCNH2,SCN2B,YWHAH,HCN4	grey	VSMC_M10
GO:0007173	0.36	0.72	50	90	epidermal growth factor receptor signaling pathway	BP	1	PTK2,NCK2,EPGN,AREG,TGFA,EGFR,GAB1,BRAF,RAB7A,SOX9,NEU3,SNX5,HBEGF,PDE6G,FER,HIP1R,PLCE1,FBXW7,PTPRJ,WDR54,PTK2B,NEURL1,ADRA2A,SLC30A10,RNF115,NCF1,EGF,GPER1,DGKD,IFI6,AGT,CBL,REPS2,PDE6H,SNX6,ZFYVE28,SOCS4,HAP1,PSEN1,RPS6KA5,GAREM1,PTPN2,PDPK1,RNF126,MVB12B,DAB2IP,MIR29A,MIR21,GRB2,EREG	grey	VSMC_M10
GO:0009134	0.36	0.84	7	11	nucleoside diphosphate catabolic process	BP	1	NT5E,ENTPD6,NUDT5,NUDT16,ENTPD4,NUDT18,ENTPD5	grey	VSMC_M10
GO:0060761	0.36	0.73	35	61	negative regulation of response to cytokine stimulus	BP	1	IL1R2,PXDN,NR1H2,GAS6,OTUD4,ROBO1,NR1H3,OAS3,NLRC5,F2RL1,YTHDF2,PARP14,PELI3,MUL1,PADI2,GPS2,USP18,PYDC1,DCST1,MAP2K5,STAT2,CCL5,MAPK7,PTPN2,PIAS4,MIR21,MIR125B1,MIR27B,MIR24-1,MIR27A,OAS1,NOL3,MIR125A,CCDC3,HIST1H2BJ	grey	VSMC_M10
GO:0008652	0.36	0.74	32	56	cellular amino acid biosynthetic process	BP	1	GLS2,SHMT2,PYCR3,PSAT1,MTAP,ALDH1A1,BCAT2,DHFR,APIP,SERINC5,ENOPH1,SDSL,ASNSD1,GLS,MTRR,ASL,MTR,CBSL,OAT,GLUL,UPB1,PLOD3,CPS1,AASDHPPT,AC138894.1,SHMT1,ABAT,PARK7,ASS1,MTHFD2L,PHGDH,PCBD2	grey	VSMC_M10
GO:0003382	0.36	0.78	14	24	epithelial cell morphogenesis	BP	1	HOXA13,MET,SPINT2,RILPL1,RILPL2,FLNB,ARHGEF26,STC1,CLIC4,PLOD3,RAB25,TNMD,CCDC88C,BCL11B	grey	VSMC_M10
GO:0039531	0.36	0.79	12	20	regulation of viral-induced cytoplasmic pattern recognition receptor signaling pathway	BP	1	USP15,ANKRD17,PUM2,SEC14L1,NPLOC4,OASL,NLRX1,BIRC3,TKFC,TSPAN6,RIOK3,ZCCHC3	grey	VSMC_M10
GO:0097205	0.36	0.79	13	22	renal filtration	BP	1	XPNPEP3,GAS6,F2RL1,F2R,SULF2,MCAM,PTPRO,ITGA3,TMEM63C,CORO2B,TBC1D8B,CYBA,JCHAIN	grey	VSMC_M10
GO:0007411	0.36	0.70	98	181	axon guidance	BP	1	LRP1,SEMA6A,KIF5A,GFRA3,PTK2,PTCH1,PLXNA4,RAC2,SMO,SEMA4D,DLX5,ETV1,SEMA4C,HOXA2,EPHA7,EPHA1,NRCAM,PLXNA1,GLI2,MEGF8,MYCBP2,KIF5C,FEZ2,SEMA3D,PLXNC1,SEMA3A,CHL1,UNC5D,RYK,EPHB1,RET,ROBO1,ROBO2,FZD3,SEMA4F,DPYSL2,SLIT1,TRIO,SEMA3G,CYFIP1,ALCAM,SEMA4G,SIAH1,BMPR1B,BDNF,LGR6,SEMA3F,GAP43,DAG1,LHX9,GBX2,KALRN,LAMA5,YTHDF1,PTPRO,EPHB3,WNT3,KLF7,MYPN,SEMA6D,EPHB4,TNR,CRMP1,EPHA4,LRTM2,UNC5A,SEMA4A,NGFR,EFNA1,NTN1,RPS6KA5,RNF165,BCL11B,LHX1,LAMA3,CDK5R1,ISL1,EMB,LAMA1,L1CAM,NTN3,PLXNA3,BSG,TUBB3,DPYSL5,EFNA4,CNTN2,VASP,FOXG1,ISL2,NOG,WNT5A,CHN1,ARTN,NRXN3,LYPLA2,EFNB3,ISPD	grey	VSMC_M10
GO:1901016	0.36	0.76	20	35	regulation of potassium ion transmembrane transporter activity	BP	1	NEDD4L,KCNRG,OXSR1,KCNE2,CASQ2,KCNIP2,KCNG1,ANK2,KLHL24,NOS1AP,DLG1,ATP1B1,KCNA1,ATP1B2,DNM2,RNF207,AMIGO1,WWP2,NETO2,CRBN	grey	VSMC_M10
GO:0030318	0.36	0.79	12	20	melanocyte differentiation	BP	1	SOX10,KIT,MITF,MEF2C,ADAMTS20,OCA2,USP13,HPS4,BLOC1S6,MREG,LRMDA,CITED1	grey	VSMC_M10
GO:0045109	0.36	0.84	7	11	intermediate filament organization	BP	1	BFSP1,NEFM,DSP,PKP2,KLHL24,DES,MTM1	grey	VSMC_M10
GO:0006754	0.36	0.75	23	39	ATP biosynthetic process	BP	1	ATP5MGL,PPARA,SLC25A13,SPHK2,PRKAG2,ATP5F1C,ATP5F1A,DGUOK,FAM173B,DNAJC30,ATP5S,PINK1,STAT3,PARP1,PKLR,ATP6V0C,ATP5PB,VPS9D1,MT-ATP8,MT-ATP6,ATP5ME,ATP5MF,NUDT2	grey	VSMC_M10
GO:0007625	0.36	0.84	7	11	grooming behavior	BP	1	SLC1A1,PPT1,DDO,DLG4,HOXB8,NMUR2,CNTNAP4	grey	VSMC_M10
GO:0009081	0.36	0.80	11	18	branched-chain amino acid metabolic process	BP	1	BCAT2,SDSL,IVD,HIBCH,ACAD8,ACADSB,HSD17B10,SLC25A44,ALDH6A1,HIBADH,AUH	grey	VSMC_M10

GO:0044281	0.36	0.66	705	1301	small molecule metabolic process	BP	1	NME1,RDH5,KDSR,APOF,ATP8B1,GLS2,RDH16,HSD17B4,SHMT2,TBPL1,ALDH8A1,MARS,PDE7B,PEX7,CYP27B1,AIG1,FUCA2,LTC4S,ENPP3,ATP5MGL,GFPT2,TPH2,RIDA,MIOX,CPT1B,SYNJ2,DPYS,SLC27A6,TNXB,PPT2,PRKN,QKI,PPARA,PDE10A,ACSL6,PNPLA3,MCAT,HSD17B8,SMPDL3A,PMM1,ECHDC1,ACO2,PRXL2C,HSD17B3,AKR1B1,ATF4,PYCR3,APOBEC3H,GTPBP1,MAPK14,PLA2G6,TTC39B,TXN2,PSAT1,OARD1,GUCA1B,GPT,IMPDH1,HACD4,MTAP,NMRK1,CARNMT1,GNMT,ALDH1A1,CRY1,GDA,CROT,ABCC10,GNPDA2,PGM5,GALT,SLC35B2,ENPP4,KIT,PLPP6,CA9,SRD5A3,UXS1,TFAP2B,SPTLC1,SLC25A13,GAPDH,AASDH,PPAT,IPPK,LIPT1,HMGCLL1,INPP4A,ADH5,RAB23,CAT,PPCS,RIMKLA,TMEM86B,PRKCE,RDH13,ETFB,PPP4R3B,SLC4A4,ME1,NT5E,PPT1,ZNF692,BPGM,PLEK,NR1H2,DDC,IL4I1,FAM3C,LPIN1,INPP4B,DKKL1,MGST2,QRSL1,SLC7A11,DLD,DHDH,DDO,APOB,BCAT2,NANP,PRKAR2B,SPHK2,DHFR,CMPK1,ADCY3,MTMR2,NAPEPLD,FAAH,PRTFDC1,HADHA,EBP,GAD2,ENTPD6,SOX9,CDADC1,NUDT15,ABHD5,ESD,RUBCNL,DLG2,AKR1A1,CUBN,MMACHC,LACC1,APOC1,HPDL,GLB1,KHK,EPM2AIP1,NUDT5,LIPE,DHTKD1,ECHDC3,MBD4,PRKAG2,ACOX1,SLC22A13,ATP5F1C,XYLB,PIBF1,ITPKC,CYGB,GCKR,HYI,HNMT,RBKS,ACER3,TSKU,PPP1CB,ELOVL1,TGDS,CSGALNACT1,AKT2,PIP4P1,PFKP,UGGT2,PGM2L1,C1QTNF1,SPTLC3,IDO2,IDO1,ACSS3,MDH2,SIRT7,HARS,HARS2,BTD,HACL1,SLC25A15,SLC23A2,CARS2,NUDT16,PANK2,PDK1,FUT10,AMDHD1,LARS,SUCLG2,PPP1R3B,NR1H4,PDHX,APIP,MTMR7,SLC5A8,TRIB3,LYPLA1,SDR16C5,ACOXL,CSAD,SOAT2,CYP7A1,ELOVL7,ASAH1,PANK1,POFUT1,CYP7B1,ACOX2,GPI,ABHD6,CEBPA,THNSL2,N[illegible]	grey	VSMC_M10
GO:0098962	0.36	0.82	8	13	regulation of postsynaptic neurotransmitter receptor activity	BP	1	DLGAP3,DLGAP4,HOMER1,CNIH2,NPTX2,BEGAIN,DLGAP1,CACNG5	grey	VSMC_M10
GO:2000831	0.36	0.83	7	11	regulation of steroid hormone secretion	BP	1	GDF9,CRY1,C1QTNF1,NKX3-1,CRY2,AGT,CRH	grey	VSMC_M10
GO:0090336	0.36	0.82	8	13	positive regulation of brown fat cell differentiation	BP	1	MAPK14,PIM1,NAPEPLD,RREB1,SOX13,TFE3,ZBTB7B,SIX1	grey	VSMC_M10
GO:0021510	0.36	0.73	39	70	spinal cord development	BP	1	PTCH1,SMO,DAAM2,PTN,INTU,VSTM5,EED,ERCC2,GLI2,IFT172,MNX1,SOX12,ROBO2,SOX6,PKD2,SOX13,SUFU,SRD5A1,ADARB1,GIGYF2,BAG3,CHRD,MDGA2,SCYL3,TULP3,CLN8,PLXDC1,HOXB8,LHX1,ISL1,SUZ12,DAB1,LRP8,PKD1,SOX4,ISL2,NOG,LBX1,PHGDH	grey	VSMC_M10
GO:0060347	0.36	0.83	7	11	heart trabecula formation	BP	1	ADGRG6,TEK,SRF,FHL2,OVOL2,ADAMTS1,HEY2	grey	VSMC_M10
GO:2000345	0.36	0.83	7	11	regulation of hepatocyte proliferation	BP	1	GLI1,TNFAIP3,PTN,FBXW7,SULF2,MED1,FGF1	grey	VSMC_M10
GO:2000463	0.36	0.78	14	24	positive regulation of excitatory postsynaptic potential	BP	1	RIMS2,RIMS1,SHANK1,CUX2,PTK2B,IGSF11,SSH1,STX1A,DLG4,NLGN2,STX1B,NLGN3,PRKCZ,RGS4	grey	VSMC_M10
GO:1901889	0.36	0.77	15	26	negative regulation of cell junction assembly	BP	1	LRP1,EPHA7,ITGB1BP1,CLASP2,IKBKB,FAM107A,ROBO2,SLIT1,DMTN,ARHGAP6,APOD,BCAS3,ACVRL1,WNT5A,DUSP22	grey	VSMC_M10
GO:0080182	0.36	0.81	9	15	histone H3-K4 trimethylation	BP	1	KMT2D,RTF1,WDR82,ZNF335,WDR61,SETD1A,OGT,ARID4A,BEND3	grey	VSMC_M10
GO:0010658	0.36	0.75	22	38	striated muscle cell apoptotic process	BP	1	MAP3K5,PPP1R10,RGL2,HSF1,PAX8,HAND2,SIRT5,SIRT4,AGT,BAG3,NFE2L2,CXCR2,CAMK2A,GNB1,PDPK1,MIR199A1,MIR21,MIR24-1,NOL3,TIGAR,NKX2-5,HEY2	grey	VSMC_M10
GO:0017062	0.36	0.85	6	9	respiratory chain complex III assembly	BP	1	LYRM7,UQCC1,BCS1L,SLC25A33,C12orf73,UQCC2	grey	VSMC_M10
GO:0034551	0.36	0.85	6	9	mitochondrial respiratory chain complex III assembly	BP	1	LYRM7,UQCC1,BCS1L,SLC25A33,C12orf73,UQCC2	grey	VSMC_M10
GO:0070102	0.36	0.79	12	20	interleukin-6-mediated signaling pathway	BP	1	ST18,CEBPA,FER,RIPK1,STAT3,IL6R,JAK1,PTPN2,GFI1,MIR125B1,MIR125A,C1QTNF4	grey	VSMC_M10
GO:1990748	0.36	0.72	45	79	cellular detoxification	BP	1	LTC4S,APOM,PRXL2C,KDM3B,FANCC,PIM1,ALDH1A1,TXNRD1,PRDX1,ADH5,CAT,ALB,PXDN,MGST2,TXNRD3,DHFR,ESD,AKR1A1,GSTK1,PRDX3,CYGB,PXDNL,GLRX,GPX8,TP53INP1,ABCG2,FBLN5,MARC2,MARC1,NFE2L2,DUOX1,GCH1,ABCB6,RDH11,SOD3,GSTZ1,MPO,TXNDC2,PARK7,NQO1,MIR21,HBA2,TXN,PTGES,ADH4	grey	VSMC_M10
GO:0140131	0.36	0.83	7	11	positive regulation of lymphocyte chemotaxis	BP	1	CCL27,OXSR1,PTK2B,TMEM102,CCL5,CCL7,WNT5A	grey	VSMC_M10
GO:0099590	0.36	0.82	8	13	neurotransmitter receptor internalization	BP	1	USP46,EPS15,AP2M1,CALY,DNM3,AP2B1,CACNG7,CACNG5	grey	VSMC_M10
GO:0044764	0.36	0.82	8	13	multi-organism cellular process	BP	1	EPS15,NECTIN2,FCHSD2,FCHSD1,GAS6,TPCN1,PIKFYVE,PI4KA	grey	VSMC_M10
GO:0071625	0.36	0.82	8	13	vocalization behavior	BP	1	SHANK1,MYH14,FOXP2,NLGN4Y,NRXN2,DLG4,NLGN3,NRXN3	grey	VSMC_M10
GO:0051645	0.36	0.82	8	13	Golgi localization	BP	1	YWHAZ,ARHGAP21,COPG1,HOOK3,TBCCD1,PDCD10,DAB1,RIPOR1	grey	VSMC_M10
GO:0071902	0.37	0.69	93	170	positive regulation of protein serine/threonine kinase activity	BP	1	MAP3K5,STK3,MAP3K4,AGER,TAB1,CCND3,KIT,ROR2,EPGN,ETAA1,TGFA,EGFR,ARHGEF5,TLR4,CCNY,SLC8A2,MAPRE3,RALB,SPRY2,NEK10,RPTOR,TCIM,FLT1,TRAF6,ROBO1,PTK2B,RASGRP1,RAPGEF2,TIAM1,PKD2,STK4,NOD2,TAOK3,CD40,IGF2,BMPR1B,CCND1,MAP2K6,ADRA2A,FAM20A,AJUBA,KRAS,EGF,SPATC1L,CIB1,DVL3,SYAP1,STRADA,CDKN1B,MAP3K11,ADAM8,FZD5,CEMIP,DBF4B,MAP4K2,DDX3X,DUSP19,PDCD10,SLC27A1,EPHA4,MAGED1,DVL2,BMPR1A,PSEN1,FGF18,MAP2K1,PIK3R5,MAP2K5,CALM1,PSMD10,INSR,CSF1R,CDK5R1,GHR,TAOK1,MLST8,PKD1,TRAF2,MTCP1,PSRC1,DAB2IP,RHOA,S100A12,IRGM,WNT5A,SPDYA,RGCC,DAZAP2,HRAS,MAP2K7,C3orf58,FGF1,PDGFD	grey	VSMC_M10
GO:0031018	0.37	0.76	18	31	endocrine pancreas development	BP	1	ONECUT2,BAK1,SMO,CDK6,RFX3,SOX9,GIPR,MNX1,ARNTL,NKX6-1,ZBED6,SIDT2,MEN1,IL6R,GATA6,PDPK1,SOX4,WNT5A	grey	VSMC_M10
GO:0030888	0.37	0.74	24	42	regulation of B cell proliferation	BP	1	IL5,TNFRSF13C,TNFRSF21,PELI1,TLR4,BMI1,PAWR,NCKAP1L,CASP3,MEF2C,IL7,CD40,BCL6,RC3H1,CD38,BST1,CARD11,CHRNB2,BTK,CD74,CD320,TICAM1,VAV3,TNFSF13B	grey	VSMC_M10
GO:0015937	0.37	0.83	7	11	coenzyme A biosynthetic process	BP	1	PPCS,PANK2,PANK1,SLC25A16,COASY,PANK3,PANK4	grey	VSMC_M10
GO:0002504	0.37	0.79	11	18	antigen processing and presentation of peptide or polysaccharide antigen via MHC class II	BP	1	HLA-DRA,HLA-DOB,HLA-DMA,HLA-DPB1,TRAF6,MARCH8,FCER1G,PIKFYVE,CTSV,DNM2,CD74	grey	VSMC_M10

GO:0001913	0.37	0.79	12	20	T cell mediated cytotoxicity	BP	1	STX7,AGER,HLA-DRA,NECTIN2,PVR,SLC22A13,NCKAP1L,CTSH,IL12A,PPP3CB,IL7R,FADD	grey	VSMC_M10
GO:1901096	0.37	0.82	8	13	regulation of autophagosome maturation	BP	1	TMEM39A,RUBCN,TBC1D25,PHF23,AC138894.1,SMCR8,CLEC16A,IRGM	grey	VSMC_M10
GO:0007185	0.37	0.88	4	6	transmembrane receptor protein tyrosine phosphatase signaling pathway	BP	1	FRS2,PTN,PTPRF,TRIO	grey	VSMC_M10
GO:0032494	0.37	0.83	7	11	response to peptidoglycan	BP	1	IRF5,C5AR1,MYD88,RIPK2,NOD2,DEFB124,INAVA	grey	VSMC_M10
GO:0001502	0.37	0.80	9	15	cartilage condensation	BP	1	MAPK14,ROR2,MYCN,SOX9,SOX6,BMPR1B,THRA,PKD1,OTOR	grey	VSMC_M10
GO:0006399	0.37	0.69	90	162	tRNA metabolic process	BP	1	MARS,ADAT2,RPP21,TRMU,TRMT11,C9orf64,EXOSC3,MTO1,TSEN34,ELP1,PUS10,TRIT1,QRSL1,DUS4L,NSUN6,THUMPD3,DPH3,HARS,HARS2,TRMT6,TRNT1,CARS2,LARS,THUMPD2,TRMT9B,ELP4,POP4,CLP1,ELP3,BCDIN3D,TPRKB,NSUN3,OSGEPL1,RPP40,WARS2,CDKAL1,GATC,POP5,RPUSD4,YARS2,TRMT10A,ZBTB8OS,QTRT2,TYW1B,TYW5,TRUB1,RTRAF,TARS2,DALRD3,LCMT2,METTL8,ELP6,METTL2B,KIAA0391,TRMT1L,LARS2,POP7,DARS2,TRPT1,ALKBH8,TRMT5,FARSB,HSD17B10,MTFMT,ALKBH1,THG1L,QTRT1,EARS2,ELP2,THUMPD1,METTL6,PUSL1,LRRC47,POLR3K,KARS,ADAT1,AARS,DUS2,CTU1,MARS2,RPP38,TRMT10B,DTD1,TP53RK,TRMT61A,DTD2,TRMT10C,RPP30,TRMT61B,TYW3	grey	VSMC_M10
GO:0045050	0.37	0.85	6	9	protein insertion into ER membrane by stop-transfer membrane-anchor sequence	BP	1	EMC2,EMC10,EMC3,EMC4,EMC6,EMC7	grey	VSMC_M10
GO:0031345	0.37	0.69	82	150	negative regulation of cell projection organization	BP	1	LRP1,SEMA6A,TBC1D30,FSTL4,SYNGAP1,SEMA4D,SEMA4C,RTN4,RUFY3,ACP4,LPAR1,EPHA7,HDAC2,TSKU,SPRY2,PRNP,SEMA3D,SPART,SEMA3A,ULK1,DPYSL3,RYK,CBFA2T2,SEMA4F,TLX2,RAPGEF2,DGUOK,PAQR3,ARHGAP24,SEMA3G,SPP1,SEMA4G,MYLIP,SRGAP2C,KIAA0319,PPP3CA,TRPV4,SEMA3F,KREMEN1,CIB1,CERS2,NFATC4,PTPRO,INPP5J,LIMK2,LRIG2,WNT3,SEMA6D,TNR,DNM3,RAP1GAP,CD38,CRMP1,EPHA4,GAK,STX1B,PSEN1,SEMA4A,ITGA3,TRIM46,EFNA1,NTN1,SNAPIN,DNM2,RIT2,ARHGAP44,EVL,CDK5R1,THOC2,DAB1,ARHGAP4,PLXNA3,CDK10,TSC1,GFI1,ODF2L,RHOA,RTN4RL1,THY1,WNT5A,YWHAH,EFNB3	grey	VSMC_M10
GO:0008347	0.37	0.74	23	40	glial cell migration	BP	1	LRP1,TSPO,SUN2,MBOAT7,RTN4,P2RY1,TIAM1,SRGAP2C,TGFB2,DISC1,CERS2,EPHA4,ATP1B2,CCL2,CDK5R1,DAB1,LRP8,DAB2IP,MIR222,ADGRG1,FOXG1,LAMB1,CSPG4	grey	VSMC_M10
GO:0090322	0.37	0.77	16	27	regulation of superoxide metabolic process	BP	1	FPR2,EGFR,DHFR,HVCN1,F2RL1,FBLN5,AGT,NFE2L2,MAPT,GCH1,BST1,ITGAM,GNAI3,CYBA,MIR21,MIR27B	grey	VSMC_M10
GO:0033539	0.37	0.83	7	11	fatty acid beta-oxidation using acyl-CoA dehydrogenase	BP	1	ETFB,ETFDH,IVD,ACADL,ACADVL,GCDH,ACADM	grey	VSMC_M10
GO:1900037	0.37	0.81	8	13	regulation of cellular response to hypoxia	BP	1	TMBIM6,AJUBA,USP19,HYOU1,KCNK2,PINK1,MIR21,NOL3	grey	VSMC_M10
GO:0062125	0.37	0.76	16	27	regulation of mitochondrial gene expression	BP	1	SHMT2,TSFM,RMND1,LRPPRC,C6orf203,MRPS27,NSUN3,RPUSD4,RCC1L,MTG2,COA3,ALKBH1,METTL4,RPUSD3,UQCC2,TRMT10C	grey	VSMC_M10
GO:0001964	0.37	0.81	8	13	startle response	BP	1	PRKN,GRIN2D,CHD8,SLC6A3,BACE1,KCNA1,MECP2,GRID2	grey	VSMC_M10
GO:0070373	0.37	0.73	30	53	negative regulation of ERK1 and ERK2 cascade	BP	1	SEMA6A,FBLN1,WNK2,ITGB1BP1,VRK3,TLR4,SPRY1,PHB,SPRY2,ARRB1,SPRED3,DUSP6,SIRT3,TBC1D10C,GPER1,DLG1,DUSP10,RGS14,SYNJ2BP,TNIP1,PTPN2,DAB2IP,MIR21,CHRNA10,DUSP26,DUSP1,DUSP4,C3orf33,LIF,C1QL4	grey	VSMC_M10
GO:0009650	0.37	0.83	7	11	UV protection	BP	1	CAT,ERCC1,ERCC2,ERCC3,SCARA3,ERCC4,SDF4	grey	VSMC_M10
GO:0072243	0.37	0.80	9	15	metanephric nephron epithelium development	BP	1	POU3F3,OSR1,SOX9,PAX8,PKD2,PAX2,PKD1,SOX8,LIF	grey	VSMC_M10
GO:0044088	0.37	0.74	24	42	regulation of vacuole organization	BP	1	PIP4K2C,RNF5,PIP4K2A,RALB,TMEM106B,RAB5A,FEZ2,ULK1,TBC1D12,SEC22B,TMEM39A,ADPRH,MTMR3,RAB1B,PIKFYVE,TBC1D14,PINK1,PHF23,ANXA2,SMCR8,IFT20,MTM1,KIAA1324,SH3GLB1	grey	VSMC_M10
GO:0014046	0.37	0.78	12	20	dopamine secretion	BP	1	PRKN,SYT1,SYT9,DTNBP1,SYT8,GRM2,RAB3A,PINK1,SYT7,SYT11,CHRNB2,ABAT	grey	VSMC_M10
GO:0014059	0.37	0.78	12	20	regulation of dopamine secretion	BP	1	PRKN,SYT1,SYT9,DTNBP1,SYT8,GRM2,RAB3A,PINK1,SYT7,SYT11,CHRNB2,ABAT	grey	VSMC_M10
GO:0086070	0.37	0.85	5	8	SA node cell to atrial cardiac muscle cell communication	BP	1	SCN5A,RYR2,ANK2,CACNA1G,HCN4	grey	VSMC_M10
GO:0031114	0.37	0.76	16	28	regulation of microtubule depolymerization	BP	1	CLASP2,TRIM54,NAV3,SPEF1,ATXN7,KATNB1,TRPV4,TTBK2,CIB1,CAMSAP3,TAOK1,APC2,CAMSAP1,APC,MAP1A,MAP6D1	grey	VSMC_M10
GO:0097479	0.37	0.73	26	46	synaptic vesicle localization	BP	1	AP3S1,KIF5A,PRKN,BRSK1,AP3M2,KIF5C,LIN7A,TMEM230,SYNJ1,AP3B1,DTNBP1,CNIH2,SPG11,BLOC1S6,RAB3A,PINK1,SYN1,AP3M1,SLC2A4,NLGN2,TRIM46,SNAPIN,DNM2,BLOC1S3,BORCS5,AP3S2	grey	VSMC_M10
GO:0043506	0.37	0.73	26	46	regulation of JUN kinase activity	BP	1	MAP3K5,TNXB,MAP3K4,AGER,ROR2,DNAJA1,ARHGEF5,TRAF6,PTK2B,TIAM1,DTNBP1,TAOK3,PDCD4,DVL3,MAP3K11,AIDA,FZD5,DUSP10,MAP4K2,DUSP19,EPHA4,DVL2,TRAF2,DAB2IP,WNT5A,MAP2K7	grey	VSMC_M10
GO:0050671	0.37	0.71	46	82	positive regulation of lymphocyte proliferation	BP	1	AGER,IL5,HLA-DPB1,TNFRSF13C,NCK2,PELI1,TLR4,IL15,BMI1,MYD88,TGFBR2,NCKAP1L,SLC7A1,TRAF6,IL1A,MEF2C,IL7,RIPK2,ZNF335,CD40,IGF2,ICOSLG,GPAM,CD28,BCL6,IL12A,CD38,BST1,STAT5B,FOXP3,RASAL3,CARD11,CHRNB2,CD74,CCL5,CD320,TICAM1,DNAJA3,VAV3,MIR21,FADD,CD46,CD70,TNFSF9,TNFSF13B,HAVCR2	grey	VSMC_M10
GO:0007141	0.37	0.78	12	20	male meiosis I	BP	1	MOV10L1,DMC1,FOXJ3,ING2,HSF2BP,SPO11,RAD51C,MEIOB,UBB,SYCP3,FOXJ2,ZSCAN21	grey	VSMC_M10
GO:0045651	0.37	0.81	8	13	positive regulation of macrophage differentiation	BP	1	TRIB1,HSF1,ROR2,RIPK1,HCLS1,FADD,CASP8,LIF	grey	VSMC_M10

GO:0043269	0.37	0.66	240	444	regulation of ion transport	BP	1	NEDD4L,ATP8B1,STAC3,MAPK8IP2,SLC12A2,KCNV1,PRRT1,PRKN,TSPO,GOPC,BAK1,CATSPER3,MCHR1,KCNJ4,PLA2G6,CHRNA3,ABCB1,CLIC5,WNK2,TSPAN13,PRKCE,KCNQ4,SHANK1,SLC31A2,HECW1,TCAF2,CLCN1,SFRP4,KEL,ATG5,OSR1,SPHK2,KCNJ14,TRPC3,SLC26A5,KCNRG,CACNB2,STAC,OXSR1,SCN5A,SCN1A,GPD1L,SYT1,CACNB4,EFHB,NALCN,PRNP,DIAPH1,P2RX2,CLIC1,ARL6IP5,SYT9,KCNIP3,PXK,SLMAP,SLC43A1,GLRX,P2RX3,ORAI1,MEF2C,TMBIM6,P2RX7,HVCN1,KCNH3,P2RY1,PTK2B,KCNJ11,TPCN1,SLC8B1,HOMER1,IFNGR2,STC1,KCNJ3,F2R,TESC,RIPK1,ANO6,CRY2,KCNK15,ABCC2,KCNE2,PKD2,PLA2R1,CASQ2,KCNJ15,CNTN1,DTNBP1,KCNIP2,CHRM3,RYR2,SYT8,FAM173B,GRM2,PM20D1,CALHM1,PKP2,SIK1,HECW2,KCNJ2,SLC38A3,MAP2K6,ADRA2A,SLC30A10,TCIRG1,COX17,SCN2A,ISCU,KCNB1,EGF,KCNG1,SCN7A,EPB41,OPRD1,UBASH3B,ANK2,GPER1,KLHL24,ABCB11,AGT,ACE2,ATP1A2,SLC9A1,PLPP4,PER2,C4orf3,CASQ1,SNX4,NTSR1,SCN4B,CNIH2,CLIC4,DNAJC30,NOS1AP,HOMER2,CATSPER2,DLG1,KCNK7,PLA2G4A,IL16,CEMIP,SESTD1,CASK,PTPN6,KCNK4,HTT,RAB3A,PINK1,CAMK2G,ATP1B1,KCNA1,ARL6IP1,KCNMA1,CHRM1,KCNH4,GNB5,KCND1,MINK1,HAP1,DLG4,PSEN2,SYT7,NLGN2,CNIH3,WNK3,STAC2,CACNB1,ATP1B2,PSEN1,KCNAB3,CACNA1A,SYT11,KCNIP1,HCN3,NLGN3,CACNA1G,CHRNB2,ATP2A1,CD19,CALM1,DNM2,VMP1,BDKRB1,TRPV2,CAMK2A,CCL5,CCL2,ASIC2,KCNJ18,ABAT,FMR1,GABRE,INPP5K,TRPV3,PDPK1,P2RX1,CACNA1B,RNF207,CACNA1H,PARK7,KCNC4,AMIGO1,CYBA,JPH3,ATP2C2,LCN2,STXBP1,WWP2,RRAD,MIR21,MIR24-1,RHOA,DMD,GPR35,JPH2,CA2,KCNH2,FGF11,THY1,S[illegible]	grey	VSMC_M10
GO:0035137	0.37	0.77	14	24	hindlimb morphogenesis	BP	1	OSR2,PITX1,PTCH1,TFAP2B,OSR1,RARB,FMN1,RPGRIP1L,WNT3,BMPR1A,MED1,TBX4,GPC3,ALX3	grey	VSMC_M10
GO:0050999	0.37	0.75	18	31	regulation of nitric-oxide synthase activity	BP	1	ZDHHC21,NOD1,EGFR,NOSIP,DHFR,ACVR2A,IL1A,PIK3CB,PTK2B,TERT,KRAS,NOS1AP,GCH1,CALM1,GLA,TERF2,MIR27B,FCER2	grey	VSMC_M10
GO:0043507	0.37	0.75	20	35	positive regulation of JUN kinase activity	BP	1	MAP3K5,MAP3K4,AGER,ROR2,ARHGEF5,TRAF6,PTK2B,TIAM1,TAOK3,DVL3,MAP3K11,FZD5,MAP4K2,DUSP19,EPHA4,DVL2,TRAF2,DAB2IP,WNT5A,MAP2K7	grey	VSMC_M10
GO:0035909	0.37	0.76	16	28	aorta morphogenesis	BP	1	LRP1,FOXH1,SRF,TFAP2B,JAG1,EYA1,TGFB2,EFEMP2,BMPR1A,RBPJ,TBX2,ENG,SOX4,ACVRL1,SIX1,HEY2	grey	VSMC_M10
GO:0060045	0.37	0.77	14	24	positive regulation of cardiac muscle cell proliferation	BP	1	GLI1,MAPK14,PIM1,MEF2C,TBX5,MAPK1,BMPR1A,RBPJ,TBX2,GATA6,MIR199A1,MIR222,HEY2,WNT2	grey	VSMC_M10
GO:0045779	0.37	0.83	7	11	negative regulation of bone resorption	BP	1	TNFAIP3,TNFRSF11B,P2RX7,GPR137B,UBASH3B,GPR137,CD38	grey	VSMC_M10
GO:0005977	0.37	0.72	32	57	glycogen metabolic process	BP	1	GNMT,PYGB,RUBCNL,KHK,EPM2AIP1,PRKAG2,PPP1CB,AKT2,PPP1R3B,GBE1,SORBS1,PFKM,IGF2,NHLRC1,PHKB,IRS1,PPP1R3E,PPP1R3D,PER2,PASK,PHKA2,PYGM,PYGL,PRKAG3,PPP1R3F,PHKA1,INSR,LEPR,INPP5K,AGL,ACADM,PPP1R1A	grey	VSMC_M10
GO:0007042	0.37	0.83	7	11	lysosomal lumen acidification	BP	1	PPT1,TMEM106B,TMEM9,AC138894.1,CLN6,SNAPIN,TMEM199	grey	VSMC_M10
GO:0033292	0.37	0.87	4	6	T-tubule organization	BP	1	MYOF,DYSF,ANK2,SYPL2	grey	VSMC_M10
GO:0071286	0.37	0.87	4	6	cellular response to magnesium ion	BP	1	RYR3,SLC41A1,KCNA1,SLFN14	grey	VSMC_M10
GO:0010694	0.38	0.87	4	6	positive regulation of alkaline phosphatase activity	BP	1	MEF2C,GPLD1,SMAD3,PPARGC1B	grey	VSMC_M10
GO:0051150	0.38	0.75	18	31	regulation of smooth muscle cell differentiation	BP	1	NFATC1,SRF,KIT,ANKRD17,PDCD4,GPER1,EFEMP2,FOXO4,RBPMS2,MECP2,ENG,NFATC3,MIR21,MIR125B1,MIR22,PRDM6,EREG,HEY2	grey	VSMC_M10
GO:0010661	0.38	0.77	13	22	positive regulation of muscle cell apoptotic process	BP	1	MAP3K5,CDKN2A,ARRB1,STK4,PDCD4,AGT,IL12A,CXCR2,RBM10,MFN2,CAMK2A,MIR24-1,TIGAR	grey	VSMC_M10
GO:0061323	0.38	0.80	9	15	cell proliferation involved in heart morphogenesis	BP	1	PIM1,SOX9,HAND2,EYA1,TBX5,RBPJ,MKS1,ISL1,SIX1	grey	VSMC_M10
GO:2000136	0.38	0.80	9	15	regulation of cell proliferation involved in heart morphogenesis	BP	1	PIM1,SOX9,HAND2,EYA1,TBX5,RBPJ,MKS1,ISL1,SIX1	grey	VSMC_M10
GO:0002544	0.38	0.82	7	11	chronic inflammatory response	BP	1	TNFAIP3,UNC13D,AHCY,FOXP3,S100A8,CCL5,PTGES	grey	VSMC_M10
GO:0062208	0.38	0.75	19	33	positive regulation of pattern recognition receptor signaling pathway	BP	1	USP15,HSPA1B,NINJ1,RTN4,ANKRD17,SLC46A2,PELI1,RSAD2,TLR4,PUM2,MFHAS1,NR1H3,F2RL1,OASL,TLR5,DDX3X,WDFY1,CYBA,ZCCHC3	grey	VSMC_M10
GO:0018149	0.38	0.80	9	15	peptide cross-linking	BP	1	NDNF,EVPL,DCN,DSP,TGM1,FN1,SPOCK2,COL3A1,ABCA7	grey	VSMC_M10
GO:0002793	0.38	0.71	37	66	positive regulation of peptide secretion	BP	1	LRP1,PLA2G6,PRKCE,EGFR,TRPM4,GIPR,AIMP1,SIRT3,OSBP,NNAT,PFKM,NKX6-1,GPLD1,TCF7L2,PFKFB2,GPER1,GLUL,MCU,PPP3CB,CD38,MPC2,NLGN2,TARDBP,STX4,RAB8B,GPR68,S100A8,ISL1,OXCT1,ABAT,NADK,BAIAP3,LRRC8A,FGA,SOX4,CRH,DOC2B	grey	VSMC_M10
GO:0042276	0.38	0.87	4	6	error-prone translesion synthesis	BP	1	POLI,REV1,REV3L,PRIMPOL	grey	VSMC_M10
GO:0032872	0.38	0.68	81	148	regulation of stress-activated MAPK cascade	BP	1	PHLPP1,MAP3K5,STK3,MAPK8IP2,MAP3K4,PRKN,AGER,MYC,GADD45G,KLHL31,PRDX1,SEMA4C,DNAJA1,FKTN,EMC10,MTURN,NOD1,EGFR,TLR4,TRAF1,SDCBP,MYD88,ZMYND11,PLCB1,EIF2AK2,RELL2,SEMA3A,SH3RF2,ARL6IP5,MFHAS1,TRAF6,EPHB1,HAND2,PTK2B,RASGRP1,RIPK2,F2RL1,RIPK1,NOD2,TAOK3,TRPV4,PDCD4,TGFB2,NCF1,TRAF5,FZD7,MAP3K3,MAGI3,DLG1,MAP3K11,AIDA,DUSP10,MBIP,MEN1,MAP4K2,DUSP19,PDCD10,PINK1,LTBR,MAPK1,MINK1,GPS2,RELL1,TAOK2,MAPK3,MAP2K1,NCOR1,TRAF3,TAOK1,SERPINF2,MAPK8IP3,TRAF2,MARVELD3,DAB2IP,ZNF622,WNT5A,GADD45A,INAVA,DUSP1,DUSP22,HRAS	grey	VSMC_M10
GO:0060013	0.38	0.87	4	6	righting reflex	BP	1	USP46,SLC1A1,SHANK1,ALDH1A3	grey	VSMC_M10
GO:1901607	0.38	0.72	28	49	alpha-amino acid biosynthetic process	BP	1	GLS2,SHMT2,PYCR3,PSAT1,MTAP,DHFR,APIP,SERINC5,ENOPH1,SDSL,ASNSD1,GLS,MTRR,ASL,MTR,CBSL,OAT,GLUL,PLOD3,CPS1,AASDHPPT,AC138894.1,SHMT1,PARK7,ASS1,MTHFD2L,PHGDH,PCBD2	grey	VSMC_M10
GO:2001223	0.38	0.87	4	6	negative regulation of neuron migration	BP	1	NRG1,COL3A1,STAT3,ADGRG1	grey	VSMC_M10
GO:0034471	0.38	0.78	12	20	ncRNA 5'-end processing	BP	1	RPP21,POP4,RPP40,POP5,NOP9,KIAA0391,POP7,HSD17B10,THG1L,RPP38,TRMT10C,RPP30	grey	VSMC_M10

GO:0032365	0.38	0.74	20	35	intracellular lipid transport	BP	1	CPT1B,SERAC1,PIP4K2A,OSBP,COL4A3BP,CPT1A,SLC25A20,OSBPL2,ABCA12,SYT7,ANXA2,ABCD4,LDLR,NPC1,ABCD3,VPS4A,MIR27B,LDLRAP1,STARD4,SGPP1	grey	VSMC_M10
GO:0008211	0.38	0.81	8	13	glucocorticoid metabolic process	BP	1	CYP21A2,DKK3,WNT4,DGKQ,MECP2,CACNA1H,HSD11B2,YWHAH	grey	VSMC_M10
GO:0007267	0.38	0.64	614	1146	cell-cell signaling	BP	1	NFATC1,LRP1,RPS12,STAC3,GLI1,KIF5A,PDE7B,TNFAIP3,STK3,SNCAIP,MAPK8IP2,SLC12A2,FZD6,RIMS2,PRRT1,PRKN,AGER,GDF9,TCF7,SYNGAP1,UBE2B,RSPO3,RNF146,CXCL14,AKAP7,GRM4,LY6E,GRAP2,EXOC4,ATF4,SYNGR1,MAPK14,NTRK2,CBY1,CSNK1E,PLA2G6,PICK1,SOX10,BTBD9,SMO,DAAM2,MDFI,GUCA1B,CHRNA3,TEK,LRRC4,GRM8,CHRNA5,CRY1,PTK7,SRF,GABRA1,APBA1,CDK14,FZD1,CCL27,PHF24,USP46,RFX3,KIT,SLC1A1,TFAP2B,ROR2,DLX5,WNK2,SOSTDC1,GABBR2,IL11,BRSK1,PRKCE,RIMS1,PPP2R1A,HMGN3,RIMS3,ACP4,SHANK1,CYB5R4,PPT1,AREG,BMP8A,CRHR2,GJA10,SLC1A4,RSPO1,PTN,DGKI,GRB10,TNC,ATP6V1C2,EGFR,HECW1,FUZ,NCDN,GRIK2,DLGAP3,GJB5,AMPH,IL15,SFRP4,CCNY,DKKL1,VPS35,SDCBP,TRPM4,SLC7A11,FOXO3,PPFIA3,LIN7B,NTF4,FGF21,PRKAR2B,WISP3,GRIN2D,MTMR2,NAPA,SLC8A2,PANX1,PTGIR,GAD2,SOX9,GIPR,PPM1N,CACNB2,DLG2,LRRFIP2,GLI2,PCDH8,GRIK5,RAF1,KLF5,SCN5A,NAPB,SCEL,TSKU,PLAT,WNT11,KPNA2,ABCC4,CHRNA6,RBMS3,C1QTNF1,FARP1,CHD8,FCHSD2,SYT1,CACNB4,UBAC2,RAB5A,LIN7A,PLCB1,PLCL2,FERMT1,RAC3,PRNP,ADRA1D,SPEF1,FCHSD1,PCDH1,P2RX2,AIMP1,NRG1,SIRT3,PTPRA,MITF,STX2,CALCOCO1,RYK,SYT9,ADRA2B,SNPH,NR1H4,EPHB1,PLK2,PFDN5,NDUFAF2,FAM3D,HTR7,FAM107A,PAX8,FGFBP3,NR4A1,PXK,ABHD6,OSBP,CLSTN2,CTNND1,DKK3,TRABD2A,PCSK1,P2RX3,CHRM5,ARNTL,ERC2,SCG5,FZD3,MEF2C,TMEM131L,ASIC1,SEMA4F,IL7,P2RX7,DLGAP4,CSNK2A2,GUCY1A1,GUCY1B1,CUX2,P2RY1,PTK2B,LMBR1L,MME,RAPGEF2,KCNJ11,CCL17,EIF2AK4,NNAT,TIAM1,FGF5,RAB11FIP5,OTULIN,PFKM,SYNJ1,SLC8B1,HOMER1,NKX3-1,NKX6-[illegible]	grey	VSMC_M10
GO:0017158	0.38	0.76	15	26	regulation of calcium ion-dependent exocytosis	BP	1	SYT1,SYT9,SYT8,HYAL3,ADRA2A,KCNB1,STX1A,RAP1B,SYT7,SYT11,CACNA1G,SCAMP5,CACNA1H,STXBP1,DOC2B	grey	VSMC_M10
GO:0030514	0.38	0.73	22	39	negative regulation of BMP signaling pathway	BP	1	LEMD2,SOSTDC1,BMPER,SPART,SORL1,HTRA1,CHRD,DLX1,CTDSPL2,HTRA3,FBN1,GREM2,RBPMS2,TOB1,CHRDL1,TWSG1,SKI,FSTL3,LEMD3,NOG,WNT5A,UBE2D1	grey	VSMC_M10
GO:0003211	0.38	0.87	4	6	cardiac ventricle formation	BP	1	HAND2,MEF2C,TBX5,SOX4	grey	VSMC_M10
GO:0021522	0.38	0.77	13	22	spinal cord motor neuron differentiation	BP	1	PTCH1,GLI2,IFT172,MNX1,SUFU,GIGYF2,MDGA2,SCYL3,CLN8,ISL1,SOX4,ISL2,LBX1	grey	VSMC_M10
GO:0045948	0.38	0.77	13	22	positive regulation of translational initiation	BP	1	HABP4,CDC123,EIF2AK4,RPS6KB2,YTHDF2,YTHDF1,UHMK1,DDX3X,POLR2G,LARP1,CCL5,IMPACT,PPP1R15A	grey	VSMC_M10
GO:0035092	0.38	0.87	4	6	sperm chromatin condensation	BP	1	SYCP1,CHD5,SYCP3,RNF8	grey	VSMC_M10
GO:0021602	0.38	0.76	14	24	cranial nerve morphogenesis	BP	1	ATP8B1,PLXNA4,SEMA3A,EPHB1,TFAP2A,PAX2,SEMA3F,ADARB1,CHRNB2,PLXNA3,MAFB,HOXB3,SIX1,HOXA3	grey	VSMC_M10
GO:1900006	0.38	0.79	9	15	positive regulation of dendrite development	BP	1	PTN,ALK,TMEM106B,OPA1,CYFIP1,CRTC1,ZDHHC15,LRP8,DAB2IP	grey	VSMC_M10
GO:0045588	0.38	0.87	4	6	positive regulation of gamma-delta T cell differentiation	BP	1	NCKAP1L,SOX13,STAT5B,SOX4	grey	VSMC_M10
GO:0030593	0.38	0.71	34	60	neutrophil chemotaxis	BP	1	RAC2,CXCL8,C5AR2,C5AR1,NCKAP1L,CCL17,NOD2,GBF1,DNM1L,TGFB2,MOSPD2,FCER1G,PIKFYVE,MCU,CXCR2,BST1,DAPK2,S100A8,CD74,CCL14,CCL5,CCL7,CCL2,CCL25,VAV1,PIP5K1C,MPP1,BSG,VAV3,CCL16,S100A12,DPP4,CXCL6,CCL22	grey	VSMC_M10
GO:0099509	0.38	0.87	4	6	regulation of presynaptic cytosolic calcium ion concentration	BP	1	CACNB2,P2RY1,ATP2B3,P2RX1	grey	VSMC_M10
GO:0001865	0.38	0.87	4	6	NK T cell differentiation	BP	1	TGFBR2,TOX,AP3B1,ZBTB7B	grey	VSMC_M10
GO:0097062	0.38	0.79	9	15	dendritic spine maintenance	BP	1	PICK1,ABHD17B,VPS35,MTMR2,PRNP,HOMER1,ITPKA,ITGA3,INSR	grey	VSMC_M10
GO:0048853	0.38	0.87	4	6	forebrain morphogenesis	BP	1	SMO,SLIT1,WNT5A,OTX1	grey	VSMC_M10
GO:1990264	0.38	0.87	4	6	peptidyl-tyrosine dephosphorylation involved in inactivation of protein kinase activity	BP	1	ACP4,PTPRQ,PTPRO,DUSP10	grey	VSMC_M10
GO:0032927	0.38	0.87	4	6	positive regulation of activin receptor signaling pathway	BP	1	ZC3H3,ACVR2B,ACVR2A,ACVR1B	grey	VSMC_M10
GO:1903779	0.38	0.78	11	19	regulation of cardiac conduction	BP	1	TRPM4,SLC8A2,TBX5,RYR2,ANK2,AGT,ACE2,ATP2A1,ATP2B3,TMEM65,NKX2-5	grey	VSMC_M10
GO:0009209	0.38	0.82	7	11	pyrimidine ribonucleoside triphosphate biosynthetic process	BP	1	NME1,NME4,CTPS2,NME7,NME3,UCK1,NME6	grey	VSMC_M10
GO:0098937	0.38	0.87	4	6	anterograde dendritic transport	BP	1	KIF5A,KIF5C,KIF3B,KIF17	grey	VSMC_M10
GO:0038007	0.38	0.87	4	6	netrin-activated signaling pathway	BP	1	PTK2,UNC5D,UNC5A,TUBB3	grey	VSMC_M10
GO:0007605	0.38	0.69	56	102	sensory perception of sound	BP	1	ATP8B1,MYO1A,EYA4,ALDH7A1,COL11A2,CLIC5,KIT,KCNQ4,GSDME,MYH14,WHRN,SOBP,SLC26A5,MYO3A,MYO7B,EML2,USH1G,MYO7A,SPRY2,MKKS,LRTOMT,AP000812.5,TMTC4,DIAPH1,P2RX2,TUB,CASP3,MARVELD2,EYA1,ADGRV1,USH1C,TFAP2A,PGAP1,MYO3B,COCH,STRC,HOMER2,CDKN1B,LRIG2,CEMIP,CDH23,CHRNB2,POU3F4,OTOA,CRYM,ASIC2,CDC14A,SRRM4,PJVK,CHRNA10,EPS8L2,SIX1,DDIT3,OTOR,TIMM8B,OTOS	grey	VSMC_M10
GO:0036466	0.39	0.87	4	6	synaptic vesicle recycling via endosome	BP	1	AP3B1,DNM1L,GRIPAP1,BTBD8	grey	VSMC_M10
GO:0090493	0.39	0.82	7	11	catecholamine uptake	BP	1	PRKN,MAPK15,SLC6A2,SLC6A3,ACTB,SLC29A4,PARK7	grey	VSMC_M10
GO:0001738	0.39	0.69	46	84	morphogenesis of a polarized epithelium	BP	1	FZD6,RSPO3,PTK7,ROR2,BRSK1,FUZ,INTU,RAB10,WNT11,ACTG1,SPEF1,SYNE4,RYK,TCF15,NPHP1,FZD3,TIAM1,RPGRIP1L,VANGL1,PRICKLE1,NKD1,JHY,ZNRF3,DVL3,FZD7,TRAF3IP1,LAMA5,GRHL3,EXOC5,DAAM1,ARHGEF19,ACTB,DVL2,MKS1,MYO9A,LAMA3,CAMSAP3,LAMA1,IFT20,GPC3,SAPCD2,CELSR2,FOXF1,RHOA,WNT5A,CELSR3	grey	VSMC_M10
GO:0008207	0.39	0.76	13	22	C21-steroid hormone metabolic process	BP	1	TSPO,AKR1B1,DKK3,SRD5A1,DHRS9,WNT4,STAT5B,HSD17B10,DGKQ,SCNN1B,CYP46A1,CACNA1H,DHRS2	grey	VSMC_M10
GO:0033522	0.39	0.75	15	26	histone H2A ubiquitination	BP	1	SKP1,UBE2B,BMI1,RYBP,DDB2,PCGF6,DTX3L,RNF168,BCOR,DDB1,PCGF3,TRIM37,OTUB2,CUL4B,RNF8	grey	VSMC_M10

GO:0045776	0.39	0.76	14	24	negative regulation of blood pressure	BP	1	ADM2,PPARA,SNX5,MKKS,VEGFC,NTSR1,GCH1,SCPEP1,BDKRB1,NOS2,ABAT,TRPV1,GPR37L1,CRH	grey	VSMC_M10
GO:0095500	0.39	0.76	13	22	acetylcholine receptor signaling pathway	BP	1	LY6E,CHRNA3,PLCB1,CHRM5,CHRM4,CHRM3,GRK2,CHRM1,CHRNB2,HRH4,CDK5R1,GNB1,GNA15	grey	VSMC_M10
GO:0032793	0.39	0.78	10	17	positive regulation of CREB transcription factor activity	BP	1	ADGRF1,PRKD2,CD200,OPRD1,TSSK4,CRTC1,CRTC2,RPS6KA5,LRP8,RPS6KA4	grey	VSMC_M10
GO:0033209	0.39	0.70	42	75	tumor necrosis factor-mediated signaling pathway	BP	1	TNFAIP3,HSPA1B,PRKN,TNFRSF13C,TRIM32,TRAF1,FOXO3,TRAF3IP2,IKBKB,GAS6,ST18,TRAF6,PTK2B,OTULIN,F2RL1,RIPK1,PIAS3,SPATA2,TRAF5,PELI3,TNFSF18,BIRC3,SPPL2A,GPS2,TNFRSF1B,PYDC1,TRAF3,PTPN2,PIAS4,CDIP1,SPPL2B,TRAF2,TNFRSF25,MIR27B,MIR24-1,NOL3,CASP8,CD70,COMMD7,CCDC3,TNFSF13B,HIST1H2BJ	grey	VSMC_M10
GO:1904294	0.39	0.80	8	13	positive regulation of ERAD pathway	BP	1	RNFT2,USP13,RNF185,NFE2L2,BCAP31,SGTA,TMEM259,RNFT1	grey	VSMC_M10
GO:1900151	0.39	0.78	10	17	regulation of nuclear-transcribed mRNA catabolic process, deadenylation-dependent decay	BP	1	PABPC1,AGO2,TNRC6B,TNRC6C,CNOT1,TENT4B,RC3H1,TOB1,NANOS1,ZFP36L2	grey	VSMC_M10
GO:0086012	0.39	0.77	11	19	membrane depolarization during cardiac muscle cell action potential	BP	1	TRPM4,CACNB2,SCN5A,SLMAP,KCNJ2,ANK2,ATP1A2,SCN4B,CACNA1G,SCN2B,HCN4	grey	VSMC_M10
GO:0030903	0.39	0.79	9	15	notochord development	BP	1	GLI1,TEAD2,SOX9,GLI2,WNT11,COL13A1,EFNA1,NOG,WNT5A	grey	VSMC_M10
GO:0036158	0.39	0.79	9	15	outer dynein arm assembly	BP	1	LRRC6,ARMC4,DNAH5,ZMYND10,DNAAF2,TTC25,DNAAF4,CCDC151,C16orf71	grey	VSMC_M10
GO:0097485	0.39	0.67	98	182	neuron projection guidance	BP	1	LRP1,SEMA6A,KIF5A,GFRA3,PTK2,PTCH1,PLXNA4,RAC2,SMO,SEMA4D,DLX5,ETV1,SEMA4C,HOXA2,EPHA7,EPHA1,NRCAM,PLXNA1,GLI2,MEGF8,MYCBP2,KIF5C,FEZ2,SEMA3D,PLXNC1,SEMA3A,CHL1,UNC5D,RYK,EPHB1,RET,ROBO1,ROBO2,FZD3,SEMA4F,DPYSL2,SLIT1,TRIO,SEMA3G,CYFIP1,ALCAM,SEMA4G,SIAH1,BMPR1B,BDNF,LGR6,SEMA3F,GAP43,DAG1,LHX9,GBX2,KALRN,LAMA5,YTHDF1,PTPRO,EPHB3,WNT3,KLF7,MYPN,SEMA6D,EPHB4,TNR,CRMP1,EPHA4,LRTM2,UNC5A,SEMA4A,NGFR,EFNA1,NTN1,RPS6KA5,RNF165,BCL11B,LHX1,LAMA3,CDK5R1,ISL1,EMB,LAMA1,L1CAM,NTN3,PLXNA3,BSG,TUBB3,DPYSL5,EFNA4,CNTN2,VASP,FOXG1,ISL2,NOG,WNT5A,CHN1,ARTN,NRXN3,LYPLA2,EFNB3,ISPD	grey	VSMC_M10
GO:0090209	0.39	0.87	4	6	negative regulation of triglyceride metabolic process	BP	1	FBXW7,GPLD1,SIK1,SORL1	grey	VSMC_M10
GO:0003417	0.39	0.79	9	15	growth plate cartilage development	BP	1	COL27A1,SOX9,TSKU,TGFBR2,RARB,STC1,POR,MMP13,THBS3	grey	VSMC_M10
GO:0002790	0.39	0.67	94	172	peptide secretion	BP	1	LRP1,RIMS2,PRKN,PLA2G6,PICK1,RFX3,TFAP2B,PRKCE,HMGN3,CYB5R4,BMP8A,EGFR,TRPM4,GIPR,RAF1,AIMP1,SIRT3,SYT9,NR1H4,NDUFAF2,FAM3D,PAX8,OSBP,ARNTL,KCNJ11,NNAT,TIAM1,RAB11FIP5,PFKM,SLC8B1,NKX6-1,ACVR1C,ZBED6,SIRT4,GPLD1,IRS1,HDAC1,PPP3CA,CPT1A,TRPV4,SLC9B2,ADRA2A,TCIRG1,TCF7L2,KCNB1,PFKFB2,GPER1,RAB11FIP2,PER2,STX1A,PASK,GLUL,SIDT2,SLC16A1,KLF7,VGF,ENY2,ABCA12,MCU,PPP3CB,CAMK2G,PTPRN,CD38,MPC2,ILDR2,SYT7,NLGN2,CPLX1,TARDBP,STX4,RAB8B,UBE2Q1,SYTL4,GPR68,S100A8,CD74,CCL5,SREBF1,ISL1,OXCT1,NOS2,ABAT,NADK,TRPV1,BAIAP3,PARK7,LRRC8A,FGA,CHGA,SOX4,DPP4,CRH,DOC2B,UQCC2	grey	VSMC_M10
GO:0046486	0.39	0.65	172	317	glycerolipid metabolic process	BP	1	PIP4K2C,AVIL,ABHD16A,SYNJ2,SERAC1,TNXB,AGPAT4,AGPAT1,SLC22A4,PNPLA3,PNPLA5,PLA2G6,GPAA1,CWH43,INPP4A,CAT,TMEM246,TMEM86B,PIGF,MBOAT7,PLEK,NR1H2,MBOAT2,DGKI,LPIN1,INPP4B,FIG4,APOB,AGK,FGF21,MTMR2,PIP4K2A,NAPEPLD,FAAH,HADHA,SELENOI,ABHD5,APOC1,LIPE,TAMM41,ITPKC,PIP4P1,MTMR14,PLCB1,PGAP2,CRLS1,CDS2,DDHD2,PANK2,NR1H4,MTMR7,ABHD6,PIGU,PIK3CB,PITPNM2,PIK3R1,TMEM150A,GK5,PLCE1,CHRM5,MTMR9,FBXW7,GPD1,SLA2,NR1H3,SERINC5,SYNJ1,LPIN3,CDS1,FITM2,ITPKA,PIGT,PLA2G4B,GPLD1,LPCAT1,SIK1,PGAP1,CPT1A,GPAM,CHKA,PLA1A,ACP6,INPP5A,ABHD4,AJUBA,PITPNM1,ABHD2,MTMR11,SORL1,GNPAT,DGKD,PIGM,BMX,FAM126B,IP6K2,LPGAT1,PIGV,MTMR3,THEM5,PCYT1A,INPP5J,PIGX,PIGZ,PISD,DGKG,PIKFYVE,PLA2G4A,MOGAT3,CPS1,SACM1L,LPCAT3,ABHD12B,GNB3,PIGC,PLCD4,SMPD4,SOCS4,PLA2G16,PI4KA,SLC27A1,ABHD8,DAGLB,PGAP3,PIGH,PI4K2B,LIPC,SOCS7,DGKQ,GDPD3,APOBR,AC138894.1,SEL1L,DGKE,TTC7B,LDLR,GDPD1,PIGL,GDE1,CSF1R,ABHD3,PNPLA6,SREBF1,LPIN2,C3,MPPE1,IMPA2,MTMR12,SOCS1,MTM1,MTMR1,INPP5K,PIP5K1C,ABCA3,MECP2,AGPAT2,LMF1,PIP5KL1,PLA2G15,FABP5,PNPLA4,PEMT,CNEP1R1,PYURF,LYPLA2,LCLAT1,INSIG2,PIGB,PIGA,INPP1,PTPMT1,DNAJC19,CDIPT	grey	VSMC_M10
GO:0050909	0.39	0.76	13	22	sensory perception of taste	BP	1	ASIC3,P2RX2,P2RX3,ASIC1,CALHM1,RTP4,SCNN1B,ASIC2,GNB1,GNG13,GNAT2,PKD1L3,TAS2R3	grey	VSMC_M10
GO:0099612	0.39	0.87	4	6	protein localization to axon	BP	1	CNTNAP1,TRIM46,EPB41L3,CNTN2	grey	VSMC_M10
GO:0072319	0.39	0.87	4	6	vesicle uncoating	BP	1	SYNJ1,HSPA8,GAK,VPS4A	grey	VSMC_M10
GO:0098532	0.39	0.87	4	6	histone H3-K27 trimethylation	BP	1	SUZ12,CHD5,BEND3,ZNF304	grey	VSMC_M10
GO:0010985	0.39	0.83	6	9	negative regulation of lipoprotein particle clearance	BP	1	APOC1,MYLIP,LRPAP1,MIR199A1,MIR27B,MIR27A	grey	VSMC_M10
GO:1905005	0.39	0.87	4	6	regulation of epithelial to mesenchymal transition involved in endocardial cushion formation	BP	1	TGFBR2,TGFB2,ENG,NOG	grey	VSMC_M10
GO:0086013	0.39	0.79	9	15	membrane repolarization during cardiac muscle cell action potential	BP	1	KCNJ3,KCNE2,KCNIP2,KCNJ2,NOS1AP,ATP1B1,RNF207,KCNH2,KCNJ8	grey	VSMC_M10
GO:0002076	0.39	0.79	9	15	osteoblast development	BP	1	HOXA2,GLI2,PTHLH,SATB2,HDAC4,PTH1R,MEN1,SMAD3,BGLAP	grey	VSMC_M10

GO:0022406	0.39	0.69	42	76	membrane docking	BP	1	STX7,TSNARE1,EXOC4,SCFD2,STX17,PLEK,BVES,PPFIA3,RAB7A,RAB10,SYTL2,RALB,SYT1,CEP290,STX2,SNPH,PEX16,VPS18,KCNB1,PDZD8,STX1A,STX6,NSF,BLOC1S6,MOSPD3,RAB3A,SNAP29,STX5,RAB8A,PSEN2,RAB15,STX1B,STX4,RAB8B,AC138894.1,STX8,RAB3D,CALM1,VMP1,STXBP2,STXBP1,VAMP3	grey	VSMC_M10
GO:0045621	0.39	0.70	40	72	positive regulation of lymphocyte differentiation	BP	1	HLA-DRA,XRCC6,IL4I1,IL15,GLI2,IL15RA,TGFBR2,NCKAP1L,GAS6,PRKDC,SOX12,TOX,IL7,RASGRP1,RIPK2,AP3B1,SOX13,HLX,RUNX3,PPP2R3C,ADAM8,DUSP10,BCL6,VSIR,STAT5B,FOXP3,ZBTB1,ZBTB7B,IL4R,BTK,CD74,NKAP,IL7R,SOCS1,PRKCZ,MIR21,RHOA,SOX4,CD46,TNFSF9	grey	VSMC_M10
GO:0046949	0.39	0.75	14	24	fatty-acyl-CoA biosynthetic process	BP	1	PPT2,ACSL6,PPT1,ELOVL1,ELOVL7,ACSL5,HACD2,ACSBG1,TECR,GCDH,ACACA,ACSBG2,ACSF3,ELOVL3	grey	VSMC_M10
GO:0070327	0.39	0.87	4	6	thyroid hormone transport	BP	1	ABCC2,SLCO1C1,CRYM,SLC7A5	grey	VSMC_M10
GO:0001960	0.39	0.71	33	58	negative regulation of cytokine-mediated signaling pathway	BP	1	IL1R2,PXDN,NR1H2,GAS6,OTUD4,ROBO1,NR1H3,OAS3,NLRC5,F2RL1,YTHDF2,PARP14,PELI3,MUL1,PADI2,GPS2,USP18,PYDC1,DCST1,STAT2,CCL5,PTPN2,PIAS4,MIR21,MIR125B1,MIR27B,MIR24-1,MIR27A,OAS1,NOL3,MIR125A,CCDC3,HIST1H2BJ	grey	VSMC_M10
GO:0006750	0.39	0.80	8	13	glutathione biosynthetic process	BP	1	SLC1A1,CHAC2,MGST2,SLC7A11,GSS,NFE2L2,GGT6,GGT5	grey	VSMC_M10
GO:0032933	0.39	0.79	9	15	SREBP signaling pathway	BP	1	C12orf49,FBXW7,AMFR,ERLIN1,SCAP,LPCAT3,ZBTB7B,SREBF1,INSIG2	grey	VSMC_M10
GO:0071501	0.39	0.79	9	15	cellular response to sterol depletion	BP	1	C12orf49,FBXW7,AMFR,ERLIN1,SCAP,LPCAT3,ZBTB7B,SREBF1,INSIG2	grey	VSMC_M10
GO:0090596	0.39	0.66	107	198	sensory organ morphogenesis	BP	1	FRS2,OSR2,FZD6,BAK1,ATF4,AGTPBP1,NTRK2,RORB,PTK7,SLC1A1,TFAP2B,ROR2,DLX5,TDRD7,SIX2,RDH13,KCNQ4,HOXA2,WHRN,PTN,TSPAN12,SOBP,OSR1,INTU,FOXE3,HDAC2,MYO3A,SOX9,USH1G,GLI2,IFT172,MYO7A,TSKU,SPRY2,DZANK1,JAG1,RARB,FSCN2,CEP290,EPHB1,PAX8,EYA1,PLS1,TENM3,FZD3,USH1C,RPGRIP1L,TFAP2A,PAX2,NKD1,NECTIN3,HDAC1,ALDH1A3,SHROOM2,NRL,GBX2,MYO3B,GRHL3,SP3,STRC,FZD5,STOX1,PRKRA,FBN1,COL5A2,STAT3,NKX3-2,MAPK1,OBSL1,ROM1,MFAP2,DVL2,WDR19,SDK1,TCAP,MFN2,MAPK3,MEGF11,LCTL,POU3F4,NTN1,GSC,TBX2,LHX1,STRA6,YY1,SKI,SOX8,COL5A1,OLFM3,FJX1,CHRNA10,FOXG1,MAFB,CNTF,NOG,THY1,WNT5A,DIO3,OTX1,SIX1,TBC1D20,INSIG2,PITX3,WNT2,COL8A2,ZIC1	grey	VSMC_M10
GO:0007223	0.39	0.86	4	6	Wnt signaling pathway, calcium modulating pathway	BP	1	ROR2,WNT11,NLK,WNT5A	grey	VSMC_M10
GO:0015074	0.39	0.86	4	6	DNA integration	BP	1	GIN1,THAP9,NYNRIN,KRBA2	grey	VSMC_M10
GO:2000300	0.39	0.72	22	39	regulation of synaptic vesicle exocytosis	BP	1	CHRNA5,APBA1,RIMS3,CACNB2,SYT1,RAB5A,P2RY1,VPS18,DTNBP1,DNM1L,ADRA2A,BACE1,CSPG5,RAP1B,CASK,RAB3A,SYN1,CALM1,SNAPIN,PRKCB,FMR1,P2RX1	grey	VSMC_M10
GO:0006651	0.39	0.86	4	6	diacylglycerol biosynthetic process	BP	1	AVIL,PLCE1,GPAM,PLA2G15	grey	VSMC_M10
GO:2000773	0.39	0.79	9	15	negative regulation of cellular senescence	BP	1	CDK6,PRKDC,PLK2,TERT,SLC30A10,MAP3K3,BCL6,TERF2,YBX1	grey	VSMC_M10
GO:1901880	0.39	0.70	34	62	negative regulation of protein depolymerization	BP	1	AVIL,LMOD2,KIAA1211,TMOD1,PLEKHH2,SPTBN1,ADD2,CLASP2,TRIM54,NAV3,SPEF1,LMOD3,ATXN7,LIMA1,KATNB1,DMTN,CARMIL1,LMOD1,ADD3,TTBK2,CIB1,SPTBN2,TMOD4,EPS8,CAPZA1,TMOD2,SPTB,CAMSAP3,TAOK1,APC2,CAMSAP1,CARMIL2,APC,MAP6D1	grey	VSMC_M10
GO:0098751	0.39	0.74	17	30	bone cell development	BP	1	MPIG6B,SRF,KIT,PIP4K2A,ABI1,FOXP1,FBXW7,LRRK1,SLC9B2,WASF2,THPO,PTPN6,FBN1,MED1,ANXA2,GPR68,ATP6AP1	grey	VSMC_M10
GO:0060134	0.39	0.86	4	6	prepulse inhibition	BP	1	CHD8,SLC6A3,BACE1,GRID2	grey	VSMC_M10
GO:0006352	0.39	0.68	58	105	DNA-templated transcription, initiation	BP	1	BAZ2A,TBPL1,ESR1,TFB1M,MED30,TAF2,TAF8,SRF,ERCC1,ERCC3,GTF2F2,RSF1,PAXIP1,TAF7,BRF2,GTF2E2,MITF,TAF10,POLR2I,ERCC6,NCOA6,CDK7,TAF9,CCNH,TAF5,CRCP,GTF2E1,TFAM,TAF4,E2F2,CREB1,MED14,TAF6,POLR2G,THRA,MED24,MED1,TAF1,TAF9B,SNAPC5,MED7,TAF4B,RRN3,JUN,TAF13,GTF3C4,MED27,TAF1C,TAF12,HIST1H4B,HIST2H4A,HIST1H4H,HIST1H4D,HIST1H4A,NKX2-5,HIST1H4F,HEY2,GTF2H5	grey	VSMC_M10
GO:0048861	0.39	0.86	4	6	leukemia inhibitory factor signaling pathway	BP	1	CTF1,OSMR,LIFR,LIF	grey	VSMC_M10

GO:1901615	0.39	0.64	207	382	organic hydroxy compound metabolic process	BP	1	RDH5,APOF,ATP8B1,RDH16,CYP27B1,TPH2,SNCAIP,MIOX,SYNJ2,CYP21A2,PRKN,ACO2,HSD17B3,LY6E,AKR1B1,BTBD9,TTC39B,PSAT1,GPR37,ALDH1A1,KIT,SLC1A1,PLPP6,SRD5A3,SPTLC1,IPPK,INPP4A,ADH5,CAT,EPAS1,RDH13,PLEK,DDC,INPP4B,DKKL1,SLC7A11,APOB,SPHK2,DHFR,MTMR2,NAPEPLD,EBP,EBPL,AKR1A1,CUBN,LIPE,PRKAG2,ITPKC,ACER3,TSKU,PIP4P1,SPTLC3,LRTOMT,AP000812.5,PANK2,NR1H4,MTMR7,SDR16C5,SOAT2,CYP7A1,ASAH1,CYP7B1,PAX8,CEBPA,CBR4,OSBP,GK5,RETSAT,DKK3,HAND2,LIMA1,GPD1,EPHX2,CYP2R1,P2RY1,PTK2B,RAPGEF2,SPR,SYNJ1,COQ2,NR4A2,ITPKA,ERLIN1,PLTP,OCA2,SRD5A1,CYB5R1,SLC6A3,ALDH1A3,ABCA5,TGFB2,FMO5,CHKA,ITPK1,INPP5A,NR5A2,ALDH3B2,SNAI1,PFKFB2,MSMO1,THTPA,GPER1,SC5D,ABCB11,DHRS9,IP6K2,DPAGT1,SLCO1C1,PER2,ACTN3,OSBPL2,NTSR1,INPP5J,SCAP,HSD17B7,POR,PTH1R,SORD,DUOX1,PLA2G4A,OSBPL6,ACADL,SOAT1,MOGAT3,TM7SF2,FAH,OSBPL1A,IDH3A,FDX1,COMT,PECR,LPCAT3,PNKD,GNB3,WNT4,CYP27A1,GCH1,HDC,CH25H,LIPA,ACADVL,TKFC,HSD17B10,CLN8,DHRS3,MED1,RDH11,ALDH1A2,FGFR4,LIPC,DGKQ,OSBPL7,PNPO,ITGAM,ERG28,GDPD3,APOBR,CITED1,CHRNB2,CLN6,LDLR,GDPD1,SCNN1B,CRYM,CYP46A1,GDE1,NPC1,SREBF1,LEPR,IMPA2,SLC45A2,ACAA2,ABAT,PIAS4,INPP5K,MECP2,TKTL1,CACNA1H,PARK7,ALDH2,RXRA,CEL,ABCD3,GFI1,NFE2L1,TPK1,PNPLA4,DHDDS,MINPP1,WNT5A,TIGAR,LDLRAP1,DIO3,AMACR,ACER2,LRAT,STARD4,AOC2,INSIG2,RDH14,INPP1,ADH4,PDXP,FGF1,SGPP1,DIO2,PCBD2	grey	VSMC_M10
GO:0051891	0.39	0.86	4	6	positive regulation of cardioblast differentiation	BP	1	TBX5,TGFB2,GATA6,NKX2-5	grey	VSMC_M10
GO:0060232	0.40	0.86	4	6	delamination	BP	1	AKNA,TRPV4,YTHDF2,ABCA12	grey	VSMC_M10
GO:2000197	0.40	0.86	4	6	regulation of ribonucleoprotein complex localization	BP	1	IWS1,SETD2,AKAP8L,SUPT6H	grey	VSMC_M10
GO:0019532	0.40	0.86	4	6	oxalate transport	BP	1	SLC26A10,SLC26A5,SLC26A1,SLC26A2	grey	VSMC_M10
GO:0000245	0.40	0.71	29	51	spliceosomal complex assembly	BP	1	SF3A3,PUF60,YTHDC1,SRSF10,CELF2,CRNKL1,USP39,GCFC2,CELF1,SRSF6,SCAF11,RBM5,ZRSR2,CELF3,GEMIN2,PRPF39,PRPF19,KHDC4,LUC7L3,SLU7,CELF5,LUC7L,RNVU1-6,NOL3,RNU4ATAC,RF00003,SNRPC,RNU2-1,SRSF12	grey	VSMC_M10
GO:0003433	0.40	0.86	4	6	chondrocyte development involved in endochondral bone morphogenesis	BP	1	COL27A1,SOX9,TSKU,TGFBR2	grey	VSMC_M10
GO:0070729	0.40	0.86	4	6	cyclic nucleotide transport	BP	1	ABCC4,ABCC5,LRRC8E,LRRC8A	grey	VSMC_M10
GO:0043376	0.40	0.86	4	6	regulation of CD8-positive, alpha-beta T cell differentiation	BP	1	NCKAP1L,RUNX3,ZBTB7B,SOCS1	grey	VSMC_M10
GO:0010232	0.40	0.69	39	71	vascular transport	BP	1	LRP1,SLC16A7,SLC12A2,AGER,ABCB1,SLC1A1,SLC4A4,SLC1A4,SLC8A2,SLC24A3,ABCC4,SLC7A1,SLC38A2,SLC38A1,ABCC2,ABCG2,SLC2A13,SLC2A10,SLC38A3,SLCO3A1,ABCC5,ATP1A2,SLCO1C1,SLC29A2,SLC16A1,SLC27A1,SLC38A5,SLC4A3,SLC29A4,SLC2A4,ATP1B2,SLC7A3,ABCC3,INSR,LEPR,SLC7A5,FABP5,SLC22A5,KCNJ8	grey	VSMC_M10
GO:0097064	0.40	0.86	4	6	ncRNA export from nucleus	BP	1	PHAX,NOL6,NCBP2,TSC1	grey	VSMC_M10
GO:0008063	0.40	0.86	4	6	Toll signaling pathway	BP	1	PELI1,MYD88,PELI3,TRAF3	grey	VSMC_M10
GO:0051055	0.40	0.71	25	44	negative regulation of lipid biosynthetic process	BP	1	ORMDL2,CYP27B1,DKKL1,APOC1,PIBF1,TRIB3,CYP7A1,DKK3,FBXW7,ORMDL1,ERLIN1,PRMT3,LPCAT1,SIK1,SNAI1,GPER1,SCAP,ACADL,WNT4,SLC27A1,ACADVL,ASXL3,GFI1,INSIG2,CCDC3	grey	VSMC_M10
GO:0097090	0.40	0.83	5	8	presynaptic membrane organization	BP	1	NLGN4Y,NLGN2,NLGN3,CEL,CNTN2	grey	VSMC_M10
GO:0072525	0.40	0.76	13	22	pyridine-containing compound biosynthetic process	BP	1	PSAT1,NMRK1,SLC22A13,IDO2,IDO1,SLC5A8,NUDT12,PARP9,PNPO,NADK,NNMT,NAPRT,NMNAT1	grey	VSMC_M10
GO:0016556	0.40	0.75	14	24	mRNA modification	BP	1	CMTR1,TRMT6,BAG4,WTAP,RPUSD2,TRUB1,METTL8,METTL16,CMTR2,METTL14,TRMT61A,TRMT10C,TRMT61B,CBLL1	grey	VSMC_M10
GO:0051895	0.40	0.77	10	17	negative regulation of focal adhesion assembly	BP	1	LRP1,ITGB1BP1,CLASP2,FAM107A,DMTN,ARHGAP6,APOD,BCAS3,ACVRL1,DUSP22	grey	VSMC_M10
GO:0150118	0.40	0.77	10	17	negative regulation of cell-substrate junction organization	BP	1	LRP1,ITGB1BP1,CLASP2,FAM107A,DMTN,ARHGAP6,APOD,BCAS3,ACVRL1,DUSP22	grey	VSMC_M10
GO:0002707	0.40	0.74	15	26	negative regulation of lymphocyte mediated immunity	BP	1	IL4I1,FOXJ1,NCKAP1L,NOD2,CD96,BCL6,PTPN6,PPP3CB,FOXP3,TBX21,IL7R,CRK,CD46,SERPINB9,HAVCR2	grey	VSMC_M10
GO:0060317	0.40	0.74	16	28	cardiac epithelial to mesenchymal transition	BP	1	RTN4,SPRY1,TGFBR2,JAG1,PDCD4,TGFB2,SNAI1,RBPJ,EFNA1,TMEM100,ENG,MIR21,NOG,HEY2,OLFM1,WNT2	grey	VSMC_M10
GO:0002716	0.40	0.86	4	6	negative regulation of natural killer cell mediated immunity	BP	1	CD96,CRK,SERPINB9,HAVCR2	grey	VSMC_M10
GO:0010719	0.40	0.74	15	26	negative regulation of epithelial to mesenchymal transition	BP	1	FUZ,SPRY1,SPRY2,OVOL2,SPRED3,IL17RD,TBX5,USF3,TGFB2,EFNA1,DAB2IP,NOG,DACT3,SDHAF2,LDLRAD4	grey	VSMC_M10
GO:1903147	0.40	0.86	4	6	negative regulation of autophagy of mitochondrion	BP	1	TSPO,USP30,PINK1,TIGAR	grey	VSMC_M10
GO:0048671	0.40	0.83	5	8	negative regulation of collateral sprouting	BP	1	FSTL4,EPHA7,SPART,ULK1,SPP1	grey	VSMC_M10
GO:0002322	0.40	0.86	4	6	B cell proliferation involved in immune response	BP	1	TLR4,PLCL2,GAPT,CD19	grey	VSMC_M10
GO:0090400	0.40	0.86	4	6	stress-induced premature senescence	BP	1	MAPK14,ARNTL,MAPKAPK5,PLA2R1	grey	VSMC_M10
GO:2000738	0.40	0.77	10	17	positive regulation of stem cell differentiation	BP	1	PTN,SOX9,TCF15,SOX6,TBX5,RBM24,TGFB2,GATA6,SP7,NKX2-5	grey	VSMC_M10
GO:0009208	0.40	0.79	8	13	pyrimidine ribonucleoside triphosphate metabolic process	BP	1	NME1,ENTPD4,NME4,CTPS2,NME7,NME3,UCK1,NME6	grey	VSMC_M10
GO:0072283	0.40	0.78	9	15	metanephric renal vesicle morphogenesis	BP	1	SMO,SIX2,PAX8,FMN1,PAX2,SALL1,CITED1,LHX1,LIF	grey	VSMC_M10
GO:0015015	0.40	0.86	4	6	heparan sulfate proteoglycan biosynthetic process, enzymatic modification	BP	1	HS3ST5,HS6ST2,HS6ST3,HS2ST1	grey	VSMC_M10
GO:0046532	0.40	0.86	4	6	regulation of photoreceptor cell differentiation	BP	1	SOX9,DLX1,SOX8,CNTF	grey	VSMC_M10
GO:0046533	0.40	0.86	4	6	negative regulation of photoreceptor cell differentiation	BP	1	SOX9,DLX1,SOX8,CNTF	grey	VSMC_M10
GO:0038031	0.40	0.86	4	6	non-canonical Wnt signaling pathway via JNK cascade	BP	1	NKD1,DVL3,FZD7,WNT5A	grey	VSMC_M10

GO:0006661	0.40	0.67	63	115	phosphatidylinositol biosynthetic process	BP	1	PIP4K2C,SYNJ2,GPAA1,CWH43,INPP4A,TMEM246,PIGF,MBOAT7,INPP4B,FIG4,MTMR2,PIP4K2A,ITPKC,MTMR14,PGAP2,MTMR7,PIGU,PIK3CB,PITPNM2,PIK3R1,TMEM150A,SLA2,SYNJ1,CDS1,ITPKA,PIGT,PGAP1,PITPNM1,PIGM,BMX,FAM126B,IP6K2,PIGV,MTMR3,INPP5J,PIGX,PIGZ,PIKFYVE,SACM1L,PIGC,SOCS4,PI4KA,SLC27A1,PGAP3,PIGH,PI4K2B,SOCS7,DGKE,TTC7B,PIGL,MPPE1,IMPA2,SOCS1,MTM1,MTMR1,INPP5K,PIP5K1C,PIP5KL1,PYURF,PIGB,PIGA,INPP1,CDIPT	grey	VSMC_M10
GO:0060745	0.40	0.86	4	6	mammary gland branching involved in pregnancy	BP	1	ESR1,VDR,WNT4,MED1	grey	VSMC_M10
GO:0009064	0.40	0.70	33	59	glutamine family amino acid metabolic process	BP	1	GLS2,GFPT2,PYCR3,PPAT,RIMKLA,SLC7A11,GAD2,AMDHD1,NR1H4,GMPS,NIT2,ASNSD1,GLS,SIRT4,ASL,ALDH5A1,SLC7A7,CTPS2,OAT,GLUL,FAH,CPS1,MTHFS,PRODH,ALDH4A1,ASRGL1,AC138894.1,ADSSL1,NOS2,MECP2,ASS1,MIR21,PHGDH	grey	VSMC_M10
GO:0070873	0.40	0.74	15	26	regulation of glycogen metabolic process	BP	1	RUBCNL,KHK,EPM2AIP1,PPP1CB,AKT2,PPP1R3B,SORBS1,IGF2,IRS1,PPP1R3E,PPP1R3D,PASK,PPP1R3F,INSR,INPP5K	grey	VSMC_M10
GO:1903209	0.40	0.78	9	15	positive regulation of oxidative stress-induced cell death	BP	1	TLR4,FOXO3,PAWR,FBXW7,RIPK1,MCL1,MMP3,SFPQ,PARK7	grey	VSMC_M10
GO:0007586	0.40	0.69	35	63	digestion	BP	1	ADM2,KIAA1211,ABCG5,ABCG8,AQP1,NR1H2,TLR4,SOX9,CCKBR,SOAT2,HIP1R,PLS1,CHRM5,LIMA1,NR1H3,VDR,NOD2,CHRM3,ADRA2A,EPB41,UCN2,COPA,PBLD,LPCAT3,CHRM1,CAPN8,HRH2,LDLR,SLC46A1,TRPV1,CEL,SLC22A5,SNX10,INAVA,CRH	grey	VSMC_M10
GO:0033085	0.40	0.86	4	6	negative regulation of T cell differentiation in thymus	BP	1	FOXJ1,ZC3H8,TMEM131L,PTPN2	grey	VSMC_M10
GO:0006112	0.40	0.69	36	65	energy reserve metabolic process	BP	1	GFPT2,MYC,GNMT,PYGB,RUBCNL,KHK,EPM2AIP1,PRKAG2,PPP1CB,AKT2,KL,PPP1R3B,GBE1,SORBS1,PFKM,IGF2,NHLRC1,PHKB,IRS1,PPP1R3E,PPP1R3D,PER2,PASK,PHKA2,PYGM,PYGL,PRKAG3,PPP1R3F,PHKA1,INSR,LEPR,INPP5K,AGL,ACADM,PPP1R1A,MRAP2	grey	VSMC_M10
GO:0019530	0.40	0.86	4	6	taurine metabolic process	BP	1	CSAD,STAT5B,GHR,PHGDH	grey	VSMC_M10
GO:0019694	0.40	0.86	4	6	alkanesulfonate metabolic process	BP	1	CSAD,STAT5B,GHR,PHGDH	grey	VSMC_M10
GO:0006605	0.40	0.65	143	262	protein targeting	BP	1	TIMM21,PEX7,PEX3,ZDHHC14,PRKN,TSPO,HSPA4,SIL1,SYNGR1,TOMM22,MICALL1,RAC2,ZDHHC21,PEX6,FBXO7,PEX1,SLC1A1,TRAM2,SEC61B,ITGB1BP1,YWHAZ,SEC61G,TCAF2,VPS41,SDCBP,MGARP,AGK,RAB7A,DNAJC15,AQP11,ZFAND6,SEC62,AKT2,PITRM1,TIMM50,USP36,HACL1,PRNP,BAG4,FUT10,SRP14,NCOA4,MIPEP,ZDHHC20,IMMP1L,FBXW7,PEX2,HTRA2,CSNK2A2,NUP54,KATNB1,SSR3,SPCS1,AP3B1,GOLGA7B,AP4B1,PEX16,TOMM34,DMTN,LONP2,SIRT4,SRPRA,ZDHHC23,MON1A,ZDHHC6,KCNB1,NCF1,HSPA8,HPS4,SORL1,C2CD5,ARIH2,CIB1,BAG3,ZDHHC18,PEX19,ZDHHC24,RNF215,TOMM40L,VPS37D,RPL11,FZD5,YWHAG,PIKFYVE,FIS1,CEMIP,RTP4,GRPEL1,C11orf65,AP4M1,PINK1,ZFAND2B,AP3M1,ARL6IP1,TIMM17B,ZDHHC4,HAP1,PEX26,VPS13D,MFN2,NPEPPS,VPS13C,PEX14,ITGAM,SSR2,RAB8B,TAOK2,HAX1,ZDHHC15,TIMM29,AP1M2,MIEF2,CDK5R1,SREBF1,LAMP2,INPP5K,PAM16,BCAP31,SGTA,PEX10,ZDHHC12,HSPA5,SH3GLB1,VPS4A,ZDHHC1,MICALL2,RASSF9,ZDHHC11,BNIP3L,TIMM10B,TIMM23B,TRAM1L1,SNX16,HRAS,TIMM8B,MIEF1,RHOU,LEPROT,GRPEL2,PEX12,SPCS2,DNAJC19,SEC61A2	grey	VSMC_M10
GO:0032000	0.40	0.86	4	6	positive regulation of fatty acid beta-oxidation	BP	1	PPARA,AKT2,IRS1,CPT1A	grey	VSMC_M10
GO:0043470	0.40	0.71	23	41	regulation of carbohydrate catabolic process	BP	1	PPARA,PRXL2C,SLC4A4,PRKAG2,PPP1CB,PPP1R3B,GPD1,P2RX7,PRKAG1,ZBTB20,ARNT,PPP1R3D,ACTN3,HDAC4,MLXIPL,STAT3,PSEN1,OGT,NCOR1,INSR,SLC2A6,ENTPD5,TIGAR	grey	VSMC_M10
GO:0009649	0.40	0.74	15	26	entrainment of circadian clock	BP	1	PHLPP1,FBXL6,CRY1,PPP1CB,BHLHE40,CRY2,SIK1,ARNTL2,USP2,PER2,CRTC1,FBXL22,PER3,FBXL8,FBXL12	grey	VSMC_M10
GO:0043570	0.40	0.86	4	6	maintenance of DNA repeat elements	BP	1	MSH2,TCF7L2,AXIN2,RMI2	grey	VSMC_M10
GO:1902803	0.40	0.86	4	6	regulation of synaptic vesicle transport	BP	1	PRKN,CNIH2,PINK1,BORCS5	grey	VSMC_M10
GO:0043501	0.40	0.77	10	17	skeletal muscle adaptation	BP	1	IL15,TNNC1,PPP3CA,TNNI1,MYH7,GTF2IRD1,MYOZ2,ACTN3,MYOZ1,CAMK2G	grey	VSMC_M10
GO:0044805	0.40	0.86	4	6	late nucleophagy	BP	1	ULK1,ATG2A,ATG9A,ATG2B	grey	VSMC_M10
GO:0032534	0.40	0.86	4	6	regulation of microvillus assembly	BP	1	ATP8B1,PODXL,KLF5,RAP1GAP	grey	VSMC_M10
GO:0090150	0.40	0.65	115	210	establishment of protein localization to membrane	BP	1	PEX3,ZDHHC14,EMC2,GOPC,HSPA4,SIL1,TOMM22,MICALL1,ZDHHC21,SLC1A1,TRAM2,SEC61B,SPTBN1,EMC10,ITGB1BP1,SEC61G,EGFR,TCAF2,VPS35,SDCBP,AGK,BRAF,RAB7A,RAB10,OPTN,GOLGA4,AQP11,EMC3,SEC62,AKT2,PRNP,SRP14,LYPLA1,ZDHHC20,RILPL1,RILPL2,VAMP5,EMC4,SSR3,AP3B1,GOLGA7B,PEX16,DMTN,KIF13A,NECTIN3,SRPRA,ZDHHC23,ZDHHC6,OXA1L,GET4,KCNB1,NCF1,C2CD5,CIB1,ZDHHC18,PEX19,ZDHHC24,BBS1,GORASP2,VPS37D,NSF,PIKFYVE,FIS1,CEMIP,NMT1,RTP4,BCS1L,ZFAND2B,ATP1B1,ARL6IP1,NSG1,GRIPAP1,ZDHHC4,RAB8A,CNST,RSC1A1,PEX26,CPLX1,STX1B,ITGAM,SSR2,RAB8B,TAOK2,AC138894.1,ZDHHC15,RAB11A,CALM1,TIMM29,GGA2,ARHGAP44,NPC1,MIEF2,CDK5R1,MAIP1,INPP5K,EMC6,SGTA,RAB26,UBL4A,SEC16A,ZDHHC12,HSPA5,ZDHHC1,C16orf70,ZDHHC11,WDR83OS,TIMM10B,CAMLG,EMC7,TRAM1L1,HRAS,TIMM8B,MIEF1,VAMP3,SEC61A2	grey	VSMC_M10
GO:0060440	0.40	0.86	4	6	trachea formation	BP	1	TGFBR2,MAPK1,MAPK3,MAP2K1	grey	VSMC_M10
GO:0035754	0.40	0.86	4	6	B cell chemotaxis	BP	1	GAS6,CYP7B1,PTK2B,CH25H	grey	VSMC_M10
GO:0023019	0.40	0.78	9	15	signal transduction involved in regulation of gene expression	BP	1	EPCAM,TRAF3IP2,P2RY1,FGF5,TRAF5,PARP1,SMAD3,GSC,TRAF2	grey	VSMC_M10
GO:0016255	0.40	0.86	4	6	attachment of GPI anchor to protein	BP	1	GPAA1,PIGU,PIGT,PGAP1	grey	VSMC_M10
GO:0021885	0.40	0.71	24	43	forebrain cell migration	BP	1	SUN2,SRF,EFHC1,MBOAT7,RTN4,POU3F3,NRG1,ROBO1,SRGAP2C,DISC1,DIXDC1,TNR,PSEN1,NDEL1,CDK5R1,DAB1,LRP8,DAB2IP,RHOA,ADGRG1,CNTN2,FOXG1,LAMB1,POU3F2	grey	VSMC_M10

GO:0006081	0.40	0.70	26	46	cellular aldehyde metabolic process	BP	1	ALDH8A1,ALDH7A1,ALDH1A1,ADH5,RDH13,ESD,AKR1A1,HYI,SDR16C5,DKK3,HOGA1,GLYCTK,ALDH1A3,ALDH3B2,BCO2,PNKD,WNT4,RDH11,ALDH1A2,PNPO,SCNN1B,CACNA1H,PARK7,GLO1,ADH4,PDXP	grey	VSMC_M10
GO:0046543	0.41	0.86	4	6	development of secondary female sexual characteristics	BP	1	AREG,STAT5B,MED1,WNT5A	grey	VSMC_M10
GO:1903358	0.41	0.77	10	17	regulation of Golgi organization	BP	1	RBSN,ARMH3,PDCD10,MAPK1,STX5,STX18,MAPK3,MAP2K1,DNM2,CAMSAP3	grey	VSMC_M10
GO:0042473	0.41	0.86	4	6	outer ear morphogenesis	BP	1	EYA1,PRKRA,MAPK1,MAPK3	grey	VSMC_M10
GO:0032526	0.41	0.68	46	84	response to retinoic acid	BP	1	ADNP2,SNW1,IGF2R,RXRB,PTCH1,RORB,PTK7,HOXA2,AQP1,TNC,TEAD2,OSR1,HDAC2,SOX9,WNT11,RET,ASXL1,HTRA2,PTK2B,SYNJ1,TESC,PAX2,GDAP2,FZD7,RXRG,WNT3,CREB1,ABL2,CTSH,BRINP2,DNAAF2,CD38,ALDH1A2,GATA6,YES1,SREBF1,SUZ12,RXRA,OVCA2,WNT5A,ACER2,DUSP1,LRAT,PTGES,ZNF35,WNT2	grey	VSMC_M10
GO:0035066	0.41	0.74	15	26	positive regulation of histone acetylation	BP	1	SPHK2,PAXIP1,ARRB1,AUTS2,TADA2B,FOXP3,KAT7,MUC1,MAPK3,NAP1L2,RPS6KA5,ISL1,GLYR1,LIF,RPS6KA4	grey	VSMC_M10
GO:0072330	0.41	0.66	79	144	monocarboxylic acid biosynthetic process	BP	1	LTC4S,QKI,MCAT,HSD17B8,HACD4,NR1H2,APOC1,PRKAG2,PIBF1,ELOVL1,NR1H4,TRIB3,CYP7A1,ELOVL7,CYP7B1,THNSL2,CBR4,OSBP,PRKAG1,NR1H3,HOGA1,ERLIN1,PRMT3,PRKAB1,ALDH1A3,ABHD2,MECR,ABCB11,DHRS9,HACD2,LPGAT1,PER2,OSBPL2,MLXIPL,SCAP,PLA2G4A,OSBPL6,ACADL,OSBPL1A,ACSBG1,PECR,CYP27A1,PRKAG3,DAGLB,FADS3,LIAS,ACADVL,TECR,HSD17B10,DEGS1,ALDH1A2,FGFR4,LIPC,OSBPL7,ALOXE3,PKLR,HACD3,NDUFAB1,CYP46A1,ACSM3,ACACA,CD74,ABHD3,ACSBG2,GAMT,PARK7,DECR2,ACSF3,MLYCD,PTGES2,ABCD3,ACOT4,FABP5,FADS6,ELOVL3,AMACR,STARD4,PTGES,INSIG2	grey	VSMC_M10
GO:0046137	0.41	0.86	4	6	negative regulation of vitamin metabolic process	BP	1	CYP27B1,PRMT3,SNAI1,GFI1	grey	VSMC_M10
GO:0042755	0.41	0.78	9	15	eating behavior	BP	1	NAPEPLD,TCF15,P2RY1,CPT1A,TTC21B,OPRD1,GDF15,STAT3,NMUR2	grey	VSMC_M10
GO:0006777	0.41	0.86	4	6	Mo-molybdopterin cofactor biosynthetic process	BP	1	MOCS1,GPHN,MOCOS,MOCS2	grey	VSMC_M10
GO:0019720	0.41	0.86	4	6	Mo-molybdopterin cofactor metabolic process	BP	1	MOCS1,GPHN,MOCOS,MOCS2	grey	VSMC_M10
GO:0043545	0.41	0.86	4	6	molybdopterin cofactor metabolic process	BP	1	MOCS1,GPHN,MOCOS,MOCS2	grey	VSMC_M10
GO:0051189	0.41	0.86	4	6	prosthetic group metabolic process	BP	1	MOCS1,GPHN,MOCOS,MOCS2	grey	VSMC_M10
GO:0015727	0.41	0.86	4	6	lactate transport	BP	1	SLC16A7,SLC5A8,SLC16A1,EMB	grey	VSMC_M10
GO:0035873	0.41	0.86	4	6	lactate transmembrane transport	BP	1	SLC16A7,SLC5A8,SLC16A1,EMB	grey	VSMC_M10
GO:0048741	0.41	0.73	16	28	skeletal muscle fiber development	BP	1	STAC3,SMO,KEL,KLHL40,RYR1,P2RX2,LMOD3,HOMER1,BIN3,PPP3CA,ACTN3,HDAC4,FBXO22,SKI,SIX1,MYORG	grey	VSMC_M10
GO:0046329	0.41	0.73	17	30	negative regulation of JNK cascade	BP	1	PRKN,KLHL31,DNAJA1,FKTN,ZMYND11,SH3RF2,F2RL1,TAOK3,PDCD4,AIDA,DUSP10,MEN1,DUSP19,PINK1,GPS2,NCOR1,MARVELD3	grey	VSMC_M10
GO:0048846	0.41	0.72	19	34	axon extension involved in axon guidance	BP	1	SEMA6A,PLXNA4,SEMA4D,SEMA4C,MEGF8,SEMA3D,SEMA3A,RYK,SEMA4F,SLIT1,SEMA3G,ALCAM,SEMA4G,SEMA3F,WNT3,SEMA6D,SEMA4A,PLXNA3,WNT5A	grey	VSMC_M10
GO:1902284	0.41	0.72	19	34	neuron projection extension involved in neuron projection guidance	BP	1	SEMA6A,PLXNA4,SEMA4D,SEMA4C,MEGF8,SEMA3D,SEMA3A,RYK,SEMA4F,SLIT1,SEMA3G,ALCAM,SEMA4G,SEMA3F,WNT3,SEMA6D,SEMA4A,PLXNA3,WNT5A	grey	VSMC_M10
GO:2000698	0.41	0.86	4	6	positive regulation of epithelial cell differentiation involved in kidney development	BP	1	PAX8,PAX2,LHX1,LIF	grey	VSMC_M10
GO:0002230	0.41	0.76	10	17	positive regulation of defense response to virus by host	BP	1	TRAF3IP2,TMEM173,MAVS,TRIM44,EIF2AK4,PARP9,DTX3L,SIN3A,ZDHHC1,ZDHHC11	grey	VSMC_M10
GO:0062042	0.41	0.83	5	8	regulation of cardiac epithelial to mesenchymal transition	BP	1	TGFBR2,JAG1,TGFB2,ENG,NOG	grey	VSMC_M10
GO:0032892	0.41	0.74	15	26	positive regulation of organic acid transport	BP	1	SLC12A2,PLA2G6,PLA2R1,SLC38A3,MAP2K6,ACSL5,AGT,ACE2,NTSR1,PLA2G4A,ARL6IP1,PSEN1,ABAT,STXBP1,PTGES	grey	VSMC_M10
GO:0060920	0.41	0.83	5	8	cardiac pacemaker cell differentiation	BP	1	MAML1,BVES,TBX5,BMPR1A,ISL1	grey	VSMC_M10
GO:0060926	0.41	0.83	5	8	cardiac pacemaker cell development	BP	1	MAML1,BVES,TBX5,BMPR1A,ISL1	grey	VSMC_M10
GO:0042104	0.41	0.79	8	13	positive regulation of activated T cell proliferation	BP	1	AGER,IGF2,ICOSLG,GPAM,STAT5B,MIR21,FADD,TNFSF9	grey	VSMC_M10
GO:2000323	0.41	0.86	4	6	negative regulation of glucocorticoid receptor signaling pathway	BP	1	CRY1,PHB,ARNTL,CRY2	grey	VSMC_M10
GO:0006090	0.41	0.68	42	76	pyruvate metabolic process	BP	1	PPARA,PRXL2C,GAPDH,SLC4A4,ME1,BPGM,DLD,DHTKD1,PRKAG2,PFKP,PDK1,PDHX,GPI,GPD1,P2RX7,PRKAG1,PFKM,HOGA1,ZBTB20,PFKFB2,ENO4,PC,ARNT,ACTN3,HDAC4,MLXIPL,PDK3,HK1,PRKAG3,STAT3,MPC2,ENO3,PSEN1,PKLR,OGT,ADPGK,NCOR1,INSR,ALDOC,SLC2A6,ENTPD5,TIGAR	grey	VSMC_M10
GO:0031112	0.41	0.72	18	32	positive regulation of microtubule polymerization or depolymerization	BP	1	HSPA1B,SLAIN2,MET,CDK5RAP2,MAPRE1,SLAIN1,NAV3,CLIP1,KATNB1,TRPV4,CDKN1B,MAPT,TOGARAM1,NIN,CDK5R1,MECP2,PSRC1,DRG1	grey	VSMC_M10
GO:1903286	0.41	0.83	5	8	regulation of potassium ion import	BP	1	WNK2,DLG1,ATP1B1,WNK3,ATP1B2	grey	VSMC_M10
GO:0090190	0.41	0.76	10	17	positive regulation of branching involved in ureteric bud morphogenesis	BP	1	SMO,SOX9,PAX8,PAX2,AGT,MAGED1,LHX1,SOX8,NOG,SIX1	grey	VSMC_M10
GO:0006689	0.41	0.86	4	6	ganglioside catabolic process	BP	1	NEU3,HEXA,GM2A,NEU2	grey	VSMC_M10
GO:0050906	0.41	0.69	29	52	detection of stimulus involved in sensory perception	BP	1	PRPH2,PHF24,KIT,GJA10,WHRN,GRIK2,ASIC3,SCN1A,MKKS,EPHB1,CEP250,CCDC66,ADGRV1,NTSR1,BACE1,STRC,RTP4,KCNK4,KCNA1,ROM1,ASIC2,TRPV1,GNAT2,PKD1L3,REEP6,PJVK,CHRNA10,TAS2R3,OR5AN1	grey	VSMC_M10
GO:0014744	0.41	0.86	4	6	positive regulation of muscle adaptation	BP	1	FOXO3,TRPC3,PPP3CA,MIR199A1	grey	VSMC_M10
GO:1990535	0.41	0.83	5	8	neuron projection maintenance	BP	1	ATP1A3,PRNP,PSEN1,INSR,MAP1A	grey	VSMC_M10
GO:0048665	0.41	0.77	9	15	neuron fate specification	BP	1	GLI2,MNX1,POU4F1,EYA1,SUFU,ISL1,ISL2,POU3F2,SIX1	grey	VSMC_M10
GO:0090166	0.41	0.86	4	6	Golgi disassembly	BP	1	PLK3,GBF1,STX5,PLK5	grey	VSMC_M10
GO:1904892	0.41	0.69	33	59	regulation of receptor signaling pathway via STAT	BP	1	IL5,AKR1B1,OCIAD1,KIT,PPP2R1A,PIBF1,RET,PIGU,PTK2B,F2R,MST1,EGF,PARP14,AGT,IL10RA,IL6R,ELP2,CCL5,CDK5R1,SUZ12,GHR,NLK,IL7R,PTPN2,SOCS1,DAB1,DOT1L,MIR125B1,MIR125A,GADD45A,LIF,TSLP,LEPROT	grey	VSMC_M10
GO:0033129	0.41	0.85	4	6	positive regulation of histone phosphorylation	BP	1	MAPK3,RPS6KA5,FMR1,RPS6KA4	grey	VSMC_M10
GO:0015693	0.41	0.76	10	17	magnesium ion transport	BP	1	NIPAL2,SLC41A2,CNNM4,ZDHHC13,NIPA1,SLC41A1,KCNJ2,NIPAL3,NIPAL4,NIPAL1	grey	VSMC_M10

GO:0033084	0.41	0.85	4	6	regulation of immature T cell proliferation in thymus	BP	1	BMI1,IL1A,TMEM131L,FOXP3	grey	VSMC_M10
GO:0062012	0.41	0.64	132	243	regulation of small molecule metabolic process	BP	1	CYP27B1,PRKN,PPARA,PRXL2C,TTC39B,GUCA1B,GPT,GNMT,CRY1,KIT,PRKCE,PPP4R3B,SLC4A4,ZNF692,PLEK,NR1H2,FAM3C,DKKL1,SLC7A11,APOB,SPHK2,SOX9,RUBCNL,LACC1,APOC1,KHK,EPM2AIP1,PRKAG2,SLC22A13,PIBF1,GCKR,PPP1CB,AKT2,C1QTNF1,SIRT7,PANK2,PDK1,PPP1R3B,NR1H4,TRIB3,CYP7A1,NLN,NCOA2,DKK3,SORBS1,GPD1,P2RX7,EPHX2,P2RY1,PTK2B,PRKAG1,NR1H3,ERLIN1,PRMT3,SIRT5,LONP2,FAM173B,IGF2,SIRT4,GPLD1,IRS1,SIK1,CPT1A,ZBTB20,FMO5,SLC7A7,MST1,PPP1R3E,SNAI1,PFKFB2,GPER1,ABCB11,ARNT,LPGAT1,PPP1R3D,ACTN3,HDAC4,NTSR1,PASK,DNAJC30,MLXIPL,DUSP12,SCAP,POR,PDK3,PTH1R,ACADL,TYSND1,COMT,LPCAT3,GNB3,WNT4,PINK1,STAT3,PPP1R3F,ACADVL,IGFBP4,PARP1,DDB1,FGFR4,DGKQ,PSEN1,OGT,AC138894.1,COX11,LCMT1,LDLR,NCOR1,CD74,INSR,SREBF1,LEPR,NOS2,PTPN2,INPP5K,PARK7,SLC2A6,MLYCD,GFI1,ACADM,MIR21,FABP5,ENTPD5,PDZD3,TIGAR,LDLRAP1,NNMT,STARD4,INSIG2,CLYBL,FGF1,ARPP19	grey	VSMC_M10
GO:0043371	0.41	0.76	10	17	negative regulation of CD4-positive, alpha-beta T cell differentiation	BP	1	HLX,RUNX3,BCL6,RC3H1,TNFSF18,FOXP3,TBX21,ZBTB7B,IL4R,RC3H2	grey	VSMC_M10
GO:0060430	0.41	0.85	4	6	lung saccule development	BP	1	ASXL1,CREB1,STRA6,GATA6	grey	VSMC_M10
GO:0009838	0.41	0.85	4	6	abscission	BP	1	SPART,CHMP4C,ZFYVE19,VPS4A	grey	VSMC_M10
GO:0007638	0.41	0.83	5	8	mechanosensory behavior	BP	1	ETV1,NRXN2,HTT,STRA6,STRBP	grey	VSMC_M10
GO:0043525	0.41	0.70	26	46	positive regulation of neuron apoptotic process	BP	1	ATF4,TFAP2B,EPHA7,FOXO3,GRIK5,PRNP,CASP3,FBXW7,NQO2,TFAP2A,TGFB2,MCL1,HDAC4,BACE1,MAP3K11,FIS1,ITGAM,CDK5R1,JUN,LCN2,NQO1,MIR125B1,RHOA,DDIT3,BCL2L11,PITX3	grey	VSMC_M10
GO:1902525	0.41	0.85	4	6	regulation of protein monoubiquitination	BP	1	WDR48,PEF1,PDCD6,UBB	grey	VSMC_M10
GO:2000369	0.41	0.75	11	19	regulation of clathrin-dependent endocytosis	BP	1	SMAP1,AAK1,NEU3,PIK3CB,HIP1R,BMP2K,DGKD,SH3GL3,SYT11,DNM2,UNC119	grey	VSMC_M10
GO:0038092	0.41	0.82	5	8	nodal signaling pathway	BP	1	FOXH1,ACVR1B,ACVR1C,SMAD3,TGIF2	grey	VSMC_M10
GO:0030856	0.41	0.66	58	106	regulation of epithelial cell differentiation	BP	1	CYP27B1,PTCH1,SMO,ZDHHC21,RFX3,S1PR3,CD109,HOXA7,MYCL,GRHL1,SFRP4,MYCN,OSR1,SPRY1,FOXE3,SOX9,PTCH2,FOXJ1,SPRY2,OVOL2,IKBKB,JAG1,PLCB1,MAFG,SPRED3,BTG1,PAX8,VDR,NKX6-1,SRSF6,PAX2,CCND1,PRKX,KDF1,SFN,APOLD1,CDKN1B,KLF7,H2AFY2,ABCA12,VCL,STAT5B,MED1,CITED1,TMEM100,VEZF1,KEAP1,LHX1,MIR21,MIR125B1,ACVRL1,FOXJ2,MIR99B,ZBED2,LIF,CEBPB,SGPP1,HEY2	grey	VSMC_M10
GO:0006091	0.41	0.63	215	392	generation of precursor metabolites and energy	BP	1	FECH,RDH16,SHMT2,GFPT2,LYRM7,PPARA,P4HA2,UQCRQ,NDUFA6,ACO2,MYC,MCHR1,PRXL2C,AKR1B1,GNMT,CROT,SLC25A13,GAPDH,HMGCLL1,ADH5,CAT,SDHAF4,MSH2,ETFB,PNPT1,SLC4A4,ME1,CYB5R4,BPGM,DLD,NDUFB2,SPHK2,PYGB,GIPR,NDUFB6,RUBCNL,AKR1A1,DNAJC15,KHK,EPM2AIP1,DHTKD1,NDUFC2,PRKAG2,ACOX1,ATP5F1C,XYLB,RBKS,PPP1CB,AKT2,PFKP,IDO1,ACSS3,MDH2,NDUFS8,PANK2,NDUFA12,KL,SIRT3,COX7A1,SUCLG2,PPP1R3B,COX4I2,ASPH,CYB5A,GPI,CEBPA,IDE,GBE1,ATP5F1A,SORBS1,GPD1,P2RX7,NDUFS3,ETFDH,CIAPIN1,DGUOK,PRKAG1,PFKM,LOXL2,NQO2,UQCRB,COX15,FAM173B,IGF2,NHLRC1,COX6B1,PHKB,SRD5A1,IRS1,NDUFV3,GLRX2,TRPV4,ZBTB20,COQ10B,COX17,OXA1L,NDUFB4,ISCU,NCF1,PPP1R3E,COA6,PFKFB2,THTPA,POLG2,ENO4,PDIA5,NDUFB3,ARNT,CISD1,PPP1R3D,NFATC4,PER2,ACTN3,HDAC4,PASK,NDUFS2,PHKA2,DNAJC30,MLXIPL,SDHC,HMGCL,NCF2,MDH1B,POR,COX6A1,RPE,VGF,HK1,IDH3A,FDX1,PYGM,PYGL,COX8A,PRKAG3,PINK1,STAT3,NDUFA9,ALDH4A1,ENO3,SDHB,PPP1R3F,ACADVL,DHRS3,DEGS1,PSEN1,COX6A2,PKLR,OGT,PHKA1,COX11,NDUFAB1,ADPGK,UQCRC2,COX10,COX5A,NCOR1,INSR,TEFM,NDUFA7,OXCT1,LEPR,NOS2,SLC25A23,NDUFV2,ALDOC,SLC25A14,ENOX2,INPP5K,PRDM16,NDUFB10,UQCR11,PARK7,SLC25A33,ATP5PB,ALDH2,SLC2A6,CYBA,AGL,SLC25A25,ACADM,MT-ND1,MT-ND2,MT-ATP8,MT-ATP6,MT-CO3,MT-ND6,MT-CYB,RHOA,ATP5ME,ATP5MF,NDUFA5,ENTPD5,DHRS2,IFNLR1,NDUFC1,COX7A2,TIGAR,FXN,PPP1R1A,ETFRF1,NDUFA3,NDUFB5,NDUFS6,NDUFA8,SDHAF2,NDUFS5,AOC2,PHGDH,APOPT1,UQCC2,ADH4,MRAP2,CO[illegible]	grey	VSMC_M10
GO:0003162	0.41	0.82	5	8	atrioventricular node development	BP	1	MAML1,TBX5,BMPR1A,GATA6,NKX2-5	grey	VSMC_M10
GO:0007585	0.41	0.69	28	50	respiratory gaseous exchange by respiratory system	BP	1	ZFAND5,ADH5,BPGM,YWHAZ,DACH1,HNMT,NEK10,NDUFA12,TCF15,AP3B1,NR4A2,TNNC1,FTO,GLS,COX15,CFAP43,ATP1A2,MTG2,NTSR1,PASK,SPAG16,RAB3A,TTC25,NLGN2,NLGN3,JAG2,MECP2,MAFB	grey	VSMC_M10
GO:1902884	0.41	0.85	4	6	positive regulation of response to oxidative stress	BP	1	FOXO3,PAWR,ACOX2,RIPK1	grey	VSMC_M10
GO:0042148	0.41	0.85	4	6	strand invasion	BP	1	DMC1,XRCC2,WRN,RAD51D	grey	VSMC_M10
GO:2001033	0.41	0.85	4	6	negative regulation of double-strand break repair via nonhomologous end joining	BP	1	HSF1,CYREN,ERCC6,NUDT16L1	grey	VSMC_M10
GO:0034144	0.41	0.85	4	6	negative regulation of toll-like receptor 4 signaling pathway	BP	1	TNFAIP3,MFHAS1,RAB7B,DAB2IP	grey	VSMC_M10
GO:0047496	0.41	0.70	25	45	vesicle transport along microtubule	BP	1	AP3S1,KIF5A,KIF3A,AP3M2,KIF3B,RASGRP1,AP3B1,DTNBP1,KIF13A,BICDL1,CNIH2,BLOC1S6,HTT,AP3M1,KIF1C,HAP1,TRIM46,NDEL1,AC138894.1,SNAPIN,BICDL2,PRKCZ,BLOC1S3,BORCS5,AP3S2	grey	VSMC_M10
GO:0006991	0.41	0.76	10	17	response to sterol depletion	BP	1	PIP4P1,C12orf49,FBXW7,AMFR,ERLIN1,SCAP,LPCAT3,ZBTB7B,SREBF1,INSIG2	grey	VSMC_M10
GO:2000772	0.41	0.71	21	37	regulation of cellular senescence	BP	1	CDKN2A,NUAK1,CDK6,PNPT1,ZNF277,PAWR,PRKDC,PLK2,ING2,ARNTL,TERT,SLC30A10,KRAS,MAP3K3,BCL6,BMPR1A,YPEL3,VASH1,TERF2,MIR22,YBX1	grey	VSMC_M10

GO:0048878	0.41	0.61	446	832	chemical homeostasis	BP	1	ZNF236,NME1,ORMDL2,SLC39A5,NEDD4L,LRP1,CYP27B1,ESR1,APOM,SLC12A2,PRKN,AGER,PNPLA3,PNPLA5,TSPO,SMDT1,BAK1,MYC,KLHL3,MCHR1,GPR20,PTCH1,AKR1B1,PLA2G6,CKB,BTBD9,TMPRSS6,TTC39B,SLC39A4,CRY1,SRF,STEAP2,SLC25A27,SLC1A1,TFAP2B,S1PR3,WNK2,CNNM4,ABCG5,ABCG8,PRKCE,TNNI3,EPAS1,RTN4,FPR2,HMGN3,IBTK,SLC4A4,CYB5R4,LPAR1,PPT1,AQP1,SLC31A2,NR1H2,GRHL1,MET,POU3F3,EGFR,SFRP4,KEL,ATG5,TRPM4,SLC7A11,FOXO3,APOB,FGF21,GRIN2D,TRPC3,SESN3,SLC8A2,RAB7A,C5AR2,C5AR1,HEPHL1,ATP7B,PTGIR,CNBP,FOXA3,GIPR,ABHD5,CACNB2,SLC39A12,ATP6V0E2,LACC1,SLC4A2,STEAP3,AQP11,GRIK5,RAF1,ATP1A3,ACOX1,SCN5A,GCKR,TSKU,ELOVL1,ACKR2,SLC24A3,GPD1L,SNX5,C1QTNF1,SLC4A7,NR1D2,TMEM106B,ASPSCR1,TMTC4,MAFG,RYR1,LETM2,PRNP,ADRA1D,DCN,DIAPH1,P2RX2,KL,CCKBR,GAS6,HPX,RASA3,NCOA4,COMMD9,NR1H4,MICU3,ATP6V1H,USF2,ACOXL,SOAT2,LSR,CYP7A1,TM9SF4,NDUFAF2,IL1A,CYP7B1,ACOX2,GPI,CEBPA,IMMT,OSBP,C19orf12,PIK3R1,SLMAP,P2RX3,PLCE1,SLC9A9,RYR3,FBXW7,LIMA1,TMBIM6,P2RX7,EPHX2,HVCN1,P2RY1,PTK2B,SLC39A13,KCNJ11,NR1H3,TIAM1,RAB11FIP5,PFKM,TPCN1,SLC8B1,HOMER1,VDR,STC1,SLC25A37,OPA1,TBX5,AP3B1,RMDN3,F2RL1,F2R,SLC40A1,FITM2,CRY2,ORMDL1,COL4A3BP,GLS,CUTC,ABCC2,ZBED6,PKD2,PAX2,TWNK,CASQ2,MCUR1,ITGB6,MYLIP,SLC12A5,TENT4B,CD40,RYR2,GRM2,SLC41A1,SLC4A10,RAP1GDS1,GPLD1,TRPM1,IRS1,SPPL3,LPCAT1,LCK,PPP3CA,KCNJ2,TESMIN,SLC9B1,SLC12A7,TRPV4,SLC9B2,ABCA5,BDH2,ZBTB20,TMEM9,ADRA2A,SLC30A10,GPAM,TCIRG1,FAM20A,TCF7L2,GNA13,ABHD4,NR5A2,[illegible]	grey	VSMC_M10
GO:0097468	0.41	0.78	8	13	programmed cell death in response to reactive oxygen species	BP	1	MET,FOXO3,PAWR,PDCD10,PINK1,PARK7,MIR21,PJVK	grey	VSMC_M10
GO:0071499	0.42	0.85	4	6	cellular response to laminar fluid shear stress	BP	1	NFE2L2,MAP2K5,MAPK7,ASS1	grey	VSMC_M10
GO:2000109	0.42	0.85	4	6	regulation of macrophage apoptotic process	BP	1	CDKN2A,IRF7,MEF2C,NOD2	grey	VSMC_M10
GO:0032930	0.42	0.77	9	15	positive regulation of superoxide anion generation	BP	1	FPR2,EGFR,HVCN1,F2RL1,AGT,MAPT,ITGAM,GNAI3,CYBA	grey	VSMC_M10
GO:0031573	0.42	0.77	9	15	mitotic intra-S DNA damage checkpoint signaling	BP	1	MDC1,MSH2,XPC,NEK11,RAD17,RAD9B,CHEK2,EME1,TIPIN	grey	VSMC_M10
GO:2000425	0.42	0.85	4	6	regulation of apoptotic cell clearance	BP	1	CD300LF,CCL2,C3,ABCA7	grey	VSMC_M10
GO:0051590	0.42	0.76	10	17	positive regulation of neurotransmitter transport	BP	1	PRKN,CACNB2,SYT1,DTNBP1,DNM1L,GPER1,STX1A,STX1B,BAIAP3,STXBP1	grey	VSMC_M10
GO:0099527	0.42	0.82	5	8	postsynapse to nucleus signaling pathway	BP	1	KPNA2,NRG1,CRTC1,STAT3,PRR7	grey	VSMC_M10
GO:0043302	0.42	0.76	10	17	positive regulation of leukocyte degranulation	BP	1	SPHK2,GAB2,F2RL1,FGR,SNX4,STX4,ITGAM,IL4R,STXBP1,AP1G1	grey	VSMC_M10
GO:0030432	0.42	0.85	4	6	peristalsis	BP	1	P2RX2,P2RX3,DLG1,TBX2	grey	VSMC_M10
GO:0090277	0.42	0.68	36	65	positive regulation of peptide hormone secretion	BP	1	LRP1,PLA2G6,PRKCE,EGFR,TRPM4,GIPR,AIMP1,SIRT3,OSBP,NNAT,PFKM,NKX6-1,GPLD1,TCF7L2,PFKFB2,GPER1,GLUL,MCU,PPP3CB,CD38,MPC2,NLGN2,TARDBP,STX4,RAB8B,GPR68,ISL1,OXCT1,ABAT,NADK,BAIAP3,LRRC8A,FGA,SOX4,CRH,DOC2B	grey	VSMC_M10
GO:0045346	0.42	0.82	5	8	regulation of MHC class II biosynthetic process	BP	1	NFX1,TLR4,HDAC2,TAF7,CIITA	grey	VSMC_M10
GO:0090118	0.42	0.85	4	6	receptor-mediated endocytosis involved in cholesterol transport	BP	1	ANXA2,LDLR,MIR27B,LDLRAP1	grey	VSMC_M10
GO:0044003	0.42	0.77	9	15	modulation by symbiont of host process	BP	1	ATG5,ULK1,ANO6,DAG1,TNIP1,INSR,RXRA,IRGM,SERPINB9	grey	VSMC_M10
GO:0045586	0.42	0.82	5	8	regulation of gamma-delta T cell differentiation	BP	1	TCF7,NCKAP1L,SOX13,STAT5B,SOX4	grey	VSMC_M10
GO:0019227	0.42	0.85	4	6	neuronal action potential propagation	BP	1	NTRK2,CLDN19,CLCN1,CNTNAP1	grey	VSMC_M10
GO:0098870	0.42	0.85	4	6	action potential propagation	BP	1	NTRK2,CLDN19,CLCN1,CNTNAP1	grey	VSMC_M10
GO:0009111	0.42	0.85	4	6	vitamin catabolic process	BP	1	CYP27B1,CBR3,MTHFS,PDXP	grey	VSMC_M10
GO:0010226	0.42	0.76	10	17	response to lithium ion	BP	1	TPH2,CEBPA,XRCC4,PTK2B,ADCY7,FABP4,NFATC4,CDKN1B,NFE2L2,PKLR	grey	VSMC_M10
GO:0015838	0.42	0.85	4	6	amino-acid betaine transport	BP	1	SLC22A4,SLC38A2,SLC25A20,SLC22A5	grey	VSMC_M10
GO:0061371	0.42	0.69	26	47	determination of heart left/right asymmetry	BP	1	SMO,FOXH1,SRF,BBS7,SETDB2,MEGF8,IFT172,TGFBR2,OVOL2,MKKS,HAND2,MEF2C,IFT52,PKD2,SUFU,MMP21,PSEN1,SMAD3,TBX2,MIB1,RNF207,ENG,LBX1,WNT5A,C2CD3,NKX2-5	grey	VSMC_M10
GO:0048340	0.42	0.82	5	8	paraxial mesoderm morphogenesis	BP	1	EXOC4,WNT11,BMPR1A,SMAD3,WNT5A	grey	VSMC_M10
GO:0060331	0.42	0.85	4	6	negative regulation of response to interferon-gamma	BP	1	NR1H2,NR1H3,PARP14,PTPN2	grey	VSMC_M10
GO:0060336	0.42	0.85	4	6	negative regulation of interferon-gamma-mediated signaling pathway	BP	1	NR1H2,NR1H3,PARP14,PTPN2	grey	VSMC_M10
GO:0043504	0.42	0.85	4	6	mitochondrial DNA repair	BP	1	MGME1,PRIMPOL,PARP1,LIG3	grey	VSMC_M10
GO:0032946	0.42	0.67	46	83	positive regulation of mononuclear cell proliferation	BP	1	AGER,IL5,HLA-DPB1,TNFRSF13C,NCK2,PELI1,TLR4,IL15,BMI1,MYD88,TGFBR2,NCKAP1L,SLC7A1,TRAF6,IL1A,MEF2C,IL7,RIPK2,ZNF335,CD40,IGF2,ICOSLG,GPAM,CD28,BCL6,IL12A,CD38,BST1,STAT5B,FOXP3,RASAL3,CARD11,CHRNB2,CD74,CCL5,CD320,TICAM1,DNAJA3,VAV3,MIR21,FADD,CD46,CD70,TNFSF9,TNFSF13B,HAVCR2	grey	VSMC_M10
GO:0007286	0.42	0.66	55	100	spermatid development	BP	1	TBPL1,YTHDC2,PACRG,QKI,UBE2B,CATSPER3,H3F3C,DMC1,CFAP69,FANCG,SMARCA2,KIT,ARMC2,DLD,TTC26,RSPH6A,TOPAZ1,NECTIN2,TTC21A,CCNB1IP1,MKKS,PANK2,ING2,CELF1,JAM2,CDYL,SYCP1,DNAH1,OCA2,ROPN1L,NECTIN3,NEURL1,DDX25,CFAP43,ABHD2,SPO11,TSSK4,CIB1,OSBP2,CATSPER2,TTC12,SPAG16,ZMYND15,MNS1,SOX30,CCDC42,SPEF2,CATSPERD,CHD5,TPGS1,MFSD14A,STRBP,SYCP3,RNF8,TBC1D20	grey	VSMC_M10
GO:0090383	0.42	0.85	4	6	phagosome acidification	BP	1	RAB7A,TCIRG1,SLAMF8,RAB39A	grey	VSMC_M10

GO:0015833	0.42	0.64	101	186	peptide transport	BP	1	LRP1,RIMS2,PRKN,TAP1,PLA2G6,PICK1,RFX3,TFAP2B,PRKCE,HMGN3,CYB5R4,BMP8A,EGFR,TRPM4,SLC7A11,GIPR,RAF1,ABCC4,AIMP1,SIRT3,SYT9,NR1H4,NDUFAF2,FAM3D,PAX8,OSBP,ABCB9,ARNTL,KCNJ11,NNAT,TIAM1,RAB11FIP5,PFKM,SLC8B1,CDH17,NKX6-1,ACVR1C,ZBED6,SIRT4,GPLD1,IRS1,HDAC1,PPP3CA,CPT1A,TRPV4,SLC9B2,ADRA2A,TCIRG1,TCF7L2,KCNB1,PFKFB2,GPER1,RAB11FIP2,ABCC5,PER2,STX1A,PASK,GLUL,SIDT2,SLC16A1,KLF7,VGF,ENY2,ABCA12,MCU,PPP3CB,CAMK2G,PTPRN,CD38,MPC2,ILDR2,SYT7,NLGN2,CPLX1,TARDBP,STX4,RAB8B,UBE2Q1,SYTL4,GPR68,S100A8,CD74,CCL5,SREBF1,ISL1,OXCT1,NOS2,ABAT,NADK,TRPV1,BAIAP3,PARK7,LRRC8A,FGA,CHGA,SOX4,DPP4,CA2,CRH,DOC2B,UQCC2	grey	VSMC_M10
GO:0002886	0.42	0.70	23	41	regulation of myeloid leukocyte mediated immunity	BP	1	RAC2,SPHK2,GAB2,UNC13D,MAVS,FER,F2RL1,FGR,SNX4,C12orf4,STX4,ITGAM,IL4R,BTK,STXBP2,PRAM1,C3,TICAM1,DNASE1,PDPK1,FOXF1,STXBP1,CXCL6	grey	VSMC_M10
GO:0052651	0.42	0.85	4	6	monoacylglycerol catabolic process	BP	1	ABHD16A,FAAH,ABHD6,ABHD12B	grey	VSMC_M10
GO:0030949	0.42	0.82	5	8	positive regulation of vascular endothelial growth factor receptor signaling pathway	BP	1	GRB10,PRKD2,ARNT,FGF18,PRKCB	grey	VSMC_M10
GO:0097006	0.42	0.68	30	53	regulation of plasma lipoprotein particle levels	BP	1	APOM,NR1H2,APOB,FGF21,APOC1,NR1H4,SOAT2,PLTP,MYLIP,GPLD1,ABCA5,AGT,SOAT1,EHD1,ZDHHC8,LPCAT3,LRPAP1,LIPA,LIPC,ANXA2,MPO,LDLR,ABCA7,MIR199A1,MIR27B,MIR27A,MIR33B,NCEH1,LDLRAP1,PLAGL2	grey	VSMC_M10
GO:0006575	0.42	0.65	75	136	cellular modified amino acid metabolic process	BP	1	SHMT2,CPT1B,ALDH7A1,ABHD16A,SLC22A4,CKB,GNMT,CROT,CLIC5,SLC1A1,CHAC2,MBOAT2,MGST2,SLC7A11,DHFR,MMACHC,GSTK1,GGACT,PANK2,CLIC1,ARL6IP5,PAX8,AHCY,GSS,CHDH,CKMT2,MTHFD2,SERINC5,HOGA1,MTRR,GLRX2,CPT1A,PLA1A,SLCO1C1,CKMT1B,POR,NFE2L2,DUOX1,ACADL,PLOD3,GGT2,MTHFS,PRODH,LPCAT3,ABHD12B,AASDHPPT,GCH1,GGT6,SLC27A1,ALDH4A1,MED1,GSTZ1,CRYM,SHMT1,PCYOX1L,SLC46A1,PIPOX,GAMT,TMLHE,PARK7,SARDH,ASS1,KYAT1,PTGES2,PLA2G15,ACADM,GSTT4,MTHFD2L,PEMT,SLC25A32,GLO1,DIO3,ALDH1L1,GGT5,DIO2	grey	VSMC_M10
GO:0071356	0.42	0.64	89	163	cellular response to tumor necrosis factor	BP	1	MAP3K5,TNFAIP3,HSPA1B,PRKN,TNFRSF13C,MAPK14,TNFRSF21,GSDME,CXCL8,TRIM32,TRAF1,FOXO3,APOB,TRAF3IP2,ZFAND6,IKBKB,BAG4,GAS6,ST18,TRAF6,ASAH1,CEBPA,GPD1,PTK2B,KCNJ11,CCL17,OTULIN,NKX3-1,F2RL1,RIPK1,ABCC2,CD40,HDAC1,HYAL1,HYAL3,PIAS3,FABP4,SPATA2,GPER1,CIB1,TRAF5,HDAC4,PELI3,YBX3,NFE2L2,TNFSF18,SMPD4,BIRC3,SPPL2A,MAPK1,SGMS1,SLC2A4,GPS2,TNFRSF1B,PYDC1,MAPK3,TRAF3,CCL14,CCL5,CCL7,CCL2,CCL25,ADAMTS12,PTPN2,PIAS4,INPP5K,CDIP1,TRPV1,SPPL2B,TRAF2,TNFRSF25,ADAMTS13,CYBA,ASS1,LCN2,DAB2IP,MIR27B,MIR24-1,CCL16,NOL3,CASP8,CLDN1,CD70,COMMD7,CCDC3,ZFP36L2,TNFSF13B,HIST1H2BJ,CCL22	grey	VSMC_M10
GO:0006560	0.42	0.85	4	6	proline metabolic process	BP	1	PYCR3,OAT,PRODH,ALDH4A1	grey	VSMC_M10
GO:0072070	0.42	0.82	5	8	loop of Henle development	BP	1	POU3F3,JAG1,PKD2,IRX2,PKD1	grey	VSMC_M10
GO:0046761	0.42	0.85	4	6	viral budding from plasma membrane	BP	1	VPS4B,CHMP2A,CHMP4C,VPS4A	grey	VSMC_M10
GO:0048148	0.42	0.82	5	8	behavioral response to cocaine	BP	1	HOMER1,HOMER2,SDK1,ABAT,FADD	grey	VSMC_M10
GO:2000678	0.42	0.76	9	15	negative regulation of transcription regulatory region DNA binding	BP	1	FBXW7,PER2,MSX2,PSEN1,TAF1,SIN3A,DDIT3,ZNF593,HEY2	grey	VSMC_M10
GO:0097396	0.42	0.82	5	8	response to interleukin-17	BP	1	TRIM32,TRAF3IP2,TRAF6,TRAF5,TRAF2	grey	VSMC_M10
GO:0097398	0.42	0.82	5	8	cellular response to interleukin-17	BP	1	TRIM32,TRAF3IP2,TRAF6,TRAF5,TRAF2	grey	VSMC_M10
GO:0097400	0.42	0.82	5	8	interleukin-17-mediated signaling pathway	BP	1	TRIM32,TRAF3IP2,TRAF6,TRAF5,TRAF2	grey	VSMC_M10
GO:0006568	0.42	0.85	4	6	tryptophan metabolic process	BP	1	IL4I1,IDO2,IDO1,GCDH	grey	VSMC_M10
GO:0051291	0.42	0.74	11	19	protein heterooligomerization	BP	1	CPSF6,HSD17B8,PRPH2,SYT1,CBR4,TMEM120B,PKD2,CALHM1,ROM1,FARSB,PKD1	grey	VSMC_M10
GO:0098810	0.42	0.75	10	17	neurotransmitter reuptake	BP	1	PRKN,SLC6A2,SLC6A3,ATP1A2,PER2,SLC29A2,SLC29A4,CLN8,SLC1A7,PARK7	grey	VSMC_M10
GO:0050931	0.42	0.72	16	28	pigment cell differentiation	BP	1	SOX10,KIT,MITF,ANKRD27,MEF2C,ADAMTS20,OCA2,USP13,HPS4,BLOC1S6,MREG,LRMDA,CITED1,AP1G1,BLOC1S3,RAB32	grey	VSMC_M10
GO:0035902	0.42	0.75	10	17	response to immobilization stress	BP	1	BRD1,FOXO3,ERCC1,HNMT,GPI,PTK2B,FOXO4,FOS,CRH,PITX3	grey	VSMC_M10
GO:0015936	0.42	0.75	10	17	coenzyme A metabolic process	BP	1	CROT,PPCS,PANK2,PANK1,MCCC2,SLC25A16,COASY,PANK3,PANK4,NUDT19	grey	VSMC_M10
GO:0016081	0.42	0.82	5	8	synaptic vesicle docking	BP	1	PPFIA3,SNPH,STX1A,BLOC1S6,STX1B	grey	VSMC_M10
GO:0070427	0.42	0.85	4	6	nucleotide-binding oligomerization domain containing 1 signaling pathway	BP	1	TNFAIP3,SLC46A2,TLR4,RIPK2	grey	VSMC_M10
GO:1902747	0.42	0.85	4	6	negative regulation of lens fiber cell differentiation	BP	1	SPRY1,FOXE3,SPRY2,SPRED3	grey	VSMC_M10
GO:0033632	0.42	0.82	5	8	regulation of cell-cell adhesion mediated by integrin	BP	1	PODXL,FERMT3,CCL5,PIEZO1,DPP4	grey	VSMC_M10
GO:1904464	0.42	0.85	4	6	regulation of matrix metallopeptidase secretion	BP	1	SLC12A2,TLR4,CD200,MIR199A1	grey	VSMC_M10
GO:1990773	0.42	0.85	4	6	matrix metallopeptidase secretion	BP	1	SLC12A2,TLR4,CD200,MIR199A1	grey	VSMC_M10
GO:0034138	0.42	0.76	9	15	toll-like receptor 3 signaling pathway	BP	1	TNFAIP3,PELI1,F2RL1,UNC93B1,TNIP2,WDFY1,COLEC12,OAS1,HAVCR2	grey	VSMC_M10
GO:0014866	0.42	0.82	5	8	skeletal myofibril assembly	BP	1	LMOD3,ACTC1,TTN,TCAP,MYH11	grey	VSMC_M10
GO:0009948	0.42	0.71	18	32	anterior/posterior axis specification	BP	1	FRS2,SRF,SIX2,FAM208A,LDB1,PGAP1,WNT3,HOXD8,FZD5,LHX1,CDX1,GPC3,SKI,WDR77,WNT5A,BASP1,DDIT3,HEY2	grey	VSMC_M10
GO:0019731	0.42	0.77	8	13	antibacterial humoral response	BP	1	PLA2G6,NOD2,SPON2,FGA,HIST1H2BK,JCHAIN,HIST1H2BF,HIST1H2BJ	grey	VSMC_M10
GO:0033081	0.42	0.74	11	19	regulation of T cell differentiation in thymus	BP	1	CLPTM1,GLI2,FOXJ1,TOX,ZC3H8,CAMK4,TMEM131L,RASGRP1,ADAM8,IL7R,PTPN2	grey	VSMC_M10

GO:0043433	0.42	0.64	69	126	negative regulation of DNA-binding transcription factor activity	BP	1	TNFAIP3,ESR1,FZD6,TRIB1,PTCH1,PIM1,MDFI,FOXH1,LRRC14,CDKN2A,NFKBIE,CAT,MTURN,PELI1,ANXA4,SFRP4,FAF1,HDAC2,TAF3,FOXJ1,PAXIP1,IRAK2,ARRB1,EOMES,BHLHE40,NFKBIB,PURA,PRNP,GAS6,HAND2,SETD6,EIF2AK4,NLRC5,OTULIN,PKD2,NOD2,SUFU,CD200,SIK1,SP100,TCF7L2,CMKLR1,HES6,HDAC4,MEN1,NWD1,FOXP3,PEX14,PYDC1,TAF1,MAP2K5,TRIM37,KEAP1,PSMD10,TRAF3,PIAS4,PKD1,GFI1,WWP2,DAB2IP,MIR199A1,MIR21,MIR27A,MIR125A,RWDD3,COMMD6,DDIT3,COMMD7,HAVCR2	grey	VSMC_M10
GO:0070943	0.42	0.85	4	6	neutrophil-mediated killing of symbiont cell	BP	1	MYD88,F2RL1,SCNN1B,CXCL6	grey	VSMC_M10
GO:0070570	0.42	0.72	16	28	regulation of neuron projection regeneration	BP	1	LRP1,PTN,PTPRF,SPP1,KIAA0319,KREMEN1,CERS2,LRIG2,TNR,EPHA4,NDEL1,MAP2K1,MIR222,CNTF,RTN4RL1,THY1	grey	VSMC_M10
GO:0002823	0.43	0.71	17	30	negative regulation of adaptive immune response based on somatic recombination of immune receptors built from immunoglobulin superfamily domains	BP	1	IL4I1,FOXJ1,NCKAP1L,NOD2,BCL6,RC3H1,PTPN6,TNFSF18,PPP3CB,FOXP3,TBX21,ZBTB7B,IL4R,IL7R,CD46,HAVCR2,RC3H2	grey	VSMC_M10
GO:0015810	0.43	0.82	5	8	aspartate transmembrane transport	BP	1	SLC25A13,SLC3A1,LRRC8E,LRRC8A,LRRC8D	grey	VSMC_M10
GO:0061085	0.43	0.82	5	8	regulation of histone H3-K27 methylation	BP	1	PHF1,OGT,SUPT6H,MTF2,ZNF304	grey	VSMC_M10
GO:0002024	0.43	0.82	5	8	diet induced thermogenesis	BP	1	BMP8A,UCP1,TRPV4,SORL1,TRPV1	grey	VSMC_M10
GO:0050680	0.43	0.65	59	108	negative regulation of epithelial cell proliferation	BP	1	RIDA,MTSS1,PTCH1,IFT74,CDK6,CD109,PTN,INTU,SOX9,AQP11,IFT172,AIMP1,FLT1,ATP5F1A,ROBO1,FBXW7,MEF2C,PEX2,VDR,NKX3-1,IFT52,TGFB2,SOX2,KDF1,SFN,RUNX3,CDKN1B,DLG1,DUSP10,MEN1,IL12A,CASK,SAV1,PDCD10,RAP1GAP,WDR13,MAGED1,MED1,SYNJ2BP,NGFR,VASH1,TNMD,CCL2,ISL1,SUZ12,GPC3,WDR77,MARVELD3,DAB2IP,MIR21,MIR222,MIR125B1,MIR22,MIR24-1,ACVRL1,TINF2,WNT5A,RGCC,EREG	grey	VSMC_M10
GO:0023061	0.43	0.62	175	325	signal release	BP	1	LRP1,SNCAIP,RIMS2,PRKN,GDF9,GRM4,PLA2G6,PICK1,CHRNA3,CHRNA5,CRY1,APBA1,RFX3,TFAP2B,IL11,BRSK1,PRKCE,RIMS1,HMGN3,RIMS3,CYB5R4,PPT1,BMP8A,EGFR,TRPM4,PPFIA3,LIN7B,NAPA,GIPR,CACNB2,GRIK5,RAF1,NAPB,ABCC4,C1QTNF1,SYT1,RAB5A,LIN7A,AIMP1,SIRT3,STX2,SYT9,ADRA2B,SNPH,NR1H4,NDUFAF2,FAM3D,PAX8,NR4A1,OSBP,ARNTL,ERC2,SCG5,MEF2C,ASIC1,P2RY1,KCNJ11,NNAT,TIAM1,RAB11FIP5,PFKM,SYNJ1,SLC8B1,NKX3-1,NKX6-1,ACVR1C,F2RL1,F2R,CRY2,VPS18,SPP1,ZBED6,DTNBP1,SYT8,GRM2,SIRT4,GPLD1,IRS1,DNM1L,HDAC1,PPP3CA,CPT1A,MAP2K6,TRPV4,SLC9B2,ADRA2A,TCIRG1,TCF7L2,SNAP23,KCNB1,PFKFB2,GPER1,RAB11FIP2,AGT,PER2,OSM,STX1A,SNX4,PASK,BACE1,DNAJC5,GLUL,CSPG5,SIDT2,SLC16A1,KLF7,CREB1,RAP1B,BLOC1S6,PLA2G4A,VGF,ENY2,ABCA12,NRXN2,CASK,MCU,ADRA2C,PNKD,PPP3CB,RAB3A,PINK1,SYN1,CAMK2G,SNAP29,PTPRN,CD38,MPC2,ILDR2,INHA,SYT7,NLGN2,CPLX1,TARDBP,STX1B,STX4,PSEN1,SYT11,RAB8B,CHRNB2,UBE2Q1,CALM1,SYTL4,SNAPIN,GPR68,PRKCB,BTK,CAMK2A,CCL5,STXBP2,SREBF1,ISL1,OXCT1,NOS2,ABAT,FMR1,PIP5K1C,NADK,P2RX1,BAIAP3,PARK7,LRRC8A,STXBP1,FGA,CHRNB4,CHGA,SOX4,DPP4,SNAP47,PDZD11,CRH,DOC2B,PTGES,LIF,UQCC2,KCNMB4	grey	VSMC_M10
GO:0070544	0.43	0.79	6	10	histone H3-K36 demethylation	BP	1	KDM4C,KDM7A,KDM4A,PHF8,RIOX1,KDM8	grey	VSMC_M10
GO:0046164	0.43	0.69	23	41	alcohol catabolic process	BP	1	MIOX,SYNJ2,SRD5A3,INPP4B,MTMR2,MTMR7,CYP7A1,GK5,SYNJ1,INPP5A,ALDH3B2,NTSR1,INPP5J,SORD,CYP27A1,TKFC,CYP46A1,IMPA2,INPP5K,ALDH2,CEL,INPP1,ADH4	grey	VSMC_M10
GO:1904950	0.43	0.65	55	100	negative regulation of establishment of protein localization	BP	1	PRKN,GOPC,RAB23,DNAJA1,BMP8A,RSAD2,ITGB1BP1,FRMD4A,UBAC2,DPH3,FERMT1,BAG4,LYPLA1,NDUFAF2,FAM3D,PKIA,KCNJ11,NR1H3,RAB11FIP5,ACVR1C,F2R,DMTN,ADTRP,SUFU,SIRT4,IRS1,CABP1,CD200,HDAC1,PPP3CA,SP100,ADRA2A,KCNB1,YOD1,BAG3,APOD,KLF7,MAPT,DERL3,IL12A,ENY2,C11orf65,RSC1A1,SNX12,CORO2B,SYTL4,SREBF1,INPP5K,PARK7,WWP2,MIR199A1,CHGA,TXN,UBE2G2,DERL2	grey	VSMC_M10
GO:0060219	0.43	0.74	11	19	camera-type eye photoreceptor cell differentiation	BP	1	NTRK2,RORB,PTN,SOX9,USH1C,RPGRIP1L,NRL,ROM1,SOX8,THY1,DIO3	grey	VSMC_M10
GO:1903960	0.43	0.81	5	8	negative regulation of anion transmembrane transport	BP	1	GOPC,TCAF2,ARL6IP5,SLC43A1,RGS4	grey	VSMC_M10
GO:1905063	0.43	0.75	10	17	regulation of vascular associated smooth muscle cell differentiation	BP	1	NFATC1,KIT,PDCD4,GPER1,EFEMP2,ENG,NFATC3,MIR21,MIR125B1,HEY2	grey	VSMC_M10
GO:0090189	0.43	0.74	11	19	regulation of branching involved in ureteric bud morphogenesis	BP	1	SMO,SIX2,SOX9,PAX8,PAX2,AGT,MAGED1,LHX1,SOX8,NOG,SIX1	grey	VSMC_M10
GO:0071051	0.43	0.84	4	6	polyadenylation-dependent snoRNA 3'-end processing	BP	1	EXOSC4,EXOSC3,PARN,EXOSC5	grey	VSMC_M10
GO:0070306	0.43	0.71	17	30	lens fiber cell differentiation	BP	1	FRS2,ATF4,TDRD7,TMOD1,SLC7A11,SPRY1,FOXE3,SPRY2,BFSP1,SPRED3,CDKN1B,PLA2G16,SMAD3,FZR1,HSF4,TBC1D20,PITX3	grey	VSMC_M10
GO:0051968	0.43	0.74	11	19	positive regulation of synaptic transmission, glutamatergic	BP	1	ROR2,EGFR,PTK2B,GLUL,TNR,NLGN2,NLGN3,CCL2,STXBP1,CACNG7,CACNG5	grey	VSMC_M10
GO:0006626	0.43	0.66	48	86	protein targeting to mitochondrion	BP	1	TIMM21,PRKN,TSPO,HSPA4,TOMM22,RAC2,FBXO7,MGARP,AGK,DNAJC15,PITRM1,TIMM50,USP36,BAG4,MIPEP,IMMP1L,FBXW7,HTRA2,CSNK2A2,TOMM34,SIRT4,HPS4,ARIH2,BAG3,TOMM40L,FZD5,FIS1,GRPEL1,C11orf65,PINK1,TIMM17B,MFN2,NPEPPS,HAX1,TIMM29,SREBF1,PAM16,BCAP31,SH3GLB1,MICALL2,BNIP3L,TIMM10B,TIMM23B,TIMM8B,RHOU,LEPROT,GRPEL2,DNAJC19	grey	VSMC_M10
GO:1903232	0.43	0.84	4	6	melanosome assembly	BP	1	HPS4,ABCB6,RAB32,TRAPPC6A	grey	VSMC_M10
GO:0033625	0.43	0.81	5	8	positive regulation of integrin activation	BP	1	PLEK,FERMT1,CDH17,RAP1B,PIEZO1	grey	VSMC_M10
GO:0061687	0.43	0.84	4	6	detoxification of inorganic compound	BP	1	ABCC2,ABCB6,PARK7,MT1X	grey	VSMC_M10
GO:0019432	0.43	0.70	18	32	triglyceride biosynthetic process	BP	1	PNPLA3,NR1H2,LPIN1,FBXW7,NR1H3,LPIN3,FITM2,GPLD1,SIK1,GPAM,LPGAT1,MOGAT3,SLC27A1,LDLR,SREBF1,LPIN2,C3,CNEP1R1	grey	VSMC_M10
GO:0089709	0.43	0.84	4	6	L-histidine transmembrane transport	BP	1	SLC7A1,SLC38A3,SLC38A5,SLC25A29	grey	VSMC_M10

GO:0072584	0.43	0.81	5	8	caveolin-mediated endocytosis	BP	1	NEDD4L,MAPK1,MAPK3,AC138894.1,UNC119	grey	VSMC_M10
GO:0006558	0.43	0.84	4	6	L-phenylalanine metabolic process	BP	1	IL4I1,HGD,FAH,GSTZ1	grey	VSMC_M10
GO:1902221	0.43	0.84	4	6	erythrose 4-phosphate/phosphoenolpyruvate family amino acid metabolic process	BP	1	IL4I1,HGD,FAH,GSTZ1	grey	VSMC_M10
GO:0036465	0.43	0.67	35	64	synaptic vesicle recycling	BP	1	CANX,SYNJ2,BTBD9,AMPH,ACTG1,SYT1,RAB5A,FCHO2,SYNJ1,AP3B1,CYFIP1,SYT8,DNM1L,NLGN4Y,AP2M1,STX1A,DNM3,PPP3CB,RAB3A,GRIPAP1,ACTB,SYT7,SLC2A4,NLGN2,DGKQ,GAK,STX1B,SYT11,NLGN3,CALM1,DNM2,PIP5K1C,CEL,BTBD8,DENND1A	grey	VSMC_M10
GO:0006564	0.43	0.84	4	6	L-serine biosynthetic process	BP	1	SHMT2,PSAT1,SERINC5,PHGDH	grey	VSMC_M10
GO:0140627	0.43	0.81	5	8	ubiquitin-dependent protein catabolic process via the C-end degron rule pathway	BP	1	FEM1C,KLHDC3,DCAF12,TRPC4AP,KLHDC1	grey	VSMC_M10
GO:0021702	0.43	0.79	6	10	cerebellar Purkinje cell differentiation	BP	1	AGTPBP1,FAIM2,LDB1,TTC21B,LHX1,CEND1	grey	VSMC_M10
GO:0051096	0.43	0.84	4	6	positive regulation of helicase activity	BP	1	POT1,MSH2,SSBP1,CHTOP	grey	VSMC_M10
GO:0061339	0.43	0.72	13	23	establishment or maintenance of monopolar cell polarity	BP	1	PTK7,FOXJ1,SYNE4,TCF15,GBF1,MYO9A,CAMSAP3,LAMA1,IFT20,FOXF1,CARMIL2,RHOA,WNT5A	grey	VSMC_M10
GO:1904153	0.43	0.81	5	8	negative regulation of retrograde protein transport, ER to cytosol	BP	1	UBAC2,YOD1,DERL3,UBE2G2,DERL2	grey	VSMC_M10
GO:0060392	0.43	0.81	5	8	negative regulation of SMAD protein signal transduction	BP	1	LRP1,VEPH1,SLC2A10,PBLD,CILP	grey	VSMC_M10
GO:0051665	0.43	0.84	4	6	membrane raft localization	BP	1	MAL,NAXE,DOCK2,RALA	grey	VSMC_M10
GO:0070255	0.43	0.84	4	6	regulation of mucus secretion	BP	1	PRKCE,EGFR,ATG5,CYBA	grey	VSMC_M10
GO:2001204	0.43	0.81	5	8	regulation of osteoclast development	BP	1	FBXW7,SLC9B2,FBN1,GPR68,ATP6AP1	grey	VSMC_M10
GO:0003214	0.43	0.79	6	10	cardiac left ventricle morphogenesis	BP	1	TGFBR2,TBX5,RYR2,RBPJ,FOXF1,HEY2	grey	VSMC_M10
GO:1902473	0.43	0.74	11	19	regulation of protein localization to synapse	BP	1	ABHD17B,DAG1,DLG1,MAPT,GRIPAP1,IQSEC2,NLGN2,ARHGAP44,PRKCZ,WNT5A,HRAS	grey	VSMC_M10
GO:0035511	0.43	0.84	4	6	oxidative DNA demethylation	BP	1	ALKBH3,FTO,ALKBH2,ALKBH1	grey	VSMC_M10
GO:0072574	0.43	0.75	10	17	hepatocyte proliferation	BP	1	GLI1,TNFAIP3,TGFA,PTN,FBXW7,SULF2,RPS6KA1,MED1,CEBPB,FGF1	grey	VSMC_M10
GO:0072575	0.43	0.75	10	17	epithelial cell proliferation involved in liver morphogenesis	BP	1	GLI1,TNFAIP3,TGFA,PTN,FBXW7,SULF2,RPS6KA1,MED1,CEBPB,FGF1	grey	VSMC_M10
GO:0034312	0.43	0.75	10	17	diol biosynthetic process	BP	1	SPTLC1,SPHK2,DHFR,ACER3,SPTLC3,ASAH1,SPR,GCH1,ACER2,PCBD2	grey	VSMC_M10
GO:0101023	0.43	0.74	11	19	vascular endothelial cell proliferation	BP	1	FLT1,MEF2C,IGF2,ITGA4,STAT3,CCL2,PDPK1,MIR29A,MIR21,MIR24-1,MIR27A	grey	VSMC_M10
GO:1905562	0.43	0.74	11	19	regulation of vascular endothelial cell proliferation	BP	1	FLT1,MEF2C,IGF2,ITGA4,STAT3,CCL2,PDPK1,MIR29A,MIR21,MIR24-1,MIR27A	grey	VSMC_M10
GO:1990253	0.43	0.79	6	10	cellular response to leucine starvation	BP	1	RRAGD,SESN3,LARS,EIF2AK4,RRAGB,IMPACT	grey	VSMC_M10
GO:0045141	0.43	0.84	4	6	meiotic telomere clustering	BP	1	UBE2B,MLH1,SPO11,MAJIN	grey	VSMC_M10
GO:0032020	0.43	0.84	4	6	ISG15-protein conjugation	BP	1	UBE2E2,UBE2E1,UBE2L6,HERC5	grey	VSMC_M10
GO:0036119	0.43	0.73	12	21	response to platelet-derived growth factor	BP	1	MARS,HAS1,PTN,TLR4,FER,HYAL1,CBL,SYAP1,CREB1,YES1,MTSS1L,PDGFD	grey	VSMC_M10
GO:0002456	0.43	0.67	30	54	T cell mediated immunity	BP	1	STX7,AGER,HLA-DRA,RSAD2,IL4I1,TRPM4,NECTIN2,PVR,SLC22A13,JAG1,NCKAP1L,TRAF6,NOD2,DENND1B,FZD5,CTSH,IL12A,PPP3CB,WAS,FOXP3,ZBTB1,TNFRSF1B,TBX21,FBXO38,IL7R,PRKCZ,TRAF2,FADD,CD46,CD70	grey	VSMC_M10
GO:0009131	0.43	0.84	4	6	pyrimidine nucleoside monophosphate catabolic process	BP	1	DPYS,UPB1,NT5M,UPP1	grey	VSMC_M10
GO:0009178	0.43	0.84	4	6	pyrimidine deoxyribonucleoside monophosphate catabolic process	BP	1	DPYS,UPB1,NT5M,UPP1	grey	VSMC_M10
GO:0046079	0.43	0.84	4	6	dUMP catabolic process	BP	1	DPYS,UPB1,NT5M,UPP1	grey	VSMC_M10
GO:1901299	0.43	0.84	4	6	negative regulation of hydrogen peroxide-mediated programmed cell death	BP	1	MET,PINK1,PARK7,MIR21	grey	VSMC_M10
GO:0098884	0.43	0.79	6	10	postsynaptic neurotransmitter receptor internalization	BP	1	USP46,EPS15,AP2M1,CALY,DNM3,AP2B1	grey	VSMC_M10
GO:0140239	0.43	0.79	6	10	postsynaptic endocytosis	BP	1	USP46,EPS15,AP2M1,CALY,DNM3,AP2B1	grey	VSMC_M10
GO:1902932	0.43	0.74	10	17	positive regulation of alcohol biosynthetic process	BP	1	CYP7A1,P2RY1,GPER1,NTSR1,SCAP,POR,PTH1R,WNT4,SREBF1,FGF1	grey	VSMC_M10
GO:0046394	0.43	0.62	116	213	carboxylic acid biosynthetic process	BP	1	GLS2,SHMT2,LTC4S,QKI,MCAT,HSD17B8,PYCR3,PSAT1,HACD4,MTAP,ALDH1A1,NR1H2,MGST2,BCAT2,NANP,DHFR,AKR1A1,APOC1,PRKAG2,PIBF1,ELOVL1,NR1H4,APIP,TRIB3,CYP7A1,ELOVL7,CYP7B1,THNSL2,CBR4,OSBP,PRKAG1,NR1H3,SERINC5,ENOPH1,SDSL,HOGA1,ASNSD1,GLS,ERLIN1,PRMT3,PRKAB1,MTRR,ASL,MTR,CBSL,ALDH1A3,ABHD2,MECR,ABCB11,DHRS9,HACD2,LPGAT1,PER2,OSBPL2,OAT,MLXIPL,GLUL,SCAP,PLA2G4A,OSBPL6,UPB1,ACADL,PLOD3,GGT2,CPS1,OSBPL1A,ACSBG1,PECR,AASDHPPT,CYP27A1,PRKAG3,GGT6,DAGLB,FADS3,LIAS,ACADVL,TECR,HSD17B10,DEGS1,ALDH1A2,FGFR4,LIPC,OSBPL7,ALOXE3,PKLR,AC138894.1,HACD3,NDUFAB1,CYP46A1,ACSM3,ACACA,CD74,ABHD3,SHMT1,ACSBG2,ABAT,GAMT,PARK7,DECR2,ACSF3,ASS1,MLYCD,PTGES2,ABCD3,ACOT4,FABP5,FADS6,ELOVL3,MTHFD2L,AMACR,GGT5,STARD4,PTGES,INSIG2,PHGDH,PCBD2	grey	VSMC_M10
GO:0001708	0.43	0.66	34	62	cell fate specification	BP	1	PTCH1,SMO,SIX2,HOXA13,SOX9,PTCH2,GLI2,MNX1,POU4F1,WNT11,EOMES,EYA1,TBX5,MGA,PAX2,SUFU,SOX2,FZD7,FKBP8,TBX19,BMPR1A,RBPJ,TBX21,PSEN1,GSC,TBX4,TBX2,ISL1,APC2,ISL2,WNT5A,POU3F2,SIX1,APC	grey	VSMC_M10
GO:1905719	0.43	0.81	5	8	protein localization to perinuclear region of cytoplasm	BP	1	HOOK3,FAM160A2,FAM160A1,RNF26,HOOK2	grey	VSMC_M10
GO:0034316	0.43	0.84	4	6	negative regulation of Arp2/3 complex-mediated actin nucleation	BP	1	PICK1,HIP1R,WDR63,GMFG	grey	VSMC_M10
GO:0010721	0.43	0.63	70	130	negative regulation of cell development	BP	1	SEMA6A,FBLN1,TSPO,FSTL4,SYNGAP1,SOX10,DAAM2,SEMA4D,S1PR3,SEMA4C,RTN4,RUFY3,EPHA7,PTN,ITGB1BP1,MYCN,KDM4A,HOOK3,SIRT2,SEMA3D,SPART,SEMA3A,ULK1,RYK,FBXW7,SEMA4F,TLX2,RAPGEF2,NKX6-1,SEMA3G,SPP1,DMTN,SEMA4G,RNF10,KIAA0319,PPP3CA,SEMA3F,PTHLH,AP1AR,SORL1,CERS2,NFATC4,DLX1,MELTF,WNT3,DUSP10,SEMA6D,TNR,FBN1,BMPR1A,PSEN1,SEMA4A,TRIM46,NTN1,LDLR,CDK5R1,MBD1,DAB1,RFLNB,TRPV1,SKI,ARHGAP4,PLXNA3,GPR37L1,NOG,THY1,WNT5A,YWHAH,EFNB3,PITX3	grey	VSMC_M10

GO:0016575	0.43	0.66	39	71	histone deacetylation	BP	1	BAZ2A,C6orf89,SPHK2,HDAC2,PRKD2,PHB,HDAC11,TADA3,SIRT2,SIRT7,SIRT3,MTA3,SDR16C5,ING2,HDAC7,SUDS3,ELK4,HDAC1,RCOR3,PER2,HDAC4,BCL6,RCOR2,PINK1,TBL1XR1,KDM5A,FOXP3,AKAP8L,AKAP8,JDP2,ZBTB7B,SFPQ,SIN3A,RCOR1,SREBF1,SKI,MIER2,PRMT6,SAP30BP	grey	VSMC_M10
GO:0044346	0.43	0.73	11	19	fibroblast apoptotic process	BP	1	BAK1,MYC,XRCC2,CHD8,BTG1,GAS6,PRDM11,STK17B,CFDP1,MIR24-1,BCL2L11	grey	VSMC_M10
GO:0003413	0.43	0.79	6	10	chondrocyte differentiation involved in endochondral bone morphogenesis	BP	1	COL27A1,SOX9,TSKU,TGFBR2,TRIP11,AXIN2	grey	VSMC_M10
GO:0002087	0.43	0.81	5	8	regulation of respiratory gaseous exchange by nervous system process	BP	1	GLS,ATP1A2,NLGN2,NLGN3,MECP2	grey	VSMC_M10
GO:0009221	0.43	0.84	4	6	pyrimidine deoxyribonucleotide biosynthetic process	BP	1	TBPL1,DTYMK,CMPK2,DCTD	grey	VSMC_M10
GO:0033615	0.43	0.84	4	6	mitochondrial proton-transporting ATP synthase complex assembly	BP	1	ATP23,ATPAF1,OXA1L,ATPAF2	grey	VSMC_M10
GO:0043461	0.43	0.84	4	6	proton-transporting ATP synthase complex assembly	BP	1	ATP23,ATPAF1,OXA1L,ATPAF2	grey	VSMC_M10
GO:0035900	0.44	0.84	4	6	response to isolation stress	BP	1	ACO2,NAPEPLD,KRAS,HRAS	grey	VSMC_M10
GO:0042590	0.44	0.84	4	6	antigen processing and presentation of exogenous peptide antigen via MHC class I	BP	1	TAP1,IKBKB,MFSD6,FCER1G	grey	VSMC_M10
GO:0060027	0.44	0.81	5	8	convergent extension involved in gastrulation	BP	1	WNT11,MKKS,DVL2,WNT5A,LBX2	grey	VSMC_M10
GO:1900542	0.44	0.67	31	56	regulation of purine nucleotide metabolic process	BP	1	PPARA,PRXL2C,GUCA1B,SLC4A4,SPHK2,LACC1,PRKAG2,PDK1,GPD1,P2RX7,PRKAG1,FAM173B,ZBTB20,ARNT,ACTN3,HDAC4,DNAJC30,MLXIPL,PDK3,PINK1,STAT3,PARP1,PSEN1,OGT,NCOR1,INSR,NOS2,SLC2A6,ENTPD5,PDZD3,TIGAR	grey	VSMC_M10
GO:0000455	0.44	0.84	4	6	enzyme-directed rRNA pseudouridine synthesis	BP	1	RPUSD2,RPUSD4,RPUSD1,RPUSD3	grey	VSMC_M10
GO:0043457	0.44	0.69	22	39	regulation of cellular respiration	BP	1	SHMT2,PNPT1,DNAJC15,COX7A1,IDE,TRPV4,ISCU,CISD1,ACTN3,MLXIPL,PINK1,NOS2,SLC25A23,PRDM16,PARK7,SLC25A33,RHOA,IFNLR1,COX7A2,UQCC2,COX7A2L,PPIF	grey	VSMC_M10
GO:0051000	0.44	0.73	11	19	positive regulation of nitric-oxide synthase activity	BP	1	NOD1,DHFR,PIK3CB,PTK2B,TERT,KRAS,NOS1AP,GCH1,CALM1,TERF2,FCER2	grey	VSMC_M10
GO:0019885	0.44	0.81	5	8	antigen processing and presentation of endogenous peptide antigen via MHC class I	BP	1	TAP1,IDE,ABCB9,ERAP1,ERAP2	grey	VSMC_M10
GO:0003094	0.44	0.73	12	21	glomerular filtration	BP	1	XPNPEP3,GAS6,F2RL1,F2R,SULF2,MCAM,PTPRO,TMEM63C,CORO2B,TBC1D8B,CYBA,JCHAIN	grey	VSMC_M10
GO:0043467	0.44	0.64	57	104	regulation of generation of precursor metabolites and energy	BP	1	SHMT2,PPARA,PRXL2C,PNPT1,SLC4A4,SPHK2,RUBCNL,DNAJC15,KHK,EPM2AIP1,PRKAG2,PPP1CB,AKT2,COX7A1,PPP1R3B,IDE,SORBS1,GPD1,P2RX7,PRKAG1,IGF2,IRS1,TRPV4,ZBTB20,COX17,ISCU,PPP1R3E,ARNT,CISD1,PPP1R3D,ACTN3,HDAC4,PASK,MLXIPL,PINK1,STAT3,PPP1R3F,PSEN1,OGT,NCOR1,INSR,NOS2,SLC25A23,INPP5K,PRDM16,PARK7,SLC25A33,SLC2A6,RHOA,ENTPD5,IFNLR1,COX7A2,TIGAR,APOPT1,UQCC2,COX7A2L,PPIF	grey	VSMC_M10
GO:0071600	0.44	0.81	5	8	otic vesicle morphogenesis	BP	1	SOX9,CEP290,EYA1,STOX1,TCAP	grey	VSMC_M10
GO:0006721	0.44	0.67	31	56	terpenoid metabolic process	BP	1	RDH5,RDH16,LRP1,ALDH8A1,AKR1B1,ALDH1A1,ADH5,RDH13,EGFR,NAPEPLD,LIPE,SDR16C5,RETSAT,HTRA2,PRMT3,SRD5A1,ALDH1A3,GGPS1,DHRS9,BCO2,PECR,DHRS3,RDH11,ALDH1A2,SCPEP1,LRP8,PNPLA4,CRH,LRAT,RDH14,ADH4	grey	VSMC_M10
GO:0060379	0.44	0.79	6	10	cardiac muscle cell myoblast differentiation	BP	1	SRF,NRG1,PRICKLE1,RBPJ,TBX2,ISL1	grey	VSMC_M10
GO:1903533	0.44	0.66	38	69	regulation of protein targeting	BP	1	PRKN,RAC2,SLC1A1,ITGB1BP1,TCAF2,AKT2,USP36,PRNP,BAG4,FBXW7,HTRA2,CSNK2A2,DMTN,KCNB1,HPS4,C2CD5,ARIH2,CIB1,BAG3,FZD5,FIS1,CEMIP,C11orf65,PINK1,NPEPPS,ITGAM,HAX1,MIEF2,CDK5R1,SREBF1,INPP5K,SH3GLB1,MICALL2,BNIP3L,HRAS,MIEF1,RHOU,LEPROT	grey	VSMC_M10
GO:0030072	0.44	0.62	91	168	peptide hormone secretion	BP	1	LRP1,RIMS2,PRKN,PLA2G6,PICK1,RFX3,TFAP2B,PRKCE,HMGN3,CYB5R4,BMP8A,EGFR,TRPM4,GIPR,RAF1,AIMP1,SIRT3,SYT9,NR1H4,NDUFAF2,FAM3D,PAX8,OSBP,ARNTL,KCNJ11,NNAT,TIAM1,RAB11FIP5,PFKM,SLC8B1,NKX6-1,ACVR1C,ZBED6,SIRT4,GPLD1,IRS1,HDAC1,PPP3CA,CPT1A,TRPV4,SLC9B2,ADRA2A,TCIRG1,TCF7L2,KCNB1,PFKFB2,GPER1,RAB11FIP2,PER2,STX1A,PASK,GLUL,SIDT2,SLC16A1,KLF7,VGF,ENY2,ABCA12,MCU,PPP3CB,CAMK2G,PTPRN,CD38,MPC2,ILDR2,SYT7,NLGN2,CPLX1,TARDBP,STX4,RAB8B,UBE2Q1,SYTL4,GPR68,CCL5,SREBF1,ISL1,OXCT1,NOS2,ABAT,NADK,BAIAP3,PARK7,LRRC8A,FGA,CHGA,SOX4,DPP4,CRH,DOC2B,UQCC2	grey	VSMC_M10
GO:0016053	0.44	0.62	117	215	organic acid biosynthetic process	BP	1	GLS2,SHMT2,LTC4S,QKI,MCAT,HSD17B8,PYCR3,PSAT1,HACD4,MTAP,ALDH1A1,NR1H2,MGST2,BCAT2,NANP,DHFR,AKR1A1,APOC1,PRKAG2,PIBF1,ELOVL1,NR1H4,APIP,TRIB3,CSAD,CYP7A1,ELOVL7,CYP7B1,THNSL2,CBR4,OSBP,PRKAG1,NR1H3,SERINC5,ENOPH1,SDSL,HOGA1,ASNSD1,GLS,ERLIN1,PRMT3,PRKAB1,MTRR,ASL,MTR,CBSL,ALDH1A3,ABHD2,MECR,ABCB11,DHRS9,HACD2,LPGAT1,PER2,OSBPL2,OAT,MLXIPL,GLUL,SCAP,PLA2G4A,OSBPL6,UPB1,ACADL,PLOD3,GGT2,CPS1,OSBPL1A,ACSBG1,PECR,AASDHPPT,CYP27A1,PRKAG3,GGT6,DAGLB,FADS3,LIAS,ACADVL,TECR,HSD17B10,DEGS1,ALDH1A2,FGFR4,LIPC,OSBPL7,ALOXE3,PKLR,AC138894.1,HACD3,NDUFAB1,CYP46A1,ACSM3,ACACA,CD74,ABHD3,SHMT1,ACSBG2,ABAT,GAMT,PARK7,DECR2,ACSF3,ASS1,MLYCD,PTGES2,ABCD3,ACOT4,FABP5,FADS6,ELOVL3,MTHFD2L,AMACR,GGT5,STARD4,PTGES,INSIG2,PHGDH,PCBD2	grey	VSMC_M10
GO:1903747	0.44	0.68	24	43	regulation of establishment of protein localization to mitochondrion	BP	1	PRKN,RAC2,DNAJA1,USP36,BAG4,FBXW7,HTRA2,CSNK2A2,HPS4,ARIH2,BAG3,FZD5,MAPT,NMT1,C11orf65,PINK1,NPEPPS,HAX1,SREBF1,SH3GLB1,MICALL2,BNIP3L,RHOU,LEPROT	grey	VSMC_M10
GO:0042415	0.44	0.81	5	8	norepinephrine metabolic process	BP	1	PRKN,LY6E,AKR1B1,EPAS1,HAND2	grey	VSMC_M10
GO:0046639	0.44	0.73	11	19	negative regulation of alpha-beta T cell differentiation	BP	1	HLX,RUNX3,BCL6,RC3H1,TNFSF18,FOXP3,TBX21,ZBTB7B,IL4R,SOCS1,RC3H2	grey	VSMC_M10
GO:0030656	0.44	0.81	5	8	regulation of vitamin metabolic process	BP	1	CYP27B1,PRMT3,SNAI1,GFI1,CLYBL	grey	VSMC_M10
GO:0099639	0.44	0.78	6	10	neurotransmitter receptor transport, endosome to plasma membrane	BP	1	VPS35,NSG1,GRIPAP1,RAB8A,RAB11A,ARHGAP44	grey	VSMC_M10

GO:0030225	0.44	0.69	18	32	macrophage differentiation	BP	1	TRIB1,L3MBTL3,HSF1,ROR2,IL15,CEBPA,RIPK1,TSPAN2,HCLS1,ZBTB46,INHA,PARP1,CSF1R,PTPN2,FADD,CASP8,NRROS,LIF	grey	VSMC_M10
GO:0010960	0.44	0.77	7	12	magnesium ion homeostasis	BP	1	TFAP2B,CNNM4,EGFR,KEL,SLC41A1,TRPM7,KCNA1	grey	VSMC_M10
GO:0048515	0.44	0.63	58	106	spermatid differentiation	BP	1	TBPL1,YTHDC2,PACRG,QKI,UBE2B,CATSPER3,H3F3C,DMC1,CFAP69,FANCG,SMARCA2,KIT,ARMC2,DLD,TTC26,RSPH6A,TOPAZ1,NECTIN2,TTC21A,CCNB1IP1,MKKS,PANK2,NPHP1,ING2,CELF1,JAM2,CDYL,SYCP1,DNAH1,OCA2,ROPN1L,NECTIN3,RHBDD1,NEURL1,DDX25,CFAP43,ABHD2,SPO11,TSSK4,CIB1,DDX6,OSBP2,CATSPER2,TTC12,SPAG16,ZMYND15,MNS1,SOX30,CCDC42,SPEF2,CATSPERD,CHD5,TPGS1,MFSD14A,STRBP,SYCP3,RNF8,TBC1D20	grey	VSMC_M10
GO:0048635	0.44	0.81	5	8	negative regulation of muscle organ development	BP	1	SIRT2,SFMBT1,USP19,YBX3,SOX15	grey	VSMC_M10
GO:0042402	0.44	0.74	10	17	cellular biogenic amine catabolic process	BP	1	ALDH7A1,IL4I1,HNMT,IDO2,IDO1,LRTOMT,AP000812.5,CHDH,SLC6A3,COMT	grey	VSMC_M10
GO:0007606	0.44	0.67	24	43	sensory perception of chemical stimulus	BP	1	CFAP69,ADCY3,C5AR1,ASIC3,MKKS,P2RX2,P2RX3,ASIC1,NCAM2,CALHM1,SLC6A3,BBS1,RTP4,SCNN1B,PDE4A,ASIC2,GNAL,GNB1,TRPV1,GNG13,GNAT2,PKD1L3,TAS2R3,OR5AN1	grey	VSMC_M10
GO:0031642	0.44	0.80	5	8	negative regulation of myelination	BP	1	TNFRSF21,LPIN1,FIG4,MTMR2,JAM2	grey	VSMC_M10
GO:1902373	0.44	0.67	27	49	negative regulation of mRNA catabolic process	BP	1	PABPC1,MAPK14,TRAF3IP2,DHX34,MYD88,PKP3,TENT5C,TENT4B,RBM24,AXIN2,MAPKAPK2,TRAF5,ANGEL2,EIF4ENIF1,YBX3,SLC11A1,RBM10,TARDBP,TOB1,TENT5D,LARP1,FMR1,METTL16,CIRBP,TRAF2,YBX1,TENT5A	grey	VSMC_M10
GO:0033152	0.44	0.80	5	8	immunoglobulin V(D)J recombination	BP	1	CYREN,PRKDC,XRCC4,YY1,NHEJ1	grey	VSMC_M10
GO:0048675	0.44	0.63	57	106	axon extension	BP	1	LRP1,SEMA6A,RAB21,PLXNA4,SRF,SEMA4D,SEMA4C,RTN4,RUFY3,NRCAM,GOLGA4,MEGF8,SEMA3D,SEMA3A,ULK1,RYK,TTL,SEMA4F,DPYSL2,SLIT1,NKX6-1,ZFYVE27,SEMA3G,CYFIP1,ALCAM,SEMA4G,KIAA0319,SEMA3F,AUTS2,ADNP,DISC1,CDKL5,WNT3,SPG11,MAPT,SEMA6D,TNR,FN1,PPP3CB,VCL,SEMA4A,NLGN3,TRIM46,NDEL1,RAB11A,NTN1,DNM2,SIN3A,TRPV2,CDK5R1,SLC9A6,L1CAM,ARHGAP4,PLXNA3,BARHL2,WNT5A,OLFM1	grey	VSMC_M10
GO:0002934	0.44	0.78	6	10	desmosome organization	BP	1	GRHL1,PKP3,DSP,PKP2,DSG2,PERP	grey	VSMC_M10
GO:0090162	0.44	0.70	15	27	establishment of epithelial cell polarity	BP	1	PTK7,FRMD4A,FBF1,FERMT1,FRMD4B,SYNE4,TCF15,CYTH3,MYO9A,CAMSAP3,LAMA1,IFT20,FOXF1,RHOA,WNT5A	grey	VSMC_M10
GO:0045860	0.44	0.60	164	305	positive regulation of protein kinase activity	BP	1	ERBB3,MAP3K5,AGAP2,MRNIP,STK3,MAP3K4,AGER,XRCC6,PTK2,TAB1,CCND3,CHRNA3,TTBK1,KIT,SLC1A1,ROR2,EPGN,AREG,ETAA1,TNFSF15,TGFA,ITGB1BP1,EGFR,ARHGEF5,TLR4,CCNY,SLC8A2,PHB,ABI1,MAPRE3,RALB,PRKAG2,PIBF1,SPRY2,WNT11,TGFBR2,NEK10,RPTOR,TCIM,HBEGF,PRNP,NRG1,NCKAP1L,GAS6,ERCC6,FLT1,ADRA2B,TRAF6,ZFP91,ROBO1,FBXW7,VEGFC,MADD,PTK2B,RASGRP1,RAPGEF2,PRKAG1,TIAM1,PKD2,STK4,NOD2,TAOK3,CD40,IGF2,BMPR1B,NEURL1,CCND1,MAP2K6,TGFB2,ADRA2A,FAM20A,DAG1,AJUBA,NCF1,KRAS,EGF,ADNP,CASS4,SPATC1L,CACUL1,AGT,CIB1,DVL3,STRADB,SYAP1,STRADA,GPRC5D,CDKN1B,DLG1,PPP2R3C,MAP3K11,ADAM8,FZD5,STOX1,CEMIP,DBF4B,MAP4K2,DDX3X,XRCC5,DUSP19,PDCD10,ADRA2C,DOK7,SLC11A1,MARK2,PINK1,SLC27A1,EPHA4,DLG4,MAGED1,DVL2,BMPR1A,RPLP1,DGKQ,PSEN1,FGF18,LCP2,TAOK2,EFNA1,IL6R,MAP2K1,LAT,PIK3R5,MAP2K5,CALM1,PSMD10,INSR,CCL5,CSF1R,CDK5R1,GHR,UNC119,TAOK1,SOCS1,DAB1,LRP8,PDPK1,CAMKK1,PRKCZ,MLST8,PKD1,TRAF2,MTCP1,PARK7,PSRC1,DAB2IP,MIR21,MIR125B1,RHOA,S100A12,IRGM,WNT5A,MMD,SPDYA,TXN,COPS8,RGCC,DAZAP2,HRAS,EREG,MAP2K7,C3orf58,FGF1,PDGFD	grey	VSMC_M10
GO:0048710	0.44	0.72	12	21	regulation of astrocyte differentiation	BP	1	TTBK1,MYCN,KDM4A,EPHA4,PRPF19,LDLR,MBD1,DAB1,CNTN2,GPR37L1,NOG,LIF	grey	VSMC_M10
GO:0043046	0.44	0.80	5	8	DNA methylation involved in gamete generation	BP	1	MOV10L1,PICK1,DNMT3A,TDRKH,PRMT7	grey	VSMC_M10
GO:0035094	0.44	0.69	18	32	response to nicotine	BP	1	PPARA,CHRNA3,CHRNA5,SLC7A11,HDAC2,CASP3,KCNJ11,NKX6-1,GRM2,SLC6A3,KCNK1,ATP1A2,CREB1,MAPK1,CHRNB1,CHRNB2,ABAT,MT-ND6	grey	VSMC_M10
GO:0043374	0.44	0.78	6	10	CD8-positive, alpha-beta T cell differentiation	BP	1	EOMES,NCKAP1L,TOX,RUNX3,ZBTB7B,SOCS1	grey	VSMC_M10
GO:0140052	0.44	0.80	5	8	cellular response to oxidised low-density lipoprotein particle stimulus	BP	1	TLR4,MYD88,ADTRP,MIA3,TICAM1	grey	VSMC_M10
GO:0016254	0.44	0.80	5	8	preassembly of GPI anchor in ER membrane	BP	1	PIGV,PIGZ,PIGL,PIGB,PIGA	grey	VSMC_M10
GO:0001523	0.44	0.67	25	45	retinoid metabolic process	BP	1	RDH5,RDH16,LRP1,ALDH8A1,AKR1B1,ALDH1A1,ADH5,RDH13,NAPEPLD,LIPE,SDR16C5,RETSAT,PRMT3,ALDH1A3,DHRS9,BCO2,DHRS3,RDH11,ALDH1A2,SCPEP1,LRP8,PNPLA4,LRAT,RDH14,ADH4	grey	VSMC_M10
GO:0071636	0.44	0.73	11	19	positive regulation of transforming growth factor beta production	BP	1	WNT11,FERMT1,LUM,CD200,CREB1,FOXP3,BMPR1A,SMAD3,GATA6,SERPINF2,CD46	grey	VSMC_M10
GO:1901856	0.44	0.80	5	8	negative regulation of cellular respiration	BP	1	DNAJC15,ACTN3,MLXIPL,RHOA,PPIF	grey	VSMC_M10
GO:0002643	0.44	0.78	6	10	regulation of tolerance induction	BP	1	PHLPP1,FOXJ1,TGFBR2,MARCH7,FOXP3,HAVCR2	grey	VSMC_M10
GO:0009435	0.44	0.73	10	17	NAD biosynthetic process	BP	1	NMRK1,SLC22A13,IDO2,IDO1,SLC5A8,NUDT12,PARP9,NNMT,NAPRT,NMNAT1	grey	VSMC_M10
GO:0032092	0.44	0.65	40	73	positive regulation of protein binding	BP	1	LRP1,STK3,PRKN,DERL1,MFNG,HSF1,ADD2,MAPRE1,MAPRE3,RALB,TMEM173,PLCL2,PPP2CB,TRIB3,PLK2,IDE,HIP1R,RAPGEF2,TIAM1,RIPK2,AMFR,STK4,BDNF,SPPL3,CAPRIN2,TERT,TCF7L2,EPB41,DTX3L,RPL11,MEN1,RAMP2,NVL,ANXA2,PSEN1,TAF1,MARK3,TICAM1,PKD1,WNT5A	grey	VSMC_M10

GO:0031056	0.45	0.62	67	124	regulation of histone modification	BP	1	SNW1,PHF1,UBE2B,C6orf89,RNF20,ATG5,SETD7,SPHK2,PRKD2,IWS1,KDM4A,PAXIP1,TADA3,ARRB1,CHTOP,NSD3,TAF7,SDR16C5,DNMT3B,HPF1,ING2,RTF1,ZNF335,JARID2,IGF2,AUTS2,SART3,TET1,BCL6,BCOR,WDR61,TADA2B,NELFA,PINK1,KDM5A,FOXP3,AKAP8L,AKAP8,NSD1,KAT7,MUC1,JDP2,MAPK3,ZBTB7B,OGT,NAP1L2,RPS6KA5,OTUB2,SIN3A,SREBF1,ISL1,SUPT6H,FMR1,GLYR1,SKI,MECP2,PARK7,MTF2,GFI1,CTCF,PRMT6,SAP30BP,ZNF304,NNMT,UBE2N,LIF,RPS6KA4	grey	VSMC_M10
GO:0016056	0.45	0.80	5	8	rhodopsin mediated signaling pathway	BP	1	CAMKMT,SAG,NMT1,GRK4,GUCY2D	grey	VSMC_M10
GO:0003334	0.45	0.76	7	12	keratinocyte development	BP	1	IFT74,FLNB,KDF1,SFN,ABCA12,BCL11B,DNASE1L2	grey	VSMC_M10
GO:0070091	0.45	0.80	5	8	glucagon secretion	BP	1	AIMP1,FAM3D,PASK,SYT7,CRH	grey	VSMC_M10
GO:0070092	0.45	0.80	5	8	regulation of glucagon secretion	BP	1	AIMP1,FAM3D,PASK,SYT7,CRH	grey	VSMC_M10
GO:0035176	0.45	0.69	17	31	social behavior	BP	1	MAPK8IP2,SHANK1,CHD8,MKKS,RPTOR,ATXN1,NLGN4Y,NPAS4,EIF4EBP2,NRXN2,DLG4,CLN8,NLGN3,CHRNB2,MECP2,ATXN1L,NRXN3	grey	VSMC_M10
GO:0006704	0.45	0.78	6	10	glucocorticoid biosynthetic process	BP	1	CYP21A2,DKK3,WNT4,DGKQ,CACNA1H,HSD11B2	grey	VSMC_M10
GO:0051965	0.45	0.67	25	46	positive regulation of synapse assembly	BP	1	NTRK2,LINGO2,SEMA4D,LRRN3,VSTM5,EPHB1,CLSTN2,CUX2,AMIGO2,BDNF,AMIGO3,ADNP,EPHB3,LRTM2,NLGN2,SEMA4A,NLGN3,GPRASP2,BHLHB9,ASIC2,SLITRK2,AMIGO1,ADGRL2,GRID2,LRRTM2	grey	VSMC_M10
GO:0046365	0.45	0.69	17	30	monosaccharide catabolic process	BP	1	GALT,DHDH,GLB1,NUDT5,XYLB,RBKS,PFKP,FUT10,PFKM,GLYCTK,PFKFB2,ACTN3,RPE,HK1,TKTL1,FUT4,TIGAR	grey	VSMC_M10
GO:0009953	0.45	0.65	33	60	dorsal/ventral pattern formation	BP	1	GLI1,TBC1D32,PTCH1,SMO,MDFI,SOSTDC1,HOXA2,INTU,GLI2,IFT172,OVOL2,IFT52,BMPR1B,FBXL15,SUFU,TTC21B,CHRD,WNT3,AIDA,FKBP8,GORAB,GPR161,TULP3,BMPR1A,WDR19,PSEN1,MKS1,GSC,LHX1,FOXG1,ACVRL1,NOG,DDIT3	grey	VSMC_M10
GO:0016926	0.45	0.80	5	8	protein desumoylation	BP	1	SENP1,SENP5,SENP2,SENP7,HINT1	grey	VSMC_M10
GO:0035672	0.45	0.78	6	10	oligopeptide transmembrane transport	BP	1	SLC7A11,ABCC4,ABCB9,CDH17,ABCC5,CA2	grey	VSMC_M10
GO:0009994	0.45	0.68	19	34	oocyte differentiation	BP	1	YTHDC2,GDF9,WASHC5,TUT7,DMC1,TUT4,FOXO3,SIRT2,SOHLH2,PTK2B,KMT2D,NPM2,YTHDF2,WNT4,YBX2,WDR77,AURKC,EREG,ZGLP1	grey	VSMC_M10
GO:0008088	0.45	0.65	37	68	axo-dendritic transport	BP	1	AP3S1,KIF5A,RAB21,KIF3A,AGTPBP1,APBA1,MGARP,WASF1,KLC3,DLG2,AP3M2,KIF5C,PURA,KIF3B,OPA1,AP3B1,DTNBP1,HSPA8,CNIH2,CALY,SPG11,MAPT,BLOC1S6,KIF17,AP3M1,KIF1C,HAP1,TRIM46,NDEL1,SNAPIN,SFPQ,MAPK8IP3,TERF2,BLOC1S3,BORCS5,AP3S2,MAP1A	grey	VSMC_M10
GO:0021860	0.45	0.78	6	10	pyramidal neuron development	BP	1	UQCRQ,DCLK2,SLC4A10,DISC1,PLXNA3,FOXG1	grey	VSMC_M10
GO:1904044	0.45	0.83	4	6	response to aldosterone	BP	1	RPL27,PARP1,SCNN1B,CYBA	grey	VSMC_M10
GO:0072524	0.45	0.69	16	28	pyridine-containing compound metabolic process	BP	1	PSAT1,NMRK1,SLC22A13,IDO2,IDO1,SLC5A8,NUDT12,NUDT17,PARP9,NAXE,PNPO,NADK,NNMT,NAPRT,PDXP,NMNAT1	grey	VSMC_M10
GO:0002699	0.45	0.62	77	142	positive regulation of immune effector process	BP	1	CD226,STX7,ENPP3,HLA-DRA,IL5,RAC2,KIT,EXOSC3,MSH2,ANKRD17,RSAD2,TLR4,SPHK2,HMCES,PHB,LACC1,NECTIN2,PVR,GAB2,MLH1,UNC13D,SLC22A13,PAXIP1,SHLD1,MAVS,HPX,TRAF6,GPI,FER,PTPRJ,RASGRP1,RIPK2,F2RL1,NOD2,CD40,HLX,DENND1B,FGR,SNX4,CD28,CADM1,FZD5,HK1,IL12A,LAG3,C12orf4,STAT5B,FOXP3,ZBTB1,SHLD2,SPON2,TBX21,STX4,ITGAM,IL4R,BTK,CD74,STXBP2,FBXO38,VAV1,C3,NOS2,TICAM1,PDPK1,PRKCZ,TRAF2,SLC7A5,FOXF1,STXBP1,AP1G1,BCL10,MIR21,FADD,CD46,WNT5A,INAVA,FCER2	grey	VSMC_M10
GO:0046135	0.45	0.83	4	6	pyrimidine nucleoside catabolic process	BP	1	APOBEC3H,CDADC1,UPB1,UPP1	grey	VSMC_M10
GO:2000317	0.45	0.78	6	10	negative regulation of T-helper 17 type immune response	BP	1	RC3H1,TNFSF18,FOXP3,TBX21,ZBTB7B,RC3H2	grey	VSMC_M10
GO:0070932	0.45	0.73	10	17	histone H3 deacetylation	BP	1	HDAC2,HDAC11,SIRT2,SIRT7,HDAC7,ELK4,HDAC1,PER2,HDAC4,SFPQ	grey	VSMC_M10
GO:1901203	0.45	0.78	6	10	positive regulation of extracellular matrix assembly	BP	1	SOX9,CLASP2,DAG1,PHLDB1,SMAD3,RGCC	grey	VSMC_M10
GO:1902410	0.45	0.72	11	19	mitotic cytokinetic process	BP	1	VPS4B,MITD1,CHMP2A,CHMP2B,CHMP4C,CHMP7,KLHDC8B,CNTROB,CHMP1A,VPS4A,RHOA	grey	VSMC_M10
GO:0090312	0.45	0.71	13	23	positive regulation of protein deacetylation	BP	1	C6orf89,PRKD2,SDR16C5,ING2,BCL6,PINK1,AKAP8L,AKAP8,JDP2,ZBTB7B,SREBF1,SAP30BP,NNMT	grey	VSMC_M10
GO:2000786	0.45	0.78	6	10	positive regulation of autophagosome assembly	BP	1	PIP4K2C,PIP4K2A,RALB,ULK1,KIAA1324,SH3GLB1	grey	VSMC_M10
GO:0009065	0.45	0.71	12	21	glutamine family amino acid catabolic process	BP	1	GLS2,PPAT,GAD2,GLS,OAT,GLUL,FAH,PRODH,ALDH4A1,ASRGL1,NOS2,MIR21	grey	VSMC_M10
GO:0021694	0.45	0.76	7	12	cerebellar Purkinje cell layer formation	BP	1	AGTPBP1,WHRN,FAIM2,LDB1,TTC21B,LHX1,CEND1	grey	VSMC_M10
GO:0006312	0.45	0.71	12	21	mitotic recombination	BP	1	DMC1,ERCC1,ERCC2,XRCC1,MLH1,TPRKB,RAD54B,RAD52,RAD51C,RAD51D,ERCC4,TERF2	grey	VSMC_M10
GO:0032755	0.45	0.65	34	62	positive regulation of interleukin-6 production	BP	1	AGER,MAPK13,NOD1,AKIRIN2,TLR4,SPHK2,MYD88,MAVS,TRAF6,IL1A,RIPK2,F2RL1,F2R,NOD2,RAB7B,IFIH1,TRPV4,IL16,STAT3,SPON2,IL6R,CD74,ISL1,NOS2,TICAM1,CYBA,BCL10,WNT5A,INAVA,IL33,C1QTNF4,TSLP,EREG,IL17D	grey	VSMC_M10
GO:0099022	0.45	0.78	6	10	vesicle tethering	BP	1	FAM91A1,EXOC4,STARD3NL,EXOC6B,TRIP11,C17orf75	grey	VSMC_M10
GO:0006573	0.45	0.80	5	8	valine metabolic process	BP	1	BCAT2,HIBCH,ACAD8,ALDH6A1,HIBADH	grey	VSMC_M10
GO:0034142	0.45	0.69	17	30	toll-like receptor 4 signaling pathway	BP	1	TNFAIP3,NINJ1,PRKCE,PELI1,TLR4,NMI,MFHAS1,NR1H4,NR1H3,RIPK2,F2RL1,RAB7B,RAB11FIP2,WDFY1,S100A14,DAB2IP,OAS1	grey	VSMC_M10
GO:0072710	0.45	0.78	6	10	response to hydroxyurea	BP	1	SPIDR,RTF2,DDX11,DDI2,KAT7,FMR1	grey	VSMC_M10
GO:0097152	0.45	0.78	6	10	mesenchymal cell apoptotic process	BP	1	HOXA13,SOX9,PAX8,PAX2,ETV6,POU3F4	grey	VSMC_M10
GO:0043124	0.45	0.68	19	34	negative regulation of I-kappaB kinase/NF-kappaB signaling	BP	1	TNFAIP3,TRIM39,ESR1,PPM1N,OPTN,ZMYND11,NR1H4,RIPK1,MAPKBP1,OTUD7A,HDAC1,OTUD7B,NLRX1,PPM1A,TNIP1,RIOK3,DAB2IP,RHOA,CASP8	grey	VSMC_M10
GO:0072540	0.45	0.80	5	8	T-helper 17 cell lineage commitment	BP	1	TNFSF18,STAT3,TBX21,BATF,IL6R	grey	VSMC_M10
GO:0060253	0.45	0.78	6	10	negative regulation of glial cell proliferation	BP	1	TSPO,SOX10,PTN,RNF10,CERS2,SKI	grey	VSMC_M10
GO:0045606	0.45	0.71	12	21	positive regulation of epidermal cell differentiation	BP	1	CYP27B1,PTCH1,SFRP4,PTCH2,OVOL2,VDR,KDF1,SFN,H2AFY2,MED1,MIR125B1,ZBED2	grey	VSMC_M10
GO:1905666	0.45	0.76	7	12	regulation of protein localization to endosome	BP	1	ABHD17B,ANKRD13A,EGF,SORL1,DTX3L,ABHD17A,ABHD17C	grey	VSMC_M10
GO:0006553	0.45	0.80	5	8	lysine metabolic process	BP	1	AASDHPPT,CRYM,PIPOX,SLC25A21,HYKK	grey	VSMC_M10

GO:0009310	0.45	0.72	11	19	amine catabolic process	BP	1	ALDH7A1,IL4I1,HNMT,IDO2,IDO1,LRTOMT,AP000812.5,CHDH,SLC6A3,COMT,MIR21	grey	VSMC_M10
GO:0060706	0.45	0.71	12	21	cell differentiation involved in embryonic placenta development	BP	1	STK3,MDFI,GJB5,EOMES,SPINT2,STK4,SNAI1,FZD5,SOX15,KRT19,CASP8,LIF	grey	VSMC_M10
GO:0043584	0.45	0.76	7	12	nose development	BP	1	DLX5,RPGRIP1L,ALDH1A3,STRA6,SMCHD1,SKI,SIX1	grey	VSMC_M10
GO:0032147	0.45	0.62	57	105	activation of protein kinase activity	BP	1	AGAP2,CHRNA3,SLC1A1,TNFSF15,ITGB1BP1,PHB,PRKAG2,PIBF1,WNT11,TGFBR2,RPTOR,PRNP,NRG1,NCKAP1L,GAS6,ADRA2B,TRAF6,ZFP91,MADD,PTK2B,TGFB2,ADRA2A,EGF,ADNP,AGT,STRADB,STRADA,GPRC5D,DLG1,PPP2R3C,DBF4B,DUSP19,ADRA2C,SLC11A1,MARK2,PINK1,RPLP1,DGKQ,TAOK2,IL6R,MAP2K1,INSR,CCL5,CDK5R1,GHR,SOCS1,PDPK1,PRKCZ,TRAF2,PARK7,MIR21,WNT5A,SPDYA,TXN,COPS8,RGCC,FGF1	grey	VSMC_M10
GO:0106035	0.45	0.83	4	6	protein maturation by [4Fe-4S] cluster transfer	BP	1	LTO1,CIAO2B,GLRX5,ISCA2	grey	VSMC_M10
GO:0035904	0.45	0.66	27	50	aorta development	BP	1	LRP1,TAB1,FOXH1,SRF,TFAP2B,CNTRL,JAG1,EYA1,ROBO1,ROBO2,NKX3-1,PKD2,PRICKLE1,SUFU,TGFB2,EFEMP2,BMPR1A,RBPJ,NGFR,DCTN5,TBX2,AP2B1,ENG,SOX4,ACVRL1,SIX1,HEY2	grey	VSMC_M10
GO:0032530	0.45	0.77	6	10	regulation of microvillus organization	BP	1	ATP8B1,PODXL,KLF5,PLS1,USH1C,RAP1GAP	grey	VSMC_M10
GO:1902746	0.45	0.80	5	8	regulation of lens fiber cell differentiation	BP	1	SPRY1,FOXE3,SPRY2,SPRED3,CDKN1B	grey	VSMC_M10
GO:0050802	0.45	0.80	5	8	circadian sleep/wake cycle, sleep	BP	1	BTBD9,SRD5A1,CHRNB2,PER3,CRH	grey	VSMC_M10
GO:0033131	0.45	0.80	5	8	regulation of glucokinase activity	BP	1	PRKN,GCKR,PFKFB2,DUSP12,COX11	grey	VSMC_M10
GO:0010817	0.46	0.59	178	331	regulation of hormone levels	BP	1	RDH5,RDH16,HSD17B4,LRP1,ALDH8A1,CYP27B1,ESR1,RIMS2,CYP21A2,PRKN,TSPO,GDF9,HSD17B8,HSD17B3,AKR1B1,PLA2G6,PICK1,ALDH1A1,CRY1,RFX3,SRD5A3,TFAP2B,IL11,PRKCE,RDH13,HMGN3,CYB5R4,BMP8A,IL4I1,EGFR,TRPM4,DDO,BCAT2,GIPR,LIPE,RAF1,AANAT,C1QTNF1,AIMP1,SIRT3,SYT9,NR1H4,SDR16C5,NDUFAF2,FAM3D,PAX8,IDE,OSBP,RETSAT,DKK3,PCSK1,ARNTL,SCG5,P2RY1,MME,KCNJ11,NNAT,TIAM1,RAB11FIP5,PFKM,SLC8B1,NKX3-1,NKX6-1,ACVR1C,CRY2,ABCC2,SPP1,ZBED6,PRMT3,SIRT4,GPLD1,SRD5A1,IRS1,HDAC1,ALDH1A3,PPP3CA,CPT1A,TRPV4,SLC9B2,ADRA2A,TCIRG1,TCF7L2,SNAP23,KCNB1,PFKFB2,ENPEP,GPER1,RAB11FIP2,DHRS9,AGT,ACE2,ARNT,SLCO1C1,PER2,OSM,ECE2,STX1A,SNX4,PASK,GLUL,PCSK7,SIDT2,HSD17B7,SLC16A1,POR,KLF7,CREB1,DUOX1,VGF,BCO2,FDX1,COMT,ENY2,ABCA12,MCU,GNB3,WNT4,PPP3CB,ECE1,CAMK2G,PTPRN,CD38,MPC2,STAT5B,ILDR2,INHA,SYT7,HSD17B10,NLGN2,DHRS3,MED1,RDH11,ALDH1A2,DGKQ,CPLX1,TARDBP,STX4,RAB8B,UBE2Q1,SCPEP1,SYTL4,GPR68,BTK,SCNN1B,CRYM,CYP46A1,DHRS11,SIN3A,CCL5,SREBF1,ISL1,GHR,OXCT1,NOS2,ABAT,NADK,BAIAP3,CACNA1H,SOX8,PARK7,SLC7A5,LRRC8A,GFI1,FGA,CHGA,SOX4,DPP4,DHRS2,PNPLA4,DIO3,CRH,LRAT,DOC2B,LIF,RDH14,UQCC2,ADH4,DIO2	grey	VSMC_M10
GO:0005979	0.46	0.70	13	23	regulation of glycogen biosynthetic process	BP	1	EPM2AIP1,PPP1CB,AKT2,PPP1R3B,SORBS1,IGF2,IRS1,PPP1R3E,PPP1R3D,PASK,PPP1R3F,INSR,INPP5K	grey	VSMC_M10
GO:0010962	0.46	0.70	13	23	regulation of glucan biosynthetic process	BP	1	EPM2AIP1,PPP1CB,AKT2,PPP1R3B,SORBS1,IGF2,IRS1,PPP1R3E,PPP1R3D,PASK,PPP1R3F,INSR,INPP5K	grey	VSMC_M10
GO:1990961	0.46	0.80	5	8	xenobiotic detoxification by transmembrane export across the plasma membrane	BP	1	ABCB1,ATP7B,ABCC2,RALBP1,SLC22A5	grey	VSMC_M10
GO:0045638	0.46	0.64	35	64	negative regulation of myeloid cell differentiation	BP	1	NME1,TRIB1,MYC,TOB2,CDK6,HOXA7,TLR4,PIK3R1,FBXW7,PTK2B,LDB1,GPR137B,PIAS3,TCTA,UBASH3B,CIB1,ZBTB46,WDR61,GPR137,FBN1,STAT5B,LEO1,INHA,HOXB8,GPR68,PTPN2,MIR125B1,FSTL3,MAFB,HIST1H4B,HIST2H4A,HIST1H4H,HIST1H4D,HIST1H4A,HIST1H4F	grey	VSMC_M10
GO:0051452	0.46	0.67	20	36	intracellular pH reduction	BP	1	PPT1,RAB7A,ATP6V0E2,AQP11,TMEM106B,ATP6V1H,TMEM9,TCIRG1,SLAMF8,CLIC4,SLC11A1,SLC9A7,ATP6V0A1,DMXL2,AC138894.1,CLN6,SNAPIN,TMEM199,CA2,RAB39A	grey	VSMC_M10
GO:0046883	0.46	0.60	94	174	regulation of hormone secretion	BP	1	LRP1,PRKN,GDF9,PLA2G6,PICK1,CRY1,RFX3,TFAP2B,IL11,PRKCE,HMGN3,BMP8A,EGFR,TRPM4,GIPR,C1QTNF1,AIMP1,SIRT3,SYT9,NR1H4,NDUFAF2,FAM3D,PAX8,OSBP,ARNTL,SCG5,P2RY1,KCNJ11,NNAT,TIAM1,RAB11FIP5,PFKM,SLC8B1,NKX3-1,NKX6-1,ACVR1C,CRY2,SPP1,ZBED6,SIRT4,GPLD1,IRS1,HDAC1,PPP3CA,CPT1A,SLC9B2,ADRA2A,TCIRG1,TCF7L2,KCNB1,PFKFB2,GPER1,AGT,PER2,OSM,STX1A,SNX4,PASK,GLUL,SIDT2,SLC16A1,KLF7,CREB1,ENY2,ABCA12,MCU,PPP3CB,CD38,MPC2,INHA,SYT7,NLGN2,TARDBP,STX4,RAB8B,SYTL4,GPR68,CCL5,SREBF1,ISL1,OXCT1,NOS2,ABAT,NADK,BAIAP3,LRRC8A,FGA,CHGA,SOX4,DPP4,CRH,DOC2B,LIF,UQCC2	grey	VSMC_M10
GO:0051205	0.46	0.66	25	44	protein insertion into membrane	BP	1	EMC2,HSPA4,TOMM22,TRAM2,EMC10,EGFR,AGK,EMC3,EMC4,OXA1L,GET4,NMT1,RTP4,BCS1L,TIMM29,MAIP1,EMC6,SGTA,UBL4A,WDR83OS,TIMM10B,CAMLG,EMC7,TRAM1L1,TIMM8B	grey	VSMC_M10
GO:0045023	0.46	0.77	6	10	G0 to G1 transition	BP	1	PPP2R5B,RHNO1,MED1,SOX15,FOXO4,DAB2IP	grey	VSMC_M10
GO:0006702	0.46	0.79	5	8	androgen biosynthetic process	BP	1	HSD17B3,SRD5A3,SRD5A1,WNT4,MED1	grey	VSMC_M10
GO:0009593	0.46	0.68	18	32	detection of chemical stimulus	BP	1	CYB5R4,TLR4,ASIC3,SYT1,P2RX2,NKX6-1,CASQ2,NOD2,KCNIP2,RYR2,RTP4,CALM1,TRPV1,GNAT2,PKD1L3,TAS2R3,OR5AN1,KCNMB4	grey	VSMC_M10
GO:0016601	0.46	0.67	20	36	Rac protein signal transduction	BP	1	RAC2,HACE1,WASF1,KBTBD6,PIK3CB,TIAM1,ARHGAP24,CYFIP1,AUTS2,KRAS,WASF2,EPS8,FARP2,NCKAP1,OGT,HACD3,DNM2,ARHGAP44,CRK,CADM4	grey	VSMC_M10
GO:0072376	0.46	0.79	5	8	protein activation cascade	BP	1	FBLN1,FN1,SERPINC1,F12,FGA	grey	VSMC_M10
GO:0014916	0.46	0.83	4	6	regulation of lung blood pressure	BP	1	GCH1,SMAD3,MIR199A1,MIR27B	grey	VSMC_M10
GO:1900087	0.46	0.68	18	32	positive regulation of G1/S transition of mitotic cell cycle	BP	1	CCND3,ANKRD17,LSM10,EGFR,UBE2E2,RPTOR,PLCB1,TFDP1,ADAMTS1,HYAL1,CCND1,TERT,DDX3X,MEPCE,CDK10,MIR29A,MIR222,RGCC	grey	VSMC_M10

GO:0046831	0.46	0.75	7	12	regulation of RNA export from nucleus	BP	1	CPSF6,IWS1,NCBP2,SETD2,AKAP8L,NRDE2,SUPT6H	grey	VSMC_M10
GO:1902188	0.46	0.77	6	10	positive regulation of viral release from host cell	BP	1	CHMP2A,CHMP2B,PC,DDB1,VPS4A,ZNF502	grey	VSMC_M10
GO:0019359	0.46	0.71	11	19	nicotinamide nucleotide biosynthetic process	BP	1	NMRK1,SLC22A13,IDO2,IDO1,SLC5A8,NUDT12,PARP9,NADK,NNMT,NAPRT,NMNAT1	grey	VSMC_M10
GO:0019363	0.46	0.71	11	19	pyridine nucleotide biosynthetic process	BP	1	NMRK1,SLC22A13,IDO2,IDO1,SLC5A8,NUDT12,PARP9,NADK,NNMT,NAPRT,NMNAT1	grey	VSMC_M10
GO:2000243	0.46	0.65	26	47	positive regulation of reproductive process	BP	1	YTHDC2,UBE2B,AGO2,MAPK15,CFAP69,HDAC2,SOX9,TOPAZ1,SIRT2,PLCB1,SEMA3A,DHX37,ACVR1B,P2RY1,NPM2,HYAL3,CIB1,CCDC87,HDAC4,OVOL1,WNT4,SYDE1,CACNA1H,OOEP,WNT5A,LHFPL2	grey	VSMC_M10
GO:0036159	0.46	0.77	6	10	inner dynein arm assembly	BP	1	LRRC6,DNAH1,ZMYND10,DNAAF2,DNAAF4,WDR63	grey	VSMC_M10
GO:0032471	0.46	0.79	5	8	negative regulation of endoplasmic reticulum calcium ion concentration	BP	1	BAK1,RAP1GDS1,FIS1,ATP2A1,BCAP31	grey	VSMC_M10
GO:0098840	0.46	0.75	7	12	protein transport along microtubule	BP	1	KIF5A,DLG2,KIF5C,BAG3,CAMSAP3,MAPK8IP3,MAP1A	grey	VSMC_M10
GO:0099118	0.46	0.75	7	12	microtubule-based protein transport	BP	1	KIF5A,DLG2,KIF5C,BAG3,CAMSAP3,MAPK8IP3,MAP1A	grey	VSMC_M10
GO:0048548	0.46	0.77	6	10	regulation of pinocytosis	BP	1	PPT1,NR1H2,NR1H3,ANKFY1,STX1B,AC138894.1	grey	VSMC_M10
GO:0036065	0.46	0.77	6	10	fucosylation	BP	1	FUT10,POFUT1,FUOM,FUT4,POFUT2,SLC35C1	grey	VSMC_M10
GO:0070189	0.46	0.79	5	8	kynurenine metabolic process	BP	1	ALDH8A1,IDO2,IDO1,KYAT1,KYAT3	grey	VSMC_M10
GO:0033860	0.46	0.79	5	8	regulation of NAD(P)H oxidase activity	BP	1	SLAMF8,AGT,PARK7,GNAI3,CYBA	grey	VSMC_M10
GO:0006616	0.46	0.79	5	8	SRP-dependent cotranslational protein targeting to membrane, translocation	BP	1	TRAM2,SEC61B,ZFAND2B,TRAM1L1,SEC61A2	grey	VSMC_M10
GO:2000781	0.46	0.67	20	36	positive regulation of double-strand break repair	BP	1	MRNIP,WDR48,SHLD1,PRKDC,ERCC6,SLF1,SPIDR,SLF2,PARP9,DTX3L,MGMT,ZCWPW1,WAS,DDX11,PARP1,SHLD2,WRAP53,SMCHD1,SPIRE2,OOEP	grey	VSMC_M10
GO:0042574	0.46	0.75	7	12	retinal metabolic process	BP	1	ALDH8A1,RDH13,SDR16C5,ALDH1A3,BCO2,RDH11,ALDH1A2	grey	VSMC_M10
GO:0051589	0.46	0.77	6	10	negative regulation of neurotransmitter transport	BP	1	PRKN,ASIC1,RAP1B,PNKD,SYT11,FMR1	grey	VSMC_M10
GO:0061384	0.46	0.69	15	27	heart trabecula morphogenesis	BP	1	ADGRG6,FOXH1,TEK,SRF,FHL2,OVOL2,NRG1,ADAMTS1,TGFB2,BMPR1A,RBPJ,MED1,ENG,NOG,HEY2	grey	VSMC_M10
GO:0046512	0.46	0.77	6	10	sphingosine biosynthetic process	BP	1	SPTLC1,SPHK2,ACER3,SPTLC3,ASAH1,ACER2	grey	VSMC_M10
GO:0046520	0.46	0.77	6	10	sphingoid biosynthetic process	BP	1	SPTLC1,SPHK2,ACER3,SPTLC3,ASAH1,ACER2	grey	VSMC_M10
GO:0043153	0.46	0.70	13	23	entrainment of circadian clock by photoperiod	BP	1	FBXL6,CRY1,PPP1CB,BHLHE40,CRY2,SIK1,USP2,PER2,CRTC1,FBXL22,PER3,FBXL8,FBXL12	grey	VSMC_M10
GO:0048066	0.46	0.67	18	32	developmental pigmentation	BP	1	SOX10,KIT,MITF,ANKRD27,MEF2C,ADAMTS20,OCA2,USP13,HPS4,BLOC1S6,MREG,LRMDA,CITED1,SLC45A2,AP1G1,BLOC1S3,RAB32,BCL2L11	grey	VSMC_M10
GO:0046078	0.46	0.79	5	8	dUMP metabolic process	BP	1	DPYS,UPB1,NT5M,DCTD,UPP1	grey	VSMC_M10
GO:0005513	0.46	0.77	6	10	detection of calcium ion	BP	1	SYT1,CASQ2,KCNIP2,RYR2,CALM1,KCNMB4	grey	VSMC_M10
GO:0002224	0.46	0.62	50	92	toll-like receptor signaling pathway	BP	1	TNFAIP3,ESR1,GRAMD4,LRRC14,NINJ1,PRKCE,RTN4,PELI1,RSAD2,TLR4,CD300LF,MYD88,IRAK2,NMI,IRF7,MFHAS1,TRAF6,NR1H4,OTUD4,NR1H3,RIPK2,AP3B1,F2RL1,NOD2,CD40,RAB7B,MAPKAPK3,UNC93B1,TLR5,MAPKAPK2,RAB11FIP2,DDX3X,TNIP2,BIRC3,GPS2,WDFY1,S100A14,BTK,TNIP1,TRAF3,COLEC12,GPR108,TICAM1,PDPK1,CYBA,GFI1,BCL10,DAB2IP,OAS1,HAVCR2	grey	VSMC_M10
GO:0032438	0.46	0.69	13	23	melanosome organization	BP	1	AP3B1,DTNBP1,KIF13A,SHROOM2,HPS4,PIKFYVE,BLOC1S6,ABCB6,SNAPIN,AP1G1,BLOC1S3,RAB32,TRAPPC6A	grey	VSMC_M10
GO:2000273	0.46	0.66	20	36	positive regulation of signaling receptor activity	BP	1	EPGN,AREG,TGFA,HDAC2,HBEGF,ADRA2B,FBXW7,IFNGR2,NEURL1,HDAC1,ADRA2A,NCF1,EGF,ADRA2C,PINK1,NLGN3,TAF1,CCL2,PARK7,EREG	grey	VSMC_M10
GO:0032049	0.46	0.79	5	8	cardiolipin biosynthetic process	BP	1	PLA2G6,TAMM41,CRLS1,SLC27A1,PTPMT1	grey	VSMC_M10
GO:1904646	0.46	0.67	19	34	cellular response to amyloid-beta	BP	1	LRP1,AGER,FPR2,TLR4,FOXO3,ATP1A3,PRNP,BACE1,SNX6,ITGA4,EPHA4,PARP1,CACNB1,PSEN1,CACNA1A,NGFR,LCN2,BCL2L11,BCL2L2	grey	VSMC_M10
GO:0043270	0.46	0.59	98	182	positive regulation of ion transport	BP	1	STAC3,SLC12A2,TSPO,BAK1,MCHR1,PLA2G6,ABCB1,WNK2,SPHK2,TRPC3,CACNB2,STAC,SCN5A,GPD1L,SYT1,P2RX2,GLRX,P2RX3,ORAI1,P2RX7,P2RY1,KCNJ11,HOMER1,IFNGR2,STC1,F2R,TESC,ANO6,KCNE2,PKD2,PLA2R1,CNTN1,KCNIP2,RYR2,FAM173B,PKP2,KCNJ2,SLC38A3,MAP2K6,ADRA2A,COX17,ANK2,GPER1,ABCB11,AGT,ACE2,SLC9A1,CASQ1,SNX4,NTSR1,SCN4B,NOS1AP,DLG1,PLA2G4A,CEMIP,CASK,HTT,PINK1,ATP1B1,KCNA1,ARL6IP1,CHRM1,HAP1,WNK3,STAC2,ATP1B2,PSEN1,NLGN3,CHRNB2,ATP2A1,CD19,CALM1,DNM2,VMP1,BDKRB1,TRPV2,CAMK2A,CCL5,CCL2,ABAT,TRPV3,PDPK1,P2RX1,RNF207,AMIGO1,ATP2C2,LCN2,STXBP1,MIR21,DMD,JPH2,KCNH2,THY1,CRH,PTGES,APOPT1,CEBPB,NKX2-5	grey	VSMC_M10
GO:0048147	0.46	0.69	13	23	negative regulation of fibroblast proliferation	BP	1	B4GALT7,MYC,MED25,TRIM32,DACH1,PAWR,PEX2,TP53INP1,MORC3,FTH1,SKI,DAB2IP,C1QL4	grey	VSMC_M10
GO:0090382	0.46	0.69	13	23	phagosome maturation	BP	1	RAB7A,MYO7A,RAB7B,TCIRG1,SLAMF8,SPG11,PIKFYVE,MREG,SYT7,SYT11,AC138894.1,RAB32,RAB39A	grey	VSMC_M10
GO:1902306	0.46	0.75	7	12	negative regulation of sodium ion transmembrane transport	BP	1	NEDD4L,PRKCE,HECW1,OSR1,HECW2,ATP1A2,MIR24-1	grey	VSMC_M10
GO:0031340	0.46	0.77	6	10	positive regulation of vesicle fusion	BP	1	AKT2,SYT1,SYT9,C2CD5,ANXA2,DOC2B	grey	VSMC_M10
GO:0018195	0.46	0.71	11	19	peptidyl-arginine modification	BP	1	PRDM4,NDUFAF7,NR1H4,PRMT9,ART3,PRMT3,PADI2,KRTCAP2,PARK7,PRMT7,PRMT6	grey	VSMC_M10
GO:0032305	0.47	0.77	6	10	positive regulation of icosanoid secretion	BP	1	PLA2G6,PLA2R1,MAP2K6,NTSR1,PLA2G4A,PTGES	grey	VSMC_M10
GO:0010765	0.47	0.68	15	27	positive regulation of sodium ion transport	BP	1	WNK2,SCN5A,GPD1L,GLRX,TESC,CNTN1,PKP2,SLC9A1,SCN4B,ATP1B1,WNK3,ATP1B2,DNM2,DMD,NKX2-5	grey	VSMC_M10
GO:1903313	0.47	0.61	57	105	positive regulation of mRNA metabolic process	BP	1	PAN2,CNOT2,CNOT6,RIDA,PABPC1,SNW1,PNLDC1,AGO2,TNRC6B,TUT7,GTPBP1,HSF1,EXOSC3,TRA2A,PNPT1,TUT4,ZC3HAV1,MLH1,TNRC6C,NUP98,LSM1,PAN3,CNOT1,CELF1,CNOT6L,FTO,TENT4B,RBM24,DIS3L2,GIGYF2,HSPA8,YTHDF2,YTHDF1,CELF3,NCBP2,EIF4ENIF1,RC3H1,UPF1,SAMD4A,POLR2G,LEO1,NT5C3B,DCP1B,PRPF19,TARDBP,TOB1,TNRC6A,CNOT8,PARN,METTL16,PLEKHN1,CIRBP,METTL14,NANOS1,ZFP36L2,RC3H2,EXOSC5	grey	VSMC_M10
GO:0021859	0.47	0.75	7	12	pyramidal neuron differentiation	BP	1	UQCRQ,UNC5D,DCLK2,SLC4A10,DISC1,PLXNA3,FOXG1	grey	VSMC_M10

GO:0046834	0.47	0.75	7	12	lipid phosphorylation	BP	1	DGKI,AGK,SPHK2,DGKD,DGKG,DGKQ,DGKE	grey	VSMC_M10
GO:0038203	0.47	0.75	7	12	TORC2 signaling	BP	1	SESN3,NCKAP1L,SYAP1,SIK3,PINK1,OTUD5,MAPKAP1	grey	VSMC_M10
GO:2000651	0.47	0.73	8	14	positive regulation of sodium ion transmembrane transporter activity	BP	1	WNK2,GLRX,TESC,SLC9A1,ATP1B1,WNK3,DNM2,DMD	grey	VSMC_M10
GO:0031116	0.47	0.68	16	29	positive regulation of microtubule polymerization	BP	1	HSPA1B,SLAIN2,MET,CDK5RAP2,MAPRE1,SLAIN1,NAV3,CLIP1,CDKN1B,MAPT,TOGARAM1,NIN,CDK5R1,MECP2,PSRC1,DRG1	grey	VSMC_M10
GO:0038127	0.47	0.61	55	102	ERBB signaling pathway	BP	1	PTK2,NCK2,RTN4,ACP4,EPGN,AREG,TGFA,EGFR,GAB1,BRAF,RAB7A,SOX9,NEU3,SNX5,HBEGF,PDE6G,NRG1,FER,HIP1R,PLCE1,FBXW7,PTPRJ,WDR54,PTK2B,NEURL1,ADRA2A,SLC30A10,RNF115,NCF1,EGF,GPER1,DGKD,IFI6,AGT,CBL,REPS2,PDE6H,SNX6,ZFYVE28,SOCS4,MAPK1,HAP1,RBPJ,PSEN1,RPS6KA5,GAREM1,PTPN2,PDPK1,RNF126,MVB12B,DAB2IP,MIR29A,MIR21,GRB2,EREG	grey	VSMC_M10
GO:0010470	0.47	0.75	7	12	regulation of gastrulation	BP	1	OSR1,CLASP2,POGLUT1,DAG1,PHLDB1,LHX1,TGIF2	grey	VSMC_M10
GO:1903729	0.47	0.73	8	14	regulation of plasma membrane organization	BP	1	ATP8B1,ASAP1,MYH9,RAB3A,NOX5,SYTL4,DNM2,SH3GLB1	grey	VSMC_M10
GO:0071108	0.47	0.67	17	31	protein K48-linked deubiquitination	BP	1	TNFAIP3,USP15,MINDY4,OTUD4,USP25,OTUD7A,USP13,YOD1,USP19,OTUD7B,USP37,OTUD3,OTUD5,MINDY2,OTUB2,PARK7,USP20	grey	VSMC_M10
GO:0072583	0.47	0.65	25	46	clathrin-dependent endocytosis	BP	1	CANX,SMAP1,AAK1,AP2S1,NEU3,FCHSD2,PIK3CB,HIP1R,FCHO2,BMP2K,SLC9B2,DGKD,AP2M1,SH3GL3,ITGA4,GAK,SYT11,DNM2,AP2B1,SGIP1,UNC119,PIP5K1C,GPR107,FNBP1L,BTBD8	grey	VSMC_M10
GO:0010919	0.47	0.76	6	10	regulation of inositol phosphate biosynthetic process	BP	1	PLEK,P2RY1,PTK2B,GPER1,NTSR1,PTH1R	grey	VSMC_M10
GO:0048563	0.47	0.79	5	8	post-embryonic animal organ morphogenesis	BP	1	BAK1,MYO7A,FBN1,MFAP2,BCL2L11	grey	VSMC_M10
GO:0009147	0.47	0.70	11	19	pyrimidine nucleoside triphosphate metabolic process	BP	1	NME1,TBPL1,ENTPD4,NME4,CTPS2,DTYMK,NME7,NME3,UCK1,CMPK2,NME6	grey	VSMC_M10
GO:0000266	0.47	0.65	22	40	mitochondrial fission	BP	1	PRKN,VPS35,DDHD2,DCN,OPA1,AP3B1,DNM1L,MTFP1,MAPT,FIS1,C11orf65,MCU,DDHD1,PINK1,MUL1,STAT2,COX10,MIEF2,RALBP1,DHODH,RALA,MIEF1	grey	VSMC_M10
GO:0071347	0.47	0.62	41	75	cellular response to interleukin-1	BP	1	MAPK13,IL1R2,VRK2,CXCL8,MYLK3,BMI1,SOX9,MYD88,IRAK2,IKBKB,PLCB1,ST18,TRAF6,OTUD4,CCL17,NKX3- 1,ABCC2,TOLLIP,CD40,HYAL1,HYAL3,RC3H1,UPF1,TNIP2,MAPK3,RPS6KA5,YY1,CCL14,CCL5,CCL7,CCL2,CCL25,ADAMTS12,LCN2,DAB2IP,MIR21,MIR27A,CCL16,CEBPB,RPS6KA4,CCL22	grey	VSMC_M10
GO:0045725	0.47	0.74	7	12	positive regulation of glycogen biosynthetic process	BP	1	EPM2AIP1,AKT2,SORBS1,IGF2,IRS1,PPP1R3E,INSR	grey	VSMC_M10
GO:0045654	0.47	0.76	6	10	positive regulation of megakaryocyte differentiation	BP	1	ZNF16,MTURN,TESC,RAB7B,THPO,RCOR1	grey	VSMC_M10
GO:0090370	0.47	0.82	4	6	negative regulation of cholesterol efflux	BP	1	EGF,MIR27B,MIR27A,MIR33B	grey	VSMC_M10
GO:1902033	0.47	0.76	6	10	regulation of hematopoietic stem cell proliferation	BP	1	PIM1,EIF2AK2,THPO,KAT7,MIR222,ATXN1L	grey	VSMC_M10
GO:0048339	0.47	0.73	8	14	paraxial mesoderm development	BP	1	EXOC4,TEAD2,WNT11,TCF15,POGLUT1,BMPR1A,SMAD3,WNT5A	grey	VSMC_M10
GO:0006613	0.47	0.70	12	21	cotranslational protein targeting to membrane	BP	1	SIL1,TRAM2,SEC61B,SEC62,SRP14,SSR3,SRPRA,ZFAND2B,ARL6IP1,SSR2,TRAM1L1,SEC61A2	grey	VSMC_M10
GO:0038202	0.47	0.65	22	40	TORC1 signaling	BP	1	RRAGD,MFSD8,KPTN,SESN3,SZT2,PIP4P1,RPTOR,LARS,GPR137B,DYRK3,SIK3,GPR137,OTUD5,CARD11,RRAGB,DGKQ,LARP1,SMCR8,CLEC16A,MLST8,LAMTOR5,RNF152	grey	VSMC_M10
GO:1902622	0.47	0.66	19	34	regulation of neutrophil migration	BP	1	RAC2,CXCL8,C5AR2,C5AR1,MYD88,NCKAP1L,NOD2,DNM1L,CD99,SLAMF8,MOSPD2,ADAM8,MCU,CXCR2,BST1,DAPK2,CD74,MPP1,DPP4	grey	VSMC_M10
GO:0098787	0.47	0.74	7	12	mRNA cleavage involved in mRNA processing	BP	1	CPSF6,CPSF1,CPSF2,NCBP2,CPSF4,AP002990.1,CSTF2	grey	VSMC_M10
GO:0046462	0.47	0.76	6	10	monoacylglycerol metabolic process	BP	1	ABHD16A,FAAH,ABHD6,PLA2G4A,MOGAT3,ABHD12B	grey	VSMC_M10
GO:0035282	0.47	0.62	40	74	segmentation	BP	1	FRS2,ROR2,MEOX2,HOXA2,OSR1,BMI1,ABI1,PCDH8,XRCC2,SEMA3A,PRKDC,TCF15,POFUT1,FAM208A,NKX3- 1,NKD1,IRX2,SEMA3F,POGLUT1,AXIN2,HOXD8,FZD5,MEOX1,DVL2,BMPR1A,TCAP,RBPJ,ALDH1A2,PSEN1,SMAD3,LHX1,CDX1,MIB1,NLE1,WDR77,FOXF1,MTF2,MAFB,WNT5A,BASP1	grey	VSMC_M10
GO:0032328	0.47	0.76	6	10	alanine transport	BP	1	SLC1A4,SLC38A2,SLC38A3,SLC38A5,SLC3A2,SLC36A1	grey	VSMC_M10
GO:0033628	0.47	0.65	22	40	regulation of cell adhesion mediated by integrin	BP	1	PTK2,PODXL,IFT74,ITGB1BP1,FERMT1,RAC3,NCKAP1L,RET,LPXN,TESC,TGFB2,CIB1,PTPN6,FERMT3,MUC1,EFNA1,CCL5,CRK,PIEZO1,DPP4,ACER2,LIF	grey	VSMC_M10
GO:2000403	0.47	0.70	12	21	positive regulation of lymphocyte migration	BP	1	CCL27,OXSR1,JAM2,PTK2B,ADAM8,ITGA4,TMEM102,CCL5,CCL7,RHOA,FADD,WNT5A	grey	VSMC_M10
GO:0021895	0.47	0.73	8	14	cerebral cortex neuron differentiation	BP	1	RAC3,TOX,ZNF335,DLX1,PSEN1,SIN3A,CHD5,CNTN2	grey	VSMC_M10
GO:0098703	0.47	0.73	8	14	calcium ion import across plasma membrane	BP	1	SLC8A2,PRNP,TRPV4,FAM155B,TRPV2,TRPV3,TRPV1,P2RX1	grey	VSMC_M10
GO:1902656	0.47	0.73	8	14	calcium ion import into cytosol	BP	1	SLC8A2,PRNP,TRPV4,FAM155B,TRPV2,TRPV3,TRPV1,P2RX1	grey	VSMC_M10
GO:0033006	0.47	0.68	14	25	regulation of mast cell activation involved in immune response	BP	1	ENPP3,RAC2,SPHK2,GAB2,UNC13D,FER,FGR,SNX4,C12orf4,IL4R,STXBP2,PDPK1,FOXF1,STXBP1	grey	VSMC_M10
GO:0045722	0.47	0.74	7	12	positive regulation of gluconeogenesis	BP	1	PPARA,GPT,PPP4R3B,DDB1,PTPN2,NNMT,ARPP19	grey	VSMC_M10
GO:2000659	0.47	0.78	5	8	regulation of interleukin-1-mediated signaling pathway	BP	1	IL1R2,VRK2,OTUD4,MIR21,MIR27A	grey	VSMC_M10
GO:0044090	0.47	0.74	7	12	positive regulation of vacuole organization	BP	1	PIP4K2C,PIP4K2A,RALB,ULK1,ANXA2,KIAA1324,SH3GLB1	grey	VSMC_M10
GO:0070933	0.47	0.76	6	10	histone H4 deacetylation	BP	1	HDAC2,SIRT2,SIRT7,HDAC1,HDAC4,RCOR1	grey	VSMC_M10
GO:0045911	0.47	0.66	20	36	positive regulation of DNA recombination	BP	1	MRNIP,UBE2B,EXOSC3,MSH2,HMCES,ERCC2,MLH1,PAXIP1,WDR48,SHLD1,ERCC6,CD40,CD28,ZCWPW1,WAS,PARP1,SHLD2,TBX21,WRAP53,OOEP	grey	VSMC_M10
GO:0045913	0.47	0.63	31	57	positive regulation of carbohydrate metabolic process	BP	1	PPARA,PRXL2C,GPT,PRKCE,PPP4R3B,SLC4A4,EPM2AIP1,AKT2,SORBS1,GPD1,P2RX7,P2RY1,IGF2,GPLD1,IRS1,ZBTB20,PPP1R3E,EGF,GPER1,ARNT,ACTN3,NTSR1,MLXIPL,PTH1R,DDB1,PSEN1,INSR,PTPN2,ENTPD5,NNMT,ARPP19	grey	VSMC_M10
GO:0030431	0.47	0.76	6	10	sleep	BP	1	BTBD9,SRD5A1,FOS,CHRNB2,PER3,CRH	grey	VSMC_M10

GO:0009648	0.47	0.68	14	25	photoperiodism	BP	1	FBXL6,CRY1,AANAT,PPP1CB,BHLHE40,CRY2,SIK1,USP2,PER2,CRTC1,FBXL22,PER3,FBXL8,FBXL12	grey	VSMC_M10
GO:0072132	0.47	0.64	24	44	mesenchyme morphogenesis	BP	1	MYC,EXOC4,OSR1,SOX9,WNT11,TGFBR2,ROBO1,ROBO2,ACTG2,TGFB2,SNAI1,ACTC1,BMPR1A,RBPJ,TMEM100,SMAD3,TBX2,ISL1,FOXF1,ENG,ACVRL1,NOG,WNT5A,HEY2	grey	VSMC_M10
GO:0070234	0.47	0.76	6	10	positive regulation of T cell apoptotic process	BP	1	IDO1,ZC3H8,PDCD1,ADAM8,CCL5,WNT5A	grey	VSMC_M10
GO:0032543	0.47	0.62	40	72	mitochondrial translation	BP	1	SHMT2,TSFM,RMND1,LRPPRC,MTIF2,QRSL1,MTRF1,MRPS12,HARS,MRPS27,MRPS28,NSUN3,WARS2,MRPS17,GATC,RPUSD4,YARS2,MRPS35,MRPL52,RCC1L,MRPS11,MRPS21,TARS2,MTG2,LARS2,DARS2,COA3,MRPL44,ALKBH1,EARS2,NDUFA7,MRPS34,MRPS2,MRPS6,RPUSD3,MRPL57,MRPL51,UQCC2,TRMT10C,MRPL16	grey	VSMC_M10
GO:0010842	0.47	0.70	10	18	retina layer formation	BP	1	SLC1A1,TFAP2B,RDH13,TSPAN12,TFAP2A,OBSL1,SDK1,MEGF11,LHX1,FJX1	grey	VSMC_M10
GO:0009086	0.47	0.76	6	10	methionine biosynthetic process	BP	1	MTAP,APIP,ENOPH1,MTRR,MTR,MTHFD2L	grey	VSMC_M10
GO:0002710	0.47	0.74	7	12	negative regulation of T cell mediated immunity	BP	1	IL4I1,NCKAP1L,NOD2,PPP3CB,FOXP3,TBX21,IL7R	grey	VSMC_M10
GO:0044068	0.47	0.76	6	10	modulation by symbiont of host cellular process	BP	1	ATG5,ULK1,ANO6,TNIP1,IRGM,SERPINB9	grey	VSMC_M10
GO:0042953	0.47	0.74	7	12	lipoprotein transport	BP	1	LRP1,APOB,CUBN,MIA3,PRKCB,VMP1,UNC119	grey	VSMC_M10
GO:0030149	0.47	0.69	13	23	sphingolipid catabolic process	BP	1	PPT1,SMPD2,ACER3,NEU3,ASAH1,SMPD4,GALC,GLA,HEXA,GM2A,CEL,ACER2,NEU2	grey	VSMC_M10
GO:0045454	0.47	0.66	18	32	cell redox homeostasis	BP	1	TXN2,TXNRD1,PRDX1,ERP44,DLD,TXNRD3,PRDX3,EGLN2,HVCN1,GLRX2,SLC2A10,NFE2L2,SLC11A1,NOS2,TXNDC2,NQO1,DDIT3,TXN	grey	VSMC_M10
GO:0003198	0.48	0.72	8	14	epithelial to mesenchymal transition involved in endocardial cushion formation	BP	1	TGFBR2,TGFB2,SNAI1,RBPJ,TMEM100,ENG,NOG,HEY2	grey	VSMC_M10
GO:0043647	0.48	0.64	23	42	inositol phosphate metabolic process	BP	1	SYNJ2,IPPK,INPP4A,PLEK,INPP4B,MTMR2,ITPKC,MTMR7,P2RY1,PTK2B,SYNJ1,ITPKA,ITPK1,INPP5A,GPER1,IP6K2,NTSR1,INPP5J,PTH1R,IMPA2,INPP5K,MINPP1,INPP1	grey	VSMC_M10
GO:2001032	0.48	0.68	14	25	regulation of double-strand break repair via nonhomologous end joining	BP	1	MRNIP,HSF1,POT1,CYREN,SIRT7,SHLD1,PRKDC,ERCC6,PARP9,DTX3L,SHLD2,WRAP53,SMCHD1,NUDT16L1	grey	VSMC_M10
GO:0072677	0.48	0.76	6	10	eosinophil migration	BP	1	ADAM8,DAPK2,CCL5,CCL7,CCL2,PTGER4	grey	VSMC_M10
GO:0098743	0.48	0.71	9	16	cell aggregation	BP	1	MAPK14,ROR2,MYCN,SOX9,SOX6,BMPR1B,THRA,PKD1,OTOR	grey	VSMC_M10
GO:1900153	0.48	0.72	8	14	positive regulation of nuclear-transcribed mRNA catabolic process, deadenylation-dependent decay	BP	1	PABPC1,AGO2,TNRC6B,TNRC6C,CNOT1,TOB1,NANOS1,ZFP36L2	grey	VSMC_M10
GO:0042130	0.48	0.65	22	40	negative regulation of T cell proliferation	BP	1	TNFRSF21,PELI1,IL4I1,FOXJ1,PAWR,PRNP,CASP3,TMEM131L,MARCH7,BTN2A2,PRKAR1A,DLG1,VSIR,RC3H1,PTPN6,SCGB1A1,FOXP3,ZBTB7B,TWSG1,GLMN,CEBPB,HAVCR2	grey	VSMC_M10
GO:0051013	0.48	0.74	7	12	microtubule severing	BP	1	VPS4B,KATNA1,KATNAL1,KATNB1,FIGN,TTLL11,FIGNL2	grey	VSMC_M10
GO:0061709	0.48	0.72	8	14	reticulophagy	BP	1	TMEM173,DDRGK1,ULK1,TEX264,UFC1,ATG2A,RETREG3,ATG2B	grey	VSMC_M10
GO:0021953	0.48	0.60	62	116	central nervous system neuron differentiation	BP	1	UQCRQ,PTCH1,AGTPBP1,PLXNA4,NTRK2,SMO,NDNF,GLI2,IFT172,MYCBP2,MNX1,POU4F1,TSKU,SZT2,RAC3,SEMA3A,EPHB1,TOX,ZSWIM6,DCLK2,FAIM2,RAPGEF2,NKX6-1,NR4A2,ZNF335,LDB1,SUFU,SLC4A10,SATB2,ADARB1,GIGYF2,TTC21B,DISC1,GBX2,DLX1,EPHB3,MAPT,MDGA2,NIN,SCYL3,TULP3,EPHA4,CLN8,MPP5,PSEN1,NDEL1,CHRNB2,BCL11B,LHX1,SIN3A,ISL1,CHD5,PLXNA3,LHX8,UBB,CNTN2,FOXG1,SOX4,GRID2,ISL2,LBX1,CEND1	grey	VSMC_M10
GO:0070989	0.48	0.74	7	12	oxidative demethylation	BP	1	ALKBH3,FTO,ALKBH2,TET1,CYP3A43,ALKBH1,ALKBH5	grey	VSMC_M10
GO:0060396	0.48	0.71	9	16	growth hormone receptor signaling pathway	BP	1	PTK2,LEPROTL1,PIK3R1,PXN,GDF15,STAT3,STAT5B,GHR,LEPROT	grey	VSMC_M10
GO:0071378	0.48	0.71	9	16	cellular response to growth hormone stimulus	BP	1	PTK2,LEPROTL1,PIK3R1,PXN,GDF15,STAT3,STAT5B,GHR,LEPROT	grey	VSMC_M10
GO:0000028	0.48	0.72	9	15	ribosomal small subunit assembly	BP	1	RRP7A,RPS14,RPSA,PRKDC,MRPS11,RPS27,XRCC5,ERAL1,RPS27L	grey	VSMC_M10
GO:0043523	0.48	0.58	91	169	regulation of neuron apoptotic process	BP	1	ERBB3,LRP1,AGAP2,PRKN,SYNGAP1,GRM4,ATF4,NTRK2,NRBP2,CNTFR,SLC1A1,TFAP2B,MSH2,PPT1,EPHA7,FOXO3,NTF4,NDNF,C5AR1,GRIK5,XRCC2,EGLN2,POU4F1,ARRB1,PRNP,GPI,CASP3,GBE1,FBXW7,TRIM2,MEF2C,FAIM2,PTK2B,NR4A2,F2R,NQO2,TFAP2A,BDNF,HDAC1,TERT,TGFB2,SLC30A10,KCNB1,KRAS,ADNP,MCL1,HYOU1,NFATC4,HDAC4,DLX1,BACE1,DNAJC5,FZD9,EGLN3,SNX6,MAP3K11,ADAM8,NONO,FIS1,PINK1,PARP1,PSEN1,ITGAM,AC138894.1,CITED1,ALKBH1,GPRASP2,BHLHB9,CCL2,CDK5R1,ISL1,JUN,PDPK1,MECP2,PARK7,LCN2,STXBP1,AARS,NQO1,NAE1,MIR125B1,RHOA,GRID2,CNTF,SIX1,DDIT3,BCL2L11,HRAS,CEBPB,PITX3,NMNAT1	grey	VSMC_M10
GO:0002690	0.48	0.61	39	71	positive regulation of leukocyte chemotaxis	BP	1	GPSM3,PTK2,RAC2,CCL27,FPR2,CXCL8,AKIRIN1,PTN,C5AR1,OXSR1,NCKAP1L,GAS6,VEGFC,PTK2B,F2RL1,ANO6,DNM1L,TRPV4,PRSS56,CMKLR1,MOSPD2,IL12A,MCU,TNFSF18,CXCR2,MAPK1,TMEM102,PGF,DAPK2,MAPK3,IL6R,S100A14,CD74,CCL5,CSF1R,CCL7,DEFB124,ZNF580,WNT5A	grey	VSMC_M10
GO:0098732	0.48	0.60	55	102	macromolecule deacylation	BP	1	BAZ2A,ABHD16A,PPT2,C6orf89,ABHD17B,PPT1,SPHK2,HDAC2,PRKD2,PHB,HDAC11,BEX4,TADA3,SIRT2,SIRT7,SIRT3,MTA3,LYPLA1,SDR16C5,ING2,HDAC7,SIRT5,SUDS3,ELK4,SIRT4,HDAC1,LYPLAL1,RCOR3,PER2,HDAC4,MAPT,BCL6,ABHD12B,RCOR2,PINK1,TBL1XR1,KDM5A,FOXP3,AKAP8L,AKAP8,JDP2,ZBTB7B,SFPQ,SIN3A,RCOR1,SREBF1,SKI,ABHD17A,MIER2,PRMT6,ABHD10,SAP30BP,ABHD17C,NNMT,LYPLA2	grey	VSMC_M10
GO:0043392	0.48	0.64	22	40	negative regulation of DNA binding	BP	1	HABP4,C6orf106,SMO,MDFI,ZNF462,HDAC2,RSF1,HAND2,FBXW7,SP100,PER2,MSX2,PSEN1,TAF1,WFIKKN2,SIN3A,ERCC4,JUN,WFIKKN1,DDIT3,ZNF593,HEY2	grey	VSMC_M10
GO:0016574	0.48	0.64	24	44	histone ubiquitination	BP	1	SKP1,UBE2B,RNF20,BMI1,UBE2E1,RYBP,DDB2,PCGF6,DTX3L,RNF168,PHC1,BCOR,ATXN7L3,LEO1,DDB1,PCGF3,TRIM37,OTUB2,CUL4B,PARK7,RNF8,UBE2N,UBE2U,CBX8	grey	VSMC_M10

GO:0048596	0.48	0.70	10	18	embryonic camera-type eye morphogenesis	BP	1	FRS2,IFT172,TFAP2A,PAX2,ALDH1A3,SP3,FZD5,TBX2,STRA6,WNT5A	grey	VSMC_M10
GO:0050922	0.48	0.64	25	46	negative regulation of chemotaxis	BP	1	SEMA6A,SEMA4D,SEMA4C,C5AR2,SEMA3D,SEMA3A,RYK,ROBO1,ROBO2,SEMA4F,SEMA3G,SEMA4G,SEMA3F,SLAMF8,PTPRO,WNT3,SEMA6D,PADI2,SEMA4A,CCL2,PTPN2,PLXNA3,DPP4,WNT5A,DUSP1	grey	VSMC_M10
GO:0031122	0.48	0.63	28	52	cytoplasmic microtubule organization	BP	1	TUBGCP6,SLAIN2,PLK3,IFT172,SLAIN1,CLIP4,CCDC13,HOOK3,CLIP1,TUBGCP5,SLK,TRPV4,TUBGCP4,CIB1,WDR73,LIMK2,DLG1,MAPT,TUBG1,CEP126,KPNB1,HOOK2,RAB11A,CCDC88C,CAMSAP3,FSD1,CAMSAP1,RHOA	grey	VSMC_M10
GO:0046475	0.48	0.67	14	25	glycerophospholipid catabolic process	BP	1	ABHD16A,PLA2G6,APOC1,PLA2G4B,PLA2G4A,ABHD12B,SMPD4,LIPC,GDPD3,LDLR,GDPD1,GDE1,PNPLA6,PLA2G15	grey	VSMC_M10
GO:0062013	0.48	0.60	52	96	positive regulation of small molecule metabolic process	BP	1	PPARA,PRXL2C,GUCA1B,GPT,PRKCE,PPP4R3B,SLC4A4,NR1H2,EPM2AIP1,AKT2,NR1H4,CYP7A1,SORBS1,GPD1,P2RX7,P2RY1,NR1H3,IGF2,GPLD1,IRS1,CPT1A,ZBTB20,PPP1R3E,GPER1,ARNT,LPGAT1,ACTN3,NTSR1,MLXIPL,SCAP,POR,PTH1R,COMT,WNT4,PINK1,STAT3,DDB1,PSEN1,CD74,INSR,SREBF1,NOS2,PTPN2,PARK7,MLYCD,ENTPD5,LDLRAP1,NNMT,STARD4,CLYBL,FGF1,ARPP19	grey	VSMC_M10
GO:0070302	0.48	0.58	81	151	regulation of stress-activated protein kinase signaling cascade	BP	1	PHLPP1,MAP3K5,STK3,MAPK8IP2,MAP3K4,PRKN,AGER,MYC,GADD45G,KLHL31,PRDX1,SEMA4C,DNAJA1,FKTN,EMC10,MTURN,NOD1,EGFR,TLR4,TRAF1,SDCBP,MYD88,ZMYND11,PLCB1,EIF2AK2,RELL2,SEMA3A,SH3RF2,ARL6IP5,MFHAS1,TRAF6,EPHB1,HAND2,PTK2B,RASGRP1,RIPK2,F2RL1,RIPK1,NOD2,TAOK3,TRPV4,PDCD4,TGFB2,NCF1,TRAF5,FZD7,MAP3K3,MAGI3,DLG1,MAP3K11,AIDA,DUSP10,MBIP,MEN1,MAP4K2,DUSP19,PDCD10,PINK1,LTBR,MAPK1,MINK1,GPS2,RELL1,TAOK2,MAPK3,MAP2K1,NCOR1,TRAF3,TAOK1,SERPINF2,MAPK8IP3,TRAF2,MARVELD3,DAB2IP,ZNF622,WNT5A,GADD45A,INAVA,DUSP1,DUSP22,HRAS	grey	VSMC_M10
GO:0019730	0.48	0.67	16	28	antimicrobial humoral response	BP	1	CXCL14,PLA2G6,GAPDH,CXCL8,NOD2,PPP2R3C,SLC11A1,SPON2,GATA6,FGA,S100A12,HIST1H2BK,CXCL6,JCHAIN,HIST1H2BF,HIST1H2BJ	grey	VSMC_M10
GO:0042761	0.48	0.73	7	12	very long-chain fatty acid biosynthetic process	BP	1	HACD4,ELOVL1,ELOVL7,HACD2,TECR,HACD3,ELOVL3	grey	VSMC_M10
GO:0003337	0.48	0.73	7	12	mesenchymal to epithelial transition involved in metanephros morphogenesis	BP	1	SMO,SIX2,PAX8,PAX2,SALL1,CITED1,LIF	grey	VSMC_M10
GO:0003143	0.48	0.63	27	50	embryonic heart tube morphogenesis	BP	1	SMO,FOXH1,SRF,TEAD2,BBS7,SETDB2,MEGF8,IFT172,TGFBR2,OVOL2,MKKS,HAND2,MEF2C,IFT52,PKD2,RYR2,SUFU,PSEN1,SMAD3,TBX2,MIB1,RNF207,ENG,LBX1,WNT5A,C2CD3,NKX2-5	grey	VSMC_M10
GO:1901569	0.48	0.75	6	10	fatty acid derivative catabolic process	BP	1	ABHD16A,HPGD,FITM2,OXCT1,NUDT19,LYPLA2	grey	VSMC_M10
GO:0006929	0.48	0.69	11	20	substrate-dependent cell migration	BP	1	ANKS1A,ITGB1BP1,SDCBP,ROBO1,VEGFC,STK4,ADAM8,FN1,NTN1,ITGA11,CSPG4	grey	VSMC_M10
GO:0001510	0.48	0.61	40	73	RNA methylation	BP	1	RNMT,TFB1M,TRMT11,CMTR1,MTO1,NSUN6,THUMPD3,TRMT6,WTAP,THUMPD2,TRMT9B,TGS1,DIMT1,BCDIN3D,NSUN3,TRMT10A,DALRD3,LCMT2,METTL8,NSUN5,METTL2B,TRMT1L,MEPCE,ALKBH8,TRMT5,HSD17B10,MRM2,ZCCHC4,METTL4,METTL6,MRM3,METTL16,CMTR2,METTL14,TRMT10B,TRMT61A,TRMT10C,TRMT61B,CBLL1,TYW3	grey	VSMC_M10
GO:0048670	0.48	0.71	9	16	regulation of collateral sprouting	BP	1	LRP1,FSTL4,SEMA4D,EPHA7,SPART,ULK1,SPP1,BDNF,WNT3	grey	VSMC_M10
GO:0090494	0.48	0.75	6	10	dopamine uptake	BP	1	PRKN,MAPK15,SLC6A2,SLC6A3,SLC29A4,PARK7	grey	VSMC_M10
GO:0071472	0.48	0.73	7	12	cellular response to salt stress	BP	1	XRCC6,AKR1B1,AQP1,TRPV4,XRCC5,MICU1,SLC25A23	grey	VSMC_M10
GO:0000722	0.48	0.73	7	12	telomere maintenance via recombination	BP	1	ERCC1,XRCC1,TPRKB,RAD51C,RAD51D,ERCC4,TERF2	grey	VSMC_M10
GO:2000402	0.48	0.73	7	12	negative regulation of lymphocyte migration	BP	1	ADTRP,CD200,APOD,MIA3,PADI2,CCL2,GCSAM	grey	VSMC_M10
GO:0034427	0.48	0.75	6	10	nuclear-transcribed mRNA catabolic process, exonucleolytic, 3'-5'	BP	1	SKIV2L,EXOSC4,EXOSC3,TTC37,DIS3L2,EXOSC5	grey	VSMC_M10
GO:0048246	0.49	0.66	15	27	macrophage chemotaxis	BP	1	PTK2,AKIRIN1,C5AR1,FAM19A4,MTUS1,PTK2B,TRPV4,CMKLR1,SLAMF8,TNFSF18,MAPK1,MAPK3,CCL5,CSF1R,CCL2	grey	VSMC_M10
GO:0045604	0.49	0.63	24	44	regulation of epidermal cell differentiation	BP	1	CYP27B1,PTCH1,CD109,HOXA7,MYCL,GRHL1,SFRP4,MYCN,PTCH2,OVOL2,MAFG,VDR,SRSF6,KDF1,SFN,KLF7,H2AFY2,ABCA12,MED1,KEAP1,MIR125B1,ZBED2,SGPP1,HEY2	grey	VSMC_M10
GO:0033623	0.49	0.72	8	14	regulation of integrin activation	BP	1	PLEK,FERMT1,CDH17,FARP2,RAP1B,FBLIM1,PTGER4,PIEZO1	grey	VSMC_M10
GO:0010867	0.49	0.73	7	12	positive regulation of triglyceride biosynthetic process	BP	1	NR1H2,NR1H3,GPLD1,SLC27A1,LDLR,SREBF1,CNEP1R1	grey	VSMC_M10
GO:1903861	0.49	0.69	10	18	positive regulation of dendrite extension	BP	1	NEDD4L,RIMS2,PRKN,CPNE5,RIMS1,CPNE9,SYT1,SLC23A2,MUL1,CACNG7	grey	VSMC_M10
GO:1901298	0.49	0.75	6	10	regulation of hydrogen peroxide-mediated programmed cell death	BP	1	MET,FOXO3,PAWR,PINK1,PARK7,MIR21	grey	VSMC_M10
GO:0030852	0.49	0.73	7	12	regulation of granulocyte differentiation	BP	1	IL5,TRIB1,TESC,HCLS1,ZBTB46,HAX1,EVI2B	grey	VSMC_M10
GO:0046006	0.49	0.67	14	25	regulation of activated T cell proliferation	BP	1	AGER,PRNP,CASP3,IGF2,ICOSLG,BTN2A2,GPAM,PRKAR1A,RC3H1,STAT5B,FOXP3,MIR21,FADD,TNFSF9	grey	VSMC_M10
GO:0032941	0.49	0.67	14	25	secretion by tissue	BP	1	PRKCE,AQP1,NR1H2,EGFR,ATG5,TRAF3IP2,NR1H3,CHRM3,COPA,CHRM1,SCNN1B,CELSR2,CEL,CYBA	grey	VSMC_M10
GO:0000492	0.49	0.75	6	10	box C/D snoRNP assembly	BP	1	C12orf45,TAF9,ZNHIT3,SNU13,ZNHIT6,PIH1D2	grey	VSMC_M10
GO:0048488	0.49	0.62	28	52	synaptic vesicle endocytosis	BP	1	CANX,SYNJ2,BTBD9,AMPH,ACTG1,SYT1,FCHO2,SYNJ1,SYT8,DNM1L,NLGN4Y,AP2M1,STX1A,DNM3,PPP3CB,ACTB,SLC2A4,NLGN2,DGKQ,GAK,SYT11,NLGN3,CALM1,DNM2,PIP5K1C,CEL,BTBD8,DENND1A	grey	VSMC_M10
GO:0140238	0.49	0.62	28	52	presynaptic endocytosis	BP	1	CANX,SYNJ2,BTBD9,AMPH,ACTG1,SYT1,FCHO2,SYNJ1,SYT8,DNM1L,NLGN4Y,AP2M1,STX1A,DNM3,PPP3CB,ACTB,SLC2A4,NLGN2,DGKQ,GAK,SYT11,NLGN3,CALM1,DNM2,PIP5K1C,CEL,BTBD8,DENND1A	grey	VSMC_M10
GO:0050855	0.49	0.71	8	14	regulation of B cell receptor signaling pathway	BP	1	PLCL2,FOXP1,LPXN,GPS2,CD19,PRKCB,CMTM3,GCSAM	grey	VSMC_M10

GO:0021879	0.49	0.65	16	29	forebrain neuron differentiation	BP	1	UQCRQ,NDNF,SEMA3A,ZSWIM6,DCLK2,RAPGEF2,SLC4A10,SATB2,DISC1,GBX2,DLX1,BCL11B,PLXNA3,LHX8,UBB,FOXG1	grey	VSMC_M10
GO:0003128	0.49	0.70	9	16	heart field specification	BP	1	FOXH1,WNT11,ROBO1,ROBO2,MEF2C,AXIN2,RBPJ,ISL1,WNT5A	grey	VSMC_M10
GO:0008334	0.49	0.70	9	16	histone mRNA metabolic process	BP	1	EXOSC4,LSM10,MTPAP,LSM1,TENT2,TENT4B,CPSF2,UPF1,ZNF473	grey	VSMC_M10
GO:0033151	0.49	0.71	8	14	V(D)J recombination	BP	1	TCF7,CYREN,PRKDC,XRCC4,DCAF1,BCL11B,YY1,NHEJ1	grey	VSMC_M10
GO:0002507	0.49	0.71	8	14	tolerance induction	BP	1	PHLPP1,TNFAIP3,FOXJ1,TGFBR2,MARCH7,FOXP3,HAVCR2,XKR8	grey	VSMC_M10
GO:0001845	0.49	0.71	8	14	phagolysosome assembly	BP	1	RAB7A,MYO7A,RAB7B,SPG11,PIKFYVE,SYT7,AC138894.1,RAB39A	grey	VSMC_M10
GO:0042180	0.49	0.57	81	150	cellular ketone metabolic process	BP	1	KDSR,ALDH8A1,PPARA,HSD17B3,AKR1B1,PRKCE,NR1H2,DKKL1,SLC7A11,SOX9,AKR1A1,APOC1,PRKAG2,SLC22A13,PIBF1,AKT2,IDO2,IDO1,PANK2,PDK1,NR1H4,TRIB3,CYP7A1,CBR4,DKK3,GPD1,NR1H3,COQ2,NQO2,CRYZL1,ERLIN1,CBR3,PRMT3,SIRT5,LONP2,SIRT4,SRD5A1,COQ5,IRS1,CPT1A,COQ10B,SLC7A7,ABCB11,DHRS9,LPGAT1,MLXIPL,SCAP,PDK3,ACADL,NMT1,TYSND1,COMT,PNKD,WNT4,NDUFA9,STAT5B,ACADVL,COQ8A,HSD17B10,FGFR4,DGKQ,AC138894.1,SCNN1B,CYP46A1,NCOR1,CD74,SREBF1,OXCT1,CACNA1H,PARK7,MLYCD,KYAT1,COQ4,KYAT3,MIR21,FABP5,GLO1,PDSS2,STARD4,INSIG2,ADH4	grey	VSMC_M10
GO:0032770	0.49	0.66	15	27	positive regulation of monooxygenase activity	BP	1	CYP27B1,NOD1,DHFR,PIK3CB,PTK2B,VDR,TERT,KRAS,NOS1AP,POR,GCH1,CALM1,PARK7,TERF2,FCER2	grey	VSMC_M10
GO:0030220	0.49	0.70	9	16	platelet formation	BP	1	MPIG6B,MYH9,SRF,PRKDC,CASP3,MEF2C,CIB1,NBEAL2,PTPN6	grey	VSMC_M10
GO:0036344	0.49	0.70	9	16	platelet morphogenesis	BP	1	MPIG6B,MYH9,SRF,PRKDC,CASP3,MEF2C,CIB1,NBEAL2,PTPN6	grey	VSMC_M10
GO:0044788	0.49	0.66	14	25	modulation by host of viral process	BP	1	YTHDC2,IGF2R,PRKN,PHB,FBXL2,EIF2AK4,CFL1,HSPA8,PC,PSMC3,FMR1,MIR222,TBC1D20,ZNF502	grey	VSMC_M10
GO:0086019	0.49	0.67	13	24	cell-cell signaling involved in cardiac conduction	BP	1	TRPM4,CACNB2,SCN5A,KCNJ3,TBX5,CASQ2,RYR2,PKP2,ANK2,SCN4B,CACNA1G,RNF207,HCN4	grey	VSMC_M10
GO:0050770	0.49	0.58	70	132	regulation of axonogenesis	BP	1	LRP1,SEMA6A,TIAM2,FSTL4,SYNGAP1,PLXNA4,NTRK2,SRF,SEMA4D,SEMA4C,BRSK1,RTN4,RUFY3,EPHA7,KEL,PLXNA1,GOLGA4,MEGF8,SEMA3D,SPART,PLXNC1,SEMA3A,ULK1,RYK,RET,ROBO1,ROBO2,SEMA4F,TIAM1,ZFYVE27,SEMA3G,SPP1,CYFIP1,SEMA4G,BDNF,KIAA0319,SEMA3F,ADNP,DISC1,CDKL5,EPHB3,WNT3,MAPT,SEMA6D,TNR,FN1,NIN,MARK2,EPHA4,PSEN1,SEMA4A,TRIM46,EFNA1,NDEL1,RAB11A,MAP2K1,NTN1,TRPV2,CDK5R1,DAB1,L1CAM,ARHGAP4,PLXNA3,AMIGO1,CNTN2,THY1,WNT5A,POU3F2,CHN1,EFNB3	grey	VSMC_M10
GO:0048311	0.49	0.70	9	16	mitochondrion distribution	BP	1	ARMC1,OPA1,DNM1L,MAPT,BHLHA15,HAP1,MSTO1,MTM1,CLUH	grey	VSMC_M10
GO:0009070	0.49	0.72	7	12	serine family amino acid biosynthetic process	BP	1	SHMT2,PSAT1,DHFR,SERINC5,CBSL,SHMT1,PHGDH	grey	VSMC_M10
GO:0090239	0.49	0.72	7	12	regulation of histone H4 acetylation	BP	1	ATG5,IWS1,ARRB1,TAF7,AUTS2,KAT7,MUC1	grey	VSMC_M10
GO:0034638	0.49	0.72	7	12	phosphatidylcholine catabolic process	BP	1	PLA2G6,APOC1,PLA2G4A,LIPC,GDPD3,LDLR,PLA2G15	grey	VSMC_M10
GO:0097028	0.49	0.65	16	29	dendritic cell differentiation	BP	1	AGER,TGFBR2,GAS6,TRAF6,CAMK4,F2RL1,ITGB6,ZBTB46,BATF2,LTBR,RBPJ,PSEN1,BATF,DHRS2,CEBPB,BATF3	grey	VSMC_M10
GO:0051148	0.49	0.61	30	55	negative regulation of muscle cell differentiation	BP	1	NFATC1,FRS2,PPARA,PI16,ANKRD17,SOX6,ZBED6,IGF2,BDNF,PDCD4,FZD7,HDAC4,BHLHA15,BMPR1A,FOXO4,RBPMS2,YY1,MECP2,PLPP7,NFATC3,MIR199A1,MIR21,MIR222,PRDM6,RGS4,TCF23,YBX1,EREG,NKX2-5,HEY2	grey	VSMC_M10
GO:0019362	0.49	0.67	13	23	pyridine nucleotide metabolic process	BP	1	NMRK1,SLC22A13,IDO2,IDO1,SLC5A8,NUDT12,NUDT17,PARP9,NAXE,NADK,NNMT,NAPRT,NMNAT1	grey	VSMC_M10
GO:0046496	0.49	0.67	13	23	nicotinamide nucleotide metabolic process	BP	1	NMRK1,SLC22A13,IDO2,IDO1,SLC5A8,NUDT12,NUDT17,PARP9,NAXE,NADK,NNMT,NAPRT,NMNAT1	grey	VSMC_M10
GO:0033540	0.49	0.72	7	12	fatty acid beta-oxidation using acyl-CoA oxidase	BP	1	CROT,ACOX1,ACOXL,ACOX2,EHHADH,DECR2,AMACR	grey	VSMC_M10
GO:0072189	0.50	0.70	9	16	ureter development	BP	1	OSR1,SOX9,RET,PAX2,ALDH1A2,LHX1,SOX8,FOXF1,SIX1	grey	VSMC_M10
GO:0006140	0.50	0.61	31	57	regulation of nucleotide metabolic process	BP	1	PPARA,PRXL2C,GUCA1B,SLC4A4,SPHK2,LACC1,PRKAG2,PDK1,GPD1,P2RX7,PRKAG1,FAM173B,ZBTB20,ARNT,ACTN3,HDAC4,DNAJC30,MLXIPL,PDK3,PINK1,STAT3,PARP1,PSEN1,OGT,NCOR1,INSR,NOS2,SLC2A6,ENTPD5,PDZD3,TIGAR	grey	VSMC_M10
GO:0090342	0.50	0.63	23	42	regulation of cell aging	BP	1	CDKN2A,NUAK1,CDK6,PNPT1,ZNF277,PAWR,PRKDC,PLK2,ING2,ARNTL,TERT,SLC30A10,KRAS,MAP3K3,BCL6,BMPR1A,YPEL3,VASH1,FZR1,TERF2,MIR21,MIR22,YBX1	grey	VSMC_M10
GO:0031060	0.50	0.61	28	52	regulation of histone methylation	BP	1	SNW1,PHF1,RNF20,SETD7,IWS1,KDM4A,PAXIP1,CHTOP,NSD3,DNMT3B,RTF1,ZNF335,JARID2,AUTS2,TET1,BCOR,WDR61,NELFA,NSD1,OGT,SUPT6H,MECP2,MTF2,GFI1,CTCF,PRMT6,ZNF304,NNMT	grey	VSMC_M10
GO:0060546	0.50	0.71	8	14	negative regulation of necroptotic process	BP	1	PELI1,RIPK1,FZD9,YBX3,BIRC3,MIR22,FADD,CASP8	grey	VSMC_M10
GO:0046676	0.50	0.64	17	31	negative regulation of insulin secretion	BP	1	PRKN,BMP8A,NDUFAF2,FAM3D,KCNJ11,ACVR1C,SIRT4,IRS1,HDAC1,PPP3CA,ADRA2A,KCNB1,KLF7,ENY2,SYTL4,SREBF1,CHGA	grey	VSMC_M10
GO:0043627	0.50	0.61	31	57	response to estrogen	BP	1	CYP27B1,ESR1,TPH2,TNFRSF11B,TEK,TGFBR2,SRA1,ASH2L,ARID5A,NCOA4,ZNF366,KMT2D,ABCC2,RBBP5,SRD5A1,CCND1,DTYMK,COMT,PTPRN,MAPK1,CITED1,IL4R,BCAS3,GATA6,KRT19,TRIM24,CA2,BGLAP,CRH,SERPINB9,SFR1	grey	VSMC_M10
GO:0048535	0.50	0.71	8	14	lymph node development	BP	1	IL15,TRAF3IP2,TOX,RC3H1,IL7R,FADD,CD248,RC3H2	grey	VSMC_M10
GO:0048520	0.50	0.72	7	12	positive regulation of behavior	BP	1	HDAC2,MEF2C,CFAP20,HDAC4,STRA6,SGIP1,CRH	grey	VSMC_M10
GO:0048532	0.50	0.69	9	16	anatomical structure arrangement	BP	1	PLXNA4,SEMA3A,TFAP2A,PAX2,SEMA3F,BMPR1A,DAB1,PLXNA3,HSPA5	grey	VSMC_M10
GO:0046189	0.50	0.65	16	29	phenol-containing compound biosynthetic process	BP	1	TPH2,GPR37,DDC,SLC7A11,HAND2,RAPGEF2,NR4A2,OCA2,SLC6A3,TGFB2,GCH1,HDC,CITED1,SLC45A2,PARK7,WNT5A	grey	VSMC_M10

GO:0034762	0.50	0.54	211	396	regulation of transmembrane transport	BP	1	NEDD4L,STAC3,MAPK8IP2,KCNV1,PRRT1,GOPC,CATSPER3,MAPK14,KCNJ4,PLA2G6,PIM1,ABCB1,CLIC5,SLC25A27,WNK2,TSPAN13,PRKCE,KCNQ4,SHANK1,SLC31A2,GRB10,HECW1,TCAF2,CLCN1,KEL,ATG5,OSR1,FGF21,BRAF,SPHK2,KCNJ14,SLC26A5,KCNRG,CACNB2,STAC,PRKAG2,OXSR1,SCN5A,SCN1A,AKT2,GPD1L,CACNB4,ASPSCR1,NALCN,PRNP,DIAPH1,CLIC1,ARL6IP5,KCNIP3,TRIB3,PIK3R1,SLMAP,SLC43A1,GLRX,SORBS1,MEF2C,P2RX7,HVCN1,KCNH3,PTK2B,KCNJ11,TPCN1,HOMER1,IFNGR2,KCNJ3,F2R,TESC,RIPK1,ANO6,KCNK15,KCNE2,PKD2,CASQ2,KCNJ15,KCNIP2,CHRM3,RYR2,FAM173B,PM20D1,AZIN2,IRS1,CALHM1,HECW2,KCNJ2,TERT,ADRA2A,ACSL5,TCIRG1,COX17,SCN2A,ISCU,KCNB1,KCNG1,SCN7A,UBASH3B,ANK2,C2CD5,GPER1,KLHL24,AGT,ACE2,ATP1A2,SLC9A1,PER2,C4orf3,CASQ1,PEA15,NTSR1,SCN4B,CNIH2,CLIC4,DNAJC30,NOS1AP,CATSPER2,DLG1,KCNK7,NFE2L2,CTTNBP2NL,CEMIP,SESTD1,PTPN6,KCNK4,HTT,CLTCL1,PINK1,ATP1B1,KCNA1,ARL6IP1,KCNMA1,KCNH4,GNB5,KCND1,MINK1,HAP1,DLG4,ACTB,PSEN2,RSC1A1,NLGN2,CNIH3,WNK3,STAC2,CACNB1,ATP1B2,PSEN1,KCNAB3,CACNA1A,KCNIP1,HCN3,NLGN3,CACNA1G,ATP2A1,CD19,CALM1,PRKCB,DNM2,VMP1,BDKRB1,INSR,CCL2,ASIC2,YES1,KCNJ18,C3,GPC3,FMR1,INPP5K,PDPK1,P2RX1,CACNA1B,OAZ1,RNF207,CACNA1H,PARK7,KCNC4,AMIGO1,CYBA,JPH3,LCN2,WWP2,RRAD,MIR21,MIR24-1,DMD,GPR35,FABP5,JPH2,CA2,KCNH2,FGF11,THY1,SCN2B,KCNK5,NETO2,CACNG7,CRBN,RGS4,CRH,KCNA6,APOPT1,YWHAH,TMEM109,SHISA8,KCNJ8,CACNG5,HCN4,GEM,PPIF,KCNG2,KCNJ9,ARPP19	grey	VSMC_M10
GO:0051224	0.50	0.58	52	96	negative regulation of protein transport	BP	1	PRKN,GOPC,RAB23,BMP8A,RSAD2,ITGB1BP1,FRMD4A,UBAC2,DPH3,FERMT1,BAG4,LYPLA1,NDUFAF2,FAM3D,PKIA,KCNJ11,NR1H3,RAB11FIP5,ACVR1C,F2R,DMTN,ADTRP,SUFU,SIRT4,IRS1,CABP1,CD200,HDAC1,PPP3CA,SP100,ADRA2A,KCNB1,YOD1,BAG3,APOD,KLF7,DERL3,IL12A,ENY2,C11orf65,RSC1A1,SNX12,SYTL4,SREBF1,INPP5K,PARK7,WWP2,MIR199A1,CHGA,TXN,UBE2G2,DERL2	grey	VSMC_M10
GO:0071391	0.50	0.71	8	14	cellular response to estrogen stimulus	BP	1	ESR1,SRA1,ARID5A,NCOA4,BCAS3,TRIM24,SERPINB9,SFR1	grey	VSMC_M10
GO:0019370	0.50	0.74	6	10	leukotriene biosynthetic process	BP	1	LTC4S,MGST2,PLA2G4A,GGT2,GGT6,GGT5	grey	VSMC_M10
GO:0086002	0.50	0.62	22	41	cardiac muscle cell action potential involved in contraction	BP	1	NEDD4L,TRPM4,CACNB2,SCN5A,SCN1A,GPD1L,KCNJ3,KCNE2,DSP,RYR2,PKP2,KCNJ2,ANK2,SCN4B,NOS1AP,DLG1,CACNA1G,DSG2,RNF207,KCNH2,SCN2B,KCNJ8	grey	VSMC_M10
GO:0050795	0.50	0.64	18	33	regulation of behavior	BP	1	HDAC2,NAPEPLD,MEF2C,CFAP20,EIF2AK4,TTC21B,HDAC4,STAT3,DLG4,CHRNB2,STRA6,SGIP1,LEPR,PER3,CNTNAP4,ZFHX3,HTR1D,CRH	grey	VSMC_M10
GO:2000010	0.50	0.69	9	16	positive regulation of protein localization to cell surface	BP	1	GPD1L,TM9SF4,LRIG2,ABCA12,STX4,ABCA7,CD247,TMEM35A,MAP1A	grey	VSMC_M10
GO:0002283	0.50	0.72	7	12	neutrophil activation involved in immune response	BP	1	MYD88,FCER1G,ITGAM,SCNN1B,STXBP2,PRAM1,DNASE1	grey	VSMC_M10
GO:0009268	0.50	0.64	17	31	response to pH	BP	1	ASIC3,ASIC1,HVCN1,RAB11FIP5,GPLD1,HYAL1,SLC38A3,SLC9A1,KCNK4,GPR68,ASIC2,TRPV1,CLCN7,PKD1L3,INSRR,CA2,IMPACT	grey	VSMC_M10
GO:0045947	0.50	0.69	9	16	negative regulation of translational initiation	BP	1	EIF3E,AGO2,ATF4,EIF2AK4,PAIP2B,BANK1,EIF4EBP2,LARP1,FMR1	grey	VSMC_M10
GO:0030859	0.50	0.67	11	20	polarized epithelial cell differentiation	BP	1	PTK7,RAB10,SYNE4,TCF15,MYO9A,CAMSAP3,LAMA1,IFT20,FOXF1,RHOA,WNT5A	grey	VSMC_M10
GO:0002688	0.50	0.58	50	92	regulation of leukocyte chemotaxis	BP	1	GPSM3,PTK2,RAC2,CCL27,FPR2,CXCL8,AKIRIN1,PTN,C5AR2,C5AR1,OXSR1,NCKAP1L,GAS6,MTUS1,VEGFC,PTK2B,SLC8B1,F2RL1,ANO6,NOD2,DNM1L,TRPV4,PRSS56,CMKLR1,SLAMF8,MOSPD2,IL12A,MCU,TNFSF18,CXCR2,BST1,MAPK1,PADI2,TMEM102,PGF,DAPK2,MAPK3,IL6R,S100A14,CD74,CCL5,CSF1R,CCL7,CCL2,MPP1,DEFB124,DPP4,ZNF580,WNT5A,DUSP1	grey	VSMC_M10
GO:0014904	0.50	0.64	17	31	myotube cell development	BP	1	STAC3,SMO,KEL,KLHL40,RYR1,P2RX2,LMOD3,HOMER1,BIN3,PPP3CA,ACTN3,HDAC4,FBXO22,SKI,SMYD3,SIX1,MYORG	grey	VSMC_M10
GO:0007039	0.50	0.68	10	18	protein catabolic process in the vacuole	BP	1	LRP1,MFSD8,TMEM106B,TCIRG1,ATP13A2,AC138894.1,LDLR,RNF128,LAMP2,TMEM199	grey	VSMC_M10
GO:0090022	0.50	0.65	15	27	regulation of neutrophil chemotaxis	BP	1	RAC2,CXCL8,C5AR2,C5AR1,NCKAP1L,NOD2,DNM1L,MOSPD2,MCU,CXCR2,BST1,DAPK2,CD74,MPP1,DPP4	grey	VSMC_M10
GO:2000269	0.50	0.69	9	16	regulation of fibroblast apoptotic process	BP	1	XRCC2,CHD8,BTG1,GAS6,PRDM11,STK17B,CFDP1,MIR24-1,BCL2L11	grey	VSMC_M10
GO:0033077	0.50	0.60	33	61	T cell differentiation in thymus	BP	1	SRF,ATG5,BMI1,CLPTM1,GLI2,FOXJ1,PRKDC,TOX,ZC3H8,IL1A,CAMK4,TMEM131L,RASGRP1,RABL3,FZD7,CD28,ADAM8,FZD5,WNT4,STAT5B,FOXP3,CARD11,DOCK2,BCL11B,CD74,JAG2,IL7R,PTPN2,DNAJA3,MAFB,FADD,ZFP36L2,PRR7	grey	VSMC_M10
GO:0042472	0.50	0.59	39	73	inner ear morphogenesis	BP	1	FZD6,PTK7,ROR2,DLX5,KCNQ4,WHRN,SOBP,MYO3A,SOX9,USH1G,GLI2,MYO7A,SPRY2,CEP290,PAX8,EYA1,PLS1,FZD3,USH1C,TFAP2A,PAX2,ALDH1A3,GBX2,MYO3B,GRHL3,STRC,STOX1,DVL2,TCAP,POU3F4,NTN1,CHRNA10,FOXG1,MAFB,WNT5A,OTX1,SIX1,INSIG2,ZIC1	grey	VSMC_M10
GO:0021517	0.50	0.64	17	31	ventral spinal cord development	BP	1	PTCH1,VSTM5,GLI2,IFT172,MNX1,SUFU,GIGYF2,MDGA2,SCYL3,CLN8,LHX1,ISL1,DAB1,LRP8,SOX4,ISL2,LBX1	grey	VSMC_M10
GO:0050954	0.50	0.57	64	120	sensory perception of mechanical stimulus	BP	1	ATP8B1,MYO1A,EYA4,ALDH7A1,COL11A2,PHF24,CLIC5,KIT,KCNQ4,GSDME,MYH14,WHRN,SOBP,SLC26A5,MYO3A,MYO7B,EML2,USH1G,ASIC3,SCN1A,MYO7A,SPRY2,MKKS,LRTOMT,AP000812.5,TMTC4,DIAPH1,P2RX2,TUB,CASP3,MARVELD2,EYA1,ADGRV1,USH1C,TFAP2A,PGAP1,MYO3B,COCH,BACE1,STRC,HOMER2,CDKN1B,LRIG2,CEMIP,CDH23,KCNK4,RAB3A,KCNA1,CHRNB2,POU3F4,OTOA,CRYM,ASIC2,TRPV1,CDC14A,SRRM4,PJVK,CHRNA10,EPS8L2,SIX1,DDIT3,OTOR,TIMM8B,OTOS	grey	VSMC_M10
GO:0090344	0.50	0.68	10	18	negative regulation of cell aging	BP	1	CDK6,PRKDC,PLK2,TERT,SLC30A10,MAP3K3,BCL6,FZR1,TERF2,YBX1	grey	VSMC_M10
GO:1903830	0.50	0.69	9	16	magnesium ion transmembrane transport	BP	1	NIPAL2,SLC41A2,CNNM4,ZDHHC13,NIPA1,SLC41A1,NIPAL3,NIPAL4,NIPAL1	grey	VSMC_M10

GO:0048569	0.50	0.70	8	14	post-embryonic animal organ development	BP	1	BAK1,ERCC1,MYO7A,FZD5,FBN1,MFAP2,BCL11B,BCL2L11	grey	VSMC_M10
GO:0042476	0.51	0.58	51	95	odontogenesis	BP	1	OSR2,PPARA,TNFRSF11B,SMO,CNNM4,SOSTDC1,KLK4,ACP4,AQP1,TNC,OSR1,HDAC2,SLC4A2,GLI2,SCN5A,ACVR2A,RELT,TRAF6,HAND2,TFAP2A,ITGB6,HDAC1,TGFB2,TCIRG1,FAM20A,FAM20C,AXIN2,AMELX,HTRA1,LAMA5,DLX1,BCOR,ALPL,BMPR1A,SP6,NGFR,DLX3,BCL11B,JAG2,ROGDI,BSG,ANKRD11,LHX8,RHOA,LAMB1,CA2,BGLAP,ZNF22,SNX10,BCL2L11,PERP	grey	VSMC_M10
GO:0001909	0.51	0.60	33	61	leukocyte mediated cytotoxicity	BP	1	CD226,STX7,AGER,HLA-DRA,PRDX1,NECTIN2,PVR,UNC13D,MYD88,SLC22A13,NCKAP1L,RASGRP1,F2RL1,CADM1,CTSH,IL12A,PTPN6,LAG3,PPP3CB,STAT5B,ITGAM,SCNN1B,STXBP2,VAV1,NOS2,IL7R,CRK,DNASE1,AP1G1,FADD,CXCL6,SERPINB9,HAVCR2	grey	VSMC_M10
GO:0042490	0.51	0.60	28	52	mechanoreceptor differentiation	BP	1	ATP8B1,NTRK2,GSDME,MYCL,WHRN,MYCN,NTF4,USH1G,MYO7A,TSKU,SLC4A7,JAG1,LRTOMT,AP000812.5,PLS1,ADGRV1,USH1C,TRIP11,STRC,CDH23,RBPJ,MKS1,TMEM132E,JAG2,IFT20,MCOLN3,PJVK,HEY2	grey	VSMC_M10
GO:0032722	0.51	0.61	26	48	positive regulation of chemokine production	BP	1	LRP1,AGER,TRIM32,TLR4,MYD88,EIF2AK2,MAVS,IL7,OAS3,RIPK2,F2RL1,NOD2,HDAC1,TRPV4,DDX3X,IL6R,IL4R,CD74,CSF1R,TICAM1,OAS1,DEFB124,WNT5A,IL33,TSLP,HAVCR2	grey	VSMC_M10
GO:0034694	0.51	0.66	12	22	response to prostaglandin	BP	1	PRKCE,TNC,APOB,AANAT,PTGER2,AKAP8,YY1,ACACA,PTGER4,GNB1,PTGFR,GNG2	grey	VSMC_M10
GO:0043267	0.51	0.67	10	18	negative regulation of potassium ion transport	BP	1	NEDD4L,KEL,KCNRG,OXSR1,PTK2B,KCNE2,CASQ2,KCNH2,CRBN,RGS4	grey	VSMC_M10
GO:0009620	0.51	0.64	15	27	response to fungus	BP	1	USP15,BAK1,GAPDH,TLR4,ATG5,MYD88,SPON2,MPO,S100A8,BTK,CRK,BCL10,S100A12,CHGA,COTL1	grey	VSMC_M10
GO:0045010	0.51	0.61	24	44	actin nucleation	BP	1	ARPC3,WASHC5,PICK1,LMOD2,WASF1,FCHSD2,ARFIP2,LMOD3,HIP1R,FMN1,IQGAP2,CYFIP1,MAGEL2,LMOD1,AP1AR,WASF2,WHAMM,NCKAP1,ARPC1A,WAS,EVL,SPIRE2,WDR63,GMFG	grey	VSMC_M10
GO:0042769	0.51	0.71	7	12	DNA damage response, detection of DNA damage	BP	1	MRPS9,DNAJA1,MRPS26,MRPS35,MRPS11,PARP1,SOX4	grey	VSMC_M10
GO:0019395	0.51	0.58	47	87	fatty acid oxidation	BP	1	HSD17B4,CPT1B,PPARA,MCAT,ECHDC1,MAPK14,CROT,ADH5,ETFB,HADHA,SOX9,PRKAG2,ACOX1,CYGB,AKT2,HACL1,ACOXL,ACOX2,PEX2,ETFDH,IVD,ECI2,LONP2,SIRT4,IRS1,CPT1A,BDH2,ABCB11,EHHADH,POR,ACADL,TYSND1,ACADVL,HSD17B10,ABCD4,GCDH,ACAA2,DECR2,MLYCD,ABCD3,ACADM,PHYH,HADHB,AMACR,ECI1,AUH,ADH4	grey	VSMC_M10
GO:0046580	0.51	0.61	23	43	negative regulation of Ras protein signal transduction	BP	1	SYNGAP1,MET,SPRY1,SPRY2,PPP2CB,RASA3,RASA2,ARHGAP24,TGFB2,RASA4,BCL6,ABL2,LZTR1,RASAL3,MFN2,ITGA3,KCTD13,ARHGAP44,TNFAIP1,MAPKAP1,SCAI,DAB2IP,STAMBP	grey	VSMC_M10
GO:0036499	0.51	0.68	9	16	PERK-mediated unfolded protein response	BP	1	ATF4,NCK2,QRICH1,NFE2L2,PTPN2,ABCA7,HSPA5,DDIT3,PPP1R15A	grey	VSMC_M10
GO:0050849	0.51	0.67	10	18	negative regulation of calcium-mediated signaling	BP	1	FHL2,PLEK,PRNP,SLA2,TBC1D10C,MYOZ2,ACTN3,HOMER2,MYOZ1,MAPK7	grey	VSMC_M10
GO:0061162	0.51	0.66	12	22	establishment of monopolar cell polarity	BP	1	PTK7,FOXJ1,SYNE4,TCF15,GBF1,MYO9A,CAMSAP3,LAMA1,IFT20,FOXF1,RHOA,WNT5A	grey	VSMC_M10
GO:0035089	0.51	0.66	11	20	establishment of apical/basal cell polarity	BP	1	PTK7,FOXJ1,SYNE4,TCF15,MYO9A,CAMSAP3,LAMA1,IFT20,FOXF1,RHOA,WNT5A	grey	VSMC_M10
GO:0046500	0.51	0.69	8	14	S-adenosylmethionine metabolic process	BP	1	GNMT,APIP,AHCY,ENOPH1,MTRR,MAT1A,METTL16,PEMT	grey	VSMC_M10
GO:1903427	0.51	0.69	8	14	negative regulation of reactive oxygen species biosynthetic process	BP	1	PPARA,SLC30A10,STAT3,PARK7,MIR21,RHOA,APOPT1,AC140504.1	grey	VSMC_M10
GO:0046683	0.51	0.57	50	94	response to organophosphorus	BP	1	NME1,TEK,PNPT1,AREG,AQP1,TRPM4,TRPC3,PANX1,AANAT,THBD,CRLS1,P2RX2,P2RX3,RYR3,GPD1,P2RX7,P2RY1,PTK2B,RAPGEF2,KCNJ11,STC1,RAPGEF3,PKD2,DMTN,SRD5A1,SLC6A3,SSH1,RAP1B,DUOX1,VGF,CPS1,FDX1,PTPRN,DGKQ,PKLR,FOS,TAF1,CITED1,PPARGC1B,SREBF1,JUN,INPP5K,TRPV1,P2RX1,BSG,ASS1,HSPA5,DUSP1,HCN4,PDXP	grey	VSMC_M10
GO:0021854	0.51	0.69	8	14	hypothalamus development	BP	1	UQCRQ,NDNF,SEMA3A,SRD5A1,HAP1,UBB,POU3F2,CRH	grey	VSMC_M10
GO:0044273	0.51	0.65	14	25	sulfur compound catabolic process	BP	1	CHAC2,GLB1,CSAD,AHCY,FITM2,MTRR,CBSL,HYAL1,GGT2,MAT1A,IDUA,MLYCD,NUDT19,GGT5	grey	VSMC_M10
GO:0046621	0.51	0.63	16	29	negative regulation of organ growth	BP	1	STK3,PPARA,PI16,TGFBR2,TBX5,STK4,JARID2,KCNK2,SAV1,WWC1,YY1,MIR199A1,MIR25,NOG,FXN,RGS4	grey	VSMC_M10
GO:0035020	0.51	0.67	10	18	regulation of Rac protein signal transduction	BP	1	KBTBD6,PIK3CB,ARHGAP24,AUTS2,KRAS,OGT,DNM2,ARHGAP44,CRK,CADM4	grey	VSMC_M10
GO:0001662	0.51	0.67	10	18	behavioral fear response	BP	1	MAPK8IP2,USP46,SLC1A1,MEF2C,ASIC1,ATP1A2,ASIC4,MECP2,DPP4,CRH	grey	VSMC_M10
GO:0045061	0.51	0.68	9	16	thymic T cell selection	BP	1	SRF,ATG5,TOX,CD28,CARD11,DOCK2,CD74,JAG2,PTPN2	grey	VSMC_M10
GO:0016239	0.51	0.59	30	56	positive regulation of macroautophagy	BP	1	PIP4K2C,EPM2A,SPTLC1,NOD1,SCOC,SESN3,PIP4K2A,OPTN,RALB,TMEM173,DCN,ULK1,RIPK2,NOD2,BAG3,HTT,PINK1,VPS13D,MAPK3,LARP1,SMCR8,IKBKG,GPSM1,GNAI3,KIAA1324,TSC1,SH3GLB1,BNIP3L,IRGM,HSPB8	grey	VSMC_M10
GO:0045058	0.51	0.62	18	33	T cell selection	BP	1	SRF,IL15,ATG5,TOX,CD28,TNFSF18,STAT3,FOXP3,CARD11,TBX21,DOCK2,BATF,IL6R,BCL11B,CD74,JAG2,PTPN2,RHOA	grey	VSMC_M10
GO:0036120	0.51	0.66	11	20	cellular response to platelet-derived growth factor stimulus	BP	1	MARS,HAS1,PTN,TLR4,HYAL1,CBL,SYAP1,CREB1,YES1,MTSS1L,PDGFD	grey	VSMC_M10
GO:0002053	0.51	0.66	11	20	positive regulation of mesenchymal cell proliferation	BP	1	MYC,SMO,MYCN,SOX9,TGFBR2,CHRD,BMPR1A,FOXF1,WNT5A,SIX1,WNT2	grey	VSMC_M10
GO:0006970	0.51	0.58	38	71	response to osmotic stress	BP	1	MAP7,SLC12A2,TSPO,XRCC6,AKR1B1,MAPK13,ABCB1,AQP1,EGFR,PLK3,OXSR1,HNMT,CASP3,PTK2B,PKD2,SLC12A5,ICOSLG,TRPV4,SCN2A,YBX3,SORD,DDX3X,XRCC5,MICU1,KCNMA1,SLC2A4,WNK3,AC138894.1,LRRC8E,SLC25A23,PDPK1,LRRC8A,MARVELD3,MT-CYB,LRRC8D,CLDN1,TSC22D3,MAP2K7	grey	VSMC_M10

GO:0051650	0.51	0.55	76	143	establishment of vesicle localization	BP	1	MYO1A,AP3S1,KIF5A,PRKN,KIF3A,FAM91A1,EXOC4,MAPK15,STARD3NL,BBS7,MYO7B,CLASP2,MYO7A,AP3M2,KIF5C,MKKS,LIN7A,TMEM230,KIF3B,RASGRP1,ARFGAP2,EXOC6B,SYNJ1,AP3B1,DTNBP1,KIF13A,GBF1,BICDL1,KLHL12,TRIP11,PEF1,TCIRG1,PDCD6,SNAP23,SHROOM2,AP1AR,MLPH,RAB17,CNIH2,LIMK2,TANC2,SPG11,BLOC1S6,ITGA4,MAP4K2,MREG,HTT,RAB3A,PINK1,SNAP29,AP3M1,KIF1C,HAP1,SLC2A4,PSEN1,SYT11,TMED10,TRIM46,NDEL1,AC138894.1,RAB11A,SNAPIN,DNM2,AP1M2,MYO1D,C17orf75,PPP6C,MYO1F,MYO5B,BICDL2,PRKCZ,IKBKG,FNBP1L,BLOC1S3,BORCS5,AP3S2	grey	VSMC_M10
GO:0010421	0.51	0.71	7	12	hydrogen peroxide-mediated programmed cell death	BP	1	MET,FOXO3,PAWR,PDCD10,PINK1,PARK7,MIR21	grey	VSMC_M10
GO:0048483	0.51	0.62	20	37	autonomic nervous system development	BP	1	GFRA3,PLXNA4,SOX10,TFAP2B,SEMA3A,RET,HAND2,FZD3,TLX2,TFAP2A,SEMA3F,ADARB1,HLX,GBX2,FN1,RHOXF1,PLXNA3,SOX8,SOX4,SIX1	grey	VSMC_M10
GO:0050871	0.52	0.59	33	61	positive regulation of B cell activation	BP	1	IL5,TNFRSF13C,EXOSC3,MSH2,PELI1,TLR4,HMCES,BMI1,MLH1,PAXIP1,SHLD1,NCKAP1L,MEF2C,IL7,NOD2,CD40,CD28,PPP2R3C,BCL6,TNIP2,CD38,BST1,STAT5B,SHLD2,CARD11,TBX21,CHRNB2,BTK,CD74,CD320,TICAM1,VAV3,TNFSF13B	grey	VSMC_M10
GO:0021680	0.52	0.64	13	24	cerebellar Purkinje cell layer development	BP	1	UQCRQ,AGTPBP1,WHRN,B4GALT2,FAIM2,LDB1,TTC21B,SPTBN2,LHX1,SEZ6,AARS,HSPA5,CEND1	grey	VSMC_M10
GO:1905523	0.52	0.67	10	18	positive regulation of macrophage migration	BP	1	PTK2,AKIRIN1,C5AR1,TRPV4,CMKLR1,TNFSF18,MAPK1,MAPK3,CCL5,CSF1R	grey	VSMC_M10
GO:0032731	0.52	0.62	18	33	positive regulation of interleukin-1 beta production	BP	1	AGER,NOD1,TLR4,PANX1,MYD88,P2RX7,RIPK2,F2RL1,NOD2,FZD5,HK1,STAT3,PYDC1,SMAD3,ISL1,CASP8,WNT5A,INAVA	grey	VSMC_M10
GO:0050864	0.52	0.57	50	93	regulation of B cell activation	BP	1	TNFAIP3,IL5,TNFRSF13C,TNFRSF21,EXOSC3,MSH2,PELI1,IL4I1,TLR4,HMCES,BMI1,MLH1,FOXJ1,PAXIP1,PAWR,SHLD1,NCKAP1L,CASP3,MEF2C,IL7,NOD2,CD40,BANK1,TBC1D10C,SLAMF8,THEMIS2,CD28,PPP2R3C,BCL6,RC3H1,PTPN6,TNIP2,CD38,BST1,STAT5B,INHA,FOXP3,SHLD2,CARD11,TBX21,CHRNB2,CD19,BTK,CD74,CD320,SUPT6H,TICAM1,VAV3,ZFP36L2,TNFSF13B	grey	VSMC_M10
GO:0061952	0.52	0.69	8	14	midbody abscission	BP	1	VPS4B,MITD1,CHMP2A,CHMP2B,CHMP4C,CHMP7,CHMP1A,VPS4A	grey	VSMC_M10
GO:0002792	0.52	0.62	19	35	negative regulation of peptide secretion	BP	1	PRKN,BMP8A,NDUFAF2,FAM3D,KCNJ11,ACVR1C,SIRT4,IRS1,HDAC1,PPP3CA,ADRA2A,KCNB1,KLF7,ENY2,SYTL4,CD74,SREBF1,CHGA,CRH	grey	VSMC_M10
GO:0060547	0.52	0.68	9	16	negative regulation of necrotic cell death	BP	1	PELI1,RIPK1,FZD9,YBX3,BIRC3,MIR22,NOL3,FADD,CASP8	grey	VSMC_M10
GO:0032780	0.52	0.70	7	12	negative regulation of ATPase activity	BP	1	TNNI3,PXK,OXA1L,VCPKMT,PAM16,TSC1,PPIF	grey	VSMC_M10
GO:0032259	0.52	0.53	155	290	methylation	BP	1	BAZ2A,RNMT,ARMT1,SNW1,TFB1M,MOV10L1,PHF1,TRMT11,MYC,PICK1,CMTR1,MTAP,CARNMT1,GNMT,PRDM4,CAMKMT,RNF20,STPG4,MTO1,SETD7,DNMT3A,BMI1,SETDB2,NSUN6,EED,IWS1,SUV39H2,KDM4A,PAXIP1,METTL23,HNMT,METTL17,THUMPD3,DPY30,LRTOMT,AP000812.5,SIRT7,METTL25,CHTOP,TRMT6,NSD3,NDUFAF7,ASH2L,BTG1,WTAP,TEX15,THUMPD2,TRMT9B,NR1H4,SETD9,TGS1,DIMT1,EEF1AKMT1,DNMT3B,SMYD1,PRMT9,BCDIN3D,SETD6,KMT2D,NSUN3,PRDM11,RTF1,PRMT3,WDR82,ZNF335,JARID2,RBBP5,FAM173B,MTRR,TPMT,PRDM15,HEMK1,COQ5,MTR,TRMT10A,AUTS2,DALRD3,LCMT2,ECE2,METTL8,EEF1AKMT2,TDRKH,NSUN5,MGMT,METTL2B,SETD2,TRMT1L,TET1,SNRPD3,BCOR,MEN1,WDR61,VCPKMT,COMT,MEPCE,ALKBH8,NELFA,TRMT5,DYDC1,NSUN7,PARP1,HSD17B10,PRDM2,MRM2,ZCCHC4,NSD1,SETD1A,PAGR1,FOS,OGT,RAB3D,LCMT1,SETD3,METTL4,SUZ12,METTL6,SUPT6H,CXXC1,MBD1,MRM3,SMYD4,CREBBP,METTL16,DOT1L,MECP2,PRDM16,GAMT,CHD5,SPOUT1,MTF2,GFI1,CMTR2,PRMT7,CTCF,MIR29A,PRMT6,METTL18,SETDB1,PRDM6,ARID4A,METTL14,TRMT10B,PEMT,BEND3,SMYD3,METTL21A,TRMT61A,ZNF304,NNMT,N6AMT1,TRMT10C,TRMT61B,CBLL1,TYW3,METTL7A	grey	VSMC_M10
GO:0046037	0.52	0.67	9	16	GMP metabolic process	BP	1	IMPDH1,GDA,DLG2,GMPS,MAGI3,DLG1,CASK,CARD11,MPP1	grey	VSMC_M10
GO:0006066	0.52	0.53	138	258	alcohol metabolic process	BP	1	RDH5,APOF,RDH16,CYP27B1,MIOX,SYNJ2,ACO2,AKR1B1,TTC39B,ALDH1A1,PLPP6,SRD5A3,SPTLC1,IPPK,INPP4A,ADH5,CAT,RDH13,PLEK,INPP4B,APOB,SPHK2,DHFR,MTMR2,NAPEPLD,EBP,AKR1A1,CUBN,LIPE,ITPKC,ACER3,TSKU,PIP4P1,SPTLC3,NR1H4,MTMR7,SDR16C5,SOAT2,CYP7A1,ASAH1,CYP7B1,CEBPA,CBR4,GK5,RETSAT,DKK3,LIMA1,GPD1,EPHX2,CYP2R1,P2RY1,PTK2B,SPR,SYNJ1,COQ2,ITPKA,ERLIN1,ALDH1A3,ABCA5,FMO5,CHKA,ITPK1,INPP5A,ALDH3B2,MSMO1,THTPA,GPER1,SC5D,DHRS9,IP6K2,DPAGT1,NTSR1,INPP5J,SCAP,HSD17B7,POR,PTH1R,SORD,PLA2G4A,ACADL,SOAT1,MOGAT3,TM7SF2,OSBPL1A,IDH3A,FDX1,PECR,LPCAT3,GNB3,WNT4,CYP27A1,GCH1,CH25H,ACADVL,TKFC,CLN8,DHRS3,RDH11,ALDH1A2,LIPC,DGKQ,GDPD3,APOBR,CLN6,LDLR,GDPD1,SCNN1B,CYP46A1,GDE1,NPC1,SREBF1,LEPR,IMPA2,ACAA2,INPP5K,MECP2,TKTL1,CACNA1H,PARK7,ALDH2,RXRA,CEL,GFI1,NFE2L1,TPK1,PNPLA4,DHDDS,MINPP1,LDLRAP1,ACER2,LRAT,INSIG2,RDH14,INPP1,ADH4,FGF1,SGPP1,PCBD2	grey	VSMC_M10
GO:0002360	0.52	0.66	10	18	T cell lineage commitment	BP	1	PRKDC,TOX,IL7,TNFSF18,STAT3,FOXP3,TBX21,BATF,IL6R,RHOA	grey	VSMC_M10
GO:0071305	0.52	0.65	11	20	cellular response to vitamin D	BP	1	CYP27B1,SNW1,PIM1,PTN,TNC,IL15,VDR,MED1,MIR125B1,TRIM24,BGLAP	grey	VSMC_M10
GO:0045494	0.52	0.63	14	26	photoreceptor cell maintenance	BP	1	USP45,USH1G,MKKS,ERCC6,TUB,ADGRV1,USH1C,BBS1,CDH23,CLN8,ATP1B2,ESRRB,DRAM2,BBS10	grey	VSMC_M10
GO:0097502	0.52	0.62	17	31	mannosylation	BP	1	RXYLT1,ALG12,LARGE1,ALG2,FKTN,ALG11,TMTC4,PIGM,PIGV,ALG3,PIGZ,ALG9,POMT2,ALG1,POMGNT2,PIGB,ISPD	grey	VSMC_M10

GO:0050858	0.52	0.66	10	18	negative regulation of antigen receptor-mediated signaling pathway	BP	1	PAWR,PLCL2,PRNP,LPXN,PTPRJ,SLA2,PTPN6,GPS2,PTPN2,THY1	grey	VSMC_M10
GO:0031644	0.52	0.56	53	100	regulation of nervous system process	BP	1	RIMS2,SOX10,TNFRSF21,RIMS1,SHANK1,DLGAP3,LPIN1,FIG4,GRIN2D,MTMR2,SLC8A2,CST7,FAM19A4,P2RX3,JAM2,DLGAP4,CUX2,PTK2B,HOMER1,F2R,FAM173B,RNF10,IGSF11,DAG1,SSH1,ZFHX2,OPRD1,AGT,SLC9A1,STX1A,TMEM25,NTSR1,CNIH2,COMT,TNR,ADRA2C,NPTX2,DLG4,NLGN2,TNFRSF1B,STX1B,NLGN3,TMEM100,NMUR2,BEGAIN,DLGAP1,ABAT,FMR1,PRKCZ,GPR35,FABP5,RGS4,CACNG5	grey	VSMC_M10
GO:0140374	0.52	0.68	8	14	antiviral innate immune response	BP	1	PHB,CLPB,AKAP1,RNF135,ATAD3A,ZDHHC1,OAS1,ZDHHC11	grey	VSMC_M10
GO:1903038	0.52	0.56	47	87	negative regulation of leukocyte cell-cell adhesion	BP	1	PPARA,TNFRSF21,PELI1,IL4I1,FOXJ1,PAWR,PRNP,NCKAP1L,ZC3H8,CASP3,TMEM131L,PAG1,MARCH7,ADTRP,BTN2A2,PRKAR1A,HLX,RUNX3,DLG1,MIA3,BCL6,VSIR,RC3H1,PTPN6,TNFSF18,LAG3,SCGB1A1,FOXP3,TBX21,ZBTB7B,IL4R,CD74,CCL25,TWSG1,PTPN2,SOCS1,ASS1,GLMN,MIR21,MIR222,MIR27A,MIR125A,FGL2,LAX1,CEBPB,HAVCR2,RC3H2	grey	VSMC_M10
GO:0048265	0.52	0.65	11	20	response to pain	BP	1	TSPO,P2RX2,RET,P2RX3,SLC6A2,COMT,GCH1,NMUR2,VWA1,TRPV1,CRH	grey	VSMC_M10
GO:0051703	0.52	0.62	17	32	biological process involved in intraspecies interaction between organisms	BP	1	MAPK8IP2,SHANK1,CHD8,MKKS,RPTOR,ATXN1,NLGN4Y,NPAS4,EIF4EBP2,NRXN2,DLG4,CLN8,NLGN3,CHRNB2,MECP2,ATXN1L,NRXN3	grey	VSMC_M10
GO:0042596	0.52	0.65	11	20	fear response	BP	1	MAPK8IP2,USP46,SLC1A1,MEF2C,ASIC1,ADRA2A,ATP1A2,ASIC4,MECP2,DPP4,CRH	grey	VSMC_M10
GO:0010822	0.52	0.58	32	59	positive regulation of mitochondrion organization	BP	1	PRKN,BAK1,PLA2G6,FBXO7,VPS35,MGARP,DDHD2,DCN,HIP1R,HTRA2,OPA1,DNM1L,ZDHHC6,GPER1,CIDEB,FIS1,NMT1,MCU,HTT,DDHD1,PINK1,MUL1,VPS13D,ADCK1,MIEF2,RALBP1,PARK7,MLLT11,BCL2L11,RALA,MIEF1,PPIF	grey	VSMC_M10
GO:0060972	0.52	0.65	11	20	left/right pattern formation	BP	1	SMO,SETDB2,MEGF8,IFT172,FOXJ1,DPCD,SNAI1,PKD1L1,MNS1,HSPB11,PIFO	grey	VSMC_M10
GO:0001736	0.52	0.58	34	64	establishment of planar polarity	BP	1	FZD6,RSPO3,PTK7,ROR2,BRSK1,FUZ,INTU,WNT11,SPEF1,RYK,NPHP1,FZD3,TIAM1,RPGRIP1L,VANGL1,PRICKLE1,NKD1,JHY,ZNRF3,DVL3,FZD7,GRHL3,EXOC5,DAAM1,ARHGEF19,DVL2,MKS1,IFT20,GPC3,SAPCD2,CELSR2,RHOA,WNT5A,CELSR3	grey	VSMC_M10
GO:0007164	0.52	0.58	34	64	establishment of tissue polarity	BP	1	FZD6,RSPO3,PTK7,ROR2,BRSK1,FUZ,INTU,WNT11,SPEF1,RYK,NPHP1,FZD3,TIAM1,RPGRIP1L,VANGL1,PRICKLE1,NKD1,JHY,ZNRF3,DVL3,FZD7,GRHL3,EXOC5,DAAM1,ARHGEF19,DVL2,MKS1,IFT20,GPC3,SAPCD2,CELSR2,RHOA,WNT5A,CELSR3	grey	VSMC_M10
GO:0042886	0.52	0.53	112	210	amide transport	BP	1	LRP1,RIMS2,PRKN,TAP1,PLA2G6,PICK1,ABCB1,RFX3,TFAP2B,PRKCE,HMGN3,CYB5R4,BMP8A,EGFR,TRPM4,SLC7A11,GIPR,RAF1,ABCC4,AIMP1,SIRT3,SYT9,NR1H4,NDUFAF2,FAM3D,PAX8,OSBP,ABCB9,ARNTL,KCNJ11,SLC33A1,NNAT,TIAM1,RAB11FIP5,PFKM,SLC8B1,CDH17,NKX6-1,ACVR1C,COL4A3BP,ZBED6,ABCG2,PLTP,SIRT4,GPLD1,IRS1,HDAC1,PPP3CA,CPT1A,TRPV4,SLC9B2,ADRA2A,TCIRG1,TCF7L2,KCNB1,PFKFB2,GPER1,RAB11FIP2,ABCC5,PER2,STX1A,PASK,GLUL,SIDT2,SLC16A1,KLF7,VGF,ENY2,ABCA12,MCU,PPP3CB,CAMK2G,PTPRN,CD38,SLC27A1,SLC38A5,MPC2,ILDR2,SYT7,NLGN2,CPLX1,TARDBP,STX4,RAB8B,UBE2Q1,SYTL4,GPR68,S100A8,CD74,CCL5,SREBF1,ISL1,OXCT1,NOS2,SLC46A1,ABAT,NADK,TRPV1,BAIAP3,PARK7,LRRC8A,FGA,CHGA,SOX4,DPP4,CA2,SLC25A32,CRH,CPTP,DOC2B,UQCC2,SGPP1	grey	VSMC_M10
GO:0043032	0.53	0.66	10	18	positive regulation of macrophage activation	BP	1	TTBK1,TLR4,CEBPA,PLA2G4A,TNIP2,IL4R,KARS,WNT5A,IL33,HAVCR2	grey	VSMC_M10
GO:1902254	0.53	0.66	10	18	negative regulation of intrinsic apoptotic signaling pathway by p53 class mediator	BP	1	PRKN,ARMC10,TAF9,ING2,MARCH7,MUC1,TAF9B,CD74,MIR21,PTTG1IP	grey	VSMC_M10
GO:0031016	0.53	0.59	27	50	pancreas development	BP	1	ONECUT2,BAK1,SMO,CDK6,RFX3,MET,SOX9,GIPR,MNX1,ARNTL,NKX6-1,ZBED6,IGF2,TCF7L2,NR5A2,SIDT2,MEN1,NKX3-2,ILDR2,ALDH1A2,IL6R,GATA6,ISL1,PDPK1,FOXF1,SOX4,WNT5A	grey	VSMC_M10
GO:0034612	0.53	0.53	97	181	response to tumor necrosis factor	BP	1	YTHDC2,MAP3K5,TNFAIP3,HSPA1B,PRKN,TNFRSF13C,MAPK14,TNFRSF21,GSDME,CXCL8,TRIM32,TRAF1,FOXO3,APOB,TRAF3IP2,ZFAND6,IKBKB,BAG4,GAS6,ST18,TRAF6,ASAH1,CEBPA,GSS,CASP3,GPD1,PTK2B,KCNJ11,CCL17,OTULIN,NKX3-1,F2RL1,RIPK1,ABCC2,CD40,HDAC1,HYAL1,HYAL3,PIAS3,FABP4,SPATA2,GPER1,CIB1,TRAF5,HDAC4,PELI3,YBX3,NFE2L2,GGT2,TNFSF18,GCH1,SMPD4,BIRC3,SPPL2A,MAPK1,SGMS1,CXCL16,SLC2A4,GPS2,TNFRSF1B,PYDC1,MAPK3,TRAF3,CCL14,CCL5,CCL7,CCL2,CCL25,ADAMTS12,PTPN2,PIAS4,INPP5K,CDIP1,TRPV1,SPPL2B,TRAF2,TNFRSF25,ADAMTS13,CYBA,ASS1,LCN2,DAB2IP,MIR27B,MIR24-1,CCL16,NOL3,CASP8,CLDN1,GGT5,CD70,COMMD7,CCDC3,ZFP36L2,MAP2K7,TNFSF13B,HIST1H2BJ,CCL22	grey	VSMC_M10
GO:1901028	0.53	0.67	9	16	regulation of mitochondrial outer membrane permeabilization involved in apoptotic signaling pathway	BP	1	TMEM14A,HIP1R,FZD9,NMT1,MUL1,TMEM102,ACAA2,SLC35F6,AC140504.1	grey	VSMC_M10
GO:0042059	0.53	0.63	13	24	negative regulation of epidermal growth factor receptor signaling pathway	BP	1	EGFR,RAB7A,SNX5,PTPRJ,RNF115,CBL,SNX6,ZFYVE28,SOCS4,PSEN1,PTPN2,RNF126,DAB2IP	grey	VSMC_M10
GO:0002474	0.53	0.67	9	16	antigen processing and presentation of peptide antigen via MHC class I	BP	1	TAP1,IKBKB,IDE,ABCB9,ERAP1,MFSD6,FCER1G,TAPBPL,ERAP2	grey	VSMC_M10
GO:0032740	0.53	0.68	8	14	positive regulation of interleukin-17 production	BP	1	IL15,PHB,MYD88,NR1H4,NOD2,OSM,ZBTB7B,SLC7A5	grey	VSMC_M10
GO:0007156	0.53	0.56	46	88	homophilic cell adhesion via plasma membrane adhesion molecules	BP	1	PCDH10,FAT4,NECTIN2,PVR,PCDH8,PCDHGC5,PCDH1,RET,CLSTN2,ROBO1,ROBO2,TENM3,CDH17,AMIGO2,NECTIN3,IGSF11,CDH24,NECTIN4,CADM1,MYPN,HMCN1,CDH23,OBSL1,SDK1,PCDH7,PCDH11X,PCDH19,DSG2,DSC3,EMB,CDH6,CDH10,L1CAM,PKD1,BSG,AMIGO1,CELSR2,CDH15,CDH16,PCDHAC2,PCDHA6,CNTN2,CDHR4,CELSR3,PCDHGA2,PCDHB15	grey	VSMC_M10

GO:0060416	0.53	0.63	13	24	response to growth hormone	BP	1	PTK2,PNPT1,LEPROTL1,PIK3R1,PXN,SRD5A1,CPS1,GDF15,STAT3,STAT5B,GHR,ASS1,LEPROT	grey	VSMC_M10
GO:0030104	0.53	0.60	21	39	water homeostasis	BP	1	NEDD4L,AKR1B1,SRF,TFAP2B,AQP1,GRHL1,MET,AQP11,ELOVL1,LSR,TRPV4,KDF1,SFN,GRHL3,ABCA12,ALOXE3,AC138894.1,SCNN1B,MYO5B,INPP5K,CLDN1	grey	VSMC_M10
GO:0086091	0.53	0.62	16	30	regulation of heart rate by cardiac conduction	BP	1	TRPM4,CACNB2,SCN5A,KCNJ3,KCNE2,DSP,PKP2,KCNJ2,ANK2,SCN4B,CACNA1G,DSG2,ISL1,KCNH2,SCN2B,HCN4	grey	VSMC_M10
GO:1904377	0.53	0.58	31	58	positive regulation of protein localization to cell periphery	BP	1	LRP1,STAC3,EPB41L2,PRKCE,SPTBN1,EGFR,STAC,PRNP,PIK3R1,PLS1,SORBS1,KCNJ11,EPB41,GPER1,RAB11FIP2,CIB1,DLG1,ZDHHC8,CNPY4,CNST,WNK3,STAC2,STX4,ITGA3,AC138894.1,RAB11A,EFCAB7,PDPK1,RER1,ARHGEF16,GPSM2	grey	VSMC_M10
GO:0022898	0.53	0.53	99	186	regulation of transmembrane transporter activity	BP	1	NEDD4L,STAC3,MAPK8IP2,PRRT1,GOPC,PLA2G6,PIM1,ABCB1,WNK2,PRKCE,SHANK1,HECW1,TCAF2,OSR1,SPHK2,KCNRG,CACNB2,STAC,OXSR1,GPD1L,CACNB4,PRNP,SLMAP,GLRX,MEF2C,PTK2B,KCNJ11,HOMER1,IFNGR2,TESC,RIPK1,KCNE2,PKD2,CASQ2,KCNIP2,CHRM3,RYR2,FAM173B,PM20D1,HECW2,ADRA2A,COX17,KCNG1,ANK2,KLHL24,ACE2,ATP1A2,SLC9A1,C4orf3,CASQ1,NTSR1,SCN4B,CNIH2,NOS1AP,DLG1,HTT,PINK1,ATP1B1,KCNA1,GNB5,MINK1,HAP1,DLG4,ACTB,NLGN2,CNIH3,WNK3,STAC2,CACNB1,ATP1B2,NLGN3,ATP2A1,CALM1,DNM2,VMP1,CCL2,FMR1,RNF207,PARK7,AMIGO1,JPH3,WWP2,RRAD,MIR24-1,DMD,GPR35,JPH2,FGF11,SCN2B,NETO2,CACNG7,CRBN,CRH,APOPT1,YWHAH,SHISA8,CACNG5,GEM,PPIF	grey	VSMC_M10
GO:0003401	0.53	0.63	13	24	axis elongation	BP	1	ESR1,PTK7,AREG,TNC,SPRY1,SOX9,SPRY2,WNT11,NKD1,MED1,WNT5A,SIX1,FGF1	grey	VSMC_M10
GO:0044262	0.53	0.52	122	229	cellular carbohydrate metabolic process	BP	1	MIOX,SYNJ2,PRKN,PPARA,AKR1B1,GPT,GNMT,CRY1,IPPK,PRKCE,HAS1,PPP4R3B,ZNF692,PLEK,FAM3C,INPP4B,MGAM,BRAF,MTMR2,PYGB,RUBCNL,GLB1,KHK,EPM2AIP1,PRKAG2,XYLB,GCKR,RBKS,PPP1CB,CSGALNACT1,AKT2,C1QTNF1,SIRT7,PDK1,PPP1R3B,MTMR7,NLN,GK5,NCOA2,GBE1,B3GNT4,SORBS1,P2RY1,PTK2B,PFKM,SYNJ1,COQ2,ITPKA,IGF2,GANC,NHLRC1,PHKB,GPLD1,IRS1,SIK1,ITPK1,INPP5A,MST1,PPP1R3E,PFKFB2,GPER1,PPP1R3D,PER2,TREH,PFKFB4,ACTN3,HDAC4,NTSR1,PASK,PHKA2,INPP5J,DUSP12,GAL3ST3,PDK3,PTH1R,SORD,PLA2G4A,RPE,MOGAT3,HK1,PYGM,PYGL,GNB3,PRKAG3,AC022400.6,STAT3,PPP1R3F,IGFBP4,TKFC,DDB1,IDUA,DGKQ,OGT,PHKA1,COX11,LCMT1,NCOR1,INSR,ENOSF1,LEPR,IMPA2,PTPN2,FGGY,INPP5K,MECP2,GNPTG,MLYCD,AGL,FUK,HAS3,ACADM,FBP2,ABHD10,TIGAR,PPP1R1A,IDNK,POMK,NNMT,NEU2,INPP1,HS2ST1,ARPP19	grey	VSMC_M10
GO:0044827	0.53	0.67	9	16	modulation by host of viral genome replication	BP	1	YTHDC2,PRKN,PHB,FBXL2,EIF2AK4,HSPA8,FMR1,MIR222,TBC1D20	grey	VSMC_M10
GO:0006123	0.53	0.66	10	17	mitochondrial electron transport, cytochrome c to oxygen	BP	1	COX7A1,COX4I2,COX15,COX6A1,COX8A,COX6A2,COX5A,MT-CO3,COX7A2,COX7A2L	grey	VSMC_M10
GO:0045577	0.53	0.65	11	20	regulation of B cell differentiation	BP	1	IL4I1,NCKAP1L,IL7,SLAMF8,PPP2R3C,PTPN6,STAT5B,INHA,CARD11,BTK,ZFP36L2	grey	VSMC_M10
GO:0044264	0.53	0.56	40	75	cellular polysaccharide metabolic process	BP	1	GNMT,HAS1,MGAM,PYGB,RUBCNL,KHK,EPM2AIP1,PRKAG2,PPP1CB,CSGALNACT1,AKT2,PPP1R3B,GBE1,B3GNT4,SORBS1,PFKM,IGF2,NHLRC1,PHKB,IRS1,PPP1R3E,PPP1R3D,PER2,PASK,PHKA2,GAL3ST3,PYGM,PYGL,PRKAG3,AC022400.6,PPP1R3F,PHKA1,INSR,LEPR,INPP5K,AGL,HAS3,ACADM,PPP1R1A,HS2ST1	grey	VSMC_M10
GO:0060425	0.53	0.60	21	39	lung morphogenesis	BP	1	SRF,TNC,SPRY1,SOX9,SPRY2,TGFBR2,AP3B1,SRSF6,DAG1,KRAS,PLOD3,CTSH,MAPK1,MAPK3,MAP2K1,LAMA1,FOXF1,ESRP2,NOG,LIF,WNT2	grey	VSMC_M10
GO:0051928	0.53	0.56	46	86	positive regulation of calcium ion transport	BP	1	STAC3,TSPO,BAK1,MCHR1,SPHK2,TRPC3,CACNB2,STAC,P2RX2,P2RX3,ORAI1,P2RX7,HOMER1,STC1,F2R,KCNE2,PKD2,RYR2,ANK2,GPER1,SLC9A1,CASQ1,NTSR1,CEMIP,CASK,HTT,ATP1B1,HAP1,WNK3,STAC2,ATP2A1,CD19,CALM1,VMP1,BDKRB1,TRPV2,CAMK2A,CCL5,CCL2,TRPV3,PDPK1,P2RX1,ATP2C2,JPH2,THY1,CRH	grey	VSMC_M10
GO:0044060	0.53	0.65	11	20	regulation of endocrine process	BP	1	GDF9,CRY1,C1QTNF1,NKX3-1,F2RL1,F2R,CRY2,AGT,INHA,RAB8B,CRH	grey	VSMC_M10
GO:1900078	0.53	0.64	12	22	positive regulation of cellular response to insulin stimulus	BP	1	GKAP1,ECHDC3,SNX5,SORBS1,OPA1,IGF2,IRS1,SORL1,AGT,ESRRA,ZBTB7B,PRKCZ	grey	VSMC_M10
GO:0006730	0.53	0.61	17	31	one-carbon metabolic process	BP	1	SHMT2,GNMT,CA9,DHFR,AHCY,MTHFD2,CA14,MTHFS,MAT1A,CA4,SHMT1,CA2,CA5B,MTHFD2L,ALDH1L1,CA13,CA10	grey	VSMC_M10
GO:0060261	0.53	0.64	11	20	positive regulation of transcription initiation from RNA polymerase II promoter	BP	1	ESR1,MED30,SRF,ERCC1,PAXIP1,ERCC6,CREB1,MED14,MED24,MED1,NKX2-5	grey	VSMC_M10
GO:0001947	0.53	0.59	24	45	heart looping	BP	1	SMO,FOXH1,SRF,BBS7,SETDB2,IFT172,TGFBR2,OVOL2,MKKS,HAND2,MEF2C,IFT52,PKD2,SUFU,PSEN1,SMAD3,TBX2,MIB1,RNF207,ENG,LBX1,WNT5A,C2CD3,NKX2-5	grey	VSMC_M10
GO:0034122	0.53	0.63	13	24	negative regulation of toll-like receptor signaling pathway	BP	1	TNFAIP3,GRAMD4,LRRC14,CD300LF,MFHAS1,OTUD4,F2RL1,NOD2,RAB7B,GPS2,GPR108,PDPK1,DAB2IP	grey	VSMC_M10
GO:0070423	0.53	0.64	12	22	nucleotide-binding oligomerization domain containing signaling pathway	BP	1	TNFAIP3,HSPA1B,SLC46A2,TLR4,LACC1,RIPK2,OTULIN,NOD2,MAP2K6,BIRC3,IRGM,INAVA	grey	VSMC_M10
GO:0043586	0.53	0.65	10	18	tongue development	BP	1	BNC2,KIT,EGFR,NTF4,INTU,HDAC2,HAND2,KCNE2,HDAC1,SIX1	grey	VSMC_M10
GO:0097530	0.53	0.55	49	91	granulocyte migration	BP	1	PTK2,RAC2,CXCL8,AKIRIN1,C5AR2,C5AR1,MYD88,NCKAP1L,CCL17,NOD2,GBF1,DNM1L,TRPV4,TGFB2,CD99,CMKLR1,SLAMF8,MOSPD2,FCER1G,ADAM8,PIKFYVE,MCU,TNFSF18,CXCR2,BST1,MAPK1,DAPK2,MAPK3,S100A14,S100A8,UMOD,CD74,CCL14,CCL5,CSF1R,CCL7,CCL2,CCL25,VAV1,PTGER4,PIP5K1C,MPP1,BSG,VAV3,CCL16,S100A12,DPP4,CXCL6,CCL22	grey	VSMC_M10
GO:0097503	0.53	0.65	10	18	sialylation	BP	1	ST6GAL2,ST6GALNAC2,ST8SIA4,ST3GAL6,ST8SIA2,ST8SIA5,ST6GALNAC4,ST3GAL2,ST6GALNAC5,ST8SIA6	grey	VSMC_M10

GO:0006220	0.53	0.59	23	42	pyrimidine nucleotide metabolic process	BP	1	NME1,TBPL1,ENPP3,DPYS,CMPK1,MBD4,ENTPD4,NME4,CTPS2,DTYMK,UPB1,NME7,UPRT,NEIL1,NT5M,NTHL1,NME3,UCK1,DHODH,CMPK2,NME6,DCTD,UPP1	grey	VSMC_M10
GO:2000515	0.53	0.63	12	22	negative regulation of CD4-positive, alpha-beta T cell activation	BP	1	HLX,RUNX3,BCL6,VSIR,RC3H1,TNFSF18,FOXP3,TBX21,ZBTB7B,IL4R,TWSG1,RC3H2	grey	VSMC_M10
GO:0072576	0.53	0.65	10	18	liver morphogenesis	BP	1	GLI1,TNFAIP3,TGFA,PTN,FBXW7,SULF2,RPS6KA1,MED1,CEBPB,FGF1	grey	VSMC_M10
GO:0008344	0.53	0.57	30	56	adult locomotory behavior	BP	1	PRKN,AGTPBP1,BTBD9,ADAM22,TMOD1,PPT1,NTF4,GRIN2D,LGI4,PREX2,HTRA2,NR4A2,GIGYF2,OPRD1,ATP1A2,NTSR1,EPS8,EPHA4,CLN8,HOXB8,NPC1,SEZ6,DAB1,MECP2,PARK7,TSC1,CNTN2,CEND1,FXN,EFNB3	grey	VSMC_M10
GO:0070665	0.53	0.55	50	93	positive regulation of leukocyte proliferation	BP	1	AGER,IL5,HLA-DPB1,TNFRSF13C,PTK2,NCK2,PELI1,TLR4,IL15,BMI1,MYD88,TGFBR2,NCKAP1L,SLC7A1,TRAF6,IL1A,MEF2C,IL7,RIPK2,ZNF335,CD40,IGF2,ICOSLG,GPAM,CD28,BCL6,IL12A,CD38,BST1,MAPK1,STAT5B,FOXP3,RASAL3,CARD11,MAPK3,CHRNB2,CD74,CCL5,CSF1R,CD320,TICAM1,DNAJA3,VAV3,MIR21,FADD,CD46,CD70,TNFSF9,TNFSF13B,HAVCR2	grey	VSMC_M10
GO:0048255	0.53	0.59	23	43	mRNA stabilization	BP	1	PABPC1,MAPK14,TRAF3IP2,MYD88,TENT5C,TENT4B,RBM24,AXIN2,MAPKAPK2,TRAF5,ANGEL2,EIF4ENIF1,YBX3,SLC11A1,RBM10,TARDBP,TENT5D,LARP1,METTL16,CIRBP,TRAF2,YBX1,TENT5A	grey	VSMC_M10
GO:0006198	0.53	0.75	4	7	cAMP catabolic process	BP	1	PDE7B,PDE10A,PDE8A,PDE4A	grey	VSMC_M10
GO:0035337	0.53	0.60	19	35	fatty-acyl-CoA metabolic process	BP	1	HSD17B4,PPT2,ACSL6,PPT1,ELOVL1,ELOVL7,FITM2,FAR2,ACSL5,HACD2,THEM5,ACSBG1,TECR,GCDH,ACACA,ACSBG2,ACSF3,ELOVL3,NUDT19	grey	VSMC_M10
GO:0000291	0.54	0.65	10	18	nuclear-transcribed mRNA catabolic process, exonucleolytic	BP	1	CNOT6,SKIV2L,EXOSC4,EXOSC3,TTC37,DIS3L2,POLR2G,NT5C3B,CNOT8,EXOSC5	grey	VSMC_M10
GO:0072386	0.54	0.75	4	7	plus-end-directed organelle transport along microtubule	BP	1	KIF3A,KIF3B,KIF13A,MAPT	grey	VSMC_M10
GO:0006379	0.54	0.64	11	20	mRNA cleavage	BP	1	CPSF6,AGO2,CPSF1,PCF11,POLR2I,CSTF3,CPSF2,NCBP2,CPSF4,AP002990.1,CSTF2	grey	VSMC_M10
GO:0014888	0.54	0.59	20	37	striated muscle adaptation	BP	1	IL15,TRPC3,KDM4A,TNNC1,PPP3CA,TNNI1,MYH7,GTF2IRD1,MYOZ2,ACTN3,HDAC4,MYOZ1,CAMK2G,CAMTA2,TCAP,SMAD3,GATA6,MIR199A1,MIR25,HEY2	grey	VSMC_M10
GO:0140467	0.54	0.64	11	20	integrated stress response signaling	BP	1	ATF4,NCK2,EIF2AK4,QRICH1,NFE2L2,PTPN2,ABCA7,HSPA5,IMPACT,DDIT3,PPP1R15A	grey	VSMC_M10
GO:0140331	0.54	0.74	4	7	aminophospholipid translocation	BP	1	ATP8B1,TMEM30A,TMEM30B,ATP11C	grey	VSMC_M10
GO:1903051	0.54	0.58	27	50	negative regulation of proteolysis involved in cellular protein catabolic process	BP	1	TRIM39,PDCL3,SDCBP,AQP11,KLHL40,DDRGK1,RYBP,PSMF1,TAF9,USP25,CSNK2A2,FAM192A,MARCH7,SUFU,RPL23,USP19,RPL11,EPHA4,PSEN1,EFNA1,TAF1,OGT,SGTA,PARK7,CAMLG,STYX,MAP1A	grey	VSMC_M10
GO:0061462	0.54	0.59	22	41	protein localization to lysosome	BP	1	RTN4,KPTN,RAB7A,SZT2,NCOA4,AP3B1,GPR137B,HSPA8,SORL1,SH3BP4,MFSD1,AP4M1,AP3M1,ATP13A2,RNF128,LAMP2,LAMTOR5,TLDC1,VPS4A,CACNG7,SNX16,CACNG5	grey	VSMC_M10
GO:0097105	0.54	0.74	4	7	presynaptic membrane assembly	BP	1	NLGN4Y,NLGN2,NLGN3,CEL	grey	VSMC_M10
GO:0062043	0.54	0.74	4	7	positive regulation of cardiac epithelial to mesenchymal transition	BP	1	TGFBR2,JAG1,TGFB2,ENG	grey	VSMC_M10
GO:0010524	0.54	0.59	20	37	positive regulation of calcium ion transport into cytosol	BP	1	BAK1,TRPC3,P2RX2,P2RX3,P2RX7,F2R,PKD2,GPER1,CASQ1,NTSR1,CEMIP,HTT,HAP1,CD19,CALM1,BDKRB1,PDPK1,P2RX1,JPH2,THY1	grey	VSMC_M10
GO:0048814	0.54	0.57	30	57	regulation of dendrite morphogenesis	BP	1	NEDD4L,RAB21,CHRNA3,SEMA4D,HECW1,SS18L2,ANKRD27,TLX2,CUX2,RAPGEF2,TIAM1,OPA1,ITPKA,LZTS1,DNM1L,PPP3CA,HECW2,CAPRIN2,NFATC4,SS18L1,CDKL5,OBSL1,EPHA4,CHRNB2,PARP6,GPRASP2,BHLHB9,LRP8,FBXO31,YWHAH	grey	VSMC_M10
GO:0009068	0.54	0.66	9	16	aspartate family amino acid catabolic process	BP	1	RIDA,DDO,SDSL,MAT1A,ASRGL1,CRYM,PIPOX,SLC25A21,HYKK	grey	VSMC_M10
GO:0097428	0.54	0.67	8	14	protein maturation by iron-sulfur cluster transfer	BP	1	ISCA1,CIAO1,LTO1,HSCB,CIAO2B,GLRX5,CISD3,ISCA2	grey	VSMC_M10
GO:2001222	0.54	0.60	17	32	regulation of neuron migration	BP	1	SEMA6A,SEMA3A,UNC5D,NRG1,IGSF10,RAPGEF2,NKX6-1,SRGAP2C,LRIG2,COL3A1,STAT3,ZNF609,CAMK2A,NTNG2,FBXO31,DAB2IP,ADGRG1	grey	VSMC_M10
GO:0040036	0.54	0.62	13	24	regulation of fibroblast growth factor receptor signaling pathway	BP	1	FUZ,SPRY1,PRKD2,SPRY2,FGFBP3,DSTYK,SULF2,LRIT3,WNT4,NGFR,NOG,WNT5A,SHISA2	grey	VSMC_M10
GO:0007423	0.54	0.50	219	415	sensory organ development	BP	1	SLC39A5,ATP8B1,EYA4,MDM1,FRS2,OSR2,FZD6,TBC1D32,BAK1,ATF4,AGTPBP1,NTRK2,BNC2,PRPH2,RORB,PTK7,SRF,SLC25A27,KIT,SLC1A1,TFAP2B,ROR2,DLX5,TDRD7,TMOD1,SIX2,RDH13,ABCB5,KCNQ4,GSDME,HOXA2,HOXA13,MYCL,BMPER,WHRN,PTN,EGFR,TSPAN12,USP45,ATG5,MYCN,SOBP,SLC7A11,OSR1,NTF4,INTU,SPRY1,FOXE3,HDAC2,BBS7,SLC26A5,MYO3A,SOX9,MYO7B,USH1G,GLI2,CRYGN,ZNF513,ACVR2B,IFT172,MYO7A,TSKU,SPRY2,DZANK1,TGFBR2,BFSP1,SLC4A7,JAG1,RARB,FSCN2,LRTOMT,AP000812.5,LIN7A,SPRED3,FREM2,CEP290,KERA,MAB21L1,MITF,FLT1,TUB,TCF15,EPHB1,PFDN5,NPHP1,RET,PAX8,CEBPA,FOXL2,HAND2,EYA1,PLS1,MAB21L2,TENM3,ADGRV1,FZD3,USH1C,TENT2,OPA1,RPGRIP1L,KCNE2,TFAP2A,PAX2,NKD1,BMPR1B,MYH15,NECTIN3,LPCAT1,PKNOX1,SLC6A3,HDAC1,ALDH1A3,TGFB2,TRIP11,TCIRG1,PRSS56,SHROOM2,MAPKAPK2,SOX2,CRYBA4,NRL,GBX2,MYO3B,KCNK2,CLIC4,DLX1,GRHL3,LIMK2,SP3,STRC,CDKN1B,DLG1,FZD5,STOX1,PRKRA,CDH23,FBN1,FKBP8,COL5A2,ECE1,PLA2G16,STAT3,NKX3-2,MAPK1,TULP3,OBSL1,RHOJ,ROM1,MFAP2,EPHA4,DVL2,WDR19,SDK1,MAX,CLN8,TCAP,RBPJ,MED1,ALDH1A2,MFN2,PSEN1,TTLL5,MAPK3,ESRRB,MEGF11,LCTL,POU3F4,SMAD3,NTN1,MKS1,GSC,TBX2,BCL11B,LHX1,STRA6,YY1,CHRDL1,UNC45B,TMEM132E,JAG2,SMCHD1,LAMA1,IFT20,TWSG1,GNB1,FZR1,SKI,SOX8,COL5A1,LAMC3,OLFM3,TMEM231,SLC25A25,MCOLN3,HSF4,KCNK3,BLOC1S3,FJX1,PJVK,CHRNA10,FOXG1,MAFB,CNTF,ACVRL1,NOG,THY1,WNT5A,DIO3,OTX1,SIX1,BCL2L11,TGIF2,TBC1D20,INSIG2,PITX3,HEY2,WNT2,COL8A2,ZIC1	grey	VSMC_M10
GO:1903288	0.54	0.74	4	7	positive regulation of potassium ion import across plasma membrane	BP	1	WNK2,ATP1B1,WNK3,ATP1B2	grey	VSMC_M10

GO:0022904	0.54	0.54	55	99	respiratory electron transport chain	BP	1	UQCRQ,NDUFA6,SLC25A13,DLD,NDUFB2,NDUFB6,DNAJC15,NDUFC2,NDUFS8,NDUFA12,COX7A1,COX4I2,GPD1,NDUFS3,ETFDH,DGUOK,UQCRB,COX15,NDUFV3,NDUFB4,ISCU,COA6,POLG2,NDUFB3,NDUFS2,SDHC,COX6A1,COX8A,PINK1,NDUFA9,SDHB,COX6A2,NDUFAB1,COX5A,NDUFA7,NDUFV2,NDUFB10,UQCR11,PARK7,MT-ND1,MT-ND2,MT-CO3,MT-ND6,MT-CYB,NDUFA5,NDUFC1,COX7A2,ETFRF1,NDUFA3,NDUFB5,NDUFS6,NDUFA8,SDHAF2,NDUFS5,COX7A2L	grey	VSMC_M10
GO:0090175	0.54	0.57	27	51	regulation of establishment of planar polarity	BP	1	FZD6,RSPO3,PTK7,ROR2,WNT11,SPEF1,RYK,FZD3,TIAM1,VANGL1,PRICKLE1,NKD1,JHY,ZNRF3,DVL3,FZD7,GRHL3,DAAM1,ARHGEF19,DVL2,MKS1,GPC3,SAPCD2,CELSR2,RHOA,WNT5A,CELSR3	grey	VSMC_M10
GO:0050974	0.54	0.61	14	26	detection of mechanical stimulus involved in sensory perception	BP	1	PHF24,KIT,WHRN,ASIC3,SCN1A,MKKS,ADGRV1,BACE1,STRC,KCNK4,KCNA1,ASIC2,PJVK,CHRNA10	grey	VSMC_M10
GO:0006536	0.54	0.63	12	22	glutamate metabolic process	BP	1	GLS2,SLC7A11,GAD2,AMDHD1,NR1H4,GLS,ALDH5A1,OAT,GLUL,MTHFS,PRODH,ALDH4A1	grey	VSMC_M10
GO:0046653	0.54	0.65	9	16	tetrahydrofolate metabolic process	BP	1	SHMT2,DHFR,MTHFD2,MTHFS,GCH1,SHMT1,PIPOX,MTHFD2L,ALDH1L1	grey	VSMC_M10
GO:0006171	0.54	0.71	5	9	cAMP biosynthetic process	BP	1	ADCY3,ADCY7,ADCY2,ADCY4,ADCY10	grey	VSMC_M10
GO:0042730	0.54	0.65	9	16	fibrinolysis	BP	1	SERPINB2,THBD,PLAT,FAP,USF1,F12,ANXA2,SERPINF2,FGA	grey	VSMC_M10
GO:0003081	0.54	0.65	9	16	regulation of systemic arterial blood pressure by renin-angiotensin	BP	1	MME,F2RL1,F2R,ENPEP,AGT,ACE2,SERPINF2,CYBA,RHOA	grey	VSMC_M10
GO:0070371	0.54	0.51	121	227	ERK1 and ERK2 cascade	BP	1	LRP1,SEMA6A,FRS2,AGER,FBLN1,ROS1,MYC,PRXL2C,TEK,WNK2,FPR2,MTURN,NOD1,BMPER,EPHA7,ITGB1BP1,VRK3,GRB10,YWHAZ,EGFR,TLR4,FGF21,BRAF,SPRY1,DNAJC27,C5AR2,C5AR1,PRKD2,PHB,SOX9,SPRY2,ARRB1,NEK10,SPRED3,DUSP6,NRG1,GAS6,SIRT3,MFHAS1,APIP,ST5,EPHB1,HAND2,FBXW7,P2RY1,PTK2B,RASGRP1,RAPGEF2,CCL17,TIAM1,RIPK2,F2RL1,F2R,NQO2,GCNT2,NOD2,DSTYK,TRPV4,SLC30A10,EGF,TBC1D10C,GPER1,AGT,CIB1,THPO,PDE8A,DLG1,DUSP10,RAP1B,CTSH,FN1,PTPN6,GPBAR1,FGFR3,MAPK1,RGS14,FGFR4,SYNJ2BP,FGF18,MAPK3,MAP2K1,TNIP1,GAREM1,CD74,CCL14,CCL5,CSF1R,CCL7,CCL2,CCL25,PTGER4,PTPN2,SERPINF2,PRKCZ,ATP6AP1,ABCA7,KARS,DAB2IP,MIR21,MIR222,MIR23A,MIR27B,MIR24-1,MIR27A,FGA,CCL16,CHRNA10,DUSP26,DUSP1,DUSP4,C3orf33,LIF,HRAS,ZFP36L2,STYX,MAP2K7,FGF1,C1QL4,HAVCR2,CCL22,PDGFD	grey	VSMC_M10
GO:1902369	0.54	0.56	32	60	negative regulation of RNA catabolic process	BP	1	PABPC1,PHAX,MAPK14,LRPPRC,TRAF3IP2,DHX34,MYD88,PKP3,TENT2,TENT5C,TENT4B,RBM24,AXIN2,MAPKAPK2,TRAF5,ANGEL2,EIF4ENIF1,YBX3,SLC11A1,RBM10,TARDBP,TOB1,TENT5D,NRDE2,LARP1,PARN,FMR1,METTL16,CIRBP,TRAF2,YBX1,TENT5A	grey	VSMC_M10
GO:0045661	0.54	0.58	21	39	regulation of myoblast differentiation	BP	1	MAPK14,SOSTDC1,AKIRIN1,SOX9,SRA1,PLCB1,BTG1,SMYD1,MEF2C,CCL17,HIF1AN,PRICKLE1,RBM24,CDON,RANBP3L,MBNL3,SOX8,LRRC8A,MIR199A1,DDIT3,XKR8	grey	VSMC_M10
GO:0006829	0.54	0.62	13	24	zinc ion transport	BP	1	SLC39A5,SLC39A4,SLC1A1,SLC39A12,TMEM163,SLC39A13,SLC30A10,SLC30A4,SLC39A9,SLC39A6,SLC30A7,SLC39A11,SLC39A3	grey	VSMC_M10
GO:2000649	0.54	0.58	23	43	regulation of sodium ion transmembrane transporter activity	BP	1	NEDD4L,WNK2,PRKCE,HECW1,OSR1,GPD1L,SLMAP,GLRX,TESC,HECW2,KLHL24,ATP1A2,SLC9A1,SCN4B,ATP1B1,WNK3,DNM2,MIR24-1,DMD,FGF11,SCN2B,NETO2,YWHAH	grey	VSMC_M10
GO:0051693	0.54	0.59	18	34	actin filament capping	BP	1	AVIL,LMOD2,KIAA1211,TMOD1,SPTBN1,ADD2,LMOD3,DMTN,CARMIL1,LMOD1,ADD3,SPTBN2,TMOD4,EPS8,CAPZA1,TMOD2,SPTB,CARMIL2	grey	VSMC_M10
GO:0044773	0.54	0.55	38	71	mitotic DNA damage checkpoint signaling	BP	1	MRNIP,TRIM39,PPP1R10,MDC1,BRSK1,MSH2,XPC,PLK3,NEK11,PRKDC,STK33,PLK2,RAD17,RAD9B,NBN,ABRAXAS1,DONSON,PIDD1,TAOK3,CCND1,GIGYF2,CHEK2,SYF2,CDKN1B,UIMC1,FOXO4,EME1,MUC1,TAOK2,TIPIN,FOXN3,TAOK1,FZR1,FBXO31,RPS27L,SOX4,BRCC3,CRADD	grey	VSMC_M10
GO:0043618	0.54	0.58	23	43	regulation of transcription from RNA polymerase II promoter in response to stress	BP	1	ATF4,HSF1,NCK2,EPAS1,SIRT2,TMBIM6,BACH1,HIF1AN,ARNT,BAG3,NFE2L2,STOX1,DNAJB1,RBPJ,MUC1,SIN3A,MAPK7,JUN,CREB3L3,HSPA5,IMPACT,DDIT3,CEBPB	grey	VSMC_M10
GO:0045582	0.54	0.56	32	60	positive regulation of T cell differentiation	BP	1	HLA-DRA,IL4I1,GLI2,TGFBR2,NCKAP1L,SOX12,IL7,RASGRP1,RIPK2,AP3B1,SOX13,HLX,RUNX3,ADAM8,DUSP10,BCL6,VSIR,STAT5B,FOXP3,ZBTB1,ZBTB7B,IL4R,CD74,NKAP,IL7R,SOCS1,PRKCZ,MIR21,RHOA,SOX4,CD46,TNFSF9	grey	VSMC_M10
GO:0042100	0.54	0.56	30	56	B cell proliferation	BP	1	IL5,TNFRSF13C,TNFRSF21,PELI1,TLR4,BMI1,PAWR,PLCL2,NCKAP1L,GAPT,CASP3,MEF2C,IL7,RASGRP1,CD40,BCL6,RC3H1,CD38,BST1,CARD11,CHRNB2,CD19,BTK,CD74,CD320,IL7R,TICAM1,VAV3,CD70,TNFSF13B	grey	VSMC_M10
GO:0043304	0.55	0.62	13	24	regulation of mast cell degranulation	BP	1	RAC2,SPHK2,GAB2,UNC13D,FER,FGR,SNX4,C12orf4,IL4R,STXBP2,PDPK1,FOXF1,STXBP1	grey	VSMC_M10
GO:0050820	0.55	0.64	10	18	positive regulation of coagulation	BP	1	ENPP4,THBD,F2R,ANO6,DMTN,USF1,NFE2L2,F12,PSEN1,SERPINF2	grey	VSMC_M10
GO:0045940	0.55	0.64	10	18	positive regulation of steroid metabolic process	BP	1	APOC1,CYP7A1,AGT,SCAP,POR,WNT4,SREBF1,LDLRAP1,STARD4,FGF1	grey	VSMC_M10

GO:0072507	0.55	0.50	171	323	divalent inorganic cation homeostasis	BP	1	SLC39A5,LRP1,CYP27B1,ESR1,PRKN,SMDT1,BAK1,MCHR1,GPR20,PLA2G6,SLC39A4,SLC25A27,SLC1A1,TFAP2B,S1PR3,CNNM4,PRKCE,TNNI3,FPR2,IBTK,LPAR1,EGFR,KEL,ATG5,TRPM4,GRIN2D,TRPC3,SLC8A2,C5AR2,C5AR1,ATP7B,PTGIR,GIPR,CACNB2,SLC39A12,ACKR2,SLC24A3,C1QTNF1,TMTC4,RYR1,PRNP,ADRA1D,DIAPH1,P2RX2,KL,CCKBR,RASA3,MICU3,IMMT,C19orf12,P2RX3,PLCE1,RYR3,TMBIM6,P2RX7,P2RY1,PTK2B,SLC39A13,TPCN1,SLC8B1,VDR,STC1,AP3B1,RMDN3,F2RL1,F2R,PKD2,CASQ2,MCUR1,CD40,RYR2,SLC41A1,RAP1GDS1,TRPM1,SPPL3,LCK,TRPV4,SLC30A10,TCIRG1,FAM20A,GNA13,CMKLR1,MCUB,UBASH3B,DISC1,ANK2,GPER1,TRPM8,PDZD8,AGT,ATP1A2,CASQ1,NTSR1,FZD9,TMCO1,PTH1R,SLC30A4,FIS1,CEMIP,ABL2,CDH23,PTPN6,MICU1,MCU,CXCR2,GNG3,SLC11A1,HTT,PTGDR,PTGER2,TRPM7,ATP1B1,KCNA1,CD38,ATP13A2,HAP1,DLG4,FAM155B,PSEN1,CACNA1A,AC138894.1,ATP2A1,CD19,SMAD3,CALM1,S100A14,S100A8,PRKCB,BDKRB1,NMUR2,SLC39A6,TRPV2,HRH4,CCL14,CCL5,CCL7,PTGER4,SLC25A23,MAIP1,GNB1,ATP2B3,BCAP31,GNA15,TRPV3,TRPV1,PDPK1,P2RX1,PKD1,TMEM203,GPR157,SYPL2,CYBA,JPH3,ATP2C2,SLC30A7,MCOLN3,PTGFR,MIR199A1,KCNK3,DMD,MT1X,GPR35,NOL3,CHRNA10,TRIM24,CCR10,JPH2,SNX10,THY1,WNT5A,DDIT3	grey	VSMC_M10
GO:0072348	0.55	0.58	22	41	sulfur compound transport	BP	1	SLC26A10,SLC35B2,SLC1A1,SLC3A1,SLC1A4,SLC13A4,SLC7A11,SLC26A5,SLC25A30,ABCC4,SLC25A26,SLC33A1,ABCG2,ABCC5,SLC27A1,SLC25A11,SLC26A1,SLC26A2,SLC25A14,LRRC8A,SLC36A1,LRRC8D	grey	VSMC_M10
GO:0001840	0.55	0.74	4	7	neural plate development	BP	1	PTCH1,DVL2,NOG,C2CD3	grey	VSMC_M10
GO:1901570	0.55	0.58	21	39	fatty acid derivative biosynthetic process	BP	1	PPT2,ACSL6,HMGCLL1,PPT1,ELOVL1,ACSS3,ELOVL7,FAR2,ACSL5,HACD2,HMGCL,GGT2,ACSBG1,GGT6,TECR,GCDH,ACACA,ACSBG2,ACSF3,ELOVL3,GGT5	grey	VSMC_M10
GO:0048753	0.55	0.61	13	24	pigment granule organization	BP	1	AP3B1,DTNBP1,KIF13A,SHROOM2,HPS4,PIKFYVE,BLOC1S6,ABCB6,SNAPIN,AP1G1,BLOC1S3,RAB32,TRAPPC6A	grey	VSMC_M10
GO:1903214	0.55	0.58	21	39	regulation of protein targeting to mitochondrion	BP	1	PRKN,RAC2,USP36,BAG4,FBXW7,HTRA2,CSNK2A2,HPS4,ARIH2,BAG3,FZD5,C11orf65,PINK1,NPEPPS,HAX1,SREBF1,SH3GLB1,MICALL2,BNIP3L,RHOU,LEPROT	grey	VSMC_M10
GO:2000677	0.55	0.58	20	37	regulation of transcription regulatory region DNA binding	BP	1	POU4F1,DDRGK1,TRAF6,HAND2,FBXW7,PER2,PARP1,NSD1,MSX2,PSEN1,TAF1,SIN3A,ZBTB7C,DOT1L,PARK7,LAMTOR5,DDIT3,ZNF593,LIF,HEY2	grey	VSMC_M10
GO:0032056	0.55	0.74	4	7	positive regulation of translation in response to stress	BP	1	NCK2,EIF2AK4,DDX3X,IMPACT	grey	VSMC_M10
GO:0008306	0.55	0.56	28	53	associative learning	BP	1	SYNGAP1,SRF,KIT,SLC1A1,BRSK1,SHANK1,PPT1,SLC7A11,ADCY3,B4GALT2,DDHD2,ASIC1,KRAS,AGT,ATP1A2,CREB1,TNR,NPTX2,CLN8,RGS14,FOS,AC138894.1,CHRNB2,IFT20,MECP2,ABCA7,CRH,MAP1A	grey	VSMC_M10
GO:0003299	0.55	0.62	12	22	muscle hypertrophy in response to stress	BP	1	TRPC3,KDM4A,PPP3CA,MYH7,HDAC4,CAMTA2,TCAP,SMAD3,GATA6,MIR199A1,MIR25,HEY2	grey	VSMC_M10
GO:0014887	0.55	0.62	12	22	cardiac muscle adaptation	BP	1	TRPC3,KDM4A,PPP3CA,MYH7,HDAC4,CAMTA2,TCAP,SMAD3,GATA6,MIR199A1,MIR25,HEY2	grey	VSMC_M10
GO:0014898	0.55	0.62	12	22	cardiac muscle hypertrophy in response to stress	BP	1	TRPC3,KDM4A,PPP3CA,MYH7,HDAC4,CAMTA2,TCAP,SMAD3,GATA6,MIR199A1,MIR25,HEY2	grey	VSMC_M10
GO:0030835	0.55	0.58	20	38	negative regulation of actin filament depolymerization	BP	1	AVIL,LMOD2,KIAA1211,TMOD1,PLEKHH2,SPTBN1,ADD2,LMOD3,LIMA1,DMTN,CARMIL1,LMOD1,ADD3,SPTBN2,TMOD4,EPS8,CAPZA1,TMOD2,SPTB,CARMIL2	grey	VSMC_M10
GO:0007129	0.55	0.58	21	39	homologous chromosome pairing at meiosis	BP	1	UBE2B,DMC1,CCDC155,MLH1,CCNB1IP1,SIRT7,TEX15,SYCP1,CCDC36,SPO11,TEX12,MAJIN,ZCWPW1,STAG3,P3H4,SYCE2,MEIOB,MSH4,RNF212B,AGO4,SYCE1L	grey	VSMC_M10
GO:0019430	0.55	0.64	10	18	removal of superoxide radicals	BP	1	FANCC,PRDX1,DHFR,FBLN5,NFE2L2,GCH1,SOD3,MPO,NQO1,MIR21	grey	VSMC_M10
GO:0032906	0.55	0.70	5	9	transforming growth factor beta2 production	BP	1	WNT11,TGFB2,BMPR1A,SMAD3,GATA6	grey	VSMC_M10
GO:0032909	0.55	0.70	5	9	regulation of transforming growth factor beta2 production	BP	1	WNT11,TGFB2,BMPR1A,SMAD3,GATA6	grey	VSMC_M10
GO:0045622	0.55	0.61	13	24	regulation of T-helper cell differentiation	BP	1	HLA-DRA,RIPK2,HLX,BCL6,RC3H1,TNFSF18,FOXP3,TBX21,ZBTB7B,IL4R,PRKCZ,MIR21,RC3H2	grey	VSMC_M10
GO:0045922	0.55	0.62	12	22	negative regulation of fatty acid metabolic process	BP	1	SOX9,APOC1,SLC22A13,PIBF1,TRIB3,CYP7A1,ERLIN1,SIRT4,ACADL,ACADVL,NCOR1,INSIG2	grey	VSMC_M10
GO:0051481	0.55	0.70	5	9	negative regulation of cytosolic calcium ion concentration	BP	1	LRP1,RYR3,ATP1A2,SMAD3,KCNK3	grey	VSMC_M10
GO:0008053	0.55	0.61	13	24	mitochondrial fusion	BP	1	PRKN,BAK1,OPA1,DNM1L,ZDHHC6,USP30,MFN1,FIS1,MUL1,MFN2,ADCK1,THG1L,MIGA2	grey	VSMC_M10
GO:0098693	0.55	0.73	4	7	regulation of synaptic vesicle cycle	BP	1	BSN,DNAJC5,SYN1,CDK5R1	grey	VSMC_M10
GO:1902883	0.55	0.63	10	18	negative regulation of response to oxidative stress	BP	1	NCOA7,GPR37,MET,PDE8A,NFE2L2,PINK1,PARK7,MIR21,GPR37L1,TXN	grey	VSMC_M10
GO:1901568	0.55	0.56	29	54	fatty acid derivative metabolic process	BP	1	HSD17B4,ABHD16A,PPT2,ACSL6,HMGCLL1,PPT1,ELOVL1,ACSS3,ELOVL7,HPGD,FITM2,FAR2,ACSL5,HACD2,THEM5,HMGCL,GGT2,ACSBG1,GGT6,TECR,GCDH,ACACA,OXCT1,ACSBG2,ACSF3,ELOVL3,NUDT19,GGT5,LYPLA2	grey	VSMC_M10
GO:0035050	0.55	0.56	29	55	embryonic heart tube development	BP	1	SMO,FOXH1,SRF,TEAD2,BBS7,SETDB2,MEGF8,IFT172,TGFBR2,OVOL2,MKKS,HAND2,MEF2C,IFT52,PKD2,RYR2,SUFU,MED1,PSEN1,SMAD3,TBX2,MIB1,RNF207,ENG,LBX1,WNT5A,C2CD3,NKX2-5,PIFO	grey	VSMC_M10
GO:0090314	0.55	0.60	14	26	positive regulation of protein targeting to membrane	BP	1	ITGB1BP1,TCAF2,AKT2,PRNP,KCNB1,C2CD5,CIB1,FIS1,CEMIP,ITGAM,MIEF2,CDK5R1,HRAS,MIEF1	grey	VSMC_M10

GO:0048592	0.55	0.52	62	118	eye morphogenesis	BP	1	FRS2,BAK1,ATF4,AGTPBP1,NTRK2,RORB,SLC1A1,TFAP2B,TDRD7,RDH13,PTN,TSPAN12,FOXE3,SOX9,IFT172,MYO7A,TSKU,DZANK1,JAG1,RARB,FSCN2,CEP290,EPHB1,TENM3,USH1C,RPGRIP1L,TFAP2A,PAX2,NKD1,NECTIN3,ALDH1A3,SHROOM2,NRL,SP3,FZD5,FBN1,COL5A2,STAT3,OBSL1,ROM1,MFAP2,SDK1,MFN2,MEGF11,LCTL,TBX2,LHX1,STRA6,YY1,SKI,SOX8,COL5A1,OLFM3,FJX1,CNTF,THY1,WNT5A,DIO3,TBC1D20,PITX3,WNT2,COL8A2	grey	VSMC_M10
GO:0003418	0.55	0.73	4	7	growth plate cartilage chondrocyte differentiation	BP	1	COL27A1,SOX9,TSKU,TGFBR2	grey	VSMC_M10
GO:0046548	0.55	0.73	4	7	retinal rod cell development	BP	1	NTRK2,RORB,RPGRIP1L,NRL	grey	VSMC_M10
GO:0031128	0.55	0.60	15	28	developmental induction	BP	1	FRS2,SPRY1,SOX9,WNT11,ROBO1,ROBO2,SALL1,WNT3,FZD5,WNT4,SOX8,WNT5A,SIX1,FGF1,WNT2	grey	VSMC_M10
GO:0099532	0.55	0.70	5	9	synaptic vesicle endosomal processing	BP	1	AP3B1,DNM1L,STX6,GRIPAP1,BTBD8	grey	VSMC_M10
GO:0034650	0.55	0.73	4	7	cortisol metabolic process	BP	1	DKK3,WNT4,DGKQ,CACNA1H	grey	VSMC_M10
GO:0034651	0.55	0.73	4	7	cortisol biosynthetic process	BP	1	DKK3,WNT4,DGKQ,CACNA1H	grey	VSMC_M10
GO:1902645	0.55	0.73	4	7	tertiary alcohol biosynthetic process	BP	1	DKK3,WNT4,DGKQ,CACNA1H	grey	VSMC_M10
GO:0050854	0.55	0.57	23	43	regulation of antigen receptor-mediated signaling pathway	BP	1	CD226,PRKD2,NECTIN2,PAWR,PLCL2,PRNP,FOXP1,LPXN,PTPRJ,SLA2,LCK,RC3H1,PTPN6,GPS2,CARD11,CD19,PRKCB,PTPN2,IKBKG,BCL10,CMTM3,THY1,GCSAM	grey	VSMC_M10
GO:0060947	0.55	0.73	4	7	cardiac vascular smooth muscle cell differentiation	BP	1	SRF,GPER1,GATA6,HEY2	grey	VSMC_M10
GO:0006688	0.55	0.61	13	24	glycosphingolipid biosynthetic process	BP	1	B4GALNT1,A4GALT,LARGE1,ST8SIA4,A3GALT2,ST8SIA2,B3GALT1,ST8SIA5,ST6GALNAC4,ST3GAL2,ST6GALNAC5,B4GALT3,ST8SIA6	grey	VSMC_M10
GO:0034454	0.55	0.68	6	11	microtubule anchoring at centrosome	BP	1	CCDC68,KIF3A,NINL,HOOK3,NIN,CCDC120	grey	VSMC_M10
GO:0030174	0.55	0.73	4	7	regulation of DNA-dependent DNA replication initiation	BP	1	NBN,WRNIP1,KAT7,WDR18	grey	VSMC_M10
GO:0030224	0.56	0.59	15	28	monocyte differentiation	BP	1	MYC,MYH9,CDK6,HOXA7,FOXP1,IRF7,ACIN1,ZBTB46,SP3,MED1,GPR68,CD74,CSF1R,JUN,MIR125B1	grey	VSMC_M10
GO:1905144	0.56	0.61	13	24	response to acetylcholine	BP	1	LY6E,CHRNA3,PLCB1,CHRM5,CHRM4,CHRM3,GRK2,CHRM1,CHRNB2,HRH4,CDK5R1,GNB1,GNA15	grey	VSMC_M10
GO:1905145	0.56	0.61	13	24	cellular response to acetylcholine	BP	1	LY6E,CHRNA3,PLCB1,CHRM5,CHRM4,CHRM3,GRK2,CHRM1,CHRNB2,HRH4,CDK5R1,GNB1,GNA15	grey	VSMC_M10
GO:1990456	0.56	0.73	4	7	mitochondrion-endoplasmic reticulum membrane tethering	BP	1	PDZD8,PSEN2,CALM1,VMP1	grey	VSMC_M10
GO:0061724	0.56	0.73	4	7	lipophagy	BP	1	SPTLC1,RAB7A,AUP1,HTT	grey	VSMC_M10
GO:1904645	0.56	0.57	22	41	response to amyloid-beta	BP	1	LRP1,AGER,FPR2,TLR4,FOXO3,ATP1A3,PRNP,BACE1,SNX6,ITGA4,MMP13,MMP3,EPHA4,PARP1,CACNB1,PSEN1,CACNA1A,NGFR,CACNA1B,LCN2,BCL2L11,BCL2L2	grey	VSMC_M10
GO:1990700	0.56	0.70	5	9	nucleolar chromatin organization	BP	1	BAZ2A,SIRT2,DDX11,PHF8,BEND3	grey	VSMC_M10
GO:0072393	0.56	0.66	7	13	microtubule anchoring at microtubule organizing center	BP	1	CCDC68,KIF3A,BICD2,NINL,HOOK3,NIN,CCDC120	grey	VSMC_M10
GO:1901725	0.56	0.70	5	9	regulation of histone deacetylase activity	BP	1	C6orf89,SPHK2,PRKD2,PINK1,KDM5A	grey	VSMC_M10
GO:0097104	0.56	0.70	5	9	postsynaptic membrane assembly	BP	1	NLGN4Y,NRXN2,NLGN2,NLGN3,CEL	grey	VSMC_M10
GO:0046427	0.56	0.60	13	24	positive regulation of receptor signaling pathway via JAK-STAT	BP	1	IL5,AKR1B1,OCIAD1,KIT,PIBF1,PTK2B,F2R,AGT,IL10RA,IL6R,CCL5,GHR,SOCS1	grey	VSMC_M10
GO:0003207	0.56	0.70	5	9	cardiac chamber formation	BP	1	HAND2,MEF2C,TBX5,TBX2,SOX4	grey	VSMC_M10
GO:0060700	0.56	0.73	4	7	regulation of ribonuclease activity	BP	1	TMBIM6,OAS3,OASL,OAS1	grey	VSMC_M10
GO:0039529	0.56	0.60	13	24	RIG-I signaling pathway	BP	1	USP15,ANKRD17,PUM2,PHB,SEC14L1,CLPB,NPLOC4,OAS3,OASL,NLRX1,BIRC3,RNF135,ZCCHC3	grey	VSMC_M10
GO:2000401	0.56	0.57	22	41	regulation of lymphocyte migration	BP	1	CCL27,OXSR1,JAM2,PTK2B,SLC8B1,ADTRP,CD200,APOD,ADAM8,MIA3,ITGA4,PADI2,TMEM102,STK10,CCL5,CCL7,CCL2,CRK,RHOA,FADD,WNT5A,GCSAM	grey	VSMC_M10
GO:0071389	0.56	0.73	4	7	cellular response to mineralocorticoid stimulus	BP	1	FOXO3,GPER1,NPAS4,SCNN1B	grey	VSMC_M10
GO:0070562	0.56	0.73	4	7	regulation of vitamin D receptor signaling pathway	BP	1	CYP27B1,SNW1,MED1,TRIM24	grey	VSMC_M10
GO:0045619	0.56	0.52	62	117	regulation of lymphocyte differentiation	BP	1	HLA-DRA,TCF7,XRCC6,FBXO7,SLC46A2,IL4I1,IL15,CLPTM1,GLI2,FOXJ1,IL15RA,TGFBR2,NCKAP1L,GAS6,PRKDC,SOX12,TOX,ZC3H8,CAMK4,TMEM131L,IL7,RASGRP1,RIPK2,AP3B1,SOX13,HLX,SLAMF8,CD28,RUNX3,PPP2R3C,ADAM8,DUSP10,BCL6,VSIR,RC3H1,PTPN6,TNFSF18,LAG3,STAT5B,INHA,FOXP3,ZBTB1,CARD11,TBX21,ZBTB7B,IL4R,BTK,CD74,NKAP,IL7R,PTPN2,SOCS1,DROSHA,PRKCZ,MIR21,RHOA,SOX4,CD46,FGL2,TNFSF9,ZFP36L2,RC3H2	grey	VSMC_M10
GO:1903897	0.56	0.70	5	9	regulation of PERK-mediated unfolded protein response	BP	1	NCK2,PTPN2,ABCA7,HSPA5,PPP1R15A	grey	VSMC_M10
GO:0045876	0.56	0.73	4	7	positive regulation of sister chromatid cohesion	BP	1	SLF1,SLF2,DDX11,SFPQ	grey	VSMC_M10
GO:0021515	0.56	0.59	16	30	cell differentiation in spinal cord	BP	1	PTCH1,GLI2,IFT172,MNX1,SOX6,SOX13,SUFU,GIGYF2,MDGA2,SCYL3,CLN8,LHX1,ISL1,SOX4,ISL2,LBX1	grey	VSMC_M10
GO:0030002	0.56	0.70	5	9	cellular anion homeostasis	BP	1	SLC12A2,CKB,ABCC2,SLC12A5,XPR1	grey	VSMC_M10
GO:0043406	0.56	0.52	49	93	positive regulation of MAP kinase activity	BP	1	MAP3K5,MAP3K4,AGER,TAB1,KIT,ROR2,EPGN,TGFA,EGFR,ARHGEF5,TLR4,NEK10,FLT1,TRAF6,ROBO1,PTK2B,RASGRP1,TIAM1,NOD2,TAOK3,CD40,ADRA2A,AJUBA,KRAS,EGF,DVL3,MAP3K11,ADAM8,FZD5,MAP4K2,DUSP19,PDCD10,EPHA4,MAGED1,DVL2,PSEN1,FGF18,PIK3R5,MAP2K5,INSR,GHR,TRAF2,DAB2IP,S100A12,WNT5A,HRAS,MAP2K7,FGF1,PDGFD	grey	VSMC_M10
GO:0002832	0.56	0.53	41	77	negative regulation of response to biotic stimulus	BP	1	TNFAIP3,USP15,TRIB1,C6orf106,NR1H2,IL4I1,SEC14L1,SIRT2,NMI,NPLOC4,PCBP2,NR1H3,TRAFD1,OAS3,NLRC5,MAPKBP1,CD96,YTHDF2,SLAMF8,PARP14,RNF26,NLRX1,TRAF3IP1,HTRA1,DUSP10,MUL1,FOXP3,TKFC,USP18,DCST1,TSPAN6,STAT2,RIOK3,PTPN2,CRK,MIR21,OAS1,A2M,FGL2,SERPINB9,HAVCR2	grey	VSMC_M10

GO:0016579	0.56	0.52	57	108	protein deubiquitination	BP	1	SHMT2,TNFAIP3,USP15,PRKN,JOSD1,USP49,SUPT3H,USP46,MINDY4,USP45,USP38,OTUD1,USP35,WDR48,USP36,USP44,TAF10,ATXN7,ASXL1,OTUD4,USP39,USP25,USP16,OTULIN,ABRAXAS1,OTUD7A,USP30,SART3,USP13,SPATA2,YOD1,USP19,OTUD7B,USP2,ABRAXAS2,USP21,ENY2,ATXN7L3,USP37,USP48,OTUD3,OTUD5,STAMBPL1,USP18,UIMC1,MINDY2,EPOP,OTUB2,USP31,TNIP1,USP24,PARK7,USP20,STAMBP,UCHL3,USP40,BRCC3	grey	VSMC_M10
GO:0001953	0.56	0.58	17	32	negative regulation of cell-matrix adhesion	BP	1	LRP1,CDKN2A,HOXA7,ITGB1BP1,CLASP2,JAG1,FAM107A,PIK3R1,DMTN,ARHGAP6,APOD,BCL6,CASK,BCAS3,ACVRL1,ACER2,DUSP22	grey	VSMC_M10
GO:0030836	0.56	0.67	6	11	positive regulation of actin filament depolymerization	BP	1	KIAA1211,PLEK,F2RL1,CARMIL1,CARMIL2,PDXP	grey	VSMC_M10
GO:0035735	0.56	0.73	4	7	intraciliary transport involved in cilium assembly	BP	1	IFT74,DYNC2LI1,TTC26,IFT81	grey	VSMC_M10
GO:0071772	0.56	0.51	66	126	response to BMP	BP	1	SLC39A5,USP15,GDF9,SMAD5,LEMD2,KCP,TFAP2B,ROR2,DLX5,SOSTDC1,HOXA13,BMP8A,BMPER,SFRP4,SOX9,MEGF8,ACVR2B,UBE2O,ACVR2A,SPINT2,SPART,SLC33A1,SPINT1,HIVEP1,ZNF423,BMPR1B,FBXL15,PDCD4,TGFB2,HJV,SORL1,HTRA1,CHRD,DLX1,CTDSPL2,HTRA3,FBN1,FKBP8,GDF15,GREM2,BMPR1A,RBPJ,ITGA3,RBPMS2,MAPK3,TOB1,TMEM100,SMAD3,TNMD,RNF165,CHRDL1,GATA6,FAM83G,TWSG1,ADAMTS12,GPC3,SKI,ENG,MIR21,FSTL3,LEMD3,ACVRL1,NOG,WNT5A,RGMB,UBE2D1	grey	VSMC_M10
GO:0071773	0.56	0.51	66	126	cellular response to BMP stimulus	BP	1	SLC39A5,USP15,GDF9,SMAD5,LEMD2,KCP,TFAP2B,ROR2,DLX5,SOSTDC1,HOXA13,BMP8A,BMPER,SFRP4,SOX9,MEGF8,ACVR2B,UBE2O,ACVR2A,SPINT2,SPART,SLC33A1,SPINT1,HIVEP1,ZNF423,BMPR1B,FBXL15,PDCD4,TGFB2,HJV,SORL1,HTRA1,CHRD,DLX1,CTDSPL2,HTRA3,FBN1,FKBP8,GDF15,GREM2,BMPR1A,RBPJ,ITGA3,RBPMS2,MAPK3,TOB1,TMEM100,SMAD3,TNMD,RNF165,CHRDL1,GATA6,FAM83G,TWSG1,ADAMTS12,GPC3,SKI,ENG,MIR21,FSTL3,LEMD3,ACVRL1,NOG,WNT5A,RGMB,UBE2D1	grey	VSMC_M10
GO:0006686	0.56	0.73	4	7	sphingomyelin biosynthetic process	BP	1	SGMS2,SGMS1,ABCA8,PEMT	grey	VSMC_M10
GO:1900115	0.56	0.70	5	9	extracellular regulation of signal transduction	BP	1	FBN1,LRPAP1,GREM2,CD46,NRROS	grey	VSMC_M10
GO:1900116	0.56	0.70	5	9	extracellular negative regulation of signal transduction	BP	1	FBN1,LRPAP1,GREM2,CD46,NRROS	grey	VSMC_M10
GO:0021535	0.56	0.66	7	13	cell migration in hindbrain	BP	1	RBFOX2,SEMA4C,POU4F1,TTBK2,ATP1B2,DAB1,CEND1	grey	VSMC_M10
GO:0090308	0.56	0.67	6	11	regulation of DNA methylation-dependent heterochromatin assembly	BP	1	SETDB2,FAM208A,PPHLN1,TET1,SETDB1,ZNF304	grey	VSMC_M10
GO:0045089	0.56	0.53	44	83	positive regulation of innate immune response	BP	1	CD226,XRCC6,FPR2,AKIRIN2,TLR4,NECTIN2,PVR,IKBKB,NMI,TMEM173,MAVS,HPX,PRKDC,IRF7,TRAF6,HCK,RASGRP1,NLRC5,POLR3D,NOD2,POLR3C,PARP9,COCH,ADAM8,CADM1,NONO,IL12A,XRCC5,LAG3,STAT5B,MED1,SFPQ,SIN3A,CCL5,RIOK3,VAV1,CREBBP,AP1G1,FADD,ZCCHC3,IRGM,WNT5A,EREG,HAVCR2	grey	VSMC_M10
GO:0042074	0.56	0.70	5	9	cell migration involved in gastrulation	BP	1	MEGF8,WNT11,AMOT,GPC3,WNT5A	grey	VSMC_M10
GO:0030101	0.56	0.56	23	43	natural killer cell activation	BP	1	NFIL3,PRDX1,IL15,UNC13D,PIBF1,IL15RA,GAS6,TOX,RASGRP1,RABL3,FGR,SNX27,SP3,BLOC1S6,IL12A,STAT5B,ZBTB1,KAT7,TICAM1,AP1G1,BLOC1S3,CASP8,HAVCR2	grey	VSMC_M10
GO:0048477	0.56	0.54	33	62	oogenesis	BP	1	YTHDC2,GDF9,WASHC5,H3F3C,TUT7,DMC1,YTHDC1,PTN,NOBOX,CCDC155,TUT4,FOXO3,PANX1,ERCC1,MLH1,SIRT2,SOHLH2,PTK2B,KMT2D,NPM2,BMPR1B,CCDC36,YTHDF2,SPO11,WNT4,YBX2,PAQR5,TAF4B,WDR77,AURKC,NANOS1,EREG,ZGLP1	grey	VSMC_M10
GO:0035315	0.56	0.58	18	34	hair cell differentiation	BP	1	MYCL,WHRN,MYCN,ERCC2,ERCC3,MYO7A,SLC4A7,JAG1,LRTOMT,AP000812.5,PLS1,USH1C,STRC,RBPJ,TMEM132E,JAG2,MCOLN3,HEY2	grey	VSMC_M10
GO:0032757	0.56	0.56	22	41	positive regulation of interleukin-8 production	BP	1	HSPA1B,NOD1,TLR4,PRKD2,MYD88,MAVS,F2RL1,F2R,RIPK1,NOD2,TLR5,STAT3,CD74,NOS2,PARK7,LAMTOR5,BCL10,FADD,ZNF580,WNT5A,DDIT3,IL17D	grey	VSMC_M10
GO:0150003	0.56	0.73	4	7	regulation of spontaneous synaptic transmission	BP	1	SLC12A2,PRKN,AGER,STX1B	grey	VSMC_M10
GO:0032536	0.56	0.72	4	7	regulation of cell projection size	BP	1	KEL,PLS1,USH1C,CNTN2	grey	VSMC_M10
GO:1905383	0.56	0.69	5	9	protein localization to presynapse	BP	1	KIF5A,DLG2,KIF5C,MAPK8IP3,MAP1A	grey	VSMC_M10
GO:0043410	0.56	0.48	189	356	positive regulation of MAPK cascade	BP	1	LRP1,MAP3K5,FRS2,STK3,MAPK8IP2,MAP3K4,AGER,PRXL2C,GRM4,TAB1,NTRK2,TEK,GPR37,KIT,ROR2,GADD45G,SEMA4C,IL11,PRKCE,FPR2,GSDME,EMC10,LPAR1,MTURN,EPGN,NOD1,BMPER,TGFA,EGFR,ARHGEF5,TLR4,TRAF1,SDCBP,FGF21,BRAF,DNAJC27,C5AR2,C5AR1,PRKD2,PHB,RAF1,MYD88,SPRY2,ALK,ARRB1,C1QTNF1,NEK10,PDE6G,PLCB1,EIF2AK2,ADRA1D,RELL2,SEMA3A,KL,NRG1,SH3RF2,GAS6,ARL6IP5,MFHAS1,FLT1,RYK,ADRA2B,TRAF6,ST5,MOS,RET,HAND2,ROBO1,PLCE1,FBXW7,PTPRJ,P2RY1,MADD,PTK2B,RASGRP1,RAPGEF2,CCL17,TIAM1,RIPK2,F2RL1,F2R,NQO2,RIPK1,GCNT2,NOD2,TAOK3,CD40,DSTYK,IGF2,BANK1,MAP2K6,TRPV4,TGFB2,ADRA2A,SLC30A10,AJUBA,NCF1,KRAS,EGF,SOX2,GPER1,DOK5,CIB1,TRAF5,DVL3,FZD7,OSM,THPO,PDE6H,MAP3K3,PDE8A,MAP3K11,ADAM8,FZD5,MBIP,RAP1B,MAP4K2,DUSP19,PDCD10,ADRA2C,GPBAR1,GDF15,FGFR3,LTBR,MINK1,EPHA4,MAGED1,DVL2,IGFBP4,RELL1,FGFR4,PSEN1,FGF18,TAOK2,EFNA1,WWC1,MAPK3,MAP2K1,PIK3R5,MAP2K5,RIT2,GAREM1,TRAF3,CD74,CCL14,INSR,CCL5,CSF1R,CCL7,CCL2,CCL25,GHR,KSR1,TAOK1,SERPINF2,PRKCZ,MAPK8IP3,TRAF2,ATP6AP1,ABCA7,CDK10,DAB2IP,MIR21,MIR222,MIR23A,MIR27B,MIR24-1,MIR27A,FGA,CCL16,S100A12,ZNF622,GPR37L1,AVPI1,DOK4,WNT5A,GADD45A,LAMTOR2,INAVA,CSPG4,LIF,DUSP22,HRAS,MAP2K7,FGF1,HAVCR2,NMNAT1,CCL22,PDGFD	grey	VSMC_M10

GO:0032635	0.56	0.52	51	96	interleukin-6 production	BP	1	TNFAIP3,AGER,MAPK13,NOD1,AKIRIN2,TLR4,SPHK2,C5AR2,MYD88,FOXJ1,ARRB1,MAVS,NCKAP1L,GAS6,TRAF6,IL1A,IL37,RIPK2,F2RL1,F2R,NOD2,RAB7B,IFIH1,CD200,BANK1,TRPV4,MAPKAPK2,NLRX1,IL16,PTPN6,STAT3,FOXP3,SPON2,SYT11,IL6R,CD74,ISL1,NOS2,TICAM1,CYBA,BCL10,MIR125A,WNT5A,INAVA,IL33,C1QTNF4,TSLP,CEBPB,EREG,HAVCR2,IL17D	grey	VSMC_M10
GO:0032675	0.56	0.52	51	96	regulation of interleukin-6 production	BP	1	TNFAIP3,AGER,MAPK13,NOD1,AKIRIN2,TLR4,SPHK2,C5AR2,MYD88,FOXJ1,ARRB1,MAVS,NCKAP1L,GAS6,TRAF6,IL1A,IL37,RIPK2,F2RL1,F2R,NOD2,RAB7B,IFIH1,CD200,BANK1,TRPV4,MAPKAPK2,NLRX1,IL16,PTPN6,STAT3,FOXP3,SPON2,SYT11,IL6R,CD74,ISL1,NOS2,TICAM1,CYBA,BCL10,MIR125A,WNT5A,INAVA,IL33,C1QTNF4,TSLP,CEBPB,EREG,HAVCR2,IL17D	grey	VSMC_M10
GO:0030595	0.56	0.50	80	150	leukocyte chemotaxis	BP	1	SLC12A2,GPSM3,PTK2,RAC2,CCL27,KIT,NINJ1,FPR2,CXCL8,AKIRIN1,PTN,TRPM4,C5AR2,C5AR1,OXSR1,NCKAP1L,GAS6,FAM19A4,FLT1,MTUS1,CYP7B1,VEGFC,PTK2B,CCL17,SLC8B1,F2RL1,ANO6,NOD2,GBF1,DNM1L,TRPV4,SBDS,TGFB2,PRSS56,CMKLR1,SLAMF8,MOSPD2,PTPRO,FCER1G,ADAM8,PIKFYVE,IL16,IL12A,MCU,TNFSF18,CXCR2,BST1,MAPK1,CH25H,PADI2,CXCL16,TMEM102,PGF,DAPK2,MAPK3,IL6R,S100A14,S100A8,CD74,CCL14,CCL5,CSF1R,CCL7,CCL2,CCL25,VAV1,PIP5K1C,MPP1,BSG,VAV3,CCL16,S100A12,CHGA,DEFB124,DPP4,CXCL6,ZNF580,WNT5A,DUSP1,CCL22	grey	VSMC_M10
GO:0021819	0.56	0.69	5	9	layer formation in cerebral cortex	BP	1	MBOAT7,CDK5R1,LRP8,DAB2IP,ADGRG1	grey	VSMC_M10
GO:0098887	0.56	0.69	5	9	neurotransmitter receptor transport, endosome to postsynaptic membrane	BP	1	NSG1,GRIPAP1,RAB8A,RAB11A,ARHGAP44	grey	VSMC_M10
GO:0043620	0.56	0.55	26	49	regulation of DNA-templated transcription in response to stress	BP	1	ATF4,HSF1,NCK2,EPAS1,SIRT2,TCIM,TMBIM6,BACH1,HIF1AN,ARNT,BAG3,RPS6KA1,NFE2L2,STOX1,DNAJB1,RBPJ,RGS14,MUC1,SIN3A,MAPK7,JUN,CREB3L3,HSPA5,IMPACT,DDIT3,CEBPB	grey	VSMC_M10
GO:0001408	0.56	0.72	4	7	guanine nucleotide transport	BP	1	ABCC4,ABCC5,LRRC8E,LRRC8A	grey	VSMC_M10
GO:2000758	0.56	0.58	16	30	positive regulation of peptidyl-lysine acetylation	BP	1	SPHK2,PAXIP1,ARRB1,AUTS2,TADA2B,FOXP3,KAT7,MUC1,MAPK3,NAP1L2,RPS6KA5,ISL1,GLYR1,SOX4,LIF,RPS6KA4	grey	VSMC_M10
GO:0099558	0.56	0.65	7	13	maintenance of synapse structure	BP	1	SYNGAP1,ERC2,BSN,DLG1,C1QL1,RAB3A,SDF4	grey	VSMC_M10
GO:0032689	0.56	0.60	13	24	negative regulation of interferon-gamma production	BP	1	TLR4,PRNP,GAS6,NR1H4,NOD2,CD96,VSIR,INHA,SCGB1A1,FOXP3,MIR24-1,DDIT3,HAVCR2	grey	VSMC_M10
GO:1903201	0.56	0.54	30	56	regulation of oxidative stress-induced cell death	BP	1	PRKN,NCOA7,ATF4,GPR37,FBXO7,MET,TLR4,SLC7A11,FOXO3,PAWR,FBXW7,HTRA2,RIPK1,MCL1,PDE8A,NFE2L2,NONO,PINK1,MMP3,PARP1,SFPQ,MAPK7,STK26,PARK7,TSC1,TLDC1,MIR21,NOL3,GPR37L1,TXN	grey	VSMC_M10
GO:0007320	0.56	0.72	4	7	insemination	BP	1	KLK14,DDO,ACVR2A,P2RX1	grey	VSMC_M10
GO:0002696	0.56	0.49	116	218	positive regulation of leukocyte activation	BP	1	CD226,AGER,HLA-DRA,HLA-DOB,HLA-DMA,IL5,HLA-DPB1,TNFRSF13C,XRCC6,TTBK1,NCK2,EXOSC3,MSH2,PELI1,IL4I1,TLR4,IL15,SPHK2,HMCES,BMI1,NECTIN2,GAB2,MLH1,GLI2,MYD88,PAXIP1,IL15RA,TGFBR2,SHLD1,NCKAP1L,GAS6,PRKDC,SLC7A1,TRAF6,SOX12,TOX,IL1A,CEBPA,MEF2C,IL7,RASGRP1,RIPK2,AP3B1,F2RL1,SOX13,NOD2,ZNF335,CD40,IGF2,LCK,ICOSLG,GPAM,HLX,FGR,SNX4,CD28,RUNX3,PPP2R3C,ADAM8,DUSP10,BLOC1S6,PLA2G4A,BCL6,IL12A,VSIR,PTPN6,TNIP2,CD38,BST1,STAT5B,FOXP3,RASAL3,ZBTB1,SHLD2,CARD11,TBX21,STX4,ITGAM,ZBTB7B,CHRNB2,IL4R,BTK,CD74,CCL5,CCL2,YES1,NKAP,CD320,VAV1,IL7R,SOCS1,TICAM1,DNAJA3,PRKCZ,VAV3,KARS,STXBP1,AP1G1,BCL10,MIR21,RHOA,HAVCR1,BLOC1S3,SOX4,FADD,DPP4,CD46,THY1,WNT5A,IL33,CD70,TNFSF9,TSLP,EFNB3,TNFSF13B,HAVCR2	grey	VSMC_M10
GO:0016240	0.56	0.72	4	7	autophagosome membrane docking	BP	1	STX17,SNAP29,CALM1,VMP1	grey	VSMC_M10
GO:0051969	0.56	0.72	4	7	regulation of transmission of nerve impulse	BP	1	AGT,TNR,FMR1,GPR35	grey	VSMC_M10
GO:2001054	0.56	0.72	4	7	negative regulation of mesenchymal cell apoptotic process	BP	1	SOX9,PAX8,PAX2,POU3F4	grey	VSMC_M10
GO:0018022	0.56	0.51	57	108	peptidyl-lysine methylation	BP	1	SNW1,PHF1,CAMKMT,SETD7,SETDB2,EED,IWS1,SUV39H2,KDM4A,PAXIP1,DPY30,NSD3,ASH2L,EEF1AKMT1,DNMT3B,SMYD1,SETD6,KMT2D,RTF1,WDR82,ZNF335,JARID2,RBBP5,FAM173B,AUTS2,EEF1AKMT2,SETD2,BCOR,MEN1,WDR61,VCPKMT,NELFA,DYDC1,NSD1,SETD1A,PAGR1,OGT,SETD3,SUZ12,SUPT6H,CXXC1,DOT1L,MECP2,PRDM16,CHD5,MTF2,GFI1,PRMT6,METTL18,SETDB1,ARID4A,BEND3,SMYD3,METTL21A,ZNF304,NNMT,N6AMT1	grey	VSMC_M10
GO:0034440	0.56	0.52	48	90	lipid oxidation	BP	1	HSD17B4,CPT1B,PPARA,MCAT,ECHDC1,MAPK14,CROT,ADH5,ETFB,HADHA,SOX9,PRKAG2,ACOX1,CYGB,AKT2,HACL1,ACOXL,ACOX2,PEX2,ETFDH,IVD,ECI2,LONP2,SIRT4,IRS1,CPT1A,BDH2,ABCB11,EHHADH,APOD,POR,ACADL,TYSND1,ACADVL,HSD17B10,ABCD4,GCDH,ACAA2,DECR2,MLYCD,ABCD3,ACADM,PHYH,HADHB,AMACR,ECI1,AUH,ADH4	grey	VSMC_M10
GO:0033089	0.56	0.72	4	7	positive regulation of T cell differentiation in thymus	BP	1	GLI2,RASGRP1,ADAM8,IL7R	grey	VSMC_M10
GO:0051956	0.56	0.72	4	7	negative regulation of amino acid transport	BP	1	ARL6IP5,SLC43A1,ABAT,RGS4	grey	VSMC_M10
GO:1905331	0.56	0.72	4	7	negative regulation of morphogenesis of an epithelium	BP	1	NKD1,TBX2,MIR21,WNT5A	grey	VSMC_M10
GO:0032768	0.56	0.56	23	43	regulation of monooxygenase activity	BP	1	CYP27B1,ZDHHC21,NOD1,EGFR,NOSIP,DHFR,ACVR2A,IL1A,PIK3CB,PTK2B,VDR,TERT,KRAS,NOS1AP,POR,GCH1,CALM1,GLA,PARK7,GFI1,TERF2,MIR27B,FCER2	grey	VSMC_M10
GO:1905668	0.56	0.67	6	11	positive regulation of protein localization to endosome	BP	1	ABHD17B,EGF,SORL1,DTX3L,ABHD17A,ABHD17C	grey	VSMC_M10
GO:0071450	0.56	0.61	11	20	cellular response to oxygen radical	BP	1	FANCC,PRDX1,DHFR,GLRX2,FBLN5,NFE2L2,GCH1,SOD3,MPO,NQO1,MIR21	grey	VSMC_M10
GO:0071451	0.56	0.61	11	20	cellular response to superoxide	BP	1	FANCC,PRDX1,DHFR,GLRX2,FBLN5,NFE2L2,GCH1,SOD3,MPO,NQO1,MIR21	grey	VSMC_M10

GO:0045806	0.56	0.56	22	41	negative regulation of endocytosis	BP	1	NR1H2,SDCBP,MTMR2,APOC1,NEU3,WDR54,NR1H3,ANKRD13A,ANKRD13D,RUBCN,SH3GL3,LRPAP1,DLG4,STX1B,SYT11,SCAMP5,UNC119,ABCA7,MIR199A1,MIR27B,MIR27A,LRRTM2	grey	VSMC_M10
GO:0046031	0.57	0.53	35	66	ADP metabolic process	BP	1	PPARA,PRXL2C,GAPDH,SLC4A4,NT5E,BPGM,DHTKD1,PRKAG2,PFKP,GPI,GPD1,P2RX7,PRKAG1,PFKM,ZBTB20,PFKFB2,ENO4,ARNT,ACTN3,HDAC4,MLXIPL,HK1,PRKAG3,STAT3,ENO3,PSEN1,PKLR,OGT,ADPGK,NCOR1,INSR,ALDOC,SLC2A6,ENTPD5,TIGAR	grey	VSMC_M10
GO:1900038	0.57	0.72	4	7	negative regulation of cellular response to hypoxia	BP	1	TMBIM6,HYOU1,PINK1,NOL3	grey	VSMC_M10
GO:1901214	0.57	0.49	133	251	regulation of neuron death	BP	1	ERBB3,LRP1,MAP3K5,AGAP2,PRKN,PPARA,SYNGAP1,NCOA7,GRM4,ATF4,NTRK2,NRBP2,HSF1,FBXO7,CNTFR,SLC1A1,TFAP2B,MSH2,REL,PPT1,EPHA7,TLR4,VPS35,SLC7A11,FOXO3,NTF4,FGF21,NDNF,C5AR1,ZNF746,GRIK5,XRCC2,EGLN2,POU4F1,ARRB1,SLC23A2,PRNP,EPHB1,GPI,RILPL1,CASP3,GBE1,FBXW7,TRIM2,MEF2C,FAIM2,HTRA2,PTK2B,NR4A2,F2R,NQO2,TFAP2A,PM20D1,BDNF,CD200,HDAC1,TERT,TGFB2,SLC30A10,INPP5A,SSH1,KCNB1,KRAS,ADNP,SORL1,MCL1,CBL,HYOU1,NFATC4,HDAC4,DLX1,BACE1,DNAJC5,FZD9,EGLN3,SNX6,MAP3K11,ADAM8,CREB1,MAPT,NONO,FIS1,PINK1,STAT3,ATP13A2,PARP1,TNFRSF1B,PSEN1,ITGAM,FOS,AC138894.1,CITED1,ALKBH1,GPRASP2,BHLHB9,CCL5,AP2B1,PPP1R13B,CCL2,CDK5R1,ISL1,GHR,JUN,PDPK1,MECP2,TRAF2,IKBKG,TMEM259,PARK7,TSC1,TLDC1,LCN2,STXBP1,AARS,NQO1,NAE1,MIR125B1,RHOA,UBB,CHGA,GRID2,CNTF,CASP8,PSMC1,WNT5A,TIGAR,SIX1,DDIT3,BCL2L11,HRAS,CEBPB,PITX3,NMNAT1	grey	VSMC_M10
GO:0006760	0.57	0.60	12	22	folic acid-containing compound metabolic process	BP	1	SHMT2,DHFR,MTHFD2,MTRR,MTHFS,GCH1,SHMT1,SLC46A1,PIPOX,MTHFD2L,SLC25A32,ALDH1L1	grey	VSMC_M10
GO:0048385	0.57	0.69	5	9	regulation of retinoic acid receptor signaling pathway	BP	1	SNW1,ASXL1,ZNF536,CNOT1,DHRS3	grey	VSMC_M10
GO:0060856	0.57	0.72	4	7	establishment of blood-brain barrier	BP	1	LSR,FOXP3,MXRA8,TRPV1	grey	VSMC_M10
GO:0060433	0.57	0.72	4	7	bronchus development	BP	1	SRF,SOX9,TGFBR2,TULP3	grey	VSMC_M10
GO:0034154	0.57	0.72	4	7	toll-like receptor 7 signaling pathway	BP	1	RSAD2,UNC93B1,DDX3X,HAVCR2	grey	VSMC_M10
GO:2000826	0.57	0.69	5	9	regulation of heart morphogenesis	BP	1	SMO,WNT11,ROBO1,ROBO2,WNT5A	grey	VSMC_M10
GO:0062237	0.57	0.58	16	30	protein localization to postsynapse	BP	1	ADAM22,DAG1,DLG1,NSG1,GRIPAP1,RAB8A,DLG4,IQSEC2,CPLX1,STX1B,ZDHHC15,RAB11A,ARHGAP44,PRKCZ,CACNG7,HRAS	grey	VSMC_M10
GO:0090288	0.57	0.52	41	78	negative regulation of cellular response to growth factor stimulus	BP	1	SEMA6A,LEMD2,SOSTDC1,BMPER,FUZ,SPRY1,SPRY2,DCN,SPART,PIK3CB,SULF2,PDCD6,SORL1,AGT,HTRA1,CHRD,DLX1,CTDSPL2,IL12A,CASK,HTRA3,FBN1,WNT4,GREM2,NGFR,RBPMS2,TOB1,TNMD,CHRDL1,TWSG1,ADAMTS12,SKI,DAB2IP,MIR199A1,FSTL3,LEMD3,NOG,WNT5A,SHISA2,CADM4,UBE2D1	grey	VSMC_M10
GO:0046321	0.57	0.69	5	9	positive regulation of fatty acid oxidation	BP	1	PPARA,AKT2,IRS1,CPT1A,MLYCD	grey	VSMC_M10
GO:0071044	0.57	0.67	6	11	histone mRNA catabolic process	BP	1	EXOSC4,MTPAP,LSM1,TENT2,TENT4B,UPF1	grey	VSMC_M10
GO:0061004	0.57	0.72	4	7	pattern specification involved in kidney development	BP	1	OSR1,PAX8,PAX2,IRX2	grey	VSMC_M10
GO:0072048	0.57	0.72	4	7	renal system pattern specification	BP	1	OSR1,PAX8,PAX2,IRX2	grey	VSMC_M10
GO:0048368	0.57	0.69	5	9	lateral mesoderm development	BP	1	SMO,FOXH1,TEAD2,BMPR1A,FOXF1	grey	VSMC_M10
GO:0034163	0.57	0.72	4	7	regulation of toll-like receptor 9 signaling pathway	BP	1	GRAMD4,RTN4,RSAD2,RAB7B	grey	VSMC_M10
GO:0002697	0.57	0.49	109	206	regulation of immune effector process	BP	1	CD226,STX7,ENPP3,AGER,HLA-DRA,IL5,RAC2,KIT,EXOSC3,MSH2,ANKRD17,RSAD2,IL4I1,TLR4,ATG5,TRPM4,SPHK2,HMCES,PHB,LACC1,NECTIN2,PVR,GAB2,MLH1,UNC13D,SLC22A13,FOXJ1,PAXIP1,SHLD1,MAVS,NCKAP1L,HPX,PRKDC,TRAF6,GPI,FER,PTPRJ,TMBIM6,RASGRP1,RIPK2,F2RL1,NOD2,CD40,CD96,TGFB2,HLX,DENND1B,NCF1,SLAMF8,FGR,SNX4,CD28,CADM1,FZD5,DUSP10,BCL6,HK1,IL12A,RC3H1,PTPN6,TNFSF18,LAG3,PPP3CB,C12orf4,STAT5B,WAS,FOXP3,ZBTB1,SHLD2,TNFRSF1B,SPON2,TBX21,STX4,ITGAM,ZBTB7B,IL4R,BTK,CD74,STXBP2,FBXO38,PRAM1,VAV1,C3,NOS2,IL7R,SUPT6H,TICAM1,CRK,DNASE1,PDPK1,PRKCZ,TRAF2,SLC7A5,FOXF1,STXBP1,AP1G1,BCL10,MIR21,FADD,CXCL6,CD46,A2M,WNT5A,INAVA,FGL2,FCER2,SERPINB9,HAVCR2,RC3H2	grey	VSMC_M10
GO:0051402	0.57	0.49	105	198	neuron apoptotic process	BP	1	ERBB3,LRP1,AGAP2,PRKN,SYNGAP1,GRM4,ATF4,NTRK2,NRBP2,CNTFR,SLC1A1,TNFRSF21,TFAP2B,GAPDH,MSH2,PPT1,EPHA7,FOXO3,NTF4,NDNF,C5AR1,GRIK5,XRCC2,EGLN2,POU4F1,AKT2,ARRB1,TMTC4,PRNP,GPI,CASP3,GBE1,DIABLO,FBXW7,TRIM2,MEF2C,FAIM2,PTK2B,NR4A2,F2R,NQO2,TFAP2A,PIGT,SIAH1,BDNF,HDAC1,TERT,TGFB2,SLC30A10,ADARB1,SCN2A,KCNB1,KRAS,ADNP,MCL1,HYOU1,NFATC4,HDAC4,DLX1,BACE1,DNAJC5,FZD9,EGLN3,SNX6,MAP3K11,ADAM8,NONO,FIS1,PINK1,PARP1,MAX,PSEN1,ITGAM,NGFR,AC138894.1,CITED1,ALKBH1,GPRASP2,BHLHB9,CCL2,CDK5R1,ISL1,JUN,PDPK1,MECP2,PARK7,LCN2,STXBP1,AARS,HSPA5,NQO1,NAE1,MIR125B1,RHOA,FADD,GRID2,CNTF,DIO3,SIX1,DDIT3,BCL2L11,HRAS,CEBPB,PITX3,NMNAT1	grey	VSMC_M10
GO:0016553	0.57	0.72	4	7	base conversion or substitution editing	BP	1	ADAT2,APOBEC3H,NUDT16,ADARB1	grey	VSMC_M10
GO:0048663	0.57	0.57	18	34	neuron fate commitment	BP	1	GLI2,MNX1,POU4F1,NRG1,EYA1,SUFU,SATB2,DLX1,RBPJ,EPOP,BCL11B,JAG2,ISL1,FOXG1,ISL2,LBX1,POU3F2,SIX1	grey	VSMC_M10
GO:0007614	0.57	0.69	5	9	short-term memory	BP	1	CUX2,ADNP,NPAS4,COMT,SLC2A4	grey	VSMC_M10

GO:0070646	0.57	0.50	66	125	protein modification by small protein removal	BP	1	SHMT2,TNFAIP3,USP15,PRKN,JOSD1,USP49,SUPT3H,USP46,MINDY4,USP45,USP38,OTUD1,USP35,WDR48,USP36,GPS1,USP44,TAF10,ATXN7,ASXL1,OTUD4,USP39,USP25,USP16,OTULIN,SENP1,ABRAXAS1,OTUD7A,COPS7B,USP30,SART3,USP13,SPATA2,YOD1,USP19,OTUD7B,USP2,ABRAXAS2,USP21,SENP5,SENP2,ENY2,ATXN7L3,COPS7A,USP37,USP48,OTUD3,OTUD5,STAMBPL1,USP18,UIMC1,MINDY2,EPOP,OTUB2,USP31,TNIP1,USP24,PARK7,USP20,STAMBP,UCHL3,SENP7,COPS8,USP40,BRCC3,HINT1	grey	VSMC_M10
GO:1901879	0.57	0.52	39	75	regulation of protein depolymerization	BP	1	AVIL,LMOD2,KIAA1211,TMOD1,PLEKHH2,SPTBN1,PLEK,ADD2,CLASP2,TRIM54,NAV3,SPEF1,LMOD3,ATXN7,LIMA1,KATNB1,F2RL1,DMTN,CARMIL1,LMOD1,ADD3,TRPV4,TTBK2,CIB1,SPTBN2,TMOD4,EPS8,CAPZA1,TMOD2,SPTB,CAMSAP3,TAOK1,APC2,CAMSAP1,CARMIL2,APC,PDXP,MAP1A,MAP6D1	grey	VSMC_M10
GO:0032412	0.57	0.49	94	178	regulation of ion transmembrane transporter activity	BP	1	NEDD4L,STAC3,MAPK8IP2,PRRT1,GOPC,PLA2G6,ABCB1,WNK2,PRKCE,SHANK1,HECW1,TCAF2,OSR1,SPHK2,KCNRG,CACNB2,STAC,OXSR1,GPD1L,CACNB4,PRNP,SLMAP,GLRX,MEF2C,PTK2B,KCNJ11,HOMER1,IFNGR2,TESC,RIPK1,KCNE2,PKD2,CASQ2,KCNIP2,CHRM3,RYR2,FAM173B,HECW2,ADRA2A,COX17,KCNG1,ANK2,KLHL24,ATP1A2,SLC9A1,C4orf3,CASQ1,NTSR1,SCN4B,CNIH2,NOS1AP,DLG1,HTT,PINK1,ATP1B1,KCNA1,GNB5,MINK1,HAP1,DLG4,NLGN2,CNIH3,WNK3,STAC2,CACNB1,ATP1B2,NLGN3,ATP2A1,CALM1,DNM2,VMP1,CCL2,FMR1,RNF207,AMIGO1,JPH3,WWP2,RRAD,MIR24-1,DMD,GPR35,JPH2,FGF11,SCN2B,NETO2,CACNG7,CRBN,CRH,APOPT1,YWHAH,SHISA8,CACNG5,GEM,PPIF	grey	VSMC_M10
GO:0019614	0.57	0.72	4	7	catechol-containing compound catabolic process	BP	1	LRTOMT,AP000812.5,SLC6A3,COMT	grey	VSMC_M10
GO:0042424	0.57	0.72	4	7	catecholamine catabolic process	BP	1	LRTOMT,AP000812.5,SLC6A3,COMT	grey	VSMC_M10
GO:0048599	0.57	0.57	17	32	oocyte development	BP	1	YTHDC2,GDF9,WASHC5,TUT7,DMC1,TUT4,FOXO3,SIRT2,PTK2B,KMT2D,YTHDF2,WNT4,YBX2,WDR77,AURKC,EREG,ZGLP1	grey	VSMC_M10
GO:0072522	0.57	0.49	84	157	purine-containing compound biosynthetic process	BP	1	NME1,SHMT2,ATP5MGL,PPT2,PPARA,ACSL6,GUCA1B,IMPDH1,MTAP,SLC35B2,SLC25A13,PPAT,PPCS,NT5E,PPT1,DLD,SPHK2,ADCY3,PRTFDC1,PRKAG2,ATP5F1C,ELOVL1,PANK2,PDK1,PDHX,ELOVL7,PANK1,AMPD3,ATP5F1A,GUCY1A1,GUCY1B1,DGUOK,GMPS,AMPD1,ADCY7,FAM173B,ADCY2,NME4,ACSL5,PRPS2,HACD2,ADCY4,DNAJC30,PDK3,SLC25A16,ACSBG1,ATP5S,COASY,PINK1,STAT3,NME7,ADK,MPC2,ADCY10,PARP1,TECR,SLC26A1,GUCY2D,PKLR,PANK3,GCDH,ACACA,SLC26A2,PRPSAP2,SHMT1,ADSSL1,NOS2,ACSBG2,ATP6V0C,PANK4,NME3,ATP5PB,VPS9D1,ACSF3,MLYCD,MT-ATP8,MT-ATP6,ATP5ME,ATP5MF,PDZD3,ELOVL3,MTHFD2L,NME6,NUDT2	grey	VSMC_M10
GO:0090179	0.57	0.69	5	9	planar cell polarity pathway involved in neural tube closure	BP	1	PTK7,DVL3,GRHL3,DVL2,WNT5A	grey	VSMC_M10
GO:0031098	0.57	0.49	104	197	stress-activated protein kinase signaling cascade	BP	1	PHLPP1,MAP3K5,STK3,MAPK8IP2,MAP3K4,PRKN,AGER,TRIB1,MYC,AKR1B1,MAPK14,MAPK13,ROR2,GADD45G,KLHL31,PRDX1,SEMA4C,DNAJA1,FKTN,EMC10,MTURN,NOD1,EGFR,TLR4,TRAF1,SDCBP,MYD88,IKBKB,ZMYND11,PLCB1,MAP4K1,GPS1,EIF2AK2,RELL2,SEMA3A,SH3RF2,ARL6IP5,ERCC6,MFHAS1,TRAF6,EPHB1,HAND2,PTK2B,RASGRP1,RIPK2,F2RL1,RIPK1,NOD2,TAOK3,MAP2K6,TRPV4,PDCD4,TGFB2,NCF1,MAPKAPK2,AGT,TRAF5,STRADB,FZD7,MAP3K3,MAGI3,DLG1,MAP3K11,AIDA,DUSP10,MBIP,MEN1,MAP4K2,DUSP19,PDCD10,PINK1,LTBR,MAPK1,MINK1,GPS2,RELL1,KAT7,TAOK2,MAPK3,HACD3,MAP2K1,SMAD3,CCDC88C,NCOR1,TRAF3,ADORA2B,SH2D3A,PTGER4,TAOK1,ARHGEF6,SERPINF2,MAPK8IP3,TRAF2,MARVELD3,DAB2IP,RHOA,ZNF622,WNT5A,GADD45A,INAVA,DUSP1,DUSP22,HRAS,MAP2K7	grey	VSMC_M10
GO:0008038	0.57	0.55	22	42	neuron recognition	BP	1	TNFRSF21,RTN4,YWHAZ,NRCAM,MEGF8,SEMA3A,CASP3,ROBO1,ROBO2,NCAM2,CRTAC1,GAP43,EPHB3,MYPN,EPHA4,CDK5R1,EMB,BSG,AMIGO1,CNTN2,FOXG1,EFNB3	grey	VSMC_M10
GO:1902950	0.57	0.72	4	7	regulation of dendritic spine maintenance	BP	1	ABHD17B,VPS35,PRNP,HOMER1	grey	VSMC_M10
GO:0006072	0.57	0.72	4	7	glycerol-3-phosphate metabolic process	BP	1	GPD1L,GK5,GPD1,GPAM	grey	VSMC_M10
GO:0140289	0.57	0.66	6	11	protein mono-ADP-ribosylation	BP	1	PARP4,PARP9,PARP15,PARP14,PARP11,PARP6	grey	VSMC_M10
GO:0043471	0.57	0.72	4	7	regulation of cellular carbohydrate catabolic process	BP	1	PPP1CB,PPP1R3B,PPP1R3D,TIGAR	grey	VSMC_M10
GO:0061053	0.57	0.53	33	63	somite development	BP	1	PTCH1,SMO,ROR2,MEOX2,ABI1,PCDH8,XRCC2,WNT11,PRKDC,TCF15,POFUT1,NKX3-1,NKD1,POGLUT1,AXIN2,MEOX1,WNT4,BMPR1A,WDR19,TCAP,RBPJ,ALDH1A2,RAD51B,PSEN1,SMAD3,LHX1,CDX1,MIB1,NLE1,FOXF1,NOG,WNT5A,SIX1	grey	VSMC_M10
GO:0072210	0.57	0.57	18	34	metanephric nephron development	BP	1	SMO,TFAP2B,SIX2,OSR1,SOX9,RET,PAX8,FMN1,PKD2,PAX2,SALL1,IRX2,WNT4,CITED1,LHX1,PKD1,SOX8,LIF	grey	VSMC_M10
GO:1904894	0.57	0.58	15	28	positive regulation of receptor signaling pathway via STAT	BP	1	IL5,AKR1B1,OCIAD1,KIT,PIBF1,PTK2B,F2R,AGT,IL10RA,IL6R,CCL5,GHR,IL7R,SOCS1,TSLP	grey	VSMC_M10
GO:1903579	0.57	0.59	13	24	negative regulation of ATP metabolic process	BP	1	PPARA,TSPO,DNAJC15,ACTN3,HDAC4,MLXIPL,FIS1,STAT3,PARP1,NCOR1,RHOA,TIGAR,PPIF	grey	VSMC_M10
GO:0055022	0.57	0.60	12	22	negative regulation of cardiac muscle tissue growth	BP	1	PPARA,PI16,TGFBR2,TBX5,JARID2,KCNK2,SAV1,YY1,MIR199A1,MIR25,NOG,RGS4	grey	VSMC_M10
GO:0061117	0.57	0.60	12	22	negative regulation of heart growth	BP	1	PPARA,PI16,TGFBR2,TBX5,JARID2,KCNK2,SAV1,YY1,MIR199A1,MIR25,NOG,RGS4	grey	VSMC_M10
GO:0010831	0.57	0.66	6	11	positive regulation of myotube differentiation	BP	1	MAML1,MAPK14,MAMSTR,SMYD1,RBM24,PIEZO1	grey	VSMC_M10

GO:0043648	0.57	0.52	39	73	dicarboxylic acid metabolic process	BP	1	GLS2,SHMT2,ME1,SLC7A11,DLD,DDO,DHFR,GAD2,MDH2,AMDHD1,SUCLG2,NR1H4,MTHFD2,HOGA1,NIT2,GLS,MTRR,ALDH5A1,OAT,GLUL,D2HGDH,MDH1B,MTHFS,PRODH,L2HGDH,MRPS36,ALDH4A1,SDHB,SHMT1,ADSSL1,SLC46A1,ACSF3,ASS1,KYAT3,PHYH,ACOT4,MTHFD2L,SLC25A32,ALDH1L1	grey	VSMC_M10
GO:1905064	0.57	0.69	5	9	negative regulation of vascular associated smooth muscle cell differentiation	BP	1	NFATC1,PDCD4,NFATC3,MIR21,HEY2	grey	VSMC_M10
GO:0071361	0.57	0.66	6	11	cellular response to ethanol	BP	1	PRKCE,DNMT3A,SLC23A2,TP53INP1,ADCY7,CCL7	grey	VSMC_M10
GO:0002138	0.57	0.72	4	7	retinoic acid biosynthetic process	BP	1	PRMT3,ALDH1A3,DHRS9,ALDH1A2	grey	VSMC_M10
GO:0016102	0.57	0.72	4	7	diterpenoid biosynthetic process	BP	1	PRMT3,ALDH1A3,DHRS9,ALDH1A2	grey	VSMC_M10
GO:0071605	0.57	0.66	6	11	monocyte chemotactic protein-1 production	BP	1	AGER,OAS3,NOD2,TRPV4,APOD,OAS1	grey	VSMC_M10
GO:0071637	0.57	0.66	6	11	regulation of monocyte chemotactic protein-1 production	BP	1	AGER,OAS3,NOD2,TRPV4,APOD,OAS1	grey	VSMC_M10
GO:0030150	0.57	0.61	10	18	protein import into mitochondrial matrix	BP	1	TIMM21,DNAJC15,TIMM50,TOMM40L,GRPEL1,TIMM17B,PAM16,TIMM23B,GRPEL2,DNAJC19	grey	VSMC_M10
GO:0072160	0.57	0.64	7	13	nephron tubule epithelial cell differentiation	BP	1	MTSS1,OSR1,PAX8,MEF2C,PAX2,LHX1,LIF	grey	VSMC_M10
GO:0046058	0.57	0.61	10	19	cAMP metabolic process	BP	1	PDE7B,PDE10A,ADCY3,ADCY7,ADCY2,PTHLH,ADCY4,PDE8A,ADCY10,PDE4A	grey	VSMC_M10
GO:0061515	0.57	0.53	31	59	myeloid cell development	BP	1	MPIG6B,BRD1,L3MBTL3,SRF,KIT,PTBP3,BPGM,PIP4K2A,ABI1,ERCC2,NCKAP1L,FOXP1,FBXW7,TSPAN2,DMTN,ALAS1,LRRK1,SLC9B2,WASF2,THPO,BCL6,PTPN6,FBN1,MED1,ANXA2,GPR68,EVI2B,ATP6AP1,ARID4A,NRROS,FAM210B	grey	VSMC_M10
GO:0060439	0.57	0.72	4	7	trachea morphogenesis	BP	1	TGFBR2,MAPK1,MAPK3,MAP2K1	grey	VSMC_M10
GO:0060020	0.57	0.72	4	7	Bergmann glial cell differentiation	BP	1	TTC21B,MAPK1,MAPK3,MAP2K1	grey	VSMC_M10
GO:0000303	0.57	0.60	12	22	response to superoxide	BP	1	FANCC,PRDX1,DHFR,ERCC6,GLRX2,FBLN5,NFE2L2,GCH1,SOD3,MPO,NQO1,MIR21	grey	VSMC_M10
GO:0046415	0.57	0.72	4	7	urate metabolic process	BP	1	GCKR,ABCG2,CARMIL1,SLC2A9	grey	VSMC_M10
GO:0050913	0.57	0.72	4	7	sensory perception of bitter taste	BP	1	CALHM1,RTP4,GNAT2,TAS2R3	grey	VSMC_M10
GO:0070862	0.57	0.68	5	9	negative regulation of protein exit from endoplasmic reticulum	BP	1	UBAC2,YOD1,DERL3,UBE2G2,DERL2	grey	VSMC_M10
GO:0032612	0.57	0.53	33	62	interleukin-1 production	BP	1	TNFAIP3,AGER,IL1R2,S1PR3,NOD1,TLR4,HDAC2,PANX1,MYD88,GAS6,FOXP1,P2RX7,RIPK2,SERPINB1,F2RL1,F2R,NOD2,HDAC1,FZD5,IL16,HK1,STAT3,PYDC1,TMED10,SMAD3,S100A13,ISL1,MIR27B,CASP8,WNT5A,INAVA,CPTP,HAVCR2	grey	VSMC_M10
GO:0032652	0.57	0.53	33	62	regulation of interleukin-1 production	BP	1	TNFAIP3,AGER,IL1R2,S1PR3,NOD1,TLR4,HDAC2,PANX1,MYD88,GAS6,FOXP1,P2RX7,RIPK2,SERPINB1,F2RL1,F2R,NOD2,HDAC1,FZD5,IL16,HK1,STAT3,PYDC1,TMED10,SMAD3,S100A13,ISL1,MIR27B,CASP8,WNT5A,INAVA,CPTP,HAVCR2	grey	VSMC_M10
GO:0060296	0.57	0.72	4	7	regulation of cilium beat frequency involved in ciliary motility	BP	1	MKKS,CFAP20,CFAP45,CCSAP	grey	VSMC_M10
GO:0010826	0.57	0.66	6	11	negative regulation of centrosome duplication	BP	1	MDM1,CDK5RAP2,TMEM67,CCDC84,TRIM37,NUBP1	grey	VSMC_M10
GO:0046606	0.57	0.66	6	11	negative regulation of centrosome cycle	BP	1	MDM1,CDK5RAP2,TMEM67,CCDC84,TRIM37,NUBP1	grey	VSMC_M10
GO:0046950	0.57	0.71	4	7	cellular ketone body metabolic process	BP	1	HMGCLL1,ACSS3,HMGCL,OXCT1	grey	VSMC_M10
GO:1902224	0.57	0.71	4	7	ketone body metabolic process	BP	1	HMGCLL1,ACSS3,HMGCL,OXCT1	grey	VSMC_M10
GO:0071872	0.57	0.68	5	9	cellular response to epinephrine stimulus	BP	1	SIRT2,RYR2,SRD5A1,SLC9A1,PKLR	grey	VSMC_M10
GO:0021554	0.57	0.68	5	9	optic nerve development	BP	1	EPHB1,PAX2,TCIRG1,CHRNB2,TMEM126A	grey	VSMC_M10
GO:1901881	0.57	0.63	8	15	positive regulation of protein depolymerization	BP	1	KIAA1211,PLEK,KATNB1,F2RL1,CARMIL1,TRPV4,CARMIL2,PDXP	grey	VSMC_M10
GO:0046466	0.57	0.58	14	26	membrane lipid catabolic process	BP	1	PPT1,MGST2,SMPD2,ACER3,NEU3,ASAH1,SMPD4,GALC,GLA,HEXA,GM2A,CEL,ACER2,NEU2	grey	VSMC_M10
GO:0033700	0.57	0.71	4	7	phospholipid efflux	BP	1	APOC1,ABCA12,ABCA3,ABCA7	grey	VSMC_M10
GO:1990144	0.57	0.71	4	7	intrinsic apoptotic signaling pathway in response to hypoxia	BP	1	TMBIM6,HYOU1,PINK1,NOL3	grey	VSMC_M10
GO:0022400	0.57	0.71	4	7	regulation of rhodopsin mediated signaling pathway	BP	1	CAMKMT,NMT1,GRK4,GUCY2D	grey	VSMC_M10
GO:0051132	0.57	0.71	4	7	NK T cell activation	BP	1	IL15,IL12A,RASAL3,ZBTB7B	grey	VSMC_M10
GO:0032988	0.57	0.71	4	7	ribonucleoprotein complex disassembly	BP	1	ZFAND1,DYRK3,DHX8,KLC1	grey	VSMC_M10
GO:0072711	0.57	0.68	5	9	cellular response to hydroxyurea	BP	1	SPIDR,RTF2,DDX11,DDI2,FMR1	grey	VSMC_M10
GO:0045059	0.57	0.68	5	9	positive thymic T cell selection	BP	1	SRF,TOX,DOCK2,CD74,PTPN2	grey	VSMC_M10
GO:0045869	0.57	0.71	4	7	negative regulation of single stranded viral RNA replication via double stranded DNA intermediate	BP	1	APOBEC3H,FAM208A,INPP5K,SETDB1	grey	VSMC_M10
GO:0006975	0.57	0.71	4	7	DNA damage induced protein phosphorylation	BP	1	CRY1,MAP2K6,CHEK2,MAPK3	grey	VSMC_M10
GO:0015791	0.57	0.71	4	7	polyol transport	BP	1	AQP1,AQP11,SLC2A13,SLC5A11	grey	VSMC_M10
GO:0070857	0.57	0.71	4	7	regulation of bile acid biosynthetic process	BP	1	NR1H4,CYP7A1,FGFR4,STARD4	grey	VSMC_M10
GO:0043252	0.57	0.68	5	9	sodium-independent organic anion transport	BP	1	SLCO6A1,SLCO3A1,SLCO1A2,SLCO1C1,MFSD10	grey	VSMC_M10
GO:0010454	0.57	0.71	4	7	negative regulation of cell fate commitment	BP	1	SOSTDC1,FZD7,TNFSF18,TBX21	grey	VSMC_M10
GO:2001053	0.57	0.68	5	9	regulation of mesenchymal cell apoptotic process	BP	1	HOXA13,SOX9,PAX8,PAX2,POU3F4	grey	VSMC_M10
GO:0033353	0.58	0.71	4	7	S-adenosylmethionine cycle	BP	1	APIP,AHCY,ENOPH1,MTRR	grey	VSMC_M10
GO:0018904	0.58	0.59	13	24	ether metabolic process	BP	1	PEX7,PLA2G6,TXN2,TMEM86B,LIPE,EPHX2,GNPAT,PLA2G4A,PLA2G16,EPHX1,NCEH1,EPHX3,TXN	grey	VSMC_M10
GO:0032370	0.58	0.53	29	55	positive regulation of lipid transport	BP	1	LRP1,PTCH1,PLA2G6,TMEM30A,NR1H2,C1QTNF1,NR1H3,NKX3-1,SPP1,PLA2R1,PLTP,MAP2K6,ABCA5,ACSL5,NTSR1,PLA2G4A,ZDHHC8,ABCA12,LPCAT3,GPS2,ANXA2,ABCA3,ABCA7,RXRA,ABCA8,LDLRAP1,CRH,LRAT,PTGES	grey	VSMC_M10
GO:0070828	0.58	0.55	21	40	heterochromatin organization	BP	1	BAZ2A,H3F3C,SETD7,DNMT3A,SETDB2,SIRT2,FAM208A,BAHD1,LOXL2,PPHLN1,HDAC1,ZNFX1,SNAI1,TET1,PHF8,NRDE2,SMCHD1,PRDM16,SETDB1,BEND3,ZNF304	grey	VSMC_M10
GO:0008209	0.58	0.59	12	22	androgen metabolic process	BP	1	HSD17B4,ESR1,HSD17B8,HSD17B3,SRD5A3,SPP1,SRD5A1,DHRS9,HSD17B7,WNT4,HSD17B10,MED1	grey	VSMC_M10
GO:0006013	0.58	0.71	4	7	mannose metabolic process	BP	1	PMM1,HK1,MPI,PMM2	grey	VSMC_M10
GO:0046719	0.58	0.71	4	7	regulation by virus of viral protein levels in host cell	BP	1	STAU1,DDB1,HACD3,TBC1D20	grey	VSMC_M10
GO:0071380	0.58	0.64	7	13	cellular response to prostaglandin E stimulus	BP	1	PRKCE,PTGER2,AKAP8,ACACA,PTGER4,GNB1,GNG2	grey	VSMC_M10
GO:0046007	0.58	0.66	6	11	negative regulation of activated T cell proliferation	BP	1	PRNP,CASP3,BTN2A2,PRKAR1A,RC3H1,FOXP3	grey	VSMC_M10
GO:0099638	0.58	0.64	7	13	endosome to plasma membrane protein transport	BP	1	VPS35,RAB7A,NSG1,GRIPAP1,RAB8A,RAB11A,ARHGAP44	grey	VSMC_M10

GO:0044819	0.58	0.58	14	26	mitotic G1/S transition checkpoint signaling	BP	1	PLK3,PRKDC,PLK2,PIDD1,CCND1,GIGYF2,CHEK2,CDKN1B,MUC1,FBXO31,PLK5,RPS27L,SOX4,CRADD	grey	VSMC_M10
GO:0061951	0.58	0.53	29	55	establishment of protein localization to plasma membrane	BP	1	GOPC,SPTBN1,VPS35,RAB7A,RAB10,OPTN,GOLGA4,LYPLA1,RILPL1,RILPL2,VAMP5,KIF13A,NECTIN3,BBS1,GORASP2,NSF,NSG1,GRIPAP1,RAB8A,CNST,RSC1A1,AC138894.1,RAB11A,GGA2,ARHGAP44,RAB26,SEC16A,C16orf70,VAMP3	grey	VSMC_M10
GO:0015816	0.58	0.71	4	7	glycine transport	BP	1	SLC25A38,SLC38A5,SLC36A1,RGS4	grey	VSMC_M10
GO:0017121	0.58	0.66	6	11	plasma membrane phospholipid scrambling	BP	1	ANO4,XKR6,PLSCR4,P2RX7,ANO6,XKR8	grey	VSMC_M10
GO:0006805	0.58	0.55	22	41	xenobiotic metabolic process	BP	1	NUDT15,CYP2R1,BPHL,ABCC2,CBR3,CMBL,TPMT,FMO5,CYP2U1,ABCB11,POR,EPHX1,CYP46A1,CYP2J2,NQO1,CRYZ,S100A12,SULT1C3,AHRR,NCEH1,AOC2,N6AMT1	grey	VSMC_M10
GO:0002704	0.58	0.57	16	30	negative regulation of leukocyte mediated immunity	BP	1	IL4I1,FOXJ1,NCKAP1L,NOD2,CD96,BCL6,PTPN6,PPP3CB,FOXP3,TBX21,IL7R,CRK,FOXF1,CD46,SERPINB9,HAVCR2	grey	VSMC_M10
GO:0048103	0.58	0.68	5	9	somatic stem cell division	BP	1	KIT,BMI1,TGFB2,FZD7,ZFP36L2	grey	VSMC_M10
GO:0032873	0.58	0.55	20	38	negative regulation of stress-activated MAPK cascade	BP	1	PRKN,MYC,KLHL31,DNAJA1,FKTN,ZMYND11,SH3RF2,F2RL1,TAOK3,PDCD4,DLG1,AIDA,DUSP10,MEN1,DUSP19,PINK1,GPS2,NCOR1,MARVELD3,DUSP1	grey	VSMC_M10
GO:0070303	0.58	0.55	20	38	negative regulation of stress-activated protein kinase signaling cascade	BP	1	PRKN,MYC,KLHL31,DNAJA1,FKTN,ZMYND11,SH3RF2,F2RL1,TAOK3,PDCD4,DLG1,AIDA,DUSP10,MEN1,DUSP19,PINK1,GPS2,NCOR1,MARVELD3,DUSP1	grey	VSMC_M10
GO:0072384	0.58	0.51	41	78	organelle transport along microtubule	BP	1	AP3S1,KIF5A,KIF3A,AGTPBP1,SUN2,COPG2,BICD2,LRPPRC,MGARP,WASF1,KIF3C,ARHGAP21,COPG1,AP3M2,KIF3B,RASGRP1,OPA1,AP3B1,DTNBP1,KIF13A,BICDL1,CNIH2,MAPT,BLOC1S6,MREG,HTT,UXT,AP3M1,KIF1C,HAP1,PEX14,TRIM46,NDEL1,AC138894.1,SNAPIN,BICDL2,PRKCZ,UBB,BLOC1S3,BORCS5,AP3S2	grey	VSMC_M10
GO:1902259	0.58	0.68	5	9	regulation of delayed rectifier potassium channel activity	BP	1	KCNRG,KCNE2,KCNG1,NOS1AP,RNF207	grey	VSMC_M10
GO:0086069	0.58	0.66	6	11	bundle of His cell to Purkinje myocyte communication	BP	1	TRPM4,SCN5A,TBX5,DSP,PKP2,DSG2	grey	VSMC_M10
GO:0042448	0.58	0.68	5	9	progesterone metabolic process	BP	1	SRD5A1,DHRS9,STAT5B,DGKQ,CYP46A1	grey	VSMC_M10
GO:0009798	0.58	0.53	31	59	axis specification	BP	1	FRS2,PTCH1,SMO,MDFI,SRF,SIX2,SETDB2,IFT172,FAM208A,STC1,LDB1,PGAP1,WNT3,HOXD8,FZD5,BCOR,PKD1L1,MNS1,BMPR1A,CITED1,LHX1,CDX1,GPC3,HSPB11,SKI,WDR77,WNT5A,BASP1,DDIT3,C2CD3,HEY2	grey	VSMC_M10
GO:0040015	0.58	0.68	5	9	negative regulation of multicellular organism growth	BP	1	PTCH1,GDF15,RAI1,FXN,PLAC8	grey	VSMC_M10
GO:0060042	0.58	0.54	22	42	retina morphogenesis in camera-type eye	BP	1	NTRK2,RORB,SLC1A1,TFAP2B,RDH13,PTN,TSPAN12,SOX9,USH1C,RPGRIP1L,TFAP2A,NECTIN3,NRL,OBSL1,ROM1,SDK1,MEGF11,LHX1,SOX8,FJX1,THY1,DIO3	grey	VSMC_M10
GO:0008212	0.58	0.68	5	9	mineralocorticoid metabolic process	BP	1	CYP21A2,DKK3,WNT4,SCNN1B,CACNA1H	grey	VSMC_M10
GO:0051085	0.58	0.58	14	26	chaperone cofactor-dependent protein refolding	BP	1	HSPA1B,BAG1,HSPE1,DNAJC18,HSPA8,DNAJB12,SDF2L1,DNAJB1,GAK,CD74,TOR1B,TOR2A,HSPA5,SDF2	grey	VSMC_M10
GO:0042632	0.58	0.52	33	62	cholesterol homeostasis	BP	1	APOM,TTC39B,ABCG5,ABCG8,NR1H2,APOB,CNBP,TSKU,COMMD9,SOAT2,CYP7A1,CYP7B1,LIMA1,EPHX2,NR1H3,MYLIP,ABCA5,NR5A2,FABP4,GRAMD1B,ABCB11,ABCG4,SOAT1,EHD1,FGFR4,LIPC,LDLR,ACSM3,NPC1,TMEM97,MIR27B,MIR33B,LDLRAP1	grey	VSMC_M10
GO:0009914	0.58	0.48	112	212	hormone transport	BP	1	LRP1,RIMS2,PRKN,GDF9,PLA2G6,PICK1,CRY1,RFX3,TFAP2B,IL11,PRKCE,HMGN3,CYB5R4,BMP8A,EGFR,TRPM4,GIPR,RAF1,C1QTNF1,AIMP1,SIRT3,SYT9,NR1H4,NDUFAF2,FAM3D,PAX8,OSBP,ARNTL,SCG5,P2RY1,KCNJ11,NNAT,TIAM1,RAB11FIP5,PFKM,SLC8B1,NKX3-1,NKX6-1,ACVR1C,CRY2,ABCC2,SPP1,ZBED6,SIRT4,GPLD1,IRS1,HDAC1,PPP3CA,CPT1A,TRPV4,SLC9B2,ADRA2A,TCIRG1,TCF7L2,SNAP23,KCNB1,PFKFB2,GPER1,RAB11FIP2,AGT,SLCO1C1,PER2,OSM,STX1A,SNX4,PASK,GLUL,SIDT2,SLC16A1,KLF7,CREB1,VGF,ENY2,ABCA12,MCU,PPP3CB,CAMK2G,PTPRN,CD38,MPC2,ILDR2,INHA,SYT7,NLGN2,CPLX1,TARDBP,STX4,RAB8B,UBE2Q1,SYTL4,GPR68,BTK,CRYM,CCL5,SREBF1,ISL1,OXCT1,NOS2,ABAT,NADK,BAIAP3,PARK7,SLC7A5,LRRC8A,FGA,CHGA,SOX4,DPP4,CRH,DOC2B,LIF,UQCC2	grey	VSMC_M10
GO:0150093	0.58	0.71	4	7	amyloid-beta clearance by transcytosis	BP	1	LRP1,RAB5A,LRPAP1,RAB11A	grey	VSMC_M10
GO:0045824	0.58	0.54	25	47	negative regulation of innate immune response	BP	1	TNFAIP3,USP15,NR1H2,NMI,NR1H3,TRAFD1,OAS3,NLRC5,CD96,YTHDF2,SLAMF8,PARP14,NLRX1,DUSP10,MUL1,USP18,DCST1,STAT2,PTPN2,CRK,MIR21,OAS1,A2M,SERPINB9,HAVCR2	grey	VSMC_M10
GO:0007620	0.58	0.66	6	11	copulation	BP	1	KLK14,DDO,ACVR2A,P2RY1,ABAT,P2RX1	grey	VSMC_M10
GO:2001214	0.58	0.68	5	9	positive regulation of vasculogenesis	BP	1	RIN2,RAPGEF2,HIF1AN,RAMP2,TMEM100	grey	VSMC_M10
GO:0042670	0.58	0.71	4	7	retinal cone cell differentiation	BP	1	RORB,USH1C,THY1,DIO3	grey	VSMC_M10
GO:0046549	0.58	0.71	4	7	retinal cone cell development	BP	1	RORB,USH1C,THY1,DIO3	grey	VSMC_M10
GO:0000096	0.58	0.57	15	28	sulfur amino acid metabolic process	BP	1	MTAP,GNMT,SLC7A11,APIP,CSAD,MSRA,ENOPH1,MTRR,MTR,CBSL,SMS,CPS1,COMT,MAT1A,MTHFD2L	grey	VSMC_M10
GO:0071677	0.58	0.55	22	41	positive regulation of mononuclear cell migration	BP	1	AGER,CCL27,FPR2,OXSR1,GAS6,JAM2,PTK2B,ANO6,MOSPD2,ADAM8,ITGA4,IL12A,TNFSF18,TMEM102,S100A14,CCL5,CCL7,RHOA,DEFB124,FADD,WNT5A,PDGFD	grey	VSMC_M10
GO:0090278	0.58	0.56	18	34	negative regulation of peptide hormone secretion	BP	1	PRKN,BMP8A,NDUFAF2,FAM3D,KCNJ11,ACVR1C,SIRT4,IRS1,HDAC1,PPP3CA,ADRA2A,KCNB1,KLF7,ENY2,SYTL4,SREBF1,CHGA,CRH	grey	VSMC_M10
GO:0002444	0.58	0.52	37	70	myeloid leukocyte mediated immunity	BP	1	RAC2,KIT,TRAF3IP2,SPHK2,GAB2,UNC13D,MYD88,MAVS,FER,RASGRP1,F2RL1,SNAP23,LAT2,FGR,CBL,SNX4,PTGDR,C12orf4,SPON2,STX4,ITGAM,LAT,IL4R,S100A13,BTK,SCNN1B,STXBP2,PRAM1,C3,TICAM1,DNASE1,PDPK1,FOXF1,STXBP1,CHGA,CXCL6,SERPINB9	grey	VSMC_M10
GO:0010766	0.58	0.61	9	17	negative regulation of sodium ion transport	BP	1	NEDD4L,WNK2,PRKCE,HECW1,OSR1,HECW2,ATP1A2,WNK3,MIR24-1	grey	VSMC_M10

GO:0042554	0.58	0.57	16	30	superoxide anion generation	BP	1	FPR2,EGFR,FAM19A4,HVCN1,F2RL1,SH3PXD2A,NCF1,AGT,NCF2,MAPT,DUOX1,ITGAM,NOX5,NOXA1,GNAI3,CYBA	grey	VSMC_M10
GO:0051898	0.58	0.57	16	30	negative regulation of protein kinase B signaling	BP	1	PHLPP1,PPARA,LEMD2,PIK3CB,PTPRJ,BANK1,PDCD6,DAG1,GPER1,CIB1,DLG1,MUL1,OTUD3,INPP5K,MIR29A,DDIT3	grey	VSMC_M10
GO:1902774	0.58	0.71	4	7	late endosome to lysosome transport	BP	1	VPS41,VPS35,VPS39,SNAPIN	grey	VSMC_M10
GO:0097720	0.58	0.55	21	40	calcineurin-mediated signaling	BP	1	NFATC1,FHL2,SLC8A2,EFHB,PRNP,NRG1,SPPL3,PPP3CA,NR5A2,TBC1D10C,CIB1,SLC9A1,NFATC4,MYOZ2,ACTN3,HOMER2,PPP3CB,MYOZ1,MAPK7,CAMTA1,NFATC3	grey	VSMC_M10
GO:0070914	0.58	0.68	5	9	UV-damage excision repair	BP	1	ERCC1,XPC,DDB2,DDB1,CUL4B	grey	VSMC_M10
GO:0006957	0.58	0.71	4	7	complement activation, alternative pathway	BP	1	CFP,C7,C3,CFD	grey	VSMC_M10
GO:0045843	0.58	0.71	4	7	negative regulation of striated muscle tissue development	BP	1	SIRT2,USP19,YBX3,SOX15	grey	VSMC_M10
GO:0090713	0.58	0.68	5	9	immunological memory process	BP	1	HLA-DRA,BCL6,TSC1,CD46,FGL2	grey	VSMC_M10
GO:0071340	0.58	0.68	5	9	skeletal muscle acetylcholine-gated channel clustering	BP	1	PTN,FZD9,DNAJA3,RER1,MESD	grey	VSMC_M10
GO:1902959	0.58	0.68	5	9	regulation of aspartic-type endopeptidase activity involved in amyloid precursor protein catabolic process	BP	1	AGER,SORL1,EPHA4,TMED10,EFNA1	grey	VSMC_M10
GO:0010992	0.58	0.68	5	9	ubiquitin recycling	BP	1	PRKN,IDE,FBXW7,PINK1,RNF135	grey	VSMC_M10
GO:0045054	0.58	0.71	4	7	constitutive secretory pathway	BP	1	TRAPPC11,RAB3A,TMEM167A,TMEM167B	grey	VSMC_M10
GO:1905146	0.58	0.62	8	15	lysosomal protein catabolic process	BP	1	LRP1,MFSD8,TMEM106B,ATP13A2,AC138894.1,LDLR,LAMP2,TMEM199	grey	VSMC_M10
GO:1902514	0.58	0.68	5	9	regulation of calcium ion transmembrane transport via high voltage-gated calcium channel	BP	1	CACNB2,NOS1AP,SESTD1,CACNB1,MIR21	grey	VSMC_M10
GO:1901385	0.58	0.57	15	28	regulation of voltage-gated calcium channel activity	BP	1	STAC3,CACNB2,STAC,CACNB4,KCNE2,NOS1AP,GNB5,STAC2,CACNB1,CALM1,FMR1,RRAD,DMD,GPR35,GEM	grey	VSMC_M10
GO:0090084	0.58	0.68	5	9	negative regulation of inclusion body assembly	BP	1	HSPA1B,HSF1,SORL1,DNAJB2,DNAJB1	grey	VSMC_M10
GO:0032351	0.58	0.68	5	9	negative regulation of hormone metabolic process	BP	1	CYP27B1,DKK3,PRMT3,WNT4,GFI1	grey	VSMC_M10
GO:0009222	0.58	0.71	4	7	pyrimidine ribonucleotide catabolic process	BP	1	DPYS,ENTPD4,UPB1,UPP1	grey	VSMC_M10
GO:0071599	0.58	0.64	7	13	otic vesicle development	BP	1	SOX9,CEP290,PAX8,EYA1,STOX1,TCAP,SIX1	grey	VSMC_M10
GO:2000271	0.58	0.71	4	7	positive regulation of fibroblast apoptotic process	BP	1	BTG1,PRDM11,STK17B,BCL2L11	grey	VSMC_M10
GO:0021772	0.58	0.60	10	19	olfactory bulb development	BP	1	AGTPBP1,SRF,DLX5,SEMA3A,ROBO1,ROBO2,RPGRIP1L,SALL1,SKI,WNT5A	grey	VSMC_M10
GO:0033004	0.58	0.71	4	7	negative regulation of mast cell activation	BP	1	ENPP3,CD300LF,FER,FOXF1	grey	VSMC_M10
GO:1903432	0.58	0.56	17	32	regulation of TORC1 signaling	BP	1	RRAGD,KPTN,SESN3,SZT2,PIP4P1,LARS,GPR137B,DYRK3,SIK3,GPR137,OTUD5,RRAGB,DGKQ,SMCR8,CLEC16A,LAMTOR5,RNF152	grey	VSMC_M10
GO:0021545	0.58	0.55	20	38	cranial nerve development	BP	1	ERBB3,ATP8B1,PLXNA4,POU4F1,SEMA3A,EPHB1,TFAP2A,PAX2,SALL1,SEMA3F,TCIRG1,ADARB1,CHRNB2,ISL1,PLXNA3,MAFB,HOXB3,SIX1,TMEM126A,HOXA3	grey	VSMC_M10
GO:1903409	0.58	0.53	25	47	reactive oxygen species biosynthetic process	BP	1	PPARA,TLR4,UCP1,FOXO3,SPHK2,WISP3,ACOX1,GBF1,SLC30A10,NCF1,HDAC4,PIKFYVE,DUOX1,STAT3,MPO,PARK7,SLC25A33,CYBA,LCN2,MIR21,MIR24-1,RHOA,INAVA,APOPT1,AC140504.1	grey	VSMC_M10
GO:1902416	0.58	0.71	4	7	positive regulation of mRNA binding	BP	1	EIF3E,EIF3C,LARP6,FMR1	grey	VSMC_M10
GO:0051290	0.58	0.65	6	11	protein heterotetramerization	BP	1	CPSF6,HSD17B8,CBR4,PKD2,FARSB,PKD1	grey	VSMC_M10
GO:0060155	0.58	0.71	4	7	platelet dense granule organization	BP	1	AP3B1,F2R,DTNBP1,BLOC1S3	grey	VSMC_M10
GO:0042364	0.58	0.71	4	7	water-soluble vitamin biosynthetic process	BP	1	PSAT1,AKR1A1,PNPO,TPK1	grey	VSMC_M10
GO:0032790	0.58	0.71	4	7	ribosome disassembly	BP	1	MTIF2,EIF2D,MCTS1,MRRF	grey	VSMC_M10
GO:0031998	0.58	0.62	8	15	regulation of fatty acid beta-oxidation	BP	1	PPARA,AKT2,LONP2,IRS1,CPT1A,ABCB11,TYSND1,MLYCD	grey	VSMC_M10
GO:0071675	0.58	0.50	43	81	regulation of mononuclear cell migration	BP	1	AGER,PTK2,CCL27,FPR2,AKIRIN1,C5AR1,OXSR1,PLCB1,GAS6,JAM2,PTK2B,SLC8B1,ANO6,ADTRP,CD200,TRPV4,CMKLR1,SLAMF8,MOSPD2,APOD,ADAM8,MIA3,ITGA4,IL12A,TNFSF18,MAPK1,PADI2,TMEM102,STK10,MAPK3,S100A14,CCL5,CSF1R,CCL7,CCL2,CRK,RHOA,DEFB124,FADD,WNT5A,DUSP1,GCSAM,PDGFD	grey	VSMC_M10
GO:0046753	0.58	0.71	4	7	non-lytic viral release	BP	1	VPS4B,CHMP2A,CHMP4C,VPS4A	grey	VSMC_M10
GO:0021988	0.58	0.59	11	21	olfactory lobe development	BP	1	AGTPBP1,SRF,DLX5,SEMA3A,ROBO1,ROBO2,RPGRIP1L,SALL1,ATP1A2,SKI,WNT5A	grey	VSMC_M10
GO:0046339	0.58	0.60	10	19	diacylglycerol metabolic process	BP	1	AVIL,DGKI,LIPE,PLCE1,GPAM,DGKD,DGKG,DGKQ,DGKE,PLA2G15	grey	VSMC_M10
GO:1904948	0.58	0.63	7	13	midbrain dopaminergic neuron differentiation	BP	1	CSNK1E,FZD1,RYK,NR4A2,WNT3,WNT5A,WNT2	grey	VSMC_M10
GO:0070839	0.58	0.54	21	40	metal ion export	BP	1	SLC4A4,SLC8A2,ATP7B,ATP1A3,SLC8B1,SLC40A1,ANO6,KCNE2,KCNIP2,ATP1A2,SLC9A1,DLG1,SLC11A1,ATP1B1,ATP1B2,CALM1,ATP2B3,MIR21,KCNH2,KCNK5,KCNT2	grey	VSMC_M10
GO:0032196	0.58	0.63	7	13	transposition	BP	1	TUT7,APOBEC3H,TUT4,SIRT7,TEX15,THAP9,PGBD5	grey	VSMC_M10
GO:1902307	0.58	0.61	9	17	positive regulation of sodium ion transmembrane transport	BP	1	WNK2,GLRX,TESC,SLC9A1,ATP1B1,WNK3,ATP1B2,DNM2,DMD	grey	VSMC_M10
GO:0150079	0.58	0.67	5	9	negative regulation of neuroinflammatory response	BP	1	CST7,CD200,TNFRSF1B,SYT11,LDLR	grey	VSMC_M10
GO:0043383	0.59	0.70	4	7	negative T cell selection	BP	1	ATG5,CD28,DOCK2,CD74	grey	VSMC_M10
GO:0045060	0.59	0.70	4	7	negative thymic T cell selection	BP	1	ATG5,CD28,DOCK2,CD74	grey	VSMC_M10
GO:0098696	0.59	0.70	4	7	regulation of neurotransmitter receptor localization to postsynaptic specialization membrane	BP	1	DAG1,IQSEC2,PRKCZ,HRAS	grey	VSMC_M10
GO:0060159	0.59	0.67	5	9	regulation of dopamine receptor signaling pathway	BP	1	VPS35,ALK,DTNBP1,PALM,RGS4	grey	VSMC_M10

GO:0051403	0.59	0.47	100	190	stress-activated MAPK cascade	BP	1	PHLPP1,MAP3K5,STK3,MAPK8IP2,MAP3K4,PRKN,AGER,TRIB1,MYC,MAPK14,MAPK13,ROR2,GADD45G,KLHL31,PRDX1,SEMA4C,DNAJA1,FKTN,EMC10,MTURN,NOD1,EGFR,TLR4,TRAF1,SDCBP,MYD88,IKBKB,ZMYND11,PLCB1,MAP4K1,GPS1,EIF2AK2,RELL2,SEMA3A,SH3RF2,ARL6IP5,ERCC6,MFHAS1,TRAF6,EPHB1,HAND2,PTK2B,RASGRP1,RIPK2,F2RL1,RIPK1,NOD2,TAOK3,MAP2K6,TRPV4,PDCD4,TGFB2,NCF1,MAPKAPK2,AGT,TRAF5,STRADB,FZD7,MAP3K3,MAGI3,DLG1,MAP3K11,AIDA,DUSP10,MBIP,MEN1,MAP4K2,DUSP19,PDCD10,PINK1,LTBR,MAPK1,MINK1,GPS2,RELL1,TAOK2,MAPK3,HACD3,MAP2K1,SMAD3,NCOR1,TRAF3,ADORA2B,SH2D3A,PTGER4,TAOK1,ARHGEF6,SERPINF2,MAPK8IP3,TRAF2,MARVELD3,DAB2IP,ZNF622,WNT5A,GADD45A,INAVA,DUSP1,DUSP22,HRAS,MAP2K7	grey	VSMC_M10
GO:0010764	0.59	0.67	5	9	negative regulation of fibroblast migration	BP	1	HAS1,ITGB1BP1,CYGB,ARHGAP4,C5orf30	grey	VSMC_M10
GO:0033008	0.59	0.65	6	11	positive regulation of mast cell activation involved in immune response	BP	1	SPHK2,GAB2,FGR,SNX4,IL4R,STXBP1	grey	VSMC_M10
GO:0043306	0.59	0.65	6	11	positive regulation of mast cell degranulation	BP	1	SPHK2,GAB2,FGR,SNX4,IL4R,STXBP1	grey	VSMC_M10
GO:0009119	0.59	0.58	12	22	ribonucleoside metabolic process	BP	1	APOBEC3H,MTAP,GNMT,ENPP4,NT5E,PRTFDC1,CDADC1,AHCY,DGUOK,ADK,PEMT,UPP1	grey	VSMC_M10
GO:0035627	0.59	0.65	6	11	ceramide transport	BP	1	ABCB1,COL4A3BP,PLTP,ABCA12,CPTP,SGPP1	grey	VSMC_M10
GO:0034162	0.59	0.63	7	13	toll-like receptor 9 signaling pathway	BP	1	GRAMD4,RTN4,RSAD2,RAB7B,UNC93B1,TNIP2,HAVCR2	grey	VSMC_M10
GO:0036152	0.59	0.70	4	7	phosphatidylethanolamine acyl-chain remodeling	BP	1	MBOAT2,ABHD4,LPCAT3,PLA2G16	grey	VSMC_M10
GO:0006020	0.59	0.65	6	11	inositol metabolic process	BP	1	MIOX,IPPK,ITPKA,ITPK1,IMPA2,MECP2	grey	VSMC_M10
GO:0035855	0.59	0.61	9	17	megakaryocyte development	BP	1	MPIG6B,SRF,KIT,PIP4K2A,ABI1,WASF2,THPO,PTPN6,MED1	grey	VSMC_M10
GO:0070734	0.59	0.60	9	17	histone H3-K27 methylation	BP	1	PHF1,EED,OGT,SUZ12,SUPT6H,CHD5,MTF2,BEND3,ZNF304	grey	VSMC_M10
GO:1905461	0.59	0.70	4	7	positive regulation of vascular associated smooth muscle cell apoptotic process	BP	1	STK4,PDCD4,MFN2,MIR24-1	grey	VSMC_M10
GO:0002250	0.59	0.46	129	244	adaptive immune response	BP	1	CD226,STX7,TNFAIP3,AGER,HLA-DRA,HLA-DOB,TAP1,HLA-DMA,HLA-DPB1,TNFRSF13C,TFEB,TEC,TNFRSF21,EXOSC3,MSH2,LAIR1,BACH2,RSAD2,IL4I1,TLR4,TRPM4,TRAF3IP2,HMCES,PRKD2,PHB,ERCC1,NECTIN2,PVR,MLH1,UNC13D,MYD88,SLC22A13,FOXJ1,PAXIP1,EOMES,JAG1,SHLD1,NCKAP1L,HPX,IRF7,TRAF6,MARCH8,GAPT,CAMK4,ERAP1,ORAI1,MEF2C,PAG1,SLA2,PTK2B,EIF2AK4,RIPK2,NBN,RAPGEF3,ALCAM,NOD2,ADCY7,CD40,NFKB2,ICOSLG,TCIRG1,UNC93B1,HLX,DENND1B,OTUD7B,LAT2,BMX,CD28,FCER1G,RNF168,PDCD1,FZD5,DUSP10,PLA2G4A,BCL6,CTSH,IL12A,RC3H1,PTPN6,TNFSF18,SLC11A1,LAG3,PPP3CB,RAP1GAP,STAT3,WAS,TFE3,FOXP3,ZBTB1,SHLD2,TNFRSF1B,TBX21,SEMA4A,BATF,ZBTB7B,IL6R,CD19,LAT,IL4R,PRKCB,BTK,CD74,FBXO38,C7,C3,IL7R,SUPT6H,RAP1GAP2,PRKCZ,TRAF2,TSC1,BCL10,MIR21,FGA,ERAP2,FADD,CD46,JCHAIN,CD247,TRDV3,FCER2,RNF8,CD70,HRAS,LAX1,PRR7,TNFSF13B,HAVCR2,RC3H2	grey	VSMC_M10
GO:0070233	0.59	0.63	7	13	negative regulation of T cell apoptotic process	BP	1	SLC46A2,GPAM,EFNA1,CCL5,IL7R,FADD,TSC22D3	grey	VSMC_M10
GO:0048484	0.59	0.65	6	11	enteric nervous system development	BP	1	SOX10,RET,TLX2,HLX,RHOXF1,SOX8	grey	VSMC_M10
GO:0010820	0.59	0.67	5	9	positive regulation of T cell chemotaxis	BP	1	CCL27,OXSR1,TMEM102,CCL5,WNT5A	grey	VSMC_M10
GO:0070988	0.59	0.52	27	52	demethylation	BP	1	UBE2B,KDM3B,APOBEC3H,KDM4C,STPG4,KDM7A,MMACHC,KDM4A,PPME1,ALKBH3,TOX,OTUD4,FTO,JARID2,ALKBH2,POR,TET1,CYP3A43,KDM5A,KDM5C,PHF8,KDM6B,RIOX1,ALKBH1,KDM8,ALKBH5,HSF4	grey	VSMC_M10
GO:0002713	0.59	0.67	5	9	negative regulation of B cell mediated immunity	BP	1	FOXJ1,BCL6,PTPN6,FOXP3,CD46	grey	VSMC_M10
GO:0002890	0.59	0.67	5	9	negative regulation of immunoglobulin mediated immune response	BP	1	FOXJ1,BCL6,PTPN6,FOXP3,CD46	grey	VSMC_M10
GO:0033299	0.59	0.70	4	7	secretion of lysosomal enzymes	BP	1	NR1H2,NR1H3,BLOC1S6,BLOC1S3	grey	VSMC_M10
GO:0060037	0.59	0.58	11	21	pharyngeal system development	BP	1	PTCH1,EYA1,NKX3-1,RIPPLY3,TGFB2,ECE1,BMPR1A,ISL1,NOG,SIX1,NKX2-5	grey	VSMC_M10
GO:0031000	0.59	0.62	8	15	response to caffeine	BP	1	HDAC2,RYR1,DNMT3B,RYR3,CASQ2,RYR2,HDAC1,DHODH	grey	VSMC_M10
GO:0006554	0.59	0.70	4	7	lysine catabolic process	BP	1	CRYM,PIPOX,SLC25A21,HYKK	grey	VSMC_M10
GO:0045989	0.59	0.67	5	9	positive regulation of striated muscle contraction	BP	1	TRPV4,ACE2,ACTN3,ATP2A1,CHGA	grey	VSMC_M10
GO:0106074	0.59	0.65	6	11	aminoacyl-tRNA metabolism involved in translational fidelity	BP	1	LARS,TARS2,LARS2,AARS,DTD1,DTD2	grey	VSMC_M10
GO:0003339	0.59	0.70	4	7	regulation of mesenchymal to epithelial transition involved in metanephros morphogenesis	BP	1	PAX8,PAX2,CITED1,LIF	grey	VSMC_M10
GO:0009152	0.59	0.48	73	137	purine ribonucleotide biosynthetic process	BP	1	NME1,ATP5MGL,PPT2,PPARA,ACSL6,IMPDH1,SLC35B2,SLC25A13,PPAT,PPCS,PPT1,DLD,SPHK2,ADCY3,PRKAG2,ATP5F1C,ELOVL1,PANK2,PDK1,PDHX,ELOVL7,PANK1,AMPD3,ATP5F1A,GUCY1A1,GUCY1B1,DGUOK,GMPS,AMPD1,ADCY7,FAM173B,ADCY2,NME4,ACSL5,HACD2,ADCY4,DNAJC30,PDK3,SLC25A16,ACSBG1,ATP5S,COASY,PINK1,STAT3,NME7,ADK,MPC2,ADCY10,PARP1,TECR,SLC26A1,GUCY2D,PKLR,PANK3,GCDH,ACACA,SLC26A2,ADSSL1,ACSBG2,ATP6V0C,PANK4,NME3,ATP5PB,VPS9D1,ACSF3,MLYCD,MT-ATP8,MT-ATP6,ATP5ME,ATP5MF,ELOVL3,NME6,NUDT2	grey	VSMC_M10
GO:0001756	0.59	0.53	25	48	somitogenesis	BP	1	ROR2,MEOX2,ABI1,PCDH8,XRCC2,PRKDC,TCF15,POFUT1,NKX3-1,NKD1,POGLUT1,AXIN2,MEOX1,BMPR1A,TCAP,RBPJ,ALDH1A2,PSEN1,SMAD3,LHX1,CDX1,MIB1,NLE1,FOXF1,WNT5A	grey	VSMC_M10
GO:0071397	0.59	0.61	8	15	cellular response to cholesterol	BP	1	PTCH1,SMO,CYP7A1,GPLD1,DAG1,GRAMD1B,OSBPL7,LRP8	grey	VSMC_M10
GO:0042149	0.59	0.53	23	44	cellular response to glucose starvation	BP	1	ATF4,PICK1,NUAK1,BRSK1,FOXO3,KPTN,SESN3,PRKAG2,SZT2,PRKAG1,NUAK2,SIK1,MTMR3,TBL2,NFE2L2,SIK2,PRKAG3,BHLHA15,NIM1K,HSPA5,SH3GLB1,IMPACT,UPP1	grey	VSMC_M10

GO:0098877	0.59	0.60	9	17	neurotransmitter receptor transport to plasma membrane	BP	1	SLC1A1,VPS35,NSG1,GRIPAP1,RAB8A,CPLX1,STX1B,RAB11A,ARHGAP44	grey	VSMC_M10
GO:0021546	0.59	0.70	4	7	rhombomere development	BP	1	HOXA2,GBX2,MAFB,HOXB3	grey	VSMC_M10
GO:2000347	0.59	0.70	4	7	positive regulation of hepatocyte proliferation	BP	1	TNFAIP3,PTN,MED1,FGF1	grey	VSMC_M10
GO:0035930	0.59	0.67	5	9	corticosteroid hormone secretion	BP	1	CRY1,C1QTNF1,CRY2,AGT,CRH	grey	VSMC_M10
GO:2000846	0.59	0.67	5	9	regulation of corticosteroid hormone secretion	BP	1	CRY1,C1QTNF1,CRY2,AGT,CRH	grey	VSMC_M10
GO:0021952	0.59	0.58	12	23	central nervous system projection neuron axonogenesis	BP	1	PLXNA4,GLI2,MYCBP2,TSKU,SZT2,EPHB1,NR4A2,ADARB1,EPHB3,NIN,EPHA4,CHRNB2	grey	VSMC_M10
GO:0009313	0.59	0.67	5	9	oligosaccharide catabolic process	BP	1	NEU3,TREH,GM2A,CTBS,NEU2	grey	VSMC_M10
GO:0035601	0.59	0.49	52	99	protein deacylation	BP	1	BAZ2A,C6orf89,ABHD17B,PPT1,SPHK2,HDAC2,PRKD2,PHB,HDAC11,BEX4,TADA3,SIRT2,SIRT7,SIRT3,MTA3,LYPLA1,SDR16C5,ING2,HDAC7,SIRT5,SUDS3,ELK4,SIRT4,HDAC1,LYPLAL1,RCOR3,PER2,HDAC4,MAPT,BCL6,RCOR2,PINK1,TBL1XR1,KDM5A,FOXP3,AKAP8L,AKAP8,JDP2,ZBTB7B,SFPQ,SIN3A,RCOR1,SREBF1,SKI,ABHD17A,MIER2,PRMT6,ABHD10,SAP30BP,ABHD17C,NNMT,LYPLA2	grey	VSMC_M10
GO:0045603	0.59	0.65	6	11	positive regulation of endothelial cell differentiation	BP	1	BTG1,TMEM100,VEZF1,MIR21,ACVRL1,MIR99B	grey	VSMC_M10
GO:2000018	0.59	0.70	4	7	regulation of male gonad development	BP	1	SOX9,SEMA3A,DHX37,WNT4	grey	VSMC_M10
GO:1903799	0.59	0.63	7	13	negative regulation of production of miRNAs involved in gene silencing by miRNA	BP	1	ZC3H10,ESR1,BCDIN3D,PPP3CA,TERT,STAT3,NCOR1	grey	VSMC_M10
GO:0043476	0.59	0.65	6	11	pigment accumulation	BP	1	ANKRD27,SHROOM2,BLOC1S6,AP1G1,BLOC1S3,RAB32	grey	VSMC_M10
GO:0043482	0.59	0.65	6	11	cellular pigment accumulation	BP	1	ANKRD27,SHROOM2,BLOC1S6,AP1G1,BLOC1S3,RAB32	grey	VSMC_M10
GO:0098779	0.59	0.67	5	9	positive regulation of mitophagy in response to mitochondrial depolarization	BP	1	PRKN,OPTN,PINK1,MFN2,VPS13C	grey	VSMC_M10
GO:0044794	0.59	0.63	7	13	positive regulation by host of viral process	BP	1	YTHDC2,IGF2R,CFL1,HSPA8,PC,TBC1D20,ZNF502	grey	VSMC_M10
GO:0090102	0.59	0.54	21	40	cochlea development	BP	1	PTK7,SOBP,SLC26A5,MYO3A,SOX9,GLI2,EYA1,OPA1,RPGRIP1L,PAX2,MYO3B,KCNK2,GRHL3,EPHA4,DVL2,POU3F4,IFT20,KCNK3,WNT5A,SIX1,HEY2	grey	VSMC_M10
GO:0009309	0.59	0.56	14	26	amine biosynthetic process	BP	1	GPR37,DDC,AANAT,HAND2,NR4A2,AZIN2,SLC6A3,TGFB2,SMS,GCH1,HDC,OAZ1,PARK7,NNMT	grey	VSMC_M10
GO:0042401	0.59	0.56	14	26	cellular biogenic amine biosynthetic process	BP	1	GPR37,DDC,AANAT,HAND2,NR4A2,AZIN2,SLC6A3,TGFB2,SMS,GCH1,HDC,OAZ1,PARK7,NNMT	grey	VSMC_M10
GO:0050709	0.59	0.52	27	51	negative regulation of protein secretion	BP	1	PRKN,BMP8A,RSAD2,FRMD4A,DPH3,NDUFAF2,FAM3D,KCNJ11,NR1H3,RAB11FIP5,ACVR1C,F2R,ADTRP,SIRT4,IRS1,CD200,HDAC1,PPP3CA,ADRA2A,KCNB1,KLF7,IL12A,ENY2,SYTL4,SREBF1,MIR199A1,CHGA	grey	VSMC_M10
GO:0006729	0.59	0.70	4	7	tetrahydrobiopterin biosynthetic process	BP	1	DHFR,SPR,GCH1,PCBD2	grey	VSMC_M10
GO:0046146	0.59	0.70	4	7	tetrahydrobiopterin metabolic process	BP	1	DHFR,SPR,GCH1,PCBD2	grey	VSMC_M10
GO:0090240	0.59	0.70	4	7	positive regulation of histone H4 acetylation	BP	1	ARRB1,AUTS2,KAT7,MUC1	grey	VSMC_M10
GO:0035646	0.59	0.67	5	9	endosome to melanosome transport	BP	1	ANKRD27,BLOC1S6,AP1G1,BLOC1S3,RAB32	grey	VSMC_M10
GO:0043485	0.59	0.67	5	9	endosome to pigment granule transport	BP	1	ANKRD27,BLOC1S6,AP1G1,BLOC1S3,RAB32	grey	VSMC_M10
GO:0048757	0.59	0.67	5	9	pigment granule maturation	BP	1	ANKRD27,BLOC1S6,AP1G1,BLOC1S3,RAB32	grey	VSMC_M10
GO:0035088	0.59	0.53	22	42	establishment or maintenance of apical/basal cell polarity	BP	1	PTK7,LIN7B,DLG2,FOXJ1,WNT11,ANK1,LIN7A,SYNE4,TCF15,CLIC4,DLG1,MARK2,SYNJ2BP,MYO9A,CAMSAP3,LAMA1,IFT20,FOXF1,RHOA,OOEP,PDZD11,WNT5A	grey	VSMC_M10
GO:0061245	0.59	0.53	22	42	establishment or maintenance of bipolar cell polarity	BP	1	PTK7,LIN7B,DLG2,FOXJ1,WNT11,ANK1,LIN7A,SYNE4,TCF15,CLIC4,DLG1,MARK2,SYNJ2BP,MYO9A,CAMSAP3,LAMA1,IFT20,FOXF1,RHOA,OOEP,PDZD11,WNT5A	grey	VSMC_M10
GO:0035376	0.59	0.70	4	7	sterol import	BP	1	LRP1,STARD5,LDLR,STARD4	grey	VSMC_M10
GO:0070508	0.59	0.70	4	7	cholesterol import	BP	1	LRP1,STARD5,LDLR,STARD4	grey	VSMC_M10
GO:0046688	0.59	0.56	14	26	response to copper ion	BP	1	HSF1,AQP1,ATP7B,AANAT,PRNP,IL1A,LOXL2,BACE1,NFE2L2,SORD,SOD3,PARK7,MT-CYB,MT1X	grey	VSMC_M10
GO:0045017	0.59	0.46	113	214	glycerolipid biosynthetic process	BP	1	PIP4K2C,AVIL,SYNJ2,AGPAT4,AGPAT1,PNPLA3,PLA2G6,GPAA1,CWH43,INPP4A,TMEM246,PIGF,MBOAT7,NR1H2,LPIN1,INPP4B,FIG4,MTMR2,PIP4K2A,SELENOI,ABHD5,TAMM41,ITPKC,MTMR14,PGAP2,CRLS1,CDS2,NR1H4,MTMR7,PIGU,PIK3CB,PITPNM2,PIK3R1,TMEM150A,PLCE1,FBXW7,SLA2,NR1H3,SYNJ1,LPIN3,CDS1,FITM2,ITPKA,PIGT,GPLD1,LPCAT1,SIK1,PGAP1,GPAM,CHKA,ACP6,ABHD4,AJUBA,PITPNM1,GNPAT,DGKD,PIGM,BMX,FAM126B,IP6K2,LPGAT1,PIGV,MTMR3,PCYT1A,INPP5J,PIGX,PIGZ,PISD,DGKG,PIKFYVE,PLA2G4A,MOGAT3,SACM1L,LPCAT3,PIGC,SOCS4,PI4KA,SLC27A1,ABHD8,PGAP3,PIGH,PI4K2B,SOCS7,DGKQ,AC138894.1,DGKE,TTC7B,LDLR,PIGL,ABHD3,SREBF1,LPIN2,C3,MPPE1,IMPA2,SOCS1,MTM1,MTMR1,INPP5K,PIP5K1C,AGPAT2,PIP5KL1,PLA2G15,FABP5,PEMT,CNEP1R1,PYURF,LCLAT1,PIGB,PIGA,INPP1,PTPMT1,CDIPT	grey	VSMC_M10
GO:0060071	0.59	0.52	25	48	Wnt signaling pathway, planar cell polarity pathway	BP	1	FZD6,RSPO3,PTK7,ROR2,WNT11,SPEF1,RYK,FZD3,TIAM1,VANGL1,PRICKLE1,NKD1,ZNRF3,DVL3,FZD7,GRHL3,DAAM1,ARHGEF19,DVL2,MKS1,GPC3,CELSR2,RHOA,WNT5A,CELSR3	grey	VSMC_M10
GO:0007588	0.59	0.54	19	36	excretion	BP	1	NEDD4L,CHRNA3,ABCG5,ABCG8,POU3F3,SNX5,NPHP1,STC1,ABCG2,AGT,COMT,KCNMA1,AC138894.1,CHRNB2,SCNN1B,ADORA2B,TRPV1,CHRNB4,KCNK5	grey	VSMC_M10
GO:0097494	0.59	0.64	6	11	regulation of vesicle size	BP	1	WASHC5,RAB5A,RAB22A,AP2M1,RAB11A,RILP	grey	VSMC_M10
GO:0009215	0.59	0.70	4	7	purine deoxyribonucleoside triphosphate metabolic process	BP	1	NUDT15,NUDT16,DGUOK,ADK	grey	VSMC_M10
GO:0060026	0.59	0.62	7	13	convergent extension	BP	1	PTK7,WNT11,MKKS,NKD1,DVL2,WNT5A,LBX2	grey	VSMC_M10

GO:0033674	0.59	0.45	196	373	positive regulation of kinase activity	BP	1	ERBB3,MAP3K5,AGAP2,MRNIP,STK3,MAP3K4,AGER,ROS1,XRCC6,PTK2,TAB1,NTRK2,CCND3,ZNF16,CHRNA3,TEK,TTBK1,KIT,SLC1A1,ROR2,FPR2,MOB1B,EPGN,AREG,ETAA1,EPHA7,TNFSF15,TGFA,ITGB1BP1,MET,EGFR,ARHGEF5,TLR4,EPHA1,CCNY,SLC8A2,PHB,ABI1,MAPRE3,RALB,PRKAG2,PIBF1,SPRY2,WNT11,ALK,TGFBR2,NEK10,RPTOR,TCIM,HBEGF,PRNP,NRG1,NCKAP1L,GAS6,ERCC6,FLT1,RYK,ADRA2B,TRAF6,EPHB1,RET,ZFP91,ROBO1,FBXW7,VEGFC,MADD,PTK2B,RASGRP1,RAPGEF2,PRKAG1,TIAM1,NBN,PKD2,STK4,NOD2,TAOK3,CD40,DSTYK,IGF2,BMPR1B,IRS1,NEURL1,CCND1,MAP2K6,TGFB2,ADRA2A,FAM20A,DAG1,AJUBA,NCF1,KRAS,EGF,PFKFB2,ADNP,CASS4,SPATC1L,CACUL1,AGT,FGR,CIB1,DVL3,STRADB,SYAP1,STRADA,EPHB3,DUSP12,GPRC5D,CDKN1B,DLG1,PPP2R3C,MAP3K11,ADAM8,FZD5,MAPT,STOX1,CEMIP,DBF4B,EPHB4,MAP4K2,DDX3X,XRCC5,DUSP19,PDCD10,ADRA2C,DOK7,SLC11A1,MARK2,FGFR3,PINK1,SLC27A1,EPHA4,DLG4,MAGED1,DVL2,BMPR1A,FGFR4,RPLP1,DGKQ,PSEN1,FGF18,LCP2,TAOK2,EFNA1,IL6R,MAP2K1,CD19,LAT,PIK3R5,MAP2K5,CALM1,PSMD10,CD74,INSR,CCL5,CSF1R,CDK5R1,GHR,UNC119,TAOK1,SOCS1,DAB1,LRP8,PDPK1,CAMKK1,PRKCZ,MLST8,PKD1,TRAF2,MTCP1,PARK7,PSRC1,VAV3,BCL10,DAB2IP,MIR21,MIR125B1,RHOA,S100A12,ZNF622,INSRR,IRGM,WNT5A,TIGAR,MMD,SPDYA,TXN,COPS8,RGCC,DAZAP2,HRAS,EREG,MAP2K7,C3orf58,FGF1,PIFO,PDGFD	grey	VSMC_M10
GO:0042762	0.59	0.64	6	11	regulation of sulfur metabolic process	BP	1	SLC7A11,PDK1,PDK3,NFE2L2,COMT,MIR21	grey	VSMC_M10
GO:0005978	0.59	0.54	19	36	glycogen biosynthetic process	BP	1	EPM2AIP1,PPP1CB,AKT2,PPP1R3B,GBE1,SORBS1,IGF2,NHLRC1,IRS1,PPP1R3E,PPP1R3D,PER2,PASK,PRKAG3,PPP1R3F,INSR,INPP5K,AGL,ACADM	grey	VSMC_M10
GO:0009250	0.59	0.54	19	36	glucan biosynthetic process	BP	1	EPM2AIP1,PPP1CB,AKT2,PPP1R3B,GBE1,SORBS1,IGF2,NHLRC1,IRS1,PPP1R3E,PPP1R3D,PER2,PASK,PRKAG3,PPP1R3F,INSR,INPP5K,AGL,ACADM	grey	VSMC_M10
GO:0070875	0.59	0.62	7	13	positive regulation of glycogen metabolic process	BP	1	EPM2AIP1,AKT2,SORBS1,IGF2,IRS1,PPP1R3E,INSR	grey	VSMC_M10
GO:0034356	0.59	0.67	5	9	NAD biosynthesis via nicotinamide riboside salvage pathway	BP	1	SLC22A13,SLC5A8,NUDT12,PARP9,NNMT	grey	VSMC_M10
GO:0034116	0.59	0.67	5	9	positive regulation of heterotypic cell-cell adhesion	BP	1	AGER,GCNT2,LCK,FGA,THY1	grey	VSMC_M10
GO:0032715	0.59	0.55	16	30	negative regulation of interleukin-6 production	BP	1	TNFAIP3,TLR4,C5AR2,FOXJ1,ARRB1,NCKAP1L,GAS6,IL37,CD200,BANK1,NLRX1,PTPN6,FOXP3,SYT11,MIR125A,HAVCR2	grey	VSMC_M10
GO:0071732	0.59	0.62	7	13	cellular response to nitric oxide	BP	1	AQP1,GUCY1B1,MTR,MMP3,DNM2,CRK,TRAF2	grey	VSMC_M10
GO:0010675	0.59	0.48	60	114	regulation of cellular carbohydrate metabolic process	BP	1	PRKN,PPARA,GPT,GNMT,CRY1,PRKCE,PPP4R3B,ZNF692,PLEK,FAM3C,RUBCNL,KHK,EPM2AIP1,GCKR,PPP1CB,AKT2,C1QTNF1,SIRT7,PDK1,PPP1R3B,NLN,NCOA2,SORBS1,P2RY1,PTK2B,IGF2,GPLD1,IRS1,SIK1,MST1,PPP1R3E,PFKFB2,GPER1,PPP1R3D,ACTN3,HDAC4,NTSR1,PASK,DUSP12,PDK3,PTH1R,GNB3,STAT3,PPP1R3F,IGFBP4,DDB1,DGKQ,OGT,COX11,LCMT1,NCOR1,INSR,LEPR,PTPN2,INPP5K,MLYCD,ACADM,TIGAR,NNMT,ARPP19	grey	VSMC_M10
GO:0035563	0.59	0.62	7	13	positive regulation of chromatin binding	BP	1	ZNF618,MED25,HMBOX1,EBF2,PARP9,DTX3L,DDX11	grey	VSMC_M10
GO:0036010	0.59	0.57	12	23	protein localization to endosome	BP	1	MICALL1,ABHD17B,TMEM30A,VPS35,WASHC2A,TOLLIP,ANKRD13A,EGF,SORL1,DTX3L,ABHD17A,ABHD17C	grey	VSMC_M10
GO:0039532	0.59	0.64	6	11	negative regulation of viral-induced cytoplasmic pattern recognition receptor signaling pathway	BP	1	SEC14L1,NPLOC4,NLRX1,TKFC,TSPAN6,RIOK3	grey	VSMC_M10
GO:0090659	0.59	0.58	11	21	walking behavior	BP	1	AGTPBP1,HTRA2,SPG11,EPHA4,CLN8,NPC1,DAB1,CNTN2,CEND1,FXN,EFNB3	grey	VSMC_M10
GO:0006549	0.59	0.70	4	7	isoleucine metabolic process	BP	1	BCAT2,SDSL,ACADSB,HSD17B10	grey	VSMC_M10
GO:0006359	0.59	0.60	9	17	regulation of transcription by RNA polymerase III	BP	1	CHD8,RPTOR,BRF2,ZC3H8,ZNF143,CEBPA,POLR3C,ELL,ICE2	grey	VSMC_M10
GO:0070741	0.59	0.54	18	34	response to interleukin-6	BP	1	PHB,ST18,CEBPA,FER,ST3GAL6,RIPK1,ABCC2,SELPLG,STAT3,CITED1,IL6R,JAK1,PTPN2,LCN2,GFI1,MIR125B1,MIR125A,C1QTNF4	grey	VSMC_M10
GO:0034379	0.59	0.66	5	9	very-low-density lipoprotein particle assembly	BP	1	APOB,APOC1,SOAT2,SOAT1,LPCAT3	grey	VSMC_M10
GO:0043373	0.59	0.62	7	13	CD4-positive, alpha-beta T cell lineage commitment	BP	1	TOX,TNFSF18,STAT3,FOXP3,TBX21,BATF,IL6R	grey	VSMC_M10
GO:0021561	0.59	0.64	6	11	facial nerve development	BP	1	PLXNA4,SEMA3A,SEMA3F,ADARB1,PLXNA3,SIX1	grey	VSMC_M10
GO:0021610	0.59	0.64	6	11	facial nerve morphogenesis	BP	1	PLXNA4,SEMA3A,SEMA3F,ADARB1,PLXNA3,SIX1	grey	VSMC_M10
GO:0044872	0.59	0.62	7	13	lipoprotein localization	BP	1	LRP1,APOB,CUBN,MIA3,PRKCB,VMP1,UNC119	grey	VSMC_M10
GO:0003272	0.59	0.57	12	23	endocardial cushion formation	BP	1	TGFBR2,ROBO1,ROBO2,TGFB2,SNAI1,BMPR1A,RBPJ,TMEM100,TBX2,ENG,NOG,HEY2	grey	VSMC_M10
GO:0050766	0.59	0.53	20	38	positive regulation of phagocytosis	BP	1	LRP1,FPR2,IL15,CD300LF,IL15RA,NCKAP1L,GAS6,TUB,PTPRJ,F2RL1,ANO6,NOD2,FCER1G,SLC11A1,DOCK2,DNM2,CCL2,C3,ABCA7,CYBA	grey	VSMC_M10
GO:0060438	0.60	0.61	8	15	trachea development	BP	1	SRF,SOX9,TGFBR2,MAPK1,MAPK3,MAP2K1,FOXF1,HYDIN	grey	VSMC_M10
GO:0032273	0.60	0.48	61	116	positive regulation of protein polymerization	BP	1	ARPC3,HSPA1B,WASHC5,BAIAP2L2,PICK1,CDC42EP1,LMOD2,TTBK1,SLAIN2,NCK2,KIAA1211,PRKCE,MET,CDK5RAP2,MAPRE1,WASF1,SLAIN1,FCHSD2,NAV3,BAG4,FCHSD1,NCKAP1L,ARFIP2,LMOD3,HCK,FER,HIP1R,CLIP1,FMN1,PTK2B,IQGAP2,CYFIP1,MAGEL2,CARMIL1,LMOD1,AP1AR,WASF2,WHAMM,CDKN1B,DLG1,CDC42EP2,MAPT,TOGARAM1,NCKAP1,NIN,PINK1,ARPC1A,WAS,EVL,CDK5R1,MLST8,MECP2,SPIRE2,PSRC1,WDR63,CARMIL2,RHOA,VASP,GMFG,GRB2,DRG1	grey	VSMC_M10
GO:0008090	0.60	0.59	10	19	retrograde axonal transport	BP	1	KIF5A,AGTPBP1,MGARP,DLG2,CALY,KIF1C,HAP1,NDEL1,SNAPIN,MAP1A	grey	VSMC_M10

GO:0007018	0.60	0.45	149	283	microtubule-based movement	BP	1	AP3S1,KIF5A,RAB21,KIF3A,UBE2B,CATSPER3,LRRC6,AGTPBP1,SUN2,COPG2,IFT74,CFAP69,APBA1,RFX3,BICD2,DYNC2LI1,LRPPRC,DNAJA1,DNAH11,CABS1,MGARP,ARMC2,WASF1,ARMC4,APOB,TCTE3,ADCY3,TTC26,KIF3C,ARHGAP21,COPG1,DRC1,RSPH6A,KLC3,DLG2,TCTEX1D4,IFT172,TTC21A,WDR60,CFAP61,TTLL3,DYNC1LI1,AP3M2,KIF5C,MKKS,NEK10,PURA,SPEF1,TUB,DNAH10,KIF3B,ING2,DNAH6,WDR66,IFT81,CFAP20,KIFC3,KATNB1,RASGRP1,CCDC65,OPA1,AP3B1,IFT52,DNAH1,PLTP,DNAH5,DTNBP1,DPCD,ROPN1L,KIF13A,BICDL1,KIF21A,NEURL1,CFAP43,ZMYND10,SLC9B1,SLC9B2,CFAP46,INTS13,TTC21B,HSPA8,JHY,OFD1,CFAP45,ENO4,TSSK4,BAG3,TRAF3IP1,CELF3,CNIH2,CATSPER2,KIF9,CALY,TTC30A,SPG11,MAPT,SORD,BLOC1S6,TTC12,DNAAF2,SPAG16,MREG,HTT,CFAP70,STK36,KIF17,UXT,AP3M1,ADCY10,KIF1C,TTC25,HAP1,DNAAF4,MNS1,WDR19,KPNB1,DNAH2,PEX14,TRIM46,NDEL1,AC138894.1,CCDC151,SNAPIN,SFPQ,DNAH3,TEKT3,CAMSAP3,IFT20,SPEF2,CATSPERD,CFAP53,HSPB11,BICDL2,PRKCZ,MAPK8IP3,TPGS1,CELSR2,HYDIN,TERF2,WDR63,DYNC1LI2,UBB,BLOC1S3,KIF21B,BORCS5,CCSAP,AP3S2,MAP1A,SPA17	grey	VSMC_M10
GO:0042558	0.60	0.55	15	28	pteridine-containing compound metabolic process	BP	1	SHMT2,DHFR,MTHFD2,SPR,MTRR,MTR,MTHFS,GCH1,SHMT1,SLC46A1,PIPOX,MTHFD2L,SLC25A32,ALDH1L1,PCBD2	grey	VSMC_M10
GO:0080111	0.60	0.60	9	17	DNA demethylation	BP	1	APOBEC3H,STPG4,ALKBH3,TOX,OTUD4,FTO,ALKBH2,TET1,ALKBH1	grey	VSMC_M10
GO:2000479	0.60	0.61	8	15	regulation of cAMP-dependent protein kinase activity	BP	1	PRKAR2B,PRKAG2,PKIA,RAPGEF2,PRKAR1A,MST1,SPATC1L,HTT	grey	VSMC_M10
GO:0048538	0.60	0.53	20	38	thymus development	BP	1	SRF,SLC46A2,RAF1,PRKDC,ASXL1,HAND2,JARID2,EPHB3,MAPK1,ZBTB1,PSEN1,CTC1,MAPK3,MAP2K1,BCL11B,MAFB,FADD,SIX1,BCL2L11,HOXA3	grey	VSMC_M10
GO:0007200	0.60	0.51	28	53	phospholipase C-activating G protein-coupled receptor signaling pathway	BP	1	ESR1,GPR20,FPR2,LPAR1,C5AR2,C5AR1,ADRA1D,CCKBR,PLCE1,P2RY1,HOMER1,F2RL1,F2R,ADRA2A,CMKLR1,OPRD1,AGT,PTH1R,CXCR2,CHRM1,NMUR2,GNB1,GNA15,GNG13,GPR157,GPR35,CHGA,GNA14	grey	VSMC_M10
GO:1902166	0.60	0.64	6	11	negative regulation of intrinsic apoptotic signaling pathway in response to DNA damage by p53 class mediator	BP	1	TAF9,ING2,MARCH7,MUC1,TAF9B,CD74	grey	VSMC_M10
GO:0006109	0.60	0.47	73	139	regulation of carbohydrate metabolic process	BP	1	PRKN,PPARA,PRXL2C,GPT,GNMT,CRY1,PRKCE,PPP4R3B,SLC4A4,ZNF692,PLEK,FAM3C,RUBCNL,KHK,EPM2AIP1,PRKAG2,GCKR,PPP1CB,AKT2,C1QTNF1,SIRT7,PDK1,PPP1R3B,NLN,NCOA2,SORBS1,GPD1,P2RX7,P2RY1,PTK2B,PRKAG1,IGF2,GPLD1,IRS1,SIK1,ZBTB20,MST1,PPP1R3E,EGF,PFKFB2,GPER1,ARNT,PPP1R3D,ACTN3,HDAC4,NTSR1,PASK,MLXIPL,DUSP12,PDK3,PTH1R,GNB3,STAT3,PPP1R3F,IGFBP4,DDB1,DGKQ,PSEN1,OGT,COX11,LCMT1,NCOR1,INSR,LEPR,PTPN2,INPP5K,SLC2A6,MLYCD,ACADM,ENTPD5,TIGAR,NNMT,ARPP19	grey	VSMC_M10
GO:0072079	0.60	0.61	8	15	nephron tubule formation	BP	1	OSR1,SOX9,PAX8,PAX2,IRX2,SOX8,NOG,SIX1	grey	VSMC_M10
GO:0035929	0.60	0.62	7	13	steroid hormone secretion	BP	1	GDF9,CRY1,C1QTNF1,NKX3-1,CRY2,AGT,CRH	grey	VSMC_M10
GO:0032488	0.60	0.64	6	11	Cdc42 protein signal transduction	BP	1	WAS,RHOJ,NTN1,RIT2,RALBP1,RHOU	grey	VSMC_M10
GO:0022410	0.60	0.66	5	9	circadian sleep/wake cycle process	BP	1	BTBD9,SRD5A1,CHRNB2,PER3,CRH	grey	VSMC_M10
GO:0086001	0.60	0.51	30	58	cardiac muscle cell action potential	BP	1	NEDD4L,TRPM4,CACNB2,SCN5A,SCN1A,GPD1L,SLMAP,KCNJ3,KCNE2,DSP,KCNIP2,RYR2,PKP2,KCNJ2,ANK2,ATP1A2,SCN4B,NOS1AP,DLG1,ATP1B1,SLC4A3,CACNA1G,CALM1,DSG2,RNF207,DMD,KCNH2,SCN2B,KCNJ8,HCN4	grey	VSMC_M10
GO:0070885	0.60	0.61	8	15	negative regulation of calcineurin-NFAT signaling cascade	BP	1	FHL2,PRNP,TBC1D10C,MYOZ2,ACTN3,HOMER2,MYOZ1,MAPK7	grey	VSMC_M10
GO:0106057	0.60	0.61	8	15	negative regulation of calcineurin-mediated signaling	BP	1	FHL2,PRNP,TBC1D10C,MYOZ2,ACTN3,HOMER2,MYOZ1,MAPK7	grey	VSMC_M10
GO:0048048	0.60	0.56	13	25	embryonic eye morphogenesis	BP	1	FRS2,IFT172,RARB,TFAP2A,PAX2,ALDH1A3,SP3,FZD5,FBN1,MFAP2,TBX2,STRA6,WNT5A	grey	VSMC_M10
GO:0044774	0.60	0.49	39	74	mitotic DNA integrity checkpoint signaling	BP	1	MRNIP,TRIM39,PPP1R10,MDC1,BRSK1,MSH2,XPC,PLK3,NEK11,PRKDC,STK33,PLK2,RAD17,RAD9B,NBN,ABRAXAS1,DONSON,PIDD1,TAOK3,CCND1,GIGYF2,CHEK2,SYF2,CDKN1B,UIMC1,FOXO4,EME1,MUC1,TAOK2,TIPIN,FOXN3,TAOK1,FZR1,FBXO31,NAE1,RPS27L,SOX4,BRCC3,CRADD	grey	VSMC_M10
GO:1900272	0.60	0.66	5	9	negative regulation of long-term synaptic potentiation	BP	1	AGER,PTN,PRNP,FAM107A,EPHA4	grey	VSMC_M10
GO:0010890	0.60	0.69	4	7	positive regulation of sequestering of triglyceride	BP	1	PLIN2,FITM2,FITM1,PLIN3	grey	VSMC_M10
GO:0033864	0.60	0.69	4	7	positive regulation of NAD(P)H oxidase activity	BP	1	AGT,PARK7,GNAI3,CYBA	grey	VSMC_M10
GO:0048485	0.60	0.61	8	15	sympathetic nervous system development	BP	1	GFRA3,PLXNA4,TFAP2B,SEMA3A,HAND2,FZD3,SEMA3F,SOX4	grey	VSMC_M10
GO:1905477	0.60	0.49	46	88	positive regulation of protein localization to membrane	BP	1	LRP1,STAC3,PRKN,PRKCE,SPTBN1,PTN,ITGB1BP1,EGFR,TCAF2,STAC,AKT2,PRNP,PIK3R1,PLS1,SORBS1,KCNJ11,SSH1,KCNB1,C2CD5,GPER1,RAB11FIP2,CIB1,DLG1,FIS1,CEMIP,NMT1,ZDHHC8,CNPY4,CNST,WNK3,STAC2,STX4,ITGAM,ITGA3,AC138894.1,RAB11A,AP2B1,MIEF2,CDK5R1,EFCAB7,PDPK1,RER1,ARHGEF16,HRAS,MIEF1,MESD	grey	VSMC_M10
GO:0031146	0.60	0.52	23	44	SCF-dependent proteasomal ubiquitin-dependent protein catabolic process	BP	1	SKP1,FBXL6,FBXO48,FBXL13,FBXL2,DMAC2,FBXO17,FBXW7,FBXW4,FBXL15,AMN1,FBXO33,FBXL14,FBXL18,FBXO2,FBXL22,FBXO38,CUL4B,FBXL16,FBXO31,FBXL8,FBXL12,STYX	grey	VSMC_M10
GO:0007021	0.60	0.69	4	7	tubulin complex assembly	BP	1	TBCE,TBCEL,VBP1,TBCA	grey	VSMC_M10
GO:0048854	0.60	0.55	14	27	brain morphogenesis	BP	1	FZD6,FANCC,SMO,FOXO3,MKKS,FZD3,SLIT1,PAX2,ZNF335,SLC4A10,PSEN1,SPEF2,WNT5A,OTX1	grey	VSMC_M10
GO:0031050	0.60	0.52	24	46	dsRNA processing	BP	1	ZC3H10,ESR1,AGO2,TUT7,PUS10,SNIP1,AGO1,EGFR,MYCN,TUT4,PUM2,BCDIN3D,RIPK1,PPP3CA,TERT,PRKRA,DDX3X,STAT3,MAP2K1,SMAD3,NCOR1,DROSHA,SPOUT1,AGO4	grey	VSMC_M10
GO:0035196	0.60	0.52	24	46	production of miRNAs involved in gene silencing by miRNA	BP	1	ZC3H10,ESR1,AGO2,TUT7,PUS10,SNIP1,AGO1,EGFR,MYCN,TUT4,PUM2,BCDIN3D,RIPK1,PPP3CA,TERT,PRKRA,DDX3X,STAT3,MAP2K1,SMAD3,NCOR1,DROSHA,SPOUT1,AGO4	grey	VSMC_M10

GO:0070918	0.60	0.52	24	46	production of small RNA involved in gene silencing by RNA	BP	1	ZC3H10,ESR1,AGO2,TUT7,PUS10,SNIP1,AGO1,EGFR,MYCN,TUT4,PUM2,BCDIN3D,RIPK1,PPP3CA,TERT,PRKRA,DDX3X,STAT3,MAP2K1,SMAD3,NCOR1,DROSHA,SPOUT1,AGO4	grey	VSMC_M10
GO:0007009	0.60	0.48	49	94	plasma membrane organization	BP	1	ATP8B1,A4GALT,FER1L6,MTSS1,ASAP1,BAIAP2L2,MICALL1,MYH9,SPTBN1,SPACA6,IZUMO1,PRX,AKT2,STX2,ANO4,MYOF,XKR6,PLSCR4,P2RX7,DYSF,BIN3,ANO6,TGFB2,ANK2,OSBPL2,WHAMM,LYZL6,NSF,RAB3A,SYT7,MPP5,DEGS1,STX4,SYT11,AC138894.1,SPESP1,NOX5,SYTL4,DNM2,EPB41L3,ABCA7,SYPL2,COL5A1,FNBP1L,MTSS1L,SH3GLB1,TLCD1,XKR8,CAVIN2	grey	VSMC_M10
GO:0016338	0.60	0.66	5	9	calcium-independent cell-cell adhesion via plasma membrane cell-adhesion molecules	BP	1	CLDN12,CLDN19,CLDN7,CLDN15,CLDN1	grey	VSMC_M10
GO:0001892	0.60	0.50	35	67	embryonic placenta development	BP	1	STK3,RSPO3,MDFI,TFEB,EPAS1,GJB5,OVOL2,EOMES,SPINT2,CEBPA,SPINT1,PKD2,STK4,IGF2,SNAI1,ARNT,VASH2,SP3,SETD2,FZD5,MAPK1,RBPJ,MED1,SOX15,VASH1,CITED1,MAP2K1,PKD1,KRT19,CASP8,GRB2,LIF,CEBPB,HEY2,WNT2	grey	VSMC_M10
GO:0032332	0.60	0.59	9	17	positive regulation of chondrocyte differentiation	BP	1	SOX9,ZNF219,SOX6,LOXL2,BMPR1B,POR,FGF18,SMAD3,ACVRL1	grey	VSMC_M10
GO:0006303	0.60	0.50	31	59	double-strand break repair via nonhomologous end joining	BP	1	ATP23,MRNIP,XRCC6,HSF1,POT1,CYREN,HMCES,ERCC1,XRCC1,MLH1,SIRT7,SHLD1,PRKDC,ERCC6,ERCC8,XRCC4,POLL,PARP9,DTX3L,XRCC5,IFFO1,SHLD2,PRPF19,WRAP53,LIG3,SMCHD1,ERCC4,NUDT16L1,PAXX,RNF8,NHEJ1	grey	VSMC_M10
GO:1901380	0.60	0.59	9	17	negative regulation of potassium ion transmembrane transport	BP	1	NEDD4L,KEL,KCNRG,OXSR1,KCNE2,CASQ2,KCNH2,CRBN,RGS4	grey	VSMC_M10
GO:0099637	0.60	0.57	11	21	neurotransmitter receptor transport	BP	1	SLC1A1,VPS35,NSG1,GRIPAP1,RAB8A,CPLX1,STX1B,RAB11A,ARHGAP44,CACNG7,CACNG5	grey	VSMC_M10
GO:1902237	0.60	0.69	4	7	positive regulation of endoplasmic reticulum stress-induced intrinsic apoptotic signaling pathway	BP	1	NCK2,PTPN2,DDIT3,BCL2L11	grey	VSMC_M10
GO:0030497	0.60	0.62	7	13	fatty acid elongation	BP	1	HACD4,ELOVL1,ELOVL7,HACD2,TECR,HACD3,ELOVL3	grey	VSMC_M10
GO:0018026	0.60	0.64	6	11	peptidyl-lysine monomethylation	BP	1	SETD7,SETD6,EEF1AKMT2,SETD2,SETD3,METTL18	grey	VSMC_M10
GO:0046716	0.60	0.62	7	13	muscle cell cellular homeostasis	BP	1	LARGE1,SRF,TRIM32,PFKM,BAG3,MTM1,DMD	grey	VSMC_M10
GO:0042780	0.60	0.69	4	7	tRNA 3'-end processing	BP	1	TRNT1,HSD17B10,POLR3K,TRMT10C	grey	VSMC_M10
GO:0010676	0.60	0.52	21	40	positive regulation of cellular carbohydrate metabolic process	BP	1	PPARA,GPT,PRKCE,PPP4R3B,EPM2AIP1,AKT2,SORBS1,P2RY1,IGF2,GPLD1,IRS1,PPP1R3E,GPER1,ACTN3,NTSR1,PTH1R,DDB1,INSR,PTPN2,NNMT,ARPP19	grey	VSMC_M10
GO:0040037	0.60	0.60	8	15	negative regulation of fibroblast growth factor receptor signaling pathway	BP	1	FUZ,SPRY1,SPRY2,SULF2,WNT4,NGFR,WNT5A,SHISA2	grey	VSMC_M10
GO:0061052	0.60	0.66	5	9	negative regulation of cell growth involved in cardiac muscle cell development	BP	1	PPARA,PI16,YY1,MIR199A1,RGS4	grey	VSMC_M10
GO:0002363	0.60	0.60	8	15	alpha-beta T cell lineage commitment	BP	1	TOX,TNFSF18,STAT3,FOXP3,TBX21,BATF,IL6R,RHOA	grey	VSMC_M10
GO:0046348	0.60	0.66	5	9	amino sugar catabolic process	BP	1	ALDH1A1,GNPDA2,MGAT1,OVGP1,CTBS	grey	VSMC_M10
GO:0010893	0.60	0.62	7	13	positive regulation of steroid biosynthetic process	BP	1	CYP7A1,SCAP,POR,WNT4,SREBF1,STARD4,FGF1	grey	VSMC_M10
GO:0002720	0.60	0.52	22	42	positive regulation of cytokine production involved in immune response	BP	1	CD226,KIT,RSAD2,TLR4,SPHK2,LACC1,MAVS,TRAF6,F2RL1,NOD2,DENND1B,FZD5,HK1,SPON2,CD74,TICAM1,PRKCZ,TRAF2,SLC7A5,BCL10,WNT5A,INAVA	grey	VSMC_M10
GO:0007130	0.60	0.59	9	17	synaptonemal complex assembly	BP	1	TEX15,SYCP1,SPO11,TEX12,STAG3,P3H4,SYCE2,AGO4,SYCE1L	grey	VSMC_M10
GO:0070314	0.60	0.66	5	9	G1 to G0 transition	BP	1	SLC39A5,CYP27B1,RIDA,CDK5R1,PHGDH	grey	VSMC_M10
GO:0002209	0.60	0.58	10	19	behavioral defense response	BP	1	MAPK8IP2,USP46,SLC1A1,MEF2C,ASIC1,ATP1A2,ASIC4,MECP2,DPP4,CRH	grey	VSMC_M10
GO:0032769	0.60	0.66	5	9	negative regulation of monooxygenase activity	BP	1	CYP27B1,NOSIP,GLA,GFI1,MIR27B	grey	VSMC_M10
GO:0071801	0.60	0.63	6	11	regulation of podosome assembly	BP	1	IL5,ASAP1,LCP1,HCK,KIF9,RHOA	grey	VSMC_M10
GO:1902902	0.60	0.61	7	13	negative regulation of autophagosome assembly	BP	1	FEZ2,SEC22B,TMEM39A,PINK1,PHF23,SMCR8,MTM1	grey	VSMC_M10
GO:0002407	0.60	0.60	8	15	dendritic cell chemotaxis	BP	1	TRPM4,GAS6,ANO6,SLAMF8,IL12A,TNFSF18,CXCR2,CCL5	grey	VSMC_M10
GO:0022011	0.60	0.56	13	25	myelination in peripheral nervous system	BP	1	ADGRG6,NTRK2,ADAM22,PRX,AKT2,SIRT2,NRG1,LGI4,DAG1,CNTNAP1,MPP5,SKI,POU3F2	grey	VSMC_M10
GO:0032292	0.60	0.56	13	25	peripheral nervous system axon ensheathment	BP	1	ADGRG6,NTRK2,ADAM22,PRX,AKT2,SIRT2,NRG1,LGI4,DAG1,CNTNAP1,MPP5,SKI,POU3F2	grey	VSMC_M10
GO:0033866	0.60	0.50	30	57	nucleoside bisphosphate biosynthetic process	BP	1	PPT2,ACSL6,SLC35B2,PPCS,PPT1,DLD,ELOVL1,PANK2,PDK1,PDHX,ELOVL7,PANK1,ACSL5,HACD2,PDK3,SLC25A16,ACSBG1,COASY,MPC2,TECR,SLC26A1,PANK3,GCDH,ACACA,SLC26A2,ACSBG2,PANK4,ACSF3,MLYCD,ELOVL3	grey	VSMC_M10
GO:0034030	0.60	0.50	30	57	ribonucleoside bisphosphate biosynthetic process	BP	1	PPT2,ACSL6,SLC35B2,PPCS,PPT1,DLD,ELOVL1,PANK2,PDK1,PDHX,ELOVL7,PANK1,ACSL5,HACD2,PDK3,SLC25A16,ACSBG1,COASY,MPC2,TECR,SLC26A1,PANK3,GCDH,ACACA,SLC26A2,ACSBG2,PANK4,ACSF3,MLYCD,ELOVL3	grey	VSMC_M10
GO:0034033	0.60	0.50	30	57	purine nucleoside bisphosphate biosynthetic process	BP	1	PPT2,ACSL6,SLC35B2,PPCS,PPT1,DLD,ELOVL1,PANK2,PDK1,PDHX,ELOVL7,PANK1,ACSL5,HACD2,PDK3,SLC25A16,ACSBG1,COASY,MPC2,TECR,SLC26A1,PANK3,GCDH,ACACA,SLC26A2,ACSBG2,PANK4,ACSF3,MLYCD,ELOVL3	grey	VSMC_M10
GO:0032042	0.60	0.61	7	13	mitochondrial DNA metabolic process	BP	1	MGME1,PRIMPOL,POLG2,STOX1,PARP1,LIG3,LONP1	grey	VSMC_M10
GO:0030325	0.60	0.59	9	17	adrenal gland development	BP	1	TSPO,LY6E,WNT11,DKK3,SALL1,WNT4,SMAD3,STRA6,FSTL3	grey	VSMC_M10

GO:0005996	0.60	0.45	105	199	monosaccharide metabolic process	BP	1	FUCA2,PRKN,PPARA,PMM1,AKR1B1,ATF4,MAPK14,GPT,GNMT,CRY1,PGM5,GALT,UXS1,TFAP2B,SLC25A13,GAPDH,PPP4R3B,ZNF692,FAM3C,DHDH,RUBCNL,AKR1A1,GLB1,KHK,EPM2AIP1,NUDT5,XYLB,GCKR,RBKS,PPP1CB,AKT2,PFKP,PGM2L1,C1QTNF1,SIRT7,SLC23A2,PDK1,FUT10,PPP1R3B,POFUT1,GPI,NLN,NCOA2,SORBS1,GPD1,KCNJ11,PFKM,CHST1,GLYCTK,IGF2,GPLD1,IRS1,SIK1,CPT1A,MST1,PPP1R3E,PFKFB2,PC,PPP1R3D,SLC37A4,PER2,PFKFB4,ACTN3,USF1,PASK,DUSP12,GAL3ST3,APOD,FUOM,PDK3,SORD,RPE,HK1,GNB3,SLC35A2,PPP1R3F,IGFBP4,DDB1,DGKQ,OGT,COX11,CRTC2,LCMT1,ADPGK,MPI,INSR,LEPR,ALDOC,PTPN2,FGGY,PMM2,INPP5K,TKTL1,MLYCD,ACADM,FBP2,OAS1,FABP5,FUT4,FPGT,POFUT2,TIGAR,NNMT,ARPP19,RPIA	grey	VSMC_M10
GO:1901386	0.60	0.63	6	11	negative regulation of voltage-gated calcium channel activity	BP	1	GNB5,CALM1,FMR1,RRAD,GPR35,GEM	grey	VSMC_M10
GO:0043687	0.60	0.54	16	30	post-translational protein modification	BP	1	ATG12,AGTPBP1,ATG5,CRTAP,TTL,RNF7,GPLD1,RHBDD1,FAM20C,ATG16L1,WIPI2,VIPAS39,LCMT1,FOLH1,IRGM,UCHL3	grey	VSMC_M10
GO:0002830	0.60	0.63	6	11	positive regulation of type 2 immune response	BP	1	RSAD2,NOD2,DENND1B,IL4R,CD74,PRKCZ	grey	VSMC_M10
GO:1901673	0.60	0.60	8	15	regulation of mitotic spindle assembly	BP	1	VPS4B,HSPA1B,CHMP2A,CHMP2B,CHMP4C,CHMP1A,CCSAP,DRG1	grey	VSMC_M10
GO:0045646	0.60	0.52	22	42	regulation of erythrocyte differentiation	BP	1	HSPA1B,BRD1,MAPK14,ZNF16,RBFOX2,CDK6,SMAP1,FOXO3,ACVR2A,NCKAP1L,PRKDC,ACVR1B,LDB1,ARNT,STAT3,STAT5B,MED1,KAT7,MIR222,ANKRD54,MAFB,FAM210B	grey	VSMC_M10
GO:0046628	0.60	0.58	10	19	positive regulation of insulin receptor signaling pathway	BP	1	GKAP1,SNX5,SORBS1,OPA1,IGF2,IRS1,SORL1,AGT,ZBTB7B,PRKCZ	grey	VSMC_M10
GO:0048680	0.60	0.65	5	9	positive regulation of axon regeneration	BP	1	LRP1,PTN,NDEL1,MIR222,CNTF	grey	VSMC_M10
GO:1905939	0.61	0.65	5	9	regulation of gonad development	BP	1	GDF9,SOX9,SEMA3A,DHX37,WNT4	grey	VSMC_M10
GO:0002831	0.61	0.45	118	224	regulation of response to biotic stimulus	BP	1	CD226,TNFAIP3,USP15,XRCC6,TRIB1,C6orf106,FPR2,ANKRD17,NOD1,AKIRIN2,NR1H2,IL4I1,TLR4,IL15,PUM2,TRAF3IP2,NECTIN2,PVR,OPTN,SLC22A13,SEC14L1,IKBKB,SIRT2,NMI,TMEM173,NPLOC4,MAVS,HPX,FOXP1,PRKDC,IRF7,TRAF6,PCBP2,TRIM44,HCK,ERAP1,RASGRP1,EIF2AK4,NR1H3,TRAFD1,OAS3,NLRC5,RIPK2,F2RL1,POLR3D,MAPKBP1,HERC5,NOD2,CD96,OASL,POLR3C,NCF1,YTHDF2,SLAMF8,PARP9,DTX3L,PARP14,RNF26,FGR,NLRX1,TRAF3IP1,HTRA1,COCH,PPP2R3C,ADAM8,CADM1,DUSP10,NONO,IL12A,XRCC5,PTPN6,LAG3,MUL1,BIRC3,STAT5B,FOXP3,RNF216,TKFC,USP18,MED1,SYT11,MAPK3,DCST1,TSPAN6,SFPQ,STAT2,SIN3A,TRAF3,CCL5,RIOK3,GATA6,FBXO38,RNF135,VAV1,PTPN2,SOCS1,LRP8,CRK,CREBBP,CYBA,AP1G1,ZDHHC1,MIR21,OAS1,ZDHHC11,FADD,CXCL6,CASP8,ZCCHC3,A2M,IFNLR1,IRGM,WNT5A,TIGAR,FGL2,SERPINB9,EREG,HAVCR2	grey	VSMC_M10
GO:0007416	0.61	0.46	68	132	synapse assembly	BP	1	NTRK2,LRRC4,LINGO2,GABRA1,FZD1,ADGRF1,SEMA4D,ADGRL3,GJA10,LRRN3,EPHA7,ADD2,VPS35,SDCBP,NRCAM,VSTM5,POU4F1,SLITRK5,LRFN1,FARP1,NRG1,LRFN3,RYK,EPHB1,CLSTN2,ROBO2,MEF2C,CUX2,SLIT1,AMIGO2,BDNF,GAP43,AMIGO3,NLGN4Y,BSN,ADNP,GNPAT,RAB17,SPTBN2,NPAS4,EPHB3,FZD5,NRXN2,SPOCK2,DNM3,OBSL1,LRTM2,SDK1,NLGN2,LGI2,SEMA4A,NLGN3,CHRNB2,NTN1,GPRASP2,BHLHB9,ASIC2,SLITRK2,MECP2,AMIGO1,CEL,NTNG2,ZDHHC12,ADGRL2,GRID2,WNT5A,SIX1,LRRTM2	grey	VSMC_M10
GO:0009176	0.61	0.65	5	9	pyrimidine deoxyribonucleoside monophosphate metabolic process	BP	1	DPYS,UPB1,NT5M,DCTD,UPP1	grey	VSMC_M10
GO:0009642	0.61	0.65	5	9	response to light intensity	BP	1	CAT,RDH13,AKT2,TENT2,GNB5	grey	VSMC_M10
GO:0042745	0.61	0.63	6	11	circadian sleep/wake cycle	BP	1	BTBD9,AHCY,SRD5A1,CHRNB2,PER3,CRH	grey	VSMC_M10
GO:0006222	0.61	0.65	5	9	UMP biosynthetic process	BP	1	CMPK1,UPRT,UCK1,DHODH,UPP1	grey	VSMC_M10
GO:0009174	0.61	0.65	5	9	pyrimidine ribonucleoside monophosphate biosynthetic process	BP	1	CMPK1,UPRT,UCK1,DHODH,UPP1	grey	VSMC_M10
GO:0051702	0.61	0.49	32	61	biological process involved in interaction with symbiont	BP	1	YTHDC2,SNW1,IGF2R,PRKN,GAPDH,NAPEPLD,PHB,FBXL2,MYD88,SAP30,EIF2AK4,F2RL1,HDAC1,ZNF639,CFL1,HSPA8,POU2F3,PC,FN1,DDB1,PSMC3,TARDBP,SCN1B,CCL5,JUN,FMR1,INPP5K,MIR222,CXCL6,SAP30BP,TBC1D20,ZNF502	grey	VSMC_M10
GO:0010447	0.61	0.58	9	17	response to acidic pH	BP	1	ASIC3,ASIC1,RAB11FIP5,SLC38A3,SLC9A1,ASIC2,TRPV1,PKD1L3,IMPACT	grey	VSMC_M10
GO:0060512	0.61	0.57	11	21	prostate gland morphogenesis	BP	1	ESR1,FRS2,HOXA13,TNC,SOX9,GLI2,CYP7B1,NKX3-1,SERPINB5,NOG,WNT5A	grey	VSMC_M10
GO:0060351	0.61	0.55	13	25	cartilage development involved in endochondral bone morphogenesis	BP	1	COL27A1,SOX9,TSKU,TGFBR2,RARB,STC1,TRPV4,TRIP11,AXIN2,POR,MMP13,THBS3,GHR	grey	VSMC_M10
GO:0050773	0.61	0.48	45	87	regulation of dendrite development	BP	1	NEDD4L,RAB21,CHRNA3,SEMA4D,PTN,HECW1,RTN4IP1,SS18L2,ALK,TMEM106B,ANKRD27,TLX2,CUX2,RAPGEF2,TIAM1,OPA1,ITPKA,CYFIP1,LZTS1,KIAA0319,DNM1L,PPP3CA,HECW2,CAPRIN2,MARK1,RAB17,NFATC4,SS18L1,CDKL5,DGKG,CRTC1,OBSL1,EPHA4,CHRNB2,ZDHHC15,PARP6,GPRASP2,BHLHB9,SEZ6,LRP8,CRK,FBXO31,DAB2IP,RHOA,YWHAH	grey	VSMC_M10
GO:0046717	0.61	0.56	11	21	acid secretion	BP	1	APBA1,CCKBR,HIP1R,CHRM5,ABCC2,ABCB11,NTSR1,HRH2,ABAT,TRPV1,SNX10	grey	VSMC_M10
GO:0060113	0.61	0.51	25	48	inner ear receptor cell differentiation	BP	1	ATP8B1,GSDME,MYCL,WHRN,MYCN,USH1G,MYO7A,TSKU,SLC4A7,JAG1,LRTOMT,AP000812.5,PLS1,ADGRV1,USH1C,TRIP11,STRC,CDH23,RBPJ,MKS1,JAG2,IFT20,MCOLN3,PJVK,HEY2	grey	VSMC_M10
GO:0071300	0.61	0.50	27	52	cellular response to retinoic acid	BP	1	ADNP2,SNW1,RORB,PTK7,HOXA2,AQP1,TNC,TEAD2,OSR1,HDAC2,SOX9,WNT11,RET,HTRA2,PTK2B,TESC,PAX2,FZD7,WNT3,CREB1,ABL2,BRINP2,ALDH1A2,YES1,WNT5A,ZNF35,WNT2	grey	VSMC_M10
GO:0035116	0.61	0.57	10	19	embryonic hindlimb morphogenesis	BP	1	OSR2,PITX1,OSR1,RARB,RPGRIP1L,WNT3,MED1,TBX4,GPC3,ALX3	grey	VSMC_M10
GO:0046851	0.61	0.61	7	13	negative regulation of bone remodeling	BP	1	TNFAIP3,TNFRSF11B,P2RX7,GPR137B,UBASH3B,GPR137,CD38	grey	VSMC_M10
GO:0051917	0.61	0.65	5	9	regulation of fibrinolysis	BP	1	THBD,FAP,USF1,F12,SERPINF2	grey	VSMC_M10

GO:0035493	0.61	0.61	7	13	SNARE complex assembly	BP	1	SEPT8,ANKRD27,STX1A,VAMP1,STX4,STXBP1,VAMP3	grey	VSMC_M10
GO:0035999	0.61	0.65	5	9	tetrahydrofolate interconversion	BP	1	SHMT2,MTHFD2,MTHFS,SHMT1,MTHFD2L	grey	VSMC_M10
GO:0034384	0.61	0.68	4	7	high-density lipoprotein particle clearance	BP	1	APOM,GPLD1,LDLR,MIR33B	grey	VSMC_M10
GO:0035090	0.61	0.65	5	9	maintenance of apical/basal cell polarity	BP	1	LIN7B,WNT11,ANK1,LIN7A,PDZD11	grey	VSMC_M10
GO:0045199	0.61	0.65	5	9	maintenance of epithelial cell apical/basal polarity	BP	1	LIN7B,WNT11,ANK1,LIN7A,PDZD11	grey	VSMC_M10
GO:0006620	0.61	0.63	6	11	posttranslational protein targeting to endoplasmic reticulum membrane	BP	1	SEC61B,SEC61G,SEC62,SGTA,HSPA5,SEC61A2	grey	VSMC_M10
GO:1902683	0.61	0.59	8	15	regulation of receptor localization to synapse	BP	1	STX7,DAG1,CNIH2,GRIPAP1,IQSEC2,ARHGAP44,PRKCZ,HRAS	grey	VSMC_M10
GO:0050868	0.61	0.48	40	76	negative regulation of T cell activation	BP	1	TNFRSF21,PELI1,IL4I1,FOXJ1,PAWR,PRNP,NCKAP1L,ZC3H8,CASP3,TMEM131L,PAG1,MARCH7,BTN2A2,PRKAR1A,HLX,RUNX3,DLG1,BCL6,VSIR,RC3H1,PTPN6,TNFSF18,LAG3,SCGB1A1,FOXP3,TBX21,ZBTB7B,IL4R,CD74,TWSG1,PTPN2,SOCS1,GLMN,MIR21,MIR27A,FGL2,LAX1,CEBPB,HAVCR2,RC3H2	grey	VSMC_M10
GO:0030099	0.61	0.44	154	293	myeloid cell differentiation	BP	1	NME1,MPIG6B,HSPA1B,BRD1,IL5,TRIB1,MYC,TOB2,SMAD5,L3MBTL3,MAPK14,HSF1,MYH9,ZNF16,RBFOX2,SRF,CDK6,KIT,ROR2,RPS14,IL11,SMAP1,EPAS1,CD109,HOXA7,PTBP3,MTURN,BPGM,CNOT4,TLR4,IL15,OSTM1,FOXO3,PIP4K2A,ABI1,ERCC2,GAB2,PRDX3,SLC25A38,POU4F1,TGFBR2,ACVR2A,JAG1,RASGRP4,NCKAP1L,FOXP1,MITF,PRKDC,IRF7,MFHAS1,TRAF6,ACVR1B,CAMK4,CEBPA,NCOA6,PIK3R1,CASP3,FBXW7,MEF2C,PTK2B,AP3B1,F2RL1,TESC,RIPK1,TMEM14C,TSPAN2,DMTN,ITGB6,ALAS1,LDB1,RAB7B,SH3PXD2A,PKNOX1,LRRK1,SLC9B2,TCIRG1,GPR137B,PRKX,PIAS3,ACIN1,DYRK3,TCTA,HCLS1,UBASH3B,CIB1,WASF2,ARNT,THPO,FARP2,ZBTB46,SP3,CREB1,NBEAL2,BCL6,BATF2,WDR61,ESRRA,PTPN6,GPR137,FBN1,LTBR,STAT3,STAT5B,LEO1,INHA,TFE3,PARP1,THRA,RBPJ,MED1,ANXA2,PSEN1,HOXB8,AHSP,KAT7,FOS,BATF,DNASE2,HAX1,GPR68,RCOR1,CD74,CSF1R,PPARGC1B,EVI2B,PTPN2,GPC3,JUN,ATP6AP1,MIR222,MIR125B1,PRMT6,FSTL3,ANKRD54,ARID4A,MAFB,FADD,CASP8,CA2,DHRS2,BGLAP,SNX10,HIST1H4B,HIST2H4A,HIST1H4H,GLO1,NRROS,HIST1H4D,FAM210B,LIF,CEBPB,HIST1H4A,BATF3,HIST1H4F	grey	VSMC_M10
GO:0030520	0.61	0.51	24	46	intracellular estrogen receptor signaling pathway	BP	1	ESR1,CNOT2,FOXH1,RBFOX2,EGLN2,TADA3,TAF7,DDRGK1,NCOA4,CYP7B1,UFSP2,ZNF366,CNOT1,KMT2D,DDX54,VPS18,ARID1A,PADI2,DNAAF4,PARP1,MED1,PAGR1,PPARGC1B,ISL1	grey	VSMC_M10
GO:1905168	0.61	0.61	7	13	positive regulation of double-strand break repair via homologous recombination	BP	1	MRNIP,WDR48,ERCC6,WAS,PARP1,WRAP53,OOEP	grey	VSMC_M10
GO:0062099	0.61	0.59	8	15	negative regulation of programmed necrotic cell death	BP	1	PELI1,RIPK1,FZD9,YBX3,BIRC3,MIR22,FADD,CASP8	grey	VSMC_M10
GO:0032205	0.61	0.53	16	31	negative regulation of telomere maintenance	BP	1	POT1,ERCC1,XRCC1,NBN,TENT4B,STN1,RTEL1,PARP1,GNL3L,CTC1,PIF1,ERCC4,SLX4,TERF2,TINF2,TEN1	grey	VSMC_M10
GO:0000097	0.61	0.61	7	13	sulfur amino acid biosynthetic process	BP	1	MTAP,APIP,ENOPH1,MTRR,MTR,CBSL,MTHFD2L	grey	VSMC_M10
GO:0051646	0.61	0.51	24	46	mitochondrion localization	BP	1	AGTPBP1,LRPPRC,ALB,MGARP,WASF1,NECTIN2,ARMC1,OPA1,DNM1L,MARK1,MFN1,MAPT,MARK2,MUL1,UXT,BHLHA15,HAP1,MFN2,MSTO1,ATP2A1,MTM1,TLE6,CLUH,UBB	grey	VSMC_M10
GO:0018216	0.61	0.61	7	13	peptidyl-arginine methylation	BP	1	PRDM4,NDUFAF7,NR1H4,PRMT9,PRMT3,PRMT7,PRMT6	grey	VSMC_M10
GO:0010660	0.61	0.49	30	57	regulation of muscle cell apoptotic process	BP	1	MAP3K5,PPP1R10,RGL2,HSF1,CDKN2A,ARRB1,PAX8,HAND2,PTK2B,STK4,SIRT5,SIRT4,PDCD4,AGT,BAG3,NFE2L2,IL12A,CXCR2,RBM10,MFN2,CAMK2A,PDPK1,MIR199A1,MIR21,MIR24-1,NOL3,TIGAR,C3orf58,NKX2-5,HEY2	grey	VSMC_M10
GO:0090169	0.61	0.56	11	21	regulation of spindle assembly	BP	1	VPS4B,HSPA1B,MAPK15,CHMP2A,CHMP2B,CHMP4C,RNF4,CHMP1A,GPSM2,CCSAP,DRG1	grey	VSMC_M10
GO:0016074	0.61	0.61	7	13	sno(s)RNA metabolic process	BP	1	EXOSC4,EXOSC3,NUDT16,TENT4B,RNF113B,PARN,EXOSC5	grey	VSMC_M10
GO:0039535	0.61	0.58	9	17	regulation of RIG-I signaling pathway	BP	1	USP15,ANKRD17,PUM2,SEC14L1,NPLOC4,OASL,NLRX1,BIRC3,ZCCHC3	grey	VSMC_M10
GO:0043144	0.61	0.62	6	11	sno(s)RNA processing	BP	1	EXOSC4,EXOSC3,TENT4B,RNF113B,PARN,EXOSC5	grey	VSMC_M10
GO:0042532	0.61	0.61	7	13	negative regulation of tyrosine phosphorylation of STAT protein	BP	1	PPP2R1A,PIBF1,PARP14,SUZ12,PTPN2,SOCS1,MIR125A	grey	VSMC_M10
GO:0032480	0.61	0.56	11	21	negative regulation of type I interferon production	BP	1	C6orf106,REL,NMI,NPLOC4,NLRX1,TRAF3IP1,OTUD5,RNF216,YY1,MIR21,HAVCR2	grey	VSMC_M10
GO:0007616	0.61	0.54	14	27	long-term memory	BP	1	BTBD9,SRF,SHANK1,NTF4,PRNP,CAMK4,EIF2AK4,NFATC4,NPAS4,SLC2A4,RGS14,LDLR,PRKCZ,MECP2	grey	VSMC_M10
GO:0001881	0.61	0.54	14	27	receptor recycling	BP	1	BVES,EPS15,OPTN,TBC1D16,AP1AR,SORL1,NSF,ECE1,NSG1,ANXA2,PSEN1,PHETA1,VAMP3,PLEKHJ1	grey	VSMC_M10
GO:1900543	0.61	0.59	8	15	negative regulation of purine nucleotide metabolic process	BP	1	PPARA,ACTN3,HDAC4,STAT3,PARP1,NCOR1,PDZD3,TIGAR	grey	VSMC_M10
GO:0036462	0.61	0.68	4	7	TRAIL-activated apoptotic signaling pathway	BP	1	PARK7,MIR222,FADD,CASP8	grey	VSMC_M10
GO:0035338	0.61	0.57	10	19	long-chain fatty-acyl-CoA biosynthetic process	BP	1	ACSL6,ELOVL1,ELOVL7,ACSL5,HACD2,ACSBG1,TECR,ACSBG2,ACSF3,ELOVL3	grey	VSMC_M10
GO:1990138	0.61	0.45	76	147	neuron projection extension	BP	1	NEDD4L,LRP1,SEMA6A,RAB21,RIMS2,PRKN,PLXNA4,CPNE5,SRF,SEMA4D,SEMA4C,RIMS1,RTN4,RUFY3,NRCAM,WASF1,PLXNA1,GOLGA4,MEGF8,CPNE9,SYT1,SLC23A2,SEMA3D,SEMA3A,ULK1,RYK,TTL,SEMA4F,DPYSL2,EIF2AK4,TIAM1,SLIT1,NKX6-1,ZFYVE27,SEMA3G,CYFIP1,ALCAM,SEMA4G,KIAA0319,SEMA3F,AUTS2,ADNP,DISC1,CDKL5,WNT3,SPG11,MAPT,SEMA6D,ITGA4,TNR,FN1,FLRT1,PPP3CB,MUL1,VCL,SEMA4A,NLGN3,TRIM46,NDEL1,RAB11A,NTN1,DNM2,SIN3A,TRPV2,CDK5R1,SLC9A6,L1CAM,PRKCZ,ARHGAP4,PLXNA3,BARHL2,IMPACT,WNT5A,CACNG7,LPH,OLFM1	grey	VSMC_M10

GO:0050808	0.61	0.43	171	329	synapse organization	BP	1	PRRT1,SYNGAP1,MAPK14,NTRK2,PICK1,LRRC4,LINGO2,ABHD17B,GABRA1,FZD1,SLC1A1,ADGRF1,SEMA4D,ADGRL3,SHANK1,GJA10,LRRN3,EPHA7,PTN,ADD2,TNC,CTTNBP2,YWHAZ,DLGAP3,VPS35,SDCBP,NRCAM,SLC7A11,PPFIA3,WASF1,MTMR2,SLC8A2,C5AR1,VSTM5,CACNB2,PCDH8,PTPRF,POU4F1,SLITRK5,LRFN1,FARP1,ACTG1,CACNB4,PRNP,PCDHGC5,P2RX2,NRG1,LRFN3,RYK,EPHB1,CLSTN2,CHMP2B,ROBO2,ERC2,MEF2C,CUX2,SEPT11,TIAM1,SLIT1,HOMER1,OPA1,AMIGO2,F2R,SPARCL1,ITPKA,CYFIP1,TUBA1A,BDNF,DOCK10,PPFIA4,INA,NEURL1,DNM1L,PPFIA1,CAPRIN2,SEMA3F,GAP43,PPFIBP1,CAMKV,DAG1,AMIGO3,NLGN4Y,SSH1,CFL1,STAU1,BSN,ADNP,HSPA8,DISC1,GNPAT,WASF2,RAB17,SPTBN2,NFATC4,NPAS4,PTPRO,CDKL5,IL10RA,EPHB3,TANC2,NOS1AP,FZD9,DLG1,FZD5,MAPT,SRGN,C1QL1,TNR,NRXN2,SPOCK2,DOK7,DNM3,RAB3A,SYN1,OBSL1,GRIPAP1,EPHA4,LRTM2,DLG4,ACTB,SDK1,NLGN2,GPHN,CHRNB1,LGI2,CACNB1,PSEN1,SEMA4A,ITGAM,ITGA3,NLGN3,EFNA1,AC138894.1,CHRNB2,ZDHHC15,MYH10,NTN1,SNAPIN,DRP2,GPRASP2,BHLHB9,ARHGAP44,INSR,ASIC2,CDK5R1,C3,SEZ6,SLITRK2,LRP8,DNAJA3,SDF4,L1CAM,RER1,MECP2,ABHD17A,PALM,AMIGO1,CEL,TSC1,NTNG2,ZDHHC12,DAB2IP,ADGRL2,RHOA,CNTN2,GRID2,ABHD17C,WNT5A,SIX1,MESD,LRRTM2	grey	VSMC_M10
GO:0050776	0.61	0.42	284	538	regulation of immune response	BP	1	CD226,STX7,TNFAIP3,USP15,ENPP3,ESR1,HSPA1B,GRAMD4,AGER,HLA-DRA,HLA-DPB1,XRCC6,PTK2,C6orf106,MAPK14,PLA2G6,RAC2,LRRC14,TEC,KIT,TNFRSF21,NINJ1,EXOSC3,PRKCE,MSH2,RTN4,FPR2,ANKRD17,SLC46A2,NOD1,AKIRIN2,PELI1,RSAD2,NR1H2,IL4I1,TLR4,IL15,TRPM4,PUM2,TRAF3IP2,SPHK2,HMCES,C5AR2,C5AR1,PRKD2,PHB,CD300LF,LACC1,NECTIN2,PVR,EIF2B3,GAB2,MLH1,UNC13D,MYD88,SLC22A13,FOXJ1,PAXIP1,IRAK2,SEC14L1,IKBKB,SIRT2,NMI,TMEM173,CLPB,NPLOC4,PAWR,PLCL2,SHLD1,PRNP,MAVS,NCKAP1L,HPX,FOXP1,PRKDC,IRF7,MFHAS1,TRAF6,PCBP2,NR1H4,TRIM44,PLK2,HCK,FER,OTUD4,LPXN,ERAP1,PTPRJ,MEF2C,SLA2,RASGRP1,EIF2AK4,NR1H3,TRAFD1,OAS3,NLRC5,RIPK2,OTULIN,AP3B1,F2RL1,POLR3D,MAPKBP1,HERC5,NOD2,ADCY7,CD40,RAB7B,MAPKAPK3,GPLD1,CD96,IFIH1,CD200,SPPL3,OASL,LCK,BANK1,ICOSLG,MAP2K6,TGFB2,BTN2A2,UNC93B1,POLR3C,HLX,DENND1B,TLR5,CMKLR1,NCF1,MAPKAPK2,TIFA,ALPK1,YTHDF2,SLAMF8,PARP9,DTX3L,PARP14,RAB11FIP2,THEMIS2,LAT2,BMX,RNF26,FGR,NLRX1,PLPP4,TRAF3IP1,HTRA1,SNX4,COCH,CD28,FCER1G,PDCD1,PPP2R3C,ADAM8,CADM1,FZD5,DUSP10,NONO,PLA2G4A,BCL6,CTSH,IL12A,DDX3X,VSIR,XRCC5,RC3H1,PTPN6,TNFSF18,SLC11A1,LAG3,COL3A1,PPP3CB,TNIP2,MUL1,BIRC3,CFP,CD38,SPPL2A,C12orf4,MAPK1,STAT5B,WAS,FOXP3,RNF216,ZBTB1,SHLD2,GPS2,TKFC,WDFY1,USP18,CARD11,TNFRSF1B,MED1,TBX21,STX4,PSEN1,ITGAM,SYT11,LCP2,DCST1,DNASE2,ZBTB7B,CD19,SMAD3,IL4R,TSPAN6,S100A14,SFPQ,PRKCB,BTK,STAT2,TNIP1,SIN3A,TRAF3,CD74,CCL5,RIOK3,GATA6,STXBP2,COLEC12,FBXO38,YES1,RNF13[illegible]	grey	VSMC_M10
GO:0032409	0.61	0.44	104	198	regulation of transporter activity	BP	1	NEDD4L,STAC3,MAPK8IP2,PRRT1,GOPC,PLA2G6,PIM1,ABCB1,WNK2,PRKCE,SHANK1,HECW1,TCAF2,OSR1,SPHK2,KCNRG,CACNB2,STAC,OXSR1,GPD1L,CACNB4,PRNP,SLMAP,GLRX,MEF2C,PTK2B,KCNJ11,NR1H3,HOMER1,IFNGR2,TESC,RIPK1,KCNE2,PKD2,CASQ2,KCNIP2,CHRM3,RYR2,FAM173B,PM20D1,HECW2,ADRA2A,COX17,KCNG1,ANK2,KLHL24,ACE2,ATP1A2,SLC9A1,C4orf3,CASQ1,NTSR1,SCN4B,CNIH2,NOS1AP,DLG1,CTTNBP2NL,HTT,PINK1,ATP1B1,KCNA1,GNB5,MINK1,HAP1,DLG4,ACTB,NLGN2,CNIH3,WNK3,STAC2,CACNB1,ATP1B2,NLGN3,ATP2A1,SLC5A11,CALM1,DNM2,VMP1,CCL2,FMR1,RNF207,PARK7,AMIGO1,RXRA,JPH3,WWP2,RRAD,MIR24-1,DMD,GPR35,JPH2,MIR33B,FGF11,SCN2B,NETO2,CACNG7,CRBN,CRH,APOPT1,YWHAH,SHISA8,CACNG5,GEM,PPIF	grey	VSMC_M10
GO:0034446	0.61	0.47	46	89	substrate adhesion-dependent cell spreading	BP	1	FBLN1,PARVG,PTK2,TEK,ITGB1BP1,EPHA1,BVES,UNC13D,RAC3,ARHGEF7,AKIP1,FER,PIK3R1,LPXN,PARVA,DOCK5,RREB1,DMTN,PXN,CARMIL1,AP1AR,LAMB3,CASS4,CIB1,FZD7,LAMA5,EPHB3,DOCK1,MELTF,CSPG5,LAMC1,ITGA4,FN1,FERMT3,RADIL,EFNA1,DNM2,CRK,NTNG2,LAMC3,MICALL2,RHOA,FGA,LAMB1,PEAK1,VAMP3	grey	VSMC_M10
GO:0043368	0.61	0.55	12	23	positive T cell selection	BP	1	SRF,TOX,TNFSF18,STAT3,FOXP3,TBX21,DOCK2,BATF,IL6R,BCL11B,CD74,PTPN2	grey	VSMC_M10
GO:1902036	0.61	0.60	7	13	regulation of hematopoietic stem cell differentiation	BP	1	EIF2AK2,PRKDC,TCF15,YTHDF2,OSM,NFE2L2,SETD1A	grey	VSMC_M10
GO:0043173	0.61	0.60	7	13	nucleotide salvage	BP	1	AMPD3,AMPD1,ADK,UCK1,FUK,NAPRT,UPP1	grey	VSMC_M10
GO:0006164	0.61	0.45	79	149	purine nucleotide biosynthetic process	BP	1	NME1,ATP5MGL,PPT2,PPARA,ACSL6,GUCA1B,IMPDH1,SLC35B2,SLC25A13,PPAT,PPCS,PPT1,DLD,SPHK2,ADCY3,PRKAG2,ATP5F1C,ELOVL1,PANK2,PDK1,PDHX,ELOVL7,PANK1,AMPD3,ATP5F1A,GUCY1A1,GUCY1B1,DGUOK,GMPS,AMPD1,ADCY7,FAM173B,ADCY2,NME4,ACSL5,PRPS2,HACD2,ADCY4,DNAJC30,PDK3,SLC25A16,ACSBG1,ATP5S,COASY,PINK1,STAT3,NME7,ADK,MPC2,ADCY10,PARP1,TECR,SLC26A1,GUCY2D,PKLR,PANK3,GCDH,ACACA,SLC26A2,PRPSAP2,ADSSL1,NOS2,ACSBG2,ATP6V0C,PANK4,NME3,ATP5PB,VPS9D1,ACSF3,MLYCD,MT-ATP8,MT-ATP6,ATP5ME,ATP5MF,PDZD3,ELOVL3,MTHFD2L,NME6,NUDT2	grey	VSMC_M10
GO:0000462	0.61	0.52	18	34	maturation of SSU-rRNA from tricistronic rRNA transcript (SSU-rRNA, 5.8S rRNA, LSU-rRNA)	BP	1	HELB,UTP23,TSR1,RPS14,UTP3,ERCC2,RPS8,DHX37,BMS1,RPP40,HEATR1,FAM207A,MRPS11,NOP9,TSR2,KRI1,UTP6,GTF2H5	grey	VSMC_M10

GO:0030838	0.61	0.47	43	82	positive regulation of actin filament polymerization	BP	1	ARPC3,WASHC5,BAIAP2L2,PICK1,CDC42EP1,LMOD2,NCK2,KIAA1211,PRKCE,WASF1,FCHSD2,BAG4,FCHSD1,NCKAP1L,ARFIP2,LMOD3,HCK,FER,HIP1R,FMN1,PTK2B,IQGAP2,CYFIP1,MAGEL2,CARMIL1,LMOD1,AP1AR,WASF2,WHAMM,DLG1,CDC42EP2,NCKAP1,ARPC1A,WAS,EVL,MLST8,SPIRE2,WDR63,CARMIL2,RHOA,VASP,GMFG,GRB2	grey	VSMC_M10
GO:0030509	0.62	0.46	60	116	BMP signaling pathway	BP	1	SLC39A5,USP15,GDF9,SMAD5,LEMD2,KCP,TFAP2B,ROR2,DLX5,SOSTDC1,HOXA13,BMP8A,BMPER,SFRP4,MEGF8,ACVR2B,UBE2O,ACVR2A,SPART,SLC33A1,HIVEP1,ZNF423,BMPR1B,FBXL15,PDCD4,TGFB2,HJV,SORL1,HTRA1,CHRD,DLX1,CTDSPL2,HTRA3,FBN1,FKBP8,GDF15,GREM2,BMPR1A,RBPJ,ITGA3,RBPMS2,MAPK3,TOB1,TMEM100,SMAD3,RNF165,CHRDL1,FAM83G,TWSG1,GPC3,SKI,ENG,MIR21,FSTL3,LEMD3,ACVRL1,NOG,WNT5A,RGMB,UBE2D1	grey	VSMC_M10
GO:0009260	0.62	0.45	79	149	ribonucleotide biosynthetic process	BP	1	NME1,ATP5MGL,PPT2,PPARA,ACSL6,IMPDH1,SLC35B2,SLC25A13,PPAT,PPCS,PPT1,DLD,SPHK2,CMPK1,ADCY3,PRKAG2,ATP5F1C,ELOVL1,PANK2,PDK1,PDHX,ELOVL7,PANK1,AMPD3,ATP5F1A,GUCY1A1,GUCY1B1,DGUOK,GMPS,AMPD1,ADCY7,FAM173B,ADCY2,NME4,ACSL5,HACD2,ADCY4,CTPS2,DNAJC30,PDK3,SLC25A16,ACSBG1,ATP5S,COASY,PINK1,STAT3,NME7,ADK,MPC2,ADCY10,PARP1,TECR,SLC26A1,GUCY2D,PKLR,PANK3,GCDH,UPRT,ACACA,SLC26A2,ADSSL1,ACSBG2,ATP6V0C,PANK4,NME3,ATP5PB,VPS9D1,ACSF3,UCK1,MLYCD,DHODH,MT-ATP8,MT-ATP6,ATP5ME,ATP5MF,ELOVL3,NME6,NUDT2,UPP1	grey	VSMC_M10
GO:0019915	0.62	0.48	34	65	lipid storage	BP	1	B4GALNT1,PPARA,MEST,TTC39B,PLIN2,CRY1,NR1H2,LDAH,APOB,ABHD5,CDS2,ASXL1,FBXW7,AUP1,NR1H3,CDS1,ACVR1C,FITM2,CRY2,FTO,CPT1A,FITM1,SOAT1,EHD1,STAT5B,ZFYVE1,TMEM159,GM2A,SREBF1,C3,PTPN2,PLIN3,BSCL2,STARD4	grey	VSMC_M10
GO:0050775	0.62	0.52	18	35	positive regulation of dendrite morphogenesis	BP	1	RAB21,SS18L2,ANKRD27,CUX2,TIAM1,OPA1,ITPKA,DNM1L,CAPRIN2,SS18L1,CDKL5,OBSL1,EPHA4,PARP6,GPRASP2,BHLHB9,LRP8,FBXO31	grey	VSMC_M10
GO:1903078	0.62	0.49	27	52	positive regulation of protein localization to plasma membrane	BP	1	LRP1,STAC3,PRKCE,SPTBN1,EGFR,STAC,PRNP,PIK3R1,PLS1,SORBS1,KCNJ11,GPER1,RAB11FIP2,CIB1,DLG1,ZDHHC8,CNPY4,CNST,WNK3,STAC2,STX4,ITGA3,AC138894.1,RAB11A,PDPK1,RER1,ARHGEF16	grey	VSMC_M10
GO:1903008	0.62	0.46	53	101	organelle disassembly	BP	1	ATG12,PRKN,TSPO,FBXO7,MTIF2,ATG5,PLK3,OPTN,USP36,TMEM173,DDRGK1,ULK1,FBXW7,HTRA2,ZFAND1,CSNK2A2,TEX264,GBF1,EIF2D,DNM1L,CISD2,USP30,DYRK3,UFC1,KIF9,FZD5,FIS1,ATG2A,FUNDC1,HTT,RETREG3,MARK2,PINK1,MUL1,ATG9A,PLA2G16,STX5,ATP13A2,WIPI2,VPS13D,MFN2,VPS13C,HAX1,ATG2B,KLC1,SREBF1,MCTS1,FUNDC2,PARK7,PLK5,BNIP3L,TIGAR,MRRF	grey	VSMC_M10
GO:0042491	0.62	0.53	15	29	inner ear auditory receptor cell differentiation	BP	1	MYCL,WHRN,MYCN,MYO7A,SLC4A7,JAG1,LRTOMT,AP000812.5,PLS1,USH1C,STRC,RBPJ,JAG2,MCOLN3,HEY2	grey	VSMC_M10
GO:0060263	0.62	0.60	7	13	regulation of respiratory burst	BP	1	RAC2,NCF1,SLAMF8,DUSP10,INSR,NOXA1,JCHAIN	grey	VSMC_M10
GO:0032924	0.62	0.52	16	31	activin receptor signaling pathway	BP	1	ZC3H3,FOXH1,ACVR2B,TGFBR2,ACVR2A,AMHR2,ACVR1B,ACVR1C,HJV,MEN1,SMAD3,SKI,FSTL3,LEMD3,ACVRL1,TGIF2	grey	VSMC_M10
GO:0001906	0.62	0.47	41	78	cell killing	BP	1	CD226,STX7,AGER,HLA-DRA,GAPDH,PRDX1,NECTIN2,PVR,UNC13D,MYD88,SLC22A13,NCKAP1L,P2RX7,RASGRP1,F2RL1,CADM1,CTSH,IL12A,PTPN6,LAG3,PPP3CB,STAT5B,ITGAM,SCNN1B,STXBP2,VAV1,C3,NOS2,IL7R,CRK,DNASE1,AP1G1,S100A12,HIST1H2BK,CHGA,FADD,CXCL6,FCER2,SERPINB9,HIST1H2BJ,HAVCR2	grey	VSMC_M10
GO:0002534	0.62	0.51	21	40	cytokine production involved in inflammatory response	BP	1	PPARA,GPSM3,MAPK14,IL1R2,TLR4,MYD88,CHID1,NOD2,ADCY7,PDCD4,APOD,STAT3,PLD4,NOS2,TICAM1,MIR21,MIR222,C5orf30,MIR125A,IL17D,IL17B	grey	VSMC_M10
GO:1900015	0.62	0.51	21	40	regulation of cytokine production involved in inflammatory response	BP	1	PPARA,GPSM3,MAPK14,IL1R2,TLR4,MYD88,CHID1,NOD2,ADCY7,PDCD4,APOD,STAT3,PLD4,NOS2,TICAM1,MIR21,MIR222,C5orf30,MIR125A,IL17D,IL17B	grey	VSMC_M10
GO:0030003	0.62	0.42	228	434	cellular cation homeostasis	BP	1	SLC39A5,NEDD4L,LRP1,ESR1,SLC12A2,SMDT1,BAK1,MYC,MCHR1,GPR20,PLA2G6,TMPRSS6,SLC39A4,STEAP2,SLC25A27,SLC1A1,TFAP2B,S1PR3,PRKCE,TNNI3,FPR2,IBTK,SLC4A4,LPAR1,PPT1,SLC31A2,KEL,ATG5,TRPM4,GRIN2D,TRPC3,SLC8A2,RAB7A,C5AR2,C5AR1,HEPHL1,ATP7B,PTGIR,GIPR,CACNB2,SLC39A12,ATP6V0E2,LACC1,SLC4A2,AQP11,ATP1A3,ACKR2,SLC24A3,C1QTNF1,SLC4A7,TMEM106B,TMTC4,MAFG,RYR1,LETM2,PRNP,ADRA1D,DIAPH1,P2RX2,CCKBR,HPX,RASA3,NCOA4,MICU3,ATP6V1H,TM9SF4,IL1A,IMMT,C19orf12,P2RX3,PLCE1,SLC9A9,RYR3,TMBIM6,P2RX7,HVCN1,P2RY1,PTK2B,SLC39A13,TPCN1,SLC8B1,VDR,STC1,AP3B1,RMDN3,F2RL1,F2R,SLC40A1,PKD2,CASQ2,MCUR1,CD40,RYR2,SLC41A1,SLC4A10,RAP1GDS1,TRPM1,SPPL3,LCK,KCNJ2,TESMIN,SLC9B1,TRPV4,TMEM9,SLC30A10,TCIRG1,GNA13,ISCU,CMKLR1,MCUB,HJV,SLC9A8,UBASH3B,DISC1,ANK2,GPER1,SLAMF8,TRPM8,PDZD8,AGT,ATP1A2,SLC9A1,CASQ1,NTSR1,CLIC4,FZD9,TMCO1,PTH1R,SLC30A4,FIS1,CEMIP,ABL2,CDH23,PTPN6,MICU1,MCU,CXCR2,GNG3,SLC11A1,HTT,PTGDR,PTGER2,SLC9A7,ATP6V0A1,ABCB6,TRPM7,ATP1B1,CD38,MAPK1,DMXL2,KCNMA1,SLC4A3,ATP13A2,FTH1,HAP1,DLG4,CYB561A3,ATP1B2,FAM155B,PSEN1,CACNA1A,MAPK3,AC138894.1,ATP2A1,CD19,SMAD3,CLN6,NOX5,CALM1,SNAPIN,S100A8,PRKCB,BDKRB1,NMUR2,SLC39A6,TRPV2,HRH4,CCL14,CCL5,CCL7,C7,PTGER4,SLC25A23,MAIP1,TMEM199,SLC46A1,SLC9A6,NUBP1,GNB1,ATP2B3,BCAP31,GNA15,TRPV3,TRPV1,PDPK1,P2RX1,PKD1,TMEM203,ATP6AP1,GPR157,SYPL2,CYBA,JPH3,ATP2C2,SLC30A7,FRRS1,LCN2,MCOLN3,PTGFR,SLC9A5,MIR199A1,KCNK3,DMD,MT1X,GPR35,NOL3,[illegible]	grey	VSMC_M10

GO:2000108	0.62	0.56	10	19	positive regulation of leukocyte apoptotic process	BP	1	CDKN2A,IDO1,ZC3H8,PIK3CB,MEF2C,RAPGEF2,PDCD1,ADAM8,CCL5,WNT5A	grey	VSMC_M10
GO:0035872	0.62	0.55	12	23	nucleotide-binding domain, leucine rich repeat containing receptor signaling pathway	BP	1	TNFAIP3,HSPA1B,SLC46A2,TLR4,LACC1,RIPK2,OTULIN,NOD2,MAP2K6,BIRC3,IRGM,INAVA	grey	VSMC_M10
GO:0007219	0.62	0.45	72	138	Notch signaling pathway	BP	1	MAML1,PTP4A3,MFNG,IFT74,DLK2,KIT,S1PR3,BEND6,AAK1,ANXA4,ITGB1BP1,MAML2,SOX9,LLGL2,GALNT11,TIMP4,IFT172,ARRB1,TGFBR2,JAG1,TCIM,POFUT1,CEBPA,CBFA2T2,DTX4,ROBO1,ROBO2,FBXW7,BMP2K,HIF1AN,NOD2,CNTN1,ZNF423,GAS2,NEURL1,NEPRO,TGFB2,POGLUT1,SNAI1,YTHDF2,DTX3L,WDR12,DLX1,CDKN1B,HOXD3,BCL6,PDCD10,STAT3,PSEN2,SPEN,RBPJ,SYNJ2BP,PSEN1,NEURL1B,SEL1L,TMEM100,TBX2,MIB1,NLE1,JAG2,NKAP,CDH6,MIB2,ARRDC1,WWP2,KRT19,CD46,CFD,PERP,APH1B,SLC35C1,HEY2	grey	VSMC_M10
GO:0051251	0.62	0.44	98	186	positive regulation of lymphocyte activation	BP	1	AGER,HLA-DRA,HLA-DOB,HLA-DMA,IL5,HLA-DPB1,TNFRSF13C,XRCC6,NCK2,EXOSC3,MSH2,PELI1,IL4I1,TLR4,IL15,HMCES,BMI1,MLH1,GLI2,MYD88,PAXIP1,IL15RA,TGFBR2,SHLD1,NCKAP1L,GAS6,PRKDC,SLC7A1,TRAF6,SOX12,TOX,IL1A,MEF2C,IL7,RASGRP1,RIPK2,AP3B1,SOX13,NOD2,ZNF335,CD40,IGF2,LCK,ICOSLG,GPAM,HLX,CD28,RUNX3,PPP2R3C,ADAM8,DUSP10,BLOC1S6,BCL6,IL12A,VSIR,PTPN6,TNIP2,CD38,BST1,STAT5B,FOXP3,RASAL3,ZBTB1,SHLD2,CARD11,TBX21,ZBTB7B,CHRNB2,IL4R,BTK,CD74,CCL5,CCL2,YES1,NKAP,CD320,VAV1,IL7R,SOCS1,TICAM1,DNAJA3,PRKCZ,VAV3,AP1G1,BCL10,MIR21,RHOA,BLOC1S3,SOX4,FADD,DPP4,CD46,THY1,CD70,TNFSF9,EFNB3,TNFSF13B,HAVCR2	grey	VSMC_M10
GO:0031507	0.62	0.52	17	33	heterochromatin assembly	BP	1	BAZ2A,H3F3C,DNMT3A,SETDB2,SIRT2,FAM208A,BAHD1,PPHLN1,HDAC1,ZNFX1,TET1,PHF8,NRDE2,SMCHD1,SETDB1,BEND3,ZNF304	grey	VSMC_M10
GO:0043649	0.62	0.60	7	13	dicarboxylic acid catabolic process	BP	1	DDO,GAD2,GLUL,MTHFS,ACSF3,ACOT4,ALDH1L1	grey	VSMC_M10
GO:0060044	0.62	0.60	7	13	negative regulation of cardiac muscle cell proliferation	BP	1	TGFBR2,TBX5,JARID2,KCNK2,SAV1,MIR199A1,NOG	grey	VSMC_M10
GO:0051152	0.62	0.60	7	13	positive regulation of smooth muscle cell differentiation	BP	1	KIT,GPER1,EFEMP2,ENG,MIR21,MIR125B1,MIR22	grey	VSMC_M10
GO:0034983	0.62	0.67	4	7	peptidyl-lysine deacetylation	BP	1	SIRT2,SIRT3,SIRT4,HDAC4	grey	VSMC_M10
GO:0051875	0.62	0.56	10	19	pigment granule localization	BP	1	BBS7,MYO7A,MKKS,SHROOM2,MLPH,RAB17,BLOC1S6,MREG,RAB11A,BLOC1S3	grey	VSMC_M10
GO:0051905	0.62	0.56	10	19	establishment of pigment granule localization	BP	1	BBS7,MYO7A,MKKS,SHROOM2,MLPH,RAB17,BLOC1S6,MREG,RAB11A,BLOC1S3	grey	VSMC_M10
GO:1902305	0.62	0.49	26	50	regulation of sodium ion transmembrane transport	BP	1	NEDD4L,WNK2,PRKCE,HECW1,OSR1,SCN5A,GPD1L,SLMAP,GLRX,TESC,HECW2,KLHL24,ATP1A2,SLC9A1,SCN4B,DLG1,ATP1B1,WNK3,ATP1B2,DNM2,MIR24-1,DMD,FGF11,SCN2B,NETO2,YWHAH	grey	VSMC_M10
GO:0006953	0.62	0.55	11	21	acute-phase response	BP	1	IL1A,CD163,FN1,STAT3,IL6R,SERPINF2,TRPV1,ASS1,LCN2,PTGES,CEBPB	grey	VSMC_M10
GO:0031268	0.62	0.60	7	13	pseudopodium organization	BP	1	CDC42EP1,KIT,F2RL1,SRGAP2C,CDC42EP2,RAB25,APC	grey	VSMC_M10
GO:0051220	0.62	0.55	11	21	cytoplasmic sequestering of protein	BP	1	GOPC,MDFI,NFKBIE,FAF1,NFKBIB,PKD2,SUFU,GET4,KEAP1,PSMD10,PKD1	grey	VSMC_M10
GO:0002764	0.62	0.43	146	278	immune response-regulating signaling pathway	BP	1	CD226,TNFAIP3,USP15,ESR1,HSPA1B,GRAMD4,HLA-DPB1,PTK2,PLA2G6,LRRC14,TEC,KIT,TNFRSF21,NINJ1,PRKCE,RTN4,FPR2,ANKRD17,SLC46A2,NOD1,PELI1,RSAD2,TLR4,PUM2,C5AR2,C5AR1,PRKD2,PHB,CD300LF,LACC1,NECTIN2,EIF2B3,MYD88,IRAK2,SEC14L1,IKBKB,NMI,CLPB,NPLOC4,PAWR,PLCL2,PRNP,NCKAP1L,FOXP1,IRF7,MFHAS1,TRAF6,NR1H4,HCK,FER,OTUD4,LPXN,PTPRJ,MEF2C,SLA2,NR1H3,OAS3,RIPK2,OTULIN,AP3B1,F2RL1,NOD2,CD40,RAB7B,MAPKAPK3,GPLD1,IFIH1,SPPL3,OASL,LCK,BANK1,ICOSLG,MAP2K6,BTN2A2,UNC93B1,DENND1B,TLR5,CMKLR1,MAPKAPK2,TIFA,ALPK1,RAB11FIP2,THEMIS2,LAT2,BMX,FGR,NLRX1,PLPP4,CD28,FCER1G,CTSH,DDX3X,RC3H1,PTPN6,TNIP2,BIRC3,CD38,MAPK1,FOXP3,GPS2,TKFC,WDFY1,CARD11,PSEN1,LCP2,CD19,TSPAN6,S100A14,PRKCB,BTK,TNIP1,TRAF3,RIOK3,COLEC12,YES1,RNF135,PRAM1,VAV1,GPR108,PTPN2,TICAM1,CREBBP,PDPK1,IKBKG,CYBA,VAV3,GFI1,BCL10,DAB2IP,CMTM3,FYB1,OAS1,BTN1A1,ZCCHC3,IRGM,CD247,THY1,INAVA,UBE2N,GCSAM,HRAS,LAX1,SH2B2,MAP2K7,HAVCR2,RC3H2	grey	VSMC_M10
GO:0015980	0.62	0.43	139	259	energy derivation by oxidation of organic compounds	BP	1	SHMT2,GFPT2,LYRM7,UQCRQ,NDUFA6,ACO2,MYC,GNMT,SLC25A13,CAT,SDHAF4,MSH2,PNPT1,DLD,NDUFB2,PYGB,NDUFB6,RUBCNL,DNAJC15,KHK,EPM2AIP1,DHTKD1,NDUFC2,PRKAG2,ATP5F1C,PPP1CB,AKT2,MDH2,NDUFS8,PANK2,NDUFA12,KL,SIRT3,COX7A1,SUCLG2,PPP1R3B,COX4I2,IDE,GBE1,ATP5F1A,SORBS1,GPD1,NDUFS3,ETFDH,DGUOK,PFKM,UQCRB,COX15,FAM173B,IGF2,NHLRC1,COX6B1,PHKB,IRS1,NDUFV3,TRPV4,COQ10B,OXA1L,NDUFB4,ISCU,PPP1R3E,COA6,POLG2,NDUFB3,CISD1,PPP1R3D,NFATC4,PER2,ACTN3,PASK,NDUFS2,PHKA2,DNAJC30,MLXIPL,SDHC,MDH1B,COX6A1,IDH3A,PYGM,PYGL,COX8A,PRKAG3,PINK1,NDUFA9,SDHB,PPP1R3F,ACADVL,COX6A2,PHKA1,NDUFAB1,UQCRC2,COX10,COX5A,INSR,TEFM,NDUFA7,LEPR,NOS2,SLC25A23,NDUFV2,SLC25A14,INPP5K,PRDM16,NDUFB10,UQCR11,PARK7,SLC25A33,ATP5PB,AGL,SLC25A25,ACADM,MT-ND1,MT-ND2,MT-ATP8,MT-ATP6,MT-CO3,MT-ND6,MT-CYB,RHOA,ATP5ME,ATP5MF,NDUFA5,IFNLR1,NDUFC1,COX7A2,TIGAR,FXN,PPP1R1A,ETFRF1,NDUFA3,NDUFB5,NDUFS6,NDUFA8,SDHAF2,NDUFS5,UQCC2,MRAP2,COX7A2L,PPIF	grey	VSMC_M10
GO:0009265	0.62	0.62	6	11	2'-deoxyribonucleotide biosynthetic process	BP	1	TBPL1,DGUOK,DTYMK,ADK,CMPK2,DCTD	grey	VSMC_M10
GO:0046385	0.62	0.62	6	11	deoxyribose phosphate biosynthetic process	BP	1	TBPL1,DGUOK,DTYMK,ADK,CMPK2,DCTD	grey	VSMC_M10

GO:0042102	0.62	0.49	29	55	positive regulation of T cell proliferation	BP	1	AGER,HLA-DPB1,TNFRSF13C,NCK2,IL15,BMI1,TGFBR2,NCKAP1L,SLC7A1,TRAF6,IL1A,RIPK2,IGF2,ICOSLG,GPAM,CD28,STAT5B,FOXP3,RASAL3,CARD11,CCL5,DNAJA3,MIR21,FADD,CD46,CD70,TNFSF9,TNFSF13B,HAVCR2	grey	VSMC_M10
GO:0046888	0.62	0.49	25	48	negative regulation of hormone secretion	BP	1	PRKN,CRY1,IL11,BMP8A,NDUFAF2,FAM3D,KCNJ11,RAB11FIP5,ACVR1C,CRY2,SIRT4,IRS1,HDAC1,PPP3CA,ADRA2A,KCNB1,OSM,KLF7,ENY2,INHA,SYTL4,SREBF1,CHGA,CRH,LIF	grey	VSMC_M10
GO:0001706	0.62	0.50	22	43	endoderm formation	BP	1	ITGA7,EOMES,MMP15,RTF1,SOX2,LAMB3,COL7A1,ITGB5,SETD2,ITGA4,FN1,COL5A2,LEO1,LHX1,LAMA3,GATA6,COL5A1,DUSP2,LAMB1,NOG,DUSP1,DUSP4	grey	VSMC_M10
GO:0021587	0.62	0.52	17	33	cerebellum morphogenesis	BP	1	GLI1,AGTPBP1,SMO,WHRN,XRCC1,GLI2,FAIM2,LDB1,TTC21B,GNPAT,SPTBN2,MAP2K1,LHX1,DAB1,HSPA5,GRID2,CEND1	grey	VSMC_M10
GO:0060339	0.62	0.56	10	19	negative regulation of type I interferon-mediated signaling pathway	BP	1	OAS3,NLRC5,YTHDF2,MUL1,USP18,DCST1,STAT2,PTPN2,MIR21,OAS1	grey	VSMC_M10
GO:0042537	0.62	0.60	7	13	benzene-containing compound metabolic process	BP	1	ALDH8A1,IDO2,IDO1,EPHX2,FAH,KYAT1,KYAT3	grey	VSMC_M10
GO:0009165	0.62	0.43	105	198	nucleotide biosynthetic process	BP	1	NME1,TBPL1,ATP5MGL,PPT2,PPARA,ACSL6,GUCA1B,IMPDH1,NMRK1,SLC35B2,SLC25A13,PPAT,PPCS,ME1,PPT1,DLD,SPHK2,CMPK1,ADCY3,PRKAG2,SLC22A13,ATP5F1C,ELOVL1,IDO2,IDO1,PANK2,PDK1,PDHX,SLC5A8,ELOVL7,PANK1,NUDT12,AMPD3,ATP5F1A,GUCY1A1,GUCY1B1,DGUOK,GMPS,AMPD1,ADCY7,FAM173B,ADCY2,NME4,ACSL5,PRPS2,PARP9,HACD2,ADCY4,CTPS2,DNAJC30,DTYMK,PDK3,SLC25A16,ACSBG1,ATP5S,COASY,PINK1,STAT3,NME7,ADK,MPC2,ADCY10,MAPK1,PARP1,TECR,SLC26A1,GUCY2D,PKLR,PANK3,GCDH,UPRT,ACACA,SLC26A2,PRPSAP2,ADSSL1,NOS2,ACSBG2,NADK,ATP6V0C,PANK4,NME3,ATP5PB,VPS9D1,ACSF3,AK8,UCK1,MLYCD,KARS,FUK,DHODH,MT-ATP8,MT-ATP6,ATP5ME,ATP5MF,PDZD3,ELOVL3,MTHFD2L,CMPK2,NNMT,NME6,DCTD,NUDT2,NAPRT,UPP1,NMNAT1	grey	VSMC_M10
GO:2000144	0.62	0.54	13	25	positive regulation of DNA-templated transcription, initiation	BP	1	ESR1,MED30,SRF,ERCC1,PAXIP1,MITF,ERCC6,CREB1,MED14,MED24,MED1,JUN,NKX2-5	grey	VSMC_M10
GO:1903202	0.62	0.51	20	38	negative regulation of oxidative stress-induced cell death	BP	1	PRKN,NCOA7,ATF4,GPR37,FBXO7,MET,SLC7A11,HTRA2,PDE8A,NFE2L2,NONO,PINK1,MAPK7,PARK7,TSC1,TLDC1,MIR21,NOL3,GPR37L1,TXN	grey	VSMC_M10
GO:0048009	0.62	0.53	14	27	insulin-like growth factor receptor signaling pathway	BP	1	IGF2R,PHIP,GRB10,PLCB1,PIK3R1,NKX3-1,IRS1,GIGYF2,GIGYF1,ZFAND2B,IGFBP4,CILP,GHR,MYORG	grey	VSMC_M10
GO:0005975	0.62	0.41	242	458	carbohydrate metabolic process	BP	1	B4GALT7,B4GALNT1,FUCA2,MIOX,SYNJ2,PRKN,PPARA,PMM1,PRXL2C,AKR1B1,ATF4,MAPK14,GPT,GNMT,CRY1,GNPDA2,PGM5,GALT,ST6GAL2,UXS1,TFAP2B,SLC25A13,GAPDH,CHST10,IPPK,SLC3A1,PRKCE,HAS1,PPP4R3B,SLC4A4,ME1,SLC35A1,ZNF692,BPGM,PLEK,LANCL2,FAM3C,INPP4B,MGAM,DHDH,BRAF,NANP,MTMR2,PYGB,RUBCNL,AKR1A1,GLB1,KHK,EPM2AIP1,NUDT5,DHTKD1,B4GALT2,PRKAG2,XYLB,LCT,GCKR,RBKS,PPP1CB,CSGALNACT1,AKT2,GPD1L,NEU3,PFKP,PGM2L1,C1QTNF1,MDH2,SIRT7,SLC23A2,PDK1,KL,FUT10,PPP1R3B,MTMR7,POFUT1,GPI,NLN,ST8SIA4,GK5,NCOA2,GBE1,B3GNT4,SORBS1,GPD1,P2RX7,MOGS,P2RY1,PTK2B,KCNJ11,PRKAG1,PFKM,SYNJ1,COQ2,ST3GAL6,CHST1,ITPKA,CHID1,GLYCTK,IGF2,GANC,NHLRC1,PHKB,GPLD1,A3GALT2,IRS1,MLEC,SIK1,HYAL1,HYAL3,CPT1A,ZBTB20,ITPK1,INPP5A,ST8SIA2,MST1,PPP1R3E,EGF,PFKFB2,B3GALT1,GPER1,ENO4,PC,ARNT,PPP1R3D,SLC37A4,PER2,TREH,PFKFB4,ACTN3,HDAC4,USF1,NTSR1,MAN1C1,PASK,PHKA2,INPP5J,MLXIPL,DUSP12,GAL3ST3,APOD,FUOM,MDH1B,PDK3,EDEM3,PTH1R,SORD,OVGP1,PLA2G4A,RPE,LANCL3,MOGAT3,HK1,IDH3A,MGAT2,PYGM,B3GALNT1,CHST3,GAL3ST4,PYGL,GNB3,CHST7,PRKAG3,SLC2A2,AC022400.6,GLB1L,STAT3,YDJC,SLC3A2,SLC35A2,ENO3,PPP1R3F,IGFBP4,SLC2A4,TKFC,DDB1,CHST12,IDUA,DGKQ,PSEN1,SLC5A2,PKLR,OGT,PHKA1,LCTL,COX11,CRTC2,LCMT1,ST8SIA5,GLA,HEXA,ADPGK,GM2A,MPI,NCOR1,INSR,ENOSF1,LEPR,ALDOC,IMPA2,PTPN2,FGGY,PMM2,INPP5K,MECP2,TKTL1,GNPTG,ABO,ALDH2,SLC2A6,GBGT1,MLYCD,AGL,CHST5,ST6GALNAC4,ST3GAL2,FUK,HAS3,CTBS,ST6GALNAC5,ACADM,FBP2,OAS1,FABP5,FUT4,ENTPD[illegible]	grey	VSMC_M10
GO:0055010	0.62	0.50	20	39	ventricular cardiac muscle tissue morphogenesis	BP	1	LY6E,FOXH1,TNNI3,POU4F1,NRG1,TNNC1,DSP,RYR2,PKP2,TNNI1,TGFB2,MYH7,BMPR1A,RBPJ,MED1,ISL1,ENG,NOG,NKX2-5,HEY2	grey	VSMC_M10
GO:0003309	0.62	0.55	11	21	type B pancreatic cell differentiation	BP	1	SMO,CDK6,RFX3,ARNTL,NKX6-1,ZBED6,SIDT2,MEN1,GATA6,PDPK1,WNT5A	grey	VSMC_M10
GO:0035883	0.62	0.55	11	21	enteroendocrine cell differentiation	BP	1	SMO,CDK6,RFX3,ARNTL,NKX6-1,ZBED6,SIDT2,MEN1,GATA6,PDPK1,WNT5A	grey	VSMC_M10
GO:0071676	0.62	0.56	10	19	negative regulation of mononuclear cell migration	BP	1	PLCB1,ADTRP,CD200,SLAMF8,APOD,MIA3,PADI2,CCL2,DUSP1,GCSAM	grey	VSMC_M10
GO:0031579	0.62	0.57	9	17	membrane raft organization	BP	1	PPT1,MAL,DLG1,ANXA2,NAXE,DOCK2,AC138894.1,NPC1,ABCA7	grey	VSMC_M10
GO:0032366	0.62	0.54	12	23	intracellular sterol transport	BP	1	SERAC1,PIP4K2A,OSBP,OSBPL2,SYT7,ANXA2,LDLR,NPC1,VPS4A,MIR27B,LDLRAP1,STARD4	grey	VSMC_M10
GO:0032367	0.62	0.54	12	23	intracellular cholesterol transport	BP	1	SERAC1,PIP4K2A,OSBP,OSBPL2,SYT7,ANXA2,LDLR,NPC1,VPS4A,MIR27B,LDLRAP1,STARD4	grey	VSMC_M10
GO:0051339	0.62	0.52	15	29	regulation of lyase activity	BP	1	GUCA1B,GABBR2,ADGRV1,ORAI1,CAP2,GRM2,MTRR,ISCU,NOS2,PARK7,PALM,GNAI3,CAP1,PDZD3,FXN	grey	VSMC_M10
GO:0071577	0.62	0.55	11	21	zinc ion transmembrane transport	BP	1	SLC39A5,SLC39A4,SLC1A1,SLC39A12,TMEM163,SLC39A13,SLC30A10,SLC30A4,SLC39A6,SLC39A11,SLC39A3	grey	VSMC_M10
GO:1990266	0.62	0.47	39	74	neutrophil migration	BP	1	RAC2,CXCL8,C5AR2,C5AR1,MYD88,NCKAP1L,CCL17,NOD2,GBF1,DNM1L,TGFB2,CD99,SLAMF8,MOSPD2,FCER1G,ADAM8,PIKFYVE,MCU,CXCR2,BST1,DAPK2,S100A8,UMOD,CD74,CCL14,CCL5,CCL7,CCL2,CCL25,VAV1,PIP5K1C,MPP1,BSG,VAV3,CCL16,S100A12,DPP4,CXCL6,CCL22	grey	VSMC_M10
GO:1901185	0.63	0.53	14	27	negative regulation of ERBB signaling pathway	BP	1	ACP4,EGFR,RAB7A,SNX5,PTPRJ,RNF115,CBL,SNX6,ZFYVE28,SOCS4,PSEN1,PTPN2,RNF126,DAB2IP	grey	VSMC_M10

GO:0050867	0.63	0.43	119	226	positive regulation of cell activation	BP	1	CD226,AGER,HLA-DRA,HLA-DOB,HLA-DMA,IL5,HLA-DPB1,TNFRSF13C,XRCC6,TTBK1,NCK2,EXOSC3,MSH2,PELI1,PLEK,IL4I1,TLR4,IL15,SPHK2,HMCES,BMI1,NECTIN2,GAB2,MLH1,GLI2,MYD88,PAXIP1,IL15RA,TGFBR2,SHLD1,NCKAP1L,GAS6,PRKDC,SLC7A1,TRAF6,SOX12,TOX,IL1A,CEBPA,MEF2C,IL7,RASGRP1,RIPK2,AP3B1,F2RL1,SOX13,NOD2,ZNF335,CD40,IGF2,LCK,ICOSLG,GPAM,HLX,FGR,RPS6KA1,SNX4,CD28,RUNX3,PPP2R3C,ADAM8,DUSP10,BLOC1S6,PLA2G4A,BCL6,IL12A,VSIR,PTPN6,TNIP2,CD38,BST1,STAT5B,FOXP3,RASAL3,ZBTB1,SHLD2,CARD11,SOX15,TBX21,STX4,ITGAM,ZBTB7B,CHRNB2,IL4R,BTK,CD74,CCL5,CCL2,YES1,NKAP,CD320,VAV1,IL7R,SOCS1,TICAM1,DNAJA3,PRKCZ,VAV3,KARS,STXBP1,AP1G1,BCL10,MIR21,RHOA,HAVCR1,BLOC1S3,SOX4,FADD,DPP4,CD46,THY1,WNT5A,IL33,CD70,TNFSF9,TSLP,EFNB3,TNFSF13B,HAVCR2	grey	VSMC_M10
GO:0001504	0.63	0.55	11	21	neurotransmitter uptake	BP	1	PRKN,SLC38A1,SLC6A2,SLC6A3,ATP1A2,PER2,SLC29A2,SLC29A4,CLN8,SLC1A7,PARK7	grey	VSMC_M10
GO:0046879	0.63	0.43	108	206	hormone secretion	BP	1	LRP1,RIMS2,PRKN,GDF9,PLA2G6,PICK1,CRY1,RFX3,TFAP2B,IL11,PRKCE,HMGN3,CYB5R4,BMP8A,EGFR,TRPM4,GIPR,RAF1,C1QTNF1,AIMP1,SIRT3,SYT9,NR1H4,NDUFAF2,FAM3D,PAX8,OSBP,ARNTL,SCG5,P2RY1,KCNJ11,NNAT,TIAM1,RAB11FIP5,PFKM,SLC8B1,NKX3-1,NKX6-1,ACVR1C,CRY2,SPP1,ZBED6,SIRT4,GPLD1,IRS1,HDAC1,PPP3CA,CPT1A,TRPV4,SLC9B2,ADRA2A,TCIRG1,TCF7L2,SNAP23,KCNB1,PFKFB2,GPER1,RAB11FIP2,AGT,PER2,OSM,STX1A,SNX4,PASK,GLUL,SIDT2,SLC16A1,KLF7,CREB1,VGF,ENY2,ABCA12,MCU,PPP3CB,CAMK2G,PTPRN,CD38,MPC2,ILDR2,INHA,SYT7,NLGN2,CPLX1,TARDBP,STX4,RAB8B,UBE2Q1,SYTL4,GPR68,BTK,CCL5,SREBF1,ISL1,OXCT1,NOS2,ABAT,NADK,BAIAP3,PARK7,LRRC8A,FGA,CHGA,SOX4,DPP4,CRH,DOC2B,LIF,UQCC2	grey	VSMC_M10
GO:0070098	0.63	0.50	21	40	chemokine-mediated signaling pathway	BP	1	CXCL8,OXSR1,ACKR2,ROBO1,PTK2B,CCL17,CMKLR1,CIB1,THPO,CXCR2,PADI2,CCL14,CCL5,CCL7,CCL2,CCL25,CCL16,GPR35,CCR10,CXCL6,CCL22	grey	VSMC_M10
GO:0009173	0.63	0.59	7	13	pyrimidine ribonucleoside monophosphate metabolic process	BP	1	DPYS,CMPK1,UPB1,UPRT,UCK1,DHODH,UPP1	grey	VSMC_M10
GO:0046049	0.63	0.59	7	13	UMP metabolic process	BP	1	DPYS,CMPK1,UPB1,UPRT,UCK1,DHODH,UPP1	grey	VSMC_M10
GO:0034104	0.63	0.58	8	15	negative regulation of tissue remodeling	BP	1	TNFAIP3,TNFRSF11B,P2RX7,GPR137B,UBASH3B,AGT,GPR137,CD38	grey	VSMC_M10
GO:0070372	0.63	0.43	111	211	regulation of ERK1 and ERK2 cascade	BP	1	LRP1,SEMA6A,FRS2,AGER,FBLN1,ROS1,PRXL2C,TEK,WNK2,FPR2,MTURN,NOD1,BMPER,EPHA7,ITGB1BP1,VRK3,YWHAZ,EGFR,TLR4,FGF21,BRAF,SPRY1,DNAJC27,C5AR2,C5AR1,PRKD2,PHB,SPRY2,ARRB1,NEK10,SPRED3,DUSP6,NRG1,GAS6,SIRT3,MFHAS1,APIP,ST5,EPHB1,HAND2,FBXW7,P2RY1,PTK2B,RASGRP1,RAPGEF2,CCL17,TIAM1,RIPK2,F2RL1,F2R,NQO2,GCNT2,NOD2,DSTYK,TRPV4,SLC30A10,TBC1D10C,GPER1,CIB1,THPO,PDE8A,DLG1,DUSP10,RAP1B,FN1,PTPN6,GPBAR1,FGFR3,RGS14,FGFR4,SYNJ2BP,FGF18,MAPK3,MAP2K1,TNIP1,GAREM1,CD74,CCL14,CCL5,CSF1R,CCL7,CCL2,CCL25,PTPN2,SERPINF2,PRKCZ,ATP6AP1,ABCA7,DAB2IP,MIR21,MIR222,MIR23A,MIR27B,MIR24-1,MIR27A,FGA,CCL16,CHRNA10,DUSP26,DUSP1,DUSP4,C3orf33,LIF,HRAS,STYX,MAP2K7,FGF1,C1QL4,HAVCR2,CCL22,PDGFD	grey	VSMC_M10
GO:0098900	0.63	0.50	20	39	regulation of action potential	BP	1	TRPM4,SLC8A2,SCN5A,DSP,RYR2,PKP2,KCNJ2,KCNB1,ANK2,SLC9A1,NTSR1,NOS1AP,DLG1,CHRNB2,CALM1,DSG2,FMR1,RNF207,GPR35,HCN4	grey	VSMC_M10
GO:0006873	0.63	0.41	233	444	cellular ion homeostasis	BP	1	SLC39A5,NEDD4L,LRP1,ESR1,SLC12A2,SMDT1,BAK1,MYC,MCHR1,GPR20,PLA2G6,CKB,TMPRSS6,SLC39A4,STEAP2,SLC25A27,SLC1A1,TFAP2B,S1PR3,PRKCE,TNNI3,FPR2,IBTK,SLC4A4,LPAR1,PPT1,SLC31A2,KEL,ATG5,TRPM4,GRIN2D,TRPC3,SLC8A2,RAB7A,C5AR2,C5AR1,HEPHL1,ATP7B,PTGIR,GIPR,CACNB2,SLC39A12,ATP6V0E2,LACC1,SLC4A2,AQP11,ATP1A3,ACKR2,SLC24A3,C1QTNF1,SLC4A7,TMEM106B,TMTC4,MAFG,RYR1,LETM2,PRNP,ADRA1D,DIAPH1,P2RX2,CCKBR,HPX,RASA3,NCOA4,MICU3,ATP6V1H,TM9SF4,IL1A,IMMT,C19orf12,P2RX3,PLCE1,SLC9A9,RYR3,TMBIM6,P2RX7,HVCN1,P2RY1,PTK2B,SLC39A13,TPCN1,SLC8B1,VDR,STC1,AP3B1,RMDN3,F2RL1,F2R,SLC40A1,ABCC2,PKD2,CASQ2,MCUR1,SLC12A5,CD40,RYR2,SLC41A1,SLC4A10,RAP1GDS1,TRPM1,SPPL3,LCK,KCNJ2,TESMIN,SLC9B1,TRPV4,TMEM9,SLC30A10,TCIRG1,GNA13,ISCU,CMKLR1,MCUB,HJV,SLC9A8,UBASH3B,DISC1,ANK2,GPER1,SLAMF8,RHCG,TRPM8,PDZD8,AGT,ATP1A2,SLC9A1,CASQ1,NTSR1,XPR1,CLIC4,FZD9,TMCO1,PTH1R,SLC30A4,FIS1,CEMIP,ABL2,CDH23,PTPN6,MICU1,MCU,CXCR2,GNG3,SLC11A1,HTT,PTGDR,PTGER2,SLC9A7,ATP6V0A1,ABCB6,TRPM7,ATP1B1,CD38,MAPK1,DMXL2,KCNMA1,SLC4A3,ATP13A2,FTH1,HAP1,DLG4,CYB561A3,ATP1B2,FAM155B,PSEN1,CACNA1A,MAPK3,AC138894.1,ATP2A1,CD19,SMAD3,CLN6,NOX5,CALM1,SNAPIN,S100A8,PRKCB,BDKRB1,NMUR2,SLC39A6,TRPV2,HRH4,CCL14,CCL5,CCL7,C7,PTGER4,SLC25A23,MAIP1,TMEM199,SLC46A1,SLC9A6,NUBP1,GNB1,ATP2B3,BCAP31,GNA15,TRPV3,TRPV1,PDPK1,P2RX1,PKD1,TMEM203,ATP6AP1,GPR157,SYPL2,CYBA,JPH3,ATP2C2,SLC30A7,FRRS1,LCN2,MCOLN3,PTGFR,SLC9A5,MIR199[illegible]	grey	VSMC_M10
GO:0043248	0.63	0.61	6	11	proteasome assembly	BP	1	PSMD5,ECPAS,POMP,PSMD10,PSMG2,PSMG4	grey	VSMC_M10
GO:0019751	0.63	0.46	46	88	polyol metabolic process	BP	1	CYP27B1,MIOX,SYNJ2,AKR1B1,SPTLC1,IPPK,INPP4A,PLEK,INPP4B,SPHK2,DHFR,MTMR2,ITPKC,ACER3,SPTLC3,MTMR7,ASAH1,GK5,CYP2R1,P2RY1,PTK2B,SPR,SYNJ1,COQ2,ITPKA,ITPK1,INPP5A,GPER1,IP6K2,NTSR1,INPP5J,PTH1R,SORD,PLA2G4A,MOGAT3,CYP27A1,GCH1,TKFC,IMPA2,INPP5K,MECP2,MINPP1,ACER2,INPP1,SGPP1,PCBD2	grey	VSMC_M10
GO:0019674	0.63	0.54	11	21	NAD metabolic process	BP	1	NMRK1,GPD1L,PFKP,GPD1,PFKM,ACTN3,HK1,CD38,NADK,NQO1,TIGAR	grey	VSMC_M10

GO:0060271	0.63	0.42	139	266	cilium assembly	BP	1	ONECUT2,AVIL,TBC1D30,ATAT1,KIF3A,TBC1D32,UBE2B,ASAP1,LRRC6,MAPK15,IFT74,CFAP69,RFX3,PTPDC1,RAB23,DYNC2LI1,CEP162,TMEM17,WDR92,FUZ,IQUB,ATG5,ARMC2,MAPRE1,ARMC4,INTU,SPATA6,TTC26,BBS7,DRC1,RSPH6A,GALNT11,FBF1,PIBF1,IFT172,FOXJ1,WDR60,TTLL3,CCDC13,ABCC4,MKKS,CEP290,SPEF1,TMEM80,KIF3B,RILPL1,RILPL2,CEP70,PARVA,CEP250,CCDC66,TCTN3,IFT81,CCDC113,CFAP20,CCDC65,TMEM67,IFT52,RPGRIP1L,DNAH1,DNAH5,ZNF423,GBF1,NEURL1,CFAP43,ZMYND10,ARMC9,CFAP46,TTBK2,CLCN4,ALPK1,JHY,DISC1,OFD1,RAB17,TMEM237,TRAF3IP1,BBS1,LAMA5,CDKL5,SEPT2,LIMK2,CFAP161,TTC12,TOGARAM1,EHD1,DNAAF2,SPAG16,FAM149B1,HTT,CFAP70,STK36,GORAB,CEP126,SNAP29,EXOC5,TTC25,RAB8A,SDCCAG8,HAP1,DNAAF4,CPLANE2,MNS1,WDR19,TMEM138,ATP6V1D,BBOF1,CNTROB,TMEM107,CCDC151,DYNLL2,MKS1,DNM2,TEKT3,IFT20,SPEF2,CFAP53,C16orf71,RILP,CLUAP1,WRAP73,TPGS1,CDK10,CELSR2,CDC14A,TMEM231,FNBP1L,HYDIN,ODF2L,WDR63,C5orf30,TCTN2,HYLS1,SNX10,C2CD3,CC2D2B,C7orf43,ATXN10,BBS10	grey	VSMC_M10
GO:0051648	0.63	0.43	80	154	vesicle localization	BP	1	MYO1A,AP3S1,KIF5A,PRKN,KIF3A,FAM91A1,EXOC4,MAPK15,BRSK1,STARD3NL,BBS7,MYO7B,CLASP2,MYO7A,AP3M2,KIF5C,MKKS,LIN7A,TMEM230,KIF3B,RASGRP1,ARFGAP2,EXOC6B,SYNJ1,AP3B1,DTNBP1,KIF13A,GBF1,BICDL1,KLHL12,TRIP11,PEF1,TCIRG1,PDCD6,SNAP23,SHROOM2,AP1AR,MLPH,RAB17,CNIH2,LIMK2,TANC2,SPG11,BLOC1S6,ITGA4,MAP4K2,MREG,HTT,RAB3A,PINK1,SYN1,SNAP29,AP3M1,KIF1C,HAP1,SLC2A4,NLGN2,PSEN1,SYT11,TMED10,TRIM46,NDEL1,AC138894.1,RAB11A,SNAPIN,DNM2,AP1M2,MYO1D,C17orf75,PPP6C,MYO1F,MYO5B,BICDL2,PRKCZ,IKBKG,TPGS1,FNBP1L,BLOC1S3,BORCS5,AP3S2	grey	VSMC_M10
GO:0070266	0.63	0.52	15	29	necroptotic process	BP	1	PELI1,RIPK1,DNM1L,ITPK1,SPATA2,FZD9,YBX3,PYGL,BIRC3,TRPM7,OGT,MIR22,FADD,CASP8,PPIF	grey	VSMC_M10
GO:1905048	0.63	0.57	8	15	regulation of metallopeptidase activity	BP	1	LRP1,SVBP,DDRGK1,SORL1,STAT3,MIR199A1,MIR21,MIR24-1	grey	VSMC_M10
GO:0043551	0.63	0.49	24	47	regulation of phosphatidylinositol 3-kinase activity	BP	1	AGAP2,PTK2,TEK,KIT,FPR2,WDR91,CCKBR,FLT1,PIK3R1,SLA2,PTK2B,NOD2,IRS1,FGR,RUBCN,PIK3IP1,FGFR3,SOCS4,SOCS7,CD19,PIK3R5,SOCS1,VAV3,DAB2IP	grey	VSMC_M10
GO:0010906	0.63	0.45	50	96	regulation of glucose metabolic process	BP	1	PRKN,PPARA,GPT,GNMT,CRY1,PPP4R3B,ZNF692,FAM3C,RUBCNL,KHK,EPM2AIP1,GCKR,PPP1CB,AKT2,C1QTNF1,SIRT7,PDK1,PPP1R3B,NLN,NCOA2,SORBS1,IGF2,GPLD1,IRS1,SIK1,MST1,PPP1R3E,PFKFB2,PPP1R3D,ACTN3,PASK,DUSP12,PDK3,GNB3,PPP1R3F,IGFBP4,DDB1,DGKQ,OGT,COX11,LCMT1,INSR,LEPR,PTPN2,INPP5K,MLYCD,ACADM,TIGAR,NNMT,ARPP19	grey	VSMC_M10
GO:0051591	0.63	0.46	35	68	response to cAMP	BP	1	NME1,TEK,PNPT1,AREG,AQP1,AANAT,THBD,GPD1,PTK2B,RAPGEF2,STC1,RAPGEF3,PKD2,DMTN,SRD5A1,SLC6A3,RAP1B,DUOX1,VGF,CPS1,FDX1,PTPRN,DGKQ,PKLR,FOS,CITED1,PPARGC1B,SREBF1,JUN,INPP5K,BSG,ASS1,HSPA5,DUSP1,HCN4	grey	VSMC_M10
GO:0015695	0.63	0.54	11	21	organic cation transport	BP	1	SLC22A4,CHRNA3,SEC14L1,SLC25A26,SLC38A2,SLC25A20,SLC25A42,SLC29A4,PSEN1,RALBP1,SLC22A5	grey	VSMC_M10
GO:0055092	0.63	0.47	33	63	sterol homeostasis	BP	1	APOM,TTC39B,ABCG5,ABCG8,NR1H2,APOB,CNBP,TSKU,COMMD9,SOAT2,CYP7A1,CYP7B1,LIMA1,EPHX2,NR1H3,MYLIP,ABCA5,NR5A2,FABP4,GRAMD1B,ABCB11,ABCG4,SOAT1,EHD1,FGFR4,LIPC,LDLR,ACSM3,NPC1,TMEM97,MIR27B,MIR33B,LDLRAP1	grey	VSMC_M10
GO:0031365	0.63	0.53	13	25	N-terminal protein amino acid modification	BP	1	AANAT,HHAT,METAP1D,NMT1,NAA40,NAA30,PPM1A,CREBBP,NAA60,NAA10,SOX4,PDF,MAP6D1	grey	VSMC_M10
GO:0021695	0.63	0.49	20	39	cerebellar cortex development	BP	1	GLI1,UQCRQ,AGTPBP1,SMO,WHRN,GLI2,B4GALT2,CLP1,FAIM2,LDB1,TTBK2,TTC21B,SPTBN2,MAP2K1,LHX1,SEZ6,AARS,HSPA5,GRID2,CEND1	grey	VSMC_M10
GO:1903539	0.63	0.51	15	29	protein localization to postsynaptic membrane	BP	1	ADAM22,DAG1,DLG1,NSG1,GRIPAP1,RAB8A,DLG4,IQSEC2,CPLX1,STX1B,RAB11A,ARHGAP44,PRKCZ,CACNG7,HRAS	grey	VSMC_M10
GO:0010714	0.63	0.54	11	21	positive regulation of collagen metabolic process	BP	1	HDAC2,F2R,AMELX,VSIR,WNT4,SUCO,LARP6,SERPINF2,ENG,RGCC,CBX8	grey	VSMC_M10
GO:0035567	0.63	0.46	35	68	non-canonical Wnt signaling pathway	BP	1	FZD6,RSPO3,CSNK1E,DAAM2,PTK7,FZD1,ROR2,SFRP4,WNT11,SPEF1,RYK,FZD3,TIAM1,VANGL1,PRICKLE1,NKD1,ZNRF3,DVL3,FZD7,GRHL3,FZD9,FZD5,WNT4,DAAM1,ARHGEF19,DVL2,MKS1,CCDC88C,NLK,GPC3,CELSR2,RHOA,WNT5A,LBX2,CELSR3	grey	VSMC_M10
GO:0006633	0.63	0.44	56	107	fatty acid biosynthetic process	BP	1	LTC4S,QKI,MCAT,HSD17B8,HACD4,NR1H2,APOC1,PRKAG2,PIBF1,ELOVL1,TRIB3,CYP7A1,ELOVL7,THNSL2,CBR4,PRKAG1,NR1H3,ERLIN1,PRKAB1,ABHD2,MECR,HACD2,LPGAT1,MLXIPL,SCAP,PLA2G4A,ACADL,ACSBG1,PECR,PRKAG3,DAGLB,FADS3,LIAS,ACADVL,TECR,DEGS1,LIPC,ALOXE3,HACD3,NDUFAB1,ACSM3,ACACA,CD74,ABHD3,ACSBG2,DECR2,ACSF3,MLYCD,PTGES2,ABCD3,ACOT4,FABP5,FADS6,ELOVL3,PTGES,INSIG2	grey	VSMC_M10

GO:0006650	0.63	0.42	135	257	glycerophospholipid metabolic process	BP	1	PIP4K2C,ABHD16A,SYNJ2,SERAC1,AGPAT4,AGPAT1,PNPLA3,PLA2G6,GPAA1,CWH43,INPP4A,TMEM246,TMEM86B,PIGF,MBOAT7,PLEK,NR1H2,MBOAT2,LPIN1,INPP4B,FIG4,MTMR2,PIP4K2A,NAPEPLD,HADHA,SELENOI,ABHD5,APOC1,TAMM41,ITPKC,PIP4P1,MTMR14,PLCB1,PGAP2,CRLS1,CDS2,NR1H4,MTMR7,ABHD6,PIGU,PIK3CB,PITPNM2,PIK3R1,TMEM150A,CHRM5,MTMR9,SLA2,NR1H3,SERINC5,SYNJ1,CDS1,ITPKA,PIGT,PLA2G4B,GPLD1,LPCAT1,PGAP1,GPAM,CHKA,PLA1A,ACP6,INPP5A,ABHD4,AJUBA,PITPNM1,MTMR11,GNPAT,DGKD,PIGM,BMX,FAM126B,IP6K2,LPGAT1,PIGV,MTMR3,THEM5,PCYT1A,INPP5J,PIGX,PIGZ,PISD,DGKG,PIKFYVE,PLA2G4A,SACM1L,LPCAT3,ABHD12B,PIGC,PLCD4,SMPD4,SOCS4,PLA2G16,PI4KA,SLC27A1,ABHD8,PGAP3,PIGH,PI4K2B,LIPC,SOCS7,DGKQ,GDPD3,AC138894.1,DGKE,TTC7B,LDLR,GDPD1,PIGL,GDE1,CSF1R,ABHD3,PNPLA6,MPPE1,IMPA2,MTMR12,SOCS1,MTM1,MTMR1,INPP5K,PIP5K1C,ABCA3,MECP2,AGPAT2,PIP5KL1,PLA2G15,FABP5,PEMT,PYURF,LCLAT1,PIGB,PIGA,INPP1,PTPMT1,DNAJC19,CDIPT	grey	VSMC_M10
GO:0045580	0.63	0.45	51	98	regulation of T cell differentiation	BP	1	HLA-DRA,TCF7,SLC46A2,IL4I1,IL15,CLPTM1,GLI2,FOXJ1,TGFBR2,NCKAP1L,SOX12,TOX,ZC3H8,CAMK4,TMEM131L,IL7,RASGRP1,RIPK2,AP3B1,SOX13,HLX,CD28,RUNX3,ADAM8,DUSP10,BCL6,VSIR,RC3H1,TNFSF18,LAG3,STAT5B,FOXP3,ZBTB1,CARD11,TBX21,ZBTB7B,IL4R,CD74,NKAP,IL7R,PTPN2,SOCS1,DROSHA,PRKCZ,MIR21,RHOA,SOX4,CD46,FGL2,TNFSF9,RC3H2	grey	VSMC_M10
GO:0033144	0.63	0.51	16	31	negative regulation of intracellular steroid hormone receptor signaling pathway	BP	1	CNOT2,FOXH1,CRY1,PHB,FOXP1,CYP7B1,ZNF366,ARNTL,CNOT1,CRY2,VPS18,HDAC1,PIAS2,NCOR1,ISL1,RHOA	grey	VSMC_M10
GO:0005976	0.63	0.45	43	83	polysaccharide metabolic process	BP	1	GNMT,HAS1,MGAM,PYGB,RUBCNL,KHK,EPM2AIP1,PRKAG2,PPP1CB,CSGALNACT1,AKT2,PPP1R3B,GBE1,B3GNT4,SORBS1,PFKM,CHST1,IGF2,NHLRC1,PHKB,IRS1,PPP1R3E,EGF,PPP1R3D,PER2,PASK,PHKA2,GAL3ST3,PYGM,PYGL,CHST7,PRKAG3,AC022400.6,PPP1R3F,PHKA1,INSR,LEPR,INPP5K,AGL,HAS3,ACADM,PPP1R1A,HS2ST1	grey	VSMC_M10
GO:0034377	0.63	0.55	10	19	plasma lipoprotein particle assembly	BP	1	APOM,NR1H2,APOB,APOC1,SOAT2,SOAT1,ZDHHC8,LPCAT3,ABCA7,PLAGL2	grey	VSMC_M10
GO:0070131	0.63	0.57	8	15	positive regulation of mitochondrial translation	BP	1	RMND1,MRPS27,RPUSD4,RCC1L,COA3,RPUSD3,UQCC2,TRMT10C	grey	VSMC_M10
GO:0045981	0.63	0.51	15	29	positive regulation of nucleotide metabolic process	BP	1	PPARA,PRXL2C,GUCA1B,SLC4A4,GPD1,P2RX7,ZBTB20,ARNT,MLXIPL,PINK1,STAT3,PSEN1,INSR,NOS2,ENTPD5	grey	VSMC_M10
GO:1900544	0.63	0.51	15	29	positive regulation of purine nucleotide metabolic process	BP	1	PPARA,PRXL2C,GUCA1B,SLC4A4,GPD1,P2RX7,ZBTB20,ARNT,MLXIPL,PINK1,STAT3,PSEN1,INSR,NOS2,ENTPD5	grey	VSMC_M10
GO:0007212	0.63	0.51	16	31	dopamine receptor signaling pathway	BP	1	SLC1A1,VPS35,ALK,DTNBP1,CALY,GNB5,NSG1,GNAL,GNB1,GNA15,PALM,GNAI3,RGS4,GNA14,GNG2,ARPP19	grey	VSMC_M10
GO:0045453	0.63	0.48	26	50	bone resorption	BP	1	TNFAIP3,TNFRSF11B,RAC2,EGFR,NCDN,TRAF6,IL7,P2RX7,PTK2B,SPP1,LRRK1,TCIRG1,GPR137B,UBASH3B,ADAM8,PTH1R,GPR137,CD38,RAB3D,CSF1R,PPARGC1B,ATP6AP1,DEF8,CA2,BGLAP,SNX10	grey	VSMC_M10
GO:0010989	0.63	0.66	4	7	negative regulation of low-density lipoprotein particle clearance	BP	1	MYLIP,MIR199A1,MIR27B,MIR27A	grey	VSMC_M10
GO:1903580	0.63	0.51	15	29	positive regulation of ATP metabolic process	BP	1	PPARA,PRXL2C,SLC4A4,GPD1,P2RX7,ZBTB20,ARNT,ACTN3,MLXIPL,PINK1,STAT3,PSEN1,INSR,ENTPD5,BEND3	grey	VSMC_M10
GO:0035774	0.63	0.52	13	25	positive regulation of insulin secretion involved in cellular response to glucose stimulus	BP	1	LRP1,PLA2G6,TRPM4,OSBP,GPLD1,PPP3CB,MPC2,STX4,GPR68,OXCT1,NADK,BAIAP3,CRH	grey	VSMC_M10
GO:0034367	0.63	0.54	11	21	protein-containing complex remodeling	BP	1	APOM,TAF8,APOB,APOC1,NR1H4,PLTP,ABCA5,GTF2E1,AGT,LIPC,MPO	grey	VSMC_M10
GO:0030194	0.64	0.55	9	17	positive regulation of blood coagulation	BP	1	ENPP4,THBD,F2R,ANO6,DMTN,USF1,NFE2L2,F12,SERPINF2	grey	VSMC_M10
GO:1900048	0.64	0.55	9	17	positive regulation of hemostasis	BP	1	ENPP4,THBD,F2R,ANO6,DMTN,USF1,NFE2L2,F12,SERPINF2	grey	VSMC_M10
GO:0007205	0.64	0.54	11	21	protein kinase C-activating G protein-coupled receptor signaling pathway	BP	1	LRP1,PICK1,DGKI,F2R,GAP43,DGKD,DGKG,CHRM1,DGKQ,DGKE,BDKRB1	grey	VSMC_M10
GO:0001893	0.64	0.51	15	29	maternal placenta development	BP	1	CYP27B1,PTN,PRDX3,VDR,STC1,SPP1,DEDD,MEN1,CTSV,MAPK1,MAPK3,EPOR,BSG,TCF23,LIF	grey	VSMC_M10
GO:0062207	0.64	0.45	40	77	regulation of pattern recognition receptor signaling pathway	BP	1	TNFAIP3,USP15,ESR1,HSPA1B,GRAMD4,LRRC14,NINJ1,RTN4,ANKRD17,SLC46A2,PELI1,RSAD2,TLR4,PUM2,CD300LF,SEC14L1,NPLOC4,IRF7,MFHAS1,OTUD4,NR1H3,F2RL1,NOD2,RAB7B,OASL,TLR5,NLRX1,DDX3X,BIRC3,GPS2,TKFC,WDFY1,TSPAN6,RIOK3,GPR108,PDPK1,CYBA,GFI1,DAB2IP,ZCCHC3	grey	VSMC_M10
GO:0061013	0.64	0.43	75	144	regulation of mRNA catabolic process	BP	1	PAN2,CNOT2,CNOT6,RIDA,PABPC1,PNLDC1,CSDC2,AGO2,TNRC6B,TUT7,GTPBP1,MAPK14,EXOSC3,PNPT1,TUT4,PUM2,TRAF3IP2,ZC3HAV1,DHX34,FASTK,MLH1,MYD88,TNRC6C,LSM1,PKP3,PAN3,CNOT1,CELF1,CNOT6L,FTO,TENT5C,TENT4B,RBM24,DIS3L2,AXIN2,MAPKAPK2,GIGYF2,YTHDF2,TRAF5,FASTKD1,ANGEL2,YTHDF1,EIF4ENIF1,YBX3,RC3H1,UPF1,SLC11A1,RBM10,SAMD4A,POLR2G,SCGB1A1,NT5C3B,DCP1B,FXR2,TARDBP,TOB1,TENT5D,TNRC6A,CNOT8,LARP1,ALKBH5,PARN,FMR1,METTL16,PLEKHN1,CIRBP,TRAF2,METTL14,NANOS1,CACNG7,YBX1,ZFP36L2,TENT5A,RC3H2,EXOSC5	grey	VSMC_M10
GO:0002381	0.64	0.48	25	48	immunoglobulin production involved in immunoglobulin-mediated immune response	BP	1	HLA-DRA,HLA-DOB,HLA-DMA,HLA-DPB1,EXOSC3,MSH2,TRAF3IP2,HMCES,ERCC1,MLH1,PAXIP1,SHLD1,GAPT,NBN,CD40,CD28,RNF168,BCL6,FOXP3,SHLD2,TBX21,BATF,BTK,SUPT6H,RNF8	grey	VSMC_M10
GO:0048566	0.64	0.52	13	25	embryonic digestive tract development	BP	1	VPS52,SIX2,CXCL8,GLI2,OVOL2,RARB,SALL1,TGFB2,HLX,ALDH1A2,RBPMS2,STRA6,FOXF1	grey	VSMC_M10
GO:0045197	0.64	0.49	19	37	establishment or maintenance of epithelial cell apical/basal polarity	BP	1	PTK7,LIN7B,DLG2,WNT11,ANK1,LIN7A,SYNE4,TCF15,DLG1,MARK2,SYNJ2BP,MYO9A,CAMSAP3,LAMA1,IFT20,FOXF1,RHOA,PDZD11,WNT5A	grey	VSMC_M10

GO:0060291	0.64	0.46	32	62	long-term synaptic potentiation	BP	1	PRRT1,AGER,NTRK2,SLC1A1,CRHR2,PTN,GRIN2D,SLC8A2,PRNP,PLK2,FAM107A,PTK2B,MME,EIF2AK4,IGSF11,STAU1,NFATC4,YTHDF1,CREB1,TNR,MPP2,CRTC1,MAPK1,NSG1,EPHA4,RGS14,STX4,CYP46A1,PRKCZ,MECP2,CRH,LRRTM2	grey	VSMC_M10
GO:0032088	0.64	0.46	35	67	negative regulation of NF-kappaB transcription factor activity	BP	1	TNFAIP3,LRRC14,CDKN2A,CAT,MTURN,PELI1,ANXA4,FAF1,FOXJ1,IRAK2,ARRB1,NFKBIB,SETD6,NLRC5,OTULIN,NOD2,CD200,CMKLR1,NWD1,FOXP3,PYDC1,MAP2K5,TRIM37,PSMD10,TRAF3,PIAS4,GFI1,DAB2IP,MIR21,MIR27A,RWDD3,COMMD6,DDIT3,COMMD7,HAVCR2	grey	VSMC_M10
GO:1905521	0.64	0.51	15	29	regulation of macrophage migration	BP	1	PTK2,AKIRIN1,C5AR1,MTUS1,PTK2B,CD200,TRPV4,CMKLR1,SLAMF8,TNFSF18,MAPK1,MAPK3,CCL5,CSF1R,MIR24-1	grey	VSMC_M10
GO:0090026	0.64	0.58	7	13	positive regulation of monocyte chemotaxis	BP	1	FPR2,ANO6,MOSPD2,TNFSF18,S100A14,CCL5,DEFB124	grey	VSMC_M10
GO:0071398	0.64	0.51	15	29	cellular response to fatty acid	BP	1	NME1,UCP1,NR1H4,SMARCD1,IRS1,CPT1A,PDK3,CREB1,CPS1,XRCC5,KCNK4,LDLR,SREBF1,ASS1,CLDN1	grey	VSMC_M10
GO:0009913	0.64	0.43	62	120	epidermal cell differentiation	BP	1	GLI1,CYP27B1,PTCH1,IFT74,CD109,HOXA7,MYCL,WHRN,GRHL1,SFRP4,MYCN,INTU,HDAC2,ERCC2,ERCC3,PTCH2,EVPL,MYO7A,SCEL,OVOL2,SLC4A7,JAG1,LRTOMT,AP000812.5,MAFG,ASAH1,FLNB,CASP3,PLS1,USH1C,VDR,SRSF6,DSP,STK4,PPHLN1,HDAC1,TGM1,KDF1,SFN,CLIC4,STRC,OVOL1,KLF7,H2AFY2,ABCA12,SAV1,RBPJ,MED1,MAP2K1,KEAP1,BCL11B,GATA6,TMEM132E,JAG2,MCOLN3,MIR125B1,WNT5A,DNASE1L2,ZBED2,EREG,SGPP1,HEY2	grey	VSMC_M10
GO:0048512	0.64	0.51	14	27	circadian behavior	BP	1	BTBD9,NR1D2,AHCY,NCOA2,SRD5A1,CIART,USP2,C3orf70,CHRNB2,NCOR1,PER3,GPR157,ZFHX3,CRH	grey	VSMC_M10
GO:0010592	0.64	0.52	12	23	positive regulation of lamellipodium assembly	BP	1	AVIL,RAC2,AKIRIN1,PIK3R1,PLCE1,CYFIP1,AUTS2,WASF2,HDAC4,NCKAP1,DNM2,CARMIL2	grey	VSMC_M10
GO:0072503	0.64	0.40	160	306	cellular divalent inorganic cation homeostasis	BP	1	SLC39A5,LRP1,ESR1,SMDT1,BAK1,MCHR1,GPR20,PLA2G6,SLC39A4,SLC25A27,SLC1A1,S1PR3,PRKCE,TNNI3,FPR2,IBTK,LPAR1,KEL,ATG5,TRPM4,GRIN2D,TRPC3,SLC8A2,C5AR2,C5AR1,ATP7B,PTGIR,GIPR,CACNB2,SLC39A12,ACKR2,SLC24A3,C1QTNF1,TMTC4,RYR1,PRNP,ADRA1D,DIAPH1,P2RX2,CCKBR,RASA3,MICU3,IMMT,C19orf12,P2RX3,PLCE1,RYR3,TMBIM6,P2RX7,P2RY1,PTK2B,SLC39A13,TPCN1,SLC8B1,VDR,STC1,AP3B1,RMDN3,F2RL1,F2R,PKD2,CASQ2,MCUR1,CD40,RYR2,SLC41A1,RAP1GDS1,TRPM1,SPPL3,LCK,TRPV4,SLC30A10,TCIRG1,GNA13,CMKLR1,MCUB,UBASH3B,DISC1,ANK2,GPER1,TRPM8,PDZD8,AGT,ATP1A2,CASQ1,NTSR1,FZD9,TMCO1,PTH1R,SLC30A4,FIS1,CEMIP,ABL2,CDH23,PTPN6,MICU1,MCU,CXCR2,GNG3,SLC11A1,HTT,PTGDR,PTGER2,TRPM7,ATP1B1,CD38,ATP13A2,HAP1,DLG4,FAM155B,PSEN1,CACNA1A,AC138894.1,ATP2A1,CD19,SMAD3,CALM1,S100A8,PRKCB,BDKRB1,NMUR2,SLC39A6,TRPV2,HRH4,CCL14,CCL5,CCL7,PTGER4,SLC25A23,MAIP1,GNB1,ATP2B3,BCAP31,GNA15,TRPV3,TRPV1,PDPK1,P2RX1,PKD1,TMEM203,GPR157,SYPL2,CYBA,JPH3,ATP2C2,SLC30A7,MCOLN3,PTGFR,MIR199A1,KCNK3,DMD,MT1X,GPR35,NOL3,CHRNA10,CCR10,JPH2,THY1,WNT5A,DDIT3	grey	VSMC_M10
GO:0009129	0.64	0.55	9	17	pyrimidine nucleoside monophosphate metabolic process	BP	1	DPYS,CMPK1,UPB1,UPRT,NT5M,UCK1,DHODH,DCTD,UPP1	grey	VSMC_M10
GO:0071624	0.64	0.53	11	21	positive regulation of granulocyte chemotaxis	BP	1	RAC2,CXCL8,C5AR1,NCKAP1L,DNM1L,MOSPD2,MCU,CXCR2,DAPK2,S100A14,CD74	grey	VSMC_M10
GO:0007204	0.64	0.41	101	194	positive regulation of cytosolic calcium ion concentration	BP	1	LRP1,ESR1,BAK1,MCHR1,GPR20,PLA2G6,S1PR3,PRKCE,FPR2,IBTK,LPAR1,ATG5,TRPM4,GRIN2D,TRPC3,SLC8A2,C5AR2,C5AR1,PTGIR,GIPR,CACNB2,ACKR2,C1QTNF1,RYR1,PRNP,ADRA1D,DIAPH1,P2RX2,CCKBR,RASA3,P2RX3,PLCE1,RYR3,TMBIM6,P2RX7,P2RY1,PTK2B,TPCN1,SLC8B1,F2RL1,F2R,PKD2,CASQ2,RYR2,TRPM1,SPPL3,LCK,TRPV4,GNA13,CMKLR1,UBASH3B,ANK2,GPER1,AGT,ATP1A2,CASQ1,NTSR1,PTH1R,FIS1,CEMIP,ABL2,PTPN6,CXCR2,GNG3,HTT,PTGDR,PTGER2,CD38,HAP1,DLG4,FAM155B,CACNA1A,CD19,CALM1,BDKRB1,NMUR2,TRPV2,HRH4,PTGER4,GNB1,BCAP31,GNA15,TRPV3,TRPV1,PDPK1,P2RX1,PKD1,GPR157,CYBA,JPH3,MCOLN3,PTGFR,MIR199A1,DMD,GPR35,NOL3,CHRNA10,CCR10,JPH2,THY1,DDIT3	grey	VSMC_M10
GO:0006479	0.64	0.42	77	148	protein methylation	BP	1	ARMT1,SNW1,PHF1,PRDM4,CAMKMT,RNF20,SETD7,SETDB2,EED,IWS1,SUV39H2,KDM4A,PAXIP1,DPY30,SIRT7,CHTOP,NSD3,NDUFAF7,ASH2L,BTG1,NR1H4,EEF1AKMT1,DNMT3B,SMYD1,PRMT9,SETD6,KMT2D,RTF1,PRMT3,WDR82,ZNF335,JARID2,RBBP5,FAM173B,HEMK1,AUTS2,EEF1AKMT2,SETD2,TET1,SNRPD3,BCOR,MEN1,WDR61,VCPKMT,NELFA,DYDC1,PRDM2,NSD1,SETD1A,PAGR1,OGT,RAB3D,LCMT1,SETD3,SUZ12,SUPT6H,CXXC1,CREBBP,DOT1L,MECP2,PRDM16,CHD5,MTF2,GFI1,PRMT7,CTCF,PRMT6,METTL18,SETDB1,PRDM6,ARID4A,BEND3,SMYD3,METTL21A,ZNF304,NNMT,N6AMT1	grey	VSMC_M10
GO:0008213	0.64	0.42	77	148	protein alkylation	BP	1	ARMT1,SNW1,PHF1,PRDM4,CAMKMT,RNF20,SETD7,SETDB2,EED,IWS1,SUV39H2,KDM4A,PAXIP1,DPY30,SIRT7,CHTOP,NSD3,NDUFAF7,ASH2L,BTG1,NR1H4,EEF1AKMT1,DNMT3B,SMYD1,PRMT9,SETD6,KMT2D,RTF1,PRMT3,WDR82,ZNF335,JARID2,RBBP5,FAM173B,HEMK1,AUTS2,EEF1AKMT2,SETD2,TET1,SNRPD3,BCOR,MEN1,WDR61,VCPKMT,NELFA,DYDC1,PRDM2,NSD1,SETD1A,PAGR1,OGT,RAB3D,LCMT1,SETD3,SUZ12,SUPT6H,CXXC1,CREBBP,DOT1L,MECP2,PRDM16,CHD5,MTF2,GFI1,PRMT7,CTCF,PRMT6,METTL18,SETDB1,PRDM6,ARID4A,BEND3,SMYD3,METTL21A,ZNF304,NNMT,N6AMT1	grey	VSMC_M10

GO:0010830	0.64	0.49	17	33	regulation of myotube differentiation	BP	1	MAML1,MAPK14,MAMSTR,LMOD3,SMYD1,NLN,RBM24,BDNF,SIK1,ACTN3,HDAC4,BHLHA15,FBXO22,PIEZO1,PLPP7,NKX2-5,RBM38	grey	VSMC_M10
GO:0034656	0.64	0.54	10	19	nucleobase-containing small molecule catabolic process	BP	1	APOBEC3H,GDA,GALT,ENPP4,CDADC1,AHCY,ENTPD4,MGAT1,UPB1,UPP1	grey	VSMC_M10
GO:0001835	0.64	0.52	12	23	blastocyst hatching	BP	1	CCDC62,ZBED6,PLPP4,BCOR,AKAP3,ARHGDIG,SLC25A34,PPP4R4,CMTM3,PEMT,C6orf141,ST8SIA6	grey	VSMC_M10
GO:0035188	0.64	0.52	12	23	hatching	BP	1	CCDC62,ZBED6,PLPP4,BCOR,AKAP3,ARHGDIG,SLC25A34,PPP4R4,CMTM3,PEMT,C6orf141,ST8SIA6	grey	VSMC_M10
GO:0071684	0.64	0.52	12	23	organism emergence from protective structure	BP	1	CCDC62,ZBED6,PLPP4,BCOR,AKAP3,ARHGDIG,SLC25A34,PPP4R4,CMTM3,PEMT,C6orf141,ST8SIA6	grey	VSMC_M10
GO:0035821	0.64	0.45	39	75	modulation of process of other organism	BP	1	YTHDC2,SNW1,IGF2R,PRKN,ATG5,NAPEPLD,PHB,FBXL2,ULK1,SAP30,P2RX7,EIF2AK4,F2RL1,ANO6,HDAC1,DAG1,ZNF639,CFL1,HSPA8,POU2F3,PC,PSMC3,TARDBP,TNIP1,INSR,CCL5,NOS2,JUN,FMR1,INPP5K,RXRA,MIR222,CXCL6,IRGM,SAP30BP,FCER2,SERPINB9,TBC1D20,ZNF502	grey	VSMC_M10
GO:0010970	0.64	0.42	72	139	transport along microtubule	BP	1	AP3S1,KIF5A,RAB21,KIF3A,AGTPBP1,SUN2,COPG2,IFT74,APBA1,BICD2,DYNC2LI1,LRPPRC,MGARP,WASF1,TTC26,KIF3C,ARHGAP21,COPG1,KLC3,DLG2,IFT172,TTC21A,WDR60,AP3M2,KIF5C,PURA,TUB,KIF3B,IFT81,RASGRP1,OPA1,AP3B1,IFT52,DTNBP1,KIF13A,BICDL1,TTC21B,HSPA8,BAG3,TRAF3IP1,CNIH2,CALY,TTC30A,SPG11,MAPT,BLOC1S6,MREG,HTT,KIF17,UXT,AP3M1,KIF1C,HAP1,WDR19,PEX14,TRIM46,NDEL1,AC138894.1,SNAPIN,SFPQ,CAMSAP3,IFT20,HSPB11,BICDL2,PRKCZ,MAPK8IP3,TERF2,UBB,BLOC1S3,BORCS5,AP3S2,MAP1A	grey	VSMC_M10
GO:0003407	0.64	0.47	25	49	neural retina development	BP	1	NTRK2,RORB,SLC1A1,TFAP2B,RDH13,PTN,TSPAN12,USP45,SOX9,USH1C,RPGRIP1L,TFAP2A,TGFB2,NRL,DLX1,OBSL1,ROM1,SDK1,PSEN1,MEGF11,LHX1,SOX8,FJX1,THY1,DIO3	grey	VSMC_M10
GO:0006691	0.64	0.55	9	17	leukotriene metabolic process	BP	1	LTC4S,ABCC10,MGST2,MAPKAPK2,PLA2G4A,GGT2,GGT6,DPEP2,GGT5	grey	VSMC_M10
GO:0055074	0.64	0.40	152	291	calcium ion homeostasis	BP	1	LRP1,CYP27B1,ESR1,SMDT1,BAK1,MCHR1,GPR20,PLA2G6,SLC25A27,TFAP2B,S1PR3,PRKCE,TNNI3,FPR2,IBTK,LPAR1,KEL,ATG5,TRPM4,GRIN2D,TRPC3,SLC8A2,C5AR2,C5AR1,ATP7B,PTGIR,GIPR,CACNB2,ACKR2,SLC24A3,C1QTNF1,TMTC4,RYR1,PRNP,ADRA1D,DIAPH1,P2RX2,KL,CCKBR,RASA3,MICU3,IMMT,C19orf12,P2RX3,PLCE1,RYR3,TMBIM6,P2RX7,P2RY1,PTK2B,TPCN1,SLC8B1,VDR,STC1,RMDN3,F2RL1,F2R,PKD2,CASQ2,MCUR1,CD40,RYR2,RAP1GDS1,TRPM1,SPPL3,LCK,TRPV4,TCIRG1,FAM20A,GNA13,CMKLR1,MCUB,UBASH3B,DISC1,ANK2,GPER1,TRPM8,PDZD8,AGT,ATP1A2,CASQ1,NTSR1,FZD9,TMCO1,PTH1R,FIS1,CEMIP,ABL2,CDH23,PTPN6,MICU1,MCU,CXCR2,GNG3,HTT,PTGDR,PTGER2,ATP1B1,CD38,ATP13A2,HAP1,DLG4,FAM155B,PSEN1,CACNA1A,AC138894.1,ATP2A1,CD19,SMAD3,CALM1,S100A14,PRKCB,BDKRB1,NMUR2,TRPV2,HRH4,CCL14,CCL5,CCL7,PTGER4,SLC25A23,MAIP1,GNB1,ATP2B3,BCAP31,GNA15,TRPV3,TRPV1,PDPK1,P2RX1,PKD1,TMEM203,GPR157,SYPL2,CYBA,JPH3,ATP2C2,MCOLN3,PTGFR,MIR199A1,KCNK3,DMD,GPR35,NOL3,CHRNA10,TRIM24,CCR10,JPH2,SNX10,THY1,WNT5A,DDIT3	grey	VSMC_M10
GO:1901985	0.64	0.48	20	39	positive regulation of protein acetylation	BP	1	SPHK2,PAXIP1,ARRB1,ARNTL,RAPGEF3,AUTS2,DDX3X,TADA2B,FOXP3,KAT7,MUC1,MAPK3,NAP1L2,RPS6KA5,ISL1,TAOK1,GLYR1,SOX4,LIF,RPS6KA4	grey	VSMC_M10
GO:0042744	0.64	0.56	8	15	hydrogen peroxide catabolic process	BP	1	PRDX1,CAT,PXDN,PRDX3,PXDNL,DUOX1,MPO,HBA2	grey	VSMC_M10
GO:0008202	0.65	0.41	114	217	steroid metabolic process	BP	1	RDH5,APOF,ATP8B1,RDH16,HSD17B4,CYP27B1,ESR1,CYP21A2,SULT4A1,TSPO,HSD17B8,HSD17B3,AKR1B1,TTC39B,KIT,PLPP6,SRD5A3,HINT2,CAT,DKKL1,APOB,HSD17B14,EBP,EBPL,CUBN,APOC1,LIPE,PRKAG2,TSKU,PIP4P1,PANK2,NR1H4,SOAT2,CYP7A1,ASAH1,CYP7B1,CEBPA,OSBP,DKK3,LIMA1,EPHX2,CYP2R1,SPP1,ERLIN1,SRD5A1,CYB5R1,ABCA5,FMO5,NR5A2,SNAI1,MSMO1,SC5D,HSD11B1,ABCB11,DHRS9,AGT,OSBPL2,SCAP,HSD17B7,POR,OSBPL6,ACADL,SOAT1,TM7SF2,OSBPL1A,FDX1,COMT,LPCAT3,GNB3,WNT4,CYP3A43,CYP27A1,STAT5B,CH25H,LIPA,ACADVL,HSD17B10,CLN8,MED1,FGFR4,LIPC,DGKQ,OSBPL7,ERG28,APOBR,CLN6,LDLR,SCNN1B,CYP46A1,DHRS11,NPC1,SREBF1,LEPR,ACAA2,PIAS4,MECP2,CACNA1H,RXRA,CEL,ABCD3,GFI1,HSD11B2,MIR27B,MIR27A,NFE2L1,DHRS2,SDR42E1,LDLRAP1,AMACR,CRH,STARD4,INSIG2,YWHAH,FGF1	grey	VSMC_M10
GO:0045730	0.65	0.52	12	23	respiratory burst	BP	1	RAC2,HCK,NCF1,SLAMF8,NCF2,DUSP10,SLC11A1,MPO,INSR,NOXA1,CYBA,JCHAIN	grey	VSMC_M10
GO:0016571	0.65	0.42	61	118	histone methylation	BP	1	SNW1,PHF1,PRDM4,RNF20,SETD7,SETDB2,EED,IWS1,SUV39H2,KDM4A,PAXIP1,DPY30,SIRT7,CHTOP,NSD3,ASH2L,NR1H4,DNMT3B,SMYD1,SETD6,KMT2D,RTF1,WDR82,ZNF335,JARID2,RBBP5,AUTS2,SETD2,TET1,BCOR,MEN1,WDR61,NELFA,DYDC1,PRDM2,NSD1,SETD1A,PAGR1,OGT,SETD3,SUZ12,SUPT6H,CXXC1,CREBBP,DOT1L,MECP2,PRDM16,CHD5,MTF2,GFI1,PRMT7,CTCF,PRMT6,SETDB1,PRDM6,ARID4A,BEND3,SMYD3,ZNF304,NNMT,N6AMT1	grey	VSMC_M10

GO:0019725	0.65	0.38	335	638	cellular homeostasis	BP	1	ZNF236,NME1,ORMDL2,SLC39A5,NEDD4L,LRP1,ESR1,SLC12A2,PRKN,AGER,SMDT1,BAK1,MYC,MCHR1,GPR20,PLA2G6,CKB,TMPRSS6,TXN2,SLC39A4,LARGE1,SRF,STEAP2,TXNRD1,SLC25A27,SLC1A1,TFAP2B,S1PR3,PRDX1,ERP44,PRKCE,TNNI3,RTN4,FPR2,HMGN3,IBTK,SLC4A4,LPAR1,PPT1,AQP1,SLC31A2,TRIM32,KEL,ATG5,TRPM4,FOXO3,DLD,TXNRD3,FGF21,GRIN2D,TRPC3,SLC8A2,RAB7A,C5AR2,C5AR1,HEPHL1,ATP7B,PTGIR,FOXA3,GIPR,CACNB2,SLC39A12,ATP6V0E2,LACC1,SLC4A2,DEDD2,CLNS1A,AQP11,GRIK5,RAF1,ATP1A3,PRDX3,EGLN2,ACKR2,SLC24A3,C1QTNF1,SLC4A7,TMEM106B,TMTC4,MAFG,RYR1,LETM2,PRNP,ADRA1D,DIAPH1,P2RX2,CCKBR,GAS6,HPX,RASA3,NCOA4,NR1H4,MICU3,ATP6V1H,CYP7A1,TM9SF4,NDUFAF2,IL1A,IDE,IMMT,OSBP,C19orf12,PIK3R1,CHMP2B,P2RX3,PLCE1,SLC9A9,RYR3,FBXW7,TMBIM6,P2RX7,HVCN1,P2RY1,PTK2B,SLC39A13,KCNJ11,TIAM1,RAB11FIP5,PFKM,TPCN1,SLC8B1,VDR,STC1,OPA1,AP3B1,RMDN3,F2RL1,F2R,SLC40A1,ANO6,FITM2,ORMDL1,SLC6A2,ABCC2,ZBED6,NELL2,PKD2,PAX2,TWNK,CASQ2,MCUR1,SLC12A5,CD40,RYR2,SLC41A1,SLC4A10,RAP1GDS1,GPLD1,TRPM1,SPPL3,GLRX2,LCK,SLC2A10,PPP3CA,KCNJ2,TESMIN,SLC9B1,SLC12A7,TRPV4,SLC9B2,ZBTB20,TMEM9,ADRA2A,SLC30A10,TCIRG1,GNA13,ISCU,KCNB1,CMKLR1,MCUB,HJV,SLC9A8,UBASH3B,DISC1,POLG,ANK2,GPER1,SLAMF8,RHCG,TRPM8,PDZD8,RAB11FIP2,MCL1,AGT,ATP1A2,BAG3,SLC9A1,CASQ1,SLC12A8,NTSR1,XPR1,CLIC4,MLXIPL,FZD9,SIDT2,TMCO1,PDK3,KLF7,NFE2L2,PTH1R,SLC30A4,FIS1,CEMIP,HK1,ABL2,SLC12A9,MEN1,CDH23,ENY2,ABCA12,PTPN6,MICU1,MCU,CXCR2,GNG3,SLC11A1,GNB3,HTT,PTGDR,PTGER2,SLC9A[illegible]	grey	VSMC_M10
GO:0043616	0.65	0.48	18	35	keratinocyte proliferation	BP	1	PTCH1,CD109,AREG,INTU,OVOL2,FERMT1,SDR16C5,VDR,SRSF6,TGM1,KDF1,SFN,CASK,MED1,BCL11B,MIR21,MIR125B1,EREG	grey	VSMC_M10
GO:0051607	0.65	0.41	91	174	defense response to virus	BP	1	TNFAIP3,USP15,C6orf106,APOBEC3H,EXOSC4,IRF5,ANKRD17,RSAD2,IL15,PUM2,TRAF3IP2,ZC3HAV1,PHB,UNC13D,MYD88,SEC14L1,ZMYND11,TMEM173,CLPB,NPLOC4,EIF2AK2,MAVS,AIMP1,IRF7,PCBP2,TRIM44,IRF2,EIF2AK4,SERINC5,OAS3,NLRC5,IFNGR2,F2RL1,POLR3D,IFIT2,HERC5,CD40,MX2,IFIH1,OASL,CRCP,GPAM,ADARB1,UNC93B1,POLR3C,PARP9,DTX3L,IRF9,IFI6,RNF26,NLRX1,ABCF3,TRAF3IP1,HTRA1,TRIM52,SETD2,RTP4,MUL1,BIRC3,POLR3A,FOXP3,RNF216,TKFC,SPON2,AKAP1,TSPAN6,STAT2,POLR3E,G3BP1,SIN3A,TRAF3,RIOK3,RNF135,TICAM1,TTC4,ATAD3A,NCBP3,POLR3K,IFI44L,ZDHHC1,OAS1,ZDHHC11,FADD,TRIM34,BNIP3L,ZCCHC3,IFNLR1,FGL2,KCNJ8,IFIT3,EXOSC5	grey	VSMC_M10
GO:0140546	0.65	0.41	91	174	defense response to symbiont	BP	1	TNFAIP3,USP15,C6orf106,APOBEC3H,EXOSC4,IRF5,ANKRD17,RSAD2,IL15,PUM2,TRAF3IP2,ZC3HAV1,PHB,UNC13D,MYD88,SEC14L1,ZMYND11,TMEM173,CLPB,NPLOC4,EIF2AK2,MAVS,AIMP1,IRF7,PCBP2,TRIM44,IRF2,EIF2AK4,SERINC5,OAS3,NLRC5,IFNGR2,F2RL1,POLR3D,IFIT2,HERC5,CD40,MX2,IFIH1,OASL,CRCP,GPAM,ADARB1,UNC93B1,POLR3C,PARP9,DTX3L,IRF9,IFI6,RNF26,NLRX1,ABCF3,TRAF3IP1,HTRA1,TRIM52,SETD2,RTP4,MUL1,BIRC3,POLR3A,FOXP3,RNF216,TKFC,SPON2,AKAP1,TSPAN6,STAT2,POLR3E,G3BP1,SIN3A,TRAF3,RIOK3,RNF135,TICAM1,TTC4,ATAD3A,NCBP3,POLR3K,IFI44L,ZDHHC1,OAS1,ZDHHC11,FADD,TRIM34,BNIP3L,ZCCHC3,IFNLR1,FGL2,KCNJ8,IFIT3,EXOSC5	grey	VSMC_M10
GO:1904037	0.65	0.50	15	29	positive regulation of epithelial cell apoptotic process	BP	1	SFRP4,FOXO3,ANO6,PLA2R1,CD40,HDAC1,PDCD4,GPER1,ITGA4,CCL2,PIAS4,MIR24-1,MIR125A,CD248,RGCC	grey	VSMC_M10
GO:0060043	0.65	0.48	19	37	regulation of cardiac muscle cell proliferation	BP	1	GLI1,MAPK14,PIM1,TGFBR2,MEF2C,TBX5,JARID2,KCNK2,SAV1,MAPK1,BMPR1A,RBPJ,TBX2,GATA6,MIR199A1,MIR222,NOG,HEY2,WNT2	grey	VSMC_M10
GO:0031571	0.65	0.51	13	25	mitotic G1 DNA damage checkpoint signaling	BP	1	PLK3,PRKDC,PLK2,PIDD1,CCND1,GIGYF2,CHEK2,CDKN1B,MUC1,FBXO31,RPS27L,SOX4,CRADD	grey	VSMC_M10
GO:0086014	0.65	0.58	6	12	atrial cardiac muscle cell action potential	BP	1	TRPM4,CACNB2,SCN5A,KCNJ3,RYR2,ANK2	grey	VSMC_M10
GO:0086026	0.65	0.58	6	12	atrial cardiac muscle cell to AV node cell signaling	BP	1	TRPM4,CACNB2,SCN5A,KCNJ3,RYR2,ANK2	grey	VSMC_M10
GO:0086066	0.65	0.58	6	12	atrial cardiac muscle cell to AV node cell communication	BP	1	TRPM4,CACNB2,SCN5A,KCNJ3,RYR2,ANK2	grey	VSMC_M10
GO:0050798	0.65	0.50	14	27	activated T cell proliferation	BP	1	AGER,PRNP,CASP3,IGF2,ICOSLG,BTN2A2,GPAM,PRKAR1A,RC3H1,STAT5B,FOXP3,MIR21,FADD,TNFSF9	grey	VSMC_M10
GO:0006720	0.65	0.44	38	73	isoprenoid metabolic process	BP	1	RDH5,RDH16,LRP1,ALDH8A1,AKR1B1,ALDH1A1,SRD5A3,ADH5,RDH13,EGFR,NAPEPLD,LIPE,SDR16C5,RETSAT,HTRA2,COQ2,PRMT3,SRD5A1,ALDH1A3,GGPS1,DHRS9,DPAGT1,BCO2,PECR,DHRS3,RDH11,ALDH1A2,SCPEP1,LRP8,PHYH,PNPLA4,DHDDS,PDSS2,CRH,LRAT,RDH14,ADH4,ISPD	grey	VSMC_M10
GO:0016226	0.65	0.51	12	23	iron-sulfur cluster assembly	BP	1	ISCA1,CIAO1,CIAPIN1,ISCU,HSCB,NUBPL,NUBP1,CIAO3,CIAO2B,GLRX5,FXN,ISCA2	grey	VSMC_M10
GO:0031163	0.65	0.51	12	23	metallo-sulfur cluster assembly	BP	1	ISCA1,CIAO1,CIAPIN1,ISCU,HSCB,NUBPL,NUBP1,CIAO3,CIAO2B,GLRX5,FXN,ISCA2	grey	VSMC_M10
GO:1901293	0.65	0.40	105	199	nucleoside phosphate biosynthetic process	BP	1	NME1,TBPL1,ATP5MGL,PPT2,PPARA,ACSL6,GUCA1B,IMPDH1,NMRK1,SLC35B2,SLC25A13,PPAT,PPCS,ME1,PPT1,DLD,SPHK2,CMPK1,ADCY3,PRKAG2,SLC22A13,ATP5F1C,ELOVL1,IDO2,IDO1,PANK2,PDK1,PDHX,SLC5A8,ELOVL7,PANK1,NUDT12,AMPD3,ATP5F1A,GUCY1A1,GUCY1B1,DGUOK,GMPS,AMPD1,ADCY7,FAM173B,ADCY2,NME4,ACSL5,PRPS2,PARP9,HACD2,ADCY4,CTPS2,DNAJC30,DTYMK,PDK3,SLC25A16,ACSBG1,ATP5S,COASY,PINK1,STAT3,NME7,ADK,MPC2,ADCY10,MAPK1,PARP1,TECR,SLC26A1,GUCY2D,PKLR,PANK3,GCDH,UPRT,ACACA,SLC26A2,PRPSAP2,ADSSL1,NOS2,ACSBG2,NADK,ATP6V0C,PANK4,NME3,ATP5PB,VPS9D1,ACSF3,AK8,UCK1,MLYCD,KARS,FUK,DHODH,MT-ATP8,MT-ATP6,ATP5ME,ATP5MF,PDZD3,ELOVL3,MTHFD2L,CMPK2,NNMT,NME6,DCTD,NUDT2,NAPRT,UPP1,NMNAT1	grey	VSMC_M10

GO:0006471	0.65	0.49	16	31	protein ADP-ribosylation	BP	1	XRCC1,SIRT2,SIRT3,PARP4,HPF1,ART3,SIRT5,SIRT4,ADNP,PARP9,PARP15,PARP14,PARP11,PARP1,PARP6,TINF2	grey	VSMC_M10
GO:0034661	0.65	0.48	18	35	ncRNA catabolic process	BP	1	EXOSC4,EXOSC3,PNPT1,TUT4,DEDD2,NUDT16,ZCCHC8,TENT2,TENT4B,DIS3L2,RBM7,DDX49,ZSWIM8,MRPL44,SLFN14,PARN,DROSHA,EXOSC5	grey	VSMC_M10
GO:0014074	0.65	0.42	56	109	response to purine-containing compound	BP	1	NME1,TEK,PNPT1,AREG,AQP1,TRPM4,HDAC2,TRPC3,PANX1,AANAT,THBD,RYR1,P2RX2,DNMT3B,P2RX3,RYR3,GPD1,P2RX7,P2RY1,PTK2B,RAPGEF2,KCNJ11,STC1,RAPGEF3,PKD2,DMTN,CASQ2,RYR2,SRD5A1,SLC6A3,HDAC1,SSH1,RAP1B,DUOX1,VGF,CPS1,FDX1,PTPRN,DGKQ,PKLR,FOS,TAF1,CITED1,PPARGC1B,SREBF1,JUN,INPP5K,TRPV1,P2RX1,BSG,ASS1,HSPA5,DHODH,DUSP1,HCN4,PDXP	grey	VSMC_M10
GO:0002820	0.65	0.48	17	33	negative regulation of adaptive immune response	BP	1	IL4I1,FOXJ1,NCKAP1L,NOD2,BCL6,RC3H1,PTPN6,TNFSF18,PPP3CB,FOXP3,TBX21,ZBTB7B,IL4R,IL7R,CD46,HAVCR2,RC3H2	grey	VSMC_M10
GO:1902175	0.65	0.51	12	23	regulation of oxidative stress-induced intrinsic apoptotic signaling pathway	BP	1	PRKN,FBXW7,HTRA2,MCL1,NFE2L2,NONO,PINK1,PARP1,SFPQ,MAPK7,PARK7,NOL3	grey	VSMC_M10
GO:0071354	0.65	0.48	17	33	cellular response to interleukin-6	BP	1	PHB,ST18,CEBPA,FER,ST3GAL6,RIPK1,ABCC2,SELPLG,STAT3,IL6R,JAK1,PTPN2,LCN2,GFI1,MIR125B1,MIR125A,C1QTNF4	grey	VSMC_M10
GO:1905898	0.65	0.49	15	29	positive regulation of response to endoplasmic reticulum stress	BP	1	BAK1,NCK2,PIK3R1,RNFT2,USP13,RNF185,NFE2L2,PTPN2,BCAP31,SGTA,TMEM259,DAB2IP,RNFT1,DDIT3,BCL2L11	grey	VSMC_M10
GO:0097352	0.65	0.47	19	37	autophagosome maturation	BP	1	STX17,SNX14,MFSD8,RUBCNL,VPS16,LIX1,TMEM39A,RUBCN,SNAP29,TBC1D25,PHF23,AC138894.1,CALM1,SNAPIN,VMP1,SMCR8,LAMP2,CLEC16A,IRGM	grey	VSMC_M10
GO:0070374	0.65	0.41	76	145	positive regulation of ERK1 and ERK2 cascade	BP	1	LRP1,AGER,PRXL2C,TEK,FPR2,MTURN,NOD1,BMPER,EGFR,TLR4,FGF21,BRAF,DNAJC27,C5AR2,C5AR1,PRKD2,PHB,SPRY2,ARRB1,NRG1,GAS6,MFHAS1,ST5,HAND2,FBXW7,P2RY1,PTK2B,RASGRP1,RAPGEF2,CCL17,RIPK2,F2RL1,F2R,NQO2,GCNT2,NOD2,DSTYK,TRPV4,SLC30A10,GPER1,CIB1,THPO,PDE8A,RAP1B,GPBAR1,FGFR3,FGFR4,FGF18,MAPK3,MAP2K1,GAREM1,CD74,CCL14,CCL5,CSF1R,CCL7,CCL2,CCL25,SERPINF2,PRKCZ,ATP6AP1,ABCA7,MIR21,MIR222,MIR23A,MIR27B,MIR24-1,MIR27A,FGA,CCL16,HRAS,MAP2K7,FGF1,HAVCR2,CCL22,PDGFD	grey	VSMC_M10
GO:1902930	0.65	0.47	19	37	regulation of alcohol biosynthetic process	BP	1	CYP27B1,PLEK,APOB,CYP7A1,DKK3,P2RY1,PTK2B,ERLIN1,GPER1,NTSR1,SCAP,POR,PTH1R,LPCAT3,WNT4,DGKQ,SREBF1,GFI1,FGF1	grey	VSMC_M10
GO:0007140	0.65	0.48	18	35	male meiotic nuclear division	BP	1	MOV10L1,DMC1,FOXJ3,MLH1,TEX15,ING2,M1AP,HSF2BP,SPO11,TDRKH,RAD51C,MEIOB,UBB,SYCP3,AGO4,FOXJ2,ZSCAN21,SPDYA	grey	VSMC_M10
GO:0030970	0.65	0.49	14	27	retrograde protein transport, ER to cytosol	BP	1	DERL1,SEC61B,NPLOC4,UBAC2,AUP1,RHBDD1,YOD1,SYVN1,DERL3,TMEM129,SEL1L,BCAP31,UBE2G2,DERL2	grey	VSMC_M10
GO:1903513	0.65	0.49	14	27	endoplasmic reticulum to cytosol transport	BP	1	DERL1,SEC61B,NPLOC4,UBAC2,AUP1,RHBDD1,YOD1,SYVN1,DERL3,TMEM129,SEL1L,BCAP31,UBE2G2,DERL2	grey	VSMC_M10
GO:0042304	0.66	0.49	15	29	regulation of fatty acid biosynthetic process	BP	1	NR1H2,APOC1,PIBF1,TRIB3,CYP7A1,NR1H3,ERLIN1,LPGAT1,MLXIPL,SCAP,ACADL,ACADVL,CD74,FABP5,INSIG2	grey	VSMC_M10
GO:0007189	0.66	0.43	41	80	adenylate cyclase-activating G protein-coupled receptor signaling pathway	BP	1	ADGRG6,ADM2,ADGRF1,S1PR3,ADGRL3,LPAR1,ADCY3,PTGIR,GIPR,ADRA1D,ADRA2B,ADGRG5,RAPGEF2,ADCY7,ADCY2,ADRA2A,PTHLH,GNA13,GPER1,GPR3,ADCY4,UCN2,PTH1R,ADRA2C,PTGER2,CALCRL,RAMP2,GPR161,ADGRE5,RIT2,PTGER4,GNAL,ADGRG4,GNB1,ADGRL2,PTGFR,ADGRE3,ADGRG1,CHGA,GNG2,MRAP2	grey	VSMC_M10
GO:0044782	0.66	0.39	150	288	cilium organization	BP	1	ONECUT2,AVIL,TBC1D30,ATAT1,KIF3A,TBC1D32,UBE2B,ASAP1,LRRC6,MAPK15,IFT74,CFAP69,RFX3,PTPDC1,RAB23,DYNC2LI1,CEP162,TMEM17,WDR92,FUZ,IQUB,ATG5,ARMC2,MAPRE1,ARMC4,INTU,SPATA6,TTC26,BBS7,DRC1,RSPH6A,NECTIN2,GALNT11,FBF1,PIBF1,IFT172,FOXJ1,TTC21A,WDR60,CFAP61,TTLL3,CCDC13,ABCC4,MKKS,CEP290,SPEF1,TMEM80,TTC17,TUB,KIF3B,RILPL1,RILPL2,CEP70,PARVA,CEP250,CCDC66,TCTN3,IFT81,CCDC113,CFAP20,CCDC65,TMEM67,IFT52,RPGRIP1L,PKD2,DNAH1,DNAH5,ZNF423,GBF1,NEURL1,CFAP43,ZMYND10,ARMC9,CFAP46,TTBK2,CLCN4,TTC21B,ALPK1,JHY,DISC1,OFD1,ENO4,RAB17,TMEM237,TRAF3IP1,BBS1,LAMA5,CDKL5,SEPT2,LIMK2,TTC30A,CFAP161,TTC12,TOGARAM1,EHD1,DNAAF2,SPAG16,FAM149B1,HTT,CFAP70,STK36,GORAB,CEP126,SNAP29,EXOC5,TTC25,RAB8A,SDCCAG8,HAP1,DNAAF4,CPLANE2,MNS1,WDR19,TMEM138,ATP6V1D,BBOF1,CNTROB,TMEM107,CCDC151,DYNLL2,MKS1,DNM2,TEKT3,IFT20,SPEF2,CFAP53,HSPB11,C16orf71,RILP,CLUAP1,WRAP73,TPGS1,CDK10,CELSR2,CDC14A,TMEM231,FNBP1L,HYDIN,ODF2L,WDR63,C5orf30,TCTN2,HYLS1,SNX10,C2CD3,CC2D2B,C7orf43,ATXN10,BBS10,PIFO	grey	VSMC_M10
GO:0010155	0.66	0.52	10	19	regulation of proton transport	BP	1	SPHK2,TESC,FAM173B,PM20D1,TCIRG1,COX17,DNAJC30,PARK7,APOPT1,PPIF	grey	VSMC_M10
GO:0000305	0.66	0.51	12	23	response to oxygen radical	BP	1	FANCC,PRDX1,DHFR,ERCC6,GLRX2,FBLN5,NFE2L2,GCH1,SOD3,MPO,NQO1,MIR21	grey	VSMC_M10
GO:0085029	0.66	0.47	20	39	extracellular matrix assembly	BP	1	TNXB,HAS1,PXDN,SOX9,CLASP2,GAS6,FBLN5,DAG1,LAMB3,AGT,PHLDB1,EFEMP2,PLOD3,RAMP2,SMAD3,MYH11,NTNG2,HAS3,LAMB1,RGCC	grey	VSMC_M10
GO:0021872	0.66	0.47	18	35	forebrain generation of neurons	BP	1	UQCRQ,NDNF,SEMA3A,ZSWIM6,DCLK2,RAPGEF2,SLC4A10,SATB2,DISC1,GBX2,DLX1,BCL11B,PLXNA3,LHX8,RHOA,UBB,FOXG1,METTL14	grey	VSMC_M10
GO:0035313	0.66	0.54	8	16	wound healing, spreading of epidermal cells	BP	1	CLASP2,HBEGF,FERMT1,ARHGAP24,RREB1,AJUBA,COL5A1,ACVRL1	grey	VSMC_M10

GO:0050801	0.66	0.37	284	542	ion homeostasis	BP	1	SLC39A5,NEDD4L,LRP1,CYP27B1,ESR1,SLC12A2,PRKN,TSPO,SMDT1,BAK1,MYC,KLHL3,MCHR1,GPR20,PLA2G6,CKB,BTBD9,TMPRSS6,SLC39A4,STEAP2,SLC25A27,SLC1A1,TFAP2B,S1PR3,WNK2,CNNM4,PRKCE,TNNI3,EPAS1,FPR2,IBTK,SLC4A4,LPAR1,PPT1,SLC31A2,EGFR,SFRP4,KEL,ATG5,TRPM4,SLC7A11,GRIN2D,TRPC3,SLC8A2,RAB7A,C5AR2,C5AR1,HEPHL1,ATP7B,PTGIR,GIPR,CACNB2,SLC39A12,ATP6V0E2,LACC1,SLC4A2,STEAP3,AQP11,ATP1A3,SCN5A,ACKR2,SLC24A3,GPD1L,SNX5,C1QTNF1,SLC4A7,TMEM106B,TMTC4,MAFG,RYR1,LETM2,PRNP,ADRA1D,DCN,DIAPH1,P2RX2,KL,CCKBR,HPX,RASA3,NCOA4,MICU3,ATP6V1H,TM9SF4,IL1A,IMMT,C19orf12,SLMAP,P2RX3,PLCE1,SLC9A9,RYR3,TMBIM6,P2RX7,HVCN1,P2RY1,PTK2B,SLC39A13,TPCN1,SLC8B1,VDR,STC1,SLC25A37,TBX5,AP3B1,RMDN3,F2RL1,F2R,SLC40A1,GLS,CUTC,ABCC2,PKD2,CASQ2,MCUR1,SLC12A5,CD40,RYR2,GRM2,SLC41A1,SLC4A10,RAP1GDS1,TRPM1,SPPL3,LCK,KCNJ2,TESMIN,SLC9B1,SLC12A7,TRPV4,BDH2,TMEM9,SLC30A10,TCIRG1,FAM20A,GNA13,ISCU,CMKLR1,MCUB,HJV,SLC9A8,SCN7A,UBASH3B,DISC1,ANK2,GPER1,SLAMF8,RHCG,TRPM8,PDZD8,IFI6,AGT,ATP1A2,SLC9A1,CASQ1,SLC12A8,NTSR1,XPR1,CLIC4,MELTF,FZD9,PPP2R3C,TMCO1,PTH1R,SLC30A4,UNC80,FIS1,CEMIP,ABL2,CPS1,SLC12A9,CDH23,COMT,PTPN6,MICU1,MCU,CXCR2,GNG3,SLC11A1,HTT,PTGDR,PTGER2,SLC9A7,ATP6V0A1,ABCB6,TRPM7,ATP1B1,KCNA1,CD38,MAPK1,DMXL2,KCNMA1,SLC4A3,ATP13A2,FTH1,HAP1,DLG4,PARP1,CYB561A3,WNK3,FGFR4,ATP1B2,FAM155B,PSEN1,CACNA1A,MAPK3,CACNA1G,AC138894.1,ATP2A1,CD19,COX11,SMAD3,CLN6,NOX5,CALM1,SNAPIN,S100A14,S100A8[illegible]	grey	VSMC_M10
GO:0016241	0.66	0.41	66	128	regulation of macroautophagy	BP	1	PIP4K2C,EPM2A,EXOC4,NRBP2,SPTLC1,GAPDH,ATP6V1E2,NOD1,ATP6V1C2,VPS35,SCOC,SESN3,PIP4K2A,ATP6V0E2,OPTN,RALB,ATP6V0B,SNX5,USP36,TMEM173,FEZ2,DCN,ULK1,ATP6V1H,CASP3,RIPK2,NOD2,LZTS1,ATP6V1B2,SEC22B,TMEM39A,USP30,BAG3,RUBCN,SNX6,SNX32,VPS26A,HTT,ATP6V0A1,PINK1,TBC1D25,ATP13A2,PHF23,ATP6V1D,VPS13D,VPS13C,MAPK3,AC138894.1,LARP1,NPC1,SMCR8,CDK5R1,CLEC16A,MTM1,ATP6V0C,IKBKG,GPSM1,GNAI3,KIAA1324,TSC1,SH3GLB1,BNIP3L,IRGM,TIGAR,HSPB8,CAPNS1	grey	VSMC_M10
GO:0051963	0.66	0.43	40	79	regulation of synapse assembly	BP	1	NTRK2,LINGO2,SEMA4D,LRRN3,EPHA7,VPS35,VSTM5,SLITRK5,LRFN1,FARP1,LRFN3,EPHB1,CLSTN2,ROBO2,MEF2C,CUX2,SLIT1,AMIGO2,BDNF,AMIGO3,ADNP,RAB17,EPHB3,LRTM2,NLGN2,SEMA4A,NLGN3,CHRNB2,NTN1,GPRASP2,BHLHB9,ASIC2,SLITRK2,AMIGO1,NTNG2,ADGRL2,GRID2,WNT5A,SIX1,LRRTM2	grey	VSMC_M10
GO:0043488	0.66	0.41	71	137	regulation of mRNA stability	BP	1	PAN2,CNOT2,CNOT6,RIDA,PABPC1,PNLDC1,CSDC2,AGO2,TNRC6B,TUT7,MAPK14,EXOSC3,PNPT1,TUT4,PUM2,TRAF3IP2,FASTK,MLH1,MYD88,TNRC6C,LSM1,PAN3,CNOT1,CELF1,CNOT6L,FTO,TENT5C,TENT4B,RBM24,DIS3L2,AXIN2,MAPKAPK2,GIGYF2,YTHDF2,TRAF5,FASTKD1,ANGEL2,YTHDF1,EIF4ENIF1,YBX3,RC3H1,UPF1,SLC11A1,RBM10,SAMD4A,POLR2G,SCGB1A1,NT5C3B,DCP1B,FXR2,TARDBP,TOB1,TENT5D,TNRC6A,CNOT8,LARP1,ALKBH5,PARN,FMR1,METTL16,PLEKHN1,CIRBP,TRAF2,METTL14,NANOS1,CACNG7,YBX1,ZFP36L2,TENT5A,RC3H2,EXOSC5	grey	VSMC_M10
GO:0019217	0.66	0.44	32	62	regulation of fatty acid metabolic process	BP	1	PPARA,NR1H2,SOX9,APOC1,PRKAG2,SLC22A13,PIBF1,AKT2,PANK2,PDK1,TRIB3,CYP7A1,NR1H3,ERLIN1,LONP2,SIRT4,IRS1,CPT1A,ABCB11,LPGAT1,MLXIPL,SCAP,PDK3,ACADL,TYSND1,ACADVL,NCOR1,CD74,SREBF1,MLYCD,FABP5,INSIG2	grey	VSMC_M10
GO:0035987	0.66	0.47	17	34	endodermal cell differentiation	BP	1	ITGA7,EOMES,MMP15,RTF1,SOX2,LAMB3,COL7A1,ITGB5,SETD2,ITGA4,FN1,COL5A2,LEO1,LAMA3,GATA6,COL5A1,LAMB1	grey	VSMC_M10
GO:0070972	0.66	0.44	31	60	protein localization to endoplasmic reticulum	BP	1	GPAA1,TRAM2,SEC61B,RTN4,SEC61G,BCAP29,RAB10,GRIK5,SEC62,UBAC2,DDRGK1,SRP14,SSR3,SPCS1,RYR2,GBF1,SRPRA,ANK2,MIA3,SEC16B,ZFAND2B,BCAP31,SGTA,RER1,SEC16A,HSPA5,TRAM1L1,PPP1R15A,INSIG2,SPCS2,SEC61A2	grey	VSMC_M10
GO:0033962	0.66	0.52	9	18	P-body assembly	BP	1	PAN2,CNOT2,CNOT6,PAN3,CNOT1,CNOT6L,DDX6,EIF4ENIF1,RC3H1	grey	VSMC_M10
GO:0050853	0.66	0.46	21	41	B cell receptor signaling pathway	BP	1	TEC,PLCL2,NCKAP1L,FOXP1,LPXN,MEF2C,LCK,BANK1,LAT2,BMX,PTPN6,CD38,MAPK1,GPS2,CD19,PRKCB,BTK,VAV3,CMTM3,GCSAM,SH2B2	grey	VSMC_M10
GO:0046390	0.66	0.40	82	156	ribose phosphate biosynthetic process	BP	1	NME1,ATP5MGL,PPT2,PPARA,ACSL6,IMPDH1,SLC35B2,SLC25A13,PPAT,PPCS,PPT1,DLD,SPHK2,CMPK1,ADCY3,PRKAG2,ATP5F1C,ELOVL1,PANK2,PDK1,PDHX,ELOVL7,PANK1,AMPD3,ATP5F1A,GUCY1A1,GUCY1B1,DGUOK,GMPS,AMPD1,ADCY7,FAM173B,ADCY2,NME4,ACSL5,PRPS2,HACD2,ADCY4,CTPS2,DNAJC30,PDK3,SLC25A16,ACSBG1,ATP5S,PYGL,COASY,PINK1,STAT3,NME7,ADK,MPC2,ADCY10,PARP1,TECR,SLC26A1,GUCY2D,PKLR,PANK3,GCDH,UPRT,ACACA,SLC26A2,PRPSAP2,ADSSL1,ACSBG2,ATP6V0C,PANK4,NME3,ATP5PB,VPS9D1,ACSF3,UCK1,MLYCD,DHODH,MT-ATP8,MT-ATP6,ATP5ME,ATP5MF,ELOVL3,NME6,NUDT2,UPP1	grey	VSMC_M10
GO:1903363	0.66	0.44	31	60	negative regulation of cellular protein catabolic process	BP	1	TRIM39,RGP1,PDCL3,SDCBP,AQP11,KLHL40,DDRGK1,RYBP,PSMF1,TAF9,USP25,CSNK2A2,FAM192A,MARCH7,SUFU,RPL23,USP19,RPL11,ATP13A2,EPHA4,ANXA2,PSEN1,EFNA1,TAF1,OGT,SGTA,PARK7,DAB2IP,CAMLG,STYX,MAP1A	grey	VSMC_M10

GO:0002683	0.66	0.38	152	290	negative regulation of immune system process	BP	1	NME1,TNFAIP3,USP15,ENPP3,HLA-DOB,TRIB1,MYC,TOB2,C6orf106,MAPK14,FBXO7,CDK6,TNFRSF21,HOXA7,PELI1,NR1H2,IL4I1,TLR4,C5AR2,CST7,SOX9,CD300LF,PIBF1,FOXJ1,SEC14L1,SIRT2,NMI,NPLOC4,PAWR,PLCB1,PLCL2,PRNP,NCKAP1L,PRKDC,PCBP2,PLK2,HCK,ZC3H8,FER,PIK3R1,LPXN,CASP3,FBXW7,PTPRJ,TMEM131L,TMBIM6,PAG1,SLA2,NR1H3,TRAFD1,OAS3,NLRC5,MARCH7,MAPKBP1,ADTRP,NOD2,CD96,CD200,BANK1,TGFB2,BTN2A2,GPAM,PRKAR1A,GPR137B,PIAS3,HLX,TCTA,TBC1D10C,YTHDF2,UBASH3B,GPER1,SLAMF8,PARP14,RNF26,FGR,NLRX1,TRAF3IP1,HTRA1,RUNX3,ZBTB46,PDCD1,DLG1,APOD,MIA3,DUSP10,BCL6,VSIR,RC3H1,PTPN6,GPR137,FBN1,TNFSF18,LAG3,COL3A1,PPP3CB,MUL1,TAPBPL,INHA,PADI2,SCGB1A1,FOXP3,GPS2,TKFC,USP18,TBX21,SYT11,DCST1,ZBTB7B,IL4R,TSPAN6,LDLR,GPR68,BTK,STAT2,CD74,RIOK3,CCL2,CCL25,YES1,PTGER4,TWSG1,IL7R,PTPN2,SOCS1,CRK,FOXF1,GLMN,MIR21,MIR222,MIR125B1,MIR24-1,MIR27A,FSTL3,OAS1,LST1,MAFB,FADD,DPP4,CD46,A2M,MIR125A,THY1,DUSP1,TSC22D3,FGL2,SERPINB9,GCSAM,LAX1,CEBPB,HAVCR2,IL17D,RC3H2	grey	VSMC_M10
GO:0051568	0.66	0.45	26	51	histone H3-K4 methylation	BP	1	SNW1,PAXIP1,DPY30,ASH2L,DNMT3B,KMT2D,RTF1,WDR82,ZNF335,RBBP5,AUTS2,BCOR,MEN1,WDR61,NELFA,DYDC1,SETD1A,PAGR1,OGT,SETD3,CXXC1,GFI1,PRMT6,ARID4A,BEND3,NNMT	grey	VSMC_M10
GO:0009219	0.66	0.51	11	21	pyrimidine deoxyribonucleotide metabolic process	BP	1	TBPL1,DPYS,MBD4,DTYMK,UPB1,NEIL1,NT5M,NTHL1,CMPK2,DCTD,UPP1	grey	VSMC_M10
GO:0019229	0.66	0.45	24	47	regulation of vasoconstriction	BP	1	ZDHHC21,EGFR,TRPM4,ADRA1D,ADRA2B,DOCK5,F2R,CHRM3,ADRA2A,AGT,ACE2,ATP1A2,PER2,ADRA2C,ECE1,SMTNL2,CD38,HRH2,ASIC2,P2RX1,MIR21,RHOA,FGA,KCNMB4	grey	VSMC_M10
GO:0021954	0.66	0.44	31	61	central nervous system neuron development	BP	1	UQCRQ,AGTPBP1,PLXNA4,NTRK2,NDNF,GLI2,MYCBP2,TSKU,SZT2,RAC3,SEMA3A,EPHB1,DCLK2,RAPGEF2,NR4A2,SLC4A10,ADARB1,DISC1,GBX2,EPHB3,MAPT,NIN,EPHA4,MPP5,NDEL1,CHRNB2,PLXNA3,LHX8,UBB,CNTN2,FOXG1	grey	VSMC_M10
GO:0046677	0.66	0.46	21	41	response to antibiotic	BP	1	CYB5R4,PPP2CB,ZC3H8,CASP3,MEF2C,SRD5A1,RPL23,HYAL1,HYAL3,CPT1A,CBL,SLC9A1,CDKN1B,PLA2G4A,ALPL,KDM6B,KAT7,JAK1,CIITA,HSPA5,CASP8	grey	VSMC_M10
GO:0032211	0.66	0.52	9	18	negative regulation of telomere maintenance via telomerase	BP	1	POT1,TENT4B,STN1,GNL3L,CTC1,PIF1,TERF2,TINF2,TEN1	grey	VSMC_M10
GO:0003223	0.66	0.61	4	8	ventricular compact myocardium morphogenesis	BP	1	POU4F1,DSP,BMPR1A,NOG	grey	VSMC_M10
GO:0051445	0.66	0.47	18	35	regulation of meiotic cell cycle	BP	1	YTHDC2,FBXO43,UBE2B,MAPK15,TOPAZ1,SIRT2,PLCB1,MOS,NPM2,PRKAR1A,GPR3,OVOL1,WNT4,FZR1,OOEP,WNT5A,DUSP1,LIF	grey	VSMC_M10
GO:0006968	0.66	0.48	14	27	cellular defense response	BP	1	RAB23,C5AR1,PTK2B,TCIRG1,NCF1,FCMR,NCF2,CXCR2,VEZF1,UMOD,ADORA2B,BCL10,KLRG1,IL33	grey	VSMC_M10
GO:0099518	0.66	0.43	33	65	vesicle cytoskeletal trafficking	BP	1	MYO1A,AP3S1,KIF5A,KIF3A,MYO7B,MYO7A,AP3M2,KIF3B,RASGRP1,AP3B1,DTNBP1,KIF13A,BICDL1,CNIH2,TANC2,BLOC1S6,HTT,AP3M1,KIF1C,HAP1,TRIM46,NDEL1,AC138894.1,SNAPIN,MYO1D,MYO1F,MYO5B,BICDL2,PRKCZ,FNBP1L,BLOC1S3,BORCS5,AP3S2	grey	VSMC_M10
GO:0045602	0.67	0.61	4	8	negative regulation of endothelial cell differentiation	BP	1	S1PR3,JAG1,ACVRL1,FOXJ2	grey	VSMC_M10
GO:0050994	0.67	0.46	20	39	regulation of lipid catabolic process	BP	1	PPARA,PRKCE,FGF21,ABHD5,APOC1,ALK,AKT2,LONP2,GPLD1,IRS1,CPT1A,ADRA2A,SORL1,ABCB11,TYSND1,DAGLB,THRA,LDLR,MLYCD,BSCL2	grey	VSMC_M10
GO:0034314	0.67	0.47	17	33	Arp2/3 complex-mediated actin nucleation	BP	1	ARPC3,PICK1,WASF1,FCHSD2,ARFIP2,HIP1R,IQGAP2,CYFIP1,MAGEL2,AP1AR,WASF2,WHAMM,NCKAP1,ARPC1A,WAS,WDR63,GMFG	grey	VSMC_M10
GO:1903859	0.67	0.51	10	20	regulation of dendrite extension	BP	1	NEDD4L,RIMS2,PRKN,CPNE5,RIMS1,CPNE9,SYT1,SLC23A2,MUL1,CACNG7	grey	VSMC_M10
GO:0010657	0.67	0.43	31	60	muscle cell apoptotic process	BP	1	MAP3K5,PPP1R10,RGL2,HSF1,CDKN2A,ARRB1,PAX8,HAND2,PTK2B,STK4,SIRT5,SIRT4,PDCD4,AGT,BAG3,NFE2L2,IL12A,CXCR2,RBM10,MFN2,CAMK2A,GNB1,PDPK1,MIR199A1,MIR21,MIR24-1,NOL3,TIGAR,C3orf58,NKX2-5,HEY2	grey	VSMC_M10
GO:0007626	0.67	0.40	68	132	locomotory behavior	BP	1	PRKN,AGTPBP1,BTBD9,CHRNA3,GPR37,ADAM22,APBA1,SLC1A1,TMOD1,PRKCE,PPT1,SOBP,NTF4,FIG4,GRIN2D,B4GALT2,ALK,DDHD2,NRG1,LGI4,PREX2,NCOA2,HTRA2,NR4A2,SLC4A10,SLC6A3,ALDH1A3,GIGYF2,OPRD1,CIART,USP2,ATP1A2,NTSR1,EPS8,SPG11,C1QL1,ZDHHC8,CDH23,ANKH,TNR,PPP3CB,EPHA4,DLG4,NLGN2,CLN8,HOXB8,CHRNB2,CLN6,NCOR1,NPC1,SEZ6,DAB1,ABAT,MECP2,PARK7,TSC1,ZFHX3,MCOLN3,CNTN2,DPP4,CEND1,FXN,CRBN,GPR88,APH1B,EFNB3,PITX3,RASD2	grey	VSMC_M10

GO:0007005	0.67	0.37	232	436	mitochondrion organization	BP	1	TIMM21,ATG12,HEBP2,ATP23,LYRM7,PRKN,TSPO,HSPA4,NDUFA6,BAK1,AGTPBP1,TOMM22,PLA2G6,RAC2,FBXO7,FANCG,TMEM14A,COA5,CAMKMT,SDHAF4,EPAS1,PNPT1,GGCT,MYH14,VPS35,ATG5,MGARP,WASF1,SSBP1,AGK,CHCHD6,NDUFB2,HIGD1A,ATPAF1,PHB,NDUFB6,TMEM126B,NECTIN2,DNAJC15,OPTN,NDUFC2,DMAC2,PRDX3,PITRM1,TIMM50,MGME1,USP36,SIRT7,NDUFS8,NDUFAF7,DDHD2,BAG4,DCN,PANK2,NDUFA12,ULK1,PPP2CB,COX7A1,MIPEP,NDUFAF2,ARMC1,CEBPA,IMMP1L,IMMT,PRIMPOL,HIP1R,UQCC1,FBXW7,HTRA2,NDUFS3,CSNK2A2,MTX3,OPA1,AP3B1,NDUFAF6,COL4A3BP,TOMM34,IFIT2,SIRT5,SIRT4,FOXRED1,DNM1L,TERT,CISD2,ZDHHC6,COX17,OXA1L,USP30,NDUFB4,MFN1,RCC1L,POLG2,HPS4,GPER1,ARIH2,IFI6,NDUFB3,BAG3,SLC9A1,CIDEB,SFN,TFAM,MTFP1,NDUFS2,TOMM40L,THEM4,STPG1,HMGCL,NUBPL,FZD9,PISD,FZD5,MAPT,STOX1,FIS1,SUPV3L1,NMT1,ATG2A,GRPEL1,C11orf65,FUNDC1,MCU,HTT,COA3,NDUFB11,DDHD1,BCS1L,RAB3A,MARK2,PINK1,MUL1,ATG9A,STAT3,NDUFA9,BHLHA15,TIMM17B,ATP13A2,HAP1,WIPI2,PARP1,HSD17B10,TMEM102,CLN8,VPS13D,MFN2,NPEPPS,VPS13C,MSTO1,PIF1,HAX1,ADCK1,ATP2A1,THG1L,CALM1,TIMM29,NDUFAB1,STAT2,ATG2B,COX10,PSMD10,CAMK2A,MIEF2,LIG3,ATPAF2,SREBF1,MAIP1,RALBP1,LONP1,C19orf70,JUN,ACAA2,MTM1,DNAJA3,PAM16,BCAP31,ATAD3A,CLUH,NDUFB10,FUNDC2,PARK7,SLC25A33,MIGA2,MFSD14A,NAIF1,DHODH,SH3GLB1,MIR29A,MT-ND1,MT-ND2,MT-ND6,MICALL2,PRMT6,MLLT11,NOL3,MINOS1,BNIP3L,NDUFA5,NDUFC1,COX16,NDUFAF8,COX7A2,TIGAR,TIMM10B,TIMM23B,DMAC1,FXN,NDUFA3,NDUFB5,N[illegible]	grey	VSMC_M10
GO:0007193	0.67	0.45	23	45	adenylate cyclase-inhibiting G protein-coupled receptor signaling pathway	BP	1	MCHR1,GRM4,GRM8,GPR37,S1PR3,OXER1,FPR2,LPAR1,CHRM5,P2RY1,NPY1R,CHRM4,CHRM3,GRM2,RGS1,ADRA2A,OPRD1,CHRM1,HRH4,PALM,GNAI3,HTR1D,GPR37L1	grey	VSMC_M10
GO:0021692	0.67	0.53	8	16	cerebellar Purkinje cell layer morphogenesis	BP	1	AGTPBP1,WHRN,FAIM2,LDB1,TTC21B,SPTBN2,LHX1,CEND1	grey	VSMC_M10
GO:2000378	0.67	0.46	19	37	negative regulation of reactive oxygen species metabolic process	BP	1	PRKN,PPARA,ATG5,MYCN,SIRT2,SIRT3,TFAP2A,PAX2,SIRT5,SLC30A10,PINK1,MMP3,STAT3,PARK7,MIR21,RHOA,TIGAR,APOPT1,AC140504.1	grey	VSMC_M10
GO:0051341	0.67	0.42	41	79	regulation of oxidoreductase activity	BP	1	CYP27B1,PRKN,ZDHHC21,SDHAF4,NOD1,EGFR,NOSIP,SPHK2,DHFR,SZT2,ACVR2A,SIRT3,IL1A,PIK3CB,PTK2B,VDR,NOD2,SIRT4,TERT,COX17,OXA1L,KRAS,SLAMF8,AGT,NOS1AP,POR,ABL2,GCH1,ECSIT,CALM1,GLA,PARK7,GNAI3,CYBA,GFI1,TERF2,MIR21,MIR27B,FXN,FCER2,APOPT1	grey	VSMC_M10
GO:0006888	0.67	0.40	55	106	endoplasmic reticulum to Golgi vesicle-mediated transport	BP	1	GOPC,MAPK15,NRBP2,COPG2,STX17,TRAPPC12,TRAPPC3,BCAP29,COPG1,SEC22C,ANK1,YIPF4,TRAPPC11,SEC31A,SEC31B,GBF1,KLHL12,SEC22B,PGAP1,ERGIC2,TRIP11,PEF1,VTI1A,PDCD6,SORL1,TRAPPC2,SEC22A,HYOU1,SEC23IP,TRAPPC4,COPA,RAB1B,WHAMM,SCAP,MIA3,TRAPPC6B,CTAGE9,SEC16B,SEC24C,STX5,STX18,CNIH4,TMED10,LMAN1L,CXorf56,PPP6C,MPPE1,BCAP31,SEC16A,TMED5,YIF1A,TBC1D20,TMED3,TRAPPC6A,TRAPPC3L	grey	VSMC_M10
GO:0006895	0.67	0.52	8	16	Golgi to endosome transport	BP	1	EPS15,DOP1B,GBF1,AP1AR,KLHL20,AP4M1,VPS13C,CORO7	grey	VSMC_M10
GO:0035372	0.67	0.51	9	18	protein localization to microtubule	BP	1	ABHD17B,MAPRE1,MAPRE3,DIAPH1,CHAMP1,TTBK2,ABHD17A,ABHD17C,MAP1A	grey	VSMC_M10
GO:0031646	0.67	0.48	13	26	positive regulation of nervous system process	BP	1	RIMS2,SOX10,RIMS1,CST7,P2RX3,FAM173B,RNF10,DAG1,NTSR1,TNR,NLGN2,TNFRSF1B,ABAT	grey	VSMC_M10
GO:0048644	0.67	0.43	31	61	muscle organ morphogenesis	BP	1	LY6E,FOXH1,TNNI3,POU4F1,NRG1,TCF15,TNNC1,DSP,RYR2,PKP2,TNNI1,TGFB2,ADARB1,MYH7,ACTC1,EFEMP2,TTN,BMPR1A,TCAP,RBPJ,MED1,GSC,ISL1,ENG,CNTF,NOG,WNT5A,LIF,NKX2-5,HEY2,WNT2	grey	VSMC_M10
GO:0010770	0.67	0.42	33	65	positive regulation of cell morphogenesis involved in differentiation	BP	1	RAB21,UNC13D,SS18L2,RAC3,ARHGEF7,PRKDC,ANKRD27,CUX2,TIAM1,DOCK5,OPA1,RREB1,ITPKA,DMTN,DNM1L,CARMIL1,CAPRIN2,CASS4,CIB1,SS18L1,CDKL5,DOCK1,CSPG5,OBSL1,EPHA4,DNM2,PARP6,GPRASP2,BHLHB9,LRP8,CRK,FBXO31,FGA	grey	VSMC_M10
GO:1904357	0.67	0.49	12	24	negative regulation of telomere maintenance via telomere lengthening	BP	1	POT1,TENT4B,STN1,PARP1,GNL3L,CTC1,PIF1,ERCC4,SLX4,TERF2,TINF2,TEN1	grey	VSMC_M10
GO:0051639	0.67	0.55	6	12	actin filament network formation	BP	1	LCP1,PLS1,CARMIL1,SPIRE2,CARMIL2,FHOD1	grey	VSMC_M10
GO:0061097	0.67	0.42	38	74	regulation of protein tyrosine kinase activity	BP	1	ERBB3,NCK2,IBTK,EPGN,AREG,TGFA,ABI1,HBEGF,PRNP,NRG1,GAS6,ERCC6,FBXW7,NEURL1,ADRA2A,NCF1,EGF,CASS4,AGT,CBL,SNX6,DOK7,ZFYVE28,SOCS4,EPHA4,DLG4,DVL2,PSEN1,EFNA1,CCL5,CSF1R,UNC119,PTPN2,LRP8,THY1,DUSP22,EREG,RAP2B	grey	VSMC_M10
GO:0030326	0.67	0.40	49	96	embryonic limb morphogenesis	BP	1	OSR2,FZD6,TBC1D32,PITX1,PTCH1,ROR2,DLX5,HOXA13,MYCN,OSR1,INTU,HDAC2,GLI2,MEGF8,LMBR1,RARB,FREM2,HAND2,FRAS1,TBX5,IFT52,RPGRIP1L,TFAP2A,SALL1,FBXW4,HDAC1,TGFB2,SP9,WNT3,ECE1,TULP3,BMPR1A,WDR19,MED1,ALDH1A2,PSEN1,TMEM107,MKS1,TBX4,TBX2,GPC3,CREBBP,SKI,ALX3,TMEM231,NOG,WNT5A,C2CD3,BCL2L11	grey	VSMC_M10
GO:0035113	0.67	0.40	49	96	embryonic appendage morphogenesis	BP	1	OSR2,FZD6,TBC1D32,PITX1,PTCH1,ROR2,DLX5,HOXA13,MYCN,OSR1,INTU,HDAC2,GLI2,MEGF8,LMBR1,RARB,FREM2,HAND2,FRAS1,TBX5,IFT52,RPGRIP1L,TFAP2A,SALL1,FBXW4,HDAC1,TGFB2,SP9,WNT3,ECE1,TULP3,BMPR1A,WDR19,MED1,ALDH1A2,PSEN1,TMEM107,MKS1,TBX4,TBX2,GPC3,CREBBP,SKI,ALX3,TMEM231,NOG,WNT5A,C2CD3,BCL2L11	grey	VSMC_M10

GO:0002460	0.67	0.38	90	173	adaptive immune response based on somatic recombination of immune receptors built from immunoglobulin superfamily domains	BP	1	CD226,STX7,TNFAIP3,AGER,HLA-DRA,HLA-DOB,HLA-DMA,HLA-DPB1,EXOSC3,MSH2,BACH2,RSAD2,IL4I1,TLR4,TRPM4,TRAF3IP2,HMCES,PHB,ERCC1,NECTIN2,PVR,MLH1,UNC13D,MYD88,SLC22A13,FOXJ1,PAXIP1,JAG1,SHLD1,NCKAP1L,HPX,IRF7,TRAF6,GAPT,MEF2C,SLA2,RIPK2,NBN,NOD2,CD40,NFKB2,TCIRG1,HLX,DENND1B,CD28,FCER1G,RNF168,FZD5,PLA2G4A,BCL6,CTSH,IL12A,RC3H1,PTPN6,TNFSF18,SLC11A1,PPP3CB,STAT3,WAS,FOXP3,ZBTB1,SHLD2,TNFRSF1B,TBX21,SEMA4A,BATF,ZBTB7B,IL6R,CD19,IL4R,BTK,CD74,FBXO38,C7,C3,IL7R,SUPT6H,PRKCZ,TRAF2,BCL10,MIR21,FADD,CD46,FCER2,RNF8,CD70,HRAS,TNFSF13B,HAVCR2,RC3H2	grey	VSMC_M10
GO:0002377	0.67	0.41	42	81	immunoglobulin production	BP	1	HLA-DRA,HLA-DOB,HLA-DMA,IL5,HLA-DPB1,EXOSC3,MSH2,CYREN,TRAF3IP2,HMCES,PHB,ERCC1,MLH1,PAXIP1,SHLD1,HPX,PRKDC,TRAF6,GAPT,GPI,TMBIM6,XRCC4,NBN,POLL,CD40,CD28,RNF168,BCL6,PMS2,FOXP3,SHLD2,TBX21,STX4,BATF,IL4R,BTK,YY1,SUPT6H,TRAF2,TRDV3,RNF8,NHEJ1	grey	VSMC_M10
GO:0031445	0.67	0.53	7	14	regulation of heterochromatin assembly	BP	1	SETDB2,FAM208A,PPHLN1,TET1,PHF8,SETDB1,ZNF304	grey	VSMC_M10
GO:0120261	0.67	0.53	7	14	regulation of heterochromatin organization	BP	1	SETDB2,FAM208A,PPHLN1,TET1,PHF8,SETDB1,ZNF304	grey	VSMC_M10
GO:0042445	0.67	0.39	69	133	hormone metabolic process	BP	1	RDH5,RDH16,HSD17B4,ALDH8A1,CYP27B1,ESR1,CYP21A2,TSPO,HSD17B8,HSD17B3,AKR1B1,ALDH1A1,SRD5A3,RDH13,IL4I1,DDO,LIPE,AANAT,SDR16C5,PAX8,IDE,RETSAT,DKK3,PCSK1,SCG5,MME,SPP1,PRMT3,SRD5A1,ALDH1A3,TCF7L2,ENPEP,DHRS9,ACE2,ARNT,SLCO1C1,ECE2,PCSK7,HSD17B7,POR,DUOX1,BCO2,FDX1,COMT,GNB3,WNT4,ECE1,STAT5B,HSD17B10,DHRS3,MED1,RDH11,ALDH1A2,DGKQ,SCPEP1,SCNN1B,CRYM,CYP46A1,DHRS11,GHR,CACNA1H,GFI1,DHRS2,PNPLA4,DIO3,LRAT,RDH14,ADH4,DIO2	grey	VSMC_M10
GO:0045739	0.68	0.42	34	66	positive regulation of DNA repair	BP	1	EYA4,MRNIP,EGFR,XRCC1,WDR48,FAM168A,SHLD1,PRKDC,ERCC6,EYA1,SLF1,ABRAXAS1,SPIDR,SLF2,EYA3,PARP9,DTX3L,RNF168,MGMT,ZCWPW1,WAS,DDX11,PARP1,SHLD2,UIMC1,WRAP53,SMCHD1,SPIRE2,OOEP,TIGAR,RNF8,UBE2N,BRCC3,CBX8	grey	VSMC_M10
GO:0021675	0.68	0.42	30	59	nerve development	BP	1	ERBB3,ATP8B1,PLXNA4,NTF4,POU4F1,SEMA3A,EPHB1,RET,TFAP2A,PAX2,SALL1,BDNF,SEMA3F,SULF2,TCIRG1,ADARB1,DAG1,LRIG2,ITGA4,CTSV,NGFR,CHRNB2,RNF165,ISL1,PLXNA3,MAFB,HOXB3,SIX1,TMEM126A,HOXA3	grey	VSMC_M10
GO:0002327	0.68	0.60	4	8	immature B cell differentiation	BP	1	KIT,TRAF3IP2,SYVN1,LRRC8A	grey	VSMC_M10
GO:1901524	0.68	0.60	4	8	regulation of mitophagy	BP	1	USP30,VPS13D,VPS13C,TIGAR	grey	VSMC_M10
GO:0060384	0.68	0.50	10	20	innervation	BP	1	NTF4,POU4F1,SEMA3A,RET,SULF2,ADARB1,LRIG2,ITGA4,RNF165,ISL1	grey	VSMC_M10
GO:0009267	0.68	0.39	67	130	cellular response to starvation	BP	1	MARS,MAP3K5,PPARA,ATF4,PICK1,TFEB,SLC39A4,NUAK1,BRSK1,ALB,RRAGD,VPS41,ATG5,FOXO3,KPTN,SESN3,FOXA3,DNAJC15,RALB,PRKAG2,SZT2,RPTOR,EIF2AK2,WRN,GAS6,LARS,PRKAG1,EIF2AK4,SLC38A2,NUAK2,NUPR2,SRD5A1,SIK1,SLC38A3,HSPA8,MTMR3,XPR1,TBL2,GLUL,NFE2L2,SIK2,COMT,WNT4,PRKAG3,CTSV,MAPK1,BHLHA15,WIPI2,MAX,RRAGB,ZFYVE1,MAPK3,TNRC6A,SREBF1,LAMP2,NIM1K,STK26,JUN,CLEC16A,KIAA1324,WDR59,HSPA5,SH3GLB1,RIPOR1,IMPACT,UPP1,RNF152	grey	VSMC_M10
GO:0098911	0.68	0.55	6	12	regulation of ventricular cardiac muscle cell action potential	BP	1	TRPM4,DSP,RYR2,PKP2,DLG1,DSG2	grey	VSMC_M10
GO:0006346	0.68	0.52	8	16	DNA methylation-dependent heterochromatin assembly	BP	1	DNMT3A,SETDB2,FAM208A,PPHLN1,HDAC1,TET1,SETDB1,ZNF304	grey	VSMC_M10
GO:0042634	0.68	0.49	10	20	regulation of hair cycle	BP	1	SMO,TSKU,FERMT1,ARNTL,TERT,TGFB2,NGFR,TRPV3,EPS8L3,WNT5A	grey	VSMC_M10
GO:2000737	0.68	0.50	9	18	negative regulation of stem cell differentiation	BP	1	JAG1,TCF15,PRICKLE1,YTHDF2,NFE2L2,STAT3,CDK12,ESRRB,ZFP36L2	grey	VSMC_M10
GO:0072396	0.68	0.59	4	8	response to cell cycle checkpoint signaling	BP	1	FBH1,EME1,SLX4,TIGAR	grey	VSMC_M10
GO:0072402	0.68	0.59	4	8	response to DNA integrity checkpoint signaling	BP	1	FBH1,EME1,SLX4,TIGAR	grey	VSMC_M10
GO:0072423	0.68	0.59	4	8	response to DNA damage checkpoint signaling	BP	1	FBH1,EME1,SLX4,TIGAR	grey	VSMC_M10
GO:0003163	0.68	0.59	4	8	sinoatrial node development	BP	1	BVES,CACNA1G,ISL1,HCN4	grey	VSMC_M10
GO:0007019	0.68	0.44	20	40	microtubule depolymerization	BP	1	CLASP2,TRIM54,NCKAP5,NAV3,SPEF1,ATXN7,NCKAP5L,KATNB1,STMND1,TRPV4,TTBK2,CIB1,CAMSAP3,TAOK1,APC2,CAMSAP1,CCSAP,APC,MAP1A,MAP6D1	grey	VSMC_M10
GO:0098815	0.68	0.45	17	34	modulation of excitatory postsynaptic potential	BP	1	RIMS2,RIMS1,SHANK1,MTMR2,SLC8A2,CUX2,PTK2B,IGSF11,SSH1,STX1A,TMEM25,DLG4,NLGN2,STX1B,NLGN3,PRKCZ,RGS4	grey	VSMC_M10
GO:0021756	0.68	0.53	7	14	striatum development	BP	1	FOXP2,SLC7A11,MKKS,RARB,ZSWIM6,ALDH1A3,BCL11B	grey	VSMC_M10
GO:0036289	0.68	0.59	4	8	peptidyl-serine autophosphorylation	BP	1	RIPK1,PIKFYVE,PINK1,MARK3	grey	VSMC_M10
GO:0007628	0.68	0.49	10	20	adult walking behavior	BP	1	AGTPBP1,HTRA2,EPHA4,CLN8,NPC1,DAB1,CNTN2,CEND1,FXN,EFNB3	grey	VSMC_M10
GO:0031280	0.68	0.54	6	12	negative regulation of cyclase activity	BP	1	GABBR2,ADGRV1,GRM2,PALM,GNAI3,PDZD3	grey	VSMC_M10
GO:0061912	0.68	0.42	32	63	selective autophagy	BP	1	PRKN,KLHL3,SPTLC1,NOD1,ATG5,RAB7A,OPTN,TMEM173,DDRGK1,ULK1,HTRA2,AUP1,RIPK2,NOD2,TEX264,USP30,ATG16L1,BAG3,UFC1,ATG2A,HTT,RETREG3,PINK1,WIPI2,VPS13D,MFN2,VPS13C,MAPK3,ATG2B,PJVK,TIGAR,HSPB8	grey	VSMC_M10
GO:0071313	0.68	0.59	4	8	cellular response to caffeine	BP	1	RYR1,RYR3,CASQ2,RYR2	grey	VSMC_M10
GO:0007398	0.68	0.51	8	16	ectoderm development	BP	1	VPS52,STX2,FZD7,GRHL3,BMPR1A,ITGAM,ZBTB7B,LHX1	grey	VSMC_M10
GO:0071498	0.68	0.50	9	18	cellular response to fluid shear stress	BP	1	MTSS1,MEF2C,PTK2B,PKD2,NFE2L2,MAP2K5,MAPK7,ASS1,CA2	grey	VSMC_M10
GO:0051785	0.68	0.44	21	41	positive regulation of nuclear division	BP	1	UBE2B,PHIP,HOXA13,EPGN,TGFA,SIRT2,PLCB1,IL1A,ANAPC5,ANAPC7,NPM2,IGF2,EGF,CD28,WNT4,ANAPC4,INSR,OOEP,WNT5A,RGCC,EREG	grey	VSMC_M10
GO:0060788	0.68	0.56	5	10	ectodermal placode formation	BP	1	FRS2,HDAC2,HDAC1,TBX2,SIX1	grey	VSMC_M10
GO:0071697	0.68	0.56	5	10	ectodermal placode morphogenesis	BP	1	FRS2,HDAC2,HDAC1,TBX2,SIX1	grey	VSMC_M10
GO:0010501	0.68	0.59	4	8	RNA secondary structure unwinding	BP	1	AGO2,AGO1,DDX3X,AGO4	grey	VSMC_M10

GO:0006383	0.68	0.44	20	39	transcription by RNA polymerase III	BP	1	SNAPC3,GTF3C6,CHD8,RPTOR,BRF2,ZC3H8,ZNF143,CEBPA,POLR3D,GTF3C3,CRCP,POLR3C,IVNS1ABP,ELL,ICE2,SNAPC5,SNAPC4,POLR3K,GTF3C4,POLR2K	grey	VSMC_M10
GO:0032026	0.68	0.52	7	14	response to magnesium ion	BP	1	TNFRSF11B,ENTPD6,RYR3,SLC41A1,CCND1,KCNA1,SLFN14	grey	VSMC_M10
GO:0030834	0.68	0.43	23	46	regulation of actin filament depolymerization	BP	1	AVIL,LMOD2,KIAA1211,TMOD1,PLEKHH2,SPTBN1,PLEK,ADD2,LMOD3,LIMA1,F2RL1,DMTN,CARMIL1,LMOD1,ADD3,SPTBN2,TMOD4,EPS8,CAPZA1,TMOD2,SPTB,CARMIL2,PDXP	grey	VSMC_M10
GO:1903426	0.68	0.44	19	37	regulation of reactive oxygen species biosynthetic process	BP	1	PPARA,TLR4,UCP1,FOXO3,SPHK2,WISP3,SLC30A10,HDAC4,PIKFYVE,STAT3,PARK7,SLC25A33,CYBA,LCN2,MIR21,MIR24-1,RHOA,APOPT1,AC140504.1	grey	VSMC_M10
GO:0003215	0.68	0.50	9	18	cardiac right ventricle morphogenesis	BP	1	FOXH1,JAG1,HAND2,TGFB2,BMPR1A,ISL1,SOX4,NKX2-5,HEY2	grey	VSMC_M10
GO:0051350	0.68	0.52	7	14	negative regulation of lyase activity	BP	1	GABBR2,ADGRV1,GRM2,MTRR,PALM,GNAI3,PDZD3	grey	VSMC_M10
GO:0090309	0.68	0.56	5	10	positive regulation of DNA methylation-dependent heterochromatin assembly	BP	1	SETDB2,FAM208A,PPHLN1,SETDB1,ZNF304	grey	VSMC_M10
GO:2000310	0.68	0.50	9	18	regulation of NMDA receptor activity	BP	1	MAPK8IP2,MEF2C,PTK2B,IFNGR2,CNIH2,PINK1,DLG4,CCL2,CRH	grey	VSMC_M10
GO:1902287	0.68	0.54	6	12	semaphorin-plexin signaling pathway involved in axon guidance	BP	1	PLXNA4,PLXNA1,PLXNC1,SEMA3A,SEMA3F,PLXNA3	grey	VSMC_M10
GO:0097484	0.68	0.46	15	30	dendrite extension	BP	1	NEDD4L,RIMS2,PRKN,CPNE5,RIMS1,WASF1,CPNE9,SYT1,SLC23A2,CYFIP1,AUTS2,MUL1,SLC9A6,CACNG7,LLPH	grey	VSMC_M10
GO:0055080	0.68	0.35	258	494	cation homeostasis	BP	1	SLC39A5,NEDD4L,LRP1,CYP27B1,ESR1,SLC12A2,PRKN,SMDT1,BAK1,MYC,MCHR1,GPR20,PLA2G6,BTBD9,TMPRSS6,SLC39A4,STEAP2,SLC25A27,SLC1A1,TFAP2B,S1PR3,CNNM4,PRKCE,TNNI3,EPAS1,FPR2,IBTK,SLC4A4,LPAR1,PPT1,SLC31A2,EGFR,KEL,ATG5,TRPM4,GRIN2D,TRPC3,SLC8A2,RAB7A,C5AR2,C5AR1,HEPHL1,ATP7B,PTGIR,GIPR,CACNB2,SLC39A12,ATP6V0E2,LACC1,SLC4A2,STEAP3,AQP11,ATP1A3,ACKR2,SLC24A3,SNX5,C1QTNF1,SLC4A7,TMEM106B,TMTC4,MAFG,RYR1,LETM2,PRNP,ADRA1D,DIAPH1,P2RX2,KL,CCKBR,HPX,RASA3,NCOA4,MICU3,ATP6V1H,TM9SF4,IL1A,IMMT,C19orf12,P2RX3,PLCE1,SLC9A9,RYR3,TMBIM6,P2RX7,HVCN1,P2RY1,PTK2B,SLC39A13,TPCN1,SLC8B1,VDR,STC1,SLC25A37,AP3B1,RMDN3,F2RL1,F2R,SLC40A1,CUTC,PKD2,CASQ2,MCUR1,SLC12A5,CD40,RYR2,SLC41A1,SLC4A10,RAP1GDS1,TRPM1,SPPL3,LCK,KCNJ2,TESMIN,SLC9B1,SLC12A7,TRPV4,BDH2,TMEM9,SLC30A10,TCIRG1,FAM20A,GNA13,ISCU,CMKLR1,MCUB,HJV,SLC9A8,SCN7A,UBASH3B,DISC1,ANK2,GPER1,SLAMF8,RHCG,TRPM8,PDZD8,AGT,ATP1A2,SLC9A1,CASQ1,SLC12A8,NTSR1,CLIC4,MELTF,FZD9,TMCO1,PTH1R,SLC30A4,UNC80,FIS1,CEMIP,ABL2,SLC12A9,CDH23,COMT,PTPN6,MICU1,MCU,CXCR2,GNG3,SLC11A1,HTT,PTGDR,PTGER2,SLC9A7,ATP6V0A1,ABCB6,TRPM7,ATP1B1,KCNA1,CD38,MAPK1,DMXL2,KCNMA1,SLC4A3,ATP13A2,FTH1,HAP1,DLG4,CYB561A3,ATP1B2,FAM155B,PSEN1,CACNA1A,MAPK3,AC138894.1,ATP2A1,CD19,COX11,SMAD3,CLN6,NOX5,CALM1,SNAPIN,S100A14,S100A8,PRKCB,SCNN1B,BDKRB1,NMUR2,SLC39A6,TRPV2,HRH4,CCL14,CCL5,CCL7,C7,PTGER4,SLC25A23,MAIP1,TMEM199,SLC46A1,SLC9A6,NUBP1,GNB1,AT[illegible]	grey	VSMC_M10
GO:0036376	0.68	0.54	6	12	sodium ion export across plasma membrane	BP	1	SLC4A4,ATP1A3,ATP1A2,SLC9A1,ATP1B1,ATP1B2	grey	VSMC_M10
GO:0006687	0.68	0.43	24	47	glycosphingolipid metabolic process	BP	1	B4GALNT1,A4GALT,LARGE1,KIT,GLB1,NEU3,ST8SIA4,A3GALT2,ST8SIA2,B3GALT1,B3GALNT1,GALC,CLN6,ST8SIA5,GLA,HEXA,GM2A,ST6GALNAC4,ST3GAL2,ST6GALNAC5,CPTP,NEU2,B4GALT3,ST8SIA6	grey	VSMC_M10
GO:0035973	0.68	0.59	4	8	aggrephagy	BP	1	ATG5,BAG3,HTT,HSPB8	grey	VSMC_M10
GO:0031111	0.68	0.44	18	36	negative regulation of microtubule polymerization or depolymerization	BP	1	MAPRE1,EML2,CLASP2,TRIM54,NAV3,ARHGEF7,SPEF1,ATXN7,KATNB1,TTBK2,CIB1,INPP5J,CAMSAP3,TAOK1,APC2,CAMSAP1,APC,MAP6D1	grey	VSMC_M10
GO:0002833	0.68	0.39	52	101	positive regulation of response to biotic stimulus	BP	1	CD226,USP15,XRCC6,FPR2,ANKRD17,AKIRIN2,TLR4,PUM2,NECTIN2,PVR,SLC22A13,IKBKB,NMI,TMEM173,MAVS,HPX,PRKDC,IRF7,TRAF6,HCK,RASGRP1,NLRC5,F2RL1,POLR3D,NOD2,OASL,POLR3C,PARP9,COCH,ADAM8,CADM1,NONO,IL12A,XRCC5,LAG3,STAT5B,MED1,SFPQ,SIN3A,CCL5,RIOK3,FBXO38,VAV1,CREBBP,CYBA,AP1G1,FADD,ZCCHC3,IRGM,WNT5A,EREG,HAVCR2	grey	VSMC_M10
GO:0034968	0.68	0.39	50	98	histone lysine methylation	BP	1	SNW1,PHF1,SETD7,SETDB2,EED,IWS1,SUV39H2,KDM4A,PAXIP1,DPY30,NSD3,ASH2L,DNMT3B,SMYD1,SETD6,KMT2D,RTF1,WDR82,ZNF335,JARID2,RBBP5,AUTS2,SETD2,BCOR,MEN1,WDR61,NELFA,DYDC1,NSD1,SETD1A,PAGR1,OGT,SETD3,SUZ12,SUPT6H,CXXC1,DOT1L,MECP2,PRDM16,CHD5,MTF2,GFI1,PRMT6,SETDB1,ARID4A,BEND3,SMYD3,ZNF304,NNMT,N6AMT1	grey	VSMC_M10
GO:0014032	0.68	0.41	35	69	neural crest cell development	BP	1	SEMA6A,SOX10,SMO,SEMA4D,SEMA4C,SOX9,OVOL2,JAG1,ALX1,SEMA3D,SEMA3A,NRG1,KBTBD8,RET,HAND2,SEMA4F,SEMA3G,SEMA4G,KLHL12,SEMA3F,PEF1,PDCD6,GBX2,LAMA5,SEMA6D,FN1,MAPK1,BMPR1A,RADIL,ALDH1A2,SEMA4A,MAPK3,TCOF1,ISL1,SOX8	grey	VSMC_M10
GO:0099625	0.68	0.50	9	18	ventricular cardiac muscle cell membrane repolarization	BP	1	SCN5A,KCNJ3,KCNE2,ANK2,SCN4B,NOS1AP,RNF207,KCNH2,KCNJ8	grey	VSMC_M10
GO:1900407	0.68	0.41	34	66	regulation of cellular response to oxidative stress	BP	1	PRKN,NCOA7,ATF4,GPR37,FBXO7,MET,TLR4,SLC7A11,FOXO3,DHFR,PAWR,FBXW7,HTRA2,RIPK1,FBLN5,MCL1,PDE8A,NFE2L2,NONO,GCH1,PINK1,MMP3,PARP1,SFPQ,MAPK7,STK26,PARK7,TSC1,TLDC1,MAPKAP1,MIR21,NOL3,GPR37L1,TXN	grey	VSMC_M10
GO:0021762	0.68	0.44	18	35	substantia nigra development	BP	1	CKB,TTBK1,SIRT2,NDUFS3,SUDS3,COX6B1,INA,PADI2,ACTB,CALM1,SEC16A,ATP5PB,SYPL2,MAPKAP1,HSPA5,RHOA,BASP1,YWHAH	grey	VSMC_M10

GO:0050803	0.68	0.37	87	170	regulation of synapse structure or activity	BP	1	SYNGAP1,MAPK14,NTRK2,LINGO2,ABHD17B,SEMA4D,PPT1,LRRN3,EPHA7,PTN,CTTNBP2,YWHAZ,VPS35,SLC7A11,VSTM5,PCDH8,SLITRK5,LRFN1,FARP1,PRNP,LRFN3,EPHB1,CLSTN2,CHMP2B,ROBO2,MEF2C,CUX2,SEPT11,TIAM1,SLIT1,HOMER1,OPA1,AMIGO2,ITPKA,CYFIP1,TUBA1A,BDNF,NEURL1,DNM1L,CAPRIN2,SEMA3F,CAMKV,DAG1,AMIGO3,CFL1,ADNP,HSPA8,DISC1,RAB17,NFATC4,PTPRO,CDKL5,IL10RA,EPHB3,TANC2,SRGN,DNM3,GRIPAP1,EPHA4,LRTM2,NLGN2,STX1B,SEMA4A,NLGN3,EFNA1,AC138894.1,CHRNB2,ZDHHC15,NTN1,GPRASP2,BHLHB9,ARHGAP44,ASIC2,CDK5R1,SLITRK2,LRP8,ABHD17A,AMIGO1,NTNG2,DAB2IP,ADGRL2,RHOA,GRID2,ABHD17C,WNT5A,SIX1,LRRTM2	grey	VSMC_M10
GO:0007289	0.68	0.52	7	14	spermatid nucleus differentiation	BP	1	TBPL1,NECTIN2,SYCP1,CHD5,MFSD14A,SYCP3,RNF8	grey	VSMC_M10
GO:0021575	0.68	0.45	17	34	hindbrain morphogenesis	BP	1	GLI1,AGTPBP1,SMO,WHRN,XRCC1,GLI2,FAIM2,LDB1,TTC21B,GNPAT,SPTBN2,MAP2K1,LHX1,DAB1,HSPA5,GRID2,CEND1	grey	VSMC_M10
GO:1902808	0.68	0.43	21	41	positive regulation of cell cycle G1/S phase transition	BP	1	GLI1,CCND3,ANKRD17,LSM10,EGFR,UBE2E2,RPTOR,PLCB1,TFDP1,DDRGK1,ADAMTS1,HYAL1,CCND1,TERT,DDX3X,MEPCE,PAGR1,CDK10,MIR29A,MIR222,RGCC	grey	VSMC_M10
GO:0035581	0.68	0.59	4	8	sequestering of extracellular ligand from receptor	BP	1	FBN1,GREM2,CD46,NRROS	grey	VSMC_M10
GO:0032890	0.68	0.43	24	47	regulation of organic acid transport	BP	1	SLC12A2,PPARA,PLA2G6,AKT2,ARL6IP5,SLC43A1,PLA2R1,GRM2,SLC38A3,MAP2K6,ACSL5,AGT,ACE2,ATP1A2,PER2,NTSR1,PLA2G4A,FIS1,ARL6IP1,PSEN1,ABAT,STXBP1,RGS4,PTGES	grey	VSMC_M10
GO:0048820	0.68	0.54	6	12	hair follicle maturation	BP	1	ERCC2,FERMT1,TRPC4AP,TGFB2,RBPJ,WNT5A	grey	VSMC_M10
GO:0019724	0.68	0.40	45	87	B cell mediated immunity	BP	1	CD226,HLA-DRA,HLA-DOB,HLA-DMA,HLA-DPB1,EXOSC3,MSH2,TRAF3IP2,HMCES,ERCC1,NECTIN2,MLH1,MYD88,FOXJ1,PAXIP1,SHLD1,HPX,IRF7,GAPT,SLA2,NBN,NOD2,CD40,TCIRG1,CD28,FCER1G,RNF168,BCL6,PTPN6,FOXP3,SHLD2,TBX21,BATF,CD19,IL4R,BTK,CD74,C7,C3,SUPT6H,BCL10,CD46,FCER2,RNF8,CD70	grey	VSMC_M10
GO:0002682	0.68	0.34	482	915	regulation of immune system process	BP	1	CD226,NME1,PHLPP1,STX7,TNFAIP3,USP15,ENPP3,ESR1,HSPA1B,BRD1,GRAMD4,AGER,GPSM3,HLA-DRA,HLA-DOB,HLA-DMA,IL5,HLA-DPB1,TCF7,TNFRSF13C,XRCC6,TRIB1,MYC,TOB2,PTK2,C6orf106,MAPK14,PLA2G6,RAC2,HSF1,LRRC14,ZNF16,RBFOX2,FBXO7,TTBK1,TEC,CCL27,CDK6,KIT,TNFRSF21,NCK2,ROR2,NINJ1,EXOSC3,PRKCE,SMAP1,MSH2,RTN4,FPR2,HOXA7,ANKRD17,CXCL8,SLC46A2,MTURN,NOD1,AKIRIN2,PELI1,AKIRIN1,CNOT4,RSAD2,NR1H2,PTN,IL4I1,TLR4,IL15,ATG5,TRPM4,SLC7A11,FOXO3,PUM2,TRAF3IP2,SPHK2,HMCES,BMI1,C5AR2,C5AR1,PRKD2,CST7,PHB,SOX9,CD300LF,LACC1,CLPTM1,NECTIN2,PVR,EIF2B3,GAB2,MLH1,GLI2,UNC13D,MYD88,OXSR1,SLC22A13,PIBF1,FOXJ1,PAXIP1,IRAK2,SEC14L1,POU4F1,IL15RA,TGFBR2,ACVR2A,IKBKB,JAG1,SIRT2,NMI,TMEM173,CLPB,NPLOC4,PAWR,PLCB1,PLCL2,SHLD1,PRNP,MAVS,NCKAP1L,GAS6,HPX,FOXP1,MITF,PRKDC,IRF7,SLC7A1,MFHAS1,TRAF6,PCBP2,NR1H4,TRIM44,SOX12,PLK2,MTUS1,HCK,TOX,ZC3H8,IL1A,GPI,ACVR1B,CAMK4,CEBPA,FER,PIK3CB,PIK3R1,OTUD4,LPXN,CASP3,ERAP1,FBXW7,PTPRJ,MEF2C,TMEM131L,IL7,TMBIM6,VEGFC,PAG1,JAM2,SLA2,PTK2B,RASGRP1,EIF2AK4,NR1H3,TRAFD1,OAS3,NLRC5,RIPK2,OTULIN,SLC8B1,AP3B1,F2RL1,TESC,RIPK1,ANO6,POLR3D,MARCH7,SOX13,MAPKBP1,ADTRP,HERC5,NOD2,ZNF335,ADCY7,CD40,LDB1,IGF2,RAB7B,MAPKAPK3,GPLD1,CD96,IFIH1,CD200,SPPL3,OASL,DNM1L,LCK,BANK1,ICOSLG,MAP2K6,TRPV4,SLC9B2,TGFB2,BTN2A2,GPAM,PRKAR1A,CD99,GPR137B,UNC93B1,PRSS56,POLR3C,PIAS3,HLX,DENND1B,TLR5,CMKLR1,NCF1,ACIN1,MAPKAPK2,TCTA,HCLS1,TBC1D10C,TIFA,ALPK1,YTHDF2,UBAS[illegible]	grey	VSMC_M10
GO:0016242	0.68	0.45	16	32	negative regulation of macroautophagy	BP	1	NRBP2,USP36,FEZ2,LZTS1,SEC22B,TMEM39A,USP30,RUBCN,PINK1,PHF23,NPC1,SMCR8,CLEC16A,MTM1,TSC1,TIGAR	grey	VSMC_M10
GO:0072539	0.68	0.49	10	20	T-helper 17 cell differentiation	BP	1	RC3H1,TNFSF18,STAT3,FOXP3,TBX21,BATF,ZBTB7B,IL6R,MIR21,RC3H2	grey	VSMC_M10
GO:0010996	0.69	0.51	8	16	response to auditory stimulus	BP	1	PTN,SLC26A5,XPC,KIAA0319,ATP1A2,NRXN2,HTT,STRA6	grey	VSMC_M10
GO:0003283	0.69	0.49	10	20	atrial septum development	BP	1	SMO,WNT11,TBX5,TGFB2,ANK2,ISL1,SOX4,WNT5A,NKX2-5,HEY2	grey	VSMC_M10
GO:0050731	0.69	0.38	70	136	positive regulation of peptidyl-tyrosine phosphorylation	BP	1	ERBB3,IL5,HSF1,TEC,KIT,SEMA4D,IL11,EPGN,AREG,TGFA,IL15,HDAC2,ABI1,PIBF1,HBEGF,PRNP,NRG1,GAS6,HPX,ERCC6,OSBP,FBXW7,PTPRJ,PTK2B,ARL2BP,RIPK2,NOD2,CNTN1,CD40,IGF2,NEURL1,LRRK1,HDAC1,BANK1,ADRA2A,NCF1,EGF,HCLS1,ADNP,CASS4,PARP9,PARP14,AGT,OSM,IL12A,TNFSF18,DOK7,FGFR3,EPHA4,DLG4,DVL2,DGKQ,CTF1,EFNA1,IL6R,HAX1,CD74,CCL5,CSF1R,YES1,ISL1,GHR,UNC119,LRP8,MLST8,CNTF,CSPG4,LIF,TSLP,EREG	grey	VSMC_M10
GO:0060766	0.69	0.54	6	12	negative regulation of androgen receptor signaling pathway	BP	1	FOXH1,PHB,FOXP1,HDAC1,PIAS2,NCOR1	grey	VSMC_M10
GO:0006611	0.69	0.42	25	49	protein export from nucleus	BP	1	XPO5,XPO1,DNAJC27,SIRT7,GAS6,RAPGEF3,XPO7,SP100,TCF7L2,BAG3,STRADB,SFN,STRADA,UHMK1,DUSP16,CTDSPL2,RANBP17,XPO6,RANBP3L,PKD1,PARK7,NUP214,NUPL2,TXN,STYX	grey	VSMC_M10
GO:0060740	0.69	0.49	10	20	prostate gland epithelium morphogenesis	BP	1	ESR1,FRS2,HOXA13,TNC,SOX9,GLI2,CYP7B1,NKX3-1,NOG,WNT5A	grey	VSMC_M10
GO:0032094	0.69	0.48	11	22	response to food	BP	1	PPARA,MKKS,SLC16A1,CPS1,MPO,SCNN1B,SREBF1,GHR,SLC25A25,BCL10,HSD11B2	grey	VSMC_M10
GO:0010823	0.69	0.43	22	43	negative regulation of mitochondrion organization	BP	1	PRKN,TSPO,TMEM14A,OPA1,DNM1L,USP30,FZD9,MAPT,C11orf65,PINK1,MUL1,ADCK1,PSMD10,ACAA2,PAM16,NOL3,TIGAR,FXN,SLC35F6,BCL2L2,PPIF,AC140504.1	grey	VSMC_M10
GO:1902337	0.69	0.56	5	10	regulation of apoptotic process involved in morphogenesis	BP	1	PAX8,VDR,PAX2,TGFB2,TNFRSF1B	grey	VSMC_M10
GO:1904748	0.69	0.56	5	10	regulation of apoptotic process involved in development	BP	1	PAX8,VDR,PAX2,TGFB2,TNFRSF1B	grey	VSMC_M10

GO:0046474	0.69	0.37	96	185	glycerophospholipid biosynthetic process	BP	1	PIP4K2C,SYNJ2,AGPAT4,AGPAT1,PNPLA3,PLA2G6,GPAA1,CWH43,INPP4A,TMEM246,PIGF,MBOAT7,LPIN1,INPP4B,FIG4,MTMR2,PIP4K2A,SELENOI,ABHD5,TAMM41,ITPKC,MTMR14,PGAP2,CRLS1,CDS2,NR1H4,MTMR7,PIGU,PIK3CB,PITPNM2,PIK3R1,TMEM150A,SLA2,SYNJ1,CDS1,ITPKA,PIGT,LPCAT1,PGAP1,GPAM,CHKA,ACP6,ABHD4,AJUBA,PITPNM1,GNPAT,DGKD,PIGM,BMX,FAM126B,IP6K2,PIGV,MTMR3,PCYT1A,INPP5J,PIGX,PIGZ,PISD,DGKG,PIKFYVE,PLA2G4A,SACM1L,LPCAT3,PIGC,SOCS4,PI4KA,SLC27A1,ABHD8,PGAP3,PIGH,PI4K2B,SOCS7,DGKQ,AC138894.1,DGKE,TTC7B,PIGL,ABHD3,MPPE1,IMPA2,SOCS1,MTM1,MTMR1,INPP5K,PIP5K1C,AGPAT2,PIP5KL1,FABP5,PEMT,PYURF,LCLAT1,PIGB,PIGA,INPP1,PTPMT1,CDIPT	grey	VSMC_M10
GO:0031649	0.69	0.56	5	10	heat generation	BP	1	IL1A,SLC27A1,ABAT,TRPV1,PTGES	grey	VSMC_M10
GO:2000270	0.69	0.58	4	8	negative regulation of fibroblast apoptotic process	BP	1	CHD8,GAS6,CFDP1,MIR24-1	grey	VSMC_M10
GO:0046173	0.69	0.42	25	49	polyol biosynthetic process	BP	1	CYP27B1,AKR1B1,SPTLC1,IPPK,PLEK,SPHK2,DHFR,ITPKC,ACER3,SPTLC3,ASAH1,CYP2R1,P2RY1,PTK2B,SPR,ITPKA,GPER1,IP6K2,NTSR1,PTH1R,CYP27A1,GCH1,IMPA2,ACER2,PCBD2	grey	VSMC_M10
GO:0042921	0.69	0.53	6	12	glucocorticoid receptor signaling pathway	BP	1	CRY1,PHB,ARNTL,CRY2,ARID1A,YWHAH	grey	VSMC_M10
GO:0036315	0.69	0.49	9	18	cellular response to sterol	BP	1	PTCH1,SMO,CYP7A1,GPLD1,DAG1,GRAMD1B,OSBPL7,LRP8,INSIG2	grey	VSMC_M10
GO:0050892	0.69	0.47	12	24	intestinal absorption	BP	1	ABCG5,ABCG8,SOAT2,PLS1,LIMA1,VDR,ADRA2A,EPB41,LPCAT3,LDLR,SLC46A1,CEL	grey	VSMC_M10
GO:0000183	0.69	0.58	4	8	rDNA heterochromatin assembly	BP	1	BAZ2A,SIRT2,PHF8,BEND3	grey	VSMC_M10
GO:0035584	0.69	0.50	8	16	calcium-mediated signaling using intracellular calcium source	BP	1	PRNP,PTPRJ,DMTN,RYR2,HOMER2,FIS1,BCAP31,PTGFR	grey	VSMC_M10
GO:0098771	0.69	0.35	262	502	inorganic ion homeostasis	BP	1	SLC39A5,NEDD4L,LRP1,CYP27B1,ESR1,SLC12A2,PRKN,SMDT1,BAK1,MYC,MCHR1,GPR20,PLA2G6,CKB,BTBD9,TMPRSS6,SLC39A4,STEAP2,SLC25A27,SLC1A1,TFAP2B,S1PR3,CNNM4,PRKCE,TNNI3,EPAS1,FPR2,IBTK,SLC4A4,LPAR1,PPT1,SLC31A2,EGFR,SFRP4,KEL,ATG5,TRPM4,GRIN2D,TRPC3,SLC8A2,RAB7A,C5AR2,C5AR1,HEPHL1,ATP7B,PTGIR,GIPR,CACNB2,SLC39A12,ATP6V0E2,LACC1,SLC4A2,STEAP3,AQP11,ATP1A3,ACKR2,SLC24A3,SNX5,C1QTNF1,SLC4A7,TMEM106B,TMTC4,MAFG,RYR1,LETM2,PRNP,ADRA1D,DIAPH1,P2RX2,KL,CCKBR,HPX,RASA3,NCOA4,MICU3,ATP6V1H,TM9SF4,IL1A,IMMT,C19orf12,P2RX3,PLCE1,SLC9A9,RYR3,TMBIM6,P2RX7,HVCN1,P2RY1,PTK2B,SLC39A13,TPCN1,SLC8B1,VDR,STC1,SLC25A37,AP3B1,RMDN3,F2RL1,F2R,SLC40A1,CUTC,ABCC2,PKD2,CASQ2,MCUR1,SLC12A5,CD40,RYR2,SLC41A1,SLC4A10,RAP1GDS1,TRPM1,SPPL3,LCK,KCNJ2,TESMIN,SLC9B1,SLC12A7,TRPV4,BDH2,TMEM9,SLC30A10,TCIRG1,FAM20A,GNA13,ISCU,CMKLR1,MCUB,HJV,SLC9A8,SCN7A,UBASH3B,DISC1,ANK2,GPER1,SLAMF8,RHCG,TRPM8,PDZD8,AGT,ATP1A2,SLC9A1,CASQ1,SLC12A8,NTSR1,XPR1,CLIC4,MELTF,FZD9,TMCO1,PTH1R,SLC30A4,FIS1,CEMIP,ABL2,SLC12A9,CDH23,COMT,PTPN6,MICU1,MCU,CXCR2,GNG3,SLC11A1,HTT,PTGDR,PTGER2,SLC9A7,ATP6V0A1,ABCB6,TRPM7,ATP1B1,KCNA1,CD38,MAPK1,DMXL2,KCNMA1,SLC4A3,ATP13A2,FTH1,HAP1,DLG4,CYB561A3,FGFR4,ATP1B2,FAM155B,PSEN1,CACNA1A,MAPK3,AC138894.1,ATP2A1,CD19,COX11,SMAD3,CLN6,NOX5,CALM1,SNAPIN,S100A14,S100A8,PRKCB,SCNN1B,BDKRB1,NMUR2,SLC39A6,TRPV2,HRH4,CCL14,CCL5,CCL7,C7,PTGER4,SLC25A23,MAIP1,TMEM199,SLC46A[illegible]	grey	VSMC_M10
GO:0033523	0.69	0.56	5	10	histone H2B ubiquitination	BP	1	RNF20,UBE2E1,DTX3L,LEO1,RNF8	grey	VSMC_M10
GO:0090178	0.69	0.56	5	10	regulation of establishment of planar polarity involved in neural tube closure	BP	1	PTK7,DVL3,GRHL3,DVL2,WNT5A	grey	VSMC_M10
GO:0090657	0.69	0.55	5	10	telomeric loop disassembly	BP	1	POT1,WRN,RTEL1,SLX4,TERF2	grey	VSMC_M10
GO:0030032	0.69	0.41	31	61	lamellipodium assembly	BP	1	AVIL,RAC2,KIT,NCK2,AKIRIN1,ARHGEF4,ARHGEF7,SPEF1,ARFIP2,AL359736.1,FER,PIK3R1,PLCE1,BIN3,CYFIP1,DMTN,CARMIL1,AUTS2,AJUBA,WASF2,HDAC4,PTPRO,WHAMM,NCKAP1,ABLIM2,VCL,WAS,DNM2,ARHGEF6,VAV3,CARMIL2	grey	VSMC_M10
GO:0046320	0.69	0.46	13	26	regulation of fatty acid oxidation	BP	1	PPARA,SOX9,PRKAG2,AKT2,LONP2,SIRT4,IRS1,CPT1A,ABCB11,ACADL,TYSND1,ACADVL,MLYCD	grey	VSMC_M10
GO:1904350	0.69	0.55	5	10	regulation of protein catabolic process in the vacuole	BP	1	LRP1,MFSD8,ATP13A2,LDLR,RNF128	grey	VSMC_M10
GO:0061383	0.69	0.43	20	40	trabecula morphogenesis	BP	1	ADGRG6,FOXH1,TEK,SRF,FHL2,SEMA4D,OVOL2,NRG1,ADAMTS1,TGFB2,BMPR1A,RBPJ,MED1,THBS3,CHAD,PPARGC1B,ENG,RHOA,NOG,HEY2	grey	VSMC_M10
GO:1905165	0.69	0.58	4	8	regulation of lysosomal protein catabolic process	BP	1	LRP1,MFSD8,ATP13A2,LDLR	grey	VSMC_M10
GO:1904754	0.69	0.50	8	16	positive regulation of vascular associated smooth muscle cell migration	BP	1	LRP1,ADAMTS1,DOCK5,TERT,SSH1,AGT,DOCK7,MIR21	grey	VSMC_M10
GO:0002755	0.69	0.49	9	18	MyD88-dependent toll-like receptor signaling pathway	BP	1	TLR4,CD300LF,MYD88,IRAK2,IRF7,TRAF6,TLR5,BTK,TNIP1	grey	VSMC_M10
GO:0072520	0.69	0.53	6	12	seminiferous tubule development	BP	1	WDR48,ING2,SPATA2,UBB,ADGRG1,TBC1D20	grey	VSMC_M10
GO:0044546	0.69	0.53	6	12	NLRP3 inflammasome complex assembly	BP	1	ATAT1,TLR4,MYD88,EIF2AK2,DDX3X,CPTP	grey	VSMC_M10
GO:0140632	0.69	0.53	6	12	inflammasome complex assembly	BP	1	ATAT1,TLR4,MYD88,EIF2AK2,DDX3X,CPTP	grey	VSMC_M10
GO:1900225	0.69	0.53	6	12	regulation of NLRP3 inflammasome complex assembly	BP	1	ATAT1,TLR4,MYD88,EIF2AK2,DDX3X,CPTP	grey	VSMC_M10
GO:0044804	0.69	0.52	7	14	autophagy of nucleus	BP	1	ATG12,ATG5,ULK1,ATG2A,ATG9A,WIPI2,ATG2B	grey	VSMC_M10
GO:0045780	0.69	0.52	7	14	positive regulation of bone resorption	BP	1	EGFR,SPP1,ADAM8,PPARGC1B,ATP6AP1,DEF8,CA2	grey	VSMC_M10
GO:0032528	0.69	0.48	10	20	microvillus organization	BP	1	ATP8B1,MYO1A,PODXL,KLF5,PLS1,RAPGEF2,USH1C,RAP1GAP,GLDN,STK26	grey	VSMC_M10
GO:0060717	0.69	0.58	4	8	chorion development	BP	1	MAP3K4,PAXIP1,HTRA1,FZD5	grey	VSMC_M10
GO:0000712	0.69	0.52	7	14	resolution of meiotic recombination intermediates	BP	1	MLH1,EME1,ERCC4,SLX4,MEIOB,MSH4,CENPX	grey	VSMC_M10
GO:0060998	0.69	0.41	27	54	regulation of dendritic spine development	BP	1	FSTL4,ASAP1,SHANK1,HDAC2,PLK2,MEF2C,CUX2,TIAM1,OPA1,ITPKA,SRGAP2C,NEURL1,DNM1L,CAPRIN2,DISC1,CDKL5,TANC2,DNM3,SDK1,PSEN1,EFNA1,GPRASP2,BHLHB9,FMR1,LRP8,LLPH,FOXO6	grey	VSMC_M10

GO:0090630	0.69	0.39	47	93	activation of GTPase activity	BP	1	TBC1D30,TBC1D9B,TIAM2,TBC1D22B,TBC1D8,ARHGEF5,EPHA1,TBC1D9,TBC1D4,FOXJ1,AKT2,TBC1D16,TBC1D12,ARHGEF26,PTK2B,RASGRP1,TIAM1,ARHGAP24,TBCK,ADPRH,TBC1D10C,TBC1D2B,GMIP,TBC1D14,RABGAP1L,RUNDC1,RAP1GAP,TBC1D25,ARHGEF19,SYDE1,TBC1D1,TBC1D10B,NDEL1,BCAS3,TBC1D8B,DOCK7,ABR,CRK,RAP1GAP2,TAX1BP3,ARHGEF16,APC2,ARHGAP45,TSC1,MTSS1L,RABGAP1,WNT5A	grey	VSMC_M10
GO:0098868	0.69	0.47	12	24	bone growth	BP	1	BNC2,COL27A1,SOX9,TSKU,TGFBR2,RARB,STC1,POR,FGFR3,MMP13,THBS3,LEPR	grey	VSMC_M10
GO:0060343	0.69	0.48	10	20	trabecula formation	BP	1	ADGRG6,TEK,SRF,FHL2,OVOL2,ADAMTS1,THBS3,CHAD,PPARGC1B,HEY2	grey	VSMC_M10
GO:0070997	0.69	0.35	150	288	neuron death	BP	1	ERBB3,LRP1,MAP3K5,AGAP2,PRKN,PPARA,SYNGAP1,NCOA7,GRM4,ATF4,NTRK2,NRBP2,HSF1,FBXO7,CNTFR,SLC25A27,SLC1A1,TNFRSF21,TFAP2B,GAPDH,MSH2,REL,PPT1,EPHA7,TLR4,VPS35,SLC7A11,FOXO3,NTF4,FGF21,NDNF,C5AR1,ZNF746,GRIK5,XRCC2,EGLN2,POU4F1,AKT2,ARRB1,TMTC4,SLC23A2,PRNP,ARL6IP5,EPHB1,GPI,RILPL1,CASP3,GBE1,DIABLO,FBXW7,TRIM2,MEF2C,FAIM2,HTRA2,PTK2B,NR4A2,F2R,NQO2,TFAP2A,PIGT,SIAH1,PM20D1,BDNF,CD200,HDAC1,TERT,TGFB2,SLC30A10,ADARB1,SCN2A,INPP5A,SSH1,KCNB1,KRAS,ADNP,SORL1,MCL1,CBL,HYOU1,SLC9A1,NFATC4,HDAC4,DLX1,BACE1,DNAJC5,FZD9,EGLN3,SNX6,MAP3K11,ADAM8,CREB1,MAPT,NONO,FIS1,PINK1,STAT3,ATP13A2,PARP1,MAX,TNFRSF1B,PSEN1,ITGAM,NGFR,FOS,AC138894.1,CITED1,ALKBH1,GPRASP2,BHLHB9,CCL5,AP2B1,PPP1R13B,CCL2,CDK5R1,ISL1,GHR,JUN,PDPK1,MECP2,TRAF2,IKBKG,TMEM259,PARK7,TSC1,TLDC1,LCN2,STXBP1,AARS,HSPA5,NQO1,NAE1,MIR125B1,RHOA,UBB,CHGA,FADD,GRID2,CNTF,CASP8,PSMC1,WNT5A,TIGAR,DIO3,SIX1,DDIT3,BCL2L11,HRAS,CEBPB,PITX3,NMNAT1	grey	VSMC_M10
GO:0010225	0.69	0.53	6	12	response to UV-C	BP	1	MAP3K4,BAK1,WRN,DCUN1D3,YY1,IMPACT	grey	VSMC_M10
GO:2000319	0.69	0.51	7	14	regulation of T-helper 17 cell differentiation	BP	1	RC3H1,TNFSF18,FOXP3,TBX21,ZBTB7B,MIR21,RC3H2	grey	VSMC_M10
GO:0061820	0.69	0.58	4	8	telomeric D-loop disassembly	BP	1	POT1,WRN,SLX4,TERF2	grey	VSMC_M10
GO:0003416	0.69	0.47	11	22	endochondral bone growth	BP	1	BNC2,COL27A1,SOX9,TSKU,TGFBR2,RARB,STC1,POR,FGFR3,MMP13,THBS3	grey	VSMC_M10
GO:0016188	0.69	0.55	5	10	synaptic vesicle maturation	BP	1	RAB3A,DLG4,ZDHHC15,SNAPIN,STXBP1	grey	VSMC_M10
GO:0017085	0.69	0.58	4	8	response to insecticide	BP	1	FECH,DDC,UBA52,SCN2B	grey	VSMC_M10
GO:0014047	0.69	0.53	6	12	glutamate secretion	BP	1	NTRK2,APBA1,GRM2,NTSR1,TRPV1,STXBP1	grey	VSMC_M10
GO:0015732	0.69	0.51	7	14	prostaglandin transport	BP	1	ABCC4,ABCC2,MAP2K6,SLCO3A1,PLA2G4A,NOS2,PTGES	grey	VSMC_M10
GO:0016070	0.69	0.32	1852	3480	RNA metabolic process	BP	1	ADNP2,NFATC1,ZNF236,ZNF407,ZC3H10,SMARCC2,PAN2,ATP8B1,ONECUT2,BAZ2A,TCF4,RNMT,TBPL1,GLI1,MARS,CLK4,MAP3K5,TNFAIP3,TSFM,HIVEP2,ADAT2,PLAGL1,MAML1,HELB,RPP21,ZBTB2,CPSF6,YEATS4,ESR1,MYRFL,CNOT2,ZFC3H1,CNOT6,RIDA,STK3,OSR2,PABPC1,SNW1,TFB1M,ZNF706,PHAX,ARID1B,HSPA1B,FZD6,ZBTB12,SKIV2L,SF3A3,DXO,MOV10L1,EIF3E,ZBED4,PNLDC1,PRKN,QKI,AGER,TRMU,PPARA,PDE10A,UTP23,MED30,IL5,BRD2,TAF2,RXRB,VGLL2,RRP7A,HSF2,TCF7,PHF1,XRCC6,NCOA7,CSDC2,TRMT11,PITX1,TRIB1,MYC,TOB2,SMAD5,L3MBTL3,L3MBTL2,AGO2,HABP4,KDM3B,PTP4A3,PTCH1,TNRC6B,ZC3H3,ATF4,TUT7,RPAP2,CBX7,APOBEC3H,GTPBP1,MAPK14,CBY1,PUF60,C9orf64,KDM4C,SOX10,PIM1,CMTR1,NRF1,TMPRSS6,EXOSC4,SMO,SNAPC3,BNC2,HSF1,NFYA,FOXP4,MDFI,TFEB,CPSF1,USP49,IRF5,MED20,CCND3,TAF8,FOXH1,LRRC14,RBM28,CDKN2A,ZNF16,RBFOX2,IFT74,BICRAL,RORB,ZNF300,PRDM4,CRY1,DMTF1,SRF,SMU1,ZNF318,NFX1,NOL6,XPO5,CDK14,FZD1,MTERF1,NFXL1,NFKBIE,SUPT3H,SMARCA2,RFX3,KIT,ADGRF1,NCK2,FHL2,TSR1,TGFBRAP1,TFAP2B,ROR2,NFIL3,SEMA4D,RFX8,DLX5,CNOT11,PHF14,IPPK,ETV1,ZNF135,EXOSC3,RPS14,ZNF154,MEOX2,ZNF550,KANSL3,BEND6,ZNF268,ZNF667,ZNF787,PHF3,ZNF691,CAT,MACC1,LRPPRC,SIX2,FOXJ3,ZNF865,HIVEP3,IL11,SCMH1,ZNF189,RNF20,EPAS1,EPCAM,MTO1,SP4,TSEN34,PRPF31,IGF2BP3,PPP2R1A,ZNF462,TRA2A,PHIP,HMGN3,ELP1,YTHDC1,NFYC,NFE2L3,PNPT1,UTP3,HOXA2,TAF1D,HOXA4,HOXA6,HOXA7,HOXA13,REL,PUS10,ZNF483,JAZF1,CREB5,PTBP3,MYCL,TRIT1,XPO1,MTURN,NOD1,AKIRIN2,RNGTT,SRSF10,MDN1,CASP8AP2,BACH2,ZN[illegible]	grey	VSMC_M10
GO:0001709	0.69	0.45	15	30	cell fate determination	BP	1	PTCH1,HOXA2,NTF4,PTCH2,JAG1,MEF2C,EBF2,PAX2,MCL1,TBX2,GATA6,ISL1,BARHL2,FOXG1,LBX1	grey	VSMC_M10
GO:0061517	0.69	0.55	5	10	macrophage proliferation	BP	1	PTK2,MAPK1,MAPK3,CSF1R,IL33	grey	VSMC_M10
GO:0070229	0.69	0.47	11	22	negative regulation of lymphocyte apoptotic process	BP	1	SLC46A2,FOXP1,GPAM,BCL6,EFNA1,CD74,CCL5,IL7R,BCL10,FADD,TSC22D3	grey	VSMC_M10

GO:0007283	0.69	0.35	187	357	spermatogenesis	BP	1	TBPL1,GLI1,YTHDC2,B4GALNT1,ATAT1,TSPAN8,IGF2R,MOV10L1,PACRG,QKI,BRD2,ROS1,HSF2,UBE2B,CATSPER3,H3F3C,DMC1,SPATA31D3,CFAP69,FANCG,SMARCA2,TDRD6,KIT,CCIN,TSGA10,TDRD7,DNAJA1,FOXJ3,SCMH1,RAD23B,YTHDC1,CABS1,HERPUD2,GMCL1,CCDC155,CREM,ARMC2,DLD,TXNRD3,APOB,SPATA6,SPATA5,TTC26,DNMT3A,FOXA3,SOX9,RSPH6A,TOPAZ1,ERCC1,SLC4A2,NECTIN2,CRTAP,MLH1,ACOX1,FOXJ1,WDR48,TTC21A,CCNB1IP1,ACVR2A,MKKS,SPATA24,ADAM18,GOLGA3,PANK2,BTG1,SOHLH2,TEX15,KATNAL1,NPHP1,ING2,SPATA9,ARNTL,SPAG4,M1AP,IFT81,CELF1,JAM2,CSNK2A2,PRKAG1,SFMBT1,AP3B1,CDYL,SYCP1,DNAH1,DPCD,OCA2,ROPN1L,SIAH1,ACTR1A,NECTIN3,AZIN2,RHBDD1,NEURL1,DDX25,CFAP43,HSF2BP,TESMIN,SPATA2,RNF114,ABHD2,CCDC36,YTHDF2,SPATC1L,SPO11,TSSK4,CIB1,CCDC87,DDX6,GORASP2,DEDD,CELF3,OSBP2,TDRKH,ZSCAN2,SEPT2,LIMK2,PATZ1,CATSPER2,OVOL1,YBX3,CADM1,SPATA32,GGT2,TTC12,BCL6,RPL10L,SPAG16,ZCWPW1,CTSV,BIRC3,ADCY10,PARP11,CALR3,KDM5A,ZMYND15,MNS1,YBX2,MYCBPAP,VIPAS39,SOX30,CCDC42,CREB3L4,RAD51C,DNM2,YY1,TAF4B,ALKBH5,JAG2,IFT20,ACSBG2,TXNDC2,SPEF2,CATSPERD,ZNF449,HSPB11,BCAP31,RNF151,TMEM203,GAMT,CHD5,SOX8,TPGS1,MFSD14A,LRRC8A,STRBP,TSNAXIP1,MSH4,FSTL3,AURKC,SYCP3,ARID4A,TCFL5,NKAPL,METTL14,FOXJ2,TDRP,ZSCAN21,ZFP41,GGT5,RNF8,BCL2L11,TBC1D20,ZNF35,BCL2L2,ZGLP1,SPA17,ZNF628	grey	VSMC_M10
GO:0090311	0.69	0.42	21	42	regulation of protein deacetylation	BP	1	C6orf89,SPHK2,PRKD2,BEX4,TADA3,SDR16C5,ING2,MAPT,BCL6,PINK1,KDM5A,FOXP3,AKAP8L,AKAP8,JDP2,ZBTB7B,SREBF1,SKI,PRMT6,SAP30BP,NNMT	grey	VSMC_M10
GO:2001014	0.69	0.49	9	18	regulation of skeletal muscle cell differentiation	BP	1	AKIRIN1,NR1D2,EPHB1,NLN,ARNTL,MEF2C,RBM24,SIX1,UQCC2	grey	VSMC_M10
GO:0010800	0.69	0.47	11	22	positive regulation of peptidyl-threonine phosphorylation	BP	1	PRKAG2,RPTOR,RIPK2,EGF,STOX1,CEMIP,MAPK1,WNK3,CALM1,IRGM,WNT5A	grey	VSMC_M10
GO:0001771	0.69	0.55	5	10	immunological synapse formation	BP	1	NCK2,EPHB1,DLG1,DOCK2,HAVCR2	grey	VSMC_M10
GO:0043369	0.69	0.51	7	14	CD4-positive or CD8-positive, alpha-beta T cell lineage commitment	BP	1	TOX,TNFSF18,STAT3,FOXP3,TBX21,BATF,IL6R	grey	VSMC_M10
GO:0150012	0.69	0.58	4	8	positive regulation of neuron projection arborization	BP	1	DVL3,DLG4,DVL2,WNT5A	grey	VSMC_M10
GO:0048096	0.69	0.55	5	10	chromatin-mediated maintenance of transcription	BP	1	ARID1B,SMARCD1,ARID1A,PADI2,SERTAD2	grey	VSMC_M10
GO:0061314	0.69	0.55	5	10	Notch signaling involved in heart development	BP	1	GALNT11,JAG1,SNAI1,RBPJ,HEY2	grey	VSMC_M10
GO:0021549	0.69	0.39	39	77	cerebellum development	BP	1	GLI1,UQCRQ,AGTPBP1,CKB,SMO,SEMA4C,WHRN,PTN,XRCC1,GLI2,B4GALT2,SCN5A,CLP1,FAIM2,RPGRIP1L,CNTN1,LDB1,TTBK2,TTC21B,GNPAT,GBX2,SPTBN2,HAP1,PSEN1,MAP2K1,LHX1,CDK5R1,SEZ6,DAB1,ABAT,CRK,SDF4,MECP2,AARS,HSPA5,GRID2,NANOS1,CEND1,PDSS2	grey	VSMC_M10
GO:0043508	0.69	0.55	5	10	negative regulation of JUN kinase activity	BP	1	DNAJA1,PDCD4,AIDA,DUSP10,DUSP19	grey	VSMC_M10
GO:0040019	0.69	0.50	8	16	positive regulation of embryonic development	BP	1	NLRP5,OSR1,PLCB1,CFL1,WNT4,LHX1,AMOT,TLE6	grey	VSMC_M10
GO:0014829	0.69	0.46	12	24	vascular associated smooth muscle contraction	BP	1	ZDHHC21,MKKS,ADRA2B,DOCK5,CHRM3,RAP1GDS1,AGT,CD38,SCNN1B,P2RX1,MIR21,RHOA	grey	VSMC_M10
GO:0034309	0.69	0.55	5	10	primary alcohol biosynthetic process	BP	1	DKK3,WNT4,DGKQ,CACNA1H,PARK7	grey	VSMC_M10
GO:0086011	0.69	0.49	9	18	membrane repolarization during action potential	BP	1	KCNJ3,KCNE2,KCNIP2,KCNJ2,NOS1AP,ATP1B1,RNF207,KCNH2,KCNJ8	grey	VSMC_M10
GO:0050714	0.69	0.38	51	100	positive regulation of protein secretion	BP	1	LRP1,PLA2G6,PRKCE,NR1H2,EGFR,TLR4,TRPM4,GIPR,FRMD4A,APBB3,SIRT3,IL1A,OSBP,VEGFC,NNAT,PFKM,NKX6-1,F2RL1,GPLD1,DNM1L,TGFB2,TCF7L2,PFKFB2,GPER1,GLUL,ADAM8,TTN,MCU,PPP3CB,CD38,MPC2,ATP13A2,NLGN2,TARDBP,STX4,TMED10,MYH10,SYTL4,GPR68,ISL1,OXCT1,ABAT,RPH3AL,NADK,BAIAP3,LRRC8A,FGA,SOX4,CRH,DOC2B,PARD6A	grey	VSMC_M10
GO:0072075	0.69	0.51	7	14	metanephric mesenchyme development	BP	1	MYC,OSR1,PKD2,PAX2,WNT4,BASP1,SIX1	grey	VSMC_M10
GO:0001711	0.69	0.55	5	10	endodermal cell fate commitment	BP	1	EOMES,RTF1,SOX2,LEO1,GATA6	grey	VSMC_M10
GO:0006378	0.69	0.43	20	40	mRNA polyadenylation	BP	1	CPSF6,PABPC1,ZC3H3,HSF1,CPSF1,RNF20,YTHDC1,PNPT1,MTPAP,SYMPK,PCF11,CSTF3,CLP1,TENT2,TENT4B,CPSF2,LEO1,AP002990.1,CSTF2,PAPOLA	grey	VSMC_M10
GO:0043619	0.69	0.55	5	10	regulation of transcription from RNA polymerase II promoter in response to oxidative stress	BP	1	ATF4,EPAS1,ARNT,NFE2L2,SIN3A	grey	VSMC_M10
GO:0070664	0.69	0.41	27	53	negative regulation of leukocyte proliferation	BP	1	TNFAIP3,ENPP3,TNFRSF21,PELI1,IL4I1,FOXJ1,PAWR,PRNP,CASP3,TMEM131L,MARCH7,BTN2A2,PRKAR1A,DLG1,VSIR,RC3H1,PTPN6,SCGB1A1,FOXP3,ZBTB7B,BTK,TWSG1,GLMN,LST1,IL33,CEBPB,HAVCR2	grey	VSMC_M10
GO:0035994	0.69	0.48	10	20	response to muscle stretch	BP	1	PTK2,MAPK14,RAF1,GPI,RYR2,SLC9A1,TTN,TCAP,FOS,DMD	grey	VSMC_M10
GO:0060544	0.69	0.48	10	20	regulation of necroptotic process	BP	1	PELI1,RIPK1,SPATA2,FZD9,YBX3,BIRC3,OGT,MIR22,FADD,CASP8	grey	VSMC_M10
GO:1901894	0.69	0.58	4	8	regulation of ATPase-coupled calcium transmembrane transporter activity	BP	1	RYR2,C4orf3,ATP2A1,VMP1	grey	VSMC_M10
GO:0032957	0.69	0.58	4	8	inositol trisphosphate metabolic process	BP	1	P2RY1,PTK2B,ITPK1,GPER1	grey	VSMC_M10
GO:0036336	0.69	0.49	9	18	dendritic cell migration	BP	1	TRPM4,GAS6,ANO6,SLAMF8,EPS8,IL12A,TNFSF18,CXCR2,CCL5	grey	VSMC_M10
GO:1904683	0.69	0.58	4	8	regulation of metalloendopeptidase activity	BP	1	SORL1,STAT3,MIR21,MIR24-1	grey	VSMC_M10
GO:0010663	0.69	0.55	5	10	positive regulation of striated muscle cell apoptotic process	BP	1	MAP3K5,AGT,CXCR2,CAMK2A,TIGAR	grey	VSMC_M10
GO:0010666	0.69	0.55	5	10	positive regulation of cardiac muscle cell apoptotic process	BP	1	MAP3K5,AGT,CXCR2,CAMK2A,TIGAR	grey	VSMC_M10
GO:0055062	0.69	0.58	4	8	phosphate ion homeostasis	BP	1	TFAP2B,SFRP4,XPR1,FGFR4	grey	VSMC_M10
GO:0072506	0.69	0.58	4	8	trivalent inorganic anion homeostasis	BP	1	TFAP2B,SFRP4,XPR1,FGFR4	grey	VSMC_M10

GO:0042592	0.69	0.33	681	1295	homeostatic process	BP	1	ZNF236,NME1,ORMDL2,SLC39A5,NEDD4L,LRP1,TNFAIP3,CYP27B1,ESR1,APOM,MPIG6B,SLC12A2,HSPA1B,BRD1,PRKN,AGER,TNFRSF11B,PNPLA3,PNPLA5,TSPO,SMDT1,BAK1,MYC,SMAD5,L3MBTL3,KLHL3,MCHR1,GPR20,PTCH1,FANCC,AKR1B1,ATF4,MAPK14,PLA2G6,CKB,BTBD9,RAC2,TMPRSS6,SMO,TTC39B,TXN2,HSF1,SLC39A4,ZNF16,RBFOX2,LARGE1,ALDH1A1,CRY1,SRF,STEAP2,CLDN12,TXNRD1,OCIAD1,CDK6,SLC25A27,KIT,SLC1A1,TFAP2B,GADD45G,KIAA1211,S1PR3,WNK2,PRDX1,RPS14,CNNM4,ABCG5,ABCG8,ERP44,PRKCE,SMAP1,TNNI3,EPAS1,RTN4,FPR2,HMGN3,IBTK,SLC4A4,CYB5R4,HOXA13,LPAR1,PPT1,PTBP3,ALB,SLC46A2,BMP8A,AQP1,BPGM,SLC31A2,MTF1,NR1H2,WHRN,GRHL1,MET,GRB10,POU3F3,EGFR,USP45,TRIM32,TLR4,NCDN,LPIN1,IL15,SFRP4,KEL,UCP1,ATG5,TRPM4,SLC7A11,FOXO3,DLD,TXNRD3,APOB,FGF21,TRAF3IP2,GRIN2D,LAMA4,TRPC3,SESN3,SLC8A2,RAB7A,C5AR2,C5AR1,HEPHL1,NAPEPLD,ATP7B,PTGIR,CNBP,FOXA3,SOX9,GIPR,ABHD5,CACNB2,ERCC2,SLC39A12,ATP6V0E2,CUBN,LACC1,SLC4A2,USH1G,DEDD2,CLNS1A,STEAP3,AQP11,GRIK5,RAF1,ATP1A3,ACOX1,PRDX3,ACVR2B,SCN5A,WDR48,EGLN2,SLC25A38,GCKR,TSKU,ELOVL1,ALK,ACKR2,SLC24A3,GPD1L,SNX5,ACVR2A,C1QTNF1,SLC4A7,MKKS,ACTG1,NR1D2,TMEM106B,ASPSCR1,PLCL2,TMTC4,MAFG,RYR1,RAC3,LETM2,PRNP,ADRA1D,DCN,DIAPH1,P2RX2,KL,CCKBR,NCKAP1L,GAS6,HPX,RASA3,PRKDC,ERCC6,MFHAS1,NCOA4,TRAF6,COMMD9,NR1H4,MICU3,TUB,ATP6V1H,GAPT,USF2,ACOXL,SOAT2,LSR,CYP7A1,TM9SF4,ZC3H8,NDUFAF2,ASXL1,IL1A,CYP7B1,ACOX2,GPI,ACVR1B,ABHD6,CEBPA,IDE,IMMT,PIK3CB,OSBP,C19orf12,PIK3R1,SLMAP,CASP3,CH[illegible]	grey	VSMC_M10
GO:0002888	0.69	0.51	7	14	positive regulation of myeloid leukocyte mediated immunity	BP	1	MAVS,F2RL1,STX4,ITGAM,BTK,C3,TICAM1	grey	VSMC_M10
GO:0070193	0.69	0.47	10	20	synaptonemal complex organization	BP	1	UBE2B,TEX15,SYCP1,SPO11,TEX12,STAG3,P3H4,SYCE2,AGO4,SYCE1L	grey	VSMC_M10
GO:0060677	0.69	0.58	4	8	ureteric bud elongation	BP	1	FMN1,SALL1,SIX1,FGF1	grey	VSMC_M10
GO:1903524	0.69	0.45	13	26	positive regulation of blood circulation	BP	1	SLC1A1,TRPM4,ADRA1D,RYR2,TGFB2,ACE2,ATP1A2,PTGER2,GCH1,CHGA,RGS4,NKX2-5,HEY2	grey	VSMC_M10
GO:1905564	0.69	0.51	7	14	positive regulation of vascular endothelial cell proliferation	BP	1	IGF2,ITGA4,STAT3,PDPK1,MIR29A,MIR21,MIR27A	grey	VSMC_M10
GO:0010635	0.69	0.53	6	12	regulation of mitochondrial fusion	BP	1	PRKN,OPA1,DNM1L,ZDHHC6,MUL1,ADCK1	grey	VSMC_M10
GO:0043524	0.69	0.37	59	115	negative regulation of neuron apoptotic process	BP	1	ERBB3,LRP1,AGAP2,PRKN,SYNGAP1,NTRK2,NRBP2,CNTFR,SLC1A1,TFAP2B,MSH2,PPT1,NTF4,NDNF,C5AR1,XRCC2,POU4F1,ARRB1,GPI,GBE1,MEF2C,FAIM2,PTK2B,NR4A2,F2R,BDNF,HDAC1,TERT,SLC30A10,KRAS,ADNP,HYOU1,NFATC4,DLX1,DNAJC5,FZD9,SNX6,ADAM8,NONO,PINK1,PSEN1,AC138894.1,CITED1,ALKBH1,GPRASP2,BHLHB9,CCL2,ISL1,PDPK1,MECP2,PARK7,STXBP1,AARS,RHOA,CNTF,SIX1,HRAS,CEBPB,NMNAT1	grey	VSMC_M10
GO:1900017	0.70	0.48	9	18	positive regulation of cytokine production involved in inflammatory response	BP	1	GPSM3,TLR4,MYD88,NOD2,STAT3,TICAM1,MIR21,IL17D,IL17B	grey	VSMC_M10
GO:1904353	0.70	0.46	12	24	regulation of telomere capping	BP	1	MAP3K4,MAPK15,ERCC1,XRCC1,MAPKAPK5,NBN,RTEL1,MAPK1,MAPK3,ERCC4,TERF2,MAP2K7	grey	VSMC_M10
GO:0016322	0.70	0.55	5	10	neuron remodeling	BP	1	ANKS1A,RND1,FARP2,C1QL1,C3	grey	VSMC_M10
GO:0021783	0.70	0.51	7	14	preganglionic parasympathetic fiber development	BP	1	PLXNA4,SEMA3A,TFAP2A,SEMA3F,ADARB1,PLXNA3,SIX1	grey	VSMC_M10
GO:0010464	0.70	0.45	13	26	regulation of mesenchymal cell proliferation	BP	1	MYC,SMO,PTN,MYCN,SOX9,WNT11,TGFBR2,CHRD,BMPR1A,FOXF1,WNT5A,SIX1,WNT2	grey	VSMC_M10
GO:0010829	0.70	0.52	6	12	negative regulation of glucose transmembrane transport	BP	1	GRB10,PEA15,RSC1A1,PRKCB,INPP5K,FABP5	grey	VSMC_M10
GO:0006857	0.70	0.52	6	12	oligopeptide transport	BP	1	SLC7A11,ABCC4,ABCB9,CDH17,ABCC5,CA2	grey	VSMC_M10
GO:0043414	0.70	0.35	132	254	macromolecule methylation	BP	1	BAZ2A,RNMT,ARMT1,SNW1,TFB1M,MOV10L1,PHF1,TRMT11,MYC,PICK1,CMTR1,PRDM4,CAMKMT,RNF20,STPG4,MTO1,SETD7,DNMT3A,BMI1,SETDB2,NSUN6,EED,IWS1,SUV39H2,KDM4A,PAXIP1,THUMPD3,DPY30,SIRT7,CHTOP,TRMT6,NSD3,NDUFAF7,ASH2L,BTG1,WTAP,TEX15,THUMPD2,TRMT9B,NR1H4,TGS1,DIMT1,EEF1AKMT1,DNMT3B,SMYD1,PRMT9,BCDIN3D,SETD6,KMT2D,NSUN3,RTF1,PRMT3,WDR82,ZNF335,JARID2,RBBP5,FAM173B,MTRR,HEMK1,TRMT10A,AUTS2,DALRD3,LCMT2,METTL8,EEF1AKMT2,TDRKH,NSUN5,MGMT,METTL2B,SETD2,TRMT1L,TET1,SNRPD3,BCOR,MEN1,WDR61,VCPKMT,MEPCE,ALKBH8,NELFA,TRMT5,DYDC1,PARP1,HSD17B10,PRDM2,MRM2,ZCCHC4,NSD1,SETD1A,PAGR1,FOS,OGT,RAB3D,LCMT1,SETD3,METTL4,SUZ12,METTL6,SUPT6H,CXXC1,MBD1,MRM3,CREBBP,METTL16,DOT1L,MECP2,PRDM16,CHD5,MTF2,GFI1,CMTR2,PRMT7,CTCF,MIR29A,PRMT6,METTL18,SETDB1,PRDM6,ARID4A,METTL14,TRMT10B,BEND3,SMYD3,METTL21A,TRMT61A,ZNF304,NNMT,N6AMT1,TRMT10C,TRMT61B,CBLL1,TYW3	grey	VSMC_M10
GO:0043249	0.70	0.55	5	10	erythrocyte maturation	BP	1	BRD1,L3MBTL3,PTBP3,ERCC2,FAM210B	grey	VSMC_M10
GO:0003129	0.70	0.57	4	8	heart induction	BP	1	WNT11,ROBO1,ROBO2,WNT5A	grey	VSMC_M10

GO:1904062	0.70	0.35	125	241	regulation of cation transmembrane transport	BP	1	NEDD4L,STAC3,MAPK8IP2,PRRT1,PLA2G6,WNK2,TSPAN13,PRKCE,SHANK1,SLC31A2,HECW1,KEL,ATG5,OSR1,SPHK2,KCNRG,CACNB2,STAC,OXSR1,SCN5A,GPD1L,CACNB4,PRNP,DIAPH1,KCNIP3,SLMAP,SLC43A1,GLRX,MEF2C,PTK2B,KCNJ11,HOMER1,IFNGR2,F2R,TESC,ANO6,KCNE2,PKD2,CASQ2,KCNIP2,RYR2,FAM173B,PM20D1,HECW2,KCNJ2,ADRA2A,TCIRG1,COX17,ISCU,KCNG1,UBASH3B,ANK2,GPER1,KLHL24,AGT,ACE2,ATP1A2,SLC9A1,C4orf3,CASQ1,NTSR1,SCN4B,CNIH2,DNAJC30,NOS1AP,DLG1,CEMIP,SESTD1,PTPN6,HTT,PINK1,ATP1B1,KCNA1,GNB5,MINK1,HAP1,DLG4,PSEN2,NLGN2,CNIH3,WNK3,STAC2,CACNB1,ATP1B2,KCNAB3,KCNIP1,NLGN3,ATP2A1,CD19,CALM1,DNM2,VMP1,BDKRB1,CCL2,FMR1,PDPK1,P2RX1,RNF207,PARK7,AMIGO1,CYBA,JPH3,LCN2,WWP2,RRAD,MIR21,MIR24-1,DMD,GPR35,JPH2,KCNH2,FGF11,THY1,SCN2B,NETO2,CACNG7,CRBN,RGS4,CRH,APOPT1,YWHAH,SHISA8,CACNG5,GEM,PPIF	grey	VSMC_M10
GO:0007188	0.70	0.37	66	129	adenylate cyclase-modulating G protein-coupled receptor signaling pathway	BP	1	ADGRG6,ADM2,MCHR1,GRM4,GRM8,GPR37,ADGRF1,S1PR3,ADGRL3,OXER1,FPR2,LPAR1,CRHR2,ADCY3,PTGIR,GIPR,ADRA1D,ADRA2B,CHRM5,P2RY1,ADGRG5,RAPGEF2,NPY1R,CHRM4,ADCY7,CHRM3,GRM2,ADCY2,RGS1,ADRA2A,PTHLH,GNA13,OPRD1,GPER1,WASF2,GPR3,ADCY4,UCN2,PTH1R,ADRA2C,PTGER2,CALCRL,RAMP2,GPR161,CHRM1,ADGRE5,RIT2,HRH4,PTGER4,GNAL,ADGRG4,GNB1,GNA15,PALM,GNAT2,GNAI3,ADGRL2,PTGFR,HTR1D,ADGRE3,ADGRG1,CHGA,GPR37L1,GNA14,GNG2,MRAP2	grey	VSMC_M10
GO:0007618	0.70	0.46	11	22	mating	BP	1	MAPK8IP2,KLK14,DDO,HDAC2,ACVR2A,P2RY1,HDAC4,THRA,UBE2Q1,ABAT,P2RX1	grey	VSMC_M10
GO:0033173	0.70	0.42	19	38	calcineurin-NFAT signaling cascade	BP	1	NFATC1,FHL2,EFHB,PRNP,NRG1,SPPL3,PPP3CA,TBC1D10C,CIB1,SLC9A1,NFATC4,MYOZ2,ACTN3,HOMER2,PPP3CB,MYOZ1,MAPK7,CAMTA1,NFATC3	grey	VSMC_M10
GO:0006089	0.70	0.52	6	12	lactate metabolic process	BP	1	PFKFB2,PER2,ACTN3,PNKD,PARK7,TIGAR	grey	VSMC_M10
GO:0033147	0.70	0.51	7	14	negative regulation of intracellular estrogen receptor signaling pathway	BP	1	CNOT2,FOXH1,CYP7B1,ZNF366,CNOT1,VPS18,ISL1	grey	VSMC_M10
GO:0045540	0.70	0.49	8	16	regulation of cholesterol biosynthetic process	BP	1	APOB,CYP7A1,ERLIN1,SCAP,POR,LPCAT3,SREBF1,FGF1	grey	VSMC_M10
GO:0106118	0.70	0.49	8	16	regulation of sterol biosynthetic process	BP	1	APOB,CYP7A1,ERLIN1,SCAP,POR,LPCAT3,SREBF1,FGF1	grey	VSMC_M10
GO:1905953	0.70	0.43	19	37	negative regulation of lipid localization	BP	1	PPARA,TTC39B,CRY1,ABCG5,ABCG8,NR1H2,ABHD5,APOC1,AKT2,ASXL1,NR1H3,CRY2,PLA2R1,EGF,FIS1,PTPN2,MIR27B,MIR27A,MIR33B	grey	VSMC_M10
GO:0007512	0.70	0.57	4	8	adult heart development	BP	1	HAND2,MYH7,TCAP,NKX2-5	grey	VSMC_M10
GO:0045161	0.70	0.54	5	10	neuronal ion channel clustering	BP	1	PICK1,NRCAM,KCNIP2,GLDN,CNTN2	grey	VSMC_M10
GO:2001034	0.70	0.51	7	14	positive regulation of double-strand break repair via nonhomologous end joining	BP	1	SHLD1,PRKDC,PARP9,DTX3L,SHLD2,WRAP53,SMCHD1	grey	VSMC_M10
GO:0047484	0.70	0.52	6	12	regulation of response to osmotic stress	BP	1	ABCB1,TRPV4,YBX3,MICU1,AC138894.1,SLC25A23	grey	VSMC_M10
GO:0042359	0.70	0.52	6	12	vitamin D metabolic process	BP	1	CYP27B1,CYP2R1,SNAI1,CYP27A1,PIAS4,GFI1	grey	VSMC_M10
GO:1903599	0.70	0.54	5	10	positive regulation of autophagy of mitochondrion	BP	1	PRKN,FBXO7,HTT,VPS13D,PARK7	grey	VSMC_M10
GO:0001573	0.70	0.46	12	24	ganglioside metabolic process	BP	1	B4GALNT1,NEU3,ST8SIA4,ST8SIA2,CLN6,HEXA,GM2A,ST6GALNAC4,ST3GAL2,ST6GALNAC5,NEU2,ST8SIA6	grey	VSMC_M10
GO:0045091	0.70	0.54	5	10	regulation of single stranded viral RNA replication via double stranded DNA intermediate	BP	1	APOBEC3H,CXCL8,FAM208A,INPP5K,SETDB1	grey	VSMC_M10
GO:0042403	0.70	0.51	7	14	thyroid hormone metabolic process	BP	1	PAX8,SLCO1C1,DUOX1,MED1,CRYM,DIO3,DIO2	grey	VSMC_M10
GO:0045820	0.70	0.52	6	12	negative regulation of glycolytic process	BP	1	PPARA,ACTN3,HDAC4,STAT3,NCOR1,TIGAR	grey	VSMC_M10
GO:0006590	0.70	0.54	5	10	thyroid hormone generation	BP	1	PAX8,SLCO1C1,DUOX1,MED1,DIO2	grey	VSMC_M10
GO:0072697	0.70	0.54	5	10	protein localization to cell cortex	BP	1	EPB41L2,EPB41,NUBP1,SAPCD2,GPSM2	grey	VSMC_M10
GO:0060707	0.70	0.54	5	10	trophoblast giant cell differentiation	BP	1	MDFI,GJB5,SNAI1,SOX15,LIF	grey	VSMC_M10
GO:2000696	0.70	0.51	7	14	regulation of epithelial cell differentiation involved in kidney development	BP	1	OSR1,PAX8,PAX2,PRKX,CITED1,LHX1,LIF	grey	VSMC_M10
GO:1904152	0.70	0.52	6	12	regulation of retrograde protein transport, ER to cytosol	BP	1	UBAC2,YOD1,DERL3,BCAP31,UBE2G2,DERL2	grey	VSMC_M10
GO:0046519	0.70	0.49	8	16	sphingoid metabolic process	BP	1	KDSR,SPTLC1,SPHK2,ACER3,SPTLC3,ASAH1,ACER2,SGPP1	grey	VSMC_M10
GO:0030213	0.70	0.52	6	12	hyaluronan biosynthetic process	BP	1	HAS1,HYAL1,EGF,ABCC5,CEMIP,HAS3	grey	VSMC_M10
GO:0030157	0.70	0.54	5	10	pancreatic juice secretion	BP	1	AQP1,NR1H2,NR1H3,COPA,CEL	grey	VSMC_M10
GO:0070920	0.70	0.46	11	22	regulation of production of small RNA involved in gene silencing by RNA	BP	1	ZC3H10,ESR1,EGFR,MYCN,BCDIN3D,RIPK1,PPP3CA,TERT,STAT3,MAP2K1,NCOR1	grey	VSMC_M10
GO:1903798	0.70	0.46	11	22	regulation of production of miRNAs involved in gene silencing by miRNA	BP	1	ZC3H10,ESR1,EGFR,MYCN,BCDIN3D,RIPK1,PPP3CA,TERT,STAT3,MAP2K1,NCOR1	grey	VSMC_M10
GO:0046479	0.70	0.52	6	12	glycosphingolipid catabolic process	BP	1	NEU3,GALC,GLA,HEXA,GM2A,NEU2	grey	VSMC_M10
GO:0006684	0.70	0.52	6	12	sphingomyelin metabolic process	BP	1	SMPD2,SGMS2,SMPD4,SGMS1,ABCA8,PEMT	grey	VSMC_M10
GO:0006670	0.70	0.50	7	14	sphingosine metabolic process	BP	1	SPTLC1,SPHK2,ACER3,SPTLC3,ASAH1,ACER2,SGPP1	grey	VSMC_M10
GO:2001256	0.70	0.54	5	10	regulation of store-operated calcium entry	BP	1	EFHB,SLC8B1,HOMER1,CASQ1,HOMER2	grey	VSMC_M10
GO:0060122	0.70	0.45	13	26	inner ear receptor cell stereocilium organization	BP	1	WHRN,USH1G,MYO7A,TSKU,PLS1,ADGRV1,USH1C,TRIP11,STRC,CDH23,MKS1,IFT20,PJVK	grey	VSMC_M10
GO:0002228	0.70	0.43	17	34	natural killer cell mediated immunity	BP	1	CD226,PRDX1,NECTIN2,PVR,UNC13D,RASGRP1,CD96,CADM1,IL12A,PTPN6,LAG3,STAT5B,VAV1,CRK,AP1G1,SERPINB9,HAVCR2	grey	VSMC_M10
GO:0032225	0.70	0.57	4	8	regulation of synaptic transmission, dopaminergic	BP	1	PNKD,PINK1,CHRNB2,CNTNAP4	grey	VSMC_M10
GO:0031645	0.70	0.52	6	12	negative regulation of nervous system process	BP	1	TNFRSF21,LPIN1,FIG4,MTMR2,JAM2,GPR35	grey	VSMC_M10
GO:0071236	0.70	0.52	6	12	cellular response to antibiotic	BP	1	MEF2C,RPL23,SLC9A1,CDKN1B,PLA2G4A,HSPA5	grey	VSMC_M10
GO:0036473	0.70	0.38	39	76	cell death in response to oxidative stress	BP	1	MAP3K5,PRKN,NCOA7,ATF4,GPR37,FBXO7,SLC1A1,MET,TLR4,SLC7A11,FOXO3,PAWR,PDK1,ARL6IP5,DIABLO,FBXW7,HTRA2,RIPK1,MCL1,PDE8A,NFE2L2,NONO,PRODH,PDCD10,PINK1,MMP3,PARP1,SFPQ,MAPK7,STK26,PARK7,TSC1,TLDC1,MIR21,ZNF622,PJVK,NOL3,GPR37L1,TXN	grey	VSMC_M10
GO:0009312	0.70	0.49	8	16	oligosaccharide biosynthetic process	BP	1	ST3GAL6,B3GALT1,MGAT2,B3GALNT1,ST3GAL2,ST6GALNAC5,FBP2,FUT4	grey	VSMC_M10
GO:2000726	0.70	0.57	4	8	negative regulation of cardiac muscle cell differentiation	BP	1	FRS2,SOX6,FZD7,MIR222	grey	VSMC_M10

GO:1904749	0.70	0.57	4	8	regulation of protein localization to nucleolus	BP	1	POLR1A,TERT,GLUL,NVL	grey	VSMC_M10
GO:0007501	0.70	0.57	4	8	mesodermal cell fate specification	BP	1	SIX2,EYA1,PAX2,BMPR1A	grey	VSMC_M10
GO:0055008	0.70	0.40	25	50	cardiac muscle tissue morphogenesis	BP	1	LY6E,FOXH1,TNNI3,POU4F1,NRG1,TNNC1,DSP,RYR2,PKP2,TNNI1,TGFB2,MYH7,ACTC1,TTN,BMPR1A,TCAP,RBPJ,MED1,ISL1,ENG,NOG,WNT5A,NKX2-5,HEY2,WNT2	grey	VSMC_M10
GO:1904776	0.70	0.57	4	8	regulation of protein localization to cell cortex	BP	1	EPB41L2,EPB41,SAPCD2,GPSM2	grey	VSMC_M10
GO:0019221	0.70	0.34	160	306	cytokine-mediated signaling pathway	BP	1	TNFAIP3,HSPA1B,PRKN,AGPAT1,IL5,TNFRSF13C,IRF5,CNTFR,KIT,SLC1A1,IL1R2,VRK2,CXCL8,PXDN,NR1H2,TRIM32,TRAF1,IL15,FOXO3,TRAF3IP2,CD300LF,MYD88,OXSR1,NUMBL,IRAK2,IL15RA,IL17RE,ACKR2,IKBKB,MKKS,NMI,PLCB1,MAVS,GAS6,HPX,IRF7,ST18,TRAF6,TRIM44,HCK,IL1A,IL37,CEBPA,FER,OTUD4,ROBO1,IL17RD,IL17RB,PTPRJ,IL7,PTK2B,CCL17,NR1H3,OAS3,NLRC5,RIPK2,OTULIN,IFNGR2,F2RL1,RIPK1,TOLLIP,OASL,SP100,PIAS3,CMKLR1,KRAS,SPATA2,YTHDF2,PARP9,PARP14,CIB1,CBL,TRAF5,OSM,PELI3,THPO,IL10RA,FCER1G,DUOX1,PTPN6,TNFSF18,CXCR2,TNIP2,MUL1,BIRC3,STAT3,PTPRN,SPPL2A,SLC27A1,STAT5B,GREM2,PADI2,GPS2,USP18,TNFRSF1B,MED1,PYDC1,MAPK3,DCST1,IL6R,EPOR,IL4R,RPS6KA5,STAT2,TYK2,TRAF3,CD74,CCL14,CCL5,CSF1R,CCL7,CCL2,CCL25,GHR,LEPR,JAK1,OSMR,LIFR,IL7R,PTPN2,SOCS1,LRP8,PIAS4,CDIP1,SPPL2B,TRAF2,AGPAT2,TNFRSF25,GFI1,MIR21,MIR125B1,MIR27B,MIR24-1,MIR27A,CCL16,OAS1,GPR35,NOL3,CCR10,FADD,CXCL6,CNTF,CASP8,MIR125A,IFNLR1,IRGM,IL36G,WNT5A,IL33,CD70,COMMD7,C1QTNF4,CCDC3,TSLP,SH2B2,EREG,TNFSF13B,RPS6KA4,HIST1H2BJ,CCL22	grey	VSMC_M10
GO:0021781	0.70	0.57	4	8	glial cell fate commitment	BP	1	SOX9,NRG1,SOX2,SOX8	grey	VSMC_M10
GO:0036124	0.70	0.52	6	12	histone H3-K9 trimethylation	BP	1	SUV39H2,KDM4A,MECP2,ARID4A,BEND3,ZNF304	grey	VSMC_M10
GO:0042573	0.70	0.50	7	14	retinoic acid metabolic process	BP	1	ALDH8A1,PRMT3,ALDH1A3,DHRS9,BCO2,ALDH1A2,SCPEP1	grey	VSMC_M10
GO:0071731	0.70	0.49	8	16	response to nitric oxide	BP	1	AQP1,GUCY1B1,MTR,MMP3,DNM2,CRK,TRAF2,TXN	grey	VSMC_M10
GO:2000193	0.70	0.50	7	14	positive regulation of fatty acid transport	BP	1	PLA2G6,PLA2R1,MAP2K6,ACSL5,NTSR1,PLA2G4A,PTGES	grey	VSMC_M10
GO:0001325	0.70	0.50	7	14	formation of extrachromosomal circular DNA	BP	1	ERCC1,WRN,NBN,RTEL1,XRCC5,SLX4,TERF2	grey	VSMC_M10
GO:0090656	0.70	0.50	7	14	t-circle formation	BP	1	ERCC1,WRN,NBN,RTEL1,XRCC5,SLX4,TERF2	grey	VSMC_M10
GO:0090737	0.70	0.50	7	14	telomere maintenance via telomere trimming	BP	1	ERCC1,WRN,NBN,RTEL1,XRCC5,SLX4,TERF2	grey	VSMC_M10
GO:0051457	0.70	0.47	10	20	maintenance of protein location in nucleus	BP	1	SKP1,SUN2,TAF8,TAF3,ARL2BP,MORC3,SP100,PARK7,SUPT7L,TXN	grey	VSMC_M10
GO:0045907	0.70	0.46	11	22	positive regulation of vasoconstriction	BP	1	EGFR,TRPM4,ADRA1D,F2R,ADRA2C,SMTNL2,CD38,HRH2,MIR21,RHOA,FGA	grey	VSMC_M10
GO:0014821	0.70	0.52	6	12	phasic smooth muscle contraction	BP	1	KIT,P2RX2,P2RX3,DLG1,TBX2,HTR1D	grey	VSMC_M10
GO:0045830	0.70	0.47	10	20	positive regulation of isotype switching	BP	1	EXOSC3,MSH2,HMCES,MLH1,PAXIP1,SHLD1,CD40,CD28,SHLD2,TBX21	grey	VSMC_M10
GO:0002921	0.70	0.57	4	8	negative regulation of humoral immune response	BP	1	FOXJ1,PTPN6,CD46,A2M	grey	VSMC_M10
GO:0003254	0.70	0.43	17	34	regulation of membrane depolarization	BP	1	NEDD4L,TSPO,TRPM4,SCN5A,GPD1L,DCN,SLMAP,TBX5,IFI6,NTSR1,FZD9,PPP2R3C,PARP1,CACNA1G,MLLT11,SCN2B,HCN4	grey	VSMC_M10
GO:0070863	0.70	0.52	6	12	positive regulation of protein exit from endoplasmic reticulum	BP	1	TMEM30A,TM9SF4,SORL1,SEC16B,TMEM30B,BCAP31	grey	VSMC_M10
GO:2000008	0.70	0.43	17	34	regulation of protein localization to cell surface	BP	1	NEDD4L,GOPC,GPD1L,TM9SF4,RAB11FIP5,GBF1,BDNF,EGF,LRIG2,ABCA12,STX4,TAX1BP3,ABCA7,CD247,TMEM35A,LEPROT,MAP1A	grey	VSMC_M10
GO:0060148	0.70	0.44	14	28	positive regulation of posttranscriptional gene silencing	BP	1	AGO2,TNRC6B,XPO5,EGFR,MYCN,PUM2,EIF4E2,RIPK1,AJUBA,DDX6,EIF4ENIF1,STAT3,MAP2K1,FMR1	grey	VSMC_M10
GO:0070424	0.70	0.54	5	10	regulation of nucleotide-binding oligomerization domain containing signaling pathway	BP	1	TNFAIP3,HSPA1B,SLC46A2,TLR4,BIRC3	grey	VSMC_M10
GO:0072530	0.70	0.50	7	14	purine-containing compound transmembrane transport	BP	1	SLC35B2,ABCC4,SLC29A2,SLC25A42,LRRC8E,SLC25A41,LRRC8A	grey	VSMC_M10
GO:0010907	0.70	0.43	16	32	positive regulation of glucose metabolic process	BP	1	PPARA,GPT,PPP4R3B,EPM2AIP1,AKT2,SORBS1,IGF2,GPLD1,IRS1,PPP1R3E,ACTN3,DDB1,INSR,PTPN2,NNMT,ARPP19	grey	VSMC_M10
GO:1905245	0.70	0.54	5	10	regulation of aspartic-type peptidase activity	BP	1	AGER,SORL1,EPHA4,TMED10,EFNA1	grey	VSMC_M10
GO:0032341	0.70	0.57	4	8	aldosterone metabolic process	BP	1	DKK3,WNT4,SCNN1B,CACNA1H	grey	VSMC_M10
GO:0060502	0.70	0.57	4	8	epithelial cell proliferation involved in lung morphogenesis	BP	1	SOX9,SRSF6,MAP2K1,WNT2	grey	VSMC_M10
GO:0046850	0.70	0.42	19	38	regulation of bone remodeling	BP	1	TNFAIP3,TNFRSF11B,EGFR,P2RX7,SPP1,GPR137B,UBASH3B,ADAM8,GPR137,SUCO,CD38,SYT7,CSF1R,PPARGC1B,LEPR,ATP6AP1,DEF8,CA2,BGLAP	grey	VSMC_M10
GO:0034114	0.70	0.50	7	14	regulation of heterotypic cell-cell adhesion	BP	1	AGER,GCNT2,LCK,MAP2K5,MAPK7,FGA,THY1	grey	VSMC_M10
GO:0000002	0.70	0.46	10	20	mitochondrial genome maintenance	BP	1	MGME1,PRIMPOL,OPA1,POLG2,STOX1,PARP1,PIF1,LIG3,LONP1,SLC25A33	grey	VSMC_M10
GO:0002675	0.70	0.50	7	14	positive regulation of acute inflammatory response	BP	1	OSM,ADAM8,BTK,C3,OSMR,CREB3L3,PARK7	grey	VSMC_M10
GO:0048478	0.70	0.57	4	8	replication fork protection	BP	1	ZRANB3,FBH1,DONSON,TIPIN	grey	VSMC_M10
GO:0034390	0.70	0.46	10	20	smooth muscle cell apoptotic process	BP	1	CDKN2A,ARRB1,STK4,PDCD4,IL12A,RBM10,MFN2,MIR21,MIR24-1,C3orf58	grey	VSMC_M10
GO:0034391	0.70	0.46	10	20	regulation of smooth muscle cell apoptotic process	BP	1	CDKN2A,ARRB1,STK4,PDCD4,IL12A,RBM10,MFN2,MIR21,MIR24-1,C3orf58	grey	VSMC_M10
GO:0006705	0.70	0.57	4	8	mineralocorticoid biosynthetic process	BP	1	CYP21A2,DKK3,WNT4,CACNA1H	grey	VSMC_M10
GO:0032329	0.70	0.54	5	10	serine transport	BP	1	SLC1A4,SERINC5,SLC38A2,SFXN2,SLC38A5	grey	VSMC_M10
GO:0010092	0.70	0.43	16	32	specification of animal organ identity	BP	1	FRS2,FOXH1,SPRY1,WNT11,PAX8,ROBO1,ROBO2,MEF2C,PAX2,AXIN2,RBPJ,ISL1,WNT5A,SIX1,FGF1,WNT2	grey	VSMC_M10
GO:0019062	0.70	0.57	4	8	virion attachment to host cell	BP	1	NECTIN2,GAS6,ACE2,LRRC15	grey	VSMC_M10
GO:0007603	0.70	0.54	5	10	phototransduction, visible light	BP	1	CAMKMT,SAG,NMT1,GRK4,GUCY2D	grey	VSMC_M10
GO:0035886	0.70	0.44	13	26	vascular associated smooth muscle cell differentiation	BP	1	NFATC1,SRF,KIT,PDCD4,GPER1,EFEMP2,RAMP2,GATA6,ENG,NFATC3,MIR21,MIR125B1,HEY2	grey	VSMC_M10
GO:0070243	0.70	0.56	4	8	regulation of thymocyte apoptotic process	BP	1	ZC3H8,ADAM8,EFNA1,WNT5A	grey	VSMC_M10
GO:0015865	0.70	0.46	11	22	purine nucleotide transport	BP	1	SLC35B2,ABCC4,SLC33A1,CALHM1,ABCC5,SLC25A42,LRRC8E,SLC25A23,SLC25A41,LRRC8A,SLC25A25	grey	VSMC_M10

GO:0015868	0.70	0.46	11	22	purine ribonucleotide transport	BP	1	SLC35B2,ABCC4,SLC33A1,CALHM1,ABCC5,SLC25A42,LRRC8E,SLC25A23,SLC25A41,LRRC8A,SLC25A25	grey	VSMC_M10
GO:0051503	0.70	0.46	11	22	adenine nucleotide transport	BP	1	SLC35B2,ABCC4,SLC33A1,CALHM1,ABCC5,SLC25A42,LRRC8E,SLC25A23,SLC25A41,LRRC8A,SLC25A25	grey	VSMC_M10
GO:0030214	0.70	0.56	4	8	hyaluronan catabolic process	BP	1	CEMIP2,HYAL1,HYAL3,CEMIP	grey	VSMC_M10
GO:0050869	0.70	0.45	12	24	negative regulation of B cell activation	BP	1	TNFAIP3,TNFRSF21,FOXJ1,PAWR,CASP3,BANK1,TBC1D10C,BCL6,RC3H1,INHA,FOXP3,BTK	grey	VSMC_M10
GO:0071425	0.70	0.45	12	24	hematopoietic stem cell proliferation	BP	1	PIM1,EIF2AK2,SART3,YTHDF2,ARIH2,THPO,ETV6,KAT7,CTC1,MIR222,ATXN1L,WNT5A	grey	VSMC_M10
GO:2000169	0.70	0.56	4	8	regulation of peptidyl-cysteine S-nitrosylation	BP	1	NCOA7,NOS1AP,DMD,TXN	grey	VSMC_M10
GO:0070206	0.70	0.54	5	10	protein trimerization	BP	1	PNPT1,P2RX3,ASIC1,CD74,CLYBL	grey	VSMC_M10
GO:0070232	0.70	0.45	12	24	regulation of T cell apoptotic process	BP	1	SLC46A2,IDO1,ZC3H8,GPAM,PDCD1,ADAM8,EFNA1,CCL5,IL7R,FADD,WNT5A,TSC22D3	grey	VSMC_M10
GO:0003330	0.70	0.56	4	8	regulation of extracellular matrix constituent secretion	BP	1	ADTRP,AGT,TNFRSF1B,RGCC	grey	VSMC_M10
GO:0051047	0.71	0.34	109	211	positive regulation of secretion	BP	1	VPS4B,LRP1,SLC12A2,PLA2G6,CHMP2A,PRKCE,AQP1,NR1H2,EGFR,TLR4,ATG5,SDCBP,TRPM4,SPHK2,RAB7A,GIPR,CACNB2,CLASP2,FRMD4A,GAB2,UNC13D,SNX5,C1QTNF1,SYT1,RAB5A,APBB3,AIMP1,SIRT3,SYT9,IL1A,OSBP,VEGFC,P2RY1,NNAT,PFKM,NKX3-1,NKX6-1,F2RL1,SPP1,PLA2R1,DTNBP1,GPLD1,DNM1L,HYAL3,MAP2K6,TGFB2,TCF7L2,KCNB1,PFKFB2,GRK2,GPER1,ABCB11,AGT,FGR,STX1A,SNX4,NTSR1,GLUL,ADAM8,CREB1,PLA2G4A,TTN,MCU,PPP3CB,RAB3A,PINK1,CD38,MPC2,INHA,ATP13A2,SYT7,RAB15,NLGN2,TARDBP,STX1B,STX4,ITGAM,RAB8B,TMED10,CACNA1G,CHRNB2,MYH10,RAB3D,IL4R,SYTL4,GPR68,S100A8,SCAMP5,ISL1,OXCT1,ABAT,RPH3AL,NADK,TRPV1,ATP6AP1,BAIAP3,CACNA1H,CYBA,LRRC8A,STXBP1,AP1G1,VPS4A,FGA,SOX4,CRH,RGCC,DOC2B,PTGES,PARD6A	grey	VSMC_M10
GO:0006067	0.71	0.56	4	8	ethanol metabolic process	BP	1	ADH5,ALDH3B2,ALDH2,ADH4	grey	VSMC_M10
GO:0010837	0.71	0.44	14	28	regulation of keratinocyte proliferation	BP	1	CD109,AREG,INTU,OVOL2,VDR,SRSF6,TGM1,KDF1,SFN,CASK,MED1,BCL11B,MIR21,MIR125B1	grey	VSMC_M10
GO:0043372	0.71	0.46	10	20	positive regulation of CD4-positive, alpha-beta T cell differentiation	BP	1	HLA-DRA,NCKAP1L,RIPK2,HLX,FOXP3,ZBTB7B,IL4R,SOCS1,PRKCZ,MIR21	grey	VSMC_M10
GO:0006901	0.71	0.48	8	16	vesicle coating	BP	1	MAPK15,ARFGAP2,GBF1,KLHL12,PEF1,PDCD6,TMED10,PPP6C	grey	VSMC_M10
GO:2000757	0.71	0.48	8	16	negative regulation of peptidyl-lysine acetylation	BP	1	HINT2,ATG5,HDAC2,TAF7,SDR16C5,HDAC1,FOXP3,SIN3A	grey	VSMC_M10
GO:1902931	0.71	0.51	6	12	negative regulation of alcohol biosynthetic process	BP	1	CYP27B1,PLEK,DKK3,ERLIN1,SCAP,GFI1	grey	VSMC_M10
GO:0051904	0.71	0.47	9	18	pigment granule transport	BP	1	BBS7,MYO7A,MKKS,MLPH,RAB17,BLOC1S6,MREG,RAB11A,BLOC1S3	grey	VSMC_M10
GO:0061418	0.71	0.51	6	12	regulation of transcription from RNA polymerase II promoter in response to hypoxia	BP	1	SIRT2,BACH1,HIF1AN,NFE2L2,STOX1,RBPJ	grey	VSMC_M10
GO:0050807	0.71	0.35	84	165	regulation of synapse organization	BP	1	MAPK14,NTRK2,LINGO2,ABHD17B,SEMA4D,LRRN3,EPHA7,PTN,CTTNBP2,YWHAZ,VPS35,SLC7A11,VSTM5,PCDH8,SLITRK5,LRFN1,FARP1,PRNP,LRFN3,EPHB1,CLSTN2,CHMP2B,ROBO2,MEF2C,CUX2,SEPT11,TIAM1,SLIT1,HOMER1,OPA1,AMIGO2,ITPKA,CYFIP1,TUBA1A,BDNF,NEURL1,DNM1L,CAPRIN2,SEMA3F,CAMKV,DAG1,AMIGO3,CFL1,ADNP,HSPA8,DISC1,RAB17,NFATC4,PTPRO,CDKL5,IL10RA,EPHB3,TANC2,SRGN,DNM3,GRIPAP1,EPHA4,LRTM2,NLGN2,SEMA4A,NLGN3,EFNA1,AC138894.1,CHRNB2,ZDHHC15,NTN1,GPRASP2,BHLHB9,ARHGAP44,ASIC2,CDK5R1,SLITRK2,LRP8,ABHD17A,AMIGO1,NTNG2,DAB2IP,ADGRL2,RHOA,GRID2,ABHD17C,WNT5A,SIX1,LRRTM2	grey	VSMC_M10
GO:1902745	0.71	0.43	15	30	positive regulation of lamellipodium organization	BP	1	AVIL,RAC2,AKIRIN1,ARHGEF7,PIK3R1,PLCE1,RREB1,CYFIP1,CARMIL1,AUTS2,WASF2,HDAC4,NCKAP1,DNM2,CARMIL2	grey	VSMC_M10
GO:0002523	0.71	0.53	5	10	leukocyte migration involved in inflammatory response	BP	1	NINJ1,PTN,SLAMF8,ADAM8,S100A8	grey	VSMC_M10
GO:2000322	0.71	0.56	4	8	regulation of glucocorticoid receptor signaling pathway	BP	1	CRY1,PHB,ARNTL,CRY2	grey	VSMC_M10
GO:0048240	0.71	0.51	6	12	sperm capacitation	BP	1	CATSPER3,DLD,ROPN1L,ABHD2,CATSPER2,CATSPERD	grey	VSMC_M10
GO:0032891	0.71	0.50	7	14	negative regulation of organic acid transport	BP	1	AKT2,ARL6IP5,SLC43A1,PLA2R1,FIS1,ABAT,RGS4	grey	VSMC_M10
GO:0001562	0.71	0.50	7	14	response to protozoan	BP	1	MYD88,CD40,BATF2,SLC11A1,BATF,IL4R,HRAS	grey	VSMC_M10
GO:0042832	0.71	0.50	7	14	defense response to protozoan	BP	1	MYD88,CD40,BATF2,SLC11A1,BATF,IL4R,HRAS	grey	VSMC_M10
GO:1903532	0.71	0.35	101	196	positive regulation of secretion by cell	BP	1	VPS4B,LRP1,SLC12A2,PLA2G6,CHMP2A,PRKCE,NR1H2,EGFR,TLR4,SDCBP,TRPM4,SPHK2,RAB7A,GIPR,CACNB2,CLASP2,FRMD4A,GAB2,UNC13D,C1QTNF1,SYT1,RAB5A,APBB3,AIMP1,SIRT3,SYT9,IL1A,OSBP,VEGFC,P2RY1,NNAT,PFKM,NKX3-1,NKX6-1,F2RL1,SPP1,DTNBP1,GPLD1,DNM1L,HYAL3,MAP2K6,TGFB2,TCF7L2,KCNB1,PFKFB2,GRK2,GPER1,AGT,FGR,STX1A,SNX4,NTSR1,GLUL,ADAM8,CREB1,PLA2G4A,TTN,MCU,PPP3CB,RAB3A,PINK1,CD38,MPC2,INHA,ATP13A2,SYT7,RAB15,NLGN2,TARDBP,STX1B,STX4,ITGAM,RAB8B,TMED10,CACNA1G,CHRNB2,MYH10,RAB3D,IL4R,SYTL4,GPR68,SCAMP5,ISL1,OXCT1,ABAT,RPH3AL,NADK,ATP6AP1,BAIAP3,CACNA1H,LRRC8A,STXBP1,AP1G1,VPS4A,FGA,SOX4,CRH,RGCC,DOC2B,PTGES,PARD6A	grey	VSMC_M10
GO:0010878	0.71	0.48	8	16	cholesterol storage	BP	1	PPARA,TTC39B,NR1H2,APOB,NR1H3,SOAT1,EHD1,STARD4	grey	VSMC_M10
GO:0032400	0.71	0.47	9	18	melanosome localization	BP	1	BBS7,MKKS,SHROOM2,MLPH,RAB17,BLOC1S6,MREG,RAB11A,BLOC1S3	grey	VSMC_M10
GO:0032401	0.71	0.47	9	18	establishment of melanosome localization	BP	1	BBS7,MKKS,SHROOM2,MLPH,RAB17,BLOC1S6,MREG,RAB11A,BLOC1S3	grey	VSMC_M10
GO:0032303	0.71	0.51	6	12	regulation of icosanoid secretion	BP	1	PLA2G6,PLA2R1,MAP2K6,NTSR1,PLA2G4A,PTGES	grey	VSMC_M10
GO:0043518	0.71	0.51	6	12	negative regulation of DNA damage response, signal transduction by p53 class mediator	BP	1	MARCH7,DYRK3,SNAI1,PSMD10,CD74,PTTG1IP	grey	VSMC_M10

GO:0045124	0.71	0.42	16	32	regulation of bone resorption	BP	1	TNFAIP3,TNFRSF11B,EGFR,P2RX7,SPP1,GPR137B,UBASH3B,ADAM8,GPR137,CD38,CSF1R,PPARGC1B,ATP6AP1,DEF8,CA2,BGLAP	grey	VSMC_M10
GO:1902916	0.71	0.50	7	14	positive regulation of protein polyubiquitination	BP	1	PRKN,NMI,SPSB4,MARCH7,NOD2,DDX3X,UBE2D1	grey	VSMC_M10
GO:0050891	0.71	0.42	18	36	multicellular organismal water homeostasis	BP	1	AKR1B1,SRF,TFAP2B,AQP1,GRHL1,MET,ELOVL1,LSR,TRPV4,KDF1,SFN,GRHL3,ABCA12,ALOXE3,SCNN1B,MYO5B,INPP5K,CLDN1	grey	VSMC_M10
GO:1904491	0.71	0.56	4	8	protein localization to ciliary transition zone	BP	1	MAPK15,TMEM107,TCTN2,CC2D2B	grey	VSMC_M10
GO:2000343	0.71	0.56	4	8	positive regulation of chemokine (C-X-C motif) ligand 2 production	BP	1	LRP1,TLR4,HDAC1,CD74	grey	VSMC_M10
GO:1903265	0.71	0.56	4	8	positive regulation of tumor necrosis factor-mediated signaling pathway	BP	1	HSPA1B,PRKN,TRIM32,TRAF2	grey	VSMC_M10
GO:0034143	0.71	0.47	9	18	regulation of toll-like receptor 4 signaling pathway	BP	1	TNFAIP3,NINJ1,PELI1,MFHAS1,NR1H3,F2RL1,RAB7B,WDFY1,DAB2IP	grey	VSMC_M10
GO:0032353	0.71	0.56	4	8	negative regulation of hormone biosynthetic process	BP	1	CYP27B1,DKK3,WNT4,GFI1	grey	VSMC_M10
GO:0019856	0.71	0.56	4	8	pyrimidine nucleobase biosynthetic process	BP	1	CMPK1,CTPS2,CPS1,DHODH	grey	VSMC_M10
GO:0045445	0.71	0.38	34	67	myoblast differentiation	BP	1	MAML1,PITX1,MAPK14,SRF,SOSTDC1,EPAS1,AKIRIN1,SOX9,JAG1,SRA1,PLCB1,BTG1,NRG1,SMYD1,MEF2C,CCL17,HIF1AN,PRICKLE1,RBM24,CDON,TCF7L2,DTYMK,PLEKHM3,RBPJ,SOX15,TBX2,ISL1,RANBP3L,MBNL3,SOX8,LRRC8A,MIR199A1,DDIT3,XKR8	grey	VSMC_M10
GO:0021604	0.71	0.53	5	10	cranial nerve structural organization	BP	1	PLXNA4,SEMA3A,PAX2,SEMA3F,PLXNA3	grey	VSMC_M10
GO:0007271	0.71	0.47	9	18	synaptic transmission, cholinergic	BP	1	CHRNA3,CHRNA5,CHRNA6,CHRNB1,CHRNB2,NQO1,CHRNB4,CHRNA10,CHRNG	grey	VSMC_M10
GO:0034113	0.71	0.42	18	36	heterotypic cell-cell adhesion	BP	1	ITGA7,AGER,NINJ1,IZUMO1,PARVA,DSP,GCNT2,CD200,PKP2,LCK,ITGA4,GLDN,MAP2K5,DSG2,MAPK7,FGA,THY1,PERP	grey	VSMC_M10
GO:0002347	0.71	0.45	11	22	response to tumor cell	BP	1	CD226,IL4I1,NECTIN2,PVR,SLC22A13,HNMT,RBMS3,IL12A,FBXO38,PLK5,HAVCR2	grey	VSMC_M10
GO:0050857	0.71	0.46	10	20	positive regulation of antigen receptor-mediated signaling pathway	BP	1	CD226,PRKD2,NECTIN2,FOXP1,LCK,CARD11,PRKCB,IKBKG,BCL10,CMTM3	grey	VSMC_M10
GO:0060600	0.71	0.56	4	8	dichotomous subdivision of an epithelial terminal unit	BP	1	AREG,PLXNA1,SEMA3A,CTSH	grey	VSMC_M10
GO:0045136	0.71	0.56	4	8	development of secondary sexual characteristics	BP	1	AREG,STAT5B,MED1,WNT5A	grey	VSMC_M10
GO:0043487	0.71	0.35	76	148	regulation of RNA stability	BP	1	PAN2,CNOT2,CNOT6,RIDA,PABPC1,PHAX,DXO,PNLDC1,CSDC2,AGO2,TNRC6B,TUT7,MAPK14,EXOSC3,PNPT1,TUT4,PUM2,TRAF3IP2,FASTK,MLH1,MYD88,TNRC6C,LSM1,PAN3,CNOT1,CELF1,CNOT6L,TENT2,FTO,TENT5C,TENT4B,RBM24,DIS3L2,AXIN2,MAPKAPK2,GIGYF2,YTHDF2,TRAF5,FASTKD1,ANGEL2,YTHDF1,EIF4ENIF1,YBX3,RC3H1,DDX49,UPF1,SLC11A1,RBM10,SAMD4A,POLR2G,SCGB1A1,NT5C3B,DCP1B,TNFRSF1B,FXR2,TARDBP,TOB1,TENT5D,TNRC6A,CNOT8,LARP1,ALKBH5,PARN,FMR1,METTL16,PLEKHN1,CIRBP,TRAF2,METTL14,NANOS1,CACNG7,YBX1,ZFP36L2,TENT5A,RC3H2,EXOSC5	grey	VSMC_M10
GO:0120009	0.71	0.41	19	38	intermembrane lipid transfer	BP	1	TSPO,ABCG5,ABCG8,APOB,OSBP,PITPNM2,COL4A3BP,PLTP,PITPNM1,PITPNC1,GRAMD1B,OSBPL2,STARD5,ATG2B,PRELID3A,PITPNA,ABCA3,CPTP,STARD4	grey	VSMC_M10
GO:0007622	0.71	0.43	14	28	rhythmic behavior	BP	1	BTBD9,NR1D2,AHCY,NCOA2,SRD5A1,CIART,USP2,C3orf70,CHRNB2,NCOR1,PER3,GPR157,ZFHX3,CRH	grey	VSMC_M10
GO:0042743	0.71	0.42	18	35	hydrogen peroxide metabolic process	BP	1	RAC2,PRDX1,CAT,PXDN,EGFR,ACOX1,PRDX3,PXDNL,DUOX1,PINK1,MMP3,STAT3,MPO,NOXA1,PARK7,CYBA,HBA2,AC140504.1	grey	VSMC_M10
GO:0045664	0.71	0.35	74	145	regulation of neuron differentiation	BP	1	TCF4,CSNK1E,KDM4C,BEND6,HOXA2,MYCL,TRIM32,CDK5RAP2,MYCN,NRCAM,FOXO3,NTF4,SOX9,GLI2,KDM4A,ALK,JAG1,RAC3,LSM1,ADRA2B,RET,DNMT3B,ZNF536,EYA1,MEF2C,DPYSL2,EIF2AK4,TIAM1,OPA1,NKX6-1,BDNF,CDON,NEPRO,SOX2,ZFHX2,DDX6,DLX1,EIF4ENIF1,SH3GL3,HOXD3,YWHAG,BCL6,DIXDC1,BRINP2,ADRA2C,RAP1GAP,CYB5D2,PRPF19,MED1,CASZ1,NAP1L2,MOSMO,BCL11B,SIN3A,MIB1,CDK5R1,ISL1,DAB1,SOX8,ZFHX3,RHOA,CNTN2,FOXG1,CNTF,GPR37L1,ISL2,IMPACT,LBX1,MMD,SIX1,TGIF2,YWHAH,NKX2-5,HEY2	grey	VSMC_M10
GO:0061307	0.71	0.56	4	8	cardiac neural crest cell differentiation involved in heart development	BP	1	JAG1,HAND2,MAPK1,MAPK3	grey	VSMC_M10
GO:0061308	0.71	0.56	4	8	cardiac neural crest cell development involved in heart development	BP	1	JAG1,HAND2,MAPK1,MAPK3	grey	VSMC_M10
GO:0070486	0.71	0.53	5	10	leukocyte aggregation	BP	1	RAC2,SEMA4D,JAM2,STK10,S100A8	grey	VSMC_M10
GO:1903955	0.71	0.43	14	28	positive regulation of protein targeting to mitochondrion	BP	1	RAC2,USP36,FBXW7,HTRA2,CSNK2A2,HPS4,ARIH2,FZD5,PINK1,NPEPPS,SH3GLB1,MICALL2,RHOU,LEPROT	grey	VSMC_M10
GO:0071715	0.71	0.43	15	30	icosanoid transport	BP	1	PLA2G6,ABCC10,ABCC4,ABCC2,PLA2R1,MAP2K6,SLCO3A1,NTSR1,PLA2G4A,ABCC3,NMUR2,NOS2,PROCA1,PLA2G2C,PTGES	grey	VSMC_M10
GO:0010835	0.71	0.56	4	8	regulation of protein ADP-ribosylation	BP	1	XRCC1,HPF1,ADNP,TINF2	grey	VSMC_M10
GO:1900745	0.71	0.44	13	26	positive regulation of p38MAPK cascade	BP	1	MAP3K5,MAP3K4,AGER,GADD45G,EMC10,RELL2,MFHAS1,HAND2,NCF1,MAP3K3,MINK1,RELL1,GADD45A	grey	VSMC_M10
GO:0034134	0.71	0.49	7	14	toll-like receptor 2 signaling pathway	BP	1	TNFAIP3,MFHAS1,RIPK2,F2RL1,NOD2,TNIP2,CYBA	grey	VSMC_M10
GO:0032688	0.71	0.56	4	8	negative regulation of interferon-beta production	BP	1	REL,NMI,NLRX1,YY1	grey	VSMC_M10

GO:0046777	0.71	0.34	104	204	protein autophosphorylation	BP	1	CLK4,PTK2,MAPK15,NTRK2,PIM1,TEK,TEC,KIT,WNK2,MOB1B,VRK2,AAK1,EPHA7,EGFR,EPHA1,MKNK1,PRKD2,MYO3A,OXSR1,ALK,DYRK1B,NEK10,AATK,MAP4K1,EIF2AK2,ULK1,FLT1,STK33,EPHB1,MOS,HCK,ACVR1B,CAMK4,FER,ATR,VEGFC,PTK2B,MAPKAPK5,EIF2AK4,NBN,RIPK1,STK4,TAOK3,MAPKAPK3,SLK,SIK1,STK17B,PRKX,MAPKAPK2,MAP3K21,CHEK2,BMX,FGR,TSSK4,CDKL5,PASK,MAP3K3,EPHB3,UHMK1,MAP3K11,PIKFYVE,PPP2R5B,EPHB4,SIK2,DDX3X,MARK2,FGFR3,PINK1,STK16,TRPM7,PIM2,MINK1,ATP13A2,EPHA4,TNK1,CDK12,WNK3,FGFR4,MAP3K9,STK10,DAPK2,TAOK2,TAF1,MAPK3,CALM1,BTK,CLK3,CAMK2A,INSR,CSF1R,MARK3,YES1,NLK,STK26,TAOK1,PDPK1,ENG,INSRR,TRIM24,PEAK1,IMPACT,THY1,RAP2B,PDGFD	grey	VSMC_M10
GO:1904923	0.71	0.49	7	14	regulation of autophagy of mitochondrion in response to mitochondrial depolarization	BP	1	PRKN,OPTN,HTRA2,PINK1,MUL1,MFN2,VPS13C	grey	VSMC_M10
GO:0048207	0.71	0.51	6	12	vesicle targeting, rough ER to cis-Golgi	BP	1	MAPK15,KLHL12,PEF1,PDCD6,TMED10,PPP6C	grey	VSMC_M10
GO:0048208	0.71	0.51	6	12	COPII vesicle coating	BP	1	MAPK15,KLHL12,PEF1,PDCD6,TMED10,PPP6C	grey	VSMC_M10
GO:0015825	0.71	0.56	4	8	L-serine transport	BP	1	SLC1A4,SERINC5,SLC38A2,SLC38A5	grey	VSMC_M10
GO:0071474	0.71	0.49	7	14	cellular hyperosmotic response	BP	1	XRCC6,AKR1B1,AQP1,YBX3,XRCC5,MICU1,SLC25A23	grey	VSMC_M10
GO:0009060	0.71	0.35	86	161	aerobic respiration	BP	1	SHMT2,UQCRQ,NDUFA6,ACO2,CAT,MSH2,DLD,NDUFB2,NDUFB6,DNAJC15,DHTKD1,NDUFC2,ATP5F1C,MDH2,NDUFS8,PANK2,NDUFA12,SIRT3,COX7A1,SUCLG2,COX4I2,IDE,ATP5F1A,NDUFS3,DGUOK,UQCRB,COX15,FAM173B,COX6B1,NDUFV3,TRPV4,OXA1L,NDUFB4,ISCU,COA6,NDUFB3,ACTN3,NDUFS2,DNAJC30,MLXIPL,SDHC,MDH1B,COX6A1,IDH3A,COX8A,PINK1,NDUFA9,SDHB,COX6A2,NDUFAB1,UQCRC2,COX10,COX5A,TEFM,NDUFA7,SLC25A23,NDUFV2,SLC25A14,NDUFB10,UQCR11,PARK7,SLC25A33,ATP5PB,MT-ND1,MT-ND2,MT-ATP8,MT-ATP6,MT-CO3,MT-ND6,MT-CYB,RHOA,ATP5ME,ATP5MF,NDUFA5,NDUFC1,COX7A2,FXN,NDUFA3,NDUFB5,NDUFS6,NDUFA8,SDHAF2,NDUFS5,UQCC2,COX7A2L,PPIF	grey	VSMC_M10
GO:0048672	0.71	0.56	4	8	positive regulation of collateral sprouting	BP	1	LRP1,SEMA4D,BDNF,WNT3	grey	VSMC_M10
GO:0090407	0.71	0.32	237	452	organophosphate biosynthetic process	BP	1	NME1,TBPL1,PIP4K2C,ATP5MGL,SYNJ2,SERAC1,AGPAT4,PPT2,AGPAT1,PPARA,ACSL6,PNPLA3,PLA2G6,CKB,GPAA1,MOCS1,GUCA1B,IMPDH1,NMRK1,SLC35B2,CWH43,SRD5A3,SLC25A13,PPAT,IPPK,INPP4A,PPCS,TMEM246,PIGF,MBOAT7,ME1,PPT1,PLEK,MBOAT2,LPIN1,INPP4B,DLD,FIG4,SPHK2,CMPK1,ADCY3,MTMR2,PIP4K2A,SELENOI,ABHD5,AKR1A1,PRKAG2,TAMM41,SLC22A13,ATP5F1C,XYLB,ITPKC,ELOVL1,MTMR14,IDO2,IDO1,PGAP2,CRLS1,CDS2,PANK2,PDK1,NR1H4,PDHX,MTMR7,SLC5A8,ELOVL7,PANK1,NUDT12,AMPD3,PIGU,PIK3CB,PITPNM2,PIK3R1,TMEM150A,GK5,ATP5F1A,GUCY1A1,GUCY1B1,SLA2,P2RY1,PTK2B,CKMT2,DGUOK,GMPS,SERINC5,SYNJ1,CDS1,FITM2,AMPD1,ITPKA,PIGT,ADCY7,FAM173B,ADCY2,NME4,LPCAT1,PGAP1,GPAM,ACSL5,CHKA,ACP6,ABHD4,SGMS2,AJUBA,GGPS1,PITPNM1,PRPS2,GPER1,GNPAT,DGKD,PIGM,FITM1,PARP9,BMX,HACD2,FAM126B,IP6K2,LPGAT1,ADCY4,PIGV,DPAGT1,CTPS2,MTMR3,NTSR1,DNAJC30,PCYT1A,INPP5J,PIGX,DTYMK,PIGZ,CKMT1B,PISD,PDK3,DGKG,PTH1R,PIKFYVE,SLC25A16,PLA2G4A,CPS1,SACM1L,ACSBG1,ATP5S,LPCAT3,PYGL,PIGC,COASY,GCH1,SOCS4,PINK1,PLA2G16,STAT3,PI4KA,NME7,SLC27A1,ADK,MPC2,ADCY10,MAPK1,ABHD8,SGMS1,PARP1,TECR,GPHN,PGAP3,PIGH,PI4K2B,SOCS7,SLC26A1,DGKQ,PNPO,GUCY2D,PKLR,PANK3,GCDH,AC138894.1,UPRT,DGKE,TTC7B,MOCOS,ACACA,PIGL,SLC26A2,ABHD3,PRPSAP2,ADSSL1,MOCS2,NOS2,ACSBG2,MPPE1,IMPA2,SOCS1,MTM1,MTMR1,INPP5K,PIP5K1C,NADK,ATP6V0C,PANK4,NME3,AGPAT2,ATP5PB,VPS9D1,ACSF3,AK8,UCK1,MLYCD,KARS,PIP5KL1,FUK,DHODH,MT-ATP8,MT-[illegible]	grey	VSMC_M10
GO:1901862	0.71	0.56	4	8	negative regulation of muscle tissue development	BP	1	SIRT2,USP19,YBX3,SOX15	grey	VSMC_M10
GO:0061635	0.71	0.53	5	10	regulation of protein complex stability	BP	1	SCOC,TRAPPC11,HSPA8,ECSIT,IRGM	grey	VSMC_M10
GO:0070230	0.71	0.51	6	12	positive regulation of lymphocyte apoptotic process	BP	1	IDO1,ZC3H8,PDCD1,ADAM8,CCL5,WNT5A	grey	VSMC_M10
GO:0032967	0.71	0.46	10	20	positive regulation of collagen biosynthetic process	BP	1	HDAC2,F2R,AMELX,WNT4,SUCO,LARP6,SERPINF2,ENG,RGCC,CBX8	grey	VSMC_M10
GO:1903749	0.71	0.42	15	30	positive regulation of establishment of protein localization to mitochondrion	BP	1	RAC2,USP36,FBXW7,HTRA2,CSNK2A2,HPS4,ARIH2,FZD5,NMT1,PINK1,NPEPPS,SH3GLB1,MICALL2,RHOU,LEPROT	grey	VSMC_M10
GO:0045980	0.71	0.48	8	16	negative regulation of nucleotide metabolic process	BP	1	PPARA,ACTN3,HDAC4,STAT3,PARP1,NCOR1,PDZD3,TIGAR	grey	VSMC_M10
GO:1901984	0.71	0.47	9	18	negative regulation of protein acetylation	BP	1	HINT2,ATG5,HDAC2,TAF7,SDR16C5,HDAC1,FOXP3,SIN3A,PARK7	grey	VSMC_M10
GO:0070207	0.71	0.56	4	8	protein homotrimerization	BP	1	PNPT1,P2RX3,ASIC1,CLYBL	grey	VSMC_M10
GO:0000381	0.71	0.39	26	52	regulation of alternative mRNA splicing, via spliceosome	BP	1	ZC3H10,PUF60,RBFOX2,SMU1,YTHDC1,CELF2,WTAP,CELF1,SRSF6,RBM24,RBM5,CELF3,RBM7,RBM10,FXR2,RBM25,RAVER2,MBNL3,NSRP1,FMR1,CELF5,SRRM4,NOVA1,RBM11,SRSF12,SAP18	grey	VSMC_M10
GO:0045628	0.71	0.56	4	8	regulation of T-helper 2 cell differentiation	BP	1	HLX,BCL6,IL4R,PRKCZ	grey	VSMC_M10
GO:2000508	0.71	0.56	4	8	regulation of dendritic cell chemotaxis	BP	1	GAS6,SLAMF8,IL12A,TNFSF18	grey	VSMC_M10
GO:0071260	0.71	0.38	35	69	cellular response to mechanical stimulus	BP	1	BAK1,HABP4,AQP1,CASP8AP2,EGFR,TLR4,MKX,SOX9,MYD88,WNT11,CNN2,TMEM150C,SLC38A2,CD40,KCNJ2,DAG1,TLR5,AGT,ATP1A2,BAG3,SLC9A1,HDAC4,KCNK4,LTBR,TNFRSF8,MAPK3,PTGER4,PIEZO1,CYBA,ENG,BCL10,FADD,CASP8,GADD45A,CRADD	grey	VSMC_M10
GO:0002638	0.71	0.56	4	8	negative regulation of immunoglobulin production	BP	1	PRKDC,TMBIM6,BCL6,FOXP3	grey	VSMC_M10
GO:0051180	0.71	0.43	13	26	vitamin transport	BP	1	CUBN,SLC23A2,ABCG2,SLC2A10,ABCC5,SLC2A2,SLC27A1,ABCD4,STRA6,CD320,SLC46A1,SLC2A6,SLC25A32	grey	VSMC_M10
GO:0030449	0.71	0.56	4	8	regulation of complement activation	BP	1	PHB,C3,CD46,A2M	grey	VSMC_M10
GO:0006924	0.71	0.56	4	8	activation-induced cell death of T cells	BP	1	GPAM,DNAJA3,FADD,TSC22D3	grey	VSMC_M10

GO:0008643	0.71	0.36	57	112	carbohydrate transport	BP	1	SLC2A12,SLC45A4,MAPK14,SLC25A27,SLC35A1,AQP1,GRB10,FGF21,BRAF,MFSD4B,MFSD2B,SLC26A5,AQP11,PRKAG2,AKT2,ASPSCR1,SLC23A2,TRIB3,SLC35A4,PIK3R1,PLS1,SORBS1,TMEM144,IRS1,SLC37A1,SLC35A5,SLC2A10,TERT,SLC37A2,C2CD5,SLC37A4,PEA15,NFE2L2,CLTCL1,SLC2A2,SLC27A1,SLC2A9,SLC35A2,SLC2A4,RSC1A1,SLC5A2,SLC35B1,SLC50A1,SLC5A11,PRKCB,INSR,YES1,C3,SLC45A2,GPC3,INPP5K,SLC45A1,SLC2A6,TSC1,FABP5,SLC35C1,ARPP19	grey	VSMC_M10
GO:0006977	0.71	0.48	8	16	DNA damage response, signal transduction by p53 class mediator resulting in cell cycle arrest	BP	1	PLK3,PLK2,PIDD1,CHEK2,CDKN1B,MUC1,SOX4,CRADD	grey	VSMC_M10
GO:2000272	0.71	0.42	16	32	negative regulation of signaling receptor activity	BP	1	PPARA,LY6E,DKKL1,DKK3,DKK2,CBL,ACE2,SNX6,LRPAP1,ZFYVE28,SOCS4,PSEN1,WFIKKN2,WFIKKN1,MIR125A,CNRIP1	grey	VSMC_M10
GO:0003352	0.71	0.45	10	20	regulation of cilium movement	BP	1	CFAP69,DNAH11,ARMC4,DRC1,MKKS,CFAP20,CCDC65,CFAP43,CFAP45,CCSAP	grey	VSMC_M10
GO:2000232	0.71	0.48	8	16	regulation of rRNA processing	BP	1	USP36,SIRT7,NUDT16,DIMT1,RIOK1,HEATR1,YTHDF2,METTL18	grey	VSMC_M10
GO:0009067	0.71	0.46	9	18	aspartate family amino acid biosynthetic process	BP	1	MTAP,APIP,ENOPH1,ASNSD1,MTRR,MTR,PLOD3,AASDHPPT,MTHFD2L	grey	VSMC_M10
GO:0044342	0.71	0.48	8	16	type B pancreatic cell proliferation	BP	1	NR4A1,NKX6-1,SERPINB1,SIDT2,MEN1,PTPRN,WDR13,IGFBP4	grey	VSMC_M10
GO:0036353	0.71	0.56	4	8	histone H2A-K119 monoubiquitination	BP	1	BMI1,PCGF6,PCGF3,TRIM37	grey	VSMC_M10
GO:0032928	0.71	0.46	9	18	regulation of superoxide anion generation	BP	1	FPR2,EGFR,HVCN1,F2RL1,AGT,MAPT,ITGAM,GNAI3,CYBA	grey	VSMC_M10
GO:0034311	0.71	0.45	11	22	diol metabolic process	BP	1	SPTLC1,SPHK2,DHFR,ACER3,SPTLC3,ASAH1,SPR,GCH1,ACER2,SGPP1,PCBD2	grey	VSMC_M10
GO:0035751	0.71	0.49	7	14	regulation of lysosomal lumen pH	BP	1	PPT1,TMEM106B,TMEM9,AC138894.1,CLN6,SNAPIN,TMEM199	grey	VSMC_M10
GO:0071578	0.71	0.56	4	8	zinc ion import across plasma membrane	BP	1	SLC39A5,SLC39A4,SLC39A12,SLC39A6	grey	VSMC_M10
GO:0042996	0.71	0.53	5	10	regulation of Golgi to plasma membrane protein transport	BP	1	GOPC,LYPLA1,CNST,RSC1A1,AC138894.1	grey	VSMC_M10
GO:0034135	0.71	0.53	5	10	regulation of toll-like receptor 2 signaling pathway	BP	1	TNFAIP3,MFHAS1,F2RL1,NOD2,CYBA	grey	VSMC_M10
GO:0015780	0.71	0.50	6	12	nucleotide-sugar transmembrane transport	BP	1	SLC35A1,SLC35A4,SLC35A5,SLC35A2,SLC35B1,SLC35C1	grey	VSMC_M10
GO:0014824	0.71	0.55	4	8	artery smooth muscle contraction	BP	1	MKKS,AGT,CD38,SCNN1B	grey	VSMC_M10
GO:0006487	0.71	0.38	28	55	protein N-linked glycosylation	BP	1	B4GALT7,GFPT2,ALG12,PMM1,SRD5A3,ALG2,ALG11,MGAT5B,UGGT2,MOGS,RPN2,ALG10,DAD1,DPAGT1,ALG3,MAN1C1,MGAT1,DERL3,ALG9,MGAT2,DDOST,KRTCAP2,ALG6,PMM2,ALG1,ENTPD5,DHDDS,TMEM258	grey	VSMC_M10
GO:0046847	0.71	0.38	28	56	filopodium assembly	BP	1	SRF,FGD3,VSTM5,ARHGEF4,RAB5A,SPEF1,FGD6,DPYSL3,AL359736.1,PIK3R1,DMTN,SRGAP2C,NEURL1,FGD4,GAP43,RAB17,DNM3,MIEN1,FGD1,FXR2,TENM2,AC138894.1,BCAS3,ARHGAP44,FMR1,PALM,FNBP1L,RALA	grey	VSMC_M10
GO:0071800	0.71	0.49	7	14	podosome assembly	BP	1	IL5,ASAP1,LCP1,HCK,FARP2,KIF9,RHOA	grey	VSMC_M10
GO:0034145	0.71	0.53	5	10	positive regulation of toll-like receptor 4 signaling pathway	BP	1	NINJ1,PELI1,NR1H3,F2RL1,WDFY1	grey	VSMC_M10
GO:1905288	0.71	0.53	5	10	vascular associated smooth muscle cell apoptotic process	BP	1	STK4,PDCD4,MFN2,MIR21,MIR24-1	grey	VSMC_M10
GO:1905459	0.71	0.53	5	10	regulation of vascular associated smooth muscle cell apoptotic process	BP	1	STK4,PDCD4,MFN2,MIR21,MIR24-1	grey	VSMC_M10
GO:0001991	0.71	0.53	5	10	regulation of systemic arterial blood pressure by circulatory renin-angiotensin	BP	1	MME,F2RL1,F2R,ENPEP,ACE2	grey	VSMC_M10
GO:0010224	0.71	0.49	7	14	response to UV-B	BP	1	MSH2,XPC,ERCC6,MME,HYAL1,HYAL3,IL12A	grey	VSMC_M10
GO:0050862	0.71	0.49	7	14	positive regulation of T cell receptor signaling pathway	BP	1	CD226,PRKD2,NECTIN2,LCK,CARD11,IKBKG,BCL10	grey	VSMC_M10
GO:2000352	0.71	0.44	12	24	negative regulation of endothelial cell apoptotic process	BP	1	TNFAIP3,TEK,FGF21,NDNF,GAS6,TERT,NFE2L2,TNIP2,RAMP2,MAPK7,PDPK1,FGA	grey	VSMC_M10
GO:0035246	0.71	0.52	5	10	peptidyl-arginine N-methylation	BP	1	NDUFAF7,PRMT9,PRMT3,PRMT7,PRMT6	grey	VSMC_M10
GO:1902074	0.71	0.49	7	14	response to salt	BP	1	RIDA,MAPK13,HSF1,ZNF16,SLC26A5,HSPA5,PITX3	grey	VSMC_M10
GO:0090023	0.71	0.45	10	20	positive regulation of neutrophil chemotaxis	BP	1	RAC2,CXCL8,C5AR1,NCKAP1L,DNM1L,MOSPD2,MCU,CXCR2,DAPK2,CD74	grey	VSMC_M10
GO:0010819	0.71	0.52	5	10	regulation of T cell chemotaxis	BP	1	CCL27,OXSR1,TMEM102,CCL5,WNT5A	grey	VSMC_M10
GO:1902165	0.71	0.50	6	12	regulation of intrinsic apoptotic signaling pathway in response to DNA damage by p53 class mediator	BP	1	TAF9,ING2,MARCH7,MUC1,TAF9B,CD74	grey	VSMC_M10
GO:0010677	0.71	0.41	17	34	negative regulation of cellular carbohydrate metabolic process	BP	1	PRKN,PPARA,CRY1,PLEK,FAM3C,GCKR,SIK1,MST1,ACTN3,HDAC4,PASK,STAT3,COX11,NCOR1,LEPR,INPP5K,TIGAR	grey	VSMC_M10
GO:0090650	0.71	0.55	4	8	cellular response to oxygen-glucose deprivation	BP	1	DNM1L,CBL,TSC1,LCN2	grey	VSMC_M10
GO:0071888	0.71	0.55	4	8	macrophage apoptotic process	BP	1	CDKN2A,IRF7,MEF2C,NOD2	grey	VSMC_M10
GO:2000406	0.71	0.46	9	18	positive regulation of T cell migration	BP	1	CCL27,OXSR1,ADAM8,ITGA4,TMEM102,CCL5,RHOA,FADD,WNT5A	grey	VSMC_M10
GO:0006903	0.71	0.40	21	42	vesicle targeting	BP	1	FAM91A1,EXOC4,MAPK15,STARD3NL,CLASP2,ARFGAP2,EXOC6B,GBF1,KLHL12,TRIP11,PEF1,PDCD6,SNAP23,AP1AR,MAP4K2,SNAP29,PSEN1,TMED10,AP1M2,C17orf75,PPP6C	grey	VSMC_M10
GO:0051817	0.71	0.37	34	67	modulation of process of other organism involved in symbiotic interaction	BP	1	YTHDC2,SNW1,IGF2R,PRKN,ATG5,NAPEPLD,PHB,FBXL2,ULK1,SAP30,EIF2AK4,ANO6,HDAC1,DAG1,ZNF639,CFL1,HSPA8,POU2F3,PC,PSMC3,TARDBP,TNIP1,INSR,CCL5,JUN,FMR1,INPP5K,RXRA,MIR222,IRGM,SAP30BP,SERPINB9,TBC1D20,ZNF502	grey	VSMC_M10
GO:0072161	0.72	0.55	4	8	mesenchymal cell differentiation involved in kidney development	BP	1	SIX2,OSR1,PAX2,WNT4	grey	VSMC_M10
GO:2001012	0.72	0.55	4	8	mesenchymal cell differentiation involved in renal system development	BP	1	SIX2,OSR1,PAX2,WNT4	grey	VSMC_M10
GO:0016137	0.72	0.50	6	12	glycoside metabolic process	BP	1	FUCA2,AKR1B1,AKR1A1,CBR4,GLA,ABHD10	grey	VSMC_M10
GO:0045292	0.72	0.44	12	24	mRNA cis splicing, via spliceosome	BP	1	WBP4,CLNS1A,CWC15,PRPF4B,WBP11,NCBP2,PRPF39,RNVU1-6,RNU4ATAC,RF00003,SNRPC,SRSF12	grey	VSMC_M10
GO:1905216	0.72	0.55	4	8	positive regulation of RNA binding	BP	1	EIF3E,EIF3C,LARP6,FMR1	grey	VSMC_M10
GO:0045737	0.72	0.43	13	26	positive regulation of cyclin-dependent protein serine/threonine kinase activity	BP	1	CCND3,EGFR,CCNY,MAPRE3,PKD2,CCND1,CDKN1B,PSMD10,CDK5R1,PKD1,PSRC1,SPDYA,RGCC	grey	VSMC_M10
GO:0001963	0.72	0.46	9	18	synaptic transmission, dopaminergic	BP	1	PRKN,SLC6A2,SLC6A3,PNKD,PINK1,CHRNB2,PARK7,CNTNAP4,CRH	grey	VSMC_M10

GO:0051354	0.72	0.47	8	16	negative regulation of oxidoreductase activity	BP	1	CYP27B1,PRKN,NOSIP,OXA1L,GLA,GFI1,MIR21,MIR27B	grey	VSMC_M10
GO:0010944	0.72	0.52	5	10	negative regulation of transcription by competitive promoter binding	BP	1	PHB,DACH1,TFAP2A,CREB1,MUC1	grey	VSMC_M10
GO:0000463	0.72	0.48	7	14	maturation of LSU-rRNA from tricistronic rRNA transcript (SSU-rRNA, 5.8S rRNA, LSU-rRNA)	BP	1	URB1,WDR12,RPL7L1,ZNHIT3,NOL9,RPL35,ZNHIT6	grey	VSMC_M10
GO:0032703	0.72	0.45	10	20	negative regulation of interleukin-2 production	BP	1	TNFAIP3,NAV3,PRNP,HDAC7,NOD2,HOMER2,LAG3,FOXP3,TBX21,HAVCR2	grey	VSMC_M10
GO:0051775	0.72	0.48	7	14	response to redox state	BP	1	ADH5,SLC7A11,SIRT2,RNF7,ARNTL,RYR2,GLRX2	grey	VSMC_M10
GO:0045088	0.72	0.34	76	148	regulation of innate immune response	BP	1	CD226,TNFAIP3,USP15,XRCC6,FPR2,AKIRIN2,NR1H2,TLR4,NECTIN2,PVR,IKBKB,NMI,TMEM173,MAVS,HPX,PRKDC,IRF7,TRAF6,HCK,ERAP1,RASGRP1,NR1H3,TRAFD1,OAS3,NLRC5,POLR3D,NOD2,CD96,POLR3C,NCF1,YTHDF2,SLAMF8,PARP9,PARP14,FGR,NLRX1,COCH,ADAM8,CADM1,DUSP10,NONO,IL12A,XRCC5,PTPN6,LAG3,MUL1,BIRC3,STAT5B,TKFC,USP18,MED1,DCST1,SFPQ,STAT2,SIN3A,CCL5,RIOK3,RNF135,VAV1,PTPN2,SOCS1,LRP8,CRK,CREBBP,AP1G1,MIR21,OAS1,FADD,CASP8,ZCCHC3,A2M,IRGM,WNT5A,SERPINB9,EREG,HAVCR2	grey	VSMC_M10
GO:0031397	0.72	0.37	37	72	negative regulation of protein ubiquitination	BP	1	TNFAIP3,HSPA1B,TSPO,CRY1,SVBP,DNAJA1,PRKCE,ATG5,SPOPL,SPRY2,KLHL40,ARRB1,SIRT7,USP44,SH3RF2,TRIM44,MARCH7,PRMT3,RPL23,DTX3L,PER2,RPL11,SENP2,IVNS1ABP,GPS2,GNL3L,PSEN1,TAF1,OGT,OTUB2,PARK7,GLMN,SOX4,BAG2,CAMLG,PRR7,RASD2	grey	VSMC_M10
GO:0048070	0.72	0.55	4	8	regulation of developmental pigmentation	BP	1	ADAMTS20,HPS4,BLOC1S6,BCL2L11	grey	VSMC_M10
GO:0120305	0.72	0.55	4	8	regulation of pigmentation	BP	1	ADAMTS20,HPS4,BLOC1S6,BCL2L11	grey	VSMC_M10
GO:1901099	0.72	0.43	13	26	negative regulation of signal transduction in absence of ligand	BP	1	EYA4,HSPA1B,ZC3HC1,IL1A,EYA1,IL7,TERT,EYA3,IFI6,MCL1,STRADB,MAP2K5,MAPK7	grey	VSMC_M10
GO:2001240	0.72	0.43	13	26	negative regulation of extrinsic apoptotic signaling pathway in absence of ligand	BP	1	EYA4,HSPA1B,ZC3HC1,IL1A,EYA1,IL7,TERT,EYA3,IFI6,MCL1,STRADB,MAP2K5,MAPK7	grey	VSMC_M10
GO:0060401	0.72	0.35	64	126	cytosolic calcium ion transport	BP	1	SMDT1,BAK1,PRKCE,IBTK,ATG5,GRIN2D,TRPC3,SLC8A2,RYR1,PRNP,DIAPH1,P2RX2,RASA3,MICU3,P2RX3,RYR3,TMBIM6,P2RX7,PTK2B,TPCN1,SLC8B1,OPA1,F2R,PKD2,CASQ2,MCUR1,RYR2,TRPM1,LCK,TRPV4,MCUB,UBASH3B,ANK2,GPER1,ATP1A2,CASQ1,NTSR1,CEMIP,PTPN6,MICU1,MCU,HTT,HAP1,PSEN2,FAM155B,CD19,CALM1,BDKRB1,TRPV2,SLC25A23,MAIP1,ATP2B3,TRPV3,TRPV1,PDPK1,P2RX1,CYBA,JPH3,MCOLN3,DMD,NOL3,JPH2,THY1,DDIT3	grey	VSMC_M10
GO:1904181	0.72	0.52	5	10	positive regulation of membrane depolarization	BP	1	TSPO,TRPM4,DCN,PARP1,MLLT11	grey	VSMC_M10
GO:0017185	0.72	0.55	4	8	peptidyl-lysine hydroxylation	BP	1	PLOD3,P3H4,VIPAS39,P3H3	grey	VSMC_M10
GO:0031269	0.72	0.50	6	12	pseudopodium assembly	BP	1	CDC42EP1,KIT,F2RL1,SRGAP2C,CDC42EP2,APC	grey	VSMC_M10
GO:0048742	0.72	0.55	4	8	regulation of skeletal muscle fiber development	BP	1	LMOD3,ACTN3,HDAC4,FBXO22	grey	VSMC_M10
GO:0051899	0.72	0.37	33	66	membrane depolarization	BP	1	NEDD4L,TSPO,TRPM4,CACNB2,SCN5A,SCN1A,GPD1L,DCN,SLMAP,P2RX7,TBX5,KCNJ2,SCN2A,SCN7A,ANK2,IFI6,ATP1A2,NTSR1,SCN4B,FZD9,PPP2R3C,PARP1,CACNA1G,CHRNB2,JUN,PRKCZ,CACNA1H,PARK7,MLLT11,KCNH2,SCN2B,YWHAH,HCN4	grey	VSMC_M10
GO:0000098	0.72	0.55	4	8	sulfur amino acid catabolic process	BP	1	CSAD,MTRR,CBSL,MAT1A	grey	VSMC_M10
GO:0045665	0.72	0.38	26	52	negative regulation of neuron differentiation	BP	1	HOXA2,CDK5RAP2,FOXO3,SOX9,JAG1,LSM1,ZNF536,EIF2AK4,NEPRO,SOX2,DDX6,DLX1,EIF4ENIF1,DIXDC1,RAP1GAP,MED1,MIB1,ISL1,SOX8,RHOA,CNTN2,FOXG1,CNTF,GPR37L1,ISL2,LBX1	grey	VSMC_M10
GO:0070076	0.72	0.43	12	25	histone lysine demethylation	BP	1	UBE2B,KDM3B,KDM4C,KDM7A,KDM4A,KDM5A,KDM5C,PHF8,KDM6B,RIOX1,KDM8,HSF4	grey	VSMC_M10
GO:0061469	0.72	0.55	4	8	regulation of type B pancreatic cell proliferation	BP	1	NR4A1,MEN1,PTPRN,WDR13	grey	VSMC_M10
GO:0060732	0.72	0.55	4	8	positive regulation of inositol phosphate biosynthetic process	BP	1	P2RY1,GPER1,NTSR1,PTH1R	grey	VSMC_M10
GO:0071803	0.72	0.55	4	8	positive regulation of podosome assembly	BP	1	IL5,ASAP1,LCP1,RHOA	grey	VSMC_M10
GO:0051385	0.72	0.43	13	26	response to mineralocorticoid	BP	1	FOXO3,AANAT,CCND1,KRAS,GPER1,NPAS4,RPL27,PARP1,FOS,CALM1,SCNN1B,CYBA,CRH	grey	VSMC_M10
GO:0032964	0.72	0.40	19	38	collagen biosynthetic process	BP	1	TRAM2,ADAMTS3,HDAC2,CYGB,CYP7A1,F2R,AMELX,WNT4,SUCO,P3H4,LARP6,CIITA,SERPINF2,COL5A1,ENG,MIR29A,RGCC,P3H3,CBX8	grey	VSMC_M10
GO:0032608	0.72	0.39	22	44	interferon-beta production	BP	1	IRF5,REL,TLR4,NMI,TMEM173,MAVS,IRF7,OAS3,RIPK2,POLR3D,IFIH1,POLR3C,NLRX1,DDX3X,POLR3A,RNF216,YY1,TRAF3,RIOK3,RNF135,TICAM1,OAS1	grey	VSMC_M10
GO:0032648	0.72	0.39	22	44	regulation of interferon-beta production	BP	1	IRF5,REL,TLR4,NMI,TMEM173,MAVS,IRF7,OAS3,RIPK2,POLR3D,IFIH1,POLR3C,NLRX1,DDX3X,POLR3A,RNF216,YY1,TRAF3,RIOK3,RNF135,TICAM1,OAS1	grey	VSMC_M10
GO:1905517	0.72	0.40	18	36	macrophage migration	BP	1	PTK2,ROR2,AKIRIN1,C5AR1,FAM19A4,MTUS1,PTK2B,CD200,TRPV4,CMKLR1,SLAMF8,TNFSF18,MAPK1,MAPK3,CCL5,CSF1R,CCL2,MIR24-1	grey	VSMC_M10
GO:0043101	0.72	0.52	5	10	purine-containing compound salvage	BP	1	MTAP,PRTFDC1,AMPD3,AMPD1,ADK	grey	VSMC_M10
GO:0010887	0.72	0.55	4	8	negative regulation of cholesterol storage	BP	1	PPARA,TTC39B,NR1H2,NR1H3	grey	VSMC_M10
GO:0031124	0.72	0.38	28	56	mRNA 3'-end processing	BP	1	CPSF6,PABPC1,ZC3H3,HSF1,CPSF1,RNF20,YTHDC1,PNPT1,LSM10,MTPAP,SYMPK,PCF11,CSTF3,CLP1,RPRD1B,TENT2,TENT4B,CPSF2,RPRD2,CSTF1,NCBP2,CPSF4,LEO1,AP002990.1,CSTF2,PAPOLA,RPRD1A,ZNF473	grey	VSMC_M10
GO:0032230	0.72	0.55	4	8	positive regulation of synaptic transmission, GABAergic	BP	1	PRKCE,HAP1,NLGN2,ZDHHC12	grey	VSMC_M10
GO:0035247	0.72	0.55	4	8	peptidyl-arginine omega-N-methylation	BP	1	NDUFAF7,PRMT9,PRMT7,PRMT6	grey	VSMC_M10
GO:0042594	0.72	0.34	81	158	response to starvation	BP	1	MARS,MAP3K5,PPARA,ATF4,PICK1,TFEB,SLC39A4,GPT,NUAK1,BRSK1,ALB,RRAGD,VPS41,ATG5,FOXO3,KPTN,SESN3,FOXA3,DNAJC15,RALB,PRKAG2,SZT2,RPTOR,EIF2AK2,WRN,GAS6,LARS,ULK1,PRKAG1,EIF2AK4,SLC38A2,NUAK2,NUPR2,SRD5A1,SIK1,SLC38A3,HSPA8,GNPAT,CBL,MTMR3,XPR1,TBL2,HMGCL,GLUL,NFE2L2,CPS1,SIK2,COMT,WNT4,PRKAG3,CTSV,MAPK1,BHLHA15,WIPI2,MAX,RRAGB,BNIP1,ZFYVE1,MAPK3,TNRC6A,LARP1,BCAS3,ADSSL1,SREBF1,LAMP2,NIM1K,OXCT1,STK26,JUN,CLEC16A,KIAA1324,WDR59,HSPA5,DHODH,SH3GLB1,RIPOR1,ACADM,IMPACT,DDIT3,UPP1,RNF152	grey	VSMC_M10

GO:1901264	0.72	0.37	31	61	carbohydrate derivative transport	BP	1	SLC35B2,SLC35A1,ABCC4,SLC35A4,SLC33A1,PLTP,SLC37A1,CALHM1,SLC35A5,SLC37A2,ABCC5,SLC37A4,SLC29A2,SLC29A3,SLC25A42,SLC35A2,SLC29A4,RSC1A1,SLC5A2,SLC35B1,SLC50A1,AC138894.1,ABCC3,LRRC8E,SLC25A23,SLC25A41,RALBP1,LRRC8A,SLC25A25,SLC35E3,SLC35C1	grey	VSMC_M10
GO:0020027	0.72	0.48	7	14	hemoglobin metabolic process	BP	1	CAT,HPX,SLC25A37,ALAS1,LDB1,INHA,AHSP	grey	VSMC_M10
GO:0032693	0.72	0.55	4	8	negative regulation of interleukin-10 production	BP	1	AGER,TNFRSF21,VSIR,FOXP3	grey	VSMC_M10
GO:0014068	0.72	0.37	33	66	positive regulation of phosphatidylinositol 3-kinase signaling	BP	1	ERBB3,RGL2,PTK2,NTRK2,TEK,KIT,ROR2,SEMA4D,CAT,SOX9,DCN,FLT1,NKX3-1,F2RL1,F2R,TGFB2,NCF1,EGF,HCLS1,GPER1,AGT,FGR,CBL,OSM,CD28,FN1,PTPN6,HAX1,INSR,CCL5,MIR21,HCST,PDGFD	grey	VSMC_M10
GO:0061512	0.72	0.38	27	54	protein localization to cilium	BP	1	TBC1D32,LRRC6,MAPK15,DNAH11,TULP2,TTC26,TTC21A,TUB,CCDC66,ZNF423,ZMYND10,TTC21B,BBS1,LZTFL1,EHD1,FAM149B1,TULP3,TTC25,ROM1,WDR19,ATP6V1D,TMEM107,IFT20,EFCAB7,TCTN2,SNX10,CC2D2B	grey	VSMC_M10
GO:0048280	0.72	0.55	4	8	vesicle fusion with Golgi apparatus	BP	1	YIPF4,SEC22B,VTI1A,STX5	grey	VSMC_M10
GO:0001937	0.72	0.41	17	34	negative regulation of endothelial cell proliferation	BP	1	AIMP1,FLT1,ATP5F1A,MEF2C,IL12A,PDCD10,SYNJ2BP,NGFR,VASH1,TNMD,CCL2,MIR21,MIR222,MIR22,MIR24-1,ACVRL1,RGCC	grey	VSMC_M10
GO:0006662	0.72	0.45	10	20	glycerol ether metabolic process	BP	1	PEX7,PLA2G6,TXN2,TMEM86B,LIPE,GNPAT,PLA2G4A,PLA2G16,NCEH1,TXN	grey	VSMC_M10
GO:0031647	0.72	0.32	129	249	regulation of protein stability	BP	1	NEDD4L,SERF2,TRIM39,RAB21,STK3,ZNF207,HSPA1B,PRKN,RNF5,DERL1,UBE2B,PIM1,SMO,PEX6,FBXO7,BAG1,CDC37L1,GAPDH,PDCL3,ISOC2,RTN4,AAK1,VPS35,QRSL1,PHB,CRTAP,TADA3,USP36,MCM8,PRNP,BAG4,DDRGK1,PRKDC,TRIM44,FAM107A,PIK3R1,CDK7,CASP3,HIP1R,TAF9,CTNND1,CDKN2AIP,PARVA,MTMR9,FBXW7,ADGRV1,PEX2,CCNH,TESC,MARCH7,STK4,MORC3,MYLIP,SIAH1,RPL23,TERT,DAD1,TBRG1,RABL3,GET4,KRAS,USP13,USP19,HSPA8,HPS4,ANK2,CHEK2,USP2,DVL3,BAG3,PEX19,TMEM25,RPL11,SENP2,CREB1,PRKRA,SYVN1,CTSH,MFSD1,SAV1,PDCD10,HTT,TNIP2,DDOST,PINK1,MUL1,OTUD3,ATP1B1,MAPK1,PIM2,EPHA4,DDI2,GNL3L,ATP1B2,TARDBP,TMEM88,AHSP,EFNA1,TAF1,TAF9B,SEL1L,SMAD3,SWSAP1,KDM8,CCDC88C,COG7,MOSMO,RNF128,SREBF1,LAMP2,NLK,NAPG,CREBBP,HCFC1,MAPK8IP3,SEC16A,PER3,PARK7,TSC1,STXBP1,SH3GLB1,TRIM24,SOX4,BAG2,IRGM,SUGT1,CAMLG,USP40,APOPT1	grey	VSMC_M10
GO:0035745	0.72	0.55	4	8	T-helper 2 cell cytokine production	BP	1	RSAD2,DENND1B,TBX21,PRKCZ	grey	VSMC_M10
GO:2000551	0.72	0.55	4	8	regulation of T-helper 2 cell cytokine production	BP	1	RSAD2,DENND1B,TBX21,PRKCZ	grey	VSMC_M10
GO:0098930	0.72	0.37	29	58	axonal transport	BP	1	AP3S1,KIF5A,RAB21,KIF3A,AGTPBP1,MGARP,DLG2,AP3M2,KIF5C,KIF3B,OPA1,AP3B1,DTNBP1,CNIH2,CALY,MAPT,BLOC1S6,AP3M1,KIF1C,HAP1,TRIM46,NDEL1,SNAPIN,MAPK8IP3,TERF2,BLOC1S3,BORCS5,AP3S2,MAP1A	grey	VSMC_M10
GO:0031126	0.72	0.52	5	10	sno(s)RNA 3'-end processing	BP	1	EXOSC4,EXOSC3,TENT4B,PARN,EXOSC5	grey	VSMC_M10
GO:1902624	0.72	0.43	12	24	positive regulation of neutrophil migration	BP	1	RAC2,CXCL8,C5AR1,NCKAP1L,DNM1L,CD99,MOSPD2,ADAM8,MCU,CXCR2,DAPK2,CD74	grey	VSMC_M10
GO:1902176	0.72	0.47	8	16	negative regulation of oxidative stress-induced intrinsic apoptotic signaling pathway	BP	1	PRKN,HTRA2,NFE2L2,NONO,PINK1,MAPK7,PARK7,NOL3	grey	VSMC_M10
GO:0006706	0.72	0.44	10	20	steroid catabolic process	BP	1	CYP27B1,HSD17B14,CYP7A1,SPP1,SRD5A1,HSD11B1,CYP27A1,CYP46A1,CEL,YWHAH	grey	VSMC_M10
GO:2000251	0.72	0.47	8	16	positive regulation of actin cytoskeleton reorganization	BP	1	BAIAP2L2,TEK,CCL27,HCK,HCLS1,HAX1,BCAS3,HRAS	grey	VSMC_M10
GO:0045117	0.72	0.55	4	8	azole transmembrane transport	BP	1	SLC7A1,SLC38A3,SLC38A5,SLC25A29	grey	VSMC_M10
GO:2000480	0.72	0.55	4	8	negative regulation of cAMP-dependent protein kinase activity	BP	1	PRKAR2B,PRKAG2,PKIA,PRKAR1A	grey	VSMC_M10
GO:0032377	0.72	0.55	4	8	regulation of intracellular lipid transport	BP	1	ABCA12,ANXA2,MIR27B,LDLRAP1	grey	VSMC_M10
GO:0043330	0.72	0.41	15	30	response to exogenous dsRNA	BP	1	RALB,TMEM173,MAVS,RIPK2,NOD2,IFIH1,RFTN2,MUL1,MAPK1,SLC3A2,MAPK3,COLEC12,TICAM1,ZCCHC3,KCNJ8	grey	VSMC_M10
GO:1904031	0.72	0.41	15	30	positive regulation of cyclin-dependent protein kinase activity	BP	1	CCND3,TTBK1,EGFR,CCNY,MAPRE3,PKD2,CCND1,CDKN1B,STOX1,PSMD10,CDK5R1,PKD1,PSRC1,SPDYA,RGCC	grey	VSMC_M10
GO:0006940	0.72	0.39	23	46	regulation of smooth muscle contraction	BP	1	ZDHHC21,CHRNA3,SRF,KIT,TNNI3,ADRA2B,PLCE1,GUCY1A1,DOCK5,F2R,CHRM3,ADRA2A,GPER1,ATP1A2,ADRA2C,CALCRL,KCNMA1,NMUR2,SETD3,ABAT,P2RX1,MIR21,RHOA	grey	VSMC_M10
GO:0018126	0.72	0.42	13	26	protein hydroxylation	BP	1	P4HA2,JMJD4,CRTAP,EGLN2,ASPH,HIF1AN,EGLN3,PLOD3,P3H2,P3H4,VIPAS39,P3H3,JMJD7	grey	VSMC_M10
GO:0050930	0.72	0.49	6	12	induction of positive chemotaxis	BP	1	AGER,CXCL8,VEGFC,IL16,PGF,ARTN	grey	VSMC_M10
GO:0071280	0.72	0.49	6	12	cellular response to copper ion	BP	1	HSF1,AQP1,PRNP,BACE1,NFE2L2,MT1X	grey	VSMC_M10
GO:1901659	0.72	0.46	8	16	glycosyl compound biosynthetic process	BP	1	MTAP,NT5E,PRTFDC1,TYW1B,TYW5,LCMT2,DTYMK,ADK	grey	VSMC_M10
GO:0000491	0.72	0.49	6	12	small nucleolar ribonucleoprotein complex assembly	BP	1	C12orf45,TAF9,ZNHIT3,SNU13,ZNHIT6,PIH1D2	grey	VSMC_M10
GO:0015846	0.72	0.54	4	8	polyamine transport	BP	1	TAF7,AZIN2,ATP13A2,OAZ1	grey	VSMC_M10
GO:0060193	0.72	0.38	23	46	positive regulation of lipase activity	BP	1	ESR1,KIT,LPAR1,NR1H2,EGFR,C5AR1,PHB,ABHD5,RASGRP4,FLT1,NR1H3,GNA13,ARHGAP6,AGT,ABL2,FGFR3,NMUR2,CCL5,GNA15,PDPK1,PRKCZ,RHOA,HRAS	grey	VSMC_M10
GO:0009130	0.72	0.49	6	12	pyrimidine nucleoside monophosphate biosynthetic process	BP	1	CMPK1,UPRT,UCK1,DHODH,DCTD,UPP1	grey	VSMC_M10
GO:0034139	0.72	0.54	4	8	regulation of toll-like receptor 3 signaling pathway	BP	1	TNFAIP3,PELI1,F2RL1,WDFY1	grey	VSMC_M10
GO:0010889	0.72	0.49	6	12	regulation of sequestering of triglyceride	BP	1	PPARA,PLIN2,ABHD5,FITM2,FITM1,PLIN3	grey	VSMC_M10
GO:1901799	0.72	0.39	19	38	negative regulation of proteasomal protein catabolic process	BP	1	TRIM39,SDCBP,AQP11,KLHL40,DDRGK1,RYBP,PSMF1,TAF9,USP25,FAM192A,MARCH7,USP19,RPL11,OGT,SGTA,PARK7,CAMLG,STYX,MAP1A	grey	VSMC_M10
GO:0006614	0.72	0.46	8	16	SRP-dependent cotranslational protein targeting to membrane	BP	1	TRAM2,SEC61B,SRP14,SSR3,SRPRA,ZFAND2B,TRAM1L1,SEC61A2	grey	VSMC_M10
GO:0071827	0.72	0.41	16	32	plasma lipoprotein particle organization	BP	1	APOM,NR1H2,APOB,APOC1,NR1H4,SOAT2,PLTP,ABCA5,AGT,SOAT1,ZDHHC8,LPCAT3,LIPC,MPO,ABCA7,PLAGL2	grey	VSMC_M10
GO:0046128	0.72	0.45	9	18	purine ribonucleoside metabolic process	BP	1	MTAP,GNMT,ENPP4,NT5E,PRTFDC1,AHCY,DGUOK,ADK,PEMT	grey	VSMC_M10

GO:0009395	0.72	0.40	18	36	phospholipid catabolic process	BP	1	ABHD16A,PLA2G6,SMPD2,NAPEPLD,APOC1,ABHD6,PLA2G4B,PLBD1,PLA2G4A,ABHD12B,SMPD4,LIPC,GDPD3,LDLR,GDPD1,GDE1,PNPLA6,PLA2G15	grey	VSMC_M10
GO:0060632	0.72	0.41	16	32	regulation of microtubule-based movement	BP	1	CFAP69,DNAH11,ARMC4,DRC1,MKKS,CFAP20,CCDC65,CFAP43,TTC21B,CFAP45,CNIH2,CALY,HAP1,TRIM46,BORCS5,CCSAP	grey	VSMC_M10
GO:0006086	0.72	0.51	5	10	acetyl-CoA biosynthetic process from pyruvate	BP	1	DLD,PDK1,PDHX,PDK3,MPC2	grey	VSMC_M10
GO:0002437	0.72	0.39	20	40	inflammatory response to antigenic stimulus	BP	1	MAPK14,PLK2,HCK,IL37,AHCY,RASGRP1,NOD2,FGR,CD28,SERPINC1,CXCR2,RBPJ,KDM6B,PNMA1,BTK,YES1,C3,PARK7,KARS,IL36G	grey	VSMC_M10
GO:0060563	0.72	0.47	7	14	neuroepithelial cell differentiation	BP	1	WNT11,FAM20C,WNT4,DLX3,TMEM231,SOX4,CEBPB	grey	VSMC_M10
GO:0051084	0.72	0.41	15	30	'de novo' posttranslational protein folding	BP	1	HSPA1B,BAG1,HSPE1,DNAJC18,HSPA8,DNAJB12,SDF2L1,DNAJB1,GAK,CD74,TOR1B,TOR2A,HSPA5,ENTPD5,SDF2	grey	VSMC_M10
GO:0043489	0.72	0.37	26	52	RNA stabilization	BP	1	PABPC1,PHAX,MAPK14,TRAF3IP2,MYD88,TENT2,TENT5C,TENT4B,RBM24,AXIN2,MAPKAPK2,TRAF5,ANGEL2,EIF4ENIF1,YBX3,SLC11A1,RBM10,TARDBP,TENT5D,LARP1,PARN,METTL16,CIRBP,TRAF2,YBX1,TENT5A	grey	VSMC_M10
GO:0060218	0.72	0.41	14	28	hematopoietic stem cell differentiation	BP	1	SRF,ERCC2,EIF2AK2,PRKDC,TCF15,LMBR1L,YTHDF2,OSM,NFE2L2,XRCC5,MEOX1,SETD1A,BATF,SP7	grey	VSMC_M10
GO:0048864	0.72	0.36	36	72	stem cell development	BP	1	SEMA6A,SOX10,SMO,SEMA4D,SEMA4C,SOX9,OVOL2,JAG1,ALX1,SEMA3D,SEMA3A,NRG1,KBTBD8,RET,HAND2,SEMA4F,SEMA3G,SEMA4G,KLHL12,SEMA3F,PEF1,PDCD6,GBX2,LAMA5,SETD2,SEMA6D,FN1,MAPK1,BMPR1A,RADIL,ALDH1A2,SEMA4A,MAPK3,TCOF1,ISL1,SOX8	grey	VSMC_M10
GO:0098760	0.72	0.49	6	12	response to interleukin-7	BP	1	RAD23B,PDIA3,STIP1,BTK,IL7R,YBX1	grey	VSMC_M10
GO:0098761	0.72	0.49	6	12	cellular response to interleukin-7	BP	1	RAD23B,PDIA3,STIP1,BTK,IL7R,YBX1	grey	VSMC_M10
GO:0009886	0.73	0.51	5	10	post-embryonic animal morphogenesis	BP	1	BAK1,MYO7A,FBN1,MFAP2,BCL2L11	grey	VSMC_M10
GO:0042454	0.73	0.51	5	10	ribonucleoside catabolic process	BP	1	APOBEC3H,ENPP4,CDADC1,AHCY,UPP1	grey	VSMC_M10
GO:0070572	0.73	0.51	5	10	positive regulation of neuron projection regeneration	BP	1	LRP1,PTN,NDEL1,MIR222,CNTF	grey	VSMC_M10
GO:2001257	0.73	0.34	59	116	regulation of cation channel activity	BP	1	STAC3,MAPK8IP2,PRRT1,PLA2G6,SHANK1,KCNRG,CACNB2,STAC,CACNB4,SLMAP,MEF2C,PTK2B,KCNJ11,HOMER1,IFNGR2,KCNE2,PKD2,CASQ2,KCNIP2,FAM173B,KCNG1,ANK2,KLHL24,CASQ1,NTSR1,CNIH2,NOS1AP,DLG1,HTT,PINK1,KCNA1,GNB5,MINK1,HAP1,DLG4,NLGN2,CNIH3,STAC2,CACNB1,NLGN3,CALM1,CCL2,FMR1,RNF207,AMIGO1,JPH3,RRAD,MIR24-1,DMD,GPR35,JPH2,FGF11,NETO2,CACNG7,CRBN,CRH,SHISA8,CACNG5,GEM	grey	VSMC_M10
GO:0071712	0.73	0.49	6	12	ER-associated misfolded protein catabolic process	BP	1	RNF5,DERL1,UGGT2,AUP1,RNF185,SDF2L1	grey	VSMC_M10
GO:0048245	0.73	0.54	4	8	eosinophil chemotaxis	BP	1	DAPK2,CCL5,CCL7,CCL2	grey	VSMC_M10
GO:0051583	0.73	0.54	4	8	dopamine uptake involved in synaptic transmission	BP	1	PRKN,SLC6A2,SLC6A3,PARK7	grey	VSMC_M10
GO:0051934	0.73	0.54	4	8	catecholamine uptake involved in synaptic transmission	BP	1	PRKN,SLC6A2,SLC6A3,PARK7	grey	VSMC_M10
GO:0009636	0.73	0.33	90	174	response to toxic substance	BP	1	FECH,LTC4S,APOM,PRKN,BAK1,PRXL2C,KDM3B,FANCC,PIM1,TRPM6,ALDH1A1,SRF,ABCB1,TXNRD1,PRDX1,ADH5,CAT,ALB,PXDN,DDC,MGST2,SLC7A11,TXNRD3,DHFR,DNMT3A,ATP7B,ESD,AKR1A1,GSTK1,PRDX3,CYGB,ERCC6,PXDNL,ABTB2,DNMT3B,GLRX,HTRA2,EPHX2,GPX8,BPHL,TP53INP1,ABCC2,ABCG2,SRD5A1,SLC30A10,FBLN5,CHKA,MARC2,MARC1,OPRD1,MGMT,NFE2L2,DUOX1,SLC30A4,FIS1,CPS1,UBA52,GCH1,PINK1,ABCB6,MAPK1,KDM5C,EPHX1,RDH11,SOD3,KDM6B,FOS,MAPK3,GSTZ1,MPO,CCL5,RALBP1,TXNDC2,PARK7,ADAMTS13,ASS1,LCN2,NQO1,MIR21,MT-CYB,MT1X,SLC22A5,DHRS2,PDZD3,CLDN1,SCN2B,HBA2,TXN,PTGES,ADH4	grey	VSMC_M10
GO:0006108	0.73	0.54	4	8	malate metabolic process	BP	1	ME1,MDH2,D2HGDH,MDH1B	grey	VSMC_M10
GO:0070555	0.73	0.34	50	98	response to interleukin-1	BP	1	YTHDC2,MAPK13,IL1R2,VRK2,CXCL8,MYLK3,BMI1,SOX9,MYD88,IRAK2,HNMT,IKBKB,PLCB1,ST18,TRAF6,OTUD4,TAF9,CCL17,RIPK2,NKX3-1,ABCC2,TOLLIP,CD40,HYAL1,HYAL3,HDAC4,RC3H1,UPF1,TNIP2,CD38,MAPK3,CITED1,RPS6KA5,YY1,CCL14,CCL5,CCL7,CCL2,CCL25,GHR,ADAMTS12,CYBA,LCN2,DAB2IP,MIR21,MIR27A,CCL16,CEBPB,RPS6KA4,CCL22	grey	VSMC_M10
GO:0035107	0.73	0.34	58	115	appendage morphogenesis	BP	1	OSR2,FZD6,TBC1D32,BAK1,PITX1,PTCH1,TFAP2B,ROR2,DLX5,HOXA13,MYCN,OSR1,INTU,HDAC2,SOX9,GLI2,MEGF8,LMBR1,ZNF219,RARB,FREM2,HAND2,FMN1,FRAS1,TBX5,IFT52,RPGRIP1L,TFAP2A,SALL1,FBXW4,BMPR1B,HDAC1,TGFB2,SP9,WNT3,ECE1,TULP3,BMPR1A,WDR19,IQCE,MED1,ALDH1A2,PSEN1,TMEM107,MKS1,RNF165,TBX4,TBX2,GPC3,CREBBP,SKI,ALX3,TMEM231,SOX4,NOG,WNT5A,C2CD3,BCL2L11	grey	VSMC_M10
GO:0035108	0.73	0.34	58	115	limb morphogenesis	BP	1	OSR2,FZD6,TBC1D32,BAK1,PITX1,PTCH1,TFAP2B,ROR2,DLX5,HOXA13,MYCN,OSR1,INTU,HDAC2,SOX9,GLI2,MEGF8,LMBR1,ZNF219,RARB,FREM2,HAND2,FMN1,FRAS1,TBX5,IFT52,RPGRIP1L,TFAP2A,SALL1,FBXW4,BMPR1B,HDAC1,TGFB2,SP9,WNT3,ECE1,TULP3,BMPR1A,WDR19,IQCE,MED1,ALDH1A2,PSEN1,TMEM107,MKS1,RNF165,TBX4,TBX2,GPC3,CREBBP,SKI,ALX3,TMEM231,SOX4,NOG,WNT5A,C2CD3,BCL2L11	grey	VSMC_M10
GO:0002702	0.73	0.35	38	75	positive regulation of production of molecular mediator of immune response	BP	1	CD226,IL5,KIT,EXOSC3,MSH2,RSAD2,TLR4,SPHK2,HMCES,PHB,LACC1,MLH1,PAXIP1,SHLD1,MAVS,HPX,TRAF6,GPI,F2RL1,NOD2,CD40,DENND1B,CD28,FZD5,HK1,SHLD2,SPON2,TBX21,STX4,IL4R,CD74,TICAM1,PRKCZ,TRAF2,SLC7A5,BCL10,WNT5A,INAVA	grey	VSMC_M10
GO:0042308	0.73	0.49	6	12	negative regulation of protein import into nucleus	BP	1	RAB23,FERMT1,PKIA,SUFU,CABP1,APOD	grey	VSMC_M10
GO:1904590	0.73	0.49	6	12	negative regulation of protein import	BP	1	RAB23,FERMT1,PKIA,SUFU,CABP1,APOD	grey	VSMC_M10
GO:2000774	0.73	0.51	5	10	positive regulation of cellular senescence	BP	1	CDKN2A,PAWR,KRAS,YPEL3,MIR22	grey	VSMC_M10

GO:0017148	0.73	0.33	81	158	negative regulation of translation	BP	1	PAN2,SHMT2,CNOT2,CNOT6,RIDA,PABPC1,EIF3E,PNLDC1,MRPL13,AGO2,TNRC6B,ATF4,TUT7,GAPDH,CNOT11,EXOSC3,IGF2BP3,PNPT1,AGO1,TUT4,DHFR,MLH1,TNRC6C,PURA,EIF2AK2,LSM1,PAN3,ENC1,CNOT1,CELF1,CNOT6L,EIF2AK4,EIF4E2,PAIP2B,FTO,TENT4B,RBM24,BANK1,CAPRIN2,DIS3L2,GIGYF2,YTHDF2,DDX6,YTHDF1,EIF4ENIF1,IGF2BP2,EIF4EBP2,DDX3X,RC3H1,UPF1,SAMD4A,STAT3,POLR2G,NT5C3B,DCP1B,FXR2,TARDBP,TOB1,TNRC6A,CNOT8,LARP1,SHMT1,PARN,FMR1,METTL16,PLEKHN1,CIRBP,TSC1,MIR29A,MIR21,MIR222,MIR125B1,MIR27B,MIR27A,AGO4,METTL14,NANOS1,YBX1,ZFP36L2,RC3H2,EXOSC5	grey	VSMC_M10
GO:0046940	0.73	0.54	4	8	nucleoside monophosphate phosphorylation	BP	1	CMPK1,DTYMK,AK8,CMPK2	grey	VSMC_M10
GO:0032008	0.73	0.39	19	38	positive regulation of TOR signaling	BP	1	RRAGD,PIP4P1,RPTOR,NCKAP1L,GAS6,LARS,SLC38A9,GPR137B,SIK3,GPR137,OTUD5,RRAGB,SMCR8,CLEC16A,MLST8,LAMTOR5,WDR59,MIR199A1,LAMTOR2	grey	VSMC_M10
GO:0046825	0.73	0.41	13	26	regulation of protein export from nucleus	BP	1	XPO1,DNAJC27,SIRT7,GAS6,RAPGEF3,SP100,TCF7L2,BAG3,SFN,UHMK1,CTDSPL2,PARK7,TXN	grey	VSMC_M10
GO:0032945	0.73	0.37	24	48	negative regulation of mononuclear cell proliferation	BP	1	TNFRSF21,PELI1,IL4I1,FOXJ1,PAWR,PRNP,CASP3,TMEM131L,MARCH7,BTN2A2,PRKAR1A,DLG1,VSIR,RC3H1,PTPN6,SCGB1A1,FOXP3,ZBTB7B,BTK,TWSG1,GLMN,LST1,CEBPB,HAVCR2	grey	VSMC_M10
GO:0050672	0.73	0.37	24	48	negative regulation of lymphocyte proliferation	BP	1	TNFRSF21,PELI1,IL4I1,FOXJ1,PAWR,PRNP,CASP3,TMEM131L,MARCH7,BTN2A2,PRKAR1A,DLG1,VSIR,RC3H1,PTPN6,SCGB1A1,FOXP3,ZBTB7B,BTK,TWSG1,GLMN,LST1,CEBPB,HAVCR2	grey	VSMC_M10
GO:1903530	0.73	0.31	207	399	regulation of secretion by cell	BP	1	VPS4B,LRP1,RAB21,SNCAIP,SLC12A2,RIMS2,PRKN,GDF9,PLA2G6,PICK1,RAC2,CHRNA3,CHRNA5,CRY1,APBA1,RFX3,TFAP2B,CHMP2A,IL11,PRKCE,RIMS1,HMGN3,RIMS3,BMP8A,RSAD2,NR1H2,EGFR,TLR4,SDCBP,TRPM4,SPHK2,RAB7A,RAB10,GIPR,CACNB2,CLASP2,FRMD4A,GAB2,LLGL2,UNC13D,C1QTNF1,SYT1,RAB5A,APBB3,DPH3,AIMP1,NCKAP1L,SIRT3,SYT9,ADRA2B,NR1H4,NDUFAF2,FAM3D,IL1A,PAX8,FER,OSBP,ARNTL,SCG5,MEF2C,ASIC1,VEGFC,P2RY1,KCNJ11,NNAT,NR1H3,TIAM1,RAB11FIP5,PFKM,SLC8B1,NKX3-1,NKX6-1,ACVR1C,F2RL1,F2R,CRY2,VPS18,SPP1,ZBED6,LGI3,ADTRP,DTNBP1,SYT8,GRM2,SIRT4,GPLD1,IRS1,CD200,DNM1L,HDAC1,PPP3CA,HYAL3,CPT1A,MAP2K6,SLC9B2,TGFB2,ADRA2A,TCIRG1,TCF7L2,KCNB1,PFKFB2,GRK2,GPER1,AGT,FGR,PER2,OSM,STX1A,SNX4,NTSR1,PASK,SEPT2,BACE1,GLUL,CSPG5,SIDT2,SLC16A1,ADAM8,KLF7,NSF,CREB1,RAP1B,PLA2G4A,TTN,IL12A,ENY2,ABCA12,CASK,MCU,ADRA2C,PNKD,PPP3CB,RAB3A,PINK1,SYN1,CD38,C12orf4,MPC2,INHA,RAB8A,ATP13A2,HAP1,CPLANE2,SYT7,RAB15,RSC1A1,NLGN2,TNFRSF1B,SEPT1,CPLX1,TARDBP,STX1B,STX4,ITGAM,SYT11,RAB8B,TMED10,CACNA1G,CHRNB2,MYH10,RAB3D,IL4R,CALM1,SYTL4,SNAPIN,GPR68,PRKCB,SCAMP5,CAMK2A,CCL5,STXBP2,SREBF1,ISL1,PRAM1,OXCT1,NOS2,ABAT,FMR1,RPH3AL,NADK,PDPK1,P2RX1,RAB26,ATP6AP1,BAIAP3,CACNA1H,FOXF1,LRRC8A,STXBP1,AP1G1,VPS4A,MIR199A1,FGA,CHRNB4,CHGA,SOX4,DPP4,CRH,RGCC,DOC2B,PTGES,RALA,LIF,UQCC2,VAMP3,PARD6A,KCNMB4	grey	VSMC_M10
GO:0010656	0.73	0.39	18	36	negative regulation of muscle cell apoptotic process	BP	1	PPP1R10,RGL2,HSF1,PAX8,HAND2,PTK2B,SIRT5,SIRT4,BAG3,NFE2L2,PDPK1,MIR199A1,MIR21,MIR24-1,NOL3,C3orf58,NKX2-5,HEY2	grey	VSMC_M10
GO:0140352	0.73	0.30	322	618	export from cell	BP	1	VPS4B,LRP1,CANX,RAB21,SNCAIP,SLC12A2,RIMS2,PRKN,GDF9,GRM4,LY6E,EXOC4,SYNGR1,NTRK2,PLA2G6,COPG2,PICK1,RAC2,MYH9,CHRNA3,CHRNA5,CRY1,ABCB1,STEAP2,APBA1,SCFD2,RFX3,KIT,TFAP2B,CHMP2A,NLRP5,SVBP,STX17,IL11,BRSK1,PRKCE,RIMS1,HMGN3,RIMS3,SLC4A4,CYB5R4,PPT1,SCRN1,BMP8A,PLEK,RSAD2,NR1H2,EGFR,TLR4,VPS41,SDCBP,TRPM4,PPFIA3,LIN7B,SPHK2,NAPA,SLC8A2,RAB7A,RAB10,COPG1,ATP7B,GIPR,CACNB2,SYTL2,CLASP2,FRMD4A,GAB2,LLGL2,RALB,STEAP3,GRIK5,UNC13D,RAF1,ATP1A3,NAPB,ABCC4,C1QTNF1,ANK1,SYT1,RAB5A,LIN7A,APBB3,DPH3,AIMP1,NCKAP1L,SIRT3,STX2,SYT9,ADRA2B,SNPH,NR1H4,HCK,NDUFAF2,FAM3D,IL1A,PAX8,NR4A1,FER,OSBP,TRAPPC11,ARNTL,ERC2,SCG5,MEF2C,ASIC1,VEGFC,P2RY1,RASGRP1,KCNJ11,NNAT,NR1H3,TIAM1,RAB11FIP5,EXOC6B,PFKM,SYNJ1,SLC8B1,NKX3-1,NKX6-1,ACVR1C,F2RL1,F2R,SLC40A1,ANO6,CRY2,VPS18,ABCC2,KCNE2,SPP1,ZBED6,LGI3,ABCG2,DMTN,ADTRP,DTNBP1,KCNIP2,SYT8,GRM2,SIRT4,GPLD1,IRS1,CD200,DNM1L,HDAC1,PPP3CA,HYAL3,CPT1A,MAP2K6,TRPV4,SLC9B2,TGFB2,ADRA2A,MON1A,TCIRG1,TCF7L2,SNAP23,KCNB1,PFKFB2,GRK2,GPER1,RAB11FIP2,LAT2,AGT,FGR,ABCC5,CBL,SYTL1,ATP1A2,SLC9A1,PRSS12,PER2,OSM,STX1A,SNX4,NTSR1,PASK,SEPT2,BACE1,DNAJC5,GLUL,CSPG5,SIDT2,DLG1,SLC16A1,ADAM8,KLF7,NSF,CREB1,MIA3,RAP1B,BLOC1S6,PLA2G4A,CTAGE9,VGF,TTN,IL12A,TANGO2,ENY2,ABCA12,NRXN2,CASK,MCU,ADRA2C,PNKD,PTGDR,PPP3CB,RAB3A,PINK1,SYN1,CAMK2G,SNAP29,EXOC5,PTPRN,ATP1B1,CD38,SLC27A1,C12orf4,MPC2,ILDR2,INHA,RAB8A,ATP13A2,HAP1,CPLANE2,SLC29A4,SYT7,RAB15,RSC1A1,NLGN2,T[illegible]	grey	VSMC_M10
GO:0050832	0.73	0.44	9	18	defense response to fungus	BP	1	USP15,GAPDH,SPON2,MPO,S100A8,BCL10,S100A12,CHGA,COTL1	grey	VSMC_M10
GO:0030808	0.73	0.42	12	24	regulation of nucleotide biosynthetic process	BP	1	PPARA,GUCA1B,SPHK2,PDK1,FAM173B,DNAJC30,PDK3,PINK1,STAT3,PARP1,NOS2,PDZD3	grey	VSMC_M10
GO:1900371	0.73	0.42	12	24	regulation of purine nucleotide biosynthetic process	BP	1	PPARA,GUCA1B,SPHK2,PDK1,FAM173B,DNAJC30,PDK3,PINK1,STAT3,PARP1,NOS2,PDZD3	grey	VSMC_M10
GO:0051126	0.73	0.53	4	8	negative regulation of actin nucleation	BP	1	PICK1,HIP1R,WDR63,GMFG	grey	VSMC_M10
GO:0007157	0.73	0.40	14	29	heterophilic cell-cell adhesion via plasma membrane cell adhesion molecules	BP	1	FAT4,PVR,TENM3,AMIGO2,ALCAM,NECTIN3,AMIGO3,NECTIN4,CADM1,HMCN1,TENM2,UMOD,AMIGO1,GRID2	grey	VSMC_M10

GO:1902414	0.73	0.35	41	82	protein localization to cell junction	BP	1	KIF5A,KIF3A,ABHD17B,ADAM22,SHANK1,DLG2,KIF5C,ACTG1,LSR,DSP,NECTIN3,DAG1,BSN,MPP4,DLG1,MAPT,NRXN2,VCL,NSG1,GRIPAP1,RAB8A,DLG4,ACTB,IQSEC2,NLGN2,CPLX1,STX1B,ZDHHC15,RAB11A,ARHGAP44,ASIC2,JAK1,PRKCZ,MAPK8IP3,ZDHHC12,MARVELD3,WNT5A,LAMTOR2,CACNG7,HRAS,MAP1A	grey	VSMC_M10
GO:0035384	0.73	0.38	21	42	thioester biosynthetic process	BP	1	PPT2,ACSL6,PPCS,PPT1,DLD,ELOVL1,PDK1,PDHX,ELOVL7,ACSL5,HACD2,PDK3,ACSBG1,MPC2,TECR,GCDH,ACACA,ACSBG2,ACSF3,MLYCD,ELOVL3	grey	VSMC_M10
GO:0071616	0.73	0.38	21	42	acyl-CoA biosynthetic process	BP	1	PPT2,ACSL6,PPCS,PPT1,DLD,ELOVL1,PDK1,PDHX,ELOVL7,ACSL5,HACD2,PDK3,ACSBG1,MPC2,TECR,GCDH,ACACA,ACSBG2,ACSF3,MLYCD,ELOVL3	grey	VSMC_M10
GO:0070231	0.73	0.39	18	36	T cell apoptotic process	BP	1	BAK1,SLC46A2,IDO1,ZC3H8,LMBR1L,RIPK1,GPAM,PDCD1,ADAM8,EFNA1,CCL5,IL7R,DNAJA3,BCL10,FADD,WNT5A,TSC22D3,BCL2L11	grey	VSMC_M10
GO:0051901	0.73	0.53	4	8	positive regulation of mitochondrial depolarization	BP	1	TSPO,DCN,PARP1,MLLT11	grey	VSMC_M10
GO:0009069	0.73	0.41	13	26	serine family amino acid metabolic process	BP	1	SHMT2,RIDA,PSAT1,SLC7A11,DHFR,CSAD,THNSL2,SERINC5,CBSL,AMT,SHMT1,GCSH,PHGDH	grey	VSMC_M10
GO:0042742	0.73	0.33	56	109	defense response to bacterium	BP	1	PLA2G6,FPR2,ANKRD17,NOD1,AQP1,LYPD8,TLR4,C5AR1,OPTN,MYD88,SIRT2,MAVS,FOXP1,OAS3,RIPK2,F2RL1,MAPKBP1,NOD2,TLR5,SLAMF8,FGR,COCH,FCER1G,LYZL6,VGF,IL12A,SLC11A1,CFP,RBPJ,SPON2,SYT11,IL6R,MPO,S100A14,S100A8,SCNN1B,COLEC12,NOS2,IL7R,DROSHA,CYBA,LCN2,FGA,S100A12,OAS1,HIST1H2BK,CHGA,DEFB124,CXCL6,JCHAIN,IRGM,PLAC8,CEBPB,HIST1H2BF,HIST1H2BJ,HAVCR2	grey	VSMC_M10
GO:0006289	0.73	0.37	25	50	nucleotide-excision repair	BP	1	RPA3,FANCC,RAD23B,USP45,ERCC1,XPC,ERCC2,ERCC3,POLR2I,ERCC6,ERCC8,GTF2H2C,DDB2,POLL,FAN1,RAD52,DDB1,RNF111,KAT7,XAB2,ERCC4,SLX4,NTHL1,RPA4,GTF2H5	grey	VSMC_M10
GO:0061003	0.73	0.43	9	19	positive regulation of dendritic spine morphogenesis	BP	1	CUX2,TIAM1,OPA1,ITPKA,DNM1L,CAPRIN2,GPRASP2,BHLHB9,LRP8	grey	VSMC_M10
GO:0072527	0.73	0.36	32	63	pyrimidine-containing compound metabolic process	BP	1	NME1,TBPL1,ENPP3,DPYS,APOBEC3H,CMPK1,CDADC1,MBD4,ENTPD4,NME4,THTPA,CTPS2,DTYMK,TET1,UPB1,CPS1,NME7,MAPK1,ALDH6A1,UPRT,NEIL1,NT5M,NTHL1,TKTL1,NME3,UCK1,DHODH,TPK1,CMPK2,NME6,DCTD,UPP1	grey	VSMC_M10
GO:0072665	0.73	0.36	30	60	protein localization to vacuole	BP	1	TNFAIP3,RTN4,VPS41,KPTN,RAB7A,SZT2,NCOA4,AP3B1,MON1A,GPR137B,HSPA8,SORL1,SH3BP4,RNF215,VPS37D,MFSD1,AP4M1,AP3M1,ATP13A2,VPS13D,VPS13C,RNF128,LAMP2,LAMTOR5,TLDC1,SH3GLB1,VPS4A,CACNG7,SNX16,CACNG5	grey	VSMC_M10
GO:0006290	0.73	0.53	4	8	pyrimidine dimer repair	BP	1	ERCC1,XPC,ERCC6,DDB2	grey	VSMC_M10
GO:0019318	0.73	0.32	95	185	hexose metabolic process	BP	1	FUCA2,PRKN,PPARA,PMM1,AKR1B1,ATF4,MAPK14,GPT,GNMT,CRY1,PGM5,GALT,TFAP2B,SLC25A13,GAPDH,PPP4R3B,ZNF692,FAM3C,RUBCNL,AKR1A1,GLB1,KHK,EPM2AIP1,GCKR,PPP1CB,AKT2,PFKP,PGM2L1,C1QTNF1,SIRT7,PDK1,FUT10,PPP1R3B,POFUT1,GPI,NLN,NCOA2,SORBS1,GPD1,KCNJ11,PFKM,CHST1,GLYCTK,IGF2,GPLD1,IRS1,SIK1,CPT1A,MST1,PPP1R3E,PFKFB2,PC,PPP1R3D,SLC37A4,PER2,PFKFB4,ACTN3,USF1,PASK,DUSP12,APOD,FUOM,PDK3,SORD,HK1,GNB3,SLC35A2,PPP1R3F,IGFBP4,DDB1,DGKQ,OGT,COX11,CRTC2,LCMT1,ADPGK,MPI,INSR,LEPR,ALDOC,PTPN2,PMM2,INPP5K,TKTL1,MLYCD,ACADM,FBP2,OAS1,FABP5,FUT4,FPGT,POFUT2,TIGAR,NNMT,ARPP19	grey	VSMC_M10
GO:0060041	0.73	0.33	54	108	retina development in camera-type eye	BP	1	MDM1,TBC1D32,AGTPBP1,NTRK2,PRPH2,RORB,SLC1A1,TFAP2B,RDH13,PTN,TSPAN12,USP45,SOX9,ZNF513,ACVR2B,TUB,PFDN5,NPHP1,RET,USH1C,TENT2,OPA1,RPGRIP1L,TFAP2A,PAX2,BMPR1B,NECTIN3,LPCAT1,TGFB2,TCIRG1,NRL,CLIC4,DLX1,OBSL1,RHOJ,ROM1,SDK1,MAX,CLN8,MED1,PSEN1,TTLL5,MEGF11,LHX1,LAMA1,GNB1,SKI,SOX8,LAMC3,FJX1,ACVRL1,THY1,DIO3,TGIF2	grey	VSMC_M10
GO:0051058	0.73	0.37	24	49	negative regulation of small GTPase mediated signal transduction	BP	1	SYNGAP1,MET,SPRY1,ARHGAP12,SPRY2,PPP2CB,RASA3,RASA2,ARHGAP24,TGFB2,RASA4,BCL6,ABL2,LZTR1,RASAL3,MFN2,ITGA3,KCTD13,ARHGAP44,TNFAIP1,MAPKAP1,SCAI,DAB2IP,STAMBP	grey	VSMC_M10
GO:0043393	0.73	0.32	83	162	regulation of protein binding	BP	1	LRP1,STK3,PRKN,PPARA,DERL1,LRRC6,CSNK1E,MFNG,HSF1,TTBK1,MITD1,ITGB1BP1,ADD2,MAPRE1,SYMPK,MAPRE3,RALB,PTPRF,TMEM173,EFHB,PLCL2,PPP2CB,TRIB3,PLK2,IDE,HIP1R,TMBIM6,RAPGEF2,FAM192A,TIAM1,RIPK2,AMFR,IFIT2,STK4,DTNBP1,BDNF,SPPL3,CAPRIN2,TERT,TCF7L2,TTBK2,ADNP,EPB41,UBASH3B,DISC1,SORL1,DTX3L,NFATC4,HDAC4,BTBD1,RPL11,ITGA4,MEN1,LRPAP1,MARK2,RAMP2,DNAJB2,GREM2,DDX11,CRMP1,ACTB,NVL,GNL3L,ANXA2,PEX14,PSEN1,TAF1,MAPK3,WFIKKN2,SNAPIN,MARK3,TICAM1,CRK,PKD1,WFIKKN1,PARK7,PRMT7,MIR27B,BAG2,NOG,WNT5A,LDLRAP1,STYX	grey	VSMC_M10
GO:0061178	0.73	0.37	25	50	regulation of insulin secretion involved in cellular response to glucose stimulus	BP	1	LRP1,PLA2G6,PRKCE,HMGN3,TRPM4,NR1H4,NDUFAF2,OSBP,TIAM1,ZBED6,GPLD1,SLC9B2,ADRA2A,SIDT2,KLF7,ENY2,ABCA12,PPP3CB,MPC2,STX4,GPR68,OXCT1,NADK,BAIAP3,CRH	grey	VSMC_M10
GO:0001522	0.74	0.44	9	18	pseudouridine synthesis	BP	1	PUS10,RPUSD2,PUS7L,RPUSD4,GAR1,TRUB1,PUSL1,RPUSD1,RPUSD3	grey	VSMC_M10
GO:0006388	0.74	0.50	5	10	tRNA splicing, via endonucleolytic cleavage and ligation	BP	1	TSEN34,CLP1,ZBTB8OS,RTRAF,TRPT1	grey	VSMC_M10
GO:0042129	0.74	0.33	52	102	regulation of T cell proliferation	BP	1	AGER,HLA-DPB1,TNFRSF13C,RAC2,TNFRSF21,NCK2,PELI1,IL4I1,IL15,BMI1,FOXJ1,TGFBR2,PAWR,PRNP,NCKAP1L,SLC7A1,TRAF6,IL1A,CASP3,TMEM131L,RIPK2,MARCH7,IGF2,ICOSLG,BTN2A2,GPAM,PRKAR1A,CD28,DLG1,VSIR,RC3H1,PTPN6,TNFSF18,STAT5B,SCGB1A1,FOXP3,RASAL3,CARD11,TNFRSF1B,ZBTB7B,CCL5,TWSG1,DNAJA3,GLMN,MIR21,FADD,CD46,CD70,TNFSF9,CEBPB,TNFSF13B,HAVCR2	grey	VSMC_M10

GO:0019218	0.74	0.35	37	73	regulation of steroid metabolic process	BP	1	CYP27B1,TSPO,TTC39B,KIT,DKKL1,APOB,APOC1,PANK2,NR1H4,CYP7A1,ASAH1,DKK3,EPHX2,ERLIN1,FMO5,SNAI1,ABCB11,AGT,SCAP,POR,ACADL,LPCAT3,GNB3,WNT4,STAT5B,ACADVL,FGFR4,DGKQ,LDLR,SREBF1,GFI1,MIR27B,MIR27A,LDLRAP1,STARD4,INSIG2,FGF1	grey	VSMC_M10
GO:1903587	0.74	0.44	9	18	regulation of blood vessel endothelial cell proliferation involved in sprouting angiogenesis	BP	1	IL12A,PDCD10,NGFR,MIR21,MIR222,MIR23A,MIR27B,MIR24-1,MIR27A	grey	VSMC_M10
GO:0045333	0.74	0.31	101	191	cellular respiration	BP	1	SHMT2,LYRM7,UQCRQ,NDUFA6,ACO2,SLC25A13,CAT,SDHAF4,MSH2,PNPT1,DLD,NDUFB2,NDUFB6,DNAJC15,DHTKD1,NDUFC2,ATP5F1C,MDH2,NDUFS8,PANK2,NDUFA12,SIRT3,COX7A1,SUCLG2,COX4I2,IDE,ATP5F1A,GPD1,NDUFS3,ETFDH,DGUOK,UQCRB,COX15,FAM173B,COX6B1,NDUFV3,TRPV4,COQ10B,OXA1L,NDUFB4,ISCU,COA6,POLG2,NDUFB3,CISD1,NFATC4,ACTN3,NDUFS2,DNAJC30,MLXIPL,SDHC,MDH1B,COX6A1,IDH3A,COX8A,PINK1,NDUFA9,SDHB,COX6A2,NDUFAB1,UQCRC2,COX10,COX5A,TEFM,NDUFA7,NOS2,SLC25A23,NDUFV2,SLC25A14,PRDM16,NDUFB10,UQCR11,PARK7,SLC25A33,ATP5PB,SLC25A25,MT-ND1,MT-ND2,MT-ATP8,MT-ATP6,MT-CO3,MT-ND6,MT-CYB,RHOA,ATP5ME,ATP5MF,NDUFA5,IFNLR1,NDUFC1,COX7A2,FXN,ETFRF1,NDUFA3,NDUFB5,NDUFS6,NDUFA8,SDHAF2,NDUFS5,UQCC2,COX7A2L,PPIF	grey	VSMC_M10
GO:0002698	0.74	0.35	31	62	negative regulation of immune effector process	BP	1	ENPP3,IL4I1,FOXJ1,NCKAP1L,PRKDC,FER,TMBIM6,NOD2,CD96,TGFB2,HLX,SLAMF8,DUSP10,BCL6,RC3H1,PTPN6,TNFSF18,PPP3CB,FOXP3,TBX21,ZBTB7B,IL4R,IL7R,CRK,FOXF1,CD46,A2M,FGL2,SERPINB9,HAVCR2,RC3H2	grey	VSMC_M10
GO:0045601	0.74	0.40	14	29	regulation of endothelial cell differentiation	BP	1	ZDHHC21,S1PR3,IKBKB,JAG1,PLCB1,BTG1,APOLD1,VCL,TMEM100,VEZF1,MIR21,ACVRL1,FOXJ2,MIR99B	grey	VSMC_M10
GO:0032602	0.74	0.35	32	64	chemokine production	BP	1	LRP1,AGER,TRIM32,TLR4,MYD88,EIF2AK2,MAVS,FOXP1,IL7,OAS3,RIPK2,F2RL1,NOD2,HDAC1,TRPV4,APOD,DDX3X,MUL1,IL6R,IL4R,MAP2K5,CD74,CSF1R,SUZ12,TICAM1,OAS1,DEFB124,CXCL6,WNT5A,IL33,TSLP,HAVCR2	grey	VSMC_M10
GO:0032642	0.74	0.35	32	64	regulation of chemokine production	BP	1	LRP1,AGER,TRIM32,TLR4,MYD88,EIF2AK2,MAVS,FOXP1,IL7,OAS3,RIPK2,F2RL1,NOD2,HDAC1,TRPV4,APOD,DDX3X,MUL1,IL6R,IL4R,MAP2K5,CD74,CSF1R,SUZ12,TICAM1,OAS1,DEFB124,CXCL6,WNT5A,IL33,TSLP,HAVCR2	grey	VSMC_M10
GO:0016125	0.74	0.33	58	114	sterol metabolic process	BP	1	APOF,CYP21A2,TTC39B,PLPP6,CAT,APOB,EBP,EBPL,CUBN,LIPE,PRKAG2,TSKU,PIP4P1,NR1H4,SOAT2,CYP7A1,CYP7B1,CEBPA,LIMA1,EPHX2,ERLIN1,CYB5R1,ABCA5,FMO5,MSMO1,SC5D,SCAP,HSD17B7,POR,ACADL,SOAT1,TM7SF2,OSBPL1A,FDX1,LPCAT3,GNB3,CYP27A1,CH25H,LIPA,ACADVL,CLN8,LIPC,DGKQ,ERG28,APOBR,CLN6,LDLR,CYP46A1,NPC1,SREBF1,LEPR,ACAA2,RXRA,CEL,NFE2L1,LDLRAP1,INSIG2,FGF1	grey	VSMC_M10
GO:0006163	0.74	0.30	157	302	purine nucleotide metabolic process	BP	1	NME1,HSD17B4,PDE7B,ATP5MGL,PPT2,PPARA,PDE10A,ACSL6,PRXL2C,GTPBP1,GUCA1B,IMPDH1,GDA,CROT,SLC35B2,SLC25A13,GAPDH,PPAT,RAB23,PPCS,SLC4A4,NT5E,PPT1,BPGM,DLD,SPHK2,ADCY3,NUDT15,DLG2,LACC1,DHTKD1,PRKAG2,ATP5F1C,ELOVL1,PFKP,NUDT16,PANK2,PDK1,SUCLG2,PDHX,ELOVL7,PANK1,GPI,AMPD3,ATP5F1A,MCCC2,GPD1,P2RX7,GUCY1A1,GUCY1B1,ACOT12,DGUOK,PRKAG1,GMPS,PFKM,OPA1,ENTPD4,FITM2,AMPD1,NUDT18,ADCY7,GMPR,FAM173B,ADCY2,MCEE,NME4,PDE9A,TPST1,ZBTB20,GPAM,FAR2,ACSL5,PTHLH,MFN1,PFKFB2,PRPS2,ENO4,HACD2,ARNT,ADCY4,ACTN3,HDAC4,THEM5,DNAJC30,MLXIPL,PDE8A,HMGCL,MAGI3,DLG1,ACOT9,PDK3,EFL1,SLC25A16,HK1,ACSBG1,ATP5S,CASK,COASY,PRKAG3,PINK1,STAT3,NME7,ADK,MPC2,ADCY10,ENO3,PARP1,TECR,CARD11,SLC26A1,PSEN1,ACOT2,GUCY2D,PKLR,PANK3,GCDH,OGT,ADPGK,PDE4A,ACSM3,ACACA,NCOR1,INSR,SLC26A2,PRPSAP2,ADSSL1,NOS2,ACSBG2,ALDOC,PIPOX,ACOT11,ATP6V0C,PANK4,NME3,MPP1,ATP5PB,SLC2A6,VPS9D1,GNAI3,ACSF3,MLYCD,MT-ATP8,MT-ATP6,RHOA,SULT1C3,ATP5ME,ACOT4,ATP5MF,ENTPD5,PDZD3,ELOVL3,MTHFD2L,TIGAR,NUDT19,NME6,NUDT2,HINT1	grey	VSMC_M10
GO:1902108	0.74	0.38	17	34	regulation of mitochondrial membrane permeability involved in apoptotic process	BP	1	TMEM14A,HIP1R,THEM4,STPG1,FZD9,NMT1,MUL1,TMEM102,CAMK2A,ACAA2,NAIF1,MIR29A,NOL3,BNIP3L,BCL2L11,SLC35F6,AC140504.1	grey	VSMC_M10
GO:0099515	0.74	0.43	9	19	actin filament-based transport	BP	1	MYO1A,SUN2,MYO7B,MYO7A,SYNE2,MYO1D,MYO1F,MYO5B,FNBP1L	grey	VSMC_M10
GO:0048013	0.74	0.37	22	45	ephrin receptor signaling pathway	BP	1	PTK2,ANKS1A,NCK2,EPHA7,EPHA1,ARHGEF7,ANKS1B,EPHB1,ARHGEF28,TIAM1,KALRN,EPHB3,EPHB4,EPHA4,RBPJ,EFNA1,YES1,CDK5R1,CRK,EFNA4,CHN1,EFNB3	grey	VSMC_M10
GO:0051048	0.74	0.33	56	110	negative regulation of secretion	BP	1	VPS4B,ERBB3,PRKN,CRY1,IL11,BMP8A,RSAD2,RAB7A,FRMD4A,DPH3,NRG1,NCKAP1L,ADRA2B,NDUFAF2,FAM3D,ASIC1,P2RY1,KCNJ11,NR1H3,RAB11FIP5,ACVR1C,F2R,CRY2,PLA2R1,ADTRP,SIRT4,IRS1,CD200,HDAC1,PPP3CA,ADRA2A,KCNB1,EGF,OSM,KLF7,RAP1B,IL12A,COMT,ENY2,ADRA2C,PNKD,INHA,RSC1A1,TNFRSF1B,SYT11,SYTL4,CD74,SREBF1,ABAT,FMR1,FOXF1,MIR199A1,CHGA,CRH,LIF,VAMP3	grey	VSMC_M10
GO:1901661	0.74	0.40	14	28	quinone metabolic process	BP	1	AKR1B1,AKR1A1,CBR4,COQ2,NQO2,CRYZL1,CBR3,COQ5,COQ10B,NDUFA9,COQ8A,COQ4,PDSS2,ADH4	grey	VSMC_M10

GO:0002684	0.74	0.29	283	542	positive regulation of immune system process	BP	1	CD226,STX7,ENPP3,AGER,GPSM3,HLA-DRA,HLA-DOB,HLA-DMA,IL5,HLA-DPB1,TNFRSF13C,XRCC6,TRIB1,PTK2,PLA2G6,RAC2,HSF1,TTBK1,TEC,CCL27,KIT,TNFRSF21,NCK2,ROR2,EXOSC3,PRKCE,MSH2,FPR2,ANKRD17,CXCL8,NOD1,AKIRIN2,PELI1,AKIRIN1,RSAD2,PTN,IL4I1,TLR4,IL15,SPHK2,HMCES,BMI1,C5AR2,C5AR1,PRKD2,PHB,LACC1,NECTIN2,PVR,EIF2B3,GAB2,MLH1,GLI2,UNC13D,MYD88,OXSR1,SLC22A13,FOXJ1,PAXIP1,POU4F1,IL15RA,TGFBR2,IKBKB,NMI,TMEM173,PAWR,PLCL2,SHLD1,PRNP,MAVS,NCKAP1L,GAS6,HPX,FOXP1,PRKDC,IRF7,SLC7A1,TRAF6,SOX12,HCK,TOX,IL1A,GPI,CEBPA,FER,PIK3CB,LPXN,PTPRJ,MEF2C,IL7,VEGFC,JAM2,SLA2,PTK2B,RASGRP1,EIF2AK4,NLRC5,RIPK2,AP3B1,F2RL1,TESC,RIPK1,ANO6,POLR3D,SOX13,NOD2,ZNF335,CD40,IGF2,GPLD1,SPPL3,DNM1L,LCK,BANK1,ICOSLG,TRPV4,SLC9B2,TGFB2,BTN2A2,GPAM,CD99,PRSS56,POLR3C,HLX,DENND1B,CMKLR1,ACIN1,HCLS1,MOSPD2,PARP9,THEMIS2,LAT2,BMX,FGR,PLPP4,SNX4,COCH,CD28,FCER1G,RUNX3,ZBTB46,PPP2R3C,ADAM8,CADM1,CREB1,FZD5,MIA3,DUSP10,BLOC1S6,NONO,PLA2G4A,BCL6,HK1,ITGA2B,ITGA4,IL12A,VSIR,XRCC5,RC3H1,PTPN6,MCU,TNFSF18,CXCR2,SLC11A1,LAG3,TNIP2,CFP,CD38,BST1,C12orf4,MAPK1,STAT5B,FOXP3,RASAL3,ZBTB1,SHLD2,GPS2,CARD11,TMEM102,MED1,SPON2,TBX21,STX4,PSEN1,ITGAM,LCP2,PGF,DAPK2,FOS,MAPK3,ZBTB7B,CHRNB2,IL6R,HAX1,CD19,IL4R,S100A14,GPR68,SFPQ,PRKCB,BTK,BDKRB1,SIN3A,CD74,CCL5,RIOK3,CSF1R,PPARGC1B,STXBP2,CCL7,CCL2,FBXO38,YES1,NKAP,CD320,PRAM1,VAV1,C7,EVI2B,C3,NOS2,IL7R,PTPN2,JUN,SOCS1,TICAM1,DNAJA3,CREBBP,PDPK1,PRKCZ,T[illegible]	grey	VSMC_M10
GO:0014044	0.74	0.40	13	27	Schwann cell development	BP	1	ADGRG6,NTRK2,ADAM22,PRX,AKT2,SIRT2,NRG1,LGI4,DAG1,CNTNAP1,MPP5,SKI,POU3F2	grey	VSMC_M10
GO:0030866	0.74	0.38	17	35	cortical actin cytoskeleton organization	BP	1	EPB41L2,TLN1,PLEK,LCP1,LLGL2,IKBKB,NCKAP1L,PLS1,EPB41L1,RTKN,EPB41,DLG1,NCKAP1,EPB41L3,STRIP1,VPS4A,FHOD1	grey	VSMC_M10
GO:1905330	0.74	0.35	28	56	regulation of morphogenesis of an epithelium	BP	1	ESR1,SMO,SIX2,SOX9,FOXP1,PAX8,PAX2,NKD1,EGF,AGT,STOX1,WNT4,MAGED1,BTBD7,TBX2,LHX1,ADAMTS12,RNF207,SOX8,LCN2,MIR21,NOG,WNT5A,LBX2,SIX1,LIF,FGF1,WNT2	grey	VSMC_M10
GO:1903557	0.74	0.34	36	72	positive regulation of tumor necrosis factor superfamily cytokine production	BP	1	AGER,NOD1,TLR4,SPHK2,HDAC2,MYD88,MAVS,IL1A,PIK3R1,PTPRJ,RASGRP1,OAS3,RIPK2,RIPK1,NOD2,IFIH1,HDAC1,MAPKAPK2,NFATC4,ADAM8,FZD5,STAT3,TNFRSF8,SPON2,PSEN1,ISL1,TICAM1,CYBA,BCL10,MIR27B,OAS1,FADD,WNT5A,IL33,C1QTNF4,HAVCR2	grey	VSMC_M10
GO:0003148	0.74	0.41	11	23	outflow tract septum morphogenesis	BP	1	TGFBR2,PARVA,ROBO1,ROBO2,TGFB2,BMPR1A,TBX2,GATA6,ISL1,ENG,NKX2-5	grey	VSMC_M10
GO:0031076	0.74	0.40	13	27	embryonic camera-type eye development	BP	1	FRS2,IFT172,TFAP2A,PAX2,ALDH1A3,SP3,FZD5,TULP3,WDR19,ALDH1A2,TBX2,STRA6,WNT5A	grey	VSMC_M10
GO:0072521	0.74	0.30	166	319	purine-containing compound metabolic process	BP	1	NME1,HSD17B4,SHMT2,PDE7B,ATP5MGL,PPT2,PPARA,PDE10A,ACSL6,PRXL2C,GTPBP1,OARD1,GUCA1B,IMPDH1,MTAP,GNMT,GDA,CROT,SLC35B2,ENPP4,SLC25A13,GAPDH,PPAT,RAB23,PPCS,SLC4A4,NT5E,PPT1,BPGM,DLD,SPHK2,ADCY3,PRTFDC1,NUDT15,DLG2,LACC1,DHTKD1,PRKAG2,ATP5F1C,ELOVL1,PFKP,NUDT16,PANK2,PDK1,SUCLG2,PDHX,ELOVL7,PANK1,GPI,AHCY,AMPD3,ATP5F1A,MCCC2,GPD1,P2RX7,GUCY1A1,GUCY1B1,ACOT12,DGUOK,PRKAG1,GMPS,PFKM,OPA1,ENTPD4,FITM2,AMPD1,NUDT18,ADCY7,GMPR,FAM173B,ADCY2,MCEE,NME4,PDE9A,TPST1,ZBTB20,GPAM,FAR2,ACSL5,PTHLH,MFN1,PFKFB2,PRPS2,ENO4,HACD2,ARNT,ADCY4,ACTN3,HDAC4,THEM5,DNAJC30,MLXIPL,PDE8A,HMGCL,MAGI3,DLG1,ACOT9,PDK3,EFL1,SLC25A16,HK1,ACSBG1,ATP5S,CASK,COASY,PRKAG3,PINK1,STAT3,NME7,ADK,MPC2,ADCY10,ENO3,PARP1,TECR,CARD11,SLC26A1,PSEN1,ACOT2,GUCY2D,PKLR,PANK3,GCDH,OGT,ADPGK,PDE4A,ACSM3,ACACA,NCOR1,INSR,SLC26A2,PRPSAP2,SHMT1,ADSSL1,NOS2,ACSBG2,ALDOC,PIPOX,ACOT11,ATP6V0C,PANK4,NME3,MPP1,ATP5PB,SLC2A6,VPS9D1,GNAI3,ACSF3,MLYCD,MT-ATP8,MT-ATP6,RHOA,SULT1C3,ATP5ME,ACOT4,ATP5MF,ENTPD5,PDZD3,ELOVL3,MTHFD2L,PEMT,TIGAR,NUDT19,NME6,NUDT2,HINT1	grey	VSMC_M10
GO:0086009	0.74	0.38	16	33	membrane repolarization	BP	1	NEDD4L,SCN5A,KCNJ3,KCNE2,CASQ2,KCNIP2,KCNJ2,ANK2,ATP1A2,SCN4B,NOS1AP,ATP1B1,ATP1B2,RNF207,KCNH2,KCNJ8	grey	VSMC_M10
GO:0099173	0.74	0.32	69	137	postsynapse organization	BP	1	SYNGAP1,PICK1,LRRC4,ABHD17B,SHANK1,EPHA7,PTN,VPS35,SLC7A11,MTMR2,LRFN1,FARP1,ACTG1,PRNP,EPHB1,CHMP2B,CUX2,TIAM1,HOMER1,OPA1,ITPKA,DOCK10,INA,DNM1L,CAPRIN2,SEMA3F,GAP43,NLGN4Y,SSH1,CFL1,HSPA8,WASF2,SPTBN2,CDKL5,EPHB3,TANC2,NOS1AP,FZD9,DLG1,SRGN,NRXN2,DNM3,EPHA4,DLG4,ACTB,NLGN2,GPHN,CHRNB1,ITGA3,NLGN3,EFNA1,ZDHHC15,GPRASP2,BHLHB9,ARHGAP44,INSR,CDK5R1,LRP8,DNAJA3,RER1,ABHD17A,CEL,NTNG2,ZDHHC12,GRID2,ABHD17C,WNT5A,MESD,LRRTM2	grey	VSMC_M10

GO:0072657	0.74	0.29	242	466	protein localization to membrane	BP	1	LRP1,STX7,STAC3,MAP7,PEX3,ZDHHC14,EMC2,PRKN,AGER,GOPC,HSPA4,SYNGAP1,PTCH1,SIL1,TOMM22,PICK1,MICALL1,ZDHHC21,TSPAN33,LRRC4,ADAM22,SLC1A1,TRAM2,SEC61B,PRKCE,ASB3,SPTBN1,EMC10,PTN,ITGB1BP1,SEC61G,EGFR,TCAF2,VPS35,SDCBP,SLC7A11,LIN7B,AGK,BRAF,RAB7A,RAB10,ARL5B,CACNB2,CLASP2,DLG2,STAC,OPTN,GOLGA4,AQP11,GRIK5,EMC3,SEC62,MYL12A,AKT2,IKBKB,ANK1,ARL5A,LIN7A,PRNP,BAG4,GAS6,PKP3,ARL6IP5,SRP14,TUB,KCNIP3,LYPLA1,TM9SF4,ZDHHC20,RILPL1,RILPL2,PIK3R1,TMEM150A,VAMP5,SLMAP,PLS1,EMC4,FCHO2,SORBS1,NUP54,P2RY1,RAPGEF2,KCNJ11,TIAM1,SSR3,AP3B1,ZFYVE27,GOLGA7B,TESC,PEX16,DMTN,KIF13A,NHLRC1,NECTIN3,SRPRA,PKP2,PPFIA1,ZDHHC23,ZDHHC6,OXA1L,DAG1,SSH1,GET4,KCNB1,NCF1,ANK2,C2CD5,GPER1,RAB11FIP2,FAM126B,CIB1,AP2M1,ZDHHC18,PPP2R5A,PEX19,ZDHHC24,BBS1,GORASP2,STX1A,LAMA5,VPS37D,PACS1,FZD9,DLG1,LRRC15,NSF,PIKFYVE,FIS1,CEMIP,NMT1,LZTFL1,RTP4,EHD1,ZDHHC8,ITGA4,ABCA12,NRXN2,SAV1,CNPY4,AP4M1,BCS1L,RAB3A,RAMP2,ZFAND2B,EXOC5,ATP1B1,PPIL2,ARL6IP1,NSG1,GRIPAP1,ZDHHC4,RAB8A,CNST,DLG4,ACTB,WDR19,RAB15,IQSEC2,RSC1A1,PEX26,NLGN2,GPHN,MPP5,WNK3,STAC2,ARL5C,SYNJ2BP,TSPAN17,ANXA2,GAK,CPLX1,STX1B,STX4,TMEM88,ITGAM,SSR2,RAB8B,ITGA3,TAOK2,AC138894.1,ZDHHC15,RAB11A,STX8,RAB3D,CALM1,TTC7B,TIMM29,GGA2,CYP46A1,ARHGAP44,NPC1,AP2B1,MIEF2,CDK5R1,PRAM1,EPB41L3,MAIP1,IFT20,EFCAB7,GPC3,INPP5K,DNAJA3,EMC6,PDPK1,PRKCZ,SGTA,RAB26,RER1,ABHD17A,ARHGEF16,UBL4A,SEC16A,PALM,BSG,ATP2C2,ZDHHC12,STXBP1,HSPA5,SH3GLB[illegible]	grey	VSMC_M10
GO:0003161	0.74	0.39	15	31	cardiac conduction system development	BP	1	MAML1,SMAD5,BVES,SCN5A,NRG1,TBX5,BMPR1A,MSX2,CACNA1G,DSG2,GATA6,ISL1,HCN4,NKX2-5,HEY2	grey	VSMC_M10
GO:0032535	0.74	0.30	167	325	regulation of cellular component size	BP	1	ARPC3,LRP1,SEMA6A,AVIL,RAB21,PRR16,SLC12A2,FSTL4,WASHC5,PLXNA4,BAIAP2L2,PICK1,CDC42EP1,LMOD2,SRF,NCK2,SEMA4D,KIAA1211,SEMA4C,TMOD1,PLEKHH2,PRKCE,SPTBN1,RTN4,RUFY3,AQP1,PLEK,EPHA7,ADD2,KEL,FAM49A,NRCAM,WASF1,SLC26A5,GOLGA4,MEGF8,CLNS1A,AQP11,PLEKHG2,FCHSD2,MKKS,RPTOR,RAB5A,BAG4,SEMA3D,SPART,FCHSD1,SEMA3A,NCKAP1L,LARS,ULK1,ARFIP2,LMOD3,RYK,RET,HCK,TTL,FER,HIP1R,PLS1,FMN1,LIMA1,SEMA4F,P2RX7,PTK2B,DPYSL2,USH1C,NKX6-1,ZFYVE27,F2RL1,IQGAP2,ANO6,SEMA3G,SPP1,CYFIP1,DMTN,SEMA4G,SLC12A5,MAGEL2,BDNF,KIAA0319,CARMIL1,LMOD1,ADD3,SLC12A7,TRPV4,SEMA3F,SSH1,PEX11B,HCLS1,ADNP,AP1AR,DISC1,RAB22A,WASF2,AP2M1,SPTBN2,TMOD4,SLC12A8,EPS8,CDKL5,WHAMM,DLG1,WNT3,CAPZA1,CREB1,CDC42EP2,MAPT,SEMA6D,SLC12A9,TNR,FN1,NCKAP1,GNB3,ARPC1A,KCNMA1,WAS,TMOD2,ATP13A2,SPTB,CLN8,PFN3,WNK3,SEMA4A,TRIM46,NDEL1,AC138894.1,HAX1,RAB11A,NTN1,DNM2,EVL,SIN3A,TRPV2,PEX11G,LRRC8E,CDK5R1,VAV1,IL7R,SSH2,RILP,RAP1GAP2,L1CAM,MLST8,ARHGAP4,PLXNA3,SPIRE2,TSC1,LAMTOR5,VAV3,LRRC8A,BARHL2,WDR63,SLC12A4,CARMIL2,RHOA,CNTN2,VASP,GMFG,WNT5A,PEX11A,GRB2,PPP1R15A,PFN4,PDXP,OLFM1,COTL1	grey	VSMC_M10
GO:1990868	0.74	0.36	24	48	response to chemokine	BP	1	SLC12A2,CXCL8,OXSR1,ACKR2,ROBO1,PTK2B,CCL17,CMKLR1,CIB1,THPO,CXCR2,PADI2,CCL14,CCL5,CCL7,CCL2,CCL25,RHOA,CCL16,GPR35,CCR10,CXCL6,DUSP1,CCL22	grey	VSMC_M10
GO:1990869	0.74	0.36	24	48	cellular response to chemokine	BP	1	SLC12A2,CXCL8,OXSR1,ACKR2,ROBO1,PTK2B,CCL17,CMKLR1,CIB1,THPO,CXCR2,PADI2,CCL14,CCL5,CCL7,CCL2,CCL25,RHOA,CCL16,GPR35,CCR10,CXCL6,DUSP1,CCL22	grey	VSMC_M10
GO:0003177	0.74	0.42	9	19	pulmonary valve development	BP	1	NFATC1,JAG1,ROBO1,ROBO2,TGFB2,RBPJ,TNFRSF1B,STRA6,HEY2	grey	VSMC_M10
GO:0031118	0.74	0.49	5	10	rRNA pseudouridine synthesis	BP	1	RPUSD2,RPUSD4,GAR1,RPUSD1,RPUSD3	grey	VSMC_M10
GO:0034453	0.74	0.40	12	25	microtubule anchoring	BP	1	CCDC68,KIF3A,BICD2,NINL,CLASP2,HOOK3,DAG1,SAXO2,NIN,CCDC120,PEX14,CAMSAP3	grey	VSMC_M10
GO:0043631	0.74	0.36	20	41	RNA polyadenylation	BP	1	CPSF6,PABPC1,ZC3H3,HSF1,CPSF1,RNF20,YTHDC1,PNPT1,MTPAP,SYMPK,PCF11,CSTF3,CLP1,TENT2,TENT4B,CPSF2,LEO1,AP002990.1,CSTF2,PAPOLA	grey	VSMC_M10
GO:0035773	0.74	0.35	29	58	insulin secretion involved in cellular response to glucose stimulus	BP	1	LRP1,PLA2G6,PRKCE,HMGN3,TRPM4,RAF1,NR1H4,NDUFAF2,OSBP,TIAM1,RAB11FIP5,ZBED6,GPLD1,SLC9B2,ADRA2A,RAB11FIP2,SIDT2,KLF7,ENY2,ABCA12,PPP3CB,PTPRN,MPC2,STX4,GPR68,OXCT1,NADK,BAIAP3,CRH	grey	VSMC_M10
GO:1903828	0.75	0.32	50	99	negative regulation of cellular protein localization	BP	1	NEDD4L,GOPC,C6orf106,ABHD17B,RAB23,DNAJA1,ITGB1BP1,UBAC2,FERMT1,BAG4,MFHAS1,NSFL1C,LYPLA1,POLR1A,DCLK2,PKIA,DMTN,SUFU,CABP1,PPFIA1,ANKRD13A,SP100,TTBK2,YOD1,OTUD7B,BAG3,AP2M1,PPP2R5A,FZD9,APOD,LRRC15,MAPT,LZTFL1,DERL3,C11orf65,MAGED1,RSC1A1,SIN3A,INPP5K,TAX1BP3,ABHD17A,SAPCD2,PARK7,ABHD17C,TXN,LEPROT,UBE2G2,MRAP2,MAP1A,DERL2	grey	VSMC_M10
GO:0010565	0.75	0.33	48	95	regulation of cellular ketone metabolic process	BP	1	PPARA,PRKCE,NR1H2,DKKL1,SLC7A11,SOX9,APOC1,PRKAG2,SLC22A13,PIBF1,AKT2,PANK2,PDK1,NR1H4,TRIB3,CYP7A1,DKK3,NR1H3,ERLIN1,PRMT3,SIRT5,LONP2,SIRT4,IRS1,CPT1A,SLC7A7,ABCB11,LPGAT1,MLXIPL,SCAP,PDK3,ACADL,TYSND1,COMT,WNT4,ACADVL,FGFR4,DGKQ,AC138894.1,NCOR1,CD74,SREBF1,PARK7,MLYCD,MIR21,FABP5,STARD4,INSIG2	grey	VSMC_M10

GO:0048593	0.75	0.32	48	96	camera-type eye morphogenesis	BP	1	FRS2,BAK1,ATF4,NTRK2,RORB,SLC1A1,TFAP2B,TDRD7,RDH13,PTN,TSPAN12,FOXE3,SOX9,IFT172,TSKU,JAG1,EPHB1,TENM3,USH1C,RPGRIP1L,TFAP2A,PAX2,NECTIN3,ALDH1A3,SHROOM2,NRL,SP3,FZD5,OBSL1,ROM1,SDK1,MFN2,MEGF11,LCTL,TBX2,LHX1,STRA6,YY1,SKI,SOX8,FJX1,THY1,WNT5A,DIO3,TBC1D20,PITX3,WNT2,COL8A2	grey	VSMC_M10
GO:0050670	0.75	0.31	70	137	regulation of lymphocyte proliferation	BP	1	AGER,IL5,HLA-DPB1,TNFRSF13C,RAC2,TNFRSF21,NCK2,PELI1,IL4I1,TLR4,IL15,BMI1,MYD88,FOXJ1,TGFBR2,PAWR,PRNP,NCKAP1L,SLC7A1,TRAF6,IL1A,CASP3,MEF2C,TMEM131L,IL7,RIPK2,MARCH7,ZNF335,CD40,IGF2,ICOSLG,BTN2A2,GPAM,PRKAR1A,CD28,DLG1,BCL6,IL12A,VSIR,RC3H1,PTPN6,TNFSF18,CD38,BST1,STAT5B,SCGB1A1,FOXP3,RASAL3,CARD11,TNFRSF1B,ZBTB7B,CHRNB2,BTK,CD74,CCL5,CD320,TWSG1,TICAM1,DNAJA3,VAV3,GLMN,MIR21,LST1,FADD,CD46,CD70,TNFSF9,CEBPB,TNFSF13B,HAVCR2	grey	VSMC_M10
GO:0048799	0.75	0.40	12	25	animal organ maturation	BP	1	SEMA4D,RYR1,RET,PAX2,ACTN3,FGFR3,ALDH1A2,THBS3,RFLNB,RHOA,SNX10,CCDC154	grey	VSMC_M10
GO:0050777	0.75	0.31	65	128	negative regulation of immune response	BP	1	TNFAIP3,USP15,ENPP3,C6orf106,MAPK14,NR1H2,IL4I1,FOXJ1,SEC14L1,SIRT2,NMI,NPLOC4,NCKAP1L,PCBP2,PLK2,HCK,FER,NR1H3,TRAFD1,OAS3,NLRC5,MAPKBP1,NOD2,CD96,HLX,YTHDF2,SLAMF8,PARP14,RNF26,FGR,NLRX1,TRAF3IP1,HTRA1,PDCD1,DUSP10,BCL6,RC3H1,PTPN6,TNFSF18,COL3A1,PPP3CB,MUL1,FOXP3,TKFC,USP18,TBX21,DCST1,ZBTB7B,IL4R,TSPAN6,STAT2,RIOK3,YES1,IL7R,PTPN2,CRK,FOXF1,MIR21,OAS1,CD46,A2M,FGL2,SERPINB9,HAVCR2,RC3H2	grey	VSMC_M10
GO:0099068	0.75	0.39	12	25	postsynapse assembly	BP	1	LRFN1,GAP43,NLGN4Y,NRXN2,NLGN2,NLGN3,CEL,NTNG2,ZDHHC12,GRID2,WNT5A,LRRTM2	grey	VSMC_M10
GO:0099622	0.75	0.39	12	25	cardiac muscle cell membrane repolarization	BP	1	SCN5A,KCNJ3,KCNE2,KCNIP2,KCNJ2,ANK2,SCN4B,NOS1AP,ATP1B1,RNF207,KCNH2,KCNJ8	grey	VSMC_M10
GO:0031057	0.75	0.37	18	37	negative regulation of histone modification	BP	1	PHF1,UBE2B,ATG5,SPHK2,KDM4A,TAF7,SDR16C5,DNMT3B,JARID2,BCOR,KDM5A,FOXP3,OTUB2,SIN3A,SUPT6H,SKI,MTF2,PRMT6	grey	VSMC_M10
GO:0003203	0.75	0.38	15	31	endocardial cushion morphogenesis	BP	1	SOX9,TGFBR2,ROBO1,ROBO2,TGFB2,SNAI1,BMPR1A,RBPJ,TMEM100,TBX2,ISL1,ENG,ACVRL1,NOG,HEY2	grey	VSMC_M10
GO:0007292	0.75	0.32	51	101	female gamete generation	BP	1	YTHDC2,GDF9,MCM9,WASHC5,TOB2,H3F3C,TUT7,DMC1,YTHDC1,PTN,NOBOX,CCDC155,TUT4,FOXO3,PANX1,ERCC1,MLH1,SIRT2,MCM8,SOHLH2,HPGD,M1AP,ADAMTS1,PTK2B,KMT2D,NPM2,BMPR1B,GAS2,HSF2BP,PRKAR1A,CCDC36,YTHDF2,SPO11,ETV6,WNT4,YBX2,SEPT1,IL4R,PAQR5,RAD51C,TAF4B,PARN,MEIOB,WDR77,SPIRE2,MSH4,AURKC,UBB,NANOS1,EREG,ZGLP1	grey	VSMC_M10
GO:0090398	0.75	0.33	34	68	cellular senescence	BP	1	MAP3K5,MAPK14,CDKN2A,SRF,NUAK1,CDK6,PNPT1,ZNF277,PAWR,PRKDC,PLK2,ING2,ARNTL,MAPKAPK5,OPA1,PLA2R1,TERT,MAP2K6,SLC30A10,KRAS,MAP3K3,CDKN1B,BCL6,BMPR1A,YPEL3,VASH1,MAP2K1,TBX2,TERF2,MIR22,PRMT6,YBX1,HRAS,MAP2K7	grey	VSMC_M10
GO:0043255	0.75	0.33	39	78	regulation of carbohydrate biosynthetic process	BP	1	PPARA,GPT,GNMT,CRY1,PPP4R3B,ZNF692,PLEK,FAM3C,EPM2AIP1,PPP1CB,AKT2,SIRT7,PPP1R3B,NLN,SORBS1,P2RY1,PTK2B,IGF2,IRS1,SIK1,MST1,PPP1R3E,EGF,GPER1,PPP1R3D,NTSR1,PASK,PTH1R,PPP1R3F,DDB1,DGKQ,OGT,INSR,LEPR,PTPN2,INPP5K,ACADM,NNMT,ARPP19	grey	VSMC_M10
GO:0051353	0.75	0.35	25	50	positive regulation of oxidoreductase activity	BP	1	CYP27B1,SDHAF4,NOD1,DHFR,SIRT3,PIK3CB,PTK2B,VDR,NOD2,TERT,COX17,KRAS,AGT,NOS1AP,POR,ABL2,GCH1,CALM1,PARK7,GNAI3,CYBA,TERF2,FXN,FCER2,APOPT1	grey	VSMC_M10
GO:0030857	0.75	0.38	15	31	negative regulation of epithelial cell differentiation	BP	1	SMO,S1PR3,HOXA7,OSR1,SPRY1,FOXE3,SOX9,SPRY2,JAG1,SPRED3,SRSF6,CCND1,CITED1,ACVRL1,FOXJ2	grey	VSMC_M10
GO:0021533	0.75	0.42	8	17	cell differentiation in hindbrain	BP	1	AGTPBP1,FAIM2,LDB1,TTC21B,LHX1,GRID2,NOG,CEND1	grey	VSMC_M10
GO:0097242	0.75	0.38	14	29	amyloid-beta clearance	BP	1	LRP1,SRF,C5AR1,RAB5A,IDE,MME,HDAC1,LRPAP1,ITGAM,RAB11A,LDLR,INSR,C3,ABCA7	grey	VSMC_M10
GO:0090151	0.75	0.39	12	24	establishment of protein localization to mitochondrial membrane	BP	1	HSPA4,TOMM22,AGK,AP3B1,OXA1L,NMT1,BCS1L,CALM1,TIMM29,MAIP1,TIMM10B,TIMM8B	grey	VSMC_M10
GO:0001702	0.75	0.42	8	17	gastrulation with mouth forming second	BP	1	FRS2,SRF,MEGF8,ACVR2A,LDB1,LHX1,AMOT,WNT5A	grey	VSMC_M10
GO:0036092	0.75	0.44	7	15	phosphatidylinositol-3-phosphate biosynthetic process	BP	1	SYNJ2,INPP4A,INPP4B,FIG4,PIK3CB,SYNJ1,PIKFYVE	grey	VSMC_M10
GO:0010812	0.75	0.34	26	53	negative regulation of cell-substrate adhesion	BP	1	LRP1,FBLN1,CDKN2A,HOXA7,ITGB1BP1,CLASP2,JAG1,FAM107A,PIK3R1,GCNT2,DMTN,ARHGAP6,AP1AR,FZD7,PTPRO,MELTF,APOD,BCL6,MEN1,CASK,CORO2B,BCAS3,RHOA,ACVRL1,ACER2,DUSP22	grey	VSMC_M10
GO:2001224	0.75	0.43	7	15	positive regulation of neuron migration	BP	1	SEMA6A,SEMA3A,RAPGEF2,SRGAP2C,ZNF609,FBXO31,DAB2IP	grey	VSMC_M10
GO:0043113	0.75	0.35	21	43	receptor clustering	BP	1	SYNGAP1,PICK1,PTN,ITGB1BP1,SLC7A11,DLG2,GRIK5,SSH1,FZD9,DLG1,ITGA4,NRXN2,SAV1,DLG4,WDR19,GPHN,SYNJ2BP,DNAJA3,RER1,THY1,MESD	grey	VSMC_M10
GO:0032607	0.75	0.40	10	21	interferon-alpha production	BP	1	IRF5,TLR4,NMI,MAVS,IRF7,RIPK2,IFIH1,SETD2,DDX3X,HAVCR2	grey	VSMC_M10
GO:0032647	0.75	0.40	10	21	regulation of interferon-alpha production	BP	1	IRF5,TLR4,NMI,MAVS,IRF7,RIPK2,IFIH1,SETD2,DDX3X,HAVCR2	grey	VSMC_M10
GO:0030048	0.75	0.32	48	97	actin filament-based movement	BP	1	NEDD4L,MYO1A,SUN2,MYH9,MYH14,TRPM4,MYO7B,CACNB2,SCN5A,SCN1A,MYO7A,GPD1L,PARVA,STC1,KCNJ3,TNNC1,KCNE2,DSP,RYR2,PKP2,KCNJ2,MYH7,ANK2,WASF2,ATP1A2,ACTC1,SLC9A1,SCN4B,NOS1AP,DLG1,WAS,MYO9B,SYNE2,WIPF2,CACNA1G,ATP2A1,MYH10,CCDC88C,DSG2,MYO1D,MYO1F,MYO5B,RNF207,FNBP1L,KCNH2,SCN2B,KCNJ8,HCN4	grey	VSMC_M10
GO:0003184	0.75	0.43	7	15	pulmonary valve morphogenesis	BP	1	NFATC1,JAG1,ROBO1,ROBO2,TGFB2,STRA6,HEY2	grey	VSMC_M10

GO:1990778	0.75	0.29	143	279	protein localization to cell periphery	BP	1	LRP1,STX7,STAC3,MAP7,GOPC,EPB41L2,PTCH1,TSPAN33,ADAM22,SLC1A1,PRKCE,ASB3,SPTBN1,ITGB1BP1,EGFR,VPS35,LIN7B,RAB7A,RAB10,CACNB2,CLASP2,STAC,OPTN,GOLGA4,MYL12A,AKT2,IKBKB,ANK1,LIN7A,PRNP,BAG4,GAS6,PKP3,ARL6IP5,TUB,KCNIP3,LYPLA1,RILPL1,RILPL2,PIK3R1,TMEM150A,VAMP5,SLMAP,PLS1,FCHO2,SORBS1,P2RY1,RAPGEF2,KCNJ11,ZFYVE27,TESC,KIF13A,NHLRC1,NECTIN3,PKP2,PPFIA1,ZDHHC23,DAG1,KCNB1,EPB41,ANK2,C2CD5,GPER1,RAB11FIP2,FAM126B,CIB1,AP2M1,PPP2R5A,BBS1,GORASP2,LAMA5,PACS1,DLG1,LRRC15,NSF,LZTFL1,EHD1,ZDHHC8,ABCA12,CNPY4,AP4M1,RAB3A,RAMP2,EXOC5,ATP1B1,PPIL2,NSG1,GRIPAP1,RAB8A,CNST,DLG4,ACTB,WDR19,RAB15,IQSEC2,RSC1A1,MPP5,WNK3,STAC2,TSPAN17,ANXA2,GAK,CPLX1,STX1B,STX4,TMEM88,RAB8B,ITGA3,AC138894.1,RAB11A,STX8,RAB3D,TTC7B,GGA2,ARHGAP44,PRAM1,EPB41L3,IFT20,EFCAB7,NUBP1,INPP5K,PDPK1,PRKCZ,RAB26,RER1,ARHGEF16,SAPCD2,SEC16A,PALM,BSG,GPSM2,ATP2C2,STXBP1,VPS4A,C16orf70,FYB1,PDZD3,PDZD11,LDLRAP1,CACNG7,HRAS,VAMP3,MRAP2	grey	VSMC_M10
GO:0021782	0.75	0.32	43	86	glial cell development	BP	1	LRP1,ADGRG6,AGER,NTRK2,PICK1,SOX10,SMO,TTBK1,ADAM22,CDK6,ROR2,FPR2,EGFR,TLR4,C5AR1,HDAC11,ERCC2,EIF2B3,PRX,AKT2,SIRT2,NRG1,LGI4,TSPAN2,DAG1,KRAS,CSPG5,NSUN5,MAPT,CNTNAP1,MPP5,PSEN1,LDLR,S100A8,MXRA8,SKI,LAMC3,CNTN2,SOX4,CNTF,POU3F2,NRROS,PHGDH	grey	VSMC_M10
GO:0032288	0.75	0.41	9	19	myelin assembly	BP	1	FIG4,MTMR2,ERCC2,ANK2,GNPAT,PIKFYVE,CNTNAP1,MPP5,EPB41L3	grey	VSMC_M10
GO:0016064	0.75	0.32	43	85	immunoglobulin mediated immune response	BP	1	CD226,HLA-DRA,HLA-DOB,HLA-DMA,HLA-DPB1,EXOSC3,MSH2,TRAF3IP2,HMCES,ERCC1,NECTIN2,MLH1,MYD88,FOXJ1,PAXIP1,SHLD1,HPX,IRF7,GAPT,NBN,NOD2,CD40,TCIRG1,CD28,FCER1G,RNF168,BCL6,PTPN6,FOXP3,SHLD2,TBX21,BATF,CD19,IL4R,BTK,CD74,C7,C3,SUPT6H,BCL10,CD46,FCER2,RNF8	grey	VSMC_M10
GO:0009190	0.75	0.42	8	17	cyclic nucleotide biosynthetic process	BP	1	ADCY3,GUCY1A1,GUCY1B1,ADCY7,ADCY2,ADCY4,ADCY10,GUCY2D	grey	VSMC_M10
GO:0052652	0.75	0.42	8	17	cyclic purine nucleotide metabolic process	BP	1	ADCY3,GUCY1A1,GUCY1B1,ADCY7,ADCY2,ADCY4,ADCY10,GUCY2D	grey	VSMC_M10
GO:1905207	0.75	0.38	13	27	regulation of cardiocyte differentiation	BP	1	FRS2,EGFR,MEF2C,SOX6,TBX5,PRICKLE1,TGFB2,GPER1,FZD7,GATA6,MIR222,NKX2-5,HEY2	grey	VSMC_M10
GO:0001578	0.75	0.32	43	86	microtubule bundle formation	BP	1	ZNF207,UBE2B,LRRC6,CFAP69,WDR92,CDK5RAP2,ARMC2,MAPRE1,ARMC4,TTC26,DRC1,RSPH6A,NCKAP5,FOXJ1,TTLL3,SPEF1,CLIP1,CCDC66,NCKAP5L,CCDC65,DNAH1,DNAH5,NEURL1,CFAP43,ZMYND10,CFAP46,JHY,TTC12,TOGARAM1,DNAAF2,SPAG16,TTC25,DNAAF4,MNS1,TRIM46,CCDC151,CAPN6,SPEF2,C16orf71,TPGS1,PSRC1,HYDIN,WDR63	grey	VSMC_M10
GO:0051480	0.75	0.29	108	212	regulation of cytosolic calcium ion concentration	BP	1	LRP1,ESR1,BAK1,MCHR1,GPR20,PLA2G6,S1PR3,PRKCE,FPR2,IBTK,LPAR1,ATG5,TRPM4,GRIN2D,TRPC3,SLC8A2,C5AR2,C5AR1,PTGIR,GIPR,CACNB2,ACKR2,C1QTNF1,RYR1,PRNP,ADRA1D,DIAPH1,P2RX2,CCKBR,RASA3,P2RX3,PLCE1,RYR3,TMBIM6,P2RX7,P2RY1,PTK2B,TPCN1,SLC8B1,F2RL1,F2R,PKD2,CASQ2,RYR2,TRPM1,SPPL3,LCK,TRPV4,GNA13,CMKLR1,UBASH3B,ANK2,GPER1,AGT,ATP1A2,CASQ1,NTSR1,FZD9,PTH1R,FIS1,CEMIP,ABL2,CDH23,PTPN6,CXCR2,GNG3,HTT,PTGDR,PTGER2,CD38,HAP1,DLG4,FAM155B,CACNA1A,AC138894.1,CD19,SMAD3,CALM1,BDKRB1,NMUR2,TRPV2,HRH4,PTGER4,GNB1,ATP2B3,BCAP31,GNA15,TRPV3,TRPV1,PDPK1,P2RX1,PKD1,GPR157,CYBA,JPH3,MCOLN3,PTGFR,MIR199A1,KCNK3,DMD,GPR35,NOL3,CHRNA10,CCR10,JPH2,THY1,WNT5A,DDIT3	grey	VSMC_M10
GO:0006886	0.75	0.27	386	739	intracellular protein transport	BP	1	TIMM21,STX7,AP3S1,KIF5A,PEX7,PEX3,TBC1D30,TBC1D9B,PPP1R10,RAB21,SNX31,ZDHHC14,RIMS2,PRKN,TSPO,HSPA4,DERL1,FAM91A1,FAM53C,TSNARE1,SIL1,SYNGR1,MAPK14,TOMM22,COPG2,PICK1,MICALL1,TBC1D22B,RAC2,SMO,ZDHHC21,PEX6,FBXO7,XPO5,APBA1,PEX1,SCFD2,SLC1A1,TGFBRAP1,TBC1D8,TRAM2,RAB23,SEC61B,STX17,SIX2,RIMS1,TMEM30A,RUFY3,XPO1,ITGB1BP1,YWHAZ,SEC61G,TCAF2,VPS41,TBC1D9,VPS35,SDCBP,MGARP,AGK,BCAP29,TRAF3IP2,DNAJC27,NAPA,RAB7A,AP2S1,COPG1,KPNA3,ARL5B,LCP1,SYTL2,DLG2,DNAJC15,PLK3,IPO13,AQP11,ZFAND6,TBC1D4,MYO7A,NAPB,GCKR,SEC62,KPNA2,AKT2,PITRM1,TIMM50,SNX5,USP36,TBC1D16,AP3M2,KIF5C,NPLOC4,ARL5A,UBAC2,RAB5A,ASPSCR1,TMCO6,NUP98,SIRT7,FERMT1,HACL1,CEP290,PRNP,ING1,BAG4,MAVS,FUT10,GAS6,ARFIP2,VPS16,COPZ1,STX2,SRP14,NCOA4,KCNIP3,NUP58,MIPEP,TM9SF4,ZDHHC20,IPO11,RILPL1,RILPL2,IMMP1L,PIK3R1,TBC1D12,TNPO1,FBXW7,SCG5,PEX2,PKIA,HTRA2,AUP1,ZFAND1,CSNK2A2,NUP54,KATNB1,SSR3,SPCS1,EXOC6B,SEC31A,RAPGEF3,AP3B1,GOLGA7B,AP4B1,PEX16,CRY2,VPS18,TOMM34,SEC31B,DMTN,PRICKLE1,VPS26C,HERC2,XPO7,LONP2,VPS39,KIF13A,SUFU,SIRT4,EIF2D,CABP1,RHBDD1,SRPRA,RPL23,PPP3CA,IPO9,SP100,ZDHHC23,MON1A,TBCK,ZDHHC6,VTI1A,TCF7L2,ADPRH,PDCD6,OXA1L,UNC93B1,RABL3,KCNB1,NCF1,YOD1,HCLS1,TBC1D10C,HSPA8,VPS45,HPS4,SORL1,C2CD5,ARIH2,AP1B1,AGT,CIB1,RAB22A,SYTL1,BAG3,AP1S2,MLPH,RAB17,AP2M1,SEC23IP,STRADB,SFN,ZDHHC18,PEX19,COPA,ZDHHC24,STX1A,RNF215,SNX27,STX6,RAB1B,TOMM40L,STRADA,VPS37D,UHMK1,EIF4ENIF1,RPL11,SNX6,[illegible]	grey	VSMC_M10
GO:0060038	0.75	0.35	22	45	cardiac muscle cell proliferation	BP	1	GLI1,MAPK14,PIM1,TGFBR2,MEF2C,TBX5,JARID2,TGFB2,PRKAR1A,KCNK2,SAV1,MAPK1,BMPR1A,RBPJ,TBX2,GATA6,MIR199A1,MIR222,NOG,C3orf58,HEY2,WNT2	grey	VSMC_M10

GO:0032368	0.75	0.32	48	95	regulation of lipid transport	BP	1	LRP1,PRKN,PPARA,TSPO,GDF9,PTCH1,PLA2G6,TTC39B,CRY1,ABCG5,ABCG8,TMEM30A,NR1H2,APOC1,AKT2,C1QTNF1,NR1H3,NKX3-1,CRY2,SPP1,PLA2R1,PLTP,MAP2K6,ABCA5,ACSL5,EGF,AGT,NTSR1,PLA2G4A,FIS1,OSBPL6,ZDHHC8,ABCA12,LPCAT3,GPS2,ANXA2,NAXE,ABCA3,ABCA7,RXRA,MIR27B,MIR27A,ABCA8,MIR33B,LDLRAP1,CRH,LRAT,PTGES	grey	VSMC_M10
GO:0034260	0.75	0.37	15	31	negative regulation of GTPase activity	BP	1	SPRY1,SPRY2,ARRB1,MKKS,FICD,SH3BP4,RASA4,GMIP,BCAS3,AMOT,GPSM1,TSC1,DAB2IP,MIR21,HRAS	grey	VSMC_M10
GO:0001755	0.75	0.35	23	47	neural crest cell migration	BP	1	SEMA6A,SOX10,SMO,SEMA4D,SEMA4C,OVOL2,ALX1,SEMA3D,SEMA3A,RET,HAND2,SEMA4F,SEMA3G,SEMA4G,SEMA3F,GBX2,LAMA5,SEMA6D,FN1,RADIL,SEMA4A,ISL1,SOX8	grey	VSMC_M10
GO:0006476	0.75	0.32	44	88	protein deacetylation	BP	1	BAZ2A,C6orf89,SPHK2,HDAC2,PRKD2,PHB,HDAC11,BEX4,TADA3,SIRT2,SIRT7,SIRT3,MTA3,SDR16C5,ING2,HDAC7,SIRT5,SUDS3,ELK4,SIRT4,HDAC1,RCOR3,PER2,HDAC4,MAPT,BCL6,RCOR2,PINK1,TBL1XR1,KDM5A,FOXP3,AKAP8L,AKAP8,JDP2,ZBTB7B,SFPQ,SIN3A,RCOR1,SREBF1,SKI,MIER2,PRMT6,SAP30BP,NNMT	grey	VSMC_M10
GO:0032677	0.75	0.34	28	56	regulation of interleukin-8 production	BP	1	HSPA1B,NOD1,ANXA4,TLR4,C5AR2,PRKD2,MYD88,ARRB1,MAVS,F2RL1,F2R,RIPK1,MAPKBP1,NOD2,TLR5,OTUD7B,STAT3,MAP2K5,CD74,NOS2,PARK7,LAMTOR5,BCL10,FADD,ZNF580,WNT5A,DDIT3,IL17D	grey	VSMC_M10
GO:0060074	0.75	0.42	8	17	synapse maturation	BP	1	SHANK1,YWHAZ,NEURL1,DISC1,NFATC4,SEZ6,PALM,DAB2IP	grey	VSMC_M10
GO:0051261	0.75	0.31	49	99	protein depolymerization	BP	1	VPS4B,AVIL,LMOD2,KIAA1211,TMOD1,PLEKHH2,SPTBN1,PLEK,ADD2,CLASP2,TRIM54,NCKAP5,NAV3,SPEF1,LMOD3,ATXN7,LIMA1,NCKAP5L,KATNB1,SYNJ1,F2RL1,DMTN,STMND1,CARMIL1,LMOD1,ADD3,TRPV4,TTBK2,CFL1,HSPA8,CIB1,SPTBN2,TMOD4,EPS8,CAPZA1,TMOD2,SPTB,GAK,CAMSAP3,TAOK1,APC2,CAMSAP1,VPS4A,CARMIL2,CCSAP,APC,PDXP,MAP1A,MAP6D1	grey	VSMC_M10
GO:0010165	0.75	0.37	14	29	response to X-ray	BP	1	XRCC6,MSH2,ERCC1,XRCC2,THBD,XRRA1,ERCC6,CASP3,XRCC4,CCND1,XRCC5,DNM2,KARS,BRCC3	grey	VSMC_M10
GO:0033169	0.75	0.45	6	13	histone H3-K9 demethylation	BP	1	KDM3B,KDM4C,KDM7A,KDM4A,PHF8,HSF4	grey	VSMC_M10
GO:0035459	0.75	0.41	9	19	vesicle cargo loading	BP	1	SEC31A,SEC31B,KIF13A,SCAP,MIA3,CTAGE9,SEC24C,TMED10,TBC1D20	grey	VSMC_M10
GO:0045879	0.76	0.37	14	29	negative regulation of smoothened signaling pathway	BP	1	PTCH1,PTCH2,KCTD21,GLI2,MEGF8,IFT172,KCTD6,SUFU,GPR161,TULP3,MOSMO,GPC3,MGRN1,GPR37L1	grey	VSMC_M10
GO:0048384	0.76	0.39	11	23	retinoic acid receptor signaling pathway	BP	1	SNW1,RXRB,NR1H2,RARB,ASXL1,ZNF536,CNOT1,RXRG,DHRS3,ALDH1A2,RXRA	grey	VSMC_M10
GO:0032387	0.76	0.35	23	46	negative regulation of intracellular transport	BP	1	RAB23,ITGB1BP1,UBAC2,FERMT1,BAG4,PKIA,DMTN,SUFU,CABP1,SP100,YOD1,BAG3,CNIH2,APOD,DERL3,C11orf65,SNX12,INPP5K,PARK7,MIR27B,TXN,UBE2G2,DERL2	grey	VSMC_M10
GO:0007041	0.76	0.31	50	100	lysosomal transport	BP	1	VPS4B,LRP1,IGF2R,PRKN,VPS52,TGFBRAP1,VPS41,VPS35,RAB7A,RBSN,HGSNAT,HOOK3,TMEM106B,FAM160A2,VPS16,NCOA4,CHMP2B,AP3B1,VPS18,VPS39,KIF13A,RAB7B,CCDC91,HSPA8,SORL1,DTX3L,SNX27,AP4M1,PINK1,ANKFY1,AP3M1,ATP13A2,GAK,AC138894.1,HOOK2,VIPAS39,STX8,SNAPIN,NPC1,LAMP2,CLEC16A,VPS53,MTM1,MGRN1,RILP,AP1G1,VPS4A,CACNG7,SNX16,CACNG5	grey	VSMC_M10
GO:0008203	0.76	0.31	52	103	cholesterol metabolic process	BP	1	APOF,TTC39B,PLPP6,CAT,APOB,EBP,CUBN,LIPE,TSKU,PIP4P1,NR1H4,SOAT2,CYP7A1,CYP7B1,CEBPA,LIMA1,EPHX2,ERLIN1,ABCA5,FMO5,MSMO1,SC5D,SCAP,HSD17B7,POR,ACADL,SOAT1,TM7SF2,OSBPL1A,FDX1,LPCAT3,GNB3,CYP27A1,CH25H,ACADVL,CLN8,LIPC,DGKQ,APOBR,CLN6,LDLR,CYP46A1,NPC1,SREBF1,LEPR,ACAA2,RXRA,CEL,NFE2L1,LDLRAP1,INSIG2,FGF1	grey	VSMC_M10
GO:0016233	0.76	0.36	18	37	telomere capping	BP	1	MAP3K4,MAPK15,POT1,ERCC1,XRCC1,PRKDC,MAPKAPK5,NBN,STN1,RTEL1,MAPK1,CTC1,MAPK3,ERCC4,TERF2,TINF2,TEN1,MAP2K7	grey	VSMC_M10
GO:0032486	0.76	0.44	6	13	Rap protein signal transduction	BP	1	PLK2,RAPGEF2,RAPGEF3,CBL,RAP1B,RAP2B	grey	VSMC_M10
GO:0106056	0.76	0.37	15	31	regulation of calcineurin-mediated signaling	BP	1	FHL2,SLC8A2,EFHB,PRNP,NRG1,SPPL3,TBC1D10C,CIB1,SLC9A1,MYOZ2,ACTN3,HOMER2,MYOZ1,MAPK7,CAMTA1	grey	VSMC_M10
GO:0036503	0.76	0.31	50	99	ERAD pathway	BP	1	CANX,PRKN,RNF5,DERL1,FOXRED2,SEC61B,ECPAS,AQP11,UBXN4,UGGT2,FBXO17,DNAJC18,NPLOC4,UBAC2,UBXN8,USP25,AUP1,TMEM67,AMFR,RNFT2,ERLIN1,RHBDD1,GET4,USP13,YOD1,USP19,RNF185,RHBDD2,EDEM3,NFE2L2,SYVN1,DERL3,TMUB2,DNAJC10,DNAJB12,TMEM129,ANKZF1,DNAJB2,SDF2L1,CALR3,FBXO2,SEL1L,BCAP31,SGTA,TMEM259,HSPA5,RNFT1,FAM8A1,UBE2G2,DERL2	grey	VSMC_M10
GO:0034101	0.76	0.31	56	111	erythrocyte homeostasis	BP	1	MPIG6B,HSPA1B,BRD1,SMAD5,L3MBTL3,MAPK14,ZNF16,RBFOX2,SRF,CDK6,KIT,PRDX1,RPS14,SMAP1,EPAS1,PTBP3,BPGM,FOXO3,ERCC2,SLC25A38,ACVR2A,NCKAP1L,PRKDC,MFHAS1,GPI,ACVR1B,CASP3,TMEM14C,DMTN,ALAS1,LDB1,PKNOX1,RPS17,ACIN1,DYRK3,HCLS1,ARNT,SP3,BCL6,STAT3,STAT5B,INHA,THRA,MED1,HOXB6,AHSP,KAT7,DNASE2,SCNN1B,RCOR1,PTPN2,MIR222,ANKRD54,ARID4A,MAFB,FAM210B	grey	VSMC_M10
GO:0051897	0.76	0.31	44	88	positive regulation of protein kinase B signaling	BP	1	STK3,PTK2,TEK,RTN4,ITGB1BP1,MET,EGFR,PHB,SPRY2,C1QTNF1,HBEGF,BAG4,NRG1,GAS6,MFHAS1,RET,PIK3CB,PTPRJ,GCNT2,IGF2,TCF7L2,EGF,HCLS1,CASS4,OSM,THPO,CD28,ADAM8,STOX1,GDF15,PINK1,HAX1,PIK3R5,INSR,PARK7,ENG,MIR29A,MIR199A1,MIR21,MIR222,TXN,MYORG,TSPYL5,RASD2	grey	VSMC_M10
GO:0034433	0.76	0.43	7	14	steroid esterification	BP	1	APOC1,SOAT2,AGT,SOAT1,MIR27B,MIR27A,STARD4	grey	VSMC_M10
GO:0034434	0.76	0.43	7	14	sterol esterification	BP	1	APOC1,SOAT2,AGT,SOAT1,MIR27B,MIR27A,STARD4	grey	VSMC_M10
GO:0034435	0.76	0.43	7	14	cholesterol esterification	BP	1	APOC1,SOAT2,AGT,SOAT1,MIR27B,MIR27A,STARD4	grey	VSMC_M10

GO:0009150	0.76	0.28	148	286	purine ribonucleotide metabolic process	BP	1	NME1,HSD17B4,PDE7B,ATP5MGL,PPT2,PPARA,PDE10A,ACSL6,PRXL2C,GTPBP1,IMPDH1,GDA,CROT,SLC35B2,SLC25A13,GAPDH,PPAT,RAB23,PPCS,SLC4A4,NT5E,PPT1,BPGM,DLD,SPHK2,ADCY3,DLG2,DHTKD1,PRKAG2,ATP5F1C,ELOVL1,PFKP,NUDT16,PANK2,PDK1,SUCLG2,PDHX,ELOVL7,PANK1,GPI,AMPD3,ATP5F1A,MCCC2,GPD1,P2RX7,GUCY1A1,GUCY1B1,ACOT12,DGUOK,PRKAG1,GMPS,PFKM,OPA1,ENTPD4,FITM2,AMPD1,NUDT18,ADCY7,FAM173B,ADCY2,MCEE,NME4,PDE9A,TPST1,ZBTB20,GPAM,FAR2,ACSL5,PTHLH,MFN1,PFKFB2,ENO4,HACD2,ARNT,ADCY4,ACTN3,HDAC4,THEM5,DNAJC30,MLXIPL,PDE8A,HMGCL,MAGI3,DLG1,ACOT9,PDK3,EFL1,SLC25A16,HK1,ACSBG1,ATP5S,CASK,COASY,PRKAG3,PINK1,STAT3,NME7,ADK,MPC2,ADCY10,ENO3,PARP1,TECR,CARD11,SLC26A1,PSEN1,ACOT2,GUCY2D,PKLR,PANK3,GCDH,OGT,ADPGK,PDE4A,ACSM3,ACACA,NCOR1,INSR,SLC26A2,ADSSL1,ACSBG2,ALDOC,PIPOX,ACOT11,ATP6V0C,PANK4,NME3,MPP1,ATP5PB,SLC2A6,VPS9D1,GNAI3,ACSF3,MLYCD,MT-ATP8,MT-ATP6,RHOA,SULT1C3,ATP5ME,ACOT4,ATP5MF,ENTPD5,ELOVL3,TIGAR,NUDT19,NME6,NUDT2,HINT1	grey	VSMC_M10
GO:0072538	0.76	0.38	12	25	T-helper 17 type immune response	BP	1	TRAF3IP2,PHB,RC3H1,TNFSF18,STAT3,FOXP3,TBX21,BATF,ZBTB7B,IL6R,MIR21,RC3H2	grey	VSMC_M10
GO:0042267	0.76	0.36	16	33	natural killer cell mediated cytotoxicity	BP	1	CD226,PRDX1,NECTIN2,PVR,UNC13D,RASGRP1,CADM1,IL12A,PTPN6,LAG3,STAT5B,VAV1,CRK,AP1G1,SERPINB9,HAVCR2	grey	VSMC_M10
GO:0048011	0.76	0.39	11	23	neurotrophin TRK receptor signaling pathway	BP	1	WASF1,SPRY1,RAF1,SPRY2,CASP3,ZFYVE27,CYFIP1,DOK5,AGT,PPP2R5B,HAP1	grey	VSMC_M10
GO:0030178	0.76	0.30	72	142	negative regulation of Wnt signaling pathway	BP	1	NFATC1,LRP1,GLI1,STK3,FZD6,PRKN,MAPK14,CBY1,SOX10,MDFI,ROR2,SOSTDC1,GRB10,HECW1,FUZ,SFRP4,DKKL1,FOXO3,SOX9,TSKU,WNT11,RBMS3,CHD8,UBAC2,FERMT1,PFDN5,CTNND1,DKK3,TRABD2A,TMEM131L,LMBR1L,AMFR,SOX13,STK4,PRICKLE1,NKD1,HDAC1,TCF7L2,DKK2,AXIN2,SOX2,ZNRF3,KREMEN1,BICC1,NFATC4,PTPRO,OTUD5,IGFBP4,TMEM88,CITED1,SOX30,AC004687.2,CCDC88C,GSC,G3BP1,ISL1,NLK,GPC3,TLE6,TAX1BP3,APC2,DAB2IP,MIR199A1,TRABD2B,NOG,WNT5A,DDIT3,DACT3,SDHAF2,SHISA2,APC,NKX2-5	grey	VSMC_M10
GO:0006874	0.76	0.28	145	283	cellular calcium ion homeostasis	BP	1	LRP1,ESR1,SMDT1,BAK1,MCHR1,GPR20,PLA2G6,SLC25A27,S1PR3,PRKCE,TNNI3,FPR2,IBTK,LPAR1,KEL,ATG5,TRPM4,GRIN2D,TRPC3,SLC8A2,C5AR2,C5AR1,ATP7B,PTGIR,GIPR,CACNB2,ACKR2,SLC24A3,C1QTNF1,TMTC4,RYR1,PRNP,ADRA1D,DIAPH1,P2RX2,CCKBR,RASA3,MICU3,IMMT,C19orf12,P2RX3,PLCE1,RYR3,TMBIM6,P2RX7,P2RY1,PTK2B,TPCN1,SLC8B1,VDR,STC1,RMDN3,F2RL1,F2R,PKD2,CASQ2,MCUR1,CD40,RYR2,RAP1GDS1,TRPM1,SPPL3,LCK,TRPV4,TCIRG1,GNA13,CMKLR1,MCUB,UBASH3B,DISC1,ANK2,GPER1,TRPM8,PDZD8,AGT,ATP1A2,CASQ1,NTSR1,FZD9,TMCO1,PTH1R,FIS1,CEMIP,ABL2,CDH23,PTPN6,MICU1,MCU,CXCR2,GNG3,HTT,PTGDR,PTGER2,ATP1B1,CD38,ATP13A2,HAP1,DLG4,FAM155B,PSEN1,CACNA1A,AC138894.1,ATP2A1,CD19,SMAD3,CALM1,PRKCB,BDKRB1,NMUR2,TRPV2,HRH4,CCL14,CCL5,CCL7,PTGER4,SLC25A23,MAIP1,GNB1,ATP2B3,BCAP31,GNA15,TRPV3,TRPV1,PDPK1,P2RX1,PKD1,TMEM203,GPR157,SYPL2,CYBA,JPH3,ATP2C2,MCOLN3,PTGFR,MIR199A1,KCNK3,DMD,GPR35,NOL3,CHRNA10,CCR10,JPH2,THY1,WNT5A,DDIT3	grey	VSMC_M10
GO:0034695	0.76	0.41	8	17	response to prostaglandin E	BP	1	PRKCE,AANAT,PTGER2,AKAP8,ACACA,PTGER4,GNB1,GNG2	grey	VSMC_M10
GO:0018023	0.76	0.35	19	39	peptidyl-lysine trimethylation	BP	1	IWS1,SUV39H2,KDM4A,NSD3,KMT2D,RTF1,WDR82,ZNF335,FAM173B,SETD2,WDR61,VCPKMT,SETD1A,OGT,SETD3,MECP2,ARID4A,BEND3,ZNF304	grey	VSMC_M10
GO:0010799	0.76	0.36	17	35	regulation of peptidyl-threonine phosphorylation	BP	1	PRKAG2,SPRY2,SIRT2,RPTOR,RIPK2,DMTN,EGF,STOX1,CEMIP,MAPK1,WNK3,DGKQ,CALM1,INPP5K,IRGM,WNT5A,CADM4	grey	VSMC_M10
GO:0048821	0.76	0.37	13	27	erythrocyte development	BP	1	BRD1,L3MBTL3,SRF,PTBP3,BPGM,ERCC2,NCKAP1L,DMTN,ALAS1,BCL6,MED1,ARID4A,FAM210B	grey	VSMC_M10
GO:0010463	0.76	0.36	17	35	mesenchymal cell proliferation	BP	1	MYC,SMO,SIX2,PTN,MYCN,OSR1,SOX9,WNT11,TGFBR2,HAND2,CHRD,BMPR1A,GPC3,FOXF1,WNT5A,SIX1,WNT2	grey	VSMC_M10
GO:0030168	0.76	0.31	48	96	platelet activation	BP	1	FZD6,MAPK14,MYH9,SRF,TEC,TLN1,PLEK,DGKI,TLR4,SLC7A11,THBD,MYL12A,C1QTNF1,ACTG1,CLIC1,ADRA2B,PIK3CB,P2RY1,F2R,DMTN,CD40,LCK,ADRA2A,UBASH3B,DGKD,FCER1G,DGKG,PLA2G4A,ITGA2B,FN1,PTPN6,ADRA2C,FERMT3,COL3A1,VCL,ACTB,PEAR1,DGKQ,DGKE,VAV1,ABAT,P2RX1,ADAMTS13,VAV3,STXBP1,FGA,BLOC1S3,RAP2B	grey	VSMC_M10
GO:0098698	0.76	0.41	8	17	postsynaptic specialization assembly	BP	1	LRFN1,GAP43,NRXN2,NLGN2,NTNG2,ZDHHC12,GRID2,LRRTM2	grey	VSMC_M10
GO:0060119	0.76	0.36	17	35	inner ear receptor cell development	BP	1	ATP8B1,WHRN,USH1G,MYO7A,TSKU,SLC4A7,LRTOMT,AP000812.5,PLS1,ADGRV1,USH1C,TRIP11,STRC,CDH23,MKS1,IFT20,PJVK	grey	VSMC_M10
GO:0045823	0.76	0.38	12	25	positive regulation of heart contraction	BP	1	SLC1A1,TRPM4,ADRA1D,RYR2,TGFB2,ACE2,ATP1A2,GCH1,CHGA,RGS4,NKX2-5,HEY2	grey	VSMC_M10
GO:1903531	0.76	0.31	50	99	negative regulation of secretion by cell	BP	1	VPS4B,PRKN,CRY1,IL11,BMP8A,RSAD2,RAB7A,FRMD4A,DPH3,NCKAP1L,ADRA2B,NDUFAF2,FAM3D,ASIC1,P2RY1,KCNJ11,NR1H3,RAB11FIP5,ACVR1C,F2R,CRY2,ADTRP,SIRT4,IRS1,CD200,HDAC1,PPP3CA,ADRA2A,KCNB1,OSM,KLF7,RAP1B,IL12A,ENY2,ADRA2C,PNKD,INHA,RSC1A1,TNFRSF1B,SYT11,SYTL4,SREBF1,ABAT,FMR1,FOXF1,MIR199A1,CHGA,CRH,LIF,VAMP3	grey	VSMC_M10
GO:0032728	0.76	0.36	16	33	positive regulation of interferon-beta production	BP	1	IRF5,TLR4,TMEM173,MAVS,IRF7,OAS3,RIPK2,POLR3D,IFIH1,POLR3C,DDX3X,POLR3A,RIOK3,RNF135,TICAM1,OAS1	grey	VSMC_M10

GO:0061333	0.76	0.32	31	63	renal tubule morphogenesis	BP	1	MTSS1,MYC,KLHL3,PTCH1,SMO,SIX2,OSR1,SOX9,GZF1,WNT11,PAX8,EYA1,FMN1,MEF2C,PKD2,PAX2,SALL1,IRX2,AGT,LAMA5,DLG1,WNT4,MAGED1,CITED1,LHX1,GPC3,PKD1,SOX8,NOG,SIX1,FGF1	grey	VSMC_M10
GO:0060969	0.76	0.40	9	19	negative regulation of gene silencing	BP	1	ZC3H10,ESR1,BCDIN3D,PPP3CA,TERT,TET1,STAT3,PHF8,NCOR1	grey	VSMC_M10
GO:0014850	0.76	0.39	10	21	response to muscle activity	BP	1	ABCG5,ABCG8,OPA1,RYR2,SRD5A1,AGT,MTFP1,FIS1,PRKAG3,ADSSL1	grey	VSMC_M10
GO:0010821	0.76	0.30	63	124	regulation of mitochondrion organization	BP	1	PRKN,TSPO,BAK1,PLA2G6,FBXO7,TMEM14A,VPS35,MGARP,USP36,SIRT7,DDHD2,DCN,HIP1R,FBXW7,HTRA2,CSNK2A2,OPA1,DNM1L,ZDHHC6,USP30,GPER1,CIDEB,FZD9,PISD,FZD5,MAPT,STOX1,FIS1,NMT1,C11orf65,MCU,HTT,DDHD1,PINK1,MUL1,ATP13A2,TMEM102,VPS13D,VPS13C,HAX1,ADCK1,STAT2,PSMD10,MIEF2,SREBF1,RALBP1,ACAA2,PAM16,PARK7,DHODH,PRMT6,MLLT11,NOL3,BNIP3L,TIGAR,FXN,BCL2L11,SLC35F6,RALA,BCL2L2,MIEF1,PPIF,AC140504.1	grey	VSMC_M10
GO:0001658	0.76	0.34	23	47	branching involved in ureteric bud morphogenesis	BP	1	MYC,PTCH1,SMO,SIX2,SOX9,GZF1,PAX8,EYA1,PKD2,PAX2,SALL1,AGT,LAMA5,DLG1,WNT4,MAGED1,CITED1,LHX1,GPC3,SOX8,NOG,SIX1,FGF1	grey	VSMC_M10
GO:0002931	0.76	0.33	25	51	response to ischemia	BP	1	MAP3K5,BVES,SLC8A2,NDNF,PANX1,SLC26A5,P2RX2,PIK3CB,FAIM2,P2RX7,KCNJ11,MAP2K6,CIB1,HYOU1,PER2,ABRAXAS2,PINK1,CAMK2A,CSF1R,MLYCD,NQO1,NOL3,TIGAR,BCL2L2,PPIF	grey	VSMC_M10
GO:0001556	0.76	0.40	9	19	oocyte maturation	BP	1	WASHC5,TUT7,DMC1,TUT4,FOXO3,SIRT2,PTK2B,YTHDF2,EREG	grey	VSMC_M10
GO:0002695	0.76	0.30	60	118	negative regulation of leukocyte activation	BP	1	TNFAIP3,ENPP3,FBXO7,TNFRSF21,PELI1,IL4I1,CST7,CD300LF,PIBF1,FOXJ1,PAWR,PRNP,NCKAP1L,ZC3H8,FER,CASP3,TMEM131L,PAG1,NR1H3,MARCH7,CD200,BANK1,BTN2A2,PRKAR1A,HLX,TBC1D10C,GPER1,FGR,RUNX3,DLG1,BCL6,VSIR,RC3H1,PTPN6,TNFSF18,LAG3,INHA,SCGB1A1,FOXP3,TBX21,SYT11,ZBTB7B,IL4R,LDLR,BTK,CD74,TWSG1,PTPN2,SOCS1,FOXF1,GLMN,MIR21,MIR27A,LST1,MIR125A,FGL2,LAX1,CEBPB,HAVCR2,RC3H2	grey	VSMC_M10
GO:0032925	0.76	0.41	8	17	regulation of activin receptor signaling pathway	BP	1	ZC3H3,ACVR2B,ACVR2A,ACVR1B,MEN1,SKI,FSTL3,LEMD3	grey	VSMC_M10
GO:0060147	0.76	0.34	20	41	regulation of posttranscriptional gene silencing	BP	1	ZC3H10,ESR1,AGO2,TNRC6B,XPO5,EGFR,MYCN,PUM2,BCDIN3D,EIF4E2,RIPK1,PPP3CA,TERT,AJUBA,DDX6,EIF4ENIF1,STAT3,MAP2K1,NCOR1,FMR1	grey	VSMC_M10
GO:0032958	0.76	0.39	10	21	inositol phosphate biosynthetic process	BP	1	IPPK,PLEK,ITPKC,P2RY1,PTK2B,ITPKA,GPER1,IP6K2,NTSR1,PTH1R	grey	VSMC_M10
GO:0060561	0.76	0.39	10	21	apoptotic process involved in morphogenesis	BP	1	BAK1,PAX8,HAND2,VDR,PAX2,TGFB2,FZD5,TNFRSF1B,JAG2,BCL2L11	grey	VSMC_M10
GO:0043370	0.76	0.36	16	33	regulation of CD4-positive, alpha-beta T cell differentiation	BP	1	HLA-DRA,NCKAP1L,RIPK2,HLX,RUNX3,BCL6,RC3H1,TNFSF18,FOXP3,TBX21,ZBTB7B,IL4R,SOCS1,PRKCZ,MIR21,RC3H2	grey	VSMC_M10
GO:0032881	0.76	0.36	16	33	regulation of polysaccharide metabolic process	BP	1	RUBCNL,KHK,EPM2AIP1,PPP1CB,AKT2,PPP1R3B,SORBS1,IGF2,IRS1,PPP1R3E,EGF,PPP1R3D,PASK,PPP1R3F,INSR,INPP5K	grey	VSMC_M10
GO:0060413	0.76	0.42	7	15	atrial septum morphogenesis	BP	1	SMO,TBX5,TGFB2,ISL1,SOX4,NKX2-5,HEY2	grey	VSMC_M10
GO:2000050	0.76	0.38	12	25	regulation of non-canonical Wnt signaling pathway	BP	1	RSPO3,CSNK1E,DAAM2,SFRP4,SPEF1,TIAM1,NKD1,ZNRF3,MKS1,GPC3,WNT5A,LBX2	grey	VSMC_M10
GO:0014031	0.76	0.31	36	73	mesenchymal cell development	BP	1	SEMA6A,SOX10,SMO,SEMA4D,SEMA4C,SOX9,OVOL2,JAG1,ALX1,SEMA3D,SEMA3A,NRG1,KBTBD8,RET,HAND2,SEMA4F,SEMA3G,SEMA4G,KLHL12,SEMA3F,PEF1,PDCD6,GBX2,LAMA5,SEMA6D,FN1,MAPK1,BMPR1A,RADIL,ALDH1A2,SEMA4A,MAPK3,TCOF1,ISL1,SOX8,HEY2	grey	VSMC_M10
GO:0061081	0.76	0.41	8	17	positive regulation of myeloid leukocyte cytokine production involved in immune response	BP	1	KIT,TLR4,MAVS,SPON2,CD74,TICAM1,BCL10,WNT5A	grey	VSMC_M10
GO:0060964	0.76	0.35	19	39	regulation of gene silencing by miRNA	BP	1	ZC3H10,ESR1,AGO2,TNRC6B,EGFR,MYCN,PUM2,BCDIN3D,EIF4E2,RIPK1,PPP3CA,TERT,AJUBA,DDX6,EIF4ENIF1,STAT3,MAP2K1,NCOR1,FMR1	grey	VSMC_M10
GO:0070169	0.76	0.34	20	41	positive regulation of biomineral tissue development	BP	1	OSR2,PTN,OSR1,SLC4A2,ACVR2B,ACVR2A,KL,ADGRV1,MEF2C,P2RX7,ANO6,TFAP2A,BMPR1B,AMELX,ACTN3,FZD9,WNT4,BMPR1A,SMAD3,CEBPB	grey	VSMC_M10
GO:0010872	0.76	0.46	5	10	regulation of cholesterol esterification	BP	1	APOC1,AGT,MIR27B,MIR27A,STARD4	grey	VSMC_M10
GO:2000637	0.76	0.37	13	27	positive regulation of gene silencing by miRNA	BP	1	AGO2,TNRC6B,EGFR,MYCN,PUM2,EIF4E2,RIPK1,AJUBA,DDX6,EIF4ENIF1,STAT3,MAP2K1,FMR1	grey	VSMC_M10
GO:1904738	0.76	0.37	13	27	vascular associated smooth muscle cell migration	BP	1	LRP1,GRB10,MEF2C,ADAMTS1,DOCK5,TERT,GNA13,SSH1,AGT,NFE2L2,DOCK7,MIR21,RHOA	grey	VSMC_M10
GO:0045599	0.77	0.34	21	43	negative regulation of fat cell differentiation	BP	1	MKX,JAG1,SIRT2,TRIB3,ASXL1,ARNTL,RUNX1T1,TRIO,TRPV4,GPER1,MMP11,GPS2,TGFB1I1,JDP2,SMAD3,MIR27B,MIR27A,WNT5A,DDIT3,ZFP36L2,C1QL4	grey	VSMC_M10
GO:0098869	0.77	0.32	34	67	cellular oxidant detoxification	BP	1	LTC4S,APOM,PRXL2C,KDM3B,FANCC,TXNRD1,PRDX1,CAT,ALB,PXDN,MGST2,TXNRD3,DHFR,GSTK1,PRDX3,CYGB,PXDNL,GLRX,GPX8,TP53INP1,FBLN5,NFE2L2,DUOX1,GCH1,SOD3,GSTZ1,MPO,TXNDC2,PARK7,NQO1,MIR21,HBA2,TXN,PTGES	grey	VSMC_M10
GO:0016266	0.77	0.35	16	33	O-glycan processing	BP	1	C1GALT1,POMGNT1,GALNT11,ST6GALNAC2,GALNT15,GXYLT2,POFUT1,B3GNT4,GALNT5,B3GNT5,GALNT2,XXYLT1,GALNT8,GALNT1,ST6GALNAC4,ST3GAL2	grey	VSMC_M10
GO:0072171	0.77	0.33	26	53	mesonephric tubule morphogenesis	BP	1	MYC,PTCH1,SMO,SIX2,OSR1,SOX9,GZF1,WNT11,PAX8,EYA1,FMN1,PKD2,PAX2,SALL1,AGT,LAMA5,DLG1,WNT4,MAGED1,CITED1,LHX1,GPC3,SOX8,NOG,SIX1,FGF1	grey	VSMC_M10
GO:0043409	0.77	0.29	66	131	negative regulation of MAPK cascade	BP	1	SEMA6A,PRKN,FBLN1,MYC,LEMD2,STK38,WNK2,KLHL31,DNAJA1,FKTN,ITGB1BP1,VRK3,TLR4,SPRY1,PHB,SPRY2,ARRB1,ZMYND11,SPRED3,DUSP6,SH3RF2,SIRT3,PIK3CB,PTPRJ,P2RX7,PAQR3,F2RL1,TAOK3,DUSP8,PRDM15,PDCD4,DAG1,TBC1D10C,SORL1,GPER1,AGT,DUSP16,DLG1,AIDA,DUSP10,MEN1,DUSP19,PTPN6,PINK1,GPS2,RGS14,SYNJ2BP,EFNA1,TNIP1,PSMD10,NCOR1,PTPN2,INPP5K,ABCA7,MARVELD3,DAB2IP,MIR21,DUSP2,CHRNA10,DUSP26,DUSP1,DUSP4,C3orf33,LIF,LAX1,C1QL4	grey	VSMC_M10
GO:0060716	0.77	0.40	8	17	labyrinthine layer blood vessel development	BP	1	OVOL2,VASH2,FZD5,MAPK1,RBPJ,VASH1,HEY2,WNT2	grey	VSMC_M10

GO:0060968	0.77	0.32	29	59	regulation of gene silencing	BP	1	ZC3H10,ESR1,AGO2,TNRC6B,XPO5,EGFR,MYCN,PUM2,SETDB2,FAM208A,BCDIN3D,EIF4E2,RIPK1,PPHLN1,PPP3CA,TERT,AJUBA,DDX6,EIF4ENIF1,TET1,STAT3,PHF8,MAP2K1,NCOR1,FMR1,SETDB1,HIST1H3F,HIST1H3H,ZNF304	grey	VSMC_M10
GO:0006891	0.77	0.36	14	29	intra-Golgi vesicle-mediated transport	BP	1	COPG2,TRAPPC3,NAPA,COPG1,COG1,COPZ1,VTI1A,COG2,COPA,NSF,NAPG,COG8,RAB6B,TRAPPC3L	grey	VSMC_M10
GO:0070861	0.77	0.38	11	23	regulation of protein exit from endoplasmic reticulum	BP	1	TMEM30A,UBAC2,TM9SF4,YOD1,SORL1,DERL3,SEC16B,TMEM30B,BCAP31,UBE2G2,DERL2	grey	VSMC_M10
GO:0043001	0.77	0.35	18	37	Golgi to plasma membrane protein transport	BP	1	GOPC,SPTBN1,RAB10,OPTN,GOLGA4,LYPLA1,VAMP5,KIF13A,BBS1,NSF,CNST,RSC1A1,AC138894.1,GGA2,RAB26,SEC16A,C16orf70,VAMP3	grey	VSMC_M10
GO:0060307	0.77	0.42	7	15	regulation of ventricular cardiac muscle cell membrane repolarization	BP	1	SCN5A,KCNE2,ANK2,SCN4B,NOS1AP,RNF207,KCNH2	grey	VSMC_M10
GO:0043984	0.77	0.40	8	17	histone H4-K16 acetylation	BP	1	KANSL3,ATG5,PHF20,KANSL2,AUTS2,MSL1,OGT,HCFC1	grey	VSMC_M10
GO:1901071	0.77	0.39	9	19	glucosamine-containing compound metabolic process	BP	1	LARGE1,GNPDA2,NANP,CHST1,OVGP1,CHST3,CHST7,CHST5,CTBS	grey	VSMC_M10
GO:0060441	0.77	0.38	11	23	epithelial tube branching involved in lung morphogenesis	BP	1	TNC,SPRY1,SOX9,SPRY2,DAG1,KRAS,CTSH,LAMA1,FOXF1,ESRP2,WNT2	grey	VSMC_M10
GO:0072676	0.77	0.31	36	72	lymphocyte migration	BP	1	SLC12A2,CCL27,OXSR1,GAS6,RET,CYP7B1,JAM2,PTK2B,CCL17,SLC8B1,ADTRP,CD200,CD99,APOD,ADAM8,MIA3,ITGA4,CH25H,PADI2,CXCL16,TMEM102,TBX21,STK10,CCL14,CCL5,CCL7,CCL2,CCL25,CRK,RHOA,CCL16,FADD,WNT5A,ARTN,GCSAM,CCL22	grey	VSMC_M10
GO:2000514	0.77	0.34	20	41	regulation of CD4-positive, alpha-beta T cell activation	BP	1	AGER,HLA-DRA,TGFBR2,NCKAP1L,RIPK2,HLX,RUNX3,BCL6,VSIR,RC3H1,TNFSF18,FOXP3,TBX21,ZBTB7B,IL4R,TWSG1,SOCS1,PRKCZ,MIR21,RC3H2	grey	VSMC_M10
GO:0098969	0.77	0.41	7	15	neurotransmitter receptor transport to postsynaptic membrane	BP	1	NSG1,GRIPAP1,RAB8A,CPLX1,STX1B,RAB11A,ARHGAP44	grey	VSMC_M10
GO:0051307	0.77	0.38	10	21	meiotic chromosome separation	BP	1	MAPK15,MLH1,MOS,M1AP,EME1,ERCC4,SLX4,MEIOB,MSH4,CENPX	grey	VSMC_M10
GO:0008277	0.77	0.30	48	96	regulation of G protein-coupled receptor signaling pathway	BP	1	CRY1,RIC8B,PHF24,CAMKMT,KLK14,CXCL8,PLEK,MET,PHB,GIPR,ARRB1,PDE6G,PLCB1,RASGRP4,TUB,RGS20,ABHD6,PLCE1,RPGRIP1L,RGS1,GIT2,GRK2,GPER1,SAG,PDE6H,HOMER2,NMT1,GRK4,RAMP2,TMOD2,TULP3,GRK6,RGS14,DGKQ,GUCY2D,DNM2,CAMK2A,CCL5,C3,MGRN1,GNG7,USP20,RGS11,CNTN2,CHGA,RGS12,RGS4,MRAP2	grey	VSMC_M10
GO:0060135	0.77	0.33	24	49	maternal process involved in female pregnancy	BP	1	CYP27B1,ESR1,AKR1B1,PLA2G6,PPAT,PTN,PRDX3,VDR,STC1,SPP1,ADCY7,DEDD,MEN1,WNT4,CTSV,MAPK1,MED1,ITGA3,MAPK3,EPOR,BSG,TCF23,LIF,HAVCR2	grey	VSMC_M10
GO:0010042	0.77	0.39	9	19	response to manganese ion	BP	1	PRKN,TSPO,A3GALT2,SLC30A10,BACE1,D2HGDH,ATP13A2,SLFN14,HSPA5	grey	VSMC_M10
GO:0046622	0.77	0.34	20	41	positive regulation of organ growth	BP	1	GLI1,MAPK14,PIM1,SMO,MEF2C,IL7,TBX5,IGF2,HLX,YBX3,MAPK1,BMPR1A,RBPJ,TBX2,GATA6,MIR199A1,MIR222,BASP1,HEY2,WNT2	grey	VSMC_M10
GO:0033363	0.77	0.36	14	29	secretory granule organization	BP	1	TBPL1,NECTIN2,OSBP,AP3B1,F2R,DTNBP1,CREB1,SRGN,PTPRN,SOX30,BAIAP3,MFSD14A,BLOC1S3,TBC1D20	grey	VSMC_M10
GO:1902652	0.77	0.29	56	111	secondary alcohol metabolic process	BP	1	APOF,CYP27B1,ACO2,TTC39B,PLPP6,CAT,APOB,EBP,CUBN,LIPE,TSKU,PIP4P1,NR1H4,SOAT2,CYP7A1,CYP7B1,CEBPA,LIMA1,EPHX2,CYP2R1,ERLIN1,ABCA5,FMO5,MSMO1,SC5D,SCAP,HSD17B7,POR,ACADL,SOAT1,TM7SF2,OSBPL1A,IDH3A,FDX1,LPCAT3,GNB3,CYP27A1,CH25H,ACADVL,CLN8,LIPC,DGKQ,APOBR,CLN6,LDLR,CYP46A1,NPC1,SREBF1,LEPR,ACAA2,RXRA,CEL,NFE2L1,LDLRAP1,INSIG2,FGF1	grey	VSMC_M10
GO:0051782	0.77	0.41	7	15	negative regulation of cell division	BP	1	MYC,PTCH1,INTU,CHMP4C,ZFYVE19,SUSD2,VPS4A	grey	VSMC_M10
GO:0033032	0.77	0.40	8	17	regulation of myeloid cell apoptotic process	BP	1	CDKN2A,SLC7A11,IRF7,PIK3CB,MEF2C,NOD2,CXCR2,THRA	grey	VSMC_M10
GO:0035336	0.77	0.37	12	25	long-chain fatty-acyl-CoA metabolic process	BP	1	ACSL6,ELOVL1,ELOVL7,FAR2,ACSL5,HACD2,THEM5,ACSBG1,TECR,ACSBG2,ACSF3,ELOVL3	grey	VSMC_M10
GO:0001825	0.77	0.35	15	31	blastocyst formation	BP	1	CNOT2,PNLDC1,RRP7A,MFNG,SRF,RTN4,CCNB1IP1,CNOT1,RPL7L1,SP3,TET1,WDR74,MFN2,NLE1,SUPT6H	grey	VSMC_M10
GO:0062098	0.77	0.37	11	23	regulation of programmed necrotic cell death	BP	1	PELI1,ASAH1,RIPK1,SPATA2,FZD9,YBX3,BIRC3,OGT,MIR22,FADD,CASP8	grey	VSMC_M10
GO:0043981	0.77	0.41	7	15	histone H4-K5 acetylation	BP	1	KANSL3,PHF20,KANSL2,ING4,KAT7,OGT,HCFC1	grey	VSMC_M10
GO:0043982	0.77	0.41	7	15	histone H4-K8 acetylation	BP	1	KANSL3,PHF20,KANSL2,ING4,KAT7,OGT,HCFC1	grey	VSMC_M10
GO:0050848	0.77	0.32	27	55	regulation of calcium-mediated signaling	BP	1	FHL2,PLEK,SLC8A2,EFHB,PRNP,P2RX2,NRG1,P2RX3,SLA2,PTK2B,SPPL3,CMKLR1,TBC1D10C,CIB1,SLC9A1,MYOZ2,ACTN3,HOMER2,MAPT,HTT,MYOZ1,BST1,TMEM100,CALM1,RIT2,MAPK7,CAMTA1	grey	VSMC_M10
GO:0021544	0.77	0.40	8	17	subpallium development	BP	1	FOXP2,SLC7A11,MKKS,RARB,ZSWIM6,ALDH1A3,DLX1,BCL11B	grey	VSMC_M10
GO:0051571	0.77	0.41	7	15	positive regulation of histone H3-K4 methylation	BP	1	SNW1,PAXIP1,DNMT3B,RTF1,AUTS2,WDR61,NELFA	grey	VSMC_M10
GO:0045684	0.77	0.37	12	25	positive regulation of epidermis development	BP	1	CYP27B1,PTCH1,SFRP4,PTCH2,OVOL2,VDR,KDF1,SFN,H2AFY2,MED1,MIR125B1,ZBED2	grey	VSMC_M10
GO:0097300	0.77	0.34	17	35	programmed necrotic cell death	BP	1	PELI1,ASAH1,RIPK1,DNM1L,ITPK1,SPATA2,FZD9,YBX3,PYGL,BIRC3,TRPM7,OGT,TRAF2,MIR22,FADD,CASP8,PPIF	grey	VSMC_M10
GO:0046887	0.77	0.30	43	86	positive regulation of hormone secretion	BP	1	LRP1,PLA2G6,PRKCE,EGFR,TRPM4,GIPR,C1QTNF1,AIMP1,SIRT3,OSBP,P2RY1,NNAT,PFKM,NKX3-1,NKX6-1,SPP1,GPLD1,TCF7L2,PFKFB2,GPER1,SNX4,GLUL,CREB1,MCU,PPP3CB,CD38,MPC2,INHA,NLGN2,TARDBP,STX4,RAB8B,GPR68,ISL1,OXCT1,ABAT,NADK,BAIAP3,LRRC8A,FGA,SOX4,CRH,DOC2B	grey	VSMC_M10
GO:0050870	0.77	0.29	66	130	positive regulation of T cell activation	BP	1	AGER,HLA-DRA,HLA-DOB,HLA-DMA,HLA-DPB1,TNFRSF13C,NCK2,IL4I1,IL15,BMI1,GLI2,TGFBR2,NCKAP1L,SLC7A1,TRAF6,SOX12,IL1A,IL7,RASGRP1,RIPK2,AP3B1,SOX13,NOD2,IGF2,LCK,ICOSLG,GPAM,HLX,CD28,RUNX3,ADAM8,DUSP10,BCL6,IL12A,VSIR,PTPN6,STAT5B,FOXP3,RASAL3,ZBTB1,CARD11,ZBTB7B,IL4R,CD74,CCL5,CCL2,YES1,NKAP,VAV1,IL7R,SOCS1,DNAJA3,PRKCZ,BCL10,MIR21,RHOA,SOX4,FADD,DPP4,CD46,THY1,CD70,TNFSF9,EFNB3,TNFSF13B,HAVCR2	grey	VSMC_M10

GO:0030502	0.77	0.43	6	13	negative regulation of bone mineralization	BP	1	TRPM4,SOX9,PTK2B,SRGN,BCOR,RFLNB	grey	VSMC_M10
GO:0060260	0.77	0.36	13	27	regulation of transcription initiation from RNA polymerase II promoter	BP	1	ESR1,MED30,SRF,ERCC1,PAXIP1,ERCC6,CREB1,MED14,THRA,MED24,MED1,NKX2-5,HEY2	grey	VSMC_M10
GO:0033631	0.77	0.43	6	13	cell-cell adhesion mediated by integrin	BP	1	PODXL,ITGA4,FERMT3,CCL5,PIEZO1,DPP4	grey	VSMC_M10
GO:1902285	0.77	0.43	6	13	semaphorin-plexin signaling pathway involved in neuron projection guidance	BP	1	PLXNA4,PLXNA1,PLXNC1,SEMA3A,SEMA3F,PLXNA3	grey	VSMC_M10
GO:0051281	0.77	0.36	14	29	positive regulation of release of sequestered calcium ion into cytosol	BP	1	F2R,PKD2,GPER1,CASQ1,NTSR1,CEMIP,HTT,HAP1,CD19,CALM1,BDKRB1,PDPK1,JPH2,THY1	grey	VSMC_M10
GO:0001895	0.77	0.33	21	43	retina homeostasis	BP	1	PRDX1,ALB,WHRN,USP45,USH1G,MKKS,ACTG1,ERCC6,TUB,CCDC66,ADGRV1,USH1C,BBS1,CDH23,ACTB,CLN8,ATP1B2,ESRRB,JCHAIN,DRAM2,BBS10	grey	VSMC_M10
GO:0071514	0.77	0.41	7	15	genetic imprinting	BP	1	DNMT3A,IGF2,PCGF3,MECP2,PRMT7,CTCF,ARID4A	grey	VSMC_M10
GO:0045056	0.77	0.40	8	17	transcytosis	BP	1	LRP1,AGER,FCGRT,VPS35,RAB5A,RAB17,LRPAP1,RAB11A	grey	VSMC_M10
GO:0050951	0.77	0.40	8	17	sensory perception of temperature stimulus	BP	1	BTBD9,GRIK2,ASIC3,EPHB1,TRPM8,NTSR1,KCNK4,TRPV1	grey	VSMC_M10
GO:0030218	0.77	0.29	51	102	erythrocyte differentiation	BP	1	MPIG6B,HSPA1B,BRD1,SMAD5,L3MBTL3,MAPK14,ZNF16,RBFOX2,SRF,CDK6,KIT,RPS14,SMAP1,EPAS1,PTBP3,BPGM,FOXO3,ERCC2,SLC25A38,ACVR2A,NCKAP1L,PRKDC,MFHAS1,ACVR1B,CASP3,TMEM14C,DMTN,ALAS1,LDB1,PKNOX1,ACIN1,DYRK3,HCLS1,ARNT,SP3,BCL6,STAT3,STAT5B,INHA,THRA,MED1,AHSP,KAT7,DNASE2,RCOR1,PTPN2,MIR222,ANKRD54,ARID4A,MAFB,FAM210B	grey	VSMC_M10
GO:1900181	0.77	0.36	13	27	negative regulation of protein localization to nucleus	BP	1	C6orf106,RAB23,FERMT1,MFHAS1,POLR1A,DCLK2,PKIA,SUFU,CABP1,OTUD7B,APOD,MAGED1,SIN3A	grey	VSMC_M10
GO:0048232	0.77	0.26	192	371	male gamete generation	BP	1	TBPL1,GLI1,YTHDC2,B4GALNT1,ATAT1,TSPAN8,IGF2R,MOV10L1,PACRG,QKI,BRD2,ROS1,HSF2,UBE2B,CATSPER3,H3F3C,DMC1,SPATA31D3,CFAP69,FANCG,SMARCA2,TDRD6,KIT,CCIN,TSGA10,TDRD7,DNAJA1,FOXJ3,SCMH1,RAD23B,YTHDC1,CABS1,HERPUD2,GMCL1,CCDC155,CREM,ARMC2,DLD,TXNRD3,APOB,SPATA6,SPATA5,TTC26,DNMT3A,FOXA3,SOX9,RSPH6A,TOPAZ1,ERCC1,SLC4A2,NECTIN2,CRTAP,MLH1,ACOX1,FOXJ1,WDR48,TTC21A,CCNB1IP1,ACVR2A,MKKS,SPATA24,ADAM18,MCM8,GOLGA3,PANK2,BTG1,SOHLH2,TEX15,KATNAL1,NPHP1,ING2,SPATA9,ARNTL,SPAG4,M1AP,IFT81,CELF1,JAM2,CSNK2A2,PRKAG1,SFMBT1,AP3B1,CDYL,SYCP1,DNAH1,DPCD,OCA2,ROPN1L,SIAH1,ACTR1A,NECTIN3,AZIN2,RHBDD1,NEURL1,DDX25,CFAP43,HSF2BP,TESMIN,SPATA2,RNF114,ABHD2,CCDC36,YTHDF2,SPATC1L,SPO11,TSSK4,CIB1,CCDC87,DDX6,GORASP2,DEDD,CELF3,OSBP2,TDRKH,ZSCAN2,SEPT2,LIMK2,PATZ1,CATSPER2,OVOL1,YBX3,CADM1,SPATA32,GGT2,TTC12,BCL6,RPL10L,SPAG16,ZCWPW1,CTSV,BIRC3,ADCY10,PARP11,CALR3,KDM5A,ZMYND15,MNS1,YBX2,MYCBPAP,VIPAS39,SOX30,CCDC42,CREB3L4,RAD51C,DNM2,YY1,TAF4B,ALKBH5,JAG2,IFT20,ACSBG2,TXNDC2,SPEF2,CATSPERD,ZNF449,HSPB11,BCAP31,RNF151,TMEM203,MEIOB,GAMT,CHD5,SOX8,TPGS1,MFSD14A,LRRC8A,STRBP,TSNAXIP1,MSH4,FSTL3,AURKC,UBB,SYCP3,ARID4A,AGO4,TCFL5,NKAPL,METTL14,FOXJ2,TDRP,ZSCAN21,ZFP41,SPDYA,GGT5,RNF8,BCL2L11,TBC1D20,ZNF35,BCL2L2,ZGLP1,SPA17,ZNF628	grey	VSMC_M10
GO:0072074	0.77	0.40	8	17	kidney mesenchyme development	BP	1	MYC,SIX2,OSR1,PKD2,PAX2,WNT4,BASP1,SIX1	grey	VSMC_M10
GO:0009566	0.77	0.29	57	113	fertilization	BP	1	TRIM36,H3F3C,MYH9,CFAP69,NLRP5,RIMS1,SPACA6,KLK14,DKKL1,APOB,IZUMO1,ADCY3,TRPC3,NECTIN2,WDR48,PLAT,ADAM18,PLCB1,FUT10,UBXN8,STX2,AP3B1,NPM2,NECTIN3,HYAL3,SLC9B1,INTS13,ABHD2,TSSK4,CCDC87,TDRKH,CLIC4,CATSPER2,YBX3,LYZL6,OVGP1,PLCD4,RAB3A,AKAP3,ADAM21,ADAM20,UBE2Q1,UBAP2L,SPESP1,NOX5,ZP2,SNU13,MEIOB,CACNA1H,PARK7,STXBP1,CD46,GLIPR1L2,FAM170A,LHFPL2,SPA17,TMPRSS12	grey	VSMC_M10
GO:0061484	0.77	0.40	8	17	hematopoietic stem cell homeostasis	BP	1	OCIAD1,FOXA3,TCIRG1,UBAP2L,GPRASP2,NLE1,ADGRG1,SOX4	grey	VSMC_M10
GO:1902170	0.77	0.41	7	15	cellular response to reactive nitrogen species	BP	1	AQP1,GUCY1B1,MTR,MMP3,DNM2,CRK,TRAF2	grey	VSMC_M10
GO:0090128	0.77	0.44	5	11	regulation of synapse maturation	BP	1	YWHAZ,NEURL1,DISC1,NFATC4,DAB2IP	grey	VSMC_M10
GO:0019320	0.77	0.36	12	25	hexose catabolic process	BP	1	GALT,GLB1,PFKP,FUT10,PFKM,GLYCTK,PFKFB2,ACTN3,HK1,TKTL1,FUT4,TIGAR	grey	VSMC_M10
GO:0043331	0.77	0.34	17	35	response to dsRNA	BP	1	PELI1,RALB,TMEM173,MAVS,RIPK2,NOD2,IFIH1,RFTN2,MUL1,MAPK1,SLC3A2,MAPK3,RIOK3,COLEC12,TICAM1,ZCCHC3,KCNJ8	grey	VSMC_M10
GO:1900744	0.77	0.34	18	37	regulation of p38MAPK cascade	BP	1	PHLPP1,MAP3K5,MAP3K4,AGER,GADD45G,EMC10,RELL2,MFHAS1,HAND2,NCF1,MAP3K3,DLG1,DUSP10,MINK1,RELL1,DAB2IP,GADD45A,DUSP1	grey	VSMC_M10
GO:0030278	0.77	0.30	45	91	regulation of ossification	BP	1	CYP27B1,OSR2,TOB2,MAPK14,OMD,SIX2,PTN,TRPM4,OSR1,MKX,INTU,SOX9,ACVR2B,ACVR2A,GFRA4,KL,ADGRV1,MEF2C,P2RX7,PTK2B,BMP2K,ANO6,TFAP2A,BMPR1B,TGFB2,SGMS2,KREMEN1,ACTN3,TXLNG,FZD9,SRGN,BCOR,ANKH,ESRRA,WNT4,MAPK1,BMPR1A,DHRS3,RBPJ,MAPK3,SMAD3,PTGER4,RFLNB,BGLAP,WNT5A	grey	VSMC_M10
GO:0032495	0.77	0.40	8	17	response to muramyl dipeptide	BP	1	TNFAIP3,MAPK14,IRF5,NOD1,JAG1,RIPK2,NOD2,INAVA	grey	VSMC_M10
GO:0120254	0.77	0.30	43	86	olefinic compound metabolic process	BP	1	RDH5,RDH16,ALDH8A1,HSD17B3,AKR1B1,ALDH1A1,RDH13,DKKL1,FAAH,LIPE,ELOVL1,SDR16C5,ABHD6,RETSAT,DKK3,EPHX2,PLA2G4B,SRD5A1,ALDH1A3,CYP2U1,DHRS9,PLA2G4A,BCO2,WNT4,STAT5B,DAGLB,EPHX1,DHRS3,RDH11,ALDH1A2,DGKQ,ALOXE3,MAPK3,SCNN1B,CYP46A1,CYP2J2,CACNA1H,PTGES2,PNPLA4,ELOVL3,LRAT,RDH14,ADH4	grey	VSMC_M10
GO:0050856	0.77	0.35	15	31	regulation of T cell receptor signaling pathway	BP	1	CD226,PRKD2,NECTIN2,PAWR,PRNP,PTPRJ,SLA2,LCK,RC3H1,PTPN6,CARD11,PTPN2,IKBKG,BCL10,THY1	grey	VSMC_M10
GO:0031958	0.77	0.42	6	13	corticosteroid receptor signaling pathway	BP	1	CRY1,PHB,ARNTL,CRY2,ARID1A,YWHAH	grey	VSMC_M10

GO:2000756	0.77	0.32	26	53	regulation of peptidyl-lysine acetylation	BP	1	HINT2,ATG5,SPHK2,HDAC2,IWS1,PAXIP1,ARRB1,TAF7,SDR16C5,HDAC1,AUTS2,TADA2B,FOXP3,KAT7,MUC1,MAPK3,NAP1L2,RPS6KA5,SIN3A,ISL1,GLYR1,PARK7,CTCF,SOX4,LIF,RPS6KA4	grey	VSMC_M10
GO:0002548	0.77	0.33	20	40	monocyte chemotaxis	BP	1	FPR2,FLT1,CCL17,ANO6,SLAMF8,MOSPD2,PTPRO,TNFSF18,IL6R,S100A14,CCL14,CCL5,CCL7,CCL2,CCL25,CCL16,S100A12,DEFB124,DUSP1,CCL22	grey	VSMC_M10
GO:0033059	0.77	0.33	21	43	cellular pigmentation	BP	1	BBS7,MYO7A,MKKS,ANKRD27,AP3B1,DTNBP1,KIF13A,SHROOM2,HPS4,MLPH,RAB17,PIKFYVE,BLOC1S6,MREG,ABCB6,RAB11A,SNAPIN,AP1G1,BLOC1S3,RAB32,TRAPPC6A	grey	VSMC_M10
GO:0032944	0.77	0.28	70	138	regulation of mononuclear cell proliferation	BP	1	AGER,IL5,HLA-DPB1,TNFRSF13C,RAC2,TNFRSF21,NCK2,PELI1,IL4I1,TLR4,IL15,BMI1,MYD88,FOXJ1,TGFBR2,PAWR,PRNP,NCKAP1L,SLC7A1,TRAF6,IL1A,CASP3,MEF2C,TMEM131L,IL7,RIPK2,MARCH7,ZNF335,CD40,IGF2,ICOSLG,BTN2A2,GPAM,PRKAR1A,CD28,DLG1,BCL6,IL12A,VSIR,RC3H1,PTPN6,TNFSF18,CD38,BST1,STAT5B,SCGB1A1,FOXP3,RASAL3,CARD11,TNFRSF1B,ZBTB7B,CHRNB2,BTK,CD74,CCL5,CD320,TWSG1,TICAM1,DNAJA3,VAV3,GLMN,MIR21,LST1,FADD,CD46,CD70,TNFSF9,CEBPB,TNFSF13B,HAVCR2	grey	VSMC_M10
GO:0000041	0.77	0.30	43	86	transition metal ion transport	BP	1	SLC39A5,SLC39A4,STEAP2,SLC1A1,SLC31A2,PGRMC2,TRPC3,HEPHL1,ATP7B,SLC39A12,ASIC3,STEAP3,TMEM163,HPX,SLC39A13,SLC25A37,SLC48A1,SLC40A1,CUTC,ABCC2,SLC30A10,COX17,ISCU,ABCC5,MELTF,SLC30A4,SLC11A1,ABCB6,FTH1,SLC39A9,FLVCR2,NOX5,DNM2,SLC39A6,SLC46A1,C1orf194,ATP2C2,SLC30A7,LCN2,SLC22A17,FTL,SLC39A11,SLC39A3	grey	VSMC_M10
GO:0002718	0.77	0.31	31	63	regulation of cytokine production involved in immune response	BP	1	CD226,KIT,RSAD2,TLR4,ATG5,TRPM4,SPHK2,LACC1,MAVS,TRAF6,F2RL1,NOD2,CD96,TGFB2,DENND1B,FZD5,BCL6,HK1,FOXP3,TNFRSF1B,SPON2,TBX21,BTK,CD74,TICAM1,PRKCZ,TRAF2,SLC7A5,BCL10,WNT5A,INAVA	grey	VSMC_M10
GO:0051851	0.77	0.32	25	51	modulation by host of symbiont process	BP	1	YTHDC2,SNW1,IGF2R,PRKN,NAPEPLD,PHB,FBXL2,SAP30,EIF2AK4,HDAC1,ZNF639,CFL1,HSPA8,POU2F3,PC,PSMC3,TARDBP,CCL5,JUN,FMR1,INPP5K,MIR222,SAP30BP,TBC1D20,ZNF502	grey	VSMC_M10
GO:0035065	0.77	0.32	22	45	regulation of histone acetylation	BP	1	ATG5,SPHK2,IWS1,PAXIP1,ARRB1,TAF7,SDR16C5,AUTS2,TADA2B,FOXP3,KAT7,MUC1,MAPK3,NAP1L2,RPS6KA5,SIN3A,ISL1,GLYR1,PARK7,CTCF,LIF,RPS6KA4	grey	VSMC_M10
GO:0009595	0.77	0.39	8	17	detection of biotic stimulus	BP	1	TSPO,SMO,NOD1,TLR4,DACH1,PTPRJ,NOD2,FAP	grey	VSMC_M10
GO:0002286	0.77	0.31	32	65	T cell activation involved in immune response	BP	1	HLA-DRA,LCP1,EOMES,NCKAP1L,FOXP1,EIF2AK4,RIPK2,F2RL1,HLX,FCER1G,BCL6,RC3H1,TNFSF18,SLC11A1,STAT3,FOXP3,TBX21,PSEN1,SEMA4A,BATF,ZBTB7B,IL6R,IL4R,CD74,PTGER4,PRKCZ,TSC1,MIR21,CD46,FGL2,HAVCR2,RC3H2	grey	VSMC_M10
GO:0051046	0.77	0.26	224	434	regulation of secretion	BP	1	VPS4B,ERBB3,LRP1,RAB21,SNCAIP,SLC12A2,RIMS2,PRKN,GDF9,PLA2G6,PICK1,RAC2,CHRNA3,CHRNA5,CRY1,APBA1,RFX3,TFAP2B,CHMP2A,IL11,PRKCE,RIMS1,HMGN3,RIMS3,BMP8A,AQP1,RSAD2,NR1H2,EGFR,TLR4,ATG5,SDCBP,TRPM4,SPHK2,RAB7A,RAB10,GIPR,CACNB2,CLASP2,FRMD4A,GAB2,LLGL2,UNC13D,SNX5,C1QTNF1,SYT1,RAB5A,APBB3,DPH3,AIMP1,NRG1,NCKAP1L,SIRT3,SYT9,ADRA2B,NR1H4,NDUFAF2,FAM3D,IL1A,PAX8,FER,OSBP,HIP1R,ARNTL,SCG5,MEF2C,ASIC1,VEGFC,P2RY1,KCNJ11,NNAT,NR1H3,TIAM1,RAB11FIP5,PFKM,SLC8B1,STC1,NKX3-1,NKX6-1,ACVR1C,F2RL1,F2R,CRY2,VPS18,ABCC2,SPP1,ZBED6,PLA2R1,LGI3,ADTRP,DTNBP1,SYT8,GRM2,SIRT4,GPLD1,IRS1,CD200,DNM1L,HDAC1,PPP3CA,HYAL3,CPT1A,MAP2K6,SLC9B2,TGFB2,ADRA2A,TCIRG1,TCF7L2,KCNB1,EGF,PFKFB2,GRK2,GPER1,ABCB11,AGT,FGR,PER2,OSM,STX1A,SNX4,NTSR1,PASK,SEPT2,BACE1,GLUL,CSPG5,SIDT2,SLC16A1,ADAM8,KLF7,NSF,CREB1,RAP1B,PLA2G4A,TTN,IL12A,COMT,ENY2,ABCA12,CASK,MCU,ADRA2C,PNKD,PPP3CB,RAB3A,PINK1,SYN1,CD38,C12orf4,MPC2,INHA,RAB8A,ATP13A2,HAP1,CPLANE2,SYT7,RAB15,RSC1A1,NLGN2,TNFRSF1B,SEPT1,CPLX1,TARDBP,STX1B,STX4,ITGAM,SYT11,RAB8B,TMED10,CACNA1G,CHRNB2,MYH10,RAB3D,IL4R,CALM1,SYTL4,SNAPIN,GPR68,S100A8,PRKCB,SCAMP5,CD74,CAMK2A,CCL5,STXBP2,SREBF1,ISL1,PRAM1,OXCT1,NOS2,ABAT,FMR1,RPH3AL,NADK,TRPV1,PDPK1,P2RX1,RAB26,ATP6AP1,BAIAP3,CACNA1H,CYBA,FOXF1,LRRC8A,STXBP1,AP1G1,VPS4A,MIR199A1,FGA,CHRNB4,CHGA,SOX4,DPP4,POFUT2,CRH,RGCC,DOC2B,PTGES,RALA,LIF,UQCC2,VAMP3,PARD6A,KCNMB4	grey	VSMC_M10

GO:0009259	0.77	0.26	156	302	ribonucleotide metabolic process	BP	1	NME1,HSD17B4,PDE7B,ATP5MGL,DPYS,PPT2,PPARA,PDE10A,ACSL6,PRXL2C,GTPBP1,IMPDH1,GDA,CROT,SLC35B2,SLC25A13,GAPDH,PPAT,RAB23,PPCS,SLC4A4,NT5E,PPT1,BPGM,DLD,SPHK2,CMPK1,ADCY3,DLG2,DHTKD1,PRKAG2,ATP5F1C,ELOVL1,PFKP,NUDT16,PANK2,PDK1,SUCLG2,PDHX,ELOVL7,PANK1,GPI,AMPD3,ATP5F1A,MCCC2,GPD1,P2RX7,GUCY1A1,GUCY1B1,ACOT12,DGUOK,PRKAG1,GMPS,PFKM,OPA1,ENTPD4,FITM2,AMPD1,NUDT18,ADCY7,FAM173B,ADCY2,MCEE,NME4,PDE9A,TPST1,ZBTB20,GPAM,FAR2,ACSL5,PTHLH,MFN1,PFKFB2,ENO4,HACD2,ARNT,ADCY4,CTPS2,ACTN3,HDAC4,THEM5,DNAJC30,MLXIPL,PDE8A,HMGCL,MAGI3,DLG1,ACOT9,PDK3,EFL1,SLC25A16,UPB1,HK1,ACSBG1,ATP5S,CASK,COASY,PRKAG3,PINK1,STAT3,NME7,ADK,MPC2,ADCY10,ENO3,PARP1,TECR,CARD11,SLC26A1,PSEN1,ACOT2,GUCY2D,PKLR,PANK3,GCDH,OGT,UPRT,ADPGK,PDE4A,ACSM3,ACACA,NCOR1,INSR,SLC26A2,ADSSL1,ACSBG2,ALDOC,PIPOX,ACOT11,ATP6V0C,PANK4,NME3,MPP1,ATP5PB,SLC2A6,VPS9D1,GNAI3,ACSF3,UCK1,MLYCD,DHODH,MT-ATP8,MT-ATP6,RHOA,SULT1C3,ATP5ME,ACOT4,ATP5MF,ENTPD5,ELOVL3,TIGAR,NUDT19,NME6,NUDT2,HINT1,UPP1	grey	VSMC_M10
GO:0006351	0.77	0.24	1325	2521	transcription, DNA-templated	BP	1	ADNP2,NFATC1,ZNF236,ZNF407,SMARCC2,ATP8B1,ONECUT2,BAZ2A,TCF4,TBPL1,GLI1,MARS,MAP3K5,TNFAIP3,TSFM,HIVEP2,PLAGL1,MAML1,ZBTB2,YEATS4,ESR1,MYRFL,CNOT2,CNOT6,STK3,OSR2,SNW1,TFB1M,ZNF706,ARID1B,HSPA1B,FZD6,ZBTB12,ZBED4,PRKN,AGER,PPARA,PDE10A,MED30,IL5,BRD2,TAF2,RXRB,VGLL2,HSF2,TCF7,PHF1,XRCC6,NCOA7,PITX1,TRIB1,MYC,TOB2,SMAD5,L3MBTL3,L3MBTL2,AGO2,KDM3B,PTP4A3,PTCH1,ATF4,RPAP2,CBX7,MAPK14,CBY1,KDM4C,SOX10,PIM1,NRF1,TMPRSS6,SMO,SNAPC3,BNC2,HSF1,NFYA,FOXP4,MDFI,TFEB,IRF5,MED20,CCND3,TAF8,FOXH1,LRRC14,CDKN2A,ZNF16,RBFOX2,IFT74,BICRAL,RORB,ZNF300,PRDM4,CRY1,DMTF1,SRF,ZNF318,NFX1,CDK14,FZD1,MTERF1,NFXL1,NFKBIE,SUPT3H,SMARCA2,RFX3,KIT,ADGRF1,NCK2,FHL2,TGFBRAP1,TFAP2B,ROR2,NFIL3,SEMA4D,RFX8,DLX5,PHF14,IPPK,ETV1,ZNF135,RPS14,ZNF154,MEOX2,ZNF550,KANSL3,BEND6,ZNF268,ZNF667,ZNF787,PHF3,ZNF691,CAT,MACC1,SIX2,FOXJ3,ZNF865,HIVEP3,IL11,SCMH1,ZNF189,RNF20,EPAS1,EPCAM,SP4,PPP2R1A,ZNF462,PHIP,HMGN3,NFYC,NFE2L3,HOXA2,TAF1D,HOXA4,HOXA6,HOXA7,HOXA13,REL,ZNF483,JAZF1,CREB5,MYCL,XPO1,MTURN,NOD1,AKIRIN2,SRSF10,CASP8AP2,BACH2,ZNF692,PELI1,AKIRIN1,MTF1,ZNF618,SNIP1,ANXA4,PCBP1,NR1H2,AKNA,ZNF350,ITGB1BP1,FOXP2,GRHL1,MED25,KLF11,MET,IL4I1,AGO1,POU3F3,EGFR,LANCL2,ING3,NOBOX,ARHGEF5,TRIM32,TLR4,CCNC,CDK5RAP2,TRAF1,BCL2L12,SCAF1,LPIN1,SFRP4,UCP1,CREM,TEAD2,SETD7,SDCBP,MYCN,C6orf203,ELF2,OSR1,FOXO3,ZXDC,ZBTB24,MKX,FAF1,MAMSTR,GTF3C6,ATAD2B,BEND5,CDCA7,KDM7A,ZNF438,FOXE3,HDAC2,PHF10,M[illegible]	grey	VSMC_M10
GO:1901201	0.77	0.40	7	15	regulation of extracellular matrix assembly	BP	1	SOX9,CLASP2,DAG1,AGT,PHLDB1,SMAD3,RGCC	grey	VSMC_M10
GO:0034123	0.77	0.37	11	23	positive regulation of toll-like receptor signaling pathway	BP	1	NINJ1,RTN4,PELI1,RSAD2,MFHAS1,NR1H3,F2RL1,TLR5,DDX3X,WDFY1,CYBA	grey	VSMC_M10
GO:0030033	0.77	0.40	7	15	microvillus assembly	BP	1	ATP8B1,MYO1A,PODXL,KLF5,RAPGEF2,RAP1GAP,STK26	grey	VSMC_M10
GO:0010038	0.78	0.26	143	279	response to metal ion	BP	1	NEDD4L,FECH,TPH2,SLC12A2,PRKN,TNFRSF11B,TSPO,CPNE5,HSF1,KIT,SLC1A1,SLC25A13,CAT,ALG2,AQP1,MTF1,EGFR,BRAF,TRPC3,DNMT3A,SLC26A5,ATP7B,ENTPD6,GIPR,ERCC1,DLG2,KHK,SCN5A,AANAT,CPNE9,SYT1,RYR1,PRNP,PPP2CB,SYT9,CYB5A,IL1A,GPI,CEBPA,GSS,CASP3,RYR3,ADGRV1,MEF2C,CYP2R1,HVCN1,XRCC4,PTK2B,SLC39A13,CPNE2,SEC31A,HOMER1,LOXL2,TNNC1,ABCC2,PKD2,TFAP2A,DMTN,CASQ2,ADCY7,KCNIP2,RYR2,SYT8,SLC41A1,GPLD1,A3GALT2,SLC6A3,PPP3CA,CCND1,TERT,TESMIN,SLC30A10,PEF1,TCIRG1,PDCD6,NCF1,FABP4,AMELX,NFATC4,CARF,CLIC4,BACE1,DTYMK,D2HGDH,CDKN1B,NFE2L2,CREB1,RASA4,MAPT,SORD,SLC30A4,CPS1,TTN,RASGRP2,ANXA7,ABCB6,KCNA1,MAPK1,KCNMA1,ATP13A2,DLG4,PARP1,SYT7,GPHN,SOD3,SYT11,PKLR,FOS,MAPK3,CACNA1G,CALM1,S100A8,NPC1,SLFN14,SLC25A23,LONP1,JUN,ABAT,CACNA1H,PARK7,BSG,ADAMTS13,CPNE7,ASS1,LCN2,HSPA5,NQO1,MT-CYB,FGA,FSTL3,KCNK3,MT1X,CASP8,CA2,BGLAP,CLDN1,WNT5A,TIGAR,FXN,DUSP1,PTGES,PPIF,KCNMB4	grey	VSMC_M10
GO:1902882	0.78	0.30	37	74	regulation of response to oxidative stress	BP	1	PRKN,NCOA7,ATF4,GPR37,FBXO7,MET,TLR4,SLC7A11,FOXO3,DHFR,SESN3,PAWR,ACOX2,FBXW7,HTRA2,RIPK1,FBLN5,MCL1,PDE8A,NFE2L2,NONO,STOX1,GCH1,PINK1,MMP3,PARP1,SFPQ,MAPK7,STK26,PARK7,TSC1,TLDC1,MAPKAP1,MIR21,NOL3,GPR37L1,TXN	grey	VSMC_M10
GO:0002687	0.78	0.29	51	101	positive regulation of leukocyte migration	BP	1	AGER,GPSM3,PTK2,RAC2,CCL27,FPR2,CXCL8,AKIRIN1,PTN,C5AR1,OXSR1,NCKAP1L,GAS6,VEGFC,JAM2,PTK2B,F2RL1,ANO6,DNM1L,TRPV4,CD99,PRSS56,CMKLR1,MOSPD2,ADAM8,MIA3,ITGA2B,ITGA4,IL12A,MCU,TNFSF18,CXCR2,MAPK1,TMEM102,PGF,DAPK2,MAPK3,IL6R,S100A14,BDKRB1,CD74,CCL5,CSF1R,CCL7,RHOA,DEFB124,FADD,ZNF580,THY1,WNT5A,PDGFD	grey	VSMC_M10
GO:0010818	0.78	0.40	7	15	T cell chemotaxis	BP	1	SLC12A2,CCL27,OXSR1,CXCL16,TMEM102,CCL5,WNT5A	grey	VSMC_M10
GO:0007029	0.78	0.30	40	80	endoplasmic reticulum organization	BP	1	EMC2,MAPK15,TRAM2,RTN4,EMC10,RAB10,EMC3,ATL2,EMC4,SEC31A,ZFYVE27,COL4A3BP,DOP1B,SEC31B,GET4,CASQ1,MIA3,SEC16B,MOSPD3,LPCAT3,ATL1,SMPD4,ARL6IP1,STX18,GAK,BNIP1,LMAN1L,CXorf56,TLE6,EMC6,SGTA,UBL4A,SEC16A,TOR1B,REEP6,WDR83OS,CAMLG,EMC7,TRAM1L1,POLR2M	grey	VSMC_M10

GO:0032760	0.78	0.30	34	69	positive regulation of tumor necrosis factor production	BP	1	AGER,NOD1,TLR4,SPHK2,HDAC2,MYD88,MAVS,IL1A,PIK3R1,PTPRJ,RASGRP1,OAS3,RIPK2,RIPK1,NOD2,IFIH1,HDAC1,MAPKAPK2,NFATC4,FZD5,STAT3,TNFRSF8,SPON2,PSEN1,ISL1,TICAM1,CYBA,MIR27B,OAS1,FADD,WNT5A,IL33,C1QTNF4,HAVCR2	grey	VSMC_M10
GO:0016577	0.78	0.35	13	28	histone demethylation	BP	1	UBE2B,KDM3B,KDM4C,KDM7A,KDM4A,JARID2,KDM5A,KDM5C,PHF8,KDM6B,RIOX1,KDM8,HSF4	grey	VSMC_M10
GO:2000516	0.78	0.37	11	23	positive regulation of CD4-positive, alpha-beta T cell activation	BP	1	HLA-DRA,TGFBR2,NCKAP1L,RIPK2,HLX,FOXP3,ZBTB7B,IL4R,SOCS1,PRKCZ,MIR21	grey	VSMC_M10
GO:0090161	0.78	0.44	5	11	Golgi ribbon formation	BP	1	OPTN,TRIP11,VTI1A,STX6,TMED5	grey	VSMC_M10
GO:0070986	0.78	0.42	6	13	left/right axis specification	BP	1	SMO,SETDB2,IFT172,PKD1L1,MNS1,HSPB11	grey	VSMC_M10
GO:0006474	0.78	0.40	7	15	N-terminal protein amino acid acetylation	BP	1	AANAT,NAA40,NAA30,CREBBP,NAA60,NAA10,SOX4	grey	VSMC_M10
GO:0006482	0.78	0.35	14	30	protein demethylation	BP	1	UBE2B,KDM3B,KDM4C,KDM7A,KDM4A,PPME1,JARID2,KDM5A,KDM5C,PHF8,KDM6B,RIOX1,KDM8,HSF4	grey	VSMC_M10
GO:0008214	0.78	0.35	14	30	protein dealkylation	BP	1	UBE2B,KDM3B,KDM4C,KDM7A,KDM4A,PPME1,JARID2,KDM5A,KDM5C,PHF8,KDM6B,RIOX1,KDM8,HSF4	grey	VSMC_M10
GO:0071415	0.78	0.44	5	11	cellular response to purine-containing compound	BP	1	RYR1,RYR3,P2RY1,CASQ2,RYR2	grey	VSMC_M10
GO:0006525	0.78	0.39	8	17	arginine metabolic process	BP	1	ASL,SLC7A7,OAT,FAH,AC138894.1,NOS2,ASS1,MIR21	grey	VSMC_M10
GO:0070841	0.78	0.37	10	21	inclusion body assembly	BP	1	SNCAIP,HSPA1B,PRKN,HSF1,SORL1,BAG3,MAPT,DNAJB2,DNAJB1,TRIM37	grey	VSMC_M10
GO:1902001	0.78	0.39	8	17	fatty acid transmembrane transport	BP	1	CPT1B,AKT2,CPT1A,ACSL5,SLC25A20,SLC27A1,ABCD4,ABCD3	grey	VSMC_M10
GO:0007194	0.78	0.44	5	11	negative regulation of adenylate cyclase activity	BP	1	GABBR2,ADGRV1,GRM2,PALM,GNAI3	grey	VSMC_M10
GO:0051349	0.78	0.42	6	13	positive regulation of lyase activity	BP	1	GUCA1B,ORAI1,ISCU,NOS2,PARK7,FXN	grey	VSMC_M10
GO:0032965	0.78	0.35	14	29	regulation of collagen biosynthetic process	BP	1	HDAC2,CYGB,CYP7A1,F2R,AMELX,WNT4,SUCO,LARP6,CIITA,SERPINF2,ENG,MIR29A,RGCC,CBX8	grey	VSMC_M10
GO:0060965	0.78	0.40	7	15	negative regulation of gene silencing by miRNA	BP	1	ZC3H10,ESR1,BCDIN3D,PPP3CA,TERT,STAT3,NCOR1	grey	VSMC_M10
GO:0009651	0.78	0.38	9	19	response to salt stress	BP	1	XRCC6,AKR1B1,AQP1,TRPV4,XRCC5,MICU1,SLC25A23,MT-CYB,CLDN1	grey	VSMC_M10
GO:0007608	0.78	0.39	8	17	sensory perception of smell	BP	1	CFAP69,ADCY3,MKKS,NCAM2,SLC6A3,BBS1,PDE4A,OR5AN1	grey	VSMC_M10
GO:0050730	0.78	0.27	98	193	regulation of peptidyl-tyrosine phosphorylation	BP	1	ERBB3,IL5,HSF1,TEC,KIT,NCK2,SEMA4D,PDCL3,IL11,PRKCE,PPP2R1A,IBTK,EPGN,AREG,EPHA7,TGFA,EGFR,IL15,HDAC2,ABI1,PIBF1,HBEGF,PRNP,NRG1,GAS6,HPX,ERCC6,OSBP,FBXW7,PTPRJ,IL7,PTK2B,ARL2BP,RIPK2,DMTN,NOD2,CNTN1,CD40,IGF2,NEURL1,LRRK1,HDAC1,BANK1,ADRA2A,NCF1,EGF,HCLS1,ADNP,CASS4,PARP9,PARP14,AGT,CBL,OSM,SNX6,PPP2R5B,IL12A,PTPN6,TNFSF18,DOK7,ZFYVE28,FGFR3,SOCS4,BST1,EPHA4,DLG4,DVL2,TMEM102,DGKQ,CTF1,PSEN1,EFNA1,IL6R,HAX1,CD74,CCL5,CSF1R,YES1,ISL1,SUZ12,GHR,UNC119,PTPN2,SOCS1,LRP8,PRKCZ,MLST8,CNTF,MIR125A,THY1,CSPG4,LIF,DUSP22,TSLP,CADM4,EREG,RAP2B,PDGFD	grey	VSMC_M10
GO:0034472	0.78	0.38	9	19	snRNA 3'-end processing	BP	1	EXOSC4,EXOSC3,USB1,INTS8,INTS12,INTS5,AP002990.1,INTS2,EXOSC5	grey	VSMC_M10
GO:0043038	0.78	0.33	20	41	amino acid activation	BP	1	MARS,AASDH,QRSL1,HARS,HARS2,CARS2,LARS,WARS2,GATC,YARS2,TARS2,DALRD3,LARS2,DARS2,FARSB,EARS2,LRRC47,KARS,AARS,MARS2	grey	VSMC_M10
GO:0051412	0.78	0.40	7	15	response to corticosterone	BP	1	FOXO3,AANAT,CCND1,NPAS4,FOS,CALM1,CRH	grey	VSMC_M10
GO:0001574	0.78	0.40	7	15	ganglioside biosynthetic process	BP	1	B4GALNT1,ST8SIA4,ST8SIA2,ST6GALNAC4,ST3GAL2,ST6GALNAC5,ST8SIA6	grey	VSMC_M10
GO:0051145	0.78	0.31	28	57	smooth muscle cell differentiation	BP	1	NFATC1,SRF,KIT,ANKRD17,SOX9,MEF2C,PDCD4,GPER1,NFATC4,EFEMP2,WNT4,RAMP2,FOXO4,RBPMS2,TBX2,GATA6,MRTFB,MECP2,FOXF1,ENG,NFATC3,MIR21,MIR125B1,MIR22,PRDM6,SIX1,EREG,HEY2	grey	VSMC_M10
GO:0071696	0.78	0.43	5	11	ectodermal placode development	BP	1	FRS2,HDAC2,HDAC1,TBX2,SIX1	grey	VSMC_M10
GO:2001056	0.78	0.28	53	105	positive regulation of cysteine-type endopeptidase activity	BP	1	MAP3K5,GRAMD4,BAK1,MYC,HSF1,TTBK1,SLC1A1,NOD1,CASP8AP2,TNFSF15,HSPE1,ARRB1,ARL6IP5,ST18,ASPH,FOXL2,HIP1R,ROBO1,DIABLO,HTRA2,RIPK2,SENP1,NKX3-1,ACVR1C,F2R,PIDD1,LCK,PDCD6,GPER1,CIDEB,EGLN3,MAPT,FIS1,CTSH,DDX3X,MUL1,BCL2L13,NGFR,SMAD3,S100A8,BCAP31,P2RX1,BCL10,MIR27B,RHOA,RPS27L,FADD,CASP8,ACER2,BCL2L11,PERP,PRR7,CRADD	grey	VSMC_M10
GO:0045727	0.78	0.28	55	109	positive regulation of translation	BP	1	RMND1,PRR16,PABPC1,EIF3E,JMJD4,AGO2,HABP4,NCK2,CNBP,CDC123,UPF3A,PRKDC,MRPS27,USP16,PTK2B,EIF2AK4,TENT4B,RPUSD4,RCC1L,RPS6KB2,YTHDF2,RPS4X,YTHDF1,PASK,CD28,UHMK1,NSUN5,YBX3,DDX3X,COA3,SAMD4A,PINK1,MAPK1,POLR2G,ZCCHC4,FXR2,MAPK3,TOB1,EIF3C,LARP6,LARP1,CCL5,UPF3B,FMR1,CIRBP,BARHL2,RHOA,RPS27L,SOX4,METTL14,IMPACT,RPUSD3,PPP1R15A,UQCC2,TRMT10C	grey	VSMC_M10
GO:0030522	0.78	0.26	110	217	intracellular receptor signaling pathway	BP	1	TNFAIP3,CYP27B1,USP15,ESR1,CNOT2,SNW1,HSPA1B,PPARA,RXRB,KDM4C,PIM1,FOXH1,RBFOX2,RORB,CRY1,DNAJA1,ANKRD17,SLC46A2,NR1H2,TLR4,PUM2,PHB,LACC1,EGLN2,SEC14L1,TADA3,CLPB,RARB,NR1D2,NPLOC4,TAF7,RNF14,DDRGK1,FOXP1,IRF7,CALCOCO1,NCOA4,TRAF6,NR1H4,ASXL1,CYP7B1,NR4A1,UFSP2,ZNF536,ZNF366,ARNTL,HNF4G,CNOT1,KMT2D,NR1H3,OAS3,RIPK2,OTULIN,DDX54,VDR,NKX3-1,NR4A2,CRY2,VPS18,NOD2,IFIH1,OASL,HDAC1,MAP2K6,NR5A2,TIFA,ALPK1,GPER1,ARNT,NLRX1,ARID1A,NR1I3,RXRG,PDK3,ESRRA,BIRC3,STAT3,PADI2,DNAAF4,PARP1,GPS2,THRA,TKFC,DHRS3,MED1,ALDH1A2,ALOXE3,PAGR1,ESRRB,TSPAN6,PIAS2,NCOR1,RIOK3,PPARGC1B,SREBF1,ISL1,RHOXF1,RNF135,PARK7,RXRA,NR6A1,RHOA,FABP5,TRIM24,TRIM68,ZCCHC3,AHRR,IRGM,INAVA,YWHAH	grey	VSMC_M10
GO:0035721	0.78	0.43	5	11	intraciliary retrograde transport	BP	1	DYNC2LI1,TTC21A,WDR60,TTC21B,WDR19	grey	VSMC_M10
GO:2001212	0.78	0.41	6	13	regulation of vasculogenesis	BP	1	RIN2,RAPGEF2,HIF1AN,RAMP2,TMEM100,HEY2	grey	VSMC_M10

GO:0048562	0.78	0.26	114	225	embryonic organ morphogenesis	BP	1	GLI1,FRS2,OSR2,FZD6,SMO,MDFI,FOXH1,PTK7,SRF,ROR2,DLX5,SIX2,KCNQ4,HOXA2,HOXA4,HOXA6,HOXA7,WHRN,FUZ,TEAD2,MYCN,SOBP,OSR1,BBS7,BMI1,MYO3A,SETDB2,SOX9,USH1G,GLI2,MEGF8,IFT172,MYO7A,WDR60,SPRY2,WNT11,TGFBR2,OVOL2,MKKS,RARB,ALX1,CEP290,PAX8,HAND2,EYA1,PLS1,FZD3,MEF2C,USH1C,IFT52,PKD2,TFAP2A,PAX2,RYR2,SUFU,HYAL1,ALDH1A3,SATB2,HLX,GBX2,MYO3B,GRHL3,SP3,STRC,DLG1,SETD2,HOXD4,HOXD3,FZD5,STOX1,PRKRA,FBN1,NKX3-2,MAPK1,TULP3,MFAP2,DVL2,WDR19,TCAP,PSEN1,HOXB5,HOXB6,HOXB8,HOXB9,EFNA1,RBPMS2,MAPK3,POU3F4,SMAD3,NTN1,GSC,TBX2,LHX1,STRA6,MIB1,RNF207,ALX3,FOXF1,ENG,CHRNA10,FOXG1,MAFB,HOXB3,NOG,LBX1,WNT5A,OTX1,SIX1,C2CD3,INSIG2,SLC39A3,NKX2-5,HOXA3,ZIC1	grey	VSMC_M10
GO:0032968	0.78	0.43	5	11	positive regulation of transcription elongation from RNA polymerase II promoter	BP	1	EAPP,ELL,LEO1,CDK12,SUPT6H	grey	VSMC_M10
GO:2000059	0.78	0.33	19	39	negative regulation of ubiquitin-dependent protein catabolic process	BP	1	TRIM39,PDCL3,SDCBP,KLHL40,DDRGK1,RYBP,TAF9,CSNK2A2,SUFU,RPL23,RPL11,PSEN1,TAF1,OGT,SGTA,PARK7,CAMLG,STYX,MAP1A	grey	VSMC_M10
GO:0030705	0.78	0.27	87	173	cytoskeleton-dependent intracellular transport	BP	1	MYO1A,AP3S1,KIF5A,RAB21,KIF3A,AGTPBP1,SUN2,COPG2,IFT74,APBA1,BICD2,DYNC2LI1,LRPPRC,MGARP,WASF1,TTC26,KIF3C,ARHGAP21,COPG1,MYO7B,KLC3,DLG2,IFT172,MYO7A,TTC21A,WDR60,HOOK3,AP3M2,KIF5C,PURA,TUB,KIF3B,IFT81,RASGRP1,OPA1,AP3B1,IFT52,DTNBP1,TUBA1A,KIF13A,BICDL1,TTC21B,HSPA8,BAG3,TRAF3IP1,CNIH2,TANC2,CALY,TTC30A,SPG11,MAPT,BLOC1S6,MREG,HTT,KIF17,UXT,AP3M1,KIF1C,SYNE2,HAP1,WDR19,PEX14,TRIM46,NDEL1,AC138894.1,HOOK2,SNAPIN,CCDC88C,SFPQ,CAMSAP3,MYO1D,MYO1F,IFT20,MYO5B,HSPB11,BICDL2,PRKCZ,MAPK8IP3,FNBP1L,TERF2,UBB,BLOC1S3,BORCS5,AP3S2,TUBA1C,MAP1A,MAP6D1	grey	VSMC_M10
GO:0015917	0.78	0.46	4	9	aminophospholipid transport	BP	1	ATP8B1,TMEM30A,TMEM30B,ATP11C	grey	VSMC_M10
GO:0003159	0.78	0.38	9	19	morphogenesis of an endothelium	BP	1	PRKD2,FOXP1,RBPJ,ADAMTS12,LCN2,MIR21,RHOA,ACVRL1,FGF1	grey	VSMC_M10
GO:0061154	0.78	0.38	9	19	endothelial tube morphogenesis	BP	1	PRKD2,FOXP1,RBPJ,ADAMTS12,LCN2,MIR21,RHOA,ACVRL1,FGF1	grey	VSMC_M10
GO:0060251	0.78	0.35	12	25	regulation of glial cell proliferation	BP	1	TSPO,SOX10,PTN,EGFR,PLAG1,RNF10,CERS2,CREB1,CHRM1,SKI,MIR222,MIR125B1	grey	VSMC_M10
GO:1903909	0.78	0.43	5	11	regulation of receptor clustering	BP	1	PTN,SLC7A11,SSH1,FZD9,MESD	grey	VSMC_M10
GO:0070922	0.78	0.46	4	9	small RNA loading onto RISC	BP	1	AGO2,AGO1,CLP1,AGO4	grey	VSMC_M10
GO:1901797	0.78	0.35	13	27	negative regulation of signal transduction by p53 class mediator	BP	1	PRKN,ARMC10,TAF9,ING2,MARCH7,DYRK3,SNAI1,MUC1,TAF9B,PSMD10,CD74,MIR21,PTTG1IP	grey	VSMC_M10
GO:0051093	0.78	0.24	350	676	negative regulation of developmental process	BP	1	NFATC1,NME1,SEMA6A,FRS2,STK3,ZNF706,SLC12A2,PRKN,PPARA,FBLN1,TNFRSF11B,TSPO,FSTL4,SYNGAP1,TRIB1,MYC,TOB2,ASAP1,PTCH1,SOX10,PI16,SMO,DAAM2,TEK,FBXO7,RORB,CDK6,SEMA4D,S1PR3,SEMA4C,SOSTDC1,SIX2,RTN4,RUFY3,HOXA2,HOXA7,ANKRD17,AREG,AKIRIN1,EPHA7,NR1H2,PTN,ITGB1BP1,AGO1,EGFR,FUZ,TLR4,CDK5RAP2,MYCN,TRPM4,OSR1,FOXO3,MKX,SPRY1,WISP3,FOXE3,HDAC2,SOX9,GLI2,KDM4A,FOXJ1,TSKU,SPRY2,WNT11,HOOK3,TGFBR2,OVOL2,ISM1,JAG1,SIRT2,RARB,SRA1,FERMT1,SPRED3,LSM1,SEMA3D,DCN,SPART,SEMA3A,GAS6,ULK1,PRKDC,RYK,TCF15,TRIB3,EPHB1,PLK2,ZC3H8,ASXL1,PAX8,PIK3CB,ZNF536,PIK3R1,HAND2,ROBO2,IL17RD,ARNTL,FBXW7,ADGRV1,TMEM131L,SEMA4F,VEGFC,TLX2,ADAMTS1,SOX6,TLL2,PTK2B,RAPGEF2,CCL17,SFMBT1,EIF2AK4,NR1H3,SLIT1,RUNX1T1,NKX3-1,HDAC7,NKX6-1,TRIO,RAPGEF3,LOXL2,TBX5,SRSF6,TP53INP1,SEMA3G,SPP1,ZBED6,STK4,PAX2,DMTN,SEMA4G,PRICKLE1,NKD1,JARID2,LDB1,IGF2,BDNF,SUFU,SRGAP2C,RNF10,KIAA0319,PKP2,DNM1L,NEPRO,PPP3CA,HYAL3,SINHCAF,USF3,CCND1,TERT,TRPV4,SEMA3F,PDCD4,ABCA5,TGFB2,SLC30A10,PTHLH,GPR137B,AXIN2,PIAS3,HLX,KRAS,TCTA,SNAI1,USP19,SOX2,AP1AR,YTHDF2,UBASH3B,SORL1,GPER1,KREMEN1,AGT,CIB1,CERS2,NFATC4,FZD7,DDX6,HDAC4,KCNK2,CHRD,RUNX3,MAP3K3,DLX1,ZBTB46,MELTF,EIF4ENIF1,CDKN1B,E2F2,LRIG2,YBX3,WNT3,KLF7,NFE2L2,DUSP10,MAPT,SRGN,BCL6,SEMA6D,DIXDC1,BCOR,MMP11,MEN1,WDR61,TNR,C11orf65,RC3H1,GPR137,SAV1,FBN1,TNFSF18,WNT4,LAG3,HSPG2,DNM3,COL5A2,MYOZ1,RAP1GAP,GDF[illegible]	grey	VSMC_M10
GO:0120178	0.78	0.34	14	29	steroid hormone biosynthetic process	BP	1	CYP21A2,TSPO,HSD17B8,HSD17B3,AKR1B1,SRD5A3,DKK3,SRD5A1,HSD17B7,WNT4,MED1,DGKQ,DHRS11,CACNA1H	grey	VSMC_M10
GO:0031453	0.78	0.43	5	11	positive regulation of heterochromatin assembly	BP	1	SETDB2,FAM208A,PPHLN1,SETDB1,ZNF304	grey	VSMC_M10
GO:0120263	0.78	0.43	5	11	positive regulation of heterochromatin organization	BP	1	SETDB2,FAM208A,PPHLN1,SETDB1,ZNF304	grey	VSMC_M10
GO:1905954	0.78	0.29	36	73	positive regulation of lipid localization	BP	1	LRP1,PTCH1,PLA2G6,PLIN2,TMEM30A,NR1H2,APOB,C1QTNF1,NR1H3,NKX3-1,FITM2,SPP1,PLA2R1,PLTP,MAP2K6,ABCA5,ACSL5,FITM1,NTSR1,PLA2G4A,EHD1,ZDHHC8,ABCA12,LPCAT3,GPS2,ANXA2,C3,PLIN3,ABCA3,ABCA7,RXRA,ABCA8,LDLRAP1,CRH,LRAT,PTGES	grey	VSMC_M10
GO:1901017	0.78	0.41	6	13	negative regulation of potassium ion transmembrane transporter activity	BP	1	NEDD4L,KCNRG,OXSR1,KCNE2,CASQ2,CRBN	grey	VSMC_M10
GO:0030011	0.78	0.39	8	17	maintenance of cell polarity	BP	1	LIN7B,WNT11,ANK1,LIN7A,NCKAP1L,SLC9A1,CARMIL2,PDZD11	grey	VSMC_M10
GO:1901679	0.78	0.38	8	17	nucleotide transmembrane transport	BP	1	SLC35B2,ABCC4,SLC25A42,LRRC8E,SLC25A41,SLC25A33,LRRC8A,SLC25A32	grey	VSMC_M10

GO:0055082	0.78	0.25	291	564	cellular chemical homeostasis	BP	1	ZNF236,NME1,ORMDL2,SLC39A5,NEDD4L,LRP1,ESR1,SLC12A2,AGER,SMDT1,BAK1,MYC,MCHR1,GPR20,PLA2G6,CKB,TMPRSS6,SLC39A4,SRF,STEAP2,SLC25A27,SLC1A1,TFAP2B,S1PR3,PRKCE,TNNI3,RTN4,FPR2,HMGN3,IBTK,SLC4A4,LPAR1,PPT1,AQP1,SLC31A2,KEL,ATG5,TRPM4,FOXO3,FGF21,GRIN2D,TRPC3,SLC8A2,RAB7A,C5AR2,C5AR1,HEPHL1,ATP7B,PTGIR,FOXA3,GIPR,CACNB2,SLC39A12,ATP6V0E2,LACC1,SLC4A2,AQP11,GRIK5,RAF1,ATP1A3,ACKR2,SLC24A3,C1QTNF1,SLC4A7,TMEM106B,TMTC4,MAFG,RYR1,LETM2,PRNP,ADRA1D,DIAPH1,P2RX2,CCKBR,GAS6,HPX,RASA3,NCOA4,NR1H4,MICU3,ATP6V1H,CYP7A1,TM9SF4,NDUFAF2,IL1A,IMMT,OSBP,C19orf12,PIK3R1,P2RX3,PLCE1,SLC9A9,RYR3,TMBIM6,P2RX7,HVCN1,P2RY1,PTK2B,SLC39A13,KCNJ11,TIAM1,RAB11FIP5,TPCN1,SLC8B1,VDR,STC1,OPA1,AP3B1,RMDN3,F2RL1,F2R,SLC40A1,FITM2,ORMDL1,ABCC2,ZBED6,PKD2,PAX2,TWNK,CASQ2,MCUR1,SLC12A5,CD40,RYR2,SLC41A1,SLC4A10,RAP1GDS1,GPLD1,TRPM1,SPPL3,LCK,PPP3CA,KCNJ2,TESMIN,SLC9B1,TRPV4,SLC9B2,ZBTB20,TMEM9,ADRA2A,SLC30A10,TCIRG1,GNA13,ISCU,KCNB1,CMKLR1,MCUB,HJV,SLC9A8,UBASH3B,DISC1,POLG,ANK2,GPER1,SLAMF8,RHCG,TRPM8,PDZD8,RAB11FIP2,AGT,ATP1A2,SLC9A1,CASQ1,NTSR1,XPR1,CLIC4,MLXIPL,FZD9,SIDT2,TMCO1,PDK3,KLF7,PTH1R,SLC30A4,FIS1,CEMIP,HK1,ABL2,MEN1,CDH23,ENY2,ABCA12,PTPN6,MICU1,MCU,CXCR2,GNG3,SLC11A1,HTT,PTGDR,PTGER2,SLC9A7,PPP3CB,ATP6V0A1,ABCB6,TRPM7,PTPRN,ATP1B1,CD38,MPC2,MAPK1,DMXL2,KCNMA1,SLC4A3,ATP13A2,FTH1,HAP1,DLG4,CYB561A3,ATP1B2,FAM155B,STX4,PSEN1,CACNA1A,NGFR,MAPK3,AC138894.1,ATP2A [illegible]	grey	VSMC_M10
GO:0002686	0.78	0.33	16	33	negative regulation of leukocyte migration	BP	1	HOXA7,C5AR2,PLCB1,ADTRP,CD200,SLAMF8,APOD,MIA3,PADI2,CCL2,CCL25,PTGER4,MIR24-1,DPP4,DUSP1,GCSAM	grey	VSMC_M10
GO:1900120	0.78	0.40	7	15	regulation of receptor binding	BP	1	PTPRF,PLCL2,BDNF,GREM2,ANXA2,MIR27B,NOG	grey	VSMC_M10
GO:0006825	0.78	0.40	7	15	copper ion transport	BP	1	STEAP2,SLC31A2,HEPHL1,ATP7B,STEAP3,CUTC,COX17	grey	VSMC_M10
GO:0097659	0.78	0.23	1325	2522	nucleic acid-templated transcription	BP	1	ADNP2,NFATC1,ZNF236,ZNF407,SMARCC2,ATP8B1,ONECUT2,BAZ2A,TCF4,TBPL1,GLI1,MARS,MAP3K5,TNFAIP3,TSFM,HIVEP2,PLAGL1,MAML1,ZBTB2,YEATS4,ESR1,MYRFL,CNOT2,CNOT6,STK3,OSR2,SNW1,TFB1M,ZNF706,ARID1B,HSPA1B,FZD6,ZBTB12,ZBED4,PRKN,AGER,PPARA,PDE10A,MED30,IL5,BRD2,TAF2,RXRB,VGLL2,HSF2,TCF7,PHF1,XRCC6,NCOA7,PITX1,TRIB1,MYC,TOB2,SMAD5,L3MBTL3,L3MBTL2,AGO2,KDM3B,PTP4A3,PTCH1,ATF4,RPAP2,CBX7,MAPK14,CBY1,KDM4C,SOX10,PIM1,NRF1,TMPRSS6,SMO,SNAPC3,BNC2,HSF1,NFYA,FOXP4,MDFI,TFEB,IRF5,MED20,CCND3,TAF8,FOXH1,LRRC14,CDKN2A,ZNF16,RBFOX2,IFT74,BICRAL,RORB,ZNF300,PRDM4,CRY1,DMTF1,SRF,ZNF318,NFX1,CDK14,FZD1,MTERF1,NFXL1,NFKBIE,SUPT3H,SMARCA2,RFX3,KIT,ADGRF1,NCK2,FHL2,TGFBRAP1,TFAP2B,ROR2,NFIL3,SEMA4D,RFX8,DLX5,PHF14,IPPK,ETV1,ZNF135,RPS14,ZNF154,MEOX2,ZNF550,KANSL3,BEND6,ZNF268,ZNF667,ZNF787,PHF3,ZNF691,CAT,MACC1,SIX2,FOXJ3,ZNF865,HIVEP3,IL11,SCMH1,ZNF189,RNF20,EPAS1,EPCAM,SP4,PPP2R1A,ZNF462,PHIP,HMGN3,NFYC,NFE2L3,HOXA2,TAF1D,HOXA4,HOXA6,HOXA7,HOXA13,REL,ZNF483,JAZF1,CREB5,MYCL,XPO1,MTURN,NOD1,AKIRIN2,SRSF10,CASP8AP2,BACH2,ZNF692,PELI1,AKIRIN1,MTF1,ZNF618,SNIP1,ANXA4,PCBP1,NR1H2,AKNA,ZNF350,ITGB1BP1,FOXP2,GRHL1,MED25,KLF11,MET,IL4I1,AGO1,POU3F3,EGFR,LANCL2,ING3,NOBOX,ARHGEF5,TRIM32,TLR4,CCNC,CDK5RAP2,TRAF1,BCL2L12,SCAF1,LPIN1,SFRP4,UCP1,CREM,TEAD2,SETD7,SDCBP,MYCN,C6orf203,ELF2,OSR1,FOXO3,ZXDC,ZBTB24,MKX,FAF1,MAMSTR,GTF3C6,ATAD2B,BEND5,CDCA7,KDM7A,ZNF438,FOXE3,HDAC2,PHF10,M [illegible]	grey	VSMC_M10
GO:0045618	0.78	0.40	7	15	positive regulation of keratinocyte differentiation	BP	1	CYP27B1,OVOL2,VDR,H2AFY2,MED1,MIR125B1,ZBED2	grey	VSMC_M10
GO:0034767	0.78	0.28	57	114	positive regulation of ion transmembrane transport	BP	1	STAC3,ABCB1,WNK2,CACNB2,STAC,GLRX,P2RX7,KCNJ11,IFNGR2,F2R,TESC,ANO6,KCNE2,PKD2,KCNIP2,RYR2,FAM173B,KCNJ2,COX17,ANK2,GPER1,AGT,ACE2,SLC9A1,CASQ1,NTSR1,NOS1AP,CEMIP,HTT,PINK1,ATP1B1,KCNA1,ARL6IP1,HAP1,WNK3,STAC2,ATP1B2,PSEN1,NLGN3,ATP2A1,CD19,CALM1,DNM2,VMP1,BDKRB1,CCL2,PDPK1,P2RX1,RNF207,AMIGO1,LCN2,MIR21,DMD,JPH2,KCNH2,THY1,APOPT1	grey	VSMC_M10
GO:0031214	0.78	0.27	63	126	biomineral tissue development	BP	1	CYP27B1,OSR2,PPARA,ATF4,ROR2,OMD,CNNM4,KLK4,PTN,ITGB1BP1,TRPM4,OSR1,SOX9,ERCC2,SLC4A2,ACVR2B,WNT11,SLC24A3,ACVR2A,KL,GAS6,ADGRV1,MEF2C,P2RX7,PTK2B,BMP2K,ANO6,SPP1,TFAP2A,ITGB6,BMPR1B,FBXL15,SBDS,PTHLH,TCIRG1,FAM20A,FAM20C,SGMS2,AXIN2,AMELX,FGR,ACTN3,TXLNG,FZD9,PTH1R,SRGN,BCOR,ANKH,ZBTB40,WNT4,ALPL,FGFR3,MMP13,BMPR1A,SMAD3,GPC3,RFLNB,BGLAP,MINPP1,SNX10,CCDC154,CEBPB,HEY2	grey	VSMC_M10
GO:0043550	0.78	0.30	28	58	regulation of lipid kinase activity	BP	1	PIP4K2C,AGAP2,PTK2,TEK,KIT,FPR2,WDR91,PIP4K2A,CCKBR,FLT1,PIK3R1,SLA2,PTK2B,NOD2,IRS1,FGR,PPP2R5A,RUBCN,PIK3IP1,FGFR3,SOCS4,SOCS7,CD19,PIK3R5,SOCS1,VAV3,DAB2IP,NRBF2	grey	VSMC_M10
GO:0042446	0.78	0.31	23	47	hormone biosynthetic process	BP	1	CYP27B1,CYP21A2,TSPO,HSD17B8,HSD17B3,AKR1B1,SRD5A3,AANAT,DKK3,SRD5A1,ARNT,HSD17B7,POR,DUOX1,FDX1,WNT4,MED1,DGKQ,DHRS11,CACNA1H,GFI1,DIO3,DIO2	grey	VSMC_M10
GO:0071233	0.78	0.45	4	9	cellular response to leucine	BP	1	RRAGD,SESN3,RPTOR,LARS	grey	VSMC_M10
GO:0000478	0.78	0.40	7	15	endonucleolytic cleavage involved in rRNA processing	BP	1	UTP23,TSR1,BMS1,RPP40,NOP9,KRI1,NOL9	grey	VSMC_M10
GO:0000479	0.78	0.40	7	15	endonucleolytic cleavage of tricistronic rRNA transcript (SSU-rRNA, 5.8S rRNA, LSU-rRNA)	BP	1	UTP23,TSR1,BMS1,RPP40,NOP9,KRI1,NOL9	grey	VSMC_M10
GO:0010522	0.78	0.29	35	71	regulation of calcium ion transport into cytosol	BP	1	BAK1,PRKCE,ATG5,TRPC3,PRNP,DIAPH1,P2RX2,P2RX3,TMBIM6,P2RX7,PTK2B,F2R,PKD2,CASQ2,RYR2,UBASH3B,ANK2,GPER1,ATP1A2,CASQ1,NTSR1,CEMIP,PTPN6,HTT,HAP1,CD19,CALM1,BDKRB1,PDPK1,P2RX1,CYBA,JPH3,DMD,JPH2,THY1	grey	VSMC_M10

GO:0006810	0.78	0.23	1740	3300	transport	BP	1	ATP9B,TIMM21,NME1,VPS4B,ERBB3,SLC39A5,NEDD4L,APOF,ATP8B1,MIP,STARD6,MYO1A,LRP1,STX7,SLC18B1,STAC3,AP3S1,SLC2A12,KIF5A,SLC26A10,PEX7,AGAP2,HEBP2,REPS1,CYP27B1,PEX3,SLC16A7,CANX,TBC1D30,ATP5MGL,TBC1D9B,PPP1R10,CPSF6,RAB21,NIPAL2,SNCAIP,SNX24,SNX31,MAPK8IP2,CPT1B,PHAX,MARCH3,APOM,ZDHHC14,SLC12A2,HSPA1B,SYNJ2,SERAC1,RIMS2,IGF2R,SLC27A6,CDC42SE2,KCNV1,PRRT1,PRKN,QKI,RNF5,AGER,PPARA,TAP1,SLC22A4,CALHM4,KIF3A,SEPT8,TSPO,GDF9,GOPC,SLC35F1,VPS52,HSPA4,DERL1,SMDT1,BAK1,FAM91A1,CATSPER3,WASHC5,MYC,LRRC6,KLHL3,MCHR1,PKD2L2,KCNK9,GRM4,PTK2,FAM53C,SLC45A4,PTCH1,TSNARE1,LY6E,ZC3H3,EXOC4,SIL1,SYNGR1,MAPK15,AGTPBP1,SUN2,MAPK14,NTRK2,TOMM22,NRBP2,KCNJ4,CSNK1E,TMEM184B,PLA2G6,COPG2,PICK1,MICALL1,PIM1,TBC1D22B,BTBD9,RAC2,SMO,ZDHHC21,TTC39B,HSF1,PLIN2,TOMM6,SLC39A4,ATP6V1F,MYH9,GUCA1B,MFSD3,APOL4,APOL6,IFT74,CHRNA3,CHRNA5,PEX6,TRPM6,FBXO7,CRY1,CROT,ABCB1,SLC25A40,ABCC10,NOL6,STEAP2,GABRA1,XPO5,WASHC4,APBA1,CNGA1,SLC41A2,TMEM63B,PEX1,SLC35B2,OCIAD1,USP46,CLIC5,VPS50,SLC25A27,SCFD2,RFX3,KIT,SLC1A1,CA9,TGFBRAP1,TFAP2B,SLC25A13,TBC1D8,TRAM2,PPAT,TMEM14A,RGP1,WNK2,BICD2,MFSD14C,CHMP2A,CNNM4,TSPAN13,RAB23,DYNC2LI1,NLRP5,ABCG5,SVBP,ABCG8,LRPPRC,SEC61B,SLC3A1,STX17,CLDN19,SIX2,DNAJA1,IL11,BRSK1,PRKCE,SMAP1,ATP6V1E2,RIMS1,ABCB5,TMC4,TMEM30A,DNAH11,SPTBN1,IGF2BP3,FPR2,HMGN3,KCNQ4,YTHDC1,RIMS3,IBTK,PNPT1,RUFY3,SHANK1,SLC4A4,CYB5R4,PPT1,SNX14,ALB,CXCL8,SCRN1,XPO1,[illegible]	grey	VSMC_M10
GO:1902018	0.78	0.43	5	11	negative regulation of cilium assembly	BP	1	TBC1D30,LIMK2,DNM2,CDK10,ODF2L	grey	VSMC_M10
GO:0055012	0.78	0.41	6	13	ventricular cardiac muscle cell differentiation	BP	1	FHL2,ATG5,RARB,MEF2C,NKX2-5,HEY2	grey	VSMC_M10
GO:0010939	0.78	0.34	15	31	regulation of necrotic cell death	BP	1	HEBP2,TSPO,PELI1,ASAH1,RIPK1,SPATA2,FZD9,YBX3,BIRC3,OGT,MIR22,NOL3,FADD,CASP8,PPIF	grey	VSMC_M10
GO:0060402	0.78	0.28	53	107	calcium ion transport into cytosol	BP	1	BAK1,PRKCE,IBTK,ATG5,GRIN2D,TRPC3,SLC8A2,RYR1,PRNP,DIAPH1,P2RX2,RASA3,P2RX3,RYR3,TMBIM6,P2RX7,PTK2B,TPCN1,SLC8B1,F2R,PKD2,CASQ2,RYR2,TRPM1,LCK,TRPV4,UBASH3B,ANK2,GPER1,ATP1A2,CASQ1,NTSR1,CEMIP,PTPN6,HTT,HAP1,FAM155B,CD19,CALM1,BDKRB1,TRPV2,TRPV3,TRPV1,PDPK1,P2RX1,CYBA,JPH3,MCOLN3,DMD,NOL3,JPH2,THY1,DDIT3	grey	VSMC_M10
GO:0021932	0.78	0.45	4	9	hindbrain radial glia guided cell migration	BP	1	RBFOX2,ATP1B2,DAB1,CEND1	grey	VSMC_M10
GO:0150065	0.78	0.43	5	11	regulation of deacetylase activity	BP	1	C6orf89,SPHK2,PRKD2,PINK1,KDM5A	grey	VSMC_M10
GO:2001244	0.78	0.31	23	46	positive regulation of intrinsic apoptotic signaling pathway	BP	1	NCK2,GSDME,FBH1,FBXW7,NKX3-1,SIAH1,DNM1L,LCK,MCL1,RPL11,FIS1,PRKRA,S100A8,SFPQ,PTPN2,PIAS4,BCAP31,PARK7,MIR27B,UBB,DDIT3,BCL2L11,PLAGL2	grey	VSMC_M10
GO:0009306	0.78	0.25	136	266	protein secretion	BP	1	LRP1,CANX,SLC12A2,RIMS2,PRKN,PLA2G6,COPG2,PICK1,RFX3,TFAP2B,SVBP,PRKCE,HMGN3,CYB5R4,BMP8A,PLEK,RSAD2,NR1H2,EGFR,TLR4,TRPM4,RAB10,COPG1,GIPR,FRMD4A,LLGL2,STEAP3,RAF1,APBB3,DPH3,SIRT3,SYT9,NR1H4,NDUFAF2,FAM3D,IL1A,OSBP,ARNTL,VEGFC,KCNJ11,NNAT,NR1H3,TIAM1,RAB11FIP5,PFKM,SLC8B1,NKX6-1,ACVR1C,F2RL1,F2R,ZBED6,DMTN,ADTRP,SIRT4,GPLD1,IRS1,CD200,DNM1L,HDAC1,PPP3CA,CPT1A,SLC9B2,TGFB2,ADRA2A,MON1A,TCIRG1,TCF7L2,KCNB1,PFKFB2,GPER1,RAB11FIP2,PER2,STX1A,GLUL,SIDT2,SLC16A1,ADAM8,KLF7,MIA3,BLOC1S6,CTAGE9,VGF,TTN,IL12A,TANGO2,ENY2,ABCA12,MCU,PPP3CB,RAB3A,CAMK2G,PTPRN,CD38,MPC2,ILDR2,RAB8A,ATP13A2,SYT7,RAB15,NLGN2,CPLX1,TARDBP,STX4,RAB8B,TMED10,UBE2Q1,MYH10,SEL1L,RAB3D,SYTL4,GPR68,TVP23C,CCL5,SREBF1,ISL1,OXCT1,NOS2,TVP23A,ABAT,RPH3AL,NADK,BAIAP3,PARK7,LRRC8A,TANGO6,MIR199A1,FGA,BLOC1S3,CHGA,SOX4,DPP4,TMEM167A,CRH,DOC2B,UQCC2,PARD6A	grey	VSMC_M10
GO:0035592	0.78	0.25	136	266	establishment of protein localization to extracellular region	BP	1	LRP1,CANX,SLC12A2,RIMS2,PRKN,PLA2G6,COPG2,PICK1,RFX3,TFAP2B,SVBP,PRKCE,HMGN3,CYB5R4,BMP8A,PLEK,RSAD2,NR1H2,EGFR,TLR4,TRPM4,RAB10,COPG1,GIPR,FRMD4A,LLGL2,STEAP3,RAF1,APBB3,DPH3,SIRT3,SYT9,NR1H4,NDUFAF2,FAM3D,IL1A,OSBP,ARNTL,VEGFC,KCNJ11,NNAT,NR1H3,TIAM1,RAB11FIP5,PFKM,SLC8B1,NKX6-1,ACVR1C,F2RL1,F2R,ZBED6,DMTN,ADTRP,SIRT4,GPLD1,IRS1,CD200,DNM1L,HDAC1,PPP3CA,CPT1A,SLC9B2,TGFB2,ADRA2A,MON1A,TCIRG1,TCF7L2,KCNB1,PFKFB2,GPER1,RAB11FIP2,PER2,STX1A,GLUL,SIDT2,SLC16A1,ADAM8,KLF7,MIA3,BLOC1S6,CTAGE9,VGF,TTN,IL12A,TANGO2,ENY2,ABCA12,MCU,PPP3CB,RAB3A,CAMK2G,PTPRN,CD38,MPC2,ILDR2,RAB8A,ATP13A2,SYT7,RAB15,NLGN2,CPLX1,TARDBP,STX4,RAB8B,TMED10,UBE2Q1,MYH10,SEL1L,RAB3D,SYTL4,GPR68,TVP23C,CCL5,SREBF1,ISL1,OXCT1,NOS2,TVP23A,ABAT,RPH3AL,NADK,BAIAP3,PARK7,LRRC8A,TANGO6,MIR199A1,FGA,BLOC1S3,CHGA,SOX4,DPP4,TMEM167A,CRH,DOC2B,UQCC2,PARD6A	grey	VSMC_M10
GO:0050860	0.78	0.39	7	15	negative regulation of T cell receptor signaling pathway	BP	1	PAWR,PRNP,PTPRJ,SLA2,PTPN6,PTPN2,THY1	grey	VSMC_M10
GO:0072182	0.78	0.43	5	11	regulation of nephron tubule epithelial cell differentiation	BP	1	OSR1,PAX8,PAX2,LHX1,LIF	grey	VSMC_M10
GO:0019319	0.78	0.30	30	61	hexose biosynthetic process	BP	1	PPARA,AKR1B1,ATF4,GPT,GNMT,CRY1,SLC25A13,PPP4R3B,ZNF692,FAM3C,SIRT7,GPI,NLN,GPD1,SIK1,MST1,PC,SLC37A4,PER2,SORD,DDB1,DGKQ,OGT,CRTC2,LEPR,PTPN2,ACADM,FBP2,NNMT,ARPP19	grey	VSMC_M10
GO:0006026	0.78	0.37	9	19	aminoglycan catabolic process	BP	1	CEMIP2,GLB1,HYAL1,HYAL3,OVGP1,CEMIP,IDUA,IDS,CTBS	grey	VSMC_M10
GO:0048486	0.78	0.39	7	15	parasympathetic nervous system development	BP	1	PLXNA4,SEMA3A,TFAP2A,SEMA3F,ADARB1,PLXNA3,SIX1	grey	VSMC_M10
GO:0097094	0.78	0.41	6	13	craniofacial suture morphogenesis	BP	1	RAB23,MEGF8,MSX2,TMEM107,FOXN3,INSIG2	grey	VSMC_M10

GO:0098801	0.78	0.36	11	23	regulation of renal system process	BP	1	SNX5,GAS6,STC1,F2RL1,F2R,AGT,PTPRO,COMT,CORO2B,INPP5K,CYBA	grey	VSMC_M10
GO:0006206	0.78	0.39	7	15	pyrimidine nucleobase metabolic process	BP	1	DPYS,CMPK1,CTPS2,CPS1,MAPK1,ALDH6A1,DHODH	grey	VSMC_M10
GO:0000083	0.78	0.38	8	17	regulation of transcription involved in G1/S transition of mitotic cell cycle	BP	1	CDK14,KLF11,BACH1,CDK18,CDK15,ESRRB,GFI1,E2F6	grey	VSMC_M10
GO:1901890	0.78	0.29	40	82	positive regulation of cell junction assembly	BP	1	NTRK2,TEK,LINGO2,SEMA4D,CLDN19,LRRN3,ITGB1BP1,VSTM5,EPHB1,NPHP1,CLSTN2,FMN1,PTPRJ,CUX2,TBX5,AMIGO2,BDNF,COL16A1,AMIGO3,ADNP,AGT,ACE2,EPHB3,WNT4,LRTM2,NLGN2,SEMA4A,NLGN3,SMAD3,GPRASP2,BHLHB9,ASIC2,SLITRK2,AMIGO1,TSC1,ADGRL2,GRID2,THY1,CLDN1,LRRTM2	grey	VSMC_M10
GO:0034368	0.79	0.37	9	19	protein-lipid complex remodeling	BP	1	APOM,APOB,APOC1,NR1H4,PLTP,ABCA5,AGT,LIPC,MPO	grey	VSMC_M10
GO:0034369	0.79	0.37	9	19	plasma lipoprotein particle remodeling	BP	1	APOM,APOB,APOC1,NR1H4,PLTP,ABCA5,AGT,LIPC,MPO	grey	VSMC_M10
GO:0019933	0.79	0.32	19	40	cAMP-mediated signaling	BP	1	PDE7B,ADGRG6,RIMS2,UBE2B,SOX9,GIPR,RAPGEF2,RAPGEF3,CAP2,ADCY2,EIF4EBP2,DGKQ,PDE4A,MAPK7,KSR1,MGRN1,CAP1,CRH,RASD2	grey	VSMC_M10
GO:1903036	0.79	0.30	26	53	positive regulation of response to wounding	BP	1	LRP1,PTK2,ENPP4,PRKCE,PTN,THBD,ACTG1,HBEGF,FERMT1,F2R,ANO6,RREB1,DMTN,ADRA2A,USF1,NFE2L2,DUOX1,F12,NDEL1,SERPINF2,MIR29A,MIR21,MIR222,CNTF,CLDN1,HRAS	grey	VSMC_M10
GO:0032527	0.79	0.31	21	43	protein exit from endoplasmic reticulum	BP	1	DERL1,SEC61B,TMEM30A,NPLOC4,UBAC2,TM9SF4,AUP1,RHBDD1,YOD1,SORL1,SYVN1,DERL3,SEC16B,TMEM129,TMEM30B,SEL1L,TECPR2,BCAP31,SEC16A,UBE2G2,DERL2	grey	VSMC_M10
GO:0043279	0.79	0.28	40	81	response to alkaloid	BP	1	SLC1A1,PRKCE,DDC,HDAC2,DNMT3A,HNMT,RYR1,DNMT3B,GPI,CASP3,RYR3,PTK2B,TIAM1,HOMER1,SPIDR,CASQ2,RYR2,GRM2,SLC6A3,HDAC1,CPT1A,PEA15,HOMER2,BCHE,SDK1,TMED10,CHRNB2,DNM2,GHR,MBD1,ABAT,TRPV1,HSPA5,DHODH,NQO1,MT-ND6,FADD,RGS4,CRH,PITX3	grey	VSMC_M10
GO:0072529	0.79	0.35	12	25	pyrimidine-containing compound catabolic process	BP	1	DPYS,APOBEC3H,CDADC1,MBD4,ENTPD4,TET1,UPB1,ALDH6A1,NEIL1,NT5M,NTHL1,UPP1	grey	VSMC_M10
GO:2001267	0.79	0.39	7	15	regulation of cysteine-type endopeptidase activity involved in apoptotic signaling pathway	BP	1	ST18,HTRA2,SMAD3,NLE1,PARK7,CAAP1,PRR7	grey	VSMC_M10
GO:0032753	0.79	0.39	7	15	positive regulation of interleukin-4 production	BP	1	CD28,FCER1G,FOXP3,PRKCZ,SLC7A5,CEBPB,HAVCR2	grey	VSMC_M10
GO:0035461	0.79	0.41	6	13	vitamin transmembrane transport	BP	1	SLC23A2,ABCC5,SLC27A1,STRA6,SLC46A1,SLC25A32	grey	VSMC_M10
GO:0016075	0.79	0.38	8	17	rRNA catabolic process	BP	1	EXOSC4,EXOSC3,DEDD2,DDX49,MRPL44,SLFN14,DROSHA,EXOSC5	grey	VSMC_M10
GO:0000422	0.79	0.29	35	71	autophagy of mitochondrion	BP	1	ATG12,PRKN,TSPO,FBXO7,ATG5,OPTN,USP36,ULK1,FBXW7,HTRA2,CSNK2A2,DNM1L,CISD2,USP30,FZD5,FIS1,ATG2A,FUNDC1,HTT,MARK2,PINK1,MUL1,ATG9A,ATP13A2,WIPI2,VPS13D,MFN2,VPS13C,HAX1,ATG2B,SREBF1,FUNDC2,PARK7,BNIP3L,TIGAR	grey	VSMC_M10
GO:0061726	0.79	0.29	35	71	mitochondrion disassembly	BP	1	ATG12,PRKN,TSPO,FBXO7,ATG5,OPTN,USP36,ULK1,FBXW7,HTRA2,CSNK2A2,DNM1L,CISD2,USP30,FZD5,FIS1,ATG2A,FUNDC1,HTT,MARK2,PINK1,MUL1,ATG9A,ATP13A2,WIPI2,VPS13D,MFN2,VPS13C,HAX1,ATG2B,SREBF1,FUNDC2,PARK7,BNIP3L,TIGAR	grey	VSMC_M10
GO:0030510	0.79	0.29	36	74	regulation of BMP signaling pathway	BP	1	LEMD2,KCP,TFAP2B,SOSTDC1,HOXA13,BMPER,SFRP4,UBE2O,SPART,ZNF423,FBXL15,SORL1,HTRA1,CHRD,DLX1,CTDSPL2,HTRA3,FBN1,FKBP8,GREM2,RBPJ,ITGA3,RBPMS2,TOB1,RNF165,CHRDL1,TWSG1,GPC3,SKI,ENG,FSTL3,LEMD3,ACVRL1,NOG,WNT5A,UBE2D1	grey	VSMC_M10
GO:0007635	0.79	0.42	5	11	chemosensory behavior	BP	1	CFAP69,SHANK1,ADCY3,P2RX3,TRPV1	grey	VSMC_M10
GO:0034497	0.79	0.42	5	11	protein localization to phagophore assembly site	BP	1	STX17,ATG16L1,ATG9A,WIPI2,MFN2	grey	VSMC_M10
GO:0032402	0.79	0.38	8	17	melanosome transport	BP	1	BBS7,MKKS,MLPH,RAB17,BLOC1S6,MREG,RAB11A,BLOC1S3	grey	VSMC_M10
GO:1903671	0.79	0.45	4	9	negative regulation of sprouting angiogenesis	BP	1	SEMA6A,PIK3CB,E2F2,SYNJ2BP	grey	VSMC_M10
GO:0060004	0.79	0.45	4	9	reflex	BP	1	USP46,SLC1A1,SHANK1,ALDH1A3	grey	VSMC_M10
GO:0019693	0.79	0.25	161	312	ribose phosphate metabolic process	BP	1	NME1,HSD17B4,PDE7B,ATP5MGL,DPYS,PPT2,PPARA,PDE10A,ACSL6,PRXL2C,GTPBP1,IMPDH1,GDA,CROT,SLC35B2,SLC25A13,GAPDH,PPAT,RAB23,PPCS,SLC4A4,NT5E,PPT1,BPGM,DLD,SPHK2,CMPK1,ADCY3,DLG2,NUDT5,DHTKD1,PRKAG2,ATP5F1C,ELOVL1,PFKP,NUDT16,PANK2,PDK1,SUCLG2,PDHX,ELOVL7,PANK1,GPI,AMPD3,ATP5F1A,MCCC2,GPD1,P2RX7,GUCY1A1,GUCY1B1,ACOT12,DGUOK,PRKAG1,GMPS,PFKM,OPA1,ENTPD4,FITM2,AMPD1,NUDT18,ADCY7,FAM173B,ADCY2,MCEE,NME4,PDE9A,TPST1,ZBTB20,GPAM,FAR2,ACSL5,PTHLH,MFN1,PFKFB2,PRPS2,ENO4,HACD2,ARNT,ADCY4,CTPS2,ACTN3,HDAC4,THEM5,DNAJC30,MLXIPL,PDE8A,HMGCL,MAGI3,DLG1,ACOT9,PDK3,EFL1,SLC25A16,UPB1,HK1,ACSBG1,ATP5S,CASK,PYGL,COASY,PRKAG3,PINK1,STAT3,NME7,ADK,MPC2,ADCY10,ENO3,PARP1,TECR,CARD11,SLC26A1,PSEN1,ACOT2,GUCY2D,PKLR,PANK3,GCDH,OGT,UPRT,ADPGK,PDE4A,ACSM3,ACACA,NCOR1,INSR,SLC26A2,PRPSAP2,ADSSL1,ACSBG2,ALDOC,PIPOX,ACOT11,ATP6V0C,PANK4,NME3,MPP1,ATP5PB,SLC2A6,VPS9D1,GNAI3,ACSF3,UCK1,MLYCD,DHODH,MT-ATP8,MT-ATP6,RHOA,SULT1C3,ATP5ME,ACOT4,ATP5MF,ENTPD5,ELOVL3,TIGAR,NUDT19,NME6,NUDT2,HINT1,UPP1,RPIA	grey	VSMC_M10
GO:0030889	0.79	0.42	5	11	negative regulation of B cell proliferation	BP	1	TNFRSF21,PAWR,CASP3,RC3H1,BTK	grey	VSMC_M10
GO:0035455	0.79	0.40	6	13	response to interferon-alpha	BP	1	EIF2AK2,GAS6,IFIT2,MX2,KLHL20,IFIT3	grey	VSMC_M10
GO:0001990	0.79	0.35	11	23	regulation of systemic arterial blood pressure by hormone	BP	1	MME,F2RL1,F2R,ENPEP,AGT,ACE2,ECE1,SERPINF2,CYBA,HSD11B2,RHOA	grey	VSMC_M10

GO:0051924	0.79	0.26	87	172	regulation of calcium ion transport	BP	1	STAC3,TSPO,BAK1,MCHR1,PLA2G6,TSPAN13,PRKCE,ATG5,SPHK2,TRPC3,CACNB2,STAC,CACNB4,EFHB,PRNP,DIAPH1,P2RX2,P2RX3,ORAI1,TMBIM6,P2RX7,PTK2B,SLC8B1,HOMER1,STC1,F2R,KCNE2,PKD2,CASQ2,RYR2,ADRA2A,EGF,EPB41,OPRD1,UBASH3B,ANK2,GPER1,AGT,ATP1A2,SLC9A1,PLPP4,C4orf3,CASQ1,NTSR1,NOS1AP,HOMER2,IL16,CEMIP,SESTD1,CASK,PTPN6,HTT,CAMK2G,ATP1B1,GNB5,HAP1,PSEN2,WNK3,STAC2,CACNB1,ATP2A1,CD19,CALM1,VMP1,BDKRB1,TRPV2,CAMK2A,CCL5,CCL2,FMR1,INPP5K,TRPV3,PDPK1,P2RX1,CYBA,JPH3,ATP2C2,RRAD,MIR21,RHOA,DMD,GPR35,JPH2,THY1,RGS4,CRH,GEM	grey	VSMC_M10
GO:0060670	0.79	0.42	5	11	branching involved in labyrinthine layer morphogenesis	BP	1	RSPO3,SPINT2,SPINT1,FZD5,GRB2	grey	VSMC_M10
GO:0019377	0.79	0.40	6	13	glycolipid catabolic process	BP	1	NEU3,GALC,GLA,HEXA,GM2A,NEU2	grey	VSMC_M10
GO:0002029	0.79	0.40	6	13	desensitization of G protein-coupled receptor signaling pathway	BP	1	GIPR,ARRB1,GRK2,SAG,GRK4,DNM2	grey	VSMC_M10
GO:0022401	0.79	0.40	6	13	negative adaptation of signaling pathway	BP	1	GIPR,ARRB1,GRK2,SAG,GRK4,DNM2	grey	VSMC_M10
GO:0023058	0.79	0.40	6	13	adaptation of signaling pathway	BP	1	GIPR,ARRB1,GRK2,SAG,GRK4,DNM2	grey	VSMC_M10
GO:0002732	0.79	0.45	4	9	positive regulation of dendritic cell cytokine production	BP	1	KIT,MAVS,NOD2,TICAM1	grey	VSMC_M10
GO:0043552	0.79	0.34	13	28	positive regulation of phosphatidylinositol 3-kinase activity	BP	1	AGAP2,PTK2,TEK,KIT,FPR2,FLT1,PTK2B,NOD2,IRS1,FGR,FGFR3,CD19,VAV3	grey	VSMC_M10
GO:0002693	0.79	0.40	6	13	positive regulation of cellular extravasation	BP	1	AGER,CD99,ADAM8,FADD,THY1,PDGFD	grey	VSMC_M10
GO:0045662	0.79	0.38	8	17	negative regulation of myoblast differentiation	BP	1	SOSTDC1,SOX9,SRA1,CCL17,PRICKLE1,MBNL3,SOX8,DDIT3	grey	VSMC_M10
GO:0008631	0.79	0.32	19	39	intrinsic apoptotic signaling pathway in response to oxidative stress	BP	1	MAP3K5,PRKN,PDK1,ARL6IP5,DIABLO,FBXW7,HTRA2,MCL1,NFE2L2,NONO,PRODH,PDCD10,PINK1,PARP1,SFPQ,MAPK7,PARK7,ZNF622,NOL3	grey	VSMC_M10
GO:0051100	0.79	0.27	65	129	negative regulation of binding	BP	1	PPARA,HABP4,C6orf106,CSNK1E,SMO,MDFI,TTBK1,MITD1,ZNF462,ITGB1BP1,HDAC2,SYMPK,RALB,RSF1,PTPRF,FBH1,EFHB,BTAF1,HAND2,FBXW7,TMBIM6,P2RY1,FAM192A,IFIT2,DTNBP1,PPP3CA,SP100,TTBK2,ADNP,DISC1,SORL1,NFATC4,PER2,C4orf3,PEX19,GEMIN2,ITGA4,MEPCE,LRPAP1,RAP1GAP,DNAJB2,DDX11,CRMP1,ACTB,GNL3L,MSX2,PEX14,PSEN1,TAF1,MAPK3,WFIKKN2,SIN3A,ERCC4,JUN,WFIKKN1,PARK7,MIR27B,MIR27A,BAG2,NOG,DDIT3,ZNF304,ZNF593,STYX,HEY2	grey	VSMC_M10
GO:0032411	0.79	0.28	42	85	positive regulation of transporter activity	BP	1	STAC3,ABCB1,WNK2,CACNB2,STAC,GLRX,KCNJ11,NR1H3,IFNGR2,TESC,KCNE2,PKD2,KCNIP2,RYR2,FAM173B,COX17,ANK2,SLC9A1,CASQ1,NTSR1,NOS1AP,HTT,PINK1,ATP1B1,KCNA1,HAP1,WNK3,STAC2,ATP1B2,NLGN3,ATP2A1,CALM1,DNM2,VMP1,CCL2,RNF207,PARK7,AMIGO1,RXRA,DMD,JPH2,APOPT1	grey	VSMC_M10
GO:0071871	0.79	0.42	5	11	response to epinephrine	BP	1	SIRT2,RYR2,SRD5A1,SLC9A1,PKLR	grey	VSMC_M10
GO:0006002	0.79	0.42	5	11	fructose 6-phosphate metabolic process	BP	1	GFPT2,PFKP,PFKM,HK1,FBP2	grey	VSMC_M10
GO:0051881	0.79	0.29	33	66	regulation of mitochondrial membrane potential	BP	1	HEBP2,PRKN,TSPO,BAK1,SLC25A27,WISP3,PRDX3,AKT2,DCN,PANK2,SPART,RNF122,CLIC1,ARL6IP5,PPA2,MFN1,OPRD1,IFI6,FZD9,PPP2R3C,MAPT,STOX1,BCO2,PINK1,MUL1,PARP1,TRPV1,PARK7,SLC25A33,PIP5KL1,UBB,MLLT11,BNIP3L	grey	VSMC_M10
GO:0034755	0.79	0.38	8	17	iron ion transmembrane transport	BP	1	STEAP2,SLC25A37,SLC40A1,ISCU,ABCC5,SLC11A1,ABCB6,LCN2	grey	VSMC_M10
GO:0070265	0.79	0.31	22	45	necrotic cell death	BP	1	HEBP2,TSPO,NINJ1,GSDME,PELI1,ASAH1,RIPK1,DNM1L,ITPK1,SPATA2,FZD9,YBX3,PYGL,BIRC3,TRPM7,OGT,TRAF2,MIR22,NOL3,FADD,CASP8,PPIF	grey	VSMC_M10
GO:0033500	0.79	0.26	93	184	carbohydrate homeostasis	BP	1	ZNF236,NME1,LRP1,AGER,PTCH1,PLA2G6,CRY1,SRF,TFAP2B,PRKCE,HMGN3,CYB5R4,TRPM4,FOXO3,FGF21,SESN3,FOXA3,GRIK5,RAF1,GCKR,ASPSCR1,GAS6,NR1H4,CYP7A1,NDUFAF2,GPI,CEBPA,OSBP,PIK3R1,KCNJ11,TIAM1,RAB11FIP5,PFKM,SLC8B1,OPA1,CRY2,ZBED6,PAX2,TWNK,TENT4B,GPLD1,IRS1,PPP3CA,TRPV4,SLC9B2,ZBTB20,ADRA2A,TCF7L2,KCNB1,POLG,GPER1,RAB11FIP2,USF1,MLXIPL,SIDT2,SLC16A1,PDK3,KLF7,FIS1,VGF,HK1,MEN1,ENY2,ABCA12,MCU,PYGL,PPP3CB,STAT3,PTPRN,MPC2,BHLHA15,SLC2A4,FGFR4,STX4,NGFR,CRTC2,GPR68,SIN3A,INSR,OXCT1,LEPR,PTPN2,INPP5K,NADK,BAIAP3,PARK7,CYBA,LRRC8A,OAS1,FABP5,LRRC8D,SOX4,CRH	grey	VSMC_M10
GO:0097531	0.79	0.40	6	13	mast cell migration	BP	1	RAC2,KIT,VEGFC,STAT5B,PGF,CHGA	grey	VSMC_M10
GO:1902894	0.79	0.39	7	15	negative regulation of pri-miRNA transcription by RNA polymerase II	BP	1	PPARA,SRF,SOX9,NFATC4,HDAC4,YY1,NFATC3	grey	VSMC_M10
GO:0035637	0.79	0.27	56	114	multicellular organismal signaling	BP	1	NTRK2,CLDN19,MYH14,CLCN1,TRPM4,SLC8A2,CACNB2,ATP1A3,SCN5A,SCN1A,P2RX3,CHRM5,KCNJ3,TBX5,KCNE2,DSP,CASQ2,RYR2,PKP2,KCNJ2,SCN2A,SCN7A,ANK2,GPER1,AGT,ACE2,ATP1A2,SLC9A1,SCN4B,TNR,CNTNAP1,ATP1B1,KCNA1,SLC4A3,ATP1B2,CACNA1G,ATP2A1,CALM1,OTOA,DSG2,ISL1,FMR1,ATP2B3,P2RX1,RNF207,CACNA1H,GPR35,TMEM65,KCNH2,SCN2B,CACNG7,GPR88,CACNG5,HCN4,NKX2-5,KCNMB4	grey	VSMC_M10
GO:0002639	0.79	0.32	16	33	positive regulation of immunoglobulin production	BP	1	IL5,EXOSC3,MSH2,HMCES,PHB,MLH1,PAXIP1,SHLD1,HPX,GPI,CD40,CD28,SHLD2,TBX21,STX4,IL4R	grey	VSMC_M10
GO:0071679	0.79	0.42	5	11	commissural neuron axon guidance	BP	1	PTCH1,SMO,RYK,FZD3,DAG1	grey	VSMC_M10
GO:0071360	0.79	0.39	7	15	cellular response to exogenous dsRNA	BP	1	RALB,TMEM173,MAVS,IFIH1,MUL1,COLEC12,ZCCHC3	grey	VSMC_M10
GO:0030282	0.79	0.27	48	97	bone mineralization	BP	1	CYP27B1,OSR2,ATF4,ROR2,OMD,PTN,TRPM4,OSR1,SOX9,ERCC2,ACVR2B,WNT11,SLC24A3,ACVR2A,KL,ADGRV1,MEF2C,P2RX7,PTK2B,BMP2K,ANO6,TFAP2A,BMPR1B,FBXL15,SBDS,PTHLH,SGMS2,AXIN2,FGR,ACTN3,TXLNG,FZD9,PTH1R,SRGN,BCOR,ANKH,ZBTB40,WNT4,FGFR3,MMP13,BMPR1A,SMAD3,GPC3,RFLNB,BGLAP,MINPP1,SNX10,CCDC154	grey	VSMC_M10
GO:0090177	0.79	0.42	5	11	establishment of planar polarity involved in neural tube closure	BP	1	PTK7,DVL3,GRHL3,DVL2,WNT5A	grey	VSMC_M10
GO:0006878	0.79	0.42	5	11	cellular copper ion homeostasis	BP	1	SLC31A2,ATP7B,PRNP,ABCB6,SCO1	grey	VSMC_M10
GO:0003157	0.79	0.42	5	11	endocardium development	BP	1	STK3,OVOL2,NRG1,STK4,RBPJ	grey	VSMC_M10

GO:0000212	0.79	0.42	5	11	meiotic spindle organization	BP	1	WASHC5,MYH9,MOS,TUBG1,SEPT1	grey	VSMC_M10
GO:0010528	0.79	0.42	5	11	regulation of transposition	BP	1	TUT7,APOBEC3H,TUT4,SIRT7,TEX15	grey	VSMC_M10
GO:0010529	0.79	0.42	5	11	negative regulation of transposition	BP	1	TUT7,APOBEC3H,TUT4,SIRT7,TEX15	grey	VSMC_M10
GO:0060415	0.79	0.29	27	56	muscle tissue morphogenesis	BP	1	LY6E,FOXH1,TNNI3,POU4F1,NRG1,TNNC1,DSP,RYR2,PKP2,TNNI1,TGFB2,ADARB1,MYH7,ACTC1,EFEMP2,TTN,BMPR1A,TCAP,RBPJ,MED1,ISL1,ENG,NOG,WNT5A,NKX2-5,HEY2,WNT2	grey	VSMC_M10
GO:0061577	0.79	0.42	5	11	calcium ion transmembrane transport via high voltage-gated calcium channel	BP	1	CACNB2,NOS1AP,SESTD1,CACNB1,MIR21	grey	VSMC_M10
GO:1902043	0.79	0.42	5	11	positive regulation of extrinsic apoptotic signaling pathway via death domain receptors	BP	1	STK3,FAF1,STK4,BMPR1B,PEA15	grey	VSMC_M10
GO:0055091	0.79	0.42	5	11	phospholipid homeostasis	BP	1	ITGB6,GPAM,ABCB11,ABCA3,TLCD1	grey	VSMC_M10
GO:0045475	0.79	0.42	5	11	locomotor rhythm	BP	1	NCOA2,CIART,USP2,NCOR1,ZFHX3	grey	VSMC_M10
GO:0060560	0.79	0.25	100	200	developmental growth involved in morphogenesis	BP	1	NEDD4L,LRP1,SEMA6A,ESR1,RAB21,RIMS2,PRKN,FSTL4,PLXNA4,CPNE5,PTK7,SRF,SEMA4D,SEMA4C,RIMS1,RTN4,RUFY3,AREG,EPHA7,TNC,NRCAM,WASF1,SPRY1,PLXNA1,SOX9,GOLGA4,MEGF8,SPRY2,WNT11,CPNE9,TGFBR2,SYT1,SLC23A2,SEMA3D,SPART,SEMA3A,ULK1,RYK,TTL,FMN1,SEMA4F,DPYSL2,EIF2AK4,TIAM1,SLIT1,NKX6-1,ZFYVE27,BIN3,SEMA3G,SPP1,CYFIP1,ALCAM,SEMA4G,SALL1,NKD1,BDNF,KIAA0319,SEMA3F,AUTS2,ADNP,DISC1,CDKL5,WNT3,SPG11,MAPT,SEMA6D,ITGA4,TNR,FN1,NIN,FLRT1,PPP3CB,MUL1,VCL,MED1,SEMA4A,NLGN3,TRIM46,NDEL1,RAB11A,NTN1,DNM2,TBX2,LHX1,SIN3A,TRPV2,CDK5R1,SLC9A6,L1CAM,PRKCZ,ARHGAP4,PLXNA3,BARHL2,IMPACT,WNT5A,CACNG7,SIX1,LLPH,FGF1,OLFM1	grey	VSMC_M10
GO:0045579	0.79	0.42	5	11	positive regulation of B cell differentiation	BP	1	NCKAP1L,IL7,PPP2R3C,STAT5B,BTK	grey	VSMC_M10
GO:0043473	0.79	0.28	37	75	pigmentation	BP	1	SOX10,KIT,FIG4,BBS7,MYO7A,SZT2,MKKS,MITF,ANKRD27,MEF2C,HPS3,HPS5,AP3B1,ADAMTS20,VANGL1,DTNBP1,OCA2,KIF13A,SHROOM2,USP13,HPS4,MLPH,RAB17,PIKFYVE,BLOC1S6,MREG,ABCB6,LRMDA,CITED1,RAB11A,SNAPIN,SLC45A2,AP1G1,BLOC1S3,RAB32,BCL2L11,TRAPPC6A	grey	VSMC_M10
GO:2000104	0.79	0.40	6	13	negative regulation of DNA-dependent DNA replication	BP	1	ZRANB3,FBH1,DONSON,BCL6,TIPIN,LIG3	grey	VSMC_M10
GO:1902253	0.79	0.34	12	25	regulation of intrinsic apoptotic signaling pathway by p53 class mediator	BP	1	PRKN,ARMC10,TAF9,ING2,MARCH7,RPL11,MUC1,TAF9B,CD74,MIR21,UBB,PTTG1IP	grey	VSMC_M10
GO:0016973	0.79	0.37	8	17	poly(A)+ mRNA export from nucleus	BP	1	IWS1,DDX25,ENY2,NXF1,THOC2,DDX19A,SARNP,DDX19B	grey	VSMC_M10
GO:1903894	0.79	0.40	6	13	regulation of IRE1-mediated unfolded protein response	BP	1	BAK1,DDRGK1,FICD,HSPA5,DAB2IP,BCL2L11	grey	VSMC_M10
GO:0044154	0.79	0.41	5	11	histone H3-K14 acetylation	BP	1	BRD1,PHF14,ING5,KAT7,NAP1L2	grey	VSMC_M10
GO:0030262	0.79	0.34	11	23	apoptotic nuclear changes	BP	1	HSF1,DEDD2,FOXL2,ACVR1C,ACIN1,GPER1,DNASE2,PAM16,DFFB,BLCAP,NMNAT1	grey	VSMC_M10
GO:0039692	0.79	0.41	5	11	single stranded viral RNA replication via double stranded DNA intermediate	BP	1	APOBEC3H,CXCL8,FAM208A,INPP5K,SETDB1	grey	VSMC_M10
GO:1901657	0.79	0.29	30	60	glycosyl compound metabolic process	BP	1	FUCA2,AKR1B1,APOBEC3H,OARD1,MTAP,GNMT,GDA,ENPP4,PPAT,NT5E,PRTFDC1,CDADC1,AKR1A1,AHCY,CBR4,DGUOK,NME4,TYW1B,TYW5,PRPS2,LCMT2,DTYMK,UPB1,ADK,UPRT,GLA,PRPSAP2,ABHD10,PEMT,UPP1	grey	VSMC_M10
GO:0007190	0.79	0.39	6	13	activation of adenylate cyclase activity	BP	1	GIPR,RAF1,CAP2,CALM1,ADORA2B,CAP1	grey	VSMC_M10
GO:0002043	0.79	0.34	12	25	blood vessel endothelial cell proliferation involved in sprouting angiogenesis	BP	1	BMPER,ITGB1BP1,IL12A,PDCD10,NGFR,MIR21,MIR222,MIR23A,MIR27B,MIR24-1,MIR27A,ACVRL1	grey	VSMC_M10
GO:0072659	0.79	0.25	121	239	protein localization to plasma membrane	BP	1	LRP1,STX7,STAC3,MAP7,GOPC,PTCH1,TSPAN33,PRKCE,ASB3,SPTBN1,ITGB1BP1,EGFR,VPS35,RAB7A,RAB10,CACNB2,CLASP2,STAC,OPTN,GOLGA4,MYL12A,AKT2,IKBKB,ANK1,PRNP,BAG4,GAS6,PKP3,ARL6IP5,KCNIP3,LYPLA1,RILPL1,RILPL2,PIK3R1,TMEM150A,VAMP5,SLMAP,PLS1,FCHO2,SORBS1,P2RY1,RAPGEF2,KCNJ11,ZFYVE27,TESC,KIF13A,NHLRC1,NECTIN3,PKP2,PPFIA1,ZDHHC23,KCNB1,ANK2,C2CD5,GPER1,RAB11FIP2,FAM126B,CIB1,AP2M1,PPP2R5A,BBS1,GORASP2,LAMA5,PACS1,DLG1,LRRC15,NSF,EHD1,ZDHHC8,ABCA12,CNPY4,RAB3A,RAMP2,EXOC5,ATP1B1,PPIL2,NSG1,GRIPAP1,RAB8A,CNST,ACTB,RAB15,RSC1A1,MPP5,WNK3,STAC2,TSPAN17,ANXA2,GAK,STX4,TMEM88,RAB8B,ITGA3,AC138894.1,RAB11A,STX8,RAB3D,TTC7B,GGA2,ARHGAP44,PRAM1,EPB41L3,IFT20,INPP5K,PDPK1,PRKCZ,RAB26,RER1,ARHGEF16,SEC16A,PALM,BSG,ATP2C2,STXBP1,VPS4A,C16orf70,FYB1,PDZD3,LDLRAP1,VAMP3,MRAP2	grey	VSMC_M10
GO:0016114	0.79	0.41	5	11	terpenoid biosynthetic process	BP	1	PRMT3,ALDH1A3,GGPS1,DHRS9,ALDH1A2	grey	VSMC_M10
GO:0032637	0.79	0.29	28	57	interleukin-8 production	BP	1	HSPA1B,NOD1,ANXA4,TLR4,C5AR2,PRKD2,MYD88,ARRB1,MAVS,F2RL1,F2R,RIPK1,MAPKBP1,NOD2,TLR5,OTUD7B,STAT3,MAP2K5,CD74,NOS2,PARK7,LAMTOR5,BCL10,FADD,ZNF580,WNT5A,DDIT3,IL17D	grey	VSMC_M10
GO:0032201	0.79	0.44	4	9	telomere maintenance via semi-conservative replication	BP	1	WRN,RTEL1,UPF1,TERF2	grey	VSMC_M10
GO:0070307	0.79	0.41	5	11	lens fiber cell development	BP	1	FRS2,ATF4,TMOD1,BFSP1,TBC1D20	grey	VSMC_M10
GO:0010769	0.79	0.28	39	80	regulation of cell morphogenesis involved in differentiation	BP	1	RAB21,FBLN1,PTK2,ITGB1BP1,UNC13D,SS18L2,RAC3,ARHGEF7,PRKDC,ANKRD27,CUX2,TIAM1,DOCK5,OPA1,RREB1,ITPKA,DMTN,DNM1L,CARMIL1,CAPRIN2,AP1AR,CASS4,CIB1,SS18L1,CDKL5,DOCK1,MELTF,CSPG5,OBSL1,EPHA4,DNM2,PARP6,GPRASP2,BHLHB9,LRP8,CRK,FBXO31,FGA,CNTN2	grey	VSMC_M10
GO:0032905	0.79	0.41	5	11	transforming growth factor beta1 production	BP	1	TSKU,LUM,FOXP3,GATA6,NRROS	grey	VSMC_M10
GO:0002825	0.79	0.39	6	13	regulation of T-helper 1 type immune response	BP	1	RIPK2,HLX,PLA2G4A,SLC11A1,IL4R,HAVCR2	grey	VSMC_M10
GO:0002281	0.79	0.41	5	11	macrophage activation involved in immune response	BP	1	PRKCE,NMI,DYSF,TICAM1,HAVCR2	grey	VSMC_M10

GO:0008610	0.79	0.23	282	544	lipid biosynthetic process	BP	1	KDSR,ORMDL2,PIP4K2C,PEX7,B4GALNT1,CYP27B1,AVIL,LTC4S,SYNJ2,SERAC1,CYP21A2,AGPAT4,AGPAT1,QKI,PPARA,ACSL6,PNPLA3,TSPO,MCAT,HSD17B8,A4GALT,HSD17B3,AKR1B1,PLA2G6,GPAA1,HACD4,LARGE1,CWH43,PLPP6,SRD5A3,SPTLC1,HINT2,INPP4A,TMEM246,PIGF,MBOAT7,NR1H2,MBOAT2,LPIN1,INPP4B,DKKL1,SMPD2,FIG4,APOB,AGK,SPHK2,MTMR2,PIP4K2A,PRKD2,EBP,SELENOI,ABHD5,APOC1,PRKAG2,TAMM41,PIBF1,ITPKC,ACER3,ELOVL1,MTMR14,SPTLC3,PGAP2,CRLS1,CDS2,SIRT3,NR1H4,MTMR7,TRIB3,CYP7A1,ELOVL7,ASAH1,CYP7B1,ABHD6,THNSL2,CBR4,PIGU,PIK3CB,OSBP,PITPNM2,ST8SIA4,PIK3R1,TMEM150A,DKK3,PLCE1,FBXW7,SORBS1,CYP2R1,SLA2,PRKAG1,NR1H3,SERINC5,SYNJ1,COQ2,LPIN3,CDS1,FITM2,ORMDL1,HSD17B13,ITPKA,ERLIN1,PIGT,PRMT3,PRKAB1,SIRT4,GPLD1,SRD5A1,CYB5R1,A3GALT2,LPCAT1,SIK1,ALDH1A3,PGAP1,ZBTB20,GPAM,ACSL5,CHKA,ACP6,ABHD4,ST8SIA2,SGMS2,ALDH3B2,AJUBA,GGPS1,PITPNM1,SNAI1,ABHD2,MSMO1,MECR,B3GALT1,GPER1,SC5D,GNPAT,DGKD,B3GNT5,CERS6,PIGM,FITM1,ABCB11,DHRS9,BMX,HACD2,FAM126B,IP6K2,LPGAT1,CERS2,PIGV,DPAGT1,MTMR3,OSBPL2,PCYT1A,INPP5J,MLXIPL,PIGX,GAL3ST3,PIGZ,SCAP,HSD17B7,PISD,POR,DGKG,CREB1,PIKFYVE,PLA2G4A,OSBPL6,ACADL,MOGAT3,TM7SF2,SACM1L,OSBPL1A,ACSBG1,FDX1,PPM1L,PECR,LPCAT3,GAL3ST4,WNT4,PIGC,CYP27A1,PRKAG3,SMPD4,SOCS4,PLA2G16,PI4KA,SLC27A1,ABHD8,SGMS1,CH25H,DAGLB,SCCPDH,FADS3,LIAS,ACADVL,TECR,HSD17B10,CLN8,PGAP3,DEGS1,PIGH,PRPF19,MED1,PI4K2B,ALDH1A2,FGFR4,LIPC,SOCS7,DGKQ,OSBPL7,ALOXE3,ERG28,AC138894.1,HACD3,DGKE,TTC7B,LDLR,[illegible]	grey	VSMC_M10
GO:0090527	0.79	0.44	4	9	actin filament reorganization	BP	1	DMTN,CASS4,WHAMM,MCU	grey	VSMC_M10
GO:0048678	0.79	0.28	32	65	response to axon injury	BP	1	LRP1,TSPO,TXN2,SLC1A1,PTN,TNC,DHFR,GIPR,PTPRF,CHL1,DPYSL3,MORN4,SPP1,KIAA0319,MTR,GAP43,DAG1,KREMEN1,CERS2,KCNK2,LRIG2,APOD,TNR,EPHA4,MAX,NDEL1,MAP2K1,ISL1,MAPK8IP3,MIR222,CNTF,RTN4RL1	grey	VSMC_M10
GO:0046854	0.79	0.29	26	54	phosphatidylinositol phosphate biosynthetic process	BP	1	PIP4K2C,SYNJ2,INPP4A,INPP4B,FIG4,PIP4K2A,ITPKC,PIK3CB,PIK3R1,TMEM150A,SLA2,SYNJ1,ITPKA,FAM126B,IP6K2,PIKFYVE,SOCS4,PI4KA,PI4K2B,SOCS7,TTC7B,IMPA2,SOCS1,PIP5K1C,PIP5KL1,INPP1	grey	VSMC_M10
GO:0060174	0.79	0.44	4	9	limb bud formation	BP	1	SOX9,ZNF219,WNT3,SOX4	grey	VSMC_M10
GO:0032253	0.80	0.44	4	9	dense core granule localization	BP	1	KIF5A,TANC2,KIF1C,TRIM46	grey	VSMC_M10
GO:0099519	0.80	0.44	4	9	dense core granule cytoskeletal transport	BP	1	KIF5A,TANC2,KIF1C,TRIM46	grey	VSMC_M10
GO:1901950	0.80	0.44	4	9	dense core granule transport	BP	1	KIF5A,TANC2,KIF1C,TRIM46	grey	VSMC_M10
GO:1900103	0.80	0.41	5	11	positive regulation of endoplasmic reticulum unfolded protein response	BP	1	BAK1,PIK3R1,PTPN2,DAB2IP,BCL2L11	grey	VSMC_M10
GO:1903867	0.80	0.44	4	9	extraembryonic membrane development	BP	1	MAP3K4,PAXIP1,HTRA1,FZD5	grey	VSMC_M10
GO:0002295	0.80	0.41	5	11	T-helper cell lineage commitment	BP	1	TNFSF18,STAT3,TBX21,BATF,IL6R	grey	VSMC_M10
GO:1900227	0.80	0.43	4	9	positive regulation of NLRP3 inflammasome complex assembly	BP	1	ATAT1,TLR4,MYD88,DDX3X	grey	VSMC_M10
GO:0048266	0.80	0.41	5	11	behavioral response to pain	BP	1	TSPO,P2RX2,P2RX3,VWA1,TRPV1	grey	VSMC_M10
GO:0043094	0.80	0.34	11	23	cellular metabolic compound salvage	BP	1	MTAP,PRTFDC1,APIP,AMPD3,ENOPH1,AMPD1,ADK,UCK1,FUK,NAPRT,UPP1	grey	VSMC_M10
GO:0000737	0.80	0.35	10	21	DNA catabolic process, endonucleolytic	BP	1	HSF1,FBH1,FOXL2,DNASE2,PAM16,DNASE1,DFFB,DNASE1L1,DNASE1L2,NMNAT1	grey	VSMC_M10
GO:1904064	0.80	0.26	51	103	positive regulation of cation transmembrane transport	BP	1	STAC3,WNK2,CACNB2,STAC,GLRX,KCNJ11,IFNGR2,F2R,TESC,ANO6,KCNE2,PKD2,KCNIP2,RYR2,FAM173B,KCNJ2,ANK2,GPER1,AGT,ACE2,SLC9A1,CASQ1,NTSR1,NOS1AP,CEMIP,HTT,PINK1,ATP1B1,KCNA1,HAP1,WNK3,STAC2,ATP1B2,NLGN3,ATP2A1,CD19,CALM1,DNM2,VMP1,BDKRB1,CCL2,PDPK1,P2RX1,RNF207,AMIGO1,LCN2,MIR21,DMD,JPH2,KCNH2,THY1	grey	VSMC_M10
GO:0009158	0.80	0.39	6	13	ribonucleoside monophosphate catabolic process	BP	1	DPYS,GDA,NT5E,AMPD3,UPB1,UPP1	grey	VSMC_M10
GO:0006919	0.80	0.29	28	57	activation of cysteine-type endopeptidase activity involved in apoptotic process	BP	1	BAK1,SLC1A1,NOD1,CASP8AP2,TNFSF15,HSPE1,HIP1R,ROBO1,DIABLO,SENP1,NKX3-1,F2R,PIDD1,LCK,PDCD6,EGLN3,MAPT,CTSH,MUL1,BCL2L13,NGFR,SMAD3,S100A8,P2RX1,RPS27L,CASP8,ACER2,CRADD	grey	VSMC_M10
GO:1904925	0.80	0.39	6	13	positive regulation of autophagy of mitochondrion in response to mitochondrial depolarization	BP	1	PRKN,OPTN,PINK1,MUL1,MFN2,VPS13C	grey	VSMC_M10
GO:0009125	0.80	0.38	7	15	nucleoside monophosphate catabolic process	BP	1	DPYS,GDA,NT5E,AMPD3,UPB1,NT5M,UPP1	grey	VSMC_M10
GO:0003197	0.80	0.30	19	40	endocardial cushion development	BP	1	SOX9,TGFBR2,JAG1,ROBO1,ROBO2,TBX5,RBM24,TGFB2,SNAI1,BMPR1A,RBPJ,TMEM100,TBX2,ISL1,FOXF1,ENG,ACVRL1,NOG,HEY2	grey	VSMC_M10
GO:0032310	0.80	0.41	5	11	prostaglandin secretion	BP	1	ABCC4,MAP2K6,PLA2G4A,NOS2,PTGES	grey	VSMC_M10
GO:0044848	0.80	0.43	4	9	biological phase	BP	1	FERMT1,SYCP2L,TGFB2,WNT5A	grey	VSMC_M10
GO:0019216	0.80	0.24	128	251	regulation of lipid metabolic process	BP	1	ORMDL2,PIP4K2C,AGAP2,CYP27B1,AVIL,PPARA,TSPO,PTK2,GOLM1,PLA2G6,TTC39B,TEK,KIT,PRKCE,MBOAT7,FPR2,NR1H2,LPIN1,DKKL1,APOB,FGF21,SPHK2,MTMR2,PIP4K2A,SOX9,OPA3,ABHD5,RUBCNL,APOC1,DNAJC15,PRKAG2,SLC22A13,PIBF1,ALK,AKT2,NR1D2,PANK2,PDK1,SIRT3,FLT1,NR1H4,TRIB3,CYP7A1,ASAH1,ABHD6,DKK3,CHRM5,MTMR9,FBXW7,SORBS1,EPHX2,PTK2B,NR1H3,FITM2,ORMDL1,HSD17B13,ERLIN1,NOD2,PRMT3,LONP2,SIRT4,GPLD1,IRS1,LPCAT1,SIK1,CPT1A,ZBTB20,ADRA2A,FMO5,SNAI1,SORL1,GPER1,ABCB11,AGT,FGR,LPGAT1,CERS2,PPP2R5A,MTMR3,RUBCN,MLXIPL,PIK3IP1,SCAP,APOD,POR,PDK3,CREB1,ACADL,TYSND1,LPCAT3,GNB3,WNT4,FGFR3,SLC27A1,STAT5B,DAGLB,ACADVL,GPS2,THRA,FGFR4,DGKQ,CD19,LDLR,BCL11B,ASXL3,NCOR1,CD74,SREBF1,C3,MTMR1,ABCA3,ABCA7,VAV3,MLYCD,GFI1,DAB2IP,MIR27B,MIR27A,BSCL2,FABP5,LDLRAP1,CNEP1R1,C1QTNF2,STARD4,INSIG2,CCDC3,FGF1,DNAJC19	grey	VSMC_M10
GO:1903541	0.80	0.37	7	15	regulation of exosomal secretion	BP	1	VPS4B,PRKN,CHMP2A,SDCBP,RAB7A,ATP13A2,VPS4A	grey	VSMC_M10
GO:0060442	0.80	0.43	4	9	branching involved in prostate gland morphogenesis	BP	1	ESR1,FRS2,HOXA13,NKX3-1	grey	VSMC_M10

GO:0042278	0.80	0.34	11	23	purine nucleoside metabolic process	BP	1	OARD1,MTAP,GNMT,GDA,ENPP4,NT5E,PRTFDC1,AHCY,DGUOK,ADK,PEMT	grey	VSMC_M10
GO:0010506	0.80	0.24	136	268	regulation of autophagy	BP	1	PIP4K2C,EPM2A,PRKN,RNF5,TSPO,AL096711.2,EXOC4,MAPK15,NRBP2,TFEB,FBXO7,DCAF12,SPTLC1,GAPDH,ATP6V1E2,NOD1,RRAGD,MET,ATP6V1C2,VPS35,SCOC,FOXO3,MFSD8,SESN3,PIP4K2A,ATP6V0E2,FBXL2,PLK3,OPTN,RALB,ATP6V0B,KDM4A,SNX5,USP36,SIRT2,RPTOR,TMEM173,FEZ2,DCN,ULK1,ATP6V1H,TRIB3,PLK2,DEPP1,DRAM1,PIK3CB,CASP3,MTMR9,FBXW7,HTRA2,CSNK2A2,RIPK2,TPCN1,WDR41,TP53INP1,NOD2,LZTS1,ATP6V1B2,DNM1L,SEC22B,CISD2,TMEM39A,DHRSX,GPR137B,USP30,USP13,ATG16L1,SH3BP4,MCL1,BAG3,CISD1,MTMR3,RUBCN,IL10RA,SNX6,SNX32,FZD5,MAPT,TAB3,VPS26A,ABL2,TBC1D14,GPR137,HTT,ATP6V0A1,PINK1,STAT3,TBC1D25,PIM2,RAB8A,ATP13A2,PHF23,ATP6V1D,VPS13D,RRAGB,OSBPL7,VPS13C,DAPK2,MAPK3,AC138894.1,HAX1,LARP1,KEAP1,NPC1,RMC1,SMCR8,CDK5R1,SREBF1,LEPR,ERCC4,CLEC16A,TICAM1,MTM1,ATP6V0C,IKBKG,PARK7,GPSM1,GNAI3,KIAA1324,TSC1,SH3GLB1,MIR199A1,TRIM68,TRIM34,BNIP3L,TRIM65,IRGM,TIGAR,ACER2,DDIT3,DRAM2,CPTP,BCL2L11,HSPB8,CAPNS1,RNF152	grey	VSMC_M10
GO:1990440	0.80	0.41	5	11	positive regulation of transcription from RNA polymerase II promoter in response to endoplasmic reticulum stress	BP	1	ATF4,CREB3L3,HSPA5,DDIT3,CEBPB	grey	VSMC_M10
GO:0048638	0.80	0.24	130	257	regulation of developmental growth	BP	1	NEDD4L,LRP1,SEMA6A,GLI1,RAB21,STK3,RIMS2,PRKN,PPARA,FSTL4,PTCH1,PLXNA4,MAPK14,CPNE5,PI16,PIM1,SMO,SRF,SEMA4D,SEMA4C,RIMS1,RTN4,RUFY3,EPHA7,NRCAM,GOLGA4,MEGF8,CPNE9,TGFBR2,MKKS,SYT1,PLCB1,SLC23A2,DUSP6,SEMA3D,SPART,SEMA3A,ULK1,PRKDC,RYK,TTL,PLS1,MEF2C,SEMA4F,IL7,HTRA2,CELF1,TLL2,DPYSL2,NKX6-1,TBX5,ZFYVE27,FTO,SEMA3G,SPP1,STK4,CYFIP1,SEMA4G,NKD1,JARID2,IGF2,BDNF,KIAA0319,SLC6A3,SEMA3F,GPAM,HLX,ADNP,DISC1,ACTN3,KCNK2,CDKL5,CDKN1B,YBX3,WNT3,CREB1,DUSP10,MAPT,SEMA6D,TNR,FN1,SAV1,MYOZ1,GDF15,FGFR3,MUL1,STAT3,MAPK1,STAT5B,BMPR1A,RBPJ,SOX15,SEMA4A,TRIM46,NDEL1,WWC1,RAB11A,NTN1,DNM2,TBX2,YY1,SIN3A,TRPV2,INSR,GATA6,CDK5R1,RAI1,GHR,L1CAM,ARHGAP4,GAMT,PLXNA3,BARHL2,MIR199A1,MIR222,MIR25,NOG,WNT5A,BASP1,DIO3,POU3F2,FXN,CACNG7,SIX1,RGS4,PLAC8,BCL2L11,HEY2,OLFM1,WNT2	grey	VSMC_M10
GO:0030204	0.80	0.33	12	26	chondroitin sulfate metabolic process	BP	1	NDNF,CHPF2,CSGALNACT1,DCN,HYAL1,CHST3,SPOCK2,CHST7,CHST12,IDUA,B3GALT6,CHSY3	grey	VSMC_M10
GO:0060487	0.80	0.37	7	15	lung epithelial cell differentiation	BP	1	SOX9,FOXJ1,CREB1,SAV1,THRA,RBPJ,GATA6	grey	VSMC_M10
GO:0002315	0.80	0.43	4	9	marginal zone B cell differentiation	BP	1	MFNG,PTK2B,CDH17,DOCK10	grey	VSMC_M10
GO:0006875	0.80	0.23	195	381	cellular metal ion homeostasis	BP	1	SLC39A5,NEDD4L,LRP1,ESR1,SLC12A2,SMDT1,BAK1,MYC,MCHR1,GPR20,PLA2G6,TMPRSS6,SLC39A4,STEAP2,SLC25A27,SLC1A1,S1PR3,PRKCE,TNNI3,FPR2,IBTK,LPAR1,SLC31A2,KEL,ATG5,TRPM4,GRIN2D,TRPC3,SLC8A2,C5AR2,C5AR1,HEPHL1,ATP7B,PTGIR,GIPR,CACNB2,SLC39A12,ATP1A3,ACKR2,SLC24A3,C1QTNF1,TMTC4,RYR1,LETM2,PRNP,ADRA1D,DIAPH1,P2RX2,CCKBR,HPX,RASA3,NCOA4,MICU3,IL1A,IMMT,C19orf12,P2RX3,PLCE1,RYR3,TMBIM6,P2RX7,P2RY1,PTK2B,SLC39A13,TPCN1,SLC8B1,VDR,STC1,AP3B1,RMDN3,F2RL1,F2R,SLC40A1,PKD2,CASQ2,MCUR1,CD40,RYR2,SLC41A1,RAP1GDS1,TRPM1,SPPL3,LCK,KCNJ2,TESMIN,TRPV4,SLC30A10,TCIRG1,GNA13,ISCU,CMKLR1,MCUB,HJV,UBASH3B,DISC1,ANK2,GPER1,TRPM8,PDZD8,AGT,ATP1A2,SLC9A1,CASQ1,NTSR1,FZD9,TMCO1,PTH1R,SLC30A4,FIS1,CEMIP,ABL2,CDH23,PTPN6,MICU1,MCU,CXCR2,GNG3,SLC11A1,HTT,PTGDR,PTGER2,ABCB6,TRPM7,ATP1B1,CD38,KCNMA1,ATP13A2,FTH1,HAP1,DLG4,CYB561A3,ATP1B2,FAM155B,PSEN1,CACNA1A,AC138894.1,ATP2A1,CD19,SMAD3,NOX5,CALM1,S100A8,PRKCB,BDKRB1,NMUR2,SLC39A6,TRPV2,HRH4,CCL14,CCL5,CCL7,C7,PTGER4,SLC25A23,MAIP1,TMEM199,SLC46A1,NUBP1,GNB1,ATP2B3,BCAP31,GNA15,TRPV3,TRPV1,PDPK1,P2RX1,PKD1,TMEM203,ATP6AP1,GPR157,SYPL2,CYBA,JPH3,ATP2C2,SLC30A7,FRRS1,LCN2,MCOLN3,PTGFR,MIR199A1,KCNK3,DMD,MT1X,GPR35,NOL3,CHRNA10,CCR10,JPH2,SLC22A17,THY1,WNT5A,FXN,FTL,DDIT3,SCO1	grey	VSMC_M10
GO:0043031	0.80	0.40	5	11	negative regulation of macrophage activation	BP	1	CST7,NR1H3,CD200,SYT11,LDLR	grey	VSMC_M10
GO:0010561	0.80	0.40	5	11	negative regulation of glycoprotein biosynthetic process	BP	1	AGO2,HBEGF,SLC2A10,ABCA7,ACER2	grey	VSMC_M10
GO:0022602	0.80	0.31	15	32	ovulation cycle process	BP	1	ESR1,GDF9,FOXO3,ARRB1,CASP3,ROBO2,ADAMTS1,BMPR1B,GAS2,MAP2K6,TGFB2,PTPRN,STAT5B,HSPA5,EREG	grey	VSMC_M10
GO:0071526	0.80	0.30	18	38	semaphorin-plexin signaling pathway	BP	1	SEMA6A,PLXNA4,SEMA4D,SEMA4C,MET,PLXNA1,SEMA3D,PLXNC1,SEMA3A,HAND2,SEMA4F,SEMA3G,SEMA4G,SEMA3F,FARP2,SEMA6D,SEMA4A,PLXNA3	grey	VSMC_M10
GO:0045471	0.80	0.27	43	87	response to ethanol	BP	1	FECH,PPARA,BAK1,USP46,CAT,PRKCE,TNC,SETD7,HDAC2,DNMT3A,SLC23A2,CYP7A1,HPGD,PTK2B,TP53INP1,ADCY7,SLC6A3,CCND1,CPT1A,CBL,ACTC1,EPS8,MGMT,STAT3,SLC2A4,CHRNB2,S100A8,CCL7,SREBF1,OXCT1,ABAT,SDF4,NQO1,MT-CYB,RHOA,SETDB1,CASP8,BGLAP,PEMT,CLDN1,EEF1B2,RGS4,CRH	grey	VSMC_M10
GO:0030500	0.80	0.28	30	62	regulation of bone mineralization	BP	1	CYP27B1,OSR2,OMD,PTN,TRPM4,OSR1,SOX9,ACVR2B,ACVR2A,KL,ADGRV1,MEF2C,P2RX7,PTK2B,BMP2K,ANO6,TFAP2A,BMPR1B,SGMS2,ACTN3,TXLNG,FZD9,SRGN,BCOR,ANKH,WNT4,BMPR1A,SMAD3,RFLNB,BGLAP	grey	VSMC_M10
GO:0097049	0.80	0.43	4	9	motor neuron apoptotic process	BP	1	SLC1A1,ADARB1,KCNB1,FADD	grey	VSMC_M10

GO:1902513	0.80	0.43	4	9	regulation of organelle transport along microtubule	BP	1	CNIH2,HAP1,TRIM46,BORCS5	grey	VSMC_M10
GO:0043587	0.80	0.43	4	9	tongue morphogenesis	BP	1	INTU,HDAC2,HDAC1,SIX1	grey	VSMC_M10
GO:0021696	0.80	0.32	13	28	cerebellar cortex morphogenesis	BP	1	GLI1,AGTPBP1,SMO,WHRN,GLI2,FAIM2,LDB1,TTC21B,SPTBN2,MAP2K1,LHX1,GRID2,CEND1	grey	VSMC_M10
GO:0051640	0.80	0.23	229	447	organelle localization	BP	1	VPS4B,MYO1A,STX7,AP3S1,KIF5A,PRKN,KIF3A,UBE2B,FAM91A1,TSNARE1,EXOC4,MAPK15,AGTPBP1,SUN2,COPG2,RAC2,TFEB,MYH9,PEX1,SCFD2,KIT,BICD2,CHMP2A,NLRP5,LRPPRC,STX17,BRSK1,ALB,XPO1,TRAPPC12,PLEK,YWHAZ,CDK5RAP2,BVES,STARD3NL,CCDC155,MGARP,PPFIA3,WASF1,SPHK2,SPRY1,BBS7,KIF3C,ARHGAP21,RAB10,COPG1,MYO7B,SYTL2,CLASP2,NECTIN2,GAB2,MLH1,LLGL2,RALB,UNC13D,PIBF1,MYO7A,SPRY2,HOOK3,PIP4P1,AP3M2,KIF5C,MKKS,SYT1,TMEM106B,LIN7A,CEP290,TMEM230,CHAMP1,STX2,NSFL1C,SNPH,MOS,TTL,KIF3B,ARMC1,FER,CHMP2B,CHMP4C,SDAD1,MCPH1,KATNB1,RASGRP1,ARFGAP2,SLIT1,EXOC6B,SYNJ1,OPA1,AP3B1,CHMP7,VPS18,DTNBP1,KIF13A,GBF1,BICDL1,KLHL12,DNM1L,AC112128.1,TRIP11,PEF1,TCIRG1,PDCD6,SNAP23,MARK1,MFN1,CFL1,KCNB1,SHROOM2,AP1AR,PDZD8,LAT2,SPO11,FGR,CBL,NOP9,MLPH,RAB17,STX1A,SNX4,CNIH2,STX6,KLC2,LIMK2,TANC2,DLG1,NSF,TBCCD1,SPG11,MAPT,BLOC1S6,MAJIN,ITGA4,MOSPD3,MAP4K2,MREG,PDCD10,HTT,PTGDR,KXD1,RAB3A,MARK2,PINK1,IFFO1,MUL1,SYN1,UXT,SNAP29,AP3M1,C12orf4,BHLHA15,STX5,KIF1C,RAB8A,SYNE2,HAP1,PSEN2,SLC2A4,RAB15,NLGN2,MFN2,SEPT1,KPNB1,STX1B,STX4,PEX14,PSEN1,SYT11,MSTO1,RAB8B,BORCS6,TMED10,TRIM46,NDEL1,AC138894.1,ATP2A1,RAB11A,LAT,NTN1,STX8,RAB3D,IL4R,CALM1,SYTL4,SNAPIN,S100A13,DNM2,VMP1,BTK,CLMN,AP1M2,STXBP2,MYO1D,C17orf75,PPP6C,MYO1F,DOCK7,DAB1,MYO5B,MTM1,TLE6,CLUH,BICDL2,PDPK1,PRKCZ,SAPCD2,ATP6AP1,IKBKG,TPGS1,DEF8,SPIRE2,CHMP1A,PSRC1,GPSM2,FOXF1,SPOUT1,STXBP1,FNBP1L,VPS4A,RIPOR1,FHOD1,UBB,BLOC1S3,O[illegible]	grey	VSMC_M10
GO:0060390	0.80	0.32	13	28	regulation of SMAD protein signal transduction	BP	1	LRP1,SPTBN1,OVOL2,RBPMS,VEPH1,KIAA0319,SLC2A10,PBLD,BMPR1A,PARP1,CILP,TOB1,SH2B1	grey	VSMC_M10
GO:0098927	0.80	0.30	19	40	vesicle-mediated transport between endosomal compartments	BP	1	RAB21,WDR91,MTMR2,RAB7A,HOOK3,RAB5A,DNAJC13,FAM160A2,ANKRD27,SORL1,MAPK1,SNX12,MAPK3,HOOK2,MAP2K1,STX8,MYO1D,RILP,SNX16	grey	VSMC_M10
GO:0030730	0.80	0.38	6	13	sequestering of triglyceride	BP	1	PPARA,PLIN2,ABHD5,FITM2,FITM1,PLIN3	grey	VSMC_M10
GO:0030901	0.80	0.27	34	69	midbrain development	BP	1	FZD6,UQCRQ,CSNK1E,CKB,TTBK1,FZD1,KDM7A,SIRT2,RYK,FZD3,NDUFS3,NR4A2,SUDS3,COX6B1,INA,WNT3,CXCR2,PADI2,ACTB,TAF1,CALM1,SEC16A,ATP5PB,SYPL2,MAPKAP1,HSPA5,RHOA,WNT5A,BASP1,OTX1,YWHAH,PITX3,TAL2,WNT2	grey	VSMC_M10
GO:0045778	0.80	0.30	20	42	positive regulation of ossification	BP	1	OSR2,TOB2,PTN,OSR1,ACVR2B,ACVR2A,KL,ADGRV1,MEF2C,P2RX7,ANO6,TFAP2A,BMPR1B,TGFB2,ACTN3,FZD9,WNT4,BMPR1A,SMAD3,WNT5A	grey	VSMC_M10
GO:0042310	0.80	0.28	30	62	vasoconstriction	BP	1	ZDHHC21,EGFR,KEL,TRPM4,MKKS,ADRA1D,ADRA2B,HTR7,DOCK5,F2R,CHRM3,RAP1GDS1,ADRA2A,AGT,ACE2,ATP1A2,PER2,ADRA2C,ECE1,SMTNL2,CD38,HRH2,SCNN1B,ASIC2,P2RX1,MIR21,RHOA,HTR1D,FGA,KCNMB4	grey	VSMC_M10
GO:0072078	0.80	0.28	29	60	nephron tubule morphogenesis	BP	1	MYC,KLHL3,PTCH1,SMO,SIX2,OSR1,SOX9,GZF1,WNT11,PAX8,EYA1,FMN1,PKD2,PAX2,SALL1,IRX2,AGT,LAMA5,DLG1,WNT4,MAGED1,CITED1,LHX1,GPC3,PKD1,SOX8,NOG,SIX1,FGF1	grey	VSMC_M10
GO:0030217	0.80	0.24	90	179	T cell differentiation	BP	1	HLA-DRA,TCF7,SRF,KIT,SLC46A2,RSAD2,IL4I1,IL15,ATG5,TRAF3IP2,BMI1,CLPTM1,GLI2,FOXJ1,TGFBR2,EOMES,NCKAP1L,FOXP1,PRKDC,SOX12,TOX,ZC3H8,IL1A,CAMK4,TMEM131L,IL7,RASGRP1,LMBR1L,RIPK2,AP3B1,SOX13,PKNOX1,LCK,TCIRG1,RABL3,HLX,FZD7,CD28,FCER1G,RUNX3,PATZ1,SP3,ADAM8,FZD5,DUSP10,BCL6,VSIR,RC3H1,TNFSF18,WNT4,LAG3,PPP3CB,STAT3,STAT5B,FOXP3,ZBTB1,CARD11,TBX21,SEMA4A,DOCK2,BATF,ZBTB7B,IL6R,IL4R,BCL11B,CD74,JAG2,NKAP,VAV1,LEPR,PTGER4,IL7R,PTPN2,SOCS1,DROSHA,DNAJA3,PRKCZ,TSC1,MIR21,RHOA,SOX4,MAFB,FADD,CD46,FGL2,TNFSF9,NHEJ1,ZFP36L2,PRR7,RC3H2	grey	VSMC_M10
GO:0009394	0.80	0.30	17	35	2'-deoxyribonucleotide metabolic process	BP	1	TBPL1,DPYS,GDA,NUDT15,MBD4,NUDT16,DGUOK,NUDT18,DTYMK,UPB1,ADK,NEIL1,NT5M,NTHL1,CMPK2,DCTD,UPP1	grey	VSMC_M10
GO:2000736	0.80	0.29	24	50	regulation of stem cell differentiation	BP	1	OCIAD1,PTN,TEAD2,SOX9,JAG1,EIF2AK2,PRKDC,TCF15,SOX6,TBX5,PRICKLE1,RBM24,TGFB2,YTHDF2,OSM,NFE2L2,STAT3,CDK12,SETD1A,ESRRB,GATA6,SP7,ZFP36L2,NKX2-5	grey	VSMC_M10
GO:0001941	0.80	0.31	16	34	postsynaptic membrane organization	BP	1	LRRC4,PTN,SLC7A11,NLGN4Y,SSH1,FZD9,NRXN2,DLG4,NLGN2,GPHN,CHRNB1,NLGN3,DNAJA3,RER1,CEL,MESD	grey	VSMC_M10
GO:0060837	0.80	0.43	4	9	blood vessel endothelial cell differentiation	BP	1	RBPJ,TMEM100,FOXJ2,HEY2	grey	VSMC_M10
GO:0046823	0.80	0.35	9	19	negative regulation of nucleocytoplasmic transport	BP	1	RAB23,FERMT1,PKIA,SUFU,CABP1,SP100,APOD,PARK7,TXN	grey	VSMC_M10
GO:0014066	0.80	0.26	42	86	regulation of phosphatidylinositol 3-kinase signaling	BP	1	ERBB3,RGL2,PTK2,NTRK2,TEK,KIT,ROR2,SEMA4D,CAT,EGFR,SOX9,FBXL2,DCN,FLT1,RASGRP1,NKX3-1,F2RL1,F2R,TGFB2,NCF1,EGF,HCLS1,GPER1,AGT,FGR,CBL,OSM,CD28,PIK3IP1,FN1,PTPN6,HAX1,INSR,CCL5,PIP5K1C,DAB2IP,MIR21,ENTPD5,STAMBP,C3orf58,HCST,PDGFD	grey	VSMC_M10

GO:0050708	0.80	0.24	101	200	regulation of protein secretion	BP	1	LRP1,SLC12A2,PRKN,PLA2G6,PICK1,RFX3,TFAP2B,PRKCE,HMGN3,BMP8A,RSAD2,NR1H2,EGFR,TLR4,TRPM4,GIPR,FRMD4A,LLGL2,APBB3,DPH3,SIRT3,SYT9,NR1H4,NDUFAF2,FAM3D,IL1A,OSBP,ARNTL,VEGFC,KCNJ11,NNAT,NR1H3,TIAM1,RAB11FIP5,PFKM,SLC8B1,NKX6-1,ACVR1C,F2RL1,F2R,ZBED6,ADTRP,SIRT4,GPLD1,IRS1,CD200,DNM1L,HDAC1,PPP3CA,CPT1A,SLC9B2,TGFB2,ADRA2A,TCIRG1,TCF7L2,KCNB1,PFKFB2,GPER1,PER2,STX1A,GLUL,SIDT2,SLC16A1,ADAM8,KLF7,TTN,IL12A,ENY2,ABCA12,MCU,PPP3CB,CD38,MPC2,ATP13A2,SYT7,NLGN2,TARDBP,STX4,TMED10,MYH10,SYTL4,GPR68,CCL5,SREBF1,ISL1,OXCT1,NOS2,ABAT,RPH3AL,NADK,BAIAP3,LRRC8A,MIR199A1,FGA,CHGA,SOX4,DPP4,CRH,DOC2B,UQCC2,PARD6A	grey	VSMC_M10
GO:0045064	0.80	0.40	5	11	T-helper 2 cell differentiation	BP	1	HLX,BCL6,BATF,IL4R,PRKCZ	grey	VSMC_M10
GO:0071692	0.80	0.23	138	271	protein localization to extracellular region	BP	1	LRP1,CANX,SLC12A2,RIMS2,PRKN,PLA2G6,COPG2,PICK1,RFX3,TFAP2B,SVBP,PRKCE,HMGN3,CYB5R4,BMP8A,PLEK,RSAD2,NR1H2,EGFR,TLR4,TRPM4,RAB10,COPG1,GIPR,FRMD4A,LLGL2,STEAP3,RAF1,APBB3,DPH3,SIRT3,SYT9,NR1H4,NDUFAF2,FAM3D,IL1A,OSBP,ARNTL,VEGFC,KCNJ11,NNAT,NR1H3,TIAM1,RAB11FIP5,PFKM,SLC8B1,NKX6-1,ACVR1C,F2RL1,F2R,ZBED6,DMTN,ADTRP,SIRT4,GPLD1,IRS1,CD200,DNM1L,HDAC1,PPP3CA,CPT1A,SLC9B2,TGFB2,ADRA2A,MON1A,TCIRG1,TCF7L2,KCNB1,PFKFB2,GPER1,RAB11FIP2,PER2,STX1A,GLUL,SIDT2,SLC16A1,ADAM8,KLF7,MIA3,BLOC1S6,CTAGE9,VGF,TTN,IL12A,TANGO2,ENY2,ABCA12,MCU,FBN1,PPP3CB,RAB3A,CAMK2G,PTPRN,CD38,MPC2,ILDR2,RAB8A,ATP13A2,SYT7,RAB15,NLGN2,CPLX1,TARDBP,STX4,RAB8B,TMED10,UBE2Q1,MYH10,SEL1L,RAB3D,SYTL4,GPR68,TVP23C,CCL5,SREBF1,ISL1,OXCT1,NOS2,TVP23A,ABAT,RPH3AL,NADK,BAIAP3,PARK7,LRRC8A,TANGO6,MIR199A1,FGA,BLOC1S3,CHGA,SOX4,DPP4,TMEM167A,CRH,NRROS,DOC2B,UQCC2,PARD6A	grey	VSMC_M10
GO:0072088	0.80	0.27	30	62	nephron epithelium morphogenesis	BP	1	MYC,KLHL3,PTCH1,SMO,SIX2,OSR1,SOX9,GZF1,WNT11,PAX8,EYA1,FMN1,PKD2,PAX2,SALL1,IRX2,AGT,LAMA5,DLG1,WNT4,MAGED1,CITED1,LHX1,GPC3,PKD1,SOX8,NOG,SIX1,LIF,FGF1	grey	VSMC_M10
GO:2000009	0.80	0.42	4	9	negative regulation of protein localization to cell surface	BP	1	NEDD4L,GOPC,TAX1BP3,LEPROT	grey	VSMC_M10
GO:0015749	0.80	0.26	41	84	monosaccharide transmembrane transport	BP	1	SLC2A12,MAPK14,SLC25A27,GRB10,FGF21,BRAF,MFSD4B,SLC26A5,PRKAG2,AKT2,ASPSCR1,SLC23A2,TRIB3,PIK3R1,SORBS1,IRS1,SLC2A10,TERT,C2CD5,PEA15,NFE2L2,CLTCL1,SLC2A2,SLC27A1,SLC2A9,SLC2A4,RSC1A1,SLC5A2,SLC50A1,SLC5A11,PRKCB,INSR,YES1,C3,GPC3,INPP5K,SLC45A1,SLC2A6,TSC1,FABP5,ARPP19	grey	VSMC_M10
GO:0033148	0.80	0.42	4	9	positive regulation of intracellular estrogen receptor signaling pathway	BP	1	KMT2D,PARP1,MED1,PAGR1	grey	VSMC_M10
GO:0072203	0.80	0.42	4	9	cell proliferation involved in metanephros development	BP	1	MYC,PTCH1,OSR1,GPC3	grey	VSMC_M10
GO:0044650	0.80	0.42	4	9	adhesion of symbiont to host cell	BP	1	NECTIN2,GAS6,ACE2,LRRC15	grey	VSMC_M10
GO:2001169	0.80	0.37	7	15	regulation of ATP biosynthetic process	BP	1	PPARA,SPHK2,FAM173B,DNAJC30,PINK1,STAT3,PARP1	grey	VSMC_M10
GO:0046686	0.80	0.29	23	47	response to cadmium ion	BP	1	HSF1,KIT,CAT,MTF1,EGFR,ERCC1,PRNP,CYB5A,GPI,GSS,TERT,NCF1,DTYMK,CDKN1B,SORD,ABCB6,MAPK1,FOS,MAPK3,NPC1,JUN,MT-CYB,MT1X	grey	VSMC_M10
GO:0097084	0.80	0.42	4	9	vascular associated smooth muscle cell development	BP	1	EFEMP2,RAMP2,ENG,HEY2	grey	VSMC_M10
GO:0002292	0.80	0.28	24	50	T cell differentiation involved in immune response	BP	1	HLA-DRA,EOMES,FOXP1,RIPK2,HLX,FCER1G,BCL6,RC3H1,TNFSF18,STAT3,FOXP3,TBX21,SEMA4A,BATF,ZBTB7B,IL6R,IL4R,PTGER4,PRKCZ,TSC1,MIR21,CD46,FGL2,RC3H2	grey	VSMC_M10
GO:1904753	0.80	0.40	5	11	negative regulation of vascular associated smooth muscle cell migration	BP	1	MEF2C,GNA13,NFE2L2,MIR21,RHOA	grey	VSMC_M10
GO:0046470	0.80	0.28	27	55	phosphatidylcholine metabolic process	BP	1	PLA2G6,TMEM86B,MBOAT7,NR1H2,MBOAT2,APOC1,NR1H3,CDS1,GPLD1,LPCAT1,CHKA,ABHD4,PCYT1A,PLA2G4A,LPCAT3,PLA2G16,SLC27A1,LIPC,GDPD3,LDLR,ABHD3,PNPLA6,ABCA3,MECP2,PLA2G15,FABP5,PEMT	grey	VSMC_M10
GO:0060674	0.80	0.32	12	26	placenta blood vessel development	BP	1	OVOL2,SPINT1,PKD2,VASH2,FZD5,MAPK1,RBPJ,VASH1,MAP2K1,PKD1,HEY2,WNT2	grey	VSMC_M10
GO:0035019	0.80	0.31	15	32	somatic stem cell population maintenance	BP	1	MYC,KIT,SIX2,SOX9,RAF1,PAX8,PAX2,LDB1,SOX2,VPS72,RBPJ,SKI,SOX4,NOG,ZFP36L2	grey	VSMC_M10
GO:0032616	0.80	0.40	5	11	interleukin-13 production	BP	1	TNFRSF21,SPHK2,SCGB1A1,PRKCZ,TSLP	grey	VSMC_M10
GO:0032656	0.80	0.40	5	11	regulation of interleukin-13 production	BP	1	TNFRSF21,SPHK2,SCGB1A1,PRKCZ,TSLP	grey	VSMC_M10
GO:0034219	0.80	0.26	42	86	carbohydrate transmembrane transport	BP	1	SLC2A12,MAPK14,SLC25A27,GRB10,FGF21,BRAF,MFSD4B,SLC26A5,PRKAG2,AKT2,ASPSCR1,SLC23A2,TRIB3,PIK3R1,SORBS1,TMEM144,IRS1,SLC2A10,TERT,C2CD5,PEA15,NFE2L2,CLTCL1,SLC2A2,SLC27A1,SLC2A9,SLC2A4,RSC1A1,SLC5A2,SLC50A1,SLC5A11,PRKCB,INSR,YES1,C3,GPC3,INPP5K,SLC45A1,SLC2A6,TSC1,FABP5,ARPP19	grey	VSMC_M10

GO:0050767	0.80	0.23	144	285	regulation of neurogenesis	BP	1	LRP1,SEMA6A,RAB21,SNW1,TIAM2,TSPO,FSTL4,SYNGAP1,PLXNA4,NTRK2,SOX10,SMO,DAAM2,SRF,TTBK1,KIT,SEMA4D,SEMA4C,RTN4,RUFY3,EPHA7,PTN,EGFR,TRIM32,MYCN,PLXNA1,HDAC2,GOLGA4,MEGF8,KDM4A,XRCC2,NUMBL,POU4F1,SS18L2,HOOK3,BHLHE40,SIRT2,SEMA3D,SPART,PLXNC1,SEMA3A,ULK1,RYK,PLAG1,ANKRD27,FAIM,ROBO1,ROBO2,ARNTL,FZD3,SEMA4F,VEGFC,TLX2,CUX2,MME,RAPGEF2,TIAM1,SYNJ1,OPA1,NKX6-1,ZFYVE27,SPINT1,SEMA3G,ITPKA,SPP1,CYFIP1,SEMA4G,ZNF335,BDNF,RNF10,KIAA0319,NEURL1,DNM1L,HDAC1,PPP3CA,CAPRIN2,SEMA3F,DAG1,ADNP,YTHDF2,DISC1,SORL1,GPER1,CERS2,NFATC4,HES6,PER2,SS18L1,CDKL5,DLX1,WNT3,DUSP10,MAPT,SEMA6D,TNR,FN1,XRCC5,NIN,OBSL1,HAP1,EPHA4,BMPR1A,SPEN,TNFRSF1B,RGS14,PRPF19,PSEN1,SEMA4A,TRIM46,NDEL1,RAB11A,MAP2K1,NTN1,LDLR,PARP6,GPRASP2,BHLHB9,TRPV2,CDK5R1,DOCK7,MBD1,DAB1,LRP8,L1CAM,SKI,ARHGAP4,PLXNA3,SOX8,AMIGO1,FBXO31,MIR222,MIR125B1,FOXG1,GPR37L1,HOXB3,NOG,THY1,WNT5A,LIF,YWHAH,EFNB3,PITX3,HEY2,WNT2	grey	VSMC_M10
GO:0010637	0.80	0.42	4	9	negative regulation of mitochondrial fusion	BP	1	PRKN,DNM1L,MUL1,ADCK1	grey	VSMC_M10
GO:0002521	0.80	0.23	196	383	leukocyte differentiation	BP	1	NME1,AGER,HLA-DRA,IL5,TCF7,BAK1,XRCC6,TRIB1,MYC,TOB2,L3MBTL3,MAPK14,MFNG,HSF1,MYH9,FBXO7,SRF,CDK6,KIT,ROR2,NFIL3,IL11,MSH2,CD109,HOXA7,SLC46A2,RSAD2,IL4I1,TLR4,IL15,ATG5,OSTM1,TRAF3IP2,BMI1,CLPTM1,GAB2,GLI2,FOXJ1,POU4F1,IL15RA,KLF6,CMTM7,TGFBR2,EOMES,PLCL2,NCKAP1L,GAS6,FOXP1,MITF,PRKDC,IRF7,TRAF6,SOX12,TOX,ZC3H8,IL1A,CAMK4,CEBPA,PIK3R1,FBXW7,PTPRJ,TMEM131L,IL7,TPD52,PTK2B,RASGRP1,LMBR1L,RIPK2,CDH17,AP3B1,F2RL1,TESC,RIPK1,SOX13,TSPAN2,ITGB6,DCAF1,DOCK10,SH3PXD2A,PKNOX1,LRRK1,LCK,SLC9B2,TCIRG1,GPR137B,RABL3,PIAS3,HLX,ACIN1,TCTA,HCLS1,UBASH3B,SLAMF8,FZD7,HDAC4,CD28,FCER1G,RUNX3,FARP2,ZBTB46,PATZ1,FZD9,SP3,PPP2R3C,ADAM8,CREB1,FZD5,DUSP10,BCL6,SYVN1,BATF2,ITGA4,VSIR,RC3H1,ESRRA,PTPN6,GPR137,FBN1,TNFSF18,WNT4,LAG3,PPP3CB,LTBR,STAT3,STAT5B,INHA,TFE3,FOXP3,ZBTB1,PARP1,GPS2,CARD11,RBPJ,MED1,ANXA2,TBX21,PSEN1,SEMA4A,KAT7,DOCK2,FOS,BATF,ZBTB7B,IL6R,HAX1,CD19,IL4R,GPR68,BTK,BCL11B,YY1,CD74,CSF1R,PPARGC1B,JAG2,NKAP,VAV1,LEPR,PTGER4,EVI2B,IL7R,PTPN2,GPC3,JUN,SOCS1,DROSHA,DNAJA3,PRKCZ,ATP6AP1,TSC1,LRRC8A,MIR21,MIR125B1,RHOA,FSTL3,SOX4,MAFB,FADD,CD46,CASP8,CA2,DHRS2,BGLAP,SNX10,GLO1,FGL2,NRROS,TNFSF9,NHEJ1,LIF,ZFP36L2,CEBPB,PRR7,TNFSF13B,BATF3,RC3H2	grey	VSMC_M10
GO:0060675	0.80	0.28	25	52	ureteric bud morphogenesis	BP	1	MYC,PTCH1,SMO,SIX2,SOX9,GZF1,WNT11,PAX8,EYA1,FMN1,PKD2,PAX2,SALL1,AGT,LAMA5,DLG1,WNT4,MAGED1,CITED1,LHX1,GPC3,SOX8,NOG,SIX1,FGF1	grey	VSMC_M10
GO:0032940	0.80	0.22	296	574	secretion by cell	BP	1	VPS4B,LRP1,CANX,RAB21,SNCAIP,SLC12A2,RIMS2,PRKN,GDF9,GRM4,LY6E,EXOC4,SYNGR1,NTRK2,PLA2G6,COPG2,PICK1,RAC2,MYH9,CHRNA3,CHRNA5,CRY1,STEAP2,APBA1,SCFD2,RFX3,KIT,TFAP2B,CHMP2A,NLRP5,SVBP,STX17,IL11,BRSK1,PRKCE,RIMS1,HMGN3,RIMS3,CYB5R4,PPT1,SCRN1,BMP8A,PLEK,RSAD2,NR1H2,EGFR,TLR4,VPS41,SDCBP,TRPM4,PPFIA3,LIN7B,SPHK2,NAPA,RAB7A,RAB10,COPG1,GIPR,CACNB2,SYTL2,CLASP2,FRMD4A,GAB2,LLGL2,RALB,STEAP3,GRIK5,UNC13D,RAF1,NAPB,ABCC4,C1QTNF1,ANK1,SYT1,RAB5A,LIN7A,APBB3,DPH3,AIMP1,NCKAP1L,SIRT3,STX2,SYT9,ADRA2B,SNPH,NR1H4,HCK,NDUFAF2,FAM3D,IL1A,PAX8,NR4A1,FER,OSBP,TRAPPC11,ARNTL,ERC2,SCG5,MEF2C,ASIC1,VEGFC,P2RY1,RASGRP1,KCNJ11,NNAT,NR1H3,TIAM1,RAB11FIP5,EXOC6B,PFKM,SYNJ1,SLC8B1,NKX3-1,NKX6-1,ACVR1C,F2RL1,F2R,CRY2,VPS18,SPP1,ZBED6,LGI3,DMTN,ADTRP,DTNBP1,SYT8,GRM2,SIRT4,GPLD1,IRS1,CD200,DNM1L,HDAC1,PPP3CA,HYAL3,CPT1A,MAP2K6,TRPV4,SLC9B2,TGFB2,ADRA2A,MON1A,TCIRG1,TCF7L2,SNAP23,KCNB1,PFKFB2,GRK2,GPER1,RAB11FIP2,LAT2,AGT,FGR,CBL,SYTL1,PRSS12,PER2,OSM,STX1A,SNX4,NTSR1,PASK,SEPT2,BACE1,DNAJC5,GLUL,CSPG5,SIDT2,SLC16A1,ADAM8,KLF7,NSF,CREB1,MIA3,RAP1B,BLOC1S6,PLA2G4A,CTAGE9,VGF,TTN,IL12A,TANGO2,ENY2,ABCA12,NRXN2,CASK,MCU,ADRA2C,PNKD,PTGDR,PPP3CB,RAB3A,PINK1,SYN1,CAMK2G,SNAP29,EXOC5,PTPRN,CD38,C12orf4,MPC2,ILDR2,INHA,RAB8A,ATP13A2,HAP1,CPLANE2,SYT7,RAB15,RSC1A1,NLGN2,TNFRSF1B,SEPT1,WASHC3,CPLX1,TARDBP,STX1B,STX4,PSEN1,RAB25,ITGAM,SYT11,RAB8B,TMED10,CA[illegible]	grey	VSMC_M10
GO:0009263	0.80	0.38	6	13	deoxyribonucleotide biosynthetic process	BP	1	TBPL1,DGUOK,DTYMK,ADK,CMPK2,DCTD	grey	VSMC_M10
GO:0070242	0.80	0.38	6	13	thymocyte apoptotic process	BP	1	BAK1,ZC3H8,ADAM8,EFNA1,WNT5A,BCL2L11	grey	VSMC_M10
GO:1901993	0.81	0.42	4	9	regulation of meiotic cell cycle phase transition	BP	1	MAPK15,TOPAZ1,MOS,OVOL1	grey	VSMC_M10
GO:0060907	0.81	0.42	4	9	positive regulation of macrophage cytokine production	BP	1	TLR4,SPON2,CD74,WNT5A	grey	VSMC_M10
GO:2000303	0.81	0.40	5	11	regulation of ceramide biosynthetic process	BP	1	ORMDL2,PLA2G6,SPHK2,SIRT3,ORMDL1	grey	VSMC_M10
GO:0007567	0.81	0.42	4	9	parturition	BP	1	HPGD,PLA2G4B,PTGFR,CRH	grey	VSMC_M10

GO:0008544	0.81	0.24	101	201	epidermis development	BP	1	GLI1,CYP27B1,FZD6,PPARA,PTCH1,SMO,ZDHHC21,IFT74,SOSTDC1,CD109,KLK14,HOXA7,MYCL,WHRN,GRHL1,EGFR,FUZ,GJB5,SFRP4,MYCN,NTF4,INTU,HDAC2,SOX9,ERCC2,ERCC3,PTCH2,GLI2,EVPL,IFT172,MYO7A,SCEL,OVOL2,SLC4A7,JAG1,LRTOMT,AP000812.5,MAFG,FERMT1,TFDP1,ASAH1,ACVR1B,FLNB,CASP3,PLS1,TRPC4AP,FZD3,USH1C,VDR,AP3B1,SRSF6,DSP,STK4,PPHLN1,LDB1,COL17A1,HDAC1,TGFB2,PTHLH,SNAI1,LAMB3,POU2F3,TGM1,KDF1,SFN,COL7A1,LAMA5,CLIC4,GRHL3,CST6,STRC,OVOL1,KLF7,PLOD3,H2AFY2,ABCA12,SAV1,GORAB,WAS,RBPJ,MED1,NGFR,MAP2K1,KEAP1,BCL11B,LAMA3,GATA6,TMEM132E,JAG2,AGPAT2,MCOLN3,MIR125B1,FABP5,WNT5A,POU3F2,DNASE1L2,ZBED2,YBX1,EREG,SGPP1,HEY2	grey	VSMC_M10
GO:0031272	0.81	0.40	5	11	regulation of pseudopodium assembly	BP	1	CDC42EP1,KIT,F2RL1,CDC42EP2,APC	grey	VSMC_M10
GO:0031274	0.81	0.40	5	11	positive regulation of pseudopodium assembly	BP	1	CDC42EP1,KIT,F2RL1,CDC42EP2,APC	grey	VSMC_M10
GO:0090649	0.81	0.40	5	11	response to oxygen-glucose deprivation	BP	1	DNM1L,CBL,BNIP1,TSC1,LCN2	grey	VSMC_M10
GO:0034063	0.81	0.32	11	24	stress granule assembly	BP	1	PUM2,YTHDF2,DDX6,YTHDF1,MAPT,DDX3X,PRRC2C,ATXN2L,UBAP2L,G3BP1,CIRBP	grey	VSMC_M10
GO:0010642	0.81	0.40	5	11	negative regulation of platelet-derived growth factor receptor signaling pathway	BP	1	LRP1,PTGIR,PTPRJ,APOD,PTPN2	grey	VSMC_M10
GO:0120163	0.81	0.29	21	44	negative regulation of cold-induced thermogenesis	BP	1	ATF4,ALDH1A1,NR1H2,TLR4,IL15,LAMA4,ACVR2B,PLCL2,ABHD6,ARNTL,NR1H3,ZNF423,MAP2K6,ACTN3,TFE3,RBPJ,PCTP,DOCK7,ACOT11,NOVA1,DDIT3	grey	VSMC_M10
GO:0097050	0.81	0.42	4	9	type B pancreatic cell apoptotic process	BP	1	SRSF6,RYR2,HDAC1,TCF7L2	grey	VSMC_M10
GO:0010612	0.81	0.40	5	11	regulation of cardiac muscle adaptation	BP	1	TRPC3,PPP3CA,SMAD3,MIR199A1,MIR25	grey	VSMC_M10
GO:1903242	0.81	0.40	5	11	regulation of cardiac muscle hypertrophy in response to stress	BP	1	TRPC3,PPP3CA,SMAD3,MIR199A1,MIR25	grey	VSMC_M10
GO:0009200	0.81	0.36	7	15	deoxyribonucleoside triphosphate metabolic process	BP	1	TBPL1,NUDT15,NUDT16,DGUOK,DTYMK,ADK,CMPK2	grey	VSMC_M10
GO:0002532	0.81	0.27	29	59	production of molecular mediator involved in inflammatory response	BP	1	PPARA,GPSM3,MAPK14,IL1R2,TLR4,MYD88,CHID1,NOD2,ADCY7,PDCD4,SNAP23,NCF1,SLAMF8,SNX4,APOD,DUSP10,STAT3,IL4R,BTK,PLD4,NOS2,TICAM1,P2RX1,MIR21,MIR222,C5orf30,MIR125A,IL17D,IL17B	grey	VSMC_M10
GO:0042073	0.81	0.30	16	34	intraciliary transport	BP	1	IFT74,DYNC2LI1,TTC26,IFT172,TTC21A,WDR60,TUB,KIF3B,IFT81,IFT52,TTC21B,TRAF3IP1,TTC30A,WDR19,IFT20,HSPB11	grey	VSMC_M10
GO:1901387	0.81	0.40	5	11	positive regulation of voltage-gated calcium channel activity	BP	1	STAC3,CACNB2,STAC,KCNE2,STAC2	grey	VSMC_M10
GO:0010248	0.81	0.42	4	9	establishment or maintenance of transmembrane electrochemical gradient	BP	1	BAK1,ATP1A3,ATP1A2,ATP1B1	grey	VSMC_M10
GO:0006833	0.81	0.40	5	11	water transport	BP	1	MIP,AQP1,AQP11,INPP5K,PDZD3	grey	VSMC_M10
GO:0009164	0.81	0.36	7	15	nucleoside catabolic process	BP	1	APOBEC3H,GDA,ENPP4,CDADC1,AHCY,UPB1,UPP1	grey	VSMC_M10
GO:0051974	0.81	0.42	4	9	negative regulation of telomerase activity	BP	1	POT1,MEN1,PIF1,TEN1	grey	VSMC_M10
GO:0071470	0.81	0.29	19	40	cellular response to osmotic stress	BP	1	TSPO,XRCC6,AKR1B1,AQP1,OXSR1,CASP3,PKD2,TRPV4,SCN2A,YBX3,DDX3X,XRCC5,MICU1,SLC2A4,WNK3,AC138894.1,LRRC8E,SLC25A23,LRRC8D	grey	VSMC_M10
GO:0001890	0.81	0.25	58	117	placenta development	BP	1	CYP27B1,STK3,MAP3K4,CCDC134,RSPO3,PTK2,MAPK14,MDFI,TFEB,EPAS1,PTN,GJB5,PRDX3,OVOL2,EOMES,SPINT2,DCN,CEBPA,MME,VDR,STC1,SPINT1,SPP1,PKD2,STK4,IGF2,SNAI1,ARNT,VASH2,HTRA1,DEDD,SP3,CDKN1B,SETD2,FZD5,MEN1,CTSV,MAPK1,RBPJ,MED1,SOX15,DLX3,MAPK3,VASH1,CITED1,ALKBH1,MAP2K1,EPOR,PKD1,BSG,KRT19,CASP8,TCF23,GRB2,LIF,CEBPB,HEY2,WNT2	grey	VSMC_M10
GO:0150115	0.81	0.25	46	95	cell-substrate junction organization	BP	1	LRP1,PTK2,TEK,TLN1,ITGB1BP1,IQSEC1,CLASP2,ACTG1,ARHGEF7,PTPRA,FAM107A,PIK3R1,FMN1,SORBS1,PTPRJ,PTK2B,DMTN,LDB1,SLK,COL17A1,COL16A1,AJUBA,ARHGAP6,SLC9A1,ACTN3,WHAMM,LAMC1,APOD,TRIP6,FN1,WNT4,VCL,RAB8B,TAOK2,SMAD3,CORO2B,BCAS3,CAMSAP3,PDPK1,TSC1,RHOA,ACVRL1,PEAK1,THY1,LAMTOR2,DUSP22	grey	VSMC_M10
GO:0007095	0.81	0.30	15	32	mitotic G2 DNA damage checkpoint signaling	BP	1	MRNIP,TRIM39,BRSK1,NBN,ABRAXAS1,DONSON,TAOK3,SYF2,UIMC1,FOXO4,TAOK2,FOXN3,TAOK1,FZR1,BRCC3	grey	VSMC_M10
GO:0070141	0.81	0.39	5	11	response to UV-A	BP	1	OPN1SW,EGFR,MME,CCND1,MMP3	grey	VSMC_M10
GO:0014820	0.81	0.42	4	9	tonic smooth muscle contraction	BP	1	MKKS,AGT,CD38,SCNN1B	grey	VSMC_M10
GO:1903543	0.81	0.38	6	13	positive regulation of exosomal secretion	BP	1	VPS4B,CHMP2A,SDCBP,RAB7A,ATP13A2,VPS4A	grey	VSMC_M10
GO:0051642	0.81	0.31	14	30	centrosome localization	BP	1	SUN2,BICD2,INTS13,DLG1,TBCCD1,NIN,SYNE2,NDEL1,IFT20,NUBP1,GPSM2,SPOUT1,FHOD1,DYNC1LI2	grey	VSMC_M10
GO:0090343	0.81	0.38	6	13	positive regulation of cell aging	BP	1	CDKN2A,PAWR,KRAS,YPEL3,MIR21,MIR22	grey	VSMC_M10
GO:0110148	0.81	0.24	63	127	biomineralization	BP	1	CYP27B1,OSR2,PPARA,ATF4,ROR2,OMD,CNNM4,KLK4,PTN,ITGB1BP1,TRPM4,OSR1,SOX9,ERCC2,SLC4A2,ACVR2B,WNT11,SLC24A3,ACVR2A,KL,GAS6,ADGRV1,MEF2C,P2RX7,PTK2B,BMP2K,ANO6,SPP1,TFAP2A,ITGB6,BMPR1B,FBXL15,SBDS,PTHLH,TCIRG1,FAM20A,FAM20C,SGMS2,AXIN2,AMELX,FGR,ACTN3,TXLNG,FZD9,PTH1R,SRGN,BCOR,ANKH,ZBTB40,WNT4,ALPL,FGFR3,MMP13,BMPR1A,SMAD3,GPC3,RFLNB,BGLAP,MINPP1,SNX10,CCDC154,CEBPB,HEY2	grey	VSMC_M10
GO:0010745	0.81	0.42	4	9	negative regulation of macrophage derived foam cell differentiation	BP	1	PPARA,NR1H2,NR1H3,ABCA5	grey	VSMC_M10
GO:0010968	0.81	0.42	4	9	regulation of microtubule nucleation	BP	1	HSPA1B,EML2,ARHGEF7,MECP2	grey	VSMC_M10
GO:0090481	0.81	0.39	5	11	pyrimidine nucleotide-sugar transmembrane transport	BP	1	SLC35A1,SLC35A4,SLC35A5,SLC35A2,SLC35B1	grey	VSMC_M10
GO:0055015	0.81	0.42	4	9	ventricular cardiac muscle cell development	BP	1	FHL2,ATG5,NKX2-5,HEY2	grey	VSMC_M10
GO:0031279	0.81	0.30	15	32	regulation of cyclase activity	BP	1	MAPK14,GUCA1B,GABBR2,RAF1,ADGRV1,ORAI1,CAP2,GRM2,MAPK3,CALM1,NOS2,PALM,GNAI3,CAP1,PDZD3	grey	VSMC_M10
GO:0060966	0.81	0.29	20	42	regulation of gene silencing by RNA	BP	1	ZC3H10,ESR1,AGO2,TNRC6B,XPO5,EGFR,MYCN,PUM2,BCDIN3D,EIF4E2,RIPK1,PPP3CA,TERT,AJUBA,DDX6,EIF4ENIF1,STAT3,MAP2K1,NCOR1,FMR1	grey	VSMC_M10
GO:0002679	0.81	0.39	5	11	respiratory burst involved in defense response	BP	1	HCK,NCF1,SLAMF8,DUSP10,MPO	grey	VSMC_M10
GO:1900247	0.81	0.42	4	9	regulation of cytoplasmic translational elongation	BP	1	DPH2,DPH3,DPH1,AARS	grey	VSMC_M10

GO:1903975	0.81	0.39	5	11	regulation of glial cell migration	BP	1	LRP1,TIAM1,CERS2,ATP1B2,MIR222	grey	VSMC_M10
GO:0006360	0.81	0.28	24	50	transcription by RNA polymerase I	BP	1	BAZ2A,MARS,ATF4,IPPK,ERCC2,ERCC3,POU4F1,SIRT7,ERCC6,RASL11A,POLR1A,HEATR1,DEDD,TAF1A,H2AFY2,DDX11,PHF8,TAF1,TCOF1,RRN3,TTF1,TAF1C,POLR2K,GTF2H5	grey	VSMC_M10
GO:0050673	0.81	0.22	161	316	epithelial cell proliferation	BP	1	NME1,GLI1,TNFAIP3,ESR1,FRS2,RIDA,OSR2,MTSS1,MYC,PTCH1,C6orf89,SMO,IFT74,TEK,CDK6,KIT,DLX5,PDCL3,CD109,RTN4,EMC10,EPGN,AREG,BMPER,TGFA,PTN,ITGB1BP1,EGFR,OSR1,INTU,FOXE3,C5AR2,C5AR1,PRKD2,SOX9,AQP11,IFT172,SCN5A,WDR48,THAP1,OVOL2,ACVR2A,RPTOR,PURA,FERMT1,AIMP1,PRKDC,FLT1,SDR16C5,CYP7B1,NR4A1,ATP5F1A,EYA1,ROBO1,FBXW7,MEF2C,PEX2,VEGFC,VDR,NKX3-1,NKX6-1,LOXL2,AGGF1,SERPINB1,SRSF6,IFT52,RREB1,PAX2,NOD2,IGF2,FAP,HYAL1,CCND1,TGFB2,SULF2,TCF7L2,PDCD6,PRKX,EGF,SOX2,ARNT,TGM1,KDF1,SFN,VASH2,FZD7,RPS6KA1,HTRA1,RUNX3,GLUL,SIDT2,LAMC1,CDKN1B,DLG1,DUSP10,STOX1,ITGA4,MEN1,IL12A,CASK,SAV1,PDCD10,GPBAR1,RAP1GAP,STAT3,PTPRN,MAPK1,WDR13,MAGED1,IGFBP4,BMPR1A,MED1,ALDH1A2,SYNJ2BP,PSEN1,NGFR,PGF,VASH1,MAP2K1,SMAD3,MAP2K5,NOX5,TNMD,BCL11B,CCL2,ISL1,SUZ12,GPC3,PDPK1,WDR77,CYBA,MARVELD3,DAB2IP,ESRP2,MIR29A,MIR21,MIR222,MIR23A,MIR125B1,MIR22,MIR27B,MIR24-1,MIR27A,SERPINB5,LAMB1,ACVRL1,TINF2,ZNF580,NOG,CLDN1,WNT5A,SIX1,ZNF304,RGCC,HRAS,BCL2L2,CEBPB,EREG,FGF1,WNT2,COL8A2	grey	VSMC_M10
GO:0002449	0.81	0.24	80	159	lymphocyte mediated immunity	BP	1	CD226,STX7,AGER,HLA-DRA,HLA-DOB,HLA-DMA,HLA-DPB1,PRDX1,EXOSC3,MSH2,RSAD2,IL4I1,TRPM4,TRAF3IP2,HMCES,ERCC1,NECTIN2,PVR,MLH1,UNC13D,MYD88,SLC22A13,FOXJ1,PAXIP1,JAG1,SHLD1,NCKAP1L,HPX,IRF7,TRAF6,GAPT,SLA2,RASGRP1,NBN,NOD2,CD40,CD96,TCIRG1,DENND1B,CD28,FCER1G,RNF168,CADM1,FZD5,BCL6,CTSH,IL12A,PTPN6,LAG3,PPP3CB,STAT5B,WAS,FOXP3,ZBTB1,SHLD2,TNFRSF1B,TBX21,BATF,CD19,IL4R,BTK,CD74,FBXO38,VAV1,C7,C3,IL7R,SUPT6H,CRK,PRKCZ,TRAF2,AP1G1,BCL10,FADD,CD46,FCER2,RNF8,CD70,SERPINB9,HAVCR2	grey	VSMC_M10
GO:0007492	0.81	0.27	28	59	endoderm development	BP	1	ITGA7,EOMES,MMP15,RTF1,LDB1,HDAC1,SOX2,LAMB3,COL7A1,ITGB5,LAMC1,SETD2,ITGA4,FN1,COL5A2,LEO1,BMPR1A,SMAD3,LHX1,LAMA3,GATA6,COL5A1,DUSP2,LAMB1,HOXC11,NOG,DUSP1,DUSP4	grey	VSMC_M10
GO:0000209	0.81	0.23	102	203	protein polyubiquitination	BP	1	NEDD4L,TNFAIP3,PRKN,RNF5,SKP1,UBE2B,RNF217,RNF146,KLHL3,RNF20,UBE3D,PELI1,HECW1,TRIM32,TRAF1,HACE1,TRAF3IP2,KBTBD6,UBE2E2,UBE2O,UBE3C,UBE2E1,NMI,TPP2,NHLRC3,RNF14,UBOX5,TRIM3,TRAF6,TRIM44,MARCH8,ERCC8,SPSB4,ZFP91,UBE2L6,TRIM2,ANAPC5,ANAPC7,DDB2,AMFR,MARCH7,TTC3,NOD2,MAGEL2,NHLRC1,RNF144B,DZIP3,HECW2,KLHL42,RNF115,RNF114,DTX3L,ARIH2,RNF26,CBL,TRAF5,PELI3,RNF168,SYVN1,DDX3X,RC3H1,KLHL20,RNF4,TMEM129,MUL1,PPIL2,RNF167,RNF216,GPS2,PRPF19,ANAPC4,RNF111,NPEPPS,AREL1,TAF1,UBE2Q1,OTUB2,RNF165,TRAF3,FBXO22,UBE2Q2,FBXO38,RNF135,MGRN1,FZR1,TRAF2,RNF126,WWP2,TRIM68,TRIM34,UBE2G1,RNF8,UBE2QL1,UBE2N,UBE2U,CDC26,DDA1,UBE2G2,UBE2R2,RNF152,UBE2D1,RC3H2	grey	VSMC_M10
GO:0045682	0.81	0.28	24	50	regulation of epidermis development	BP	1	CYP27B1,PTCH1,CD109,HOXA7,MYCL,GRHL1,SFRP4,MYCN,PTCH2,OVOL2,MAFG,VDR,SRSF6,KDF1,SFN,KLF7,H2AFY2,ABCA12,MED1,KEAP1,MIR125B1,ZBED2,SGPP1,HEY2	grey	VSMC_M10
GO:2000291	0.81	0.42	4	9	regulation of myoblast proliferation	BP	1	SOX15,ZNF609,GATA6,MIR199A1	grey	VSMC_M10
GO:1904375	0.81	0.25	53	107	regulation of protein localization to cell periphery	BP	1	LRP1,STX7,STAC3,GOPC,EPB41L2,PRKCE,SPTBN1,EGFR,STAC,PRNP,LYPLA1,PIK3R1,PLS1,SORBS1,KCNJ11,NHLRC1,PPFIA1,DAG1,EPB41,GPER1,RAB11FIP2,CIB1,AP2M1,PPP2R5A,DLG1,LRRC15,LZTFL1,ZDHHC8,CNPY4,GRIPAP1,CNST,ACTB,IQSEC2,RSC1A1,WNK3,STAC2,STX4,ITGA3,AC138894.1,RAB11A,STX8,ARHGAP44,EFCAB7,PDPK1,PRKCZ,RER1,ARHGEF16,SAPCD2,GPSM2,VPS4A,LDLRAP1,HRAS,MRAP2	grey	VSMC_M10
GO:0110151	0.81	0.29	20	42	positive regulation of biomineralization	BP	1	OSR2,PTN,OSR1,SLC4A2,ACVR2B,ACVR2A,KL,ADGRV1,MEF2C,P2RX7,ANO6,TFAP2A,BMPR1B,AMELX,ACTN3,FZD9,WNT4,BMPR1A,SMAD3,CEBPB	grey	VSMC_M10
GO:0032966	0.81	0.41	4	9	negative regulation of collagen biosynthetic process	BP	1	CYGB,CYP7A1,CIITA,MIR29A	grey	VSMC_M10
GO:0051865	0.81	0.27	30	62	protein autoubiquitination	BP	1	PRKN,UBE2B,RNF146,UHRF2,NFX1,UBE3D,CNOT4,RNF122,SH3RF2,TRAF6,ERCC8,RNF181,DDB2,AMFR,MARCH7,RNF10,RNF115,KLHL24,DTX3L,TRIM52,RNF185,RNF4,TAF1,TRIM37,RNF208,TRAF2,WWP2,TRIM68,RNFT1,RNF8	grey	VSMC_M10
GO:0045616	0.81	0.31	13	28	regulation of keratinocyte differentiation	BP	1	CYP27B1,CD109,HOXA7,GRHL1,OVOL2,VDR,SRSF6,H2AFY2,ABCA12,MED1,MIR125B1,ZBED2,SGPP1	grey	VSMC_M10
GO:0060840	0.81	0.26	39	81	artery development	BP	1	LRP1,TAB1,FOXH1,SRF,TFAP2B,HOXA13,CNTRL,APOB,ACVR2B,WNT11,JAG1,HAND2,HPGD,EYA1,ROBO1,ROBO2,NKX3-1,PKD2,PRICKLE1,SUFU,SLC2A10,TGFB2,EFEMP2,LUZP1,BMPR1A,RBPJ,NGFR,LDLR,DCTN5,TBX2,STRA6,AP2B1,FOXF1,ENG,SOX4,ACVRL1,NOG,SIX1,HEY2	grey	VSMC_M10

GO:0042593	0.81	0.23	92	183	glucose homeostasis	BP	1	ZNF236,NME1,LRP1,AGER,PTCH1,PLA2G6,CRY1,SRF,TFAP2B,PRKCE,HMGN3,CYB5R4,TRPM4,FOXO3,FGF21,SESN3,FOXA3,GRIK5,RAF1,GCKR,ASPSCR1,GAS6,NR1H4,CYP7A1,NDUFAF2,GPI,CEBPA,OSBP,PIK3R1,KCNJ11,TIAM1,RAB11FIP5,PFKM,SLC8B1,OPA1,CRY2,ZBED6,PAX2,TWNK,GPLD1,IRS1,PPP3CA,TRPV4,SLC9B2,ZBTB20,ADRA2A,TCF7L2,KCNB1,POLG,GPER1,RAB11FIP2,USF1,MLXIPL,SIDT2,SLC16A1,PDK3,KLF7,FIS1,VGF,HK1,MEN1,ENY2,ABCA12,MCU,PYGL,PPP3CB,STAT3,PTPRN,MPC2,BHLHA15,SLC2A4,FGFR4,STX4,NGFR,CRTC2,GPR68,SIN3A,INSR,OXCT1,LEPR,PTPN2,INPP5K,NADK,BAIAP3,PARK7,CYBA,LRRC8A,OAS1,FABP5,LRRC8D,SOX4,CRH	grey	VSMC_M10
GO:0043244	0.81	0.25	52	107	regulation of protein-containing complex disassembly	BP	1	AVIL,JMJD4,LMOD2,KIAA1211,TMOD1,PLEKHH2,SPTBN1,PLEK,ADD2,CLASP2,MTRF1,TRIM54,NAV3,SPEF1,LMOD3,ATXN7,LIMA1,KATNB1,SCAF4,F2RL1,DMTN,CARMIL1,LMOD1,ADD3,TRPV4,TMEM39A,TTBK2,CIB1,SPTBN2,TMOD4,EPS8,RUBCN,CAPZA1,UPF1,TBC1D25,TMOD2,PHF23,SPTB,AC138894.1,INSR,SMCR8,CAMSAP3,TAOK1,CLEC16A,APC2,CAMSAP1,CARMIL2,IRGM,APC,PDXP,MAP1A,MAP6D1	grey	VSMC_M10
GO:0031641	0.81	0.29	17	36	regulation of myelination	BP	1	SOX10,TNFRSF21,PTN,LPIN1,FIG4,MTMR2,CST7,SIRT2,RARB,LGI4,JAM2,CYFIP1,RNF10,DAG1,NSUN5,DLG1,TNFRSF1B	grey	VSMC_M10
GO:0002031	0.81	0.41	4	9	G protein-coupled receptor internalization	BP	1	ARRB1,SAG,GRK4,DNM2	grey	VSMC_M10
GO:0090090	0.81	0.24	56	113	negative regulation of canonical Wnt signaling pathway	BP	1	GLI1,STK3,FZD6,PRKN,MAPK14,SOX10,ROR2,SOSTDC1,HECW1,FUZ,SFRP4,DKKL1,FOXO3,SOX9,WNT11,RBMS3,CHD8,UBAC2,FERMT1,PFDN5,CTNND1,DKK3,TMEM131L,LMBR1L,AMFR,SOX13,STK4,PRICKLE1,NKD1,HDAC1,TCF7L2,DKK2,AXIN2,SOX2,ZNRF3,KREMEN1,BICC1,PTPRO,OTUD5,IGFBP4,TMEM88,CCDC88C,G3BP1,ISL1,GPC3,TLE6,APC2,DAB2IP,MIR199A1,NOG,WNT5A,DDIT3,DACT3,SDHAF2,APC,NKX2-5	grey	VSMC_M10
GO:0032774	0.81	0.20	1329	2534	RNA biosynthetic process	BP	1	ADNP2,NFATC1,ZNF236,ZNF407,SMARCC2,ATP8B1,ONECUT2,BAZ2A,TCF4,TBPL1,GLI1,MARS,MAP3K5,TNFAIP3,TSFM,HIVEP2,PLAGL1,MAML1,HELB,ZBTB2,YEATS4,ESR1,MYRFL,CNOT2,CNOT6,STK3,OSR2,SNW1,TFB1M,ZNF706,ARID1B,HSPA1B,FZD6,ZBTB12,ZBED4,PRKN,AGER,PPARA,PDE10A,MED30,IL5,BRD2,TAF2,RXRB,VGLL2,HSF2,TCF7,PHF1,XRCC6,NCOA7,PITX1,TRIB1,MYC,TOB2,SMAD5,L3MBTL3,L3MBTL2,AGO2,KDM3B,PTP4A3,PTCH1,ATF4,RPAP2,CBX7,MAPK14,CBY1,KDM4C,SOX10,PIM1,NRF1,TMPRSS6,SMO,SNAPC3,BNC2,HSF1,NFYA,FOXP4,MDFI,TFEB,IRF5,MED20,CCND3,TAF8,FOXH1,LRRC14,CDKN2A,ZNF16,RBFOX2,IFT74,BICRAL,RORB,ZNF300,PRDM4,CRY1,DMTF1,SRF,ZNF318,NFX1,CDK14,FZD1,MTERF1,NFXL1,NFKBIE,SUPT3H,SMARCA2,RFX3,KIT,ADGRF1,NCK2,FHL2,TGFBRAP1,TFAP2B,ROR2,NFIL3,SEMA4D,RFX8,DLX5,PHF14,IPPK,ETV1,ZNF135,RPS14,ZNF154,MEOX2,ZNF550,KANSL3,BEND6,ZNF268,ZNF667,ZNF787,PHF3,ZNF691,CAT,MACC1,SIX2,FOXJ3,ZNF865,HIVEP3,IL11,SCMH1,ZNF189,RNF20,EPAS1,EPCAM,SP4,PPP2R1A,ZNF462,PHIP,HMGN3,NFYC,NFE2L3,HOXA2,TAF1D,HOXA4,HOXA6,HOXA7,HOXA13,REL,ZNF483,JAZF1,CREB5,MYCL,XPO1,MTURN,NOD1,AKIRIN2,SRSF10,CASP8AP2,BACH2,ZNF692,PELI1,AKIRIN1,MTF1,ZNF618,SNIP1,ANXA4,PCBP1,NR1H2,AKNA,ZNF350,ITGB1BP1,FOXP2,GRHL1,MED25,KLF11,MET,IL4I1,AGO1,POU3F3,EGFR,LANCL2,ING3,NOBOX,ARHGEF5,TRIM32,TLR4,CCNC,CDK5RAP2,TRAF1,BCL2L12,SCAF1,LPIN1,SFRP4,UCP1,CREM,TEAD2,SETD7,SDCBP,MYCN,C6orf203,ELF2,OSR1,FOXO3,ZXDC,ZBTB24,MKX,FAF1,MAMSTR,GTF3C6,ATAD2B,BEND5,CDCA7,KDM7A,ZNF438,FOXE3,HDAC2,[illegible]	grey	VSMC_M10
GO:0098901	0.81	0.32	10	22	regulation of cardiac muscle cell action potential	BP	1	TRPM4,DSP,RYR2,PKP2,ANK2,NOS1AP,DLG1,CALM1,DSG2,HCN4	grey	VSMC_M10
GO:0006622	0.81	0.32	10	22	protein targeting to lysosome	BP	1	RAB7A,NCOA4,AP3B1,HSPA8,SORL1,AP4M1,AP3M1,LAMP2,VPS4A,SNX16	grey	VSMC_M10
GO:0033692	0.81	0.27	25	52	cellular polysaccharide biosynthetic process	BP	1	HAS1,EPM2AIP1,PPP1CB,CSGALNACT1,AKT2,PPP1R3B,GBE1,B3GNT4,SORBS1,IGF2,NHLRC1,IRS1,PPP1R3E,PPP1R3D,PER2,PASK,PRKAG3,AC022400.6,PPP1R3F,INSR,INPP5K,AGL,HAS3,ACADM,HS2ST1	grey	VSMC_M10
GO:0006959	0.81	0.25	42	84	humoral immune response	BP	1	CXCL14,PLA2G6,TFEB,TNFRSF21,GAPDH,CXCL8,TRAF3IP2,BMI1,PHB,FOXJ1,HPX,GPI,MEF2C,IL7,NOD2,YTHDF2,CD28,PDCD1,PPP2R3C,PTPN6,SLC11A1,CFP,BST1,TFE3,RBPJ,SPON2,GATA6,CCL2,C7,C3,FGA,S100A12,HIST1H2BK,CXCL6,CD46,A2M,JCHAIN,CFD,FCER2,RGCC,HIST1H2BF,HIST1H2BJ	grey	VSMC_M10
GO:2000036	0.81	0.31	12	26	regulation of stem cell population maintenance	BP	1	CNOT2,ZNF706,MYC,SMO,PTN,PAX8,CNOT1,LOXL2,PAX2,SAV1,ESRRB,ZNF322	grey	VSMC_M10
GO:0003002	0.81	0.22	127	251	regionalization	BP	1	GLI1,FRS2,TBC1D32,PTCH1,SMO,MDFI,FOXH1,SRF,ROR2,MEOX2,SOSTDC1,SIX2,SCMH1,HOXA2,HOXA4,HOXA6,HOXA7,FUZ,OSR1,NTF4,INTU,SPRY1,BMI1,SETDB2,ABI1,GLI2,MEGF8,PCDH8,XRCC2,ACVR2B,IFT172,FOXJ1,WNT11,OVOL2,ACVR2A,BHLHE40,SEMA3A,PRKDC,HOXC6,TCF15,POFUT1,PAX8,ROBO1,ROBO2,FAM208A,MEF2C,NKX3-1,IFT52,RPGRIP1L,PAX2,NKD1,DPCD,LDB1,BMPR1B,FBXL15,SUFU,IRX2,PGAP1,SEMA3F,POGLUT1,AXIN2,SNAI1,TTC21B,GBX2,HES6,CHRD,DLX1,WNT3,HOXD8,HOXD4,HOXD3,AIDA,FZD5,MEOX1,PKD1L1,FKBP8,GORAB,GPR161,TULP3,MNS1,DVL2,BMPR1A,WDR19,TCAP,RBPJ,ALDH1A2,PSEN1,HOXB5,HOXB6,HOXB8,HOXB9,ITGAM,TMEM107,SMAD3,MKS1,GSC,LHX1,YY1,CDX1,MIB1,NLE1,ISL1,C3,GPC3,HSPB11,HES4,SKI,WDR77,CELSR2,FOXF1,MTF2,ADGRG1,FOXG1,MAFB,ACVRL1,HOXB3,NOG,WNT5A,BASP1,OTX1,SIX1,DDIT3,FGF1,PIFO,HEY2,WNT2,HOXA3	grey	VSMC_M10

GO:0002367	0.81	0.26	31	64	cytokine production involved in immune response	BP	1	CD226,KIT,RSAD2,TLR4,ATG5,TRPM4,SPHK2,LACC1,MAVS,TRAF6,F2RL1,NOD2,CD96,TGFB2,DENND1B,FZD5,BCL6,HK1,FOXP3,TNFRSF1B,SPON2,TBX21,BTK,CD74,TICAM1,PRKCZ,TRAF2,SLC7A5,BCL10,WNT5A,INAVA	grey	VSMC_M10
GO:1900026	0.81	0.30	14	30	positive regulation of substrate adhesion-dependent cell spreading	BP	1	UNC13D,RAC3,ARHGEF7,DOCK5,RREB1,DMTN,CARMIL1,CASS4,CIB1,DOCK1,CSPG5,DNM2,CRK,FGA	grey	VSMC_M10
GO:0032885	0.81	0.30	14	30	regulation of polysaccharide biosynthetic process	BP	1	EPM2AIP1,PPP1CB,AKT2,PPP1R3B,SORBS1,IGF2,IRS1,PPP1R3E,EGF,PPP1R3D,PASK,PPP1R3F,INSR,INPP5K	grey	VSMC_M10
GO:0070884	0.81	0.30	14	30	regulation of calcineurin-NFAT signaling cascade	BP	1	FHL2,EFHB,PRNP,NRG1,SPPL3,TBC1D10C,CIB1,SLC9A1,MYOZ2,ACTN3,HOMER2,MYOZ1,MAPK7,CAMTA1	grey	VSMC_M10
GO:0002263	0.81	0.23	93	185	cell activation involved in immune response	BP	1	LRP1,ENPP3,HLA-DRA,MFNG,RAC2,KIT,EXOSC3,PRKCE,MSH2,TLR4,SPHK2,HMCES,ERCC1,LCP1,GAB2,MLH1,UNC13D,MYD88,PAXIP1,EOMES,NMI,TCIM,PLCL2,SHLD1,NCKAP1L,FOXP1,GAPT,FER,PTK2B,RASGRP1,EIF2AK4,RIPK2,NBN,DYSF,CDH17,F2RL1,CD40,GBF1,DOCK10,SNAP23,HLX,LAT2,FGR,CBL,SNX4,CD28,FCER1G,RNF168,BCL6,RC3H1,TNFSF18,SLC11A1,PTGDR,STAT3,C12orf4,FOXP3,SHLD2,TBX21,STX4,PSEN1,SEMA4A,ITGAM,DOCK2,BATF,ZBTB7B,IL6R,CD19,LAT,IL4R,S100A13,BTK,SCNN1B,CD74,STXBP2,PRAM1,PTGER4,SUPT6H,TICAM1,DNASE1,PDPK1,PRKCZ,TSC1,FOXF1,KARS,STXBP1,AP1G1,MIR21,CHGA,CD46,FGL2,RNF8,HAVCR2,RC3H2	grey	VSMC_M10
GO:0070663	0.81	0.23	77	153	regulation of leukocyte proliferation	BP	1	TNFAIP3,ENPP3,AGER,IL5,HLA-DPB1,TNFRSF13C,PTK2,RAC2,TNFRSF21,NCK2,PELI1,IL4I1,TLR4,IL15,BMI1,MYD88,FOXJ1,TGFBR2,PAWR,PRNP,NCKAP1L,SLC7A1,TRAF6,IL1A,CASP3,MEF2C,TMEM131L,IL7,RIPK2,MARCH7,ZNF335,CD40,IGF2,ICOSLG,BTN2A2,GPAM,PRKAR1A,CD28,DLG1,BCL6,IL12A,VSIR,RC3H1,PTPN6,TNFSF18,CD38,BST1,MAPK1,STAT5B,SCGB1A1,FOXP3,RASAL3,CARD11,TNFRSF1B,MAPK3,ZBTB7B,CHRNB2,BTK,CD74,CCL5,CSF1R,CD320,TWSG1,TICAM1,DNAJA3,VAV3,GLMN,MIR21,LST1,FADD,CD46,IL33,CD70,TNFSF9,CEBPB,TNFSF13B,HAVCR2	grey	VSMC_M10
GO:0006111	0.81	0.28	19	40	regulation of gluconeogenesis	BP	1	PPARA,GPT,GNMT,CRY1,PPP4R3B,ZNF692,FAM3C,SIRT7,NLN,SIK1,MST1,DDB1,DGKQ,OGT,LEPR,PTPN2,ACADM,NNMT,ARPP19	grey	VSMC_M10
GO:0007220	0.81	0.41	4	9	Notch receptor processing	BP	1	GALNT11,PSEN2,PSEN1,APH1B	grey	VSMC_M10
GO:0051569	0.81	0.31	12	26	regulation of histone H3-K4 methylation	BP	1	SNW1,PAXIP1,DNMT3B,RTF1,ZNF335,AUTS2,BCOR,WDR61,NELFA,GFI1,PRMT6,NNMT	grey	VSMC_M10
GO:0034121	0.81	0.27	26	54	regulation of toll-like receptor signaling pathway	BP	1	TNFAIP3,ESR1,GRAMD4,LRRC14,NINJ1,RTN4,PELI1,RSAD2,CD300LF,IRF7,MFHAS1,OTUD4,NR1H3,F2RL1,NOD2,RAB7B,TLR5,DDX3X,BIRC3,GPS2,WDFY1,GPR108,PDPK1,CYBA,GFI1,DAB2IP	grey	VSMC_M10
GO:0034375	0.81	0.38	5	11	high-density lipoprotein particle remodeling	BP	1	APOM,APOC1,PLTP,ABCA5,LIPC	grey	VSMC_M10
GO:1902415	0.81	0.41	4	9	regulation of mRNA binding	BP	1	EIF3E,EIF3C,LARP6,FMR1	grey	VSMC_M10
GO:0032414	0.81	0.25	39	80	positive regulation of ion transmembrane transporter activity	BP	1	STAC3,ABCB1,WNK2,CACNB2,STAC,GLRX,KCNJ11,IFNGR2,TESC,KCNE2,PKD2,KCNIP2,RYR2,FAM173B,COX17,ANK2,SLC9A1,CASQ1,NTSR1,NOS1AP,HTT,PINK1,ATP1B1,KCNA1,HAP1,WNK3,STAC2,ATP1B2,NLGN3,ATP2A1,CALM1,DNM2,VMP1,CCL2,RNF207,AMIGO1,DMD,JPH2,APOPT1	grey	VSMC_M10
GO:0071702	0.81	0.20	991	1891	organic substance transport	BP	1	ATP9B,TIMM21,VPS4B,APOF,ATP8B1,STARD6,LRP1,STX7,AP3S1,SLC2A12,KIF5A,SLC26A10,PEX7,AGAP2,PEX3,SLC16A7,CANX,TBC1D30,TBC1D9B,PPP1R10,CPSF6,RAB21,SNX24,SNX31,CPT1B,PHAX,APOM,ZDHHC14,SLC12A2,SERAC1,RIMS2,SLC27A6,PRKN,QKI,PPARA,TAP1,SLC22A4,KIF3A,TSPO,GDF9,GOPC,VPS52,HSPA4,DERL1,FAM91A1,WASHC5,FAM53C,SLC45A4,PTCH1,TSNARE1,LY6E,ZC3H3,EXOC4,SIL1,SYNGR1,MAPK15,MAPK14,NTRK2,TOMM22,PLA2G6,COPG2,PICK1,MICALL1,TBC1D22B,RAC2,SMO,ZDHHC21,TTC39B,HSF1,PLIN2,TOMM6,MYH9,APOL4,APOL6,CHRNA3,PEX6,FBXO7,CRY1,CROT,ABCB1,ABCC10,NOL6,XPO5,WASHC4,APBA1,PEX1,SLC35B2,VPS50,SLC25A27,SCFD2,RFX3,SLC1A1,TGFBRAP1,TFAP2B,SLC25A13,TBC1D8,TRAM2,BICD2,CHMP2A,RAB23,ABCG5,SVBP,ABCG8,LRPPRC,SEC61B,SLC3A1,STX17,SIX2,DNAJA1,PRKCE,RIMS1,TMEM30A,SPTBN1,IGF2BP3,HMGN3,YTHDC1,PNPT1,RUFY3,SLC4A4,CYB5R4,PPT1,SNX14,XPO1,SNX30,SLC35A1,BMP8A,AQP1,EHBP1,AFTPH,SLC1A4,PLEK,RSAD2,NR1H2,ITGB1BP1,DDC,GRB10,YWHAZ,SEC61G,EGFR,FUZ,ARHGEF5,TLR4,TCAF2,VPS41,TBC1D9,VPS35,SDCBP,MGARP,TRPM4,SLC7A11,PGRMC2,LIN7B,EPS15,APOB,AGK,FGF21,BRAF,BCAP29,MFSD4B,MFSD2B,TRAF3IP2,ANKRD50,TTC26,DNAJC27,NAPA,BBS7,RAB7A,SLC26A5,PIP4K2A,AP2S1,RAB10,COPG1,KPNA3,GIPR,ARL5B,IWS1,LCP1,SYTL2,COG1,SLC25A30,DLG2,CUBN,RBSN,APOC1,SLC4A2,DNAJC15,ASIC3,PLK3,FRMD4A,OPTN,GOLGA4,LLGL2,IPO13,STEAP3,AQP11,PRKAG2,CCDC93,RAF1,ZFAND6,SLC22A13,TBC1D4,MYO7A,SLC25A38,NAPB,GCKR,SEC14L1,TSKU,SEC62,SEC22C,KPNA2,AKT2,PITRM1,LRP1B,HOOK3,ARRB1,ABCC4,[illegible]	grey	VSMC_M10
GO:0048668	0.81	0.32	10	22	collateral sprouting	BP	1	LRP1,FSTL4,SEMA4D,EPHA7,SPART,ULK1,SPP1,BDNF,WNT3,NIN	grey	VSMC_M10
GO:0002562	0.81	0.27	26	54	somatic diversification of immune receptors via germline recombination within a single locus	BP	1	TCF7,EXOSC3,MSH2,CYREN,HMCES,ERCC1,MLH1,PAXIP1,SHLD1,PRKDC,XRCC4,NBN,CD40,DCAF1,CD28,RNF168,BCL6,FOXP3,SHLD2,TBX21,BATF,BCL11B,YY1,SUPT6H,RNF8,NHEJ1	grey	VSMC_M10
GO:0016444	0.81	0.27	26	54	somatic cell DNA recombination	BP	1	TCF7,EXOSC3,MSH2,CYREN,HMCES,ERCC1,MLH1,PAXIP1,SHLD1,PRKDC,XRCC4,NBN,CD40,DCAF1,CD28,RNF168,BCL6,FOXP3,SHLD2,TBX21,BATF,BCL11B,YY1,SUPT6H,RNF8,NHEJ1	grey	VSMC_M10
GO:0009159	0.81	0.38	5	11	deoxyribonucleoside monophosphate catabolic process	BP	1	DPYS,GDA,UPB1,NT5M,UPP1	grey	VSMC_M10
GO:2000107	0.81	0.29	16	34	negative regulation of leukocyte apoptotic process	BP	1	SLC46A2,GAS6,FOXP1,IRF7,NOD2,GPAM,HCLS1,BCL6,CXCR2,EFNA1,CD74,CCL5,IL7R,BCL10,FADD,TSC22D3	grey	VSMC_M10

GO:0072089	0.81	0.27	26	54	stem cell proliferation	BP	1	FBLN1,PIM1,ABCB1,SIX2,EPCAM,OVOL2,FERMT1,EIF2AK2,VEGFC,TERT,SART3,YTHDF2,ZNRF3,ARIH2,KDF1,THPO,OVOL1,ETV6,WNT3,KAT7,CTC1,CITED1,AC004687.2,MIR222,ATXN1L,WNT5A	grey	VSMC_M10
GO:2000142	0.81	0.29	15	32	regulation of DNA-templated transcription, initiation	BP	1	ESR1,MED30,SRF,ERCC1,PAXIP1,MITF,ERCC6,CREB1,MED14,THRA,MED24,MED1,JUN,NKX2-5,HEY2	grey	VSMC_M10
GO:0034969	0.81	0.41	4	9	histone arginine methylation	BP	1	PRDM4,NR1H4,PRMT7,PRMT6	grey	VSMC_M10
GO:0002438	0.81	0.38	5	11	acute inflammatory response to antigenic stimulus	BP	1	SERPINC1,CXCR2,BTK,C3,PARK7	grey	VSMC_M10
GO:0006984	0.81	0.28	21	44	ER-nucleus signaling pathway	BP	1	ATF4,NCK2,C12orf49,FBXW7,ATG10,AMFR,ERLIN1,QRICH1,SCAP,TMCO1,NFE2L2,LPCAT3,ZBTB7B,SREBF1,PTPN2,ABCA7,HSPA5,DDIT3,BCL2L11,PPP1R15A,INSIG2	grey	VSMC_M10
GO:0042255	0.81	0.28	22	45	ribosome assembly	BP	1	RRP7A,RPS14,MDN1,RPSA,PRKDC,DHX37,RPL6,SBDS,MRPS11,RPS27,DHX30,RPL11,EFL1,RPL10L,DDX3X,XRCC5,MRPL22,NLE1,RPL23A,ERAL1,MRPS2,RPS27L	grey	VSMC_M10
GO:1901021	0.81	0.29	17	36	positive regulation of calcium ion transmembrane transporter activity	BP	1	STAC3,CACNB2,STAC,KCNE2,PKD2,RYR2,ANK2,SLC9A1,CASQ1,HTT,ATP1B1,HAP1,STAC2,ATP2A1,CALM1,VMP1,JPH2	grey	VSMC_M10
GO:0051791	0.81	0.41	4	9	medium-chain fatty acid metabolic process	BP	1	CROT,ABHD2,ABHD3,ACADM	grey	VSMC_M10
GO:0021612	0.82	0.41	4	9	facial nerve structural organization	BP	1	PLXNA4,SEMA3A,SEMA3F,PLXNA3	grey	VSMC_M10
GO:1903332	0.82	0.38	5	11	regulation of protein folding	BP	1	PDCL3,DNAJB2,SGTA,HSPA5,POFUT2	grey	VSMC_M10
GO:0021697	0.82	0.33	9	20	cerebellar cortex formation	BP	1	AGTPBP1,WHRN,FAIM2,LDB1,TTC21B,MAP2K1,LHX1,GRID2,CEND1	grey	VSMC_M10
GO:0016447	0.82	0.27	23	48	somatic recombination of immunoglobulin gene segments	BP	1	EXOSC3,MSH2,CYREN,HMCES,ERCC1,MLH1,PAXIP1,SHLD1,PRKDC,XRCC4,NBN,CD40,CD28,RNF168,BCL6,FOXP3,SHLD2,TBX21,BATF,YY1,SUPT6H,RNF8,NHEJ1	grey	VSMC_M10
GO:0048880	0.82	0.22	144	286	sensory system development	BP	1	SLC39A5,MDM1,FRS2,OSR2,TBC1D32,BAK1,ATF4,AGTPBP1,NTRK2,PRPH2,RORB,SRF,SLC1A1,TFAP2B,TDRD7,TMOD1,RDH13,ABCB5,PTN,TSPAN12,USP45,SLC7A11,SPRY1,FOXE3,HDAC2,BBS7,SOX9,CRYGN,ZNF513,ACVR2B,IFT172,MYO7A,POU4F1,TSKU,SPRY2,DZANK1,TGFBR2,BFSP1,JAG1,RARB,FSCN2,SPRED3,FREM2,CEP290,KERA,SEMA3A,MAB21L1,MITF,FLT1,TUB,EPHB1,PFDN5,NPHP1,RET,FOXL2,MAB21L2,TENM3,USH1C,TENT2,OPA1,RPGRIP1L,TFAP2A,PAX2,NKD1,BMPR1B,MYH15,NECTIN3,LPCAT1,PKNOX1,SLC6A3,HDAC1,ALDH1A3,TGFB2,TCIRG1,PRSS56,SHROOM2,SOX2,CRYBA4,NRL,CLIC4,DLX1,GRHL3,LIMK2,SP3,CDKN1B,DLG1,FZD5,FBN1,FKBP8,COL5A2,PLA2G16,STAT3,TULP3,OBSL1,RHOJ,ROM1,MFAP2,WDR19,SDK1,MAX,CLN8,MED1,ALDH1A2,MFN2,PSEN1,ITGAM,TTLL5,MEGF11,LCTL,SMAD3,TBX2,BCL11B,LHX1,STRA6,YY1,CHRDL1,UNC45B,TMEM132E,ISL1,LAMA1,C3,TWSG1,GNB1,FZR1,SKI,SOX8,COL5A1,LAMC3,OLFM3,TMEM231,SLC25A25,HSF4,BLOC1S3,FJX1,CNTF,ACVRL1,THY1,WNT5A,DIO3,TGIF2,TBC1D20,PITX3,WNT2,COL8A2	grey	VSMC_M10
GO:0034341	0.82	0.24	47	95	response to interferon-gamma	BP	1	CYP27B1,GAPDH,NR1H2,TLR4,NMI,ACTG1,HPX,HCK,FLNB,CCL17,NR1H3,NLRC5,IFNGR2,CD40,RAB7B,SP100,PARP9,PARP14,CDC42EP2,SLC11A1,GCH1,WAS,CXCL16,MED1,STX4,CITED1,STX8,CD74,CCL14,CCL5,CCL7,CCL2,CCL25,JAK1,NOS2,PTPN2,SOCS1,CIITA,ADAMTS13,ASS1,STXBP1,CCL16,IRGM,CLDN1,WNT5A,VAMP3,CCL22	grey	VSMC_M10
GO:0007281	0.82	0.23	89	177	germ cell development	BP	1	TBPL1,YTHDC2,MOV10L1,PACRG,QKI,GDF9,UBE2B,CATSPER3,WASHC5,SMAD5,H3F3C,FANCC,TUT7,DMC1,CFAP69,FANCG,SMARCA2,KIT,MSH2,YTHDC1,GMCL1,PTN,NOBOX,CCDC155,TUT4,FOXO3,ARMC2,DLD,TTC26,PANX1,RSPH6A,TOPAZ1,ERCC1,NECTIN2,MLH1,TTC21A,CCNB1IP1,SIRT2,MKKS,PANK2,SOHLH2,ING2,CELF1,JAM2,PTK2B,KMT2D,CDYL,SYCP1,DNAH1,NPM2,OCA2,ROPN1L,BMPR1B,NECTIN3,NEURL1,DDX25,CFAP43,ABHD2,CCDC36,YTHDF2,SPO11,TSSK4,CIB1,OSBP2,CATSPER2,TTC12,SPAG16,WNT4,ZMYND15,MNS1,YBX2,SOX30,CCDC42,PAQR5,TAF4B,SPEF2,CATSPERD,CHD5,TPGS1,WDR77,MFSD14A,STRBP,AURKC,SYCP3,NANOS1,RNF8,TBC1D20,EREG,ZGLP1	grey	VSMC_M10
GO:0006040	0.82	0.29	15	32	amino sugar metabolic process	BP	1	GFPT2,LARGE1,ALDH1A1,GNPDA2,NANP,CSGALNACT1,CHST1,CMAS,DPAGT1,MGAT1,OVGP1,CHST3,CHST7,CHST5,CTBS	grey	VSMC_M10
GO:0051602	0.82	0.31	12	26	response to electrical stimulus	BP	1	BRD1,NR4A1,FZD3,OPA1,KCNJ3,CYFIP1,DISC1,SLC9A1,RAB3A,ADSSL1,PALM,NQO1	grey	VSMC_M10
GO:0090503	0.82	0.28	20	42	RNA phosphodiester bond hydrolysis, exonucleolytic	BP	1	PAN2,CNOT2,CNOT6,PNLDC1,EXOSC4,EXOSC3,PNPT1,PAN3,CNOT1,USB1,CNOT6L,DIS3L2,ANGEL2,EXD2,ISG20L2,ANGEL1,CNOT8,ERI2,PARN,EXOSC5	grey	VSMC_M10
GO:0086003	0.82	0.26	27	57	cardiac muscle cell contraction	BP	1	NEDD4L,TRPM4,CACNB2,SCN5A,SCN1A,GPD1L,STC1,KCNJ3,KCNE2,DSP,RYR2,PKP2,KCNJ2,ANK2,ATP1A2,SLC9A1,SCN4B,NOS1AP,DLG1,CACNA1G,ATP2A1,DSG2,RNF207,KCNH2,SCN2B,KCNJ8,HCN4	grey	VSMC_M10
GO:0150116	0.82	0.26	31	65	regulation of cell-substrate junction organization	BP	1	LRP1,PTK2,TEK,ITGB1BP1,IQSEC1,CLASP2,ACTG1,PTPRA,FAM107A,PIK3R1,FMN1,PTPRJ,DMTN,LDB1,SLK,COL16A1,ARHGAP6,SLC9A1,APOD,WNT4,VCL,SMAD3,BCAS3,CAMSAP3,TSC1,RHOA,ACVRL1,PEAK1,THY1,LAMTOR2,DUSP22	grey	VSMC_M10
GO:0060976	0.82	0.28	18	39	coronary vasculature development	BP	1	TAB1,PTK7,SRF,CNTRL,MEGF8,HAND2,TBX5,PRICKLE1,SUFU,GPER1,MMP21,SETD2,DCTN5,GATA6,AP2B1,ZBTB14,GPC3,HEY2	grey	VSMC_M10
GO:0043030	0.82	0.28	18	38	regulation of macrophage activation	BP	1	TTBK1,TLR4,CST7,MFHAS1,CEBPA,NR1H3,CD200,GPR137B,PLA2G4A,TNIP2,SYT11,IL4R,LDLR,CD74,KARS,WNT5A,IL33,HAVCR2	grey	VSMC_M10

GO:0045926	0.82	0.22	98	195	negative regulation of growth	BP	1	SEMA6A,CYP27B1,STK3,HSPA1B,PPARA,GDF9,FSTL4,PTCH1,PI16,CDKN2A,SMARCA2,SEMA4D,SEMA4C,RTN4,PPP2R1A,PNPT1,PPT1,EPHA7,SPHK2,PHB,WNT11,SERTAD3,TGFBR2,ING1,SEMA3D,BTG1,SPART,SEMA3A,ULK1,RYK,ACVR1B,CDKN2AIP,PTPRJ,SEMA4F,NDUFS3,TLL2,TBX5,PRDM11,SEMA3G,SPP1,STK4,SEMA4G,NKD1,JARID2,KIAA0319,HYAL1,CAPRIN2,SEMA3F,TGFB2,EI24,SH3BP4,AGT,IP6K2,KCNK2,ING5,CDKN1B,WNT3,DUSP10,BCL6,SEMA6D,DDX3X,TNR,SAV1,ING4,MYOZ1,GDF15,FGFR3,MUL1,DNAJB2,TSPYL2,SEMA4A,TRIM46,WWC1,SMAD3,NTN1,BDKRB1,DCUN1D3,YY1,CDK5R1,RAI1,GPC3,ARHGAP4,PLXNA3,PSRC1,NAIF1,MIR199A1,MIR25,SERTAD2,MT1X,ACVRL1,NOG,WNT5A,FXN,RGS4,PLAC8,DACT3,NME6,CGRRF1	grey	VSMC_M10
GO:0090303	0.82	0.27	20	42	positive regulation of wound healing	BP	1	PTK2,ENPP4,PRKCE,THBD,ACTG1,HBEGF,FERMT1,F2R,ANO6,RREB1,DMTN,ADRA2A,USF1,NFE2L2,DUOX1,F12,SERPINF2,MIR29A,CLDN1,HRAS	grey	VSMC_M10
GO:0019748	0.82	0.29	14	30	secondary metabolic process	BP	1	AKR1B1,DDC,SLC7A11,AKR1A1,CBR4,RAPGEF2,ABCC2,OCA2,BDH2,NFE2L2,CITED1,SLC45A2,WNT5A,N6AMT1	grey	VSMC_M10
GO:0071711	0.82	0.29	14	30	basement membrane organization	BP	1	PXDN,CLASP2,FERMT1,SPINT2,GAS2,DAG1,LAMB3,PHLDB1,PLOD3,MMP11,NID2,RAMP2,NTNG2,LAMB1	grey	VSMC_M10
GO:0072595	0.82	0.28	18	38	maintenance of protein localization in organelle	BP	1	TSPO,SKP1,SUN2,GPAA1,TAF8,GRIK5,TAF3,ARL2BP,MORC3,SP100,HK1,PINK1,RER1,PARK7,HSPA5,SUPT7L,TXN,INSIG2	grey	VSMC_M10
GO:0000394	0.82	0.38	5	11	RNA splicing, via endonucleolytic cleavage and ligation	BP	1	TSEN34,CLP1,ZBTB8OS,RTRAF,TRPT1	grey	VSMC_M10
GO:0006094	0.82	0.26	28	58	gluconeogenesis	BP	1	PPARA,ATF4,GPT,GNMT,CRY1,SLC25A13,PPP4R3B,ZNF692,FAM3C,SIRT7,GPI,NLN,GPD1,SIK1,MST1,PC,SLC37A4,PER2,DDB1,DGKQ,OGT,CRTC2,LEPR,PTPN2,ACADM,FBP2,NNMT,ARPP19	grey	VSMC_M10
GO:0000956	0.82	0.24	50	102	nuclear-transcribed mRNA catabolic process	BP	1	PAN2,CNOT2,CNOT6,PABPC1,SKIV2L,DXO,EIF3E,PNLDC1,AGO2,TNRC6B,TUT7,EXOSC4,EXOSC3,AGO1,TUT4,MTPAP,DHX34,MLH1,TNRC6C,LSM1,UPF3A,PAN3,TTC37,CNOT1,CNOT6L,TENT2,TENT4B,DIS3L2,NCBP2,EIF4ENIF1,RC3H1,UPF1,SAMD4A,POLR2G,NT5C3B,DCP1B,CASC3,SMG5,TOB1,TNRC6A,CNOT8,UPF3B,PARN,LSM7,EDC4,NANOS1,ZFP36L2,MAGOHB,RC3H2,EXOSC5	grey	VSMC_M10
GO:0140353	0.82	0.30	13	28	lipid export from cell	BP	1	GDF9,CRY1,ABCC4,C1QTNF1,NKX3-1,CRY2,SPP1,MAP2K6,AGT,PLA2G4A,NOS2,CRH,PTGES	grey	VSMC_M10
GO:1905332	0.82	0.28	16	34	positive regulation of morphogenesis of an epithelium	BP	1	SMO,SOX9,PAX8,PAX2,EGF,AGT,STOX1,WNT4,MAGED1,LHX1,SOX8,LCN2,NOG,LBX2,SIX1,LIF	grey	VSMC_M10
GO:0033280	0.82	0.29	14	30	response to vitamin D	BP	1	CYP27B1,SNW1,PIM1,PTN,TNC,IL15,VDR,STC1,SPP1,ALPL,MED1,MIR125B1,TRIM24,BGLAP	grey	VSMC_M10
GO:0030042	0.82	0.26	24	51	actin filament depolymerization	BP	1	AVIL,LMOD2,KIAA1211,TMOD1,PLEKHH2,SPTBN1,PLEK,ADD2,LMOD3,LIMA1,F2RL1,DMTN,CARMIL1,LMOD1,ADD3,CFL1,SPTBN2,TMOD4,EPS8,CAPZA1,TMOD2,SPTB,CARMIL2,PDXP	grey	VSMC_M10
GO:0038094	0.82	0.31	11	24	Fc-gamma receptor signaling pathway	BP	1	PTK2,PLA2G6,PRKCE,HCK,PTPRJ,FGR,PLPP4,FCER1G,YES1,VAV1,VAV3	grey	VSMC_M10
GO:0043408	0.82	0.20	258	502	regulation of MAPK cascade	BP	1	PHLPP1,LRP1,SEMA6A,MAP3K5,FRS2,STK3,MAPK8IP2,MAP3K4,PRKN,AGER,FBLN1,ROS1,SYNGAP1,MYC,LEMD2,PRXL2C,GRM4,TAB1,NTRK2,STK38,TEK,GPR37,KIT,ROR2,GADD45G,WNK2,KLHL31,PRDX1,SEMA4C,DNAJA1,IL11,PRKCE,FKTN,FPR2,GSDME,VRK2,EMC10,LPAR1,MTURN,EPGN,NOD1,BMPER,EPHA7,TGFA,ITGB1BP1,VRK3,YWHAZ,EGFR,ARHGEF5,TLR4,TRAF1,SDCBP,FGF21,BRAF,SPRY1,DNAJC27,C5AR2,C5AR1,PRKD2,PHB,RAF1,MYD88,SPRY2,ALK,ARRB1,C1QTNF1,NEK10,ZMYND11,PDE6G,PLCB1,SPRED3,EIF2AK2,ADRA1D,DUSP6,RELL2,SEMA3A,KL,NRG1,SH3RF2,GAS6,SIRT3,ARL6IP5,MFHAS1,FLT1,RYK,ADRA2B,TRAF6,APIP,ST5,EPHB1,MOS,RET,PIK3CB,HAND2,ROBO1,PLCE1,FBXW7,PTPRJ,P2RX7,WDR54,P2RY1,MADD,PTK2B,RASGRP1,RAPGEF2,CCL17,TIAM1,PAQR3,RIPK2,F2RL1,F2R,NQO2,RIPK1,PRDM11,STK4,GCNT2,NOD2,TAOK3,CD40,DSTYK,DUSP8,IGF2,PRDM15,BANK1,MAP2K6,TRPV4,PDCD4,TGFB2,ADRA2A,SLC30A10,DAG1,AJUBA,NCF1,KRAS,EGF,TBC1D10C,SOX2,SORL1,GPER1,DOK5,AGT,CIB1,TRAF5,DVL3,FZD7,OSM,THPO,PDE6H,MAP3K3,PDE8A,MAGI3,DUSP16,DLG1,MAP3K11,ADAM8,AIDA,FZD5,DUSP10,MBIP,RAP1B,MEN1,MAP4K2,FN1,DUSP19,PTPN6,PDCD10,ADRA2C,GPBAR1,GDF15,FGFR3,PINK1,LTBR,MAPK1,MINK1,EPHA4,MAGED1,DVL2,IGFBP4,GPS2,RELL1,RGS14,FGFR4,SYNJ2BP,PSEN1,FGF18,TAOK2,EFNA1,WWC1,MAPK3,MAP2K1,PIK3R5,MAP2K5,RIT2,TNIP1,PSMD10,GAREM1,NCOR1,TRAF3,CD74,HRH4,CCL14,INSR,CCL5,CSF1R,CCL7,CCL2,CCL25,GHR,KSR1,PTPN2,TAOK1,INPP5K,SERPINF2,PRKCZ,MAPK8IP3,TRAF2,ATP6AP1,ABCA7,CDK10,MARVELD3,DAB2IP,MIR21,MIR222,MIR23A,MIR27B,MIR24-[illegible]	grey	VSMC_M10
GO:0034766	0.82	0.25	33	68	negative regulation of ion transmembrane transport	BP	1	NEDD4L,GOPC,PRKCE,HECW1,TCAF2,KEL,OSR1,SLC26A5,KCNRG,OXSR1,ARL6IP5,SLC43A1,KCNE2,PKD2,CASQ2,HECW2,ADRA2A,ISCU,ATP1A2,C4orf3,NTSR1,SESTD1,GNB5,CALM1,FMR1,RRAD,MIR24-1,GPR35,KCNH2,CRBN,RGS4,GEM,PPIF	grey	VSMC_M10
GO:0045933	0.82	0.29	14	30	positive regulation of muscle contraction	BP	1	SRF,KIT,ADRA2B,F2R,CHRM3,TRPV4,GPER1,ACE2,ACTN3,ATP2A1,ABAT,MIR21,RHOA,CHGA	grey	VSMC_M10
GO:0110110	0.82	0.29	14	30	positive regulation of animal organ morphogenesis	BP	1	FRS2,SPRY1,SOX9,WNT11,ROBO1,ROBO2,STOX1,WNT4,NGFR,SOX8,WNT5A,SIX1,FGF1,WNT2	grey	VSMC_M10
GO:0034380	0.82	0.40	4	9	high-density lipoprotein particle assembly	BP	1	APOM,NR1H2,ZDHHC8,ABCA7	grey	VSMC_M10
GO:0048102	0.82	0.40	4	9	autophagic cell death	BP	1	TP53INP1,CDKN1B,CTSV,SH3GLB1	grey	VSMC_M10
GO:0051250	0.82	0.24	49	99	negative regulation of lymphocyte activation	BP	1	TNFAIP3,FBXO7,TNFRSF21,PELI1,IL4I1,PIBF1,FOXJ1,PAWR,PRNP,NCKAP1L,ZC3H8,CASP3,TMEM131L,PAG1,MARCH7,BANK1,BTN2A2,PRKAR1A,HLX,TBC1D10C,FGR,RUNX3,DLG1,BCL6,VSIR,RC3H1,PTPN6,TNFSF18,LAG3,INHA,SCGB1A1,FOXP3,TBX21,ZBTB7B,IL4R,BTK,CD74,TWSG1,PTPN2,SOCS1,GLMN,MIR21,MIR27A,LST1,FGL2,LAX1,CEBPB,HAVCR2,RC3H2	grey	VSMC_M10

GO:1901857	0.82	0.40	4	9	positive regulation of cellular respiration	BP	1	ISCU,PINK1,PARK7,IFNLR1	grey	VSMC_M10
GO:0014009	0.82	0.28	18	38	glial cell proliferation	BP	1	SNW1,TSPO,SOX10,AREG,PTN,EGFR,PLAG1,LGI4,PTK2B,RNF10,CERS2,CREB1,CHRM1,LEPR,SKI,MIR222,MIR125B1,SOX4	grey	VSMC_M10
GO:1904861	0.82	0.31	10	22	excitatory synapse assembly	BP	1	LRRC4,VSTM5,LRFN1,NRXN2,NLGN2,SEMA4A,ZDHHC12,GRID2,WNT5A,LRRTM2	grey	VSMC_M10
GO:0001776	0.82	0.25	34	70	leukocyte homeostasis	BP	1	TNFAIP3,BAK1,SLC46A2,SLC7A11,TRAF3IP2,NCKAP1L,GAPT,ZC3H8,PIK3CB,CASP3,MEF2C,DOCK10,GPAM,TCIRG1,PPP2R3C,RC3H1,CXCR2,PPP3CB,STAT5B,FOXP3,LAT,CD74,DNAJA3,BCL10,FADD,CXCL6,CAMLG,TSC22D3,BCL2L11,SH2B2,SLC39A3,TNFSF13B,XKR8,RC3H2	grey	VSMC_M10
GO:0071674	0.82	0.23	63	126	mononuclear cell migration	BP	1	SLC12A2,AGER,PTK2,CCL27,FPR2,AKIRIN1,TRPM4,C5AR1,OXSR1,PLCB1,GAS6,FLT1,RET,CYP7B1,JAM2,PTK2B,CCL17,SLC8B1,ANO6,ADTRP,CD200,TRPV4,CD99,CMKLR1,SLAMF8,MOSPD2,EPS8,PTPRO,APOD,ADAM8,MIA3,ITGA4,IL12A,TNFSF18,CXCR2,MAPK1,CH25H,PADI2,CXCL16,TMEM102,TBX21,STK10,MAPK3,IL6R,S100A14,CCL14,CCL5,CSF1R,CCL7,CCL2,CCL25,CRK,RHOA,CCL16,S100A12,DEFB124,FADD,WNT5A,DUSP1,ARTN,GCSAM,CCL22,PDGFD	grey	VSMC_M10
GO:0045191	0.82	0.29	13	28	regulation of isotype switching	BP	1	EXOSC3,MSH2,HMCES,MLH1,PAXIP1,SHLD1,CD40,CD28,BCL6,FOXP3,SHLD2,TBX21,SUPT6H	grey	VSMC_M10
GO:1905049	0.82	0.40	4	9	negative regulation of metallopeptidase activity	BP	1	LRP1,SORL1,MIR199A1,MIR24-1	grey	VSMC_M10
GO:0009612	0.82	0.22	88	176	response to mechanical stimulus	BP	1	BAK1,PKD2L2,HABP4,PTK2,PTCH1,MAPK14,PHF24,KIT,ETV1,AQP1,CASP8AP2,WHRN,PTN,TNC,EGFR,TLR4,SMPD2,MKX,SLC26A5,SOX9,XPC,ASIC3,RAF1,MYD88,SCN1A,SCEL,WNT11,TGFBR2,MKKS,CNN2,DCN,GPI,P2RX3,ADGRV1,P2RY1,PTK2B,TMEM150C,SLC38A2,PKD2,CD40,RYR2,KIAA0319,KCNJ2,TRPV4,DAG1,TLR5,AGT,ATP1A2,BAG3,SLC9A1,HDAC4,KCNK2,BACE1,STRC,TTN,NRXN2,KCNK4,HTT,PKD1L1,COL3A1,LTBR,KCNA1,TCAP,TNFRSF8,FOS,MAPK3,MPO,BDKRB1,STRA6,ASIC2,PTGER4,JUN,PKD1,PIEZO1,CYBA,ENG,PKD1L3,STRBP,BCL10,RHOA,DMD,PJVK,CHRNA10,FADD,CASP8,BGLAP,GADD45A,CRADD	grey	VSMC_M10
GO:0032727	0.82	0.33	8	18	positive regulation of interferon-alpha production	BP	1	IRF5,TLR4,MAVS,IRF7,RIPK2,IFIH1,SETD2,DDX3X	grey	VSMC_M10
GO:0006862	0.82	0.29	13	28	nucleotide transport	BP	1	SLC35B2,ABCC4,SLC33A1,CALHM1,ABCC5,SLC25A42,LRRC8E,SLC25A23,SLC25A41,SLC25A33,LRRC8A,SLC25A25,SLC25A32	grey	VSMC_M10
GO:1903421	0.82	0.30	11	24	regulation of synaptic vesicle recycling	BP	1	BTBD9,CYFIP1,DNM1L,AP2M1,DNM3,PPP3CB,GRIPAP1,DGKQ,STX1B,SYT11,CALM1	grey	VSMC_M10
GO:0031669	0.82	0.22	91	182	cellular response to nutrient levels	BP	1	MARS,MAP3K5,CYP27B1,SNW1,PPARA,ATF4,PICK1,PIM1,TXN2,TFEB,SLC39A4,SRF,NUAK1,BRSK1,ALB,RRAGD,PTN,TNC,VPS41,IL15,ATG5,FOXO3,KPTN,SESN3,FOXA3,DNAJC15,RALB,PRKAG2,SZT2,RPTOR,EIF2AK2,WRN,GAS6,LARS,ULK1,NR1H4,USF2,FAM107A,PRKAG1,EIF2AK4,VDR,SLC38A2,NUAK2,NUPR2,SRD5A1,DNM1L,SIK1,SLC38A3,KCNB1,HSPA8,CBL,MTMR3,UCN2,USF1,XPR1,TBL2,GLUL,NFE2L2,SIK2,COMT,WNT4,PRKAG3,CTSV,MAPK1,BHLHA15,WIPI2,MAX,MED1,RRAGB,ZFYVE1,MAPK3,TNRC6A,SREBF1,LAMP2,NIM1K,STK26,JUN,CLEC16A,KIAA1324,TSC1,LCN2,WDR59,HSPA5,SH3GLB1,RIPOR1,MIR125B1,TRIM24,BGLAP,IMPACT,UPP1,RNF152	grey	VSMC_M10
GO:0003208	0.82	0.25	27	57	cardiac ventricle morphogenesis	BP	1	LY6E,FOXH1,TNNI3,POU4F1,TGFBR2,JAG1,NRG1,HAND2,MEF2C,TBX5,TNNC1,DSP,RYR2,PKP2,TNNI1,TGFB2,MYH7,BMPR1A,RBPJ,MED1,ISL1,FOXF1,ENG,SOX4,NOG,NKX2-5,HEY2	grey	VSMC_M10
GO:0097320	0.82	0.32	8	18	plasma membrane tubulation	BP	1	ASAP1,MICALL1,BIN3,WHAMM,DNM2,FNBP1L,SH3GLB1,CAVIN2	grey	VSMC_M10
GO:0043039	0.82	0.27	19	40	tRNA aminoacylation	BP	1	MARS,QRSL1,HARS,HARS2,CARS2,LARS,WARS2,GATC,YARS2,TARS2,DALRD3,LARS2,DARS2,FARSB,EARS2,LRRC47,KARS,AARS,MARS2	grey	VSMC_M10
GO:0001764	0.82	0.23	55	113	neuron migration	BP	1	SEMA6A,CEP85L,GFRA3,NTRK2,SRF,ADGRL3,NRCAM,NDNF,SEMA3A,UNC5D,NRG1,GAS6,FZD3,MEF2C,ELP3,IGSF10,RAPGEF2,NKX6-1,NR4A2,TUBB2A,SRGAP2C,KIAA0319,SATB2,AUTS2,MARK1,DISC1,CDKL5,LRIG2,COL3A1,MARK2,STAT3,SDCCAG8,DNAAF4,PSEN1,ITGA3,TRIM46,NDEL1,ZNF609,ALKBH1,NTN1,LHX1,CAMK2A,CDK5R1,DAB1,CRK,CELSR2,NTNG2,FBXO31,BARHL2,DAB2IP,ADGRG1,CNTN2,FOXG1,POMK,POMGNT2	grey	VSMC_M10
GO:0006826	0.82	0.26	23	48	iron ion transport	BP	1	STEAP2,PGRMC2,HEPHL1,ASIC3,STEAP3,HPX,SLC25A37,SLC48A1,SLC40A1,ISCU,ABCC5,MELTF,SLC11A1,ABCB6,FTH1,FLVCR2,NOX5,DNM2,SLC46A1,C1orf194,LCN2,SLC22A17,FTL	grey	VSMC_M10
GO:1904752	0.82	0.30	12	26	regulation of vascular associated smooth muscle cell migration	BP	1	LRP1,MEF2C,ADAMTS1,DOCK5,TERT,GNA13,SSH1,AGT,NFE2L2,DOCK7,MIR21,RHOA	grey	VSMC_M10
GO:0120162	0.82	0.24	38	78	positive regulation of cold-induced thermogenesis	BP	1	HSF1,GADD45G,EPAS1,GRB10,LPIN1,UCP1,FGF21,ALMS1,EBF2,KSR2,PRKAB1,CMKLR1,FABP4,TRPM8,GPR3,PER2,ACADL,MFAP2,YBX2,THRA,MFN2,KDM6B,ZBTB7B,OGT,IL4R,TRPV2,PPARGC1B,LEPR,PRDM16,LCN2,BSCL2,FABP5,ELOVL3,PEMT,PLAC8,APC,CEBPB,DIO2	grey	VSMC_M10
GO:0033138	0.82	0.24	36	74	positive regulation of peptidyl-serine phosphorylation	BP	1	NTRK2,IL11,EGFR,NTF4,BRAF,PRKD2,RAF1,SPRY2,RPTOR,BAG4,GAS6,RET,FNIP2,RIPK2,RAPGEF3,STK4,BDNF,CAPRIN2,HCLS1,OPRD1,OSM,STOX1,PDCD10,PINK1,ARAF,HAX1,SH2D3A,DOCK7,MTCP1,PARK7,MAPKAP1,IRGM,SMYD3,WNT5A,TXN,LIF	grey	VSMC_M10
GO:0015718	0.82	0.25	32	66	monocarboxylic acid transport	BP	1	ATP8B1,SLC16A7,PLA2G6,SLC22A13,ABCC4,SLC5A8,CYP7A1,SLC10A6,ABCC2,PLA2R1,ABCG2,MAP2K6,SLC16A6,SLCO3A1,ABCB11,SLCO1A2,SLCO1C1,NTSR1,SLC16A1,PLA2G4A,SLC27A1,MPC2,SLC16A13,ABCC3,NMUR2,EMB,NOS2,PROCA1,SLC16A4,ABCD3,PLA2G2C,PTGES	grey	VSMC_M10
GO:1903035	0.82	0.25	31	64	negative regulation of response to wounding	BP	1	SERPINB2,TSPAN8,SLC12A2,CLDN19,CD109,CLASP2,THBD,PLAT,C1QTNF1,SPP1,ADTRP,FAP,KIAA0319,UBASH3B,KREMEN1,CERS2,USF1,LRIG2,TNR,CASK,WNT4,MYOZ1,EPHA4,F12,ANXA2,SMAD3,ABAT,CRK,SERPINF2,FGA,RTN4RL1	grey	VSMC_M10

GO:1905476	0.82	0.29	13	28	negative regulation of protein localization to membrane	BP	1	GOPC,ITGB1BP1,LYPLA1,DMTN,PPFIA1,AP2M1,PPP2R5A,FZD9,LRRC15,LZTFL1,RSC1A1,INPP5K,MRAP2	grey	VSMC_M10
GO:0055065	0.82	0.20	224	438	metal ion homeostasis	BP	1	SLC39A5,NEDD4L,LRP1,CYP27B1,ESR1,SLC12A2,PRKN,SMDT1,BAK1,MYC,MCHR1,GPR20,PLA2G6,BTBD9,TMPRSS6,SLC39A4,STEAP2,SLC25A27,SLC1A1,TFAP2B,S1PR3,CNNM4,PRKCE,TNNI3,EPAS1,FPR2,IBTK,LPAR1,SLC31A2,EGFR,KEL,ATG5,TRPM4,GRIN2D,TRPC3,SLC8A2,C5AR2,C5AR1,HEPHL1,ATP7B,PTGIR,GIPR,CACNB2,SLC39A12,STEAP3,ATP1A3,ACKR2,SLC24A3,SNX5,C1QTNF1,TMTC4,RYR1,LETM2,PRNP,ADRA1D,DIAPH1,P2RX2,KL,CCKBR,HPX,RASA3,NCOA4,MICU3,IL1A,IMMT,C19orf12,P2RX3,PLCE1,RYR3,TMBIM6,P2RX7,P2RY1,PTK2B,SLC39A13,TPCN1,SLC8B1,VDR,STC1,SLC25A37,AP3B1,RMDN3,F2RL1,F2R,SLC40A1,CUTC,PKD2,CASQ2,MCUR1,SLC12A5,CD40,RYR2,SLC41A1,RAP1GDS1,TRPM1,SPPL3,LCK,KCNJ2,TESMIN,SLC12A7,TRPV4,BDH2,SLC30A10,TCIRG1,FAM20A,GNA13,ISCU,CMKLR1,MCUB,HJV,SCN7A,UBASH3B,DISC1,ANK2,GPER1,TRPM8,PDZD8,AGT,ATP1A2,SLC9A1,CASQ1,SLC12A8,NTSR1,MELTF,FZD9,TMCO1,PTH1R,SLC30A4,FIS1,CEMIP,ABL2,SLC12A9,CDH23,COMT,PTPN6,MICU1,MCU,CXCR2,GNG3,SLC11A1,HTT,PTGDR,PTGER2,ABCB6,TRPM7,ATP1B1,KCNA1,CD38,KCNMA1,ATP13A2,FTH1,HAP1,DLG4,CYB561A3,ATP1B2,FAM155B,PSEN1,CACNA1A,AC138894.1,ATP2A1,CD19,COX11,SMAD3,NOX5,CALM1,S100A14,S100A8,PRKCB,SCNN1B,BDKRB1,NMUR2,SLC39A6,TRPV2,HRH4,CCL14,CCL5,CCL7,C7,PTGER4,SLC25A23,MAIP1,TMEM199,SLC46A1,NUBP1,GNB1,ATP2B3,BCAP31,GNA15,TRPV3,TRPV1,PDPK1,P2RX1,PKD1,TMEM203,ATP6AP1,GPR157,SYPL2,CYBA,JPH3,ATP2C2,SLC30A7,FRRS1,LCN2,MCOLN3,SLC12A4,PTGFR,MIR199A1,KCNK3,DMD,MT1X,GPR35,NOL3,CHRNA10,TRIM24,CCR10,JPH2,SLC22A17,KCN[illegible]	grey	VSMC_M10
GO:0030239	0.82	0.26	24	51	myofibril assembly	BP	1	OBSCN,LMOD2,SRF,PGM5,TMOD1,MYLK3,ACTG1,LMOD3,LMOD1,PRKAR1A,ACTC1,MYOZ2,CASQ1,TMOD4,MYPN,TTN,MYOZ1,TMOD2,OBSL1,LDB3,TCAP,MYH11,CSRP2,KRT19	grey	VSMC_M10
GO:0048639	0.82	0.23	66	134	positive regulation of developmental growth	BP	1	NEDD4L,LRP1,GLI1,RIMS2,PRKN,MAPK14,CPNE5,PIM1,SMO,SRF,SEMA4D,RIMS1,RUFY3,GOLGA4,MEGF8,CPNE9,TGFBR2,MKKS,SYT1,PLCB1,SLC23A2,PRKDC,PLS1,MEF2C,IL7,CELF1,TBX5,ZFYVE27,CYFIP1,IGF2,BDNF,SLC6A3,GPAM,HLX,ADNP,DISC1,ACTN3,CDKL5,YBX3,WNT3,CREB1,MAPT,FN1,MUL1,MAPK1,STAT5B,BMPR1A,RBPJ,SOX15,NDEL1,RAB11A,NTN1,TBX2,TRPV2,INSR,GATA6,GHR,L1CAM,MIR199A1,MIR222,BASP1,DIO3,POU3F2,CACNG7,HEY2,WNT2	grey	VSMC_M10
GO:0003229	0.82	0.26	22	47	ventricular cardiac muscle tissue development	BP	1	LY6E,FOXH1,TNNI3,POU4F1,NRG1,TBX5,TNNC1,DSP,RYR2,PKP2,TNNI1,TGFB2,MYH7,BMPR1A,RBPJ,MED1,DSG2,ISL1,ENG,NOG,NKX2-5,HEY2	grey	VSMC_M10
GO:0006493	0.82	0.24	36	74	protein O-linked glycosylation	BP	1	RXYLT1,LARGE1,C1GALT1,FKTN,IL15,POMGNT1,GALNT11,ST6GALNAC2,MGAT5B,TMTC4,GALNT15,GXYLT2,EOGT,POFUT1,B3GNT4,GALNT5,POGLUT1,B3GNT5,GALNT2,XXYLT1,TET1,PLOD3,KDELC2,GALNT8,GALNT16,OGT,POMT2,GALNT1,ST6GALNAC4,ST3GAL2,POFUT2,POMK,POMGNT2,ISPD,SLC35C1,ST8SIA6	grey	VSMC_M10
GO:0001659	0.82	0.22	70	141	temperature homeostasis	BP	1	ATF4,HSF1,ALDH1A1,GADD45G,EPAS1,NR1H2,GRB10,TLR4,LPIN1,IL15,UCP1,FGF21,LAMA4,NAPEPLD,ACVR2B,PLCL2,IL1A,ABHD6,ARNTL,NR1H3,ALMS1,EBF2,FTO,KSR2,ZNF423,PRKAB1,MAP2K6,CMKLR1,FABP4,TRPM8,GPR3,PER2,ACTN3,NTSR1,ACADL,STAT3,SLC27A1,TFE3,MFAP2,ACADVL,YBX2,THRA,RBPJ,MFN2,KDM6B,SLC25A44,ZBTB7B,OGT,PCTP,IL4R,TRPV2,PPARGC1B,LEPR,DOCK7,ACOT11,ABAT,TRPV1,PRDM16,LCN2,BSCL2,FABP5,ELOVL3,PEMT,NOVA1,PLAC8,DDIT3,PTGES,APC,CEBPB,DIO2	grey	VSMC_M10
GO:0051235	0.82	0.21	125	248	maintenance of location	BP	1	B4GALNT1,PPARA,TSPO,GOPC,SKP1,SUN2,MEST,GPAA1,TTC39B,MDFI,PLIN2,TAF8,CRY1,NFKBIE,PRKCE,IBTK,ALB,NR1H2,ATG5,LDAH,APOB,FAF1,ARHGAP21,ATP7B,ABHD5,GRIK5,TAF3,NFKBIB,RYR1,CDS2,DIAPH1,RASA3,ASXL1,RYR3,FBXW7,AUP1,PTK2B,ARL2BP,NR1H3,TPCN1,SLC8B1,CDS1,ACVR1C,F2R,FITM2,CRY2,FTO,PKD2,MORC3,CASQ2,RYR2,SUFU,LCK,SP100,CPT1A,IGSF11,GET4,UBASH3B,SORL1,ANK2,GPER1,FITM1,ATP1A2,CASQ1,NTSR1,TBCCD1,SLC30A4,SRGN,CEMIP,SOAT1,HK1,EHD1,PTPN6,FBN1,HTT,PINK1,STAT5B,FTH1,HAP1,VPS13D,VPS13C,ZFYVE1,PSEN1,ATP2A1,CD19,CALM1,S100A8,RIT2,BDKRB1,KEAP1,TMEM159,GM2A,PSMD10,SREBF1,C3,SLC25A23,PTPN2,PLIN3,TRPV1,PDPK1,RER1,PKD1,PARK7,GPSM2,CYBA,JPH3,SLC30A7,SPOUT1,LCN2,HSPA5,MCOLN3,DMD,BSCL2,NOL3,JPH2,THY1,SUPT7L,FTL,DDIT3,TXN,NRROS,STARD4,INSIG2,PFN4,POLR2M	grey	VSMC_M10
GO:0055003	0.82	0.35	6	14	cardiac myofibril assembly	BP	1	SRF,MYLK3,ACTC1,TTN,OBSL1,TCAP	grey	VSMC_M10
GO:2000241	0.82	0.23	52	105	regulation of reproductive process	BP	1	YTHDC2,ESR1,STK3,FBXO43,GDF9,UBE2B,AGO2,MAPK15,MYH9,CFAP69,NLRP5,HDAC2,SOX9,TOPAZ1,PLAT,SIRT2,PLCB1,SEMA3A,DHX37,MOS,ACVR1B,P2RY1,NKX3-1,ACVR1C,STK4,NPM2,HYAL3,PRKAR1A,INTS13,SNAI1,CCDC36,YTHDF2,CIB1,GPR3,CCDC87,HDAC4,CDKN1B,OVOL1,OVGP1,WNT4,SYDE1,NOX5,ZP2,FZR1,CACNA1H,WDR77,STXBP1,OOEP,WNT5A,DUSP1,LIF,LHFPL2	grey	VSMC_M10
GO:0032611	0.82	0.26	25	52	interleukin-1 beta production	BP	1	TNFAIP3,AGER,S1PR3,NOD1,TLR4,PANX1,MYD88,FOXP1,P2RX7,RIPK2,SERPINB1,F2RL1,F2R,NOD2,FZD5,HK1,STAT3,PYDC1,SMAD3,ISL1,MIR27B,CASP8,WNT5A,INAVA,CPTP	grey	VSMC_M10
GO:0032651	0.82	0.26	25	52	regulation of interleukin-1 beta production	BP	1	TNFAIP3,AGER,S1PR3,NOD1,TLR4,PANX1,MYD88,FOXP1,P2RX7,RIPK2,SERPINB1,F2RL1,F2R,NOD2,FZD5,HK1,STAT3,PYDC1,SMAD3,ISL1,MIR27B,CASP8,WNT5A,INAVA,CPTP	grey	VSMC_M10

GO:0002691	0.83	0.29	12	26	regulation of cellular extravasation	BP	1	AGER,PLCB1,CD99,ADAM8,ITGA4,BST1,CCL25,PTGER4,FADD,FUT4,THY1,PDGFD	grey	VSMC_M10
GO:0002861	0.83	0.30	11	24	regulation of inflammatory response to antigenic stimulus	BP	1	MAPK14,PLK2,HCK,NOD2,FGR,CD28,BTK,YES1,C3,PARK7,KARS	grey	VSMC_M10
GO:0034249	0.83	0.22	92	183	negative regulation of cellular amide metabolic process	BP	1	ORMDL2,PAN2,SHMT2,CNOT2,CNOT6,RIDA,PABPC1,EIF3E,PNLDC1,MRPL13,AGO2,TNRC6B,ATF4,TUT7,NTRK2,GAPDH,CNOT11,EXOSC3,IGF2BP3,RTN4,PNPT1,AGO1,TUT4,DHFR,MLH1,TNRC6C,PURA,EIF2AK2,PRNP,LSM1,PAN3,ENC1,CNOT1,CELF1,CNOT6L,EIF2AK4,EIF4E2,PAIP2B,ORMDL1,FTO,TENT4B,RBM24,BANK1,CAPRIN2,DIS3L2,GIGYF2,YTHDF2,SORL1,DDX6,YTHDF1,EIF4ENIF1,IGF2BP2,EIF4EBP2,DDX3X,RC3H1,UPF1,SAMD4A,STAT3,POLR2G,RTN1,NT5C3B,HAP1,DCP1B,FXR2,TARDBP,TOB1,TNRC6A,CNOT8,LARP1,SHMT1,PARN,FMR1,METTL16,PLEKHN1,CIRBP,ABCA7,TSC1,MIR29A,MIR21,MIR222,MIR125B1,MIR27B,MIR24-1,MIR27A,AGO4,METTL14,NANOS1,MIR339,YBX1,ZFP36L2,RC3H2,EXOSC5	grey	VSMC_M10
GO:0019048	0.83	0.39	4	9	modulation by virus of host process	BP	1	ANO6,DAG1,INSR,RXRA	grey	VSMC_M10
GO:0010876	0.83	0.21	158	311	lipid localization	BP	1	ATP9B,VPS4B,APOF,ATP8B1,STARD6,LRP1,B4GALNT1,CPT1B,APOM,SERAC1,SLC27A6,PRKN,PPARA,TSPO,GDF9,PTCH1,PLA2G6,MEST,TTC39B,PLIN2,APOL4,APOL6,CRY1,CROT,ABCB1,ABCG5,ABCG8,TMEM30A,NR1H2,LDAH,APOB,MFSD2B,PIP4K2A,ABHD5,APOC1,TSKU,AKT2,ABCC4,C1QTNF1,CDS2,ANO4,SLC5A8,SOAT2,CYP7A1,ASXL1,OSBP,PITPNM2,XKR6,FBXW7,PLSCR4,LIMA1,P2RX7,AUP1,NR1H3,NKX3-1,CDS1,ACVR1C,ANO6,FITM2,CRY2,SLC10A6,FTO,COL4A3BP,ABCC2,SPP1,PLA2R1,PLTP,NME4,CPT1A,MAP2K6,ABCA5,ABCA9,ACSL5,CHKA,SLCO3A1,PITPNM1,FABP4,PITPNC1,EGF,GRAMD1B,TEX2,FITM1,PDZD8,ABCB11,AGT,SLC25A20,ABCG4,SLCO1A2,SLCO1C1,OSBPL2,NTSR1,OSBP2,APOLD1,APOD,PLA2G4A,STARD5,FIS1,OSBPL6,SOAT1,OSBPL1A,EHD1,ZDHHC8,ABCA12,LPCAT3,SPNS3,SLC27A1,STAT5B,TMEM30B,SYT7,GPS2,CLN8,LIPC,ANXA2,OSBPL7,NAXE,ZFYVE1,ABCD4,APOBR,AC138894.1,ABCC3,PCTP,LDLR,ATG2B,NMUR2,TMEM159,STRA6,GM2A,NPC1,SREBF1,C3,NOS2,PROCA1,PRELID3A,PTPN2,PLIN3,ATP11C,PITPNA,ABCA3,ABCA7,RXRA,CEL,ABCD3,VPS4A,MIR27B,MIR27A,BSCL2,FABP5,ABCA8,MIR33B,PLA2G2C,LDLRAP1,CRH,CPTP,LRAT,STARD4,PTGES,SGPP1,XKR8	grey	VSMC_M10
GO:0000045	0.83	0.23	43	88	autophagosome assembly	BP	1	ATG12,PIP4K2C,RNF5,RAB23,STX17,ATG5,RAB7A,PIP4K2A,RALB,ATG16L2,TMEM173,RAB5A,FEZ2,ULK1,NSFL1C,TBC1D12,TP53INP1,SEC22B,TMEM39A,TCIRG1,ADPRH,ATG16L1,BAG3,MTMR3,RAB1B,PIKFYVE,ATG2A,TBC1D14,PINK1,ATG9A,WIPI2,PHF23,MFN2,PSEN1,VMP1,ATG2B,SMCR8,IFT20,MTM1,EMC6,KIAA1324,SH3GLB1,IRGM	grey	VSMC_M10
GO:0030206	0.83	0.33	7	16	chondroitin sulfate biosynthetic process	BP	1	CHPF2,CSGALNACT1,CHST3,CHST7,CHST12,B3GALT6,CHSY3	grey	VSMC_M10
GO:0036003	0.83	0.30	10	22	positive regulation of transcription from RNA polymerase II promoter in response to stress	BP	1	ATF4,HSF1,NFE2L2,RBPJ,MUC1,MAPK7,CREB3L3,HSPA5,DDIT3,CEBPB	grey	VSMC_M10
GO:0006355	0.83	0.19	1262	2411	regulation of transcription, DNA-templated	BP	1	ADNP2,NFATC1,ZNF236,ZNF407,SMARCC2,ATP8B1,ONECUT2,BAZ2A,TCF4,GLI1,MARS,MAP3K5,TNFAIP3,TSFM,HIVEP2,PLAGL1,MAML1,ZBTB2,YEATS4,ESR1,MYRFL,CNOT2,CNOT6,STK3,OSR2,SNW1,ZNF706,ARID1B,HSPA1B,FZD6,ZBTB12,ZBED4,PRKN,AGER,PPARA,MED30,IL5,BRD2,RXRB,VGLL2,HSF2,TCF7,PHF1,XRCC6,NCOA7,PITX1,TRIB1,MYC,TOB2,SMAD5,L3MBTL3,L3MBTL2,AGO2,KDM3B,PTP4A3,PTCH1,ATF4,RPAP2,CBX7,MAPK14,CBY1,KDM4C,SOX10,PIM1,NRF1,TMPRSS6,SMO,BNC2,HSF1,NFYA,FOXP4,MDFI,TFEB,IRF5,MED20,CCND3,FOXH1,LRRC14,CDKN2A,ZNF16,RBFOX2,IFT74,BICRAL,RORB,ZNF300,PRDM4,CRY1,DMTF1,SRF,ZNF318,NFX1,CDK14,FZD1,MTERF1,NFXL1,NFKBIE,SUPT3H,SMARCA2,RFX3,KIT,ADGRF1,NCK2,FHL2,TGFBRAP1,TFAP2B,ROR2,NFIL3,SEMA4D,RFX8,DLX5,PHF14,IPPK,ETV1,ZNF135,RPS14,ZNF154,MEOX2,ZNF550,KANSL3,BEND6,ZNF268,ZNF667,ZNF787,ZNF691,CAT,MACC1,SIX2,FOXJ3,ZNF865,HIVEP3,IL11,SCMH1,ZNF189,RNF20,EPAS1,EPCAM,SP4,PPP2R1A,ZNF462,PHIP,HMGN3,NFYC,NFE2L3,HOXA2,TAF1D,HOXA4,HOXA6,HOXA7,HOXA13,REL,ZNF483,JAZF1,CREB5,MYCL,XPO1,MTURN,NOD1,AKIRIN2,SRSF10,CASP8AP2,BACH2,ZNF692,PELI1,AKIRIN1,MTF1,ZNF618,SNIP1,ANXA4,PCBP1,NR1H2,AKNA,ZNF350,ITGB1BP1,FOXP2,GRHL1,MED25,KLF11,MET,IL4I1,AGO1,POU3F3,EGFR,LANCL2,ING3,NOBOX,ARHGEF5,TRIM32,TLR4,CCNC,CDK5RAP2,TRAF1,BCL2L12,LPIN1,SFRP4,UCP1,CREM,TEAD2,SETD7,SDCBP,MYCN,C6orf203,ELF2,OSR1,FOXO3,ZXDC,ZBTB24,MKX,FAF1,MAMSTR,ATAD2B,BEND5,CDCA7,KDM7A,ZNF438,FOXE3,HDAC2,PHF10,MAML2,BBS7,MLLT10,DNMT3A,BMI1,ZC3H4,PRKD2,CNBP,SET[illegible]	grey	VSMC_M10
GO:0051351	0.83	0.39	4	9	positive regulation of ligase activity	BP	1	XRCC1,XRCC4,FAM173B,NHEJ1	grey	VSMC_M10
GO:0007218	0.83	0.27	17	36	neuropeptide signaling pathway	BP	1	TAC3,MCHR1,GPR37,CYSLTR2,SCG5,RAPGEF2,NPY1R,SORCS3,CRCP,OPRD1,SORL1,NTSR1,PYY,NMUR2,NPB,NXPH4,GPR83	grey	VSMC_M10
GO:1905037	0.83	0.23	45	92	autophagosome organization	BP	1	ATG12,PIP4K2C,RNF5,RAB23,STX17,ATG5,RAB7A,PIP4K2A,RALB,ATG16L2,TMEM173,RAB5A,FEZ2,ULK1,NSFL1C,TBC1D12,TP53INP1,SEC22B,TMEM39A,TCIRG1,ADPRH,ATG16L1,BAG3,MTMR3,RAB1B,PIKFYVE,ATG2A,TBC1D14,PINK1,ATG9A,ATP13A2,WIPI2,PHF23,MFN2,PSEN1,AC138894.1,VMP1,ATG2B,SMCR8,IFT20,MTM1,EMC6,KIAA1324,SH3GLB1,IRGM	grey	VSMC_M10
GO:0048268	0.83	0.32	8	18	clathrin coat assembly	BP	1	EPS15,AP2S1,HIP1R,FCHO2,CALY,NSG1,AP2B1,SGIP1	grey	VSMC_M10
GO:0072599	0.83	0.27	16	34	establishment of protein localization to endoplasmic reticulum	BP	1	TRAM2,SEC61B,SEC61G,RAB10,SEC62,SRP14,SSR3,SPCS1,RYR2,SRPRA,ZFAND2B,SGTA,HSPA5,TRAM1L1,SPCS2,SEC61A2	grey	VSMC_M10

GO:0010591	0.83	0.27	16	34	regulation of lamellipodium assembly	BP	1	AVIL,RAC2,AKIRIN1,FER,PIK3R1,PLCE1,BIN3,CYFIP1,DMTN,AUTS2,WASF2,HDAC4,NCKAP1,WAS,DNM2,CARMIL2	grey	VSMC_M10
GO:0051154	0.83	0.28	15	32	negative regulation of striated muscle cell differentiation	BP	1	FRS2,PPARA,PI16,SOX6,BDNF,FZD7,HDAC4,BHLHA15,YY1,PLPP7,MIR199A1,MIR222,RGS4,YBX1,NKX2-5	grey	VSMC_M10
GO:0010712	0.83	0.27	16	34	regulation of collagen metabolic process	BP	1	HDAC2,CYGB,CYP7A1,F2R,FAP,AMELX,VSIR,WNT4,SUCO,LARP6,CIITA,SERPINF2,ENG,MIR29A,RGCC,CBX8	grey	VSMC_M10
GO:0061179	0.83	0.39	4	9	negative regulation of insulin secretion involved in cellular response to glucose stimulus	BP	1	NDUFAF2,ADRA2A,KLF7,ENY2	grey	VSMC_M10
GO:0016180	0.83	0.29	11	24	snRNA processing	BP	1	EXOSC4,EXOSC3,INTS10,USB1,INTS8,INTS12,INTS5,AP002990.1,INTS2,INTS11,EXOSC5	grey	VSMC_M10
GO:0014033	0.83	0.24	37	77	neural crest cell differentiation	BP	1	SEMA6A,SOX10,SMO,SEMA4D,SEMA4C,SOX9,OVOL2,JAG1,ALX1,SEMA3D,SEMA3A,NRG1,KBTBD8,RET,HAND2,MEF2C,SEMA4F,SEMA3G,SEMA4G,KLHL12,SEMA3F,PEF1,PDCD6,GBX2,LAMA5,SEMA6D,FN1,MAPK1,BMPR1A,RADIL,ALDH1A2,SEMA4A,MAPK3,GSC,TCOF1,ISL1,SOX8	grey	VSMC_M10
GO:0060428	0.83	0.29	12	26	lung epithelium development	BP	1	SOX9,FOXJ1,SRSF6,CREB1,SAV1,THRA,RBPJ,MAP2K1,STRA6,GATA6,PKD1,WNT2	grey	VSMC_M10
GO:0048857	0.83	0.26	22	46	neural nucleus development	BP	1	CKB,TTBK1,FOXP2,SIRT2,NDUFS3,SUDS3,COX6B1,INA,ALDH1A3,PADI2,ACTB,CHRNB2,CALM1,CDK5R1,SEC16A,ATP5PB,SYPL2,MAPKAP1,HSPA5,RHOA,BASP1,YWHAH	grey	VSMC_M10
GO:0030433	0.83	0.24	38	78	ubiquitin-dependent ERAD pathway	BP	1	CANX,RNF5,DERL1,FOXRED2,SEC61B,ECPAS,UBXN4,FBXO17,DNAJC18,NPLOC4,UBXN8,AUP1,TMEM67,AMFR,ERLIN1,YOD1,USP19,RNF185,RHBDD2,EDEM3,NFE2L2,SYVN1,DERL3,TMUB2,DNAJC10,DNAJB12,TMEM129,ANKZF1,DNAJB2,CALR3,FBXO2,SEL1L,BCAP31,SGTA,HSPA5,FAM8A1,UBE2G2,DERL2	grey	VSMC_M10
GO:0032482	0.83	0.32	8	18	Rab protein signal transduction	BP	1	RAB21,MADD,RAB4A,RAB15,DENND4A,RAB33A,DENND1A,RAB39A	grey	VSMC_M10
GO:0033574	0.83	0.27	16	34	response to testosterone	BP	1	NME1,TSPO,HSF1,MTAP,CA9,FOXP2,NCOA4,GPI,KCNJ11,NKX3-1,SPP1,SRD5A1,CBL,NQO1,BGLAP,DUSP1	grey	VSMC_M10
GO:0006775	0.83	0.30	10	22	fat-soluble vitamin metabolic process	BP	1	CYP27B1,CYP2R1,CBR3,PLTP,SNAI1,CYP27A1,ALDH1A2,PIAS4,GFI1,LRAT	grey	VSMC_M10
GO:0060021	0.83	0.24	34	71	roof of mouth development	BP	1	OSR2,COL11A2,TBC1D32,BNC2,DLX5,MEOX2,OSR1,INTU,BBS7,IFT172,CSRNP1,WNT11,TGFBR2,HAND2,FRAS1,TFAP2A,ITGB6,TGFB2,SATB2,SNAI1,EPHB3,DLG1,BCOR,BMPR1A,DHRS3,TMEM107,WFIKKN2,TBX2,JAG2,MMP25,SKI,WFIKKN1,WNT5A,INSIG2	grey	VSMC_M10
GO:0072080	0.83	0.23	36	75	nephron tubule development	BP	1	MTSS1,MYC,KLHL3,PTCH1,SMO,TFAP2B,SIX2,POU3F3,OSR1,SOX9,AQP11,GZF1,WNT11,JAG1,PAX8,EYA1,FMN1,MEF2C,PKD2,PAX2,SALL1,IRX2,AGT,LAMA5,DLG1,WNT4,MAGED1,CITED1,LHX1,GPC3,PKD1,SOX8,NOG,SIX1,LIF,FGF1	grey	VSMC_M10
GO:0009117	0.83	0.20	196	381	nucleotide metabolic process	BP	1	NME1,HSD17B4,TBPL1,PDE7B,ENPP3,ATP5MGL,DPYS,PPT2,PPARA,PDE10A,ACSL6,PRXL2C,GTPBP1,GUCA1B,IMPDH1,NMRK1,GDA,CROT,SLC35B2,SLC25A13,GAPDH,PPAT,RAB23,PPCS,SLC4A4,ME1,NT5E,PPT1,BPGM,DLD,SPHK2,CMPK1,ADCY3,NUDT15,DLG2,LACC1,NUDT5,DHTKD1,MBD4,PRKAG2,SLC22A13,ATP5F1C,ELOVL1,PFKP,IDO2,IDO1,NUDT16,PANK2,PDK1,SUCLG2,PDHX,SLC5A8,ELOVL7,PANK1,GPI,NUDT12,AMPD3,ATP5F1A,MCCC2,GPD1,P2RX7,GUCY1A1,GUCY1B1,ACOT12,DGUOK,PRKAG1,GMPS,PFKM,OPA1,ENTPD4,FITM2,AMPD1,NUDT18,ADCY7,GMPR,FAM173B,ADCY2,MCEE,NME4,PDE9A,TPST1,ZBTB20,GPAM,FAR2,ACSL5,PTHLH,PUDP,NUDT17,MFN1,PFKFB2,PRPS2,ENO4,PARP9,HACD2,ARNT,ADCY4,CTPS2,ACTN3,HDAC4,THEM5,DNAJC30,MLXIPL,PDE8A,DTYMK,HMGCL,MAGI3,DLG1,ACOT9,PDK3,EFL1,SLC25A16,UPB1,HK1,ACSBG1,ATP5S,CASK,COASY,PRKAG3,PINK1,STAT3,NME7,ADK,MPC2,ADCY10,MAPK1,ENO3,NT5C3B,PARP1,TECR,CARD11,SLC26A1,NAXE,PSEN1,ACOT2,GUCY2D,PKLR,PANK3,GCDH,OGT,UPRT,ADPGK,PDE4A,ACSM3,ACACA,NEIL1,NCOR1,INSR,SLC26A2,PRPSAP2,ADSSL1,NT5M,NOS2,ACSBG2,ALDOC,PIPOX,ACOT11,NADK,ATP6V0C,PANK4,NTHL1,NME3,MPP1,PARK7,ATP5PB,SLC2A6,VPS9D1,GNAI3,ACSF3,AK8,UCK1,MLYCD,KARS,FUK,DHODH,MT-ATP8,MT-ATP6,RHOA,SULT1C3,ATP5ME,ACOT4,ATP5MF,ENTPD5,PDZD3,ELOVL3,MTHFD2L,CMPK2,TIGAR,NUDT19,NNMT,NME6,DCTD,NUDT2,NAPRT,HINT1,UPP1,NMNAT1	grey	VSMC_M10
GO:0002637	0.83	0.25	23	48	regulation of immunoglobulin production	BP	1	IL5,EXOSC3,MSH2,HMCES,PHB,MLH1,PAXIP1,SHLD1,HPX,PRKDC,TRAF6,GPI,TMBIM6,CD40,CD28,BCL6,FOXP3,SHLD2,TBX21,STX4,IL4R,SUPT6H,TRAF2	grey	VSMC_M10
GO:0032309	0.83	0.29	11	24	icosanoid secretion	BP	1	PLA2G6,ABCC4,PLA2R1,MAP2K6,NTSR1,PLA2G4A,NMUR2,NOS2,PROCA1,PLA2G2C,PTGES	grey	VSMC_M10
GO:0032922	0.83	0.25	26	55	circadian regulation of gene expression	BP	1	PPARA,ATF4,CSNK1E,CRY1,HDAC2,MYCBP2,PPP1CB,BHLHE40,NUDT12,NCOA2,ARNTL,TOP1,CRY2,HDAC1,CIART,USP2,PER2,KDM5A,MAGED1,NGFR,OGT,KDM8,RAI1,PER3,ZFHX3,PASD1	grey	VSMC_M10
GO:0097553	0.83	0.23	46	95	calcium ion transmembrane import into cytosol	BP	1	PRKCE,IBTK,ATG5,GRIN2D,SLC8A2,RYR1,PRNP,DIAPH1,RASA3,RYR3,PTK2B,TPCN1,SLC8B1,F2R,PKD2,CASQ2,RYR2,LCK,TRPV4,UBASH3B,ANK2,GPER1,ATP1A2,CASQ1,NTSR1,CEMIP,PTPN6,HTT,HAP1,FAM155B,CD19,CALM1,BDKRB1,TRPV2,TRPV3,TRPV1,PDPK1,P2RX1,CYBA,JPH3,MCOLN3,DMD,NOL3,JPH2,THY1,DDIT3	grey	VSMC_M10

GO:0007186	0.83	0.19	234	458	G protein-coupled receptor signaling pathway	BP	1	TAC3,LRP1,ADGRG6,ESR1,FRS2,ADM2,FZD6,IGF2R,MCHR1,GRM4,GPR20,PICK1,RAC2,SMO,ZDHHC21,OPN1SW,GRM8,GPR37,RORB,CRY1,RIC8B,FZD1,PHF24,ADGRF1,S1PR3,ADGRL3,OXER1,GABBR2,CAMKMT,FPR2,KLK14,LPAR1,CXCL8,AREG,CRHR2,PLEK,GPR85,DGKI,MET,SPHK2,TPRA1,ADCY3,C5AR2,C5AR1,PTGIR,PHB,GIPR,CYSLTR2,TMEM145,GPR180,ACKR2,ARRB1,ZNF219,PDE6G,PLCB1,RASGRP4,ADRA1D,CCKBR,ADRA2B,TUB,PDGFRL,RGS20,HTR7,RGS7BP,PAX8,ABHD6,NLN,PIK3CB,PREX2,HPGD,PLCE1,CHRM5,ADGRV1,SCG5,FZD3,P2RY1,PTK2B,ADGRG5,RAPGEF2,CCL17,NPY1R,HOMER1,CHRM4,F2RL1,F2R,IQGAP2,RPGRIP1L,ADCY7,CHRM3,GRM2,APLP2,ADCY2,TRPM1,LGR6,RGS1,SORCS3,GIT2,CRCP,ADRA2A,GAP43,PTHLH,GNA13,GPR156,CMKLR1,RRH,OPRD1,GRK2,SORL1,GPER1,SAG,DGKD,AGT,WASF2,GPR3,ADCY4,FZD7,UCN2,ECE2,KCNK2,NTSR1,XPR1,PDE6H,FZD9,HOMER2,GPRC5D,GPR19,DGKG,FZD5,PTH1R,NMT1,PYY,PTPN6,GNB1L,ADRA2C,CXCR2,GPBAR1,GNG3,GNB3,PTGDR,GRK4,PTGER2,CALCRL,ECE1,RAMP2,GPR161,BHLHA15,CHRM1,TMOD2,TULP3,GNB5,DAGLB,GPR173,ADGRE5,GRK6,RGS14,ARHGEF11,ADGRA3,AGTRAP,DGKQ,HRH2,GUCY2D,DGKE,PIK3R5,CALM1,GPR68,DNM2,RIT2,BDKRB1,PDE4A,NMUR2,ADORA2B,HRH4,CAMK2A,CCL14,INSR,CCL5,CCL7,CCL2,CCL25,CDK5R1,RASD1,VAV1,PTGER4,C3,GNAL,ADGRG4,INPP5K,MGRN1,GNB1,GNA15,GNG10,GNG7,GPR153,BAIAP3,GNG13,PALM,GPR157,GNAT2,GNAI3,CELSR2,GPSM2,USP20,ADGRL2,PTGFR,HTR1D,CCL16,GPR35,RGS11,ADGRE3,ADGRG1,GPR182,CNTN2,TAS2R3,CHGA,CCR10,GPR37L1,CA2,GNG12,NPB,NXPH4,RGS12,CELSR3,RGS4,OR5AN1,GPR88,MLNR,G[illegible]	grey	VSMC_M10
GO:0045987	0.83	0.30	9	20	positive regulation of smooth muscle contraction	BP	1	SRF,KIT,ADRA2B,F2R,CHRM3,GPER1,ABAT,MIR21,RHOA	grey	VSMC_M10
GO:0032743	0.83	0.30	10	22	positive regulation of interleukin-2 production	BP	1	SPTBN1,PRKD2,TRAF6,IL1A,RIPK2,CD28,STAT5B,CARD11,TRAF2,GLMN	grey	VSMC_M10
GO:0006734	0.83	0.29	11	24	NADH metabolic process	BP	1	PFKP,MDH2,NUDT12,GPD1,PFKM,NUDT17,ACTN3,MDH1B,HK1,NQO1,TIGAR	grey	VSMC_M10
GO:0071346	0.83	0.23	39	80	cellular response to interferon-gamma	BP	1	GAPDH,NR1H2,TLR4,NMI,ACTG1,HPX,HCK,FLNB,CCL17,NR1H3,NLRC5,IFNGR2,RAB7B,SP100,PARP9,PARP14,CDC42EP2,WAS,MED1,STX4,STX8,CCL14,CCL5,CCL7,CCL2,CCL25,JAK1,NOS2,PTPN2,SOCS1,ADAMTS13,ASS1,STXBP1,CCL16,IRGM,CLDN1,WNT5A,VAMP3,CCL22	grey	VSMC_M10
GO:0060353	0.83	0.38	4	9	regulation of cell adhesion molecule production	BP	1	CXCL8,COLEC12,MIR222,MIR27B	grey	VSMC_M10
GO:0016925	0.83	0.26	20	42	protein sumoylation	BP	1	UHRF2,TRPM4,SUMO2,CBX4,EYA1,HDAC7,TOLLIP,IFIH1,PIAS3,ARNT,HDAC4,STX1A,MUL1,GNL3L,PIAS2,PIAS4,PARK7,RNF212B,RWDD3,RASD2	grey	VSMC_M10
GO:0060019	0.83	0.38	4	9	radial glial cell differentiation	BP	1	STAT3,GPR157,RHOA,METTL14	grey	VSMC_M10
GO:1903506	0.83	0.18	1262	2412	regulation of nucleic acid-templated transcription	BP	1	ADNP2,NFATC1,ZNF236,ZNF407,SMARCC2,ATP8B1,ONECUT2,BAZ2A,TCF4,GLI1,MARS,MAP3K5,TNFAIP3,TSFM,HIVEP2,PLAGL1,MAML1,ZBTB2,YEATS4,ESR1,MYRFL,CNOT2,CNOT6,STK3,OSR2,SNW1,ZNF706,ARID1B,HSPA1B,FZD6,ZBTB12,ZBED4,PRKN,AGER,PPARA,MED30,IL5,BRD2,RXRB,VGLL2,HSF2,TCF7,PHF1,XRCC6,NCOA7,PITX1,TRIB1,MYC,TOB2,SMAD5,L3MBTL3,L3MBTL2,AGO2,KDM3B,PTP4A3,PTCH1,ATF4,RPAP2,CBX7,MAPK14,CBY1,KDM4C,SOX10,PIM1,NRF1,TMPRSS6,SMO,BNC2,HSF1,NFYA,FOXP4,MDFI,TFEB,IRF5,MED20,CCND3,FOXH1,LRRC14,CDKN2A,ZNF16,RBFOX2,IFT74,BICRAL,RORB,ZNF300,PRDM4,CRY1,DMTF1,SRF,ZNF318,NFX1,CDK14,FZD1,MTERF1,NFXL1,NFKBIE,SUPT3H,SMARCA2,RFX3,KIT,ADGRF1,NCK2,FHL2,TGFBRAP1,TFAP2B,ROR2,NFIL3,SEMA4D,RFX8,DLX5,PHF14,IPPK,ETV1,ZNF135,RPS14,ZNF154,MEOX2,ZNF550,KANSL3,BEND6,ZNF268,ZNF667,ZNF787,ZNF691,CAT,MACC1,SIX2,FOXJ3,ZNF865,HIVEP3,IL11,SCMH1,ZNF189,RNF20,EPAS1,EPCAM,SP4,PPP2R1A,ZNF462,PHIP,HMGN3,NFYC,NFE2L3,HOXA2,TAF1D,HOXA4,HOXA6,HOXA7,HOXA13,REL,ZNF483,JAZF1,CREB5,MYCL,XPO1,MTURN,NOD1,AKIRIN2,SRSF10,CASP8AP2,BACH2,ZNF692,PELI1,AKIRIN1,MTF1,ZNF618,SNIP1,ANXA4,PCBP1,NR1H2,AKNA,ZNF350,ITGB1BP1,FOXP2,GRHL1,MED25,KLF11,MET,IL4I1,AGO1,POU3F3,EGFR,LANCL2,ING3,NOBOX,ARHGEF5,TRIM32,TLR4,CCNC,CDK5RAP2,TRAF1,BCL2L12,LPIN1,SFRP4,UCP1,CREM,TEAD2,SETD7,SDCBP,MYCN,C6orf203,ELF2,OSR1,FOXO3,ZXDC,ZBTB24,MKX,FAF1,MAMSTR,ATAD2B,BEND5,CDCA7,KDM7A,ZNF438,FOXE3,HDAC2,PHF10,MAML2,BBS7,MLLT10,DNMT3A,BMI1,ZC3H4,PRKD2,CNBP,SET[illegible]	grey	VSMC_M10
GO:0043491	0.83	0.21	73	147	protein kinase B signaling	BP	1	PHLPP1,STK3,PPARA,LEMD2,PTK2,NTRK2,TEK,RTN4,ITGB1BP1,MET,EGFR,SESN3,PHB,SOX9,PLK3,SPRY2,C1QTNF1,SIRT2,HBEGF,BAG4,NRG1,GAS6,MFHAS1,RET,PIK3CB,PTPRJ,NKX3-1,GCNT2,PAX2,ADTRP,CD40,IGF2,GRM2,BANK1,TCF7L2,PDCD6,DAG1,EGF,HCLS1,RPS6KB2,GPER1,CASS4,CIB1,OSM,THPO,CD28,THEM4,DLG1,ADAM8,STOX1,GDF15,PINK1,MUL1,OTUD3,PEAR1,HAX1,TMEM100,PIK3R5,INSR,CCL5,CCL2,INPP5K,PARK7,ENG,MIR29A,MIR199A1,MIR21,MIR222,DDIT3,TXN,MYORG,TSPYL5,RASD2	grey	VSMC_M10
GO:0071333	0.83	0.22	54	110	cellular response to glucose stimulus	BP	1	ZNF236,NME1,LRP1,AGER,PLA2G6,SRF,PRKCE,HMGN3,TRPM4,FOXO3,FGF21,GRIK5,RAF1,GAS6,NR1H4,CYP7A1,NDUFAF2,OSBP,KCNJ11,TIAM1,RAB11FIP5,OPA1,ZBED6,PAX2,TWNK,GPLD1,PPP3CA,SLC9B2,ZBTB20,ADRA2A,KCNB1,POLG,GPER1,RAB11FIP2,MLXIPL,SIDT2,PDK3,KLF7,FIS1,MEN1,ENY2,ABCA12,PPP3CB,PTPRN,MPC2,STX4,GPR68,SIN3A,OXCT1,NADK,BAIAP3,CYBA,SOX4,CRH	grey	VSMC_M10
GO:0008645	0.83	0.23	40	83	hexose transmembrane transport	BP	1	SLC2A12,MAPK14,SLC25A27,GRB10,FGF21,BRAF,MFSD4B,SLC26A5,PRKAG2,AKT2,ASPSCR1,TRIB3,PIK3R1,SORBS1,IRS1,SLC2A10,TERT,C2CD5,PEA15,NFE2L2,CLTCL1,SLC2A2,SLC27A1,SLC2A9,SLC2A4,RSC1A1,SLC5A2,SLC50A1,SLC5A11,PRKCB,INSR,YES1,C3,GPC3,INPP5K,SLC45A1,SLC2A6,TSC1,FABP5,ARPP19	grey	VSMC_M10
GO:0072202	0.83	0.29	10	22	cell differentiation involved in metanephros development	BP	1	SMO,SIX2,POU3F3,OSR1,PAX8,PAX2,SALL1,WNT4,CITED1,LIF	grey	VSMC_M10

GO:1904707	0.83	0.27	16	34	positive regulation of vascular associated smooth muscle cell proliferation	BP	1	MAP3K5,HPGD,ADAMTS1,NQO2,TERT,AGT,BMPR1A,MFN2,JUN,GNAI3,MIR21,MIR222,MIR27B,MIR27A,FOXJ2,LDLRAP1	grey	VSMC_M10
GO:0030212	0.83	0.29	10	22	hyaluronan metabolic process	BP	1	PIM1,CEMIP2,HAS1,IL15,HYAL1,HYAL3,EGF,ABCC5,CEMIP,HAS3	grey	VSMC_M10
GO:0007160	0.83	0.20	95	192	cell-matrix adhesion	BP	1	ITGA7,ONECUT2,LRP1,TNXB,PARVG,PTK2,CDKN2A,TEK,SRF,CDK6,SGCE,HOXA7,ITGB1BP1,EPHA1,CLASP2,BCAM,RIN2,JAG1,ACTG1,FERMT1,ARHGEF7,PTPRA,FAM107A,PIK3CB,PIK3R1,FMN1,SORBS1,PTPRJ,PTK2B,TIAM1,DMTN,ITGB6,LDB1,PXN,CD96,SLK,PPFIA1,COL17A1,FBLN5,COL16A1,DAG1,AJUBA,ITGA10,ARHGAP6,DISC1,CIB1,SLC9A1,ITGB5,ACTN3,WHAMM,STRC,EFEMP2,APOD,HOXD3,BCL6,COL13A1,TRIP6,ITGA2B,ITGA4,FN1,CASK,FERMT3,WNT4,NID2,COL3A1,TRPM7,VCL,BST1,MINK1,ITGA3,THBS3,TAOK2,SMAD3,ITGA11,CORO2B,OTOA,BCAS3,CAMSAP3,CCL25,ADAMTS12,PDPK1,L1CAM,PRKCZ,PKD1,ADAMTS13,TSC1,RHOA,FGA,ACVRL1,PEAK1,THY1,TIMM10B,ACER2,BCL2L11,DUSP22	grey	VSMC_M10
GO:0010640	0.83	0.30	9	20	regulation of platelet-derived growth factor receptor signaling pathway	BP	1	LRP1,PTGIR,HIP1R,PTPRJ,CBL,LRIG2,APOD,IFT20,PTPN2	grey	VSMC_M10
GO:0006623	0.83	0.26	16	35	protein targeting to vacuole	BP	1	VPS41,RAB7A,NCOA4,AP3B1,MON1A,HSPA8,SORL1,RNF215,VPS37D,AP4M1,AP3M1,VPS13D,VPS13C,LAMP2,VPS4A,SNX16	grey	VSMC_M10
GO:0001759	0.83	0.30	9	20	organ induction	BP	1	FRS2,SPRY1,WNT11,ROBO1,ROBO2,WNT5A,SIX1,FGF1,WNT2	grey	VSMC_M10
GO:0002369	0.83	0.29	10	22	T cell cytokine production	BP	1	RSAD2,TRPM4,TRAF6,DENND1B,FZD5,FOXP3,TNFRSF1B,TBX21,PRKCZ,TRAF2	grey	VSMC_M10
GO:0002724	0.83	0.29	10	22	regulation of T cell cytokine production	BP	1	RSAD2,TRPM4,TRAF6,DENND1B,FZD5,FOXP3,TNFRSF1B,TBX21,PRKCZ,TRAF2	grey	VSMC_M10
GO:0045595	0.84	0.18	597	1153	regulation of cell differentiation	BP	1	NFATC1,NME1,TCF4,LRP1,SEMA6A,GLI1,CYP27B1,MAML1,ATAT1,FRS2,RAB21,STK3,SNW1,TIAM2,HSPA1B,BRD1,AGER,PPARA,HLA-DRA,FBLN1,IL5,TSPO,FSTL4,TCF7,SYNGAP1,XRCC6,TRIB1,MYC,TOB2,SMAD5,PTK2,PTCH1,ATF4,PLXNA4,MAPK14,NTRK2,CSNK1E,KDM4C,SOX10,PI16,PIM1,SMO,ZDHHC21,HSF1,DAAM2,TAF8,ZNF16,RBFOX2,FBXO7,RORB,SRF,TTBK1,DLK2,OCIAD1,CDK6,RFX3,KIT,TNFRSF21,TFAP2B,ROR2,SEMA4D,S1PR3,SEMA4C,SOSTDC1,BEND6,ZNF268,SIX2,SMAP1,CD109,RTN4,PPP2R1A,RUFY3,HOXA2,HOXA7,ANKRD17,PTBP3,MYCL,SLC46A2,MTURN,AREG,AKIRIN1,CNOT4,EPHA7,NR1H2,MBOAT2,PTN,ITGB1BP1,GRHL1,ST7,IL4I1,EGFR,FUZ,TRIM32,TLR4,CDK5RAP2,IL15,SFRP4,MYLK3,DKKL1,TEAD2,SDCBP,MYCN,NRCAM,TRPM4,OSR1,FOXO3,NTF4,MKX,APOB,MAMSTR,SPRY1,PLXNA1,FOXE3,HDAC2,NAPEPLD,SOX9,CLASP2,CLPTM1,PTCH2,GOLGA4,GLI2,MEGF8,AAMDC,KDM4A,UNC13D,RAF1,XRCC2,ACVR2B,KLF5,FOXJ1,NUMBL,POU4F1,TSKU,IL15RA,SPRY2,SS18L2,ALK,RIN2,HOOK3,TGFBR2,OVOL2,ZNF219,ACVR2A,IKBKB,PLEKHB1,BHLHE40,JAG1,SIRT2,RARB,NR1D2,PTPRQ,SRA1,PLCB1,MAFG,SPRED3,RAC3,ALX1,EIF2AK2,LSM1,ARHGEF7,SEMA3D,BTG1,SPART,PLXNC1,SEMA3A,CLIC1,NRG1,NCKAP1L,GAS6,ULK1,FOXP1,MITF,LMOD3,PRKDC,IRF7,RYK,ADRA2B,TRAF6,TCF15,TRIB3,EPHB1,SOX12,PLAG1,RET,TOX,ZC3H8,ASXL1,DNMT3B,PAX8,ACVR1B,CAMK4,CEBPA,SMYD1,NLN,ANKRD27,FAIM,ZNF536,PIK3R1,HAND2,EYA1,ROBO1,ROBO2,IL17RD,ARNTL,FBXW7,ADGRV1,FZD3,MEF2C,TMEM131L,SEMA4F,IL7,VEGFC,TLX2,CUX2,SOX6,PTK2B,RASGRP1,MME,RAPGEF2,DPYSL2,CCL17,EIF2AK4,NR[illegible]	grey	VSMC_M10
GO:0031113	0.84	0.25	23	49	regulation of microtubule polymerization	BP	1	HSPA1B,SLAIN2,MET,CDK5RAP2,MAPRE1,EML2,SLC39A12,MAPRE3,SLAIN1,NAV3,ARHGEF7,CLIP1,INPP5J,CDKN1B,MAPT,TOGARAM1,NIN,CAMSAP3,CDK5R1,MECP2,CAMSAP1,PSRC1,DRG1	grey	VSMC_M10
GO:1903540	0.84	0.32	7	16	establishment of protein localization to postsynaptic membrane	BP	1	NSG1,GRIPAP1,RAB8A,CPLX1,STX1B,RAB11A,ARHGAP44	grey	VSMC_M10
GO:0002052	0.84	0.32	7	16	positive regulation of neuroblast proliferation	BP	1	SOX10,SMO,FZD3,VEGFC,ZNF335,DISC1,FOXG1	grey	VSMC_M10
GO:0045773	0.84	0.26	17	37	positive regulation of axon extension	BP	1	LRP1,SRF,RUFY3,GOLGA4,MEGF8,ZFYVE27,CYFIP1,ADNP,DISC1,CDKL5,MAPT,FN1,NDEL1,RAB11A,NTN1,TRPV2,L1CAM	grey	VSMC_M10
GO:0006892	0.84	0.22	48	99	post-Golgi vesicle-mediated transport	BP	1	AP3S1,GOPC,VPS52,EXOC4,STEAP2,SPTBN1,EPS15,RAB10,RBSN,OPTN,GOLGA4,CCDC93,LYPLA1,VAMP5,EXOC6B,DOP1B,KIF13A,GBF1,CCDC91,VTI1A,SNAP23,AP1AR,SORL1,BBS1,RNF215,NSF,SCAMP3,KLHL20,AP4M1,ANKFY1,EXOC5,CNST,RSC1A1,GAK,VPS13C,AC138894.1,DNM2,GGA2,SCAMP2,VPS35L,CORO7,RAB26,SEC16A,AP1G1,C16orf70,AP3S2,RABIF,VAMP3	grey	VSMC_M10
GO:0006007	0.84	0.32	7	16	glucose catabolic process	BP	1	PFKP,PFKM,PFKFB2,ACTN3,HK1,TKTL1,TIGAR	grey	VSMC_M10
GO:0007338	0.84	0.22	42	86	single fertilization	BP	1	TRIM36,H3F3C,MYH9,NLRP5,RIMS1,SPACA6,DKKL1,IZUMO1,ADCY3,TRPC3,WDR48,PLAT,ADAM18,PLCB1,UBXN8,STX2,AP3B1,NPM2,HYAL3,SLC9B1,ABHD2,CCDC87,LYZL6,OVGP1,PLCD4,RAB3A,AKAP3,ADAM21,ADAM20,UBAP2L,SPESP1,NOX5,ZP2,SNU13,CACNA1H,PARK7,STXBP1,CD46,GLIPR1L2,LHFPL2,SPA17,TMPRSS12	grey	VSMC_M10
GO:0048199	0.84	0.30	9	20	vesicle targeting, to, from or within Golgi	BP	1	MAPK15,ARFGAP2,GBF1,KLHL12,PEF1,PDCD6,AP1AR,TMED10,PPP6C	grey	VSMC_M10
GO:0010457	0.84	0.33	6	14	centriole-centriole cohesion	BP	1	KIF3A,CNTLN,CEP68,CEP44,CEP250,NIN	grey	VSMC_M10
GO:0035335	0.84	0.23	37	77	peptidyl-tyrosine dephosphorylation	BP	1	EYA4,PTP4A3,PTPDC1,ACP4,RNGTT,MTMR2,PTPRF,TIMM50,MTMR14,PTPRQ,DUSP6,PTPRA,MTMR7,EYA1,PTPRJ,PTPN5,SSH1,UBASH3B,EYA3,MTMR3,PTPRO,DUSP18,DUSP12,DUSP10,DUSP19,PTPN6,PTPN21,DUSP14,PTPN2,SSH2,MTM1,MTMR1,CDC14A,DUSP26,DUSP1,DUSP22,PTPMT1	grey	VSMC_M10

GO:0043405	0.84	0.21	72	146	regulation of MAP kinase activity	BP	1	MAP3K5,TNXB,MAP3K4,AGER,TRIB1,TAB1,STK38,KIT,ROR2,DNAJA1,EPGN,TGFA,EGFR,ARHGEF5,TLR4,SPRY1,SPRY2,NEK10,FLT1,TRAF6,TRIB3,PIK3CB,ROBO1,PTPRJ,PTK2B,RASGRP1,TIAM1,PAQR3,NOD2,DTNBP1,TAOK3,CD40,PDCD4,ADRA2A,AJUBA,KRAS,EGF,SORL1,AGT,DVL3,MAP3K11,ADAM8,AIDA,FZD5,DUSP10,MAP4K2,DUSP19,PTPN6,PDCD10,EPHA4,MAGED1,DVL2,CDK12,RGS14,PSEN1,FGF18,PIK3R5,MAP2K5,INSR,GHR,KSR1,INPP5K,TRAF2,DAB2IP,S100A12,WNT5A,DUSP1,HRAS,LAX1,MAP2K7,FGF1,PDGFD	grey	VSMC_M10
GO:0030865	0.84	0.24	26	55	cortical cytoskeleton organization	BP	1	EPB41L2,RAC2,TLN1,PLEK,LCP1,LLGL2,IKBKB,RAC3,NCKAP1L,PLS1,EPB41L1,RTKN,RND1,RHOBTB2,TRPV4,EPB41,RHOBTB1,DLG1,PPP2R3C,NCKAP1,EPB41L3,STRIP1,VPS4A,FHOD1,RHOA,RND3	grey	VSMC_M10
GO:0006006	0.84	0.21	77	155	glucose metabolic process	BP	1	PRKN,PPARA,ATF4,MAPK14,GPT,GNMT,CRY1,PGM5,TFAP2B,SLC25A13,GAPDH,PPP4R3B,ZNF692,FAM3C,RUBCNL,AKR1A1,KHK,EPM2AIP1,GCKR,PPP1CB,AKT2,PFKP,PGM2L1,C1QTNF1,SIRT7,PDK1,PPP1R3B,GPI,NLN,NCOA2,SORBS1,GPD1,KCNJ11,PFKM,IGF2,GPLD1,IRS1,SIK1,CPT1A,MST1,PPP1R3E,PFKFB2,PC,PPP1R3D,SLC37A4,PER2,ACTN3,USF1,PASK,DUSP12,APOD,PDK3,SORD,HK1,GNB3,PPP1R3F,IGFBP4,DDB1,DGKQ,OGT,COX11,CRTC2,LCMT1,ADPGK,INSR,LEPR,PTPN2,INPP5K,TKTL1,MLYCD,ACADM,FBP2,OAS1,FABP5,TIGAR,NNMT,ARPP19	grey	VSMC_M10
GO:0072163	0.84	0.22	38	79	mesonephric epithelium development	BP	1	MYC,SMAD5,PTCH1,SMO,CAT,SIX2,EPCAM,BMPER,OSR1,SPRY1,SOX9,FOXJ1,GZF1,WNT11,RARB,RET,PAX8,EYA1,ROBO2,FMN1,PKD2,PAX2,SALL1,AGT,LAMA5,DLG1,WNT4,MAGED1,CITED1,SMAD3,LHX1,GPC3,PKD1,SOX8,NOG,BASP1,SIX1,FGF1	grey	VSMC_M10
GO:0072164	0.84	0.22	38	79	mesonephric tubule development	BP	1	MYC,SMAD5,PTCH1,SMO,CAT,SIX2,EPCAM,BMPER,OSR1,SPRY1,SOX9,FOXJ1,GZF1,WNT11,RARB,RET,PAX8,EYA1,ROBO2,FMN1,PKD2,PAX2,SALL1,AGT,LAMA5,DLG1,WNT4,MAGED1,CITED1,SMAD3,LHX1,GPC3,PKD1,SOX8,NOG,BASP1,SIX1,FGF1	grey	VSMC_M10
GO:1905153	0.84	0.33	6	14	regulation of membrane invagination	BP	1	NCKAP1L,F2RL1,ANO6,SYT11,C3,ABCA7	grey	VSMC_M10
GO:0045047	0.84	0.27	14	30	protein targeting to ER	BP	1	TRAM2,SEC61B,SEC61G,SEC62,SRP14,SSR3,SPCS1,SRPRA,ZFAND2B,SGTA,HSPA5,TRAM1L1,SPCS2,SEC61A2	grey	VSMC_M10
GO:0034105	0.84	0.37	4	9	positive regulation of tissue remodeling	BP	1	IL15,PPP3CA,MIR199A1,MIR27B	grey	VSMC_M10
GO:0021987	0.84	0.22	42	87	cerebral cortex development	BP	1	LRP1,SUN2,NTRK2,SMO,EFHC1,MBOAT7,RTN4,FOXP2,POU3F3,FAT4,MKKS,PLCB1,ROBO1,CELF1,CSNK2A2,MCPH1,SLC38A2,SRGAP2C,SRD5A1,DISC1,NSUN5,BCL2A1,DIXDC1,COL3A1,MPP5,PSEN1,NDEL1,XAB2,CDK5R1,DAB1,LRP8,CRK,TSC1,DAB2IP,RHOA,ADGRG1,FOXG1,LAMB1,GNG12,CCDC85C,POU3F2,BTBD3	grey	VSMC_M10
GO:0150011	0.84	0.33	6	14	regulation of neuron projection arborization	BP	1	DVL3,DLG4,DVL2,MYO9A,NTNG2,WNT5A	grey	VSMC_M10
GO:0097107	0.84	0.33	6	14	postsynaptic density assembly	BP	1	LRFN1,NRXN2,NLGN2,ZDHHC12,GRID2,LRRTM2	grey	VSMC_M10
GO:0098742	0.84	0.20	74	152	cell-cell adhesion via plasma-membrane adhesion molecules	BP	1	MAPK14,LRRC4,CLDN12,ADGRL3,CLDN19,EPCAM,PCDH10,FAT4,NECTIN2,PVR,PCDH8,PTPRF,TGFBR2,PCDHGC5,PCDH1,UNC5D,LRFN3,RET,CLSTN2,ROBO1,ROBO2,TENM3,CDH17,AMIGO2,SPARCL1,ALCAM,NECTIN3,TGFB2,IGSF11,AMIGO3,AJUBA,CDH24,NECTIN4,CADM1,MYPN,HMCN1,CDH23,ARVCF,OBSL1,CLDN7,SDK1,PCDH7,ITGAM,TENM2,MAP2K5,PCDH11X,PCDH19,UMOD,DSG2,DSC3,MAPK7,EMB,DAB1,CDH6,CDH10,SLITRK2,L1CAM,PKD1,BSG,AMIGO1,CELSR2,CDH15,NTNG2,CDH16,PCDHAC2,PCDHA6,CLDN15,CNTN2,GRID2,CLDN1,CDHR4,CELSR3,PCDHGA2,PCDHB15	grey	VSMC_M10
GO:0006044	0.84	0.31	7	16	N-acetylglucosamine metabolic process	BP	1	LARGE1,GNPDA2,NANP,CHST1,CHST3,CHST7,CHST5	grey	VSMC_M10
GO:1990845	0.84	0.21	65	132	adaptive thermogenesis	BP	1	ATF4,HSF1,ALDH1A1,GADD45G,EPAS1,BMP8A,NR1H2,GRB10,TLR4,LPIN1,IL15,UCP1,FGF21,LAMA4,ACVR2B,PLCL2,ABHD6,ARNTL,NR1H3,ALMS1,EBF2,KSR2,ZNF423,PRKAB1,PM20D1,MAP2K6,TRPV4,CMKLR1,FABP4,SORL1,TRPM8,GPR3,PER2,ACTN3,ACADL,TFE3,MFAP2,YBX2,THRA,RBPJ,MFN2,KDM6B,SLC25A44,ZBTB7B,OGT,PCTP,IL4R,TRPV2,PPARGC1B,LEPR,DOCK7,ACOT11,TRPV1,PRDM16,LCN2,BSCL2,FABP5,ELOVL3,PEMT,NOVA1,PLAC8,DDIT3,APC,CEBPB,DIO2	grey	VSMC_M10
GO:0009156	0.84	0.27	14	30	ribonucleoside monophosphate biosynthetic process	BP	1	IMPDH1,PPAT,CMPK1,AMPD3,GMPS,AMPD1,PRPS2,ADK,UPRT,ADSSL1,UCK1,DHODH,NUDT2,UPP1	grey	VSMC_M10
GO:1902992	0.84	0.28	11	24	negative regulation of amyloid precursor protein catabolic process	BP	1	NTRK2,RTN4,PRNP,SORL1,RTN1,HAP1,TMED10,ABCA7,MIR29A,MIR24-1,MIR339	grey	VSMC_M10
GO:0086004	0.84	0.27	13	29	regulation of cardiac muscle cell contraction	BP	1	TRPM4,SCN5A,STC1,DSP,RYR2,PKP2,KCNJ2,ANK2,ATP1A2,DLG1,ATP2A1,DSG2,HCN4	grey	VSMC_M10
GO:0060249	0.84	0.20	109	218	anatomical structure homeostasis	BP	1	TNFAIP3,SLC12A2,PRKN,TNFRSF11B,AKR1B1,RAC2,SMO,LARGE1,ALDH1A1,SRF,CLDN12,SLC1A1,KIAA1211,PRDX1,EPAS1,HOXA13,ALB,MTF1,WHRN,EGFR,USP45,TRIM32,TLR4,NCDN,SLC8A2,SOX9,CACNB2,CUBN,USH1G,MKKS,ACTG1,RAC3,ERCC6,TRAF6,TUB,LSR,CHMP2B,CCDC66,ADGRV1,IL7,P2RX7,JAM2,P2RY1,PTK2B,USH1C,PFKM,HOMER1,F2R,SLC6A2,SPP1,NELL2,GCNT2,NOD2,ITGB6,LDB1,LPCAT1,LRRK1,TCIRG1,GPR137B,KRAS,GIGYF2,UBASH3B,BAG3,BBS1,HOMER2,LAMC1,ADAM8,PTH1R,PBLD,CTSH,CDH23,ABCA12,GPR137,DNAJB2,VCL,CD38,ILDR2,LIPA,ACTB,CLN8,WNK3,ATP1B2,ESRRB,RAB3D,MKS1,ACACA,CSF1R,PPARGC1B,FGGY,TAOK1,MTM1,ATP2B3,P2RX1,ABCA3,ATP6AP1,DEF8,OAS1,DMD,SLC22A5,CA2,NANOS1,JCHAIN,BGLAP,SNX10,CLDN1,INAVA,DRAM2,BBS10,MAP1A	grey	VSMC_M10
GO:0016486	0.84	0.30	8	18	peptide hormone processing	BP	1	PCSK1,SCG5,MME,ENPEP,ACE2,ECE2,PCSK7,ECE1	grey	VSMC_M10
GO:0140448	0.84	0.30	8	18	signaling receptor ligand precursor processing	BP	1	PCSK1,SCG5,MME,ENPEP,ACE2,ECE2,PCSK7,ECE1	grey	VSMC_M10
GO:0060149	0.84	0.31	7	16	negative regulation of posttranscriptional gene silencing	BP	1	ZC3H10,ESR1,BCDIN3D,PPP3CA,TERT,STAT3,NCOR1	grey	VSMC_M10

GO:0060967	0.84	0.31	7	16	negative regulation of gene silencing by RNA	BP	1	ZC3H10,ESR1,BCDIN3D,PPP3CA,TERT,STAT3,NCOR1	grey	VSMC_M10
GO:0045176	0.84	0.32	6	14	apical protein localization	BP	1	ATP8B1,GOPC,NAPA,MAL,SHROOM2,ACTB	grey	VSMC_M10
GO:0007263	0.84	0.30	8	18	nitric oxide mediated signal transduction	BP	1	EGFR,NDNF,GUCY1A1,GUCY1B1,AGT,NOS1AP,RASD1,NOS2	grey	VSMC_M10
GO:0071359	0.84	0.30	8	18	cellular response to dsRNA	BP	1	RALB,TMEM173,MAVS,IFIH1,MUL1,RIOK3,COLEC12,ZCCHC3	grey	VSMC_M10
GO:2000316	0.84	0.31	7	16	regulation of T-helper 17 type immune response	BP	1	RC3H1,TNFSF18,FOXP3,TBX21,ZBTB7B,MIR21,RC3H2	grey	VSMC_M10
GO:0045165	0.84	0.20	85	172	cell fate commitment	BP	1	ONECUT2,PITX1,SMAD5,PTCH1,SMO,ROR2,SOSTDC1,SIX2,EPAS1,HOXA2,HOXA13,NTF4,RAB10,SOX9,PTCH2,GLI2,MNX1,POU4F1,SPRY2,WNT11,EOMES,JAG1,NRG1,MITF,PRKDC,SOX12,TOX,CASP3,EYA1,MEF2C,IL7,SOX6,EBF2,TBX5,RTF1,SOX13,MGA,PAX2,SUFU,GAP43,SATB2,TCF7L2,SOX2,SLAMF8,MCL1,FZD7,DLX1,WNT3,TNFSF18,WNT4,FKBP8,STAT3,TBX19,LEO1,FOXP3,BMPR1A,RBPJ,EPOP,TBX21,PSEN1,KDM6B,TGFB1I1,BATF,IL6R,GSC,TBX4,TBX2,BCL11B,GATA6,JAG2,ISL1,DOCK7,APC2,SOX8,BARHL2,RHOA,FOXG1,ISL2,LBX1,WNT5A,POU3F2,SIX1,APC,HEY2,WNT2	grey	VSMC_M10
GO:0001823	0.84	0.22	39	81	mesonephros development	BP	1	OSR2,MYC,SMAD5,PTCH1,SMO,CAT,SIX2,EPCAM,BMPER,OSR1,SPRY1,SOX9,FOXJ1,GZF1,WNT11,RARB,RET,PAX8,EYA1,ROBO2,FMN1,PKD2,PAX2,SALL1,AGT,LAMA5,DLG1,WNT4,MAGED1,CITED1,SMAD3,LHX1,GPC3,PKD1,SOX8,NOG,BASP1,SIX1,FGF1	grey	VSMC_M10
GO:0065005	0.84	0.28	10	22	protein-lipid complex assembly	BP	1	APOM,NR1H2,APOB,APOC1,SOAT2,SOAT1,ZDHHC8,LPCAT3,ABCA7,PLAGL2	grey	VSMC_M10
GO:0033233	0.84	0.28	10	22	regulation of protein sumoylation	BP	1	TOLLIP,PIAS3,ARNT,HDAC4,MUL1,GNL3L,PIAS4,PARK7,RWDD3,RASD2	grey	VSMC_M10
GO:0031529	0.84	0.24	23	49	ruffle organization	BP	1	EPS8L1,PLEK,BAG4,ARFIP2,LIMA1,ARHGEF26,ARHGAP24,CYFIP1,CARMIL1,TCIRG1,EPS8,NDEL1,EVL,CSF1R,INPP5K,EPS8L3,DEF8,MTSS1L,CARMIL2,EPS8L2,SNX10,CSPG4,HRAS	grey	VSMC_M10
GO:0002027	0.84	0.22	35	73	regulation of heart rate	BP	1	SLC1A1,EPAS1,BVES,TRPM4,CACNB2,SCN5A,GPD1L,ADRA1D,SEMA3A,KCNJ3,KCNE2,DSP,CASQ2,RYR2,PKP2,KCNJ2,MYH7,ANK2,AGT,SCN4B,NOS1AP,GCH1,CACNA1G,CALM1,DSG2,SREBF1,ISL1,TRPV1,DMD,KCNH2,SCN2B,RGS4,POPDC2,HCN4,HEY2	grey	VSMC_M10
GO:0090110	0.84	0.32	6	14	COPII-coated vesicle cargo loading	BP	1	SEC31A,SEC31B,SCAP,MIA3,SEC24C,TBC1D20	grey	VSMC_M10
GO:0035794	0.84	0.26	16	34	positive regulation of mitochondrial membrane permeability	BP	1	HEBP2,TMEM14A,HIP1R,SLC9A1,STPG1,FZD9,NMT1,MUL1,TMEM102,ACAA2,MIR29A,BNIP3L,BCL2L11,SLC35F6,PPIF,AC140504.1	grey	VSMC_M10
GO:0032682	0.84	0.31	7	16	negative regulation of chemokine production	BP	1	OAS3,F2RL1,APOD,MUL1,MAP2K5,SUZ12,OAS1	grey	VSMC_M10
GO:0003323	0.84	0.30	8	18	type B pancreatic cell development	BP	1	SMO,CDK6,RFX3,ARNTL,NKX6-1,SIDT2,PDPK1,WNT5A	grey	VSMC_M10
GO:0071604	0.84	0.25	16	35	transforming growth factor beta production	BP	1	TSKU,WNT11,FERMT1,LUM,ITGB6,CD200,TGFB2,CREB1,FN1,FOXP3,BMPR1A,SMAD3,GATA6,SERPINF2,CD46,NRROS	grey	VSMC_M10
GO:0045814	0.84	0.24	20	43	negative regulation of gene expression, epigenetic	BP	1	BAZ2A,H3F3C,DNMT3A,BMI1,SETDB2,SIRT2,FAM208A,BAHD1,PPHLN1,HDAC1,ZNFX1,TET1,H2AFY2,PHF8,NRDE2,SMCHD1,SETDB1,CREBZF,BEND3,ZNF304	grey	VSMC_M10
GO:0010518	0.84	0.25	19	41	positive regulation of phospholipase activity	BP	1	ESR1,KIT,LPAR1,EGFR,C5AR1,PHB,RASGRP4,FLT1,GNA13,ARHGAP6,AGT,ABL2,FGFR3,NMUR2,CCL5,GNA15,PDPK1,PRKCZ,HRAS	grey	VSMC_M10
GO:0006122	0.84	0.37	4	9	mitochondrial electron transport, ubiquinol to cytochrome c	BP	1	UQCRQ,UQCRB,UQCR11,MT-CYB	grey	VSMC_M10
GO:0045600	0.84	0.23	25	53	positive regulation of fat cell differentiation	BP	1	STK3,MAPK14,PIM1,DKKL1,TRPM4,NAPEPLD,AAMDC,KLF5,CEBPA,CDS1,RREB1,SOX13,STK4,CMKLR1,SYAP1,CREB1,SAV1,TFE3,ZBTB7B,ZBTB7C,MIR21,SIX1,CCDC3,CEBPB,CCDC71L	grey	VSMC_M10
GO:0009214	0.84	0.33	5	12	cyclic nucleotide catabolic process	BP	1	PDE7B,PDE10A,PDE9A,PDE8A,PDE4A	grey	VSMC_M10
GO:0046426	0.84	0.29	9	20	negative regulation of receptor signaling pathway via JAK-STAT	BP	1	PPP2R1A,PIBF1,PARP14,SUZ12,PTPN2,SOCS1,DAB1,MIR125A,LEPROT	grey	VSMC_M10
GO:0070849	0.84	0.24	19	41	response to epidermal growth factor	BP	1	MARS,EGFR,SOX9,BAG4,PAX2,CBL,SYAP1,PDE8A,SNX6,MAPK1,STAT5B,MED1,MAPK3,GAREM1,INPP5K,PDPK1,DAB2IP,DUSP22,ZFP36L2	grey	VSMC_M10
GO:0098780	0.84	0.30	8	18	response to mitochondrial depolarisation	BP	1	PRKN,OPTN,HTRA2,PINK1,MUL1,GPS2,MFN2,VPS13C	grey	VSMC_M10
GO:0045666	0.84	0.23	29	61	positive regulation of neuron differentiation	BP	1	TCF4,CSNK1E,KDM4C,BEND6,TRIM32,NRCAM,GLI2,KDM4A,ADRA2B,DNMT3B,MEF2C,NKX6-1,DLX1,SH3GL3,HOXD3,BCL6,BRINP2,ADRA2C,CYB5D2,PRPF19,NAP1L2,SIN3A,DAB1,RHOA,FOXG1,IMPACT,MMD,TGIF2,NKX2-5	grey	VSMC_M10
GO:1901983	0.84	0.23	30	63	regulation of protein acetylation	BP	1	HINT2,ATG5,SPHK2,HDAC2,IWS1,PAXIP1,ARRB1,TAF7,SDR16C5,ARNTL,RAPGEF3,HDAC1,AUTS2,DDX3X,TADA2B,FOXP3,KAT7,MUC1,MAPK3,NAP1L2,RPS6KA5,SIN3A,ISL1,TAOK1,GLYR1,PARK7,CTCF,SOX4,LIF,RPS6KA4	grey	VSMC_M10
GO:0021955	0.84	0.26	13	29	central nervous system neuron axonogenesis	BP	1	PLXNA4,GLI2,MYCBP2,TSKU,SZT2,EPHB1,NR4A2,ADARB1,EPHB3,NIN,EPHA4,NDEL1,CHRNB2	grey	VSMC_M10
GO:0110154	0.84	0.31	7	16	RNA decapping	BP	1	DXO,LSM1,NUDT16,NUDT12,EIF4ENIF1,DCP1B,EDC4	grey	VSMC_M10
GO:0045747	0.84	0.25	15	33	positive regulation of Notch signaling pathway	BP	1	MFNG,KIT,AAK1,ITGB1BP1,JAG1,ROBO1,ROBO2,BMP2K,NOD2,NEPRO,TGFB2,POGLUT1,PDCD10,STAT3,JAG2	grey	VSMC_M10
GO:0002088	0.84	0.22	30	63	lens development in camera-type eye	BP	1	FRS2,TBC1D32,ATF4,TDRD7,TMOD1,SLC7A11,SPRY1,FOXE3,CRYGN,SPRY2,TGFBR2,BFSP1,SPRED3,NECTIN3,SHROOM2,CRYBA4,CDKN1B,DLG1,PLA2G16,MED1,LCTL,SMAD3,UNC45B,FZR1,SKI,HSF4,WNT5A,TBC1D20,PITX3,WNT2	grey	VSMC_M10
GO:1903020	0.84	0.29	9	20	positive regulation of glycoprotein metabolic process	BP	1	IL15,PAWR,PLCB1,SLC2A10,TCF7L2,RAB1B,SOAT1,POMT2,IL33	grey	VSMC_M10
GO:0031638	0.84	0.25	18	38	zymogen activation	BP	1	BAK1,PLGRKT,PLAT,ASPH,RIPK2,CIDEB,PRSS12,MELTF,CTSH,VSIR,HGFAC,F12,SERPINF2,HPR,FGA,FADD,CASP8,PERP	grey	VSMC_M10
GO:1903319	0.84	0.28	10	22	positive regulation of protein maturation	BP	1	MYH9,PLGRKT,RHBDD1,MELTF,ADAM8,LPCAT3,F12,CNTN2,SOX4,BAG2	grey	VSMC_M10
GO:1902430	0.84	0.28	10	22	negative regulation of amyloid-beta formation	BP	1	NTRK2,RTN4,PRNP,SORL1,RTN1,HAP1,ABCA7,MIR29A,MIR24-1,MIR339	grey	VSMC_M10

GO:0071634	0.84	0.25	15	33	regulation of transforming growth factor beta production	BP	1	TSKU,WNT11,FERMT1,LUM,ITGB6,CD200,TGFB2,CREB1,FN1,FOXP3,BMPR1A,SMAD3,GATA6,SERPINF2,CD46	grey	VSMC_M10
GO:0014823	0.84	0.23	27	57	response to activity	BP	1	TPH2,HSF1,CRY1,ABCG5,CAT,ABCG8,PTN,FGF21,PRKDC,DNMT3B,OPA1,CRY2,RYR2,SRD5A1,AGT,CBL,MTFP1,CREB1,FIS1,PRKAG3,BNIP1,KDM6B,ADSSL1,OXCT1,CYBA,SLC25A25,BGLAP	grey	VSMC_M10
GO:0046903	0.84	0.17	346	673	secretion	BP	1	NME1,VPS4B,ERBB3,LRP1,CANX,RAB21,SNCAIP,SLC12A2,RIMS2,PRKN,SLC22A4,GDF9,GRM4,LY6E,EXOC4,SYNGR1,NTRK2,PLA2G6,COPG2,PICK1,RAC2,MYH9,GUCA1B,CHRNA3,CHRNA5,CRY1,STEAP2,APBA1,SCFD2,RFX3,KIT,CA9,TFAP2B,PPAT,CHMP2A,NLRP5,SVBP,STX17,IL11,BRSK1,PRKCE,RIMS1,HMGN3,RIMS3,CYB5R4,PPT1,SCRN1,BMP8A,AQP1,PLEK,RSAD2,NR1H2,EGFR,TLR4,VPS41,ATG5,SDCBP,TRPM4,PPFIA3,LIN7B,TRAF3IP2,SPHK2,NAPA,RAB7A,RAB10,COPG1,GIPR,CACNB2,SYTL2,CLASP2,FRMD4A,GAB2,LLGL2,RALB,STEAP3,GRIK5,UNC13D,RAF1,NAPB,ABCC4,SNX5,C1QTNF1,ANK1,SYT1,RAB5A,LIN7A,APBB3,DPH3,AIMP1,NRG1,CCKBR,NCKAP1L,SIRT3,STX2,SYT9,ADRA2B,SNPH,NR1H4,USF2,HCK,NDUFAF2,FAM3D,IL1A,PAX8,NR4A1,FER,OSBP,HIP1R,TRAPPC11,CHRM5,ARNTL,ERC2,SCG5,MEF2C,ASIC1,TPD52,VEGFC,P2RY1,RASGRP1,KCNJ11,NNAT,NR1H3,TIAM1,RAB11FIP5,EXOC6B,PFKM,SYNJ1,SLC8B1,VDR,STC1,NKX3-1,NKX6-1,ACVR1C,F2RL1,F2R,CRY2,VPS18,ABCC2,SPP1,ZBED6,PLA2R1,LGI3,DMTN,ADTRP,DTNBP1,CHRM3,SYT8,GRM2,SIRT4,GPLD1,IRS1,NEURL1,CD200,DNM1L,SLC6A3,HDAC1,PPP3CA,HYAL3,CCND1,CPT1A,MAP2K6,TRPV4,SLC9B2,TGFB2,ADRA2A,FBLN5,MON1A,TCIRG1,TCF7L2,SNAP23,KCNB1,EGF,PFKFB2,GRK2,GPER1,ABCB11,RAB11FIP2,LAT2,AGT,FGR,CBL,SYTL1,PRSS12,PER2,OSM,COPA,STX1A,SNX4,NTSR1,PASK,SEPT2,BACE1,DNAJC5,GLUL,CSPG5,SIDT2,SLC16A1,ADAM8,KLF7,NSF,CREB1,MIA3,RAP1B,BLOC1S6,PLA2G4A,CTAGE9,VGF,TTN,IL12A,TANGO2,COMT,ENY2,ABCA12,NRXN2,CASK,SERPINC1,MCU,ADRA2C,PNKD,PTGDR,PPP3CB,RAB3A,PINK1,SYN1,CAMK2G,SNAP29,EXOC5,PTPRN,[illegible]	grey	VSMC_M10
GO:0046485	0.85	0.29	8	18	ether lipid metabolic process	BP	1	PEX7,PLA2G6,TMEM86B,LIPE,GNPAT,PLA2G4A,PLA2G16,NCEH1	grey	VSMC_M10
GO:2001141	0.85	0.17	1263	2416	regulation of RNA biosynthetic process	BP	1	ADNP2,NFATC1,ZNF236,ZNF407,SMARCC2,ATP8B1,ONECUT2,BAZ2A,TCF4,GLI1,MARS,MAP3K5,TNFAIP3,TSFM,HIVEP2,PLAGL1,MAML1,ZBTB2,YEATS4,ESR1,MYRFL,CNOT2,CNOT6,STK3,OSR2,SNW1,ZNF706,ARID1B,HSPA1B,FZD6,ZBTB12,ZBED4,PRKN,AGER,PPARA,MED30,IL5,BRD2,RXRB,VGLL2,HSF2,TCF7,PHF1,XRCC6,NCOA7,PITX1,TRIB1,MYC,TOB2,SMAD5,L3MBTL3,L3MBTL2,AGO2,KDM3B,PTP4A3,PTCH1,ATF4,RPAP2,CBX7,MAPK14,CBY1,KDM4C,SOX10,PIM1,NRF1,TMPRSS6,SMO,BNC2,HSF1,NFYA,FOXP4,MDFI,TFEB,IRF5,MED20,CCND3,FOXH1,LRRC14,CDKN2A,ZNF16,RBFOX2,IFT74,BICRAL,RORB,ZNF300,PRDM4,CRY1,DMTF1,SRF,ZNF318,NFX1,CDK14,FZD1,MTERF1,NFXL1,NFKBIE,SUPT3H,SMARCA2,RFX3,KIT,ADGRF1,NCK2,FHL2,TGFBRAP1,TFAP2B,ROR2,NFIL3,SEMA4D,RFX8,DLX5,PHF14,IPPK,ETV1,ZNF135,RPS14,ZNF154,MEOX2,ZNF550,KANSL3,BEND6,ZNF268,ZNF667,ZNF787,ZNF691,CAT,MACC1,SIX2,FOXJ3,ZNF865,HIVEP3,IL11,SCMH1,ZNF189,RNF20,EPAS1,EPCAM,SP4,PPP2R1A,ZNF462,PHIP,HMGN3,NFYC,NFE2L3,HOXA2,TAF1D,HOXA4,HOXA6,HOXA7,HOXA13,REL,ZNF483,JAZF1,CREB5,MYCL,XPO1,MTURN,NOD1,AKIRIN2,SRSF10,CASP8AP2,BACH2,ZNF692,PELI1,AKIRIN1,MTF1,ZNF618,SNIP1,ANXA4,PCBP1,NR1H2,AKNA,ZNF350,ITGB1BP1,FOXP2,GRHL1,MED25,KLF11,MET,IL4I1,AGO1,POU3F3,EGFR,LANCL2,ING3,NOBOX,ARHGEF5,TRIM32,TLR4,CCNC,CDK5RAP2,TRAF1,BCL2L12,LPIN1,SFRP4,UCP1,CREM,TEAD2,SETD7,SDCBP,MYCN,C6orf203,ELF2,OSR1,FOXO3,ZXDC,ZBTB24,MKX,FAF1,MAMSTR,ATAD2B,BEND5,CDCA7,KDM7A,ZNF438,FOXE3,HDAC2,PHF10,MAML2,BBS7,MLLT10,DNMT3A,BMI1,ZC3H4,PRKD2,CNBP,SET[illegible]	grey	VSMC_M10
GO:1901550	0.85	0.33	5	12	regulation of endothelial cell development	BP	1	ZDHHC21,S1PR3,IKBKB,PLCB1,VCL	grey	VSMC_M10
GO:1903140	0.85	0.33	5	12	regulation of establishment of endothelial barrier	BP	1	ZDHHC21,S1PR3,IKBKB,PLCB1,VCL	grey	VSMC_M10
GO:0006921	0.85	0.27	11	24	cellular component disassembly involved in execution phase of apoptosis	BP	1	HSF1,DEDD2,FOXL2,ACVR1C,ACIN1,GPER1,DNASE2,PAM16,DFFB,BLCAP,NMNAT1	grey	VSMC_M10
GO:0045022	0.85	0.24	17	37	early endosome to late endosome transport	BP	1	RAB21,WDR91,MTMR2,RAB7A,HOOK3,RAB5A,DNAJC13,FAM160A2,ANKRD27,MAPK1,SNX12,MAPK3,HOOK2,MAP2K1,STX8,RILP,SNX16	grey	VSMC_M10
GO:0010560	0.85	0.29	8	18	positive regulation of glycoprotein biosynthetic process	BP	1	IL15,PAWR,PLCB1,SLC2A10,TCF7L2,SOAT1,POMT2,IL33	grey	VSMC_M10
GO:0002443	0.85	0.19	111	221	leukocyte mediated immunity	BP	1	CD226,STX7,AGER,HLA-DRA,HLA-DOB,HLA-DMA,HLA-DPB1,RAC2,KIT,PRDX1,EXOSC3,MSH2,RSAD2,IL4I1,TLR4,TRPM4,TRAF3IP2,SPHK2,HMCES,ERCC1,NECTIN2,PVR,GAB2,MLH1,UNC13D,MYD88,SLC22A13,FOXJ1,PAXIP1,JAG1,SHLD1,MAVS,NCKAP1L,HPX,IRF7,TRAF6,GAPT,FER,SLA2,RASGRP1,NBN,F2RL1,NOD2,CD40,CD96,TCIRG1,SNAP23,DENND1B,LAT2,FGR,CBL,SNX4,CD28,FCER1G,RNF168,CADM1,FZD5,BCL6,CTSH,IL12A,PTPN6,LAG3,PTGDR,PPP3CB,C12orf4,STAT5B,WAS,FOXP3,ZBTB1,SHLD2,TNFRSF1B,SPON2,TBX21,STX4,ITGAM,BATF,CD19,LAT,IL4R,S100A13,BTK,SCNN1B,CD74,STXBP2,FBXO38,PRAM1,VAV1,C7,C3,NOS2,IL7R,SUPT6H,TICAM1,CRK,DNASE1,PDPK1,PRKCZ,TRAF2,FOXF1,STXBP1,AP1G1,BCL10,CHGA,FADD,CXCL6,CD46,FCER2,RNF8,CD70,SERPINB9,HAVCR2	grey	VSMC_M10
GO:0043271	0.85	0.21	45	93	negative regulation of ion transport	BP	1	NEDD4L,GOPC,WNK2,PRKCE,HECW1,TCAF2,SFRP4,KEL,OSR1,SLC26A5,KCNRG,OXSR1,ARL6IP5,PXK,SLC43A1,TMBIM6,PTK2B,STC1,KCNE2,PKD2,PLA2R1,CASQ2,HECW2,ADRA2A,ISCU,ATP1A2,C4orf3,NTSR1,SESTD1,GNB5,WNK3,SYT11,CALM1,ABAT,FMR1,GABRE,INPP5K,RRAD,MIR24-1,GPR35,KCNH2,CRBN,RGS4,GEM,PPIF	grey	VSMC_M10
GO:0043652	0.85	0.31	6	14	engulfment of apoptotic cell	BP	1	RAC2,XKR6,RHOBTB2,RHOBTB1,ABCA7,XKR8	grey	VSMC_M10

GO:0045214	0.85	0.25	15	33	sarcomere organization	BP	1	OBSCN,LMOD2,SRF,MYLK3,ACTG1,PRKAR1A,MYOZ2,CASQ1,MYPN,TTN,MYOZ1,LDB3,TCAP,CSRP2,KRT19	grey	VSMC_M10
GO:0006749	0.85	0.24	20	42	glutathione metabolic process	BP	1	CLIC5,SLC1A1,CHAC2,MGST2,SLC7A11,MMACHC,GSTK1,CLIC1,ARL6IP5,GSS,GLRX2,NFE2L2,GGT2,GGT6,GSTZ1,PARK7,PTGES2,GSTT4,GLO1,GGT5	grey	VSMC_M10
GO:0007000	0.85	0.31	6	14	nucleolus organization	BP	1	BAZ2A,USP36,SIRT2,DDX11,PHF8,BEND3	grey	VSMC_M10
GO:0051770	0.85	0.31	6	14	positive regulation of nitric-oxide synthase biosynthetic process	BP	1	TLR4,NOD2,MAP2K6,CCL2,FCER2,IL33	grey	VSMC_M10
GO:0009127	0.85	0.28	9	20	purine nucleoside monophosphate biosynthetic process	BP	1	IMPDH1,PPAT,AMPD3,DGUOK,GMPS,AMPD1,ADK,ADSSL1,NUDT2	grey	VSMC_M10
GO:0050772	0.85	0.22	32	68	positive regulation of axonogenesis	BP	1	LRP1,TIAM2,PLXNA4,NTRK2,SRF,SEMA4D,RUFY3,PLXNA1,GOLGA4,MEGF8,PLXNC1,ROBO1,ROBO2,TIAM1,ZFYVE27,CYFIP1,BDNF,ADNP,DISC1,CDKL5,WNT3,MAPT,FN1,NIN,NDEL1,RAB11A,MAP2K1,NTN1,TRPV2,L1CAM,PLXNA3,AMIGO1	grey	VSMC_M10
GO:0010738	0.85	0.33	5	12	regulation of protein kinase A signaling	BP	1	AKAP7,TCIM,ADGRV1,SPATC1L,PDE4A	grey	VSMC_M10
GO:1901019	0.85	0.22	32	67	regulation of calcium ion transmembrane transporter activity	BP	1	STAC3,PLA2G6,CACNB2,STAC,CACNB4,KCNE2,PKD2,CASQ2,RYR2,ADRA2A,ANK2,ATP1A2,SLC9A1,C4orf3,CASQ1,NOS1AP,HTT,ATP1B1,GNB5,HAP1,STAC2,CACNB1,ATP2A1,CALM1,VMP1,FMR1,JPH3,RRAD,DMD,GPR35,JPH2,GEM	grey	VSMC_M10
GO:0034620	0.85	0.21	42	86	cellular response to unfolded protein	BP	1	HSPA1B,PRKN,PACRG,DERL1,BAK1,ATF4,HSF1,NCK2,ERMP1,HERPUD2,FGF21,OPTN,TMTC4,EIF2AK2,DDRGK1,PIK3R1,TMBIM6,AMFR,RHBDD1,CCND1,FICD,YOD1,HSPA8,QRICH1,BAG3,TBL2,RHBDD2,NFE2L2,DERL3,BHLHA15,CREB3L4,PTPN2,CREB3L3,ABCA7,HSPA5,DAB2IP,MIR199A1,DDIT3,BCL2L11,HSPB8,PPP1R15A,DERL2	grey	VSMC_M10
GO:1904355	0.85	0.30	7	16	positive regulation of telomere capping	BP	1	MAP3K4,MAPK15,MAPKAPK5,RTEL1,MAPK1,MAPK3,MAP2K7	grey	VSMC_M10
GO:0006949	0.85	0.25	16	35	syncytium formation	BP	1	MAPK14,MYH9,CD109,ERCC1,DYRK1B,MYOF,JAM2,RAPGEF3,ADAMTS15,SH3PXD2A,TCTA,VASH2,EHD1,GDF15,IL4R,CACNA1H	grey	VSMC_M10
GO:0046112	0.85	0.29	8	18	nucleobase biosynthetic process	BP	1	SHMT2,PPAT,CMPK1,GMPS,CTPS2,CPS1,SHMT1,DHODH	grey	VSMC_M10
GO:0032252	0.85	0.33	5	12	secretory granule localization	BP	1	KIF5A,RASGRP1,TANC2,KIF1C,TRIM46	grey	VSMC_M10
GO:0030728	0.85	0.31	6	14	ovulation	BP	1	FOXO3,HPGD,ADAMTS1,GAS2,IL4R,EREG	grey	VSMC_M10
GO:0002366	0.85	0.19	90	181	leukocyte activation involved in immune response	BP	1	ENPP3,HLA-DRA,MFNG,RAC2,KIT,EXOSC3,PRKCE,MSH2,TLR4,SPHK2,HMCES,ERCC1,LCP1,GAB2,MLH1,UNC13D,MYD88,PAXIP1,EOMES,NMI,PLCL2,SHLD1,NCKAP1L,FOXP1,GAPT,FER,PTK2B,RASGRP1,EIF2AK4,RIPK2,NBN,DYSF,CDH17,F2RL1,CD40,DOCK10,SNAP23,HLX,LAT2,FGR,CBL,SNX4,CD28,FCER1G,RNF168,BCL6,RC3H1,TNFSF18,SLC11A1,PTGDR,STAT3,C12orf4,FOXP3,SHLD2,TBX21,STX4,PSEN1,SEMA4A,ITGAM,DOCK2,BATF,ZBTB7B,IL6R,CD19,LAT,IL4R,S100A13,BTK,SCNN1B,CD74,STXBP2,PRAM1,PTGER4,SUPT6H,TICAM1,DNASE1,PDPK1,PRKCZ,TSC1,FOXF1,KARS,STXBP1,AP1G1,MIR21,CHGA,CD46,FGL2,RNF8,HAVCR2,RC3H2	grey	VSMC_M10
GO:0010894	0.85	0.28	9	20	negative regulation of steroid biosynthetic process	BP	1	CYP27B1,DKKL1,DKK3,ERLIN1,SNAI1,SCAP,WNT4,GFI1,INSIG2	grey	VSMC_M10
GO:0051893	0.85	0.22	28	60	regulation of focal adhesion assembly	BP	1	LRP1,PTK2,TEK,ITGB1BP1,CLASP2,ACTG1,PTPRA,FAM107A,FMN1,PTPRJ,DMTN,LDB1,SLK,COL16A1,ARHGAP6,SLC9A1,APOD,WNT4,VCL,SMAD3,BCAS3,CAMSAP3,TSC1,RHOA,ACVRL1,PEAK1,THY1,DUSP22	grey	VSMC_M10
GO:0090109	0.85	0.22	28	60	regulation of cell-substrate junction assembly	BP	1	LRP1,PTK2,TEK,ITGB1BP1,CLASP2,ACTG1,PTPRA,FAM107A,FMN1,PTPRJ,DMTN,LDB1,SLK,COL16A1,ARHGAP6,SLC9A1,APOD,WNT4,VCL,SMAD3,BCAS3,CAMSAP3,TSC1,RHOA,ACVRL1,PEAK1,THY1,DUSP22	grey	VSMC_M10
GO:0032413	0.85	0.23	23	49	negative regulation of ion transmembrane transporter activity	BP	1	NEDD4L,GOPC,PRKCE,HECW1,TCAF2,OSR1,KCNRG,OXSR1,KCNE2,PKD2,CASQ2,HECW2,ADRA2A,ATP1A2,C4orf3,GNB5,CALM1,FMR1,RRAD,MIR24-1,GPR35,CRBN,GEM	grey	VSMC_M10
GO:1904427	0.85	0.22	26	55	positive regulation of calcium ion transmembrane transport	BP	1	STAC3,CACNB2,STAC,F2R,KCNE2,PKD2,RYR2,ANK2,GPER1,SLC9A1,CASQ1,NTSR1,CEMIP,HTT,ATP1B1,HAP1,STAC2,ATP2A1,CD19,CALM1,VMP1,BDKRB1,PDPK1,P2RX1,JPH2,THY1	grey	VSMC_M10
GO:0030501	0.85	0.24	17	37	positive regulation of bone mineralization	BP	1	OSR2,PTN,OSR1,ACVR2B,ACVR2A,KL,ADGRV1,MEF2C,P2RX7,ANO6,TFAP2A,BMPR1B,ACTN3,FZD9,WNT4,BMPR1A,SMAD3	grey	VSMC_M10
GO:0006925	0.85	0.30	7	16	inflammatory cell apoptotic process	BP	1	CDKN2A,SLC7A11,IRF7,PIK3CB,MEF2C,NOD2,CXCR2	grey	VSMC_M10
GO:0051788	0.85	0.27	10	22	response to misfolded protein	BP	1	RNF5,DERL1,UGGT2,DNAJC18,AUP1,RNF185,DNAJB12,SDF2L1,F12,RNF126	grey	VSMC_M10
GO:1904380	0.85	0.31	6	14	endoplasmic reticulum mannose trimming	BP	1	RNF5,UGGT2,AMFR,RNF185,EDEM3,SYVN1	grey	VSMC_M10
GO:0050778	0.85	0.18	155	306	positive regulation of immune response	BP	1	CD226,STX7,HLA-DRA,HLA-DPB1,XRCC6,PTK2,PLA2G6,TEC,TNFRSF21,EXOSC3,PRKCE,MSH2,FPR2,AKIRIN2,RSAD2,TLR4,IL15,SPHK2,HMCES,C5AR2,C5AR1,PRKD2,PHB,NECTIN2,PVR,EIF2B3,GAB2,MLH1,SLC22A13,PAXIP1,IKBKB,NMI,TMEM173,PAWR,PLCL2,SHLD1,PRNP,MAVS,NCKAP1L,HPX,FOXP1,PRKDC,IRF7,TRAF6,HCK,LPXN,PTPRJ,MEF2C,SLA2,RASGRP1,EIF2AK4,NLRC5,RIPK2,F2RL1,POLR3D,NOD2,CD40,GPLD1,SPPL3,LCK,BANK1,ICOSLG,TGFB2,BTN2A2,POLR3C,HLX,DENND1B,CMKLR1,PARP9,THEMIS2,LAT2,BMX,FGR,PLPP4,SNX4,COCH,CD28,FCER1G,ADAM8,CADM1,FZD5,NONO,PLA2G4A,IL12A,XRCC5,RC3H1,PTPN6,SLC11A1,LAG3,CFP,CD38,MAPK1,STAT5B,FOXP3,ZBTB1,SHLD2,GPS2,CARD11,MED1,TBX21,STX4,PSEN1,ITGAM,LCP2,CD19,IL4R,SFPQ,PRKCB,BTK,SIN3A,CD74,CCL5,RIOK3,FBXO38,YES1,PRAM1,VAV1,C7,C3,PTPN2,CREBBP,PRKCZ,TRAF2,IKBKG,PARK7,CYBA,VAV3,KARS,STXBP1,AP1G1,BCL10,CMTM3,MIR21,FYB1,FADD,BTN1A1,CD46,ZCCHC3,A2M,CFD,IRGM,CD247,THY1,WNT5A,FCER2,RGCC,UBE2N,GCSAM,HRAS,LAX1,SH2B2,EREG,TNFSF13B,HAVCR2,RC3H2	grey	VSMC_M10

GO:0009416	0.85	0.18	120	239	response to light stimulus	BP	1	FECH,CPT1B,MAP3K4,SYNGAP1,UBE2B,BAK1,MYC,ATF4,MAPK13,FBXL6,OPN1SW,PRPH2,CRY1,KIT,REV1,CAT,CAMKMT,BRSK1,RDH13,MSH2,AQP1,GJA10,PTN,EGFR,TRIM32,SLC7A11,TRPC3,ERCC1,XPC,ERCC2,ERCC3,B4GALT2,ZRANB3,AANAT,PPP1CB,AKT2,PLEKHB1,BHLHE40,DDHD2,WRN,ERCC6,NPHP1,ERCC8,PIK3R1,PRIMPOL,CASP3,ATR,CEP250,CCDC66,SCARA3,MME,EIF2AK4,DDB2,TENT2,CDS1,CRY2,TP53INP1,SLC4A10,TRPM1,RHBDD1,SIK1,HYAL1,HYAL3,CCND1,KRAS,PITPNM1,RRH,POLG,SPRTN,SAG,USP2,ATP1A2,NFATC4,PER2,USF1,CREB1,NMT1,MEN1,IL12A,CRTC1,GRK4,MMP3,RHNO1,GNB5,ROM1,ZBTB1,PARP1,DDB1,RGS14,GUCY2D,FBXL22,FOS,TAF1,CHRNB2,TIPIN,DNM2,DCUN1D3,YY1,ASIC2,CUL4B,IFT20,UNC119,ERCC4,TP53I13,FMR1,CREBBP,SDF4,MECP2,CIRBP,ABCA7,PER3,GNAT2,LCN2,FBXL8,FBXL12,REEP6,IMPACT,DUSP1,GPR88,MAP2K7	grey	VSMC_M10
GO:0033865	0.85	0.20	50	102	nucleoside bisphosphate metabolic process	BP	1	HSD17B4,PPT2,ACSL6,CROT,SLC35B2,PPCS,PPT1,DLD,ELOVL1,PANK2,PDK1,SUCLG2,PDHX,ELOVL7,PANK1,MCCC2,ACOT12,FITM2,MCEE,TPST1,GPAM,FAR2,ACSL5,HACD2,THEM5,HMGCL,ACOT9,PDK3,SLC25A16,ACSBG1,COASY,MPC2,TECR,SLC26A1,ACOT2,PANK3,GCDH,ACSM3,ACACA,SLC26A2,ACSBG2,PIPOX,ACOT11,PANK4,ACSF3,MLYCD,SULT1C3,ACOT4,ELOVL3,NUDT19	grey	VSMC_M10
GO:0033875	0.85	0.20	50	102	ribonucleoside bisphosphate metabolic process	BP	1	HSD17B4,PPT2,ACSL6,CROT,SLC35B2,PPCS,PPT1,DLD,ELOVL1,PANK2,PDK1,SUCLG2,PDHX,ELOVL7,PANK1,MCCC2,ACOT12,FITM2,MCEE,TPST1,GPAM,FAR2,ACSL5,HACD2,THEM5,HMGCL,ACOT9,PDK3,SLC25A16,ACSBG1,COASY,MPC2,TECR,SLC26A1,ACOT2,PANK3,GCDH,ACSM3,ACACA,SLC26A2,ACSBG2,PIPOX,ACOT11,PANK4,ACSF3,MLYCD,SULT1C3,ACOT4,ELOVL3,NUDT19	grey	VSMC_M10
GO:0034032	0.85	0.20	50	102	purine nucleoside bisphosphate metabolic process	BP	1	HSD17B4,PPT2,ACSL6,CROT,SLC35B2,PPCS,PPT1,DLD,ELOVL1,PANK2,PDK1,SUCLG2,PDHX,ELOVL7,PANK1,MCCC2,ACOT12,FITM2,MCEE,TPST1,GPAM,FAR2,ACSL5,HACD2,THEM5,HMGCL,ACOT9,PDK3,SLC25A16,ACSBG1,COASY,MPC2,TECR,SLC26A1,ACOT2,PANK3,GCDH,ACSM3,ACACA,SLC26A2,ACSBG2,PIPOX,ACOT11,PANK4,ACSF3,MLYCD,SULT1C3,ACOT4,ELOVL3,NUDT19	grey	VSMC_M10
GO:1902116	0.85	0.25	15	33	negative regulation of organelle assembly	BP	1	TBC1D30,MDM1,CDK5RAP2,FEZ2,SEC22B,TMEM39A,LIMK2,PINK1,PHF23,TRIM37,DNM2,SMCR8,MTM1,CDK10,ODF2L	grey	VSMC_M10
GO:0010950	0.85	0.20	63	127	positive regulation of endopeptidase activity	BP	1	MAP3K5,GRAMD4,AGER,BAK1,MYC,HSF1,TTBK1,SLC1A1,NOD1,AKIRIN2,CASP8AP2,TNFSF15,HSPE1,ARRB1,ARL6IP5,ST18,ASPH,FOXL2,HIP1R,ROBO1,DIABLO,HTRA2,RIPK2,SENP1,NKX3-1,ACVR1C,F2R,PIDD1,LCK,PDCD6,GPER1,PSME1,CIDEB,EGLN3,MAPT,FIS1,CTSH,DDX3X,VSIR,PSME3,MUL1,STAT3,EPHA4,BCL2L13,NGFR,EFNA1,SMAD3,S100A8,BCAP31,P2RX1,BCL10,MIR21,MIR27B,RHOA,RPS27L,FADD,CASP8,ACER2,BCL2L11,PERP,APH1B,PRR7,CRADD	grey	VSMC_M10
GO:1905269	0.85	0.32	5	12	positive regulation of chromatin organization	BP	1	SETDB2,FAM208A,PPHLN1,SETDB1,ZNF304	grey	VSMC_M10
GO:0009168	0.85	0.29	8	18	purine ribonucleoside monophosphate biosynthetic process	BP	1	IMPDH1,PPAT,AMPD3,GMPS,AMPD1,ADK,ADSSL1,NUDT2	grey	VSMC_M10
GO:0060100	0.85	0.32	5	12	positive regulation of phagocytosis, engulfment	BP	1	NCKAP1L,F2RL1,ANO6,C3,ABCA7	grey	VSMC_M10
GO:1905155	0.85	0.32	5	12	positive regulation of membrane invagination	BP	1	NCKAP1L,F2RL1,ANO6,C3,ABCA7	grey	VSMC_M10
GO:0010801	0.85	0.29	7	16	negative regulation of peptidyl-threonine phosphorylation	BP	1	SPRY2,SIRT2,DMTN,DGKQ,CALM1,INPP5K,CADM4	grey	VSMC_M10
GO:0072310	0.85	0.34	4	10	glomerular epithelial cell development	BP	1	PODXL,FOXJ1,JAG1,ASXL1	grey	VSMC_M10
GO:0010954	0.85	0.28	9	20	positive regulation of protein processing	BP	1	MYH9,PLGRKT,RHBDD1,MELTF,ADAM8,LPCAT3,F12,CNTN2,BAG2	grey	VSMC_M10
GO:0051482	0.85	0.29	7	16	positive regulation of cytosolic calcium ion concentration involved in phospholipase C-activating G protein-coupled signaling pathway	BP	1	GPR20,LPAR1,F2RL1,F2R,GNA15,GPR157,GPR35	grey	VSMC_M10
GO:0050920	0.85	0.19	83	167	regulation of chemotaxis	BP	1	SEMA6A,AGER,GPSM3,PTK2,PLXNA4,RAC2,CCL27,SEMA4D,SEMA4C,FPR2,CXCL8,AKIRIN1,PTN,MET,C5AR2,C5AR1,PRKD2,MEGF8,OXSR1,MYCBP2,SEMA3D,SEMA3A,NCKAP1L,GAS6,RYK,MTUS1,ROBO1,ROBO2,SEMA4F,VEGFC,PTK2B,TIAM1,SLC8B1,F2RL1,ANO6,SEMA3G,SEMA4G,NOD2,DNM1L,TRPV4,SEMA3F,PRSS56,CMKLR1,SLAMF8,MOSPD2,YTHDF1,PTPRO,WNT3,IL16,SEMA6D,IL12A,MCU,TNFSF18,CXCR2,BST1,MAPK1,PADI2,TMEM102,STX4,SEMA4A,FGF18,PGF,DAPK2,MAPK3,IL6R,SMAD3,S100A14,CD74,CCL5,CSF1R,CCL7,CCL2,PTPN2,PLXNA3,MPP1,DEFB124,DPP4,ZNF580,WNT5A,DUSP1,ARTN,FGF1,PDGFD	grey	VSMC_M10
GO:1903670	0.85	0.25	13	29	regulation of sprouting angiogenesis	BP	1	SEMA6A,CEMIP2,BMPER,SLC39A12,PIK3CB,GLUL,E2F2,RHOJ,SYNJ2BP,JAK1,PDPK1,MIR125A,FGF1	grey	VSMC_M10
GO:0034198	0.85	0.23	18	39	cellular response to amino acid starvation	BP	1	MAP3K5,ATF4,TFEB,RRAGD,KPTN,SESN3,SZT2,EIF2AK2,LARS,EIF2AK4,SLC38A2,MAPK1,RRAGB,MAPK3,WDR59,SH3GLB1,IMPACT,RNF152	grey	VSMC_M10
GO:1905952	0.85	0.19	58	118	regulation of lipid localization	BP	1	LRP1,PRKN,PPARA,TSPO,GDF9,PTCH1,PLA2G6,TTC39B,PLIN2,CRY1,ABCG5,ABCG8,TMEM30A,NR1H2,APOB,ABHD5,APOC1,AKT2,C1QTNF1,ASXL1,NR1H3,NKX3-1,FITM2,CRY2,SPP1,PLA2R1,PLTP,MAP2K6,ABCA5,ACSL5,EGF,FITM1,AGT,NTSR1,PLA2G4A,FIS1,OSBPL6,EHD1,ZDHHC8,ABCA12,LPCAT3,GPS2,ANXA2,NAXE,C3,PTPN2,PLIN3,ABCA3,ABCA7,RXRA,MIR27B,MIR27A,ABCA8,MIR33B,LDLRAP1,CRH,LRAT,PTGES	grey	VSMC_M10
GO:0002730	0.85	0.32	5	12	regulation of dendritic cell cytokine production	BP	1	KIT,TLR4,MAVS,NOD2,TICAM1	grey	VSMC_M10
GO:0045844	0.85	0.30	6	14	positive regulation of striated muscle tissue development	BP	1	LMOD3,MEF2C,IGF2,ACTN3,CREB1,MRTFB	grey	VSMC_M10
GO:0048636	0.85	0.30	6	14	positive regulation of muscle organ development	BP	1	LMOD3,MEF2C,IGF2,ACTN3,CREB1,MRTFB	grey	VSMC_M10

GO:0043280	0.85	0.20	47	96	positive regulation of cysteine-type endopeptidase activity involved in apoptotic process	BP	1	MAP3K5,GRAMD4,BAK1,MYC,HSF1,SLC1A1,NOD1,CASP8AP2,TNFSF15,HSPE1,ARRB1,ARL6IP5,ST18,FOXL2,HIP1R,ROBO1,DIABLO,HTRA2,SENP1,NKX3-1,ACVR1C,F2R,PIDD1,LCK,PDCD6,GPER1,EGLN3,MAPT,FIS1,CTSH,DDX3X,MUL1,BCL2L13,NGFR,SMAD3,S100A8,BCAP31,P2RX1,BCL10,MIR27B,RHOA,RPS27L,CASP8,ACER2,BCL2L11,PRR7,CRADD	grey	VSMC_M10
GO:0010332	0.85	0.22	25	53	response to gamma radiation	BP	1	BAK1,XRCC6,MYC,HSF1,XRCC2,WRN,PRKDC,ERCC6,ATR,POLG,CHEK2,CBL,MEN1,XRCC5,PARP1,DCUN1D3,CCL7,CYBA,HSPA5,MIR21,TIGAR,HRAS,TMEM109,TSPYL5,GTF2H5	grey	VSMC_M10
GO:1990182	0.85	0.28	8	18	exosomal secretion	BP	1	VPS4B,PRKN,CHMP2A,SDCBP,RAB7A,ATP13A2,RAB11A,VPS4A	grey	VSMC_M10
GO:0061842	0.85	0.25	14	31	microtubule organizing center localization	BP	1	SUN2,BICD2,INTS13,DLG1,TBCCD1,NIN,SYNE2,NDEL1,IFT20,NUBP1,GPSM2,SPOUT1,FHOD1,DYNC1LI2	grey	VSMC_M10
GO:0046364	0.85	0.21	31	65	monosaccharide biosynthetic process	BP	1	PPARA,AKR1B1,ATF4,GPT,GNMT,CRY1,SLC25A13,PPP4R3B,ZNF692,FAM3C,AKR1A1,SIRT7,GPI,NLN,GPD1,SIK1,MST1,PC,SLC37A4,PER2,SORD,DDB1,DGKQ,OGT,CRTC2,LEPR,PTPN2,ACADM,FBP2,NNMT,ARPP19	grey	VSMC_M10
GO:0045598	0.85	0.20	53	109	regulation of fat cell differentiation	BP	1	ATAT1,STK3,MAPK14,PIM1,TAF8,DLK2,DKKL1,TRPM4,MKX,NAPEPLD,AAMDC,KLF5,JAG1,SIRT2,PTPRQ,TRIB3,ASXL1,CEBPA,ARNTL,RUNX1T1,TRIO,CDS1,FTO,RREB1,SOX13,STK4,TRPV4,CMKLR1,GPER1,SYAP1,CREB1,DUSP10,MMP11,SAV1,GNB3,TFE3,GPS2,TGFB1I1,JDP2,ZBTB7B,SMAD3,ZBTB7C,MIR21,MIR27B,MIR27A,WNT5A,SIX1,DDIT3,CCDC3,ZFP36L2,CEBPB,CCDC71L,C1QL4	grey	VSMC_M10
GO:0048705	0.85	0.18	92	186	skeletal system morphogenesis	BP	1	OSR2,MAPK14,MDFI,TEK,ZFAND5,ROR2,DLX5,RAB23,SIX2,HOXA2,HOXA4,HOXA6,HOXA7,COL27A1,FUZ,SFRP4,MYCN,OSR1,BMI1,SOX9,MEGF8,WDR48,CSRNP1,TSKU,WDR60,TGFBR2,RARB,ALX1,RYK,TCF15,EYA1,FMN1,MEF2C,SOX6,STC1,TFAP2A,BMPR1B,HYAL1,TRPV4,TRIP11,SATB2,AXIN2,FGR,ACTN3,DLG1,SETD2,HOXD8,HOXD4,HOXD3,POR,PRKRA,COL13A1,ALPL,FGFR3,MMP13,NKX3-2,TULP3,WDR19,THRA,DHRS3,MSX2,PSEN1,HOXB5,HOXB6,HOXB8,HOXB9,FGF18,TMEM107,THBS3,CHAD,SMAD3,FOXN3,GSC,TBX4,LHX1,CDX1,PPARGC1B,NLE1,GHR,SPEF2,RFLNB,SKI,PKD1,ALX3,ANKRD11,HOXB3,NOG,SIX1,OTOR,INSIG2,SLC39A3,HOXA3	grey	VSMC_M10
GO:0036297	0.85	0.23	17	37	interstrand cross-link repair	BP	1	MCM9,ERCC6L2,FANCC,FANCG,FANCL,ERCC1,MCM8,ATR,FAN1,RNF168,POLN,RAD51D,ERCC4,SLX4,FAAP24,FANCF,RNF8	grey	VSMC_M10
GO:0060337	0.85	0.23	19	41	type I interferon signaling pathway	BP	1	MYD88,MAVS,IRF7,OAS3,NLRC5,SP100,YTHDF2,PTPN6,MUL1,USP18,DCST1,STAT2,TYK2,JAK1,PTPN2,MIR21,OAS1,FADD,WNT5A	grey	VSMC_M10
GO:0071218	0.85	0.27	9	20	cellular response to misfolded protein	BP	1	RNF5,DERL1,UGGT2,DNAJC18,AUP1,RNF185,DNAJB12,SDF2L1,RNF126	grey	VSMC_M10
GO:0062009	0.85	0.27	9	21	secondary palate development	BP	1	COL11A2,WNT11,TGFBR2,ITGB6,TGFB2,DLG1,JAG2,MMP25,WNT5A	grey	VSMC_M10
GO:0007175	0.85	0.32	5	12	negative regulation of epidermal growth factor-activated receptor activity	BP	1	CBL,SNX6,ZFYVE28,SOCS4,PSEN1	grey	VSMC_M10
GO:0050966	0.85	0.32	5	12	detection of mechanical stimulus involved in sensory perception of pain	BP	1	PHF24,ASIC3,SCN1A,BACE1,KCNA1	grey	VSMC_M10
GO:0051491	0.85	0.25	12	27	positive regulation of filopodium assembly	BP	1	SRF,DPYSL3,PIK3R1,NEURL1,DNM3,MIEN1,TENM2,BCAS3,FMR1,PALM,FNBP1L,RALA	grey	VSMC_M10
GO:0010715	0.85	0.29	7	16	regulation of extracellular matrix disassembly	BP	1	LRP1,CLASP2,FAP,MELTF,FGFR4,CARMIL2,DPP4	grey	VSMC_M10
GO:0030324	0.85	0.19	69	141	lung development	BP	1	GLI1,RIDA,TAB1,PTK7,SRF,EPAS1,PTN,TNC,YWHAZ,EGFR,MYCN,SLC7A11,SPRY1,SOX9,GLI2,FOXJ1,SPRY2,WNT11,TGFBR2,ASXL1,CEBPA,FBXW7,MME,TBX5,AP3B1,SRSF6,ITGB6,DAG1,KRAS,HSD11B1,LAMA5,SP3,CREB1,PLOD3,CTSH,ABCA12,SAV1,RAB3A,MAPK1,LIPA,TTC25,BMPR1A,THRA,RBPJ,ALDH1A2,FGF18,ITGA3,MAPK3,MAP2K1,TBX4,STRA6,GATA6,SREBF1,LAMA1,GPC3,HSPB11,ABCA3,PKD1,FOXF1,ESRP2,FSTL3,ATXN1L,NOG,WNT5A,HIKESHI,LIF,FGF1,WNT2,RC3H2	grey	VSMC_M10
GO:0051797	0.85	0.32	5	12	regulation of hair follicle development	BP	1	SMO,FERMT1,TGFB2,NGFR,WNT5A	grey	VSMC_M10
GO:0006244	0.85	0.28	8	18	pyrimidine nucleotide catabolic process	BP	1	DPYS,MBD4,ENTPD4,UPB1,NEIL1,NT5M,NTHL1,UPP1	grey	VSMC_M10
GO:0050482	0.85	0.29	7	16	arachidonic acid secretion	BP	1	PLA2G6,PLA2R1,NTSR1,PLA2G4A,NMUR2,PROCA1,PLA2G2C	grey	VSMC_M10
GO:1903963	0.85	0.29	7	16	arachidonate transport	BP	1	PLA2G6,PLA2R1,NTSR1,PLA2G4A,NMUR2,PROCA1,PLA2G2C	grey	VSMC_M10
GO:0000387	0.85	0.23	18	38	spliceosomal snRNP assembly	BP	1	PRPF31,SRSF10,CLNS1A,TGS1,AAR2,SART3,GEMIN2,SNRPD3,PRPF19,COIL,PRMT7,RNU5E-1,RNU5B-1,RNU4ATAC,SNRPC,RNU4-2,SRSF12,RNU4-1	grey	VSMC_M10
GO:0039702	0.85	0.28	8	18	viral budding via host ESCRT complex	BP	1	VPS4B,CHMP2A,CHMP2B,CHMP4C,CHMP7,VPS37D,CHMP1A,VPS4A	grey	VSMC_M10
GO:0051451	0.85	0.32	5	12	myoblast migration	BP	1	SMO,AKIRIN1,ITGB1BP1,BIN3,SIX1	grey	VSMC_M10
GO:0001836	0.85	0.22	23	48	release of cytochrome c from mitochondria	BP	1	PRKN,BAK1,PLA2G6,GGCT,TIMM50,OPA1,DNM1L,GPER1,IFI6,CIDEB,SFN,FZD9,FIS1,PINK1,PSMD10,JUN,PAM16,MLLT11,NOL3,FXN,BCL2L11,BCL2L2,PPIF	grey	VSMC_M10
GO:0098586	0.85	0.21	30	63	cellular response to virus	BP	1	USP15,MAPK14,GSDME,ANKRD17,PUM2,PHB,SEC14L1,CLPB,NPLOC4,IRF7,OAS3,GBF1,IFIH1,OASL,IFI6,NLRX1,IL12A,DDX3X,BIRC3,TKFC,SMAD3,TSPAN6,CCL5,RIOK3,RNF135,FMR1,MIR21,OAS1,ZCCHC3,IRGM	grey	VSMC_M10
GO:0006700	0.86	0.30	6	14	C21-steroid hormone biosynthetic process	BP	1	TSPO,AKR1B1,DKK3,WNT4,DGKQ,CACNA1H	grey	VSMC_M10
GO:0060306	0.86	0.26	10	23	regulation of membrane repolarization	BP	1	NEDD4L,SCN5A,KCNE2,CASQ2,KCNJ2,ANK2,SCN4B,NOS1AP,RNF207,KCNH2	grey	VSMC_M10
GO:0045744	0.86	0.23	17	37	negative regulation of G protein-coupled receptor signaling pathway	BP	1	CRY1,KLK14,CXCL8,PLEK,MET,GIPR,ARRB1,RPGRIP1L,GRK2,SAG,GRK4,RGS14,DNM2,CCL5,MGRN1,RGS4,MRAP2	grey	VSMC_M10
GO:0035269	0.86	0.29	7	16	protein O-linked mannosylation	BP	1	RXYLT1,LARGE1,FKTN,TMTC4,POMT2,POMGNT2,ISPD	grey	VSMC_M10
GO:0090025	0.86	0.27	9	20	regulation of monocyte chemotaxis	BP	1	FPR2,ANO6,SLAMF8,MOSPD2,TNFSF18,S100A14,CCL5,DEFB124,DUSP1	grey	VSMC_M10

GO:0031167	0.86	0.27	9	20	rRNA methylation	BP	1	TFB1M,DIMT1,NSUN3,NSUN5,MRM2,ZCCHC4,MRM3,METTL16,TRMT61B	grey	VSMC_M10
GO:0019827	0.86	0.19	51	106	stem cell population maintenance	BP	1	CNOT2,ZNF706,MED30,MYC,FANCC,SMO,KIT,SIX2,PTN,TUT4,FOXO3,SOX9,RAF1,HOOK3,JAG1,LSM1,TCF15,PAX8,CNOT1,SETD6,MCPH1,LOXL2,RTF1,PAX2,LDB1,DIS3L2,SOX2,FZD7,DDX6,VPS72,EIF4ENIF1,TET1,MED14,SAV1,STAT3,LEO1,BMPR1A,RBPJ,MED6,ESRRB,MED7,SKI,MED27,MTF2,SOX4,METTL14,NOG,ZNF322,LIF,ZFP36L2,MED21	grey	VSMC_M10
GO:0030216	0.86	0.20	35	74	keratinocyte differentiation	BP	1	CYP27B1,IFT74,CD109,HOXA7,GRHL1,INTU,EVPL,SCEL,OVOL2,JAG1,ASAH1,FLNB,CASP3,VDR,SRSF6,DSP,STK4,PPHLN1,TGM1,KDF1,SFN,CLIC4,H2AFY2,ABCA12,SAV1,RBPJ,MED1,MAP2K1,BCL11B,MIR125B1,WNT5A,DNASE1L2,ZBED2,EREG,SGPP1	grey	VSMC_M10
GO:0000578	0.86	0.25	11	25	embryonic axis specification	BP	1	FRS2,PTCH1,FAM208A,HOXD8,FZD5,CITED1,LHX1,WDR77,WNT5A,BASP1,C2CD3	grey	VSMC_M10
GO:0030323	0.86	0.18	71	145	respiratory tube development	BP	1	GLI1,HECA,RIDA,TAB1,PTK7,SRF,EPAS1,PTN,TNC,YWHAZ,EGFR,MYCN,SLC7A11,SPRY1,SOX9,GLI2,FOXJ1,SPRY2,WNT11,TGFBR2,ASXL1,CEBPA,FBXW7,MME,TBX5,AP3B1,SRSF6,ITGB6,DAG1,KRAS,HSD11B1,LAMA5,SP3,CREB1,PLOD3,CTSH,ABCA12,SAV1,RAB3A,MAPK1,TULP3,LIPA,TTC25,BMPR1A,THRA,RBPJ,ALDH1A2,FGF18,ITGA3,MAPK3,MAP2K1,TBX4,STRA6,GATA6,SREBF1,LAMA1,GPC3,HSPB11,ABCA3,PKD1,FOXF1,ESRP2,FSTL3,ATXN1L,NOG,WNT5A,HIKESHI,LIF,FGF1,WNT2,RC3H2	grey	VSMC_M10
GO:0051447	0.86	0.30	6	14	negative regulation of meiotic cell cycle	BP	1	FBXO43,MOS,PRKAR1A,OVOL1,DUSP1,LIF	grey	VSMC_M10
GO:0000077	0.86	0.19	51	105	DNA damage checkpoint signaling	BP	1	MRNIP,TRIM39,PPP1R10,MDC1,MAPK14,CRY1,BRSK1,MSH2,ETAA1,XPC,PLK3,NEK11,PRKDC,ERCC6,CEP63,STK33,PLK2,RAD17,ATR,RAD9B,EIF2AK4,NBN,ABRAXAS1,DONSON,PIDD1,TAOK3,CCND1,GIGYF2,CHEK2,ATRIP,SYF2,CDKN1B,TIPRL,RHNO1,PRPF19,UIMC1,FOXO4,EME1,MUC1,TAOK2,TIPIN,FOXN3,TAOK1,FZR1,DOT1L,FBXO31,RPA4,RPS27L,SOX4,BRCC3,CRADD	grey	VSMC_M10
GO:0032410	0.86	0.21	27	57	negative regulation of transporter activity	BP	1	NEDD4L,GOPC,PRKCE,HECW1,TCAF2,OSR1,KCNRG,OXSR1,KCNE2,PKD2,CASQ2,HECW2,ADRA2A,ATP1A2,C4orf3,CTTNBP2NL,GNB5,CALM1,FMR1,WWP2,RRAD,MIR24-1,GPR35,MIR33B,CRBN,GEM,PPIF	grey	VSMC_M10
GO:0022037	0.86	0.20	40	84	metencephalon development	BP	1	GLI1,UQCRQ,AGTPBP1,CKB,SMO,SEMA4C,WHRN,PTN,XRCC1,GLI2,B4GALT2,SCN5A,CLP1,FAIM2,RPGRIP1L,CNTN1,LDB1,TTBK2,TTC21B,GNPAT,GBX2,SPTBN2,HAP1,PSEN1,MAP2K1,LHX1,CDK5R1,SEZ6,DAB1,ABAT,CRK,SDF4,MECP2,AARS,HSPA5,GRID2,NANOS1,CEND1,OTX1,PDSS2	grey	VSMC_M10
GO:0072497	0.86	0.33	4	10	mesenchymal stem cell differentiation	BP	1	FZD1,SOX9,SOX6,WNT3	grey	VSMC_M10
GO:0001675	0.86	0.31	5	12	acrosome assembly	BP	1	TBPL1,NECTIN2,SOX30,MFSD14A,TBC1D20	grey	VSMC_M10
GO:0007097	0.86	0.27	8	19	nuclear migration	BP	1	SUN2,HOOK3,SLIT1,SYNE2,NTN1,CLMN,DOCK7,FHOD1	grey	VSMC_M10
GO:0006085	0.86	0.28	7	16	acetyl-CoA biosynthetic process	BP	1	PPCS,DLD,PDK1,PDHX,PDK3,MPC2,MLYCD	grey	VSMC_M10
GO:0060644	0.86	0.30	6	14	mammary gland epithelial cell differentiation	BP	1	PTCH1,SMO,AKT2,MGMT,FOXF1,CEBPB	grey	VSMC_M10
GO:0055002	0.86	0.21	24	52	striated muscle cell development	BP	1	OBSCN,LMOD2,SRF,PGM5,TMOD1,MYLK3,ACTG1,LMOD3,LMOD1,PRKAR1A,ACTC1,MYOZ2,CASQ1,TMOD4,MYPN,TTN,MYOZ1,TMOD2,OBSL1,LDB3,TCAP,MYH11,CSRP2,KRT19	grey	VSMC_M10
GO:0007044	0.86	0.20	42	89	cell-substrate junction assembly	BP	1	LRP1,PTK2,TEK,TLN1,ITGB1BP1,CLASP2,ACTG1,ARHGEF7,PTPRA,FAM107A,FMN1,SORBS1,PTPRJ,PTK2B,DMTN,LDB1,SLK,COL17A1,COL16A1,AJUBA,ARHGAP6,SLC9A1,ACTN3,WHAMM,LAMC1,APOD,TRIP6,FN1,WNT4,VCL,TAOK2,SMAD3,CORO2B,BCAS3,CAMSAP3,PDPK1,TSC1,RHOA,ACVRL1,PEAK1,THY1,DUSP22	grey	VSMC_M10
GO:0060541	0.86	0.18	80	163	respiratory system development	BP	1	GLI1,RIDA,TAB1,PTK7,SRF,DLX5,EPAS1,PTN,TNC,YWHAZ,EGFR,MYCN,SLC7A11,SPRY1,SOX9,GLI2,FOXJ1,SPRY2,WNT11,TGFBR2,ASXL1,CEBPA,FBXW7,MME,TBX5,AP3B1,SRSF6,RPGRIP1L,ITGB6,ALDH1A3,DAG1,KRAS,HSD11B1,LAMA5,SP3,CREB1,PLOD3,CTSH,ABCA12,SAV1,RAB3A,MAPK1,TULP3,LIPA,TTC25,BMPR1A,THRA,RBPJ,ALDH1A2,FGF18,ITGA3,MAPK3,MAP2K1,TBX4,STRA6,GATA6,SMCHD1,SREBF1,LAMA1,SPEF2,GPC3,HSPB11,ABCA3,SKI,PKD1,FOXF1,ASS1,HYDIN,ESRP2,FSTL3,ATXN1L,NOG,WNT5A,BASP1,SIX1,HIKESHI,LIF,FGF1,WNT2,RC3H2	grey	VSMC_M10
GO:0010885	0.86	0.30	6	14	regulation of cholesterol storage	BP	1	PPARA,TTC39B,NR1H2,APOB,NR1H3,EHD1	grey	VSMC_M10
GO:0030953	0.86	0.33	4	10	astral microtubule organization	BP	1	LIMK2,DLG1,KPNB1,RAB11A	grey	VSMC_M10
GO:0071248	0.86	0.18	69	141	cellular response to metal ion	BP	1	TPH2,SLC12A2,PRKN,TSPO,CPNE5,HSF1,SLC1A1,AQP1,MTF1,EGFR,BRAF,DLG2,SCN5A,CPNE9,SYT1,RYR1,PRNP,SYT9,CEBPA,RYR3,ADGRV1,MEF2C,HVCN1,XRCC4,CPNE2,PKD2,TFAP2A,DMTN,ADCY7,SYT8,SLC41A1,GPLD1,A3GALT2,SLC30A10,NCF1,FABP4,NFATC4,CARF,CLIC4,BACE1,CDKN1B,NFE2L2,CREB1,RASA4,RASGRP2,ABCB6,KCNA1,MAPK1,ATP13A2,DLG4,PARP1,SYT7,SYT11,FOS,MAPK3,SLFN14,SLC25A23,JUN,CACNA1H,CPNE7,HSPA5,NQO1,FSTL3,KCNK3,MT1X,CLDN1,WNT5A,TIGAR,PPIF	grey	VSMC_M10
GO:0055070	0.86	0.30	6	14	copper ion homeostasis	BP	1	SLC31A2,ATP7B,PRNP,CUTC,ABCB6,SCO1	grey	VSMC_M10
GO:0033262	0.86	0.31	5	12	regulation of nuclear cell cycle DNA replication	BP	1	DACH1,NUGGC,BCL6,DBF4B,TIPIN	grey	VSMC_M10
GO:1904659	0.86	0.20	38	80	glucose transmembrane transport	BP	1	SLC2A12,MAPK14,SLC25A27,GRB10,FGF21,BRAF,MFSD4B,PRKAG2,AKT2,ASPSCR1,TRIB3,PIK3R1,SORBS1,IRS1,SLC2A10,TERT,C2CD5,PEA15,NFE2L2,CLTCL1,SLC2A2,SLC27A1,SLC2A9,SLC2A4,RSC1A1,SLC5A2,SLC5A11,PRKCB,INSR,YES1,C3,GPC3,INPP5K,SLC45A1,SLC2A6,TSC1,FABP5,ARPP19	grey	VSMC_M10
GO:0010883	0.86	0.22	19	41	regulation of lipid storage	BP	1	PPARA,MEST,TTC39B,PLIN2,NR1H2,APOB,ABHD5,ASXL1,FBXW7,NR1H3,FITM2,FTO,CPT1A,FITM1,EHD1,SREBF1,C3,PTPN2,PLIN3	grey	VSMC_M10
GO:0033013	0.86	0.22	23	49	tetrapyrrole metabolic process	BP	1	FECH,TSPO,MMACHC,SLC25A38,HPX,MMAA,CPOX,COX15,ABCC2,TMEM14C,ALAS1,MTR,BDH2,MMAB,ABCB6,ABCD4,COX10,CD320,SLC46A1,NFE2L1,FXN,MMADHC,CLYBL	grey	VSMC_M10

GO:0010863	0.86	0.24	13	29	positive regulation of phospholipase C activity	BP	1	ESR1,KIT,LPAR1,EGFR,C5AR1,PHB,RASGRP4,FLT1,ARHGAP6,AGT,ABL2,GNA15,HRAS	grey	VSMC_M10
GO:0043567	0.86	0.29	6	14	regulation of insulin-like growth factor receptor signaling pathway	BP	1	PHIP,NKX3-1,ZFAND2B,IGFBP4,CILP,MYORG	grey	VSMC_M10
GO:0046434	0.86	0.19	62	126	organophosphate catabolic process	BP	1	PDE7B,ENPP3,ABHD16A,SYNJ2,DPYS,PDE10A,SMPDL3A,PLA2G6,GDA,NT5E,INPP4B,SMPD2,MTMR2,NAPEPLD,ENTPD6,NUDT15,APOC1,NUDT5,MBD4,GPD1L,NUDT16,MTMR7,ABHD6,NUDT12,AMPD3,GPD1,SYNJ1,ENTPD4,FITM2,NUDT18,PLA2G4B,PDE9A,INPP5A,NUDT17,NTSR1,INPP5J,PDE8A,PLBD1,PLA2G4A,UPB1,ABHD12B,SMPD4,LIPC,GDPD3,LDLR,GDPD1,PDE4A,NEIL1,GDE1,PNPLA6,NT5M,IMPA2,INPP5K,NTHL1,MLYCD,PLA2G15,ENTPD5,NUDT19,INPP1,PDXP,HINT1,UPP1	grey	VSMC_M10
GO:2000679	0.86	0.28	7	16	positive regulation of transcription regulatory region DNA binding	BP	1	POU4F1,DDRGK1,TRAF6,HAND2,PARP1,PARK7,LAMTOR5	grey	VSMC_M10
GO:0071322	0.86	0.19	59	121	cellular response to carbohydrate stimulus	BP	1	ZNF236,NME1,LRP1,AGER,MAPK13,PLA2G6,SRF,PRKCE,HMGN3,TRPM4,FOXO3,FGF21,GRIK5,RAF1,GAS6,NR1H4,CYP7A1,NDUFAF2,OSBP,KCNJ11,TIAM1,RAB11FIP5,OPA1,ZBED6,PAX2,TWNK,GPLD1,PPP3CA,MAP2K6,SLC9B2,ZBTB20,ADRA2A,KCNB1,POLG,GPER1,RAB11FIP2,MLXIPL,SIDT2,PDK3,KLF7,FIS1,MEN1,ENY2,ABCA12,CALCRL,PPP3CB,PTPRN,MPC2,STX4,GPR68,PRKCB,SIN3A,COLEC12,OXCT1,NADK,BAIAP3,CYBA,SOX4,CRH	grey	VSMC_M10
GO:0002689	0.86	0.29	6	14	negative regulation of leukocyte chemotaxis	BP	1	C5AR2,SLAMF8,PADI2,CCL2,DPP4,DUSP1	grey	VSMC_M10
GO:0045746	0.86	0.23	15	33	negative regulation of Notch signaling pathway	BP	1	DLK2,BEND6,ARRB1,TCIM,CBFA2T2,FBXW7,HIF1AN,NEURL1,YTHDF2,DLX1,BCL6,ARRDC1,WWP2,SLC35C1,HEY2	grey	VSMC_M10
GO:0006753	0.86	0.16	198	387	nucleoside phosphate metabolic process	BP	1	NME1,HSD17B4,TBPL1,PDE7B,ENPP3,ATP5MGL,DPYS,PPT2,PPARA,PDE10A,ACSL6,SMPDL3A,PRXL2C,GTPBP1,GUCA1B,IMPDH1,NMRK1,GDA,CROT,SLC35B2,SLC25A13,GAPDH,PPAT,RAB23,PPCS,SLC4A4,ME1,NT5E,PPT1,BPGM,DLD,SPHK2,CMPK1,ADCY3,ENTPD6,NUDT15,DLG2,LACC1,NUDT5,DHTKD1,MBD4,PRKAG2,SLC22A13,ATP5F1C,ELOVL1,PFKP,IDO2,IDO1,NUDT16,PANK2,PDK1,SUCLG2,PDHX,SLC5A8,ELOVL7,PANK1,GPI,NUDT12,AMPD3,ATP5F1A,MCCC2,GPD1,P2RX7,GUCY1A1,GUCY1B1,ACOT12,DGUOK,PRKAG1,GMPS,PFKM,OPA1,ENTPD4,FITM2,AMPD1,NUDT18,ADCY7,GMPR,FAM173B,ADCY2,MCEE,NME4,PDE9A,TPST1,ZBTB20,GPAM,FAR2,ACSL5,PTHLH,PUDP,NUDT17,MFN1,PFKFB2,PRPS2,ENO4,PARP9,HACD2,ARNT,ADCY4,CTPS2,ACTN3,HDAC4,THEM5,DNAJC30,MLXIPL,PDE8A,DTYMK,HMGCL,MAGI3,DLG1,ACOT9,PDK3,EFL1,SLC25A16,UPB1,HK1,ACSBG1,ATP5S,CASK,COASY,PRKAG3,PINK1,STAT3,NME7,ADK,MPC2,ADCY10,MAPK1,ENO3,NT5C3B,PARP1,TECR,CARD11,SLC26A1,NAXE,PSEN1,ACOT2,GUCY2D,PKLR,PANK3,GCDH,OGT,UPRT,ADPGK,PDE4A,ACSM3,ACACA,NEIL1,NCOR1,INSR,SLC26A2,PRPSAP2,ADSSL1,NT5M,NOS2,ACSBG2,ALDOC,PIPOX,ACOT11,NADK,ATP6V0C,PANK4,NTHL1,NME3,MPP1,PARK7,ATP5PB,SLC2A6,VPS9D1,GNAI3,ACSF3,AK8,UCK1,MLYCD,KARS,FUK,DHODH,MT-ATP8,MT-ATP6,RHOA,SULT1C3,ATP5ME,ACOT4,ATP5MF,ENTPD5,PDZD3,ELOVL3,MTHFD2L,CMPK2,TIGAR,NUDT19,NNMT,NME6,DCTD,NUDT2,NAPRT,HINT1,UPP1,NMNAT1	grey	VSMC_M10
GO:0014855	0.86	0.21	26	56	striated muscle cell proliferation	BP	1	GLI1,MAPK14,PIM1,AKIRIN1,TGFBR2,EPHB1,MEF2C,TBX5,JARID2,TGFB2,PRKAR1A,KCNK2,SAV1,STAT3,MAPK1,BMPR1A,RBPJ,TBX2,GATA6,MIR199A1,MIR222,NOG,SIX1,C3orf58,HEY2,WNT2	grey	VSMC_M10
GO:0106106	0.86	0.18	60	123	cold-induced thermogenesis	BP	1	ATF4,HSF1,ALDH1A1,GADD45G,EPAS1,NR1H2,GRB10,TLR4,LPIN1,IL15,UCP1,FGF21,LAMA4,ACVR2B,PLCL2,ABHD6,ARNTL,NR1H3,ALMS1,EBF2,KSR2,ZNF423,PRKAB1,MAP2K6,CMKLR1,FABP4,TRPM8,GPR3,PER2,ACTN3,ACADL,TFE3,MFAP2,YBX2,THRA,RBPJ,MFN2,KDM6B,SLC25A44,ZBTB7B,OGT,PCTP,IL4R,TRPV2,PPARGC1B,LEPR,DOCK7,ACOT11,PRDM16,LCN2,BSCL2,FABP5,ELOVL3,PEMT,NOVA1,PLAC8,DDIT3,APC,CEBPB,DIO2	grey	VSMC_M10
GO:0120161	0.86	0.18	60	123	regulation of cold-induced thermogenesis	BP	1	ATF4,HSF1,ALDH1A1,GADD45G,EPAS1,NR1H2,GRB10,TLR4,LPIN1,IL15,UCP1,FGF21,LAMA4,ACVR2B,PLCL2,ABHD6,ARNTL,NR1H3,ALMS1,EBF2,KSR2,ZNF423,PRKAB1,MAP2K6,CMKLR1,FABP4,TRPM8,GPR3,PER2,ACTN3,ACADL,TFE3,MFAP2,YBX2,THRA,RBPJ,MFN2,KDM6B,SLC25A44,ZBTB7B,OGT,PCTP,IL4R,TRPV2,PPARGC1B,LEPR,DOCK7,ACOT11,PRDM16,LCN2,BSCL2,FABP5,ELOVL3,PEMT,NOVA1,PLAC8,DDIT3,APC,CEBPB,DIO2	grey	VSMC_M10
GO:0060479	0.86	0.28	7	16	lung cell differentiation	BP	1	SOX9,FOXJ1,CREB1,SAV1,THRA,RBPJ,GATA6	grey	VSMC_M10
GO:1990416	0.86	0.33	4	10	cellular response to brain-derived neurotrophic factor stimulus	BP	1	NTRK2,WASF1,MAPT,PDPK1	grey	VSMC_M10
GO:0055023	0.86	0.24	14	31	positive regulation of cardiac muscle tissue growth	BP	1	GLI1,MAPK14,PIM1,MEF2C,TBX5,MAPK1,BMPR1A,RBPJ,TBX2,GATA6,MIR199A1,MIR222,HEY2,WNT2	grey	VSMC_M10
GO:0060340	0.86	0.31	5	12	positive regulation of type I interferon-mediated signaling pathway	BP	1	MAVS,IRF7,NLRC5,FADD,WNT5A	grey	VSMC_M10
GO:0048634	0.86	0.24	13	29	regulation of muscle organ development	BP	1	SIRT2,LMOD3,MEF2C,SFMBT1,IGF2,USP19,ACTN3,YBX3,CREB1,SOX15,SMAD3,MRTFB,JPH2	grey	VSMC_M10
GO:0033627	0.86	0.20	28	60	cell adhesion mediated by integrin	BP	1	PTK2,PODXL,IFT74,ITGB1BP1,FERMT1,RAC3,NCKAP1L,RET,LPXN,TESC,ITGB6,TGFB2,COL16A1,CIB1,ITGB5,ITGA4,PTPN6,FBN1,FERMT3,MUC1,EFNA1,ITGA11,CCL5,CRK,PIEZO1,DPP4,ACER2,LIF	grey	VSMC_M10
GO:0090201	0.86	0.27	8	18	negative regulation of release of cytochrome c from mitochondria	BP	1	PRKN,OPA1,PSMD10,PAM16,NOL3,FXN,BCL2L2,PPIF	grey	VSMC_M10
GO:0043247	0.86	0.31	5	12	telomere maintenance in response to DNA damage	BP	1	ERCC1,XRCC1,RTEL1,ERCC4,TERF2	grey	VSMC_M10
GO:0043558	0.86	0.31	5	12	regulation of translational initiation in response to stress	BP	1	NCK2,EIF2AK2,EIF2AK4,IMPACT,PPP1R15A	grey	VSMC_M10

GO:0072091	0.86	0.23	15	33	regulation of stem cell proliferation	BP	1	FBLN1,PIM1,EPCAM,OVOL2,FERMT1,EIF2AK2,VEGFC,TERT,KDF1,THPO,OVOL1,KAT7,CITED1,MIR222,ATXN1L	grey	VSMC_M10
GO:0070167	0.86	0.20	35	74	regulation of biomineral tissue development	BP	1	CYP27B1,OSR2,OMD,PTN,TRPM4,OSR1,SOX9,SLC4A2,ACVR2B,ACVR2A,KL,GAS6,ADGRV1,MEF2C,P2RX7,PTK2B,BMP2K,ANO6,TFAP2A,BMPR1B,SGMS2,AMELX,ACTN3,TXLNG,FZD9,SRGN,BCOR,ANKH,WNT4,BMPR1A,SMAD3,RFLNB,BGLAP,CEBPB,HEY2	grey	VSMC_M10
GO:0051764	0.86	0.31	5	12	actin crosslink formation	BP	1	BAIAP2L2,LCP1,DPYSL3,GAS2,EPS8	grey	VSMC_M10
GO:0060350	0.86	0.21	21	46	endochondral bone morphogenesis	BP	1	TEK,DLX5,COL27A1,SOX9,TSKU,TGFBR2,RARB,MEF2C,STC1,BMPR1B,TRPV4,TRIP11,AXIN2,POR,COL13A1,ALPL,FGFR3,MMP13,FGF18,THBS3,GHR	grey	VSMC_M10
GO:0048863	0.86	0.18	84	171	stem cell differentiation	BP	1	SEMA6A,ESR1,SOX10,SMO,SRF,FZD1,OCIAD1,KIT,SEMA4D,SEMA4C,EPCAM,HOXA7,PTN,TEAD2,OSR1,SOX9,ERCC2,OVOL2,JAG1,ALX1,EIF2AK2,SEMA3D,SEMA3A,NRG1,PRKDC,KBTBD8,TCF15,RET,HAND2,MEF2C,SEMA4F,SETD6,SOX6,LMBR1L,TBX5,SEMA3G,PAX2,SEMA4G,PRICKLE1,JARID2,RBM24,KLHL12,SEMA3F,TGFB2,PEF1,PDCD6,YTHDF2,GBX2,OSM,LAMA5,SETD2,WNT3,NFE2L2,SEMA6D,FN1,XRCC5,MEOX1,SHC4,STAT3,MAPK1,BMPR1A,RADIL,RBPJ,CDK12,ALDH1A2,EPOP,SETD1A,SEMA4A,FOXO4,BATF,MAPK3,ESRRB,GSC,TBX2,TCOF1,GATA6,ISL1,SOX8,MTF2,SP7,A2M,LIF,ZFP36L2,NKX2-5	grey	VSMC_M10
GO:0019722	0.86	0.18	72	147	calcium-mediated signaling	BP	1	NFATC1,RCAN2,FHL2,TNNI3,FPR2,CXCL8,PLEK,EGFR,TRPM4,GRIN2D,SLC8A2,ACKR2,EFHB,PRNP,P2RX2,NRG1,P2RX3,PLCE1,PTPRJ,P2RX7,SLA2,PTK2B,PLA2G4B,DMTN,CASQ2,KSR2,CHRM3,RYR2,SPPL3,PPP3CA,NR5A2,CMKLR1,TBC1D10C,ANK2,TRPM8,LAT2,CIB1,ATP1A2,SLC9A1,NFATC4,MYOZ2,CASQ1,ACTN3,HDAC4,RCAN3,HOMER2,MAPT,FIS1,MCU,CXCR2,HTT,PPP3CB,MYOZ1,ATP1B1,BST1,BHLHA15,LAT,TMEM100,CALM1,BTK,RIT2,NMUR2,MAPK7,BCAP31,PDPK1,CAMTA1,NFATC3,PTGFR,DMD,CCR10,IRGM,SAMD14	grey	VSMC_M10
GO:0031349	0.86	0.17	88	177	positive regulation of defense response	BP	1	CD226,GPSM3,XRCC6,MAPK13,TTBK1,NINJ1,FPR2,NOD1,AKIRIN2,EGFR,TLR4,IL15,MGST2,CDK19,NAPEPLD,NECTIN2,PVR,OPTN,IKBKB,NMI,TMEM173,MAVS,HPX,PRKDC,IRF7,TRAF6,HCK,CEBPA,MEF2C,RASGRP1,NLRC5,RIPK2,F2RL1,RIPK1,POLR3D,NOD2,TRPV4,PDCD4,POLR3C,FABP4,PARP9,AGT,OSM,SNX4,COCH,CD28,ADAM8,CADM1,NONO,IL16,IL12A,XRCC5,TNFSF18,LAG3,STAT5B,MED1,MAPK3,LDLR,S100A8,SFPQ,BTK,TNIP1,SIN3A,CCL5,RIOK3,VAV1,PTGER4,C3,OSMR,CREB3L3,CREBBP,PARK7,CYBA,KARS,AP1G1,MIR21,MIR22,S100A12,FADD,ZCCHC3,IRGM,WNT5A,IL33,CRH,TSLP,CEBPB,EREG,HAVCR2	grey	VSMC_M10
GO:0043010	0.86	0.17	120	242	camera-type eye development	BP	1	MDM1,FRS2,OSR2,TBC1D32,BAK1,ATF4,AGTPBP1,NTRK2,PRPH2,RORB,SRF,SLC1A1,TFAP2B,TDRD7,TMOD1,RDH13,PTN,TSPAN12,USP45,SLC7A11,SPRY1,FOXE3,HDAC2,SOX9,CRYGN,ZNF513,ACVR2B,IFT172,TSKU,SPRY2,TGFBR2,BFSP1,JAG1,SPRED3,CEP290,KERA,MAB21L1,MITF,FLT1,TUB,EPHB1,PFDN5,NPHP1,RET,FOXL2,MAB21L2,TENM3,USH1C,TENT2,OPA1,RPGRIP1L,TFAP2A,PAX2,BMPR1B,MYH15,NECTIN3,LPCAT1,PKNOX1,SLC6A3,HDAC1,ALDH1A3,TGFB2,TCIRG1,PRSS56,SHROOM2,CRYBA4,NRL,CLIC4,DLX1,GRHL3,LIMK2,SP3,CDKN1B,DLG1,FZD5,FBN1,FKBP8,PLA2G16,TULP3,OBSL1,RHOJ,ROM1,WDR19,SDK1,MAX,CLN8,MED1,ALDH1A2,MFN2,PSEN1,TTLL5,MEGF11,LCTL,SMAD3,TBX2,BCL11B,LHX1,STRA6,YY1,UNC45B,LAMA1,TWSG1,GNB1,FZR1,SKI,SOX8,LAMC3,TMEM231,SLC25A25,HSF4,FJX1,ACVRL1,THY1,WNT5A,DIO3,TGIF2,TBC1D20,PITX3,WNT2,COL8A2	grey	VSMC_M10
GO:0031053	0.86	0.30	5	12	primary miRNA processing	BP	1	PUS10,DDX3X,STAT3,SMAD3,DROSHA	grey	VSMC_M10
GO:0032623	0.86	0.22	20	43	interleukin-2 production	BP	1	TNFAIP3,SPTBN1,PRKD2,NAV3,PRNP,TRAF6,IL1A,RIPK2,HDAC7,NOD2,CD28,HOMER2,LAG3,STAT5B,FOXP3,CARD11,TBX21,TRAF2,GLMN,HAVCR2	grey	VSMC_M10
GO:0032663	0.86	0.22	20	43	regulation of interleukin-2 production	BP	1	TNFAIP3,SPTBN1,PRKD2,NAV3,PRNP,TRAF6,IL1A,RIPK2,HDAC7,NOD2,CD28,HOMER2,LAG3,STAT5B,FOXP3,CARD11,TBX21,TRAF2,GLMN,HAVCR2	grey	VSMC_M10
GO:0019079	0.86	0.19	48	99	viral genome replication	BP	1	YTHDC2,PABPC1,PRKN,PPARA,APOBEC3H,CXCL8,RSAD2,PCBP1,ATG5,ZC3HAV1,PHB,FBXL2,ATG16L2,RAB5A,EIF2AK2,MAVS,GAS6,PCBP2,FAM208A,EIF2AK4,OAS3,IFIH1,OASL,TMEM39A,ADARB1,NR5A2,STAU1,HSPA8,GRK2,ATG16L1,CD28,DDX3X,PI4KA,DDB1,HACD3,LARP1,TNIP1,CCL5,CCL2,FMR1,INPP5K,LAMTOR5,MIR222,HTATSF1,SETDB1,OAS1,TBC1D20,FAM111A	grey	VSMC_M10
GO:0050851	0.86	0.18	57	117	antigen receptor-mediated signaling pathway	BP	1	CD226,HLA-DPB1,TEC,TNFRSF21,PRKD2,NECTIN2,EIF2B3,IKBKB,PAWR,PLCL2,PRNP,NCKAP1L,FOXP1,TRAF6,LPXN,PTPRJ,MEF2C,SLA2,RIPK2,SPPL3,LCK,BANK1,ICOSLG,BTN2A2,DENND1B,THEMIS2,LAT2,BMX,CD28,RC3H1,PTPN6,CD38,MAPK1,FOXP3,GPS2,CARD11,PSEN1,LCP2,CD19,PRKCB,BTK,PRAM1,PTPN2,IKBKG,VAV3,BCL10,CMTM3,FYB1,BTN1A1,CD247,THY1,UBE2N,GCSAM,HRAS,LAX1,SH2B2,RC3H2	grey	VSMC_M10
GO:0045721	0.86	0.30	5	12	negative regulation of gluconeogenesis	BP	1	CRY1,FAM3C,SIK1,MST1,LEPR	grey	VSMC_M10
GO:0071168	0.86	0.24	12	27	protein localization to chromatin	BP	1	MRNIP,ESR1,MCM9,LEMD2,MSH2,MCM8,FAM208A,CHMP7,PPHLN1,SETD2,H2AFY2,MAU2	grey	VSMC_M10
GO:0014842	0.86	0.32	4	10	regulation of skeletal muscle satellite cell proliferation	BP	1	AKIRIN1,EPHB1,STAT3,SIX1	grey	VSMC_M10
GO:0014857	0.86	0.32	4	10	regulation of skeletal muscle cell proliferation	BP	1	AKIRIN1,EPHB1,STAT3,SIX1	grey	VSMC_M10

GO:0048736	0.86	0.18	68	140	appendage development	BP	1	OSR2,FZD6,TBC1D32,BAK1,PITX1,PTCH1,TFAP2B,ROR2,DLX5,MEOX2,HOXA13,MYCN,SLC7A11,OSR1,INTU,HDAC2,BBS7,SOX9,GLI2,MEGF8,IFT172,LMBR1,ZNF219,RARB,FREM2,HAND2,FMN1,FRAS1,TBX5,IFT52,RPGRIP1L,TFAP2A,SALL1,FBXW4,PRKAB1,BMPR1B,HDAC1,TGFB2,ZNRF3,KREMEN1,KDF1,SP9,WNT3,ECE1,TULP3,BMPR1A,WDR19,IQCE,MED1,ALDH1A2,PSEN1,TMEM107,MKS1,RNF165,TBX4,TBX2,GPC3,CREBBP,SKI,ALX3,TMEM231,SOX4,NOG,WNT5A,C2CD3,BCL2L11,SLC39A3,RC3H2	grey	VSMC_M10
GO:0060173	0.86	0.18	68	140	limb development	BP	1	OSR2,FZD6,TBC1D32,BAK1,PITX1,PTCH1,TFAP2B,ROR2,DLX5,MEOX2,HOXA13,MYCN,SLC7A11,OSR1,INTU,HDAC2,BBS7,SOX9,GLI2,MEGF8,IFT172,LMBR1,ZNF219,RARB,FREM2,HAND2,FMN1,FRAS1,TBX5,IFT52,RPGRIP1L,TFAP2A,SALL1,FBXW4,PRKAB1,BMPR1B,HDAC1,TGFB2,ZNRF3,KREMEN1,KDF1,SP9,WNT3,ECE1,TULP3,BMPR1A,WDR19,IQCE,MED1,ALDH1A2,PSEN1,TMEM107,MKS1,RNF165,TBX4,TBX2,GPC3,CREBBP,SKI,ALX3,TMEM231,SOX4,NOG,WNT5A,C2CD3,BCL2L11,SLC39A3,RC3H2	grey	VSMC_M10
GO:0038034	0.86	0.21	23	49	signal transduction in absence of ligand	BP	1	ERBB3,EYA4,HSPA1B,BAK1,ZC3HC1,FOXO3,RET,IL1A,EYA1,IL7,HTRA2,TERT,EYA3,IFI6,MCL1,BAG3,STRADB,BCL2A1,MAP2K5,MAPK7,FADD,BCL2L11,BCL2L2	grey	VSMC_M10
GO:0097192	0.86	0.21	23	49	extrinsic apoptotic signaling pathway in absence of ligand	BP	1	ERBB3,EYA4,HSPA1B,BAK1,ZC3HC1,FOXO3,RET,IL1A,EYA1,IL7,HTRA2,TERT,EYA3,IFI6,MCL1,BAG3,STRADB,BCL2A1,MAP2K5,MAPK7,FADD,BCL2L11,BCL2L2	grey	VSMC_M10
GO:0042178	0.86	0.29	6	14	xenobiotic catabolic process	BP	1	NUDT15,CYP2R1,ABCC2,CYP2U1,CYP2J2,CRYZ	grey	VSMC_M10
GO:0030397	0.86	0.30	5	12	membrane disassembly	BP	1	LPIN1,PLA2G16,AKAP8L,NDEL1,PRKCB	grey	VSMC_M10
GO:0008654	0.86	0.17	111	222	phospholipid biosynthetic process	BP	1	PIP4K2C,SYNJ2,SERAC1,AGPAT4,AGPAT1,PNPLA3,PLA2G6,GPAA1,CWH43,SRD5A3,INPP4A,TMEM246,PIGF,MBOAT7,MBOAT2,LPIN1,INPP4B,FIG4,SPHK2,MTMR2,PIP4K2A,SELENOI,ABHD5,TAMM41,ITPKC,MTMR14,PGAP2,CRLS1,CDS2,NR1H4,MTMR7,PIGU,PIK3CB,PITPNM2,PIK3R1,TMEM150A,SLA2,SERINC5,SYNJ1,CDS1,FITM2,ITPKA,PIGT,LPCAT1,PGAP1,GPAM,CHKA,ACP6,ABHD4,SGMS2,AJUBA,GGPS1,PITPNM1,GNPAT,DGKD,PIGM,FITM1,BMX,FAM126B,IP6K2,LPGAT1,PIGV,DPAGT1,MTMR3,PCYT1A,INPP5J,PIGX,PIGZ,PISD,DGKG,PIKFYVE,PLA2G4A,SACM1L,LPCAT3,PIGC,SOCS4,PLA2G16,PI4KA,SLC27A1,ABHD8,SGMS1,PGAP3,PIGH,PI4K2B,SOCS7,DGKQ,AC138894.1,DGKE,TTC7B,PIGL,ABHD3,MPPE1,IMPA2,SOCS1,MTM1,MTMR1,INPP5K,PIP5K1C,AGPAT2,PIP5KL1,FABP5,ABCA8,DHDDS,PEMT,PYURF,LCLAT1,PIGB,PIGA,INPP1,PTPMT1,CDIPT	grey	VSMC_M10
GO:0030947	0.86	0.25	10	23	regulation of vascular endothelial growth factor receptor signaling pathway	BP	1	GRB10,PRKD2,VEGFC,PDCD6,ARNT,FGF18,TNMD,PRKCB,DAB2IP,CADM4	grey	VSMC_M10
GO:0045986	0.86	0.30	5	12	negative regulation of smooth muscle contraction	BP	1	GUCY1A1,DOCK5,ADRA2C,CALCRL,KCNMA1	grey	VSMC_M10
GO:0007030	0.86	0.18	59	122	Golgi organization	BP	1	ATP8B1,TJAP1,STX17,TRAPPC12,YWHAZ,HACE1,ARHGAP21,COG1,CLASP2,RBSN,PLK3,OPTN,YIPF4,NPLOC4,ARHGEF7,ATL2,NSFL1C,TRAPPC11,KIFC3,ARMH3,GBF1,SEC22B,TRIP11,VTI1A,COG2,SEC23IP,GORASP2,STX6,SEC16B,TANGO2,PDCD10,HTT,MAPK1,BHLHA15,STX5,OBSL1,STX18,PI4K2B,GAK,TMED10,MAPK3,MAP2K1,DNM2,VMP1,COG7,BCAS3,LMAN1L,CAMSAP3,SEC16A,SURF4,GOLGA8M,TMED5,COG4,PLK5,HIKESHI,TBC1D20,FAM174B,TMED3,ZFP69B	grey	VSMC_M10
GO:0021681	0.86	0.32	4	10	cerebellar granular layer development	BP	1	FAIM2,TTBK2,GRID2,CEND1	grey	VSMC_M10
GO:0014902	0.86	0.19	41	86	myotube differentiation	BP	1	STAC3,MAML1,MAPK14,SMO,MYH9,KEL,MAMSTR,KLF5,KLHL40,DYRK1B,RYR1,P2RX2,LMOD3,SMYD1,NLN,MYOF,MEF2C,JAM2,HOMER1,BIN3,RBM24,ADAMTS15,BDNF,SIK1,PPP3CA,ACTN3,HDAC4,EHD1,GDF15,BHLHA15,IL4R,FBXO22,SKI,CACNA1H,PIEZO1,PLPP7,SMYD3,SIX1,MYORG,NKX2-5,RBM38	grey	VSMC_M10
GO:0006418	0.86	0.22	17	37	tRNA aminoacylation for protein translation	BP	1	MARS,HARS,HARS2,CARS2,LARS,WARS2,YARS2,TARS2,DALRD3,LARS2,DARS2,FARSB,EARS2,LRRC47,KARS,AARS,MARS2	grey	VSMC_M10
GO:0007617	0.86	0.30	5	12	mating behavior	BP	1	MAPK8IP2,HDAC2,HDAC4,THRA,UBE2Q1	grey	VSMC_M10
GO:0032011	0.86	0.27	7	17	ARF protein signal transduction	BP	1	IQSEC1,PSD3,FBXO8,GBF1,PSD,CYTH3,IQSEC2	grey	VSMC_M10
GO:0032012	0.86	0.27	7	17	regulation of ARF protein signal transduction	BP	1	IQSEC1,PSD3,FBXO8,GBF1,PSD,CYTH3,IQSEC2	grey	VSMC_M10
GO:0002431	0.86	0.24	12	27	Fc receptor mediated stimulatory signaling pathway	BP	1	CD226,PTK2,PLA2G6,PRKCE,HCK,PTPRJ,FGR,PLPP4,FCER1G,YES1,VAV1,VAV3	grey	VSMC_M10
GO:0008593	0.86	0.19	36	76	regulation of Notch signaling pathway	BP	1	MFNG,DLK2,KIT,BEND6,AAK1,ITGB1BP1,LLGL2,GALNT11,ARRB1,JAG1,TCIM,POFUT1,CBFA2T2,ROBO1,ROBO2,FBXW7,BMP2K,HIF1AN,NOD2,GAS2,NEURL1,NEPRO,TGFB2,POGLUT1,YTHDF2,DLX1,BCL6,PDCD10,STAT3,SYNJ2BP,JAG2,ARRDC1,WWP2,CD46,SLC35C1,HEY2	grey	VSMC_M10
GO:1903573	0.86	0.22	18	39	negative regulation of response to endoplasmic reticulum stress	BP	1	PRKN,NCK2,NR1H2,AQP11,DDRGK1,USP25,TMBIM6,NR1H3,OPA1,HYOU1,SYVN1,LPCAT3,SGTA,ABCA7,PARK7,HSPA5,MIR199A1,PPP1R15A	grey	VSMC_M10
GO:1901888	0.86	0.17	80	165	regulation of cell junction assembly	BP	1	LRP1,PTK2,NTRK2,TEK,LINGO2,SEMA4D,CLDN19,LRRN3,EPHA7,ITGB1BP1,VPS35,VSTM5,CLASP2,SLITRK5,IKBKB,LRFN1,FARP1,ACTG1,PTPRA,LRFN3,EPHB1,NPHP1,FAM107A,CLSTN2,ROBO2,FMN1,PTPRJ,MEF2C,CUX2,RAPGEF2,SLIT1,TBX5,AMIGO2,DMTN,LDB1,BDNF,SLK,COL16A1,AMIGO3,SNAI1,ADNP,ARHGAP6,AGT,ACE2,SLC9A1,RAB17,EPHB3,APOD,FZD5,RAP1B,GPBAR1,WNT4,VCL,LRTM2,NLGN2,SEMA4A,NLGN3,CHRNB2,SMAD3,NTN1,BCAS3,GPRASP2,BHLHB9,CAMSAP3,ASIC2,SLITRK2,AMIGO1,TSC1,NTNG2,ADGRL2,RHOA,GRID2,ACVRL1,PEAK1,THY1,CLDN1,WNT5A,SIX1,DUSP22,LRRTM2	grey	VSMC_M10
GO:0035067	0.86	0.30	5	12	negative regulation of histone acetylation	BP	1	ATG5,TAF7,SDR16C5,FOXP3,SIN3A	grey	VSMC_M10
GO:0007530	0.86	0.30	5	12	sex determination	BP	1	MAP3K4,SOX9,SRD5A1,WNT4,INSRR	grey	VSMC_M10

GO:0070886	0.86	0.30	5	12	positive regulation of calcineurin-NFAT signaling cascade	BP	1	NRG1,SPPL3,CIB1,SLC9A1,CAMTA1	grey	VSMC_M10
GO:0106058	0.86	0.30	5	12	positive regulation of calcineurin-mediated signaling	BP	1	NRG1,SPPL3,CIB1,SLC9A1,CAMTA1	grey	VSMC_M10
GO:0048016	0.86	0.21	22	48	inositol phosphate-mediated signaling	BP	1	NFATC1,FHL2,EFHB,PRNP,NRG1,PLCE1,SPPL3,PPP3CA,INPP5A,TBC1D10C,CIB1,SLC9A1,NFATC4,MYOZ2,ACTN3,HOMER2,PPP3CB,MYOZ1,NMUR2,MAPK7,CAMTA1,NFATC3	grey	VSMC_M10
GO:1904779	0.87	0.32	4	10	regulation of protein localization to centrosome	BP	1	NSFL1C,CEP250,APC,PARD6A	grey	VSMC_M10
GO:0009223	0.87	0.27	7	16	pyrimidine deoxyribonucleotide catabolic process	BP	1	DPYS,MBD4,UPB1,NEIL1,NT5M,NTHL1,UPP1	grey	VSMC_M10
GO:0001657	0.87	0.19	37	78	ureteric bud development	BP	1	MYC,SMAD5,PTCH1,SMO,CAT,SIX2,EPCAM,BMPER,OSR1,SPRY1,SOX9,FOXJ1,GZF1,WNT11,RARB,RET,PAX8,EYA1,ROBO2,FMN1,PKD2,PAX2,SALL1,AGT,LAMA5,DLG1,WNT4,MAGED1,CITED1,SMAD3,LHX1,GPC3,SOX8,NOG,BASP1,SIX1,FGF1	grey	VSMC_M10
GO:0071825	0.87	0.22	16	35	protein-lipid complex subunit organization	BP	1	APOM,NR1H2,APOB,APOC1,NR1H4,SOAT2,PLTP,ABCA5,AGT,SOAT1,ZDHHC8,LPCAT3,LIPC,MPO,ABCA7,PLAGL2	grey	VSMC_M10
GO:0019637	0.87	0.15	384	743	organophosphate metabolic process	BP	1	NME1,HSD17B4,TBPL1,PDE7B,PIP4K2C,ENPP3,ATP5MGL,GFPT2,ABHD16A,SYNJ2,SERAC1,DPYS,AGPAT4,PPT2,AGPAT1,PPARA,PDE10A,ACSL6,PNPLA3,SMPDL3A,PRXL2C,GTPBP1,PLA2G6,CKB,GPAA1,MOCS1,GUCA1B,IMPDH1,NMRK1,GDA,CROT,SLC35B2,CWH43,SRD5A3,SLC25A13,GAPDH,PPAT,IPPK,INPP4A,RAB23,PPCS,PLPPR1,TMEM246,TMEM86B,PIGF,MBOAT7,SLC4A4,ME1,NT5E,PPT1,BPGM,PLEK,NR1H2,MBOAT2,LPIN1,INPP4B,SMPD2,DLD,FIG4,SPHK2,CMPK1,ADCY3,MTMR2,PIP4K2A,NAPEPLD,HADHA,SELENOI,ENTPD6,NUDT15,ABHD5,DLG2,AKR1A1,LACC1,APOC1,NUDT5,DHTKD1,MBD4,PRKAG2,TAMM41,SLC22A13,ATP5F1C,XYLB,ITPKC,RBKS,ELOVL1,GPD1L,PIP4P1,MTMR14,PFKP,IDO2,IDO1,PLCB1,PGAP2,CRLS1,CDS2,PLPP5,NUDT16,PANK2,PDK1,SUCLG2,NR1H4,PDHX,MTMR7,SLC5A8,ELOVL7,PANK1,GPI,ABHD6,NUDT12,AMPD3,PIGU,PIK3CB,PITPNM2,PIK3R1,TMEM150A,GK5,ATP5F1A,MCCC2,CHRM5,MTMR9,GPD1,P2RX7,GUCY1A1,GUCY1B1,SLA2,P2RY1,PTK2B,ACOT12,CKMT2,DGUOK,PRKAG1,GMPS,NR1H3,SERINC5,PFKM,SYNJ1,OPA1,ENTPD4,CDS1,FITM2,AMPD1,ITPKA,NUDT18,PIGT,PLA2G4B,GLYCTK,ADCY7,GMPR,FAM173B,ADCY2,GPLD1,MCEE,NME4,PDE9A,LPCAT1,PGAP1,TPST1,ZBTB20,GPAM,FAR2,ACSL5,CHKA,PTHLH,PLA1A,ITPK1,ACP6,INPP5A,ABHD4,SGMS2,AJUBA,PUDP,NUDT17,GGPS1,MFN1,PITPNM1,PFKFB2,THTPA,MTMR11,PRPS2,GPER1,GNPAT,DGKD,PIGM,ENO4,FITM1,PARP9,BMX,HACD2,FAM126B,IP6K2,ARNT,LPGAT1,ADCY4,PIGV,DPAGT1,PLPP4,CTPS2,MTMR3,PFKFB4,ACTN3,HDAC4,NTSR1,THEM5,DNAJC30,PCYT1A,INPP5J,MLXIPL,PDE8A,PIGX,DTYMK,HMGCL,PLBD1,PIGZ,MAGI3,CKMT1B,PISD,DL[illegible]	grey	VSMC_M10
GO:0002433	0.87	0.25	10	23	immune response-regulating cell surface receptor signaling pathway involved in phagocytosis	BP	1	PTK2,PLA2G6,PRKCE,HCK,PTPRJ,FGR,PLPP4,YES1,VAV1,VAV3	grey	VSMC_M10
GO:0038096	0.87	0.25	10	23	Fc-gamma receptor signaling pathway involved in phagocytosis	BP	1	PTK2,PLA2G6,PRKCE,HCK,PTPRJ,FGR,PLPP4,YES1,VAV1,VAV3	grey	VSMC_M10
GO:0010717	0.87	0.19	38	80	regulation of epithelial to mesenchymal transition	BP	1	FUZ,SDCBP,SPRY1,HDAC2,CLASP2,SPRY2,TGFBR2,OVOL2,JAG1,SPRED3,ALX1,IL17RD,TIAM1,LOXL2,TBX5,GCNT2,USF3,TGFB2,TCF7L2,DAG1,AXIN2,SNAI1,PHLDB1,TGFB1I1,EFNA1,SMAD3,ISL1,ENG,DAB2IP,MIR21,MIR222,POFUT2,NOG,DACT3,SDHAF2,RGCC,LDLRAD4,OLFM1	grey	VSMC_M10
GO:0002551	0.87	0.30	5	12	mast cell chemotaxis	BP	1	RAC2,KIT,VEGFC,PGF,CHGA	grey	VSMC_M10
GO:0035458	0.87	0.28	6	14	cellular response to interferon-beta	BP	1	PNPT1,TMEM173,HTRA2,OAS1,IRGM,UBE2G2	grey	VSMC_M10
GO:0043624	0.87	0.18	57	119	cellular protein complex disassembly	BP	1	VPS4B,AVIL,JMJD4,LMOD2,KIAA1211,TMOD1,PLEKHH2,SPTBN1,PLEK,ADD2,NAPA,CLASP2,MTRF1,TRIM54,NCKAP5,NAPB,NAV3,SPEF1,LMOD3,ATXN7,C12orf65,LIMA1,NCKAP5L,KATNB1,SYNJ1,F2RL1,DMTN,STMND1,CARMIL1,LMOD1,ADD3,TRPV4,TTBK2,CFL1,HSPA8,CIB1,SPTBN2,TMOD4,EPS8,CAPZA1,NSF,UPF1,TMOD2,SPTB,GAK,PEX14,CAMSAP3,TAOK1,APC2,CAMSAP1,VPS4A,CARMIL2,CCSAP,APC,PDXP,MAP1A,MAP6D1	grey	VSMC_M10
GO:0034086	0.87	0.32	4	10	maintenance of sister chromatid cohesion	BP	1	SLF1,SLF2,MAU2,NAA10	grey	VSMC_M10
GO:0034088	0.87	0.32	4	10	maintenance of mitotic sister chromatid cohesion	BP	1	SLF1,SLF2,MAU2,NAA10	grey	VSMC_M10
GO:0071241	0.87	0.17	81	165	cellular response to inorganic substance	BP	1	TPH2,SLC12A2,PRKN,TSPO,CPNE5,HSF1,SLC1A1,AQP1,MTF1,EGFR,BRAF,DLG2,SCN5A,CPNE9,SYT1,RYR1,PRNP,SYT9,CEBPA,RYR3,ADGRV1,MEF2C,HVCN1,GUCY1B1,XRCC4,CPNE2,SPIDR,PKD2,TFAP2A,DMTN,ADCY7,SYT8,SLC41A1,GPLD1,A3GALT2,MTR,SLC30A10,NCF1,FABP4,RTF2,NFATC4,CARF,CLIC4,BACE1,CDKN1B,NFE2L2,CREB1,RASA4,RASGRP2,MMP3,ABCB6,KCNA1,ADCY10,MAPK1,DDX11,ATP13A2,DLG4,PARP1,SYT7,DDI2,SYT11,FOS,MAPK3,DNM2,SLFN14,SLC25A23,JUN,FMR1,CRK,TRAF2,CACNA1H,CPNE7,HSPA5,NQO1,FSTL3,KCNK3,MT1X,CLDN1,WNT5A,TIGAR,PPIF	grey	VSMC_M10
GO:0016073	0.87	0.22	16	35	snRNA metabolic process	BP	1	EXOSC4,EXOSC3,INTS10,ZCCHC8,USB1,INTS8,INTS12,RBM7,MEPCE,INTS5,AP002990.1,INTS2,METTL4,INTS11,METTL16,EXOSC5	grey	VSMC_M10
GO:0034250	0.87	0.18	64	130	positive regulation of cellular amide metabolic process	BP	1	RMND1,PRR16,PABPC1,EIF3E,JMJD4,AGO2,HABP4,PLA2G6,NCK2,SPHK2,CNBP,CDC123,SIRT3,UPF3A,PRKDC,CASP3,MRPS27,USP16,PTK2B,EIF2AK4,NOD2,TENT4B,SLC2A13,RPUSD4,RCC1L,RPS6KB2,YTHDF2,RPS4X,YTHDF1,PASK,CD28,UHMK1,NSUN5,YBX3,NFE2L2,DDX3X,COA3,SAMD4A,PINK1,MAPK1,POLR2G,EPHA4,ZCCHC4,FXR2,EFNA1,MAPK3,TOB1,EIF3C,LARP6,LARP1,CCL5,UPF3B,FMR1,CIRBP,BARHL2,RHOA,RPS27L,SOX4,METTL14,IMPACT,RPUSD3,PPP1R15A,UQCC2,TRMT10C	grey	VSMC_M10
GO:0048041	0.87	0.19	38	81	focal adhesion assembly	BP	1	LRP1,PTK2,TEK,ITGB1BP1,CLASP2,ACTG1,ARHGEF7,PTPRA,FAM107A,FMN1,SORBS1,PTPRJ,PTK2B,DMTN,LDB1,SLK,COL16A1,AJUBA,ARHGAP6,SLC9A1,ACTN3,WHAMM,APOD,TRIP6,WNT4,VCL,TAOK2,SMAD3,CORO2B,BCAS3,CAMSAP3,PDPK1,TSC1,RHOA,ACVRL1,PEAK1,THY1,DUSP22	grey	VSMC_M10
GO:0046033	0.87	0.28	6	14	AMP metabolic process	BP	1	NT5E,AMPD3,AMPD1,ADK,ADSSL1,NUDT2	grey	VSMC_M10

GO:0001767	0.87	0.32	4	10	establishment of lymphocyte polarity	BP	1	MYH9,TRAF3IP2,SNX27,DOCK2	grey	VSMC_M10
GO:0001704	0.87	0.18	44	93	formation of primary germ layer	BP	1	ITGA7,EXOC4,SRF,TXNRD1,SIX2,WNT11,EOMES,GPI,EYA1,TLX2,MMP15,RTF1,PAX2,PRKAR1A,SNAI1,SOX2,LAMB3,COL7A1,FZD7,ITGB5,SETD2,WNT3,ITGA4,FN1,COL5A2,TBX19,LEO1,BMPR1A,KDM6B,ITGA3,SMAD3,LHX1,LAMA3,GATA6,TWSG1,COL5A1,FOXF1,DUSP2,LAMB1,POFUT2,NOG,WNT5A,DUSP1,DUSP4	grey	VSMC_M10
GO:0045429	0.87	0.23	12	27	positive regulation of nitric oxide biosynthetic process	BP	1	TLR4,PTK2B,PKD2,AGT,NOS1AP,SMAD3,DNM2,INSR,TICAM1,TRPV1,ASS1,MIR99B	grey	VSMC_M10
GO:0030851	0.87	0.24	11	25	granulocyte differentiation	BP	1	IL5,TRIB1,L3MBTL3,CEBPA,AP3B1,TESC,HCLS1,ZBTB46,SP3,HAX1,EVI2B	grey	VSMC_M10
GO:0070278	0.87	0.30	5	12	extracellular matrix constituent secretion	BP	1	ADTRP,AGT,TNFRSF1B,ENG,RGCC	grey	VSMC_M10
GO:1905475	0.87	0.17	72	147	regulation of protein localization to membrane	BP	1	LRP1,STX7,STAC3,PRKN,GOPC,SLC1A1,PRKCE,SPTBN1,PTN,ITGB1BP1,EGFR,TCAF2,SLC7A11,STAC,AKT2,PRNP,LYPLA1,PIK3R1,PLS1,SORBS1,KCNJ11,DMTN,NHLRC1,PPFIA1,DAG1,SSH1,KCNB1,C2CD5,GPER1,RAB11FIP2,CIB1,AP2M1,PPP2R5A,FZD9,DLG1,LRRC15,FIS1,CEMIP,NMT1,LZTFL1,ZDHHC8,CNPY4,GRIPAP1,CNST,ACTB,IQSEC2,RSC1A1,WNK3,STAC2,STX4,ITGAM,ITGA3,AC138894.1,RAB11A,STX8,ARHGAP44,AP2B1,MIEF2,CDK5R1,EFCAB7,GPC3,INPP5K,PDPK1,PRKCZ,RER1,ARHGEF16,VPS4A,LDLRAP1,HRAS,MIEF1,MRAP2,MESD	grey	VSMC_M10
GO:0001656	0.87	0.19	35	74	metanephros development	BP	1	OSR2,MYC,PTCH1,AKR1B1,SMO,TFAP2B,SIX2,POU3F3,OSR1,SPRY1,SOX9,FOXJ1,RET,PAX8,EYA1,ROBO2,FMN1,FRAS1,NKX3-1,PKD2,PAX2,SALL1,IRX2,CTSH,FBN1,CXCR2,WNT4,CITED1,LHX1,GPC3,PKD1,SOX8,BASP1,SIX1,LIF	grey	VSMC_M10
GO:0010832	0.87	0.29	5	12	negative regulation of myotube differentiation	BP	1	BDNF,HDAC4,BHLHA15,PLPP7,NKX2-5	grey	VSMC_M10
GO:2001239	0.87	0.22	15	33	regulation of extrinsic apoptotic signaling pathway in absence of ligand	BP	1	EYA4,HSPA1B,ZC3HC1,RET,IL1A,EYA1,IL7,HTRA2,TERT,EYA3,IFI6,MCL1,STRADB,MAP2K5,MAPK7	grey	VSMC_M10
GO:0048251	0.87	0.31	4	10	elastic fiber assembly	BP	1	TNXB,FBLN5,EFEMP2,MYH11	grey	VSMC_M10
GO:0018158	0.87	0.29	5	12	protein oxidation	BP	1	PRDX3,TRABD2A,LOXL2,LOXL4,TRABD2B	grey	VSMC_M10
GO:0032435	0.87	0.23	13	29	negative regulation of proteasomal ubiquitin-dependent protein catabolic process	BP	1	TRIM39,SDCBP,KLHL40,DDRGK1,RYBP,TAF9,RPL11,OGT,SGTA,PARK7,CAMLG,STYX,MAP1A	grey	VSMC_M10
GO:0048557	0.87	0.29	5	12	embryonic digestive tract morphogenesis	BP	1	SIX2,OVOL2,HLX,RBPMS2,FOXF1	grey	VSMC_M10
GO:0061311	0.87	0.24	11	25	cell surface receptor signaling pathway involved in heart development	BP	1	GALNT11,WNT11,JAG1,HAND2,SNAI1,BMPR1A,RBPJ,MKS1,NOG,WNT5A,HEY2	grey	VSMC_M10
GO:0051592	0.87	0.18	55	114	response to calcium ion	BP	1	TPH2,CPNE5,SLC25A13,ALG2,EGFR,BRAF,TRPC3,ENTPD6,GIPR,SCN5A,AANAT,CPNE9,SYT1,RYR1,SYT9,RYR3,ADGRV1,MEF2C,PTK2B,CPNE2,SEC31A,HOMER1,PKD2,DMTN,CASQ2,KCNIP2,RYR2,SYT8,GPLD1,PPP3CA,CCND1,PEF1,PDCD6,AMELX,CARF,CLIC4,RASA4,TTN,RASGRP2,ANXA7,KCNMA1,SYT7,SYT11,FOS,CALM1,SLC25A23,CPNE7,HSPA5,MT-CYB,FGA,WNT5A,DUSP1,PTGES,PPIF,KCNMB4	grey	VSMC_M10
GO:0015867	0.87	0.29	5	12	ATP transport	BP	1	CALHM1,SLC25A42,SLC25A23,SLC25A41,SLC25A25	grey	VSMC_M10
GO:0032634	0.87	0.29	5	12	interleukin-5 production	BP	1	TNFRSF21,SCGB1A1,FOXP3,PRKCZ,TSLP	grey	VSMC_M10
GO:0032674	0.87	0.29	5	12	regulation of interleukin-5 production	BP	1	TNFRSF21,SCGB1A1,FOXP3,PRKCZ,TSLP	grey	VSMC_M10
GO:0003205	0.87	0.17	65	135	cardiac chamber development	BP	1	ADGRG6,MAML1,FRS2,LY6E,TAB1,SMO,FOXH1,TEK,PTK7,SRF,FHL2,TNNI3,DNAH11,HOXA13,CNTRL,SCN5A,POU4F1,WNT11,TGFBR2,OVOL2,JAG1,NRG1,PAX8,HAND2,PARVA,ROBO1,ROBO2,MEF2C,ADAMTS1,TBX5,TNNC1,DSP,SALL1,RYR2,SUFU,PKP2,TNNI1,TGFB2,TRIP11,MYH7,ANK2,KCNK2,LUZP1,SAV1,BMPR1A,DHRS3,RBPJ,MED1,MKS1,DCTN5,TBX2,STRA6,GATA6,AP2B1,ISL1,ZBTB14,FOXF1,ENG,SOX4,TMEM65,NOG,WNT5A,NKX2-5,HEY2,WNT2	grey	VSMC_M10
GO:0051960	0.87	0.16	172	344	regulation of nervous system development	BP	1	LRP1,SEMA6A,RAB21,SNW1,TIAM2,TSPO,FSTL4,SYNGAP1,PLXNA4,NTRK2,SOX10,SMO,DAAM2,LINGO2,SRF,TTBK1,KIT,TNFRSF21,SEMA4D,SEMA4C,RTN4,RUFY3,LRRN3,EPHA7,PTN,EGFR,TRIM32,LPIN1,MYCN,FIG4,PLXNA1,HDAC2,MTMR2,VSTM5,CST7,GOLGA4,MEGF8,KDM4A,XRCC2,NUMBL,POU4F1,SS18L2,HOOK3,BHLHE40,SIRT2,RARB,SEMA3D,SPART,PLXNC1,SEMA3A,ULK1,RYK,EPHB1,PLAG1,LGI4,ANKRD27,FAIM,CLSTN2,ROBO1,ROBO2,ARNTL,FZD3,SEMA4F,VEGFC,TLX2,JAM2,CUX2,MME,RAPGEF2,TIAM1,SLIT1,SYNJ1,OPA1,NKX6-1,AMIGO2,ZFYVE27,SPINT1,SEMA3G,ITPKA,SPP1,CYFIP1,SEMA4G,ZNF335,BDNF,RNF10,KIAA0319,NEURL1,DNM1L,HDAC1,PPP3CA,CAPRIN2,SEMA3F,DAG1,AMIGO3,ADNP,YTHDF2,DISC1,SORL1,GPER1,GBX2,CERS2,NFATC4,HES6,PER2,SS18L1,CDKL5,DLX1,EPHB3,NSUN5,DLG1,WNT3,DUSP10,MAPT,SEMA6D,TNR,FN1,XRCC5,NIN,OBSL1,HAP1,EPHA4,LRTM2,BMPR1A,SPEN,NLGN2,TNFRSF1B,RGS14,PRPF19,PSEN1,SEMA4A,NLGN3,TRIM46,NDEL1,RAB11A,MAP2K1,NTN1,LDLR,PARP6,GPRASP2,BHLHB9,TRPV2,ASIC2,CDK5R1,DOCK7,MBD1,DAB1,SLITRK2,LRP8,L1CAM,SKI,ARHGAP4,PLXNA3,SOX8,AMIGO1,FBXO31,ADGRL2,MIR222,MIR125B1,FOXG1,GRID2,GPR37L1,HOXB3,NOG,THY1,WNT5A,LIF,YWHAH,EFNB3,PITX3,HEY2,WNT2,LRRTM2	grey	VSMC_M10
GO:0008360	0.87	0.17	65	134	regulation of cell shape	BP	1	ITGA7,CDC42SE2,PTK2,PLXNA4,CDC42EP1,RAC2,STRIP2,MYH9,KIT,SEMA4D,FGD3,PHIP,LPAR1,MYH14,PTN,BVES,PLXNA1,SLC26A5,MYL12A,RAC3,DIAPH1,PLXNC1,FGD6,HCK,PARVA,P2RY1,PTK2B,RND1,RHOBTB2,CYFIP1,DMTN,BRWD1,GAS2,FGD4,DAG1,EPB41,FGR,EPS8,COCH,RHOBTB1,DLG1,TBCCD1,CDC42EP2,FN1,MARK2,RHOJ,FBLIM1,FGD1,SEMA4A,TAOK2,MYH10,BRWD3,CSF1R,CCL7,CCL2,EPB41L3,CRK,PLXNA3,PALM,CFDP1,MIR21,RHOA,LST1,RND3,RHOU	grey	VSMC_M10
GO:0060009	0.87	0.31	4	10	Sertoli cell development	BP	1	HSD17B4,SOX9,SOX8,ARID4A	grey	VSMC_M10
GO:0002028	0.87	0.19	31	66	regulation of sodium ion transport	BP	1	NEDD4L,WNK2,PRKCE,HECW1,OSR1,SCN5A,GPD1L,SLMAP,GLRX,P2RX7,TESC,CNTN1,PKP2,SIK1,HECW2,KLHL24,ATP1A2,SLC9A1,SCN4B,DLG1,ATP1B1,WNK3,ATP1B2,DNM2,MIR24-1,DMD,FGF11,SCN2B,NETO2,YWHAH,NKX2-5	grey	VSMC_M10

GO:0019068	0.87	0.22	14	31	virion assembly	BP	1	VPS4B,CHMP2A,CHMP2B,CHMP4C,SPCS1,CHMP7,PC,DDX6,RAB1B,VPS37D,CHMP1A,MVB12B,VPS4A,TBC1D20	grey	VSMC_M10
GO:1903441	0.87	0.31	4	10	protein localization to ciliary membrane	BP	1	TUB,LZTFL1,WDR19,EFCAB7	grey	VSMC_M10
GO:0060795	0.87	0.24	10	23	cell fate commitment involved in formation of primary germ layer	BP	1	SIX2,EOMES,EYA1,RTF1,PAX2,SOX2,FZD7,LEO1,BMPR1A,GATA6	grey	VSMC_M10
GO:1903725	0.87	0.22	14	31	regulation of phospholipid metabolic process	BP	1	LPIN1,MTMR2,APOC1,NR1H4,CHRM5,MTMR9,LPCAT1,MTMR3,GNB3,SLC27A1,LDLR,MTMR1,ABCA3,DNAJC19	grey	VSMC_M10
GO:0060295	0.87	0.29	5	12	regulation of cilium movement involved in cell motility	BP	1	CFAP69,MKKS,CFAP20,CFAP45,CCSAP	grey	VSMC_M10
GO:1902019	0.87	0.29	5	12	regulation of cilium-dependent cell motility	BP	1	CFAP69,MKKS,CFAP20,CFAP45,CCSAP	grey	VSMC_M10
GO:0046636	0.87	0.22	13	29	negative regulation of alpha-beta T cell activation	BP	1	HLX,RUNX3,BCL6,VSIR,RC3H1,TNFSF18,FOXP3,TBX21,ZBTB7B,IL4R,TWSG1,SOCS1,RC3H2	grey	VSMC_M10
GO:0006942	0.87	0.19	32	68	regulation of striated muscle contraction	BP	1	TNNI3,ASB3,TRPM4,SCN5A,NR4A1,STC1,DSP,CASQ2,RYR2,PKP2,KCNJ2,TNNI1,TRPV4,MYH7,GRK2,ANK2,ACE2,ATP1A2,SLC9A1,CASQ1,ACTN3,HDAC4,DLG1,ATP1B1,ATP2A1,CALM1,DSG2,RNF207,DMD,CHGA,HCN4,NKX2-5	grey	VSMC_M10
GO:2000644	0.87	0.31	4	10	regulation of receptor catabolic process	BP	1	MTMR2,MYLIP,DTX3L,ANXA2	grey	VSMC_M10
GO:0046626	0.87	0.20	23	50	regulation of insulin receptor signaling pathway	BP	1	PIP4K2C,GKAP1,CCND3,GRB10,SESN3,PIP4K2A,SNX5,PIK3R1,SORBS1,OPA1,IGF2,IRS1,GRB14,SORL1,AGT,SIK2,ZBTB7B,OGT,PRKCB,PTPN2,SOCS1,INPP5K,PRKCZ	grey	VSMC_M10
GO:0150063	0.87	0.16	140	282	visual system development	BP	1	SLC39A5,MDM1,FRS2,OSR2,TBC1D32,BAK1,ATF4,AGTPBP1,NTRK2,PRPH2,RORB,SRF,SLC1A1,TFAP2B,TDRD7,TMOD1,RDH13,ABCB5,PTN,TSPAN12,USP45,SLC7A11,SPRY1,FOXE3,HDAC2,BBS7,SOX9,CRYGN,ZNF513,ACVR2B,IFT172,MYO7A,TSKU,SPRY2,DZANK1,TGFBR2,BFSP1,JAG1,RARB,FSCN2,SPRED3,FREM2,CEP290,KERA,MAB21L1,MITF,FLT1,TUB,EPHB1,PFDN5,NPHP1,RET,FOXL2,MAB21L2,TENM3,USH1C,TENT2,OPA1,RPGRIP1L,TFAP2A,PAX2,NKD1,BMPR1B,MYH15,NECTIN3,LPCAT1,PKNOX1,SLC6A3,HDAC1,ALDH1A3,TGFB2,TCIRG1,PRSS56,SHROOM2,SOX2,CRYBA4,NRL,CLIC4,DLX1,GRHL3,LIMK2,SP3,CDKN1B,DLG1,FZD5,FBN1,FKBP8,COL5A2,PLA2G16,STAT3,TULP3,OBSL1,RHOJ,ROM1,MFAP2,WDR19,SDK1,MAX,CLN8,MED1,ALDH1A2,MFN2,PSEN1,ITGAM,TTLL5,MEGF11,LCTL,SMAD3,TBX2,BCL11B,LHX1,STRA6,YY1,CHRDL1,UNC45B,LAMA1,C3,TWSG1,GNB1,FZR1,SKI,SOX8,COL5A1,LAMC3,OLFM3,TMEM231,SLC25A25,HSF4,BLOC1S3,FJX1,CNTF,ACVRL1,THY1,WNT5A,DIO3,TGIF2,TBC1D20,PITX3,WNT2,COL8A2	grey	VSMC_M10
GO:0070977	0.87	0.24	9	21	bone maturation	BP	1	SEMA4D,RYR1,ACTN3,FGFR3,THBS3,RFLNB,RHOA,SNX10,CCDC154	grey	VSMC_M10
GO:0010927	0.87	0.18	41	87	cellular component assembly involved in morphogenesis	BP	1	TBPL1,OBSCN,LMOD2,SRF,PGM5,TMOD1,MYLK3,FIG4,MTMR2,ERCC2,CLASP2,NECTIN2,ACTG1,LMOD3,LMOD1,PRKAR1A,DAG1,ANK2,GNPAT,ACTC1,MYOZ2,CASQ1,TMOD4,PHLDB1,MYPN,PIKFYVE,TTN,MYOZ1,CNTNAP1,TMOD2,OBSL1,LDB3,MPP5,TCAP,SOX30,MYH11,EPB41L3,MFSD14A,CSRP2,KRT19,TBC1D20	grey	VSMC_M10
GO:0010623	0.87	0.31	4	10	programmed cell death involved in cell development	BP	1	KIT,PRKDC,IL1A,CNTF	grey	VSMC_M10
GO:0000122	0.87	0.15	355	694	negative regulation of transcription by RNA polymerase II	BP	1	PLAGL1,ZBTB2,ESR1,CNOT2,OSR2,SNW1,ZBTB12,PRKN,PPARA,MYC,SMAD5,PTCH1,ATF4,CBX7,SOX10,TMPRSS6,SMO,HSF1,FOXP4,MDFI,CCND3,FOXH1,CRY1,SRF,NFX1,SMARCA2,NCK2,FHL2,TFAP2B,NFIL3,SEMA4D,RPS14,ZNF550,ZNF268,NFE2L3,HOXA2,HOXA7,JAZF1,XPO1,AKIRIN2,BACH2,ZNF692,NR1H2,ZNF350,FOXP2,MED25,KLF11,CREM,SDCBP,OSR1,FOXO3,HDAC2,DNMT3A,BMI1,CNBP,PHB,SOX9,ZNF746,EED,SUV39H2,ZNF404,PLK3,GLI2,DACH1,TAF3,ACVR2B,KLF5,KLF12,FOXJ1,GZF1,POU4F1,THAP1,EID2,OVOL2,ZNF219,EOMES,CBX4,BHLHE40,CHD8,SIRT2,RARB,PURA,NR1D2,PAWR,SIRT7,ALX1,TAF7,RYBP,FOXP1,MITF,MTA3,ARID5A,IRF7,TRAF6,LIN37,NR1H4,KCNIP3,TRIB3,SDR16C5,ZC3H8,DNMT3B,CEBPA,CBFA2T2,ZNF536,IRF2,NCOA2,SAP30,ZNF366,HMBOX1,MEF2C,PEX2,PKIA,TMBIM6,CNOT1,SLA2,CUX2,MCPH1,FNIP2,BACH1,NR1H3,SCAF4,LCOR,VDR,HDAC7,NKX6-1,LOXL2,NR4A2,PHF21A,CRY2,RREB1,RTF1,ZBED6,SOX13,ZNF248,TFAP2A,RIPPLY3,HIF1AN,SALL1,HIVEP1,JARID2,ATXN1,SUDS3,LDB1,IGF2,MAGEL2,ELK4,NUPR2,DCAF1,SUFU,TAGLN3,PCGF6,IRX2,RPL23,HDAC1,CCND1,SP100,TRPV4,ZBTB20,SATB2,TCF7L2,AJUBA,PIAS3,HJV,SNAI1,ZNF202,HCLS1,SOX2,OTUD7B,GTF2IRD1,ZNF217,PARP9,PARP15,TFAP2C,USP2,ORC2,BAG3,NFATC4,HES6,MYOZ2,PER2,AEBP2,VPS72,HDAC4,RUNX3,NR1I3,DLX1,MLXIPL,PATZ1,SP3,DLG1,OVOL1,ETV6,YBX3,HOXD8,KLF7,HEXIM2,BCL6,H2AFY2,BCOR,MEN1,MEPCE,ZBTB37,MYOZ1,RBM10,UXT,STAT3,NKX3-2,TBL1XR1,PPM1A,SCGB1A1,FOXP3,ZBTB25,ZBTB1,MAGED1,MAGED4B,PARP1,SPEN,GPS2,THRA,MAX,DNAJB1,PRDM2,RBPJ,MED1,NSD1,MAGEH1,MAFK,KLF8,C[illegible]	grey	VSMC_M10
GO:0060324	0.87	0.21	18	40	face development	BP	1	ZFAND5,SRF,DLX5,RAF1,CSRNP1,MKKS,CRISPLD1,ALDH1A3,MAPK1,ALDH1A2,MAPK3,MAP2K1,STRA6,SKI,ANKRD11,HOXB3,NOG,WNT5A	grey	VSMC_M10
GO:0006527	0.87	0.31	4	10	arginine catabolic process	BP	1	OAT,FAH,NOS2,MIR21	grey	VSMC_M10
GO:0071480	0.87	0.22	13	29	cellular response to gamma radiation	BP	1	XRCC6,HSF1,WRN,ATR,CHEK2,XRCC5,CYBA,HSPA5,MIR21,HRAS,TMEM109,TSPYL5,GTF2H5	grey	VSMC_M10
GO:0090153	0.87	0.29	5	12	regulation of sphingolipid biosynthetic process	BP	1	ORMDL2,PLA2G6,SPHK2,SIRT3,ORMDL1	grey	VSMC_M10
GO:1905038	0.87	0.29	5	12	regulation of membrane lipid metabolic process	BP	1	ORMDL2,PLA2G6,SPHK2,SIRT3,ORMDL1	grey	VSMC_M10
GO:1904019	0.87	0.18	41	86	epithelial cell apoptotic process	BP	1	MAP3K5,TNFAIP3,STK3,PPARA,TEK,SFRP4,FOXO3,FGF21,DNMT3A,NDNF,TGFBR2,TMTC4,GAS6,SRSF6,ANO6,PLA2R1,STK4,CD40,RYR2,HDAC1,TERT,PDCD4,TCF7L2,GPER1,E2F2,NFE2L2,ITGA4,TNIP2,RAMP2,EXOC5,MAPK7,CCL2,JAG2,PIAS4,PDPK1,DAB2IP,MIR24-1,FGA,MIR125A,CD248,RGCC	grey	VSMC_M10
GO:0051489	0.87	0.20	20	44	regulation of filopodium assembly	BP	1	SRF,RAB5A,DPYSL3,PIK3R1,DMTN,SRGAP2C,NEURL1,GAP43,RAB17,DNM3,MIEN1,FXR2,TENM2,AC138894.1,BCAS3,ARHGAP44,FMR1,PALM,FNBP1L,RALA	grey	VSMC_M10

GO:0009161	0.87	0.20	24	51	ribonucleoside monophosphate metabolic process	BP	1	DPYS,IMPDH1,GDA,PPAT,NT5E,CMPK1,DLG2,AMPD3,GMPS,AMPD1,PRPS2,MAGI3,DLG1,UPB1,CASK,ADK,CARD11,UPRT,ADSSL1,MPP1,UCK1,DHODH,NUDT2,UPP1	grey	VSMC_M10
GO:0045185	0.87	0.18	38	80	maintenance of protein location	BP	1	TSPO,GOPC,SKP1,SUN2,GPAA1,MDFI,TAF8,NFKBIE,FAF1,GRIK5,TAF3,NFKBIB,ARL2BP,PKD2,MORC3,SUFU,SP100,IGSF11,GET4,SORL1,SRGN,HK1,FBN1,PINK1,VPS13D,VPS13C,RIT2,KEAP1,PSMD10,RER1,PKD1,PARK7,HSPA5,SUPT7L,TXN,NRROS,INSIG2,PFN4	grey	VSMC_M10
GO:0031061	0.87	0.25	8	19	negative regulation of histone methylation	BP	1	PHF1,KDM4A,DNMT3B,JARID2,BCOR,SUPT6H,MTF2,PRMT6	grey	VSMC_M10
GO:0072337	0.87	0.23	11	25	modified amino acid transport	BP	1	SLC22A4,SLC3A1,SLC1A4,SLC7A11,ABCC4,SLC38A2,SLC25A20,ABCC5,SLC46A1,SLC22A5,SLC25A32	grey	VSMC_M10
GO:0021846	0.87	0.25	8	19	cell proliferation in forebrain	BP	1	AKNA,POU3F3,NUMBL,HOOK3,DISC1,DIXDC1,DOCK7,POU3F2	grey	VSMC_M10
GO:1903034	0.87	0.17	60	123	regulation of response to wounding	BP	1	SERPINB2,LRP1,TNFAIP3,TSPAN8,SLC12A2,PTK2,ENPP4,CLDN19,PRKCE,CD109,PTN,CLASP2,PTPRF,THBD,PLAT,C1QTNF1,ACTG1,HBEGF,FERMT1,F2RL1,F2R,SRSF6,ANO6,RREB1,SPP1,DMTN,ADTRP,FAP,KIAA0319,ADRA2A,UBASH3B,KREMEN1,CERS2,USF1,LRIG2,NFE2L2,DUOX1,TNR,CASK,SERPINC1,WNT4,MYOZ1,EPHA4,F12,ANXA2,NDEL1,MAP2K1,SMAD3,ABAT,CRK,SERPINF2,MIR29A,MIR21,MIR222,FGA,CNTF,RTN4RL1,CLDN1,HRAS,CADM4	grey	VSMC_M10
GO:1905214	0.87	0.29	5	12	regulation of RNA binding	BP	1	EIF3E,GEMIN2,EIF3C,LARP6,FMR1	grey	VSMC_M10
GO:0009743	0.87	0.16	92	186	response to carbohydrate	BP	1	ZNF236,NME1,LRP1,APOM,AGER,PNPLA3,MAPK13,PLA2G6,TXN2,SRF,CAT,PRKCE,HMGN3,ME1,TRPM4,FOXO3,APOB,FGF21,GIPR,ERCC1,KHK,EIF2B3,GRIK5,RAF1,GCKR,TGFBR2,P2RX2,GAS6,NR1H4,CYP7A1,NDUFAF2,OSBP,CASP3,P2RX3,PTK2B,KCNJ11,TIAM1,RAB11FIP5,OPA1,NKX6-1,ACVR1C,ZBED6,PAX2,TWNK,GPLD1,PPP3CA,MAP2K6,SLC9B2,ZBTB20,ADRA2A,GPAM,TCF7L2,KCNB1,PFKFB2,ADNP,POLG,GPER1,RAB11FIP2,MLXIPL,GLUL,SIDT2,CDKN1B,PDK3,KLF7,RAP1B,FIS1,MEN1,ENY2,ABCA12,CALCRL,PPP3CB,CTSV,PTPRN,NDUFA9,MPC2,ILDR2,STX4,KAT7,PKLR,GPR68,PRKCB,SIN3A,COLEC12,SREBF1,OXCT1,NADK,BAIAP3,CYBA,NQO1,RHOA,SOX4,CRH	grey	VSMC_M10
GO:0045923	0.87	0.24	10	23	positive regulation of fatty acid metabolic process	BP	1	PPARA,NR1H2,AKT2,NR1H3,IRS1,CPT1A,LPGAT1,MLXIPL,CD74,MLYCD	grey	VSMC_M10
GO:0051252	0.87	0.14	1386	2654	regulation of RNA metabolic process	BP	1	ADNP2,NFATC1,ZNF236,ZNF407,ZC3H10,SMARCC2,PAN2,ATP8B1,ONECUT2,BAZ2A,TCF4,GLI1,MARS,CLK4,MAP3K5,TNFAIP3,TSFM,HIVEP2,PLAGL1,MAML1,ZBTB2,CPSF6,YEATS4,ESR1,MYRFL,CNOT2,CNOT6,RIDA,STK3,OSR2,PABPC1,SNW1,ZNF706,PHAX,ARID1B,HSPA1B,FZD6,ZBTB12,DXO,ZBED4,PNLDC1,PRKN,QKI,AGER,PPARA,MED30,IL5,BRD2,RXRB,VGLL2,HSF2,TCF7,PHF1,XRCC6,NCOA7,CSDC2,PITX1,TRIB1,MYC,TOB2,SMAD5,L3MBTL3,L3MBTL2,AGO2,HABP4,KDM3B,PTP4A3,PTCH1,TNRC6B,ZC3H3,ATF4,TUT7,RPAP2,CBX7,GTPBP1,MAPK14,CBY1,PUF60,KDM4C,SOX10,PIM1,NRF1,TMPRSS6,SMO,BNC2,HSF1,NFYA,FOXP4,MDFI,TFEB,IRF5,MED20,CCND3,FOXH1,LRRC14,CDKN2A,ZNF16,RBFOX2,IFT74,BICRAL,RORB,ZNF300,PRDM4,CRY1,DMTF1,SRF,SMU1,ZNF318,NFX1,CDK14,FZD1,MTERF1,NFXL1,NFKBIE,SUPT3H,SMARCA2,RFX3,KIT,ADGRF1,NCK2,FHL2,TGFBRAP1,TFAP2B,ROR2,NFIL3,SEMA4D,RFX8,DLX5,PHF14,IPPK,ETV1,ZNF135,EXOSC3,RPS14,ZNF154,MEOX2,ZNF550,KANSL3,BEND6,ZNF268,ZNF667,ZNF787,ZNF691,CAT,MACC1,LRPPRC,SIX2,FOXJ3,ZNF865,HIVEP3,IL11,SCMH1,ZNF189,RNF20,EPAS1,EPCAM,SP4,IGF2BP3,PPP2R1A,ZNF462,TRA2A,PHIP,HMGN3,YTHDC1,NFYC,NFE2L3,PNPT1,HOXA2,TAF1D,HOXA4,HOXA6,HOXA7,HOXA13,REL,ZNF483,JAZF1,CREB5,PTBP3,MYCL,XPO1,MTURN,NOD1,AKIRIN2,SRSF10,CASP8AP2,BACH2,ZNF692,PELI1,AKIRIN1,MTF1,ZNF618,SNIP1,ANXA4,PCBP1,NR1H2,AKNA,ZNF350,ITGB1BP1,FOXP2,GRHL1,MED25,KLF11,MET,IL4I1,AGO1,POU3F3,EGFR,LANCL2,ING3,NOBOX,ARHGEF5,TRIM32,TLR4,CCNC,CDK5RAP2,TRAF1,BCL2L12,LPIN1,SFRP4,UCP1,CREM,TEAD2,SETD7,SDCBP,MYCN,C6orf203,E[illegible]	grey	VSMC_M10
GO:1903076	0.87	0.18	43	90	regulation of protein localization to plasma membrane	BP	1	LRP1,STX7,STAC3,GOPC,PRKCE,SPTBN1,EGFR,STAC,PRNP,LYPLA1,PIK3R1,PLS1,SORBS1,KCNJ11,NHLRC1,PPFIA1,GPER1,RAB11FIP2,CIB1,AP2M1,PPP2R5A,DLG1,LRRC15,ZDHHC8,CNPY4,GRIPAP1,CNST,ACTB,RSC1A1,WNK3,STAC2,STX4,ITGA3,AC138894.1,RAB11A,STX8,ARHGAP44,PDPK1,RER1,ARHGEF16,VPS4A,LDLRAP1,MRAP2	grey	VSMC_M10
GO:2001269	0.87	0.31	4	10	positive regulation of cysteine-type endopeptidase activity involved in apoptotic signaling pathway	BP	1	ST18,HTRA2,SMAD3,PRR7	grey	VSMC_M10
GO:0010517	0.87	0.20	23	50	regulation of phospholipase activity	BP	1	LRP1,ESR1,KIT,LPAR1,PPT1,EGFR,C5AR1,PHB,RASGRP4,FLT1,PLA2R1,GNA13,ARHGAP6,AGT,ABL2,FGFR3,ANXA8,NMUR2,CCL5,GNA15,PDPK1,PRKCZ,HRAS	grey	VSMC_M10
GO:0021537	0.87	0.16	90	184	telencephalon development	BP	1	NME1,LRP1,ATAT1,UQCRQ,AGTPBP1,PLXNA4,SUN2,NTRK2,SMO,SRF,CDK6,DLX5,EFHC1,MBOAT7,RTN4,AQP1,FOXP2,POU3F3,SLC7A11,FAT4,EIF2B3,XRCC1,SCN5A,NUMBL,TSKU,SZT2,ALK,MKKS,RARB,PLCB1,SEMA3A,NRG1,RYK,ZSWIM6,CASP3,DCLK2,ROBO1,ROBO2,CELF1,CSNK2A2,MCPH1,TENT2,SLC38A2,RPGRIP1L,SALL1,DNAH5,DPCD,SRGAP2C,SRD5A1,HDAC1,ALDH1A3,DISC1,ATP1A2,DLX1,EPHB3,NSUN5,BCL2A1,DIXDC1,TNR,NIN,COL3A1,KCNA1,MPP5,ATP1B2,PSEN1,KDM6B,NDEL1,BCL11B,LHX1,XAB2,CDK5R1,DAB1,LRP8,CRK,SKI,PLXNA3,TSC1,PHLPP2,DAB2IP,RHOA,ADGRG1,CNTN2,FOXG1,LAMB1,RTN4RL1,GNG12,CCDC85C,WNT5A,POU3F2,BTBD3	grey	VSMC_M10

GO:0007569	0.87	0.17	47	98	cell aging	BP	1	MAP3K5,MAPK14,CDKN2A,SRF,NUAK1,CDK6,PNPT1,ZNF277,ERCC1,PAWR,WRN,PRKDC,PLK2,ATR,ING2,ARNTL,MME,MAPKAPK5,OPA1,PRELP,PLA2R1,MORC3,TERT,MAP2K6,PDCD4,SLC30A10,KRAS,CHEK2,MAP3K3,CDKN1B,BCL6,BMPR1A,CTC1,YPEL3,VASH1,MAP2K1,TBX2,DNAJA3,FZR1,TERF2,MIR21,MIR22,PRMT6,BGLAP,YBX1,HRAS,MAP2K7	grey	VSMC_M10
GO:0030050	0.87	0.26	7	17	vesicle transport along actin filament	BP	1	MYO1A,MYO7B,MYO7A,MYO1D,MYO1F,MYO5B,FNBP1L	grey	VSMC_M10
GO:0006458	0.87	0.21	15	33	'de novo' protein folding	BP	1	HSPA1B,BAG1,HSPE1,DNAJC18,HSPA8,DNAJB12,SDF2L1,DNAJB1,GAK,CD74,TOR1B,TOR2A,HSPA5,ENTPD5,SDF2	grey	VSMC_M10
GO:1900101	0.87	0.22	12	27	regulation of endoplasmic reticulum unfolded protein response	BP	1	BAK1,NCK2,DDRGK1,PIK3R1,FICD,PTPN2,ABCA7,HSPA5,DAB2IP,MIR199A1,BCL2L11,PPP1R15A	grey	VSMC_M10
GO:0051279	0.87	0.19	27	58	regulation of release of sequestered calcium ion into cytosol	BP	1	PRKCE,ATG5,DIAPH1,PTK2B,F2R,PKD2,CASQ2,RYR2,UBASH3B,ANK2,GPER1,ATP1A2,CASQ1,NTSR1,CEMIP,PTPN6,HTT,HAP1,CD19,CALM1,BDKRB1,PDPK1,CYBA,JPH3,DMD,JPH2,THY1	grey	VSMC_M10
GO:0014889	0.87	0.30	4	10	muscle atrophy	BP	1	IL15,FOXO3,ACTN3,NOL3	grey	VSMC_M10
GO:0006298	0.87	0.22	12	27	mismatch repair	BP	1	RPA3,MCM9,MSH2,ERCC1,XPC,MLH1,PMS1,AXIN2,SETD2,PMS2,RNASEH2A,MSH4	grey	VSMC_M10
GO:2001259	0.87	0.19	24	52	positive regulation of cation channel activity	BP	1	STAC3,CACNB2,STAC,KCNJ11,IFNGR2,KCNE2,PKD2,KCNIP2,FAM173B,ANK2,CASQ1,NTSR1,NOS1AP,HTT,PINK1,KCNA1,HAP1,STAC2,NLGN3,CALM1,CCL2,RNF207,AMIGO1,JPH2	grey	VSMC_M10
GO:1903170	0.87	0.22	13	29	negative regulation of calcium ion transmembrane transport	BP	1	PKD2,CASQ2,ADRA2A,ATP1A2,C4orf3,NTSR1,SESTD1,GNB5,CALM1,FMR1,RRAD,GPR35,GEM	grey	VSMC_M10
GO:0006304	0.87	0.18	40	84	DNA modification	BP	1	BAZ2A,MOV10L1,MYC,APOBEC3H,PICK1,EXOSC4,EXOSC3,STPG4,ASCC3,DNMT3A,BMI1,CDADC1,MBD4,TEX15,ALKBH3,TOX,DNMT3B,OTUD4,FTO,MTRR,HEMK1,ALKBH2,TDRKH,MGMT,TET1,PARP1,FOS,ALKBH1,NEIL1,METTL4,MBD1,NTHL1,MECP2,MPG,PRMT7,CTCF,MIR29A,BEND3,N6AMT1,EXOSC5	grey	VSMC_M10
GO:0061615	0.87	0.30	4	10	glycolytic process through fructose-6-phosphate	BP	1	PFKP,PFKM,HK1,ADPGK	grey	VSMC_M10
GO:0061620	0.87	0.30	4	10	glycolytic process through glucose-6-phosphate	BP	1	PFKP,PFKM,HK1,ADPGK	grey	VSMC_M10
GO:0048730	0.87	0.23	10	23	epidermis morphogenesis	BP	1	SMO,SOSTDC1,KLK14,INTU,GLI2,TGFB2,SNAI1,PLOD3,GORAB,NGFR	grey	VSMC_M10
GO:0097529	0.87	0.16	70	142	myeloid leukocyte migration	BP	1	AGER,PTK2,RAC2,KIT,ROR2,FPR2,CXCL8,AKIRIN1,C5AR2,C5AR1,MYD88,PLCB1,NCKAP1L,FAM19A4,FLT1,MTUS1,VEGFC,PTK2B,CCL17,ANO6,NOD2,GBF1,CD200,DNM1L,TRPV4,TGFB2,CD99,CMKLR1,SLAMF8,MOSPD2,PTPRO,FCER1G,ADAM8,PIKFYVE,MCU,TNFSF18,CXCR2,BST1,MAPK1,STAT5B,PGF,DAPK2,MAPK3,IL6R,S100A14,S100A8,UMOD,CD74,CCL14,CCL5,CSF1R,CCL7,CCL2,CCL25,VAV1,PTGER4,PIP5K1C,MPP1,BSG,VAV3,MIR24-1,CCL16,S100A12,CHGA,DEFB124,DPP4,CXCL6,DUSP1,CCL22,PDGFD	grey	VSMC_M10
GO:0002903	0.87	0.30	4	10	negative regulation of B cell apoptotic process	BP	1	FOXP1,BCL6,CD74,BCL10	grey	VSMC_M10
GO:0090085	0.87	0.28	5	12	regulation of protein deubiquitination	BP	1	WDR48,OTUD4,SART3,TNIP1,PARK7	grey	VSMC_M10
GO:0002685	0.88	0.16	75	152	regulation of leukocyte migration	BP	1	AGER,GPSM3,PTK2,RAC2,CCL27,FPR2,HOXA7,CXCL8,AKIRIN1,PTN,C5AR2,C5AR1,MYD88,OXSR1,PLCB1,NCKAP1L,GAS6,MTUS1,VEGFC,JAM2,PTK2B,SLC8B1,F2RL1,ANO6,ADTRP,NOD2,CD200,DNM1L,TRPV4,CD99,PRSS56,CMKLR1,SLAMF8,MOSPD2,APOD,ADAM8,MIA3,ITGA2B,ITGA4,IL12A,MCU,TNFSF18,CXCR2,BST1,MAPK1,PADI2,TMEM102,STK10,PGF,DAPK2,MAPK3,IL6R,S100A14,BDKRB1,CD74,CCL5,CSF1R,CCL7,CCL2,CCL25,PTGER4,CRK,MPP1,MIR24-1,RHOA,DEFB124,FADD,DPP4,FUT4,ZNF580,THY1,WNT5A,DUSP1,GCSAM,PDGFD	grey	VSMC_M10
GO:0034975	0.88	0.30	4	10	protein folding in endoplasmic reticulum	BP	1	CANX,PDIA3,DNAJC10,HSPA5	grey	VSMC_M10
GO:0008333	0.88	0.19	23	50	endosome to lysosome transport	BP	1	VPS4B,TGFBRAP1,VPS41,RAB7A,HOOK3,FAM160A2,VPS16,CHMP2B,VPS18,VPS39,KIF13A,RAB7B,DTX3L,SNX27,HOOK2,VIPAS39,STX8,SNAPIN,CLEC16A,MTM1,MGRN1,RILP,SNX16	grey	VSMC_M10
GO:0042118	0.88	0.30	4	10	endothelial cell activation	BP	1	BMPER,TCIM,FOXP1,APOLD1	grey	VSMC_M10
GO:0051284	0.88	0.28	5	12	positive regulation of sequestering of calcium ion	BP	1	PKD2,CASQ2,RYR2,NTSR1,CALM1	grey	VSMC_M10
GO:0070816	0.88	0.30	4	10	phosphorylation of RNA polymerase II C-terminal domain	BP	1	CDK7,CCNH,CDK12,GTF2H5	grey	VSMC_M10
GO:0006465	0.88	0.28	5	12	signal peptide processing	BP	1	SEC11C,IMMP1L,SPCS1,SPPL3,SPCS2	grey	VSMC_M10
GO:0023035	0.88	0.28	5	12	CD40 signaling pathway	BP	1	TNFAIP3,TRAF3IP2,PHB,CD40,TNIP2	grey	VSMC_M10
GO:0001678	0.88	0.16	60	124	cellular glucose homeostasis	BP	1	ZNF236,NME1,LRP1,AGER,PLA2G6,SRF,PRKCE,HMGN3,TRPM4,FOXO3,FGF21,FOXA3,GRIK5,RAF1,GAS6,NR1H4,CYP7A1,NDUFAF2,OSBP,PIK3R1,KCNJ11,TIAM1,RAB11FIP5,OPA1,ZBED6,PAX2,TWNK,GPLD1,PPP3CA,SLC9B2,ZBTB20,ADRA2A,KCNB1,POLG,GPER1,RAB11FIP2,MLXIPL,SIDT2,PDK3,KLF7,FIS1,HK1,MEN1,ENY2,ABCA12,PPP3CB,PTPRN,MPC2,STX4,NGFR,GPR68,SIN3A,OXCT1,NADK,BAIAP3,CYBA,LRRC8A,LRRC8D,SOX4,CRH	grey	VSMC_M10
GO:0032908	0.88	0.30	4	10	regulation of transforming growth factor beta1 production	BP	1	TSKU,LUM,FOXP3,GATA6	grey	VSMC_M10
GO:0090101	0.88	0.17	48	101	negative regulation of transmembrane receptor protein serine/threonine kinase signaling pathway	BP	1	ONECUT2,LRP1,PPARA,LEMD2,SOSTDC1,CD109,BMPER,SPRY1,SPRY2,EID2,SPRED3,SPART,SNX25,IL17RD,VEPH1,SLC2A10,SORL1,HTRA1,CHRD,DLX1,SNX6,CTDSPL2,PBLD,HTRA3,FBN1,PPM1A,GREM2,TGFB1I1,RBPMS2,CILP,TOB1,WFIKKN2,DNM2,CHRDL1,TWSG1,PDPK1,SKI,PRDM16,WFIKKN1,HSPA5,FSTL3,LEMD3,NOG,WNT5A,NRROS,LDLRAD4,UBE2D1,PEG10	grey	VSMC_M10
GO:0046322	0.88	0.30	4	10	negative regulation of fatty acid oxidation	BP	1	SOX9,SIRT4,ACADL,ACADVL	grey	VSMC_M10
GO:0035404	0.88	0.30	4	10	histone-serine phosphorylation	BP	1	MST1,RPS6KA5,AURKC,RPS6KA4	grey	VSMC_M10
GO:0106049	0.88	0.30	4	10	regulation of cellular response to osmotic stress	BP	1	YBX3,MICU1,AC138894.1,SLC25A23	grey	VSMC_M10
GO:0072666	0.88	0.20	19	42	establishment of protein localization to vacuole	BP	1	TNFAIP3,VPS41,RAB7A,NCOA4,AP3B1,MON1A,HSPA8,SORL1,RNF215,VPS37D,AP4M1,AP3M1,VPS13D,VPS13C,LAMP2,VPS4A,CACNG7,SNX16,CACNG5	grey	VSMC_M10
GO:0090030	0.88	0.30	4	10	regulation of steroid hormone biosynthetic process	BP	1	DKK3,POR,WNT4,DGKQ	grey	VSMC_M10

GO:0097306	0.88	0.18	33	71	cellular response to alcohol	BP	1	PTCH1,SMO,PRKCE,TNC,LANCL2,FOXO3,SPHK2,ADCY3,DNMT3A,MAP4K1,SLC23A2,CYP7A1,SPIDR,TP53INP1,ADCY7,ADCY2,GPLD1,DAG1,GRAMD1B,NPAS4,FDX1,PTGER2,AKAP8,OSBPL7,LARP1,SCNN1B,ACACA,CCL7,PTGER4,LRP8,GNB1,PTGFR,GNG2	grey	VSMC_M10
GO:1901617	0.88	0.16	88	178	organic hydroxy compound biosynthetic process	BP	1	CYP27B1,TPH2,HSD17B3,AKR1B1,PSAT1,GPR37,PLPP6,SRD5A3,SPTLC1,IPPK,PLEK,DDC,DKKL1,SLC7A11,APOB,SPHK2,DHFR,EBP,PRKAG2,ITPKC,ACER3,SPTLC3,NR1H4,CYP7A1,ASAH1,CYP7B1,OSBP,DKK3,HAND2,CYP2R1,P2RY1,PTK2B,RAPGEF2,SPR,NR4A2,ITPKA,ERLIN1,PLTP,OCA2,CYB5R1,SLC6A3,TGFB2,SNAI1,MSMO1,GPER1,SC5D,ABCB11,IP6K2,PER2,OSBPL2,NTSR1,SCAP,HSD17B7,POR,PTH1R,OSBPL6,TM7SF2,OSBPL1A,LPCAT3,WNT4,CYP27A1,GCH1,HDC,CH25H,HSD17B10,FGFR4,DGKQ,OSBPL7,PNPO,ERG28,CITED1,CYP46A1,SREBF1,IMPA2,SLC45A2,ACAA2,CACNA1H,PARK7,ABCD3,GFI1,DHDDS,WNT5A,AMACR,ACER2,STARD4,INSIG2,FGF1,PCBD2	grey	VSMC_M10
GO:0006935	0.88	0.14	215	426	chemotaxis	BP	1	LRP1,SEMA6A,KIF5A,SLC12A2,AGER,GPSM3,CXCL14,GFRA3,PTK2,PTCH1,PLXNA4,MAPK14,PLA2G6,RAC2,SMO,CCL27,KIT,PLGRKT,SEMA4D,DLX5,NINJ1,ETV1,SEMA4C,FPR2,HOXA2,LPAR1,CXCL8,AKIRIN1,EPHA7,PTN,MET,EPHA1,GAB1,NRCAM,TRPM4,PLXNA1,C5AR2,C5AR1,PRKD2,GLI2,MEGF8,OXSR1,MYCBP2,ACKR2,CMTM7,CMTM8,KIF5C,HBEGF,FEZ2,SEMA3D,PLXNC1,SEMA3A,CHL1,UNC5D,NRG1,NCKAP1L,GAS6,FAM19A4,FLT1,RYK,EPHB1,MTUS1,RET,CYP7B1,NR4A1,FER,PIK3CB,PARVA,ROBO1,ROBO2,PTPRJ,FZD3,SEMA4F,VEGFC,PTK2B,DPYSL2,CCL17,TIAM1,SLIT1,SLC8B1,TRIO,F2RL1,ANO6,SEMA3G,CYFIP1,ALCAM,SEMA4G,NOD2,SIAH1,BMPR1B,GBF1,BDNF,ELMO2,LGR6,DNM1L,TRPV4,SBDS,SEMA3F,TGFB2,GAP43,DAG1,PRSS56,LHX9,CMKLR1,SLAMF8,MOSPD2,GBX2,KALRN,LAMA5,YTHDF1,PTPRO,MACO1,FCER1G,EPHB3,ADAM8,WNT3,KLF7,CREB1,MYPN,PIKFYVE,IL16,SEMA6D,EPHB4,IL12A,TNR,MCU,TNFSF18,CXCR2,BST1,MAPK1,CH25H,CRMP1,PADI2,CXCL16,EPHA4,LRTM2,TMEM102,UNC5A,STX4,SEMA4A,HOXB9,FGF18,NGFR,DOCK2,PGF,DAPK2,EFNA1,MAPK3,IL6R,ALKBH1,MAP2K1,SMAD3,NTN1,RPS6KA5,S100A14,S100A8,RNF165,BCL11B,LHX1,CD74,CCL14,CCL5,LAMA3,AMOT,CSF1R,CCL7,CCL2,CCL25,CDK5R1,ISL1,EMB,VAV1,LAMA1,RALBP1,PTPN2,CRK,PIP5K1C,L1CAM,NTN3,ARHGEF16,PLXNA3,MPP1,BSG,TUBB3,VAV3,CMTM3,RHOA,CCL16,DPYSL5,S100A12,EFNA4,CNTN2,VASP,FOXG1,CHGA,DEFB124,CCR10,DPP4,CXCL6,ZNF580,ISL2,NOG,WNT5A,CHN1,DUSP1,ARTN,NRXN3,LYPLA2,RALA,HRAS,EFNB3,ISPD,FGF1,CCL22,PDGFD	grey	VSMC_M10
GO:0044771	0.88	0.30	4	10	meiotic cell cycle phase transition	BP	1	MAPK15,TOPAZ1,MOS,OVOL1	grey	VSMC_M10
GO:0071025	0.88	0.28	5	12	RNA surveillance	BP	1	DXO,EXOSC4,EXOSC3,SUPV3L1,EXOSC5	grey	VSMC_M10
GO:0009749	0.88	0.16	75	153	response to glucose	BP	1	ZNF236,NME1,LRP1,APOM,AGER,PLA2G6,TXN2,SRF,PRKCE,HMGN3,TRPM4,FOXO3,FGF21,GIPR,KHK,EIF2B3,GRIK5,RAF1,TGFBR2,GAS6,NR1H4,CYP7A1,NDUFAF2,OSBP,CASP3,PTK2B,KCNJ11,TIAM1,RAB11FIP5,OPA1,NKX6-1,ACVR1C,ZBED6,PAX2,TWNK,GPLD1,PPP3CA,SLC9B2,ZBTB20,ADRA2A,GPAM,TCF7L2,KCNB1,PFKFB2,POLG,GPER1,RAB11FIP2,MLXIPL,GLUL,SIDT2,CDKN1B,PDK3,KLF7,FIS1,MEN1,ENY2,ABCA12,PPP3CB,CTSV,PTPRN,NDUFA9,MPC2,ILDR2,STX4,PKLR,GPR68,SIN3A,SREBF1,OXCT1,NADK,BAIAP3,CYBA,RHOA,SOX4,CRH	grey	VSMC_M10
GO:0031848	0.88	0.30	4	10	protection from non-homologous end joining at telomere	BP	1	ERCC1,XRCC1,ERCC4,TERF2	grey	VSMC_M10
GO:0045927	0.88	0.15	98	199	positive regulation of growth	BP	1	ADNP2,NEDD4L,LRP1,GLI1,RIMS2,PRKN,H3F3C,MAPK14,CPNE5,PIM1,EXOSC4,SMO,SRF,SEMA4D,RIMS1,RUFY3,EGFR,TRIM32,SDCBP,GOLGA4,MEGF8,CPNE9,TGFBR2,MKKS,RPTOR,HBEGF,SYT1,PLCB1,SLC23A2,PRKDC,CDKN2AIP,PLS1,MEF2C,IL7,CELF1,PTK2B,TBX5,ZFYVE27,CYFIP1,IGF2,BDNF,SLC6A3,HYAL1,TGFB2,GPAM,ZNF639,HLX,ADNP,DISC1,CIB1,SLC9A1,SFN,RPS6KA1,ACTN3,CDKL5,YBX3,WNT3,CREB1,MAPT,SUPV3L1,DDX3X,FN1,DDX49,MUL1,CD38,MAPK1,STAT5B,CXCL16,BMPR1A,RBPJ,SOX15,NDEL1,RAB11A,TAF9B,NTN1,MAP2K5,S100A8,TBX2,PSMD10,TRPV2,INSR,GATA6,GHR,L1CAM,SLC25A33,CYBA,MIR199A1,MIR222,RHOA,BASP1,DIO3,POU3F2,FXN,CACNG7,N6AMT1,HEY2,DERL2,WNT2	grey	VSMC_M10
GO:0071331	0.88	0.17	54	112	cellular response to hexose stimulus	BP	1	ZNF236,NME1,LRP1,AGER,PLA2G6,SRF,PRKCE,HMGN3,TRPM4,FOXO3,FGF21,GRIK5,RAF1,GAS6,NR1H4,CYP7A1,NDUFAF2,OSBP,KCNJ11,TIAM1,RAB11FIP5,OPA1,ZBED6,PAX2,TWNK,GPLD1,PPP3CA,SLC9B2,ZBTB20,ADRA2A,KCNB1,POLG,GPER1,RAB11FIP2,MLXIPL,SIDT2,PDK3,KLF7,FIS1,MEN1,ENY2,ABCA12,PPP3CB,PTPRN,MPC2,STX4,GPR68,SIN3A,OXCT1,NADK,BAIAP3,CYBA,SOX4,CRH	grey	VSMC_M10

GO:0016050	0.88	0.15	127	255	vesicle organization	BP	1	VPS4B,STX7,TBPL1,PRKN,SEPT8,WASHC5,TSNARE1,MAPK15,WASHC4,TGFBRAP1,CHMP2A,STX17,VPS41,SDCBP,EPS15,RAB7A,PIP4K2A,RUBCNL,NECTIN2,AQP11,GRIK5,TBC1D4,MYO7A,AKT2,HOOK3,YIPF4,SYT1,DNAJC13,FAM160A2,STX2,SYT9,TMEM127,ANKRD27,OSBP,CHMP2B,CHMP4C,ARFGAP2,SEC31A,DYSF,SYNJ1,AP3B1,CHMP7,F2R,VPS18,SEC31B,DTNBP1,VPS39,KIF13A,GBF1,RAB7B,KLHL12,SEC22B,TMEM9,PEF1,VTI1A,PDCD6,SNAP23,SHROOM2,HPS4,C2CD5,RNF26,RAB22A,AP2M1,STX1A,STX6,VPS37D,VPS8,SCAP,CREB1,MIA3,SPG11,PIKFYVE,ALS2CL,BLOC1S6,SRGN,SEC24C,RAB3A,ABCB6,VAMP1,SNAP29,ANKFY1,PTPRN,STX5,RAB8A,ATP13A2,CPLANE2,DLG4,SYT7,SLC2A4,ANXA8,PI4K2B,ANXA2,CPLX1,STX1B,STX4,SYT11,RAB8B,TMED10,AC138894.1,HOOK2,ZDHHC15,RAB11A,SOX30,STX8,SNAPIN,PPP6C,RILP,BAIAP3,CHMP1A,GNAI3,VAV3,MFSD14A,STXBP1,FNBP1L,AP1G1,VPS4A,PHETA1,BLOC1S3,SNAP47,SNX10,RAB32,DOC2B,TBC1D20,VAMP3,PLEKHJ1,TRAPPC6A,RAB39A	grey	VSMC_M10
GO:0042551	0.88	0.20	17	38	neuron maturation	BP	1	ANKS1A,PICK1,NRCAM,RAC3,RET,LGI4,RND1,OPA1,NR4A2,KCNIP2,FARP2,C1QL1,GLDN,C3,MECP2,SRRM4,CNTN2	grey	VSMC_M10
GO:0060068	0.88	0.30	4	10	vagina development	BP	1	ESR1,BAK1,LHX1,WNT5A	grey	VSMC_M10
GO:0031123	0.88	0.17	45	94	RNA 3'-end processing	BP	1	CPSF6,PABPC1,ZC3H3,TUT7,EXOSC4,HSF1,CPSF1,EXOSC3,RNF20,YTHDC1,PNPT1,LSM10,TUT4,MTPAP,SYMPK,PCF11,TRNT1,CSTF3,CLP1,USB1,RPRD1B,TENT2,INTS8,TENT4B,INTS12,CPSF2,RPRD2,CSTF1,NCBP2,SUPV3L1,CPSF4,LEO1,INTS5,AP002990.1,HSD17B10,CSTF2,INTS2,PAPOLA,RPRD1A,ERI2,PARN,POLR3K,TRMT10C,ZNF473,EXOSC5	grey	VSMC_M10
GO:0031348	0.88	0.16	73	149	negative regulation of defense response	BP	1	TNFAIP3,USP15,ENPP3,PPARA,C6orf106,MAPK14,TEK,FPR2,NT5E,NR1H2,VPS35,CST7,FNDC4,SEC14L1,SIRT2,NMI,NR1D2,NPLOC4,MFHAS1,PCBP2,NR1H4,PLK2,HCK,NR1H3,TRAFD1,OAS3,NLRC5,OTULIN,MAPKBP1,NOD2,CD96,CD200,YTHDF2,GPER1,SLAMF8,PARP14,RNF26,FGR,NLRX1,TRAF3IP1,HTRA1,DUSP10,LPCAT3,CALCRL,MUL1,FOXP3,GPS2,TKFC,USP18,TNFRSF1B,SYT11,DCST1,SMAD3,TSPAN6,LDLR,STAT2,RIOK3,MAPK7,YES1,ISL1,PTGER4,PTPN2,CRK,FOXF1,MIR199A1,MIR21,MIR222,C5orf30,OAS1,A2M,FGL2,SERPINB9,HAVCR2	grey	VSMC_M10
GO:0043087	0.88	0.15	141	285	regulation of GTPase activity	BP	1	ARHGAP9,TBC1D30,TBC1D9B,TIAM2,SYNGAP1,ASAP1,PTK2,PLXNA4,NTRK2,TBC1D22B,SEMA4D,TBC1D8,RGP1,FGD3,ASAP2,ITGB1BP1,MET,ARHGEF5,EPHA1,BVES,TBC1D9,SPRY1,PLXNA1,ARHGAP12,IQSEC1,RSU1,TBC1D4,FOXJ1,SPRY2,WNT11,AKT2,ARRB1,TBC1D16,MKKS,ARHGEF7,PLXNC1,FGD6,LARS,RASA3,SBF2,RASA2,TBC1D12,ARHGEF26,PTK2B,RASGRP1,RAPGEF2,CCL17,TIAM1,RAPGEF3,F2RL1,F2R,IQGAP2,ARHGAP24,CD40,RAP1GDS1,DOCK10,FGD4,RGS1,TBCK,ADPRH,GPR137B,AJUBA,ADAP1,DENND1B,FICD,ARHGAP6,TBC1D10C,RAB11FIP2,SH3BP4,DVL3,CDKL5,GRHL3,EPHB3,RASA4,TRAPPC6B,BCL6,RASAL2,TBC1D2B,GMIP,RASGRP2,TBC1D14,RABGAP1L,RUNDC1,WNT4,ELMOD1,RAP1GAP,TBC1D25,GNB5,EPHA4,ARHGEF19,RASAL3,DVL2,SYDE1,TBC1D1,ARAP2,RGS14,FGD1,TBC1D10B,NDEL1,MYO9A,BCAS3,ARHGAP44,TBC1D8B,CCL14,CCL5,AMOT,CCL7,CCL2,CCL25,ADAP2,VAV1,RALBP1,DOCK7,ABR,CRK,RAP1GAP2,TAX1BP3,MLST8,ARHGEF16,APC2,ARHGAP45,PLXNA3,GPSM1,TSC1,VAV3,MTSS1L,GAPVD1,DENND1A,RABGAP1,DAB2IP,MIR21,CCL16,ASAP3,THY1,WNT5A,CHN1,RGS4,TBC1D20,HRAS,TRAPPC6A,CCL22	grey	VSMC_M10
GO:0001952	0.88	0.17	50	105	regulation of cell-matrix adhesion	BP	1	ONECUT2,LRP1,PTK2,CDKN2A,TEK,CDK6,HOXA7,ITGB1BP1,EPHA1,CLASP2,RIN2,JAG1,ACTG1,FERMT1,PTPRA,FAM107A,PIK3CB,PIK3R1,FMN1,PTPRJ,PTK2B,DMTN,LDB1,SLK,COL16A1,DAG1,ARHGAP6,DISC1,CIB1,SLC9A1,EFEMP2,APOD,BCL6,CASK,WNT4,VCL,BST1,MINK1,SMAD3,BCAS3,CAMSAP3,CCL25,PRKCZ,TSC1,RHOA,ACVRL1,PEAK1,THY1,ACER2,DUSP22	grey	VSMC_M10
GO:0002694	0.88	0.14	178	352	regulation of leukocyte activation	BP	1	CD226,TNFAIP3,ENPP3,AGER,HLA-DRA,HLA-DOB,HLA-DMA,IL5,HLA-DPB1,TCF7,TNFRSF13C,XRCC6,RAC2,FBXO7,TTBK1,TNFRSF21,NCK2,EXOSC3,MSH2,SLC46A2,PELI1,IL4I1,TLR4,IL15,SPHK2,HMCES,BMI1,CST7,CD300LF,CLPTM1,NECTIN2,GAB2,MLH1,GLI2,UNC13D,MYD88,PIBF1,FOXJ1,PAXIP1,IL15RA,TGFBR2,PAWR,SHLD1,PRNP,NCKAP1L,GAS6,PRKDC,SLC7A1,MFHAS1,TRAF6,SOX12,TOX,ZC3H8,IL1A,CAMK4,CEBPA,FER,CASP3,MEF2C,TMEM131L,IL7,PAG1,RASGRP1,NR1H3,RIPK2,AP3B1,F2RL1,MARCH7,SOX13,NOD2,ZNF335,CD40,IGF2,CD200,LCK,BANK1,ICOSLG,BTN2A2,GPAM,PRKAR1A,GPR137B,HLX,TBC1D10C,GPER1,SLAMF8,THEMIS2,FGR,SNX4,CD28,RUNX3,DLG1,PPP2R3C,ADAM8,DUSP10,BLOC1S6,PLA2G4A,BCL6,IL12A,VSIR,RC3H1,PTPN6,TNFSF18,LAG3,TNIP2,CD38,BST1,C12orf4,STAT5B,INHA,SCGB1A1,FOXP3,RASAL3,ZBTB1,SHLD2,CARD11,TNFRSF1B,TBX21,STX4,ITGAM,SYT11,ZBTB7B,CHRNB2,CD19,LAT,IL4R,LDLR,BTK,CD74,CCL5,STXBP2,CCL2,YES1,NKAP,CD320,PRAM1,VAV1,TWSG1,IL7R,SUPT6H,PTPN2,SOCS1,DROSHA,TICAM1,DNAJA3,PDPK1,PRKCZ,FOXF1,VAV3,KARS,STXBP1,GLMN,AP1G1,BCL10,MIR21,MIR27A,RHOA,HAVCR1,LST1,BLOC1S3,SOX4,FADD,DPP4,CD46,MIR125A,THY1,WNT5A,FGL2,IL33,CD70,TNFSF9,TSLP,ZFP36L2,LAX1,EFNB3,CEBPB,TNFSF13B,HAVCR2,RC3H2	grey	VSMC_M10

GO:0044091	0.88	0.19	20	44	membrane biogenesis	BP	1	PEX3,CHMP2A,SPTBN1,SIRT2,STX2,NSFL1C,CHMP7,PEX16,NLGN4Y,NRXN2,NLGN2,ANXA2,GAK,STX4,SYT11,NLGN3,CEL,VPS4A,TLCD1,PEX11A	grey	VSMC_M10
GO:1902065	0.88	0.30	4	10	response to L-glutamate	BP	1	TMBIM6,OPA1,CREB1,AMIGO1	grey	VSMC_M10
GO:0060272	0.88	0.30	4	10	embryonic skeletal joint morphogenesis	BP	1	OSR2,OSR1,HYAL1,NOG	grey	VSMC_M10
GO:0034374	0.88	0.30	4	10	low-density lipoprotein particle remodeling	BP	1	APOB,AGT,LIPC,MPO	grey	VSMC_M10
GO:0048844	0.88	0.18	26	57	artery morphogenesis	BP	1	LRP1,FOXH1,SRF,TFAP2B,HOXA13,APOB,WNT11,JAG1,HAND2,HPGD,EYA1,PKD2,TGFB2,EFEMP2,BMPR1A,RBPJ,LDLR,TBX2,STRA6,FOXF1,ENG,SOX4,ACVRL1,NOG,SIX1,HEY2	grey	VSMC_M10
GO:0002089	0.88	0.24	8	19	lens morphogenesis in camera-type eye	BP	1	ATF4,TDRD7,NECTIN3,SHROOM2,LCTL,SKI,TBC1D20,PITX3	grey	VSMC_M10
GO:1904063	0.88	0.18	29	62	negative regulation of cation transmembrane transport	BP	1	NEDD4L,PRKCE,HECW1,KEL,OSR1,KCNRG,OXSR1,SLC43A1,KCNE2,PKD2,CASQ2,HECW2,ADRA2A,ISCU,ATP1A2,C4orf3,NTSR1,SESTD1,GNB5,CALM1,FMR1,RRAD,MIR24-1,GPR35,KCNH2,CRBN,RGS4,GEM,PPIF	grey	VSMC_M10
GO:0099623	0.88	0.25	7	17	regulation of cardiac muscle cell membrane repolarization	BP	1	SCN5A,KCNE2,ANK2,SCN4B,NOS1AP,RNF207,KCNH2	grey	VSMC_M10
GO:1903169	0.88	0.16	53	110	regulation of calcium ion transmembrane transport	BP	1	STAC3,PLA2G6,TSPAN13,PRKCE,ATG5,CACNB2,STAC,CACNB4,PRNP,DIAPH1,PTK2B,F2R,KCNE2,PKD2,CASQ2,RYR2,ADRA2A,UBASH3B,ANK2,GPER1,ATP1A2,SLC9A1,C4orf3,CASQ1,NTSR1,NOS1AP,CEMIP,SESTD1,PTPN6,HTT,ATP1B1,GNB5,HAP1,PSEN2,STAC2,CACNB1,ATP2A1,CD19,CALM1,VMP1,BDKRB1,FMR1,PDPK1,P2RX1,CYBA,JPH3,RRAD,MIR21,DMD,GPR35,JPH2,THY1,GEM	grey	VSMC_M10
GO:0032025	0.88	0.29	4	10	response to cobalt ion	BP	1	CASP3,D2HGDH,CASP8,TIGAR	grey	VSMC_M10
GO:0010596	0.88	0.19	22	48	negative regulation of endothelial cell migration	BP	1	MEOX2,SVBP,ITGB1BP1,DCN,MEF2C,STC1,SP100,PATZ1,PDCD10,SYNJ2BP,VASH1,MAP2K5,MECP2,DAB2IP,MIR199A1,MIR21,MIR22,MIR24-1,RHOA,ACVRL1,GADD45A,RGCC	grey	VSMC_M10
GO:0043931	0.88	0.24	8	19	ossification involved in bone maturation	BP	1	SEMA4D,RYR1,ACTN3,THBS3,RFLNB,RHOA,SNX10,CCDC154	grey	VSMC_M10
GO:0051208	0.88	0.17	41	87	sequestering of calcium ion	BP	1	PRKCE,IBTK,ATG5,ATP7B,RYR1,DIAPH1,RASA3,RYR3,PTK2B,TPCN1,SLC8B1,F2R,PKD2,CASQ2,RYR2,LCK,UBASH3B,ANK2,GPER1,ATP1A2,CASQ1,NTSR1,CEMIP,PTPN6,HTT,HAP1,PSEN1,CD19,CALM1,BDKRB1,SLC25A23,TRPV1,PDPK1,CYBA,JPH3,MCOLN3,DMD,NOL3,JPH2,THY1,DDIT3	grey	VSMC_M10
GO:0016445	0.88	0.18	25	54	somatic diversification of immunoglobulins	BP	1	EXOSC3,MSH2,CYREN,HMCES,ERCC1,MLH1,PAXIP1,SHLD1,PRKDC,XRCC4,NBN,POLL,CD40,CD28,RNF168,BCL6,PMS2,FOXP3,SHLD2,TBX21,BATF,YY1,SUPT6H,RNF8,NHEJ1	grey	VSMC_M10
GO:0045944	0.88	0.13	463	904	positive regulation of transcription by RNA polymerase II	BP	1	NFATC1,ZNF407,ONECUT2,TCF4,GLI1,PLAGL1,MAML1,ESR1,OSR2,SNW1,ZBED4,PRKN,PPARA,MED30,RXRB,VGLL2,HSF2,XRCC6,NCOA7,PITX1,MYC,SMAD5,AGO2,ATF4,MAPK14,SOX10,NRF1,TMPRSS6,SMO,HSF1,TFEB,IRF5,FOXH1,IFT74,RORB,ZNF300,PRDM4,DMTF1,SRF,SMARCA2,RFX3,NCK2,TFAP2B,DLX5,ETV1,MEOX2,KANSL3,ZNF268,MACC1,SIX2,FOXJ3,IL11,EPAS1,EPCAM,ZNF462,PHIP,HMGN3,NFYC,HOXA2,HOXA7,HOXA13,REL,AKIRIN2,AKIRIN1,MTF1,PCBP1,NR1H2,AKNA,ITGB1BP1,GRHL1,MED25,MET,IL4I1,AGO1,POU3F3,EGFR,TLR4,CCNC,BCL2L12,LPIN1,TEAD2,MYCN,OSR1,FOXO3,MAMSTR,ATAD2B,ZNF438,HDAC2,PHF10,MAML2,MLLT10,PRKD2,CNBP,FOXA3,SOX9,ZNF786,ZNF746,ERCC1,UTF1,SUMO2,GLI2,RSF1,ZNF513,ZNF574,RAF1,KLF5,KLF12,FOXJ1,PAXIP1,CSRNP1,METTL23,POU4F1,SS18L2,KLF6,ARRB1,OVOL2,ZNF219,ACVR2A,EOMES,IKBKB,CHD8,JAG1,SIRT2,TMEM173,RARB,NR1D2,SIRT7,MAFG,DPF1,ALX1,TAF7,LUM,DCN,MAVS,TFDP1,DDRGK1,MITF,PRKDC,IRF7,ERCC6,CALCOCO1,ST18,TRAF6,NR1H4,TCF15,SOX12,PLAG1,USF2,LMO2,ASXL1,IL1A,PAX8,NR4A1,ACVR1B,ZNF143,CEBPA,NCOA6,PIK3R1,TFDP2,CDK7,IRF2,TAF9,NCOA2,MED19,CCDC62,TFCP2,HAND2,CSRNP2,EYA1,MLXIP,ARNTL,HLTF,HNF4G,ZNF395,MEF2C,TLX2,ZNF319,P2RY1,USP16,SSBP2,KMT2D,BACH1,NR1H3,RPRD1B,EAPP,EBF2,NLRC5,RIPK2,PFKM,SENP1,VDR,NKX3-1,LPIN3,NKX6-1,TBX5,AP3B1,NR4A2,F2RL1,RIPK1,RREB1,PKD2,TFAP2A,PAX2,SALL1,HIVEP1,NOD2,ZNF335,CD40,LDB1,IGF2,BMPR1B,ELK4,NFKB2,PRDM15,MED10,RNF10,PKNOX1,HDAC1,ELF3,PPP3CA,CAPRIN2,TERT,SLC38A3,TGFB2,SATB2,TCF7L2,ARNTL2,NR5A2,AUTS2,ZNF639,[illegible]	grey	VSMC_M10
GO:0019692	0.88	0.20	17	37	deoxyribose phosphate metabolic process	BP	1	TBPL1,DPYS,GDA,NUDT15,MBD4,NUDT16,DGUOK,NUDT18,DTYMK,UPB1,ADK,NEIL1,NT5M,NTHL1,CMPK2,DCTD,UPP1	grey	VSMC_M10
GO:1903115	0.88	0.21	14	32	regulation of actin filament-based movement	BP	1	TRPM4,SCN5A,STC1,TNNC1,DSP,RYR2,PKP2,KCNJ2,ANK2,ATP1A2,DLG1,ATP2A1,DSG2,HCN4	grey	VSMC_M10
GO:0006336	0.88	0.22	11	25	DNA replication-independent nucleosome assembly	BP	1	ASF1A,UBN2,RBBP4,HIRA,HIST1H4B,HIST2H4A,HIST1H4H,ITGB3BP,HIST1H4D,HIST1H4A,HIST1H4F	grey	VSMC_M10
GO:0071364	0.88	0.20	17	38	cellular response to epidermal growth factor stimulus	BP	1	MARS,EGFR,SOX9,BAG4,PAX2,CBL,SYAP1,PDE8A,SNX6,STAT5B,MED1,GAREM1,INPP5K,PDPK1,DAB2IP,DUSP22,ZFP36L2	grey	VSMC_M10
GO:0090594	0.88	0.29	4	10	inflammatory response to wounding	BP	1	TLR4,IL1A,F2R,MIR21	grey	VSMC_M10
GO:1902686	0.88	0.21	14	31	mitochondrial outer membrane permeabilization involved in programmed cell death	BP	1	TMEM14A,HIP1R,STPG1,FZD9,NMT1,MUL1,TMEM102,ACAA2,MIR29A,BNIP3L,BCL2L11,SLC35F6,PPIF,AC140504.1	grey	VSMC_M10
GO:2000279	0.88	0.20	14	32	negative regulation of DNA biosynthetic process	BP	1	POT1,DACH1,TENT4B,STN1,KCNK2,MEN1,GNL3L,CTC1,PIF1,TERF2,ACVRL1,TINF2,TEN1,DUSP1	grey	VSMC_M10
GO:1901163	0.88	0.29	4	10	regulation of trophoblast cell migration	BP	1	AGO2,ACVR1B,ACVR1C,SYDE1	grey	VSMC_M10
GO:0035967	0.88	0.16	50	103	cellular response to topologically incorrect protein	BP	1	HSPA1B,PRKN,PACRG,RNF5,DERL1,BAK1,ATF4,HSF1,NCK2,ERMP1,HERPUD2,FGF21,OPTN,UGGT2,DNAJC18,TMTC4,EIF2AK2,DDRGK1,PIK3R1,TMBIM6,AUP1,AMFR,RHBDD1,CCND1,FICD,YOD1,HSPA8,QRICH1,BAG3,RNF185,TBL2,RHBDD2,NFE2L2,DERL3,DNAJB12,SDF2L1,BHLHA15,CREB3L4,PTPN2,CREB3L3,ABCA7,RNF126,HSPA5,DAB2IP,MIR199A1,DDIT3,BCL2L11,HSPB8,PPP1R15A,DERL2	grey	VSMC_M10
GO:0046637	0.88	0.19	20	44	regulation of alpha-beta T cell differentiation	BP	1	HLA-DRA,TGFBR2,NCKAP1L,RIPK2,AP3B1,HLX,RUNX3,BCL6,RC3H1,TNFSF18,FOXP3,TBX21,ZBTB7B,IL4R,NKAP,SOCS1,PRKCZ,MIR21,RHOA,RC3H2	grey	VSMC_M10

GO:1901020	0.88	0.22	11	25	negative regulation of calcium ion transmembrane transporter activity	BP	1	PKD2,CASQ2,ADRA2A,ATP1A2,C4orf3,GNB5,CALM1,FMR1,RRAD,GPR35,GEM	grey	VSMC_M10
GO:0031110	0.88	0.17	37	79	regulation of microtubule polymerization or depolymerization	BP	1	HSPA1B,SLAIN2,MET,CDK5RAP2,MAPRE1,EML2,SLC39A12,CLASP2,MAPRE3,TRIM54,SLAIN1,NAV3,ARHGEF7,SPEF1,ATXN7,CLIP1,KATNB1,STMND1,TRPV4,TTBK2,CIB1,INPP5J,CDKN1B,MAPT,TOGARAM1,NIN,CAMSAP3,CDK5R1,TAOK1,MECP2,APC2,CAMSAP1,PSRC1,APC,MAP1A,DRG1,MAP6D1	grey	VSMC_M10
GO:0019985	0.88	0.24	7	17	translesion synthesis	BP	1	POLI,REV1,REV3L,PRIMPOL,SPRTN,POLN,ZBTB1	grey	VSMC_M10
GO:0097009	0.88	0.20	14	32	energy homeostasis	BP	1	BMP8A,TSKU,ALK,NR1D2,PM20D1,TRPV4,PASK,MLXIPL,CRTC1,STAT3,SGIP1,LEPR,UBB,MRAP2	grey	VSMC_M10
GO:0061326	0.88	0.17	36	77	renal tubule development	BP	1	MTSS1,MYC,KLHL3,PTCH1,SMO,TFAP2B,SIX2,POU3F3,OSR1,SOX9,AQP11,GZF1,WNT11,JAG1,PAX8,EYA1,FMN1,MEF2C,PKD2,PAX2,SALL1,IRX2,AGT,LAMA5,DLG1,WNT4,MAGED1,CITED1,LHX1,GPC3,PKD1,SOX8,NOG,SIX1,LIF,FGF1	grey	VSMC_M10
GO:0060767	0.88	0.29	4	10	epithelial cell proliferation involved in prostate gland development	BP	1	SOX9,NKX3-1,CDKN1B,WDR77	grey	VSMC_M10
GO:0008299	0.88	0.22	10	23	isoprenoid biosynthetic process	BP	1	SRD5A3,COQ2,PRMT3,ALDH1A3,GGPS1,DHRS9,ALDH1A2,DHDDS,PDSS2,ISPD	grey	VSMC_M10
GO:1901741	0.88	0.29	4	10	positive regulation of myoblast fusion	BP	1	MAPK14,EHD1,GDF15,IL4R	grey	VSMC_M10
GO:0046931	0.88	0.23	8	19	pore complex assembly	BP	1	TSPAN33,RTN4,NUP98,P2RX7,PLEKHA7,ANO6,AHCTF1,PDZD11	grey	VSMC_M10
GO:1903902	0.88	0.23	9	21	positive regulation of viral life cycle	BP	1	CHMP2A,KPNA2,CHMP2B,PC,DDB1,CD74,FMR1,VPS4A,ZNF502	grey	VSMC_M10
GO:0045740	0.88	0.20	16	36	positive regulation of DNA replication	BP	1	GLI1,EGFR,SSBP1,FAF1,GLI2,NPM2,STN1,EGF,DBF4B,KAT7,KCTD13,CTC1,TNFAIP1,JUN,HRAS,EREG	grey	VSMC_M10
GO:0046916	0.88	0.17	41	86	cellular transition metal ion homeostasis	BP	1	SLC39A5,MYC,TMPRSS6,SLC39A4,STEAP2,SLC1A1,SLC31A2,HEPHL1,ATP7B,SLC39A12,PRNP,HPX,NCOA4,SLC39A13,AP3B1,SLC40A1,LCK,SLC30A10,ISCU,HJV,SLC30A4,SLC11A1,ABCB6,ATP13A2,FTH1,CYB561A3,NOX5,S100A8,SLC39A6,TMEM199,SLC46A1,NUBP1,ATP6AP1,SLC30A7,FRRS1,LCN2,MT1X,SLC22A17,FXN,FTL,SCO1	grey	VSMC_M10
GO:2000191	0.88	0.22	10	23	regulation of fatty acid transport	BP	1	PPARA,PLA2G6,AKT2,PLA2R1,MAP2K6,ACSL5,NTSR1,PLA2G4A,FIS1,PTGES	grey	VSMC_M10
GO:1902110	0.88	0.21	13	29	positive regulation of mitochondrial membrane permeability involved in apoptotic process	BP	1	TMEM14A,HIP1R,STPG1,FZD9,NMT1,MUL1,TMEM102,ACAA2,MIR29A,BNIP3L,BCL2L11,SLC35F6,AC140504.1	grey	VSMC_M10
GO:1903055	0.88	0.22	10	23	positive regulation of extracellular matrix organization	BP	1	SOX9,CLASP2,DAG1,AGT,PHLDB1,MELTF,EFEMP2,SMAD3,CARMIL2,RGCC	grey	VSMC_M10
GO:0010882	0.88	0.22	10	23	regulation of cardiac muscle contraction by calcium ion signaling	BP	1	TNNI3,CASQ2,RYR2,ANK2,ATP1A2,SLC9A1,HDAC4,ATP1B1,CALM1,DMD	grey	VSMC_M10
GO:1902117	0.88	0.18	28	61	positive regulation of organelle assembly	BP	1	VPS4B,PAN2,PIP4K2C,CNOT2,CNOT6,IL5,ASAP1,MAPK15,FUZ,SDCBP,PIP4K2A,LCP1,RALB,ULK1,PAN3,CNOT1,CNOT6L,ZMYND10,HTT,STX18,HAP1,MNS1,G3BP1,WRAP73,KIAA1324,GPSM2,SH3GLB1,RHOA	grey	VSMC_M10
GO:0007020	0.88	0.20	14	32	microtubule nucleation	BP	1	HSPA1B,TUBGCP6,SLAIN2,EML2,CLASP2,SLAIN1,ARHGEF7,TUBGCP5,TUBGCP4,NIN,TUBG1,NDEL1,CEP192,MECP2	grey	VSMC_M10
GO:1902914	0.88	0.22	10	23	regulation of protein polyubiquitination	BP	1	PRKN,NMI,TRIM44,SPSB4,MARCH7,NOD2,DDX3X,GPS2,OTUB2,UBE2D1	grey	VSMC_M10
GO:0010713	0.88	0.29	4	10	negative regulation of collagen metabolic process	BP	1	CYGB,CYP7A1,CIITA,MIR29A	grey	VSMC_M10
GO:0007131	0.88	0.19	20	44	reciprocal meiotic recombination	BP	1	UBE2B,DMC1,KLHDC3,MLH1,CCNB1IP1,RAD54B,SYCP1,HSF2BP,SPO11,C11orf80,RAD51B,EME1,RAD51C,RAD51D,ERCC4,SLX4,MEIOB,MSH4,RNF212B,CENPX	grey	VSMC_M10
GO:0140527	0.88	0.19	20	44	reciprocal homologous recombination	BP	1	UBE2B,DMC1,KLHDC3,MLH1,CCNB1IP1,RAD54B,SYCP1,HSF2BP,SPO11,C11orf80,RAD51B,EME1,RAD51C,RAD51D,ERCC4,SLX4,MEIOB,MSH4,RNF212B,CENPX	grey	VSMC_M10
GO:0060993	0.88	0.17	36	77	kidney morphogenesis	BP	1	MYC,KLHL3,PTCH1,SMO,SIX2,OSR1,SOX9,FOXJ1,GZF1,WNT11,ASXL1,PAX8,EYA1,FMN1,FRAS1,PKD2,PAX2,SALL1,IRX2,PRKX,AGT,LAMA5,DLG1,WNT4,MAGED1,CITED1,LHX1,GPC3,PKD1,SOX8,SOX4,NOG,BASP1,SIX1,LIF,FGF1	grey	VSMC_M10
GO:0043588	0.88	0.15	83	171	skin development	BP	1	CYP27B1,FZD6,SMO,ZDHHC21,IFT74,SRF,TFAP2B,SOSTDC1,CD109,HOXA7,GRHL1,MET,EGFR,FUZ,INTU,TRAF3IP2,HDAC2,SOX9,ERCC2,PTCH2,GLI2,EVPL,WDR48,SCEL,ELOVL1,OVOL2,JAG1,RYR1,FERMT1,TCF15,LSR,ASAH1,ACVR1B,FLNB,CASP3,TRPC4AP,FZD3,FRAS1,VDR,AP3B1,SRSF6,DSP,STK4,PPHLN1,ITGB6,LDB1,SUFU,HDAC1,SLC2A10,TGFB2,SNAI1,TGM1,KDF1,SFN,LAMA5,CLIC4,GRHL3,OVOL1,H2AFY2,ABCA12,SAV1,COL3A1,COL5A2,GORAB,ABCB6,RBPJ,MED1,PSEN1,NGFR,ALOXE3,ITGA3,MAP2K1,BCL11B,GATA6,PKD1,COL5A1,MIR125B1,CLDN1,WNT5A,DNASE1L2,ZBED2,EREG,SGPP1	grey	VSMC_M10
GO:0140059	0.88	0.29	4	10	dendrite arborization	BP	1	SULT4A1,PTN,SEMA3A,TAOK2	grey	VSMC_M10
GO:0002673	0.88	0.22	10	23	regulation of acute inflammatory response	BP	1	OSM,ADAM8,F12,BTK,C3,OSMR,CREB3L3,DNASE1,PARK7,PTGES	grey	VSMC_M10
GO:0097178	0.88	0.19	17	38	ruffle assembly	BP	1	EPS8L1,BAG4,ARHGEF26,ARHGAP24,CYFIP1,EPS8,NDEL1,EVL,INPP5K,EPS8L3,DEF8,MTSS1L,CARMIL2,EPS8L2,SNX10,CSPG4,HRAS	grey	VSMC_M10
GO:0038179	0.88	0.20	15	34	neurotrophin signaling pathway	BP	1	NTRK2,NTF4,WASF1,SPRY1,RAF1,SPRY2,CASP3,RAPGEF2,ZFYVE27,CYFIP1,BDNF,DOK5,AGT,PPP2R5B,HAP1	grey	VSMC_M10
GO:1990928	0.88	0.19	19	42	response to amino acid starvation	BP	1	MAP3K5,ATF4,TFEB,RRAGD,KPTN,SESN3,SZT2,EIF2AK2,LARS,EIF2AK4,SLC38A2,MAPK1,RRAGB,MAPK3,LARP1,WDR59,SH3GLB1,IMPACT,RNF152	grey	VSMC_M10
GO:0043462	0.88	0.19	17	37	regulation of ATPase activity	BP	1	TNNI3,NAPA,CHTOP,PXK,TNNC1,OXA1L,TOR1AIP1,VCPKMT,DNAJC10,DNAJB2,ATP1B1,DNAJB1,ATP1B2,PAM16,TSC1,VMA21,PPIF	grey	VSMC_M10

GO:0010959	0.88	0.14	131	263	regulation of metal ion transport	BP	1	NEDD4L,STAC3,TSPO,BAK1,MCHR1,PLA2G6,WNK2,TSPAN13,PRKCE,SLC31A2,HECW1,KEL,ATG5,OSR1,SPHK2,TRPC3,KCNRG,CACNB2,STAC,OXSR1,SCN5A,GPD1L,CACNB4,EFHB,PRNP,DIAPH1,P2RX2,KCNIP3,SLMAP,GLRX,P2RX3,ORAI1,TMBIM6,P2RX7,PTK2B,SLC8B1,HOMER1,STC1,F2R,TESC,ANO6,KCNE2,PKD2,CASQ2,CNTN1,KCNIP2,RYR2,PKP2,SIK1,HECW2,KCNJ2,ADRA2A,SLC30A10,ISCU,EGF,KCNG1,EPB41,OPRD1,UBASH3B,ANK2,GPER1,KLHL24,AGT,ATP1A2,SLC9A1,PLPP4,C4orf3,CASQ1,NTSR1,SCN4B,NOS1AP,HOMER2,DLG1,IL16,CEMIP,SESTD1,CASK,PTPN6,HTT,CAMK2G,ATP1B1,KCNA1,GNB5,HAP1,PSEN2,WNK3,STAC2,CACNB1,ATP1B2,KCNAB3,KCNIP1,ATP2A1,CD19,CALM1,DNM2,VMP1,BDKRB1,TRPV2,CAMK2A,CCL5,CCL2,FMR1,INPP5K,TRPV3,PDPK1,P2RX1,RNF207,AMIGO1,CYBA,JPH3,ATP2C2,LCN2,WWP2,RRAD,MIR21,MIR24-1,RHOA,DMD,GPR35,JPH2,KCNH2,FGF11,THY1,SCN2B,NETO2,CRBN,RGS4,CRH,YWHAH,GEM,NKX2-5	grey	VSMC_M10
GO:0051099	0.88	0.15	69	141	positive regulation of binding	BP	1	NME1,LRP1,STK3,EIF3E,PRKN,DERL1,MFNG,HSF1,ZNF618,MED25,MET,ADD2,MAPRE1,ERCC2,MAPRE3,RALB,POU4F1,SIRT2,TMEM173,PLCL2,DPH3,DDRGK1,PPP2CB,TRAF6,TRIB3,PLK2,IDE,HIP1R,HAND2,HMBOX1,RAPGEF2,TIAM1,EBF2,RIPK2,AMFR,STK4,BDNF,SPPL3,CAPRIN2,TERT,TCF7L2,EGF,EPB41,PARP9,DTX3L,RPL11,MEN1,RAMP2,DDX11,EPHA4,PARP1,NVL,ANXA2,PSEN1,TAF1,EIF3C,CALM1,LARP6,MARK3,ISL1,TICAM1,FMR1,SKI,PKD1,PARK7,GPSM1,LAMTOR5,WNT5A,TXN	grey	VSMC_M10
GO:0019835	0.88	0.29	4	10	cytolysis	BP	1	NINJ1,LYZL6,C7,MMD	grey	VSMC_M10
GO:0051668	0.88	0.13	273	537	localization within membrane	BP	1	LRP1,STX7,STAC3,MAP7,PEX3,ZDHHC14,EMC2,PRKN,AGER,GOPC,VPS52,HSPA4,SYNGAP1,PTCH1,EXOC4,SIL1,TOMM22,PICK1,MICALL1,ZDHHC21,TSPAN33,LRRC4,ADAM22,STEAP2,VPS50,SLC1A1,TRAM2,SEC61B,PRKCE,ASB3,SPTBN1,EMC10,PTN,ITGB1BP1,SEC61G,EGFR,TCAF2,BVES,VPS35,SDCBP,SLC7A11,LIN7B,EPS15,AGK,BRAF,ANKRD50,RAB7A,RAB10,ARL5B,CACNB2,CLASP2,DLG2,STAC,OPTN,GOLGA4,AQP11,CCDC93,GRIK5,EMC3,SEC62,MYL12A,AKT2,IKBKB,ANK1,ARL5A,LIN7A,PRNP,BAG4,GAS6,PKP3,ARL6IP5,SRP14,TUB,KCNIP3,LYPLA1,MAL,TM9SF4,ZDHHC20,RILPL1,RILPL2,ANKRD27,PIK3R1,TMEM150A,VAMP5,SLMAP,PLS1,EMC4,FCHO2,SORBS1,NUP54,P2RY1,RAPGEF2,KCNJ11,TIAM1,SSR3,EXOC6B,AP3B1,ZFYVE27,GOLGA7B,TESC,PEX16,DMTN,VPS26C,VPS39,KIF13A,NHLRC1,NECTIN3,SRPRA,PKP2,PPFIA1,ZDHHC23,ZDHHC6,OXA1L,DAG1,SSH1,GET4,DENND1B,KCNB1,NCF1,SORL1,ANK2,C2CD5,GPER1,RAB11FIP2,FAM126B,CIB1,RAB17,AP2M1,ZDHHC18,PPP2R5A,PEX19,ZDHHC24,BBS1,GORASP2,STX1A,SNX4,LAMA5,SNX27,CNIH2,STX6,VPS37D,PACS1,FZD9,DLG1,LRRC15,NSF,PIKFYVE,FIS1,VPS26A,CEMIP,NMT1,LZTFL1,RTP4,EHD1,ZDHHC8,ITGA4,ABCA12,NRXN2,SAV1,CNPY4,AP4M1,BCS1L,RAB3A,RAMP2,ZFAND2B,EXOC5,ATP1B1,PPIL2,ARL6IP1,NSG1,GRIPAP1,ZDHHC4,RAB8A,CNST,DLG4,ACTB,WDR19,RAB15,IQSEC2,RSC1A1,PEX26,NLGN2,GPHN,MPP5,WNK3,STAC2,ARL5C,SYNJ2BP,TSPAN17,ANXA2,GAK,CPLX1,STX1B,STX4,NAXE,TMEM88,ITGAM,SSR2,SNX12,DOCK2,RAB8B,ITGA3,TAOK2,AC138894.1,ZDHHC15,RAB11A,STX8,RAB3D,CALM1,TTC7B,TIMM29,DNM2,GGA2,CYP46A1,ARHGAP44,VPS35L,NPC1,AP2B1,MIEF2,CDK5[illegible]	grey	VSMC_M10
GO:0000768	0.88	0.20	15	34	syncytium formation by plasma membrane fusion	BP	1	MAPK14,MYH9,CD109,DYRK1B,MYOF,JAM2,RAPGEF3,ADAMTS15,SH3PXD2A,TCTA,VASH2,EHD1,GDF15,IL4R,CACNA1H	grey	VSMC_M10
GO:0140253	0.88	0.20	15	34	cell-cell fusion	BP	1	MAPK14,MYH9,CD109,DYRK1B,MYOF,JAM2,RAPGEF3,ADAMTS15,SH3PXD2A,TCTA,VASH2,EHD1,GDF15,IL4R,CACNA1H	grey	VSMC_M10
GO:0036037	0.88	0.24	7	17	CD8-positive, alpha-beta T cell activation	BP	1	EOMES,NCKAP1L,TOX,RUNX3,VSIR,ZBTB7B,SOCS1	grey	VSMC_M10
GO:0050732	0.88	0.18	21	46	negative regulation of peptidyl-tyrosine phosphorylation	BP	1	SEMA4D,PPP2R1A,IBTK,PIBF1,DMTN,LRRK1,PARP14,CBL,SNX6,PTPN6,ZFYVE28,SOCS4,PSEN1,SUZ12,PTPN2,SOCS1,PRKCZ,MIR125A,THY1,DUSP22,CADM4	grey	VSMC_M10
GO:0038066	0.89	0.18	20	44	p38MAPK cascade	BP	1	PHLPP1,MAP3K5,MAP3K4,AGER,MAPK14,GADD45G,EMC10,RELL2,MFHAS1,HAND2,NCF1,MAPKAPK2,MAP3K3,DLG1,DUSP10,MINK1,RELL1,DAB2IP,GADD45A,DUSP1	grey	VSMC_M10
GO:0035743	0.89	0.28	4	10	CD4-positive, alpha-beta T cell cytokine production	BP	1	RSAD2,DENND1B,TBX21,PRKCZ	grey	VSMC_M10
GO:0051608	0.89	0.28	4	10	histamine transport	BP	1	SNAP23,SNX4,SLC29A4,BTK	grey	VSMC_M10
GO:0002182	0.89	0.28	4	10	cytoplasmic translational elongation	BP	1	DPH2,DPH3,DPH1,AARS	grey	VSMC_M10
GO:0034763	0.89	0.16	42	88	negative regulation of transmembrane transport	BP	1	NEDD4L,GOPC,PRKCE,GRB10,HECW1,TCAF2,KEL,OSR1,SLC26A5,KCNRG,OXSR1,AKT2,ARL6IP5,SLC43A1,KCNE2,PKD2,CASQ2,HECW2,ADRA2A,ISCU,ATP1A2,C4orf3,PEA15,NTSR1,CTTNBP2NL,SESTD1,GNB5,RSC1A1,CALM1,PRKCB,FMR1,INPP5K,OAZ1,RRAD,MIR24-1,GPR35,FABP5,KCNH2,CRBN,RGS4,GEM,PPIF	grey	VSMC_M10
GO:0110149	0.89	0.17	35	75	regulation of biomineralization	BP	1	CYP27B1,OSR2,OMD,PTN,TRPM4,OSR1,SOX9,SLC4A2,ACVR2B,ACVR2A,KL,GAS6,ADGRV1,MEF2C,P2RX7,PTK2B,BMP2K,ANO6,TFAP2A,BMPR1B,SGMS2,AMELX,ACTN3,TXLNG,FZD9,SRGN,BCOR,ANKH,WNT4,BMPR1A,SMAD3,RFLNB,BGLAP,CEBPB,HEY2	grey	VSMC_M10
GO:0072683	0.89	0.28	4	10	T cell extravasation	BP	1	CD99,CCL2,CRK,FADD	grey	VSMC_M10
GO:0009155	0.89	0.28	4	10	purine deoxyribonucleotide catabolic process	BP	1	GDA,NUDT15,NUDT16,NUDT18	grey	VSMC_M10
GO:0016048	0.89	0.24	7	17	detection of temperature stimulus	BP	1	GRIK2,ASIC3,EPHB1,TRPM8,NTSR1,NGFR,TRPV1	grey	VSMC_M10
GO:0060049	0.89	0.28	4	10	regulation of protein glycosylation	BP	1	FKTN,IL15,POMT2,ACER2	grey	VSMC_M10
GO:0031648	0.89	0.19	19	42	protein destabilization	BP	1	SERF2,PRKN,RNF5,DERL1,ISOC2,PRNP,PRKDC,FBXW7,PEX2,MYLIP,SIAH1,SENP2,CTSH,HTT,MUL1,KDM8,CCDC88C,CREBBP,IRGM	grey	VSMC_M10
GO:0001783	0.89	0.23	8	19	B cell apoptotic process	BP	1	BAK1,TNFRSF21,TRAF3IP2,FOXP1,BCL6,BTK,CD74,BCL10	grey	VSMC_M10

GO:0070227	0.89	0.18	24	52	lymphocyte apoptotic process	BP	1	BAK1,TNFRSF21,SLC46A2,TRAF3IP2,IDO1,FOXP1,ZC3H8,LMBR1L,RIPK1,GPAM,PDCD1,ADAM8,BCL6,EFNA1,BTK,CD74,CCL5,IL7R,DNAJA3,BCL10,FADD,WNT5A,TSC22D3,BCL2L11	grey	VSMC_M10
GO:0071357	0.89	0.18	19	42	cellular response to type I interferon	BP	1	MYD88,MAVS,IRF7,OAS3,NLRC5,SP100,YTHDF2,PTPN6,MUL1,USP18,DCST1,STAT2,TYK2,JAK1,PTPN2,MIR21,OAS1,FADD,WNT5A	grey	VSMC_M10
GO:0060612	0.89	0.20	14	32	adipose tissue development	BP	1	TRPM4,PGRMC2,PAXIP1,DYRK1B,SPART,EBF2,FTO,SORL1,KLF7,PLA2G16,OXCT1,SOX8,SLC25A25,UBB	grey	VSMC_M10
GO:0042330	0.89	0.13	215	427	taxis	BP	1	LRP1,SEMA6A,KIF5A,SLC12A2,AGER,GPSM3,CXCL14,GFRA3,PTK2,PTCH1,PLXNA4,MAPK14,PLA2G6,RAC2,SMO,CCL27,KIT,PLGRKT,SEMA4D,DLX5,NINJ1,ETV1,SEMA4C,FPR2,HOXA2,LPAR1,CXCL8,AKIRIN1,EPHA7,PTN,MET,EPHA1,GAB1,NRCAM,TRPM4,PLXNA1,C5AR2,C5AR1,PRKD2,GLI2,MEGF8,OXSR1,MYCBP2,ACKR2,CMTM7,CMTM8,KIF5C,HBEGF,FEZ2,SEMA3D,PLXNC1,SEMA3A,CHL1,UNC5D,NRG1,NCKAP1L,GAS6,FAM19A4,FLT1,RYK,EPHB1,MTUS1,RET,CYP7B1,NR4A1,FER,PIK3CB,PARVA,ROBO1,ROBO2,PTPRJ,FZD3,SEMA4F,VEGFC,PTK2B,DPYSL2,CCL17,TIAM1,SLIT1,SLC8B1,TRIO,F2RL1,ANO6,SEMA3G,CYFIP1,ALCAM,SEMA4G,NOD2,SIAH1,BMPR1B,GBF1,BDNF,ELMO2,LGR6,DNM1L,TRPV4,SBDS,SEMA3F,TGFB2,GAP43,DAG1,PRSS56,LHX9,CMKLR1,SLAMF8,MOSPD2,GBX2,KALRN,LAMA5,YTHDF1,PTPRO,MACO1,FCER1G,EPHB3,ADAM8,WNT3,KLF7,CREB1,MYPN,PIKFYVE,IL16,SEMA6D,EPHB4,IL12A,TNR,MCU,TNFSF18,CXCR2,BST1,MAPK1,CH25H,CRMP1,PADI2,CXCL16,EPHA4,LRTM2,TMEM102,UNC5A,STX4,SEMA4A,HOXB9,FGF18,NGFR,DOCK2,PGF,DAPK2,EFNA1,MAPK3,IL6R,ALKBH1,MAP2K1,SMAD3,NTN1,RPS6KA5,S100A14,S100A8,RNF165,BCL11B,LHX1,CD74,CCL14,CCL5,LAMA3,AMOT,CSF1R,CCL7,CCL2,CCL25,CDK5R1,ISL1,EMB,VAV1,LAMA1,RALBP1,PTPN2,CRK,PIP5K1C,L1CAM,NTN3,ARHGEF16,PLXNA3,MPP1,BSG,TUBB3,VAV3,CMTM3,RHOA,CCL16,DPYSL5,S100A12,EFNA4,CNTN2,VASP,FOXG1,CHGA,DEFB124,CCR10,DPP4,CXCL6,ZNF580,ISL2,NOG,WNT5A,CHN1,DUSP1,ARTN,NRXN3,LYPLA2,RALA,HRAS,EFNB3,ISPD,FGF1,CCL22,PDGFD	grey	VSMC_M10
GO:0051095	0.89	0.28	4	10	regulation of helicase activity	BP	1	POT1,MSH2,SSBP1,CHTOP	grey	VSMC_M10
GO:0055090	0.89	0.22	9	21	acylglycerol homeostasis	BP	1	SLC25A27,ABCG5,ABCG8,GCKR,NR1H4,NR1H3,FITM2,MLXIPL,LIPC	grey	VSMC_M10
GO:0070328	0.89	0.22	9	21	triglyceride homeostasis	BP	1	SLC25A27,ABCG5,ABCG8,GCKR,NR1H4,NR1H3,FITM2,MLXIPL,LIPC	grey	VSMC_M10
GO:0060575	0.89	0.23	7	17	intestinal epithelial cell differentiation	BP	1	KLF5,CBFA2T2,PYY,SAV1,NKX3-2,GATA6,TIGAR	grey	VSMC_M10
GO:0006869	0.89	0.14	136	273	lipid transport	BP	1	ATP9B,VPS4B,APOF,ATP8B1,STARD6,LRP1,CPT1B,APOM,SERAC1,SLC27A6,PRKN,PPARA,TSPO,GDF9,PTCH1,PLA2G6,TTC39B,PLIN2,APOL4,APOL6,CRY1,CROT,ABCB1,ABCG5,ABCG8,TMEM30A,NR1H2,APOB,MFSD2B,PIP4K2A,APOC1,TSKU,AKT2,ABCC4,C1QTNF1,ANO4,SLC5A8,SOAT2,CYP7A1,OSBP,PITPNM2,XKR6,PLSCR4,LIMA1,P2RX7,NR1H3,NKX3-1,ANO6,CRY2,SLC10A6,COL4A3BP,ABCC2,SPP1,PLA2R1,PLTP,NME4,CPT1A,MAP2K6,ABCA5,ABCA9,ACSL5,CHKA,SLCO3A1,PITPNM1,FABP4,PITPNC1,EGF,GRAMD1B,TEX2,PDZD8,ABCB11,AGT,SLC25A20,ABCG4,SLCO1A2,SLCO1C1,OSBPL2,NTSR1,OSBP2,APOLD1,APOD,PLA2G4A,STARD5,FIS1,OSBPL6,SOAT1,OSBPL1A,ZDHHC8,ABCA12,LPCAT3,SPNS3,SLC27A1,TMEM30B,SYT7,GPS2,CLN8,LIPC,ANXA2,OSBPL7,NAXE,ABCD4,APOBR,AC138894.1,ABCC3,PCTP,LDLR,ATG2B,NMUR2,STRA6,GM2A,NPC1,NOS2,PROCA1,PRELID3A,ATP11C,PITPNA,ABCA3,ABCA7,RXRA,CEL,ABCD3,VPS4A,MIR27B,MIR27A,FABP5,ABCA8,MIR33B,PLA2G2C,LDLRAP1,CRH,CPTP,LRAT,STARD4,PTGES,SGPP1,XKR8	grey	VSMC_M10
GO:0150172	0.89	0.28	4	10	regulation of phosphatidylcholine metabolic process	BP	1	APOC1,LPCAT1,LDLR,ABCA3	grey	VSMC_M10
GO:0030031	0.89	0.13	223	443	cell projection assembly	BP	1	ATP8B1,ONECUT2,MYO1A,AVIL,TBC1D30,ATAT1,PARVG,KIF3A,TBC1D32,UBE2B,MTSS1,ASAP1,LRRC6,MAPK15,PODXL,CDC42EP1,RAC2,IFT74,SRF,CFAP69,RFX3,KIT,NCK2,PTPDC1,FGD3,RAB23,DYNC2LI1,EPS8L1,CEP162,TMEM17,AKIRIN1,WDR92,FUZ,IQUB,ATG5,SDCBP,ARMC2,MAPRE1,ARMC4,INTU,SPATA6,TTC26,BBS7,VSTM5,ARHGEF4,DRC1,RSPH6A,GALNT11,FBF1,PIBF1,KLF5,IFT172,FOXJ1,WDR60,TTLL3,CCDC13,ABCC4,MKKS,RAB5A,RAC3,CEP290,BAG4,ARHGEF7,SPEF1,FGD6,NCKAP1L,ARFIP2,TMEM80,DPYSL3,AL359736.1,KIF3B,FER,RILPL1,RILPL2,CEP70,PIK3R1,PARVA,PLCE1,CEP250,CCDC66,P2RX7,TCTN3,IFT81,CCDC113,CFAP20,ARHGEF26,RAPGEF2,CCDC65,TMEM67,BIN3,F2RL1,IFT52,ARHGAP24,ANO6,RPGRIP1L,DNAH1,CYFIP1,DMTN,DNAH5,ZNF423,GBF1,SRGAP2C,NEURL1,CARMIL1,CFAP43,FGD4,ZMYND10,GAP43,ARMC9,CFAP46,TTBK2,AUTS2,AJUBA,CLCN4,ALPK1,JHY,DISC1,OFD1,WASF2,RAB17,TMEM237,TRAF3IP1,HDAC4,BBS1,LAMA5,EPS8,PTPRO,CDKL5,SEPT2,LIMK2,WHAMM,CDC42EP2,CFAP161,TTC12,TOGARAM1,EHD1,DNAAF2,SPAG16,NCKAP1,ABLIM2,FAM149B1,HTT,CFAP70,DNM3,RAP1GAP,STK36,GORAB,CEP126,SNAP29,EXOC5,VCL,WAS,RHOJ,TTC25,RAB8A,SDCCAG8,HAP1,DNAAF4,CPLANE2,MNS1,WDR19,TMEM138,MIEN1,ATP6V1D,PLEK2,FGD1,FXR2,BBOF1,CNTROB,TMEM107,NDEL1,TENM2,AC138894.1,CCDC151,NTN1,DYNLL2,MKS1,DNM2,BCAS3,ARHGAP44,EVL,TEKT3,IFT20,SPEF2,STK26,CFAP53,ARHGEF6,FMR1,INPP5K,C16orf71,RILP,CLUAP1,WRAP73,PALM,TPGS1,EPS8L3,DEF8,CDK10,CELSR2,VAV3,CDC14A,TMEM231,FNBP1L,MTSS1L,HYDIN,ODF2L,WDR63,CARMIL2,C5orf30,TCTN2,EPS8L2,HYLS1 [illegible]	grey	VSMC_M10
GO:0009113	0.89	0.28	4	10	purine nucleobase biosynthetic process	BP	1	SHMT2,PPAT,GMPS,SHMT1	grey	VSMC_M10
GO:0003230	0.89	0.20	13	30	cardiac atrium development	BP	1	SMO,WNT11,TBX5,TGFB2,ANK2,ISL1,ENG,SOX4,NOG,WNT5A,NKX2-5,HEY2,WNT2	grey	VSMC_M10
GO:0006646	0.89	0.28	4	10	phosphatidylethanolamine biosynthetic process	BP	1	SELENOI,CHKA,PISD,SLC27A1	grey	VSMC_M10

GO:0045104	0.89	0.20	13	30	intermediate filament cytoskeleton organization	BP	1	RAF1,EVPL,BFSP1,NEFM,DSP,INA,PKP2,SYNM,KLHL24,DES,CLN8,NDEL1,MTM1	grey	VSMC_M10
GO:0010884	0.89	0.23	8	19	positive regulation of lipid storage	BP	1	PLIN2,NR1H2,APOB,FITM2,FITM1,EHD1,C3,PLIN3	grey	VSMC_M10
GO:1905897	0.89	0.17	33	70	regulation of response to endoplasmic reticulum stress	BP	1	PRKN,BAK1,NCK2,NR1H2,AQP11,DDRGK1,PIK3R1,USP25,TMBIM6,NR1H3,OPA1,RNFT2,FICD,USP13,USP19,HYOU1,RNF185,NFE2L2,SYVN1,LPCAT3,PTPN2,BCAP31,SGTA,ABCA7,TMEM259,PARK7,HSPA5,DAB2IP,MIR199A1,RNFT1,DDIT3,BCL2L11,PPP1R15A	grey	VSMC_M10
GO:0098773	0.89	0.17	30	65	skin epidermis development	BP	1	FZD6,SMO,ZDHHC21,SOSTDC1,CD109,EGFR,FUZ,INTU,HDAC2,SOX9,ERCC2,GLI2,FERMT1,ACVR1B,TRPC4AP,FZD3,AP3B1,LDB1,HDAC1,TGFB2,SNAI1,KDF1,LAMA5,SAV1,GORAB,RBPJ,NGFR,GATA6,WNT5A,DNASE1L2	grey	VSMC_M10
GO:0019935	0.89	0.17	26	57	cyclic-nucleotide-mediated signaling	BP	1	PDE7B,ADGRG6,RIMS2,PDE10A,UBE2B,AQP1,SOX9,GIPR,GUCY1B1,PTK2B,RAPGEF2,RAPGEF3,CAP2,ADCY2,PDE9A,ADNP,EIF4EBP2,DGKQ,PDE4A,MAPK7,KSR1,MGRN1,CAP1,PDZD3,CRH,RASD2	grey	VSMC_M10
GO:0002204	0.89	0.18	18	40	somatic recombination of immunoglobulin genes involved in immune response	BP	1	EXOSC3,MSH2,HMCES,ERCC1,MLH1,PAXIP1,SHLD1,NBN,CD40,CD28,RNF168,BCL6,FOXP3,SHLD2,TBX21,BATF,SUPT6H,RNF8	grey	VSMC_M10
GO:0002208	0.89	0.18	18	40	somatic diversification of immunoglobulins involved in immune response	BP	1	EXOSC3,MSH2,HMCES,ERCC1,MLH1,PAXIP1,SHLD1,NBN,CD40,CD28,RNF168,BCL6,FOXP3,SHLD2,TBX21,BATF,SUPT6H,RNF8	grey	VSMC_M10
GO:0045190	0.89	0.18	18	40	isotype switching	BP	1	EXOSC3,MSH2,HMCES,ERCC1,MLH1,PAXIP1,SHLD1,NBN,CD40,CD28,RNF168,BCL6,FOXP3,SHLD2,TBX21,BATF,SUPT6H,RNF8	grey	VSMC_M10
GO:0006707	0.89	0.28	4	10	cholesterol catabolic process	BP	1	CYP7A1,CYP27A1,CYP46A1,CEL	grey	VSMC_M10
GO:0016127	0.89	0.28	4	10	sterol catabolic process	BP	1	CYP7A1,CYP27A1,CYP46A1,CEL	grey	VSMC_M10
GO:0055086	0.89	0.13	224	439	nucleobase-containing small molecule metabolic process	BP	1	NME1,HSD17B4,SHMT2,TBPL1,PDE7B,ENPP3,ATP5MGL,GFPT2,DPYS,PPT2,PPARA,PDE10A,ACSL6,SMPDL3A,PMM1,PRXL2C,APOBEC3H,GTPBP1,OARD1,GUCA1B,IMPDH1,MTAP,NMRK1,GNMT,GDA,CROT,GNPDA2,GALT,SLC35B2,ENPP4,UXS1,SLC25A13,GAPDH,PPAT,RAB23,PPCS,SLC4A4,ME1,NT5E,PPT1,BPGM,DLD,SPHK2,CMPK1,ADCY3,PRTFDC1,ENTPD6,CDADC1,NUDT15,DLG2,LACC1,NUDT5,DHTKD1,MBD4,PRKAG2,SLC22A13,ATP5F1C,ELOVL1,TGDS,CSGALNACT1,PFKP,UGGT2,IDO2,IDO1,NUDT16,PANK2,PDK1,SUCLG2,PDHX,SLC5A8,ELOVL7,PANK1,GPI,NUDT12,AHCY,AMPD3,ATP5F1A,MCCC2,GLRX,GPD1,P2RX7,GUCY1A1,GUCY1B1,ACOT12,DGUOK,PRKAG1,GMPS,PFKM,OPA1,ENTPD4,FITM2,AMPD1,NUDT18,ADCY7,GMPR,FAM173B,ADCY2,MCEE,NME4,PDE9A,TPST1,ZBTB20,GPAM,FAR2,ACSL5,PTHLH,PUDP,NUDT17,MFN1,PFKFB2,PRPS2,ENO4,PARP9,HACD2,ARNT,ADCY4,DPAGT1,CTPS2,ACTN3,HDAC4,THEM5,MGAT1,DNAJC30,MLXIPL,PDE8A,DTYMK,HMGCL,MAGI3,DLG1,ACOT9,PDK3,EFL1,SLC25A16,UPB1,HK1,CPS1,ACSBG1,ATP5S,CASK,COASY,PRKAG3,PINK1,STAT3,NME7,ADK,MPC2,ADCY10,MAPK1,ENO3,NT5C3B,PARP1,TECR,CARD11,SLC26A1,NAXE,PSEN1,ACOT2,ALDH6A1,GUCY2D,PKLR,PANK3,GCDH,OGT,UPRT,ADPGK,PDE4A,ACSM3,MPI,ACACA,NEIL1,NCOR1,INSR,SLC26A2,PRPSAP2,SHMT1,ADSSL1,NT5M,NOS2,ACSBG2,ALDOC,PIPOX,PMM2,ACOT11,NADK,ATP6V0C,PANK4,NTHL1,NME3,MPP1,PARK7,ATP5PB,SLC2A6,VPS9D1,GNAI3,ACSF3,AK8,UCK1,MLYCD,KARS,FUK,DHODH,MT-ATP8,MT-ATP6,RHOA,SULT1C3,ATP5ME,ACOT4,ATP5MF,ENTPD5,PDZD3,ELOVL3,MTHFD2L,PEMT,CMPK2,TIGAR,N[illegible]	grey	VSMC_M10
GO:0050654	0.89	0.20	12	28	chondroitin sulfate proteoglycan metabolic process	BP	1	NDNF,CHPF2,CSGALNACT1,DCN,HYAL1,CHST3,SPOCK2,CHST7,CHST12,IDUA,B3GALT6,CHSY3	grey	VSMC_M10
GO:0002262	0.89	0.15	65	134	myeloid cell homeostasis	BP	1	MPIG6B,HSPA1B,BRD1,BAK1,SMAD5,L3MBTL3,FANCC,MAPK14,ZNF16,RBFOX2,SRF,CDK6,KIT,PRDX1,RPS14,SMAP1,EPAS1,PTBP3,BPGM,SLC7A11,FOXO3,TRAF3IP2,ERCC2,SLC25A38,ACVR2A,NCKAP1L,PRKDC,MFHAS1,GPI,ACVR1B,PIK3CB,CASP3,TMEM14C,DMTN,ALAS1,LDB1,PKNOX1,RPS17,ACIN1,DYRK3,HCLS1,ARNT,SP3,BCL6,CXCR2,STAT3,STAT5B,INHA,FOXP3,THRA,MED1,HOXB6,AHSP,KAT7,DNASE2,SCNN1B,RCOR1,PTPN2,MIR222,ANKRD54,ARID4A,MAFB,BCL2L11,FAM210B,XKR8	grey	VSMC_M10
GO:2000648	0.89	0.23	7	17	positive regulation of stem cell proliferation	BP	1	EPCAM,VEGFC,TERT,THPO,KAT7,CITED1,ATXN1L	grey	VSMC_M10
GO:0006213	0.89	0.26	5	12	pyrimidine nucleoside metabolic process	BP	1	APOBEC3H,CDADC1,DTYMK,UPB1,UPP1	grey	VSMC_M10
GO:0000470	0.89	0.21	10	23	maturation of LSU-rRNA	BP	1	URB1,WDR12,RPL7L1,LAS1L,ZNHIT3,SNU13,NOL9,RPL35,ZNHIT6,NSA2	grey	VSMC_M10
GO:0009116	0.89	0.18	21	45	nucleoside metabolic process	BP	1	APOBEC3H,OARD1,MTAP,GNMT,GDA,ENPP4,PPAT,NT5E,PRTFDC1,CDADC1,AHCY,DGUOK,NME4,PRPS2,DTYMK,UPB1,ADK,UPRT,PRPSAP2,PEMT,UPP1	grey	VSMC_M10
GO:0060117	0.89	0.23	7	17	auditory receptor cell development	BP	1	WHRN,MYO7A,SLC4A7,LRTOMT,AP000812.5,PLS1,STRC	grey	VSMC_M10
GO:0002429	0.89	0.15	72	148	immune response-activating cell surface receptor signaling pathway	BP	1	CD226,HLA-DPB1,PTK2,PLA2G6,TEC,TNFRSF21,PRKCE,FPR2,C5AR2,C5AR1,PRKD2,NECTIN2,EIF2B3,IKBKB,PAWR,PLCL2,PRNP,NCKAP1L,FOXP1,TRAF6,HCK,LPXN,PTPRJ,MEF2C,SLA2,RIPK2,GPLD1,SPPL3,LCK,BANK1,ICOSLG,BTN2A2,DENND1B,CMKLR1,THEMIS2,LAT2,BMX,FGR,PLPP4,CD28,FCER1G,RC3H1,PTPN6,CD38,MAPK1,FOXP3,GPS2,CARD11,PSEN1,LCP2,CD19,PRKCB,BTK,YES1,PRAM1,VAV1,PTPN2,CREBBP,IKBKG,VAV3,BCL10,CMTM3,FYB1,BTN1A1,CD247,THY1,UBE2N,GCSAM,HRAS,LAX1,SH2B2,RC3H2	grey	VSMC_M10
GO:0002757	0.89	0.15	72	148	immune response-activating signal transduction	BP	1	CD226,HLA-DPB1,PTK2,PLA2G6,TEC,TNFRSF21,PRKCE,FPR2,C5AR2,C5AR1,PRKD2,NECTIN2,EIF2B3,IKBKB,PAWR,PLCL2,PRNP,NCKAP1L,FOXP1,TRAF6,HCK,LPXN,PTPRJ,MEF2C,SLA2,RIPK2,GPLD1,SPPL3,LCK,BANK1,ICOSLG,BTN2A2,DENND1B,CMKLR1,THEMIS2,LAT2,BMX,FGR,PLPP4,CD28,FCER1G,RC3H1,PTPN6,CD38,MAPK1,FOXP3,GPS2,CARD11,PSEN1,LCP2,CD19,PRKCB,BTK,YES1,PRAM1,VAV1,PTPN2,CREBBP,IKBKG,VAV3,BCL10,CMTM3,FYB1,BTN1A1,CD247,THY1,UBE2N,GCSAM,HRAS,LAX1,SH2B2,RC3H2	grey	VSMC_M10

GO:0031334	0.89	0.14	98	199	positive regulation of protein-containing complex assembly	BP	1	ARPC3,ATAT1,ESR1,HSPA1B,IL5,BAK1,WASHC5,ASAP1,BAIAP2L2,PICK1,CDC42EP1,LMOD2,TTBK1,SLAIN2,NCK2,KIAA1211,PRKCE,PLEK,NR1H2,MED25,MET,TLR4,CDK5RAP2,MAPRE1,WASF1,FAF1,LCP1,MYD88,SLAIN1,FCHSD2,NAV3,FERMT1,BAG4,FCHSD1,NRG1,NCKAP1L,ARFIP2,LMOD3,HCK,FER,HIP1R,ATR,TRABD2A,CLIP1,SLF1,FMN1,PTK2B,FNIP2,SPIDR,CDH17,IQGAP2,CYFIP1,SLF2,MAGEL2,CARMIL1,LMOD1,AJUBA,AP1AR,WASF2,WHAMM,CDKN1B,DLG1,CALY,CREB1,CDC42EP2,MAPT,RAP1B,TOGARAM1,PPP2R5B,DDX3X,NCKAP1,NIN,PINK1,MMP3,ARPC1A,WAS,GNL3L,EVL,CDK5R1,ABCA3,MLST8,MECP2,PARK7,SPIRE2,PSRC1,PIEZO1,SH3GLB1,DAB2IP,WDR63,CARMIL2,RHOA,TRABD2B,VASP,GMFG,CRBN,GRB2,BCL2L11,DRG1	grey	VSMC_M10
GO:0006637	0.89	0.16	37	78	acyl-CoA metabolic process	BP	1	HSD17B4,PPT2,ACSL6,PPCS,PPT1,DLD,ELOVL1,PDK1,SUCLG2,PDHX,ELOVL7,ACOT12,FITM2,MCEE,GPAM,FAR2,ACSL5,HACD2,THEM5,HMGCL,ACOT9,PDK3,ACSBG1,MPC2,TECR,ACOT2,GCDH,ACSM3,ACACA,ACSBG2,PIPOX,ACOT11,ACSF3,MLYCD,ACOT4,ELOVL3,NUDT19	grey	VSMC_M10
GO:0035383	0.89	0.16	37	78	thioester metabolic process	BP	1	HSD17B4,PPT2,ACSL6,PPCS,PPT1,DLD,ELOVL1,PDK1,SUCLG2,PDHX,ELOVL7,ACOT12,FITM2,MCEE,GPAM,FAR2,ACSL5,HACD2,THEM5,HMGCL,ACOT9,PDK3,ACSBG1,MPC2,TECR,ACOT2,GCDH,ACSM3,ACACA,ACSBG2,PIPOX,ACOT11,ACSF3,MLYCD,ACOT4,ELOVL3,NUDT19	grey	VSMC_M10
GO:0051004	0.89	0.28	4	10	regulation of lipoprotein lipase activity	BP	1	NR1H2,ABHD5,APOC1,NR1H3	grey	VSMC_M10
GO:0006012	0.89	0.28	4	10	galactose metabolic process	BP	1	GALT,GLB1,CHST1,SLC35A2	grey	VSMC_M10
GO:0010460	0.89	0.23	7	17	positive regulation of heart rate	BP	1	SLC1A1,TRPM4,ADRA1D,RYR2,GCH1,RGS4,HEY2	grey	VSMC_M10
GO:0036507	0.89	0.23	7	17	protein demannosylation	BP	1	RNF5,UGGT2,AMFR,MAN1C1,RNF185,EDEM3,SYVN1	grey	VSMC_M10
GO:0036508	0.89	0.23	7	17	protein alpha-1,2-demannosylation	BP	1	RNF5,UGGT2,AMFR,MAN1C1,RNF185,EDEM3,SYVN1	grey	VSMC_M10
GO:0055078	0.89	0.19	15	34	sodium ion homeostasis	BP	1	NEDD4L,SLC12A2,TFAP2B,ATP1A3,SNX5,IL1A,SCN7A,AGT,ATP1A2,SLC9A1,COMT,ATP1B1,ATP1B2,SCNN1B,C7	grey	VSMC_M10
GO:0030279	0.89	0.20	11	26	negative regulation of ossification	BP	1	TRPM4,MKX,SOX9,GFRA4,MEF2C,PTK2B,KREMEN1,SRGN,BCOR,RBPJ,RFLNB	grey	VSMC_M10
GO:0072028	0.89	0.16	30	65	nephron morphogenesis	BP	1	MYC,KLHL3,PTCH1,SMO,SIX2,OSR1,SOX9,GZF1,WNT11,PAX8,EYA1,FMN1,PKD2,PAX2,SALL1,IRX2,AGT,LAMA5,DLG1,WNT4,MAGED1,CITED1,LHX1,GPC3,PKD1,SOX8,NOG,SIX1,LIF,FGF1	grey	VSMC_M10
GO:0032479	0.89	0.16	35	75	regulation of type I interferon production	BP	1	C6orf106,IRF5,GAPDH,REL,TLR4,MYD88,NMI,TMEM173,NPLOC4,MAVS,IRF7,DTX4,OAS3,RIPK2,POLR3D,IFIH1,POLR3C,RNF26,NLRX1,TRAF3IP1,SETD2,DDX3X,POLR3A,OTUD5,RNF216,G3BP1,YY1,TRAF3,RIOK3,RNF135,TICAM1,MIR21,OAS1,ZCCHC3,HAVCR2	grey	VSMC_M10
GO:0032606	0.89	0.16	35	75	type I interferon production	BP	1	C6orf106,IRF5,GAPDH,REL,TLR4,MYD88,NMI,TMEM173,NPLOC4,MAVS,IRF7,DTX4,OAS3,RIPK2,POLR3D,IFIH1,POLR3C,RNF26,NLRX1,TRAF3IP1,SETD2,DDX3X,POLR3A,OTUD5,RNF216,G3BP1,YY1,TRAF3,RIOK3,RNF135,TICAM1,MIR21,OAS1,ZCCHC3,HAVCR2	grey	VSMC_M10
GO:0044728	0.89	0.16	31	67	DNA methylation or demethylation	BP	1	BAZ2A,MOV10L1,MYC,APOBEC3H,PICK1,STPG4,DNMT3A,BMI1,TEX15,ALKBH3,TOX,DNMT3B,OTUD4,FTO,MTRR,HEMK1,ALKBH2,TDRKH,MGMT,TET1,PARP1,FOS,ALKBH1,METTL4,MBD1,MECP2,PRMT7,CTCF,MIR29A,BEND3,N6AMT1	grey	VSMC_M10
GO:0006515	0.89	0.21	11	25	protein quality control for misfolded or incompletely synthesized proteins	BP	1	RNF5,DERL1,ZYG11B,UGGT2,AUP1,LONP2,RNF185,SDF2L1,LONP1,RNF126,ZER1	grey	VSMC_M10
GO:0042113	0.89	0.14	95	193	B cell activation	BP	1	TNFAIP3,IL5,TNFRSF13C,BAK1,MFNG,KIT,TNFRSF21,EXOSC3,IL11,MSH2,SKAP2,PELI1,IL4I1,TLR4,TRAF3IP2,HMCES,BMI1,PHB,ERCC1,MLH1,FOXJ1,PAXIP1,KLF6,CMTM7,PAWR,PLCL2,SHLD1,NCKAP1L,PRKDC,GAPT,PIK3R1,CASP3,PTPRJ,MEF2C,IL7,TPD52,PTK2B,RASGRP1,NBN,CDH17,NOD2,CD40,DCAF1,DOCK10,BANK1,ICOSLG,TCIRG1,RABL3,TBC1D10C,SLAMF8,THEMIS2,LAT2,HDAC4,CD28,RNF168,FZD9,SP3,PPP2R3C,BCL6,SYVN1,ITGA4,RC3H1,PTPN6,TNIP2,CD38,BST1,STAT5B,INHA,FOXP3,SHLD2,GPS2,CARD11,RBPJ,TBX21,BATF,CHRNB2,CD19,PRKCB,BTK,YY1,CD74,CD320,IL7R,SUPT6H,PTPN2,TICAM1,VAV3,LRRC8A,CASP8,RNF8,CD70,NHEJ1,ZFP36L2,LAX1,TNFSF13B	grey	VSMC_M10
GO:0006308	0.89	0.20	11	25	DNA catabolic process	BP	1	HSF1,FBH1,FOXL2,DNASE2,TREX2,PAM16,DNASE1,DFFB,DNASE1L1,DNASE1L2,NMNAT1	grey	VSMC_M10
GO:2000234	0.89	0.28	4	10	positive regulation of rRNA processing	BP	1	SIRT7,DIMT1,RIOK1,HEATR1	grey	VSMC_M10

GO:0061564	0.89	0.13	189	380	axon development	BP	1	LRP1,SEMA6A,KIF5A,RAB21,TIAM2,TSPO,FSTL4,SYNGAP1,GFRA3,PTK2,PTCH1,PLXNA4,NTRK2,RAC2,SMO,SRF,TNFRSF21,SEMA4D,DLX5,ETV1,SEMA4C,SVBP,BRSK1,RTN4,RUFY3,HOXA2,EPHA7,PTN,TNC,TRIM32,EPHA1,KEL,NRCAM,DHFR,PLXNA1,RAB10,GOLGA4,GLI2,MEGF8,PTPRF,MYCBP2,NUMBL,POU4F1,TSKU,SZT2,SLITRK5,KIF5C,FEZ2,SEMA3D,SPART,PLXNC1,SEMA3A,CHL1,UNC5D,ULK1,RYK,EPHB1,RET,TTL,CASP3,ROBO1,ROBO2,FZD3,SEMA4F,NCAM2,DPYSL2,TIAM1,SLIT1,NKX6-1,TRIO,NR4A2,ZFYVE27,CRTAC1,SEMA3G,SPP1,PAX2,CYFIP1,ALCAM,TSPAN2,SEMA4G,SIAH1,BMPR1B,BDNF,KIAA0319,MTR,LGR6,SEMA3F,GAP43,ADARB1,DAG1,AUTS2,LHX9,ADNP,DISC1,KREMEN1,GBX2,KALRN,CERS2,VASH2,LAMA5,YTHDF1,PTPRO,CDKL5,EPHB3,LRIG2,APOD,WNT3,KLF7,CREB1,MYPN,SPG11,MAPT,SEMA6D,EPHB4,ITGA4,TNR,FN1,ATL1,NIN,PPP3CB,RAB3A,MARK2,VCL,RAB8A,CRMP1,EPHA4,LRTM2,ACTB,UNC5A,PSEN1,SEMA4A,NGFR,NLGN3,TAOK2,TRIM46,EFNA1,NDEL1,CHRNB2,RAB11A,MAP2K1,NTN1,RPS6KA5,RNF165,DNM2,BCL11B,LHX1,SIN3A,TRPV2,LAMA3,CDK5R1,ISL1,EMB,LAMA1,DOCK7,SLC9A6,DAB1,SLITRK2,L1CAM,NTN3,ARHGAP4,MAPK8IP3,PLXNA3,BSG,TUBB3,AMIGO1,NTNG2,STXBP1,BARHL2,MIR222,DPYSL5,EFNA4,CNTN2,VASP,FOXG1,CNTF,RTN4RL1,ISL2,NOG,THY1,WNT5A,POU3F2,CHN1,ARTN,NRXN3,LYPLA2,EFNB3,ISPD,MAP1A,OLFM1	grey	VSMC_M10
GO:0007369	0.89	0.15	66	138	gastrulation	BP	1	ITGA7,FRS2,EXOC4,SRF,TXNRD1,SIX2,OSR1,DLD,CLASP2,MEGF8,WNT11,TGFBR2,ACVR2A,EOMES,MKKS,GPI,EYA1,FAM208A,TLX2,MMP15,RTF1,PAX2,LDB1,POGLUT1,PRKAR1A,DAG1,SNAI1,SOX2,LAMB3,COL7A1,FZD7,ITGB5,PHLDB1,SYF2,SETD2,WNT3,ITGA4,FN1,COL5A2,TBX19,LEO1,DVL2,BMPR1A,KDM6B,ITGA3,SMAD3,MAP2K5,GSC,LHX1,LAMA3,MAPK7,AMOT,GATA6,TWSG1,GPC3,COL5A1,FOXF1,DUSP2,LAMB1,POFUT2,NOG,WNT5A,LBX2,DUSP1,DUSP4,TGIF2	grey	VSMC_M10
GO:0032633	0.89	0.21	9	21	interleukin-4 production	BP	1	CD28,FCER1G,SCGB1A1,FOXP3,PRKCZ,SLC7A5,DDIT3,CEBPB,HAVCR2	grey	VSMC_M10
GO:0032673	0.89	0.21	9	21	regulation of interleukin-4 production	BP	1	CD28,FCER1G,SCGB1A1,FOXP3,PRKCZ,SLC7A5,DDIT3,CEBPB,HAVCR2	grey	VSMC_M10
GO:0006690	0.89	0.17	29	62	icosanoid metabolic process	BP	1	LTC4S,AKR1B1,ABCC10,MGST2,FAAH,ACOX1,PIBF1,ABHD6,HPGD,PLA2G4B,CYP2U1,MAPKAPK2,PLA2G4A,GGT2,GGT6,DAGLB,EPHX1,AC005520.1,ALOXE3,MAPK3,CD74,CYP2J2,PTGES2,CES2,CYP4F22,FABP5,DPEP2,GGT5,PTGES	grey	VSMC_M10
GO:0031330	0.89	0.14	102	207	negative regulation of cellular catabolic process	BP	1	TRIM39,PABPC1,PHAX,RNF5,TSPO,MAPK14,NRBP2,RGP1,PDCL3,LRPPRC,MET,SDCBP,TRAF3IP2,MTMR2,DHX34,APOC1,KDM4A,AQP11,MYD88,TIMP4,KLHL40,USP36,SIRT2,FEZ2,DDRGK1,RYBP,PKP3,PSMF1,TAF9,MTMR9,USP25,CSNK2A2,FAM192A,TENT2,MARCH7,TENT5C,TENT4B,RBM24,LZTS1,SUFU,GPLD1,RPL23,SEC22B,TMEM39A,USP30,AXIN2,MAPKAPK2,USP19,SORL1,MCL1,TRAF5,ANGEL2,RUBCN,IL10RA,EIF4ENIF1,RPL11,LRIG2,YBX3,TAB3,TBC1D14,SLC11A1,RBM10,PINK1,STAT3,ATP13A2,EPHA4,PHF23,ANXA2,TARDBP,PSEN1,EFNA1,TAF1,OGT,TOB1,TENT5D,NRDE2,LARP1,NPC1,SMCR8,LEPR,PARN,CLEC16A,FMR1,MTM1,PAM16,METTL16,SGTA,CIRBP,TRAF2,PARK7,TSC1,DAB2IP,MIR199A1,MIR21,TIGAR,CAMLG,CPTP,YBX1,STYX,TENT5A,MAP1A,NMNAT1	grey	VSMC_M10
GO:0048588	0.89	0.14	95	195	developmental cell growth	BP	1	NEDD4L,LRP1,SEMA6A,RAB21,RIMS2,PRKN,PPARA,GDF9,FSTL4,PLXNA4,CPNE5,PI16,SRF,SEMA4D,SEMA4C,RIMS1,RTN4,RUFY3,EPHA7,NRCAM,WASF1,PLXNA1,SOX9,GOLGA4,MEGF8,CPNE9,TGFBR2,SYT1,SLC23A2,SEMA3D,SPART,SEMA3A,ULK1,RYK,TTL,SEMA4F,KMT2D,DPYSL2,EIF2AK4,TIAM1,SLIT1,NKX6-1,ZFYVE27,SEMA3G,SPP1,CYFIP1,ALCAM,SEMA4G,BDNF,KIAA0319,SEMA3F,AUTS2,ADNP,DISC1,ARIH2,AGT,CDKL5,WNT3,SPG11,MAPT,SEMA6D,ITGA4,TNR,FN1,NIN,FLRT1,PPP3CB,MUL1,VCL,SEMA4A,NLGN3,TRIM46,NDEL1,RAB11A,NTN1,DNM2,YY1,SIN3A,TRPV2,CDK5R1,SLC9A6,L1CAM,PRKCZ,ARHGAP4,PLXNA3,BARHL2,MIR199A1,MIR23A,MIR24-1,IMPACT,WNT5A,CACNG7,RGS4,LLPH,OLFM1	grey	VSMC_M10
GO:0000154	0.89	0.19	14	31	rRNA modification	BP	1	TFB1M,DIMT1,NSUN3,RPUSD2,RPUSD4,GAR1,NSUN5,MRM2,ZCCHC4,MRM3,METTL16,RPUSD1,RPUSD3,TRMT61B	grey	VSMC_M10
GO:0070228	0.89	0.18	17	38	regulation of lymphocyte apoptotic process	BP	1	SLC46A2,IDO1,FOXP1,ZC3H8,GPAM,PDCD1,ADAM8,BCL6,EFNA1,BTK,CD74,CCL5,IL7R,BCL10,FADD,WNT5A,TSC22D3	grey	VSMC_M10
GO:0022408	0.89	0.15	63	130	negative regulation of cell-cell adhesion	BP	1	PPARA,PTK2,PODXL,TNFRSF21,EPCAM,PELI1,AKNA,IL4I1,FOXJ1,C1QTNF1,JAG1,PAWR,SPINT2,PRNP,NCKAP1L,ZC3H8,CASP3,TMEM131L,PAG1,MARCH7,ADTRP,TRPV4,BTN2A2,PRKAR1A,HLX,YTHDF2,UBASH3B,RUNX3,DLG1,MIA3,BCL6,ABCA12,TNR,VSIR,RC3H1,PTPN6,TNFSF18,LAG3,SCGB1A1,FOXP3,TBX21,ZBTB7B,IL4R,MAP2K5,CD74,MAPK7,CCL25,TWSG1,PTPN2,SOCS1,ABAT,ASS1,GLMN,MIR21,MIR222,MIR27A,MIR125A,FGL2,RGCC,LAX1,CEBPB,HAVCR2,RC3H2	grey	VSMC_M10
GO:0140112	0.89	0.21	9	21	extracellular vesicle biogenesis	BP	1	VPS4B,PRKN,CHMP2A,SDCBP,RAB7A,ATP13A2,RAB11A,ARRDC1,VPS4A	grey	VSMC_M10

GO:0030900	0.89	0.13	139	282	forebrain development	BP	1	NME1,LRP1,GLI1,ATAT1,FRS2,UQCRQ,PITX1,AGTPBP1,PLXNA4,SUN2,NTRK2,SMO,SRF,CDK6,DLX5,EFHC1,MBOAT7,RTN4,AQP1,AKNA,PTN,FOXP2,POU3F3,SLC7A11,FAT4,NDNF,EIF2B3,XRCC1,GLI2,SCN5A,NUMBL,POU4F1,TSKU,SZT2,ALK,HOOK3,MKKS,RARB,PLCB1,RAC3,SEMA3A,NRG1,RYK,TOX,ZSWIM6,CASP3,DCLK2,ROBO1,ROBO2,HTRA2,CELF1,CSNK2A2,MCPH1,RAPGEF2,SLIT1,TENT2,NR4A2,SLC38A2,RPGRIP1L,SALL1,DNAH5,ZNF335,DPCD,SRGAP2C,SLC4A10,SRD5A1,SLC6A3,HDAC1,ALDH1A3,PGAP1,SATB2,KRAS,TTC21B,SOX2,DISC1,GBX2,ATP1A2,DLX1,EPHB3,NSUN5,SETD2,CREB1,BCL2A1,DIXDC1,TNR,NIN,WNT4,COL3A1,TBX19,KCNA1,HAP1,BMPR1A,DNAJB1,MPP5,RBPJ,ALDH1A2,ATP1B2,PSEN1,KDM6B,ITGAM,NDEL1,CHRNB2,GSC,BCL11B,LHX1,SIN3A,XAB2,MYO1D,CDK5R1,ISL1,TWSG1,DOCK7,DAB1,LRP8,CRK,SKI,CHD5,PLXNA3,TSC1,PHLPP2,DAB2IP,LHX8,RHOA,UBB,ADGRG1,CNTN2,FOXG1,LAMB1,METTL14,RTN4RL1,GNG12,CCDC85C,NOG,WNT5A,POU3F2,OTX1,CRH,BTBD3,TAL2	grey	VSMC_M10
GO:0000338	0.89	0.27	4	10	protein deneddylation	BP	1	GPS1,COPS7B,COPS7A,COPS8	grey	VSMC_M10
GO:0033119	0.89	0.22	8	19	negative regulation of RNA splicing	BP	1	PTBP3,SRSF10,TMBIM6,SRSF6,SRSF4,RBM10,SRSF12,SAP18	grey	VSMC_M10
GO:1900242	0.89	0.23	7	17	regulation of synaptic vesicle endocytosis	BP	1	BTBD9,DNM1L,AP2M1,PPP3CB,DGKQ,SYT11,CALM1	grey	VSMC_M10
GO:0009261	0.89	0.18	18	40	ribonucleotide catabolic process	BP	1	PDE7B,DPYS,PDE10A,GDA,NT5E,NUDT16,AMPD3,ENTPD4,FITM2,NUDT18,PDE9A,PDE8A,UPB1,PDE4A,MLYCD,NUDT19,HINT1,UPP1	grey	VSMC_M10
GO:0003209	0.89	0.20	11	26	cardiac atrium morphogenesis	BP	1	SMO,TBX5,TGFB2,ISL1,ENG,SOX4,NOG,WNT5A,NKX2-5,HEY2,WNT2	grey	VSMC_M10
GO:0042451	0.89	0.27	4	10	purine nucleoside biosynthetic process	BP	1	MTAP,NT5E,PRTFDC1,ADK	grey	VSMC_M10
GO:0042455	0.89	0.27	4	10	ribonucleoside biosynthetic process	BP	1	MTAP,NT5E,PRTFDC1,ADK	grey	VSMC_M10
GO:0046129	0.89	0.27	4	10	purine ribonucleoside biosynthetic process	BP	1	MTAP,NT5E,PRTFDC1,ADK	grey	VSMC_M10
GO:0006396	0.89	0.12	500	950	RNA processing	BP	1	ZC3H10,PAN2,RNMT,CLK4,ADAT2,HELB,RPP21,CPSF6,ESR1,ZFC3H1,PABPC1,SNW1,TFB1M,SF3A3,QKI,TRMU,UTP23,RRP7A,CSDC2,TRMT11,AGO2,HABP4,ZC3H3,TUT7,PUF60,C9orf64,CMTR1,EXOSC4,HSF1,CPSF1,USP49,RBM28,RBFOX2,SMU1,NOL6,TSR1,EXOSC3,RPS14,RNF20,MTO1,TSEN34,PRPF31,PPP2R1A,TRA2A,ELP1,YTHDC1,PNPT1,UTP3,PUS10,PTBP3,TRIT1,RNGTT,SRSF10,MDN1,PRPF4,SNIP1,SNRNP27,LSM10,AGO1,EGFR,FCF1,SCAF1,MYCN,TUT4,PRPF38A,PUM2,DUS4L,MTPAP,SYMPK,NSUN6,IWS1,ERCC2,CLASRP,PCF11,FASTK,RPS8,WBP4,DEDD2,CLNS1A,CELF2,KIN,RPL14,RBM26,CRNKL1,PRX,INTS10,THUMPD3,USP36,ESF1,KRR1,IK,NUP98,DPH3,SIRT7,ZMAT2,CWC15,TRMT6,TRNT1,LSM1,NUDT16,WTAP,PPP4R2,THUMPD2,RBPMS,PHRF1,PRKDC,PAN3,TRMT9B,THOC7,DHX37,TGS1,BMS1,DIMT1,CSTF3,SREK1IP1,GPATCH1,ELP4,SREK1,PIK3R1,POP4,USP39,PRMT9,CLP1,ZCCHC8,GCFC2,ELP3,RBM39,M1AP,BCDIN3D,TMBIM6,AAR2,CELF1,USB1,CNOT6L,TPRKB,RPRD1B,SCAF4,URB1,DDX54,NSUN3,RRP12,DHX35,TENT2,HELQ,RBM12B,RPUSD2,SLC38A2,SRSF6,SCAF11,RIPK1,OSGEPL1,PRPF4B,INTS8,RPP40,BCAS2,RIOK1,ATXN1,TENT4B,RBM24,CDKAL1,POP5,PDCD11,RPUSD4,TRMT10A,ZBTB8OS,PPP3CA,RPS17,SMNDC1,TERT,QTRT2,SBDS,RBM5,TYW1B,INTS12,CPSF2,HEATR1,TYW5,FAM207A,ADARB1,SART3,ACIN1,MRPS11,GAR1,HSPA8,SRSF4,TRUB1,YTHDF2,RTRAF,RPRD2,CSTF1,DALRD3,YBEY,LCMT2,ZRSR2,NOP9,RPS27,ZMAT5,FASTKD1,PPIG,WDR12,METTL8,CELF3,RPL7L1,SYF2,WBP11,NCBP2,ELP6,NSUN5,DDX47,RPL11,METTL2B,KIAA0391,BUD13,MFAP1,RBM7,TRMT1L,IVNS1ABP,NONO,SUPV3L1,PRKRA,GEMIN2,SNRPD3,PRP[illegible]	grey	VSMC_M10
GO:0002440	0.89	0.14	67	138	production of molecular mediator of immune response	BP	1	CD226,HLA-DRA,HLA-DOB,HLA-DMA,IL5,HLA-DPB1,KIT,EXOSC3,MSH2,CYREN,RSAD2,TLR4,ATG5,TRPM4,TRAF3IP2,SPHK2,HMCES,PHB,ERCC1,LACC1,MLH1,PAXIP1,SHLD1,MAVS,HPX,PRKDC,TRAF6,GAPT,GPI,TMBIM6,XRCC4,NBN,F2RL1,NOD2,POLL,CD40,CD96,TGFB2,DENND1B,CD28,RNF168,FZD5,BCL6,HK1,PMS2,FOXP3,SHLD2,TNFRSF1B,SPON2,TBX21,STX4,BATF,IL4R,BTK,YY1,CD74,SUPT6H,TICAM1,PRKCZ,TRAF2,SLC7A5,BCL10,WNT5A,TRDV3,INAVA,RNF8,NHEJ1	grey	VSMC_M10
GO:0071276	0.89	0.21	9	21	cellular response to cadmium ion	BP	1	HSF1,EGFR,NCF1,ABCB6,MAPK1,FOS,MAPK3,JUN,MT1X	grey	VSMC_M10
GO:0044766	0.89	0.23	7	17	multi-organism transport	BP	1	EPS15,RAB7A,KPNA3,KPNA2,TPCN1,PIKFYVE,FMR1	grey	VSMC_M10
GO:1902579	0.89	0.23	7	17	multi-organism localization	BP	1	EPS15,RAB7A,KPNA3,KPNA2,TPCN1,PIKFYVE,FMR1	grey	VSMC_M10
GO:0051767	0.89	0.23	7	17	nitric-oxide synthase biosynthetic process	BP	1	PGGT1B,TLR4,NOD2,MAP2K6,CCL2,FCER2,IL33	grey	VSMC_M10
GO:0051769	0.89	0.23	7	17	regulation of nitric-oxide synthase biosynthetic process	BP	1	PGGT1B,TLR4,NOD2,MAP2K6,CCL2,FCER2,IL33	grey	VSMC_M10
GO:0010508	0.89	0.15	50	105	positive regulation of autophagy	BP	1	PIP4K2C,EPM2A,PRKN,TFEB,FBXO7,SPTLC1,NOD1,SCOC,FOXO3,SESN3,PIP4K2A,PLK3,OPTN,RALB,TMEM173,DCN,ULK1,PLK2,PIK3CB,RIPK2,TPCN1,TP53INP1,NOD2,DHRSX,ATG16L1,SH3BP4,BAG3,HTT,PINK1,PIM2,VPS13D,MAPK3,LARP1,SMCR8,TICAM1,IKBKG,PARK7,GPSM1,GNAI3,KIAA1324,TSC1,SH3GLB1,TRIM68,TRIM34,BNIP3L,TRIM65,IRGM,BCL2L11,HSPB8,RNF152	grey	VSMC_M10
GO:0010453	0.89	0.23	7	17	regulation of cell fate commitment	BP	1	SOSTDC1,WNT11,FZD7,TNFSF18,BMPR1A,TBX21,WNT5A	grey	VSMC_M10
GO:0045939	0.89	0.21	9	21	negative regulation of steroid metabolic process	BP	1	CYP27B1,DKKL1,DKK3,ERLIN1,SNAI1,SCAP,WNT4,GFI1,INSIG2	grey	VSMC_M10
GO:0032069	0.89	0.23	7	17	regulation of nuclease activity	BP	1	TMBIM6,OAS3,OASL,DDX11,NEIL1,TERF2,OAS1	grey	VSMC_M10
GO:1901658	0.89	0.20	10	23	glycosyl compound catabolic process	BP	1	FUCA2,APOBEC3H,GDA,ENPP4,CDADC1,AHCY,UPB1,GLA,ABHD10,UPP1	grey	VSMC_M10
GO:0061158	0.89	0.23	6	15	3'-UTR-mediated mRNA destabilization	BP	1	RBM24,RC3H1,UPF1,TARDBP,PLEKHN1,ZFP36L2	grey	VSMC_M10
GO:0000731	0.89	0.20	11	26	DNA synthesis involved in DNA repair	BP	1	POLI,REV1,REV3L,WRN,PRIMPOL,WRNIP1,SYCP1,SPRTN,TEX12,POLN,ZBTB1	grey	VSMC_M10
GO:0044550	0.89	0.22	7	17	secondary metabolite biosynthetic process	BP	1	SLC7A11,RAPGEF2,OCA2,BDH2,CITED1,SLC45A2,WNT5A	grey	VSMC_M10

GO:0035590	0.89	0.22	8	19	purinergic nucleotide receptor signaling pathway	BP	1	P2RX2,P2RX3,P2RX7,P2RY1,ANO6,ADORA2B,P2RX1,CNTN2	grey	VSMC_M10
GO:1903037	0.89	0.13	102	206	regulation of leukocyte cell-cell adhesion	BP	1	AGER,PPARA,HLA-DRA,HLA-DOB,HLA-DMA,HLA-DPB1,TNFRSF13C,ZDHHC21,TNFRSF21,NCK2,PELI1,IL4I1,IL15,BMI1,GLI2,FOXJ1,TGFBR2,PAWR,PRNP,NCKAP1L,SLC7A1,TRAF6,SOX12,ZC3H8,IL1A,CASP3,TMEM131L,IL7,PAG1,RASGRP1,RIPK2,AP3B1,MARCH7,SOX13,ADTRP,NOD2,IGF2,LCK,ICOSLG,BTN2A2,GPAM,PRKAR1A,HLX,CD28,RUNX3,DLG1,ADAM8,MIA3,DUSP10,BCL6,ITGA4,IL12A,VSIR,RC3H1,PTPN6,TNFSF18,LAG3,STAT5B,SCGB1A1,FOXP3,RASAL3,ZBTB1,CARD11,TBX21,ZBTB7B,IL4R,CD74,CCL5,CCL2,CCL25,YES1,NKAP,VAV1,TWSG1,IL7R,PTPN2,SOCS1,DNAJA3,PRKCZ,ASS1,GLMN,BCL10,MIR21,MIR222,MIR27A,RHOA,SOX4,FADD,DPP4,CD46,FUT4,MIR125A,THY1,FGL2,CD70,TNFSF9,LAX1,EFNB3,CEBPB,TNFSF13B,HAVCR2,RC3H2	grey	VSMC_M10
GO:1905209	0.89	0.23	6	15	positive regulation of cardiocyte differentiation	BP	1	MEF2C,TBX5,TGFB2,GPER1,GATA6,NKX2-5	grey	VSMC_M10
GO:1904994	0.89	0.21	9	21	regulation of leukocyte adhesion to vascular endothelial cell	BP	1	ZDHHC21,TRAF6,ITGA4,CCL25,MIR21,MIR222,RHOA,FUT4,MIR125A	grey	VSMC_M10
GO:0048259	0.89	0.15	40	85	regulation of receptor-mediated endocytosis	BP	1	RAB21,PICK1,USP46,SMAP1,PPT1,RSPO1,AAK1,SFRP4,SDCBP,MTMR2,APOC1,ARRB1,NEU3,PIK3CB,HIP1R,WDR54,BMP2K,SYNJ1,ANKRD13A,EGF,ANKRD13D,DGKD,CBL,AP2M1,SH3GL3,LRPAP1,DLG4,ANXA2,SYT11,DNM2,INSR,SGIP1,C3,UNC119,FMR1,MIR199A1,MIR27B,MIR27A,LDLRAP1,LRRTM2	grey	VSMC_M10
GO:0001654	0.89	0.13	138	280	eye development	BP	1	SLC39A5,MDM1,FRS2,OSR2,TBC1D32,BAK1,ATF4,AGTPBP1,NTRK2,PRPH2,RORB,SRF,SLC1A1,TFAP2B,TDRD7,TMOD1,RDH13,ABCB5,PTN,TSPAN12,USP45,SLC7A11,SPRY1,FOXE3,HDAC2,BBS7,SOX9,CRYGN,ZNF513,ACVR2B,IFT172,MYO7A,TSKU,SPRY2,DZANK1,TGFBR2,BFSP1,JAG1,RARB,FSCN2,SPRED3,FREM2,CEP290,KERA,MAB21L1,MITF,FLT1,TUB,EPHB1,PFDN5,NPHP1,RET,FOXL2,MAB21L2,TENM3,USH1C,TENT2,OPA1,RPGRIP1L,TFAP2A,PAX2,NKD1,BMPR1B,MYH15,NECTIN3,LPCAT1,PKNOX1,SLC6A3,HDAC1,ALDH1A3,TGFB2,TCIRG1,PRSS56,SHROOM2,SOX2,CRYBA4,NRL,CLIC4,DLX1,GRHL3,LIMK2,SP3,CDKN1B,DLG1,FZD5,FBN1,FKBP8,COL5A2,PLA2G16,STAT3,TULP3,OBSL1,RHOJ,ROM1,MFAP2,WDR19,SDK1,MAX,CLN8,MED1,ALDH1A2,MFN2,PSEN1,TTLL5,MEGF11,LCTL,SMAD3,TBX2,BCL11B,LHX1,STRA6,YY1,CHRDL1,UNC45B,LAMA1,TWSG1,GNB1,FZR1,SKI,SOX8,COL5A1,LAMC3,OLFM3,TMEM231,SLC25A25,HSF4,BLOC1S3,FJX1,CNTF,ACVRL1,THY1,WNT5A,DIO3,TGIF2,TBC1D20,PITX3,WNT2,COL8A2	grey	VSMC_M10
GO:0046638	0.89	0.18	15	34	positive regulation of alpha-beta T cell differentiation	BP	1	HLA-DRA,TGFBR2,NCKAP1L,RIPK2,AP3B1,HLX,RUNX3,FOXP3,ZBTB7B,IL4R,NKAP,SOCS1,PRKCZ,MIR21,RHOA	grey	VSMC_M10
GO:0034764	0.89	0.14	75	155	positive regulation of transmembrane transport	BP	1	STAC3,MAPK14,ABCB1,WNK2,FGF21,BRAF,CACNB2,STAC,AKT2,PIK3R1,GLRX,SORBS1,P2RX7,KCNJ11,IFNGR2,F2R,TESC,ANO6,KCNE2,PKD2,KCNIP2,RYR2,FAM173B,AZIN2,IRS1,KCNJ2,TERT,ACSL5,COX17,ANK2,C2CD5,GPER1,AGT,ACE2,SLC9A1,CASQ1,NTSR1,NOS1AP,NFE2L2,CEMIP,HTT,CLTCL1,PINK1,ATP1B1,KCNA1,ARL6IP1,HAP1,WNK3,STAC2,ATP1B2,PSEN1,NLGN3,ATP2A1,CD19,CALM1,DNM2,VMP1,BDKRB1,INSR,CCL2,C3,GPC3,PDPK1,P2RX1,RNF207,AMIGO1,LCN2,MIR21,DMD,JPH2,CA2,KCNH2,THY1,APOPT1,ARPP19	grey	VSMC_M10
GO:0060603	0.89	0.19	11	26	mammary gland duct morphogenesis	BP	1	ESR1,PTCH1,SOSTDC1,AREG,GLI2,VDR,WNT4,MED1,NTN1,CSF1R,WNT5A	grey	VSMC_M10
GO:1904893	0.90	0.20	10	23	negative regulation of receptor signaling pathway via STAT	BP	1	PPP2R1A,PIBF1,PARP14,SUZ12,PTPN2,SOCS1,DAB1,MIR125B1,MIR125A,LEPROT	grey	VSMC_M10
GO:0071326	0.90	0.14	54	113	cellular response to monosaccharide stimulus	BP	1	ZNF236,NME1,LRP1,AGER,PLA2G6,SRF,PRKCE,HMGN3,TRPM4,FOXO3,FGF21,GRIK5,RAF1,GAS6,NR1H4,CYP7A1,NDUFAF2,OSBP,KCNJ11,TIAM1,RAB11FIP5,OPA1,ZBED6,PAX2,TWNK,GPLD1,PPP3CA,SLC9B2,ZBTB20,ADRA2A,KCNB1,POLG,GPER1,RAB11FIP2,MLXIPL,SIDT2,PDK3,KLF7,FIS1,MEN1,ENY2,ABCA12,PPP3CB,PTPRN,MPC2,STX4,GPR68,SIN3A,OXCT1,NADK,BAIAP3,CYBA,SOX4,CRH	grey	VSMC_M10
GO:0090051	0.90	0.22	7	17	negative regulation of cell migration involved in sprouting angiogenesis	BP	1	MEOX2,ITGB1BP1,PDCD10,MAP2K5,MIR199A1,MIR22,RHOA	grey	VSMC_M10
GO:0070262	0.90	0.23	6	15	peptidyl-serine dephosphorylation	BP	1	PPP2CB,PPP3CA,PPP2R2D,DUSP26,DUSP1,PPP1R15A	grey	VSMC_M10
GO:0046849	0.90	0.15	32	69	bone remodeling	BP	1	TNFAIP3,TNFRSF11B,RAC2,PTN,EGFR,NCDN,MITF,TRAF6,IL7,P2RX7,PTK2B,SPP1,LRRK1,TCIRG1,GPR137B,UBASH3B,ADAM8,PTH1R,GPR137,SUCO,CD38,P3H4,SYT7,RAB3D,CSF1R,PPARGC1B,LEPR,ATP6AP1,DEF8,CA2,BGLAP,SNX10	grey	VSMC_M10
GO:0034394	0.90	0.16	27	59	protein localization to cell surface	BP	1	NEDD4L,PRRT1,GOPC,WNT11,GPD1L,TM9SF4,PIGU,RAB11FIP5,AP3B1,GBF1,BDNF,FBLN5,EGF,ANK2,LRIG2,ABCA12,VCL,STX4,RAB11A,GGA2,TAX1BP3,ABCA7,CD247,TMEM35A,LEPROT,MESD,MAP1A	grey	VSMC_M10
GO:0071320	0.90	0.18	16	37	cellular response to cAMP	BP	1	AQP1,AANAT,GPD1,RAPGEF2,STC1,RAPGEF3,PKD2,DMTN,SRD5A1,RAP1B,CPS1,FDX1,INPP5K,ASS1,HSPA5,HCN4	grey	VSMC_M10
GO:0048565	0.90	0.15	43	92	digestive tract development	BP	1	GLI1,VPS52,SOX10,SMO,KIT,SIX2,CXCL8,EGFR,BBS7,SLC4A2,GLI2,MEGF8,KLF5,IFT172,WNT11,TGFBR2,OVOL2,CHD8,RARB,CCKBR,RET,CBFA2T2,SALL1,TGFB2,HLX,EPHB3,CPS1,PYY,SAV1,NKX3-2,ALDH1A2,RBPMS2,SMAD3,TBX2,STRA6,GATA6,DAB1,PKD1,FOXL1,FOXF1,ASS1,WNT5A,TIGAR	grey	VSMC_M10
GO:0045840	0.90	0.18	14	32	positive regulation of mitotic nuclear division	BP	1	PHIP,HOXA13,EPGN,TGFA,IL1A,ANAPC5,ANAPC7,IGF2,EGF,CD28,ANAPC4,INSR,RGCC,EREG	grey	VSMC_M10
GO:0033146	0.90	0.18	14	32	regulation of intracellular estrogen receptor signaling pathway	BP	1	CNOT2,FOXH1,DDRGK1,CYP7B1,UFSP2,ZNF366,CNOT1,KMT2D,VPS18,DNAAF4,PARP1,MED1,PAGR1,ISL1	grey	VSMC_M10
GO:0046337	0.90	0.20	9	21	phosphatidylethanolamine metabolic process	BP	1	PLA2G6,NAPEPLD,SELENOI,CHKA,ABHD4,PISD,PLA2G16,SLC27A1,PLA2G15	grey	VSMC_M10

GO:0045070	0.90	0.19	12	28	positive regulation of viral genome replication	BP	1	PABPC1,PPARA,TMEM39A,ADARB1,NR5A2,STAU1,CD28,DDX3X,DDB1,HACD3,LARP1,CCL5	grey	VSMC_M10
GO:0060338	0.90	0.18	15	34	regulation of type I interferon-mediated signaling pathway	BP	1	MAVS,IRF7,OAS3,NLRC5,YTHDF2,PTPN6,MUL1,USP18,DCST1,STAT2,PTPN2,MIR21,OAS1,FADD,WNT5A	grey	VSMC_M10
GO:0046902	0.90	0.16	23	50	regulation of mitochondrial membrane permeability	BP	1	HEBP2,BAK1,TMEM14A,HIP1R,SLC9A1,THEM4,STPG1,FZD9,NMT1,MUL1,STAT3,TMEM102,CAMK2A,ACAA2,NAIF1,MIR29A,NOL3,BNIP3L,BCL2L11,SLC35F6,BCL2L2,PPIF,AC140504.1	grey	VSMC_M10
GO:0050995	0.90	0.23	6	15	negative regulation of lipid catabolic process	BP	1	APOC1,ALK,GPLD1,ADRA2A,SORL1,BSCL2	grey	VSMC_M10
GO:0006900	0.90	0.16	25	55	vesicle budding from membrane	BP	1	PRKN,MAPK15,RAB7A,CHMP4C,ARFGAP2,SEC31A,AP3B1,CHMP7,SEC31B,GBF1,KLHL12,PEF1,PDCD6,AP2M1,SCAP,MIA3,SEC24C,SLC2A4,ANXA2,TMED10,PPP6C,RILP,FNBP1L,VPS4A,TBC1D20	grey	VSMC_M10
GO:2000242	0.90	0.17	19	42	negative regulation of reproductive process	BP	1	FBXO43,MYH9,NLRP5,PLAT,MOS,NKX3-1,ACVR1C,HYAL3,PRKAR1A,SNAI1,CDKN1B,OVOL1,OVGP1,WNT4,ZP2,WDR77,WNT5A,DUSP1,LIF	grey	VSMC_M10
GO:0071763	0.90	0.18	14	32	nuclear membrane organization	BP	1	NEMP1,CHMP2A,LPIN1,SIRT2,NSFL1C,CHMP7,GPER1,TOR1AIP1,AKAP8L,TARDBP,NDEL1,PRKCB,TOR1B,VPS4A	grey	VSMC_M10
GO:0032104	0.90	0.23	6	15	regulation of response to extracellular stimulus	BP	1	CYP27B1,SNW1,PPARA,MKKS,MED1,TRIM24	grey	VSMC_M10
GO:0032107	0.90	0.23	6	15	regulation of response to nutrient levels	BP	1	CYP27B1,SNW1,PPARA,MKKS,MED1,TRIM24	grey	VSMC_M10
GO:0098727	0.90	0.14	51	108	maintenance of cell number	BP	1	CNOT2,ZNF706,MED30,MYC,FANCC,SMO,KIT,SIX2,PTN,TUT4,FOXO3,SOX9,RAF1,HOOK3,JAG1,LSM1,TCF15,PAX8,CNOT1,SETD6,MCPH1,LOXL2,RTF1,PAX2,LDB1,DIS3L2,SOX2,FZD7,DDX6,VPS72,EIF4ENIF1,TET1,MED14,SAV1,STAT3,LEO1,BMPR1A,RBPJ,MED6,ESRRB,MED7,SKI,MED27,MTF2,SOX4,METTL14,NOG,ZNF322,LIF,ZFP36L2,MED21	grey	VSMC_M10
GO:0097734	0.90	0.21	8	19	extracellular exosome biogenesis	BP	1	VPS4B,PRKN,CHMP2A,SDCBP,RAB7A,ATP13A2,RAB11A,VPS4A	grey	VSMC_M10
GO:0030490	0.90	0.17	21	46	maturation of SSU-rRNA	BP	1	HELB,UTP23,TSR1,RPS14,UTP3,ERCC2,RPS8,DHX37,BMS1,RPP40,RIOK1,HEATR1,FAM207A,MRPS11,NOP9,TSR2,KRI1,RIOK3,UTP6,SNU13,GTF2H5	grey	VSMC_M10
GO:0002115	0.90	0.23	6	15	store-operated calcium entry	BP	1	EFHB,ORAI1,SLC8B1,HOMER1,CASQ1,HOMER2	grey	VSMC_M10
GO:0010888	0.90	0.22	7	17	negative regulation of lipid storage	BP	1	PPARA,TTC39B,NR1H2,ABHD5,ASXL1,NR1H3,PTPN2	grey	VSMC_M10
GO:0045039	0.90	0.26	4	10	protein insertion into mitochondrial inner membrane	BP	1	AGK,TIMM29,TIMM10B,TIMM8B	grey	VSMC_M10
GO:1903522	0.90	0.13	87	179	regulation of blood circulation	BP	1	AGER,ZDHHC21,SLC1A1,TNNI3,EPAS1,ASB3,EGFR,BVES,TRPM4,SLC8A2,CACNB2,ATP1A3,CELF2,SCN5A,GPD1L,HBEGF,ADRA1D,SEMA3A,ADRA2B,DOCK5,STC1,KCNJ3,TBX5,F2R,KCNE2,DSP,CASQ2,KCNIP2,CHRM3,RYR2,PKP2,KCNJ2,TGFB2,ADRA2A,MYH7,GRK2,ANK2,AGT,ACE2,ATP1A2,SLC9A1,PER2,HDAC4,SCN4B,NOS1AP,DLG1,ADRA2C,PTGER2,ECE1,GCH1,ATP1B1,SMTNL2,CD38,DES,SLC4A3,THRA,ATP1B2,HRH2,CACNA1G,ATP2A1,CALM1,TBX2,DSG2,ASIC2,SREBF1,ISL1,CYP2J2,ATP2B3,TRPV1,P2RX1,RNF207,CACNA1H,MIR21,RHOA,FGA,DMD,CHGA,TMEM65,KCNH2,SCN2B,RGS4,POPDC2,HCN4,NKX2-5,KCNMB4,HEY2,KCNG2	grey	VSMC_M10
GO:0051123	0.90	0.21	8	19	RNA polymerase II preinitiation complex assembly	BP	1	ESR1,TAF7,TAF9,CREB1,TAF6,THRA,TAF1,TAF12	grey	VSMC_M10
GO:0070252	0.90	0.15	32	70	actin-mediated cell contraction	BP	1	NEDD4L,TRPM4,CACNB2,SCN5A,SCN1A,GPD1L,PARVA,STC1,KCNJ3,TNNC1,KCNE2,DSP,RYR2,PKP2,KCNJ2,MYH7,ANK2,ATP1A2,ACTC1,SLC9A1,SCN4B,NOS1AP,DLG1,CACNA1G,ATP2A1,CCDC88C,DSG2,RNF207,KCNH2,SCN2B,KCNJ8,HCN4	grey	VSMC_M10
GO:0044272	0.90	0.14	61	127	sulfur compound biosynthetic process	BP	1	PPT2,ACSL6,MTAP,SLC35B2,SLC1A1,PPCS,CHAC2,PPT1,MGST2,SLC7A11,DLD,AKR1A1,CHPF2,ELOVL1,CSGALNACT1,PDK1,PDHX,APIP,CSAD,ELOVL7,GSS,B3GNT4,ENOPH1,ST3GAL6,CHST1,MTRR,MTR,CBSL,ACSL5,HACD2,PDK3,NFE2L2,GGT2,ACSBG1,CHST3,CHST7,AC022400.6,GGT6,MPC2,MAT1A,LIAS,TECR,CHST12,SLC26A1,GCDH,GLCE,ACACA,SLC26A2,ACSBG2,METTL16,B3GALT6,ACSF3,MLYCD,CHST5,ST3GAL2,MIR21,TPK1,ELOVL3,MTHFD2L,GGT5,CHSY3	grey	VSMC_M10
GO:1903589	0.90	0.26	4	10	positive regulation of blood vessel endothelial cell proliferation involved in sprouting angiogenesis	BP	1	MIR21,MIR23A,MIR27B,MIR27A	grey	VSMC_M10
GO:1901863	0.90	0.23	6	15	positive regulation of muscle tissue development	BP	1	LMOD3,MEF2C,IGF2,ACTN3,CREB1,MRTFB	grey	VSMC_M10
GO:0051896	0.90	0.14	60	125	regulation of protein kinase B signaling	BP	1	PHLPP1,STK3,PPARA,LEMD2,PTK2,NTRK2,TEK,RTN4,ITGB1BP1,MET,EGFR,SESN3,PHB,SPRY2,C1QTNF1,HBEGF,BAG4,NRG1,GAS6,MFHAS1,RET,PIK3CB,PTPRJ,GCNT2,IGF2,GRM2,BANK1,TCF7L2,PDCD6,DAG1,EGF,HCLS1,GPER1,CASS4,CIB1,OSM,THPO,CD28,DLG1,ADAM8,STOX1,GDF15,PINK1,MUL1,OTUD3,HAX1,PIK3R5,INSR,INPP5K,PARK7,ENG,MIR29A,MIR199A1,MIR21,MIR222,DDIT3,TXN,MYORG,TSPYL5,RASD2	grey	VSMC_M10
GO:0090114	0.90	0.19	12	28	COPII-coated vesicle budding	BP	1	MAPK15,SEC31A,SEC31B,KLHL12,PEF1,PDCD6,SCAP,MIA3,SEC24C,TMED10,PPP6C,TBC1D20	grey	VSMC_M10
GO:0050764	0.90	0.16	27	59	regulation of phagocytosis	BP	1	LRP1,FPR2,IL15,ATG5,CD300LF,IL15RA,CNN2,NCKAP1L,GAS6,TUB,HCK,PTPRJ,DYSF,F2RL1,ANO6,NOD2,FGR,FCER1G,SLC11A1,SYT7,SYT11,DOCK2,DNM2,CCL2,C3,ABCA7,CYBA	grey	VSMC_M10
GO:0009880	0.90	0.16	21	47	embryonic pattern specification	BP	1	FRS2,SMAD5,PTCH1,MEOX2,SEMA3A,FAM208A,RIPPLY3,PGAP1,SEMA3F,SATB2,HOXD8,FZD5,MEOX1,CITED1,SMAD3,LHX1,WDR77,OOEP,WNT5A,BASP1,C2CD3	grey	VSMC_M10

GO:0044089	0.90	0.12	221	441	positive regulation of cellular component biogenesis	BP	1	VPS4B,PAN2,ARPC3,PIP4K2C,AVIL,ATAT1,ESR1,CNOT2,CNOT6,HSPA1B,IL5,BAK1,MTSS1,WASHC5,ASAP1,MAPK15,NTRK2,BAIAP2L2,PICK1,CDC42EP1,RAC2,HSF1,TEK,LINGO2,LMOD2,SRF,TTBK1,SLAIN2,KIT,NCK2,SEMA4D,KIAA1211,CHMP2A,CLDN19,PRKCE,EPS8L1,LPAR1,AKIRIN1,PLEK,LRRN3,NR1H2,ITGB1BP1,MED25,MET,FUZ,ARHGEF5,TLR4,CDK5RAP2,EPHA1,SDCBP,MAPRE1,WASF1,FAF1,RAB7A,PIP4K2A,VSTM5,SETDB2,SOX9,ERCC1,LCP1,CLASP2,RALB,MYD88,SLAIN1,WNT11,FCHSD2,NAV3,FERMT1,BAG4,FCHSD1,NRG1,NCKAP1L,ULK1,ARFIP2,LMOD3,DPYSL3,PAN3,EPHB1,NPHP1,HCK,FER,PIK3R1,CLSTN2,HIP1R,ATR,TRABD2A,CLIP1,PLCE1,SLF1,FAM208A,FMN1,PTPRJ,P2RX7,CNOT1,CUX2,PTK2B,CNOT6L,FNIP2,SPIDR,CDH17,RAPGEF3,TBX5,AMIGO2,F2RL1,IQGAP2,CYFIP1,SLF2,PPHLN1,MAGEL2,BDNF,PXN,NEURL1,CARMIL1,HYAL1,ZMYND10,LMOD1,COL16A1,DAG1,AUTS2,AMIGO3,AJUBA,ADNP,AP1AR,AGT,WASF2,ACE2,PHLDB1,HDAC4,EPS8,EPHB3,WHAMM,CDKN1B,DLG1,CALY,CREB1,CDC42EP2,MAPT,RAP1B,TOGARAM1,PPP2R5B,DDX3X,NCKAP1,NIN,WNT4,HTT,DNM3,PINK1,MMP3,ARPC1A,WAS,STX18,ATP13A2,HAP1,LRTM2,MNS1,NLGN2,MIEN1,PLEK2,PFN3,GNL3L,SEMA4A,NLGN3,NDEL1,TENM2,SMAD3,DNM2,BCAS3,GPRASP2,BHLHB9,G3BP1,EVL,ASIC2,CDK5R1,SLITRK2,FMR1,SERPINF2,SLX4,ABCA3,MLST8,MECP2,WRAP73,PARK7,PALM,EPS8L3,DEF8,SPIRE2,AMIGO1,PSRC1,KIAA1324,PIEZO1,GPSM2,TSC1,FNBP1L,SH3GLB1,VPS4A,DAB2IP,WDR63,HAS3,ADGRL2,CARMIL2,FHOD1,RHOA,SETDB1,TRABD2B,VASP,CHGA,GRID2,EPS8L2,GMFG,THY1,CLDN1,CRBN,ZNF304,GRB2,BCL2L11,RGCC,APC,RALA,HRAS,DRG1,LRRT[illegible]	grey	VSMC_M10
GO:0035728	0.90	0.22	6	15	response to hepatocyte growth factor	BP	1	GAB1,SIRT2,CREB1,MED1,ADAMTS12,CRK	grey	VSMC_M10
GO:0002200	0.90	0.15	28	61	somatic diversification of immune receptors	BP	1	TCF7,EXOSC3,MSH2,CYREN,HMCES,ERCC1,MLH1,PAXIP1,SHLD1,PRKDC,XRCC4,NBN,POLL,CD40,DCAF1,CD28,RNF168,BCL6,PMS2,FOXP3,SHLD2,TBX21,BATF,BCL11B,YY1,SUPT6H,RNF8,NHEJ1	grey	VSMC_M10
GO:1990089	0.90	0.16	21	47	response to nerve growth factor	BP	1	ARPC3,NTRK2,MICALL1,PTN,FOXO3,NTF4,WASF1,RAPGEF2,BDNF,CIB1,CBL,CREB1,MAPT,EHD1,CSTF2,CRK,TRPV1,PDPK1,LCN2,HSPA5,DYNC1LI2	grey	VSMC_M10
GO:0072111	0.90	0.21	7	17	cell proliferation involved in kidney development	BP	1	MYC,PTCH1,OSR1,IL6R,GPC3,MIR125A,PDGFD	grey	VSMC_M10
GO:0046794	0.90	0.22	6	15	transport of virus	BP	1	EPS15,KPNA3,KPNA2,TPCN1,PIKFYVE,FMR1	grey	VSMC_M10
GO:1904036	0.90	0.17	15	34	negative regulation of epithelial cell apoptotic process	BP	1	TNFAIP3,PPARA,TEK,FGF21,NDNF,GAS6,SRSF6,TERT,TCF7L2,NFE2L2,TNIP2,RAMP2,MAPK7,PDPK1,FGA	grey	VSMC_M10
GO:0099172	0.90	0.17	16	37	presynapse organization	BP	1	FZD1,VPS35,SDCBP,C5AR1,SLITRK5,FARP1,LRFN3,NLGN4Y,BSN,NLGN2,NLGN3,SNAPIN,SLITRK2,CEL,NTNG2,WNT5A	grey	VSMC_M10
GO:0006027	0.90	0.21	7	17	glycosaminoglycan catabolic process	BP	1	CEMIP2,GLB1,HYAL1,HYAL3,CEMIP,IDUA,IDS	grey	VSMC_M10
GO:0019400	0.90	0.21	7	17	alditol metabolic process	BP	1	AKR1B1,GK5,COQ2,SORD,PLA2G4A,MOGAT3,TKFC	grey	VSMC_M10
GO:0071705	0.90	0.11	835	1609	nitrogen compound transport	BP	1	TIMM21,VPS4B,ATP8B1,LRP1,STX7,AP3S1,KIF5A,PEX7,AGAP2,PEX3,CANX,TBC1D30,TBC1D9B,PPP1R10,CPSF6,RAB21,SNX24,SNX31,PHAX,ZDHHC14,SLC12A2,RIMS2,PRKN,QKI,TAP1,SLC22A4,KIF3A,TSPO,GOPC,VPS52,HSPA4,DERL1,FAM91A1,WASHC5,FAM53C,TSNARE1,LY6E,ZC3H3,EXOC4,SIL1,SYNGR1,MAPK15,MAPK14,NTRK2,TOMM22,PLA2G6,COPG2,PICK1,MICALL1,TBC1D22B,RAC2,SMO,ZDHHC21,HSF1,TOMM6,MYH9,CHRNA3,PEX6,FBXO7,ABCB1,NOL6,XPO5,WASHC4,APBA1,PEX1,SLC35B2,VPS50,SCFD2,RFX3,SLC1A1,TGFBRAP1,TFAP2B,SLC25A13,TBC1D8,TRAM2,BICD2,CHMP2A,RAB23,SVBP,LRPPRC,SEC61B,SLC3A1,STX17,SIX2,DNAJA1,PRKCE,RIMS1,TMEM30A,SPTBN1,IGF2BP3,HMGN3,YTHDC1,PNPT1,RUFY3,CYB5R4,PPT1,SNX14,XPO1,SNX30,SLC35A1,BMP8A,AQP1,EHBP1,AFTPH,SLC1A4,PLEK,RSAD2,NR1H2,ITGB1BP1,DDC,YWHAZ,SEC61G,EGFR,FUZ,ARHGEF5,TLR4,TCAF2,VPS41,TBC1D9,VPS35,SDCBP,MGARP,TRPM4,SLC7A11,PGRMC2,LIN7B,EPS15,APOB,AGK,BCAP29,TRAF3IP2,ANKRD50,TTC26,DNAJC27,NAPA,BBS7,RAB7A,AP2S1,RAB10,COPG1,KPNA3,GIPR,ARL5B,IWS1,LCP1,SYTL2,COG1,DLG2,CUBN,RBSN,DNAJC15,PLK3,FRMD4A,OPTN,GOLGA4,LLGL2,IPO13,STEAP3,AQP11,CCDC93,RAF1,ZFAND6,SLC22A13,TBC1D4,MYO7A,SLC25A38,NAPB,GCKR,SEC14L1,SEC62,SEC22C,KPNA2,AKT2,PITRM1,LRP1B,HOOK3,ARRB1,ABCC4,TIMM50,TGFBR2,SNX5,USP36,TBC1D16,AP3M2,KIF5C,FCHSD2,RINL,ATG16L2,SLC38A10,NPLOC4,ARL5A,SYT1,UBAC2,RAB5A,LIN7A,APBB3,ASPSCR1,TMCO6,NUP98,DPH3,SIRT7,FERMT1,CHTOP,SLC6A15,HACL1,SLC25A15,HEATR5B,CEP290,PRNP,ING1,BAG4,TAF7,MAVS,AP5S1,DNAJC13,AIMP1,FUT1[illegible]	grey	VSMC_M10
GO:0042339	0.90	0.22	6	15	keratan sulfate metabolic process	BP	1	GLB1,B3GNT4,ST3GAL6,CHST1,CHST5,ST3GAL2	grey	VSMC_M10
GO:0016202	0.90	0.18	12	28	regulation of striated muscle tissue development	BP	1	SIRT2,LMOD3,MEF2C,IGF2,USP19,ACTN3,YBX3,CREB1,SOX15,SMAD3,MRTFB,JPH2	grey	VSMC_M10
GO:0021915	0.90	0.13	62	130	neural tube development	BP	1	STK3,FZD6,BRD2,TBC1D32,PTCH1,SMO,PTK7,SEMA4C,FUZ,TEAD2,INTU,BMI1,GLI2,IFT172,OVOL2,SPINT2,TRAF6,CASP3,FZD3,OPA1,IFT52,SPINT1,RPGRIP1L,PKD2,STK4,PAX2,PRICKLE1,SUFU,TGFB2,ITPK1,GBX2,DVL3,CHRD,GRHL3,LUZP1,SETD2,PLOD3,FKBP8,GORAB,GPR161,TULP3,LIAS,DVL2,WDR19,ALDH1A2,PSEN1,TMEM107,MKS1,GSC,MIB1,SKI,PKD1,TSC1,GLMN,BCL10,VASP,SOX4,NOG,WNT5A,C2CD3,PHGDH,RALA	grey	VSMC_M10
GO:1903321	0.90	0.14	39	82	negative regulation of protein modification by small protein conjugation or removal	BP	1	TNFAIP3,HSPA1B,TSPO,CRY1,SVBP,DNAJA1,PRKCE,ATG5,SPOPL,SPRY2,KLHL40,ARRB1,SIRT7,USP44,SH3RF2,TRIM44,MARCH7,PRMT3,RPL23,PIAS3,DTX3L,PER2,RPL11,SENP2,IVNS1ABP,GPS2,GNL3L,PSEN1,TAF1,OGT,OTUB2,DCUN1D3,PARK7,GLMN,SOX4,BAG2,CAMLG,PRR7,RASD2	grey	VSMC_M10
GO:0032613	0.90	0.18	14	32	interleukin-10 production	BP	1	AGER,TNFRSF21,TLR4,PIBF1,F2RL1,NOD2,CD28,VSIR,STAT3,FOXP3,PRKCZ,CD46,INAVA,TSLP	grey	VSMC_M10
GO:0032653	0.90	0.18	14	32	regulation of interleukin-10 production	BP	1	AGER,TNFRSF21,TLR4,PIBF1,F2RL1,NOD2,CD28,VSIR,STAT3,FOXP3,PRKCZ,CD46,INAVA,TSLP	grey	VSMC_M10
GO:2000106	0.90	0.15	27	59	regulation of leukocyte apoptotic process	BP	1	CDKN2A,SLC46A2,SLC7A11,IDO1,GAS6,FOXP1,IRF7,ZC3H8,PIK3CB,MEF2C,RAPGEF2,NOD2,GPAM,HCLS1,PDCD1,ADAM8,BCL6,CXCR2,EFNA1,BTK,CD74,CCL5,IL7R,BCL10,FADD,WNT5A,TSC22D3	grey	VSMC_M10

GO:0120031	0.90	0.12	218	435	plasma membrane bounded cell projection assembly	BP	1	ATP8B1,ONECUT2,MYO1A,AVIL,TBC1D30,ATAT1,KIF3A,TBC1D32,UBE2B,MTSS1,ASAP1,LRRC6,MAPK15,PODXL,CDC42EP1,RAC2,IFT74,SRF,CFAP69,RFX3,KIT,NCK2,PTPDC1,FGD3,RAB23,DYNC2LI1,EPS8L1,CEP162,TMEM17,AKIRIN1,WDR92,FUZ,IQUB,ATG5,SDCBP,ARMC2,MAPRE1,ARMC4,INTU,SPATA6,TTC26,BBS7,VSTM5,ARHGEF4,DRC1,RSPH6A,GALNT11,FBF1,PIBF1,KLF5,IFT172,FOXJ1,WDR60,TTLL3,CCDC13,ABCC4,MKKS,RAB5A,CEP290,BAG4,ARHGEF7,SPEF1,FGD6,ARFIP2,TMEM80,DPYSL3,AL359736.1,KIF3B,FER,RILPL1,RILPL2,CEP70,PIK3R1,PARVA,PLCE1,CEP250,CCDC66,P2RX7,TCTN3,IFT81,CCDC113,CFAP20,ARHGEF26,RAPGEF2,CCDC65,TMEM67,BIN3,F2RL1,IFT52,ARHGAP24,ANO6,RPGRIP1L,DNAH1,CYFIP1,DMTN,DNAH5,ZNF423,GBF1,SRGAP2C,NEURL1,CARMIL1,CFAP43,FGD4,ZMYND10,GAP43,ARMC9,CFAP46,TTBK2,AUTS2,AJUBA,CLCN4,ALPK1,JHY,DISC1,OFD1,WASF2,RAB17,TMEM237,TRAF3IP1,HDAC4,BBS1,LAMA5,EPS8,PTPRO,CDKL5,SEPT2,LIMK2,WHAMM,CDC42EP2,CFAP161,TTC12,TOGARAM1,EHD1,DNAAF2,SPAG16,NCKAP1,ABLIM2,FAM149B1,HTT,CFAP70,DNM3,RAP1GAP,STK36,GORAB,CEP126,SNAP29,EXOC5,VCL,WAS,TTC25,RAB8A,SDCCAG8,HAP1,DNAAF4,CPLANE2,MNS1,WDR19,TMEM138,MIEN1,ATP6V1D,PLEK2,FGD1,FXR2,BBOF1,CNTROB,TMEM107,NDEL1,TENM2,AC138894.1,CCDC151,NTN1,DYNLL2,MKS1,DNM2,BCAS3,ARHGAP44,EVL,TEKT3,IFT20,SPEF2,STK26,CFAP53,ARHGEF6,FMR1,INPP5K,C16orf71,RILP,CLUAP1,WRAP73,PALM,TPGS1,EPS8L3,DEF8,CDK10,CELSR2,VAV3,CDC14A,TMEM231,FNBP1L,MTSS1L,HYDIN,ODF2L,WDR63,CARMIL2,C5orf30,TCTN2,EPS8L2,HYLS1,SNX10,C2CD3,CSPG4,CC2[illegible]	grey	VSMC_M10
GO:0035850	0.90	0.16	18	41	epithelial cell differentiation involved in kidney development	BP	1	MTSS1,PODXL,SMO,SIX2,OSR1,FOXJ1,JAG1,ASXL1,PAX8,MEF2C,PAX2,SALL1,PRKX,PTPRO,CITED1,LHX1,BASP1,LIF	grey	VSMC_M10
GO:0033189	0.90	0.23	5	13	response to vitamin A	BP	1	CAT,DNMT3A,DNMT3B,ALDH1A2,LRAT	grey	VSMC_M10
GO:0006644	0.90	0.12	157	314	phospholipid metabolic process	BP	1	PIP4K2C,ABHD16A,SYNJ2,SERAC1,AGPAT4,AGPAT1,PNPLA3,PLA2G6,GPAA1,CWH43,SRD5A3,INPP4A,PLPPR1,TMEM246,TMEM86B,PIGF,MBOAT7,PLEK,NR1H2,MBOAT2,LPIN1,INPP4B,SMPD2,FIG4,SPHK2,MTMR2,PIP4K2A,NAPEPLD,HADHA,SELENOI,ABHD5,APOC1,TAMM41,ITPKC,PIP4P1,MTMR14,PLCB1,PGAP2,CRLS1,CDS2,PLPP5,NR1H4,MTMR7,ABHD6,PIGU,PIK3CB,PITPNM2,PIK3R1,TMEM150A,CHRM5,MTMR9,SLA2,NR1H3,SERINC5,SYNJ1,CDS1,FITM2,ITPKA,PIGT,PLA2G4B,GPLD1,LPCAT1,PGAP1,GPAM,CHKA,PLA1A,ACP6,INPP5A,ABHD4,SGMS2,AJUBA,GGPS1,PITPNM1,MTMR11,GNPAT,DGKD,PIGM,FITM1,BMX,FAM126B,IP6K2,LPGAT1,PIGV,DPAGT1,PLPP4,MTMR3,THEM5,PCYT1A,INPP5J,PIGX,PLBD1,PIGZ,PISD,DGKG,PIKFYVE,PLA2G4A,SACM1L,LPCAT3,ABHD12B,GNB3,PIGC,PLCD4,SMPD4,SOCS4,PLA2G16,PI4KA,SLC27A1,ABHD8,SGMS1,CLN8,PGAP3,PIGH,PI4K2B,LIPC,SOCS7,DGKQ,GDPD3,AC138894.1,DGKE,PLPPR2,TTC7B,LDLR,GDPD1,PIGL,GDE1,CSF1R,GATA6,ABHD3,PNPLA6,PROCA1,MPPE1,IMPA2,MTMR12,SOCS1,MTM1,MTMR1,INPP5K,PIP5K1C,ABCA3,MECP2,AGPAT2,PIP5KL1,PLA2G15,FABP5,ABCA8,DHDDS,PEMT,PLA2G2C,PYURF,LCLAT1,PIGB,PIGA,INPP1,SGPP1,PTPMT1,DNAJC19,CDIPT	grey	VSMC_M10
GO:0009410	0.90	0.12	157	314	response to xenobiotic stimulus	BP	1	NME1,FECH,TNFRSF11B,TSPO,UBE2B,BAK1,MYC,PTCH1,SOX10,TXN2,PLIN2,ABCB1,FZD1,CA9,TFAP2B,PPAT,ABCG5,CAT,ABCG8,XPO1,TGFA,PTN,DDC,EGFR,ATG5,FOXO3,FGF21,TRAF3IP2,HDAC2,DNMT3A,SLC26A5,HADHA,GAD2,NUDT15,XPC,XRCC1,TIMP4,ARRB1,ABCC4,TGFBR2,RPTOR,PRNP,DUSP6,NCKAP1L,GAS6,RET,DNMT3B,PARP4,GSS,CASP3,ATR,FZD3,MEF2C,VEGFC,CYP2R1,PTK2B,KCNJ11,RPN2,NKX6-1,RAD54B,BPHL,PMS1,SLC6A2,ABCC2,KCNE2,CBR3,SLC12A5,CMBL,GRM2,TPMT,GPLD1,SRD5A1,SLC6A3,HDAC1,LCK,CPT1A,MAP2K6,TGFB2,FMO5,DAD1,CYP2U1,AMELX,SLAMF8,GNPAT,CHEK2,ABCB11,DVL3,ACTC1,HDAC4,CDKN1B,MGMT,APOD,POR,NFE2L2,CREB1,SORD,RAP1B,CPS1,COMT,XRCC5,BCHE,STAT3,CD38,PMS2,ENO3,SCGB1A1,EPHX1,ITGA3,PGF,FOS,SCNN1B,UQCRC2,PDE4A,CYP46A1,NPC1,GATA6,ADSSL1,SREBF1,OXCT1,NOS2,CYP2J2,JUN,MBD1,DAB1,ABAT,RPH3AL,LRP8,ABCA3,AGPAT2,CYBA,ASS1,VAV3,LCN2,ENG,ABCD3,HSPA5,DHODH,NQO1,HSD11B2,CRYZ,MT-ND1,MT-CYB,RHOA,S100A12,KCNK3,SULT1C3,BLOC1S3,AHRR,KCNH2,BGLAP,PEMT,NCEH1,TIGAR,CRH,NNMT,AOC2,N6AMT1	grey	VSMC_M10
GO:0051282	0.90	0.14	39	84	regulation of sequestering of calcium ion	BP	1	PRKCE,IBTK,ATG5,RYR1,DIAPH1,RASA3,RYR3,PTK2B,TPCN1,SLC8B1,F2R,PKD2,CASQ2,RYR2,LCK,UBASH3B,ANK2,GPER1,ATP1A2,CASQ1,NTSR1,CEMIP,PTPN6,HTT,HAP1,CD19,CALM1,BDKRB1,SLC25A23,TRPV1,PDPK1,CYBA,JPH3,MCOLN3,DMD,NOL3,JPH2,THY1,DDIT3	grey	VSMC_M10
GO:0033598	0.90	0.19	10	24	mammary gland epithelial cell proliferation	BP	1	ESR1,RTN4,AREG,ROBO1,RREB1,CCND1,MAPK1,MED1,WNT5A,CEBPB	grey	VSMC_M10
GO:0051209	0.90	0.14	38	82	release of sequestered calcium ion into cytosol	BP	1	PRKCE,IBTK,ATG5,RYR1,DIAPH1,RASA3,RYR3,PTK2B,TPCN1,SLC8B1,F2R,PKD2,CASQ2,RYR2,LCK,UBASH3B,ANK2,GPER1,ATP1A2,CASQ1,NTSR1,CEMIP,PTPN6,HTT,HAP1,CD19,CALM1,BDKRB1,TRPV1,PDPK1,CYBA,JPH3,MCOLN3,DMD,NOL3,JPH2,THY1,DDIT3	grey	VSMC_M10
GO:0030540	0.90	0.22	6	15	female genitalia development	BP	1	ESR1,BAK1,SRD5A1,LHX1,STRA6,WNT5A	grey	VSMC_M10

GO:2000027	0.90	0.14	51	108	regulation of animal organ morphogenesis	BP	1	FRS2,FZD6,TNFRSF11B,RSPO3,SMO,PTK7,ROR2,SIX2,SPRY1,SOX9,WNT11,SPEF1,RYK,PAX8,ROBO1,ROBO2,FZD3,TIAM1,PAX2,VANGL1,PRICKLE1,NKD1,JHY,ZNRF3,AGT,DVL3,FZD7,GRHL3,STOX1,WNT4,DAAM1,ARHGEF19,MAGED1,DVL2,BMPR1A,SP6,NGFR,MKS1,BTBD7,LHX1,GPC3,SAPCD2,SOX8,CELSR2,RHOA,NOG,WNT5A,SIX1,CELSR3,FGF1,WNT2	grey	VSMC_M10
GO:0006450	0.90	0.21	7	17	regulation of translational fidelity	BP	1	LARS,GATC,TARS2,LARS2,AARS,DTD1,DTD2	grey	VSMC_M10
GO:0006266	0.90	0.22	6	15	DNA ligation	BP	1	XRCC6,XRCC1,XRCC4,MGMT,LIG3,NHEJ1	grey	VSMC_M10
GO:0043486	0.90	0.21	7	17	histone exchange	BP	1	ACTR6,SPTY2D1,VPS72,CHD5,SYCP3,ITGB3BP,RNF8	grey	VSMC_M10
GO:1904407	0.90	0.18	12	28	positive regulation of nitric oxide metabolic process	BP	1	TLR4,PTK2B,PKD2,AGT,NOS1AP,SMAD3,DNM2,INSR,TICAM1,TRPV1,ASS1,MIR99B	grey	VSMC_M10
GO:1905907	0.90	0.25	4	10	negative regulation of amyloid fibril formation	BP	1	PFDN5,PFDN4,LDLR,VBP1	grey	VSMC_M10
GO:0060099	0.90	0.23	5	13	regulation of phagocytosis, engulfment	BP	1	NCKAP1L,F2RL1,ANO6,C3,ABCA7	grey	VSMC_M10
GO:0090200	0.90	0.20	9	21	positive regulation of release of cytochrome c from mitochondria	BP	1	BAK1,PLA2G6,DNM1L,GPER1,CIDEB,PINK1,MLLT11,BCL2L11,PPIF	grey	VSMC_M10
GO:0031281	0.90	0.23	5	13	positive regulation of cyclase activity	BP	1	MAPK14,GUCA1B,ORAI1,MAPK3,NOS2	grey	VSMC_M10
GO:0060749	0.90	0.22	6	15	mammary gland alveolus development	BP	1	ESR1,AREG,TGFA,CCND1,EGF,FOXF1	grey	VSMC_M10
GO:0061377	0.90	0.22	6	15	mammary gland lobule development	BP	1	ESR1,AREG,TGFA,CCND1,EGF,FOXF1	grey	VSMC_M10
GO:0043547	0.90	0.12	98	202	positive regulation of GTPase activity	BP	1	TBC1D30,TBC1D9B,TIAM2,ASAP1,TBC1D22B,SEMA4D,TBC1D8,RGP1,ASAP2,ARHGEF5,EPHA1,TBC1D9,IQSEC1,RSU1,TBC1D4,FOXJ1,WNT11,AKT2,TBC1D16,ARHGEF7,LARS,TBC1D12,ARHGEF26,PTK2B,RASGRP1,RAPGEF2,CCL17,TIAM1,RAPGEF3,F2RL1,F2R,ARHGAP24,CD40,RAP1GDS1,DOCK10,RGS1,TBCK,ADPRH,ADAP1,DENND1B,ARHGAP6,TBC1D10C,RAB11FIP2,DVL3,CDKL5,GRHL3,TBC1D2B,GMIP,RASGRP2,TBC1D14,RABGAP1L,RUNDC1,WNT4,ELMOD1,RAP1GAP,TBC1D25,GNB5,EPHA4,ARHGEF19,DVL2,SYDE1,TBC1D1,ARAP2,RGS14,TBC1D10B,NDEL1,MYO9A,BCAS3,TBC1D8B,CCL14,CCL5,CCL7,CCL2,CCL25,ADAP2,VAV1,RALBP1,DOCK7,ABR,CRK,RAP1GAP2,TAX1BP3,ARHGEF16,APC2,ARHGAP45,TSC1,MTSS1L,DENND1A,RABGAP1,CCL16,ASAP3,THY1,WNT5A,CHN1,RGS4,TBC1D20,HRAS,CCL22	grey	VSMC_M10
GO:0060191	0.90	0.15	29	63	regulation of lipase activity	BP	1	LRP1,ESR1,KIT,LPAR1,PPT1,NR1H2,EGFR,C5AR1,PHB,ABHD5,APOC1,RASGRP4,FLT1,NR1H3,PLA2R1,GNA13,ARHGAP6,AGT,POR,ABL2,FGFR3,ANXA8,NMUR2,CCL5,GNA15,PDPK1,PRKCZ,RHOA,HRAS	grey	VSMC_M10
GO:0010718	0.90	0.16	20	45	positive regulation of epithelial to mesenchymal transition	BP	1	SDCBP,HDAC2,TGFBR2,JAG1,ALX1,TIAM1,LOXL2,GCNT2,TGFB2,TCF7L2,AXIN2,SNAI1,TGFB1I1,SMAD3,ISL1,ENG,MIR21,MIR222,RGCC,OLFM1	grey	VSMC_M10
GO:1903428	0.90	0.21	7	17	positive regulation of reactive oxygen species biosynthetic process	BP	1	TLR4,FOXO3,HDAC4,PARK7,CYBA,LCN2,MIR24-1	grey	VSMC_M10
GO:0090083	0.90	0.22	6	15	regulation of inclusion body assembly	BP	1	SNCAIP,HSPA1B,HSF1,SORL1,DNAJB2,DNAJB1	grey	VSMC_M10
GO:2000095	0.90	0.22	6	15	regulation of Wnt signaling pathway, planar cell polarity pathway	BP	1	RSPO3,SPEF1,NKD1,ZNRF3,MKS1,GPC3	grey	VSMC_M10
GO:0061082	0.90	0.19	10	24	myeloid leukocyte cytokine production	BP	1	KIT,TLR4,MAVS,TGFB2,BCL6,SPON2,CD74,TICAM1,BCL10,WNT5A	grey	VSMC_M10
GO:0046068	0.90	0.23	5	13	cGMP metabolic process	BP	1	PDE10A,GUCY1A1,GUCY1B1,PDE9A,GUCY2D	grey	VSMC_M10
GO:0051147	0.90	0.13	55	115	regulation of muscle cell differentiation	BP	1	NFATC1,MAML1,FRS2,PPARA,MAPK14,PI16,SRF,KIT,ANKRD17,TRIM32,MYLK3,MAMSTR,NRG1,LMOD3,SMYD1,NLN,MEF2C,SOX6,ZBED6,RBM24,IGF2,BDNF,SIK1,PDCD4,GPER1,FZD7,ACTN3,HDAC4,EFEMP2,BHLHA15,BMPR1A,FOXO4,RBPMS2,SETD3,YY1,FBXO22,SUPT6H,MECP2,PIEZO1,PLPP7,ENG,NFATC3,MIR199A1,MIR21,MIR222,MIR125B1,MIR22,PRDM6,RGS4,TCF23,YBX1,EREG,NKX2-5,RBM38,HEY2	grey	VSMC_M10
GO:1903651	0.90	0.23	5	13	positive regulation of cytoplasmic transport	BP	1	RAB21,PRKN,MTMR2,SORL1,TRIM46	grey	VSMC_M10
GO:0043628	0.90	0.17	16	36	ncRNA 3'-end processing	BP	1	EXOSC4,EXOSC3,TRNT1,USB1,INTS8,TENT4B,INTS12,INTS5,AP002990.1,HSD17B10,INTS2,ERI2,PARN,POLR3K,TRMT10C,EXOSC5	grey	VSMC_M10
GO:0031424	0.90	0.23	5	13	keratinization	BP	1	EVPL,PPHLN1,TGM1,SFN,ABCA12	grey	VSMC_M10
GO:0048026	0.90	0.21	7	17	positive regulation of mRNA splicing, via spliceosome	BP	1	SNW1,TRA2A,NUP98,HSPA8,CELF3,PRPF19,CIRBP	grey	VSMC_M10
GO:2000179	0.90	0.16	17	39	positive regulation of neural precursor cell proliferation	BP	1	GLI1,SOX10,SMO,GLI2,TOX,FZD3,VEGFC,ZNF335,ZNF423,EGF,DISC1,FZD9,LHX1,ADGRG1,FOXG1,GPR37L1,PITX3	grey	VSMC_M10
GO:0007183	0.90	0.23	5	13	SMAD protein complex assembly	BP	1	EID2,PPM1A,PARP1,SMAD3,LDLRAD4	grey	VSMC_M10
GO:0003231	0.90	0.13	47	101	cardiac ventricle development	BP	1	FRS2,LY6E,FOXH1,PTK7,TNNI3,HOXA13,CNTRL,SCN5A,POU4F1,WNT11,TGFBR2,JAG1,NRG1,PAX8,HAND2,ROBO1,ROBO2,MEF2C,TBX5,TNNC1,DSP,SALL1,RYR2,SUFU,PKP2,TNNI1,TGFB2,TRIP11,MYH7,KCNK2,LUZP1,SAV1,BMPR1A,RBPJ,MED1,DCTN5,STRA6,AP2B1,ISL1,FOXF1,ENG,SOX4,TMEM65,NOG,WNT5A,NKX2-5,HEY2	grey	VSMC_M10
GO:0051044	0.90	0.23	5	13	positive regulation of membrane protein ectodomain proteolysis	BP	1	SH3D19,GPLD1,ADRA2A,ADAM8,TNFRSF1B	grey	VSMC_M10
GO:0009167	0.90	0.16	16	36	purine ribonucleoside monophosphate metabolic process	BP	1	IMPDH1,GDA,PPAT,NT5E,DLG2,AMPD3,GMPS,AMPD1,MAGI3,DLG1,CASK,ADK,CARD11,ADSSL1,MPP1,NUDT2	grey	VSMC_M10
GO:0040014	0.91	0.15	21	47	regulation of multicellular organism growth	BP	1	PTCH1,SMO,MKKS,PLS1,HTRA2,CELF1,FTO,IGF2,SLC6A3,GPAM,CREB1,GDF15,STAT3,STAT5B,RAI1,GHR,GAMT,DIO3,POU3F2,FXN,PLAC8	grey	VSMC_M10
GO:0030512	0.91	0.14	29	64	negative regulation of transforming growth factor beta receptor signaling pathway	BP	1	ONECUT2,PPARA,CD109,SPRY1,SPRY2,EID2,SPRED3,SNX25,IL17RD,VEPH1,SLC2A10,HTRA1,SNX6,PBLD,HTRA3,FBN1,PPM1A,TGFB1I1,WFIKKN2,DNM2,PDPK1,SKI,PRDM16,WFIKKN1,HSPA5,LEMD3,NRROS,LDLRAD4,PEG10	grey	VSMC_M10
GO:0038093	0.91	0.16	17	39	Fc receptor signaling pathway	BP	1	PTK2,PLA2G6,KIT,PRKCE,IKBKB,TRAF6,HCK,FER,PTPRJ,FGR,PLPP4,FCER1G,BTK,YES1,VAV1,VAV3,MAP2K7	grey	VSMC_M10
GO:2000052	0.91	0.21	6	15	positive regulation of non-canonical Wnt signaling pathway	BP	1	RSPO3,CSNK1E,NKD1,GPC3,WNT5A,LBX2	grey	VSMC_M10

GO:0051051	0.91	0.12	157	314	negative regulation of transport	BP	1	VPS4B,ERBB3,NEDD4L,PRKN,GOPC,CRY1,WNK2,RAB23,ABCG5,ABCG8,IL11,PRKCE,BMP8A,RSAD2,NR1H2,ITGB1BP1,GRB10,HECW1,TCAF2,SFRP4,KEL,VPS35,ATG5,SDCBP,OSR1,MTMR2,RAB7A,SLC26A5,KCNRG,CD300LF,APOC1,FRMD4A,OXSR1,TBC1D4,AKT2,NEU3,UBAC2,DPH3,FERMT1,CNN2,BAG4,NRG1,NCKAP1L,ARL6IP5,ADRA2B,LYPLA1,NDUFAF2,FAM3D,PXK,ANKRD27,SLC43A1,ASIC1,PKIA,TMBIM6,WDR54,P2RY1,PTK2B,KCNJ11,NR1H3,RAB11FIP5,DYSF,STC1,ACVR1C,F2R,CRY2,KCNE2,PKD2,PLA2R1,CYFIP1,DMTN,CASQ2,ADTRP,SUFU,SIRT4,IRS1,CABP1,CD200,HDAC1,ANKRD13A,PPP3CA,HECW2,SP100,ADRA2A,ISCU,KCNB1,YOD1,EGF,ANKRD13D,ATP1A2,BAG3,C4orf3,OSM,PEA15,NTSR1,RUBCN,CNIH2,SH3GL3,APOD,KLF7,CTTNBP2NL,RAP1B,FIS1,DERL3,SESTD1,IL12A,COMT,ENY2,C11orf65,ADRA2C,LRPAP1,PNKD,INHA,GNB5,DLG4,RSC1A1,TNFRSF1B,WNK3,STX1B,SYT11,SNX12,CALM1,SYTL4,PRKCB,SCAMP5,CD74,SREBF1,UNC119,ABAT,FMR1,GABRE,INPP5K,OAZ1,ABCA7,PARK7,FOXF1,WWP2,RRAD,MIR199A1,MIR27B,MIR24-1,MIR27A,GPR35,FABP5,CHGA,KCNH2,MIR33B,CRBN,RGS4,CRH,TXN,LIF,VAMP3,UBE2G2,GEM,PPIF,DERL2,LRRTM2	grey	VSMC_M10
GO:0001894	0.91	0.12	92	189	tissue homeostasis	BP	1	TNFAIP3,SLC12A2,TNFRSF11B,AKR1B1,RAC2,SMO,ALDH1A1,SRF,CLDN12,SLC1A1,KIAA1211,PRDX1,EPAS1,HOXA13,ALB,MTF1,WHRN,EGFR,USP45,TRIM32,TLR4,NCDN,SOX9,CUBN,USH1G,MKKS,ACTG1,RAC3,ERCC6,TRAF6,TUB,LSR,CCDC66,ADGRV1,IL7,P2RX7,JAM2,PTK2B,USH1C,HOMER1,F2R,SPP1,GCNT2,NOD2,ITGB6,LDB1,LPCAT1,LRRK1,TCIRG1,GPR137B,KRAS,GIGYF2,UBASH3B,BBS1,HOMER2,LAMC1,ADAM8,PTH1R,PBLD,CTSH,CDH23,ABCA12,GPR137,VCL,CD38,ILDR2,LIPA,ACTB,CLN8,WNK3,ATP1B2,ESRRB,RAB3D,MKS1,ACACA,CSF1R,PPARGC1B,ABCA3,ATP6AP1,DEF8,OAS1,DMD,SLC22A5,CA2,NANOS1,JCHAIN,BGLAP,SNX10,CLDN1,INAVA,DRAM2,BBS10	grey	VSMC_M10
GO:0009187	0.91	0.17	14	33	cyclic nucleotide metabolic process	BP	1	PDE7B,PDE10A,ADCY3,GUCY1A1,GUCY1B1,ADCY7,ADCY2,PDE9A,PTHLH,ADCY4,PDE8A,ADCY10,GUCY2D,PDE4A	grey	VSMC_M10
GO:0046827	0.91	0.21	6	15	positive regulation of protein export from nucleus	BP	1	GAS6,RAPGEF3,TCF7L2,BAG3,SFN,CTDSPL2	grey	VSMC_M10
GO:0010771	0.91	0.22	5	13	negative regulation of cell morphogenesis involved in differentiation	BP	1	FBLN1,ITGB1BP1,DMTN,AP1AR,MELTF	grey	VSMC_M10
GO:1900025	0.91	0.22	5	13	negative regulation of substrate adhesion-dependent cell spreading	BP	1	FBLN1,ITGB1BP1,DMTN,AP1AR,MELTF	grey	VSMC_M10
GO:0060765	0.91	0.18	10	24	regulation of androgen receptor signaling pathway	BP	1	KDM4C,FOXH1,PHB,RNF14,FOXP1,HDAC1,PIAS2,NCOR1,PARK7,TRIM68	grey	VSMC_M10
GO:1900102	0.91	0.21	6	15	negative regulation of endoplasmic reticulum unfolded protein response	BP	1	NCK2,DDRGK1,ABCA7,HSPA5,MIR199A1,PPP1R15A	grey	VSMC_M10
GO:0099560	0.91	0.19	8	20	synaptic membrane adhesion	BP	1	MAPK14,LRRC4,PCDH8,PTPRF,LRFN3,SPARCL1,SLITRK2,NTNG2	grey	VSMC_M10
GO:0060628	0.91	0.22	5	13	regulation of ER to Golgi vesicle-mediated transport	BP	1	MAPK15,PGAP1,SORL1,STX18,TBC1D20	grey	VSMC_M10
GO:0051101	0.91	0.13	43	91	regulation of DNA binding	BP	1	NME1,PRKN,AGER,HABP4,C6orf106,SMO,MDFI,ZNF462,HDAC2,ERCC2,RSF1,POU4F1,SIRT2,DDRGK1,TAF10,TRAF6,HAND2,FBXW7,SP100,EGF,PER2,PARP1,NSD1,MSX2,PSEN1,TAF1,WFIKKN2,CALM1,SIN3A,ISL1,ERCC4,JUN,ZBTB7C,SKI,DOT1L,WFIKKN1,PARK7,LAMTOR5,DDIT3,TXN,ZNF593,LIF,HEY2	grey	VSMC_M10
GO:0038084	0.91	0.16	18	41	vascular endothelial growth factor signaling pathway	BP	1	SEMA6A,PTP4A3,ADAMTS3,TSPAN12,GAB1,PRKD2,SPRY2,DCN,FLT1,PIK3CB,ROBO1,VEGFC,IL12A,PGF,DAB2IP,MIR199A1,MIR21,CADM4	grey	VSMC_M10
GO:0002371	0.91	0.22	5	13	dendritic cell cytokine production	BP	1	KIT,TLR4,MAVS,NOD2,TICAM1	grey	VSMC_M10
GO:0035025	0.91	0.19	9	22	positive regulation of Rho protein signal transduction	BP	1	GPR20,LPAR1,ARRB1,ROBO1,F2RL1,F2R,COL3A1,GPR35,ADGRG1	grey	VSMC_M10
GO:0090231	0.91	0.21	6	15	regulation of spindle checkpoint	BP	1	CDK5RAP2,DYNC1LI1,ANAPC15,USP44,LCMT1,DUSP1	grey	VSMC_M10
GO:0090266	0.91	0.21	6	15	regulation of mitotic cell cycle spindle assembly checkpoint	BP	1	CDK5RAP2,DYNC1LI1,ANAPC15,USP44,LCMT1,DUSP1	grey	VSMC_M10
GO:1903504	0.91	0.21	6	15	regulation of mitotic spindle checkpoint	BP	1	CDK5RAP2,DYNC1LI1,ANAPC15,USP44,LCMT1,DUSP1	grey	VSMC_M10
GO:0006402	0.91	0.12	98	201	mRNA catabolic process	BP	1	PAN2,CNOT2,CNOT6,RIDA,PABPC1,HSPA1B,SKIV2L,DXO,EIF3E,PNLDC1,CSDC2,AGO2,TNRC6B,TUT7,GTPBP1,MAPK14,EXOSC4,CNOT11,EXOSC3,PNPT1,AGO1,TUT4,PUM2,TRAF3IP2,MTPAP,ZC3HAV1,DHX34,FASTK,MLH1,MYD88,TNRC6C,LSM1,NUDT16,PKP3,UPF3A,PAN3,NUDT12,TTC37,CNOT1,CELF1,CNOT6L,TENT2,FTO,TENT5C,TENT4B,RBM24,DIS3L2,AXIN2,MAPKAPK2,GIGYF2,YTHDF2,TRAF5,FASTKD1,ANGEL2,YTHDF1,NCBP2,EIF4ENIF1,YBX3,SUPV3L1,RC3H1,UPF1,SLC11A1,RBM10,SAMD4A,POLR2G,SCGB1A1,NT5C3B,DCP1B,CASC3,FXR2,TARDBP,SMG5,TOB1,TENT5D,TNRC6A,CNOT8,LARP1,SLFN14,ALKBH5,UPF3B,PARN,FMR1,METTL16,PLEKHN1,LSM7,CIRBP,TRAF2,EDC4,AGO4,METTL14,NANOS1,CACNG7,YBX1,ZFP36L2,TENT5A,MAGOHB,RC3H2,EXOSC5	grey	VSMC_M10
GO:1904035	0.91	0.14	30	65	regulation of epithelial cell apoptotic process	BP	1	TNFAIP3,PPARA,TEK,SFRP4,FOXO3,FGF21,NDNF,GAS6,SRSF6,ANO6,PLA2R1,CD40,HDAC1,TERT,PDCD4,TCF7L2,GPER1,NFE2L2,ITGA4,TNIP2,RAMP2,MAPK7,CCL2,PIAS4,PDPK1,MIR24-1,FGA,MIR125A,CD248,RGCC	grey	VSMC_M10
GO:0032735	0.91	0.18	11	26	positive regulation of interleukin-12 production	BP	1	AGER,MAPK14,IRF5,TLR4,PLCB1,TRAF6,RIPK2,NOD2,CD40,IL16,ISL1	grey	VSMC_M10
GO:0006939	0.91	0.14	39	84	smooth muscle contraction	BP	1	PLA2G6,ZDHHC21,CHRNA3,SRF,KIT,TNNI3,MKKS,P2RX2,ADRA2B,HTR7,P2RX3,PLCE1,GUCY1A1,DOCK5,F2R,CHRM3,RAP1GDS1,ADRA2A,SULF2,GPER1,AGT,ATP1A2,DLG1,ADRA2C,CALCRL,CD38,KCNMA1,CHRNB2,SCNN1B,TBX2,NMUR2,SETD3,MYH11,ABAT,TRPV1,P2RX1,MIR21,RHOA,HTR1D	grey	VSMC_M10

GO:0070723	0.91	0.19	9	22	response to cholesterol	BP	1	PTCH1,SMO,TGFBR2,CYP7A1,GPLD1,DAG1,GRAMD1B,OSBPL7,LRP8	grey	VSMC_M10
GO:1900027	0.91	0.18	11	26	regulation of ruffle assembly	BP	1	EPS8L1,ARHGAP24,CYFIP1,EPS8,NDEL1,EVL,EPS8L3,DEF8,CARMIL2,EPS8L2,HRAS	grey	VSMC_M10
GO:0051249	0.91	0.11	143	287	regulation of lymphocyte activation	BP	1	TNFAIP3,AGER,HLA-DRA,HLA-DOB,HLA-DMA,IL5,HLA-DPB1,TCF7,TNFRSF13C,XRCC6,RAC2,FBXO7,TNFRSF21,NCK2,EXOSC3,MSH2,SLC46A2,PELI1,IL4I1,TLR4,IL15,HMCES,BMI1,CLPTM1,MLH1,GLI2,MYD88,PIBF1,FOXJ1,PAXIP1,IL15RA,TGFBR2,PAWR,SHLD1,PRNP,NCKAP1L,GAS6,PRKDC,SLC7A1,TRAF6,SOX12,TOX,ZC3H8,IL1A,CAMK4,CASP3,MEF2C,TMEM131L,IL7,PAG1,RASGRP1,RIPK2,AP3B1,MARCH7,SOX13,NOD2,ZNF335,CD40,IGF2,LCK,BANK1,ICOSLG,BTN2A2,GPAM,PRKAR1A,HLX,TBC1D10C,SLAMF8,THEMIS2,FGR,CD28,RUNX3,DLG1,PPP2R3C,ADAM8,DUSP10,BLOC1S6,BCL6,IL12A,VSIR,RC3H1,PTPN6,TNFSF18,LAG3,TNIP2,CD38,BST1,STAT5B,INHA,SCGB1A1,FOXP3,RASAL3,ZBTB1,SHLD2,CARD11,TNFRSF1B,TBX21,ZBTB7B,CHRNB2,CD19,LAT,IL4R,BTK,CD74,CCL5,CCL2,YES1,NKAP,CD320,VAV1,TWSG1,IL7R,SUPT6H,PTPN2,SOCS1,DROSHA,TICAM1,DNAJA3,PRKCZ,VAV3,GLMN,AP1G1,BCL10,MIR21,MIR27A,RHOA,LST1,BLOC1S3,SOX4,FADD,DPP4,CD46,THY1,FGL2,CD70,TNFSF9,ZFP36L2,LAX1,EFNB3,CEBPB,TNFSF13B,HAVCR2,RC3H2	grey	VSMC_M10
GO:0051973	0.91	0.17	13	30	positive regulation of telomerase activity	BP	1	MAP3K4,MYC,MAPK15,POT1,HMBOX1,MAPKAPK5,XRCC5,MAPK1,NVL,WRAP53,MAPK3,PARN,MAP2K7	grey	VSMC_M10
GO:0051656	0.91	0.11	166	334	establishment of organelle localization	BP	1	VPS4B,MYO1A,AP3S1,KIF5A,PRKN,KIF3A,UBE2B,FAM91A1,EXOC4,MAPK15,AGTPBP1,SUN2,COPG2,RAC2,MYH9,KIT,BICD2,CHMP2A,NLRP5,LRPPRC,XPO1,TRAPPC12,YWHAZ,CDK5RAP2,STARD3NL,CCDC155,MGARP,WASF1,SPHK2,SPRY1,BBS7,KIF3C,ARHGAP21,COPG1,MYO7B,CLASP2,NECTIN2,GAB2,MLH1,LLGL2,UNC13D,PIBF1,MYO7A,SPRY2,HOOK3,AP3M2,KIF5C,MKKS,LIN7A,TMEM230,CHAMP1,NSFL1C,MOS,TTL,KIF3B,FER,CHMP2B,CHMP4C,SDAD1,MCPH1,KATNB1,RASGRP1,ARFGAP2,SLIT1,EXOC6B,SYNJ1,OPA1,AP3B1,CHMP7,DTNBP1,KIF13A,GBF1,BICDL1,KLHL12,AC112128.1,TRIP11,PEF1,TCIRG1,PDCD6,SNAP23,MARK1,CFL1,SHROOM2,AP1AR,LAT2,SPO11,FGR,CBL,NOP9,MLPH,RAB17,SNX4,CNIH2,LIMK2,TANC2,DLG1,SPG11,MAPT,BLOC1S6,MAJIN,ITGA4,MAP4K2,MREG,PDCD10,HTT,PTGDR,RAB3A,PINK1,UXT,SNAP29,AP3M1,C12orf4,KIF1C,SYNE2,HAP1,SLC2A4,SEPT1,KPNB1,PEX14,PSEN1,SYT11,TMED10,TRIM46,NDEL1,AC138894.1,RAB11A,LAT,NTN1,IL4R,SNAPIN,S100A13,DNM2,BTK,CLMN,AP1M2,STXBP2,MYO1D,C17orf75,PPP6C,MYO1F,DOCK7,MYO5B,TLE6,BICDL2,PDPK1,PRKCZ,SAPCD2,ATP6AP1,IKBKG,SPIRE2,CHMP1A,PSRC1,GPSM2,FOXF1,STXBP1,FNBP1L,VPS4A,RIPOR1,FHOD1,UBB,BLOC1S3,OOEP,CHGA,BORCS5,AP3S2,GEM	grey	VSMC_M10
GO:1904889	0.91	0.22	5	13	regulation of excitatory synapse assembly	BP	1	VSTM5,LRFN1,SEMA4A,GRID2,LRRTM2	grey	VSMC_M10
GO:0071542	0.91	0.18	10	24	dopaminergic neuron differentiation	BP	1	CSNK1E,FZD1,CDNF,RYK,TIAM1,NR4A2,WNT3,WNT5A,PITX3,WNT2	grey	VSMC_M10
GO:0062014	0.91	0.14	34	73	negative regulation of small molecule metabolic process	BP	1	CYP27B1,PPARA,CRY1,PLEK,FAM3C,DKKL1,SOX9,APOC1,SLC22A13,PIBF1,TRIB3,CYP7A1,DKK3,ERLIN1,PRMT3,SIRT4,SIK1,MST1,SNAI1,ACTN3,HDAC4,SCAP,ACADL,WNT4,STAT3,ACADVL,PARP1,NCOR1,LEPR,GFI1,MIR21,PDZD3,TIGAR,INSIG2	grey	VSMC_M10
GO:0097581	0.91	0.14	36	78	lamellipodium organization	BP	1	AVIL,RAC2,KIT,NCK2,AKIRIN1,WASF1,ARHGEF4,ABI1,ARHGEF7,SPEF1,ARFIP2,AL359736.1,FER,PIK3R1,PLCE1,BIN3,RREB1,CYFIP1,DMTN,CARMIL1,AUTS2,AJUBA,WASF2,HDAC4,PTPRO,WHAMM,NCKAP1,ABLIM2,VCL,WAS,SNX1,DNM2,ARHGEF6,VAV3,MTSS1L,CARMIL2	grey	VSMC_M10
GO:2001235	0.91	0.13	47	98	positive regulation of apoptotic signaling pathway	BP	1	TRIM39,STK3,NCK2,GSDME,FAF1,PDIA3,DEDD2,FBH1,RET,FBXW7,HTRA2,NKX3-1,RIPK1,STK4,SIAH1,BMPR1B,DNM1L,LCK,GPER1,MCL1,AGT,NFATC4,PEA15,ING5,RPL11,FIS1,PRKRA,CTSH,LTBR,MAGED1,NGFR,S100A8,SFPQ,PTPN2,PIAS4,BCAP31,TRAF2,PARK7,BCL10,DAB2IP,MIR27B,UBB,FADD,DDIT3,BCL2L11,PLAGL2,CRADD	grey	VSMC_M10
GO:1905710	0.91	0.16	16	36	positive regulation of membrane permeability	BP	1	HEBP2,TMEM14A,HIP1R,SLC9A1,STPG1,FZD9,NMT1,MUL1,TMEM102,ACAA2,MIR29A,BNIP3L,BCL2L11,SLC35F6,PPIF,AC140504.1	grey	VSMC_M10
GO:0050678	0.91	0.11	136	275	regulation of epithelial cell proliferation	BP	1	NME1,GLI1,TNFAIP3,FRS2,RIDA,OSR2,MTSS1,MYC,PTCH1,SMO,IFT74,TEK,CDK6,DLX5,PDCL3,CD109,RTN4,EMC10,EPGN,AREG,TGFA,PTN,EGFR,OSR1,INTU,FOXE3,C5AR2,C5AR1,PRKD2,SOX9,AQP11,IFT172,SCN5A,WDR48,OVOL2,RPTOR,AIMP1,PRKDC,FLT1,CYP7B1,NR4A1,ATP5F1A,EYA1,ROBO1,FBXW7,MEF2C,PEX2,VEGFC,VDR,NKX3-1,AGGF1,SRSF6,IFT52,RREB1,PAX2,NOD2,IGF2,HYAL1,CCND1,TGFB2,SULF2,TCF7L2,PDCD6,EGF,SOX2,ARNT,TGM1,KDF1,SFN,VASH2,FZD7,HTRA1,RUNX3,GLUL,LAMC1,CDKN1B,DLG1,DUSP10,STOX1,ITGA4,MEN1,IL12A,CASK,SAV1,PDCD10,GPBAR1,RAP1GAP,STAT3,PTPRN,WDR13,MAGED1,BMPR1A,MED1,ALDH1A2,SYNJ2BP,NGFR,PGF,VASH1,SMAD3,MAP2K5,TNMD,BCL11B,CCL2,ISL1,SUZ12,GPC3,PDPK1,WDR77,CYBA,MARVELD3,DAB2IP,ESRP2,MIR29A,MIR21,MIR222,MIR23A,MIR125B1,MIR22,MIR27B,MIR24-1,MIR27A,SERPINB5,LAMB1,ACVRL1,TINF2,ZNF580,NOG,CLDN1,WNT5A,SIX1,ZNF304,RGCC,HRAS,EREG,FGF1,WNT2	grey	VSMC_M10

GO:0006941	0.91	0.13	61	128	striated muscle contraction	BP	1	NEDD4L,STAC3,SMAD5,TNNI3,ASB3,MYH14,TRPM4,CACNB2,STAC,SCN5A,SCN1A,GPD1L,LMOD3,NR4A1,HOMER1,STC1,KCNJ3,TNNC1,KCNE2,DSP,CASQ2,RYR2,PKP2,KCNJ2,MAP2K6,TNNI1,TRPV4,SYNM,MYH7,GRK2,ANK2,ACE2,ATP1A2,ACTC1,SLC9A1,CASQ1,ACTN3,HDAC4,SCN4B,NOS1AP,DLG1,TTN,ATP1B1,CHRNB1,TCAP,STAC2,ARHGEF11,CACNA1G,ATP2A1,CALM1,DTNA,DSG2,RNF207,DMD,CHGA,KCNH2,TNNC2,SCN2B,KCNJ8,HCN4,NKX2-5	grey	VSMC_M10
GO:0002726	0.91	0.21	6	15	positive regulation of T cell cytokine production	BP	1	RSAD2,TRAF6,DENND1B,FZD5,PRKCZ,TRAF2	grey	VSMC_M10
GO:0009896	0.91	0.11	211	420	positive regulation of catabolic process	BP	1	NEDD4L,PAN2,LRP1,PIP4K2C,TNFAIP3,EPM2A,CNOT2,CNOT6,RIDA,PABPC1,HSPA1B,DXO,PNLDC1,PRKN,PPARA,RNF217,TRIB1,AGO2,PRXL2C,PTK2,TNRC6B,TUT7,AGTPBP1,GTPBP1,CSNK1E,HSF1,TFEB,FBXO7,SPTLC1,EXOSC3,PRKCE,PNPT1,SLC4A4,NOD1,HECW1,TRIM32,HACE1,VPS35,SCOC,ZYG11B,TUT4,FOXO3,FAF1,FGF21,ZC3HAV1,SESN3,BBS7,RAB7A,PIP4K2A,SOX9,ABHD5,PLK3,SUMO2,OPTN,MLH1,RALB,EGLN2,TNRC6C,KLHL40,AKT2,SIRT2,TMEM173,LSM1,DCN,RNF14,DDRGK1,SH3RF2,ULK1,PAN3,TRIB3,PLK2,CEBPA,IDE,PIK3CB,SH3D19,FBXW7,GPD1,P2RX7,CNOT1,CELF1,PTK2B,CNOT6L,RIPK2,TPCN1,RNFT2,TP53INP1,FTO,KCNE2,PRICKLE1,NOD2,NKD1,MYLIP,TENT4B,RBM24,RNF144B,GPLD1,IRS1,LPCAT1,HECW2,SEC22B,CPT1A,ZBTB20,ADRA2A,DHRSX,DIS3L2,USP13,GIGYF2,AC013489.1,EGF,YTHDF2,DISC1,SORL1,ATG16L1,ASB11,DTX3L,SH3BP4,ARIH2,ARNT,BAG3,ACTN3,YTHDF1,MLXIPL,RNF185,EIF4ENIF1,CDKN1B,ADAM8,NSF,NFE2L2,SUPV3L1,COP1,VSIR,RC3H1,UPF1,HTT,SAMD4A,ZSWIM8,SOCS4,PINK1,DNAJB2,POLR2G,STX5,PIM2,DAGLB,NT5C3B,DCP1B,DDB1,VPS13D,TNFRSF1B,PSMC3,TARDBP,OSBPL7,PSEN1,AREL1,SNX1,TAF1,MAPK3,TOB1,TNRC6A,LDLR,CNOT8,LARP1,KEAP1,RNF128,PSMD10,FBXO22,INSR,SMCR8,PARN,GPC3,TICAM1,FMR1,RILP,FZR1,BCAP31,METTL16,PLEKHN1,SGTA,OAZ1,APC2,IKBKG,TMEM259,PARK7,GPSM1,GNAI3,KIAA1324,TSC1,ZER1,SH3GLB1,WWP2,DAB2IP,TRIM68,TRIM34,BNIP3L,ENTPD5,METTL14,PSMC1,TRIM65,NANOS1,BAG2,RNFT1,IRGM,WNT5A,BCL2L11,HSPB8,APC,PTTG1IP,ZFP36L2,DDA1,RNF152,RC3H2,EXOSC5	grey	VSMC_M10
GO:0055021	0.91	0.15	24	53	regulation of cardiac muscle tissue growth	BP	1	GLI1,PPARA,MAPK14,PI16,PIM1,TGFBR2,MEF2C,TBX5,JARID2,KCNK2,SAV1,MAPK1,BMPR1A,RBPJ,TBX2,YY1,GATA6,MIR199A1,MIR222,MIR25,NOG,RGS4,HEY2,WNT2	grey	VSMC_M10
GO:0055057	0.91	0.24	4	11	neuroblast division	BP	1	AKNA,RAB10,NUMBL,DOCK7	grey	VSMC_M10
GO:0018146	0.91	0.22	5	13	keratan sulfate biosynthetic process	BP	1	B3GNT4,ST3GAL6,CHST1,CHST5,ST3GAL2	grey	VSMC_M10
GO:0009262	0.91	0.16	17	38	deoxyribonucleotide metabolic process	BP	1	TBPL1,DPYS,GDA,NUDT15,MBD4,NUDT16,DGUOK,NUDT18,DTYMK,UPB1,ADK,NEIL1,NT5M,NTHL1,CMPK2,DCTD,UPP1	grey	VSMC_M10
GO:0006744	0.91	0.20	7	17	ubiquinone biosynthetic process	BP	1	COQ2,COQ5,COQ10B,NDUFA9,COQ8A,COQ4,PDSS2	grey	VSMC_M10
GO:1901663	0.91	0.20	7	17	quinone biosynthetic process	BP	1	COQ2,COQ5,COQ10B,NDUFA9,COQ8A,COQ4,PDSS2	grey	VSMC_M10
GO:0046599	0.91	0.19	8	20	regulation of centriole replication	BP	1	VPS4B,MDM1,CDK5RAP2,CEP295NL,PLK2,ALMS1,AC112128.1,TRIM37	grey	VSMC_M10
GO:0046889	0.91	0.14	28	61	positive regulation of lipid biosynthetic process	BP	1	PPARA,PLA2G6,NR1H2,SPHK2,SIRT3,NR1H4,CYP7A1,ABHD6,SORBS1,NR1H3,HSD17B13,SIRT4,GPLD1,ZBTB20,LPGAT1,MLXIPL,SCAP,POR,CREB1,WNT4,SLC27A1,LDLR,CD74,SREBF1,CNEP1R1,STARD4,CCDC3,FGF1	grey	VSMC_M10
GO:0003091	0.91	0.22	5	13	renal water homeostasis	BP	1	AKR1B1,TFAP2B,AQP1,MYO5B,INPP5K	grey	VSMC_M10
GO:2001185	0.91	0.22	5	13	regulation of CD8-positive, alpha-beta T cell activation	BP	1	NCKAP1L,RUNX3,VSIR,ZBTB7B,SOCS1	grey	VSMC_M10
GO:0050961	0.91	0.22	5	13	detection of temperature stimulus involved in sensory perception	BP	1	GRIK2,ASIC3,EPHB1,NTSR1,TRPV1	grey	VSMC_M10
GO:0043201	0.91	0.23	4	11	response to leucine	BP	1	RRAGD,SESN3,RPTOR,LARS	grey	VSMC_M10
GO:0045761	0.91	0.19	8	20	regulation of adenylate cyclase activity	BP	1	GABBR2,ADGRV1,ORAI1,CAP2,GRM2,PALM,GNAI3,CAP1	grey	VSMC_M10
GO:0120034	0.91	0.13	43	92	positive regulation of plasma membrane bounded cell projection assembly	BP	1	AVIL,CDC42EP1,RAC2,SRF,KIT,EPS8L1,AKIRIN1,FUZ,DPYSL3,PIK3R1,PLCE1,P2RX7,F2RL1,CYFIP1,NEURL1,ZMYND10,AUTS2,WASF2,HDAC4,EPS8,CDC42EP2,NCKAP1,HTT,DNM3,HAP1,MNS1,MIEN1,PLEK2,NDEL1,TENM2,DNM2,BCAS3,FMR1,WRAP73,PALM,EPS8L3,DEF8,FNBP1L,CARMIL2,EPS8L2,APC,RALA,HRAS	grey	VSMC_M10
GO:0090218	0.91	0.16	13	31	positive regulation of lipid kinase activity	BP	1	AGAP2,PTK2,TEK,KIT,FPR2,FLT1,PTK2B,NOD2,IRS1,FGR,FGFR3,CD19,VAV3	grey	VSMC_M10
GO:1901836	0.91	0.21	6	15	regulation of transcription of nucleolar large rRNA by RNA polymerase I	BP	1	MARS,IPPK,SIRT7,DEDD,H2AFY2,DDX11	grey	VSMC_M10
GO:0008210	0.91	0.21	6	15	estrogen metabolic process	BP	1	HSD17B4,HSD17B8,HSD17B7,COMT,HSD17B10,DHRS11	grey	VSMC_M10
GO:0060395	0.91	0.14	28	62	SMAD protein signal transduction	BP	1	LRP1,GDF9,SMAD5,ROR2,SPTBN1,BMP8A,OVOL2,RBPMS,SLC33A1,VEPH1,ITGB6,KIAA0319,SLC2A10,TGFB2,PBLD,GDF15,INHA,BMPR1A,PARP1,FOS,CILP,TOB1,CITED1,SH2B1,SMAD3,JUN,SKI,NCEH1	grey	VSMC_M10
GO:0071549	0.91	0.18	10	24	cellular response to dexamethasone stimulus	BP	1	FECH,AQP1,EGFR,BMI1,DNMT3B,ABCC2,SRD5A1,ASS1,SMYD3,CRH	grey	VSMC_M10
GO:0019369	0.91	0.17	11	26	arachidonic acid metabolic process	BP	1	FAAH,ABHD6,PLA2G4B,CYP2U1,PLA2G4A,DAGLB,EPHX1,ALOXE3,MAPK3,CYP2J2,PTGES2	grey	VSMC_M10
GO:0006103	0.91	0.21	6	15	2-oxoglutarate metabolic process	BP	1	DLD,D2HGDH,L2HGDH,MRPS36,KYAT3,PHYH	grey	VSMC_M10
GO:0010828	0.91	0.16	15	35	positive regulation of glucose transmembrane transport	BP	1	MAPK14,FGF21,BRAF,AKT2,PIK3R1,SORBS1,IRS1,TERT,C2CD5,NFE2L2,CLTCL1,INSR,C3,GPC3,ARPP19	grey	VSMC_M10
GO:0019511	0.91	0.21	6	15	peptidyl-proline hydroxylation	BP	1	P4HA2,CRTAP,EGLN2,EGLN3,P3H2,P3H3	grey	VSMC_M10
GO:0035970	0.91	0.22	5	13	peptidyl-threonine dephosphorylation	BP	1	PPP2CB,DUSP18,DUSP10,PPM1A,DUSP1	grey	VSMC_M10
GO:0070542	0.91	0.15	22	49	response to fatty acid	BP	1	NME1,CAT,UCP1,FOXO3,SOX9,GIPR,NR1H4,SMARCD1,IRS1,CPT1A,GNPAT,HMGCL,PDK3,CREB1,CPS1,XRCC5,KCNK4,LDLR,SREBF1,ASS1,CLDN1,INSIG2	grey	VSMC_M10
GO:0034340	0.91	0.15	21	47	response to type I interferon	BP	1	SHMT2,MYD88,MAVS,IRF7,OAS3,NLRC5,SP100,YTHDF2,SETD2,PTPN6,MUL1,USP18,DCST1,STAT2,TYK2,JAK1,PTPN2,MIR21,OAS1,FADD,WNT5A	grey	VSMC_M10

GO:0110156	0.91	0.22	5	13	methylguanosine-cap decapping	BP	1	LSM1,NUDT12,EIF4ENIF1,DCP1B,EDC4	grey	VSMC_M10
GO:0018212	0.91	0.11	143	290	peptidyl-tyrosine modification	BP	1	ERBB3,CLK4,IL5,ROS1,PTK2,NTRK2,HSF1,TEK,TTBK1,TEC,KIT,NCK2,ROR2,SEMA4D,PDCL3,IL11,PRKCE,PPP2R1A,IBTK,EPGN,AREG,EPHA7,TGFA,MET,EGFR,EPHA1,IL15,HDAC2,ABI1,PIBF1,ALK,DYRK1B,AATK,HBEGF,EIF2AK2,PRNP,NRG1,GAS6,HPX,ERCC6,FLT1,EPHB1,RET,HCK,TTL,FER,OSBP,FBXW7,PTPRJ,IL7,PTK2B,ARL2BP,RIPK2,DMTN,NOD2,CNTN1,CD40,DSTYK,IGF2,NEURL1,LRRK1,HDAC1,LCK,BANK1,MAP2K6,TPST1,ADRA2A,NCF1,DYRK3,EGF,HCLS1,ADNP,CASS4,PARP9,PARP14,BMX,AGT,FGR,CBL,OSM,EPHB3,BAZ1B,SNX6,ABL2,TTN,PPP2R5B,EPHB4,IL12A,PTPN6,TNFSF18,DOK7,ZFYVE28,FGFR3,SOCS4,STK16,BST1,EPHA4,DLG4,DVL2,TNK1,TMEM102,FGFR4,DGKQ,CTF1,PSEN1,EFNA1,MAPK3,IL6R,HAX1,MAP2K1,MAP2K5,BTK,TYK2,CLK3,CD74,INSR,CCL5,CSF1R,YES1,ISL1,SUZ12,GHR,JAK1,UNC119,PTPN2,SOCS1,LRP8,PRKCZ,MLST8,INSRR,CNTF,PEAK1,MIR125A,THY1,CSPG4,LIF,DUSP22,TSLP,CADM4,EREG,MAP2K7,RAP2B,PDGFD	grey	VSMC_M10
GO:0032352	0.91	0.22	5	13	positive regulation of hormone metabolic process	BP	1	PAX8,ARNT,SLCO1C1,POR,WNT4	grey	VSMC_M10
GO:0008156	0.91	0.18	10	24	negative regulation of DNA replication	BP	1	ZRANB3,FBH1,RAD17,ATR,DONSON,BCL6,TSPYL2,TIPIN,LIG3,TTF1	grey	VSMC_M10
GO:2000404	0.91	0.17	12	28	regulation of T cell migration	BP	1	CCL27,OXSR1,CD200,APOD,ADAM8,ITGA4,TMEM102,CCL5,CRK,RHOA,FADD,WNT5A	grey	VSMC_M10
GO:1903131	0.91	0.11	147	297	mononuclear cell differentiation	BP	1	AGER,HLA-DRA,TCF7,BAK1,XRCC6,MYC,MFNG,MYH9,FBXO7,SRF,CDK6,KIT,NFIL3,IL11,MSH2,HOXA7,SLC46A2,RSAD2,IL4I1,IL15,ATG5,TRAF3IP2,BMI1,CLPTM1,GLI2,FOXJ1,IL15RA,KLF6,CMTM7,TGFBR2,EOMES,PLCL2,NCKAP1L,GAS6,FOXP1,PRKDC,IRF7,TRAF6,SOX12,TOX,ZC3H8,IL1A,CAMK4,PIK3R1,PTPRJ,TMEM131L,IL7,TPD52,PTK2B,RASGRP1,LMBR1L,RIPK2,CDH17,AP3B1,F2RL1,SOX13,ITGB6,DCAF1,DOCK10,PKNOX1,LCK,TCIRG1,RABL3,HLX,ACIN1,SLAMF8,FZD7,HDAC4,CD28,FCER1G,RUNX3,ZBTB46,PATZ1,FZD9,SP3,PPP2R3C,ADAM8,FZD5,DUSP10,BCL6,SYVN1,BATF2,ITGA4,VSIR,RC3H1,PTPN6,TNFSF18,WNT4,LAG3,PPP3CB,LTBR,STAT3,STAT5B,INHA,FOXP3,ZBTB1,GPS2,CARD11,RBPJ,MED1,TBX21,PSEN1,SEMA4A,KAT7,DOCK2,BATF,ZBTB7B,IL6R,CD19,IL4R,GPR68,BTK,BCL11B,YY1,CD74,CSF1R,JAG2,NKAP,VAV1,LEPR,PTGER4,IL7R,PTPN2,JUN,SOCS1,DROSHA,DNAJA3,PRKCZ,TSC1,LRRC8A,MIR21,MIR125B1,RHOA,SOX4,MAFB,FADD,CD46,DHRS2,FGL2,TNFSF9,NHEJ1,ZFP36L2,CEBPB,PRR7,TNFSF13B,BATF3,RC3H2	grey	VSMC_M10
GO:0042274	0.91	0.14	30	64	ribosomal small subunit biogenesis	BP	1	HELB,UTP23,RRP7A,TSR1,RPS14,UTP3,ERCC2,RPS8,RPSA,METTL17,PRKDC,DHX37,BMS1,RPP40,RIOK1,RPS17,HEATR1,FAM207A,MRPS11,NOP9,RPS27,XRCC5,TSR2,KRI1,RIOK3,UTP6,SNU13,ERAL1,RPS27L,GTF2H5	grey	VSMC_M10
GO:0071709	0.91	0.15	17	39	membrane assembly	BP	1	CHMP2A,SPTBN1,SIRT2,STX2,NSFL1C,CHMP7,NLGN4Y,NRXN2,NLGN2,ANXA2,GAK,STX4,SYT11,NLGN3,CEL,VPS4A,TLCD1	grey	VSMC_M10
GO:0051204	0.91	0.19	8	19	protein insertion into mitochondrial membrane	BP	1	HSPA4,TOMM22,AGK,NMT1,TIMM29,MAIP1,TIMM10B,TIMM8B	grey	VSMC_M10
GO:0009952	0.91	0.12	72	150	anterior/posterior pattern specification	BP	1	FRS2,SMO,FOXH1,SRF,ROR2,MEOX2,SIX2,SCMH1,HOXA2,HOXA4,HOXA6,HOXA7,FUZ,OSR1,BMI1,ABI1,GLI2,PCDH8,XRCC2,ACVR2B,ACVR2A,BHLHE40,PRKDC,HOXC6,TCF15,POFUT1,FAM208A,NKX3-1,NKD1,LDB1,PGAP1,POGLUT1,AXIN2,GBX2,HES6,WNT3,HOXD8,HOXD4,HOXD3,FZD5,MEOX1,TULP3,BMPR1A,TCAP,RBPJ,ALDH1A2,PSEN1,HOXB5,HOXB6,HOXB8,HOXB9,SMAD3,LHX1,YY1,CDX1,MIB1,NLE1,GPC3,HES4,SKI,WDR77,CELSR2,FOXF1,HOXB3,NOG,WNT5A,BASP1,OTX1,DDIT3,HEY2,WNT2,HOXA3	grey	VSMC_M10
GO:0031468	0.91	0.21	5	13	nuclear membrane reassembly	BP	1	CHMP2A,SIRT2,NSFL1C,CHMP7,VPS4A	grey	VSMC_M10
GO:0000165	0.91	0.10	293	579	MAPK cascade	BP	1	PHLPP1,LRP1,SEMA6A,MAP3K5,FRS2,STK3,MAPK8IP2,MAP3K4,PRKN,AGER,FBLN1,ROS1,SYNGAP1,TRIB1,MYC,LEMD2,PRXL2C,GRM4,TAB1,MAPK15,MAPK14,NTRK2,MAPK13,STK38,HSF1,TEK,GPR37,KIT,ROR2,GADD45G,WNK2,KLHL31,PRDX1,SEMA4C,DNAJA1,IL11,PRKCE,FKTN,FPR2,GSDME,VRK2,EMC10,LPAR1,MTURN,EPGN,NOD1,BMPER,EPHA7,TGFA,ITGB1BP1,VRK3,GRB10,YWHAZ,EGFR,ARHGEF5,TLR4,TRAF1,SDCBP,FGF21,BRAF,SPRY1,DNAJC27,C5AR2,C5AR1,PRKD2,PHB,SOX9,RAF1,MYD88,PPP1CB,SPRY2,ALK,ARRB1,IKBKB,C1QTNF1,NEK10,ZMYND11,PDE6G,PLCB1,MAP4K1,SPRED3,GPS1,EIF2AK2,ADRA1D,DUSP6,RELL2,SEMA3A,KL,NRG1,SH3RF2,GAS6,SIRT3,ARL6IP5,ERCC6,MFHAS1,FLT1,RYK,ADRA2B,TRAF6,APIP,ST5,EPHB1,MOS,RET,PIK3CB,HAND2,ROBO1,PLCE1,FBXW7,PTPRJ,MEF2C,P2RX7,WDR54,P2RY1,MADD,PTK2B,RASGRP1,RAPGEF2,MAPKAPK5,CCL17,TIAM1,PAQR3,RIPK2,F2RL1,F2R,NQO2,RIPK1,PRDM11,STK4,GCNT2,NOD2,TAOK3,CD40,DSTYK,DUSP8,IGF2,PRDM15,MAPKAPK3,BANK1,MAP2K6,TRPV4,PDCD4,TGFB2,ADRA2A,SLC30A10,DAG1,AJUBA,NCF1,KRAS,MAPKAPK2,EGF,MAP3K21,TBC1D10C,SOX2,SORL1,GPER1,DOK5,AGT,CIB1,TRAF5,MAP3K6,DVL3,STRADB,FZD7,OSM,PEA15,THPO,PDE6H,MAP3K3,PDE8A,MAP3K15,MAGI3,DUSP16,DLG1,MAP3K11,ADAM8,AIDA,FZD5,DUSP10,MBIP,RAP1B,CTSH,MEN1,MAP4K2,PPM1L,FN1,DUSP19,PTPN6,PDCD10,ADRA2C,GPBAR1,GDF15,FGFR3,PINK1,ARAF,LTBR,MAPK1,MINK1,EPHA4,MAGED1,DVL2,IGFBP4,GPS2,RELL1,RGS14,FGFR4,SYNJ2BP,MAP3K9,PSEN1,FGF18,TAOK2,EFNA1,WWC1,MAPK3,HACD3,MAP2K1,PIK3R5,SMAD3,MAP2K5,RIT2,TNIP1,PSMD10,GAREM1,N[illegible]	grey	VSMC_M10

GO:0016485	0.91	0.12	83	170	protein processing	BP	1	SEC11C,ATP23,CPM,MYRFL,BAK1,XPNPEP3,PTCH1,MYH9,PLGRKT,IL1R2,ACP4,ADAMTS3,BCL2L12,KEL,CST7,IFT172,PLAT,PITRM1,PRNP,CPXM1,MIPEP,ASPH,IMMP1L,CASP3,PCSK1,SCG5,HTRA2,MME,SPCS1,RIPK2,IFT52,PIDD1,CPN1,LONP2,SIRT4,RHBDD1,SPPL3,HJV,ENPEP,SPRTN,ACE2,CIDEB,PRSS12,ECE2,CPXM2,BACE1,MELTF,PCSK7,PISD,ADAM8,SRGN,TYSND1,CTSH,VSIR,LPCAT3,CPZ,HGFAC,ECE1,PSEN2,PARP1,DDI2,F12,PSEN1,SNX12,OGT,AC138894.1,SERPINF2,ADAMTS13,HPR,TMEM208,FGA,NOL3,CNTN2,FADD,CASP8,BAG2,FXN,C2CD3,PERP,APH1B,FAM111A,TMPRSS12,SPCS2	grey	VSMC_M10
GO:0009151	0.91	0.20	6	15	purine deoxyribonucleotide metabolic process	BP	1	GDA,NUDT15,NUDT16,DGUOK,NUDT18,ADK	grey	VSMC_M10
GO:0060996	0.91	0.13	40	87	dendritic spine development	BP	1	FSTL4,ASAP1,NCK2,SHANK1,HDAC2,IQSEC1,EPHB1,PLK2,MEF2C,CUX2,TIAM1,OPA1,ITPKA,SLC12A5,SRGAP2C,DOCK10,NEURL1,DNM1L,CAPRIN2,CFL1,DISC1,CDKL5,EPHB3,TANC2,DNM3,EPHA4,DLG4,SDK1,PSEN1,EFNA1,ZDHHC15,GPRASP2,BHLHB9,ARHGAP44,CAMK2A,CDK5R1,FMR1,LRP8,LLPH,FOXO6	grey	VSMC_M10
GO:0045892	0.91	0.10	482	943	negative regulation of transcription, DNA-templated	BP	1	SMARCC2,ATP8B1,PLAGL1,ZBTB2,ESR1,CNOT2,OSR2,SNW1,ZNF706,ZBTB12,PRKN,PPARA,TCF7,XRCC6,PITX1,MYC,TOB2,SMAD5,L3MBTL3,L3MBTL2,PTCH1,ATF4,CBX7,CBY1,SOX10,TMPRSS6,SMO,HSF1,FOXP4,MDFI,CCND3,FOXH1,CDKN2A,RBFOX2,RORB,CRY1,SRF,ZNF318,NFX1,SMARCA2,RFX3,NCK2,FHL2,TFAP2B,NFIL3,SEMA4D,RPS14,ZNF550,BEND6,ZNF268,SCMH1,NFE2L3,HOXA2,HOXA7,JAZF1,XPO1,AKIRIN2,CASP8AP2,BACH2,ZNF692,NR1H2,ZNF350,FOXP2,MED25,KLF11,POU3F3,LANCL2,CREM,SDCBP,OSR1,FOXO3,BEND5,HDAC2,PHF10,DNMT3A,BMI1,ZC3H4,CNBP,SETDB2,PHB,SOX9,ZNF746,EED,SUV39H2,ZNF404,PLK3,GLI2,DEDD2,RSF1,KDM4A,DACH1,TAF3,ACVR2B,KLF5,KLF12,FOXJ1,SFMBT2,GZF1,POU4F1,WNT11,THAP1,EID2,OVOL2,ZNF219,EOMES,CBX4,BHLHE40,CHD8,SIRT2,ZMYND11,RARB,PURA,NR1D2,PAWR,PLCB1,SIRT7,DPF1,ALX1,TAF7,NRG1,GAS6,RYBP,FOXP1,MITF,MTA3,ARID5A,IRF7,TRAF6,LIN37,NR1H4,KCNIP3,TRIB3,PFDN5,SDR16C5,ZC3H8,DNMT3B,BTAF1,CEBPA,CBFA2T2,SMYD1,FOXL2,ZNF536,TFDP2,IRF2,NCOA2,DKK3,SAP30,ZNF366,ARNTL,GCFC2,HMBOX1,MEF2C,PEX2,PKIA,TMBIM6,CNOT1,SLA2,CUX2,MCPH1,SOX6,FNIP2,BACH1,SFMBT1,NR1H3,EAPP,SCAF4,LCOR,DDX54,RUNX1T1,VDR,NKX3-1,BAHD1,HDAC7,NKX6-1,LOXL2,NR4A2,PHF21A,CRY2,PRDM11,CDYL,RREB1,RTF1,ZBED6,SOX13,ZNF248,TFAP2A,RIPPLY3,HIF1AN,PAX2,SALL1,PPHLN1,PRICKLE1,HIVEP1,JARID2,ATXN1,SUDS3,ZNF423,LDB1,IGF2,MAGEL2,ELK4,NUPR2,DCAF1,SUFU,TAGLN3,PCGF6,IRX2,RPL23,HDAC1,ELF3,CCND1,SP100,TRPV4,PDCD4,ZBTB20,SATB2,TCF7L2,AJUBA,ZNF639,PIAS3,LHX9,HJV,FABP4,SNAI1,ZNF202,H[illegible]	grey	VSMC_M10
GO:0050829	0.91	0.16	14	32	defense response to Gram-negative bacterium	BP	1	AQP1,LYPD8,TLR4,OPTN,F2RL1,SLC11A1,IL6R,NOS2,DROSHA,HIST1H2BK,CHGA,CXCL6,IRGM,HIST1H2BJ	grey	VSMC_M10
GO:0034724	0.91	0.17	11	26	DNA replication-independent nucleosome organization	BP	1	ASF1A,UBN2,RBBP4,HIRA,HIST1H4B,HIST2H4A,HIST1H4H,ITGB3BP,HIST1H4D,HIST1H4A,HIST1H4F	grey	VSMC_M10
GO:0042092	0.91	0.17	11	26	type 2 immune response	BP	1	RSAD2,TRAF3IP2,NOD2,HLX,DENND1B,BCL6,TBX21,BATF,IL4R,CD74,PRKCZ	grey	VSMC_M10
GO:1900221	0.91	0.21	5	13	regulation of amyloid-beta clearance	BP	1	LRP1,SRF,HDAC1,LRPAP1,ABCA7	grey	VSMC_M10
GO:0010259	0.91	0.17	10	24	multicellular organism aging	BP	1	MSH2,DDC,ERCC1,WRN,IDE,RAD54B,CISD2,CTSV,PRDM2,RNF165	grey	VSMC_M10
GO:0030968	0.91	0.13	32	69	endoplasmic reticulum unfolded protein response	BP	1	DERL1,BAK1,ATF4,NCK2,ERMP1,HERPUD2,FGF21,TMTC4,EIF2AK2,DDRGK1,PIK3R1,AMFR,CCND1,FICD,YOD1,QRICH1,TBL2,RHBDD2,NFE2L2,DERL3,BHLHA15,CREB3L4,PTPN2,CREB3L3,ABCA7,HSPA5,DAB2IP,MIR199A1,DDIT3,BCL2L11,PPP1R15A,DERL2	grey	VSMC_M10
GO:0062149	0.91	0.18	8	20	detection of stimulus involved in sensory perception of pain	BP	1	PHF24,ASIC3,SCN1A,EPHB1,NTSR1,BACE1,KCNA1,TRPV1	grey	VSMC_M10
GO:0007420	0.91	0.10	295	587	brain development	BP	1	NME1,LRP1,GLI1,ATAT1,FRS2,RIDA,SYNJ2,FZD6,UQCRQ,CCDC134,BAK1,XRCC6,PITX1,PTCH1,FANCC,AGTPBP1,PLXNA4,SUN2,NTRK2,CSNK1E,CKB,SMO,RBFOX2,SRF,TTBK1,FZD1,CDK6,SLC1A1,DLX5,EFHC1,CCIN,ADGRL3,SEMA4C,MBOAT7,RTN4,UTP3,HOXA2,PPT1,AQP1,EPHA7,AKNA,WHRN,PTN,FOXP2,CTTNBP2,POU3F3,CDK5RAP2,SLC7A11,FOXO3,ARMC4,SPHK2,FAT4,SPATA5,KDM7A,GRIN2D,NAPA,BBS7,BMI1,NDNF,C5AR1,EIF2B3,XRCC1,GLI2,B4GALT2,IFT172,FOXJ1,SCN5A,NUMBL,HNMT,POU4F1,TSKU,SZT2,ALK,HOOK3,TGFBR2,EOMES,CHD8,SIRT2,MKKS,AATK,RARB,SYT1,PLCB1,RAC3,CEP290,SEMA3A,NRG1,WRN,PRKDC,RYK,SNPH,DHX37,TOX,ZSWIM6,NCOA6,CASP3,CTNND1,CLP1,DCLK2,ROBO1,ROBO2,FZD3,FAIM2,HTRA2,NDUFS3,CELF1,CSNK2A2,MCPH1,SOX6,RAPGEF2,DPYSL2,NNAT,SLIT1,SYNJ1,TENT2,NR4A2,AMIGO2,SLC38A2,RPGRIP1L,PAX2,TSPAN2,SALL1,DNAH5,CNTN1,ZNF335,DPCD,ATXN1,SUDS3,ZNF423,LDB1,COX6B1,SRGAP2C,SLC4A10,SRD5A1,INA,NEURL1,CFAP43,SLC6A3,HDAC1,ALDH1A3,PPP3CA,PGAP1,SLC38A3,SATB2,COX17,TTBK2,AMIGO3,SHROOM2,KRAS,PITPNM1,EGF,TTC21B,SOX2,ZFHX2,JHY,DISC1,GNPAT,B3GNT5,GBX2,ATP1A2,BAG3,SPTBN2,UFC1,MACO1,DLX1,DNAJC30,EPHB3,NSUN5,APOD,SETD2,WNT3,CREB1,SEMA6D,BCL2A1,H2AFY2,DIXDC1,MEN1,TNR,XRCC5,NIN,CXCR2,WNT4,HSPG2,COL3A1,STK36,ABCB6,TBX19,KCNA1,TULP3,TTC25,PADI2,HAP1,ACTB,SHROOM4,BMPR1A,DNAJB1,MPP5,RBPJ,PHF8,MED1,ALDH1A2,ATP1B2,SETD1A,PSEN1,KDM6B,ITGAM,NDEL1,TAF1,CITED1,CHRNB2,MAP2K1,POU3F4,EPOR,PCDH19,CALM1,GSC,BCL11B,LHX1,SIN3A,XAB2,MYO1D,CDK5R1,ISL1,OXCT1,TWSG1,[illegible]	grey	VSMC_M10

GO:1905114	0.91	0.10	224	448	cell surface receptor signaling pathway involved in cell-cell signaling	BP	1	NFATC1,LRP1,RPS12,GLI1,TNFAIP3,STK3,MAPK8IP2,FZD6,RIMS2,PRKN,TCF7,UBE2B,RSPO3,RNF146,MAPK14,CBY1,CSNK1E,SOX10,SMO,DAAM2,MDFI,CHRNA3,CHRNA5,PTK7,CDK14,FZD1,ROR2,DLX5,WNK2,SOSTDC1,RIMS1,PPP2R1A,SHANK1,RSPO1,GRB10,ATP6V1C2,EGFR,HECW1,FUZ,GRIK2,SFRP4,CCNY,DKKL1,VPS35,TRPM4,FOXO3,GRIN2D,MTMR2,SLC8A2,SOX9,PPM1N,LRRFIP2,GLI2,GRIK5,SCEL,TSKU,WNT11,CHRNA6,RBMS3,CHD8,UBAC2,RAB5A,FERMT1,RAC3,SPEF1,P2RX2,MITF,CALCOCO1,RYK,PFDN5,CTNND1,DKK3,TRABD2A,P2RX3,ARNTL,FZD3,MEF2C,TMEM131L,P2RX7,CSNK2A2,CUX2,PTK2B,LMBR1L,TIAM1,OTULIN,AMFR,NR4A2,RTF1,PKD2,SOX13,STK4,SALL1,VANGL1,PRICKLE1,NKD1,FBXW4,ZNF423,LDB1,PRDM15,KLHL12,LGR6,LRRK1,HDAC1,PPP3CA,CCND1,CAPRIN2,TERT,TMEM9,SULF2,IGSF11,TCF7L2,DKK2,AXIN2,SSH1,MARK1,EGF,TTC21B,SOX2,DISC1,ZNRF3,KREMEN1,DVL3,TMEM237,BICC1,NFATC4,FZD7,STX1A,TMEM25,NPAS4,NTSR1,GID8,PTPRO,GRHL3,FZD9,SENP2,WNT3,CCNYL1,FZD5,DIXDC1,WDR61,DDX3X,MPP2,CPZ,WNT4,MARK2,DAAM1,TBL1XR1,PPM1A,LEO1,OTUD5,ARHGEF19,DLG4,DVL2,IGFBP4,DDB1,NLGN2,CHRNB1,RBPJ,GRK6,STX1B,PSEN1,TMEM88,ITGAM,TGFB1I1,ITGA3,NLGN3,CSNK1G1,CITED1,CHRNB2,SOX30,SMAD3,MKS1,AC004687.2,CCDC88C,GSC,G3BP1,NLE1,ISL1,C3,NLK,IFT20,SEZ6,GPC3,ABAT,PIAS4,TLE6,TRPV1,TAX1BP3,P2RX1,ATP6V0C,PRKCZ,SKI,PKD1,MECP2,APC2,CELSR2,FOXL1,DAB2IP,MIR199A1,MIR222,MIR32,RHOA,ZBTB33,BCL7B,CHRNB4,TRABD2B,CHRNA10,CHRNG,SOX4,GRID2,NOG,WNT5A,LBX2,CELSR3,RGS4,DDIT3,DACT3,SDHAF2,SHISA2,APC,INSYN1,NKX2-5,MESD,WNT2	grey	VSMC_M10
GO:0038095	0.91	0.18	8	20	Fc-epsilon receptor signaling pathway	BP	1	IKBKB,TRAF6,FER,FCER1G,BTK,VAV1,VAV3,MAP2K7	grey	VSMC_M10
GO:1903312	0.91	0.13	34	74	negative regulation of mRNA metabolic process	BP	1	PABPC1,MAPK14,RNF20,SRSF10,TRAF3IP2,DHX34,MYD88,BAG4,PKP3,SRSF6,TENT5C,TENT4B,RBM24,AXIN2,MAPKAPK2,SRSF4,TRAF5,ANGEL2,EIF4ENIF1,YBX3,SLC11A1,RBM10,TARDBP,TOB1,TENT5D,LARP1,FMR1,METTL16,CIRBP,TRAF2,SRSF12,YBX1,TENT5A,SAP18	grey	VSMC_M10
GO:0051016	0.91	0.18	8	20	barbed-end actin filament capping	BP	1	AVIL,KIAA1211,ADD2,CARMIL1,ADD3,EPS8,CAPZA1,CARMIL2	grey	VSMC_M10
GO:0001541	0.91	0.15	17	39	ovarian follicle development	BP	1	ESR1,DMC1,FANCG,KIT,FOXO3,ARRB1,FOXL2,BMPR1B,GAS2,HYAL3,SPO11,TAF4,VGF,INHA,MSH4,CEBPB,EREG	grey	VSMC_M10
GO:0006998	0.91	0.15	19	43	nuclear envelope organization	BP	1	NEMP1,LEMD2,SUN2,CHMP2A,LPIN1,SIRT2,NSFL1C,SPAG4,CHMP7,GPER1,TOR1AIP1,PARP11,AKAP8L,TARDBP,NDEL1,PRKCB,TOR1B,VPS4A,LEMD3	grey	VSMC_M10
GO:0010827	0.91	0.14	26	58	regulation of glucose transmembrane transport	BP	1	MAPK14,SLC25A27,GRB10,FGF21,BRAF,PRKAG2,AKT2,ASPSCR1,TRIB3,PIK3R1,SORBS1,IRS1,TERT,C2CD5,PEA15,NFE2L2,CLTCL1,RSC1A1,PRKCB,INSR,YES1,C3,GPC3,INPP5K,FABP5,ARPP19	grey	VSMC_M10
GO:0072498	0.91	0.21	5	13	embryonic skeletal joint development	BP	1	OSR2,OSR1,HYAL1,SLC2A10,NOG	grey	VSMC_M10
GO:0060538	0.91	0.12	63	132	skeletal muscle organ development	BP	1	STAC3,VGLL2,PITX1,LEMD2,MAPK14,SMO,LARGE1,CNTFR,MEOX2,HIVEP3,MYH14,AKIRIN1,BVES,KEL,KLF5,KLHL40,NR1D2,RYR1,DCN,P2RX2,LMOD3,EPHB1,NR4A1,SMYD1,NLN,FOXL2,FLNB,VAMP5,SAP30,ARNTL,MEF2C,TLL2,HOMER1,BIN3,RBM24,IGF2,MYH15,NEURL1,PPP3CA,DAG1,HLX,USP19,MYOZ2,CASQ1,ACTN3,HDAC4,YBX3,MYOZ1,MYLPF,ZNF689,FOS,STRA6,FBXO22,SKI,SOX8,ASS1,RHOA,DMD,BASP1,SIX1,POPDC2,MYORG,UQCC2	grey	VSMC_M10
GO:0072593	0.91	0.11	86	176	reactive oxygen species metabolic process	BP	1	GLS2,PRKN,PPARA,TSPO,FANCC,MAPK14,RAC2,SLC1A1,PRDX1,CAT,FPR2,CYB5R4,PXDN,EGFR,TLR4,UCP1,ATG5,MYCN,FOXO3,SPHK2,DHFR,WISP3,ACOX1,PRDX3,TGFBR2,SIRT2,SIRT3,FAM19A4,PXDNL,NDUFS3,HVCN1,PTK2B,F2RL1,NQO2,RIPK1,PLA2R1,TFAP2A,PAX2,SIRT5,GBF1,SH3PXD2A,SLC30A10,FBLN5,NCF1,IFI6,AGT,ACE2,HDAC4,NCF2,PDK3,NFE2L2,MAPT,PIKFYVE,DUOX1,BCO2,GCH1,PINK1,MMP3,STAT3,BST1,SOD3,ITGAM,MPO,NOX5,VAV1,NOS2,NOXA1,PARK7,SLC25A33,GNAI3,CYBA,LCN2,NQO1,MIR21,MIR27B,MIR24-1,MT-ND2,RHOA,TIGAR,GADD45A,INAVA,HBA2,NRROS,GRB2,APOPT1,AC140504.1	grey	VSMC_M10
GO:0051923	0.91	0.21	5	13	sulfation	BP	1	SULT4A1,HS3ST5,TPST1,CHST5,SULT1C3	grey	VSMC_M10
GO:0042759	0.91	0.21	5	13	long-chain fatty acid biosynthetic process	BP	1	LTC4S,QKI,ACSBG1,ALOXE3,ACSBG2	grey	VSMC_M10
GO:0036490	0.91	0.22	4	11	regulation of translation in response to endoplasmic reticulum stress	BP	1	NCK2,EIF2AK4,DDX3X,PPP1R15A	grey	VSMC_M10
GO:0060711	0.91	0.15	16	37	labyrinthine layer development	BP	1	RSPO3,GJB5,OVOL2,SPINT2,SPINT1,VASH2,FZD5,MAPK1,RBPJ,VASH1,CITED1,MAP2K1,CASP8,GRB2,HEY2,WNT2	grey	VSMC_M10
GO:0035437	0.91	0.21	5	13	maintenance of protein localization in endoplasmic reticulum	BP	1	GPAA1,GRIK5,RER1,HSPA5,INSIG2	grey	VSMC_M10
GO:0045143	0.91	0.14	21	47	homologous chromosome segregation	BP	1	UBE2B,DMC1,CCDC155,MLH1,CCNB1IP1,SIRT7,TEX15,SYCP1,CCDC36,SPO11,TEX12,MAJIN,ZCWPW1,STAG3,P3H4,SYCE2,MEIOB,MSH4,RNF212B,AGO4,SYCE1L	grey	VSMC_M10
GO:0007171	0.91	0.22	4	11	activation of transmembrane receptor protein tyrosine kinase activity	BP	1	CHRNA3,NRG1,EGF,DGKQ	grey	VSMC_M10
GO:0008542	0.91	0.16	13	31	visual learning	BP	1	SYNGAP1,KIT,SLC7A11,B4GALT2,DDHD2,KRAS,ATP1A2,CREB1,RGS14,CHRNB2,IFT20,MECP2,ABCA7	grey	VSMC_M10
GO:0099150	0.91	0.22	4	11	regulation of postsynaptic specialization assembly	BP	1	LRFN1,GAP43,GRID2,LRRTM2	grey	VSMC_M10
GO:0055123	0.91	0.12	46	99	digestive system development	BP	1	GLI1,VPS52,SOX10,SMO,KIT,SIX2,CXCL8,EGFR,BBS7,SLC4A2,GLI2,MEGF8,KLF5,IFT172,WNT11,TGFBR2,OVOL2,CHD8,RARB,CCKBR,RET,CBFA2T2,HIP1R,SALL1,IGF2,TGFB2,HLX,EPHB3,CPS1,PYY,SAV1,NKX3-2,WDR19,ALDH1A2,RBPMS2,SMAD3,TBX2,STRA6,GATA6,DAB1,PKD1,FOXL1,FOXF1,ASS1,WNT5A,TIGAR	grey	VSMC_M10
GO:0045624	0.91	0.21	5	13	positive regulation of T-helper cell differentiation	BP	1	RIPK2,HLX,IL4R,PRKCZ,MIR21	grey	VSMC_M10
GO:0006361	0.91	0.22	4	11	transcription initiation from RNA polymerase I promoter	BP	1	BAZ2A,TAF1,RRN3,TAF1C	grey	VSMC_M10

GO:0045912	0.91	0.15	17	39	negative regulation of carbohydrate metabolic process	BP	1	PRKN,PPARA,CRY1,PLEK,FAM3C,GCKR,SIK1,MST1,ACTN3,HDAC4,PASK,STAT3,COX11,NCOR1,LEPR,INPP5K,TIGAR	grey	VSMC_M10
GO:0030202	0.91	0.22	4	11	heparin metabolic process	BP	1	CSGALNACT1,AC022400.6,IDUA,GLCE	grey	VSMC_M10
GO:0061298	0.92	0.19	6	16	retina vasculature development in camera-type eye	BP	1	ACVR2B,CLIC4,RHOJ,ROM1,LAMA1,ACVRL1	grey	VSMC_M10
GO:0010458	0.92	0.16	11	26	exit from mitosis	BP	1	CHMP2A,CLASP2,SIRT2,SIRT7,CHMP7,NPM2,PPP2R2D,EPS8,CDKN1B,CDC14A,RGCC	grey	VSMC_M10
GO:0050863	0.92	0.11	101	206	regulation of T cell activation	BP	1	AGER,HLA-DRA,HLA-DOB,HLA-DMA,HLA-DPB1,TCF7,TNFRSF13C,RAC2,TNFRSF21,NCK2,SLC46A2,PELI1,IL4I1,IL15,BMI1,CLPTM1,GLI2,FOXJ1,TGFBR2,PAWR,PRNP,NCKAP1L,SLC7A1,TRAF6,SOX12,TOX,ZC3H8,IL1A,CAMK4,CASP3,TMEM131L,IL7,PAG1,RASGRP1,RIPK2,AP3B1,MARCH7,SOX13,NOD2,IGF2,LCK,ICOSLG,BTN2A2,GPAM,PRKAR1A,HLX,CD28,RUNX3,DLG1,ADAM8,DUSP10,BCL6,IL12A,VSIR,RC3H1,PTPN6,TNFSF18,LAG3,STAT5B,SCGB1A1,FOXP3,RASAL3,ZBTB1,CARD11,TNFRSF1B,TBX21,ZBTB7B,LAT,IL4R,CD74,CCL5,CCL2,YES1,NKAP,VAV1,TWSG1,IL7R,PTPN2,SOCS1,DROSHA,DNAJA3,PRKCZ,GLMN,BCL10,MIR21,MIR27A,RHOA,SOX4,FADD,DPP4,CD46,THY1,FGL2,CD70,TNFSF9,LAX1,EFNB3,CEBPB,TNFSF13B,HAVCR2,RC3H2	grey	VSMC_M10
GO:0072009	0.92	0.12	41	89	nephron epithelium development	BP	1	MTSS1,MYC,KLHL3,PTCH1,PODXL,SMO,TFAP2B,SIX2,POU3F3,OSR1,SOX9,AQP11,FOXJ1,GZF1,WNT11,JAG1,ASXL1,PAX8,EYA1,FMN1,MEF2C,PKD2,PAX2,SALL1,IRX2,AGT,LAMA5,PTPRO,DLG1,WNT4,MAGED1,CITED1,LHX1,GPC3,PKD1,SOX8,NOG,BASP1,SIX1,LIF,FGF1	grey	VSMC_M10
GO:0046635	0.92	0.14	18	41	positive regulation of alpha-beta T cell activation	BP	1	HLA-DRA,TGFBR2,NCKAP1L,RIPK2,AP3B1,HLX,CD28,RUNX3,IL12A,FOXP3,RASAL3,ZBTB7B,IL4R,NKAP,SOCS1,PRKCZ,MIR21,RHOA	grey	VSMC_M10
GO:0021543	0.92	0.12	60	127	pallium development	BP	1	NME1,LRP1,ATAT1,UQCRQ,SUN2,NTRK2,SMO,SRF,CDK6,EFHC1,MBOAT7,RTN4,FOXP2,POU3F3,FAT4,EIF2B3,XRCC1,TSKU,ALK,MKKS,PLCB1,CASP3,DCLK2,ROBO1,CELF1,CSNK2A2,MCPH1,TENT2,SLC38A2,SRGAP2C,SRD5A1,HDAC1,DISC1,DLX1,NSUN5,BCL2A1,DIXDC1,COL3A1,KCNA1,MPP5,PSEN1,KDM6B,NDEL1,XAB2,CDK5R1,DAB1,LRP8,CRK,PLXNA3,TSC1,PHLPP2,DAB2IP,RHOA,ADGRG1,FOXG1,LAMB1,GNG12,CCDC85C,POU3F2,BTBD3	grey	VSMC_M10
GO:0006309	0.92	0.19	6	15	apoptotic DNA fragmentation	BP	1	HSF1,FOXL2,DNASE2,PAM16,DFFB,NMNAT1	grey	VSMC_M10
GO:0043243	0.92	0.16	12	29	positive regulation of protein-containing complex disassembly	BP	1	JMJD4,KIAA1211,PLEK,KATNB1,F2RL1,CARMIL1,TRPV4,INSR,SMCR8,CARMIL2,IRGM,PDXP	grey	VSMC_M10
GO:0002768	0.92	0.11	76	158	immune response-regulating cell surface receptor signaling pathway	BP	1	CD226,HLA-DPB1,PTK2,PLA2G6,TEC,KIT,TNFRSF21,PRKCE,FPR2,C5AR2,C5AR1,PRKD2,NECTIN2,EIF2B3,IKBKB,PAWR,PLCL2,PRNP,NCKAP1L,FOXP1,TRAF6,HCK,FER,LPXN,PTPRJ,MEF2C,SLA2,RIPK2,CD40,GPLD1,SPPL3,LCK,BANK1,ICOSLG,BTN2A2,DENND1B,CMKLR1,THEMIS2,LAT2,BMX,FGR,PLPP4,CD28,FCER1G,RC3H1,PTPN6,CD38,MAPK1,FOXP3,GPS2,CARD11,PSEN1,LCP2,CD19,PRKCB,BTK,YES1,PRAM1,VAV1,PTPN2,CREBBP,IKBKG,VAV3,BCL10,CMTM3,FYB1,BTN1A1,CD247,THY1,UBE2N,GCSAM,HRAS,LAX1,SH2B2,MAP2K7,RC3H2	grey	VSMC_M10
GO:0043928	0.92	0.21	5	13	exonucleolytic catabolism of deadenylated mRNA	BP	1	CNOT6,EXOSC3,NT5C3B,CNOT8,EXOSC5	grey	VSMC_M10
GO:0014911	0.92	0.15	15	35	positive regulation of smooth muscle cell migration	BP	1	LRP1,TLR4,ADAMTS1,DOCK5,TERT,SSH1,AGT,HDAC4,SEMA6D,FOXO4,CCL5,DOCK7,CRK,MIR21,PDGFD	grey	VSMC_M10
GO:0045103	0.92	0.15	13	31	intermediate filament-based process	BP	1	RAF1,EVPL,BFSP1,NEFM,DSP,INA,PKP2,SYNM,KLHL24,DES,CLN8,NDEL1,MTM1	grey	VSMC_M10
GO:0051567	0.92	0.16	12	29	histone H3-K9 methylation	BP	1	SETD7,SETDB2,SUV39H2,KDM4A,DNMT3B,JARID2,MECP2,PRDM16,SETDB1,ARID4A,BEND3,ZNF304	grey	VSMC_M10
GO:0030850	0.92	0.15	16	37	prostate gland development	BP	1	GLI1,ESR1,FRS2,PTCH1,HOXA13,TNC,SOX9,GLI2,PLAG1,CYP7B1,NKX3-1,CDKN1B,WDR77,SERPINB5,NOG,WNT5A	grey	VSMC_M10
GO:0031069	0.92	0.18	8	20	hair follicle morphogenesis	BP	1	SMO,SOSTDC1,INTU,GLI2,TGFB2,SNAI1,GORAB,NGFR	grey	VSMC_M10
GO:0032729	0.92	0.15	15	35	positive regulation of interferon-gamma production	BP	1	CD226,HLA-DPB1,TLR4,RASGRP1,RIPK2,F2RL1,FZD5,IL12A,SLC11A1,ISL1,SLC7A5,FADD,WNT5A,HRAS,HAVCR2	grey	VSMC_M10
GO:0045116	0.92	0.19	6	15	protein neddylation	BP	1	EPAS1,RNF7,NEDD8,RPL11,DCUN1D3,NAE1	grey	VSMC_M10
GO:0006983	0.92	0.20	5	13	ER overload response	BP	1	ATG10,TMCO1,HSPA5,DDIT3,BCL2L11	grey	VSMC_M10
GO:0007250	0.92	0.20	5	13	activation of NF-kappaB-inducing kinase activity	BP	1	TNFSF15,TRAF6,ZFP91,TRAF2,COPS8	grey	VSMC_M10
GO:0006893	0.92	0.13	25	56	Golgi to plasma membrane transport	BP	1	GOPC,EXOC4,STEAP2,SPTBN1,RAB10,OPTN,GOLGA4,CCDC93,LYPLA1,VAMP5,EXOC6B,KIF13A,BBS1,NSF,EXOC5,CNST,RSC1A1,AC138894.1,DNM2,GGA2,VPS35L,RAB26,SEC16A,C16orf70,VAMP3	grey	VSMC_M10
GO:0006898	0.92	0.11	93	192	receptor-mediated endocytosis	BP	1	LRP1,REPS1,CANX,RAB21,IGF2R,PICK1,MICALL1,USP46,SMAP1,FPR2,PPT1,CXCL8,RSPO1,AAK1,SFRP4,SDCBP,WASF1,EPS15,MTMR2,AP2S1,CUBN,APOC1,LRP1B,ARRB1,TGFBR2,NEU3,FCHSD2,RAB5A,DNAJC13,PIK3CB,HIP1R,FCHO2,WDR54,LMBR1L,BMP2K,SYNJ1,PLA2R1,ANKRD13A,SLC9B2,EGF,ANKRD13D,GRK2,SORL1,SAG,DGKD,CBL,AP2M1,FCER1G,SH3GL3,CALY,PIKFYVE,ITGA4,LRPAP1,CXCR2,GRK4,CLTCL1,CALCRL,HSPG2,DNM3,RAMP2,CXCL16,DLG4,PDLIM7,ANXA2,GAK,ITGAM,SYT11,NLGN3,SNX1,AC138894.1,LDLR,DNM2,INSR,AP2B1,SGIP1,C3,UNC119,FMR1,PIP5K1C,GPR107,FNBP1L,BTBD8,AP1G1,CAP1,MIR199A1,MIR27B,MIR27A,CNTN2,LDLRAP1,CACNG7,GRB2,CACNG5,LRRTM2	grey	VSMC_M10
GO:0030513	0.92	0.16	11	27	positive regulation of BMP signaling pathway	BP	1	KCP,UBE2O,ZNF423,FBXL15,FKBP8,RBPJ,RNF165,TWSG1,GPC3,ENG,ACVRL1	grey	VSMC_M10
GO:0046456	0.92	0.16	12	28	icosanoid biosynthetic process	BP	1	LTC4S,MGST2,PIBF1,PLA2G4A,GGT2,GGT6,DAGLB,CD74,PTGES2,FABP5,GGT5,PTGES	grey	VSMC_M10
GO:1903019	0.92	0.20	5	13	negative regulation of glycoprotein metabolic process	BP	1	AGO2,HBEGF,SLC2A10,ABCA7,ACER2	grey	VSMC_M10

GO:0018108	0.92	0.10	141	287	peptidyl-tyrosine phosphorylation	BP	1	ERBB3,CLK4,IL5,ROS1,PTK2,NTRK2,HSF1,TEK,TTBK1,TEC,KIT,NCK2,ROR2,SEMA4D,PDCL3,IL11,PRKCE,PPP2R1A,IBTK,EPGN,AREG,EPHA7,TGFA,MET,EGFR,EPHA1,IL15,HDAC2,ABI1,PIBF1,ALK,DYRK1B,AATK,HBEGF,EIF2AK2,PRNP,NRG1,GAS6,HPX,ERCC6,FLT1,EPHB1,RET,HCK,FER,OSBP,FBXW7,PTPRJ,IL7,PTK2B,ARL2BP,RIPK2,DMTN,NOD2,CNTN1,CD40,DSTYK,IGF2,NEURL1,LRRK1,HDAC1,LCK,BANK1,MAP2K6,ADRA2A,NCF1,DYRK3,EGF,HCLS1,ADNP,CASS4,PARP9,PARP14,BMX,AGT,FGR,CBL,OSM,EPHB3,BAZ1B,SNX6,ABL2,TTN,PPP2R5B,EPHB4,IL12A,PTPN6,TNFSF18,DOK7,ZFYVE28,FGFR3,SOCS4,STK16,BST1,EPHA4,DLG4,DVL2,TNK1,TMEM102,FGFR4,DGKQ,CTF1,PSEN1,EFNA1,MAPK3,IL6R,HAX1,MAP2K1,MAP2K5,BTK,TYK2,CLK3,CD74,INSR,CCL5,CSF1R,YES1,ISL1,SUZ12,GHR,JAK1,UNC119,PTPN2,SOCS1,LRP8,PRKCZ,MLST8,INSRR,CNTF,PEAK1,MIR125A,THY1,CSPG4,LIF,DUSP22,TSLP,CADM4,EREG,MAP2K7,RAP2B,PDGFD	grey	VSMC_M10
GO:0034035	0.92	0.20	5	13	purine ribonucleoside bisphosphate metabolic process	BP	1	SLC35B2,TPST1,SLC26A1,SLC26A2,SULT1C3	grey	VSMC_M10
GO:0050427	0.92	0.20	5	13	3'-phosphoadenosine 5'-phosphosulfate metabolic process	BP	1	SLC35B2,TPST1,SLC26A1,SLC26A2,SULT1C3	grey	VSMC_M10
GO:1902115	0.92	0.11	77	161	regulation of organelle assembly	BP	1	VPS4B,PAN2,PIP4K2C,TBC1D30,MDM1,CNOT2,CNOT6,HSPA1B,RNF5,IL5,ASAP1,MAPK15,CHMP2A,DYNC2LI1,TRAPPC12,FUZ,CDK5RAP2,ATG5,SDCBP,PIP4K2A,LCP1,RALB,CEP295NL,LRFN1,RAB5A,FEZ2,ULK1,PAN3,PLK2,HCK,CHMP2B,TBC1D12,CNOT1,CHMP4C,CNOT6L,ALMS1,ZMYND10,AC112128.1,SEC22B,GAP43,TMEM39A,ADPRH,MTMR3,CDKL5,RAB1B,LIMK2,KIF9,PIKFYVE,TBC1D14,HTT,RNF4,PINK1,STX18,SDCCAG8,HAP1,MNS1,PHF23,CNTROB,CCDC151,TRIM37,DNM2,G3BP1,SMCR8,IFT20,MTM1,WRAP73,CDK10,CHMP1A,KIAA1324,GPSM2,SH3GLB1,ODF2L,RHOA,GRID2,CCSAP,DRG1,LRRTM2	grey	VSMC_M10
GO:0008637	0.92	0.12	41	87	apoptotic mitochondrial changes	BP	1	PRKN,BAK1,PLA2G6,TMEM14A,GGCT,VPS35,TIMM50,PPP2CB,HIP1R,OPA1,IFIT2,DNM1L,GPER1,IFI6,CIDEB,SFN,THEM4,STPG1,FZD9,FIS1,NMT1,PINK1,MUL1,TMEM102,ATP2A1,PSMD10,CAMK2A,JUN,ACAA2,PAM16,NAIF1,MIR29A,MLLT11,NOL3,BNIP3L,FXN,BCL2L11,SLC35F6,BCL2L2,PPIF,AC140504.1	grey	VSMC_M10
GO:0060421	0.92	0.15	15	35	positive regulation of heart growth	BP	1	GLI1,MAPK14,PIM1,MEF2C,TBX5,MAPK1,BMPR1A,RBPJ,TBX2,GATA6,MIR199A1,MIR222,BASP1,HEY2,WNT2	grey	VSMC_M10
GO:0086065	0.92	0.14	20	46	cell communication involved in cardiac conduction	BP	1	TRPM4,CACNB2,ATP1A3,SCN5A,KCNJ3,TBX5,DSP,CASQ2,RYR2,PKP2,ANK2,ATP1A2,SCN4B,ATP1B1,ATP1B2,CACNA1G,CALM1,DSG2,RNF207,HCN4	grey	VSMC_M10
GO:0050650	0.92	0.18	7	18	chondroitin sulfate proteoglycan biosynthetic process	BP	1	CHPF2,CSGALNACT1,CHST3,CHST7,CHST12,B3GALT6,CHSY3	grey	VSMC_M10
GO:0032232	0.92	0.15	12	29	negative regulation of actin filament bundle assembly	BP	1	PRKN,SHANK1,MET,CLASP2,PIK3R1,PPFIA1,ARHGAP6,WASF2,WAS,CORO2B,INPP5K,MIR21	grey	VSMC_M10
GO:0032102	0.92	0.10	131	266	negative regulation of response to external stimulus	BP	1	SERPINB2,SEMA6A,TNFAIP3,USP15,ENPP3,TSPAN8,SLC12A2,PPARA,TRIB1,C6orf106,MAPK14,TEK,SEMA4D,SEMA4C,CLDN19,CD109,FPR2,NT5E,NR1H2,VPS35,C5AR2,CST7,CLASP2,FNDC4,THBD,SEC14L1,PLAT,C1QTNF1,CHD8,SIRT2,MKKS,NMI,NR1D2,NPLOC4,SEMA3D,SEMA3A,MFHAS1,RYK,PCBP2,NR1H4,PLK2,HCK,ROBO1,ROBO2,SEMA4F,NR1H3,TRAFD1,OAS3,NLRC5,OTULIN,SEMA3G,SPP1,MAPKBP1,SEMA4G,ADTRP,NOD2,CD96,FAP,KIAA0319,CD200,SLC6A3,SEMA3F,YTHDF2,UBASH3B,GPER1,SLAMF8,PARP14,KREMEN1,RNF26,FGR,NLRX1,CERS2,TRAF3IP1,HTRA1,USF1,PTPRO,BACE1,LRIG2,WNT3,DUSP10,SEMA6D,TNR,CASK,LPCAT3,WNT4,CALCRL,MYOZ1,MUL1,PADI2,FOXP3,EPHA4,GPS2,TKFC,USP18,F12,TNFRSF1B,ANXA2,SEMA4A,SYT11,DCST1,SMAD3,TSPAN6,LDLR,STAT2,RIOK3,MAPK7,CCL2,YES1,ISL1,PTGER4,PTPN2,ABAT,CRK,SERPINF2,PLXNA3,FOXF1,MIR199A1,MIR21,MIR222,FGA,C5orf30,OAS1,GRID2,DPP4,A2M,RTN4RL1,WNT5A,DUSP1,FGL2,SERPINB9,HAVCR2	grey	VSMC_M10
GO:0071466	0.92	0.12	43	92	cellular response to xenobiotic stimulus	BP	1	NME1,MYC,SOX10,PPAT,DDC,EGFR,FGF21,NUDT15,PRNP,GAS6,MEF2C,CYP2R1,BPHL,ABCC2,KCNE2,CBR3,CMBL,TPMT,GPLD1,FMO5,CYP2U1,SLAMF8,CHEK2,ABCB11,POR,NFE2L2,RAP1B,EPHX1,PDE4A,CYP46A1,ADSSL1,NOS2,CYP2J2,HSPA5,NQO1,CRYZ,S100A12,SULT1C3,AHRR,KCNH2,NCEH1,AOC2,N6AMT1	grey	VSMC_M10
GO:0036005	0.92	0.22	4	11	response to macrophage colony-stimulating factor	BP	1	TLR4,FER,CSF1R,PTPN2	grey	VSMC_M10
GO:0036006	0.92	0.22	4	11	cellular response to macrophage colony-stimulating factor stimulus	BP	1	TLR4,FER,CSF1R,PTPN2	grey	VSMC_M10
GO:0003188	0.92	0.21	4	11	heart valve formation	BP	1	SOX9,EFNA1,HEY2,OLFM1	grey	VSMC_M10
GO:0042659	0.92	0.21	4	11	regulation of cell fate specification	BP	1	WNT11,FZD7,BMPR1A,WNT5A	grey	VSMC_M10
GO:0051283	0.92	0.12	38	83	negative regulation of sequestering of calcium ion	BP	1	PRKCE,IBTK,ATG5,RYR1,DIAPH1,RASA3,RYR3,PTK2B,TPCN1,SLC8B1,F2R,PKD2,CASQ2,RYR2,LCK,UBASH3B,ANK2,GPER1,ATP1A2,CASQ1,NTSR1,CEMIP,PTPN6,HTT,HAP1,CD19,CALM1,BDKRB1,TRPV1,PDPK1,CYBA,JPH3,MCOLN3,DMD,NOL3,JPH2,THY1,DDIT3	grey	VSMC_M10
GO:0009123	0.92	0.12	30	65	nucleoside monophosphate metabolic process	BP	1	DPYS,IMPDH1,GDA,PPAT,NT5E,CMPK1,DLG2,AMPD3,DGUOK,GMPS,AMPD1,PRPS2,DTYMK,MAGI3,DLG1,UPB1,CASK,ADK,CARD11,UPRT,ADSSL1,NT5M,MPP1,AK8,UCK1,DHODH,CMPK2,DCTD,NUDT2,UPP1	grey	VSMC_M10
GO:0006971	0.92	0.21	4	11	hypotonic response	BP	1	TSPO,OXSR1,SLC12A5,TRPV4	grey	VSMC_M10
GO:0046620	0.92	0.12	36	78	regulation of organ growth	BP	1	GLI1,STK3,PPARA,MAPK14,PI16,PIM1,SMO,TGFBR2,DUSP6,MEF2C,IL7,TBX5,STK4,JARID2,IGF2,HLX,KCNK2,YBX3,SAV1,MAPK1,BMPR1A,RBPJ,WWC1,TBX2,YY1,GATA6,MIR199A1,MIR222,MIR25,NOG,BASP1,FXN,RGS4,BCL2L11,HEY2,WNT2	grey	VSMC_M10

GO:0002218	0.92	0.15	14	33	activation of innate immune response	BP	1	XRCC6,TLR4,IKBKB,TMEM173,MAVS,PRKDC,TRAF6,HCK,NONO,XRCC5,SFPQ,SIN3A,CREBBP,ZCCHC3	grey	VSMC_M10
GO:0060394	0.92	0.21	4	11	negative regulation of pathway-restricted SMAD protein phosphorylation	BP	1	LRP1,SNX25,NOG,LDLRAD4	grey	VSMC_M10
GO:0000271	0.92	0.13	26	58	polysaccharide biosynthetic process	BP	1	HAS1,EPM2AIP1,PPP1CB,CSGALNACT1,AKT2,PPP1R3B,GBE1,B3GNT4,SORBS1,IGF2,NHLRC1,IRS1,PPP1R3E,EGF,PPP1R3D,PER2,PASK,PRKAG3,AC022400.6,PPP1R3F,INSR,INPP5K,AGL,HAS3,ACADM,HS2ST1	grey	VSMC_M10
GO:0003279	0.92	0.12	40	88	cardiac septum development	BP	1	MAML1,FRS2,TAB1,SMO,PTK7,DNAH11,HOXA13,CNTRL,WNT11,TGFBR2,JAG1,PAX8,PARVA,ROBO1,ROBO2,TBX5,SALL1,SUFU,TGFB2,TRIP11,ANK2,LUZP1,SAV1,BMPR1A,DHRS3,RBPJ,MKS1,DCTN5,TBX2,STRA6,GATA6,AP2B1,ISL1,ZBTB14,ENG,SOX4,NOG,WNT5A,NKX2-5,HEY2	grey	VSMC_M10
GO:0071482	0.92	0.12	44	94	cellular response to light stimulus	BP	1	BAK1,MYC,ATF4,MAPK13,OPN1SW,CRY1,CAMKMT,AQP1,PTN,ERCC1,XPC,AKT2,PIK3R1,ATR,MME,EIF2AK4,DDB2,TENT2,CRY2,TP53INP1,TRPM1,RHBDD1,HYAL1,HYAL3,RRH,SAG,NFATC4,NMT1,GRK4,MMP3,RHNO1,GNB5,ZBTB1,PARP1,DDB1,GUCY2D,TAF1,YY1,CUL4B,ERCC4,FMR1,CREBBP,IMPACT,GPR88	grey	VSMC_M10
GO:0045444	0.92	0.10	91	188	fat cell differentiation	BP	1	ATAT1,STK3,PSMB8,PNPLA3,MAPK14,CBY1,PIM1,TAF8,DLK2,TFAP2B,IL11,TRIM32,DKKL1,UCP1,TRPM4,MKX,BBS7,NAPEPLD,AAMDC,KLF5,AKT2,JAG1,SIRT2,MKKS,PTPRQ,PLCB1,TRIB3,ASXL1,NR4A1,CEBPA,ERAP1,NOC3L,ARNTL,TMEM120B,EBF2,RUNX1T1,TRIO,CDS1,NR4A2,FTO,RREB1,SOX13,STK4,CCND1,TRPV4,TCF7L2,CMKLR1,FABP4,GPER1,LAMB3,PER2,SYAP1,SENP2,CREB1,DUSP10,MMP11,SAV1,GNB3,TFE3,SLC2A4,GPS2,MED1,TGFB1I1,ALDH6A1,ALOXE3,JDP2,ZBTB7B,SMAD3,SREBF1,SOCS1,ZBTB7C,SDF4,PRDM16,SOX8,MIR21,MIR27B,MIR27A,BSCL2,MAFB,WNT5A,SIX1,PLAC8,DDIT3,PEX11A,CCDC3,ZFP36L2,SH2B2,CEBPB,CCDC71L,C1QL4,DIO2	grey	VSMC_M10
GO:0050665	0.92	0.20	5	13	hydrogen peroxide biosynthetic process	BP	1	ACOX1,DUOX1,STAT3,CYBA,AC140504.1	grey	VSMC_M10
GO:0002828	0.92	0.16	9	22	regulation of type 2 immune response	BP	1	RSAD2,NOD2,HLX,DENND1B,BCL6,TBX21,IL4R,CD74,PRKCZ	grey	VSMC_M10
GO:0048873	0.92	0.16	9	22	homeostasis of number of cells within a tissue	BP	1	SMO,SOX9,RAC3,IL7,F2R,KRAS,GIGYF2,ILDR2,LIPA	grey	VSMC_M10
GO:0009264	0.92	0.15	11	26	deoxyribonucleotide catabolic process	BP	1	DPYS,GDA,NUDT15,MBD4,NUDT16,NUDT18,UPB1,NEIL1,NT5M,NTHL1,UPP1	grey	VSMC_M10
GO:0045472	0.92	0.21	4	11	response to ether	BP	1	OPA1,FOXP3,LARP1,CRH	grey	VSMC_M10
GO:0010523	0.92	0.20	5	13	negative regulation of calcium ion transport into cytosol	BP	1	TMBIM6,PKD2,CASQ2,NTSR1,CALM1	grey	VSMC_M10
GO:0034333	0.92	0.21	4	11	adherens junction assembly	BP	1	RAMP2,VCL,ACTB,PIP5K1C	grey	VSMC_M10
GO:0048012	0.92	0.20	5	13	hepatocyte growth factor receptor signaling pathway	BP	1	MET,SIRT2,ESM1,ADAMTS12,PTPN2	grey	VSMC_M10
GO:0150077	0.92	0.16	9	22	regulation of neuroinflammatory response	BP	1	TTBK1,CST7,CD200,CD200R1L,MMP3,TNFRSF1B,SYT11,LDLR,IL33	grey	VSMC_M10
GO:0051081	0.92	0.21	4	11	nuclear membrane disassembly	BP	1	LPIN1,AKAP8L,NDEL1,PRKCB	grey	VSMC_M10
GO:1904705	0.92	0.13	24	53	regulation of vascular associated smooth muscle cell proliferation	BP	1	MAP3K5,HPGD,MEF2C,ADAMTS1,NQO2,TERT,PDCD4,GNA13,GPER1,AGT,EFEMP2,CDKN1B,BMPR1A,MFN2,JUN,GNAI3,MIR21,MIR222,MIR27B,MIR27A,RHOA,FOXJ2,MIR339,LDLRAP1	grey	VSMC_M10
GO:0060088	0.92	0.21	4	11	auditory receptor cell stereocilium organization	BP	1	WHRN,MYO7A,PLS1,STRC	grey	VSMC_M10
GO:0090066	0.92	0.09	208	418	regulation of anatomical structure size	BP	1	ARPC3,LRP1,SEMA6A,AVIL,RAB21,PRR16,SLC12A2,FSTL4,WASHC5,PLXNA4,PLA2G6,BAIAP2L2,PICK1,CDC42EP1,ZDHHC21,LMOD2,SRF,NCK2,SEMA4D,KIAA1211,SEMA4C,TMOD1,PLEKHH2,PRKCE,SPTBN1,RTN4,RUFY3,AQP1,PLEK,EPHA7,ITGB1BP1,ADD2,EGFR,KEL,ATG5,FAM49A,NRCAM,TRPM4,WASF1,SLC26A5,GOLGA4,MEGF8,CLNS1A,AQP11,PLEKHG2,FCHSD2,MKKS,RPTOR,RAB5A,ADRA1D,BAG4,SEMA3D,SPART,FCHSD1,SEMA3A,NCKAP1L,LARS,ULK1,ARFIP2,LMOD3,RYK,ADRA2B,RET,HCK,TTL,ASXL1,HTR7,FER,HIP1R,PLS1,FMN1,LIMA1,SEMA4F,P2RX7,GUCY1A1,P2RY1,PTK2B,DPYSL2,USH1C,DOCK5,NKX6-1,ZFYVE27,F2RL1,F2R,IQGAP2,ANO6,SEMA3G,SPP1,PAX2,CYFIP1,DMTN,SEMA4G,SLC12A5,CHRM3,MAGEL2,BDNF,RAP1GDS1,KIAA0319,CARMIL1,LMOD1,ADD3,SLC12A7,TRPV4,SEMA3F,ADRA2A,SSH1,PEX11B,HCLS1,ADNP,AP1AR,DISC1,GPER1,AGT,RAB22A,WASF2,ACE2,ATP1A2,AP2M1,SPTBN2,PER2,TMOD4,SLC12A8,EPS8,CDKL5,WHAMM,DLG1,WNT3,CAPZA1,CREB1,CDC42EP2,MAPT,PLOD3,SEMA6D,CPS1,SLC12A9,TNR,FN1,NCKAP1,ADRA2C,GNB3,ECE1,GCH1,ARPC1A,SMTNL2,CD38,KCNMA1,WAS,TMOD2,ATP13A2,SPTB,CLN8,PFN3,WNK3,SOD3,HRH2,SEMA4A,TRIM46,NDEL1,AC138894.1,HAX1,RAB11A,NTN1,SCPEP1,DNM2,SCNN1B,EVL,SIN3A,TRPV2,PEX11G,LRRC8E,ASIC2,CDK5R1,VAV1,IL7R,SSH2,RILP,SERPINF2,RAP1GAP2,P2RX1,L1CAM,MLST8,ARHGAP4,PLXNA3,SPIRE2,TSC1,LAMTOR5,VAV3,LRRC8A,BARHL2,WDR63,SLC12A4,CARMIL2,MIR21,RHOA,HTR1D,FGA,CNTN2,VASP,GMFG,WNT5A,PEX11A,GRB2,PPP1R15A,PFN4,PDXP,KCNMB4,OLFM1,COTL1	grey	VSMC_M10

GO:0007409	0.92	0.10	167	341	axonogenesis	BP	1	LRP1,SEMA6A,KIF5A,RAB21,TIAM2,FSTL4,SYNGAP1,GFRA3,PTK2,PTCH1,PLXNA4,NTRK2,RAC2,SMO,SRF,SEMA4D,DLX5,ETV1,SEMA4C,BRSK1,RTN4,RUFY3,HOXA2,EPHA7,EPHA1,KEL,NRCAM,PLXNA1,RAB10,GOLGA4,GLI2,MEGF8,MYCBP2,NUMBL,POU4F1,TSKU,SZT2,SLITRK5,KIF5C,FEZ2,SEMA3D,SPART,PLXNC1,SEMA3A,CHL1,UNC5D,ULK1,RYK,EPHB1,RET,TTL,ROBO1,ROBO2,FZD3,SEMA4F,DPYSL2,TIAM1,SLIT1,NKX6-1,TRIO,NR4A2,ZFYVE27,SEMA3G,SPP1,PAX2,CYFIP1,ALCAM,SEMA4G,SIAH1,BMPR1B,BDNF,KIAA0319,LGR6,SEMA3F,GAP43,ADARB1,DAG1,AUTS2,LHX9,ADNP,DISC1,GBX2,KALRN,LAMA5,YTHDF1,PTPRO,CDKL5,EPHB3,WNT3,KLF7,CREB1,MYPN,SPG11,MAPT,SEMA6D,EPHB4,ITGA4,TNR,FN1,ATL1,NIN,PPP3CB,RAB3A,MARK2,VCL,RAB8A,CRMP1,EPHA4,LRTM2,ACTB,UNC5A,PSEN1,SEMA4A,NGFR,NLGN3,TAOK2,TRIM46,EFNA1,NDEL1,CHRNB2,RAB11A,MAP2K1,NTN1,RPS6KA5,RNF165,DNM2,BCL11B,LHX1,SIN3A,TRPV2,LAMA3,CDK5R1,ISL1,EMB,LAMA1,DOCK7,SLC9A6,DAB1,SLITRK2,L1CAM,NTN3,ARHGAP4,PLXNA3,BSG,TUBB3,AMIGO1,NTNG2,STXBP1,BARHL2,DPYSL5,EFNA4,CNTN2,VASP,FOXG1,ISL2,NOG,THY1,WNT5A,POU3F2,CHN1,ARTN,NRXN3,LYPLA2,EFNB3,ISPD,MAP1A,OLFM1	grey	VSMC_M10
GO:0007034	0.92	0.11	65	137	vacuolar transport	BP	1	VPS4B,LRP1,AP3S1,IGF2R,PRKN,VPS52,TGFBRAP1,CHMP2A,VPS41,VPS35,RAB7A,RBSN,HGSNAT,HOOK3,TMEM106B,FAM160A2,LEPROTL1,VPS16,SLC46A3,NCOA4,CHMP2B,CHMP4C,AP3B1,CHMP7,VPS18,VPS39,KIF13A,RAB7B,MON1A,CCDC91,VTI1A,HSPA8,SORL1,DTX3L,RNF215,SNX27,VPS37D,AP4M1,PINK1,ANKFY1,AP3M1,ATP13A2,VPS13D,GAK,VPS13C,AC138894.1,HOOK2,VIPAS39,STX8,SNAPIN,NPC1,LAMP2,CLEC16A,VPS53,MTM1,MGRN1,RILP,CHMP1A,AP1G1,VPS4A,CACNG7,AP3S2,SNX16,LEPROT,CACNG5	grey	VSMC_M10
GO:0090501	0.92	0.11	59	124	RNA phosphodiester bond hydrolysis	BP	1	PAN2,RPP21,CPSF6,CNOT2,CNOT6,RIDA,PNLDC1,UTP23,AGO2,EXOSC4,CPSF1,TSR1,EXOSC3,TSEN34,PNPT1,RNASEH1,PCF11,NUDT16,POLR2I,PAN3,BMS1,CSTF3,NUDT12,POP4,CNOT1,USB1,CNOT6L,RPP40,POP5,CPSF2,DIS3L2,YBEY,NOP9,ANGEL2,NYNRIN,NCBP2,KIAA0391,POP7,ZC3H12C,CPSF4,AP002990.1,MRPL44,EXD2,ISG20L2,ANGEL1,RNASEH2A,CSTF2,CNOT8,KRI1,ERI2,SLFN14,PARN,DROSHA,INTS11,NOL9,POLR3K,RPP38,RPP30,EXOSC5	grey	VSMC_M10
GO:0001832	0.92	0.17	7	18	blastocyst growth	BP	1	TAF8,NBN,ACVR1C,RTF1,SBDS,RAD51B,NDEL1	grey	VSMC_M10
GO:0009409	0.92	0.14	17	39	response to cold	BP	1	SLC25A27,UCP1,P2RX3,EIF2AK4,GMPR,TRPM8,SLC9A1,VGF,SLC27A1,ACADVL,THRA,FOS,ACOT11,CIRBP,ACADM,CASP8,PLAC8	grey	VSMC_M10
GO:0099084	0.92	0.15	11	27	postsynaptic specialization organization	BP	1	SYNGAP1,LRRC4,LRFN1,GAP43,DLG1,NRXN2,NLGN2,NTNG2,ZDHHC12,GRID2,LRRTM2	grey	VSMC_M10
GO:0034330	0.92	0.09	269	541	cell junction organization	BP	1	LRP1,PRRT1,SYNGAP1,MTSS1,PTK2,MAPK14,NTRK2,PICK1,MPDZ,TEK,LRRC4,LINGO2,ABHD17B,SRF,GABRA1,FZD1,SLC1A1,ADGRF1,TLN1,SEMA4D,ADGRL3,CLDN19,SHANK1,GJA10,LRRN3,EPHA7,WHRN,PTN,ITGB1BP1,GRHL1,ADD2,TNC,CTTNBP2,YWHAZ,DLGAP3,VPS35,SDCBP,NRCAM,SLC7A11,PPFIA3,WASF1,MTMR2,SLC8A2,C5AR1,VSTM5,IQSEC1,CACNB2,CLASP2,PCDH8,FBF1,PTPRF,NUMBL,POU4F1,WNT11,SLITRK5,IKBKB,LRFN1,FARP1,ACTG1,CACNB4,PRNP,PCDHGC5,ARHGEF7,P2RX2,NRG1,PTPRA,PKP3,LRFN3,RYK,EPHB1,NPHP1,LSR,FAM107A,PIK3R1,CLSTN2,CTNND1,CHMP2B,MARVELD2,ROBO2,ERC2,FMN1,SORBS1,PTPRJ,MEF2C,CUX2,KIFC3,PLEKHA7,SEPT11,PTK2B,RAPGEF2,TIAM1,SLIT1,HOMER1,OPA1,HDAC7,TBX5,AMIGO2,F2RL1,F2R,SPARCL1,ITPKA,DSP,CYFIP1,DMTN,TUBA1A,LDB1,BDNF,DOCK10,PPFIA4,INA,NEURL1,PKP2,SLK,DNM1L,PPFIA1,COL17A1,CAPRIN2,TRPV4,SEMA3F,TGFB2,GAP43,COL16A1,PPFIBP1,CAMKV,DAG1,AMIGO3,NLGN4Y,SSH1,AJUBA,CFL1,STAU1,CLDN34,CDH24,BSN,SNAI1,ADNP,ARHGAP6,HSPA8,DISC1,ANK2,GNPAT,AGT,WASF2,ACE2,SLC9A1,RAB17,SPTBN2,NFATC4,ACTN3,NPAS4,PTPRO,CDKL5,IL10RA,EPHB3,TANC2,NOS1AP,WHAMM,FZD9,LAMC1,DLG1,APOD,CAPZA1,FZD5,MAPT,RAP1B,SRGN,C1QL1,TRIP6,ARVCF,TNR,NRXN2,FN1,SPOCK2,DOK7,GPBAR1,WNT4,DNM3,CNTNAP1,RAB3A,RAMP2,SYN1,VCL,OBSL1,GRIPAP1,EPHA4,LRTM2,DLG4,ACTB,CLDN7,SDK1,NLGN2,GPHN,CHRNB1,LGI2,CACNB1,PSEN1,SEMA4A,ITGAM,TLN2,RAB8B,ITGA3,NLGN3,TAOK2,EFNA1,AC138894.1,CHRNB2,ZDHHC15,MYH10,SMAD3,NTN1,CORO2B,SNAPIN,DRP2,MYO9A,VMP1,BCAS3,GPRASP2,BHLHB9,ARHGAP44,DSG [illegible]	grey	VSMC_M10
GO:0035357	0.92	0.17	7	18	peroxisome proliferator activated receptor signaling pathway	BP	1	ASXL1,PDK3,GPS2,MED1,ALOXE3,RXRA,FABP5	grey	VSMC_M10
GO:0045071	0.92	0.14	15	35	negative regulation of viral genome replication	BP	1	APOBEC3H,RSAD2,ZC3HAV1,EIF2AK2,MAVS,FAM208A,OAS3,IFIH1,OASL,TNIP1,CCL5,INPP5K,SETDB1,OAS1,FAM111A	grey	VSMC_M10
GO:0021761	0.92	0.12	34	75	limbic system development	BP	1	NME1,ATAT1,UQCRQ,SMO,SRF,CDK6,NDNF,EIF2B3,XRCC1,TSKU,ALK,MKKS,SEMA3A,CASP3,DCLK2,TENT2,SRD5A1,HDAC1,ALDH1A3,ATP1A2,DLX1,KCNA1,HAP1,KDM6B,CDK5R1,DAB1,LRP8,CRK,PLXNA3,TSC1,PHLPP2,UBB,POU3F2,CRH	grey	VSMC_M10
GO:0033145	0.92	0.21	4	11	positive regulation of intracellular steroid hormone receptor signaling pathway	BP	1	KMT2D,PARP1,MED1,PAGR1	grey	VSMC_M10
GO:0042693	0.92	0.21	4	11	muscle cell fate commitment	BP	1	MEF2C,TBX5,RBPJ,TBX2	grey	VSMC_M10
GO:0097091	0.92	0.21	4	11	synaptic vesicle clustering	BP	1	BRSK1,RAB3A,SYN1,NLGN2	grey	VSMC_M10
GO:1900274	0.92	0.14	13	31	regulation of phospholipase C activity	BP	1	ESR1,KIT,LPAR1,EGFR,C5AR1,PHB,RASGRP4,FLT1,ARHGAP6,AGT,ABL2,GNA15,HRAS	grey	VSMC_M10
GO:0032695	0.92	0.21	4	11	negative regulation of interleukin-12 production	BP	1	PIBF1,NOD2,CMKLR1,MIR21	grey	VSMC_M10

GO:0007589	0.92	0.12	31	68	body fluid secretion	BP	1	NME1,SLC22A4,GUCA1B,PPAT,PRKCE,AQP1,NR1H2,EGFR,ATG5,TRAF3IP2,USF2,NR1H3,VDR,CHRM3,NEURL1,SLC6A3,CCND1,COPA,CREB1,SERPINC1,CHRM1,STAT5B,MED1,ANXA2,ZBTB7B,UPRT,SCNN1B,CELSR2,CEL,CYBA,DHODH	grey	VSMC_M10
GO:0099175	0.92	0.12	33	73	regulation of postsynapse organization	BP	1	ABHD17B,EPHA7,VPS35,LRFN1,PRNP,CHMP2B,CUX2,TIAM1,HOMER1,OPA1,ITPKA,DNM1L,CAPRIN2,SEMA3F,CFL1,HSPA8,CDKL5,TANC2,SRGN,DNM3,EPHA4,EFNA1,ZDHHC15,GPRASP2,BHLHB9,ARHGAP44,CDK5R1,LRP8,ABHD17A,GRID2,ABHD17C,WNT5A,LRRTM2	grey	VSMC_M10
GO:0001824	0.92	0.11	40	86	blastocyst development	BP	1	CNOT2,PNLDC1,RRP7A,MFNG,TAF8,SRF,RTN4,CCNB1IP1,CCDC62,CNOT1,NBN,ACVR1C,RTF1,ZBED6,NPM2,ELF3,SBDS,DAD1,PLPP4,RPL7L1,SP3,TET1,BCOR,TTLL4,AKAP3,ARHGDIG,WDR74,SLC25A34,MFN2,RAD51B,NDEL1,PPP4R4,NLE1,XAB2,SUPT6H,CMTM3,PEMT,C6orf141,MED21,ST8SIA6	grey	VSMC_M10
GO:1901841	0.92	0.19	5	13	regulation of high voltage-gated calcium channel activity	BP	1	CACNB2,NOS1AP,CALM1,RRAD,GEM	grey	VSMC_M10
GO:0071496	0.92	0.10	131	267	cellular response to external stimulus	BP	1	MARS,MAP3K5,CYP27B1,SNW1,PPARA,BAK1,HABP4,ATF4,PICK1,PIM1,TXN2,TFEB,SLC39A4,SRF,NUAK1,BRSK1,ALB,AQP1,RRAGD,CASP8AP2,PTN,TNC,EGFR,TLR4,VPS41,IL15,ATG5,FOXO3,MKX,KPTN,SESN3,FOXA3,SOX9,NUDT15,DNAJC15,RALB,PRKAG2,MYD88,SZT2,WNT11,RPTOR,CNN2,EIF2AK2,WRN,GAS6,LARS,ULK1,NR1H4,USF2,FAM107A,PRKAG1,EIF2AK4,TMEM150C,VDR,NR4A2,SLC38A2,CD40,NUAK2,NUPR2,SRD5A1,DNM1L,CBSL,GLRX2,SIK1,KCNJ2,SLC38A3,DAG1,KCNB1,TLR5,ADNP,HSPA8,AGT,CBL,ATP1A2,BAG3,SLC9A1,MTMR3,UCN2,HDAC4,USF1,XPR1,TBL2,GLUL,NFE2L2,ITGA4,SIK2,COMT,KCNK4,WNT4,PRKAG3,CTSV,LTBR,MAPK1,BHLHA15,WIPI2,MAX,TNFRSF8,MED1,RRAGB,ZFYVE1,FOS,MAPK3,TNRC6A,SREBF1,LAMP2,NIM1K,PTGER4,STK26,JUN,CLEC16A,KIAA1324,PIEZO1,CYBA,TSC1,LCN2,WDR59,ENG,HSPA5,SH3GLB1,BCL10,RIPOR1,MIR125B1,TRIM24,FADD,CASP8,BGLAP,IMPACT,GADD45A,CRADD,UPP1,RNF152	grey	VSMC_M10
GO:0043949	0.92	0.17	7	18	regulation of cAMP-mediated signaling	BP	1	UBE2B,GIPR,RAPGEF2,PDE4A,MGRN1,CRH,RASD2	grey	VSMC_M10
GO:0046755	0.92	0.16	9	22	viral budding	BP	1	VPS4B,CHMP2A,CHMP2B,CHMP4C,CHMP7,VPS37D,CHMP1A,MVB12B,VPS4A	grey	VSMC_M10
GO:0034660	0.92	0.09	208	412	ncRNA metabolic process	BP	1	MARS,ADAT2,HELB,RPP21,TFB1M,MOV10L1,TRMU,UTP23,RRP7A,TRMT11,AGO2,TUT7,C9orf64,EXOSC4,NOL6,XPO5,TSR1,EXOSC3,RPS14,MTO1,TSEN34,ELP1,PNPT1,UTP3,PUS10,TRIT1,MDN1,AGO1,FCF1,QRSL1,TUT4,DUS4L,NSUN6,ERCC2,RPS8,DEDD2,RPL14,INTS10,THUMPD3,USP36,ESF1,KRR1,DPH3,SIRT7,HARS,HARS2,TRMT6,TRNT1,CARS2,NUDT16,LARS,THUMPD2,PRKDC,TRMT9B,DHX37,BMS1,DIMT1,ELP4,POP4,CLP1,ZCCHC8,ELP3,BCDIN3D,USB1,TPRKB,URB1,DDX54,NSUN3,RRP12,TENT2,HELQ,RPUSD2,OSGEPL1,INTS8,RPP40,EXD1,RIOK1,TENT4B,WARS2,CDKAL1,GATC,POP5,PDCD11,RPUSD4,YARS2,TRMT10A,ZBTB8OS,RPS17,QTRT2,SBDS,TYW1B,INTS12,HEATR1,TYW5,FAM207A,DIS3L2,MRPS11,GAR1,TRUB1,YTHDF2,RTRAF,TARS2,DALRD3,YBEY,LCMT2,NOP9,RPS27,WDR12,METTL8,RPL7L1,TDRKH,WBP11,ELP6,NSUN5,DDX47,RPL11,METTL2B,KIAA0391,RBM7,TRMT1L,MAPT,PRKRA,LARS2,POP7,DDX3X,DDX10,RC3H1,MEPCE,DDX49,DARS2,TRPT1,RPL27,ALKBH8,ZSWIM8,STAT3,WDR74,TRMT5,INTS5,AP002990.1,FARSB,MRPL44,HSD17B10,NVL,MRM2,TSR2,ZCCHC4,LAS1L,MTFMT,ALKBH1,UTP18,THG1L,SMAD3,QTRT1,EARS2,INTS2,KRI1,ZNHIT3,ELP2,ERI2,THUMPD1,RIOK3,SLFN14,METTL4,RNF113B,UTP6,METTL6,SNU13,UTP14A,PARN,DROSHA,MRM3,INTS11,PUSL1,METTL16,LRRC47,NOL9,RPUSD1,WDR18,POLR3K,KARS,ADAT1,AARS,RPL35,ZNHIT6,DUS2,METTL18,CTU1,AGO4,MARS2,RPP38,MPHOSPH6,TRMT10B,DTD1,TP53RK,TRMT61A,RPUSD3,NSA2,C1D,DTD2,HRAS,PIH1D2,TRMT10C,RPP30,TRMT61B,TYW3,RC3H2,EXOSC5,GTF2H5	grey	VSMC_M10
GO:0044406	0.92	0.21	4	11	adhesion of symbiont to host	BP	1	NECTIN2,GAS6,ACE2,LRRC15	grey	VSMC_M10
GO:1901216	0.92	0.12	36	78	positive regulation of neuron death	BP	1	MAP3K5,ATF4,TFAP2B,EPHA7,TLR4,FOXO3,ZNF746,GRIK5,PRNP,CASP3,FBXW7,NQO2,TFAP2A,TGFB2,SSH1,MCL1,HDAC4,BACE1,MAP3K11,MAPT,FIS1,PARP1,ITGAM,FOS,PPP1R13B,CDK5R1,JUN,LCN2,NQO1,MIR125B1,RHOA,CASP8,WNT5A,DDIT3,BCL2L11,PITX3	grey	VSMC_M10

GO:1903507	0.92	0.09	482	945	negative regulation of nucleic acid-templated transcription	BP	1	SMARCC2,ATP8B1,PLAGL1,ZBTB2,ESR1,CNOT2,OSR2,SNW1,ZNF706,ZBTB12,PRKN,PPARA,TCF7,XRCC6,PITX1,MYC,TOB2,SMAD5,L3MBTL3,L3MBTL2,PTCH1,ATF4,CBX7,CBY1,SOX10,TMPRSS6,SMO,HSF1,FOXP4,MDFI,CCND3,FOXH1,CDKN2A,RBFOX2,RORB,CRY1,SRF,ZNF318,NFX1,SMARCA2,RFX3,NCK2,FHL2,TFAP2B,NFIL3,SEMA4D,RPS14,ZNF550,BEND6,ZNF268,SCMH1,NFE2L3,HOXA2,HOXA7,JAZF1,XPO1,AKIRIN2,CASP8AP2,BACH2,ZNF692,NR1H2,ZNF350,FOXP2,MED25,KLF11,POU3F3,LANCL2,CREM,SDCBP,OSR1,FOXO3,BEND5,HDAC2,PHF10,DNMT3A,BMI1,ZC3H4,CNBP,SETDB2,PHB,SOX9,ZNF746,EED,SUV39H2,ZNF404,PLK3,GLI2,DEDD2,RSF1,KDM4A,DACH1,TAF3,ACVR2B,KLF5,KLF12,FOXJ1,SFMBT2,GZF1,POU4F1,WNT11,THAP1,EID2,OVOL2,ZNF219,EOMES,CBX4,BHLHE40,CHD8,SIRT2,ZMYND11,RARB,PURA,NR1D2,PAWR,PLCB1,SIRT7,DPF1,ALX1,TAF7,NRG1,GAS6,RYBP,FOXP1,MITF,MTA3,ARID5A,IRF7,TRAF6,LIN37,NR1H4,KCNIP3,TRIB3,PFDN5,SDR16C5,ZC3H8,DNMT3B,BTAF1,CEBPA,CBFA2T2,SMYD1,FOXL2,ZNF536,TFDP2,IRF2,NCOA2,DKK3,SAP30,ZNF366,ARNTL,GCFC2,HMBOX1,MEF2C,PEX2,PKIA,TMBIM6,CNOT1,SLA2,CUX2,MCPH1,SOX6,FNIP2,BACH1,SFMBT1,NR1H3,EAPP,SCAF4,LCOR,DDX54,RUNX1T1,VDR,NKX3-1,BAHD1,HDAC7,NKX6-1,LOXL2,NR4A2,PHF21A,CRY2,PRDM11,CDYL,RREB1,RTF1,ZBED6,SOX13,ZNF248,TFAP2A,RIPPLY3,HIF1AN,PAX2,SALL1,PPHLN1,PRICKLE1,HIVEP1,JARID2,ATXN1,SUDS3,ZNF423,LDB1,IGF2,MAGEL2,ELK4,NUPR2,DCAF1,SUFU,TAGLN3,PCGF6,IRX2,RPL23,HDAC1,ELF3,CCND1,SP100,TRPV4,PDCD4,ZBTB20,SATB2,TCF7L2,AJUBA,ZNF639,PIAS3,LHX9,HJV,FABP4,SNAI1,ZNF202,H[illegible]	grey	VSMC_M10
GO:0032506	0.92	0.14	15	35	cytokinetic process	BP	1	VPS4B,MYH9,MITD1,CHMP2A,CHMP2B,CHMP4C,RTKN,CHMP7,BIN3,KLHDC8B,CNTROB,SPIRE2,CHMP1A,VPS4A,RHOA	grey	VSMC_M10
GO:0009163	0.92	0.19	5	13	nucleoside biosynthetic process	BP	1	MTAP,NT5E,PRTFDC1,DTYMK,ADK	grey	VSMC_M10
GO:0034404	0.92	0.19	5	13	nucleobase-containing small molecule biosynthetic process	BP	1	MTAP,NT5E,PRTFDC1,DTYMK,ADK	grey	VSMC_M10
GO:0051651	0.92	0.10	78	163	maintenance of location in cell	BP	1	TSPO,SKP1,SUN2,GPAA1,TAF8,PRKCE,IBTK,ALB,ATG5,ARHGAP21,ATP7B,GRIK5,TAF3,RYR1,DIAPH1,RASA3,RYR3,PTK2B,ARL2BP,TPCN1,SLC8B1,F2R,PKD2,MORC3,CASQ2,RYR2,LCK,SP100,UBASH3B,SORL1,ANK2,GPER1,ATP1A2,CASQ1,NTSR1,TBCCD1,SLC30A4,SRGN,CEMIP,HK1,PTPN6,HTT,PINK1,FTH1,HAP1,VPS13D,VPS13C,PSEN1,ATP2A1,CD19,CALM1,S100A8,RIT2,BDKRB1,SLC25A23,TRPV1,PDPK1,RER1,PARK7,GPSM2,CYBA,JPH3,SLC30A7,SPOUT1,LCN2,HSPA5,MCOLN3,DMD,NOL3,JPH2,THY1,SUPT7L,FTL,DDIT3,TXN,INSIG2,PFN4,POLR2M	grey	VSMC_M10
GO:1990090	0.92	0.13	20	46	cellular response to nerve growth factor stimulus	BP	1	ARPC3,NTRK2,MICALL1,FOXO3,NTF4,WASF1,RAPGEF2,BDNF,CIB1,CBL,CREB1,MAPT,EHD1,CSTF2,CRK,TRPV1,PDPK1,LCN2,HSPA5,DYNC1LI2	grey	VSMC_M10
GO:0048872	0.92	0.10	112	229	homeostasis of number of cells	BP	1	TNFAIP3,MPIG6B,HSPA1B,BRD1,BAK1,SMAD5,L3MBTL3,FANCC,MAPK14,SMO,ZNF16,RBFOX2,SRF,OCIAD1,CDK6,KIT,PRDX1,RPS14,SMAP1,EPAS1,PTBP3,SLC46A2,BPGM,SLC7A11,FOXO3,TRAF3IP2,NAPEPLD,FOXA3,SOX9,ERCC2,WDR48,SLC25A38,ACVR2A,RAC3,NCKAP1L,PRKDC,MFHAS1,GAPT,ZC3H8,ASXL1,GPI,ACVR1B,PIK3CB,CASP3,MEF2C,IL7,AP3B1,F2R,TMEM14C,DMTN,ALAS1,LDB1,DOCK10,PKNOX1,RPS17,GPAM,TCIRG1,SART3,ACIN1,KRAS,DYRK3,GIGYF2,HCLS1,ARNT,SP3,PPP2R3C,BCL6,LPCAT3,RC3H1,CXCR2,PPP3CB,STAT3,STAT5B,ILDR2,INHA,LIPA,FOXP3,THRA,CARD11,MED1,HOXB6,AHSP,KAT7,DNASE2,UBAP2L,LAT,SCNN1B,GPRASP2,RCOR1,CD74,NLE1,IL7R,PTPN2,DNAJA3,BCL10,MIR222,ANKRD54,ARID4A,ADGRG1,SOX4,MAFB,FADD,CXCL6,CAMLG,TSC22D3,BCL2L11,FAM210B,SH2B2,SLC39A3,TNFSF13B,XKR8,RC3H2	grey	VSMC_M10
GO:0009154	0.92	0.14	15	35	purine ribonucleotide catabolic process	BP	1	PDE7B,PDE10A,GDA,NT5E,NUDT16,AMPD3,ENTPD4,FITM2,NUDT18,PDE9A,PDE8A,PDE4A,MLYCD,NUDT19,HINT1	grey	VSMC_M10
GO:0043555	0.92	0.17	7	18	regulation of translation in response to stress	BP	1	NCK2,EIF2AK2,EIF2AK4,RPS6KA1,DDX3X,IMPACT,PPP1R15A	grey	VSMC_M10
GO:0071548	0.92	0.14	13	31	response to dexamethasone	BP	1	FECH,AQP1,EGFR,FOXO3,BMI1,DNMT3B,ABCC2,SRD5A1,CPS1,ASS1,SMYD3,CLDN1,CRH	grey	VSMC_M10
GO:0003085	0.92	0.21	4	11	negative regulation of systemic arterial blood pressure	BP	1	NTSR1,TRPV1,GPR37L1,CRH	grey	VSMC_M10
GO:0061744	0.92	0.21	4	11	motor behavior	BP	1	SLC1A1,ADARB1,ATP1B2,DPP4	grey	VSMC_M10
GO:0042303	0.92	0.12	34	75	molting cycle	BP	1	FZD6,SMO,ZDHHC21,SOSTDC1,CD109,EGFR,FUZ,INTU,HDAC2,SOX9,ERCC2,PTCH2,GLI2,TSKU,FERMT1,ACVR1B,TRPC4AP,ARNTL,FZD3,LDB1,HDAC1,TERT,TGFB2,SNAI1,LAMA5,FARP2,SAV1,GORAB,RBPJ,NGFR,TRPV3,EPS8L3,WNT5A,DNASE1L2	grey	VSMC_M10
GO:0042633	0.92	0.12	34	75	hair cycle	BP	1	FZD6,SMO,ZDHHC21,SOSTDC1,CD109,EGFR,FUZ,INTU,HDAC2,SOX9,ERCC2,PTCH2,GLI2,TSKU,FERMT1,ACVR1B,TRPC4AP,ARNTL,FZD3,LDB1,HDAC1,TERT,TGFB2,SNAI1,LAMA5,FARP2,SAV1,GORAB,RBPJ,NGFR,TRPV3,EPS8L3,WNT5A,DNASE1L2	grey	VSMC_M10
GO:0007063	0.92	0.17	7	18	regulation of sister chromatid cohesion	BP	1	SLF1,SLF2,AXIN2,DDX11,SFPQ,NAA10,CTCF	grey	VSMC_M10
GO:0072673	0.92	0.18	6	16	lamellipodium morphogenesis	BP	1	WASF1,ABI1,ARHGEF7,RREB1,WASF2,SNX1	grey	VSMC_M10

GO:0007159	0.92	0.10	115	234	leukocyte cell-cell adhesion	BP	1	AGER,PPARA,HLA-DRA,HLA-DOB,HLA-DMA,HLA-DPB1,TNFRSF13C,RAC2,ZDHHC21,TNFRSF21,NCK2,SEMA4D,NT5E,PELI1,ADD2,IL4I1,IL15,PODXL2,BMI1,GLI2,FOXJ1,TGFBR2,PAWR,PRNP,NCKAP1L,SLC7A1,TRAF6,SOX12,ZC3H8,IL1A,CASP3,TMEM131L,IL7,PAG1,JAM2,RASGRP1,RIPK2,AP3B1,MARCH7,SOX13,ADTRP,NOD2,IGF2,LCK,ICOSLG,BTN2A2,GPAM,PRKAR1A,SELPLG,HLX,CD28,RUNX3,DLG1,ADAM8,OLR1,MIA3,DUSP10,BCL6,ITGA4,IL12A,VSIR,RC3H1,PTPN6,TNFSF18,FERMT3,LAG3,STAT5B,SCGB1A1,FOXP3,RASAL3,ZBTB1,CARD11,TBX21,STK10,ZBTB7B,IL4R,S100A8,TNIP1,UMOD,CD74,CCL5,CCL2,CCL25,YES1,NKAP,VAV1,TWSG1,IL7R,PTPN2,SOCS1,DNAJA3,PRKCZ,ASS1,GLMN,BCL10,MIR21,MIR222,MIR27A,RHOA,SOX4,FADD,DPP4,CD46,FUT4,MIR125A,THY1,FGL2,CD70,TNFSF9,LAX1,EFNB3,CEBPB,TNFSF13B,HAVCR2,RC3H2	grey	VSMC_M10
GO:0006368	0.92	0.12	26	58	transcription elongation from RNA polymerase II promoter	BP	1	ARID1B,SOX10,ERCC3,GTF2F2,ZMYND11,POLR2I,ELP4,SMARCD1,EAPP,RTF1,LDB1,ARID1A,RNF168,SETD2,WDR61,ENY2,ELL,NELFA,LEO1,PADI2,CDK12,ELP2,SUPT6H,RF00100,SERTAD2,RNF8	grey	VSMC_M10
GO:0061450	0.92	0.20	4	11	trophoblast cell migration	BP	1	AGO2,ACVR1B,ACVR1C,SYDE1	grey	VSMC_M10
GO:0051293	0.92	0.13	19	44	establishment of spindle localization	BP	1	MYH9,NLRP5,CDK5RAP2,SPRY1,CLASP2,LLGL2,SPRY2,NSFL1C,MOS,MCPH1,CFL1,HTT,KPNB1,NDEL1,TLE6,SAPCD2,SPIRE2,GPSM2,OOEP	grey	VSMC_M10
GO:0007229	0.92	0.11	42	92	integrin-mediated signaling pathway	BP	1	ITGA7,MPIG6B,PTK2,MYH9,TEC,TLN1,PLEK,PTN,ITGB1BP1,FERMT1,PTPRA,HCK,ADAMTS1,PTK2B,CDH17,DMTN,ITGB6,SLC2A10,COL16A1,ITGA10,FGR,ITGB5,LAMA5,DOCK1,ITGA2B,ITGA4,FN1,FERMT3,COL3A1,BST1,ITGAM,ITGA3,LAT,ITGA11,LAMA3,PRAM1,VAV1,ADAMTS13,VAV3,FYB1,THY1,ZNF304	grey	VSMC_M10
GO:0060999	0.92	0.13	16	38	positive regulation of dendritic spine development	BP	1	SHANK1,CUX2,TIAM1,OPA1,ITPKA,NEURL1,DNM1L,CAPRIN2,CDKL5,PSEN1,GPRASP2,BHLHB9,FMR1,LRP8,LLPH,FOXO6	grey	VSMC_M10
GO:0003151	0.92	0.12	26	59	outflow tract morphogenesis	BP	1	FOXH1,WNT11,TGFBR2,JAG1,RYR1,HAND2,EYA1,PARVA,ROBO1,ROBO2,MEF2C,NPY1R,TGFB2,DVL2,BMPR1A,DHRS3,RBPJ,TBX2,GATA6,ISL1,ENG,NOG,WNT5A,SIX1,NKX2-5,HEY2	grey	VSMC_M10
GO:0042983	0.92	0.20	4	11	amyloid precursor protein biosynthetic process	BP	1	AGO2,PAWR,SOAT1,ABCA7	grey	VSMC_M10
GO:0042984	0.92	0.20	4	11	regulation of amyloid precursor protein biosynthetic process	BP	1	AGO2,PAWR,SOAT1,ABCA7	grey	VSMC_M10
GO:0007006	0.92	0.11	47	99	mitochondrial membrane organization	BP	1	HEBP2,HSPA4,BAK1,TOMM22,TMEM14A,AGK,CHCHD6,TIMM50,IMMT,HIP1R,MTX3,OPA1,AP3B1,OXA1L,MFN1,RCC1L,SLC9A1,THEM4,STPG1,FZD9,NMT1,BCS1L,PINK1,MUL1,STAT3,TMEM102,CLN8,MFN2,ADCK1,CALM1,TIMM29,CAMK2A,MAIP1,C19orf70,ACAA2,NAIF1,MIR29A,NOL3,MINOS1,BNIP3L,TIMM10B,BCL2L11,SLC35F6,BCL2L2,TIMM8B,PPIF,AC140504.1	grey	VSMC_M10
GO:0071295	0.92	0.14	12	29	cellular response to vitamin	BP	1	CYP27B1,SNW1,PIM1,PTN,TNC,IL15,GAS6,VDR,MED1,MIR125B1,TRIM24,BGLAP	grey	VSMC_M10
GO:0032481	0.92	0.13	21	48	positive regulation of type I interferon production	BP	1	IRF5,GAPDH,TLR4,MYD88,TMEM173,MAVS,IRF7,OAS3,RIPK2,POLR3D,IFIH1,POLR3C,SETD2,DDX3X,POLR3A,G3BP1,RIOK3,RNF135,TICAM1,OAS1,ZCCHC3	grey	VSMC_M10
GO:0050927	0.92	0.16	8	20	positive regulation of positive chemotaxis	BP	1	AGER,CXCL8,VEGFC,F2RL1,IL16,PGF,SMAD3,ARTN	grey	VSMC_M10
GO:2000641	0.92	0.17	7	18	regulation of early endosome to late endosome transport	BP	1	RAB21,MTMR2,DNAJC13,MAPK1,SNX12,MAPK3,MAP2K1	grey	VSMC_M10
GO:1900044	0.93	0.20	4	11	regulation of protein K63-linked ubiquitination	BP	1	NOD2,DDX3X,GPS2,OTUB2	grey	VSMC_M10
GO:0045738	0.93	0.14	13	31	negative regulation of DNA repair	BP	1	HELB,HSF1,CYREN,FBH1,RNF169,SHLD1,ERCC6,SHLD2,OTUB2,SMCHD1,RMI2,NUDT16L1,TERF2	grey	VSMC_M10
GO:0060420	0.93	0.12	26	58	regulation of heart growth	BP	1	GLI1,PPARA,MAPK14,PI16,PIM1,TGFBR2,DUSP6,MEF2C,TBX5,JARID2,KCNK2,SAV1,MAPK1,BMPR1A,RBPJ,TBX2,YY1,GATA6,MIR199A1,MIR222,MIR25,NOG,BASP1,RGS4,HEY2,WNT2	grey	VSMC_M10
GO:0007422	0.93	0.12	31	69	peripheral nervous system development	BP	1	ERBB3,ONECUT2,ADGRG6,GFRA3,PLXNA4,NTRK2,SOX10,ADAM22,ETV1,NTF4,POU4F1,PRX,AKT2,SIRT2,NRG1,LGI4,HAND2,BDNF,DAG1,RUNX3,SERPINI1,CNTNAP1,MPP5,ASIC2,ISL1,SKI,SOX8,ISL2,CLDN1,POU3F2,ARTN	grey	VSMC_M10
GO:0048144	0.93	0.12	30	66	fibroblast proliferation	BP	1	B4GALT7,ESR1,MYC,CDK6,AQP1,MED25,TRIM32,BMI1,DACH1,PAWR,GAS6,PRKDC,PEX2,TP53INP1,MORC3,AGT,CREB1,FN1,FTH1,ANXA2,NGFR,CTC1,CD74,SKI,DAB2IP,WNT5A,EREG,C1QL4,WNT2,PDGFD	grey	VSMC_M10
GO:0048145	0.93	0.12	30	66	regulation of fibroblast proliferation	BP	1	B4GALT7,ESR1,MYC,CDK6,AQP1,MED25,TRIM32,BMI1,DACH1,PAWR,GAS6,PRKDC,PEX2,TP53INP1,MORC3,AGT,CREB1,FN1,FTH1,ANXA2,NGFR,CTC1,CD74,SKI,DAB2IP,WNT5A,EREG,C1QL4,WNT2,PDGFD	grey	VSMC_M10
GO:1900271	0.93	0.14	14	33	regulation of long-term synaptic potentiation	BP	1	AGER,PTN,PRNP,FAM107A,MME,EIF2AK4,IGSF11,STAU1,YTHDF1,CREB1,CRTC1,NSG1,EPHA4,CYP46A1	grey	VSMC_M10
GO:2001238	0.93	0.13	16	37	positive regulation of extrinsic apoptotic signaling pathway	BP	1	STK3,FAF1,PDIA3,DEDD2,RET,HTRA2,RIPK1,STK4,BMPR1B,GPER1,AGT,PEA15,LTBR,TRAF2,BCL10,FADD	grey	VSMC_M10
GO:0002287	0.93	0.13	20	46	alpha-beta T cell activation involved in immune response	BP	1	HLA-DRA,EOMES,FOXP1,RIPK2,HLX,BCL6,RC3H1,TNFSF18,STAT3,FOXP3,TBX21,SEMA4A,BATF,ZBTB7B,IL6R,IL4R,PTGER4,PRKCZ,MIR21,RC3H2	grey	VSMC_M10
GO:0002293	0.93	0.13	20	46	alpha-beta T cell differentiation involved in immune response	BP	1	HLA-DRA,EOMES,FOXP1,RIPK2,HLX,BCL6,RC3H1,TNFSF18,STAT3,FOXP3,TBX21,SEMA4A,BATF,ZBTB7B,IL6R,IL4R,PTGER4,PRKCZ,MIR21,RC3H2	grey	VSMC_M10
GO:0043537	0.93	0.14	14	33	negative regulation of blood vessel endothelial cell migration	BP	1	MEOX2,ITGB1BP1,MEF2C,PDCD10,VASH1,MAP2K5,MECP2,MIR199A1,MIR22,MIR24-1,RHOA,ACVRL1,GADD45A,RGCC	grey	VSMC_M10
GO:0021871	0.93	0.20	4	11	forebrain regionalization	BP	1	PGAP1,TTC21B,LHX1,ADGRG1	grey	VSMC_M10

GO:0031929	0.93	0.11	49	105	TOR signaling	BP	1	EPM2A,ROS1,RRAGD,MFSD8,KPTN,SESN3,SZT2,PIP4P1,RPTOR,NCKAP1L,GAS6,LARS,TMEM127,SLC38A9,ARNTL,RHEBL1,MAPKAPK5,TTI1,TBCK,PDCD6,GPR137B,DYRK3,RPS6KB2,DISC1,SH3BP4,SYAP1,SIK3,EIF4EBP2,GPR137,PINK1,ARAF,TIPRL,OTUD5,CARD11,RRAGB,DGKQ,LARP1,SMCR8,CLEC16A,TNFAIP8L1,MLST8,TSC1,LAMTOR5,TLDC1,WDR59,MAPKAP1,MIR199A1,LAMTOR2,RNF152	grey	VSMC_M10
GO:0046641	0.93	0.20	4	11	positive regulation of alpha-beta T cell proliferation	BP	1	TGFBR2,RIPK2,CD28,RASAL3	grey	VSMC_M10
GO:0060571	0.93	0.17	7	18	morphogenesis of an epithelial fold	BP	1	SOSTDC1,GLI2,OVOL2,LUZP1,NOG,WNT5A,WNT2	grey	VSMC_M10
GO:0018206	0.93	0.20	4	11	peptidyl-methionine modification	BP	1	METAP1D,NAA30,NAA60,PDF	grey	VSMC_M10
GO:0007549	0.93	0.17	6	16	dosage compensation	BP	1	YTHDC1,CDYL,H2AFY2,UPF1,PCGF3,SMCHD1	grey	VSMC_M10
GO:0045932	0.93	0.17	7	18	negative regulation of muscle contraction	BP	1	GUCY1A1,DOCK5,GRK2,ATP1A2,ADRA2C,CALCRL,KCNMA1	grey	VSMC_M10
GO:0007202	0.93	0.16	8	20	activation of phospholipase C activity	BP	1	LPAR1,EGFR,C5AR1,PHB,RASGRP4,ARHGAP6,AGT,GNA15	grey	VSMC_M10
GO:0030308	0.93	0.10	73	153	negative regulation of cell growth	BP	1	SEMA6A,CYP27B1,HSPA1B,PPARA,GDF9,FSTL4,PI16,CDKN2A,SMARCA2,SEMA4D,SEMA4C,RTN4,PPP2R1A,PPT1,EPHA7,SPHK2,PHB,WNT11,SERTAD3,ING1,SEMA3D,BTG1,SPART,SEMA3A,ULK1,RYK,ACVR1B,CDKN2AIP,PTPRJ,SEMA4F,NDUFS3,PRDM11,SEMA3G,SPP1,SEMA4G,KIAA0319,HYAL1,CAPRIN2,SEMA3F,TGFB2,EI24,SH3BP4,AGT,IP6K2,CDKN1B,WNT3,BCL6,SEMA6D,DDX3X,TNR,MUL1,DNAJB2,TSPYL2,SEMA4A,TRIM46,SMAD3,NTN1,BDKRB1,DCUN1D3,YY1,CDK5R1,ARHGAP4,PLXNA3,PSRC1,NAIF1,MIR199A1,SERTAD2,ACVRL1,WNT5A,RGS4,DACT3,NME6,CGRRF1	grey	VSMC_M10
GO:1900024	0.93	0.13	19	44	regulation of substrate adhesion-dependent cell spreading	BP	1	FBLN1,PTK2,ITGB1BP1,UNC13D,RAC3,ARHGEF7,DOCK5,RREB1,DMTN,CARMIL1,AP1AR,CASS4,CIB1,DOCK1,MELTF,CSPG5,DNM2,CRK,FGA	grey	VSMC_M10
GO:0015031	0.93	0.08	670	1301	protein transport	BP	1	TIMM21,VPS4B,LRP1,STX7,AP3S1,KIF5A,PEX7,AGAP2,PEX3,CANX,TBC1D30,TBC1D9B,PPP1R10,RAB21,SNX24,SNX31,PHAX,ZDHHC14,SLC12A2,RIMS2,PRKN,TAP1,KIF3A,TSPO,GOPC,VPS52,HSPA4,DERL1,FAM91A1,WASHC5,FAM53C,TSNARE1,EXOC4,SIL1,SYNGR1,MAPK14,TOMM22,PLA2G6,COPG2,PICK1,MICALL1,TBC1D22B,RAC2,SMO,ZDHHC21,TOMM6,MYH9,PEX6,FBXO7,XPO5,WASHC4,APBA1,PEX1,VPS50,SCFD2,RFX3,SLC1A1,TGFBRAP1,TFAP2B,TBC1D8,TRAM2,BICD2,CHMP2A,RAB23,SVBP,SEC61B,STX17,SIX2,DNAJA1,PRKCE,RIMS1,TMEM30A,SPTBN1,HMGN3,RUFY3,CYB5R4,PPT1,SNX14,XPO1,SNX30,BMP8A,EHBP1,AFTPH,PLEK,RSAD2,NR1H2,ITGB1BP1,YWHAZ,SEC61G,EGFR,FUZ,ARHGEF5,TLR4,TCAF2,VPS41,TBC1D9,VPS35,SDCBP,MGARP,TRPM4,SLC7A11,LIN7B,EPS15,APOB,AGK,BCAP29,TRAF3IP2,ANKRD50,TTC26,DNAJC27,NAPA,BBS7,RAB7A,AP2S1,RAB10,COPG1,KPNA3,GIPR,ARL5B,LCP1,SYTL2,COG1,DLG2,CUBN,RBSN,DNAJC15,PLK3,FRMD4A,OPTN,GOLGA4,LLGL2,IPO13,STEAP3,AQP11,CCDC93,RAF1,ZFAND6,TBC1D4,MYO7A,NAPB,GCKR,SEC62,SEC22C,KPNA2,AKT2,PITRM1,LRP1B,HOOK3,ARRB1,TIMM50,SNX5,USP36,TBC1D16,AP3M2,KIF5C,FCHSD2,RINL,ATG16L2,NPLOC4,ARL5A,UBAC2,RAB5A,LIN7A,APBB3,ASPSCR1,TMCO6,NUP98,DPH3,SIRT7,FERMT1,HACL1,HEATR5B,CEP290,PRNP,ING1,BAG4,MAVS,AP5S1,DNAJC13,FUT10,FAM160A2,GAS6,BET1L,TRIM3,SIRT3,ARFIP2,VPS16,COPZ1,ARL6IP5,STX2,SYT9,SRP14,NCOA4,NR1H4,WASHC2A,KCNIP3,LYPLA1,NUP58,MIPEP,TM9SF4,NDUFAF2,ZDHHC20,KIF3B,FAM3D,IPO11,IL1A,RILPL1,RILPL2,IMMP1L,ANKRD27,SNX25,OSBP,PIK3R1,ABCB9,VAMP5,CHMP2B,TBC1D12,TNP[illegible]	grey	VSMC_M10
GO:0001676	0.93	0.11	30	66	long-chain fatty acid metabolic process	BP	1	AIG1,LTC4S,SLC27A6,QKI,ACSL6,PNPLA3,FAAH,ELOVL1,HACL1,ABHD6,PLA2G4B,ADTRP,CPT1A,ACSL5,CYP2U1,PLA2G4A,ACADL,ACSBG1,SLC27A1,DAGLB,EPHX1,ACOT2,ALOXE3,MAPK3,ACSBG2,CYP2J2,PTGES2,ABCD3,ACOT4,ELOVL3	grey	VSMC_M10
GO:1903054	0.93	0.20	4	11	negative regulation of extracellular matrix organization	BP	1	ADTRP,FAP,TNFRSF1B,DPP4	grey	VSMC_M10
GO:0071880	0.93	0.17	6	16	adenylate cyclase-activating adrenergic receptor signaling pathway	BP	1	ADRA1D,ADRA2B,RAPGEF2,ADRA2A,ADRA2C,CHGA	grey	VSMC_M10
GO:1900034	0.93	0.17	6	16	regulation of cellular response to heat	BP	1	HSF1,ATR,MAPKAPK2,MAPT,DNAJB1,CREBBP	grey	VSMC_M10
GO:2000780	0.93	0.14	12	29	negative regulation of double-strand break repair	BP	1	HELB,HSF1,CYREN,FBH1,RNF169,SHLD1,ERCC6,SHLD2,OTUB2,SMCHD1,RMI2,NUDT16L1	grey	VSMC_M10
GO:0050866	0.93	0.10	65	136	negative regulation of cell activation	BP	1	TNFAIP3,ENPP3,FBXO7,TNFRSF21,PELI1,IL4I1,CST7,CD300LF,PIBF1,FOXJ1,CYGB,THBD,C1QTNF1,PAWR,PRNP,NCKAP1L,ZC3H8,FER,CASP3,TMEM131L,PAG1,NR1H3,MARCH7,CD200,BANK1,BTN2A2,PRKAR1A,HLX,TBC1D10C,UBASH3B,GPER1,FGR,RUNX3,DLG1,BCL6,VSIR,RC3H1,PTPN6,TNFSF18,LAG3,INHA,SCGB1A1,FOXP3,TBX21,SYT11,ZBTB7B,IL4R,LDLR,BTK,CD74,TWSG1,PTPN2,SOCS1,ABAT,FOXF1,GLMN,MIR21,MIR27A,LST1,MIR125A,FGL2,LAX1,CEBPB,HAVCR2,RC3H2	grey	VSMC_M10
GO:0120192	0.93	0.12	21	48	tight junction assembly	BP	1	MPDZ,SRF,CLDN19,WNT11,IKBKB,ACTG1,NPHP1,LSR,MARVELD2,CLDN34,SNAI1,DLG1,FZD5,GPBAR1,RAMP2,CLDN7,MARVELD3,MICALL2,CLDN15,CLDN1,APC	grey	VSMC_M10
GO:0035630	0.93	0.20	4	11	bone mineralization involved in bone maturation	BP	1	ACTN3,RFLNB,SNX10,CCDC154	grey	VSMC_M10
GO:0010288	0.93	0.16	7	18	response to lead ion	BP	1	FECH,CAT,DNMT3A,PPP2CB,BACE1,MAPT,CLDN1	grey	VSMC_M10
GO:0090502	0.93	0.12	28	62	RNA phosphodiester bond hydrolysis, endonucleolytic	BP	1	RPP21,RIDA,UTP23,AGO2,TSR1,TSEN34,RNASEH1,NUDT16,BMS1,NUDT12,POP4,RPP40,POP5,YBEY,NOP9,NYNRIN,KIAA0391,POP7,ZC3H12C,MRPL44,RNASEH2A,KRI1,SLFN14,DROSHA,INTS11,NOL9,RPP38,RPP30	grey	VSMC_M10
GO:0031570	0.93	0.10	52	111	DNA integrity checkpoint signaling	BP	1	MRNIP,TRIM39,PPP1R10,MDC1,MAPK14,CRY1,BRSK1,MSH2,ETAA1,XPC,PLK3,NEK11,PRKDC,ERCC6,CEP63,STK33,PLK2,RAD17,ATR,RAD9B,EIF2AK4,NBN,ABRAXAS1,DONSON,PIDD1,TAOK3,CCND1,GIGYF2,CHEK2,ATRIP,SYF2,CDKN1B,TIPRL,RHNO1,PRPF19,UIMC1,FOXO4,EME1,MUC1,TAOK2,TIPIN,FOXN3,TAOK1,FZR1,DOT1L,FBXO31,NAE1,RPA4,RPS27L,SOX4,BRCC3,CRADD	grey	VSMC_M10

GO:0042093	0.93	0.12	19	44	T-helper cell differentiation	BP	1	HLA-DRA,FOXP1,RIPK2,HLX,BCL6,RC3H1,TNFSF18,STAT3,FOXP3,TBX21,SEMA4A,BATF,ZBTB7B,IL6R,IL4R,PTGER4,PRKCZ,MIR21,RC3H2	grey	VSMC_M10
GO:0033157	0.93	0.09	95	196	regulation of intracellular protein transport	BP	1	PRKN,MAPK14,RAC2,SMO,SLC1A1,RAB23,TMEM30A,RUFY3,XPO1,ITGB1BP1,TCAF2,DNAJC27,LCP1,PLK3,AKT2,USP36,UBAC2,SIRT7,FERMT1,CEP290,PRNP,BAG4,MAVS,GAS6,NUP58,TM9SF4,PIK3R1,FBXW7,PKIA,HTRA2,ZFAND1,CSNK2A2,NUP54,RAPGEF3,DMTN,SUFU,CABP1,SP100,TCF7L2,KCNB1,YOD1,HCLS1,HPS4,SORL1,C2CD5,ARIH2,CIB1,BAG3,SFN,UHMK1,APOD,CTDSPL2,FZD5,FIS1,CEMIP,NMT1,DERL3,SEC16B,C11orf65,PDCD10,PINK1,MAPK1,CHRM1,TMEM30B,GRIPAP1,ATP13A2,NPEPPS,TARDBP,PSEN1,ITGAM,NDEL1,HAX1,SMAD3,BCAS3,ARHGAP44,MIEF2,CDK5R1,SREBF1,EFCAB7,INPP5K,BCAP31,OAZ1,PARK7,SH3GLB1,RIPOR1,MICALL2,BNIP3L,TXN,HRAS,MIEF1,RHOU,LEPROT,UBE2G2,DERL2,ZIC1	grey	VSMC_M10
GO:2000377	0.93	0.10	57	120	regulation of reactive oxygen species metabolic process	BP	1	PRKN,PPARA,TSPO,MAPK14,RAC2,FPR2,EGFR,TLR4,UCP1,ATG5,MYCN,FOXO3,SPHK2,DHFR,WISP3,TGFBR2,SIRT2,SIRT3,HVCN1,PTK2B,F2RL1,NQO2,RIPK1,TFAP2A,PAX2,SIRT5,SLC30A10,FBLN5,AGT,ACE2,HDAC4,PDK3,NFE2L2,MAPT,PIKFYVE,BCO2,GCH1,PINK1,MMP3,STAT3,BST1,ITGAM,NOXA1,PARK7,SLC25A33,GNAI3,CYBA,LCN2,MIR21,MIR27B,MIR24-1,RHOA,TIGAR,GADD45A,GRB2,APOPT1,AC140504.1	grey	VSMC_M10
GO:0050667	0.93	0.20	4	11	homocysteine metabolic process	BP	1	MTRR,CBSL,CPS1,COMT	grey	VSMC_M10
GO:0048536	0.93	0.14	13	31	spleen development	BP	1	TRAF3IP2,PRKDC,CDH17,JARID2,NFKB2,PPP2R3C,RC3H1,NKX3-2,CTC1,FADD,BCL2L11,NKX2-5,RC3H2	grey	VSMC_M10
GO:1903800	0.93	0.20	4	11	positive regulation of production of miRNAs involved in gene silencing by miRNA	BP	1	EGFR,MYCN,RIPK1,MAP2K1	grey	VSMC_M10
GO:0042559	0.93	0.18	5	13	pteridine-containing compound biosynthetic process	BP	1	DHFR,SPR,MTHFS,GCH1,PCBD2	grey	VSMC_M10
GO:0006937	0.93	0.10	57	121	regulation of muscle contraction	BP	1	ZDHHC21,CHRNA3,SRF,KIT,TNNI3,ASB3,TRPM4,SCN5A,ADRA2B,NR4A1,PLCE1,GUCY1A1,DOCK5,STC1,F2R,TNNC1,DSP,CASQ2,CHRM3,RYR2,PKP2,KCNJ2,TNNI1,TRPV4,ADRA2A,MYH7,GRK2,ANK2,GPER1,ACE2,ATP1A2,SLC9A1,CASQ1,ACTN3,HDAC4,DLG1,ADRA2C,CALCRL,ATP1B1,KCNA1,KCNMA1,MYL5,ATP2A1,CALM1,NMUR2,SETD3,DSG2,ABAT,P2RX1,RNF207,MIR21,RHOA,DMD,CHGA,TNNC2,HCN4,NKX2-5	grey	VSMC_M10
GO:0021766	0.93	0.12	26	59	hippocampus development	BP	1	NME1,ATAT1,UQCRQ,SMO,SRF,CDK6,EIF2B3,XRCC1,TSKU,ALK,MKKS,CASP3,DCLK2,TENT2,SRD5A1,HDAC1,DLX1,KCNA1,KDM6B,CDK5R1,DAB1,LRP8,CRK,PLXNA3,TSC1,PHLPP2	grey	VSMC_M10
GO:1902679	0.93	0.08	482	946	negative regulation of RNA biosynthetic process	BP	1	SMARCC2,ATP8B1,PLAGL1,ZBTB2,ESR1,CNOT2,OSR2,SNW1,ZNF706,ZBTB12,PRKN,PPARA,TCF7,XRCC6,PITX1,MYC,TOB2,SMAD5,L3MBTL3,L3MBTL2,PTCH1,ATF4,CBX7,CBY1,SOX10,TMPRSS6,SMO,HSF1,FOXP4,MDFI,CCND3,FOXH1,CDKN2A,RBFOX2,RORB,CRY1,SRF,ZNF318,NFX1,SMARCA2,RFX3,NCK2,FHL2,TFAP2B,NFIL3,SEMA4D,RPS14,ZNF550,BEND6,ZNF268,SCMH1,NFE2L3,HOXA2,HOXA7,JAZF1,XPO1,AKIRIN2,CASP8AP2,BACH2,ZNF692,NR1H2,ZNF350,FOXP2,MED25,KLF11,POU3F3,LANCL2,CREM,SDCBP,OSR1,FOXO3,BEND5,HDAC2,PHF10,DNMT3A,BMI1,ZC3H4,CNBP,SETDB2,PHB,SOX9,ZNF746,EED,SUV39H2,ZNF404,PLK3,GLI2,DEDD2,RSF1,KDM4A,DACH1,TAF3,ACVR2B,KLF5,KLF12,FOXJ1,SFMBT2,GZF1,POU4F1,WNT11,THAP1,EID2,OVOL2,ZNF219,EOMES,CBX4,BHLHE40,CHD8,SIRT2,ZMYND11,RARB,PURA,NR1D2,PAWR,PLCB1,SIRT7,DPF1,ALX1,TAF7,NRG1,GAS6,RYBP,FOXP1,MITF,MTA3,ARID5A,IRF7,TRAF6,LIN37,NR1H4,KCNIP3,TRIB3,PFDN5,SDR16C5,ZC3H8,DNMT3B,BTAF1,CEBPA,CBFA2T2,SMYD1,FOXL2,ZNF536,TFDP2,IRF2,NCOA2,DKK3,SAP30,ZNF366,ARNTL,GCFC2,HMBOX1,MEF2C,PEX2,PKIA,TMBIM6,CNOT1,SLA2,CUX2,MCPH1,SOX6,FNIP2,BACH1,SFMBT1,NR1H3,EAPP,SCAF4,LCOR,DDX54,RUNX1T1,VDR,NKX3-1,BAHD1,HDAC7,NKX6-1,LOXL2,NR4A2,PHF21A,CRY2,PRDM11,CDYL,RREB1,RTF1,ZBED6,SOX13,ZNF248,TFAP2A,RIPPLY3,HIF1AN,PAX2,SALL1,PPHLN1,PRICKLE1,HIVEP1,JARID2,ATXN1,SUDS3,ZNF423,LDB1,IGF2,MAGEL2,ELK4,NUPR2,DCAF1,SUFU,TAGLN3,PCGF6,IRX2,RPL23,HDAC1,ELF3,CCND1,SP100,TRPV4,PDCD4,ZBTB20,SATB2,TCF7L2,AJUBA,ZNF639,PIAS3,LHX9,HJV,FABP4,SNAI1,ZNF202,H[illegible]	grey	VSMC_M10

GO:1901137	0.93	0.08	249	494	carbohydrate derivative biosynthetic process	BP	1	NME1,TBPL1,B4GALT7,B4GALNT1,RXYLT1,ATP5MGL,GFPT2,ALG12,PPT2,PPARA,ACSL6,A4GALT,PMM1,AGO2,GPAA1,IMPDH1,MTAP,LARGE1,GNPDA2,SLC35B2,CWH43,ST6GAL2,SRD5A3,UXS1,SLC25A13,PPAT,C1GALT1,CHST10,ALG2,PPCS,TMEM246,PIGF,FKTN,HAS1,NT5E,PPT1,IL15,DLD,NANP,SPHK2,CMPK1,HS3ST5,ADCY3,ALG11,PRTFDC1,POMGNT1,AKR1A1,B4GALT2,CHPF2,AQP11,PRKAG2,GALNT11,ATP5F1C,XYLB,ST6GALNAC2,MGAT5B,ELOVL1,CSGALNACT1,UGGT2,HBEGF,PAWR,PLCB1,TMTC4,PGAP2,GALNT15,PANK2,PDK1,FUT10,GXYLT2,EOGT,PDHX,ELOVL7,PANK1,POFUT1,AMPD3,PIGU,ST8SIA4,GK5,ATP5F1A,B3GNT4,MOGS,GUCY1A1,GUCY1B1,RPN2,DGUOK,GMPS,ST3GAL6,GALNT5,CHST1,AMPD1,GCNT2,PIGT,ADCY7,FAM173B,BMPR1B,ADCY2,NME4,A3GALT2,ALG10,HYAL1,SLC2A10,PGAP1,TYW1B,TRIP11,ACSL5,POGLUT1,TYW5,TCF7L2,DAD1,ST8SIA2,EGF,PRPS2,B3GALT1,B3GNT5,PIGM,LCMT2,ABCC5,HACD2,GALNT2,ADCY4,PIGV,DPAGT1,CTPS2,ALG3,MAN1C1,MGAT1,DNAJC30,PIGX,GAL3ST3,DTYMK,PIGZ,XXYLT1,PDK3,EDEM3,SLC25A16,TET1,PLOD3,CEMIP,SOAT1,DERL3,ALG9,MGAT2,ACSBG1,ATP5S,B3GALNT1,KDELC2,CHST3,GAL3ST4,PYGL,PIGC,CHST7,COASY,DDOST,PINK1,AC022400.6,STAT3,NME7,GALNT8,ADK,MPC2,ADCY10,HS3ST1,SCCPDH,PARP1,SLC2A4,TECR,PGAP3,CHST12,PIGH,GALNT16,SLC26A1,PSEN1,GUCY2D,PKLR,PANK3,KRTCAP2,GCDH,OGT,POMT2,UPRT,GLCE,ST8SIA5,COG7,HEXA,GALNT1,TNIP1,MPI,ACACA,PIGL,NPC1,SLC26A2,PRPSAP2,ADSSL1,ALG6,ACSBG2,MPPE1,HS6ST2,PMM2,ALG1,B3GALT6,ATP6V0C,PANK4,NME3,ABCA7,ATP5PB,ABO,VPS9D1,ACSF3,GBGT1,UCK1,FOXL1,MLYCD,CHST5,ST6[illegible]	grey	VSMC_M10
GO:1905906	0.93	0.18	5	13	regulation of amyloid fibril formation	BP	1	PFDN5,PFDN4,PSEN1,LDLR,VBP1	grey	VSMC_M10
GO:0042541	0.93	0.20	4	11	hemoglobin biosynthetic process	BP	1	SLC25A37,ALAS1,LDB1,INHA	grey	VSMC_M10
GO:0045834	0.93	0.10	51	109	positive regulation of lipid metabolic process	BP	1	AGAP2,PPARA,PTK2,PLA2G6,TEK,KIT,PRKCE,FPR2,NR1H2,FGF21,SPHK2,ABHD5,APOC1,AKT2,SIRT3,FLT1,NR1H4,CYP7A1,ABHD6,SORBS1,PTK2B,NR1H3,HSD17B13,NOD2,SIRT4,GPLD1,IRS1,CPT1A,ZBTB20,AGT,FGR,LPGAT1,MLXIPL,SCAP,POR,CREB1,WNT4,FGFR3,SLC27A1,DAGLB,CD19,LDLR,CD74,SREBF1,VAV3,MLYCD,LDLRAP1,CNEP1R1,STARD4,CCDC3,FGF1	grey	VSMC_M10
GO:0048704	0.93	0.11	38	83	embryonic skeletal system morphogenesis	BP	1	OSR2,MDFI,SIX2,HOXA2,HOXA4,HOXA6,HOXA7,FUZ,MYCN,OSR1,BMI1,MEGF8,WDR60,TGFBR2,ALX1,EYA1,TFAP2A,HYAL1,SATB2,DLG1,SETD2,HOXD4,HOXD3,TULP3,WDR19,HOXB5,HOXB6,HOXB8,HOXB9,SMAD3,GSC,LHX1,ALX3,HOXB3,NOG,SIX1,SLC39A3,HOXA3	grey	VSMC_M10
GO:0097709	0.93	0.19	4	11	connective tissue replacement	BP	1	IL1A,F2R,PPP3CA,MIR199A1	grey	VSMC_M10
GO:1902003	0.93	0.13	15	35	regulation of amyloid-beta formation	BP	1	NTRK2,RTN4,PRNP,CASP3,SLC2A13,SORL1,RTN1,HAP1,EPHA4,TMED10,EFNA1,ABCA7,MIR29A,MIR24-1,MIR339	grey	VSMC_M10
GO:0000729	0.93	0.17	6	16	DNA double-strand break processing	BP	1	HELB,SPO11,RAD52,EXD2,SLX4,UBE2N	grey	VSMC_M10
GO:0009126	0.93	0.12	17	39	purine nucleoside monophosphate metabolic process	BP	1	IMPDH1,GDA,PPAT,NT5E,DLG2,AMPD3,DGUOK,GMPS,AMPD1,MAGI3,DLG1,CASK,ADK,CARD11,ADSSL1,MPP1,NUDT2	grey	VSMC_M10
GO:0050872	0.93	0.19	4	11	white fat cell differentiation	BP	1	PNPLA3,CEBPA,FABP4,PER2	grey	VSMC_M10
GO:0061337	0.93	0.11	34	76	cardiac conduction	BP	1	TRPM4,SLC8A2,CACNB2,ATP1A3,SCN5A,KCNJ3,TBX5,KCNE2,DSP,CASQ2,RYR2,PKP2,KCNJ2,ANK2,AGT,ACE2,ATP1A2,SLC9A1,SCN4B,ATP1B1,SLC4A3,ATP1B2,CACNA1G,ATP2A1,CALM1,DSG2,ISL1,ATP2B3,RNF207,TMEM65,KCNH2,SCN2B,HCN4,NKX2-5	grey	VSMC_M10
GO:0071428	0.93	0.19	4	11	rRNA-containing ribonucleoprotein complex export from nucleus	BP	1	XPO1,SDAD1,NOP9,TSC1	grey	VSMC_M10
GO:0016446	0.93	0.19	4	11	somatic hypermutation of immunoglobulin genes	BP	1	MSH2,MLH1,POLL,PMS2	grey	VSMC_M10
GO:0055001	0.93	0.09	71	150	muscle cell development	BP	1	STAC3,MAML1,PPARA,OBSCN,PI16,SMO,FLNC,LMOD2,SRF,PGM5,FHL2,TMOD1,BVES,KEL,MYLK3,ATG5,KLHL40,ACTG1,RYR1,P2RX2,LMOD3,MYOF,DYSF,HOMER1,TBX5,BIN3,LMOD1,PPP3CA,PRKAR1A,ANK2,AGT,ACTC1,NFATC4,MYOZ2,CASQ1,ACTN3,TMOD4,HDAC4,ALPK3,EFEMP2,MYPN,TTN,MYOZ1,RAMP2,TMOD2,OBSL1,LDB3,BMPR1A,CHRNB1,TCAP,WFIKKN2,YY1,FBXO22,ISL1,MYH11,SKI,WFIKKN1,SYPL2,ENG,MIR199A1,MIR23A,MIR24-1,DMD,CSRP2,KRT19,SMYD3,SIX1,RGS4,MYORG,NKX2-5,HEY2	grey	VSMC_M10
GO:0022404	0.93	0.11	28	63	molting cycle process	BP	1	FZD6,SMO,ZDHHC21,SOSTDC1,CD109,EGFR,FUZ,INTU,HDAC2,SOX9,ERCC2,GLI2,FERMT1,ACVR1B,TRPC4AP,FZD3,LDB1,HDAC1,TGFB2,SNAI1,LAMA5,FARP2,SAV1,GORAB,RBPJ,NGFR,WNT5A,DNASE1L2	grey	VSMC_M10
GO:0022405	0.93	0.11	28	63	hair cycle process	BP	1	FZD6,SMO,ZDHHC21,SOSTDC1,CD109,EGFR,FUZ,INTU,HDAC2,SOX9,ERCC2,GLI2,FERMT1,ACVR1B,TRPC4AP,FZD3,LDB1,HDAC1,TGFB2,SNAI1,LAMA5,FARP2,SAV1,GORAB,RBPJ,NGFR,WNT5A,DNASE1L2	grey	VSMC_M10
GO:0009124	0.93	0.13	16	37	nucleoside monophosphate biosynthetic process	BP	1	IMPDH1,PPAT,CMPK1,AMPD3,DGUOK,GMPS,AMPD1,PRPS2,ADK,UPRT,ADSSL1,UCK1,DHODH,DCTD,NUDT2,UPP1	grey	VSMC_M10
GO:0001942	0.93	0.11	27	61	hair follicle development	BP	1	FZD6,SMO,ZDHHC21,SOSTDC1,CD109,EGFR,FUZ,INTU,HDAC2,SOX9,ERCC2,GLI2,FERMT1,ACVR1B,TRPC4AP,FZD3,LDB1,HDAC1,TGFB2,SNAI1,LAMA5,SAV1,GORAB,RBPJ,NGFR,WNT5A,DNASE1L2	grey	VSMC_M10
GO:0006334	0.93	0.11	31	68	nucleosome assembly	BP	1	BRD2,ASF1A,UBN2,SOX9,RSF1,SPTY2D1,NAP1L5,RBBP4,SART3,H2AFY2,HIRA,TSPYL2,NAP1L2,NAA60,TSPYL4,HIST1H2BK,HIST1H4B,HIST2H4A,HIST1H4H,SMYD3,HIST1H3F,ITGB3BP,HIST1H3H,HIST1H4D,HIST2H2BF,HIST1H2BF,HIST1H2BM,HIST1H2BJ,HIST1H4A,HIST1H4F,TSPYL5	grey	VSMC_M10
GO:0045777	0.93	0.15	8	20	positive regulation of blood pressure	BP	1	TAC3,ADH5,ADRA1D,ADRA2B,AGT,CYBA,HSD11B2,RHOA	grey	VSMC_M10
GO:0048332	0.93	0.11	24	55	mesoderm morphogenesis	BP	1	EXOC4,SRF,TXNRD1,SIX2,WNT11,EOMES,GPI,EYA1,TLX2,PAX2,PRKAR1A,SNAI1,SETD2,WNT3,TBX19,BMPR1A,KDM6B,ITGA3,SMAD3,TWSG1,FOXF1,POFUT2,NOG,WNT5A	grey	VSMC_M10

GO:0010507	0.93	0.11	30	67	negative regulation of autophagy	BP	1	RNF5,TSPO,NRBP2,MET,KDM4A,USP36,SIRT2,FEZ2,MTMR9,LZTS1,SEC22B,TMEM39A,USP30,MCL1,RUBCN,IL10RA,TAB3,TBC1D14,PINK1,STAT3,PHF23,NPC1,SMCR8,LEPR,CLEC16A,MTM1,TSC1,MIR199A1,TIGAR,CPTP	grey	VSMC_M10
GO:0032570	0.93	0.13	12	29	response to progesterone	BP	1	TSPO,SOX10,PTN,GPI,NCOA2,NR1H3,TGFB2,ABHD2,RAMP2,CD38,FOS,SREBF1	grey	VSMC_M10
GO:0036314	0.93	0.14	10	25	response to sterol	BP	1	PTCH1,SMO,TGFBR2,CYP7A1,GPLD1,DAG1,GRAMD1B,OSBPL7,LRP8,INSIG2	grey	VSMC_M10
GO:0060445	0.93	0.16	7	18	branching involved in salivary gland morphogenesis	BP	1	PLXNA1,SEMA3A,DAG1,LAMA5,BTBD7,LAMA1,ESRP2	grey	VSMC_M10
GO:0050900	0.93	0.09	120	245	leukocyte migration	BP	1	SLC12A2,AGER,GPSM3,PTK2,RAC2,MYH9,CCL27,KIT,ROR2,NINJ1,FPR2,HOXA7,CXCL8,AKIRIN1,PTN,ADD2,TRPM4,PODXL2,C5AR2,C5AR1,MYD88,OXSR1,FOXJ1,PLCB1,AIMP1,NCKAP1L,GAS6,FAM19A4,FLT1,MTUS1,RET,HCK,CYP7B1,FER,VEGFC,JAM2,PTK2B,CCL17,SLC8B1,F2RL1,ANO6,ADTRP,NOD2,GBF1,CD200,DNM1L,LCK,TRPV4,SBDS,TGFB2,CD99,PRSS56,SELPLG,CMKLR1,SLAMF8,MOSPD2,EPS8,PTPRO,FCER1G,APOD,ADAM8,MIA3,PIKFYVE,IL16,ITGA2B,ITGA4,IL12A,MCU,TNFSF18,CXCR2,BST1,MAPK1,STAT5B,CH25H,PADI2,CXCL16,TMEM102,TBX21,STK10,PGF,DAPK2,MAPK3,IL6R,S100A14,S100A8,BDKRB1,UMOD,CD74,CCL14,CCL5,CSF1R,CCL7,CCL2,CCL25,YES1,VAV1,PTGER4,CRK,PIP5K1C,MPP1,BSG,VAV3,MIR24-1,RHOA,CCL16,S100A12,CHGA,DEFB124,FADD,DPP4,CXCL6,FUT4,ZNF580,THY1,WNT5A,DUSP1,ARTN,GCSAM,CCL22,PDGFD	grey	VSMC_M10
GO:1901653	0.93	0.09	132	270	cellular response to peptide	BP	1	LRP1,AP3S1,MARS,PIP4K2C,AGER,PNPLA3,PTK2,AKR1B1,GKAP1,HSF1,CCND3,GPT,PPAT,PHIP,FPR2,NOD1,CRHR2,KLF11,GRB10,TLR4,GAB1,LPIN1,MGARP,FOXO3,FGF21,SESN3,PIP4K2A,RAB10,CYSLTR2,ECHDC3,ATP1A3,KLF5,TBC1D4,AKT2,SNX5,PRNP,KL,PTPRA,LEPROTL1,PRKDC,TRIB3,NR4A1,FER,IDE,PIK3R1,SORBS1,RIPK2,OPA1,LPIN3,NKX6-1,NR4A2,CYFIP1,NOD2,IGF2,PXN,GPLD1,SRD5A1,IRS1,RPL23,GRB14,SLC30A10,SORL1,C2CD5,GPER1,AGT,SLC9A1,SYAP1,USF1,BACE1,SNX6,POR,NFE2L2,CREB1,YWHAG,RAP1B,FIS1,CPS1,EIF4EBP2,ITGA4,MEN1,SIK2,ESRRA,FBN1,GDF15,STAT3,SLC27A1,STAT5B,DDX11,RAB8A,EPHA4,GPR173,PARP1,SLC2A4,RAB15,MAX,CACNB1,AGTRAP,PSEN1,CACNA1A,FOXO4,NGFR,PKLR,RAB8B,NDEL1,ZBTB7B,OGT,PRKCB,CAMK2A,INSR,SREBF1,LPIN2,GHR,PTPN2,SOCS1,CRK,INPP5K,PDPK1,PRKCZ,SLC25A33,CYBA,ASS1,LCN2,INSRR,CA2,BGLAP,GRB2,BCL2L11,INSIG2,APC,BCL2L2,SH2B2,LEPROT	grey	VSMC_M10
GO:0061045	0.93	0.11	23	52	negative regulation of wound healing	BP	1	SERPINB2,TSPAN8,SLC12A2,CLDN19,CD109,CLASP2,THBD,PLAT,C1QTNF1,ADTRP,FAP,UBASH3B,USF1,CASK,WNT4,MYOZ1,F12,ANXA2,SMAD3,ABAT,CRK,SERPINF2,FGA	grey	VSMC_M10
GO:0051647	0.93	0.15	8	21	nucleus localization	BP	1	SUN2,HOOK3,SLIT1,SYNE2,NTN1,CLMN,DOCK7,FHOD1	grey	VSMC_M10
GO:0002526	0.93	0.11	26	58	acute inflammatory response	BP	1	MYLK3,IL1A,ANO6,OSM,ADAM8,CD163,FN1,SERPINC1,CXCR2,STAT3,F12,IL6R,S100A8,BTK,C3,OSMR,CREB3L3,SERPINF2,DNASE1,TRPV1,PARK7,ASS1,LCN2,HPR,PTGES,CEBPB	grey	VSMC_M10
GO:0008284	0.93	0.08	345	681	positive regulation of cell population proliferation	BP	1	NME1,ERBB3,SHMT2,PGGT1B,GLI1,MAP3K5,TNFAIP3,TSPAN31,ESR1,CNOT6,OSR2,AGER,IL5,HLA-DPB1,TSPO,GDF9,TNFRSF13C,MYC,PTK2,AKR1B1,MAPK15,MAPK14,NTRK2,KDM4C,SOX10,PIM1,RAC2,SMO,HSF1,ZNF16,TEK,CNTFR,CDK6,SLC25A27,KIT,NCK2,TFAP2B,DLX5,S1PR3,TBC1D8,PDCL3,ZNF268,SIX2,IL11,EPCAM,RTN4,PHIP,EMC10,EPGN,AREG,AKIRIN2,AQP1,PELI1,TGFA,PTN,ITGB1BP1,TNC,POU3F3,EGFR,TLR4,EPHA1,IL15,SDCBP,MYCN,TRPM4,OSR1,FGF21,SPHK2,FOXE3,HDAC2,BMI1,C5AR2,C5AR1,PRKD2,CNBP,PHB,SOX9,GAB2,CDC123,GLI2,AQP11,MYD88,PRDX3,KLF5,SCN5A,WDR48,ACER3,AKT2,ARRB1,ABCC4,TGFBR2,RPTOR,PURA,HBEGF,CHTOP,ADRA1D,ASH2L,MAB21L1,NRG1,CCKBR,DDRGK1,NCKAP1L,GAS6,FOXP1,MTA3,PRKDC,SLC7A1,FLT1,CIAO1,TRAF6,PLAG1,HCK,TOX,IL1A,CYP7B1,NR4A1,FER,HPGD,EYA1,MAB21L2,FZD3,MEF2C,IL7,VEGFC,ADAMTS1,PTK2B,CNOT6L,KMT2D,RPRD1B,EAPP,TIAM1,FGF5,RIPK2,NKX3-1,TBX5,AGGF1,F2R,SRSF6,NQO2,MARCH7,RREB1,GCNT2,PAX2,NOD2,ZNF335,ZNF423,CD40,IGF2,IRS1,RPL23,HDAC1,HYAL1,SINHCAF,CCND1,ICOSLG,TERT,TGFB2,ADRA2A,GPAM,PTHLH,TCIRG1,TCF7L2,COX17,PDCD6,HLX,KRAS,EGF,HCLS1,DISC1,GPER1,CACUL1,AGT,CIB1,TRAF5,ARNT,TGM1,VASH2,FZD7,OSM,RPS4X,HDAC4,HTRA1,GID8,THPO,CHRD,CD28,SYF2,MAP3K3,MLXIPL,RTKN2,GLUL,FZD9,LAMC1,CDKN1B,DLG1,PTH1R,TET1,CCAR1,STOX1,BCL6,CTSH,DBF4B,ITGA4,IL12A,FN1,PTPN6,PDCD10,CXCR2,GPBAR1,CALCRL,SHC4,FGFR3,STAT3,PTPRN,CD38,BST1,MAPK1,STAT5B,FOXP3,RASAL3,BMPR1A,CARD11,RBPJ,MED1,ALDH1A2,MFN2,RAD51B,FGFR4,SOX15,ANXA2,CTF1,STX4,RAB[illegible]	grey	VSMC_M10
GO:0009112	0.93	0.13	13	31	nucleobase metabolic process	BP	1	SHMT2,DPYS,GDA,PPAT,CMPK1,GMPS,GMPR,CTPS2,CPS1,MAPK1,ALDH6A1,SHMT1,DHODH	grey	VSMC_M10
GO:0035710	0.93	0.11	29	65	CD4-positive, alpha-beta T cell activation	BP	1	AGER,HLA-DRA,RSAD2,TGFBR2,NCKAP1L,FOXP1,TOX,RIPK2,TCIRG1,HLX,RUNX3,BCL6,VSIR,RC3H1,TNFSF18,STAT3,FOXP3,TBX21,SEMA4A,BATF,ZBTB7B,IL6R,IL4R,PTGER4,TWSG1,SOCS1,PRKCZ,MIR21,RC3H2	grey	VSMC_M10
GO:0046627	0.93	0.14	10	25	negative regulation of insulin receptor signaling pathway	BP	1	PIP4K2C,GRB10,PIP4K2A,IRS1,GRB14,PRKCB,PTPN2,SOCS1,INPP5K,PRKCZ	grey	VSMC_M10

GO:0070085	0.93	0.09	94	194	glycosylation	BP	1	B4GALT7,B4GALNT1,RXYLT1,GFPT2,ALG12,PMM1,LARGE1,ST6GAL2,SRD5A3,C1GALT1,ALG2,FKTN,IL15,ALG11,POMGNT1,B4GALT2,AQP11,GALNT11,ST6GALNAC2,MGAT5B,UGGT2,TMTC4,GALNT15,FUT10,GXYLT2,EOGT,POFUT1,ST8SIA4,B3GNT4,MOGS,RPN2,ST3GAL6,GALNT5,GCNT2,A3GALT2,ALG10,TRIP11,POGLUT1,DAD1,ST8SIA2,B3GALT1,B3GNT5,PIGM,GALNT2,PIGV,DPAGT1,ALG3,MAN1C1,MGAT1,PIGZ,FUOM,XXYLT1,EDEM3,TET1,PLOD3,DERL3,ALG9,MGAT2,B3GALNT1,KDELC2,DDOST,GALNT8,GALNT16,PSEN1,KRTCAP2,OGT,POMT2,ST8SIA5,COG7,GALNT1,MPI,NPC1,ALG6,PMM2,ALG1,B3GALT6,ABO,GBGT1,ST6GALNAC4,ST3GAL2,ST6GALNAC5,FUT4,ENTPD5,DHDDS,TMEM258,POFUT2,ACER2,POMK,POMGNT2,PIGB,B4GALT3,ISPD,SLC35C1,ST8SIA6	grey	VSMC_M10
GO:0050852	0.93	0.10	39	85	T cell receptor signaling pathway	BP	1	CD226,HLA-DPB1,TEC,TNFRSF21,PRKD2,NECTIN2,EIF2B3,IKBKB,PAWR,PRNP,TRAF6,PTPRJ,SLA2,RIPK2,SPPL3,LCK,ICOSLG,BTN2A2,DENND1B,THEMIS2,CD28,RC3H1,PTPN6,MAPK1,FOXP3,CARD11,PSEN1,LCP2,PRAM1,PTPN2,IKBKG,BCL10,FYB1,BTN1A1,CD247,THY1,UBE2N,HRAS,RC3H2	grey	VSMC_M10
GO:0045838	0.93	0.19	4	11	positive regulation of membrane potential	BP	1	AKT2,GLRX,MFN1,PIAS3	grey	VSMC_M10
GO:0060192	0.93	0.19	4	11	negative regulation of lipase activity	BP	1	APOC1,PLA2R1,POR,ANXA8	grey	VSMC_M10
GO:0035825	0.93	0.12	20	46	homologous recombination	BP	1	UBE2B,DMC1,KLHDC3,MLH1,CCNB1IP1,RAD54B,SYCP1,HSF2BP,SPO11,C11orf80,RAD51B,EME1,RAD51C,RAD51D,ERCC4,SLX4,MEIOB,MSH4,RNF212B,CENPX	grey	VSMC_M10
GO:0044793	0.93	0.19	4	11	negative regulation by host of viral process	BP	1	PRKN,EIF2AK4,PSMC3,MIR222	grey	VSMC_M10
GO:0006301	0.93	0.14	11	27	postreplication repair	BP	1	POLI,UBE2B,REV1,MSH2,REV3L,PRIMPOL,SPRTN,POLN,ZBTB1,NSMCE1,UBE2N	grey	VSMC_M10
GO:0031623	0.93	0.10	39	85	receptor internalization	BP	1	LRP1,PICK1,USP46,CXCL8,RSPO1,SFRP4,SDCBP,EPS15,MTMR2,ARRB1,RAB5A,WDR54,ANKRD13A,EGF,ANKRD13D,GRK2,SAG,AP2M1,FCER1G,CALY,LRPAP1,CXCR2,GRK4,CALCRL,DNM3,RAMP2,DLG4,SNX1,DNM2,INSR,AP2B1,FMR1,MIR199A1,CNTN2,LDLRAP1,CACNG7,GRB2,CACNG5,LRRTM2	grey	VSMC_M10
GO:0061036	0.93	0.14	10	25	positive regulation of cartilage development	BP	1	SOX9,ZNF219,SOX6,LOXL2,BMPR1B,POR,FGF18,SMAD3,ACVRL1,WNT5A	grey	VSMC_M10
GO:1903322	0.93	0.10	57	121	positive regulation of protein modification by small protein conjugation or removal	BP	1	PRKN,DERL1,SKP1,CRY1,RNF20,PELI1,BMI1,PAXIP1,WDR48,MYCBP2,KLHL40,ARRB1,NMI,TRAF6,TRIB3,FAM107A,SPSB4,FBXW7,RIPK2,MARCH7,PRICKLE1,NOD2,TOLLIP,NHLRC1,PEF1,PDCD6,PIAS3,SART3,SPRTN,ARNT,HDAC4,SENP2,FBXO33,DDX3X,PINK1,MUL1,BIRC3,PHF23,RNF111,DCUN1D3,TNIP1,PSMD10,TICAM1,PIAS4,FZR1,HSPA5,BCL10,UBB,RWDD3,INAVA,RASSF5,UBE2N,PTTG1IP,CENPX,UBE2D1,TSPYL5,RASD2	grey	VSMC_M10
GO:0045815	0.93	0.16	6	16	positive regulation of gene expression, epigenetic	BP	1	ARID1B,SPHK2,SMARCD1,ARID1A,PADI2,SERTAD2	grey	VSMC_M10
GO:0007032	0.93	0.11	32	71	endosome organization	BP	1	VPS4B,PRKN,WASHC5,WASHC4,CHMP2A,RAB7A,AQP11,HOOK3,DNAJC13,FAM160A2,TMEM127,CHMP2B,CHMP4C,SYNJ1,CHMP7,VPS18,TMEM9,RNF26,RAB22A,STX6,VPS37D,ALS2CL,ANXA8,PI4K2B,HOOK2,RAB11A,RILP,CHMP1A,VPS4A,PHETA1,SNX10,PLEKHJ1	grey	VSMC_M10
GO:0006692	0.93	0.13	12	29	prostanoid metabolic process	BP	1	AKR1B1,ACOX1,PIBF1,HPGD,PLA2G4A,DAGLB,AC005520.1,CD74,PTGES2,CES2,FABP5,PTGES	grey	VSMC_M10
GO:0006693	0.93	0.13	12	29	prostaglandin metabolic process	BP	1	AKR1B1,ACOX1,PIBF1,HPGD,PLA2G4A,DAGLB,AC005520.1,CD74,PTGES2,CES2,FABP5,PTGES	grey	VSMC_M10
GO:0051654	0.93	0.13	11	27	establishment of mitochondrion localization	BP	1	AGTPBP1,LRPPRC,MGARP,WASF1,NECTIN2,OPA1,MARK1,MAPT,UXT,HAP1,UBB	grey	VSMC_M10
GO:2000035	0.93	0.19	4	11	regulation of stem cell division	BP	1	PRDM15,ESRRB,NAP1L2,EVI2B	grey	VSMC_M10
GO:0071027	0.93	0.19	4	11	nuclear RNA surveillance	BP	1	DXO,EXOSC4,EXOSC3,EXOSC5	grey	VSMC_M10
GO:0071028	0.93	0.19	4	11	nuclear mRNA surveillance	BP	1	DXO,EXOSC4,EXOSC3,EXOSC5	grey	VSMC_M10
GO:0007565	0.93	0.09	66	139	female pregnancy	BP	1	TAC3,CYP27B1,ESR1,FBLN1,AGO2,H3F3C,AKR1B1,PLA2G6,CLIC5,RLN1,PPAT,PTN,SPHK2,PSG3,PRDX3,THBD,TGFBR2,IDO1,ADRA2B,ACVR1B,HPGD,VDR,STC1,SLC38A2,ACVR1C,SLC38A1,ABCC2,SPP1,ADCY7,SLC38A3,COL16A1,PTHLH,KRAS,AGT,DEDD,SP3,OVGP1,MEN1,COMT,ADRA2C,WNT4,RAMP2,CTSV,CD38,MAPK1,STAT5B,SCGB1A1,SYDE1,MED1,ITGA3,PGF,FOS,MAPK3,UBE2Q1,UPRT,EPOR,VMP1,TLE6,BSG,DHODH,HSD11B2,MIR21,CRH,TCF23,LIF,HAVCR2	grey	VSMC_M10
GO:0090075	0.93	0.14	10	25	relaxation of muscle	BP	1	GUCY1A1,P2RY1,KCNJ2,GRK2,ATP1A2,ACTN3,ATP1B1,KCNMA1,ATP2A1,CHGA	grey	VSMC_M10
GO:0006285	0.93	0.19	4	11	base-excision repair, AP site formation	BP	1	MBD4,NEIL1,NTHL1,MPG	grey	VSMC_M10
GO:2001022	0.93	0.10	44	95	positive regulation of response to DNA damage stimulus	BP	1	EYA4,MRNIP,MYC,EGFR,XRCC1,PAXIP1,WDR48,FBH1,FAM168A,SHLD1,PRKDC,ERCC6,ATR,EYA1,SLF1,NKX3-1,ABRAXAS1,SPIDR,PLA2R1,SLF2,EYA3,PARP9,DTX3L,RNF168,MGMT,ZCWPW1,WAS,DDX11,PARP1,SHLD2,UIMC1,FXR2,WRAP53,NSMCE1,SMCHD1,FMR1,PIAS4,SPIRE2,OOEP,TIGAR,RNF8,UBE2N,BRCC3,CBX8	grey	VSMC_M10
GO:0009226	0.93	0.15	7	18	nucleotide-sugar biosynthetic process	BP	1	GFPT2,PMM1,GNPDA2,UXS1,MPI,PMM2,FUK	grey	VSMC_M10
GO:0010559	0.93	0.13	13	31	regulation of glycoprotein biosynthetic process	BP	1	AGO2,FKTN,IL15,HBEGF,PAWR,PLCB1,SLC2A10,TCF7L2,SOAT1,POMT2,ABCA7,ACER2,IL33	grey	VSMC_M10
GO:0010952	0.93	0.09	68	142	positive regulation of peptidase activity	BP	1	MAP3K5,GRAMD4,AGER,FBLN1,BAK1,MYC,HSF1,TTBK1,SLC1A1,NOD1,AKIRIN2,CASP8AP2,TNFSF15,HSPE1,ARRB1,DDRGK1,ARL6IP5,ST18,ASPH,FOXL2,HIP1R,ROBO1,PCOLCE2,DIABLO,HTRA2,RIPK2,SENP1,NKX3-1,ACVR1C,F2R,PIDD1,LCK,PDCD6,GPER1,PSME1,CIDEB,EGLN3,MAPT,FIS1,CTSH,DDX3X,FN1,VSIR,PSME3,PINK1,MUL1,STAT3,EPHA4,BCL2L13,NGFR,EFNA1,SMAD3,S100A8,BCAP31,P2RX1,BCL10,MIR21,MIR27B,RHOA,RPS27L,FADD,CASP8,ACER2,BCL2L11,PERP,APH1B,PRR7,CRADD	grey	VSMC_M10
GO:0001516	0.93	0.15	7	18	prostaglandin biosynthetic process	BP	1	PIBF1,PLA2G4A,DAGLB,CD74,PTGES2,FABP5,PTGES	grey	VSMC_M10

GO:0046457	0.93	0.15	7	18	prostanoid biosynthetic process	BP	1	PIBF1,PLA2G4A,DAGLB,CD74,PTGES2,FABP5,PTGES	grey	VSMC_M10
GO:0051900	0.93	0.15	7	18	regulation of mitochondrial depolarization	BP	1	TSPO,DCN,IFI6,FZD9,PPP2R3C,PARP1,MLLT11	grey	VSMC_M10
GO:0001707	0.93	0.11	23	53	mesoderm formation	BP	1	EXOC4,SRF,TXNRD1,SIX2,WNT11,EOMES,GPI,EYA1,TLX2,PAX2,PRKAR1A,SNAI1,WNT3,TBX19,BMPR1A,KDM6B,ITGA3,SMAD3,TWSG1,FOXF1,POFUT2,NOG,WNT5A	grey	VSMC_M10
GO:1901722	0.93	0.19	4	11	regulation of cell proliferation involved in kidney development	BP	1	MYC,IL6R,MIR125A,PDGFD	grey	VSMC_M10
GO:0042790	0.93	0.15	7	18	nucleolar large rRNA transcription by RNA polymerase I	BP	1	MARS,IPPK,SIRT7,DEDD,H2AFY2,DDX11,TCOF1	grey	VSMC_M10
GO:0009746	0.93	0.09	76	159	response to hexose	BP	1	ZNF236,NME1,LRP1,APOM,AGER,PLA2G6,TXN2,SRF,PRKCE,HMGN3,TRPM4,FOXO3,FGF21,GIPR,KHK,EIF2B3,GRIK5,RAF1,GCKR,TGFBR2,GAS6,NR1H4,CYP7A1,NDUFAF2,OSBP,CASP3,PTK2B,KCNJ11,TIAM1,RAB11FIP5,OPA1,NKX6-1,ACVR1C,ZBED6,PAX2,TWNK,GPLD1,PPP3CA,SLC9B2,ZBTB20,ADRA2A,GPAM,TCF7L2,KCNB1,PFKFB2,POLG,GPER1,RAB11FIP2,MLXIPL,GLUL,SIDT2,CDKN1B,PDK3,KLF7,FIS1,MEN1,ENY2,ABCA12,PPP3CB,CTSV,PTPRN,NDUFA9,MPC2,ILDR2,STX4,PKLR,GPR68,SIN3A,SREBF1,OXCT1,NADK,BAIAP3,CYBA,RHOA,SOX4,CRH	grey	VSMC_M10
GO:0051098	0.93	0.08	146	296	regulation of binding	BP	1	NME1,LRP1,STK3,EIF3E,PRKN,AGER,PPARA,DERL1,LRRC6,HABP4,C6orf106,CSNK1E,MFNG,SMO,HSF1,MDFI,TTBK1,MITD1,ZNF462,ZNF618,ITGB1BP1,MED25,MET,ADD2,MAPRE1,HDAC2,SYMPK,ERCC2,MAPRE3,RALB,RSF1,PTPRF,POU4F1,FBH1,SIRT2,TMEM173,EFHB,PLCL2,DPH3,ARHGEF7,DDRGK1,PPP2CB,TAF10,TRAF6,TRIB3,PLK2,BTAF1,IDE,HIP1R,HAND2,FBXW7,HMBOX1,TMBIM6,P2RY1,RAPGEF2,FAM192A,TIAM1,EBF2,RIPK2,AMFR,IFIT2,STK4,DTNBP1,BDNF,SPPL3,PPP3CA,CAPRIN2,SP100,TERT,TCF7L2,TTBK2,EGF,ADNP,EPB41,UBASH3B,DISC1,SORL1,PARP9,DTX3L,NFATC4,PER2,C4orf3,PEX19,HDAC4,BTBD1,RPL11,GEMIN2,ITGA4,MEN1,MEPCE,LRPAP1,RAP1GAP,MARK2,RAMP2,DNAJB2,GREM2,DDX11,CRMP1,EPHA4,ACTB,PARP1,NVL,GNL3L,NSD1,ANXA2,MSX2,PEX14,PSEN1,TAF1,MAPK3,EIF3C,WFIKKN2,SMAD3,CALM1,SNAPIN,LARP6,SIN3A,MARK3,ISL1,ERCC4,JUN,TICAM1,ZBTB7C,FMR1,CRK,SKI,DOT1L,PKD1,WFIKKN1,PARK7,GPSM1,LAMTOR5,PRMT7,MIR27B,MIR27A,BAG2,NOG,WNT5A,LDLRAP1,DDIT3,TXN,ZNF304,ZNF593,LIF,STYX,HEY2	grey	VSMC_M10
GO:0042116	0.93	0.10	31	69	macrophage activation	BP	1	AGER,TTBK1,PRKCE,FPR2,TLR4,C5AR1,CST7,NMI,FOXP1,MFHAS1,CEBPA,NR1H3,DYSF,IFNGR2,CD200,GPR137B,MAPT,PLA2G4A,SLC11A1,TNIP2,ITGAM,SYT11,IL4R,LDLR,CD74,TICAM1,TRPV1,KARS,WNT5A,IL33,HAVCR2	grey	VSMC_M10
GO:0030097	0.93	0.08	311	618	hemopoiesis	BP	1	NME1,STK3,MPIG6B,HSPA1B,BRD1,AGER,HLA-DRA,IL5,TCF7,CCDC134,BAK1,XRCC6,TRIB1,MYC,TOB2,SMAD5,L3MBTL3,ATF4,MAPK14,PIM1,MFNG,HSF1,KCP,MYH9,ZNF16,RBFOX2,TEK,FBXO7,SRF,CDK6,KIT,ROR2,NFIL3,RPS14,TMEM190,IL11,SMAP1,EPAS1,MSH2,CD109,HOXA7,PTBP3,SLC46A2,MTURN,BPGM,CNOT4,PLEK,RSAD2,PTN,ADD2,IL4I1,TLR4,BVES,IL15,ATG5,OSTM1,FOXO3,ZBTB24,TRAF3IP2,BMI1,PIP4K2A,ABI1,ERCC1,ERCC2,CLPTM1,GAB2,GLI2,DHTKD1,PRDX3,FOXJ1,SLC25A38,POU4F1,IL15RA,KLF6,CMTM7,TGFBR2,ACVR2A,EOMES,JAG1,PLCL2,RASGRP4,CNN2,C12orf29,EIF2AK2,ASH2L,FUT10,NCKAP1L,GAS6,FOXP1,MITF,PRKDC,IRF7,MFHAS1,FLT1,TRAF6,TCF15,SOX12,TOX,ZC3H8,ASXL1,IL1A,ACVR1B,CAMK4,CEBPA,NCOA6,PIK3R1,CASP3,FBXW7,PTPRJ,MEF2C,TMEM131L,IL7,TPD52,PTK2B,RASGRP1,LMBR1L,CIAPIN1,RIPK2,TENT2,CDH17,AP3B1,F2RL1,TESC,RIPK1,SOX13,STK4,TMEM14C,TSPAN2,DMTN,ITGB6,ALAS1,LDB1,DCAF1,RAB7B,DOCK10,SH3PXD2A,PKNOX1,LRRK1,LCK,SBDS,SLC9B2,TGFB2,TCIRG1,ACP6,GPR137B,PRKX,RABL3,SELPLG,PIAS3,HLX,SART3,ACIN1,DYRK3,TCTA,HCLS1,YTHDF2,UBASH3B,SLAMF8,ARIH2,CIB1,WASF2,ARNT,FZD7,OSM,HDAC4,THPO,MMP21,CD28,FCER1G,RUNX3,FARP2,RTKN2,ZBTB46,PATZ1,FZD9,SP3,PPP2R3C,ETV6,ADAM8,NFE2L2,CREB1,NBEAL2,FZD5,DUSP10,BCL6,SYVN1,BATF2,ITGA4,WDR61,VSIR,XRCC5,ARMC6,RC3H1,ESRRA,PTPN6,GPR137,MEOX1,FBN1,TNFSF18,WNT4,LAG3,PPP3CB,PRRC2C,LTBR,STAT3,STAT5B,LEO1,INHA,TFE3,FOXP3,ZBTB1,PARP1,GPS2,THRA,CARD11,RBPJ,MED1,ANXA2,TBX21,SETD1A,PSEN1,SEMA4A,HOXB8,AHSP,KAT7,DOCK2,CTC1,FOS,BATF,DNASE [illegible]	grey	VSMC_M10
GO:1904706	0.93	0.15	8	20	negative regulation of vascular associated smooth muscle cell proliferation	BP	1	MEF2C,PDCD4,GNA13,GPER1,EFEMP2,CDKN1B,RHOA,MIR339	grey	VSMC_M10
GO:0060352	0.93	0.18	4	11	cell adhesion molecule production	BP	1	CXCL8,COLEC12,MIR222,MIR27B	grey	VSMC_M10
GO:0050830	0.93	0.12	14	33	defense response to Gram-positive bacterium	BP	1	NOD1,C5AR1,MYD88,RIPK2,NOD2,FGR,IL12A,IL7R,DROSHA,HIST1H2BK,CHGA,HIST1H2BF,HIST1H2BJ,HAVCR2	grey	VSMC_M10
GO:0051340	0.93	0.18	4	11	regulation of ligase activity	BP	1	XRCC1,XRCC4,FAM173B,NHEJ1	grey	VSMC_M10
GO:0009048	0.93	0.17	5	14	dosage compensation by inactivation of X chromosome	BP	1	YTHDC1,CDYL,UPF1,PCGF3,SMCHD1	grey	VSMC_M10

GO:0048534	0.93	0.08	327	649	hematopoietic or lymphoid organ development	BP	1	NME1,STK3,MPIG6B,HSPA1B,BRD1,AGER,HLA-DRA,IL5,TCF7,CCDC134,BAK1,XRCC6,TRIB1,MYC,TOB2,SMAD5,L3MBTL3,ATF4,MAPK14,PIM1,MFNG,HSF1,KCP,MYH9,ZNF16,RBFOX2,TEK,FBXO7,SRF,CDK6,KIT,ROR2,NFIL3,RPS14,TMEM190,IL11,SMAP1,EPAS1,MSH2,CD109,HOXA7,PTBP3,SLC46A2,MTURN,BPGM,CNOT4,PLEK,RSAD2,PTN,ADD2,IL4I1,TLR4,BVES,IL15,ATG5,OSTM1,FOXO3,ZBTB24,TRAF3IP2,BMI1,PIP4K2A,ABI1,ERCC1,ERCC2,CLPTM1,GAB2,GLI2,DHTKD1,RAF1,PRDX3,FOXJ1,SLC25A38,POU4F1,IL15RA,KLF6,CMTM7,TGFBR2,ACVR2A,EOMES,JAG1,PLCL2,RASGRP4,CNN2,C12orf29,EIF2AK2,ASH2L,FUT10,NCKAP1L,GAS6,FOXP1,MITF,PRKDC,IRF7,MFHAS1,FLT1,TRAF6,TCF15,SOX12,RET,TOX,ZC3H8,ASXL1,IL1A,ACVR1B,CAMK4,CEBPA,NCOA6,PIK3R1,CASP3,HAND2,FBXW7,PTPRJ,MEF2C,TMEM131L,IL7,TPD52,PTK2B,RASGRP1,LMBR1L,CIAPIN1,RIPK2,TENT2,CDH17,AP3B1,F2RL1,TESC,RIPK1,SOX13,STK4,TMEM14C,TSPAN2,DMTN,ITGB6,ALAS1,JARID2,LDB1,NFKB2,DCAF1,RAB7B,DOCK10,SH3PXD2A,PKNOX1,LRRK1,LCK,SBDS,SLC9B2,TGFB2,TCIRG1,ACP6,GPR137B,PRKX,RABL3,SELPLG,PIAS3,HLX,SART3,ACIN1,DYRK3,TCTA,HCLS1,YTHDF2,UBASH3B,SLAMF8,ARIH2,CIB1,WASF2,ARNT,FZD7,OSM,HDAC4,THPO,MMP21,CD28,FCER1G,RUNX3,FARP2,EPHB3,RTKN2,ZBTB46,PATZ1,FZD9,SP3,PPP2R3C,ETV6,ADAM8,NFE2L2,CREB1,NBEAL2,FZD5,DUSP10,BCL6,SYVN1,BATF2,ITGA4,WDR61,VSIR,XRCC5,ARMC6,RC3H1,ESRRA,PTPN6,GPR137,MEOX1,FBN1,TNFSF18,WNT4,LAG3,PPP3CB,PRRC2C,LTBR,STAT3,NKX3-2,MAPK1,STAT5B,LEO1,INHA,TFE3,FOXP3,ZBTB1,PARP1,GPS2,THRA,CARD11,RBPJ,MED1,ANXA2,TBX21,SE[illegible]	grey	VSMC_M10
GO:1903672	0.93	0.16	6	16	positive regulation of sprouting angiogenesis	BP	1	BMPER,SLC39A12,JAK1,PDPK1,MIR125A,FGF1	grey	VSMC_M10
GO:0060349	0.93	0.10	34	76	bone morphogenesis	BP	1	OSR2,TEK,DLX5,RAB23,COL27A1,SFRP4,SOX9,MEGF8,TSKU,TGFBR2,RARB,MEF2C,STC1,TFAP2A,BMPR1B,TRPV4,TRIP11,AXIN2,ACTN3,POR,COL13A1,ALPL,FGFR3,MMP13,DHRS3,MSX2,FGF18,TMEM107,THBS3,FOXN3,CDX1,GHR,SKI,INSIG2	grey	VSMC_M10
GO:0014013	0.93	0.10	33	73	regulation of gliogenesis	BP	1	TSPO,SOX10,DAAM2,TTBK1,PTN,EGFR,MYCN,HDAC2,KDM4A,SIRT2,PLAG1,SYNJ1,NKX6-1,SPINT1,RNF10,HDAC1,DAG1,CERS2,DLX1,DUSP10,TNFRSF1B,PRPF19,LDLR,MBD1,DAB1,SKI,SOX8,MIR222,MIR125B1,GPR37L1,NOG,LIF,PITX3	grey	VSMC_M10
GO:0019932	0.93	0.08	105	218	second-messenger-mediated signaling	BP	1	NFATC1,PDE7B,ADGRG6,RIMS2,PDE10A,UBE2B,RCAN2,FHL2,TNNI3,PPP2R1A,FPR2,CXCL8,AQP1,PLEK,EGFR,TRPM4,GRIN2D,SLC8A2,NDNF,SOX9,GIPR,ACKR2,EFHB,PRNP,P2RX2,NRG1,P2RX3,PLCE1,PTPRJ,P2RX7,GUCY1A1,GUCY1B1,SLA2,PTK2B,RAPGEF2,RAPGEF3,PLA2G4B,DMTN,CASQ2,KSR2,CHRM3,CAP2,RYR2,ADCY2,SPPL3,PDE9A,PPP3CA,INPP5A,NR5A2,CMKLR1,ADNP,TBC1D10C,ANK2,DGKD,TRPM8,LAT2,AGT,CIB1,ATP1A2,SLC9A1,NFATC4,MYOZ2,CASQ1,ACTN3,HDAC4,RCAN3,NOS1AP,HOMER2,MAPT,FIS1,EIF4EBP2,MCU,CXCR2,HTT,PPP3CB,MYOZ1,ATP1B1,BST1,BHLHA15,DGKQ,LAT,TMEM100,CALM1,BTK,RIT2,PDE4A,NMUR2,MAPK7,RASD1,KSR1,NOS2,MGRN1,BCAP31,PDPK1,CAMTA1,NFATC3,PTGFR,CAP1,DMD,CCR10,PDZD3,IRGM,SAMD14,CRH,RASD2	grey	VSMC_M10
GO:0009615	0.93	0.08	124	253	response to virus	BP	1	TNFAIP3,USP15,C6orf106,APOBEC3H,MAPK14,EXOSC4,IRF5,CDK6,GSDME,ANKRD17,RSAD2,IL15,PUM2,APOB,TRAF3IP2,ZC3HAV1,PHB,UNC13D,MYD88,SEC14L1,IKBKB,NMI,ZMYND11,TMEM173,CLPB,NPLOC4,EIF2AK2,MAVS,AIMP1,IRF7,PCBP2,TRIM44,IRF2,AUP1,EIF2AK4,SERINC5,OAS3,NLRC5,IFNGR2,F2RL1,POLR3D,IFIT2,HERC5,ITGB6,CD40,GBF1,MX2,IFIH1,OASL,HYAL1,HYAL3,CRCP,GPAM,ADARB1,UNC93B1,POLR3C,CFL1,PARP9,DTX3L,IRF9,IFI6,RNF26,FGR,NLRX1,ABCF3,TRAF3IP1,HTRA1,TRIM52,XPR1,SETD2,IVNS1ABP,PRKRA,RTP4,IL12A,DDX3X,MUL1,BIRC3,POLR3A,PIM2,FOXP3,RNF216,TKFC,CCDC130,SPON2,TBX21,SMAD3,AKAP1,TSPAN6,STAT2,POLR3E,G3BP1,SIN3A,TRAF3,CCL5,RIOK3,RNF135,TICAM1,TTC4,FMR1,ATAD3A,NCBP3,IKBKG,POLR3K,LAMTOR5,IFI44L,ZDHHC1,MIR21,OAS1,RPS15A,ZDHHC11,FADD,TRIM34,BNIP3L,ZCCHC3,CREBZF,IFNLR1,IRGM,SAP30BP,FGL2,KCNJ8,IFIT3,BATF3,CCL22,EXOSC5	grey	VSMC_M10
GO:0046890	0.93	0.09	60	127	regulation of lipid biosynthetic process	BP	1	ORMDL2,CYP27B1,AVIL,PPARA,TSPO,PLA2G6,NR1H2,LPIN1,DKKL1,APOB,SPHK2,APOC1,PIBF1,SIRT3,NR1H4,TRIB3,CYP7A1,ASAH1,ABHD6,DKK3,FBXW7,SORBS1,NR1H3,FITM2,ORMDL1,HSD17B13,ERLIN1,PRMT3,SIRT4,GPLD1,LPCAT1,SIK1,ZBTB20,SNAI1,GPER1,LPGAT1,MLXIPL,SCAP,POR,CREB1,ACADL,LPCAT3,WNT4,SLC27A1,ACADVL,FGFR4,DGKQ,LDLR,ASXL3,CD74,SREBF1,C3,ABCA3,GFI1,FABP5,CNEP1R1,STARD4,INSIG2,CCDC3,FGF1	grey	VSMC_M10
GO:1903039	0.93	0.09	68	143	positive regulation of leukocyte cell-cell adhesion	BP	1	AGER,HLA-DRA,HLA-DOB,HLA-DMA,HLA-DPB1,TNFRSF13C,NCK2,IL4I1,IL15,BMI1,GLI2,TGFBR2,NCKAP1L,SLC7A1,TRAF6,SOX12,IL1A,IL7,RASGRP1,RIPK2,AP3B1,SOX13,NOD2,IGF2,LCK,ICOSLG,GPAM,HLX,CD28,RUNX3,ADAM8,DUSP10,BCL6,ITGA4,IL12A,VSIR,PTPN6,STAT5B,FOXP3,RASAL3,ZBTB1,CARD11,ZBTB7B,IL4R,CD74,CCL5,CCL2,YES1,NKAP,VAV1,IL7R,SOCS1,DNAJA3,PRKCZ,BCL10,MIR21,RHOA,SOX4,FADD,DPP4,CD46,FUT4,THY1,CD70,TNFSF9,EFNB3,TNFSF13B,HAVCR2	grey	VSMC_M10
GO:0051280	0.93	0.18	4	11	negative regulation of release of sequestered calcium ion into cytosol	BP	1	PKD2,CASQ2,NTSR1,CALM1	grey	VSMC_M10

GO:0030307	0.94	0.09	63	134	positive regulation of cell growth	BP	1	ADNP2,NEDD4L,LRP1,RIMS2,PRKN,H3F3C,CPNE5,EXOSC4,SRF,SEMA4D,RIMS1,RUFY3,EGFR,TRIM32,SDCBP,GOLGA4,MEGF8,CPNE9,RPTOR,HBEGF,SYT1,SLC23A2,CDKN2AIP,PTK2B,ZFYVE27,CYFIP1,BDNF,HYAL1,TGFB2,ZNF639,ADNP,DISC1,CIB1,SLC9A1,SFN,RPS6KA1,CDKL5,WNT3,MAPT,SUPV3L1,DDX3X,FN1,DDX49,MUL1,CD38,CXCL16,NDEL1,RAB11A,TAF9B,NTN1,MAP2K5,S100A8,PSMD10,TRPV2,L1CAM,SLC25A33,CYBA,MIR199A1,RHOA,FXN,CACNG7,N6AMT1,DERL2	grey	VSMC_M10
GO:0042438	0.94	0.16	6	16	melanin biosynthetic process	BP	1	SLC7A11,RAPGEF2,OCA2,CITED1,SLC45A2,WNT5A	grey	VSMC_M10
GO:0007520	0.94	0.13	10	25	myoblast fusion	BP	1	MAPK14,MYH9,DYRK1B,MYOF,JAM2,ADAMTS15,EHD1,GDF15,IL4R,CACNA1H	grey	VSMC_M10
GO:1902751	0.94	0.13	11	27	positive regulation of cell cycle G2/M phase transition	BP	1	VPS4B,MTA3,CCND1,DYRK3,STOX1,DBF4B,RAD51B,RAB11A,RAD51C,SIN3A,MECP2	grey	VSMC_M10
GO:0032781	0.94	0.14	8	20	positive regulation of ATPase activity	BP	1	NAPA,CHTOP,TOR1AIP1,DNAJC10,DNAJB2,ATP1B1,DNAJB1,ATP1B2	grey	VSMC_M10
GO:0120033	0.94	0.13	10	25	negative regulation of plasma membrane bounded cell projection assembly	BP	1	TBC1D30,ARHGAP24,SRGAP2C,LIMK2,RAP1GAP,DNM2,ARHGAP44,EVL,CDK10,ODF2L	grey	VSMC_M10
GO:0030166	0.94	0.11	21	49	proteoglycan biosynthetic process	BP	1	B4GALT7,CHST10,HS3ST5,CHPF2,CSGALNACT1,BMPR1B,SLC2A10,TCF7L2,GAL3ST3,CHST3,GAL3ST4,CHST7,AC022400.6,CHST12,GLCE,HS6ST2,B3GALT6,FOXL1,HS6ST3,CHSY3,HS2ST1	grey	VSMC_M10
GO:0030575	0.94	0.16	5	14	nuclear body organization	BP	1	HABP4,DYRK3,CELF3,WRAP53,AFF2	grey	VSMC_M10
GO:0030837	0.94	0.11	23	53	negative regulation of actin filament polymerization	BP	1	AVIL,LMOD2,KIAA1211,TMOD1,SPTBN1,ADD2,MKKS,LMOD3,HIP1R,DMTN,CARMIL1,LMOD1,ADD3,SSH1,SPTBN2,TMOD4,EPS8,CAPZA1,TMOD2,SPTB,SSH2,CARMIL2,PFN4	grey	VSMC_M10
GO:0000184	0.94	0.12	14	34	nuclear-transcribed mRNA catabolic process, nonsense-mediated decay	BP	1	PABPC1,SKIV2L,EIF3E,PNLDC1,DHX34,UPF3A,NCBP2,UPF1,DCP1B,CASC3,SMG5,UPF3B,PARN,MAGOHB	grey	VSMC_M10
GO:0150146	0.94	0.16	5	14	cell junction disassembly	BP	1	IQSEC1,PIK3R1,EPHA4,ITGAM,C3	grey	VSMC_M10
GO:0003281	0.94	0.10	26	60	ventricular septum development	BP	1	FRS2,PTK7,HOXA13,CNTRL,WNT11,TGFBR2,PAX8,ROBO1,ROBO2,TBX5,SALL1,SUFU,TGFB2,TRIP11,LUZP1,SAV1,BMPR1A,RBPJ,DCTN5,STRA6,AP2B1,SOX4,NOG,WNT5A,NKX2-5,HEY2	grey	VSMC_M10
GO:0040013	0.94	0.08	127	261	negative regulation of locomotion	BP	1	LRP1,SEMA6A,SRGAP1,FBLN1,TRIB1,MAPK15,SRF,SEMA4D,SEMA4C,MEOX2,SVBP,CLDN19,RNF20,HAS1,HOXA7,PTN,ITGB1BP1,FUZ,EPHA1,FOXO3,C5AR2,CLASP2,DACH1,CYGB,WNT11,SRGAP3,JAG1,NAV3,PLCB1,SPINT2,CNN2,SEMA3D,DCN,SEMA3A,NRG1,MITF,DPYSL3,RYK,ABHD6,ROBO1,ROBO2,PTPRJ,MEF2C,SEMA4F,STC1,TBX5,ACVR1C,TP53INP1,SEMA3G,SEMA4G,ADTRP,SRGAP2C,CD200,SINHCAF,SP100,SEMA3F,ADARB1,GNA13,DAG1,ABHD2,AP1AR,SLAMF8,CERS2,PTPRO,CHRD,CLIC4,PATZ1,APOD,WNT3,FRMD5,NFE2L2,MIA3,DUSP10,SEMA6D,PDCD10,WNT4,COL3A1,STAT3,VCL,WAS,PADI2,EPHA4,BMPR1A,SYNJ2BP,ATP1B2,SEMA4A,NGFR,VASH1,MAP2K5,EVL,CD74,CCL2,CCL25,PTGER4,STK26,PTPN2,CRK,ARHGAP4,MECP2,PLXNA3,PIP5KL1,ENG,MARVELD3,SCAI,DAB2IP,WDR63,MIR29A,MIR199A1,MIR21,MIR22,MIR24-1,MIR491,RHOA,C5orf30,ADGRG1,DPP4,ACVRL1,NOG,THY1,WNT5A,GADD45A,DUSP1,RGCC,GCSAM,DUSP22,LDLRAD4,RAP2B	grey	VSMC_M10
GO:0051223	0.94	0.08	205	412	regulation of protein transport	BP	1	LRP1,SLC12A2,PRKN,GOPC,MAPK14,PLA2G6,PICK1,RAC2,SMO,RFX3,SLC1A1,TFAP2B,RAB23,DNAJA1,PRKCE,TMEM30A,HMGN3,RUFY3,XPO1,BMP8A,RSAD2,NR1H2,ITGB1BP1,EGFR,ARHGEF5,TLR4,TCAF2,TRPM4,SLC7A11,DNAJC27,GIPR,LCP1,PLK3,FRMD4A,LLGL2,AKT2,USP36,UBAC2,APBB3,DPH3,SIRT7,FERMT1,CEP290,PRNP,BAG4,MAVS,GAS6,SIRT3,SYT9,NR1H4,LYPLA1,NUP58,TM9SF4,NDUFAF2,FAM3D,IL1A,OSBP,PIK3R1,ARNTL,FBXW7,PKIA,HTRA2,VEGFC,ZFAND1,CSNK2A2,NUP54,KCNJ11,NNAT,NR1H3,TIAM1,RAB11FIP5,PFKM,SLC8B1,NKX6-1,RAPGEF3,ACVR1C,F2RL1,F2R,ZBED6,DMTN,ADTRP,SUFU,SIRT4,GPLD1,IRS1,CABP1,CD200,DNM1L,HDAC1,PPP3CA,SP100,CPT1A,SLC9B2,TGFB2,ADRA2A,TCIRG1,TCF7L2,KCNB1,YOD1,PFKFB2,HCLS1,HSPA8,HPS4,SORL1,C2CD5,GPER1,ARIH2,CIB1,BAG3,SFN,PER2,STX1A,UHMK1,GLUL,SIDT2,SLC16A1,APOD,ADAM8,KLF7,CTDSPL2,FZD5,FIS1,CEMIP,NMT1,TTN,DERL3,SEC16B,IL12A,ENY2,ABCA12,C11orf65,MCU,PDCD10,PPP3CB,PINK1,CD38,MPC2,MAPK1,CHRM1,TMEM30B,GRIPAP1,RAB8A,ATP13A2,CNST,SYT7,RSC1A1,NLGN2,NPEPPS,TARDBP,STX4,PSEN1,ITGAM,SNX12,TMED10,NDEL1,AC138894.1,HAX1,RAB11A,MYH10,SMAD3,SYTL4,GPR68,BCAS3,ARHGAP44,CCL5,MIEF2,CDK5R1,SREBF1,ISL1,OXCT1,NOS2,EFCAB7,ABAT,RPH3AL,INPP5K,NADK,BCAP31,PRKCZ,OAZ1,BAIAP3,PARK7,LRRC8A,GAPVD1,SH3GLB1,WWP2,RIPOR1,MIR199A1,MICALL2,FGA,CHGA,SOX4,DPP4,BNIP3L,CRH,TXN,DOC2B,HRAS,UQCC2,MIEF1,RHOU,LEPROT,PARD6A,UBE2G2,DERL2,ZIC1	grey	VSMC_M10
GO:0007519	0.94	0.09	57	122	skeletal muscle tissue development	BP	1	STAC3,VGLL2,PITX1,LEMD2,MAPK14,SMO,MEOX2,HIVEP3,MYH14,AKIRIN1,BVES,KEL,KLF5,KLHL40,NR1D2,RYR1,DCN,P2RX2,LMOD3,EPHB1,NR4A1,SMYD1,NLN,FOXL2,FLNB,VAMP5,SAP30,ARNTL,MEF2C,TLL2,HOMER1,BIN3,RBM24,IGF2,MYH15,NEURL1,PPP3CA,HLX,USP19,MYOZ2,CASQ1,ACTN3,HDAC4,YBX3,MYOZ1,MYLPF,ZNF689,FOS,FBXO22,SKI,SOX8,RHOA,DMD,SIX1,POPDC2,MYORG,UQCC2	grey	VSMC_M10
GO:0007040	0.94	0.10	27	61	lysosome organization	BP	1	WASHC5,TFEB,ACP4,PPT1,MFSD8,RAB7A,MYO7A,HOOK3,TMEM106B,FAM160A2,CHMP2B,ACP2,AP3B1,VPS18,RAB7B,TMEM9,HPS4,SPG11,PIKFYVE,SYT7,CLN8,AC138894.1,HOOK2,CLN6,SNAPIN,TMEM199,RAB39A	grey	VSMC_M10

GO:0080171	0.94	0.10	27	61	lytic vacuole organization	BP	1	WASHC5,TFEB,ACP4,PPT1,MFSD8,RAB7A,MYO7A,HOOK3,TMEM106B,FAM160A2,CHMP2B,ACP2,AP3B1,VPS18,RAB7B,TMEM9,HPS4,SPG11,PIKFYVE,SYT7,CLN8,AC138894.1,HOOK2,CLN6,SNAPIN,TMEM199,RAB39A	grey	VSMC_M10
GO:0050679	0.94	0.09	73	154	positive regulation of epithelial cell proliferation	BP	1	NME1,TNFAIP3,OSR2,MYC,SMO,TEK,DLX5,PDCL3,RTN4,EMC10,EPGN,AREG,TGFA,PTN,EGFR,OSR1,FOXE3,C5AR2,C5AR1,PRKD2,SOX9,SCN5A,WDR48,RPTOR,CYP7B1,NR4A1,EYA1,VEGFC,AGGF1,SRSF6,RREB1,PAX2,NOD2,IGF2,HYAL1,CCND1,TCF7L2,PDCD6,EGF,ARNT,TGM1,VASH2,FZD7,HTRA1,GLUL,LAMC1,STOX1,ITGA4,GPBAR1,STAT3,PTPRN,BMPR1A,MED1,PGF,MAP2K5,PDPK1,CYBA,ESRP2,MIR29A,MIR21,MIR23A,MIR27B,MIR27A,LAMB1,ACVRL1,ZNF580,NOG,CLDN1,WNT5A,ZNF304,HRAS,FGF1,WNT2	grey	VSMC_M10
GO:0071243	0.94	0.15	7	18	cellular response to arsenic-containing substance	BP	1	ATF4,MAPK13,HSF1,ZFAND1,SLC38A2,DDX3X,PPIF	grey	VSMC_M10
GO:0042098	0.94	0.09	60	127	T cell proliferation	BP	1	AGER,HLA-DPB1,TNFRSF13C,RAC2,CCND3,TNFRSF21,NCK2,PELI1,IL4I1,IL15,BMI1,FOXJ1,TGFBR2,PAWR,PRNP,NCKAP1L,SLC7A1,TRAF6,IL1A,CASP3,TMEM131L,RASGRP1,LMBR1L,RIPK2,MARCH7,IGF2,ICOSLG,BTN2A2,GPAM,PRKAR1A,CD28,DLG1,VSIR,RC3H1,PTPN6,TNFSF18,SLC11A1,WNT4,PPP3CB,STAT5B,SCGB1A1,FOXP3,RASAL3,CARD11,TNFRSF1B,DOCK2,ZBTB7B,CCL5,TWSG1,DNAJA3,GLMN,MIR21,FADD,CD46,CD70,TNFSF9,CEBPB,TNFSF13B,HAVCR2,RC3H2	grey	VSMC_M10
GO:0034982	0.94	0.18	4	11	mitochondrial protein processing	BP	1	ATP23,MIPEP,IMMP1L,SIRT4	grey	VSMC_M10
GO:0000027	0.94	0.15	7	18	ribosomal large subunit assembly	BP	1	MDN1,RPL6,DHX30,RPL11,RPL10L,NLE1,RPL23A	grey	VSMC_M10
GO:0002065	0.94	0.10	27	61	columnar/cuboidal epithelial cell differentiation	BP	1	ROS1,SMO,CDK6,RFX3,KLF5,WNT11,RARB,CBFA2T2,ARNTL,NKX6-1,ZBED6,FAM20C,SIDT2,MEN1,PYY,SAV1,WNT4,NKX3-2,DLX3,GATA6,PDPK1,WDR77,TMEM231,SOX4,WNT5A,TIGAR,CEBPB	grey	VSMC_M10
GO:0034329	0.94	0.08	154	318	cell junction assembly	BP	1	LRP1,PTK2,NTRK2,MPDZ,TEK,LRRC4,LINGO2,SRF,GABRA1,FZD1,ADGRF1,TLN1,SEMA4D,ADGRL3,CLDN19,GJA10,LRRN3,EPHA7,ITGB1BP1,ADD2,VPS35,SDCBP,NRCAM,VSTM5,CLASP2,FBF1,POU4F1,WNT11,SLITRK5,IKBKB,LRFN1,FARP1,ACTG1,ARHGEF7,NRG1,PTPRA,PKP3,LRFN3,RYK,EPHB1,NPHP1,LSR,FAM107A,CLSTN2,CTNND1,MARVELD2,ROBO2,FMN1,SORBS1,PTPRJ,MEF2C,CUX2,PTK2B,RAPGEF2,SLIT1,HDAC7,TBX5,AMIGO2,DMTN,LDB1,BDNF,PKP2,SLK,COL17A1,TRPV4,GAP43,COL16A1,AMIGO3,NLGN4Y,AJUBA,CLDN34,CDH24,BSN,SNAI1,ADNP,ARHGAP6,ANK2,GNPAT,AGT,ACE2,SLC9A1,RAB17,SPTBN2,ACTN3,NPAS4,PTPRO,EPHB3,WHAMM,LAMC1,DLG1,APOD,CAPZA1,FZD5,RAP1B,TRIP6,ARVCF,NRXN2,FN1,SPOCK2,GPBAR1,WNT4,DNM3,CNTNAP1,RAMP2,VCL,OBSL1,LRTM2,ACTB,CLDN7,SDK1,NLGN2,LGI2,SEMA4A,TLN2,NLGN3,TAOK2,CHRNB2,SMAD3,NTN1,CORO2B,MYO9A,VMP1,BCAS3,GPRASP2,BHLHB9,CAMSAP3,ASIC2,EPB41L3,CDH6,CDH10,SLITRK2,PIP5K1C,PDPK1,MECP2,AMIGO1,CEL,TSC1,NTNG2,ZDHHC12,MARVELD3,ADGRL2,MICALL2,RHOA,CLDN15,GRID2,ACVRL1,PEAK1,THY1,CLDN1,WNT5A,SIX1,APC,DUSP22,LRRTM2	grey	VSMC_M10
GO:0045324	0.94	0.14	7	18	late endosome to vacuole transport	BP	1	CHMP2A,LEPROTL1,CHMP2B,CHMP4C,CHMP7,CHMP1A,LEPROT	grey	VSMC_M10
GO:0007143	0.94	0.13	10	25	female meiotic nuclear division	BP	1	WASHC5,MLH1,HSF2BP,PRKAR1A,SEPT1,RAD51C,MEIOB,SPIRE2,UBB,EREG	grey	VSMC_M10
GO:0034637	0.94	0.10	28	63	cellular carbohydrate biosynthetic process	BP	1	AKR1B1,HAS1,EPM2AIP1,PPP1CB,CSGALNACT1,AKT2,PPP1R3B,GBE1,B3GNT4,SORBS1,IGF2,NHLRC1,IRS1,PPP1R3E,PPP1R3D,PER2,PASK,PRKAG3,AC022400.6,PPP1R3F,INSR,IMPA2,INPP5K,AGL,HAS3,ACADM,FBP2,HS2ST1	grey	VSMC_M10
GO:1904376	0.94	0.13	10	25	negative regulation of protein localization to cell periphery	BP	1	GOPC,LYPLA1,PPFIA1,AP2M1,PPP2R5A,LRRC15,LZTFL1,RSC1A1,SAPCD2,MRAP2	grey	VSMC_M10
GO:0040029	0.94	0.09	39	86	regulation of gene expression, epigenetic	BP	1	BAZ2A,ARID1B,H3F3C,YTHDC1,SPHK2,DNMT3A,BMI1,SETDB2,SIRT2,FAM208A,SMARCD1,BAHD1,CDYL,PPHLN1,ZNF335,IGF2,HDAC1,ZNFX1,TFAP2C,ARID1A,HDAC4,TET1,H2AFY2,UPF1,PADI2,PHF8,PCGF3,NRDE2,SMCHD1,MECP2,GLMN,PRMT7,CTCF,SERTAD2,SETDB1,ARID4A,CREBZF,BEND3,ZNF304	grey	VSMC_M10
GO:0006305	0.94	0.10	23	53	DNA alkylation	BP	1	BAZ2A,MOV10L1,MYC,PICK1,STPG4,DNMT3A,BMI1,TEX15,DNMT3B,MTRR,HEMK1,TDRKH,MGMT,PARP1,FOS,METTL4,MBD1,MECP2,PRMT7,CTCF,MIR29A,BEND3,N6AMT1	grey	VSMC_M10
GO:0006306	0.94	0.10	23	53	DNA methylation	BP	1	BAZ2A,MOV10L1,MYC,PICK1,STPG4,DNMT3A,BMI1,TEX15,DNMT3B,MTRR,HEMK1,TDRKH,MGMT,PARP1,FOS,METTL4,MBD1,MECP2,PRMT7,CTCF,MIR29A,BEND3,N6AMT1	grey	VSMC_M10
GO:1900076	0.94	0.10	22	51	regulation of cellular response to insulin stimulus	BP	1	PIP4K2C,GKAP1,GRB10,PIP4K2A,ECHDC3,SNX5,SORBS1,OPA1,IGF2,GPLD1,IRS1,GRB14,SORL1,AGT,ESRRA,ZBTB7B,PRKCB,PTPN2,SOCS1,INPP5K,PRKCZ,BGLAP	grey	VSMC_M10
GO:0006448	0.94	0.15	6	16	regulation of translational elongation	BP	1	DPH2,DPH3,USP16,ALKBH1,DPH1,AARS	grey	VSMC_M10
GO:0042147	0.94	0.09	36	80	retrograde transport, endosome to Golgi	BP	1	PRKN,VPS52,RGP1,VPS35,RAB7A,RBSN,UBE2O,SNX5,HEATR5B,BET1L,WASHC2A,TMEM87B,GBF1,MAGEL2,RAB7B,VTI1A,TBC1D10C,STX6,SNX6,SNX32,PIKFYVE,VPS26A,TBC1D14,CLTCL1,ANKFY1,STX5,SNX12,TBC1D10B,SNX1,VPS53,BAIAP3,RNF126,PHETA1,RAB6B,VAMP3,PLEKHJ1	grey	VSMC_M10

GO:0018205	0.94	0.08	154	313	peptidyl-lysine modification	BP	1	ATAT1,YEATS4,SNW1,BRD1,PHF1,UHRF2,SUPT3H,HINT2,PHF14,LIPT1,KANSL3,CAMKMT,ING3,ATG5,SETD7,TRPM4,SPHK2,HDAC2,BMI1,SETDB2,EED,IWS1,SUV39H2,SUMO2,KDM4A,PAXIP1,TADA3,ARRB1,CBX4,SIRT2,DPY30,SIRT7,NSD3,TAF7,ASH2L,EP400,SIRT3,TAF10,SDR16C5,EEF1AKMT1,DNMT3B,SMYD1,TAF9,EYA1,PHF20,SETD6,KMT2D,NNAT,KANSL2,SENP1,HDAC7,LOXL2,LOXL4,RTF1,SIRT5,WDR82,ZNF335,JARID2,TOLLIP,RBBP5,LDB1,FAM173B,SIRT4,IFIH1,HDAC1,AUTS2,PIAS3,ARNT,HDAC4,STX1A,MRGBP,EEF1AKMT2,ING5,SENP5,SENP2,SETD2,POR,MBIP,PLOD3,BCOR,MEN1,WDR61,VCPKMT,TADA2B,NAA40,ING4,NELFA,MUL1,TADA1,DYDC1,P3H4,FOXP3,LIAS,MSL1,MED24,GNL3L,NSD1,SETD1A,KAT7,PAGR1,MUC1,TAF1,MAPK3,OGT,NAP1L2,VIPAS39,CRTC2,RPS6KA5,PIAS2,NDUFAB1,SETD3,SIN3A,ISL1,SUZ12,SUPT6H,CXXC1,GLYR1,PIAS4,DOHH,CREBBP,NAA60,DOT1L,HCFC1,MECP2,PRDM16,CHD5,PARK7,GTF3C4,GCSH,MTF2,GFI1,CTCF,PRMT6,METTL18,SETDB1,RNF212B,ARID4A,TAF12,SOX4,CPA4,RWDD3,BEND3,SMYD3,METTL21A,SUPT7L,SENP7,ZNF304,NNMT,LIF,N6AMT1,P3H3,RPS6KA4,HINT1,RASD2	grey	VSMC_M10
GO:0016331	0.94	0.09	59	127	morphogenesis of embryonic epithelium	BP	1	STK3,FZD6,BRD2,PTCH1,PTK7,SEMA4C,FUZ,TEAD2,OSR1,SOX9,GLI2,PCDH8,IFT172,OVOL2,SPINT2,TRAF6,RET,PAX8,CASP3,FZD3,VEGFC,OPA1,IFT52,SPINT1,STK4,TFAP2A,PAX2,PRICKLE1,SUFU,IRX2,ALDH1A3,TGFB2,DVL3,KDF1,LAMA5,GRHL3,LUZP1,SETD2,WNT4,TULP3,LIAS,DVL2,ALDH1A2,TGFB1I1,MKS1,MIB1,JAG2,SKI,SOX8,TSC1,GLMN,BCL10,VASP,SOX4,NOG,WNT5A,SIX1,RALA,WNT2	grey	VSMC_M10
GO:0044057	0.94	0.08	197	398	regulation of system process	BP	1	RIMS2,AGER,PPARA,GDF9,SOX10,PI16,ZDHHC21,CHRNA3,CRY1,SRF,KIT,SLC1A1,TNFRSF21,ABCG5,ABCG8,TNNI3,EPAS1,RIMS1,ASB3,SHANK1,AQP1,NR1H2,EGFR,BVES,DLGAP3,LPIN1,TRPM4,FOXO3,FIG4,GRIN2D,MTMR2,TRPC3,SLC8A2,CST7,CACNB2,ATP1A3,CELF2,SCN5A,GPD1L,SNX5,C1QTNF1,HBEGF,ADRA1D,SEMA3A,GAS6,FAM19A4,ADRA2B,NR4A1,HIP1R,HAND2,P2RX3,PLCE1,JAM2,DLGAP4,GUCY1A1,CUX2,PTK2B,NR1H3,DOCK5,HOMER1,STC1,NKX3-1,KCNJ3,TBX5,F2RL1,F2R,CRY2,TNNC1,FTO,GLS,KCNE2,DSP,CASQ2,JARID2,KCNIP2,CHRM3,RYR2,FAM173B,RNF10,PKP2,PDE9A,PPP3CA,KCNJ2,TNNI1,TRPV4,TGFB2,ADRA2A,IGSF11,DAG1,SSH1,MYH7,ZFHX2,EPB41,OPRD1,GRK2,GTF2IRD1,ANK2,GPER1,AGT,ACE2,ATP1A2,SLC9A1,PER2,CASQ1,ACTN3,HDAC4,MTG2,STX1A,TMEM25,NTSR1,PTPRO,SCN4B,CNIH2,NOS1AP,DLG1,COMT,TNR,LPCAT3,ADRA2C,PTGER2,CALCRL,ECE1,PRKAG3,GCH1,CAMK2G,ATP1B1,KCNA1,SMTNL2,CD38,NPTX2,KCNMA1,DES,INHA,SLC4A3,DLG4,PARP1,THRA,NLGN2,TNFRSF1B,ATP1B2,HRH2,MYL5,STX1B,RAB8B,NLGN3,CACNA1G,ATP2A1,TMEM100,SMAD3,CORO2B,CALM1,TBX2,NMUR2,SETD3,YY1,BEGAIN,DSG2,ASIC2,SREBF1,ISL1,DLGAP1,CYP2J2,ABAT,FMR1,INPP5K,ATP2B3,TRPV1,P2RX1,PRKCZ,MECP2,RNF207,CACNA1H,CYBA,MIR199A1,MIR21,MIR25,RHOA,FGA,DMD,GPR35,FABP5,NOL3,CHGA,SLC22A5,TMEM65,KCNH2,TNNC2,SCN2B,RGS4,CRH,POPDC2,CACNG5,HCN4,NKX2-5,KCNMB4,HEY2,KCNG2	grey	VSMC_M10
GO:0006509	0.94	0.11	15	36	membrane protein ectodomain proteolysis	BP	1	MYH9,TIMP4,ERAP1,SH3D19,GPLD1,ADRA2A,DAG1,BACE1,LRIG2,ADAM8,SPPL2A,PSEN2,TNFRSF1B,PSEN1,SPPL2B	grey	VSMC_M10
GO:0003206	0.94	0.09	46	101	cardiac chamber morphogenesis	BP	1	ADGRG6,LY6E,SMO,FOXH1,TEK,SRF,FHL2,TNNI3,DNAH11,POU4F1,WNT11,TGFBR2,OVOL2,JAG1,NRG1,HAND2,PARVA,ROBO1,ROBO2,MEF2C,ADAMTS1,TBX5,TNNC1,DSP,RYR2,PKP2,TNNI1,TGFB2,MYH7,SAV1,BMPR1A,DHRS3,RBPJ,MED1,MKS1,TBX2,GATA6,ISL1,FOXF1,ENG,SOX4,NOG,WNT5A,NKX2-5,HEY2,WNT2	grey	VSMC_M10
GO:0030183	0.94	0.09	44	96	B cell differentiation	BP	1	BAK1,MFNG,KIT,IL11,MSH2,IL4I1,TRAF3IP2,KLF6,CMTM7,PLCL2,NCKAP1L,PRKDC,PIK3R1,PTPRJ,IL7,TPD52,PTK2B,CDH17,DCAF1,DOCK10,TCIRG1,RABL3,SLAMF8,HDAC4,FZD9,SP3,PPP2R3C,BCL6,SYVN1,ITGA4,PTPN6,STAT5B,INHA,GPS2,CARD11,RBPJ,CD19,BTK,YY1,PTPN2,LRRC8A,NHEJ1,ZFP36L2,TNFSF13B	grey	VSMC_M10
GO:0061005	0.94	0.10	21	49	cell differentiation involved in kidney development	BP	1	MTSS1,PTCH1,PODXL,SMO,SIX2,POU3F3,OSR1,FOXJ1,JAG1,ASXL1,PAX8,MEF2C,PAX2,SALL1,PRKX,PTPRO,WNT4,CITED1,LHX1,BASP1,LIF	grey	VSMC_M10
GO:0072073	0.94	0.09	53	115	kidney epithelium development	BP	1	MTSS1,MYC,SMAD5,KLHL3,PTCH1,PODXL,SMO,TFAP2B,CAT,SIX2,EPCAM,BMPER,EPHA7,POU3F3,OSR1,SPRY1,SOX9,AQP11,FOXJ1,GZF1,WNT11,JAG1,RARB,RET,ASXL1,PAX8,EYA1,ROBO2,FMN1,MEF2C,PKD2,PAX2,SALL1,IRX2,AGT,LAMA5,PTPRO,DLG1,CXCR2,WNT4,EPHA4,MAGED1,CITED1,SMAD3,LHX1,GPC3,PKD1,SOX8,NOG,BASP1,SIX1,LIF,FGF1	grey	VSMC_M10
GO:0031643	0.94	0.16	5	14	positive regulation of myelination	BP	1	SOX10,CST7,RNF10,DAG1,TNFRSF1B	grey	VSMC_M10
GO:1990874	0.94	0.10	24	54	vascular associated smooth muscle cell proliferation	BP	1	MAP3K5,HPGD,MEF2C,ADAMTS1,NQO2,TERT,PDCD4,GNA13,GPER1,AGT,EFEMP2,CDKN1B,BMPR1A,MFN2,JUN,GNAI3,MIR21,MIR222,MIR27B,MIR27A,RHOA,FOXJ2,MIR339,LDLRAP1	grey	VSMC_M10
GO:0010633	0.94	0.10	26	59	negative regulation of epithelial cell migration	BP	1	MEOX2,SVBP,ITGB1BP1,DCN,SEMA3A,MEF2C,STC1,SP100,PATZ1,DUSP10,PDCD10,SYNJ2BP,VASH1,MAP2K5,EVL,MECP2,MARVELD3,DAB2IP,MIR199A1,MIR21,MIR22,MIR24-1,RHOA,ACVRL1,GADD45A,RGCC	grey	VSMC_M10
GO:0034205	0.94	0.11	19	44	amyloid-beta formation	BP	1	NTRK2,RTN4,PRNP,CASP3,SLC2A13,SORL1,BACE1,RTN1,HAP1,EPHA4,PSEN2,PSEN1,TMED10,EFNA1,ABCA7,MIR29A,MIR24-1,MIR339,APH1B	grey	VSMC_M10

GO:0044283	0.94	0.07	207	414	small molecule biosynthetic process	BP	1	GLS2,SHMT2,CYP27B1,LTC4S,QKI,PPARA,MCAT,HSD17B8,HSD17B3,AKR1B1,ATF4,PYCR3,PSAT1,GPT,HACD4,MTAP,GNMT,ALDH1A1,CRY1,PLPP6,SRD5A3,SPTLC1,SLC25A13,IPPK,HMGCLL1,PPP4R3B,NT5E,ZNF692,PLEK,NR1H2,FAM3C,DKKL1,MGST2,APOB,BCAT2,NANP,SPHK2,DHFR,PRTFDC1,EBP,AKR1A1,APOC1,PRKAG2,PIBF1,ITPKC,ACER3,ELOVL1,SPTLC3,ACSS3,SIRT7,NR1H4,APIP,TRIB3,CSAD,CYP7A1,ELOVL7,ASAH1,CYP7B1,GPI,THNSL2,NLN,CBR4,OSBP,DKK3,GPD1,CYP2R1,P2RY1,PTK2B,PRKAG1,NR1H3,SERINC5,SPR,ENOPH1,COQ2,SDSL,HOGA1,ASNSD1,GLS,ITPKA,ERLIN1,PRMT3,PLTP,SIRT5,PRKAB1,MTRR,ASL,COQ5,MTR,CBSL,SIK1,ALDH1A3,COQ10B,MST1,SNAI1,ABHD2,MSMO1,MECR,GPER1,SC5D,ABCB11,DHRS9,PC,HACD2,IP6K2,LPGAT1,SLC37A4,PER2,OSBPL2,NTSR1,OAT,MLXIPL,DTYMK,HMGCL,GLUL,SCAP,HSD17B7,POR,PTH1R,SORD,PLA2G4A,OSBPL6,UPB1,ACADL,PLOD3,GGT2,TM7SF2,CPS1,OSBPL1A,ACSBG1,PECR,LPCAT3,WNT4,AASDHPPT,CYP27A1,PRKAG3,GCH1,GGT6,NDUFA9,ADK,DAGLB,FADS3,LIAS,ACADVL,COQ8A,DDB1,TECR,HSD17B10,DEGS1,ALDH1A2,FGFR4,LIPC,DGKQ,OSBPL7,PNPO,ALOXE3,PKLR,OGT,AC138894.1,HACD3,CRTC2,MPO,NDUFAB1,CYP46A1,ACSM3,ACACA,CD74,ABHD3,SHMT1,SREBF1,LEPR,ACSBG2,IMPA2,PTPN2,ACAA2,ABAT,GAMT,CACNA1H,PARK7,DECR2,ACSF3,ASS1,MLYCD,COQ4,PTGES2,ABCD3,GFI1,ACADM,FBP2,ACOT4,FABP5,FADS6,TPK1,ELOVL3,DHDDS,MTHFD2L,AMACR,ACER2,GGT5,PDSS2,NNMT,STARD4,PTGES,INSIG2,PHGDH,FGF1,ARPP19,PCBD2	grey	VSMC_M10
GO:0042088	0.94	0.13	10	25	T-helper 1 type immune response	BP	1	TLR4,TRAF6,RIPK2,HLX,PLA2G4A,SLC11A1,SEMA4A,IL4R,HRAS,HAVCR2	grey	VSMC_M10
GO:0042249	0.94	0.16	5	14	establishment of planar polarity of embryonic epithelium	BP	1	PTK7,DVL3,GRHL3,DVL2,WNT5A	grey	VSMC_M10
GO:0048025	0.94	0.15	6	16	negative regulation of mRNA splicing, via spliceosome	BP	1	SRSF10,SRSF6,SRSF4,RBM10,SRSF12,SAP18	grey	VSMC_M10
GO:0033135	0.94	0.09	48	104	regulation of peptidyl-serine phosphorylation	BP	1	BAK1,NTRK2,NCK2,IL11,EGFR,NTF4,BRAF,PRKD2,RAF1,SPRY2,GPD1L,RPTOR,PLCL2,BAG4,GAS6,RET,FNIP2,PAQR3,RIPK2,RAPGEF3,STK4,DMTN,BDNF,CAPRIN2,HCLS1,OPRD1,OSM,INPP5J,MLXIPL,STOX1,PDCD10,PINK1,ARAF,NSD1,HAX1,SH2D3A,DOCK7,INPP5K,MTCP1,PARK7,MAPKAP1,DMD,IRGM,SMYD3,WNT5A,GADD45A,TXN,LIF	grey	VSMC_M10
GO:0006024	0.94	0.10	29	66	glycosaminoglycan biosynthetic process	BP	1	B4GALT7,HAS1,HS3ST5,CHPF2,CSGALNACT1,B3GNT4,ST3GAL6,GALNT5,CHST1,GCNT2,HYAL1,EGF,ABCC5,CEMIP,CHST3,CHST7,AC022400.6,HS3ST1,CHST12,GLCE,HEXA,HS6ST2,B3GALT6,CHST5,ST3GAL2,HAS3,HS3ST2,CHSY3,HS2ST1	grey	VSMC_M10
GO:0002068	0.94	0.13	9	23	glandular epithelial cell development	BP	1	SMO,CDK6,RFX3,RARB,ARNTL,NKX6-1,SIDT2,PDPK1,WNT5A	grey	VSMC_M10
GO:0070527	0.94	0.10	22	51	platelet aggregation	BP	1	MYH9,TLN1,PLEK,SLC7A11,MYL12A,C1QTNF1,ACTG1,CLIC1,PIK3CB,DMTN,UBASH3B,ITGA2B,FN1,PTPN6,FERMT3,VCL,ACTB,PEAR1,ABAT,STXBP1,FGA,RAP2B	grey	VSMC_M10
GO:0032609	0.94	0.10	26	59	interferon-gamma production	BP	1	CD226,HLA-DPB1,TLR4,PRNP,GAS6,NR1H4,RASGRP1,RIPK2,F2RL1,NOD2,CD96,FZD5,IL12A,VSIR,SLC11A1,INHA,SCGB1A1,FOXP3,ISL1,SLC7A5,MIR24-1,FADD,WNT5A,DDIT3,HRAS,HAVCR2	grey	VSMC_M10
GO:0032649	0.94	0.10	26	59	regulation of interferon-gamma production	BP	1	CD226,HLA-DPB1,TLR4,PRNP,GAS6,NR1H4,RASGRP1,RIPK2,F2RL1,NOD2,CD96,FZD5,IL12A,VSIR,SLC11A1,INHA,SCGB1A1,FOXP3,ISL1,SLC7A5,MIR24-1,FADD,WNT5A,DDIT3,HRAS,HAVCR2	grey	VSMC_M10
GO:0010971	0.94	0.12	10	25	positive regulation of G2/M transition of mitotic cell cycle	BP	1	VPS4B,MTA3,CCND1,STOX1,DBF4B,RAD51B,RAB11A,RAD51C,SIN3A,MECP2	grey	VSMC_M10
GO:0060008	0.94	0.15	5	14	Sertoli cell differentiation	BP	1	HSD17B4,SOX9,CTSV,SOX8,ARID4A	grey	VSMC_M10
GO:0010862	0.94	0.11	13	32	positive regulation of pathway-restricted SMAD protein phosphorylation	BP	1	GDF9,BMP8A,SDCBP,ACVR2A,RBPMS,ACVR1B,TGFB2,GDF15,INHA,BMPR1A,TWSG1,ENG,ACVRL1	grey	VSMC_M10
GO:1902017	0.94	0.10	19	45	regulation of cilium assembly	BP	1	TBC1D30,MAPK15,DYNC2LI1,FUZ,ATG5,ZMYND10,CDKL5,LIMK2,HTT,SDCCAG8,HAP1,MNS1,CNTROB,CCDC151,DNM2,IFT20,WRAP73,CDK10,ODF2L	grey	VSMC_M10
GO:1902692	0.94	0.13	8	21	regulation of neuroblast proliferation	BP	1	SOX10,SMO,PTN,FZD3,VEGFC,ZNF335,DISC1,FOXG1	grey	VSMC_M10
GO:0051234	0.94	0.06	1789	3429	establishment of localization	BP	1	ATP9B,TIMM21,NME1,VPS4B,ERBB3,SLC39A5,NEDD4L,APOF,ATP8B1,MIP,STARD6,MYO1A,LRP1,STX7,SLC18B1,STAC3,AP3S1,SLC2A12,KIF5A,SLC26A10,PEX7,TNFAIP3,AGAP2,HEBP2,REPS1,CYP27B1,PEX3,SLC16A7,CANX,TBC1D30,ATP5MGL,TBC1D9B,PPP1R10,CPSF6,RAB21,NIPAL2,SNCAIP,SNX24,SNX31,MAPK8IP2,CPT1B,PHAX,MARCH3,APOM,ZDHHC14,SLC12A2,HSPA1B,SYNJ2,SERAC1,RIMS2,IGF2R,SLC27A6,EMC2,CDC42SE2,KCNV1,PRRT1,PRKN,QKI,RNF5,AGER,PPARA,TAP1,SLC22A4,CALHM4,KIF3A,SEPT8,TSPO,GDF9,GOPC,SLC35F1,VPS52,HSPA4,DERL1,SMDT1,UBE2B,BAK1,FAM91A1,CATSPER3,WASHC5,MYC,LRRC6,KLHL3,MCHR1,PKD2L2,KCNK9,GRM4,PTK2,FAM53C,SLC45A4,PTP4A3,PTCH1,TSNARE1,LY6E,ZC3H3,EXOC4,SIL1,SYNGR1,MAPK15,AGTPBP1,SUN2,MAPK14,NTRK2,TOMM22,NRBP2,KCNJ4,CSNK1E,TMEM184B,PLA2G6,COPG2,PICK1,MICALL1,PIM1,TBC1D22B,BTBD9,RAC2,SMO,ZDHHC21,TTC39B,HSF1,PLIN2,TOMM6,SLC39A4,ATP6V1F,MYH9,GUCA1B,MFSD3,APOL4,APOL6,IFT74,CHRNA3,CHRNA5,PEX6,TRPM6,POT1,FBXO7,CRY1,CROT,ABCB1,SLC25A40,ABCC10,NOL6,STEAP2,GABRA1,XPO5,WASHC4,APBA1,CNGA1,SLC41A2,TMEM63B,PEX1,SLC35B2,OCIAD1,USP46,CLIC5,VPS50,SLC25A27,SCFD2,RFX3,KIT,SLC1A1,CA9,TGFBRAP1,TFAP2B,SLC25A13,TBC1D8,TRAM2,PPAT,TMEM14A,RGP1,WNK2,BICD2,MFSD14C,CHMP2A,CNNM4,TSPAN13,RAB23,DYNC2LI1,NLRP5,ABCG5,SVBP,ABCG8,LRPPRC,SEC61B,SLC3A1,STX17,CLDN19,SIX2,DNAJA1,IL11,BRSK1,PRKCE,SMAP1,ATP6V1E2,RIMS1,ABCB5,TMC4,TMEM30A,DNAH11,SPTBN1,IGF2BP3,FPR2,HMGN3,KCNQ4,YTHDC1,RIMS3,IBTK,PNPT1,RUFY3,SHANK1,SLC4A4,CYB5R[illegible]	grey	VSMC_M10

GO:0032733	0.94	0.12	10	25	positive regulation of interleukin-10 production	BP	1	TLR4,PIBF1,F2RL1,NOD2,CD28,STAT3,PRKCZ,CD46,INAVA,TSLP	grey	VSMC_M10
GO:0010934	0.94	0.15	5	14	macrophage cytokine production	BP	1	TLR4,TGFB2,SPON2,CD74,WNT5A	grey	VSMC_M10
GO:0010935	0.94	0.15	5	14	regulation of macrophage cytokine production	BP	1	TLR4,TGFB2,SPON2,CD74,WNT5A	grey	VSMC_M10
GO:0006739	0.94	0.12	11	27	NADP metabolic process	BP	1	ME1,TP53I3,RBKS,NUDT12,FMO5,NUDT17,RPE,NADK,NQO1,TIGAR,RPIA	grey	VSMC_M10
GO:0043217	0.94	0.15	5	14	myelin maintenance	BP	1	PRX,AKT2,MPP5,DEGS1,EPB41L3	grey	VSMC_M10
GO:0019953	0.94	0.07	284	565	sexual reproduction	BP	1	TBPL1,TRIM36,GLI1,YTHDC2,B4GALNT1,ATAT1,TSPAN8,MAPK8IP2,IGF2R,MOV10L1,PACRG,QKI,BRD2,GDF9,ROS1,MCM9,HSF2,UBE2B,CATSPER3,WASHC5,TOB2,SMAD5,H3F3C,FANCC,TUT7,DMC1,PICK1,SPATA31D3,MYH9,CFAP69,FANCG,SMARCA2,TDRD6,KIT,CCIN,TSGA10,TDRD7,NLRP5,DNAJA1,FOXJ3,SCMH1,RIMS1,MSH2,RAD23B,SPACA6,YTHDC1,KLK14,CABS1,FANCL,GJA10,HERPUD2,GMCL1,PTN,NOBOX,CCDC155,DKKL1,CREM,TUT4,FOXO3,ARMC2,DLD,DDO,TXNRD3,APOB,IZUMO1,SPATA6,SPATA5,HDAC2,ADCY3,TTC26,TRPC3,DNMT3A,PANX1,FOXA3,SOX9,RSPH6A,TOPAZ1,ERCC1,SLC4A2,NECTIN2,CRTAP,MLH1,ACOX1,FOXJ1,WDR48,TTC21A,PLAT,CCNB1IP1,ACVR2A,SIRT2,MKKS,SPATA24,ADAM18,PLCB1,MCM8,GOLGA3,PANK2,BTG1,SOHLH2,FUT10,TEX15,UBXN8,KATNAL1,STX2,NPHP1,ING2,HPGD,SPATA9,ARNTL,SPAG4,M1AP,IFT81,CELF1,JAM2,CSNK2A2,ADAMTS1,P2RY1,PTK2B,KMT2D,PRKAG1,SFMBT1,AP3B1,CDYL,SYCP1,DNAH1,NPM2,DPCD,OCA2,ROPN1L,SIAH1,BMPR1B,GAS2,ACTR1A,NECTIN3,AZIN2,RHBDD1,NEURL1,DDX25,CFAP43,HSF2BP,HYAL3,TESMIN,SLC9B1,PRKAR1A,INTS13,SPATA2,RNF114,ABHD2,CCDC36,YTHDF2,SPATC1L,SPO11,TSSK4,CIB1,CCDC87,DDX6,HDAC4,GORASP2,DEDD,CELF3,OSBP2,TDRKH,ZSCAN2,CLIC4,SEPT2,LIMK2,PATZ1,CATSPER2,OVOL1,ETV6,YBX3,LYZL6,WNT3,CADM1,OVGP1,SPATA32,GGT2,TTC12,BCL6,VGF,RPL10L,DDX3X,SPAG16,ZCWPW1,WNT4,PLCD4,RAB3A,CTSV,BIRC3,STAT3,AKAP3,ADCY10,PARP11,CALR3,KDM5A,ZMYND15,MNS1,YBX2,THRA,SPIN2A,SEPT1,ADAM21,ADAM20,MYCBPAP,UBE2Q1,VIPAS39,UBAP2L,SOX30,CCDC42,CREB3L4,IL4R,SPESP1,NOX5,PAQR5,RAD51C[illegible]	grey	VSMC_M10
GO:0045920	0.94	0.13	9	23	negative regulation of exocytosis	BP	1	VPS4B,PRKN,RAB7A,NCKAP1L,ADRA2A,RAP1B,RSC1A1,FMR1,FOXF1	grey	VSMC_M10
GO:0050685	0.94	0.12	10	25	positive regulation of mRNA processing	BP	1	SNW1,HSF1,TRA2A,NUP98,HSPA8,CELF3,NCBP2,LEO1,PRPF19,CIRBP	grey	VSMC_M10
GO:0090224	0.94	0.11	14	34	regulation of spindle organization	BP	1	VPS4B,HSPA1B,MAPK15,CHMP2A,CHMP2B,CHMP4C,RNF4,FSD1,PKD1,CHMP1A,PSRC1,GPSM2,CCSAP,DRG1	grey	VSMC_M10
GO:1902742	0.94	0.11	13	32	apoptotic process involved in development	BP	1	BAK1,PAX8,HAND2,ROBO2,XKR6,VDR,PAX2,TGFB2,FZD5,TNFRSF1B,JAG2,BCL2L11,XKR8	grey	VSMC_M10
GO:0060389	0.94	0.10	20	47	pathway-restricted SMAD protein phosphorylation	BP	1	LRP1,USP15,GDF9,BMP8A,BMPER,SDCBP,TGFBR2,ACVR2A,RBPMS,ACVR1B,SNX25,TGFB2,GDF15,INHA,BMPR1A,TWSG1,ENG,ACVRL1,NOG,LDLRAD4	grey	VSMC_M10
GO:0010763	0.94	0.15	5	14	positive regulation of fibroblast migration	BP	1	PTK2,PRKCE,BAG4,ARHGEF7,DMTN	grey	VSMC_M10
GO:0010256	0.94	0.07	209	422	endomembrane system organization	BP	1	VPS4B,ATP8B1,NEMP1,TBPL1,EMC2,PRKN,A4GALT,FER1L6,MTSS1,WASHC5,ASAP1,LEMD2,SYNGR1,MAPK15,SUN2,BAIAP2L2,MICALL1,MYH9,TJAP1,WASHC4,TRAM2,CHMP2A,STX17,SPTBN1,RTN4,SPACA6,EMC10,TRAPPC12,YWHAZ,HACE1,LPIN1,IZUMO1,RAB7A,ARHGAP21,RAB10,COG1,CLASP2,RBSN,NECTIN2,PLK3,OPTN,AQP11,GRIK5,EMC3,PRX,AKT2,HOOK3,SIRT2,YIPF4,NPLOC4,SYT1,ARHGEF7,ATL2,DNAJC13,FAM160A2,STX2,SYT9,TMEM127,NSFL1C,ANO4,OSBP,MYOF,CHMP2B,TRAPPC11,EMC4,XKR6,PLSCR4,SPAG4,P2RX7,CHMP4C,KIFC3,SEC31A,DYSF,SYNJ1,AP3B1,CHMP7,ZFYVE27,BIN3,F2R,ANO6,VPS18,COL4A3BP,DOP1B,SEC31B,DTNBP1,ARMH3,GBF1,SEC22B,TGFB2,TMEM9,TRIP11,VTI1A,SNAP23,GET4,ANK2,GPER1,RNF26,COG2,RAB22A,SEC23IP,CASQ1,OSBPL2,GORASP2,STX1A,TOR1AIP1,STX6,VPS37D,WHAMM,LYZL6,NSF,CREB1,MIA3,ALS2CL,SRGN,SEC16B,MOSPD3,TANGO2,LPCAT3,ATL1,PDCD10,HTT,RAB3A,SMPD4,SNAP29,PTPRN,PARP11,MAPK1,BHLHA15,ARL6IP1,STX5,OBSL1,STX18,AKAP8L,SYT7,ANXA8,MPP5,DEGS1,PI4K2B,GAK,CPLX1,TARDBP,STX1B,STX4,BNIP1,SYT11,TMED10,NDEL1,MAPK3,AC138894.1,HOOK2,RAB11A,MAP2K1,SOX30,SPESP1,NOX5,SYTL4,SNAPIN,PRKCB,DNM2,VMP1,COG7,BCAS3,LMAN1L,CAMSAP3,CXorf56,EPB41L3,RILP,TLE6,EMC6,SGTA,BAIAP3,UBL4A,ABCA7,SEC16A,SURF4,CHMP1A,SYPL2,COL5A1,TOR1B,MFSD14A,GOLGA8M,STXBP1,FNBP1L,TMED5,COG4,MTSS1L,SH3GLB1,VPS4A,REEP6,PHETA1,LEMD3,PLK5,BLOC1S3,SNAP47,WDR83OS,SNX10,CAMLG,TLCD1,EMC7,HIKESHI,TRAM1L1,TBC1D20,FAM174B,TMED3,PLEKHJ1,POLR2M,XKR8,CAVIN2,ZFP69B	grey	VSMC_M10
GO:0033137	0.94	0.12	10	25	negative regulation of peptidyl-serine phosphorylation	BP	1	BAK1,NCK2,GPD1L,PAQR3,DMTN,INPP5J,MLXIPL,INPP5K,DMD,GADD45A	grey	VSMC_M10
GO:0002700	0.94	0.08	49	106	regulation of production of molecular mediator of immune response	BP	1	CD226,IL5,KIT,EXOSC3,MSH2,RSAD2,TLR4,ATG5,TRPM4,SPHK2,HMCES,PHB,LACC1,MLH1,PAXIP1,SHLD1,MAVS,HPX,PRKDC,TRAF6,GPI,TMBIM6,F2RL1,NOD2,CD40,CD96,TGFB2,DENND1B,CD28,FZD5,BCL6,HK1,FOXP3,SHLD2,TNFRSF1B,SPON2,TBX21,STX4,IL4R,BTK,CD74,SUPT6H,TICAM1,PRKCZ,TRAF2,SLC7A5,BCL10,WNT5A,INAVA	grey	VSMC_M10
[truncated: 670,845 more chars]
