# Supplementary material for: Locational memory of macrovessel vascular cells is transcriptionally imprinted
Source: Sci Rep. 2023 Aug 10;13:13028. doi: 10.1038/s41598-023-38880-6 (PMC10415317; doi:10.1038/s41598-023-38880-6)
Supplement: Supplementary file 13 — Supplementary Table 4. [file 41598_2023_38880_MOESM13_ESM.pdf]

Supplemental Table 4. Matrix of significantly differentially expressed genes (Padj < 0.05) in all sample groups. Up-regulated gene = 1, down-regulated gene = -1. ACOR, coronary artery; APUL, pulmonary artery; VPUL, pulmonary vein; VPOR, portal vein; VCAV, caval vein; AORT, aorta; AFEM, femoral artery; VFEM, femoral vein; VSAP, saphenous vein.

Gene	ACOR_EC	VSAP_EC	AORT_EC	APUL_EC	VCAV_EC	VPOR_EC	VFEM_EC	VPUL_EC	AFEM_EC	ACOR_VSMC	VSAP_VSMC	AORT_VSMC	APUL_VSMC	VCAV_VSMC	VPOR_VSMC	VFEM_VSMC	VPUL_VSMC	AFEM_VSMC
DOK6	1	0	1	0	0	0	0	0	0	1	0	0	0	0	0	0	0	0
GDF11	1	-1	0	0	0	0	0	0	0	1	0	0	0	0	0	0	0	0
BCL2	1	0	0	0	0	0	0	0	0	1	0	0	0	0	0	0	0	0
DDR1	1	0	0	0	0	0	0	0	0	1	0	0	0	0	0	0	0	0
VARS2	1	0	0	0	0	0	0	0	0	-1	0	0	0	0	0	0	0	0
MAPK8IP2	1	0	0	0	0	0	0	0	0	0	0	0	0	0	0	0	0	0
PANX2	1	0	0	0	0	0	0	0	0	0	0	0	0	0	0	0	0	0
TRPS1	1	0	0	0	0	0	0	0	0	1	0	0	0	0	0	0	0	0
COL11A2	1	0	0	0	0	0	0	0	0	0	0	0	0	0	0	0	0	0
TAPBP	1	0	0	0	0	0	0	0	0	0	0	0	0	0	0	0	0	0
TEF	1	0	0	0	0	0	0	0	0	0	0	0	0	0	0	0	0	0
PTPRK	1	-1	0	0	1	0	0	0	0	0	0	0	0	0	0	0	0	0
OBSCN	1	0	0	0	0	0	0	0	0	0	0	0	0	0	0	0	0	0
PTP4A3	1	0	0	0	0	0	0	0	0	0	0	0	0	0	0	0	0	0
CDC42EP1	1	-1	0	0	0	0	0	0	0	0	0	0	0	0	0	0	0	0
UBE2H	1	0	0	0	0	0	0	0	0	1	0	0	0	0	0	0	0	0
SH3GL2	1	0	0	0	0	0	0	0	0	0	0	0	0	0	0	0	0	0
LRRC4	1	0	0	0	0	0	0	0	0	1	0	0	0	0	0	0	0	0
TRPM3	1	0	0	0	0	0	0	0	0	1	0	0	0	0	0	0	0	0
IL11RA	1	0	0	0	0	0	0	0	0	0	0	0	0	0	0	0	0	0
SH3RF3	1	-1	0	0	0	0	0	0	0	1	0	0	0	0	0	0	0	0
PON2	1	-1	0	0	0	0	0	0	0	1	0	0	0	0	0	0	0	0
DYNC1I1	1	0	0	0	0	0	0	0	0	1	0	0	0	0	0	0	0	0
ENSCAFG00000002230	1	0	0	1	0	0	0	0	0	0	0	0	0	0	0	0	0	0
ADGRL3	1	-1	0	0	0	0	0	1	0	1	0	0	0	1	0	0	0	0
TMOD1	1	0	0	0	1	0	0	0	0	0	0	0	0	0	0	0	0	0
TMEM150B	1	0	0	0	0	0	0	0	0	0	0	0	0	0	0	0	0	0
BRSK1	1	0	0	0	-1	0	0	0	0	0	0	0	0	0	0	0	0	0
EFEMP1	1	0	0	0	0	0	0	0	0	0	0	0	0	0	0	0	0	0
TBX18	1	0	0	0	0	0	0	0	0	1	0	0	0	0	0	0	0	0
AQP1	1	0	0	0	0	0	0	0	0	0	0	0	0	0	0	0	0	0
MYH14	1	-1	0	0	0	0	0	0	0	0	0	0	0	0	0	0	0	0
FIGNL1	1	0	0	0	1	0	0	0	0	0	0	0	0	0	0	0	0	0
GPR63	1	0	0	0	0	0	0	0	0	1	0	0	0	0	1	0	0	0
HECW1	1	0	0	0	0	0	0	0	0	0	0	0	0	0	0	0	0	0
KIAA0319L	1	-1	0	0	0	0	0	0	0	1	0	0	0	0	0	0	0	0
FLT3LG	1	1	0	0	0	0	0	0	0	0	0	0	0	0	0	0	0	0
EPHB6	1	0	0	0	0	0	0	0	0	1	0	0	0	0	0	0	0	0
NRP1	1	-1	0	0	0	0	0	0	0	1	0	0	0	0	0	0	0	0
OTUD1	1	0	0	0	0	0	0	0	0	0	0	0	0	0	0	0	0	0
KIAA1217	1	-1	0	0	0	0	0	0	0	0	0	0	0	0	0	0	0	0
ATP7B	1	0	0	0	0	0	0	0	0	0	0	0	0	0	0	0	0	0
PTGIR	1	0	0	0	0	0	0	0	0	1	0	0	0	0	0	0	0	0
ENSCAFG00000004331	1	0	0	0	0	0	0	0	0	0	0	0	0	0	0	0	0	0
MAGI2	1	0	0	0	0	0	0	0	0	0	0	0	0	0	0	0	0	0
SOX9	1	0	0	0	0	0	0	0	0	0	0	0	0	0	0	0	0	0
AGBL5	1	0	0	0	0	0	0	0	0	0	0	0	0	0	0	0	0	0
RARRES2	1	0	0	0	0	0	0	0	0	0	0	0	0	0	0	0	0	0
PREB	1	0	0	0	0	0	0	0	0	-1	0	0	0	0	0	0	0	0
SLC16A5	1	0	0	0	0	0	0	0	0	-1	0	0	0	0	0	0	0	0
TENM4	1	0	0	0	-1	0	0	0	0	0	0	0	0	0	0	0	0	0
INHBB	1	0	0	0	0	0	0	0	0	1	0	0	0	0	0	0	0	0
WDR86	1	0	0	0	0	0	0	0	0	1	0	0	0	0	0	0	0	0
CYP2S1	1	0	0	0	0	0	0	0	0	0	0	0	0	0	0	0	0	0
KCTD12	1	0	0	0	0	0	0	0	0	0	0	0	0	0	0	0	0	0
SFMBT2	1	0	0	0	0	0	0	0	0	0	0	0	0	0	0	0	0	0
FNDC4	1	0	0	0	0	0	0	0	0	0	0	0	0	0	0	0	0	0
PRRT3	1	0	0	0	0	0	0	0	0	0	0	0	0	0	0	0	0	0
SYNGR2	1	0	0	0	0	0	0	0	0	1	0	0	0	0	0	0	0	0
CAMK1	1	1	0	0	0	0	0	0	0	0	0	0	0	0	0	0	0	0
PLD3	1	0	0	0	0	0	0	0	0	1	0	0	0	0	0	0	0	0
RIN2	1	0	0	0	0	0	0	0	0	0	0	0	0	0	0	0	0	0
SLC24A3	1	0	0	0	0	0	0	0	0	0	0	0	0	0	0	0	0	0
NDRG2	1	0	0	0	0	0	0	0	0	1	0	0	0	0	0	0	0	0
ATG16L2	1	0	0	0	0	0	0	0	0	1	0	0	0	0	0	0	0	0
SALL2	1	0	0	0	0	0	0	0	0	1	0	0	0	0	0	0	0	0
ENSCAFG00000005758	1	0	0	0	0	0	0	0	0	1	0	0	0	0	0	0	0	0
ACSS3	1	0	0	0	0	0	0	0	0	0	0	0	0	0	0	0	0	0
PLCL2	1	-1	0	0	0	0	0	1	0	0	0	0	0	0	0	0	0	0
RFTN1	1	-1	0	1	0	0	-1	0	0	1	0	0	0	0	0	0	0	0
TMTC2	1	-1	0	0	0	0	0	0	0	0	0	0	0	0	0	0	0	0
DCXR	1	0	0	0	0	0	0	0	0	0	0	0	0	0	0	0	0	0
BTD	1	0	0	0	-1	0	0	0	0	0	0	0	0	0	0	0	0	0
POC1B	1	0	0	0	1	0	0	0	0	0	0	0	0	0	0	0	0	0
MAVS	1	1	0	0	-1	0	0	0	0	0	0	0	0	0	0	0	0	0
GAS6	1	1	0	0	0	0	0	0	0	0	0	0	0	0	0	0	0	0
VPS16	1	0	0	0	0	0	0	0	0	0	0	0	0	0	0	0	0	0
ENSCAFG00000006540	1	0	0	0	0	0	0	0	0	1	0	0	0	0	0	0	0	0
OLFML1	1	0	0	0	0	0	0	0	0	1	0	0	0	0	0	0	0	0
PPFIBP2	1	0	0	0	0	0	0	0	0	0	0	0	0	0	0	0	0	0
RASL11A	1	0	0	0	0	0	0	0	0	0	0	0	0	0	0	0	0	0
NR1H4	1	-1	0	1	0	0	0	1	0	0	0	0	0	0	0	0	0	0
PLPP1	1	0	0	0	-1	0	0	0	0	0	0	0	0	0	0	0	0	0
ENSCAFG00000006873	1	0	0	0	0	0	0	0	0	1	0	0	0	0	0	0	0	0
ABTB2	1	0	0	0	0	0	0	0	0	0	0	0	0	0	0	0	0	0
HM13	1	-1	0	0	-1	0	0	0	0	-1	0	0	0	0	0	0	0	0
PDRG1	1	0	0	0	0	0	0	0	0	0	0	0	0	0	0	0	0	0
LMO2	1	1	0	0	0	0	0	0	0	0	0	0	0	0	0	0	0	0
TNFRSF19	1	0	0	0	0	0	0	0	0	0	0	0	0	0	0	0	0	0
FBLN7	1	0	0	0	0	0	0	0	0	1	0	0	0	0	0	0	0	0
CHPT1	1	0	0	0	0	0	0	0	0	1	0	0	0	0	0	0	0	0
THNSL2	1	0	0	0	-1	0	0	0	0	0	0	0	0	0	0	0	0	0
EFNA5	1	-1	0	0	0	0	0	1	0	0	0	0	0	0	0	0	0	0
CHMP4B	1	0	0	0	0	0	0	0	0	0	0	0	0	0	0	0	0	0
TP53INP2	1	0	0	0	0	0	0	0	0	0	0	0	0	0	0	0	0	0
ATOH8	1	0	0	0	0	0	0	0	0	1	0	0	0	0	0	0	0	0
ABCB9	1	0	0	0	0	0	0	0	0	0	0	0	0	0	0	0	0	0
GRK7	1	0	0	0	0	0	0	0	0	1	0	0	0	0	0	0	0	0
SERPING1	1	1	0	0	0	0	0	0	1	1	0	0	0	0	0	0	0	0
DCLK2	1	0	0	0	0	0	0	0	0	0	0	0	0	0	0	0	0	0
PLS1	1	0	0	0	0	0	0	0	0	0	0	0	0	0	0	0	0	0
ARSK	1	0	0	0	0	0	0	0	0	0	0	0	0	0	0	0	0	0
EDEM2	1	0	0	0	0	0	0	0	0	0	0	0	0	0	0	0	0	0
SLC35G1	1	0	0	0	0	0	0	0	0	1	0	0	0	0	0	0	0	0
PLCE1	1	0	0	0	0	0	0	0	0	0	0	0	0	0	0	0	0	0
SLC9A9	1	0	0	0	0	0	0	0	0	0	0	0	0	0	0	0	0	0
PKIA	1	0	0	0	0	0	0	0	0	0	0	0	0	0	0	0	0	0
PHF20	1	0	0	0	0	0	0	0	0	1	0	0	0	0	0	0	0	0
CLU	1	0	0	0	0	0	0	0	0	0	0	0	0	0	0	0	0	0
EPB41L1	1	0	0	0	-1	0	0	0	0	0	0	0	0	0	0	0	0	0
GUCY1B1	1	0	0	0	1	0	0	0	0	0	0	0	0	0	0	0	0	0
ARHGEF26	1	0	0	1	0	0	0	0	0	1	0	0	0	0	0	0	0	0
PRKCD	1	0	0	0	0	0	0	0	0	0	0	0	0	0	0	0	0	0
EPHA3	1	0	0	0	0	0	0	0	0	0	0	0	0	0	0	0	0	0
SRC	1	0	0	0	-1	0	0	0	0	0	0	0	0	0	0	0	0	0
FKBP11	1	-1	0	0	-1	0	0	0	0	-1	0	0	0	0	0	0	0	0
ANXA3	1	0	0	0	0	0	0	0	0	1	0	0	0	0	0	0	0	0
ENSCAFG00000008917	1	0	0	0	0	0	0	0	0	1	0	0	0	0	0	0	0	0
LRP4	1	1	0	0	0	0	0	0	0	0	0	0	0	0	0	0	0	0
CCDC9B	1	0	0	0	0	0	0	0	0	0	0	0	0	0	0	0	0	0
RIOX2	1	0	0	0	0	0	0	0	0	0	0	0	0	0	0	0	0	0
TBX5	1	0	0	1	0	0	0	1	0	1	0	0	1	0	0	0	1	0
GNAO1	1	0	0	0	0	0	0	0	0	0	0	0	0	0	0	0	0	0
GALNT5	1	0	0	0	0	0	0	0	0	0	0	0	0	0	0	0	0	0
NIT2	1	0	0	0	0	0	0	0	0	1	0	0	0	0	0	0	0	0
SLC40A1	1	0	0	0	0	0	0	0	0	0	0	0	0	0	0	0	0	0

PYROXD2	1	0	0	0	0	0	0	0	0	1	0	0	0	0	0	0	0	0
MAPK8IP1	1	0	0	0	0	0	0	0	0	0	0	0	0	0	0	0	0	0
PTPN5	1	0	0	0	0	0	0	0	0	0	0	0	0	0	0	0	0	0
FMOD	1	0	0	0	0	0	0	0	0	1	0	0	0	0	0	0	0	0
PRELP	1	1	0	0	-1	0	0	0	0	1	0	0	0	0	0	0	0	0
CPQ	1	0	0	0	0	0	0	0	0	1	0	0	0	0	0	0	0	0
IFIT2	1	0	0	0	0	0	0	0	0	0	0	0	0	0	0	0	0	0
NXPE3	1	0	0	0	0	0	0	0	0	0	0	0	0	0	0	0	0	0
PI3	1	0	0	0	0	0	0	0	0	0	0	0	0	0	0	0	0	0
DBNDD2	1	0	0	0	0	0	0	0	0	0	0	0	0	0	0	0	0	0
PIGT	1	0	0	0	-1	0	0	0	0	1	0	0	0	0	0	0	0	0
ZSWIM1	1	0	0	0	0	0	0	0	0	0	0	0	0	0	0	0	0	0
NOD2	1	0	0	0	0	0	0	0	0	1	0	0	0	0	0	0	0	0
NFASC	1	0	0	0	0	0	0	0	0	1	0	0	0	0	0	0	0	0
ADAMTS15	1	0	0	0	0	0	0	0	0	1	0	0	0	0	0	0	0	0
CMBL	1	0	0	0	0	0	0	0	1	0	0	0	0	0	0	0	0	0
CD81	1	0	0	0	-1	0	0	0	0	1	0	0	0	0	0	0	0	0
PM20D1	1	0	0	0	0	0	0	0	0	0	0	0	0	0	0	0	0	0
PHKG1	1	0	0	0	0	0	0	0	0	0	0	0	0	0	0	0	0	0
TRIM8	1	0	0	0	-1	0	0	0	0	0	0	0	0	0	0	0	0	0
ELMO2	1	0	0	0	0	0	0	0	0	0	0	0	0	0	0	0	0	0
AZIN2	1	1	0	0	0	0	0	0	0	1	0	0	0	0	0	0	0	0
ENSCAFG00000010493	1	0	0	0	0	0	0	0	0	0	0	0	0	0	0	0	0	0
BOC	1	0	0	0	-1	0	0	0	0	0	0	0	0	0	0	0	0	0
RGS1	1	0	0	0	0	0	0	1	0	0	0	0	0	0	0	0	0	0
BICD1	1	0	1	0	0	0	0	0	0	0	0	0	0	0	0	0	0	0
EYA2	1	1	0	0	-1	0	0	0	0	1	0	0	0	0	0	0	0	0
MRGPRF	1	1	0	0	0	0	0	0	0	0	0	0	0	0	0	0	0	0
ZMYND8	1	-1	0	0	0	0	0	0	0	0	0	0	0	0	0	0	0	0
RBM20	1	0	0	0	0	0	0	0	0	1	0	0	0	0	0	0	0	0
ANKRD44	1	-1	0	0	0	0	0	0	0	1	0	0	0	0	0	0	0	0
PDE4DIP	1	0	0	0	-1	0	0	0	1	1	0	0	0	0	0	0	0	0
ACTN2	1	0	0	0	0	0	0	0	0	1	0	0	0	0	0	0	0	0
PKNOX2	1	0	0	0	0	0	0	0	0	1	0	0	0	0	0	0	0	0
PLCL1	1	-1	0	1	-1	0	-1	0	0	1	0	0	0	0	0	0	0	0
RGMA	1	0	0	0	-1	0	0	0	0	0	0	0	0	0	0	0	0	0
CCDC15	1	-1	0	0	0	0	0	0	0	0	0	0	0	0	0	0	0	0
B3GNT7	1	0	0	0	0	0	0	0	0	1	0	0	0	0	0	0	0	0
STK32C	1	0	0	0	0	0	0	0	0	0	0	0	0	0	0	0	0	0
ROBO3	1	-1	0	0	0	0	0	0	0	0	0	0	0	0	0	0	0	0
LHX9	1	0	0	0	0	0	0	0	0	1	0	0	0	0	0	0	0	0
AFAP1L2	1	0	0	0	0	0	0	0	0	1	0	0	0	0	0	0	0	0
KCNB1	1	1	0	0	0	0	0	0	0	0	0	0	0	0	0	0	0	0
KLHL25	1	0	0	0	0	0	0	0	0	0	0	0	0	0	0	0	0	0
CFI	1	0	0	0	0	0	0	0	0	1	0	0	0	0	0	0	0	0
RNF114	1	0	0	0	0	0	0	0	0	0	0	0	0	0	0	0	0	0
NGEF	1	0	0	0	0	0	0	0	0	0	0	0	0	0	0	0	0	0
ARHGAP6	1	0	0	0	0	0	0	0	0	0	0	0	0	0	0	0	0	0
ENSCAFG00000011635	1	0	0	0	0	0	0	0	0	1	0	0	0	0	0	0	0	0
OPRD1	1	0	0	0	0	0	0	0	0	0	0	0	0	0	0	0	0	0
KLHDC8B	1	1	0	0	0	0	0	0	1	1	0	0	0	0	0	0	0	0
UBASH3B	1	0	0	0	0	0	0	0	0	0	0	0	0	0	0	0	0	0
MOSPD2	1	0	0	0	0	0	0	0	0	0	0	0	0	0	0	0	0	0
TFAP2C	1	0	0	0	0	0	0	0	0	0	0	0	0	0	0	0	0	0
IFI6	1	0	0	0	0	0	0	0	0	0	0	0	0	0	0	0	0	0
GABBR1	1	0	0	0	0	0	0	0	0	1	0	0	0	0	0	0	0	0
CASP10	1	0	0	0	1	0	0	0	0	1	0	0	0	0	0	0	0	0
CTSZ	1	0	0	0	0	0	0	0	1	1	0	0	0	0	0	0	0	0
TNS3	1	0	0	1	0	0	0	0	0	1	0	0	0	0	0	0	0	0
ANXA9	1	0	0	0	0	0	0	0	0	0	0	0	0	0	0	0	0	0
PDE3A	1	0	0	0	0	0	0	0	0	-1	0	0	0	0	0	0	0	0
SYNPO2	1	0	0	0	0	0	0	0	0	1	0	0	0	0	0	0	0	0
MYOZ2	1	0	0	0	0	0	0	0	0	1	0	0	0	0	0	0	0	0
USP53	1	0	0	0	0	0	0	0	0	1	0	0	0	0	0	0	0	0
ENSCAFG00000012643	1	0	0	0	0	0	0	0	0	0	0	0	0	0	0	0	0	0
ENSCAFG00000012668	1	0	0	0	0	0	0	0	0	0	0	0	0	0	0	0	0	0
TRIM63	1	0	0	0	0	0	0	0	0	1	0	0	0	0	0	0	0	0
SNED1	1	0	0	0	0	0	0	0	0	1	0	0	0	0	0	0	0	0
KIAA1614	1	0	0	0	0	0	0	0	0	0	0	0	0	0	0	0	0	0
ARHGDIB	1	0	0	0	1	0	0	0	0	0	0	0	0	0	0	0	0	0
MAP4	1	0	0	0	0	0	0	0	0	0	0	0	0	0	0	0	0	0
CABCOCO1	1	0	0	0	0	0	0	0	0	0	0	0	0	0	0	0	0	0
GMEB2	1	0	0	0	0	0	0	0	0	0	0	0	0	0	0	0	0	0
NOS1AP	1	0	0	0	0	0	0	0	0	1	0	0	0	0	0	0	0	0
RAPGEF4	1	0	0	0	0	0	0	0	0	0	0	0	0	0	0	0	0	0
CSPG5	1	0	0	0	0	0	0	0	0	1	0	0	0	0	0	0	0	0
ENSCAFG00000013221	1	0	0	0	0	0	0	0	0	0	0	0	0	0	0	0	0	0
NMNAT2	1	-1	0	0	0	0	0	0	0	0	0	0	0	0	0	0	0	0
CALY	1	0	0	0	0	0	0	0	0	0	0	0	0	0	0	0	0	0
LRRC15	1	0	0	0	0	0	0	0	1	0	0	0	0	0	0	0	0	0
CCNYL1	1	-1	0	0	0	0	0	0	0	0	0	0	0	0	0	0	0	0
FZD5	1	0	0	0	0	0	0	0	0	0	0	0	0	0	0	0	0	0
PTH1R	1	0	0	0	0	0	0	0	0	0	0	0	0	0	0	0	0	0
HMCN1	1	0	0	0	0	0	0	0	0	0	0	0	0	0	0	0	0	0
GNAZ	1	0	0	0	-1	0	0	0	0	0	0	0	0	0	0	0	0	0
MAPT	1	0	0	0	0	0	0	0	0	0	0	0	0	0	0	0	0	0
ENSCAFG00000013855	1	0	0	0	0	0	0	0	0	0	0	0	0	0	0	0	0	0
LRRC75B	1	0	0	0	0	0	0	0	0	0	0	0	0	0	0	0	0	0
ENSCAFG00000013899	1	0	0	0	0	0	0	0	0	0	0	0	0	0	0	0	0	0
SUSD2	1	0	0	0	0	0	0	0	0	0	0	0	0	0	0	0	0	0
SEMA6D	1	0	0	0	0	0	0	0	0	1	0	0	0	0	0	0	0	0
DIXDC1	1	0	0	0	0	0	0	0	0	0	0	0	0	0	0	0	0	0
C1RL	1	1	0	0	0	0	0	0	0	1	0	0	0	0	0	0	0	0
GMIP	1	1	0	0	0	0	0	0	0	0	0	0	0	0	0	0	0	0
PDE1A	1	-1	0	1	0	0	0	0	0	1	0	0	0	0	0	0	0	0
ANKH	1	0	0	0	0	0	0	0	0	0	0	0	0	0	0	0	0	0
SLC25A42	1	0	0	0	0	0	0	0	0	0	0	0	0	0	0	0	0	0
ANKRD45	1	0	0	0	0	0	0	0	0	0	0	0	0	0	0	0	0	0
HGFAC	1	0	0	0	0	0	0	0	0	0	0	0	0	0	0	0	0	0
PNKD	1	0	0	0	0	0	0	0	0	0	0	0	0	0	0	0	0	0
CNTNAP1	1	0	0	0	0	0	0	0	0	0	0	0	0	0	0	0	0	0
ZFYVE28	1	0	0	0	0	0	0	0	0	1	0	0	0	0	0	0	0	0
FMO3	1	0	0	0	0	0	0	0	0	1	0	0	0	0	0	0	0	0
FAM129C	1	0	0	0	0	0	0	0	0	0	0	0	0	0	0	0	0	0
ATP1B1	1	0	0	0	0	0	0	0	0	0	0	0	0	0	0	0	0	0
CD38	1	0	0	0	0	0	0	0	0	0	0	0	0	0	0	0	0	0
DACT1	1	-1	0	0	-1	0	0	0	0	1	0	0	0	0	0	0	0	0
PORCN	1	0	0	0	0	0	0	0	0	0	0	0	0	0	0	0	0	0
SPEG	1	0	0	0	0	0	0	0	0	1	0	0	0	0	0	0	0	0
CPAMD8	1	0	0	0	0	0	0	0	0	1	0	0	0	0	0	0	0	0
PCSK1N	1	0	0	1	0	0	0	0	0	0	0	0	0	0	0	0	0	0
PLD2	1	0	0	0	1	0	0	0	0	0	0	0	0	0	0	0	0	0
LDB3	1	0	0	0	0	0	0	0	0	0	0	0	0	0	0	0	0	0
MYRF	1	0	0	1	0	0	0	0	0	1	-1	0	0	1	1	-1	1	-1
CECR2	1	0	0	0	0	0	0	0	0	0	0	0	0	0	0	0	0	0
ARAP2	1	-1	0	0	0	0	0	0	0	1	0	0	0	0	0	0	0	0
ADGRL1	1	0	0	0	0	0	0	0	0	1	0	0	0	0	0	0	0	0
PRKACA	1	0	0	0	0	0	0	0	0	0	0	0	0	0	0	0	0	0
DLX3	1	1	0	0	0	0	0	0	0	0	0	0	0	0	0	0	0	0
ACSF2	1	0	0	0	0	0	0	0	0	0	0	0	0	0	0	0	0	0
SLC16A2	1	0	0	0	0	0	0	0	0	0	0	0	0	0	0	0	0	0
RNF145	1	0	0	0	0	0	0	0	0	1	0	0	0	0	0	0	0	0
SMAD6	1	0	0	0	0	0	0	0	0	1	0	0	0	0	0	0	0	0
NTN1	1	0	0	0	0	0	0	0	0	0	0	0	0	0	0	0	0	0
EPOR	1	0	0	0	0	0	0	0	0	0	0	0	0	0	0	0	0	0
PIAS1	1	0	0	0	0	0	0	0	0	0	0	0	0	0	0	0	0	0
SEPT4	1	0	0	0	0	0	0	0	0	0	0	0	0	0	0	0	0	0
C20H19orf38	1	0	0	0	0	0	0	0	0	0	0	0	0	0	0	0	0	0
AK7	1	0	0	0	0	0	0	0	0	0	0	0	0	0	0	0	0	0
PDE4A	1	0	0	0	0	0	0	0	0	0	0	0	0	0	0	0	0	0

CA4	1	0	0	0	0	0	0	0	0	0	0	0	0	0	0	0	0	0
PAK3	1	0	0	0	0	0	0	0	0	0	0	0	0	0	0	0	0	0
MMP28	1	-1	0	0	1	0	0	0	0	0	0	0	0	0	0	0	0	0
ENSCAFG00000018235	1	0	0	0	0	0	0	0	0	1	0	0	0	0	0	0	0	0
SH3TC2	1	0	0	0	0	0	0	0	0	1	0	0	0	0	0	0	0	0
HTR4	1	0	0	1	0	0	0	0	0	0	0	0	0	0	0	0	0	0
ESM1	1	-1	0	0	0	0	0	0	0	0	0	0	0	0	0	0	0	0
FST	1	-1	0	0	-1	0	0	0	0	1	0	0	0	0	0	0	0	0
ARHGAP28	1	0	0	0	1	0	0	0	0	1	0	0	0	0	0	0	0	0
C7	1	0	0	0	0	0	0	0	0	1	0	0	0	0	0	0	0	0
PTGER4	1	0	0	0	0	0	0	0	0	0	0	0	0	0	0	0	0	0
C3	1	0	0	0	0	0	0	0	0	0	0	0	0	0	0	0	0	0
TUBB4A	1	0	0	1	0	0	0	0	0	1	0	0	0	0	0	0	0	0
SMARCA1	1	0	0	0	0	0	0	0	0	1	0	0	0	0	0	0	0	0
PIGS	1	0	0	0	-1	0	0	0	0	1	0	0	0	0	0	0	0	0
ALDOC	1	-1	0	0	0	0	0	0	0	1	0	0	0	0	0	0	0	0
TLE2	1	1	0	0	0	0	0	0	0	0	0	0	0	0	0	0	0	0
AGRN	1	-1	0	0	0	0	0	0	0	1	0	0	0	0	0	0	0	0
L1CAM	1	0	0	0	0	0	0	0	0	1	0	0	0	0	0	0	0	0
ADAMTSL5	1	1	0	0	-1	0	0	0	0	0	0	0	0	0	0	0	0	0
PTGDS	1	0	0	0	0	0	0	0	0	0	0	0	0	0	0	0	0	0
AGPAT2	1	0	0	0	0	0	0	0	1	0	0	0	0	0	0	0	0	0
TNFRSF25	1	0	0	0	1	0	0	0	0	1	0	0	0	0	0	0	0	0
CCDC78	1	0	0	0	0	0	0	0	0	0	0	0	0	0	0	0	0	0
CLSTN1	1	0	0	0	-1	0	0	0	0	1	0	0	0	0	0	0	0	0
ADAMTS13	1	0	0	0	0	0	0	0	0	0	0	0	0	0	0	0	0	0
SARDH	1	0	0	0	0	0	0	0	0	1	0	0	0	0	0	0	0	0
PPP1R26	1	0	0	0	0	0	0	0	0	1	0	0	0	0	0	0	0	0
FOXC2	1	0	0	0	0	0	0	0	0	0	0	0	0	0	0	0	0	0
OSGIN1	1	0	0	0	0	0	0	0	0	0	0	0	0	0	0	0	0	0
S1PR1	1	0	0	0	1	0	0	0	0	0	0	0	0	0	0	0	0	0
VCAM1	1	0	0	0	0	0	0	0	0	1	0	0	0	0	0	0	0	0
VAT1L	1	0	0	0	0	0	0	0	0	0	0	0	0	0	0	0	0	0
CNTNAP4	1	0	0	0	0	0	0	0	0	0	0	0	0	0	0	0	0	0
CDH3	1	0	0	0	0	0	0	0	0	0	0	0	0	0	0	0	0	0
EXOC3L1	1	0	0	0	1	0	0	0	0	0	0	0	0	0	0	0	0	0
HSF4	1	1	0	0	0	0	0	0	0	0	0	0	0	0	0	0	0	0
CES2	1	1	0	0	0	0	0	0	0	1	0	0	0	0	0	0	0	0
CCDC160	1	0	0	0	0	0	0	0	0	1	0	0	0	0	0	0	0	0
ADAMTSL2	1	0	0	0	0	0	0	0	0	0	0	0	0	0	0	0	0	0
TRABD2B	1	0	0	0	0	0	0	0	0	1	0	0	0	1	0	0	1	0
ARL16	1	0	0	0	0	0	0	0	0	0	0	0	0	0	0	0	0	0
TCFL5	1	0	0	0	0	0	0	0	0	0	0	0	0	0	0	0	0	0
EPS8L2	1	0	0	0	0	0	0	0	0	1	0	0	0	0	0	0	0	0
CD59	1	0	0	0	0	0	0	0	0	1	0	0	0	0	0	0	0	0
CST3	1	1	-1	0	-1	-1	0	0	0	1	0	0	0	0	0	0	0	0
RNF150	1	0	0	0	0	0	0	0	0	0	0	0	0	0	0	0	0	0
ENSCAFG00000028814	1	-1	0	1	0	0	0	1	0	1	-1	0	0	1	0	0	0	0
PDLIM3	1	0	0	1	0	0	0	0	0	1	0	0	0	0	0	0	0	0
RAB6B	1	0	0	0	0	0	0	0	0	1	0	0	0	0	0	0	0	0
GATA4	1	0	0	0	0	0	0	0	0	1	0	0	0	0	0	0	0	0
SAT1	1	0	0	0	0	0	-1	0	0	1	0	0	0	0	0	0	0	0
ENSCAFG00000029292	1	0	0	0	0	0	0	0	0	0	0	0	0	0	0	0	0	0
ENSCAFG00000029378	1	0	0	0	0	0	0	0	0	0	0	0	0	0	0	0	0	0
ENSCAFG00000029395	1	0	0	0	-1	0	0	0	0	1	0	0	0	0	0	0	0	0
DUSP26	1	0	0	0	0	0	0	0	0	1	0	0	0	0	0	0	0	0
ENSCAFG00000029897	1	0	0	0	0	0	0	0	0	0	0	0	0	0	1	0	0	0
CLIC3	1	0	0	0	0	0	0	0	0	0	0	0	0	0	0	0	0	0
PKIB	1	0	0	0	0	0	0	0	0	0	0	0	0	0	0	0	0	0
RASSF3	1	-1	0	0	0	0	0	0	0	0	0	0	0	0	0	0	0	0
ENSCAFG00000030031	1	0	0	0	0	0	0	0	0	0	0	0	0	0	0	0	0	0
IL33	1	0	0	0	0	0	0	0	0	0	0	0	0	0	0	0	0	0
ZNF229	1	0	0	0	0	0	0	0	0	0	0	0	0	0	0	0	0	0
KCTD1	1	0	0	0	0	0	0	0	0	1	0	0	0	0	0	0	0	0
MFAP4	1	0	0	0	0	0	0	0	1	1	0	0	0	0	0	0	0	0
TNFSF9	1	0	0	0	0	0	0	0	0	0	0	0	0	0	0	0	0	0
ENSCAFG00000030404	1	0	0	0	0	0	0	0	0	0	0	0	0	0	0	0	0	0
RNLS	1	0	0	0	0	0	0	0	0	1	0	0	0	0	0	0	0	0
INSYN1	1	0	0	0	0	0	0	0	0	1	0	0	0	0	0	0	0	0
CCDC3	1	0	0	0	0	0	0	0	0	1	0	0	0	0	0	0	0	0
OCIAD2	1	0	0	0	-1	0	0	0	0	0	0	0	0	0	0	0	0	0
RHOU	1	0	0	0	0	0	0	0	0	0	0	0	0	0	0	0	0	0
PYGO2	1	0	0	0	0	0	0	0	0	0	0	0	0	0	0	0	0	0
C1QTNF7	1	0	0	0	0	0	0	0	0	1	0	0	0	0	0	0	0	0
ZNF853	1	0	0	0	0	0	0	0	0	1	0	0	0	0	0	0	0	0
PRAP1	1	0	0	0	0	0	0	0	1	0	0	0	0	0	0	0	0	0
FBXO42	1	0	0	0	0	0	0	0	0	0	0	0	0	0	0	0	0	0
C23H3orf58	1	0	0	0	0	0	0	0	0	0	0	0	0	0	0	0	0	0
RBM38	1	0	0	0	-1	0	0	0	0	0	0	0	0	0	0	0	0	0
EVA1A	1	0	0	0	0	0	0	0	0	0	0	0	0	0	0	0	0	0
FAM83H	1	0	0	0	0	0	0	0	0	1	0	0	0	0	0	0	0	0
PARD6G	0	0	0	0	1	0	0	0	0	0	0	0	0	0	0	0	0	0
MBP	0	0	0	0	0	0	0	0	0	1	0	0	0	0	0	0	0	0
TSHZ1	0	1	0	0	0	0	0	0	0	0	0	0	0	0	0	0	0	0
ZADH2	0	1	0	0	0	0	0	0	0	0	0	0	0	0	0	0	0	0
NETO1	0	0	0	0	0	0	0	0	0	0	1	0	0	0	0	1	0	0
TMX3	0	0	0	0	0	0	0	0	0	1	0	0	0	0	0	0	0	0
DSEL	0	0	0	0	0	0	0	0	0	1	0	0	0	0	0	0	0	0
SERPINB8	0	1	0	0	0	0	0	0	0	0	0	0	0	0	0	0	0	0
KDSR	0	0	0	0	0	0	0	0	0	1	0	0	0	0	0	0	0	0
ENSCAFG00000000071	0	1	0	0	0	0	0	0	0	0	0	0	0	0	0	0	0	0
PYM1	0	1	0	0	-1	0	0	0	0	0	0	0	0	0	0	0	0	0
PIGN	0	0	0	0	0	0	0	0	0	1	0	0	0	0	0	0	0	0
SUOX	0	1	0	0	-1	0	0	0	0	0	0	0	0	0	0	0	0	0
CCBE1	0	1	0	0	0	0	0	0	0	0	0	0	0	0	0	0	0	0
ZC3H10	0	1	0	0	0	0	0	0	0	0	0	0	0	0	0	0	0	0
MYL6B	0	0	0	1	-1	0	0	0	0	0	0	0	0	0	0	0	0	0
RNF41	0	0	0	0	0	0	0	0	0	1	0	0	0	0	0	0	0	0
TIMELESS	0	0	0	0	1	0	0	0	0	-1	0	0	0	0	0	0	0	0
FECH	0	1	0	0	-1	0	0	0	0	0	0	0	0	0	0	0	0	0
TCF4	0	0	0	0	1	0	0	0	0	0	0	0	0	0	0	0	0	0
HSD17B6	0	0	0	0	0	0	0	0	0	1	0	0	0	0	0	0	0	0
STARD6	0	1	0	0	0	0	0	0	0	0	0	0	0	0	0	0	0	0
STAT6	0	1	0	0	0	0	0	0	0	0	0	0	0	0	0	0	0	0
TNFAIP8	0	0	0	0	1	0	0	0	0	0	0	0	0	0	0	0	0	0
ELAC1	0	1	0	0	0	0	0	0	0	0	0	0	0	0	0	0	0	0
DMXL1	0	0	0	0	0	0	0	0	0	1	0	0	0	0	0	0	0	0
MOXD1	0	0	0	0	0	0	0	0	1	1	0	0	0	0	0	0	0	0
SLC18B1	0	0	0	0	0	0	0	0	0	1	0	0	0	0	0	0	0	0
EYA4	0	0	1	1	0	1	0	1	0	0	-1	1	0	1	0	-1	0	0
NDUFA4L2	0	-1	0	0	0	0	0	0	0	1	0	0	0	0	0	0	0	0
TCF21	0	0	0	1	0	1	0	1	0	1	0	0	0	0	0	0	0	0
CDO1	0	0	0	0	0	0	0	0	1	0	0	0	0	0	0	0	0	0
FEM1C	0	0	0	0	0	0	0	0	1	0	0	0	0	0	0	0	0	0
SLC2A12	0	0	0	0	0	0	0	0	0	1	0	0	0	0	0	0	0	0
N4BP3	0	0	0	0	1	0	0	0	0	0	0	0	0	0	0	0	0	0
PDE7B	0	0	0	0	1	0	0	0	0	0	0	0	0	0	0	0	0	0
MAP3K5	0	1	0	0	0	0	0	0	0	0	0	0	0	0	0	0	0	0
PEX7	0	1	0	0	0	0	0	0	0	0	0	0	0	0	0	0	0	0
OS9	0	0	0	0	0	0	0	0	0	1	0	0	0	0	0	0	0	0
IFNGR1	0	0	0	0	1	0	0	0	0	1	0	0	0	0	0	0	0	0
TNFAIP3	0	0	0	0	1	0	0	0	0	0	0	0	0	0	0	0	0	0
ABRACL	0	-1	0	0	1	0	0	0	0	0	0	0	0	0	0	0	0	0
CITED2	0	1	0	0	0	-1	0	0	0	1	0	0	0	0	0	0	0	0
ENSCAFG00000000288	0	0	0	0	1	1	0	0	0	0	0	0	0	0	0	0	0	0
LRIG3	0	1	0	0	0	0	0	0	0	0	0	0	0	0	0	0	0	0
PHACTR2	0	0	0	0	1	0	0	0	0	0	0	0	0	0	0	0	0	0
SLC16A7	0	1	0	0	0	0	0	0	0	0	0	0	0	0	0	0	0	0
STX11	0	0	0	0	1	0	0	0	0	1	0	0	0	0	0	0	0	0
FBXO30	0	0	0	0	1	0	0	0	0	0	0	0	0	0	0	0	0	0

SHPRH	0	0	0	0	0	0	0	0	0	1	0	0	0	0	0	0	0	0
HNRNPH1	0	0	0	0	1	0	0	0	0	-1	0	0	0	0	0	0	0	0
AVPR1A	0	0	0	0	0	0	1	0	1	0	0	0	0	0	0	0	0	0
XPOT	0	-1	0	0	0	1	0	0	0	0	0	0	0	0	0	0	0	0
TBK1	0	0	0	0	1	0	0	0	0	0	0	0	0	0	0	0	0	0
WIF1	0	0	0	1	0	0	0	1	0	0	0	0	0	0	0	0	0	0
SASH1	0	1	0	0	-1	0	0	0	0	1	0	0	0	0	0	0	0	0
ENSCAFG00000000370	0	0	0	0	0	0	0	0	0	1	0	0	0	0	0	0	0	0
IRAK3	0	1	0	0	0	0	0	0	0	1	0	0	0	0	0	0	0	0
MED23	0	0	0	0	0	0	0	0	0	1	0	0	0	0	0	0	0	0
GRIP1	0	0	0	0	0	0	0	0	0	1	0	0	0	0	0	0	0	0
PCMT1	0	0	0	0	0	0	0	0	0	1	0	0	0	0	0	0	0	0
PLEKHG1	0	0	0	0	1	0	0	0	0	0	0	0	0	0	0	0	0	0
MDM1	0	1	0	0	0	0	0	0	0	-1	0	0	0	0	0	0	0	0
C12H6orf136	0	1	0	0	0	0	0	0	0	0	0	0	0	0	0	0	0	0
AKAP12	0	0	0	0	1	0	0	0	0	0	0	0	0	0	0	0	0	0
ZBTB2	0	1	0	0	0	0	0	0	0	0	0	0	0	0	0	0	0	0
LYZ	0	0	0	0	1	0	0	0	0	0	0	0	0	0	0	0	0	0
CCDC170	0	0	0	0	0	0	0	0	1	0	0	0	0	0	0	0	0	0
ESR1	0	0	0	0	0	0	0	0	0	0	0	1	0	0	0	0	0	0
FRS2	0	0	0	0	0	0	0	0	0	1	0	0	0	0	0	0	0	0
PPP1R18	0	0	0	0	1	0	0	0	0	-1	0	0	0	0	0	0	0	0
FLOT1	0	1	0	0	0	0	0	0	0	0	0	0	0	0	0	0	0	0
PTPRB	0	0	0	0	1	0	0	0	1	0	0	0	0	0	0	0	0	0
TSPAN8	0	0	0	0	1	0	0	0	0	0	0	0	0	0	0	0	0	0
ENSCAFG00000000451	0	0	0	0	0	0	0	0	0	-1	0	0	0	0	0	0	1	0
RAB21	0	0	0	0	1	0	0	0	0	0	0	0	0	0	0	0	0	0
TBC1D15	0	0	0	0	1	0	0	0	0	0	0	0	0	0	0	0	0	0
LAPTM4B	0	0	0	0	1	0	0	0	0	1	0	0	0	0	0	0	0	0
CNOT6	0	0	0	0	1	0	0	0	0	0	0	0	0	0	0	0	0	0
FLT4	0	0	0	0	1	0	0	0	0	0	0	0	0	0	0	0	0	0
DLA88	0	0	0	0	0	0	0	0	0	1	0	0	0	0	0	0	0	0
OSR2	0	1	0	0	0	0	0	0	0	0	0	0	0	0	0	0	0	0
VPS13B	0	0	0	0	0	0	0	0	0	1	0	0	0	0	0	0	0	0
PRR16	0	1	0	0	0	0	0	0	1	0	1	0	0	0	0	0	0	0
SNCAIP	0	0	0	0	1	0	0	0	0	0	0	0	0	0	0	0	0	0
RGS22	0	1	0	0	0	0	0	0	0	1	0	0	0	0	0	0	0	0
SNX2	0	0	0	0	1	0	0	0	0	0	0	0	0	0	0	0	0	0
MYCT1	0	0	0	0	1	0	0	0	0	0	0	0	0	0	0	0	0	0
CEP120	0	0	0	0	1	0	0	0	0	0	0	0	0	0	0	0	0	0
FBXO5	0	0	0	0	1	0	0	0	0	-1	0	0	0	0	0	0	0	0
RGS17	0	1	0	0	0	0	0	0	0	0	0	0	0	0	0	0	0	0
RNF19A	0	0	0	0	0	0	0	0	0	1	0	0	0	0	0	0	0	0
CSNK1G3	0	0	0	0	1	0	0	0	0	0	0	0	0	0	0	0	0	0
ARSA	0	1	0	0	-1	0	0	0	0	0	0	0	0	0	0	0	0	0
ALDH7A1	0	1	0	0	0	0	0	0	0	0	0	0	0	0	0	0	0	0
RRM2B	0	0	0	0	0	1	0	0	0	0	0	0	0	0	0	0	0	0
ABHD16A	0	1	0	0	0	0	0	0	0	0	0	0	0	0	0	0	0	0
DDAH2	0	1	0	0	0	0	0	0	0	0	0	0	0	0	0	0	0	0
ZDHHC14	0	0	0	0	1	0	0	0	0	0	0	0	0	0	0	0	0	0
ENSCAFG00000000626	0	1	0	0	0	0	0	0	0	0	0	0	0	0	0	0	0	0
ENSCAFG00000000640	0	1	0	0	0	0	0	0	0	0	0	0	0	0	0	0	0	0
SERAC1	0	0	0	0	1	0	0	0	0	0	0	0	0	0	0	0	0	0
TMEM181	0	0	0	0	0	0	0	0	0	1	0	0	0	0	0	0	0	0
CTHRC1	0	0	0	0	1	0	0	0	0	0	0	0	0	0	0	0	0	0
RIMS2	0	0	0	0	1	0	0	0	0	0	0	0	0	0	0	0	0	0
SOD2	0	1	0	0	0	-1	0	0	1	0	0	0	0	0	0	0	0	0
PLXNB2	0	0	0	0	-1	0	0	0	0	1	0	0	0	0	0	0	0	0
OXR1	0	0	0	0	0	0	0	0	0	1	0	0	0	0	0	0	0	0
FBN2	0	0	0	0	0	0	0	0	0	-1	0	1	0	0	0	0	0	0
HDAC10	0	1	0	0	0	0	0	0	0	0	0	0	0	0	0	0	0	0
SELENOO	0	1	0	0	0	0	0	0	0	0	0	0	0	0	0	0	0	0
ANGPT1	0	0	0	0	-1	0	0	0	0	1	0	0	0	0	0	0	0	0
ENSCAFG00000000701	0	0	0	0	0	0	0	0	0	1	0	0	0	0	0	0	0	0
SLC27A6	0	1	0	0	0	0	0	0	0	0	0	0	0	0	0	0	0	0
FAM19A5	0	0	0	0	0	0	0	0	0	1	0	0	0	0	0	0	0	0
SYBU	0	1	0	0	0	0	0	0	0	0	0	0	0	0	0	0	0	0
TNXB	0	1	0	0	0	0	0	0	1	0	0	0	0	0	0	0	0	0
CERK	0	1	0	0	0	0	0	0	0	0	0	0	0	0	0	0	0	0
CELSR1	0	0	0	0	1	0	0	0	0	0	0	0	0	0	0	0	0	0
QKI	0	0	0	0	1	0	0	0	0	0	0	0	0	0	0	0	0	0
NOTCH4	0	0	0	0	1	0	0	0	0	0	0	0	0	0	0	0	0	0
ACSL6	0	0	0	0	0	0	0	0	0	1	0	0	0	0	0	0	0	0
FBLN1	0	1	0	0	0	0	0	0	0	0	0	0	0	0	0	0	0	0
RAD21	0	0	0	0	1	0	0	0	0	0	0	0	0	0	0	0	0	0
RPS6KA2	0	1	0	0	-1	0	0	0	0	0	0	0	0	0	0	0	0	0
TNFRSF11B	0	0	0	0	0	0	0	0	0	1	0	0	0	0	0	0	0	0
ENSCAFG00000000842	0	0	0	0	0	0	0	0	0	1	0	0	0	0	0	0	0	0
AFDN	0	0	0	0	1	0	0	0	0	0	0	0	0	0	0	0	0	0
ENPP2	0	1	0	0	0	0	0	0	0	0	0	0	0	0	0	0	0	0
SMOC2	0	0	0	0	0	0	0	0	0	1	0	0	0	0	0	0	0	0
WDR27	0	1	0	0	0	0	0	0	0	0	0	0	0	0	0	0	0	0
DSE	0	1	0	0	-1	0	0	0	0	0	0	0	0	0	0	0	0	0
CALHM5	0	0	0	0	0	0	0	0	1	1	0	0	0	0	0	0	0	0
EFCAB6	0	0	0	0	0	0	1	0	0	0	0	0	0	0	0	0	0	0
DCBLD1	0	0	0	0	1	0	0	0	0	0	0	0	0	0	0	0	0	0
HSD17B8	0	1	0	0	0	0	0	0	0	0	0	0	0	0	0	0	0	0
AFF4	0	0	0	0	1	0	0	0	0	1	0	0	0	0	0	0	0	0
COL14A1	0	1	0	0	0	0	0	0	0	0	0	0	0	0	0	0	0	0
CEP85L	0	0	0	0	1	0	0	0	0	0	0	0	0	0	0	0	0	0
PACSIN2	0	-1	0	0	1	0	0	0	0	0	0	0	0	0	0	0	0	0
ASF1A	0	0	0	0	1	0	0	0	0	0	0	0	0	0	0	0	0	0
A4GALT	0	1	0	0	-1	0	0	0	1	0	0	0	0	0	0	0	0	0
B3GALT4	0	1	0	0	0	0	0	0	0	0	0	0	0	0	0	0	0	0
CYB5R3	0	1	0	0	-1	0	0	0	0	0	0	0	0	0	0	0	0	0
GJA1	0	0	0	0	0	0	0	0	0	1	0	0	0	0	0	0	0	0
RGL2	0	0	0	0	1	0	0	0	0	0	0	0	0	0	0	0	0	0
SERHL2	0	0	0	0	0	0	0	0	0	1	0	0	0	0	0	0	0	0
ZBTB22	0	1	0	0	0	0	0	0	0	0	0	0	0	0	0	0	0	0
SERINC1	0	0	0	0	0	0	0	0	0	1	0	0	0	0	0	0	0	0
TCF7	0	0	0	0	0	0	0	0	0	0	1	0	0	0	0	0	0	0
PHF1	0	1	0	0	0	0	0	0	0	0	0	0	0	0	0	0	0	0
ZHX1	0	0	0	0	0	0	0	0	0	1	0	0	0	0	0	0	0	0
SMPDL3A	0	1	0	0	0	0	0	0	0	0	0	0	0	0	0	0	0	0
PPP2CA	0	0	0	0	1	0	0	0	0	0	0	0	0	0	0	0	0	0
JADE2	0	1	0	0	0	0	0	0	0	0	0	0	0	0	0	0	0	0
FAM91A1	0	-1	0	0	1	0	0	0	0	0	0	0	0	0	0	0	0	0
TPD52L1	0	0	0	0	0	0	0	0	0	1	0	0	0	0	0	0	0	0
TATDN1	0	0	0	0	0	0	0	0	0	1	0	0	0	0	0	0	0	0
DESI1	0	-1	0	0	1	0	0	0	0	-1	0	0	0	0	0	0	0	0
NCOA7	0	0	0	0	1	0	0	0	0	0	0	0	0	0	0	0	0	0
PMM1	0	1	0	0	0	0	0	0	0	0	0	0	0	0	0	0	0	0
MTSS1	0	0	0	0	1	0	1	0	0	0	0	0	0	0	0	0	0	0
CSDC2	0	0	0	0	0	0	0	0	1	0	0	0	0	0	0	0	0	0
PITX1	0	0	0	0	0	0	0	0	0	0	1	0	0	0	0	0	0	0
MYC	0	1	0	0	0	0	0	0	0	0	0	0	0	0	0	0	0	0
TGFBI	0	0	0	0	0	0	0	0	0	0	-1	0	0	1	0	0	0	0
TOB2	0	1	0	0	0	0	0	0	0	0	0	0	0	0	0	0	0	0
ASAP1	0	0	0	0	0	0	0	0	0	1	0	0	0	0	0	0	0	0
TMEM71	0	0	0	0	1	0	0	0	0	-1	0	0	0	0	0	0	0	0
ARHGAP18	0	0	0	0	1	0	0	0	0	1	0	0	0	0	0	0	0	0
L3MBTL3	0	0	0	0	1	0	0	0	0	0	0	0	0	0	0	0	0	0
TMEM200A	0	0	0	0	0	0	0	0	0	1	0	0	0	0	0	0	0	0
EP300	0	0	0	0	0	0	0	0	0	1	0	0	0	0	0	0	0	0
KLHL3	0	-1	0	0	1	0	0	0	0	0	0	0	0	0	0	0	0	0
ENSCAFG00000001129	0	0	0	0	1	1	0	0	0	0	0	0	0	0	0	0	0	0
ITPR3	0	1	0	0	0	0	0	0	0	-1	0	0	0	0	0	0	0	0
WISP1	0	0	1	0	0	0	0	0	1	-1	0	0	0	0	0	0	0	0
CDC14B	0	1	0	0	0	0	0	0	0	0	0	0	0	0	0	0	0	0
ZNF367	0	1	0	0	0	0	0	0	0	-1	0	0	0	0	0	0	0	0

PTK2	0	0	0	0	1	0	0	0	0	0	0	0	0	0	0	0	0	0
ERCC6L2	0	1	0	0	0	0	0	0	0	0	0	0	0	0	0	0	0	0
FAM53C	0	0	0	0	0	0	0	0	0	1	0	0	0	0	0	0	0	0
DENND3	0	0	0	0	1	0	0	0	0	0	0	0	0	0	0	0	0	0
KDM3B	0	1	0	0	0	0	0	0	0	0	0	0	0	0	0	0	0	0
REEP2	0	0	0	0	0	0	0	0	1	0	0	0	0	0	0	0	0	0
PTCH1	0	1	0	0	0	0	0	0	0	0	0	0	0	0	0	0	0	0
ADGRB1	0	0	0	0	0	0	0	0	0	1	0	0	0	0	0	0	0	0
ENSCAFG00000001272	0	0	0	0	0	0	0	0	0	1	0	0	0	0	0	0	0	0
ANKS1A	0	0	0	0	1	0	0	0	0	0	0	0	0	0	0	0	0	0
LY6E	0	0	0	0	0	0	0	0	0	1	0	0	0	0	0	0	0	0
RHPN1	0	1	0	0	0	0	0	0	0	0	0	0	0	0	0	0	0	0
ZNF76	0	1	0	0	0	0	0	0	0	0	0	0	0	0	0	0	0	0
FBP1	0	0	0	0	0	1	0	0	0	0	0	0	0	0	0	1	0	0
DEF6	0	1	0	0	0	0	0	0	0	-1	0	0	0	0	0	0	0	0
CTNNA1	0	0	0	0	1	0	0	0	0	0	0	0	0	0	0	0	0	0
DAPK1	0	0	0	0	0	0	0	0	0	1	0	0	0	0	0	0	0	0
TUT7	0	0	0	0	1	0	0	0	0	0	0	0	0	0	0	0	0	0
TAB1	0	1	0	0	0	0	0	0	0	0	0	0	0	0	0	0	0	0
ISCA1	0	0	0	0	0	0	0	0	0	1	0	0	0	0	0	0	0	0
PDGFB	0	0	0	0	1	0	0	0	0	0	0	0	0	0	0	0	0	0
CBX7	0	1	0	0	0	0	0	0	0	1	0	0	0	0	0	0	0	0
PLXNA4	0	1	0	0	0	0	0	0	1	0	0	0	0	0	0	0	0	0
SRPK1	0	0	0	0	1	0	0	0	0	-1	0	0	0	0	0	0	0	0
ENSCAFG00000001367	0	1	0	0	0	0	0	0	0	0	0	0	0	0	0	0	0	0
GTPBP1	0	1	0	0	0	0	0	0	0	0	0	0	0	0	0	0	0	0
NTRK2	0	0	0	1	0	0	0	0	0	0	0	0	0	0	0	0	0	0
UHRF2	0	0	0	0	1	0	0	0	0	0	0	0	0	0	0	0	0	0
MAPK13	0	0	0	0	1	0	0	0	0	0	0	0	0	0	0	0	0	0
BRPF3	0	0	0	0	1	0	0	0	0	0	0	0	0	0	0	0	0	0
DMC1	0	0	0	0	0	0	0	0	0	1	0	0	0	0	0	0	0	0
PODXL	0	0	0	0	1	0	0	0	0	0	0	0	0	0	0	0	0	0
NRBP2	0	1	0	0	-1	0	0	0	0	1	0	0	0	0	0	0	0	0
MKLN1	0	-1	0	0	1	0	0	0	0	0	0	0	0	0	0	0	0	0
KCNJ4	0	0	0	0	0	0	0	0	0	1	0	0	0	0	0	0	0	0
KIF27	0	1	0	0	0	0	0	0	0	0	0	0	0	0	0	0	0	0
PLA2G6	0	1	0	0	0	0	0	0	0	0	0	0	0	0	0	0	0	0
PI16	0	0	0	0	0	0	0	0	1	0	0	0	0	0	0	0	0	0
C10H22orf23	0	1	0	0	0	0	0	0	0	0	0	0	0	0	0	0	0	0
MICALL1	0	0	0	0	1	0	0	0	0	0	0	0	0	0	0	0	0	0
EIF3L	0	1	0	0	0	0	0	0	0	0	0	0	0	0	0	0	0	0
PIM1	0	0	0	0	1	0	0	0	0	0	0	0	0	0	0	0	0	0
ZFAND3	0	0	0	0	0	0	0	0	0	1	0	0	0	0	0	0	0	0
MPDZ	0	0	0	0	0	0	0	0	0	1	0	0	0	0	0	0	0	0
OPLAH	0	1	0	0	0	0	0	0	0	0	0	0	0	0	0	0	0	0
MFNG	0	0	0	0	1	0	0	0	0	0	0	0	0	0	0	0	0	0
MPST	0	1	0	0	0	0	0	0	0	0	0	0	0	0	0	0	0	0
NFIB	0	1	0	0	0	0	0	0	0	0	0	0	0	0	0	0	0	0
AHCYL2	0	0	0	0	0	0	0	0	0	1	0	0	0	0	0	0	0	0
ENSCAFG00000001530	0	0	1	0	1	1	0	0	0	0	0	0	0	0	0	0	0	0
ZDHHC21	0	0	0	0	0	0	0	0	0	1	0	0	0	0	0	0	0	0
TLE4	0	0	0	0	0	0	0	0	0	1	0	0	0	0	0	0	0	0
ENSCAFG00000001540	0	1	0	0	0	0	0	0	0	0	0	0	0	0	0	0	0	0
EIF3D	0	1	0	0	0	0	0	0	0	0	0	0	0	0	0	0	0	0
FOXRED2	0	1	0	0	0	0	0	0	0	0	0	0	0	0	0	0	0	0
DAAM2	0	0	1	0	0	0	0	0	0	0	0	0	0	0	0	0	0	0
FBXL6	0	1	0	0	0	0	0	0	0	0	0	0	0	0	0	0	0	0
GNAQ	0	-1	0	0	0	0	0	0	0	1	0	0	0	0	0	0	0	0
NFYA	0	0	0	0	1	0	0	0	0	0	0	0	0	0	0	0	0	0
MDFI	0	0	0	0	1	0	0	0	0	0	0	0	0	0	0	0	0	0
PLIN2	0	0	0	0	0	0	0	0	0	1	0	0	0	0	0	0	0	0
ENSCAFG00000001604	0	1	0	0	0	0	0	0	0	0	0	0	0	0	0	0	0	0
ENSCAFG00000001608	0	0	0	0	1	0	0	0	0	0	0	0	0	0	0	0	0	0
CCND3	0	1	0	0	-1	0	0	0	0	0	0	0	0	0	0	0	0	0
TAF8	0	1	0	0	0	0	0	0	0	0	0	0	0	0	0	0	0	0
MLLT3	0	0	0	0	0	0	0	0	0	1	0	0	0	0	0	0	0	0
TRERF1	0	1	0	0	0	0	0	0	0	0	0	0	0	0	0	0	0	0
FAM71F2	0	-1	0	0	0	0	0	0	1	1	0	0	0	0	0	0	0	0
HACD4	0	1	0	0	0	0	0	0	0	0	0	0	0	0	0	0	0	0
MFSD3	0	1	0	0	-1	0	0	0	0	0	0	0	0	0	0	0	0	0
LEP	0	0	0	0	0	0	0	0	1	1	0	0	0	0	0	0	0	0
COMMD5	0	1	0	0	0	0	0	0	0	0	0	0	0	0	0	0	0	0
CDKN2A	0	0	0	0	-1	0	0	0	0	1	0	0	0	0	0	0	0	0
CDKN2B	0	0	0	0	0	0	0	0	0	1	0	0	0	0	0	0	0	0
C8orf33	0	1	0	0	0	0	0	0	0	0	0	0	0	0	0	0	0	0
OSTF1	0	0	0	0	1	0	0	0	0	0	0	0	0	0	0	0	0	0
NMRK1	0	0	0	0	0	0	0	0	0	1	0	0	0	0	0	0	0	0
APOL6	0	0	0	0	0	0	0	0	0	1	0	0	0	0	0	0	0	0
HMOX1	0	1	0	0	0	0	0	0	0	0	0	0	0	0	0	0	0	0
TEK	0	0	0	0	1	0	0	0	0	0	0	0	0	0	0	0	0	0
MOB3B	0	1	0	0	0	0	0	0	0	0	0	0	0	0	0	0	0	0
TOM1	0	1	0	0	-1	0	0	0	0	0	0	0	0	0	0	0	0	0
BICRAL	0	1	0	0	0	0	0	0	0	0	0	0	0	0	0	0	0	0
ZNF800	0	0	0	0	1	0	0	0	0	0	0	0	0	0	0	0	0	0
GRM8	0	0	0	0	1	0	0	0	0	0	0	0	0	0	0	0	0	0
PEX6	0	1	0	0	0	0	0	0	0	0	0	0	0	0	0	0	0	0
FBXO7	0	1	0	0	0	0	0	0	0	0	0	0	0	0	0	0	0	0
WASL	0	0	0	0	0	0	0	0	0	1	0	0	0	0	0	0	0	0
WFS1	0	0	0	0	0	0	0	0	0	1	0	0	0	0	0	0	0	0
LIMCH1	0	0	0	0	1	0	0	1	0	1	0	0	0	0	0	0	0	0
RIC8B	0	0	0	0	1	0	0	0	0	1	0	0	0	0	0	0	0	0
DDX58	0	0	0	0	0	0	0	0	0	1	0	0	0	0	0	0	0	0
ZFAND5	0	1	0	0	0	0	0	0	0	0	0	0	0	0	0	0	0	0
PTK7	0	-1	0	0	0	1	0	0	0	0	0	0	0	0	0	0	0	0
GDA	0	1	0	0	0	0	0	0	0	0	0	0	0	0	0	0	0	0
ATP8A1	0	0	0	0	1	0	0	0	0	0	0	0	0	0	0	0	0	0
ABHD17B	0	1	0	0	0	0	0	0	0	0	0	0	0	0	0	0	0	0
ENSCAFG00000001830	0	0	0	0	1	0	0	0	0	0	0	0	0	0	0	0	0	0
ABCB4	0	0	0	0	0	0	0	0	1	0	0	0	0	0	0	0	0	0
TTBK1	0	0	0	0	0	0	0	0	1	0	0	0	0	0	0	0	0	0
SLC25A40	0	1	0	0	0	0	0	0	0	0	0	0	0	0	0	0	0	0
ENSCAFG00000001846	0	0	0	0	0	0	0	0	0	1	0	0	0	0	0	0	0	0
ADAM22	0	0	1	0	0	0	0	0	0	1	0	0	0	0	0	0	0	0
SRI	0	1	0	0	0	0	0	0	0	0	0	0	0	0	0	0	0	0
ENSCAFG00000001866	0	1	0	0	0	0	0	0	0	0	0	0	0	0	0	0	0	0
KLF9	0	1	0	0	0	0	0	0	0	0	0	0	0	0	0	0	0	0
TCP11L2	0	1	0	0	0	0	0	0	0	1	0	0	0	0	0	0	0	0
CFAP69	0	1	0	0	0	0	0	0	0	0	0	0	0	0	0	0	0	0
SMC5	0	0	0	0	1	0	0	0	0	0	0	0	0	0	0	0	0	0
MAMDC2	0	0	0	0	1	0	0	0	0	0	0	0	0	0	0	0	0	0
TJP2	0	0	0	0	1	0	0	0	0	0	0	0	0	0	0	0	0	0
FZD1	0	1	0	0	0	0	0	0	0	0	0	0	0	0	0	0	0	0
PIP5K1B	0	0	0	0	1	0	0	0	0	1	0	0	0	0	0	0	0	0
FAM122A	0	0	0	0	0	0	0	0	0	1	0	0	0	0	0	0	0	0
AKAP9	0	0	0	0	1	0	0	0	0	0	0	0	0	0	0	0	0	0
ANKIB1	0	0	0	0	0	0	0	0	0	1	0	0	0	0	0	0	0	0
KANK1	0	0	0	0	0	0	0	0	1	0	0	0	0	0	0	0	0	0
DMRT2	0	1	0	0	0	0	0	0	0	0	1	0	0	0	0	0	0	0
CDK6	0	0	0	0	0	0	0	0	0	1	0	0	0	0	0	0	0	0
DCUN1D4	0	0	0	0	0	0	0	0	0	1	0	0	0	0	0	0	0	0
SGCB	0	0	0	0	0	0	0	0	0	1	0	0	0	0	0	0	0	0
SMARCA2	0	1	0	0	0	0	0	0	0	0	0	0	0	0	0	0	0	0
ENSCAFG00000002015	0	-1	0	0	1	1	0	0	0	0	0	0	0	0	0	0	0	0
CLIC5	0	1	0	0	0	0	0	0	0	0	0	0	0	0	0	0	0	0
FAM214B	0	0	0	0	0	0	0	0	0	1	0	0	0	0	0	0	0	0
RASL11B	0	0	0	0	0	0	0	0	0	1	0	0	0	0	0	0	0	0
ENPP4	0	0	0	0	0	0	0	0	0	1	0	0	0	0	0	0	0	0
RCAN2	0	0	0	0	0	0	0	0	0	1	0	0	0	0	0	0	0	0
GCC2	0	0	0	0	1	1	0	0	0	0	0	0	0	0	0	0	0	0
SCFD2	0	1	0	0	0	0	0	0	0	0	0	0	0	0	0	0	0	0

PUM3	0	-1	0	0	0	1	0	0	0	-1	0	0	0	0	0	0	0	0
TFPI2	0	0	0	0	0	0	0	0	1	-1	0	0	0	0	0	0	0	0
ENSCAFG00000002042	0	0	0	0	0	0	0	0	0	0	0	1	0	0	0	0	0	0
GNG11	0	0	0	0	1	0	0	0	0	0	0	0	0	0	0	0	0	0
RUSC2	0	0	0	0	0	0	0	0	1	1	0	0	0	0	0	0	0	0
CHIC2	0	0	0	0	1	0	0	0	0	0	0	0	0	0	0	0	0	0
ADGRF5	0	0	0	0	1	0	0	0	0	0	0	0	0	0	0	0	0	0
KIT	0	0	0	0	1	0	0	0	0	0	0	0	0	0	0	0	0	0
SLC1A1	0	0	0	0	0	0	0	0	0	1	0	1	0	0	0	0	0	0
SPATA6L	0	0	0	0	0	0	0	0	0	1	0	0	0	0	0	0	0	0
PLPP6	0	1	0	0	0	0	0	0	0	0	0	0	0	0	0	0	0	0
CA9	0	0	0	0	0	0	1	0	0	0	0	0	0	0	0	0	0	0
ENSCAFG00000002078	0	0	0	0	1	0	0	0	0	0	0	0	0	0	0	0	0	0
KDR	0	0	0	0	1	0	0	0	0	0	0	0	0	0	0	0	0	0
ENSCAFG00000002083	0	1	0	0	0	0	0	0	0	0	0	0	0	0	0	0	0	0
CD2AP	0	0	0	0	1	0	0	0	0	0	0	0	0	0	0	0	0	0
RCL1	0	1	0	0	0	0	0	0	0	0	0	0	0	0	0	0	0	0
CASD1	0	0	0	0	0	0	0	0	0	1	0	0	0	0	0	0	0	0
PPP1R9A	0	0	0	0	0	0	0	0	0	1	0	0	0	0	0	0	0	0
C10H2orf49	0	0	0	0	0	0	0	0	0	1	0	0	0	0	0	0	0	0
CD274	0	1	0	0	0	0	0	0	0	1	0	0	0	0	0	0	0	0
PDCD1LG2	0	1	0	0	0	0	0	0	0	0	0	0	0	0	0	0	0	0
RIC1	0	1	0	0	0	0	0	0	1	1	0	0	0	0	0	0	0	0
IL18R1	0	0	0	0	0	0	0	0	0	1	0	0	0	0	0	0	0	0
ENSCAFG00000002139	0	0	1	0	1	1	0	0	0	0	0	0	0	0	0	0	0	0
ENSCAFG00000002155	0	0	0	0	1	0	0	0	0	0	0	0	0	0	0	0	0	0
ENSCAFG00000002166	0	0	0	0	1	1	0	0	0	0	0	0	0	0	0	0	0	0
RFX8	0	1	0	0	0	0	0	0	0	0	0	0	0	0	0	0	0	0
RNF149	0	0	0	0	1	0	0	0	0	1	0	0	0	0	0	0	0	0
CNOT11	0	1	0	0	0	0	0	0	0	0	0	0	0	0	0	0	0	0
KIAA1211	0	0	0	0	0	0	0	0	0	0	0	0	0	0	1	0	0	0
CREB3	0	0	0	0	0	0	0	0	0	1	0	0	0	0	0	0	0	0
TBC1D8	0	1	0	0	0	0	0	0	0	0	0	0	0	0	0	0	0	0
NPAS2	0	1	0	0	0	0	0	0	0	0	0	0	0	0	0	0	0	0
BARX1	0	0	0	0	0	1	0	0	0	0	0	0	0	0	1	0	0	0
HINT2	0	1	0	0	0	0	0	0	0	0	0	0	0	0	0	0	0	0
GLCCI1	0	0	0	0	0	0	0	0	0	1	0	0	0	0	0	0	0	0
ARL9	0	0	0	0	0	0	0	0	0	1	0	0	0	0	0	0	0	0
ICA1	0	0	0	0	1	0	0	0	0	0	0	0	0	0	0	0	0	0
PHF14	0	0	0	0	1	0	0	0	0	0	0	0	0	0	0	0	0	0
THSD7A	0	0	0	0	1	0	0	0	0	0	0	0	0	0	0	0	0	0
RECK	0	1	0	0	-1	0	0	0	1	1	0	0	0	0	0	0	0	0
ENSCAFG00000002290	0	0	0	0	0	1	0	0	0	0	0	0	0	0	0	0	0	0
MITD1	0	0	0	0	0	0	0	0	0	1	0	0	0	0	0	0	0	0
ECM2	0	1	0	0	-1	0	0	0	0	1	0	0	0	0	0	0	0	0
ENSCAFG00000002306	0	0	0	0	0	0	0	0	0	1	0	0	0	0	0	0	0	0
OMD	0	1	0	0	-1	0	0	0	0	0	0	0	0	0	0	0	0	0
OGN	0	1	0	0	0	0	0	0	0	0	0	0	0	0	0	0	0	0
ARL4A	0	1	0	0	0	0	0	0	0	0	0	0	0	0	0	0	0	0
MGAT4A	0	0	0	0	1	0	0	0	0	0	0	0	0	0	0	0	0	0
NOL8	0	0	0	0	1	0	0	0	0	-1	0	0	0	0	0	0	0	0
IGFBP7	0	-1	0	0	0	0	0	0	0	1	0	0	0	0	0	0	0	0
ENSCAFG00000002336	0	0	1	0	0	0	0	0	0	0	0	0	0	0	0	0	0	0
ENSCAFG00000002360	0	1	0	0	0	0	0	0	0	0	0	0	0	0	0	0	0	0
FBXO10	0	0	0	0	0	0	1	0	0	0	0	0	0	0	0	0	0	0
FRMPD1	0	0	0	0	0	0	0	0	0	1	0	0	0	0	0	0	0	0
ZSCAN18	0	0	0	0	0	0	0	0	0	1	0	0	0	0	0	0	0	0
CCDC180	0	0	0	0	0	0	0	0	0	1	0	0	0	0	0	0	0	0
CNNM4	0	0	0	0	0	0	0	0	1	0	0	0	0	0	0	0	0	0
LMAN2L	0	0	0	0	0	0	0	0	0	1	0	0	0	0	0	0	0	0
TDRD7	0	0	0	0	0	0	0	0	0	1	0	0	0	0	0	0	0	0
MEOX2	0	1	0	0	1	0	1	0	0	0	0	0	0	0	0	0	0	0
ZNF550	0	0	0	0	0	0	0	0	0	1	0	0	0	0	0	0	0	0
TSPAN13	0	0	0	0	1	0	0	0	0	0	0	0	0	0	0	0	0	0
AHR	0	0	0	0	1	0	0	0	0	0	0	0	0	0	0	0	0	0
TRMO	0	1	0	0	0	0	0	0	0	0	0	0	0	0	0	0	0	0
SNX13	0	0	0	0	0	0	0	0	0	1	0	0	0	0	0	0	0	0
GABBR2	0	1	0	0	0	0	0	0	0	0	0	0	0	0	0	0	0	0
PTP4A1	0	-1	0	0	1	0	0	0	0	0	0	0	0	0	0	0	0	0
PHF3	0	0	0	0	1	0	0	0	0	0	0	0	0	0	0	0	0	0
HDAC9	0	0	0	0	0	0	0	0	1	-1	0	0	0	0	0	0	0	0
C15H1orf50	0	1	0	0	0	0	0	0	0	1	0	0	0	0	0	0	0	0
ENSCAFG00000002523	0	0	0	0	1	1	0	0	0	0	0	0	0	0	0	0	0	0
TGFBR1	0	-1	0	0	0	0	0	0	0	1	-1	0	0	0	0	0	0	0
PREPL	0	0	0	0	0	0	0	0	0	1	0	0	0	0	0	0	0	0
LMBRD1	0	0	0	0	0	0	0	0	0	1	0	0	0	0	0	0	0	0
SSC5D	0	1	0	0	-1	0	0	0	0	0	0	0	0	0	0	0	0	0
MSANTD3-TMEFF1	0	0	0	0	1	0	0	0	0	0	0	0	0	0	0	0	0	0
FAM135A	0	0	0	0	0	1	0	0	0	0	0	0	0	0	0	0	0	0
PRKCE	0	0	0	0	0	0	0	0	0	1	0	0	0	0	0	0	0	0
DNAAF3	0	1	0	0	-1	0	0	0	0	0	0	0	0	0	0	0	0	0
EPAS1	0	0	0	0	0	0	0	0	1	0	0	0	0	0	0	0	0	0
SLC17A5	0	0	0	0	0	0	0	0	1	1	0	0	0	0	0	0	0	0
GTF2A1L	0	0	0	0	0	0	0	0	0	1	0	0	0	0	0	0	0	0
ENSCAFG00000002703	0	1	0	0	-1	0	1	0	0	0	0	0	0	0	0	0	0	0
FILIP1	0	0	0	0	1	0	0	0	0	1	0	0	0	0	0	0	0	0
SENP6	0	0	0	0	1	0	0	0	0	0	0	0	0	0	0	0	0	0
ABCA1	0	0	0	0	1	0	0	0	0	1	0	0	0	0	0	0	0	0
PSME4	0	0	0	0	1	0	0	0	0	0	0	0	0	0	0	0	0	0
IL6	0	0	0	0	1	0	0	0	0	0	0	0	0	0	0	0	0	0
ZNF331	0	0	0	0	0	0	0	0	0	1	0	0	0	0	0	0	0	0
KLHL7	0	0	0	0	0	0	0	0	0	1	0	0	0	0	0	0	0	0
CENPC	0	0	0	0	1	0	0	0	0	0	0	0	0	0	0	0	0	0
SLC44A1	0	1	0	0	0	0	0	0	0	0	0	0	0	0	0	0	0	0
EML6	0	0	0	0	0	0	0	0	0	1	0	0	0	0	0	0	0	0
IRAK1BP1	0	1	0	0	0	0	0	0	0	0	0	0	0	0	0	0	0	0
KLF4	0	1	0	0	0	0	0	0	0	0	0	0	0	0	0	0	0	0
ENSCAFG00000002800	0	0	0	0	0	0	0	0	0	1	0	0	0	0	0	0	0	0
ACTL7A	0	1	0	0	0	0	0	0	0	0	0	0	0	0	0	0	0	0
HAS1	0	0	0	0	0	0	0	1	0	0	0	0	0	0	0	0	0	0
YTHDC1	0	0	0	0	1	0	0	0	0	0	0	0	0	0	0	0	0	0
LCA5	0	0	0	0	1	0	0	0	0	0	0	0	0	0	0	0	0	0
SH3BGRL2	0	0	0	0	1	0	0	0	0	0	0	0	0	0	0	0	0	0
CTNNAL1	0	0	1	0	1	0	0	0	0	0	0	0	0	0	0	0	0	0
TMEM245	0	0	0	0	0	0	0	0	0	1	0	0	0	0	0	0	0	0
BCKDHB	0	1	0	0	-1	0	0	0	0	0	0	0	0	0	0	0	0	0
PPP4R3B	0	0	0	0	1	0	0	0	0	0	0	0	0	0	0	0	0	0
NFE2L3	0	0	0	0	1	0	0	0	0	1	0	0	0	0	0	0	0	0
PTPN3	0	0	0	0	0	0	0	0	0	1	0	0	0	0	0	0	0	0
ENSCAFG00000002897	0	0	0	0	1	0	0	0	0	0	0	0	0	0	0	0	0	0
SHANK1	0	0	0	0	0	0	0	0	1	0	0	0	0	0	0	0	0	0
SVEP1	0	1	0	0	-1	0	0	0	1	1	0	0	0	0	0	0	0	0
DCK	0	0	0	0	1	0	0	0	0	-1	0	0	0	0	0	0	0	0
SLC4A4	0	1	0	0	0	0	0	0	0	1	0	0	0	0	0	0	0	0
HOXA2	0	0	0	1	0	1	0	1	0	0	0	0	1	0	0	0	1	0
SNAP91	0	0	0	0	1	0	0	0	0	1	0	0	0	0	0	0	0	0
NPFFR2	0	0	0	0	0	0	0	0	1	0	0	0	0	0	0	0	0	0
HOXA6	0	1	0	0	0	0	0	0	1	0	0	0	0	0	0	0	0	0
HOXA9	0	1	1	0	0	0	1	0	1	-1	1	1	0	0	0	1	0	1
HOXA10	0	1	0	0	0	0	1	0	1	-1	1	0	0	0	0	1	0	1
HOXA11	0	1	0	0	0	0	0	0	0	0	1	0	0	0	0	1	0	0
HOXA13	0	1	0	0	0	0	0	0	0	1	1	0	0	0	0	0	0	0
PAPOLG	0	0	0	0	0	0	0	0	0	1	0	0	0	0	0	0	0	0
ENSCAFG00000002977	0	1	0	0	-1	0	0	0	0	1	0	0	0	0	0	0	0	0
LPAR1	0	1	0	0	0	0	0	0	0	0	0	0	0	0	0	0	0	0
MYBPC2	0	-1	0	0	0	0	0	1	0	0	0	0	0	0	0	0	0	0
CREB5	0	0	0	0	1	0	0	0	0	0	0	0	0	0	0	0	0	0
SNX14	0	0	0	0	0	0	0	0	0	1	0	0	0	0	0	0	0	0
UGCG	0	-1	0	0	0	0	0	0	0	1	0	0	0	0	0	0	0	0
SUSD1	0	0	0	0	0	0	0	0	0	1	0	0	0	0	0	0	0	0

CHN2	0	0	0	0	1	0	0	0	0	1	0	0	0	0	0	0	0	0
CXCL8	0	0	0	0	1	0	0	0	0	0	0	0	0	0	0	0	0	0
PPIE	0	0	0	0	1	0	0	0	0	-1	0	0	0	0	0	0	0	0
ZNF292	0	0	0	0	1	0	0	0	0	0	0	0	0	0	0	0	0	0
SLC46A2	0	0	0	0	0	0	0	0	1	0	0	0	0	0	0	0	0	0
SLC35A1	0	0	0	0	1	0	0	0	0	0	0	0	0	0	0	0	0	0
GARS	0	-1	0	0	0	1	0	0	0	-1	0	0	0	0	0	0	0	0
UBE2J1	0	0	0	0	1	0	0	0	0	0	0	0	0	0	0	0	0	0
ANKRD6	0	0	0	0	0	0	0	0	0	0	0	0	0	0	1	0	0	0
TMEM17	0	1	0	0	0	0	0	0	0	0	0	0	0	0	0	0	0	0
MACF1	0	0	0	0	0	0	0	0	0	1	0	0	0	0	0	0	0	0
MAP3K7	0	0	0	0	0	0	0	0	0	1	0	0	0	0	0	0	0	0
PDE1C	0	0	0	0	0	1	0	0	0	0	0	0	0	0	0	0	0	0
AVL9	0	0	0	0	1	0	0	0	0	0	0	0	0	0	0	0	0	0
KBTBD2	0	0	0	0	1	0	0	0	0	0	0	0	0	0	0	0	0	0
VPS54	0	0	0	0	1	0	0	0	0	0	0	0	0	0	0	0	0	0
PELI1	0	0	0	0	1	0	0	0	0	0	0	0	0	0	0	0	0	0
TMEM140	0	0	0	0	1	0	0	0	0	0	0	0	0	0	0	0	0	0
CEP68	0	0	0	0	1	0	0	0	0	0	0	0	0	0	0	0	0	0
HERPUD2	0	1	0	0	0	0	0	0	0	1	0	0	0	0	0	0	0	0
FKBP15	0	0	0	0	1	0	0	0	0	0	0	0	0	0	0	0	0	0
CNOT4	0	0	0	0	0	0	0	0	0	1	0	0	0	0	0	0	0	0
PRPF4	0	0	0	0	1	0	0	0	0	0	0	0	0	0	0	0	0	0
ETAA1	0	0	0	0	1	0	0	0	0	0	0	0	0	0	0	0	0	0
MTF1	0	1	0	0	0	0	0	0	0	0	0	0	0	0	0	0	0	0
MANEAL	0	1	0	0	-1	0	0	0	0	0	0	0	0	0	0	0	0	0
COLEC11	0	0	0	1	0	0	0	0	0	0	0	0	0	0	0	0	0	0
ARHGAP25	0	0	0	0	1	0	0	0	0	0	0	0	0	0	0	0	0	0
ELMO1	0	0	0	0	1	0	0	0	0	0	0	0	0	0	0	0	0	0
LRRN3	0	1	0	0	0	0	0	0	0	0	0	0	0	0	0	0	0	0
RNF144A	0	1	0	0	0	0	0	0	0	0	0	0	0	0	0	0	0	0
DOCK4	0	0	0	0	1	0	0	0	0	0	0	0	0	0	0	0	0	0
ANXA4	0	1	0	0	0	0	0	0	0	0	0	0	0	0	0	0	0	0
IFRD1	0	-1	0	0	1	0	0	0	0	0	0	0	0	0	0	0	0	0
KIDINS220	0	0	0	0	0	0	0	0	0	1	0	0	0	0	0	0	0	0
PCYOX1	0	0	0	0	0	0	0	0	0	1	0	0	0	0	0	0	0	0
CHRM2	0	0	0	0	0	0	0	0	1	0	0	0	0	0	0	0	0	0
ASAP2	0	0	0	0	1	0	0	0	0	0	0	0	0	0	0	0	0	0
OSCP1	0	1	0	0	0	0	0	0	0	0	0	0	0	0	0	0	0	0
LSM10	0	1	0	0	-1	0	0	0	0	0	0	0	0	0	0	0	0	0
ADAM17	0	-1	0	0	0	0	0	0	1	0	0	0	0	0	0	0	0	0
FOXP2	0	0	0	0	1	0	0	0	0	1	0	0	0	1	0	0	0	0
MED25	0	1	0	0	0	0	0	0	0	0	0	0	0	0	0	0	0	0
CAV2	0	0	0	0	1	0	0	0	0	0	0	0	0	0	0	0	0	0
CAV1	0	0	0	0	0	0	0	0	0	-1	1	0	0	0	0	0	0	0
KLF11	0	0	0	0	0	0	0	0	0	1	0	0	0	0	0	0	0	0
ODC1	0	0	0	0	1	0	0	0	0	0	0	0	0	0	0	0	0	0
ENSCAFG00000003424	0	0	0	0	1	0	0	0	0	0	0	0	0	0	0	0	0	0
TNC	0	0	0	0	0	0	0	0	1	0	0	0	0	0	0	0	0	0
CTTNBP2	0	1	0	0	0	0	0	0	0	0	0	0	0	0	0	0	0	0
COBL	0	0	0	0	0	0	0	0	0	1	0	0	0	0	0	0	0	0
ENSCAFG00000003455	0	0	1	0	0	0	0	0	0	0	0	0	0	0	0	0	0	0
TBC1D17	0	1	0	0	0	0	0	0	0	0	0	0	0	0	0	0	0	0
POU3F3	0	1	0	0	0	0	0	0	0	0	0	0	0	0	0	0	0	0
FBXL4	0	0	0	0	0	0	0	0	0	1	0	0	0	0	0	0	0	0
TSPAN12	0	0	0	0	1	0	0	0	0	0	0	0	0	0	0	0	0	0
BLVRA	0	0	0	0	1	0	0	0	0	0	0	0	0	0	0	0	0	0
STK17A	0	1	0	0	0	0	0	0	0	0	0	0	0	0	0	0	0	0
CPED1	0	1	0	0	0	0	0	0	0	0	0	0	0	0	0	0	0	0
FAM3C	0	0	0	0	1	0	0	0	0	0	0	0	0	0	0	0	0	0
FUZ	0	1	0	0	0	0	0	0	0	0	0	0	0	0	0	0	0	0
ARHGEF5	0	1	0	0	0	0	0	0	0	0	0	0	0	0	0	0	0	0
TLR4	0	0	0	0	1	0	0	0	0	0	0	0	0	0	0	0	0	0
TCAF1	0	0	0	0	0	0	0	0	0	1	0	0	0	0	0	0	0	0
ENSCAFG00000003530	0	0	0	0	1	0	0	0	0	1	0	0	0	0	0	0	0	0
C15H1orf216	0	0	0	0	0	0	0	0	0	1	0	0	0	0	0	0	0	0
ENSCAFG00000003544	0	0	0	0	0	1	0	0	0	0	0	0	0	0	0	0	0	0
CADPS2	0	0	0	0	1	0	0	0	0	0	0	0	0	0	0	0	0	0
FBXW2	0	0	0	0	0	0	0	0	0	1	0	0	0	0	0	0	0	0
GREB1	0	0	0	0	0	0	0	0	0	0	1	0	0	0	0	0	0	0
ZMYM4	0	0	0	0	0	0	0	0	0	1	0	0	0	0	0	0	0	0
IQUB	0	0	0	0	1	0	0	0	0	0	0	0	0	0	0	0	0	0
IRF3	0	1	0	0	0	0	0	0	0	0	0	0	0	0	0	0	0	0
BVES	0	0	0	0	0	0	0	0	1	1	0	0	0	0	0	0	0	0
PRR12	0	1	0	0	0	0	0	0	0	0	0	0	0	0	0	0	0	0
GJB5	0	1	0	0	0	0	0	0	0	0	1	0	0	0	0	0	0	0
NOSIP	0	1	0	0	0	0	0	0	0	0	0	0	0	0	0	0	0	0
AMPH	0	0	0	0	0	0	0	0	0	0	0	0	0	0	0	1	0	0
USP38	0	0	0	0	1	0	0	0	0	0	0	0	0	0	0	0	0	0
FCGRT	0	0	0	0	0	0	0	0	0	1	0	0	0	0	0	0	0	0
ENSCAFG00000003645	0	0	0	0	1	1	0	0	0	0	0	0	0	0	0	0	0	0
PREP	0	0	0	0	0	0	0	0	0	1	0	0	0	0	0	0	0	0
TBC1D9	0	-1	0	0	1	0	0	0	0	0	0	0	0	0	0	0	0	0
GSN	0	1	0	0	-1	0	0	0	0	0	0	0	0	0	0	0	0	0
ENSCAFG00000003668	0	0	0	0	1	0	0	0	0	0	0	0	0	0	0	0	0	0
FZD8	0	0	0	0	0	0	0	0	0	1	0	0	0	0	0	0	0	0
DKKL1	0	0	0	0	0	0	0	0	0	1	0	0	0	0	0	0	0	0
CREM	0	0	0	0	1	0	0	0	0	0	0	0	0	0	0	0	0	0
STOM	0	0	0	0	0	0	0	0	0	1	0	0	0	0	0	0	0	0
ENSCAFG00000003714	0	0	0	0	1	0	0	0	0	0	0	0	0	0	0	0	0	0
ENSCAFG00000003715	0	0	0	0	1	0	0	0	0	0	0	0	0	0	0	0	0	0
FAM49A	0	1	0	0	0	0	1	0	0	0	0	0	0	0	0	0	0	0
NRCAM	0	0	0	0	1	0	0	0	0	0	0	0	0	0	0	0	0	0
SLC7A11	0	-1	0	0	0	1	0	0	0	0	0	0	0	0	0	0	0	0
OSR1	0	1	0	0	0	0	0	0	0	0	0	0	0	0	0	0	0	0
ENSCAFG00000003792	0	1	0	0	0	0	0	0	0	0	0	0	0	0	0	0	0	0
SESN1	0	1	0	0	0	0	0	0	0	1	0	0	0	0	0	0	0	0
NRDC	0	0	0	0	1	0	0	0	0	0	0	0	0	0	0	0	0	0
KLF15	0	1	0	0	0	0	0	0	0	0	0	0	0	0	0	0	0	0
PPIL6	0	1	0	0	0	0	0	0	0	0	0	0	0	0	0	0	0	0
SMPD2	0	1	0	0	-1	0	0	0	0	0	0	0	0	0	0	0	0	0
MICAL1	0	1	0	0	-1	0	0	0	0	0	0	0	0	0	0	0	0	0
ZXDC	0	1	0	0	0	0	0	0	0	0	0	0	0	0	0	0	0	0
LAPTM4A	0	1	0	0	-1	0	0	0	0	1	0	0	0	0	0	0	0	0
DLD	0	1	0	0	0	0	0	0	0	0	0	0	0	0	0	0	0	0
OSBPL9	0	-1	0	0	1	0	0	0	0	0	0	0	0	0	0	0	0	0
HS1BP3	0	1	0	0	0	0	0	0	0	0	0	0	0	0	0	0	0	0
GYS1	0	0	0	0	0	0	0	0	0	1	0	0	0	0	0	0	0	0
FTL	0	1	0	0	0	0	0	0	0	0	0	0	0	0	0	0	0	0
DHDH	0	1	0	0	0	0	0	0	0	0	0	0	0	0	0	0	0	0
EPS15	0	0	0	0	0	0	0	0	0	1	0	0	0	0	0	0	0	0
NUCB1	0	0	0	0	0	0	0	0	0	1	0	0	0	0	0	0	0	0
KIAA1147	0	0	0	0	1	0	0	0	0	0	0	0	0	0	0	0	0	0
MPP7	0	0	0	0	0	0	0	0	0	1	0	0	0	0	0	0	0	0
HSD17B14	0	1	0	0	0	0	0	0	0	0	0	0	0	0	0	0	0	0
BCAT2	0	1	0	0	-1	0	0	0	0	0	0	0	0	0	0	0	0	0
RASIP1	0	0	0	0	1	0	0	0	0	0	0	0	0	0	0	0	0	0
ATAD2B	0	0	0	0	1	0	0	0	0	0	0	0	0	0	0	0	0	0
ENSCAFG00000003936	0	0	0	0	1	0	0	0	0	0	0	0	0	0	0	0	0	0
SPATA6	0	0	0	0	1	0	0	0	1	0	0	0	0	0	0	0	0	0
DENND2A	0	0	0	0	0	0	0	0	0	1	0	0	0	0	0	0	0	0
ENSCAFG00000003956	0	-1	0	0	0	1	0	0	0	0	0	0	0	0	0	0	0	0
ANKRD50	0	-1	0	0	1	0	0	0	-1	0	0	0	0	0	0	0	0	0
SLC37A3	0	-1	0	0	1	0	0	0	0	0	0	0	0	0	0	0	0	0
SPRY1	0	1	0	0	0	0	0	0	0	0	0	0	0	0	0	0	0	0
MAP3K8	0	0	0	0	0	0	0	0	0	1	0	0	0	0	0	0	0	0
JCAD	0	-1	0	0	1	0	0	0	0	0	0	0	0	0	0	0	0	0
ITSN2	0	0	0	0	1	0	0	0	0	1	0	0	0	0	0	0	0	0
ZEB1	0	1	0	0	1	0	0	0	0	0	0	0	0	0	0	0	0	0
TMEM143	0	1	0	0	0	0	0	0	0	0	0	0	0	0	0	0	0	0

EMP3	0	1	0	0	0	0	0	0	0	0	0	0	0	0	0	0	0	0
HIPK2	0	-1	0	0	0	0	0	0	0	1	0	0	0	0	0	0	0	0
TPRA1	0	1	0	0	0	0	0	0	0	0	0	0	0	0	0	0	0	0
LAMA4	0	0	0	0	1	0	0	0	0	0	0	0	0	0	0	0	0	0
ARHGAP12	0	1	0	0	0	0	0	0	0	0	0	0	0	0	0	0	0	0
NOP53	0	1	0	0	0	0	0	0	0	0	0	0	0	0	0	0	0	0
JRKL	0	0	0	0	0	0	0	0	0	1	0	0	0	0	0	0	0	0
ABTB1	0	1	0	0	0	0	0	0	0	0	0	0	0	0	0	0	0	0
ERMARD	0	0	0	0	0	0	0	0	0	1	0	0	0	0	0	0	0	0
MAML2	0	1	0	0	0	0	0	0	0	0	0	0	0	0	0	0	0	0
ADCY3	0	0	0	0	0	0	1	0	0	0	0	0	0	0	0	0	0	0
ENSCAFG00000004091	0	0	1	0	1	1	0	0	0	0	0	0	0	0	0	0	0	0
MTMR2	0	0	0	0	1	0	0	0	0	0	0	0	0	0	0	0	0	0
DLL1	0	1	0	0	0	0	0	0	1	0	0	0	0	0	0	0	0	0
ZC3HAV1	0	1	0	0	0	0	0	0	0	0	0	0	0	0	0	0	0	0
KBTBD12	0	1	0	0	0	0	0	0	0	0	0	0	0	0	0	0	0	0
PLXDC2	0	0	0	0	0	0	0	0	1	0	0	0	0	0	0	0	0	0
SESN3	0	0	0	0	0	0	0	0	0	1	0	0	0	0	0	0	0	0
NEBL	0	0	0	0	0	0	0	0	0	1	0	0	0	0	0	0	0	0
GATA2	0	0	0	0	1	0	0	0	0	0	0	0	0	0	0	0	0	0
RAB7A	0	0	0	0	0	0	0	0	1	0	0	0	0	0	0	0	0	0
MEIS3	0	1	0	0	-1	0	0	0	1	0	0	0	0	0	0	0	0	0
C5AR2	0	1	0	0	0	0	0	0	0	0	0	0	0	0	0	0	0	0
HEPHL1	0	0	0	0	0	0	0	0	0	1	0	0	0	0	0	0	0	0
ENSCAFG00000004186	0	0	0	0	0	0	0	0	0	1	0	0	0	0	0	0	0	0
FKRP	0	1	0	0	0	0	0	0	0	0	0	0	0	0	0	0	0	0
EFCAB14	0	0	0	0	0	0	0	0	0	1	0	0	0	0	0	0	0	0
ARHGEF4	0	0	0	0	0	0	0	0	1	0	0	0	0	0	0	0	0	0
ATPAF1	0	1	0	0	0	0	0	0	0	1	0	0	0	0	0	0	0	0
MOB3C	0	0	0	0	0	0	0	0	0	1	0	0	0	0	0	0	0	0
ENSCAFG00000004223	0	0	0	0	0	0	0	0	0	1	0	0	0	0	0	0	0	0
PLEKHB2	0	1	0	0	0	0	0	0	0	1	0	0	0	0	0	0	0	0
HS6ST1	0	0	0	0	0	0	0	0	0	1	0	0	0	0	0	0	0	0
CEP295	0	0	0	0	1	0	0	0	0	0	0	0	0	0	0	0	0	0
DNAJC2	0	0	0	0	0	1	0	0	0	-1	0	0	0	0	0	0	0	0
NAPEPLD	0	1	0	0	-1	0	0	0	1	0	0	0	0	0	0	0	0	0
KIF3C	0	0	0	0	1	0	0	0	0	0	0	0	0	0	0	0	0	0
ARHGAP21	0	0	0	0	1	0	0	0	0	0	0	0	0	0	0	0	0	0
FAAH	0	1	0	0	0	0	0	0	0	0	0	0	0	0	0	0	0	0
PRKD2	0	0	0	0	1	0	0	0	0	0	0	0	0	0	0	0	0	0
ENSCAFG00000004260	0	1	0	0	0	0	0	0	0	0	0	0	0	0	0	0	0	0
THNSL1	0	0	0	0	0	0	0	0	0	1	0	0	0	0	0	0	0	0
LRRC17	0	0	0	0	0	0	0	0	0	0	0	0	0	1	0	0	0	0
MYO3A	0	0	0	0	0	1	0	0	0	0	0	0	0	0	0	0	0	0
NAALAD2	0	1	0	0	0	0	0	0	0	0	0	0	0	0	0	0	0	0
GSAP	0	-1	0	0	1	0	0	0	0	0	0	0	0	0	0	0	0	0
RSBN1L	0	0	0	0	0	0	0	0	0	1	0	0	0	0	0	0	0	0
TMEM60	0	1	0	0	0	0	0	0	0	0	0	0	0	0	0	0	0	0
ADGRF3	0	1	0	0	0	0	0	0	0	0	0	0	0	0	0	0	0	0
SETDB2	0	0	0	0	1	0	0	0	0	0	0	0	0	0	0	0	0	0
PHTF2	0	0	0	0	0	0	0	0	1	0	0	0	0	0	0	0	0	0
LIMS2	0	1	0	0	0	0	0	0	0	1	0	0	0	0	0	0	0	0
IQSEC1	0	1	0	0	0	0	0	0	0	0	0	0	0	0	0	0	0	0
NOVA2	0	0	0	0	1	0	0	0	0	0	0	0	0	0	0	0	0	0
ACSS1	0	1	0	0	0	0	0	0	0	0	0	0	0	0	0	0	0	0
IRF2BP1	0	1	0	0	0	0	0	0	0	0	0	0	0	0	0	0	0	0
CTSC	0	0	0	0	1	0	0	0	0	0	0	0	0	0	0	0	0	0
RAB38	0	0	0	0	1	0	0	0	0	0	0	0	0	0	0	0	0	0
ABHD12	0	0	0	0	0	0	0	0	0	1	0	0	0	0	0	0	0	0
DMPK	0	0	0	0	0	0	0	0	1	1	0	0	0	0	0	0	0	0
SIX5	0	1	0	0	0	0	0	0	0	0	0	0	0	0	0	0	0	0
FZD4	0	1	0	0	0	0	0	0	0	1	0	0	0	0	0	0	0	0
NINL	0	0	0	0	1	0	0	0	0	0	0	0	0	0	0	0	0	0
CAB39L	0	0	0	0	1	0	0	0	0	0	0	0	0	0	0	0	0	0
GPR4	0	0	0	0	1	0	0	0	0	0	0	0	0	0	0	0	0	0
TCAIM	0	0	0	0	0	0	0	0	0	1	0	0	0	0	0	0	0	0
ZNF398	0	0	0	0	0	0	0	0	0	1	0	0	0	0	0	0	0	0
RCBTB2	0	0	0	0	0	0	0	0	0	1	0	0	0	0	0	0	0	0
ENSCAFG00000004444	0	1	0	0	0	0	0	0	0	0	0	0	0	0	0	0	0	0
TMEM43	0	1	0	0	-1	0	0	0	0	0	0	0	0	0	0	0	0	0
ITM2B	0	0	0	0	0	0	0	0	0	1	0	0	0	0	0	0	0	0
ABHD5	0	-1	0	0	0	1	0	0	0	-1	0	0	0	0	0	0	0	0
IWS1	0	-1	0	0	1	0	0	0	0	0	0	0	0	0	0	0	0	0
LRCH1	0	0	0	0	1	0	0	0	0	1	0	0	0	0	0	0	0	0
RUBCNL	0	0	0	0	0	0	0	0	1	0	0	0	0	0	0	0	0	0
SLC6A6	0	0	0	0	0	0	0	0	1	0	0	0	0	0	0	0	0	0
SNRK	0	0	0	0	1	0	0	0	0	0	0	0	0	0	0	0	0	0
CCDC17	0	1	0	0	0	0	0	0	0	0	0	0	0	0	0	0	0	0
MRC1	0	0	0	0	0	0	0	1	0	0	0	0	0	0	0	0	0	0
ZC3H13	0	0	0	0	1	0	0	0	0	0	0	0	0	0	0	0	0	0
CCDC89	0	0	0	0	0	0	0	0	0	1	0	0	0	0	0	0	0	0
NASP	0	0	0	0	1	0	0	0	0	0	0	0	0	0	0	0	0	0
FGD5	0	0	0	0	1	0	0	0	0	0	0	0	0	0	0	0	0	0
TPT1	0	0	0	0	0	0	0	0	0	1	0	0	0	0	0	0	0	0
EXOC3L2	0	0	0	0	1	0	0	0	0	0	0	0	0	0	0	0	0	0
DLG2	0	1	0	0	0	0	0	0	0	0	0	0	0	0	0	0	0	0
ENSCAFG00000004573	0	0	0	0	0	0	0	0	0	1	0	0	0	0	0	0	0	0
TSC22D1	0	1	0	0	0	0	0	0	0	0	0	0	0	0	0	0	0	0
TMEM176B	0	0	0	0	0	0	0	0	0	1	0	0	0	0	0	0	1	0
TESK2	0	0	0	0	0	0	0	0	0	1	0	0	0	0	0	0	0	0
TMCC1	0	0	0	0	1	0	0	0	0	1	0	0	0	0	0	0	0	0
BIN1	0	0	0	0	0	0	0	0	0	0	0	0	0	1	0	0	0	0
RAB30	0	1	0	0	0	0	0	0	0	0	0	0	0	0	0	0	0	0
NECTIN2	0	0	0	0	1	0	0	0	0	0	0	0	0	0	0	0	0	0
PRCP	0	-1	0	0	1	0	0	0	0	-1	0	0	0	0	0	0	0	0
FBXL2	0	0	0	0	0	0	0	0	0	1	0	0	0	0	0	0	0	0
BCAM	0	0	0	0	1	0	0	0	0	0	0	0	0	0	0	0	0	0
SUSD5	0	0	0	0	0	0	0	0	0	0	0	0	0	0	0	0	0	1
ENOX1	0	1	0	0	0	0	1	0	0	0	0	0	0	0	0	0	0	0
ENSCAFG00000004650	0	-1	0	0	1	1	0	0	0	0	0	0	0	0	0	0	0	0
ITGA8	0	0	0	0	0	0	0	0	0	1	0	0	0	0	0	0	0	0
KHK	0	1	0	0	0	0	0	0	0	0	0	0	0	0	0	0	0	0
ENSCAFG00000004676	0	0	0	0	0	0	0	0	1	0	0	0	0	0	0	0	0	0
CNOT10	0	0	0	0	0	1	0	0	0	0	0	0	0	0	0	0	0	0
NOS3	0	0	0	0	1	0	0	0	0	0	0	0	0	0	0	0	0	0
CNTNAP5	0	0	0	0	0	0	0	0	0	1	0	0	0	0	0	0	0	0
ENSCAFG00000004690	0	-1	1	0	1	1	0	0	0	-1	0	0	0	0	0	0	0	0
CDNF	0	1	0	0	0	0	0	0	0	0	0	0	0	0	0	0	0	0
KCNN4	0	1	0	0	0	0	0	0	0	0	0	0	0	0	0	0	0	0
SMG9	0	1	0	0	0	0	0	0	0	-1	0	0	0	0	0	0	0	0
FRMD4A	0	-1	0	0	1	0	0	0	0	0	0	0	0	0	0	0	0	0
DGKH	0	0	0	0	1	0	0	0	0	0	0	0	0	0	0	0	0	0
MIF4GD	0	1	0	0	0	0	0	0	0	0	0	0	0	0	0	0	0	0
TMEM94	0	1	0	0	0	0	0	0	0	0	0	0	0	0	0	0	0	0
TRANK1	0	0	0	0	1	0	0	0	0	1	0	0	0	0	0	0	0	0
CASKIN2	0	0	0	0	1	0	0	0	0	0	0	0	0	0	0	0	0	0
RNF220	0	1	0	0	0	0	0	0	0	0	0	0	0	0	0	0	0	0
GOLGA4	0	0	0	0	1	0	0	0	0	0	0	0	0	0	0	0	0	0
LIPE	0	1	0	0	0	0	0	0	0	1	0	0	0	0	0	0	0	0
ELF1	0	0	0	0	0	0	0	0	0	1	0	0	0	0	0	0	0	0
ITGA9	0	0	0	0	1	0	0	0	0	0	0	0	0	0	0	0	0	0
UPF2	0	0	0	0	1	0	0	0	0	0	0	0	0	0	0	0	0	0
RALB	0	0	0	0	1	0	0	0	0	0	0	0	0	0	0	0	0	0
PAFAH1B3	0	1	0	0	0	0	0	0	0	0	0	0	0	0	0	0	0	0
ATP6V0B	0	0	0	0	0	0	0	0	1	0	0	0	0	0	0	0	0	0
PROSER2	0	0	0	0	0	0	0	0	0	1	0	0	0	0	0	0	0	0
SMARCD3	0	0	0	0	0	0	0	0	0	1	0	0	0	0	0	0	0	0
USP6NL	0	0	0	0	1	0	0	0	0	1	0	0	0	0	0	0	0	0
DEDD2	0	1	0	0	0	0	0	0	0	0	0	0	0	0	0	0	0	0
TMEM37	0	1	0	0	0	0	0	0	0	0	0	0	0	0	0	0	0	0

STEAP3	0	1	0	0	0	0	0	0	0	0	0	0	0	0	0	0	0	0
CAND2	0	0	0	0	0	0	0	0	0	1	0	0	0	0	0	0	0	0
ENSCAFG00000004905	0	0	0	0	1	0	0	0	0	0	0	0	0	0	0	0	0	0
GALK1	0	1	0	0	0	0	0	0	0	0	0	0	0	0	0	0	0	0
CCDC93	0	1	0	0	0	0	0	0	0	0	0	0	0	0	0	0	0	0
TMEM40	0	0	0	0	0	0	0	0	0	1	0	0	0	0	0	0	0	0
GALNT11	0	1	0	0	0	0	0	0	0	0	0	0	0	0	0	0	0	0
RAF1	0	1	0	0	0	0	0	0	0	0	0	0	0	0	0	0	0	0
TRIM47	0	1	0	0	0	0	0	0	0	0	0	0	0	0	0	0	0	0
ACAA1	0	1	0	0	-1	0	0	0	0	0	0	0	0	0	0	0	0	0
MRPL38	0	1	0	0	0	0	0	0	0	-1	0	0	0	0	0	0	0	0
MYD88	0	1	0	0	0	0	0	0	0	0	0	0	0	0	0	0	0	0
MKRN2	0	0	0	0	0	0	0	0	0	1	0	0	0	0	0	0	0	0
CELF2	0	1	0	0	0	0	0	0	0	0	0	0	0	0	0	0	0	0
DACH1	0	0	0	0	1	0	0	0	0	0	0	0	0	0	0	0	0	0
SLC35F5	0	0	0	0	0	0	0	0	0	1	0	0	0	0	0	0	0	0
PPARG	0	0	0	0	1	0	0	0	0	0	0	0	0	0	0	0	0	0
DMAC2	0	1	0	0	0	0	0	0	0	0	0	0	0	0	0	0	0	0
GPR39	0	0	0	0	0	0	0	0	0	1	0	0	0	0	0	0	0	0
ENSCAFG00000005006	0	1	0	0	-1	0	0	0	0	0	0	0	0	0	0	0	0	0
CCDC97	0	1	0	0	0	0	0	0	0	0	0	0	0	0	0	0	0	0
GATA3	0	0	0	0	1	0	0	0	0	0	0	0	0	0	0	0	0	0
HRH1	0	0	0	0	0	0	0	0	0	1	0	0	0	0	0	0	0	0
TBC1D4	0	-1	0	0	1	0	0	0	0	0	0	0	0	0	0	0	0	0
RNF157	0	1	0	0	0	0	0	0	0	-1	0	0	0	0	0	0	0	0
WDR48	0	0	0	0	0	0	0	0	0	1	0	0	0	0	0	0	0	0
SPHK1	0	0	0	0	0	0	0	0	0	-1	0	1	0	0	0	0	0	0
TTC21A	0	1	0	0	0	0	0	0	0	0	0	0	0	0	0	0	0	0
ITIH5	0	0	0	0	0	0	0	0	0	1	0	0	0	0	0	0	0	0
ENSCAFG00000005099	0	0	0	0	1	0	0	0	0	0	0	0	0	0	0	0	0	0
MYCBP2	0	1	0	0	0	0	0	0	0	0	0	0	0	0	0	0	0	0
CXCR4	0	0	0	0	1	0	0	0	0	0	0	0	0	0	0	0	0	0
ENTPD3	0	1	0	0	-1	0	0	0	0	1	0	0	0	0	0	0	0	0
LTBP4	0	1	0	0	0	0	0	0	0	0	0	0	0	0	0	0	0	0
VHL	0	0	0	0	1	0	0	0	0	0	0	0	0	0	0	0	0	0
BRK1	0	0	0	0	0	0	0	0	0	1	0	0	0	0	0	0	0	0
CD93	0	0	0	0	1	0	0	0	0	0	0	0	0	0	0	0	0	0
PAX1	0	0	0	0	0	0	1	0	0	0	0	0	0	0	0	0	0	0
DNAJB6	0	0	0	0	0	0	0	0	0	1	0	0	0	0	0	0	0	0
SCEL	0	0	0	0	0	0	0	0	0	1	0	0	0	0	0	0	0	0
SEC14L1	0	0	0	0	1	0	0	0	0	0	0	0	0	0	0	0	0	0
CAPN5	0	1	0	0	0	0	0	0	0	0	0	0	0	0	0	0	0	0
SLAIN1	0	0	0	0	0	0	1	0	0	0	0	0	0	0	0	0	0	0
EDNRB	0	0	0	0	1	0	0	0	0	0	0	0	0	0	0	0	0	0
NXPH2	0	0	0	0	0	0	0	0	0	1	0	0	0	0	0	0	0	0
BABAM2	0	0	0	0	-1	0	0	0	1	1	0	0	0	0	0	0	0	0
FOSL2	0	0	0	0	0	0	0	0	1	1	0	0	0	0	0	0	0	0
IL17RC	0	1	0	0	0	0	0	0	0	0	0	0	0	0	0	0	0	0
ANKRD16	0	1	0	0	0	0	0	0	0	1	0	0	0	0	0	0	0	0
TNRC6C	0	1	0	0	0	0	0	0	0	0	0	0	0	0	0	0	0	0
PLB1	0	0	0	0	0	0	0	0	0	0	0	0	0	0	0	0	1	0
ASB13	0	1	0	0	0	0	0	0	0	0	0	0	0	0	0	0	0	0
PPP1CB	0	0	0	0	1	0	0	0	0	0	0	0	0	0	0	0	0	0
NET1	0	0	0	0	1	0	0	0	0	-1	0	0	0	0	0	0	0	0
LRRC32	0	0	0	0	1	0	0	0	1	0	0	0	0	0	0	0	0	0
RALGAPA2	0	0	0	0	1	0	0	0	0	0	0	0	0	0	0	0	0	0
SPTBN4	0	0	0	0	1	0	0	0	0	0	0	0	0	0	0	0	0	0
TIE1	0	0	0	0	1	0	0	0	0	0	0	0	0	0	0	0	0	0
WNT11	0	1	0	0	0	0	0	0	0	0	0	0	0	0	0	0	0	0
SLITRK5	0	0	0	1	0	0	0	0	0	1	0	0	0	1	0	0	0	0
ENSCAFG00000005298	0	0	0	0	1	0	0	0	0	0	0	0	0	0	0	0	0	0
LBH	0	1	0	0	0	0	0	0	1	1	0	0	0	0	0	0	0	0
SH2D4A	0	1	0	0	0	0	0	0	0	0	0	0	0	0	0	0	0	0
NKTR	0	0	0	0	1	0	0	0	0	0	0	0	0	0	0	0	0	0
DGAT2	0	0	0	0	-1	0	0	0	1	0	0	0	0	0	0	0	0	0
ACKR2	0	1	0	0	0	0	0	0	0	0	0	0	0	0	0	0	0	0
ZNF662	0	1	0	0	0	0	0	0	0	0	0	0	0	0	0	0	0	0
FAM198A	0	1	0	0	0	0	0	0	0	0	0	0	0	0	0	0	0	0
GDPD5	0	1	0	0	1	0	0	0	0	0	0	0	0	0	0	0	0	0
HOOK3	0	-1	0	0	1	1	0	0	0	0	0	0	0	0	0	0	0	0
ARRB1	0	1	0	0	1	0	0	0	0	0	0	0	0	0	0	0	0	0
PIP4P1	0	1	0	0	0	0	0	0	0	0	0	0	0	0	0	0	0	0
PNP	0	1	0	0	0	0	0	0	0	0	0	0	0	0	0	0	0	0
STT3B	0	0	0	0	0	0	0	0	0	1	0	0	0	0	0	0	0	0
DYRK1B	0	0	0	0	0	0	0	0	0	1	0	0	0	0	0	0	0	0
ABCC4	0	1	0	0	0	0	0	0	0	0	0	0	0	0	0	0	0	0
TGFBR2	0	1	0	0	0	0	0	0	0	0	0	0	0	0	0	0	0	0
NEU3	0	0	0	0	0	0	0	0	0	1	0	0	0	0	0	0	0	0
ENSCAFG00000005456	0	0	0	0	1	0	0	0	0	0	0	0	0	0	0	0	0	0
XRRA1	0	0	0	0	0	0	0	0	0	1	0	0	0	0	0	0	0	0
SLC20A2	0	-1	0	0	0	0	0	0	0	0	0	1	0	0	0	0	0	0
LIPT2	0	0	0	0	0	0	0	0	0	1	0	0	0	0	0	0	0	0
KCNE3	0	1	0	0	0	0	0	0	0	1	0	0	0	0	0	0	0	0
ZNF219	0	1	0	0	0	0	1	0	0	0	0	0	0	0	0	0	0	0
ENSCAFG00000005494	0	0	1	0	0	1	0	0	0	0	0	0	0	0	0	0	0	0
PGM2L1	0	1	0	0	0	0	0	0	0	0	0	0	0	0	0	0	0	0
CYTH1	0	0	0	0	1	0	0	0	0	0	0	0	0	0	0	0	0	0
ACVR2A	0	0	0	0	0	1	0	0	0	0	0	0	0	0	0	0	0	0
TIMP2	0	1	0	0	-1	0	0	0	0	0	0	0	0	0	0	0	0	0
MED29	0	1	0	0	0	0	0	0	0	0	0	0	0	0	0	0	0	0
MBD5	0	0	0	0	0	0	0	0	0	1	0	0	0	0	0	0	0	0
ENSCAFG00000005538	0	0	0	0	-1	0	1	0	0	0	0	0	0	0	0	0	0	0
C1QTNF1	0	0	0	0	-1	0	0	0	1	0	0	0	0	0	0	0	0	0
FLRT3	0	0	0	0	0	0	0	1	0	0	0	0	0	0	0	0	0	0
SAMD4B	0	1	0	0	-1	0	0	0	1	0	0	0	0	0	0	0	0	0
LMCD1	0	0	0	0	0	0	0	0	1	0	0	0	0	0	0	0	0	0
CBX4	0	0	0	0	0	0	0	0	0	1	0	0	0	0	0	0	0	0
TBC1D16	0	0	0	0	0	0	0	0	1	1	0	0	0	0	0	0	0	0
GAA	0	0	0	0	0	0	0	0	0	1	0	0	0	0	0	0	0	0
XDH	0	1	0	0	0	0	0	0	0	0	0	0	0	0	0	0	0	0
SLC4A7	0	-1	0	0	1	0	0	0	0	0	0	0	0	0	0	0	0	0
STK24	0	1	0	0	0	0	0	0	0	0	0	0	0	0	0	0	0	0
CHD8	0	0	0	0	1	0	0	0	0	0	0	0	0	0	0	0	0	0
JAG1	0	0	0	0	1	0	0	0	0	0	0	0	0	0	0	0	0	0
ARAP1	0	1	0	0	0	0	0	0	0	0	0	0	0	0	0	0	0	0
PHLDA1	0	0	0	0	0	0	0	1	1	0	0	0	0	0	0	0	0	0
PDE2A	0	0	0	0	1	0	0	0	0	0	0	0	0	0	0	0	0	0
DNAJC18	0	0	0	0	0	0	0	0	0	1	0	0	0	0	0	0	0	0
TOP2B	0	0	0	0	1	0	0	0	0	0	0	0	0	0	0	0	0	0
TMEM173	0	0	0	0	1	0	0	0	0	0	0	0	0	0	0	0	0	0
GINS4	0	0	0	0	1	0	0	0	0	-1	0	0	0	0	0	0	0	0
DOCK9	0	1	0	0	0	0	0	0	0	-1	0	0	0	0	0	0	0	0
NRG2	0	1	0	0	0	0	0	0	0	0	0	0	0	0	0	0	0	0
BAHCC1	0	1	0	0	0	0	0	0	0	0	0	0	0	0	0	0	0	0
THRB	0	1	0	0	-1	0	0	0	1	0	0	0	0	0	0	0	0	0
IDO2	0	1	0	0	0	0	0	0	0	0	0	0	0	0	0	0	0	0
NR1D2	0	0	0	0	1	0	0	0	0	0	0	0	0	0	0	0	0	0
YIPF4	0	0	0	0	0	0	0	0	0	1	0	0	0	0	0	0	0	0
RPL15	0	1	0	0	0	0	0	0	0	0	0	0	0	0	0	0	0	0
BIRC6	0	0	0	0	0	0	0	0	0	1	0	0	0	0	0	0	0	0
ANKHD1	0	1	0	0	0	0	0	0	0	0	0	0	0	0	0	0	0	0
FMNL2	0	1	0	0	-1	0	0	0	0	1	0	0	0	0	0	0	0	0
LIN7A	0	0	0	0	0	0	0	0	0	0	0	0	0	0	0	1	0	0
SRA1	0	1	0	0	0	0	0	0	0	0	0	0	0	0	0	0	0	0
APBB3	0	1	0	0	0	0	0	0	0	0	0	0	0	0	0	0	0	0
IL18BP	0	0	0	0	0	0	0	1	0	0	0	0	0	0	0	0	0	0
ZIC2	0	1	0	0	0	0	0	0	0	0	0	0	0	0	0	0	0	0
ENSCAFG00000005837	0	1	0	0	0	0	0	0	0	0	0	0	0	0	0	0	0	0
SATB1	0	0	0	0	0	0	0	0	0	1	0	0	0	0	0	0	0	0
ARL6IP6	0	0	0	0	1	0	0	0	0	-1	0	0	0	0	0	0	0	0

TMCO6	0	1	0	0	0	0	0	0	0	0	0	0	0	0	0	0	0	0
TMTC4	0	0	0	0	1	0	0	0	0	0	0	0	0	0	0	0	0	0
EIF3K	0	1	0	0	-1	0	0	0	0	0	0	0	0	0	0	0	0	0
RASGRP3	0	0	0	0	1	0	0	0	0	0	0	0	0	0	0	0	0	0
NUP98	0	1	0	0	0	0	0	0	0	0	0	0	0	0	0	0	0	0
TACC1	0	0	0	0	0	0	0	0	0	1	0	0	0	0	0	0	0	0
TMX4	0	0	0	0	0	0	0	0	0	1	0	0	0	0	0	0	0	0
CRIM1	0	0	0	0	0	0	0	0	0	1	0	0	0	0	0	0	0	0
GALNT15	0	1	0	0	0	0	0	0	0	0	0	0	0	0	0	0	0	0
STIM1	0	0	0	0	0	0	0	0	0	0	1	0	0	0	0	0	0	0
FEZ2	0	0	0	0	0	0	0	0	0	1	0	0	0	0	0	0	0	0
METTL25	0	0	0	0	1	0	0	0	0	0	0	0	0	0	0	0	0	0
FERMT1	0	0	0	0	1	0	0	0	0	1	0	0	0	0	0	0	0	0
ITGBL1	0	0	0	0	0	0	0	0	0	1	0	0	0	0	0	0	0	0
YIF1B	0	0	0	0	0	0	0	0	1	0	0	0	0	0	0	0	0	0
VIT	0	1	0	0	0	0	0	0	0	0	1	0	0	0	0	0	0	0
VPS36	0	1	0	0	0	0	0	0	0	0	0	0	0	0	0	0	0	0
CRLS1	0	0	0	0	0	0	0	0	0	1	0	0	0	0	0	0	0	0
STRN	0	-1	0	0	1	0	0	0	0	-1	0	0	0	0	0	0	0	0
THSD1	0	0	0	0	1	0	0	0	0	0	0	0	0	0	0	0	0	0
SPINT2	0	0	0	0	0	0	0	0	0	1	0	0	0	0	0	0	0	0
HACL1	0	1	0	0	0	0	0	0	0	0	0	0	0	0	0	0	0	0
SUMF1	0	0	0	0	0	0	0	0	0	1	0	0	0	0	0	0	0	0
SETMAR	0	1	0	0	0	0	0	0	0	0	0	0	0	0	0	0	0	0
FOXO1	0	1	0	0	0	0	0	0	0	1	0	0	0	0	0	0	0	0
HEATR5B	0	0	0	0	0	0	0	0	0	1	0	0	0	0	0	0	0	0
LHFPL6	0	0	0	0	0	0	0	0	0	1	0	0	0	0	0	0	0	0
NHLRC3	0	0	0	0	0	0	0	0	0	1	0	0	0	0	0	0	0	0
EAF1	0	0	0	0	0	0	0	0	1	0	0	0	0	0	0	0	0	0
FREM2	0	0	0	0	0	0	0	0	0	1	0	0	0	0	0	0	0	0
EFNB2	0	0	0	0	0	0	0	0	0	1	0	0	0	0	0	0	0	0
SH3BP5	0	0	0	0	1	0	0	0	0	0	0	0	0	0	0	0	0	0
CEP290	0	0	0	0	1	0	0	0	0	0	0	0	0	0	0	0	0	0
TRPC4	0	0	0	0	0	0	0	0	0	0	0	0	0	0	1	0	0	0
RASSF2	0	0	0	0	0	0	0	0	0	1	0	0	0	0	0	0	0	0
ATP2C1	0	0	0	0	0	0	0	0	0	1	0	0	0	0	0	0	0	0
NSD3	0	0	0	0	1	0	0	0	0	0	0	0	0	0	0	0	0	0
IRS2	0	0	0	0	-1	0	0	0	1	0	0	0	0	0	0	0	0	0
CSNK1D	0	1	0	0	-1	0	0	0	0	0	0	0	0	0	0	0	0	0
COL4A1	0	-1	0	0	0	0	0	0	0	1	0	0	0	0	0	0	0	0
COL4A2	0	0	0	0	0	0	0	0	0	1	0	0	0	0	0	0	0	0
RAB20	0	0	0	0	0	0	0	0	1	0	0	0	0	0	0	0	0	0
POSTN	0	0	0	0	0	0	0	0	1	0	0	0	0	0	0	0	0	0
NAXD	0	0	0	0	0	0	0	0	0	1	0	0	0	0	0	0	0	0
PLPP5	0	1	0	0	0	0	0	0	0	0	0	0	0	0	0	0	0	0
DDHD2	0	1	0	0	0	0	0	0	0	0	0	0	0	0	0	0	0	0
PCDHB4	0	0	0	0	0	0	0	0	0	1	0	0	0	0	0	0	0	0
BAG4	0	1	0	0	0	0	0	0	0	0	0	0	0	0	0	0	0	0
ARHGEF7	0	0	0	0	1	0	0	0	0	0	0	0	0	0	0	0	0	0
ATP2B1	0	-1	0	0	1	0	0	0	0	0	0	0	0	0	0	0	0	0
SEMA3D	0	0	0	0	0	1	0	0	0	0	0	0	0	0	0	0	0	0
EPYC	0	0	0	0	0	0	0	0	0	1	0	0	0	0	0	0	0	0
RNF24	0	1	0	0	0	0	0	0	0	1	0	0	0	0	0	0	0	0
QPCT	0	0	0	0	0	0	0	0	0	1	0	0	0	0	0	0	0	0
BTG1	0	1	0	0	0	0	0	0	0	0	0	0	0	0	0	0	0	0
EEA1	0	1	0	0	0	0	0	0	0	0	0	0	0	0	0	0	0	0
CDC25B	0	1	0	0	0	0	0	0	0	0	0	0	0	0	0	0	0	0
ENSCAFG00000006187	0	1	0	0	-1	0	0	0	0	-1	0	0	0	0	0	0	0	0
ADGRA2	0	1	0	0	1	0	0	0	0	0	0	0	0	0	0	0	0	0
CPNE4	0	0	0	1	0	0	0	0	0	1	0	0	0	0	0	0	0	0
SEMA3A	0	0	0	0	0	1	0	0	0	0	0	0	0	0	0	0	0	0
ARAP3	0	0	0	0	1	0	0	0	0	0	0	0	0	0	0	0	0	0
TMCC3	0	-1	0	0	1	0	0	0	0	0	0	0	0	0	0	0	0	0
PCDH1	0	0	0	0	1	0	0	0	0	0	0	0	0	0	0	0	0	0
ADAM33	0	1	0	0	0	0	0	0	0	0	0	0	0	0	0	0	0	0
ACPP	0	0	0	0	1	0	0	0	0	0	0	0	0	0	0	0	0	0
ENSCAFG00000006238	0	0	0	0	0	0	0	0	0	1	0	0	0	0	0	0	0	0
PCDH12	0	0	0	0	1	0	0	0	0	0	0	0	0	0	0	0	0	0
GNPDA1	0	1	0	0	0	0	0	0	0	0	0	0	0	0	0	0	0	0
CHL1	0	0	0	0	0	0	0	0	0	0	0	0	0	0	1	0	0	0
NDFIP1	0	1	0	0	0	0	0	0	0	0	1	0	0	0	-1	0	0	0
SPRY4	0	1	0	0	-1	0	0	0	0	0	0	0	0	0	0	0	0	0
MAB21L1	0	1	0	0	0	0	1	0	1	0	1	0	0	0	0	0	0	0
DNAJC13	0	0	0	0	1	0	0	0	0	0	0	0	0	0	0	0	0	0
PCLO	0	0	0	0	0	0	0	0	0	1	0	0	0	0	0	0	0	0
ARHGAP26	0	0	0	0	1	0	0	0	0	0	0	0	0	0	0	0	0	0
P2RX2	0	0	0	0	1	0	0	0	0	0	0	0	0	0	0	0	0	0
FGD6	0	0	0	0	1	0	0	0	0	0	0	0	0	0	0	0	0	0
ATRN	0	0	0	0	0	0	0	0	0	1	0	0	0	0	0	0	0	0
CUL4A	0	0	0	0	1	0	0	0	0	0	0	0	0	0	0	0	0	0
NR3C1	0	0	0	0	-1	0	0	0	1	1	0	0	0	0	0	0	0	0
VEZT	0	-1	0	0	0	0	0	0	0	1	0	0	0	0	0	0	0	0
CACNA2D1	0	0	0	0	1	0	0	0	0	0	0	0	0	0	0	0	0	0
ENSCAFG00000006373	0	0	1	0	1	1	0	0	0	0	0	0	0	0	0	0	0	0
C24H20orf194	0	0	0	0	0	0	0	0	0	1	0	0	0	0	0	0	0	0
N4BP2L1	0	1	0	0	0	0	0	0	0	0	0	0	0	0	0	0	0	0
NTN4	0	1	0	0	1	0	0	0	0	0	0	0	0	0	0	0	0	0
ACKR4	0	1	0	0	0	0	0	0	1	0	0	0	0	0	0	0	0	0
CCKBR	0	0	0	0	0	0	0	0	1	0	0	0	0	0	0	0	0	0
AMDHD1	0	1	0	0	0	0	0	0	0	0	0	0	0	0	0	0	0	0
SH3RF2	0	0	0	0	0	0	0	0	0	1	0	0	0	0	0	0	0	0
SMPD1	0	0	0	0	0	0	0	0	0	1	0	0	0	0	0	0	0	0
TMEM108	0	0	0	0	0	0	0	0	0	1	0	0	0	0	0	0	0	0
PPP4R2	0	-1	0	0	1	1	0	0	0	0	0	0	0	0	0	0	0	0
APBB1	0	1	0	0	-1	0	0	0	0	0	0	0	0	0	0	0	0	0
GXYLT2	0	0	0	0	0	1	0	1	0	0	0	0	0	0	0	0	0	0
MAP4K3	0	0	0	0	0	0	0	0	0	1	0	0	0	0	0	0	0	0
TRIM3	0	1	0	0	0	0	0	0	0	0	0	0	0	0	0	0	0	0
EIF4E3	0	0	0	0	0	0	0	0	0	1	0	0	0	0	0	0	0	0
ULK1	0	1	0	0	0	0	0	0	0	0	0	0	0	0	0	0	0	0
TOPBP1	0	0	0	0	1	0	0	0	0	-1	0	0	0	0	0	0	0	0
CDK17	0	0	0	0	1	0	0	0	0	0	0	0	0	0	0	0	0	0
TMEM178A	0	0	0	0	0	0	0	0	0	1	0	0	0	0	0	0	0	0
ENSCAFG00000006478	0	1	0	0	0	0	0	0	0	-1	0	0	0	0	0	0	0	0
TCERG1	0	0	0	0	1	0	0	0	0	-1	0	0	0	0	0	0	0	0
SIGIRR	0	1	0	0	0	0	1	0	0	0	0	0	0	0	0	0	0	0
EML4	0	-1	0	0	1	0	0	0	0	1	0	0	0	0	0	0	0	0
FRMD4B	0	0	0	0	1	0	0	0	0	0	0	0	0	0	0	0	0	0
HSPH1	0	0	0	0	1	0	0	0	0	0	0	0	0	0	0	0	0	0
ARID5A	0	0	0	0	1	0	0	0	0	0	0	0	0	0	0	0	0	0
NEURL3	0	0	0	0	1	0	0	0	0	0	0	0	0	0	0	0	0	0
TMF1	0	0	0	0	1	0	0	0	0	0	0	0	0	0	0	0	0	0
APAF1	0	0	0	0	1	0	0	0	0	0	0	0	0	0	0	0	0	0
EOGT	0	0	0	0	0	0	0	0	0	1	0	0	0	0	0	0	0	0
WDFY4	0	-1	0	0	0	0	0	1	0	0	0	0	0	0	0	0	0	0
ENSCAFG00000006577	0	-1	0	0	1	0	0	0	0	-1	0	0	0	0	0	0	0	0
COX7A1	0	0	0	0	0	0	0	0	1	0	0	0	0	0	0	0	0	0
TMEM80	0	1	0	0	0	0	0	0	0	0	0	0	0	0	0	0	0	0
HMGB1	0	0	0	0	1	0	0	0	0	-1	0	0	0	0	0	0	0	0
SMUG1	0	1	0	0	0	0	0	0	0	0	0	0	0	0	0	0	0	0
VSTM4	0	1	0	0	0	0	0	0	0	0	0	0	0	0	0	0	0	0
CPXM1	0	0	0	0	0	0	0	0	0	1	0	0	0	0	0	0	0	0
HOXC6	0	0	1	0	0	0	0	0	0	-1	0	0	0	1	0	0	0	0
EBF4	0	0	0	0	0	0	0	0	0	1	0	0	0	0	0	0	0	0
HOXC10	0	1	1	0	0	0	1	0	1	-1	1	1	0	0	0	1	0	1
ENSCAFG00000006639	0	1	0	0	0	0	0	0	0	0	0	0	0	0	0	0	0	0
ADGRD1	0	1	0	0	0	0	0	0	0	1	0	0	0	0	0	0	0	0
ALKBH6	0	1	0	0	0	0	0	0	0	0	0	0	0	0	0	0	0	0
PPP1R3B	0	0	0	0	0	0	0	0	0	1	1	0	0	0	0	0	0	0
SPINK5	0	0	0	0	0	0	0	0	0	0	0	0	0	0	0	1	0	0

MFHAS1	0	1	0	0	0	0	0	0	1	0	0	0	0	0	0	0	0	0
LRFN3	0	1	0	0	0	0	0	0	0	0	0	0	0	0	0	0	0	0
SLCO2A1	0	0	0	0	1	0	0	0	0	0	0	0	0	0	0	0	0	0
APLP1	0	0	0	0	0	0	0	0	0	1	0	0	0	0	0	0	0	0
AMOTL2	0	1	0	0	0	0	0	0	0	1	0	0	0	0	0	0	0	0
RIMBP2	0	0	0	0	0	0	0	0	1	0	0	0	0	0	0	0	0	0
SYT9	0	0	0	0	0	0	0	0	0	0	1	0	0	0	0	0	0	0
TTC17	0	0	0	0	0	0	0	0	0	1	0	0	0	0	0	0	0	0
PAN3	0	0	0	0	1	0	0	0	0	0	0	0	0	0	0	0	0	0
PRAG1	0	0	0	0	1	0	0	0	0	0	0	0	0	0	0	0	0	0
TRMT9B	0	0	0	0	1	0	0	0	0	1	0	0	0	0	0	0	0	0
ENSCAFG00000006763	0	1	0	0	0	0	0	0	0	0	0	0	0	0	0	0	0	0
DLC1	0	1	0	0	0	0	0	0	0	0	0	0	0	0	0	0	0	0
ADAMTS9	0	0	0	0	1	0	0	0	0	0	0	0	0	0	0	0	0	0
C16H8orf48	0	0	0	0	0	0	0	0	0	1	0	0	0	0	0	0	0	0
LNX2	0	0	0	0	1	0	0	0	0	0	0	0	0	0	0	0	0	0
ENSCAFG00000006785	0	1	0	0	0	0	0	0	0	-1	0	0	0	0	0	0	0	0
PRICKLE2	0	0	0	0	-1	0	0	0	1	1	0	0	0	0	0	0	0	0
NCOA4	0	1	0	0	0	0	0	0	0	0	0	0	0	0	0	0	0	0
PRR5L	0	0	0	0	1	0	0	0	0	0	0	0	0	0	0	0	0	0
WASF3	0	1	0	0	0	0	0	0	0	1	0	0	0	0	0	0	0	0
ENSCAFG00000006847	0	0	0	0	1	0	0	0	0	0	0	0	0	0	0	0	0	0
SLC38A9	0	0	0	0	0	0	0	0	0	1	0	0	0	0	0	0	0	0
TMEM132B	0	1	0	0	0	0	0	0	0	0	0	0	0	0	0	0	0	0
ANGPT4	0	0	0	0	0	0	0	0	1	0	0	0	0	0	0	0	0	0
PAMR1	0	1	0	0	0	0	0	0	0	0	0	0	0	0	0	0	0	0
TARBP2	0	1	0	0	0	0	0	0	0	-1	0	0	0	0	0	0	0	0
MARCH8	0	0	0	0	0	0	0	0	0	1	0	0	0	0	0	0	0	0
GAS2L3	0	0	0	0	1	0	0	0	0	-1	0	0	0	0	0	0	0	0
EIF3F	0	1	0	0	-1	0	0	0	0	0	0	0	0	0	0	0	0	0
CNOT7	0	-1	0	0	1	0	0	0	0	0	0	0	0	0	0	0	0	0
IL6ST	0	0	0	0	0	0	0	0	0	1	0	0	0	0	0	0	0	0
ENSCAFG00000006899	0	0	0	0	1	0	0	0	0	0	0	0	0	0	0	0	0	0
RNF6	0	0	0	0	0	1	0	0	0	0	0	0	0	0	0	0	0	0
MAP3K1	0	0	0	0	0	0	0	0	0	1	0	0	0	0	0	0	0	0
RBCK1	0	1	0	0	0	0	0	0	0	0	0	0	0	0	0	0	0	0
ENSCAFG00000006937	0	0	1	0	1	0	0	0	0	0	0	0	0	0	0	0	0	0
CAT	0	0	0	0	0	0	0	0	0	1	0	0	0	0	0	0	0	0
GPBP1	0	0	0	0	1	0	0	0	0	0	0	0	0	0	0	0	0	0
PDGFRL	0	1	0	0	0	0	0	0	0	0	0	0	0	0	0	0	0	0
ST5	0	0	0	0	0	0	0	0	1	0	0	0	0	0	0	0	0	0
SCARB1	0	-1	0	0	1	0	0	0	0	0	0	0	0	0	0	0	0	0
TCEA1	0	0	0	0	0	1	0	0	0	-1	0	0	0	0	0	0	0	0
EPHB1	0	0	0	0	1	0	0	0	0	0	0	0	0	0	0	0	0	0
PLK2	0	0	0	0	1	0	0	0	0	0	0	0	0	0	0	0	0	0
MTUS1	0	1	0	0	0	0	0	0	0	0	0	0	0	0	0	0	0	0
NCOR2	0	1	0	0	0	0	0	0	0	0	0	0	0	0	0	0	0	0
ZNF664	0	0	0	0	1	0	0	0	0	0	0	0	0	0	0	0	0	0
AKIP1	0	0	0	0	0	0	0	0	0	1	0	0	0	0	0	0	0	0
TMEM68	0	0	0	0	0	1	0	0	0	0	0	0	0	0	0	0	0	0
NRIP3	0	0	0	0	1	0	0	0	0	0	0	0	0	0	0	0	0	0
PFDN5	0	0	0	0	0	0	0	0	0	1	0	0	0	0	0	0	0	0
DEPP1	0	0	0	0	1	0	0	0	0	0	0	0	0	0	0	0	0	0
RARG	0	1	0	0	0	0	0	0	0	0	0	0	0	0	0	0	0	0
MALL	0	0	0	0	1	0	0	0	0	0	0	0	0	0	0	0	0	0
CSGALNACT2	0	0	0	0	0	0	0	0	1	0	0	0	0	0	0	0	0	0
SDR16C5	0	1	0	0	0	0	0	0	0	0	0	0	0	0	0	0	0	0
PPP2R3A	0	1	0	0	0	0	0	0	0	0	0	0	0	0	0	0	0	0
ITGB7	0	0	0	0	0	0	0	0	1	0	0	0	0	0	0	0	0	0
RET	0	1	0	0	1	0	0	0	0	0	0	0	0	0	0	0	0	0
FAM110B	0	0	0	0	0	0	0	0	0	1	0	0	0	0	0	0	0	0
SCUBE2	0	1	0	0	0	0	0	0	0	0	0	0	0	0	0	0	0	0
FXYD1	0	1	0	0	-1	0	0	0	1	1	0	0	0	0	0	0	0	0
PDE4D	0	0	0	0	1	0	0	0	0	0	0	0	0	0	0	0	0	0
SPRYD3	0	1	0	0	0	0	0	0	0	0	0	0	0	0	0	0	0	0
TNS2	0	1	0	0	0	0	0	0	0	0	0	0	0	0	0	0	0	0
LGI4	0	0	0	0	0	0	0	0	1	0	0	0	0	0	0	0	0	0
ENSCAFG00000007112	0	0	0	0	1	0	0	0	0	0	0	0	0	0	0	0	0	0
CCM2L	0	0	0	0	1	0	0	0	0	0	0	0	0	0	0	0	0	0
HCK	0	0	0	0	0	0	0	0	1	0	0	0	0	0	0	0	0	0
C20H3orf14	0	0	0	0	0	0	0	0	0	1	0	0	0	0	0	0	0	0
MERTK	0	0	0	0	1	0	0	0	0	0	0	0	0	0	0	0	0	0
TOX	0	1	0	0	0	0	1	0	0	-1	0	0	0	0	0	0	0	0
TMEM87B	0	-1	0	0	0	1	0	0	0	0	0	0	0	0	0	0	0	0
ELOVL7	0	0	0	0	1	0	0	0	0	1	0	0	0	0	0	0	0	0
FBXO3	0	0	0	0	0	0	0	0	0	1	0	0	0	0	0	0	0	0
ENSCAFG00000007154	0	-1	0	0	0	0	0	1	0	1	-1	0	0	0	0	-1	0	0
DENND5A	0	1	0	0	0	0	0	0	0	0	0	0	0	0	0	0	0	0
SLC16A12	0	1	0	0	0	0	0	0	0	0	1	0	0	0	0	1	0	1
SLC35A4	0	1	0	0	0	0	0	0	0	0	0	0	0	0	0	0	0	0
CHD7	0	0	0	0	1	0	0	0	0	1	0	0	0	0	0	0	0	0
ZSWIM6	0	0	0	0	1	0	0	0	0	0	0	0	0	0	0	0	0	0
TTL	0	1	0	0	0	0	0	0	0	0	0	0	0	0	0	0	0	0
MICU2	0	0	0	0	0	0	0	0	0	1	0	0	0	0	0	0	0	0
ZNF772	0	1	0	0	0	0	0	0	0	0	0	0	0	0	0	0	0	0
LATS2	0	1	0	0	0	0	0	0	0	1	0	0	0	0	0	0	0	0
HTR7	0	-1	0	1	0	1	0	1	0	0	0	0	0	0	0	0	0	0
FAM107A	0	1	0	0	0	0	0	0	0	1	0	0	0	0	0	0	0	0
ENSCAFG00000007230	0	0	0	0	0	0	0	0	0	1	0	0	0	0	0	0	0	0
NOL4L	0	0	0	0	1	0	0	0	0	0	0	0	0	0	0	0	0	0
HIPK3	0	0	0	0	0	0	0	0	0	1	0	0	0	0	0	0	0	0
TMEM41B	0	0	0	0	1	1	0	0	0	-1	0	0	0	0	0	0	0	0
IPO11	0	0	0	0	1	0	0	0	0	0	0	0	0	0	0	0	0	0
ANKRD1	0	0	0	0	0	0	0	0	0	1	0	0	0	0	0	0	0	0
PCGF5	0	0	0	0	0	0	0	0	0	1	0	0	0	0	0	0	0	0
DRAM1	0	1	0	0	0	0	0	0	0	0	0	0	0	0	0	0	0	0
DNMT3B	0	0	0	0	1	0	0	0	0	0	0	0	0	0	0	0	0	0
ENSCAFG00000007270	0	1	0	0	0	0	0	0	0	0	0	0	0	0	0	0	0	0
NUP37	0	1	0	0	0	0	0	0	0	0	0	0	0	0	0	0	0	0
PSD4	0	1	0	0	0	0	0	0	0	-1	0	0	0	0	0	0	0	0
ENSCAFG00000007307	0	1	0	0	0	0	0	0	0	0	0	0	0	0	0	0	0	0
DEPDC7	0	0	0	0	0	0	0	0	0	1	0	0	0	0	0	0	0	0
QSER1	0	0	0	0	1	0	0	0	0	0	0	0	0	0	0	0	0	0
ADHFE1	0	1	0	0	0	0	0	0	0	0	0	0	0	0	0	0	0	0
NR4A1	0	-1	0	0	1	0	0	0	0	0	0	0	0	0	0	0	0	0
KIAA0355	0	0	0	0	1	0	0	0	0	0	0	0	0	0	0	0	0	0
ZMYM2	0	0	0	0	1	0	0	0	0	1	0	0	0	0	0	0	0	0
ENSCAFG00000007343	0	0	0	0	0	0	0	0	0	1	0	0	0	0	0	0	0	0
MYBL1	0	0	0	0	0	0	0	0	1	0	0	0	0	0	0	0	0	0
LSM14A	0	0	0	0	1	0	0	0	0	0	0	0	0	0	0	0	0	0
ACVR1B	0	0	0	0	0	0	0	0	1	0	0	0	0	0	0	0	0	0
EPB41L4A	0	0	0	0	0	0	0	0	0	1	0	0	0	0	0	0	0	0
KLKB1	0	1	0	0	1	0	0	0	0	0	0	0	0	0	0	0	0	0
PARP4	0	0	0	0	1	0	0	0	0	0	0	0	0	0	0	0	0	0
CYP4V2	0	1	0	0	0	0	0	0	0	0	0	0	0	0	0	0	0	0
RPP14	0	0	0	0	0	0	0	0	0	1	0	0	0	0	0	0	0	0
FAM149A	0	1	0	0	0	0	0	0	0	1	0	0	0	0	0	0	0	0
TLR3	0	0	0	0	1	0	0	0	0	1	0	0	0	0	0	0	0	0
SWAP70	0	0	0	0	1	0	0	0	0	0	0	0	0	0	0	0	0	0
PJA2	0	0	0	0	1	0	0	0	0	0	0	0	0	0	0	0	0	0
WT1	0	0	0	0	0	0	0	0	0	0	0	0	0	1	0	0	0	0
MARCH5	0	0	0	0	0	0	0	0	0	1	0	0	0	0	0	0	0	0
NECAB3	0	1	0	0	-1	0	0	0	0	-1	0	0	0	0	0	0	0	0
PXMP4	0	1	0	0	0	0	0	0	0	0	0	0	0	0	0	0	0	0
TRIM23	0	0	0	0	0	0	0	0	0	1	0	0	0	0	0	0	0	0
RMND5A	0	1	0	0	0	0	0	0	0	0	0	0	0	0	0	0	0	0
NCK1	0	1	0	0	1	0	0	0	0	0	0	0	0	0	0	0	0	0
KRCC1	0	1	0	0	0	0	0	0	0	0	0	0	0	0	0	0	0	0
GLT8D2	0	1	0	0	-1	0	0	0	0	0	0	0	0	0	0	0	0	0
SORBS2	0	0	0	0	-1	0	0	0	1	1	0	0	0	0	0	0	0	0

RILPL2	0	1	0	0	0	0	0	0	0	0	0	0	0	0	0	0	0	0
SBF2	0	1	0	0	0	0	0	0	0	1	0	0	0	0	0	0	0	0
HHEX	0	0	0	0	1	0	0	0	0	0	0	0	0	0	0	0	0	0
NLN	0	0	0	0	0	0	0	0	0	1	0	0	0	0	0	0	0	0
KDM3A	0	-1	0	0	1	0	0	0	0	0	0	0	0	0	0	0	0	0
ITCH	0	0	0	0	1	0	0	0	0	0	0	0	0	0	0	0	0	0
EXOC6	0	0	0	0	1	0	0	0	0	0	0	0	0	0	0	0	0	0
ADM	0	1	0	0	0	0	0	0	1	1	0	0	0	0	0	0	0	0
UFSP2	0	0	0	0	1	0	0	0	0	0	0	0	0	0	0	0	0	0
AMPD3	0	1	0	0	0	0	0	0	1	0	0	0	0	0	0	0	0	0
ANKRD37	0	-1	0	0	0	0	0	0	0	1	0	0	0	0	0	0	0	0
RNF141	0	0	0	0	0	0	0	0	0	1	0	0	0	0	0	0	0	0
LYVE1	0	0	0	0	1	0	0	0	0	0	0	0	0	0	0	0	0	0
PAM	0	1	0	0	0	0	0	0	0	1	0	0	0	0	0	0	0	0
SCN8A	0	0	0	0	0	0	0	0	0	1	0	0	0	0	0	0	0	0
FLNB	0	-1	0	0	1	0	0	0	0	-1	0	0	0	0	0	0	0	0
SH3RF1	0	0	0	0	0	0	0	0	0	1	0	0	0	0	0	0	0	0
PITPNM2	0	0	0	0	1	0	0	0	0	0	0	0	0	0	0	0	0	0
ST8SIA4	0	-1	0	0	1	0	0	0	0	0	0	0	0	0	0	0	0	0
PREX2	0	0	0	0	1	0	0	0	0	0	0	0	0	0	0	0	0	0
PIK3R1	0	1	0	0	0	0	0	0	0	0	0	0	0	0	0	0	0	0
PLEKHF1	0	1	0	0	0	0	0	0	0	0	0	0	0	0	0	0	0	0
OTUD4	0	0	0	0	1	0	0	0	0	0	0	0	0	0	0	0	0	0
UQCRFS1	0	1	0	0	0	0	0	0	0	0	0	0	0	0	0	0	0	0
ACSS2	0	1	0	0	-1	0	0	0	0	0	0	0	0	0	0	0	0	0
NEK1	0	-1	0	0	1	0	0	0	0	0	0	0	0	0	0	0	0	0
DTX4	0	0	0	0	0	0	0	0	0	1	0	0	0	0	0	0	0	0
ST3GAL5	0	0	0	0	0	0	0	0	0	1	0	0	0	0	0	0	0	0
SMAD1	0	0	0	0	1	0	0	0	0	0	0	0	0	0	0	0	0	0
ACSL1	0	1	0	0	0	0	0	0	0	-1	0	0	0	0	0	0	0	0
ZBTB38	0	0	0	0	1	0	0	0	0	0	0	0	0	0	0	0	0	0
CLCN3	0	-1	0	0	0	0	0	0	0	1	0	0	0	0	0	0	0	0
CHD1	0	0	0	0	1	0	0	0	0	0	0	0	0	0	0	0	0	0
RASA2	0	0	0	0	1	0	0	0	0	0	0	0	0	0	0	0	0	0
ENSCAFG00000007675	0	0	0	0	1	0	0	0	0	0	0	0	0	0	0	0	0	0
TMEM150A	0	0	0	0	-1	0	0	0	0	1	0	0	0	0	0	0	0	0
AADAT	0	0	0	0	1	0	0	0	0	-1	0	0	0	0	0	0	0	0
RNF181	0	0	0	0	0	0	0	0	1	1	0	0	0	0	0	0	0	0
GALNT18	0	0	0	0	1	0	0	0	0	0	0	0	0	0	0	0	0	0
LPXN	0	0	0	0	1	0	0	0	0	0	0	0	0	0	0	0	0	0
GGCX	0	0	0	0	-1	0	0	0	1	0	0	0	0	0	0	0	0	0
ATP1B3	0	0	0	0	1	0	0	0	0	-1	0	0	0	0	0	0	0	0
SLC10A7	0	0	0	0	0	1	0	0	0	0	0	0	0	0	0	0	0	0
GK5	0	0	0	0	1	0	0	0	0	-1	0	0	0	0	0	0	0	0
CCDC125	0	0	0	0	1	0	0	0	0	0	0	0	0	0	0	0	0	0
LNPEP	0	0	1	0	0	0	0	0	0	0	0	0	0	0	0	0	0	0
RAD17	0	0	0	0	1	0	0	0	0	0	0	0	0	0	0	0	0	0
NCOA2	0	1	0	0	0	0	0	0	0	0	0	0	0	0	0	0	0	0
ARHGAP10	0	1	0	0	-1	0	0	0	0	0	0	0	0	0	0	0	0	0
ELMOD3	0	1	0	0	0	0	0	0	0	0	0	0	0	0	0	0	0	0
OCLN	0	0	0	0	1	0	0	0	0	0	0	0	0	0	0	0	0	0
STOX2	0	0	0	0	1	0	0	0	0	0	0	0	0	0	0	0	0	0
RETSAT	0	1	0	0	-1	0	0	0	0	0	0	0	0	0	0	0	0	0
ERAP1	0	0	0	0	0	0	0	0	0	1	0	0	0	0	0	0	0	0
LPCAT4	0	1	0	0	0	0	0	0	0	0	0	0	0	0	0	0	0	0
BDP1	0	0	0	0	1	0	0	0	0	0	0	0	0	0	0	0	0	0
ENSCAFG00000007840	0	0	0	0	1	0	0	0	0	0	0	0	0	0	0	0	0	0
RSRC2	0	0	0	0	1	0	0	0	0	0	0	0	0	0	0	0	0	0
HAND2	0	1	0	0	0	1	0	0	0	1	1	0	0	0	1	0	0	0
SLC43A1	0	1	0	0	0	0	0	0	1	0	0	0	0	0	0	0	0	0
ENSCAFG00000007869	0	0	0	0	1	1	0	0	0	0	0	0	0	0	0	0	0	0
FBXO8	0	1	0	0	0	0	0	0	0	0	0	0	0	0	0	0	0	0
SLC43A3	0	1	0	0	0	0	0	0	0	0	0	0	0	0	0	0	0	0
SLC12A6	0	1	0	0	0	0	0	0	0	0	0	0	0	0	0	0	0	0
HPGD	0	1	0	0	0	0	0	0	0	1	0	0	0	0	0	0	0	0
RBP4	0	0	0	0	0	0	0	0	1	-1	0	0	0	0	0	0	0	0
EYA1	0	0	0	0	0	0	1	0	0	0	0	0	0	0	0	1	0	1
ELL2	0	0	0	0	0	0	0	0	0	1	0	0	0	0	0	0	0	0
WWC2	0	0	0	0	0	0	0	0	0	1	0	0	0	0	0	0	0	0
LRBA	0	0	0	0	0	0	0	0	0	1	0	0	0	0	0	0	0	0
PCOLCE2	0	1	0	0	-1	0	0	0	0	0	0	0	0	0	0	0	0	0
CTSB	0	0	0	0	0	0	0	0	0	1	0	0	0	0	0	0	0	0
PROCR	0	0	0	0	1	0	0	0	0	0	0	0	0	0	0	0	0	0
NEIL2	0	1	0	0	0	0	0	0	0	0	0	0	0	0	0	0	0	0
TNKS1BP1	0	1	0	0	0	0	0	0	0	0	0	0	0	0	0	0	0	0
APLNR	0	0	0	0	1	0	0	0	0	0	0	0	0	0	0	0	0	0
MMP24	0	0	0	0	1	0	0	0	0	0	0	0	0	0	0	0	0	0
FAM167A	0	1	0	0	0	0	0	0	0	0	0	0	0	0	0	0	0	0
U2SURP	0	0	0	0	1	0	0	0	0	0	0	0	0	0	0	0	0	0
TEAD1	0	0	0	0	-1	0	0	0	0	1	0	0	0	0	0	0	0	0
ENSCAFG00000007984	0	0	0	0	1	0	0	0	0	0	0	0	0	0	0	0	0	0
WDR66	0	1	0	0	-1	0	0	0	1	0	0	0	0	0	0	0	0	0
IL17RD	0	1	0	0	0	0	0	0	0	0	0	0	0	0	0	0	0	0
PSMD9	0	1	0	0	0	0	0	0	0	0	0	0	0	0	0	0	0	0
KIAA0825	0	0	0	0	0	0	0	0	0	1	0	0	0	0	0	0	0	0
RYR3	0	0	0	0	0	0	0	0	0	1	0	0	0	0	0	0	0	0
ARHGEF3	0	0	0	0	1	0	0	0	0	1	0	0	0	0	0	0	0	0
ENSCAFG00000008063	0	0	0	-1	1	0	0	0	0	0	0	0	0	0	0	0	0	0
FAM172A	0	0	0	0	0	0	0	0	0	1	0	0	0	0	0	0	0	0
ERGIC3	0	0	0	0	-1	0	0	0	0	1	0	0	0	0	0	0	0	0
ENSCAFG00000008104	0	0	0	0	1	0	0	0	0	0	0	0	0	0	0	0	0	0
TENM3	0	0	0	0	0	0	0	0	0	-1	0	0	0	0	1	0	0	0
TMEM154	0	0	0	0	0	0	0	0	0	1	0	0	0	0	0	0	0	0
PI15	0	0	0	0	0	0	0	0	1	0	0	0	0	0	0	0	0	0
KIF13B	0	1	0	0	1	0	0	0	0	1	0	0	0	0	0	0	0	0
AGTR1	0	1	0	0	0	0	0	0	0	0	0	0	0	0	0	0	0	0
GREM1	0	0	0	0	-1	0	0	0	1	-1	0	0	0	0	0	0	0	0
SCG5	0	0	0	0	0	0	0	0	0	1	0	0	0	0	0	0	0	0
HMBOX1	0	0	0	0	1	0	0	0	0	1	0	0	0	0	0	0	0	0
ENSCAFG00000008243	0	1	0	0	0	0	0	0	0	0	0	0	0	0	0	0	0	0
FAR1	0	0	0	0	0	0	0	0	0	1	0	0	0	0	0	0	0	0
GYG1	0	0	0	0	0	0	0	0	0	1	0	0	0	0	0	0	0	0
ACTA1	0	0	0	0	0	0	0	0	0	1	0	0	0	0	0	0	0	0
KDM2B	0	1	0	0	0	0	0	0	0	0	0	0	0	0	0	0	0	0
INTS9	0	0	0	0	0	0	0	0	0	1	0	0	0	0	0	0	0	0
ARHGEF28	0	0	0	0	1	0	0	0	0	0	0	0	0	0	0	0	0	0
LYSMD3	0	0	0	0	0	0	0	0	0	1	0	0	0	0	0	0	0	0
PTPRJ	0	0	0	0	-1	0	0	0	0	1	0	0	0	0	0	0	0	0
EXTL3	0	1	0	0	0	0	0	0	0	1	0	0	0	0	0	0	0	0
ENC1	0	0	0	0	1	0	0	0	0	0	0	0	0	0	0	0	0	0
HEXB	0	0	0	0	1	0	0	0	0	1	0	0	0	0	0	0	0	0
FZD3	0	0	0	0	0	0	0	0	0	1	0	0	0	0	0	0	0	0
CHDH	0	1	0	0	0	0	0	0	0	0	0	0	0	0	0	0	0	0
MEF2C	0	0	0	0	1	0	0	0	0	0	0	0	0	0	0	0	0	0
SMARCD1	0	0	0	0	1	0	0	0	0	0	0	0	0	0	0	0	0	0
ZFHX4	0	1	0	0	-1	0	0	0	1	0	0	0	0	0	0	0	0	0
CP	0	0	0	0	1	0	0	0	0	0	0	0	0	0	0	0	0	0
TM4SF18	0	0	0	0	1	0	0	0	0	0	0	0	0	0	0	0	0	0
PEX2	0	1	0	0	0	0	0	0	0	0	0	0	0	0	0	0	0	0
CAMKK2	0	1	0	0	0	0	0	0	0	0	0	0	0	0	0	0	0	0
THAP6	0	0	0	0	0	0	0	0	0	1	0	0	0	0	0	0	0	0
ENSCAFG00000008335	0	0	1	0	0	0	0	0	0	0	0	0	0	0	0	0	0	0
AGBL2	0	1	0	0	0	0	0	0	0	1	0	0	0	0	0	0	0	0
CDKL2	0	0	0	0	-1	0	0	0	0	1	0	0	0	0	0	0	0	0
TLR2	0	0	0	0	0	0	0	0	0	0	1	0	0	0	0	0	0	1
SCARA5	0	1	0	0	-1	0	0	0	0	0	0	0	0	0	0	0	0	0
SCAND1	0	1	0	0	0	0	0	0	0	0	0	0	0	0	0	0	0	0
G3BP2	0	0	0	0	0	0	0	0	0	1	0	0	0	0	0	0	0	0
FAIM2	0	-1	0	0	0	0	0	0	1	0	0	0	0	0	0	0	0	0
HTRA2	0	1	0	0	0	0	0	0	0	0	0	0	0	0	0	0	0	0

NCKAP5L	0	1	0	0	0	0	0	0	0	0	0	0	0	0	0	0	0	0
SCARA3	0	1	0	0	-1	0	0	0	0	-1	0	0	0	0	0	0	0	0
P2RX7	0	0	0	0	1	0	0	0	0	1	0	0	0	0	0	0	0	0
VEGFC	0	0	0	0	1	0	0	0	0	0	0	0	0	0	0	0	0	0
NDUFS3	0	1	0	0	0	0	0	0	0	0	0	0	0	0	0	0	0	0
ZBTB10	0	0	0	0	0	1	0	0	0	0	0	0	0	0	0	0	0	0
NCAM2	0	1	0	0	0	0	0	0	1	0	0	0	0	0	0	0	0	0
PAG1	0	0	0	0	0	0	0	0	0	1	0	0	0	0	0	0	0	0
ENTPD1	0	0	0	0	1	0	0	0	0	0	0	0	0	0	0	0	0	0
CCNJ	0	-1	0	0	1	0	0	0	0	0	0	0	0	0	0	0	0	0
ENSCAFG00000008456	0	-1	0	0	1	1	0	0	0	0	0	0	0	0	0	0	0	0
SETD6	0	1	0	0	0	0	0	0	0	0	0	0	0	0	0	0	0	0
FMNL3	0	-1	0	0	1	0	0	0	0	-1	0	0	0	0	0	0	0	0
BLNK	0	0	0	0	0	0	0	0	0	1	0	0	0	0	0	0	0	0
NDRG4	0	0	0	0	1	0	0	0	0	0	0	0	0	0	0	0	0	0
WBP1	0	0	0	0	0	0	0	0	0	1	0	0	0	0	0	0	0	0
GPM6A	0	0	0	0	0	0	0	0	0	1	0	0	0	0	0	0	0	0
MEIS2	0	-1	0	0	0	0	0	0	0	1	-1	0	0	0	0	0	0	0
GUCY1A1	0	0	0	0	1	0	0	0	0	0	0	0	0	0	0	0	0	0
APP	0	0	0	0	0	0	0	0	0	1	0	0	0	0	0	0	0	0
ZNF319	0	1	0	0	0	0	0	0	0	0	0	0	0	0	0	0	0	0
KCNH3	0	1	0	0	0	0	0	0	0	0	0	0	0	0	0	0	0	0
MCPH1	0	1	0	0	0	0	0	0	0	0	0	0	0	0	0	0	0	0
SPATS2	0	-1	0	0	1	0	0	0	-1	0	0	0	0	0	0	0	0	0
ART3	0	0	0	0	0	0	0	0	1	0	0	0	0	0	0	0	0	0
ANGPT2	0	0	0	0	1	0	0	0	0	0	0	0	0	0	0	0	0	0
ATXN2	0	0	0	0	0	0	0	0	0	1	0	0	0	0	0	0	0	0
SOX6	0	0	0	0	0	0	0	0	0	0	0	0	0	0	1	0	0	0
SCARB2	0	0	0	0	0	0	0	0	0	1	0	0	0	0	0	0	0	0
PDGFC	0	0	0	0	-1	0	0	0	1	1	0	0	0	0	0	0	0	0
RWDD2B	0	1	0	0	0	0	0	0	0	0	0	0	0	0	0	0	0	0
GLRB	0	0	0	0	0	0	0	0	0	1	0	0	0	0	0	0	0	0
PRPH	0	0	0	0	0	0	0	0	0	1	0	0	0	0	0	0	0	0
STBD1	0	0	0	0	0	0	0	0	0	1	0	0	0	0	0	0	0	0
CCDC158	0	0	0	0	0	0	0	0	0	1	0	0	0	0	0	0	0	0
SHROOM3	0	0	0	0	0	0	0	0	0	1	0	0	0	0	0	0	0	0
ENSCAFG00000008655	0	0	0	0	1	0	0	0	0	0	0	0	0	0	0	0	0	0
MADD	0	-1	0	0	1	0	0	0	0	0	0	0	0	0	0	0	0	0
SEPT11	0	0	0	0	1	0	0	0	0	0	0	0	0	0	0	0	0	0
RXFP1	0	0	0	0	0	0	0	0	0	1	0	0	0	0	0	0	0	0
SSBP2	0	1	0	0	-1	0	0	0	0	1	0	0	0	0	0	0	0	0
CCNG2	0	0	0	0	0	0	0	0	0	1	0	0	0	0	0	0	0	0
CNOT6L	0	0	0	0	0	0	0	1	0	0	0	0	0	0	0	0	0	0
FNIP2	0	1	0	0	0	0	0	0	0	0	0	0	0	0	0	0	0	0
MME	0	0	0	0	0	0	1	0	0	0	0	0	0	0	0	0	0	0
RAPGEF2	0	0	0	0	1	0	0	0	0	0	0	0	0	0	0	0	0	0
COQ9	0	1	0	0	0	0	0	0	0	0	0	0	0	0	0	0	0	0
DPYSL2	0	1	0	0	0	0	0	0	0	0	0	0	0	0	0	0	0	0
WWP1	0	0	0	0	1	0	0	0	0	0	0	0	0	0	0	0	0	0
RND1	0	0	0	0	0	0	0	0	0	1	0	0	0	0	0	0	0	0
TRAFD1	0	1	0	0	0	0	0	0	0	0	0	0	0	0	0	0	0	0
RPRD1B	0	0	0	0	0	0	0	0	0	1	0	0	0	0	0	0	0	0
ACP2	0	0	0	0	0	0	0	0	0	1	0	0	0	0	0	0	0	0
KLHL2	0	0	0	0	1	0	0	0	0	0	0	0	0	0	0	0	0	0
PPP2R2A	0	-1	0	0	1	0	0	0	0	0	0	0	0	0	0	0	0	0
HECTD4	0	0	0	0	0	0	0	0	0	1	0	0	0	0	0	0	0	0
DDB2	0	1	0	0	0	0	0	0	0	0	0	0	0	0	0	0	0	0
TIAM1	0	1	0	0	0	0	1	0	0	0	0	0	0	0	0	0	0	0
CACNB3	0	0	0	0	-1	0	0	0	0	1	0	0	0	0	0	0	0	0
BMP2K	0	0	0	0	1	0	0	0	0	0	0	0	0	0	0	0	0	0
SOD1	0	0	0	0	0	0	0	0	1	0	0	0	0	0	0	0	0	0
CPE	0	0	0	0	0	0	0	0	0	1	0	0	0	0	0	0	0	0
TIPARP	0	0	0	0	1	0	0	0	0	0	0	0	0	0	0	0	0	0
TLL1	0	0	0	0	1	0	0	0	0	0	0	0	0	0	1	0	0	0
CCNL1	0	0	0	0	1	0	0	0	0	0	0	0	0	0	0	0	0	0
FGF5	0	0	0	0	0	0	0	0	0	-1	1	0	0	0	1	0	0	0
SERINC5	0	0	0	0	1	0	0	0	0	0	0	0	0	0	0	0	0	0
VEPH1	0	0	0	0	0	0	0	0	0	1	0	0	0	0	0	0	0	0
BPI	0	0	1	1	-1	0	0	0	1	0	0	0	0	0	0	0	0	0
PTX3	0	0	0	0	-1	0	0	0	0	1	0	0	0	0	0	0	0	0
SHOX2	0	1	0	0	0	0	0	0	0	0	1	0	0	0	0	1	0	0
ADCY6	0	0	0	0	-1	0	0	0	0	1	0	0	0	0	0	0	0	0
EBF2	0	1	0	0	0	0	0	0	0	0	0	0	0	0	0	0	0	0
RASGEF1B	0	0	0	0	0	0	0	0	1	0	0	0	0	0	0	0	0	0
CCNT1	0	0	0	0	0	0	0	0	0	1	0	0	0	0	0	0	0	0
OAS3	0	0	0	0	1	1	0	0	0	0	0	0	0	0	0	0	0	0
NLRC5	0	0	0	0	1	0	0	0	0	0	0	0	0	0	0	0	0	0
ENSCAFG00000008934	0	0	0	0	1	0	0	0	0	0	0	0	0	0	0	0	0	0
RAB11FIP5	0	1	0	0	-1	0	0	0	0	0	0	0	0	0	0	0	0	0
TMEM150C	0	0	0	0	1	0	0	0	0	0	0	0	0	0	0	0	0	0
HERPUD1	0	0	0	0	0	0	0	0	0	1	0	0	0	0	0	0	0	0
ENSCAFG00000008984	0	0	0	0	1	0	0	0	0	0	0	0	0	0	0	0	0	0
PROS1	0	0	0	0	-1	0	0	0	0	1	0	0	0	0	0	0	0	0
DOCK5	0	0	0	0	0	0	0	0	0	1	0	0	0	0	0	0	0	0
CYP26B1	0	0	0	0	0	0	1	0	0	0	0	0	0	0	1	0	0	0
ZNF641	0	0	0	0	-1	0	0	0	0	1	0	0	0	0	0	0	0	0
PPP1R16B	0	0	0	0	1	0	0	0	0	0	0	0	0	0	0	0	0	0
TMEM64	0	-1	0	0	0	0	0	0	0	1	0	0	0	0	0	0	0	0
ASB8	0	1	0	0	0	0	0	0	0	0	0	0	0	0	0	0	0	0
DYSF	0	0	0	0	1	0	0	0	0	0	0	0	0	0	0	0	0	0
PFKM	0	0	0	0	0	0	0	0	0	1	0	0	0	0	0	0	0	0
TENT2	0	0	0	0	1	0	0	0	0	0	0	0	0	0	0	0	0	0
SLC26A7	0	0	0	0	0	0	0	0	0	1	0	0	0	0	0	0	0	0
JMY	0	0	0	0	0	0	0	0	0	1	0	0	0	0	0	0	0	0
TOP1	0	0	0	0	1	0	0	0	0	0	0	0	0	0	0	0	0	0
SLC8B1	0	1	0	0	0	0	0	0	0	0	0	0	0	0	0	0	0	0
PLBD2	0	1	0	0	-1	0	0	0	0	0	0	0	0	0	0	0	0	0
PLCG1	0	0	0	0	1	0	0	0	0	0	0	0	0	0	0	0	0	0
ZDHHC16	0	0	0	1	0	0	0	0	0	0	0	0	0	0	0	0	0	0
HPSE	0	1	0	0	0	0	0	0	0	0	0	0	0	0	0	0	0	0
ZHX3	0	1	0	0	0	0	0	0	0	0	0	0	0	0	0	0	0	0
VDR	0	1	0	0	-1	0	0	0	0	0	0	0	0	0	0	0	0	0
STC1	0	0	0	0	1	0	0	0	0	0	0	0	0	0	0	0	0	0
ARL6	0	0	0	0	0	0	0	0	0	1	0	0	0	0	0	0	0	0
ENSCAFG00000009113	0	1	0	0	0	0	0	0	1	0	0	0	0	0	0	0	0	0
IVD	0	1	0	0	0	0	0	0	0	0	0	0	0	0	0	0	0	0
ENSCAFG00000009117	0	1	0	0	0	0	0	0	0	0	0	0	0	0	0	0	0	0
BBS2	0	1	0	0	0	0	0	0	0	0	0	0	0	0	0	0	0	0
BAHD1	0	0	0	0	0	0	0	0	0	1	0	0	0	0	0	0	0	0
SAA1	0	1	0	0	0	0	0	0	0	0	0	0	0	0	0	0	0	0
LPIN3	0	1	0	0	0	0	0	0	0	0	0	0	0	0	0	0	0	0
CHST14	0	1	0	0	0	0	0	0	0	0	0	0	0	0	0	0	0	0
HDAC7	0	1	0	0	0	-1	0	0	0	0	0	0	0	0	0	0	0	0
TRIO	0	0	0	0	1	0	0	0	0	0	0	0	0	0	0	0	0	0
SLC48A1	0	1	0	0	0	0	0	0	0	0	0	0	0	0	0	0	0	0
UBTD1	0	-1	0	0	1	0	0	0	0	0	0	0	0	0	0	0	0	0
RAPGEF3	0	1	0	0	1	0	0	0	0	0	0	0	0	0	0	0	0	0
CPOX	0	1	0	0	0	0	0	0	0	0	0	0	0	0	0	0	0	0
KCNJ3	0	0	0	0	1	0	0	0	0	0	0	0	0	0	0	0	0	0
ST3GAL6	0	0	0	0	1	0	0	0	0	0	0	0	0	0	0	0	0	0
AMBRA1	0	1	0	0	0	0	0	0	0	0	0	0	0	0	0	0	0	0
FOXF2	0	1	0	0	0	0	0	0	0	0	0	0	0	0	0	0	0	0
NR4A2	0	-1	0	0	1	0	0	1	0	0	0	0	0	0	0	0	0	0
DCBLD2	0	0	0	0	0	0	0	0	0	1	0	0	0	0	0	0	0	0
TBX3	0	0	0	0	0	0	0	1	0	0	0	0	0	0	0	0	0	0
WDR41	0	0	0	0	0	1	0	0	0	0	0	0	0	0	0	0	0	0
CHD6	0	0	0	0	0	0	0	0	0	1	0	0	0	0	0	0	0	0
PTPN22	0	0	0	0	0	0	0	0	0	1	0	0	0	0	0	0	0	0
MED13L	0	1	0	-1	0	0	0	0	1	1	0	0	0	0	0	0	0	0
STAB1	0	0	0	0	1	0	0	0	0	0	0	0	0	0	0	0	0	0

F2RL1	0	1	0	0	0	0	0	0	0	1	0	0	0	0	0	0	0	0
F2R	0	1	0	0	0	0	0	0	0	0	0	0	0	0	0	0	0	0
SLC38A1	0	-1	0	0	1	0	0	0	0	0	0	0	0	0	0	0	0	0
RNFT2	0	1	0	0	-1	0	0	0	0	1	0	0	0	0	0	0	0	0
L3MBTL1	0	1	0	0	0	0	0	0	0	0	0	0	0	0	0	0	0	0
IQGAP2	0	0	0	0	1	0	0	0	0	-1	0	0	0	0	0	0	0	0
SCAF11	0	0	0	0	1	0	0	0	0	0	0	0	0	0	0	0	0	0
F2RL2	0	1	0	0	0	0	0	0	0	0	0	0	0	0	0	0	0	0
ENSCAFG00000009307	0	1	0	0	0	0	0	0	0	1	0	0	0	0	0	0	0	0
RIPK1	0	0	0	0	1	0	0	0	0	0	0	0	0	0	0	0	0	0
PDLIM2	0	1	0	0	-1	0	0	0	0	1	0	0	0	0	0	0	0	0
SV2C	0	0	0	0	0	1	0	0	0	0	0	0	0	0	0	0	0	0
DPY19L4	0	-1	0	0	0	0	0	0	0	1	0	0	0	0	0	0	0	0
SLC22A23	0	1	0	0	0	0	0	0	0	1	0	0	0	0	0	0	0	0
TOX2	0	0	0	0	1	0	0	0	0	0	0	0	0	0	0	0	0	0
PKP4	0	0	0	0	0	0	0	0	0	1	0	0	0	0	0	0	0	0
SPINT1	0	0	0	0	0	0	0	0	0	1	0	0	0	0	0	0	0	0
TANC1	0	0	0	0	1	0	0	0	0	1	0	0	0	0	0	0	0	0
WDSUB1	0	1	0	0	0	0	0	0	0	0	0	0	0	0	0	0	0	0
CRY2	0	0	0	0	0	0	0	0	1	0	0	0	0	0	0	0	0	0
OSGEPL1	0	0	0	0	0	0	0	0	0	1	0	0	0	0	0	0	0	0
PRPF4B	0	0	0	0	1	0	0	0	0	0	0	0	0	0	0	0	0	0
POLK	0	0	0	0	0	0	0	0	0	1	0	0	0	0	0	0	0	0
CHST1	0	0	0	0	1	0	0	0	0	0	0	0	0	0	0	0	0	0
MAPK10	0	-1	0	1	0	0	0	0	0	0	0	0	0	0	0	0	0	0
PRDM11	0	0	0	0	0	0	0	0	0	1	0	0	0	0	0	0	0	0
DLL4	0	0	0	0	1	0	0	0	0	0	0	0	0	0	0	0	0	0
HNF4A	0	1	0	0	0	0	0	0	0	0	0	0	0	0	0	0	0	0
CDYL	0	-1	0	0	1	0	0	0	0	0	0	0	0	0	0	0	0	0
TP53INP1	0	0	0	0	0	0	0	0	0	1	0	0	0	0	0	0	0	0
SLC39A14	0	-1	0	0	0	0	0	0	1	0	0	0	0	0	0	0	0	0
TTPAL	0	0	0	0	0	0	0	0	1	0	0	0	0	0	0	0	0	0
IRX5	0	1	0	0	-1	0	0	0	1	0	1	0	0	0	0	0	0	1
SLC10A6	0	1	0	0	0	0	0	0	0	0	0	0	0	0	0	0	0	0
GOT1	0	1	0	0	0	0	0	0	0	0	0	0	0	0	0	0	0	0
NKX2-3	0	0	0	0	0	1	0	0	0	0	0	0	0	0	1	0	0	0
SERINC3	0	0	0	0	0	0	0	0	0	1	0	0	0	0	0	0	0	0
AFF1	0	1	0	0	0	0	0	0	0	0	0	0	0	0	0	0	0	0
EXT2	0	0	0	0	0	0	0	0	0	1	0	0	0	0	0	0	0	0
SEMA3G	0	1	0	0	0	0	0	0	0	0	0	0	0	0	0	0	0	0
FARS2	0	1	0	0	0	0	0	0	0	0	0	0	0	0	0	0	0	0
MFSD6	0	0	0	0	0	0	0	0	0	1	0	0	0	0	0	0	0	0
MTDH	0	-1	0	0	0	1	0	0	0	-1	0	0	0	0	0	0	0	0
NAB1	0	0	0	0	0	0	0	0	0	1	0	0	0	0	0	0	0	0
ADA	0	0	0	0	0	0	0	0	0	1	0	0	0	0	0	0	0	0
AKTIP	0	0	0	0	0	0	0	0	0	1	0	0	0	0	0	0	0	0
ABI3BP	0	1	0	0	0	0	0	0	0	0	0	0	0	0	0	0	0	0
WISP2	0	1	0	0	0	0	0	0	0	0	0	0	0	0	0	0	0	0
RREB1	0	0	0	0	0	0	0	0	0	1	0	0	0	0	0	0	0	0
NRAS	0	0	0	0	1	0	0	0	0	-1	0	0	0	0	0	0	0	0
GCNT4	0	0	0	0	0	0	0	0	1	0	0	0	0	0	0	0	0	0
SPARCL1	0	0	0	0	1	0	0	0	0	0	0	0	0	0	0	0	0	0
PNPLA2	0	1	0	0	-1	-1	0	0	0	0	0	0	0	0	0	0	0	0
CRACR2B	0	1	0	0	0	0	0	0	0	0	0	0	0	0	0	0	0	0
CLIC6	0	0	0	0	1	0	0	0	0	0	0	0	0	0	0	0	0	0
SNRNP48	0	-1	0	0	1	0	0	0	0	0	0	0	0	0	0	0	0	0
ZBED6	0	0	0	0	1	0	0	0	0	0	0	0	0	0	0	0	0	0
TXNDC5	0	0	0	0	0	0	0	0	0	1	0	0	0	0	0	0	0	0
PKD2	0	0	0	0	-1	0	0	0	0	1	0	0	0	0	0	0	0	0
PPM1K	0	1	0	0	1	0	0	0	0	0	0	0	0	0	0	0	0	0
SOX13	0	0	0	0	1	0	0	0	0	0	0	0	0	0	0	0	0	0
CBR3	0	1	0	0	0	0	0	0	0	0	0	0	0	0	0	0	0	0
ABCG2	0	0	0	0	1	0	0	0	0	0	0	0	0	0	0	0	0	0
TWF1	0	-1	0	0	0	1	0	0	0	0	0	0	0	0	0	0	0	0
DOP1B	0	1	0	0	1	0	0	0	0	0	0	0	0	0	0	0	0	0
RBL2	0	1	0	0	0	0	0	0	0	0	0	0	0	0	0	0	0	0
MORC3	0	1	0	0	0	0	0	0	0	0	0	0	0	0	0	0	0	0
PIK3C2B	0	0	0	0	1	0	0	0	0	0	0	0	0	0	0	0	0	0
MATN4	0	0	0	0	0	0	0	0	0	1	0	0	0	0	0	0	0	0
LRRN2	0	0	0	0	0	0	0	0	1	0	0	0	0	0	0	0	0	0
MAPKBP1	0	1	0	0	0	0	0	0	0	0	0	0	0	0	0	0	0	0
SLF2	0	0	0	0	1	0	0	0	0	0	0	0	0	0	0	0	0	0
DMTN	0	0	0	0	0	0	0	0	1	0	0	0	0	0	0	0	0	0
NGF	0	0	0	0	0	0	0	0	0	1	0	0	0	0	0	0	0	0
ADAMTS20	0	0	0	0	1	0	0	0	0	0	0	0	0	0	0	0	0	0
SALL1	0	0	0	0	0	0	0	0	0	0	1	0	0	0	0	0	0	0
VANGL1	0	1	0	0	0	0	0	0	0	0	0	0	0	0	0	0	0	0
TTC3	0	-1	1	0	1	0	0	0	0	0	0	0	0	0	0	0	0	0
NEDD9	0	0	0	0	1	0	0	0	0	0	0	0	0	0	0	0	0	0
NAV2	0	0	0	0	1	0	0	0	0	-1	0	0	0	0	0	0	0	0
HERC6	0	0	0	0	1	0	0	0	0	0	0	0	0	0	0	0	0	0
HIVEP1	0	0	0	0	0	0	0	0	0	1	0	0	0	0	0	0	0	0
VPS26C	0	0	0	0	0	0	0	0	1	0	0	0	0	0	0	0	0	0
EDN1	0	0	0	0	1	0	0	0	0	1	0	0	0	0	0	0	0	0
CYLD	0	0	0	0	0	0	0	0	1	0	0	0	0	0	0	0	0	0
PHACTR1	0	1	0	0	0	0	0	0	0	0	0	0	0	0	0	0	0	0
JAM3	0	0	0	0	0	0	0	0	0	1	0	0	0	0	0	0	0	0
IGSF3	0	0	0	0	1	0	0	0	0	0	0	0	0	0	0	0	0	0
GFOD1	0	0	0	0	0	0	0	0	1	0	0	0	0	0	0	0	0	0
FAM13A	0	0	0	0	0	0	0	0	1	0	0	0	0	0	0	0	0	0
WDR82	0	1	0	0	0	0	0	0	0	0	0	0	0	0	0	0	0	0
TRIM45	0	1	0	0	0	0	0	0	0	1	0	0	0	0	0	0	0	0
PPM1M	0	1	0	0	0	0	0	0	0	0	0	0	0	0	0	0	0	0
GPRIN3	0	0	0	0	0	0	0	0	0	1	0	0	0	0	0	0	0	0
BBX	0	0	0	0	0	0	0	0	0	1	0	0	0	0	0	0	0	0
EHD4	0	0	0	0	1	0	0	0	0	0	0	0	0	0	0	0	0	0
TENT5C	0	1	0	0	0	0	0	0	1	0	0	0	0	0	0	0	0	0
MMRN1	0	0	0	0	1	0	0	0	0	0	0	0	0	0	0	0	0	0
CD47	0	0	0	0	0	0	0	0	0	1	0	0	0	0	0	0	0	0
MYLIP	0	0	0	0	1	0	0	0	0	0	0	0	0	0	0	0	0	0
OCA2	0	0	0	0	0	0	0	0	0	1	0	0	0	0	0	0	0	0
FBXW4	0	1	0	0	0	0	0	0	0	0	0	0	0	0	0	0	0	0
ERG	0	0	0	0	1	0	0	0	0	0	0	0	0	0	0	0	0	0
NPM3	0	0	0	0	0	0	1	0	0	0	0	0	0	0	0	0	0	0
OGA	0	0	0	0	0	1	0	0	0	0	0	0	0	0	0	0	0	0
ABHD14B	0	1	0	0	0	0	0	0	0	0	0	0	0	0	0	0	0	0
ENSCAFG00000009957	0	0	1	0	1	0	0	0	0	0	0	0	0	0	0	0	0	0
RBBP5	0	0	0	0	1	0	0	0	0	0	0	0	0	0	0	0	0	0
ARMH3	0	0	0	0	0	1	0	0	0	0	0	0	0	0	0	0	0	0
ADAMTS8	0	0	0	1	0	0	0	0	0	0	0	0	0	0	0	0	0	0
TBX15	0	1	0	0	-1	-1	1	0	1	-1	1	0	0	0	0	1	0	1
ZNF423	0	1	0	0	0	0	0	0	0	0	0	0	0	0	0	0	0	0
PRKAB1	0	1	0	0	0	0	0	0	0	0	0	0	0	0	0	0	0	0
CD40	0	0	0	0	0	0	0	0	1	0	0	0	0	0	0	0	0	0
WARS2	0	1	0	0	0	0	0	0	0	0	0	0	0	0	0	0	0	0
ANKRD33B	0	1	0	0	0	0	0	0	1	0	0	0	0	0	0	0	0	0
ENSCAFG00000009998	0	0	0	0	1	0	0	0	0	0	0	0	0	0	0	0	0	0
N4BP1	0	0	0	0	0	0	0	0	0	1	0	0	0	0	0	0	0	0
SIAH1	0	0	0	0	0	0	0	0	0	1	0	0	0	0	0	0	0	0
LONP2	0	1	0	0	-1	0	0	0	0	0	0	0	0	0	0	0	0	0
BRWD1	0	0	0	0	1	0	0	0	0	0	0	0	0	0	0	0	0	0
NUAK2	0	0	0	0	1	0	0	0	0	0	0	0	0	0	0	0	0	0
ANO5	0	0	0	0	1	0	0	0	0	1	0	0	0	0	0	0	0	0
LZTS1	0	0	0	1	0	0	0	0	0	0	0	0	0	0	0	0	0	0
MYO1B	0	1	0	0	-1	0	0	0	0	1	0	0	0	0	0	0	0	0
ATP10A	0	0	0	0	0	0	0	0	1	0	0	0	0	0	0	0	0	0
ATP6V1B2	0	0	0	0	0	0	0	0	1	0	0	0	0	0	0	0	0	0
TEX264	0	0	0	0	0	0	0	0	0	1	0	0	0	0	0	0	0	0
SEMA5A	0	1	0	0	0	0	0	0	0	1	0	0	0	0	0	0	0	0
UBE3A	0	0	0	0	1	0	0	0	0	0	0	0	0	0	0	0	0	0

RAD54L2	0	0	0	0	1	0	0	0	0	0	0	0	0	0	0	0	0	0
APLP2	0	0	0	0	0	0	0	0	0	1	0	0	0	0	0	0	0	0
MFSD4A	0	0	0	0	1	0	0	0	0	0	0	0	0	0	0	0	0	0
BICDL1	0	0	0	0	0	0	0	0	1	0	0	0	0	0	0	0	0	0
SLC39A10	0	-1	1	0	0	0	0	0	0	0	0	0	0	0	0	0	0	0
NHLRC1	0	1	0	0	0	0	0	0	0	0	0	0	0	0	0	0	0	0
ANO3	0	0	0	0	1	0	0	0	0	0	0	0	0	0	0	0	0	0
LPL	0	1	0	0	0	0	0	0	0	1	0	0	0	0	0	0	0	0
MX2	0	0	0	0	1	0	0	0	0	0	0	0	0	0	0	0	0	0
FIBIN	0	0	0	0	0	0	0	0	1	0	0	0	0	0	0	0	0	0
MX1	0	0	0	0	0	0	0	0	0	1	0	0	0	0	0	0	0	0
SLC41A1	0	0	0	0	0	0	0	0	0	1	0	0	0	0	0	0	0	0
RAB29	0	0	0	0	0	0	0	0	0	1	0	0	0	0	0	0	0	0
LGR4	0	1	0	0	-1	0	0	0	0	0	0	0	0	0	0	0	0	0
FBXO25	0	0	0	0	0	0	0	0	0	1	0	0	0	0	0	0	0	0
BDNF	0	0	0	0	0	0	0	0	1	0	0	0	0	0	1	0	0	0
ENSCAFG00000010209	0	0	0	0	1	0	0	0	0	0	0	0	0	0	0	0	0	0
ENSCAFG00000010224	0	0	0	0	1	0	0	0	0	-1	0	0	0	0	0	0	0	0
DOCK3	0	0	0	0	0	0	0	0	0	1	0	0	0	0	0	0	0	0
CUL3	0	0	0	0	1	1	0	0	0	0	0	0	0	0	0	0	0	0
ITFG1	0	0	0	0	0	0	0	0	0	1	0	0	0	0	0	0	0	0
WBP1L	0	0	0	0	0	0	0	0	0	1	0	0	0	0	0	0	0	0
DZIP3	0	0	0	0	0	0	0	0	0	1	0	0	0	0	0	0	0	0
ABCG1	0	0	0	0	1	0	0	0	0	1	0	0	0	0	0	0	0	0
OTUD7A	0	1	0	0	0	0	0	0	0	0	0	0	0	0	0	0	0	0
TSPAN5	0	1	0	0	0	0	0	0	0	0	0	0	0	0	0	0	0	0
ENSCAFG00000010290	0	0	0	0	0	0	0	0	0	1	0	0	0	0	0	0	0	0
CISH	0	1	0	0	0	0	0	0	0	0	0	0	0	0	0	0	0	0
ETS1	0	0	0	0	1	0	0	0	0	-1	0	0	0	0	0	0	0	0
CNNM2	0	0	0	0	0	0	0	0	0	1	0	0	0	0	0	0	0	0
PPFIA4	0	0	0	0	0	0	0	0	1	0	0	0	0	0	0	0	0	0
OSBPL5	0	1	0	0	0	0	0	0	0	0	0	0	0	0	0	0	0	0
MTMR10	0	0	0	0	1	0	0	0	0	1	0	0	0	0	0	0	0	0
NECTIN3	0	-1	0	0	0	1	0	0	0	-1	0	0	0	0	0	0	0	0
MED10	0	0	0	0	0	0	0	0	0	1	0	0	0	0	0	0	0	0
FAN1	0	0	0	0	0	0	0	0	0	1	0	0	0	0	0	0	0	0
CYB5R1	0	0	0	0	-1	0	0	0	0	1	0	0	0	0	0	0	0	0
NT5C2	0	1	0	0	-1	0	0	0	0	1	0	0	0	0	0	0	0	0
ICE1	0	1	0	0	0	0	0	0	0	0	0	0	0	0	0	0	0	0
ALDH5A1	0	1	0	0	0	0	0	0	0	0	0	0	0	0	0	0	0	0
ENSCAFG00000010373	0	0	0	0	1	0	0	0	0	0	0	0	0	0	0	0	0	0
DNAH7	0	0	0	0	0	0	0	0	0	1	0	0	0	0	0	0	0	0
FAP	0	0	0	0	0	0	0	0	1	0	0	0	0	0	0	0	0	0
NADSYN1	0	1	0	0	0	0	0	0	0	0	0	0	0	0	0	0	0	0
FAM189A1	0	0	0	0	0	0	0	0	1	0	0	0	0	0	0	0	0	0
IRS1	0	1	0	0	-1	0	0	0	0	0	0	0	0	0	0	0	0	0
RNF19B	0	0	0	0	1	0	0	0	0	-1	0	0	0	0	0	0	0	0
TJP1	0	0	0	0	1	0	0	0	0	0	0	0	0	0	0	0	0	0
ENSCAFG00000010422	0	1	0	0	0	0	0	0	0	0	0	0	0	0	0	0	0	0
TIRAP	0	0	0	0	0	0	0	0	0	1	0	0	0	0	0	0	0	0
FNDC5	0	0	0	0	0	0	0	0	0	1	0	0	0	0	0	0	0	0
ACADS	0	1	0	0	0	0	0	0	0	0	0	0	0	0	0	0	0	0
CALHM2	0	1	0	0	0	0	0	0	0	0	0	0	0	0	0	0	0	0
KDM5B	0	-1	0	0	1	0	0	0	0	0	0	0	0	0	0	0	0	0
TARSL2	0	0	0	0	1	0	0	0	0	1	0	0	0	0	0	0	0	0
S100PBP	0	0	0	0	0	0	0	0	0	1	0	0	0	0	0	0	0	0
CD200	0	0	0	0	1	0	0	0	0	0	0	0	0	0	0	0	0	0
STN1	0	0	0	0	0	0	0	0	0	1	0	0	0	0	0	0	0	0
PDE9A	0	0	0	0	0	0	0	0	0	1	0	0	0	0	0	0	0	0
RPUSD4	0	1	0	0	0	0	0	0	0	0	0	0	0	0	0	0	0	0
PPP1R12B	0	0	0	0	0	0	0	0	0	1	0	0	0	0	0	0	0	0
CDON	0	1	0	0	1	0	0	0	0	0	0	0	0	0	0	0	0	0
TM2D3	0	0	0	0	0	0	0	0	0	1	0	0	0	0	0	0	0	0
SLK	0	0	0	0	1	0	0	0	0	0	0	0	0	0	0	0	0	0
SLC35A5	0	0	0	0	0	0	0	0	0	1	0	0	0	0	0	0	0	0
LGR6	0	0	0	0	0	0	0	0	1	1	0	0	0	0	0	0	0	0
FIGN	0	1	0	0	0	0	0	0	0	1	0	0	0	0	0	0	0	0
PCSK6	0	0	0	0	-1	0	0	0	0	1	0	0	0	0	0	0	0	0
CARMIL1	0	1	0	0	0	0	0	0	0	0	0	0	0	0	0	0	0	0
RNPEP	0	1	0	0	-1	0	0	0	0	0	0	0	0	0	0	0	0	0
TROVE2	0	-1	0	0	1	0	0	0	0	0	0	0	0	0	0	0	0	0
DAPP1	0	0	0	0	0	0	0	0	0	1	0	0	0	0	0	0	0	0
MARCKSL1	0	-1	0	0	0	0	0	0	0	1	0	0	0	0	0	0	0	0
FGD4	0	1	0	0	0	0	0	0	0	0	0	0	0	0	0	0	0	0
RGS2	0	-1	0	0	1	0	0	0	0	1	0	0	0	0	0	0	0	0
ENSCAFG00000010596	0	0	0	0	1	0	0	0	0	0	0	0	0	0	0	0	0	0
ALDH1A3	0	0	0	0	1	0	0	0	0	0	0	0	0	0	0	0	0	0
DDIT4L	0	1	0	0	0	0	0	0	0	0	0	0	0	0	0	0	0	0
SORCS1	0	0	0	0	0	1	0	0	0	0	0	1	0	0	1	0	0	0
KIAA1551	0	0	0	0	1	0	0	0	0	0	0	0	0	0	0	0	0	0
CSTB	0	0	0	0	-1	0	0	0	1	0	0	0	0	0	0	0	0	0
PPP3CA	0	0	0	0	1	0	0	0	0	0	0	0	0	0	0	0	0	0
STK17B	0	0	0	0	0	1	0	0	0	0	0	0	0	0	0	0	0	0
AGPAT3	0	1	0	0	0	0	0	0	1	1	0	0	0	0	0	0	0	0
FAM167B	0	0	0	0	1	0	0	0	0	0	0	0	0	0	0	0	0	0
HYAL2	0	0	0	0	1	0	0	0	0	0	0	0	0	0	0	0	0	0
ANO1	0	0	0	0	1	0	0	0	0	0	0	0	0	0	0	0	0	0
HYAL3	0	1	0	0	0	0	0	0	0	0	0	0	0	0	0	0	0	0
HECW2	0	0	0	0	1	0	0	0	0	0	0	0	0	0	0	0	0	0
SLC39A8	0	0	0	0	1	0	0	0	0	0	0	0	0	0	0	0	0	0
ADD3	0	1	0	0	0	0	0	0	0	0	0	0	0	0	0	0	0	0
MXI1	0	0	0	0	0	0	0	0	0	1	0	0	0	0	0	0	0	0
ADAMTS17	0	0	0	0	0	0	0	0	0	0	1	0	0	0	0	0	0	0
CAPRIN2	0	0	0	0	0	0	0	0	0	1	0	0	0	0	0	0	0	0
TPCN2	0	1	0	0	0	0	0	0	0	0	0	0	0	0	0	0	0	0
ENSCAFG00000010717	0	0	0	0	0	0	0	0	0	1	0	0	0	0	0	0	0	0
SEMA3B	0	1	0	0	0	0	0	0	0	0	0	0	0	0	0	0	0	0
GIT2	0	0	0	0	1	0	0	0	0	1	0	0	0	0	0	0	0	0
KCNJ2	0	0	0	0	1	0	0	0	0	0	0	0	0	0	0	0	0	0
KPNA6	0	0	0	0	1	0	0	0	0	0	0	0	0	0	0	0	0	0
MAP2K6	0	0	0	0	0	0	1	0	0	0	0	0	0	0	0	0	0	0
TPST1	0	1	0	0	0	0	0	0	0	1	0	0	0	0	0	0	0	0
GPATCH2	0	0	0	0	0	0	0	0	0	1	0	0	0	0	0	0	0	0
SLC12A7	0	0	0	0	1	0	0	0	0	1	0	0	0	0	0	0	0	0
TNNT2	0	0	0	0	0	0	0	0	0	1	0	0	0	0	0	0	0	0
SEMA3F	0	0	0	0	1	0	0	0	0	0	0	0	0	0	0	0	0	0
PDCD4	0	0	0	0	0	0	0	0	0	1	0	0	0	0	0	0	0	0
LRRC28	0	1	0	0	0	0	0	0	0	0	0	0	0	0	0	0	0	0
PPP6R3	0	0	0	0	1	0	0	0	0	0	0	0	0	0	0	0	0	0
TC2N	0	0	0	0	1	0	0	0	0	0	0	0	0	0	0	0	0	0
MVK	0	1	0	0	0	0	0	0	0	0	0	0	0	0	0	0	0	0
RRP15	0	0	0	0	0	1	0	0	0	0	0	0	0	0	0	0	0	0
LYPLAL1	0	0	0	0	0	0	0	0	0	1	0	0	0	0	0	0	0	0
ADRA2A	0	0	0	0	0	0	0	0	1	0	0	0	0	0	0	0	0	0
PFKL	0	0	0	0	0	0	0	0	0	1	0	0	0	0	0	0	0	0
SYNM	0	0	0	0	1	0	0	0	0	0	0	0	0	0	0	0	0	0
LSAMP	0	0	0	0	1	0	0	0	0	0	0	0	0	0	0	0	0	0
ENSCAFG00000010861	0	0	1	0	1	1	0	0	0	0	0	0	0	0	0	0	0	0
SCN3A	0	0	0	0	1	0	0	0	0	0	0	0	0	0	1	0	0	0
GPAM	0	0	0	0	-1	0	0	0	1	0	0	0	0	0	0	0	0	0
LRP5	0	0	0	0	0	0	0	0	0	1	0	0	0	0	0	0	0	0
SULF2	0	1	0	0	0	0	0	0	0	0	0	0	0	0	0	0	0	0
TET2	0	0	0	0	1	0	0	0	0	0	0	0	0	0	0	0	0	0
ABCA9	0	1	0	0	0	0	0	0	0	0	0	0	0	0	0	0	0	0
PPP2R2D	0	0	0	0	1	0	0	0	0	0	0	0	0	0	0	0	0	0
FMO5	0	1	0	0	0	0	0	0	0	0	0	0	0	0	0	0	0	0
ENSCAFG00000010893	0	0	0	0	1	0	0	0	0	0	0	0	0	0	0	0	0	0
INTS12	0	1	0	0	0	0	0	0	0	0	0	0	0	0	0	0	0	0
FEZ1	0	0	0	0	0	0	0	0	0	1	0	0	0	0	0	0	0	0
RFTN2	0	0	0	0	1	0	0	0	0	0	0	0	0	0	0	0	0	0

NPNT	0	0	0	1	0	0	0	0	0	1	0	0	0	0	0	0	0	0
SDHA	0	1	0	0	0	0	0	0	0	0	0	0	0	0	0	0	0	0
PTHLH	0	0	0	0	0	0	0	0	1	0	0	0	0	0	0	0	0	0
RIN3	0	0	0	0	1	0	0	0	0	0	0	0	0	0	0	0	0	0
ZDHHC6	0	0	0	0	1	0	0	0	0	0	0	0	0	0	0	0	0	0
HTR2B	0	0	0	0	0	0	0	0	0	1	0	0	0	0	0	0	0	0
MST1R	0	0	0	0	0	0	0	0	0	1	0	0	0	0	0	0	0	0
SLC25A6	0	1	0	0	-1	0	0	0	0	0	0	0	0	0	0	0	0	0
ACACB	0	1	0	0	0	0	0	0	1	0	0	0	0	0	0	0	0	0
CHD2	0	0	0	0	0	0	0	0	0	1	0	0	0	0	0	0	0	0
TINAGL1	0	0	0	0	0	0	0	0	0	1	0	0	0	0	0	0	0	0
TCF7L2	0	1	0	0	0	0	0	0	0	0	0	0	0	0	0	0	0	0
ROBO4	0	0	0	0	1	0	0	0	0	0	0	0	0	0	0	0	0	0
EDARADD	0	1	0	0	0	0	0	0	0	0	0	0	0	0	0	0	0	0
IL3RA	0	0	0	0	1	0	0	0	0	0	0	0	0	0	0	0	0	0
KCTD18	0	1	0	0	0	0	0	0	0	0	0	0	0	0	0	0	0	0
SERINC2	0	0	0	0	0	0	0	0	0	1	0	0	0	0	0	0	0	0
ERO1B	0	0	0	0	0	0	0	0	0	1	0	0	0	0	0	0	0	0
CD99	0	0	0	0	-1	0	0	0	0	1	0	0	0	0	0	0	0	0
ZMAT3	0	0	0	0	-1	0	0	0	0	1	0	0	0	0	0	0	0	0
DKK2	0	0	1	0	0	0	0	0	0	0	0	0	0	0	0	0	0	0
XG	0	1	0	0	0	0	0	0	0	0	0	0	0	0	0	0	0	0
AMZ2	0	0	0	0	-1	0	0	0	0	1	0	0	0	0	0	0	0	0
GPR137B	0	0	0	0	0	0	0	0	0	1	0	0	0	0	0	0	0	0
PDE6D	0	1	0	0	0	0	0	0	0	0	0	0	0	0	0	0	0	0
SNAP23	0	0	0	0	1	0	0	0	0	0	0	0	0	0	0	0	0	0
NDUFV1	0	1	0	0	0	0	0	0	0	0	0	0	0	0	0	0	0	0
GYG2	0	0	0	0	0	0	0	0	0	1	0	0	0	0	0	0	0	0
ENSCAFG00000011121	0	1	0	0	0	0	0	0	0	0	0	0	0	0	0	0	0	0
ARSD	0	0	0	0	0	0	0	0	0	1	0	0	0	0	0	0	0	0
ABHD4	0	1	0	0	0	0	0	0	0	0	0	0	0	0	0	0	0	0
ESAM	0	0	0	0	1	0	0	0	0	0	0	0	0	0	0	0	0	0
GALNT17	0	0	0	1	0	0	0	0	0	0	1	0	0	0	0	0	0	0
UBA7	0	1	0	0	0	0	0	0	0	0	0	0	0	0	0	0	0	0
ALDH3B1	0	1	0	0	0	0	0	0	1	0	0	0	0	0	0	0	0	0
NR5A2	0	0	0	0	1	0	0	0	0	0	0	0	0	0	0	0	0	0
MXRA5	0	0	0	0	0	0	0	1	0	1	0	-1	0	0	0	0	0	0
TM7SF3	0	0	0	0	0	0	0	0	0	1	0	0	0	0	0	0	0	0
SIAE	0	0	0	0	0	0	0	0	0	1	0	0	0	0	0	0	0	0
PRKX	0	0	0	0	1	0	0	0	0	0	0	0	0	0	0	0	0	0
GALNT3	0	0	0	0	0	0	0	0	0	1	0	0	0	0	0	0	0	0
NID1	0	0	0	0	0	0	0	0	0	1	0	0	0	0	0	0	0	0
CYP2U1	0	0	0	0	0	0	0	0	0	1	0	0	0	0	0	0	0	0
DAG1	0	0	0	0	0	0	0	0	0	1	0	0	0	0	0	0	0	0
UNC93B1	0	1	0	0	0	0	0	0	0	0	0	0	0	0	0	0	0	0
HADH	0	0	0	0	0	0	0	0	0	1	0	0	0	0	0	0	0	0
LYST	0	0	0	0	1	0	0	0	0	1	0	0	0	0	0	0	0	0
AXIN2	0	1	0	0	0	0	0	0	1	0	0	0	0	0	0	0	0	0
SLCO3A1	0	0	0	0	1	0	0	0	0	0	0	0	0	0	0	0	0	0
LAPTM5	0	0	0	0	1	0	0	0	0	0	0	0	0	0	0	0	0	0
B3GALNT2	0	0	0	0	0	0	0	0	0	1	0	0	0	0	0	0	0	0
SUN1	0	0	0	0	0	0	0	0	0	1	0	0	0	0	0	0	0	0
PUDP	0	1	0	0	0	0	0	0	0	0	0	0	0	0	0	0	0	0
C8H14orf93	0	1	0	0	0	0	0	0	0	0	0	0	0	0	0	0	0	0
OSTC	0	0	0	0	0	1	0	0	0	0	0	0	0	0	0	0	0	0
GGPS1	0	0	0	0	0	0	0	0	0	1	0	0	0	0	0	0	0	0
ANOS1	0	0	0	0	0	0	0	0	0	0	1	0	0	0	0	1	0	0
PRKCA	0	0	0	0	-1	0	0	0	0	1	0	0	0	0	0	0	0	0
ENSCAFG00000011327	0	0	0	0	1	0	0	0	0	0	0	0	0	0	0	0	0	0
GNB4	0	0	0	0	0	0	0	0	0	1	0	0	0	0	0	0	0	0
MARC1	0	1	0	0	0	0	0	0	0	0	0	0	0	0	0	0	0	0
GSTP1	0	1	0	0	-1	-1	0	0	0	0	0	0	0	0	0	0	0	0
VWA5A	0	0	0	0	1	0	0	0	0	0	0	0	0	0	0	0	0	0
ENSCAFG00000011368	0	0	0	0	0	0	0	0	1	1	0	0	0	0	0	0	0	0
CMKLR1	0	0	0	0	0	0	0	0	1	0	0	0	0	0	0	0	0	0
SHROOM2	0	-1	0	0	1	0	0	0	0	0	0	0	0	0	0	0	0	0
CLDN34	0	0	0	0	1	0	0	0	0	1	0	0	0	0	0	0	0	0
CDH24	0	0	0	0	1	0	0	0	0	0	0	0	0	0	0	0	0	0
SEC24B	0	0	0	0	0	0	0	0	0	1	0	0	0	0	0	0	0	0
MCUB	0	1	0	0	0	0	0	0	0	0	0	0	0	0	0	0	0	0
HELZ	0	0	0	0	0	0	0	0	0	1	0	0	0	0	0	0	0	0
UBR1	0	0	0	0	0	0	0	0	0	1	0	0	0	0	0	0	0	0
ARID4B	0	0	0	0	1	0	0	0	0	0	0	0	0	0	0	0	0	0
USP13	0	0	0	0	1	0	0	0	0	0	0	0	0	0	0	0	0	0
PSMD12	0	0	0	0	1	0	0	0	0	0	0	0	0	0	0	0	0	0
BCAT1	0	-1	0	0	1	0	0	0	0	-1	0	0	0	0	0	0	0	0
SGSM1	0	0	0	0	-1	0	0	0	1	0	0	0	0	0	0	0	0	0
DET1	0	1	0	0	0	0	0	0	0	0	0	0	0	0	0	0	0	0
ABLIM1	0	0	0	0	1	0	0	0	1	1	0	0	0	0	0	0	0	0
TTC14	0	-1	0	0	0	1	0	0	0	0	0	0	0	0	0	0	0	0
TARBP1	0	0	0	0	1	0	0	0	0	-1	0	0	0	0	0	0	0	0
BPTF	0	0	0	0	0	0	0	0	0	1	0	0	0	0	0	0	0	0
MFGE8	0	0	0	0	0	0	0	0	0	1	0	0	0	0	0	0	0	0
RIPOR3	0	1	0	0	0	0	0	0	0	0	0	0	0	0	0	0	0	0
HCLS1	0	0	0	0	1	0	0	0	0	0	0	0	0	0	0	0	0	0
PPP1CA	0	1	0	0	0	0	0	0	0	0	0	0	0	0	0	0	0	0
USP19	0	1	0	0	0	0	0	0	0	0	0	0	0	0	0	0	0	0
PARD6B	0	0	0	0	0	0	0	0	0	1	0	0	0	0	0	0	0	0
MID1	0	-1	0	0	0	0	0	0	0	1	0	0	0	0	0	0	0	0
HIST2H2AC	0	0	0	0	1	0	0	0	0	-1	0	0	0	0	0	0	0	0
TBC1D10C	0	1	0	0	0	0	0	0	0	0	0	0	0	0	0	0	0	0
GRK3	0	0	0	0	0	0	0	0	1	0	0	0	0	0	0	0	0	0
EFS	0	1	0	0	-1	0	0	0	1	-1	0	0	0	0	0	0	0	0
NFATC2	0	0	0	0	1	0	0	0	0	0	0	0	0	0	0	0	0	0
INPP5D	0	0	0	0	1	0	0	0	0	0	0	0	0	0	0	0	0	0
MTMR11	0	0	0	0	-1	0	0	0	1	0	0	0	0	0	0	0	0	0
SSH3	0	0	0	0	0	0	0	0	0	1	0	0	0	0	0	0	0	0
ENSCAFG00000011658	0	0	1	0	1	1	0	0	0	0	0	0	0	0	0	0	0	0
ATP9A	0	0	0	0	1	0	0	0	0	1	0	0	0	0	0	0	0	0
MYO18B	0	0	0	0	0	0	0	0	0	0	0	1	0	0	1	0	0	0
DHRS4	0	1	0	0	0	0	0	0	0	0	0	0	0	0	0	0	0	0
SIPA1L2	0	0	0	0	0	0	0	0	0	1	0	0	0	0	0	0	0	0
HSPA8	0	-1	0	0	1	0	0	0	0	0	0	0	0	0	0	0	0	0
ALPK1	0	0	0	0	0	0	0	0	0	1	0	0	0	0	0	0	0	0
TMEM200B	0	1	0	0	0	0	0	0	0	0	0	0	0	0	0	0	0	0
EPB41	0	0	0	0	1	0	0	0	0	0	0	0	0	0	0	0	0	0
SEZ6L	0	1	0	0	0	0	0	0	0	0	0	0	0	0	0	0	0	0
CCDC71	0	1	0	0	0	0	0	0	0	0	0	0	0	0	0	0	0	0
DDX5	0	0	0	0	1	0	0	0	0	0	0	0	0	0	0	0	0	0
CD34	0	0	0	0	1	0	0	0	0	0	0	0	0	0	0	0	0	0
ATRNL1	0	0	0	0	0	0	0	0	0	1	0	0	0	0	0	0	0	0
HPS4	0	1	0	0	0	0	0	0	0	0	0	0	0	0	0	0	0	0
CADM3	0	0	0	0	1	0	0	0	0	0	0	0	0	0	0	0	0	0
DISC1	0	-1	0	0	0	0	0	0	0	1	0	0	0	0	0	0	0	0
PECAM1	0	0	0	0	1	0	0	0	0	0	0	0	0	0	0	0	0	0
PLEKHO1	0	0	0	0	1	0	0	0	0	0	0	0	0	0	0	0	0	0
LAMB2	0	1	0	0	0	0	0	0	0	0	0	0	0	0	0	0	0	0
ANP32E	0	0	0	0	1	0	0	0	0	0	0	0	0	0	0	0	0	0
CD86	0	0	0	0	1	0	0	0	0	0	0	0	0	0	0	0	0	0
PLXNA2	0	0	0	0	0	-1	0	0	0	1	0	0	0	0	0	0	0	0
TPST2	0	0	0	0	0	0	0	0	1	1	0	0	0	0	0	0	0	0
EGLN1	0	0	0	0	0	0	0	0	0	1	0	0	0	0	0	0	0	0
GPR146	0	0	0	0	1	0	0	0	0	0	0	0	0	0	0	0	0	0
ICAM2	0	0	0	0	1	0	0	0	0	0	0	0	0	0	0	0	0	0
ZNF217	0	1	0	0	0	0	0	0	0	0	0	0	0	0	0	0	0	0
B3GNT5	0	0	0	0	1	0	0	0	0	0	0	0	0	0	0	0	0	0
QARS	0	1	0	0	0	0	0	0	0	0	0	0	0	0	0	0	0	0
TTC28	0	1	0	0	0	0	0	0	0	0	0	0	0	0	0	0	0	0
KLHL24	0	1	0	0	0	0	0	0	0	0	0	0	0	0	0	0	0	0
PCK2	0	0	0	0	0	0	1	0	0	0	0	0	0	0	0	0	0	0

BZW1	0	-1	0	0	1	0	0	0	0	0	0	0	0	0	0	0	0	0
ASB9	0	0	0	0	1	0	0	0	0	0	0	0	0	0	0	0	0	0
TMEM62	0	1	0	0	0	0	0	0	0	0	0	0	0	0	0	0	0	0
HSD11B1	0	1	0	0	0	0	0	0	1	0	0	0	0	0	0	0	0	0
CCNDBP1	0	1	0	0	0	0	0	0	0	1	0	0	0	0	0	0	0	0
TTC13	0	0	0	0	0	0	0	0	0	1	0	0	0	0	0	0	0	0
ARHGEF12	0	0	0	0	1	0	0	0	0	0	0	0	0	0	0	0	0	0
PARP9	0	0	0	0	0	0	0	0	0	1	0	0	0	0	0	0	0	0
VEGFD	0	0	0	0	0	0	0	0	0	1	0	0	0	0	0	0	0	0
NDUFAF3	0	1	0	0	0	0	0	0	0	0	0	0	0	0	0	0	0	0
TMEM136	0	0	0	0	0	0	0	0	0	1	0	0	0	0	0	0	0	0
GCNT7	0	1	0	0	0	0	0	0	0	1	0	0	0	0	0	0	0	0
DALRD3	0	1	0	0	0	0	0	0	0	0	0	0	0	0	0	0	0	0
PARP14	0	0	0	0	0	0	0	0	0	1	0	0	0	0	0	0	0	0
PDZD8	0	0	0	0	1	0	0	0	0	0	0	0	0	0	0	0	0	0
ZNRF3	0	0	0	0	0	0	0	0	0	1	0	0	0	0	0	0	0	0
PIR	0	1	0	0	0	0	0	0	0	0	0	0	0	0	0	0	0	0
KIF7	0	1	0	0	0	0	0	0	0	0	0	0	0	0	0	0	0	0
ABCB11	0	1	0	0	0	0	0	0	0	0	0	0	0	0	0	0	0	0
EMX2	0	1	0	0	0	0	0	0	0	0	1	0	0	0	0	1	0	0
DIRC2	0	0	0	0	0	0	0	0	0	1	0	0	0	0	0	0	0	0
RAB11FIP2	0	1	0	0	0	0	0	0	0	0	0	0	0	0	0	0	0	0
ENSCAFG00000011993	0	1	0	0	0	0	0	0	0	0	0	0	0	0	0	0	0	0
KREMEN1	0	1	0	0	-1	0	0	0	1	1	0	0	0	0	0	0	0	0
IRF9	0	0	0	0	0	0	0	0	0	1	0	0	0	0	0	0	0	0
SH3BP4	0	0	0	0	0	0	0	0	0	1	0	0	0	0	0	0	0	0
P4HTM	0	0	0	0	0	0	0	0	0	1	0	0	0	0	0	0	0	0
ENSCAFG00000012022	0	1	0	0	-1	0	0	0	0	0	0	0	0	0	0	0	0	0
REC8	0	1	0	0	0	0	0	0	0	0	0	0	0	0	0	0	0	0
DHRS9	0	1	0	0	0	0	0	0	0	0	0	0	0	0	0	0	0	0
HHAT	0	0	0	0	1	0	0	0	0	0	0	0	0	0	0	0	0	0
ADAMTSL4	0	1	0	0	-1	0	0	0	0	0	0	0	0	0	0	0	0	0
PDIA5	0	0	0	0	1	0	0	0	0	0	0	0	0	0	0	0	0	0
CAMK2D	0	-1	0	0	-1	0	0	0	0	1	0	0	0	0	0	0	0	0
BMX	0	0	0	0	1	0	0	0	0	0	0	0	0	0	0	0	0	0
ABCC9	0	0	0	0	0	0	0	0	0	1	0	0	0	0	0	0	0	0
LIMK1	0	0	0	0	0	0	0	0	0	0	0	0	0	1	0	0	0	0
SLC25A20	0	0	0	0	0	0	0	0	0	1	0	0	0	0	0	0	0	0
MCAM	0	0	0	0	1	0	0	0	0	0	0	0	0	0	0	0	0	0
PC	0	1	0	0	0	0	0	0	0	0	0	0	0	0	0	0	0	0
CTSS	0	1	0	0	0	0	0	0	1	1	0	0	0	0	0	0	0	0
AHDC1	0	1	0	0	0	0	0	0	0	0	0	0	0	0	0	0	0	0
FAM126B	0	0	0	0	1	0	0	0	0	0	0	0	0	0	0	0	0	0
NGRN	0	0	0	0	0	0	0	0	0	1	0	0	0	0	0	0	0	0
LRP2	0	0	0	0	0	0	0	0	0	1	0	0	0	0	0	0	0	0
CFLAR	0	-1	0	0	1	0	0	0	0	1	0	0	0	0	0	0	0	0
CA5B	0	1	0	0	0	0	0	0	0	0	0	0	0	0	0	0	0	0
NUP133	0	0	0	0	1	0	0	0	0	0	0	0	0	0	0	0	0	0
SYTL1	0	0	0	0	-1	0	0	0	0	1	0	0	0	0	0	0	0	0
SLC26A6	0	0	0	0	1	0	0	0	0	-1	0	0	0	0	0	0	0	0
TMEM222	0	0	0	0	0	0	0	0	0	1	0	0	0	0	0	0	0	0
NLRX1	0	1	0	0	0	0	0	0	0	0	0	0	0	0	0	0	0	0
INPP5F	0	0	0	0	0	0	0	0	0	1	0	0	0	0	0	0	0	0
ACKR3	0	1	0	0	0	0	0	0	0	0	0	0	0	0	0	0	0	0
PRMT2	0	1	0	0	0	0	0	0	0	0	0	0	0	0	0	0	0	0
HDDC3	0	1	0	0	0	0	0	0	0	0	0	0	0	0	0	0	0	0
NEFH	0	0	0	0	0	0	0	0	1	0	0	0	0	0	0	0	0	0
GOLT1B	0	-1	0	0	0	1	0	0	0	0	0	0	0	0	0	0	0	0
MAN2A2	0	0	0	0	0	0	0	0	0	1	0	0	0	0	0	0	0	0
ADCY4	0	0	0	0	1	0	0	0	0	0	0	0	0	0	0	0	0	0
FES	0	0	0	0	1	0	0	0	0	0	0	0	0	0	0	0	0	0
PPP2R5A	0	0	0	0	1	0	0	0	0	0	0	0	0	0	0	0	0	0
TMEM206	0	0	0	0	1	0	0	0	0	0	0	0	0	0	0	0	0	0
ATF3	0	-1	0	0	1	0	0	0	0	0	0	0	0	0	0	0	0	0
ALS2	0	0	0	0	1	0	0	0	0	0	0	0	0	0	0	0	0	0
VPS11	0	1	0	0	0	0	0	0	0	0	0	0	0	0	0	0	0	0
ENSCAFG00000012367	0	1	0	0	0	0	0	0	0	0	0	0	0	0	0	0	0	0
SLCO1A2	0	0	0	0	0	0	0	0	0	1	0	0	0	0	0	0	0	0
PPIG	0	0	0	0	1	0	0	0	0	0	0	0	0	0	0	0	0	0
FGFR2	0	0	0	0	0	0	0	0	0	1	0	0	0	0	0	0	0	0
NFATC4	0	1	0	0	0	0	0	0	0	-1	0	0	0	0	0	0	0	0
RBM4B	0	0	0	0	0	0	0	0	0	1	0	0	0	0	0	0	0	0
BLM	0	0	0	0	1	0	0	0	0	0	0	0	0	0	0	0	0	0
PHYHIPL	0	1	0	0	0	0	1	0	1	0	0	0	0	0	0	0	0	0
CCS	0	1	0	0	0	0	0	0	0	0	0	0	0	0	0	0	0	0
VWA5B2	0	0	0	0	0	0	0	0	1	0	0	0	0	0	0	0	0	0
PER2	0	1	0	0	0	0	0	0	0	0	0	0	0	0	0	0	0	0
CTSF	0	1	0	0	0	0	0	0	0	1	0	0	0	0	0	0	0	0
SDR39U1	0	1	0	0	0	0	0	0	0	0	0	0	0	0	0	0	0	0
PROX1	0	0	0	0	1	0	0	0	0	0	0	0	0	0	0	0	0	0
ENSCAFG00000012457	0	0	0	0	0	0	0	0	1	1	0	0	0	0	0	0	0	0
TWIST2	0	1	0	0	-1	0	0	0	1	-1	0	0	0	0	0	0	0	0
CASQ1	0	0	0	0	0	0	0	0	1	0	0	0	0	0	0	0	0	0
PDE5A	0	0	0	0	0	0	0	0	0	1	0	0	0	0	0	0	0	0
SEMA6C	0	1	0	0	0	0	0	0	0	0	0	0	0	0	0	0	0	0
PLEKHA5	0	1	0	0	0	0	0	0	0	0	0	0	0	0	0	0	0	0
CASTOR1	0	1	0	0	0	0	0	0	0	1	0	0	0	0	0	0	0	0
ENSCAFG00000012507	0	0	0	0	1	0	0	0	0	0	0	0	0	0	0	0	0	0
TP53BP1	0	0	0	0	0	0	0	0	0	1	0	0	0	0	0	0	0	0
TAF4	0	1	0	0	0	0	0	0	0	0	0	0	0	0	0	0	0	0
ENSCAFG00000012527	0	0	1	0	0	0	0	0	0	0	0	0	0	0	0	0	0	0
PTPN14	0	-1	0	0	0	0	0	0	0	1	0	0	0	0	0	0	0	0
HEG1	0	1	0	0	0	0	0	0	0	1	0	0	0	0	0	0	0	0
HDAC4	0	1	0	0	0	0	0	0	0	0	0	0	0	0	0	0	0	0
PRKD1	0	0	0	0	1	0	0	0	0	0	0	0	0	0	0	0	0	0
NCSTN	0	0	0	0	0	0	0	0	0	1	0	0	0	0	0	0	0	0
SH3BGRL3	0	1	0	0	0	0	0	0	0	0	0	0	0	0	0	0	0	0
CEP85	0	1	0	0	0	0	0	0	0	0	0	0	0	0	0	0	0	0
G2E3	0	0	0	0	1	0	0	0	0	0	0	0	0	0	0	0	0	0
MGST1	0	1	0	0	0	0	0	0	0	0	0	0	0	0	0	0	0	0
PI4KB	0	0	0	0	0	0	0	0	0	1	0	0	0	0	0	0	0	0
NBEAL1	0	0	0	0	0	0	0	0	0	1	0	0	0	0	0	0	0	0
FAM110D	0	0	0	0	1	0	0	0	0	0	0	0	0	0	0	0	0	0
F11R	0	0	0	0	1	0	0	0	0	1	0	0	0	0	0	0	0	0
CYBRD1	0	0	0	0	-1	0	0	0	0	1	0	0	0	0	0	0	0	0
SELENBP1	0	1	0	0	0	0	0	0	0	0	0	0	0	0	0	0	0	0
PELI3	0	1	0	0	0	0	0	0	0	0	0	0	0	0	0	0	0	0
TSTD1	0	0	0	0	0	0	0	0	0	1	0	0	0	0	0	0	0	0
RBBP7	0	-1	0	0	0	1	0	0	0	0	0	0	0	0	0	0	0	0
ANK3	0	0	0	0	0	0	0	0	0	-1	1	0	0	0	0	0	0	0
DYNC1I2	0	0	0	0	0	0	0	0	0	1	0	0	0	0	0	0	0	0
SMARCD2	0	1	0	0	0	0	0	0	0	0	0	0	0	0	0	0	0	0
GATA5	0	0	0	0	0	0	0	0	0	1	-1	0	0	1	0	-1	1	-1
SLCO4A1	0	0	0	0	1	0	0	0	0	0	0	0	0	0	0	0	0	0
KIF1A	0	0	0	0	0	0	0	0	1	1	0	0	0	0	0	0	0	0
PAFAH2	0	1	0	0	0	0	0	0	0	0	0	0	0	0	0	0	0	0
LRCH3	0	0	0	0	0	0	0	0	0	1	0	0	0	0	0	0	0	0
ENSCAFG00000012747	0	1	0	0	0	0	0	0	0	0	0	0	0	0	0	0	0	0
FAM53B	0	1	0	0	0	0	0	0	1	0	0	0	0	0	0	0	0	0
MTFR1L	0	1	0	0	-1	0	0	0	0	0	0	0	0	0	0	0	0	0
COCH	0	0	0	0	0	0	0	0	0	1	0	0	0	0	0	0	0	0
ENSCAFG00000012763	0	0	0	0	0	0	0	0	0	1	0	0	0	0	0	0	0	0
CLCN2	0	1	0	0	0	0	0	0	0	0	0	0	0	0	0	0	0	0
CYP20A1	0	0	0	0	0	0	0	0	0	1	0	0	0	0	0	0	0	0
ZRANB1	0	0	0	0	0	0	0	0	0	1	0	0	0	0	0	0	0	0
MAN1C1	0	1	0	0	0	0	0	0	1	0	0	0	0	0	0	0	0	0
REPS2	0	0	0	0	0	0	0	0	1	0	0	0	0	0	0	0	0	0
BEND2	0	0	0	0	0	0	0	0	0	0	0	0	0	0	0	0	0	1
SLC25A12	0	1	0	0	0	0	0	0	0	1	0	0	0	0	0	0	0	0
PPOX	0	1	0	0	0	0	0	0	0	0	0	0	0	0	0	0	0	0

STRN3	0	0	0	0	0	0	0	0	0	1	0	0	0	0	0	0	0	0
TCN2	0	0	0	0	0	0	0	0	0	1	0	0	0	0	0	0	0	0
ENSCAFG00000012839	0	0	0	0	1	0	0	0	0	0	0	0	0	0	0	0	0	0
PASK	0	1	0	0	0	0	0	0	0	0	0	0	0	0	0	0	0	0
NDUFS2	0	1	0	0	0	0	0	0	0	0	0	0	0	0	0	0	0	0
RERG	0	0	0	0	0	0	0	0	0	1	0	0	0	0	0	0	0	0
TDRKH	0	1	0	0	0	0	0	0	0	0	0	0	0	0	0	0	0	0
RAPH1	0	1	0	0	0	0	0	0	0	1	0	0	0	0	0	0	0	0
CHRD	0	1	0	0	0	0	0	0	0	0	0	0	0	0	0	0	0	0
CCDC47	0	0	0	0	1	1	0	0	0	0	0	0	0	0	0	0	0	0
PARD3B	0	1	0	0	-1	0	0	0	0	0	0	0	0	0	0	0	0	0
HECTD1	0	0	0	0	1	0	0	0	0	1	0	0	0	0	0	0	0	0
CLIC4	0	0	0	0	-1	0	0	0	1	0	0	0	0	0	0	0	0	0
DSCAML1	0	1	0	0	0	0	0	0	0	0	0	0	0	0	0	0	0	0
PHKA2	0	1	0	0	0	0	0	0	0	0	0	0	0	0	0	0	0	0
ALPK3	0	0	0	0	0	0	0	0	0	1	0	0	0	0	0	0	0	0
MAP3K3	0	0	0	0	1	0	0	0	0	0	0	0	0	0	0	0	0	0
DNAJC30	0	1	0	0	0	0	0	0	0	0	0	0	0	0	0	0	0	0
NIPAL3	0	1	0	0	0	0	0	0	0	1	0	0	0	0	0	0	0	0
HELZ2	0	0	0	0	1	0	0	0	0	0	0	0	0	0	0	0	0	0
ADGRG2	0	1	0	0	0	0	1	0	1	0	1	0	0	0	0	0	0	0
H2AFJ	0	1	0	0	0	0	0	0	0	0	0	0	0	0	0	0	0	0
ARID5B	0	0	0	0	0	0	0	0	0	1	0	0	0	0	0	0	0	0
ACE	0	0	0	0	1	0	0	0	0	0	0	0	0	0	0	0	0	0
PPIP5K1	0	1	0	0	0	0	0	0	0	1	0	0	0	0	0	0	0	0
ITGA6	0	0	0	0	1	0	0	0	0	0	0	0	0	0	0	0	0	0
RTKN2	0	0	0	0	0	0	0	0	0	1	0	0	0	0	0	0	0	0
STMN3	0	1	0	0	0	0	0	0	0	0	0	0	0	0	0	0	0	0
ZNF365	0	0	0	0	0	0	0	0	0	1	0	0	0	0	0	0	0	0
CEP19	0	1	0	0	0	0	0	0	0	0	0	0	0	0	0	0	0	0
TANC2	0	-1	0	0	1	0	0	0	0	0	0	0	0	0	0	0	0	0
SLC2A4RG	0	1	0	0	0	0	0	0	0	0	0	0	0	0	0	0	0	0
ENSCAFG00000013060	0	-1	0	0	0	0	0	0	0	1	0	0	0	0	0	0	0	0
DOCK1	0	0	0	0	1	0	0	0	0	0	0	0	0	0	0	0	0	0
PAK2	0	-1	0	0	1	0	0	0	0	0	0	0	0	0	0	0	0	0
PIK3IP1	0	1	0	0	0	0	0	0	0	0	0	0	0	0	0	0	0	0
JMJD1C	0	0	0	0	1	0	0	0	0	0	0	0	0	0	0	0	0	0
PATZ1	0	1	0	0	0	0	0	0	0	0	0	0	0	0	0	0	0	0
PLBD1	0	1	0	0	0	0	0	0	0	0	0	0	0	0	0	0	0	0
MAP7D2	0	0	0	0	0	0	0	0	0	1	0	0	0	0	0	0	0	0
GLUL	0	1	0	0	0	0	0	0	0	0	0	0	0	0	0	0	0	0
AP3B2	0	1	0	0	0	0	0	0	0	1	0	0	0	0	0	0	0	0
BAZ1B	0	0	0	0	1	0	0	0	0	0	0	0	0	0	0	0	0	0
WHAMM	0	1	0	0	0	0	0	0	0	0	0	0	0	0	0	0	0	0
ARHGAP5	0	0	0	0	0	0	0	0	0	1	0	0	0	0	0	0	0	0
GALE	0	-1	0	0	1	0	0	0	0	-1	0	0	0	0	0	0	0	0
NPL	0	0	0	0	1	0	0	0	0	0	0	0	0	0	0	0	0	0
AKAP6	0	0	0	0	0	0	0	0	0	1	0	0	0	0	0	0	0	0
RPS6KA3	0	-1	0	0	1	0	0	0	0	0	0	0	0	0	0	0	0	0
HOMER2	0	0	0	0	0	0	0	0	0	1	0	0	0	0	0	0	0	0
NPAS3	0	0	0	0	0	0	0	0	0	1	0	0	0	0	0	0	0	0
DDR2	0	1	0	0	-1	0	0	0	0	1	0	0	0	0	0	0	0	0
RAMAC	0	0	0	0	0	0	0	0	0	1	0	0	0	0	0	0	0	0
MAGEF1	0	1	0	0	0	0	0	0	0	0	0	0	0	0	0	0	0	0
EFEMP2	0	1	0	0	-1	0	0	0	0	0	0	0	0	0	0	0	0	0
TM6SF1	0	0	0	0	1	0	0	0	0	0	0	0	0	0	0	0	0	0
HEBP1	0	0	0	0	-1	0	0	0	1	1	0	0	0	0	0	0	0	0
GPRC5A	0	0	0	0	0	0	0	0	0	1	0	0	0	0	0	0	0	0
EHHADH	0	1	0	0	0	0	0	0	0	0	0	0	0	0	0	0	0	0
BNC1	0	0	0	0	0	0	0	0	0	1	0	0	0	0	1	0	1	0
LAMC2	0	0	0	0	1	0	0	0	0	0	0	0	0	0	0	0	0	0
PRR14L	0	0	0	0	0	0	0	0	0	1	0	0	0	0	0	0	0	0
CREBL2	0	0	0	0	0	0	0	0	0	1	0	0	0	0	0	0	0	0
ENSCAFG00000013249	0	0	0	0	0	0	0	0	1	0	0	0	0	0	0	0	0	0
GPR155	0	0	1	0	0	0	0	0	0	1	0	0	0	0	0	0	0	0
PCMTD2	0	0	0	0	0	0	0	0	0	1	0	0	0	0	0	0	0	0
EBF3	0	1	0	0	0	0	0	0	0	0	0	0	0	0	0	0	0	0
ID3	0	0	0	0	0	0	0	0	0	1	0	0	0	0	0	0	0	0
WIPF1	0	1	0	0	-1	0	0	0	0	0	0	0	0	0	0	0	0	0
DUSP16	0	0	0	0	1	0	0	0	0	0	0	0	0	0	0	0	0	0
SMG7	0	-1	0	0	1	0	0	0	0	0	0	0	0	0	0	0	0	0
OVOL1	0	0	0	0	0	0	0	0	1	0	0	0	0	0	0	0	0	0
ADAMTSL3	0	1	0	0	0	0	1	0	1	1	0	0	0	0	0	0	0	0
TCEA3	0	1	0	0	0	0	0	0	0	0	0	0	0	0	0	0	0	0
BAZ1A	0	0	0	0	1	0	0	0	0	0	0	0	0	0	0	0	0	0
PHEX	0	0	0	0	0	0	0	0	0	0	0	0	0	0	0	0	1	0
HTR1D	0	0	0	0	0	0	0	0	1	0	0	0	0	0	0	0	0	0
LUZP1	0	0	0	0	1	0	0	0	1	0	0	0	0	0	0	0	0	0
SRP54	0	-1	0	0	0	1	0	0	0	0	0	0	0	0	0	0	0	0
NDUFS1	0	1	0	0	0	0	0	0	0	0	0	0	0	0	0	0	0	0
SIPA1	0	0	0	0	1	0	0	0	0	-1	0	0	0	0	0	0	0	0
PTCHD1	0	0	0	0	1	0	0	0	0	0	0	0	0	0	0	0	0	0
MGST3	0	1	0	0	0	0	0	0	0	0	0	0	0	0	0	0	0	0
KIF9	0	0	0	0	0	0	0	0	0	1	0	0	0	0	0	0	0	0
GPR1	0	1	0	0	0	0	0	0	1	0	0	0	0	0	0	0	0	0
ENSCAFG00000013366	0	1	0	0	0	0	0	0	0	-1	0	0	0	0	0	0	0	0
APOD	0	0	0	0	1	0	0	0	0	0	0	0	0	0	0	0	0	0
PRDX4	0	0	0	0	0	0	0	0	0	1	0	0	0	0	0	0	0	0
LNPK	0	0	0	0	0	0	0	0	0	1	0	0	0	0	0	0	0	0
CCL24	0	0	0	0	0	0	0	1	0	0	0	0	0	0	0	0	0	0
RGL1	0	1	0	0	0	0	0	0	0	0	0	0	0	0	0	0	0	0
MAP3K11	0	0	0	0	1	0	0	0	0	0	0	0	0	0	0	0	0	0
ALDH9A1	0	1	0	0	0	0	0	0	0	0	0	0	0	0	0	0	0	0
FAM43A	0	0	0	0	1	0	0	0	0	0	0	0	0	0	0	0	0	0
COLGALT2	0	0	0	0	0	0	0	0	0	1	0	0	0	0	0	0	0	0
HOXD8	0	1	0	0	1	0	1	0	1	0	1	0	0	1	0	0	0	0
ENSCAFG00000013452	0	1	0	0	0	0	0	0	0	0	0	0	0	0	0	0	0	0
MOV10	0	0	0	0	1	0	0	0	0	0	0	0	0	0	0	0	0	0
C7H1orf21	0	0	0	0	0	0	0	0	0	1	0	0	0	0	0	0	0	0
HOXD3	0	1	0	0	0	0	0	0	0	0	0	0	0	0	0	0	0	0
FRMD5	0	0	0	0	0	0	0	0	0	1	0	0	0	0	0	0	0	0
GABARAPL1	0	0	0	0	0	0	0	0	0	1	0	0	0	0	0	0	0	0
KLF7	0	0	0	0	1	0	0	0	0	-1	0	0	0	0	0	0	0	0
FAM129A	0	0	0	0	0	0	0	0	0	1	0	0	0	0	0	0	0	0
FAM78B	0	1	0	0	0	0	0	0	0	0	0	0	0	0	0	0	0	0
OLR1	0	0	0	0	0	0	0	0	0	0	0	0	0	0	1	0	0	0
ENSCAFG00000013496	0	1	0	0	0	0	0	0	0	1	0	0	0	0	0	0	0	0
RNF2	0	0	0	0	1	0	0	0	0	0	0	0	0	0	0	0	0	0
NFE2L2	0	1	0	0	0	0	0	0	0	0	0	0	0	0	0	0	0	0
CASC4	0	0	0	0	0	0	0	0	0	1	0	0	0	0	0	0	0	0
CREB1	0	0	0	0	1	0	0	0	0	0	0	0	0	0	0	0	0	0
NBEAL2	0	0	0	0	1	0	0	0	0	0	0	0	0	0	0	0	0	0
TRMT1L	0	0	0	0	0	0	0	0	0	1	0	0	0	0	0	0	0	0
CTDSPL2	0	0	0	0	1	0	0	0	0	0	0	0	0	0	0	0	0	0
ENSCAFG00000013536	0	0	0	0	0	0	0	0	0	1	0	0	0	0	0	0	0	0
SRRM3	0	0	0	0	0	0	0	0	1	0	0	0	0	0	0	0	0	0
ZBTB16	0	1	0	0	-1	0	0	0	0	0	0	0	0	0	0	0	0	0
IVNS1ABP	0	0	0	0	1	0	0	0	-1	0	0	0	0	0	0	0	0	0
ZNF280B	0	0	0	0	0	0	0	0	0	1	0	0	0	0	0	0	0	0
ST7L	0	0	0	0	0	0	0	0	0	1	0	0	0	0	0	0	0	0
DTX2	0	1	0	0	0	0	0	0	0	0	0	0	0	0	0	0	0	0
FRMD8	0	0	0	0	0	0	0	0	1	0	0	0	0	0	0	0	0	0
IDH1	0	1	0	0	0	0	0	0	0	0	0	0	0	0	0	0	0	0
B2M	0	0	0	0	0	0	0	0	0	1	0	0	0	0	0	0	0	0
CTTNBP2NL	0	0	0	0	1	0	0	0	0	0	0	0	0	0	0	0	0	0
ENSCAFG00000013651	0	1	0	0	0	0	0	0	0	1	0	0	0	0	0	0	0	0
EIF4A2	0	0	0	0	0	0	0	0	0	1	0	0	0	0	0	0	0	0
CDC42EP2	0	1	0	0	0	0	0	0	0	0	0	0	0	0	0	0	0	0
RAP1A	0	0	0	0	1	0	0	0	0	0	0	0	0	0	0	0	0	0
PRG4	0	0	0	0	0	0	0	0	1	0	0	0	0	0	0	0	0	0

ALS2CL	0	0	0	0	1	0	0	0	0	0	0	0	0	0	0	0	0	0
ST6GAL1	0	0	0	0	1	0	0	0	0	0	0	0	0	0	0	0	0	0
ADGRA1	0	0	0	0	1	0	0	0	0	0	0	0	0	0	0	0	0	0
ENSCAFG00000013713	0	0	0	0	0	1	0	0	0	-1	0	0	0	0	0	0	0	0
TPR	0	0	0	0	1	0	0	0	0	0	0	0	0	0	0	0	0	0
ZW10	0	0	0	0	1	0	0	0	0	-1	0	0	0	0	0	0	0	0
SHF	0	0	0	0	0	0	0	0	0	1	0	0	0	0	0	0	0	0
SLC25A16	0	-1	0	0	0	1	0	0	0	-1	0	0	0	0	0	0	0	0
OVGP1	0	1	0	0	0	0	0	0	0	0	0	0	0	0	0	0	0	0
GATM	0	0	0	0	0	0	0	0	0	0	1	0	0	0	0	1	0	0
ITGB3	0	-1	0	0	1	0	0	0	0	-1	0	0	0	0	0	0	0	0
CLEC14A	0	0	0	0	1	0	0	0	0	0	0	0	0	0	0	0	0	0
CCAR1	0	0	0	0	1	1	0	0	0	0	0	0	0	0	0	0	0	0
PTGS2	0	-1	0	0	1	0	0	0	0	0	0	0	0	0	0	0	0	0
ENSCAFG00000013770	0	0	0	0	1	0	0	0	0	0	0	0	0	0	0	0	0	0
STOX1	0	0	0	0	0	0	0	0	0	1	0	0	0	1	0	0	0	0
BCR	0	0	0	0	1	0	0	0	0	0	0	0	0	0	0	0	0	0
MAP3K14	0	1	0	0	0	0	0	0	0	0	0	0	0	0	0	0	0	0
OSBPL6	0	-1	0	0	1	0	0	0	0	0	0	0	0	0	0	0	0	0
ENSCAFG00000013802	0	0	0	0	0	1	0	0	0	0	0	0	0	0	0	0	0	0
TMEM47	0	0	0	0	1	0	0	0	0	1	0	0	0	0	0	0	0	0
FMNL1	0	1	0	0	0	0	0	0	0	0	0	0	0	0	0	0	0	0
SPECC1L	0	0	0	0	0	0	0	0	0	1	0	0	0	0	0	0	0	0
PHC1	0	1	0	0	0	0	0	0	0	0	0	0	0	0	0	0	0	0
VPS26A	0	0	0	0	0	0	0	0	0	1	0	0	0	0	0	0	0	0
IL16	0	1	0	0	0	0	0	0	0	0	0	0	0	0	0	0	0	0
MASP1	0	0	0	0	0	0	0	0	0	0	1	0	0	0	0	0	0	0
MATN2	0	0	0	0	0	0	0	0	0	1	0	0	0	0	0	0	0	0
TLNRD1	0	1	0	0	0	0	0	0	0	0	0	0	0	0	0	0	0	0
CEMIP	0	0	1	0	0	0	0	0	1	0	0	0	0	0	0	0	0	0
PRRG1	0	0	0	0	1	0	0	0	0	0	0	0	0	0	0	0	0	0
RIMKLB	0	0	0	0	0	0	0	0	0	1	0	0	0	0	0	0	0	0
SOAT1	0	1	0	0	-1	0	0	0	0	0	0	0	0	0	0	0	0	0
ACBD4	0	1	0	0	0	0	0	0	0	0	0	0	0	0	0	0	0	0
PLET1	0	0	0	0	0	0	0	0	0	1	0	0	0	0	0	0	0	0
PTS	0	0	0	0	0	0	0	0	0	1	0	0	0	0	0	0	0	0
SLC2A3	0	-1	0	0	1	0	0	0	0	0	0	0	0	0	0	0	0	0
LPP	0	-1	1	0	0	0	0	0	0	0	0	0	0	0	0	0	0	0
ARNT2	0	0	0	0	1	0	0	0	0	0	0	0	0	0	0	0	0	0
PLCD3	0	1	0	0	0	0	0	0	0	0	0	0	0	0	0	0	0	0
TSPAN15	0	0	0	0	1	0	0	0	0	0	0	0	0	0	0	0	0	0
ENSCAFG00000013972	0	0	0	0	0	0	0	0	0	1	0	0	0	0	0	0	0	0
ENSCAFG00000013974	0	0	0	0	1	0	0	0	0	0	0	0	0	0	0	0	0	0
C8H14orf28	0	1	0	0	0	0	0	0	0	0	0	0	0	0	0	0	0	0
KLHL28	0	0	0	0	0	1	0	0	0	0	0	0	0	0	0	0	0	0
BCL2A1	0	1	0	0	0	0	1	0	0	0	0	0	0	0	0	0	0	0
ENSCAFG00000013986	0	1	0	0	0	0	0	0	0	0	0	0	0	0	0	0	0	0
MYEF2	0	0	0	0	1	0	0	0	0	0	0	0	0	0	0	0	0	0
LIMD1	0	1	0	0	0	0	0	0	0	0	0	0	0	0	0	0	0	0
BATF2	0	1	0	0	0	0	0	0	0	0	0	0	0	0	0	0	0	0
IL1RAP	0	0	0	0	1	0	0	0	0	0	0	0	0	0	0	0	0	0
LARS2	0	1	0	0	0	0	0	0	0	0	0	0	0	0	0	0	0	0
PPA1	0	0	0	0	0	0	0	0	0	1	0	0	0	0	0	0	0	0
PRPF39	0	0	0	0	1	0	0	0	0	0	0	0	0	0	0	0	0	0
PPP2R5B	0	1	0	0	0	0	0	0	0	0	0	0	0	0	0	0	0	0
CLEC3B	0	1	0	0	0	0	0	0	0	0	0	0	0	1	0	0	0	0
TSPAN7	0	0	0	0	0	0	0	0	0	0	1	0	0	0	0	0	0	0
ACHE	0	0	0	0	0	0	0	0	0	1	0	0	0	0	0	0	0	0
EXOSC7	0	1	0	0	0	0	0	0	0	0	0	0	0	0	0	0	0	0
CCDC50	0	0	0	0	1	0	0	0	0	0	0	0	0	0	0	0	0	0
ENSCAFG00000014066	0	0	0	0	0	0	0	0	0	1	0	0	0	0	0	0	0	0
PALD1	0	0	0	0	1	0	0	0	0	0	0	0	0	0	0	0	0	0
TMEM42	0	1	0	0	-1	0	0	0	0	0	0	0	0	0	0	0	0	0
CRYAB	0	-1	0	0	0	0	0	0	1	0	0	0	0	0	0	0	0	0
CTSH	0	1	0	0	-1	0	0	0	0	0	0	0	0	0	0	0	0	0
CCDC141	0	0	0	0	0	0	0	0	0	1	0	0	0	0	0	0	0	0
ADAMTS14	0	0	0	0	1	0	0	0	0	-1	0	0	0	0	0	0	0	0
BCOR	0	0	0	0	1	0	0	0	0	0	0	0	0	0	0	0	0	0
SESTD1	0	1	0	0	0	0	0	0	0	0	0	0	0	0	0	0	0	0
PCBD1	0	1	0	0	0	0	0	0	0	1	0	0	0	0	0	0	0	0
ATG2A	0	1	0	0	0	0	0	0	0	0	0	0	0	0	0	0	0	0
OSBPL1A	0	0	0	0	0	0	0	0	0	1	0	0	0	0	0	0	0	0
ATP6AP2	0	0	0	0	-1	0	0	0	0	1	0	0	0	0	0	0	0	0
ENSCAFG00000014120	0	0	0	0	1	0	0	0	0	0	0	0	0	0	0	0	0	0
CHCHD10	0	0	0	0	-1	0	0	0	1	0	0	0	0	0	0	0	0	0
C18H7orf57	0	1	0	0	0	0	0	0	0	0	0	0	0	0	0	0	0	0
FZD2	0	0	0	0	0	0	0	0	1	0	0	0	0	0	0	0	0	0
MED14	0	0	0	0	0	0	0	0	0	1	0	0	0	0	0	0	0	0
ZNF385B	0	0	0	0	0	0	0	0	0	0	-1	0	0	1	0	0	0	0
RPL10L	0	0	0	0	0	1	0	0	0	0	0	0	0	0	0	0	0	0
ADAMTS7	0	0	0	0	1	0	0	0	0	0	0	0	0	0	0	0	0	0
ENSCAFG00000014154	0	1	0	0	0	0	0	0	0	0	0	0	0	0	0	0	0	0
TBC1D2B	0	1	0	0	0	0	0	0	0	0	0	0	0	0	0	0	0	0
EPHB4	0	0	0	0	1	0	0	0	0	0	0	0	0	0	0	0	0	0
MDGA2	0	0	0	0	0	0	0	0	0	1	0	0	0	0	0	0	0	0
IDH3A	0	1	0	0	0	0	0	0	0	0	0	0	0	0	0	0	0	0
ZDHHC8	0	1	0	0	0	0	0	0	0	0	0	0	0	0	0	0	0	0
ASTN1	0	0	0	0	0	1	0	1	0	0	0	0	0	0	0	0	0	0
PPP2R1B	0	0	0	0	1	0	0	0	0	-1	0	0	0	0	0	0	0	0
TRMT2A	0	1	0	0	0	0	0	0	0	0	0	0	0	0	0	0	0	0
ENSCAFG00000014207	0	0	0	0	0	0	0	0	0	1	0	0	0	0	0	0	0	0
KLHDC1	0	1	0	0	0	0	0	0	0	0	0	0	0	0	0	0	0	0
TANGO2	0	0	0	0	0	0	0	0	0	1	0	0	0	0	0	0	0	0
ARHGAP20	0	0	0	0	0	0	0	0	0	1	0	0	0	0	0	0	0	0
IKZF2	0	1	0	0	0	0	0	0	0	0	0	0	0	0	0	0	0	0
PAPPA2	0	0	0	0	0	0	0	0	0	1	0	0	0	0	0	0	0	0
ENSCAFG00000014227	0	0	0	0	1	0	0	0	0	0	0	0	0	0	0	0	0	0
CDH23	0	0	0	0	1	0	0	0	0	0	0	0	0	0	0	0	0	0
ARVCF	0	0	0	0	0	0	0	0	0	1	0	0	0	0	0	0	0	0
ENSCAFG00000014234	0	0	0	0	1	1	0	0	0	0	0	0	0	0	0	0	0	0
CEACAM1	0	0	0	0	0	0	0	0	0	1	0	0	0	0	0	0	0	0
RDX	0	0	0	0	0	0	0	0	0	1	0	0	0	0	0	0	0	0
ABCA12	0	0	0	0	0	0	0	0	0	1	0	0	0	0	0	0	0	0
PYGM	0	0	0	0	0	0	0	0	0	1	0	0	0	0	0	0	0	0
ZC3H12C	0	0	0	0	1	0	0	0	0	0	0	0	0	0	0	0	0	0
RASGRP2	0	1	0	0	0	0	0	0	0	1	0	0	0	0	0	0	0	0
UNC5B	0	0	0	0	0	0	0	0	0	1	0	0	0	0	0	0	0	0
EXPH5	0	0	0	0	0	0	0	0	0	1	0	0	0	0	0	0	0	0
S100P	0	1	0	0	0	0	0	0	0	0	0	0	0	0	0	0	0	0
BLOC1S4	0	1	0	0	0	0	0	0	0	0	0	0	0	0	0	0	0	0
C1S	0	0	0	0	0	0	0	0	0	1	0	0	0	0	0	0	0	0
KDELC2	0	1	0	0	0	0	0	0	0	0	0	0	0	0	0	0	0	0
NCKAP1	0	0	0	0	1	0	0	0	0	0	0	0	0	0	0	0	0	0
CASK	0	1	0	0	0	0	0	0	0	0	0	0	0	0	0	0	0	0
ENSCAFG00000014378	0	0	1	-1	1	1	0	0	0	0	0	0	0	0	0	0	0	0
GPR82	0	0	0	0	0	0	0	0	1	0	0	0	0	0	0	0	0	0
ASCC1	0	1	0	0	0	0	0	0	0	0	0	0	0	0	0	0	0	0
MAOA	0	0	0	0	-1	0	0	0	0	1	0	0	0	0	0	0	0	0
TSC22D4	0	1	0	0	0	0	0	0	0	0	0	0	0	0	0	0	0	0
PPP1R35	0	1	0	0	0	0	0	0	0	0	0	0	0	0	0	0	0	0
MAOB	0	0	0	0	0	0	0	0	0	1	0	0	0	0	0	0	0	0
ESRRA	0	1	0	0	0	0	0	0	0	0	0	0	0	0	0	0	0	0
HOMER3	0	0	0	0	1	0	0	0	0	0	0	0	0	0	0	0	0	0
MEOX1	0	0	0	0	1	0	0	0	0	0	0	0	0	0	0	0	0	0
SAV1	0	1	0	0	0	0	0	0	0	0	0	0	0	0	0	0	0	0
ETV4	0	-1	0	0	1	0	0	1	0	0	0	0	0	0	0	0	0	0
ZNF445	0	0	0	0	0	0	0	0	0	1	0	0	0	0	0	0	0	0
IGFBP2	0	0	0	0	0	0	0	0	1	0	0	0	0	0	0	0	0	0
TBX1	0	0	0	0	1	0	0	0	0	0	0	0	0	0	0	0	0	0
MCU	0	0	0	0	1	0	0	0	0	0	0	0	0	0	0	0	0	0

ABLIM2	0	1	0	0	0	0	0	0	1	0	0	0	0	0	0	0	0	0
SH3TC1	0	0	0	0	1	0	0	0	0	0	0	0	0	0	0	0	0	0
HTRA3	0	1	0	0	0	0	1	0	0	-1	0	0	0	0	0	0	0	0
KLHL20	0	0	0	0	0	0	0	0	0	1	0	0	0	0	0	0	0	0
NIN	0	1	0	0	0	0	0	0	0	0	0	0	0	0	0	0	0	0
ENSCAFG00000014560	0	0	0	0	1	0	0	0	0	0	0	0	0	0	0	0	0	0
PDCD10	0	-1	0	0	1	0	0	0	0	0	0	0	0	0	0	0	0	0
P4HA1	0	-1	0	0	0	0	0	0	0	1	0	0	0	0	0	0	0	0
DNAJC4	0	1	0	0	0	0	0	0	0	0	0	0	0	0	0	0	0	0
SERPINI1	0	0	1	0	0	0	0	0	0	0	0	0	0	0	0	0	0	0
NBR1	0	0	0	0	0	0	0	0	0	1	0	0	0	0	0	0	0	0
ABHD12B	0	1	0	0	0	0	0	0	0	0	0	0	0	0	0	0	0	0
GOLIM4	0	1	0	0	0	0	0	0	0	0	0	0	0	0	0	0	0	0
TRIM9	0	0	0	0	0	0	1	0	0	0	0	0	0	0	0	0	0	0
NUDT13	0	1	0	0	0	0	0	0	0	0	0	0	0	0	0	0	0	0
LRPAP1	0	0	0	0	-1	0	0	0	0	1	0	0	0	0	0	0	0	0
COMP	0	0	0	0	0	0	0	0	1	0	1	0	0	0	0	0	0	0
MECOM	0	0	0	0	1	0	0	0	0	0	0	0	0	0	0	0	0	0
DOK7	0	0	0	0	0	0	0	0	0	1	0	0	0	0	0	0	0	0
ENSCAFG00000014627	0	0	0	0	1	1	0	0	0	0	0	0	0	0	0	0	0	0
FRMD6	0	1	0	0	0	0	0	0	0	0	0	0	0	0	0	0	0	0
ZBTB40	0	0	0	0	0	0	0	0	0	1	0	0	0	0	0	0	0	0
CTDSP1	0	1	0	0	0	0	0	0	0	0	0	0	0	0	0	0	0	0
ENSCAFG00000014664	0	0	0	0	1	1	0	0	0	0	0	0	0	0	0	0	0	0
PLEKHH3	0	1	0	0	0	0	0	0	0	0	0	0	0	0	0	0	0	0
SUCO	0	0	0	0	0	1	0	0	0	0	0	0	0	0	0	0	0	0
ENSCAFG00000014689	0	0	0	0	1	0	0	0	0	0	0	0	0	0	0	0	0	0
PTGDR	0	0	0	0	0	0	0	0	0	1	0	0	0	0	0	0	0	0
PTGER2	0	0	0	0	0	0	0	1	0	0	0	0	0	0	0	0	0	0
TXNDC16	0	1	0	0	0	0	0	0	0	0	0	0	0	0	0	0	0	0
MACROD1	0	1	0	0	0	0	0	0	0	0	0	0	0	0	0	0	0	0
PIANP	0	0	0	0	0	0	0	0	0	1	0	0	0	0	0	0	0	0
UBA52	0	1	0	0	-1	0	0	0	0	1	0	0	0	0	0	0	0	0
KXD1	0	0	0	0	0	0	0	0	1	0	0	0	0	0	0	0	0	0
CALCRL	0	0	0	0	1	0	0	0	0	0	0	0	0	0	0	0	0	0
COX8A	0	1	0	0	-1	0	0	0	0	0	0	0	0	0	0	0	0	0
TFPI	0	0	0	0	1	0	0	0	0	-1	0	0	0	0	0	0	0	0
CHST7	0	1	0	0	1	0	1	0	0	0	0	0	0	0	0	0	0	0
USP37	0	0	0	0	1	0	0	0	0	0	0	0	0	0	0	0	0	0
KBTBD3	0	1	0	0	0	0	0	0	0	1	0	0	0	0	0	0	0	0
ENSCAFG00000014799	0	0	0	0	1	0	0	0	0	0	0	0	0	0	0	0	0	0
DNM3	0	0	0	0	1	0	0	0	0	0	0	0	0	0	0	0	0	0
JADE3	0	0	0	0	1	0	0	0	0	0	0	0	0	0	0	0	0	0
PPP3CB	0	1	0	0	0	0	0	0	0	0	0	0	0	0	0	0	0	0
SECISBP2L	0	0	0	0	1	0	0	0	0	0	0	0	0	0	0	0	0	0
GNPNAT1	0	0	0	0	0	1	0	0	0	0	0	0	0	0	0	0	0	0
EZH1	0	1	0	0	0	0	0	0	0	1	0	0	0	0	0	0	0	0
USP54	0	0	0	0	0	0	0	0	1	0	0	0	0	0	0	0	0	0
ENSCAFG00000014844	0	0	0	0	0	0	0	0	0	1	0	0	0	0	0	0	0	0
SKIL	0	0	0	0	1	0	0	0	0	0	0	0	0	0	0	0	0	0
SYNPO2L	0	0	0	0	0	0	0	0	0	1	0	0	0	0	0	0	0	0
SSBP4	0	1	0	0	0	-1	0	0	0	0	0	0	0	0	0	0	0	0
CASP12	0	1	0	0	0	0	0	0	0	0	0	0	0	0	0	0	0	0
DDHD1	0	1	0	0	0	0	0	0	0	0	0	0	0	0	0	0	0	0
FUT11	0	0	0	0	0	0	0	0	0	1	0	0	0	0	0	0	0	0
MXD4	0	1	0	0	0	0	0	0	0	1	0	0	0	0	0	0	0	0
BMP4	0	0	0	0	0	0	0	0	0	1	0	0	0	0	0	0	0	0
HAUS3	0	0	0	0	1	0	0	0	0	0	0	0	0	0	0	0	0	0
PGPEP1	0	0	0	0	0	0	0	0	1	0	0	0	0	0	0	0	0	0
GALK2	0	1	0	0	0	0	0	0	0	0	0	0	0	0	0	0	0	0
FGF7	0	1	0	0	0	0	0	0	0	0	0	0	0	0	0	0	0	0
CHD4	0	-1	0	0	1	0	0	0	0	-1	0	0	0	0	0	0	0	0
TTLL4	0	1	0	0	0	0	0	0	0	0	0	0	0	0	0	0	0	0
CYP27A1	0	1	0	0	0	0	0	0	0	0	0	0	0	0	0	0	0	0
RAB3A	0	0	0	0	0	0	0	0	0	1	0	0	0	0	0	0	0	0
NSD2	0	0	0	0	1	0	0	0	0	-1	0	0	0	0	0	0	0	0
IFI30	0	0	0	0	0	0	0	0	1	0	0	0	0	0	0	0	0	0
PRKAG3	0	0	0	0	0	0	0	0	0	1	0	0	0	0	0	0	0	0
ATP8B4	0	0	0	0	0	0	0	0	0	0	0	0	0	0	0	1	0	0
GCH1	0	0	0	0	1	0	0	0	0	0	0	0	0	0	0	0	0	0
ENSCAFG00000014970	0	0	0	0	1	0	0	0	0	0	0	0	0	0	0	0	0	0
EIF4G3	0	0	0	0	0	0	0	0	0	1	0	0	0	0	0	0	0	0
FMO4	0	0	0	0	0	0	0	0	0	1	0	0	0	0	0	0	0	0
HP1BP3	0	0	0	0	1	0	0	0	0	0	0	0	0	0	0	0	0	0
LGALS3	0	1	0	0	-1	0	0	0	0	-1	0	0	0	0	0	0	0	0
FBXO34	0	1	0	0	0	0	0	0	0	1	0	0	0	0	0	0	0	0
ATG14	0	1	0	0	0	0	0	0	0	1	0	0	0	0	0	0	0	0
IFFO1	0	1	0	0	0	0	0	0	0	0	0	0	0	0	0	0	0	0
CDA	0	0	0	0	1	0	0	0	0	0	0	0	0	0	0	0	0	0
MMP1	0	0	0	1	0	0	0	0	0	1	0	0	0	0	0	0	0	0
MMP27	0	1	0	0	0	0	0	0	0	0	0	0	0	0	0	0	0	0
ATG9A	0	1	0	0	0	0	0	0	0	0	0	0	0	0	0	0	0	0
PLA2G16	0	0	0	0	0	0	0	0	1	1	0	0	0	0	0	0	0	0
ARRDC2	0	1	0	0	0	0	0	0	0	0	0	0	0	0	0	0	0	0
LAP3	0	0	0	0	-1	0	0	0	1	1	0	0	0	0	0	0	0	0
GABPB1	0	-1	0	0	1	0	0	0	0	0	0	0	0	0	0	0	0	0
ARAF	0	0	0	0	0	0	0	0	0	1	0	0	0	0	0	0	0	0
CFAP300	0	0	0	0	0	0	0	0	0	1	0	0	0	0	0	0	0	0
LDB2	0	0	0	0	1	0	0	0	0	0	0	0	0	0	0	0	0	0
TAPBPL	0	0	0	0	0	0	0	0	0	1	0	0	0	0	0	0	0	0
KTN1	0	-1	0	0	1	0	0	0	0	0	0	0	0	0	0	0	0	0
SELE	0	0	0	0	1	0	0	0	0	0	0	0	0	0	0	0	0	0
SELL	0	0	0	0	1	0	0	0	0	0	0	0	0	0	0	0	0	0
SELP	0	0	0	0	1	0	0	0	0	0	0	0	0	0	0	0	0	0
GLB1L	0	1	0	0	-1	0	0	0	0	1	0	0	0	0	0	0	0	0
CRKL	0	0	0	0	0	0	0	0	0	1	0	0	0	0	0	0	0	0
STK16	0	1	0	0	0	0	0	0	0	0	0	0	0	0	0	0	0	0
ENSCAFG00000015206	0	0	0	0	0	0	0	0	0	1	0	0	0	0	0	0	0	0
F5	0	0	0	0	0	0	0	0	1	0	1	0	0	0	0	0	0	0
CYB5D2	0	1	0	0	0	0	0	0	0	0	0	0	0	0	0	0	0	0
VWF	0	0	0	0	1	0	0	0	0	0	0	0	0	0	0	0	0	0
ANKFY1	0	1	0	0	0	0	0	0	0	0	0	0	0	0	0	0	0	0
EXOC5	0	-1	0	0	1	0	0	0	0	0	0	0	0	0	0	0	0	0
PI4KA	0	1	0	0	0	0	0	0	0	0	0	0	0	0	0	0	0	0
SPNS3	0	0	0	0	1	0	0	0	0	0	0	0	0	0	0	0	0	0
ANO2	0	0	0	0	0	0	1	0	0	0	0	0	0	0	0	0	0	0
DPT	0	1	0	0	0	0	0	0	0	0	0	0	0	0	0	0	0	0
SFT2D2	0	0	0	0	0	0	0	0	1	0	0	0	0	0	0	0	0	0
SMTNL2	0	1	0	0	0	0	0	0	0	0	0	0	0	0	0	0	0	0
SPPL2A	0	0	0	0	1	0	0	0	0	0	0	0	0	0	0	0	0	0
BST1	0	1	0	0	-1	0	0	0	0	0	0	0	0	0	0	0	0	0
ENSCAFG00000015313	0	1	0	0	0	0	0	0	0	0	0	0	0	0	0	0	0	0
AP4E1	0	0	0	0	1	0	0	0	0	0	0	0	0	0	0	0	0	0
TNFAIP8L3	0	0	0	0	1	0	0	0	0	0	0	0	0	0	0	0	0	0
MVB12A	0	1	0	0	0	0	0	0	0	0	0	0	0	0	0	0	0	0
AP3M1	0	1	0	0	0	0	0	0	0	0	0	0	0	0	0	0	0	0
PLVAP	0	0	0	0	1	0	0	0	0	0	0	0	0	0	0	0	0	0
STAT5A	0	1	0	0	0	0	0	0	0	0	0	0	0	0	0	0	0	0
BAIAP2L1	0	0	0	0	1	0	0	0	0	0	0	0	0	0	0	0	0	0
KAT6B	0	1	0	0	0	0	0	0	0	0	0	0	0	0	0	0	0	0
FGF23	0	0	0	0	1	0	0	0	0	0	0	0	0	0	0	0	0	0
CPEB2	0	0	0	0	1	0	0	0	0	0	0	0	0	0	0	0	0	0
CCND2	0	0	0	0	-1	0	0	0	0	1	0	0	0	0	0	0	0	0
TNFSF10	0	0	0	0	0	0	0	0	0	1	0	0	0	0	0	0	0	0
SAMD8	0	0	0	0	1	0	0	0	0	0	0	0	0	0	0	0	0	0
RCSD1	0	0	0	1	0	0	0	0	0	0	0	0	0	0	0	0	0	0
COMTD1	0	1	0	0	0	0	1	0	0	0	0	0	0	0	0	0	0	0
LMTK2	0	0	0	0	1	0	0	0	0	1	0	0	0	0	0	0	0	0
PPM1F	0	0	0	0	1	0	0	0	0	0	0	0	0	0	0	0	0	0
ENSCAFG00000015445	0	0	1	0	1	1	0	0	0	0	0	0	0	0	0	0	0	0

TSPAN11	0	1	0	0	0	0	0	0	1	0	0	0	0	0	0	0	0	0
USHBP1	0	0	0	0	1	0	0	0	0	0	0	0	0	0	0	0	0	0
ZNF518B	0	0	0	0	1	0	0	0	0	0	0	0	0	0	0	0	0	0
PIMREG	0	0	0	0	0	0	1	0	0	-1	0	0	0	0	0	0	0	0
TSPAN9	0	0	0	0	1	0	0	0	0	0	0	0	0	0	0	0	0	0
CHRM1	0	1	0	0	0	0	0	0	0	0	0	0	0	0	0	0	0	0
DES	0	0	0	0	0	0	0	0	0	1	0	0	0	0	0	0	0	0
WSCD1	0	0	0	0	1	0	0	0	0	0	0	0	0	0	0	0	0	0
NR2F6	0	1	0	0	0	0	0	0	0	0	0	0	0	0	0	0	0	0
SCG3	0	0	0	0	1	0	0	0	0	0	0	0	0	0	0	0	0	0
STAT5B	0	0	0	0	0	0	0	0	0	1	0	0	0	0	0	0	0	0
USE1	0	1	0	0	0	0	0	0	0	0	0	0	0	0	0	0	0	0
ENSCAFG00000015518	0	0	0	0	0	0	0	0	0	1	0	0	0	0	0	0	0	0
NAALADL2	0	0	0	0	0	1	0	0	0	1	0	0	0	0	0	0	0	0
RPAIN	0	0	0	0	0	0	0	0	0	1	0	0	0	0	0	0	0	0
L3HYPDH	0	1	0	0	0	0	0	0	0	0	0	0	0	0	0	0	0	0
JKAMP	0	0	0	0	0	0	0	0	0	1	0	0	0	0	0	0	0	0
CSTF2T	0	1	0	0	0	0	0	0	0	0	0	0	0	0	0	0	0	0
RABEP1	0	0	0	0	0	0	0	0	0	1	0	0	0	0	0	0	0	0
PCNX4	0	1	0	0	0	0	0	0	0	1	0	0	0	0	0	0	0	0
DHRS7	0	1	0	0	0	-1	0	0	0	1	0	0	0	0	0	0	0	0
GHDC	0	1	0	0	0	0	0	0	0	0	0	0	0	0	0	0	0	0
SIN3B	0	0	0	0	-1	0	0	0	0	1	0	0	0	0	0	0	0	0
HDAC6	0	1	0	0	0	0	0	0	0	0	0	0	0	0	0	0	0	0
RAB5C	0	0	0	0	0	0	0	0	0	1	0	0	0	0	0	0	0	0
GREM2	0	0	0	0	0	0	0	0	1	0	0	0	0	0	0	0	0	0
SLC2A9	0	0	0	0	1	0	0	0	0	1	0	0	0	0	0	0	0	0
TMEM179B	0	1	0	0	0	0	0	0	0	0	0	0	0	0	0	0	0	0
MAPK6	0	-1	0	0	1	0	0	0	0	-1	0	0	0	0	0	0	0	0
PQBP1	0	1	0	0	0	0	0	0	0	0	0	0	0	0	0	0	0	0
RGS7	0	1	0	0	-1	0	0	0	0	0	0	0	0	0	0	0	0	0
STK11IP	0	1	0	0	0	0	0	0	0	0	0	0	0	0	0	0	0	0
C20H19orf44	0	1	0	0	0	0	0	0	0	0	0	0	0	0	0	0	0	0
CALR3	0	1	0	0	0	0	0	0	0	1	0	0	0	0	0	0	0	0
PRKCH	0	0	0	0	1	0	0	0	0	0	0	0	0	0	0	0	0	0
KCND1	0	0	0	0	0	0	0	0	1	0	0	0	0	0	0	0	0	0
CYTH3	0	0	0	0	-1	0	0	0	0	1	0	0	0	0	0	0	0	0
LRRN4CL	0	1	0	0	0	0	0	0	0	0	0	0	0	0	0	0	0	0
MSX1	0	1	0	0	0	0	0	0	0	-1	0	0	0	0	0	0	0	0
UBXN1	0	1	0	0	0	0	0	0	0	0	0	0	0	0	0	0	0	0
HIF1A	0	-1	0	0	1	0	0	0	0	0	0	0	0	0	0	0	0	0
DHX58	0	0	0	0	0	0	0	0	0	1	0	0	0	0	0	0	0	0
LIPA	0	0	0	0	1	0	0	0	0	0	0	0	0	0	0	0	0	0
STK32B	0	0	0	0	0	0	0	1	0	0	0	0	0	0	0	0	0	0
SYT16	0	0	0	0	0	0	0	0	0	1	0	0	0	0	0	0	0	0
OPN3	0	1	0	0	-1	0	0	0	0	1	0	0	0	0	0	0	0	0
DAGLB	0	1	0	0	0	0	0	0	0	0	0	0	0	0	0	0	0	0
EPS15L1	0	1	0	0	0	0	0	0	0	0	0	0	0	0	0	0	0	0
ANXA11	0	0	0	0	-1	0	0	0	1	0	0	0	0	0	0	0	0	0
ENSCAFG00000015742	0	1	0	0	0	0	0	0	0	0	0	0	0	0	0	0	0	0
C6H7orf26	0	1	0	0	0	0	0	0	0	0	0	0	0	0	0	0	0	0
TFE3	0	1	0	0	-1	0	0	0	0	0	0	0	0	0	0	0	0	0
CCDC120	0	1	0	0	0	0	0	0	0	0	0	0	0	0	0	0	0	0
KDM5A	0	0	0	0	1	0	0	0	0	0	0	0	0	0	0	0	0	0
PPP2R5E	0	0	0	0	0	0	0	0	0	1	0	0	0	0	0	0	0	0
EML3	0	1	0	0	0	0	0	0	0	0	0	0	0	0	0	0	0	0
WNK1	0	0	0	0	0	0	0	0	0	1	0	0	0	0	0	0	0	0
MTA2	0	0	0	0	1	0	0	0	0	0	0	0	0	0	0	0	0	0
ZBTB18	0	0	0	0	0	0	0	0	0	1	0	0	0	0	0	0	0	0
ADSS	0	0	0	0	1	0	0	0	0	0	0	0	0	0	0	0	0	0
SYNE2	0	0	0	0	1	0	0	0	0	0	0	0	0	0	0	0	0	0
CHRNE	0	0	0	0	0	0	0	0	0	1	0	0	0	0	0	0	0	0
ATP13A2	0	1	0	0	0	0	0	0	0	0	0	0	0	0	0	0	0	0
MFAP2	0	1	0	0	0	0	0	0	1	0	0	0	0	0	0	0	0	0
FAM214A	0	0	0	0	0	0	0	0	0	1	0	0	0	0	0	0	0	0
TSPAN14	0	0	0	0	1	0	0	0	0	0	0	0	0	0	0	0	0	0
ERC1	0	0	1	0	1	0	0	0	0	0	0	0	0	0	0	0	0	0
ENSCAFG00000015899	0	0	1	0	1	1	0	0	0	0	0	0	0	0	0	0	0	0
RSL24D1	0	-1	0	0	0	1	0	0	0	0	0	0	0	0	0	0	0	0
JUP	0	0	0	0	0	0	0	0	0	1	0	0	0	0	0	-1	0	0
RAB27A	0	0	0	0	1	0	0	0	0	0	0	0	0	0	0	0	0	0
CCPG1	0	0	0	0	0	0	0	0	0	1	0	0	0	0	0	0	0	0
EPHA4	0	0	0	0	0	0	0	0	1	1	0	0	0	0	0	0	0	0
AHCTF1	0	1	0	0	0	0	0	0	0	0	0	0	0	0	0	0	0	0
ADIPOR2	0	0	0	0	1	0	0	0	0	-1	0	0	0	0	0	0	0	0
ARHGEF19	0	0	0	0	0	0	0	0	0	0	1	0	0	0	0	0	0	0
FADS3	0	1	0	0	-1	-1	0	0	0	0	0	0	0	0	0	0	0	0
BCL6B	0	0	0	0	1	0	0	0	0	0	0	0	0	0	0	0	0	0
RNF216	0	1	0	0	-1	0	0	0	0	0	0	0	0	0	0	0	0	0
DCP1B	0	0	0	0	0	0	0	0	0	1	0	0	0	0	0	0	0	0
KRT23	0	0	0	0	1	0	0	0	0	0	0	0	0	0	0	0	0	0
FSCN1	0	-1	0	0	1	0	0	0	-1	-1	0	0	0	0	0	0	0	0
EPHA2	0	-1	0	0	1	0	0	0	0	0	0	0	0	0	0	0	0	0
COQ8A	0	1	0	0	0	0	0	0	0	0	0	0	0	0	0	0	0	0
FBXL18	0	1	0	0	0	0	0	0	0	0	0	0	0	0	0	0	0	0
ZBTB25	0	1	0	0	0	0	0	0	0	0	0	0	0	0	0	0	0	0
SHROOM4	0	0	0	0	1	0	0	0	0	0	0	0	0	0	0	0	0	0
PSEN2	0	0	0	0	0	0	0	0	0	1	0	0	0	0	0	0	0	0
CACNA1C	0	0	0	0	0	0	0	0	1	0	0	0	0	0	0	0	0	0
ITPKB	0	0	0	0	1	0	0	0	0	0	0	0	0	0	0	0	0	0
MMRN2	0	0	0	0	1	0	0	0	0	0	0	0	0	0	0	0	0	0
SNCG	0	0	0	0	1	0	0	0	0	0	0	0	0	0	0	0	0	0
RADIL	0	1	0	0	0	0	1	0	0	-1	0	0	0	0	0	0	0	0
AP5Z1	0	1	0	0	0	0	0	0	0	0	0	0	0	0	0	0	0	0
HSPB7	0	0	0	0	0	0	0	0	1	0	0	0	0	0	0	0	0	0
SYT7	0	0	0	0	1	0	0	0	0	0	0	0	0	0	0	0	0	0
ENSCAFG00000016140	0	1	0	0	0	0	0	0	0	0	0	0	0	0	0	0	0	0
KLHL5	0	-1	0	0	0	1	0	0	0	0	0	0	0	0	0	0	0	0
ENSCAFG00000016148	0	1	0	0	0	0	0	0	0	0	0	0	0	0	0	0	0	0
TMEM63A	0	0	0	0	0	0	0	0	0	1	0	0	0	0	0	0	0	0
TMEM156	0	-1	0	0	1	1	0	0	0	0	0	0	0	0	0	0	0	0
TLR6	0	0	0	0	1	0	0	0	0	0	0	0	0	0	0	0	0	0
AP1S3	0	0	0	0	1	0	0	0	0	1	0	0	0	0	0	0	0	0
TKFC	0	1	0	0	0	0	0	0	0	0	0	0	0	0	0	0	0	0
TCF12	0	1	0	0	0	0	0	0	0	0	0	0	0	0	0	0	0	0
TBC1D1	0	0	0	0	1	0	0	0	0	0	0	0	0	0	0	0	0	0
RSC1A1	0	0	0	0	1	0	0	0	0	0	0	0	0	0	0	0	0	0
MICAL3	0	0	0	0	0	0	0	0	0	1	0	0	0	0	0	0	0	0
FUT8	0	0	0	0	-1	0	0	0	0	1	0	0	0	0	0	0	0	0
DCP2	0	0	0	0	1	0	0	0	0	0	0	0	0	0	0	0	0	0
LBR	0	0	0	0	1	0	0	0	0	0	0	0	0	0	0	0	0	0
PGM2	0	0	0	0	1	0	0	0	0	0	0	0	0	0	0	0	0	0
MCC	0	0	0	0	1	0	0	0	0	0	0	0	0	0	0	0	0	0
CSF3	0	0	0	0	1	0	1	0	0	0	0	0	0	0	0	0	0	0
ENSCAFG00000016285	0	0	0	0	0	0	0	0	0	1	0	0	0	0	0	0	0	0
VWCE	0	1	0	0	0	0	0	0	0	0	0	0	0	0	0	0	0	0
ENSCAFG00000016321	0	0	1	0	1	0	0	0	0	0	0	0	0	0	0	0	0	0
CLN8	0	0	0	0	0	0	0	0	0	1	0	0	0	0	0	0	0	0
TMEM51	0	0	0	0	0	0	0	0	0	0	0	0	0	0	1	0	0	0
WDR26	0	0	0	0	0	0	0	0	0	1	0	0	0	0	0	0	0	0
ZBTB4	0	1	0	0	0	0	0	0	0	1	0	0	0	0	0	0	0	0
DBN1	0	1	0	0	0	0	0	0	0	-1	0	0	0	0	0	0	0	0
ENSCAFG00000016371	0	0	0	0	1	0	0	0	0	0	0	0	0	0	0	0	0	0
SMIM20	0	1	0	0	0	0	0	0	0	0	0	0	0	0	0	0	0	0
PLEKHH1	0	0	0	0	0	0	0	0	0	1	0	0	0	0	0	0	0	0
GNAI1	0	0	0	0	1	0	0	0	0	1	0	0	0	0	0	0	0	0
TNFRSF1B	0	0	0	0	1	0	0	0	0	0	0	0	0	0	0	0	0	0
ARG2	0	0	0	0	1	0	0	0	0	-1	0	1	0	0	0	0	0	0
FBXL20	0	0	0	0	0	0	0	0	0	1	0	0	0	0	0	0	0	0
MFN2	0	1	0	0	0	0	0	0	0	0	0	0	0	0	0	0	0	0

NSD1	0	0	0	0	1	0	0	0	0	0	0	0	0	0	0	0	0	0
ZFP36L1	0	1	0	0	-1	0	0	0	0	0	0	0	0	0	0	0	0	0
SOD3	0	1	0	0	0	0	0	0	0	0	0	0	0	0	0	0	0	0
UBQLN2	0	1	0	0	0	0	0	0	0	0	0	0	0	0	0	0	0	0
PEAR1	0	0	0	0	1	0	0	0	0	0	0	0	0	0	0	0	0	0
PPARGC1A	0	0	0	0	0	0	0	0	1	1	0	0	0	0	0	0	0	0
NPPB	0	0	0	0	0	0	0	0	0	1	0	0	0	0	0	0	0	0
DCAF5	0	0	0	0	0	0	0	0	0	1	0	0	0	0	0	0	0	0
MTHFR	0	1	0	0	0	0	0	0	0	0	0	0	0	0	0	0	0	0
ZC4H2	0	0	0	0	1	0	0	0	0	0	0	0	0	0	0	0	0	0
LIPC	0	1	0	0	0	0	0	0	0	-1	0	0	0	0	0	0	0	0
ADAM10	0	0	0	0	1	0	0	0	0	0	0	0	0	0	0	0	0	0
ENSCAFG00000016569	0	0	0	0	0	0	0	0	0	1	0	0	0	0	0	0	0	0
SLC39A9	0	0	0	0	0	0	0	0	0	1	0	0	0	0	0	0	0	0
PIP4K2B	0	1	0	0	0	0	0	0	0	0	0	0	0	0	0	0	0	0
ENSCAFG00000016577	0	0	1	0	0	0	0	0	0	-1	0	0	0	0	0	0	0	0
MINDY2	0	1	0	0	0	0	0	0	0	1	0	0	0	0	0	0	0	0
SLTM	0	0	0	0	1	0	0	0	0	0	0	0	0	0	0	0	0	0
MLLT6	0	1	0	0	0	0	0	0	0	0	0	0	0	0	0	0	0	0
SMOC1	0	0	0	0	1	0	0	0	0	0	0	0	0	0	0	0	0	0
SLIT2	0	1	0	0	0	0	0	0	0	0	0	0	0	0	0	0	1	0
TNFSF13	0	1	0	0	0	0	0	0	0	0	0	0	0	0	0	0	0	0
SYNJ2BP	0	0	0	0	0	0	0	0	0	1	0	0	0	0	0	0	0	0
ARHGAP23	0	0	0	0	1	0	0	0	0	0	0	0	0	0	0	0	0	0
SOCS7	0	1	0	0	0	0	0	0	0	0	0	0	0	0	0	0	0	0
HEPH	0	0	0	0	1	0	0	0	0	0	0	0	0	0	0	0	0	0
CD68	0	0	0	0	0	0	0	0	1	0	0	0	0	0	0	0	0	0
MTOR	0	0	0	0	1	0	0	0	0	0	0	0	0	0	0	0	0	0
FAM81A	0	1	0	0	0	0	1	0	0	-1	0	0	0	0	0	1	0	0
AR	0	0	0	0	0	0	0	0	0	1	0	0	0	0	0	0	0	0
BNIP2	0	0	0	0	1	0	0	0	0	0	0	0	0	0	0	0	0	0
FAF2	0	0	0	0	0	0	0	0	0	1	0	0	0	0	0	0	0	0
CTBP1	0	1	0	0	0	0	0	0	0	0	0	0	0	0	0	0	0	0
ENSCAFG00000016695	0	1	0	0	0	0	0	0	0	0	0	0	0	0	0	0	0	0
FGFRL1	0	0	0	0	0	0	0	0	0	1	0	0	0	0	0	0	0	0
RORA	0	0	0	0	0	1	0	0	0	1	0	0	0	0	0	0	0	0
KIAA1191	0	0	0	0	0	0	0	0	0	1	0	0	0	0	0	0	0	0
HRH2	0	0	0	0	0	0	0	0	0	1	0	0	0	0	0	0	0	0
SIPA1L1	0	-1	0	0	0	0	0	0	0	1	0	0	0	0	0	0	0	0
CRABP2	0	1	0	0	0	0	0	0	0	0	0	0	0	0	0	0	0	0
CPEB4	0	0	0	0	0	0	0	0	1	1	0	0	0	0	0	0	0	0
PCGF3	0	0	0	0	1	0	0	0	0	0	0	0	0	0	0	0	0	0
SLC49A3	0	0	0	0	1	0	0	0	0	0	0	0	0	0	0	0	0	0
OSBPL7	0	1	0	0	0	0	0	0	0	0	0	0	0	0	0	0	0	0
NES	0	-1	0	0	1	0	0	0	0	-1	0	0	0	0	0	0	0	0
HSD3B7	0	1	0	0	-1	-1	0	0	0	0	0	0	0	0	0	0	0	0
CASZ1	0	0	0	0	0	0	0	0	0	1	0	0	0	0	0	0	0	0
NAXE	0	1	0	0	0	0	0	0	0	0	0	0	0	0	0	0	0	0
PIGG	0	0	0	0	0	0	0	0	0	1	0	0	0	0	0	0	0	0
CREBRF	0	1	0	0	0	0	0	0	0	1	0	0	0	0	0	0	0	0
MRPL10	0	1	0	0	0	0	0	0	0	0	0	0	0	0	0	0	0	0
ZFYVE1	0	0	0	0	0	0	0	0	0	1	0	0	0	0	0	0	0	0
SCRN2	0	0	0	0	0	0	0	0	0	1	0	0	0	0	0	0	0	0
ENSCAFG00000016809	0	0	0	0	1	0	0	0	0	0	0	0	0	0	0	0	0	0
SP2	0	1	0	0	0	0	0	0	0	0	0	0	0	0	0	0	0	0
ERGIC1	0	1	0	0	-1	-1	0	0	0	0	0	0	0	0	0	0	0	0
PNPO	0	1	0	0	0	0	0	0	0	0	0	0	0	0	0	0	0	0
PSEN1	0	0	0	0	0	0	0	0	0	1	0	0	0	0	0	0	0	0
PAPLN	0	0	0	0	1	0	0	0	0	0	0	0	0	0	0	0	0	0
COPZ2	0	0	0	0	0	0	0	0	1	0	0	0	0	0	0	0	0	0
NUMB	0	1	0	0	0	0	0	0	0	1	0	0	0	0	0	0	0	0
PAQR6	0	1	0	0	-1	0	0	0	0	0	0	0	0	0	0	0	0	0
HOXB5	0	0	0	0	1	0	0	0	0	-1	0	0	1	0	0	0	1	0
HOXB6	0	-1	0	0	1	0	0	0	0	0	0	0	0	0	0	0	0	0
HOXB7	0	-1	0	0	1	0	1	0	0	-1	0	0	0	0	0	0	0	0
STK10	0	0	0	0	1	0	0	0	0	0	0	0	0	0	0	0	0	0
DNAL1	0	0	0	0	0	0	0	0	1	0	0	0	0	0	0	0	0	0
HOXB8	0	0	0	0	1	0	0	0	0	0	0	0	0	0	0	0	0	0
PNMA1	0	0	0	0	0	0	0	0	1	0	0	0	0	0	0	0	0	0
ZNF410	0	0	0	0	1	0	0	0	0	0	0	0	0	0	0	0	0	0
ARHGEF2	0	-1	0	0	1	1	0	1	0	-1	0	0	0	0	0	0	0	0
ALDH6A1	0	1	0	0	0	0	0	0	0	0	0	0	0	0	0	0	0	0
FGF18	0	0	0	0	0	0	0	0	0	1	0	0	0	0	0	0	0	0
GNGT2	0	0	0	0	1	0	0	0	0	0	0	0	0	0	0	0	0	0
ABI3	0	0	0	0	1	0	0	0	0	0	0	0	0	0	0	0	0	0
ASH1L	0	0	0	0	0	0	0	0	0	1	0	0	0	0	0	0	0	0
FAM117A	0	1	0	0	0	0	0	0	0	0	0	0	0	0	0	0	0	0
KCNIP1	0	1	0	0	0	0	0	0	0	0	0	0	0	0	0	0	0	0
PDK2	0	1	0	0	0	0	0	0	0	0	0	0	0	0	0	0	0	0
LACTB	0	1	0	0	0	0	0	0	0	0	0	0	0	0	0	0	0	0
DLST	0	1	0	0	0	0	0	0	0	0	0	0	0	0	0	0	0	0
CA12	0	0	0	0	0	0	0	0	0	0	1	0	0	0	0	0	0	0
RPS6KL1	0	0	0	0	1	0	0	0	0	0	0	0	0	0	0	0	0	0
PGF	0	0	0	0	1	0	0	0	0	0	0	0	0	0	0	0	0	0
EIF2B2	0	0	0	0	1	0	0	0	0	0	0	0	0	0	0	0	0	0
ENSCAFG00000017001	0	1	0	0	0	0	0	0	0	0	0	0	0	0	0	0	0	0
BORCS6	0	1	0	0	0	0	0	0	0	0	0	0	0	0	0	0	0	0
MLH3	0	1	0	0	0	0	0	0	0	0	0	0	0	0	0	0	0	0
SGCA	0	0	0	0	0	0	0	0	0	1	0	0	0	0	0	0	0	0
SLIT3	0	1	0	0	-1	0	0	0	0	1	0	0	0	0	0	0	0	0
NEK9	0	0	0	0	0	0	0	0	0	1	0	0	0	0	0	0	0	0
TAOK2	0	0	0	0	1	0	0	0	0	0	0	0	0	0	0	0	0	0
FLVCR2	0	1	0	0	0	0	0	0	0	0	0	0	0	0	0	0	0	0
PANK3	0	0	0	0	1	0	0	0	0	0	0	0	0	0	0	0	0	0
ARHGEF15	0	0	0	0	1	0	0	0	0	0	0	0	0	0	0	0	0	0
KRBA2	0	0	0	0	0	0	0	0	0	1	0	0	0	0	0	0	0	0
ITGB1BP2	0	-1	0	0	0	0	0	0	1	0	0	0	0	0	0	0	0	0
WWC1	0	0	0	0	0	0	0	0	0	1	0	0	0	0	0	0	0	0
ZNF609	0	0	0	0	1	0	0	0	0	0	0	0	0	0	0	0	0	0
YPEL3	0	1	0	0	0	0	0	0	0	0	0	0	0	0	0	0	0	0
ADAM15	0	1	0	0	0	0	0	0	0	1	0	0	0	0	0	0	0	0
ENSCAFG00000017103	0	0	0	0	1	0	0	0	0	-1	0	0	0	0	0	0	0	0
GCDH	0	1	0	0	0	0	0	0	0	0	0	0	0	0	0	0	0	0
SPG21	0	1	0	0	0	0	0	0	0	0	0	0	0	0	0	0	0	0
IFT43	0	0	0	0	0	0	0	0	0	1	0	0	0	0	0	0	0	0
SULT1A1	0	1	0	0	0	0	0	0	0	0	0	0	0	0	0	0	0	0
ZBTB7B	0	0	0	0	-1	0	0	0	1	0	0	0	0	0	0	0	0	0
SGF29	0	1	0	0	0	0	0	0	0	0	0	0	0	0	0	0	0	0
GPATCH2L	0	0	0	0	0	0	0	0	0	1	0	0	0	0	0	0	0	0
VASH1	0	0	0	0	1	0	0	0	0	0	0	0	0	0	0	0	0	0
IRF2BPL	0	1	0	0	-1	0	0	0	0	0	0	0	0	0	0	0	0	0
MAT2B	0	0	0	0	0	0	0	0	0	1	0	0	0	0	0	0	0	0
SHE	0	0	0	0	1	0	0	0	0	0	0	0	0	0	0	0	0	0
IL6R	0	1	0	0	0	0	0	0	0	0	0	0	0	0	0	0	0	0
ATP8B2	0	1	0	0	-1	0	0	0	0	0	0	0	0	0	0	0	0	0
SH2B1	0	1	0	0	0	0	0	0	0	0	0	0	0	0	0	0	0	0
SPTLC2	0	0	0	0	1	0	0	0	0	0	0	0	0	0	0	0	0	0
ZDHHC15	0	0	0	0	0	0	0	0	0	1	0	0	0	0	0	0	0	0
ENSCAFG00000017231	0	0	0	0	0	1	0	0	0	0	0	0	0	0	0	0	0	0
DENND4A	0	0	0	0	1	0	0	0	0	0	0	0	0	0	0	0	0	0
ATRX	0	0	0	0	1	0	0	0	0	1	0	0	0	0	0	0	0	0
ATP2A1	0	0	0	0	1	0	0	0	0	0	0	0	0	0	0	0	0	0
RAB11A	0	0	0	0	1	0	0	0	0	0	0	0	0	0	0	0	0	0
MAGT1	0	0	0	0	0	0	0	0	0	1	0	0	0	0	0	0	0	0
MYH10	0	0	0	0	1	0	0	0	0	0	0	0	0	0	0	0	0	0
ATP7A	0	0	0	0	0	0	0	0	0	1	0	0	0	0	0	0	0	0
SPAG9	0	0	0	0	1	0	0	0	0	-1	0	0	0	0	0	0	0	0
C7H1orf43	0	1	0	0	0	0	0	0	0	0	0	0	0	0	0	0	0	0
TAF9B	0	1	0	0	-1	0	0	0	0	0	0	0	0	0	0	0	0	0
ADRA1B	0	0	0	0	0	0	0	0	0	1	0	0	0	0	0	0	0	0

STON2	0	1	0	0	0	0	0	0	0	0	0	0	0	0	0	0	0	0
EBF1	0	1	0	0	0	0	0	0	0	0	0	0	0	0	0	0	0	0
TMEM100	0	0	0	0	0	0	0	0	1	0	0	0	0	0	0	0	0	0
KIAA0556	0	0	0	0	-1	0	0	0	1	1	0	0	0	0	0	0	0	0
ZNF711	0	0	0	0	1	0	0	0	0	0	0	0	0	0	0	0	0	0
CRTC2	0	1	0	0	0	0	0	0	0	0	0	0	0	0	0	0	0	0
GTF3C1	0	0	0	0	0	0	0	0	0	1	0	0	0	0	0	0	0	0
DENND4B	0	1	0	0	0	0	0	0	0	0	0	0	0	0	0	0	0	0
VEZF1	0	1	0	0	0	0	0	0	0	0	0	0	0	0	0	0	0	0
CCDC159	0	0	0	0	0	0	0	0	0	1	0	0	0	0	0	0	0	0
RAB3D	0	0	0	0	1	0	0	0	0	0	0	0	0	0	0	0	0	0
KLHL4	0	0	0	0	1	0	0	0	0	0	0	0	0	0	0	0	0	0
PABPC5	0	1	0	0	0	0	0	0	0	0	0	0	0	0	0	0	0	0
GAS7	0	1	0	0	0	0	0	0	0	0	0	0	0	0	0	0	0	0
TTC8	0	1	0	0	-1	0	0	0	0	0	0	0	0	0	0	0	0	0
TSPOAP1	0	1	0	0	0	0	0	0	0	0	0	0	0	0	0	0	0	0
CORO2B	0	1	0	0	0	0	0	0	0	0	0	0	0	0	0	0	0	0
DOCK6	0	0	0	0	1	0	0	0	0	0	0	0	0	0	0	0	0	0
NPR1	0	0	0	0	1	0	0	0	0	0	0	0	0	0	0	0	0	0
PCDH19	0	1	0	0	0	0	0	0	0	0	0	0	0	0	0	0	0	0
ILF2	0	0	0	0	1	0	0	0	0	-1	0	0	0	0	0	0	0	0
RPS6KA5	0	0	0	0	1	0	0	0	0	0	0	0	0	0	0	0	0	0
S100A4	0	1	0	0	0	0	0	0	0	0	0	0	0	0	0	0	0	0
S100A5	0	0	0	0	1	0	0	0	0	0	0	0	0	0	0	0	0	0
GPR68	0	0	0	0	1	0	0	0	0	0	0	0	0	0	0	0	0	0
CCDC88C	0	0	0	0	1	0	0	0	0	0	0	0	0	0	0	0	0	0
RBBP6	0	0	0	0	1	0	0	0	0	0	0	0	0	0	0	0	0	0
PPP4R3A	0	0	0	0	1	0	0	0	0	0	0	0	0	0	0	0	0	0
SMARCA4	0	-1	0	0	1	0	0	0	0	0	0	0	0	0	0	0	0	0
SGCD	0	0	1	0	0	0	0	0	0	0	0	0	0	0	0	0	0	0
TMEM220	0	0	0	0	0	0	0	0	0	1	0	0	0	0	0	0	0	0
BTBD7	0	0	0	0	1	0	0	0	0	0	0	0	0	0	0	0	0	0
ST8SIA5	0	1	0	0	0	0	0	0	0	0	1	0	0	0	0	0	0	0
LARP6	0	1	0	0	-1	0	0	0	0	0	0	0	0	0	0	0	0	0
FAXDC2	0	1	0	0	0	0	0	0	0	1	0	0	0	0	0	0	0	0
THSD4	0	0	0	0	1	0	0	0	0	0	0	0	0	0	0	0	0	0
C7H18orf25	0	0	0	0	1	0	0	0	0	0	0	0	0	0	0	0	0	0
GALNT10	0	0	0	0	0	0	0	0	0	1	0	0	0	0	0	0	0	0
MYO9A	0	0	0	0	1	0	0	0	0	0	0	0	0	0	0	0	0	0
EPG5	0	0	0	0	0	0	0	0	0	1	0	0	0	0	0	0	0	0
SLC14A1	0	0	0	0	0	0	0	1	0	1	0	0	0	0	0	0	0	0
VMP1	0	-1	0	0	0	0	0	0	1	0	0	0	0	0	0	0	0	0
PARP6	0	1	0	0	0	0	0	0	0	0	0	0	0	0	0	0	0	0
SETBP1	0	1	0	0	0	0	0	0	0	0	0	0	0	0	0	0	0	0
USP31	0	0	0	0	1	0	0	0	0	0	0	0	0	0	0	0	0	0
ENSCAFG00000017697	0	1	0	0	0	0	0	0	0	0	0	0	0	0	0	0	0	0
SYNE3	0	1	0	0	0	0	0	0	0	0	0	0	0	0	0	0	0	0
SLC44A2	0	0	0	0	0	0	0	0	0	1	0	0	0	0	0	0	0	0
EEF2K	0	1	0	0	-1	0	0	0	0	0	0	0	0	0	0	0	0	0
BBS4	0	0	0	0	0	0	0	0	0	1	0	0	0	0	0	0	0	0
PIK3C3	0	0	0	0	0	0	0	0	0	1	0	0	0	0	0	0	0	0
BCAS3	0	0	0	0	0	0	0	0	0	1	0	0	0	0	0	0	0	0
KIAA1328	0	0	0	0	1	0	0	0	0	0	0	0	0	0	0	0	0	0
GPRASP2	0	0	0	0	0	0	0	0	0	1	0	0	0	0	0	0	0	0
NEO1	0	-1	0	0	0	0	0	1	0	0	0	0	0	0	0	0	0	0
KEAP1	0	1	0	0	0	0	0	0	0	0	0	0	0	0	0	0	0	0
USP32	0	0	0	0	0	0	0	0	0	1	0	0	0	0	0	0	0	0
ENSCAFG00000017795	0	-1	0	0	0	0	0	0	0	1	0	0	0	0	0	0	0	0
MOCOS	0	1	0	0	0	0	0	0	0	0	0	0	0	0	0	0	0	0
HHIPL1	0	0	0	0	0	0	0	0	1	0	0	0	0	0	0	0	0	0
STOML1	0	0	0	0	0	0	0	0	0	1	0	0	0	0	0	0	0	0
ISLR2	0	0	1	0	0	0	0	0	0	0	0	0	0	0	0	0	0	0
ISLR	0	1	0	0	0	0	0	0	0	0	0	0	0	0	0	0	0	0
ENSCAFG00000017868	0	0	0	0	0	0	0	0	0	0	0	0	0	1	0	0	0	0
ICAM5	0	-1	0	0	0	0	0	0	1	0	0	0	0	0	0	0	0	0
DCUN1D3	0	0	0	0	0	0	0	0	1	0	0	0	0	0	0	0	0	0
MRPL4	0	1	0	0	0	0	0	0	0	0	0	0	0	0	0	0	0	0
EVL	0	0	0	0	0	0	0	0	0	1	0	0	0	0	0	0	0	0
ENSCAFG00000017892	0	1	0	0	0	0	0	0	0	-1	0	0	0	0	0	0	0	0
EIF3G	0	1	0	0	0	0	0	0	0	0	0	0	0	0	0	0	0	0
CCDC69	0	0	0	0	1	0	0	0	0	0	0	0	0	0	0	0	0	0
CXHXorf57	0	0	0	0	0	1	0	0	0	0	0	0	0	0	0	0	0	0
UBL7	0	1	0	0	0	0	0	0	0	0	0	0	0	0	0	0	0	0
ARID3B	0	0	0	0	1	0	0	0	0	0	0	0	0	0	0	0	0	0
CYP1A1	0	0	0	0	0	0	0	0	0	0	0	0	0	1	0	0	0	0
ENSCAFG00000017940	0	0	0	0	1	0	0	0	0	0	0	0	0	0	0	0	0	0
MAPRE2	0	1	0	0	0	0	0	0	0	1	0	0	0	0	0	0	0	0
ACSM3	0	1	0	0	0	0	0	0	0	0	0	0	0	0	0	0	0	0
TNIP1	0	0	0	0	1	0	0	0	0	0	0	0	0	0	0	0	0	0
SCAMP2	0	1	0	0	-1	0	0	0	0	0	0	0	0	0	0	0	0	0
ENSCAFG00000017975	0	1	0	0	0	0	0	0	0	0	0	0	0	0	0	0	0	0
SCAMP5	0	0	0	0	0	0	0	0	0	1	0	0	0	0	0	0	0	0
GAREM1	0	1	0	0	0	0	0	0	0	0	0	0	0	0	0	0	0	0
ATG4A	0	0	0	0	0	0	0	0	0	1	0	0	0	0	0	0	0	0
LRRC75A	0	1	0	0	-1	0	1	0	1	0	0	0	0	0	0	0	0	0
COL4A5	0	0	0	0	1	0	0	0	0	0	0	0	0	0	1	0	0	0
GP2	0	0	0	0	0	0	0	0	0	1	0	0	0	0	0	0	0	0
GPRC5B	0	0	0	0	0	0	0	1	0	1	0	0	0	0	0	0	1	0
PTPN9	0	1	0	0	-1	0	0	0	0	0	0	0	0	0	0	0	0	0
SNUPN	0	1	0	0	-1	0	0	0	0	0	0	0	0	0	0	0	0	0
B4GALT6	0	0	0	0	1	0	0	0	0	0	0	0	0	0	0	0	0	0
SNX33	0	1	0	0	0	0	0	0	0	0	0	0	0	0	0	0	0	0
DSG2	0	0	0	0	1	0	0	0	0	0	0	0	0	0	0	0	0	0
TECPR2	0	0	0	0	0	0	0	0	0	1	0	0	0	0	0	0	0	0
ANKRD9	0	1	0	0	0	0	0	0	1	0	0	0	0	0	0	0	0	0
HMG20A	0	0	0	0	1	0	0	0	0	0	0	0	0	0	0	0	0	0
TSPAN3	0	0	0	0	0	0	0	0	0	1	0	0	0	0	0	0	0	0
ZNF317	0	0	0	0	1	0	0	0	0	0	0	0	0	0	0	0	0	0
PSTPIP1	0	0	0	0	0	0	0	0	1	0	0	0	0	0	0	0	0	0
TTC19	0	0	0	0	0	0	0	0	0	1	0	0	0	0	0	0	0	0
TMEM164	0	1	0	0	0	0	0	0	0	0	0	0	0	0	0	0	0	0
DSC2	0	0	0	0	1	0	0	0	0	0	0	0	0	0	0	0	0	0
CHST9	0	0	0	0	1	0	0	0	0	0	0	0	0	0	0	0	0	0
ARSI	0	0	0	0	0	0	0	0	0	-1	0	0	0	0	0	0	1	0
ULK2	0	0	0	0	0	0	0	0	0	1	0	0	0	0	0	0	0	0
TMC7	0	0	0	0	1	0	0	0	0	0	0	0	0	0	0	0	0	0
SYNRG	0	1	0	0	0	0	0	0	0	0	0	0	0	0	0	0	0	0
ENSCAFG00000018136	0	1	0	0	0	0	0	0	0	0	0	0	0	0	0	0	0	0
ENSCAFG00000018144	0	0	1	0	1	0	0	0	0	0	0	0	0	0	0	0	0	0
HEATR6	0	0	0	0	1	0	0	0	0	0	0	0	0	0	0	0	0	0
ZNF521	0	0	0	0	1	0	0	0	0	0	0	0	0	0	0	0	0	0
CAMK2A	0	0	0	0	0	0	0	0	1	0	0	0	0	0	0	0	0	0
CCL5	0	0	0	0	0	0	0	0	1	1	0	0	0	0	0	0	0	0
LAMA3	0	0	0	0	-1	0	0	0	0	1	0	0	0	0	0	0	0	0
XYLT1	0	0	0	0	0	0	0	0	1	-1	0	0	0	0	0	0	0	0
DCX	0	0	0	0	0	0	1	0	1	0	0	0	0	0	0	0	0	0
ANKRD29	0	0	0	0	0	0	0	0	0	1	0	0	0	0	0	0	0	0
ENSCAFG00000018181	0	0	0	0	0	0	0	0	0	1	0	0	0	0	0	0	0	0
TNFAIP2	0	1	0	0	0	0	0	0	0	0	0	0	0	0	0	0	0	0
ENSCAFG00000018194	0	0	0	0	-1	0	1	0	1	0	0	0	0	0	0	0	0	0
RIOK3	0	0	0	0	1	0	0	0	0	0	0	0	0	0	0	0	0	0
MAPK7	0	0	0	0	0	0	0	0	0	1	0	0	0	0	0	0	0	0
CABLES1	0	1	0	0	0	0	0	0	0	0	0	0	0	0	0	0	0	0
EPN2	0	1	0	0	0	0	0	0	0	0	0	0	0	0	0	0	0	0
EIF5	0	-1	0	0	0	1	0	0	0	0	0	0	0	0	0	0	0	0
LHFPL1	0	0	0	0	1	0	0	0	0	0	0	0	0	0	0	0	0	0
AMOT	0	0	0	0	1	0	0	0	0	0	0	0	0	0	0	0	0	0
ZNF358	0	1	0	0	0	0	0	0	0	0	0	0	0	0	0	0	0	0
LRCH2	0	0	0	0	0	1	0	0	0	0	0	0	0	0	0	0	0	0
MIB1	0	0	0	0	1	0	0	0	0	0	0	0	0	0	0	0	0	0

AP2B1	0	0	0	0	0	0	0	0	0	1	0	0	0	0	0	0	0	0
ESCO1	0	0	0	0	1	0	0	0	0	0	0	0	0	0	0	0	0	0
ENSCAFG00000018258	0	0	0	0	1	1	0	0	0	0	0	0	0	0	0	0	0	0
PNPLA6	0	0	0	0	0	0	0	0	0	1	0	0	0	0	0	0	0	0
PPARGC1B	0	1	0	0	0	0	0	0	0	0	0	0	0	0	0	0	0	0
UNC45B	0	0	0	0	0	0	0	0	1	0	0	0	0	0	0	0	0	0
ENSCAFG00000018285	0	0	0	0	1	0	0	0	0	0	0	0	0	0	0	0	0	0
ZFYVE21	0	1	0	0	0	0	0	0	0	0	0	0	0	0	0	0	0	0
PPP1R13B	0	0	0	0	1	0	0	0	0	0	0	0	0	0	0	0	0	0
AFAP1L1	0	0	0	0	1	0	0	0	0	0	0	0	0	0	0	0	0	0
EVI5L	0	0	0	0	-1	0	0	0	0	1	0	0	0	0	0	0	0	0
ABLIM3	0	0	0	0	1	0	0	0	0	1	0	0	0	0	0	0	0	0
THOC1	0	0	0	0	1	0	0	0	0	0	0	0	0	0	0	0	0	0
COLEC12	0	1	0	0	0	0	0	0	1	0	0	0	0	0	0	0	0	0
ENOSF1	0	0	0	0	0	0	0	0	0	1	0	0	0	0	0	0	0	0
IL13RA1	0	-1	0	0	1	0	0	0	0	0	0	0	0	0	0	0	0	0
ENSCAFG00000018360	0	-1	0	0	0	0	0	0	0	1	0	0	0	0	0	0	0	0
YES1	0	-1	0	0	1	0	0	0	0	0	0	0	0	0	0	0	0	0
PGRMC1	0	0	0	0	0	0	0	0	0	1	0	0	0	0	0	0	0	0
ENSCAFG00000018377	0	0	0	0	0	1	0	0	0	0	0	0	0	0	0	0	0	0
SLC25A43	0	1	0	0	0	0	0	0	0	0	0	0	0	0	0	0	0	0
ARL15	0	0	0	0	1	0	0	0	0	0	0	0	0	0	0	0	0	0
C9H17orf75	0	1	0	0	0	0	0	0	0	0	0	0	0	0	0	0	0	0
RHBDL3	0	1	0	0	0	0	0	0	0	0	0	0	0	0	0	0	0	0
CRIP2	0	1	0	0	1	0	0	0	0	0	0	0	0	0	0	0	0	0
CRIP1	0	1	0	0	0	0	0	0	0	0	0	0	0	0	0	0	0	0
RHOT1	0	0	0	0	0	0	0	0	0	1	0	0	0	0	0	0	0	0
ISL1	0	0	0	1	0	1	0	0	0	0	0	0	0	0	1	0	0	0
TMEM255A	0	0	0	0	0	0	0	0	0	1	0	0	0	0	0	0	0	0
RNF135	0	1	0	0	0	0	0	0	0	1	0	0	0	0	0	0	0	0
CERS4	0	1	0	0	0	0	0	0	0	0	0	0	0	0	0	0	0	0
CRLF3	0	0	0	0	0	0	0	0	0	1	0	0	0	0	0	0	0	0
ANGPTL4	0	1	0	0	0	0	0	0	0	1	0	0	0	0	0	0	0	0
LAMP2	0	0	0	0	0	0	0	0	0	1	0	0	0	0	0	0	0	0
MARCH2	0	0	0	0	0	0	0	0	0	1	0	0	0	0	0	0	0	0
WDR78	0	0	0	0	0	0	0	0	0	1	0	0	0	0	0	0	0	0
EPB41L3	0	0	0	1	0	0	0	0	0	0	0	0	0	0	0	0	0	0
PDE4B	0	0	0	0	1	0	0	0	0	0	0	0	0	0	0	0	0	0
FBXO4	0	1	0	0	0	0	0	0	0	0	0	0	0	0	0	0	0	0
L3MBTL4	0	1	0	0	0	0	0	0	1	1	0	0	0	0	0	0	0	0
OXCT1	0	1	0	0	0	0	0	0	0	0	0	0	0	0	0	0	0	0
NF1	0	0	0	0	0	0	0	0	0	1	0	0	0	0	0	0	0	0
C6	0	0	0	0	0	0	0	0	0	0	0	0	0	1	0	0	0	0
DNAJC6	0	0	0	0	0	0	0	0	0	1	0	0	0	0	0	0	0	0
TRIP10	0	1	0	0	0	0	0	0	0	0	0	0	0	0	0	0	0	0
ENSCAFG00000018611	0	0	0	0	0	0	0	0	0	1	0	0	0	0	0	0	0	0
JAK1	0	0	0	0	1	0	0	0	0	0	0	0	0	0	0	0	0	0
GPR108	0	1	0	0	-1	0	0	0	0	1	0	0	0	0	0	0	0	0
DAB2	0	0	0	0	0	0	0	0	0	1	0	0	0	0	0	0	0	0
EVI2B	0	-1	0	0	0	0	0	1	1	0	0	0	0	0	0	0	0	0
WSB1	0	-1	0	0	1	0	0	0	0	0	0	0	0	0	0	0	0	0
LGALS9	0	0	0	0	0	0	0	0	0	1	0	0	0	0	0	0	0	0
NOS2	0	0	0	0	1	0	0	0	0	0	0	0	0	0	0	0	0	0
TNFAIP1	0	-1	0	0	1	0	0	0	0	0	0	0	0	0	0	0	0	0
POLDIP2	0	1	0	0	-1	0	0	0	0	0	0	0	0	0	0	0	0	0
OCRL	0	0	0	0	0	0	0	0	0	1	0	0	0	0	0	0	0	0
ANKRD12	0	0	0	0	1	0	0	0	0	0	0	0	0	0	0	0	0	0
TWSG1	0	0	0	0	0	0	0	0	0	1	0	0	0	0	0	0	0	0
XPNPEP2	0	1	0	0	0	0	0	0	0	0	0	0	0	0	0	0	0	0
ENSCAFG00000018701	0	0	0	0	0	0	0	0	0	1	0	0	0	0	0	0	0	0
RALBP1	0	0	0	0	1	0	0	0	0	0	0	0	0	0	0	0	0	0
SKP2	0	0	0	0	0	0	0	0	0	1	0	0	0	0	0	0	0	0
RFX2	0	1	0	0	0	0	0	0	0	0	0	0	0	0	0	0	0	0
LMBRD2	0	0	0	0	0	0	0	0	0	1	0	0	0	0	0	0	0	0
MARF1	0	0	0	0	0	0	0	0	0	1	0	0	0	0	0	0	0	0
ELF4	0	0	0	0	1	0	0	0	0	-1	0	0	0	0	0	0	0	0
ENSCAFG00000018740	0	0	0	0	1	1	0	0	0	0	0	0	0	0	0	0	0	0
IL7R	0	0	0	0	1	0	0	0	0	0	0	0	0	0	0	0	0	0
PIEZO2	0	0	0	0	1	0	0	0	0	1	0	0	0	0	0	0	0	0
LONP1	0	-1	0	0	1	0	0	0	0	0	0	0	0	0	0	0	0	0
ENOX2	0	0	0	0	0	0	0	0	0	1	0	0	0	0	0	0	0	0
ENSCAFG00000018792	0	1	0	0	0	0	0	0	0	0	0	0	0	0	0	0	0	0
C20H19orf70	0	1	0	0	-1	0	0	0	0	0	0	0	0	0	0	0	0	0
RAI14	0	-1	0	0	1	0	0	0	0	-1	0	0	0	0	0	0	0	0
PATJ	0	0	0	0	0	0	0	0	0	1	0	0	0	0	0	0	0	0
SLC45A2	0	0	0	0	0	0	0	0	0	1	0	0	0	0	0	0	0	0
TM2D1	0	0	0	0	0	0	0	0	0	1	0	0	0	0	0	0	0	0
MBNL3	0	0	0	0	0	0	0	0	0	0	0	0	1	0	1	0	1	0
SPIRE1	0	0	0	0	1	0	0	0	0	0	0	0	0	0	0	0	0	0
PHF6	0	0	0	0	0	0	0	0	0	1	0	0	0	0	0	0	0	0
MYO18A	0	0	0	0	1	0	0	0	0	0	0	0	0	0	0	0	0	0
CYP2J2	0	0	0	0	0	0	0	0	0	1	0	0	0	0	0	0	0	0
NPR3	0	0	0	0	0	0	0	0	0	1	0	0	0	0	0	0	0	0
PIPOX	0	0	0	0	1	0	0	0	0	0	0	0	0	0	0	0	0	0
CXXC1	0	1	0	0	0	0	0	0	0	0	0	0	0	0	0	0	0	0
TAOK1	0	0	1	0	1	0	0	0	0	0	0	0	0	0	0	0	0	0
ZNF449	0	1	0	0	0	0	0	0	0	0	0	0	0	0	0	0	0	0
INTS6L	0	0	0	0	0	0	0	0	0	1	0	0	0	0	0	0	0	0
SOCS1	0	1	0	0	0	0	0	0	0	0	0	0	0	0	0	0	0	0
CLEC16A	0	0	0	0	-1	0	0	0	1	0	0	0	0	0	0	0	0	0
ABHD15	0	1	0	0	-1	0	0	0	0	0	0	0	0	0	0	0	0	0
DEXI	0	0	0	0	-1	0	0	0	1	0	0	0	0	0	0	0	0	0
GIT1	0	0	0	0	1	0	0	0	0	0	0	0	0	0	0	0	0	0
ADGRG4	0	1	0	0	0	0	0	0	0	0	0	0	0	0	0	0	0	0
ARHGEF6	0	1	0	0	0	0	0	0	0	0	0	0	0	0	0	0	0	0
EMP2	0	1	0	0	0	0	0	0	0	-1	0	0	0	0	0	0	0	0
PRKAA2	0	0	0	0	0	0	0	0	0	1	0	0	0	0	0	0	0	0
ATF7IP2	0	0	0	0	1	0	0	0	0	0	0	0	0	0	0	0	0	0
SLC6A4	0	1	0	0	0	0	0	0	0	0	0	0	0	0	0	0	0	0
CARHSP1	0	1	0	0	0	0	0	0	0	0	0	0	0	0	0	0	0	0
TNFAIP8L1	0	0	0	0	1	0	0	0	0	0	0	0	0	0	0	0	0	0
SEMA6B	0	0	0	0	1	0	1	0	0	0	0	0	0	0	0	0	0	0
ACAA2	0	0	0	0	0	0	0	0	0	1	0	0	0	0	0	0	0	0
ATP11C	0	0	0	0	1	0	0	0	0	0	0	0	0	0	0	0	0	0
ABAT	0	1	0	0	0	0	0	0	0	0	0	0	0	0	0	0	0	0
MRPL37	0	1	0	0	-1	0	0	0	0	-1	0	0	0	0	0	0	0	0
CDH18	0	0	0	0	0	0	0	0	1	1	0	0	0	0	0	0	0	0
UBXN6	0	1	0	0	0	0	0	0	0	0	0	0	0	0	0	0	0	0
TMEM59	0	0	0	0	0	0	0	0	0	1	0	0	0	0	0	0	0	0
CTIF	0	0	0	0	0	0	0	0	1	1	0	0	0	0	0	0	0	0
MPND	0	1	0	0	0	0	0	0	0	0	0	0	0	0	0	0	0	0
ENSCAFG00000019092	0	0	0	0	-1	0	1	0	0	0	0	0	0	0	0	0	0	0
RETREG1	0	1	0	0	-1	0	0	0	0	0	0	0	0	0	0	0	0	0
MAMLD1	0	0	0	0	1	0	0	0	0	0	0	0	0	0	0	0	0	0
NAGPA	0	1	0	0	0	0	0	0	0	0	0	0	0	0	0	0	0	0
RPH3AL	0	0	0	0	1	0	0	0	0	0	0	0	0	0	0	0	0	0
MTMR1	0	1	0	0	0	0	0	0	0	0	0	0	0	0	0	0	0	0
MAP2K2	0	1	0	0	0	0	0	0	0	0	0	0	0	0	0	0	0	0
UBN1	0	0	0	0	1	0	0	0	0	-1	0	0	0	0	0	0	0	0
ENSCAFG00000019141	0	1	0	0	0	0	0	0	0	0	0	0	0	0	0	0	0	0
GABRE	0	1	0	0	0	0	0	0	0	0	0	0	0	0	0	0	0	0
EEF2	0	1	0	0	-1	0	0	0	0	0	0	0	0	0	0	0	0	0
C5H1orf123	0	1	0	0	0	0	0	0	0	0	0	0	0	0	0	0	0	0
TBXA2R	0	1	0	0	0	0	0	0	1	0	0	0	0	0	0	0	0	0
ZNF185	0	0	0	0	0	0	0	0	0	-1	1	0	0	0	0	1	0	0
INPP5K	0	0	0	0	0	0	0	0	0	1	0	0	0	0	0	0	0	0
PODN	0	1	0	0	-1	0	0	0	0	0	0	0	0	0	0	0	0	0
MATK	0	0	0	0	1	0	0	0	0	0	0	0	0	0	0	0	0	0
SLC43A2	0	0	0	0	1	0	0	0	0	1	0	0	0	0	0	0	0	0
TJP3	0	1	0	0	0	0	0	0	1	0	0	0	0	0	0	0	0	0

ENSCAFG00000019200	0	0	0	0	1	0	0	0	0	0	0	0	0	0	0	0	0	0
RILP	0	1	0	0	0	0	0	0	0	0	0	0	0	0	0	0	0	0
PIP5K1C	0	1	0	0	0	0	0	0	0	0	0	0	0	0	0	0	0	0
CALML6	0	0	0	0	1	0	0	0	0	0	0	0	0	0	0	0	0	0
NADK	0	1	0	0	0	0	0	0	0	0	0	0	0	0	0	0	0	0
ENSCAFG00000019220	0	1	0	0	0	0	0	0	0	0	0	0	0	0	0	0	0	0
GLIS2	0	1	0	0	-1	0	0	0	0	0	0	0	0	0	0	0	0	0
MMP23	0	0	0	0	-1	0	0	0	0	1	0	0	0	0	0	0	0	0
CCNQ	0	1	0	0	0	0	0	0	0	0	0	0	0	0	0	0	0	0
PNCK	0	0	0	0	0	0	0	0	1	0	0	0	0	0	0	0	0	0
DPH1	0	1	0	0	0	0	0	0	0	-1	0	0	0	0	0	0	0	0
HIC1	0	1	0	0	0	0	0	0	0	0	0	0	0	0	0	0	0	0
VWA1	0	0	0	0	0	0	0	0	1	0	0	0	0	0	0	0	0	0
AURKAIP1	0	1	0	0	0	0	0	0	0	0	0	0	0	0	0	0	0	0
PLXNB3	0	0	0	0	0	0	0	0	0	1	0	0	0	0	0	0	0	0
CLUAP1	0	1	0	0	0	0	0	0	0	0	0	0	0	0	0	0	0	0
NAA60	0	1	0	0	0	0	0	0	0	0	0	0	0	0	0	0	0	0
SGSM2	0	0	0	0	0	0	0	0	0	1	0	0	0	0	0	0	0	0
ENSCAFG00000019300	0	1	0	0	0	0	0	0	0	0	0	0	0	0	0	0	0	0
CLUH	0	1	0	0	0	0	0	0	0	0	0	0	0	0	0	0	0	0
HCFC1R1	0	1	0	0	0	0	1	0	0	0	0	0	0	0	0	0	0	0
TRPV3	0	1	0	0	0	0	0	0	0	0	0	0	0	0	0	0	0	0
ENSCAFG00000019344	0	1	0	0	0	0	0	0	0	0	0	0	0	0	0	0	0	0
CTNS	0	1	0	0	0	0	0	0	0	0	0	0	0	0	0	0	0	0
ISG15	0	0	0	0	0	0	0	0	0	1	0	0	0	0	0	0	0	0
SKI	0	1	0	0	0	0	0	0	0	0	0	0	0	0	0	0	0	0
OAZ1	0	1	0	0	0	0	0	0	0	0	0	0	0	0	0	0	0	0
PNPLA7	0	1	0	0	0	0	0	0	0	0	0	0	0	0	0	0	0	0
TRAF7	0	1	0	0	0	0	0	0	0	0	0	0	0	0	0	0	0	0
RENBP	0	1	0	0	0	0	0	0	0	0	0	0	0	0	0	0	0	0
MKNK2	0	-1	0	0	0	1	0	0	-1	-1	0	0	0	0	0	0	0	0
ZNF598	0	1	0	0	0	0	0	0	0	-1	0	0	0	0	0	0	0	0
TTC34	0	0	0	0	0	0	0	0	1	0	0	0	0	0	0	0	0	0
ENSCAFG00000019472	0	1	0	0	-1	0	0	0	0	0	0	0	0	0	0	0	0	0
NDOR1	0	1	0	0	0	0	0	0	0	0	0	0	0	0	0	0	0	0
NDUFB10	0	1	0	0	0	0	0	0	0	0	0	0	0	0	0	0	0	0
ENSCAFG00000019479	0	1	0	0	0	0	0	0	0	0	0	0	0	0	0	0	0	0
ANAPC2	0	1	0	0	0	0	0	0	0	0	0	0	0	0	0	0	0	0
IGFALS	0	1	0	0	0	0	0	0	0	0	0	0	0	0	0	0	0	0
SPSB3	0	1	0	0	0	0	0	0	0	0	0	0	0	0	0	0	0	0
CEP104	0	0	0	0	0	0	0	0	0	1	0	0	0	0	0	0	0	0
GAMT	0	0	0	0	0	0	0	0	0	1	0	0	0	0	0	0	0	0
DFFB	0	1	0	0	0	0	0	0	0	0	0	0	0	0	0	0	0	0
NPDC1	0	1	0	0	0	0	0	0	0	0	0	0	0	0	0	0	0	0
NDUFS7	0	1	0	0	0	0	0	0	0	0	0	0	0	0	0	0	0	0
FBXW5	0	1	0	0	0	0	0	0	0	0	0	0	0	0	0	0	0	0
TMEM204	0	0	0	0	1	0	0	0	0	0	0	0	0	0	0	0	0	0
AJM1	0	1	0	0	0	0	0	0	0	0	0	0	0	0	0	0	0	0
TAZ	0	1	0	0	0	0	0	0	0	0	0	0	0	0	0	0	0	0
EGFL7	0	0	0	0	1	0	0	0	0	0	0	0	0	0	0	0	0	0
UNKL	0	1	0	0	0	0	0	0	0	0	0	0	0	0	0	0	0	0
G6PD	0	1	0	0	0	0	0	0	0	-1	0	0	0	0	0	0	0	0
PLEKHG5	0	0	0	0	1	0	0	0	0	0	0	0	0	0	0	0	0	0
LMF1	0	1	0	0	0	0	0	0	0	1	0	0	0	0	0	0	0	0
MPP1	0	1	0	0	0	0	0	0	0	0	0	0	0	0	0	0	0	0
F8	0	0	0	0	1	0	0	0	0	0	0	0	0	0	0	0	0	0
NOTCH1	0	0	0	0	1	0	0	0	0	0	0	0	0	0	0	0	0	0
FUNDC2	0	1	0	0	0	0	0	0	0	0	0	0	0	0	0	0	0	0
MTCP1	0	1	0	0	0	0	0	0	0	0	0	0	0	0	0	0	0	0
RHOT2	0	1	0	0	0	0	0	0	0	0	0	0	0	0	0	0	0	0
PIGQ	0	1	0	0	0	0	0	0	0	0	0	0	0	0	0	0	0	0
INPP5E	0	1	0	0	0	0	0	0	0	0	0	0	0	0	0	0	0	0
CAPN15	0	1	0	0	0	0	0	0	0	0	0	0	0	0	0	0	0	0
RAB11FIP3	0	1	0	0	0	0	0	0	0	0	0	0	0	0	0	0	0	0
PARK7	0	0	0	0	-1	0	0	0	0	1	0	0	0	0	0	0	0	0
AXIN1	0	1	0	0	0	0	0	0	0	0	0	0	0	0	0	0	0	0
ENSCAFG00000019687	0	0	0	0	-1	0	0	0	0	1	0	0	0	0	0	0	0	0
NPRL3	0	1	0	0	0	0	0	0	0	0	0	0	0	0	0	0	0	0
PALM	0	1	0	0	0	0	0	0	0	0	0	0	0	0	0	0	0	0
UBAC1	0	1	0	0	0	0	0	0	0	0	0	0	0	0	0	0	0	0
SLC2A5	0	0	0	0	0	0	0	0	0	1	0	0	0	0	0	0	0	0
BSG	0	0	0	0	0	0	0	0	0	1	0	0	0	0	0	0	0	0
GPR157	0	0	0	0	0	0	0	0	1	0	0	0	0	0	0	0	0	0
SPSB1	0	0	0	0	0	0	0	0	1	0	0	0	0	0	0	0	0	0
PLPP2	0	0	0	0	0	0	0	0	0	1	0	0	0	0	0	0	0	0
KCNC4	0	1	0	0	0	0	0	0	1	1	0	0	0	0	0	0	0	0
AHCYL1	0	1	0	0	-1	0	0	0	0	1	0	0	0	0	0	0	0	0
CSF1	0	0	0	0	-1	0	0	0	0	1	0	0	0	0	0	0	0	0
GSTM4	0	1	0	0	-1	0	0	0	0	0	0	0	0	0	0	0	0	0
SPATA2L	0	1	0	0	-1	0	0	0	0	0	0	0	0	0	0	0	0	0
AMIGO1	0	1	0	0	0	0	0	0	0	0	0	0	0	0	0	0	0	0
CPNE7	0	0	0	0	0	0	0	0	1	0	0	0	0	0	0	0	0	0
SORT1	0	1	0	0	-1	0	0	0	1	1	0	0	0	0	0	0	0	0
RXRA	0	1	0	0	0	0	0	0	0	0	0	0	0	0	0	0	0	0
RPL13	0	1	0	0	-1	0	0	0	0	0	0	0	0	0	0	0	0	0
CELSR2	0	0	0	0	0	0	0	0	0	1	0	0	0	0	0	0	0	0
WDR47	0	0	0	0	0	0	0	0	0	1	0	0	0	0	0	0	0	0
GTF3C5	0	1	0	0	0	0	0	0	0	0	0	0	0	0	0	0	0	0
RNF166	0	1	0	0	0	0	0	0	0	0	0	0	0	0	0	0	0	0
CYBA	0	1	0	0	-1	0	0	0	0	0	0	0	0	0	0	0	0	0
TSC1	0	0	0	0	0	0	0	0	0	1	0	0	0	0	0	0	0	0
RAPGEF1	0	0	0	0	1	0	0	0	0	0	0	0	0	0	0	0	0	0
PRRC2B	0	0	0	0	0	0	0	0	0	1	0	0	0	0	0	0	0	0
ENSCAFG00000019909	0	0	0	0	0	0	0	0	0	1	0	0	0	0	0	0	0	0
ENSCAFG00000019914	0	1	0	0	0	0	0	0	0	0	0	0	0	0	0	0	0	0
FOXF1	0	0	0	0	0	1	0	1	0	0	0	0	0	0	1	0	0	0
IRF8	0	0	0	0	1	0	0	0	0	0	0	0	0	0	0	0	0	0
LAMC3	0	1	0	0	0	0	0	0	0	0	0	0	0	0	0	0	0	0
ABL1	0	1	0	0	0	0	0	0	0	0	0	0	0	0	0	0	0	0
ASS1	0	1	0	0	0	0	0	0	0	0	0	0	0	0	0	0	0	0
KLHL36	0	1	0	0	0	0	0	0	0	1	0	0	0	0	0	0	0	0
VAV3	0	1	0	0	0	0	0	0	0	0	0	0	0	0	0	0	0	0
FNBP1	0	0	0	0	0	0	0	0	0	1	0	0	0	0	0	0	0	0
HSDL1	0	0	0	0	0	0	0	0	0	1	0	0	0	0	0	0	0	0
MLYCD	0	0	0	0	0	0	0	0	0	1	0	0	0	0	0	0	0	0
CDH13	0	1	0	0	0	0	0	0	0	0	0	0	0	0	0	0	0	0
USP20	0	0	0	0	0	0	0	0	0	1	0	0	0	0	0	0	0	0
ENSCAFG00000019976	0	0	0	0	1	1	0	0	0	0	0	0	0	0	0	0	0	0
PRRX2	0	1	0	0	0	0	0	0	0	0	0	0	0	0	0	0	0	0
ASB6	0	1	0	0	-1	0	0	0	0	0	0	0	0	0	0	0	0	0
PLCG2	0	0	0	0	1	0	0	0	0	1	0	0	0	0	0	0	0	0
IER5L	0	0	0	0	-1	0	0	1	0	0	0	0	0	0	0	0	0	0
DPH5	0	0	0	0	1	0	0	0	0	0	0	0	0	0	0	0	0	0
CRAT	0	1	0	0	0	0	0	0	0	0	0	0	0	0	0	0	0	0
CMIP	0	0	0	0	1	0	0	0	0	1	0	0	0	0	0	0	0	0
EXTL2	0	0	0	0	0	0	0	0	-1	1	0	0	0	0	0	0	0	0
CDC14A	0	1	0	0	0	0	0	0	0	0	0	0	0	0	0	0	0	0
SASS6	0	0	0	0	1	0	0	0	0	-1	0	0	0	0	0	0	0	0
ENSCAFG00000020027	0	0	0	0	0	0	0	0	0	1	0	0	0	0	0	0	0	0
SLC35A3	0	0	0	0	1	0	0	0	0	0	0	0	0	0	0	0	0	0
TBC1D13	0	0	0	0	0	0	0	0	0	1	0	0	0	0	0	0	0	0
ZER1	0	1	0	0	0	0	0	0	0	0	0	0	0	0	0	0	0	0
MON1B	0	1	0	0	0	0	0	0	0	0	0	0	0	0	0	0	0	0
PKN3	0	1	0	0	0	0	0	0	0	-1	0	0	0	0	0	0	0	0
SPTAN1	0	0	0	0	0	0	0	0	0	1	0	0	0	0	0	0	0	0
FRRS1	0	0	0	0	0	0	0	0	0	1	0	0	0	0	0	0	0	0
PALMD	0	0	0	0	1	0	0	0	0	0	0	0	0	0	0	0	0	0
ENSCAFG00000020060	0	0	0	0	1	0	0	0	0	0	0	0	0	0	0	0	0	0
DPYD	0	1	0	0	-1	0	0	0	0	0	0	0	0	0	0	0	0	0
DNM1	0	1	0	0	0	0	0	0	0	0	0	0	0	0	0	0	0	0

CIZ1	0	1	0	0	0	0	0	0	0	0	0	0	0	0	0	0	0	0
PTGES2	0	1	0	0	0	0	0	0	0	0	0	0	0	0	0	0	0	0
LDHD	0	1	0	0	0	0	0	0	0	0	0	0	0	0	0	0	0	0
SLC25A25	0	0	0	0	1	0	0	0	0	0	0	0	0	0	0	0	0	0
NAIF1	0	0	0	0	0	0	0	0	0	1	0	0	0	0	0	0	0	0
ENG	0	0	0	0	1	0	0	0	0	0	0	0	0	0	0	0	0	0
F3	0	0	0	0	0	0	0	0	1	0	0	0	0	0	0	0	0	0
ARHGAP29	0	0	0	0	1	0	0	0	0	1	0	0	0	0	0	0	0	0
SH2D3C	0	0	0	0	1	0	0	0	0	0	0	0	0	0	0	0	0	0
PDPR	0	1	0	0	0	0	0	0	0	0	0	0	0	0	0	0	0	0
ABCA4	0	0	0	0	0	0	0	0	0	1	0	0	0	0	0	0	0	0
DNTTIP2	0	0	0	0	1	0	0	0	0	-1	0	0	0	0	0	0	0	0
FNBP1L	0	0	0	0	1	0	0	0	0	0	0	0	0	0	0	0	0	0
DR1	0	1	0	0	0	0	0	0	0	0	0	0	0	0	0	0	0	0
EVI5	0	0	0	0	1	0	0	0	0	0	0	0	0	0	0	0	0	0
CDC7	0	0	0	0	0	0	0	0	0	1	0	0	0	0	0	0	0	0
ZNF326	0	-1	0	0	1	0	0	0	0	0	0	0	0	0	0	0	0	0
LRRC8C	0	0	0	0	1	0	0	0	0	1	0	0	0	0	0	0	0	0
GOLGA1	0	0	0	0	0	0	0	0	0	1	0	0	0	0	0	0	0	0
ZNF821	0	0	0	0	0	0	0	0	0	1	0	0	0	0	0	0	0	0
LMO4	0	0	0	0	-1	0	0	0	0	1	0	0	0	0	0	0	0	0
SH3GLB1	0	-1	0	0	1	0	0	0	0	0	0	0	0	0	0	0	0	0
RABGAP1	0	0	0	0	1	0	0	0	0	0	0	0	0	0	0	0	0	0
NQO1	0	1	0	0	0	0	0	0	0	0	0	0	0	0	0	0	0	0
NFAT5	0	0	0	0	1	0	0	0	0	1	0	0	0	0	0	0	0	0
PTGS1	0	0	0	0	1	0	0	0	0	0	0	0	0	0	0	0	0	0
ENSCAFG00000020269	0	0	0	0	0	0	0	0	0	1	0	0	0	0	0	0	0	0
LHX6	0	0	0	0	1	0	0	0	0	0	0	0	0	0	0	0	0	0
ENSCAFG00000020278	0	1	0	0	0	0	0	0	0	0	0	0	0	0	0	0	0	0
BCL10	0	0	0	0	1	0	0	0	0	0	0	0	0	0	0	0	0	0
SYDE2	0	-1	0	0	0	0	0	0	0	1	0	0	0	0	0	0	0	0
SNTB2	0	0	0	0	0	0	0	0	0	1	0	0	0	0	0	0	0	0
DAB2IP	0	0	0	0	0	0	0	0	0	1	0	0	0	0	0	0	0	0
CDH1	0	1	0	0	0	0	0	0	0	0	0	0	0	0	0	0	0	0
ZFP90	0	0	0	0	0	0	0	0	0	1	0	0	0	0	0	0	0	0
ADGRL2	0	0	0	0	1	0	0	0	0	0	0	0	0	0	0	0	0	0
LCAT	0	1	0	0	-1	0	0	0	0	1	0	0	0	0	0	0	0	0
PSMB10	0	0	0	0	0	0	0	1	0	0	0	0	0	0	0	0	0	0
PSKH1	0	0	0	0	0	0	0	0	0	1	0	0	0	0	0	0	0	0
ADGRL4	0	0	0	0	1	0	0	0	0	0	0	0	0	0	0	0	0	0
IFI44	0	0	0	0	0	1	0	0	0	1	0	0	0	0	0	0	0	0
IFI44L	0	0	0	0	0	0	0	0	0	1	0	0	0	0	0	0	0	0
THAP11	0	1	0	0	0	0	0	0	0	0	0	0	0	0	0	0	0	0
PTGFR	0	1	0	0	0	0	0	0	0	0	0	0	0	0	0	0	0	0
ENKD1	0	1	0	0	0	0	0	0	0	0	0	0	0	0	0	0	0	0
CTCF	0	0	0	0	1	0	0	0	0	0	0	0	0	0	0	0	0	0
FUBP1	0	0	0	0	1	0	0	0	0	0	0	0	0	0	0	0	0	0
ZDHHC1	0	1	0	0	-1	0	0	0	0	0	0	0	0	0	0	0	0	0
KCTD19	0	0	0	0	0	0	0	0	0	1	0	0	0	0	0	0	0	0
USP33	0	0	0	0	1	1	0	0	-1	0	0	0	0	0	0	0	0	0
E2F4	0	1	0	0	0	0	0	0	0	0	0	0	0	0	0	0	0	0
AK5	0	-1	0	0	0	1	0	0	0	0	-1	0	0	0	0	0	0	0
TRADD	0	1	0	0	0	0	0	0	0	0	0	0	0	0	0	0	0	0
B3GNT9	0	0	0	0	0	0	0	0	1	0	0	0	0	0	0	0	0	0
LHX8	0	0	0	0	0	0	0	0	0	0	1	0	0	0	0	0	0	0
CRYZ	0	1	0	0	0	0	0	0	0	0	0	0	0	0	0	0	0	0
ZRANB2	0	0	0	0	1	0	0	0	0	0	0	0	0	0	0	0	0	0
CDH5	0	0	0	0	1	0	0	0	0	0	0	0	0	0	0	0	0	0
TK2	0	0	0	0	0	0	0	0	0	1	0	0	0	0	0	0	0	0
ENSCAFG00000020536	0	0	0	0	0	1	0	0	0	0	0	0	0	0	0	0	0	0
ENSCAFG00000023004	0	0	0	0	1	0	0	0	0	0	0	0	0	0	0	0	0	0
ENSCAFG00000023039	0	0	1	0	1	1	0	0	0	0	0	0	0	0	0	0	0	0
RANBP2	0	0	0	0	1	0	0	0	0	0	0	0	0	0	0	0	0	0
APOL5	0	1	0	0	0	0	0	0	0	0	0	0	0	0	0	0	0	0
ACYP2	0	0	0	0	0	0	0	0	0	1	0	0	0	0	0	0	0	0
AFF3	0	0	0	0	0	0	0	0	0	1	0	0	0	0	0	0	0	0
NFIA	0	0	0	0	0	0	0	0	0	1	0	0	0	0	0	0	0	0
NFE2L1	0	0	0	0	0	0	0	0	1	0	0	0	0	0	0	0	0	0
ENSCAFG00000023151	0	0	0	0	0	0	0	0	0	1	0	0	0	0	0	0	0	0
ENSCAFG00000023188	0	0	1	0	1	1	0	0	-1	0	0	0	0	0	0	0	0	0
WTAP	0	0	0	0	1	0	0	0	0	-1	0	0	0	0	0	0	0	0
ENSCAFG00000023230	0	0	0	0	0	0	0	0	1	0	0	0	0	0	0	0	0	0
CILP2	0	0	0	0	0	0	1	0	0	0	0	0	0	0	0	0	0	0
GAS1	0	1	0	0	0	0	0	0	1	0	0	0	0	0	0	0	0	0
KRT39	0	0	0	0	1	0	0	0	0	0	0	0	0	0	0	0	0	0
DUSP2	0	0	1	0	0	0	0	0	0	0	0	0	0	0	0	0	0	0
RASSF9	0	0	0	0	1	0	0	0	0	0	0	0	0	0	0	0	0	0
ENSCAFG00000023349	0	1	0	0	0	0	0	0	0	0	0	0	0	0	0	0	0	0
ENSCAFG00000023364	0	1	0	0	0	0	0	0	0	0	0	0	0	0	0	0	0	0
CTXN1	0	1	0	0	0	0	0	0	0	0	0	0	0	0	0	0	0	0
C5H11orf87	0	0	0	0	0	0	0	0	0	0	0	0	0	0	1	0	0	0
MPP3	0	0	0	0	1	0	0	0	0	0	0	0	0	0	0	0	0	0
CNNM3	0	1	0	0	0	0	0	0	0	0	0	0	0	0	0	0	0	0
KCNE4	0	0	0	0	0	0	0	0	0	1	0	0	0	0	0	0	0	0
SOWAHC	0	0	0	0	0	0	0	0	0	1	0	0	0	0	0	0	0	0
ITPR1	0	0	0	0	0	0	0	0	0	1	0	0	0	0	0	0	0	0
GLRX5	0	1	0	0	0	0	0	0	0	0	0	0	0	0	0	0	0	0
CNPPD1	0	1	0	0	0	0	0	0	0	0	0	0	0	0	0	0	0	0
DMD	0	-1	1	0	1	0	0	0	0	0	0	0	0	0	0	0	0	0
ENSCAFG00000023591	0	0	0	0	0	0	0	0	0	1	0	0	0	0	0	0	0	0
SLC8A1	0	0	0	0	0	0	0	0	0	1	0	0	0	0	0	0	0	0
MFSD9	0	0	0	0	0	0	0	0	0	1	0	0	0	0	0	0	0	0
ENSCAFG00000023602	0	0	0	0	1	0	0	0	0	0	0	0	0	0	0	0	0	0
ITPRIPL2	0	1	0	0	-1	0	0	0	0	0	0	0	0	0	0	0	0	0
ZNF622	0	0	0	0	1	0	0	0	0	0	0	0	0	0	0	0	0	0
ANKRD54	0	1	0	0	-1	0	0	0	0	0	0	0	0	0	0	0	0	0
PRDM6	0	0	0	0	0	0	0	0	0	0	0	1	0	0	0	0	0	0
ENSCAFG00000023674	0	1	0	0	0	0	0	0	0	0	0	0	0	0	0	0	0	0
PCDHGA1	0	0	0	0	0	0	0	0	1	1	0	0	0	0	0	0	0	0
ENSCAFG00000023721	0	0	0	0	0	0	0	0	0	1	0	0	0	0	0	0	0	0
CCDC136	0	0	0	0	0	0	0	0	0	1	0	0	0	0	0	0	0	0
MT2A	0	1	0	0	-1	0	0	0	1	0	0	0	0	0	0	0	0	0
ENSCAFG00000023760	0	0	0	0	1	0	0	0	0	-1	0	0	0	0	0	0	0	0
ARMCX1	0	1	0	0	0	0	0	0	0	0	0	0	0	0	0	0	0	0
ENSCAFG00000023820	0	1	0	0	0	0	0	0	0	0	0	0	0	0	0	0	0	0
PMAIP1	0	0	0	0	1	0	0	0	0	0	0	0	0	0	0	0	0	0
ENSCAFG00000023832	0	0	0	0	0	0	0	0	0	1	0	0	0	0	0	0	0	0
ACAD11	0	0	0	0	0	0	0	0	0	1	0	0	0	0	0	0	0	0
ZNF205	0	1	0	0	0	0	0	0	0	0	0	0	0	0	0	0	0	0
BCAS4	0	1	0	0	0	0	0	0	0	0	0	0	0	0	0	0	0	0
ZSCAN30	0	0	0	0	0	0	0	0	0	1	0	0	0	0	0	0	0	0
SLC8A3	0	0	0	0	1	0	0	0	0	0	0	0	0	1	0	0	0	0
HSPB2	0	0	0	0	0	0	0	0	1	0	0	0	0	0	0	0	0	0
NIPSNAP3B	0	0	0	0	0	0	0	0	0	1	0	0	0	0	0	0	0	0
TLR1	0	0	0	0	0	0	0	0	0	1	0	0	0	0	0	0	0	0
ENSCAFG00000024028	0	1	0	0	0	0	0	0	0	0	0	0	0	0	0	0	0	0
BLOC1S3	0	1	0	0	0	0	0	0	0	0	0	0	0	0	0	0	0	0
HEXIM1	0	1	0	0	0	0	0	0	0	0	0	0	0	0	0	0	0	0
FJX1	0	1	0	0	0	0	0	0	0	0	0	0	0	0	0	0	0	0
SPINK2	0	-1	0	0	0	0	0	0	0	1	0	0	0	0	0	0	0	0
ENSCAFG00000024183	0	0	0	0	1	0	0	0	0	0	0	0	0	0	0	0	0	0
ZNF444	0	1	0	0	0	0	0	0	0	0	0	0	0	0	0	0	0	0
MLLT11	0	0	0	0	0	0	0	0	1	0	0	0	0	0	0	0	0	0
LRRC8D	0	0	0	0	1	0	0	0	0	0	0	0	0	0	0	0	0	0
ENSCAFG00000024295	0	0	0	0	1	0	0	0	0	0	0	0	0	0	0	0	0	0
AGO4	0	1	0	0	0	0	0	0	0	0	0	0	0	0	0	0	0	0
HIST1H1A	0	0	0	0	1	0	0	0	0	-1	0	0	0	0	0	0	0	0
FXYD5	0	0	0	0	0	0	0	0	1	-1	0	0	0	0	0	0	0	0
ENSCAFG00000024323	0	0	0	0	1	0	0	0	0	0	0	0	0	0	0	0	0	0
ZBTB7A	0	1	0	0	0	0	0	0	0	0	0	0	0	0	0	0	0	0

CENPP	0	1	0	0	0	0	0	0	0	-1	0	0	0	0	0	0	0	0
ADGRG1	0	0	0	0	1	0	0	0	0	0	0	0	0	0	0	0	0	0
ENSCAFG00000024474	0	0	0	0	1	0	0	0	0	0	0	0	0	0	0	0	0	0
GBP5	0	-1	0	0	1	0	0	0	0	0	0	0	0	0	0	0	0	0
PCDHB2	0	0	0	0	0	0	0	0	0	1	0	0	0	0	0	0	0	0
KIAA1671	0	1	0	0	0	0	0	0	0	0	0	0	0	0	0	0	0	0
BNIP3	0	-1	0	0	0	0	0	0	0	1	0	0	0	0	0	0	0	0
ERAP2	0	0	0	0	1	0	0	0	0	0	0	0	0	0	0	0	0	0
SERPINB5	0	0	0	0	0	0	0	0	0	1	0	0	0	0	0	0	0	0
ENSCAFG00000024681	0	0	1	0	1	1	0	0	0	0	0	0	0	0	0	0	0	0
TRIM24	0	0	0	0	1	0	0	0	0	0	0	0	0	0	0	0	0	0
CD55	0	0	0	0	0	0	0	0	0	1	0	0	0	0	0	0	0	0
ADAMTS5	0	1	0	0	0	0	0	0	1	1	0	0	0	0	0	0	0	0
ENSCAFG00000024769	0	0	0	0	1	0	0	0	0	1	0	0	0	0	0	0	0	0
PKHD1L1	0	0	0	0	1	0	0	0	0	0	0	0	0	0	0	0	0	0
ZNF654	0	0	0	0	0	0	0	0	0	1	0	0	0	0	0	0	0	0
KIF21B	0	0	0	0	1	0	0	0	0	-1	0	0	0	0	0	0	0	0
ADAMDEC1	0	0	0	0	1	0	0	0	0	0	0	0	0	0	0	0	0	0
FOLH1	0	1	0	0	0	0	0	0	0	-1	1	0	0	0	0	0	0	0
PLA2G4E	0	0	0	0	1	0	0	0	0	0	0	0	0	0	0	0	0	0
LRRN4	0	0	0	0	0	0	0	0	0	1	0	0	0	0	0	0	1	0
FRZB	0	0	0	0	0	0	0	0	0	1	0	0	0	0	0	0	0	0
TMOD3	0	0	0	0	1	0	0	0	0	0	0	0	0	0	0	0	0	0
ATP6AP1L	0	0	0	0	0	0	0	0	0	0	1	0	0	0	0	1	0	0
TRIM6	0	0	0	0	1	0	0	0	0	1	0	0	0	0	0	0	0	0
ENSCAFG00000025044	0	0	0	0	0	0	0	0	0	1	0	0	0	0	0	0	0	0
ENSCAFG00000025063	0	0	0	0	1	0	0	0	0	0	0	0	0	0	0	0	0	0
ENSCAFG00000025105	0	0	0	0	0	0	0	0	0	1	0	0	0	0	0	0	0	0
ENSCAFG00000025115	0	1	0	0	0	0	0	0	0	0	0	0	0	0	0	0	0	0
ENSCAFG00000025131	0	0	0	0	0	0	0	0	0	1	0	0	0	0	0	0	0	0
AP5B1	0	1	0	0	0	0	0	0	0	0	0	0	0	0	0	0	0	0
ENSCAFG00000025142	0	0	0	0	1	0	0	0	0	0	0	0	0	0	0	0	0	0
ENSCAFG00000025170	0	0	0	0	1	1	0	0	0	0	0	0	0	0	0	0	0	0
CASP8	0	0	0	0	1	0	0	0	0	0	0	0	0	0	0	0	0	0
CA2	0	0	0	0	0	0	0	0	0	0	0	0	0	1	0	0	0	0
ZCCHC3	0	1	0	0	0	0	0	0	0	0	0	0	0	0	0	0	0	0
PEAK1	0	0	0	0	0	0	0	0	0	1	0	0	0	0	0	0	0	0
SOX17	0	0	0	0	1	0	0	0	0	0	0	0	0	0	0	0	0	0
ENSCAFG00000025332	0	1	0	0	-1	0	0	0	0	0	0	0	0	0	0	0	0	0
PTPN12	0	-1	0	0	1	0	0	0	0	0	0	0	0	0	0	0	0	0
RELN	0	0	0	0	1	0	0	0	0	0	0	0	0	0	0	0	0	0
ACKR1	0	0	0	0	1	0	0	0	0	0	0	0	0	0	0	0	0	0
SLC25A28	0	1	0	0	0	0	0	0	0	0	0	0	0	0	0	0	0	0
EXO5	0	0	0	0	0	0	0	0	0	1	0	0	0	0	0	0	0	0
AHRR	0	1	0	0	0	0	0	0	0	0	0	0	0	0	0	0	0	0
SLC22A17	0	0	0	0	0	0	0	0	1	0	0	0	0	0	0	0	0	0
VXN	0	0	0	0	0	0	0	0	1	0	0	0	0	0	0	0	0	0
METTL14	0	0	0	0	0	0	0	0	0	1	0	0	0	0	0	0	0	0
ENSCAFG00000025410	0	0	0	0	1	0	0	0	0	0	0	0	0	0	0	0	0	0
ALKAL1	0	0	0	0	0	0	0	0	0	0	0	0	0	0	1	0	0	0
C3AR1	0	0	0	0	0	1	0	0	0	0	0	0	0	0	1	0	0	0
TRIM65	0	1	0	0	0	0	0	0	0	0	0	0	0	0	0	0	0	0
DHRS2	0	0	0	0	0	0	0	0	1	1	0	0	0	0	0	0	0	0
C9H17orf58	0	1	0	0	0	0	0	0	0	0	0	0	0	0	0	0	0	0
ENSCAFG00000025524	0	1	0	0	0	0	0	0	0	-1	0	0	0	0	0	0	0	0
NT5C	0	1	0	0	0	0	0	0	0	0	0	0	0	0	0	0	0	0
ENSCAFG00000028127	0	1	0	0	0	0	0	0	0	0	0	0	0	0	0	0	0	0
ENSCAFG00000028156	0	1	0	0	0	0	0	0	0	0	0	0	0	0	0	0	0	0
ENSCAFG00000028195	0	1	0	0	0	0	0	0	0	0	0	0	0	0	0	0	0	0
ENSCAFG00000028442	0	1	0	0	0	0	0	0	0	0	0	0	0	0	0	0	0	0
CD83	0	1	0	0	0	0	0	0	0	1	0	0	0	0	0	0	0	0
MT3	0	1	0	0	0	0	0	0	1	0	0	0	0	0	0	0	0	0
LOX	0	1	0	0	-1	0	0	0	0	0	0	0	0	0	0	0	0	0
ENSCAFG00000028464	0	0	0	0	0	0	0	0	0	1	0	0	0	0	0	0	0	0
TDRP	0	0	0	0	1	0	0	0	0	0	0	0	0	0	0	0	0	0
HOXB3	0	-1	0	1	0	0	0	1	0	0	0	0	0	0	0	0	1	0
TBX20	0	0	0	0	0	0	0	0	0	1	0	0	0	0	0	0	0	0
ZFP30	0	0	0	0	0	0	0	0	0	1	0	0	0	0	0	0	0	0
ENSCAFG00000028550	0	0	0	0	0	0	0	0	0	1	0	0	0	0	0	0	0	0
TENT5B	0	0	0	0	0	0	1	0	0	0	0	0	0	0	0	0	0	0
JCHAIN	0	1	0	0	0	0	0	0	0	0	0	0	0	0	0	0	0	0
DUSP3	0	0	0	0	1	0	0	0	0	0	0	0	0	0	0	0	0	0
ENSCAFG00000028578	0	0	0	0	0	0	0	0	0	1	0	0	0	0	0	0	0	0
RTN4RL1	0	1	0	0	0	0	0	0	0	0	0	0	0	0	0	0	0	0
ENSCAFG00000028589	0	1	0	0	0	0	0	0	0	1	0	0	0	0	0	0	0	0
ENSCAFG00000028601	0	0	0	0	0	0	0	0	0	1	0	0	0	0	0	0	0	0
GMFG	0	0	0	0	1	0	0	0	0	0	0	0	0	0	0	0	0	0
ZNF174	0	0	0	0	0	0	0	0	0	1	0	0	0	0	0	0	0	0
OLFML2A	0	0	0	0	1	0	0	0	0	0	0	0	0	0	0	0	0	0
ENSCAFG00000028627	0	1	0	0	0	0	0	0	0	0	0	0	0	0	0	0	0	0
TRIM7	0	1	0	0	0	0	0	0	0	0	0	0	0	0	0	0	0	0
IGFBP6	0	1	0	0	-1	0	0	0	0	0	0	0	0	0	0	0	0	0
SMIM1	0	0	0	0	0	0	0	0	0	1	0	0	0	0	0	0	0	0
BGLAP	0	1	0	0	0	0	0	0	0	0	0	0	0	0	0	0	0	0
LRRC24	0	0	0	0	0	0	0	0	0	1	0	0	0	0	0	0	0	0
ENSCAFG00000028699	0	0	0	0	0	0	0	0	0	1	0	0	0	0	0	0	0	0
ITM2C	0	0	0	0	0	0	0	0	0	1	0	0	0	0	0	0	0	0
ZNF580	0	1	0	0	0	0	0	0	0	0	0	0	0	0	0	0	0	0
FPGT	0	1	0	0	0	0	0	0	0	0	0	0	0	0	0	0	0	0
ZFP3	0	0	0	0	0	0	0	0	0	1	0	0	0	0	0	0	0	0
C28H10orf71	0	0	0	0	0	0	0	0	0	1	0	0	0	0	0	0	0	0
PLPP3	0	1	0	0	0	0	0	0	0	0	0	0	0	0	0	0	0	0
RNFT1	0	1	0	0	0	0	0	0	0	0	0	0	0	0	0	0	0	0
ARL14EP	0	0	0	0	0	0	0	0	0	1	0	0	0	0	0	0	0	0
ENSCAFG00000028801	0	1	0	0	0	0	1	0	1	0	0	1	0	0	0	0	0	0
ENSCAFG00000028808	0	0	0	0	1	0	0	0	0	0	0	0	0	0	0	0	0	0
CFD	0	1	0	0	-1	0	0	0	0	0	0	0	0	0	0	0	0	0
XIRP1	0	0	0	0	0	0	0	0	0	1	0	0	0	0	0	0	0	0
RAB6A	0	-1	0	0	1	0	0	0	0	0	0	0	0	0	0	0	0	0
ENSCAFG00000028871	0	0	0	0	0	0	0	0	1	0	0	1	0	0	0	0	0	0
ENSCAFG00000028878	0	1	0	0	0	0	0	0	0	0	0	0	0	0	0	0	0	0
SDR42E1	0	0	0	0	0	0	0	0	1	0	0	0	0	0	0	0	0	0
RWDD3	0	1	0	0	0	0	0	0	0	0	0	0	0	0	0	0	0	0
ENSCAFG00000028936	0	1	0	0	0	0	0	0	0	0	0	0	0	0	0	0	0	0
IL2RG	0	0	0	0	1	0	0	0	0	0	0	0	0	0	0	0	0	0
ENSCAFG00000028993	0	0	0	0	0	0	0	0	0	0	0	0	0	0	1	0	0	0
NREP	0	0	0	0	0	0	0	0	0	0	0	0	0	0	1	0	0	0
ZNF226	0	0	0	0	0	0	0	0	0	1	0	0	0	0	0	0	0	0
ENSCAFG00000029058	0	0	0	0	0	0	0	0	0	1	0	0	0	0	0	0	0	0
ENSCAFG00000029067	0	0	0	0	0	0	0	0	0	1	0	0	0	0	0	0	0	0
CCDC126	0	0	0	0	0	0	0	0	0	1	0	0	0	0	0	0	0	0
PTGES3	0	0	0	0	1	0	0	0	0	0	0	0	0	0	0	0	0	0
HRCT1	0	1	0	0	0	0	0	0	0	0	0	0	0	0	0	0	0	0
ENSCAFG00000029083	0	0	0	0	0	0	0	0	1	0	0	0	0	0	0	0	0	0
LYPD6	0	0	0	0	1	0	0	0	0	1	0	0	0	0	0	0	0	0
FAM76A	0	1	0	0	0	0	0	0	0	0	0	0	0	0	0	0	0	0
FAM78A	0	0	0	0	0	0	0	0	0	1	0	0	0	0	0	0	0	0
ENSCAFG00000029124	0	0	0	0	1	0	0	0	0	0	0	0	0	0	0	0	0	0
ENSCAFG00000029129	0	-1	1	0	1	1	0	0	0	0	0	0	0	0	0	0	0	0
HAPLN1	0	0	0	0	0	0	0	0	0	0	1	0	0	0	0	0	0	0
ZNF629	0	0	0	0	0	0	0	0	0	1	0	0	0	0	0	0	0	0
ZNF385A	0	1	0	0	0	0	0	0	0	0	0	0	0	0	0	0	0	0
ENSCAFG00000029170	0	0	0	0	1	0	1	0	0	0	0	0	0	0	0	0	0	0
SCAMP4	0	1	-1	0	-1	0	0	0	0	0	0	0	0	0	0	0	0	0
STMN1	0	0	0	0	1	0	0	0	0	-1	0	0	0	0	0	0	0	0
ZNF354C	0	0	0	0	0	0	0	0	0	1	0	0	0	0	0	0	0	0
PPP1R14A	0	0	0	0	0	0	0	0	1	0	0	0	0	0	0	0	0	0
CDC42EP5	0	1	0	0	0	0	1	0	1	0	0	0	0	0	0	0	0	0
SOX18	0	0	0	0	1	0	0	0	0	0	0	0	0	0	0	0	0	0
CLDN1	0	1	0	0	0	0	0	0	0	0	0	0	0	0	0	0	0	0

ZNF484	0	0	0	0	0	0	0	0	0	1	0	0	0	0	0	0	0	0
ENSCAFG00000029346	0	0	0	0	1	0	0	0	0	0	0	0	0	0	0	0	0	0
NCEH1	0	1	0	0	0	0	0	0	0	1	0	0	0	0	0	0	0	0
KCNK5	0	0	0	0	1	0	0	0	0	0	0	0	0	0	0	0	0	0
COL28A1	0	1	0	0	0	0	0	0	0	0	0	0	0	0	0	0	0	0
BASP1	0	0	0	0	1	0	0	0	0	0	0	0	0	0	0	0	0	0
ENSCAFG00000029405	0	-1	0	0	0	0	0	1	0	0	0	0	0	0	0	0	0	0
REPIN1	0	1	0	0	0	0	0	0	0	1	0	0	0	0	0	0	0	0
ZNF688	0	1	0	0	0	0	0	0	0	0	0	0	0	0	0	0	0	0
OSGIN2	0	0	0	0	1	0	0	0	0	1	0	0	0	0	0	0	0	0
ZNF432	0	0	0	0	0	0	0	0	0	1	0	0	0	0	0	0	0	0
LRRC7	0	0	0	0	0	0	0	0	0	0	0	0	0	0	0	0	1	0
ENSCAFG00000029553	0	0	0	0	0	0	0	0	0	1	0	0	0	0	0	0	0	0
BMF	0	0	0	0	1	0	0	0	0	0	0	0	0	0	0	0	0	0
KANK3	0	0	0	0	1	0	0	0	0	0	0	0	0	0	0	0	0	0
FAM8A1	0	0	0	0	0	0	0	0	0	1	0	0	0	0	0	0	0	0
LHFPL4	0	0	0	0	0	0	0	0	0	0	0	0	0	0	1	0	0	0
FOXN2	0	0	0	0	1	0	0	0	0	0	0	0	0	0	0	0	0	0
ZNF425	0	0	0	0	0	0	0	0	0	1	0	0	0	0	0	0	0	0
TM2D2	0	0	0	0	0	0	0	0	0	1	0	0	0	0	0	0	0	0
FXN	0	1	0	0	0	0	0	0	0	0	0	0	0	0	0	0	0	0
ENSCAFG00000029677	0	0	0	0	1	0	0	0	0	0	0	0	0	0	0	0	0	0
ZNF517	0	1	0	0	0	0	0	0	0	0	0	0	0	0	0	0	0	0
ENSCAFG00000029731	0	0	0	0	1	0	0	0	0	0	0	0	0	0	0	0	0	0
ENSCAFG00000029756	0	0	0	0	0	0	0	0	1	0	0	0	0	0	0	0	0	0
MMD	0	-1	0	0	1	0	0	0	0	0	0	0	0	0	0	0	0	0
ENSCAFG00000029758	0	1	0	0	-1	0	0	0	0	0	0	0	0	0	0	0	0	0
SIX1	0	0	0	0	0	0	1	0	0	0	0	0	0	0	0	0	0	0
ZNF175	0	0	0	0	0	0	0	0	0	1	0	0	0	0	0	0	0	0
DUSP1	0	1	0	0	0	0	0	0	0	0	0	0	0	0	0	0	0	0
MAFF	0	0	0	0	1	0	0	0	0	0	0	0	0	0	0	0	0	0
ZNF48	0	1	0	0	0	0	0	0	0	0	0	0	0	0	0	0	0	0
CDKN1A	0	-1	0	0	0	0	0	0	0	1	0	0	0	0	0	0	0	0
ENSCAFG00000029877	0	0	0	0	0	0	0	0	0	1	0	0	0	0	0	0	0	0
C1QTNF2	0	1	0	0	0	0	0	0	0	0	0	0	0	0	0	0	0	0
SENP7	0	0	0	0	0	0	0	0	0	1	0	0	0	0	0	0	0	0
EDA	0	0	0	0	0	0	0	0	0	1	0	0	0	0	0	0	0	0
ZFP14	0	0	0	0	0	0	0	0	0	1	0	0	0	0	0	0	0	0
ENSCAFG00000029966	0	1	0	0	0	0	0	0	0	1	0	0	0	0	0	0	0	0
ENSCAFG00000029967	0	1	0	0	0	0	0	0	1	0	0	0	0	0	0	0	0	0
TMEM250	0	1	0	0	0	0	0	0	0	0	0	0	0	0	0	0	0	0
IMPA1	0	0	0	0	1	0	0	0	0	0	0	0	0	0	0	0	0	0
ENSCAFG00000030021	0	1	0	0	0	0	0	0	0	0	0	0	0	0	0	0	0	0
RBM18	0	-1	0	0	1	0	0	0	0	0	0	0	0	0	0	0	0	0
MFAP3	0	0	0	0	1	0	0	0	0	0	0	0	0	0	0	0	0	0
PLAC8B	0	0	0	0	0	0	0	0	0	1	0	0	0	0	0	0	0	0
LMX1B	0	1	0	0	0	0	0	0	1	0	1	0	0	0	0	1	0	1
XKRX	0	0	0	1	0	0	0	0	1	0	0	0	0	0	0	0	0	0
ENSCAFG00000030097	0	0	0	0	0	0	1	0	0	0	0	0	0	0	0	0	0	0
C1D	0	0	0	0	0	1	0	0	0	0	0	0	0	0	0	0	0	0
TPC3	0	0	0	0	-1	0	0	0	0	1	0	0	0	0	0	0	0	0
EVA1B	0	1	0	0	0	0	0	0	0	0	0	0	0	0	0	0	0	0
POMK	0	0	0	0	0	0	0	0	0	1	0	0	0	0	0	0	0	0
NCALD	0	0	0	0	0	0	0	0	0	1	0	0	0	0	0	0	0	0
ZNF572	0	0	0	0	0	0	0	0	0	1	0	0	0	0	0	0	0	0
ENSCAFG00000030144	0	1	0	0	0	0	0	0	0	0	0	0	0	0	0	0	0	0
RFK	0	0	0	0	1	0	0	0	0	1	0	0	0	0	0	0	0	0
CDK9	0	1	0	0	0	0	0	0	0	0	0	0	0	0	0	0	0	0
HS3ST2	0	0	0	0	0	0	0	0	0	-1	0	1	0	0	0	1	0	0
FAM131C	0	0	0	0	1	0	0	0	0	0	0	0	0	0	0	0	0	0
BAMBI	0	0	0	0	0	0	0	0	0	0	0	0	0	0	1	0	0	0
ENSCAFG00000030283	0	0	0	0	0	0	0	0	0	1	0	0	0	0	0	0	0	0
ENSCAFG00000030310	0	0	0	0	0	0	0	0	0	1	0	0	0	0	0	0	0	0
ENSCAFG00000030317	0	0	0	0	0	0	0	0	0	1	0	0	0	0	0	0	0	0
SERPINB9	0	0	0	0	1	0	0	0	0	0	0	0	0	0	0	0	0	0
C33H3orf52	0	0	0	0	1	0	0	0	0	0	0	0	0	0	0	0	0	0
ENSCAFG00000030359	0	1	-1	0	-1	-1	1	1	0	0	0	0	0	0	0	0	0	0
TSPAN4	0	1	0	0	0	0	0	0	0	0	0	0	0	0	0	0	0	0
RAB32	0	0	0	0	1	0	0	0	0	0	0	0	0	0	0	0	0	0
NACC2	0	0	0	0	0	0	0	0	0	1	0	0	0	0	0	0	0	0
RGS16	0	0	0	0	1	0	0	0	0	0	0	0	0	0	0	0	0	0
ENSCAFG00000030425	0	-1	1	0	1	1	0	0	0	0	0	0	0	0	0	0	0	0
ZNF74	0	0	0	0	0	0	0	0	0	1	0	0	0	0	0	0	0	0
ENSCAFG00000030466	0	0	0	0	0	0	0	0	1	0	0	0	0	0	0	0	0	0
ENSCAFG00000030498	0	0	0	0	1	0	0	0	0	0	0	0	0	0	0	0	0	0
HSPB8	0	1	0	0	-1	0	0	0	0	0	0	0	0	0	0	0	0	0
ENSCAFG00000030548	0	0	0	0	0	0	0	0	0	1	0	0	0	0	0	0	0	0
ENSCAFG00000030568	0	0	0	0	0	0	0	0	0	1	0	0	0	0	0	0	0	0
ZNF782	0	0	0	0	0	0	0	0	0	1	0	0	0	0	0	0	0	0
ENSCAFG00000030587	0	0	0	0	0	0	0	0	1	0	0	0	0	0	0	0	0	0
HYKK	0	1	0	0	0	0	0	0	0	0	0	0	0	0	0	0	0	0
MZT1	0	-1	0	0	1	1	0	0	0	0	0	0	0	0	0	0	0	0
GATAD1	0	1	0	0	0	0	0	0	0	0	0	0	0	0	0	0	0	0
DSTN	0	0	0	0	0	0	0	0	0	1	0	0	0	0	0	0	0	0
CMTM4	0	0	0	0	0	0	0	0	0	1	0	0	0	0	0	0	0	0
PID1	0	1	0	0	0	0	0	0	0	0	0	0	0	0	0	0	0	0
TTC33	0	1	0	0	0	0	0	0	0	0	0	0	0	0	0	0	0	0
ENSCAFG00000030662	0	1	0	0	0	0	0	0	0	0	0	0	0	0	0	0	0	0
ENSCAFG00000030682	0	1	0	0	0	0	0	0	1	0	0	0	0	0	0	0	0	0
ADPRHL2	0	1	0	0	0	0	0	0	0	0	0	0	0	0	0	0	0	0
KIAA0040	0	0	0	0	0	0	0	0	0	1	0	0	0	0	0	0	0	0
TMEM38A	0	0	0	0	0	0	0	0	0	1	0	0	0	0	0	0	0	0
IL22RA1	0	0	0	0	0	0	0	0	0	0	0	0	0	0	0	0	1	0
PSMB9	0	0	0	0	1	0	0	0	0	0	0	0	0	0	0	0	0	0
ENSCAFG00000030734	0	-1	0	0	1	0	0	0	0	0	0	0	0	0	0	0	0	0
ENSCAFG00000030758	0	0	0	0	0	0	0	0	1	0	0	0	0	0	0	0	0	0
FAM210B	0	0	0	0	0	0	0	0	0	1	0	0	0	0	0	0	0	0
SLC25A29	0	1	0	0	0	0	0	0	0	0	0	0	0	0	0	0	0	0
MSMP	0	0	0	0	1	0	0	0	0	0	0	0	0	0	0	0	0	0
ZNF250	0	1	0	0	0	0	0	0	0	0	0	0	0	0	0	0	0	0
NUDT8	0	1	0	0	0	0	0	0	0	0	0	0	0	0	0	0	0	0
DOC2B	0	0	0	0	1	0	0	0	0	0	0	0	0	0	0	0	0	0
PCDHB15	0	0	0	0	0	0	0	0	0	1	0	0	0	0	0	0	0	0
ENSCAFG00000030822	0	0	0	0	0	0	0	0	0	1	0	0	0	0	0	0	0	0
PTGES	0	1	0	0	0	0	0	0	0	0	0	0	0	0	0	0	0	0
PNRC1	0	0	0	0	0	0	0	0	0	1	0	0	0	0	0	0	0	0
INSIG2	0	1	0	0	0	0	0	0	0	0	0	0	0	0	0	0	0	0
CREG2	0	1	0	0	-1	0	0	0	1	0	0	0	0	0	0	0	0	0
ENSCAFG00000030873	0	0	0	0	0	0	0	0	0	1	0	0	0	0	0	0	0	0
BMT2	0	0	0	0	0	0	0	0	0	1	0	0	0	0	0	0	0	0
ZBTB47	0	1	0	0	-1	-1	0	0	0	0	0	0	0	0	0	0	0	0
PERP	0	0	0	0	0	0	0	0	0	1	0	0	0	0	0	0	0	0
GIPC2	0	0	0	0	0	0	0	0	1	0	0	0	0	0	0	0	0	0
TMEM218	0	0	0	0	-1	0	0	0	0	1	0	0	0	0	0	0	0	0
ENSCAFG00000030939	0	0	0	0	0	0	0	0	0	1	0	0	0	0	0	0	0	0
ZNF524	0	1	0	0	0	0	0	0	0	0	0	0	0	0	0	0	0	0
GYPC	0	0	0	0	1	0	0	0	0	0	0	0	0	0	0	0	0	0
ZNF789	0	0	0	0	1	0	0	0	0	0	0	0	0	0	0	0	0	0
TSNAX-DISC1	0	0	0	0	1	0	0	0	0	0	0	0	0	0	0	0	0	0
DUSP22	0	0	0	0	0	0	0	0	0	1	0	0	0	0	0	0	0	0
N6AMT1	0	1	0	0	0	0	0	0	0	0	0	0	0	0	0	0	0	0
ZNF354A	0	0	0	0	1	0	0	0	0	0	0	0	0	0	0	0	0	0
ENSCAFG00000031003	0	0	0	0	0	0	0	0	0	1	0	0	0	0	0	0	0	0
FAM180A	0	1	0	0	0	0	0	0	0	0	0	0	0	0	0	0	0	0
CDC26	0	1	0	0	0	0	0	0	0	0	0	0	0	0	0	0	0	0
EMP1	0	0	0	0	0	0	0	0	0	0	1	0	0	0	0	0	0	0
PLAGL2	0	0	0	0	0	0	0	0	1	0	0	0	0	0	0	0	0	0
ZFP36L2	0	1	0	0	0	0	0	0	0	0	0	0	0	0	0	0	0	0
ZNF79	0	0	0	0	0	0	0	0	0	1	0	0	0	0	0	0	0	0
ENSCAFG00000031105	0	1	0	0	0	0	0	0	0	0	0	0	0	0	0	0	0	0

COA1	0	1	0	0	-1	0	0	0	0	0	0	0	0	0	0	0	0	0
APH1B	0	1	0	0	0	0	0	0	0	1	0	0	0	0	0	0	0	0
EPDR1	0	1	0	0	0	0	0	0	1	0	1	0	0	0	0	0	0	0
ZNF454	0	0	0	0	0	0	0	0	0	1	0	0	0	0	0	0	0	0
CADM4	0	0	0	0	1	0	0	0	0	0	0	0	0	0	0	0	0	0
ST6GALNAC6	0	1	0	0	0	0	0	0	0	0	0	0	0	0	0	0	0	0
SERTAD4	0	1	0	0	0	0	0	0	0	0	0	0	0	0	0	0	0	0
CXHXorf36	0	0	0	0	1	0	0	0	0	0	0	0	0	0	0	0	0	0
ENSCAFG00000031217	0	0	0	0	0	0	0	0	0	1	0	0	0	0	0	0	0	0
ZNF252	0	0	0	0	0	0	0	0	0	1	0	0	0	0	0	0	0	0
CYYR1	0	0	0	0	1	0	0	0	0	0	0	0	0	0	0	0	0	0
ZKSCAN1	0	0	0	0	0	0	0	0	0	1	0	0	0	0	0	0	0	0
ENSCAFG00000031302	0	1	0	0	0	0	0	0	0	0	0	0	0	0	0	0	0	0
ENSCAFG00000031306	0	1	0	0	-1	0	0	0	1	1	0	0	0	0	0	0	0	0
TMEM236	0	-1	0	0	1	0	0	0	0	0	0	0	0	0	0	0	0	0
CEBPB	0	0	0	0	0	0	0	0	1	0	0	0	0	0	0	0	0	0
SARAF	0	0	0	0	0	0	0	0	0	1	0	0	0	0	0	0	0	0
TMEM176A	0	0	0	0	0	0	0	0	0	1	0	0	0	0	0	0	1	0
ENSCAFG00000031423	0	0	0	0	0	0	0	0	1	0	0	0	0	0	0	0	0	0
GNA14	0	0	0	0	0	0	0	0	0	1	0	0	0	0	0	0	0	0
ZNF791	0	0	0	0	0	0	0	0	0	1	0	0	0	0	0	0	0	0
ENSCAFG00000031488	0	0	0	0	1	0	0	0	0	0	0	0	0	0	0	0	0	0
ZNF461	0	0	0	0	0	0	0	0	0	1	0	0	0	0	0	0	0	0
BMP2	0	0	0	0	1	0	0	0	0	0	0	0	0	0	0	0	0	0
C15orf48	0	0	0	0	0	0	0	0	0	1	0	0	0	0	0	0	0	0
LEPROT	0	0	0	0	0	0	0	0	0	1	0	0	0	0	0	0	0	0
PIK3C2A	0	-1	0	0	1	0	0	0	0	1	0	0	0	0	0	0	0	0
CLYBL	0	1	0	0	0	0	0	0	0	0	0	0	0	0	0	0	0	0
ENSCAFG00000031583	0	1	0	0	0	0	0	0	0	0	0	0	0	0	0	0	0	0
LGALSL	0	0	0	0	1	0	0	0	0	0	0	0	0	0	0	0	0	0
NUDT2	0	1	0	0	0	0	0	0	0	0	0	0	0	0	0	0	0	0
IFIT3	0	0	0	0	1	0	0	0	0	0	0	0	0	0	0	0	0	0
ORMDL3	0	0	0	0	-1	0	0	0	0	1	0	0	0	0	0	0	0	0
ZNF529	0	0	0	0	0	0	0	0	0	1	0	0	0	0	0	0	0	0
GNG4	0	0	0	0	1	0	0	0	0	0	0	0	0	0	0	0	0	0
MARCH9	0	0	0	0	0	0	0	0	1	0	0	0	0	0	0	0	0	0
SDC2	0	0	0	0	-1	0	0	0	0	1	0	0	0	0	0	0	0	0
ENSCAFG00000031664	0	0	0	0	0	0	0	0	0	1	0	0	0	0	0	0	0	0
CNRIP1	0	1	0	0	0	0	0	0	0	0	0	0	0	0	0	0	0	0
POLR2M	0	0	0	0	0	0	0	0	0	1	0	0	0	0	0	0	0	0
CRADD	0	1	0	0	0	0	0	0	0	0	0	0	0	0	0	0	0	0
ATXN10	0	0	0	0	0	0	0	0	0	1	0	0	0	0	0	0	0	0
PHETA2	0	1	0	0	0	0	0	0	0	0	0	0	0	0	0	0	0	0
STC2	0	0	0	0	0	0	0	0	0	1	0	0	0	0	0	0	0	0
YAP1	0	0	0	0	0	0	0	0	0	1	0	0	0	0	0	0	0	0
NAPRT	0	1	0	0	0	0	0	0	0	0	0	0	0	0	0	0	0	0
RAP2B	0	1	0	0	0	0	0	0	0	0	0	0	0	0	0	0	0	0
NIPAL1	0	1	0	0	0	0	0	0	1	0	0	0	0	0	0	0	0	0
ZNF146	0	0	0	0	1	0	0	0	0	0	0	0	0	0	0	0	0	0
ENSCAFG00000031808	0	0	0	0	1	0	0	0	0	0	0	0	0	0	0	0	0	0
HCN4	0	0	0	0	0	0	0	0	0	1	0	0	0	1	0	0	1	0
ENSCAFG00000031827	0	0	0	0	0	0	0	0	0	1	0	0	0	0	0	0	0	0
ENSCAFG00000031828	0	0	0	0	0	0	0	0	0	1	0	0	0	0	0	0	0	0
ENSCAFG00000031848	0	0	0	0	1	0	0	0	0	0	0	0	0	0	0	0	0	0
ZNF329	0	0	0	0	0	0	0	0	0	1	0	0	0	0	0	0	0	0
ENSCAFG00000031865	0	0	0	0	1	0	0	0	0	0	0	0	0	0	0	0	0	0
ZNF518A	0	0	0	0	1	0	0	0	0	0	0	0	0	0	0	0	0	0
HPCAL1	0	1	0	0	0	0	0	0	0	-1	0	0	0	0	0	0	0	0
ATP6V0E1	0	0	0	0	0	0	0	0	0	1	0	0	0	0	0	0	0	0
ENSCAFG00000031903	0	0	0	0	1	0	0	0	0	0	0	0	0	0	0	0	0	0
ENSCAFG00000031912	0	1	0	0	0	0	0	0	0	0	0	0	0	0	0	0	0	0
TMEM26	0	0	0	0	0	0	0	0	0	0	1	0	0	0	0	1	0	1
ENSCAFG00000031933	0	1	0	0	0	0	0	0	0	0	0	0	0	0	0	0	0	0
ENSCAFG00000031956	0	0	0	0	1	0	0	0	0	0	0	0	0	0	0	0	0	0
NKX2-5	0	0	0	0	0	0	0	0	0	0	0	0	0	0	1	0	0	0
C9H9orf16	0	1	0	0	-1	0	0	0	0	0	0	0	0	0	0	0	0	0
SAR1B	0	0	0	0	0	0	0	0	0	1	0	0	0	0	0	0	0	0
HKR1	0	0	0	0	0	0	0	0	0	1	0	0	0	0	0	0	0	0
ENSCAFG00000032016	0	0	0	0	0	0	0	0	1	1	0	0	0	0	0	0	0	0
TRAPPC3L	0	0	0	0	0	0	0	0	1	0	0	0	0	0	0	0	0	0
ENSCAFG00000032029	0	0	0	0	0	0	0	0	0	1	0	0	0	0	0	0	0	0
TMEM119	0	0	0	0	-1	0	0	0	1	0	0	0	0	0	0	0	0	0
ENSCAFG00000032037	0	0	0	0	0	0	0	0	0	1	0	0	0	0	0	0	0	0
RAB11B	0	1	0	0	0	-1	0	0	0	1	0	0	0	0	0	0	0	0
FAM180B	0	1	0	0	0	0	0	0	0	0	0	0	0	0	0	0	0	0
GON7	0	0	0	0	-1	0	0	0	1	0	0	0	0	0	0	0	0	0
ENSCAFG00000032123	0	0	0	0	0	0	0	0	0	1	0	0	0	0	0	0	0	0
BAALC	0	0	0	0	1	0	0	0	0	1	0	0	0	0	0	0	0	0
ZNF792	0	0	0	0	1	0	0	0	0	0	0	0	0	0	0	0	0	0
TMEM240	0	1	0	0	0	0	0	0	0	0	0	0	0	0	0	0	0	0
ENSCAFG00000032225	0	0	0	0	0	0	0	0	0	1	0	0	0	0	0	0	0	0
NMNAT1	0	0	0	0	0	0	0	0	0	1	0	0	0	0	0	0	0	0
DHRS1	0	1	0	0	0	0	0	0	0	0	0	0	0	0	0	0	0	0
CLEC2L	0	1	0	0	0	0	0	0	0	0	0	0	0	0	0	0	0	0
FXYD6	0	0	0	0	1	0	0	0	0	0	0	0	0	0	0	0	0	0
XKR8	0	1	0	0	0	0	0	0	0	0	0	0	0	0	0	0	0	0
BMP6	0	0	0	0	1	0	0	0	0	1	0	0	0	0	0	0	0	0
ENSCAFG00000032311	0	0	0	0	1	0	0	0	0	0	0	0	0	0	0	0	0	0
FGL1	0	1	0	0	0	0	0	0	0	0	0	0	0	0	0	0	0	0
ENSCAFG00000032314	0	0	0	0	0	0	0	0	0	1	0	0	0	0	0	0	0	0
PDGFD	0	1	0	0	1	0	0	0	0	0	0	0	0	0	0	0	0	0
ENSCAFG00000032347	0	1	0	0	-1	0	0	0	0	0	0	0	0	0	0	0	0	0
ENSCAFG00000032349	0	0	0	0	1	1	0	0	0	0	0	0	0	0	0	0	0	0
UBE2D1	0	0	0	0	1	0	0	0	0	0	0	0	0	0	0	0	0	0
ENSCAFG00000032355	0	0	0	0	1	0	0	0	0	0	0	0	0	0	0	0	0	0
ZDHHC22	0	0	0	0	1	0	0	0	0	0	0	0	0	0	0	0	0	0
ENSCAFG00000032375	0	1	0	0	0	0	0	0	1	0	0	0	0	0	0	0	0	0
DENR	0	-1	0	0	0	1	0	0	0	0	0	0	0	0	0	0	0	0
DCAKD	0	1	0	0	0	0	0	0	0	0	0	0	0	0	0	0	0	0
SCAMP1	0	-1	0	0	1	0	0	0	0	0	0	0	0	0	0	0	0	0
CCDC117	0	0	0	0	1	0	0	0	0	0	0	0	0	0	0	0	0	0
NPAS1	0	0	0	0	0	0	0	0	1	1	0	0	0	0	0	0	0	0
TMEM225B	0	1	0	0	0	0	0	0	0	0	0	0	0	0	0	0	0	0
FLI1	0	0	0	0	1	0	0	0	0	0	0	0	0	0	0	0	0	0
PLEKHA2	0	-1	0	0	0	0	0	0	0	1	0	0	0	0	0	0	0	0
RARRES1	0	0	0	0	-1	0	0	0	0	1	0	0	0	0	0	0	0	0
SCP2	0	0	0	0	-1	0	0	0	0	1	0	0	0	0	0	0	0	0
PTPMT1	0	1	0	0	0	0	0	0	0	0	0	0	0	0	0	0	0	0
ENSCAFG00000032445	0	0	0	0	0	0	0	0	0	0	1	0	0	0	0	0	0	0
ARPC5L	0	0	0	0	1	0	0	0	0	0	0	0	0	0	0	0	0	0
ZNF621	0	1	0	0	0	0	0	0	0	0	0	0	0	0	0	0	0	0
COL8A2	0	0	0	0	0	0	0	0	1	0	0	0	0	0	0	0	0	0
DDIT4	0	1	0	0	0	0	0	0	0	1	0	0	0	0	0	0	0	0
EN1	0	1	0	0	0	0	0	0	0	0	1	0	0	0	0	0	0	0
GINM1	0	0	0	0	0	0	0	0	0	1	0	0	0	0	0	0	0	0
ENSCAFG00000032488	0	-1	0	0	0	0	0	0	0	1	0	0	0	0	0	0	0	0
CRELD1	0	0	0	0	0	0	0	0	0	1	0	0	0	0	0	0	0	0
VGLL3	0	1	0	0	-1	0	0	0	1	0	0	0	0	0	0	0	0	0
TSPYL5	0	0	0	0	0	0	0	0	0	1	0	0	0	0	0	0	0	0
REEP3	0	0	0	0	0	0	0	0	0	1	0	0	0	0	0	0	0	0
ENSCAFG00000032541	0	1	0	0	0	0	0	0	0	0	0	0	0	0	0	0	0	0
RBP1	0	0	0	0	1	0	0	0	0	0	0	0	0	0	0	0	0	0
IRX3	0	1	0	0	0	0	0	0	1	0	0	0	0	0	0	0	0	0
METTL7A	0	1	0	0	0	0	0	0	0	0	0	0	0	0	0	0	0	0
ENSCAFG00000032585	0	0	0	0	1	0	0	0	0	0	0	0	0	0	0	0	0	0
RASD2	0	1	0	0	0	0	1	0	0	0	0	0	0	0	0	0	0	0
C4orf48	0	0	0	0	0	0	0	0	1	0	0	0	0	0	0	0	0	0
ENSCAFG00000032618	0	0	0	0	0	0	0	0	1	0	0	0	0	0	0	0	0	0
ENSCAFG00000032625	0	0	1	0	0	0	0	0	0	-1	0	0	0	0	0	0	0	0
WNT7A	0	0	0	0	0	0	0	0	0	0	0	1	0	0	0	0	0	0

ENSCAFG00000032635	0	0	0	0	0	0	0	0	0	1	0	0	0	0	0	0	0	0
MRRF	0	0	0	0	1	0	0	0	0	0	0	0	0	0	0	0	0	0
PPP1R2	0	0	0	0	1	0	0	0	0	0	0	0	0	0	0	0	0	0
SPOCK1	0	0	0	0	0	0	0	0	1	0	0	0	0	0	0	0	0	0
FGF13	0	0	0	0	0	0	0	0	0	1	0	0	0	0	0	0	0	0
C1QTNF5	0	0	0	0	0	0	0	0	1	0	0	0	0	0	0	0	0	0
MAP6D1	0	0	0	0	-1	0	0	0	1	0	0	0	0	0	0	0	0	0
ST8SIA6	0	0	0	0	1	0	0	0	0	0	0	0	0	0	0	0	0	0
EMCN	0	0	0	0	1	0	0	0	0	0	0	0	0	0	0	0	0	0
ENSCAFG00000032728	0	0	0	0	1	0	0	0	0	-1	0	0	0	0	0	0	0	0
ENSCAFG00000032731	0	0	0	0	0	1	0	0	0	0	0	0	0	0	0	0	0	0
MXD1	0	1	0	0	0	0	0	0	0	0	0	0	0	0	0	0	0	0
PHLPP1	-1	1	0	0	0	0	0	0	0	0	0	0	0	0	0	0	0	0
SYNE1	-1	1	0	0	0	0	0	0	0	0	0	0	0	0	0	0	0	0
STEAP2	-1	1	0	0	0	0	0	0	0	0	0	0	0	0	0	0	0	0
ASPN	-1	0	0	0	-1	1	0	0	0	0	0	0	0	0	0	0	0	0
HOXA1	-1	0	0	1	0	0	0	0	0	0	0	0	0	0	0	0	0	0
HOXA5	-1	1	0	0	0	0	0	1	0	-1	0	0	0	0	0	0	0	0
HOXA7	-1	1	0	0	0	0	1	0	1	-1	0	0	0	0	0	0	0	0
EHBP1	-1	1	0	0	0	0	0	0	0	0	0	0	0	0	0	0	0	0
PLAUR	-1	0	0	0	0	0	0	0	1	-1	0	0	0	0	0	0	0	0
RAB3GAP1	-1	0	0	0	0	1	0	0	0	0	0	0	0	0	0	0	0	0
ELK3	-1	0	0	0	1	0	0	0	0	-1	0	0	0	0	0	0	0	0
HOXC4	-1	1	0	0	-1	0	0	0	0	-1	0	0	0	0	0	0	0	0
USP47	-1	0	0	0	0	1	0	0	0	0	0	0	0	0	0	0	0	0
COBLL1	-1	0	0	0	1	0	0	0	1	-1	0	0	0	-1	0	0	0	0
YEATS2	-1	1	0	0	0	0	0	0	1	1	0	0	0	0	0	0	0	0
ANPEP	-1	1	0	0	-1	0	0	0	0	-1	0	0	0	0	0	0	0	0
ATRIP	-1	1	0	0	0	0	0	0	0	-1	0	0	0	0	0	0	0	0
YBX3	-1	1	0	0	0	0	1	0	0	-1	0	0	0	0	0	0	0	0
USP28	-1	0	0	0	1	0	0	0	0	0	0	0	0	0	0	0	0	0
PAPSS2	-1	0	0	0	0	0	0	0	1	0	0	0	0	0	0	0	0	0
RCC2	-1	-1	0	0	1	0	0	0	0	-1	0	0	0	0	0	0	0	0
STX4	-1	1	0	0	0	0	0	0	0	0	0	0	0	0	0	0	0	0
ENSCAFG00000017317	-1	0	1	0	1	1	0	0	0	0	0	0	0	0	0	0	0	0
ENSCAFG00000017848	-1	0	1	0	1	1	0	0	0	0	0	0	0	0	0	0	0	0
MID2	-1	1	0	0	0	0	0	0	1	0	0	0	0	0	0	0	0	0
MYO1D	-1	0	0	0	0	0	0	0	1	0	0	0	0	0	0	0	0	0
ADAMTS12	-1	0	0	0	0	0	1	0	0	0	0	0	0	0	0	0	0	0
LRP8	-1	1	0	0	0	0	0	0	0	0	0	0	0	0	0	0	0	0
ACVRL1	-1	1	0	0	0	0	0	0	0	0	0	0	0	0	0	0	0	0
LCLAT1	-1	0	0	0	0	0	0	0	1	0	0	0	0	0	0	0	0	0
FBXL7	-1	1	0	0	0	0	0	0	0	-1	1	0	0	0	0	0	0	0
HOXA3	-1	0	0	1	0	1	0	1	0	-1	0	0	1	0	1	0	1	0
